# Supplementary material for: Epitope Classification and RBD Binding Properties of Neutralizing Antibodies Against SARS-CoV-2 Variants of Concern
Source: Front Immunol. 2021 Jun 4;12:691715. doi: 10.3389/fimmu.2021.691715 (PMC8212047; doi:10.3389/fimmu.2021.691715)
Supplement: Supplementary file 1 [file DataSheet_1.pdf]

Supplementary Material

Epitope classification and RBD binding properties of neutralizing antibodies against SARS-CoV-2 variants of concern

Ashlesha Deshpande[1], Bethany D. Harris[1], Luis Martinez-Sobrido[2], James J. Kobie[3], and Mark R. Walter[1,4*]

* correspondence Mark R. Walter (walter@uab.edu)

1. Supplementary Figure 1
2. Supplementary Table 1, listing of GISAID S sequence headers: four separate files ST1a.pdf,ST1b.pdf,ST1c.pdf, ST1d.pdf

345**I**
|
MRPTLLWSLLLLLGVFAAAAAG*TNLCPFGEVFNATRFASVYAWNRKRISNCVADYSVLYNSASFSTFKCYGVSPTKLNDLC*

417**T** 440**Y** 446**V** 452**R**
| | | |
FTNVYADSFVIRGDEVRQIAPGQTGKIADYNYKLPDDFTGCVIAWNSNNLDSKVGGNYNYLYRLFRKSNLKPFERDISTEI

475**V** 484**R** 501**Y**505**W**
| | | |
*YQAGSTPCNGVEGFNCYFPLQSYGFQPTNGVGYQPYRVVVLSFELLHAPATVCGPKKST*GSGGGGPSVFIFPPKIKDVLMI
SLSPIVTCVVVDVSEDDPDVQISWFVNNVEVHTAQTQTHREDYNSTLRVVSALPIQHQDWMSGKEFKCKVNNKDLPAPIER
TISKPKGSVRAPQVYVLPPPEEEMTKKQVTLTCMVTDFMPEDIYVEWTNNGKTELNYKNTEPVLDSDGSYFMTSKLRVEKK
NWVERNSYSCSVVHEGLHNHHTTKSFGGCGTPASGGLNDIFEAQKIEWHE

Supplementary Figure 1. Amino acid sequence of RBD-FC and location of variants
Amino acid sequence of the SARS-CoV-2 RBD-FC used for variant binding analysis as previously described (1). RBD (WA-1 strain) residues 333-531 are shown in italics, followed by the murine IgG1 FC and a C-terminal biotinylation tag. The heterologous signal sequence is underlined. The location of RBD variants studied are shown on the RBD sequence with residue numbers and the variant amino acid in bold.

1. Piepenbrink, M. S., Park, J. G., Oladunni, F. S., Deshpande, A., Basu, M., Sarkar, S., Loos, A., Woo, J., Lovalenti, P., Sloan, D., Ye, C., Chiem, K., Bates, C. W., Burch, R. E., Erdmann, N. B., Goepfert, P. A., Truong, V. L., Walter, M. R., Martinez-Sobrido, L., and Kobie, J. J. (2021) Therapeutic activity of an inhaled potent SARS-CoV-2 neutralizing human monoclonal antibody in hamsters. *Cell reports medicine* **2**, 100218
