# Supplementary material for: Epitope Classification and RBD Binding Properties of Neutralizing Antibodies Against SARS-CoV-2 Variants of Concern
Source: Front Immunol. 2021 Jun 4;12:691715. doi: 10.3389/fimmu.2021.691715 (PMC8212047; doi:10.3389/fimmu.2021.691715)
Supplement: Supplementary file 2 [file DataSheet_2.pdf]

Supplementary Table 1. GISAID sequence headers (1-75,000)for each SARS-CoV-2 S amino acid sequence used in the frequency analysis.

>Spike|hCoV-19/Wuhan/WIV04/2019|2019-12-30|EPI_ISL_402124|Original|hCoV-19^^Hubei|Human|Wuhan Jinyintan Hospital|Wuhan Institute of Virology|Shi|China
>Spike|hCoV-19/Shanghai/IVDC-SH-001/2020|2020-01-20|EPI_ISL_408483|Original|hCoV-19^^Shanghai|Human|National Institute for Viral Disease Control and Prevention|National Institute for Viral Disease Control & Prevention|Tan|China
>Spike|hCoV-19/Beijing/WH-09/2020|2020-01-08|EPI_ISL_411957|Vero E6|hCoV-19^^China|Human|Key Laboratory of Human Diseases|Key Laboratory of Human Diseases|Submitter|China
>Spike|hCoV-19/Wuhan/HBCDC-HB-04/2019|2019-12-30|EPI_ISL_412900|Original|hCoV-19^^Hubei|Human|Wuhan Jinyintan Hospital|Hubei Provincial Center for Disease Control and Prevention|Fang|China
>Spike|hCoV-19/Guangzhou/IQTC02/2020|2020-01-29|EPI_ISL_412967|Vero|hCoV-19^^Guangzhou|Human|Technology Centre|Technology Centre|Submitter|China
>Spike|hCoV-19/Japan/Hu_DP_Kng_19-027/2020|2020-02-10|EPI_ISL_412969|unknown|hCoV-19^^Japan|Human|Takayuki Hishiki Kanagawa Prefectural Institute of Public Health|Takayuki Hishiki Kanagawa Prefectural Institute of Public Health|Submitter|Japan
>Spike|hCoV-19/Switzerland/ZH-UZH-1000477102/2020|2020-02-26|EPI_ISL_413019|Original|hCoV-19^^Zurich|Human|Department of Internal Medicine|Institute of Medical Virology|Huber|Switzerland
>Spike|hCoV-19/USA/CA-CDPH-UC11/2020|2020-03-05|EPI_ISL_413931|Original|hCoV-19^^California|Human|California Department of Public Health|Chiu Laboratory|Chiu|USA
>Spike|hCoV-19/USA/MN1-MDH1/2020|2020-03-05|EPI_ISL_414588|Original|hCoV-19^^Minnesota|Human|Minnesota Department of Health|Minnesota Department of Health|Wang|USA
>Spike|hCoV-19/Shandong/LY002-2/2020|2020-01-21|EPI_ISL_414935|Original|hCoV-19^^Shandong|Human|Shandong Provincial Center for Disease Control and Prevention|Beijing Institute of Microbiology and Epidemiology|Ma|China
>Spike|hCoV-19/Denmark/SSI-101/2020|2020-03-03|EPI_ISL_415646|Original|hCoV-19^^Denmark|Human|Department of Virus and Microbiological Special diagnostics|ViFU|Rasmussen|Denmark
>Spike|hCoV-19/Denmark/SSI-102/2020|2020-03-03|EPI_ISL_415647|Original|hCoV-19^^Copenhagen|Human|Department of Virus and Microbiological Special diagnostics|Statens Serum Institute|Rasmussen|Denmark
>Spike|hCoV-19/Hangzhou/ZJU-05/2020|2020-01-22|EPI_ISL_415711|Vero|hCoV-19^^Hangzhou|Human|State Key Laboratory for Diagnosis and Treatment of Infectious Diseases|State Key Laboratory for Diagnosis and Treatment of Infectious Diseases|Jiang|China
>Spike|hCoV-19/Hangzhou/ZJU-02/2020|2020-01-26|EPI_ISL_416042|Vero|hCoV-19^^Hangzhou|Human|State Key Laboratory for Diagnosis and Treatment of Infectious Diseases|State Key Laboratory for Diagnosis and Treatment of Infectious Diseases|Jiang|China
>Spike|hCoV-19/Hangzhou/ZJU-03/2020|2020-01-25|EPI_ISL_416044|Vero|hCoV-19^^Hangzhou|Human|State Key Laboratory for Diagnosis and Treatment of Infectious Diseases|State Key Laboratory for Diagnosis and Treatment of Infectious Diseases|Jiang|China
>Spike|hCoV-19/Hangzhou/ZJU-04/2020|2020-01-24|EPI_ISL_416046|Vero|hCoV-19^^Hangzhou|Human|State Key Laboratory for Diagnosis and Treatment of Infectious Diseases|State Key Laboratory for Diagnosis and Treatment of Infectious Diseases|Jiang|China
>Spike|hCoV-19/Hangzhou/ZJU-06/2020|2020-02-02|EPI_ISL_416047|Vero|hCoV-19^^Hangzhou|Human|State Key Laboratory for Diagnosis and Treatment of Infectious Diseases|State Key Laboratory for Diagnosis and Treatment of Infectious Diseases|Jiang|China
>Spike|hCoV-19/Hangzhou/ZJU-07/2020|2020-02-03|EPI_ISL_416425|Vero|hCoV-19^^Hangzhou|Human|State Key Laboratory for Diagnosis and Treatment of Infectious Diseases|State Key Laboratory for Diagnosis and Treatment of Infectious Diseases|Jiang|China

>Spike|hCoV-19/Saudi Arabia/KAIMRC-Alghoribi/2020|2020-03-07|EPI_ISL_416432|Original|hCoV-19^^Riyadh|Human|Clinical Microbiology Lab|Infectious Disease Research Department|Alswaji|Saudi Arabia
>Spike|hCoV-19/USA/WA-UW95/2020|2020-03-10|EPI_ISL_416451|Original|hCoV-19^^Washington|Human|UW Virology Lab|UW Virology Lab|Roychoudhury|USA
>Spike|hCoV-19/Hangzhou/ZJU-09/2020|2020-01-28|EPI_ISL_416474|Vero|hCoV-19^^Hangzhou|Human|State Key Laboratory for Diagnosis and Treatment of Infectious Diseases|State Key Laboratory for Diagnosis and Treatment of Infectious Diseases|Jiang|China
>Spike|hCoV-19/Saudi Arabia/SCDC-3321/2020|2020-03-10|EPI_ISL_416521|Original|hCoV-19^^Saudi Arabia|Human|Public Health Laboratory|Public Health Laboratory|Albarrag|Saudi Arabia
>Spike|hCoV-19/Saudi Arabia/SCDC-3324/2020|2020-03-10|EPI_ISL_416522|Original|hCoV-19^^Saudi Arabia|Human|Public Health Laboratory|Public Health Laboratory|Albarrag|Saudi Arabia
>Spike|hCoV-19/USA/WA-UW106/2020|2020-03-11|EPI_ISL_416644|Original|hCoV-19^^Washington|Human|UW Virology Lab|UW Virology Lab|Roychoudhury|USA
>Spike|hCoV-19/USA/WA-UW116/2020|2020-03-11|EPI_ISL_416654|Original|hCoV-19^^Washington|Human|UW Virology Lab|UW Virology Lab|Roychoudhury|USA
>Spike|hCoV-19/USA/WA-UW117/2020|2020-03-11|EPI_ISL_416655|Original|hCoV-19^^Washington|Human|UW Virology Lab|UW Virology Lab|Roychoudhury|USA
>Spike|hCoV-19/USA/WA-UW123/2020|2020-03-12|EPI_ISL_416661|Original|hCoV-19^^Washington|Human|UW Virology Lab|UW Virology Lab|Roychoudhury|USA
>Spike|hCoV-19/USA/WA-UW143/2020|2020-03-11|EPI_ISL_416681|Original|hCoV-19^^Washington|Human|UW Virology Lab|UW Virology Lab|Roychoudhury|USA
>Spike|hCoV-19/USA/WA-UW152/2020|2020-03-13|EPI_ISL_416690|Original|hCoV-19^^Washington|Human|UW Virology Lab|UW Virology Lab|Roychoudhury|USA
>Spike|hCoV-19/USA/MN5-MDH5/2020|2020-03-10|EPI_ISL_417191|Original|hCoV-19^^Minnesota|Human|Minnesota Department of Health|Minnesota Department of Health|Wang|USA
>Spike|hCoV-19/USA/MN30-MDH30/2020|2020-03-12|EPI_ISL_417198|Original|hCoV-19^^Minnesota|Human|Minnesota Department of Health|Minnesota Department of Health|Wang|USA
>Spike|hCoV-19/England/20106005103/2020|2020-03-03|EPI_ISL_417242|Original|hCoV-19^^England|Human|Respiratory Virus Unit|Respiratory Virus Unit|Platt|United Kingdom
>Spike|hCoV-19/England/20108006603/2020|2020-03-03|EPI_ISL_417253|Original|hCoV-19^^England|Human|Respiratory Virus Unit|Respiratory Virus Unit|Platt|United Kingdom
>Spike|hCoV-19/USA/CA-SCCPHD-UC14/2020|2020-02-29|EPI_ISL_417318|Original|hCoV-19^^California|Human|Santa Clara County Public Health Department|Chiu Laboratory|Chiu|USA
>Spike|hCoV-19/USA/CA-CDPH-UC20/2020|2020-03-04|EPI_ISL_417324|Original|hCoV-19^^California|Human|California Department of Public Health|Chiu Laboratory|Chiu|USA
>Spike|hCoV-19/USA/CA-CDPH-UC28/2020|2020-03-14|EPI_ISL_417332|Original|hCoV-19^^California|Human|Chiu Laboratory|Chiu Laboratory|Chiu|USA
>Spike|hCoV-19/USA/WA-UW192/2020|2020-03-13|EPI_ISL_417341|Original|hCoV-19^^Washington|Human|UW Virology Lab|UW Virology Lab|Roychoudhury|USA
>Spike|hCoV-19/Australia/NSW31/2020|2020-03-10|EPI_ISL_417392|Original|hCoV-19^^New South Wales|Human|Centre for Infectious Diseases and Microbiology Public Health|NSW Health Pathology - Institute of Clinical Pathology and Medical Research; Westmead Hospital; University of Sydney|Eden|Australia
>Spike|hCoV-19/USA/WA-UW236/2020|2020-03-14|EPI_ISL_417450|Original|hCoV-19^^Washington|Human|UW Virology Lab|UW Virology Lab|Roychoudhury|USA
>Spike|hCoV-19/USA/MN34-MDH34/2020|2020-03-12|EPI_ISL_417471|Original|hCoV-19^^Minnesota|Human|Minnesota Department of Health|Minnesota Department of Health|Wang|USA
>Spike|hCoV-19/USA/MN47-MDH47/2020|2020-03-13|EPI_ISL_417494|Original|hCoV-19^^Minnesota|Human|Minnesota Department of Health|Minnesota Department of Health|Wang|USA

>Spike|hCoV-19/USA/MN51-MDH51/2020|2020-03-13|EPI_ISL_417496|Original|hCoV-19^^Minnesota|Human|Minnesota Department of Health|Minnesota Department of Health|Wang|USA
>Spike|hCoV-19/Iceland/122/2020|2020-03-17|EPI_ISL_417547|Original|hCoV-19^^Reykjavik|Human|deCODE genetics|deCODE genetics|J\u00f3nsson|Iceland
>Spike|hCoV-19/Iceland/250/2020|2020-03-17|EPI_ISL_417578|Original|hCoV-19^^Reykjavik|Human|The National University Hospital of Iceland|deCODE genetics|J\u00f3nsson|Iceland
>Spike|hCoV-19/Iceland/342/2020|2020-03-15|EPI_ISL_417671|Original|hCoV-19^^Reykjavik|Human|deCODE genetics|deCODE genetics|J\u00f3nsson|Iceland
>Spike|hCoV-19/Iceland/207/2020|2020-03-16|EPI_ISL_417733|Original|hCoV-19^^Reykjavik|Human|The National University Hospital of Iceland|deCODE genetics|J\u00f3nsson|Iceland
>Spike|hCoV-19/Iceland/68/2020|2020-03-08|EPI_ISL_417858|Original|hCoV-19^^Reykjavik|Human|The National University Hospital of Iceland|deCODE genetics|J\u00f3nsson|Iceland
>Spike|hCoV-19/USA/CT-UW250/2020|2020-03-14|EPI_ISL_418035|Original|hCoV-19^^Connecticut|Human|UW Virology Lab|UW Virology Lab|Roychoudhury|USA
>Spike|hCoV-19/USA/CT-UW258/2020|2020-03-14|EPI_ISL_418043|Original|hCoV-19^^Connecticut|Human|UW Virology Lab|UW Virology Lab|Roychoudhury|USA
>Spike|hCoV-19/USA/WA-UW278/2020|2020-03-16|EPI_ISL_418063|Original|hCoV-19^^Washington|Human|UW Virology Lab|UW Virology Lab|Roychoudhury|USA
>Spike|hCoV-19/USA/WA-UW283/2020|2020-03-14|EPI_ISL_418068|Original|hCoV-19^^Washington|Human|UW Virology Lab|UW Virology Lab|Roychoudhury|USA
>Spike|hCoV-19/USA/WA-UW289/2020|2020-03-15|EPI_ISL_418074|Original|hCoV-19^^Washington|Human|UW Virology Lab|UW Virology Lab|Roychoudhury|USA
>Spike|hCoV-19/USA/WA-UW295/2020|2020-03-15|EPI_ISL_418080|Original|hCoV-19^^Washington|Human|UW Virology Lab|UW Virology Lab|Roychoudhury|USA
>Spike|hCoV-19/Wales/PHWC-23DB5/2020|2020-03-13|EPI_ISL_418113|Original|hCoV-19^^Wales|Human|Wales Specialist Virology Centre|Public Health Wales Microbiology Cardiff|Moore|United Kingdom
>Spike|hCoV-19/Canada/ON-PHL-2294/2020|2020-02-00|EPI_ISL_418384|Original|hCoV-19^^Ontario|Human|Public Health Ontario Laboratories|Public Health Ontario Laboratories|Eshaghi|Canada
>Spike|hCoV-19/England/20124001702/2020|2020-03-17|EPI_ISL_418685|Original|hCoV-19^^England|Human|Respiratory Virus Unit|Respiratory Virus Unit|Platt|United Kingdom
>Spike|hCoV-19/England/20124094702/2020|2020-03-18|EPI_ISL_418725|Original|hCoV-19^^England|Human|Respiratory Virus Unit|Respiratory Virus Unit|Platt|United Kingdom
>Spike|hCoV-19/England/20124095802/2020|2020-03-13|EPI_ISL_418731|Original|hCoV-19^^England|Human|Respiratory Virus Unit|Respiratory Virus Unit|Platt|United Kingdom
>Spike|hCoV-19/USA/WA-UW312/2020|2020-03-14|EPI_ISL_418879|Original|hCoV-19^^Washington|Human|UW Virology Lab|UW Virology Lab|Roychoudhury|USA
>Spike|hCoV-19/USA/WA-UW337/2020|2020-03-16|EPI_ISL_418904|Original|hCoV-19^^Washington|Human|UW Virology Lab|UW Virology Lab|Roychoudhury|USA
>Spike|hCoV-19/USA/WA-UW361/2020|2020-03-15|EPI_ISL_418928|Original|hCoV-19^^Washington|Human|UW Virology Lab|UW Virology Lab|Roychoudhury|USA
>Spike|hCoV-19/USA/WA-UW368/2020|2020-03-16|EPI_ISL_418935|Original|hCoV-19^^Washington|Human|UW Virology Lab|UW Virology Lab|Roychoudhury|USA
>Spike|hCoV-19/USA/WA-UW373/2020|2020-03-17|EPI_ISL_418940|Original|hCoV-19^^Washington|Human|UW Virology Lab|UW Virology Lab|Roychoudhury|USA
>Spike|hCoV-19/Wales/PHWC-243C1/2020|2020-03-16|EPI_ISL_419405|Original|hCoV-19^^Wales|Human|Wales Specialist Virology Centre|Public Health Wales Microbiology Cardiff|Moore|United Kingdom
>Spike|hCoV-19/Wales/PHWC-24446/2020|2020-03-17|EPI_ISL_419411|Original|hCoV-19^^Wales|Human|Wales Specialist Virology Centre|Public Health Wales Microbiology Cardiff|Moore|United Kingdom
>Spike|hCoV-19/Wales/PHWC-24482/2020|2020-03-18|EPI_ISL_419415|Original|hCoV-19^^Wales|Human|Wales Specialist Virology Centre|Public Health Wales Microbiology Cardiff|Moore|United Kingdom

>Spike|hCoV-19/Wales/PHWC-24552/2020|2020-03-18|EPI_ISL_419427|Original|hCoV-19^^Wales|Human|Wales Specialist Virology Centre|Public Health Wales Microbiology Cardiff|Moore|United Kingdom
>Spike|hCoV-19/Wales/PHWC-2459E/2020|2020-03-17|EPI_ISL_419429|Original|hCoV-19^^Wales|Human|Wales Specialist Virology Centre|Public Health Wales Microbiology Cardiff|Moore|United Kingdom
>Spike|hCoV-19/Wales/PHWC-2482C/2020|2020-03-18|EPI_ISL_419451|Original|hCoV-19^^Wales|Human|Wales Specialist Virology Centre|Public Health Wales Microbiology Cardiff|Moore|United Kingdom
>Spike|hCoV-19/Wales/PHWC-24BD8/2020|2020-03-20|EPI_ISL_419497|Original|hCoV-19^^Wales|Human|Wales Specialist Virology Centre|Public Health Wales Microbiology Cardiff|Moore|United Kingdom
>Spike|hCoV-19/Wales/PHWC-24D1E/2020|2020-03-20|EPI_ISL_419502|Original|hCoV-19^^Wales|Human|Wales Specialist Virology Centre|Public Health Wales Microbiology Cardiff|Moore|United Kingdom
>Spike|hCoV-19/Australia/VIC73/2020|2020-03-14|EPI_ISL_419785|Original|hCoV-19^^Victoria|Human|Victorian Infectious Diseases Reference Laboratory (VIDRL)|Victorian Infectious Diseases Reference Laboratory and Microbiological Diagnostic Unit Public Health Laboratory|Schultz|Australia
>Spike|hCoV-19/Australia/VIC179/2020|2020-03-19|EPI_ISL_419876|Original|hCoV-19^^Victoria|Human|Victorian Infectious Diseases Reference Laboratory (VIDRL)|Victorian Infectious Diseases Reference Laboratory and Microbiological Diagnostic Unit Public Health Laboratory|Schultz|Australia
>Spike|hCoV-19/Australia/VIC211/2020|2020-03-20|EPI_ISL_419906|Original|hCoV-19^^Victoria|Human|Victorian Infectious Diseases Reference Laboratory (VIDRL)|Victorian Infectious Diseases Reference Laboratory and Microbiological Diagnostic Unit Public Health Laboratory|Schultz|Australia
>Spike|hCoV-19/England/20139061104/2020|2020-03-27|EPI_ISL_420735|Original|hCoV-19^^England|Human|Respiratory Virus Unit|Respiratory Virus Unit|Platt|United Kingdom
>Spike|hCoV-19/England/20142061304/2020|2020-03-27|EPI_ISL_420770|Original|hCoV-19^^England|Human|Respiratory Virus Unit|Respiratory Virus Unit|Platt|United Kingdom
>Spike|hCoV-19/Wales/PHWC-25032/2020|2020-03-21|EPI_ISL_420946|Original|hCoV-19^^Wales|Human|Wales Specialist Virology Centre|Public Health Wales Microbiology Cardiff|Moore|United Kingdom
>Spike|hCoV-19/Wales/PHWC-25524/2020|2020-03-23|EPI_ISL_421002|Original|hCoV-19^^Wales|Human|Wales Specialist Virology Centre|Public Health Wales Microbiology Cardiff|Moore|United Kingdom
>Spike|hCoV-19/England/20139010404/2020|2020-03-27|EPI_ISL_421871|Original|hCoV-19^^England|Human|Respiratory Virus Unit|Respiratory Virus Unit|Platt|United Kingdom
>Spike|hCoV-19/England/20139032004/2020|2020-03-27|EPI_ISL_421917|Original|hCoV-19^^England|Human|Respiratory Virus Unit|Respiratory Virus Unit|Platt|United Kingdom
>Spike|hCoV-19/England/20148002404/2020|2020-04-02|EPI_ISL_421998|Original|hCoV-19^^England|Human|Respiratory Virus Unit|Respiratory Virus Unit|Platt|United Kingdom
>Spike|hCoV-19/Wales/PHWC-26796/2020|2020-03-28|EPI_ISL_422016|Original|hCoV-19^^Wales|Human|Wales Specialist Virology Centre|Public Health Wales Microbiology Cardiff|Moore|United Kingdom
>Spike|hCoV-19/Wales/PHWC-2649F/2020|2020-03-27|EPI_ISL_422032|Original|hCoV-19^^Wales|Human|Wales Specialist Virology Centre|Public Health Wales Microbiology Cardiff|Moore|United Kingdom
>Spike|hCoV-19/Wales/PHWC-2666C/2020|2020-03-27|EPI_ISL_422046|Original|hCoV-19^^Wales|Human|Wales Specialist Virology Centre|Public Health Wales Microbiology Cardiff|Moore|United Kingdom
>Spike|hCoV-19/Wales/PHWC-25B6E/2020|2020-03-25|EPI_ISL_422063|Original|hCoV-19^^Wales|Human|Wales Specialist Virology Centre|Public Health Wales Microbiology Cardiff|Moore|United Kingdom
>Spike|hCoV-19/Wales/PHWC-26875/2020|2020-03-27|EPI_ISL_422184|Original|hCoV-19^^Wales|Human|Wales Specialist Virology Centre|Public Health Wales Microbiology Cardiff|Moore|United Kingdom

>Spike|hCoV-19/Wales/PHWC-25C4D/2020|2020-03-23|EPI_ISL_422263|Original|hCoV-19^^Wales|Human|Wales Specialist Virology Centre|Public Health Wales Microbiology Cardiff|Moore|United Kingdom
>Spike|hCoV-19/Wales/PHWC-26936/2020|2020-03-28|EPI_ISL_422300|Original|hCoV-19^^Wales|Human|Wales Specialist Virology Centre|Public Health Wales Microbiology Cardiff|Moore|United Kingdom
>Spike|hCoV-19/Wales/PHWC-26505/2020|2020-03-28|EPI_ISL_422312|Original|hCoV-19^^Wales|Human|Wales Specialist Virology Centre|Public Health Wales Microbiology Cardiff|Moore|United Kingdom
>Spike|hCoV-19/Wales/PHWC-25A9E/2020|2020-03-24|EPI_ISL_422349|Original|hCoV-19^^Wales|Human|Wales Specialist Virology Centre|Public Health Wales Microbiology Cardiff|Moore|United Kingdom
>Spike|hCoV-19/Wales/PHWC-26471/2020|2020-03-27|EPI_ISL_422360|Original|hCoV-19^^Wales|Human|Wales Specialist Virology Centre|Public Health Wales Microbiology Cardiff|Moore|United Kingdom
>Spike|hCoV-19/USA/UN-UW-1331/2020|2020-03-00|EPI_ISL_422968|Original|hCoV-19^^USA|Human|UW Virology Lab|UW Virology Lab|Roychoudhury|USA
>Spike|hCoV-19/USA/WA-UW-1593/2020|2020-03-19|EPI_ISL_423002|Original|hCoV-19^^Washington|Human|UW Virology Lab|UW Virology Lab|Roychoudhury|USA
>Spike|hCoV-19/USA/WA-UW-1426/2020|2020-03-18|EPI_ISL_423008|Original|hCoV-19^^Washington|Human|UW Virology Lab|UW Virology Lab|Roychoudhury|USA
>Spike|hCoV-19/USA/UN-UW-1449/2020|2020-03-00|EPI_ISL_423023|Original|hCoV-19^^USA|Human|UW Virology Lab|UW Virology Lab|Roychoudhury|USA
>Spike|hCoV-19/USA/WA-UW-1471/2020|2020-03-18|EPI_ISL_423029|Original|hCoV-19^^Washington|Human|UW Virology Lab|UW Virology Lab|Roychoudhury|USA
>Spike|hCoV-19/England/20134020004/2020|2020-03-23|EPI_ISL_423108|Original|hCoV-19^^England|Human|Respiratory Virus Unit|Respiratory Virus Unit|Platt|United Kingdom
>Spike|hCoV-19/England/20134020104/2020|2020-03-23|EPI_ISL_423109|Original|hCoV-19^^England|Human|Respiratory Virus Unit|Respiratory Virus Unit|Platt|United Kingdom
>Spike|hCoV-19/England/20130049804/2020|2020-03-23|EPI_ISL_423621|Original|hCoV-19^^England|Human|Respiratory Virus Unit|Respiratory Virus Unit|Platt|United Kingdom
>Spike|hCoV-19/England/20130061504/2020|2020-03-22|EPI_ISL_423640|Original|hCoV-19^^England|Human|Respiratory Virus Unit|Respiratory Virus Unit|Platt|United Kingdom
>Spike|hCoV-19/England/20132025004/2020|2020-03-22|EPI_ISL_423760|Original|hCoV-19^^England|Human|Respiratory Virus Unit|Respiratory Virus Unit|Platt|United Kingdom
>Spike|hCoV-19/England/20132027704/2020|2020-03-23|EPI_ISL_423766|Original|hCoV-19^^England|Human|Respiratory Virus Unit|Respiratory Virus Unit|Platt|United Kingdom
>Spike|hCoV-19/England/20132080404/2020|2020-03-24|EPI_ISL_423805|Original|hCoV-19^^England|Human|Respiratory Virus Unit|Respiratory Virus Unit|Platt|United Kingdom
>Spike|hCoV-19/USA/UN-UW-1486/2020|2020-03-18|EPI_ISL_424185|Original|hCoV-19^^USA|Human|UW Virology Lab|UW Virology Lab|Roychoudhury|USA
>Spike|hCoV-19/USA/WA-UW-1690/2020|2020-03-19|EPI_ISL_424216|Original|hCoV-19^^Washington|Human|UW Virology Lab|UW Virology Lab|Roychoudhury|USA
>Spike|hCoV-19/USA/WA-UW-1762/2020|2020-03-21|EPI_ISL_424245|Original|hCoV-19^^Washington|Human|UW Virology Lab|UW Virology Lab|Roychoudhury|USA
>Spike|hCoV-19/USA/UN-UW-1818/2020|2020-03-22|EPI_ISL_424266|Original|hCoV-19^^USA|Human|UW Virology Lab|UW Virology Lab|Roychoudhury|USA
>Spike|hCoV-19/Iran/HGRC-01-IPI-8206/2020|2020-03-09|EPI_ISL_424349|unknown|hCoV-19^^Iran|Human|Human Genetic Research Center|Human Genetic Research Center|Submitter|Iran
>Spike|hCoV-19/Iceland/348/2020|2020-03-00|EPI_ISL_424372|Original|hCoV-19^^Reykjavik|Human|The National University Hospital of Iceland|deCODE genetics|J\u00f3nsson|Iceland
>Spike|hCoV-19/Iceland/391/2020|2020-03-19|EPI_ISL_424415|Original|hCoV-19^^Reykjavik|Human|The National University Hospital of Iceland|deCODE genetics|J\u00f3nsson|Iceland
>Spike|hCoV-19/Iceland/533/2020|2020-03-27|EPI_ISL_424553|Original|hCoV-19^^Reykjavik|Human|deCODE genetics|deCODE genetics|J\u00f3nsson|Iceland
>Spike|hCoV-19/Iceland/550/2020|2020-03-28|EPI_ISL_424570|Original|hCoV-19^^Reykjavik|Human|deCODE genetics|deCODE genetics|J\u00f3nsson|Iceland

>Spike|hCoV-19/Iceland/559/2020|2020-03-26|EPI_ISL_424579|Original|hCoV-19^^Reykjavik|Human|The National University Hospital of Iceland|deCODE genetics|J\u00f3nsson|Iceland
>Spike|hCoV-19/Iceland/567/2020|2020-03-28|EPI_ISL_424587|Original|hCoV-19^^Reykjavik|Human|The National University Hospital of Iceland|deCODE genetics|J\u00f3nsson|Iceland
>Spike|hCoV-19/Iceland/600/2020|2020-03-28|EPI_ISL_424620|Original|hCoV-19^^Reykjavik|Human|The National University Hospital of Iceland|deCODE genetics|J\u00f3nsson|Iceland
>Spike|hCoV-19/Mexico/CMX-IMSS_01/2020|2020-03-13|EPI_ISL_424731|Original|hCoV-19^^Chihuahua|Human|Hospital General Regional No.66|Laboratorio Central de Epidemiolog\u00eda-DLVIE / Laboratorio de Secuenciaci\u00f3n-Centro de Instrumentos. Instituto Mexicano del Seguro Social|Gaytan|Mexico
>Spike|hCoV-19/Scotland/CVR138/2020|2020-03-16|EPI_ISL_425681|Original|hCoV-19^^Scotland|Human|West of Scotland Specialist Virology Centre|COVID-19 Genomics UK (COG-UK) Consortium|Robertson|United Kingdom
>Spike|hCoV-19/USA/ID-UW-4424/2020|2020-03-29|EPI_ISL_426071|Original|hCoV-19^^Idaho|Human|UW Virology Lab|UW Virology Lab|Roychoudhury|USA
>Spike|hCoV-19/USA/UN-UW-2069/2020|2020-03-21|EPI_ISL_426077|Original|hCoV-19^^USA|Human|UW Virology Lab|UW Virology Lab|Roychoudhury|USA
>Spike|hCoV-19/USA/WA-UW-2253/2020|2020-03-24|EPI_ISL_426132|Original|hCoV-19^^Washington|Human|UW Virology Lab|UW Virology Lab|Roychoudhury|USA
>Spike|hCoV-19/Slovenia/MB0329/2020|2020-03-29|EPI_ISL_426379|Original|hCoV-19^^Slovenia|Human|The National Laboratory of Health|The National Laboratory of Health|Hed\u017eet|Slovenia
>Spike|hCoV-19/Australia/NT18/2020|2020-00-00|EPI_ISL_426635|Original|hCoV-19^^Northern territory|Human|Royal Darwin Hospital Pathology|Microbiological Diagnostic Unit Public Health Laboratory and Victorian Infectious Diseases Reference Laboratory|Schultz|Australia
>Spike|hCoV-19/Australia/VIC328/2020|2020-03-17|EPI_ISL_426638|Original|hCoV-19^^Victoria|Human|Victorian Infectious Diseases Reference Laboratory (VIDRL)|Microbiological Diagnostic Unit Public Health Laboratory and Victorian Infectious Diseases Reference Laboratory|Schultz|Australia
>Spike|hCoV-19/Australia/VIC350/2020|2020-03-20|EPI_ISL_426658|Original|hCoV-19^^Victoria|Human|Victorian Infectious Diseases Reference Laboratory (VIDRL)|Microbiological Diagnostic Unit Public Health Laboratory and Victorian Infectious Diseases Reference Laboratory|Schultz|Australia
>Spike|hCoV-19/Australia/VIC353/2020|2020-03-21|EPI_ISL_426661|Original|hCoV-19^^Victoria|Human|Victorian Infectious Diseases Reference Laboratory (VIDRL)|Microbiological Diagnostic Unit Public Health Laboratory and Victorian Infectious Diseases Reference Laboratory|Schultz|Australia
>Spike|hCoV-19/Australia/VIC367/2020|2020-03-22|EPI_ISL_426675|Original|hCoV-19^^Victoria|Human|Victorian Infectious Diseases Reference Laboratory (VIDRL)|Microbiological Diagnostic Unit Public Health Laboratory and Victorian Infectious Diseases Reference Laboratory|Schultz|Australia
>Spike|hCoV-19/Australia/VIC406/2020|2020-03-25|EPI_ISL_426708|Original|hCoV-19^^Victoria|Human|Victorian Infectious Diseases Reference Laboratory (VIDRL)|Microbiological Diagnostic Unit Public Health Laboratory and Victorian Infectious Diseases Reference Laboratory|Schultz|Australia
>Spike|hCoV-19/Australia/VIC410/2020|2020-03-26|EPI_ISL_426712|Original|hCoV-19^^Victoria|Human|Victorian Infectious Diseases Reference Laboratory (VIDRL)|Microbiological Diagnostic Unit Public Health Laboratory and Victorian Infectious Diseases Reference Laboratory|Schultz|Australia
>Spike|hCoV-19/Australia/VIC426/2020|2020-03-25|EPI_ISL_426726|Original|hCoV-19^^Victoria|Human|Victorian Infectious Diseases Reference Laboratory (VIDRL)|Microbiological Diagnostic Unit Public Health Laboratory and Victorian Infectious Diseases Reference Laboratory|Schultz|Australia
>Spike|hCoV-19/Australia/VIC493/2020|2020-03-22|EPI_ISL_426766|Original|hCoV-19^^Victoria|Human|Victorian Infectious Diseases Reference Laboratory

(VIDRL)|Microbiological Diagnostic Unit Public Health Laboratory and Victorian Infectious Diseases Reference Laboratory|Schultz|Australia
>Spike|hCoV-19/Australia/VIC500/2020|2020-03-24|EPI_ISL_426772|Original|hCoV-19^^Victoria|Human|Victorian Infectious Diseases Reference Laboratory (VIDRL)|Microbiological Diagnostic Unit Public Health Laboratory and Victorian Infectious Diseases Reference Laboratory|Schultz|Australia
>Spike|hCoV-19/Australia/VIC514/2020|2020-03-23|EPI_ISL_426784|Original|hCoV-19^^Victoria|Human|Victorian Infectious Diseases Reference Laboratory (VIDRL)|Microbiological Diagnostic Unit Public Health Laboratory and Victorian Infectious Diseases Reference Laboratory|Schultz|Australia
>Spike|hCoV-19/Australia/VIC522/2020|2020-03-23|EPI_ISL_426790|Original|hCoV-19^^Victoria|Human|Victorian Infectious Diseases Reference Laboratory (VIDRL)|Microbiological Diagnostic Unit Public Health Laboratory and Victorian Infectious Diseases Reference Laboratory|Schultz|Australia
>Spike|hCoV-19/Australia/VIC527/2020|2020-03-24|EPI_ISL_426793|Original|hCoV-19^^Victoria|Human|Victorian Infectious Diseases Reference Laboratory (VIDRL)|Microbiological Diagnostic Unit Public Health Laboratory and Victorian Infectious Diseases Reference Laboratory|Schultz|Australia
>Spike|hCoV-19/Australia/VIC546/2020|2020-03-24|EPI_ISL_426809|Original|hCoV-19^^Victoria|Human|Victorian Infectious Diseases Reference Laboratory (VIDRL)|Microbiological Diagnostic Unit Public Health Laboratory and Victorian Infectious Diseases Reference Laboratory|Schultz|Australia
>Spike|hCoV-19/Australia/VIC588/2020|2020-03-21|EPI_ISL_426845|Original|hCoV-19^^Victoria|Human|Victorian Infectious Diseases Reference Laboratory (VIDRL)|Microbiological Diagnostic Unit Public Health Laboratory and Victorian Infectious Diseases Reference Laboratory|Schultz|Australia
>Spike|hCoV-19/Australia/VIC609/2020|2020-03-27|EPI_ISL_426863|Original|hCoV-19^^Victoria|Human|Victorian Infectious Diseases Reference Laboratory (VIDRL)|Microbiological Diagnostic Unit Public Health Laboratory and Victorian Infectious Diseases Reference Laboratory|Schultz|Australia
>Spike|hCoV-19/Australia/VIC615/2020|2020-03-28|EPI_ISL_426870|Original|hCoV-19^^Victoria|Human|Victorian Infectious Diseases Reference Laboratory (VIDRL)|Microbiological Diagnostic Unit Public Health Laboratory and Victorian Infectious Diseases Reference Laboratory|Schultz|Australia
>Spike|hCoV-19/Australia/VIC616/2020|2020-03-26|EPI_ISL_426871|Original|hCoV-19^^Victoria|Human|Victorian Infectious Diseases Reference Laboratory (VIDRL)|Microbiological Diagnostic Unit Public Health Laboratory and Victorian Infectious Diseases Reference Laboratory|Schultz|Australia
>Spike|hCoV-19/Australia/VIC481/2020|2020-03-29|EPI_ISL_426912|Original|hCoV-19^^Victoria|Human|Microbiological Diagnostic Unit Public Health Laboratory|Microbiological Diagnostic Unit Public Health Laboratory|Schultz|Australia
>Spike|hCoV-19/Australia/VIC483/2020|2020-03-29|EPI_ISL_426914|Original|hCoV-19^^Victoria|Human|Microbiological Diagnostic Unit Public Health Laboratory|Microbiological Diagnostic Unit Public Health Laboratory|Schultz|Australia
>Spike|hCoV-19/Australia/VIC627/2020|2020-03-29|EPI_ISL_426931|Original|hCoV-19^^Victoria|Human|Victorian Infectious Diseases Reference Laboratory (VIDRL)|Microbiological Diagnostic Unit Public Health Laboratory and Victorian Infectious Diseases Reference Laboratory|Schultz|Australia
>Spike|hCoV-19/Australia/VIC637/2020|2020-03-28|EPI_ISL_426933|Original|hCoV-19^^Victoria|Human|Victorian Infectious Diseases Reference Laboratory (VIDRL)|Microbiological Diagnostic Unit Public Health Laboratory and Victorian Infectious Diseases Reference Laboratory|Schultz|Australia
>Spike|hCoV-19/Australia/VIC652/2020|2020-03-28|EPI_ISL_426948|Original|hCoV-19^^Victoria|Human|Victorian Infectious Diseases Reference Laboratory (VIDRL)|Microbiological Diagnostic Unit Public Health Laboratory and Victorian Infectious Diseases Reference Laboratory|Schultz|Australia
>Spike|hCoV-19/Australia/VIC670/2020|2020-03-27|EPI_ISL_426966|Original|hCoV-19^^Victoria|Human|Victorian Infectious Diseases Reference Laboratory (VIDRL)|Microbiological Diagnostic Unit Public Health Laboratory and Victorian Infectious Diseases Reference Laboratory|Schultz|Australia

>Spike|hCoV-19/Australia/VIC674/2020|2020-03-27|EPI_ISL_426970|Original|hCoV-19^^Victoria|Human|Victorian Infectious Diseases Reference Laboratory (VIDRL)|Microbiological Diagnostic Unit Public Health Laboratory and Victorian Infectious Diseases Reference Laboratory|Schultz|Australia
>Spike|hCoV-19/Australia/VIC676/2020|2020-03-27|EPI_ISL_426971|Original|hCoV-19^^Victoria|Human|Victorian Infectious Diseases Reference Laboratory (VIDRL)|Microbiological Diagnostic Unit Public Health Laboratory and Victorian Infectious Diseases Reference Laboratory|Schultz|Australia
>Spike|hCoV-19/Australia/VIC680/2020|2020-03-27|EPI_ISL_426975|Original|hCoV-19^^Victoria|Human|Victorian Infectious Diseases Reference Laboratory (VIDRL)|Microbiological Diagnostic Unit Public Health Laboratory and Victorian Infectious Diseases Reference Laboratory|Schultz|Australia
>Spike|hCoV-19/Australia/VIC681/2020|2020-03-27|EPI_ISL_426976|Original|hCoV-19^^Victoria|Human|Victorian Infectious Diseases Reference Laboratory (VIDRL)|Microbiological Diagnostic Unit Public Health Laboratory and Victorian Infectious Diseases Reference Laboratory|Schultz|Australia
>Spike|hCoV-19/Australia/VIC683/2020|2020-03-29|EPI_ISL_426978|Original|hCoV-19^^Victoria|Human|Victorian Infectious Diseases Reference Laboratory (VIDRL)|Microbiological Diagnostic Unit Public Health Laboratory and Victorian Infectious Diseases Reference Laboratory|Schultz|Australia
>Spike|hCoV-19/Australia/VIC700/2020|2020-03-27|EPI_ISL_426990|Original|hCoV-19^^Victoria|Human|Victorian Infectious Diseases Reference Laboratory (VIDRL)|Microbiological Diagnostic Unit Public Health Laboratory and Victorian Infectious Diseases Reference Laboratory|Schultz|Australia
>Spike|hCoV-19/Australia/VIC721/2020|2020-03-27|EPI_ISL_427004|Original|hCoV-19^^Victoria|Human|Victorian Infectious Diseases Reference Laboratory (VIDRL)|Microbiological Diagnostic Unit Public Health Laboratory and Victorian Infectious Diseases Reference Laboratory|Schultz|Australia
>Spike|hCoV-19/Australia/VIC751/2020|2020-03-30|EPI_ISL_427028|Original|hCoV-19^^Victoria|Human|Victorian Infectious Diseases Reference Laboratory (VIDRL)|Microbiological Diagnostic Unit Public Health Laboratory and Victorian Infectious Diseases Reference Laboratory|Schultz|Australia
>Spike|hCoV-19/Australia/VIC761/2020|2020-03-30|EPI_ISL_427037|Original|hCoV-19^^Victoria|Human|Victorian Infectious Diseases Reference Laboratory (VIDRL)|Microbiological Diagnostic Unit Public Health Laboratory and Victorian Infectious Diseases Reference Laboratory|Schultz|Australia
>Spike|hCoV-19/Australia/VIC763/2020|2020-03-30|EPI_ISL_427039|Original|hCoV-19^^Victoria|Human|Victorian Infectious Diseases Reference Laboratory (VIDRL)|Microbiological Diagnostic Unit Public Health Laboratory and Victorian Infectious Diseases Reference Laboratory|Schultz|Australia
>Spike|hCoV-19/Australia/VIC774/2020|2020-03-18|EPI_ISL_427050|Original|hCoV-19^^Victoria|Human|Victorian Infectious Diseases Reference Laboratory (VIDRL)|Microbiological Diagnostic Unit Public Health Laboratory and Victorian Infectious Diseases Reference Laboratory|Schultz|Australia
>Spike|hCoV-19/Australia/VIC796/2020|2020-03-31|EPI_ISL_427070|Original|hCoV-19^^Victoria|Human|Microbiological Diagnostic Unit Public Health Laboratory|Microbiological Diagnostic Unit Public Health Laboratory|Schultz|Australia
>Spike|hCoV-19/Australia/VIC802/2020|2020-04-01|EPI_ISL_427076|Original|hCoV-19^^Victoria|Human|Microbiological Diagnostic Unit Public Health Laboratory|Microbiological Diagnostic Unit Public Health Laboratory|Schultz|Australia
>Spike|hCoV-19/Australia/VIC846/2020|2020-04-04|EPI_ISL_427106|Original|hCoV-19^^Victoria|Human|Victorian Infectious Diseases Reference Laboratory (VIDRL)|Microbiological Diagnostic Unit Public Health Laboratory and Victorian Infectious Diseases Reference Laboratory|Schultz|Australia
>Spike|hCoV-19/Australia/VIC895/2020|2020-04-06|EPI_ISL_427147|Original|hCoV-19^^Victoria|Human|Victorian Infectious Diseases Reference Laboratory (VIDRL)|Microbiological Diagnostic Unit Public Health Laboratory and Victorian Infectious Diseases Reference Laboratory|Schultz|Australia
>Spike|hCoV-19/Australia/VIC929/2020|2020-03-30|EPI_ISL_427157|Original|hCoV-19^^Victoria|Human|Victorian Infectious Diseases Reference Laboratory

(VIDRL)|Microbiological Diagnostic Unit Public Health Laboratory and Victorian Infectious Diseases Reference Laboratory|Schultz|Australia
>Spike|hCoV-19/USA/CT-UW-4251/2020|2020-03-31|EPI_ISL_427164|Original|hCoV-19^^Connecticut|Human|UW Virology Lab|UW Virology Lab|Roychoudhury|USA
>Spike|hCoV-19/USA/WA-UW-3895/2020|2020-03-27|EPI_ISL_427203|Original|hCoV-19^^Washington|Human|UW Virology Lab|UW Virology Lab|Roychoudhury|USA
>Spike|hCoV-19/USA/WA-UW-3959/2020|2020-03-27|EPI_ISL_427220|Original|hCoV-19^^Washington|Human|UW Virology Lab|UW Virology Lab|Roychoudhury|USA
>Spike|hCoV-19/USA/WA-UW-4130/2020|2020-03-31|EPI_ISL_427244|Original|hCoV-19^^Washington|Human|UW Virology Lab|UW Virology Lab|Roychoudhury|USA
>Spike|hCoV-19/USA/AZ-TG271866/2020|2020-03-23|EPI_ISL_427271|Original|hCoV-19^^Arizona|Human|AZ SPHL|TGen North|Lemmer|USA
>Spike|hCoV-19/USA/AZ-TG271868/2020|2020-03-23|EPI_ISL_427272|Original|hCoV-19^^Arizona|Human|AZ SPHL|TGen North|Lemmer|USA
>Spike|hCoV-19/Taiwan/TSGH-10/2020|2020-03-24|EPI_ISL_427397|Original|hCoV-19^^Taipei|Human|TSGH-CP molecular lab|TSGH-CP molecular lab|Shang|Taiwan
>Spike|hCoV-19/Qatar/QA19/2020|2020-03-23|EPI_ISL_427415|Original|hCoV-19^^Doha|Human|Ministry of Public Health (MoPH)|Biomedical Research Center (BRC)|Benslimane|Qatar
>Spike|hCoV-19/Australia/NSW65/2020|2020-03-04|EPI_ISL_427645|Original|hCoV-19^^New South Wales|Human|Centre for Infectious Diseases and Microbiology Public Health|NSW Health Pathology - Institute of Clinical Pathology and Medical Research; Westmead Hospital; University of Sydney|Gall|Australia
>Spike|hCoV-19/Australia/NSW66/2020|2020-03-05|EPI_ISL_427646|Original|hCoV-19^^New South Wales|Human|Centre for Infectious Diseases and Microbiology Public Health|NSW Health Pathology - Institute of Clinical Pathology and Medical Research; Westmead Hospital; University of Sydney|Gall|Australia
>Spike|hCoV-19/Spain/MD-H12-LP23-5852/2020|2020-03-08|EPI_ISL_428679|Original|hCoV-19^^Madrid|Human|Hospital Universitario La Paz|Hospital Universitario 12 de Octubre|Viedma|Spain
>Spike|hCoV-19/Spain/MD-H12-LP24-5999/2020|2020-03-09|EPI_ISL_428680|Original|hCoV-19^^Madrid|Human|Hospital Universitario La Paz|Hospital Universitario 12 de Octubre|Viedma|Spain
>Spike|hCoV-19/Spain/MD-H12-LP30-2226/2020|2020-03-11|EPI_ISL_428682|Original|hCoV-19^^Madrid|Human|Hospital Universitario La Paz|Hospital Universitario 12 de Octubre|Viedma|Spain
>Spike|hCoV-19/Spain/MD-H12-27/2020|2020-03-21|EPI_ISL_428690|Original|hCoV-19^^Madrid|Human|Hospital Universitario 12 de Octubre|Hospital Universitario 12 de Octubre|Viedma|Spain
>Spike|hCoV-19/Spain/MD-H12-33/2020|2020-03-12|EPI_ISL_428696|Original|hCoV-19^^Madrid|Human|Hospital Universitario 12 de Octubre|Hospital Universitario 12 de Octubre|Viedma|Spain
>Spike|hCoV-19/USA/WA-UW-4579/2020|2020-03-31|EPI_ISL_429606|Original|hCoV-19^^Washington|Human|UW Virology Lab|UW Virology Lab|Roychoudhury|USA
>Spike|hCoV-19/USA/WA-UW-4582/2020|2020-03-31|EPI_ISL_429607|Original|hCoV-19^^Washington|Human|UW Virology Lab|UW Virology Lab|Roychoudhury|USA
>Spike|hCoV-19/USA/VA-DCLS-0083/2020|2020-04-03|EPI_ISL_429981|Original|hCoV-19^^Virginia|Human|Virginia DCLS|Virginia DCLS|St. Jacques|USA
>Spike|hCoV-19/Jordan/SR-035/2020|2020-03-30|EPI_ISL_429995|Original|hCoV-19^^Amman|Human|Biolab Diagnostic Laboratories|Andersen lab at Scripps Research|Gangavarapu|Jordan
>Spike|hCoV-19/Jordan/SR-042/2020|2020-03-30|EPI_ISL_430000|Original|hCoV-19^^Amman|Human|Biolab Diagnostic Laboratories|Andersen lab at Scripps Research|Gangavarapu|Jordan
>Spike|hCoV-19/USA/UT-076/2020|2020-03-17|EPI_ISL_430027|Original|hCoV-19^^Utah|Human|Utah Public Health Laboratory|Utah Public Health Laboratory|Young|USA
>Spike|hCoV-19/Spain/MD-H12-LP29/2020|2020-03-11|EPI_ISL_430719|Original|hCoV-19^^Madrid|Human|Hospital Universitario La Paz|Hospital Universitario 12 de Octubre|Viedma|Spain

>Spike|hCoV-19/Spain/MD-H12-LP33/2020|2020-03-11|EPI_ISL_430721|Original|hCoV-19^^Madrid|Human|Hospital Universitario La Paz|Hospital Universitario 12 de Octubre|Viedma|Spain
>Spike|hCoV-19/Thailand/NIH-2492/2020|2020-03-17|EPI_ISL_430841|Original|hCoV-19^^Bangkok|Human|Praram 9 Hospital|National Institute of Health. Department of medical Sciences|Phuygun|Thailand
>Spike|hCoV-19/USA/WA-UW-5397/2020|2020-04-05|EPI_ISL_430905|Original|hCoV-19^^Washington|Human|UW Virology Lab|UW Virology Lab|Roychoudhury|USA
>Spike|hCoV-19/Fujian/IM3520002T/2020|2020-03-15|EPI_ISL_431180|Vero|hCoV-19^^Fujian|Human|Fujian Center for Disease Control and Prevention|Fujian Center for Disease Control and Prevention|Jianfeng|China
>Spike|hCoV-19/Fujian/IM3520004T/2020|2020-03-18|EPI_ISL_431292|Vero|hCoV-19^^Fujian|Human|Fujian Center for Disease Control and Prevention|Fujian Center for Disease Control and Prevention|Jianfeng|China
>Spike|hCoV-19/Fujian/IM3520008T/2020|2020-03-21|EPI_ISL_431779|Vero|hCoV-19^^Fujian|Human|Fujian Center for Disease Control and Prevention|Fujian Center for Disease Control and Prevention|Jianfeng|China
>Spike|hCoV-19/Fujian/IM3520010T/2020|2020-03-17|EPI_ISL_431780|Vero|hCoV-19^^Fujian|Human|Fujian Center for Disease Control and Prevention|Fujian Center for Disease Control and Prevention|Jianfeng|China
>Spike|hCoV-19/Fujian/IM3520013T/2020|2020-03-19|EPI_ISL_431781|Vero|hCoV-19^^Fujian|Human|Fujian Center for Disease Control and Prevention|Fujian Center for Disease Control and Prevention|Jianfeng|China
>Spike|hCoV-19/Fujian/IM3520017T/2020|2020-03-18|EPI_ISL_431784|Vero|hCoV-19^^Fujian|Human|Fujian Center for Disease Control and Prevention|Fujian Center for Disease Control and Prevention|Jianfeng|China
>Spike|hCoV-19/Fujian/3520022Y/2020|2020-01-23|EPI_ISL_431785|Vero|hCoV-19^^Fujian|Human|Fujian Center for Disease Control and Prevention|Fujian Center for Disease Control and Prevention|Jianfeng|China
>Spike|hCoV-19/Wales/PHWC-27838/2020|2020-04-01|EPI_ISL_431931|Original|hCoV-19^^Wales|Human|Wales Specialist Virology Centre|Public Health Wales Microbiology Cardiff|Moore|United Kingdom
>Spike|hCoV-19/Wales/PHWC-27856/2020|2020-03-25|EPI_ISL_431947|Original|hCoV-19^^Wales|Human|Wales Specialist Virology Centre|Public Health Wales Microbiology Cardiff|Moore|United Kingdom
>Spike|hCoV-19/Wales/PHWC-29FB9/2020|2020-04-03|EPI_ISL_431998|Original|hCoV-19^^Wales|Human|Wales Specialist Virology Centre|Public Health Wales Microbiology Cardiff|Moore|United Kingdom
>Spike|hCoV-19/Wales/PHWC-24E66/2020|2020-03-00|EPI_ISL_432020|Original|hCoV-19^^Wales|Human|Wales Specialist Virology Centre|Public Health Wales Microbiology Cardiff|Moore|United Kingdom
>Spike|hCoV-19/Wales/PHWC-28873/2020|2020-04-01|EPI_ISL_432082|Original|hCoV-19^^Wales|Human|Wales Specialist Virology Centre|Public Health Wales Microbiology Cardiff|Moore|United Kingdom
>Spike|hCoV-19/Wales/PHWC-28F14/2020|2020-04-01|EPI_ISL_432119|Original|hCoV-19^^Wales|Human|Wales Specialist Virology Centre|Public Health Wales Microbiology Cardiff|Moore|United Kingdom
>Spike|hCoV-19/Wales/PHWC-25603/2020|2020-03-24|EPI_ISL_432141|Original|hCoV-19^^Wales|Human|Wales Specialist Virology Centre|Public Health Wales Microbiology Cardiff|Moore|United Kingdom
>Spike|hCoV-19/Wales/PHWC-29E8F/2020|2020-04-05|EPI_ISL_432202|Original|hCoV-19^^Wales|Human|Wales Specialist Virology Centre|Public Health Wales Microbiology Cardiff|Moore|United Kingdom
>Spike|hCoV-19/Wales/PHWC-29D55/2020|2020-04-04|EPI_ISL_432244|Original|hCoV-19^^Wales|Human|Wales Specialist Virology Centre|Public Health Wales Microbiology Cardiff|Moore|United Kingdom
>Spike|hCoV-19/Wales/PHWC-29EDA/2020|2020-03-30|EPI_ISL_432319|Original|hCoV-19^^Wales|Human|Wales Specialist Virology Centre|Public Health Wales Microbiology Cardiff|Moore|United Kingdom

>Spike|hCoV-19/Wales/PHWC-28460/2020|2020-03-29|EPI_ISL_432347|Original|hCoV-19^^Wales|Human|Wales Specialist Virology Centre|Public Health Wales Microbiology Cardiff|Moore|United Kingdom
>Spike|hCoV-19/Wales/PHWC-29D46/2020|2020-04-03|EPI_ISL_432363|Original|hCoV-19^^Wales|Human|Wales Specialist Virology Centre|Public Health Wales Microbiology Cardiff|Moore|United Kingdom
>Spike|hCoV-19/Wales/PHWC-279AE/2020|2020-04-01|EPI_ISL_432438|Original|hCoV-19^^Wales|Human|Wales Specialist Virology Centre|Public Health Wales Microbiology Cardiff|Moore|United Kingdom
>Spike|hCoV-19/Wales/PHWC-27540/2020|2020-03-30|EPI_ISL_432442|Original|hCoV-19^^Wales|Human|Wales Specialist Virology Centre|Public Health Wales Microbiology Cardiff|Moore|United Kingdom
>Spike|hCoV-19/Scotland/CVR229/2020|2020-03-19|EPI_ISL_433275|Original|hCoV-19^^Scotland|Human|West of Scotland Specialist Virology Centre|COVID-19 Genomics UK (COG-UK) Consortium|Robertson|United Kingdom
>Spike|hCoV-19/Scotland/CVR544/2020|2020-03-24|EPI_ISL_433311|Original|hCoV-19^^Scotland|Human|West of Scotland Specialist Virology Centre|COVID-19 Genomics UK (COG-UK) Consortium|Robertson|United Kingdom
>Spike|hCoV-19/England/CAMB-78944/2020|2020-04-06|EPI_ISL_433794|Original|hCoV-19^^England|Human|Department of Pathology|COVID-19 Genomics UK (COG-UK) Consortium|Meredith|United Kingdom
>Spike|hCoV-19/Philippines/PGC04/2020|2020-03-26|EPI_ISL_434556|Original|hCoV-19^^Manila|Human|National Institutes of Health|Philippine Genome Center|Tablizo|Philippines
>Spike|hCoV-19/USA/VA-DCLS-0097/2020|2020-04-10|EPI_ISL_434591|Original|hCoV-19^^Virginia|Human|Virginia DCLS|Virginia DCLS|St. Jacques|USA
>Spike|hCoV-19/Thailand/NIH-2492-2/2020|2020-03-17|EPI_ISL_434704|Original|hCoV-19^^Bangkok|Human|Praram 9 Hospital|National Institute of Health. Department of medical Sciences|Okada|Thailand
>Spike|hCoV-19/United Arab Emirates/L068/2020|2020-03-14|EPI_ISL_435120|Original|hCoV-19^^United Arab Emirates|Human|Mohammed Bin Rashid University of Medicine and Health Sciences|Al Jalila Genomics Center|Ramaswamy|United Arab Emirates
>Spike|hCoV-19/USA/CA-SCCPHD-UC185/2020|2020-03-16|EPI_ISL_435664|Original|hCoV-19^^California|Human|Santa Clara County Public Health Department|Chiu Laboratory|Chiu|USA
>Spike|hCoV-19/USA/MI-MDHHS-SC20100/2020|2020-03-13|EPI_ISL_436809|Original|hCoV-19^^Michigan|Human|Michigan Department of Health and Human Services|Michigan Department of Health and Human Services|Blankenship|USA
>Spike|hCoV-19/USA/CA-CZB-1065/2020|2020-04-13|EPI_ISL_437065|Original|hCoV-19^^California|Human|County of Santa Clara Public Health|Chan-Zuckerberg Biohub|Hao|USA
>Spike|hCoV-19/USA/MI-MDHHS-SC20023/2020|2020-03-17|EPI_ISL_437110|Original|hCoV-19^^Michigan|Human|Michigan Department of Health and Human Services|Michigan Department of Health and Human Services|Blankenship|USA
>Spike|hCoV-19/DRC/2819/2020|2020-04-16|EPI_ISL_437347|Original|hCoV-19^^Kinshasa|Human|Viral Respiratory Lab|Pathogen Sequencing Lab|Muyembe|Democratic Republic of the Congo
>Spike|hCoV-19/Denmark/ALAB-SSI-1259/2020|2020-03-11|EPI_ISL_437638|Original|hCoV-19^^Denmark|Human|Department of Virus and Microbiological Special Diagnostics|Albertsen lab|Kirkegaard|Denmark
>Spike|hCoV-19/Denmark/ALAB-SSI-247/2020|2020-03-10|EPI_ISL_437662|Original|hCoV-19^^Denmark|Human|Department of Virus and Microbiological Special Diagnostics|Albertsen lab|Kirkegaard|Denmark
>Spike|hCoV-19/USA/WA-UW-6490/2020|2020-04-03|EPI_ISL_437847|Original|hCoV-19^^Washington|Human|UW Virology Lab|UW Virology Lab|Roychoudhury|USA
>Spike|hCoV-19/India/TG-GMC-RK1090/2020|2020-03-25|EPI_ISL_438139|Original|hCoV-19^^Telangana|Human|Department of Microbiology,Gandhi Medical College and Hospital,Hyderabad|Virus Research Laboratory|Muttineni|India

>Spike|hCoV-19/England/CAMB-76849/2020|2020-03-30|EPI_ISL_438269|Original|hCoV-19^^England|Human|Department of Pathology|Wellcome Sanger Institute for the COVID-19 Genomics UK (COG-UK) consortium|Amato|United Kingdom
>Spike|hCoV-19/England/CAMB-759B0/2020|2020-03-31|EPI_ISL_438282|Original|hCoV-19^^England|Human|Department of Pathology|Wellcome Sanger Institute for the COVID-19 Genomics UK (COG-UK) consortium|Amato|United Kingdom
>Spike|hCoV-19/England/CAMB-76612/2020|2020-03-29|EPI_ISL_438357|Original|hCoV-19^^England|Human|Department of Pathology|Wellcome Sanger Institute for the COVID-19 Genomics UK (COG-UK) consortium|Amato|United Kingdom
>Spike|hCoV-19/England/CAMB-81EEF/2020|2020-04-22|EPI_ISL_438593|Original|hCoV-19^^England|Human|Department of Pathology|COVID-19 Genomics UK (COG-UK) Consortium|Meredith|United Kingdom
>Spike|hCoV-19/England/CAMB-82C20/2020|2020-04-23|EPI_ISL_438671|Original|hCoV-19^^England|Human|Department of Pathology|COVID-19 Genomics UK (COG-UK) Consortium|Meredith|United Kingdom
>Spike|hCoV-19/Scotland/CVR992/2020|2020-03-28|EPI_ISL_439143|Original|hCoV-19^^Scotland|Human|West of Scotland Specialist Virology Centre|COVID-19 Genomics UK (COG-UK) Consortium|Robertson|United Kingdom
>Spike|hCoV-19/England/CAMB-7773C/2020|2020-03-30|EPI_ISL_439396|Original|hCoV-19^^England|Human|Department of Pathology|Wellcome Sanger Institute for the COVID-19 Genomics UK (COG-UK) consortium|Amato|United Kingdom
>Spike|hCoV-19/England/CAMB-77365/2020|2020-03-31|EPI_ISL_439513|Original|hCoV-19^^England|Human|Department of Pathology|Wellcome Sanger Institute for the COVID-19 Genomics UK (COG-UK) consortium|Amato|United Kingdom
>Spike|hCoV-19/England/CAMB-727E6/2020|2020-03-23|EPI_ISL_439580|Original|hCoV-19^^England|Human|Department of Pathology|Wellcome Sanger Institute for the COVID-19 Genomics UK (COG-UK) consortium|Amato|United Kingdom
>Spike|hCoV-19/England/LIVE-9A0FC/2020|2020-04-17|EPI_ISL_439846|Original|hCoV-19^^England|Human|Liverpool Clinical Laboratories|COVID-19 Genomics UK (COG-UK) Consortium|Haldenby|United Kingdom
>Spike|hCoV-19/England/CAMB-7422F/2020|2020-03-19|EPI_ISL_440345|Original|hCoV-19^^England|Human|Department of Pathology|Wellcome Sanger Institute for the COVID-19 Genomics UK (COG-UK) consortium|Amato|United Kingdom
>Spike|hCoV-19/England/CAMB-84B8B/2020|2020-03-12|EPI_ISL_440516|Original|hCoV-19^^England|Human|Department of Pathology|Wellcome Sanger Institute for the COVID-19 Genomics UK (COG-UK) consortium|Amato|United Kingdom
>Spike|hCoV-19/England/CAMB-84990/2020|2020-03-08|EPI_ISL_440520|Original|hCoV-19^^England|Human|Department of Pathology|Wellcome Sanger Institute for the COVID-19 Genomics UK (COG-UK) consortium|Amato|United Kingdom
>Spike|hCoV-19/England/CAMB-847B4/2020|2020-03-02|EPI_ISL_440548|Original|hCoV-19^^England|Human|Department of Pathology|Wellcome Sanger Institute for the COVID-19 Genomics UK (COG-UK) consortium|Amato|United Kingdom
>Spike|hCoV-19/England/LIVE-9CBDB/2020|2020-04-26|EPI_ISL_440938|Original|hCoV-19^^England|Human|Liverpool Clinical Laboratories|COVID-19 Genomics UK (COG-UK) Consortium|Haldenby|United Kingdom
>Spike|hCoV-19/England/CAMB-791A5/2020|2020-04-03|EPI_ISL_441113|Original|hCoV-19^^England|Human|Department of Pathology|Wellcome Sanger Institute for the COVID-19 Genomics UK (COG-UK) consortium|Amato|United Kingdom
>Spike|hCoV-19/England/CAMB-7970D/2020|2020-04-04|EPI_ISL_441148|Original|hCoV-19^^England|Human|Department of Pathology|Wellcome Sanger Institute for the COVID-19 Genomics UK (COG-UK) consortium|Amato|United Kingdom
>Spike|hCoV-19/England/CAMB-78D0C/2020|2020-04-03|EPI_ISL_441273|Original|hCoV-19^^England|Human|Department of Pathology|Wellcome Sanger Institute for the COVID-19 Genomics UK (COG-UK) consortium|Amato|United Kingdom
>Spike|hCoV-19/England/CAMB-7B5A7/2020|2020-04-03|EPI_ISL_441318|Original|hCoV-19^^England|Human|Department of Pathology|Wellcome Sanger Institute for the COVID-19 Genomics UK (COG-UK) consortium|Amato|United Kingdom
>Spike|hCoV-19/England/CAMB-7A02F/2020|2020-04-02|EPI_ISL_441327|Original|hCoV-19^^England|Human|Department of Pathology|Wellcome Sanger Institute for the COVID-19 Genomics UK (COG-UK) consortium|Amato|United Kingdom

>Spike|hCoV-19/Northern Ireland/NIRE-FB35A/2020|2020-03-
17|EPI_ISL_441413|Original|hCoV-19^^Northern Ireland|Human|Regional Virus
Laboratory|COVID-19 Genomics UK (COG-UK) Consortium|Fairley|United Kingdom
>Spike|hCoV-19/England/CAMB-7B98D/2020|2020-04-04|EPI_ISL_441625|Original|hCoV-
19^^England|Human|Department of Pathology|Wellcome Sanger Institute for the COVID-19
Genomics UK (COG-UK) consortium|Amato|United Kingdom
>Spike|hCoV-19/England/CAMB-7D36F/2020|2020-04-06|EPI_ISL_442301|Original|hCoV-
19^^England|Human|Department of Pathology|Wellcome Sanger Institute for the COVID-19
Genomics UK (COG-UK) consortium|Amato|United Kingdom
>Spike|hCoV-19/England/BRIS-123376/2020|2020-04-05|EPI_ISL_442696|Original|hCoV-
19^^England|Human|PHE South West Regional Laboratory|Wellcome Sanger Institute for
the COVID-19 Genomics UK (COG-UK) consortium|Amato|United Kingdom
>Spike|hCoV-19/England/BRIS-123B32/2020|2020-04-07|EPI_ISL_442699|Original|hCoV-
19^^England|Human|PHE South West Regional Laboratory|Wellcome Sanger Institute for
the COVID-19 Genomics UK (COG-UK) consortium|Amato|United Kingdom
>Spike|hCoV-19/England/BRIS-123B41/2020|2020-04-07|EPI_ISL_442769|Original|hCoV-
19^^England|Human|PHE South West Regional Laboratory|Wellcome Sanger Institute for
the COVID-19 Genomics UK (COG-UK) consortium|Amato|United Kingdom
>Spike|hCoV-19/England/CAMB-7EE03/2020|2020-04-07|EPI_ISL_442928|Original|hCoV-
19^^England|Human|Department of Pathology|Wellcome Sanger Institute for the COVID-19
Genomics UK (COG-UK) consortium|Amato|United Kingdom
>Spike|hCoV-19/England/CAMB-81447/2020|2020-04-10|EPI_ISL_443065|Original|hCoV-
19^^England|Human|Department of Pathology|Wellcome Sanger Institute for the COVID-19
Genomics UK (COG-UK) consortium|Amato|United Kingdom
>Spike|hCoV-19/France/IDF-5850/2020|2020-03-26|EPI_ISL_443297|Original|hCoV-
19^^\u00cele-de-France|Human|H\u00f4pital Necker - Enfants - Malades\nLaboratoire de
Virologie|National Reference Center for Viruses of Respiratory
Infections|Enouf|France
>Spike|hCoV-19/France/IDF-3359/2020|2020-03-23|EPI_ISL_443301|Original|hCoV-
19^^\u00cele-de-France|Human|Cabinet M\u00e9dical|National Reference Center for
Viruses of Respiratory Infections|Enouf|France
>Spike|hCoV-19/England/CAMB-82F81/2020|2020-04-13|EPI_ISL_443335|Original|hCoV-
19^^England|Human|Department of Pathology|Wellcome Sanger Institute for the COVID-19
Genomics UK (COG-UK) consortium|Amato|United Kingdom
>Spike|hCoV-19/England/CAMB-82EEE/2020|2020-04-14|EPI_ISL_443363|Original|hCoV-
19^^England|Human|Department of Pathology|Wellcome Sanger Institute for the COVID-19
Genomics UK (COG-UK) consortium|Amato|United Kingdom
>Spike|hCoV-19/England/CAMB-82DE1/2020|2020-04-13|EPI_ISL_443666|Original|hCoV-
19^^England|Human|Department of Pathology|Wellcome Sanger Institute for the COVID-19
Genomics UK (COG-UK) consortium|Amato|United Kingdom
>Spike|hCoV-19/England/BRIS-125E37/2020|2020-03-30|EPI_ISL_443716|Original|hCoV-
19^^England|Human|PHE South West Regional Laboratory|Wellcome Sanger Institute for
the COVID-19 Genomics UK (COG-UK) consortium|Amato|United Kingdom
>Spike|hCoV-19/England/BRIS-1253FC/2020|2020-03-27|EPI_ISL_443723|Original|hCoV-
19^^England|Human|PHE South West Regional Laboratory|Wellcome Sanger Institute for
the COVID-19 Genomics UK (COG-UK) consortium|Amato|United Kingdom
>Spike|hCoV-19/England/BRIS-124788/2020|2020-03-25|EPI_ISL_443742|Original|hCoV-
19^^England|Human|PHE South West Regional Laboratory|Wellcome Sanger Institute for
the COVID-19 Genomics UK (COG-UK) consortium|Amato|United Kingdom
>Spike|hCoV-19/England/BRIS-1251E3/2020|2020-03-27|EPI_ISL_443885|Original|hCoV-
19^^England|Human|PHE South West Regional Laboratory|Wellcome Sanger Institute for
the COVID-19 Genomics UK (COG-UK) consortium|Amato|United Kingdom
>Spike|hCoV-19/England/BRIS-1253ED/2020|2020-03-28|EPI_ISL_443996|Original|hCoV-
19^^England|Human|PHE South West Regional Laboratory|Wellcome Sanger Institute for
the COVID-19 Genomics UK (COG-UK) consortium|Amato|United Kingdom
>Spike|hCoV-19/Sweden/KI-CTMR-V300037768-3D/2020|2020-03-
00|EPI_ISL_444487|Original|hCoV-19^^Stockholm|Human|Karolinska
Universitetslaboratoriet|CTMR|Hu|Sweden

>Spike|hCoV-19/Sweden/KI-CTMR-V300037768-4D/2020|2020-03-00|EPI_ISL_444490|Original|hCoV-19^^Stockholm|Human|Karolinska Universitetslaboratoriet|CTMR|Hu|Sweden
>Spike|hCoV-19/Canada/QC_AB4/2020|2020-04-06|EPI_ISL_444502|Original|hCoV-19^^Quebec|Human|Laboratoire de microbiologie|Smith Laboratory|Smith|Canada
>Spike|hCoV-19/Bangladesh/DNAS-CPH-471/2020|2020-04-28|EPI_ISL_445214|Original|hCoV-19^^Dhaka|Human|DNA Solution Ltd.|DNA Solution Ltd.|Rabbi|Bangladesh
>Spike|hCoV-19/Bangladesh/Akbiomed-01/2020|2020-04-25|EPI_ISL_445244|Original|hCoV-19^^Bangladesh|Human|Akbiomed lab|Tejgaon College bmb lab|Atafat|Bangladesh
>Spike|hCoV-19/Chile/RM-ISPCH-9/2020|2020-03-09|EPI_ISL_445252|Original|hCoV-19^^Region Metropolitana de Santiago|Human|PONTIFICIA U. CATOLICA FAC. MEDICINA|Instituto de Salud Publica de Chile|Fernandez|Chile
>Spike|hCoV-19/Chile/MA-ISPCH-15/2020|2020-03-22|EPI_ISL_445299|Original|hCoV-19^^Magallanes|Human|CLINICA MAGALLANES S.A.|Instituto de Salud Publica de Chile|Fernandez|Chile
>Spike|hCoV-19/Chile/RM-ISPCH-29/2020|2020-03-16|EPI_ISL_445308|Original|hCoV-19^^Region Metropolitana de Santiago|Human|HOSPITAL EL CARMEN DR.LUIS VALENTIN F.|Instituto de Salud Publica de Chile|Fernandez|Chile
>Spike|hCoV-19/Chile/RM-ISPCH-50/2020|2020-03-23|EPI_ISL_445329|Original|hCoV-19^^Region Metropolitana de Santiago|Human|HOSPITAL DR.SOTERO DEL RIO|Instituto de Salud Publica de Chile|Fernandez|Chile
>Spike|hCoV-19/Chile/AR-ISPCH-3/2020|2020-03-25|EPI_ISL_445338|Original|hCoV-19^^La Araucania|Human|HOSPITAL DR.HERNAN HENRIQUEZ ARAVENA|Instituto de Salud Publica de Chile|Fernandez|Chile
>Spike|hCoV-19/Chile/AR-ISPCH-7/2020|2020-03-25|EPI_ISL_445342|Original|hCoV-19^^La Araucania|Human|HOSPITAL DR.HERNAN HENRIQUEZ ARAVENA|Instituto de Salud Publica de Chile|Fernandez|Chile
>Spike|hCoV-19/Chile/RM-ISPCH-63/2020|2020-03-16|EPI_ISL_445359|Original|hCoV-19^^Region Metropolitana de Santiago|Human|HOSP. SANTIAGO ORIENTE DR. LUIS TISNE B.|Instituto de Salud Publica de Chile|Fernandez|Chile
>Spike|hCoV-19/Chile/RM-ISPCH-67/2020|2020-03-20|EPI_ISL_445363|Original|hCoV-19^^Region Metropolitana de Santiago|Human|ASISTENCIA PUBLICA DR.ALEJANDRO DEL RIO|Instituto de Salud Publica de Chile|Fernandez|Chile
>Spike|hCoV-19/Wales/PHWC-28FBA/2020|2020-04-01|EPI_ISL_445464|Original|hCoV-19^^Wales|Human|Wales Specialist Virology Centre|Public Health Wales Microbiology Cardiff|Moore|United Kingdom
>Spike|hCoV-19/Wales/PHWC-290B6/2020|2020-04-03|EPI_ISL_445478|Original|hCoV-19^^Wales|Human|Wales Specialist Virology Centre|Public Health Wales Microbiology Cardiff|Moore|United Kingdom
>Spike|hCoV-19/Wales/PHWC-290D4/2020|2020-04-03|EPI_ISL_445479|Original|hCoV-19^^Wales|Human|Wales Specialist Virology Centre|Public Health Wales Microbiology Cardiff|Moore|United Kingdom
>Spike|hCoV-19/Wales/PHWC-2911D/2020|2020-04-03|EPI_ISL_445483|Original|hCoV-19^^Wales|Human|Wales Specialist Virology Centre|Public Health Wales Microbiology Cardiff|Moore|United Kingdom
>Spike|hCoV-19/Wales/PHWC-291FF/2020|2020-04-03|EPI_ISL_445494|Original|hCoV-19^^Wales|Human|Wales Specialist Virology Centre|Public Health Wales Microbiology Cardiff|Moore|United Kingdom
>Spike|hCoV-19/Wales/PHWC-2A246/2020|2020-04-05|EPI_ISL_445541|Original|hCoV-19^^Wales|Human|Wales Specialist Virology Centre|Public Health Wales Microbiology Cardiff|Moore|United Kingdom
>Spike|hCoV-19/Wales/PHWC-2A291/2020|2020-04-05|EPI_ISL_445546|Original|hCoV-19^^Wales|Human|Wales Specialist Virology Centre|Public Health Wales Microbiology Cardiff|Moore|United Kingdom
>Spike|hCoV-19/Wales/PHWC-2A6D1/2020|2020-04-05|EPI_ISL_445607|Original|hCoV-19^^Wales|Human|Wales Specialist Virology Centre|Public Health Wales Microbiology Cardiff|Moore|United Kingdom
>Spike|hCoV-19/Wales/PHWC-2A826/2020|2020-04-07|EPI_ISL_445624|Original|hCoV-19^^Wales|Human|Wales Specialist Virology Centre|Public Health Wales Microbiology Cardiff|Moore|United Kingdom

>Spike|hCoV-19/Wales/PHWC-2A914/2020|2020-04-08|EPI_ISL_445636|Original|hCoV-19^^Wales|Human|Wales Specialist Virology Centre|Public Health Wales Microbiology Cardiff|Moore|United Kingdom
>Spike|hCoV-19/Wales/PHWC-2A9BA/2020|2020-04-06|EPI_ISL_445646|Original|hCoV-19^^Wales|Human|Wales Specialist Virology Centre|Public Health Wales Microbiology Cardiff|Moore|United Kingdom
>Spike|hCoV-19/Wales/PHWC-2B5D3/2020|2020-04-10|EPI_ISL_445733|Original|hCoV-19^^Wales|Human|Wales Specialist Virology Centre|Public Health Wales Microbiology Cardiff|Moore|United Kingdom
>Spike|hCoV-19/Wales/PHWC-2B685/2020|2020-04-13|EPI_ISL_445743|Original|hCoV-19^^Wales|Human|Wales Specialist Virology Centre|Public Health Wales Microbiology Cardiff|Moore|United Kingdom
>Spike|hCoV-19/Wales/PHWC-2B9E6/2020|2020-03-28|EPI_ISL_445791|Original|hCoV-19^^Wales|Human|Wales Specialist Virology Centre|Public Health Wales Microbiology Cardiff|Moore|United Kingdom
>Spike|hCoV-19/Wales/PHWC-2BC0B/2020|2020-03-29|EPI_ISL_445820|Original|hCoV-19^^Wales|Human|Wales Specialist Virology Centre|Public Health Wales Microbiology Cardiff|Moore|United Kingdom
>Spike|hCoV-19/Wales/PHWC-2BC1A/2020|2020-03-29|EPI_ISL_445821|Original|hCoV-19^^Wales|Human|Wales Specialist Virology Centre|Public Health Wales Microbiology Cardiff|Moore|United Kingdom
>Spike|hCoV-19/Wales/PHWC-2BD44/2020|2020-04-07|EPI_ISL_445840|Original|hCoV-19^^Wales|Human|Wales Specialist Virology Centre|Public Health Wales Microbiology Cardiff|Moore|United Kingdom
>Spike|hCoV-19/Wales/PHWC-2BD80/2020|2020-04-09|EPI_ISL_445844|Original|hCoV-19^^Wales|Human|Wales Specialist Virology Centre|Public Health Wales Microbiology Cardiff|Moore|United Kingdom
>Spike|hCoV-19/Wales/PHWC-2C718/2020|2020-03-31|EPI_ISL_445901|Original|hCoV-19^^Wales|Human|Wales Specialist Virology Centre|Public Health Wales Microbiology Cardiff|Moore|United Kingdom
>Spike|hCoV-19/Wales/PHWC-2C736/2020|2020-03-31|EPI_ISL_445903|Original|hCoV-19^^Wales|Human|Wales Specialist Virology Centre|Public Health Wales Microbiology Cardiff|Moore|United Kingdom
>Spike|hCoV-19/Wales/PHWC-2C842/2020|2020-04-01|EPI_ISL_445918|Original|hCoV-19^^Wales|Human|Wales Specialist Virology Centre|Public Health Wales Microbiology Cardiff|Moore|United Kingdom
>Spike|hCoV-19/Wales/PHWC-2C9A9/2020|2020-04-01|EPI_ISL_445929|Original|hCoV-19^^Wales|Human|Wales Specialist Virology Centre|Public Health Wales Microbiology Cardiff|Moore|United Kingdom
>Spike|hCoV-19/Wales/PHWC-2D340/2020|2020-04-04|EPI_ISL_445974|Original|hCoV-19^^Wales|Human|Wales Specialist Virology Centre|Public Health Wales Microbiology Cardiff|Moore|United Kingdom
>Spike|hCoV-19/Wales/PHWC-2D4C5/2020|2020-04-04|EPI_ISL_445991|Original|hCoV-19^^Wales|Human|Wales Specialist Virology Centre|Public Health Wales Microbiology Cardiff|Moore|United Kingdom
>Spike|hCoV-19/Wales/PHWC-2D674/2020|2020-04-04|EPI_ISL_446009|Original|hCoV-19^^Wales|Human|Wales Specialist Virology Centre|Public Health Wales Microbiology Cardiff|Moore|United Kingdom
>Spike|hCoV-19/Wales/PHWC-2DF00/2020|2020-04-09|EPI_ISL_446039|Original|hCoV-19^^Wales|Human|Wales Specialist Virology Centre|Public Health Wales Microbiology Cardiff|Moore|United Kingdom
>Spike|hCoV-19/Wales/PHWC-2E2CA/2020|2020-04-16|EPI_ISL_446083|Original|hCoV-19^^Wales|Human|Wales Specialist Virology Centre|Public Health Wales Microbiology Cardiff|Moore|United Kingdom
>Spike|hCoV-19/Wales/PHWC-2EA59/2020|2020-04-07|EPI_ISL_446104|Original|hCoV-19^^Wales|Human|Wales Specialist Virology Centre|Public Health Wales Microbiology Cardiff|Moore|United Kingdom
>Spike|hCoV-19/Wales/PHWC-2EBA1/2020|2020-04-06|EPI_ISL_446122|Original|hCoV-19^^Wales|Human|Wales Specialist Virology Centre|Public Health Wales Microbiology Cardiff|Moore|United Kingdom

>Spike|hCoV-19/Wales/PHWC-2EF1E/2020|2020-04-06|EPI_ISL_446175|Original|hCoV-19^^Wales|Human|Wales Specialist Virology Centre|Public Health Wales Microbiology Cardiff|Moore|United Kingdom
>Spike|hCoV-19/Wales/PHWC-30DD3/2020|2020-04-09|EPI_ISL_446360|Original|hCoV-19^^Wales|Human|Wales Specialist Virology Centre|Public Health Wales Microbiology Cardiff|Moore|United Kingdom
>Spike|hCoV-19/Wales/PHWC-30F0A/2020|2020-04-09|EPI_ISL_446378|Original|hCoV-19^^Wales|Human|Wales Specialist Virology Centre|Public Health Wales Microbiology Cardiff|Moore|United Kingdom
>Spike|hCoV-19/Wales/PHWC-316E6/2020|2020-04-12|EPI_ISL_446475|Original|hCoV-19^^Wales|Human|Wales Specialist Virology Centre|Public Health Wales Microbiology Cardiff|Moore|United Kingdom
>Spike|hCoV-19/Wales/PHWC-31789/2020|2020-04-12|EPI_ISL_446484|Original|hCoV-19^^Wales|Human|Wales Specialist Virology Centre|Public Health Wales Microbiology Cardiff|Moore|United Kingdom
>Spike|hCoV-19/Wales/PHWC-31B05/2020|2020-04-12|EPI_ISL_446530|Original|hCoV-19^^Wales|Human|Wales Specialist Virology Centre|Public Health Wales Microbiology Cardiff|Moore|United Kingdom
>Spike|hCoV-19/Wales/PHWC-31B41/2020|2020-04-11|EPI_ISL_446534|Original|hCoV-19^^Wales|Human|Wales Specialist Virology Centre|Public Health Wales Microbiology Cardiff|Moore|United Kingdom
>Spike|hCoV-19/Wales/PHWC-31C6C/2020|2020-04-13|EPI_ISL_446550|Original|hCoV-19^^Wales|Human|Wales Specialist Virology Centre|Public Health Wales Microbiology Cardiff|Moore|United Kingdom
>Spike|hCoV-19/Wales/PHWC-31CA8/2020|2020-04-13|EPI_ISL_446554|Original|hCoV-19^^Wales|Human|Wales Specialist Virology Centre|Public Health Wales Microbiology Cardiff|Moore|United Kingdom
>Spike|hCoV-19/Wales/PHWC-31EFD/2020|2020-04-06|EPI_ISL_446583|Original|hCoV-19^^Wales|Human|Wales Specialist Virology Centre|Public Health Wales Microbiology Cardiff|Moore|United Kingdom
>Spike|hCoV-19/Wales/PHWC-32032/2020|2020-04-13|EPI_ISL_446600|Original|hCoV-19^^Wales|Human|Wales Specialist Virology Centre|Public Health Wales Microbiology Cardiff|Moore|United Kingdom
>Spike|hCoV-19/Wales/PHWC-321D5/2020|2020-04-14|EPI_ISL_446617|Original|hCoV-19^^Wales|Human|Wales Specialist Virology Centre|Public Health Wales Microbiology Cardiff|Moore|United Kingdom
>Spike|hCoV-19/Wales/PHWC-324BE/2020|2020-04-13|EPI_ISL_446652|Original|hCoV-19^^Wales|Human|Wales Specialist Virology Centre|Public Health Wales Microbiology Cardiff|Moore|United Kingdom
>Spike|hCoV-19/Wales/PHWC-32E29/2020|2020-04-14|EPI_ISL_446694|Original|hCoV-19^^Wales|Human|Wales Specialist Virology Centre|Public Health Wales Microbiology Cardiff|Moore|United Kingdom
>Spike|hCoV-19/Wales/PHWC-32F26/2020|2020-04-14|EPI_ISL_446710|Original|hCoV-19^^Wales|Human|Wales Specialist Virology Centre|Public Health Wales Microbiology Cardiff|Moore|United Kingdom
>Spike|hCoV-19/Wales/PHWC-32F35/2020|2020-04-14|EPI_ISL_446711|Original|hCoV-19^^Wales|Human|Wales Specialist Virology Centre|Public Health Wales Microbiology Cardiff|Moore|United Kingdom
>Spike|hCoV-19/Wales/PHWC-33963/2020|2020-04-20|EPI_ISL_446771|Original|hCoV-19^^Wales|Human|Wales Specialist Virology Centre|Public Health Wales Microbiology Cardiff|Moore|United Kingdom
>Spike|hCoV-19/Wales/PHWC-33F70/2020|2020-04-16|EPI_ISL_446847|Original|hCoV-19^^Wales|Human|Wales Specialist Virology Centre|Public Health Wales Microbiology Cardiff|Moore|United Kingdom
>Spike|hCoV-19/Wales/PHWC-34003/2020|2020-04-16|EPI_ISL_446856|Original|hCoV-19^^Wales|Human|Wales Specialist Virology Centre|Public Health Wales Microbiology Cardiff|Moore|United Kingdom
>Spike|hCoV-19/Wales/PHWC-34012/2020|2020-04-16|EPI_ISL_446857|Original|hCoV-19^^Wales|Human|Wales Specialist Virology Centre|Public Health Wales Microbiology Cardiff|Moore|United Kingdom

>Spike|hCoV-19/Wales/PHWC-340B8/2020|2020-04-20|EPI_ISL_446867|Original|hCoV-19^^Wales|Human|Wales Specialist Virology Centre|Public Health Wales Microbiology Cardiff|Moore|United Kingdom
>Spike|hCoV-19/Wales/PHWC-341E2/2020|2020-04-18|EPI_ISL_446886|Original|hCoV-19^^Wales|Human|Wales Specialist Virology Centre|Public Health Wales Microbiology Cardiff|Moore|United Kingdom
>Spike|hCoV-19/Wales/PHWC-34319/2020|2020-04-17|EPI_ISL_446903|Original|hCoV-19^^Wales|Human|Wales Specialist Virology Centre|Public Health Wales Microbiology Cardiff|Moore|United Kingdom
>Spike|hCoV-19/Wales/PHWC-34443/2020|2020-04-16|EPI_ISL_446922|Original|hCoV-19^^Wales|Human|Wales Specialist Virology Centre|Public Health Wales Microbiology Cardiff|Moore|United Kingdom
>Spike|hCoV-19/Wales/PHWC-345F5/2020|2020-04-20|EPI_ISL_446947|Original|hCoV-19^^Wales|Human|Wales Specialist Virology Centre|Public Health Wales Microbiology Cardiff|Moore|United Kingdom
>Spike|hCoV-19/Georgia/Tb-6598/2020|2020-04-29|EPI_ISL_447055|Original|hCoV-19^^Tbilisi|Human|Department for Virology|Department for Virology|Tomashvili|Georgia
>Spike|hCoV-19/Taiwan/TSGH-26/2020|2020-02-25|EPI_ISL_447252|Original|hCoV-19^^New Taipei City|Human|TSGH-CP molecular lab|TSGH-CP molecular lab|Shang|Taiwan
>Spike|hCoV-19/mink/Netherlands/NB04_index/2020|2020-05-06|EPI_ISL_447634|Original|hCoV-19^^Netherlands|Mustela lutreola|Virology|Virology|Oreshkova|Netherlands
>Spike|hCoV-19/England/NORW-E8D07/2020|2020-04-15|EPI_ISL_448303|Original|hCoV-19^^England|Human|Quadram Institute Bioscience|COVID-19 Genomics UK (COG-UK) Consortium|Alikhan|United Kingdom
>Spike|hCoV-19/England/NORW-E946B/2020|2020-04-22|EPI_ISL_448314|Original|hCoV-19^^England|Human|Quadram Institute Bioscience|COVID-19 Genomics UK (COG-UK) Consortium|Alikhan|United Kingdom
>Spike|hCoV-19/England/NORW-E96CF/2020|2020-04-22|EPI_ISL_448327|Original|hCoV-19^^England|Human|Quadram Institute Bioscience|COVID-19 Genomics UK (COG-UK) Consortium|Alikhan|United Kingdom
>Spike|hCoV-19/England/NORW-E9B57/2020|2020-04-30|EPI_ISL_448353|Original|hCoV-19^^England|Human|Quadram Institute Bioscience|COVID-19 Genomics UK (COG-UK) Consortium|Alikhan|United Kingdom
>Spike|hCoV-19/England/NORW-E9EB8/2020|2020-05-03|EPI_ISL_448378|Original|hCoV-19^^England|Human|Quadram Institute Bioscience|COVID-19 Genomics UK (COG-UK) Consortium|Alikhan|United Kingdom
>Spike|hCoV-19/England/NOTT-110E8A/2020|2020-04-29|EPI_ISL_448429|Original|hCoV-19^^England|Human|Queens Medical Centre|COVID-19 Genomics UK (COG-UK) Consortium|Loose|United Kingdom
>Spike|hCoV-19/England/NOTT-110EC6/2020|2020-04-29|EPI_ISL_448433|Original|hCoV-19^^England|Human|Queens Medical Centre|COVID-19 Genomics UK (COG-UK) Consortium|Loose|United Kingdom
>Spike|hCoV-19/England/OXON-AC69B/2020|2020-04-05|EPI_ISL_448474|Original|hCoV-19^^England|Human|Oxford Viromics|COVID-19 Genomics UK (COG-UK) Consortium|Golubchik|United Kingdom
>Spike|hCoV-19/England/OXON-AE12F/2020|2020-00-00|EPI_ISL_448665|Original|hCoV-19^^England|Human|Oxford Viromics|COVID-19 Genomics UK (COG-UK) Consortium|Golubchik|United Kingdom
>Spike|hCoV-19/England/NORW-E6F03/2020|2020-04-21|EPI_ISL_448991|Original|hCoV-19^^England|Human|Quadram Institute Bioscience|COVID-19 Genomics UK (COG-UK) Consortium|Alikhan|United Kingdom
>Spike|hCoV-19/England/NORW-E711B/2020|2020-04-18|EPI_ISL_449003|Original|hCoV-19^^England|Human|Quadram Institute Bioscience|COVID-19 Genomics UK (COG-UK) Consortium|Alikhan|United Kingdom
>Spike|hCoV-19/England/NORW-E76D0/2020|2020-05-04|EPI_ISL_449064|Original|hCoV-19^^England|Human|Quadram Institute Bioscience|COVID-19 Genomics UK (COG-UK) Consortium|Alikhan|United Kingdom

>Spike|hCoV-19/England/NORW-E84D5/2020|2020-04-17|EPI_ISL_449156|Original|hCoV-19^^England|Human|Quadram Institute Bioscience|COVID-19 Genomics UK (COG-UK) Consortium|Alikhan|United Kingdom
>Spike|hCoV-19/England/NORW-E852D/2020|2020-04-18|EPI_ISL_449159|Original|hCoV-19^^England|Human|Quadram Institute Bioscience|COVID-19 Genomics UK (COG-UK) Consortium|Alikhan|United Kingdom
>Spike|hCoV-19/Wuhan/WHUH001/2020|2020-01-22|EPI_ISL_449476|unknown|hCoV-19^^Wuhan|Human|unknown|Department of Respiratory and Critical Care|Submitter|China
>Spike|hCoV-19/Wuhan/WHUH002/2020|2020-01-22|EPI_ISL_449477|unknown|hCoV-19^^Wuhan|Human|unknown|Department of Respiratory and Critical Care|Submitter|China
>Spike|hCoV-19/Wuhan/WHUH003/2020|2020-01-22|EPI_ISL_449478|unknown|hCoV-19^^Wuhan|Human|unknown|Department of Respiratory and Critical Care|Submitter|China
>Spike|hCoV-19/Wuhan/WHUH004/2020|2020-01-22|EPI_ISL_449479|unknown|hCoV-19^^Wuhan|Human|unknown|Department of Respiratory and Critical Care|Submitter|China
>Spike|hCoV-19/Wuhan/WHUH005/2020|2020-01-22|EPI_ISL_449480|unknown|hCoV-19^^Wuhan|Human|unknown|Department of Respiratory and Critical Care|Submitter|China
>Spike|hCoV-19/Wuhan/WHUH006/2020|2020-01-22|EPI_ISL_449481|unknown|hCoV-19^^Wuhan|Human|unknown|Department of Respiratory and Critical Care|Submitter|China
>Spike|hCoV-19/Wuhan/WHUH008/2020|2020-01-22|EPI_ISL_449483|unknown|hCoV-19^^Wuhan|Human|unknown|Department of Respiratory and Critical Care|Submitter|China
>Spike|hCoV-19/Wuhan/WHUH012/2020|2020-01-22|EPI_ISL_449485|unknown|hCoV-19^^Wuhan|Human|unknown|Department of Respiratory and Critical Care|Submitter|China
>Spike|hCoV-19/Wuhan/WHUH021/2020|2020-01-22|EPI_ISL_449487|unknown|hCoV-19^^Wuhan|Human|unknown|Department of Respiratory and Critical Care|Submitter|China
>Spike|hCoV-19/Australia/SAP039/2020|2020-03-21|EPI_ISL_450195|Original|hCoV-19^^South Australia|Human|SA Pathology|SA Pathology|Leong|Australia
>Spike|hCoV-19/Canada/Qc-L00241205/2020|2020-03-27|EPI_ISL_450313|Original|hCoV-19^^Quebec|Human|H\u00f4pital du Suro\u00eet|Laboratoire de sant\u00e9 publique du Qu\u00e9bec|Moreira|Canada
>Spike|hCoV-19/Canada/Qc-L00241355/2020|2020-03-27|EPI_ISL_450318|Original|hCoV-19^^Quebec|Human|H\u00f4pital Pierre-Boucher|Laboratoire de sant\u00e9 publique du Qu\u00e9bec|Moreira|Canada
>Spike|hCoV-19/Bangladesh/BARJ-CVASU-CTG-502/2020|2020-05-13|EPI_ISL_450340|Original|hCoV-19^^Chittagong|Human|Bangladesh Institute of Tropical & Infectious Diseases|Basic and Applied Research on Jute Project|Ahmed|Bangladesh
>Spike|hCoV-19/Bangladesh/BARJ-CVASU-CTG-506/2020|2020-05-08|EPI_ISL_450342|Original|hCoV-19^^Chittagong|Human|Bangladesh Institute of Tropical & Infectious Diseases|Basic and Applied Research on Jute Project|Ahmed|Bangladesh
>Spike|hCoV-19/Jamaica/JM-CDC-0078/2020|2020-03-11|EPI_ISL_450792|Original|hCoV-19^^Jamaica|Human|Jamaica Ministry of Health and Wellness|Pathogen Discovery|Queen|Jamaica
>Spike|hCoV-19/USA/UT-03701/2020|2020-04-07|EPI_ISL_450838|Original|hCoV-19^^Utah|Human|Utah Public Health Laboratory|Utah Public Health Laboratory|Young|USA
>Spike|hCoV-19/Uganda/UG014/2020|2020-03-25|EPI_ISL_451196|Original|hCoV-19^^Uganda|Human|Uganda Virus Research Institute|MRC/UVRI & LSHTM Uganda\u00a0Research Unit|Cotten|Uganda
>Spike|hCoV-19/USA/LA-EVTL067/2020|2020-04-29|EPI_ISL_451228|Original|hCoV-19^^Louisiana|Human|LSUHS Emerging Viral Threat Laboratory|Microbial Genome Sequencing Center|Kamil|USA
>Spike|hCoV-19/Australia/NSW252/2020|2020-03-08|EPI_ISL_451517|Original|hCoV-19^^New South Wales|Human|South Eastern Area Laboratory Services|NSW Health Pathology - Institute of Clinical Pathology and Medical Research; Westmead Hospital; University of Sydney|Gall|Australia
>Spike|hCoV-19/Russia/SCPM-O-02/2020|2020-03-22|EPI_ISL_451964|Original|hCoV-19^^Moscow|Human|Federal Budget Institution of Science|Federal Budget Institution of Science|Bogun|Russia
>Spike|hCoV-19/Russia/SCPM-O-03/2020|2020-03-20|EPI_ISL_451965|Original|hCoV-19^^Moscow|Human|Federal Budget Institution of Science|Federal Budget Institution of Science|Bogun|Russia

>Spike|hCoV-19/Russia/SCPM-O-06/2020|2020-03-18|EPI_ISL_451968|Original|hCoV-19^^Moscow|Human|Federal Budget Institution of Science|Federal Budget Institution of Science|Bogun|Russia
>Spike|hCoV-19/Russia/SCPM-O-08/2020|2020-03-22|EPI_ISL_451970|Original|hCoV-19^^Moscow|Human|Federal Budget Institution of Science|Federal Budget Institution of Science|Bogun|Russia
>Spike|hCoV-19/Spain/AN-IBV-002032/2020|2020-03-17|EPI_ISL_452390|Original|hCoV-19^^Andalucia|Human|Servicio de Microbiolog\u00eda. HRU de M\u00e1laga. Servicio Andaluz de Salud|SeqCOVID-SPAIN consortium/IBV(CSIC)|Comas|Spain
>Spike|hCoV-19/Spain/AN-IBV-001065/2020|2020-04-01|EPI_ISL_452611|Original|hCoV-19^^Andalucia|Human|Servicio de Microbiolog\u00eda y Parasitolog\u00eda cl\u00ednica. UCEIMP. Hospital Universitario Virgen del Roc\u00edo/IBIS/CSIC/US.|SeqCOVID-SPAIN consortium/IBV(CSIC)|Comas|Spain
>Spike|hCoV-19/Spain/PV-IBV-000860/2020|2020-02-29|EPI_ISL_452692|Original|hCoV-19^^Basque Country|Human|Hospital Universitario Araba. Vitoria-Gasteiz,|SeqCOVID-SPAIN consortium/IBV(CSIC)|Comas|Spain
>Spike|hCoV-19/Scotland/CVR3151/2020|2020-04-19|EPI_ISL_453094|Original|hCoV-19^^Scotland|Human|West of Scotland Specialist Virology Centre|COVID-19 Genomics UK (COG-UK) Consortium|Robertson|United Kingdom
>Spike|hCoV-19/England/LIVE-9D554/2020|2020-04-15|EPI_ISL_453210|Original|hCoV-19^^England|Human|Liverpool Clinical Laboratories|COVID-19 Genomics UK (COG-UK) Consortium|Haldenby|United Kingdom
>Spike|hCoV-19/England/LIVE-9E16E/2020|2020-04-03|EPI_ISL_453271|Original|hCoV-19^^England|Human|Liverpool Clinical Laboratories|COVID-19 Genomics UK (COG-UK) Consortium|Haldenby|United Kingdom
>Spike|hCoV-19/Northern Ireland/NIRE-101F10/2020|2020-03-23|EPI_ISL_453478|Original|hCoV-19^^Northern Ireland|Human|Regional Virus Laboratory|COVID-19 Genomics UK (COG-UK) Consortium|Fairley|United Kingdom
>Spike|hCoV-19/England/NORW-E9058/2020|2020-04-25|EPI_ISL_453578|Original|hCoV-19^^England|Human|Quadram Institute Bioscience|COVID-19 Genomics UK (COG-UK) Consortium|Alikhan|United Kingdom
>Spike|hCoV-19/England/NORW-E9155/2020|2020-04-27|EPI_ISL_453581|Original|hCoV-19^^England|Human|Quadram Institute Bioscience|COVID-19 Genomics UK (COG-UK) Consortium|Alikhan|United Kingdom
>Spike|hCoV-19/England/NORW-E92F8/2020|2020-04-27|EPI_ISL_453590|Original|hCoV-19^^England|Human|Quadram Institute Bioscience|COVID-19 Genomics UK (COG-UK) Consortium|Alikhan|United Kingdom
>Spike|hCoV-19/Portugal/PT0533/2020|2020-04-07|EPI_ISL_454257|Original|hCoV-19^^Portugal|Human|unknown|Instituto Nacional de Saude (INSA)|Borges|Portugal
>Spike|hCoV-19/Portugal/PT0534/2020|2020-04-07|EPI_ISL_454258|Original|hCoV-19^^Portugal|Human|unknown|Instituto Nacional de Saude (INSA)|Borges|Portugal
>Spike|hCoV-19/USA/MDH-7760/2020|2020-03-12|EPI_ISL_454428|Original|hCoV-19^^Maryland|Human|Maryland Department of Health Laboratories Administration|Maryland Department of Health Laboratories Administration|Vepachedu|USA
>Spike|hCoV-19/USA/MDH-8144/2020|2020-03-13|EPI_ISL_454431|Original|hCoV-19^^Maryland|Human|Maryland Department of Health Laboratories Administration|Maryland Department of Health Laboratories Administration|Vepachedu|USA
>Spike|hCoV-19/India/OR-ILC-RMRC25/2020|2020-04-04|EPI_ISL_455308|Original|hCoV-19^^Odisha|Human|REGIONAL VRDL,ICMR-RMRC BBSR|Immunogenomics group|Raghav|India
>Spike|hCoV-19/India/OR-ILC-RMRC26/2020|2020-04-04|EPI_ISL_455309|Original|hCoV-19^^Odisha|Human|REGIONAL VRDL,ICMR-RMRC BBSR|Immunogenomics group|Raghav|India
>Spike|hCoV-19/India/OR-ILC-RMRC48/2020|2020-04-08|EPI_ISL_455310|Original|hCoV-19^^Odisha|Human|REGIONAL VRDL,ICMR-RMRC BBSR|Immunogenomics group|Raghav|India
>Spike|hCoV-19/India/OR-ILC-RMRC123/2020|2020-05-07|EPI_ISL_455311|Original|hCoV-19^^Odisha|Human|REGIONAL VRDL,ICMR-RMRC BBSR|Immunogenomics group|Raghav|India
>Spike|hCoV-19/Thailand/Nonthaburi_3472/2020|2020-03-20|EPI_ISL_455593|Vero P1|hCoV-19^^Nonthaburi|Human|National Institute of Health. Department of medical Sciences|National Institute of Health. Department of medical Sciences|Phuygun|Thailand

>Spike|hCoV-19/Australia/VIC1432/2020|2020-04-17|EPI_ISL_456439|Original|hCoV-19^^Victoria|Human|Victorian Infectious Diseases Reference Laboratory (VIDRL)|Microbiological Diagnostic Unit Public Health Laboratory and Victorian Infectious Diseases Reference Laboratory|Schultz|Australia
>Spike|hCoV-19/Scotland/CVR3405/2020|2020-05-06|EPI_ISL_456829|Original|hCoV-19^^Scotland|Human|West of Scotland Specialist Virology Centre|COVID-19 Genomics UK (COG-UK) Consortium|Robertson|United Kingdom
>Spike|hCoV-19/Scotland/CVR3425/2020|2020-05-07|EPI_ISL_456846|Original|hCoV-19^^Scotland|Human|West of Scotland Specialist Virology Centre|COVID-19 Genomics UK (COG-UK) Consortium|Robertson|United Kingdom
>Spike|hCoV-19/England/NORW-EA42E/2020|2020-05-12|EPI_ISL_457346|Original|hCoV-19^^England|Human|Quadram Institute Bioscience|COVID-19 Genomics UK (COG-UK) Consortium|Alikhan|United Kingdom
>Spike|hCoV-19/England/NORW-EAE4E/2020|2020-04-29|EPI_ISL_457384|Original|hCoV-19^^England|Human|Quadram Institute Bioscience|COVID-19 Genomics UK (COG-UK) Consortium|Alikhan|United Kingdom
>Spike|hCoV-19/England/NORW-EB8B8/2020|2020-05-07|EPI_ISL_457453|Original|hCoV-19^^England|Human|Quadram Institute Bioscience|COVID-19 Genomics UK (COG-UK) Consortium|Alikhan|United Kingdom
>Spike|hCoV-19/England/NORW-EC44A/2020|2020-05-11|EPI_ISL_457526|Original|hCoV-19^^England|Human|Quadram Institute Bioscience|COVID-19 Genomics UK (COG-UK) Consortium|Alikhan|United Kingdom
>Spike|hCoV-19/Oman/205024123/2020|2020-03-25|EPI_ISL_457703|Original|hCoV-19^^Muscat|Human|Oman-NIC|Department of\u00a0Microbiology\u00a0and Immunology-SQUH|AL Zadjali|Oman
>Spike|hCoV-19/Oman/205027055/2020|2020-04-06|EPI_ISL_458123|Original|hCoV-19^^Muscat|Human|Oman National Influenza Centre|Department of\u00a0Microbiology\u00a0and Immunology-SQUH|AL Zadjali|Oman
>Spike|hCoV-19/Oman/205033391/2020|2020-05-03|EPI_ISL_458124|Original|hCoV-19^^Muscat|Human|Oman National Influenza Centre|Department of\u00a0Microbiology\u00a0and Immunology-SQUH|AL Zadjali|Oman
>Spike|hCoV-19/England/BRIS-1286C3/2020|2020-04-14|EPI_ISL_458776|Original|hCoV-19^^England|Human|PHE South West Regional Laboratory|Wellcome Sanger Institute for the COVID-19 Genomics UK (COG-UK) consortium|Davies|United Kingdom
>Spike|hCoV-19/England/BRIS-1286D2/2020|2020-04-14|EPI_ISL_458786|Original|hCoV-19^^England|Human|PHE South West Regional Laboratory|Wellcome Sanger Institute for the COVID-19 Genomics UK (COG-UK) Consortium|Davies|United Kingdom
>Spike|hCoV-19/England/BRIS-1292A0/2020|2020-04-19|EPI_ISL_458791|Original|hCoV-19^^England|Human|PHE South West Regional Laboratory|Wellcome Sanger Institute for the COVID-19 Genomics UK (COG-UK) consortium|Davies|United Kingdom
>Spike|hCoV-19/England/BRIS-129510/2020|2020-04-19|EPI_ISL_458836|Original|hCoV-19^^England|Human|PHE South West Regional Laboratory|Wellcome Sanger Institute for the COVID-19 Genomics UK (COG-UK) consortium|Davies|United Kingdom
>Spike|hCoV-19/England/BRIS-1294C8/2020|2020-04-20|EPI_ISL_458869|Original|hCoV-19^^England|Human|PHE South West Regional Laboratory|Wellcome Sanger Institute for the COVID-19 Genomics UK (COG-UK) consortium|Davies|United Kingdom
>Spike|hCoV-19/England/BRIS-126012/2020|2020-04-09|EPI_ISL_459039|Original|hCoV-19^^England|Human|PHE South West Regional Laboratory|Wellcome Sanger Institute for the COVID-19 Genomics UK (COG-UK) Consortium|Davies|United Kingdom
>Spike|hCoV-19/England/BRIS-1263CE/2020|2020-04-10|EPI_ISL_459070|Original|hCoV-19^^England|Human|PHE South West Regional Laboratory|Wellcome Sanger Institute for the COVID-19 Genomics UK (COG-UK) consortium|Davies|United Kingdom
>Spike|hCoV-19/England/CAMB-1B1049/2020|2020-05-05|EPI_ISL_459175|Original|hCoV-19^^England|Human|Department of Pathology|Wellcome Sanger Institute for the COVID-19 Genomics UK (COG-UK) consortium|Davies|United Kingdom
>Spike|hCoV-19/England/CAMB-1AF61F/2020|2020-05-01|EPI_ISL_459241|Original|hCoV-19^^England|Human|Department of Pathology|Wellcome Sanger Institute for the COVID-19 Genomics UK (COG-UK) Consortium|Davies|United Kingdom

>Spike|hCoV-19/England/CAMB-1B03D8/2020|2020-05-01|EPI_ISL_459243|Original|hCoV-19^^England|Human|Department of Pathology|Wellcome Sanger Institute for the COVID-19 Genomics UK (COG-UK) Consortium|Davies|United Kingdom
>Spike|hCoV-19/England/CAMB-1AF6F1/2020|2020-05-01|EPI_ISL_459244|Original|hCoV-19^^England|Human|Department of Pathology|Wellcome Sanger Institute for the COVID-19 Genomics UK (COG-UK) consortium|Davies|United Kingdom
>Spike|hCoV-19/England/CAMB-1B047B/2020|2020-05-01|EPI_ISL_459250|Original|hCoV-19^^England|Human|Department of Pathology|Wellcome Sanger Institute for the COVID-19 Genomics UK (COG-UK) consortium|Davies|United Kingdom
>Spike|hCoV-19/England/CAMB-1B0411/2020|2020-05-01|EPI_ISL_459281|Original|hCoV-19^^England|Human|Department of Pathology|Wellcome Sanger Institute for the COVID-19 Genomics UK (COG-UK) consortium|Davies|United Kingdom
>Spike|hCoV-19/England/CAMB-1B03F6/2020|2020-05-01|EPI_ISL_459323|Original|hCoV-19^^England|Human|Department of Pathology|Wellcome Sanger Institute for the COVID-19 Genomics UK (COG-UK) consortium|Davies|United Kingdom
>Spike|hCoV-19/England/CAMB-1ADF07/2020|2020-04-26|EPI_ISL_459413|Original|hCoV-19^^England|Human|Department of Pathology|Wellcome Sanger Institute for the COVID-19 Genomics UK (COG-UK) consortium|Davies|United Kingdom
>Spike|hCoV-19/England/CAMB-1ADFBC/2020|2020-04-26|EPI_ISL_459414|Original|hCoV-19^^England|Human|Department of Pathology|Wellcome Sanger Institute for the COVID-19 Genomics UK (COG-UK) consortium|Davies|United Kingdom
>Spike|hCoV-19/Scotland/GCVR-17028C/2020|2020-03-24|EPI_ISL_459566|Original|hCoV-19^^Scotland|Human|NHSGGC West of Scotland Specialist Virology Centre / MRC-University of Glasgow Centre for Virus Research|Wellcome Sanger Institute for the COVID-19 Genomics UK (COG-UK) consortium|Davies|United Kingdom
>Spike|hCoV-19/Scotland/GCVR-170073/2020|2020-03-24|EPI_ISL_459574|Original|hCoV-19^^Scotland|Human|NHSGGC West of Scotland Specialist Virology Centre / MRC-University of Glasgow Centre for Virus Research|Wellcome Sanger Institute for the COVID-19 Genomics UK (COG-UK) consortium|Davies|United Kingdom
>Spike|hCoV-19/Canada/ON-QGLO-021/2020|2020-03-27|EPI_ISL_459878|Original|hCoV-19^^Ontario|Human|Kingston Health Sciences Center|Queen's Genomics Lab at Ongwanada (Q-GLO)|Sjaarda|Canada
>Spike|hCoV-19/India/DL-MaxCov0037-CSIR-IGIB/2020|2020-05-11|EPI_ISL_459939|Original|hCoV-19^^Delhi|Human|Devki Devi Foundation|CSIR-IGIB/Max|Pandey|India
>Spike|hCoV-19/Malaysia/IMR_WC2665/2020|2020-03-18|EPI_ISL_459955|Vero E6|hCoV-19^^Malaysia|Human|Institute for Medical Research|Institute for Medical Research|Suppiah|Malaysia
>Spike|hCoV-19/Malaysia/IMR_WC10180/2020|2020-04-01|EPI_ISL_459957|Original|hCoV-19^^Malaysia|Human|Institute for Medical Research|Institute for Medical Research|Suppiah|Malaysia
>Spike|hCoV-19/USA/MN-MDH-93/2020|2020-03-14|EPI_ISL_460074|Original|hCoV-19^^Minnesota|Human|Minnesota Department of Health|Minnesota Department of Health|Garfin|USA
>Spike|hCoV-19/USA/MA-MGH-00523/2020|2020-03-18|EPI_ISL_460262|Original|hCoV-19^^Massachusetts|Human|Massachusetts General Hospital|Infectious Disease Program|Lemieux|USA
>Spike|hCoV-19/USA/WA-UW-4646/2020|2020-04-06|EPI_ISL_460634|Original|hCoV-19^^Washington|Human|UW Virology Lab|UW Virology Lab|Roychoudhury|USA
>Spike|hCoV-19/USA/WA-UW-5812/2020|2020-03-31|EPI_ISL_461443|Original|hCoV-19^^Washington|Human|UW Virology Lab|UW Virology Lab|Roychoudhury|USA
>Spike|hCoV-19/Scotland/CVR3114/2020|2020-04-25|EPI_ISL_461686|Original|hCoV-19^^Scotland|Human|West of Scotland Specialist Virology Centre|COVID-19 Genomics UK (COG-UK) Consortium|Robertson|United Kingdom
>Spike|hCoV-19/England/NORW-E8DCB/2020|2020-04-28|EPI_ISL_461809|Original|hCoV-19^^England|Human|Quadram Institute Bioscience|COVID-19 Genomics UK (COG-UK) Consortium|Alikhan|United Kingdom
>Spike|hCoV-19/England/NORW-E9094/2020|2020-04-29|EPI_ISL_461816|Original|hCoV-19^^England|Human|Quadram Institute Bioscience|COVID-19 Genomics UK (COG-UK) Consortium|Alikhan|United Kingdom

>Spike|hCoV-19/England/NORW-E91BF/2020|2020-04-28|EPI_ISL_461826|Original|hCoV-19^^England|Human|Quadram Institute Bioscience|COVID-19 Genomics UK (COG-UK) Consortium|Alikhan|United Kingdom
>Spike|hCoV-19/England/NORW-E9708/2020|2020-04-29|EPI_ISL_461830|Original|hCoV-19^^England|Human|Quadram Institute Bioscience|COVID-19 Genomics UK (COG-UK) Consortium|Alikhan|United Kingdom
>Spike|hCoV-19/England/NORW-E9735/2020|2020-04-29|EPI_ISL_461831|Original|hCoV-19^^England|Human|Quadram Institute Bioscience|COVID-19 Genomics UK (COG-UK) Consortium|Alikhan|United Kingdom
>Spike|hCoV-19/England/NORW-E9771/2020|2020-04-29|EPI_ISL_461832|Original|hCoV-19^^England|Human|Quadram Institute Bioscience|COVID-19 Genomics UK (COG-UK) Consortium|Alikhan|United Kingdom
>Spike|hCoV-19/England/NORW-E9850/2020|2020-04-29|EPI_ISL_461833|Original|hCoV-19^^England|Human|Quadram Institute Bioscience|COVID-19 Genomics UK (COG-UK) Consortium|Alikhan|United Kingdom
>Spike|hCoV-19/England/NORW-E986F/2020|2020-04-29|EPI_ISL_461834|Original|hCoV-19^^England|Human|Quadram Institute Bioscience|COVID-19 Genomics UK (COG-UK) Consortium|Alikhan|United Kingdom
>Spike|hCoV-19/England/NORW-E995D/2020|2020-04-29|EPI_ISL_461838|Original|hCoV-19^^England|Human|Quadram Institute Bioscience|COVID-19 Genomics UK (COG-UK) Consortium|Alikhan|United Kingdom
>Spike|hCoV-19/England/NORW-E99A8/2020|2020-04-29|EPI_ISL_461840|Original|hCoV-19^^England|Human|Quadram Institute Bioscience|COVID-19 Genomics UK (COG-UK) Consortium|Alikhan|United Kingdom
>Spike|hCoV-19/England/NORW-E9A1E/2020|2020-04-29|EPI_ISL_461841|Original|hCoV-19^^England|Human|Quadram Institute Bioscience|COVID-19 Genomics UK (COG-UK) Consortium|Alikhan|United Kingdom
>Spike|hCoV-19/Russia/CRIE160583/2020|2020-03-30|EPI_ISL_462149|Original|hCoV-19^^Moscow|Human|Molecular diagnostic laboratory of Federal Budget Institution of Science \Central Research Institute of Epidemiology\ of The Federal Service on Customers' Rights Protection and Human Well-being Surveillance|Group of Genomics and Postgenomic Technologies of Central Research Institute of Epidemilology|Spera
>nskaya|Russia
>Spike|hCoV-19/Spain/AN-ISCIII-202659/2020|2020-03-13|EPI_ISL_462477|Original|hCoV-19^^Andalucia|Human|Hospital Costa del Sol|Instituto de Salud Carlos III|Caballero|Spain
>Spike|hCoV-19/USA/MN-MDH-1018/2020|2020-04-30|EPI_ISL_462863|Original|hCoV-19^^Minnesota|Human|Minnesota Department of Health|Minnesota Department of Health|Garfin|USA
>Spike|hCoV-19/USA/MN-MDH-1051/2020|2020-05-14|EPI_ISL_462896|Original|hCoV-19^^Minnesota|Human|Minnesota Department of Health|Minnesota Department of Health|Garfin|USA
>Spike|hCoV-19/England/201090358/2020|2020-03-05|EPI_ISL_464553|Original|hCoV-19^^England|Human|Respiratory Virus Unit|Respiratory Virus Unit|Platt|United Kingdom
>Spike|hCoV-19/England/201180067/2020|2020-03-12|EPI_ISL_464879|Original|hCoV-19^^England|Human|Respiratory Virus Unit|Respiratory Virus Unit|Platt|United Kingdom
>Spike|hCoV-19/England/20118164704/2020|2020-03-12|EPI_ISL_464989|Original|hCoV-19^^England|Human|Respiratory Virus Unit|Respiratory Virus Unit|Platt|United Kingdom
>Spike|hCoV-19/England/20120013604/2020|2020-03-15|EPI_ISL_465155|Original|hCoV-19^^England|Human|Respiratory Virus Unit|Respiratory Virus Unit|Platt|United Kingdom
>Spike|hCoV-19/England/20169042704/2020|2020-04-18|EPI_ISL_465257|Original|hCoV-19^^England|Human|Respiratory Virus Unit|Respiratory Virus Unit|Platt|United Kingdom
>Spike|hCoV-19/England/20169042802/2020|2020-04-18|EPI_ISL_465258|Original|hCoV-19^^England|Human|Respiratory Virus Unit|Respiratory Virus Unit|Platt|United Kingdom
>Spike|hCoV-19/England/20180037704/2020|2020-04-27|EPI_ISL_465438|Original|hCoV-19^^England|Human|Respiratory Virus Unit|Respiratory Virus Unit|Platt|United Kingdom
>Spike|hCoV-19/England/20180038104/2020|2020-04-24|EPI_ISL_465439|Original|hCoV-19^^England|Human|Respiratory Virus Unit|Respiratory Virus Unit|Platt|United Kingdom
>Spike|hCoV-19/England/20182008804/2020|2020-04-24|EPI_ISL_465449|Original|hCoV-19^^England|Human|Respiratory Virus Unit|Respiratory Virus Unit|Platt|United Kingdom

>Spike|hCoV-19/England/20200086504/2020|2020-05-09|EPI_ISL_465646|Original|hCoV-19^^England|Human|Respiratory Virus Unit|Respiratory Virus Unit|Platt|United Kingdom
>Spike|hCoV-19/England/20206019104/2020|2020-05-12|EPI_ISL_465654|Original|hCoV-19^^England|Human|Respiratory Virus Unit|Respiratory Virus Unit|Platt|United Kingdom
>Spike|hCoV-19/England/20124095904/2020|2020-03-13|EPI_ISL_465791|Original|hCoV-19^^England|Human|Respiratory Virus Unit|Respiratory Virus Unit|Platt|United Kingdom
>Spike|hCoV-19/England/20156001404/2020|2020-04-07|EPI_ISL_466281|Original|hCoV-19^^England|Human|Respiratory Virus Unit|Respiratory Virus Unit|Platt|United Kingdom
>Spike|hCoV-19/England/20166011404/2020|2020-04-14|EPI_ISL_466527|Original|hCoV-19^^England|Human|Respiratory Virus Unit|Respiratory Virus Unit|Platt|United Kingdom
>Spike|hCoV-19/England/20166054404/2020|2020-04-13|EPI_ISL_466547|Original|hCoV-19^^England|Human|Respiratory Virus Unit|Respiratory Virus Unit|Platt|United Kingdom
>Spike|hCoV-19/Morocco/RA85/2020|2020-05-21|EPI_ISL_467299|Original|hCoV-19^^Morocco|Human|Research and Medical Analysis Laboratory of Gendarmerie Royale|Research and Medical Analysis Laboratory of Gendarmerie Royale|Lemriss|Morocco
>Spike|hCoV-19/Mongolia/Imp3989/2020|2020-03-25|EPI_ISL_467666|Original|hCoV-19^^Mongolia|Human|Virology lab|National Centre for Communicable Deseases (NCCD)|Tsedenbal|Mongolia
>Spike|hCoV-19/USA/NY-QDX-130/2020|2020-03-16|EPI_ISL_467887|Original|hCoV-19^^New York|Human|Quest Diagnostics|Quest Diagnostics|Kagan|USA
>Spike|hCoV-19/Australia/SAP282/2020|2020-03-25|EPI_ISL_467988|Original|hCoV-19^^South Australia|Human|SA Pathology|SA Pathology|Leong|Australia
>Spike|hCoV-19/USA/CA-CZB-1312/2020|2020-05-07|EPI_ISL_468352|Original|hCoV-19^^California|Human|County of Santa Clara Public Health Department|Chan-Zuckerberg Biohub|Hao|USA
>Spike|hCoV-19/USA/CA-CZB-1359/2020|2020-04-12|EPI_ISL_468371|Original|hCoV-19^^California|Human|Alameda County Public Health Lab|Chan-Zuckerberg Biohub|Hao|USA
>Spike|hCoV-19/USA/CA-CZB-1463/2020|2020-04-23|EPI_ISL_468423|Original|hCoV-19^^California|Human|County of San Luis Obispo Public Health Laboratory|Chan-Zuckerberg Biohub|Hao|USA
>Spike|hCoV-19/USA/CA-CZB-1529/2020|2020-05-02|EPI_ISL_468474|Original|hCoV-19^^California|Human|Ventura County Public Health Lab|Chan-Zuckerberg Biohub|Hao|USA
>Spike|hCoV-19/USA/CA-CZB-1716/2020|2020-04-03|EPI_ISL_468644|Original|hCoV-19^^California|Human|Contra Costa Public Health Lab|Chan-Zuckerberg Biohub|McGeever|USA
>Spike|hCoV-19/Spain/AR-IBV-2442/2020|2020-03-24|EPI_ISL_468776|Original|hCoV-19^^Aragon|Human|Servicio de Microbiolog\u00eda|SeqCOVID-SPAIN consortium/IBV(CSIC)|Comas|Spain
>Spike|hCoV-19/Spain/AR-IBV-2385/2020|2020-03-23|EPI_ISL_468838|Original|hCoV-19^^Aragon|Human|Servicio de Microbiolog\u00eda|SeqCOVID-SPAIN consortium/IBV(CSIC)|Comas|Spain
>Spike|hCoV-19/Spain/IB-IBV-2498/2020|2020-03-20|EPI_ISL_468963|Original|hCoV-19^^Illes Balears|Human|Servicio de Microbiolog\u00eda|SeqCOVID-SPAIN consortium/IBV(CSIC)|Comas|Spain
>Spike|hCoV-19/Spain/IB-IBV-2540/2020|2020-03-28|EPI_ISL_468969|Original|hCoV-19^^Illes Balears|Human|Servicio de Microbiolog\u00eda|SeqCOVID-SPAIN consortium/IBV(CSIC)|Comas|Spain
>Spike|hCoV-19/Spain/IB-IBV-h2551/2020|2020-03-20|EPI_ISL_468999|Original|hCoV-19^^Illes Balears|Human|Servicio de Microbiolog\u00eda|SeqCOVID-SPAIN consortium/IBV(CSIC)|Comas|Spain
>Spike|hCoV-19/Sweden/20-50261/2020|2020-03-07|EPI_ISL_469078|Original|hCoV-19^^Stockholm|Human|Karolinska Universitetslaboratoriet|The Public Health Agency of Sweden|Svartstr\u00f6m|Sweden
>Spike|hCoV-19/England/BRIS-127239/2020|2020-04-24|EPI_ISL_469614|Original|hCoV-19^^England|Human|PHE South West Regional Laboratory|Wellcome Sanger Institute for the COVID-19 Genomics UK (COG-UK) consortium|Davies|United Kingdom
>Spike|hCoV-19/England/BRIS-127F50/2020|2020-04-18|EPI_ISL_469728|Original|hCoV-19^^England|Human|PHE South West Regional Laboratory|Wellcome Sanger Institute for the COVID-19 Genomics UK (COG-UK) consortium|Davies|United Kingdom

>Spike|hCoV-19/England/BRIS-128335/2020|2020-04-16|EPI_ISL_469770|Original|hCoV-19^^England|Human|PHE South West Regional Laboratory|Wellcome Sanger Institute for the COVID-19 Genomics UK (COG-UK) consortium|Davies|United Kingdom
>Spike|hCoV-19/England/CAMB-1AAC30/2020|2020-04-19|EPI_ISL_470121|Original|hCoV-19^^England|Human|Department of Pathology|Wellcome Sanger Institute for the COVID-19 Genomics UK (COG-UK) consortium|Davies|United Kingdom
>Spike|hCoV-19/England/CAMB-83B7D/2020|2020-04-16|EPI_ISL_470355|Original|hCoV-19^^England|Human|Department of Pathology|Wellcome Sanger Institute for the COVID-19 Genomics UK (COG-UK) consortium|Davies|United Kingdom
>Spike|hCoV-19/England/CAMB-7F9C5/2020|2020-04-09|EPI_ISL_470406|Original|hCoV-19^^England|Human|Department of Pathology|Wellcome Sanger Institute for the COVID-19 Genomics UK (COG-UK) consortium|Davies|United Kingdom
>Spike|hCoV-19/USA/UT-02133/2020|2020-04-13|EPI_ISL_470735|Original|hCoV-19^^Utah|Human|Utah Public Health Laboratory|Utah Public Health Laboratory|Butz|USA
>Spike|hCoV-19/England/LIVE-9FF45/2020|2020-06-08|EPI_ISL_472074|Original|hCoV-19^^England|Human|Liverpool Clinical Laboratories|COVID-19 Genomics UK (COG-UK) Consortium|Haldenby|United Kingdom
>Spike|hCoV-19/England/LIVE-A047F/2020|2020-05-25|EPI_ISL_472112|Original|hCoV-19^^England|Human|Liverpool Clinical Laboratories|COVID-19 Genomics UK (COG-UK) Consortium|Haldenby|United Kingdom
>Spike|hCoV-19/England/LIVE-A0512/2020|2020-05-26|EPI_ISL_472117|Original|hCoV-19^^England|Human|Liverpool Clinical Laboratories|COVID-19 Genomics UK (COG-UK) Consortium|Haldenby|United Kingdom
>Spike|hCoV-19/England/LIVE-A059A/2020|2020-05-26|EPI_ISL_472120|Original|hCoV-19^^England|Human|Liverpool Clinical Laboratories|COVID-19 Genomics UK (COG-UK) Consortium|Haldenby|United Kingdom
>Spike|hCoV-19/England/NORW-EB0ED/2020|2020-05-04|EPI_ISL_472300|Original|hCoV-19^^England|Human|Quadram Institute Bioscience|COVID-19 Genomics UK (COG-UK) Consortium|Alikhan|United Kingdom
>Spike|hCoV-19/England/NORW-EB77F/2020|2020-05-05|EPI_ISL_472315|Original|hCoV-19^^England|Human|Quadram Institute Bioscience|COVID-19 Genomics UK (COG-UK) Consortium|Alikhan|United Kingdom
>Spike|hCoV-19/England/NORW-EBF86/2020|2020-05-21|EPI_ISL_472326|Original|hCoV-19^^England|Human|Quadram Institute Bioscience|COVID-19 Genomics UK (COG-UK) Consortium|Alikhan|United Kingdom
>Spike|hCoV-19/England/NORW-ECA1B/2020|2020-05-26|EPI_ISL_472329|Original|hCoV-19^^England|Human|Quadram Institute Bioscience|COVID-19 Genomics UK (COG-UK) Consortium|Alikhan|United Kingdom
>Spike|hCoV-19/England/NORW-ED704/2020|2020-06-05|EPI_ISL_472380|Original|hCoV-19^^England|Human|Quadram Institute Bioscience|COVID-19 Genomics UK (COG-UK) Consortium|Alikhan|United Kingdom
>Spike|hCoV-19/Wales/PHWC-15C198/2020|2020-04-20|EPI_ISL_472447|Original|hCoV-19^^Wales|Human|Wales Specialist Virology Centre Sequencing lab|COVID-19 Genomics UK (COG-UK) Consortium|Moore|United Kingdom
>Spike|hCoV-19/Wales/PHWC-15C1E3/2020|2020-04-19|EPI_ISL_472451|Original|hCoV-19^^Wales|Human|Wales Specialist Virology Centre Sequencing lab|COVID-19 Genomics UK (COG-UK) Consortium|Moore|United Kingdom
>Spike|hCoV-19/Wales/PHWC-15C22C/2020|2020-05-05|EPI_ISL_472455|Original|hCoV-19^^Wales|Human|Wales Specialist Virology Centre Sequencing lab|COVID-19 Genomics UK (COG-UK) Consortium|Moore|United Kingdom
>Spike|hCoV-19/Wales/PHWC-15C3ED/2020|2020-05-04|EPI_ISL_472483|Original|hCoV-19^^Wales|Human|Wales Specialist Virology Centre Sequencing lab|COVID-19 Genomics UK (COG-UK) Consortium|Moore|United Kingdom
>Spike|hCoV-19/Wales/PHWC-15C462/2020|2020-03-26|EPI_ISL_472490|Original|hCoV-19^^Wales|Human|Wales Specialist Virology Centre Sequencing lab|COVID-19 Genomics UK (COG-UK) Consortium|Moore|United Kingdom
>Spike|hCoV-19/Wales/PHWC-15C6B7/2020|2020-05-09|EPI_ISL_472523|Original|hCoV-19^^Wales|Human|Wales Specialist Virology Centre Sequencing lab|COVID-19 Genomics UK (COG-UK) Consortium|Moore|United Kingdom

>Spike|hCoV-19/Wales/PHWC-15CA7F/2020|2020-05-06|EPI_ISL_472574|Original|hCoV-19^^Wales|Human|Wales Specialist Virology Centre Sequencing lab|COVID-19 Genomics UK (COG-UK) Consortium|Moore|United Kingdom
>Spike|hCoV-19/Wales/PHWC-15CACA/2020|2020-03-31|EPI_ISL_472578|Original|hCoV-19^^Wales|Human|Wales Specialist Virology Centre Sequencing lab|COVID-19 Genomics UK (COG-UK) Consortium|Moore|United Kingdom
>Spike|hCoV-19/Wales/PHWC-15CB6D/2020|2020-05-03|EPI_ISL_472587|Original|hCoV-19^^Wales|Human|Wales Specialist Virology Centre Sequencing lab|COVID-19 Genomics UK (COG-UK) Consortium|Moore|United Kingdom
>Spike|hCoV-19/Wales/PHWC-15CC1F/2020|2020-05-05|EPI_ISL_472597|Original|hCoV-19^^Wales|Human|Wales Specialist Virology Centre Sequencing lab|COVID-19 Genomics UK (COG-UK) Consortium|Moore|United Kingdom
>Spike|hCoV-19/Wales/PHWC-15CCD3/2020|2020-05-05|EPI_ISL_472607|Original|hCoV-19^^Wales|Human|Wales Specialist Virology Centre Sequencing lab|COVID-19 Genomics UK (COG-UK) Consortium|Moore|United Kingdom
>Spike|hCoV-19/Wales/PHWC-15CF16/2020|2020-05-05|EPI_ISL_472640|Original|hCoV-19^^Wales|Human|Wales Specialist Virology Centre Sequencing lab|COVID-19 Genomics UK (COG-UK) Consortium|Moore|United Kingdom
>Spike|hCoV-19/Wales/PHWC-15CFBC/2020|2020-05-08|EPI_ISL_472650|Original|hCoV-19^^Wales|Human|Wales Specialist Virology Centre Sequencing lab|COVID-19 Genomics UK (COG-UK) Consortium|Moore|United Kingdom
>Spike|hCoV-19/Wales/PHWC-15D021/2020|2020-03-30|EPI_ISL_472657|Original|hCoV-19^^Wales|Human|Wales Specialist Virology Centre Sequencing lab|COVID-19 Genomics UK (COG-UK) Consortium|Moore|United Kingdom
>Spike|hCoV-19/Wales/PHWC-15D13D/2020|2020-05-06|EPI_ISL_472671|Original|hCoV-19^^Wales|Human|Wales Specialist Virology Centre Sequencing lab|COVID-19 Genomics UK (COG-UK) Consortium|Moore|United Kingdom
>Spike|hCoV-19/Wales/PHWC-15D188/2020|2020-04-19|EPI_ISL_472676|Original|hCoV-19^^Wales|Human|Wales Specialist Virology Centre Sequencing lab|COVID-19 Genomics UK (COG-UK) Consortium|Moore|United Kingdom
>Spike|hCoV-19/Wales/PHWC-15D1D3/2020|2020-04-08|EPI_ISL_472681|Original|hCoV-19^^Wales|Human|Wales Specialist Virology Centre Sequencing lab|COVID-19 Genomics UK (COG-UK) Consortium|Moore|United Kingdom
>Spike|hCoV-19/Wales/PHWC-15D373/2020|2020-04-20|EPI_ISL_472707|Original|hCoV-19^^Wales|Human|Wales Specialist Virology Centre Sequencing lab|COVID-19 Genomics UK (COG-UK) Consortium|Moore|United Kingdom
>Spike|hCoV-19/Wales/PHWC-15E011/2020|2020-05-13|EPI_ISL_472786|Original|hCoV-19^^Wales|Human|Wales Specialist Virology Centre Sequencing lab|COVID-19 Genomics UK (COG-UK) Consortium|Moore|United Kingdom
>Spike|hCoV-19/Wales/PHWC-15E020/2020|2020-05-15|EPI_ISL_472787|Original|hCoV-19^^Wales|Human|Wales Specialist Virology Centre Sequencing lab|COVID-19 Genomics UK (COG-UK) Consortium|Moore|United Kingdom
>Spike|hCoV-19/Wales/PHWC-15E0F3/2020|2020-05-13|EPI_ISL_472794|Original|hCoV-19^^Wales|Human|Wales Specialist Virology Centre Sequencing lab|COVID-19 Genomics UK (COG-UK) Consortium|Moore|United Kingdom
>Spike|hCoV-19/Wales/PHWC-15E970/2020|2020-05-13|EPI_ISL_472843|Original|hCoV-19^^Wales|Human|Wales Specialist Virology Centre Sequencing lab|COVID-19 Genomics UK (COG-UK) Consortium|Moore|United Kingdom
>Spike|hCoV-19/Wales/PHWC-15FD28/2020|2020-04-26|EPI_ISL_472848|Original|hCoV-19^^Wales|Human|Wales Specialist Virology Centre Sequencing lab|COVID-19 Genomics UK (COG-UK) Consortium|Moore|United Kingdom
>Spike|hCoV-19/Wales/PHWC-1600C1/2020|2020-05-20|EPI_ISL_472894|Original|hCoV-19^^Wales|Human|Wales Specialist Virology Centre Sequencing lab|COVID-19 Genomics UK (COG-UK) Consortium|Moore|United Kingdom
>Spike|hCoV-19/Wales/PHWC-160304/2020|2020-05-21|EPI_ISL_472919|Original|hCoV-19^^Wales|Human|Wales Specialist Virology Centre Sequencing lab|COVID-19 Genomics UK (COG-UK) Consortium|Moore|United Kingdom
>Spike|hCoV-19/Wales/PHWC-16044D/2020|2020-05-26|EPI_ISL_472934|Original|hCoV-19^^Wales|Human|Wales Specialist Virology Centre Sequencing lab|COVID-19 Genomics UK (COG-UK) Consortium|Moore|United Kingdom

>Spike|hCoV-19/Wales/PHWC-1604A7/2020|2020-05-24|EPI_ISL_472939|Original|hCoV-19^^Wales|Human|Wales Specialist Virology Centre Sequencing lab|COVID-19 Genomics UK (COG-UK) Consortium|Moore|United Kingdom
>Spike|hCoV-19/Wales/PHWC-160577/2020|2020-05-19|EPI_ISL_472950|Original|hCoV-19^^Wales|Human|Wales Specialist Virology Centre Sequencing lab|COVID-19 Genomics UK (COG-UK) Consortium|Moore|United Kingdom
>Spike|hCoV-19/Wales/PHWC-160902/2020|2020-05-19|EPI_ISL_472992|Original|hCoV-19^^Wales|Human|Wales Specialist Virology Centre Sequencing lab|COVID-19 Genomics UK (COG-UK) Consortium|Moore|United Kingdom
>Spike|hCoV-19/Wales/PHWC-16094E/2020|2020-05-25|EPI_ISL_472996|Original|hCoV-19^^Wales|Human|Wales Specialist Virology Centre Sequencing lab|COVID-19 Genomics UK (COG-UK) Consortium|Moore|United Kingdom
>Spike|hCoV-19/Wales/PHWC-160B48/2020|2020-05-19|EPI_ISL_473018|Original|hCoV-19^^Wales|Human|Wales Specialist Virology Centre Sequencing lab|COVID-19 Genomics UK (COG-UK) Consortium|Moore|United Kingdom
>Spike|hCoV-19/Wales/PHWC-160C54/2020|2020-05-18|EPI_ISL_473032|Original|hCoV-19^^Wales|Human|Wales Specialist Virology Centre Sequencing lab|COVID-19 Genomics UK (COG-UK) Consortium|Moore|United Kingdom
>Spike|hCoV-19/Wales/PHWC-1629D3/2020|2020-05-16|EPI_ISL_473148|Original|hCoV-19^^Wales|Human|Wales Specialist Virology Centre Sequencing lab|COVID-19 Genomics UK (COG-UK) Consortium|Moore|United Kingdom
>Spike|hCoV-19/Wales/PHWC-162A2B/2020|2020-05-13|EPI_ISL_473152|Original|hCoV-19^^Wales|Human|Wales Specialist Virology Centre Sequencing lab|COVID-19 Genomics UK (COG-UK) Consortium|Moore|United Kingdom
>Spike|hCoV-19/Wales/PHWC-162D04/2020|2020-05-26|EPI_ISL_473190|Original|hCoV-19^^Wales|Human|Wales Specialist Virology Centre Sequencing lab|COVID-19 Genomics UK (COG-UK) Consortium|Moore|United Kingdom
>Spike|hCoV-19/Wales/PHWC-163019/2020|2020-05-21|EPI_ISL_473234|Original|hCoV-19^^Wales|Human|Wales Specialist Virology Centre Sequencing lab|COVID-19 Genomics UK (COG-UK) Consortium|Moore|United Kingdom
>Spike|hCoV-19/Wales/PHWC-163082/2020|2020-05-18|EPI_ISL_473241|Original|hCoV-19^^Wales|Human|Wales Specialist Virology Centre Sequencing lab|COVID-19 Genomics UK (COG-UK) Consortium|Moore|United Kingdom
>Spike|hCoV-19/Wales/PHWC-1631AD/2020|2020-05-16|EPI_ISL_473258|Original|hCoV-19^^Wales|Human|Wales Specialist Virology Centre Sequencing lab|COVID-19 Genomics UK (COG-UK) Consortium|Moore|United Kingdom
>Spike|hCoV-19/Wales/PHWC-1631F8/2020|2020-05-20|EPI_ISL_473262|Original|hCoV-19^^Wales|Human|Wales Specialist Virology Centre Sequencing lab|COVID-19 Genomics UK (COG-UK) Consortium|Moore|United Kingdom
>Spike|hCoV-19/Wales/PHWC-1634B3/2020|2020-05-06|EPI_ISL_473303|Original|hCoV-19^^Wales|Human|Wales Specialist Virology Centre Sequencing lab|COVID-19 Genomics UK (COG-UK) Consortium|Moore|United Kingdom
>Spike|hCoV-19/Scotland/CVR3664/2020|2020-05-22|EPI_ISL_473750|Original|hCoV-19^^Scotland|Human|West of Scotland Specialist Virology Centre|COVID-19 Genomics UK (COG-UK) Consortium|Robertson|United Kingdom
>Spike|hCoV-19/Wales/PHWC-163811/2020|2020-05-09|EPI_ISL_474001|Original|hCoV-19^^Wales|Human|Originating lab|COVID-19 Genomics UK (COG-UK) Consortium|Moore|United Kingdom
>Spike|hCoV-19/Wales/PHWC-1638D5/2020|2020-05-10|EPI_ISL_474013|Original|hCoV-19^^Wales|Human|Originating lab|COVID-19 Genomics UK (COG-UK) Consortium|Moore|United Kingdom
>Spike|hCoV-19/Wales/PHWC-163BCD/2020|2020-04-22|EPI_ISL_474053|Original|hCoV-19^^Wales|Human|Originating lab|COVID-19 Genomics UK (COG-UK) Consortium|Moore|United Kingdom
>Spike|hCoV-19/Wales/PHWC-163CE8/2020|2020-04-24|EPI_ISL_474069|Original|hCoV-19^^Wales|Human|Originating lab|COVID-19 Genomics UK (COG-UK) Consortium|Moore|United Kingdom
>Spike|hCoV-19/Wales/PHWC-163E5B/2020|2020-05-02|EPI_ISL_474092|Original|hCoV-19^^Wales|Human|Originating lab|COVID-19 Genomics UK (COG-UK) Consortium|Moore|United Kingdom

>Spike|hCoV-19/Wales/PHWC-16432E/2020|2020-04-29|EPI_ISL_474161|Original|hCoV-19^^Wales|Human|Originating lab|COVID-19 Genomics UK (COG-UK) Consortium|Moore|United Kingdom
>Spike|hCoV-19/Wales/PHWC-1645FB/2020|2020-05-08|EPI_ISL_474203|Original|hCoV-19^^Wales|Human|Originating lab|COVID-19 Genomics UK (COG-UK) Consortium|Moore|United Kingdom
>Spike|hCoV-19/Wales/PHWC-164704/2020|2020-05-07|EPI_ISL_474220|Original|hCoV-19^^Wales|Human|Originating lab|COVID-19 Genomics UK (COG-UK) Consortium|Moore|United Kingdom
>Spike|hCoV-19/Wales/PHWC-2C00E/2020|2020-04-02|EPI_ISL_474267|Original|hCoV-19^^Wales|Human|Originating lab|COVID-19 Genomics UK (COG-UK) Consortium|Moore|United Kingdom
>Spike|hCoV-19/Wales/PHWC-325E8/2020|2020-04-15|EPI_ISL_474332|Original|hCoV-19^^Wales|Human|Originating lab|COVID-19 Genomics UK (COG-UK) Consortium|Moore|United Kingdom
>Spike|hCoV-19/Wales/PHWC-32A8F/2020|2020-04-14|EPI_ISL_474389|Original|hCoV-19^^Wales|Human|Originating lab|COVID-19 Genomics UK (COG-UK) Consortium|Moore|United Kingdom
>Spike|hCoV-19/Wales/PHWC-32B31/2020|2020-04-15|EPI_ISL_474400|Original|hCoV-19^^Wales|Human|Originating lab|COVID-19 Genomics UK (COG-UK) Consortium|Moore|United Kingdom
>Spike|hCoV-19/Wales/PHWC-355B8/2020|2020-04-23|EPI_ISL_474448|Original|hCoV-19^^Wales|Human|Originating lab|COVID-19 Genomics UK (COG-UK) Consortium|Moore|United Kingdom
>Spike|hCoV-19/Wales/PHWC-35679/2020|2020-04-24|EPI_ISL_474459|Original|hCoV-19^^Wales|Human|Originating lab|COVID-19 Genomics UK (COG-UK) Consortium|Moore|United Kingdom
>Spike|hCoV-19/Wales/PHWC-35961/2020|2020-04-24|EPI_ISL_474502|Original|hCoV-19^^Wales|Human|Originating lab|COVID-19 Genomics UK (COG-UK) Consortium|Moore|United Kingdom
>Spike|hCoV-19/Wales/PHWC-35C68/2020|2020-04-27|EPI_ISL_474549|Original|hCoV-19^^Wales|Human|Originating lab|COVID-19 Genomics UK (COG-UK) Consortium|Moore|United Kingdom
>Spike|hCoV-19/Wales/PHWC-35FD8/2020|2020-04-27|EPI_ISL_474603|Original|hCoV-19^^Wales|Human|Originating lab|COVID-19 Genomics UK (COG-UK) Consortium|Moore|United Kingdom
>Spike|hCoV-19/Wales/PHWC-3606B/2020|2020-04-28|EPI_ISL_474612|Original|hCoV-19^^Wales|Human|Originating lab|COVID-19 Genomics UK (COG-UK) Consortium|Moore|United Kingdom
>Spike|hCoV-19/Wales/PHWC-36229/2020|2020-04-29|EPI_ISL_474638|Original|hCoV-19^^Wales|Human|Originating lab|COVID-19 Genomics UK (COG-UK) Consortium|Moore|United Kingdom
>Spike|hCoV-19/Wales/PHWC-36256/2020|2020-04-29|EPI_ISL_474641|Original|hCoV-19^^Wales|Human|Originating lab|COVID-19 Genomics UK (COG-UK) Consortium|Moore|United Kingdom
>Spike|hCoV-19/Wales/PHWC-3656C/2020|2020-05-01|EPI_ISL_474682|Original|hCoV-19^^Wales|Human|Originating lab|COVID-19 Genomics UK (COG-UK) Consortium|Moore|United Kingdom
>Spike|hCoV-19/Wales/PHWC-365E4/2020|2020-04-30|EPI_ISL_474688|Original|hCoV-19^^Wales|Human|Originating lab|COVID-19 Genomics UK (COG-UK) Consortium|Moore|United Kingdom
>Spike|hCoV-19/Wales/PHWC-3661E/2020|2020-04-30|EPI_ISL_474691|Original|hCoV-19^^Wales|Human|Originating lab|COVID-19 Genomics UK (COG-UK) Consortium|Moore|United Kingdom
>Spike|hCoV-19/Wales/PHWC-369F7/2020|2020-04-24|EPI_ISL_474745|Original|hCoV-19^^Wales|Human|Originating lab|COVID-19 Genomics UK (COG-UK) Consortium|Moore|United Kingdom
>Spike|hCoV-19/Spain/CM-IBV-003838/2020|2020-05-25|EPI_ISL_474821|Original|hCoV-19^^Castilla La Mancha|Human|Complejo Hospitalario Universitario de Albacete|SeqCOVID-SPAIN consortium/IBV(CSIC)|Comas|Spain

>Spike|hCoV-19/Spain/CM-IBV-003869/2020|2020-05-22|EPI_ISL_474905|Original|hCoV-19^^Castilla La Mancha|Human|Complejo Hospitalario Universitario de Albacete|SeqCOVID-SPAIN consortium/IBV(CSIC)|Comas|Spain
>Spike|hCoV-19/Spain/CM-IBV-003876/2020|2020-05-14|EPI_ISL_474906|Original|hCoV-19^^Castilla La Mancha|Human|Complejo Hospitalario Universitario de Albacete|SeqCOVID-SPAIN consortium/IBV(CSIC)|Comas|Spain
>Spike|hCoV-19/Israel/CVL-n-6106/2020|2020-03-15|EPI_ISL_474992|Original|hCoV-19^^Petah-Tikva|Human|Israel Central Virology laboratory|Israel Central Virology laboratory|Zuckerman|Israel
>Spike|hCoV-19/Morocco/moh100/2020|2020-05-05|EPI_ISL_476024|Original|hCoV-19^^Morocco|Human|Laboratoire de Recherche et d'Analyses M\u00e9dicales de la Gendarmerie Royale|Laboratoire de Recherche et d'Analyses M\u00e9dicales de la Gendarmerie Royale|Lemriss|Morocco
>Spike|hCoV-19/Morocco/CA24/2020|2020-05-02|EPI_ISL_476025|Original|hCoV-19^^Morocco|Human|Laboratoire de Recherche et d'Analyses M\u00e9dicales de la Gendarmerie Royale|Laboratoire de Recherche et d'Analyses M\u00e9dicales de la Gendarmerie Royale|Lemriss|Morocco
>Spike|hCoV-19/Morocco/AZ183/2020|2020-05-11|EPI_ISL_476026|Original|hCoV-19^^Morocco|Human|Laboratoire de Recherche et d'Analyses M\u00e9dicales de la Gendarmerie Royale|Laboratoire de Recherche et d'Analyses M\u00e9dicales de la Gendarmerie Royale|Lemriss|Morocco
>Spike|hCoV-19/USA/MI-MDHHS-SC20895/2020|2020-04-23|EPI_ISL_476032|Original|hCoV-19^^Michigan|Human|Michigan Department of Health and Human Services|Michigan Department of Health and Human Services|Blankenship|USA
>Spike|hCoV-19/Senegal/57481/2020|2020-06-06|EPI_ISL_476150|Original|hCoV-19^^Dakar|Human|Institut Pasteur Dakar|Institut Pasteur de Dakar|Dia|Senegal
>Spike|hCoV-19/Brazil/CE-L16-CD334/2020|2020-03-29|EPI_ISL_476283|Original|hCoV-19^^Cear\u00e1|Human|DB Diagn\u00f3sticos do Brasil|Instituto de Medicina Tropical da Univesidade de S\u00e3o Paulo|Faria|Brazil
>Spike|hCoV-19/Senegal/64914/2020|2020-06-13|EPI_ISL_476566|Original|hCoV-19^^Dakar|Human|Institut pasteur Dakar|Institut Pasteur de Dakar|Dia|Senegal
>Spike|hCoV-19/Greece/AYTH_8/2020|2020-04-04|EPI_ISL_476835|Original|hCoV-19^^Thessaloniki|Human|National Influenza Centre for Northern Greece|National Influenza Centre for Northern Greece|Christoforidi|Greece
>Spike|hCoV-19/Greece/AUTH_5/2020|2020-03-30|EPI_ISL_476838|Original|hCoV-19^^Thessaloniki|Human|National Influenza Centre for Northern Greece|National Influenza Centre for Northern Greece|Christoforidi|Greece
>Spike|hCoV-19/Greece/AYTH_3/2020|2020-04-09|EPI_ISL_476839|Original|hCoV-19^^Thessaloniki|Human|National Influenza Centre for Northern Greece|National Influenza Centre for Northern Greece|Christoforidi|Greece
>Spike|hCoV-19/Greece/Thessaloniki/2020|2020-04-20|EPI_ISL_476841|Original|hCoV-19^^Thessaloniki|Human|National Influenza Centre for Northern Greece|National Influenza Centre for Northern Greece|Christoforidi|Greece
>Spike|hCoV-19/Greece/AUTH_1/2020|2020-03-09|EPI_ISL_476843|Original|hCoV-19^^Thessaloniki|Human|National Influenza Centre for Northern Greece|National Influenza Centre for Northern Greece|Christoforidi|Greece
>Spike|hCoV-19/Greece/NIC_8/2020|2020-03-13|EPI_ISL_476845|Original|hCoV-19^^Thessaloniki|Human|National Influenza Centre for Northern Greece|National Influenza Centre for Northern Greece|Christoforidi|Greece
>Spike|hCoV-19/Greece/NIC_5/2020|2020-04-12|EPI_ISL_476847|Original|hCoV-19^^Thessaloniki|Human|National Influenza Centre for Northern Greece|National Influenza Centre for Northern Greece|Christoforidi|Greece
>Spike|hCoV-19/Senegal/64772/2020|2020-06-13|EPI_ISL_477154|Original|hCoV-19^^Dakar|Human|Institut Pasteur Dakar|Institut Pasteur de Dakar|Dia|Senegal
>Spike|hCoV-19/Senegal/63930/2020|2020-06-12|EPI_ISL_477158|Original|hCoV-19^^Dakar|Human|Institut Pasteur Dakar|Institut Pasteur de Dakar|Dia|Senegal
>Spike|hCoV-19/Italy/CAM-CEINGE-1/2020|2020-05-30|EPI_ISL_477204|Original|hCoV-19^^Campania|Human|Prof. Massimo Zollo CEINGE TASK-FORCE COVID19 - Regione Campania|Prof. Massimo Zollo CEINGE TASK-FORCE COVID19 - Regione Campania|Zollo|Italy

>Spike|hCoV-19/India/KA-InStem-NCBS-0004/2020|2020-05-29|EPI_ISL_477207|Original|hCoV-19^^Karnataka|Human|Institute for Stem Cell Science and Regenerative Medicine|National Centre for Biological Sciences|Ali|India
>Spike|hCoV-19/India/KA-InStem-NCBS-0005/2020|2020-06-01|EPI_ISL_477208|Original|hCoV-19^^Karnataka|Human|Institute for Stem Cell Science and Regenerative Medicine|National Centre for Biological Sciences|Ali|India
>Spike|hCoV-19/India/KA-InStem-NCBS-0008/2020|2020-06-02|EPI_ISL_477211|Original|hCoV-19^^Karnataka|Human|Institute for Stem Cell Science and Regenerative Medicine|National Centre for Biological Sciences|Ali|India
>Spike|hCoV-19/India/KA-InStem-NCBS-0037/2020|2020-06-16|EPI_ISL_477238|Original|hCoV-19^^Karnataka|Human|Institute for Stem Cell Science and Regenerative Medicine|National Centre for Biological Sciences|Ali|India
>Spike|hCoV-19/India/KA-InStem-NCBS-0041/2020|2020-06-17|EPI_ISL_477242|Original|hCoV-19^^Karnataka|Human|Institute for Stem Cell Science and Regenerative Medicine|National Centre for Biological Sciences|Ali|India
>Spike|hCoV-19/India/KA-InStem-NCBS-0047/2020|2020-00-00|EPI_ISL_477247|Original|hCoV-19^^Karnataka|Human|Institute for Stem Cell Science and Regenerative Medicine|National Centre for Biological Sciences|Ali|India
>Spike|hCoV-19/India/KA-InStem-NCBS-0049/2020|2020-05-07|EPI_ISL_477249|Original|hCoV-19^^Karnataka|Human|Institute for Stem Cell Science and Regenerative Medicine|National Centre for Biological Sciences|Ali|India
>Spike|hCoV-19/India/KA-InStem-NCBS-0050/2020|2020-05-07|EPI_ISL_477250|Original|hCoV-19^^Karnataka|Human|Institute for Stem Cell Science and Regenerative Medicine|National Centre for Biological Sciences|Ali|India
>Spike|hCoV-19/India/KA-InStem-NCBS-0054/2020|2020-00-00|EPI_ISL_477254|Original|hCoV-19^^Karnataka|Human|Institute for Stem Cell Science and Regenerative Medicine|National Centre for Biological Sciences|Ali|India
>Spike|hCoV-19/India/KA-InStem-NCBS-0056/2020|2020-05-16|EPI_ISL_477256|Original|hCoV-19^^Karnataka|Human|Institute for Stem Cell Science and Regenerative Medicine|National Centre for Biological Sciences|Ali|India
>Spike|hCoV-19/India/KA-InStem-NCBS-0058/2020|2020-05-16|EPI_ISL_477258|Original|hCoV-19^^Karnataka|Human|Institute for Stem Cell Science and Regenerative Medicine|National Centre for Biological Sciences|Ali|India
>Spike|hCoV-19/India/KA-InStem-NCBS-0061/2020|2020-05-18|EPI_ISL_477261|Original|hCoV-19^^Karnataka|Human|Institute for Stem Cell Science and Regenerative Medicine|National Centre for Biological Sciences|Ali|India
>Spike|hCoV-19/England/CAMB-1B0E40/2020|2020-05-11|EPI_ISL_477785|Original|hCoV-19^^England|Human|Department of Pathology|COVID-19 Genomics UK (COG-UK) Consortium|Meredith|United Kingdom
>Spike|hCoV-19/Scotland/CVR116/2020|2020-03-13|EPI_ISL_477820|Original|hCoV-19^^Scotland|Human|West of Scotland Specialist Virology Centre|COVID-19 Genomics UK (COG-UK) Consortium|Robertson|United Kingdom
>Spike|hCoV-19/Scotland/CVR2780/2020|2020-04-20|EPI_ISL_478037|Original|hCoV-19^^Scotland|Human|West of Scotland Specialist Virology Centre|COVID-19 Genomics UK (COG-UK) Consortium|Robertson|United Kingdom
>Spike|hCoV-19/Scotland/CVR2793/2020|2020-04-20|EPI_ISL_478038|Original|hCoV-19^^Scotland|Human|West of Scotland Specialist Virology Centre|COVID-19 Genomics UK (COG-UK) Consortium|Robertson|United Kingdom
>Spike|hCoV-19/Scotland/CVR2897/2020|2020-04-21|EPI_ISL_478042|Original|hCoV-19^^Scotland|Human|West of Scotland Specialist Virology Centre|COVID-19 Genomics UK (COG-UK) Consortium|Robertson|United Kingdom
>Spike|hCoV-19/Scotland/CVR3172/2020|2020-04-26|EPI_ISL_478054|Original|hCoV-19^^Scotland|Human|West of Scotland Specialist Virology Centre|COVID-19 Genomics UK (COG-UK) Consortium|Robertson|United Kingdom
>Spike|hCoV-19/Scotland/CVR3229/2020|2020-04-26|EPI_ISL_478055|Original|hCoV-19^^Scotland|Human|West of Scotland Specialist Virology Centre|COVID-19 Genomics UK (COG-UK) Consortium|Robertson|United Kingdom
>Spike|hCoV-19/Scotland/CVR3351/2020|2020-05-07|EPI_ISL_478056|Original|hCoV-19^^Scotland|Human|West of Scotland Specialist Virology Centre|COVID-19 Genomics UK (COG-UK) Consortium|Robertson|United Kingdom

>Spike|hCoV-19/Scotland/CVR3487/2020|2020-05-11|EPI_ISL_478073|Original|hCoV-19^^Scotland|Human|West of Scotland Specialist Virology Centre|COVID-19 Genomics UK (COG-UK) Consortium|Robertson|United Kingdom
>Spike|hCoV-19/Scotland/CVR3644/2020|2020-05-21|EPI_ISL_478083|Original|hCoV-19^^Scotland|Human|West of Scotland Specialist Virology Centre|COVID-19 Genomics UK (COG-UK) Consortium|Robertson|United Kingdom
>Spike|hCoV-19/Scotland/CVR3661/2020|2020-05-19|EPI_ISL_478088|Original|hCoV-19^^Scotland|Human|West of Scotland Specialist Virology Centre|COVID-19 Genomics UK (COG-UK) Consortium|Robertson|United Kingdom
>Spike|hCoV-19/Scotland/CVR3677/2020|2020-05-22|EPI_ISL_478091|Original|hCoV-19^^Scotland|Human|West of Scotland Specialist Virology Centre|COVID-19 Genomics UK (COG-UK) Consortium|Robertson|United Kingdom
>Spike|hCoV-19/Scotland/CVR3705/2020|2020-05-26|EPI_ISL_478096|Original|hCoV-19^^Scotland|Human|West of Scotland Specialist Virology Centre|COVID-19 Genomics UK (COG-UK) Consortium|Robertson|United Kingdom
>Spike|hCoV-19/Scotland/CVR3747/2020|2020-05-26|EPI_ISL_478102|Original|hCoV-19^^Scotland|Human|West of Scotland Specialist Virology Centre|COVID-19 Genomics UK (COG-UK) Consortium|Robertson|United Kingdom
>Spike|hCoV-19/Scotland/CVR3777/2020|2020-05-27|EPI_ISL_478110|Original|hCoV-19^^Scotland|Human|West of Scotland Specialist Virology Centre|COVID-19 Genomics UK (COG-UK) Consortium|Robertson|United Kingdom
>Spike|hCoV-19/Scotland/CVR3780/2020|2020-05-27|EPI_ISL_478113|Original|hCoV-19^^Scotland|Human|West of Scotland Specialist Virology Centre|COVID-19 Genomics UK (COG-UK) Consortium|Robertson|United Kingdom
>Spike|hCoV-19/Scotland/CVR477/2020|2020-03-24|EPI_ISL_478161|Original|hCoV-19^^Scotland|Human|West of Scotland Specialist Virology Centre|COVID-19 Genomics UK (COG-UK) Consortium|Robertson|United Kingdom
>Spike|hCoV-19/Scotland/CVR650/2020|2020-03-25|EPI_ISL_478162|Original|hCoV-19^^Scotland|Human|West of Scotland Specialist Virology Centre|COVID-19 Genomics UK (COG-UK) Consortium|Robertson|United Kingdom
>Spike|hCoV-19/England/LIVE-9FFAF/2020|2020-06-08|EPI_ISL_478374|Original|hCoV-19^^England|Human|Liverpool Clinical Laboratories|COVID-19 Genomics UK (COG-UK) Consortium|Haldenby|United Kingdom
>Spike|hCoV-19/England/LIVE-A00D5/2020|2020-06-04|EPI_ISL_478375|Original|hCoV-19^^England|Human|Liverpool Clinical Laboratories|COVID-19 Genomics UK (COG-UK) Consortium|Haldenby|United Kingdom
>Spike|hCoV-19/England/LIVE-A012D/2020|2020-06-04|EPI_ISL_478376|Original|hCoV-19^^England|Human|Liverpool Clinical Laboratories|COVID-19 Genomics UK (COG-UK) Consortium|Haldenby|United Kingdom
>Spike|hCoV-19/England/LIVE-A013C/2020|2020-06-04|EPI_ISL_478377|Original|hCoV-19^^England|Human|Liverpool Clinical Laboratories|COVID-19 Genomics UK (COG-UK) Consortium|Haldenby|United Kingdom
>Spike|hCoV-19/England/LIVE-A022A/2020|2020-06-03|EPI_ISL_478378|Original|hCoV-19^^England|Human|Liverpool Clinical Laboratories|COVID-19 Genomics UK (COG-UK) Consortium|Haldenby|United Kingdom
>Spike|hCoV-19/England/LIVE-A0284/2020|2020-06-02|EPI_ISL_478379|Original|hCoV-19^^England|Human|Liverpool Clinical Laboratories|COVID-19 Genomics UK (COG-UK) Consortium|Haldenby|United Kingdom
>Spike|hCoV-19/England/LIVE-A0309/2020|2020-06-02|EPI_ISL_478380|Original|hCoV-19^^England|Human|Liverpool Clinical Laboratories|COVID-19 Genomics UK (COG-UK) Consortium|Haldenby|United Kingdom
>Spike|hCoV-19/England/LIVE-A03EB/2020|2020-05-25|EPI_ISL_478381|Original|hCoV-19^^England|Human|Liverpool Clinical Laboratories|COVID-19 Genomics UK (COG-UK) Consortium|Haldenby|United Kingdom
>Spike|hCoV-19/England/LIVE-A0433/2020|2020-05-25|EPI_ISL_478382|Original|hCoV-19^^England|Human|Liverpool Clinical Laboratories|COVID-19 Genomics UK (COG-UK) Consortium|Haldenby|United Kingdom
>Spike|hCoV-19/England/LIVE-A04BB/2020|2020-05-25|EPI_ISL_478383|Original|hCoV-19^^England|Human|Liverpool Clinical Laboratories|COVID-19 Genomics UK (COG-UK) Consortium|Haldenby|United Kingdom

>Spike|hCoV-19/England/LIVE-A05A9/2020|2020-05-26|EPI_ISL_478384|Original|hCoV-19^^England|Human|Liverpool Clinical Laboratories|COVID-19 Genomics UK (COG-UK) Consortium|Haldenby|United Kingdom
>Spike|hCoV-19/England/LIVE-A05D6/2020|2020-05-26|EPI_ISL_478385|Original|hCoV-19^^England|Human|Liverpool Clinical Laboratories|COVID-19 Genomics UK (COG-UK) Consortium|Haldenby|United Kingdom
>Spike|hCoV-19/England/LIVE-A05E5/2020|2020-05-26|EPI_ISL_478386|Original|hCoV-19^^England|Human|Liverpool Clinical Laboratories|COVID-19 Genomics UK (COG-UK) Consortium|Haldenby|United Kingdom
>Spike|hCoV-19/England/LIVE-A066A/2020|2020-05-26|EPI_ISL_478388|Original|hCoV-19^^England|Human|Liverpool Clinical Laboratories|COVID-19 Genomics UK (COG-UK) Consortium|Haldenby|United Kingdom
>Spike|hCoV-19/England/LIVE-A0679/2020|2020-05-27|EPI_ISL_478389|Original|hCoV-19^^England|Human|Liverpool Clinical Laboratories|COVID-19 Genomics UK (COG-UK) Consortium|Haldenby|United Kingdom
>Spike|hCoV-19/England/LIVE-A06B5/2020|2020-05-26|EPI_ISL_478390|Original|hCoV-19^^England|Human|Liverpool Clinical Laboratories|COVID-19 Genomics UK (COG-UK) Consortium|Haldenby|United Kingdom
>Spike|hCoV-19/England/LOND-D4278/2020|2020-04-06|EPI_ISL_478404|Original|hCoV-19^^England|Human|University College London|COVID-19 Genomics UK (COG-UK) Consortium|Roy|United Kingdom
>Spike|hCoV-19/England/LOND-D5954/2020|2020-04-17|EPI_ISL_478409|Original|hCoV-19^^England|Human|University College London|COVID-19 Genomics UK (COG-UK) Consortium|Roy|United Kingdom
>Spike|hCoV-19/England/LOND-D5DD0/2020|2020-04-18|EPI_ISL_478410|Original|hCoV-19^^England|Human|University College London|COVID-19 Genomics UK (COG-UK) Consortium|Roy|United Kingdom
>Spike|hCoV-19/Turkey/KOC-IST-B303/2020|2020-03-27|EPI_ISL_478669|Original|hCoV-19^^Turkey|Human|unknown|Microbiology|Submitter|Turkey
>Spike|hCoV-19/Wales/PHWC-160E12/2020|2020-05-18|EPI_ISL_479300|Original|hCoV-19^^Wales|Human|Wales Specialist Virology Centre Sequencing lab|COVID-19 Genomics UK (COG-UK) Consortium|Moore|United Kingdom
>Spike|hCoV-19/Wales/PHWC-16479B/2020|2020-05-25|EPI_ISL_479306|Original|hCoV-19^^Wales|Human|Wales Specialist Virology Centre Sequencing lab|COVID-19 Genomics UK (COG-UK) Consortium|Moore|United Kingdom
>Spike|hCoV-19/Wales/PHWC-164A0B/2020|2020-05-08|EPI_ISL_479334|Original|hCoV-19^^Wales|Human|Wales Specialist Virology Centre Sequencing lab|COVID-19 Genomics UK (COG-UK) Consortium|Moore|United Kingdom
>Spike|hCoV-19/Wales/PHWC-164A74/2020|2020-06-02|EPI_ISL_479340|Original|hCoV-19^^Wales|Human|Wales Specialist Virology Centre Sequencing lab|COVID-19 Genomics UK (COG-UK) Consortium|Moore|United Kingdom
>Spike|hCoV-19/Wales/PHWC-164B80/2020|2020-06-04|EPI_ISL_479353|Original|hCoV-19^^Wales|Human|Wales Specialist Virology Centre Sequencing lab|COVID-19 Genomics UK (COG-UK) Consortium|Moore|United Kingdom
>Spike|hCoV-19/Wales/PHWC-164BBD/2020|2020-04-21|EPI_ISL_479356|Original|hCoV-19^^Wales|Human|Wales Specialist Virology Centre Sequencing lab|COVID-19 Genomics UK (COG-UK) Consortium|Moore|United Kingdom
>Spike|hCoV-19/Wales/PHWC-1652B7/2020|2020-04-20|EPI_ISL_479445|Original|hCoV-19^^Wales|Human|Wales Specialist Virology Centre Sequencing lab|COVID-19 Genomics UK (COG-UK) Consortium|Moore|United Kingdom
>Spike|hCoV-19/Wales/PHWC-1653B4/2020|2020-05-18|EPI_ISL_479459|Original|hCoV-19^^Wales|Human|Wales Specialist Virology Centre Sequencing lab|COVID-19 Genomics UK (COG-UK) Consortium|Moore|United Kingdom
>Spike|hCoV-19/Wales/PHWC-1653F0/2020|2020-04-21|EPI_ISL_479462|Original|hCoV-19^^Wales|Human|Wales Specialist Virology Centre Sequencing lab|COVID-19 Genomics UK (COG-UK) Consortium|Moore|United Kingdom
>Spike|hCoV-19/USA/FL-04-UMTL-97QX/2020|2020-04-08|EPI_ISL_479761|Original|hCoV-19^^Florida|Human|University of Miami Immunology and Histocompatibility Laboratory|University of Miami Immunology and Histocompatibility Laboratory|Margolles-Clark|USA

>Spike|hCoV-19/USA/FL-06-UMTL-A6U4/2020|2020-04-15|EPI_ISL_479770|Original|hCoV-19^^Florida|Human|University of Miami Immunology and Histocompatibility Laboratory|University of Miami Immunology and Histocompatibility Laboratory|Margolles-Clark|USA
>Spike|hCoV-19/Japan/PG-0293/2020|2020-03-26|EPI_ISL_480225|Original|hCoV-19^^HokurikuShinsyu|Human|Fukui Prefectural Institute of Public Health and Environmental Science|Pathogen Genomics Center|Sekizuka|Japan
>Spike|hCoV-19/Japan/PG-0198/2020|2020-03-23|EPI_ISL_480226|Original|hCoV-19^^Kanto|Human|Niigata Prefectural Institute of Public Health and Environmental Sciences|Pathogen Genomics Center|Sekizuka|Japan
>Spike|hCoV-19/England/NORW-E6FB8/2020|2020-04-08|EPI_ISL_480329|Original|hCoV-19^^England|Human|Quadram Institute Bioscience|COVID-19 Genomics UK (COG-UK) Consortium|Alikhan|United Kingdom
>Spike|hCoV-19/Australia/VIC1468/2020|2020-04-22|EPI_ISL_480594|Original|hCoV-19^^Victoria|Human|Victorian Infectious Diseases Reference Laboratory (VIDRL)|VIDRL and MDU-PHL|Schultz|Australia
>Spike|hCoV-19/Australia/VIC1896/2020|2020-05-31|EPI_ISL_480652|Original|hCoV-19^^Victoria|Human|Victorian Infectious Diseases Reference Laboratory (VIDRL)|VIDRL and MDU-PHL|Schultz|Australia
>Spike|hCoV-19/Australia/VIC2095/2020|2020-06-15|EPI_ISL_480705|Original|hCoV-19^^Victoria|Human|Victorian Infectious Diseases Reference Laboratory (VIDRL)|VIDRL and MDU-PHL|Schultz|Australia
>Spike|hCoV-19/Australia/VIC2129/2020|2020-06-19|EPI_ISL_480728|Original|hCoV-19^^Victoria|Human|Victorian Infectious Diseases Reference Laboratory (VIDRL)|VIDRL and MDU-PHL|Schultz|Australia
>Spike|hCoV-19/Australia/VIC2125/2020|2020-06-19|EPI_ISL_480734|Original|hCoV-19^^Victoria|Human|Victorian Infectious Diseases Reference Laboratory (VIDRL)|VIDRL and MDU-PHL|Schultz|Australia
>Spike|hCoV-19/Senegal/1698/2020|2020-03-31|EPI_ISL_480786|Original|hCoV-19^^Dakar|Human|Institut Pasteur Dakar|Institut Pasteur de Dakar|Dia|Senegal
>Spike|hCoV-19/Senegal/1938/2020|2020-03-31|EPI_ISL_480788|Original|hCoV-19^^Dakar|Human|Institut Pasteur Dakar|Institut Pasteur de Dakar|Dia|Senegal
>Spike|hCoV-19/Spain/MD-IBV-004978/2020|2020-05-13|EPI_ISL_481050|Original|hCoV-19^^Madrid|Human|Hospital General Universitario Gregorio Mara\u00f1\u00f3n|SeqCOVID-SPAIN consortium/IBV(CSIC)|Comas|Spain
>Spike|hCoV-19/India/OR-ILSCV35308/2020|2020-06-10|EPI_ISL_481188|Original|hCoV-19^^Odisha|Human|Immunogenomics lab|Immunogenomics lab|Raghav|India
>Spike|hCoV-19/Senegal/2840/2020|2020-04-06|EPI_ISL_481220|Original|hCoV-19^^Dakar|Human|Institut Pasteur Dakar|Institut Pasteur de Dakar|Dia|Senegal
>Spike|hCoV-19/USA/MD-MDH-0026/2020|2020-04-03|EPI_ISL_481272|Original|hCoV-19^^Maryland|Human|Maryland Department of Health|Maryland Department of Health|Keller|USA
>Spike|hCoV-19/England/BRIS-12CCFA/2020|2020-04-18|EPI_ISL_481796|Original|hCoV-19^^England|Human|PHE South West Regional Laboratory|Wellcome Sanger Institute for the COVID-19 Genomics UK (COG-UK) consortium|Davies|United Kingdom
>Spike|hCoV-19/England/BRIS-12D53A/2020|2020-05-03|EPI_ISL_481848|Original|hCoV-19^^England|Human|PHE South West Regional Laboratory|Wellcome Sanger Institute for the COVID-19 Genomics UK (COG-UK) consortium|Davies|United Kingdom
>Spike|hCoV-19/England/BRIS-12D549/2020|2020-05-04|EPI_ISL_481934|Original|hCoV-19^^England|Human|PHE South West Regional Laboratory|Wellcome Sanger Institute for the COVID-19 Genomics UK (COG-UK) consortium|Davies|United Kingdom
>Spike|hCoV-19/England/LOND-D6829/2020|2020-04-24|EPI_ISL_482172|Original|hCoV-19^^England|Human|University College London|Wellcome Sanger Institute for the COVID-19 Genomics UK (COG-UK) consortium|Davies|United Kingdom
>Spike|hCoV-19/South Africa/KRISP-0180/2020|2020-06-06|EPI_ISL_482706|Original|hCoV-19^^KwaZulu-Natal|Human|Molecular Diagnostics Services (MDS)|KRISP|de Oliveira|South Africa
>Spike|hCoV-19/South Africa/KRISP-0200/2020|2020-06-11|EPI_ISL_482715|Original|hCoV-19^^KwaZulu-Natal|Human|Molecular Diagnostics Services (MDS)|KRISP|de Oliveira|South Africa

>Spike|hCoV-19/Australia/SAP219/2020|2020-03-07|EPI_ISL_483074|Original|hCoV-19^^South Australia|Human|SA Pathology|SA Pathology|Leong|Australia
>Spike|hCoV-19/Australia/SAP230/2020|2020-03-27|EPI_ISL_483076|Original|hCoV-19^^South Australia|Human|SA Pathology|SA Pathology|Leong|Australia
>Spike|hCoV-19/Australia/SAP313/2020|2020-04-09|EPI_ISL_483095|Original|hCoV-19^^South Australia|Human|SA Pathology|SA Pathology|Leong|Australia
>Spike|hCoV-19/Australia/SAP337/2020|2020-03-22|EPI_ISL_483114|Original|hCoV-19^^South Australia|Human|SA Pathology|SA Pathology|Leong|Australia
>Spike|hCoV-19/Australia/SAP338/2020|2020-03-23|EPI_ISL_483115|Original|hCoV-19^^South Australia|Human|SA Pathology|SA Pathology|Leong|Australia
>Spike|hCoV-19/Australia/SAP347/2020|2020-03-23|EPI_ISL_483123|Original|hCoV-19^^South Australia|Human|SA Pathology|SA Pathology|Leong|Australia
>Spike|hCoV-19/Israel/n14277/2020|2020-03-23|EPI_ISL_483723|Original|hCoV-19^^Kfar Saba|Human|Israel Central Virology laboratory|Israel Central Virology laboratory|Zuckerman|Israel
>Spike|hCoV-19/England/NORW-ECD9A/2020|2020-06-18|EPI_ISL_484330|Original|hCoV-19^^England|Human|Quadram Institute Bioscience|COVID-19 Genomics UK (COG-UK) Consortium|Alikhan|United Kingdom
>Spike|hCoV-19/USA/WI-UW-593/2020|2020-06-27|EPI_ISL_484968|Original|hCoV-19^^Wisconsin|Human|University of Wisconsin-Madison AIDS Vaccine Research Laboratories|University of Wisconsin-Madison AIDS Vaccine Research Laboratories|Baczenas|USA
>Spike|hCoV-19/USA/WI-UW-602/2020|2020-06-28|EPI_ISL_484977|Original|hCoV-19^^Wisconsin|Human|University of Wisconsin-Madison AIDS Vaccine Research Laboratories|University of Wisconsin-Madison AIDS Vaccine Research Laboratories|Baczenas|USA
>Spike|hCoV-19/USA/WI-UW-605/2020|2020-06-25|EPI_ISL_484980|Original|hCoV-19^^Wisconsin|Human|University of Wisconsin-Madison AIDS Vaccine Research Laboratories|University of Wisconsin-Madison AIDS Vaccine Research Laboratories|Baczenas|USA
>Spike|hCoV-19/USA/LA-GBCL-26556577/2020|2020-04-14|EPI_ISL_485891|Original|hCoV-19^^Louisiana|Human|River Road Testing Lab|Ginkgo Bioworks Clinical Laboratory|Smith|USA
>Spike|hCoV-19/USA/LA-GBCL-26791710/2020|2020-04-09|EPI_ISL_485905|Original|hCoV-19^^Louisiana|Human|River Road Testing Lab|Ginkgo Bioworks Clinical Laboratory|Smith|USA
>Spike|hCoV-19/USA/WA-UW-688/2020|2020-03-13|EPI_ISL_486106|Original|hCoV-19^^Washington|Human|UW Virology Lab|UW Virology Lab|Roychoudhury|USA
>Spike|hCoV-19/USA/CA-CZB-1888/2020|2020-04-17|EPI_ISL_486187|Original|hCoV-19^^California|Human|Orange County Public Health Laboratory|Chan-Zuckerberg Biohub|McGeever|USA
>Spike|hCoV-19/USA/CA-CZB-2064/2020|2020-04-24|EPI_ISL_486307|Original|hCoV-19^^California|Human|San Joaquin County Public Health Lab|Chan-Zuckerberg Biohub|McGeever|USA
>Spike|hCoV-19/USA/CA-CZB-2124/2020|2020-05-22|EPI_ISL_486352|Original|hCoV-19^^California|Human|UCSF Clinical Microbiology Laboratory|Chan-Zuckerberg Biohub|McGeever|USA
>Spike|hCoV-19/India/KA-nimh-1553/2020|2020-04-16|EPI_ISL_486393|Original|hCoV-19^^Karnataka|Human|SJMCH|Department of Neurovirology|Pattabiraman|India
>Spike|hCoV-19/India/KA-InStem-NCBS-0085/2020|2020-00-00|EPI_ISL_486666|Original|hCoV-19^^Karnataka|Human|Institute for Stem Cell Science and Regenerative Medicine|National Centre for Biological Sciences|Ali|India
>Spike|hCoV-19/India/KA-InStem-NCBS-0093/2020|2020-00-00|EPI_ISL_486668|Original|hCoV-19^^Karnataka|Human|Institute for Stem Cell Science and Regenerative Medicine|National Centre for Biological Sciences|Ali|India
>Spike|hCoV-19/India/KA-InStem-NCBS-0094/2020|2020-00-00|EPI_ISL_486669|Original|hCoV-19^^Karnataka|Human|Institute for Stem Cell Science and Regenerative Medicine|National Centre for Biological Sciences|Ali|India

>Spike|hCoV-19/India/KA-InStem-NCBS-0096/2020|2020-00-
00|EPI_ISL_486671|Original|hCoV-19^^Karnataka|Human|Institute for Stem Cell Science
and Regenerative Medicine|National Centre for Biological Sciences|Ali|India
>Spike|hCoV-19/India/KA-InStem-NCBS-0098/2020|2020-00-
00|EPI_ISL_486672|Original|hCoV-19^^Karnataka|Human|Institute for Stem Cell Science
and Regenerative Medicine|National Centre for Biological Sciences|Ali|India
>Spike|hCoV-19/USA/OR-PROV-034/2020|2020-03-26|EPI_ISL_486831|Original|hCoV-
19^^Oregon|Human|Providence St. Joseph Health Molecular Genomics
Laboratory|Providence St. Joseph Health Molecular Genomics Laboratory|Dowdell|USA
>Spike|hCoV-19/Senegal/9867/2020|2020-04-20|EPI_ISL_486857|Original|hCoV-
19^^Dakar|Human|Institut Pasteur Dakar|Institut Pasteur de Dakar|Dia|Senegal
>Spike|hCoV-19/Italy/LOM-UniMI-1/2020|2020-04-04|EPI_ISL_487276|Original|hCoV-
19^^Lombardy|Human|Department of Food Safety|Department of Biomedical|Submitter|Italy
>Spike|hCoV-19/England/LOND-D65F5/2020|2020-04-22|EPI_ISL_487614|Original|hCoV-
19^^England|Human|University College London|Wellcome Sanger Institute for the COVID-
19 Genomics UK (COG-UK) consortium|Davies|United Kingdom
>Spike|hCoV-19/England/LOND-D9880/2020|2020-04-15|EPI_ISL_487645|Original|hCoV-
19^^England|Human|University College London|Wellcome Sanger Institute for the COVID-
19 Genomics UK (COG-UK) consortium|Davies|United Kingdom
>Spike|hCoV-19/Scotland/EDB5638/2020|2020-03-26|EPI_ISL_487695|Original|hCoV-
19^^Scotland|Human|Virology Department|Wellcome Sanger Institute for the COVID-19
Genomics UK (COG-UK) consortium|Davies|United Kingdom
>Spike|hCoV-19/Scotland/EDB6413/2020|2020-04-13|EPI_ISL_487842|Original|hCoV-
19^^Scotland|Human|Virology Department|Wellcome Sanger Institute for the COVID-19
Genomics UK (COG-UK) consortium|Davies|United Kingdom
>Spike|hCoV-19/Scotland/EDB6267/2020|2020-04-07|EPI_ISL_487875|Original|hCoV-
19^^Scotland|Human|Virology Department|Wellcome Sanger Institute for the COVID-19
Genomics UK (COG-UK) consortium|Davies|United Kingdom
>Spike|hCoV-19/Scotland/EDB5709/2020|2020-04-12|EPI_ISL_487973|Original|hCoV-
19^^Scotland|Human|Virology Department|Wellcome Sanger Institute for the COVID-19
Genomics UK (COG-UK) consortium|Davies|United Kingdom
>Spike|hCoV-19/England/NORT-297910/2020|2020-04-22|EPI_ISL_488027|Original|hCoV-
19^^England|Human|NU-OMICS DNA Sequencing research facility|Wellcome Sanger Institute
for the COVID-19 Genomics UK (COG-UK) consortium|Davies|United Kingdom
>Spike|hCoV-19/England/NORT-2968E7/2020|2020-04-27|EPI_ISL_488041|Original|hCoV-
19^^England|Human|NU-OMICS DNA Sequencing research facility|Wellcome Sanger Institute
for the COVID-19 Genomics UK (COG-UK) consortium|Davies|United Kingdom
>Spike|hCoV-19/England/BRIS-12E6DC/2020|2020-04-21|EPI_ISL_488235|Original|hCoV-
19^^England|Human|PHE South West Regional Laboratory|Wellcome Sanger Institute for
the COVID-19 Genomics UK (COG-UK) consortium|Davies|United Kingdom
>Spike|hCoV-19/England/NORT-295D8F/2020|2020-05-17|EPI_ISL_488542|Original|hCoV-
19^^England|Human|NU-OMICS DNA Sequencing research facility|Wellcome Sanger Institute
for the COVID-19 Genomics UK (COG-UK) consortium|Davies|United Kingdom
>Spike|hCoV-19/England/NORT-283DFD/2020|2020-04-04|EPI_ISL_488637|Original|hCoV-
19^^England|Human|NU-OMICS DNA Sequencing research facility|Wellcome Sanger Institute
for the COVID-19 Genomics UK (COG-UK) consortium|Davies|United Kingdom
>Spike|hCoV-19/England/NORT-28190A/2020|2020-03-21|EPI_ISL_488781|Original|hCoV-
19^^England|Human|NU-OMICS DNA Sequencing research facility|Wellcome Sanger Institute
for the COVID-19 Genomics UK (COG-UK) consortium|Davies|United Kingdom
>Spike|hCoV-19/Scotland/EDB3221/2020|2020-04-06|EPI_ISL_488989|Original|hCoV-
19^^Scotland|Human|Virology Department|Wellcome Sanger Institute for the COVID-19
Genomics UK (COG-UK) consortium|Davies|United Kingdom
>Spike|hCoV-19/England/CAMB-1AC621/2020|2020-04-22|EPI_ISL_489426|Original|hCoV-
19^^England|Human|Department of Pathology|Wellcome Sanger Institute for the COVID-19
Genomics UK (COG-UK) consortium|Davies|United Kingdom
>Spike|hCoV-19/England/CAMB-1AD505/2020|2020-04-26|EPI_ISL_489446|Original|hCoV-
19^^England|Human|Department of Pathology|Wellcome Sanger Institute for the COVID-19
Genomics UK (COG-UK) consortium|Davies|United Kingdom
>Spike|hCoV-19/Scotland/GCVR-1717AA/2020|2020-03-28|EPI_ISL_489581|Original|hCoV-
19^^Scotland|Human|NHSGGC West of Scotland Specialist Virology Centre / MRC-

University of Glasgow Centre for Virus Research|Wellcome Sanger Institute for the COVID-19 Genomics UK (COG-UK) Consortium|Davies|United Kingdom
>Spike|hCoV-19/Scotland/GCVR-1717B9/2020|2020-03-28|EPI_ISL_489612|Original|hCoV-19^^Scotland|Human|NHSGGC West of Scotland Specialist Virology Centre / MRC-University of Glasgow Centre for Virus Research|Wellcome Sanger Institute for the COVID-19 Genomics UK (COG-UK) consortium|Davies|United Kingdom
>Spike|hCoV-19/Malaysia/IMR-WC9174/2020|2020-03-04|EPI_ISL_489993|Vero E6|hCoV-19^^Selangor|Human|Institute for Medical Research|Institute for Medical Research|Suppiah|Malaysia
>Spike|hCoV-19/Malaysia/IMR_WC10195/2020|2020-04-10|EPI_ISL_490014|Original|hCoV-19^^Selangor|Human|Institute for Medical Research|Institute for Medical Research|Suppiah|Malaysia
>Spike|hCoV-19/Malaysia/IMR_WC12897/2020|2020-04-10|EPI_ISL_490015|Vero E6|hCoV-19^^Selangor|Human|Institute for Medical Research|Institute for Medical Research|Suppiah|Malaysia
>Spike|hCoV-19/Malaysia/IMR_WC80036/2020|2020-05-23|EPI_ISL_490091|Original|hCoV-19^^Selangor|Human|Institute for Medical Research|Institute for Medical Research|Suppiah|Malaysia
>Spike|hCoV-19/Malaysia/IMR_WC81528/2020|2020-05-23|EPI_ISL_490097|Original|hCoV-19^^Malaysia|Human|Institute for Medical Research|Institute for Medical Research|Suppiah|Malaysia
>Spike|hCoV-19/USA/CA-QRTP-Donor20/2020|2020-04-01|EPI_ISL_490222|Original|hCoV-19^^California|Human|Quest Diagnostics|Q Squared Solutions - QRTP facility|Weigman|USA
>Spike|hCoV-19/England/202691845/2020|2020-06-18|EPI_ISL_490226|Original|hCoV-19^^England|Human|Respiratory Virus Unit|Respiratory Virus Unit|Platt|United Kingdom
>Spike|hCoV-19/England/LIVE-A17EE/2020|2020-06-19|EPI_ISL_490339|Original|hCoV-19^^England|Human|Liverpool Clinical Laboratories|COVID-19 Genomics UK (COG-UK) Consortium|Haldenby|United Kingdom
>Spike|hCoV-19/England/LIVE-A2167/2020|2020-05-18|EPI_ISL_490378|Original|hCoV-19^^England|Human|Liverpool Clinical Laboratories|COVID-19 Genomics UK (COG-UK) Consortium|Haldenby|United Kingdom
>Spike|hCoV-19/England/NORW-EDA29/2020|2020-00-00|EPI_ISL_490511|Original|hCoV-19^^England|Human|Quadram Institute Bioscience|COVID-19 Genomics UK (COG-UK) Consortium|Alikhan|United Kingdom
>Spike|hCoV-19/England/NORW-EE4FD/2020|2020-04-24|EPI_ISL_490550|Original|hCoV-19^^England|Human|Quadram Institute Bioscience|COVID-19 Genomics UK (COG-UK) Consortium|Alikhan|United Kingdom
>Spike|hCoV-19/England/NORW-EE554/2020|2020-04-24|EPI_ISL_490554|Original|hCoV-19^^England|Human|Quadram Institute Bioscience|COVID-19 Genomics UK (COG-UK) Consortium|Alikhan|United Kingdom
>Spike|hCoV-19/Wales/PHWC-165EE0/2020|2020-06-22|EPI_ISL_490814|Original|hCoV-19^^Wales|Human|Wales Specialist Virology Centre Sequencing lab|COVID-19 Genomics UK (COG-UK) Consortium|Moore|United Kingdom
>Spike|hCoV-19/Wales/PHWC-1661D7/2020|2020-06-04|EPI_ISL_490848|Original|hCoV-19^^Wales|Human|Wales Specialist Virology Centre Sequencing lab|COVID-19 Genomics UK (COG-UK) Consortium|Moore|United Kingdom
>Spike|hCoV-19/Romania/ROSV_5230/2020|2020-04-28|EPI_ISL_491083|Original|hCoV-19^^Suceava|Human|Suceava County Emergency Hospital|\Stefan cel Mare\ University Metagenomics Lab|Lobiuc|Romania
>Spike|hCoV-19/Scotland/EDB1122/2020|2020-04-06|EPI_ISL_491537|Original|hCoV-19^^Scotland|Human|Virology Department|Wellcome Sanger Institute for the COVID-19 Genomics UK (COG-UK) consortium|Davies|United Kingdom
>Spike|hCoV-19/Oman/205038885/2020|2020-05-26|EPI_ISL_491999|Original|hCoV-19^^Muscat|Human|Oman-NIC|Department of\u00a0Microbiology\u00a0and Immunology-SQUH|AL Zadjali|Oman
>Spike|hCoV-19/England/BRIS-1853337/2020|2020-04-03|EPI_ISL_492257|Original|hCoV-19^^England|Human|PHE South West Regional Laboratory|Wellcome Sanger Institute for the COVID-19 Genomics UK (COG-UK) consortium|Davies|United Kingdom

>Spike|hCoV-19/England/LOND-D80D4/2020|2020-04-28|EPI_ISL_492497|Original|hCoV-19^^England|Human|University College London|Wellcome Sanger Institute for the COVID-19 Genomics UK (COG-UK) Consortium|Davies|United Kingdom
>Spike|hCoV-19/England/BRIS-12FC60/2020|2020-04-05|EPI_ISL_492596|Original|hCoV-19^^England|Human|PHE South West Regional Laboratory|Wellcome Sanger Institute for the COVID-19 Genomics UK (COG-UK) Consortium|Davies|United Kingdom
>Spike|hCoV-19/England/LOND-D8DFB/2020|2020-04-17|EPI_ISL_492746|Original|hCoV-19^^England|Human|University College London|Wellcome Sanger Institute for the COVID-19 Genomics UK (COG-UK) Consortium|Davies|United Kingdom
>Spike|hCoV-19/England/LOND-D88CD/2020|2020-04-23|EPI_ISL_492747|Original|hCoV-19^^England|Human|University College London|Wellcome Sanger Institute for the COVID-19 Genomics UK (COG-UK) consortium|Davies|United Kingdom
>Spike|hCoV-19/England/NORT-28AD05/2020|2020-04-15|EPI_ISL_492970|Original|hCoV-19^^England|Human|NU-OMICS DNA Sequencing research facility|Wellcome Sanger Institute for the COVID-19 Genomics UK (COG-UK) consortium|Davies|United Kingdom
>Spike|hCoV-19/England/NORT-28AD9C/2020|2020-04-15|EPI_ISL_492976|Original|hCoV-19^^England|Human|NU-OMICS DNA Sequencing research facility|Wellcome Sanger Institute for the COVID-19 Genomics UK (COG-UK) consortium|Davies|United Kingdom
>Spike|hCoV-19/USA/UT-03265/2020|2020-05-08|EPI_ISL_493006|Original|hCoV-19^^Utah|Human|Utah Public Health Laboratory|Utah Public Health Laboratory|Butz|USA
>Spike|hCoV-19/USA/UT-03278/2020|2020-05-08|EPI_ISL_493011|Original|hCoV-19^^Utah|Human|Utah Public Health Laboratory|Utah Public Health Laboratory|Butz|USA
>Spike|hCoV-19/USA/UT-03827/2020|2020-05-09|EPI_ISL_493023|Original|hCoV-19^^Utah|Human|Utah Public Health Laboratory|Utah Public Health Laboratory|Butz|USA
>Spike|hCoV-19/USA/MO-WUSTL069/2020|2020-06-19|EPI_ISL_493086|Original|hCoV-19^^Missouri|Human|Washington University in St. Louis|Washington University in St. Louis|Tahan|USA
>Spike|hCoV-19/USA/UT-02455/2020|2020-04-22|EPI_ISL_493096|Original|hCoV-19^^Utah|Human|Utah Public Health Laboratory|Utah Public Health Laboratory|Butz|USA
>Spike|hCoV-19/England/NORW-EEB43/2020|2020-07-04|EPI_ISL_493545|Original|hCoV-19^^England|Human|Quadram Institute Bioscience|COVID-19 Genomics UK (COG-UK) Consortium|Alikhan|United Kingdom
>Spike|hCoV-19/Scotland/CVR2017/2020|2020-04-09|EPI_ISL_493755|Original|hCoV-19^^Scotland|Human|West of Scotland Specialist Virology Centre|COVID-19 Genomics UK (COG-UK) Consortium|Robertson|United Kingdom
>Spike|hCoV-19/Scotland/CVR2143/2020|2020-04-10|EPI_ISL_493776|Original|hCoV-19^^Scotland|Human|West of Scotland Specialist Virology Centre|COVID-19 Genomics UK (COG-UK) Consortium|Robertson|United Kingdom
>Spike|hCoV-19/Scotland/CVR2157/2020|2020-04-10|EPI_ISL_493779|Original|hCoV-19^^Scotland|Human|West of Scotland Specialist Virology Centre|COVID-19 Genomics UK (COG-UK) Consortium|Robertson|United Kingdom
>Spike|hCoV-19/Scotland/CVR2197/2020|2020-04-11|EPI_ISL_493784|Original|hCoV-19^^Scotland|Human|West of Scotland Specialist Virology Centre|COVID-19 Genomics UK (COG-UK) Consortium|Robertson|United Kingdom
>Spike|hCoV-19/Scotland/CVR2392/2020|2020-04-14|EPI_ISL_493794|Original|hCoV-19^^Scotland|Human|West of Scotland Specialist Virology Centre|COVID-19 Genomics UK (COG-UK) Consortium|Robertson|United Kingdom
>Spike|hCoV-19/Scotland/CVR3336/2020|2020-05-01|EPI_ISL_493885|Original|hCoV-19^^Scotland|Human|West of Scotland Specialist Virology Centre|COVID-19 Genomics UK (COG-UK) Consortium|Robertson|United Kingdom
>Spike|hCoV-19/Scotland/CVR3864/2020|2020-06-10|EPI_ISL_493894|Original|hCoV-19^^Scotland|Human|West of Scotland Specialist Virology Centre|COVID-19 Genomics UK (COG-UK) Consortium|Robertson|United Kingdom
>Spike|hCoV-19/Wales/PHWC-165E59/2020|2020-05-15|EPI_ISL_493980|Original|hCoV-19^^Wales|Human|Originating lab|COVID-19 Genomics UK (COG-UK) Consortium|Moore|United Kingdom
>Spike|hCoV-19/Wales/PHWC-16626B/2020|2020-04-17|EPI_ISL_493983|Original|hCoV-19^^Wales|Human|Originating lab|COVID-19 Genomics UK (COG-UK) Consortium|Moore|United Kingdom

>Spike|hCoV-19/Wales/PHWC-166289/2020|2020-06-13|EPI_ISL_493984|Original|hCoV-19^^Wales|Human|Originating lab|COVID-19 Genomics UK (COG-UK) Consortium|Moore|United Kingdom
>Spike|hCoV-19/Wales/PHWC-16692A/2020|2020-06-16|EPI_ISL_494061|Original|hCoV-19^^Wales|Human|Originating lab|COVID-19 Genomics UK (COG-UK) Consortium|Moore|United Kingdom
>Spike|hCoV-19/Wales/PHWC-167DA5/2020|2020-04-20|EPI_ISL_494156|Original|hCoV-19^^Wales|Human|Originating lab|COVID-19 Genomics UK (COG-UK) Consortium|Moore|United Kingdom
>Spike|hCoV-19/Wales/PHWC-168041/2020|2020-04-02|EPI_ISL_494192|Original|hCoV-19^^Wales|Human|Originating lab|COVID-19 Genomics UK (COG-UK) Consortium|Moore|United Kingdom
>Spike|hCoV-19/Wales/PHWC-1680AB/2020|2020-06-09|EPI_ISL_494197|Original|hCoV-19^^Wales|Human|Originating lab|COVID-19 Genomics UK (COG-UK) Consortium|Moore|United Kingdom
>Spike|hCoV-19/Wales/PHWC-1680BA/2020|2020-04-01|EPI_ISL_494198|Original|hCoV-19^^Wales|Human|Originating lab|COVID-19 Genomics UK (COG-UK) Consortium|Moore|United Kingdom
>Spike|hCoV-19/Wales/PHWC-1680D8/2020|2020-05-28|EPI_ISL_494200|Original|hCoV-19^^Wales|Human|Originating lab|COVID-19 Genomics UK (COG-UK) Consortium|Moore|United Kingdom
>Spike|hCoV-19/Wales/PHWC-16815D/2020|2020-05-22|EPI_ISL_494206|Original|hCoV-19^^Wales|Human|Originating lab|COVID-19 Genomics UK (COG-UK) Consortium|Moore|United Kingdom
>Spike|hCoV-19/Wales/PHWC-168287/2020|2020-05-22|EPI_ISL_494219|Original|hCoV-19^^Wales|Human|Originating lab|COVID-19 Genomics UK (COG-UK) Consortium|Moore|United Kingdom
>Spike|hCoV-19/Wales/PHWC-16830C/2020|2020-05-18|EPI_ISL_494225|Original|hCoV-19^^Wales|Human|Originating lab|COVID-19 Genomics UK (COG-UK) Consortium|Moore|United Kingdom
>Spike|hCoV-19/Wales/PHWC-168515/2020|2020-04-17|EPI_ISL_494251|Original|hCoV-19^^Wales|Human|Originating lab|COVID-19 Genomics UK (COG-UK) Consortium|Moore|United Kingdom
>Spike|hCoV-19/Wales/PHWC-168551/2020|2020-04-01|EPI_ISL_494253|Original|hCoV-19^^Wales|Human|Originating lab|COVID-19 Genomics UK (COG-UK) Consortium|Moore|United Kingdom
>Spike|hCoV-19/Wales/PHWC-1686B8/2020|2020-04-02|EPI_ISL_494271|Original|hCoV-19^^Wales|Human|Originating lab|COVID-19 Genomics UK (COG-UK) Consortium|Moore|United Kingdom
>Spike|hCoV-19/Wales/PHWC-16872E/2020|2020-04-20|EPI_ISL_494278|Original|hCoV-19^^Wales|Human|Originating lab|COVID-19 Genomics UK (COG-UK) Consortium|Moore|United Kingdom
>Spike|hCoV-19/Wales/PHWC-168797/2020|2020-03-31|EPI_ISL_494285|Original|hCoV-19^^Wales|Human|Originating lab|COVID-19 Genomics UK (COG-UK) Consortium|Moore|United Kingdom
>Spike|hCoV-19/Wales/PHWC-168A70/2020|2020-05-21|EPI_ISL_494322|Original|hCoV-19^^Wales|Human|Originating lab|COVID-19 Genomics UK (COG-UK) Consortium|Moore|United Kingdom
>Spike|hCoV-19/USA/CA-ALSR-1276/2020|2020-05-05|EPI_ISL_494474|Original|hCoV-19^^California|Human|San Diego County Public Health Laboratory|Andersen lab at Scripps Research|Gangavarapu|USA
>Spike|hCoV-19/USA/CA-ALSR-1516/2020|2020-05-09|EPI_ISL_494638|Original|hCoV-19^^California|Human|Scripps Medical Laboratory|Andersen lab at Scripps Research|Gangavarapu|USA
>Spike|hCoV-19/USA/CA-ALSR-1534/2020|2020-05-09|EPI_ISL_494648|Original|hCoV-19^^California|Human|Scripps Medical Laboratory|Andersen lab at Scripps Research|Gangavarapu|USA
>Spike|hCoV-19/Mexico/BCN-ALSR-1471/2020|2020-04-28|EPI_ISL_496341|Original|hCoV-19^^Baja California|Human|Infectolab|Andersen lab at Scripps Research|Gangavarapu|Mexico

>Spike|hCoV-19/Mexico/BCN-ALSR-1478/2020|2020-04-28|EPI_ISL_496347|Original|hCoV-19^^Baja California|Human|Infectolab|Andersen lab at Scripps Research|Gangavarapu|Mexico
>Spike|hCoV-19/India/MH-NCCS-NR567/2020|2020-05-07|EPI_ISL_496536|Original|hCoV-19^^Maharashtra|Human|National Centre For Cell Science|National Centre For Cell Science|Paul|India
>Spike|hCoV-19/Panama/334468/2020|2020-03-30|EPI_ISL_496781|Original|hCoV-19^^Panama Center|Human|Gorgas Memorial Laboratory of Health Studies|Gorgas Memorial Laboratory of Health Studies|Martinez|Panama
>Spike|hCoV-19/Senegal/9840/2020|2020-04-25|EPI_ISL_498234|Original|hCoV-19^^Pikine|Human|Institut Pasteur de Dakar|Institut Pasteur de Dakar|Dia|Senegal
>Spike|hCoV-19/England/LIVE-A5CFA/2020|2020-03-17|EPI_ISL_499473|Original|hCoV-19^^England|Human|Liverpool Clinical Laboratories|COVID-19 Genomics UK (COG-UK) Consortium|Haldenby|United Kingdom
>Spike|hCoV-19/England/LIVE-A596C/2020|2020-03-18|EPI_ISL_499486|Original|hCoV-19^^England|Human|Liverpool Clinical Laboratories|COVID-19 Genomics UK (COG-UK) Consortium|Haldenby|United Kingdom
>Spike|hCoV-19/England/LIVE-A6716/2020|2020-03-19|EPI_ISL_499491|Original|hCoV-19^^England|Human|Liverpool Clinical Laboratories|COVID-19 Genomics UK (COG-UK) Consortium|Haldenby|United Kingdom
>Spike|hCoV-19/Spain/AR-IBV-005120/2020|2020-04-26|EPI_ISL_500364|Original|hCoV-19^^Aragon|Human|Servicio de Microbiolog\u00eda|SeqCOVID-SPAIN consortium/IBV(CSIC)|Comas|Spain
>Spike|hCoV-19/Spain/RI-IBV-006013/2020|2020-03-28|EPI_ISL_500443|Original|hCoV-19^^La Rioja|Human|Centro de Investigaci\u00f3n Biom\u00e9dica de La Rioja - Hospital San Pedro Logro\u00f1o|SeqCOVID-SPAIN consortium/IBV(CSIC)|Comas|Spain
>Spike|hCoV-19/Northern Ireland/NIRE-106CC9/2020|2020-05-19|EPI_ISL_500981|Original|hCoV-19^^Northern Ireland|Human|Regional Virus Laboratory|Wellcome Sanger Institute for the COVID-19 Genomics UK (COG-UK) consortium|Davies|United Kingdom
>Spike|hCoV-19/USA/WA-UW-9755/2020|2020-05-25|EPI_ISL_501162|Original|hCoV-19^^Washington|Human|University of Washington Virology Lab|University of Washington Virology Lab|Roychoudhury|USA
>Spike|hCoV-19/USA/TX-GCID-192000440/2020|2020-04-23|EPI_ISL_501174|Original|hCoV-19^^Texas|Human|Baylor College of Medicine|Baylor College of Medicine|Skinner|USA
>Spike|hCoV-19/Malaysia/2297/2020|2020-03-27|EPI_ISL_501193|Original|hCoV-19^^Kuala Lumpur|Human|Department of Medical Microbiology|Department of Medical Microbiology|Chan|Malaysia
>Spike|hCoV-19/Malaysia/6306/2020|2020-04-07|EPI_ISL_501219|Original|hCoV-19^^Kuala Lumpur|Human|Department of Medical Microbiology|Department of Medical Microbiology|Chan|Malaysia
>Spike|hCoV-19/South Korea/CBNU-nCoV01/2020|2020-02-00|EPI_ISL_507039|Vero P1|hCoV-19^^South Korea|Human|Department of Microbiology|Department of Microbiology|Kim|South Korea
>Spike|hCoV-19/USA/NM-UNM-00315/2020|2020-04-21|EPI_ISL_508021|Original|hCoV-19^^New Mexico|Human|New Mexico Department of Health Scientific Laboratory Division|Center for Global Health|Domman|USA
>Spike|hCoV-19/Australia/SAP434/2020|2020-03-22|EPI_ISL_508125|Original|hCoV-19^^South Australia|Human|SA Pathology|SA Pathology|Leong|Australia
>Spike|hCoV-19/India/WB-IK17/2020|2020-04-24|EPI_ISL_508346|Original|hCoV-19^^West Bengal|Human|Institute of Post Graduate Medical Education & Research|National Institute of Biomedical Genomics|Maitra|India
>Spike|hCoV-19/India/MH-MW32/2020|2020-05-25|EPI_ISL_508435|Original|hCoV-19^^Maharashtra|Human|Mahatma Gandhi Institute of Medical Sciences|National Institute of Biomedical Genomics|Maitra|India
>Spike|hCoV-19/India/WB-S35/2020|2020-05-02|EPI_ISL_508444|Original|hCoV-19^^West Bengal|Human|ICMR-National Institute of Cholera and Enteric Diseases|National Institute of Biomedical Genomics|Maitra|India

>Spike|hCoV-19/USA/OR-OHSU-0066/2020|2020-04-22|EPI_ISL_509059|Original|hCoV-19^^Oregon|Human|OHSU Lab Services Molecular Microbiology Lab|Oregon SARS-CoV-2 Genome Sequencing Center|O'Roak|USA
>Spike|hCoV-19/USA/OR-OHSU-0380/2020|2020-05-08|EPI_ISL_509121|Original|hCoV-19^^Oregon|Human|OHSU Lab Services Molecular Microbiology Lab|Oregon SARS-CoV-2 Genome Sequencing Center|O'Roak|USA
>Spike|hCoV-19/USA/OR-OHSU-0418/2020|2020-05-23|EPI_ISL_509132|Original|hCoV-19^^Oregon|Human|OHSU Lab Services Molecular Microbiology Lab|Oregon SARS-CoV-2 Genome Sequencing Center|O'Roak|USA
>Spike|hCoV-19/USA/OR-OHSU-1197/2020|2020-07-11|EPI_ISL_509160|Original|hCoV-19^^Oregon|Human|OHSU Lab Services Molecular Microbiology Lab|Oregon SARS-CoV-2 Genome Sequencing Center|O'Roak|USA
>Spike|hCoV-19/USA/OR-OHSU-1213/2020|2020-07-13|EPI_ISL_509163|Original|hCoV-19^^Oregon|Human|OHSU Lab Services Molecular Microbiology Lab|Oregon SARS-CoV-2 Genome Sequencing Center|O'Roak|USA
>Spike|hCoV-19/USA/OR-OHSU-1315/2020|2020-07-14|EPI_ISL_509181|Original|hCoV-19^^Oregon|Human|OHSU Lab Services Molecular Microbiology Lab|Oregon SARS-CoV-2 Genome Sequencing Center|O'Roak|USA
>Spike|hCoV-19/USA/OR-OHSU-1451/2020|2020-07-17|EPI_ISL_509204|Original|hCoV-19^^Oregon|Human|OHSU Lab Services Molecular Microbiology Lab|Oregon SARS-CoV-2 Genome Sequencing Center|O'Roak|USA
>Spike|hCoV-19/USA/OR-OHSU-1457/2020|2020-07-17|EPI_ISL_509207|Original|hCoV-19^^Oregon|Human|OHSU Lab Services Molecular Microbiology Lab|Oregon SARS-CoV-2 Genome Sequencing Center|O'Roak|USA
>Spike|hCoV-19/USA/OR-OHSU-1463/2020|2020-07-17|EPI_ISL_509208|Original|hCoV-19^^Oregon|Human|OHSU Lab Services Molecular Microbiology Lab|Oregon SARS-CoV-2 Genome Sequencing Center|O'Roak|USA
>Spike|hCoV-19/USA/OR-OHSU-1487/2020|2020-07-17|EPI_ISL_509214|Original|hCoV-19^^Oregon|Human|OHSU Lab Services Molecular Microbiology Lab|Oregon SARS-CoV-2 Genome Sequencing Center|O'Roak|USA
>Spike|hCoV-19/USA/MD-MDH-0061/2020|2020-05-26|EPI_ISL_509465|Original|hCoV-19^^Maryland|Human|Maryland Department of Health|Maryland Department of Health|Keller|USA
>Spike|hCoV-19/USA/MD-MDH-0080/2020|2020-05-25|EPI_ISL_509484|Original|hCoV-19^^Maryland|Human|Maryland Department of Health|Maryland Department of Health|Keller|USA
>Spike|hCoV-19/USA/UT-02883/2020|2020-04-30|EPI_ISL_509528|Original|hCoV-19^^Utah|Human|Utah Public Health Laboratory|Utah Public Health Laboratory|Butz|USA
>Spike|hCoV-19/USA/UT-02885/2020|2020-04-30|EPI_ISL_509530|Original|hCoV-19^^Utah|Human|Utah Public Health Laboratory|Utah Public Health Laboratory|Butz|USA
>Spike|hCoV-19/USA/UT-02909/2020|2020-04-30|EPI_ISL_509536|Original|hCoV-19^^Utah|Human|Utah Public Health Laboratory|Utah Public Health Laboratory|Butz|USA
>Spike|hCoV-19/USA/UT-02962/2020|2020-05-01|EPI_ISL_509554|Original|hCoV-19^^Utah|Human|Utah Public Health Laboratory|Utah Public Health Laboratory|Butz|USA
>Spike|hCoV-19/USA/IL-UW-901/2020|2020-07-10|EPI_ISL_509995|Original|hCoV-19^^Illinois|Human|University of Wisconsin-Madison AIDS Vaccine Research Laboratories|University of Wisconsin-Madison AIDS Vaccine Research Laboratories|Baczenas|USA
>Spike|hCoV-19/Spain/MD-IBV-006390/2020|2020-05-14|EPI_ISL_510115|Original|hCoV-19^^Madrid|Human|Hospital General Universitario Gregorio Mara\u00f1\u00f3n|SeqCOVID-SPAIN consortium/IBV(CSIC)|Comas|Spain
>Spike|hCoV-19/Spain/MD-IBV-006393/2020|2020-04-29|EPI_ISL_510116|Original|hCoV-19^^Madrid|Human|Hospital General Universitario Gregorio Mara\u00f1\u00f3n|SeqCOVID-SPAIN consortium/IBV(CSIC)|Comas|Spain
>Spike|hCoV-19/Spain/MD-IBV-006412/2020|2020-05-04|EPI_ISL_510127|Original|hCoV-19^^Madrid|Human|Hospital General Universitario Gregorio Mara\u00f1\u00f3n|SeqCOVID-SPAIN consortium/IBV(CSIC)|Comas|Spain
>Spike|hCoV-19/Spain/MD-IBV-006422/2020|2020-06-04|EPI_ISL_510137|Original|hCoV-19^^Madrid|Human|Hospital General Universitario Gregorio Mara\u00f1\u00f3n|SeqCOVID-SPAIN consortium/IBV(CSIC)|Comas|Spain

>Spike|hCoV-19/Spain/MD-IBV-005313/2020|2020-03-17|EPI_ISL_510193|Original|hCoV-19^^Madrid|Human|Hospital General Universitario Gregorio Mara\u00f1\u00f3n|SeqCOVID-SPAIN consortium/IBV(CSIC)|Comas|Spain
>Spike|hCoV-19/Spain/AR-IBV-005043/2020|2020-04-20|EPI_ISL_510346|Original|hCoV-19^^Aragon|Human|Servicio de Microbiolog\u00eda|SeqCOVID-SPAIN consortium/IBV(CSIC)|Comas|Spain
>Spike|hCoV-19/Spain/AR-IBV-005086/2020|2020-04-23|EPI_ISL_510369|Original|hCoV-19^^Aragon|Human|Servicio de Microbiolog\u00eda|SeqCOVID-SPAIN consortium/IBV(CSIC)|Comas|Spain
>Spike|hCoV-19/Spain/AN-IBV-006128/2020|2020-07-06|EPI_ISL_510455|Original|hCoV-19^^Andalucia|Human|Hospital Universitario Virgen de las Nieves de Granada-SAS|SeqCOVID-SPAIN consortium/IBV(CSIC)|Comas|Spain
>Spike|hCoV-19/USA/WA-UW-10030/2020|2020-05-29|EPI_ISL_511852|Original|hCoV-19^^Washington|Human|UW Virology Lab|UW Virology Lab|Addetia|USA
>Spike|hCoV-19/USA/WA-UW-10105/2020|2020-05-29|EPI_ISL_511856|Original|hCoV-19^^Washington|Human|UW Virology Lab|UW Virology Lab|Addetia|USA
>Spike|hCoV-19/USA/WA-UW-10115/2020|2020-05-29|EPI_ISL_511858|Original|hCoV-19^^Washington|Human|UW Virology Lab|UW Virology Lab|Addetia|USA
>Spike|hCoV-19/USA/WA-UW-10126/2020|2020-05-29|EPI_ISL_511860|Original|hCoV-19^^Washington|Human|UW Virology Lab|UW Virology Lab|Addetia|USA
>Spike|hCoV-19/USA/WA-UW-10133/2020|2020-05-29|EPI_ISL_511861|Original|hCoV-19^^Washington|Human|UW Virology Lab|UW Virology Lab|Addetia|USA
>Spike|hCoV-19/Vietnam/VNHN_0554/2020|2020-03-11|EPI_ISL_511892|Original|hCoV-19^^Hanoi|Human|National Hospital of Tropical Diseases|Oxford University Clinical Research Unit|Nguyen|Vietnam
>Spike|hCoV-19/Vietnam/VNHN_0847/2020|2020-03-15|EPI_ISL_511893|Original|hCoV-19^^Hanoi|Human|National Hospital of Tropical Diseases|Oxford University Clinical Research Unit|Nguyen|Vietnam
>Spike|hCoV-19/India/MH-GA91/2020|2020-06-17|EPI_ISL_511936|Original|hCoV-19^^Maharashtra|Human|Government Medical College|National Institute of Biomedical Genomics -\u00a0DBT's PAN-INDIA 1000 SARS--CoV-2 RNA Genome Sequencing Consortium|Maitra|India
>Spike|hCoV-19/India/KA-IB56/2020|2020-06-19|EPI_ISL_511947|Original|hCoV-19^^Karnataka|Human|Indian Institute of Science|National Institute of Biomedical Genomics -\u00a0DBT's PAN-INDIA 1000 SARS--CoV-2 RNA Genome Sequencing Consortium|Maitra|India
>Spike|hCoV-19/USA/CA-ALSR-1795/2020|2020-05-12|EPI_ISL_512189|Original|hCoV-19^^California|Human|San Diego County Public Health Laboratory|Andersen lab at Scripps Research|Gangavarapu|USA
>Spike|hCoV-19/England/NORW-EF131/2020|2020-05-13|EPI_ISL_512378|Original|hCoV-19^^England|Human|Quadram Institute Bioscience|COVID-19 Genomics UK (COG-UK) Consortium|Alikhan|United Kingdom
>Spike|hCoV-19/Wales/PHWC-1695BA/2020|2020-07-20|EPI_ISL_512481|Original|hCoV-19^^Wales|Human|Wales Specialist Virology Centre Sequencing lab|COVID-19 Genomics UK (COG-UK) Consortium|Moore|United Kingdom
>Spike|hCoV-19/Wales/PHWC-169963/2020|2020-07-16|EPI_ISL_512539|Original|hCoV-19^^Wales|Human|Wales Specialist Virology Centre Sequencing lab|COVID-19 Genomics UK (COG-UK) Consortium|Moore|United Kingdom
>Spike|hCoV-19/Costa Rica/INC-0047/2020|2020-03-09|EPI_ISL_512653|Original|hCoV-19^^San Jose|Human|Area De Salud Desamparados 1 - Clinica Dr. Marcial Fallas [Grifo Alto/Desampara|Inciensa|Brenes Porras|Costa Rica
>Spike|hCoV-19/SaudiArabia/KAUST-JEDDAH1015/2020|2020-06-17|EPI_ISL_512886|Original|hCoV-19^^Jeddah|Human|Pathogen Genomics Lab King Abdullah University of Science and Technology(KAUST)|Pathogen Genomics Lab King Abdullah University of Science and Technology(KAUST)|Mohamed Ghazzali|Saudi Arabia
>Spike|hCoV-19/SaudiArabia/KAUST-JEDDAH1021/2020|2020-06-23|EPI_ISL_512891|Original|hCoV-19^^Jeddah|Human|Pathogen Genomics Lab King Abdullah University of Science and Technology(KAUST)|Pathogen Genomics Lab King Abdullah University of Science and Technology(KAUST)|Mohamed Ghazzali|Saudi Arabia

>Spike|hCoV-19/SaudiArabia/KAUST-JEDDAH1027/2020|2020-06-23|EPI_ISL_512897|Original|hCoV-19^^Jeddah|Human|Pathogen Genomics Lab King Abdullah University of Science and Technology(KAUST)|Pathogen Genomics Lab King Abdullah University of Science and Technology(KAUST)|Mohamed Ghazzali|Saudi Arabia
>Spike|hCoV-19/SaudiArabia/KAUST-JEDDAH538/2020|2020-04-23|EPI_ISL_512951|Original|hCoV-19^^Jeddah|Human|Pathogen Genomics Lab King Abdullah University of Science and Technology(KAUST)|Pathogen Genomics Lab King Abdullah University of Science and Technology(KAUST)|Mohamed Ghazzali|Saudi Arabia
>Spike|hCoV-19/SaudiArabia/KAUST-JEDDAH547/2020|2020-04-21|EPI_ISL_512957|Original|hCoV-19^^Jeddah|Human|Pathogen Genomics Lab King Abdullah University of Science and Technology(KAUST)|Pathogen Genomics Lab King Abdullah University of Science and Technology(KAUST)|Mohamed Ghazzali|Saudi Arabia
>Spike|hCoV-19/Saudi Arabia/KAUST-JEDDAH570/2020|2020-04-23|EPI_ISL_512965|Original|hCoV-19^^Jeddah|Human|Pathogen Genomics Lab King Abdullah University of Science and Technology(KAUST)|Pathogen Genomics Lab King Abdullah University of Science and Technology(KAUST)|Mohamed Ghazzali|Saudi Arabia
>Spike|hCoV-19/Saudi Arabia/KAUST-JEDDAH572/2020|2020-04-30|EPI_ISL_512966|Original|hCoV-19^^Jeddah|Human|Pathogen Genomics Lab King Abdullah University of Science and Technology(KAUST)|Pathogen Genomics Lab King Abdullah University of Science and Technology(KAUST)|Mohamed Ghazzali|Saudi Arabia
>Spike|hCoV-19/SaudiArabia/KAUST-JEDDAH995/2020|2020-06-17|EPI_ISL_513068|Original|hCoV-19^^Jeddah|Human|Pathogen Genomics Lab King Abdullah University of Science and Technology(KAUST)|Pathogen Genomics Lab King Abdullah University of Science and Technology(KAUST)|Mohamed Ghazzali|Saudi Arabia
>Spike|hCoV-19/SaudiArabia/KAUST-MADINAH516/2020|2020-04-05|EPI_ISL_513165|Original|hCoV-19^^Madinah|Human|Pathogen Genomics Lab King Abdullah University of Science and Technology(KAUST)|Pathogen Genomics Lab King Abdullah University of Science and Technology(KAUST)|Mohamed Ghazzali|Saudi Arabia
>Spike|hCoV-19/SaudiArabia/KAUST-MADINAH519/2020|2020-04-05|EPI_ISL_513166|Original|hCoV-19^^Madinah|Human|Pathogen Genomics Lab King Abdullah University of Science and Technology(KAUST)|Pathogen Genomics Lab King Abdullah University of Science and Technology(KAUST)|Mohamed Ghazzali|Saudi Arabia
>Spike|hCoV-19/SaudiArabia/KAUST-MADINAH702/2020|2020-04-30|EPI_ISL_513181|Original|hCoV-19^^Madinah|Human|Pathogen Genomics Lab King Abdullah University of Science and Technology(KAUST)|Pathogen Genomics Lab King Abdullah University of Science and Technology(KAUST)|Mohamed Ghazzali|Saudi Arabia
>Spike|hCoV-19/SaudiArabia/KAUST-MADINAH975/2020|2020-05-26|EPI_ISL_513213|Original|hCoV-19^^Madinah|Human|Pathogen Genomics Lab King Abdullah University of Science and Technology(KAUST)|Pathogen Genomics Lab King Abdullah University of Science and Technology(KAUST)|Mohamed Ghazzali|Saudi Arabia
>Spike|hCoV-19/SaudiArabia/KAUST-MAKKAH766/2020|2020-04-18|EPI_ISL_513237|Original|hCoV-19^^Makkah|Human|Pathogen Genomics Lab King Abdullah University of Science and Technology(KAUST)|Pathogen Genomics Lab King Abdullah University of Science and Technology(KAUST)|Mohamed Ghazzali|Saudi Arabia
>Spike|hCoV-19/SaudiArabia/KAUST-MAKKAH769/2020|2020-04-24|EPI_ISL_513239|Original|hCoV-19^^Makkah|Human|Pathogen Genomics Lab King Abdullah University of Science and Technology(KAUST)|Pathogen Genomics Lab King Abdullah University of Science and Technology(KAUST)|Mohamed Ghazzali|Saudi Arabia
>Spike|hCoV-19/SaudiArabia/KAUST-MAKKAH771/2020|2020-04-18|EPI_ISL_513241|Original|hCoV-19^^Makkah|Human|Pathogen Genomics Lab King Abdullah University of Science and Technology(KAUST)|Pathogen Genomics Lab King Abdullah University of Science and Technology(KAUST)|Mohamed Ghazzali|Saudi Arabia
>Spike|hCoV-19/USA/TX_GCID_192000354/2020|2020-04-17|EPI_ISL_513294|Original|hCoV-19^^Texas|Human|Baylor College of Medicine|Baylor College of Medicine|Skinner|USA
>Spike|hCoV-19/USA/UT-02837/2020|2020-04-29|EPI_ISL_513647|Original|hCoV-19^^Utah|Human|Utah Public Health Laboratory|Utah Public Health Laboratory|Butz|USA
>Spike|hCoV-19/USA/UT-02853/2020|2020-04-29|EPI_ISL_513661|Original|hCoV-19^^Utah|Human|Utah Public Health Laboratory|Utah Public Health Laboratory|Butz|USA
>Spike|hCoV-19/USA/UT-02874/2020|2020-04-29|EPI_ISL_513675|Original|hCoV-19^^Utah|Human|Utah Public Health Laboratory|Utah Public Health Laboratory|Butz|USA

>Spike|hCoV-19/USA/UT-02893/2020|2020-04-29|EPI_ISL_513684|Original|hCoV-19^^Utah|Human|Utah Public Health Laboratory|Utah Public Health Laboratory|Butz|USA
>Spike|hCoV-19/USA/CA-CZB-2296/2020|2020-06-22|EPI_ISL_513787|Original|hCoV-19^^California|Human|County of Santa Clara Public Health Department|Chan-Zuckerberg Biohub|Kamm|USA
>Spike|hCoV-19/Bangladesh/NGRI-NSTU-02/2020|2020-07-22|EPI_ISL_514434|Original|hCoV-19^^Chittagong|Human|NSTU COVID-19 Diagnostic Center,|NSU Genome Research Institute (NGRI)|Hossain|Bangladesh
>Spike|hCoV-19/Bangladesh/NGRI-NSTU-05/2020|2020-07-23|EPI_ISL_514441|Original|hCoV-19^^Chittagong|Human|NSTU COVID-19 Diagnostic Center|NSU Genome Research Institute (NGRI)|Hossain|Bangladesh
>Spike|hCoV-19/Bangladesh/NGRI-NSTU-06/2020|2020-07-23|EPI_ISL_514442|Original|hCoV-19^^Chittagong|Human|NSTU COVID-19 Diagnostic Center|NSU Genome Research Institute (NGRI)|Hossain|Bangladesh
>Spike|hCoV-19/India/GJ-GBRC350a/2020|2020-06-18|EPI_ISL_514594|Original|hCoV-19^^Gujarat|Human|B.J. Medical College and Civil hospital|Gujarat Biotechnology Research Centre|Joshi|India
>Spike|hCoV-19/Bangladesh/NGRI-NSTU-18/2020|2020-07-21|EPI_ISL_514613|Original|hCoV-19^^Chittagong|Human|NSTU COVID-19 Diagnostic Center|NSU Genome Research Institute (NGRI)|Hossain|Bangladesh
>Spike|hCoV-19/Bangladesh/NGRI-NSTU-31/2020|2020-07-21|EPI_ISL_514614|Original|hCoV-19^^Chittagong|Human|NSTU COVID-19 Diagnostic Center|NSU Genome Research Institute (NGRI)|Hossain|Bangladesh
>Spike|hCoV-19/USA/NV-NSPHL-A0207/2020|2020-06-05|EPI_ISL_514674|Original|hCoV-19^^Nevada|Human|Nevada State Public Health Laboratory|Nevada State Public Health Laboratory|Sevinsky|USA
>Spike|hCoV-19/Beijing/Beijing-01/2020|2020-01-03|EPI_ISL_514752|Original|hCoV-19^^Beijing|Human|Infectious Disease Control Center|Infectious Disease Control Center|Li|China
>Spike|hCoV-19/env/USA/NV-NSPHL-A0130/2020|2020-04-29|EPI_ISL_515398|Original|hCoV-19^^Nevada|Environment|Nevada State Public Health Laboratory|Nevada State Public Health Laboratory|Sevinsky|USA
>Spike|hCoV-19/Thailand/Bangkok_2857/2020|2020-03-00|EPI_ISL_515474|Vero P2|hCoV-19^^Bangkok|Human|National Institute of Health|National Institute of Health|Phuygun|Thailand
>Spike|hCoV-19/USA/CA-CDPH001/2020|2020-03-15|EPI_ISL_515894|Original|hCoV-19^^California|Human|California Department of Public Health|California Department of Public Health|Bell|USA
>Spike|hCoV-19/USA/SC-20-1385-T10/2020|2020-07-03|EPI_ISL_516755|Original|hCoV-19^^South Carolina|Human|van Bakel Laboratory|van Bakel Laboratory|van Bakel|USA
>Spike|hCoV-19/USA/SC-20-1609-T07/2020|2020-07-07|EPI_ISL_516774|Original|hCoV-19^^South Carolina|Human|van Bakel Laboratory|van Bakel Laboratory|van Bakel|USA
>Spike|hCoV-19/USA/SC-20-1803-T07/2020|2020-07-21|EPI_ISL_516777|Original|hCoV-19^^South Carolina|Human|van Bakel Laboratory|van Bakel Laboratory|van Bakel|USA
>Spike|hCoV-19/USA/SC-20-1963-T00/2020|2020-07-15|EPI_ISL_516781|Original|hCoV-19^^South Carolina|Human|van Bakel Laboratory|van Bakel Laboratory|van Bakel|USA
>Spike|hCoV-19/England/LIVE-A7548/2020|2020-03-28|EPI_ISL_517035|Original|hCoV-19^^England|Human|Liverpool Clinical Laboratories|COVID-19 Genomics UK (COG-UK) Consortium|Haldenby|United Kingdom
>Spike|hCoV-19/England/LIVE-A7F2C/2020|2020-03-26|EPI_ISL_517092|Original|hCoV-19^^England|Human|Liverpool Clinical Laboratories|COVID-19 Genomics UK (COG-UK) Consortium|Haldenby|United Kingdom
>Spike|hCoV-19/USA/TX-DSHS-0044/2020|2020-05-09|EPI_ISL_517975|Original|hCoV-19^^Texas|Human|Texas Department of State Health Services|Texas Department of State Health Services|Rahman|USA
>Spike|hCoV-19/Oman/RESP-20-4941/2020|2020-03-23|EPI_ISL_518823|Original|hCoV-19^^Muscat|Human|Oman-National Influenza Center|Biotechnology & OMICs Laboratory|Al Mahruqi|Oman

>Spike|hCoV-19/Oman/RESP-20-B-15753/2020|2020-05-22|EPI_ISL_518834|Original|hCoV-19^^Muscat|Human|Oman-National Influenza Center|Biotechnology & OMICs Laboratory|Al Mahruqi|Oman
>Spike|hCoV-19/Australia/VIC4704/2020|2020-07-10|EPI_ISL_519092|Original|hCoV-19^^Victoria|Human|Microbiological Diagnostic Unit - Public Health Laboratory (MDU-PHL)|MDU-PHL|Schultz|Australia
>Spike|hCoV-19/Australia/VIC4083/2020|2020-07-15|EPI_ISL_519464|Original|hCoV-19^^Victoria|Human|Microbiological Diagnostic Unit - Public Health Laboratory (MDU-PHL)|MDU-PHL|Schultz|Australia
>Spike|hCoV-19/Australia/VIC2172/2020|2020-06-24|EPI_ISL_521921|Original|hCoV-19^^Victoria|Human|Victorian Infectious Diseases Reference Laboratory (VIDRL)|VIDRL and MDU-PHL|Schultz|Australia
>Spike|hCoV-19/Australia/VIC2539/2020|2020-07-02|EPI_ISL_522232|Original|hCoV-19^^Victoria|Human|Microbiological Diagnostic Unit - Public Health Laboratory (MDU-PHL)|MDU-PHL|Schultz|Australia
>Spike|hCoV-19/USA/TX-DSHS-0066/2020|2020-05-09|EPI_ISL_522352|Original|hCoV-19^^Texas|Human|Texas Department of State Health Services|Texas Department of State Health Services|Rahman|USA
>Spike|hCoV-19/South Korea/KCDC2688/2020|2020-06-30|EPI_ISL_522468|Original|hCoV-19^^South Korea|Human|Center for Laboratory Control of Infectious Diseases|Center for Laboratory Control of Infectious Diseases|Kim|South Korea
>Spike|hCoV-19/USA/TX-DSHS-0174/2020|2020-03-11|EPI_ISL_524055|Original|hCoV-19^^Texas|Human|Texas Department of State Health Services|Texas Department of State Health Services|Rahman|USA
>Spike|hCoV-19/Scotland/GCVR-173113/2020|2020-04-04|EPI_ISL_524514|Original|hCoV-19^^Scotland|Human|NHSGGC West of Scotland Specialist Virology Centre / MRC-University of Glasgow Centre for Virus Research|Wellcome Sanger Institute for the COVID-19 Genomics UK (COG-UK) consortium|Davies|United Kingdom
>Spike|hCoV-19/Scotland/GCVR-171C41/2020|2020-03-29|EPI_ISL_524540|Original|hCoV-19^^Scotland|Human|NHSGGC West of Scotland Specialist Virology Centre / MRC-University of Glasgow Centre for Virus Research|Wellcome Sanger Institute for the COVID-19 Genomics UK (COG-UK) consortium|Davies|United Kingdom
>Spike|hCoV-19/England/LOND-126085C/2020|2020-04-05|EPI_ISL_524640|Original|hCoV-19^^England|Human|North West London Pathology|Wellcome Sanger Institute for the COVID-19 Genomics UK (COG-UK) consortium|Davies|United Kingdom
>Spike|hCoV-19/England/LOND-1260D8A/2020|2020-04-04|EPI_ISL_524678|Original|hCoV-19^^England|Human|North West London Pathology|Wellcome Sanger Institute for the COVID-19 Genomics UK (COG-UK) consortium|Davies|United Kingdom
>Spike|hCoV-19/Oman/RESP-20-B-15491/2020|2020-05-21|EPI_ISL_525427|Original|hCoV-19^^Muscat|Human|Oman-National Influenza Center|Biotechnology & OMICs Laboratory|Al Mahruqi|Oman
>Spike|hCoV-19/USA/OR-OHSU-0528/2020|2020-06-15|EPI_ISL_525832|Original|hCoV-19^^Oregon|Human|OHSU Lab Services Molecular Microbiology Lab|Ginkgo Bioworks Clinical Laboratory|O'Roak|USA
>Spike|hCoV-19/USA/OR-OHSU-0536/2020|2020-06-15|EPI_ISL_525835|Original|hCoV-19^^Oregon|Human|OHSU Lab Services Molecular Microbiology Lab|Ginkgo Bioworks Clinical Laboratory|O'Roak|USA
>Spike|hCoV-19/USA/OR-OHSU-0548/2020|2020-06-16|EPI_ISL_525842|Original|hCoV-19^^Oregon|Human|OHSU Lab Services Molecular Microbiology Lab|Ginkgo Bioworks Clinical Laboratory|O'Roak|USA
>Spike|hCoV-19/USA/OR-OHSU-0626/2020|2020-06-13|EPI_ISL_525855|Original|hCoV-19^^Oregon|Human|OHSU Lab Services Molecular Microbiology Lab|Ginkgo Bioworks Clinical Laboratory|O'Roak|USA
>Spike|hCoV-19/USA/OR-OHSU-0675/2020|2020-06-17|EPI_ISL_525883|Original|hCoV-19^^Oregon|Human|OHSU Lab Services Molecular Microbiology Lab|Oregon SARS-CoV-2 Genome Sequencing Center|O'Roak|USA
>Spike|hCoV-19/USA/OR-OHSU-0827/2020|2020-06-23|EPI_ISL_525909|Original|hCoV-19^^Oregon|Human|OHSU Lab Services Molecular Microbiology Lab|Oregon SARS-CoV-2 Genome Sequencing Center|O'Roak|USA

>Spike|hCoV-19/USA/OR-OHSU-1637/2020|2020-07-22|EPI_ISL_525990|Original|hCoV-19^^Oregon|Human|OHSU Lab Services Molecular Microbiology Lab|Oregon SARS-CoV-2 Genome Sequencing Center|O'Roak|USA
>Spike|hCoV-19/USA/OR-OHSU-1815/2020|2020-07-27|EPI_ISL_526036|Original|hCoV-19^^Oregon|Human|OHSU Lab Services Molecular Microbiology Lab|Oregon SARS-CoV-2 Genome Sequencing Center|O'Roak|USA
>Spike|hCoV-19/Scotland/CVR1380/2020|2020-03-31|EPI_ISL_526454|Original|hCoV-19^^Scotland|Human|West of Scotland Specialist Virology Centre|COVID-19 Genomics UK (COG-UK) Consortium|Robertson|United Kingdom
>Spike|hCoV-19/Scotland/CVR1409/2020|2020-04-01|EPI_ISL_526456|Original|hCoV-19^^Scotland|Human|West of Scotland Specialist Virology Centre|COVID-19 Genomics UK (COG-UK) Consortium|Robertson|United Kingdom
>Spike|hCoV-19/Scotland/CVR1439/2020|2020-04-02|EPI_ISL_526457|Original|hCoV-19^^Scotland|Human|West of Scotland Specialist Virology Centre|COVID-19 Genomics UK (COG-UK) Consortium|Robertson|United Kingdom
>Spike|hCoV-19/Scotland/EDB7362/2020|2020-08-20|EPI_ISL_526535|Original|hCoV-19^^Scotland|Human|Virology Department|COVID-19 Genomics UK (COG-UK) Consortium|Williams|United Kingdom
>Spike|hCoV-19/USA/VA-DCLS-1045/2020|2020-06-10|EPI_ISL_526928|Original|hCoV-19^^Virginia|Human|Virginia DCLS|Virginia DCLS|St. Jacques|USA
>Spike|hCoV-19/Colombia/I30/2020|2020-04-12|EPI_ISL_526959|Original|hCoV-19^^Antioquia|Human|Instituto Nacional de Salud|Instituto Nacional de Salud|Franco-Mu\u00f1oz|Colombia
>Spike|hCoV-19/USA/TX-DSHS-0249/2020|2020-04-25|EPI_ISL_527825|Original|hCoV-19^^Texas|Human|Texas Department of State Health Services|Texas Department of State Health Services|Rahman|USA
>Spike|hCoV-19/Guangzhou/IQTC04/2020|2020-01-27|EPI_ISL_529150|Original|hCoV-19^^Guangdong|Human|Technology Centre|Technology Centre|Huang|China
>Spike|hCoV-19/England/BIRM-623D6/2020|2020-07-19|EPI_ISL_529237|Original|hCoV-19^^England|Human|University of Birmingham|COVID-19 Genomics UK (COG-UK) Consortium|Loman|United Kingdom
>Spike|hCoV-19/England/ALDP-989C8A/2020|2020-08-02|EPI_ISL_529270|Original|hCoV-19^^England|Human|Liverpool Clinical Laboratories|COVID-19 Genomics UK (COG-UK) Consortium|Haldenby|United Kingdom
>Spike|hCoV-19/Wales/PHWC-169C88/2020|2020-08-16|EPI_ISL_529698|Original|hCoV-19^^Wales|Human|Wales Specialist Virology Centre Sequencing lab|COVID-19 Genomics UK (COG-UK) Consortium|Moore|United Kingdom
>Spike|hCoV-19/Wales/PHWC-169FAD/2020|2020-08-20|EPI_ISL_529717|Original|hCoV-19^^Wales|Human|Wales Specialist Virology Centre Sequencing lab|COVID-19 Genomics UK (COG-UK) Consortium|Moore|United Kingdom
>Spike|hCoV-19/USA/VA-DCLS-1257/2020|2020-08-06|EPI_ISL_529947|Original|hCoV-19^^Virginia|Human|Virginia DCLS|Virginia DCLS|St. Jacques|USA
>Spike|hCoV-19/Switzerland/BS-UHB-42201286/2020|2020-03-21|EPI_ISL_530123|Original|hCoV-19^^Basel-Stadt|Human|University Hospital Basel|University Hospital Basel|Roloff Handschin|Switzerland
>Spike|hCoV-19/England/QEUH-96F6C8/2020|2020-08-17|EPI_ISL_530469|Original|hCoV-19^^England|Human|Lighthouse Lab in Glasgow|Wellcome Sanger Institute for the COVID-19 Genomics UK (COG-UK) consortium|Davies|United Kingdom
>Spike|hCoV-19/England/QEUH-96F349/2020|2020-08-17|EPI_ISL_530482|Original|hCoV-19^^England|Human|Lighthouse Lab in Glasgow|Wellcome Sanger Institute for the COVID-19 Genomics UK (COG-UK) Consortium|Davies|United Kingdom
>Spike|hCoV-19/England/QEUH-96F2D3/2020|2020-08-17|EPI_ISL_530527|Original|hCoV-19^^England|Human|Lighthouse Lab in Glasgow|Wellcome Sanger Institute for the COVID-19 Genomics UK (COG-UK) consortium|Davies|United Kingdom
>Spike|hCoV-19/England/QEUH-969BFC/2020|2020-08-12|EPI_ISL_530899|Original|hCoV-19^^England|Human|Lighthouse Lab in Glasgow|Wellcome Sanger Institute for the COVID-19 Genomics UK (COG-UK) Consortium|Davies|United Kingdom
>Spike|hCoV-19/Scotland/QEUH-969637/2020|2020-08-14|EPI_ISL_530953|Original|hCoV-19^^Scotland|Human|NHSGGC West of Scotland Specialist Virology Centre / MRC-

University of Glasgow Centre for Virus Research|Wellcome Sanger Institute for the COVID-19 Genomics UK (COG-UK) consortium|Davies|United Kingdom
>Spike|hCoV-19/England/QEUH-9699B6/2020|2020-08-12|EPI_ISL_531135|Original|hCoV-19^^England|Human|Lighthouse Lab in Glasgow|Wellcome Sanger Institute for the COVID-19 Genomics UK (COG-UK) consortium|Davies|United Kingdom
>Spike|hCoV-19/England/QEUH-962E36/2020|2020-08-08|EPI_ISL_531579|Original|hCoV-19^^England|Human|Lighthouse Lab in Glasgow|Wellcome Sanger Institute for the COVID-19 Genomics UK (COG-UK) consortium|Davies|United Kingdom
>Spike|hCoV-19/England/QEUH-962A23/2020|2020-08-10|EPI_ISL_531664|Original|hCoV-19^^England|Human|Lighthouse Lab in Glasgow|Wellcome Sanger Institute for the COVID-19 Genomics UK (COG-UK) consortium|Davies|United Kingdom
>Spike|hCoV-19/England/QEUH-96256E/2020|2020-08-09|EPI_ISL_531688|Original|hCoV-19^^England|Human|Lighthouse Lab in Glasgow|Wellcome Sanger Institute for the COVID-19 Genomics UK (COG-UK) Consortium|Davies|United Kingdom
>Spike|hCoV-19/England/QEUH-962AE7/2020|2020-08-10|EPI_ISL_531722|Original|hCoV-19^^England|Human|Lighthouse Lab in Glasgow|Wellcome Sanger Institute for the COVID-19 Genomics UK (COG-UK) consortium|Davies|United Kingdom
>Spike|hCoV-19/Scotland/QEUH-962C0F/2020|2020-08-11|EPI_ISL_531815|Original|hCoV-19^^Scotland|Human|NHSGGC West of Scotland Specialist Virology Centre / MRC-University of Glasgow Centre for Virus Research|Wellcome Sanger Institute for the COVID-19 Genomics UK (COG-UK) consortium|Davies|United Kingdom
>Spike|hCoV-19/England/QEUH-96007E/2020|2020-08-06|EPI_ISL_531872|Original|hCoV-19^^England|Human|Lighthouse Lab in Glasgow|Wellcome Sanger Institute for the COVID-19 Genomics UK (COG-UK) Consortium|Davies|United Kingdom
>Spike|hCoV-19/England/QEUH-960F71/2020|2020-08-02|EPI_ISL_531955|Original|hCoV-19^^England|Human|Lighthouse Lab in Glasgow|Wellcome Sanger Institute for the COVID-19 Genomics UK (COG-UK) consortium|Davies|United Kingdom
>Spike|hCoV-19/England/QEUH-960D86/2020|2020-08-03|EPI_ISL_531981|Original|hCoV-19^^England|Human|Lighthouse Lab in Glasgow|Wellcome Sanger Institute for the COVID-19 Genomics UK (COG-UK) consortium|Davies|United Kingdom
>Spike|hCoV-19/England/QEUH-53931F/2020|2020-05-18|EPI_ISL_532261|Original|hCoV-19^^England|Human|Lighthouse Lab in Glasgow|Wellcome Sanger Institute for the COVID-19 Genomics UK (COG-UK) consortium|Davies|United Kingdom
>Spike|hCoV-19/England/QEUH-95F379/2020|2020-08-05|EPI_ISL_532294|Original|hCoV-19^^England|Human|Lighthouse Lab in Glasgow|Wellcome Sanger Institute for the COVID-19 Genomics UK (COG-UK) Consortium|Davies|United Kingdom
>Spike|hCoV-19/England/QEUH-95EF49/2020|2020-08-06|EPI_ISL_532511|Original|hCoV-19^^England|Human|Lighthouse Lab in Glasgow|Wellcome Sanger Institute for the COVID-19 Genomics UK (COG-UK) Consortium|Davies|United Kingdom
>Spike|hCoV-19/England/QEUH-7EEE43/2020|2020-07-18|EPI_ISL_532863|Original|hCoV-19^^England|Human|Lighthouse Lab in Glasgow|Wellcome Sanger Institute for the COVID-19 Genomics UK (COG-UK) consortium|Davies|United Kingdom
>Spike|hCoV-19/England/QEUH-7B20BB/2020|2020-07-18|EPI_ISL_532870|Original|hCoV-19^^England|Human|Lighthouse Lab in Glasgow|Wellcome Sanger Institute for the COVID-19 Genomics UK (COG-UK) consortium|Davies|United Kingdom
>Spike|hCoV-19/England/QEUH-7DAB61/2020|2020-07-19|EPI_ISL_532942|Original|hCoV-19^^England|Human|Lighthouse Lab in Glasgow|Wellcome Sanger Institute for the COVID-19 Genomics UK (COG-UK) consortium|Davies|United Kingdom
>Spike|hCoV-19/Spain/MD-H12-88/2020|2020-04-18|EPI_ISL_534329|Original|hCoV-19^^Madrid|Human|Hospital Universitario 12 de Octubre|Hospital Universitario 12 de Octubre|Viedma|Spain
>Spike|hCoV-19/Scotland/QEUH-963F41/2020|2020-08-11|EPI_ISL_534395|Original|hCoV-19^^Scotland|Human|NHSGGC West of Scotland Specialist Virology Centre / MRC-University of Glasgow Centre for Virus Research|Wellcome Sanger Institute for the COVID-19 Genomics UK (COG-UK) Consortium|Davies|United Kingdom
>Spike|hCoV-19/Scotland/QEUH-96D396/2020|2020-08-20|EPI_ISL_534634|Original|hCoV-19^^Scotland|Human|NHSGGC West of Scotland Specialist Virology Centre / MRC-University of Glasgow Centre for Virus Research|Wellcome Sanger Institute for the COVID-19 Genomics UK (COG-UK) Consortium|Davies|United Kingdom

>Spike|hCoV-19/Wales/PHWC-16A100/2020|2020-08-31|EPI_ISL_535202|Original|hCoV-19^^Wales|Human|Wales Specialist Virology Centre Sequencing lab|COVID-19 Genomics UK (COG-UK) Consortium|Moore|United Kingdom
>Spike|hCoV-19/Wales/PHWC-16A1E2/2020|2020-09-03|EPI_ISL_535214|Original|hCoV-19^^Wales|Human|Wales Specialist Virology Centre Sequencing lab|COVID-19 Genomics UK (COG-UK) Consortium|Moore|United Kingdom
>Spike|hCoV-19/Wales/PHWC-16A49E/2020|2020-09-05|EPI_ISL_535256|Original|hCoV-19^^Wales|Human|Wales Specialist Virology Centre Sequencing lab|COVID-19 Genomics UK (COG-UK) Consortium|Moore|United Kingdom
>Spike|hCoV-19/Canada/QC-LSPQ-L00234922/2020|2020-03-24|EPI_ISL_536081|Original|hCoV-19^^Quebec|Human|H\u00f4pital Charles-LeMoyne|Laboratoire de sant\u00e9 publique du Qu\u00e9bec|Moreira|Canada
>Spike|hCoV-19/England/SHEF-10B2899/2020|2020-04-29|EPI_ISL_537285|Original|hCoV-19^^England|Human|Virology Department|Wellcome Sanger Institute for the COVID-19 Genomics UK (COG-UK) Consortium|Davies|United Kingdom
>Spike|hCoV-19/Spain/CL-IBV-97006144/2020|2020-04-01|EPI_ISL_537291|Original|hCoV-19^^Castilla y Leon|Human|Universidad de Le\u00f3n|SeqCOVID-SPAIN consortium/IBV(CSIC)|Comas|Spain
>Spike|hCoV-19/Spain/CL-IBV-97006170/2020|2020-04-05|EPI_ISL_537317|Original|hCoV-19^^Castilla y Leon|Human|Universidad de Le\u00f3n|SeqCOVID-SPAIN consortium/IBV(CSIC)|Comas|Spain
>Spike|hCoV-19/Spain/CN-IBV-97006487/2020|2020-03-25|EPI_ISL_537702|Original|hCoV-19^^Canary Islands|Human|Hospital Universitario de Gran Canaria Dr. Negr\u00edn|SeqCOVID-SPAIN consortium/IBV(CSIC)|Comas|Spain
>Spike|hCoV-19/Spain/AN-IBV-98001835/2020|2020-04-01|EPI_ISL_538167|Original|hCoV-19^^Andalucia|Human|Servicio de Microbiolog\u00eda y Parasitolog\u00eda cl\u00ednica. UCEIMP. Hospital Universitario Virgen del Roc\u00edo/IBIS/CSIC/US|SeqCOVID-SPAIN consortium/IBV(CSIC)|Comas|Spain
>Spike|hCoV-19/USA/SC-DHEC-0885/2020|2020-06-26|EPI_ISL_538409|Original|hCoV-19^^South Carolina|Human|Microbiology Division|Microbiology Division|Flores|USA
>Spike|hCoV-19/Indonesia/JB-NIHRD-PME4992/2020|2020-00-00|EPI_ISL_538513|Original|hCoV-19^^West Java|Human|Provincial Health Laboratory Bekasi West Java|National Institute of Health Research and Development|Setiawati|Indonesia
>Spike|hCoV-19/Spain/VC-IBV-98003607/2020|2020-03-16|EPI_ISL_538624|Original|hCoV-19^^Comunitat Valenciana|Human|Servicio de Microbiolog\u00eda. Hospital General Universitario de Castell\u00f3n|SeqCOVID-SPAIN consortium/IBV(CSIC)|Comas|Spain
>Spike|hCoV-19/England/LEED-2AA5BC/2020|2020-04-06|EPI_ISL_538851|Original|hCoV-19^^England|Human|Leeds Teaching Hospitals NHS Trust and Public Health England|Wellcome Sanger Institute for the COVID-19 Genomics UK (COG-UK) Consortium|Davies|United Kingdom
>Spike|hCoV-19/England/LEED-2AA640/2020|2020-04-05|EPI_ISL_538905|Original|hCoV-19^^England|Human|Leeds Teaching Hospitals NHS Trust and Public Health England|Wellcome Sanger Institute for the COVID-19 Genomics UK (COG-UK) consortium|Davies|United Kingdom
>Spike|hCoV-19/England/LEED-2A8712/2020|2020-03-23|EPI_ISL_539170|Original|hCoV-19^^England|Human|Leeds Teaching Hospitals NHS Trust and Public Health England|Wellcome Sanger Institute for the COVID-19 Genomics UK (COG-UK) consortium|Davies|United Kingdom
>Spike|hCoV-19/England/QEUH-998BA9/2020|2020-08-26|EPI_ISL_539906|Original|hCoV-19^^England|Human|Lighthouse Lab in Glasgow|Wellcome Sanger Institute for the COVID-19 Genomics UK (COG-UK) Consortium|Davies|United Kingdom
>Spike|hCoV-19/England/QEUH-998D49/2020|2020-08-26|EPI_ISL_540030|Original|hCoV-19^^England|Human|Lighthouse Lab in Glasgow|Wellcome Sanger Institute for the COVID-19 Genomics UK (COG-UK) Consortium|Davies|United Kingdom
>Spike|hCoV-19/England/QEUH-96F59E/2020|2020-08-17|EPI_ISL_540278|Original|hCoV-19^^England|Human|Lighthouse Lab in Glasgow|Wellcome Sanger Institute for the COVID-19 Genomics UK (COG-UK) Consortium|Davies|United Kingdom

>Spike|hCoV-19/England/QEUH-96F965/2020|2020-08-18|EPI_ISL_540281|Original|hCoV-19^^England|Human|Lighthouse Lab in Glasgow|Wellcome Sanger Institute for the COVID-19 Genomics UK (COG-UK) Consortium|Davies|United Kingdom
>Spike|hCoV-19/Wales/PHWC-16A56E/2020|2020-09-06|EPI_ISL_540905|Original|hCoV-19^^Wales|Human|Wales Specialist Virology Centre Sequencing lab|COVID-19 Genomics UK (COG-UK) Consortium|Moore|United Kingdom
>Spike|hCoV-19/USA/TX-DSHS-0476/2020|2020-06-21|EPI_ISL_542007|Original|hCoV-19^^Texas|Human|Texas Department of State Health Services|Texas Department of State Health Services|Rahman|USA
>Spike|hCoV-19/USA/TX-HMH-1266/2020|2020-04-16|EPI_ISL_542619|Original|hCoV-19^^Texas|Human|Houston Methodist Hospital|Houston Methodist Hospital|Long|USA
>Spike|hCoV-19/USA/TX-HMH-6336/2020|2020-07-04|EPI_ISL_547400|Original|hCoV-19^^Texas|Human|Houston Methodist Hospital|Houston Methodist Hospital|Long|USA
>Spike|hCoV-19/USA/WI-UW-1265/2020|2020-08-22|EPI_ISL_547581|Original|hCoV-19^^Wisconsin|Human|University of Wisconsin-Madison AIDS Vaccine Research Laboratories|University of Wisconsin-Madison AIDS Vaccine Research Laboratories|Baczenas|USA
>Spike|hCoV-19/USA/WI-UW-1320/2020|2020-08-26|EPI_ISL_547582|Original|hCoV-19^^Wisconsin|Human|University of Wisconsin-Madison AIDS Vaccine Research Laboratories|University of Wisconsin-Madison AIDS Vaccine Research Laboratories|Baczenas|USA
>Spike|hCoV-19/USA/WI-UW-1402/2020|2020-09-03|EPI_ISL_547583|Original|hCoV-19^^Wisconsin|Human|University of Wisconsin-Madison AIDS Vaccine Research Laboratories|University of Wisconsin-Madison AIDS Vaccine Research Laboratories|Baczenas|USA
>Spike|hCoV-19/USA/CA-CZB-4053/2020|2020-07-16|EPI_ISL_548631|Original|hCoV-19^^California|Human|County of San Luis Obispo Public Health Laboratory|Chan-Zuckerberg Biohub|Bhatt|USA
>Spike|hCoV-19/USA/FL-BPHL-1464/2020|2020-05-07|EPI_ISL_549246|Original|hCoV-19^^Florida|Human|Florida Bureau of Public Health Laboratories|Florida Bureau of Public Health Laboratories|Blanton|USA
>Spike|hCoV-19/England/NOTT-1135BF/2020|2020-09-03|EPI_ISL_549362|Original|hCoV-19^^England|Human|Lincolnshire Hospitals and DeepSeq Nottingham|COVID-19 Genomics UK (COG-UK) Consortium|Loose|United Kingdom
>Spike|hCoV-19/England/QEUH-9B9D33/2020|2020-09-09|EPI_ISL_549734|Original|hCoV-19^^England|Human|Lighthouse Lab in Glasgow|Wellcome Sanger Institute for the COVID-19 Genomics UK (COG-UK) consortium|Davies|United Kingdom
>Spike|hCoV-19/England/QEUH-9B9E9A/2020|2020-09-07|EPI_ISL_549772|Original|hCoV-19^^England|Human|Lighthouse Lab in Glasgow|Wellcome Sanger Institute for the COVID-19 Genomics UK (COG-UK) Consortium|Davies|United Kingdom
>Spike|hCoV-19/England/MILK-9A6DFD/2020|2020-09-01|EPI_ISL_550069|Original|hCoV-19^^England|Human|Lighthouse Lab in Milton Keynes|Wellcome Sanger Institute for the COVID-19 Genomics UK (COG-UK) Consortium|Davies|United Kingdom
>Spike|hCoV-19/England/MILK-999D0C/2020|2020-08-29|EPI_ISL_550391|Original|hCoV-19^^England|Human|Lighthouse Lab in Milton Keynes|Wellcome Sanger Institute for the COVID-19 Genomics UK (COG-UK) consortium|Davies|United Kingdom
>Spike|hCoV-19/England/MILK-9A97CF/2020|2020-08-31|EPI_ISL_551110|Original|hCoV-19^^England|Human|Lighthouse Lab in Milton Keynes|Wellcome Sanger Institute for the COVID-19 Genomics UK (COG-UK) consortium|Davies|United Kingdom
>Spike|hCoV-19/England/MILK-9AAE32/2020|2020-08-26|EPI_ISL_551158|Original|hCoV-19^^England|Human|Lighthouse Lab in Milton Keynes|Wellcome Sanger Institute for the COVID-19 Genomics UK (COG-UK) consortium|Davies|United Kingdom
>Spike|hCoV-19/England/ALDP-951EC1/2020|2020-06-10|EPI_ISL_551294|Original|hCoV-19^^England|Human|Lighthouse Lab in Alderley Park|Wellcome Sanger Institute for the COVID-19 Genomics UK (COG-UK) Consortium|Davies|United Kingdom
>Spike|hCoV-19/England/ALDP-92EF7F/2020|2020-06-20|EPI_ISL_551386|Original|hCoV-19^^England|Human|Lighthouse Lab in Alderley Park|Wellcome Sanger Institute for the COVID-19 Genomics UK (COG-UK) consortium|Davies|United Kingdom

>Spike|hCoV-19/England/ALDP-9056B5/2020|2020-06-24|EPI_ISL_551428|Original|hCoV-19^^England|Human|Lighthouse Lab in Alderley Park|Wellcome Sanger Institute for the COVID-19 Genomics UK (COG-UK) consortium|Davies|United Kingdom
>Spike|hCoV-19/England/ALDP-91BF75/2020|2020-06-23|EPI_ISL_551453|Original|hCoV-19^^England|Human|Lighthouse Lab in Alderley Park|Wellcome Sanger Institute for the COVID-19 Genomics UK (COG-UK) Consortium|Davies|United Kingdom
>Spike|hCoV-19/England/ALDP-9100E6/2020|2020-06-22|EPI_ISL_551496|Original|hCoV-19^^England|Human|Lighthouse Lab in Alderley Park|Wellcome Sanger Institute for the COVID-19 Genomics UK (COG-UK) Consortium|Davies|United Kingdom
>Spike|hCoV-19/England/ALDP-92DF34/2020|2020-06-20|EPI_ISL_551558|Original|hCoV-19^^England|Human|Lighthouse Lab in Alderley Park|Wellcome Sanger Institute for the COVID-19 Genomics UK (COG-UK) consortium|Davies|United Kingdom
>Spike|hCoV-19/England/CAMC-990901/2020|2020-08-26|EPI_ISL_551850|Original|hCoV-19^^England|Human|Lighthouse Lab in Cambridge|Wellcome Sanger Institute for the COVID-19 Genomics UK (COG-UK) Consortium|Davies|United Kingdom
>Spike|hCoV-19/England/CAMC-990840/2020|2020-08-27|EPI_ISL_551903|Original|hCoV-19^^England|Human|Lighthouse Lab in Cambridge|Wellcome Sanger Institute for the COVID-19 Genomics UK (COG-UK) consortium|Davies|United Kingdom
>Spike|hCoV-19/England/MILK-992DF4/2020|2020-08-26|EPI_ISL_551935|Original|hCoV-19^^England|Human|Lighthouse Lab in Milton Keynes|Wellcome Sanger Institute for the COVID-19 Genomics UK (COG-UK) consortium|Davies|United Kingdom
>Spike|hCoV-19/England/MILK-99527A/2020|2020-08-24|EPI_ISL_552240|Original|hCoV-19^^England|Human|Lighthouse Lab in Milton Keynes|Wellcome Sanger Institute for the COVID-19 Genomics UK (COG-UK) Consortium|Davies|United Kingdom
>Spike|hCoV-19/England/MILK-993DA8/2020|2020-08-27|EPI_ISL_552279|Original|hCoV-19^^England|Human|Lighthouse Lab in Milton Keynes|Wellcome Sanger Institute for the COVID-19 Genomics UK (COG-UK) consortium|Davies|United Kingdom
>Spike|hCoV-19/England/MILK-99518C/2020|2020-08-24|EPI_ISL_552283|Original|hCoV-19^^England|Human|Lighthouse Lab in Milton Keynes|Wellcome Sanger Institute for the COVID-19 Genomics UK (COG-UK) consortium|Davies|United Kingdom
>Spike|hCoV-19/England/ALDP-935C7E/2020|2020-06-21|EPI_ISL_552841|Original|hCoV-19^^England|Human|Lighthouse Lab in Alderley Park|Wellcome Sanger Institute for the COVID-19 Genomics UK (COG-UK) Consortium|Davies|United Kingdom
>Spike|hCoV-19/England/MILK-93DE34/2020|2020-06-21|EPI_ISL_552875|Original|hCoV-19^^England|Human|Lighthouse Lab in Milton Keynes|Wellcome Sanger Institute for the COVID-19 Genomics UK (COG-UK) consortium|Davies|United Kingdom
>Spike|hCoV-19/England/MILK-93D405/2020|2020-06-21|EPI_ISL_552880|Original|hCoV-19^^England|Human|Lighthouse Lab in Milton Keynes|Wellcome Sanger Institute for the COVID-19 Genomics UK (COG-UK) Consortium|Davies|United Kingdom
>Spike|hCoV-19/England/MILK-971836/2020|2020-08-18|EPI_ISL_553032|Original|hCoV-19^^England|Human|Lighthouse Lab in Milton Keynes|Wellcome Sanger Institute for the COVID-19 Genomics UK (COG-UK) consortium|Davies|United Kingdom
>Spike|hCoV-19/England/ALDP-92156B/2020|2020-06-23|EPI_ISL_553320|Original|hCoV-19^^England|Human|Lighthouse Lab in Alderley Park|Wellcome Sanger Institute for the COVID-19 Genomics UK (COG-UK) consortium|Davies|United Kingdom
>Spike|hCoV-19/England/ALDP-91F788/2020|2020-06-23|EPI_ISL_553327|Original|hCoV-19^^England|Human|Lighthouse Lab in Alderley Park|Wellcome Sanger Institute for the COVID-19 Genomics UK (COG-UK) consortium|Davies|United Kingdom
>Spike|hCoV-19/England/ALDP-955030/2020|2020-06-12|EPI_ISL_553382|Original|hCoV-19^^England|Human|Lighthouse Lab in Alderley Park|Wellcome Sanger Institute for the COVID-19 Genomics UK (COG-UK) consortium|Davies|United Kingdom
>Spike|hCoV-19/England/ALDP-95505E/2020|2020-06-12|EPI_ISL_553391|Original|hCoV-19^^England|Human|Lighthouse Lab in Alderley Park|Wellcome Sanger Institute for the COVID-19 Genomics UK (COG-UK) consortium|Davies|United Kingdom
>Spike|hCoV-19/England/ALDP-95D27E/2020|2020-06-16|EPI_ISL_553421|Original|hCoV-19^^England|Human|Lighthouse Lab in Alderley Park|Wellcome Sanger Institute for the COVID-19 Genomics UK (COG-UK) Consortium|Davies|United Kingdom
>Spike|hCoV-19/England/ALDP-954FDA/2020|2020-06-11|EPI_ISL_553458|Original|hCoV-19^^England|Human|Lighthouse Lab in Alderley Park|Wellcome Sanger Institute for the COVID-19 Genomics UK (COG-UK) consortium|Davies|United Kingdom

>Spike|hCoV-19/England/ALDP-95A1FC/2020|2020-06-09|EPI_ISL_553499|Original|hCoV-19^^England|Human|Lighthouse Lab in Alderley Park|Wellcome Sanger Institute for the COVID-19 Genomics UK (COG-UK) consortium|Davies|United Kingdom
>Spike|hCoV-19/England/ALDP-95504F/2020|2020-06-12|EPI_ISL_553521|Original|hCoV-19^^England|Human|Lighthouse Lab in Alderley Park|Wellcome Sanger Institute for the COVID-19 Genomics UK (COG-UK) consortium|Davies|United Kingdom
>Spike|hCoV-19/England/ALDP-954E28/2020|2020-06-13|EPI_ISL_553568|Original|hCoV-19^^England|Human|Lighthouse Lab in Alderley Park|Wellcome Sanger Institute for the COVID-19 Genomics UK (COG-UK) Consortium|Davies|United Kingdom
>Spike|hCoV-19/England/ALDP-954FE9/2020|2020-06-11|EPI_ISL_553608|Original|hCoV-19^^England|Human|Lighthouse Lab in Alderley Park|Wellcome Sanger Institute for the COVID-19 Genomics UK (COG-UK) consortium|Davies|United Kingdom
>Spike|hCoV-19/England/ALDP-95405F/2020|2020-06-12|EPI_ISL_553784|Original|hCoV-19^^England|Human|Lighthouse Lab in Alderley Park|Wellcome Sanger Institute for the COVID-19 Genomics UK (COG-UK) Consortium|Davies|United Kingdom
>Spike|hCoV-19/England/ALDP-95422C/2020|2020-06-13|EPI_ISL_553798|Original|hCoV-19^^England|Human|Lighthouse Lab in Alderley Park|Wellcome Sanger Institute for the COVID-19 Genomics UK (COG-UK) consortium|Davies|United Kingdom
>Spike|hCoV-19/England/ALDP-953928/2020|2020-06-13|EPI_ISL_553805|Original|hCoV-19^^England|Human|Lighthouse Lab in Alderley Park|Wellcome Sanger Institute for the COVID-19 Genomics UK (COG-UK) consortium|Davies|United Kingdom
>Spike|hCoV-19/England/ALDP-953867/2020|2020-06-08|EPI_ISL_553812|Original|hCoV-19^^England|Human|Lighthouse Lab in Alderley Park|Wellcome Sanger Institute for the COVID-19 Genomics UK (COG-UK) Consortium|Davies|United Kingdom
>Spike|hCoV-19/England/ALDP-953A52/2020|2020-06-08|EPI_ISL_553846|Original|hCoV-19^^England|Human|Lighthouse Lab in Alderley Park|Wellcome Sanger Institute for the COVID-19 Genomics UK (COG-UK) Consortium|Davies|United Kingdom
>Spike|hCoV-19/England/ALDP-953BD7/2020|2020-06-08|EPI_ISL_553866|Original|hCoV-19^^England|Human|Lighthouse Lab in Alderley Park|Wellcome Sanger Institute for the COVID-19 Genomics UK (COG-UK) Consortium|Davies|United Kingdom
>Spike|hCoV-19/England/ALDP-955F60/2020|2020-06-08|EPI_ISL_553874|Original|hCoV-19^^England|Human|Lighthouse Lab in Alderley Park|Wellcome Sanger Institute for the COVID-19 Genomics UK (COG-UK) consortium|Davies|United Kingdom
>Spike|hCoV-19/England/MILK-95771B/2020|2020-06-09|EPI_ISL_553880|Original|hCoV-19^^England|Human|Lighthouse Lab in Milton Keynes|Wellcome Sanger Institute for the COVID-19 Genomics UK (COG-UK) consortium|Davies|United Kingdom
>Spike|hCoV-19/England/ALDP-952561/2020|2020-06-09|EPI_ISL_553946|Original|hCoV-19^^England|Human|Lighthouse Lab in Alderley Park|Wellcome Sanger Institute for the COVID-19 Genomics UK (COG-UK) Consortium|Davies|United Kingdom
>Spike|hCoV-19/England/MILK-956CE0/2020|2020-06-10|EPI_ISL_554118|Original|hCoV-19^^England|Human|Lighthouse Lab in Milton Keynes|Wellcome Sanger Institute for the COVID-19 Genomics UK (COG-UK) consortium|Davies|United Kingdom
>Spike|hCoV-19/England/MILK-9569DA/2020|2020-06-07|EPI_ISL_554195|Original|hCoV-19^^England|Human|Lighthouse Lab in Milton Keynes|Wellcome Sanger Institute for the COVID-19 Genomics UK (COG-UK) Consortium|Davies|United Kingdom
>Spike|hCoV-19/England/MILK-956ECC/2020|2020-06-08|EPI_ISL_554246|Original|hCoV-19^^England|Human|Lighthouse Lab in Milton Keynes|Wellcome Sanger Institute for the COVID-19 Genomics UK (COG-UK) consortium|Davies|United Kingdom
>Spike|hCoV-19/England/MILK-956CD1/2020|2020-06-09|EPI_ISL_554261|Original|hCoV-19^^England|Human|Lighthouse Lab in Milton Keynes|Wellcome Sanger Institute for the COVID-19 Genomics UK (COG-UK) consortium|Davies|United Kingdom
>Spike|hCoV-19/England/MILK-956CFF/2020|2020-06-10|EPI_ISL_554281|Original|hCoV-19^^England|Human|Lighthouse Lab in Milton Keynes|Wellcome Sanger Institute for the COVID-19 Genomics UK (COG-UK) Consortium|Davies|United Kingdom
>Spike|hCoV-19/England/ALDP-950A82/2020|2020-06-14|EPI_ISL_554455|Original|hCoV-19^^England|Human|Lighthouse Lab in Alderley Park|Wellcome Sanger Institute for the COVID-19 Genomics UK (COG-UK) consortium|Davies|United Kingdom
>Spike|hCoV-19/England/ALDP-955971/2020|2020-06-12|EPI_ISL_554478|Original|hCoV-19^^England|Human|Lighthouse Lab in Alderley Park|Wellcome Sanger Institute for the COVID-19 Genomics UK (COG-UK) Consortium|Davies|United Kingdom

>Spike|hCoV-19/England/ALDP-9556B6/2020|2020-06-12|EPI_ISL_554484|Original|hCoV-19^^England|Human|Lighthouse Lab in Alderley Park|Wellcome Sanger Institute for the COVID-19 Genomics UK (COG-UK) Consortium|Davies|United Kingdom
>Spike|hCoV-19/England/ALDP-953DB3/2020|2020-06-12|EPI_ISL_554537|Original|hCoV-19^^England|Human|Lighthouse Lab in Alderley Park|Wellcome Sanger Institute for the COVID-19 Genomics UK (COG-UK) Consortium|Davies|United Kingdom
>Spike|hCoV-19/England/MILK-957E34/2020|2020-06-14|EPI_ISL_554627|Original|hCoV-19^^England|Human|Lighthouse Lab in Milton Keynes|Wellcome Sanger Institute for the COVID-19 Genomics UK (COG-UK) consortium|Davies|United Kingdom
>Spike|hCoV-19/England/MILK-957D37/2020|2020-06-14|EPI_ISL_554662|Original|hCoV-19^^England|Human|Lighthouse Lab in Milton Keynes|Wellcome Sanger Institute for the COVID-19 Genomics UK (COG-UK) Consortium|Davies|United Kingdom
>Spike|hCoV-19/England/MILK-957D64/2020|2020-06-12|EPI_ISL_554724|Original|hCoV-19^^England|Human|Lighthouse Lab in Milton Keynes|Wellcome Sanger Institute for the COVID-19 Genomics UK (COG-UK) consortium|Davies|United Kingdom
>Spike|hCoV-19/England/ALDP-944571/2020|2020-06-07|EPI_ISL_554763|Original|hCoV-19^^England|Human|Lighthouse Lab in Alderley Park|Wellcome Sanger Institute for the COVID-19 Genomics UK (COG-UK) Consortium|Davies|United Kingdom
>Spike|hCoV-19/England/MILK-957F7D/2020|2020-06-11|EPI_ISL_554793|Original|hCoV-19^^England|Human|Lighthouse Lab in Milton Keynes|Wellcome Sanger Institute for the COVID-19 Genomics UK (COG-UK) consortium|Davies|United Kingdom
>Spike|hCoV-19/England/ALDP-944580/2020|2020-06-07|EPI_ISL_554845|Original|hCoV-19^^England|Human|Lighthouse Lab in Alderley Park|Wellcome Sanger Institute for the COVID-19 Genomics UK (COG-UK) Consortium|Davies|United Kingdom
>Spike|hCoV-19/England/MILK-958747/2020|2020-06-09|EPI_ISL_554855|Original|hCoV-19^^England|Human|Lighthouse Lab in Milton Keynes|Wellcome Sanger Institute for the COVID-19 Genomics UK (COG-UK) consortium|Davies|United Kingdom
>Spike|hCoV-19/England/ALDP-95C45B/2020|2020-06-20|EPI_ISL_554929|Original|hCoV-19^^England|Human|Lighthouse Lab in Alderley Park|Wellcome Sanger Institute for the COVID-19 Genomics UK (COG-UK) Consortium|Davies|United Kingdom
>Spike|hCoV-19/England/ALDP-95C594/2020|2020-06-21|EPI_ISL_555019|Original|hCoV-19^^England|Human|Lighthouse Lab in Alderley Park|Wellcome Sanger Institute for the COVID-19 Genomics UK (COG-UK) Consortium|Davies|United Kingdom
>Spike|hCoV-19/England/ALDP-95C4E2/2020|2020-06-20|EPI_ISL_555041|Original|hCoV-19^^England|Human|Lighthouse Lab in Alderley Park|Wellcome Sanger Institute for the COVID-19 Genomics UK (COG-UK) Consortium|Davies|United Kingdom
>Spike|hCoV-19/England/ALDP-950202/2020|2020-06-16|EPI_ISL_555127|Original|hCoV-19^^England|Human|Lighthouse Lab in Alderley Park|Wellcome Sanger Institute for the COVID-19 Genomics UK (COG-UK) Consortium|Davies|United Kingdom
>Spike|hCoV-19/England/ALDP-950633/2020|2020-06-18|EPI_ISL_555148|Original|hCoV-19^^England|Human|Lighthouse Lab in Alderley Park|Wellcome Sanger Institute for the COVID-19 Genomics UK (COG-UK) Consortium|Davies|United Kingdom
>Spike|hCoV-19/England/ALDP-94FC08/2020|2020-06-16|EPI_ISL_555198|Original|hCoV-19^^England|Human|Lighthouse Lab in Alderley Park|Wellcome Sanger Institute for the COVID-19 Genomics UK (COG-UK) consortium|Davies|United Kingdom
>Spike|hCoV-19/England/ALDP-94FB92/2020|2020-06-16|EPI_ISL_555265|Original|hCoV-19^^England|Human|Lighthouse Lab in Alderley Park|Wellcome Sanger Institute for the COVID-19 Genomics UK (COG-UK) consortium|Davies|United Kingdom
>Spike|hCoV-19/England/ALDP-94FB83/2020|2020-06-16|EPI_ISL_555290|Original|hCoV-19^^England|Human|Lighthouse Lab in Alderley Park|Wellcome Sanger Institute for the COVID-19 Genomics UK (COG-UK) consortium|Davies|United Kingdom
>Spike|hCoV-19/England/ALDP-952C7B/2020|2020-06-11|EPI_ISL_555389|Original|hCoV-19^^England|Human|Lighthouse Lab in Alderley Park|Wellcome Sanger Institute for the COVID-19 Genomics UK (COG-UK) consortium|Davies|United Kingdom
>Spike|hCoV-19/England/ALDP-954848/2020|2020-06-09|EPI_ISL_555391|Original|hCoV-19^^England|Human|Lighthouse Lab in Alderley Park|Wellcome Sanger Institute for the COVID-19 Genomics UK (COG-UK) Consortium|Davies|United Kingdom
>Spike|hCoV-19/England/ALDP-95481B/2020|2020-06-09|EPI_ISL_555398|Original|hCoV-19^^England|Human|Lighthouse Lab in Alderley Park|Wellcome Sanger Institute for the COVID-19 Genomics UK (COG-UK) Consortium|Davies|United Kingdom

>Spike|hCoV-19/England/ALDP-94E401/2020|2020-06-17|EPI_ISL_555399|Original|hCoV-19^^England|Human|Lighthouse Lab in Alderley Park|Wellcome Sanger Institute for the COVID-19 Genomics UK (COG-UK) consortium|Davies|United Kingdom
>Spike|hCoV-19/England/ALDP-9448C3/2020|2020-06-07|EPI_ISL_555512|Original|hCoV-19^^England|Human|Lighthouse Lab in Alderley Park|Wellcome Sanger Institute for the COVID-19 Genomics UK (COG-UK) consortium|Davies|United Kingdom
>Spike|hCoV-19/England/ALDP-94491B/2020|2020-06-07|EPI_ISL_555522|Original|hCoV-19^^England|Human|Lighthouse Lab in Alderley Park|Wellcome Sanger Institute for the COVID-19 Genomics UK (COG-UK) consortium|Davies|United Kingdom
>Spike|hCoV-19/England/ALDP-661DB5/2020|2020-07-03|EPI_ISL_555645|Original|hCoV-19^^England|Human|Lighthouse Lab in Alderley Park|Wellcome Sanger Institute for the COVID-19 Genomics UK (COG-UK) consortium|Davies|United Kingdom
>Spike|hCoV-19/England/ALDP-9448A5/2020|2020-06-07|EPI_ISL_555647|Original|hCoV-19^^England|Human|Lighthouse Lab in Alderley Park|Wellcome Sanger Institute for the COVID-19 Genomics UK (COG-UK) consortium|Davies|United Kingdom
>Spike|hCoV-19/England/ALDP-9448D2/2020|2020-06-07|EPI_ISL_555655|Original|hCoV-19^^England|Human|Lighthouse Lab in Alderley Park|Wellcome Sanger Institute for the COVID-19 Genomics UK (COG-UK) consortium|Davies|United Kingdom
>Spike|hCoV-19/England/ALDP-94FED5/2020|2020-06-16|EPI_ISL_555815|Original|hCoV-19^^England|Human|Lighthouse Lab in Alderley Park|Wellcome Sanger Institute for the COVID-19 Genomics UK (COG-UK) Consortium|Davies|United Kingdom
>Spike|hCoV-19/England/ALDP-94CB86/2020|2020-06-16|EPI_ISL_555986|Original|hCoV-19^^England|Human|Lighthouse Lab in Alderley Park|Wellcome Sanger Institute for the COVID-19 Genomics UK (COG-UK) Consortium|Davies|United Kingdom
>Spike|hCoV-19/England/ALDP-94F224/2020|2020-06-18|EPI_ISL_556000|Original|hCoV-19^^England|Human|Lighthouse Lab in Alderley Park|Wellcome Sanger Institute for the COVID-19 Genomics UK (COG-UK) Consortium|Davies|United Kingdom
>Spike|hCoV-19/England/ALDP-6CC1AD/2020|2020-06-30|EPI_ISL_556081|Original|hCoV-19^^England|Human|Lighthouse Lab in Alderley Park|Wellcome Sanger Institute for the COVID-19 Genomics UK (COG-UK) consortium|Davies|United Kingdom
>Spike|hCoV-19/England/ALDP-6D2EAD/2020|2020-07-02|EPI_ISL_556141|Original|hCoV-19^^England|Human|Lighthouse Lab in Alderley Park|Wellcome Sanger Institute for the COVID-19 Genomics UK (COG-UK) Consortium|Davies|United Kingdom
>Spike|hCoV-19/England/ALDP-6C60B5/2020|2020-07-02|EPI_ISL_556229|Original|hCoV-19^^England|Human|Lighthouse Lab in Alderley Park|Wellcome Sanger Institute for the COVID-19 Genomics UK (COG-UK) consortium|Davies|United Kingdom
>Spike|hCoV-19/England/ALDP-94EF3D/2020|2020-06-17|EPI_ISL_556283|Original|hCoV-19^^England|Human|Lighthouse Lab in Alderley Park|Wellcome Sanger Institute for the COVID-19 Genomics UK (COG-UK) consortium|Davies|United Kingdom
>Spike|hCoV-19/England/ALDP-94A414/2020|2020-06-12|EPI_ISL_556411|Original|hCoV-19^^England|Human|Lighthouse Lab in Alderley Park|Wellcome Sanger Institute for the COVID-19 Genomics UK (COG-UK) Consortium|Davies|United Kingdom
>Spike|hCoV-19/England/ALDP-94EC09/2020|2020-06-17|EPI_ISL_556466|Original|hCoV-19^^England|Human|Lighthouse Lab in Alderley Park|Wellcome Sanger Institute for the COVID-19 Genomics UK (COG-UK) consortium|Davies|United Kingdom
>Spike|hCoV-19/England/CAMC-946F35/2020|2020-07-26|EPI_ISL_556530|Original|hCoV-19^^England|Human|Lighthouse Lab in Cambridge|Wellcome Sanger Institute for the COVID-19 Genomics UK (COG-UK) consortium|Davies|United Kingdom
>Spike|hCoV-19/England/ALDP-8F79EB/2020|2020-06-24|EPI_ISL_556591|Original|hCoV-19^^England|Human|Lighthouse Lab in Alderley Park|Wellcome Sanger Institute for the COVID-19 Genomics UK (COG-UK) consortium|Davies|United Kingdom
>Spike|hCoV-19/England/CAMC-945947/2020|2020-07-15|EPI_ISL_556801|Original|hCoV-19^^England|Human|Lighthouse Lab in Cambridge|Wellcome Sanger Institute for the COVID-19 Genomics UK (COG-UK) consortium|Davies|United Kingdom
>Spike|hCoV-19/England/ALDP-8CFAF8/2020|2020-06-23|EPI_ISL_557002|Original|hCoV-19^^England|Human|Lighthouse Lab in Alderley Park|Wellcome Sanger Institute for the COVID-19 Genomics UK (COG-UK) Consortium|Davies|United Kingdom
>Spike|hCoV-19/England/ALDP-90FAEC/2020|2020-06-24|EPI_ISL_557043|Original|hCoV-19^^England|Human|Lighthouse Lab in Alderley Park|Wellcome Sanger Institute for the COVID-19 Genomics UK (COG-UK) Consortium|Davies|United Kingdom

>Spike|hCoV-19/England/ALDP-8D78A8/2020|2020-06-24|EPI_ISL_557142|Original|hCoV-19^^England|Human|Lighthouse Lab in Alderley Park|Wellcome Sanger Institute for the COVID-19 Genomics UK (COG-UK) Consortium|Davies|United Kingdom
>Spike|hCoV-19/England/MILK-80D1BD/2020|2020-07-13|EPI_ISL_557178|Original|hCoV-19^^England|Human|Lighthouse Lab in Milton Keynes|Wellcome Sanger Institute for the COVID-19 Genomics UK (COG-UK) consortium|Davies|United Kingdom
>Spike|hCoV-19/England/ALDP-6FC186/2020|2020-06-29|EPI_ISL_557383|Original|hCoV-19^^England|Human|Lighthouse Lab in Alderley Park|Wellcome Sanger Institute for the COVID-19 Genomics UK (COG-UK) consortium|Davies|United Kingdom
>Spike|hCoV-19/England/ALDP-6FD62C/2020|2020-06-29|EPI_ISL_557482|Original|hCoV-19^^England|Human|Lighthouse Lab in Alderley Park|Wellcome Sanger Institute for the COVID-19 Genomics UK (COG-UK) consortium|Davies|United Kingdom
>Spike|hCoV-19/England/MILK-85C54A/2020|2020-07-12|EPI_ISL_557515|Original|hCoV-19^^England|Human|Lighthouse Lab in Milton Keynes|Wellcome Sanger Institute for the COVID-19 Genomics UK (COG-UK) consortium|Davies|United Kingdom
>Spike|hCoV-19/England/ALDP-70B086/2020|2020-06-30|EPI_ISL_557563|Original|hCoV-19^^England|Human|Lighthouse Lab in Alderley Park|Wellcome Sanger Institute for the COVID-19 Genomics UK (COG-UK) consortium|Davies|United Kingdom
>Spike|hCoV-19/England/ALDP-720416/2020|2020-07-01|EPI_ISL_557581|Original|hCoV-19^^England|Human|Lighthouse Lab in Alderley Park|Wellcome Sanger Institute for the COVID-19 Genomics UK (COG-UK) consortium|Davies|United Kingdom
>Spike|hCoV-19/England/ALDP-71DF66/2020|2020-07-01|EPI_ISL_557639|Original|hCoV-19^^England|Human|Lighthouse Lab in Alderley Park|Wellcome Sanger Institute for the COVID-19 Genomics UK (COG-UK) consortium|Davies|United Kingdom
>Spike|hCoV-19/England/ALDP-71D151/2020|2020-07-01|EPI_ISL_557642|Original|hCoV-19^^England|Human|Lighthouse Lab in Alderley Park|Wellcome Sanger Institute for the COVID-19 Genomics UK (COG-UK) consortium|Davies|United Kingdom
>Spike|hCoV-19/England/ALDP-720C1E/2020|2020-07-01|EPI_ISL_557651|Original|hCoV-19^^England|Human|Lighthouse Lab in Alderley Park|Wellcome Sanger Institute for the COVID-19 Genomics UK (COG-UK) consortium|Davies|United Kingdom
>Spike|hCoV-19/England/ALDP-71D160/2020|2020-07-01|EPI_ISL_557663|Original|hCoV-19^^England|Human|Lighthouse Lab in Alderley Park|Wellcome Sanger Institute for the COVID-19 Genomics UK (COG-UK) consortium|Davies|United Kingdom
>Spike|hCoV-19/England/ALDP-6C0CE4/2020|2020-07-02|EPI_ISL_557805|Original|hCoV-19^^England|Human|Lighthouse Lab in Alderley Park|Wellcome Sanger Institute for the COVID-19 Genomics UK (COG-UK) consortium|Davies|United Kingdom
>Spike|hCoV-19/England/ALDP-64B30A/2020|2020-07-04|EPI_ISL_558024|Original|hCoV-19^^England|Human|Lighthouse Lab in Alderley Park|Wellcome Sanger Institute for the COVID-19 Genomics UK (COG-UK) Consortium|Davies|United Kingdom
>Spike|hCoV-19/England/ALDP-651AEE/2020|2020-07-04|EPI_ISL_558042|Original|hCoV-19^^England|Human|Lighthouse Lab in Alderley Park|Wellcome Sanger Institute for the COVID-19 Genomics UK (COG-UK) consortium|Davies|United Kingdom
>Spike|hCoV-19/England/ALDP-651BAF/2020|2020-07-04|EPI_ISL_558182|Original|hCoV-19^^England|Human|Lighthouse Lab in Alderley Park|Wellcome Sanger Institute for the COVID-19 Genomics UK (COG-UK) consortium|Davies|United Kingdom
>Spike|hCoV-19/England/ALDP-692BFD/2020|2020-07-02|EPI_ISL_558187|Original|hCoV-19^^England|Human|Lighthouse Lab in Alderley Park|Wellcome Sanger Institute for the COVID-19 Genomics UK (COG-UK) consortium|Davies|United Kingdom
>Spike|hCoV-19/England/MILK-566B99/2020|2020-06-12|EPI_ISL_558237|Original|hCoV-19^^England|Human|Lighthouse Lab in Milton Keynes|Wellcome Sanger Institute for the COVID-19 Genomics UK (COG-UK) Consortium|Davies|United Kingdom
>Spike|hCoV-19/England/MILK-596C15/2020|2020-06-11|EPI_ISL_558249|Original|hCoV-19^^England|Human|Lighthouse Lab in Milton Keynes|Wellcome Sanger Institute for the COVID-19 Genomics UK (COG-UK) consortium|Davies|United Kingdom
>Spike|hCoV-19/England/MILK-5630D9/2020|2020-06-10|EPI_ISL_558290|Original|hCoV-19^^England|Human|Lighthouse Lab in Milton Keynes|Wellcome Sanger Institute for the COVID-19 Genomics UK (COG-UK) consortium|Davies|United Kingdom
>Spike|hCoV-19/England/ALDP-599A72/2020|2020-06-08|EPI_ISL_558291|Original|hCoV-19^^England|Human|Lighthouse Lab in Alderley Park|Wellcome Sanger Institute for the COVID-19 Genomics UK (COG-UK) consortium|Davies|United Kingdom

>Spike|hCoV-19/England/ALDP-5D65C3/2020|2020-06-19|EPI_ISL_558417|Original|hCoV-19^^England|Human|Lighthouse Lab in Alderley Park|Wellcome Sanger Institute for the COVID-19 Genomics UK (COG-UK) consortium|Davies|United Kingdom
>Spike|hCoV-19/England/ALDP-5C51E1/2020|2020-06-17|EPI_ISL_558439|Original|hCoV-19^^England|Human|Lighthouse Lab in Alderley Park|Wellcome Sanger Institute for the COVID-19 Genomics UK (COG-UK) consortium|Davies|United Kingdom
>Spike|hCoV-19/England/ALDP-5D0A42/2020|2020-06-19|EPI_ISL_558449|Original|hCoV-19^^England|Human|Lighthouse Lab in Alderley Park|Wellcome Sanger Institute for the COVID-19 Genomics UK (COG-UK) consortium|Davies|United Kingdom
>Spike|hCoV-19/England/ALDP-5C174D/2020|2020-06-19|EPI_ISL_558617|Original|hCoV-19^^England|Human|Lighthouse Lab in Alderley Park|Wellcome Sanger Institute for the COVID-19 Genomics UK (COG-UK) Consortium|Davies|United Kingdom
>Spike|hCoV-19/England/ALDP-5C2542/2020|2020-06-19|EPI_ISL_558745|Original|hCoV-19^^England|Human|Lighthouse Lab in Alderley Park|Wellcome Sanger Institute for the COVID-19 Genomics UK (COG-UK) consortium|Davies|United Kingdom
>Spike|hCoV-19/England/ALDP-5B6EEC/2020|2020-06-19|EPI_ISL_558763|Original|hCoV-19^^England|Human|Lighthouse Lab in Alderley Park|Wellcome Sanger Institute for the COVID-19 Genomics UK (COG-UK) consortium|Davies|United Kingdom
>Spike|hCoV-19/England/ALDP-5B5F71/2020|2020-06-19|EPI_ISL_558767|Original|hCoV-19^^England|Human|Lighthouse Lab in Alderley Park|Wellcome Sanger Institute for the COVID-19 Genomics UK (COG-UK) consortium|Davies|United Kingdom
>Spike|hCoV-19/England/ALDP-59EF6F/2020|2020-06-14|EPI_ISL_558783|Original|hCoV-19^^England|Human|Lighthouse Lab in Alderley Park|Wellcome Sanger Institute for the COVID-19 Genomics UK (COG-UK) consortium|Davies|United Kingdom
>Spike|hCoV-19/England/ALDP-59DC3C/2020|2020-06-14|EPI_ISL_558796|Original|hCoV-19^^England|Human|Lighthouse Lab in Alderley Park|Wellcome Sanger Institute for the COVID-19 Genomics UK (COG-UK) Consortium|Davies|United Kingdom
>Spike|hCoV-19/England/ALDP-5B0116/2020|2020-06-17|EPI_ISL_558861|Original|hCoV-19^^England|Human|Lighthouse Lab in Alderley Park|Wellcome Sanger Institute for the COVID-19 Genomics UK (COG-UK) consortium|Davies|United Kingdom
>Spike|hCoV-19/England/ALDP-4A5151/2020|2020-06-09|EPI_ISL_559616|Original|hCoV-19^^England|Human|Lighthouse Lab in Alderley Park|Wellcome Sanger Institute for the COVID-19 Genomics UK (COG-UK) Consortium|Davies|United Kingdom
>Spike|hCoV-19/England/MILK-46D753/2020|2020-06-05|EPI_ISL_559706|Original|hCoV-19^^England|Human|Lighthouse Lab in Milton Keynes|Wellcome Sanger Institute for the COVID-19 Genomics UK (COG-UK) Consortium|Davies|United Kingdom
>Spike|hCoV-19/England/MILK-4689CB/2020|2020-05-22|EPI_ISL_559733|Original|hCoV-19^^England|Human|Lighthouse Lab in Milton Keynes|Wellcome Sanger Institute for the COVID-19 Genomics UK (COG-UK) Consortium|Davies|United Kingdom
>Spike|hCoV-19/England/MILK-468406/2020|2020-05-22|EPI_ISL_559734|Original|hCoV-19^^England|Human|Lighthouse Lab in Milton Keynes|Wellcome Sanger Institute for the COVID-19 Genomics UK (COG-UK) Consortium|Davies|United Kingdom
>Spike|hCoV-19/England/MILK-46861F/2020|2020-05-23|EPI_ISL_559740|Original|hCoV-19^^England|Human|Lighthouse Lab in Milton Keynes|Wellcome Sanger Institute for the COVID-19 Genomics UK (COG-UK) Consortium|Davies|United Kingdom
>Spike|hCoV-19/Wales/PHWC-16A829/2020|2020-09-08|EPI_ISL_560110|Original|hCoV-19^^Wales|Human|Wales Specialist Virology Centre Sequencing lab|COVID-19 Genomics UK (COG-UK) Consortium|Moore|United Kingdom
>Spike|hCoV-19/Wales/PHWC-16ACF0/2020|2020-09-11|EPI_ISL_560186|Original|hCoV-19^^Wales|Human|Wales Specialist Virology Centre Sequencing lab|COVID-19 Genomics UK (COG-UK) Consortium|Moore|United Kingdom
>Spike|hCoV-19/Wales/PHWC-16AD84/2020|2020-09-08|EPI_ISL_560195|Original|hCoV-19^^Wales|Human|Wales Specialist Virology Centre Sequencing lab|COVID-19 Genomics UK (COG-UK) Consortium|Moore|United Kingdom
>Spike|hCoV-19/Wales/PHWC-16AE36/2020|2020-09-08|EPI_ISL_560206|Original|hCoV-19^^Wales|Human|Wales Specialist Virology Centre Sequencing lab|COVID-19 Genomics UK (COG-UK) Consortium|Moore|United Kingdom
>Spike|hCoV-19/Wales/PHWC-16AE63/2020|2020-09-11|EPI_ISL_560209|Original|hCoV-19^^Wales|Human|Wales Specialist Virology Centre Sequencing lab|COVID-19 Genomics UK (COG-UK) Consortium|Moore|United Kingdom

>Spike|hCoV-19/Wales/PHWC-16B275/2020|2020-09-13|EPI_ISL_560266|Original|hCoV-19^^Wales|Human|Wales Specialist Virology Centre Sequencing lab|COVID-19 Genomics UK (COG-UK) Consortium|Moore|United Kingdom
>Spike|hCoV-19/Wales/PHWC-16B2EE/2020|2020-09-11|EPI_ISL_560271|Original|hCoV-19^^Wales|Human|Wales Specialist Virology Centre Sequencing lab|COVID-19 Genomics UK (COG-UK) Consortium|Moore|United Kingdom
>Spike|hCoV-19/USA/MD-MDH-0193/2020|2020-04-20|EPI_ISL_560806|Original|hCoV-19^^Maryland|Human|Maryland Public Health Laboratory|Maryland Public Health Laboratory|Keller|USA
>Spike|hCoV-19/USA/UT-UPHL-03071/2020|2020-05-04|EPI_ISL_560908|Original|hCoV-19^^Utah|Human|Utah Public Health Laboratory|Utah Public Health Laboratory|Young|USA
>Spike|hCoV-19/England/GSTT-6636/2020|2020-03-29|EPI_ISL_561368|Original|hCoV-19^^England|Human|Centre for Clinical Infection and Diagnostics Research and Genomics Innovation Unit|Centre for Clinical Infection and Diagnostics Research and Genomics Innovation Unit|Awan|United Kingdom
>Spike|hCoV-19/England/GSTT-6697/2020|2020-03-29|EPI_ISL_561369|Original|hCoV-19^^England|Human|Centre for Clinical Infection and Diagnostics Research and Genomics Innovation Unit|Centre for Clinical Infection and Diagnostics Research and Genomics Innovation Unit|Awan|United Kingdom
>Spike|hCoV-19/Australia/VIC10692/2020|2020-09-01|EPI_ISL_562591|Original|hCoV-19^^Victoria|Human|Microbiological Diagnostic Unit - Public Health Laboratory (MDU-PHL)|MDU-PHL|Schultz|Australia
>Spike|hCoV-19/Australia/VIC10789/2020|2020-09-01|EPI_ISL_562592|Original|hCoV-19^^Victoria|Human|Microbiological Diagnostic Unit - Public Health Laboratory (MDU-PHL)|MDU-PHL|Schultz|Australia
>Spike|hCoV-19/Australia/VIC12847/2020|2020-09-11|EPI_ISL_562594|Original|hCoV-19^^Victoria|Human|Microbiological Diagnostic Unit - Public Health Laboratory (MDU-PHL)|MDU-PHL|Schultz|Australia
>Spike|hCoV-19/Australia/VIC10131/2020|2020-08-26|EPI_ISL_562597|Original|hCoV-19^^Victoria|Human|Microbiological Diagnostic Unit - Public Health Laboratory (MDU-PHL)|MDU-PHL|Schultz|Australia
>Spike|hCoV-19/Australia/VIC10355/2020|2020-09-01|EPI_ISL_562598|Original|hCoV-19^^Victoria|Human|Microbiological Diagnostic Unit - Public Health Laboratory (MDU-PHL)|MDU-PHL|Schultz|Australia
>Spike|hCoV-19/Australia/VIC10479/2020|2020-09-05|EPI_ISL_562599|Original|hCoV-19^^Victoria|Human|Microbiological Diagnostic Unit - Public Health Laboratory (MDU-PHL)|MDU-PHL|Schultz|Australia
>Spike|hCoV-19/Australia/VIC10480/2020|2020-08-13|EPI_ISL_562600|Original|hCoV-19^^Victoria|Human|Microbiological Diagnostic Unit - Public Health Laboratory (MDU-PHL)|MDU-PHL|Schultz|Australia
>Spike|hCoV-19/Australia/VIC10501/2020|2020-08-07|EPI_ISL_562601|Original|hCoV-19^^Victoria|Human|Microbiological Diagnostic Unit - Public Health Laboratory (MDU-PHL)|MDU-PHL|Schultz|Australia
>Spike|hCoV-19/Australia/VIC10778/2020|2020-08-02|EPI_ISL_562602|Original|hCoV-19^^Victoria|Human|Microbiological Diagnostic Unit - Public Health Laboratory (MDU-PHL)|MDU-PHL|Schultz|Australia
>Spike|hCoV-19/Australia/VIC10922/2020|2020-09-03|EPI_ISL_562603|Original|hCoV-19^^Victoria|Human|Microbiological Diagnostic Unit - Public Health Laboratory (MDU-PHL)|MDU-PHL|Schultz|Australia
>Spike|hCoV-19/Australia/VIC10927/2020|2020-09-07|EPI_ISL_562604|Original|hCoV-19^^Victoria|Human|Microbiological Diagnostic Unit - Public Health Laboratory (MDU-PHL)|MDU-PHL|Schultz|Australia
>Spike|hCoV-19/Australia/VIC11001/2020|2020-07-30|EPI_ISL_562606|Original|hCoV-19^^Victoria|Human|Microbiological Diagnostic Unit - Public Health Laboratory (MDU-PHL)|MDU-PHL|Schultz|Australia
>Spike|hCoV-19/Australia/VIC11027/2020|2020-07-30|EPI_ISL_562607|Original|hCoV-19^^Victoria|Human|Microbiological Diagnostic Unit - Public Health Laboratory (MDU-PHL)|MDU-PHL|Schultz|Australia

>Spike|hCoV-19/Australia/VIC11039/2020|2020-07-30|EPI_ISL_562608|Original|hCoV-19^^Victoria|Human|Microbiological Diagnostic Unit - Public Health Laboratory (MDU-PHL)|MDU-PHL|Schultz|Australia
>Spike|hCoV-19/Australia/VIC11062/2020|2020-09-08|EPI_ISL_562609|Original|hCoV-19^^Victoria|Human|Microbiological Diagnostic Unit - Public Health Laboratory (MDU-PHL)|MDU-PHL|Schultz|Australia
>Spike|hCoV-19/Australia/VIC11095/2020|2020-08-12|EPI_ISL_562610|Original|hCoV-19^^Victoria|Human|Microbiological Diagnostic Unit - Public Health Laboratory (MDU-PHL)|MDU-PHL|Schultz|Australia
>Spike|hCoV-19/Australia/VIC11167/2020|2020-08-04|EPI_ISL_562611|Original|hCoV-19^^Victoria|Human|Microbiological Diagnostic Unit - Public Health Laboratory (MDU-PHL)|MDU-PHL|Schultz|Australia
>Spike|hCoV-19/Australia/VIC11425/2020|2020-08-24|EPI_ISL_562612|Original|hCoV-19^^Victoria|Human|Microbiological Diagnostic Unit - Public Health Laboratory (MDU-PHL)|MDU-PHL|Schultz|Australia
>Spike|hCoV-19/Australia/VIC11435/2020|2020-08-29|EPI_ISL_562613|Original|hCoV-19^^Victoria|Human|Microbiological Diagnostic Unit - Public Health Laboratory (MDU-PHL)|MDU-PHL|Schultz|Australia
>Spike|hCoV-19/Australia/VIC11439/2020|2020-08-22|EPI_ISL_562614|Original|hCoV-19^^Victoria|Human|Microbiological Diagnostic Unit - Public Health Laboratory (MDU-PHL)|MDU-PHL|Schultz|Australia
>Spike|hCoV-19/Australia/VIC12465/2020|2020-09-13|EPI_ISL_562615|Original|hCoV-19^^Victoria|Human|Microbiological Diagnostic Unit - Public Health Laboratory (MDU-PHL)|MDU-PHL|Schultz|Australia
>Spike|hCoV-19/Australia/VIC13173/2020|2020-08-15|EPI_ISL_562617|Original|hCoV-19^^Victoria|Human|Microbiological Diagnostic Unit - Public Health Laboratory (MDU-PHL)|MDU-PHL|Schultz|Australia
>Spike|hCoV-19/Australia/VIC13621/2020|2020-08-02|EPI_ISL_562618|Original|hCoV-19^^Victoria|Human|Microbiological Diagnostic Unit - Public Health Laboratory (MDU-PHL)|MDU-PHL|Schultz|Australia
>Spike|hCoV-19/Australia/VIC13645/2020|2020-08-03|EPI_ISL_562619|Original|hCoV-19^^Victoria|Human|Microbiological Diagnostic Unit - Public Health Laboratory (MDU-PHL)|MDU-PHL|Schultz|Australia
>Spike|hCoV-19/Australia/VIC13664/2020|2020-09-14|EPI_ISL_562620|Original|hCoV-19^^Victoria|Human|Microbiological Diagnostic Unit - Public Health Laboratory (MDU-PHL)|MDU-PHL|Schultz|Australia
>Spike|hCoV-19/Australia/VIC13772/2020|2020-08-18|EPI_ISL_562621|Original|hCoV-19^^Victoria|Human|Microbiological Diagnostic Unit - Public Health Laboratory (MDU-PHL)|MDU-PHL|Schultz|Australia
>Spike|hCoV-19/Australia/VIC14039/2020|2020-09-17|EPI_ISL_562622|Original|hCoV-19^^Victoria|Human|Microbiological Diagnostic Unit - Public Health Laboratory (MDU-PHL)|MDU-PHL|Schultz|Australia
>Spike|hCoV-19/Australia/VIC7919/2020|2020-08-16|EPI_ISL_562623|Original|hCoV-19^^Victoria|Human|Microbiological Diagnostic Unit - Public Health Laboratory (MDU-PHL)|MDU-PHL|Schultz|Australia
>Spike|hCoV-19/Australia/VIC8076/2020|2020-08-18|EPI_ISL_562624|Original|hCoV-19^^Victoria|Human|Microbiological Diagnostic Unit - Public Health Laboratory (MDU-PHL)|MDU-PHL|Schultz|Australia
>Spike|hCoV-19/Australia/VIC8295/2020|2020-08-14|EPI_ISL_562626|Original|hCoV-19^^Victoria|Human|Microbiological Diagnostic Unit - Public Health Laboratory (MDU-PHL)|MDU-PHL|Schultz|Australia
>Spike|hCoV-19/Australia/VIC8482/2020|2020-08-17|EPI_ISL_562627|Original|hCoV-19^^Victoria|Human|Microbiological Diagnostic Unit - Public Health Laboratory (MDU-PHL)|MDU-PHL|Schultz|Australia
>Spike|hCoV-19/Australia/VIC9130/2020|2020-08-19|EPI_ISL_562629|Original|hCoV-19^^Victoria|Human|Microbiological Diagnostic Unit - Public Health Laboratory (MDU-PHL)|MDU-PHL|Schultz|Australia
>Spike|hCoV-19/Australia/VIC9282/2020|2020-00-00|EPI_ISL_562630|Original|hCoV-19^^Victoria|Human|Microbiological Diagnostic Unit - Public Health Laboratory (MDU-PHL)|MDU-PHL|Schultz|Australia

>Spike|hCoV-19/Australia/VIC9316/2020|2020-08-19|EPI_ISL_562632|Original|hCoV-19^^Victoria|Human|Microbiological Diagnostic Unit - Public Health Laboratory (MDU-PHL)|MDU-PHL|Schultz|Australia
>Spike|hCoV-19/Australia/VIC9325/2020|2020-08-21|EPI_ISL_562633|Original|hCoV-19^^Victoria|Human|Microbiological Diagnostic Unit - Public Health Laboratory (MDU-PHL)|MDU-PHL|Schultz|Australia
>Spike|hCoV-19/Australia/VIC9442/2020|2020-08-18|EPI_ISL_562634|Original|hCoV-19^^Victoria|Human|Microbiological Diagnostic Unit - Public Health Laboratory (MDU-PHL)|MDU-PHL|Schultz|Australia
>Spike|hCoV-19/Australia/VIC9994/2020|2020-08-24|EPI_ISL_562636|Original|hCoV-19^^Victoria|Human|Microbiological Diagnostic Unit - Public Health Laboratory (MDU-PHL)|MDU-PHL|Schultz|Australia
>Spike|hCoV-19/USA/MI-MDHHS-SC21811/2020|2020-07-21|EPI_ISL_565854|Original|hCoV-19^^Michigan|Human|Michigan Department of Health and Human Services|Michigan Department of Health and Human Services|Blankenship|USA
>Spike|hCoV-19/USA/MI-MDHHS-SC21823/2020|2020-03-14|EPI_ISL_565866|Original|hCoV-19^^Michigan|Human|Michigan Department of Health and Human Services|Michigan Department of Health and Human Services|Blankenship|USA
>Spike|hCoV-19/Spain/AN-IBV-98001933/2020|2020-03-12|EPI_ISL_565908|Original|hCoV-19^^Andalusia|Human|Servicio de Microbiolog\u00eda. HRU de M\u00e1laga. Servicio Andaluz de Salud|SeqCOVID-SPAIN consortium/IBV(CSIC)|Comas|Spain
>Spike|hCoV-19/Spain/AN-IBV-98001945/2020|2020-03-13|EPI_ISL_565909|Original|hCoV-19^^Andalusia|Human|Servicio de Microbiolog\u00eda. HRU de M\u00e1laga. Servicio Andaluz de Salud|SeqCOVID-SPAIN consortium/IBV(CSIC)|Comas|Spain
>Spike|hCoV-19/Spain/AN-IBV-98001966/2020|2020-03-14|EPI_ISL_565911|Original|hCoV-19^^Andalusia|Human|Servicio de Microbiolog\u00eda. HRU de M\u00e1laga. Servicio Andaluz de Salud|SeqCOVID-SPAIN consortium/IBV(CSIC)|Comas|Spain
>Spike|hCoV-19/Spain/AN-IBV-98001999/2020|2020-03-16|EPI_ISL_565914|Original|hCoV-19^^Andalusia|Human|Servicio de Microbiolog\u00eda. HRU de M\u00e1laga. Servicio Andaluz de Salud|SeqCOVID-SPAIN consortium/IBV(CSIC)|Comas|Spain
>Spike|hCoV-19/Spain/AN-IBV-98002007/2020|2020-03-14|EPI_ISL_565915|Original|hCoV-19^^Andalusia|Human|Servicio de Microbiolog\u00eda. HRU de M\u00e1laga. Servicio Andaluz de Salud|SeqCOVID-SPAIN consortium/IBV(CSIC)|Comas|Spain
>Spike|hCoV-19/Spain/AN-IBV-98002013/2020|2020-03-15|EPI_ISL_565917|Original|hCoV-19^^Andalusia|Human|Servicio de Microbiolog\u00eda. HRU de M\u00e1laga. Servicio Andaluz de Salud|SeqCOVID-SPAIN consortium/IBV(CSIC)|Comas|Spain
>Spike|hCoV-19/Spain/AN-IBV-98002035/2020|2020-03-17|EPI_ISL_565919|Original|hCoV-19^^Andalusia|Human|Servicio de Microbiolog\u00eda. HRU de M\u00e1laga. Servicio Andaluz de Salud|SeqCOVID-SPAIN consortium/IBV(CSIC)|Comas|Spain
>Spike|hCoV-19/Spain/PV-IBV-98005136/2020|2020-06-12|EPI_ISL_565920|Original|hCoV-19^^Basque Country|Human|Servicio de Microbiolog\u00eda. Hospital Universitario Donostia. OSI Donostialdea. \u00c1rea de Enfermedades Infecciosas|SeqCOVID-SPAIN consortium/IBV(CSIC)|Comas|Spain
>Spike|hCoV-19/Spain/PV-IBV-98005142/2020|2020-06-17|EPI_ISL_565922|Original|hCoV-19^^Basque Country|Human|Servicio de Microbiolog\u00eda. Hospital Universitario Donostia. OSI Donostialdea. \u00c1rea de Enfermedades Infecciosas|SeqCOVID-SPAIN consortium/IBV(CSIC)|Comas|Spain
>Spike|hCoV-19/Spain/PV-IBV-98005146/2020|2020-06-19|EPI_ISL_565923|Original|hCoV-19^^Basque Country|Human|Servicio de Microbiolog\u00eda. Hospital Universitario Donostia. OSI Donostialdea. \u00c1rea de Enfermedades Infecciosas|SeqCOVID-SPAIN consortium/IBV(CSIC)|Comas|Spain
>Spike|hCoV-19/Spain/PV-IBV-98005160/2020|2020-06-23|EPI_ISL_565924|Original|hCoV-19^^Basque Country|Human|Servicio de Microbiolog\u00eda. Hospital Universitario Donostia. OSI Donostialdea. \u00c1rea de Enfermedades Infecciosas|SeqCOVID-SPAIN consortium/IBV(CSIC)|Comas|Spain
>Spike|hCoV-19/Spain/PV-IBV-98005164/2020|2020-06-24|EPI_ISL_565925|Original|hCoV-19^^Basque Country|Human|Servicio de Microbiolog\u00eda. Hospital Universitario Donostia. OSI Donostialdea. \u00c1rea de Enfermedades Infecciosas|SeqCOVID-SPAIN consortium/IBV(CSIC)|Comas|Spain

>Spike|hCoV-19/England/CAMC-9B1BAA/2020|2020-09-04|EPI_ISL_566474|Original|hCoV-19^^England|Human|Lighthouse Lab in Cambridge|Wellcome Sanger Institute for the COVID-19 Genomics UK (COG-UK) Consortium|Davies|United Kingdom
>Spike|hCoV-19/England/QEUH-9C8F68/2020|2020-09-11|EPI_ISL_567024|Original|hCoV-19^^England|Human|Lighthouse Lab in Glasgow|Wellcome Sanger Institute for the COVID-19 Genomics UK (COG-UK) Consortium|Davies|United Kingdom
>Spike|hCoV-19/England/ALDP-9C4B59/2020|2020-09-11|EPI_ISL_567803|Original|hCoV-19^^England|Human|Lighthouse Lab in Alderley Park|Wellcome Sanger Institute for the COVID-19 Genomics UK (COG-UK) Consortium|Davies|United Kingdom
>Spike|hCoV-19/Scotland/QEUH-998CA6/2020|2020-08-26|EPI_ISL_568301|Original|hCoV-19^^Scotland|Human|Lighthouse Lab in Glasgow|Wellcome Sanger Institute for the COVID-19 Genomics UK (COG-UK) Consortium|Davies|United Kingdom
>Spike|hCoV-19/Scotland/QEUH-998D94/2020|2020-08-26|EPI_ISL_568315|Original|hCoV-19^^Scotland|Human|Lighthouse Lab in Glasgow|Wellcome Sanger Institute for the COVID-19 Genomics UK (COG-UK) Consortium|Davies|United Kingdom
>Spike|hCoV-19/Scotland/QEUH-998D67/2020|2020-08-26|EPI_ISL_568317|Original|hCoV-19^^Scotland|Human|Lighthouse Lab in Glasgow|Wellcome Sanger Institute for the COVID-19 Genomics UK (COG-UK) Consortium|Davies|United Kingdom
>Spike|hCoV-19/Scotland/QEUH-998E0A/2020|2020-08-26|EPI_ISL_568344|Original|hCoV-19^^Scotland|Human|Lighthouse Lab in Glasgow|Wellcome Sanger Institute for the COVID-19 Genomics UK (COG-UK) Consortium|Davies|United Kingdom
>Spike|hCoV-19/Scotland/QEUH-998022/2020|2020-08-27|EPI_ISL_568392|Original|hCoV-19^^Scotland|Human|Lighthouse Lab in Glasgow|Wellcome Sanger Institute for the COVID-19 Genomics UK (COG-UK) Consortium|Davies|United Kingdom
>Spike|hCoV-19/Scotland/QEUH-998013/2020|2020-08-27|EPI_ISL_568398|Original|hCoV-19^^Scotland|Human|Lighthouse Lab in Glasgow|Wellcome Sanger Institute for the COVID-19 Genomics UK (COG-UK) Consortium|Davies|United Kingdom
>Spike|hCoV-19/Scotland/ALDP-8D396D/2020|2020-06-24|EPI_ISL_568474|Original|hCoV-19^^Scotland|Human|Lighthouse Lab in Alderley Park|Wellcome Sanger Institute for the COVID-19 Genomics UK (COG-UK) consortium|Davies|United Kingdom
>Spike|hCoV-19/USA/ID-UW-6454/2020|2020-04-03|EPI_ISL_570117|Original|hCoV-19^^Idaho|Human|UW Virology Lab|UW Virology Lab|Roychoudhury|USA
>Spike|hCoV-19/USA/WA-UW-9504/2020|2020-05-18|EPI_ISL_570257|Original|hCoV-19^^Washington|Human|UW Virology Lab|UW Virology Lab|Roychoudhury|USA
>Spike|hCoV-19/USA/WA-UW-9534/2020|2020-05-19|EPI_ISL_570260|Original|hCoV-19^^Washington|Human|UW Virology Lab|UW Virology Lab|Roychoudhury|USA
>Spike|hCoV-19/USA/WA-UW-12117/2020|2020-06-24|EPI_ISL_570490|Original|hCoV-19^^Washington|Human|UW Virology Lab|UW Virology Lab|Roychoudhury|USA
>Spike|hCoV-19/USA/WA-UW-22460/2020|2020-08-05|EPI_ISL_570578|Original|hCoV-19^^Washington|Human|UW Virology Lab|UW Virology Lab|Roychoudhury|USA
>Spike|hCoV-19/USA/WA-UW-13047/2020|2020-06-30|EPI_ISL_570844|Original|hCoV-19^^Washington|Human|UW Virology Lab|UW Virology Lab|Roychoudhury|USA
>Spike|hCoV-19/USA/WA-UW-191/2020|2020-03-08|EPI_ISL_570976|Original|hCoV-19^^Washington|Human|UW Virology Lab|UW Virology Lab|Roychoudhury|USA
>Spike|hCoV-19/USA/MI-QDX-890/2020|2020-03-18|EPI_ISL_571611|Original|hCoV-19^^Michigan|Human|Quest Diagnostics|Quest Diagnostics|Kagan|USA
>Spike|hCoV-19/USA/VA-DCLS-1447/2020|2020-08-19|EPI_ISL_572314|Original|hCoV-19^^Virginia|Human|Virginia DCLS|Virginia DCLS|St. Jacques|USA
>Spike|hCoV-19/Brazil/PE-IAM391/2020|2020-04-20|EPI_ISL_572337|Original|hCoV-19^^Pernambuco|Human|LACEN/PE|WallauLab|Wallau|Brazil
>Spike|hCoV-19/Brazil/PE-IAM397/2020|2020-05-14|EPI_ISL_572339|Original|hCoV-19^^Pernambuco|Human|LACEN/PE|WallauLab|Wallau|Brazil
>Spike|hCoV-19/Brazil/PE-IAM404/2020|2020-04-20|EPI_ISL_572340|Original|hCoV-19^^Pernambuco|Human|LACEN/PE|WallauLab|Wallau|Brazil
>Spike|hCoV-19/Brazil/PE-IAM448/2020|2020-04-20|EPI_ISL_572341|Original|hCoV-19^^Pernambuco|Human|LACEN/PE|WallauLab|Wallau|Brazil
>Spike|hCoV-19/Brazil/PE-IAM464/2020|2020-04-20|EPI_ISL_572343|Original|hCoV-19^^Pernambuco|Human|LACEN/PE|WallauLab|Wallau|Brazil
>Spike|hCoV-19/Brazil/PE-IAM479/2020|2020-04-20|EPI_ISL_572344|Original|hCoV-19^^Pernambuco|Human|LACEN/PE|WallauLab|Wallau|Brazil

>Spike|hCoV-19/Brazil/PE-IAM604/2020|2020-04-22|EPI_ISL_572347|Original|hCoV-
19^^Pernambuco|Human|LACEN/PE|WallauLab|Wallau|Brazil
>Spike|hCoV-19/Brazil/PE-IAM714/2020|2020-04-26|EPI_ISL_572352|Original|hCoV-
19^^Pernambuco|Human|LACEN/PE|WallauLab|Wallau|Brazil
>Spike|hCoV-19/Brazil/PE-IAM726/2020|2020-04-26|EPI_ISL_572354|Original|hCoV-
19^^Pernambuco|Human|LACEN/PE|WallauLab|Wallau|Brazil
>Spike|hCoV-19/Brazil/PE-IAM807/2020|2020-04-27|EPI_ISL_572356|Original|hCoV-
19^^Pernambuco|Human|LACEN/PE|WallauLab|Wallau|Brazil
>Spike|hCoV-19/Brazil/PE-IAM887/2020|2020-05-03|EPI_ISL_572357|Original|hCoV-
19^^Pernambuco|Human|LACEN/PE|WallauLab|Wallau|Brazil
>Spike|hCoV-19/Brazil/PE-IAM959/2020|2020-05-03|EPI_ISL_572364|Original|hCoV-
19^^Pernambuco|Human|LACEN/PE|WallauLab|Wallau|Brazil
>Spike|hCoV-19/Brazil/PE-IAM960/2020|2020-04-29|EPI_ISL_572365|Original|hCoV-
19^^Pernambuco|Human|LACEN/PE|WallauLab|Wallau|Brazil
>Spike|hCoV-19/Brazil/PE-IAM981/2020|2020-05-06|EPI_ISL_572370|Original|hCoV-
19^^Pernambuco|Human|LACEN/PE|WallauLab|Wallau|Brazil
>Spike|hCoV-19/Brazil/PE-IAM1026/2020|2020-05-06|EPI_ISL_572373|Original|hCoV-
19^^Pernambuco|Human|LACEN/PE|WallauLab|Wallau|Brazil
>Spike|hCoV-19/Brazil/PE-IAM1092/2020|2020-05-06|EPI_ISL_572376|Original|hCoV-
19^^Pernambuco|Human|LACEN/PE|WallauLab|Wallau|Brazil
>Spike|hCoV-19/Brazil/PE-IAM1116/2020|2020-05-07|EPI_ISL_572378|Original|hCoV-
19^^Pernambuco|Human|LACEN/PE|WallauLab|Wallau|Brazil
>Spike|hCoV-19/Brazil/PE-IAM1126/2020|2020-05-07|EPI_ISL_572379|Original|hCoV-
19^^Pernambuco|Human|LACEN/PE|WallauLab|Wallau|Brazil
>Spike|hCoV-19/Brazil/PE-IAM1237/2020|2020-05-10|EPI_ISL_572383|Original|hCoV-
19^^Pernambuco|Human|LACEN/PE|WallauLab|Wallau|Brazil
>Spike|hCoV-19/Brazil/PE-IAM1314/2020|2020-05-11|EPI_ISL_572387|Original|hCoV-
19^^Pernambuco|Human|LACEN/PE|WallauLab|Wallau|Brazil
>Spike|hCoV-19/Brazil/PE-IAM1352/2020|2020-05-11|EPI_ISL_572390|Original|hCoV-
19^^Pernambuco|Human|LACEN/PE|WallauLab|Wallau|Brazil
>Spike|hCoV-19/Brazil/PE-IAM1399/2020|2020-05-14|EPI_ISL_572391|Original|hCoV-
19^^Pernambuco|Human|LACEN/PE|WallauLab|Wallau|Brazil
>Spike|hCoV-19/Brazil/PE-IAM1434/2020|2020-05-13|EPI_ISL_572392|Original|hCoV-
19^^Pernambuco|Human|LACEN/PE|WallauLab|Wallau|Brazil
>Spike|hCoV-19/Brazil/PE-IAM1451/2020|2020-05-14|EPI_ISL_572395|Original|hCoV-
19^^Pernambuco|Human|LACEN/PE|WallauLab|Wallau|Brazil
>Spike|hCoV-19/Wales/PHWC-16D352/2020|2020-09-23|EPI_ISL_572577|Original|hCoV-
19^^Wales|Human|Wales Specialist Virology Centre Sequencing lab|COVID-19 Genomics UK
(COG-UK) Consortium|Moore|United Kingdom
>Spike|hCoV-19/Wales/PHWC-16C0C5/2020|2020-09-16|EPI_ISL_572931|Original|hCoV-
19^^Wales|Human|Wales Specialist Virology Centre Sequencing lab|COVID-19 Genomics UK
(COG-UK) Consortium|Moore|United Kingdom
>Spike|hCoV-19/Wales/PHWC-16C274/2020|2020-09-25|EPI_ISL_574030|Original|hCoV-
19^^Wales|Human|Wales Specialist Virology Centre Sequencing lab|COVID-19 Genomics UK
(COG-UK) Consortium|Moore|United Kingdom
>Spike|hCoV-19/Wales/PHWC-16C441/2020|2020-09-19|EPI_ISL_574056|Original|hCoV-
19^^Wales|Human|Wales Specialist Virology Centre Sequencing lab|COVID-19 Genomics UK
(COG-UK) Consortium|Moore|United Kingdom
>Spike|hCoV-19/Wales/PHWC-16C7A2/2020|2020-09-17|EPI_ISL_574092|Original|hCoV-
19^^Wales|Human|Wales Specialist Virology Centre Sequencing lab|COVID-19 Genomics UK
(COG-UK) Consortium|Moore|United Kingdom
>Spike|hCoV-19/Wales/PHWC-16CA12/2020|2020-09-25|EPI_ISL_574121|Original|hCoV-
19^^Wales|Human|Wales Specialist Virology Centre Sequencing lab|COVID-19 Genomics UK
(COG-UK) Consortium|Moore|United Kingdom
>Spike|hCoV-19/Wales/PHWC-16CDFB/2020|2020-09-25|EPI_ISL_574171|Original|hCoV-
19^^Wales|Human|Wales Specialist Virology Centre Sequencing lab|COVID-19 Genomics UK
(COG-UK) Consortium|Moore|United Kingdom
>Spike|hCoV-19/Wales/PHWC-16CE34/2020|2020-06-12|EPI_ISL_574174|Original|hCoV-
19^^Wales|Human|Wales Specialist Virology Centre Sequencing lab|COVID-19 Genomics UK
(COG-UK) Consortium|Moore|United Kingdom

>Spike|hCoV-19/Wales/PHWC-16CFF5/2020|2020-09-23|EPI_ISL_574195|Original|hCoV-19^^Wales|Human|Wales Specialist Virology Centre Sequencing lab|COVID-19 Genomics UK (COG-UK) Consortium|Moore|United Kingdom
>Spike|hCoV-19/Wales/PHWC-16D000/2020|2020-09-19|EPI_ISL_574196|Original|hCoV-19^^Wales|Human|Wales Specialist Virology Centre Sequencing lab|COVID-19 Genomics UK (COG-UK) Consortium|Moore|United Kingdom
>Spike|hCoV-19/Wales/PHWC-16D307/2020|2020-09-22|EPI_ISL_574226|Original|hCoV-19^^Wales|Human|Wales Specialist Virology Centre Sequencing lab|COVID-19 Genomics UK (COG-UK) Consortium|Moore|United Kingdom
>Spike|hCoV-19/Wales/PHWC-16D4F5/2020|2020-09-26|EPI_ISL_574246|Original|hCoV-19^^Wales|Human|Wales Specialist Virology Centre Sequencing lab|COVID-19 Genomics UK (COG-UK) Consortium|Moore|United Kingdom
>Spike|hCoV-19/USA/UT-UPHL-04306/2020|2020-05-15|EPI_ISL_575136|Original|hCoV-19^^Utah|Human|Utah Public Health Laboratory|Utah Public Health Laboratory|Young|USA
>Spike|hCoV-19/Scotland/QEUH-9CA35B/2020|2020-09-10|EPI_ISL_576050|Original|hCoV-19^^Scotland|Human|Lighthouse Lab in Glasgow|Wellcome Sanger Institute for the COVID-19 Genomics UK (COG-UK) Consortium|Davies|United Kingdom
>Spike|hCoV-19/Scotland/QEUH-9CA25E/2020|2020-09-13|EPI_ISL_576064|Original|hCoV-19^^Scotland|Human|Lighthouse Lab in Glasgow|Wellcome Sanger Institute for the COVID-19 Genomics UK (COG-UK) Consortium|Davies|United Kingdom
>Spike|hCoV-19/Scotland/QEUH-9C96CC/2020|2020-09-14|EPI_ISL_576088|Original|hCoV-19^^Scotland|Human|Lighthouse Lab in Glasgow|Wellcome Sanger Institute for the COVID-19 Genomics UK (COG-UK) Consortium|Davies|United Kingdom
>Spike|hCoV-19/USA/UN-UW-6996/2020|2020-03-11|EPI_ISL_576494|Original|hCoV-19^^USA|Human|UW Virology Lab|UW Virology Lab|Roychoudhury|USA
>Spike|hCoV-19/England/ALDP-9D76DA/2020|2020-09-18|EPI_ISL_576580|Original|hCoV-19^^England|Human|Oxford Viromics|COVID-19 Genomics UK (COG-UK) Consortium|Golubchik|United Kingdom
>Spike|hCoV-19/England/ALDP-9D96F6/2020|2020-09-18|EPI_ISL_576755|Original|hCoV-19^^England|Human|Oxford Viromics|COVID-19 Genomics UK (COG-UK) Consortium|Golubchik|United Kingdom
>Spike|hCoV-19/Wales/MILK-9E18D7/2020|2020-09-19|EPI_ISL_577416|Original|hCoV-19^^Wales|Human|Oxford Viromics|COVID-19 Genomics UK (COG-UK) Consortium|Golubchik|United Kingdom
>Spike|hCoV-19/Wales/PHWC-16D923/2020|2020-09-27|EPI_ISL_577466|Original|hCoV-19^^Wales|Human|Wales Specialist Virology Centre Sequencing lab|COVID-19 Genomics UK (COG-UK) Consortium|Moore|United Kingdom
>Spike|hCoV-19/USA/MI-UM-10033991503/2020|2020-04-22|EPI_ISL_578185|Original|hCoV-19^^Michigan|Human|University of Michigan Clinical Microbiology Laboratory|Lauring Lab|Valesano|USA
>Spike|hCoV-19/England/QEUH-9E568E/2020|2020-09-22|EPI_ISL_579794|Original|hCoV-19^^England|Human|Lighthouse Lab in Glasgow|Wellcome Sanger Institute for the COVID-19 Genomics UK (COG-UK) Consortium|Davies|United Kingdom
>Spike|hCoV-19/England/ALDP-9E9DEF/2020|2020-09-24|EPI_ISL_580062|Original|hCoV-19^^England|Human|Lighthouse Lab in Alderley Park|Wellcome Sanger Institute for the COVID-19 Genomics UK (COG-UK) Consortium|Davies|United Kingdom
>Spike|hCoV-19/England/ALDP-9E9D94/2020|2020-09-24|EPI_ISL_580138|Original|hCoV-19^^England|Human|Lighthouse Lab in Alderley Park|Wellcome Sanger Institute for the COVID-19 Genomics UK (COG-UK) Consortium|Davies|United Kingdom
>Spike|hCoV-19/England/ALDP-9E9E37/2020|2020-09-24|EPI_ISL_580210|Original|hCoV-19^^England|Human|Lighthouse Lab in Alderley Park|Wellcome Sanger Institute for the COVID-19 Genomics UK (COG-UK) Consortium|Davies|United Kingdom
>Spike|hCoV-19/England/ALDP-9E6C7C/2020|2020-09-24|EPI_ISL_580285|Original|hCoV-19^^England|Human|Lighthouse Lab in Alderley Park|Wellcome Sanger Institute for the COVID-19 Genomics UK (COG-UK) Consortium|Davies|United Kingdom
>Spike|hCoV-19/Iraq/ICGEB-2T/2020|2020-06-30|EPI_ISL_582029|Original|hCoV-19^^Al-Muthanna Province|Human|Biology Department|International Centre for Genetic Engineering and Biotechnology (ICGEB) and ARGO Open Lab Platform|Marcello|Iraq
>Spike|hCoV-19/Spain/PV-IBV-98007621/2020|2020-09-05|EPI_ISL_582048|Original|hCoV-19^^Basque Country|Human|Servicio de Microbiolog\u00eda. Hospital Universitario

Donostia. OSI Donostialdea. \u00c1rea de Enfermedades Infecciosas|SeqCOVID-SPAIN consortium/IBV(CSIC)|Comas|Spain
>Spike|hCoV-19/Spain/CB-IBV-98007071/2020|2020-09-03|EPI_ISL_582096|Original|hCoV-19^^Cantabria|Human|Hospital Universitario Marqu\u00e9s de Valdecilla - IDIVAL (Santander|SeqCOVID-SPAIN consortium/IBV(CSIC)|Comas|Spain
>Spike|hCoV-19/Portugal/PT0954/2020|2020-04-17|EPI_ISL_582515|Original|hCoV-19^^Portugal|Human|unknown|Instituto Nacional de Saude (INSA) and Instituto Gulbenkian de Ciencia (IGC)|Borges|Portugal
>Spike|hCoV-19/Portugal/PT1295/2020|2020-03-24|EPI_ISL_582522|Original|hCoV-19^^Portugal|Human|unknown|Instituto Nacional de Saude (INSA) and Instituto Gulbenkian de Ciencia (IGC)|Borges|Portugal
>Spike|hCoV-19/USA/CA-CZB-6941/2020|2020-08-26|EPI_ISL_582893|Original|hCoV-19^^California|Human|County of Santa Clara Public Health Department|Chan-Zuckerberg Biohub|Bhatt|USA
>Spike|hCoV-19/USA/CA-CZB-5670/2020|2020-07-28|EPI_ISL_583216|Original|hCoV-19^^California|Human|UCSF Clinical Microbiology Laboratory|Chan-Zuckerberg Biohub|Bhatt|USA
>Spike|hCoV-19/USA/NY-MSK-4C/2020|2020-05-06|EPI_ISL_583440|Original|hCoV-19^^New York|Human|Memorial Sloan Kettering Cancer Center|van Bakel Laboratory|van Bakel|USA
>Spike|hCoV-19/Italy/LOM-UniMI-ICCS1/2020|2020-03-21|EPI_ISL_584048|Vero|hCoV-19^^Lombardy|Human|Laboratory of Molecular Virology|Laboratory of Molecular Virology|Delbue|Italy
>Spike|hCoV-19/Italy/LOM-UniMI-ICCS2/2020|2020-03-24|EPI_ISL_584049|Vero|hCoV-19^^Lombardy|Human|Laboratory of Molecular Virology|Laboratory of Molecular Virology|Delbue|Italy
>Spike|hCoV-19/Italy/LOM-UniMI-vir1/2020|2020-04-05|EPI_ISL_584051|Vero|hCoV-19^^Lombardy|Human|Laboratory of Molecular Virology|Laboratory of Molecular Virology|Delbue|Italy
>Spike|hCoV-19/Italy/LOM-UniMI-ICCS3/2020|2020-04-05|EPI_ISL_584052|Vero|hCoV-19^^Lombardy|Human|Laboratory of Molecular Virology|Laboratory of Molecular Virology|Delbue|Italy
>Spike|hCoV-19/USA/MI-UM-10035769699/2020|2020-09-02|EPI_ISL_584096|Original|hCoV-19^^Michigan|Human|University of Michigan Clinical Microbiology Laboratory|Lauring Lab|Valesano|USA
>Spike|hCoV-19/USA/MI-UM-10035905827/2020|2020-09-11|EPI_ISL_584097|Original|hCoV-19^^Michigan|Human|University of Michigan Clinical Microbiology Laboratory|Lauring Lab|Valesano|USA
>Spike|hCoV-19/England/QEUH-A02ACA/2020|2020-10-01|EPI_ISL_584945|Original|hCoV-19^^England|Human|Quadram Institute Bioscience|COVID-19 Genomics UK (COG-UK) Consortium|Alikhan|United Kingdom
>Spike|hCoV-19/England/QEUH-A0C3D4/2020|2020-10-01|EPI_ISL_584970|Original|hCoV-19^^England|Human|Quadram Institute Bioscience|COVID-19 Genomics UK (COG-UK) Consortium|Alikhan|United Kingdom
>Spike|hCoV-19/England/QEUH-A0C44A/2020|2020-10-03|EPI_ISL_584976|Original|hCoV-19^^England|Human|Quadram Institute Bioscience|COVID-19 Genomics UK (COG-UK) Consortium|Alikhan|United Kingdom
>Spike|hCoV-19/Scotland/QEUH-A0CA66/2020|2020-10-03|EPI_ISL_585612|Original|hCoV-19^^Scotland|Human|Quadram Institute Bioscience|COVID-19 Genomics UK (COG-UK) Consortium|Alikhan|United Kingdom
>Spike|hCoV-19/Wales/PHWC-16E193/2020|2020-09-28|EPI_ISL_585678|Original|hCoV-19^^Wales|Human|Wales Specialist Virology Centre Sequencing lab|COVID-19 Genomics UK (COG-UK) Consortium|Moore|United Kingdom
>Spike|hCoV-19/Wales/PHWC-16E8AD/2020|2020-10-01|EPI_ISL_585783|Original|hCoV-19^^Wales|Human|Wales Specialist Virology Centre Sequencing lab|COVID-19 Genomics UK (COG-UK) Consortium|Moore|United Kingdom
>Spike|hCoV-19/Wales/PHWC-16F2DB/2020|2020-10-05|EPI_ISL_585943|Original|hCoV-19^^Wales|Human|Wales Specialist Virology Centre Sequencing lab|COVID-19 Genomics UK (COG-UK) Consortium|Moore|United Kingdom

>Spike|hCoV-19/Wales/PHWC-16F4C6/2020|2020-10-06|EPI_ISL_585974|Original|hCoV-19^^Wales|Human|Wales Specialist Virology Centre Sequencing lab|COVID-19 Genomics UK (COG-UK) Consortium|Moore|United Kingdom
>Spike|hCoV-19/Wales/PHWC-16F587/2020|2020-10-01|EPI_ISL_585986|Original|hCoV-19^^Wales|Human|Wales Specialist Virology Centre Sequencing lab|COVID-19 Genomics UK (COG-UK) Consortium|Moore|United Kingdom
>Spike|hCoV-19/Wales/PHWC-16F7CD/2020|2020-10-01|EPI_ISL_586020|Original|hCoV-19^^Wales|Human|Wales Specialist Virology Centre Sequencing lab|COVID-19 Genomics UK (COG-UK) Consortium|Moore|United Kingdom
>Spike|hCoV-19/Wales/PHWC-47D4FF/2020|2020-10-05|EPI_ISL_586154|Original|hCoV-19^^Wales|Human|Wales Specialist Virology Centre Sequencing lab|COVID-19 Genomics UK (COG-UK) Consortium|Moore|United Kingdom
>Spike|hCoV-19/Wales/PHWC-47D94B/2020|2020-04-17|EPI_ISL_586212|Original|hCoV-19^^Wales|Human|Wales Specialist Virology Centre Sequencing lab|COVID-19 Genomics UK (COG-UK) Consortium|Moore|United Kingdom
>Spike|hCoV-19/USA/AK-PHL635/2020|2020-06-25|EPI_ISL_586247|Original|hCoV-19^^Alaska|Human|Alaska State Virology Laboratory|Alaska State Virology Laboratory|Chen|USA
>Spike|hCoV-19/Canada/ON-S1320/2020|2020-06-04|EPI_ISL_586353|Original|hCoV-19^^Ontario|Human|Toronto Invasive Bacterial Diseases Network|McMaster University|McArthur|Canada
>Spike|hCoV-19/England/ALDP-A2DBF3/2020|2020-10-05|EPI_ISL_586621|Original|hCoV-19^^England|Human|Lighthouse Lab in Alderley Park|Wellcome Sanger Institute for the COVID-19 Genomics UK (COG-UK) Consortium|Davies|United Kingdom
>Spike|hCoV-19/England/ALDP-A2EC86/2020|2020-10-05|EPI_ISL_587195|Original|hCoV-19^^England|Human|Lighthouse Lab in Alderley Park|Wellcome Sanger Institute for the COVID-19 Genomics UK (COG-UK) consortium|Davies|United Kingdom
>Spike|hCoV-19/England/QEUH-A1F480/2020|2020-10-01|EPI_ISL_587526|Original|hCoV-19^^England|Human|Lighthouse Lab in Glasgow|Wellcome Sanger Institute for the COVID-19 Genomics UK (COG-UK) consortium|Davies|United Kingdom
>Spike|hCoV-19/England/ALDP-9FD38C/2020|2020-10-02|EPI_ISL_587815|Original|hCoV-19^^England|Human|Lighthouse Lab in Alderley Park|Wellcome Sanger Institute for the COVID-19 Genomics UK (COG-UK) consortium|Davies|United Kingdom
>Spike|hCoV-19/England/ALDP-9FD762/2020|2020-10-01|EPI_ISL_587977|Original|hCoV-19^^England|Human|Lighthouse Lab in Alderley Park|Wellcome Sanger Institute for the COVID-19 Genomics UK (COG-UK) Consortium|Davies|United Kingdom
>Spike|hCoV-19/England/ALDP-9FDA87/2020|2020-10-01|EPI_ISL_588007|Original|hCoV-19^^England|Human|Lighthouse Lab in Alderley Park|Wellcome Sanger Institute for the COVID-19 Genomics UK (COG-UK) Consortium|Davies|United Kingdom
>Spike|hCoV-19/England/ALDP-9FDAE1/2020|2020-10-01|EPI_ISL_588026|Original|hCoV-19^^England|Human|Lighthouse Lab in Alderley Park|Wellcome Sanger Institute for the COVID-19 Genomics UK (COG-UK) Consortium|Davies|United Kingdom
>Spike|hCoV-19/England/QEUH-9FAF12/2020|2020-09-27|EPI_ISL_588167|Original|hCoV-19^^England|Human|Lighthouse Lab in Glasgow|Wellcome Sanger Institute for the COVID-19 Genomics UK (COG-UK) consortium|Davies|United Kingdom
>Spike|hCoV-19/England/QEUH-9FB148/2020|2020-09-29|EPI_ISL_588175|Original|hCoV-19^^England|Human|Lighthouse Lab in Glasgow|Wellcome Sanger Institute for the COVID-19 Genomics UK (COG-UK) Consortium|Davies|United Kingdom
>Spike|hCoV-19/England/QEUH-9FAEF7/2020|2020-09-27|EPI_ISL_588250|Original|hCoV-19^^England|Human|Lighthouse Lab in Glasgow|Wellcome Sanger Institute for the COVID-19 Genomics UK (COG-UK) consortium|Davies|United Kingdom
>Spike|hCoV-19/England/QEUH-9F368D/2020|2020-09-25|EPI_ISL_588717|Original|hCoV-19^^England|Human|Lighthouse Lab in Glasgow|Wellcome Sanger Institute for the COVID-19 Genomics UK (COG-UK) Consortium|Davies|United Kingdom
>Spike|hCoV-19/Wales/QEUH-9F6444/2020|2020-09-25|EPI_ISL_588909|Original|hCoV-19^^Wales|Human|Lighthouse Lab in Glasgow|Wellcome Sanger Institute for the COVID-19 Genomics UK (COG-UK) consortium|Davies|United Kingdom
>Spike|hCoV-19/England/MILK-9F2967/2020|2020-09-25|EPI_ISL_589050|Original|hCoV-19^^England|Human|Lighthouse Lab in Milton Keynes|Wellcome Sanger Institute for the COVID-19 Genomics UK (COG-UK) Consortium|Davies|United Kingdom

>Spike|hCoV-19/England/MILK-9F27F4/2020|2020-09-25|EPI_ISL_589130|Original|hCoV-19^^England|Human|Lighthouse Lab in Milton Keynes|Wellcome Sanger Institute for the COVID-19 Genomics UK (COG-UK) Consortium|Davies|United Kingdom
>Spike|hCoV-19/England/MILK-9F278B/2020|2020-09-25|EPI_ISL_589229|Original|hCoV-19^^England|Human|Lighthouse Lab in Milton Keynes|Wellcome Sanger Institute for the COVID-19 Genomics UK (COG-UK) consortium|Davies|United Kingdom
>Spike|hCoV-19/England/MILK-9F20F9/2020|2020-09-24|EPI_ISL_589262|Original|hCoV-19^^England|Human|Lighthouse Lab in Milton Keynes|Wellcome Sanger Institute for the COVID-19 Genomics UK (COG-UK) Consortium|Davies|United Kingdom
>Spike|hCoV-19/England/ALDP-9E5C04/2020|2020-09-24|EPI_ISL_589289|Original|hCoV-19^^England|Human|Lighthouse Lab in Alderley Park|Wellcome Sanger Institute for the COVID-19 Genomics UK (COG-UK) Consortium|Davies|United Kingdom
>Spike|hCoV-19/England/MILK-9ECC76/2020|2020-04-29|EPI_ISL_589526|Original|hCoV-19^^England|Human|Lighthouse Lab in Milton Keynes|Wellcome Sanger Institute for the COVID-19 Genomics UK (COG-UK) consortium|Davies|United Kingdom
>Spike|hCoV-19/Wales/MILK-9F0D7C/2020|2020-09-24|EPI_ISL_589644|Original|hCoV-19^^Wales|Human|Lighthouse Lab in Milton Keynes|Wellcome Sanger Institute for the COVID-19 Genomics UK (COG-UK) consortium|Davies|United Kingdom
>Spike|hCoV-19/England/QEUH-9B7227/2020|2020-09-04|EPI_ISL_590497|Original|hCoV-19^^England|Human|Lighthouse Lab in Glasgow|Wellcome Sanger Institute for the COVID-19 Genomics UK (COG-UK) Consortium|Davies|United Kingdom
>Spike|hCoV-19/England/QEUH-9B7904/2020|2020-09-08|EPI_ISL_590680|Original|hCoV-19^^England|Human|Lighthouse Lab in Glasgow|Wellcome Sanger Institute for the COVID-19 Genomics UK (COG-UK) consortium|Davies|United Kingdom
>Spike|hCoV-19/England/CAMB-8331B/2020|2020-04-14|EPI_ISL_591078|Original|hCoV-19^^England|Human|Department of Pathology|Wellcome Sanger Institute for the COVID-19 Genomics UK (COG-UK) consortium|Davies|United Kingdom
>Spike|hCoV-19/USA/UT-UPHL-04306_2/2020|2020-05-15|EPI_ISL_591297|Original|hCoV-19^^Utah|Human|Utah Public Health Laboratory|Utah Public Health Laboratory|Young|USA
>Spike|hCoV-19/Australia/VIC11783/2020|2020-08-11|EPI_ISL_591791|Original|hCoV-19^^Victoria|Human|Victorian Infectious Diseases Reference Laboratory (VIDRL)|VIDRL and MDU-PHL|Schultz|Australia
>Spike|hCoV-19/Australia/VIC11804/2020|2020-08-04|EPI_ISL_591792|Original|hCoV-19^^Victoria|Human|Victorian Infectious Diseases Reference Laboratory (VIDRL)|VIDRL and MDU-PHL|Schultz|Australia
>Spike|hCoV-19/Australia/VIC12942/2020|2020-07-30|EPI_ISL_591793|Original|hCoV-19^^Victoria|Human|Microbiological Diagnostic Unit - Public Health Laboratory (MDU-PHL)|MDU-PHL|Schultz|Australia
>Spike|hCoV-19/Australia/VIC14064/2020|2020-08-22|EPI_ISL_591794|Original|hCoV-19^^Victoria|Human|Microbiological Diagnostic Unit - Public Health Laboratory (MDU-PHL)|MDU-PHL|Schultz|Australia
>Spike|hCoV-19/Australia/VIC14134/2020|2020-09-09|EPI_ISL_591795|Original|hCoV-19^^Victoria|Human|Microbiological Diagnostic Unit - Public Health Laboratory (MDU-PHL)|MDU-PHL|Schultz|Australia
>Spike|hCoV-19/Australia/VIC14341/2020|2020-07-29|EPI_ISL_591796|Original|hCoV-19^^Victoria|Human|Microbiological Diagnostic Unit - Public Health Laboratory (MDU-PHL)|MDU-PHL|Schultz|Australia
>Spike|hCoV-19/Australia/VIC14888/2020|2020-09-03|EPI_ISL_592819|Original|hCoV-19^^Victoria|Human|Microbiological Diagnostic Unit - Public Health Laboratory (MDU-PHL)|MDU-PHL|Schultz|Australia
>Spike|hCoV-19/Australia/VIC15335/2020|2020-09-07|EPI_ISL_592820|Original|hCoV-19^^Victoria|Human|Victorian Infectious Diseases Reference Laboratory (VIDRL)|VIDRL and MDU-PHL|Schultz|Australia
>Spike|hCoV-19/Australia/VIC15338/2020|2020-09-07|EPI_ISL_592821|Original|hCoV-19^^Victoria|Human|Victorian Infectious Diseases Reference Laboratory (VIDRL)|VIDRL and MDU-PHL|Schultz|Australia
>Spike|hCoV-19/Australia/VIC14572/2020|2020-08-26|EPI_ISL_592822|Original|hCoV-19^^Victoria|Human|Microbiological Diagnostic Unit - Public Health Laboratory (MDU-PHL)|MDU-PHL|Schultz|Australia

>Spike|hCoV-19/Australia/VIC14598/2020|2020-08-28|EPI_ISL_592823|Original|hCoV-19^^Victoria|Human|Microbiological Diagnostic Unit - Public Health Laboratory (MDU-PHL)|MDU-PHL|Schultz|Australia
>Spike|hCoV-19/Australia/VIC14726/2020|2020-08-23|EPI_ISL_592824|Original|hCoV-19^^Victoria|Human|Victorian Infectious Diseases Reference Laboratory (VIDRL)|VIDRL and MDU-PHL|Schultz|Australia
>Spike|hCoV-19/Australia/VIC14752/2020|2020-08-12|EPI_ISL_592825|Original|hCoV-19^^Victoria|Human|Microbiological Diagnostic Unit - Public Health Laboratory (MDU-PHL)|MDU-PHL|Schultz|Australia
>Spike|hCoV-19/Australia/VIC14759/2020|2020-08-13|EPI_ISL_592826|Original|hCoV-19^^Victoria|Human|Microbiological Diagnostic Unit - Public Health Laboratory (MDU-PHL)|MDU-PHL|Schultz|Australia
>Spike|hCoV-19/Australia/VIC15098/2020|2020-08-28|EPI_ISL_592829|Original|hCoV-19^^Victoria|Human|Microbiological Diagnostic Unit - Public Health Laboratory (MDU-PHL)|MDU-PHL|Schultz|Australia
>Spike|hCoV-19/Australia/VIC15115/2020|2020-08-28|EPI_ISL_592830|Original|hCoV-19^^Victoria|Human|Microbiological Diagnostic Unit - Public Health Laboratory (MDU-PHL)|MDU-PHL|Schultz|Australia
>Spike|hCoV-19/Australia/VIC15151/2020|2020-08-14|EPI_ISL_592831|Original|hCoV-19^^Victoria|Human|Microbiological Diagnostic Unit - Public Health Laboratory (MDU-PHL)|MDU-PHL|Schultz|Australia
>Spike|hCoV-19/USA/TX-DSHS-0826/2020|2020-06-03|EPI_ISL_593569|Original|hCoV-19^^Texas|Human|Texas Department of State Health Services|Texas Department of State Health Services|Rahman|USA
>Spike|hCoV-19/USA/DE-DHSS-F918330/2020|2020-03-29|EPI_ISL_593946|Original|hCoV-19^^Delaware|Human|Delaware Public Health Lab|Delaware Public Health Lab|Bajwa|USA
>Spike|hCoV-19/USA/UT-UPHL-201018212/2020|2020-05-31|EPI_ISL_594011|Original|hCoV-19^^Utah|Human|Utah Public Health Laboratory|Utah Public Health Laboratory|Young|USA
>Spike|hCoV-19/USA/CT-Yale-375/2020|2020-04-24|EPI_ISL_594119|Original|hCoV-19^^Delaware|Human|Yale COVID-19 Biorepository|Grubaugh Lab - Yale School of Public Health|Grubaugh|USA
>Spike|hCoV-19/Sweden/20-52670/2020|2020-08-20|EPI_ISL_594148|Original|hCoV-19^^Halland|Human|Halmstad klinisk mikrobiologi|The Public Health Agency of Sweden|Svartstr\u00f6m|Sweden
>Spike|hCoV-19/Sweden/20-08987/2020|2020-09-16|EPI_ISL_594150|Original|hCoV-19^^Ostergotland|Human|Klinsisk mikrobiologi Linkoping|The Public Health Agency of Sweden|Svartstr\u00f6m|Sweden
>Spike|hCoV-19/Sweden/20-51969/2020|2020-06-01|EPI_ISL_594154|Original|hCoV-19^^Sormland|Human|Unilabs Eskilstuna|The Public Health Agency of Sweden|Svartstr\u00f6m|Sweden
>Spike|hCoV-19/USA/MI-MDHHS-SC22097/2020|2020-04-23|EPI_ISL_594209|Original|hCoV-19^^Michigan|Human|Michigan Department of Health and Human Services|Michigan Department of Health and Human Services|Jacob|USA
>Spike|hCoV-19/USA/UT-UPHL-201007266/2020|2020-06-22|EPI_ISL_594274|Original|hCoV-19^^Utah|Human|Utah Public Health Laboratory|Utah Public Health Laboratory|Young|USA
>Spike|hCoV-19/England/CAMC-9ECB79/2020|2020-09-23|EPI_ISL_594642|Original|hCoV-19^^England|Human|Oxford Viromics|COVID-19 Genomics UK (COG-UK) Consortium|Golubchik|United Kingdom
>Spike|hCoV-19/England/NORW-F058C/2020|2020-10-16|EPI_ISL_595183|Original|hCoV-19^^England|Human|Quadram Institute Bioscience|COVID-19 Genomics UK (COG-UK) Consortium|Alikhan|United Kingdom
>Spike|hCoV-19/England/NORW-F0CC3/2020|2020-00-00|EPI_ISL_595212|Original|hCoV-19^^England|Human|Quadram Institute Bioscience|COVID-19 Genomics UK (COG-UK) Consortium|Alikhan|United Kingdom
>Spike|hCoV-19/Wales/PHWC-47DF1C/2020|2020-09-28|EPI_ISL_595380|Original|hCoV-19^^Wales|Human|Originating lab|COVID-19 Genomics UK (COG-UK) Consortium|Moore|United Kingdom
>Spike|hCoV-19/Wales/PHWC-47E24F/2020|2020-09-27|EPI_ISL_595427|Original|hCoV-19^^Wales|Human|Originating lab|COVID-19 Genomics UK (COG-UK) Consortium|Moore|United Kingdom

>Spike|hCoV-19/Wales/PHWC-47E2F4/2020|2020-10-02|EPI_ISL_595436|Original|hCoV-19^^Wales|Human|Originating lab|COVID-19 Genomics UK (COG-UK) Consortium|Moore|United Kingdom
>Spike|hCoV-19/Wales/PHWC-47EBDB/2020|2020-10-07|EPI_ISL_595566|Original|hCoV-19^^Wales|Human|Originating lab|COVID-19 Genomics UK (COG-UK) Consortium|Moore|United Kingdom
>Spike|hCoV-19/Scotland/QEUH-9F70C7/2020|2020-09-27|EPI_ISL_595660|Original|hCoV-19^^Scotland|Human|Oxford Viromics|COVID-19 Genomics UK (COG-UK) Consortium|Golubchik|United Kingdom
>Spike|hCoV-19/England/QEUH-A31039/2020|2020-10-05|EPI_ISL_595855|Original|hCoV-19^^England|Human|Quadram Institute Bioscience|COVID-19 Genomics UK (COG-UK) Consortium|Alikhan|United Kingdom
>Spike|hCoV-19/England/QEUH-A31321/2020|2020-10-05|EPI_ISL_595879|Original|hCoV-19^^England|Human|Quadram Institute Bioscience|COVID-19 Genomics UK (COG-UK) Consortium|Alikhan|United Kingdom
>Spike|hCoV-19/England/QEUH-A3138B/2020|2020-10-05|EPI_ISL_595882|Original|hCoV-19^^England|Human|Quadram Institute Bioscience|COVID-19 Genomics UK (COG-UK) Consortium|Alikhan|United Kingdom
>Spike|hCoV-19/England/QEUH-A4CA6A/2020|2020-10-05|EPI_ISL_596012|Original|hCoV-19^^England|Human|Quadram Institute Bioscience|COVID-19 Genomics UK (COG-UK) Consortium|Alikhan|United Kingdom
>Spike|hCoV-19/Scotland/QEUH-A638DC/2020|2020-10-09|EPI_ISL_596160|Original|hCoV-19^^Scotland|Human|Quadram Institute Bioscience|COVID-19 Genomics UK (COG-UK) Consortium|Alikhan|United Kingdom
>Spike|hCoV-19/Scotland/QEUH-A639BB/2020|2020-10-09|EPI_ISL_596174|Original|hCoV-19^^Scotland|Human|Quadram Institute Bioscience|COVID-19 Genomics UK (COG-UK) Consortium|Alikhan|United Kingdom
>Spike|hCoV-19/Russia/SPE-RII-34613S/2020|2020-09-17|EPI_ISL_596236|Original|hCoV-19^^Saint Petersburg|Human|WHO National Influenza Centre Russian Federation|WHO National Influenza Centre Russian Federation|Komissarov|Russia
>Spike|hCoV-19/Russia/SPE-RII-35589S/2020|2020-09-28|EPI_ISL_596249|Original|hCoV-19^^Saint Petersburg|Human|WHO National Influenza Centre Russian Federation|WHO National Influenza Centre Russian Federation|Komissarov|Russia
>Spike|hCoV-19/Russia/SPE-RII-MH1049S/2020|2020-09-06|EPI_ISL_596256|Original|hCoV-19^^Saint Petersburg|Human|HELIX LCC|WHO National Influenza Centre Russian Federation|Komissarov|Russia
>Spike|hCoV-19/Russia/SPE-RII-MH1061S/2020|2020-09-06|EPI_ISL_596261|Original|hCoV-19^^Saint Petersburg|Human|HELIX LCC|WHO National Influenza Centre Russian Federation|Komissarov|Russia
>Spike|hCoV-19/Russia/SPE-RII-MH1512S/2020|2020-09-11|EPI_ISL_596285|Original|hCoV-19^^Saint Petersburg|Human|HELIX LCC|WHO National Influenza Centre Russian Federation|Komissarov|Russia
>Spike|hCoV-19/Russia/SPE-RII-MH1515S/2020|2020-09-11|EPI_ISL_596286|Original|hCoV-19^^Saint Petersburg|Human|HELIX LCC|WHO National Influenza Centre Russian Federation|Komissarov|Russia
>Spike|hCoV-19/Russia/SVE-RII-MH1633S/2020|2020-09-12|EPI_ISL_596303|Original|hCoV-19^^Sverdlovsk|Human|HELIX LCC|WHO National Influenza Centre Russian Federation|Komissarov|Russia
>Spike|hCoV-19/Russia/SPE-RII-MH977S/2020|2020-09-04|EPI_ISL_596369|Original|hCoV-19^^Saint Petersburg|Human|HELIX LCC|WHO National Influenza Centre Russian Federation|Komissarov|Russia
>Spike|hCoV-19/Russia/SPE-RII-MH981S/2020|2020-09-04|EPI_ISL_596370|Original|hCoV-19^^Saint Petersburg|Human|HELIX LCC|WHO National Influenza Centre Russian Federation|Komissarov|Russia
>Spike|hCoV-19/Russia/SPE-RII-MH982S/2020|2020-09-04|EPI_ISL_596371|Original|hCoV-19^^Saint Petersburg|Human|HELIX LCC|WHO National Influenza Centre Russian Federation|Komissarov|Russia
>Spike|hCoV-19/Palestine/35/2020|2020-03-04|EPI_ISL_596568|Original|hCoV-19^^Tulkarem|Human|Palestinian Ministry of Health|Molecular Genetics Lab|van Dorp|Palestine

>Spike|hCoV-19/England/MILK-A46945/2020|2020-10-06|EPI_ISL_598249|Original|hCoV-19^^England|Human|Lighthouse Lab in Milton Keynes|Wellcome Sanger Institute for the COVID-19 Genomics UK (COG-UK) Consortium|Davies|United Kingdom
>Spike|hCoV-19/England/MILK-A47AC9/2020|2020-10-06|EPI_ISL_598330|Original|hCoV-19^^England|Human|Lighthouse Lab in Milton Keynes|Wellcome Sanger Institute for the COVID-19 Genomics UK (COG-UK) Consortium|Davies|United Kingdom
>Spike|hCoV-19/England/MILK-A47188/2020|2020-10-06|EPI_ISL_598504|Original|hCoV-19^^England|Human|Lighthouse Lab in Milton Keynes|Wellcome Sanger Institute for the COVID-19 Genomics UK (COG-UK) consortium|Davies|United Kingdom
>Spike|hCoV-19/England/MILK-A4573D/2020|2020-10-06|EPI_ISL_598687|Original|hCoV-19^^England|Human|Lighthouse Lab in Milton Keynes|Wellcome Sanger Institute for the COVID-19 Genomics UK (COG-UK) consortium|Davies|United Kingdom
>Spike|hCoV-19/Scotland/QEUH-A1F8A2/2020|2020-10-01|EPI_ISL_598970|Original|hCoV-19^^Scotland|Human|Lighthouse Lab in Glasgow|Wellcome Sanger Institute for the COVID-19 Genomics UK (COG-UK) Consortium|Davies|United Kingdom
>Spike|hCoV-19/Scotland/QEUH-A1F945/2020|2020-10-01|EPI_ISL_599010|Original|hCoV-19^^Scotland|Human|Lighthouse Lab in Glasgow|Wellcome Sanger Institute for the COVID-19 Genomics UK (COG-UK) Consortium|Davies|United Kingdom
>Spike|hCoV-19/England/QEUH-A0BFA4/2020|2020-10-03|EPI_ISL_599166|Original|hCoV-19^^England|Human|Lighthouse Lab in Glasgow|Wellcome Sanger Institute for the COVID-19 Genomics UK (COG-UK) Consortium|Davies|United Kingdom
>Spike|hCoV-19/England/QEUH-A0BF95/2020|2020-10-03|EPI_ISL_599194|Original|hCoV-19^^England|Human|Lighthouse Lab in Glasgow|Wellcome Sanger Institute for the COVID-19 Genomics UK (COG-UK) Consortium|Davies|United Kingdom
>Spike|hCoV-19/Scotland/QEUH-A0BCCB/2020|2020-10-03|EPI_ISL_599378|Original|hCoV-19^^Scotland|Human|Lighthouse Lab in Glasgow|Wellcome Sanger Institute for the COVID-19 Genomics UK (COG-UK) consortium|Davies|United Kingdom
>Spike|hCoV-19/England/QEUH-9DCCE2/2020|2020-09-21|EPI_ISL_599627|Original|hCoV-19^^England|Human|Lighthouse Lab in Glasgow|Wellcome Sanger Institute for the COVID-19 Genomics UK (COG-UK) Consortium|Davies|United Kingdom
>Spike|hCoV-19/England/QEUH-9FFB0A/2020|2020-09-30|EPI_ISL_600139|Original|hCoV-19^^England|Human|Lighthouse Lab in Glasgow|Wellcome Sanger Institute for the COVID-19 Genomics UK (COG-UK) Consortium|Davies|United Kingdom
>Spike|hCoV-19/Bangladesh/202/2020|2020-04-09|EPI_ISL_600433|Original|hCoV-19^^Barishal|Human|Institute of Epidemiology Disease Control And Research|Institute for Developing Science and Health Initiatives|Cowley|Bangladesh
>Spike|hCoV-19/Bangladesh/203/2020|2020-04-09|EPI_ISL_600434|Original|hCoV-19^^Barishal|Human|Institute of Epidemiology Disease Control And Research|Institute for Developing Science and Health Initiatives|Cowley|Bangladesh
>Spike|hCoV-19/Bangladesh/G-10/2020|2020-04-14|EPI_ISL_600444|Original|hCoV-19^^Dhaka|Human|Institute of Epidemiology Disease Control And Research|Institute for Developing Science and Health Initiatives|Cowley|Bangladesh
>Spike|hCoV-19/Bangladesh/G-127/2020|2020-07-01|EPI_ISL_600471|Original|hCoV-19^^Dhaka|Human|Institute of Epidemiology Disease Control And Research|Institute for Developing Science and Health Initiatives|Cowley|Bangladesh
>Spike|hCoV-19/Bangladesh/G-129/2020|2020-07-02|EPI_ISL_600473|Original|hCoV-19^^Dhaka|Human|Institute of Epidemiology Disease Control And Research|Institute for Developing Science and Health Initiatives|Cowley|Bangladesh
>Spike|hCoV-19/Bangladesh/G-130/2020|2020-07-01|EPI_ISL_600476|Original|hCoV-19^^Dhaka|Human|Institute of Epidemiology Disease Control And Research|Institute for Developing Science and Health Initiatives|Cowley|Bangladesh
>Spike|hCoV-19/Bangladesh/G-14/2020|2020-04-14|EPI_ISL_600484|Original|hCoV-19^^Dhaka|Human|Institute of Epidemiology Disease Control And Research|Institute for Developing Science and Health Initiatives|Cowley|Bangladesh
>Spike|hCoV-19/Bangladesh/G-31/2020|2020-03-26|EPI_ISL_600559|Original|hCoV-19^^Dhaka|Human|Institute of Epidemiology Disease Control And Research|Institute for Developing Science and Health Initiatives|Cowley|Bangladesh
>Spike|hCoV-19/Scotland/QEUH-9F2D4D/2020|2020-09-25|EPI_ISL_600824|Original|hCoV-19^^Scotland|Human|Lighthouse Lab in Glasgow|Wellcome Sanger Institute for the COVID-19 Genomics UK (COG-UK) Consortium|Davies|United Kingdom

>Spike|hCoV-19/Scotland/QEUH-9DC918/2020|2020-09-22|EPI_ISL_601150|Original|hCoV-19^^Scotland|Human|Lighthouse Lab in Glasgow|Wellcome Sanger Institute for the COVID-19 Genomics UK (COG-UK) Consortium|Davies|United Kingdom
>Spike|hCoV-19/Scotland/QEUH-9DEA31/2020|2020-09-21|EPI_ISL_601192|Original|hCoV-19^^Scotland|Human|Lighthouse Lab in Glasgow|Wellcome Sanger Institute for the COVID-19 Genomics UK (COG-UK) Consortium|Davies|United Kingdom
>Spike|hCoV-19/England/QEUH-9DBDE0/2020|2020-09-21|EPI_ISL_601262|Original|hCoV-19^^England|Human|Lighthouse Lab in Glasgow|Wellcome Sanger Institute for the COVID-19 Genomics UK (COG-UK) Consortium|Davies|United Kingdom
>Spike|hCoV-19/Wales/MILK-9E08E7/2020|2020-09-21|EPI_ISL_601290|Original|hCoV-19^^Wales|Human|Lighthouse Lab in Milton Keynes|Wellcome Sanger Institute for the COVID-19 Genomics UK (COG-UK) Consortium|Davies|United Kingdom
>Spike|hCoV-19/Wales/MILK-9E0225/2020|2020-09-19|EPI_ISL_601399|Original|hCoV-19^^Wales|Human|Lighthouse Lab in Milton Keynes|Wellcome Sanger Institute for the COVID-19 Genomics UK (COG-UK) Consortium|Davies|United Kingdom
>Spike|hCoV-19/Scotland/QEUH-9D098D/2020|2020-09-16|EPI_ISL_601797|Original|hCoV-19^^Scotland|Human|Lighthouse Lab in Glasgow|Wellcome Sanger Institute for the COVID-19 Genomics UK (COG-UK) Consortium|Davies|United Kingdom
>Spike|hCoV-19/Scotland/QEUH-9D0E51/2020|2020-09-16|EPI_ISL_601834|Original|hCoV-19^^Scotland|Human|Lighthouse Lab in Glasgow|Wellcome Sanger Institute for the COVID-19 Genomics UK (COG-UK) Consortium|Davies|United Kingdom
>Spike|hCoV-19/Scotland/QEUH-9CECE3/2020|2020-09-17|EPI_ISL_601950|Original|hCoV-19^^Scotland|Human|Lighthouse Lab in Glasgow|Wellcome Sanger Institute for the COVID-19 Genomics UK (COG-UK) Consortium|Davies|United Kingdom
>Spike|hCoV-19/Scotland/QEUH-9CF532/2020|2020-09-17|EPI_ISL_602006|Original|hCoV-19^^Scotland|Human|Lighthouse Lab in Glasgow|Wellcome Sanger Institute for the COVID-19 Genomics UK (COG-UK) Consortium|Davies|United Kingdom
>Spike|hCoV-19/Scotland/QEUH-9CE603/2020|2020-09-17|EPI_ISL_602015|Original|hCoV-19^^Scotland|Human|Lighthouse Lab in Glasgow|Wellcome Sanger Institute for the COVID-19 Genomics UK (COG-UK) Consortium|Davies|United Kingdom
>Spike|hCoV-19/Scotland/QEUH-9B511D/2020|2020-09-07|EPI_ISL_602077|Original|hCoV-19^^Scotland|Human|Lighthouse Lab in Glasgow|Wellcome Sanger Institute for the COVID-19 Genomics UK (COG-UK) Consortium|Davies|United Kingdom
>Spike|hCoV-19/Scotland/QEUH-9B4B2F/2020|2020-09-02|EPI_ISL_602083|Original|hCoV-19^^Scotland|Human|Lighthouse Lab in Glasgow|Wellcome Sanger Institute for the COVID-19 Genomics UK (COG-UK) Consortium|Davies|United Kingdom
>Spike|hCoV-19/Scotland/QEUH-9B739D/2020|2020-09-09|EPI_ISL_602090|Original|hCoV-19^^Scotland|Human|Lighthouse Lab in Glasgow|Wellcome Sanger Institute for the COVID-19 Genomics UK (COG-UK) Consortium|Davies|United Kingdom
>Spike|hCoV-19/Scotland/QEUH-9B73BB/2020|2020-09-09|EPI_ISL_602091|Original|hCoV-19^^Scotland|Human|Lighthouse Lab in Glasgow|Wellcome Sanger Institute for the COVID-19 Genomics UK (COG-UK) Consortium|Davies|United Kingdom
>Spike|hCoV-19/Scotland/QEUH-9B73AC/2020|2020-09-09|EPI_ISL_602102|Original|hCoV-19^^Scotland|Human|Lighthouse Lab in Glasgow|Wellcome Sanger Institute for the COVID-19 Genomics UK (COG-UK) Consortium|Davies|United Kingdom
>Spike|hCoV-19/Scotland/QEUH-9B7403/2020|2020-09-09|EPI_ISL_602106|Original|hCoV-19^^Scotland|Human|Lighthouse Lab in Glasgow|Wellcome Sanger Institute for the COVID-19 Genomics UK (COG-UK) Consortium|Davies|United Kingdom
>Spike|hCoV-19/Scotland/QEUH-9B6ECA/2020|2020-09-09|EPI_ISL_602109|Original|hCoV-19^^Scotland|Human|Lighthouse Lab in Glasgow|Wellcome Sanger Institute for the COVID-19 Genomics UK (COG-UK) Consortium|Davies|United Kingdom
>Spike|hCoV-19/Scotland/QEUH-9B73D9/2020|2020-09-09|EPI_ISL_602122|Original|hCoV-19^^Scotland|Human|Lighthouse Lab in Glasgow|Wellcome Sanger Institute for the COVID-19 Genomics UK (COG-UK) Consortium|Davies|United Kingdom
>Spike|hCoV-19/USA/TX-DSHS-0930/2020|2020-06-15|EPI_ISL_602228|Original|hCoV-19^^Texas|Human|Texas Department of State Health Services|Texas Department of State Health Services|Rahman|USA
>Spike|hCoV-19/USA/MD-MDH-0253/2020|2020-10-05|EPI_ISL_602276|Original|hCoV-19^^Maryland|Human|MD PHL|MD PHL|Keller|USA

>Spike|hCoV-19/Russia/SVE-RII-MH1691S/2020|2020-09-13|EPI_ISL_602342|Original|hCoV-19^^Sverdlovsk|Human|HELIX LLC|WHO National Influenza Centre Russian Federation|Komissarov|Russia
>Spike|hCoV-19/Russia/PER-RII-MH1951S/2020|2020-09-16|EPI_ISL_602376|Original|hCoV-19^^Perm|Human|HELIX LLC|WHO National Influenza Centre Russian Federation|Komissarov|Russia
>Spike|hCoV-19/Russia/KLU-RII-MH2795S/2020|2020-09-16|EPI_ISL_602457|Original|hCoV-19^^Kaluga|Human|HELIX LLC|WHO National Influenza Centre Russian Federation|Komissarov|Russia
>Spike|hCoV-19/Russia/KEM-RII-MH2796S/2020|2020-09-25|EPI_ISL_602458|Original|hCoV-19^^Kemerovo|Human|HELIX LLC|WHO National Influenza Centre Russian Federation|Komissarov|Russia
>Spike|hCoV-19/Russia/MOS-RII-MH2797S/2020|2020-09-20|EPI_ISL_602459|Original|hCoV-19^^Moscow Region|Human|HELIX LLC|WHO National Influenza Centre Russian Federation|Komissarov|Russia
>Spike|hCoV-19/USA/WA-UW-251/2020|2020-03-09|EPI_ISL_603260|Original|hCoV-19^^Washington|Human|UW Virology Lab|UW Virology Lab|Roychoudhury|USA
>Spike|hCoV-19/USA/UT-QDX-1804/2020|2020-03-14|EPI_ISL_604131|Original|hCoV-19^^Utah|Human|Quest Diagnostics|Quest Diagnostics|Kagan|USA
>Spike|hCoV-19/USA/NY-QDX-2237/2020|2020-03-15|EPI_ISL_604571|Original|hCoV-19^^New York|Human|Quest Diagnostics|Quest Diagnostics|Kagan|USA
>Spike|hCoV-19/USA/TX-QDX-2554/2020|2020-03-14|EPI_ISL_604843|Original|hCoV-19^^Texas|Human|Quest Diagnostics|Quest Diagnostics|Kagan|USA
>Spike|hCoV-19/USA/CA-QDX-2615/2020|2020-03-15|EPI_ISL_604895|Original|hCoV-19^^California|Human|Quest Diagnostics|Quest Diagnostics|Kagan|USA
>Spike|hCoV-19/USA/UT-QDX-2589/2020|2020-03-14|EPI_ISL_604965|Original|hCoV-19^^Utah|Human|Quest Diagnostics|Quest Diagnostics|Kagan|USA
>Spike|hCoV-19/USA/PR-B2CY/2020|2020-08-02|EPI_ISL_605147|Original|hCoV-19^^Puerto Rico|Human|University of Miami Immunology and Histocompatibility Laboratory|University of Miami Immunology and Histocompatibility Laboratory|Margolles-Clark|USA
>Spike|hCoV-19/India/DL-ILBS-279M/2020|2020-05-01|EPI_ISL_605799|Original|hCoV-19^^Delhi|Human|Clinical Virology Laboratory|ILBS - IGIB|Rooge|India
>Spike|hCoV-19/India/DL-ILBS-9070D/2020|2020-05-01|EPI_ISL_605802|Original|hCoV-19^^Delhi|Human|Clinical Virology Laboratory|ILBS - IGIB|Rooge|India
>Spike|hCoV-19/England/ALDP-A870BC/2020|2020-10-17|EPI_ISL_605991|Original|hCoV-19^^England|Human|Lighthouse Lab in Alderley Park|Wellcome Sanger Institute for the COVID-19 Genomics UK (COG-UK) consortium|Davies|United Kingdom
>Spike|hCoV-19/England/ALDP-A87289/2020|2020-10-17|EPI_ISL_606097|Original|hCoV-19^^England|Human|Lighthouse Lab in Alderley Park|Wellcome Sanger Institute for the COVID-19 Genomics UK (COG-UK) consortium|Davies|United Kingdom
>Spike|hCoV-19/England/MILK-A7AE58/2020|2020-10-16|EPI_ISL_606311|Original|hCoV-19^^England|Human|Lighthouse Lab in Milton Keynes|Wellcome Sanger Institute for the COVID-19 Genomics UK (COG-UK) Consortium|Davies|United Kingdom
>Spike|hCoV-19/England/MILK-A7AB06/2020|2020-10-15|EPI_ISL_606314|Original|hCoV-19^^England|Human|Lighthouse Lab in Milton Keynes|Wellcome Sanger Institute for the COVID-19 Genomics UK (COG-UK) Consortium|Davies|United Kingdom
>Spike|hCoV-19/England/MILK-A7DB03/2020|2020-10-13|EPI_ISL_606353|Original|hCoV-19^^England|Human|Lighthouse Lab in Milton Keynes|Wellcome Sanger Institute for the COVID-19 Genomics UK (COG-UK) Consortium|Davies|United Kingdom
>Spike|hCoV-19/England/MILK-A7AE0D/2020|2020-10-16|EPI_ISL_606462|Original|hCoV-19^^England|Human|Lighthouse Lab in Milton Keynes|Wellcome Sanger Institute for the COVID-19 Genomics UK (COG-UK) Consortium|Davies|United Kingdom
>Spike|hCoV-19/England/MILK-A7AE1C/2020|2020-10-16|EPI_ISL_606797|Original|hCoV-19^^England|Human|Lighthouse Lab in Milton Keynes|Wellcome Sanger Institute for the COVID-19 Genomics UK (COG-UK) Consortium|Davies|United Kingdom
>Spike|hCoV-19/England/ALDP-A6E6E6/2020|2020-10-15|EPI_ISL_606960|Original|hCoV-19^^England|Human|Lighthouse Lab in Alderley Park|Wellcome Sanger Institute for the COVID-19 Genomics UK (COG-UK) Consortium|Davies|United Kingdom

>Spike|hCoV-19/England/ALDP-A6F2C3/2020|2020-10-15|EPI_ISL_607155|Original|hCoV-19^^England|Human|Lighthouse Lab in Alderley Park|Wellcome Sanger Institute for the COVID-19 Genomics UK (COG-UK) consortium|Davies|United Kingdom
>Spike|hCoV-19/England/ALDP-A6DDE2/2020|2020-10-15|EPI_ISL_607189|Original|hCoV-19^^England|Human|Lighthouse Lab in Alderley Park|Wellcome Sanger Institute for the COVID-19 Genomics UK (COG-UK) Consortium|Davies|United Kingdom
>Spike|hCoV-19/England/MILK-A7B552/2020|2020-10-15|EPI_ISL_607516|Original|hCoV-19^^England|Human|Lighthouse Lab in Milton Keynes|Wellcome Sanger Institute for the COVID-19 Genomics UK (COG-UK) Consortium|Davies|United Kingdom
>Spike|hCoV-19/England/MILK-A7BB7E/2020|2020-10-15|EPI_ISL_607550|Original|hCoV-19^^England|Human|Lighthouse Lab in Milton Keynes|Wellcome Sanger Institute for the COVID-19 Genomics UK (COG-UK) Consortium|Davies|United Kingdom
>Spike|hCoV-19/England/MILK-A7BCC6/2020|2020-10-15|EPI_ISL_607587|Original|hCoV-19^^England|Human|Lighthouse Lab in Milton Keynes|Wellcome Sanger Institute for the COVID-19 Genomics UK (COG-UK) Consortium|Davies|United Kingdom
>Spike|hCoV-19/England/ALDP-A6D201/2020|2020-10-14|EPI_ISL_608310|Original|hCoV-19^^England|Human|Lighthouse Lab in Alderley Park|Wellcome Sanger Institute for the COVID-19 Genomics UK (COG-UK) Consortium|Davies|United Kingdom
>Spike|hCoV-19/England/MILK-A6B476/2020|2020-10-09|EPI_ISL_608834|Original|hCoV-19^^England|Human|Lighthouse Lab in Milton Keynes|Wellcome Sanger Institute for the COVID-19 Genomics UK (COG-UK) Consortium|Davies|United Kingdom
>Spike|hCoV-19/England/CAMC-A42378/2020|2020-10-05|EPI_ISL_609472|Original|hCoV-19^^England|Human|Lighthouse Lab in Cambridge|Wellcome Sanger Institute for the COVID-19 Genomics UK (COG-UK) Consortium|Davies|United Kingdom
>Spike|hCoV-19/England/CAMC-A3ED44/2020|2020-10-05|EPI_ISL_609640|Original|hCoV-19^^England|Human|Lighthouse Lab in Cambridge|Wellcome Sanger Institute for the COVID-19 Genomics UK (COG-UK) consortium|Davies|United Kingdom
>Spike|hCoV-19/England/CAMC-A82989/2020|2020-10-12|EPI_ISL_610782|Original|hCoV-19^^England|Human|Lighthouse Lab in Cambridge|Wellcome Sanger Institute for the COVID-19 Genomics UK (COG-UK) consortium|Davies|United Kingdom
>Spike|hCoV-19/England/QEUH-A9FB65/2020|2020-10-20|EPI_ISL_611305|Original|hCoV-19^^England|Human|Lighthouse Lab in Glasgow|Wellcome Sanger Institute for the COVID-19 Genomics UK (COG-UK) consortium|Davies|United Kingdom
>Spike|hCoV-19/USA/UT-UPHL-201012055/2020|2020-06-22|EPI_ISL_611502|Original|hCoV-19^^Utah|Human|Utah Public Health Laboratory|Utah Public Health Laboratory|Young|USA
>Spike|hCoV-19/Wales/PHWC-4823EB/2020|2020-10-20|EPI_ISL_611625|Original|hCoV-19^^Wales|Human|Wales Specialist Virology Centre Sequencing lab|COVID-19 Genomics UK (COG-UK) Consortium|Moore|United Kingdom
>Spike|hCoV-19/England/MILK-A191AD/2020|2020-10-03|EPI_ISL_612117|Original|hCoV-19^^England|Human|Wales Specialist Virology Centre Sequencing lab|COVID-19 Genomics UK (COG-UK) Consortium|Moore|United Kingdom
>Spike|hCoV-19/Wales/PHWC-480110/2020|2020-10-07|EPI_ISL_612162|Original|hCoV-19^^Wales|Human|Wales Specialist Virology Centre Sequencing lab|COVID-19 Genomics UK (COG-UK) Consortium|Moore|United Kingdom
>Spike|hCoV-19/Wales/PHWC-480BB8/2020|2020-10-07|EPI_ISL_612171|Original|hCoV-19^^Wales|Human|Wales Specialist Virology Centre Sequencing lab|COVID-19 Genomics UK (COG-UK) Consortium|Moore|United Kingdom
>Spike|hCoV-19/Wales/PHWC-482406/2020|2020-10-20|EPI_ISL_612199|Original|hCoV-19^^Wales|Human|Wales Specialist Virology Centre Sequencing lab|COVID-19 Genomics UK (COG-UK) Consortium|Moore|United Kingdom
>Spike|hCoV-19/Wales/PHWC-481A32/2020|2020-10-19|EPI_ISL_612202|Original|hCoV-19^^Wales|Human|Wales Specialist Virology Centre Sequencing lab|COVID-19 Genomics UK (COG-UK) Consortium|Moore|United Kingdom
>Spike|hCoV-19/England/MILK-A192AA/2020|2020-10-01|EPI_ISL_612496|Original|hCoV-19^^England|Human|Wales Specialist Virology Centre Sequencing lab|COVID-19 Genomics UK (COG-UK) Consortium|Moore|United Kingdom
>Spike|hCoV-19/Wales/PHWC-47F202/2020|2020-10-14|EPI_ISL_612645|Original|hCoV-19^^Wales|Human|Wales Specialist Virology Centre Sequencing lab|COVID-19 Genomics UK (COG-UK) Consortium|Moore|United Kingdom

>Spike|hCoV-19/Wales/PHWC-47F27B/2020|2020-10-13|EPI_ISL_612652|Original|hCoV-19^^Wales|Human|Wales Specialist Virology Centre Sequencing lab|COVID-19 Genomics UK (COG-UK) Consortium|Moore|United Kingdom
>Spike|hCoV-19/Wales/PHWC-47F7D6/2020|2020-10-07|EPI_ISL_612726|Original|hCoV-19^^Wales|Human|Wales Specialist Virology Centre Sequencing lab|COVID-19 Genomics UK (COG-UK) Consortium|Moore|United Kingdom
>Spike|hCoV-19/Wales/PHWC-47F7E5/2020|2020-10-07|EPI_ISL_612727|Original|hCoV-19^^Wales|Human|Wales Specialist Virology Centre Sequencing lab|COVID-19 Genomics UK (COG-UK) Consortium|Moore|United Kingdom
>Spike|hCoV-19/Wales/PHWC-47FA37/2020|2020-10-13|EPI_ISL_612761|Original|hCoV-19^^Wales|Human|Wales Specialist Virology Centre Sequencing lab|COVID-19 Genomics UK (COG-UK) Consortium|Moore|United Kingdom
>Spike|hCoV-19/Wales/PHWC-47FC5F/2020|2020-10-08|EPI_ISL_612791|Original|hCoV-19^^Wales|Human|Wales Specialist Virology Centre Sequencing lab|COVID-19 Genomics UK (COG-UK) Consortium|Moore|United Kingdom
>Spike|hCoV-19/Wales/PHWC-47FC7D/2020|2020-10-10|EPI_ISL_612793|Original|hCoV-19^^Wales|Human|Wales Specialist Virology Centre Sequencing lab|COVID-19 Genomics UK (COG-UK) Consortium|Moore|United Kingdom
>Spike|hCoV-19/Wales/PHWC-4800D7/2020|2020-10-08|EPI_ISL_612850|Original|hCoV-19^^Wales|Human|Wales Specialist Virology Centre Sequencing lab|COVID-19 Genomics UK (COG-UK) Consortium|Moore|United Kingdom
>Spike|hCoV-19/Wales/PHWC-480329/2020|2020-10-17|EPI_ISL_612874|Original|hCoV-19^^Wales|Human|Wales Specialist Virology Centre Sequencing lab|COVID-19 Genomics UK (COG-UK) Consortium|Moore|United Kingdom
>Spike|hCoV-19/Wales/PHWC-4803FC/2020|2020-10-11|EPI_ISL_612883|Original|hCoV-19^^Wales|Human|Wales Specialist Virology Centre Sequencing lab|COVID-19 Genomics UK (COG-UK) Consortium|Moore|United Kingdom
>Spike|hCoV-19/Wales/PHWC-4806D5/2020|2020-10-08|EPI_ISL_612920|Original|hCoV-19^^Wales|Human|Wales Specialist Virology Centre Sequencing lab|COVID-19 Genomics UK (COG-UK) Consortium|Moore|United Kingdom
>Spike|hCoV-19/Wales/PHWC-480866/2020|2020-10-12|EPI_ISL_612941|Original|hCoV-19^^Wales|Human|Wales Specialist Virology Centre Sequencing lab|COVID-19 Genomics UK (COG-UK) Consortium|Moore|United Kingdom
>Spike|hCoV-19/Wales/PHWC-480B7C/2020|2020-10-01|EPI_ISL_612978|Original|hCoV-19^^Wales|Human|Wales Specialist Virology Centre Sequencing lab|COVID-19 Genomics UK (COG-UK) Consortium|Moore|United Kingdom
>Spike|hCoV-19/Wales/PHWC-481425/2020|2020-10-18|EPI_ISL_613069|Original|hCoV-19^^Wales|Human|Wales Specialist Virology Centre Sequencing lab|COVID-19 Genomics UK (COG-UK) Consortium|Moore|United Kingdom
>Spike|hCoV-19/Wales/PHWC-481A14/2020|2020-10-21|EPI_ISL_613137|Original|hCoV-19^^Wales|Human|Wales Specialist Virology Centre Sequencing lab|COVID-19 Genomics UK (COG-UK) Consortium|Moore|United Kingdom
>Spike|hCoV-19/Wales/PHWC-48214B/2020|2020-10-21|EPI_ISL_613201|Original|hCoV-19^^Wales|Human|Wales Specialist Virology Centre Sequencing lab|COVID-19 Genomics UK (COG-UK) Consortium|Moore|United Kingdom
>Spike|hCoV-19/Wales/PHWC-482239/2020|2020-10-20|EPI_ISL_613206|Original|hCoV-19^^Wales|Human|Wales Specialist Virology Centre Sequencing lab|COVID-19 Genomics UK (COG-UK) Consortium|Moore|United Kingdom
>Spike|hCoV-19/Wales/PHWC-4825A9/2020|2020-10-20|EPI_ISL_613230|Original|hCoV-19^^Wales|Human|Wales Specialist Virology Centre Sequencing lab|COVID-19 Genomics UK (COG-UK) Consortium|Moore|United Kingdom
>Spike|hCoV-19/Wales/PHWC-4825D6/2020|2020-10-19|EPI_ISL_613231|Original|hCoV-19^^Wales|Human|Wales Specialist Virology Centre Sequencing lab|COVID-19 Genomics UK (COG-UK) Consortium|Moore|United Kingdom
>Spike|hCoV-19/Wales/PHWC-4826D3/2020|2020-10-21|EPI_ISL_613237|Original|hCoV-19^^Wales|Human|Wales Specialist Virology Centre Sequencing lab|COVID-19 Genomics UK (COG-UK) Consortium|Moore|United Kingdom
>Spike|hCoV-19/Wales/PHWC-482819/2020|2020-10-21|EPI_ISL_613246|Original|hCoV-19^^Wales|Human|Wales Specialist Virology Centre Sequencing lab|COVID-19 Genomics UK (COG-UK) Consortium|Moore|United Kingdom

>Spike|hCoV-19/Wales/PHWC-482882/2020|2020-10-19|EPI_ISL_613250|Original|hCoV-19^^Wales|Human|Wales Specialist Virology Centre Sequencing lab|COVID-19 Genomics UK (COG-UK) Consortium|Moore|United Kingdom
>Spike|hCoV-19/USA/TX-DSHS-0981/2020|2020-06-16|EPI_ISL_613994|Original|hCoV-19^^Texas|Human|Texas Department of State Health Services|Texas Department of State Health Services|Rahman|USA
>Spike|hCoV-19/USA/VA-DCLS-0325/2020|2020-05-04|EPI_ISL_614106|Original|hCoV-19^^Virginia|Human|Virginia DCLS|Virginia DCLS|St. Jacques|USA
>Spike|hCoV-19/Rwanda/U66157-RD/2020|2020-07-00|EPI_ISL_614980|Original|hCoV-19^^Rwanda|Human|Rwanda National Reference Laboratory|Rwanda National Reference Laboratory|Mukantwari|Rwanda
>Spike|hCoV-19/Rwanda/U65707-RD/2020|2020-07-00|EPI_ISL_615074|Original|hCoV-19^^Rwanda|Human|Rwanda National Reference Laboratory|Rwanda National Reference Laboratory|Mukantwari|Rwanda
>Spike|hCoV-19/Denmark/DCGC-3687/2020|2020-09-07|EPI_ISL_615462|Original|hCoV-19^^Hovedstaden|Human|Department of Virus and Microbiological Special Diagnostics|Albertsen lab|Michaelsen|Denmark
>Spike|hCoV-19/Denmark/DCGC-3698/2020|2020-08-31|EPI_ISL_615476|Original|hCoV-19^^Hovedstaden|Human|Department of Virus and Microbiological Special Diagnostics|Albertsen lab|Michaelsen|Denmark
>Spike|hCoV-19/Denmark/DCGC-4006/2020|2020-09-14|EPI_ISL_615778|Original|hCoV-19^^Midtjylland|Human|Department of Virus and Microbiological Special Diagnostics|Albertsen lab|Michaelsen|Denmark
>Spike|hCoV-19/Denmark/DCGC-3928/2020|2020-09-14|EPI_ISL_615839|Original|hCoV-19^^Hovedstaden|Human|Department of Virus and Microbiological Special Diagnostics|Albertsen lab|Michaelsen|Denmark
>Spike|hCoV-19/Denmark/DCGC-6327/2020|2020-09-14|EPI_ISL_615877|Original|hCoV-19^^Hovedstaden|Human|Department of Virus and Microbiological Special Diagnostics|Albertsen lab|Michaelsen|Denmark
>Spike|hCoV-19/Denmark/DCGC-3100/2020|2020-08-31|EPI_ISL_615916|Original|hCoV-19^^Nordjylland|Human|Department of Virus and Microbiological Special Diagnostics|Albertsen lab|Michaelsen|Denmark
>Spike|hCoV-19/Denmark/DCGC-3168/2020|2020-08-31|EPI_ISL_615990|Original|hCoV-19^^Midtjylland|Human|Department of Virus and Microbiological Special Diagnostics|Albertsen lab|Michaelsen|Denmark
>Spike|hCoV-19/Denmark/DCGC-3084/2020|2020-08-31|EPI_ISL_616864|Original|hCoV-19^^Midtjylland|Human|Department of Virus and Microbiological Special Diagnostics|Albertsen lab|Michaelsen|Denmark
>Spike|hCoV-19/Denmark/DCGC-8348/2020|2020-10-26|EPI_ISL_616974|Original|hCoV-19^^Syddanmark|Human|Department of Virus and Microbiological Special Diagnostics|Albertsen lab|Michaelsen|Denmark
>Spike|hCoV-19/Denmark/DCGC-1675/2020|2020-03-09|EPI_ISL_616975|Original|hCoV-19^^Syddanmark|Human|Department of Virus and Microbiological Special Diagnostics|Albertsen lab|Michaelsen|Denmark
>Spike|hCoV-19/Denmark/DCGC-2472/2020|2020-08-03|EPI_ISL_617423|Original|hCoV-19^^NA|Human|Department of Virus and Microbiological Special Diagnostics|Albertsen lab|Michaelsen|Denmark
>Spike|hCoV-19/Denmark/DCGC-2147/2020|2020-08-10|EPI_ISL_617445|Original|hCoV-19^^Midtjylland|Human|Department of Virus and Microbiological Special Diagnostics|Albertsen lab|Michaelsen|Denmark
>Spike|hCoV-19/Denmark/DCGC-2315/2020|2020-08-10|EPI_ISL_617652|Original|hCoV-19^^Hovedstaden|Human|Department of Virus and Microbiological Special Diagnostics|Albertsen lab|Michaelsen|Denmark
>Spike|hCoV-19/Denmark/DCGC-2321/2020|2020-08-10|EPI_ISL_617658|Original|hCoV-19^^Hovedstaden|Human|Department of Virus and Microbiological Special Diagnostics|Albertsen lab|Michaelsen|Denmark
>Spike|hCoV-19/Denmark/DCGC-2323/2020|2020-08-10|EPI_ISL_617660|Original|hCoV-19^^Syddanmark|Human|Department of Virus and Microbiological Special Diagnostics|Albertsen lab|Michaelsen|Denmark

>Spike|hCoV-19/Denmark/DCGC-2648/2020|2020-08-17|EPI_ISL_617867|Original|hCoV-19^^Midtjylland|Human|Department of Virus and Microbiological Special Diagnostics|Albertsen lab|Michaelsen|Denmark
>Spike|hCoV-19/Denmark/DCGC-2097/2020|2020-07-27|EPI_ISL_618356|Original|hCoV-19^^Sjaelland|Human|Department of Virus and Microbiological Special Diagnostics|Albertsen lab|Michaelsen|Denmark
>Spike|hCoV-19/Denmark/DCGC-1634/2020|2020-04-06|EPI_ISL_618962|Original|hCoV-19^^Hovedstaden|Human|Department of Virus and Microbiological Special Diagnostics|Albertsen lab|Michaelsen|Denmark
>Spike|hCoV-19/Denmark/DCGC-6205/2020|2020-10-05|EPI_ISL_620108|Original|hCoV-19^^Nordjylland|Human|Department of Virus and Microbiological Special Diagnostics|Albertsen lab|Michaelsen|Denmark
>Spike|hCoV-19/Denmark/DCGC-1748/2020|2020-03-09|EPI_ISL_620138|Original|hCoV-19^^Midtjylland|Human|Department of Virus and Microbiological Special Diagnostics|Albertsen lab|Michaelsen|Denmark
>Spike|hCoV-19/Denmark/DCGC-6144/2020|2020-09-14|EPI_ISL_621178|Original|hCoV-19^^Midtjylland|Human|Department of Virus and Microbiological Special Diagnostics|Albertsen lab|Michaelsen|Denmark
>Spike|hCoV-19/Denmark/DCGC-5739/2020|2020-09-28|EPI_ISL_621244|Original|hCoV-19^^Hovedstaden|Human|Department of Virus and Microbiological Special Diagnostics|Albertsen lab|Michaelsen|Denmark
>Spike|hCoV-19/Denmark/DCGC-4857/2020|2020-09-21|EPI_ISL_621543|Original|hCoV-19^^Hovedstaden|Human|Department of Virus and Microbiological Special Diagnostics|Albertsen lab|Michaelsen|Denmark
>Spike|hCoV-19/Denmark/DCGC-5065/2020|2020-09-21|EPI_ISL_621603|Original|hCoV-19^^Syddanmark|Human|Department of Virus and Microbiological Special Diagnostics|Albertsen lab|Michaelsen|Denmark
>Spike|hCoV-19/Denmark/DCGC-5092/2020|2020-09-21|EPI_ISL_621630|Original|hCoV-19^^Sjaelland|Human|Department of Virus and Microbiological Special Diagnostics|Albertsen lab|Michaelsen|Denmark
>Spike|hCoV-19/Denmark/DCGC-5009/2020|2020-09-14|EPI_ISL_621762|Original|hCoV-19^^Hovedstaden|Human|Department of Virus and Microbiological Special Diagnostics|Albertsen lab|Michaelsen|Denmark
>Spike|hCoV-19/Denmark/DCGC-4201/2020|2020-09-14|EPI_ISL_622094|Original|hCoV-19^^Sjaelland|Human|Department of Virus and Microbiological Special Diagnostics|Albertsen lab|Michaelsen|Denmark
>Spike|hCoV-19/Denmark/DCGC-1722/2020|2020-03-23|EPI_ISL_622203|Original|hCoV-19^^Hovedstaden|Human|Department of Virus and Microbiological Special Diagnostics|Albertsen lab|Michaelsen|Denmark
>Spike|hCoV-19/Denmark/DCGC-1376/2020|2020-03-30|EPI_ISL_622227|Original|hCoV-19^^Sjaelland|Human|Department of Virus and Microbiological Special Diagnostics|Albertsen lab|Michaelsen|Denmark
>Spike|hCoV-19/Denmark/DCGC-1646/2020|2020-03-23|EPI_ISL_622449|Original|hCoV-19^^Hovedstaden|Human|Department of Virus and Microbiological Special Diagnostics|Albertsen lab|Michaelsen|Denmark
>Spike|hCoV-19/Denmark/DCGC-1449/2020|2020-04-27|EPI_ISL_622466|Original|hCoV-19^^Hovedstaden|Human|Department of Virus and Microbiological Special Diagnostics|Albertsen lab|Michaelsen|Denmark
>Spike|hCoV-19/Denmark/DCGC-3861/2020|2020-08-24|EPI_ISL_622733|Original|hCoV-19^^Hovedstaden|Human|Department of Virus and Microbiological Special Diagnostics|Albertsen lab|Michaelsen|Denmark
>Spike|hCoV-19/England/MILK-AB7E40/2020|2020-10-21|EPI_ISL_623389|Original|hCoV-19^^England|Human|Lighthouse Lab in Milton Keynes|Wellcome Sanger Institute for the COVID-19 Genomics UK (COG-UK) consortium|Davies|United Kingdom
>Spike|hCoV-19/England/CAMC-AAFA74/2020|2020-10-23|EPI_ISL_623477|Original|hCoV-19^^England|Human|Lighthouse Lab in Cambridge|Wellcome Sanger Institute for the COVID-19 Genomics UK (COG-UK) Consortium|Davies|United Kingdom
>Spike|hCoV-19/England/ALDP-AA34EB/2020|2020-10-22|EPI_ISL_623864|Original|hCoV-19^^England|Human|Lighthouse Lab in Alderley Park|Wellcome Sanger Institute for the COVID-19 Genomics UK (COG-UK) Consortium|Davies|United Kingdom

>Spike|hCoV-19/England/ALDP-AA1E85/2020|2020-10-22|EPI_ISL_624222|Original|hCoV-19^^England|Human|Lighthouse Lab in Alderley Park|Wellcome Sanger Institute for the COVID-19 Genomics UK (COG-UK) consortium|Davies|United Kingdom
>Spike|hCoV-19/England/ALDP-AA3D86/2020|2020-10-21|EPI_ISL_624244|Original|hCoV-19^^England|Human|Lighthouse Lab in Alderley Park|Wellcome Sanger Institute for the COVID-19 Genomics UK (COG-UK) Consortium|Davies|United Kingdom
>Spike|hCoV-19/England/ALDP-AA223D/2020|2020-10-22|EPI_ISL_625213|Original|hCoV-19^^England|Human|Lighthouse Lab in Alderley Park|Wellcome Sanger Institute for the COVID-19 Genomics UK (COG-UK) Consortium|Davies|United Kingdom
>Spike|hCoV-19/Wales/CAMC-A829E3/2020|2020-10-12|EPI_ISL_625289|Original|hCoV-19^^Wales|Human|Lighthouse Lab in Cambridge|Wellcome Sanger Institute for the COVID-19 Genomics UK (COG-UK) Consortium|Davies|United Kingdom
>Spike|hCoV-19/Denmark/DCGC-8898/2020|2020-11-02|EPI_ISL_625709|Original|hCoV-19^^Syddanmark|Human|Department of Virus and Microbiological Special Diagnostics|Albertsen lab|Michaelsen|Denmark
>Spike|hCoV-19/Mongolia/3001/2020|2020-03-18|EPI_ISL_626566|Original|hCoV-19^^Mongolia|Human|National Centre for Communication Disease (NCCD) National Influenza Center|National Centre for Communication Disease (NCCD) National Influenza Center|Tsedenbal|Mongolia
>Spike|hCoV-19/Mongolia/3622/2020|2020-03-21|EPI_ISL_626567|Original|hCoV-19^^Mongolia|Human|National Centre for Communicable Disease (NCCD) National Influenza Center|National Centre for Communicable Disease (NCCD) National Influenza Center|Tsedenbal|Mongolia
>Spike|hCoV-19/Mongolia/3623/2020|2020-03-21|EPI_ISL_626568|Original|hCoV-19^^Mongolia|Human|National Centre for Communicable Disease (NCCD) National Influenza Center|National Centre for Communicable Disease (NCCD) National Influenza Center|Tsedenbal|Mongolia
>Spike|hCoV-19/Mongolia/29432/2020|2020-08-12|EPI_ISL_626569|Original|hCoV-19^^Mongolia|Human|National Centre for Communicable Disease (NCCD) National Influenza Center|National Centre for Communicable Disease (NCCD) National Influenza Center|Tsedenbal|Mongolia
>Spike|hCoV-19/Mongolia/3624/2020|2020-03-21|EPI_ISL_626626|Original|hCoV-19^^Mongolia|Human|National Centre for Communicable Disease (NCCD) National Influenza Center|National Centre for Communicable Disease (NCCD) National Influenza Center|Tsedenbal|Mongolia
>Spike|hCoV-19/Mongolia/7908/2020|2020-04-15|EPI_ISL_626627|Original|hCoV-19^^Mongolia|Human|National Centre for Communicable Disease (NCCD) National Influenza Center|National Centre for Communicable Disease (NCCD) National Influenza Center|Tsedenbal|Mongolia
>Spike|hCoV-19/Wales/PHWC-483168/2020|2020-10-19|EPI_ISL_626787|Original|hCoV-19^^Wales|Human|Wales Specialist Virology Centre Sequencing lab|COVID-19 Genomics UK (COG-UK) Consortium|Moore|United Kingdom
>Spike|hCoV-19/Wales/PHWC-4844C8/2020|2020-10-22|EPI_ISL_626836|Original|hCoV-19^^Wales|Human|Wales Specialist Virology Centre Sequencing lab|COVID-19 Genomics UK (COG-UK) Consortium|Moore|United Kingdom
>Spike|hCoV-19/Wales/PHWC-48511B/2020|2020-10-22|EPI_ISL_626961|Original|hCoV-19^^Wales|Human|Wales Specialist Virology Centre Sequencing lab|COVID-19 Genomics UK (COG-UK) Consortium|Moore|United Kingdom
>Spike|hCoV-19/Wales/PHWC-4831D1/2020|2020-10-05|EPI_ISL_627097|Original|hCoV-19^^Wales|Human|Wales Specialist Virology Centre Sequencing lab|COVID-19 Genomics UK (COG-UK) Consortium|Moore|United Kingdom
>Spike|hCoV-19/Wales/PHWC-483B2E/2020|2020-10-11|EPI_ISL_627269|Original|hCoV-19^^Wales|Human|Wales Specialist Virology Centre Sequencing lab|COVID-19 Genomics UK (COG-UK) Consortium|Moore|United Kingdom
>Spike|hCoV-19/Wales/PHWC-48563A/2020|2020-10-21|EPI_ISL_627278|Original|hCoV-19^^Wales|Human|Wales Specialist Virology Centre Sequencing lab|COVID-19 Genomics UK (COG-UK) Consortium|Moore|United Kingdom
>Spike|hCoV-19/England/MILK-A190DD/2020|2020-10-01|EPI_ISL_627485|Original|hCoV-19^^England|Human|Wales Specialist Virology Centre Sequencing lab|COVID-19 Genomics UK (COG-UK) Consortium|Moore|United Kingdom

>Spike|hCoV-19/England/MILK-A19565/2020|2020-10-02|EPI_ISL_627489|Original|hCoV-19^^England|Human|Wales Specialist Virology Centre Sequencing lab|COVID-19 Genomics UK (COG-UK) Consortium|Moore|United Kingdom
>Spike|hCoV-19/Wales/MILK-A195A1/2020|2020-10-03|EPI_ISL_627490|Original|hCoV-19^^Wales|Human|Wales Specialist Virology Centre Sequencing lab|COVID-19 Genomics UK (COG-UK) Consortium|Moore|United Kingdom
>Spike|hCoV-19/Northern Ireland/NIRE-1089A2/2020|2020-10-11|EPI_ISL_627519|Original|hCoV-19^^Northern Ireland|Human|Regional Virus Laboratory|COVID-19 Genomics UK (COG-UK) Consortium|Fairley|United Kingdom
>Spike|hCoV-19/England/NORW-F0F33/2020|2020-10-17|EPI_ISL_627588|Original|hCoV-19^^England|Human|Quadram Institute Bioscience|COVID-19 Genomics UK (COG-UK) Consortium|Alikhan|United Kingdom
>Spike|hCoV-19/England/NORW-F161F/2020|2020-10-23|EPI_ISL_627608|Original|hCoV-19^^England|Human|Quadram Institute Bioscience|COVID-19 Genomics UK (COG-UK) Consortium|Alikhan|United Kingdom
>Spike|hCoV-19/England/NORW-F1BD4/2020|2020-10-24|EPI_ISL_627621|Original|hCoV-19^^England|Human|Quadram Institute Bioscience|COVID-19 Genomics UK (COG-UK) Consortium|Alikhan|United Kingdom
>Spike|hCoV-19/England/NORW-F1C68/2020|2020-10-24|EPI_ISL_627626|Original|hCoV-19^^England|Human|Quadram Institute Bioscience|COVID-19 Genomics UK (COG-UK) Consortium|Alikhan|United Kingdom
>Spike|hCoV-19/England/NORW-F1E17/2020|2020-10-19|EPI_ISL_627639|Original|hCoV-19^^England|Human|Quadram Institute Bioscience|COVID-19 Genomics UK (COG-UK) Consortium|Alikhan|United Kingdom
>Spike|hCoV-19/England/NORW-F2186/2020|2020-10-22|EPI_ISL_627658|Original|hCoV-19^^England|Human|Quadram Institute Bioscience|COVID-19 Genomics UK (COG-UK) Consortium|Alikhan|United Kingdom
>Spike|hCoV-19/Wales/PHWC-482F05/2020|2020-10-13|EPI_ISL_627755|Original|hCoV-19^^Wales|Human|Wales Specialist Virology Centre Sequencing lab|COVID-19 Genomics UK (COG-UK) Consortium|Moore|United Kingdom
>Spike|hCoV-19/Wales/PHWC-482F14/2020|2020-10-20|EPI_ISL_627756|Original|hCoV-19^^Wales|Human|Wales Specialist Virology Centre Sequencing lab|COVID-19 Genomics UK (COG-UK) Consortium|Moore|United Kingdom
>Spike|hCoV-19/Wales/PHWC-4830A7/2020|2020-10-20|EPI_ISL_627776|Original|hCoV-19^^Wales|Human|Wales Specialist Virology Centre Sequencing lab|COVID-19 Genomics UK (COG-UK) Consortium|Moore|United Kingdom
>Spike|hCoV-19/Wales/PHWC-4830D4/2020|2020-10-20|EPI_ISL_627779|Original|hCoV-19^^Wales|Human|Wales Specialist Virology Centre Sequencing lab|COVID-19 Genomics UK (COG-UK) Consortium|Moore|United Kingdom
>Spike|hCoV-19/Wales/PHWC-483159/2020|2020-09-18|EPI_ISL_627785|Original|hCoV-19^^Wales|Human|Wales Specialist Virology Centre Sequencing lab|COVID-19 Genomics UK (COG-UK) Consortium|Moore|United Kingdom
>Spike|hCoV-19/Wales/PHWC-4831C2/2020|2020-10-13|EPI_ISL_627788|Original|hCoV-19^^Wales|Human|Wales Specialist Virology Centre Sequencing lab|COVID-19 Genomics UK (COG-UK) Consortium|Moore|United Kingdom
>Spike|hCoV-19/Wales/PHWC-483344/2020|2020-09-22|EPI_ISL_627805|Original|hCoV-19^^Wales|Human|Wales Specialist Virology Centre Sequencing lab|COVID-19 Genomics UK (COG-UK) Consortium|Moore|United Kingdom
>Spike|hCoV-19/Wales/PHWC-48339F/2020|2020-10-13|EPI_ISL_627810|Original|hCoV-19^^Wales|Human|Wales Specialist Virology Centre Sequencing lab|COVID-19 Genomics UK (COG-UK) Consortium|Moore|United Kingdom
>Spike|hCoV-19/Wales/PHWC-483405/2020|2020-10-20|EPI_ISL_627816|Original|hCoV-19^^Wales|Human|Wales Specialist Virology Centre Sequencing lab|COVID-19 Genomics UK (COG-UK) Consortium|Moore|United Kingdom
>Spike|hCoV-19/Wales/PHWC-483414/2020|2020-10-13|EPI_ISL_627817|Original|hCoV-19^^Wales|Human|Wales Specialist Virology Centre Sequencing lab|COVID-19 Genomics UK (COG-UK) Consortium|Moore|United Kingdom
>Spike|hCoV-19/Wales/PHWC-483432/2020|2020-10-21|EPI_ISL_627819|Original|hCoV-19^^Wales|Human|Wales Specialist Virology Centre Sequencing lab|COVID-19 Genomics UK (COG-UK) Consortium|Moore|United Kingdom

>Spike|hCoV-19/Wales/PHWC-483599/2020|2020-10-11|EPI_ISL_627834|Original|hCoV-19^^Wales|Human|Wales Specialist Virology Centre Sequencing lab|COVID-19 Genomics UK (COG-UK) Consortium|Moore|United Kingdom
>Spike|hCoV-19/Wales/PHWC-483906/2020|2020-10-03|EPI_ISL_627872|Original|hCoV-19^^Wales|Human|Wales Specialist Virology Centre Sequencing lab|COVID-19 Genomics UK (COG-UK) Consortium|Moore|United Kingdom
>Spike|hCoV-19/Wales/PHWC-48397F/2020|2020-10-11|EPI_ISL_627877|Original|hCoV-19^^Wales|Human|Wales Specialist Virology Centre Sequencing lab|COVID-19 Genomics UK (COG-UK) Consortium|Moore|United Kingdom
>Spike|hCoV-19/Wales/PHWC-48398E/2020|2020-10-18|EPI_ISL_627878|Original|hCoV-19^^Wales|Human|Wales Specialist Virology Centre Sequencing lab|COVID-19 Genomics UK (COG-UK) Consortium|Moore|United Kingdom
>Spike|hCoV-19/Wales/PHWC-4839CA/2020|2020-10-21|EPI_ISL_627881|Original|hCoV-19^^Wales|Human|Wales Specialist Virology Centre Sequencing lab|COVID-19 Genomics UK (COG-UK) Consortium|Moore|United Kingdom
>Spike|hCoV-19/Wales/PHWC-483A21/2020|2020-10-20|EPI_ISL_627885|Original|hCoV-19^^Wales|Human|Wales Specialist Virology Centre Sequencing lab|COVID-19 Genomics UK (COG-UK) Consortium|Moore|United Kingdom
>Spike|hCoV-19/Wales/PHWC-483A5E/2020|2020-10-13|EPI_ISL_627887|Original|hCoV-19^^Wales|Human|Wales Specialist Virology Centre Sequencing lab|COVID-19 Genomics UK (COG-UK) Consortium|Moore|United Kingdom
>Spike|hCoV-19/Wales/PHWC-483AA9/2020|2020-10-11|EPI_ISL_627890|Original|hCoV-19^^Wales|Human|Wales Specialist Virology Centre Sequencing lab|COVID-19 Genomics UK (COG-UK) Consortium|Moore|United Kingdom
>Spike|hCoV-19/Wales/PHWC-483C1C/2020|2020-10-11|EPI_ISL_627910|Original|hCoV-19^^Wales|Human|Wales Specialist Virology Centre Sequencing lab|COVID-19 Genomics UK (COG-UK) Consortium|Moore|United Kingdom
>Spike|hCoV-19/Wales/PHWC-483FC8/2020|2020-10-20|EPI_ISL_627952|Original|hCoV-19^^Wales|Human|Wales Specialist Virology Centre Sequencing lab|COVID-19 Genomics UK (COG-UK) Consortium|Moore|United Kingdom
>Spike|hCoV-19/Wales/PHWC-4840E2/2020|2020-10-23|EPI_ISL_627965|Original|hCoV-19^^Wales|Human|Wales Specialist Virology Centre Sequencing lab|COVID-19 Genomics UK (COG-UK) Consortium|Moore|United Kingdom
>Spike|hCoV-19/Wales/PHWC-4843AD/2020|2020-10-19|EPI_ISL_627997|Original|hCoV-19^^Wales|Human|Wales Specialist Virology Centre Sequencing lab|COVID-19 Genomics UK (COG-UK) Consortium|Moore|United Kingdom
>Spike|hCoV-19/Wales/PHWC-4843CB/2020|2020-10-23|EPI_ISL_627999|Original|hCoV-19^^Wales|Human|Wales Specialist Virology Centre Sequencing lab|COVID-19 Genomics UK (COG-UK) Consortium|Moore|United Kingdom
>Spike|hCoV-19/Wales/PHWC-4845B6/2020|2020-10-23|EPI_ISL_628019|Original|hCoV-19^^Wales|Human|Wales Specialist Virology Centre Sequencing lab|COVID-19 Genomics UK (COG-UK) Consortium|Moore|United Kingdom
>Spike|hCoV-19/Wales/PHWC-48461D/2020|2020-10-22|EPI_ISL_628023|Original|hCoV-19^^Wales|Human|Wales Specialist Virology Centre Sequencing lab|COVID-19 Genomics UK (COG-UK) Consortium|Moore|United Kingdom
>Spike|hCoV-19/Wales/PHWC-4847B0/2020|2020-10-25|EPI_ISL_628043|Original|hCoV-19^^Wales|Human|Wales Specialist Virology Centre Sequencing lab|COVID-19 Genomics UK (COG-UK) Consortium|Moore|United Kingdom
>Spike|hCoV-19/Wales/PHWC-484CFD/2020|2020-10-20|EPI_ISL_628099|Original|hCoV-19^^Wales|Human|Wales Specialist Virology Centre Sequencing lab|COVID-19 Genomics UK (COG-UK) Consortium|Moore|United Kingdom
>Spike|hCoV-19/Wales/PHWC-484D54/2020|2020-10-22|EPI_ISL_628105|Original|hCoV-19^^Wales|Human|Wales Specialist Virology Centre Sequencing lab|COVID-19 Genomics UK (COG-UK) Consortium|Moore|United Kingdom
>Spike|hCoV-19/Wales/PHWC-484DEB/2020|2020-10-21|EPI_ISL_628113|Original|hCoV-19^^Wales|Human|Wales Specialist Virology Centre Sequencing lab|COVID-19 Genomics UK (COG-UK) Consortium|Moore|United Kingdom
>Spike|hCoV-19/Wales/PHWC-4850C3/2020|2020-10-21|EPI_ISL_628144|Original|hCoV-19^^Wales|Human|Wales Specialist Virology Centre Sequencing lab|COVID-19 Genomics UK (COG-UK) Consortium|Moore|United Kingdom

>Spike|hCoV-19/Wales/PHWC-48510C/2020|2020-10-21|EPI_ISL_628148|Original|hCoV-19^^Wales|Human|Wales Specialist Virology Centre Sequencing lab|COVID-19 Genomics UK (COG-UK) Consortium|Moore|United Kingdom
>Spike|hCoV-19/Wales/PHWC-485245/2020|2020-10-25|EPI_ISL_628160|Original|hCoV-19^^Wales|Human|Wales Specialist Virology Centre Sequencing lab|COVID-19 Genomics UK (COG-UK) Consortium|Moore|United Kingdom
>Spike|hCoV-19/Wales/PHWC-485324/2020|2020-10-23|EPI_ISL_628169|Original|hCoV-19^^Wales|Human|Wales Specialist Virology Centre Sequencing lab|COVID-19 Genomics UK (COG-UK) Consortium|Moore|United Kingdom
>Spike|hCoV-19/Wales/PHWC-4853AC/2020|2020-10-19|EPI_ISL_628176|Original|hCoV-19^^Wales|Human|Wales Specialist Virology Centre Sequencing lab|COVID-19 Genomics UK (COG-UK) Consortium|Moore|United Kingdom
>Spike|hCoV-19/Wales/PHWC-48546D/2020|2020-10-21|EPI_ISL_628184|Original|hCoV-19^^Wales|Human|Wales Specialist Virology Centre Sequencing lab|COVID-19 Genomics UK (COG-UK) Consortium|Moore|United Kingdom
>Spike|hCoV-19/Wales/PHWC-485667/2020|2020-10-22|EPI_ISL_628203|Original|hCoV-19^^Wales|Human|Wales Specialist Virology Centre Sequencing lab|COVID-19 Genomics UK (COG-UK) Consortium|Moore|United Kingdom
>Spike|hCoV-19/Wales/PHWC-4857CE/2020|2020-10-22|EPI_ISL_628220|Original|hCoV-19^^Wales|Human|Wales Specialist Virology Centre Sequencing lab|COVID-19 Genomics UK (COG-UK) Consortium|Moore|United Kingdom
>Spike|hCoV-19/Wales/PHWC-4857DD/2020|2020-10-22|EPI_ISL_628221|Original|hCoV-19^^Wales|Human|Wales Specialist Virology Centre Sequencing lab|COVID-19 Genomics UK (COG-UK) Consortium|Moore|United Kingdom
>Spike|hCoV-19/Wales/PHWC-485861/2020|2020-10-20|EPI_ISL_628228|Original|hCoV-19^^Wales|Human|Wales Specialist Virology Centre Sequencing lab|COVID-19 Genomics UK (COG-UK) Consortium|Moore|United Kingdom
>Spike|hCoV-19/Wales/PHWC-4858AD/2020|2020-10-23|EPI_ISL_628231|Original|hCoV-19^^Wales|Human|Wales Specialist Virology Centre Sequencing lab|COVID-19 Genomics UK (COG-UK) Consortium|Moore|United Kingdom
>Spike|hCoV-19/Wales/PHWC-4858CB/2020|2020-10-25|EPI_ISL_628232|Original|hCoV-19^^Wales|Human|Wales Specialist Virology Centre Sequencing lab|COVID-19 Genomics UK (COG-UK) Consortium|Moore|United Kingdom
>Spike|hCoV-19/Wales/PHWC-4858DA/2020|2020-10-25|EPI_ISL_628233|Original|hCoV-19^^Wales|Human|Wales Specialist Virology Centre Sequencing lab|COVID-19 Genomics UK (COG-UK) Consortium|Moore|United Kingdom
>Spike|hCoV-19/Wales/PHWC-48599B/2020|2020-10-20|EPI_ISL_628238|Original|hCoV-19^^Wales|Human|Wales Specialist Virology Centre Sequencing lab|COVID-19 Genomics UK (COG-UK) Consortium|Moore|United Kingdom
>Spike|hCoV-19/Wales/PHWC-485BA4/2020|2020-10-26|EPI_ISL_628260|Original|hCoV-19^^Wales|Human|Wales Specialist Virology Centre Sequencing lab|COVID-19 Genomics UK (COG-UK) Consortium|Moore|United Kingdom
>Spike|hCoV-19/Scotland/CVR93/2020|2020-03-14|EPI_ISL_629000|Original|hCoV-19^^Scotland|Human|West of Scotland Specialist Virology Centre|MRC-University of Glasgow Centre for Virus Research|Robertson|United Kingdom
>Spike|hCoV-19/USA/MD-MDH-0284/2020|2020-10-12|EPI_ISL_629064|Original|hCoV-19^^Maryland|Human|Maryland Public Health Laboratory|Maryland Public Health Laboratory|Keller|USA
>Spike|hCoV-19/France/ARA-SC078/2020|2020-04-30|EPI_ISL_629108|Original|hCoV-19^^Auvergne-Rh\u00f4ne-Alpes|Human|Laboratoire du Centre Hospitalier Annecy Genevois|CNR Virus des Infections Respiratoires - France SUD|Bal|France
>Spike|hCoV-19/England/MILK-ACBF19/2020|2020-10-19|EPI_ISL_629181|Original|hCoV-19^^England|Human|Lighthouse Lab in Milton Keynes|Wellcome Sanger Institute for the COVID-19 Genomics UK (COG-UK) consortium|Davies|United Kingdom
>Spike|hCoV-19/England/MILK-ACBCB8/2020|2020-10-20|EPI_ISL_629409|Original|hCoV-19^^England|Human|Lighthouse Lab in Milton Keynes|Wellcome Sanger Institute for the COVID-19 Genomics UK (COG-UK) Consortium|Davies|United Kingdom
>Spike|hCoV-19/England/MILK-ACE6C6/2020|2020-10-21|EPI_ISL_629490|Original|hCoV-19^^England|Human|Lighthouse Lab in Milton Keynes|Wellcome Sanger Institute for the COVID-19 Genomics UK (COG-UK) Consortium|Davies|United Kingdom

>Spike|hCoV-19/England/MILK-ACCE0C/2020|2020-10-21|EPI_ISL_630219|Original|hCoV-19^^England|Human|Lighthouse Lab in Milton Keynes|Wellcome Sanger Institute for the COVID-19 Genomics UK (COG-UK) Consortium|Davies|United Kingdom
>Spike|hCoV-19/England/ALDP-A670A3/2020|2020-10-12|EPI_ISL_630461|Original|hCoV-19^^England|Human|Lighthouse Lab in Alderley Park|Wellcome Sanger Institute for the COVID-19 Genomics UK (COG-UK) consortium|Davies|United Kingdom
>Spike|hCoV-19/England/MILK-ABC036/2020|2020-10-22|EPI_ISL_631102|Original|hCoV-19^^England|Human|Lighthouse Lab in Milton Keynes|Wellcome Sanger Institute for the COVID-19 Genomics UK (COG-UK) Consortium|Davies|United Kingdom
>Spike|hCoV-19/USA/NY-NYCPHL-000041/2020|2020-03-08|EPI_ISL_631536|Original|hCoV-19^^New York|Human|New York Presbyterian Lower Manhattan|New York City Public Health Laboratory|Wang|USA
>Spike|hCoV-19/USA/NY-NYCPHL-000447/2020|2020-04-03|EPI_ISL_631921|Original|hCoV-19^^New York|Human|Jamaica Hospital Medical Center|New York City Public Health Laboratory|Wang|USA
>Spike|hCoV-19/Tunisia/55006/2020|2020-10-31|EPI_ISL_632310|Original|hCoV-19^^Ben Arous|Human|1-Laboratory of Microbiology|1-Clinical and Experimental Pharmacology Lab|Boutiba-Ben Boubaker|Tunisia
>Spike|hCoV-19/USA/WI-UW-1574/2020|2020-09-16|EPI_ISL_632890|Original|hCoV-19^^Wisconsin|Human|University of Wisconsin-Madison AIDS Vaccine Research Laboratories|University of Wisconsin-Madison AIDS Vaccine Research Laboratories|Baczenas|USA
>Spike|hCoV-19/USA/WI-UW-1611/2020|2020-09-17|EPI_ISL_632891|Original|hCoV-19^^Wisconsin|Human|University of Wisconsin-Madison AIDS Vaccine Research Laboratories|University of Wisconsin-Madison AIDS Vaccine Research Laboratories|Baczenas|USA
>Spike|hCoV-19/USA/WI-UW-1613/2020|2020-09-17|EPI_ISL_632892|Original|hCoV-19^^Wisconsin|Human|University of Wisconsin-Madison AIDS Vaccine Research Laboratories|University of Wisconsin-Madison AIDS Vaccine Research Laboratories|Baczenas|USA
>Spike|hCoV-19/USA/WI-UW-1614/2020|2020-09-17|EPI_ISL_632893|Original|hCoV-19^^Wisconsin|Human|University of Wisconsin-Madison AIDS Vaccine Research Laboratories|University of Wisconsin-Madison AIDS Vaccine Research Laboratories|Baczenas|USA
>Spike|hCoV-19/USA/WI-UW-1768/2020|2020-09-16|EPI_ISL_632895|Original|hCoV-19^^Wisconsin|Human|University of Wisconsin-Madison AIDS Vaccine Research Laboratories|University of Wisconsin-Madison AIDS Vaccine Research Laboratories|Baczenas|USA
>Spike|hCoV-19/USA/WI-UW-1885/2020|2020-09-16|EPI_ISL_632898|Original|hCoV-19^^Wisconsin|Human|University of Wisconsin-Madison AIDS Vaccine Research Laboratories|University of Wisconsin-Madison AIDS Vaccine Research Laboratories|Baczenas|USA
>Spike|hCoV-19/Yunnan/02/2020|2020-09-13|EPI_ISL_632934|Original|hCoV-19^^Yunnan|Human|Department of Acute Infectious Diseases Control and Prevention,Yunnan Provincial Center for Disease Control and Prevention|Department of Acute Infectious Diseases Control and Prevention,Yunnan Provincial Center for Disease Control and Prevention|Zhang|China
>Spike|hCoV-19/Yunnan/03/2020|2020-09-13|EPI_ISL_632935|Original|hCoV-19^^Yunnan|Human|Department of Acute Infectious Diseases Control and Prevention|Department of Acute Infectious Diseases Control and Prevention|Zhang|China
>Spike|hCoV-19/USA/NY-NYCPHL-000973/2020|2020-09-23|EPI_ISL_633003|Original|hCoV-19^^New York|Human|DOHMH Jamaica|New York City Public Health Laboratory|Wang|USA
>Spike|hCoV-19/USA/NY-NYCPHL-001080/2020|2020-10-17|EPI_ISL_633081|Original|hCoV-19^^New York|Human|DOHMH Morrisania|New York City Public Health Laboratory|Wang|USA
>Spike|hCoV-19/England/QEUH-AE52D4/2020|2020-10-27|EPI_ISL_633361|Original|hCoV-19^^England|Human|Lighthouse Lab in Glasgow|Wellcome Sanger Institute for the COVID-19 Genomics UK (COG-UK) Consortium|Davies|United Kingdom
>Spike|hCoV-19/England/QEUH-AE2204/2020|2020-10-27|EPI_ISL_633577|Original|hCoV-19^^England|Human|Lighthouse Lab in Glasgow|Wellcome Sanger Institute for the COVID-19 Genomics UK (COG-UK) Consortium|Davies|United Kingdom

>Spike|hCoV-19/England/QEUH-AE1F2C/2020|2020-10-27|EPI_ISL_633653|Original|hCoV-19^^England|Human|Lighthouse Lab in Glasgow|Wellcome Sanger Institute for the COVID-19 Genomics UK (COG-UK) Consortium|Davies|United Kingdom
>Spike|hCoV-19/England/QEUH-AD1138/2020|2020-10-27|EPI_ISL_633760|Original|hCoV-19^^England|Human|Lighthouse Lab in Glasgow|Wellcome Sanger Institute for the COVID-19 Genomics UK (COG-UK) Consortium|Davies|United Kingdom
>Spike|hCoV-19/England/QEUH-AD5978/2020|2020-10-24|EPI_ISL_633799|Original|hCoV-19^^England|Human|Lighthouse Lab in Glasgow|Wellcome Sanger Institute for the COVID-19 Genomics UK (COG-UK) Consortium|Davies|United Kingdom
>Spike|hCoV-19/England/MILK-ACD5CA/2020|2020-10-21|EPI_ISL_634428|Original|hCoV-19^^England|Human|Lighthouse Lab in Milton Keynes|Wellcome Sanger Institute for the COVID-19 Genomics UK (COG-UK) Consortium|Davies|United Kingdom
>Spike|hCoV-19/England/MILK-ACC491/2020|2020-10-21|EPI_ISL_634465|Original|hCoV-19^^England|Human|Lighthouse Lab in Milton Keynes|Wellcome Sanger Institute for the COVID-19 Genomics UK (COG-UK) Consortium|Davies|United Kingdom
>Spike|hCoV-19/Tunisia/55153/2020|2020-10-31|EPI_ISL_634977|Original|hCoV-19^^Ben Arous|Human|1-Laboratory of Microbiology|1-Clinical and Experimental Pharmacology Lab|Boutiba-Ben Boubaker|Tunisia
>Spike|hCoV-19/South Africa/KRISP-K003990/2020|2020-09-25|EPI_ISL_634978|Original|hCoV-19^^KwaZulu-Natal|Human|National Health Laboratory Service - Inkosi Albert Luthuli Central Hospital (NHLS-IALCH)|KRISP|de Oliveira|South Africa
>Spike|hCoV-19/South Africa/KRISP-K004015/2020|2020-10-12|EPI_ISL_635000|Original|hCoV-19^^KwaZulu-Natal|Human|National Health Laboratory Service - Inkosi Albert Luthuli Central Hospital (NHLS-IALCH)|KRISP|de Oliveira|South Africa
>Spike|hCoV-19/South Africa/KRISP-K004056/2020|2020-10-20|EPI_ISL_635035|Original|hCoV-19^^KwaZulu-Natal|Human|National Health Laboratory Service - Inkosi Albert Luthuli Central Hospital (NHLS-IALCH)|KRISP|de Oliveira|South Africa
>Spike|hCoV-19/South Africa/KRISP-K004057/2020|2020-10-18|EPI_ISL_635036|Original|hCoV-19^^KwaZulu-Natal|Human|National Health Laboratory Service - Inkosi Albert Luthuli Central Hospital (NHLS-IALCH)|KRISP|de Oliveira|South Africa
>Spike|hCoV-19/Tunisia/55439/2020|2020-11-02|EPI_ISL_635062|Original|hCoV-19^^Tunis|Human|1-Laboratory of Microbiology|1-Clinical and Experimental Pharmacology Lab|Boutiba-Ben Boubaker|Tunisia
>Spike|hCoV-19/Slovenia/8246/2020|2020-03-16|EPI_ISL_635249|Original|hCoV-19^^Slovenia|Human|Institute of Microbiology and Immunology|Institute of Microbiology and Immunology|Zakotnik|Slovenia
>Spike|hCoV-19/Slovenia/V0-571/2020|2020-03-27|EPI_ISL_635293|Original|hCoV-19^^Slovenia|Human|Institute of Microbiology and Immunology|Institute of Microbiology and Immunology|Zakotnik|Slovenia
>Spike|hCoV-19/USA/CA-ALSR-2050/2020|2020-05-11|EPI_ISL_635317|Original|hCoV-19^^California|Human|San Diego County Public Health Laboratory|Andersen lab at Scripps Research|Gangavarapu|USA
>Spike|hCoV-19/USA/CA-ALSR-2051/2020|2020-05-11|EPI_ISL_635318|Original|hCoV-19^^California|Human|San Diego County Public Health Laboratory|Andersen lab at Scripps Research|Gangavarapu|USA
>Spike|hCoV-19/USA/CA-ALSR-2053/2020|2020-05-07|EPI_ISL_635320|Original|hCoV-19^^California|Human|San Diego County Public Health Laboratory|Andersen lab at Scripps Research|Gangavarapu|USA
>Spike|hCoV-19/Mexico/BCN-ALSR-2448/2020|2020-05-14|EPI_ISL_635482|Original|hCoV-19^^Baja California|Human|Centro de Diagn\u221a\u2265stico COVID-19 UABC Tijuana|Andersen lab at Scripps Research|Gangavarapu|Mexico
>Spike|hCoV-19/Mexico/BCN-ALSR-2547/2020|2020-06-29|EPI_ISL_635550|Original|hCoV-19^^Baja California|Human|Centro de Diagn\u221a\u2265stico COVID-19 UABC Tijuana|Andersen lab at Scripps Research|Gangavarapu|Mexico

>Spike|hCoV-19/Jordan/AM-ALSR-3913/2020|2020-09-30|EPI_ISL_636279|Original|hCoV-19^^Amman|Human|Biolab Diagnostic Laboratories|Andersen lab at Scripps Research|Gangavarapu|Jordan
>Spike|hCoV-19/Jordan/AM-ALSR-3931/2020|2020-09-30|EPI_ISL_636296|Original|hCoV-19^^Amman|Human|Biolab Diagnostic Laboratories|Andersen lab at Scripps Research|Gangavarapu|Jordan
>Spike|hCoV-19/Jordan/AM-ALSR-3940/2020|2020-09-30|EPI_ISL_636304|Original|hCoV-19^^Amman|Human|Biolab Diagnostic Laboratories|Andersen lab at Scripps Research|Gangavarapu|Jordan
>Spike|hCoV-19/Jordan/AM-ALSR-3945/2020|2020-09-30|EPI_ISL_636309|Original|hCoV-19^^Amman|Human|Biolab Diagnostic Laboratories|Andersen lab at Scripps Research|Gangavarapu|Jordan
>Spike|hCoV-19/Jordan/AM-ALSR-3950/2020|2020-09-30|EPI_ISL_636314|Original|hCoV-19^^Amman|Human|Biolab Diagnostic Laboratories|Andersen lab at Scripps Research|Gangavarapu|Jordan
>Spike|hCoV-19/Jordan/AM-ALSR-3965/2020|2020-09-30|EPI_ISL_636328|Original|hCoV-19^^Amman|Human|Biolab Diagnostic Laboratories|Andersen lab at Scripps Research|Gangavarapu|Jordan
>Spike|hCoV-19/Jordan/AM-ALSR-3966/2020|2020-10-02|EPI_ISL_636329|Original|hCoV-19^^Amman|Human|Biolab Diagnostic Laboratories|Andersen lab at Scripps Research|Gangavarapu|Jordan
>Spike|hCoV-19/Jordan/AM-ALSR-3974/2020|2020-10-03|EPI_ISL_636337|Original|hCoV-19^^Amman|Human|Biolab Diagnostic Laboratories|Andersen lab at Scripps Research|Gangavarapu|Jordan
>Spike|hCoV-19/Jordan/AM-ALSR-3980/2020|2020-10-03|EPI_ISL_636343|Original|hCoV-19^^Amman|Human|Biolab Diagnostic Laboratories|Andersen lab at Scripps Research|Gangavarapu|Jordan
>Spike|hCoV-19/Jordan/AM-ALSR-4022/2020|2020-10-03|EPI_ISL_636383|Original|hCoV-19^^Amman|Human|Biolab Diagnostic Laboratories|Andersen lab at Scripps Research|Gangavarapu|Jordan
>Spike|hCoV-19/Jordan/AM-ALSR-4032/2020|2020-10-03|EPI_ISL_636392|Original|hCoV-19^^Amman|Human|Biolab Diagnostic Laboratories|Andersen lab at Scripps Research|Gangavarapu|Jordan
>Spike|hCoV-19/Jordan/AM-ALSR-4042/2020|2020-10-03|EPI_ISL_636402|Original|hCoV-19^^Amman|Human|Biolab Diagnostic Laboratories|Andersen lab at Scripps Research|Gangavarapu|Jordan
>Spike|hCoV-19/Jordan/AM-ALSR-4078/2020|2020-10-03|EPI_ISL_636438|Original|hCoV-19^^Amman|Human|Biolab Diagnostic Laboratories|Andersen lab at Scripps Research|Gangavarapu|Jordan
>Spike|hCoV-19/France/ARA-34732/2020|2020-05-06|EPI_ISL_636480|Original|hCoV-19^^Auvergne-Rhone-Alpes|Human|Centre Hospitalier Pierre Oudot|CNR Virus des Infections Respiratoires - France SUD|Josset|France
>Spike|hCoV-19/France/ARA-37154/2020|2020-05-13|EPI_ISL_636486|Original|hCoV-19^^Auvergne-Rhone-Alpes|Human|Centre Hospitalier Pierre Oudot|CNR Virus des Infections Respiratoires - France SUD|Josset|France
>Spike|hCoV-19/England/C200031962/2020|2020-10-15|EPI_ISL_636736|Original|hCoV-19^^England|Human|Respiratory Virus Unit|Respiratory Virus Unit|Platt|United Kingdom
>Spike|hCoV-19/Brazil/RJ-0920/2020|2020-03-29|EPI_ISL_636838|Original|hCoV-19^^Rio de Janeiro|Human|Laborat\u00f3rio de Imunofarmacologia - Instituto Oswaldo Cruz|Laborat\u00f3rio de Imunofarmacologia - Instituto Oswaldo Cruz|da Silva|Brazil
>Spike|hCoV-19/Lithuania/MR-LUHS-Eilnr274/2020|2020-08-10|EPI_ISL_636858|Original|hCoV-19^^Kauno apskritis|Human|Lithuanian University of Health Sciences Hospital|Lithuanian University of Health Sciences|Zemaitis|Lithuania
>Spike|hCoV-19/Lithuania/MR-LUHS-Eilnr303/2020|2020-08-14|EPI_ISL_636862|Original|hCoV-19^^Kauno apskritis|Human|Lithuanian University of Health Sciences Hospital|Lithuanian University of Health Sciences|Zemaitis|Lithuania
>Spike|hCoV-19/Lithuania/MR-LUHS-Eilnr338/2020|2020-08-19|EPI_ISL_636868|Original|hCoV-19^^Kauno apskritis|Human|Lithuanian University of Health Sciences Hospital|Lithuanian University of Health Sciences|Zemaitis|Lithuania

>Spike|hCoV-19/Turkey/ETLKVET1/2020|2020-04-00|EPI_ISL_636969|Vero E6/P2|hCoV-19^^Ankara|Human|Etlik Veterinary Control Central Research Institute|Etlik Veterinary Control Central Research Institute|Hacioglu|Turkey
>Spike|hCoV-19/Wales/PHWC-488840/2020|2020-10-19|EPI_ISL_637256|Original|hCoV-19^^Wales|Human|Wales Specialist Virology Centre Sequencing lab|COVID-19 Genomics UK (COG-UK) Consortium|Moore|United Kingdom
>Spike|hCoV-19/Wales/PHWC-487AB4/2020|2020-10-28|EPI_ISL_637288|Original|hCoV-19^^Wales|Human|Wales Specialist Virology Centre Sequencing lab|COVID-19 Genomics UK (COG-UK) Consortium|Moore|United Kingdom
>Spike|hCoV-19/England/NORW-F3914/2020|2020-10-30|EPI_ISL_637442|Original|hCoV-19^^England|Human|Quadram Institute Bioscience|COVID-19 Genomics UK (COG-UK) Consortium|Alikhan|United Kingdom
>Spike|hCoV-19/England/NORW-F6B75/2020|2020-11-02|EPI_ISL_637588|Original|hCoV-19^^England|Human|Quadram Institute Bioscience|COVID-19 Genomics UK (COG-UK) Consortium|Alikhan|United Kingdom
>Spike|hCoV-19/Wales/PHWC-4897BB/2020|2020-11-01|EPI_ISL_637626|Original|hCoV-19^^Wales|Human|Wales Specialist Virology Centre Sequencing lab|COVID-19 Genomics UK (COG-UK) Consortium|Moore|United Kingdom
>Spike|hCoV-19/Wales/PHWC-4890B0/2020|2020-11-01|EPI_ISL_638012|Original|hCoV-19^^Wales|Human|Wales Specialist Virology Centre Sequencing lab|COVID-19 Genomics UK (COG-UK) Consortium|Moore|United Kingdom
>Spike|hCoV-19/England/QEUH-A780DC/2020|2020-10-14|EPI_ISL_638037|Original|hCoV-19^^England|Human|Oxford Viromics|COVID-19 Genomics UK (COG-UK) Consortium|Golubchik|United Kingdom
>Spike|hCoV-19/Wales/PHWC-486A6A/2020|2020-10-26|EPI_ISL_638042|Original|hCoV-19^^Wales|Human|Wales Specialist Virology Centre Sequencing lab|COVID-19 Genomics UK (COG-UK) Consortium|Moore|United Kingdom
>Spike|hCoV-19/Wales/PHWC-488682/2020|2020-10-27|EPI_ISL_638160|Original|hCoV-19^^Wales|Human|Wales Specialist Virology Centre Sequencing lab|COVID-19 Genomics UK (COG-UK) Consortium|Moore|United Kingdom
>Spike|hCoV-19/England/QEUH-A3691B/2020|2020-10-06|EPI_ISL_638682|Original|hCoV-19^^England|Human|Wales Specialist Virology Centre Sequencing lab|COVID-19 Genomics UK (COG-UK) Consortium|Moore|United Kingdom
>Spike|hCoV-19/England/QEUH-A36975/2020|2020-10-06|EPI_ISL_638685|Original|hCoV-19^^England|Human|Wales Specialist Virology Centre Sequencing lab|COVID-19 Genomics UK (COG-UK) Consortium|Moore|United Kingdom
>Spike|hCoV-19/England/QEUH-A36ADC/2020|2020-10-05|EPI_ISL_638694|Original|hCoV-19^^England|Human|Wales Specialist Virology Centre Sequencing lab|COVID-19 Genomics UK (COG-UK) Consortium|Moore|United Kingdom
>Spike|hCoV-19/England/QEUH-A36B06/2020|2020-10-05|EPI_ISL_638697|Original|hCoV-19^^England|Human|Wales Specialist Virology Centre Sequencing lab|COVID-19 Genomics UK (COG-UK) Consortium|Moore|United Kingdom
>Spike|hCoV-19/England/QEUH-A36B42/2020|2020-10-05|EPI_ISL_638700|Original|hCoV-19^^England|Human|Wales Specialist Virology Centre Sequencing lab|COVID-19 Genomics UK (COG-UK) Consortium|Moore|United Kingdom
>Spike|hCoV-19/England/QEUH-A36CE5/2020|2020-10-05|EPI_ISL_638707|Original|hCoV-19^^England|Human|Wales Specialist Virology Centre Sequencing lab|COVID-19 Genomics UK (COG-UK) Consortium|Moore|United Kingdom
>Spike|hCoV-19/England/QEUH-A36CF4/2020|2020-10-05|EPI_ISL_638708|Original|hCoV-19^^England|Human|Wales Specialist Virology Centre Sequencing lab|COVID-19 Genomics UK (COG-UK) Consortium|Moore|United Kingdom
>Spike|hCoV-19/England/QEUH-A36E3A/2020|2020-10-05|EPI_ISL_638719|Original|hCoV-19^^England|Human|Wales Specialist Virology Centre Sequencing lab|COVID-19 Genomics UK (COG-UK) Consortium|Moore|United Kingdom
>Spike|hCoV-19/England/QEUH-A37051/2020|2020-10-05|EPI_ISL_638722|Original|hCoV-19^^England|Human|Wales Specialist Virology Centre Sequencing lab|COVID-19 Genomics UK (COG-UK) Consortium|Moore|United Kingdom
>Spike|hCoV-19/England/QEUH-A37060/2020|2020-10-04|EPI_ISL_638723|Original|hCoV-19^^England|Human|Wales Specialist Virology Centre Sequencing lab|COVID-19 Genomics UK (COG-UK) Consortium|Moore|United Kingdom

>Spike|hCoV-19/Scotland/QEUH-A36984/2020|2020-10-05|EPI_ISL_638930|Original|hCoV-19^^Scotland|Human|Wales Specialist Virology Centre Sequencing lab|COVID-19 Genomics UK (COG-UK) Consortium|Moore|United Kingdom
>Spike|hCoV-19/Wales/PHWC-486156/2020|2020-10-31|EPI_ISL_639011|Original|hCoV-19^^Wales|Human|Wales Specialist Virology Centre Sequencing lab|COVID-19 Genomics UK (COG-UK) Consortium|Moore|United Kingdom
>Spike|hCoV-19/Wales/PHWC-48639C/2020|2020-10-21|EPI_ISL_639036|Original|hCoV-19^^Wales|Human|Wales Specialist Virology Centre Sequencing lab|COVID-19 Genomics UK (COG-UK) Consortium|Moore|United Kingdom
>Spike|hCoV-19/Wales/PHWC-4866A2/2020|2020-10-31|EPI_ISL_639067|Original|hCoV-19^^Wales|Human|Wales Specialist Virology Centre Sequencing lab|COVID-19 Genomics UK (COG-UK) Consortium|Moore|United Kingdom
>Spike|hCoV-19/Wales/PHWC-486B58/2020|2020-10-27|EPI_ISL_639113|Original|hCoV-19^^Wales|Human|Wales Specialist Virology Centre Sequencing lab|COVID-19 Genomics UK (COG-UK) Consortium|Moore|United Kingdom
>Spike|hCoV-19/Wales/PHWC-486C46/2020|2020-10-27|EPI_ISL_639120|Original|hCoV-19^^Wales|Human|Wales Specialist Virology Centre Sequencing lab|COVID-19 Genomics UK (COG-UK) Consortium|Moore|United Kingdom
>Spike|hCoV-19/Wales/PHWC-486D52/2020|2020-10-21|EPI_ISL_639129|Original|hCoV-19^^Wales|Human|Wales Specialist Virology Centre Sequencing lab|COVID-19 Genomics UK (COG-UK) Consortium|Moore|United Kingdom
>Spike|hCoV-19/Wales/PHWC-486EC8/2020|2020-10-27|EPI_ISL_639144|Original|hCoV-19^^Wales|Human|Wales Specialist Virology Centre Sequencing lab|COVID-19 Genomics UK (COG-UK) Consortium|Moore|United Kingdom
>Spike|hCoV-19/Wales/PHWC-486FA7/2020|2020-10-30|EPI_ISL_639155|Original|hCoV-19^^Wales|Human|Wales Specialist Virology Centre Sequencing lab|COVID-19 Genomics UK (COG-UK) Consortium|Moore|United Kingdom
>Spike|hCoV-19/Wales/PHWC-4871BF/2020|2020-10-27|EPI_ISL_639171|Original|hCoV-19^^Wales|Human|Wales Specialist Virology Centre Sequencing lab|COVID-19 Genomics UK (COG-UK) Consortium|Moore|United Kingdom
>Spike|hCoV-19/Wales/PHWC-487489/2020|2020-10-30|EPI_ISL_639199|Original|hCoV-19^^Wales|Human|Wales Specialist Virology Centre Sequencing lab|COVID-19 Genomics UK (COG-UK) Consortium|Moore|United Kingdom
>Spike|hCoV-19/Wales/PHWC-48761A/2020|2020-10-28|EPI_ISL_639220|Original|hCoV-19^^Wales|Human|Wales Specialist Virology Centre Sequencing lab|COVID-19 Genomics UK (COG-UK) Consortium|Moore|United Kingdom
>Spike|hCoV-19/Wales/PHWC-487726/2020|2020-10-27|EPI_ISL_639230|Original|hCoV-19^^Wales|Human|Wales Specialist Virology Centre Sequencing lab|COVID-19 Genomics UK (COG-UK) Consortium|Moore|United Kingdom
>Spike|hCoV-19/Wales/PHWC-487771/2020|2020-10-30|EPI_ISL_639234|Original|hCoV-19^^Wales|Human|Wales Specialist Virology Centre Sequencing lab|COVID-19 Genomics UK (COG-UK) Consortium|Moore|United Kingdom
>Spike|hCoV-19/Wales/PHWC-48779F/2020|2020-10-25|EPI_ISL_639236|Original|hCoV-19^^Wales|Human|Wales Specialist Virology Centre Sequencing lab|COVID-19 Genomics UK (COG-UK) Consortium|Moore|United Kingdom
>Spike|hCoV-19/Wales/PHWC-4877AE/2020|2020-10-28|EPI_ISL_639237|Original|hCoV-19^^Wales|Human|Wales Specialist Virology Centre Sequencing lab|COVID-19 Genomics UK (COG-UK) Consortium|Moore|United Kingdom
>Spike|hCoV-19/Wales/PHWC-4879D5/2020|2020-10-30|EPI_ISL_639261|Original|hCoV-19^^Wales|Human|Wales Specialist Virology Centre Sequencing lab|COVID-19 Genomics UK (COG-UK) Consortium|Moore|United Kingdom
>Spike|hCoV-19/Wales/PHWC-487C09/2020|2020-10-27|EPI_ISL_639287|Original|hCoV-19^^Wales|Human|Wales Specialist Virology Centre Sequencing lab|COVID-19 Genomics UK (COG-UK) Consortium|Moore|United Kingdom
>Spike|hCoV-19/Wales/PHWC-487DD9/2020|2020-10-27|EPI_ISL_639307|Original|hCoV-19^^Wales|Human|Wales Specialist Virology Centre Sequencing lab|COVID-19 Genomics UK (COG-UK) Consortium|Moore|United Kingdom
>Spike|hCoV-19/Wales/PHWC-487EB8/2020|2020-10-30|EPI_ISL_639319|Original|hCoV-19^^Wales|Human|Wales Specialist Virology Centre Sequencing lab|COVID-19 Genomics UK (COG-UK) Consortium|Moore|United Kingdom

>Spike|hCoV-19/Wales/PHWC-487F5B/2020|2020-10-30|EPI_ISL_639327|Original|hCoV-19^^Wales|Human|Wales Specialist Virology Centre Sequencing lab|COVID-19 Genomics UK (COG-UK) Consortium|Moore|United Kingdom
>Spike|hCoV-19/Wales/PHWC-488075/2020|2020-10-30|EPI_ISL_639344|Original|hCoV-19^^Wales|Human|Wales Specialist Virology Centre Sequencing lab|COVID-19 Genomics UK (COG-UK) Consortium|Moore|United Kingdom
>Spike|hCoV-19/Wales/PHWC-488127/2020|2020-10-27|EPI_ISL_639354|Original|hCoV-19^^Wales|Human|Wales Specialist Virology Centre Sequencing lab|COVID-19 Genomics UK (COG-UK) Consortium|Moore|United Kingdom
>Spike|hCoV-19/Wales/PHWC-4881DC/2020|2020-10-27|EPI_ISL_639364|Original|hCoV-19^^Wales|Human|Wales Specialist Virology Centre Sequencing lab|COVID-19 Genomics UK (COG-UK) Consortium|Moore|United Kingdom
>Spike|hCoV-19/Wales/PHWC-488251/2020|2020-10-27|EPI_ISL_639370|Original|hCoV-19^^Wales|Human|Wales Specialist Virology Centre Sequencing lab|COVID-19 Genomics UK (COG-UK) Consortium|Moore|United Kingdom
>Spike|hCoV-19/Wales/PHWC-4882AC/2020|2020-10-29|EPI_ISL_639374|Original|hCoV-19^^Wales|Human|Wales Specialist Virology Centre Sequencing lab|COVID-19 Genomics UK (COG-UK) Consortium|Moore|United Kingdom
>Spike|hCoV-19/Wales/PHWC-4882CA/2020|2020-10-28|EPI_ISL_639376|Original|hCoV-19^^Wales|Human|Wales Specialist Virology Centre Sequencing lab|COVID-19 Genomics UK (COG-UK) Consortium|Moore|United Kingdom
>Spike|hCoV-19/Wales/PHWC-48834F/2020|2020-10-29|EPI_ISL_639383|Original|hCoV-19^^Wales|Human|Wales Specialist Virology Centre Sequencing lab|COVID-19 Genomics UK (COG-UK) Consortium|Moore|United Kingdom
>Spike|hCoV-19/Wales/PHWC-4883A9/2020|2020-10-29|EPI_ISL_639387|Original|hCoV-19^^Wales|Human|Wales Specialist Virology Centre Sequencing lab|COVID-19 Genomics UK (COG-UK) Consortium|Moore|United Kingdom
>Spike|hCoV-19/Wales/PHWC-48843D/2020|2020-10-26|EPI_ISL_639391|Original|hCoV-19^^Wales|Human|Wales Specialist Virology Centre Sequencing lab|COVID-19 Genomics UK (COG-UK) Consortium|Moore|United Kingdom
>Spike|hCoV-19/Wales/PHWC-4884A6/2020|2020-11-01|EPI_ISL_639397|Original|hCoV-19^^Wales|Human|Wales Specialist Virology Centre Sequencing lab|COVID-19 Genomics UK (COG-UK) Consortium|Moore|United Kingdom
>Spike|hCoV-19/Wales/PHWC-4886DD/2020|2020-11-01|EPI_ISL_639421|Original|hCoV-19^^Wales|Human|Wales Specialist Virology Centre Sequencing lab|COVID-19 Genomics UK (COG-UK) Consortium|Moore|United Kingdom
>Spike|hCoV-19/Wales/PHWC-4886FB/2020|2020-10-28|EPI_ISL_639423|Original|hCoV-19^^Wales|Human|Wales Specialist Virology Centre Sequencing lab|COVID-19 Genomics UK (COG-UK) Consortium|Moore|United Kingdom
>Spike|hCoV-19/Wales/PHWC-4888B9/2020|2020-11-02|EPI_ISL_639440|Original|hCoV-19^^Wales|Human|Wales Specialist Virology Centre Sequencing lab|COVID-19 Genomics UK (COG-UK) Consortium|Moore|United Kingdom
>Spike|hCoV-19/Wales/PHWC-4889A7/2020|2020-10-26|EPI_ISL_639451|Original|hCoV-19^^Wales|Human|Wales Specialist Virology Centre Sequencing lab|COVID-19 Genomics UK (COG-UK) Consortium|Moore|United Kingdom
>Spike|hCoV-19/Wales/PHWC-488BDE/2020|2020-10-20|EPI_ISL_639478|Original|hCoV-19^^Wales|Human|Wales Specialist Virology Centre Sequencing lab|COVID-19 Genomics UK (COG-UK) Consortium|Moore|United Kingdom
>Spike|hCoV-19/Wales/PHWC-488DAB/2020|2020-11-02|EPI_ISL_639501|Original|hCoV-19^^Wales|Human|Wales Specialist Virology Centre Sequencing lab|COVID-19 Genomics UK (COG-UK) Consortium|Moore|United Kingdom
>Spike|hCoV-19/Wales/PHWC-488E11/2020|2020-10-30|EPI_ISL_639504|Original|hCoV-19^^Wales|Human|Wales Specialist Virology Centre Sequencing lab|COVID-19 Genomics UK (COG-UK) Consortium|Moore|United Kingdom
>Spike|hCoV-19/Wales/PHWC-488E20/2020|2020-10-26|EPI_ISL_639505|Original|hCoV-19^^Wales|Human|Wales Specialist Virology Centre Sequencing lab|COVID-19 Genomics UK (COG-UK) Consortium|Moore|United Kingdom
>Spike|hCoV-19/Wales/PHWC-488F2D/2020|2020-10-28|EPI_ISL_639517|Original|hCoV-19^^Wales|Human|Wales Specialist Virology Centre Sequencing lab|COVID-19 Genomics UK (COG-UK) Consortium|Moore|United Kingdom

>Spike|hCoV-19/Wales/PHWC-488F69/2020|2020-10-29|EPI_ISL_639520|Original|hCoV-19^^Wales|Human|Wales Specialist Virology Centre Sequencing lab|COVID-19 Genomics UK (COG-UK) Consortium|Moore|United Kingdom
>Spike|hCoV-19/Wales/PHWC-4891DB/2020|2020-11-01|EPI_ISL_639548|Original|hCoV-19^^Wales|Human|Wales Specialist Virology Centre Sequencing lab|COVID-19 Genomics UK (COG-UK) Consortium|Moore|United Kingdom
>Spike|hCoV-19/Wales/PHWC-489205/2020|2020-10-24|EPI_ISL_639550|Original|hCoV-19^^Wales|Human|Wales Specialist Virology Centre Sequencing lab|COVID-19 Genomics UK (COG-UK) Consortium|Moore|United Kingdom
>Spike|hCoV-19/Wales/PHWC-4892E7/2020|2020-10-31|EPI_ISL_639560|Original|hCoV-19^^Wales|Human|Wales Specialist Virology Centre Sequencing lab|COVID-19 Genomics UK (COG-UK) Consortium|Moore|United Kingdom
>Spike|hCoV-19/Wales/PHWC-48937B/2020|2020-11-03|EPI_ISL_639569|Original|hCoV-19^^Wales|Human|Wales Specialist Virology Centre Sequencing lab|COVID-19 Genomics UK (COG-UK) Consortium|Moore|United Kingdom
>Spike|hCoV-19/Wales/PHWC-48940F/2020|2020-10-25|EPI_ISL_639573|Original|hCoV-19^^Wales|Human|Wales Specialist Virology Centre Sequencing lab|COVID-19 Genomics UK (COG-UK) Consortium|Moore|United Kingdom
>Spike|hCoV-19/Wales/PHWC-48952A/2020|2020-10-31|EPI_ISL_639586|Original|hCoV-19^^Wales|Human|Wales Specialist Virology Centre Sequencing lab|COVID-19 Genomics UK (COG-UK) Consortium|Moore|United Kingdom
>Spike|hCoV-19/Australia/NSW1168/2020|2020-10-21|EPI_ISL_639736|Original|hCoV-19^^New South Wales|Human|South Eastern Area Laboratory Services (SEALS)|NSW Health Pathology - Institute of Clinical Pathology and Medical Research; Westmead Hospital; University of Sydney|Gall|Australia
>Spike|hCoV-19/Romania/BUCHAREST-IMT-1/2020|2020-07-20|EPI_ISL_639739|Original|hCoV-19^^Romania|Human|Centre of Nanotechnologies|Centre of Nanotechnologies|Salceanu,A.|Romania
>Spike|hCoV-19/Russia/MOS-RII-MH2800S/2020|2020-09-22|EPI_ISL_639951|Original|hCoV-19^^Moscow|Human|HELIX LLC|WHO National Influenza Centre Russian Federation|Komissarov|Russia
>Spike|hCoV-19/Russia/SVE-RII-MH2830S/2020|2020-09-26|EPI_ISL_639972|Original|hCoV-19^^Sverdlovsk|Human|HELIX LLC|WHO National Influenza Centre Russian Federation|Komissarov|Russia
>Spike|hCoV-19/France/ARA-NRC2813/2020|2020-11-02|EPI_ISL_639983|Original|hCoV-19^^Auvergne-Rhone-Alpes|Human|CNR Virus des Infections Respiratoires - France SUD|CNR Virus des Infections Respiratoires - France SUD|Josset|France
>Spike|hCoV-19/Germany/BW-MVZ-LS-07/2020|2020-04-08|EPI_ISL_640225|Original|hCoV-19^^Baden-W\u00fcrttemberg|Human|MVZ Labor\u00e4rzte Singen|MVZ Labor\u00e4rzte Singen|Schmidt|Germany
>Spike|hCoV-19/Australia/VIC12537/2020|2020-07-30|EPI_ISL_640312|Original|hCoV-19^^Victoria|Human|Microbiological Diagnostic Unit - Public Health Laboratory (MDU-PHL)|MDU-PHL|Schultz|Australia
>Spike|hCoV-19/Australia/VIC12455/2020|2020-07-30|EPI_ISL_640469|Original|hCoV-19^^Victoria|Human|Microbiological Diagnostic Unit - Public Health Laboratory (MDU-PHL)|MDU-PHL|Schultz|Australia
>Spike|hCoV-19/Australia/VIC16695/2020|2020-08-06|EPI_ISL_640578|Original|hCoV-19^^Victoria|Human|Microbiological Diagnostic Unit - Public Health Laboratory (MDU-PHL)|MDU-PHL|Schultz|Australia
>Spike|hCoV-19/Australia/VIC16158/2020|2020-08-10|EPI_ISL_640631|Original|hCoV-19^^Victoria|Human|Microbiological Diagnostic Unit - Public Health Laboratory (MDU-PHL)|MDU-PHL|Schultz|Australia
>Spike|hCoV-19/Australia/VIC12889/2020|2020-08-02|EPI_ISL_640694|Original|hCoV-19^^Victoria|Human|Microbiological Diagnostic Unit - Public Health Laboratory (MDU-PHL)|MDU-PHL|Schultz|Australia
>Spike|hCoV-19/Australia/VIC13231/2020|2020-08-01|EPI_ISL_640931|Original|hCoV-19^^Victoria|Human|Microbiological Diagnostic Unit - Public Health Laboratory (MDU-PHL)|MDU-PHL|Schultz|Australia

>Spike|hCoV-19/Australia/VIC11730/2020|2020-08-14|EPI_ISL_641196|Original|hCoV-19^^Victoria|Human|Microbiological Diagnostic Unit - Public Health Laboratory (MDU-PHL)|MDU-PHL|Schultz|Australia
>Spike|hCoV-19/England/QEUH-B0F3B2/2020|2020-11-02|EPI_ISL_641940|Original|hCoV-19^^England|Human|Lighthouse Lab in Glasgow|Wellcome Sanger Institute for the COVID-19 Genomics UK (COG-UK) Consortium|Davies|United Kingdom
>Spike|hCoV-19/England/QEUH-B0DACE/2020|2020-11-02|EPI_ISL_641995|Original|hCoV-19^^England|Human|Lighthouse Lab in Glasgow|Wellcome Sanger Institute for the COVID-19 Genomics UK (COG-UK) Consortium|Davies|United Kingdom
>Spike|hCoV-19/England/QEUH-B0FBAB/2020|2020-11-02|EPI_ISL_642077|Original|hCoV-19^^England|Human|Lighthouse Lab in Glasgow|Wellcome Sanger Institute for the COVID-19 Genomics UK (COG-UK) Consortium|Davies|United Kingdom
>Spike|hCoV-19/England/QEUH-B11B3D/2020|2020-11-02|EPI_ISL_642213|Original|hCoV-19^^England|Human|Lighthouse Lab in Glasgow|Wellcome Sanger Institute for the COVID-19 Genomics UK (COG-UK) Consortium|Davies|United Kingdom
>Spike|hCoV-19/England/QEUH-B1172A/2020|2020-11-02|EPI_ISL_642267|Original|hCoV-19^^England|Human|Lighthouse Lab in Glasgow|Wellcome Sanger Institute for the COVID-19 Genomics UK (COG-UK) Consortium|Davies|United Kingdom
>Spike|hCoV-19/England/QEUH-B10C2C/2020|2020-11-02|EPI_ISL_642673|Original|hCoV-19^^England|Human|Lighthouse Lab in Glasgow|Wellcome Sanger Institute for the COVID-19 Genomics UK (COG-UK) Consortium|Davies|United Kingdom
>Spike|hCoV-19/England/CAMC-B080EF/2020|2020-10-29|EPI_ISL_643375|Original|hCoV-19^^England|Human|Lighthouse Lab in Cambridge|Wellcome Sanger Institute for the COVID-19 Genomics UK (COG-UK) Consortium|Davies|United Kingdom
>Spike|hCoV-19/England/CAMC-B07E13/2020|2020-10-29|EPI_ISL_643572|Original|hCoV-19^^England|Human|Lighthouse Lab in Cambridge|Wellcome Sanger Institute for the COVID-19 Genomics UK (COG-UK) Consortium|Davies|United Kingdom
>Spike|hCoV-19/England/CAMC-B07639/2020|2020-10-30|EPI_ISL_643892|Original|hCoV-19^^England|Human|Lighthouse Lab in Cambridge|Wellcome Sanger Institute for the COVID-19 Genomics UK (COG-UK) Consortium|Davies|United Kingdom
>Spike|hCoV-19/France/PAC-MEPHI-2361/2020|2020-10-09|EPI_ISL_644399|Original|hCoV-19^^Provence-Alpes-Cote-d\u2019Azur|Human|MEPHI|MEPHI|Levasseur|France
>Spike|hCoV-19/England/CAMC-B3667D/2020|2020-11-05|EPI_ISL_645365|Original|hCoV-19^^England|Human|Lighthouse Lab in Cambridge|Wellcome Sanger Institute for the COVID-19 Genomics UK (COG-UK) Consortium|Davies|United Kingdom
>Spike|hCoV-19/England/CAMC-B37409/2020|2020-11-05|EPI_ISL_645434|Original|hCoV-19^^England|Human|Lighthouse Lab in Cambridge|Wellcome Sanger Institute for the COVID-19 Genomics UK (COG-UK) Consortium|Davies|United Kingdom
>Spike|hCoV-19/England/ALDP-B1B939/2020|2020-11-01|EPI_ISL_645601|Original|hCoV-19^^England|Human|Lighthouse Lab in Alderley Park|Wellcome Sanger Institute for the COVID-19 Genomics UK (COG-UK) Consortium|Davies|United Kingdom
>Spike|hCoV-19/England/ALDP-B1B6BA/2020|2020-11-01|EPI_ISL_645798|Original|hCoV-19^^England|Human|Lighthouse Lab in Alderley Park|Wellcome Sanger Institute for the COVID-19 Genomics UK (COG-UK) Consortium|Davies|United Kingdom
>Spike|hCoV-19/England/QEUH-B3200A/2020|2020-11-04|EPI_ISL_646411|Original|hCoV-19^^England|Human|Lighthouse Lab in Glasgow|Wellcome Sanger Institute for the COVID-19 Genomics UK (COG-UK) Consortium|Davies|United Kingdom
>Spike|hCoV-19/England/QEUH-B2CA8D/2020|2020-10-31|EPI_ISL_646679|Original|hCoV-19^^England|Human|Lighthouse Lab in Glasgow|Wellcome Sanger Institute for the COVID-19 Genomics UK (COG-UK) Consortium|Davies|United Kingdom
>Spike|hCoV-19/England/ALDP-B24575/2020|2020-11-04|EPI_ISL_647044|Original|hCoV-19^^England|Human|Lighthouse Lab in Alderley Park|Wellcome Sanger Institute for the COVID-19 Genomics UK (COG-UK) Consortium|Davies|United Kingdom
>Spike|hCoV-19/England/ALDP-B244A5/2020|2020-11-04|EPI_ISL_647049|Original|hCoV-19^^England|Human|Lighthouse Lab in Alderley Park|Wellcome Sanger Institute for the COVID-19 Genomics UK (COG-UK) Consortium|Davies|United Kingdom
>Spike|hCoV-19/England/QEUH-B2ED91/2020|2020-11-03|EPI_ISL_647053|Original|hCoV-19^^England|Human|Lighthouse Lab in Glasgow|Wellcome Sanger Institute for the COVID-19 Genomics UK (COG-UK) Consortium|Davies|United Kingdom

>Spike|hCoV-19/England/CAMC-B21B67/2020|2020-11-01|EPI_ISL_647255|Original|hCoV-19^^England|Human|Lighthouse Lab in Cambridge|Wellcome Sanger Institute for the COVID-19 Genomics UK (COG-UK) Consortium|Davies|United Kingdom
>Spike|hCoV-19/England/CAMC-B214D5/2020|2020-11-03|EPI_ISL_647289|Original|hCoV-19^^England|Human|Lighthouse Lab in Cambridge|Wellcome Sanger Institute for the COVID-19 Genomics UK (COG-UK) Consortium|Davies|United Kingdom
>Spike|hCoV-19/England/CAMC-B224E3/2020|2020-11-02|EPI_ISL_647295|Original|hCoV-19^^England|Human|Lighthouse Lab in Cambridge|Wellcome Sanger Institute for the COVID-19 Genomics UK (COG-UK) Consortium|Davies|United Kingdom
>Spike|hCoV-19/England/CAMC-B218AC/2020|2020-11-01|EPI_ISL_647419|Original|hCoV-19^^England|Human|Lighthouse Lab in Cambridge|Wellcome Sanger Institute for the COVID-19 Genomics UK (COG-UK) Consortium|Davies|United Kingdom
>Spike|hCoV-19/Equatorial Guinea/25536/2020|2020-06-25|EPI_ISL_648370|Original|hCoV-19^^Bioko Norte|Human|Laboratorio de Investigaciones de Baney|University Hospital Basel|Hosch|Equatorial Guinea
>Spike|hCoV-19/USA/CA-ALSR-4288/2020|2020-10-20|EPI_ISL_648940|Original|hCoV-19^^California|Human|San Diego County Public Health Laboratory|Andersen lab at Scripps Research|Gangavarapu|USA
>Spike|hCoV-19/Equatorial Guinea/22908/2020|2020-06-21|EPI_ISL_649163|Original|hCoV-19^^Bioko Norte|Human|Laboratorio de Investigaciones de Baney|University Hospital Basel|Hosch|Equatorial Guinea
>Spike|hCoV-19/Scotland/QEUH-B0E386/2020|2020-11-02|EPI_ISL_649541|Original|hCoV-19^^Scotland|Human|Lighthouse Lab in Glasgow|Wellcome Sanger Institute for the COVID-19 Genomics UK (COG-UK) Consortium|Davies|United Kingdom
>Spike|hCoV-19/England/204381381/2020|2020-10-23|EPI_ISL_649863|Original|hCoV-19^^England|Human|Respiratory Virus Unit|COVID-19 Genomics UK (COG-UK) Consortium|Platt|United Kingdom
>Spike|hCoV-19/France/ARA-SC305/2020|2020-03-23|EPI_ISL_649943|Original|hCoV-19^^Auvergne-Rh\u00f4ne-Alpes|Human|CHU de Saint-\u00c9tienne H\u00f4pital Nord|CNR Virus des Infections Respiratoires - France SUD|Bal|France
>Spike|hCoV-19/England/NORW-F7CAE/2020|2020-11-16|EPI_ISL_650459|Original|hCoV-19^^England|Human|Quadram Institute Bioscience|COVID-19 Genomics UK (COG-UK) Consortium|Alikhan|United Kingdom
>Spike|hCoV-19/England/NORW-F2933/2020|2020-11-10|EPI_ISL_650465|Original|hCoV-19^^England|Human|Quadram Institute Bioscience|COVID-19 Genomics UK (COG-UK) Consortium|Alikhan|United Kingdom
>Spike|hCoV-19/England/NORW-F7C71/2020|2020-11-13|EPI_ISL_650468|Original|hCoV-19^^England|Human|Quadram Institute Bioscience|COVID-19 Genomics UK (COG-UK) Consortium|Alikhan|United Kingdom
>Spike|hCoV-19/England/NORW-F7910/2020|2020-11-10|EPI_ISL_650686|Original|hCoV-19^^England|Human|Quadram Institute Bioscience|COVID-19 Genomics UK (COG-UK) Consortium|Alikhan|United Kingdom
>Spike|hCoV-19/England/NORW-F8830/2020|2020-11-10|EPI_ISL_650722|Original|hCoV-19^^England|Human|Quadram Institute Bioscience|COVID-19 Genomics UK (COG-UK) Consortium|Alikhan|United Kingdom
>Spike|hCoV-19/England/NORW-F5FB6/2020|2020-11-06|EPI_ISL_651326|Original|hCoV-19^^England|Human|Quadram Institute Bioscience|COVID-19 Genomics UK (COG-UK) Consortium|Alikhan|United Kingdom
>Spike|hCoV-19/Wales/MILK-B064E5/2020|2020-10-24|EPI_ISL_652235|Original|hCoV-19^^Wales|Human|Quadram Institute Bioscience|COVID-19 Genomics UK (COG-UK) Consortium|Alikhan|United Kingdom
>Spike|hCoV-19/England/NORW-F610A/2020|2020-11-07|EPI_ISL_652350|Original|hCoV-19^^England|Human|Quadram Institute Bioscience|COVID-19 Genomics UK (COG-UK) Consortium|Alikhan|United Kingdom
>Spike|hCoV-19/Wales/PHWC-48A42C/2020|2020-10-29|EPI_ISL_652553|Original|hCoV-19^^Wales|Human|Wales Specialist Virology Centre Sequencing lab|COVID-19 Genomics UK (COG-UK) Consortium|Moore|United Kingdom
>Spike|hCoV-19/Wales/PHWC-48A486/2020|2020-10-28|EPI_ISL_652557|Original|hCoV-19^^Wales|Human|Wales Specialist Virology Centre Sequencing lab|COVID-19 Genomics UK (COG-UK) Consortium|Moore|United Kingdom

>Spike|hCoV-19/Wales/QEUH-AF63DD/2020|2020-10-29|EPI_ISL_653058|Original|hCoV-
19^^Wales|Human|Quadram Institute Bioscience|COVID-19 Genomics UK (COG-UK)
Consortium|Alikhan|United Kingdom
>Spike|hCoV-19/Spain/MD-IBV-99008789/2020|2020-09-21|EPI_ISL_654140|Original|hCoV-
19^^Madrid|Human|Hospital General Universitario Gregorio Mara\u00f1\u00f3n|SeqCOVID-
SPAIN consortium/IBV(CSIC)|Comas|Spain
>Spike|hCoV-19/Spain/MD-IBV-99008721/2020|2020-10-16|EPI_ISL_654277|Original|hCoV-
19^^Madrid|Human|Hospital General Universitario Gregorio Mara\u00f1\u00f3n|SeqCOVID-
SPAIN consortium/IBV(CSIC)|Comas|Spain
>Spike|hCoV-19/Spain/MD-IBV-99009619/2020|2020-07-02|EPI_ISL_654336|Original|hCoV-
19^^Madrid|Human|Hospital General Universitario Gregorio Mara\u00f1\u00f3n|SeqCOVID-
SPAIN consortium/IBV(CSIC)|Comas|Spain
>Spike|hCoV-19/Spain/MD-IBV-99009625/2020|2020-06-15|EPI_ISL_654337|Original|hCoV-
19^^Madrid|Human|Hospital General Universitario Gregorio Mara\u00f1\u00f3n|SeqCOVID-
SPAIN consortium/IBV(CSIC)|Comas|Spain
>Spike|hCoV-19/Spain/MD-IBV-99009629/2020|2020-07-02|EPI_ISL_654338|Original|hCoV-
19^^Madrid|Human|Hospital General Universitario Gregorio Mara\u00f1\u00f3n|SeqCOVID-
SPAIN consortium/IBV(CSIC)|Comas|Spain
>Spike|hCoV-19/Spain/MD-IBV-99009630/2020|2020-08-08|EPI_ISL_654339|Original|hCoV-
19^^Madrid|Human|Hospital General Universitario Gregorio Mara\u00f1\u00f3n|SeqCOVID-
SPAIN consortium/IBV(CSIC)|Comas|Spain
>Spike|hCoV-19/Spain/MD-IBV-99009631/2020|2020-06-22|EPI_ISL_654340|Original|hCoV-
19^^Madrid|Human|Hospital General Universitario Gregorio Mara\u00f1\u00f3n|SeqCOVID-
SPAIN consortium/IBV(CSIC)|Comas|Spain
>Spike|hCoV-19/Spain/AR-IBV-98008038/2020|2020-09-29|EPI_ISL_654441|Original|hCoV-
19^^Aragon|Human|Servicio de Microbiolog\u00eda|SeqCOVID-SPAIN
consortium/IBV(CSIC)|Comas|Spain
>Spike|hCoV-19/Spain/AR-IBV-98008039/2020|2020-09-29|EPI_ISL_654442|Original|hCoV-
19^^Aragon|Human|Servicio de Microbiolog\u00eda|SeqCOVID-SPAIN
consortium/IBV(CSIC)|Comas|Spain
>Spike|hCoV-19/Spain/AR-IBV-98008041/2020|2020-09-29|EPI_ISL_654443|Original|hCoV-
19^^Aragon|Human|Servicio de Microbiolog\u00eda|SeqCOVID-SPAIN
consortium/IBV(CSIC)|Comas|Spain
>Spike|hCoV-19/Sweden/20-23680/2020|2020-10-05|EPI_ISL_654508|Original|hCoV-
19^^Sweden|Human|The Public Health Agency of Sweden|The Public Health Agency of
Sweden|Svartstr\u00f6m|Sweden
>Spike|hCoV-19/Sweden/20-52831/2020|2020-09-07|EPI_ISL_654956|Original|hCoV-
19^^Vastra Gotaland|Human|Skovde/Unilabs|The Public Health Agency of
Sweden|Svartstr\u00f6m|Sweden
>Spike|hCoV-19/England/ALDP-B5B988/2020|2020-11-08|EPI_ISL_655379|Original|hCoV-
19^^England|Human|Lighthouse Lab in Alderley Park|Wellcome Sanger Institute for the
COVID-19 Genomics UK (COG-UK) Consortium|Davies|United Kingdom
>Spike|hCoV-19/England/ALDP-B5AEE4/2020|2020-11-09|EPI_ISL_655586|Original|hCoV-
19^^England|Human|Lighthouse Lab in Alderley Park|Wellcome Sanger Institute for the
COVID-19 Genomics UK (COG-UK) Consortium|Davies|United Kingdom
>Spike|hCoV-19/England/ALDP-B5C85D/2020|2020-11-10|EPI_ISL_655717|Original|hCoV-
19^^England|Human|Lighthouse Lab in Alderley Park|Wellcome Sanger Institute for the
COVID-19 Genomics UK (COG-UK) Consortium|Davies|United Kingdom
>Spike|hCoV-19/England/QEUH-B660A3/2020|2020-11-10|EPI_ISL_655828|Original|hCoV-
19^^England|Human|Lighthouse Lab in Glasgow|Wellcome Sanger Institute for the COVID-
19 Genomics UK (COG-UK) Consortium|Davies|United Kingdom
>Spike|hCoV-19/England/ALDP-B4604F/2020|2020-11-07|EPI_ISL_656073|Original|hCoV-
19^^England|Human|Lighthouse Lab in Alderley Park|Wellcome Sanger Institute for the
COVID-19 Genomics UK (COG-UK) Consortium|Davies|United Kingdom
>Spike|hCoV-19/England/CAMC-B542C0/2020|2020-11-08|EPI_ISL_656767|Original|hCoV-
19^^England|Human|Lighthouse Lab in Cambridge|Wellcome Sanger Institute for the
COVID-19 Genomics UK (COG-UK) Consortium|Davies|United Kingdom
>Spike|hCoV-19/England/QEUH-B408F2/2020|2020-11-08|EPI_ISL_657202|Original|hCoV-
19^^England|Human|Lighthouse Lab in Glasgow|Wellcome Sanger Institute for the COVID-
19 Genomics UK (COG-UK) Consortium|Davies|United Kingdom

>Spike|hCoV-19/England/MILK-B54E9F/2020|2020-11-06|EPI_ISL_657975|Original|hCoV-19^^England|Human|Lighthouse Lab in Milton Keynes|Wellcome Sanger Institute for the COVID-19 Genomics UK (COG-UK) Consortium|Davies|United Kingdom
>Spike|hCoV-19/England/MILK-B5A585/2020|2020-11-06|EPI_ISL_658232|Original|hCoV-19^^England|Human|Lighthouse Lab in Milton Keynes|Wellcome Sanger Institute for the COVID-19 Genomics UK (COG-UK) Consortium|Davies|United Kingdom
>Spike|hCoV-19/England/MILK-B38C6A/2020|2020-11-01|EPI_ISL_659059|Original|hCoV-19^^England|Human|Lighthouse Lab in Milton Keynes|Wellcome Sanger Institute for the COVID-19 Genomics UK (COG-UK) Consortium|Davies|United Kingdom
>Spike|hCoV-19/England/CAMC-B32A2A/2020|2020-11-05|EPI_ISL_659474|Original|hCoV-19^^England|Human|Lighthouse Lab in Cambridge|Wellcome Sanger Institute for the COVID-19 Genomics UK (COG-UK) Consortium|Davies|United Kingdom
>Spike|hCoV-19/England/CAMC-B3419C/2020|2020-11-05|EPI_ISL_659566|Original|hCoV-19^^England|Human|Lighthouse Lab in Cambridge|Wellcome Sanger Institute for the COVID-19 Genomics UK (COG-UK) Consortium|Davies|United Kingdom
>Spike|hCoV-19/USA/MD-MDH-0376/2020|2020-10-25|EPI_ISL_660077|Original|hCoV-19^^Maryland|Human|MD PHL|MD PHL|Keller|USA
>Spike|hCoV-19/South Africa/KRISP-K004277-CE1/2020|2020-11-05|EPI_ISL_660161|Original|hCoV-19^^KwaZulu-Natal|Human|NHLS-IALCH|KRISP|de Oliveira|South Africa
>Spike|hCoV-19/South Africa/KRISP-K004316/2020|2020-10-26|EPI_ISL_660193|Original|hCoV-19^^KwaZulu-Natal|Human|NHLS-IALCH|KRISP|de Oliveira|South Africa
>Spike|hCoV-19/South Africa/KRISP-K004321/2020|2020-10-26|EPI_ISL_660197|Original|hCoV-19^^KwaZulu-Natal|Human|NHLS-IALCH|KRISP|de Oliveira|South Africa
>Spike|hCoV-19/South Africa/KRISP-K004725-CS1/2020|2020-11-18|EPI_ISL_660258|Original|hCoV-19^^KwaZulu-Natal|Human|KRISP|KRISP|de Oliveira|South Africa
>Spike|hCoV-19/France/ARA-SC361/2020|2020-03-07|EPI_ISL_660346|Original|hCoV-19^^Auvergne-Rh\u00f4ne-Alpes|Human|CHU de Saint-\u00c9tienne H\u00f4pital Nord|CNR Virus des Infections Respiratoires - France SUD|Bal|France
>Spike|hCoV-19/France/NAQ-SC226/2020|2020-04-28|EPI_ISL_660372|Original|hCoV-19^^Nouvelle-Aquitaine|Human|CHU Bordeaux|CNR Virus des Infections Respiratoires - France SUD|Bal|France
>Spike|hCoV-19/Ecuador/USFQ-231/2020|2020-10-01|EPI_ISL_660531|Original|hCoV-19^^Tungurahua|Human|Institute of Microbiology|Institute of Microbiology|Cardenas|Ecuador
>Spike|hCoV-19/England/204380390/2020|2020-10-21|EPI_ISL_660547|Original|hCoV-19^^England|Human|Respiratory Virus Unit|COVID-19 Genomics UK (COG-UK) Consortium|Platt|United Kingdom
>Spike|hCoV-19/England/204380637/2020|2020-10-22|EPI_ISL_660548|Original|hCoV-19^^England|Human|Respiratory Virus Unit|COVID-19 Genomics UK (COG-UK) Consortium|Platt|United Kingdom
>Spike|hCoV-19/England/204390705/2020|2020-10-23|EPI_ISL_660549|Original|hCoV-19^^England|Human|Respiratory Virus Unit|COVID-19 Genomics UK (COG-UK) Consortium|Platt|United Kingdom
>Spike|hCoV-19/England/204391077/2020|2020-10-24|EPI_ISL_660550|Original|hCoV-19^^England|Human|Respiratory Virus Unit|COVID-19 Genomics UK (COG-UK) Consortium|Platt|United Kingdom
>Spike|hCoV-19/Spain/CN-IBV-97006480/2020|2020-03-24|EPI_ISL_660600|Original|hCoV-19^^Canary_Islands|Human|Hospital Universitario de Gran Canaria Dr. Negr\u00edn|SeqCOVID-SPAIN consortium/IBV(CSIC)|Comas|Spain
>Spike|hCoV-19/England/204394007/2020|2020-10-25|EPI_ISL_660601|Original|hCoV-19^^England|Human|Respiratory Virus Unit|COVID-19 Genomics UK (COG-UK) Consortium|Platt|United Kingdom
>Spike|hCoV-19/England/204400758/2020|2020-10-26|EPI_ISL_660602|Original|hCoV-19^^England|Human|Respiratory Virus Unit|COVID-19 Genomics UK (COG-UK) Consortium|Platt|United Kingdom

>Spike|hCoV-19/England/204421003/2020|2020-10-27|EPI_ISL_660603|Original|hCoV-19^^England|Human|Respiratory Virus Unit|COVID-19 Genomics UK (COG-UK) Consortium|Platt|United Kingdom
>Spike|hCoV-19/England/204441634/2020|2020-10-28|EPI_ISL_660604|Original|hCoV-19^^England|Human|Respiratory Virus Unit|COVID-19 Genomics UK (COG-UK) Consortium|Platt|United Kingdom
>Spike|hCoV-19/France/OCC-SC509/2020|2020-04-23|EPI_ISL_660700|Original|hCoV-19^^Occitanie|Human|CHU Montpellier|CNR Virus des Infections Respiratoires - France SUD|Bal|France
>Spike|hCoV-19/France/OCC-SC518/2020|2020-04-30|EPI_ISL_660706|Original|hCoV-19^^Occitanie|Human|CHU Montpellier|CNR Virus des Infections Respiratoires - France SUD|Bal|France
>Spike|hCoV-19/France/OCC-SC552/2020|2020-04-03|EPI_ISL_660723|Original|hCoV-19^^Occitanie|Human|CHU N\u00eemes|CNR Virus des Infections Respiratoires - France SUD|Bal|France
>Spike|hCoV-19/France/OCC-SC555/2020|2020-04-04|EPI_ISL_660724|Original|hCoV-19^^Occitanie|Human|CHU N\u00eemes|CNR Virus des Infections Respiratoires - France SUD|Bal|France
>Spike|hCoV-19/England/QEUH-B69B54/2020|2020-11-11|EPI_ISL_661390|Original|hCoV-19^^England|Human|Lighthouse Lab in Glasgow|Wellcome Sanger Institute for the COVID-19 Genomics UK (COG-UK) Consortium|Davies|United Kingdom
>Spike|hCoV-19/England/QEUH-B80568/2020|2020-11-14|EPI_ISL_661862|Original|hCoV-19^^England|Human|Lighthouse Lab in Glasgow|Wellcome Sanger Institute for the COVID-19 Genomics UK (COG-UK) Consortium|Davies|United Kingdom
>Spike|hCoV-19/England/ALDP-B74321/2020|2020-11-12|EPI_ISL_662234|Original|hCoV-19^^England|Human|Lighthouse Lab in Alderley Park|Wellcome Sanger Institute for the COVID-19 Genomics UK (COG-UK) Consortium|Davies|United Kingdom
>Spike|hCoV-19/England/QEUH-AF27D5/2020|2020-10-30|EPI_ISL_662739|Original|hCoV-19^^England|Human|Lighthouse Lab in Glasgow|Wellcome Sanger Institute for the COVID-19 Genomics UK (COG-UK) Consortium|Davies|United Kingdom
>Spike|hCoV-19/England/QEUH-AF27A8/2020|2020-10-30|EPI_ISL_662850|Original|hCoV-19^^England|Human|Lighthouse Lab in Glasgow|Wellcome Sanger Institute for the COVID-19 Genomics UK (COG-UK) Consortium|Davies|United Kingdom
>Spike|hCoV-19/England/QEUH-AF4C4D/2020|2020-10-30|EPI_ISL_662906|Original|hCoV-19^^England|Human|Lighthouse Lab in Glasgow|Wellcome Sanger Institute for the COVID-19 Genomics UK (COG-UK) Consortium|Davies|United Kingdom
>Spike|hCoV-19/England/QEUH-AF407E/2020|2020-10-30|EPI_ISL_663054|Original|hCoV-19^^England|Human|Lighthouse Lab in Glasgow|Wellcome Sanger Institute for the COVID-19 Genomics UK (COG-UK) Consortium|Davies|United Kingdom
>Spike|hCoV-19/France/PDL-SC637/2020|2020-04-04|EPI_ISL_663276|Original|hCoV-19^^Pays-de-la-Loire|Human|CHU Nantes|CNR Virus des Infections Respiratoires - France SUD|Bal|France
>Spike|hCoV-19/Australia/VIC16905/2020|2020-08-06|EPI_ISL_663529|Original|hCoV-19^^Victoria|Human|Microbiological Diagnostic Unit - Public Health Laboratory (MDU-PHL)|MDU-PHL|Schultz|Australia
>Spike|hCoV-19/Australia/VIC16908/2020|2020-08-07|EPI_ISL_663530|Original|hCoV-19^^Victoria|Human|Microbiological Diagnostic Unit - Public Health Laboratory (MDU-PHL)|MDU-PHL|Schultz|Australia
>Spike|hCoV-19/Australia/VIC16924/2020|2020-08-06|EPI_ISL_663532|Original|hCoV-19^^Victoria|Human|Microbiological Diagnostic Unit - Public Health Laboratory (MDU-PHL)|MDU-PHL|Schultz|Australia
>Spike|hCoV-19/Australia/VIC16925/2020|2020-08-06|EPI_ISL_663533|Original|hCoV-19^^Victoria|Human|Microbiological Diagnostic Unit - Public Health Laboratory (MDU-PHL)|MDU-PHL|Schultz|Australia
>Spike|hCoV-19/Australia/VIC16927/2020|2020-08-06|EPI_ISL_663534|Original|hCoV-19^^Victoria|Human|Microbiological Diagnostic Unit - Public Health Laboratory (MDU-PHL)|MDU-PHL|Schultz|Australia
>Spike|hCoV-19/England/ALDP-B73F00/2020|2020-11-12|EPI_ISL_664351|Original|hCoV-19^^England|Human|Quadram Institute Bioscience|COVID-19 Genomics UK (COG-UK) Consortium|Alikhan|United Kingdom

>Spike|hCoV-19/England/ALDP-B740FD/2020|2020-11-12|EPI_ISL_664508|Original|hCoV-19^^England|Human|Quadram Institute Bioscience|COVID-19 Genomics UK (COG-UK) Consortium|Alikhan|United Kingdom
>Spike|hCoV-19/England/ALDP-B7396C/2020|2020-11-12|EPI_ISL_664741|Original|hCoV-19^^England|Human|Quadram Institute Bioscience|COVID-19 Genomics UK (COG-UK) Consortium|Alikhan|United Kingdom
>Spike|hCoV-19/England/LOND-1250628/2020|2020-03-23|EPI_ISL_664742|Original|hCoV-19^^England|Human|University College London Hospital|COVID-19 Genomics UK (COG-UK) Consortium|Byott|United Kingdom
>Spike|hCoV-19/England/ALDP-B741DC/2020|2020-11-12|EPI_ISL_664910|Original|hCoV-19^^England|Human|Quadram Institute Bioscience|COVID-19 Genomics UK (COG-UK) Consortium|Alikhan|United Kingdom
>Spike|hCoV-19/Wales/PHWC-48AB27/2020|2020-11-06|EPI_ISL_665546|Original|hCoV-19^^Wales|Human|Wales Specialist Virology Centre Sequencing lab|COVID-19 Genomics UK (COG-UK) Consortium|Moore|United Kingdom
>Spike|hCoV-19/Wales/PHWC-48B036/2020|2020-11-04|EPI_ISL_665582|Original|hCoV-19^^Wales|Human|Wales Specialist Virology Centre Sequencing lab|COVID-19 Genomics UK (COG-UK) Consortium|Moore|United Kingdom
>Spike|hCoV-19/Wales/PHWC-48AED3/2020|2020-11-03|EPI_ISL_665608|Original|hCoV-19^^Wales|Human|Wales Specialist Virology Centre Sequencing lab|COVID-19 Genomics UK (COG-UK) Consortium|Moore|United Kingdom
>Spike|hCoV-19/Wales/PHWC-48B3B5/2020|2020-11-04|EPI_ISL_665753|Original|hCoV-19^^Wales|Human|Wales Specialist Virology Centre Sequencing lab|COVID-19 Genomics UK (COG-UK) Consortium|Moore|United Kingdom
>Spike|hCoV-19/Wales/PHWC-48AA48/2020|2020-10-28|EPI_ISL_665795|Original|hCoV-19^^Wales|Human|Wales Specialist Virology Centre Sequencing lab|COVID-19 Genomics UK (COG-UK) Consortium|Moore|United Kingdom
>Spike|hCoV-19/England/NORW-F5147/2020|2020-11-05|EPI_ISL_665886|Original|hCoV-19^^England|Human|Quadram Institute Bioscience|COVID-19 Genomics UK (COG-UK) Consortium|Alikhan|United Kingdom
>Spike|hCoV-19/Wales/PHWC-48A9D2/2020|2020-11-05|EPI_ISL_666110|Original|hCoV-19^^Wales|Human|Wales Specialist Virology Centre Sequencing lab|COVID-19 Genomics UK (COG-UK) Consortium|Moore|United Kingdom
>Spike|hCoV-19/Wales/PHWC-48ABFA/2020|2020-11-03|EPI_ISL_666134|Original|hCoV-19^^Wales|Human|Wales Specialist Virology Centre Sequencing lab|COVID-19 Genomics UK (COG-UK) Consortium|Moore|United Kingdom
>Spike|hCoV-19/Wales/PHWC-48ADD6/2020|2020-11-04|EPI_ISL_666159|Original|hCoV-19^^Wales|Human|Wales Specialist Virology Centre Sequencing lab|COVID-19 Genomics UK (COG-UK) Consortium|Moore|United Kingdom
>Spike|hCoV-19/Wales/PHWC-48ADF4/2020|2020-10-28|EPI_ISL_666161|Original|hCoV-19^^Wales|Human|Wales Specialist Virology Centre Sequencing lab|COVID-19 Genomics UK (COG-UK) Consortium|Moore|United Kingdom
>Spike|hCoV-19/Wales/PHWC-48B142/2020|2020-11-01|EPI_ISL_666197|Original|hCoV-19^^Wales|Human|Wales Specialist Virology Centre Sequencing lab|COVID-19 Genomics UK (COG-UK) Consortium|Moore|United Kingdom
>Spike|hCoV-19/Wales/PHWC-48B1BB/2020|2020-11-05|EPI_ISL_666203|Original|hCoV-19^^Wales|Human|Wales Specialist Virology Centre Sequencing lab|COVID-19 Genomics UK (COG-UK) Consortium|Moore|United Kingdom
>Spike|hCoV-19/Wales/PHWC-48B379/2020|2020-10-31|EPI_ISL_666227|Original|hCoV-19^^Wales|Human|Wales Specialist Virology Centre Sequencing lab|COVID-19 Genomics UK (COG-UK) Consortium|Moore|United Kingdom
>Spike|hCoV-19/Wales/PHWC-48B388/2020|2020-11-05|EPI_ISL_666228|Original|hCoV-19^^Wales|Human|Wales Specialist Virology Centre Sequencing lab|COVID-19 Genomics UK (COG-UK) Consortium|Moore|United Kingdom
>Spike|hCoV-19/Wales/PHWC-48B397/2020|2020-10-30|EPI_ISL_666229|Original|hCoV-19^^Wales|Human|Wales Specialist Virology Centre Sequencing lab|COVID-19 Genomics UK (COG-UK) Consortium|Moore|United Kingdom
>Spike|hCoV-19/Wales/PHWC-48B50A/2020|2020-11-02|EPI_ISL_666247|Original|hCoV-19^^Wales|Human|Wales Specialist Virology Centre Sequencing lab|COVID-19 Genomics UK (COG-UK) Consortium|Moore|United Kingdom

>Spike|hCoV-19/Wales/PHWC-48B78C/2020|2020-10-30|EPI_ISL_666280|Original|hCoV-19^^Wales|Human|Wales Specialist Virology Centre Sequencing lab|COVID-19 Genomics UK (COG-UK) Consortium|Moore|United Kingdom
>Spike|hCoV-19/Wales/PHWC-48BADE/2020|2020-10-31|EPI_ISL_666321|Original|hCoV-19^^Wales|Human|Wales Specialist Virology Centre Sequencing lab|COVID-19 Genomics UK (COG-UK) Consortium|Moore|United Kingdom
>Spike|hCoV-19/Wales/PHWC-48C220/2020|2020-11-07|EPI_ISL_666408|Original|hCoV-19^^Wales|Human|Wales Specialist Virology Centre Sequencing lab|COVID-19 Genomics UK (COG-UK) Consortium|Moore|United Kingdom
>Spike|hCoV-19/Wales/PHWC-48C703/2020|2020-11-02|EPI_ISL_666460|Original|hCoV-19^^Wales|Human|Wales Specialist Virology Centre Sequencing lab|COVID-19 Genomics UK (COG-UK) Consortium|Moore|United Kingdom
>Spike|hCoV-19/Wales/PHWC-48CBAD/2020|2020-11-08|EPI_ISL_666515|Original|hCoV-19^^Wales|Human|Wales Specialist Virology Centre Sequencing lab|COVID-19 Genomics UK (COG-UK) Consortium|Moore|United Kingdom
>Spike|hCoV-19/Wales/PHWC-48D052/2020|2020-11-05|EPI_ISL_666572|Original|hCoV-19^^Wales|Human|Wales Specialist Virology Centre Sequencing lab|COVID-19 Genomics UK (COG-UK) Consortium|Moore|United Kingdom
>Spike|hCoV-19/France/ARA-SC019/2020|2020-03-16|EPI_ISL_666654|Original|hCoV-19^^Auvergne-Rh\u00f4ne-Alpes|Human|Laboratoire du Centre Hospitalier Annecy Genevois|CNR Virus des Infections Respiratoires - France SUD|Bal|France
>Spike|hCoV-19/France/ARA-SC077/2020|2020-04-30|EPI_ISL_666680|Original|hCoV-19^^Auvergne-Rh\u00f4ne-Alpes|Human|Laboratoire du Centre Hospitalier Annecy Genevois|CNR Virus des Infections Respiratoires - France SUD|Bal|France
>Spike|hCoV-19/USA/CA-ALSR-4524/2020|2020-10-22|EPI_ISL_666985|Original|hCoV-19^^California|Human|San Diego County Public Health Laboratory|Andersen lab at Scripps Research|Gangavarapu|USA
>Spike|hCoV-19/USA/OR-OHSU-2009/2020|2020-07-31|EPI_ISL_667089|Original|hCoV-19^^Oregon|Human|OHSU Lab Services Molecular Microbiology Lab|Oregon SARS-CoV-2 Genome Sequencing Center|O'Roak|USA
>Spike|hCoV-19/USA/OR-OHSU-2189/2020|2020-08-06|EPI_ISL_667141|Original|hCoV-19^^Oregon|Human|OHSU Lab Services Molecular Microbiology Lab|Oregon SARS-CoV-2 Genome Sequencing Center|O'Roak|USA
>Spike|hCoV-19/USA/OR-OHSU-2443/2020|2020-08-14|EPI_ISL_667209|Original|hCoV-19^^Oregon|Human|OHSU Lab Services Molecular Microbiology Lab|Oregon SARS-CoV-2 Genome Sequencing Center|O'Roak|USA
>Spike|hCoV-19/USA/OR-OHSU-2451/2020|2020-08-15|EPI_ISL_667212|Original|hCoV-19^^Oregon|Human|OHSU Lab Services Molecular Microbiology Lab|Oregon SARS-CoV-2 Genome Sequencing Center|O'Roak|USA
>Spike|hCoV-19/USA/OR-OHSU-2457/2020|2020-08-15|EPI_ISL_667213|Original|hCoV-19^^Oregon|Human|OHSU Lab Services Molecular Microbiology Lab|Oregon SARS-CoV-2 Genome Sequencing Center|O'Roak|USA
>Spike|hCoV-19/USA/OR-OHSU-2463/2020|2020-08-15|EPI_ISL_667216|Original|hCoV-19^^Oregon|Human|OHSU Lab Services Molecular Microbiology Lab|Oregon SARS-CoV-2 Genome Sequencing Center|O'Roak|USA
>Spike|hCoV-19/USA/OR-OHSU-2477/2020|2020-08-01|EPI_ISL_667221|Original|hCoV-19^^Oregon|Human|OHSU Lab Services Molecular Microbiology Lab|Oregon SARS-CoV-2 Genome Sequencing Center|O'Roak|USA
>Spike|hCoV-19/USA/OR-OHSU-2525/2020|2020-08-18|EPI_ISL_667238|Original|hCoV-19^^Oregon|Human|OHSU Lab Services Molecular Microbiology Lab|Oregon SARS-CoV-2 Genome Sequencing Center|O'Roak|USA
>Spike|hCoV-19/USA/OR-OHSU-2607/2020|2020-08-21|EPI_ISL_667263|Original|hCoV-19^^Oregon|Human|OHSU Lab Services Molecular Microbiology Lab|Oregon SARS-CoV-2 Genome Sequencing Center|O'Roak|USA
>Spike|hCoV-19/USA/OR-OHSU-2765/2020|2020-08-29|EPI_ISL_667304|Original|hCoV-19^^Oregon|Human|OHSU Lab Services Molecular Microbiology Lab|Oregon SARS-CoV-2 Genome Sequencing Center|O'Roak|USA
>Spike|hCoV-19/USA/OR-OHSU-2844/2020|2020-09-02|EPI_ISL_667349|Original|hCoV-19^^Oregon|Human|OHSU Lab Services Molecular Microbiology Lab|Oregon SARS-CoV-2 Genome Sequencing Center|O'Roak|USA

>Spike|hCoV-19/USA/OR-OHSU-2851/2020|2020-09-02|EPI_ISL_667354|Original|hCoV-19^^Oregon|Human|OHSU Lab Services Molecular Microbiology Lab|Oregon SARS-CoV-2 Genome Sequencing Center|O'Roak|USA
>Spike|hCoV-19/USA/OR-OHSU-2854/2020|2020-09-02|EPI_ISL_667357|Original|hCoV-19^^Oregon|Human|OHSU Lab Services Molecular Microbiology Lab|Oregon SARS-CoV-2 Genome Sequencing Center|O'Roak|USA
>Spike|hCoV-19/USA/WA-OHSU-2878/2020|2020-09-02|EPI_ISL_667379|Original|hCoV-19^^Washington|Human|OHSU Lab Services Molecular Microbiology Lab|Oregon SARS-CoV-2 Genome Sequencing Center|O'Roak|USA
>Spike|hCoV-19/USA/OR-OHSU-2889/2020|2020-09-04|EPI_ISL_667390|Original|hCoV-19^^Oregon|Human|OHSU Lab Services Molecular Microbiology Lab|Oregon SARS-CoV-2 Genome Sequencing Center|O'Roak|USA
>Spike|hCoV-19/USA/OR-OHSU-2892/2020|2020-09-04|EPI_ISL_667393|Original|hCoV-19^^Oregon|Human|OHSU Lab Services Molecular Microbiology Lab|Oregon SARS-CoV-2 Genome Sequencing Center|O'Roak|USA
>Spike|hCoV-19/USA/OR-OHSU-2923/2020|2020-09-05|EPI_ISL_667419|Original|hCoV-19^^Oregon|Human|OHSU Lab Services Molecular Microbiology Lab|Oregon SARS-CoV-2 Genome Sequencing Center|O'Roak|USA
>Spike|hCoV-19/USA/OR-OHSU-2931/2020|2020-09-07|EPI_ISL_667426|Original|hCoV-19^^Oregon|Human|OHSU Lab Services Molecular Microbiology Lab|Oregon SARS-CoV-2 Genome Sequencing Center|O'Roak|USA
>Spike|hCoV-19/USA/OR-OHSU-3052/2020|2020-09-30|EPI_ISL_667461|Original|hCoV-19^^Oregon|Human|OHSU Lab Services Molecular Microbiology Lab|Oregon SARS-CoV-2 Genome Sequencing Center|O'Roak|USA
>Spike|hCoV-19/USA/OR-OHSU-3115/2020|2020-10-02|EPI_ISL_667487|Original|hCoV-19^^Oregon|Human|OHSU Lab Services Molecular Microbiology Lab|Oregon SARS-CoV-2 Genome Sequencing Center|O'Roak|USA
>Spike|hCoV-19/USA/OR-OHSU-3119/2020|2020-10-02|EPI_ISL_667489|Original|hCoV-19^^Oregon|Human|OHSU Lab Services Molecular Microbiology Lab|Oregon SARS-CoV-2 Genome Sequencing Center|O'Roak|USA
>Spike|hCoV-19/USA/OR-OHSU-3139/2020|2020-10-03|EPI_ISL_667493|Original|hCoV-19^^Oregon|Human|OHSU Lab Services Molecular Microbiology Lab|Oregon SARS-CoV-2 Genome Sequencing Center|O'Roak|USA
>Spike|hCoV-19/USA/WA-OHSU-3149/2020|2020-10-05|EPI_ISL_667494|Original|hCoV-19^^Washington|Human|OHSU Lab Services Molecular Microbiology Lab|Oregon SARS-CoV-2 Genome Sequencing Center|O'Roak|USA
>Spike|hCoV-19/USA/OR-OHSU-3153/2020|2020-10-03|EPI_ISL_667495|Original|hCoV-19^^Oregon|Human|OHSU Lab Services Molecular Microbiology Lab|Oregon SARS-CoV-2 Genome Sequencing Center|O'Roak|USA
>Spike|hCoV-19/USA/OR-OHSU-3165/2020|2020-10-05|EPI_ISL_667496|Original|hCoV-19^^Oregon|Human|OHSU Lab Services Molecular Microbiology Lab|Oregon SARS-CoV-2 Genome Sequencing Center|O'Roak|USA
>Spike|hCoV-19/Japan/IC-0283/2020|2020-11-00|EPI_ISL_667712|Original|hCoV-19^^Japan|Human|Pathogen Genomics Center|Pathogen Genomics Center|Sekizuka|Japan
>Spike|hCoV-19/England/CAMC-B79122/2020|2020-11-12|EPI_ISL_667854|Original|hCoV-19^^England|Human|Lighthouse Lab in Cambridge|Wellcome Sanger Institute for the COVID-19 Genomics UK (COG-UK) Consortium|Davies|United Kingdom
>Spike|hCoV-19/England/CAMC-B79948/2020|2020-11-10|EPI_ISL_667865|Original|hCoV-19^^England|Human|Lighthouse Lab in Cambridge|Wellcome Sanger Institute for the COVID-19 Genomics UK (COG-UK) Consortium|Davies|United Kingdom
>Spike|hCoV-19/England/CAMC-B79140/2020|2020-11-12|EPI_ISL_667908|Original|hCoV-19^^England|Human|Lighthouse Lab in Cambridge|Wellcome Sanger Institute for the COVID-19 Genomics UK (COG-UK) Consortium|Davies|United Kingdom
>Spike|hCoV-19/England/QEUH-B819A7/2020|2020-11-14|EPI_ISL_668044|Original|hCoV-19^^England|Human|Lighthouse Lab in Glasgow|Wellcome Sanger Institute for the COVID-19 Genomics UK (COG-UK) Consortium|Davies|United Kingdom
>Spike|hCoV-19/England/CAMC-B77FB1/2020|2020-11-12|EPI_ISL_668163|Original|hCoV-19^^England|Human|Lighthouse Lab in Cambridge|Wellcome Sanger Institute for the COVID-19 Genomics UK (COG-UK) Consortium|Davies|United Kingdom

>Spike|hCoV-19/England/QEUH-B7E0F3/2020|2020-11-14|EPI_ISL_668293|Original|hCoV-
19^^England|Human|Lighthouse Lab in Glasgow|Wellcome Sanger Institute for the COVID-
19 Genomics UK (COG-UK) Consortium|Davies|United Kingdom
>Spike|hCoV-19/Denmark/DCGC-9973/2020|2020-11-09|EPI_ISL_668463|Original|hCoV-
19^^Hovedstaden|Human|Department of Virus and Microbiological Special
Diagnostics|Albertsen Lab|Michaelsen|Denmark
>Spike|hCoV-19/Denmark/DCGC-9232/2020|2020-11-16|EPI_ISL_668465|Original|hCoV-
19^^Hovedstaden|Human|Department of Virus and Microbiological Special
Diagnostics|Albertsen Lab|Michaelsen|Denmark
>Spike|hCoV-19/Denmark/DCGC-10166/2020|2020-11-09|EPI_ISL_668625|Original|hCoV-
19^^Nordjylland|Human|Department of Virus and Microbiological Special
Diagnostics|Albertsen Lab|Michaelsen|Denmark
>Spike|hCoV-19/Denmark/DCGC-11079/2020|2020-11-02|EPI_ISL_669464|Original|hCoV-
19^^Hovedstaden|Human|Department of Virus and Microbiological Special
Diagnostics|Albertsen Lab|Michaelsen|Denmark
>Spike|hCoV-19/Denmark/DCGC-10964/2020|2020-11-02|EPI_ISL_669593|Original|hCoV-
19^^Hovedstaden|Human|Department of Virus and Microbiological Special
Diagnostics|Albertsen Lab|Michaelsen|Denmark
>Spike|hCoV-19/Denmark/DCGC-10669/2020|2020-11-02|EPI_ISL_669594|Original|hCoV-
19^^Nordjylland|Human|Department of Virus and Microbiological Special
Diagnostics|Albertsen Lab|Michaelsen|Denmark
>Spike|hCoV-19/Denmark/DCGC-10666/2020|2020-11-02|EPI_ISL_669595|Original|hCoV-
19^^Sjaelland|Human|Department of Virus and Microbiological Special
Diagnostics|Albertsen Lab|Michaelsen|Denmark
>Spike|hCoV-19/Denmark/DCGC-12022/2020|2020-03-09|EPI_ISL_670461|Original|hCoV-
19^^Nordjylland|Human|Department of Virus and Microbiological Special
Diagnostics|Albertsen Lab|Michaelsen|Denmark
>Spike|hCoV-19/Canada/ON-PHL-20-02208/2020|2020-09-00|EPI_ISL_671520|Original|hCoV-
19^^Ontario|Human|Public Health Ontario Laboratory|Public Health Ontario
Laboratory|Guthrie|Canada
>Spike|hCoV-19/Canada/ON-PHL-20-02291/2020|2020-09-00|EPI_ISL_671521|Original|hCoV-
19^^Ontario|Human|Public Health Ontario Laboratory|Public Health Ontario
Laboratory|Guthrie|Canada
>Spike|hCoV-19/Canada/ON-PHL-20-02332/2020|2020-09-00|EPI_ISL_671556|Original|hCoV-
19^^Ontario|Human|Public Health Ontario Laboratory|Public Health Ontario
Laboratory|Guthrie|Canada
>Spike|hCoV-19/Canada/ON-PHL-20-02406/2020|2020-09-00|EPI_ISL_671596|Original|hCoV-
19^^Ontario|Human|Public Health Ontario Laboratory|Public Health Ontario
Laboratory|Guthrie|Canada
>Spike|hCoV-19/Canada/ON-UHTC-0315/2020|2020-09-20|EPI_ISL_671672|Original|hCoV-
19^^Ontario|Human|Unity Health Toronto|Ontario Institute for Cancer Research|de
Borja|Canada
>Spike|hCoV-19/USA/NY-NYCPHL-001108/2020|2020-10-19|EPI_ISL_671691|Original|hCoV-
19^^New York|Human|DOHMH Corona|New York City Public Health Laboratory|Wang|USA
>Spike|hCoV-19/USA/NY-NYCPHL-001126/2020|2020-10-26|EPI_ISL_671709|Original|hCoV-
19^^New York|Human|DOHMH Riverside|New York City Public Health Laboratory|Wang|USA
>Spike|hCoV-19/USA/NY-NYCPHL-001128/2020|2020-10-26|EPI_ISL_671711|Original|hCoV-
19^^New York|Human|DOHMH Corona|New York City Public Health Laboratory|Wang|USA
>Spike|hCoV-19/USA/NY-NYCPHL-001168/2020|2020-10-29|EPI_ISL_671748|Original|hCoV-
19^^New York|Human|DOHMH Riverside|New York City Public Health Laboratory|Wang|USA
>Spike|hCoV-19/USA/NY-NYCPHL-001169/2020|2020-10-29|EPI_ISL_671749|Original|hCoV-
19^^New York|Human|DOHMH Riverside|New York City Public Health Laboratory|Wang|USA
>Spike|hCoV-19/USA/NY-NYCPHL-001170/2020|2020-10-29|EPI_ISL_671750|Original|hCoV-
19^^New York|Human|DOHMH Riverside|New York City Public Health Laboratory|Wang|USA
>Spike|hCoV-19/England/QEUH-BA33FC/2020|2020-11-18|EPI_ISL_672826|Original|hCoV-
19^^England|Human|Lighthouse Lab in Glasgow|Wellcome Sanger Institute for the COVID-
19 Genomics UK (COG-UK) Consortium|Davies|United Kingdom
>Spike|hCoV-19/England/CAMC-B9C5F0/2020|2020-11-15|EPI_ISL_673501|Original|hCoV-
19^^England|Human|Lighthouse Lab in Cambridge|Wellcome Sanger Institute for the
COVID-19 Genomics UK (COG-UK) Consortium|Davies|United Kingdom

>Spike|hCoV-19/England/CAMC-B9A6E0/2020|2020-11-15|EPI_ISL_673668|Original|hCoV-19^^England|Human|Lighthouse Lab in Cambridge|Wellcome Sanger Institute for the COVID-19 Genomics UK (COG-UK) Consortium|Davies|United Kingdom
>Spike|hCoV-19/England/MILK-B94B6F/2020|2020-11-14|EPI_ISL_673686|Original|hCoV-19^^England|Human|Lighthouse Lab in Milton Keynes|Wellcome Sanger Institute for the COVID-19 Genomics UK (COG-UK) Consortium|Davies|United Kingdom
>Spike|hCoV-19/England/MILK-B8D62C/2020|2020-11-13|EPI_ISL_673988|Original|hCoV-19^^England|Human|Lighthouse Lab in Milton Keynes|Wellcome Sanger Institute for the COVID-19 Genomics UK (COG-UK) Consortium|Davies|United Kingdom
>Spike|hCoV-19/England/MILK-B8D404/2020|2020-11-13|EPI_ISL_674018|Original|hCoV-19^^England|Human|Lighthouse Lab in Milton Keynes|Wellcome Sanger Institute for the COVID-19 Genomics UK (COG-UK) Consortium|Davies|United Kingdom
>Spike|hCoV-19/England/MILK-B86526/2020|2020-11-13|EPI_ISL_674119|Original|hCoV-19^^England|Human|Lighthouse Lab in Milton Keynes|Wellcome Sanger Institute for the COVID-19 Genomics UK (COG-UK) Consortium|Davies|United Kingdom
>Spike|hCoV-19/England/MILK-B8DD45/2020|2020-11-13|EPI_ISL_674515|Original|hCoV-19^^England|Human|Lighthouse Lab in Milton Keynes|Wellcome Sanger Institute for the COVID-19 Genomics UK (COG-UK) Consortium|Davies|United Kingdom
>Spike|hCoV-19/England/MILK-B8F44E/2020|2020-11-13|EPI_ISL_674591|Original|hCoV-19^^England|Human|Lighthouse Lab in Milton Keynes|Wellcome Sanger Institute for the COVID-19 Genomics UK (COG-UK) Consortium|Davies|United Kingdom
>Spike|hCoV-19/England/MILK-B8F43F/2020|2020-11-13|EPI_ISL_674627|Original|hCoV-19^^England|Human|Lighthouse Lab in Milton Keynes|Wellcome Sanger Institute for the COVID-19 Genomics UK (COG-UK) Consortium|Davies|United Kingdom
>Spike|hCoV-19/England/MILK-B8F4E4/2020|2020-11-13|EPI_ISL_674671|Original|hCoV-19^^England|Human|Lighthouse Lab in Milton Keynes|Wellcome Sanger Institute for the COVID-19 Genomics UK (COG-UK) Consortium|Davies|United Kingdom
>Spike|hCoV-19/England/MILK-B8F47B/2020|2020-11-13|EPI_ISL_674706|Original|hCoV-19^^England|Human|Lighthouse Lab in Milton Keynes|Wellcome Sanger Institute for the COVID-19 Genomics UK (COG-UK) Consortium|Davies|United Kingdom
>Spike|hCoV-19/England/MILK-B974BC/2020|2020-11-12|EPI_ISL_675102|Original|hCoV-19^^England|Human|Lighthouse Lab in Milton Keynes|Wellcome Sanger Institute for the COVID-19 Genomics UK (COG-UK) Consortium|Davies|United Kingdom
>Spike|hCoV-19/England/MILK-B8F332/2020|2020-11-13|EPI_ISL_675134|Original|hCoV-19^^England|Human|Lighthouse Lab in Milton Keynes|Wellcome Sanger Institute for the COVID-19 Genomics UK (COG-UK) Consortium|Davies|United Kingdom
>Spike|hCoV-19/England/MILK-B8F2CC/2020|2020-11-13|EPI_ISL_675260|Original|hCoV-19^^England|Human|Lighthouse Lab in Milton Keynes|Wellcome Sanger Institute for the COVID-19 Genomics UK (COG-UK) Consortium|Davies|United Kingdom
>Spike|hCoV-19/England/ALDP-B84607/2020|2020-11-15|EPI_ISL_675392|Original|hCoV-19^^England|Human|Lighthouse Lab in Alderley Park|Wellcome Sanger Institute for the COVID-19 Genomics UK (COG-UK) Consortium|Davies|United Kingdom
>Spike|hCoV-19/England/MILK-B8C6B4/2020|2020-11-12|EPI_ISL_675765|Original|hCoV-19^^England|Human|Lighthouse Lab in Milton Keynes|Wellcome Sanger Institute for the COVID-19 Genomics UK (COG-UK) Consortium|Davies|United Kingdom
>Spike|hCoV-19/England/MILK-B88919/2020|2020-11-13|EPI_ISL_675801|Original|hCoV-19^^England|Human|Lighthouse Lab in Milton Keynes|Wellcome Sanger Institute for the COVID-19 Genomics UK (COG-UK) Consortium|Davies|United Kingdom
>Spike|hCoV-19/England/MILK-B8968A/2020|2020-11-13|EPI_ISL_675870|Original|hCoV-19^^England|Human|Lighthouse Lab in Milton Keynes|Wellcome Sanger Institute for the COVID-19 Genomics UK (COG-UK) Consortium|Davies|United Kingdom
>Spike|hCoV-19/England/MILK-B8C9BB/2020|2020-11-13|EPI_ISL_675975|Original|hCoV-19^^England|Human|Lighthouse Lab in Milton Keynes|Wellcome Sanger Institute for the COVID-19 Genomics UK (COG-UK) Consortium|Davies|United Kingdom
>Spike|hCoV-19/England/ALDP-B84E4B/2020|2020-11-14|EPI_ISL_676010|Original|hCoV-19^^England|Human|Lighthouse Lab in Alderley Park|Wellcome Sanger Institute for the COVID-19 Genomics UK (COG-UK) Consortium|Davies|United Kingdom
>Spike|hCoV-19/England/MILK-B96A24/2020|2020-11-13|EPI_ISL_676099|Original|hCoV-19^^England|Human|Lighthouse Lab in Milton Keynes|Wellcome Sanger Institute for the COVID-19 Genomics UK (COG-UK) Consortium|Davies|United Kingdom

>Spike|hCoV-19/Scotland/QEUH-AF49BF/2020|2020-10-30|EPI_ISL_676484|Original|hCoV-19^^Scotland|Human|Lighthouse Lab in Glasgow|Wellcome Sanger Institute for the COVID-19 Genomics UK (COG-UK) Consortium|Davies|United Kingdom
>Spike|hCoV-19/Sweden/20-53532/2020|2020-11-10|EPI_ISL_676505|Original|hCoV-19^^Jonkoping|Human|Klinisk mikrobiologi|The Public Health Agency of Sweden|Svartstr\u00f6m|Sweden
>Spike|hCoV-19/USA/VA-DCLS-2031/2020|2020-10-14|EPI_ISL_677175|Original|hCoV-19^^Virginia|Human|Virginia Division of Consolidated Laboratory Services|Virginia Division of Consolidated Laboratory Services|St. Jacques|USA
>Spike|hCoV-19/USA/WI-UW-2134/2020|2020-10-12|EPI_ISL_677662|Original|hCoV-19^^Wisconsin|Human|University of Wisconsin-Madison AIDS Vaccine Research Laboratories|University of Wisconsin-Madison AIDS Vaccine Research Laboratories|Baczenas|USA
>Spike|hCoV-19/USA/NY-Wadsworth-17311-01/2020|2020-03-16|EPI_ISL_677664|Original|hCoV-19^^New York|Human|Wadsworth Center|Wadsworth Center|Lasek-Nesselquist|USA
>Spike|hCoV-19/USA/NY-Wadsworth-210205-01/2020|2020-09-02|EPI_ISL_677670|Original|hCoV-19^^New York|Human|Wadsworth Center|Wadsworth Center|Lasek-Nesselquist|USA
>Spike|hCoV-19/Saudi Arabia/KAUST-MADINAH1101/2020|2020-06-13|EPI_ISL_677972|Original|hCoV-19^^Madinah|Human|Pathogen Genomics Lab King Abdullah University of Science and Technology(KAUST)|Pathogen Genomics Lab King Abdullah University of Science and Technology(KAUST)|Mohamed Ghazzali|Saudi Arabia
>Spike|hCoV-19/Saudi Arabia/KAUST-MADINAH669/2020|2020-04-01|EPI_ISL_677973|Original|hCoV-19^^Madinah|Human|Pathogen Genomics Lab King Abdullah University of Science and Technology(KAUST)|Pathogen Genomics Lab King Abdullah University of Science and Technology(KAUST)|Mohamed Ghazzali|Saudi Arabia
>Spike|hCoV-19/Saudi Arabia/KAUST-MAKKAH149/2020|2020-03-24|EPI_ISL_678057|Original|hCoV-19^^Makkah|Human|Pathogen Genomics Lab King Abdullah University of Science and Technology(KAUST)|Pathogen Genomics Lab King Abdullah University of Science and Technology(KAUST)|Mohamed Ghazzali|Saudi Arabia
>Spike|hCoV-19/Saudi Arabia/KAUST-MAKKAH1413/2020|2020-06-28|EPI_ISL_678070|Original|hCoV-19^^Makkah|Human|Pathogen Genomics Lab King Abdullah University of Science and Technology(KAUST)|Pathogen Genomics Lab King Abdullah University of Science and Technology(KAUST)|Mohamed Ghazzali|Saudi Arabia
>Spike|hCoV-19/Saudi Arabia/KAUST-MAKKAH105/2020|2020-03-24|EPI_ISL_678072|Original|hCoV-19^^Makkah|Human|Pathogen Genomics Lab King Abdullah University of Science and Technology(KAUST)|Pathogen Genomics Lab King Abdullah University of Science and Technology(KAUST)|Mohamed Ghazzali|Saudi Arabia
>Spike|hCoV-19/Saudi Arabia/KAUST-MAKKAH1596/2020|2020-04-13|EPI_ISL_678121|Original|hCoV-19^^Makkah|Human|Pathogen Genomics Lab King Abdullah University of Science and Technology(KAUST)|Pathogen Genomics Lab King Abdullah University of Science and Technology(KAUST)|Mohamed Ghazzali|Saudi Arabia
>Spike|hCoV-19/Saudi Arabia/KAUST-MAKKAH1621/2020|2020-04-26|EPI_ISL_678128|Original|hCoV-19^^Makkah|Human|Pathogen Genomics Lab King Abdullah University of Science and Technology(KAUST)|Pathogen Genomics Lab King Abdullah University of Science and Technology(KAUST)|Mohamed Ghazzali|Saudi Arabia
>Spike|hCoV-19/Saudi Arabia/KAUST-RIYADH1162/2020|2020-06-05|EPI_ISL_678188|Original|hCoV-19^^Riyadh|Human|Pathogen Genomics Lab King Abdullah University of Science and Technology(KAUST)|Pathogen Genomics Lab King Abdullah University of Science and Technology(KAUST)|Mohamed Ghazzali|Saudi Arabia
>Spike|hCoV-19/Australia/NSW2099/2020|2020-03-21|EPI_ISL_678297|Original|hCoV-19^^New South Wales|Human|Area of Virology|Virology Research Laboratory; Area of Virology|Foster|Australia
>Spike|hCoV-19/Australia/NSW2110/2020|2020-03-22|EPI_ISL_678299|Original|hCoV-19^^New South Wales|Human|Area of Virology|Virology Research Laboratory; Area of Virology|Foster|Australia
>Spike|hCoV-19/Australia/NSW2799/2020|2020-10-03|EPI_ISL_678349|Original|hCoV-19^^New South Wales|Human|Area of Virology|Virology Research Laboratory; Area of Virology|Foster|Australia

>Spike|hCoV-19/South Africa/KRISP-K005314/2020|2020-11-16|EPI_ISL_678607|Original|hCoV-19^^KwaZulu-Natal|Human|NHLS-IALCH|KRISP|de Oliveira|South Africa
>Spike|hCoV-19/South Africa/KRISP-K005316/2020|2020-11-15|EPI_ISL_678608|Original|hCoV-19^^KwaZulu-Natal|Human|NHLS-IALCH|KRISP|de Oliveira|South Africa
>Spike|hCoV-19/England/204881254/2020|2020-11-00|EPI_ISL_678796|Original|hCoV-19^^England|Human|Respiratory Virus Unit|COVID-19 Genomics UK (COG-UK) Consortium|Platt|United Kingdom
>Spike|hCoV-19/Northern Ireland/NIRE-234159/2020|2020-10-13|EPI_ISL_680345|Original|hCoV-19^^Northern Ireland|Human|Regional Virus Laboratory|COVID-19 Genomics UK (COG-UK) Consortium|Fairley|United Kingdom
>Spike|hCoV-19/Northern Ireland/NIRE-234326/2020|2020-10-15|EPI_ISL_680370|Original|hCoV-19^^Northern Ireland|Human|Regional Virus Laboratory|COVID-19 Genomics UK (COG-UK) Consortium|Fairley|United Kingdom
>Spike|hCoV-19/Northern Ireland/NIRE-234748/2020|2020-10-14|EPI_ISL_680430|Original|hCoV-19^^Northern Ireland|Human|Regional Virus Laboratory|COVID-19 Genomics UK (COG-UK) Consortium|Fairley|United Kingdom
>Spike|hCoV-19/Wales/PHWC-48D3A4/2020|2020-10-22|EPI_ISL_680616|Original|hCoV-19^^Wales|Human|Wales Specialist Virology Centre Sequencing lab|COVID-19 Genomics UK (COG-UK) Consortium|Moore|United Kingdom
>Spike|hCoV-19/Wales/PHWC-48D474/2020|2020-11-01|EPI_ISL_680628|Original|hCoV-19^^Wales|Human|Wales Specialist Virology Centre Sequencing lab|COVID-19 Genomics UK (COG-UK) Consortium|Moore|United Kingdom
>Spike|hCoV-19/Wales/PHWC-48D553/2020|2020-11-13|EPI_ISL_680642|Original|hCoV-19^^Wales|Human|Wales Specialist Virology Centre Sequencing lab|COVID-19 Genomics UK (COG-UK) Consortium|Moore|United Kingdom
>Spike|hCoV-19/Wales/PHWC-48DA18/2020|2020-11-09|EPI_ISL_680713|Original|hCoV-19^^Wales|Human|Wales Specialist Virology Centre Sequencing lab|COVID-19 Genomics UK (COG-UK) Consortium|Moore|United Kingdom
>Spike|hCoV-19/Wales/PHWC-48DA63/2020|2020-11-01|EPI_ISL_680718|Original|hCoV-19^^Wales|Human|Wales Specialist Virology Centre Sequencing lab|COVID-19 Genomics UK (COG-UK) Consortium|Moore|United Kingdom
>Spike|hCoV-19/Wales/PHWC-48DC8B/2020|2020-11-11|EPI_ISL_680742|Original|hCoV-19^^Wales|Human|Wales Specialist Virology Centre Sequencing lab|COVID-19 Genomics UK (COG-UK) Consortium|Moore|United Kingdom
>Spike|hCoV-19/Wales/PHWC-48DE94/2020|2020-11-07|EPI_ISL_680770|Original|hCoV-19^^Wales|Human|Wales Specialist Virology Centre Sequencing lab|COVID-19 Genomics UK (COG-UK) Consortium|Moore|United Kingdom
>Spike|hCoV-19/Wales/PHWC-48DEB2/2020|2020-11-09|EPI_ISL_680772|Original|hCoV-19^^Wales|Human|Wales Specialist Virology Centre Sequencing lab|COVID-19 Genomics UK (COG-UK) Consortium|Moore|United Kingdom
>Spike|hCoV-19/Wales/PHWC-48DED0/2020|2020-11-07|EPI_ISL_680774|Original|hCoV-19^^Wales|Human|Wales Specialist Virology Centre Sequencing lab|COVID-19 Genomics UK (COG-UK) Consortium|Moore|United Kingdom
>Spike|hCoV-19/Wales/PHWC-48EA62/2020|2020-11-09|EPI_ISL_680953|Original|hCoV-19^^Wales|Human|Wales Specialist Virology Centre Sequencing lab|COVID-19 Genomics UK (COG-UK) Consortium|Moore|United Kingdom
>Spike|hCoV-19/Wales/PHWC-48EAAE/2020|2020-11-10|EPI_ISL_680957|Original|hCoV-19^^Wales|Human|Wales Specialist Virology Centre Sequencing lab|COVID-19 Genomics UK (COG-UK) Consortium|Moore|United Kingdom
>Spike|hCoV-19/Wales/PHWC-48EC11/2020|2020-11-09|EPI_ISL_680979|Original|hCoV-19^^Wales|Human|Wales Specialist Virology Centre Sequencing lab|COVID-19 Genomics UK (COG-UK) Consortium|Moore|United Kingdom
>Spike|hCoV-19/Wales/PHWC-48ED69/2020|2020-11-10|EPI_ISL_681000|Original|hCoV-19^^Wales|Human|Wales Specialist Virology Centre Sequencing lab|COVID-19 Genomics UK (COG-UK) Consortium|Moore|United Kingdom
>Spike|hCoV-19/Wales/PHWC-48F1D5/2020|2020-11-07|EPI_ISL_681057|Original|hCoV-19^^Wales|Human|Wales Specialist Virology Centre Sequencing lab|COVID-19 Genomics UK (COG-UK) Consortium|Moore|United Kingdom

>Spike|hCoV-19/Wales/PHWC-48F3FD/2020|2020-11-09|EPI_ISL_681089|Original|hCoV-19^^Wales|Human|Wales Specialist Virology Centre Sequencing lab|COVID-19 Genomics UK (COG-UK) Consortium|Moore|United Kingdom
>Spike|hCoV-19/Wales/PHWC-48F454/2020|2020-11-09|EPI_ISL_681094|Original|hCoV-19^^Wales|Human|Wales Specialist Virology Centre Sequencing lab|COVID-19 Genomics UK (COG-UK) Consortium|Moore|United Kingdom
>Spike|hCoV-19/Wales/PHWC-48F533/2020|2020-11-09|EPI_ISL_681107|Original|hCoV-19^^Wales|Human|Wales Specialist Virology Centre Sequencing lab|COVID-19 Genomics UK (COG-UK) Consortium|Moore|United Kingdom
>Spike|hCoV-19/Wales/PHWC-48F982/2020|2020-11-10|EPI_ISL_681166|Original|hCoV-19^^Wales|Human|Wales Specialist Virology Centre Sequencing lab|COVID-19 Genomics UK (COG-UK) Consortium|Moore|United Kingdom
>Spike|hCoV-19/Wales/PHWC-48F991/2020|2020-11-10|EPI_ISL_681167|Original|hCoV-19^^Wales|Human|Wales Specialist Virology Centre Sequencing lab|COVID-19 Genomics UK (COG-UK) Consortium|Moore|United Kingdom
>Spike|hCoV-19/Wales/PHWC-48FD3B/2020|2020-11-11|EPI_ISL_681221|Original|hCoV-19^^Wales|Human|Wales Specialist Virology Centre Sequencing lab|COVID-19 Genomics UK (COG-UK) Consortium|Moore|United Kingdom
>Spike|hCoV-19/Wales/PHWC-48FD4A/2020|2020-11-11|EPI_ISL_681222|Original|hCoV-19^^Wales|Human|Wales Specialist Virology Centre Sequencing lab|COVID-19 Genomics UK (COG-UK) Consortium|Moore|United Kingdom
>Spike|hCoV-19/Wales/PHWC-48FE47/2020|2020-11-11|EPI_ISL_681237|Original|hCoV-19^^Wales|Human|Wales Specialist Virology Centre Sequencing lab|COVID-19 Genomics UK (COG-UK) Consortium|Moore|United Kingdom
>Spike|hCoV-19/Wales/PHWC-48FE56/2020|2020-11-11|EPI_ISL_681238|Original|hCoV-19^^Wales|Human|Wales Specialist Virology Centre Sequencing lab|COVID-19 Genomics UK (COG-UK) Consortium|Moore|United Kingdom
>Spike|hCoV-19/Wales/PHWC-48FF26/2020|2020-11-11|EPI_ISL_681250|Original|hCoV-19^^Wales|Human|Wales Specialist Virology Centre Sequencing lab|COVID-19 Genomics UK (COG-UK) Consortium|Moore|United Kingdom
>Spike|hCoV-19/England/204881261/2020|2020-11-00|EPI_ISL_681323|Original|hCoV-19^^England|Human|Respiratory Virus Unit|COVID-19 Genomics UK (COG-UK) Consortium|Platt|United Kingdom
>Spike|hCoV-19/England/MILK-BAAC45/2020|2020-11-18|EPI_ISL_681573|Original|hCoV-19^^England|Human|Lighthouse Lab in Milton Keynes|Wellcome Sanger Institute for the COVID-19 Genomics UK (COG-UK) Consortium|Davies|United Kingdom
>Spike|hCoV-19/England/MILK-BA9ED7/2020|2020-11-18|EPI_ISL_681589|Original|hCoV-19^^England|Human|Lighthouse Lab in Milton Keynes|Wellcome Sanger Institute for the COVID-19 Genomics UK (COG-UK) Consortium|Davies|United Kingdom
>Spike|hCoV-19/USA/PA-MGEL-00674/2020|2020-10-06|EPI_ISL_681937|Original|hCoV-19^^Pennsylvania|Human|UPMC Clinical Microbiology Laboratory|Microbial Genomic Epidemiology Laboratory|Griffith|USA
>Spike|hCoV-19/South Africa/UFS-VIRO-NGS-28/2020|2020-09-28|EPI_ISL_682334|Original|hCoV-19^^Free State|Human|NHLS Universitas Academic|UFS Virology|Bester|South Africa
>Spike|hCoV-19/Denmark/DCGC-12584/2020|2020-11-16|EPI_ISL_682765|Original|hCoV-19^^Hovedstaden|Human|Department of Virus and Microbiological Special Diagnostics|Albertsen Lab|Michaelsen|Denmark
>Spike|hCoV-19/France/ARA-SC039/2020|2020-03-30|EPI_ISL_683333|Original|hCoV-19^^Auvergne-Rh\u00f4ne-Alpes|Human|Laboratoire du Centre Hospitalier Annecy Genevois|CNR Virus des Infections Respiratoires - France SUD|Bal|France
>Spike|hCoV-19/USA/MN-MDH-2083/2020|2020-11-01|EPI_ISL_683712|Original|hCoV-19^^Minnesota|Human|Essentia Health-St. Mary's Medical Center|Minnesota Department of Health|Garfin|USA
>Spike|hCoV-19/USA/NY-NYCPHL-001237/2020|2020-11-06|EPI_ISL_683860|Original|hCoV-19^^New York|Human|DOHMH Corona|New York City Public Health Laboratory|Wang|USA
>Spike|hCoV-19/USA/NY-NYCPHL-001303/2020|2020-11-10|EPI_ISL_683876|Original|hCoV-19^^New York|Human|DOHMH Corona|New York City Public Health Laboratory|Wang|USA

>Spike|hCoV-19/USA/NY-NYCPHL-001343/2020|2020-11-13|EPI_ISL_683877|Original|hCoV-19^^New York|Human|DOHMH Central Harlem|New York City Public Health Laboratory|Wang|USA
>Spike|hCoV-19/USA/UT-UPHL-201211705/2020|2020-12-01|EPI_ISL_684018|Original|hCoV-19^^Utah|Human|Utah Public Health Laboratory|Utah Public Health Laboratory|Young|USA
>Spike|hCoV-19/Japan/PG-0240/2020|2020-03-00|EPI_ISL_684148|Original|hCoV-19^^Japan|Human|Saitama Prefectural Institute of Public Health|Pathogen Genomics Center|Sekizuka|Japan
>Spike|hCoV-19/Japan/PG-0790/2020|2020-04-00|EPI_ISL_684400|Original|hCoV-19^^Japan|Human|Pathogen Genomics Center|Pathogen Genomics Center|Sekizuka|Japan
>Spike|hCoV-19/Japan/PG-1140/2020|2020-04-00|EPI_ISL_684750|Original|hCoV-19^^Japan|Human|Pathogen Genomics Center|Pathogen Genomics Center|Sekizuka|Japan
>Spike|hCoV-19/Japan/PG-1217/2020|2020-04-00|EPI_ISL_684827|Original|hCoV-19^^Japan|Human|Yamagata Prefectural Institute of Public Health|Pathogen Genomics Center|Sekizuka|Japan
>Spike|hCoV-19/Japan/PG-1226/2020|2020-04-00|EPI_ISL_684836|Original|hCoV-19^^Japan|Human|Yamagata Prefectural Institute of Public Health|Pathogen Genomics Center|Sekizuka|Japan
>Spike|hCoV-19/Japan/PG-1792/2020|2020-05-00|EPI_ISL_685398|Original|hCoV-19^^Japan|Human|Pathogen Genomics Center|Pathogen Genomics Center|Sekizuka|Japan
>Spike|hCoV-19/Japan/PG-1793/2020|2020-05-00|EPI_ISL_685399|Original|hCoV-19^^Japan|Human|Pathogen Genomics Center|Pathogen Genomics Center|Sekizuka|Japan
>Spike|hCoV-19/Japan/PG-1951/2020|2020-04-00|EPI_ISL_685557|Original|hCoV-19^^Japan|Human|Pathogen Genomics Center|Pathogen Genomics Center|Sekizuka|Japan
>Spike|hCoV-19/Japan/PG-2318/2020|2020-05-00|EPI_ISL_685923|Original|hCoV-19^^Japan|Human|Fukuoka Institute of Health and Environmental Sciences|Pathogen Genomics Center|Sekizuka|Japan
>Spike|hCoV-19/Japan/PG-8066/2020|2020-07-00|EPI_ISL_687167|Original|hCoV-19^^Japan|Human|Pathogen Genomics Center|Pathogen Genomics Center|Sekizuka|Japan
>Spike|hCoV-19/Japan/PG-8067/2020|2020-07-00|EPI_ISL_687168|Original|hCoV-19^^Japan|Human|Pathogen Genomics Center|Pathogen Genomics Center|Sekizuka|Japan
>Spike|hCoV-19/Japan/PG-8068/2020|2020-07-00|EPI_ISL_687169|Original|hCoV-19^^Japan|Human|Pathogen Genomics Center|Pathogen Genomics Center|Sekizuka|Japan
>Spike|hCoV-19/Japan/PG-8069/2020|2020-07-00|EPI_ISL_687170|Original|hCoV-19^^Japan|Human|Pathogen Genomics Center|Pathogen Genomics Center|Sekizuka|Japan
>Spike|hCoV-19/Japan/PG-8070/2020|2020-07-00|EPI_ISL_687171|Original|hCoV-19^^Japan|Human|Pathogen Genomics Center|Pathogen Genomics Center|Sekizuka|Japan
>Spike|hCoV-19/Japan/PG-8071/2020|2020-07-00|EPI_ISL_687172|Original|hCoV-19^^Japan|Human|Pathogen Genomics Center|Pathogen Genomics Center|Sekizuka|Japan
>Spike|hCoV-19/Japan/PG-8074/2020|2020-08-00|EPI_ISL_687175|Original|hCoV-19^^Japan|Human|Pathogen Genomics Center|Pathogen Genomics Center|Sekizuka|Japan
>Spike|hCoV-19/Japan/PG-8075/2020|2020-08-00|EPI_ISL_687176|Original|hCoV-19^^Japan|Human|Pathogen Genomics Center|Pathogen Genomics Center|Sekizuka|Japan
>Spike|hCoV-19/Japan/PG-8077/2020|2020-08-00|EPI_ISL_687178|Original|hCoV-19^^Japan|Human|Pathogen Genomics Center|Pathogen Genomics Center|Sekizuka|Japan
>Spike|hCoV-19/Japan/PG-8079/2020|2020-08-00|EPI_ISL_687180|Original|hCoV-19^^Japan|Human|Pathogen Genomics Center|Pathogen Genomics Center|Sekizuka|Japan
>Spike|hCoV-19/Japan/PG-8082/2020|2020-08-00|EPI_ISL_687183|Original|hCoV-19^^Japan|Human|Pathogen Genomics Center|Pathogen Genomics Center|Sekizuka|Japan
>Spike|hCoV-19/Japan/PG-8086/2020|2020-08-00|EPI_ISL_687186|Original|hCoV-19^^Japan|Human|Pathogen Genomics Center|Pathogen Genomics Center|Sekizuka|Japan
>Spike|hCoV-19/Japan/PG-8087/2020|2020-08-00|EPI_ISL_687187|Original|hCoV-19^^Japan|Human|Pathogen Genomics Center|Pathogen Genomics Center|Sekizuka|Japan
>Spike|hCoV-19/Japan/PG-6564/2020|2020-09-00|EPI_ISL_687612|Original|hCoV-19^^Japan|Human|Pathogen Genomics Center|Pathogen Genomics Center|Sekizuka|Japan
>Spike|hCoV-19/Japan/PG-6563/2020|2020-09-00|EPI_ISL_687613|Original|hCoV-19^^Japan|Human|Pathogen Genomics Center|Pathogen Genomics Center|Sekizuka|Japan
>Spike|hCoV-19/Japan/PG-5744/2020|2020-08-00|EPI_ISL_688616|Original|hCoV-19^^Japan|Human|Pathogen Genomics Center|Pathogen Genomics Center|Sekizuka|Japan

>Spike|hCoV-19/Japan/PG-5984/2020|2020-08-00|EPI_ISL_688617|Original|hCoV-19^^Japan|Human|Pathogen Genomics Center|Pathogen Genomics Center|Sekizuka|Japan
>Spike|hCoV-19/Japan/PG-5985/2020|2020-08-00|EPI_ISL_688618|Original|hCoV-19^^Japan|Human|Pathogen Genomics Center|Pathogen Genomics Center|Sekizuka|Japan
>Spike|hCoV-19/Japan/PG-6221/2020|2020-08-00|EPI_ISL_689333|Original|hCoV-19^^Japan|Human|Pathogen Genomics Center|Pathogen Genomics Center|Sekizuka|Japan
>Spike|hCoV-19/Japan/PG-4525/2020|2020-08-00|EPI_ISL_689619|Original|hCoV-19^^Japan|Human|Pathogen Genomics Center|Pathogen Genomics Center|Sekizuka|Japan
>Spike|hCoV-19/Japan/PG-4805/2020|2020-08-00|EPI_ISL_689620|Original|hCoV-19^^Japan|Human|Pathogen Genomics Center|Pathogen Genomics Center|Sekizuka|Japan
>Spike|hCoV-19/Japan/PG-4857/2020|2020-07-00|EPI_ISL_689621|Original|hCoV-19^^Japan|Human|Pathogen Genomics Center|Pathogen Genomics Center|Sekizuka|Japan
>Spike|hCoV-19/Japan/PG-4975/2020|2020-08-00|EPI_ISL_689622|Original|hCoV-19^^Japan|Human|Pathogen Genomics Center|Pathogen Genomics Center|Sekizuka|Japan
>Spike|hCoV-19/Japan/PG-4977/2020|2020-08-00|EPI_ISL_689623|Original|hCoV-19^^Japan|Human|Pathogen Genomics Center|Pathogen Genomics Center|Sekizuka|Japan
>Spike|hCoV-19/Japan/PG-2570/2020|2020-06-00|EPI_ISL_690618|Original|hCoV-19^^Japan|Human|Pathogen Genomics Center|Pathogen Genomics Center|Sekizuka|Japan
>Spike|hCoV-19/Japan/PG-3972/2020|2020-07-00|EPI_ISL_691737|Original|hCoV-19^^Japan|Human|Pathogen Genomics Center|Pathogen Genomics Center|Sekizuka|Japan
>Spike|hCoV-19/Japan/PG-3970/2020|2020-07-00|EPI_ISL_691740|Original|hCoV-19^^Japan|Human|Pathogen Genomics Center|Pathogen Genomics Center|Sekizuka|Japan
>Spike|hCoV-19/Japan/PG-3974/2020|2020-07-00|EPI_ISL_691741|Original|hCoV-19^^Japan|Human|Pathogen Genomics Center|Pathogen Genomics Center|Sekizuka|Japan
>Spike|hCoV-19/Japan/PG-3973/2020|2020-07-00|EPI_ISL_691745|Original|hCoV-19^^Japan|Human|Pathogen Genomics Center|Pathogen Genomics Center|Sekizuka|Japan
>Spike|hCoV-19/Japan/PG-3975/2020|2020-07-00|EPI_ISL_691746|Original|hCoV-19^^Japan|Human|Pathogen Genomics Center|Pathogen Genomics Center|Sekizuka|Japan
>Spike|hCoV-19/Japan/PG-4280/2020|2020-07-00|EPI_ISL_691747|Original|hCoV-19^^Japan|Human|Pathogen Genomics Center|Pathogen Genomics Center|Sekizuka|Japan
>Spike|hCoV-19/USA/MA-MASPHL-01027/2020|2020-11-05|EPI_ISL_692784|Original|hCoV-19^^Massachusetts|Human|Massachusetts State Public Health Laboratory|Massachusetts State Public Health Laboratory|Lang|USA
>Spike|hCoV-19/USA/MA-MASPHL-00788/2020|2020-09-24|EPI_ISL_692910|Original|hCoV-19^^Massachusetts|Human|Massachusetts State Public Health Laboratory|Massachusetts State Public Health Laboratory|Lang|USA
>Spike|hCoV-19/USA/MA-MASPHL-00739/2020|2020-09-24|EPI_ISL_692933|Original|hCoV-19^^Massachusetts|Human|Massachusetts State Public Health Laboratory|Massachusetts State Public Health Laboratory|Lang|USA
>Spike|hCoV-19/USA/MA-MASPHL-00614/2020|2020-05-11|EPI_ISL_693007|Original|hCoV-19^^Massachusetts|Human|Massachusetts State Public Health Laboratory|Massachusetts State Public Health Laboratory|Lang|USA
>Spike|hCoV-19/USA/MA-MASPHL-00536/2020|2020-05-06|EPI_ISL_693043|Original|hCoV-19^^Massachusetts|Human|Massachusetts State Public Health Laboratory|Massachusetts State Public Health Laboratory|Lang|USA
>Spike|hCoV-19/USA/MA-MASPHL-00444/2020|2020-04-22|EPI_ISL_693115|Original|hCoV-19^^Massachusetts|Human|Massachusetts State Public Health Laboratory|Massachusetts State Public Health Laboratory|Lang|USA
>Spike|hCoV-19/Thailand/Bangkok-CONI-0291/2020|2020-03-15|EPI_ISL_693364|Original|hCoV-19^^Bangkok|Human|Ramathibodi Hospital|COVID-19 Network Investigations (CONI) Alliance|Batty|Thailand
>Spike|hCoV-19/England/204661600/2020|2020-11-11|EPI_ISL_693485|Original|hCoV-19^^England|Human|Respiratory Virus Unit|COVID-19 Genomics UK (COG-UK) Consortium|Platt|United Kingdom
>Spike|hCoV-19/France/ARA-72179/2020|2020-09-14|EPI_ISL_693497|Original|hCoV-19^^Auvergne-Rh\u00f4ne-Alpes|Human|CNR Virus des Infections Respiratoires - France SUD|CNR Virus des Infections Respiratoires - France SUD|Bal|France
>Spike|hCoV-19/USA/DE-DHSS-F1001832/2020|2020-09-18|EPI_ISL_693688|Original|hCoV-19^^Delaware|Human|Delaware Public Health Laboratory|Delaware Public Health Laboratory|Bajwa|USA

>Spike|hCoV-19/USA/AZ-TG280873/2020|2020-04-23|EPI_ISL_694204|Original|hCoV-
19^^Arizona|Human|AZ SPHL|TGen North|French|USA
>Spike|hCoV-19/USA/AZ-TG280884/2020|2020-04-24|EPI_ISL_694213|Original|hCoV-
19^^Arizona|Human|AZ SPHL|TGen North|French|USA
>Spike|hCoV-19/USA/AZ-TG321154/2020|2020-05-19|EPI_ISL_694334|Original|hCoV-
19^^Arizona|Human|TGen North|TGen North|French|USA
>Spike|hCoV-19/USA/AZ-TG341343/2020|2020-06-01|EPI_ISL_694377|Original|hCoV-
19^^Arizona|Human|TGen North|TGen North|French|USA
>Spike|hCoV-19/USA/AZ-TG346994/2020|2020-06-03|EPI_ISL_694393|Original|hCoV-
19^^Arizona|Human|TGen North|TGen North|French|USA
>Spike|hCoV-19/USA/AZ-TG378469/2020|2020-06-16|EPI_ISL_694628|Original|hCoV-
19^^Arizona|Human|TGen North|TGen North|French|USA
>Spike|hCoV-19/USA/AZ-TG389574/2020|2020-06-19|EPI_ISL_694700|Original|hCoV-
19^^Arizona|Human|TGen North|TGen North|French|USA
>Spike|hCoV-19/USA/AZ-TG396024/2020|2020-06-22|EPI_ISL_694798|Original|hCoV-
19^^Arizona|Human|TGen North|TGen North|French|USA
>Spike|hCoV-19/USA/AZ-TG396367/2020|2020-06-11|EPI_ISL_694823|Original|hCoV-
19^^Arizona|Human|AZ SPHL|TGen North|French|USA
>Spike|hCoV-19/USA/AZ-TG408380/2020|2020-06-25|EPI_ISL_694952|Original|hCoV-
19^^Arizona|Human|TGen North|TGen North|French|USA
>Spike|hCoV-19/USA/AZ-TG475585/2020|2020-07-07|EPI_ISL_695050|Original|hCoV-
19^^Arizona|Human|TGen North|TGen North|French|USA
>Spike|hCoV-19/USA/AZ-TG481122/2020|2020-07-08|EPI_ISL_695071|Original|hCoV-
19^^Arizona|Human|TGen North|TGen North|French|USA
>Spike|hCoV-19/USA/AZ-TG486083/2020|2020-07-09|EPI_ISL_695082|Original|hCoV-
19^^Arizona|Human|TGen North|TGen North|French|USA
>Spike|hCoV-19/USA/AZ-TG491565/2020|2020-06-23|EPI_ISL_695174|Original|hCoV-
19^^Arizona|Human|AZ SPHL|TGen North|French|USA
>Spike|hCoV-19/USA/AZ-TG491591/2020|2020-06-21|EPI_ISL_695183|Original|hCoV-
19^^Arizona|Human|AZ SPHL|TGen North|French|USA
>Spike|hCoV-19/USA/AZ-TG491600/2020|2020-06-24|EPI_ISL_695186|Original|hCoV-
19^^Arizona|Human|AZ SPHL|TGen North|French|USA
>Spike|hCoV-19/USA/AZ-TG491604/2020|2020-06-23|EPI_ISL_695189|Original|hCoV-
19^^Arizona|Human|AZ SPHL|TGen North|French|USA
>Spike|hCoV-19/USA/AZ-TG493511/2020|2020-07-13|EPI_ISL_695233|Original|hCoV-
19^^Arizona|Human|TGen North|TGen North|French|USA
>Spike|hCoV-19/USA/AZ-TG498366/2020|2020-07-14|EPI_ISL_695267|Original|hCoV-
19^^Arizona|Human|TGen North|TGen North|French|USA
>Spike|hCoV-19/USA/AZ-TG501918/2020|2020-07-15|EPI_ISL_695277|Original|hCoV-
19^^Arizona|Human|TGen North|TGen North|French|USA
>Spike|hCoV-19/USA/AZ-TG503714/2020|2020-07-15|EPI_ISL_695288|Original|hCoV-
19^^Arizona|Human|TGen North|TGen North|French|USA
>Spike|hCoV-19/USA/AZ-TG507595/2020|2020-07-15|EPI_ISL_695298|Original|hCoV-
19^^Arizona|Human|TGen North|TGen North|French|USA
>Spike|hCoV-19/USA/AZ-TG521374/2020|2020-07-20|EPI_ISL_695377|Original|hCoV-
19^^Arizona|Human|TGen North|TGen North|French|USA
>Spike|hCoV-19/USA/AZ-TG522907/2020|2020-07-17|EPI_ISL_695387|Original|hCoV-
19^^Arizona|Human|TGen North|TGen North|French|USA
>Spike|hCoV-19/USA/AZ-TG525764/2020|2020-07-20|EPI_ISL_695390|Original|hCoV-
19^^Arizona|Human|TGen North|TGen North|French|USA
>Spike|hCoV-19/USA/AZ-TG547229/2020|2020-07-02|EPI_ISL_695436|Original|hCoV-
19^^Arizona|Human|AZ SPHL|TGen North|French|USA
>Spike|hCoV-19/USA/AZ-TG555472/2020|2020-07-28|EPI_ISL_695540|Original|hCoV-
19^^Arizona|Human|TGen North|TGen North|French|USA
>Spike|hCoV-19/USA/AZ-TG565817/2020|2020-07-29|EPI_ISL_695557|Original|hCoV-
19^^Arizona|Human|TGen North|TGen North|French|USA
>Spike|hCoV-19/USA/AZ-TG569455/2020|2020-07-10|EPI_ISL_695565|Original|hCoV-
19^^Arizona|Human|AZ SPHL|TGen North|French|USA
>Spike|hCoV-19/USA/AZ-TG569462/2020|2020-07-08|EPI_ISL_695566|Original|hCoV-
19^^Arizona|Human|AZ SPHL|TGen North|French|USA

>Spike|hCoV-19/USA/AZ-TG569471/2020|2020-07-10|EPI_ISL_695571|Original|hCoV-19^^Arizona|Human|AZ SPHL|TGen North|French|USA
>Spike|hCoV-19/USA/AZ-TG637272/2020|2020-07-24|EPI_ISL_695712|Original|hCoV-19^^Arizona|Human|AZ SPHL|TGen North|French|USA
>Spike|hCoV-19/USA/AZ-TG637273/2020|2020-07-24|EPI_ISL_695713|Original|hCoV-19^^Arizona|Human|AZ SPHL|TGen North|French|USA
>Spike|hCoV-19/USA/AZ-TG637277/2020|2020-07-23|EPI_ISL_695715|Original|hCoV-19^^Arizona|Human|AZ SPHL|TGen North|French|USA
>Spike|hCoV-19/USA/AZ-TG637304/2020|2020-07-27|EPI_ISL_695727|Original|hCoV-19^^Arizona|Human|AZ SPHL|TGen North|French|USA
>Spike|hCoV-19/USA/AZ-TG637359/2020|2020-08-04|EPI_ISL_695750|Original|hCoV-19^^Arizona|Human|AZ SPHL|TGen North|French|USA
>Spike|hCoV-19/USA/AZ-TG640780/2020|2020-04-27|EPI_ISL_695755|Original|hCoV-19^^Arizona|Human|Sonora Quest Laboratories|TGen North|French|USA
>Spike|hCoV-19/USA/AZ-TG640795/2020|2020-04-27|EPI_ISL_695766|Original|hCoV-19^^Arizona|Human|Sonora Quest Laboratories|TGen North|French|USA
>Spike|hCoV-19/USA/AZ-TG640944/2020|2020-04-27|EPI_ISL_695822|Original|hCoV-19^^Arizona|Human|Sonora Quest Laboratories|TGen North|French|USA
>Spike|hCoV-19/USA/AZ-TG640956/2020|2020-04-27|EPI_ISL_695824|Original|hCoV-19^^Arizona|Human|Sonora Quest Laboratories|TGen North|French|USA
>Spike|hCoV-19/USA/AZ-TG640957/2020|2020-04-27|EPI_ISL_695825|Original|hCoV-19^^Arizona|Human|Sonora Quest Laboratories|TGen North|French|USA
>Spike|hCoV-19/USA/AZ-TG645823/2020|2020-08-07|EPI_ISL_695836|Original|hCoV-19^^Arizona|Human|AZ SPHL|TGen North|French|USA
>Spike|hCoV-19/USA/AZ-TG654295/2020|2020-04-10|EPI_ISL_695951|Original|hCoV-19^^Arizona|Human|Sonora Quest Laboratories|TGen North|French|USA
>Spike|hCoV-19/USA/AZ-TG654296/2020|2020-04-13|EPI_ISL_695952|Original|hCoV-19^^Arizona|Human|Sonora Quest Laboratories|TGen North|French|USA
>Spike|hCoV-19/USA/AZ-TG654302/2020|2020-04-13|EPI_ISL_695958|Original|hCoV-19^^Arizona|Human|Sonora Quest Laboratories|TGen North|French|USA
>Spike|hCoV-19/USA/AZ-TG664297/2020|2020-04-28|EPI_ISL_696091|Original|hCoV-19^^Arizona|Human|Sonora Quest Laboratories|TGen North|French|USA
>Spike|hCoV-19/USA/AZ-TG666109/2020|2020-05-01|EPI_ISL_696198|Original|hCoV-19^^Arizona|Human|Sonora Quest Laboratories|TGen North|French|USA
>Spike|hCoV-19/USA/AZ-TG666115/2020|2020-05-02|EPI_ISL_696202|Original|hCoV-19^^Arizona|Human|Sonora Quest Laboratories|TGen North|French|USA
>Spike|hCoV-19/South Africa/NHLS-UCT-GS-7041-KRISP/2020|2020-11-24|EPI_ISL_696508|Original|hCoV-19^^Western Cape|Human|Conville CDC wc CVC & NHLS/UCT|KRISP|de Oliveira|South Africa
>Spike|hCoV-19/Australia/NSW1025/2020|2020-04-01|EPI_ISL_699641|Original|hCoV-19^^New South Wales|Human|Sydney South West Pathology Service (SSWPS) - Liverpool Hospital - NSW Health Pathology|NSW Health Pathology - Institute of Clinical Pathology and Medical Research; Westmead Hospital; University of Sydney|Gall|Australia
>Spike|hCoV-19/Australia/NSW274/2020|2020-04-03|EPI_ISL_699645|Original|hCoV-19^^New South Wales|Human|Pathology West - NSW Health Pathology|NSW Health Pathology - Institute of Clinical Pathology and Medical Research; Westmead Hospital; University of Sydney|Gall|Australia
>Spike|hCoV-19/Australia/NSW313/2020|2020-03-05|EPI_ISL_699646|Original|hCoV-19^^New South Wales|Human|Pathology West - NSW Health Pathology|NSW Health Pathology - Institute of Clinical Pathology and Medical Research; Westmead Hospital; University of Sydney|Gall|Australia
>Spike|hCoV-19/Australia/NSW343/2020|2020-04-03|EPI_ISL_699648|Original|hCoV-19^^New South Wales|Human|Pathology West - NSW Health Pathology|NSW Health Pathology - Institute of Clinical Pathology and Medical Research; Westmead Hospital; University of Sydney|Gall|Australia
>Spike|hCoV-19/Australia/NSW372/2020|2020-03-21|EPI_ISL_699649|Original|hCoV-19^^New South Wales|Human|South Eastern Area Laboratory Services|NSW Health Pathology - Institute of Clinical Pathology and Medical Research; Westmead Hospital; University of Sydney|Gall|Australia

>Spike|hCoV-19/Australia/NSW746/2020|2020-07-27|EPI_ISL_699650|Original|hCoV-19^^New South Wales|Human|Douglas Hanly Moir|NSW Health Pathology - Institute of Clinical Pathology and Medical Research; Westmead Hospital; University of Sydney|Gall|Australia
>Spike|hCoV-19/Australia/NSW1202/2020|2020-11-09|EPI_ISL_699653|Original|hCoV-19^^New South Wales|Human|South Eastern Area Laboratory Services (SEALS)|NSW Health Pathology - Institute of Clinical Pathology and Medical Research; Westmead Hospital; University of Sydney|Gall|Australia
>Spike|hCoV-19/India/MH-ACTREC-274/2020|2020-07-10|EPI_ISL_699926|Original|hCoV-19^^Maharashtra|Human|Hematopathology Laboratory|Hematopathology Laboratory|Patkar|India
>Spike|hCoV-19/India/MH-ACTREC-324/2020|2020-07-17|EPI_ISL_699973|Original|hCoV-19^^Maharashtra|Human|Hematopathology Laboratory|Hematopathology Laboratory|Patkar|India
>Spike|hCoV-19/India/MH-ACTREC-334/2020|2020-07-19|EPI_ISL_699983|Original|hCoV-19^^Maharashtra|Human|Hematopathology Laboratory|Hematopathology Laboratory|Patkar|India
>Spike|hCoV-19/India/MH-ACTREC-337/2020|2020-07-19|EPI_ISL_699986|Original|hCoV-19^^Maharashtra|Human|Hematopathology Laboratory|Hematopathology Laboratory|Patkar|India
>Spike|hCoV-19/India/MH-ACTREC-341/2020|2020-07-20|EPI_ISL_699990|Original|hCoV-19^^Maharashtra|Human|Hematopathology Laboratory|Hematopathology Laboratory|Patkar|India
>Spike|hCoV-19/India/MH-ACTREC-460/2020|2020-08-10|EPI_ISL_700108|Original|hCoV-19^^Maharashtra|Human|Hematopathology Laboratory|Hematopathology Laboratory|Patkar|India
>Spike|hCoV-19/France/NAQ-SC208/2020|2020-03-17|EPI_ISL_700326|Original|hCoV-19^^Nouvelle-Aquitaine|Human|CHU Bordeaux|CNR Virus des Infections Respiratoires - France SUD|Bal|France
>Spike|hCoV-19/France/ARA-SC113/2020|2020-03-28|EPI_ISL_700377|Original|hCoV-19^^Auvergne-Rh\u00f4ne-Alpes|Human|Centre hospitalier M\u00e9tropole Savoie|CNR Virus des Infections Respiratoires - France SUD|Bal|France
>Spike|hCoV-19/France/ARA-SC128/2020|2020-04-22|EPI_ISL_700392|Original|hCoV-19^^Auvergne-Rh\u00f4ne-Alpes|Human|Centre hospitalier M\u00e9tropole Savoie|CNR Virus des Infections Respiratoires - France SUD|Bal|France
>Spike|hCoV-19/South Africa/NHLS-UCT-GS-1493/2020|2020-08-11|EPI_ISL_700421|Original|hCoV-19^^Western Cape|Human|Victoria Hospital wc VHW|NHLS/UCT|Iranzadeh|South Africa
>Spike|hCoV-19/England/ALDP-BBA77E/2020|2020-11-20|EPI_ISL_701898|Original|hCoV-19^^England|Human|Lighthouse Lab in Alderley Park|Wellcome Sanger Institute for the COVID-19 Genomics UK (COG-UK) Consortium|Davies|United Kingdom
>Spike|hCoV-19/England/CAMC-BD2312/2020|2020-11-27|EPI_ISL_702343|Original|hCoV-19^^England|Human|Lighthouse Lab in Cambridge|Wellcome Sanger Institute for the COVID-19 Genomics UK (COG-UK) Consortium|Davies|United Kingdom
>Spike|hCoV-19/England/ALDP-BB9C34/2020|2020-11-21|EPI_ISL_702460|Original|hCoV-19^^England|Human|Lighthouse Lab in Alderley Park|Wellcome Sanger Institute for the COVID-19 Genomics UK (COG-UK) Consortium|Davies|United Kingdom
>Spike|hCoV-19/England/ALDP-BB9B73/2020|2020-11-21|EPI_ISL_702482|Original|hCoV-19^^England|Human|Lighthouse Lab in Alderley Park|Wellcome Sanger Institute for the COVID-19 Genomics UK (COG-UK) Consortium|Davies|United Kingdom
>Spike|hCoV-19/England/NORW-F9082/2020|2020-11-24|EPI_ISL_702716|Original|hCoV-19^^England|Human|Quadram Institute Bioscience|COVID-19 Genomics UK (COG-UK) Consortium|Alikhan|United Kingdom
>Spike|hCoV-19/England/ALDP-BB984F/2020|2020-11-20|EPI_ISL_702946|Original|hCoV-19^^England|Human|Lighthouse Lab in Alderley Park|Wellcome Sanger Institute for the COVID-19 Genomics UK (COG-UK) Consortium|Davies|United Kingdom
>Spike|hCoV-19/England/ALDP-BB2AE6/2020|2020-11-20|EPI_ISL_703104|Original|hCoV-19^^England|Human|Lighthouse Lab in Alderley Park|Wellcome Sanger Institute for the COVID-19 Genomics UK (COG-UK) Consortium|Davies|United Kingdom

>Spike|hCoV-19/England/ALDP-BCA66F/2020|2020-11-23|EPI_ISL_703344|Original|hCoV-19^^England|Human|Lighthouse Lab in Alderley Park|Wellcome Sanger Institute for the COVID-19 Genomics UK (COG-UK) Consortium|Davies|United Kingdom
>Spike|hCoV-19/England/QEUH-BCB8B3/2020|2020-11-27|EPI_ISL_703875|Original|hCoV-19^^England|Human|Lighthouse Lab in Glasgow|Wellcome Sanger Institute for the COVID-19 Genomics UK (COG-UK) Consortium|Davies|United Kingdom
>Spike|hCoV-19/Wales/PHWC-491659/2020|2020-10-28|EPI_ISL_703902|Original|hCoV-19^^Wales|Human|Originating lab|COVID-19 Genomics UK (COG-UK) Consortium|Moore|United Kingdom
>Spike|hCoV-19/England/NORW-F8F77/2020|2020-11-24|EPI_ISL_703998|Original|hCoV-19^^England|Human|Quadram Institute Bioscience|COVID-19 Genomics UK (COG-UK) Consortium|Alikhan|United Kingdom
>Spike|hCoV-19/England/QEUH-BCC612/2020|2020-11-27|EPI_ISL_704050|Original|hCoV-19^^England|Human|Lighthouse Lab in Glasgow|Wellcome Sanger Institute for the COVID-19 Genomics UK (COG-UK) Consortium|Davies|United Kingdom
>Spike|hCoV-19/England/QEUH-BC36A2/2020|2020-11-25|EPI_ISL_704098|Original|hCoV-19^^England|Human|Lighthouse Lab in Glasgow|Wellcome Sanger Institute for the COVID-19 Genomics UK (COG-UK) Consortium|Davies|United Kingdom
>Spike|hCoV-19/England/QEUH-BC35E1/2020|2020-11-25|EPI_ISL_704101|Original|hCoV-19^^England|Human|Lighthouse Lab in Glasgow|Wellcome Sanger Institute for the COVID-19 Genomics UK (COG-UK) Consortium|Davies|United Kingdom
>Spike|hCoV-19/England/QEUH-BCD75A/2020|2020-11-26|EPI_ISL_704432|Original|hCoV-19^^England|Human|Lighthouse Lab in Glasgow|Wellcome Sanger Institute for the COVID-19 Genomics UK (COG-UK) Consortium|Davies|United Kingdom
>Spike|hCoV-19/England/QEUH-BCCB6E/2020|2020-11-25|EPI_ISL_704436|Original|hCoV-19^^England|Human|Lighthouse Lab in Glasgow|Wellcome Sanger Institute for the COVID-19 Genomics UK (COG-UK) Consortium|Davies|United Kingdom
>Spike|hCoV-19/England/QEUH-BCE1C4/2020|2020-11-26|EPI_ISL_704464|Original|hCoV-19^^England|Human|Lighthouse Lab in Glasgow|Wellcome Sanger Institute for the COVID-19 Genomics UK (COG-UK) Consortium|Davies|United Kingdom
>Spike|hCoV-19/Wales/PHWC-490326/2020|2020-10-13|EPI_ISL_704918|Original|hCoV-19^^Wales|Human|Originating lab|COVID-19 Genomics UK (COG-UK) Consortium|Moore|United Kingdom
>Spike|hCoV-19/England/CAMC-BC0238/2020|2020-11-22|EPI_ISL_705119|Original|hCoV-19^^England|Human|Lighthouse Lab in Cambridge|Wellcome Sanger Institute for the COVID-19 Genomics UK (COG-UK) Consortium|Davies|United Kingdom
>Spike|hCoV-19/Wales/PHWC-492412/2020|2020-11-17|EPI_ISL_705412|Original|hCoV-19^^Wales|Human|Originating lab|COVID-19 Genomics UK (COG-UK) Consortium|Moore|United Kingdom
>Spike|hCoV-19/Wales/PHWC-491C0C/2020|2020-11-10|EPI_ISL_705527|Original|hCoV-19^^Wales|Human|Originating lab|COVID-19 Genomics UK (COG-UK) Consortium|Moore|United Kingdom
>Spike|hCoV-19/England/NORW-F8FE0/2020|2020-11-23|EPI_ISL_706408|Original|hCoV-19^^England|Human|Quadram Institute Bioscience|COVID-19 Genomics UK (COG-UK) Consortium|Alikhan|United Kingdom
>Spike|hCoV-19/Wales/PHWC-4901A4/2020|2020-11-16|EPI_ISL_706453|Original|hCoV-19^^Wales|Human|Originating lab|COVID-19 Genomics UK (COG-UK) Consortium|Moore|United Kingdom
>Spike|hCoV-19/Wales/PHWC-4902CF/2020|2020-09-23|EPI_ISL_706467|Original|hCoV-19^^Wales|Human|Originating lab|COVID-19 Genomics UK (COG-UK) Consortium|Moore|United Kingdom
>Spike|hCoV-19/Wales/PHWC-490599/2020|2020-11-06|EPI_ISL_706489|Original|hCoV-19^^Wales|Human|Originating lab|COVID-19 Genomics UK (COG-UK) Consortium|Moore|United Kingdom
>Spike|hCoV-19/Wales/PHWC-490687/2020|2020-11-13|EPI_ISL_706499|Original|hCoV-19^^Wales|Human|Originating lab|COVID-19 Genomics UK (COG-UK) Consortium|Moore|United Kingdom
>Spike|hCoV-19/Wales/PHWC-490C49/2020|2020-11-15|EPI_ISL_706552|Original|hCoV-19^^Wales|Human|Originating lab|COVID-19 Genomics UK (COG-UK) Consortium|Moore|United Kingdom

>Spike|hCoV-19/Wales/PHWC-490D28/2020|2020-11-16|EPI_ISL_706564|Original|hCoV-19^^Wales|Human|Originating lab|COVID-19 Genomics UK (COG-UK) Consortium|Moore|United Kingdom
>Spike|hCoV-19/Wales/PHWC-490D55/2020|2020-11-16|EPI_ISL_706567|Original|hCoV-19^^Wales|Human|Originating lab|COVID-19 Genomics UK (COG-UK) Consortium|Moore|United Kingdom
>Spike|hCoV-19/Wales/PHWC-490DA0/2020|2020-11-16|EPI_ISL_706570|Original|hCoV-19^^Wales|Human|Originating lab|COVID-19 Genomics UK (COG-UK) Consortium|Moore|United Kingdom
>Spike|hCoV-19/Wales/PHWC-490F13/2020|2020-11-19|EPI_ISL_706586|Original|hCoV-19^^Wales|Human|Originating lab|COVID-19 Genomics UK (COG-UK) Consortium|Moore|United Kingdom
>Spike|hCoV-19/Wales/PHWC-491413/2020|2020-11-18|EPI_ISL_706653|Original|hCoV-19^^Wales|Human|Originating lab|COVID-19 Genomics UK (COG-UK) Consortium|Moore|United Kingdom
>Spike|hCoV-19/Wales/PHWC-49164A/2020|2020-10-28|EPI_ISL_706674|Original|hCoV-19^^Wales|Human|Originating lab|COVID-19 Genomics UK (COG-UK) Consortium|Moore|United Kingdom
>Spike|hCoV-19/Wales/PHWC-4919E7/2020|2020-11-10|EPI_ISL_706724|Original|hCoV-19^^Wales|Human|Originating lab|COVID-19 Genomics UK (COG-UK) Consortium|Moore|United Kingdom
>Spike|hCoV-19/England/204961132/2020|2020-12-03|EPI_ISL_707703|Original|hCoV-19^^England|Human|Respiratory Virus Unit|COVID-19 Genomics UK (COG-UK) Consortium|Platt|United Kingdom
>Spike|hCoV-19/USA/CA-LACPHL-AE00002/2020|2020-06-08|EPI_ISL_707927|Original|hCoV-19^^California|Human|Los Angeles County Public Health Laboratory|Los Angeles County Public Health Laboratory|Hemarajata|USA
>Spike|hCoV-19/Norway/5542/2020|2020-11-07|EPI_ISL_708199|Original|hCoV-19^^Viken|Human|Medical Microbiology Unit|Norwegian Institute of Public Health|Kopperud Riis|Norway
>Spike|hCoV-19/USA/DE-DHSS-F1009555/2020|2020-10-03|EPI_ISL_708409|Original|hCoV-19^^Delaware|Human|Delaware Public Health Lab|Delaware Public Health Lab|Bajwa|USA
>Spike|hCoV-19/USA/DE-DHSS-F1009616/2020|2020-10-06|EPI_ISL_708419|Original|hCoV-19^^Delaware|Human|Delaware Public Health Lab|Delaware Public Health Lab|Bajwa|USA
>Spike|hCoV-19/USA/DE-DHSS-F994288/2020|2020-09-15|EPI_ISL_708433|Original|hCoV-19^^Delaware|Human|Delaware Public Health Lab|Delaware Public Health Lab|Bajwa|USA
>Spike|hCoV-19/USA/DE-DHSS-F1001769/2020|2020-09-10|EPI_ISL_708434|Original|hCoV-19^^Delaware|Human|Delaware Public Health Lab|Delaware Public Health Lab|Bajwa|USA
>Spike|hCoV-19/Belgium/ULG-10969/2020|2020-09-08|EPI_ISL_708454|Original|hCoV-19^^Liege|Human|Department of Clinical Microbiology|GIGA Medical Genomics|Durkin|Belgium
>Spike|hCoV-19/England/QEUH-BEB05B/2020|2020-12-02|EPI_ISL_708921|Original|hCoV-19^^England|Human|Lighthouse Lab in Glasgow|Wellcome Sanger Institute for the COVID-19 Genomics UK (COG-UK) Consortium|Davies|United Kingdom
>Spike|hCoV-19/England/QEUH-BF0D4D/2020|2020-12-03|EPI_ISL_709098|Original|hCoV-19^^England|Human|Lighthouse Lab in Glasgow|Wellcome Sanger Institute for the COVID-19 Genomics UK (COG-UK) Consortium|Davies|United Kingdom
>Spike|hCoV-19/England/QEUH-C145A4/2020|2020-12-06|EPI_ISL_709366|Original|hCoV-19^^England|Human|Lighthouse Lab in Glasgow|Wellcome Sanger Institute for the COVID-19 Genomics UK (COG-UK) Consortium|Davies|United Kingdom
>Spike|hCoV-19/England/QEUH-C1453B/2020|2020-12-06|EPI_ISL_709541|Original|hCoV-19^^England|Human|Lighthouse Lab in Glasgow|Wellcome Sanger Institute for the COVID-19 Genomics UK (COG-UK) Consortium|Davies|United Kingdom
>Spike|hCoV-19/USA/MI-UM-10036767604/2020|2020-11-03|EPI_ISL_710037|Original|hCoV-19^^Michigan|Human|University of Michigan Clinical Microbiology Laboratory|Lauring Lab|Valesano|USA
>Spike|hCoV-19/USA/MD-MDH-0479/2020|2020-11-12|EPI_ISL_710094|Original|hCoV-19^^Maryland|Human|Maryland Public Health Laboratory|Maryland Public Health Laboratory|Keller|USA

>Spike|hCoV-19/Gibraltar/205000668/2020|2020-12-00|EPI_ISL_710536|Original|hCoV-19^^Gibraltar|Human|Microbiology|Respiratory Virus Unit|Platt|Gibraltar
>Spike|hCoV-19/Wales/ALDP-B1E22C/2020|2020-11-01|EPI_ISL_710942|Original|hCoV-19^^Wales|Human|Lighthouse Lab in Alderley Park|Wellcome Sanger Institute for the COVID-19 Genomics UK (COG-UK) Consortium|Davies|United Kingdom
>Spike|hCoV-19/Denmark/DCGC-14302/2020|2020-11-30|EPI_ISL_711186|Original|hCoV-19^^Hovedstaden|Human|Department of Virus and Microbiological Special Diagnostics|Albertsen Lab|Michaelsen|Denmark
>Spike|hCoV-19/Denmark/DCGC-14175/2020|2020-11-30|EPI_ISL_711280|Original|hCoV-19^^Nordjylland|Human|Department of Virus and Microbiological Special Diagnostics|Albertsen Lab|Michaelsen|Denmark
>Spike|hCoV-19/Denmark/DCGC-14533/2020|2020-11-30|EPI_ISL_711284|Original|hCoV-19^^Nordjylland|Human|Department of Virus and Microbiological Special Diagnostics|Albertsen Lab|Michaelsen|Denmark
>Spike|hCoV-19/Denmark/DCGC-14036/2020|2020-11-30|EPI_ISL_711285|Original|hCoV-19^^Hovedstaden|Human|Department of Virus and Microbiological Special Diagnostics|Albertsen Lab|Michaelsen|Denmark
>Spike|hCoV-19/Denmark/DCGC-13784/2020|2020-11-30|EPI_ISL_712112|Original|hCoV-19^^Hovedstaden|Human|Department of Virus and Microbiological Special Diagnostics|Albertsen Lab|Michaelsen|Denmark
>Spike|hCoV-19/Denmark/DCGC-13281/2020|2020-11-30|EPI_ISL_712247|Original|hCoV-19^^Sjaelland|Human|Department of Virus and Microbiological Special Diagnostics|Albertsen Lab|Michaelsen|Denmark
>Spike|hCoV-19/Denmark/DCGC-15825/2020|2020-11-23|EPI_ISL_713119|Original|hCoV-19^^Hovedstaden|Human|Department of Virus and Microbiological Special Diagnostics|Albertsen Lab|Michaelsen|Denmark
>Spike|hCoV-19/Denmark/DCGC-15478/2020|2020-11-23|EPI_ISL_713211|Original|hCoV-19^^Hovedstaden|Human|Department of Virus and Microbiological Special Diagnostics|Albertsen Lab|Michaelsen|Denmark
>Spike|hCoV-19/Denmark/DCGC-16590/2020|2020-11-16|EPI_ISL_714368|Original|hCoV-19^^Hovedstaden|Human|Department of Virus and Microbiological Special Diagnostics|Albertsen Lab|Michaelsen|Denmark
>Spike|hCoV-19/Slovakia/UKBA-408/2020|2020-11-30|EPI_ISL_718256|Original|hCoV-19^^Bratislava|Human|Institute of Virology|Faculty of Natural Sciences|Vinar|Slovakia
>Spike|hCoV-19/Malaysia/IMR-WI093/2020|2020-07-31|EPI_ISL_718268|Original|hCoV-19^^Malaysia|Human|Institute for Medical Research|Institute for Medical Research|Suppiah|Malaysia
>Spike|hCoV-19/Malaysia/IMR_WI100/2020|2020-07-31|EPI_ISL_718269|Original|hCoV-19^^Malaysia|Human|Institute for Medical Research|Institute for Medical Research|Suppiah|Malaysia
>Spike|hCoV-19/Malaysia/IMR-WI101/2020|2020-07-31|EPI_ISL_718270|Vero|hCoV-19^^Malaysia|Human|Institute for Medical Research|Institute for Medical Research|Suppiah|Malaysia
>Spike|hCoV-19/Malaysia/IMR-WI123/2020|2020-08-16|EPI_ISL_718277|Original|hCoV-19^^Malaysia|Human|Institute for Medical Research|Institute for Medical Research|Suppiah|Malaysia
>Spike|hCoV-19/Malaysia/IMR-WI125/2020|2020-08-16|EPI_ISL_718279|Original|hCoV-19^^Malaysia|Human|Institute for Medical Research|Institute for Medical Research|Suppiah|Malaysia
>Spike|hCoV-19/Malaysia/IMR-WI206/2020|2020-09-29|EPI_ISL_718286|Vero|hCoV-19^^Malaysia|Human|Institute for Medical Research|Institute for Medical Research|Suppiah|Malaysia
>Spike|hCoV-19/Malaysia/IMR-CV136859/2020|2020-09-01|EPI_ISL_718287|Original|hCoV-19^^Malaysia|Human|Institute for Medical Research|Institute for Medical Research|Suppiah|Malaysia
>Spike|hCoV-19/Malaysia/IMR-CV138687/2020|2020-09-01|EPI_ISL_718292|Original|hCoV-19^^Malaysia|Human|Institute for Medical Research|Institute for Medical Research|Suppiah|Malaysia

>Spike|hCoV-19/Malaysia/IMR-CV138815/2020|2020-09-01|EPI_ISL_718293|Original|hCoV-19^^Malaysia|Human|Institute for Medical Research|Institute for Medical Research|Suppiah|Malaysia
>Spike|hCoV-19/Malaysia/IMR-CV139206/2020|2020-09-01|EPI_ISL_718298|Original|hCoV-19^^Malaysia|Human|Institute for Medical Research|Institute for Medical Research|Suppiah|Malaysia
>Spike|hCoV-19/Malaysia/IMR-CV140153/2020|2020-09-01|EPI_ISL_718300|Original|hCoV-19^^Malaysia|Human|Institute for Medical Research|Institute for Medical Research|Suppiah|Malaysia
>Spike|hCoV-19/Malaysia/IMR-CV140278/2020|2020-09-01|EPI_ISL_718303|Original|hCoV-19^^Malaysia|Human|Institute for Medical Research|Institute for Medical Research|Suppiah|Malaysia
>Spike|hCoV-19/Malaysia/IMR-CV140314/2020|2020-09-01|EPI_ISL_718304|Original|hCoV-19^^Malaysia|Human|Institute for Medical Research|Institute for Medical Research|Suppiah|Malaysia
>Spike|hCoV-19/Malaysia/IMR-WI212/2020|2020-09-29|EPI_ISL_718312|Vero|hCoV-19^^Malaysia|Human|Institute for Medical Research|Institute for Medical Research|Suppiah|Malaysia
>Spike|hCoV-19/England/ALDP-C49B3B/2020|2020-12-12|EPI_ISL_718723|Original|hCoV-19^^England|Human|Lighthouse Lab in Alderley Park|Wellcome Sanger Institute for the COVID-19 Genomics UK (COG-UK) Consortium|Davies|United Kingdom
>Spike|hCoV-19/England/ALDP-C49A10/2020|2020-12-12|EPI_ISL_718975|Original|hCoV-19^^England|Human|Lighthouse Lab in Alderley Park|Wellcome Sanger Institute for the COVID-19 Genomics UK (COG-UK) Consortium|Davies|United Kingdom
>Spike|hCoV-19/England/CAMC-C4133B/2020|2020-12-10|EPI_ISL_719095|Original|hCoV-19^^England|Human|Lighthouse Lab in Cambridge|Wellcome Sanger Institute for the COVID-19 Genomics UK (COG-UK) Consortium|Davies|United Kingdom
>Spike|hCoV-19/England/CAMC-C43C5C/2020|2020-12-10|EPI_ISL_719187|Original|hCoV-19^^England|Human|Lighthouse Lab in Cambridge|Wellcome Sanger Institute for the COVID-19 Genomics UK (COG-UK) Consortium|Davies|United Kingdom
>Spike|hCoV-19/England/QEUH-BA19B0/2020|2020-11-18|EPI_ISL_719752|Original|hCoV-19^^England|Human|Lighthouse Lab in Glasgow|Wellcome Sanger Institute for the COVID-19 Genomics UK (COG-UK) Consortium|Davies|United Kingdom
>Spike|hCoV-19/England/MILK-BB0329/2020|2020-11-17|EPI_ISL_720016|Original|hCoV-19^^England|Human|Lighthouse Lab in Milton Keynes|Wellcome Sanger Institute for the COVID-19 Genomics UK (COG-UK) Consortium|Davies|United Kingdom
>Spike|hCoV-19/England/MILK-BB068A/2020|2020-11-17|EPI_ISL_720108|Original|hCoV-19^^England|Human|Lighthouse Lab in Milton Keynes|Wellcome Sanger Institute for the COVID-19 Genomics UK (COG-UK) Consortium|Davies|United Kingdom
>Spike|hCoV-19/England/MILK-BB1847/2020|2020-11-18|EPI_ISL_720320|Original|hCoV-19^^England|Human|Lighthouse Lab in Milton Keynes|Wellcome Sanger Institute for the COVID-19 Genomics UK (COG-UK) Consortium|Davies|United Kingdom
>Spike|hCoV-19/England/MILK-BAFBF8/2020|2020-11-17|EPI_ISL_720368|Original|hCoV-19^^England|Human|Lighthouse Lab in Milton Keynes|Wellcome Sanger Institute for the COVID-19 Genomics UK (COG-UK) Consortium|Davies|United Kingdom
>Spike|hCoV-19/England/MILK-BAFBE9/2020|2020-11-17|EPI_ISL_720514|Original|hCoV-19^^England|Human|Lighthouse Lab in Milton Keynes|Wellcome Sanger Institute for the COVID-19 Genomics UK (COG-UK) Consortium|Davies|United Kingdom
>Spike|hCoV-19/England/ALDP-C04C1B/2020|2020-12-04|EPI_ISL_720891|Original|hCoV-19^^England|Human|Lighthouse Lab in Alderley Park|Wellcome Sanger Institute for the COVID-19 Genomics UK (COG-UK) Consortium|Davies|United Kingdom
>Spike|hCoV-19/England/QEUH-B9FC52/2020|2020-11-18|EPI_ISL_721059|Original|hCoV-19^^England|Human|Lighthouse Lab in Glasgow|Wellcome Sanger Institute for the COVID-19 Genomics UK (COG-UK) Consortium|Davies|United Kingdom
>Spike|hCoV-19/England/ALDP-BC73F2/2020|2020-11-22|EPI_ISL_721250|Original|hCoV-19^^England|Human|Lighthouse Lab in Alderley Park|Wellcome Sanger Institute for the COVID-19 Genomics UK (COG-UK) Consortium|Davies|United Kingdom
>Spike|hCoV-19/England/ALDP-BC7AEE/2020|2020-11-23|EPI_ISL_721254|Original|hCoV-19^^England|Human|Lighthouse Lab in Alderley Park|Wellcome Sanger Institute for the COVID-19 Genomics UK (COG-UK) Consortium|Davies|United Kingdom

>Spike|hCoV-19/India/MH-NCCS-NR-10951/2020|2020-07-11|EPI_ISL_721647|Original|hCoV-19^^Maharashtra|Human|National Centre For Cell Science|National Centre For Cell Science|Paul|India
>Spike|hCoV-19/India/MH-NCCS-NR-7631/2020|2020-06-26|EPI_ISL_721662|Original|hCoV-19^^Maharashtra|Human|National Centre For Cell Science|National Centre For Cell Science|Paul|India
>Spike|hCoV-19/India/MH-NCCS-NR-8031/2020|2020-06-28|EPI_ISL_722181|Original|hCoV-19^^Maharashtra|Human|National Centre For Cell Science|National Centre For Cell Science|Paul|India
>Spike|hCoV-19/India/MH-AFCO-15380/2020|2020-07-26|EPI_ISL_722199|Original|hCoV-19^^Maharashtra|Human|Armed Forces Medical College|National Centre For Cell Science|Paul|India
>Spike|hCoV-19/India/MH-ACTREC-643/2020|2020-09-14|EPI_ISL_723069|Original|hCoV-19^^Maharashtra|Human|Hematopathology Laboratory|Hematopathology Laboratory|Patkar|India
>Spike|hCoV-19/India/MH-ACTREC-644/2020|2020-09-14|EPI_ISL_723070|Original|hCoV-19^^Maharashtra|Human|Hematopathology Laboratory|Hematopathology Laboratory|Patkar|India
>Spike|hCoV-19/India/MH-ACTREC-645/2020|2020-09-14|EPI_ISL_723071|Original|hCoV-19^^Maharashtra|Human|Hematopathology Laboratory|Hematopathology Laboratory|Patkar|India
>Spike|hCoV-19/India/MH-ACTREC-666/2020|2020-09-16|EPI_ISL_723074|Original|hCoV-19^^Maharashtra|Human|Hematopathology Laboratory|Hematopathology Laboratory|Patkar|India
>Spike|hCoV-19/India/MH-ACTREC-667/2020|2020-09-16|EPI_ISL_723075|Original|hCoV-19^^Maharashtra|Human|Hematopathology Laboratory|Hematopathology Laboratory|Patkar|India
>Spike|hCoV-19/India/MH-ACTREC-674/2020|2020-09-18|EPI_ISL_723076|Original|hCoV-19^^Maharashtra|Human|Hematopathology Laboratory|Hematopathology Laboratory|Patkar|India
>Spike|hCoV-19/Germany/BW-UKT-a045/2020|2020-03-26|EPI_ISL_723078|Original|hCoV-19^^Baden-Wurttemberg|Human|Institute of Medical Genetics and Applied Genomics|Institute of Medical Genetics and Applied Genomics|Gross|Germany
>Spike|hCoV-19/USA/MN-MDH-2186/2020|2020-12-02|EPI_ISL_723137|Original|hCoV-19^^Minnesota|Human|Minnesota Department of Health|Minnesota Department of Health|Lorentz|USA
>Spike|hCoV-19/South Africa/N00351/2020|2020-11-06|EPI_ISL_723468|Original isolate|hCoV-19^^Eastern Cape|Human|Port Elizabeth Provincial Hospital|National Institute for Communicable Diseases of the National Health Laboratory Service|Bhiman|South Africa
>Spike|hCoV-19/England/MILK-B87A26/2020|2020-11-13|EPI_ISL_724822|Original|hCoV-19^^England|Human|Oxford Viromics|COVID-19 Genomics UK (COG-UK) Consortium|Golubchik|United Kingdom
>Spike|hCoV-19/Wales/PHWC-4925D3/2020|2020-11-30|EPI_ISL_725671|Original|hCoV-19^^Wales|Human|Originating lab|COVID-19 Genomics UK (COG-UK) Consortium|Moore|United Kingdom
>Spike|hCoV-19/Wales/PHWC-4926A3/2020|2020-11-22|EPI_ISL_725683|Original|hCoV-19^^Wales|Human|Originating lab|COVID-19 Genomics UK (COG-UK) Consortium|Moore|United Kingdom
>Spike|hCoV-19/Wales/PHWC-4929D7/2020|2020-11-21|EPI_ISL_725731|Original|hCoV-19^^Wales|Human|Originating lab|COVID-19 Genomics UK (COG-UK) Consortium|Moore|United Kingdom
>Spike|hCoV-19/Wales/PHWC-492A98/2020|2020-11-24|EPI_ISL_725742|Original|hCoV-19^^Wales|Human|Originating lab|COVID-19 Genomics UK (COG-UK) Consortium|Moore|United Kingdom
>Spike|hCoV-19/Wales/PHWC-492B86/2020|2020-11-27|EPI_ISL_725757|Original|hCoV-19^^Wales|Human|Originating lab|COVID-19 Genomics UK (COG-UK) Consortium|Moore|United Kingdom

>Spike|hCoV-19/Wales/PHWC-492BD1/2020|2020-11-27|EPI_ISL_725761|Original|hCoV-19^^Wales|Human|Originating lab|COVID-19 Genomics UK (COG-UK) Consortium|Moore|United Kingdom
>Spike|hCoV-19/Wales/PHWC-492F02/2020|2020-11-28|EPI_ISL_725807|Original|hCoV-19^^Wales|Human|Originating lab|COVID-19 Genomics UK (COG-UK) Consortium|Moore|United Kingdom
>Spike|hCoV-19/Wales/PHWC-492F5D/2020|2020-11-28|EPI_ISL_725812|Original|hCoV-19^^Wales|Human|Originating lab|COVID-19 Genomics UK (COG-UK) Consortium|Moore|United Kingdom
>Spike|hCoV-19/Wales/PHWC-493578/2020|2020-11-29|EPI_ISL_725906|Original|hCoV-19^^Wales|Human|Originating lab|COVID-19 Genomics UK (COG-UK) Consortium|Moore|United Kingdom
>Spike|hCoV-19/Wales/PHWC-49396D/2020|2020-11-30|EPI_ISL_725961|Original|hCoV-19^^Wales|Human|Originating lab|COVID-19 Genomics UK (COG-UK) Consortium|Moore|United Kingdom
>Spike|hCoV-19/Wales/PHWC-493A3D/2020|2020-11-30|EPI_ISL_725974|Original|hCoV-19^^Wales|Human|Originating lab|COVID-19 Genomics UK (COG-UK) Consortium|Moore|United Kingdom
>Spike|hCoV-19/Wales/PHWC-493A4C/2020|2020-11-30|EPI_ISL_725975|Original|hCoV-19^^Wales|Human|Originating lab|COVID-19 Genomics UK (COG-UK) Consortium|Moore|United Kingdom
>Spike|hCoV-19/Wales/PHWC-493B2B/2020|2020-11-10|EPI_ISL_725987|Original|hCoV-19^^Wales|Human|Originating lab|COVID-19 Genomics UK (COG-UK) Consortium|Moore|United Kingdom
>Spike|hCoV-19/Wales/PHWC-493B85/2020|2020-11-13|EPI_ISL_725991|Original|hCoV-19^^Wales|Human|Originating lab|COVID-19 Genomics UK (COG-UK) Consortium|Moore|United Kingdom
>Spike|hCoV-19/Wales/PHWC-493C64/2020|2020-11-19|EPI_ISL_726004|Original|hCoV-19^^Wales|Human|Originating lab|COVID-19 Genomics UK (COG-UK) Consortium|Moore|United Kingdom
>Spike|hCoV-19/Wales/PHWC-493D25/2020|2020-11-19|EPI_ISL_726016|Original|hCoV-19^^Wales|Human|Originating lab|COVID-19 Genomics UK (COG-UK) Consortium|Moore|United Kingdom
>Spike|hCoV-19/Wales/PHWC-493D9E/2020|2020-11-20|EPI_ISL_726023|Original|hCoV-19^^Wales|Human|Originating lab|COVID-19 Genomics UK (COG-UK) Consortium|Moore|United Kingdom
>Spike|hCoV-19/Wales/PHWC-493E6E/2020|2020-11-08|EPI_ISL_726034|Original|hCoV-19^^Wales|Human|Originating lab|COVID-19 Genomics UK (COG-UK) Consortium|Moore|United Kingdom
>Spike|hCoV-19/Wales/PHWC-493FD4/2020|2020-11-12|EPI_ISL_726051|Original|hCoV-19^^Wales|Human|Originating lab|COVID-19 Genomics UK (COG-UK) Consortium|Moore|United Kingdom
>Spike|hCoV-19/Wales/PHWC-494085/2020|2020-11-15|EPI_ISL_726062|Original|hCoV-19^^Wales|Human|Originating lab|COVID-19 Genomics UK (COG-UK) Consortium|Moore|United Kingdom
>Spike|hCoV-19/Wales/PHWC-49410A/2020|2020-11-16|EPI_ISL_726069|Original|hCoV-19^^Wales|Human|Originating lab|COVID-19 Genomics UK (COG-UK) Consortium|Moore|United Kingdom
>Spike|hCoV-19/Wales/PHWC-49428F/2020|2020-11-17|EPI_ISL_726092|Original|hCoV-19^^Wales|Human|Originating lab|COVID-19 Genomics UK (COG-UK) Consortium|Moore|United Kingdom
>Spike|hCoV-19/Wales/PHWC-4943D7/2020|2020-11-19|EPI_ISL_726109|Original|hCoV-19^^Wales|Human|Originating lab|COVID-19 Genomics UK (COG-UK) Consortium|Moore|United Kingdom
>Spike|hCoV-19/Wales/PHWC-49450E/2020|2020-11-19|EPI_ISL_726128|Original|hCoV-19^^Wales|Human|Originating lab|COVID-19 Genomics UK (COG-UK) Consortium|Moore|United Kingdom
>Spike|hCoV-19/Wales/PHWC-494586/2020|2020-11-20|EPI_ISL_726135|Original|hCoV-19^^Wales|Human|Originating lab|COVID-19 Genomics UK (COG-UK) Consortium|Moore|United Kingdom

>Spike|hCoV-19/Wales/PHWC-4945A4/2020|2020-11-20|EPI_ISL_726137|Original|hCoV-19^^Wales|Human|Originating lab|COVID-19 Genomics UK (COG-UK) Consortium|Moore|United Kingdom
>Spike|hCoV-19/Wales/PHWC-4945E0/2020|2020-11-20|EPI_ISL_726141|Original|hCoV-19^^Wales|Human|Originating lab|COVID-19 Genomics UK (COG-UK) Consortium|Moore|United Kingdom
>Spike|hCoV-19/Wales/PHWC-49460B/2020|2020-11-20|EPI_ISL_726143|Original|hCoV-19^^Wales|Human|Originating lab|COVID-19 Genomics UK (COG-UK) Consortium|Moore|United Kingdom
>Spike|hCoV-19/Wales/PHWC-494674/2020|2020-11-20|EPI_ISL_726150|Original|hCoV-19^^Wales|Human|Originating lab|COVID-19 Genomics UK (COG-UK) Consortium|Moore|United Kingdom
>Spike|hCoV-19/Wales/PHWC-494683/2020|2020-11-20|EPI_ISL_726151|Original|hCoV-19^^Wales|Human|Originating lab|COVID-19 Genomics UK (COG-UK) Consortium|Moore|United Kingdom
>Spike|hCoV-19/Wales/PHWC-494726/2020|2020-11-20|EPI_ISL_726161|Original|hCoV-19^^Wales|Human|Originating lab|COVID-19 Genomics UK (COG-UK) Consortium|Moore|United Kingdom
>Spike|hCoV-19/Wales/PHWC-4947DB/2020|2020-11-20|EPI_ISL_726168|Original|hCoV-19^^Wales|Human|Originating lab|COVID-19 Genomics UK (COG-UK) Consortium|Moore|United Kingdom
>Spike|hCoV-19/Wales/PHWC-494C18/2020|2020-11-23|EPI_ISL_726232|Original|hCoV-19^^Wales|Human|Originating lab|COVID-19 Genomics UK (COG-UK) Consortium|Moore|United Kingdom
>Spike|hCoV-19/Wales/PHWC-494CCD/2020|2020-11-24|EPI_ISL_726241|Original|hCoV-19^^Wales|Human|Originating lab|COVID-19 Genomics UK (COG-UK) Consortium|Moore|United Kingdom
>Spike|hCoV-19/Wales/PHWC-494DAC/2020|2020-11-16|EPI_ISL_726253|Original|hCoV-19^^Wales|Human|Originating lab|COVID-19 Genomics UK (COG-UK) Consortium|Moore|United Kingdom
>Spike|hCoV-19/Wales/PHWC-494DBB/2020|2020-11-17|EPI_ISL_726254|Original|hCoV-19^^Wales|Human|Originating lab|COVID-19 Genomics UK (COG-UK) Consortium|Moore|United Kingdom
>Spike|hCoV-19/Wales/PHWC-494F4C/2020|2020-11-26|EPI_ISL_726274|Original|hCoV-19^^Wales|Human|Originating lab|COVID-19 Genomics UK (COG-UK) Consortium|Moore|United Kingdom
>Spike|hCoV-19/Wales/PHWC-4951DC/2020|2020-11-19|EPI_ISL_726310|Original|hCoV-19^^Wales|Human|Originating lab|COVID-19 Genomics UK (COG-UK) Consortium|Moore|United Kingdom
>Spike|hCoV-19/Wales/PHWC-495260/2020|2020-11-22|EPI_ISL_726318|Original|hCoV-19^^Wales|Human|Originating lab|COVID-19 Genomics UK (COG-UK) Consortium|Moore|United Kingdom
>Spike|hCoV-19/Wales/PHWC-49553A/2020|2020-11-22|EPI_ISL_726359|Original|hCoV-19^^Wales|Human|Originating lab|COVID-19 Genomics UK (COG-UK) Consortium|Moore|United Kingdom
>Spike|hCoV-19/Wales/PHWC-4955A3/2020|2020-11-23|EPI_ISL_726366|Original|hCoV-19^^Wales|Human|Originating lab|COVID-19 Genomics UK (COG-UK) Consortium|Moore|United Kingdom
>Spike|hCoV-19/Wales/PHWC-495734/2020|2020-11-23|EPI_ISL_726389|Original|hCoV-19^^Wales|Human|Originating lab|COVID-19 Genomics UK (COG-UK) Consortium|Moore|United Kingdom
>Spike|hCoV-19/Wales/PHWC-4957E9/2020|2020-11-23|EPI_ISL_726399|Original|hCoV-19^^Wales|Human|Originating lab|COVID-19 Genomics UK (COG-UK) Consortium|Moore|United Kingdom
>Spike|hCoV-19/Wales/PHWC-495B38/2020|2020-11-25|EPI_ISL_726443|Original|hCoV-19^^Wales|Human|Originating lab|COVID-19 Genomics UK (COG-UK) Consortium|Moore|United Kingdom
>Spike|hCoV-19/Wales/PHWC-495D14/2020|2020-11-25|EPI_ISL_726473|Original|hCoV-19^^Wales|Human|Originating lab|COVID-19 Genomics UK (COG-UK) Consortium|Moore|United Kingdom

>Spike|hCoV-19/Wales/PHWC-495D32/2020|2020-11-26|EPI_ISL_726475|Original|hCoV-19^^Wales|Human|Originating lab|COVID-19 Genomics UK (COG-UK) Consortium|Moore|United Kingdom
>Spike|hCoV-19/Wales/PHWC-495E6C/2020|2020-11-26|EPI_ISL_726494|Original|hCoV-19^^Wales|Human|Originating lab|COVID-19 Genomics UK (COG-UK) Consortium|Moore|United Kingdom
>Spike|hCoV-19/Wales/PHWC-4974D1/2020|2020-11-30|EPI_ISL_726838|Original|hCoV-19^^Wales|Human|Originating lab|COVID-19 Genomics UK (COG-UK) Consortium|Moore|United Kingdom
>Spike|hCoV-19/Wales/PHWC-4974FF/2020|2020-11-30|EPI_ISL_726840|Original|hCoV-19^^Wales|Human|Originating lab|COVID-19 Genomics UK (COG-UK) Consortium|Moore|United Kingdom
>Spike|hCoV-19/Wales/PHWC-497529/2020|2020-11-30|EPI_ISL_726843|Original|hCoV-19^^Wales|Human|Originating lab|COVID-19 Genomics UK (COG-UK) Consortium|Moore|United Kingdom
>Spike|hCoV-19/Wales/PHWC-497592/2020|2020-12-01|EPI_ISL_726850|Original|hCoV-19^^Wales|Human|Originating lab|COVID-19 Genomics UK (COG-UK) Consortium|Moore|United Kingdom
>Spike|hCoV-19/Wales/PHWC-4976DB/2020|2020-11-21|EPI_ISL_726868|Original|hCoV-19^^Wales|Human|Originating lab|COVID-19 Genomics UK (COG-UK) Consortium|Moore|United Kingdom
>Spike|hCoV-19/Wales/PHWC-4977AB/2020|2020-11-22|EPI_ISL_726880|Original|hCoV-19^^Wales|Human|Originating lab|COVID-19 Genomics UK (COG-UK) Consortium|Moore|United Kingdom
>Spike|hCoV-19/Wales/PHWC-4977E7/2020|2020-11-22|EPI_ISL_726884|Original|hCoV-19^^Wales|Human|Originating lab|COVID-19 Genomics UK (COG-UK) Consortium|Moore|United Kingdom
>Spike|hCoV-19/Wales/PHWC-497811/2020|2020-11-22|EPI_ISL_726887|Original|hCoV-19^^Wales|Human|Originating lab|COVID-19 Genomics UK (COG-UK) Consortium|Moore|United Kingdom
>Spike|hCoV-19/Wales/PHWC-49786C/2020|2020-11-22|EPI_ISL_726892|Original|hCoV-19^^Wales|Human|Originating lab|COVID-19 Genomics UK (COG-UK) Consortium|Moore|United Kingdom
>Spike|hCoV-19/Wales/PHWC-4978B7/2020|2020-11-22|EPI_ISL_726897|Original|hCoV-19^^Wales|Human|Originating lab|COVID-19 Genomics UK (COG-UK) Consortium|Moore|United Kingdom
>Spike|hCoV-19/Wales/PHWC-497978/2020|2020-11-23|EPI_ISL_726909|Original|hCoV-19^^Wales|Human|Originating lab|COVID-19 Genomics UK (COG-UK) Consortium|Moore|United Kingdom
>Spike|hCoV-19/Wales/PHWC-497996/2020|2020-11-23|EPI_ISL_726911|Original|hCoV-19^^Wales|Human|Originating lab|COVID-19 Genomics UK (COG-UK) Consortium|Moore|United Kingdom
>Spike|hCoV-19/Wales/PHWC-497B18/2020|2020-11-23|EPI_ISL_726935|Original|hCoV-19^^Wales|Human|Originating lab|COVID-19 Genomics UK (COG-UK) Consortium|Moore|United Kingdom
>Spike|hCoV-19/Wales/PHWC-497BCD/2020|2020-11-24|EPI_ISL_726944|Original|hCoV-19^^Wales|Human|Originating lab|COVID-19 Genomics UK (COG-UK) Consortium|Moore|United Kingdom
>Spike|hCoV-19/Wales/PHWC-497CBB/2020|2020-11-27|EPI_ISL_726959|Original|hCoV-19^^Wales|Human|Originating lab|COVID-19 Genomics UK (COG-UK) Consortium|Moore|United Kingdom
>Spike|hCoV-19/Wales/PHWC-497CCA/2020|2020-11-28|EPI_ISL_726960|Original|hCoV-19^^Wales|Human|Originating lab|COVID-19 Genomics UK (COG-UK) Consortium|Moore|United Kingdom
>Spike|hCoV-19/Wales/PHWC-497D4F/2020|2020-11-30|EPI_ISL_726968|Original|hCoV-19^^Wales|Human|Originating lab|COVID-19 Genomics UK (COG-UK) Consortium|Moore|United Kingdom
>Spike|hCoV-19/Wales/PHWC-4981D9/2020|2020-11-30|EPI_ISL_727038|Original|hCoV-19^^Wales|Human|Originating lab|COVID-19 Genomics UK (COG-UK) Consortium|Moore|United Kingdom

>Spike|hCoV-19/Wales/PHWC-498555/2020|2020-12-01|EPI_ISL_727091|Original|hCoV-19^^Wales|Human|Originating lab|COVID-19 Genomics UK (COG-UK) Consortium|Moore|United Kingdom
>Spike|hCoV-19/Wales/PHWC-4985CE/2020|2020-12-02|EPI_ISL_727097|Original|hCoV-19^^Wales|Human|Originating lab|COVID-19 Genomics UK (COG-UK) Consortium|Moore|United Kingdom
>Spike|hCoV-19/Wales/PHWC-4987D7/2020|2020-11-26|EPI_ISL_727124|Original|hCoV-19^^Wales|Human|Originating lab|COVID-19 Genomics UK (COG-UK) Consortium|Moore|United Kingdom
>Spike|hCoV-19/Wales/PHWC-4988B6/2020|2020-11-27|EPI_ISL_727137|Original|hCoV-19^^Wales|Human|Originating lab|COVID-19 Genomics UK (COG-UK) Consortium|Moore|United Kingdom
>Spike|hCoV-19/Wales/PHWC-49890E/2020|2020-11-29|EPI_ISL_727142|Original|hCoV-19^^Wales|Human|Originating lab|COVID-19 Genomics UK (COG-UK) Consortium|Moore|United Kingdom
>Spike|hCoV-19/Wales/PHWC-498A47/2020|2020-12-04|EPI_ISL_727162|Original|hCoV-19^^Wales|Human|Originating lab|COVID-19 Genomics UK (COG-UK) Consortium|Moore|United Kingdom
>Spike|hCoV-19/Wales/PHWC-498A83/2020|2020-12-05|EPI_ISL_727166|Original|hCoV-19^^Wales|Human|Originating lab|COVID-19 Genomics UK (COG-UK) Consortium|Moore|United Kingdom
>Spike|hCoV-19/Wales/PHWC-498AA1/2020|2020-12-06|EPI_ISL_727168|Original|hCoV-19^^Wales|Human|Originating lab|COVID-19 Genomics UK (COG-UK) Consortium|Moore|United Kingdom
>Spike|hCoV-19/Wales/PHWC-498ACF/2020|2020-12-06|EPI_ISL_727170|Original|hCoV-19^^Wales|Human|Originating lab|COVID-19 Genomics UK (COG-UK) Consortium|Moore|United Kingdom
>Spike|hCoV-19/Wales/PHWC-498DB7/2020|2020-11-27|EPI_ISL_727210|Original|hCoV-19^^Wales|Human|Originating lab|COVID-19 Genomics UK (COG-UK) Consortium|Moore|United Kingdom
>Spike|hCoV-19/Wales/PHWC-498DF3/2020|2020-11-28|EPI_ISL_727213|Original|hCoV-19^^Wales|Human|Originating lab|COVID-19 Genomics UK (COG-UK) Consortium|Moore|United Kingdom
>Spike|hCoV-19/Wales/PHWC-498EF0/2020|2020-11-30|EPI_ISL_727228|Original|hCoV-19^^Wales|Human|Originating lab|COVID-19 Genomics UK (COG-UK) Consortium|Moore|United Kingdom
>Spike|hCoV-19/Wales/PHWC-498F66/2020|2020-12-01|EPI_ISL_727234|Original|hCoV-19^^Wales|Human|Originating lab|COVID-19 Genomics UK (COG-UK) Consortium|Moore|United Kingdom
>Spike|hCoV-19/Wales/PHWC-498FA2/2020|2020-12-01|EPI_ISL_727238|Original|hCoV-19^^Wales|Human|Originating lab|COVID-19 Genomics UK (COG-UK) Consortium|Moore|United Kingdom
>Spike|hCoV-19/Wales/PHWC-499080/2020|2020-12-02|EPI_ISL_727252|Original|hCoV-19^^Wales|Human|Originating lab|COVID-19 Genomics UK (COG-UK) Consortium|Moore|United Kingdom
>Spike|hCoV-19/Wales/PHWC-4991C9/2020|2020-12-02|EPI_ISL_727269|Original|hCoV-19^^Wales|Human|Originating lab|COVID-19 Genomics UK (COG-UK) Consortium|Moore|United Kingdom
>Spike|hCoV-19/Wales/PHWC-499466/2020|2020-12-03|EPI_ISL_727307|Original|hCoV-19^^Wales|Human|Originating lab|COVID-19 Genomics UK (COG-UK) Consortium|Moore|United Kingdom
>Spike|hCoV-19/Wales/PHWC-499581/2020|2020-12-03|EPI_ISL_727323|Original|hCoV-19^^Wales|Human|Originating lab|COVID-19 Genomics UK (COG-UK) Consortium|Moore|United Kingdom
>Spike|hCoV-19/Wales/PHWC-4995AF/2020|2020-12-03|EPI_ISL_727325|Original|hCoV-19^^Wales|Human|Originating lab|COVID-19 Genomics UK (COG-UK) Consortium|Moore|United Kingdom
>Spike|hCoV-19/Wales/PHWC-4996F7/2020|2020-12-03|EPI_ISL_727344|Original|hCoV-19^^Wales|Human|Originating lab|COVID-19 Genomics UK (COG-UK) Consortium|Moore|United Kingdom

>Spike|hCoV-19/Wales/PHWC-4997E5/2020|2020-12-03|EPI_ISL_727359|Original|hCoV-19^^Wales|Human|Originating lab|COVID-19 Genomics UK (COG-UK) Consortium|Moore|United Kingdom
>Spike|hCoV-19/Wales/PHWC-49990D/2020|2020-12-04|EPI_ISL_727376|Original|hCoV-19^^Wales|Human|Originating lab|COVID-19 Genomics UK (COG-UK) Consortium|Moore|United Kingdom
>Spike|hCoV-19/Wales/PHWC-499B61/2020|2020-12-05|EPI_ISL_727407|Original|hCoV-19^^Wales|Human|Originating lab|COVID-19 Genomics UK (COG-UK) Consortium|Moore|United Kingdom
>Spike|hCoV-19/Wales/PHWC-499B8F/2020|2020-11-24|EPI_ISL_727409|Original|hCoV-19^^Wales|Human|Originating lab|COVID-19 Genomics UK (COG-UK) Consortium|Moore|United Kingdom
>Spike|hCoV-19/Wales/PHWC-49A1B9/2020|2020-12-01|EPI_ISL_727506|Original|hCoV-19^^Wales|Human|Originating lab|COVID-19 Genomics UK (COG-UK) Consortium|Moore|United Kingdom
>Spike|hCoV-19/England/QEUH-B7E318/2020|2020-11-14|EPI_ISL_727855|Original|hCoV-19^^England|Human|Oxford Viromics|COVID-19 Genomics UK (COG-UK) Consortium|Golubchik|United Kingdom
>Spike|hCoV-19/Malaysia/IMR-WC194446/2020|2020-10-01|EPI_ISL_728158|Original|hCoV-19^^Malaysia|Human|Institute for Medical Research|Institute for Medical Research|Suppiah|Malaysia
>Spike|hCoV-19/Malaysia/IMR-WC197307/2020|2020-09-29|EPI_ISL_728162|Original|hCoV-19^^Malaysia|Human|Institute for Medical Research|Institute for Medical Research|Suppiah|Malaysia
>Spike|hCoV-19/Malaysia/IMR-WC200594/2020|2020-09-29|EPI_ISL_728163|Original|hCoV-19^^Malaysia|Human|Institute for Medical Research|Institute for Medical Research|Suppiah|Malaysia
>Spike|hCoV-19/Ecuador/USFQ-558/2020|2020-12-09|EPI_ISL_728205|Original|hCoV-19^^Sucumbios|Human|Institute of Microbiology|Institute of Microbiology|Cardenas|Ecuador
>Spike|hCoV-19/Malaysia/IMR-WC277466/2020|2020-11-16|EPI_ISL_728249|Original|hCoV-19^^Malaysia|Human|Institute for Medical Research|Institute for Medical Research|Suppiah|Malaysia
>Spike|hCoV-19/Malaysia/IMR-WC277457/2020|2020-11-16|EPI_ISL_728250|Original|hCoV-19^^Malaysia|Human|Institute for Medical Research|Institute for Medical Research|Suppiah|Malaysia
>Spike|hCoV-19/Malaysia/IMR-WC280729/2020|2020-11-18|EPI_ISL_728251|Original|hCoV-19^^Malaysia|Human|Institute for Medical Research|Institute for Medical Research|Suppiah|Malaysia
>Spike|hCoV-19/India/MH-BJMC-22244/2020|2020-07-18|EPI_ISL_728256|Original|hCoV-19^^Maharashtra|Human|B.J. Govt. Medical College|National Centre For Cell Science|Paul|India
>Spike|hCoV-19/India/MH-BJMC-8860/2020|2020-06-23|EPI_ISL_728263|Original|hCoV-19^^Maharashtra|Human|B.J. Govt. Medical College|National Centre For Cell Science|Paul|India
>Spike|hCoV-19/France/ARA-109096/2020|2020-11-18|EPI_ISL_728526|Original|hCoV-19^^Auvergne-Rh\u00f4ne-Alpes|Human|CNR Virus des Infections Respiratoires - France SUD|CNR Virus des Infections Respiratoires - France SUD|Bal|France
>Spike|hCoV-19/France/ARA-110872/2020|2020-11-23|EPI_ISL_728540|Original|hCoV-19^^Auvergne-Rh\u00f4ne-Alpes|Human|CNR Virus des Infections Respiratoires - France SUD|CNR Virus des Infections Respiratoires - France SUD|Bal|France
>Spike|hCoV-19/USA/WI-UW-2272/2020|2020-11-18|EPI_ISL_728553|Original|hCoV-19^^Wisconsin|Human|University of Wisconsin-Madison AIDS Vaccine Research Laboratories|University of Wisconsin-Madison AIDS Vaccine Research Laboratories|Baczenas|USA
>Spike|hCoV-19/USA/WI-UW-2274/2020|2020-11-08|EPI_ISL_728554|Original|hCoV-19^^Wisconsin|Human|University of Wisconsin-Madison AIDS Vaccine Research Laboratories|University of Wisconsin-Madison AIDS Vaccine Research Laboratories|Baczenas|USA

>Spike|hCoV-19/USA/WI-UW-2378/2020|2020-11-01|EPI_ISL_728556|Original|hCoV-19^^Wisconsin|Human|University of Wisconsin-Madison AIDS Vaccine Research Laboratories|University of Wisconsin-Madison AIDS Vaccine Research Laboratories|Baczenas|USA
>Spike|hCoV-19/Germany/BE-ChVir8996/2020|2020-09-21|EPI_ISL_729408|Original|hCoV-19^^Berlin|Human|Charit\u00e9 Universit\u00e4tsmedizin Berlin|Charit\u00e9 Universit\u00e4tsmedizin Berlin|Muehlemann|Germany
>Spike|hCoV-19/Germany/SH-ChVir9283/2020|2020-09-28|EPI_ISL_729419|Original|hCoV-19^^Schleswig-Holstein|Human|A. Krumbholz|Charit\u00e9 Universit\u00e4tsmedizin Berlin|Muehlemann|Germany
>Spike|hCoV-19/Nigeria/OS-CV299/2020|2020-08-07|EPI_ISL_729977|Original|hCoV-19^^Osun State|Human|Nigeria Centre for Disease Control (NCDC)|African Centre of Excellence for Genomics of Infectious Diseases (ACEGID)|Oluniyi|Nigeria
>Spike|hCoV-19/Jordan/AM-ALSR-4961/2020|2020-10-03|EPI_ISL_730234|Original|hCoV-19^^Amman|Human|Biolab Diagnostic Laboratories|Andersen lab at Scripps Research|Gangavarapu|Jordan
>Spike|hCoV-19/Jordan/AM-ALSR-4964/2020|2020-10-03|EPI_ISL_730237|Original|hCoV-19^^Amman|Human|Biolab Diagnostic Laboratories|Andersen lab at Scripps Research|Gangavarapu|Jordan
>Spike|hCoV-19/Jordan/AM-ALSR-5015/2020|2020-10-04|EPI_ISL_730288|Original|hCoV-19^^Amman|Human|Biolab Diagnostic Laboratories|Andersen lab at Scripps Research|Gangavarapu|Jordan
>Spike|hCoV-19/Jordan/AM-ALSR-5143/2020|2020-10-12|EPI_ISL_730382|Original|hCoV-19^^Amman|Human|Biolab Diagnostic Laboratories|Andersen lab at Scripps Research|Gangavarapu|Jordan
>Spike|hCoV-19/Jordan/IR-ALSR-5200/2020|2020-10-18|EPI_ISL_730438|Original|hCoV-19^^Irbid|Human|Biolab Diagnostic Laboratories|Andersen lab at Scripps Research|Gangavarapu|Jordan
>Spike|hCoV-19/Jordan/IR-ALSR-5232/2020|2020-10-18|EPI_ISL_730470|Original|hCoV-19^^Irbid|Human|Biolab Diagnostic Laboratories|Andersen lab at Scripps Research|Gangavarapu|Jordan
>Spike|hCoV-19/Jordan/AQ-ALSR-5240/2020|2020-10-18|EPI_ISL_730478|Original|hCoV-19^^Aqaba|Human|Biolab Diagnostic Laboratories|Andersen lab at Scripps Research|Gangavarapu|Jordan
>Spike|hCoV-19/Jordan/AQ-ALSR-5247/2020|2020-10-15|EPI_ISL_730485|Original|hCoV-19^^Aqaba|Human|Biolab Diagnostic Laboratories|Andersen lab at Scripps Research|Gangavarapu|Jordan
>Spike|hCoV-19/Jordan/AQ-ALSR-5248/2020|2020-10-06|EPI_ISL_730486|Original|hCoV-19^^Aqaba|Human|Biolab Diagnostic Laboratories|Andersen lab at Scripps Research|Gangavarapu|Jordan
>Spike|hCoV-19/England/ALDP-C3D5E1/2020|2020-12-10|EPI_ISL_730900|Original|hCoV-19^^England|Human|Lighthouse Lab in Alderley Park|Wellcome Sanger Institute for the COVID-19 Genomics UK (COG-UK) Consortium|Davies|United Kingdom
>Spike|hCoV-19/England/QEUH-C35F45/2020|2020-12-11|EPI_ISL_731372|Original|hCoV-19^^England|Human|Lighthouse Lab in Glasgow|Wellcome Sanger Institute for the COVID-19 Genomics UK (COG-UK) Consortium|Davies|United Kingdom
>Spike|hCoV-19/England/QEUH-C311CB/2020|2020-12-11|EPI_ISL_731482|Original|hCoV-19^^England|Human|Lighthouse Lab in Glasgow|Wellcome Sanger Institute for the COVID-19 Genomics UK (COG-UK) Consortium|Davies|United Kingdom
>Spike|hCoV-19/England/ALDP-C3BCDF/2020|2020-12-11|EPI_ISL_731651|Original|hCoV-19^^England|Human|Lighthouse Lab in Alderley Park|Wellcome Sanger Institute for the COVID-19 Genomics UK (COG-UK) Consortium|Davies|United Kingdom
>Spike|hCoV-19/England/ALDP-C3CE7E/2020|2020-12-10|EPI_ISL_731668|Original|hCoV-19^^England|Human|Lighthouse Lab in Alderley Park|Wellcome Sanger Institute for the COVID-19 Genomics UK (COG-UK) Consortium|Davies|United Kingdom
>Spike|hCoV-19/England/ALDP-C3B04C/2020|2020-12-09|EPI_ISL_731824|Original|hCoV-19^^England|Human|Lighthouse Lab in Alderley Park|Wellcome Sanger Institute for the COVID-19 Genomics UK (COG-UK) Consortium|Davies|United Kingdom
>Spike|hCoV-19/Portugal/PT1680/2020|2020-11-13|EPI_ISL_731917|Original|hCoV-19^^Portugal|Human|Instituto Nacional de Saude (INSA) and Instituto Gulbenkian de

Ciencia (IGC)|Instituto Nacional de Saude (INSA) and Instituto Gulbenkian de Ciencia (IGC)|Borges|Portugal
>Spike|hCoV-19/Ireland/LH-NVRL-20ILR08015/2020|2020-11-10|EPI_ISL_732415|Original|hCoV-19^^Louth|Human|National Virus Reference Laboratory|National Virus Reference Laboratory|Gonzalez|Ireland
>Spike|hCoV-19/Germany/BY-IMB06496/2020|2020-05-26|EPI_ISL_732533|Original|hCoV-19^^Bavaria|Human|Bundeswehr Institute of Microbiology|Bundeswehr Institute of Microbiology|Antwerpen|Germany
>Spike|hCoV-19/Thailand/SamutSakhon-CONI-0344/2020|2020-12-19|EPI_ISL_733538|Original|hCoV-19^^Samut Sakhon|Human|Institute for Urban Disease Control and Prevention|COVID-19 Network Investigations (CONI) Alliance|Batty|Thailand
>Spike|hCoV-19/England/ALDP-C23162/2020|2020-12-06|EPI_ISL_733795|Original|hCoV-19^^England|Human|Lighthouse Lab in Alderley Park|Wellcome Sanger Institute for the COVID-19 Genomics UK (COG-UK) Consortium|Davies|United Kingdom
>Spike|hCoV-19/England/QEUH-C33FDE/2020|2020-12-11|EPI_ISL_734016|Original|hCoV-19^^England|Human|Lighthouse Lab in Glasgow|Wellcome Sanger Institute for the COVID-19 Genomics UK (COG-UK) Consortium|Davies|United Kingdom
>Spike|hCoV-19/France/BRE-IPP9997/2020|2020-11-05|EPI_ISL_734169|Original|hCoV-19^^Bretagne|Human|CHRU Pontchaillou - Laboratoire de Virologie|National Reference Center for Viruses of Respiratory Infections|Enouf|France
>Spike|hCoV-19/USA/VA-DCLS-2460/2020|2020-11-18|EPI_ISL_734205|Original|hCoV-19^^Virginia|Human|Virginia Division of Consolidated Laboratory Services (DCLS)|Virginia Division of Consolidated Laboratory Services (DCLS)|St. Jacques|USA
>Spike|hCoV-19/USA/VA-DCLS-2225/2020|2020-11-03|EPI_ISL_734209|Original|hCoV-19^^Virginia|Human|Virginia Division of Consolidated Laboratory Services (DCLS)|Virginia Division of Consolidated Laboratory Services (DCLS)|St. Jacques|USA
>Spike|hCoV-19/North Macedonia/ZMC6203/2020|2020-06-05|EPI_ISL_735392|Original|hCoV-19^^Skopje|Human|Laboratory of Genetics and Personalized Medicine|Laboratory of Genetics and Personalized Medicine|Kungulovski|North Macedonia
>Spike|hCoV-19/North Macedonia/ZMC6144/2020|2020-06-05|EPI_ISL_735393|Original|hCoV-19^^Skopje|Human|Laboratory of Genetics and Personalized Medicine|Laboratory of Genetics and Personalized Medicine|Kungulovski|North Macedonia
>Spike|hCoV-19/North Macedonia/ZMC6076/2020|2020-06-05|EPI_ISL_735394|Original|hCoV-19^^Skopje|Human|Laboratory of Genetics and Personalized Medicine|Laboratory of Genetics and Personalized Medicine|Kungulovski|North Macedonia
>Spike|hCoV-19/North Macedonia/ZMC6075/2020|2020-06-05|EPI_ISL_735395|Original|hCoV-19^^Skopje|Human|Laboratory of Genetics and Personalized Medicine|Laboratory of Genetics and Personalized Medicine|Kungulovski|North Macedonia
>Spike|hCoV-19/England/QEUH-C53E17/2020|2020-12-12|EPI_ISL_736301|Original|hCoV-19^^England|Human|Lighthouse Lab in Glasgow|Wellcome Sanger Institute for the COVID-19 Genomics UK (COG-UK) Consortium|Davies|United Kingdom
>Spike|hCoV-19/England/QEUH-C54177/2020|2020-12-12|EPI_ISL_736352|Original|hCoV-19^^England|Human|Lighthouse Lab in Glasgow|Wellcome Sanger Institute for the COVID-19 Genomics UK (COG-UK) Consortium|Davies|United Kingdom
>Spike|hCoV-19/England/QEUH-C53E35/2020|2020-12-12|EPI_ISL_736358|Original|hCoV-19^^England|Human|Lighthouse Lab in Glasgow|Wellcome Sanger Institute for the COVID-19 Genomics UK (COG-UK) Consortium|Davies|United Kingdom
>Spike|hCoV-19/England/ALDP-C487B0/2020|2020-12-12|EPI_ISL_736603|Original|hCoV-19^^England|Human|Lighthouse Lab in Alderley Park|Wellcome Sanger Institute for the COVID-19 Genomics UK (COG-UK) Consortium|Davies|United Kingdom
>Spike|hCoV-19/Italy/CAM-TIGEM-167/2020|2020-10-26|EPI_ISL_736777|Original|hCoV-19^^Campania|Human|Istituto Zooprofilattico Sperimentale del Mezzogiorno|TIGEM|Grimaldi|Italy
>Spike|hCoV-19/Italy/CAM-TIGEM-166/2020|2020-10-26|EPI_ISL_736778|Original|hCoV-19^^Campania|Human|Istituto Zooprofilattico Sperimentale del Mezzogiorno|TIGEM|Grimaldi|Italy
>Spike|hCoV-19/Italy/CAM-TIGEM-24/2020|2020-10-26|EPI_ISL_736779|Original|hCoV-19^^Campania|Human|Istituto Zooprofilattico Sperimentale del Mezzogiorno|TIGEM|Grimaldi|Italy

>Spike|hCoV-19/Italy/CAM-TIGEM-20/2020|2020-10-26|EPI_ISL_736780|Original|hCoV-19^^Campania|Human|Istituto Zooprofilattico Sperimentale del Mezzogiorno|TIGEM|Grimaldi|Italy
>Spike|hCoV-19/Italy/CAM-TIGEM-117/2020|2020-10-26|EPI_ISL_736781|Original|hCoV-19^^Campania|Human|Istituto Zooprofilattico Sperimentale del Mezzogiorno|TIGEM|Grimaldi|Italy
>Spike|hCoV-19/Italy/CAM-TIGEM-17/2020|2020-10-26|EPI_ISL_736788|Original|hCoV-19^^Campania|Human|Istituto Zooprofilattico Sperimentale del Mezzogiorno|TIGEM|Grimaldi|Italy
>Spike|hCoV-19/Italy/CAM-TIGEM-55/2020|2020-10-26|EPI_ISL_736789|Original|hCoV-19^^Campania|Human|Istituto Zooprofilattico Sperimentale del Mezzogiorno|TIGEM|Grimaldi|Italy
>Spike|hCoV-19/Italy/CAM-TIGEM-104/2020|2020-10-26|EPI_ISL_736790|Original|hCoV-19^^Campania|Human|Istituto Zooprofilattico Sperimentale del Mezzogiorno|TIGEM|Grimaldi|Italy
>Spike|hCoV-19/Italy/CAM-TIGEM-1/2020|2020-10-26|EPI_ISL_736791|Original|hCoV-19^^Campania|Human|Istituto Zooprofilattico Sperimentale del Mezzogiorno|TIGEM|Grimaldi|Italy
>Spike|hCoV-19/Italy/CAM-TIGEM-2/2020|2020-10-26|EPI_ISL_736792|Original|hCoV-19^^Campania|Human|Istituto Zooprofilattico Sperimentale del Mezzogiorno|TIGEM|Grimaldi|Italy
>Spike|hCoV-19/Italy/CAM-TIGEM-3/2020|2020-10-26|EPI_ISL_736793|Original|hCoV-19^^Campania|Human|Istituto Zooprofilattico Sperimentale del Mezzogiorno|TIGEM|Grimaldi|Italy
>Spike|hCoV-19/Italy/CAM-TIGEM-4/2020|2020-10-26|EPI_ISL_736794|Original|hCoV-19^^Campania|Human|Istituto Zooprofilattico Sperimentale del Mezzogiorno|TIGEM|Grimaldi|Italy
>Spike|hCoV-19/Italy/CAM-TIGEM-5/2020|2020-10-26|EPI_ISL_736795|Original|hCoV-19^^Campania|Human|Istituto Zooprofilattico Sperimentale del Mezzogiorno|TIGEM|Grimaldi|Italy
>Spike|hCoV-19/Italy/CAM-TIGEM-7/2020|2020-10-26|EPI_ISL_736796|Original|hCoV-19^^Campania|Human|Istituto Zooprofilattico Sperimentale del Mezzogiorno|TIGEM|Grimaldi|Italy
>Spike|hCoV-19/Italy/CAM-TIGEM-8/2020|2020-10-26|EPI_ISL_736797|Original|hCoV-19^^Campania|Human|Istituto Zooprofilattico Sperimentale del Mezzogiorno|TIGEM|Grimaldi|Italy
>Spike|hCoV-19/Italy/CAM-TIGEM-9/2020|2020-10-26|EPI_ISL_736798|Original|hCoV-19^^Campania|Human|Istituto Zooprofilattico Sperimentale del Mezzogiorno|TIGEM|Grimaldi|Italy
>Spike|hCoV-19/Italy/CAM-TIGEM-11/2020|2020-10-26|EPI_ISL_736799|Original|hCoV-19^^Campania|Human|Istituto Zooprofilattico Sperimentale del Mezzogiorno|TIGEM|Grimaldi|Italy
>Spike|hCoV-19/Italy/CAM-TIGEM-13/2020|2020-10-26|EPI_ISL_736800|Original|hCoV-19^^Campania|Human|Istituto Zooprofilattico Sperimentale del Mezzogiorno|TIGEM|Grimaldi|Italy
>Spike|hCoV-19/Italy/CAM-TIGEM-14/2020|2020-10-26|EPI_ISL_736801|Original|hCoV-19^^Campania|Human|Istituto Zooprofilattico Sperimentale del Mezzogiorno|TIGEM|Grimaldi|Italy
>Spike|hCoV-19/Italy/CAM-TIGEM-15/2020|2020-10-26|EPI_ISL_736802|Original|hCoV-19^^Campania|Human|Istituto Zooprofilattico Sperimentale del Mezzogiorno|TIGEM|Grimaldi|Italy
>Spike|hCoV-19/Italy/CAM-TIGEM-16/2020|2020-10-26|EPI_ISL_736803|Original|hCoV-19^^Campania|Human|Istituto Zooprofilattico Sperimentale del Mezzogiorno|TIGEM|Grimaldi|Italy
>Spike|hCoV-19/Italy/CAM-TIGEM-22/2020|2020-10-26|EPI_ISL_736804|Original|hCoV-19^^Campania|Human|Istituto Zooprofilattico Sperimentale del Mezzogiorno|TIGEM|Grimaldi|Italy
>Spike|hCoV-19/Italy/CAM-TIGEM-26/2020|2020-10-26|EPI_ISL_736805|Original|hCoV-19^^Campania|Human|Istituto Zooprofilattico Sperimentale del Mezzogiorno|TIGEM|Grimaldi|Italy

>Spike|hCoV-19/Italy/CAM-TIGEM-28/2020|2020-10-26|EPI_ISL_736806|Original|hCoV-19^^Campania|Human|Istituto Zooprofilattico Sperimentale del Mezzogiorno|TIGEM|Grimaldi|Italy
>Spike|hCoV-19/Italy/CAM-TIGEM-31/2020|2020-10-26|EPI_ISL_736807|Original|hCoV-19^^Campania|Human|Istituto Zooprofilattico Sperimentale del Mezzogiorno|TIGEM|Grimaldi|Italy
>Spike|hCoV-19/Italy/CAM-TIGEM-32/2020|2020-10-26|EPI_ISL_736808|Original|hCoV-19^^Campania|Human|Istituto Zooprofilattico Sperimentale del Mezzogiorno|TIGEM|Grimaldi|Italy
>Spike|hCoV-19/Italy/CAM-TIGEM-33/2020|2020-10-26|EPI_ISL_736809|Original|hCoV-19^^Campania|Human|Istituto Zooprofilattico Sperimentale del Mezzogiorno|TIGEM|Grimaldi|Italy
>Spike|hCoV-19/Italy/CAM-TIGEM-34/2020|2020-10-26|EPI_ISL_736810|Original|hCoV-19^^Campania|Human|Istituto Zooprofilattico Sperimentale del Mezzogiorno|TIGEM|Grimaldi|Italy
>Spike|hCoV-19/Italy/CAM-TIGEM-35/2020|2020-10-26|EPI_ISL_736811|Original|hCoV-19^^Campania|Human|Istituto Zooprofilattico Sperimentale del Mezzogiorno|TIGEM|Grimaldi|Italy
>Spike|hCoV-19/Italy/CAM-TIGEM-36/2020|2020-10-26|EPI_ISL_736812|Original|hCoV-19^^Campania|Human|Istituto Zooprofilattico Sperimentale del Mezzogiorno|TIGEM|Grimaldi|Italy
>Spike|hCoV-19/Italy/CAM-TIGEM-37/2020|2020-10-26|EPI_ISL_736813|Original|hCoV-19^^Campania|Human|Istituto Zooprofilattico Sperimentale del Mezzogiorno|TIGEM|Grimaldi|Italy
>Spike|hCoV-19/Italy/CAM-TIGEM-38/2020|2020-10-26|EPI_ISL_736814|Original|hCoV-19^^Campania|Human|Istituto Zooprofilattico Sperimentale del Mezzogiorno|TIGEM|Grimaldi|Italy
>Spike|hCoV-19/Italy/CAM-TIGEM-39/2020|2020-10-26|EPI_ISL_736815|Original|hCoV-19^^Campania|Human|Istituto Zooprofilattico Sperimentale del Mezzogiorno|TIGEM|Grimaldi|Italy
>Spike|hCoV-19/Italy/CAM-TIGEM-41/2020|2020-10-26|EPI_ISL_736816|Original|hCoV-19^^Campania|Human|Istituto Zooprofilattico Sperimentale del Mezzogiorno|TIGEM|Grimaldi|Italy
>Spike|hCoV-19/Italy/CAM-TIGEM-42/2020|2020-10-26|EPI_ISL_736817|Original|hCoV-19^^Campania|Human|Istituto Zooprofilattico Sperimentale del Mezzogiorno|TIGEM|Grimaldi|Italy
>Spike|hCoV-19/Italy/CAM-TIGEM-45/2020|2020-10-26|EPI_ISL_736818|Original|hCoV-19^^Campania|Human|Istituto Zooprofilattico Sperimentale del Mezzogiorno|TIGEM|Grimaldi|Italy
>Spike|hCoV-19/Italy/CAM-TIGEM-47/2020|2020-10-26|EPI_ISL_736819|Original|hCoV-19^^Campania|Human|Istituto Zooprofilattico Sperimentale del Mezzogiorno|TIGEM|Grimaldi|Italy
>Spike|hCoV-19/Italy/CAM-TIGEM-48/2020|2020-10-26|EPI_ISL_736820|Original|hCoV-19^^Campania|Human|Istituto Zooprofilattico Sperimentale del Mezzogiorno|TIGEM|Grimaldi|Italy
>Spike|hCoV-19/Italy/CAM-TIGEM-49/2020|2020-10-26|EPI_ISL_736821|Original|hCoV-19^^Campania|Human|Istituto Zooprofilattico Sperimentale del Mezzogiorno|TIGEM|Grimaldi|Italy
>Spike|hCoV-19/Italy/CAM-TIGEM-50/2020|2020-10-26|EPI_ISL_736822|Original|hCoV-19^^Campania|Human|Istituto Zooprofilattico Sperimentale del Mezzogiorno|TIGEM|Grimaldi|Italy
>Spike|hCoV-19/Italy/CAM-TIGEM-53/2020|2020-10-26|EPI_ISL_736823|Original|hCoV-19^^Campania|Human|Istituto Zooprofilattico Sperimentale del Mezzogiorno|TIGEM|Grimaldi|Italy
>Spike|hCoV-19/Italy/CAM-TIGEM-54/2020|2020-10-26|EPI_ISL_736824|Original|hCoV-19^^Campania|Human|Istituto Zooprofilattico Sperimentale del Mezzogiorno|TIGEM|Grimaldi|Italy
>Spike|hCoV-19/Italy/CAM-TIGEM-57/2020|2020-10-26|EPI_ISL_736825|Original|hCoV-19^^Campania|Human|Istituto Zooprofilattico Sperimentale del Mezzogiorno|TIGEM|Grimaldi|Italy

>Spike|hCoV-19/Italy/CAM-TIGEM-58/2020|2020-10-26|EPI_ISL_736826|Original|hCoV-19^^Campania|Human|Istituto Zooprofilattico Sperimentale del Mezzogiorno|TIGEM|Grimaldi|Italy
>Spike|hCoV-19/Italy/CAM-TIGEM-59/2020|2020-10-26|EPI_ISL_736827|Original|hCoV-19^^Campania|Human|Istituto Zooprofilattico Sperimentale del Mezzogiorno|TIGEM|Grimaldi|Italy
>Spike|hCoV-19/Italy/CAM-TIGEM-60/2020|2020-10-26|EPI_ISL_736828|Original|hCoV-19^^Campania|Human|Istituto Zooprofilattico Sperimentale del Mezzogiorno|TIGEM|Grimaldi|Italy
>Spike|hCoV-19/Italy/CAM-TIGEM-61/2020|2020-10-26|EPI_ISL_736829|Original|hCoV-19^^Campania|Human|Istituto Zooprofilattico Sperimentale del Mezzogiorno|TIGEM|Grimaldi|Italy
>Spike|hCoV-19/Italy/CAM-TIGEM-62/2020|2020-10-26|EPI_ISL_736830|Original|hCoV-19^^Campania|Human|Istituto Zooprofilattico Sperimentale del Mezzogiorno|TIGEM|Grimaldi|Italy
>Spike|hCoV-19/Italy/CAM-TIGEM-63/2020|2020-10-26|EPI_ISL_736831|Original|hCoV-19^^Campania|Human|Istituto Zooprofilattico Sperimentale del Mezzogiorno|TIGEM|Grimaldi|Italy
>Spike|hCoV-19/Italy/CAM-TIGEM-64/2020|2020-10-26|EPI_ISL_736832|Original|hCoV-19^^Campania|Human|Istituto Zooprofilattico Sperimentale del Mezzogiorno|TIGEM|Grimaldi|Italy
>Spike|hCoV-19/Italy/CAM-TIGEM-70/2020|2020-10-26|EPI_ISL_736833|Original|hCoV-19^^Campania|Human|Istituto Zooprofilattico Sperimentale del Mezzogiorno|TIGEM|Grimaldi|Italy
>Spike|hCoV-19/Italy/CAM-TIGEM-71/2020|2020-10-26|EPI_ISL_736834|Original|hCoV-19^^Campania|Human|Istituto Zooprofilattico Sperimentale del Mezzogiorno|TIGEM|Grimaldi|Italy
>Spike|hCoV-19/Italy/CAM-TIGEM-74/2020|2020-10-26|EPI_ISL_736835|Original|hCoV-19^^Campania|Human|Istituto Zooprofilattico Sperimentale del Mezzogiorno|TIGEM|Grimaldi|Italy
>Spike|hCoV-19/Italy/CAM-TIGEM-76/2020|2020-10-26|EPI_ISL_736836|Original|hCoV-19^^Campania|Human|Istituto Zooprofilattico Sperimentale del Mezzogiorno|TIGEM|Grimaldi|Italy
>Spike|hCoV-19/Italy/CAM-TIGEM-80/2020|2020-10-26|EPI_ISL_736837|Original|hCoV-19^^Campania|Human|Istituto Zooprofilattico Sperimentale del Mezzogiorno|TIGEM|Grimaldi|Italy
>Spike|hCoV-19/Italy/CAM-TIGEM-88/2020|2020-10-26|EPI_ISL_736838|Original|hCoV-19^^Campania|Human|Istituto Zooprofilattico Sperimentale del Mezzogiorno|TIGEM|Grimaldi|Italy
>Spike|hCoV-19/Italy/CAM-TIGEM-89/2020|2020-10-26|EPI_ISL_736839|Original|hCoV-19^^Campania|Human|Istituto Zooprofilattico Sperimentale del Mezzogiorno|TIGEM|Grimaldi|Italy
>Spike|hCoV-19/Italy/CAM-TIGEM-94/2020|2020-10-26|EPI_ISL_736840|Original|hCoV-19^^Campania|Human|Istituto Zooprofilattico Sperimentale del Mezzogiorno|TIGEM|Grimaldi|Italy
>Spike|hCoV-19/Italy/CAM-TIGEM-95/2020|2020-10-26|EPI_ISL_736841|Original|hCoV-19^^Campania|Human|Istituto Zooprofilattico Sperimentale del Mezzogiorno|TIGEM|Grimaldi|Italy
>Spike|hCoV-19/Italy/CAM-TIGEM-97/2020|2020-10-26|EPI_ISL_736842|Original|hCoV-19^^Campania|Human|Istituto Zooprofilattico Sperimentale del Mezzogiorno|TIGEM|Grimaldi|Italy
>Spike|hCoV-19/Italy/CAM-TIGEM-99/2020|2020-10-26|EPI_ISL_736843|Original|hCoV-19^^Campania|Human|Istituto Zooprofilattico Sperimentale del Mezzogiorno|TIGEM|Grimaldi|Italy
>Spike|hCoV-19/Italy/CAM-TIGEM-100/2020|2020-10-26|EPI_ISL_736844|Original|hCoV-19^^Campania|Human|Istituto Zooprofilattico Sperimentale del Mezzogiorno|TIGEM|Grimaldi|Italy
>Spike|hCoV-19/Italy/CAM-TIGEM-102/2020|2020-10-26|EPI_ISL_736845|Original|hCoV-19^^Campania|Human|Istituto Zooprofilattico Sperimentale del Mezzogiorno|TIGEM|Grimaldi|Italy

>Spike|hCoV-19/Italy/CAM-TIGEM-105/2020|2020-10-26|EPI_ISL_736846|Original|hCoV-19^^Campania|Human|Istituto Zooprofilattico Sperimentale del Mezzogiorno|TIGEM|Grimaldi|Italy
>Spike|hCoV-19/Italy/CAM-TIGEM-107/2020|2020-10-26|EPI_ISL_736847|Original|hCoV-19^^Campania|Human|Istituto Zooprofilattico Sperimentale del Mezzogiorno|TIGEM|Grimaldi|Italy
>Spike|hCoV-19/Italy/CAM-TIGEM-108/2020|2020-10-26|EPI_ISL_736848|Original|hCoV-19^^Campania|Human|Istituto Zooprofilattico Sperimentale del Mezzogiorno|TIGEM|Grimaldi|Italy
>Spike|hCoV-19/Italy/CAM-TIGEM-111/2020|2020-10-26|EPI_ISL_736849|Original|hCoV-19^^Campania|Human|Istituto Zooprofilattico Sperimentale del Mezzogiorno|TIGEM|Grimaldi|Italy
>Spike|hCoV-19/Italy/CAM-TIGEM-112/2020|2020-10-26|EPI_ISL_736850|Original|hCoV-19^^Campania|Human|Istituto Zooprofilattico Sperimentale del Mezzogiorno|TIGEM|Grimaldi|Italy
>Spike|hCoV-19/Italy/CAM-TIGEM-113/2020|2020-10-26|EPI_ISL_736851|Original|hCoV-19^^Campania|Human|Istituto Zooprofilattico Sperimentale del Mezzogiorno|TIGEM|Grimaldi|Italy
>Spike|hCoV-19/Italy/CAM-TIGEM-116/2020|2020-10-26|EPI_ISL_736852|Original|hCoV-19^^Campania|Human|Istituto Zooprofilattico Sperimentale del Mezzogiorno|TIGEM|Grimaldi|Italy
>Spike|hCoV-19/Italy/CAM-TIGEM-118/2020|2020-10-26|EPI_ISL_736853|Original|hCoV-19^^Campania|Human|Istituto Zooprofilattico Sperimentale del Mezzogiorno|TIGEM|Grimaldi|Italy
>Spike|hCoV-19/Italy/CAM-TIGEM-119/2020|2020-10-26|EPI_ISL_736854|Original|hCoV-19^^Campania|Human|Istituto Zooprofilattico Sperimentale del Mezzogiorno|TIGEM|Grimaldi|Italy
>Spike|hCoV-19/Italy/CAM-TIGEM-121/2020|2020-10-26|EPI_ISL_736855|Original|hCoV-19^^Campania|Human|Istituto Zooprofilattico Sperimentale del Mezzogiorno|TIGEM|Grimaldi|Italy
>Spike|hCoV-19/Italy/CAM-TIGEM-122/2020|2020-10-26|EPI_ISL_736856|Original|hCoV-19^^Campania|Human|Istituto Zooprofilattico Sperimentale del Mezzogiorno|TIGEM|Grimaldi|Italy
>Spike|hCoV-19/Italy/CAM-TIGEM-123/2020|2020-10-26|EPI_ISL_736857|Original|hCoV-19^^Campania|Human|Istituto Zooprofilattico Sperimentale del Mezzogiorno|TIGEM|Grimaldi|Italy
>Spike|hCoV-19/Italy/CAM-TIGEM-124/2020|2020-10-26|EPI_ISL_736858|Original|hCoV-19^^Campania|Human|Istituto Zooprofilattico Sperimentale del Mezzogiorno|TIGEM|Grimaldi|Italy
>Spike|hCoV-19/Italy/CAM-TIGEM-126/2020|2020-10-26|EPI_ISL_736859|Original|hCoV-19^^Campania|Human|Istituto Zooprofilattico Sperimentale del Mezzogiorno|TIGEM|Grimaldi|Italy
>Spike|hCoV-19/Italy/CAM-TIGEM-127/2020|2020-10-26|EPI_ISL_736860|Original|hCoV-19^^Campania|Human|Istituto Zooprofilattico Sperimentale del Mezzogiorno|TIGEM|Grimaldi|Italy
>Spike|hCoV-19/Italy/CAM-TIGEM-130/2020|2020-10-26|EPI_ISL_736861|Original|hCoV-19^^Campania|Human|Istituto Zooprofilattico Sperimentale del Mezzogiorno|TIGEM|Grimaldi|Italy
>Spike|hCoV-19/Italy/CAM-TIGEM-132/2020|2020-10-26|EPI_ISL_736862|Original|hCoV-19^^Campania|Human|Istituto Zooprofilattico Sperimentale del Mezzogiorno|TIGEM|Grimaldi|Italy
>Spike|hCoV-19/Italy/CAM-TIGEM-133/2020|2020-10-26|EPI_ISL_736863|Original|hCoV-19^^Campania|Human|Istituto Zooprofilattico Sperimentale del Mezzogiorno|TIGEM|Grimaldi|Italy
>Spike|hCoV-19/Italy/CAM-TIGEM-135/2020|2020-10-26|EPI_ISL_736864|Original|hCoV-19^^Campania|Human|Istituto Zooprofilattico Sperimentale del Mezzogiorno|TIGEM|Grimaldi|Italy
>Spike|hCoV-19/Italy/CAM-TIGEM-136/2020|2020-10-26|EPI_ISL_736865|Original|hCoV-19^^Campania|Human|Istituto Zooprofilattico Sperimentale del Mezzogiorno|TIGEM|Grimaldi|Italy

>Spike|hCoV-19/Italy/CAM-TIGEM-138/2020|2020-10-26|EPI_ISL_736866|Original|hCoV-
19^^Campania|Human|Istituto Zooprofilattico Sperimentale del
Mezzogiorno|TIGEM|Grimaldi|Italy
>Spike|hCoV-19/Italy/CAM-TIGEM-140/2020|2020-10-26|EPI_ISL_736867|Original|hCoV-
19^^Campania|Human|Istituto Zooprofilattico Sperimentale del
Mezzogiorno|TIGEM|Grimaldi|Italy
>Spike|hCoV-19/Italy/CAM-TIGEM-141/2020|2020-10-26|EPI_ISL_736868|Original|hCoV-
19^^Campania|Human|Istituto Zooprofilattico Sperimentale del
Mezzogiorno|TIGEM|Grimaldi|Italy
>Spike|hCoV-19/Italy/CAM-TIGEM-142/2020|2020-10-26|EPI_ISL_736869|Original|hCoV-
19^^Campania|Human|Istituto Zooprofilattico Sperimentale del
Mezzogiorno|TIGEM|Grimaldi|Italy
>Spike|hCoV-19/Italy/CAM-TIGEM-144/2020|2020-10-26|EPI_ISL_736870|Original|hCoV-
19^^Campania|Human|Istituto Zooprofilattico Sperimentale del
Mezzogiorno|TIGEM|Grimaldi|Italy
>Spike|hCoV-19/Italy/CAM-TIGEM-147/2020|2020-10-26|EPI_ISL_736871|Original|hCoV-
19^^Campania|Human|Istituto Zooprofilattico Sperimentale del
Mezzogiorno|TIGEM|Grimaldi|Italy
>Spike|hCoV-19/Italy/CAM-TIGEM-148/2020|2020-10-26|EPI_ISL_736872|Original|hCoV-
19^^Campania|Human|Istituto Zooprofilattico Sperimentale del
Mezzogiorno|TIGEM|Grimaldi|Italy
>Spike|hCoV-19/Italy/CAM-TIGEM-149/2020|2020-10-26|EPI_ISL_736873|Original|hCoV-
19^^Campania|Human|Istituto Zooprofilattico Sperimentale del
Mezzogiorno|TIGEM|Grimaldi|Italy
>Spike|hCoV-19/Italy/CAM-TIGEM-150/2020|2020-10-26|EPI_ISL_736874|Original|hCoV-
19^^Campania|Human|Istituto Zooprofilattico Sperimentale del
Mezzogiorno|TIGEM|Grimaldi|Italy
>Spike|hCoV-19/Italy/CAM-TIGEM-151/2020|2020-10-26|EPI_ISL_736875|Original|hCoV-
19^^Campania|Human|Istituto Zooprofilattico Sperimentale del
Mezzogiorno|TIGEM|Grimaldi|Italy
>Spike|hCoV-19/Italy/CAM-TIGEM-154/2020|2020-10-26|EPI_ISL_736876|Original|hCoV-
19^^Campania|Human|Istituto Zooprofilattico Sperimentale del
Mezzogiorno|TIGEM|Grimaldi|Italy
>Spike|hCoV-19/Italy/CAM-TIGEM-155/2020|2020-10-26|EPI_ISL_736877|Original|hCoV-
19^^Campania|Human|Istituto Zooprofilattico Sperimentale del
Mezzogiorno|TIGEM|Grimaldi|Italy
>Spike|hCoV-19/Italy/CAM-TIGEM-157/2020|2020-10-26|EPI_ISL_736878|Original|hCoV-
19^^Campania|Human|Istituto Zooprofilattico Sperimentale del
Mezzogiorno|TIGEM|Grimaldi|Italy
>Spike|hCoV-19/Italy/CAM-TIGEM-158/2020|2020-10-26|EPI_ISL_736879|Original|hCoV-
19^^Campania|Human|Istituto Zooprofilattico Sperimentale del
Mezzogiorno|TIGEM|Grimaldi|Italy
>Spike|hCoV-19/Italy/CAM-TIGEM-159/2020|2020-10-26|EPI_ISL_736880|Original|hCoV-
19^^Campania|Human|Istituto Zooprofilattico Sperimentale del
Mezzogiorno|TIGEM|Grimaldi|Italy
>Spike|hCoV-19/Italy/CAM-TIGEM-160/2020|2020-10-26|EPI_ISL_736881|Original|hCoV-
19^^Campania|Human|Istituto Zooprofilattico Sperimentale del
Mezzogiorno|TIGEM|Grimaldi|Italy
>Spike|hCoV-19/Italy/CAM-TIGEM-161/2020|2020-10-26|EPI_ISL_736882|Original|hCoV-
19^^Campania|Human|Istituto Zooprofilattico Sperimentale del
Mezzogiorno|TIGEM|Grimaldi|Italy
>Spike|hCoV-19/Italy/CAM-TIGEM-162/2020|2020-10-26|EPI_ISL_736883|Original|hCoV-
19^^Campania|Human|Istituto Zooprofilattico Sperimentale del
Mezzogiorno|TIGEM|Grimaldi|Italy
>Spike|hCoV-19/Italy/CAM-TIGEM-163/2020|2020-10-26|EPI_ISL_736884|Original|hCoV-
19^^Campania|Human|Istituto Zooprofilattico Sperimentale del
Mezzogiorno|TIGEM|Grimaldi|Italy
>Spike|hCoV-19/Italy/CAM-TIGEM-164/2020|2020-10-26|EPI_ISL_736885|Original|hCoV-
19^^Campania|Human|Istituto Zooprofilattico Sperimentale del
Mezzogiorno|TIGEM|Grimaldi|Italy

>Spike|hCoV-19/Italy/CAM-TIGEM-168/2020|2020-10-26|EPI_ISL_736886|Original|hCoV-19^^Campania|Human|Istituto Zooprofilattico Sperimentale del Mezzogiorno|TIGEM|Grimaldi|Italy
>Spike|hCoV-19/Italy/CAM-TIGEM-169/2020|2020-10-26|EPI_ISL_736887|Original|hCoV-19^^Campania|Human|Istituto Zooprofilattico Sperimentale del Mezzogiorno|TIGEM|Grimaldi|Italy
>Spike|hCoV-19/Italy/CAM-TIGEM-170/2020|2020-10-26|EPI_ISL_736888|Original|hCoV-19^^Campania|Human|Istituto Zooprofilattico Sperimentale del Mezzogiorno|TIGEM|Grimaldi|Italy
>Spike|hCoV-19/Italy/CAM-TIGEM-171/2020|2020-10-26|EPI_ISL_736889|Original|hCoV-19^^Campania|Human|Istituto Zooprofilattico Sperimentale del Mezzogiorno|TIGEM|Grimaldi|Italy
>Spike|hCoV-19/Italy/CAM-TIGEM-177/2020|2020-12-21|EPI_ISL_736890|Original|hCoV-19^^Campania|Human|Istituto Zooprofilattico Sperimentale del Mezzogiorno|TIGEM|Grimaldi|Italy
>Spike|hCoV-19/South Africa/KRISP-EH00430877/2020|2020-11-26|EPI_ISL_736990|Original|hCoV-19^^KwaZulu-Natal|Human|NHLS-IALCH|KRISP|de Oliveira|South Africa
>Spike|hCoV-19/Uganda/UG046/2020|2020-07-15|EPI_ISL_737939|Original|hCoV-19^^Uganda|Human|Uganda Central Public Health Lab and Uganda Virus Research Institute|MRC/UVRI & LSHTM Uganda\u00a0Research Unit|Cotten|Uganda
>Spike|hCoV-19/Uganda/UG060/2020|2020-07-28|EPI_ISL_737976|Original|hCoV-19^^Uganda|Human|Uganda Central Public Health Lab and Uganda Virus Research Institute|MRC/UVRI & LSHTM Uganda\u00a0Research Unit|Cotten|Uganda
>Spike|hCoV-19/USA/TX-DSHS-1787/2020|2020-06-13|EPI_ISL_738162|Original|hCoV-19^^Texas|Human|Texas Department of State Health Services|Texas Department of State Health Services|Rahman|USA
>Spike|hCoV-19/USA/TX-DSHS-1806/2020|2020-06-15|EPI_ISL_738176|Original|hCoV-19^^Texas|Human|Texas Department of State Health Services|Texas Department of State Health Services|Rahman|USA
>Spike|hCoV-19/Canada/BC-BCCDC-7521/2020|2020-11-00|EPI_ISL_740690|Original|hCoV-19^^British Columbia|Human|BCCDC Public Health Laboratory|BCCDC Public Health Laboratory|Prystajecky|Canada
>Spike|hCoV-19/England/CAMC-B79E76/2020|2020-11-09|EPI_ISL_741055|Original|hCoV-19^^England|Human|Oxford Viromics|COVID-19 Genomics UK (COG-UK) Consortium|Golubchik|United Kingdom
>Spike|hCoV-19/England/CAMC-B7B5BB/2020|2020-11-12|EPI_ISL_741183|Original|hCoV-19^^England|Human|Oxford Viromics|COVID-19 Genomics UK (COG-UK) Consortium|Golubchik|United Kingdom
>Spike|hCoV-19/Northern Ireland/NIRE-2356FF/2020|2020-11-12|EPI_ISL_742131|Original|hCoV-19^^Northern Ireland|Human|Regional Virus Laboratory|COVID-19 Genomics UK (COG-UK) Consortium|Fairley|United Kingdom
>Spike|hCoV-19/Wales/PHWC-49B042/2020|2020-10-27|EPI_ISL_742285|Original|hCoV-19^^Wales|Human|Wales Specialist Virology Centre Sequencing lab|COVID-19 Genomics UK (COG-UK) Consortium|Moore|United Kingdom
>Spike|hCoV-19/Wales/PHWC-49B0CA/2020|2020-12-07|EPI_ISL_742293|Original|hCoV-19^^Wales|Human|Wales Specialist Virology Centre Sequencing lab|COVID-19 Genomics UK (COG-UK) Consortium|Moore|United Kingdom
>Spike|hCoV-19/Wales/PHWC-49B279/2020|2020-10-31|EPI_ISL_742312|Original|hCoV-19^^Wales|Human|Wales Specialist Virology Centre Sequencing lab|COVID-19 Genomics UK (COG-UK) Consortium|Moore|United Kingdom
>Spike|hCoV-19/Wales/PHWC-49B31C/2020|2020-11-10|EPI_ISL_742319|Original|hCoV-19^^Wales|Human|Wales Specialist Virology Centre Sequencing lab|COVID-19 Genomics UK (COG-UK) Consortium|Moore|United Kingdom
>Spike|hCoV-19/Wales/PHWC-49B349/2020|2020-11-12|EPI_ISL_742322|Original|hCoV-19^^Wales|Human|Wales Specialist Virology Centre Sequencing lab|COVID-19 Genomics UK (COG-UK) Consortium|Moore|United Kingdom
>Spike|hCoV-19/Wales/PHWC-49B455/2020|2020-11-12|EPI_ISL_742338|Original|hCoV-19^^Wales|Human|Wales Specialist Virology Centre Sequencing lab|COVID-19 Genomics UK (COG-UK) Consortium|Moore|United Kingdom

>Spike|hCoV-19/Wales/PHWC-49B561/2020|2020-12-05|EPI_ISL_742352|Original|hCoV-19^^Wales|Human|Wales Specialist Virology Centre Sequencing lab|COVID-19 Genomics UK (COG-UK) Consortium|Moore|United Kingdom
>Spike|hCoV-19/Wales/PHWC-49B798/2020|2020-12-04|EPI_ISL_742383|Original|hCoV-19^^Wales|Human|Wales Specialist Virology Centre Sequencing lab|COVID-19 Genomics UK (COG-UK) Consortium|Moore|United Kingdom
>Spike|hCoV-19/Wales/PHWC-49B7D4/2020|2020-12-04|EPI_ISL_742386|Original|hCoV-19^^Wales|Human|Wales Specialist Virology Centre Sequencing lab|COVID-19 Genomics UK (COG-UK) Consortium|Moore|United Kingdom
>Spike|hCoV-19/Wales/PHWC-49B90B/2020|2020-12-05|EPI_ISL_742403|Original|hCoV-19^^Wales|Human|Wales Specialist Virology Centre Sequencing lab|COVID-19 Genomics UK (COG-UK) Consortium|Moore|United Kingdom
>Spike|hCoV-19/Wales/PHWC-49B992/2020|2020-12-06|EPI_ISL_742409|Original|hCoV-19^^Wales|Human|Wales Specialist Virology Centre Sequencing lab|COVID-19 Genomics UK (COG-UK) Consortium|Moore|United Kingdom
>Spike|hCoV-19/Wales/PHWC-49BD78/2020|2020-12-06|EPI_ISL_742464|Original|hCoV-19^^Wales|Human|Wales Specialist Virology Centre Sequencing lab|COVID-19 Genomics UK (COG-UK) Consortium|Moore|United Kingdom
>Spike|hCoV-19/Wales/PHWC-49BDD2/2020|2020-12-06|EPI_ISL_742467|Original|hCoV-19^^Wales|Human|Wales Specialist Virology Centre Sequencing lab|COVID-19 Genomics UK (COG-UK) Consortium|Moore|United Kingdom
>Spike|hCoV-19/Wales/PHWC-49BF54/2020|2020-12-06|EPI_ISL_742485|Original|hCoV-19^^Wales|Human|Wales Specialist Virology Centre Sequencing lab|COVID-19 Genomics UK (COG-UK) Consortium|Moore|United Kingdom
>Spike|hCoV-19/Wales/PHWC-49BF81/2020|2020-12-06|EPI_ISL_742487|Original|hCoV-19^^Wales|Human|Wales Specialist Virology Centre Sequencing lab|COVID-19 Genomics UK (COG-UK) Consortium|Moore|United Kingdom
>Spike|hCoV-19/Wales/PHWC-49C13F/2020|2020-12-07|EPI_ISL_742512|Original|hCoV-19^^Wales|Human|Wales Specialist Virology Centre Sequencing lab|COVID-19 Genomics UK (COG-UK) Consortium|Moore|United Kingdom
>Spike|hCoV-19/Wales/PHWC-49C384/2020|2020-11-18|EPI_ISL_742548|Original|hCoV-19^^Wales|Human|Wales Specialist Virology Centre Sequencing lab|COVID-19 Genomics UK (COG-UK) Consortium|Moore|United Kingdom
>Spike|hCoV-19/Wales/PHWC-49C5BB/2020|2020-12-12|EPI_ISL_742576|Original|hCoV-19^^Wales|Human|Wales Specialist Virology Centre Sequencing lab|COVID-19 Genomics UK (COG-UK) Consortium|Moore|United Kingdom
>Spike|hCoV-19/Wales/PHWC-49C5CA/2020|2020-12-13|EPI_ISL_742577|Original|hCoV-19^^Wales|Human|Wales Specialist Virology Centre Sequencing lab|COVID-19 Genomics UK (COG-UK) Consortium|Moore|United Kingdom
>Spike|hCoV-19/Wales/PHWC-49C69A/2020|2020-11-18|EPI_ISL_742589|Original|hCoV-19^^Wales|Human|Wales Specialist Virology Centre Sequencing lab|COVID-19 Genomics UK (COG-UK) Consortium|Moore|United Kingdom
>Spike|hCoV-19/Wales/PHWC-49CA61/2020|2020-12-09|EPI_ISL_742637|Original|hCoV-19^^Wales|Human|Wales Specialist Virology Centre Sequencing lab|COVID-19 Genomics UK (COG-UK) Consortium|Moore|United Kingdom
>Spike|hCoV-19/Wales/PHWC-49CB04/2020|2020-12-07|EPI_ISL_742646|Original|hCoV-19^^Wales|Human|Wales Specialist Virology Centre Sequencing lab|COVID-19 Genomics UK (COG-UK) Consortium|Moore|United Kingdom
>Spike|hCoV-19/Wales/PHWC-49CB8C/2020|2020-12-07|EPI_ISL_742653|Original|hCoV-19^^Wales|Human|Wales Specialist Virology Centre Sequencing lab|COVID-19 Genomics UK (COG-UK) Consortium|Moore|United Kingdom
>Spike|hCoV-19/Wales/PHWC-49CBF5/2020|2020-12-07|EPI_ISL_742660|Original|hCoV-19^^Wales|Human|Wales Specialist Virology Centre Sequencing lab|COVID-19 Genomics UK (COG-UK) Consortium|Moore|United Kingdom
>Spike|hCoV-19/Wales/PHWC-49D0F5/2020|2020-12-10|EPI_ISL_742734|Original|hCoV-19^^Wales|Human|Wales Specialist Virology Centre Sequencing lab|COVID-19 Genomics UK (COG-UK) Consortium|Moore|United Kingdom
>Spike|hCoV-19/Wales/PHWC-49D3ED/2020|2020-12-10|EPI_ISL_742777|Original|hCoV-19^^Wales|Human|Wales Specialist Virology Centre Sequencing lab|COVID-19 Genomics UK (COG-UK) Consortium|Moore|United Kingdom

>Spike|hCoV-19/Wales/PHWC-49D444/2020|2020-12-10|EPI_ISL_742783|Original|hCoV-19^^Wales|Human|Wales Specialist Virology Centre Sequencing lab|COVID-19 Genomics UK (COG-UK) Consortium|Moore|United Kingdom
>Spike|hCoV-19/Wales/PHWC-49D8A2/2020|2020-11-30|EPI_ISL_742842|Original|hCoV-19^^Wales|Human|Wales Specialist Virology Centre Sequencing lab|COVID-19 Genomics UK (COG-UK) Consortium|Moore|United Kingdom
>Spike|hCoV-19/Wales/PHWC-49D8C0/2020|2020-11-30|EPI_ISL_742844|Original|hCoV-19^^Wales|Human|Wales Specialist Virology Centre Sequencing lab|COVID-19 Genomics UK (COG-UK) Consortium|Moore|United Kingdom
>Spike|hCoV-19/Wales/PHWC-49DAD9/2020|2020-12-08|EPI_ISL_742873|Original|hCoV-19^^Wales|Human|Wales Specialist Virology Centre Sequencing lab|COVID-19 Genomics UK (COG-UK) Consortium|Moore|United Kingdom
>Spike|hCoV-19/Wales/PHWC-49DBE5/2020|2020-11-25|EPI_ISL_742883|Original|hCoV-19^^Wales|Human|Wales Specialist Virology Centre Sequencing lab|COVID-19 Genomics UK (COG-UK) Consortium|Moore|United Kingdom
>Spike|hCoV-19/Wales/PHWC-49DC3D/2020|2020-11-26|EPI_ISL_742888|Original|hCoV-19^^Wales|Human|Wales Specialist Virology Centre Sequencing lab|COVID-19 Genomics UK (COG-UK) Consortium|Moore|United Kingdom
>Spike|hCoV-19/Wales/PHWC-49DD3A/2020|2020-12-01|EPI_ISL_742902|Original|hCoV-19^^Wales|Human|Wales Specialist Virology Centre Sequencing lab|COVID-19 Genomics UK (COG-UK) Consortium|Moore|United Kingdom
>Spike|hCoV-19/Wales/PHWC-49DF25/2020|2020-12-06|EPI_ISL_742931|Original|hCoV-19^^Wales|Human|Wales Specialist Virology Centre Sequencing lab|COVID-19 Genomics UK (COG-UK) Consortium|Moore|United Kingdom
>Spike|hCoV-19/Wales/PHWC-49EA32/2020|2020-12-08|EPI_ISL_743084|Original|hCoV-19^^Wales|Human|Wales Specialist Virology Centre Sequencing lab|COVID-19 Genomics UK (COG-UK) Consortium|Moore|United Kingdom
>Spike|hCoV-19/Wales/PHWC-49ED57/2020|2020-12-08|EPI_ISL_743116|Original|hCoV-19^^Wales|Human|Wales Specialist Virology Centre Sequencing lab|COVID-19 Genomics UK (COG-UK) Consortium|Moore|United Kingdom
>Spike|hCoV-19/Wales/PHWC-49EF51/2020|2020-12-10|EPI_ISL_743147|Original|hCoV-19^^Wales|Human|Wales Specialist Virology Centre Sequencing lab|COVID-19 Genomics UK (COG-UK) Consortium|Moore|United Kingdom
>Spike|hCoV-19/Wales/PHWC-49EFBB/2020|2020-12-10|EPI_ISL_743151|Original|hCoV-19^^Wales|Human|Wales Specialist Virology Centre Sequencing lab|COVID-19 Genomics UK (COG-UK) Consortium|Moore|United Kingdom
>Spike|hCoV-19/Wales/PHWC-49F08A/2020|2020-11-29|EPI_ISL_743162|Original|hCoV-19^^Wales|Human|Wales Specialist Virology Centre Sequencing lab|COVID-19 Genomics UK (COG-UK) Consortium|Moore|United Kingdom
>Spike|hCoV-19/Wales/PHWC-49F169/2020|2020-12-02|EPI_ISL_743173|Original|hCoV-19^^Wales|Human|Wales Specialist Virology Centre Sequencing lab|COVID-19 Genomics UK (COG-UK) Consortium|Moore|United Kingdom
>Spike|hCoV-19/Wales/PHWC-49F2A2/2020|2020-12-07|EPI_ISL_743190|Original|hCoV-19^^Wales|Human|Wales Specialist Virology Centre Sequencing lab|COVID-19 Genomics UK (COG-UK) Consortium|Moore|United Kingdom
>Spike|hCoV-19/Wales/PHWC-49F327/2020|2020-12-07|EPI_ISL_743198|Original|hCoV-19^^Wales|Human|Wales Specialist Virology Centre Sequencing lab|COVID-19 Genomics UK (COG-UK) Consortium|Moore|United Kingdom
>Spike|hCoV-19/Wales/PHWC-49F3CD/2020|2020-12-07|EPI_ISL_743208|Original|hCoV-19^^Wales|Human|Wales Specialist Virology Centre Sequencing lab|COVID-19 Genomics UK (COG-UK) Consortium|Moore|United Kingdom
>Spike|hCoV-19/Wales/PHWC-49F424/2020|2020-12-07|EPI_ISL_743214|Original|hCoV-19^^Wales|Human|Wales Specialist Virology Centre Sequencing lab|COVID-19 Genomics UK (COG-UK) Consortium|Moore|United Kingdom
>Spike|hCoV-19/Wales/PHWC-49F521/2020|2020-12-07|EPI_ISL_743230|Original|hCoV-19^^Wales|Human|Wales Specialist Virology Centre Sequencing lab|COVID-19 Genomics UK (COG-UK) Consortium|Moore|United Kingdom
>Spike|hCoV-19/Wales/PHWC-49F54F/2020|2020-12-07|EPI_ISL_743232|Original|hCoV-19^^Wales|Human|Wales Specialist Virology Centre Sequencing lab|COVID-19 Genomics UK (COG-UK) Consortium|Moore|United Kingdom

>Spike|hCoV-19/Wales/PHWC-49F5D6/2020|2020-12-08|EPI_ISL_743239|Original|hCoV-19^^Wales|Human|Wales Specialist Virology Centre Sequencing lab|COVID-19 Genomics UK (COG-UK) Consortium|Moore|United Kingdom
>Spike|hCoV-19/Wales/PHWC-49F600/2020|2020-12-08|EPI_ISL_743242|Original|hCoV-19^^Wales|Human|Wales Specialist Virology Centre Sequencing lab|COVID-19 Genomics UK (COG-UK) Consortium|Moore|United Kingdom
>Spike|hCoV-19/Wales/PHWC-49F64C/2020|2020-12-08|EPI_ISL_743246|Original|hCoV-19^^Wales|Human|Wales Specialist Virology Centre Sequencing lab|COVID-19 Genomics UK (COG-UK) Consortium|Moore|United Kingdom
>Spike|hCoV-19/Wales/PHWC-49F66A/2020|2020-12-08|EPI_ISL_743247|Original|hCoV-19^^Wales|Human|Wales Specialist Virology Centre Sequencing lab|COVID-19 Genomics UK (COG-UK) Consortium|Moore|United Kingdom
>Spike|hCoV-19/Wales/PHWC-49F6E2/2020|2020-12-08|EPI_ISL_743255|Original|hCoV-19^^Wales|Human|Wales Specialist Virology Centre Sequencing lab|COVID-19 Genomics UK (COG-UK) Consortium|Moore|United Kingdom
>Spike|hCoV-19/Wales/PHWC-49F71C/2020|2020-12-08|EPI_ISL_743258|Original|hCoV-19^^Wales|Human|Wales Specialist Virology Centre Sequencing lab|COVID-19 Genomics UK (COG-UK) Consortium|Moore|United Kingdom
>Spike|hCoV-19/Wales/PHWC-49F749/2020|2020-12-08|EPI_ISL_743261|Original|hCoV-19^^Wales|Human|Wales Specialist Virology Centre Sequencing lab|COVID-19 Genomics UK (COG-UK) Consortium|Moore|United Kingdom
>Spike|hCoV-19/Wales/PHWC-49F7D0/2020|2020-12-08|EPI_ISL_743270|Original|hCoV-19^^Wales|Human|Wales Specialist Virology Centre Sequencing lab|COVID-19 Genomics UK (COG-UK) Consortium|Moore|United Kingdom
>Spike|hCoV-19/Wales/PHWC-49F7FE/2020|2020-12-08|EPI_ISL_743272|Original|hCoV-19^^Wales|Human|Wales Specialist Virology Centre Sequencing lab|COVID-19 Genomics UK (COG-UK) Consortium|Moore|United Kingdom
>Spike|hCoV-19/Wales/PHWC-49F828/2020|2020-12-08|EPI_ISL_743275|Original|hCoV-19^^Wales|Human|Wales Specialist Virology Centre Sequencing lab|COVID-19 Genomics UK (COG-UK) Consortium|Moore|United Kingdom
>Spike|hCoV-19/Wales/PHWC-49F846/2020|2020-12-08|EPI_ISL_743277|Original|hCoV-19^^Wales|Human|Wales Specialist Virology Centre Sequencing lab|COVID-19 Genomics UK (COG-UK) Consortium|Moore|United Kingdom
>Spike|hCoV-19/Wales/PHWC-49F873/2020|2020-12-08|EPI_ISL_743280|Original|hCoV-19^^Wales|Human|Wales Specialist Virology Centre Sequencing lab|COVID-19 Genomics UK (COG-UK) Consortium|Moore|United Kingdom
>Spike|hCoV-19/Wales/PHWC-49F952/2020|2020-12-09|EPI_ISL_743294|Original|hCoV-19^^Wales|Human|Wales Specialist Virology Centre Sequencing lab|COVID-19 Genomics UK (COG-UK) Consortium|Moore|United Kingdom
>Spike|hCoV-19/Wales/PHWC-49FA40/2020|2020-12-09|EPI_ISL_743309|Original|hCoV-19^^Wales|Human|Wales Specialist Virology Centre Sequencing lab|COVID-19 Genomics UK (COG-UK) Consortium|Moore|United Kingdom
>Spike|hCoV-19/Wales/PHWC-49FAB9/2020|2020-12-09|EPI_ISL_743316|Original|hCoV-19^^Wales|Human|Wales Specialist Virology Centre Sequencing lab|COVID-19 Genomics UK (COG-UK) Consortium|Moore|United Kingdom
>Spike|hCoV-19/Wales/PHWC-49FBA7/2020|2020-12-09|EPI_ISL_743327|Original|hCoV-19^^Wales|Human|Wales Specialist Virology Centre Sequencing lab|COVID-19 Genomics UK (COG-UK) Consortium|Moore|United Kingdom
>Spike|hCoV-19/Wales/PHWC-49FCC2/2020|2020-12-01|EPI_ISL_743342|Original|hCoV-19^^Wales|Human|Wales Specialist Virology Centre Sequencing lab|COVID-19 Genomics UK (COG-UK) Consortium|Moore|United Kingdom
>Spike|hCoV-19/Wales/PHWC-49FE35/2020|2020-11-30|EPI_ISL_743359|Original|hCoV-19^^Wales|Human|Wales Specialist Virology Centre Sequencing lab|COVID-19 Genomics UK (COG-UK) Consortium|Moore|United Kingdom
>Spike|hCoV-19/Wales/PHWC-49FFD8/2020|2020-11-29|EPI_ISL_743381|Original|hCoV-19^^Wales|Human|Wales Specialist Virology Centre Sequencing lab|COVID-19 Genomics UK (COG-UK) Consortium|Moore|United Kingdom
>Spike|hCoV-19/Wales/PHWC-4A003B/2020|2020-11-30|EPI_ISL_743386|Original|hCoV-19^^Wales|Human|Wales Specialist Virology Centre Sequencing lab|COVID-19 Genomics UK (COG-UK) Consortium|Moore|United Kingdom

>Spike|hCoV-19/Wales/PHWC-4A0086/2020|2020-12-02|EPI_ISL_743391|Original|hCoV-19^^Wales|Human|Wales Specialist Virology Centre Sequencing lab|COVID-19 Genomics UK (COG-UK) Consortium|Moore|United Kingdom
>Spike|hCoV-19/Wales/PHWC-4A0402/2020|2020-12-06|EPI_ISL_743442|Original|hCoV-19^^Wales|Human|Wales Specialist Virology Centre Sequencing lab|COVID-19 Genomics UK (COG-UK) Consortium|Moore|United Kingdom
>Spike|hCoV-19/Wales/PHWC-4A0420/2020|2020-12-06|EPI_ISL_743443|Original|hCoV-19^^Wales|Human|Wales Specialist Virology Centre Sequencing lab|COVID-19 Genomics UK (COG-UK) Consortium|Moore|United Kingdom
>Spike|hCoV-19/Wales/PHWC-4A055A/2020|2020-12-07|EPI_ISL_743462|Original|hCoV-19^^Wales|Human|Wales Specialist Virology Centre Sequencing lab|COVID-19 Genomics UK (COG-UK) Consortium|Moore|United Kingdom
>Spike|hCoV-19/Wales/PHWC-4A05F0/2020|2020-12-08|EPI_ISL_743469|Original|hCoV-19^^Wales|Human|Wales Specialist Virology Centre Sequencing lab|COVID-19 Genomics UK (COG-UK) Consortium|Moore|United Kingdom
>Spike|hCoV-19/Wales/PHWC-4A062A/2020|2020-12-08|EPI_ISL_743471|Original|hCoV-19^^Wales|Human|Wales Specialist Virology Centre Sequencing lab|COVID-19 Genomics UK (COG-UK) Consortium|Moore|United Kingdom
>Spike|hCoV-19/Wales/PHWC-4A0736/2020|2020-12-08|EPI_ISL_743487|Original|hCoV-19^^Wales|Human|Wales Specialist Virology Centre Sequencing lab|COVID-19 Genomics UK (COG-UK) Consortium|Moore|United Kingdom
>Spike|hCoV-19/Wales/PHWC-4A07EB/2020|2020-12-09|EPI_ISL_743495|Original|hCoV-19^^Wales|Human|Wales Specialist Virology Centre Sequencing lab|COVID-19 Genomics UK (COG-UK) Consortium|Moore|United Kingdom
>Spike|hCoV-19/Wales/PHWC-4A0930/2020|2020-12-10|EPI_ISL_743515|Original|hCoV-19^^Wales|Human|Wales Specialist Virology Centre Sequencing lab|COVID-19 Genomics UK (COG-UK) Consortium|Moore|United Kingdom
>Spike|hCoV-19/Wales/PHWC-4A097C/2020|2020-12-10|EPI_ISL_743519|Original|hCoV-19^^Wales|Human|Wales Specialist Virology Centre Sequencing lab|COVID-19 Genomics UK (COG-UK) Consortium|Moore|United Kingdom
>Spike|hCoV-19/Wales/PHWC-4A0A00/2020|2020-12-10|EPI_ISL_743528|Original|hCoV-19^^Wales|Human|Wales Specialist Virology Centre Sequencing lab|COVID-19 Genomics UK (COG-UK) Consortium|Moore|United Kingdom
>Spike|hCoV-19/Wales/PHWC-4A0E8C/2020|2020-12-11|EPI_ISL_743586|Original|hCoV-19^^Wales|Human|Wales Specialist Virology Centre Sequencing lab|COVID-19 Genomics UK (COG-UK) Consortium|Moore|United Kingdom
>Spike|hCoV-19/Wales/PHWC-4A1049/2020|2020-11-23|EPI_ISL_743611|Original|hCoV-19^^Wales|Human|Wales Specialist Virology Centre Sequencing lab|COVID-19 Genomics UK (COG-UK) Consortium|Moore|United Kingdom
>Spike|hCoV-19/Wales/PHWC-4A1155/2020|2020-11-25|EPI_ISL_743628|Original|hCoV-19^^Wales|Human|Wales Specialist Virology Centre Sequencing lab|COVID-19 Genomics UK (COG-UK) Consortium|Moore|United Kingdom
>Spike|hCoV-19/Wales/PHWC-4A1270/2020|2020-12-08|EPI_ISL_743645|Original|hCoV-19^^Wales|Human|Wales Specialist Virology Centre Sequencing lab|COVID-19 Genomics UK (COG-UK) Consortium|Moore|United Kingdom
>Spike|hCoV-19/Wales/PHWC-4A12E9/2020|2020-12-09|EPI_ISL_743652|Original|hCoV-19^^Wales|Human|Wales Specialist Virology Centre Sequencing lab|COVID-19 Genomics UK (COG-UK) Consortium|Moore|United Kingdom
>Spike|hCoV-19/Wales/PHWC-4A1340/2020|2020-12-09|EPI_ISL_743658|Original|hCoV-19^^Wales|Human|Wales Specialist Virology Centre Sequencing lab|COVID-19 Genomics UK (COG-UK) Consortium|Moore|United Kingdom
>Spike|hCoV-19/Wales/PHWC-4A13E6/2020|2020-12-09|EPI_ISL_743667|Original|hCoV-19^^Wales|Human|Wales Specialist Virology Centre Sequencing lab|COVID-19 Genomics UK (COG-UK) Consortium|Moore|United Kingdom
>Spike|hCoV-19/Wales/PHWC-4A145C/2020|2020-12-09|EPI_ISL_743674|Original|hCoV-19^^Wales|Human|Wales Specialist Virology Centre Sequencing lab|COVID-19 Genomics UK (COG-UK) Consortium|Moore|United Kingdom
>Spike|hCoV-19/Wales/PHWC-4A14B6/2020|2020-12-10|EPI_ISL_743680|Original|hCoV-19^^Wales|Human|Wales Specialist Virology Centre Sequencing lab|COVID-19 Genomics UK (COG-UK) Consortium|Moore|United Kingdom

>Spike|hCoV-19/Wales/PHWC-4A151D/2020|2020-12-10|EPI_ISL_743685|Original|hCoV-19^^Wales|Human|Wales Specialist Virology Centre Sequencing lab|COVID-19 Genomics UK (COG-UK) Consortium|Moore|United Kingdom
>Spike|hCoV-19/Wales/PHWC-4A1638/2020|2020-12-10|EPI_ISL_743703|Original|hCoV-19^^Wales|Human|Wales Specialist Virology Centre Sequencing lab|COVID-19 Genomics UK (COG-UK) Consortium|Moore|United Kingdom
>Spike|hCoV-19/Wales/PHWC-4A16ED/2020|2020-12-10|EPI_ISL_743713|Original|hCoV-19^^Wales|Human|Wales Specialist Virology Centre Sequencing lab|COVID-19 Genomics UK (COG-UK) Consortium|Moore|United Kingdom
>Spike|hCoV-19/Wales/PHWC-4A17F9/2020|2020-12-11|EPI_ISL_743729|Original|hCoV-19^^Wales|Human|Wales Specialist Virology Centre Sequencing lab|COVID-19 Genomics UK (COG-UK) Consortium|Moore|United Kingdom
>Spike|hCoV-19/Wales/PHWC-4A1841/2020|2020-12-10|EPI_ISL_743734|Original|hCoV-19^^Wales|Human|Wales Specialist Virology Centre Sequencing lab|COVID-19 Genomics UK (COG-UK) Consortium|Moore|United Kingdom
>Spike|hCoV-19/Wales/PHWC-4A187E/2020|2020-12-10|EPI_ISL_743736|Original|hCoV-19^^Wales|Human|Wales Specialist Virology Centre Sequencing lab|COVID-19 Genomics UK (COG-UK) Consortium|Moore|United Kingdom
>Spike|hCoV-19/Wales/PHWC-4A18C9/2020|2020-12-11|EPI_ISL_743741|Original|hCoV-19^^Wales|Human|Wales Specialist Virology Centre Sequencing lab|COVID-19 Genomics UK (COG-UK) Consortium|Moore|United Kingdom
>Spike|hCoV-19/Wales/PHWC-4A194E/2020|2020-12-11|EPI_ISL_743749|Original|hCoV-19^^Wales|Human|Wales Specialist Virology Centre Sequencing lab|COVID-19 Genomics UK (COG-UK) Consortium|Moore|United Kingdom
>Spike|hCoV-19/Wales/PHWC-4A197B/2020|2020-12-11|EPI_ISL_743752|Original|hCoV-19^^Wales|Human|Wales Specialist Virology Centre Sequencing lab|COVID-19 Genomics UK (COG-UK) Consortium|Moore|United Kingdom
>Spike|hCoV-19/Wales/PHWC-4A1999/2020|2020-12-11|EPI_ISL_743754|Original|hCoV-19^^Wales|Human|Wales Specialist Virology Centre Sequencing lab|COVID-19 Genomics UK (COG-UK) Consortium|Moore|United Kingdom
>Spike|hCoV-19/Wales/PHWC-4A19C6/2020|2020-12-11|EPI_ISL_743757|Original|hCoV-19^^Wales|Human|Wales Specialist Virology Centre Sequencing lab|COVID-19 Genomics UK (COG-UK) Consortium|Moore|United Kingdom
>Spike|hCoV-19/Wales/PHWC-4A1A2D/2020|2020-12-11|EPI_ISL_743763|Original|hCoV-19^^Wales|Human|Wales Specialist Virology Centre Sequencing lab|COVID-19 Genomics UK (COG-UK) Consortium|Moore|United Kingdom
>Spike|hCoV-19/Wales/PHWC-4A1AC3/2020|2020-12-11|EPI_ISL_743773|Original|hCoV-19^^Wales|Human|Wales Specialist Virology Centre Sequencing lab|COVID-19 Genomics UK (COG-UK) Consortium|Moore|United Kingdom
>Spike|hCoV-19/Wales/PHWC-4A1B48/2020|2020-12-11|EPI_ISL_743781|Original|hCoV-19^^Wales|Human|Wales Specialist Virology Centre Sequencing lab|COVID-19 Genomics UK (COG-UK) Consortium|Moore|United Kingdom
>Spike|hCoV-19/Wales/PHWC-4A1D42/2020|2020-12-12|EPI_ISL_743807|Original|hCoV-19^^Wales|Human|Wales Specialist Virology Centre Sequencing lab|COVID-19 Genomics UK (COG-UK) Consortium|Moore|United Kingdom
>Spike|hCoV-19/Wales/PHWC-4A1D51/2020|2020-12-12|EPI_ISL_743808|Original|hCoV-19^^Wales|Human|Wales Specialist Virology Centre Sequencing lab|COVID-19 Genomics UK (COG-UK) Consortium|Moore|United Kingdom
>Spike|hCoV-19/Wales/PHWC-4A1DAC/2020|2020-12-12|EPI_ISL_743813|Original|hCoV-19^^Wales|Human|Wales Specialist Virology Centre Sequencing lab|COVID-19 Genomics UK (COG-UK) Consortium|Moore|United Kingdom
>Spike|hCoV-19/Wales/PHWC-4A22BB/2020|2020-12-14|EPI_ISL_743869|Original|hCoV-19^^Wales|Human|Wales Specialist Virology Centre Sequencing lab|COVID-19 Genomics UK (COG-UK) Consortium|Moore|United Kingdom
>Spike|hCoV-19/Wales/PHWC-4A2A86/2020|2020-12-11|EPI_ISL_743973|Original|hCoV-19^^Wales|Human|Wales Specialist Virology Centre Sequencing lab|COVID-19 Genomics UK (COG-UK) Consortium|Moore|United Kingdom
>Spike|hCoV-19/Wales/PHWC-4A2F69/2020|2020-12-14|EPI_ISL_744036|Original|hCoV-19^^Wales|Human|Wales Specialist Virology Centre Sequencing lab|COVID-19 Genomics UK (COG-UK) Consortium|Moore|United Kingdom

>Spike|hCoV-19/Luxembourg/LNS8249021/2020|2020-11-12|EPI_ISL_744709|Original|hCoV-19^^Luxembourg|Human|Laboratoire national de sant\u00e9|Laboratoire national de sant\u00e9|Wienecke-Baldacchino|Luxembourg
>Spike|hCoV-19/Luxembourg/LNS8592787/2020|2020-12-14|EPI_ISL_744769|Original|hCoV-19^^Luxembourg|Human|Laboratoire national de sant\u00e9|Laboratoire national de sant\u00e9|Wienecke-Baldacchino|Luxembourg
>Spike|hCoV-19/Luxembourg/LNS9120473/2020|2020-11-21|EPI_ISL_744856|Original|hCoV-19^^Luxembourg|Human|Laboratoire national de sant\u00e9|Laboratoire national de sant\u00e9|Wienecke-Baldacchino|Luxembourg
>Spike|hCoV-19/Israel/CVL-44950/2020|2020-12-01|EPI_ISL_745035|Original|hCoV-19^^Northern|Human|Israel Central Virology laboratory|Israel Central Virology laboratory|Zuckerman|Israel
>Spike|hCoV-19/Israel/CVL-45590/2020|2020-12-08|EPI_ISL_745049|Original|hCoV-19^^Tel Aviv|Human|Israel Central Virology laboratory|Israel Central Virology laboratory|Zuckerman|Israel
>Spike|hCoV-19/Russia/MOS-CRIE-13386284/2020|2020-11-06|EPI_ISL_745225|Original|hCoV-19^^Moscow|Human|Molecular diagnostic laboratory of Federal Budget Institution of Science \Central Research Institute of Epidemiology\ of The Federal Service on Customers' Rights Protection and Human Well-being Surveillance|Group of Genomics and Postgenomic Technologies of Central Research Institute of Epidemilolog
>y|Korneenko|Russia
>Spike|hCoV-19/Russia/MOS-CRIE-13380492/2020|2020-11-06|EPI_ISL_745226|Original|hCoV-19^^Moscow|Human|Molecular diagnostic laboratory of Federal Budget Institution of Science \Central Research Institute of Epidemiology\ of The Federal Service on Customers' Rights Protection and Human Well-being Surveillance|Group of Genomics and Postgenomic Technologies of Central Research Institute of Epidemilolog
>y|Korneenko|Russia
>Spike|hCoV-19/Russia/MOS-CRIE-13378888/2020|2020-11-06|EPI_ISL_745227|Original|hCoV-19^^Moscow|Human|Molecular diagnostic laboratory of Federal Budget Institution of Science \Central Research Institute of Epidemiology\ of The Federal Service on Customers' Rights Protection and Human Well-being Surveillance|Group of Genomics and Postgenomic Technologies of Central Research Institute of Epidemilolog
>y|Korneenko|Russia
>Spike|hCoV-19/Russia/MOS-CRIE-13352861/2020|2020-11-06|EPI_ISL_745228|Original|hCoV-19^^Moscow|Human|Molecular diagnostic laboratory of Federal Budget Institution of Science \Central Research Institute of Epidemiology\ of The Federal Service on Customers' Rights Protection and Human Well-being Surveillance|Group of Genomics and Postgenomic Technologies of Central Research Institute of Epidemilolog
>y|Korneenko|Russia
>Spike|hCoV-19/France/ARA-11455/2020|2020-12-01|EPI_ISL_745327|Original|hCoV-19^^Auvergne-Rh\u00f4ne-Alpes|Human|CNR Virus des Infections Respiratoires - France SUD|CNR Virus des Infections Respiratoires - France SUD|Bal|France
>Spike|hCoV-19/France/ARA-113487/2020|2020-11-28|EPI_ISL_745345|Original|hCoV-19^^Auvergne-Rh\u00f4ne-Alpes|Human|CNR Virus des Infections Respiratoires - France SUD|CNR Virus des Infections Respiratoires - France SUD|Bal|France
>Spike|hCoV-19/France/ARA-115486/2020|2020-12-03|EPI_ISL_745359|Original|hCoV-19^^Auvergne-Rh\u00f4ne-Alpes|Human|CNR Virus des Infections Respiratoires - France SUD|CNR Virus des Infections Respiratoires - France SUD|Bal|France
>Spike|hCoV-19/France/ARA-117764/2020|2020-12-09|EPI_ISL_745376|Original|hCoV-19^^Auvergne-Rh\u00f4ne-Alpes|Human|CNR Virus des Infections Respiratoires - France SUD|CNR Virus des Infections Respiratoires - France SUD|Bal|France
>Spike|hCoV-19/USA/UT-UPHL-2012777849/2020|2020-06-18|EPI_ISL_746381|Original|hCoV-19^^Utah|Human|Utah Public Health Laboratory|Utah Public Health Laboratory|Gallagher|USA
>Spike|hCoV-19/USA/UT-UPHL-2012361621/2020|2020-06-19|EPI_ISL_746442|Original|hCoV-19^^Utah|Human|Utah Public Health Laboratory|Utah Public Health Laboratory|Gallagher|USA
>Spike|hCoV-19/USA/UT-UPHL-2012614705/2020|2020-06-19|EPI_ISL_746452|Original|hCoV-19^^Utah|Human|Utah Public Health Laboratory|Utah Public Health Laboratory|Gallagher|USA

>Spike|hCoV-19/Chile/RM-84488/2020|2020-05-31|EPI_ISL_746805|Original|hCoV-19^^Region Metropolitana de Santiago|Human|Genetica Molecular and Subdepartamento de Virologia ISP Chile|Instituto de Salud Publica de Chile|Tognarelli|Chile
>Spike|hCoV-19/USA/UT-UPHL-2012995113/2020|2020-11-30|EPI_ISL_746891|Original|hCoV-19^^Utah|Human|Utah Public Health Laboratory|Utah Public Health Laboratory|Gallagher|USA
>Spike|hCoV-19/USA/UT-UPHL-2012863653/2020|2020-11-30|EPI_ISL_746897|Original|hCoV-19^^Utah|Human|Utah Public Health Laboratory|Utah Public Health Laboratory|Gallagher|USA
>Spike|hCoV-19/USA/UT-UPHL-2012838733/2020|2020-11-30|EPI_ISL_746903|Original|hCoV-19^^Utah|Human|Utah Public Health Laboratory|Utah Public Health Laboratory|Gallagher|USA
>Spike|hCoV-19/USA/UT-UPHL-2012805523/2020|2020-11-27|EPI_ISL_746939|Original|hCoV-19^^Utah|Human|Utah Public Health Laboratory|Utah Public Health Laboratory|Gallagher|USA
>Spike|hCoV-19/USA/UT-UPHL-2012802557/2020|2020-12-02|EPI_ISL_746993|Original|hCoV-19^^Utah|Human|Utah Public Health Laboratory|Utah Public Health Laboratory|Gallagher|USA
>Spike|hCoV-19/USA/UT-UPHL-2012012900/2020|2020-12-03|EPI_ISL_747029|Original|hCoV-19^^Utah|Human|Utah Public Health Laboratory|Utah Public Health Laboratory|Gallagher|USA
>Spike|hCoV-19/Indonesia/BB-NIHRD-PME9185/2020|2020-10-01|EPI_ISL_747237|Original|hCoV-19^^Bangka Belitung Islands|Human|National Institute of Health Research and Development|National Institute of Health Research and Development|Setiawati|Indonesia
>Spike|hCoV-19/South Korea/KDCA0029/2020|2020-09-10|EPI_ISL_747268|Original|hCoV-19^^South Korea|Human|Division of Emerging Infectious Diseases|Division of Emerging Infectious Diseases|Park|South Korea
>Spike|hCoV-19/South Korea/KDCA0058/2020|2020-09-15|EPI_ISL_747299|Original|hCoV-19^^South Korea|Human|Division of Emerging Infectious Diseases|Division of Emerging Infectious Diseases|Park|South Korea
>Spike|hCoV-19/South Korea/KDCA0112/2020|2020-09-26|EPI_ISL_747347|Original|hCoV-19^^South Korea|Human|Division of Emerging Infectious Diseases|Division of Emerging Infectious Diseases|Park|South Korea
>Spike|hCoV-19/South Korea/KDCA0130/2020|2020-10-06|EPI_ISL_747365|Original|hCoV-19^^South Korea|Human|Division of Emerging Infectious Diseases|Division of Emerging Infectious Diseases|Park|South Korea
>Spike|hCoV-19/South Korea/KDCA0179/2020|2020-10-28|EPI_ISL_747413|Original|hCoV-19^^South Korea|Human|Division of Emerging Infectious Diseases|Division of Emerging Infectious Diseases|Park|South Korea
>Spike|hCoV-19/South Korea/KDCA0218/2020|2020-11-26|EPI_ISL_747453|Original|hCoV-19^^South Korea|Human|Division of Emerging Infectious Diseases|Division of Emerging Infectious Diseases|Park|South Korea
>Spike|hCoV-19/Germany/NW-HHU-323/2020|2020-10-12|EPI_ISL_747520|Original|hCoV-19^^North Rhine-Westphalia|Human|ZOTZ KLIMAS MVZ D\u00fcsseldorf-Centrum GbR \u00dcBAG f\u00fcr Labormedizin|Center of Medical Microbiology|Wienemann|Germany
>Spike|hCoV-19/Denmark/DCGC-19890/2020|2020-12-07|EPI_ISL_747592|Original|hCoV-19^^Hovedstaden|Human|Department of Virus and Microbiological Special Diagnostics|Albertsen Lab|Michaelsen|Denmark
>Spike|hCoV-19/Denmark/DCGC-20304/2020|2020-11-23|EPI_ISL_747609|Original|hCoV-19^^Hovedstaden|Human|Department of Virus and Microbiological Special Diagnostics|Albertsen Lab|Michaelsen|Denmark
>Spike|hCoV-19/Denmark/DCGC-19976/2020|2020-12-07|EPI_ISL_747659|Original|hCoV-19^^Sjaelland|Human|Department of Virus and Microbiological Special Diagnostics|Albertsen Lab|Michaelsen|Denmark
>Spike|hCoV-19/Denmark/DCGC-20101/2020|2020-12-07|EPI_ISL_747660|Original|hCoV-19^^Hovedstaden|Human|Department of Virus and Microbiological Special Diagnostics|Albertsen Lab|Michaelsen|Denmark

>Spike|hCoV-19/Denmark/DCGC-19588/2020|2020-12-07|EPI_ISL_748265|Original|hCoV-19^^Sjaelland|Human|Department of Virus and Microbiological Special Diagnostics|Albertsen Lab|Michaelsen|Denmark
>Spike|hCoV-19/Denmark/DCGC-18096/2020|2020-12-14|EPI_ISL_749174|Original|hCoV-19^^Hovedstaden|Human|Department of Virus and Microbiological Special Diagnostics|Albertsen Lab|Michaelsen|Denmark
>Spike|hCoV-19/Denmark/DCGC-18168/2020|2020-12-14|EPI_ISL_749175|Original|hCoV-19^^Syddanmark|Human|Department of Virus and Microbiological Special Diagnostics|Albertsen Lab|Michaelsen|Denmark
>Spike|hCoV-19/Denmark/DCGC-18461/2020|2020-12-07|EPI_ISL_749176|Original|hCoV-19^^Syddanmark|Human|Department of Virus and Microbiological Special Diagnostics|Albertsen Lab|Michaelsen|Denmark
>Spike|hCoV-19/Denmark/DCGC-17865/2020|2020-12-14|EPI_ISL_749359|Original|hCoV-19^^Hovedstaden|Human|Department of Virus and Microbiological Special Diagnostics|Albertsen Lab|Michaelsen|Denmark
>Spike|hCoV-19/Denmark/DCGC-18111/2020|2020-12-14|EPI_ISL_749378|Original|hCoV-19^^Hovedstaden|Human|Department of Virus and Microbiological Special Diagnostics|Albertsen Lab|Michaelsen|Denmark
>Spike|hCoV-19/Denmark/DCGC-17218/2020|2020-12-14|EPI_ISL_750258|Original|hCoV-19^^Hovedstaden|Human|Department of Virus and Microbiological Special Diagnostics|Albertsen Lab|Michaelsen|Denmark
>Spike|hCoV-19/Denmark/DCGC-17104/2020|2020-12-14|EPI_ISL_750261|Original|hCoV-19^^Hovedstaden|Human|Department of Virus and Microbiological Special Diagnostics|Albertsen Lab|Michaelsen|Denmark
>Spike|hCoV-19/Denmark/DCGC-17497/2020|2020-12-14|EPI_ISL_750269|Original|hCoV-19^^Midtjylland|Human|Department of Virus and Microbiological Special Diagnostics|Albertsen Lab|Michaelsen|Denmark
>Spike|hCoV-19/Denmark/DCGC-17748/2020|2020-12-14|EPI_ISL_750369|Original|hCoV-19^^Nordjylland|Human|Department of Virus and Microbiological Special Diagnostics|Albertsen Lab|Michaelsen|Denmark
>Spike|hCoV-19/Denmark/DCGC-17607/2020|2020-12-14|EPI_ISL_750392|Original|hCoV-19^^Midtjylland|Human|Department of Virus and Microbiological Special Diagnostics|Albertsen Lab|Michaelsen|Denmark
>Spike|hCoV-19/USA/TX-DSHS-1908/2020|2020-07-11|EPI_ISL_751510|Original|hCoV-19^^Texas|Human|Texas Department of State Health Services|Texas Department of State Health Services|Rahman|USA
>Spike|hCoV-19/USA/CT-CDC-2-3714059/2020|2020-11-10|EPI_ISL_751554|Original|hCoV-19^^Connecticut|Human|CT-Dr. Katherine A. Kelley State Public Health Lab|Genomics and Discovery|Queen|USA
>Spike|hCoV-19/Ireland/CO-NVRL-78IRL76954/2020|2020-11-23|EPI_ISL_752579|Original|hCoV-19^^Cork|Human|National Virus Reference Laboratory|National Virus Reference Laboratory|Gonzalez|Ireland
>Spike|hCoV-19/USA/HI-H200011/2020|2020-03-11|EPI_ISL_752609|Original|hCoV-19^^Hawaii|Human|State Laboratories Division|State Laboratories Division|Sultana|USA
>Spike|hCoV-19/USA/HI-H200319/2020|2020-08-14|EPI_ISL_752878|Original|hCoV-19^^Hawaii|Human|State Laboratories Division|State Laboratories Division|Sultana|USA
>Spike|hCoV-19/USA/HI-H200340/2020|2020-08-25|EPI_ISL_752897|Original|hCoV-19^^Hawaii|Human|State Laboratories Division|State Laboratories Division|Sultana|USA
>Spike|hCoV-19/USA/HI-H200346/2020|2020-09-08|EPI_ISL_752902|Original|hCoV-19^^Hawaii|Human|State Laboratories Division|State Laboratories Division|Sultana|USA
>Spike|hCoV-19/USA/HI-H200348/2020|2020-09-11|EPI_ISL_752904|Original|hCoV-19^^Hawaii|Human|State Laboratories Division|State Laboratories Division|Sultana|USA
>Spike|hCoV-19/USA/HI-H200361/2020|2020-09-28|EPI_ISL_752917|Original|hCoV-19^^Hawaii|Human|State Laboratories Division|State Laboratories Division|Sultana|USA
>Spike|hCoV-19/USA/HI-H200362/2020|2020-09-29|EPI_ISL_752918|Original|hCoV-19^^Hawaii|Human|State Laboratories Division|State Laboratories Division|Sultana|USA
>Spike|hCoV-19/USA/HI-H200387/2020|2020-09-28|EPI_ISL_752934|Original|hCoV-19^^Hawaii|Human|State Laboratories Division|State Laboratories Division|Sultana|USA
>Spike|hCoV-19/USA/HI-H200395/2020|2020-09-09|EPI_ISL_752941|Original|hCoV-19^^Hawaii|Human|State Laboratories Division|State Laboratories Division|Sultana|USA

>Spike|hCoV-19/USA/HI-H200398/2020|2020-09-09|EPI_ISL_752943|Original|hCoV-19^^Hawaii|Human|State Laboratories Division|State Laboratories Division|Sultana|USA
>Spike|hCoV-19/USA/HI-H200399/2020|2020-09-09|EPI_ISL_752944|Original|hCoV-19^^Hawaii|Human|State Laboratories Division|State Laboratories Division|Sultana|USA
>Spike|hCoV-19/USA/HI-H200417/2020|2020-08-10|EPI_ISL_752961|Original|hCoV-19^^Hawaii|Human|State Laboratories Division|State Laboratories Division|Sultana|USA
>Spike|hCoV-19/USA/HI-H200422/2020|2020-08-11|EPI_ISL_752966|Original|hCoV-19^^Hawaii|Human|State Laboratories Division|State Laboratories Division|Sultana|USA
>Spike|hCoV-19/USA/HI-H200425/2020|2020-09-23|EPI_ISL_752969|Original|hCoV-19^^Hawaii|Human|State Laboratories Division|State Laboratories Division|Sultana|USA
>Spike|hCoV-19/USA/HI-H200457/2020|2020-09-15|EPI_ISL_753000|Original|hCoV-19^^Hawaii|Human|State Laboratories Division|State Laboratories Division|Sultana|USA
>Spike|hCoV-19/USA/HI-H200483/2020|2020-09-22|EPI_ISL_753025|Original|hCoV-19^^Hawaii|Human|State Laboratories Division|State Laboratories Division|Sultana|USA
>Spike|hCoV-19/USA/HI-H200503/2020|2020-08-02|EPI_ISL_753042|Original|hCoV-19^^Hawaii|Human|State Laboratories Division|State Laboratories Division|Sultana|USA
>Spike|hCoV-19/USA/HI-H200504/2020|2020-08-02|EPI_ISL_753043|Original|hCoV-19^^Hawaii|Human|State Laboratories Division|State Laboratories Division|Sultana|USA
>Spike|hCoV-19/USA/HI-H200513/2020|2020-08-13|EPI_ISL_753049|Original|hCoV-19^^Hawaii|Human|State Laboratories Division|State Laboratories Division|Sultana|USA
>Spike|hCoV-19/USA/HI-H200520/2020|2020-10-30|EPI_ISL_753054|Original|hCoV-19^^Hawaii|Human|State Laboratories Division|State Laboratories Division|Sultana|USA
>Spike|hCoV-19/USA/HI-H200553/2020|2020-10-24|EPI_ISL_753082|Original|hCoV-19^^Hawaii|Human|State Laboratories Division|State Laboratories Division|Sultana|USA
>Spike|hCoV-19/USA/HI-H200566/2020|2020-10-24|EPI_ISL_753094|Original|hCoV-19^^Hawaii|Human|State Laboratories Division|State Laboratories Division|Sultana|USA
>Spike|hCoV-19/USA/HI-H200571/2020|2020-10-24|EPI_ISL_753098|Original|hCoV-19^^Hawaii|Human|State Laboratories Division|State Laboratories Division|Sultana|USA
>Spike|hCoV-19/USA/HI-H200635/2020|2020-11-23|EPI_ISL_753159|Original|hCoV-19^^Hawaii|Human|State Laboratories Division|State Laboratories Division|Sultana|USA
>Spike|hCoV-19/USA/HI-H200681/2020|2020-12-01|EPI_ISL_753204|Original|hCoV-19^^Hawaii|Human|State Laboratories Division|State Laboratories Division|Sultana|USA
>Spike|hCoV-19/Indonesia/SN-NIHRD-PME1701/2020|2020-06-17|EPI_ISL_753698|Original|hCoV-19^^South Sulawesi|Human|Balai Besar Veteriner Maros|National Institute of Health Research and Development|Setiawati|Indonesia
>Spike|hCoV-19/Germany/BE-ChVir-W2121-4431/2020|2020-10-15|EPI_ISL_754186|Original|hCoV-19^^Berlin|Human|Charit\u00e9 Universit\u00e4tsmedizin Berlin|Charit\u00e9 Universit\u00e4tsmedizin Berlin|Muehlemann|Germany
>Spike|hCoV-19/Germany/BE-ChVir-D1917-8291/2020|2020-10-06|EPI_ISL_754188|Original|hCoV-19^^Berlin|Human|Charit\u00e9 Universit\u00e4tsmedizin Berlin|Charit\u00e9 Universit\u00e4tsmedizin Berlin|Muehlemann|Germany
>Spike|hCoV-19/Germany/BE-ChVir-D1947-5915/2020|2020-10-04|EPI_ISL_754189|Original|hCoV-19^^Berlin|Human|Charit\u00e9 Universit\u00e4tsmedizin Berlin|Charit\u00e9 Universit\u00e4tsmedizin Berlin|Muehlemann|Germany
>Spike|hCoV-19/Germany/BE-ChVir-W2120-5976/2020|2020-10-15|EPI_ISL_754190|Original|hCoV-19^^Berlin|Human|Charit\u00e9 Universit\u00e4tsmedizin Berlin|Charit\u00e9 Universit\u00e4tsmedizin Berlin|Muehlemann|Germany
>Spike|hCoV-19/Germany/NW-ChVir-D1925-5947/2020|2020-10-07|EPI_ISL_754193|Original|hCoV-19^^North Rhine-Westphalia|Human|Charit\u00e9 Universit\u00e4tsmedizin Berlin|Charit\u00e9 Universit\u00e4tsmedizin Berlin|Muehlemann|Germany
>Spike|hCoV-19/Russia/MOS-CRIE-13651164/2020|2020-11-09|EPI_ISL_754204|Original|hCoV-19^^Moscow|Human|Molecular diagnostic laboratory of Federal Budget Institution of Science \Central Research Institute of Epidemiology\ of The Federal Service on Customers' Rights Protection and Human Well-being Surveillance|Group of Genomics and Postgenomic Technologies of Central Research Institute of Epidemilolog

>y|Korneenko|Russia
>Spike|hCoV-19/USA/NY-Wadsworth-264207-01/2020|2020-11-05|EPI_ISL_754518|Original|hCoV-19^^New York|Human|Wadsworth Center|Wadsworth Center|Lasek-Nesselquist|USA
>Spike|hCoV-19/France/GES-IPP10958/2020|2020-12-17|EPI_ISL_754843|Original|hCoV-19^^Grand-Est|Human|Laboratoire de Virologie \nH\u00f4pital Robert Debr\u00e9|National Reference Center for Viruses of Respiratory Infections|Enouf|France
>Spike|hCoV-19/France/GES-IPP10959/2020|2020-12-18|EPI_ISL_754844|Original|hCoV-19^^Grand-Est|Human|Laboratoire de Virologie \nH\u00f4pital Robert Debr\u00e9|National Reference Center for Viruses of Respiratory Infections|Enouf|France
>Spike|hCoV-19/France/GES-IPP10957/2020|2020-12-17|EPI_ISL_754845|Original|hCoV-19^^Grand-Est|Human|Laboratoire de Virologie \nH\u00f4pital Robert Debr\u00e9|National Reference Center for Viruses of Respiratory Infections|Enouf|France
>Spike|hCoV-19/France/GES-IPP10971/2020|2020-12-18|EPI_ISL_754846|Original|hCoV-19^^Grand-Est|Human|Laboratoire de Virologie \nH\u00f4pital Robert Debr\u00e9|National Reference Center for Viruses of Respiratory Infections|Enouf|France
>Spike|hCoV-19/USA/CA-CDPH264/2020|2020-11-28|EPI_ISL_754914|Original|hCoV-19^^California|Human|California Department of Public Health|California Department of Public Health|Bell|USA
>Spike|hCoV-19/USA/CA-CDPH233/2020|2020-11-14|EPI_ISL_754966|Original|hCoV-19^^California|Human|California Department of Public Health|California Department of Public Health|Bell|USA
>Spike|hCoV-19/USA/CA-CDPH220/2020|2020-11-15|EPI_ISL_755010|Original|hCoV-19^^California|Human|California Department of Public Health|California Department of Public Health|Bell|USA
>Spike|hCoV-19/USA/CA-CDPH267/2020|2020-11-15|EPI_ISL_755094|Original|hCoV-19^^California|Human|California Department of Public Health|California Department of Public Health|Bell|USA
>Spike|hCoV-19/Jordan/SEARCH-5444/2020|2020-08-24|EPI_ISL_755124|Original|hCoV-19^^Amman|Human|Biolab Diagnostic Laboratories|Andersen lab at Scripps Research|Gangavarapu|Jordan
>Spike|hCoV-19/USA/CA-SEARCH-5347/2020|2020-12-09|EPI_ISL_755162|Original|hCoV-19^^California|Human|UCSD EXCITE lab|Andersen lab at Scripps Research|Gangavarapu|USA
>Spike|hCoV-19/USA/CA-SEARCH-5369/2020|2020-12-16|EPI_ISL_755181|Original|hCoV-19^^California|Human|UCSD EXCITE lab|Andersen lab at Scripps Research|Gangavarapu|USA
>Spike|hCoV-19/USA/CA-SEARCH-5375/2020|2020-12-17|EPI_ISL_755187|Original|hCoV-19^^California|Human|UCSD EXCITE lab|Andersen lab at Scripps Research|Gangavarapu|USA
>Spike|hCoV-19/USA/CA-SEARCH-5419/2020|2020-11-30|EPI_ISL_755228|Original|hCoV-19^^California|Human|UCSD EXCITE lab|Andersen lab at Scripps Research|Gangavarapu|USA
>Spike|hCoV-19/Canada/ON-S2000/2020|2020-11-24|EPI_ISL_755708|Original|hCoV-19^^Ontario|Human|Toronto Invasive Bacterial Diseases Network|McMaster University|McArthur|Canada
>Spike|hCoV-19/Canada/ON-S1768/2020|2020-11-08|EPI_ISL_755769|Original|hCoV-19^^Ontario|Human|Toronto Invasive Bacterial Diseases Network|McMaster University|McArthur|Canada
>Spike|hCoV-19/Canada/ON-S1772/2020|2020-11-08|EPI_ISL_755773|Original|hCoV-19^^Ontario|Human|Toronto Invasive Bacterial Diseases Network|McMaster University|McArthur|Canada
>Spike|hCoV-19/USA/HI-H200397/2020|2020-09-07|EPI_ISL_756275|Original|hCoV-19^^Hawaii|Human|State Laboratories Division|State Laboratories Division|Sultana|USA
>Spike|hCoV-19/USA/HI-H200581/2020|2020-10-29|EPI_ISL_756277|Original|hCoV-19^^Hawaii|Human|State Laboratories Division|State Laboratories Division|Sultana|USA
>Spike|hCoV-19/USA/HI-H200624/2020|2020-09-29|EPI_ISL_756278|Original|hCoV-19^^Hawaii|Human|State Laboratories Division|State Laboratories Division|Sultana|USA
>Spike|hCoV-19/USA/HI-H200632/2020|2020-11-23|EPI_ISL_756279|Original|hCoV-19^^Hawaii|Human|State Laboratories Division|State Laboratories Division|Sultana|USA

>Spike|hCoV-19/USA/HI-H200650/2020|2020-11-25|EPI_ISL_756280|Original|hCoV-19^^Hawaii|Human|State Laboratories Division|State Laboratories Division|Sultana|USA
>Spike|hCoV-19/USA/HI-H200697/2020|2020-12-01|EPI_ISL_756281|Original|hCoV-19^^Hawaii|Human|State Laboratories Division|State Laboratories Division|Sultana|USA
>Spike|hCoV-19/Thailand/Bangkok-Si11/2020|2020-12-28|EPI_ISL_756375|Original|hCoV-19^^Bangkok|Human|Siriraj Hospital|National Institute of Health|Phuygun|Thailand
>Spike|hCoV-19/England/ALDP-C8B7B1/2020|2020-12-17|EPI_ISL_756574|Original|hCoV-19^^England|Human|Lighthouse Lab in Alderley Park|Wellcome Sanger Institute for the COVID-19 Genomics UK (COG-UK) Consortium|Davies|United Kingdom
>Spike|hCoV-19/Denmark/DCGC-22458/2020|2020-12-14|EPI_ISL_757400|Original|hCoV-19^^Denmark|Human|Department of Virus and Microbiological Special Diagnostics|Albertsen Lab|Michaelsen|Denmark
>Spike|hCoV-19/Denmark/DCGC-22594/2020|2020-12-14|EPI_ISL_757449|Original|hCoV-19^^Syddanmark|Human|Department of Virus and Microbiological Special Diagnostics|Albertsen Lab|Michaelsen|Denmark
>Spike|hCoV-19/Denmark/DCGC-21647/2020|2020-12-21|EPI_ISL_757708|Original|hCoV-19^^Hovedstaden|Human|Department of Virus and Microbiological Special Diagnostics|Albertsen Lab|Michaelsen|Denmark
>Spike|hCoV-19/Denmark/DCGC-21581/2020|2020-12-21|EPI_ISL_757715|Original|hCoV-19^^Syddanmark|Human|Department of Virus and Microbiological Special Diagnostics|Albertsen Lab|Michaelsen|Denmark
>Spike|hCoV-19/Denmark/DCGC-21644/2020|2020-12-21|EPI_ISL_757716|Original|hCoV-19^^Syddanmark|Human|Department of Virus and Microbiological Special Diagnostics|Albertsen Lab|Michaelsen|Denmark
>Spike|hCoV-19/Denmark/DCGC-21709/2020|2020-12-21|EPI_ISL_757945|Original|hCoV-19^^Midtjylland|Human|Department of Virus and Microbiological Special Diagnostics|Albertsen Lab|Michaelsen|Denmark
>Spike|hCoV-19/Denmark/DCGC-21669/2020|2020-12-21|EPI_ISL_757959|Original|hCoV-19^^Sjaelland|Human|Department of Virus and Microbiological Special Diagnostics|Albertsen Lab|Michaelsen|Denmark
>Spike|hCoV-19/Denmark/DCGC-21258/2020|2020-12-21|EPI_ISL_758968|Original|hCoV-19^^Midtjylland|Human|Department of Virus and Microbiological Special Diagnostics|Albertsen Lab|Michaelsen|Denmark
>Spike|hCoV-19/Denmark/DCGC-20621/2020|2020-12-21|EPI_ISL_758969|Original|hCoV-19^^Hovedstaden|Human|Department of Virus and Microbiological Special Diagnostics|Albertsen Lab|Michaelsen|Denmark
>Spike|hCoV-19/North Macedonia/ZMC6084/2020|2020-06-05|EPI_ISL_760059|Original|hCoV-19^^Skopje|Human|Laboratory of Genetics and Personalized Medicine|Laboratory of Genetics and Personalized Medicine|Kungulovski|North Macedonia
>Spike|hCoV-19/South Korea/KDCA0287/2020|2020-10-24|EPI_ISL_760081|Original|hCoV-19^^South Korea|Human|Division of Emerging Infectious Diseases|Division of Emerging Infectious Diseases|Park|South Korea
>Spike|hCoV-19/South Korea/KDCA0295/2020|2020-10-27|EPI_ISL_760089|Original|hCoV-19^^South Korea|Human|Division of Emerging Infectious Diseases|Division of Emerging Infectious Diseases|Park|South Korea
>Spike|hCoV-19/South Korea/KDCA0301/2020|2020-10-28|EPI_ISL_760095|Original|hCoV-19^^South Korea|Human|Division of Emerging Infectious Diseases|Division of Emerging Infectious Diseases|Park|South Korea
>Spike|hCoV-19/South Korea/KDCA0327/2020|2020-10-16|EPI_ISL_760113|Original|hCoV-19^^South Korea|Human|Division of Emerging Infectious Diseases|Division of Emerging Infectious Diseases|Park|South Korea
>Spike|hCoV-19/England/QEUH-CC310A/2020|2020-12-21|EPI_ISL_760297|Original|hCoV-19^^England|Human|Lighthouse Lab in Glasgow|Wellcome Sanger Institute for the COVID-19 Genomics UK (COG-UK) Consortium|Davies|United Kingdom
>Spike|hCoV-19/England/QEUH-CC3146/2020|2020-12-20|EPI_ISL_760374|Original|hCoV-19^^England|Human|Lighthouse Lab in Glasgow|Wellcome Sanger Institute for the COVID-19 Genomics UK (COG-UK) Consortium|Davies|United Kingdom
>Spike|hCoV-19/England/QEUH-CC7BDB/2020|2020-12-22|EPI_ISL_760451|Original|hCoV-19^^England|Human|Lighthouse Lab in Glasgow|Wellcome Sanger Institute for the COVID-19 Genomics UK (COG-UK) Consortium|Davies|United Kingdom

>Spike|hCoV-19/England/QEUH-CC23BA/2020|2020-12-21|EPI_ISL_760631|Original|hCoV-19^^England|Human|Lighthouse Lab in Glasgow|Wellcome Sanger Institute for the COVID-19 Genomics UK (COG-UK) Consortium|Davies|United Kingdom
>Spike|hCoV-19/England/QEUH-CC3DCA/2020|2020-12-21|EPI_ISL_760775|Original|hCoV-19^^England|Human|Lighthouse Lab in Glasgow|Wellcome Sanger Institute for the COVID-19 Genomics UK (COG-UK) Consortium|Davies|United Kingdom
>Spike|hCoV-19/England/QEUH-CC3D51/2020|2020-12-21|EPI_ISL_760778|Original|hCoV-19^^England|Human|Lighthouse Lab in Glasgow|Wellcome Sanger Institute for the COVID-19 Genomics UK (COG-UK) Consortium|Davies|United Kingdom
>Spike|hCoV-19/England/ALDP-CB3CEE/2020|2020-12-19|EPI_ISL_760898|Original|hCoV-19^^England|Human|Lighthouse Lab in Alderley Park|Wellcome Sanger Institute for the COVID-19 Genomics UK (COG-UK) Consortium|Davies|United Kingdom
>Spike|hCoV-19/England/QEUH-CAD0EB/2020|2020-12-20|EPI_ISL_760917|Original|hCoV-19^^England|Human|Lighthouse Lab in Glasgow|Wellcome Sanger Institute for the COVID-19 Genomics UK (COG-UK) Consortium|Davies|United Kingdom
>Spike|hCoV-19/England/QEUH-C4D87C/2020|2020-12-13|EPI_ISL_760980|Original|hCoV-19^^England|Human|Lighthouse Lab in Glasgow|Wellcome Sanger Institute for the COVID-19 Genomics UK (COG-UK) Consortium|Davies|United Kingdom
>Spike|hCoV-19/England/ALDP-CB3F9A/2020|2020-12-18|EPI_ISL_761069|Original|hCoV-19^^England|Human|Lighthouse Lab in Alderley Park|Wellcome Sanger Institute for the COVID-19 Genomics UK (COG-UK) Consortium|Davies|United Kingdom
>Spike|hCoV-19/England/ALDP-CB3E24/2020|2020-12-19|EPI_ISL_761135|Original|hCoV-19^^England|Human|Lighthouse Lab in Alderley Park|Wellcome Sanger Institute for the COVID-19 Genomics UK (COG-UK) Consortium|Davies|United Kingdom
>Spike|hCoV-19/England/ALDP-CB4342/2020|2020-12-18|EPI_ISL_761314|Original|hCoV-19^^England|Human|Lighthouse Lab in Alderley Park|Wellcome Sanger Institute for the COVID-19 Genomics UK (COG-UK) Consortium|Davies|United Kingdom
>Spike|hCoV-19/England/ALDP-CB447C/2020|2020-12-19|EPI_ISL_761734|Original|hCoV-19^^England|Human|Lighthouse Lab in Alderley Park|Wellcome Sanger Institute for the COVID-19 Genomics UK (COG-UK) Consortium|Davies|United Kingdom
>Spike|hCoV-19/England/QEUH-C93491/2020|2020-12-16|EPI_ISL_762128|Original|hCoV-19^^England|Human|Lighthouse Lab in Glasgow|Wellcome Sanger Institute for the COVID-19 Genomics UK (COG-UK) Consortium|Davies|United Kingdom
>Spike|hCoV-19/England/ALDP-C88990/2020|2020-12-17|EPI_ISL_762629|Original|hCoV-19^^England|Human|Lighthouse Lab in Alderley Park|Wellcome Sanger Institute for the COVID-19 Genomics UK (COG-UK) Consortium|Davies|United Kingdom
>Spike|hCoV-19/England/ALDP-C88769/2020|2020-12-17|EPI_ISL_762692|Original|hCoV-19^^England|Human|Lighthouse Lab in Alderley Park|Wellcome Sanger Institute for the COVID-19 Genomics UK (COG-UK) Consortium|Davies|United Kingdom
>Spike|hCoV-19/England/ALDP-C88954/2020|2020-12-17|EPI_ISL_762835|Original|hCoV-19^^England|Human|Lighthouse Lab in Alderley Park|Wellcome Sanger Institute for the COVID-19 Genomics UK (COG-UK) Consortium|Davies|United Kingdom
>Spike|hCoV-19/USA/FL-BPHL-2256/2020|2020-11-17|EPI_ISL_763339|Original|hCoV-19^^Florida|Human|Florida Bureau of Public Health Laboratories|Florida Bureau of Public Health Laboratories|Blanton|USA
>Spike|hCoV-19/Northern Ireland/NIRE-235325/2020|2020-10-19|EPI_ISL_763395|Original|hCoV-19^^Northern Ireland|Human|Regional Virus Laboratory|COVID-19 Genomics UK (COG-UK) Consortium|Fairley|United Kingdom
>Spike|hCoV-19/Wales/PHWC-4A63F0/2020|2020-12-17|EPI_ISL_763443|Original|hCoV-19^^Wales|Human|Wales Specialist Virology Centre Sequencing lab|COVID-19 Genomics UK (COG-UK) Consortium|Moore|United Kingdom
>Spike|hCoV-19/England/SHEF-10D36A7/2020|2020-12-20|EPI_ISL_763501|Original|hCoV-19^^England|Human|Virology Department|COVID-19 Genomics UK (COG-UK) Consortium|de Silva|United Kingdom
>Spike|hCoV-19/Wales/PHWC-4A400A/2020|2020-12-14|EPI_ISL_763584|Original|hCoV-19^^Wales|Human|Wales Specialist Virology Centre Sequencing lab|COVID-19 Genomics UK (COG-UK) Consortium|Moore|United Kingdom
>Spike|hCoV-19/Wales/PHWC-4A3ED4/2020|2020-12-13|EPI_ISL_763738|Original|hCoV-19^^Wales|Human|Wales Specialist Virology Centre Sequencing lab|COVID-19 Genomics UK (COG-UK) Consortium|Moore|United Kingdom

>Spike|hCoV-19/Wales/PHWC-4A5CC9/2020|2020-12-13|EPI_ISL_763787|Original|hCoV-19^^Wales|Human|Wales Specialist Virology Centre Sequencing lab|COVID-19 Genomics UK (COG-UK) Consortium|Moore|United Kingdom
>Spike|hCoV-19/Wales/PHWC-4A3E7A/2020|2020-12-12|EPI_ISL_764266|Original|hCoV-19^^Wales|Human|Wales Specialist Virology Centre Sequencing lab|COVID-19 Genomics UK (COG-UK) Consortium|Moore|United Kingdom
>Spike|hCoV-19/Wales/PHWC-4A3B91/2020|2020-12-12|EPI_ISL_764603|Original|hCoV-19^^Wales|Human|Wales Specialist Virology Centre Sequencing lab|COVID-19 Genomics UK (COG-UK) Consortium|Moore|United Kingdom
>Spike|hCoV-19/Wales/PHWC-4A3CF8/2020|2020-12-12|EPI_ISL_764620|Original|hCoV-19^^Wales|Human|Wales Specialist Virology Centre Sequencing lab|COVID-19 Genomics UK (COG-UK) Consortium|Moore|United Kingdom
>Spike|hCoV-19/Wales/PHWC-4A3D7D/2020|2020-12-12|EPI_ISL_764627|Original|hCoV-19^^Wales|Human|Wales Specialist Virology Centre Sequencing lab|COVID-19 Genomics UK (COG-UK) Consortium|Moore|United Kingdom
>Spike|hCoV-19/Wales/PHWC-4A3DE6/2020|2020-12-12|EPI_ISL_764631|Original|hCoV-19^^Wales|Human|Wales Specialist Virology Centre Sequencing lab|COVID-19 Genomics UK (COG-UK) Consortium|Moore|United Kingdom
>Spike|hCoV-19/Wales/PHWC-4A3E6B/2020|2020-12-13|EPI_ISL_764636|Original|hCoV-19^^Wales|Human|Wales Specialist Virology Centre Sequencing lab|COVID-19 Genomics UK (COG-UK) Consortium|Moore|United Kingdom
>Spike|hCoV-19/Wales/PHWC-4A3E98/2020|2020-12-12|EPI_ISL_764638|Original|hCoV-19^^Wales|Human|Wales Specialist Virology Centre Sequencing lab|COVID-19 Genomics UK (COG-UK) Consortium|Moore|United Kingdom
>Spike|hCoV-19/Wales/PHWC-4A3F59/2020|2020-12-13|EPI_ISL_764646|Original|hCoV-19^^Wales|Human|Wales Specialist Virology Centre Sequencing lab|COVID-19 Genomics UK (COG-UK) Consortium|Moore|United Kingdom
>Spike|hCoV-19/Wales/PHWC-4A4019/2020|2020-12-13|EPI_ISL_764652|Original|hCoV-19^^Wales|Human|Wales Specialist Virology Centre Sequencing lab|COVID-19 Genomics UK (COG-UK) Consortium|Moore|United Kingdom
>Spike|hCoV-19/Wales/PHWC-4A437A/2020|2020-12-16|EPI_ISL_764683|Original|hCoV-19^^Wales|Human|Wales Specialist Virology Centre Sequencing lab|COVID-19 Genomics UK (COG-UK) Consortium|Moore|United Kingdom
>Spike|hCoV-19/Wales/PHWC-4A486C/2020|2020-12-12|EPI_ISL_764739|Original|hCoV-19^^Wales|Human|Wales Specialist Virology Centre Sequencing lab|COVID-19 Genomics UK (COG-UK) Consortium|Moore|United Kingdom
>Spike|hCoV-19/Wales/PHWC-4A48A8/2020|2020-12-15|EPI_ISL_764743|Original|hCoV-19^^Wales|Human|Wales Specialist Virology Centre Sequencing lab|COVID-19 Genomics UK (COG-UK) Consortium|Moore|United Kingdom
>Spike|hCoV-19/Wales/PHWC-4A5018/2020|2020-12-13|EPI_ISL_764811|Original|hCoV-19^^Wales|Human|Wales Specialist Virology Centre Sequencing lab|COVID-19 Genomics UK (COG-UK) Consortium|Moore|United Kingdom
>Spike|hCoV-19/Wales/PHWC-4A5106/2020|2020-12-13|EPI_ISL_764822|Original|hCoV-19^^Wales|Human|Wales Specialist Virology Centre Sequencing lab|COVID-19 Genomics UK (COG-UK) Consortium|Moore|United Kingdom
>Spike|hCoV-19/Wales/PHWC-4A5133/2020|2020-12-13|EPI_ISL_764825|Original|hCoV-19^^Wales|Human|Wales Specialist Virology Centre Sequencing lab|COVID-19 Genomics UK (COG-UK) Consortium|Moore|United Kingdom
>Spike|hCoV-19/Wales/PHWC-4A524F/2020|2020-12-15|EPI_ISL_764842|Original|hCoV-19^^Wales|Human|Wales Specialist Virology Centre Sequencing lab|COVID-19 Genomics UK (COG-UK) Consortium|Moore|United Kingdom
>Spike|hCoV-19/Wales/PHWC-4A56E9/2020|2020-12-15|EPI_ISL_764907|Original|hCoV-19^^Wales|Human|Wales Specialist Virology Centre Sequencing lab|COVID-19 Genomics UK (COG-UK) Consortium|Moore|United Kingdom
>Spike|hCoV-19/Wales/PHWC-4A5B26/2020|2020-12-11|EPI_ISL_764954|Original|hCoV-19^^Wales|Human|Wales Specialist Virology Centre Sequencing lab|COVID-19 Genomics UK (COG-UK) Consortium|Moore|United Kingdom
>Spike|hCoV-19/Portugal/PT1652/2020|2020-11-08|EPI_ISL_765210|Original|hCoV-19^^Portugal|Human|Instituto Nacional de Saude (INSA)|Instituto Nacional de Saude (INSA)|Borges|Portugal

>Spike|hCoV-19/Portugal/PT1752/2020|2020-11-11|EPI_ISL_765212|Original|hCoV-19^^Portugal|Human|Instituto Nacional de Saude (INSA)|Instituto Nacional de Saude (INSA)|Borges|Portugal
>Spike|hCoV-19/France/GES-IPP10968/2020|2020-12-18|EPI_ISL_765479|Original|hCoV-19^^Grand-Est|Human|Laboratoire de Virologie|National Reference Center for Viruses of Respiratory Infections|Enouf|France
>Spike|hCoV-19/USA/MA-MGH-03013/2020|2020-07-23|EPI_ISL_765680|Original|hCoV-19^^Massachusetts|Human|Massachusetts General Hospital|Infectious Disease Program|Park|USA
>Spike|hCoV-19/USA/MA-MGH-03028/2020|2020-07-25|EPI_ISL_765684|Original|hCoV-19^^Massachusetts|Human|Massachusetts General Hospital|Infectious Disease Program|Park|USA
>Spike|hCoV-19/USA/MA-MGH-03266/2020|2020-10-20|EPI_ISL_765764|Original|hCoV-19^^Massachusetts|Human|Massachusetts General Hospital|Infectious Disease Program|Park|USA
>Spike|hCoV-19/USA/MA-MGH-03318/2020|2020-11-01|EPI_ISL_765785|Original|hCoV-19^^Massachusetts|Human|Massachusetts General Hospital|Infectious Disease Program|Park|USA
>Spike|hCoV-19/USA/WY-WPHL-20168339/2020|2020-12-18|EPI_ISL_766789|Original|hCoV-19^^Wyoming|Human|Wyoming Public Health Laboratory|Wyoming Public Health Laboratory|Hull|USA
>Spike|hCoV-19/England/204981915/2020|2020-12-04|EPI_ISL_766842|Original|hCoV-19^^England|Human|Respiratory Virus Unit|COVID-19 Genomics UK (COG-UK) Consortium|Platt|United Kingdom
>Spike|hCoV-19/England/205000381/2020|2020-12-07|EPI_ISL_766853|Original|hCoV-19^^England|Human|Respiratory Virus Unit|COVID-19 Genomics UK (COG-UK) Consortium|Platt|United Kingdom
>Spike|hCoV-19/USA/NY-Wadsworth-292062-01/2020|2020-12-20|EPI_ISL_767585|Original|hCoV-19^^New York|Human|WHITE PLAINS HOSPITAL CENTER LABORATORY|Wadsworth Center|Lasek-Nesselquist|USA
>Spike|hCoV-19/USA/NY-Wadsworth-292707-01/2020|2020-12-04|EPI_ISL_767654|Original|hCoV-19^^New York|Human|MEMORIAL SLOAN KETTERING CANCER CENTER|Wadsworth Center|Lasek-Nesselquist|USA
>Spike|hCoV-19/Australia/NSW1380/2020|2020-12-28|EPI_ISL_768602|Original|hCoV-19^^New South Wales|Human|South Eastern Area Laboratory Services (SEALS)|NSW Health Pathology - Institute of Clinical Pathology and Medical Research; Westmead Hospital; University of Sydney|Gall|Australia
>Spike|hCoV-19/Japan/IC-0427/2020|2020-11-00|EPI_ISL_768636|Original|hCoV-19^^Japan|Human|Pathogen Genomics Center|Pathogen Genomics Center|Sekizuka|Japan
>Spike|hCoV-19/Japan/IC-0440/2020|2020-10-00|EPI_ISL_768649|Original|hCoV-19^^Japan|Human|Pathogen Genomics Center|Pathogen Genomics Center|Sekizuka|Japan
>Spike|hCoV-19/Japan/IC-0482/2020|2020-12-00|EPI_ISL_768691|Original|hCoV-19^^Japan|Human|Pathogen Genomics Center|Pathogen Genomics Center|Sekizuka|Japan
>Spike|hCoV-19/Japan/IC-0513/2020|2020-12-00|EPI_ISL_768722|Original|hCoV-19^^Japan|Human|Pathogen Genomics Center|Pathogen Genomics Center|Sekizuka|Japan
>Spike|hCoV-19/Bangladesh/CHRF-0065/2020|2020-07-20|EPI_ISL_768741|Original|hCoV-19^^Bangladesh|Human|Child Health Research Foundation|Child Health Research Foundation|Saha|Bangladesh
>Spike|hCoV-19/England/QEUH-C5C3E2/2020|2020-12-14|EPI_ISL_769225|Original|hCoV-19^^England|Human|Lighthouse Lab in Glasgow|Wellcome Sanger Institute for the COVID-19 Genomics UK (COG-UK) Consortium|Davies|United Kingdom
>Spike|hCoV-19/England/QEUH-C5CA38/2020|2020-12-14|EPI_ISL_769500|Original|hCoV-19^^England|Human|Lighthouse Lab in Glasgow|Wellcome Sanger Institute for the COVID-19 Genomics UK (COG-UK) Consortium|Davies|United Kingdom
>Spike|hCoV-19/England/QEUH-C4B841/2020|2020-12-13|EPI_ISL_769652|Original|hCoV-19^^England|Human|Lighthouse Lab in Glasgow|Wellcome Sanger Institute for the COVID-19 Genomics UK (COG-UK) Consortium|Davies|United Kingdom
>Spike|hCoV-19/England/205081145/2020|2020-12-11|EPI_ISL_769869|Original|hCoV-19^^England|Human|Respiratory Virus Unit|COVID-19 Genomics UK (COG-UK) Consortium|Platt|United Kingdom

>Spike|hCoV-19/Gibraltar/205121254/2020|2020-00-00|EPI_ISL_769872|Original|hCoV-19^^Gibraltar|Human|Microbiology|Respiratory Virus Unit|Platt|Gibraltar
>Spike|hCoV-19/Costa Rica/INC-0178/2020|2020-11-16|EPI_ISL_769988|Original|hCoV-19^^San Jose|Human|Area De Salud San Francisco-San Antonio (Coopesana)|Inciensa|Soto-Garita|Costa Rica
>Spike|hCoV-19/USA/WY-WPHL-20062692/2020|2020-08-18|EPI_ISL_770122|Original|hCoV-19^^Wyoming|Human|Wyoming Public Health Laboratory|Wyoming Public Health Laboratory|Hull|USA
>Spike|hCoV-19/USA/WY-WPHL-20044859/2020|2020-07-17|EPI_ISL_770125|Original|hCoV-19^^Wyoming|Human|Wyoming Public Health Laboratory|Wyoming Public Health Laboratory|Hull|USA
>Spike|hCoV-19/USA/WY-WPHL-20044667/2020|2020-07-15|EPI_ISL_770126|Original|hCoV-19^^Wyoming|Human|Wyoming Public Health Laboratory|Wyoming Public Health Laboratory|Hull|USA
>Spike|hCoV-19/USA/CO-CDPHE-2100015138/2020|2020-11-21|EPI_ISL_771222|Original|hCoV-19^^Colorado|Human|Colorado Department of Public Health and Environment|Colorado Department of Puplic Health and Environment|Bankers|USA
>Spike|hCoV-19/USA/CO-CDPHE-2100030884/2020|2020-11-25|EPI_ISL_771223|Original|hCoV-19^^Colorado|Human|Colorado Department of Public Health and Environment|Colorado Department of Puplic Health and Environment|Bankers|USA
>Spike|hCoV-19/Canada/ON-PHL-20-03709/2020|2020-11-00|EPI_ISL_775191|Original|hCoV-19^^Ontario|Human|Public Health Ontario Laboratory|Public Health Ontario Laboratory|Guthrie|Canada
>Spike|hCoV-19/Canada/ON-PHL-20-03737/2020|2020-11-00|EPI_ISL_775207|Original|hCoV-19^^Ontario|Human|Public Health Ontario Laboratory|Public Health Ontario Laboratory|Guthrie|Canada
>Spike|hCoV-19/Norway/6238/2020|2020-11-25|EPI_ISL_775276|Original|hCoV-19^^Vestland|Human|Haukeland University Hospital|Norwegian Institute of Public Health|Vollan|Norway
>Spike|hCoV-19/Israel/CVL-32-ngs/2020|2020-12-24|EPI_ISL_776618|Original|hCoV-19^^Israel|Human|Israel Central Virology laboratory|Israel Central Virology laboratory|Zuckerman|Israel
>Spike|hCoV-19/Israel/CVL-60-ngs/2020|2020-12-24|EPI_ISL_776641|Original|hCoV-19^^Israel|Human|Israel Central Virology laboratory|Israel Central Virology laboratory|Zuckerman|Israel
>Spike|hCoV-19/Italy/CAM-TIGEM-196/2020|2020-10-26|EPI_ISL_776879|Original|hCoV-19^^Campania|Human|Istituto Zooprofilattico Sperimentale del Mezzogiorno|TIGEM|Grimaldi|Italy
>Spike|hCoV-19/Italy/CAM-TIGEM-202/2020|2020-10-26|EPI_ISL_776880|Original|hCoV-19^^Campania|Human|Istituto Zooprofilattico Sperimentale del Mezzogiorno|TIGEM|Grimaldi|Italy
>Spike|hCoV-19/Italy/CAM-TIGEM-200/2020|2020-10-26|EPI_ISL_776881|Original|hCoV-19^^Campania|Human|Istituto Zooprofilattico Sperimentale del Mezzogiorno|TIGEM|Grimaldi|Italy
>Spike|hCoV-19/Italy/CAM-TIGEM-195/2020|2020-10-26|EPI_ISL_776882|Original|hCoV-19^^Campania|Human|Istituto Zooprofilattico Sperimentale del Mezzogiorno|TIGEM|Grimaldi|Italy
>Spike|hCoV-19/Italy/CAM-TIGEM-199/2020|2020-10-26|EPI_ISL_776883|Original|hCoV-19^^Campania|Human|Istituto Zooprofilattico Sperimentale del Mezzogiorno|TIGEM|Grimaldi|Italy
>Spike|hCoV-19/Italy/CAM-TIGEM-194/2020|2020-10-26|EPI_ISL_776884|Original|hCoV-19^^Campania|Human|Istituto Zooprofilattico Sperimentale del Mezzogiorno|TIGEM|Grimaldi|Italy
>Spike|hCoV-19/Italy/CAM-TIGEM-219/2020|2020-10-26|EPI_ISL_776885|Original|hCoV-19^^Campania|Human|Istituto Zooprofilattico Sperimentale del Mezzogiorno|TIGEM|Grimaldi|Italy
>Spike|hCoV-19/Italy/CAM-TIGEM-207/2020|2020-10-26|EPI_ISL_776886|Original|hCoV-19^^Campania|Human|Istituto Zooprofilattico Sperimentale del Mezzogiorno|TIGEM|Grimaldi|Italy

>Spike|hCoV-19/Italy/CAM-TIGEM-230/2020|2020-10-26|EPI_ISL_776887|Original|hCoV-19^^Campania|Human|Istituto Zooprofilattico Sperimentale del Mezzogiorno|TIGEM|Grimaldi|Italy
>Spike|hCoV-19/Italy/CAM-TIGEM-208/2020|2020-10-26|EPI_ISL_776888|Original|hCoV-19^^Campania|Human|Istituto Zooprofilattico Sperimentale del Mezzogiorno|TIGEM|Grimaldi|Italy
>Spike|hCoV-19/Italy/CAM-TIGEM-211/2020|2020-10-26|EPI_ISL_776889|Original|hCoV-19^^Campania|Human|Istituto Zooprofilattico Sperimentale del Mezzogiorno|TIGEM|Grimaldi|Italy
>Spike|hCoV-19/Italy/CAM-TIGEM-210/2020|2020-10-26|EPI_ISL_776890|Original|hCoV-19^^Campania|Human|Istituto Zooprofilattico Sperimentale del Mezzogiorno|TIGEM|Grimaldi|Italy
>Spike|hCoV-19/Italy/CAM-TIGEM-216/2020|2020-10-26|EPI_ISL_776891|Original|hCoV-19^^Campania|Human|Istituto Zooprofilattico Sperimentale del Mezzogiorno|TIGEM|Grimaldi|Italy
>Spike|hCoV-19/Italy/CAM-TIGEM-225/2020|2020-10-26|EPI_ISL_776892|Original|hCoV-19^^Campania|Human|Istituto Zooprofilattico Sperimentale del Mezzogiorno|TIGEM|Grimaldi|Italy
>Spike|hCoV-19/Italy/CAM-TIGEM-233/2020|2020-10-26|EPI_ISL_776893|Original|hCoV-19^^Campania|Human|Istituto Zooprofilattico Sperimentale del Mezzogiorno|TIGEM|Grimaldi|Italy
>Spike|hCoV-19/Italy/CAM-TIGEM-231/2020|2020-10-26|EPI_ISL_776894|Original|hCoV-19^^Campania|Human|Istituto Zooprofilattico Sperimentale del Mezzogiorno|TIGEM|Grimaldi|Italy
>Spike|hCoV-19/Italy/CAM-TIGEM-248/2020|2020-10-26|EPI_ISL_776895|Original|hCoV-19^^Campania|Human|Istituto Zooprofilattico Sperimentale del Mezzogiorno|TIGEM|Grimaldi|Italy
>Spike|hCoV-19/Italy/CAM-TIGEM-247/2020|2020-10-26|EPI_ISL_776896|Original|hCoV-19^^Campania|Human|Istituto Zooprofilattico Sperimentale del Mezzogiorno|TIGEM|Grimaldi|Italy
>Spike|hCoV-19/Italy/CAM-TIGEM-229/2020|2020-10-26|EPI_ISL_776897|Original|hCoV-19^^Campania|Human|Istituto Zooprofilattico Sperimentale del Mezzogiorno|TIGEM|Grimaldi|Italy
>Spike|hCoV-19/Italy/CAM-TIGEM-239/2020|2020-10-26|EPI_ISL_776898|Original|hCoV-19^^Campania|Human|Istituto Zooprofilattico Sperimentale del Mezzogiorno|TIGEM|Grimaldi|Italy
>Spike|hCoV-19/Italy/CAM-TIGEM-236/2020|2020-10-26|EPI_ISL_776899|Original|hCoV-19^^Campania|Human|Istituto Zooprofilattico Sperimentale del Mezzogiorno|TIGEM|Grimaldi|Italy
>Spike|hCoV-19/Italy/CAM-TIGEM-245/2020|2020-10-26|EPI_ISL_776900|Original|hCoV-19^^Campania|Human|Istituto Zooprofilattico Sperimentale del Mezzogiorno|TIGEM|Grimaldi|Italy
>Spike|hCoV-19/Italy/CAM-TIGEM-250/2020|2020-10-26|EPI_ISL_776901|Original|hCoV-19^^Campania|Human|Istituto Zooprofilattico Sperimentale del Mezzogiorno|TIGEM|Grimaldi|Italy
>Spike|hCoV-19/Italy/CAM-TIGEM-238/2020|2020-10-26|EPI_ISL_776902|Original|hCoV-19^^Campania|Human|Istituto Zooprofilattico Sperimentale del Mezzogiorno|TIGEM|Grimaldi|Italy
>Spike|hCoV-19/Italy/CAM-TIGEM-226/2020|2020-10-26|EPI_ISL_776903|Original|hCoV-19^^Campania|Human|Istituto Zooprofilattico Sperimentale del Mezzogiorno|TIGEM|Grimaldi|Italy
>Spike|hCoV-19/Italy/CAM-TIGEM-228/2020|2020-10-26|EPI_ISL_776904|Original|hCoV-19^^Campania|Human|Istituto Zooprofilattico Sperimentale del Mezzogiorno|TIGEM|Grimaldi|Italy
>Spike|hCoV-19/Italy/CAM-TIGEM-242/2020|2020-10-26|EPI_ISL_776905|Original|hCoV-19^^Campania|Human|Istituto Zooprofilattico Sperimentale del Mezzogiorno|TIGEM|Grimaldi|Italy
>Spike|hCoV-19/Italy/CAM-TIGEM-246/2020|2020-10-26|EPI_ISL_776906|Original|hCoV-19^^Campania|Human|Istituto Zooprofilattico Sperimentale del Mezzogiorno|TIGEM|Grimaldi|Italy

>Spike|hCoV-19/Italy/CAM-TIGEM-232/2020|2020-10-26|EPI_ISL_776907|Original|hCoV-19^^Campania|Human|Istituto Zooprofilattico Sperimentale del Mezzogiorno|TIGEM|Grimaldi|Italy
>Spike|hCoV-19/Italy/CAM-TIGEM-227/2020|2020-10-26|EPI_ISL_776908|Original|hCoV-19^^Campania|Human|Istituto Zooprofilattico Sperimentale del Mezzogiorno|TIGEM|Grimaldi|Italy
>Spike|hCoV-19/Italy/CAM-TIGEM-224/2020|2020-10-26|EPI_ISL_776909|Original|hCoV-19^^Campania|Human|Istituto Zooprofilattico Sperimentale del Mezzogiorno|TIGEM|Grimaldi|Italy
>Spike|hCoV-19/Italy/CAM-TIGEM-197/2020|2020-10-26|EPI_ISL_776910|Original|hCoV-19^^Campania|Human|Istituto Zooprofilattico Sperimentale del Mezzogiorno|TIGEM|Grimaldi|Italy
>Spike|hCoV-19/Italy/CAM-TIGEM-237/2020|2020-10-26|EPI_ISL_776911|Original|hCoV-19^^Campania|Human|Istituto Zooprofilattico Sperimentale del Mezzogiorno|TIGEM|Grimaldi|Italy
>Spike|hCoV-19/Italy/CAM-TIGEM-243/2020|2020-10-26|EPI_ISL_776912|Original|hCoV-19^^Campania|Human|Istituto Zooprofilattico Sperimentale del Mezzogiorno|TIGEM|Grimaldi|Italy
>Spike|hCoV-19/Italy/CAM-TIGEM-235/2020|2020-10-26|EPI_ISL_776913|Original|hCoV-19^^Campania|Human|Istituto Zooprofilattico Sperimentale del Mezzogiorno|TIGEM|Grimaldi|Italy
>Spike|hCoV-19/Italy/CAM-TIGEM-249/2020|2020-10-26|EPI_ISL_776914|Original|hCoV-19^^Campania|Human|Istituto Zooprofilattico Sperimentale del Mezzogiorno|TIGEM|Grimaldi|Italy
>Spike|hCoV-19/Italy/CAM-TIGEM-198/2020|2020-10-26|EPI_ISL_776915|Original|hCoV-19^^Campania|Human|Istituto Zooprofilattico Sperimentale del Mezzogiorno|TIGEM|Grimaldi|Italy
>Spike|hCoV-19/Italy/CAM-TIGEM-212/2020|2020-10-26|EPI_ISL_776916|Original|hCoV-19^^Campania|Human|Istituto Zooprofilattico Sperimentale del Mezzogiorno|TIGEM|Grimaldi|Italy
>Spike|hCoV-19/Italy/CAM-TIGEM-215/2020|2020-10-26|EPI_ISL_776917|Original|hCoV-19^^Campania|Human|Istituto Zooprofilattico Sperimentale del Mezzogiorno|TIGEM|Grimaldi|Italy
>Spike|hCoV-19/Italy/CAM-TIGEM-214/2020|2020-10-26|EPI_ISL_776918|Original|hCoV-19^^Campania|Human|Istituto Zooprofilattico Sperimentale del Mezzogiorno|TIGEM|Grimaldi|Italy
>Spike|hCoV-19/Italy/CAM-TIGEM-209/2020|2020-10-26|EPI_ISL_776919|Original|hCoV-19^^Campania|Human|Istituto Zooprofilattico Sperimentale del Mezzogiorno|TIGEM|Grimaldi|Italy
>Spike|hCoV-19/Italy/CAM-TIGEM-201/2020|2020-10-26|EPI_ISL_776920|Original|hCoV-19^^Campania|Human|Istituto Zooprofilattico Sperimentale del Mezzogiorno|TIGEM|Grimaldi|Italy
>Spike|hCoV-19/Italy/CAM-TIGEM-290/2020|2020-10-26|EPI_ISL_776921|Original|hCoV-19^^Campania|Human|Istituto Zooprofilattico Sperimentale del Mezzogiorno|TIGEM|Grimaldi|Italy
>Spike|hCoV-19/Italy/CAM-TIGEM-269/2020|2020-10-26|EPI_ISL_776922|Original|hCoV-19^^Campania|Human|Istituto Zooprofilattico Sperimentale del Mezzogiorno|TIGEM|Grimaldi|Italy
>Spike|hCoV-19/Italy/CAM-TIGEM-254/2020|2020-10-26|EPI_ISL_776923|Original|hCoV-19^^Campania|Human|Istituto Zooprofilattico Sperimentale del Mezzogiorno|TIGEM|Grimaldi|Italy
>Spike|hCoV-19/Italy/CAM-TIGEM-263/2020|2020-10-26|EPI_ISL_776924|Original|hCoV-19^^Campania|Human|Istituto Zooprofilattico Sperimentale del Mezzogiorno|TIGEM|Grimaldi|Italy
>Spike|hCoV-19/Italy/CAM-TIGEM-295/2020|2020-10-26|EPI_ISL_776925|Original|hCoV-19^^Campania|Human|Istituto Zooprofilattico Sperimentale del Mezzogiorno|TIGEM|Grimaldi|Italy
>Spike|hCoV-19/Italy/CAM-TIGEM-292/2020|2020-10-26|EPI_ISL_776926|Original|hCoV-19^^Campania|Human|Istituto Zooprofilattico Sperimentale del Mezzogiorno|TIGEM|Grimaldi|Italy

>Spike|hCoV-19/Italy/CAM-TIGEM-273/2020|2020-10-26|EPI_ISL_776927|Original|hCoV-
19^^Campania|Human|Istituto Zooprofilattico Sperimentale del
Mezzogiorno|TIGEM|Grimaldi|Italy
>Spike|hCoV-19/Italy/CAM-TIGEM-252/2020|2020-10-26|EPI_ISL_776928|Original|hCoV-
19^^Campania|Human|Istituto Zooprofilattico Sperimentale del
Mezzogiorno|TIGEM|Grimaldi|Italy
>Spike|hCoV-19/Italy/CAM-TIGEM-283/2020|2020-10-26|EPI_ISL_776929|Original|hCoV-
19^^Campania|Human|Istituto Zooprofilattico Sperimentale del
Mezzogiorno|TIGEM|Grimaldi|Italy
>Spike|hCoV-19/Italy/CAM-TIGEM-274/2020|2020-10-26|EPI_ISL_776930|Original|hCoV-
19^^Campania|Human|Istituto Zooprofilattico Sperimentale del
Mezzogiorno|TIGEM|Grimaldi|Italy
>Spike|hCoV-19/Italy/CAM-TIGEM-265/2020|2020-10-26|EPI_ISL_776931|Original|hCoV-
19^^Campania|Human|Istituto Zooprofilattico Sperimentale del
Mezzogiorno|TIGEM|Grimaldi|Italy
>Spike|hCoV-19/Italy/CAM-TIGEM-284/2020|2020-10-26|EPI_ISL_776932|Original|hCoV-
19^^Campania|Human|Istituto Zooprofilattico Sperimentale del
Mezzogiorno|TIGEM|Grimaldi|Italy
>Spike|hCoV-19/Italy/CAM-TIGEM-293/2020|2020-10-26|EPI_ISL_776933|Original|hCoV-
19^^Campania|Human|Istituto Zooprofilattico Sperimentale del
Mezzogiorno|TIGEM|Grimaldi|Italy
>Spike|hCoV-19/Italy/CAM-TIGEM-281/2020|2020-10-26|EPI_ISL_776934|Original|hCoV-
19^^Campania|Human|Istituto Zooprofilattico Sperimentale del
Mezzogiorno|TIGEM|Grimaldi|Italy
>Spike|hCoV-19/Italy/CAM-TIGEM-286/2020|2020-10-26|EPI_ISL_776935|Original|hCoV-
19^^Campania|Human|Istituto Zooprofilattico Sperimentale del
Mezzogiorno|TIGEM|Grimaldi|Italy
>Spike|hCoV-19/Italy/CAM-TIGEM-253/2020|2020-10-26|EPI_ISL_776936|Original|hCoV-
19^^Campania|Human|Istituto Zooprofilattico Sperimentale del
Mezzogiorno|TIGEM|Grimaldi|Italy
>Spike|hCoV-19/Italy/CAM-TIGEM-279/2020|2020-10-26|EPI_ISL_776937|Original|hCoV-
19^^Campania|Human|Istituto Zooprofilattico Sperimentale del
Mezzogiorno|TIGEM|Grimaldi|Italy
>Spike|hCoV-19/Italy/CAM-TIGEM-258/2020|2020-10-26|EPI_ISL_776938|Original|hCoV-
19^^Campania|Human|Istituto Zooprofilattico Sperimentale del
Mezzogiorno|TIGEM|Grimaldi|Italy
>Spike|hCoV-19/Italy/CAM-TIGEM-294/2020|2020-10-26|EPI_ISL_776939|Original|hCoV-
19^^Campania|Human|Istituto Zooprofilattico Sperimentale del
Mezzogiorno|TIGEM|Grimaldi|Italy
>Spike|hCoV-19/Italy/CAM-TIGEM-259/2020|2020-10-26|EPI_ISL_776940|Original|hCoV-
19^^Campania|Human|Istituto Zooprofilattico Sperimentale del
Mezzogiorno|TIGEM|Grimaldi|Italy
>Spike|hCoV-19/Italy/CAM-TIGEM-255/2020|2020-10-26|EPI_ISL_776941|Original|hCoV-
19^^Campania|Human|Istituto Zooprofilattico Sperimentale del
Mezzogiorno|TIGEM|Grimaldi|Italy
>Spike|hCoV-19/Italy/CAM-TIGEM-278/2020|2020-10-26|EPI_ISL_776942|Original|hCoV-
19^^Campania|Human|Istituto Zooprofilattico Sperimentale del
Mezzogiorno|TIGEM|Grimaldi|Italy
>Spike|hCoV-19/Italy/CAM-TIGEM-285/2020|2020-10-26|EPI_ISL_776943|Original|hCoV-
19^^Campania|Human|Istituto Zooprofilattico Sperimentale del
Mezzogiorno|TIGEM|Grimaldi|Italy
>Spike|hCoV-19/Italy/CAM-TIGEM-268/2020|2020-10-26|EPI_ISL_776944|Original|hCoV-
19^^Campania|Human|Istituto Zooprofilattico Sperimentale del
Mezzogiorno|TIGEM|Grimaldi|Italy
>Spike|hCoV-19/Italy/CAM-TIGEM-257/2020|2020-10-26|EPI_ISL_776945|Original|hCoV-
19^^Campania|Human|Istituto Zooprofilattico Sperimentale del
Mezzogiorno|TIGEM|Grimaldi|Italy
>Spike|hCoV-19/Italy/CAM-TIGEM-264/2020|2020-10-26|EPI_ISL_776946|Original|hCoV-
19^^Campania|Human|Istituto Zooprofilattico Sperimentale del
Mezzogiorno|TIGEM|Grimaldi|Italy

>Spike|hCoV-19/Italy/CAM-TIGEM-260/2020|2020-10-26|EPI_ISL_776947|Original|hCoV-19^^Campania|Human|Istituto Zooprofilattico Sperimentale del Mezzogiorno|TIGEM|Grimaldi|Italy
>Spike|hCoV-19/Italy/CAM-TIGEM-256/2020|2020-10-26|EPI_ISL_776948|Original|hCoV-19^^Campania|Human|Istituto Zooprofilattico Sperimentale del Mezzogiorno|TIGEM|Grimaldi|Italy
>Spike|hCoV-19/Italy/CAM-TIGEM-270/2020|2020-10-26|EPI_ISL_776949|Original|hCoV-19^^Campania|Human|Istituto Zooprofilattico Sperimentale del Mezzogiorno|TIGEM|Grimaldi|Italy
>Spike|hCoV-19/Italy/CAM-TIGEM-291/2020|2020-10-26|EPI_ISL_776950|Original|hCoV-19^^Campania|Human|Istituto Zooprofilattico Sperimentale del Mezzogiorno|TIGEM|Grimaldi|Italy
>Spike|hCoV-19/Italy/CAM-TIGEM-282/2020|2020-10-26|EPI_ISL_776952|Original|hCoV-19^^Campania|Human|Istituto Zooprofilattico Sperimentale del Mezzogiorno|TIGEM|Grimaldi|Italy
>Spike|hCoV-19/Italy/CAM-TIGEM-276/2020|2020-10-26|EPI_ISL_776953|Original|hCoV-19^^Campania|Human|Istituto Zooprofilattico Sperimentale del Mezzogiorno|TIGEM|Grimaldi|Italy
>Spike|hCoV-19/Italy/CAM-TIGEM-297/2020|2020-10-26|EPI_ISL_776954|Original|hCoV-19^^Campania|Human|Istituto Zooprofilattico Sperimentale del Mezzogiorno|TIGEM|Grimaldi|Italy
>Spike|hCoV-19/Italy/CAM-TIGEM-277/2020|2020-10-26|EPI_ISL_776955|Original|hCoV-19^^Campania|Human|Istituto Zooprofilattico Sperimentale del Mezzogiorno|TIGEM|Grimaldi|Italy
>Spike|hCoV-19/Italy/CAM-TIGEM-262/2020|2020-10-26|EPI_ISL_776956|Original|hCoV-19^^Campania|Human|Istituto Zooprofilattico Sperimentale del Mezzogiorno|TIGEM|Grimaldi|Italy
>Spike|hCoV-19/Italy/CAM-TIGEM-275/2020|2020-10-26|EPI_ISL_776957|Original|hCoV-19^^Campania|Human|Istituto Zooprofilattico Sperimentale del Mezzogiorno|TIGEM|Grimaldi|Italy
>Spike|hCoV-19/Italy/CAM-TIGEM-266/2020|2020-10-26|EPI_ISL_776958|Original|hCoV-19^^Campania|Human|Istituto Zooprofilattico Sperimentale del Mezzogiorno|TIGEM|Grimaldi|Italy
>Spike|hCoV-19/Italy/CAM-TIGEM-267/2020|2020-10-26|EPI_ISL_776959|Original|hCoV-19^^Campania|Human|Istituto Zooprofilattico Sperimentale del Mezzogiorno|TIGEM|Grimaldi|Italy
>Spike|hCoV-19/Italy/CAM-TIGEM-374/2020|2020-09-29|EPI_ISL_776960|Original|hCoV-19^^Campania|Human|Istituto Zooprofilattico Sperimentale del Mezzogiorno|TIGEM|Grimaldi|Italy
>Spike|hCoV-19/Italy/CAM-TIGEM-368/2020|2020-09-29|EPI_ISL_776961|Original|hCoV-19^^Campania|Human|Istituto Zooprofilattico Sperimentale del Mezzogiorno|TIGEM|Grimaldi|Italy
>Spike|hCoV-19/Italy/CAM-TIGEM-381/2020|2020-09-29|EPI_ISL_776962|Original|hCoV-19^^Campania|Human|Istituto Zooprofilattico Sperimentale del Mezzogiorno|TIGEM|Grimaldi|Italy
>Spike|hCoV-19/Italy/CAM-TIGEM-363/2020|2020-09-29|EPI_ISL_776963|Original|hCoV-19^^Campania|Human|Istituto Zooprofilattico Sperimentale del Mezzogiorno|TIGEM|Grimaldi|Italy
>Spike|hCoV-19/Italy/CAM-TIGEM-312/2020|2020-09-28|EPI_ISL_776964|Original|hCoV-19^^Campania|Human|Istituto Zooprofilattico Sperimentale del Mezzogiorno|TIGEM|Grimaldi|Italy
>Spike|hCoV-19/Italy/CAM-TIGEM-359/2020|2020-09-28|EPI_ISL_776965|Original|hCoV-19^^Campania|Human|Istituto Zooprofilattico Sperimentale del Mezzogiorno|TIGEM|Grimaldi|Italy
>Spike|hCoV-19/Italy/CAM-TIGEM-304/2020|2020-09-28|EPI_ISL_776966|Original|hCoV-19^^Campania|Human|Istituto Zooprofilattico Sperimentale del Mezzogiorno|TIGEM|Grimaldi|Italy
>Spike|hCoV-19/Italy/CAM-TIGEM-325/2020|2020-09-28|EPI_ISL_776967|Original|hCoV-19^^Campania|Human|Istituto Zooprofilattico Sperimentale del Mezzogiorno|TIGEM|Grimaldi|Italy

>Spike|hCoV-19/Italy/CAM-TIGEM-305/2020|2020-09-01|EPI_ISL_776968|Original|hCoV-19^^Campania|Human|Istituto Zooprofilattico Sperimentale del Mezzogiorno|TIGEM|Grimaldi|Italy
>Spike|hCoV-19/Italy/CAM-TIGEM-344/2020|2020-09-02|EPI_ISL_776969|Original|hCoV-19^^Campania|Human|Istituto Zooprofilattico Sperimentale del Mezzogiorno|TIGEM|Grimaldi|Italy
>Spike|hCoV-19/Italy/CAM-TIGEM-324/2020|2020-09-02|EPI_ISL_776971|Original|hCoV-19^^Campania|Human|Istituto Zooprofilattico Sperimentale del Mezzogiorno|TIGEM|Grimaldi|Italy
>Spike|hCoV-19/Italy/CAM-TIGEM-351/2020|2020-09-02|EPI_ISL_776972|Original|hCoV-19^^Campania|Human|Istituto Zooprofilattico Sperimentale del Mezzogiorno|TIGEM|Grimaldi|Italy
>Spike|hCoV-19/Italy/CAM-TIGEM-338/2020|2020-09-02|EPI_ISL_776973|Original|hCoV-19^^Campania|Human|Istituto Zooprofilattico Sperimentale del Mezzogiorno|TIGEM|Grimaldi|Italy
>Spike|hCoV-19/Italy/CAM-TIGEM-341/2020|2020-09-03|EPI_ISL_776974|Original|hCoV-19^^Campania|Human|Istituto Zooprofilattico Sperimentale del Mezzogiorno|TIGEM|Grimaldi|Italy
>Spike|hCoV-19/Italy/CAM-TIGEM-330/2020|2020-09-03|EPI_ISL_776975|Original|hCoV-19^^Campania|Human|Istituto Zooprofilattico Sperimentale del Mezzogiorno|TIGEM|Grimaldi|Italy
>Spike|hCoV-19/Italy/CAM-TIGEM-350/2020|2020-09-03|EPI_ISL_776976|Original|hCoV-19^^Campania|Human|Istituto Zooprofilattico Sperimentale del Mezzogiorno|TIGEM|Grimaldi|Italy
>Spike|hCoV-19/Italy/CAM-TIGEM-317/2020|2020-09-04|EPI_ISL_776977|Original|hCoV-19^^Campania|Human|Istituto Zooprofilattico Sperimentale del Mezzogiorno|TIGEM|Grimaldi|Italy
>Spike|hCoV-19/Italy/CAM-TIGEM-300/2020|2020-09-07|EPI_ISL_776978|Original|hCoV-19^^Campania|Human|Istituto Zooprofilattico Sperimentale del Mezzogiorno|TIGEM|Grimaldi|Italy
>Spike|hCoV-19/Italy/CAM-TIGEM-335/2020|2020-09-08|EPI_ISL_776979|Original|hCoV-19^^Campania|Human|Istituto Zooprofilattico Sperimentale del Mezzogiorno|TIGEM|Grimaldi|Italy
>Spike|hCoV-19/Italy/CAM-TIGEM-322/2020|2020-09-08|EPI_ISL_776980|Original|hCoV-19^^Campania|Human|Istituto Zooprofilattico Sperimentale del Mezzogiorno|TIGEM|Grimaldi|Italy
>Spike|hCoV-19/Italy/CAM-TIGEM-307/2020|2020-09-08|EPI_ISL_776981|Original|hCoV-19^^Campania|Human|Istituto Zooprofilattico Sperimentale del Mezzogiorno|TIGEM|Grimaldi|Italy
>Spike|hCoV-19/Italy/CAM-TIGEM-345/2020|2020-09-10|EPI_ISL_776982|Original|hCoV-19^^Campania|Human|Istituto Zooprofilattico Sperimentale del Mezzogiorno|TIGEM|Grimaldi|Italy
>Spike|hCoV-19/Italy/CAM-TIGEM-352/2020|2020-09-10|EPI_ISL_776983|Original|hCoV-19^^Campania|Human|Istituto Zooprofilattico Sperimentale del Mezzogiorno|TIGEM|Grimaldi|Italy
>Spike|hCoV-19/Italy/CAM-TIGEM-328/2020|2020-09-10|EPI_ISL_776984|Original|hCoV-19^^Campania|Human|Istituto Zooprofilattico Sperimentale del Mezzogiorno|TIGEM|Grimaldi|Italy
>Spike|hCoV-19/Italy/CAM-TIGEM-334/2020|2020-09-10|EPI_ISL_776985|Original|hCoV-19^^Campania|Human|Istituto Zooprofilattico Sperimentale del Mezzogiorno|TIGEM|Grimaldi|Italy
>Spike|hCoV-19/Italy/CAM-TIGEM-342/2020|2020-09-10|EPI_ISL_776986|Original|hCoV-19^^Campania|Human|Istituto Zooprofilattico Sperimentale del Mezzogiorno|TIGEM|Grimaldi|Italy
>Spike|hCoV-19/Italy/CAM-TIGEM-348/2020|2020-09-10|EPI_ISL_776987|Original|hCoV-19^^Campania|Human|Istituto Zooprofilattico Sperimentale del Mezzogiorno|TIGEM|Grimaldi|Italy
>Spike|hCoV-19/Italy/CAM-TIGEM-354/2020|2020-09-10|EPI_ISL_776988|Original|hCoV-19^^Campania|Human|Istituto Zooprofilattico Sperimentale del Mezzogiorno|TIGEM|Grimaldi|Italy

>Spike|hCoV-19/Italy/CAM-TIGEM-313/2020|2020-09-10|EPI_ISL_776989|Original|hCoV-19^^Campania|Human|Istituto Zooprofilattico Sperimentale del Mezzogiorno|TIGEM|Grimaldi|Italy
>Spike|hCoV-19/Italy/CAM-TIGEM-306/2020|2020-09-10|EPI_ISL_776990|Original|hCoV-19^^Campania|Human|Istituto Zooprofilattico Sperimentale del Mezzogiorno|TIGEM|Grimaldi|Italy
>Spike|hCoV-19/Italy/CAM-TIGEM-346/2020|2020-09-14|EPI_ISL_776991|Original|hCoV-19^^Campania|Human|Istituto Zooprofilattico Sperimentale del Mezzogiorno|TIGEM|Grimaldi|Italy
>Spike|hCoV-19/Italy/CAM-TIGEM-361/2020|2020-09-14|EPI_ISL_776992|Original|hCoV-19^^Campania|Human|Istituto Zooprofilattico Sperimentale del Mezzogiorno|TIGEM|Grimaldi|Italy
>Spike|hCoV-19/Italy/CAM-TIGEM-360/2020|2020-09-14|EPI_ISL_776993|Original|hCoV-19^^Campania|Human|Istituto Zooprofilattico Sperimentale del Mezzogiorno|TIGEM|Grimaldi|Italy
>Spike|hCoV-19/Italy/CAM-TIGEM-379/2020|2020-09-15|EPI_ISL_776995|Original|hCoV-19^^Campania|Human|Istituto Zooprofilattico Sperimentale del Mezzogiorno|TIGEM|Grimaldi|Italy
>Spike|hCoV-19/Italy/CAM-TIGEM-332/2020|2020-09-16|EPI_ISL_776996|Original|hCoV-19^^Campania|Human|Istituto Zooprofilattico Sperimentale del Mezzogiorno|TIGEM|Grimaldi|Italy
>Spike|hCoV-19/Italy/CAM-TIGEM-365/2020|2020-09-20|EPI_ISL_776997|Original|hCoV-19^^Campania|Human|Istituto Zooprofilattico Sperimentale del Mezzogiorno|TIGEM|Grimaldi|Italy
>Spike|hCoV-19/Italy/CAM-TIGEM-364/2020|2020-09-20|EPI_ISL_776998|Original|hCoV-19^^Campania|Human|Istituto Zooprofilattico Sperimentale del Mezzogiorno|TIGEM|Grimaldi|Italy
>Spike|hCoV-19/Italy/CAM-TIGEM-366/2020|2020-09-20|EPI_ISL_776999|Original|hCoV-19^^Campania|Human|Istituto Zooprofilattico Sperimentale del Mezzogiorno|TIGEM|Grimaldi|Italy
>Spike|hCoV-19/Italy/CAM-TIGEM-372/2020|2020-09-20|EPI_ISL_777000|Original|hCoV-19^^Campania|Human|Istituto Zooprofilattico Sperimentale del Mezzogiorno|TIGEM|Grimaldi|Italy
>Spike|hCoV-19/Italy/CAM-TIGEM-378/2020|2020-09-20|EPI_ISL_777001|Original|hCoV-19^^Campania|Human|Istituto Zooprofilattico Sperimentale del Mezzogiorno|TIGEM|Grimaldi|Italy
>Spike|hCoV-19/Italy/CAM-TIGEM-371/2020|2020-09-20|EPI_ISL_777002|Original|hCoV-19^^Campania|Human|Istituto Zooprofilattico Sperimentale del Mezzogiorno|TIGEM|Grimaldi|Italy
>Spike|hCoV-19/Italy/CAM-TIGEM-382/2020|2020-09-20|EPI_ISL_777003|Original|hCoV-19^^Campania|Human|Istituto Zooprofilattico Sperimentale del Mezzogiorno|TIGEM|Grimaldi|Italy
>Spike|hCoV-19/Italy/CAM-TIGEM-370/2020|2020-09-20|EPI_ISL_777004|Original|hCoV-19^^Campania|Human|Istituto Zooprofilattico Sperimentale del Mezzogiorno|TIGEM|Grimaldi|Italy
>Spike|hCoV-19/Italy/CAM-TIGEM-369/2020|2020-09-20|EPI_ISL_777005|Original|hCoV-19^^Campania|Human|Istituto Zooprofilattico Sperimentale del Mezzogiorno|TIGEM|Grimaldi|Italy
>Spike|hCoV-19/Italy/CAM-TIGEM-376/2020|2020-09-20|EPI_ISL_777006|Original|hCoV-19^^Campania|Human|Istituto Zooprofilattico Sperimentale del Mezzogiorno|TIGEM|Grimaldi|Italy
>Spike|hCoV-19/Italy/CAM-TIGEM-383/2020|2020-09-20|EPI_ISL_777007|Original|hCoV-19^^Campania|Human|Istituto Zooprofilattico Sperimentale del Mezzogiorno|TIGEM|Grimaldi|Italy
>Spike|hCoV-19/Italy/CAM-TIGEM-308/2020|2020-09-21|EPI_ISL_777008|Original|hCoV-19^^Campania|Human|Istituto Zooprofilattico Sperimentale del Mezzogiorno|TIGEM|Grimaldi|Italy
>Spike|hCoV-19/Italy/CAM-TIGEM-323/2020|2020-09-21|EPI_ISL_777009|Original|hCoV-19^^Campania|Human|Istituto Zooprofilattico Sperimentale del Mezzogiorno|TIGEM|Grimaldi|Italy

>Spike|hCoV-19/England/MILK-C90D02/2020|2020-12-10|EPI_ISL_777207|Original|hCoV-19^^England|Human|Lighthouse Lab in Milton Keynes|Wellcome Sanger Institute for the COVID-19 Genomics UK (COG-UK) Consortium|Davies|United Kingdom
>Spike|hCoV-19/England/ALDP-C8EA79/2020|2020-12-14|EPI_ISL_777213|Original|hCoV-19^^England|Human|Lighthouse Lab in Alderley Park|Wellcome Sanger Institute for the COVID-19 Genomics UK (COG-UK) Consortium|Davies|United Kingdom
>Spike|hCoV-19/England/MILK-C90B9F/2020|2020-12-10|EPI_ISL_777304|Original|hCoV-19^^England|Human|Lighthouse Lab in Milton Keynes|Wellcome Sanger Institute for the COVID-19 Genomics UK (COG-UK) Consortium|Davies|United Kingdom
>Spike|hCoV-19/Spain/M-20120611/2020|2020-04-28|EPI_ISL_777490|Original|hCoV-19^^Madrid|Human|Hospital General Universitario Gregorio Mara\u00f1\u00f3n|Hospital General Universitario Gregorio Mara\u00f1\u00f3n|Garc\u00eda de Viedma|Spain
>Spike|hCoV-19/Spain/M-20157372/2020|2020-05-17|EPI_ISL_778445|Original|hCoV-19^^Madrid|Human|Hospital General Universitario Gregorio Mara\u00f1\u00f3n|Hospital General Universitario Gregorio Mara\u00f1\u00f3n|Garc\u00eda de Viedma|Spain
>Spike|hCoV-19/Spain/M-20115914/2020|2020-04-27|EPI_ISL_778644|Original|hCoV-19^^Madrid|Human|Hospital General Universitario Gregorio Mara\u00f1\u00f3n|Hospital General Universitario Gregorio Mara\u00f1\u00f3n|Garc\u00eda de Viedma|Spain
>Spike|hCoV-19/Italy/CAM-TIGEM-589/2020|2020-04-12|EPI_ISL_778646|Original|hCoV-19^^Campania|Human|Istituto Zooprofilattico Sperimentale del Mezzogiorno|TIGEM|Grimaldi|Italy
>Spike|hCoV-19/Italy/CAM-TIGEM-636/2020|2020-03-29|EPI_ISL_778647|Original|hCoV-19^^Campania|Human|Istituto Zooprofilattico Sperimentale del Mezzogiorno|TIGEM|Grimaldi|Italy
>Spike|hCoV-19/Italy/CAM-TIGEM-600/2020|2020-04-03|EPI_ISL_778648|Original|hCoV-19^^Campania|Human|Istituto Zooprofilattico Sperimentale del Mezzogiorno|TIGEM|Grimaldi|Italy
>Spike|hCoV-19/Italy/CAM-TIGEM-601/2020|2020-04-03|EPI_ISL_778649|Original|hCoV-19^^Campania|Human|Istituto Zooprofilattico Sperimentale del Mezzogiorno|TIGEM|Grimaldi|Italy
>Spike|hCoV-19/Italy/CAM-TIGEM-652/2020|2020-03-29|EPI_ISL_778650|Original|hCoV-19^^Campania|Human|Istituto Zooprofilattico Sperimentale del Mezzogiorno|TIGEM|Grimaldi|Italy
>Spike|hCoV-19/Italy/CAM-TIGEM-654/2020|2020-03-29|EPI_ISL_778651|Original|hCoV-19^^Campania|Human|Istituto Zooprofilattico Sperimentale del Mezzogiorno|TIGEM|Grimaldi|Italy
>Spike|hCoV-19/Italy/CAM-TIGEM-611/2020|2020-04-07|EPI_ISL_778652|Original|hCoV-19^^Campania|Human|Istituto Zooprofilattico Sperimentale del Mezzogiorno|TIGEM|Grimaldi|Italy
>Spike|hCoV-19/Italy/CAM-TIGEM-603/2020|2020-04-08|EPI_ISL_778653|Original|hCoV-19^^Campania|Human|Istituto Zooprofilattico Sperimentale del Mezzogiorno|TIGEM|Grimaldi|Italy
>Spike|hCoV-19/Italy/CAM-TIGEM-634/2020|2020-04-08|EPI_ISL_778654|Original|hCoV-19^^Campania|Human|Istituto Zooprofilattico Sperimentale del Mezzogiorno|TIGEM|Grimaldi|Italy
>Spike|hCoV-19/Italy/CAM-TIGEM-613/2020|2020-04-08|EPI_ISL_778655|Original|hCoV-19^^Campania|Human|Istituto Zooprofilattico Sperimentale del Mezzogiorno|TIGEM|Grimaldi|Italy
>Spike|hCoV-19/Italy/CAM-TIGEM-614/2020|2020-04-09|EPI_ISL_778656|Original|hCoV-19^^Campania|Human|Istituto Zooprofilattico Sperimentale del Mezzogiorno|TIGEM|Grimaldi|Italy
>Spike|hCoV-19/Italy/CAM-TIGEM-585/2020|2020-04-11|EPI_ISL_778657|Original|hCoV-19^^Campania|Human|Istituto Zooprofilattico Sperimentale del Mezzogiorno|TIGEM|Grimaldi|Italy
>Spike|hCoV-19/Italy/CAM-TIGEM-579/2020|2020-04-12|EPI_ISL_778658|Original|hCoV-19^^Campania|Human|Istituto Zooprofilattico Sperimentale del Mezzogiorno|TIGEM|Grimaldi|Italy
>Spike|hCoV-19/Italy/CAM-TIGEM-581/2020|2020-04-13|EPI_ISL_778659|Original|hCoV-19^^Campania|Human|Istituto Zooprofilattico Sperimentale del Mezzogiorno|TIGEM|Grimaldi|Italy

>Spike|hCoV-19/Italy/CAM-TIGEM-582/2020|2020-04-13|EPI_ISL_778660|Original|hCoV-19^^Campania|Human|Istituto Zooprofilattico Sperimentale del Mezzogiorno|TIGEM|Grimaldi|Italy
>Spike|hCoV-19/Italy/CAM-TIGEM-583/2020|2020-04-13|EPI_ISL_778661|Original|hCoV-19^^Campania|Human|Istituto Zooprofilattico Sperimentale del Mezzogiorno|TIGEM|Grimaldi|Italy
>Spike|hCoV-19/Italy/CAM-TIGEM-595/2020|2020-04-13|EPI_ISL_778662|Original|hCoV-19^^Campania|Human|Istituto Zooprofilattico Sperimentale del Mezzogiorno|TIGEM|Grimaldi|Italy
>Spike|hCoV-19/Italy/CAM-TIGEM-590/2020|2020-04-14|EPI_ISL_778663|Original|hCoV-19^^Campania|Human|Istituto Zooprofilattico Sperimentale del Mezzogiorno|TIGEM|Grimaldi|Italy
>Spike|hCoV-19/Italy/CAM-TIGEM-592/2020|2020-04-14|EPI_ISL_778665|Original|hCoV-19^^Campania|Human|Istituto Zooprofilattico Sperimentale del Mezzogiorno|TIGEM|Grimaldi|Italy
>Spike|hCoV-19/Italy/CAM-TIGEM-588/2020|2020-04-14|EPI_ISL_778666|Original|hCoV-19^^Campania|Human|Istituto Zooprofilattico Sperimentale del Mezzogiorno|TIGEM|Grimaldi|Italy
>Spike|hCoV-19/Italy/CAM-TIGEM-598/2020|2020-04-14|EPI_ISL_778667|Original|hCoV-19^^Campania|Human|Istituto Zooprofilattico Sperimentale del Mezzogiorno|TIGEM|Grimaldi|Italy
>Spike|hCoV-19/Italy/CAM-TIGEM-599/2020|2020-04-16|EPI_ISL_778669|Original|hCoV-19^^Campania|Human|Istituto Zooprofilattico Sperimentale del Mezzogiorno|TIGEM|Grimaldi|Italy
>Spike|hCoV-19/Italy/CAM-TIGEM-606/2020|2020-04-17|EPI_ISL_778670|Original|hCoV-19^^Campania|Human|Istituto Zooprofilattico Sperimentale del Mezzogiorno|TIGEM|Grimaldi|Italy
>Spike|hCoV-19/Italy/CAM-TIGEM-608/2020|2020-04-18|EPI_ISL_778671|Original|hCoV-19^^Campania|Human|Istituto Zooprofilattico Sperimentale del Mezzogiorno|TIGEM|Grimaldi|Italy
>Spike|hCoV-19/Italy/CAM-TIGEM-605/2020|2020-04-18|EPI_ISL_778672|Original|hCoV-19^^Campania|Human|Istituto Zooprofilattico Sperimentale del Mezzogiorno|TIGEM|Grimaldi|Italy
>Spike|hCoV-19/Italy/CAM-TIGEM-609/2020|2020-04-20|EPI_ISL_778673|Original|hCoV-19^^Campania|Human|Istituto Zooprofilattico Sperimentale del Mezzogiorno|TIGEM|Grimaldi|Italy
>Spike|hCoV-19/Italy/CAM-TIGEM-610/2020|2020-04-20|EPI_ISL_778674|Original|hCoV-19^^Campania|Human|Istituto Zooprofilattico Sperimentale del Mezzogiorno|TIGEM|Grimaldi|Italy
>Spike|hCoV-19/Italy/CAM-TIGEM-617/2020|2020-04-21|EPI_ISL_778675|Original|hCoV-19^^Campania|Human|Istituto Zooprofilattico Sperimentale del Mezzogiorno|TIGEM|Grimaldi|Italy
>Spike|hCoV-19/Italy/CAM-TIGEM-618/2020|2020-04-21|EPI_ISL_778676|Original|hCoV-19^^Campania|Human|Istituto Zooprofilattico Sperimentale del Mezzogiorno|TIGEM|Grimaldi|Italy
>Spike|hCoV-19/Italy/CAM-TIGEM-619/2020|2020-04-21|EPI_ISL_778677|Original|hCoV-19^^Campania|Human|Istituto Zooprofilattico Sperimentale del Mezzogiorno|TIGEM|Grimaldi|Italy
>Spike|hCoV-19/Italy/CAM-TIGEM-620/2020|2020-04-21|EPI_ISL_778678|Original|hCoV-19^^Campania|Human|Istituto Zooprofilattico Sperimentale del Mezzogiorno|TIGEM|Grimaldi|Italy
>Spike|hCoV-19/Italy/CAM-TIGEM-621/2020|2020-04-21|EPI_ISL_778679|Original|hCoV-19^^Campania|Human|Istituto Zooprofilattico Sperimentale del Mezzogiorno|TIGEM|Grimaldi|Italy
>Spike|hCoV-19/Italy/CAM-TIGEM-623/2020|2020-04-21|EPI_ISL_778680|Original|hCoV-19^^Campania|Human|Istituto Zooprofilattico Sperimentale del Mezzogiorno|TIGEM|Grimaldi|Italy
>Spike|hCoV-19/Italy/CAM-TIGEM-616/2020|2020-04-21|EPI_ISL_778681|Original|hCoV-19^^Campania|Human|Istituto Zooprofilattico Sperimentale del Mezzogiorno|TIGEM|Grimaldi|Italy

>Spike|hCoV-19/Italy/CAM-TIGEM-628/2020|2020-04-24|EPI_ISL_778682|Original|hCoV-19^^Campania|Human|Istituto Zooprofilattico Sperimentale del Mezzogiorno|TIGEM|Grimaldi|Italy
>Spike|hCoV-19/Italy/CAM-TIGEM-630/2020|2020-04-27|EPI_ISL_778683|Original|hCoV-19^^Campania|Human|Istituto Zooprofilattico Sperimentale del Mezzogiorno|TIGEM|Grimaldi|Italy
>Spike|hCoV-19/Italy/CAM-TIGEM-631/2020|2020-04-27|EPI_ISL_778684|Original|hCoV-19^^Campania|Human|Istituto Zooprofilattico Sperimentale del Mezzogiorno|TIGEM|Grimaldi|Italy
>Spike|hCoV-19/Italy/CAM-TIGEM-629/2020|2020-04-28|EPI_ISL_778685|Original|hCoV-19^^Campania|Human|Istituto Zooprofilattico Sperimentale del Mezzogiorno|TIGEM|Grimaldi|Italy
>Spike|hCoV-19/Italy/CAM-TIGEM-665/2020|2020-03-31|EPI_ISL_778686|Original|hCoV-19^^Campania|Human|Istituto Zooprofilattico Sperimentale del Mezzogiorno|TIGEM|Grimaldi|Italy
>Spike|hCoV-19/Italy/CAM-TIGEM-666/2020|2020-03-31|EPI_ISL_778687|Original|hCoV-19^^Campania|Human|Istituto Zooprofilattico Sperimentale del Mezzogiorno|TIGEM|Grimaldi|Italy
>Spike|hCoV-19/Italy/CAM-TIGEM-667/2020|2020-03-31|EPI_ISL_778688|Original|hCoV-19^^Campania|Human|Istituto Zooprofilattico Sperimentale del Mezzogiorno|TIGEM|Grimaldi|Italy
>Spike|hCoV-19/Italy/CAM-TIGEM-668/2020|2020-03-31|EPI_ISL_778689|Original|hCoV-19^^Campania|Human|Istituto Zooprofilattico Sperimentale del Mezzogiorno|TIGEM|Grimaldi|Italy
>Spike|hCoV-19/Italy/CAM-TIGEM-669/2020|2020-03-31|EPI_ISL_778690|Original|hCoV-19^^Campania|Human|Istituto Zooprofilattico Sperimentale del Mezzogiorno|TIGEM|Grimaldi|Italy
>Spike|hCoV-19/Italy/CAM-TIGEM-417/2020|2020-09-29|EPI_ISL_778691|Original|hCoV-19^^Campania|Human|Istituto Zooprofilattico Sperimentale del Mezzogiorno|TIGEM|Grimaldi|Italy
>Spike|hCoV-19/Italy/CAM-TIGEM-397/2020|2020-09-29|EPI_ISL_778692|Original|hCoV-19^^Campania|Human|Istituto Zooprofilattico Sperimentale del Mezzogiorno|TIGEM|Grimaldi|Italy
>Spike|hCoV-19/Italy/CAM-TIGEM-389/2020|2020-09-29|EPI_ISL_778693|Original|hCoV-19^^Campania|Human|Istituto Zooprofilattico Sperimentale del Mezzogiorno|TIGEM|Grimaldi|Italy
>Spike|hCoV-19/Italy/CAM-TIGEM-416/2020|2020-09-29|EPI_ISL_778694|Original|hCoV-19^^Campania|Human|Istituto Zooprofilattico Sperimentale del Mezzogiorno|TIGEM|Grimaldi|Italy
>Spike|hCoV-19/Italy/CAM-TIGEM-390/2020|2020-09-29|EPI_ISL_778695|Original|hCoV-19^^Campania|Human|Istituto Zooprofilattico Sperimentale del Mezzogiorno|TIGEM|Grimaldi|Italy
>Spike|hCoV-19/Italy/CAM-TIGEM-564/2020|2020-04-03|EPI_ISL_778696|Original|hCoV-19^^Campania|Human|Istituto Zooprofilattico Sperimentale del Mezzogiorno|TIGEM|Grimaldi|Italy
>Spike|hCoV-19/Italy/CAM-TIGEM-565/2020|2020-04-03|EPI_ISL_778697|Original|hCoV-19^^Campania|Human|Istituto Zooprofilattico Sperimentale del Mezzogiorno|TIGEM|Grimaldi|Italy
>Spike|hCoV-19/Italy/CAM-TIGEM-568/2020|2020-04-03|EPI_ISL_778699|Original|hCoV-19^^Campania|Human|Istituto Zooprofilattico Sperimentale del Mezzogiorno|TIGEM|Grimaldi|Italy
>Spike|hCoV-19/Italy/CAM-TIGEM-570/2020|2020-04-03|EPI_ISL_778700|Original|hCoV-19^^Campania|Human|Istituto Zooprofilattico Sperimentale del Mezzogiorno|TIGEM|Grimaldi|Italy
>Spike|hCoV-19/Italy/CAM-TIGEM-571/2020|2020-04-03|EPI_ISL_778701|Original|hCoV-19^^Campania|Human|Istituto Zooprofilattico Sperimentale del Mezzogiorno|TIGEM|Grimaldi|Italy
>Spike|hCoV-19/Italy/CAM-TIGEM-572/2020|2020-04-03|EPI_ISL_778702|Original|hCoV-19^^Campania|Human|Istituto Zooprofilattico Sperimentale del Mezzogiorno|TIGEM|Grimaldi|Italy

>Spike|hCoV-19/Italy/CAM-TIGEM-573/2020|2020-04-03|EPI_ISL_778703|Original|hCoV-19^^Campania|Human|Istituto Zooprofilattico Sperimentale del Mezzogiorno|TIGEM|Grimaldi|Italy
>Spike|hCoV-19/Italy/CAM-TIGEM-388/2020|2020-09-15|EPI_ISL_778704|Original|hCoV-19^^Campania|Human|Istituto Zooprofilattico Sperimentale del Mezzogiorno|TIGEM|Grimaldi|Italy
>Spike|hCoV-19/Italy/CAM-TIGEM-401/2020|2020-09-15|EPI_ISL_778705|Original|hCoV-19^^Campania|Human|Istituto Zooprofilattico Sperimentale del Mezzogiorno|TIGEM|Grimaldi|Italy
>Spike|hCoV-19/Italy/CAM-TIGEM-394/2020|2020-09-15|EPI_ISL_778706|Original|hCoV-19^^Campania|Human|Istituto Zooprofilattico Sperimentale del Mezzogiorno|TIGEM|Grimaldi|Italy
>Spike|hCoV-19/Italy/CAM-TIGEM-400/2020|2020-09-15|EPI_ISL_778707|Original|hCoV-19^^Campania|Human|Istituto Zooprofilattico Sperimentale del Mezzogiorno|TIGEM|Grimaldi|Italy
>Spike|hCoV-19/Italy/CAM-TIGEM-412/2020|2020-09-15|EPI_ISL_778708|Original|hCoV-19^^Campania|Human|Istituto Zooprofilattico Sperimentale del Mezzogiorno|TIGEM|Grimaldi|Italy
>Spike|hCoV-19/Italy/CAM-TIGEM-411/2020|2020-09-15|EPI_ISL_778709|Original|hCoV-19^^Campania|Human|Istituto Zooprofilattico Sperimentale del Mezzogiorno|TIGEM|Grimaldi|Italy
>Spike|hCoV-19/Italy/CAM-TIGEM-391/2020|2020-09-15|EPI_ISL_778710|Original|hCoV-19^^Campania|Human|Istituto Zooprofilattico Sperimentale del Mezzogiorno|TIGEM|Grimaldi|Italy
>Spike|hCoV-19/Italy/CAM-TIGEM-414/2020|2020-09-15|EPI_ISL_778711|Original|hCoV-19^^Campania|Human|Istituto Zooprofilattico Sperimentale del Mezzogiorno|TIGEM|Grimaldi|Italy
>Spike|hCoV-19/Italy/CAM-TIGEM-418/2020|2020-09-15|EPI_ISL_778712|Original|hCoV-19^^Campania|Human|Istituto Zooprofilattico Sperimentale del Mezzogiorno|TIGEM|Grimaldi|Italy
>Spike|hCoV-19/Italy/CAM-TIGEM-392/2020|2020-09-15|EPI_ISL_778713|Original|hCoV-19^^Campania|Human|Istituto Zooprofilattico Sperimentale del Mezzogiorno|TIGEM|Grimaldi|Italy
>Spike|hCoV-19/Italy/CAM-TIGEM-419/2020|2020-09-15|EPI_ISL_778714|Original|hCoV-19^^Campania|Human|Istituto Zooprofilattico Sperimentale del Mezzogiorno|TIGEM|Grimaldi|Italy
>Spike|hCoV-19/Italy/CAM-TIGEM-404/2020|2020-09-15|EPI_ISL_778715|Original|hCoV-19^^Campania|Human|Istituto Zooprofilattico Sperimentale del Mezzogiorno|TIGEM|Grimaldi|Italy
>Spike|hCoV-19/Italy/CAM-TIGEM-399/2020|2020-09-15|EPI_ISL_778716|Original|hCoV-19^^Campania|Human|Istituto Zooprofilattico Sperimentale del Mezzogiorno|TIGEM|Grimaldi|Italy
>Spike|hCoV-19/Italy/CAM-TIGEM-408/2020|2020-09-15|EPI_ISL_778717|Original|hCoV-19^^Campania|Human|Istituto Zooprofilattico Sperimentale del Mezzogiorno|TIGEM|Grimaldi|Italy
>Spike|hCoV-19/Italy/CAM-TIGEM-407/2020|2020-09-15|EPI_ISL_778718|Original|hCoV-19^^Campania|Human|Istituto Zooprofilattico Sperimentale del Mezzogiorno|TIGEM|Grimaldi|Italy
>Spike|hCoV-19/Italy/CAM-TIGEM-406/2020|2020-09-15|EPI_ISL_778719|Original|hCoV-19^^Campania|Human|Istituto Zooprofilattico Sperimentale del Mezzogiorno|TIGEM|Grimaldi|Italy
>Spike|hCoV-19/Italy/CAM-TIGEM-420/2020|2020-09-15|EPI_ISL_778720|Original|hCoV-19^^Campania|Human|Istituto Zooprofilattico Sperimentale del Mezzogiorno|TIGEM|Grimaldi|Italy
>Spike|hCoV-19/Italy/CAM-TIGEM-413/2020|2020-09-15|EPI_ISL_778721|Original|hCoV-19^^Campania|Human|Istituto Zooprofilattico Sperimentale del Mezzogiorno|TIGEM|Grimaldi|Italy
>Spike|hCoV-19/Italy/CAM-TIGEM-561/2020|2020-04-09|EPI_ISL_778722|Original|hCoV-19^^Campania|Human|Istituto Zooprofilattico Sperimentale del Mezzogiorno|TIGEM|Grimaldi|Italy

>Spike|hCoV-19/Italy/CAM-TIGEM-553/2020|2020-04-09|EPI_ISL_778723|Original|hCoV-19^^Campania|Human|Istituto Zooprofilattico Sperimentale del Mezzogiorno|TIGEM|Grimaldi|Italy
>Spike|hCoV-19/Italy/CAM-TIGEM-548/2020|2020-04-09|EPI_ISL_778724|Original|hCoV-19^^Campania|Human|Istituto Zooprofilattico Sperimentale del Mezzogiorno|TIGEM|Grimaldi|Italy
>Spike|hCoV-19/Italy/CAM-TIGEM-557/2020|2020-04-09|EPI_ISL_778725|Original|hCoV-19^^Campania|Human|Istituto Zooprofilattico Sperimentale del Mezzogiorno|TIGEM|Grimaldi|Italy
>Spike|hCoV-19/Italy/CAM-TIGEM-575/2020|2020-04-11|EPI_ISL_778728|Original|hCoV-19^^Campania|Human|Istituto Zooprofilattico Sperimentale del Mezzogiorno|TIGEM|Grimaldi|Italy
>Spike|hCoV-19/Italy/CAM-TIGEM-545/2020|2020-04-13|EPI_ISL_778729|Original|hCoV-19^^Campania|Human|Istituto Zooprofilattico Sperimentale del Mezzogiorno|TIGEM|Grimaldi|Italy
>Spike|hCoV-19/Italy/CAM-TIGEM-552/2020|2020-04-13|EPI_ISL_778730|Original|hCoV-19^^Campania|Human|Istituto Zooprofilattico Sperimentale del Mezzogiorno|TIGEM|Grimaldi|Italy
>Spike|hCoV-19/Italy/CAM-TIGEM-550/2020|2020-04-13|EPI_ISL_778732|Original|hCoV-19^^Campania|Human|Istituto Zooprofilattico Sperimentale del Mezzogiorno|TIGEM|Grimaldi|Italy
>Spike|hCoV-19/Italy/CAM-TIGEM-555/2020|2020-04-13|EPI_ISL_778733|Original|hCoV-19^^Campania|Human|Istituto Zooprofilattico Sperimentale del Mezzogiorno|TIGEM|Grimaldi|Italy
>Spike|hCoV-19/Italy/CAM-TIGEM-558/2020|2020-04-20|EPI_ISL_778735|Original|hCoV-19^^Campania|Human|Istituto Zooprofilattico Sperimentale del Mezzogiorno|TIGEM|Grimaldi|Italy
>Spike|hCoV-19/Italy/CAM-TIGEM-562/2020|2020-04-29|EPI_ISL_778736|Original|hCoV-19^^Campania|Human|Istituto Zooprofilattico Sperimentale del Mezzogiorno|TIGEM|Grimaldi|Italy
>Spike|hCoV-19/Italy/CAM-TIGEM-421/2020|2020-06-29|EPI_ISL_778737|Original|hCoV-19^^Campania|Human|Istituto Zooprofilattico Sperimentale del Mezzogiorno|TIGEM|Grimaldi|Italy
>Spike|hCoV-19/Italy/CAM-TIGEM-430/2020|2020-06-29|EPI_ISL_778738|Original|hCoV-19^^Campania|Human|Istituto Zooprofilattico Sperimentale del Mezzogiorno|TIGEM|Grimaldi|Italy
>Spike|hCoV-19/Italy/CAM-TIGEM-524/2020|2020-06-29|EPI_ISL_778739|Original|hCoV-19^^Campania|Human|Istituto Zooprofilattico Sperimentale del Mezzogiorno|TIGEM|Grimaldi|Italy
>Spike|hCoV-19/Italy/CAM-TIGEM-526/2020|2020-06-29|EPI_ISL_778740|Original|hCoV-19^^Campania|Human|Istituto Zooprofilattico Sperimentale del Mezzogiorno|TIGEM|Grimaldi|Italy
>Spike|hCoV-19/Italy/CAM-TIGEM-527/2020|2020-06-29|EPI_ISL_778741|Original|hCoV-19^^Campania|Human|Istituto Zooprofilattico Sperimentale del Mezzogiorno|TIGEM|Grimaldi|Italy
>Spike|hCoV-19/Italy/CAM-TIGEM-530/2020|2020-06-29|EPI_ISL_778742|Original|hCoV-19^^Campania|Human|Istituto Zooprofilattico Sperimentale del Mezzogiorno|TIGEM|Grimaldi|Italy
>Spike|hCoV-19/Italy/CAM-TIGEM-531/2020|2020-06-29|EPI_ISL_778743|Original|hCoV-19^^Campania|Human|Istituto Zooprofilattico Sperimentale del Mezzogiorno|TIGEM|Grimaldi|Italy
>Spike|hCoV-19/Italy/CAM-TIGEM-532/2020|2020-06-29|EPI_ISL_778744|Original|hCoV-19^^Campania|Human|Istituto Zooprofilattico Sperimentale del Mezzogiorno|TIGEM|Grimaldi|Italy
>Spike|hCoV-19/Italy/CAM-TIGEM-533/2020|2020-06-29|EPI_ISL_778745|Original|hCoV-19^^Campania|Human|Istituto Zooprofilattico Sperimentale del Mezzogiorno|TIGEM|Grimaldi|Italy
>Spike|hCoV-19/Italy/CAM-TIGEM-534/2020|2020-06-29|EPI_ISL_778746|Original|hCoV-19^^Campania|Human|Istituto Zooprofilattico Sperimentale del Mezzogiorno|TIGEM|Grimaldi|Italy

>Spike|hCoV-19/Italy/CAM-TIGEM-431/2020|2020-06-28|EPI_ISL_778747|Original|hCoV-19^^Campania|Human|Istituto Zooprofilattico Sperimentale del Mezzogiorno|TIGEM|Grimaldi|Italy
>Spike|hCoV-19/Italy/CAM-TIGEM-536/2020|2020-06-29|EPI_ISL_778748|Original|hCoV-19^^Campania|Human|Istituto Zooprofilattico Sperimentale del Mezzogiorno|TIGEM|Grimaldi|Italy
>Spike|hCoV-19/Italy/CAM-TIGEM-537/2020|2020-06-29|EPI_ISL_778749|Original|hCoV-19^^Campania|Human|Istituto Zooprofilattico Sperimentale del Mezzogiorno|TIGEM|Grimaldi|Italy
>Spike|hCoV-19/Italy/CAM-TIGEM-540/2020|2020-06-29|EPI_ISL_778750|Original|hCoV-19^^Campania|Human|Istituto Zooprofilattico Sperimentale del Mezzogiorno|TIGEM|Grimaldi|Italy
>Spike|hCoV-19/Italy/CAM-TIGEM-541/2020|2020-06-29|EPI_ISL_778751|Original|hCoV-19^^Campania|Human|Istituto Zooprofilattico Sperimentale del Mezzogiorno|TIGEM|Grimaldi|Italy
>Spike|hCoV-19/Italy/CAM-TIGEM-434/2020|2020-06-28|EPI_ISL_778752|Original|hCoV-19^^Campania|Human|Istituto Zooprofilattico Sperimentale del Mezzogiorno|TIGEM|Grimaldi|Italy
>Spike|hCoV-19/Italy/CAM-TIGEM-544/2020|2020-06-29|EPI_ISL_778753|Original|hCoV-19^^Campania|Human|Istituto Zooprofilattico Sperimentale del Mezzogiorno|TIGEM|Grimaldi|Italy
>Spike|hCoV-19/Italy/CAM-TIGEM-435/2020|2020-06-28|EPI_ISL_778754|Original|hCoV-19^^Campania|Human|Istituto Zooprofilattico Sperimentale del Mezzogiorno|TIGEM|Grimaldi|Italy
>Spike|hCoV-19/Italy/CAM-TIGEM-437/2020|2020-06-28|EPI_ISL_778756|Original|hCoV-19^^Campania|Human|Istituto Zooprofilattico Sperimentale del Mezzogiorno|TIGEM|Grimaldi|Italy
>Spike|hCoV-19/Italy/CAM-TIGEM-438/2020|2020-06-28|EPI_ISL_778757|Original|hCoV-19^^Campania|Human|Istituto Zooprofilattico Sperimentale del Mezzogiorno|TIGEM|Grimaldi|Italy
>Spike|hCoV-19/Italy/CAM-TIGEM-439/2020|2020-06-30|EPI_ISL_778758|Original|hCoV-19^^Campania|Human|Istituto Zooprofilattico Sperimentale del Mezzogiorno|TIGEM|Grimaldi|Italy
>Spike|hCoV-19/Italy/CAM-TIGEM-440/2020|2020-06-30|EPI_ISL_778759|Original|hCoV-19^^Campania|Human|Istituto Zooprofilattico Sperimentale del Mezzogiorno|TIGEM|Grimaldi|Italy
>Spike|hCoV-19/Italy/CAM-TIGEM-441/2020|2020-06-30|EPI_ISL_778760|Original|hCoV-19^^Campania|Human|Istituto Zooprofilattico Sperimentale del Mezzogiorno|TIGEM|Grimaldi|Italy
>Spike|hCoV-19/Italy/CAM-TIGEM-422/2020|2020-06-29|EPI_ISL_778761|Original|hCoV-19^^Campania|Human|Istituto Zooprofilattico Sperimentale del Mezzogiorno|TIGEM|Grimaldi|Italy
>Spike|hCoV-19/Italy/CAM-TIGEM-442/2020|2020-06-30|EPI_ISL_778762|Original|hCoV-19^^Campania|Human|Istituto Zooprofilattico Sperimentale del Mezzogiorno|TIGEM|Grimaldi|Italy
>Spike|hCoV-19/Italy/CAM-TIGEM-444/2020|2020-06-28|EPI_ISL_778763|Original|hCoV-19^^Campania|Human|Istituto Zooprofilattico Sperimentale del Mezzogiorno|TIGEM|Grimaldi|Italy
>Spike|hCoV-19/Italy/CAM-TIGEM-445/2020|2020-06-28|EPI_ISL_778764|Original|hCoV-19^^Campania|Human|Istituto Zooprofilattico Sperimentale del Mezzogiorno|TIGEM|Grimaldi|Italy
>Spike|hCoV-19/Italy/CAM-TIGEM-446/2020|2020-06-29|EPI_ISL_778765|Original|hCoV-19^^Campania|Human|Istituto Zooprofilattico Sperimentale del Mezzogiorno|TIGEM|Grimaldi|Italy
>Spike|hCoV-19/Italy/CAM-TIGEM-447/2020|2020-06-29|EPI_ISL_778766|Original|hCoV-19^^Campania|Human|Istituto Zooprofilattico Sperimentale del Mezzogiorno|TIGEM|Grimaldi|Italy
>Spike|hCoV-19/Italy/CAM-TIGEM-448/2020|2020-06-29|EPI_ISL_778767|Original|hCoV-19^^Campania|Human|Istituto Zooprofilattico Sperimentale del Mezzogiorno|TIGEM|Grimaldi|Italy

>Spike|hCoV-19/Italy/CAM-TIGEM-449/2020|2020-06-29|EPI_ISL_778768|Original|hCoV-19^^Campania|Human|Istituto Zooprofilattico Sperimentale del Mezzogiorno|TIGEM|Grimaldi|Italy
>Spike|hCoV-19/Italy/CAM-TIGEM-450/2020|2020-07-02|EPI_ISL_778769|Original|hCoV-19^^Campania|Human|Istituto Zooprofilattico Sperimentale del Mezzogiorno|TIGEM|Grimaldi|Italy
>Spike|hCoV-19/Italy/CAM-TIGEM-423/2020|2020-06-29|EPI_ISL_778770|Original|hCoV-19^^Campania|Human|Istituto Zooprofilattico Sperimentale del Mezzogiorno|TIGEM|Grimaldi|Italy
>Spike|hCoV-19/Italy/CAM-TIGEM-452/2020|2020-06-24|EPI_ISL_778771|Original|hCoV-19^^Campania|Human|Istituto Zooprofilattico Sperimentale del Mezzogiorno|TIGEM|Grimaldi|Italy
>Spike|hCoV-19/Italy/CAM-TIGEM-454/2020|2020-06-24|EPI_ISL_778772|Original|hCoV-19^^Campania|Human|Istituto Zooprofilattico Sperimentale del Mezzogiorno|TIGEM|Grimaldi|Italy
>Spike|hCoV-19/Italy/CAM-TIGEM-458/2020|2020-07-02|EPI_ISL_778773|Original|hCoV-19^^Campania|Human|Istituto Zooprofilattico Sperimentale del Mezzogiorno|TIGEM|Grimaldi|Italy
>Spike|hCoV-19/Italy/CAM-TIGEM-462/2020|2020-07-02|EPI_ISL_778774|Original|hCoV-19^^Campania|Human|Istituto Zooprofilattico Sperimentale del Mezzogiorno|TIGEM|Grimaldi|Italy
>Spike|hCoV-19/Italy/CAM-TIGEM-466/2020|2020-07-02|EPI_ISL_778775|Original|hCoV-19^^Campania|Human|Istituto Zooprofilattico Sperimentale del Mezzogiorno|TIGEM|Grimaldi|Italy
>Spike|hCoV-19/Italy/CAM-TIGEM-467/2020|2020-07-02|EPI_ISL_778776|Original|hCoV-19^^Campania|Human|Istituto Zooprofilattico Sperimentale del Mezzogiorno|TIGEM|Grimaldi|Italy
>Spike|hCoV-19/Italy/CAM-TIGEM-471/2020|2020-07-02|EPI_ISL_778778|Original|hCoV-19^^Campania|Human|Istituto Zooprofilattico Sperimentale del Mezzogiorno|TIGEM|Grimaldi|Italy
>Spike|hCoV-19/Italy/CAM-TIGEM-425/2020|2020-06-29|EPI_ISL_778779|Original|hCoV-19^^Campania|Human|Istituto Zooprofilattico Sperimentale del Mezzogiorno|TIGEM|Grimaldi|Italy
>Spike|hCoV-19/Italy/CAM-TIGEM-473/2020|2020-07-02|EPI_ISL_778780|Original|hCoV-19^^Campania|Human|Istituto Zooprofilattico Sperimentale del Mezzogiorno|TIGEM|Grimaldi|Italy
>Spike|hCoV-19/Italy/CAM-TIGEM-474/2020|2020-07-02|EPI_ISL_778781|Original|hCoV-19^^Campania|Human|Istituto Zooprofilattico Sperimentale del Mezzogiorno|TIGEM|Grimaldi|Italy
>Spike|hCoV-19/Italy/CAM-TIGEM-478/2020|2020-07-02|EPI_ISL_778782|Original|hCoV-19^^Campania|Human|Istituto Zooprofilattico Sperimentale del Mezzogiorno|TIGEM|Grimaldi|Italy
>Spike|hCoV-19/Italy/CAM-TIGEM-479/2020|2020-06-28|EPI_ISL_778783|Original|hCoV-19^^Campania|Human|Istituto Zooprofilattico Sperimentale del Mezzogiorno|TIGEM|Grimaldi|Italy
>Spike|hCoV-19/Italy/CAM-TIGEM-482/2020|2020-06-28|EPI_ISL_778784|Original|hCoV-19^^Campania|Human|Istituto Zooprofilattico Sperimentale del Mezzogiorno|TIGEM|Grimaldi|Italy
>Spike|hCoV-19/Italy/CAM-TIGEM-483/2020|2020-06-28|EPI_ISL_778785|Original|hCoV-19^^Campania|Human|Istituto Zooprofilattico Sperimentale del Mezzogiorno|TIGEM|Grimaldi|Italy
>Spike|hCoV-19/Italy/CAM-TIGEM-426/2020|2020-06-29|EPI_ISL_778786|Original|hCoV-19^^Campania|Human|Istituto Zooprofilattico Sperimentale del Mezzogiorno|TIGEM|Grimaldi|Italy
>Spike|hCoV-19/Italy/CAM-TIGEM-484/2020|2020-06-28|EPI_ISL_778787|Original|hCoV-19^^Campania|Human|Istituto Zooprofilattico Sperimentale del Mezzogiorno|TIGEM|Grimaldi|Italy
>Spike|hCoV-19/Italy/CAM-TIGEM-485/2020|2020-06-28|EPI_ISL_778788|Original|hCoV-19^^Campania|Human|Istituto Zooprofilattico Sperimentale del Mezzogiorno|TIGEM|Grimaldi|Italy

>Spike|hCoV-19/Italy/CAM-TIGEM-486/2020|2020-06-28|EPI_ISL_778789|Original|hCoV-19^^Campania|Human|Istituto Zooprofilattico Sperimentale del Mezzogiorno|TIGEM|Grimaldi|Italy
>Spike|hCoV-19/Italy/CAM-TIGEM-487/2020|2020-06-28|EPI_ISL_778790|Original|hCoV-19^^Campania|Human|Istituto Zooprofilattico Sperimentale del Mezzogiorno|TIGEM|Grimaldi|Italy
>Spike|hCoV-19/Italy/CAM-TIGEM-488/2020|2020-06-28|EPI_ISL_778791|Original|hCoV-19^^Campania|Human|Istituto Zooprofilattico Sperimentale del Mezzogiorno|TIGEM|Grimaldi|Italy
>Spike|hCoV-19/Italy/CAM-TIGEM-489/2020|2020-06-28|EPI_ISL_778792|Original|hCoV-19^^Campania|Human|Istituto Zooprofilattico Sperimentale del Mezzogiorno|TIGEM|Grimaldi|Italy
>Spike|hCoV-19/Italy/CAM-TIGEM-490/2020|2020-06-28|EPI_ISL_778793|Original|hCoV-19^^Campania|Human|Istituto Zooprofilattico Sperimentale del Mezzogiorno|TIGEM|Grimaldi|Italy
>Spike|hCoV-19/Italy/CAM-TIGEM-494/2020|2020-06-27|EPI_ISL_778794|Original|hCoV-19^^Campania|Human|Istituto Zooprofilattico Sperimentale del Mezzogiorno|TIGEM|Grimaldi|Italy
>Spike|hCoV-19/Italy/CAM-TIGEM-427/2020|2020-06-29|EPI_ISL_778795|Original|hCoV-19^^Campania|Human|Istituto Zooprofilattico Sperimentale del Mezzogiorno|TIGEM|Grimaldi|Italy
>Spike|hCoV-19/Italy/CAM-TIGEM-495/2020|2020-06-27|EPI_ISL_778796|Original|hCoV-19^^Campania|Human|Istituto Zooprofilattico Sperimentale del Mezzogiorno|TIGEM|Grimaldi|Italy
>Spike|hCoV-19/Italy/CAM-TIGEM-497/2020|2020-06-27|EPI_ISL_778797|Original|hCoV-19^^Campania|Human|Istituto Zooprofilattico Sperimentale del Mezzogiorno|TIGEM|Grimaldi|Italy
>Spike|hCoV-19/Italy/CAM-TIGEM-498/2020|2020-06-27|EPI_ISL_778798|Original|hCoV-19^^Campania|Human|Istituto Zooprofilattico Sperimentale del Mezzogiorno|TIGEM|Grimaldi|Italy
>Spike|hCoV-19/Italy/CAM-TIGEM-499/2020|2020-06-27|EPI_ISL_778799|Original|hCoV-19^^Campania|Human|Istituto Zooprofilattico Sperimentale del Mezzogiorno|TIGEM|Grimaldi|Italy
>Spike|hCoV-19/Italy/CAM-TIGEM-502/2020|2020-06-29|EPI_ISL_778800|Original|hCoV-19^^Campania|Human|Istituto Zooprofilattico Sperimentale del Mezzogiorno|TIGEM|Grimaldi|Italy
>Spike|hCoV-19/Italy/CAM-TIGEM-503/2020|2020-06-29|EPI_ISL_778801|Original|hCoV-19^^Campania|Human|Istituto Zooprofilattico Sperimentale del Mezzogiorno|TIGEM|Grimaldi|Italy
>Spike|hCoV-19/Italy/CAM-TIGEM-428/2020|2020-06-29|EPI_ISL_778802|Original|hCoV-19^^Campania|Human|Istituto Zooprofilattico Sperimentale del Mezzogiorno|TIGEM|Grimaldi|Italy
>Spike|hCoV-19/Italy/CAM-TIGEM-504/2020|2020-06-29|EPI_ISL_778803|Original|hCoV-19^^Campania|Human|Istituto Zooprofilattico Sperimentale del Mezzogiorno|TIGEM|Grimaldi|Italy
>Spike|hCoV-19/Italy/CAM-TIGEM-506/2020|2020-06-29|EPI_ISL_778804|Original|hCoV-19^^Campania|Human|Istituto Zooprofilattico Sperimentale del Mezzogiorno|TIGEM|Grimaldi|Italy
>Spike|hCoV-19/Italy/CAM-TIGEM-507/2020|2020-06-29|EPI_ISL_778805|Original|hCoV-19^^Campania|Human|Istituto Zooprofilattico Sperimentale del Mezzogiorno|TIGEM|Grimaldi|Italy
>Spike|hCoV-19/Italy/CAM-TIGEM-513/2020|2020-06-29|EPI_ISL_778806|Original|hCoV-19^^Campania|Human|Istituto Zooprofilattico Sperimentale del Mezzogiorno|TIGEM|Grimaldi|Italy
>Spike|hCoV-19/Italy/CAM-TIGEM-429/2020|2020-06-29|EPI_ISL_778807|Original|hCoV-19^^Campania|Human|Istituto Zooprofilattico Sperimentale del Mezzogiorno|TIGEM|Grimaldi|Italy
>Spike|hCoV-19/Italy/CAM-TIGEM-514/2020|2020-06-29|EPI_ISL_778808|Original|hCoV-19^^Campania|Human|Istituto Zooprofilattico Sperimentale del Mezzogiorno|TIGEM|Grimaldi|Italy

>Spike|hCoV-19/Italy/CAM-TIGEM-515/2020|2020-06-29|EPI_ISL_778809|Original|hCoV-
19^^Campania|Human|Istituto Zooprofilattico Sperimentale del
Mezzogiorno|TIGEM|Grimaldi|Italy
>Spike|hCoV-19/Italy/CAM-TIGEM-516/2020|2020-06-29|EPI_ISL_778810|Original|hCoV-
19^^Campania|Human|Istituto Zooprofilattico Sperimentale del
Mezzogiorno|TIGEM|Grimaldi|Italy
>Spike|hCoV-19/Italy/CAM-TIGEM-517/2020|2020-06-29|EPI_ISL_778811|Original|hCoV-
19^^Campania|Human|Istituto Zooprofilattico Sperimentale del
Mezzogiorno|TIGEM|Grimaldi|Italy
>Spike|hCoV-19/Italy/CAM-TIGEM-518/2020|2020-06-29|EPI_ISL_778812|Original|hCoV-
19^^Campania|Human|Istituto Zooprofilattico Sperimentale del
Mezzogiorno|TIGEM|Grimaldi|Italy
>Spike|hCoV-19/Italy/CAM-TIGEM-519/2020|2020-06-29|EPI_ISL_778813|Original|hCoV-
19^^Campania|Human|Istituto Zooprofilattico Sperimentale del
Mezzogiorno|TIGEM|Grimaldi|Italy
>Spike|hCoV-19/Italy/CAM-TIGEM-520/2020|2020-06-29|EPI_ISL_778814|Original|hCoV-
19^^Campania|Human|Istituto Zooprofilattico Sperimentale del
Mezzogiorno|TIGEM|Grimaldi|Italy
>Spike|hCoV-19/Italy/CAM-TIGEM-521/2020|2020-06-29|EPI_ISL_778815|Original|hCoV-
19^^Campania|Human|Istituto Zooprofilattico Sperimentale del
Mezzogiorno|TIGEM|Grimaldi|Italy
>Spike|hCoV-19/Italy/CAM-TIGEM-522/2020|2020-06-29|EPI_ISL_778816|Original|hCoV-
19^^Campania|Human|Istituto Zooprofilattico Sperimentale del
Mezzogiorno|TIGEM|Grimaldi|Italy
>Spike|hCoV-19/Italy/CAM-TIGEM-523/2020|2020-06-29|EPI_ISL_778817|Original|hCoV-
19^^Campania|Human|Istituto Zooprofilattico Sperimentale del
Mezzogiorno|TIGEM|Grimaldi|Italy
>Spike|hCoV-19/Canada/ON-PHL-20-03753/2020|2020-11-00|EPI_ISL_778852|Original|hCoV-
19^^Ontario|Human|Public Health Ontario Laboratory|Public Health Ontario
Laboratory|Guthrie|Canada
>Spike|hCoV-19/USA/AL-SL-01/2020|2020-00-00|EPI_ISL_778867|Original|hCoV-
19^^Alabama|Human|Synergy Laboratories|Synergy Laboratories|Cornwell|USA
>Spike|hCoV-19/USA/MD-MDH-0498/2020|2020-12-23|EPI_ISL_778879|Original|hCoV-
19^^Maryland|Human|Maryland Public Health Laboratory|Maryland Public Health
Laboratory|Keller|USA
>Spike|hCoV-19/USA/MD-MDH-0534/2020|2020-12-01|EPI_ISL_778910|Original|hCoV-
19^^Maryland|Human|Maryland Public Health Laboratory|Maryland Public Health
Laboratory|Keller|USA
>Spike|hCoV-19/USA/WY-WPHL-20153662/2020|2020-11-30|EPI_ISL_779135|Original|hCoV-
19^^Wyoming|Human|Wyoming Public Health Laboratory|Wyoming Public Health
Laboratory|Hull|USA
>Spike|hCoV-19/Brazil/RS-00621/2020|2020-11-24|EPI_ISL_779157|Original|hCoV-19^^Rio
Grande do Sul|Human|Laborat\u00f3rio de Microbiologia Molecular - Universidade
FEEVALE|Bioinformatics Laboratory / LNCC|Almeida|Brazil
>Spike|hCoV-19/Brazil/RS-00624/2020|2020-11-24|EPI_ISL_779158|Original|hCoV-19^^Rio
Grande do Sul|Human|Laborat\u00f3rio de Microbiologia Molecular - Universidade
FEEVALE|Bioinformatics Laboratory / LNCC|Almeida|Brazil
>Spike|hCoV-19/Brazil/RS-00658/2020|2020-11-26|EPI_ISL_779162|Original|hCoV-19^^Rio
Grande do Sul|Human|Laborat\u00f3rio de Microbiologia Molecular - Universidade
FEEVALE|Bioinformatics Laboratory / LNCC|Almeida|Brazil
>Spike|hCoV-19/Brazil/RS-00671/2020|2020-11-26|EPI_ISL_779164|Original|hCoV-19^^Rio
Grande do Sul|Human|Laborat\u00f3rio de Microbiologia Molecular - Universidade
FEEVALE|Bioinformatics Laboratory / LNCC|Almeida|Brazil
>Spike|hCoV-19/Japan/IC-0538/2020|2020-10-00|EPI_ISL_779221|Original|hCoV-
19^^Japan|Human|Pathogen Genomics Center|Pathogen Genomics Center|Sekizuka|Japan
>Spike|hCoV-19/Japan/IC-0547/2020|2020-11-00|EPI_ISL_779230|Original|hCoV-
19^^Japan|Human|Pathogen Genomics Center|Pathogen Genomics Center|Sekizuka|Japan
>Spike|hCoV-19/Slovakia/UKBA-401/2020|2020-11-22|EPI_ISL_779403|Original|hCoV-
19^^Bratislava|Human|Institute of Virology|Faculty of Natural Sciences|Vinar|Slovakia

>Spike|hCoV-19/Australia/VIC17883/2020|2020-08-23|EPI_ISL_779530|Original|hCoV-19^^Victoria|Human|Microbiological Diagnostic Unit - Public Health Laboratory (MDU-PHL)|MDU-PHL|Beck|Australia
>Spike|hCoV-19/France/ARA-124983/2020|2020-12-26|EPI_ISL_779780|Original|hCoV-19^^Auvergne-Rh\u00f4ne-Alpes|Human|CNR Virus des Infections Respiratoires - France SUD|CNR Virus des Infections Respiratoires - France SUD|Bal|France
>Spike|hCoV-19/Spain/MD-IBV-99012471/2020|2020-11-14|EPI_ISL_780051|Original|hCoV-19^^Madrid|Human|Hospital General Universitario Gregorio Mara\u00f1\u00f3n|SeqCOVID-SPAIN consortium/IBV(CSIC)|Comas|Spain
>Spike|hCoV-19/USA/TX-HMH-MCoV-9662/2020|2020-07-06|EPI_ISL_780097|Original|hCoV-19^^Texas|Human|Houston Methodist Hospital|Houston Methodist Hospital|Long|USA
>Spike|hCoV-19/USA/TX-HMH-MCoV-9621/2020|2020-07-05|EPI_ISL_780120|Original|hCoV-19^^Texas|Human|Houston Methodist Hospital|Houston Methodist Hospital|Long|USA
>Spike|hCoV-19/USA/TX-HMH-MCoV-9813/2020|2020-07-06|EPI_ISL_780122|Original|hCoV-19^^Texas|Human|Houston Methodist Hospital|Houston Methodist Hospital|Long|USA
>Spike|hCoV-19/USA/TX-HMH-MCoV-9909/2020|2020-07-06|EPI_ISL_780123|Original|hCoV-19^^Texas|Human|Houston Methodist Hospital|Houston Methodist Hospital|Long|USA
>Spike|hCoV-19/USA/TX-HMH-MCoV-9717/2020|2020-07-04|EPI_ISL_780124|Original|hCoV-19^^Texas|Human|Houston Methodist Hospital|Houston Methodist Hospital|Long|USA
>Spike|hCoV-19/USA/TX-HMH-MCoV-9597/2020|2020-06-22|EPI_ISL_780125|Original|hCoV-19^^Texas|Human|Houston Methodist Hospital|Houston Methodist Hospital|Long|USA
>Spike|hCoV-19/USA/TX-HMH-MCoV-9957/2020|2020-07-06|EPI_ISL_780126|Original|hCoV-19^^Texas|Human|Houston Methodist Hospital|Houston Methodist Hospital|Long|USA
>Spike|hCoV-19/USA/TX-HMH-MCoV-9719/2020|2020-07-06|EPI_ISL_780130|Original|hCoV-19^^Texas|Human|Houston Methodist Hospital|Houston Methodist Hospital|Long|USA
>Spike|hCoV-19/USA/TX-HMH-MCoV-9933/2020|2020-07-06|EPI_ISL_780133|Original|hCoV-19^^Texas|Human|Houston Methodist Hospital|Houston Methodist Hospital|Long|USA
>Spike|hCoV-19/USA/TX-HMH-MCoV-9881/2020|2020-07-06|EPI_ISL_780134|Original|hCoV-19^^Texas|Human|Houston Methodist Hospital|Houston Methodist Hospital|Long|USA
>Spike|hCoV-19/USA/TX-HMH-MCoV-8554/2020|2020-07-01|EPI_ISL_780438|Original|hCoV-19^^Texas|Human|Houston Methodist Hospital|Houston Methodist Hospital|Long|USA
>Spike|hCoV-19/USA/TX-HMH-MCoV-8517/2020|2020-07-01|EPI_ISL_780494|Original|hCoV-19^^Texas|Human|Houston Methodist Hospital|Houston Methodist Hospital|Long|USA
>Spike|hCoV-19/USA/TX-HMH-MCoV-8661/2020|2020-07-01|EPI_ISL_780495|Original|hCoV-19^^Texas|Human|Houston Methodist Hospital|Houston Methodist Hospital|Long|USA
>Spike|hCoV-19/USA/TX-HMH-MCoV-8733/2020|2020-07-01|EPI_ISL_780496|Original|hCoV-19^^Texas|Human|Houston Methodist Hospital|Houston Methodist Hospital|Long|USA
>Spike|hCoV-19/USA/TX-HMH-MCoV-8757/2020|2020-07-01|EPI_ISL_780497|Original|hCoV-19^^Texas|Human|Houston Methodist Hospital|Houston Methodist Hospital|Long|USA
>Spike|hCoV-19/USA/TX-HMH-MCoV-8781/2020|2020-07-01|EPI_ISL_780498|Original|hCoV-19^^Texas|Human|Houston Methodist Hospital|Houston Methodist Hospital|Long|USA
>Spike|hCoV-19/USA/TX-HMH-MCoV-8805/2020|2020-07-01|EPI_ISL_780499|Original|hCoV-19^^Texas|Human|Houston Methodist Hospital|Houston Methodist Hospital|Long|USA
>Spike|hCoV-19/USA/TX-HMH-MCoV-8877/2020|2020-07-02|EPI_ISL_780500|Original|hCoV-19^^Texas|Human|Houston Methodist Hospital|Houston Methodist Hospital|Long|USA
>Spike|hCoV-19/USA/TX-HMH-MCoV-8997/2020|2020-07-02|EPI_ISL_780501|Original|hCoV-19^^Texas|Human|Houston Methodist Hospital|Houston Methodist Hospital|Long|USA
>Spike|hCoV-19/USA/TX-HMH-MCoV-9093/2020|2020-07-02|EPI_ISL_780502|Original|hCoV-19^^Texas|Human|Houston Methodist Hospital|Houston Methodist Hospital|Long|USA
>Spike|hCoV-19/USA/TX-HMH-MCoV-9237/2020|2020-07-03|EPI_ISL_780503|Original|hCoV-19^^Texas|Human|Houston Methodist Hospital|Houston Methodist Hospital|Long|USA
>Spike|hCoV-19/USA/TX-HMH-MCoV-9309/2020|2020-07-03|EPI_ISL_780504|Original|hCoV-19^^Texas|Human|Houston Methodist Hospital|Houston Methodist Hospital|Long|USA
>Spike|hCoV-19/USA/TX-HMH-MCoV-9429/2020|2020-07-03|EPI_ISL_780505|Original|hCoV-19^^Texas|Human|Houston Methodist Hospital|Houston Methodist Hospital|Long|USA
>Spike|hCoV-19/USA/TX-HMH-MCoV-8973/2020|2020-07-02|EPI_ISL_780506|Original|hCoV-19^^Texas|Human|Houston Methodist Hospital|Houston Methodist Hospital|Long|USA
>Spike|hCoV-19/USA/TX-HMH-MCoV-9069/2020|2020-07-02|EPI_ISL_780507|Original|hCoV-19^^Texas|Human|Houston Methodist Hospital|Houston Methodist Hospital|Long|USA

>Spike|hCoV-19/USA/TX-HMH-MCoV-9525/2020|2020-07-07|EPI_ISL_780508|Original|hCoV-19^^Texas|Human|Houston Methodist Hospital|Houston Methodist Hospital|Long|USA
>Spike|hCoV-19/USA/TX-HMH-MCoV-8949/2020|2020-07-02|EPI_ISL_780509|Original|hCoV-19^^Texas|Human|Houston Methodist Hospital|Houston Methodist Hospital|Long|USA
>Spike|hCoV-19/USA/TX-HMH-MCoV-9213/2020|2020-07-02|EPI_ISL_780510|Original|hCoV-19^^Texas|Human|Houston Methodist Hospital|Houston Methodist Hospital|Long|USA
>Spike|hCoV-19/USA/TX-HMH-MCoV-9333/2020|2020-07-03|EPI_ISL_780511|Original|hCoV-19^^Texas|Human|Houston Methodist Hospital|Houston Methodist Hospital|Long|USA
>Spike|hCoV-19/USA/TX-HMH-MCoV-8612/2020|2020-07-01|EPI_ISL_780513|Original|hCoV-19^^Texas|Human|Houston Methodist Hospital|Houston Methodist Hospital|Long|USA
>Spike|hCoV-19/USA/TX-HMH-MCoV-9507/2020|2020-07-03|EPI_ISL_780521|Original|hCoV-19^^Texas|Human|Houston Methodist Hospital|Houston Methodist Hospital|Long|USA
>Spike|hCoV-19/USA/TX-HMH-MCoV-9045/2020|2020-07-04|EPI_ISL_780522|Original|hCoV-19^^Texas|Human|Houston Methodist Hospital|Houston Methodist Hospital|Long|USA
>Spike|hCoV-19/USA/TX-HMH-MCoV-8541/2020|2020-07-01|EPI_ISL_780523|Original|hCoV-19^^Texas|Human|Houston Methodist Hospital|Houston Methodist Hospital|Long|USA
>Spike|hCoV-19/USA/TX-HMH-MCoV-8682/2020|2020-07-01|EPI_ISL_780524|Original|hCoV-19^^Texas|Human|Houston Methodist Hospital|Houston Methodist Hospital|Long|USA
>Spike|hCoV-19/USA/TX-HMH-MCoV-9165/2020|2020-07-02|EPI_ISL_780525|Original|hCoV-19^^Texas|Human|Houston Methodist Hospital|Houston Methodist Hospital|Long|USA
>Spike|hCoV-19/USA/TX-HMH-MCoV-9500/2020|2020-07-03|EPI_ISL_780526|Original|hCoV-19^^Texas|Human|Houston Methodist Hospital|Houston Methodist Hospital|Long|USA
>Spike|hCoV-19/USA/TX-HMH-MCoV-9494/2020|2020-07-03|EPI_ISL_780527|Original|hCoV-19^^Texas|Human|Houston Methodist Hospital|Houston Methodist Hospital|Long|USA
>Spike|hCoV-19/USA/TX-HMH-MCoV-8470/2020|2020-07-01|EPI_ISL_780528|Original|hCoV-19^^Texas|Human|Houston Methodist Hospital|Houston Methodist Hospital|Long|USA
>Spike|hCoV-19/USA/TX-HMH-MCoV-8853/2020|2020-07-02|EPI_ISL_780529|Original|hCoV-19^^Texas|Human|Houston Methodist Hospital|Houston Methodist Hospital|Long|USA
>Spike|hCoV-19/USA/TX-HMH-MCoV-8925/2020|2020-07-02|EPI_ISL_780530|Original|hCoV-19^^Texas|Human|Houston Methodist Hospital|Houston Methodist Hospital|Long|USA
>Spike|hCoV-19/USA/TX-HMH-MCoV-9117/2020|2020-07-02|EPI_ISL_780531|Original|hCoV-19^^Texas|Human|Houston Methodist Hospital|Houston Methodist Hospital|Long|USA
>Spike|hCoV-19/USA/TX-HMH-MCoV-9141/2020|2020-07-02|EPI_ISL_780540|Original|hCoV-19^^Texas|Human|Houston Methodist Hospital|Houston Methodist Hospital|Long|USA
>Spike|hCoV-19/USA/TX-HMH-MCoV-8445/2020|2020-07-01|EPI_ISL_780563|Original|hCoV-19^^Texas|Human|Houston Methodist Hospital|Houston Methodist Hospital|Long|USA
>Spike|hCoV-19/USA/TX-HMH-MCoV-8468/2020|2020-07-01|EPI_ISL_780579|Original|hCoV-19^^Texas|Human|Houston Methodist Hospital|Houston Methodist Hospital|Long|USA
>Spike|hCoV-19/USA/TX-HMH-MCoV-8564/2020|2020-07-01|EPI_ISL_780628|Original|hCoV-19^^Texas|Human|Houston Methodist Hospital|Houston Methodist Hospital|Long|USA
>Spike|hCoV-19/USA/TX-HMH-MCoV-8613/2020|2020-07-01|EPI_ISL_780651|Original|hCoV-19^^Texas|Human|Houston Methodist Hospital|Houston Methodist Hospital|Long|USA
>Spike|hCoV-19/USA/TX-HMH-MCoV-8631/2020|2020-07-01|EPI_ISL_780662|Original|hCoV-19^^Texas|Human|Houston Methodist Hospital|Houston Methodist Hospital|Long|USA
>Spike|hCoV-19/USA/TX-HMH-MCoV-8637/2020|2020-07-01|EPI_ISL_780666|Original|hCoV-19^^Texas|Human|Houston Methodist Hospital|Houston Methodist Hospital|Long|USA
>Spike|hCoV-19/USA/TX-HMH-MCoV-8685/2020|2020-07-01|EPI_ISL_780698|Original|hCoV-19^^Texas|Human|Houston Methodist Hospital|Houston Methodist Hospital|Long|USA
>Spike|hCoV-19/USA/TX-HMH-MCoV-8709/2020|2020-07-01|EPI_ISL_780712|Original|hCoV-19^^Texas|Human|Houston Methodist Hospital|Houston Methodist Hospital|Long|USA
>Spike|hCoV-19/USA/TX-HMH-MCoV-8970/2020|2020-07-02|EPI_ISL_780866|Original|hCoV-19^^Texas|Human|Houston Methodist Hospital|Houston Methodist Hospital|Long|USA
>Spike|hCoV-19/USA/TX-HMH-MCoV-9048/2020|2020-07-02|EPI_ISL_780919|Original|hCoV-19^^Texas|Human|Houston Methodist Hospital|Houston Methodist Hospital|Long|USA
>Spike|hCoV-19/USA/TX-HMH-MCoV-9280/2020|2020-07-03|EPI_ISL_781031|Original|hCoV-19^^Texas|Human|Houston Methodist Hospital|Houston Methodist Hospital|Long|USA
>Spike|hCoV-19/USA/TX-HMH-MCoV-9402/2020|2020-07-03|EPI_ISL_781100|Original|hCoV-19^^Texas|Human|Houston Methodist Hospital|Houston Methodist Hospital|Long|USA

>Spike|hCoV-19/England/QEUH-CCDCD2/2020|2020-12-24|EPI_ISL_782223|Original|hCoV-19^^England|Human|Lighthouse Lab in Glasgow|Wellcome Sanger Institute for the COVID-19 Genomics UK (COG-UK) Consortium|Davies|United Kingdom
>Spike|hCoV-19/England/QEUH-CCDA8D/2020|2020-12-24|EPI_ISL_782303|Original|hCoV-19^^England|Human|Lighthouse Lab in Glasgow|Wellcome Sanger Institute for the COVID-19 Genomics UK (COG-UK) Consortium|Davies|United Kingdom
>Spike|hCoV-19/England/ALDP-CEB9F5/2020|2020-12-21|EPI_ISL_782355|Original|hCoV-19^^England|Human|Lighthouse Lab in Alderley Park|Wellcome Sanger Institute for the COVID-19 Genomics UK (COG-UK) Consortium|Davies|United Kingdom
>Spike|hCoV-19/England/QEUH-C3326C/2020|2020-12-11|EPI_ISL_782496|Original|hCoV-19^^England|Human|Lighthouse Lab in Glasgow|Wellcome Sanger Institute for the COVID-19 Genomics UK (COG-UK) Consortium|Davies|United Kingdom
>Spike|hCoV-19/England/QEUH-C322C7/2020|2020-12-11|EPI_ISL_782611|Original|hCoV-19^^England|Human|Lighthouse Lab in Glasgow|Wellcome Sanger Institute for the COVID-19 Genomics UK (COG-UK) Consortium|Davies|United Kingdom
>Spike|hCoV-19/England/QEUH-C32494/2020|2020-12-11|EPI_ISL_782648|Original|hCoV-19^^England|Human|Lighthouse Lab in Glasgow|Wellcome Sanger Institute for the COVID-19 Genomics UK (COG-UK) Consortium|Davies|United Kingdom
>Spike|hCoV-19/England/ALDP-C23681/2020|2020-12-05|EPI_ISL_782892|Original|hCoV-19^^England|Human|Lighthouse Lab in Alderley Park|Wellcome Sanger Institute for the COVID-19 Genomics UK (COG-UK) Consortium|Davies|United Kingdom
>Spike|hCoV-19/England/CAMC-B77A38/2020|2020-11-09|EPI_ISL_783429|Original|hCoV-19^^England|Human|Lighthouse Lab in Cambridge|Wellcome Sanger Institute for the COVID-19 Genomics UK (COG-UK) Consortium|Davies|United Kingdom
>Spike|hCoV-19/USA/TX-HMH-MCoV-8085/2020|2020-06-30|EPI_ISL_783574|Original|hCoV-19^^Texas|Human|Houston Methodist Hospital|Houston Methodist Hospital|Long|USA
>Spike|hCoV-19/USA/TX-HMH-MCoV-8109/2020|2020-06-30|EPI_ISL_783575|Original|hCoV-19^^Texas|Human|Houston Methodist Hospital|Houston Methodist Hospital|Long|USA
>Spike|hCoV-19/USA/TX-HMH-MCoV-8373/2020|2020-06-29|EPI_ISL_783576|Original|hCoV-19^^Texas|Human|Houston Methodist Hospital|Houston Methodist Hospital|Long|USA
>Spike|hCoV-19/USA/TX-HMH-MCoV-8349/2020|2020-07-07|EPI_ISL_783578|Original|hCoV-19^^Texas|Human|Houston Methodist Hospital|Houston Methodist Hospital|Long|USA
>Spike|hCoV-19/USA/TX-HMH-MCoV-8404/2020|2020-06-22|EPI_ISL_783579|Original|hCoV-19^^Texas|Human|Houston Methodist Hospital|Houston Methodist Hospital|Long|USA
>Spike|hCoV-19/USA/TX-HMH-MCoV-19737/2020|2020-11-25|EPI_ISL_783580|Original|hCoV-19^^Texas|Human|Houston Methodist Hospital|Houston Methodist Hospital|Long|USA
>Spike|hCoV-19/USA/TX-HMH-MCoV-19206/2020|2020-11-24|EPI_ISL_783691|Original|hCoV-19^^Texas|Human|Houston Methodist Hospital|Houston Methodist Hospital|Long|USA
>Spike|hCoV-19/USA/TX-HMH-MCoV-19341/2020|2020-11-28|EPI_ISL_783774|Original|hCoV-19^^Texas|Human|Houston Methodist Hospital|Houston Methodist Hospital|Long|USA
>Spike|hCoV-19/USA/TX-HMH-MCoV-19391/2020|2020-11-30|EPI_ISL_783812|Original|hCoV-19^^Texas|Human|Houston Methodist Hospital|Houston Methodist Hospital|Long|USA
>Spike|hCoV-19/USA/TX-HMH-MCoV-19571/2020|2020-11-17|EPI_ISL_783943|Original|hCoV-19^^Texas|Human|Houston Methodist Hospital|Houston Methodist Hospital|Long|USA
>Spike|hCoV-19/USA/TX-HMH-MCoV-19572/2020|2020-11-17|EPI_ISL_783944|Original|hCoV-19^^Texas|Human|Houston Methodist Hospital|Houston Methodist Hospital|Long|USA
>Spike|hCoV-19/USA/TX-HMH-MCoV-19624/2020|2020-11-28|EPI_ISL_783990|Original|hCoV-19^^Texas|Human|Houston Methodist Hospital|Houston Methodist Hospital|Long|USA
>Spike|hCoV-19/USA/TX-HMH-MCoV-19738/2020|2020-11-20|EPI_ISL_784083|Original|hCoV-19^^Texas|Human|Houston Methodist Hospital|Houston Methodist Hospital|Long|USA
>Spike|hCoV-19/USA/TX-HMH-MCoV-19767/2020|2020-11-24|EPI_ISL_784109|Original|hCoV-19^^Texas|Human|Houston Methodist Hospital|Houston Methodist Hospital|Long|USA
>Spike|hCoV-19/USA/TX-HMH-MCoV-19814/2020|2020-11-23|EPI_ISL_784145|Original|hCoV-19^^Texas|Human|Houston Methodist Hospital|Houston Methodist Hospital|Long|USA
>Spike|hCoV-19/USA/TX-HMH-MCoV-19860/2020|2020-11-22|EPI_ISL_784181|Original|hCoV-19^^Texas|Human|Houston Methodist Hospital|Houston Methodist Hospital|Long|USA
>Spike|hCoV-19/USA/TX-HMH-MCoV-8133/2020|2020-06-30|EPI_ISL_784223|Original|hCoV-19^^Texas|Human|Houston Methodist Hospital|Houston Methodist Hospital|Long|USA
>Spike|hCoV-19/USA/TX-HMH-MCoV-8181/2020|2020-06-30|EPI_ISL_784250|Original|hCoV-19^^Texas|Human|Houston Methodist Hospital|Houston Methodist Hospital|Long|USA

>Spike|hCoV-19/USA/TX-HMH-MCoV-8253/2020|2020-07-07|EPI_ISL_784293|Original|hCoV-19^^Texas|Human|Houston Methodist Hospital|Houston Methodist Hospital|Long|USA
>Spike|hCoV-19/USA/TX-HMH-MCoV-18148/2020|2020-11-15|EPI_ISL_784496|Original|hCoV-19^^Texas|Human|Houston Methodist Hospital|Houston Methodist Hospital|Long|USA
>Spike|hCoV-19/USA/TX-HMH-MCoV-18416/2020|2020-11-18|EPI_ISL_784764|Original|hCoV-19^^Texas|Human|Houston Methodist Hospital|Houston Methodist Hospital|Long|USA
>Spike|hCoV-19/USA/TX-HMH-MCoV-18706/2020|2020-11-20|EPI_ISL_785054|Original|hCoV-19^^Texas|Human|Houston Methodist Hospital|Houston Methodist Hospital|Long|USA
>Spike|hCoV-19/USA/TX-HMH-MCoV-18758/2020|2020-11-23|EPI_ISL_785095|Original|hCoV-19^^Texas|Human|Houston Methodist Hospital|Houston Methodist Hospital|Long|USA
>Spike|hCoV-19/USA/TX-HMH-MCoV-18794/2020|2020-11-24|EPI_ISL_785121|Original|hCoV-19^^Texas|Human|Houston Methodist Hospital|Houston Methodist Hospital|Long|USA
>Spike|hCoV-19/USA/TX-HMH-MCoV-18820/2020|2020-11-23|EPI_ISL_785144|Original|hCoV-19^^Texas|Human|Houston Methodist Hospital|Houston Methodist Hospital|Long|USA
>Spike|hCoV-19/USA/TX-HMH-MCoV-18863/2020|2020-11-25|EPI_ISL_785179|Original|hCoV-19^^Texas|Human|Houston Methodist Hospital|Houston Methodist Hospital|Long|USA
>Spike|hCoV-19/USA/TX-HMH-MCoV-18922/2020|2020-11-25|EPI_ISL_785223|Original|hCoV-19^^Texas|Human|Houston Methodist Hospital|Houston Methodist Hospital|Long|USA
>Spike|hCoV-19/USA/TX-HMH-MCoV-18964/2020|2020-11-25|EPI_ISL_785250|Original|hCoV-19^^Texas|Human|Houston Methodist Hospital|Houston Methodist Hospital|Long|USA
>Spike|hCoV-19/USA/TX-HMH-MCoV-15806/2020|2020-07-10|EPI_ISL_785490|Original|hCoV-19^^Texas|Human|Houston Methodist Hospital|Houston Methodist Hospital|Long|USA
>Spike|hCoV-19/USA/TX-HMH-MCoV-15187/2020|2020-10-20|EPI_ISL_786671|Original|hCoV-19^^Texas|Human|Houston Methodist Hospital|Houston Methodist Hospital|Long|USA
>Spike|hCoV-19/USA/TX-HMH-MCoV-15252/2020|2020-10-19|EPI_ISL_786736|Original|hCoV-19^^Texas|Human|Houston Methodist Hospital|Houston Methodist Hospital|Long|USA
>Spike|hCoV-19/USA/TX-HMH-MCoV-15322/2020|2020-10-21|EPI_ISL_786806|Original|hCoV-19^^Texas|Human|Houston Methodist Hospital|Houston Methodist Hospital|Long|USA
>Spike|hCoV-19/USA/TX-HMH-MCoV-15472/2020|2020-10-23|EPI_ISL_786956|Original|hCoV-19^^Texas|Human|Houston Methodist Hospital|Houston Methodist Hospital|Long|USA
>Spike|hCoV-19/USA/TX-HMH-MCoV-15480/2020|2020-10-23|EPI_ISL_786964|Original|hCoV-19^^Texas|Human|Houston Methodist Hospital|Houston Methodist Hospital|Long|USA
>Spike|hCoV-19/USA/TX-HMH-MCoV-15564/2020|2020-10-26|EPI_ISL_787048|Original|hCoV-19^^Texas|Human|Houston Methodist Hospital|Houston Methodist Hospital|Long|USA
>Spike|hCoV-19/USA/TX-HMH-MCoV-14212/2020|2020-10-03|EPI_ISL_787497|Original|hCoV-19^^Texas|Human|Houston Methodist Hospital|Houston Methodist Hospital|Long|USA
>Spike|hCoV-19/USA/TX-HMH-MCoV-14371/2020|2020-10-05|EPI_ISL_787656|Original|hCoV-19^^Texas|Human|Houston Methodist Hospital|Houston Methodist Hospital|Long|USA
>Spike|hCoV-19/USA/TX-HMH-MCoV-14422/2020|2020-10-07|EPI_ISL_787707|Original|hCoV-19^^Texas|Human|Houston Methodist Hospital|Houston Methodist Hospital|Long|USA
>Spike|hCoV-19/USA/TX-HMH-MCoV-14616/2020|2020-10-11|EPI_ISL_787901|Original|hCoV-19^^Texas|Human|Houston Methodist Hospital|Houston Methodist Hospital|Long|USA
>Spike|hCoV-19/USA/TX-HMH-MCoV-13316/2020|2020-08-25|EPI_ISL_788464|Original|hCoV-19^^Texas|Human|Houston Methodist Hospital|Houston Methodist Hospital|Long|USA
>Spike|hCoV-19/USA/TX-HMH-MCoV-13570/2020|2020-08-21|EPI_ISL_788681|Original|hCoV-19^^Texas|Human|Houston Methodist Hospital|Houston Methodist Hospital|Long|USA
>Spike|hCoV-19/USA/WI-UW-2536/2020|2020-12-30|EPI_ISL_788940|Original|hCoV-19^^Wisconsin|Human|University of Wisconsin-Madison AIDS Vaccine Research Laboratories|University of Wisconsin-Madison AIDS Vaccine Research Laboratories|Moreno|USA
>Spike|hCoV-19/Slovakia/UKBA-610/2020|2020-11-30|EPI_ISL_788987|Original|hCoV-19^^Bratislava|Human|Institute of Virology|Faculty of Natural Sciences|Vinar|Slovakia
>Spike|hCoV-19/USA/TX-HMH-MCoV-11194/2020|2020-09-02|EPI_ISL_789159|Original|hCoV-19^^Texas|Human|Houston Methodist Hospital|Houston Methodist Hospital|Long|USA
>Spike|hCoV-19/USA/TX-HMH-MCoV-10941/2020|2020-07-09|EPI_ISL_789173|Original|hCoV-19^^Texas|Human|Houston Methodist Hospital|Houston Methodist Hospital|Long|USA
>Spike|hCoV-19/USA/TX-HMH-MCoV-10581/2020|2020-07-08|EPI_ISL_789194|Original|hCoV-19^^Texas|Human|Houston Methodist Hospital|Houston Methodist Hospital|Long|USA
>Spike|hCoV-19/USA/TX-HMH-MCoV-10605/2020|2020-07-08|EPI_ISL_789198|Original|hCoV-19^^Texas|Human|Houston Methodist Hospital|Houston Methodist Hospital|Long|USA

>Spike|hCoV-19/USA/TX-HMH-MCoV-10461/2020|2020-07-08|EPI_ISL_789205|Original|hCoV-19^^Texas|Human|Houston Methodist Hospital|Houston Methodist Hospital|Long|USA
>Spike|hCoV-19/USA/TX-HMH-MCoV-10749/2020|2020-07-09|EPI_ISL_789206|Original|hCoV-19^^Texas|Human|Houston Methodist Hospital|Houston Methodist Hospital|Long|USA
>Spike|hCoV-19/USA/TX-HMH-MCoV-10482/2020|2020-07-08|EPI_ISL_789207|Original|hCoV-19^^Texas|Human|Houston Methodist Hospital|Houston Methodist Hospital|Long|USA
>Spike|hCoV-19/USA/TX-HMH-MCoV-10029/2020|2020-07-06|EPI_ISL_789212|Original|hCoV-19^^Texas|Human|Houston Methodist Hospital|Houston Methodist Hospital|Long|USA
>Spike|hCoV-19/USA/TX-HMH-MCoV-10293/2020|2020-07-08|EPI_ISL_789213|Original|hCoV-19^^Texas|Human|Houston Methodist Hospital|Houston Methodist Hospital|Long|USA
>Spike|hCoV-19/USA/TX-HMH-MCoV-10533/2020|2020-07-08|EPI_ISL_789214|Original|hCoV-19^^Texas|Human|Houston Methodist Hospital|Houston Methodist Hospital|Long|USA
>Spike|hCoV-19/USA/TX-HMH-MCoV-10821/2020|2020-07-05|EPI_ISL_789215|Original|hCoV-19^^Texas|Human|Houston Methodist Hospital|Houston Methodist Hospital|Long|USA
>Spike|hCoV-19/USA/TX-HMH-MCoV-10845/2020|2020-07-05|EPI_ISL_789216|Original|hCoV-19^^Texas|Human|Houston Methodist Hospital|Houston Methodist Hospital|Long|USA
>Spike|hCoV-19/USA/TX-HMH-MCoV-11061/2020|2020-09-01|EPI_ISL_789217|Original|hCoV-19^^Texas|Human|Houston Methodist Hospital|Houston Methodist Hospital|Long|USA
>Spike|hCoV-19/USA/TX-HMH-MCoV-11229/2020|2020-09-02|EPI_ISL_789218|Original|hCoV-19^^Texas|Human|Houston Methodist Hospital|Houston Methodist Hospital|Long|USA
>Spike|hCoV-19/USA/TX-HMH-MCoV-10653/2020|2020-07-08|EPI_ISL_789219|Original|hCoV-19^^Texas|Human|Houston Methodist Hospital|Houston Methodist Hospital|Long|USA
>Spike|hCoV-19/USA/TX-HMH-MCoV-10797/2020|2020-07-05|EPI_ISL_789220|Original|hCoV-19^^Texas|Human|Houston Methodist Hospital|Houston Methodist Hospital|Long|USA
>Spike|hCoV-19/USA/TX-HMH-MCoV-11349/2020|2020-09-07|EPI_ISL_789221|Original|hCoV-19^^Texas|Human|Houston Methodist Hospital|Houston Methodist Hospital|Long|USA
>Spike|hCoV-19/USA/TX-HMH-MCoV-10005/2020|2020-07-06|EPI_ISL_789222|Original|hCoV-19^^Texas|Human|Houston Methodist Hospital|Houston Methodist Hospital|Long|USA
>Spike|hCoV-19/USA/TX-HMH-MCoV-10101/2020|2020-07-06|EPI_ISL_789223|Original|hCoV-19^^Texas|Human|Houston Methodist Hospital|Houston Methodist Hospital|Long|USA
>Spike|hCoV-19/USA/TX-HMH-MCoV-10078/2020|2020-07-08|EPI_ISL_789225|Original|hCoV-19^^Texas|Human|Houston Methodist Hospital|Houston Methodist Hospital|Long|USA
>Spike|hCoV-19/USA/TX-HMH-MCoV-10488/2020|2020-07-09|EPI_ISL_789228|Original|hCoV-19^^Texas|Human|Houston Methodist Hospital|Houston Methodist Hospital|Long|USA
>Spike|hCoV-19/USA/TX-HMH-MCoV-10017/2020|2020-07-06|EPI_ISL_789234|Original|hCoV-19^^Texas|Human|Houston Methodist Hospital|Houston Methodist Hospital|Long|USA
>Spike|hCoV-19/USA/TX-HMH-MCoV-10158/2020|2020-07-06|EPI_ISL_789235|Original|hCoV-19^^Texas|Human|Houston Methodist Hospital|Houston Methodist Hospital|Long|USA
>Spike|hCoV-19/USA/TX-HMH-MCoV-10413/2020|2020-07-08|EPI_ISL_789236|Original|hCoV-19^^Texas|Human|Houston Methodist Hospital|Houston Methodist Hospital|Long|USA
>Spike|hCoV-19/USA/TX-HMH-MCoV-10592/2020|2020-07-08|EPI_ISL_789237|Original|hCoV-19^^Texas|Human|Houston Methodist Hospital|Houston Methodist Hospital|Long|USA
>Spike|hCoV-19/USA/TX-HMH-MCoV-10090/2020|2020-07-06|EPI_ISL_789238|Original|hCoV-19^^Texas|Human|Houston Methodist Hospital|Houston Methodist Hospital|Long|USA
>Spike|hCoV-19/USA/TX-HMH-MCoV-10173/2020|2020-07-06|EPI_ISL_789239|Original|hCoV-19^^Texas|Human|Houston Methodist Hospital|Houston Methodist Hospital|Long|USA
>Spike|hCoV-19/USA/TX-HMH-MCoV-10358/2020|2020-07-08|EPI_ISL_789240|Original|hCoV-19^^Texas|Human|Houston Methodist Hospital|Houston Methodist Hospital|Long|USA
>Spike|hCoV-19/USA/TX-HMH-MCoV-10197/2020|2020-07-01|EPI_ISL_789367|Original|hCoV-19^^Texas|Human|Houston Methodist Hospital|Houston Methodist Hospital|Long|USA
>Spike|hCoV-19/USA/TX-HMH-MCoV-10434/2020|2020-07-08|EPI_ISL_789474|Original|hCoV-19^^Texas|Human|Houston Methodist Hospital|Houston Methodist Hospital|Long|USA
>Spike|hCoV-19/USA/TX-HMH-MCoV-11157/2020|2020-09-02|EPI_ISL_789718|Original|hCoV-19^^Texas|Human|Houston Methodist Hospital|Houston Methodist Hospital|Long|USA
>Spike|hCoV-19/USA/TX-HMH-MCoV-11445/2020|2020-09-09|EPI_ISL_789933|Original|hCoV-19^^Texas|Human|Houston Methodist Hospital|Houston Methodist Hospital|Long|USA
>Spike|hCoV-19/USA/TX-HMH-MCoV-11565/2020|2020-09-11|EPI_ISL_789947|Original|hCoV-19^^Texas|Human|Houston Methodist Hospital|Houston Methodist Hospital|Long|USA
>Spike|hCoV-19/USA/TX-HMH-MCoV-11853/2020|2020-09-16|EPI_ISL_789948|Original|hCoV-19^^Texas|Human|Houston Methodist Hospital|Houston Methodist Hospital|Long|USA

>Spike|hCoV-19/USA/TX-HMH-MCoV-12021/2020|2020-09-19|EPI_ISL_789949|Original|hCoV-19^^Texas|Human|Houston Methodist Hospital|Houston Methodist Hospital|Long|USA
>Spike|hCoV-19/USA/TX-HMH-MCoV-12069/2020|2020-09-20|EPI_ISL_789950|Original|hCoV-19^^Texas|Human|Houston Methodist Hospital|Houston Methodist Hospital|Long|USA
>Spike|hCoV-19/USA/TX-HMH-MCoV-12093/2020|2020-09-20|EPI_ISL_789951|Original|hCoV-19^^Texas|Human|Houston Methodist Hospital|Houston Methodist Hospital|Long|USA
>Spike|hCoV-19/USA/TX-HMH-MCoV-12117/2020|2020-09-21|EPI_ISL_789952|Original|hCoV-19^^Texas|Human|Houston Methodist Hospital|Houston Methodist Hospital|Long|USA
>Spike|hCoV-19/USA/TX-HMH-MCoV-12165/2020|2020-09-21|EPI_ISL_789953|Original|hCoV-19^^Texas|Human|Houston Methodist Hospital|Houston Methodist Hospital|Long|USA
>Spike|hCoV-19/USA/TX-HMH-MCoV-12309/2020|2020-09-25|EPI_ISL_789954|Original|hCoV-19^^Texas|Human|Houston Methodist Hospital|Houston Methodist Hospital|Long|USA
>Spike|hCoV-19/USA/TX-HMH-MCoV-12189/2020|2020-09-21|EPI_ISL_789955|Original|hCoV-19^^Texas|Human|Houston Methodist Hospital|Houston Methodist Hospital|Long|USA
>Spike|hCoV-19/USA/TX-HMH-MCoV-11792/2020|2020-09-09|EPI_ISL_789964|Original|hCoV-19^^Texas|Human|Houston Methodist Hospital|Houston Methodist Hospital|Long|USA
>Spike|hCoV-19/USA/TX-HMH-MCoV-12381/2020|2020-09-25|EPI_ISL_789965|Original|hCoV-19^^Texas|Human|Houston Methodist Hospital|Houston Methodist Hospital|Long|USA
>Spike|hCoV-19/USA/TX-HMH-MCoV-12464/2020|2020-09-28|EPI_ISL_789966|Original|hCoV-19^^Texas|Human|Houston Methodist Hospital|Houston Methodist Hospital|Long|USA
>Spike|hCoV-19/USA/TX-HMH-MCoV-11709/2020|2020-09-10|EPI_ISL_789967|Original|hCoV-19^^Texas|Human|Houston Methodist Hospital|Houston Methodist Hospital|Long|USA
>Spike|hCoV-19/USA/TX-HMH-MCoV-11973/2020|2020-09-18|EPI_ISL_789968|Original|hCoV-19^^Texas|Human|Houston Methodist Hospital|Houston Methodist Hospital|Long|USA
>Spike|hCoV-19/USA/TX-HMH-MCoV-12333/2020|2020-09-25|EPI_ISL_789969|Original|hCoV-19^^Texas|Human|Houston Methodist Hospital|Houston Methodist Hospital|Long|USA
>Spike|hCoV-19/USA/TX-HMH-MCoV-12376/2020|2020-09-25|EPI_ISL_789970|Original|hCoV-19^^Texas|Human|Houston Methodist Hospital|Houston Methodist Hospital|Long|USA
>Spike|hCoV-19/USA/TX-HMH-MCoV-11997/2020|2020-09-18|EPI_ISL_789982|Original|hCoV-19^^Texas|Human|Houston Methodist Hospital|Houston Methodist Hospital|Long|USA
>Spike|hCoV-19/USA/TX-HMH-MCoV-12555/2020|2020-09-30|EPI_ISL_790388|Original|hCoV-19^^Texas|Human|Houston Methodist Hospital|Houston Methodist Hospital|Long|USA
>Spike|hCoV-19/USA/TX-HMH-MCoV-12613/2020|2020-08-31|EPI_ISL_790420|Original|hCoV-19^^Texas|Human|Houston Methodist Hospital|Houston Methodist Hospital|Long|USA
>Spike|hCoV-19/USA/TX-HMH-MCoV-12619/2020|2020-08-31|EPI_ISL_790423|Original|hCoV-19^^Texas|Human|Houston Methodist Hospital|Houston Methodist Hospital|Long|USA
>Spike|hCoV-19/Canada/ON-SK-20H-328MM0909/2020|2020-11-24|EPI_ISL_791497|Original|hCoV-19^^Ontario|Human|The Hosptial for Sick Children|The Hospital for Sick Children|Lau|Canada
>Spike|hCoV-19/USA/MA-MASPHL-00037/2020|2020-04-02|EPI_ISL_791541|Original|hCoV-19^^Massachusetts|Human|Massachusetts State Public Health Laboratory|Infectious Disease Program|Park|USA
>Spike|hCoV-19/USA/MA-MASPHL-00166/2020|2020-04-03|EPI_ISL_791631|Original|hCoV-19^^Massachusetts|Human|Massachusetts State Public Health Laboratory|Infectious Disease Program|Park|USA
>Spike|hCoV-19/USA/MA-MASPHL-00200/2020|2020-04-07|EPI_ISL_791655|Original|hCoV-19^^Massachusetts|Human|Massachusetts State Public Health Laboratory|Infectious Disease Program|Park|USA
>Spike|hCoV-19/USA/MA-MASPHL-00206/2020|2020-04-09|EPI_ISL_791659|Original|hCoV-19^^Massachusetts|Human|Massachusetts State Public Health Laboratory|Infectious Disease Program|Park|USA
>Spike|hCoV-19/USA/MA-MASPHL-00229/2020|2020-04-15|EPI_ISL_791675|Original|hCoV-19^^Massachusetts|Human|Massachusetts State Public Health Laboratory|Infectious Disease Program|Park|USA
>Spike|hCoV-19/USA/MA-MGH-00753/2020|2020-04-10|EPI_ISL_791747|Original|hCoV-19^^Massachusetts|Human|Massachusetts General Hospital|Infectious Disease Program|Park|USA
>Spike|hCoV-19/USA/MA-MGH-01033/2020|2020-04-30|EPI_ISL_791852|Original|hCoV-19^^Massachusetts|Human|Massachusetts General Hospital|Infectious Disease Program|Park|USA

>Spike|hCoV-19/USA/MA-MGH-01103/2020|2020-05-01|EPI_ISL_791874|Original|hCoV-19^^Massachusetts|Human|Massachusetts General Hospital|Infectious Disease Program|Park|USA
>Spike|hCoV-19/USA/MA-MGH-01109/2020|2020-05-01|EPI_ISL_791877|Original|hCoV-19^^Massachusetts|Human|Massachusetts General Hospital|Infectious Disease Program|Park|USA
>Spike|hCoV-19/USA/MA-MGH-01381/2020|2020-05-06|EPI_ISL_791939|Original|hCoV-19^^Massachusetts|Human|Massachusetts General Hospital|Infectious Disease Program|Park|USA
>Spike|hCoV-19/England/BRIS-122DE2/2020|2020-04-03|EPI_ISL_440749|Original|hCoV-19^^England|Human|PHE South West Regional Laboratory|Wellcome Sanger Institute for the COVID-19 Genomics UK (COG-UK) consortium|Amato|United Kingdom
>Spike|hCoV-19/England/MILK-A7A30E/2020|2020-10-15|EPI_ISL_606671|Original|hCoV-19^^England|Human|Lighthouse Lab in Milton Keynes|Wellcome Sanger Institute for the COVID-19 Genomics UK (COG-UK) consortium|Davies|United Kingdom
>Spike|hCoV-19/USA/MI-UM-2006S001053/2020|2020-04-02|EPI_ISL_484798|Original|hCoV-19^^Michigan|Human|University of Michigan Clinical Microbiology Laboratory|Lauring Lab|Valesano|USA
>Spike|hCoV-19/England/ALDP-A66CA0/2020|2020-10-12|EPI_ISL_630407|Original|hCoV-19^^England|Human|Lighthouse Lab in Alderley Park|Wellcome Sanger Institute for the COVID-19 Genomics UK (COG-UK) consortium|Davies|United Kingdom
>Spike|hCoV-19/USA/TX-HMH-3791/2020|2020-06-12|EPI_ISL_543196|Original|hCoV-19^^Texas|Human|Houston Methodist Hospital|Houston Methodist Hospital|Long|USA
>Spike|hCoV-19/England/QEUH-A8BC78/2020|2020-10-17|EPI_ISL_643079|Original|hCoV-19^^England|Human|Lighthouse Lab in Glasgow|Wellcome Sanger Institute for the COVID-19 Genomics UK (COG-UK) Consortium|Davies|United Kingdom
>Spike|hCoV-19/Finland/9A785S11A/2020|2020-04-09|EPI_ISL_481735|Original|hCoV-19^^Uusimaa|Human|Department of Virology and Immunology|Department of Virology|Smura|Finland
>Spike|hCoV-19/India/MH-NIV-7558/2020|2020-04-05|EPI_ISL_454549|Original|hCoV-19^^Maharashtra|Human|NIV Influenza|NIV Influenza|Potdar|India
>Spike|hCoV-19/Switzerland/SO-ETHZ-400048/2020|2020-12-04|EPI_ISL_729107|Original|hCoV-19^^Solothurn|Human|Viollier AG|Department of Biosystems Science and Engineering|Nadeau|Switzerland
>Spike|hCoV-19/England/ALDP-6E825D/2020|2020-06-30|EPI_ISL_557693|Original|hCoV-19^^England|Human|Lighthouse Lab in Alderley Park|Wellcome Sanger Institute for the COVID-19 Genomics UK (COG-UK) consortium|Davies|United Kingdom
>Spike|hCoV-19/Japan/PG-1202/2020|2020-04-00|EPI_ISL_684812|Original|hCoV-19^^Japan|Human|Yamagata Prefectural Institute of Public Health|Pathogen Genomics Center|Sekizuka|Japan
>Spike|hCoV-19/England/CAMC-B232F7/2020|2020-11-02|EPI_ISL_647388|Original|hCoV-19^^England|Human|Lighthouse Lab in Cambridge|Wellcome Sanger Institute for the COVID-19 Genomics UK (COG-UK) Consortium|Davies|United Kingdom
>Spike|hCoV-19/England/MILK-B3D6FE/2020|2020-11-02|EPI_ISL_645264|Original|hCoV-19^^England|Human|Lighthouse Lab in Milton Keynes|Wellcome Sanger Institute for the COVID-19 Genomics UK (COG-UK) Consortium|Davies|United Kingdom
>Spike|hCoV-19/England/ALDP-B1FA14/2020|2020-11-02|EPI_ISL_646069|Original|hCoV-19^^England|Human|Lighthouse Lab in Alderley Park|Wellcome Sanger Institute for the COVID-19 Genomics UK (COG-UK) Consortium|Davies|United Kingdom
>Spike|hCoV-19/England/20188160304/2020|2020-04-21|EPI_ISL_465523|Original|hCoV-19^^England|Human|Respiratory Virus Unit|Respiratory Virus Unit|Platt|United Kingdom
>Spike|hCoV-19/Japan/PG-3898/2020|2020-07-00|EPI_ISL_692149|Original|hCoV-19^^Japan|Human|Pathogen Genomics Center|Pathogen Genomics Center|Sekizuka|Japan
>Spike|hCoV-19/Wales/PHWC-488716/2020|2020-11-02|EPI_ISL_639424|Original|hCoV-19^^Wales|Human|Wales Specialist Virology Centre Sequencing lab|COVID-19 Genomics UK (COG-UK) Consortium|Moore|United Kingdom
>Spike|hCoV-19/England/QEUH-B2DB6B/2020|2020-11-03|EPI_ISL_646983|Original|hCoV-19^^England|Human|Lighthouse Lab in Glasgow|Wellcome Sanger Institute for the COVID-19 Genomics UK (COG-UK) Consortium|Davies|United Kingdom

>Spike|hCoV-19/England/QEUH-5474EE/2020|2020-05-14|EPI_ISL_533405|Original|hCoV-19^^England|Human|Lighthouse Lab in Glasgow|Wellcome Sanger Institute for the COVID-19 Genomics UK (COG-UK) consortium|Davies|United Kingdom
>Spike|hCoV-19/England/ALDP-A6EA26/2020|2020-10-15|EPI_ISL_607670|Original|hCoV-19^^England|Human|Lighthouse Lab in Alderley Park|Wellcome Sanger Institute for the COVID-19 Genomics UK (COG-UK) consortium|Davies|United Kingdom
>Spike|hCoV-19/USA/TX-HMH-MCoV-15992/2020|2020-07-10|EPI_ISL_785676|Original|hCoV-19^^Texas|Human|Houston Methodist Hospital|Houston Methodist Hospital|Long|USA
>Spike|hCoV-19/Australia/ACT0040/2020|2020-03-25|EPI_ISL_498507|Original|hCoV-19^^Australian Capital Territory|Human|ACT Pathology|Schwessinger Lab|Hall|Australia
>Spike|hCoV-19/Wales/ALDP-CC089F/2020|2020-12-18|EPI_ISL_760284|Original|hCoV-19^^Wales|Human|Lighthouse Lab in Alderley Park|Wellcome Sanger Institute for the COVID-19 Genomics UK (COG-UK) Consortium|Davies|United Kingdom
>Spike|hCoV-19/England/QEUH-7A7A24/2020|2020-07-13|EPI_ISL_533100|Original|hCoV-19^^England|Human|Lighthouse Lab in Glasgow|Wellcome Sanger Institute for the COVID-19 Genomics UK (COG-UK) consortium|Davies|United Kingdom
>Spike|hCoV-19/USA/TX-HMH-MCoV-10825/2020|2020-07-05|EPI_ISL_789645|Original|hCoV-19^^Texas|Human|Houston Methodist Hospital|Houston Methodist Hospital|Long|USA
>Spike|hCoV-19/England/QEUH-9F5199/2020|2020-09-25|EPI_ISL_588379|Original|hCoV-19^^England|Human|Lighthouse Lab in Glasgow|Wellcome Sanger Institute for the COVID-19 Genomics UK (COG-UK) consortium|Davies|United Kingdom
>Spike|hCoV-19/USA/TX-HMH-MCoV-19503/2020|2020-11-30|EPI_ISL_783884|Original|hCoV-19^^Texas|Human|Houston Methodist Hospital|Houston Methodist Hospital|Long|USA
>Spike|hCoV-19/Australia/VIC13293/2020|2020-07-29|EPI_ISL_563654|Original|hCoV-19^^Victoria|Human|Microbiological Diagnostic Unit - Public Health Laboratory (MDU-PHL)|MDU-PHL|Schultz|Australia
>Spike|hCoV-19/Denmark/DCGC-12177/2020|2020-11-23|EPI_ISL_682358|Original|hCoV-19^^Midtjylland|Human|Department of Virus and Microbiological Special Diagnostics|Albertsen Lab|Michaelsen|Denmark
>Spike|hCoV-19/India/GJ-GBRC72b/2020|2020-04-29|EPI_ISL_447050|Original|hCoV-19^^Gujarat|Human|GMERS Medical College and Hospital|Gujarat Biotechnology Research Centre|Joshi|India
>Spike|hCoV-19/England/CAMC-BBC3F0/2020|2020-11-21|EPI_ISL_724135|Original|hCoV-19^^England|Human|Quadram Institute Bioscience|COVID-19 Genomics UK (COG-UK) Consortium|Alikhan|United Kingdom
>Spike|hCoV-19/USA/MI-MDHHS-SC20041/2020|2020-03-19|EPI_ISL_437128|Original|hCoV-19^^Michigan|Human|Michigan Department of Health and Human Services|Michigan Department of Health and Human Services|Blankenship|USA
>Spike|hCoV-19/England/ALDP-AC12ED/2020|2020-10-10|EPI_ISL_631050|Original|hCoV-19^^England|Human|Lighthouse Lab in Alderley Park|Wellcome Sanger Institute for the COVID-19 Genomics UK (COG-UK) consortium|Davies|United Kingdom
>Spike|hCoV-19/USA/TX-HMH-4134/2020|2020-06-18|EPI_ISL_543398|Original|hCoV-19^^Texas|Human|Houston Methodist Hospital|Houston Methodist Hospital|Long|USA
>Spike|hCoV-19/Spain/PV-IBV-003223/2020|2020-03-14|EPI_ISL_480968|Original|hCoV-19^^Basque Country|Human|Servicio de Microbiolog\u00eda. Hospital Universitario Donostia. OSI Donostialdea. \u00c1rea de Enfermedades Infecciosas|SeqCOVID-SPAIN consortium/IBV(CSIC)|Comas|Spain
>Spike|hCoV-19/USA/TX-HMH-7258/2020|2020-06-29|EPI_ISL_546630|Original|hCoV-19^^Texas|Human|Houston Methodist Hospital|Houston Methodist Hospital|Long|USA
>Spike|hCoV-19/USA/UT-UPHL-2009300/2020|2020-05-17|EPI_ISL_524909|Original|hCoV-19^^Utah|Human|Utah Public Health Laboratory|Utah Public Health Laboratory|Young|USA
>Spike|hCoV-19/Wales/PHWC-164BCC/2020|2020-05-22|EPI_ISL_479357|Original|hCoV-19^^Wales|Human|Wales Specialist Virology Centre Sequencing lab|COVID-19 Genomics UK (COG-UK) Consortium|Moore|United Kingdom
>Spike|hCoV-19/Australia/VIC2026/2020|2020-06-06|EPI_ISL_480667|Original|hCoV-19^^Victoria|Human|Victorian Infectious Diseases Reference Laboratory (VIDRL)|VIDRL and MDU-PHL|Schultz|Australia
>Spike|hCoV-19/USA/CA-QDX-2405/2020|2020-03-17|EPI_ISL_604673|Original|hCoV-19^^California|Human|Quest Diagnostics|Quest Diagnostics|Kagan|USA

>Spike|hCoV-19/Scotland/EDB7887/2020|2020-09-18|EPI_ISL_585440|Original|hCoV-19^^Scotland|Human|Virology Department|COVID-19 Genomics UK (COG-UK) Consortium|McHugh|United Kingdom
>Spike|hCoV-19/England/CAMC-B7A455/2020|2020-11-12|EPI_ISL_741111|Original|hCoV-19^^England|Human|Oxford Viromics|COVID-19 Genomics UK (COG-UK) Consortium|Golubchik|United Kingdom
>Spike|hCoV-19/England/CAMC-BFB568/2020|2020-12-04|EPI_ISL_709173|Original|hCoV-19^^England|Human|Lighthouse Lab in Cambridge|Wellcome Sanger Institute for the COVID-19 Genomics UK (COG-UK) Consortium|Davies|United Kingdom
>Spike|hCoV-19/England/QEUH-B7F02F/2020|2020-11-14|EPI_ISL_661704|Original|hCoV-19^^England|Human|Lighthouse Lab in Glasgow|Wellcome Sanger Institute for the COVID-19 Genomics UK (COG-UK) Consortium|Davies|United Kingdom
>Spike|hCoV-19/England/LOND-125094D/2020|2020-09-10|EPI_ISL_664449|Original|hCoV-19^^England|Human|University College London Hospital|COVID-19 Genomics UK (COG-UK) Consortium|Byott|United Kingdom
>Spike|hCoV-19/Russia/StPetersburg-RII7488S/2020|2020-04-20|EPI_ISL_450253|Original|hCoV-19^^Saint Petersburg|Human|WHO National Influenza Centre Russian Federation|WHO National Influenza Centre Russian Federation|Komissarov|Russia
>Spike|hCoV-19/USA/WA-S1439/2020|2020-05-21|EPI_ISL_495889|Original|hCoV-19^^Washington|Human|Washington State Department of Health|Seattle Flu Study|Lee|USA
>Spike|hCoV-19/India/MH-GA21/2020|2020-06-17|EPI_ISL_508217|Original|hCoV-19^^Maharashtra|Human|Government Medical College|National Institute of Biomedical Genomics|Maitra|India
>Spike|hCoV-19/Scotland/CVR3592/2020|2020-05-19|EPI_ISL_473721|Original|hCoV-19^^Scotland|Human|West of Scotland Specialist Virology Centre|COVID-19 Genomics UK (COG-UK) Consortium|Robertson|United Kingdom
>Spike|hCoV-19/England/20129170304/2020|2020-03-20|EPI_ISL_424134|Original|hCoV-19^^England|Human|Respiratory Virus Unit|Respiratory Virus Unit|Platt|United Kingdom
>Spike|hCoV-19/England/QEUH-BA2B22/2020|2020-11-18|EPI_ISL_672817|Original|hCoV-19^^England|Human|Lighthouse Lab in Glasgow|Wellcome Sanger Institute for the COVID-19 Genomics UK (COG-UK) Consortium|Davies|United Kingdom
>Spike|hCoV-19/Japan/PG-4417/2020|2020-07-00|EPI_ISL_692642|Original|hCoV-19^^Japan|Human|Pathogen Genomics Center|Pathogen Genomics Center|Sekizuka|Japan
>Spike|hCoV-19/Scotland/QEUH-BEAF31/2020|2020-12-04|EPI_ISL_710644|Original|hCoV-19^^Scotland|Human|Lighthouse Lab in Glasgow|Wellcome Sanger Institute for the COVID-19 Genomics UK (COG-UK) Consortium|Davies|United Kingdom
>Spike|hCoV-19/Scotland/QEUH-9C9082/2020|2020-09-14|EPI_ISL_576003|Original|hCoV-19^^Scotland|Human|Lighthouse Lab in Glasgow|Wellcome Sanger Institute for the COVID-19 Genomics UK (COG-UK) consortium|Davies|United Kingdom
>Spike|hCoV-19/England/ALDP-CB3D90/2020|2020-12-19|EPI_ISL_760960|Original|hCoV-19^^England|Human|Lighthouse Lab in Alderley Park|Wellcome Sanger Institute for the COVID-19 Genomics UK (COG-UK) Consortium|Davies|United Kingdom
>Spike|hCoV-19/USA/MD-MDH-0116/2020|2020-05-26|EPI_ISL_522849|Original|hCoV-19^^Maryland|Human|Maryland Department of Health|Maryland Department of Health|Keller|USA
>Spike|hCoV-19/England/CAMB-82051/2020|2020-04-21|EPI_ISL_438613|Original|hCoV-19^^England|Human|Department of Pathology|COVID-19 Genomics UK (COG-UK) Consortium|Meredith|United Kingdom
>Spike|hCoV-19/Australia/VIC8235/2020|2020-08-13|EPI_ISL_562706|Original|hCoV-19^^Victoria|Human|Microbiological Diagnostic Unit - Public Health Laboratory (MDU-PHL)|MDU-PHL|Schultz|Australia
>Spike|hCoV-19/USA/TX-HMH-5742/2020|2020-06-29|EPI_ISL_546982|Original|hCoV-19^^Texas|Human|Houston Methodist Hospital|Houston Methodist Hospital|Long|USA
>Spike|hCoV-19/USA/WA-S1040/2020|2020-05-03|EPI_ISL_463530|Original|hCoV-19^^Washington|Human|Washington State Department of Health|Seattle Flu Study|Bedford|USA
>Spike|hCoV-19/Brazil/CE-L16-CD346/2020|2020-03-30|EPI_ISL_476289|Original|hCoV-19^^Cear\u00e1|Human|DB Diagn\u00f3sticos do Brasil|Instituto de Medicina Tropical da Univesidade de S\u00e3o Paulo|Sabino|Brazil

>Spike|hCoV-19/Australia/VIC14105/2020|2020-08-25|EPI_ISL_561959|Original|hCoV-19^^Victoria|Human|Microbiological Diagnostic Unit - Public Health Laboratory (MDU-PHL)|MDU-PHL|Schultz|Australia
>Spike|hCoV-19/Denmark/DCGC-15235/2020|2020-11-23|EPI_ISL_713714|Original|hCoV-19^^Hovedstaden|Human|Department of Virus and Microbiological Special Diagnostics|Albertsen Lab|Michaelsen|Denmark
>Spike|hCoV-19/England/MILK-B8C8EB/2020|2020-11-12|EPI_ISL_675956|Original|hCoV-19^^England|Human|Lighthouse Lab in Milton Keynes|Wellcome Sanger Institute for the COVID-19 Genomics UK (COG-UK) Consortium|Davies|United Kingdom
>Spike|hCoV-19/England/QEUH-961D85/2020|2020-07-30|EPI_ISL_537104|Original|hCoV-19^^England|Human|Lighthouse Lab in Glasgow|Wellcome Sanger Institute for the COVID-19 Genomics UK (COG-UK) consortium|Davies|United Kingdom
>Spike|hCoV-19/Italy/PIE-SLL-NZ140/2020|2020-03-31|EPI_ISL_569880|Original|hCoV-19^^Piedmont|Human|Amedeo di savoia|Crosetto lab|Harbers|Italy
>Spike|hCoV-19/Scotland/EDB401/2020|2020-03-29|EPI_ISL_425999|Original|hCoV-19^^Scotland|Human|Virology Department|COVID-19 Genomics UK (COG-UK) Consortium|Williams|United Kingdom
>Spike|hCoV-19/USA/CA-ALSR-5053/2020|2020-11-27|EPI_ISL_730320|Original|hCoV-19^^California|Human|San Diego County Public Health Laboratory|Andersen lab at Scripps Research|Gangavarapu|USA
>Spike|hCoV-19/Iceland/150/2020|2020-03-13|EPI_ISL_417711|Original|hCoV-19^^Reykjavik|Human|The National University Hospital of Iceland|deCODE genetics|J\u00f3nsson|Iceland
>Spike|hCoV-19/England/PORT-2D75F2/2020|2020-11-03|EPI_ISL_650575|Original|hCoV-19^^England|Human|Centre for Enzyme Innovation|COVID-19 Genomics UK (COG-UK) Consortium|Robson|United Kingdom
>Spike|hCoV-19/DRC/5205/2020|2020-05-01|EPI_ISL_487365|Original|hCoV-19^^Kongo Central|Human|Viral Respiratory Lab|Pathogen Sequencing Lab|Lokilo|Democratic Republic of the Congo
>Spike|hCoV-19/Australia/VIC7849/2020|2020-07-28|EPI_ISL_564700|Original|hCoV-19^^Victoria|Human|Microbiological Diagnostic Unit - Public Health Laboratory (MDU-PHL)|MDU-PHL|Schultz|Australia
>Spike|hCoV-19/England/MILK-B3C765/2020|2020-11-02|EPI_ISL_706156|Original|hCoV-19^^England|Human|Oxford Viromics|COVID-19 Genomics UK (COG-UK) Consortium|Golubchik|United Kingdom
>Spike|hCoV-19/USA/TX-DSHS-2019/2020|2020-06-18|EPI_ISL_766669|Original|hCoV-19^^Texas|Human|Texas Department of State Health Services|Texas Department of State Health Services|Rahman|USA
>Spike|hCoV-19/Canada/BC_05295317/2020|2020-04-00|EPI_ISL_466786|Original|hCoV-19^^British Columbia|Human|BCCDC Public Health Laboratory|BCCDC Public Health Laboratory|Harrigan|Canada
>Spike|hCoV-19/Canada/BC_14727596/2020|2020-04-00|EPI_ISL_468659|Original|hCoV-19^^British Columbia|Human|BCCDC Public Health Laboratory|BCCDC Public Health Laboratory|Harrigan|Canada
>Spike|hCoV-19/England/QEUH-A0C161/2020|2020-10-03|EPI_ISL_584955|Original|hCoV-19^^England|Human|Quadram Institute Bioscience|COVID-19 Genomics UK (COG-UK) Consortium|Alikhan|United Kingdom
>Spike|hCoV-19/Wales/PHWC-499A28/2020|2020-12-04|EPI_ISL_727390|Original|hCoV-19^^Wales|Human|Originating lab|COVID-19 Genomics UK (COG-UK) Consortium|Moore|United Kingdom
>Spike|hCoV-19/USA/UT-UPHL-2012608904/2020|2020-08-03|EPI_ISL_745543|Original|hCoV-19^^Utah|Human|Ginkgo Bioworks Clinical Laboratory|Utah Public Health Laboratory|Young|USA
>Spike|hCoV-19/Spain/AS-IBV-98006637/2020|2020-04-03|EPI_ISL_654684|Original|hCoV-19^^Asturias|Human|Servicio de Microbiolog\u00eda|SeqCOVID-SPAIN consortium/IBV(CSIC)|Comas|Spain
>Spike|hCoV-19/England/MILK-BB17A4/2020|2020-11-18|EPI_ISL_720131|Original|hCoV-19^^England|Human|Lighthouse Lab in Milton Keynes|Wellcome Sanger Institute for the COVID-19 Genomics UK (COG-UK) Consortium|Davies|United Kingdom

>Spike|hCoV-19/England/QEUH-B12B87/2020|2020-11-02|EPI_ISL_642688|Original|hCoV-19^^England|Human|Lighthouse Lab in Glasgow|Wellcome Sanger Institute for the COVID-19 Genomics UK (COG-UK) Consortium|Davies|United Kingdom
>Spike|hCoV-19/Wales/PHWC-481C5A/2020|2020-10-18|EPI_ISL_613160|Original|hCoV-19^^Wales|Human|Wales Specialist Virology Centre Sequencing lab|COVID-19 Genomics UK (COG-UK) Consortium|Moore|United Kingdom
>Spike|hCoV-19/England/MILK-9F267F/2020|2020-09-24|EPI_ISL_589030|Original|hCoV-19^^England|Human|Lighthouse Lab in Milton Keynes|Wellcome Sanger Institute for the COVID-19 Genomics UK (COG-UK) consortium|Davies|United Kingdom
>Spike|hCoV-19/Greece/151/2020|2020-03-13|EPI_ISL_434486|Original|hCoV-19^^Athens|Human|Laboratory of Microbiology|Laboratory of Biology|Karakasiliotis|Greece
>Spike|hCoV-19/USA/TX-HMH-2927/2020|2020-06-09|EPI_ISL_543869|Original|hCoV-19^^Texas|Human|Houston Methodist Hospital|Houston Methodist Hospital|Long|USA
>Spike|hCoV-19/England/CAMB-733A5/2020|2020-03-22|EPI_ISL_440259|Original|hCoV-19^^England|Human|Department of Pathology|Wellcome Sanger Institute for the COVID-19 Genomics UK (COG-UK) consortium|Amato|United Kingdom
>Spike|hCoV-19/England/EXET-135872/2020|2020-04-15|EPI_ISL_457109|Original|hCoV-19^^England|Human|University of Exeter|COVID-19 Genomics UK (COG-UK) Consortium|Temperton|United Kingdom
>Spike|hCoV-19/Denmark/DCGC-19001/2020|2020-12-07|EPI_ISL_748691|Original|hCoV-19^^Midtjylland|Human|Department of Virus and Microbiological Special Diagnostics|Albertsen Lab|Michaelsen|Denmark
>Spike|hCoV-19/Switzerland/BL-ETHZ-210050/2020|2020-07-19|EPI_ISL_510787|Original|hCoV-19^^Basel-Landschaft|Human|Viollier AG|Department of Biosystems Science and Engineering|Nadeau|Switzerland
>Spike|hCoV-19/England/QEUH-B129C9/2020|2020-11-01|EPI_ISL_642635|Original|hCoV-19^^England|Human|Lighthouse Lab in Glasgow|Wellcome Sanger Institute for the COVID-19 Genomics UK (COG-UK) Consortium|Davies|United Kingdom
>Spike|hCoV-19/USA/CA-CZB-4088/2020|2020-08-11|EPI_ISL_548614|Original|hCoV-19^^California|Human|Orange County Public Health Laboratory|Chan-Zuckerberg Biohub|Bhatt|USA
>Spike|hCoV-19/England/204881247/2020|2020-11-00|EPI_ISL_678790|Original|hCoV-19^^England|Human|Respiratory Virus Unit|COVID-19 Genomics UK (COG-UK) Consortium|Platt|United Kingdom
>Spike|hCoV-19/Spain/MD-IBV-99007328/2020|2020-08-18|EPI_ISL_582815|Original|hCoV-19^^Madrid|Human|Hospital General Universitario Gregorio Mara\u00f1\u00f3n|SeqCOVID-SPAIN consortium/IBV(CSIC)|Comas|Spain
>Spike|hCoV-19/England/MILK-B05219/2020|2020-10-24|EPI_ISL_637553|Original|hCoV-19^^England|Human|Virology Department|COVID-19 Genomics UK (COG-UK) Consortium|de Silva|United Kingdom
>Spike|hCoV-19/Japan/PG-3235/2020|2020-04-00|EPI_ISL_691351|Original|hCoV-19^^Japan|Human|Pathogen Genomics Center|Pathogen Genomics Center|Sekizuka|Japan
>Spike|hCoV-19/England/PORT-2CF6DC/2020|2020-05-12|EPI_ISL_461975|Original|hCoV-19^^England|Human|Centre for Enzyme Innovation|COVID-19 Genomics UK (COG-UK) Consortium|Robson|United Kingdom
>Spike|hCoV-19/Luxembourg/LNS8583405/2020|2020-12-23|EPI_ISL_771081|Original|hCoV-19^^Luxembourg|Human|Laboratoire national de sant\u00e9|Laboratoire national de sant\u00e9|Wienecke-Baldacchino|Luxembourg
>Spike|hCoV-19/England/ALDP-952E84/2020|2020-06-11|EPI_ISL_553697|Original|hCoV-19^^England|Human|Lighthouse Lab in Alderley Park|Wellcome Sanger Institute for the COVID-19 Genomics UK (COG-UK) consortium|Davies|United Kingdom
>Spike|hCoV-19/Switzerland/ZH-ETHZ-100202/2020|2020-03-17|EPI_ISL_575015|Original|hCoV-19^^Zurich|Human|Viollier AG|Department of Biosystems Science and Engineering|Nadeau|Switzerland
>Spike|hCoV-19/mink/Netherlands/NB-EMC-47-5/2020|2020-09-04|EPI_ISL_577813|Original|hCoV-19^^Noord Brabant|Neovison vison|Dutch COVID-19 response team|Erasmus Medical Center|Oude Munnink|Netherlands

>Spike|hCoV-19/Luxembourg/LNS7243430/2020|2020-11-22|EPI_ISL_744542|Original|hCoV-19^^Luxembourg|Human|Laboratoire national de sant\u00e9|Laboratoire national de sant\u00e9|Wienecke-Baldacchino|Luxembourg
>Spike|hCoV-19/England/MILK-977D7D/2020|2020-07-27|EPI_ISL_550427|Original|hCoV-19^^England|Human|Lighthouse Lab in Milton Keynes|Wellcome Sanger Institute for the COVID-19 Genomics UK (COG-UK) consortium|Davies|United Kingdom
>Spike|hCoV-19/England/QEUH-BC4410/2020|2020-11-25|EPI_ISL_703510|Original|hCoV-19^^England|Human|Lighthouse Lab in Glasgow|Wellcome Sanger Institute for the COVID-19 Genomics UK (COG-UK) Consortium|Davies|United Kingdom
>Spike|hCoV-19/England/MILK-A46875/2020|2020-10-06|EPI_ISL_598676|Original|hCoV-19^^England|Human|Lighthouse Lab in Milton Keynes|Wellcome Sanger Institute for the COVID-19 Genomics UK (COG-UK) consortium|Davies|United Kingdom
>Spike|hCoV-19/Netherlands/ZuidHolland_68/2020|2020-03-24|EPI_ISL_422936|Original|hCoV-19^^Zuid Holland|Human|Dutch COVID-19 response team|Erasmus Medical Center|Oude Munnink|Netherlands
>Spike|hCoV-19/USA/UT-UPHL-2012719632/2020|2020-08-10|EPI_ISL_745842|Original|hCoV-19^^Utah|Human|Ginkgo Bioworks Clinical Laboratory|Utah Public Health Laboratory|Young|USA
>Spike|hCoV-19/USA/MI-MDHHS-SC22311/2020|2020-11-10|EPI_ISL_708287|Original|hCoV-19^^Michigan|Human|Michigan Department of Health and Human Services|Michigan Department of Health and Human Services|Jacob|USA
>Spike|hCoV-19/England/ALDP-94CD71/2020|2020-06-15|EPI_ISL_555936|Original|hCoV-19^^England|Human|Lighthouse Lab in Alderley Park|Wellcome Sanger Institute for the COVID-19 Genomics UK (COG-UK) consortium|Davies|United Kingdom
>Spike|hCoV-19/England/ALDP-A9282F/2020|2020-10-19|EPI_ISL_611162|Original|hCoV-19^^England|Human|Lighthouse Lab in Alderley Park|Wellcome Sanger Institute for the COVID-19 Genomics UK (COG-UK) consortium|Davies|United Kingdom
>Spike|hCoV-19/Latvia/083/2020|2020-06-15|EPI_ISL_501896|Original|hCoV-19^^Latvia|Human|Centr\u0101l\u0101 laboratorija|Latvian Biomedical Research and Study Centre|Zrelovs|Latvia
>Spike|hCoV-19/Brazil/SP-L9-CD131/2020|2020-03-19|EPI_ISL_476479|Original|hCoV-19^^S\u00e3o Paulo|Human|Hospital da Cl\u00ednicas da Faculdade de Medicina da Universidade de S\u00e3o Paulo|Instituto de Medicina Tropical da Univesidade de S\u00e3o Paulo|Faria|Brazil
>Spike|hCoV-19/England/MILK-B8ED53/2020|2020-11-13|EPI_ISL_674510|Original|hCoV-19^^England|Human|Lighthouse Lab in Milton Keynes|Wellcome Sanger Institute for the COVID-19 Genomics UK (COG-UK) Consortium|Davies|United Kingdom
>Spike|hCoV-19/England/20139009004/2020|2020-03-27|EPI_ISL_421866|Original|hCoV-19^^England|Human|Respiratory Virus Unit|Respiratory Virus Unit|Platt|United Kingdom
>Spike|hCoV-19/England/ALDP-A5031D/2020|2020-10-07|EPI_ISL_597715|Original|hCoV-19^^England|Human|Lighthouse Lab in Alderley Park|Wellcome Sanger Institute for the COVID-19 Genomics UK (COG-UK) consortium|Davies|United Kingdom
>Spike|hCoV-19/Denmark/DCGC-6061/2020|2020-10-05|EPI_ISL_620255|Original|hCoV-19^^Hovedstaden|Human|Department of Virus and Microbiological Special Diagnostics|Albertsen lab|Michaelsen|Denmark
>Spike|hCoV-19/Switzerland/BL-ETHZ-330064/2020|2020-10-16|EPI_ISL_614898|Original|hCoV-19^^Basel-Landschaft|Human|Viollier AG|Department of Biosystems Science and Engineering|Nadeau|Switzerland
>Spike|hCoV-19/Germany/BE-ChVir-D754-8111/2020|2020-03-16|EPI_ISL_754058|Original|hCoV-19^^Berlin|Human|Charit\u00e9 Universit\u00e4tsmedizin Berlin|Charit\u00e9 Universit\u00e4tsmedizin Berlin|Muehlemann|Germany
>Spike|hCoV-19/England/CAMC-BC0229/2020|2020-11-21|EPI_ISL_704803|Original|hCoV-19^^England|Human|Lighthouse Lab in Cambridge|Wellcome Sanger Institute for the COVID-19 Genomics UK (COG-UK) Consortium|Davies|United Kingdom
>Spike|hCoV-19/Thailand/Bangkok-CONI-0275/2020|2020-03-14|EPI_ISL_693353|Original|hCoV-19^^Bangkok|Human|Ramathibodi Hospital|COVID-19 Network Investigations (CONI) Alliance|Batty|Thailand

>Spike|hCoV-19/Bangladesh/BCSIR-NILMRC_398/2020|2020-10-08|EPI_ISL_603222|Original|hCoV-19^^Dhaka|Human|National Institute of Laboratory Medicine and Referral Center|Genomic Research Lab|Khan|Bangladesh
>Spike|hCoV-19/England/ALDP-92BB50/2020|2020-06-22|EPI_ISL_551390|Original|hCoV-19^^England|Human|Lighthouse Lab in Alderley Park|Wellcome Sanger Institute for the COVID-19 Genomics UK (COG-UK) consortium|Davies|United Kingdom
>Spike|hCoV-19/USA/VA-DCLS-1947/2020|2020-09-27|EPI_ISL_644929|Original|hCoV-19^^Virginia|Human|Virginia DCLS|Virginia DCLS|St. Jacques|USA
>Spike|hCoV-19/India/MH-ACTREC-142/2020|2020-05-29|EPI_ISL_699798|Original|hCoV-19^^Maharashtra|Human|Hematopathology Laboratory|Hematopathology Laboratory|Patkar|India
>Spike|hCoV-19/USA/VA-DCLS-2307/2020|2020-11-04|EPI_ISL_734262|Original|hCoV-19^^Virginia|Human|Virginia Division of Consolidated Laboratory Services (DCLS)|Virginia Division of Consolidated Laboratory Services (DCLS)|St. Jacques|USA
>Spike|hCoV-19/Wales/PHWC-27935/2020|2020-04-01|EPI_ISL_432270|Original|hCoV-19^^Wales|Human|Wales Specialist Virology Centre|Public Health Wales Microbiology Cardiff|Moore|United Kingdom
>Spike|hCoV-19/USA/NY-QDX-2034/2020|2020-03-18|EPI_ISL_604363|Original|hCoV-19^^New York|Human|Quest Diagnostics|Quest Diagnostics|Kagan|USA
>Spike|hCoV-19/USA/CA-SCCPHD-UC155/2020|2020-03-10|EPI_ISL_435634|Original|hCoV-19^^California|Human|Santa Clara County Public Health Department|Chiu Laboratory|Chiu|USA
>Spike|hCoV-19/England/QEUH-9FFFB3/2020|2020-10-02|EPI_ISL_600372|Original|hCoV-19^^England|Human|Lighthouse Lab in Glasgow|Wellcome Sanger Institute for the COVID-19 Genomics UK (COG-UK) consortium|Davies|United Kingdom
>Spike|hCoV-19/Belgium/ULG-10780/2020|2020-10-30|EPI_ISL_644626|Original|hCoV-19^^Li\u00e8ge|Human|Department of Clinical Microbiology|GIGA Medical Genomics|Durkin|Belgium
>Spike|hCoV-19/USA/LA-EVTL885/2020|2020-07-07|EPI_ISL_653576|Original|hCoV-19^^Louisiana|Human|LSUHS Emerging Viral Threat Laboratory|Microbial Genome Sequencing Center|Kamil|USA
>Spike|hCoV-19/Scotland/EDB7939/2020|2020-09-25|EPI_ISL_585455|Original|hCoV-19^^Scotland|Human|Virology Department|COVID-19 Genomics UK (COG-UK) Consortium|McHugh|United Kingdom
>Spike|hCoV-19/England/CAMC-A40EA6/2020|2020-10-06|EPI_ISL_609509|Original|hCoV-19^^England|Human|Lighthouse Lab in Cambridge|Wellcome Sanger Institute for the COVID-19 Genomics UK (COG-UK) consortium|Davies|United Kingdom
>Spike|hCoV-19/Singapore/1199/2020|2020-04-20|EPI_ISL_648615|Original|hCoV-19^^Singapore|Human|Department of Laboratory Medicine|Department of Laboratory Medicine|Chen|Singapore
>Spike|hCoV-19/Wales/PHWC-16CBF1/2020|2020-09-24|EPI_ISL_574147|Original|hCoV-19^^Wales|Human|Wales Specialist Virology Centre Sequencing lab|COVID-19 Genomics UK (COG-UK) Consortium|Moore|United Kingdom
>Spike|hCoV-19/England/ALDP-904C87/2020|2020-06-22|EPI_ISL_551575|Original|hCoV-19^^England|Human|Lighthouse Lab in Alderley Park|Wellcome Sanger Institute for the COVID-19 Genomics UK (COG-UK) consortium|Davies|United Kingdom
>Spike|hCoV-19/England/CAMB-7CFA8/2020|2020-04-06|EPI_ISL_442264|Original|hCoV-19^^England|Human|Department of Pathology|Wellcome Sanger Institute for the COVID-19 Genomics UK (COG-UK) consortium|Amato|United Kingdom
>Spike|hCoV-19/Wales/PHWC-35A5F/2020|2020-04-24|EPI_ISL_474517|Original|hCoV-19^^Wales|Human|Originating lab|COVID-19 Genomics UK (COG-UK) Consortium|Moore|United Kingdom
>Spike|hCoV-19/England/LIVE-DC3E4E/2020|2020-11-08|EPI_ISL_705880|Original|hCoV-19^^England|Human|Liverpool Clinical Laboratories|COVID-19 Genomics UK (COG-UK) Consortium|Haldenby|United Kingdom
>Spike|hCoV-19/USA/CA-CZB-2368/2020|2020-05-15|EPI_ISL_513809|Original|hCoV-19^^California|Human|Orange County Public Health Laboratory|Chan-Zuckerberg Biohub|Kamm|USA

>Spike|hCoV-19/Switzerland/ZH-ETHZ-370480/2020|2020-11-16|EPI_ISL_693966|Original|hCoV-19^^Zurich|Human|Viollier AG|Department of Biosystems Science and Engineering|Nadeau|Switzerland
>Spike|hCoV-19/England/CAMC-B9C55A/2020|2020-11-15|EPI_ISL_673236|Original|hCoV-19^^England|Human|Lighthouse Lab in Cambridge|Wellcome Sanger Institute for the COVID-19 Genomics UK (COG-UK) Consortium|Davies|United Kingdom
>Spike|hCoV-19/Scotland/CVR5224/2020|2020-10-19|EPI_ISL_627396|Original|hCoV-19^^Scotland|Human|West of Scotland Specialist Virology Centre|COVID-19 Genomics UK (COG-UK) Consortium|Robertson|United Kingdom
>Spike|hCoV-19/Austria/CeMM0512/2020|2020-03-11|EPI_ISL_583597|Original|hCoV-19^^Austria|Human|Institute for Medical and Chemical Laboratory Diagnostics|Bergthaler laboratory|Bergthaler|Austria
>Spike|hCoV-19/Australia/VIC14614/2020|2020-08-05|EPI_ISL_592835|Original|hCoV-19^^Victoria|Human|Microbiological Diagnostic Unit - Public Health Laboratory (MDU-PHL)|MDU-PHL|Schultz|Australia
>Spike|hCoV-19/Wales/PHWC-494CBE/2020|2020-11-24|EPI_ISL_726240|Original|hCoV-19^^Wales|Human|Originating lab|COVID-19 Genomics UK (COG-UK) Consortium|Moore|United Kingdom
>Spike|hCoV-19/Australia/VIC1660/2020|2020-05-12|EPI_ISL_456561|Original|hCoV-19^^Victoria|Human|Victorian Infectious Diseases Reference Laboratory (VIDRL)|Microbiological Diagnostic Unit Public Health Laboratory and Victorian Infectious Diseases Reference Laboratory|Schultz|Australia
>Spike|hCoV-19/Singapore/225/2020|2020-04-20|EPI_ISL_462343|Original|hCoV-19^^Singapore|Human|National Public Health Laboratory|National Public Health Laboratory|Mak|Singapore
>Spike|hCoV-19/England/NORT-283BD5/2020|2020-04-03|EPI_ISL_488532|Original|hCoV-19^^England|Human|NU-OMICS DNA Sequencing research facility|Wellcome Sanger Institute for the COVID-19 Genomics UK (COG-UK) consortium|Davies|United Kingdom
>Spike|hCoV-19/England/MILK-973272/2020|2020-08-20|EPI_ISL_575893|Original|hCoV-19^^England|Human|Lighthouse Lab in Milton Keynes|Wellcome Sanger Institute for the COVID-19 Genomics UK (COG-UK) consortium|Davies|United Kingdom
>Spike|hCoV-19/England/QEUH-C32BBD/2020|2020-12-11|EPI_ISL_782513|Original|hCoV-19^^England|Human|Lighthouse Lab in Glasgow|Wellcome Sanger Institute for the COVID-19 Genomics UK (COG-UK) Consortium|Davies|United Kingdom
>Spike|hCoV-19/England/MILK-A19398/2020|2020-10-03|EPI_ISL_612508|Original|hCoV-19^^England|Human|Wales Specialist Virology Centre Sequencing lab|COVID-19 Genomics UK (COG-UK) Consortium|Moore|United Kingdom
>Spike|hCoV-19/England/201580039/2020|2020-04-09|EPI_ISL_466352|Original|hCoV-19^^England|Human|Respiratory Virus Unit|Respiratory Virus Unit|Platt|United Kingdom
>Spike|hCoV-19/USA/AZ-QDX-2311/2020|2020-03-15|EPI_ISL_604504|Original|hCoV-19^^Arizona|Human|Quest Diagnostics|Quest Diagnostics|Kagan|USA
>Spike|hCoV-19/Japan/PG-4360/2020|2020-07-00|EPI_ISL_692588|Original|hCoV-19^^Japan|Human|Pathogen Genomics Center|Pathogen Genomics Center|Sekizuka|Japan
>Spike|hCoV-19/England/PORT-2CF52A/2020|2020-04-27|EPI_ISL_453691|Original|hCoV-19^^England|Human|Centre for Enzyme Innovation|COVID-19 Genomics UK (COG-UK) Consortium|Robson|United Kingdom
>Spike|hCoV-19/USA/TX-HMH-MCoV-10012/2020|2020-07-08|EPI_ISL_789284|Original|hCoV-19^^Texas|Human|Houston Methodist Hospital|Houston Methodist Hospital|Long|USA
>Spike|hCoV-19/England/QEUH-B2CF9D/2020|2020-11-04|EPI_ISL_680090|Original|hCoV-19^^England|Human|Department of Pathology|COVID-19 Genomics UK (COG-UK) Consortium|Goodfellow|United Kingdom
>Spike|hCoV-19/USA/NY-Wadsworth-220236-01/2020|2020-09-17|EPI_ISL_765508|Original|hCoV-19^^New York|Human|Wadsworth Center|Wadsworth Center|Lasek-Nesselquist|USA
>Spike|hCoV-19/USA/UT-08268/2020|2020-05-19|EPI_ISL_524074|Original|hCoV-19^^Utah|Human|Utah Public Health Laboratory|Utah Public Health Laboratory|Young|USA
>Spike|hCoV-19/Mali/M002659/2020|2020-04-09|EPI_ISL_487451|Original|hCoV-19^^Bamako|Human|CICM-Mali|Bundeswehr Institut of Microbiology|Antwerpen|Mali

>Spike|hCoV-19/USA/ME-HETL-J0472/2020|2020-12-11|EPI_ISL_755563|Original|hCoV-19^^Maine|Human|Maine Health and Environmental Testing Laboratory|Tewhey Lab|Tewhey|USA
>Spike|hCoV-19/Switzerland/AG-ETHZ-270096/2020|2020-09-02|EPI_ISL_541406|Original|hCoV-19^^Aargau|Human|Viollier AG|Department of Biosystems Science and Engineering|Nadeau|Switzerland
>Spike|hCoV-19/Scotland/CVR5830/2020|2020-11-12|EPI_ISL_666037|Original|hCoV-19^^Scotland|Human|West of Scotland Specialist Virology Centre|COVID-19 Genomics UK (COG-UK) Consortium|Robertson|United Kingdom
>Spike|hCoV-19/England/CAMC-B0AF0E/2020|2020-10-30|EPI_ISL_643405|Original|hCoV-19^^England|Human|Lighthouse Lab in Cambridge|Wellcome Sanger Institute for the COVID-19 Genomics UK (COG-UK) Consortium|Davies|United Kingdom
>Spike|hCoV-19/England/ALDP-94CC29/2020|2020-06-16|EPI_ISL_555909|Original|hCoV-19^^England|Human|Lighthouse Lab in Alderley Park|Wellcome Sanger Institute for the COVID-19 Genomics UK (COG-UK) consortium|Davies|United Kingdom
>Spike|hCoV-19/USA/MA-MASPHL-00556/2020|2020-05-08|EPI_ISL_693031|Original|hCoV-19^^Massachusetts|Human|Massachusetts State Public Health Laboratory|Massachusetts State Public Health Laboratory|Lang|USA
>Spike|hCoV-19/England/BRIS-132F8F/2020|2020-06-19|EPI_ISL_511958|Original|hCoV-19^^England|Human|PHE South West Regional Laboratory|Wellcome Sanger Institute for the COVID-19 Genomics UK (COG-UK) consortium|Davies|United Kingdom
>Spike|hCoV-19/Canada/ON-S1507/2020|2020-08-30|EPI_ISL_586362|Original|hCoV-19^^Ontario|Human|Toronto Invasive Bacterial Diseases Network|McMaster University|McArthur|Canada
>Spike|hCoV-19/Belgium/rega-10141384/2020|2020-10-14|EPI_ISL_738436|Original|hCoV-19^^Wezembeek-Oppem|Human|UZ Leuven|KU Leuven|Maes|Belgium
>Spike|hCoV-19/Germany/SH-ChVir9951/2020|2020-11-02|EPI_ISL_729428|Original|hCoV-19^^Schleswig-Holstein|Human|A. Krumbholz|Charit\u00e9 Universit\u00e4tsmedizin Berlin|Muehlemann|Germany
>Spike|hCoV-19/South Africa/KRISP-0089/2020|2020-04-05|EPI_ISL_467458|Original|hCoV-19^^KwaZulu-Natal|Human|AMPATH-DBN|KRISP|de Oliveira|South Africa
>Spike|hCoV-19/USA/UT-QDX-1834/2020|2020-03-14|EPI_ISL_604152|Original|hCoV-19^^Utah|Human|Quest Diagnostics|Quest Diagnostics|Kagan|USA
>Spike|hCoV-19/Netherlands/ZE-EMC-64/2020|2020-00-00|EPI_ISL_523563|Original|hCoV-19^^Zeeland|Human|Dutch COVID-19 response team|Erasmus Medical Center|Oude Munnink|Netherlands
>Spike|hCoV-19/Wales/PHWC-348DE/2020|2020-04-17|EPI_ISL_446991|Original|hCoV-19^^Wales|Human|Wales Specialist Virology Centre|Public Health Wales Microbiology Cardiff|Moore|United Kingdom
>Spike|hCoV-19/India/TG-CCMB-L1082/2020|2020-05-13|EPI_ISL_458055|Original|hCoV-19^^Telangana|Human|CSIR-Centre for Cellular and Molecular Biology|CSIR-Centre for Cellular and Molecular Biology|Banu|India
>Spike|hCoV-19/England/LOND-DB244/2020|2020-11-08|EPI_ISL_724747|Original|hCoV-19^^England|Human|University College London|COVID-19 Genomics UK (COG-UK) Consortium|Roy|United Kingdom
>Spike|hCoV-19/Denmark/DCGC-14462/2020|2020-11-30|EPI_ISL_711781|Original|hCoV-19^^Hovedstaden|Human|Department of Virus and Microbiological Special Diagnostics|Albertsen Lab|Michaelsen|Denmark
>Spike|hCoV-19/England/QEUH-B7E9DA/2020|2020-11-14|EPI_ISL_667832|Original|hCoV-19^^England|Human|Lighthouse Lab in Glasgow|Wellcome Sanger Institute for the COVID-19 Genomics UK (COG-UK) Consortium|Davies|United Kingdom
>Spike|hCoV-19/Japan/PG-0194/2020|2020-03-16|EPI_ISL_479899|Original|hCoV-19^^Kanto|Human|Gunma Prefectural Institute of Public Health and Environmental Sciences|Pathogen Genomics Center|Sekizuka|Japan
>Spike|hCoV-19/Russia/OMS-ORINFI-61S/2020|2020-05-14|EPI_ISL_569802|Original|hCoV-19^^Omsk|Human|Omsk Research Institute of Natural Focal Infections|WHO National Influenza Centre Russian Federation|Komissarov|Russia
>Spike|hCoV-19/England/CAMB-1BCF05/2020|2020-12-04|EPI_ISL_723909|Original|hCoV-19^^England|Human|Department of Pathology|COVID-19 Genomics UK (COG-UK) Consortium|Goodfellow|United Kingdom

>Spike|hCoV-19/England/MILK-994642/2020|2020-08-25|EPI_ISL_552311|Original|hCoV-19^^England|Human|Lighthouse Lab in Milton Keynes|Wellcome Sanger Institute for the COVID-19 Genomics UK (COG-UK) consortium|Davies|United Kingdom
>Spike|hCoV-19/England/NORT-293A7B/2020|2020-07-13|EPI_ISL_512351|Original|hCoV-19^^England|Human|Northumbria University / South Tees Hospitals NHS Foundation Trust / North Cumbria Integrated Care NHS Foundation Trust / North Tees and Hartlepool NHS Foundation Trust / Newcastle Hospitals NHS Foundation Trust|COVID-19 Genomics UK (COG-UK) Consortium|Bashton|United Kingdom
>Spike|hCoV-19/USA/UT-UPHL-2012359598/2020|2020-08-17|EPI_ISL_746274|Original|hCoV-19^^Utah|Human|Ginkgo Bioworks Clinical Laboratory|Utah Public Health Laboratory|Young|USA
>Spike|hCoV-19/Germany/BE-ChVir-D909-4341/2020|2020-03-16|EPI_ISL_753922|Original|hCoV-19^^Berlin|Human|Charit\u00e9 Universit\u00e4tsmedizin Berlin|Charit\u00e9 Universit\u00e4tsmedizin Berlin|Muehlemann|Germany
>Spike|hCoV-19/Northern Ireland/NIRE-106EF0/2020|2020-05-21|EPI_ISL_501010|Original|hCoV-19^^Northern Ireland|Human|Regional Virus Laboratory|Wellcome Sanger Institute for the COVID-19 Genomics UK (COG-UK) consortium|Davies|United Kingdom
>Spike|hCoV-19/USA/IL-NM-0477/2020|2020-04-22|EPI_ISL_626421|Original|hCoV-19^^Illinois|Human|Northwestern Memorial Hospital|Ozer Lab|Ozer|USA
>Spike|hCoV-19/Denmark/DCGC-13895/2020|2020-11-30|EPI_ISL_713088|Original|hCoV-19^^Hovedstaden|Human|Department of Virus and Microbiological Special Diagnostics|Albertsen Lab|Michaelsen|Denmark
>Spike|hCoV-19/USA/CA-ALSR-2970/2020|2020-09-02|EPI_ISL_635760|Original|hCoV-19^^California|Human|San Diego County Public Health Laboratory|Andersen lab at Scripps Research|Gangavarapu|USA
>Spike|hCoV-19/England/CAMC-B98697/2020|2020-11-15|EPI_ISL_674893|Original|hCoV-19^^England|Human|Lighthouse Lab in Cambridge|Wellcome Sanger Institute for the COVID-19 Genomics UK (COG-UK) Consortium|Davies|United Kingdom
>Spike|hCoV-19/England/NORW-EB469/2020|2020-04-27|EPI_ISL_457433|Original|hCoV-19^^England|Human|Quadram Institute Bioscience|COVID-19 Genomics UK (COG-UK) Consortium|Alikhan|United Kingdom
>Spike|hCoV-19/England/ALDP-B85211/2020|2020-11-15|EPI_ISL_674179|Original|hCoV-19^^England|Human|Lighthouse Lab in Alderley Park|Wellcome Sanger Institute for the COVID-19 Genomics UK (COG-UK) Consortium|Davies|United Kingdom
>Spike|hCoV-19/England/CAMB-1B9BC8/2020|2020-11-10|EPI_ISL_664962|Original|hCoV-19^^England|Human|Department of Pathology|COVID-19 Genomics UK (COG-UK) Consortium|Goodfellow|United Kingdom
>Spike|hCoV-19/Canada/ON-S1909/2020|2020-11-24|EPI_ISL_729270|Original|hCoV-19^^Ontario|Human|Toronto Invasive Bacterial Diseases Network|McMaster University|McArthur|Canada
>Spike|hCoV-19/England/ALDP-94E45C/2020|2020-06-17|EPI_ISL_555408|Original|hCoV-19^^England|Human|Lighthouse Lab in Alderley Park|Wellcome Sanger Institute for the COVID-19 Genomics UK (COG-UK) consortium|Davies|United Kingdom
>Spike|hCoV-19/Japan/PG-3424/2020|2020-04-00|EPI_ISL_691536|Original|hCoV-19^^Japan|Human|Sakai City Institute of Public Health|Pathogen Genomics Center|Sekizuka|Japan
>Spike|hCoV-19/Denmark/DCGC-8989/2020|2020-10-26|EPI_ISL_626199|Original|hCoV-19^^Sjaelland|Human|Department of Virus and Microbiological Special Diagnostics|Albertsen lab|Michaelsen|Denmark
>Spike|hCoV-19/England/QEUH-BCC15D/2020|2020-11-27|EPI_ISL_704365|Original|hCoV-19^^England|Human|Lighthouse Lab in Glasgow|Wellcome Sanger Institute for the COVID-19 Genomics UK (COG-UK) Consortium|Davies|United Kingdom
>Spike|hCoV-19/Scotland/QEUH-C5448D/2020|2020-12-12|EPI_ISL_757064|Original|hCoV-19^^Scotland|Human|Lighthouse Lab in Glasgow|Wellcome Sanger Institute for the COVID-19 Genomics UK (COG-UK) Consortium|Davies|United Kingdom
>Spike|hCoV-19/Germany/HH-hpi-p2335/2020|2020-03-13|EPI_ISL_776447|Original|hCoV-19^^Hamburg|Human|University Medical Center Hamburg Eppendorf|Heinrich Pette Institute|Robitaille|Germany

>Spike|hCoV-19/England/CAMC-B79F73/2020|2020-11-12|EPI_ISL_741068|Original|hCoV-19^^England|Human|Oxford Viromics|COVID-19 Genomics UK (COG-UK) Consortium|Golubchik|United Kingdom
>Spike|hCoV-19/Luxembourg/LNS9018804/2020|2020-12-02|EPI_ISL_771003|Original|hCoV-19^^Luxembourg|Human|Laboratoire national de sant\u00e9|Laboratoire national de sant\u00e9|Wienecke-Baldacchino|Luxembourg
>Spike|hCoV-19/Denmark/DCGC-6484/2020|2020-10-05|EPI_ISL_620384|Original|hCoV-19^^Syddanmark|Human|Department of Virus and Microbiological Special Diagnostics|Albertsen lab|Michaelsen|Denmark
>Spike|hCoV-19/England/QEUH-9B7A2F/2020|2020-09-04|EPI_ISL_590465|Original|hCoV-19^^England|Human|Lighthouse Lab in Glasgow|Wellcome Sanger Institute for the COVID-19 Genomics UK (COG-UK) consortium|Davies|United Kingdom
>Spike|hCoV-19/England/ALDP-B5AF69/2020|2020-11-09|EPI_ISL_655607|Original|hCoV-19^^England|Human|Lighthouse Lab in Alderley Park|Wellcome Sanger Institute for the COVID-19 Genomics UK (COG-UK) Consortium|Davies|United Kingdom
>Spike|hCoV-19/England/CAMC-B54196/2020|2020-11-08|EPI_ISL_656858|Original|hCoV-19^^England|Human|Lighthouse Lab in Cambridge|Wellcome Sanger Institute for the COVID-19 Genomics UK (COG-UK) Consortium|Davies|United Kingdom
>Spike|hCoV-19/England/MILK-9C451F/2020|2020-09-07|EPI_ISL_566728|Original|hCoV-19^^England|Human|Lighthouse Lab in Milton Keynes|Wellcome Sanger Institute for the COVID-19 Genomics UK (COG-UK) consortium|Davies|United Kingdom
>Spike|hCoV-19/Mexico/MOR-InDRE-26/2020|2020-05-27|EPI_ISL_493341|Original|hCoV-19^^Morelos|Human|Instituto de Diagnostico y Referencia Epidemiologicos (INDRE)|Instituto de Diagnostico y Referencia Epidemiologicos (INDRE)|Rodriguez|Mexico
>Spike|hCoV-19/Japan/PG-7790/2020|2020-07-00|EPI_ISL_686892|Original|hCoV-19^^Japan|Human|Pathogen Genomics Center|Pathogen Genomics Center|Sekizuka|Japan
>Spike|hCoV-19/Australia/QLD981/2020|2020-03-20|EPI_ISL_530261|Original|hCoV-19^^Queensland|Human|Queensland Health Forensic and Scientific Services|Public Health Virology Laboratory|Nguyen|Australia
>Spike|hCoV-19/England/CAMB-76A61/2020|2020-03-30|EPI_ISL_438329|Original|hCoV-19^^England|Human|Department of Pathology|Wellcome Sanger Institute for the COVID-19 Genomics UK (COG-UK) consortium|Amato|United Kingdom
>Spike|hCoV-19/USA/MI-MDHHS-SC21916/2020|2020-04-13|EPI_ISL_565977|Original|hCoV-19^^Michigan|Human|Michigan Department of Health and Human Services|Michigan Department of Health and Human Services|Blankenship|USA
>Spike|hCoV-19/Japan/PG-0981/2020|2020-04-00|EPI_ISL_684591|Original|hCoV-19^^Japan|Human|Pathogen Genomics Center|Pathogen Genomics Center|Sekizuka|Japan
>Spike|hCoV-19/England/BRIS-1255C9/2020|2020-03-28|EPI_ISL_443775|Original|hCoV-19^^England|Human|PHE South West Regional Laboratory|Wellcome Sanger Institute for the COVID-19 Genomics UK (COG-UK) consortium|Amato|United Kingdom
>Spike|hCoV-19/Switzerland/BE-ETHZ-120237/2020|2020-05-01|EPI_ISL_468298|Original|hCoV-19^^Bern|Human|Viollier AG|Department of Biosystems Science and Engineering|Nadeau|Switzerland
>Spike|hCoV-19/USA/TX-HMH-MCoV-18816/2020|2020-11-26|EPI_ISL_785142|Original|hCoV-19^^Texas|Human|Houston Methodist Hospital|Houston Methodist Hospital|Long|USA
>Spike|hCoV-19/England/20186133904/2020|2020-04-30|EPI_ISL_465712|Original|hCoV-19^^England|Human|Respiratory Virus Unit|Respiratory Virus Unit|Platt|United Kingdom
>Spike|hCoV-19/England/QEUH-96F5AD/2020|2020-08-17|EPI_ISL_530484|Original|hCoV-19^^England|Human|Lighthouse Lab in Glasgow|Wellcome Sanger Institute for the COVID-19 Genomics UK (COG-UK) consortium|Davies|United Kingdom
>Spike|hCoV-19/Norway/3111/2020|2020-08-05|EPI_ISL_549134|Original|hCoV-19^^Viken|Human|Ostfold Hospital Trust - Kalnes|Norwegian Institute of Public Health|Kopperud Riis|Norway
>Spike|hCoV-19/USA/MI-MDHHS-SC22154/2020|2020-10-20|EPI_ISL_614205|Original|hCoV-19^^Michigan|Human|Michigan Department of Health and Human Services|Michigan Department of Health and Human Services|Jacob|USA
>Spike|hCoV-19/England/MILK-562ABE/2020|2020-06-10|EPI_ISL_558399|Original|hCoV-19^^England|Human|Lighthouse Lab in Milton Keynes|Wellcome Sanger Institute for the COVID-19 Genomics UK (COG-UK) consortium|Davies|United Kingdom

>Spike|hCoV-19/England/CAMC-BD15E0/2020|2020-11-27|EPI_ISL_702363|Original|hCoV-19^^England|Human|Lighthouse Lab in Cambridge|Wellcome Sanger Institute for the COVID-19 Genomics UK (COG-UK) Consortium|Davies|United Kingdom
>Spike|hCoV-19/USA/CA-IGI-0353/2020|2020-06-26|EPI_ISL_576539|Original|hCoV-19^^California|Human|Innovative Genomics Institute|Innovative Genomics Institute|Wyman|USA
>Spike|hCoV-19/USA/CA-ALSR-1636/2020|2020-04-24|EPI_ISL_494705|Original|hCoV-19^^California|Human|Scripps Medical Laboratory|Andersen lab at Scripps Research|Gangavarapu|USA
>Spike|hCoV-19/Australia/VIC5307/2020|2020-07-18|EPI_ISL_520070|Original|hCoV-19^^Victoria|Human|Microbiological Diagnostic Unit - Public Health Laboratory (MDU-PHL)|MDU-PHL|Schultz|Australia
>Spike|hCoV-19/England/QEUH-BA29CE/2020|2020-11-18|EPI_ISL_672895|Original|hCoV-19^^England|Human|Lighthouse Lab in Glasgow|Wellcome Sanger Institute for the COVID-19 Genomics UK (COG-UK) Consortium|Davies|United Kingdom
>Spike|hCoV-19/Switzerland/BS-42344759/2020|2020-07-08|EPI_ISL_581881|Original|hCoV-19^^Basel-Stadt|Human|University Hospital Basel|University Hospital Basel|Roloff Handschin|Switzerland
>Spike|hCoV-19/USA/AZ-TG290722/2020|2020-05-05|EPI_ISL_694238|Original|hCoV-19^^Arizona|Human|TGen North|TGen North|French|USA
>Spike|hCoV-19/Wales/PHWC-480F70/2020|2020-10-19|EPI_ISL_613019|Original|hCoV-19^^Wales|Human|Wales Specialist Virology Centre Sequencing lab|COVID-19 Genomics UK (COG-UK) Consortium|Moore|United Kingdom
>Spike|hCoV-19/USA/MA-MASPHL-00463/2020|2020-04-30|EPI_ISL_693099|Original|hCoV-19^^Massachusetts|Human|Massachusetts State Public Health Laboratory|Massachusetts State Public Health Laboratory|Lang|USA
>Spike|hCoV-19/Brazil/RJ-00494/2020|2020-10-29|EPI_ISL_717888|Original|hCoV-19^^Rio de Janeiro|Human|Laboratorio de Virologia Molecular / UFRJ|Bioinformatics Laboratory / LNCC|Almeida|Brazil
>Spike|hCoV-19/Brazil/RS-00687/2020|2020-11-27|EPI_ISL_779167|Original|hCoV-19^^Rio Grande do Sul|Human|Laborat\u00f3rio de Microbiologia Molecular - Universidade FEEVALE|Bioinformatics Laboratory / LNCC|Almeida|Brazil
>Spike|hCoV-19/Japan/PG-0283/2020|2020-03-23|EPI_ISL_479962|Original|hCoV-19^^Kanto|Human|Tokyo Metropolitan Institute of Public Health|Pathogen Genomics Center|Sekizuka|Japan
>Spike|hCoV-19/France/PAC-IHU-0449/2020|2020-04-04|EPI_ISL_569516|Original|hCoV-19^^Provence-Alpes-C\u00f4te-d\u2019Azur|Human|MEPHI|MEPHI|Levasseur|France
>Spike|hCoV-19/England/CAMB-7BD72/2020|2020-04-04|EPI_ISL_442191|Original|hCoV-19^^England|Human|Department of Pathology|Wellcome Sanger Institute for the COVID-19 Genomics UK (COG-UK) consortium|Amato|United Kingdom
>Spike|hCoV-19/India/OR-ILSCV31694/2020|2020-05-26|EPI_ISL_481152|Original|hCoV-19^^Odisha|Human|Immunogenomics lab|Immunogenomics lab|Raghav|India
>Spike|hCoV-19/Portugal/PT0891/2020|2020-03-20|EPI_ISL_511149|Original|hCoV-19^^Portugal|Human|Instituto Nacional de Saude (INSA)|Instituto Nacional de Saude (INSA)|Borges|Portugal
>Spike|hCoV-19/England/QEUH-9DE0E4/2020|2020-09-21|EPI_ISL_599801|Original|hCoV-19^^England|Human|Lighthouse Lab in Glasgow|Wellcome Sanger Institute for the COVID-19 Genomics UK (COG-UK) consortium|Davies|United Kingdom
>Spike|hCoV-19/USA/CT-Yale-463/2020|2020-09-12|EPI_ISL_729760|Original|hCoV-19^^Connecticut|Human|Connecticut Department of Health|Grubaugh Lab - Yale School of Public Health|Grubaugh|USA
>Spike|hCoV-19/Hong Kong/HKU-IHCE0511-011b/2020|2020-03-15|EPI_ISL_538548|Original|hCoV-19^^Hong Kong|Human|Department of Microbiology|Department of Microbiology|Chan|China
>Spike|hCoV-19/Ireland/CO-NVRL-20G32225/2020|2020-03-17|EPI_ISL_605146|Original|hCoV-19^^Cork|Human|National Virus Reference Laboratory|Irish Coronavirus Sequencing Consortium - Helixworks|Chalapati|Ireland
>Spike|hCoV-19/USA/CA-CZB-13474/2020|2020-10-22|EPI_ISL_672529|Original|hCoV-19^^California|Human|Madera County Department of Public Health|Chan-Zuckerberg Biohub|Bhatt|USA

>Spike|hCoV-19/United Arab Emirates/0865/2020|2020-05-
19|EPI_ISL_699004|Original|hCoV-19^^Abu Dhabi|Human|Group 42 (G42) Healthcare|G42
Healthcare|Ogrodzki|United Arab Emirates
>Spike|hCoV-19/Australia/VIC8060/2020|2020-08-17|EPI_ISL_564825|Original|hCoV-
19^^Victoria|Human|Microbiological Diagnostic Unit - Public Health Laboratory (MDU-
PHL)|MDU-PHL|Schultz|Australia
>Spike|hCoV-19/USA/UT-UPHL-2008848/2020|2020-06-01|EPI_ISL_524865|Original|hCoV-
19^^Utah|Human|Utah Public Health Laboratory|Utah Public Health Laboratory|Young|USA
>Spike|hCoV-19/England/ALDP-B4814A/2020|2020-11-07|EPI_ISL_649430|Original|hCoV-
19^^England|Human|Lighthouse Lab in Alderley Park|Wellcome Sanger Institute for the
COVID-19 Genomics UK (COG-UK) Consortium|Davies|United Kingdom
>Spike|hCoV-19/Spain/MD-IBV-003665/2020|2020-04-01|EPI_ISL_467215|Original|hCoV-
19^^Madrid|Human|Hospital General Universitario Gregorio Mara\u00f1\u00f3n|SeqCOVID-
SPAIN consortium/IBV(CSIC)|Comas|Spain
>Spike|hCoV-19/Australia/VIC8939/2020|2020-07-23|EPI_ISL_565293|Original|hCoV-
19^^Victoria|Human|Microbiological Diagnostic Unit - Public Health Laboratory (MDU-
PHL)|MDU-PHL|Schultz|Australia
>Spike|hCoV-19/Wales/PHWC-1604E3/2020|2020-05-24|EPI_ISL_472943|Original|hCoV-
19^^Wales|Human|Wales Specialist Virology Centre Sequencing lab|COVID-19 Genomics UK
(COG-UK) Consortium|Moore|United Kingdom
>Spike|hCoV-19/Kenya/C3980/2020|2020-05-05|EPI_ISL_568719|Original|hCoV-
19^^Mombasa|Human|KEMRI-Wellcome Trust Research Programme/KEMRI-CGMR-C Kilifi|KEMRI-
Wellcome Trust Research Programme/KEMRI-CGMR-C Kilifi|Githinji|Kenya
>Spike|hCoV-19/USA/MI-UM-10036682652/2020|2020-10-29|EPI_ISL_682087|Original|hCoV-
19^^Michigan|Human|University of Michigan Clinical Microbiology Laboratory|Lauring
Lab|Valesano|USA
>Spike|hCoV-19/Denmark/DCGC-7410/2020|2020-10-12|EPI_ISL_619364|Original|hCoV-
19^^Midtjylland|Human|Department of Virus and Microbiological Special
Diagnostics|Albertsen lab|Michaelsen|Denmark
>Spike|hCoV-19/Denmark/DCGC-18697/2020|2020-12-07|EPI_ISL_750130|Original|hCoV-
19^^Midtjylland|Human|Department of Virus and Microbiological Special
Diagnostics|Albertsen Lab|Michaelsen|Denmark
>Spike|hCoV-19/England/ALDP-650B64/2020|2020-07-04|EPI_ISL_558197|Original|hCoV-
19^^England|Human|Lighthouse Lab in Alderley Park|Wellcome Sanger Institute for the
COVID-19 Genomics UK (COG-UK) consortium|Davies|United Kingdom
>Spike|hCoV-19/USA/FL-BPHL-0153/2020|2020-03-31|EPI_ISL_480804|Original|hCoV-
19^^Florida|Human|Florida Bureau of Public Health Laboratories|Florida Bureau of
Public Health Laboratories|Blanton|USA
>Spike|hCoV-19/England/ALDP-94CB1D/2020|2020-06-15|EPI_ISL_556048|Original|hCoV-
19^^England|Human|Lighthouse Lab in Alderley Park|Wellcome Sanger Institute for the
COVID-19 Genomics UK (COG-UK) consortium|Davies|United Kingdom
>Spike|hCoV-19/USA/CA-ALSR-4172/2020|2020-07-02|EPI_ISL_648863|Original|hCoV-
19^^California|Human|San Diego County Public Health Laboratory|Andersen lab at
Scripps Research|Gangavarapu|USA
>Spike|hCoV-19/England/BIRM-66FA1/2020|2020-10-28|EPI_ISL_651603|Original|hCoV-
19^^England|Human|University of Birmingham|COVID-19 Genomics UK (COG-UK)
Consortium|Loman|United Kingdom
>Spike|hCoV-19/England/NOTT-10F853/2020|2020-04-04|EPI_ISL_433055|Original|hCoV-
19^^England|Human|Queens Medical Centre|COVID-19 Genomics UK (COG-UK)
Consortium|Loose|United Kingdom
>Spike|hCoV-19/Wales/PHWC-4A1AD2/2020|2020-12-11|EPI_ISL_743774|Original|hCoV-
19^^Wales|Human|Wales Specialist Virology Centre Sequencing lab|COVID-19 Genomics UK
(COG-UK) Consortium|Moore|United Kingdom
>Spike|hCoV-19/USA/NY-Wadsworth-281870-01/2020|2020-12-
01|EPI_ISL_754424|Original|hCoV-19^^New York|Human|Wadsworth Center|Wadsworth
Center|Lasek-Nesselquist|USA
>Spike|hCoV-19/England/ALDP-955003/2020|2020-06-12|EPI_ISL_553600|Original|hCoV-
19^^England|Human|Lighthouse Lab in Alderley Park|Wellcome Sanger Institute for the
COVID-19 Genomics UK (COG-UK) consortium|Davies|United Kingdom

>Spike|hCoV-19/Denmark/DCGC-6612/2020|2020-10-12|EPI_ISL_619661|Original|hCoV-19^^Midtjylland|Human|Department of Virus and Microbiological Special Diagnostics|Albertsen lab|Michaelsen|Denmark
>Spike|hCoV-19/Singapore/343/2020|2020-04-09|EPI_ISL_469097|Original|hCoV-19^^Singapore|Human|National Public Health Laboratory|National Public Health Laboratory|Mak|Singapore
>Spike|hCoV-19/England/NORT-282DB2/2020|2020-03-29|EPI_ISL_488474|Original|hCoV-19^^England|Human|NU-OMICS DNA Sequencing research facility|Wellcome Sanger Institute for the COVID-19 Genomics UK (COG-UK) consortium|Davies|United Kingdom
>Spike|hCoV-19/USA/TX-HMH-MCoV-14433/2020|2020-10-07|EPI_ISL_787718|Original|hCoV-19^^Texas|Human|Houston Methodist Hospital|Houston Methodist Hospital|Long|USA
>Spike|hCoV-19/USA/TX-HMH-6125/2020|2020-07-07|EPI_ISL_547258|Original|hCoV-19^^Texas|Human|Houston Methodist Hospital|Houston Methodist Hospital|Long|USA
>Spike|hCoV-19/England/MILK-AC8FD0/2020|2020-10-22|EPI_ISL_629557|Original|hCoV-19^^England|Human|Lighthouse Lab in Milton Keynes|Wellcome Sanger Institute for the COVID-19 Genomics UK (COG-UK) consortium|Davies|United Kingdom
>Spike|hCoV-19/England/MILK-AB7772/2020|2020-10-19|EPI_ISL_623499|Original|hCoV-19^^England|Human|Lighthouse Lab in Milton Keynes|Wellcome Sanger Institute for the COVID-19 Genomics UK (COG-UK) consortium|Davies|United Kingdom
>Spike|hCoV-19/USA/MN-MDH-1820/2020|2020-04-15|EPI_ISL_590822|Original|hCoV-19^^Minnesota|Human|Minnesota Department of Health|Minnesota Department of Health|Lorentz|USA
>Spike|hCoV-19/USA/TX-HMH-MCoV-17993/2020|2020-11-16|EPI_ISL_786141|Original|hCoV-19^^Texas|Human|Houston Methodist Hospital|Houston Methodist Hospital|Long|USA
>Spike|hCoV-19/Denmark/DCGC-15038/2020|2020-11-23|EPI_ISL_713598|Original|hCoV-19^^Hovedstaden|Human|Department of Virus and Microbiological Special Diagnostics|Albertsen Lab|Michaelsen|Denmark
>Spike|hCoV-19/Denmark/DCGC-8889/2020|2020-10-26|EPI_ISL_626131|Original|hCoV-19^^Syddanmark|Human|Department of Virus and Microbiological Special Diagnostics|Albertsen lab|Michaelsen|Denmark
>Spike|hCoV-19/Netherlands/NB-EMC-271/2020|2020-00-00|EPI_ISL_523393|Original|hCoV-19^^Noord Brabant|Human|Dutch COVID-19 response team|Erasmus Medical Center|Oude Munnink|Netherlands
>Spike|hCoV-19/USA/TX-HMH-MCoV-10540/2020|2020-07-08|EPI_ISL_789527|Original|hCoV-19^^Texas|Human|Houston Methodist Hospital|Houston Methodist Hospital|Long|USA
>Spike|hCoV-19/England/QEUH-B66B48/2020|2020-11-11|EPI_ISL_662465|Original|hCoV-19^^England|Human|Lighthouse Lab in Glasgow|Wellcome Sanger Institute for the COVID-19 Genomics UK (COG-UK) Consortium|Davies|United Kingdom
>Spike|hCoV-19/England/LOND-D5F16/2020|2020-04-18|EPI_ISL_444262|Original|hCoV-19^^England|Human|University College London|COVID-19 Genomics UK (COG-UK) Consortium|Roy|United Kingdom
>Spike|hCoV-19/England/BRIS-12F3A7/2020|2020-04-13|EPI_ISL_488291|Original|hCoV-19^^England|Human|PHE South West Regional Laboratory|Wellcome Sanger Institute for the COVID-19 Genomics UK (COG-UK) consortium|Davies|United Kingdom
>Spike|hCoV-19/India/GJ-GBRC245/2020|2020-06-14|EPI_ISL_483826|Original|hCoV-19^^Gujarat|Human|GMERS Medical College Himmatnagar|Gujarat Biotechnology Research Centre|Joshi|India
>Spike|hCoV-19/England/QEUH-9637FE/2020|2020-08-10|EPI_ISL_531744|Original|hCoV-19^^England|Human|Lighthouse Lab in Glasgow|Wellcome Sanger Institute for the COVID-19 Genomics UK (COG-UK) consortium|Davies|United Kingdom
>Spike|hCoV-19/Wales/PHWC-495E3F/2020|2020-11-26|EPI_ISL_726491|Original|hCoV-19^^Wales|Human|Originating lab|COVID-19 Genomics UK (COG-UK) Consortium|Moore|United Kingdom
>Spike|hCoV-19/South Korea/KCDC2326/2020|2020-05-26|EPI_ISL_510662|Original|hCoV-19^^South Korea|Human|Division of Viral Diseases|Division of Viral Diseases|Kim|South Korea
>Spike|hCoV-19/Scotland/EDB3658/2020|2020-04-28|EPI_ISL_439309|Original|hCoV-19^^Scotland|Human|Virology Department|COVID-19 Genomics UK (COG-UK) Consortium|Williams|United Kingdom

>Spike|hCoV-19/England/ALDP-9CD6AA/2020|2020-09-16|EPI_ISL_566151|Original|hCoV-19^^England|Human|Lighthouse Lab in Alderley Park|Wellcome Sanger Institute for the COVID-19 Genomics UK (COG-UK) consortium|Davies|United Kingdom
>Spike|hCoV-19/Denmark/DCGC-22382/2020|2020-12-14|EPI_ISL_757406|Original|hCoV-19^^Sjaelland|Human|Department of Virus and Microbiological Special Diagnostics|Albertsen Lab|Michaelsen|Denmark
>Spike|hCoV-19/Wales/PHWC-165CAA/2020|2020-05-26|EPI_ISL_490788|Original|hCoV-19^^Wales|Human|Wales Specialist Virology Centre Sequencing lab|COVID-19 Genomics UK (COG-UK) Consortium|Moore|United Kingdom
>Spike|hCoV-19/England/NORT-2A59D3/2020|2020-10-01|EPI_ISL_584702|Original|hCoV-19^^England|Human|Northumbria University / South Tees Hospitals NHS Foundation Trust / North Cumbria Integrated Care NHS Foundation Trust / North Tees and Hartlepool NHS Foundation Trust / Newcastle Hospitals NHS Foundation Trust|COVID-19 Genomics UK (COG-UK) Consortium|Bashton|United Kingdom
>Spike|hCoV-19/England/ALDP-AA4C6A/2020|2020-10-21|EPI_ISL_630918|Original|hCoV-19^^England|Human|Lighthouse Lab in Alderley Park|Wellcome Sanger Institute for the COVID-19 Genomics UK (COG-UK) consortium|Davies|United Kingdom
>Spike|hCoV-19/Netherlands/Utrecht_10010/2020|2020-03-13|EPI_ISL_454759|Original|hCoV-19^^Utrecht|Human|Dutch COVID-19 response team|National Institute for Public Health and the Environment (RIVM)|Meijer|Netherlands
>Spike|hCoV-19/New Zealand/20CV0209/2020|2020-08-29|EPI_ISL_548114|Original|hCoV-19^^Auckland|Human|Middlemore Hospital|Institute of Environmental Science and Research (ESR)|Wang|New Zealand
>Spike|hCoV-19/USA/TX-HMH-MCoV-11674/2020|2020-09-08|EPI_ISL_790082|Original|hCoV-19^^Texas|Human|Houston Methodist Hospital|Houston Methodist Hospital|Long|USA
>Spike|hCoV-19/USA/AZ-TG396355/2020|2020-06-10|EPI_ISL_694813|Original|hCoV-19^^Arizona|Human|AZ SPHL|TGen North|French|USA
>Spike|hCoV-19/India/WB-S54/2020|2020-05-01|EPI_ISL_508451|Original|hCoV-19^^West Bengal|Human|ICMR-National Institute of Cholera and Enteric Diseases|National Institute of Biomedical Genomics|Maitra|India
>Spike|hCoV-19/England/CAMB-1B8543/2020|2020-10-14|EPI_ISL_637859|Original|hCoV-19^^England|Human|Department of Pathology|COVID-19 Genomics UK (COG-UK) Consortium|Goodfellow|United Kingdom
>Spike|hCoV-19/England/SHEF-C62E2/2020|2020-05-02|EPI_ISL_457609|Original|hCoV-19^^England|Human|Virology Department|COVID-19 Genomics UK (COG-UK) Consortium|de Silva|United Kingdom
>Spike|hCoV-19/Wales/PHWC-27B11/2020|2020-03-30|EPI_ISL_431921|Original|hCoV-19^^Wales|Human|Wales Specialist Virology Centre|Public Health Wales Microbiology Cardiff|Moore|United Kingdom
>Spike|hCoV-19/England/MILK-CA9D33/2020|2020-12-18|EPI_ISL_762225|Original|hCoV-19^^England|Human|Lighthouse Lab in Milton Keynes|Wellcome Sanger Institute for the COVID-19 Genomics UK (COG-UK) Consortium|Davies|United Kingdom
>Spike|hCoV-19/England/QEUH-B2D275/2020|2020-11-04|EPI_ISL_659753|Original|hCoV-19^^England|Human|Lighthouse Lab in Glasgow|Wellcome Sanger Institute for the COVID-19 Genomics UK (COG-UK) Consortium|Davies|United Kingdom
>Spike|hCoV-19/France/OCC-113/2020|2020-10-22|EPI_ISL_671959|Original|hCoV-19^^Occitanie|Human|CHU Purpan - Laboratoire de Virologie - Institut F\u00e9d\u00e9ratif de Biologie|CHU Purpan - Laboratoire de Virologie - Institut F\u00e9d\u00e9ratif de Biologie|Latour|France
>Spike|hCoV-19/USA/WA-S1943/2020|2020-06-02|EPI_ISL_497371|Original|hCoV-19^^Washington|Human|Washington State Department of Health|Seattle Flu Study|Lee|USA
>Spike|hCoV-19/Australia/TAS87/2020|2020-03-21|EPI_ISL_522758|Original|hCoV-19^^Tasmania|Human|Royal Hobart Hospital Microbiology Department|MDU-PHL|Schultz|Australia
>Spike|hCoV-19/Scotland/EDB2958/2020|2020-04-02|EPI_ISL_456907|Original|hCoV-19^^Scotland|Human|Virology Department|COVID-19 Genomics UK (COG-UK) Consortium|Williams|United Kingdom

>Spike|hCoV-19/England/NORW-E6DAF/2020|2020-04-21|EPI_ISL_453545|Original|hCoV-19^^England|Human|Quadram Institute Bioscience|COVID-19 Genomics UK (COG-UK) Consortium|Alikhan|United Kingdom
>Spike|hCoV-19/Japan/PG-5538/2020|2020-08-00|EPI_ISL_688663|Original|hCoV-19^^Japan|Human|Pathogen Genomics Center|Pathogen Genomics Center|Sekizuka|Japan
>Spike|hCoV-19/Denmark/DCGC-7748/2020|2020-10-19|EPI_ISL_618712|Original|hCoV-19^^Syddanmark|Human|Department of Virus and Microbiological Special Diagnostics|Albertsen lab|Michaelsen|Denmark
>Spike|hCoV-19/Japan/PG-1040/2020|2020-04-00|EPI_ISL_684650|Original|hCoV-19^^Japan|Human|Fukuoka Institute of Health and Environmental Sciences|Pathogen Genomics Center|Sekizuka|Japan
>Spike|hCoV-19/USA/TX-HMH-MCoV-10710/2020|2020-07-08|EPI_ISL_789150|Original|hCoV-19^^Texas|Human|Houston Methodist Hospital|Houston Methodist Hospital|Long|USA
>Spike|hCoV-19/England/204742200/2020|2020-00-00|EPI_ISL_649910|Original|hCoV-19^^England|Human|Respiratory Virus Unit|COVID-19 Genomics UK (COG-UK) Consortium|Platt|United Kingdom
>Spike|hCoV-19/England/ALDP-B7642C/2020|2020-11-12|EPI_ISL_723761|Original|hCoV-19^^England|Human|Oxford Viromics|COVID-19 Genomics UK (COG-UK) Consortium|Golubchik|United Kingdom
>Spike|hCoV-19/Australia/VIC12268/2020|2020-07-23|EPI_ISL_640384|Original|hCoV-19^^Victoria|Human|Microbiological Diagnostic Unit - Public Health Laboratory (MDU-PHL)|MDU-PHL|Schultz|Australia
>Spike|hCoV-19/England/PORT-2EAAC0/2020|2020-00-00|EPI_ISL_727770|Original|hCoV-19^^England|Human|Centre for Enzyme Innovation|COVID-19 Genomics UK (COG-UK) Consortium|Robson|United Kingdom
>Spike|hCoV-19/England/202822173/2020|2020-06-15|EPI_ISL_490230|Original|hCoV-19^^England|Human|Respiratory Virus Unit|Respiratory Virus Unit|Platt|United Kingdom
>Spike|hCoV-19/Japan/PG-4119/2020|2020-07-00|EPI_ISL_692359|Original|hCoV-19^^Japan|Human|Pathogen Genomics Center|Pathogen Genomics Center|Sekizuka|Japan
>Spike|hCoV-19/Finland/5LS20C12/2020|2020-10-05|EPI_ISL_756051|Original|hCoV-19^^Uusimaa|Human|Department of Virology and Immunology|Department of Virology|Smura|Finland
>Spike|hCoV-19/USA/NJ-QDX-74/2020|2020-03-14|EPI_ISL_467883|Original|hCoV-19^^New Jersey|Human|Quest Diagnostics|Quest Diagnostics|Kagan|USA
>Spike|hCoV-19/Italy/UMB-IZSUM-48682-20/2020|2020-10-29|EPI_ISL_763097|Original|hCoV-19^^Umbria|Human|Istituto Zooprofilattico Sperimentale dell' Umbria e delle Marche - Togo Rosati|Istituto Superiore di Sanit\u00e0|De Sabato|Italy
>Spike|hCoV-19/Scotland/QEUH-B6A29A/2020|2020-11-10|EPI_ISL_676341|Original|hCoV-19^^Scotland|Human|Lighthouse Lab in Glasgow|Wellcome Sanger Institute for the COVID-19 Genomics UK (COG-UK) Consortium|Davies|United Kingdom
>Spike|hCoV-19/Australia/WA353/2020|2020-10-18|EPI_ISL_605830|Original|hCoV-19^^Western Australia|Human|PathWest Laboratory Medicine WA|PathWest Laboratory Medicine WA Microbial Surveillance Unit|Sikazwe|Australia
>Spike|hCoV-19/USA/NY-PV09097/2020|2020-03-18|EPI_ISL_421631|Original|hCoV-19^^New York|Human|MSHS Clinical Microbiology Laboratories|MSHS Pathogen Surveillance Program|van Bakel|USA
>Spike|hCoV-19/USA/FL-BPHL-0397/2020|2020-03-13|EPI_ISL_489806|Original|hCoV-19^^Florida|Human|Florida Bureau of Public Health Laboratories|Florida Bureau of Public Health Laboratories|Blanton|USA
>Spike|hCoV-19/England/BRIS-12CDE8/2020|2020-04-24|EPI_ISL_481824|Original|hCoV-19^^England|Human|PHE South West Regional Laboratory|Wellcome Sanger Institute for the COVID-19 Genomics UK (COG-UK) consortium|Davies|United Kingdom
>Spike|hCoV-19/England/ALDP-B468CF/2020|2020-11-06|EPI_ISL_655981|Original|hCoV-19^^England|Human|Lighthouse Lab in Alderley Park|Wellcome Sanger Institute for the COVID-19 Genomics UK (COG-UK) Consortium|Davies|United Kingdom
>Spike|hCoV-19/England/MILK-ABBDA9/2020|2020-10-21|EPI_ISL_631240|Original|hCoV-19^^England|Human|Lighthouse Lab in Milton Keynes|Wellcome Sanger Institute for the COVID-19 Genomics UK (COG-UK) consortium|Davies|United Kingdom

>Spike|hCoV-19/USA/CA-ALSR-1101-SAN/2020|2020-06-17|EPI_ISL_483527|Original|hCoV-19^^California|Human|UC San Diego Center for Advanced Laboratory Medicine|Andersen lab at Scripps Research|Gangavarapu|USA
>Spike|hCoV-19/England/OXON-AFBC6/2020|2020-04-09|EPI_ISL_478811|Original|hCoV-19^^England|Human|Oxford Viromics|COVID-19 Genomics UK (COG-UK) Consortium|Golubchik|United Kingdom
>Spike|hCoV-19/Wales/PHWC-4879F3/2020|2020-10-31|EPI_ISL_639262|Original|hCoV-19^^Wales|Human|Wales Specialist Virology Centre Sequencing lab|COVID-19 Genomics UK (COG-UK) Consortium|Moore|United Kingdom
>Spike|hCoV-19/Switzerland/BE-ETHZ-140048/2020|2020-05-28|EPI_ISL_476099|Original|hCoV-19^^Bern|Human|Viollier AG|Department of Biosystems Science and Engineering|Nadeau|Switzerland
>Spike|hCoV-19/Denmark/DCGC-13215/2020|2020-11-30|EPI_ISL_712658|Original|hCoV-19^^Hovedstaden|Human|Department of Virus and Microbiological Special Diagnostics|Albertsen Lab|Michaelsen|Denmark
>Spike|hCoV-19/USA/OR-OHSU-1835/2020|2020-07-27|EPI_ISL_526042|Original|hCoV-19^^Oregon|Human|OHSU Lab Services Molecular Microbiology Lab|Oregon SARS-CoV-2 Genome Sequencing Center|O'Roak|USA
>Spike|hCoV-19/Switzerland/SH-ETHZ-420734/2020|2020-12-24|EPI_ISL_768158|Original|hCoV-19^^Schaffhausen|Human|Viollier AG|Department of Biosystems Science and Engineering|Nadeau|Switzerland
>Spike|hCoV-19/Belgium/rega-0411463/2020|2020-04-11|EPI_ISL_476973|Original|hCoV-19^^Halle|Human|KU Leuven|KU Leuven|Maes|Belgium
>Spike|hCoV-19/Kenya/C7383/2020|2020-05-17|EPI_ISL_568837|Original|hCoV-19^^Mombasa|Human|KEMRI-Wellcome Trust Research Programme/KEMRI-CGMR-C Kilifi|KEMRI-Wellcome Trust Research Programme/KEMRI-CGMR-C Kilifi|Githinji|Kenya
>Spike|hCoV-19/England/LOND-124DE26/2020|2020-00-00|EPI_ISL_584651|Original|hCoV-19^^England|Human|University College London Hospital|COVID-19 Genomics UK (COG-UK) Consortium|Byott|United Kingdom
>Spike|hCoV-19/England/MILK-A05EE9/2020|2020-10-01|EPI_ISL_599820|Original|hCoV-19^^England|Human|Lighthouse Lab in Milton Keynes|Wellcome Sanger Institute for the COVID-19 Genomics UK (COG-UK) consortium|Davies|United Kingdom
>Spike|hCoV-19/England/GSTT-265D545/2020|2020-03-23|EPI_ISL_483975|Original|hCoV-19^^England|Human|Centre for Clinical Infection and Diagnostics Research and Genomics Innovation Unit|COVID-19 Genomics UK (COG-UK) Consortium|Awan|United Kingdom
>Spike|hCoV-19/England/QEUH-AE2C9D/2020|2020-10-27|EPI_ISL_633614|Original|hCoV-19^^England|Human|Lighthouse Lab in Glasgow|Wellcome Sanger Institute for the COVID-19 Genomics UK (COG-UK) consortium|Davies|United Kingdom
>Spike|hCoV-19/Russia/MOW-RII-MH2007S/2020|2020-05-15|EPI_ISL_596349|Original|hCoV-19^^Moscow|Human|Pathogenic Microorganisms Variability Laboratory|WHO National Influenza Centre Russian Federation|Komissarov|Russia
>Spike|hCoV-19/England/NORW-ED476/2020|2020-05-28|EPI_ISL_472368|Original|hCoV-19^^England|Human|Quadram Institute Bioscience|COVID-19 Genomics UK (COG-UK) Consortium|Alikhan|United Kingdom
>Spike|hCoV-19/USA/MA-MGH-00832/2020|2020-04-13|EPI_ISL_791788|Original|hCoV-19^^Massachusetts|Human|Massachusetts General Hospital|Infectious Disease Program|Park|USA
>Spike|hCoV-19/England/MILK-A46ABB/2020|2020-10-06|EPI_ISL_598473|Original|hCoV-19^^England|Human|Lighthouse Lab in Milton Keynes|Wellcome Sanger Institute for the COVID-19 Genomics UK (COG-UK) consortium|Davies|United Kingdom
>Spike|hCoV-19/Denmark/DCGC-10429/2020|2020-11-02|EPI_ISL_669964|Original|hCoV-19^^Midtjylland|Human|Department of Virus and Microbiological Special Diagnostics|Albertsen Lab|Michaelsen|Denmark
>Spike|hCoV-19/Japan/PG-6122/2020|2020-08-00|EPI_ISL_689234|Original|hCoV-19^^Japan|Human|Pathogen Genomics Center|Pathogen Genomics Center|Sekizuka|Japan
>Spike|hCoV-19/Singapore/1491/2020|2020-12-22|EPI_ISL_754100|Original|hCoV-19^^Singapore|Human|National Public Health Laboratory|National Public Health Laboratory|Mak|Singapore

>Spike|hCoV-19/India/UT-67339-CSIR-IGIB/2020|2020-08-09|EPI_ISL_636742|Original|hCoV-19^^Uttarakhand|Human|National Centre for Disease control (NCDC)|NCDC/CSIR-IGIB|Faruq|India
>Spike|hCoV-19/USA/WA1-F6/2020|2020-01-25|EPI_ISL_407215|Vero P1|hCoV-19^^Washington|Human|Washington State Department of Health|Pathogen Discovery|Queen|USA
>Spike|hCoV-19/Singapore/1144/2020|2020-04-16|EPI_ISL_648053|Original|hCoV-19^^Singapore|Human|Department of Laboratory Medicine|Department of Laboratory Medicine|Chen|Singapore
>Spike|hCoV-19/Japan/PG-0364/2020|2020-03-05|EPI_ISL_480034|Original|hCoV-19^^Kanto|Human|Tochigi Prefectural Institute of Public Health and Environmental Science|Pathogen Genomics Center|Sekizuka|Japan
>Spike|hCoV-19/England/NOTT-10E03E/2020|2020-03-17|EPI_ISL_425486|Original|hCoV-19^^England|Human|Queens Medical Centre|COVID-19 Genomics UK (COG-UK) Consortium|Loose|United Kingdom
>Spike|hCoV-19/England/ALDP-4A55CE/2020|2020-06-12|EPI_ISL_559614|Original|hCoV-19^^England|Human|Lighthouse Lab in Alderley Park|Wellcome Sanger Institute for the COVID-19 Genomics UK (COG-UK) consortium|Davies|United Kingdom
>Spike|hCoV-19/Germany/HH-hpi-p1365/2020|2020-03-28|EPI_ISL_775837|Original|hCoV-19^^Hamburg|Human|University Medical Center Hamburg Eppendorf|Heinrich Pette Institute|Robitaille|Germany
>Spike|hCoV-19/USA/UT-UPHL-201131133/2020|2020-04-01|EPI_ISL_648556|Original|hCoV-19^^Utah|Human|Utah Public Health Laboratory|Utah Public Health Laboratory|Young|USA
>Spike|hCoV-19/Luxembourg/LNS8772889/2020|2020-03-29|EPI_ISL_429755|Original|hCoV-19^^Luxembourg|Human|Laboratoire National de Sante|Laboratoire National de Sante|Wienecke-Baldacchino|Luxembourg
>Spike|hCoV-19/Denmark/DCGC-21724/2020|2020-12-21|EPI_ISL_758328|Original|hCoV-19^^Syddanmark|Human|Department of Virus and Microbiological Special Diagnostics|Albertsen Lab|Michaelsen|Denmark
>Spike|hCoV-19/England/QEUH-CC3A1E/2020|2020-12-21|EPI_ISL_760584|Original|hCoV-19^^England|Human|Lighthouse Lab in Glasgow|Wellcome Sanger Institute for the COVID-19 Genomics UK (COG-UK) Consortium|Davies|United Kingdom
>Spike|hCoV-19/Switzerland/BE-ETHZ-270109/2020|2020-09-03|EPI_ISL_541427|Original|hCoV-19^^Bern|Human|Viollier AG|Department of Biosystems Science and Engineering|Nadeau|Switzerland
>Spike|hCoV-19/USA/TX-HMH0301/2020|2020-03-28|EPI_ISL_434924|Original|hCoV-19^^Texas|Human|Houston Methodist Hospital|Houston Methodist Hospital|Long|USA
>Spike|hCoV-19/South Africa/KRISP-K002188/2020|2020-07-08|EPI_ISL_515587|Original|hCoV-19^^KwaZulu-Natal|Human|NHLS-IALCH|KRISP|de Oliveira|South Africa
>Spike|hCoV-19/England/20138077804/2020|2020-03-26|EPI_ISL_423323|Original|hCoV-19^^England|Human|Respiratory Virus Unit|Respiratory Virus Unit|Platt|United Kingdom
>Spike|hCoV-19/England/ALDP-954AE8/2020|2020-06-12|EPI_ISL_553487|Original|hCoV-19^^England|Human|Lighthouse Lab in Alderley Park|Wellcome Sanger Institute for the COVID-19 Genomics UK (COG-UK) consortium|Davies|United Kingdom
>Spike|hCoV-19/USA/CO-CDPHE-2100008783/2020|2020-11-20|EPI_ISL_771291|Original|hCoV-19^^Colorado|Human|Colorado Department of Public Health and Environment|Colorado Department of Puplic Health and Environment|Bankers|USA
>Spike|hCoV-19/England/ALDP-AA41E3/2020|2020-10-22|EPI_ISL_624107|Original|hCoV-19^^England|Human|Lighthouse Lab in Alderley Park|Wellcome Sanger Institute for the COVID-19 Genomics UK (COG-UK) consortium|Davies|United Kingdom
>Spike|hCoV-19/South Korea/KCDC2341/2020|2020-05-26|EPI_ISL_510677|Original|hCoV-19^^South Korea|Human|Division of Viral Diseases|Division of Viral Diseases|Kim|South Korea
>Spike|hCoV-19/Canada/ON-E76b/2020|2020-05-06|EPI_ISL_770827|Original|hCoV-19^^Ontario|Human|Eastern Ontario Regional Laboratory Association|McMaster University|McArthur|Canada
>Spike|hCoV-19/Scotland/EDB4584/2020|2020-05-07|EPI_ISL_473797|Original|hCoV-19^^Scotland|Human|Virology Department|COVID-19 Genomics UK (COG-UK) Consortium|Williams|United Kingdom

>Spike|hCoV-19/England/QEUH-CC3577/2020|2020-12-21|EPI_ISL_760539|Original|hCoV-19^^England|Human|Lighthouse Lab in Glasgow|Wellcome Sanger Institute for the COVID-19 Genomics UK (COG-UK) Consortium|Davies|United Kingdom
>Spike|hCoV-19/England/QEUH-A49809/2020|2020-10-05|EPI_ISL_628431|Original|hCoV-19^^England|Human|Oxford Viromics|COVID-19 Genomics UK (COG-UK) Consortium|Golubchik|United Kingdom
>Spike|hCoV-19/England/SHEF-C6F45/2020|2020-05-19|EPI_ISL_529682|Original|hCoV-19^^England|Human|Virology Department|COVID-19 Genomics UK (COG-UK) Consortium|de Silva|United Kingdom
>Spike|hCoV-19/England/ALDP-B19F39/2020|2020-11-01|EPI_ISL_645684|Original|hCoV-19^^England|Human|Lighthouse Lab in Alderley Park|Wellcome Sanger Institute for the COVID-19 Genomics UK (COG-UK) Consortium|Davies|United Kingdom
>Spike|hCoV-19/England/ALDP-C3A317/2020|2020-12-09|EPI_ISL_731131|Original|hCoV-19^^England|Human|Lighthouse Lab in Alderley Park|Wellcome Sanger Institute for the COVID-19 Genomics UK (COG-UK) Consortium|Davies|United Kingdom
>Spike|hCoV-19/Australia/VIC3570/2020|2020-07-08|EPI_ISL_520319|Original|hCoV-19^^Victoria|Human|Victorian Infectious Diseases Reference Laboratory (VIDRL)|VIDRL and MDU-PHL|Schultz|Australia
>Spike|hCoV-19/England/BIRM-646DB/2020|2020-09-23|EPI_ISL_594506|Original|hCoV-19^^England|Human|University of Birmingham|COVID-19 Genomics UK (COG-UK) Consortium|Loman|United Kingdom
>Spike|hCoV-19/Bosnia and Herzegovina/ChVir7367/2020|2020-03-27|EPI_ISL_462469|Original|hCoV-19^^Sarajevo|Human|Clinical Center|Charite Universitatsmedizin Berlin|Muehlemann|Bosnia and Herzegovina
>Spike|hCoV-19/USA/SC-DHEC-0001/2020|2020-03-06|EPI_ISL_491097|Original|hCoV-19^^South Carolina|Human|SC Department of Health and Environmental Control|SC Department of Health and Environmental Control|Flores|USA
>Spike|hCoV-19/USA/TX-HMH-MCoV-12558/2020|2020-09-30|EPI_ISL_789941|Original|hCoV-19^^Texas|Human|Houston Methodist Hospital|Houston Methodist Hospital|Long|USA
>Spike|hCoV-19/Israel/CVL-n2775/2020|2020-03-08|EPI_ISL_649101|Original|hCoV-19^^Tel-Aviv|Human|Israel Central Virology laboratory|Israel Central Virology laboratory|Zuckerman|Israel
>Spike|hCoV-19/Canada/ON-PHL-8539/2020|2020-03-00|EPI_ISL_418369|Original|hCoV-19^^Ontario|Human|Public Health Ontario Laboratories|Public Health Ontario Laboratories|Eshaghi|Canada
>Spike|hCoV-19/Scotland/EDB2994/2020|2020-04-02|EPI_ISL_487971|Original|hCoV-19^^Scotland|Human|Virology Department|Wellcome Sanger Institute for the COVID-19 Genomics UK (COG-UK) consortium|Davies|United Kingdom
>Spike|hCoV-19/USA/TX-HMH-MCoV-18761/2020|2020-11-21|EPI_ISL_785098|Original|hCoV-19^^Texas|Human|Houston Methodist Hospital|Houston Methodist Hospital|Long|USA
>Spike|hCoV-19/England/MILK-A6A4FF/2020|2020-10-08|EPI_ISL_626852|Original|hCoV-19^^England|Human|Virology Department|COVID-19 Genomics UK (COG-UK) Consortium|de Silva|United Kingdom
>Spike|hCoV-19/England/CAMC-B3630D/2020|2020-11-05|EPI_ISL_645437|Original|hCoV-19^^England|Human|Lighthouse Lab in Cambridge|Wellcome Sanger Institute for the COVID-19 Genomics UK (COG-UK) Consortium|Davies|United Kingdom
>Spike|hCoV-19/England/MILK-974C55/2020|2020-08-21|EPI_ISL_552688|Original|hCoV-19^^England|Human|Lighthouse Lab in Milton Keynes|Wellcome Sanger Institute for the COVID-19 Genomics UK (COG-UK) consortium|Davies|United Kingdom
>Spike|hCoV-19/England/MILK-B88B31/2020|2020-11-14|EPI_ISL_676009|Original|hCoV-19^^England|Human|Lighthouse Lab in Milton Keynes|Wellcome Sanger Institute for the COVID-19 Genomics UK (COG-UK) Consortium|Davies|United Kingdom
>Spike|hCoV-19/Austria/CeMM0288/2020|2020-03-26|EPI_ISL_438113|Original|hCoV-19^^Vienna|Human|Center for Virology|Bergthaler laboratory|Bergthaler|Austria
>Spike|hCoV-19/Northern Ireland/QEUH-96AC25/2020|2020-08-17|EPI_ISL_531307|Original|hCoV-19^^Northern Ireland|Human|Lighthouse Lab in Glasgow|Wellcome Sanger Institute for the COVID-19 Genomics UK (COG-UK) consortium|Davies|United Kingdom

>Spike|hCoV-19/Denmark/DCGC-3868/2020|2020-09-07|EPI_ISL_615586|Original|hCoV-19^^Hovedstaden|Human|Department of Virus and Microbiological Special Diagnostics|Albertsen lab|Michaelsen|Denmark
>Spike|hCoV-19/England/QEUH-B6C386/2020|2020-11-11|EPI_ISL_654995|Original|hCoV-19^^England|Human|Lighthouse Lab in Glasgow|Wellcome Sanger Institute for the COVID-19 Genomics UK (COG-UK) Consortium|Davies|United Kingdom
>Spike|hCoV-19/USA/AZ-TG409147/2020|2020-06-23|EPI_ISL_694955|Original|hCoV-19^^Arizona|Human|TGen North|TGen North|French|USA
>Spike|hCoV-19/Northern Ireland/NIRE-FAC7E/2020|2020-04-15|EPI_ISL_459395|Original|hCoV-19^^Northern Ireland|Human|Regional Virus Laboratory|Wellcome Sanger Institute for the COVID-19 Genomics UK (COG-UK) consortium|Davies|United Kingdom
>Spike|hCoV-19/Suriname/SR-60/2020|2020-07-16|EPI_ISL_518809|Original|hCoV-19^^Suriname|Human|Academic Hospital Paramaribo|Erasmus Medical Center|M\u00fcnger|Suriname
>Spike|hCoV-19/Luxembourg/LNS2333525/2020|2020-12-02|EPI_ISL_771022|Original|hCoV-19^^Luxembourg|Human|Laboratoire national de sant\u00e9|Laboratoire national de sant\u00e9|Wienecke-Baldacchino|Luxembourg
>Spike|hCoV-19/Scotland/EDB1473/2020|2020-04-11|EPI_ISL_491672|Original|hCoV-19^^Scotland|Human|Virology Department|Wellcome Sanger Institute for the COVID-19 Genomics UK (COG-UK) consortium|Davies|United Kingdom
>Spike|hCoV-19/Wales/PHWC-1698B1/2020|2020-07-25|EPI_ISL_512528|Original|hCoV-19^^Wales|Human|Wales Specialist Virology Centre Sequencing lab|COVID-19 Genomics UK (COG-UK) Consortium|Moore|United Kingdom
>Spike|hCoV-19/England/CAMC-B7B21E/2020|2020-11-12|EPI_ISL_723995|Original|hCoV-19^^England|Human|Oxford Viromics|COVID-19 Genomics UK (COG-UK) Consortium|Golubchik|United Kingdom
>Spike|hCoV-19/Portugal/PT1751/2020|2020-11-11|EPI_ISL_731988|Original|hCoV-19^^Portugal|Human|Instituto Nacional de Saude (INSA)|Instituto Nacional de Saude (INSA)|Borges|Portugal
>Spike|hCoV-19/Austria/CeMM0038/2020|2020-03-09|EPI_ISL_437927|Original|hCoV-19^^Ischgl|Human|Institut f\u00fcr Virologie am Department f\u00fcr Hygiene|Bergthaler laboratory|Bergthaler|Austria
>Spike|hCoV-19/India/MH-NIV-126959/2020|2020-07-21|EPI_ISL_541694|Original|hCoV-19^^Maharashtra|Human|National Institute of Virology|National Institute of Virology|Potdar|India
>Spike|hCoV-19/USA/TX-HMH-7889/2020|2020-06-30|EPI_ISL_545685|Original|hCoV-19^^Texas|Human|Houston Methodist Hospital|Houston Methodist Hospital|Long|USA
>Spike|hCoV-19/Denmark/DCGC-992/2020|2020-03-09|EPI_ISL_614587|Original|hCoV-19^^Syddanmark|Human|Department of Virus and Microbiological Special Diagnostics|Albertsen lab|Michaelsen|Denmark
>Spike|hCoV-19/USA/CA-CZB-11581/2020|2020-07-16|EPI_ISL_625550|Original|hCoV-19^^California|Human|UCSF Clinical Microbiology Laboratory|Chan-Zuckerberg Biohub|Bhatt|USA
>Spike|hCoV-19/USA/MN-MDH-1829/2020|2020-09-18|EPI_ISL_602964|Original|hCoV-19^^Minnesota|Human|Minnesota Department of Health|Minnesota Department of Health|Garfin|USA
>Spike|hCoV-19/England/LOND-12E84B2/2020|2020-12-06|EPI_ISL_741314|Original|hCoV-19^^England|Human|University College London|COVID-19 Genomics UK (COG-UK) Consortium|Roy|United Kingdom
>Spike|hCoV-19/England/QEUH-A0B751/2020|2020-10-01|EPI_ISL_599467|Original|hCoV-19^^England|Human|Lighthouse Lab in Glasgow|Wellcome Sanger Institute for the COVID-19 Genomics UK (COG-UK) consortium|Davies|United Kingdom
>Spike|hCoV-19/Poland/76302/2020|2020-11-00|EPI_ISL_732865|Original|hCoV-19^^Poland|Human|genXone SA|genXone SA|Kaszuba|Poland
>Spike|hCoV-19/Wales/PHWC-497E5B/2020|2020-11-30|EPI_ISL_726985|Original|hCoV-19^^Wales|Human|Originating lab|COVID-19 Genomics UK (COG-UK) Consortium|Moore|United Kingdom

>Spike|hCoV-19/England/ALDP-A94E49/2020|2020-10-19|EPI_ISL_624903|Original|hCoV-19^^England|Human|Lighthouse Lab in Alderley Park|Wellcome Sanger Institute for the COVID-19 Genomics UK (COG-UK) consortium|Davies|United Kingdom
>Spike|hCoV-19/South Korea/KCDC2385/2020|2020-05-31|EPI_ISL_514788|Original|hCoV-19^^South Korea|Human|Division of Viral Diseases|Division of Viral Diseases|Kim|South Korea
>Spike|hCoV-19/Denmark/DCGC-21621/2020|2020-12-21|EPI_ISL_757912|Original|hCoV-19^^Nordjylland|Human|Department of Virus and Microbiological Special Diagnostics|Albertsen Lab|Michaelsen|Denmark
>Spike|hCoV-19/USA/CA-QDX-1980/2020|2020-03-17|EPI_ISL_604293|Original|hCoV-19^^California|Human|Quest Diagnostics|Quest Diagnostics|Kagan|USA
>Spike|hCoV-19/Spain/MD-IBV-99008779/2020|2020-10-20|EPI_ISL_654322|Original|hCoV-19^^Madrid|Human|Hospital General Universitario Gregorio Mara\u00f1\u00f3n|SeqCOVID-SPAIN consortium/IBV(CSIC)|Comas|Spain
>Spike|hCoV-19/Denmark/DCGC-3724/2020|2020-08-31|EPI_ISL_615504|Original|hCoV-19^^Hovedstaden|Human|Department of Virus and Microbiological Special Diagnostics|Albertsen lab|Michaelsen|Denmark
>Spike|hCoV-19/England/204841929/2020|2020-11-25|EPI_ISL_683588|Original|hCoV-19^^England|Human|Respiratory Virus Unit|COVID-19 Genomics UK (COG-UK) Consortium|Platt|United Kingdom
>Spike|hCoV-19/England/QEUH-B108FB/2020|2020-11-02|EPI_ISL_642614|Original|hCoV-19^^England|Human|Lighthouse Lab in Glasgow|Wellcome Sanger Institute for the COVID-19 Genomics UK (COG-UK) Consortium|Davies|United Kingdom
>Spike|hCoV-19/Scotland/CVR4974/2020|2020-10-04|EPI_ISL_594737|Original|hCoV-19^^Scotland|Human|West of Scotland Specialist Virology Centre|COVID-19 Genomics UK (COG-UK) Consortium|Robertson|United Kingdom
>Spike|hCoV-19/England/BIRM-5F882/2020|2020-05-15|EPI_ISL_473343|Original|hCoV-19^^England|Human|University of Birmingham|COVID-19 Genomics UK (COG-UK) Consortium|Loman|United Kingdom
>Spike|hCoV-19/Spain/PV-IBV-000706/2020|2020-03-05|EPI_ISL_452760|Original|hCoV-19^^Basque Country|Human|Hospital Universitario Araba. Vitoria-Gasteiz,|SeqCOVID-SPAIN consortium/IBV(CSIC)|Comas|Spain
>Spike|hCoV-19/USA/TX-HMH-4749/2020|2020-06-22|EPI_ISL_544303|Original|hCoV-19^^Texas|Human|Houston Methodist Hospital|Houston Methodist Hospital|Long|USA
>Spike|hCoV-19/Netherlands/ZH-EMC-934/2020|2020-10-24|EPI_ISL_722544|Original|hCoV-19^^Zuid Holland|Human|Dutch COVID-19 response team|Erasmus Medical Center|Oude Munnink|Netherlands
>Spike|hCoV-19/England/LIVE-A7302/2020|2020-03-28|EPI_ISL_517002|Original|hCoV-19^^England|Human|Liverpool Clinical Laboratories|COVID-19 Genomics UK (COG-UK) Consortium|Haldenby|United Kingdom
>Spike|hCoV-19/South Africa/KRISP-K001319/2020|2020-07-18|EPI_ISL_515804|Original|hCoV-19^^KwaZulu-Natal|Human|Medical Disagnostics Services (MDS)|KRISP|de Oliveira|South Africa
>Spike|hCoV-19/England/QEUH-B7DDDF/2020|2020-11-12|EPI_ISL_741921|Original|hCoV-19^^England|Human|Oxford Viromics|COVID-19 Genomics UK (COG-UK) Consortium|Golubchik|United Kingdom
>Spike|hCoV-19/England/P4/2020|2020-00-00|EPI_ISL_479788|unknown|hCoV-19^^England|Human|Breuer Lab|Breuer Lab|Pang|United Kingdom
>Spike|hCoV-19/Switzerland/BS-42397123/2020|2020-08-16|EPI_ISL_581964|Original|hCoV-19^^Basel-Stadt|Human|University Hospital Basel|University Hospital Basel|Roloff Handschin|Switzerland
>Spike|hCoV-19/England/MILK-ACD80D/2020|2020-10-21|EPI_ISL_634422|Original|hCoV-19^^England|Human|Lighthouse Lab in Milton Keynes|Wellcome Sanger Institute for the COVID-19 Genomics UK (COG-UK) consortium|Davies|United Kingdom
>Spike|hCoV-19/USA/NY-NYCPHL-001059/2020|2020-10-09|EPI_ISL_633066|Original|hCoV-19^^New York|Human|DOHMH Chelsea|New York City Public Health Laboratory|Wang|USA
>Spike|hCoV-19/England/ALDP-B1B22F/2020|2020-11-02|EPI_ISL_645755|Original|hCoV-19^^England|Human|Lighthouse Lab in Alderley Park|Wellcome Sanger Institute for the COVID-19 Genomics UK (COG-UK) Consortium|Davies|United Kingdom

>Spike|hCoV-19/USA/LA-EVTL603/2020|2020-06-30|EPI_ISL_579007|Original|hCoV-19^^Louisiana|Human|LSUHS Emerging Viral Threat Laboratory|Microbial Genome Sequencing Center|Kamil|USA
>Spike|hCoV-19/Scotland/EDB6120/2020|2020-05-13|EPI_ISL_478231|Original|hCoV-19^^Scotland|Human|Virology Department|COVID-19 Genomics UK (COG-UK) Consortium|Williams|United Kingdom
>Spike|hCoV-19/USA/LA-EVTL1416/2020|2020-12-14|EPI_ISL_779082|Original|hCoV-19^^Louisiana|Human|LSUHS Emerging Viral Threat Laboratory|Microbial Genome Sequencing Center|Kamil|USA
>Spike|hCoV-19/Spain/PV-IBV-001421/2020|2020-03-22|EPI_ISL_452480|Original|hCoV-19^^Basque Country|Human|Cl\u00ednica Universidad de Navarra. Servicio de Enfermedades Infecciosas y Microbiolog\u00eda cl\u00ednica|SeqCOVID-SPAIN consortium/IBV(CSIC)|Comas|Spain
>Spike|hCoV-19/Scotland/CVR5764/2020|2020-11-11|EPI_ISL_703287|Original|hCoV-19^^Scotland|Human|West of Scotland Specialist Virology Centre|COVID-19 Genomics UK (COG-UK) Consortium|Robertson|United Kingdom
>Spike|hCoV-19/England/CAMC-B32BEB/2020|2020-11-05|EPI_ISL_659392|Original|hCoV-19^^England|Human|Lighthouse Lab in Cambridge|Wellcome Sanger Institute for the COVID-19 Genomics UK (COG-UK) Consortium|Davies|United Kingdom
>Spike|hCoV-19/England/MILK-BFCD05/2020|2020-12-02|EPI_ISL_720748|Original|hCoV-19^^England|Human|Lighthouse Lab in Milton Keynes|Wellcome Sanger Institute for the COVID-19 Genomics UK (COG-UK) Consortium|Davies|United Kingdom
>Spike|hCoV-19/England/LOND-1263877/2020|2020-11-04|EPI_ISL_702831|Original|hCoV-19^^England|Human|University College London|COVID-19 Genomics UK (COG-UK) Consortium|Roy|United Kingdom
>Spike|hCoV-19/England/ALDP-9563DC/2020|2020-06-08|EPI_ISL_554112|Original|hCoV-19^^England|Human|Lighthouse Lab in Alderley Park|Wellcome Sanger Institute for the COVID-19 Genomics UK (COG-UK) consortium|Davies|United Kingdom
>Spike|hCoV-19/England/BRIS-12C665/2020|2020-05-07|EPI_ISL_469524|Original|hCoV-19^^England|Human|PHE South West Regional Laboratory|Wellcome Sanger Institute for the COVID-19 Genomics UK (COG-UK) consortium|Davies|United Kingdom
>Spike|hCoV-19/England/CAMB-1B00A4/2020|2020-05-07|EPI_ISL_448107|Original|hCoV-19^^England|Human|Department of Pathology|COVID-19 Genomics UK (COG-UK) Consortium|Meredith|United Kingdom
>Spike|hCoV-19/Wuhan/HB-WHCM-101/2020|2020-03-02|EPI_ISL_455399|Original|hCoV-19^^Hubei|Human|Wuhan Chain Medical Labs (CMLabs)|State Key Laboratory of Biotherapy of Sichuan University|Tang|China
>Spike|hCoV-19/Japan/PG-7876/2020|2020-07-00|EPI_ISL_686977|Original|hCoV-19^^Japan|Human|Pathogen Genomics Center|Pathogen Genomics Center|Sekizuka|Japan
>Spike|hCoV-19/Australia/VIC12863/2020|2020-07-29|EPI_ISL_592092|Original|hCoV-19^^Victoria|Human|Microbiological Diagnostic Unit - Public Health Laboratory (MDU-PHL)|MDU-PHL|Schultz|Australia
>Spike|hCoV-19/South Korea/KCDC2135/2020|2020-03-16|EPI_ISL_498036|Original|hCoV-19^^South Korea|Human|Division of Viral Diseases|Division of Viral Diseases|Kim|South Korea
>Spike|hCoV-19/Japan/PG-5492/2020|2020-08-00|EPI_ISL_690594|Original|hCoV-19^^Japan|Human|Pathogen Genomics Center|Pathogen Genomics Center|Sekizuka|Japan
>Spike|hCoV-19/England/ALDP-A6DF73/2020|2020-10-14|EPI_ISL_607644|Original|hCoV-19^^England|Human|Lighthouse Lab in Alderley Park|Wellcome Sanger Institute for the COVID-19 Genomics UK (COG-UK) consortium|Davies|United Kingdom
>Spike|hCoV-19/England/204691396/2020|2020-11-14|EPI_ISL_686598|Original|hCoV-19^^England|Human|Respiratory Virus Unit|COVID-19 Genomics UK (COG-UK) Consortium|Platt|United Kingdom
>Spike|hCoV-19/Romania/USAFSAM-S551/2020|2020-11-28|EPI_ISL_738323|Original|hCoV-19^^Romania|Human|Landstuhl Regional Medical Center|United States Air Force School of Aerospace Medicine|Fries|Romania
>Spike|hCoV-19/Peru/LIM-UPCH-0188/2020|2020-10-14|EPI_ISL_729864|Original|hCoV-19^^Lima|Human|Laboratorio de Referencia Nacional de Virus Respiratorios|Laboratorio de Gen\u00f3mica Microbiana|Tsukayama|Peru

>Spike|hCoV-19/England/CAMB-7517C/2020|2020-03-18|EPI_ISL_440420|Original|hCoV-19^^England|Human|Department of Pathology|Wellcome Sanger Institute for the COVID-19 Genomics UK (COG-UK) consortium|Amato|United Kingdom
>Spike|hCoV-19/USA/MI-MDHHS-SC20266/2020|2020-04-03|EPI_ISL_447064|Original|hCoV-19^^Michigan|Human|Michigan Department of Health and Human Services|Michigan Department of Health and Human Services|Blankenship|USA
>Spike|hCoV-19/Netherlands/ZH-EMC-715/2020|2020-10-05|EPI_ISL_632562|Original|hCoV-19^^Zuid Holland|Human|Dutch COVID-19 response team|Erasmus Medical Center|Oude Munnink|Netherlands
>Spike|hCoV-19/England/CAMB-81DC4/2020|2020-04-13|EPI_ISL_443075|Original|hCoV-19^^England|Human|Department of Pathology|Wellcome Sanger Institute for the COVID-19 Genomics UK (COG-UK) consortium|Amato|United Kingdom
>Spike|hCoV-19/Canada/ON-S12/2020|2020-03-14|EPI_ISL_586292|Original|hCoV-19^^Ontario|Human|Toronto Invasive Bacterial Diseases Network|McMaster University|McArthur|Canada
>Spike|hCoV-19/USA/CA-LACPHL-AF00005/2020|2020-12-17|EPI_ISL_737240|Original|hCoV-19^^California|Human|Los Angeles County PHL|Los Angeles County PHL|Hemarajata|USA
>Spike|hCoV-19/England/MILK-C90555/2020|2020-12-10|EPI_ISL_777200|Original|hCoV-19^^England|Human|Lighthouse Lab in Milton Keynes|Wellcome Sanger Institute for the COVID-19 Genomics UK (COG-UK) Consortium|Davies|United Kingdom
>Spike|hCoV-19/Russia/KDA-RII-MH4736S/2020|2020-10-16|EPI_ISL_733251|Original|hCoV-19^^Krasnodar|Human|WHO National Influenza Centre Russian Federation|WHO National Influenza Centre Russian Federation|Komissarov|Russia
>Spike|hCoV-19/USA/MI-QDX-655/2020|2020-03-20|EPI_ISL_571378|Original|hCoV-19^^Michigan|Human|Quest Diagnostics|Quest Diagnostics|Kagan|USA
>Spike|hCoV-19/England/ALDP-AA57E0/2020|2020-10-22|EPI_ISL_625184|Original|hCoV-19^^England|Human|Lighthouse Lab in Alderley Park|Wellcome Sanger Institute for the COVID-19 Genomics UK (COG-UK) consortium|Davies|United Kingdom
>Spike|hCoV-19/USA/CA-ALSR-0532-SAN/2020|2020-05-04|EPI_ISL_483206|Original|hCoV-19^^California|Human|UC San Diego Center for Advanced Laboratory Medicine|Andersen lab at Scripps Research|Gangavarapu|USA
>Spike|hCoV-19/England/ALDP-A6FC01/2020|2020-10-15|EPI_ISL_607212|Original|hCoV-19^^England|Human|Lighthouse Lab in Alderley Park|Wellcome Sanger Institute for the COVID-19 Genomics UK (COG-UK) consortium|Davies|United Kingdom
>Spike|hCoV-19/Spain/MD-IBV-99008573/2020|2020-09-21|EPI_ISL_654113|Original|hCoV-19^^Madrid|Human|Hospital General Universitario Gregorio Mara\u00f1\u00f3n|SeqCOVID-SPAIN consortium/IBV(CSIC)|Comas|Spain
>Spike|hCoV-19/United Arab Emirates/0544/2020|2020-05-17|EPI_ISL_698705|Original|hCoV-19^^Abu Dhabi|Human|Group 42 (G42) Healthcare|G42 Healthcare|Ogrodzki|United Arab Emirates
>Spike|hCoV-19/USA/TX-HMH-MCoV-15157/2020|2020-10-20|EPI_ISL_786641|Original|hCoV-19^^Texas|Human|Houston Methodist Hospital|Houston Methodist Hospital|Long|USA
>Spike|hCoV-19/Switzerland/BE-ETHZ-420332/2020|2020-12-20|EPI_ISL_768021|Original|hCoV-19^^Bern|Human|Viollier AG|Department of Biosystems Science and Engineering|Nadeau|Switzerland
>Spike|hCoV-19/Scotland/QEUH-9F5F53/2020|2020-09-27|EPI_ISL_600656|Original|hCoV-19^^Scotland|Human|Lighthouse Lab in Glasgow|Wellcome Sanger Institute for the COVID-19 Genomics UK (COG-UK) consortium|Davies|United Kingdom
>Spike|hCoV-19/Denmark/DCGC-18589/2020|2020-12-07|EPI_ISL_749402|Original|hCoV-19^^Midtjylland|Human|Department of Virus and Microbiological Special Diagnostics|Albertsen Lab|Michaelsen|Denmark
>Spike|hCoV-19/Denmark/DCGC-1752/2020|2020-03-09|EPI_ISL_620129|Original|hCoV-19^^Syddanmark|Human|Department of Virus and Microbiological Special Diagnostics|Albertsen lab|Michaelsen|Denmark
>Spike|hCoV-19/Singapore/1218/2020|2020-04-23|EPI_ISL_648634|Original|hCoV-19^^Singapore|Human|Department of Laboratory Medicine|Department of Laboratory Medicine|Chen|Singapore
>Spike|hCoV-19/Scotland/QEUH-A8BDEE/2020|2020-10-16|EPI_ISL_649568|Original|hCoV-19^^Scotland|Human|Lighthouse Lab in Glasgow|Wellcome Sanger Institute for the COVID-19 Genomics UK (COG-UK) Consortium|Davies|United Kingdom

>Spike|hCoV-19/USA/CA-CSMC43/2020|2020-04-01|EPI_ISL_475655|Original|hCoV-19^^California|Human|Cedars-Sinai Medical Center|Cedars-Sinai Medical Center|Zhang|USA
>Spike|hCoV-19/Wales/PHWC-359DA/2020|2020-04-24|EPI_ISL_474509|Original|hCoV-19^^Wales|Human|Originating lab|COVID-19 Genomics UK (COG-UK) Consortium|Moore|United Kingdom
>Spike|hCoV-19/England/QEUH-B4227A/2020|2020-11-08|EPI_ISL_657043|Original|hCoV-19^^England|Human|Lighthouse Lab in Glasgow|Wellcome Sanger Institute for the COVID-19 Genomics UK (COG-UK) Consortium|Davies|United Kingdom
>Spike|hCoV-19/England/MILK-A46963/2020|2020-10-06|EPI_ISL_598352|Original|hCoV-19^^England|Human|Lighthouse Lab in Milton Keynes|Wellcome Sanger Institute for the COVID-19 Genomics UK (COG-UK) consortium|Davies|United Kingdom
>Spike|hCoV-19/New Zealand/20CV0601/2020|2020-11-20|EPI_ISL_682282|Original|hCoV-19^^Counties Manukau|Human|Middlemore Hospital|Institute of Environmental Science and Research (ESR)|Wang|New Zealand
>Spike|hCoV-19/England/ALDP-A938F1/2020|2020-10-20|EPI_ISL_611143|Original|hCoV-19^^England|Human|Lighthouse Lab in Alderley Park|Wellcome Sanger Institute for the COVID-19 Genomics UK (COG-UK) consortium|Davies|United Kingdom
>Spike|hCoV-19/England/OXON-B35D0/2020|2020-03-21|EPI_ISL_534871|Original|hCoV-19^^England|Human|Oxford Viromics|COVID-19 Genomics UK (COG-UK) Consortium|Golubchik|United Kingdom
>Spike|hCoV-19/England/QEUH-B9F94C/2020|2020-11-18|EPI_ISL_720914|Original|hCoV-19^^England|Human|Lighthouse Lab in Glasgow|Wellcome Sanger Institute for the COVID-19 Genomics UK (COG-UK) Consortium|Davies|United Kingdom
>Spike|hCoV-19/USA/GA-QDX-597/2020|2020-03-17|EPI_ISL_571296|Original|hCoV-19^^Georgia|Human|Quest Diagnostics|Quest Diagnostics|Kagan|USA
>Spike|hCoV-19/Singapore/1340/2020|2020-06-05|EPI_ISL_648689|Original|hCoV-19^^Singapore|Human|Department of Laboratory Medicine|Department of Laboratory Medicine|Chen|Singapore
>Spike|hCoV-19/England/ALDP-9BD9C2/2020|2020-09-09|EPI_ISL_568019|Original|hCoV-19^^England|Human|Lighthouse Lab in Alderley Park|Wellcome Sanger Institute for the COVID-19 Genomics UK (COG-UK) consortium|Davies|United Kingdom
>Spike|hCoV-19/United Arab Emirates/skmc-3594464/2020|2020-09-13|EPI_ISL_582668|Original|hCoV-19^^United Arab Emirates|Human|Sheikh Khalifa Medical City|Molecular/Surveillance lab\nSheikh Khalifa Medical City|Amirtharaj|United Arab Emirates
>Spike|hCoV-19/New Zealand/20VR3161/2020|2020-03-23|EPI_ISL_579451|Original|hCoV-19^^Canterbury|Human|Canterbury Health Laboratories|Institute of Environmental Science and Research (ESR)|Wang|New Zealand
>Spike|hCoV-19/Switzerland/VD-ETHZ-260090/2020|2020-08-25|EPI_ISL_539411|Original|hCoV-19^^Vaud|Human|Viollier AG|Department of Biosystems Science and Engineering|Nadeau|Switzerland
>Spike|hCoV-19/USA/ME-HETL-J0242/2020|2020-09-28|EPI_ISL_755398|Original|hCoV-19^^Maine|Human|Maine Health and Environmental Testing Laboratory|Tewhey Lab|Tewhey|USA
>Spike|hCoV-19/Wales/QEUH-96BF94/2020|2020-08-14|EPI_ISL_531433|Original|hCoV-19^^Wales|Human|Lighthouse Lab in Glasgow|Wellcome Sanger Institute for the COVID-19 Genomics UK (COG-UK) consortium|Davies|United Kingdom
>Spike|hCoV-19/Switzerland/GE-SNRCI-7014/2020|2020-03-00|EPI_ISL_548183|Original|hCoV-19^^Geneve|Human|Laboratoire de Virologie|Swiss National Reference Centre for Influenza|Gon\u00e7alves Cabecinhas|Switzerland
>Spike|hCoV-19/Singapore/807/2020|2020-04-10|EPI_ISL_516807|Original|hCoV-19^^Singapore|Human|National Public Health Laboratory|National Public Health Laboratory|Mak|Singapore
>Spike|hCoV-19/South Africa/KRISP-K002553/2020|2020-07-29|EPI_ISL_535422|Original|hCoV-19^^KwaZulu-Natal|Human|NHLS-IALCH|KRISP|de Oliveira|South Africa
>Spike|hCoV-19/England/QEUH-B140E0/2020|2020-11-03|EPI_ISL_641617|Original|hCoV-19^^England|Human|Lighthouse Lab in Glasgow|Wellcome Sanger Institute for the COVID-19 Genomics UK (COG-UK) Consortium|Davies|United Kingdom

>Spike|hCoV-19/USA/TX-HMH-1140/2020|2020-04-28|EPI_ISL_542548|Original|hCoV-19^^Texas|Human|Houston Methodist Hospital|Houston Methodist Hospital|Long|USA
>Spike|hCoV-19/Canada/MB-NML-1144/2020|2020-08-04|EPI_ISL_582473|Original|hCoV-19^^Manitoba|Human|Cadham Provincial Laboratory|National Microbiology Laboratory (NML)|Majer|Canada
>Spike|hCoV-19/Norway/4109/2020|2020-09-21|EPI_ISL_635133|Original|hCoV-19^^Vestlandet|Human|Haukeland University Hospital|Norwegian Institute of Public Health|Vollan|Norway
>Spike|hCoV-19/England/ALDP-A70502/2020|2020-10-15|EPI_ISL_607651|Original|hCoV-19^^England|Human|Lighthouse Lab in Alderley Park|Wellcome Sanger Institute for the COVID-19 Genomics UK (COG-UK) consortium|Davies|United Kingdom
>Spike|hCoV-19/USA/IL-QDX-198/2020|2020-04-30|EPI_ISL_494515|Original|hCoV-19^^Illinois|Human|Quest Diagnostics|Quest Diagnostics|Kagan|USA
>Spike|hCoV-19/USA/MI-QDX-1313/2020|2020-03-20|EPI_ISL_571967|Original|hCoV-19^^Michigan|Human|Quest Diagnostics|Quest Diagnostics|Kagan|USA
>Spike|hCoV-19/England/QEUH-9DE13C/2020|2020-09-21|EPI_ISL_599710|Original|hCoV-19^^England|Human|Lighthouse Lab in Glasgow|Wellcome Sanger Institute for the COVID-19 Genomics UK (COG-UK) consortium|Davies|United Kingdom
>Spike|hCoV-19/Japan/PG-0851/2020|2020-04-00|EPI_ISL_684461|Original|hCoV-19^^Japan|Human|Fukuoka Institute of Health and Environmental Sciences|Pathogen Genomics Center|Sekizuka|Japan
>Spike|hCoV-19/England/QEUH-96C84D/2020|2020-08-21|EPI_ISL_530694|Original|hCoV-19^^England|Human|Lighthouse Lab in Glasgow|Wellcome Sanger Institute for the COVID-19 Genomics UK (COG-UK) consortium|Davies|United Kingdom
>Spike|hCoV-19/USA/MI-UM-10036633055/2020|2020-10-26|EPI_ISL_650044|Original|hCoV-19^^Michigan|Human|University of Michigan Clinical Microbiology Laboratory|Lauring Lab|Valesano|USA
>Spike|hCoV-19/England/BRIS-121730/2020|2020-03-30|EPI_ISL_440130|Original|hCoV-19^^England|Human|PHE South West Regional Laboratory|Wellcome Sanger Institute for the COVID-19 Genomics UK (COG-UK) consortium|Amato|United Kingdom
>Spike|hCoV-19/USA/AZ-TG276171/2020|2020-04-10|EPI_ISL_694095|Original|hCoV-19^^Arizona|Human|AZ SPHL|TGen North|French|USA
>Spike|hCoV-19/South Korea/KCDC2407/2020|2020-06-02|EPI_ISL_514810|Original|hCoV-19^^South Korea|Human|Division of Viral Diseases|Division of Viral Diseases|Kim|South Korea
>Spike|hCoV-19/England/SHEF-10D8A1F/2020|2020-12-21|EPI_ISL_765208|Original|hCoV-19^^England|Human|Virology Department|COVID-19 Genomics UK (COG-UK) Consortium|de Silva|United Kingdom
>Spike|hCoV-19/England/QEUH-96A320/2020|2020-08-14|EPI_ISL_536866|Original|hCoV-19^^England|Human|Lighthouse Lab in Glasgow|Wellcome Sanger Institute for the COVID-19 Genomics UK (COG-UK) consortium|Davies|United Kingdom
>Spike|hCoV-19/USA/AL-MDHHS-SC20345/2020|2020-03-29|EPI_ISL_450624|Original|hCoV-19^^Alabama|Human|Michigan Department of Health and Human Services|Michigan Department of Health and Human Services|Blankenship|USA
>Spike|hCoV-19/India/TG-CCMB-J208/2020|2020-04-01|EPI_ISL_447574|Original|hCoV-19^^Telangana|Human|CSIR-Centre for Cellular and Molecular Biology|CSIR-Centre for Cellular and Molecular Biology|Banu|India
>Spike|hCoV-19/USA/NY-QDX-861/2020|2020-03-17|EPI_ISL_571668|Original|hCoV-19^^New York|Human|Quest Diagnostics|Quest Diagnostics|Kagan|USA
>Spike|hCoV-19/England/QEUH-B7E3FA/2020|2020-11-14|EPI_ISL_661676|Original|hCoV-19^^England|Human|Lighthouse Lab in Glasgow|Wellcome Sanger Institute for the COVID-19 Genomics UK (COG-UK) Consortium|Davies|United Kingdom
>Spike|hCoV-19/USA/TX-DSHS-0595/2020|2020-06-03|EPI_ISL_569706|Original|hCoV-19^^Texas|Human|Texas Department of State Health Services|Texas Department of State Health Services|Rahman|USA
>Spike|hCoV-19/England/ALDP-A6F2A5/2020|2020-10-15|EPI_ISL_607009|Original|hCoV-19^^England|Human|Lighthouse Lab in Alderley Park|Wellcome Sanger Institute for the COVID-19 Genomics UK (COG-UK) consortium|Davies|United Kingdom

>Spike|hCoV-19/Scotland/CVR5718/2020|2020-11-08|EPI_ISL_651540|Original|hCoV-19^^Scotland|Human|West of Scotland Specialist Virology Centre|COVID-19 Genomics UK (COG-UK) Consortium|Robertson|United Kingdom
>Spike|hCoV-19/Australia/VIC583/2020|2020-03-25|EPI_ISL_426840|Original|hCoV-19^^Victoria|Human|Victorian Infectious Diseases Reference Laboratory (VIDRL)|Microbiological Diagnostic Unit Public Health Laboratory and Victorian Infectious Diseases Reference Laboratory|Schultz|Australia
>Spike|hCoV-19/USA/TX-HMH-MCoV-18308/2020|2020-11-17|EPI_ISL_784656|Original|hCoV-19^^Texas|Human|Houston Methodist Hospital|Houston Methodist Hospital|Long|USA
>Spike|hCoV-19/USA/NY-MSK-14A/2020|2020-03-31|EPI_ISL_583472|Original|hCoV-19^^New York|Human|Memorial Sloan Kettering Cancer Center|van Bakel Laboratory|van Bakel|USA
>Spike|hCoV-19/England/MILK-AB736F/2020|2020-10-19|EPI_ISL_623262|Original|hCoV-19^^England|Human|Lighthouse Lab in Milton Keynes|Wellcome Sanger Institute for the COVID-19 Genomics UK (COG-UK) consortium|Davies|United Kingdom
>Spike|hCoV-19/USA/WA-UW186/2020|2020-03-13|EPI_ISL_416724|Original|hCoV-19^^Washington|Human|UW Virology Lab|UW Virology Lab|Roychoudhury|USA
>Spike|hCoV-19/USA/MI-MDHHS-SC22710/2020|2020-12-07|EPI_ISL_767359|Original|hCoV-19^^Michigan|Human|Michigan Department of Health and Human Services|Michigan Department of Health and Human Services|Jacob|USA
>Spike|hCoV-19/Denmark/DCGC-7184/2020|2020-10-12|EPI_ISL_618971|Original|hCoV-19^^Hovedstaden|Human|Department of Virus and Microbiological Special Diagnostics|Albertsen lab|Michaelsen|Denmark
>Spike|hCoV-19/England/ALDP-9CC0BC/2020|2020-09-17|EPI_ISL_575524|Original|hCoV-19^^England|Human|Lighthouse Lab in Alderley Park|Wellcome Sanger Institute for the COVID-19 Genomics UK (COG-UK) consortium|Davies|United Kingdom
>Spike|hCoV-19/India/MH-ACTREC-373/2020|2020-07-24|EPI_ISL_700022|Original|hCoV-19^^Maharashtra|Human|Hematopathology Laboratory|Hematopathology Laboratory|Patkar|India
>Spike|hCoV-19/England/QEUH-9FFA49/2020|2020-09-30|EPI_ISL_600148|Original|hCoV-19^^England|Human|Lighthouse Lab in Glasgow|Wellcome Sanger Institute for the COVID-19 Genomics UK (COG-UK) consortium|Davies|United Kingdom
>Spike|hCoV-19/Japan/PG-8995/2020|2020-09-00|EPI_ISL_686530|Original|hCoV-19^^Japan|Human|Sakai City Institute of Public Health|Pathogen Genomics Center|Sekizuka|Japan
>Spike|hCoV-19/Australia/VIC857/2020|2020-04-05|EPI_ISL_427116|Original|hCoV-19^^Victoria|Human|Victorian Infectious Diseases Reference Laboratory (VIDRL)|Microbiological Diagnostic Unit Public Health Laboratory and Victorian Infectious Diseases Reference Laboratory|Schultz|Australia
>Spike|hCoV-19/mink/Netherlands/NB02_07KS/2020|2020-04-29|EPI_ISL_447629|Original|hCoV-19^^Netherlands|Mustela lutreola|Virology|Virology|Harders|Netherlands
>Spike|hCoV-19/USA/VA-DCLS-1159/2020|2020-07-07|EPI_ISL_525190|Original|hCoV-19^^Virginia|Human|Virginia DCLS|Virginia DCLS|St. Jacques|USA
>Spike|hCoV-19/England/QEUH-AD3C80/2020|2020-10-26|EPI_ISL_634061|Original|hCoV-19^^England|Human|Lighthouse Lab in Glasgow|Wellcome Sanger Institute for the COVID-19 Genomics UK (COG-UK) consortium|Davies|United Kingdom
>Spike|hCoV-19/England/QEUH-9617D2/2020|2020-08-01|EPI_ISL_536988|Original|hCoV-19^^England|Human|Lighthouse Lab in Glasgow|Wellcome Sanger Institute for the COVID-19 Genomics UK (COG-UK) consortium|Davies|United Kingdom
>Spike|hCoV-19/USA/MI-QDX-216/2020|2020-03-14|EPI_ISL_498728|Original|hCoV-19^^Michigan|Human|Quest Diagnostics|Quest Diagnostics|Kagan|USA
>Spike|hCoV-19/Japan/PG-1927/2020|2020-04-00|EPI_ISL_685533|Original|hCoV-19^^Japan|Human|Pathogen Genomics Center|Pathogen Genomics Center|Sekizuka|Japan
>Spike|hCoV-19/Spain/MD-IBV-97010037/2020|2020-03-12|EPI_ISL_779721|Original|hCoV-19^^Madrid|Human|IdISSC/Hospital Cl\u00ednico San Carlos de Madrid\u200b|SeqCOVID-SPAIN consortium/IBV(CSIC)|Comas|Spain
>Spike|hCoV-19/USA/CA-CZB-3332/2020|2020-07-10|EPI_ISL_548276|Original|hCoV-19^^California|Human|Orange County Public Health Laboratory|Chan-Zuckerberg Biohub|Bhatt|USA

>Spike|hCoV-19/Scotland/EDB7825/2020|2020-09-21|EPI_ISL_572536|Original|hCoV-19^^Scotland|Human|Virology Department|COVID-19 Genomics UK (COG-UK) Consortium|Williams|United Kingdom
>Spike|hCoV-19/South Africa/R12643-20/2020|2020-06-16|EPI_ISL_504205|Original|hCoV-19^^North West|Human|National Institute for Communicable Diseases of the National Health Laboratory Service|National Institute for Communicable Diseases of the National Health Laboratory Service|Allam|South Africa
>Spike|hCoV-19/USA/WI-GMF-10571/2020|2020-07-13|EPI_ISL_547610|Original|hCoV-19^^Wisconsin|Human|Gundersen Clinical Microbiology Laboratory|Kabara Cancer Research Institute|Kenny|USA
>Spike|hCoV-19/Scotland/EDB10295/2020|2020-11-25|EPI_ISL_705739|Original|hCoV-19^^Scotland|Human|Virology Department|COVID-19 Genomics UK (COG-UK) Consortium|McHugh|United Kingdom
>Spike|hCoV-19/USA/UT-UPHL-2012298356/2020|2020-08-09|EPI_ISL_745779|Original|hCoV-19^^Utah|Human|Ginkgo Bioworks Clinical Laboratory|Utah Public Health Laboratory|Young|USA
>Spike|hCoV-19/USA/UT-02597/2020|2020-04-26|EPI_ISL_522262|Original|hCoV-19^^Utah|Human|Utah Public Health Laboratory|Utah Public Health Laboratory|Young|USA
>Spike|hCoV-19/South Africa/NHLS-UCT-GS-1368/2020|2020-11-12|EPI_ISL_700474|Original|hCoV-19^^Western Cape|Human|Knysna CDC wc WLC|NHLS/UCT|Iranzadeh|South Africa
>Spike|hCoV-19/South Africa/KRISP-K004347-CS1/2020|2020-11-09|EPI_ISL_660221|Original|hCoV-19^^KwaZulu-Natal|Human|KRISP|KRISP|de Oliveira|South Africa
>Spike|hCoV-19/Wales/PHWC-28A7D/2020|2020-04-02|EPI_ISL_445393|Original|hCoV-19^^Wales|Human|Wales Specialist Virology Centre|Public Health Wales Microbiology Cardiff|Moore|United Kingdom
>Spike|hCoV-19/Canada/ON-PHL-20-02493/2020|2020-09-00|EPI_ISL_671589|Original|hCoV-19^^Ontario|Human|Public Health Ontario Laboratory|Public Health Ontario Laboratory|Guthrie|Canada
>Spike|hCoV-19/Australia/VIC15937/2020|2020-08-01|EPI_ISL_641307|Original|hCoV-19^^Victoria|Human|Microbiological Diagnostic Unit - Public Health Laboratory (MDU-PHL)|MDU-PHL|Schultz|Australia
>Spike|hCoV-19/USA/LA-EVTL112/2020|2020-05-08|EPI_ISL_451273|Original|hCoV-19^^Louisiana|Human|LSUHS Emerging Viral Threat Laboratory|Microbial Genome Sequencing Center|Kamil|USA
>Spike|hCoV-19/Denmark/DCGC-3747/2020|2020-05-04|EPI_ISL_622635|Original|hCoV-19^^Hovedstaden|Human|Department of Virus and Microbiological Special Diagnostics|Albertsen lab|Michaelsen|Denmark
>Spike|hCoV-19/USA/TX-HMH-2325/2020|2020-05-29|EPI_ISL_545244|Original|hCoV-19^^Texas|Human|Houston Methodist Hospital|Houston Methodist Hospital|Long|USA
>Spike|hCoV-19/USA/MA-Yale-430/2020|2020-04-08|EPI_ISL_738265|Original|hCoV-19^^Massachusetts|Human|Connecticut Veterans' Affairs Hospital|Grubaugh Lab - Yale School of Public Health|Grubaugh|USA
>Spike|hCoV-19/England/ALDP-A856E9/2020|2020-10-17|EPI_ISL_610452|Original|hCoV-19^^England|Human|Lighthouse Lab in Alderley Park|Wellcome Sanger Institute for the COVID-19 Genomics UK (COG-UK) consortium|Davies|United Kingdom
>Spike|hCoV-19/Denmark/DCGC-6373/2020|2020-09-28|EPI_ISL_622330|Original|hCoV-19^^Hovedstaden|Human|Department of Virus and Microbiological Special Diagnostics|Albertsen lab|Michaelsen|Denmark
>Spike|hCoV-19/Wales/PHWC-498722/2020|2020-11-25|EPI_ISL_727117|Original|hCoV-19^^Wales|Human|Originating lab|COVID-19 Genomics UK (COG-UK) Consortium|Moore|United Kingdom
>Spike|hCoV-19/USA/UT-08565/2020|2020-05-26|EPI_ISL_524322|Original|hCoV-19^^Utah|Human|Utah Public Health Laboratory|Utah Public Health Laboratory|Young|USA
>Spike|hCoV-19/Wales/LIVE-DC9A08/2020|2020-12-04|EPI_ISL_705161|Original|hCoV-19^^Wales|Human|Liverpool Clinical Laboratories|COVID-19 Genomics UK (COG-UK) Consortium|Haldenby|United Kingdom
>Spike|hCoV-19/USA/TX-HMH-MCoV-12520/2020|2020-09-30|EPI_ISL_789838|Original|hCoV-19^^Texas|Human|Houston Methodist Hospital|Houston Methodist Hospital|Long|USA

>Spike|hCoV-19/USA/TX-DSHS-0574/2020|2020-06-25|EPI_ISL_574476|Original|hCoV-19^^Texas|Human|Texas Department of State Health Services|Texas Department of State Health Services|Rahman|USA
>Spike|hCoV-19/Australia/VIC7698/2020|2020-08-03|EPI_ISL_564587|Original|hCoV-19^^Victoria|Human|Microbiological Diagnostic Unit - Public Health Laboratory (MDU-PHL)|MDU-PHL|Schultz|Australia
>Spike|hCoV-19/USA/NY-QDX-1983/2020|2020-03-17|EPI_ISL_604357|Original|hCoV-19^^New York|Human|Quest Diagnostics|Quest Diagnostics|Kagan|USA
>Spike|hCoV-19/Scotland/CVR5564/2020|2020-10-28|EPI_ISL_651841|Original|hCoV-19^^Scotland|Human|West of Scotland Specialist Virology Centre|COVID-19 Genomics UK (COG-UK) Consortium|Robertson|United Kingdom
>Spike|hCoV-19/England/QEUH-9FF566/2020|2020-10-02|EPI_ISL_600346|Original|hCoV-19^^England|Human|Lighthouse Lab in Glasgow|Wellcome Sanger Institute for the COVID-19 Genomics UK (COG-UK) consortium|Davies|United Kingdom
>Spike|hCoV-19/Denmark/DCGC-22606/2020|2020-12-14|EPI_ISL_757439|Original|hCoV-19^^Hovedstaden|Human|Department of Virus and Microbiological Special Diagnostics|Albertsen Lab|Michaelsen|Denmark
>Spike|hCoV-19/USA/NY-NYCPHL-001498/2020|2020-11-30|EPI_ISL_745424|Original|hCoV-19^^New York|Human|DOHMH Corona|New York City Public Health Laboratory|Wang|USA
>Spike|hCoV-19/Netherlands/Utrecht_10017/2020|2020-03-23|EPI_ISL_454766|Original|hCoV-19^^Utrecht|Human|Dutch COVID-19 response team|National Institute for Public Health and the Environment (RIVM)|Meijer|Netherlands
>Spike|hCoV-19/USA/TX-DSHS-1509/2020|2020-06-22|EPI_ISL_683424|Original|hCoV-19^^Texas|Human|Texas Department of State Health Services|Texas Department of State Health Services|Rahman|USA
>Spike|hCoV-19/USA/CA-QDX-546/2020|2020-03-19|EPI_ISL_571269|Original|hCoV-19^^California|Human|Quest Diagnostics|Quest Diagnostics|Kagan|USA
>Spike|hCoV-19/England/201161543/2020|2020-03-11|EPI_ISL_464855|Original|hCoV-19^^England|Human|Respiratory Virus Unit|Respiratory Virus Unit|Platt|United Kingdom
>Spike|hCoV-19/England/MILK-9AAFF3/2020|2020-08-31|EPI_ISL_551013|Original|hCoV-19^^England|Human|Lighthouse Lab in Milton Keynes|Wellcome Sanger Institute for the COVID-19 Genomics UK (COG-UK) Consortium|Davies|United Kingdom
>Spike|hCoV-19/Australia/VIC13489/2020|2020-08-03|EPI_ISL_592263|Original|hCoV-19^^Victoria|Human|Microbiological Diagnostic Unit - Public Health Laboratory (MDU-PHL)|MDU-PHL|Schultz|Australia
>Spike|hCoV-19/USA/MI-MDHHS-SC20888/2020|2020-05-17|EPI_ISL_471909|Original|hCoV-19^^Michigan|Human|Michigan Department of Health and Human Services|Michigan Department of Health and Human Services|Blankenship|USA
>Spike|hCoV-19/England/CAMB-84699/2020|2020-03-02|EPI_ISL_440531|Original|hCoV-19^^England|Human|Department of Pathology|Wellcome Sanger Institute for the COVID-19 Genomics UK (COG-UK) consortium|Amato|United Kingdom
>Spike|hCoV-19/England/QEUH-B816FB/2020|2020-11-14|EPI_ISL_667980|Original|hCoV-19^^England|Human|Lighthouse Lab in Glasgow|Wellcome Sanger Institute for the COVID-19 Genomics UK (COG-UK) Consortium|Davies|United Kingdom
>Spike|hCoV-19/Denmark/DCGC-14219/2020|2020-11-30|EPI_ISL_711633|Original|hCoV-19^^Nordjylland|Human|Department of Virus and Microbiological Special Diagnostics|Albertsen Lab|Michaelsen|Denmark
>Spike|hCoV-19/Canada/QC-LSPQ-L00243646/2020|2020-03-31|EPI_ISL_536368|Original|hCoV-19^^Quebec|Human|Centre hospitalier Anna-Laberge|Laboratoire de sant\u00e9 publique du Qu\u00e9bec|Moreira|Canada
>Spike|hCoV-19/USA/NY-NYCPHL-000027/2020|2020-03-11|EPI_ISL_631523|Original|hCoV-19^^New York|Human|NYU Langone Health|New York City Public Health Laboratory|Wang|USA
>Spike|hCoV-19/Luxembourg/LNS8882011/2020|2020-10-22|EPI_ISL_744820|Original|hCoV-19^^Luxembourg|Human|Laboratoire national de sant\u00e9|Laboratoire national de sant\u00e9|Wienecke-Baldacchino|Luxembourg
>Spike|hCoV-19/England/CAMB-1B099A/2020|2020-05-05|EPI_ISL_459206|Original|hCoV-19^^England|Human|Department of Pathology|Wellcome Sanger Institute for the COVID-19 Genomics UK (COG-UK) consortium|Davies|United Kingdom

>Spike|hCoV-19/Singapore/147/2020|2020-03-17|EPI_ISL_443239|Original|hCoV-19^^Singapore|Human|National Public Health Laboratory|National Public Health Laboratory|Mak|Singapore
>Spike|hCoV-19/Australia/VIC10858/2020|2020-09-01|EPI_ISL_563129|Original|hCoV-19^^Victoria|Human|Microbiological Diagnostic Unit - Public Health Laboratory (MDU-PHL)|MDU-PHL|Schultz|Australia
>Spike|hCoV-19/Finland/21A39S11/2020|2020-04-21|EPI_ISL_481610|Original|hCoV-19^^Uusimaa|Human|Department of Virology and Immunology|Department of Virology|Smura|Finland
>Spike|hCoV-19/USA/NJ-QDX-2268/2020|2020-03-14|EPI_ISL_604558|Original|hCoV-19^^New Jersey|Human|Quest Diagnostics|Quest Diagnostics|Kagan|USA
>Spike|hCoV-19/Nigeria/ON969-CV145/2020|2020-06-17|EPI_ISL_527892|Original|hCoV-19^^Ondo|Human|Nigeria Centre for Disease Control (NCDC)|African Centre of Excellence for Genomics of Infectious Diseases (ACEGID)|Oluniyi|Nigeria
>Spike|hCoV-19/Australia/VIC3406/2020|2020-07-09|EPI_ISL_521085|Original|hCoV-19^^Victoria|Human|Microbiological Diagnostic Unit - Public Health Laboratory (MDU-PHL)|MDU-PHL|Schultz|Australia
>Spike|hCoV-19/Lithuania/C20-05-R3/2020|2020-05-18|EPI_ISL_560399|Original|hCoV-19^^Vilnius r.|Human|Vilnius University Hospital Santaros Klinikos|Institute of Biotechnology|Timinskas|Lithuania
>Spike|hCoV-19/England/QEUH-9B51A4/2020|2020-09-03|EPI_ISL_590332|Original|hCoV-19^^England|Human|Lighthouse Lab in Glasgow|Wellcome Sanger Institute for the COVID-19 Genomics UK (COG-UK) consortium|Davies|United Kingdom
>Spike|hCoV-19/Iceland/423/2020|2020-03-19|EPI_ISL_424447|Original|hCoV-19^^Reykjavik|Human|The National University Hospital of Iceland|deCODE genetics|J\u00f3nsson|Iceland
>Spike|hCoV-19/England/CAMC-AB1954/2020|2020-10-23|EPI_ISL_623663|Original|hCoV-19^^England|Human|Lighthouse Lab in Cambridge|Wellcome Sanger Institute for the COVID-19 Genomics UK (COG-UK) consortium|Davies|United Kingdom
>Spike|hCoV-19/France/ARA-32536/2020|2020-04-30|EPI_ISL_678540|Original|hCoV-19^^Auvergne-Rh\u00f4ne-Alpes|Human|CNR Virus des Infections Respiratoires - France SUD|CNR Virus des Infections Respiratoires - France SUD|Bal|France
>Spike|hCoV-19/India/WB-IK70/2020|2020-05-14|EPI_ISL_511904|Original|hCoV-19^^West Bengal|Human|Institute of Post Graduate Medical Education & Research|National Institute of Biomedical Genomics -\u00a0DBT's PAN-INDIA 1000 SARS--CoV-2 RNA Genome Sequencing Consortium|Maitra|India
>Spike|hCoV-19/England/BRIS-12854E/2020|2020-04-16|EPI_ISL_469768|Original|hCoV-19^^England|Human|PHE South West Regional Laboratory|Wellcome Sanger Institute for the COVID-19 Genomics UK (COG-UK) consortium|Davies|United Kingdom
>Spike|hCoV-19/Australia/VIC2829/2020|2020-07-03|EPI_ISL_521660|Original|hCoV-19^^Victoria|Human|Microbiological Diagnostic Unit - Public Health Laboratory (MDU-PHL)|MDU-PHL|Schultz|Australia
>Spike|hCoV-19/South Africa/KRISP-K003354/2020|2020-09-07|EPI_ISL_602745|Original|hCoV-19^^KwaZulu-Natal|Human|NHLS-IALCH|KRISP|de Oliveira|South Africa
>Spike|hCoV-19/Denmark/DCGC-16178/2020|2020-11-16|EPI_ISL_714623|Original|hCoV-19^^Hovedstaden|Human|Department of Virus and Microbiological Special Diagnostics|Albertsen Lab|Michaelsen|Denmark
>Spike|hCoV-19/Spain/IB-IBV-99010812/2020|2020-07-23|EPI_ISL_691637|Original|hCoV-19^^Balear Islands|Human|Servicio de Microbiolog\u00eda|SeqCOVID-SPAIN consortium/IBV(CSIC)|Comas|Spain
>Spike|hCoV-19/Denmark/DCGC-6850/2020|2020-10-12|EPI_ISL_619695|Original|hCoV-19^^Syddanmark|Human|Department of Virus and Microbiological Special Diagnostics|Albertsen lab|Michaelsen|Denmark
>Spike|hCoV-19/USA/WA-UW-47255/2020|2020-12-09|EPI_ISL_776746|Original|hCoV-19^^Washington|Human|UW Virology Lab|UW Virology Lab|Roychoudhury|USA
>Spike|hCoV-19/Japan/PG-2858/2020|2020-04-00|EPI_ISL_690984|Original|hCoV-19^^Japan|Human|Fukuoka Institute of Health and Environmental Sciences|Pathogen Genomics Center|Sekizuka|Japan

>Spike|hCoV-19/USA/OR-OHSU-2619/2020|2020-08-22|EPI_ISL_667264|Original|hCoV-19^^Oregon|Human|OHSU Lab Services Molecular Microbiology Lab|Oregon SARS-CoV-2 Genome Sequencing Center|O'Roak|USA
>Spike|hCoV-19/USA/MN-MDH-1221/2020|2020-06-02|EPI_ISL_477310|Original|hCoV-19^^Minnesota|Human|Mayo Clinic & Mayo Clinic Laboratories|Minnesota Department of Health|Garfin|USA
>Spike|hCoV-19/England/ALDP-B755C0/2020|2020-11-12|EPI_ISL_662129|Original|hCoV-19^^England|Human|Lighthouse Lab in Alderley Park|Wellcome Sanger Institute for the COVID-19 Genomics UK (COG-UK) Consortium|Davies|United Kingdom
>Spike|hCoV-19/England/QEUH-AD4979/2020|2020-10-26|EPI_ISL_634165|Original|hCoV-19^^England|Human|Lighthouse Lab in Glasgow|Wellcome Sanger Institute for the COVID-19 Genomics UK (COG-UK) consortium|Davies|United Kingdom
>Spike|hCoV-19/England/MILK-9758E7/2020|2020-08-20|EPI_ISL_552648|Original|hCoV-19^^England|Human|Lighthouse Lab in Milton Keynes|Wellcome Sanger Institute for the COVID-19 Genomics UK (COG-UK) consortium|Davies|United Kingdom
>Spike|hCoV-19/England/LIVE-9B3B6/2020|2020-04-03|EPI_ISL_449441|Original|hCoV-19^^England|Human|Liverpool Clinical Laboratories|COVID-19 Genomics UK (COG-UK) Consortium|Haldenby|United Kingdom
>Spike|hCoV-19/England/204441527/2020|2020-10-20|EPI_ISL_622883|Original|hCoV-19^^England|Human|Respiratory Virus Unit|Respiratory Virus Unit|Platt|United Kingdom
>Spike|hCoV-19/Canada/QC-LSPQ-L00237135/2020|2020-03-25|EPI_ISL_536166|Original|hCoV-19^^Quebec|Human|CSSS Haut-Richelieu/Rouville (H\u00f4pital)|Laboratoire de sant\u00e9 publique du Qu\u00e9bec|Moreira|Canada
>Spike|hCoV-19/USA/CA-QDX-92/2020|2020-03-13|EPI_ISL_468567|Original|hCoV-19^^California|Human|Quest Diagnostics|Quest Diagnostics|Kagan|USA
>Spike|hCoV-19/Denmark/DCGC-16671/2020|2020-11-09|EPI_ISL_714275|Original|hCoV-19^^Hovedstaden|Human|Department of Virus and Microbiological Special Diagnostics|Albertsen Lab|Michaelsen|Denmark
>Spike|hCoV-19/England/MILK-BABF69/2020|2020-11-18|EPI_ISL_720457|Original|hCoV-19^^England|Human|Lighthouse Lab in Milton Keynes|Wellcome Sanger Institute for the COVID-19 Genomics UK (COG-UK) Consortium|Davies|United Kingdom
>Spike|hCoV-19/Denmark/DCGC-13882/2020|2020-11-30|EPI_ISL_712335|Original|hCoV-19^^Midtjylland|Human|Department of Virus and Microbiological Special Diagnostics|Albertsen Lab|Michaelsen|Denmark
>Spike|hCoV-19/Wales/PHWC-48C8B5/2020|2020-10-31|EPI_ISL_666480|Original|hCoV-19^^Wales|Human|Wales Specialist Virology Centre Sequencing lab|COVID-19 Genomics UK (COG-UK) Consortium|Moore|United Kingdom
>Spike|hCoV-19/USA/UT-02518/2020|2020-04-23|EPI_ISL_493106|Original|hCoV-19^^Utah|Human|Utah Public Health Laboratory|Utah Public Health Laboratory|Butz|USA
>Spike|hCoV-19/England/CAMB-7F51C/2020|2020-04-08|EPI_ISL_443037|Original|hCoV-19^^England|Human|Department of Pathology|Wellcome Sanger Institute for the COVID-19 Genomics UK (COG-UK) consortium|Amato|United Kingdom
>Spike|hCoV-19/Wales/PHWC-49B024/2020|2020-10-27|EPI_ISL_742284|Original|hCoV-19^^Wales|Human|Wales Specialist Virology Centre Sequencing lab|COVID-19 Genomics UK (COG-UK) Consortium|Moore|United Kingdom
>Spike|hCoV-19/Austria/CeMM1064/2020|2020-03-13|EPI_ISL_583877|Original|hCoV-19^^Austria|Human|Center for Virology|Bergthaler laboratory|Bergthaler|Austria
>Spike|hCoV-19/Indonesia/SU-NIHRD-PME4760/2020|2020-00-00|EPI_ISL_538501|Original|hCoV-19^^North Sumatra|Human|RS Prima Husada Cipta Medan North Sumatra|National Institute of Health Research and Development|Setiawati|Indonesia
>Spike|hCoV-19/USA/FL-BPHL-1990/2020|2020-04-10|EPI_ISL_653143|Original|hCoV-19^^Florida|Human|Florida Bureau of Public Health Laboratories|Florida Bureau of Public Health Laboratories|Blanton|USA
>Spike|hCoV-19/Denmark/DCGC-10761/2020|2020-11-02|EPI_ISL_670189|Original|hCoV-19^^Hovedstaden|Human|Department of Virus and Microbiological Special Diagnostics|Albertsen Lab|Michaelsen|Denmark
>Spike|hCoV-19/England/SHEF-10C607C/2020|2020-12-21|EPI_ISL_764103|Original|hCoV-19^^England|Human|Virology Department|COVID-19 Genomics UK (COG-UK) Consortium|de Silva|United Kingdom

>Spike|hCoV-19/England/MILK-B8C13B/2020|2020-11-13|EPI_ISL_674198|Original|hCoV-19^^England|Human|Lighthouse Lab in Milton Keynes|Wellcome Sanger Institute for the COVID-19 Genomics UK (COG-UK) Consortium|Davies|United Kingdom
>Spike|hCoV-19/England/ALDP-B75733/2020|2020-11-12|EPI_ISL_662194|Original|hCoV-19^^England|Human|Lighthouse Lab in Alderley Park|Wellcome Sanger Institute for the COVID-19 Genomics UK (COG-UK) Consortium|Davies|United Kingdom
>Spike|hCoV-19/USA/TX-HMH-4245/2020|2020-06-20|EPI_ISL_543458|Original|hCoV-19^^Texas|Human|Houston Methodist Hospital|Houston Methodist Hospital|Long|USA
>Spike|hCoV-19/Denmark/DCGC-17304/2020|2020-12-14|EPI_ISL_750893|Original|hCoV-19^^Nordjylland|Human|Department of Virus and Microbiological Special Diagnostics|Albertsen Lab|Michaelsen|Denmark
>Spike|hCoV-19/England/CAMC-B0AA76/2020|2020-10-29|EPI_ISL_643352|Original|hCoV-19^^England|Human|Lighthouse Lab in Cambridge|Wellcome Sanger Institute for the COVID-19 Genomics UK (COG-UK) Consortium|Davies|United Kingdom
>Spike|hCoV-19/England/CAMC-B54327/2020|2020-11-08|EPI_ISL_656804|Original|hCoV-19^^England|Human|Lighthouse Lab in Cambridge|Wellcome Sanger Institute for the COVID-19 Genomics UK (COG-UK) Consortium|Davies|United Kingdom
>Spike|hCoV-19/United Arab Emirates/H10/2020|2020-04-25|EPI_ISL_528710|Original|hCoV-19^^Abu Dhabi|Human|Alsafar - Khalifa University Abu Dhabi|Alsafar - Khalifa University Abu Dhabi|Daw Elbait|United Arab Emirates
>Spike|hCoV-19/USA/CA-ALSR-0709-SAN/2020|2020-03-31|EPI_ISL_483317|Original|hCoV-19^^California|Human|UC San Diego Center for Advanced Laboratory Medicine|Andersen lab at Scripps Research|Gangavarapu|USA
>Spike|hCoV-19/USA/TX-HMH-MCoV-10137/2020|2020-07-06|EPI_ISL_789336|Original|hCoV-19^^Texas|Human|Houston Methodist Hospital|Houston Methodist Hospital|Long|USA
>Spike|hCoV-19/USA/LA-EVTL263/2020|2020-08-22|EPI_ISL_574397|Original|hCoV-19^^Louisiana|Human|LSUHS Emerging Viral Threat Laboratory|Microbial Genome Sequencing Center|Kamil|USA
>Spike|hCoV-19/England/ALDP-A85A74/2020|2020-10-17|EPI_ISL_610556|Original|hCoV-19^^England|Human|Lighthouse Lab in Alderley Park|Wellcome Sanger Institute for the COVID-19 Genomics UK (COG-UK) consortium|Davies|United Kingdom
>Spike|hCoV-19/Wales/PHWC-48B652/2020|2020-10-30|EPI_ISL_666265|Original|hCoV-19^^Wales|Human|Wales Specialist Virology Centre Sequencing lab|COVID-19 Genomics UK (COG-UK) Consortium|Moore|United Kingdom
>Spike|hCoV-19/Australia/VIC5491/2020|2020-07-20|EPI_ISL_520237|Original|hCoV-19^^Victoria|Human|Microbiological Diagnostic Unit - Public Health Laboratory (MDU-PHL)|MDU-PHL|Schultz|Australia
>Spike|hCoV-19/Colombia/BOY-GUR-0206/2020|2020-03-26|EPI_ISL_447738|Original|hCoV-19^^Boyaca|Human|Grupo de Investigaciones Microbiol\u00f3gicas-UR (GIMUR)|Grupo de Investigaciones Microbiol\u00f3gicas-UR (GIMUR)|Ram\u00edrez|Colombia
>Spike|hCoV-19/Scotland/CVR70/2020|2020-03-09|EPI_ISL_425801|Original|hCoV-19^^Scotland|Human|West of Scotland Specialist Virology Centre|COVID-19 Genomics UK (COG-UK) Consortium|Robertson|United Kingdom
>Spike|hCoV-19/USA/CA-CZB-1219/2020|2020-03-18|EPI_ISL_450468|Original|hCoV-19^^California|Human|Stanford clinical virology lab|Chan-Zuckerberg Biohub|Hao|USA
>Spike|hCoV-19/USA/IL-NM0103/2020|2020-03-21|EPI_ISL_444588|Original|hCoV-19^^Illinois|Human|Northwestern Memorial Hospital|Ozer Lab|Ozer|USA
>Spike|hCoV-19/Australia/VIC7847/2020|2020-07-30|EPI_ISL_564698|Original|hCoV-19^^Victoria|Human|Microbiological Diagnostic Unit - Public Health Laboratory (MDU-PHL)|MDU-PHL|Schultz|Australia
>Spike|hCoV-19/England/CAMC-A81F3D/2020|2020-10-12|EPI_ISL_610842|Original|hCoV-19^^England|Human|Lighthouse Lab in Cambridge|Wellcome Sanger Institute for the COVID-19 Genomics UK (COG-UK) consortium|Davies|United Kingdom
>Spike|hCoV-19/USA/TX-DSHS-0724/2020|2020-05-13|EPI_ISL_584127|Original|hCoV-19^^Texas|Human|Texas Department of State Health Services|Texas Department of State Health Services|Rahman|USA
>Spike|hCoV-19/England/CAMB-7B853/2020|2020-04-05|EPI_ISL_442170|Original|hCoV-19^^England|Human|Department of Pathology|Wellcome Sanger Institute for the COVID-19 Genomics UK (COG-UK) consortium|Amato|United Kingdom

>Spike|hCoV-19/England/MILK-B38620/2020|2020-11-01|EPI_ISL_658820|Original|hCoV-19^^England|Human|Lighthouse Lab in Milton Keynes|Wellcome Sanger Institute for the COVID-19 Genomics UK (COG-UK) Consortium|Davies|United Kingdom
>Spike|hCoV-19/Scotland/QEUH-9630D5/2020|2020-08-11|EPI_ISL_531464|Original|hCoV-19^^Scotland|Human|NHSGGC West of Scotland Specialist Virology Centre / MRC-University of Glasgow Centre for Virus Research|Wellcome Sanger Institute for the COVID-19 Genomics UK (COG-UK) consortium|Davies|United Kingdom
>Spike|hCoV-19/England/CAMC-94881A/2020|2020-07-16|EPI_ISL_556810|Original|hCoV-19^^England|Human|Lighthouse Lab in Cambridge|Wellcome Sanger Institute for the COVID-19 Genomics UK (COG-UK) consortium|Davies|United Kingdom
>Spike|hCoV-19/USA/WA-UW-12624/2020|2020-06-28|EPI_ISL_570877|Original|hCoV-19^^Washington|Human|UW Virology Lab|UW Virology Lab|Roychoudhury|USA
>Spike|hCoV-19/India/GJ-GBRC99/2020|2020-05-06|EPI_ISL_450788|Original|hCoV-19^^Gujarat|Human|Pandit Deendayal Upadhyay Government Medical College|Gujarat Biotechnology Research Centre|Joshi|India
>Spike|hCoV-19/England/QEUH-A9B729/2020|2020-10-20|EPI_ISL_611411|Original|hCoV-19^^England|Human|Lighthouse Lab in Glasgow|Wellcome Sanger Institute for the COVID-19 Genomics UK (COG-UK) consortium|Davies|United Kingdom
>Spike|hCoV-19/USA/MA-MGH-00707/2020|2020-04-07|EPI_ISL_460106|Original|hCoV-19^^Massachusetts|Human|Massachusetts General Hospital|Infectious Disease Program|Lemieux|USA
>Spike|hCoV-19/Sweden/20-52419/2020|2020-07-17|EPI_ISL_560986|Original|hCoV-19^^Vasternorrland|Human|Sundsvalls sjukhus|The Public Health Agency of Sweden|Svartstr\u00f6m|Sweden
>Spike|hCoV-19/Peru/JUN-INS-127/2020|2020-04-01|EPI_ISL_536525|Original|hCoV-19^^Junin|Human|Instituto Nacional de Salud|Laboratorio de Infecciones Respiratorias Agudas|Lopez|Peru
>Spike|hCoV-19/Australia/VIC9610/2020|2020-00-00|EPI_ISL_565624|Original|hCoV-19^^Victoria|Human|Microbiological Diagnostic Unit - Public Health Laboratory (MDU-PHL)|MDU-PHL|Schultz|Australia
>Spike|hCoV-19/USA/WI-GMF-08126/2020|2020-06-26|EPI_ISL_489928|Original|hCoV-19^^Wisconsin|Human|Gundersen Molecular Diagnostics Laboratory|Kabara Cancer Research Institute|Kenny|USA
>Spike|hCoV-19/USA/FL-BPHL-1825/2020|2020-04-08|EPI_ISL_613912|Original|hCoV-19^^Florida|Human|Florida Bureau of Public Health Laboratories|Florida Bureau of Public Health Laboratories|Blanton|USA
>Spike|hCoV-19/USA/AZ-TG268281/2020|2020-03-17|EPI_ISL_426503|Original|hCoV-19^^Arizona|Human|TGen North|TGen North|Lemmer|USA
>Spike|hCoV-19/USA/OR-OHSU-1413/2020|2020-07-16|EPI_ISL_509195|Original|hCoV-19^^Oregon|Human|OHSU Lab Services Molecular Microbiology Lab|Oregon SARS-CoV-2 Genome Sequencing Center|O'Roak|USA
>Spike|hCoV-19/Sweden/20-50259/2020|2020-03-07|EPI_ISL_455897|Original|hCoV-19^^Stockholm|Human|Karolinska Universitetslaboratoriet|The Public Health Agency of Sweden|Svartstr\u00f6m|Sweden
>Spike|hCoV-19/Denmark/DCGC-1859/2020|2020-03-30|EPI_ISL_622597|Original|hCoV-19^^Hovedstaden|Human|Department of Virus and Microbiological Special Diagnostics|Albertsen lab|Michaelsen|Denmark
>Spike|hCoV-19/Australia/VIC13581/2020|2020-08-02|EPI_ISL_563764|Original|hCoV-19^^Victoria|Human|Microbiological Diagnostic Unit - Public Health Laboratory (MDU-PHL)|MDU-PHL|Schultz|Australia
>Spike|hCoV-19/USA/UT-04141/2020|2020-05-13|EPI_ISL_493040|Original|hCoV-19^^Utah|Human|Utah Public Health Laboratory|Utah Public Health Laboratory|Butz|USA
>Spike|hCoV-19/United Arab Emirates/0594/2020|2020-05-15|EPI_ISL_698752|Original|hCoV-19^^Abu Dhabi|Human|Group 42 (G42) Healthcare|G42 Healthcare|Ogrodzki|United Arab Emirates
>Spike|hCoV-19/Scotland/QEUH-9FB4A9/2020|2020-09-29|EPI_ISL_600601|Original|hCoV-19^^Scotland|Human|Lighthouse Lab in Glasgow|Wellcome Sanger Institute for the COVID-19 Genomics UK (COG-UK) consortium|Davies|United Kingdom
>Spike|hCoV-19/India/OR-RMRC49/2020|2020-04-09|EPI_ISL_455478|Original|hCoV-19^^Odisha|Human|REGIONAL VRDL,ICMR-RMRC BBSR|Immunogenomics group|Raghav|India

>Spike|hCoV-19/Germany/HH-hpi-p1583/2020|2020-03-16|EPI_ISL_776058|Original|hCoV-19^^Hamburg|Human|University Medical Center Hamburg Eppendorf|Heinrich Pette Institute|Robitaille|Germany
>Spike|hCoV-19/England/MILK-C7B392/2020|2020-12-15|EPI_ISL_756828|Original|hCoV-19^^England|Human|Lighthouse Lab in Milton Keynes|Wellcome Sanger Institute for the COVID-19 Genomics UK (COG-UK) Consortium|Davies|United Kingdom
>Spike|hCoV-19/England/QEUH-9C9BAF/2020|2020-09-10|EPI_ISL_567495|Original|hCoV-19^^England|Human|Lighthouse Lab in Glasgow|Wellcome Sanger Institute for the COVID-19 Genomics UK (COG-UK) consortium|Davies|United Kingdom
>Spike|hCoV-19/Denmark/DCGC-13684/2020|2020-11-30|EPI_ISL_712193|Original|hCoV-19^^Hovedstaden|Human|Department of Virus and Microbiological Special Diagnostics|Albertsen Lab|Michaelsen|Denmark
>Spike|hCoV-19/USA/FL-BPHL-1453/2020|2020-04-08|EPI_ISL_549235|Original|hCoV-19^^Florida|Human|Florida Bureau of Public Health Laboratories|Florida Bureau of Public Health Laboratories|Blanton|USA
>Spike|hCoV-19/Scotland/QEUH-9B711B/2020|2020-09-09|EPI_ISL_602108|Original|hCoV-19^^Scotland|Human|Lighthouse Lab in Glasgow|Wellcome Sanger Institute for the COVID-19 Genomics UK (COG-UK) consortium|Davies|United Kingdom
>Spike|hCoV-19/Singapore/376/2020|2020-04-04|EPI_ISL_469125|Original|hCoV-19^^Singapore|Human|National Public Health Laboratory|National Public Health Laboratory|Mak|Singapore
>Spike|hCoV-19/Spain/VC-FISABIO-122/2020|2020-03-13|EPI_ISL_436217|Original|hCoV-19^^Comunitat Valenciana|Human|Servicio de Microbiolog\u00eda. Consorcio Hospital General Universitario de Valencia|Sequencing and Bioinformatics Service and Molecular Epidemiology Research Group. FISABIO-Public Health|Gonzalez-Candelas|Spain
>Spike|hCoV-19/Brazil/SP-L14-CD262/2020|2020-03-28|EPI_ISL_476257|Original|hCoV-19^^S\u00e3o Paulo|Human|Hospital da Cl\u00ednicas da Faculdade de Medicina da Universidade de S\u00e3o Paulo|Instituto de Medicina Tropical da Univesidade de S\u00e3o Paulo|Faria|Brazil
>Spike|hCoV-19/England/ALDP-AC5D33/2020|2020-10-14|EPI_ISL_631033|Original|hCoV-19^^England|Human|Lighthouse Lab in Alderley Park|Wellcome Sanger Institute for the COVID-19 Genomics UK (COG-UK) consortium|Davies|United Kingdom
>Spike|hCoV-19/Malaysia/IMR-WC206980/2020|2020-10-12|EPI_ISL_728165|Original|hCoV-19^^Malaysia|Human|Institute for Medical Research|Institute for Medical Research|Suppiah|Malaysia
>Spike|hCoV-19/England/20118042804/2020|2020-03-12|EPI_ISL_464913|Original|hCoV-19^^England|Human|Respiratory Virus Unit|Respiratory Virus Unit|Platt|United Kingdom
>Spike|hCoV-19/Luxembourg/LNS6326231/2020|2020-04-02|EPI_ISL_428960|Original|hCoV-19^^Luxembourg|Human|Laboratoire National de Sante|Laboratoire National de Sante|Wienecke-Baldacchino|Luxembourg
>Spike|hCoV-19/England/ALDP-9FD568/2020|2020-10-01|EPI_ISL_587873|Original|hCoV-19^^England|Human|Lighthouse Lab in Alderley Park|Wellcome Sanger Institute for the COVID-19 Genomics UK (COG-UK) consortium|Davies|United Kingdom
>Spike|hCoV-19/England/SHEF-CFC09/2020|2020-10-02|EPI_ISL_585075|Original|hCoV-19^^England|Human|Virology Department|COVID-19 Genomics UK (COG-UK) Consortium|de Silva|United Kingdom
>Spike|hCoV-19/Netherlands/FL-RIVM-10338/2020|2020-00-00|EPI_ISL_636524|Original|hCoV-19^^Flevoland|Human|Dutch COVID-19 response team|National Institute for Public Health and the Environment (RIVM)|Meijer|Netherlands
>Spike|hCoV-19/England/CAMB-7F118/2020|2020-04-08|EPI_ISL_443022|Original|hCoV-19^^England|Human|Department of Pathology|Wellcome Sanger Institute for the COVID-19 Genomics UK (COG-UK) consortium|Amato|United Kingdom
>Spike|hCoV-19/USA/UT-02528/2020|2020-04-23|EPI_ISL_493113|Original|hCoV-19^^Utah|Human|Utah Public Health Laboratory|Utah Public Health Laboratory|Butz|USA
>Spike|hCoV-19/England/MILK-AC84B3/2020|2020-10-21|EPI_ISL_629656|Original|hCoV-19^^England|Human|Lighthouse Lab in Milton Keynes|Wellcome Sanger Institute for the COVID-19 Genomics UK (COG-UK) consortium|Davies|United Kingdom

>Spike|hCoV-19/Spain/MD-IBV-99012329/2020|2020-11-20|EPI_ISL_779982|Original|hCoV-19^^Madrid|Human|Hospital General Universitario Gregorio Mara\u00f1\u00f3n|SeqCOVID-SPAIN consortium/IBV(CSIC)|Comas|Spain
>Spike|hCoV-19/England/ALDP-AA3EA1/2020|2020-10-22|EPI_ISL_625080|Original|hCoV-19^^England|Human|Lighthouse Lab in Alderley Park|Wellcome Sanger Institute for the COVID-19 Genomics UK (COG-UK) consortium|Davies|United Kingdom
>Spike|hCoV-19/USA/TX-HMH-MCoV-18201/2020|2020-11-14|EPI_ISL_784549|Original|hCoV-19^^Texas|Human|Houston Methodist Hospital|Houston Methodist Hospital|Long|USA
>Spike|hCoV-19/Denmark/ALAB-SSI-754/2020|2020-03-19|EPI_ISL_444889|Original|hCoV-19^^Denmark|Human|Department of Virus and Microbiological Special Diagnostics|Albertsen lab|Kirkegaard|Denmark
>Spike|hCoV-19/USA/MD-MDH-0342/2020|2020-10-15|EPI_ISL_666775|Original|hCoV-19^^Maryland|Human|Maryland Public Health Laboratory|Maryland Public Health Laboratory|Keller|USA
>Spike|hCoV-19/Netherlands/UT-RIVM-20785/2020|2020-12-20|EPI_ISL_790846|Original|hCoV-19^^Utrecht|Human|Dutch COVID-19 response team|National Institute for Public Health and the Environment (RIVM)|Meijer|Netherlands
>Spike|hCoV-19/Canada/BC_17847248/2020|2020-04-00|EPI_ISL_468692|Original|hCoV-19^^British Columbia|Human|BCCDC Public Health Laboratory|BCCDC Public Health Laboratory|Harrigan|Canada
>Spike|hCoV-19/USA/WI-WSLH-200467/2020|2020-05-18|EPI_ISL_578609|Original|hCoV-19^^Wisconsin|Human|Wisconsin State Laboratory of Hygiene Communicable Disease Division|Wisconsin State Laboratory of Hygiene Communicable Disease Division|Florek|USA
>Spike|hCoV-19/Japan/PG-2865/2020|2020-04-00|EPI_ISL_690991|Original|hCoV-19^^Japan|Human|Pathogen Genomics Center|Pathogen Genomics Center|Sekizuka|Japan
>Spike|hCoV-19/Japan/PG-1261/2020|2020-00-00|EPI_ISL_684871|Original|hCoV-19^^Japan|Human|Pathogen Genomics Center|Pathogen Genomics Center|Sekizuka|Japan
>Spike|hCoV-19/Wales/PHWC-4A2EC6/2020|2020-12-14|EPI_ISL_744031|Original|hCoV-19^^Wales|Human|Wales Specialist Virology Centre Sequencing lab|COVID-19 Genomics UK (COG-UK) Consortium|Moore|United Kingdom
>Spike|hCoV-19/England/20154002704/2020|2020-04-07|EPI_ISL_466248|Original|hCoV-19^^England|Human|Respiratory Virus Unit|Respiratory Virus Unit|Platt|United Kingdom
>Spike|hCoV-19/Canada/QC-LSPQ-L00241901/2020|2020-03-28|EPI_ISL_536293|Original|hCoV-19^^Quebec|Human|H\u00f4pital Charles-LeMoyne|Laboratoire de sant\u00e9 publique du Qu\u00e9bec|Moreira|Canada
>Spike|hCoV-19/England/MILK-B8CB5B/2020|2020-11-13|EPI_ISL_675866|Original|hCoV-19^^England|Human|Lighthouse Lab in Milton Keynes|Wellcome Sanger Institute for the COVID-19 Genomics UK (COG-UK) Consortium|Davies|United Kingdom
>Spike|hCoV-19/Wales/PHWC-16B59A/2020|2020-09-19|EPI_ISL_573874|Original|hCoV-19^^Wales|Human|Wales Specialist Virology Centre Sequencing lab|COVID-19 Genomics UK (COG-UK) Consortium|Moore|United Kingdom
>Spike|hCoV-19/Scotland/EDB7828/2020|2020-09-21|EPI_ISL_572427|Original|hCoV-19^^Scotland|Human|Virology Department|COVID-19 Genomics UK (COG-UK) Consortium|Williams|United Kingdom
>Spike|hCoV-19/USA/VA-DCLS-0547/2020|2020-03-15|EPI_ISL_485848|Original|hCoV-19^^Virginia|Human|Virginia DCLS|Virginia DCLS|St. Jacques|USA
>Spike|hCoV-19/USA/CA-CZB-2069/2020|2020-04-30|EPI_ISL_486312|Original|hCoV-19^^California|Human|San Joaquin County Public Health Lab|Chan-Zuckerberg Biohub|McGeever|USA
>Spike|hCoV-19/Netherlands/ZH-EMC-945/2020|2020-10-18|EPI_ISL_722513|Original|hCoV-19^^Zuid Holland|Human|Dutch COVID-19 response team|Erasmus Medical Center|Oude Munnink|Netherlands
>Spike|hCoV-19/Northern Ireland/NIRE-10396B/2020|2020-04-16|EPI_ISL_489191|Original|hCoV-19^^Northern Ireland|Human|Regional Virus Laboratory|Wellcome Sanger Institute for the COVID-19 Genomics UK (COG-UK) consortium|Davies|United Kingdom

>Spike|hCoV-19/Belgium/ULG-10191/2020|2020-05-11|EPI_ISL_455979|Original|hCoV-19^^Li\u00e8ge|Human|Department of Clinical Microbiology|GIGA Medical Genomics|Durkin|Belgium
>Spike|hCoV-19/USA/WA-UW-12898/2020|2020-06-29|EPI_ISL_570924|Original|hCoV-19^^Washington|Human|UW Virology Lab|UW Virology Lab|Roychoudhury|USA
>Spike|hCoV-19/Hong Kong/VM20074703/2020|2020-07-24|EPI_ISL_602603|Original|hCoV-19^^Hong Kong|Human|Queen Mary Hospital|Hong Kong Department of Health|Mak|China
>Spike|hCoV-19/Scotland/CVR5026/2020|2020-10-08|EPI_ISL_594760|Original|hCoV-19^^Scotland|Human|West of Scotland Specialist Virology Centre|COVID-19 Genomics UK (COG-UK) Consortium|Robertson|United Kingdom
>Spike|hCoV-19/USA/MI-MDHHS-SC21609/2020|2020-03-28|EPI_ISL_516336|Original|hCoV-19^^Michigan|Human|Michigan Department of Health and Human Services|Michigan Department of Health and Human Services|Blankenship|USA
>Spike|hCoV-19/New Zealand/20CHL869065O/2020|2020-03-21|EPI_ISL_483060|Original|hCoV-19^^New Zealand|Human|unknown|Microbiology|Submitter|New Zealand
>Spike|hCoV-19/Brazil/RJ-00516/2020|2020-07-28|EPI_ISL_717905|Original|hCoV-19^^Rio de Janeiro|Human|LACEN RJ - Noel Nutels|Bioinformatics Laboratory / LNCC|Almeida|Brazil
>Spike|hCoV-19/Scotland/QEUH-9DE7A3/2020|2020-09-21|EPI_ISL_601189|Original|hCoV-19^^Scotland|Human|Lighthouse Lab in Glasgow|Wellcome Sanger Institute for the COVID-19 Genomics UK (COG-UK) consortium|Davies|United Kingdom
>Spike|hCoV-19/Australia/VIC240/2020|2020-03-21|EPI_ISL_419935|Original|hCoV-19^^Victoria|Human|Victorian Infectious Diseases Reference Laboratory (VIDRL)|Victorian Infectious Diseases Reference Laboratory and Microbiological Diagnostic Unit Public Health Laboratory|Schultz|Australia
>Spike|hCoV-19/Brazil/RS-00619/2020|2020-11-25|EPI_ISL_770571|Original|hCoV-19^^Rio Grande do Sul|Human|Laborat\u00f3rio de Microbiologia Molecular - Universidade FEEVALE|Bioinformatics Laboratory / LNCC|Almeida|Brazil
>Spike|hCoV-19/Belgium/ULG-10897/2020|2020-11-14|EPI_ISL_678439|Original|hCoV-19^^Li\u00e8ge|Human|Department of Clinical Microbiology|GIGA Medical Genomics|Durkin|Belgium
>Spike|hCoV-19/England/QEUH-C3393A/2020|2020-12-11|EPI_ISL_734025|Original|hCoV-19^^England|Human|Lighthouse Lab in Glasgow|Wellcome Sanger Institute for the COVID-19 Genomics UK (COG-UK) Consortium|Davies|United Kingdom
>Spike|hCoV-19/Wales/PHWC-49CFCC/2020|2020-12-09|EPI_ISL_742716|Original|hCoV-19^^Wales|Human|Wales Specialist Virology Centre Sequencing lab|COVID-19 Genomics UK (COG-UK) Consortium|Moore|United Kingdom
>Spike|hCoV-19/England/ALDP-A8585C/2020|2020-10-18|EPI_ISL_610621|Original|hCoV-19^^England|Human|Lighthouse Lab in Alderley Park|Wellcome Sanger Institute for the COVID-19 Genomics UK (COG-UK) consortium|Davies|United Kingdom
>Spike|hCoV-19/England/NOTT-1133A6/2020|2020-09-15|EPI_ISL_549501|Original|hCoV-19^^England|Human|Queens Medical Centre|COVID-19 Genomics UK (COG-UK) Consortium|Loose|United Kingdom
>Spike|hCoV-19/England/QEUH-9FF3E4/2020|2020-09-30|EPI_ISL_600135|Original|hCoV-19^^England|Human|Lighthouse Lab in Glasgow|Wellcome Sanger Institute for the COVID-19 Genomics UK (COG-UK) consortium|Davies|United Kingdom
>Spike|hCoV-19/Australia/NSW880/2020|2020-08-12|EPI_ISL_544961|Original|hCoV-19^^New South Wales|Human|St Vincent's Pathology (SydPath)|NSW Health Pathology - Institute of Clinical Pathology and Medical Research; Westmead Hospital; University of Sydney|Gall|Australia
>Spike|hCoV-19/England/CAMB-7B48C/2020|2020-04-12|EPI_ISL_433961|Original|hCoV-19^^England|Human|Department of Pathology|COVID-19 Genomics UK (COG-UK) Consortium|Meredith|United Kingdom
>Spike|hCoV-19/Spain/IB-IBV-99011567/2020|2020-12-07|EPI_ISL_779900|Original|hCoV-19^^Balear Islands|Human|Servicio de Microbiolog\u00eda|SeqCOVID-SPAIN consortium/IBV(CSIC)|Comas|Spain
>Spike|hCoV-19/England/PORT-2D184A/2020|2020-04-11|EPI_ISL_484424|Original|hCoV-19^^England|Human|Centre for Enzyme Innovation|COVID-19 Genomics UK (COG-UK) Consortium|Robson|United Kingdom

>Spike|hCoV-19/England/LEED-2A824E/2020|2020-04-03|EPI_ISL_539092|Original|hCoV-19^^England|Human|Leeds Teaching Hospitals NHS Trust and Public Health England|Wellcome Sanger Institute for the COVID-19 Genomics UK (COG-UK) consortium|Davies|United Kingdom
>Spike|hCoV-19/England/ALDP-C3A72A/2020|2020-12-09|EPI_ISL_731530|Original|hCoV-19^^England|Human|Lighthouse Lab in Alderley Park|Wellcome Sanger Institute for the COVID-19 Genomics UK (COG-UK) Consortium|Davies|United Kingdom
>Spike|hCoV-19/Scotland/QEUH-87C2F2/2020|2020-07-17|EPI_ISL_532705|Original|hCoV-19^^Scotland|Human|Virology Department|Wellcome Sanger Institute for the COVID-19 Genomics UK (COG-UK) consortium|Davies|United Kingdom
>Spike|hCoV-19/England/LIVE-A4A97/2020|2020-03-10|EPI_ISL_500067|Original|hCoV-19^^England|Human|Liverpool Clinical Laboratories|COVID-19 Genomics UK (COG-UK) Consortium|Haldenby|United Kingdom
>Spike|hCoV-19/England/MILK-C63755/2020|2020-12-13|EPI_ISL_735521|Original|hCoV-19^^England|Human|Lighthouse Lab in Milton Keynes|Wellcome Sanger Institute for the COVID-19 Genomics UK (COG-UK) Consortium|Davies|United Kingdom
>Spike|hCoV-19/USA/TX-HMH-MCoV-12888/2020|2020-08-18|EPI_ISL_788040|Original|hCoV-19^^Texas|Human|Houston Methodist Hospital|Houston Methodist Hospital|Long|USA
>Spike|hCoV-19/England/MILK-AB8410/2020|2020-10-21|EPI_ISL_623447|Original|hCoV-19^^England|Human|Lighthouse Lab in Milton Keynes|Wellcome Sanger Institute for the COVID-19 Genomics UK (COG-UK) consortium|Davies|United Kingdom
>Spike|hCoV-19/Japan/PG-6164/2020|2020-08-00|EPI_ISL_689276|Original|hCoV-19^^Japan|Human|Pathogen Genomics Center|Pathogen Genomics Center|Sekizuka|Japan
>Spike|hCoV-19/Denmark/ALAB-SSI-563/2020|2020-03-30|EPI_ISL_436971|Original|hCoV-19^^Denmark|Human|Department of Virus and Microbiological Special Diagnostics|Albertsen lab|Kirkegaard|Denmark
>Spike|hCoV-19/England/CAMC-A41CF6/2020|2020-10-06|EPI_ISL_609413|Original|hCoV-19^^England|Human|Lighthouse Lab in Cambridge|Wellcome Sanger Institute for the COVID-19 Genomics UK (COG-UK) consortium|Davies|United Kingdom
>Spike|hCoV-19/England/ALDP-BC62D8/2020|2020-11-23|EPI_ISL_721224|Original|hCoV-19^^England|Human|Lighthouse Lab in Alderley Park|Wellcome Sanger Institute for the COVID-19 Genomics UK (COG-UK) Consortium|Davies|United Kingdom
>Spike|hCoV-19/USA/NM-DOH-2020326795/2020|2020-09-03|EPI_ISL_575301|Original|hCoV-19^^New Mexico|Human|Molecular Biology|Molecular Biology|Johnson|USA
>Spike|hCoV-19/USA/TX-HMH-MCoV-8666/2020|2020-07-02|EPI_ISL_780687|Original|hCoV-19^^Texas|Human|Houston Methodist Hospital|Houston Methodist Hospital|Long|USA
>Spike|hCoV-19/Czech Republic/NRL-10288/2020|2020-10-22|EPI_ISL_660557|Original|hCoV-19^^Prague|Human|The National Institute of Public Health|State Veterinary Institute Prague|Nagy|Czech Republic
>Spike|hCoV-19/Iceland/66/2020|2020-03-07|EPI_ISL_417857|Original|hCoV-19^^Reykjavik|Human|The National University Hospital of Iceland|deCODE genetics|J\u00f3nsson|Iceland
>Spike|hCoV-19/USA/MI-MDHHS-SC22340/2020|2020-11-16|EPI_ISL_708320|Original|hCoV-19^^Michigan|Human|Michigan Department of Health and Human Services|Michigan Department of Health and Human Services|Jacob|USA
>Spike|hCoV-19/Canada/NS-NML-1245/2020|2020-04-05|EPI_ISL_579637|Original|hCoV-19^^Nova Scotia|Human|QEII Health Sciences Centre|National Microbiology Laboratory (NML)|Majer|Canada
>Spike|hCoV-19/England/ALDP-B24144/2020|2020-11-04|EPI_ISL_647089|Original|hCoV-19^^England|Human|Lighthouse Lab in Alderley Park|Wellcome Sanger Institute for the COVID-19 Genomics UK (COG-UK) Consortium|Davies|United Kingdom
>Spike|hCoV-19/England/BRIS-126003/2020|2020-04-10|EPI_ISL_459022|Original|hCoV-19^^England|Human|PHE South West Regional Laboratory|Wellcome Sanger Institute for the COVID-19 Genomics UK (COG-UK) consortium|Davies|United Kingdom
>Spike|hCoV-19/England/NORW-F2906/2020|2020-11-10|EPI_ISL_650880|Original|hCoV-19^^England|Human|Quadram Institute Bioscience|COVID-19 Genomics UK (COG-UK) Consortium|Alikhan|United Kingdom
>Spike|hCoV-19/Wales/PHWC-496E01/2020|2020-11-24|EPI_ISL_726733|Original|hCoV-19^^Wales|Human|Originating lab|COVID-19 Genomics UK (COG-UK) Consortium|Moore|United Kingdom

>Spike|hCoV-19/England/QEUH-B6A5CE/2020|2020-11-11|EPI_ISL_662608|Original|hCoV-19^^England|Human|Lighthouse Lab in Glasgow|Wellcome Sanger Institute for the COVID-19 Genomics UK (COG-UK) Consortium|Davies|United Kingdom
>Spike|hCoV-19/South Korea/S2/2020|2020-03-30|EPI_ISL_485393|Original|hCoV-19^^Uijeongbu|Human|University of Ulsan College of Medicine and Asan Medical Center|University of Ulsan College of Medicine and Asan Medical Center|Yoon|South Korea
>Spike|hCoV-19/England/ALDP-90C86D/2020|2020-06-23|EPI_ISL_553730|Original|hCoV-19^^England|Human|Lighthouse Lab in Alderley Park|Wellcome Sanger Institute for the COVID-19 Genomics UK (COG-UK) consortium|Davies|United Kingdom
>Spike|hCoV-19/Australia/VIC3333/2020|2020-07-09|EPI_ISL_521012|Original|hCoV-19^^Victoria|Human|Microbiological Diagnostic Unit - Public Health Laboratory (MDU-PHL)|MDU-PHL|Schultz|Australia
>Spike|hCoV-19/Canada/ON-PHL-20-03633/2020|2020-11-00|EPI_ISL_775137|Original|hCoV-19^^Ontario|Human|Public Health Ontario Laboratory|Public Health Ontario Laboratory|Guthrie|Canada
>Spike|hCoV-19/Wales/PHWC-162803/2020|2020-05-18|EPI_ISL_473125|Original|hCoV-19^^Wales|Human|Wales Specialist Virology Centre Sequencing lab|COVID-19 Genomics UK (COG-UK) Consortium|Moore|United Kingdom
>Spike|hCoV-19/England/QEUH-AF5EBF/2020|2020-10-30|EPI_ISL_663051|Original|hCoV-19^^England|Human|Lighthouse Lab in Glasgow|Wellcome Sanger Institute for the COVID-19 Genomics UK (COG-UK) Consortium|Davies|United Kingdom
>Spike|hCoV-19/England/CAMB-1AFE62/2020|2020-05-02|EPI_ISL_459171|Original|hCoV-19^^England|Human|Department of Pathology|Wellcome Sanger Institute for the COVID-19 Genomics UK (COG-UK) consortium|Davies|United Kingdom
>Spike|hCoV-19/Belgium/ULG-10261/2020|2020-07-14|EPI_ISL_515068|Original|hCoV-19^^Liege|Human|Department of Clinical Microbiology|GIGA Medical Genomics|Durkin|Belgium
>Spike|hCoV-19/Denmark/DCGC-19072/2020|2020-12-07|EPI_ISL_748736|Original|hCoV-19^^Hovedstaden|Human|Department of Virus and Microbiological Special Diagnostics|Albertsen Lab|Michaelsen|Denmark
>Spike|hCoV-19/England/QEUH-7C9C08/2020|2020-07-19|EPI_ISL_532959|Original|hCoV-19^^England|Human|Lighthouse Lab in Glasgow|Wellcome Sanger Institute for the COVID-19 Genomics UK (COG-UK) consortium|Davies|United Kingdom
>Spike|hCoV-19/England/CAMC-B54A04/2020|2020-11-08|EPI_ISL_658215|Original|hCoV-19^^England|Human|Lighthouse Lab in Cambridge|Wellcome Sanger Institute for the COVID-19 Genomics UK (COG-UK) Consortium|Davies|United Kingdom
>Spike|hCoV-19/Japan/IC-0189/2020|2020-10-00|EPI_ISL_667618|Original|hCoV-19^^Japan|Human|Pathogen Genomics Center|Pathogen Genomics Center|Sekizuka|Japan
>Spike|hCoV-19/USA/PA-MGSC15-04/2020|2020-03-25|EPI_ISL_436470|Original|hCoV-19^^Pennsylvania|Human|UPMC Clinical Laboratory|Microbial Genome Sequencing Center|Cooper|USA
>Spike|hCoV-19/USA/WI-GMF-07214/2020|2020-06-21|EPI_ISL_489917|Original|hCoV-19^^Wisconsin|Human|Gundersen Molecular Diagnostics Laboratory|Kabara Cancer Research Institute|Kenny|USA
>Spike|hCoV-19/England/CAMB-1ABA44/2020|2020-04-20|EPI_ISL_470328|Original|hCoV-19^^England|Human|Department of Pathology|Wellcome Sanger Institute for the COVID-19 Genomics UK (COG-UK) consortium|Davies|United Kingdom
>Spike|hCoV-19/England/CAMC-B5312E/2020|2020-11-09|EPI_ISL_657673|Original|hCoV-19^^England|Human|Lighthouse Lab in Cambridge|Wellcome Sanger Institute for the COVID-19 Genomics UK (COG-UK) Consortium|Davies|United Kingdom
>Spike|hCoV-19/USA/CA-ALSR-1302/2020|2020-04-30|EPI_ISL_494486|Original|hCoV-19^^California|Human|San Diego County Public Health Laboratory|Andersen lab at Scripps Research|Gangavarapu|USA
>Spike|hCoV-19/Germany/ST-MD030/2020|2020-04-16|EPI_ISL_728354|Original|hCoV-19^^Saxony-Anhalt|Human|Institute of medical Microbiology and hospital Hygiene|Institute of medical Microbiology and hospital Hygiene|Tersteegen|Germany
>Spike|hCoV-19/Russia/TAM-RII-MH2855S/2020|2020-09-21|EPI_ISL_733037|Original|hCoV-19^^Tambov|Human|HELIX LLC|WHO National Influenza Centre Russian Federation|Komissarov|Russia

>Spike|hCoV-19/Germany/BE-ChVir-W1699-8236/2020|2020-07-29|EPI_ISL_753708|Original|hCoV-19^^Berlin|Human|Charit\u00e9 Universit\u00e4tsmedizin Berlin|Charit\u00e9 Universit\u00e4tsmedizin Berlin|Muehlemann|Germany
>Spike|hCoV-19/Canada/ON-SLB3106/2020|2020-04-18|EPI_ISL_717759|Original|hCoV-19^^Ontario|Human|Kingston Health Sciences Centre and Queen's University|Ontario Institute for Cancer Research|de Borja|Canada
>Spike|hCoV-19/USA/MI-MDHHS-SC20232/2020|2020-03-24|EPI_ISL_447202|Original|hCoV-19^^Michigan|Human|Michigan Department of Health and Human Services|Michigan Department of Health and Human Services|Blankenship|USA
>Spike|hCoV-19/England/QEUH-AD4478/2020|2020-10-26|EPI_ISL_634702|Original|hCoV-19^^England|Human|Lighthouse Lab in Glasgow|Wellcome Sanger Institute for the COVID-19 Genomics UK (COG-UK) consortium|Davies|United Kingdom
>Spike|hCoV-19/England/CAMB-1B5B26/2020|2020-08-13|EPI_ISL_516994|Original|hCoV-19^^England|Human|Department of Pathology|COVID-19 Genomics UK (COG-UK) Consortium|Meredith|United Kingdom
>Spike|hCoV-19/England/CAMB-77EDD/2020|2020-04-01|EPI_ISL_441204|Original|hCoV-19^^England|Human|Department of Pathology|Wellcome Sanger Institute for the COVID-19 Genomics UK (COG-UK) consortium|Amato|United Kingdom
>Spike|hCoV-19/USA/CA-LACPHL-AA00008/2020|2020-08-04|EPI_ISL_710106|Original|hCoV-19^^California|Human|Los Angeles County PHL|Los Angeles County PHL|Hemarajata|USA
>Spike|hCoV-19/Luxembourg/LNS2954433/2020|2020-12-27|EPI_ISL_771060|Original|hCoV-19^^Luxembourg|Human|Laboratoire national de sant\u00e9|Laboratoire national de sant\u00e9|Wienecke-Baldacchino|Luxembourg
>Spike|hCoV-19/Gambia/GC19-2526/2020|2020-07-21|EPI_ISL_561114|Original|hCoV-19^^Kombo|Human|MRCG at LSHTM Genomics lab|MRCG at LSHTM Genomics lab|Sesay|Gambia
>Spike|hCoV-19/Russia/MOW-RII-MH2055S/2020|2020-09-24|EPI_ISL_602391|Original|hCoV-19^^Moscow|Human|HELIX LLC|WHO National Influenza Centre Russian Federation|Komissarov|Russia
>Spike|hCoV-19/England/QEUH-A4BC83/2020|2020-10-05|EPI_ISL_628520|Original|hCoV-19^^England|Human|Oxford Viromics|COVID-19 Genomics UK (COG-UK) Consortium|Golubchik|United Kingdom
>Spike|hCoV-19/Bahrain/340861249/2020|2020-10-14|EPI_ISL_682311|Original|hCoV-19^^Bahrain|Human|Communicable Disease Laboratory|Communicable Disease Laboratory|Altaif|Bahrain
>Spike|hCoV-19/Finland/24SS4D3/2020|2020-09-24|EPI_ISL_759832|Original|hCoV-19^^Uusimaa|Human|Department of Virology and Immunology|Department of Virology|Smura|Finland
>Spike|hCoV-19/USA/LA-CDC-2147/2020|2020-05-29|EPI_ISL_535354|Original|hCoV-19^^Louisiana|Human|LA Office of Public Health Laboratories|Pathogen Discovery|Queen|USA
>Spike|hCoV-19/USA/TX-HMH-MCoV-8674/2020|2020-07-01|EPI_ISL_780692|Original|hCoV-19^^Texas|Human|Houston Methodist Hospital|Houston Methodist Hospital|Long|USA
>Spike|hCoV-19/Singapore/715/2020|2020-07-19|EPI_ISL_498613|Original|hCoV-19^^Singapore|Human|National Public Health Laboratory|National Public Health Laboratory|Mak|Singapore
>Spike|hCoV-19/England/LIVE-1DD7CA/2020|2020-10-02|EPI_ISL_611568|Original|hCoV-19^^England|Human|Liverpool Clinical Laboratories|COVID-19 Genomics UK (COG-UK) Consortium|Haldenby|United Kingdom
>Spike|hCoV-19/England/SHEF-CBF8C/2020|2020-10-26|EPI_ISL_650674|Original|hCoV-19^^England|Human|Virology Department|COVID-19 Genomics UK (COG-UK) Consortium|de Silva|United Kingdom
>Spike|hCoV-19/England/ALDP-A2E58B/2020|2020-10-05|EPI_ISL_586757|Original|hCoV-19^^England|Human|Lighthouse Lab in Alderley Park|Wellcome Sanger Institute for the COVID-19 Genomics UK (COG-UK) consortium|Davies|United Kingdom
>Spike|hCoV-19/USA/TX-HMH-6854/2020|2020-06-25|EPI_ISL_546292|Original|hCoV-19^^Texas|Human|Houston Methodist Hospital|Houston Methodist Hospital|Long|USA
>Spike|hCoV-19/Japan/PG-0641/2020|2020-04-00|EPI_ISL_684251|Original|hCoV-19^^Japan|Human|Gunma Prefectural Institute of Public Health and Environmental Sciences|Pathogen Genomics Center|Sekizuka|Japan

>Spike|hCoV-19/England/MILK-B55E8F/2020|2020-11-06|EPI_ISL_658353|Original|hCoV-19^^England|Human|Lighthouse Lab in Milton Keynes|Wellcome Sanger Institute for the COVID-19 Genomics UK (COG-UK) Consortium|Davies|United Kingdom
>Spike|hCoV-19/USA/CO-UW-MAYO36/2020|2020-03-18|EPI_ISL_501073|Original|hCoV-19^^Colorado|Human|Mayo Clinic Laboratories|University of Washington Virology Lab|Roychoudhury|USA
>Spike|hCoV-19/England/204591424/2020|2020-11-06|EPI_ISL_686558|Original|hCoV-19^^England|Human|Respiratory Virus Unit|COVID-19 Genomics UK (COG-UK) Consortium|Platt|United Kingdom
>Spike|hCoV-19/England/ALDP-B1B59F/2020|2020-11-01|EPI_ISL_645728|Original|hCoV-19^^England|Human|Lighthouse Lab in Alderley Park|Wellcome Sanger Institute for the COVID-19 Genomics UK (COG-UK) Consortium|Davies|United Kingdom
>Spike|hCoV-19/Sweden/20-08832/2020|2020-04-08|EPI_ISL_475123|Original|hCoV-19^^Halland|Human|Halmstad klinisk mikrobiologi|The Public Health Agency of Sweden|Svartstr\u00f6m|Sweden
>Spike|hCoV-19/Denmark/DCGC-5819/2020|2020-09-28|EPI_ISL_621006|Original|hCoV-19^^Hovedstaden|Human|Department of Virus and Microbiological Special Diagnostics|Albertsen lab|Michaelsen|Denmark
>Spike|hCoV-19/Jordan/AM-ALSR-3993/2020|2020-10-02|EPI_ISL_636356|Original|hCoV-19^^Amman|Human|Biolab Diagnostic Laboratories|Andersen lab at Scripps Research|Gangavarapu|Jordan
>Spike|hCoV-19/USA/MA-MASPHL-00869/2020|2020-05-21|EPI_ISL_692857|Original|hCoV-19^^Massachusetts|Human|Massachusetts State Public Health Laboratory|Massachusetts State Public Health Laboratory|Lang|USA
>Spike|hCoV-19/USA/TX-DSHS-0785/2020|2020-05-22|EPI_ISL_590854|Original|hCoV-19^^Texas|Human|Texas Department of State Health Services|Texas Department of State Health Services|Rahman|USA
>Spike|hCoV-19/USA/TX-HMH-MCoV-9793/2020|2020-07-06|EPI_ISL_780279|Original|hCoV-19^^Texas|Human|Houston Methodist Hospital|Houston Methodist Hospital|Long|USA
>Spike|hCoV-19/Wales/PHWC-49E4CB/2020|2020-12-07|EPI_ISL_743009|Original|hCoV-19^^Wales|Human|Wales Specialist Virology Centre Sequencing lab|COVID-19 Genomics UK (COG-UK) Consortium|Moore|United Kingdom
>Spike|hCoV-19/USA/UT-QDX-2024/2020|2020-03-16|EPI_ISL_604382|Original|hCoV-19^^Utah|Human|Quest Diagnostics|Quest Diagnostics|Kagan|USA
>Spike|hCoV-19/Australia/VIC7651/2020|2020-08-04|EPI_ISL_564552|Original|hCoV-19^^Victoria|Human|Victorian Infectious Diseases Reference Laboratory (VIDRL)|VIDRL and MDU-PHL|Schultz|Australia
>Spike|hCoV-19/Ireland/D-NVRL-MPW51790/2020|2020-04-06|EPI_ISL_767767|Original|hCoV-19^^Dublin|Human|National Virus Reference Laboratory|Irish Coronavirus Sequencing Consortium - Teagasc Moorepark|Walsh|Ireland
>Spike|hCoV-19/USA/SC-20-1615-T14/2020|2020-07-14|EPI_ISL_516775|Original|hCoV-19^^South Carolina|Human|van Bakel Laboratory|van Bakel Laboratory|van Bakel|USA
>Spike|hCoV-19/USA/VA-DCLS-0935/2020|2020-05-21|EPI_ISL_522800|Original|hCoV-19^^Virginia|Human|Virginia DCLS|Virginia DCLS|St. Jacques|USA
>Spike|hCoV-19/England/QEUH-9C9DA9/2020|2020-09-10|EPI_ISL_567402|Original|hCoV-19^^England|Human|Lighthouse Lab in Glasgow|Wellcome Sanger Institute for the COVID-19 Genomics UK (COG-UK) consortium|Davies|United Kingdom
>Spike|hCoV-19/Wales/PHWC-4894D2/2020|2020-10-25|EPI_ISL_639583|Original|hCoV-19^^Wales|Human|Wales Specialist Virology Centre Sequencing lab|COVID-19 Genomics UK (COG-UK) Consortium|Moore|United Kingdom
>Spike|hCoV-19/England/CAMB-1B0B2B/2020|2020-05-11|EPI_ISL_452941|Original|hCoV-19^^England|Human|Department of Pathology|COVID-19 Genomics UK (COG-UK) Consortium|Meredith|United Kingdom
>Spike|hCoV-19/Portugal/PT0728/2020|2020-03-27|EPI_ISL_510987|Original|hCoV-19^^Portugal|Human|Instituto Nacional de Saude (INSA)|Instituto Nacional de Saude (INSA)|Borges|Portugal
>Spike|hCoV-19/USA/WA-S1089/2020|2020-04-29|EPI_ISL_463579|Original|hCoV-19^^Washington|Human|Washington State Department of Health|Seattle Flu Study|Bedford|USA

>Spike|hCoV-19/Australia/VIC10831/2020|2020-00-00|EPI_ISL_563116|Original|hCoV-19^^Victoria|Human|Microbiological Diagnostic Unit - Public Health Laboratory (MDU-PHL)|MDU-PHL|Schultz|Australia
>Spike|hCoV-19/England/CAMB-A97E37/2020|2020-12-12|EPI_ISL_764332|Original|hCoV-19^^England|Human|Department of Pathology|COVID-19 Genomics UK (COG-UK) Consortium|Goodfellow|United Kingdom
>Spike|hCoV-19/England/QEUH-9C85EE/2020|2020-09-11|EPI_ISL_567107|Original|hCoV-19^^England|Human|Lighthouse Lab in Glasgow|Wellcome Sanger Institute for the COVID-19 Genomics UK (COG-UK) consortium|Davies|United Kingdom
>Spike|hCoV-19/Wales/PHWC-2B2AF/2020|2020-04-08|EPI_ISL_432291|Original|hCoV-19^^Wales|Human|Wales Specialist Virology Centre|Public Health Wales Microbiology Cardiff|Moore|United Kingdom
>Spike|hCoV-19/France/PAC-IHU-1160/2020|2020-08-12|EPI_ISL_569065|Original|hCoV-19^^Provence-Alpes-C\u00f4te-d\u2019Azur|Human|MEPHI|MEPHI|Levasseur|France
>Spike|hCoV-19/Wales/PHWC-48547C/2020|2020-10-25|EPI_ISL_627015|Original|hCoV-19^^Wales|Human|Wales Specialist Virology Centre Sequencing lab|COVID-19 Genomics UK (COG-UK) Consortium|Moore|United Kingdom
>Spike|hCoV-19/mink/Denmark/AL64-Farm1/2020|2020-06-17|EPI_ISL_626349|Original|hCoV-19^^Denmark|Neovison vison|Statens Serum Institute|Statens Serum Institute|Rasmussen|Denmark
>Spike|hCoV-19/USA/WA-UW-1729/2020|2020-03-21|EPI_ISL_424220|Original|hCoV-19^^Washington|Human|UW Virology Lab|UW Virology Lab|Roychoudhury|USA
>Spike|hCoV-19/Luxembourg/LNS6321145/2020|2020-12-28|EPI_ISL_771133|Original|hCoV-19^^Luxembourg|Human|Laboratoire national de sant\u00e9|Laboratoire national de sant\u00e9|Wienecke-Baldacchino|Luxembourg
>Spike|hCoV-19/Scotland/EDB108/2020|2020-03-22|EPI_ISL_425896|Original|hCoV-19^^Scotland|Human|Virology Department|COVID-19 Genomics UK (COG-UK) Consortium|Williams|United Kingdom
>Spike|hCoV-19/England/CAMB-72F0C/2020|2020-03-23|EPI_ISL_439954|Original|hCoV-19^^England|Human|Department of Pathology|Wellcome Sanger Institute for the COVID-19 Genomics UK (COG-UK) consortium|Amato|United Kingdom
>Spike|hCoV-19/England/QEUH-B0E474/2020|2020-11-02|EPI_ISL_642162|Original|hCoV-19^^England|Human|Lighthouse Lab in Glasgow|Wellcome Sanger Institute for the COVID-19 Genomics UK (COG-UK) Consortium|Davies|United Kingdom
>Spike|hCoV-19/England/CAMC-A64A3F/2020|2020-10-10|EPI_ISL_608906|Original|hCoV-19^^England|Human|Lighthouse Lab in Cambridge|Wellcome Sanger Institute for the COVID-19 Genomics UK (COG-UK) consortium|Davies|United Kingdom
>Spike|hCoV-19/Denmark/DCGC-12667/2020|2020-11-16|EPI_ISL_682848|Original|hCoV-19^^Nordjylland|Human|Department of Virus and Microbiological Special Diagnostics|Albertsen Lab|Michaelsen|Denmark
>Spike|hCoV-19/England/ALDP-9D7AFC/2020|2020-09-17|EPI_ISL_576612|Original|hCoV-19^^England|Human|Oxford Viromics|COVID-19 Genomics UK (COG-UK) Consortium|Golubchik|United Kingdom
>Spike|hCoV-19/Australia/NSW804/2020|2020-04-03|EPI_ISL_526146|Original|hCoV-19^^New South Wales|Human|South Eastern Area Laboratory Services (SEALS)|NSW Health Pathology - Institute of Clinical Pathology and Medical Research; Westmead Hospital; University of Sydney|Gall|Australia
>Spike|hCoV-19/USA/WI-UW-788/2020|2020-07-03|EPI_ISL_495492|Original|hCoV-19^^Wisconsin|Human|University of Wisconsin-Madison AIDS Vaccine Research Laboratories|University of Wisconsin-Madison AIDS Vaccine Research Laboratories|Baczenas|USA
>Spike|hCoV-19/USA/TX-DSHS-1005/2020|2020-07-03|EPI_ISL_613985|Original|hCoV-19^^Texas|Human|Texas Department of State Health Services|Texas Department of State Health Services|Rahman|USA
>Spike|hCoV-19/England/QEUH-A48CC2/2020|2020-10-05|EPI_ISL_595917|Original|hCoV-19^^England|Human|Quadram Institute Bioscience|COVID-19 Genomics UK (COG-UK) Consortium|Alikhan|United Kingdom
>Spike|hCoV-19/USA/CA-CSMC19/2020|2020-03-25|EPI_ISL_475628|Original|hCoV-19^^California|Human|Cedars-Sinai Medical Center|Cedars-Sinai Medical Center|Zhang|USA

>Spike|hCoV-19/Australia/NSW92/2020|2020-03-17|EPI_ISL_427675|Original|hCoV-19^^New South Wales|Human|Centre for Infectious Diseases and Microbiology Public Health|NSW Health Pathology - Institute of Clinical Pathology and Medical Research; Westmead Hospital; University of Sydney|Gall|Australia
>Spike|hCoV-19/Japan/PG-5287/2020|2020-08-00|EPI_ISL_690390|Original|hCoV-19^^Japan|Human|Pathogen Genomics Center|Pathogen Genomics Center|Sekizuka|Japan
>Spike|hCoV-19/England/BRIS-12D9D4/2020|2020-05-07|EPI_ISL_481852|Original|hCoV-19^^England|Human|PHE South West Regional Laboratory|Wellcome Sanger Institute for the COVID-19 Genomics UK (COG-UK) consortium|Davies|United Kingdom
>Spike|hCoV-19/Scotland/CVR4309/2020|2020-09-14|EPI_ISL_573770|Original|hCoV-19^^Scotland|Human|West of Scotland Specialist Virology Centre|COVID-19 Genomics UK (COG-UK) Consortium|Robertson|United Kingdom
>Spike|hCoV-19/Australia/VIC206/2020|2020-03-19|EPI_ISL_419901|Original|hCoV-19^^Victoria|Human|Victorian Infectious Diseases Reference Laboratory (VIDRL)|Victorian Infectious Diseases Reference Laboratory and Microbiological Diagnostic Unit Public Health Laboratory|Schultz|Australia
>Spike|hCoV-19/Japan/PG-2560/2020|2020-06-00|EPI_ISL_690695|Original|hCoV-19^^Japan|Human|Pathogen Genomics Center|Pathogen Genomics Center|Sekizuka|Japan
>Spike|hCoV-19/Denmark/DCGC-4356/2020|2020-09-07|EPI_ISL_615558|Original|hCoV-19^^Nordjylland|Human|Department of Virus and Microbiological Special Diagnostics|Albertsen lab|Michaelsen|Denmark
>Spike|hCoV-19/England/MILK-9C3DFA/2020|2020-09-09|EPI_ISL_566923|Original|hCoV-19^^England|Human|Lighthouse Lab in Milton Keynes|Wellcome Sanger Institute for the COVID-19 Genomics UK (COG-UK) consortium|Davies|United Kingdom
>Spike|hCoV-19/Nigeria/BO-CV331/2020|2020-04-26|EPI_ISL_730000|Original|hCoV-19^^Borno State|Human|Nigeria Centre for Disease Control (NCDC)|African Centre of Excellence for Genomics of Infectious Diseases (ACEGID)|Oluniyi|Nigeria
>Spike|hCoV-19/Denmark/DCGC-14340/2020|2020-11-30|EPI_ISL_711423|Original|hCoV-19^^Hovedstaden|Human|Department of Virus and Microbiological Special Diagnostics|Albertsen Lab|Michaelsen|Denmark
>Spike|hCoV-19/England/MILK-B38480/2020|2020-11-01|EPI_ISL_658978|Original|hCoV-19^^England|Human|Lighthouse Lab in Milton Keynes|Wellcome Sanger Institute for the COVID-19 Genomics UK (COG-UK) Consortium|Davies|United Kingdom
>Spike|hCoV-19/Denmark/DCGC-17212/2020|2020-12-14|EPI_ISL_750830|Original|hCoV-19^^Nordjylland|Human|Department of Virus and Microbiological Special Diagnostics|Albertsen Lab|Michaelsen|Denmark
>Spike|hCoV-19/Chile/MA-ISPCH-8/2020|2020-03-20|EPI_ISL_445289|Original|hCoV-19^^Magallanes|Human|HOSPITAL NAVAL PUERTO WILLIAMS|Instituto de Salud Publica de Chile|Fernandez|Chile
>Spike|hCoV-19/England/MILK-9A9D72/2020|2020-09-01|EPI_ISL_551203|Original|hCoV-19^^England|Human|Lighthouse Lab in Milton Keynes|Wellcome Sanger Institute for the COVID-19 Genomics UK (COG-UK) consortium|Davies|United Kingdom
>Spike|hCoV-19/England/LIVE-DC443C/2020|2020-11-14|EPI_ISL_705326|Original|hCoV-19^^England|Human|Liverpool Clinical Laboratories|COVID-19 Genomics UK (COG-UK) Consortium|Haldenby|United Kingdom
>Spike|hCoV-19/England/LOND-D5A60/2020|2020-04-17|EPI_ISL_457294|Original|hCoV-19^^England|Human|University College London|COVID-19 Genomics UK (COG-UK) Consortium|Roy|United Kingdom
>Spike|hCoV-19/England/210100363/2021|2021-01-04|EPI_ISL_791246|Original|hCoV-19^^England|Human|Respiratory Virus Unit|COVID-19 Genomics UK (COG-UK) Consortium|Platt|United Kingdom
>Spike|hCoV-19/Denmark/DCGC-8209/2020|2020-10-19|EPI_ISL_617251|Original|hCoV-19^^Syddanmark|Human|Department of Virus and Microbiological Special Diagnostics|Albertsen lab|Michaelsen|Denmark
>Spike|hCoV-19/USA/CA-ALSR-4211/2020|2020-06-19|EPI_ISL_648889|Original|hCoV-19^^California|Human|San Diego County Public Health Laboratory|Andersen lab at Scripps Research|Gangavarapu|USA
>Spike|hCoV-19/Canada/BC_18165111/2020|2020-03-00|EPI_ISL_462757|Original|hCoV-19^^British Columbia|Human|BCCDC Public Health Laboratory|BCCDC Public Health Laboratory|Harrigan|Canada

>Spike|hCoV-19/England/PORT-2D4FE8/2020|2020-00-00|EPI_ISL_741672|Original|hCoV-19^^England|Human|Centre for Enzyme Innovation|COVID-19 Genomics UK (COG-UK) Consortium|Robson|United Kingdom
>Spike|hCoV-19/Scotland/EDB10124/2020|2020-10-16|EPI_ISL_680484|Original|hCoV-19^^Scotland|Human|Virology Department|COVID-19 Genomics UK (COG-UK) Consortium|McHugh|United Kingdom
>Spike|hCoV-19/England/NOTT-1116CD/2020|2020-05-21|EPI_ISL_461908|Original|hCoV-19^^England|Human|Queens Medical Centre|COVID-19 Genomics UK (COG-UK) Consortium|Loose|United Kingdom
>Spike|hCoV-19/India/MH-NIV-11182/2020|2020-04-18|EPI_ISL_454521|Original|hCoV-19^^Maharashtra|Human|NIV Influenza|NIV Influenza|Potdar|India
>Spike|hCoV-19/France/IDF-10036BG/2020|2020-03-00|EPI_ISL_447660|Original|hCoV-19^^France|Human|H\u00f4pital Henri-Mondor Ap-Hp|H\u00f4pital Henri-Mondor Ap-Hp|Rodriguez|France
>Spike|hCoV-19/Australia/VIC4468/2020|2020-07-16|EPI_ISL_519764|Original|hCoV-19^^Victoria|Human|Microbiological Diagnostic Unit - Public Health Laboratory (MDU-PHL)|MDU-PHL|Schultz|Australia
>Spike|hCoV-19/Denmark/DCGC-10342/2020|2020-11-02|EPI_ISL_669912|Original|hCoV-19^^Nordjylland|Human|Department of Virus and Microbiological Special Diagnostics|Albertsen Lab|Michaelsen|Denmark
>Spike|hCoV-19/USA/CA-LACPHL-AA00038/2020|2020-10-27|EPI_ISL_710422|Original|hCoV-19^^California|Human|Los Angeles County PHL|Los Angeles County PHL|Hemarajata|USA
>Spike|hCoV-19/England/ALDP-A86008/2020|2020-10-17|EPI_ISL_606041|Original|hCoV-19^^England|Human|Lighthouse Lab in Alderley Park|Wellcome Sanger Institute for the COVID-19 Genomics UK (COG-UK) consortium|Davies|United Kingdom
>Spike|hCoV-19/USA/NY-Wadsworth-290718-01/2020|2020-12-21|EPI_ISL_767548|Original|hCoV-19^^New York|Human|URMC LABS|Wadsworth Center|Lasek-Nesselquist|USA
>Spike|hCoV-19/Spain/PV-IBV-000677/2020|2020-03-05|EPI_ISL_452781|Original|hCoV-19^^Basque Country|Human|Hospital Universitario Araba. Vitoria-Gasteiz,|SeqCOVID-SPAIN consortium/IBV(CSIC)|Comas|Spain
>Spike|hCoV-19/Australia/VIC2304/2020|2020-06-25|EPI_ISL_522025|Original|hCoV-19^^Victoria|Human|Microbiological Diagnostic Unit - Public Health Laboratory (MDU-PHL)|MDU-PHL|Schultz|Australia
>Spike|hCoV-19/Australia/VIC11343/2020|2020-08-24|EPI_ISL_563339|Original|hCoV-19^^Victoria|Human|Microbiological Diagnostic Unit - Public Health Laboratory (MDU-PHL)|MDU-PHL|Schultz|Australia
>Spike|hCoV-19/England/ALDP-9ECE43/2020|2020-05-05|EPI_ISL_589548|Original|hCoV-19^^England|Human|Lighthouse Lab in Alderley Park|Wellcome Sanger Institute for the COVID-19 Genomics UK (COG-UK) consortium|Davies|United Kingdom
>Spike|hCoV-19/Wales/PHWC-32BB9/2020|2020-04-15|EPI_ISL_446661|Original|hCoV-19^^Wales|Human|Wales Specialist Virology Centre|Public Health Wales Microbiology Cardiff|Moore|United Kingdom
>Spike|hCoV-19/England/QEUH-AA0071/2020|2020-10-20|EPI_ISL_652704|Original|hCoV-19^^England|Human|University of Exeter|COVID-19 Genomics UK (COG-UK) Consortium|Temperton|United Kingdom
>Spike|hCoV-19/England/CAMB-1ACC98/2020|2020-04-22|EPI_ISL_489422|Original|hCoV-19^^England|Human|Department of Pathology|Wellcome Sanger Institute for the COVID-19 Genomics UK (COG-UK) consortium|Davies|United Kingdom
>Spike|hCoV-19/USA/MI-QDX-900/2020|2020-03-19|EPI_ISL_571621|Original|hCoV-19^^Michigan|Human|Quest Diagnostics|Quest Diagnostics|Kagan|USA
>Spike|hCoV-19/England/204881370/2020|2020-11-25|EPI_ISL_754296|Original|hCoV-19^^England|Human|Respiratory Virus Unit|COVID-19 Genomics UK (COG-UK) Consortium|Platt|United Kingdom
>Spike|hCoV-19/Iceland/78/2020|2020-03-09|EPI_ISL_417863|Original|hCoV-19^^Reykjavik|Human|The National University Hospital of Iceland|deCODE genetics|J\u00f3nsson|Iceland
>Spike|hCoV-19/United Arab Emirates/0915/2020|2020-06-26|EPI_ISL_699053|Original|hCoV-19^^Abu Dhabi|Human|Group 42 (G42) Healthcare|G42 Healthcare|Ogrodzki|United Arab Emirates

>Spike|hCoV-19/USA/TX-DSHS-1850/2020|2020-06-23|EPI_ISL_745288|Original|hCoV-19^^Texas|Human|Texas Department of State Health Services|Texas Department of State Health Services|Rahman|USA
>Spike|hCoV-19/USA/AZ-TG664261/2020|2020-04-28|EPI_ISL_696063|Original|hCoV-19^^Arizona|Human|Sonora Quest Laboratories|TGen North|French|USA
>Spike|hCoV-19/Japan/PG-2742/2020|2020-04-00|EPI_ISL_690874|Original|hCoV-19^^Japan|Human|Pathogen Genomics Center|Pathogen Genomics Center|Sekizuka|Japan
>Spike|hCoV-19/USA/CA-CZB-3755/2020|2020-07-27|EPI_ISL_548467|Original|hCoV-19^^California|Human|Orange County Public Health Laboratory|Chan-Zuckerberg Biohub|Bhatt|USA
>Spike|hCoV-19/England/QEUH-B67594/2020|2020-11-11|EPI_ISL_662339|Original|hCoV-19^^England|Human|Lighthouse Lab in Glasgow|Wellcome Sanger Institute for the COVID-19 Genomics UK (COG-UK) Consortium|Davies|United Kingdom
>Spike|hCoV-19/England/ALDP-92D2C2/2020|2020-06-21|EPI_ISL_553704|Original|hCoV-19^^England|Human|Lighthouse Lab in Alderley Park|Wellcome Sanger Institute for the COVID-19 Genomics UK (COG-UK) consortium|Davies|United Kingdom
>Spike|hCoV-19/England/CAMB-1B7993/2020|2020-10-20|EPI_ISL_638346|Original|hCoV-19^^England|Human|Department of Pathology|COVID-19 Genomics UK (COG-UK) Consortium|Goodfellow|United Kingdom
>Spike|hCoV-19/Scotland/EDB4275/2020|2020-04-20|EPI_ISL_487841|Original|hCoV-19^^Scotland|Human|Virology Department|Wellcome Sanger Institute for the COVID-19 Genomics UK (COG-UK) consortium|Davies|United Kingdom
>Spike|hCoV-19/USA/AZ-TG491509/2020|2020-06-18|EPI_ISL_695133|Original|hCoV-19^^Arizona|Human|AZ SPHL|TGen North|French|USA
>Spike|hCoV-19/Israel/CVL-n26425/2020|2020-07-21|EPI_ISL_514300|Original|hCoV-19^^Jerusalem District|Human|Israel Central Virology laboratory|Israel Central Virology laboratory|Zuckerman|Israel
>Spike|hCoV-19/Canada/NS-NML-1374/2020|2020-04-15|EPI_ISL_579750|Original|hCoV-19^^Nova Scotia|Human|QEII Health Sciences Centre|National Microbiology Laboratory (NML)|Majer|Canada
>Spike|hCoV-19/England/20100022706/2020|2020-02-29|EPI_ISL_415136|Original|hCoV-19^^England|Human|Respiratory Virus Unit|Respiratory Virus Unit|Platt|United Kingdom
>Spike|hCoV-19/England/CAMC-A5810C/2020|2020-10-08|EPI_ISL_597130|Original|hCoV-19^^England|Human|Lighthouse Lab in Cambridge|Wellcome Sanger Institute for the COVID-19 Genomics UK (COG-UK) consortium|Davies|United Kingdom
>Spike|hCoV-19/USA/WI-UW-872/2020|2020-07-06|EPI_ISL_509874|Original|hCoV-19^^Wisconsin|Human|University of Wisconsin-Madison AIDS Vaccine Research Laboratories|University of Wisconsin-Madison AIDS Vaccine Research Laboratories|Baczenas|USA
>Spike|hCoV-19/India/GJ-GBRC356/2020|2020-06-25|EPI_ISL_514605|Original|hCoV-19^^Gujarat|Human|B.J. Medical College and Civil hospital|Gujarat Biotechnology Research Centre|Joshi|India
>Spike|hCoV-19/Canada/ON-S1493/2020|2020-08-00|EPI_ISL_770844|Original|hCoV-19^^Ontario|Human|Toronto Invasive Bacterial Diseases Network|McMaster University|McArthur|Canada
>Spike|hCoV-19/England/QEUH-5ED5D8/2020|2020-06-16|EPI_ISL_581439|Original|hCoV-19^^England|Human|Lighthouse Lab in Glasgow|Wellcome Sanger Institute for the COVID-19 Genomics UK (COG-UK) consortium|Davies|United Kingdom
>Spike|hCoV-19/USA/MI-MDHHS-SC22256/2020|2020-11-04|EPI_ISL_666922|Original|hCoV-19^^Michigan|Human|Michigan Department of Health and Human Services|Michigan Department of Health and Human Services|Jacob|USA
>Spike|hCoV-19/England/QEUH-B6800B/2020|2020-11-11|EPI_ISL_662064|Original|hCoV-19^^England|Human|Lighthouse Lab in Glasgow|Wellcome Sanger Institute for the COVID-19 Genomics UK (COG-UK) Consortium|Davies|United Kingdom
>Spike|hCoV-19/United Arab Emirates/skmc-205223/2020|2020-04-19|EPI_ISL_582634|Original|hCoV-19^^United Arab Emirates|Human|Sheikh Khalifa Medical City|Molecular/Surveillance lab\nSheikh Khalifa Medical City|Amirtharaj|United Arab Emirates

>Spike|hCoV-19/Wales/PHWC-164DD5/2020|2020-05-27|EPI_ISL_479382|Original|hCoV-19^^Wales|Human|Wales Specialist Virology Centre Sequencing lab|COVID-19 Genomics UK (COG-UK) Consortium|Moore|United Kingdom
>Spike|hCoV-19/USA/SC-20-1278-T07/2020|2020-06-30|EPI_ISL_516754|Original|hCoV-19^^South Carolina|Human|van Bakel Laboratory|van Bakel Laboratory|van Bakel|USA
>Spike|hCoV-19/England/CAMC-BBE1A9/2020|2020-11-22|EPI_ISL_705195|Original|hCoV-19^^England|Human|Virology Department|COVID-19 Genomics UK (COG-UK) Consortium|de Silva|United Kingdom
>Spike|hCoV-19/England/MILK-95D900/2020|2020-06-20|EPI_ISL_556211|Original|hCoV-19^^England|Human|Lighthouse Lab in Milton Keynes|Wellcome Sanger Institute for the COVID-19 Genomics UK (COG-UK) consortium|Davies|United Kingdom
>Spike|hCoV-19/Northern Ireland/NIRE-233FF6/2020|2020-10-14|EPI_ISL_680332|Original|hCoV-19^^Northern Ireland|Human|Regional Virus Laboratory|COVID-19 Genomics UK (COG-UK) Consortium|Fairley|United Kingdom
>Spike|hCoV-19/Portugal/PT0574/2020|2020-03-29|EPI_ISL_454297|Original|hCoV-19^^Portugal|Human|unknown|Instituto Nacional de Saude (INSA)|Borges|Portugal
>Spike|hCoV-19/Netherlands/ZH-RIVM-20365/2020|2020-12-07|EPI_ISL_728585|Original|hCoV-19^^Zuid-holland|Human|Dutch COVID-19 response team|National Institute for Public Health and the Environment (RIVM)|Meijer|Netherlands
>Spike|hCoV-19/Iceland/101/2020|2020-03-10|EPI_ISL_417682|Original|hCoV-19^^Reykjavik|Human|The National University Hospital of Iceland|deCODE genetics|J\u00f3nsson|Iceland
>Spike|hCoV-19/Wales/PHWC-34188/2020|2020-04-17|EPI_ISL_446880|Original|hCoV-19^^Wales|Human|Wales Specialist Virology Centre|Public Health Wales Microbiology Cardiff|Moore|United Kingdom
>Spike|hCoV-19/England/QEUH-B405DD/2020|2020-11-08|EPI_ISL_657342|Original|hCoV-19^^England|Human|Lighthouse Lab in Glasgow|Wellcome Sanger Institute for the COVID-19 Genomics UK (COG-UK) Consortium|Davies|United Kingdom
>Spike|hCoV-19/Australia/VIC974/2020|2020-03-28|EPI_ISL_430659|Original|hCoV-19^^Victoria|Human|Microbiological Diagnostic Unit Public Health Laboratory|Microbiological Diagnostic Unit Public Health Laboratory|Schultz|Australia
>Spike|hCoV-19/Scotland/QEUH-AD0694/2020|2020-10-24|EPI_ISL_644003|Original|hCoV-19^^Scotland|Human|Lighthouse Lab in Glasgow|Wellcome Sanger Institute for the COVID-19 Genomics UK (COG-UK) Consortium|Davies|United Kingdom
>Spike|hCoV-19/USA/WA-UW-45113/2020|2020-12-08|EPI_ISL_747222|Original|hCoV-19^^Washington|Human|UW Virology Lab|UW Virology Lab|Roychoudhury|USA
>Spike|hCoV-19/USA/WA-UW-2921/2020|2020-03-29|EPI_ISL_486051|Original|hCoV-19^^Washington|Human|UW Virology Lab|UW Virology Lab|Roychoudhury|USA
>Spike|hCoV-19/England/ALDP-A2E71C/2020|2020-10-05|EPI_ISL_587267|Original|hCoV-19^^England|Human|Lighthouse Lab in Alderley Park|Wellcome Sanger Institute for the COVID-19 Genomics UK (COG-UK) consortium|Davies|United Kingdom
>Spike|hCoV-19/Australia/NSW1093/2020|2020-08-26|EPI_ISL_593682|Original|hCoV-19^^New South Wales|Human|Pathology West - NSW Health Pathology|NSW Health Pathology - Institute of Clinical Pathology and Medical Research; Westmead Hospital; University of Sydney|Gall|Australia
>Spike|hCoV-19/Scotland/QEUH-533694/2020|2020-05-18|EPI_ISL_532221|Original|hCoV-19^^Scotland|Human|NHSGGC West of Scotland Specialist Virology Centre / MRC-University of Glasgow Centre for Virus Research|Wellcome Sanger Institute for the COVID-19 Genomics UK (COG-UK) consortium|Davies|United Kingdom
>Spike|hCoV-19/Sweden/20-22296/2020|2020-03-28|EPI_ISL_660393|Original|hCoV-19^^Ostergotland|Human|Klinsisk mikrobiologi Linkoping|The Public Health Agency of Sweden|Svartstr\u00f6m|Sweden
>Spike|hCoV-19/England/QEUH-B0F1C7/2020|2020-11-02|EPI_ISL_642500|Original|hCoV-19^^England|Human|Lighthouse Lab in Glasgow|Wellcome Sanger Institute for the COVID-19 Genomics UK (COG-UK) Consortium|Davies|United Kingdom
>Spike|hCoV-19/Japan/PG-7967/2020|2020-05-00|EPI_ISL_687068|Original|hCoV-19^^Japan|Human|Kanagawa Prefectural Institute of Public Health|Pathogen Genomics Center|Sekizuka|Japan

>Spike|hCoV-19/England/BRIS-126C0F/2020|2020-04-10|EPI_ISL_459003|Original|hCoV-19^^England|Human|PHE South West Regional Laboratory|Wellcome Sanger Institute for the COVID-19 Genomics UK (COG-UK) consortium|Davies|United Kingdom
>Spike|hCoV-19/Luxembourg/LNS3708276/2020|2020-11-24|EPI_ISL_740363|Original|hCoV-19^^Luxembourg|Human|Laboratoire national de sant\u00e9|Laboratoire national de sant\u00e9|Wienecke-Baldacchino|Luxembourg
>Spike|hCoV-19/Australia/NSW259/2020|2020-03-28|EPI_ISL_451524|Original|hCoV-19^^New South Wales|Human|Pathology West - NSW Health Pathology|NSW Health Pathology - Institute of Clinical Pathology and Medical Research; Westmead Hospital; University of Sydney|Gall|Australia
>Spike|hCoV-19/USA/NV-QDX-1393/2020|2020-03-17|EPI_ISL_572181|Original|hCoV-19^^Nevada|Human|Quest Diagnostics|Quest Diagnostics|Kagan|USA
>Spike|hCoV-19/Denmark/DCGC-12439/2020|2020-11-16|EPI_ISL_682620|Original|hCoV-19^^Hovedstaden|Human|Department of Virus and Microbiological Special Diagnostics|Albertsen Lab|Michaelsen|Denmark
>Spike|hCoV-19/USA/WA-S1991/2020|2020-06-04|EPI_ISL_497419|Original|hCoV-19^^Washington|Human|Washington State Department of Health|Seattle Flu Study|Lee|USA
>Spike|hCoV-19/USA/UT-UPHL-2008801/2020|2020-05-29|EPI_ISL_524827|Original|hCoV-19^^Utah|Human|Utah Public Health Laboratory|Utah Public Health Laboratory|Young|USA
>Spike|hCoV-19/England/CAMB-1BA58D/2020|2020-11-13|EPI_ISL_664988|Original|hCoV-19^^England|Human|Department of Pathology|COVID-19 Genomics UK (COG-UK) Consortium|Goodfellow|United Kingdom
>Spike|hCoV-19/Bahrein/BAH-03/2020|2020-03-17|EPI_ISL_483544|Original|hCoV-19^^Bahrain|Human|Kingdom of Bahrein Ministry of Health|Erasmus Medical Center|Oude Munnink|Bahrain
>Spike|hCoV-19/Denmark/DCGC-7698/2020|2020-10-19|EPI_ISL_618622|Original|hCoV-19^^Hovedstaden|Human|Department of Virus and Microbiological Special Diagnostics|Albertsen lab|Michaelsen|Denmark
>Spike|hCoV-19/Wales/ALDP-9BE466/2020|2020-09-09|EPI_ISL_568103|Original|hCoV-19^^Wales|Human|Lighthouse Lab in Alderley Park|Wellcome Sanger Institute for the COVID-19 Genomics UK (COG-UK) consortium|Davies|United Kingdom
>Spike|hCoV-19/England/ALDP-B7446A/2020|2020-11-12|EPI_ISL_662122|Original|hCoV-19^^England|Human|Lighthouse Lab in Alderley Park|Wellcome Sanger Institute for the COVID-19 Genomics UK (COG-UK) Consortium|Davies|United Kingdom
>Spike|hCoV-19/Japan/PG-5628/2020|2020-08-00|EPI_ISL_688753|Original|hCoV-19^^Japan|Human|Pathogen Genomics Center|Pathogen Genomics Center|Sekizuka|Japan
>Spike|hCoV-19/England/CAMC-B09840/2020|2020-10-29|EPI_ISL_672939|Original|hCoV-19^^England|Human|Lighthouse Lab in Cambridge|Wellcome Sanger Institute for the COVID-19 Genomics UK (COG-UK) Consortium|Davies|United Kingdom
>Spike|hCoV-19/Scotland/CVR4112/2020|2020-09-08|EPI_ISL_573765|Original|hCoV-19^^Scotland|Human|West of Scotland Specialist Virology Centre|COVID-19 Genomics UK (COG-UK) Consortium|Robertson|United Kingdom
>Spike|hCoV-19/Australia/VIC2907/2020|2020-07-07|EPI_ISL_521738|Original|hCoV-19^^Victoria|Human|Microbiological Diagnostic Unit - Public Health Laboratory (MDU-PHL)|MDU-PHL|Schultz|Australia
>Spike|hCoV-19/Scotland/CVR2973/2020|2020-04-22|EPI_ISL_448213|Original|hCoV-19^^Scotland|Human|West of Scotland Specialist Virology Centre|COVID-19 Genomics UK (COG-UK) Consortium|Robertson|United Kingdom
>Spike|hCoV-19/Wales/PHWC-48F6E5/2020|2020-11-10|EPI_ISL_681128|Original|hCoV-19^^Wales|Human|Wales Specialist Virology Centre Sequencing lab|COVID-19 Genomics UK (COG-UK) Consortium|Moore|United Kingdom
>Spike|hCoV-19/England/ALDP-BB3DA0/2020|2020-11-20|EPI_ISL_701928|Original|hCoV-19^^England|Human|Lighthouse Lab in Alderley Park|Wellcome Sanger Institute for the COVID-19 Genomics UK (COG-UK) Consortium|Davies|United Kingdom
>Spike|hCoV-19/Scotland/QEUH-9D0404/2020|2020-09-16|EPI_ISL_601842|Original|hCoV-19^^Scotland|Human|Lighthouse Lab in Glasgow|Wellcome Sanger Institute for the COVID-19 Genomics UK (COG-UK) consortium|Davies|United Kingdom
>Spike|hCoV-19/England/QEUH-B114D8/2020|2020-11-02|EPI_ISL_642358|Original|hCoV-19^^England|Human|Lighthouse Lab in Glasgow|Wellcome Sanger Institute for the COVID-19 Genomics UK (COG-UK) Consortium|Davies|United Kingdom

>Spike|hCoV-19/USA/NY-NYCPHL-000302/2020|2020-03-14|EPI_ISL_631829|Original|hCoV-19^^New York|Human|Brookdale University Hospital Medical Center|New York City Public Health Laboratory|Wang|USA
>Spike|hCoV-19/Australia/VIC4475/2020|2020-07-17|EPI_ISL_519771|Original|hCoV-19^^Victoria|Human|Microbiological Diagnostic Unit - Public Health Laboratory (MDU-PHL)|MDU-PHL|Schultz|Australia
>Spike|hCoV-19/Denmark/DCGC-17922/2020|2020-12-14|EPI_ISL_749645|Original|hCoV-19^^Hovedstaden|Human|Department of Virus and Microbiological Special Diagnostics|Albertsen Lab|Michaelsen|Denmark
>Spike|hCoV-19/Australia/VIC17407/2020|2020-08-17|EPI_ISL_663874|Original|hCoV-19^^Victoria|Human|Microbiological Diagnostic Unit - Public Health Laboratory (MDU-PHL)|MDU-PHL|Schultz|Australia
>Spike|hCoV-19/USA/CA-CZB-1414/2020|2020-03-16|EPI_ISL_468389|Original|hCoV-19^^California|Human|County of San Luis Obispo Public Health Laboratory|Chan-Zuckerberg Biohub|Hao|USA
>Spike|hCoV-19/Denmark/DCGC-9584/2020|2020-11-09|EPI_ISL_669050|Original|hCoV-19^^Midtjylland|Human|Department of Virus and Microbiological Special Diagnostics|Albertsen Lab|Michaelsen|Denmark
>Spike|hCoV-19/Peru/LIM-UPCH-0196/2020|2020-10-14|EPI_ISL_729890|Original|hCoV-19^^Lima|Human|Laboratorio de Referencia Nacional de Virus Respiratorios|Laboratorio de Gen\u00f3mica Microbiana|Tsukayama|Peru
>Spike|hCoV-19/USA/TX-HMH-3040/2020|2020-05-31|EPI_ISL_543952|Original|hCoV-19^^Texas|Human|Houston Methodist Hospital|Houston Methodist Hospital|Long|USA
>Spike|hCoV-19/England/ALDP-B8318F/2020|2020-11-14|EPI_ISL_675624|Original|hCoV-19^^England|Human|Lighthouse Lab in Alderley Park|Wellcome Sanger Institute for the COVID-19 Genomics UK (COG-UK) Consortium|Davies|United Kingdom
>Spike|hCoV-19/USA/OR-OHSU-0433/2020|2020-05-26|EPI_ISL_509136|Original|hCoV-19^^Oregon|Human|OHSU Lab Services Molecular Microbiology Lab|Oregon SARS-CoV-2 Genome Sequencing Center|O'Roak|USA
>Spike|hCoV-19/England/QEUH-B0EA45/2020|2020-11-02|EPI_ISL_641951|Original|hCoV-19^^England|Human|Lighthouse Lab in Glasgow|Wellcome Sanger Institute for the COVID-19 Genomics UK (COG-UK) Consortium|Davies|United Kingdom
>Spike|hCoV-19/USA/NM-UNM-00859/2020|2020-05-06|EPI_ISL_610256|Original|hCoV-19^^New Mexico|Human|TriCore Reference Laboratories|Center for Global Health|Domman|USA
>Spike|hCoV-19/Belgium/rega-12141409/2020|2020-12-14|EPI_ISL_738461|Original|hCoV-19^^Boechout|Human|UZ Leuven|KU Leuven|Maes|Belgium
>Spike|hCoV-19/England/QEUH-9B49CB/2020|2020-09-03|EPI_ISL_590339|Original|hCoV-19^^England|Human|Lighthouse Lab in Glasgow|Wellcome Sanger Institute for the COVID-19 Genomics UK (COG-UK) consortium|Davies|United Kingdom
>Spike|hCoV-19/England/ALDP-A6EC02/2020|2020-10-15|EPI_ISL_607106|Original|hCoV-19^^England|Human|Lighthouse Lab in Alderley Park|Wellcome Sanger Institute for the COVID-19 Genomics UK (COG-UK) consortium|Davies|United Kingdom
>Spike|hCoV-19/England/MILK-9C3DEB/2020|2020-09-09|EPI_ISL_566865|Original|hCoV-19^^England|Human|Lighthouse Lab in Milton Keynes|Wellcome Sanger Institute for the COVID-19 Genomics UK (COG-UK) consortium|Davies|United Kingdom
>Spike|hCoV-19/USA/CA-ALSR-0926-IPL/2020|2020-04-27|EPI_ISL_483417|Original|hCoV-19^^California|Human|UC San Diego Center for Advanced Laboratory Medicine|Andersen lab at Scripps Research|Gangavarapu|USA
>Spike|hCoV-19/Switzerland/BL-ETHZ-270140/2020|2020-08-28|EPI_ISL_541451|Original|hCoV-19^^Basel-Landschaft|Human|Viollier AG|Department of Biosystems Science and Engineering|Nadeau|Switzerland
>Spike|hCoV-19/USA/MI-MDHHS-SC20253/2020|2020-03-24|EPI_ISL_447223|Original|hCoV-19^^Michigan|Human|Michigan Department of Health and Human Services|Michigan Department of Health and Human Services|Blankenship|USA
>Spike|hCoV-19/England/CAMC-B33AA1/2020|2020-11-05|EPI_ISL_659288|Original|hCoV-19^^England|Human|Lighthouse Lab in Cambridge|Wellcome Sanger Institute for the COVID-19 Genomics UK (COG-UK) Consortium|Davies|United Kingdom
>Spike|hCoV-19/Bangladesh/CHRF-0062/2020|2020-07-24|EPI_ISL_625475|Original|hCoV-19^^Bangladesh|Human|Child Health Research Foundation|Child Health Research Foundation|Saha|Bangladesh

>Spike|hCoV-19/New Zealand/20VR3096/2020|2020-03-31|EPI_ISL_579418|Original|hCoV-19^^Hutt Valley|Human|Wellington SCL (WN)|Institute of Environmental Science and Research (ESR)|Wang|New Zealand
>Spike|hCoV-19/England/LIVE-DC4B46/2020|2020-11-11|EPI_ISL_705260|Original|hCoV-19^^England|Human|Liverpool Clinical Laboratories|COVID-19 Genomics UK (COG-UK) Consortium|Haldenby|United Kingdom
>Spike|hCoV-19/Japan/IC-0043/2020|2020-05-00|EPI_ISL_591385|Original|hCoV-19^^Japan|Human|Pathogen Genomics Center|Pathogen Genomics Center|Sekizuka|Japan
>Spike|hCoV-19/Netherlands/un-EMC-712/2020|2020-05-09|EPI_ISL_523467|Original|hCoV-19^^Netherlands|Human|Dutch COVID-19 response team|Erasmus Medical Center|Oude Munnink|Netherlands
>Spike|hCoV-19/Denmark/DCGC-6127/2020|2020-09-21|EPI_ISL_621161|Original|hCoV-19^^Midtjylland|Human|Department of Virus and Microbiological Special Diagnostics|Albertsen lab|Michaelsen|Denmark
>Spike|hCoV-19/Scotland/GCVR-16FBA3/2020|2020-03-22|EPI_ISL_489639|Original|hCoV-19^^Scotland|Human|NHSGGC West of Scotland Specialist Virology Centre / MRC-University of Glasgow Centre for Virus Research|Wellcome Sanger Institute for the COVID-19 Genomics UK (COG-UK) consortium|Davies|United Kingdom
>Spike|hCoV-19/Denmark/DCGC-14309/2020|2020-11-30|EPI_ISL_711685|Original|hCoV-19^^Midtjylland|Human|Department of Virus and Microbiological Special Diagnostics|Albertsen Lab|Michaelsen|Denmark
>Spike|hCoV-19/England/LIVE-DBD22D/2020|2020-10-27|EPI_ISL_665130|Original|hCoV-19^^England|Human|Liverpool Clinical Laboratories|COVID-19 Genomics UK (COG-UK) Consortium|Haldenby|United Kingdom
>Spike|hCoV-19/England/QEUH-9F764D/2020|2020-09-27|EPI_ISL_595691|Original|hCoV-19^^England|Human|Oxford Viromics|COVID-19 Genomics UK (COG-UK) Consortium|Golubchik|United Kingdom
>Spike|hCoV-19/England/QEUH-C93A53/2020|2020-12-16|EPI_ISL_777047|Original|hCoV-19^^England|Human|Lighthouse Lab in Glasgow|Wellcome Sanger Institute for the COVID-19 Genomics UK (COG-UK) Consortium|Davies|United Kingdom
>Spike|hCoV-19/Canada/ON-PHL-20-03608/2020|2020-11-00|EPI_ISL_775116|Original|hCoV-19^^Ontario|Human|Public Health Ontario Laboratory|Public Health Ontario Laboratory|Guthrie|Canada
>Spike|hCoV-19/USA/OR-PROV-163/2020|2020-05-06|EPI_ISL_482416|Original|hCoV-19^^Oregon|Human|Providence St. Joseph Health Molecular Genomics Laboratory|Providence St. Joseph Health Molecular Genomics Laboratory|Dowdell|USA
>Spike|hCoV-19/Australia/VIC8510/2020|2020-07-28|EPI_ISL_565062|Original|hCoV-19^^Victoria|Human|Microbiological Diagnostic Unit - Public Health Laboratory (MDU-PHL)|MDU-PHL|Schultz|Australia
>Spike|hCoV-19/USA/UT-UPHL-2008916/2020|2020-05-28|EPI_ISL_525022|Original|hCoV-19^^Utah|Human|Utah Public Health Laboratory|Utah Public Health Laboratory|Young|USA
>Spike|hCoV-19/Denmark/DCGC-13541/2020|2020-11-30|EPI_ISL_712867|Original|hCoV-19^^Sjaelland|Human|Department of Virus and Microbiological Special Diagnostics|Albertsen Lab|Michaelsen|Denmark
>Spike|hCoV-19/Saudi Arabia/KAUST-MAKKAH786/2020|2020-04-24|EPI_ISL_513254|Original|hCoV-19^^Makkah|Human|Pathogen Genomics Lab King Abdullah University of Science and Technology(KAUST)|Pathogen Genomics Lab King Abdullah University of Science and Technology(KAUST)|Mohamed Ghazzali|Saudi Arabia
>Spike|hCoV-19/Scotland/CVR4366/2020|2020-09-14|EPI_ISL_572637|Original|hCoV-19^^Scotland|Human|West of Scotland Specialist Virology Centre|COVID-19 Genomics UK (COG-UK) Consortium|Robertson|United Kingdom
>Spike|hCoV-19/Denmark/DCGC-15863/2020|2020-11-23|EPI_ISL_714070|Original|hCoV-19^^Midtjylland|Human|Department of Virus and Microbiological Special Diagnostics|Albertsen Lab|Michaelsen|Denmark
>Spike|hCoV-19/England/ALDP-9D7D20/2020|2020-09-18|EPI_ISL_576626|Original|hCoV-19^^England|Human|Oxford Viromics|COVID-19 Genomics UK (COG-UK) Consortium|Golubchik|United Kingdom
>Spike|hCoV-19/England/QEUH-BC44B6/2020|2020-11-25|EPI_ISL_703320|Original|hCoV-19^^England|Human|Lighthouse Lab in Glasgow|Wellcome Sanger Institute for the COVID-19 Genomics UK (COG-UK) Consortium|Davies|United Kingdom

>Spike|hCoV-19/USA/MA-MASPHL-00060/2020|2020-04-03|EPI_ISL_791558|Original|hCoV-19^^Massachusetts|Human|Massachusetts State Public Health Laboratory|Infectious Disease Program|Park|USA
>Spike|hCoV-19/England/CAMC-A3EA10/2020|2020-10-05|EPI_ISL_609561|Original|hCoV-19^^England|Human|Lighthouse Lab in Cambridge|Wellcome Sanger Institute for the COVID-19 Genomics UK (COG-UK) consortium|Davies|United Kingdom
>Spike|hCoV-19/England/QEUH-95E240/2020|2020-08-08|EPI_ISL_536969|Original|hCoV-19^^England|Human|Lighthouse Lab in Glasgow|Wellcome Sanger Institute for the COVID-19 Genomics UK (COG-UK) consortium|Davies|United Kingdom
>Spike|hCoV-19/Australia/VIC3286/2020|2020-07-07|EPI_ISL_520965|Original|hCoV-19^^Victoria|Human|Microbiological Diagnostic Unit - Public Health Laboratory (MDU-PHL)|MDU-PHL|Schultz|Australia
>Spike|hCoV-19/England/MILK-B5A752/2020|2020-11-06|EPI_ISL_658289|Original|hCoV-19^^England|Human|Lighthouse Lab in Milton Keynes|Wellcome Sanger Institute for the COVID-19 Genomics UK (COG-UK) Consortium|Davies|United Kingdom
>Spike|hCoV-19/Wales/PHWC-2F241/2020|2020-04-06|EPI_ISL_446222|Original|hCoV-19^^Wales|Human|Wales Specialist Virology Centre|Public Health Wales Microbiology Cardiff|Moore|United Kingdom
>Spike|hCoV-19/England/QEUH-9FB588/2020|2020-09-28|EPI_ISL_588257|Original|hCoV-19^^England|Human|Lighthouse Lab in Glasgow|Wellcome Sanger Institute for the COVID-19 Genomics UK (COG-UK) consortium|Davies|United Kingdom
>Spike|hCoV-19/South Korea/KCDC2686/2020|2020-06-27|EPI_ISL_522466|Original|hCoV-19^^South Korea|Human|Division of Viral Diseases|Division of Viral Diseases|Kim|South Korea
>Spike|hCoV-19/Israel/CVL-n14292/2020|2020-04-10|EPI_ISL_514315|Original|hCoV-19^^Southern District|Human|Israel Central Virology laboratory|Israel Central Virology laboratory|Zuckerman|Israel
>Spike|hCoV-19/Canada/MB-NML-1038/2020|2020-07-28|EPI_ISL_582399|Original|hCoV-19^^Manitoba|Human|Cadham Provincial Laboratory|National Microbiology Laboratory (NML)|Majer|Canada
>Spike|hCoV-19/India/MH-NCCS-NR-13749/2020|2020-07-22|EPI_ISL_721653|Original|hCoV-19^^Maharashtra|Human|National Centre For Cell Science|National Centre For Cell Science|Paul|India
>Spike|hCoV-19/Australia/VIC5225/2020|2020-07-16|EPI_ISL_519990|Original|hCoV-19^^Victoria|Human|Microbiological Diagnostic Unit - Public Health Laboratory (MDU-PHL)|MDU-PHL|Schultz|Australia
>Spike|hCoV-19/England/QEUH-9645E4/2020|2020-08-11|EPI_ISL_536956|Original|hCoV-19^^England|Human|Lighthouse Lab in Glasgow|Wellcome Sanger Institute for the COVID-19 Genomics UK (COG-UK) consortium|Davies|United Kingdom
>Spike|hCoV-19/England/ALDP-CB3E7F/2020|2020-12-19|EPI_ISL_761329|Original|hCoV-19^^England|Human|Lighthouse Lab in Alderley Park|Wellcome Sanger Institute for the COVID-19 Genomics UK (COG-UK) Consortium|Davies|United Kingdom
>Spike|hCoV-19/USA/WA-UW-2369/2020|2020-03-31|EPI_ISL_485987|Original|hCoV-19^^Washington|Human|UW Virology Lab|UW Virology Lab|Roychoudhury|USA
>Spike|hCoV-19/England/QEUH-B7EC95/2020|2020-11-14|EPI_ISL_661746|Original|hCoV-19^^England|Human|Lighthouse Lab in Glasgow|Wellcome Sanger Institute for the COVID-19 Genomics UK (COG-UK) Consortium|Davies|United Kingdom
>Spike|hCoV-19/USA/NY-PV09171/2020|2020-03-19|EPI_ISL_422533|Original|hCoV-19^^New York|Human|MSHS Clinical Microbiology Laboratories|MSHS Pathogen Surveillance Program|van Bakel|USA
>Spike|hCoV-19/England/QEUH-B2F404/2020|2020-11-03|EPI_ISL_659800|Original|hCoV-19^^England|Human|Lighthouse Lab in Glasgow|Wellcome Sanger Institute for the COVID-19 Genomics UK (COG-UK) Consortium|Davies|United Kingdom
>Spike|hCoV-19/USA/WY-WYPHL-20006826/2020|2020-03-30|EPI_ISL_493061|Original|hCoV-19^^Wyoming|Human|Wyoming Public Health Laboratory|Wyoming Public Health Laboratory|Sevinsky|USA
>Spike|hCoV-19/USA/TX-HMH-MCoV-19630/2020|2020-11-22|EPI_ISL_783995|Original|hCoV-19^^Texas|Human|Houston Methodist Hospital|Houston Methodist Hospital|Long|USA
>Spike|hCoV-19/USA/TX-DSHS-1932/2020|2020-06-21|EPI_ISL_765932|Original|hCoV-19^^Texas|Human|TXDSHS|TXDSHS|Rahman|USA

>Spike|hCoV-19/USA/AL-CDC-2-3714561/2020|2020-11-04|EPI_ISL_751647|Original|hCoV-19^^Alabama|Human|AL Dept. of Public Health Bureau of Clinical Laboratories|Genomics and Discovery|Queen|USA
>Spike|hCoV-19/USA/VA-DCLS-2149/2020|2020-10-28|EPI_ISL_734235|Original|hCoV-19^^Virginia|Human|Virginia Division of Consolidated Laboratory Services (DCLS)|Virginia Division of Consolidated Laboratory Services (DCLS)|St. Jacques|USA
>Spike|hCoV-19/India/GJ-GBRC-422b/2020|2020-07-14|EPI_ISL_586552|Original|hCoV-19^^Gujarat|Human|Government Hospital,Veraval|Gujarat Biotechnology Research Centre|Joshi|India
>Spike|hCoV-19/England/NORT-28413C/2020|2020-05-05|EPI_ISL_472267|Original|hCoV-19^^England|Human|Northumbria University / South Tees Hospitals NHS Foundation Trust / North Cumbria Integrated Care NHS Foundation Trust / North Tees and Hartlepool NHS Foundation Trust / Newcastle Hospitals NHS Foundation Trust|COVID-19 Genomics UK (COG-UK) Consortium|Bashton|United Kingdom
>Spike|hCoV-19/USA/MN-MDH-1641/2020|2020-05-21|EPI_ISL_539835|Original|hCoV-19^^Minnesota|Human|Minnesota Department of Health|Minnesota Department of Health|Garfin|USA
>Spike|hCoV-19/USA/CA-IGI-0609/2020|2020-08-01|EPI_ISL_677845|Original|hCoV-19^^California|Human|Innovative Genomics Institute|Innovative Genomics Institute|Wyman|USA
>Spike|hCoV-19/Denmark/DCGC-5013/2020|2020-09-21|EPI_ISL_621809|Original|hCoV-19^^Sjaelland|Human|Department of Virus and Microbiological Special Diagnostics|Albertsen lab|Michaelsen|Denmark
>Spike|hCoV-19/Japan/PG-1702/2020|2020-04-00|EPI_ISL_685308|Original|hCoV-19^^Japan|Human|Niigata Prefectural Institute of Public Health and Environmental Sciences|Pathogen Genomics Center|Sekizuka|Japan
>Spike|hCoV-19/England/MILK-9AAD71/2020|2020-09-01|EPI_ISL_550971|Original|hCoV-19^^England|Human|Lighthouse Lab in Milton Keynes|Wellcome Sanger Institute for the COVID-19 Genomics UK (COG-UK) consortium|Davies|United Kingdom
>Spike|hCoV-19/Denmark/DCGC-11022/2020|2020-11-02|EPI_ISL_669484|Original|hCoV-19^^Sjaelland|Human|Department of Virus and Microbiological Special Diagnostics|Albertsen Lab|Michaelsen|Denmark
>Spike|hCoV-19/England/QEUH-B68AA3/2020|2020-11-11|EPI_ISL_655239|Original|hCoV-19^^England|Human|Lighthouse Lab in Glasgow|Wellcome Sanger Institute for the COVID-19 Genomics UK (COG-UK) Consortium|Davies|United Kingdom
>Spike|hCoV-19/England/CAMC-941A39/2020|2020-07-22|EPI_ISL_556831|Original|hCoV-19^^England|Human|Lighthouse Lab in Cambridge|Wellcome Sanger Institute for the COVID-19 Genomics UK (COG-UK) consortium|Davies|United Kingdom
>Spike|hCoV-19/USA/AZ-TG276594/2020|2020-04-15|EPI_ISL_694118|Original|hCoV-19^^Arizona|Human|AZ SPHL|TGen North|French|USA
>Spike|hCoV-19/England/QEUH-96C8B6/2020|2020-08-21|EPI_ISL_530719|Original|hCoV-19^^England|Human|Lighthouse Lab in Glasgow|Wellcome Sanger Institute for the COVID-19 Genomics UK (COG-UK) consortium|Davies|United Kingdom
>Spike|hCoV-19/USA/LA-GBCL-26791709/2020|2020-04-04|EPI_ISL_485330|Original|hCoV-19^^Louisiana|Human|River Road Testing Lab|Ginkgo Bioworks Clinical Laboratory|Smith|USA
>Spike|hCoV-19/Russia/MOS-CRIE-13385401/2020|2020-11-07|EPI_ISL_732670|Original|hCoV-19^^Moscow|Human|Molecular diagnostic laboratory of Federal Budget Institution of Science \Central Research Institute of Epidemiology\ of The Federal Service on Customers' Rights Protection and Human Well-being Surveillance|Group of Genomics and Postgenomic Technologies of Central Research Institute of Epidemilolog
>y|Korneenko|Russia
>Spike|hCoV-19/USA/CA-ALSR-3304/2020|2020-09-21|EPI_ISL_635954|Original|hCoV-19^^California|Human|San Diego County Public Health Laboratory|Andersen lab at Scripps Research|Gangavarapu|USA
>Spike|hCoV-19/Netherlands/ZH-EMC-1119/2020|2020-12-11|EPI_ISL_763198|Original|hCoV-19^^Zuid Holland|Human|Dutch COVID-19 response team|Erasmus Medical Center|Oude Munnink|Netherlands

>Spike|hCoV-19/England/ALDP-9E6CB8/2020|2020-09-24|EPI_ISL_580412|Original|hCoV-
19^^England|Human|Lighthouse Lab in Alderley Park|Wellcome Sanger Institute for the
COVID-19 Genomics UK (COG-UK) consortium|Davies|United Kingdom
>Spike|hCoV-19/USA/MI-MDHHS-SC21379/2020|2020-03-26|EPI_ISL_507633|Original|hCoV-
19^^Michigan|Human|Michigan Department of Health and Human Services|Michigan
Department of Health and Human Services|Blankenship|USA
>Spike|hCoV-19/England/ALDP-9CD640/2020|2020-09-15|EPI_ISL_566250|Original|hCoV-
19^^England|Human|Lighthouse Lab in Alderley Park|Wellcome Sanger Institute for the
COVID-19 Genomics UK (COG-UK) consortium|Davies|United Kingdom
>Spike|hCoV-19/England/CAMC-A3F790/2020|2020-10-05|EPI_ISL_609603|Original|hCoV-
19^^England|Human|Lighthouse Lab in Cambridge|Wellcome Sanger Institute for the
COVID-19 Genomics UK (COG-UK) consortium|Davies|United Kingdom
>Spike|hCoV-19/USA/WA-S1202/2020|2020-05-07|EPI_ISL_463692|Original|hCoV-
19^^Washington|Human|Washington State Department of Health|Seattle Flu
Study|Bedford|USA
>Spike|hCoV-19/Switzerland/GE-SNRCI-8099/2020|2020-03-
00|EPI_ISL_548215|Original|hCoV-19^^Geneve|Human|Laboratoire de Virologie|Swiss
National Reference Centre for Influenza|Gon\u00e7alves Cabecinhas|Switzerland
>Spike|hCoV-19/Saudi Arabia/KAUST-Makkah237/2020|2020-04-
06|EPI_ISL_437732|Original|hCoV-19^^Makkah|Human|Pathogen Genomics Lab King Abdullah
University of Science and Technology(KAUST)|Pathogen Genomics Lab King Abdullah
University of Science and Technology(KAUST)|Mohamed Ghazzali|Saudi Arabia
>Spike|hCoV-19/USA/CA-ALSR-0944-SAN/2020|2020-03-28|EPI_ISL_483428|Original|hCoV-
19^^California|Human|UC San Diego Center for Advanced Laboratory Medicine|Andersen
lab at Scripps Research|Gangavarapu|USA
>Spike|hCoV-19/USA/TX-HMH-5371/2020|2020-06-25|EPI_ISL_544723|Original|hCoV-
19^^Texas|Human|Houston Methodist Hospital|Houston Methodist Hospital|Long|USA
>Spike|hCoV-19/Denmark/DCGC-3649/2020|2020-08-31|EPI_ISL_615421|Original|hCoV-
19^^Hovedstaden|Human|Department of Virus and Microbiological Special
Diagnostics|Albertsen lab|Michaelsen|Denmark
>Spike|hCoV-19/USA/TX-HMH-MCoV-18418/2020|2020-11-18|EPI_ISL_784766|Original|hCoV-
19^^Texas|Human|Houston Methodist Hospital|Houston Methodist Hospital|Long|USA
>Spike|hCoV-19/Denmark/DCGC-22300/2020|2020-12-14|EPI_ISL_758664|Original|hCoV-
19^^Hovedstaden|Human|Department of Virus and Microbiological Special
Diagnostics|Albertsen Lab|Michaelsen|Denmark
>Spike|hCoV-19/England/201420223/2020|2020-03-30|EPI_ISL_465933|Original|hCoV-
19^^England|Human|Respiratory Virus Unit|Respiratory Virus Unit|Platt|United Kingdom
>Spike|hCoV-19/France/PAC-IHU-1502/2020|2020-08-10|EPI_ISL_569180|Original|hCoV-
19^^Provence-Alpes-C\u00f4te-d\u2019Azur|Human|MEPHI|MEPHI|Levasseur|France
>Spike|hCoV-19/Spain/AN-IBV-006097/2020|2020-06-25|EPI_ISL_510430|Original|hCoV-
19^^Andalucia|Human|Hospital Universitario Virgen de las Nieves de Granada-
SAS|SeqCOVID-SPAIN consortium/IBV(CSIC)|Comas|Spain
>Spike|hCoV-19/Netherlands/GE-EMC-317/2020|2020-10-05|EPI_ISL_632649|Original|hCoV-
19^^Gelderland|Human|Dutch COVID-19 response team|Erasmus Medical Center|Oude
Munnink|Netherlands
>Spike|hCoV-19/Japan/PG-2451/2020|2020-04-00|EPI_ISL_686056|Original|hCoV-
19^^Japan|Human|Pathogen Genomics Center|Pathogen Genomics Center|Sekizuka|Japan
>Spike|hCoV-19/Wales/PHWC-49254C/2020|2020-11-18|EPI_ISL_706856|Original|hCoV-
19^^Wales|Human|Originating lab|COVID-19 Genomics UK (COG-UK) Consortium|Moore|United
Kingdom
>Spike|hCoV-19/Wales/MILK-9A6C69/2020|2020-09-03|EPI_ISL_550175|Original|hCoV-
19^^Wales|Human|Lighthouse Lab in Milton Keynes|Wellcome Sanger Institute for the
COVID-19 Genomics UK (COG-UK) consortium|Davies|United Kingdom
>Spike|hCoV-19/England/ALDP-A6D0E9/2020|2020-10-14|EPI_ISL_608109|Original|hCoV-
19^^England|Human|Lighthouse Lab in Alderley Park|Wellcome Sanger Institute for the
COVID-19 Genomics UK (COG-UK) consortium|Davies|United Kingdom
>Spike|hCoV-19/Denmark/DCGC-6639/2020|2020-10-12|EPI_ISL_619541|Original|hCoV-
19^^Sjaelland|Human|Department of Virus and Microbiological Special
Diagnostics|Albertsen lab|Michaelsen|Denmark

>Spike|hCoV-19/Wales/PHWC-160832/2020|2020-05-24|EPI_ISL_472983|Original|hCoV-19^^Wales|Human|Wales Specialist Virology Centre Sequencing lab|COVID-19 Genomics UK (COG-UK) Consortium|Moore|United Kingdom
>Spike|hCoV-19/Belgium/rega-0427424/2020|2020-04-27|EPI_ISL_476992|Original|hCoV-19^^Kersbeek-miskom|Human|KU Leuven|KU Leuven|Maes|Belgium
>Spike|hCoV-19/Wales/PHWC-28415/2020|2020-03-27|EPI_ISL_432029|Original|hCoV-19^^Wales|Human|Wales Specialist Virology Centre|Public Health Wales Microbiology Cardiff|Moore|United Kingdom
>Spike|hCoV-19/France/NAQ-SC597/2020|2020-05-12|EPI_ISL_663241|Original|hCoV-19^^Nouvelle-Aquitaine|Human|CHU Poitiers|CNR Virus des Infections Respiratoires - France SUD|Bal|France
>Spike|hCoV-19/USA/OR-PROV-040/2020|2020-03-27|EPI_ISL_482322|Original|hCoV-19^^Oregon|Human|Providence St. Joseph Health Molecular Genomics Laboratory|Providence St. Joseph Health Molecular Genomics Laboratory|Dowdell|USA
>Spike|hCoV-19/England/ALDP-B46416/2020|2020-11-07|EPI_ISL_656287|Original|hCoV-19^^England|Human|Lighthouse Lab in Alderley Park|Wellcome Sanger Institute for the COVID-19 Genomics UK (COG-UK) Consortium|Davies|United Kingdom
>Spike|hCoV-19/Australia/NSW957/2020|2020-09-08|EPI_ISL_545029|Original|hCoV-19^^New South Wales|Human|Pathology West - NSW Health Pathology|NSW Health Pathology - Institute of Clinical Pathology and Medical Research; Westmead Hospital; University of Sydney|Gall|Australia
>Spike|hCoV-19/Scotland/QEUH-98F5F2/2020|2020-08-27|EPI_ISL_568439|Original|hCoV-19^^Scotland|Human|Lighthouse Lab in Glasgow|Wellcome Sanger Institute for the COVID-19 Genomics UK (COG-UK) consortium|Davies|United Kingdom
>Spike|hCoV-19/Switzerland/BL-ETHZ-380273/2020|2020-11-22|EPI_ISL_721782|Original|hCoV-19^^Basel-Landschaft|Human|Viollier AG|Department of Biosystems Science and Engineering|Nadeau|Switzerland
>Spike|hCoV-19/USA/TX-HMH-MCoV-8321/2020|2020-07-07|EPI_ISL_784330|Original|hCoV-19^^Texas|Human|Houston Methodist Hospital|Houston Methodist Hospital|Long|USA
>Spike|hCoV-19/USA/LA-GBCL-26556546/2020|2020-04-15|EPI_ISL_485183|Original|hCoV-19^^Louisiana|Human|River Road Testing Lab|Ginkgo Bioworks Clinical Laboratory|Smith|USA
>Spike|hCoV-19/USA/TX-HMH-6863/2020|2020-06-26|EPI_ISL_546299|Original|hCoV-19^^Texas|Human|Houston Methodist Hospital|Houston Methodist Hospital|Long|USA
>Spike|hCoV-19/Japan/IC-0326/2020|2020-08-00|EPI_ISL_667755|Original|hCoV-19^^Japan|Human|Pathogen Genomics Center|Pathogen Genomics Center|Sekizuka|Japan
>Spike|hCoV-19/England/BRIS-12616A/2020|2020-04-09|EPI_ISL_459081|Original|hCoV-19^^England|Human|PHE South West Regional Laboratory|Wellcome Sanger Institute for the COVID-19 Genomics UK (COG-UK) consortium|Davies|United Kingdom
>Spike|hCoV-19/England/CAMB-79837/2020|2020-04-01|EPI_ISL_441805|Original|hCoV-19^^England|Human|Department of Pathology|Wellcome Sanger Institute for the COVID-19 Genomics UK (COG-UK) consortium|Amato|United Kingdom
>Spike|hCoV-19/England/QEUH-B67E8A/2020|2020-11-11|EPI_ISL_661615|Original|hCoV-19^^England|Human|Lighthouse Lab in Glasgow|Wellcome Sanger Institute for the COVID-19 Genomics UK (COG-UK) Consortium|Davies|United Kingdom
>Spike|hCoV-19/United Arab Emirates/0943/2020|2020-06-27|EPI_ISL_699079|Original|hCoV-19^^Abu Dhabi|Human|Group 42 (G42) Healthcare|G42 Healthcare|Ogrodzki|United Arab Emirates
>Spike|hCoV-19/England/ALDP-4F7C9B/2020|2020-06-24|EPI_ISL_559156|Original|hCoV-19^^England|Human|Lighthouse Lab in Alderley Park|Wellcome Sanger Institute for the COVID-19 Genomics UK (COG-UK) consortium|Davies|United Kingdom
>Spike|hCoV-19/England/MILK-62AE79/2020|2020-07-05|EPI_ISL_557963|Original|hCoV-19^^England|Human|Lighthouse Lab in Milton Keynes|Wellcome Sanger Institute for the COVID-19 Genomics UK (COG-UK) consortium|Davies|United Kingdom
>Spike|hCoV-19/England/MILK-AC8529/2020|2020-10-21|EPI_ISL_629677|Original|hCoV-19^^England|Human|Lighthouse Lab in Milton Keynes|Wellcome Sanger Institute for the COVID-19 Genomics UK (COG-UK) consortium|Davies|United Kingdom
>Spike|hCoV-19/Denmark/DCGC-19831/2020|2020-12-07|EPI_ISL_747817|Original|hCoV-19^^Sjaelland|Human|Department of Virus and Microbiological Special Diagnostics|Albertsen Lab|Michaelsen|Denmark

>Spike|hCoV-19/Canada/QC-AL2/2020|2020-04-08|EPI_ISL_450647|Original|hCoV-19^^Quebec|Human|Laboratoire de microbiologie|Smith Laboratory|Smith|Canada
>Spike|hCoV-19/USA/CA-CZB-9788/2020|2020-05-17|EPI_ISL_672051|Original|hCoV-19^^California|Human|The Ashley Laboratory|Chan-Zuckerberg Biohub|Bhatt|USA
>Spike|hCoV-19/USA/WI-UW-2379/2020|2020-11-01|EPI_ISL_728144|Original|hCoV-19^^Wisconsin|Human|University of Wisconsin-Madison AIDS Vaccine Research Laboratories|University of Wisconsin-Madison AIDS Vaccine Research Laboratories|Baczenas|USA
>Spike|hCoV-19/Denmark/DCGC-1062/2020|2020-04-06|EPI_ISL_614693|Original|hCoV-19^^Hovedstaden|Human|Department of Virus and Microbiological Special Diagnostics|Albertsen lab|Michaelsen|Denmark
>Spike|hCoV-19/England/MILK-B3BE61/2020|2020-11-02|EPI_ISL_706101|Original|hCoV-19^^England|Human|Oxford Viromics|COVID-19 Genomics UK (COG-UK) Consortium|Golubchik|United Kingdom
>Spike|hCoV-19/France/ARA-29628/2020|2020-04-23|EPI_ISL_684107|Original|hCoV-19^^Auvergne-Rh\u00f4ne-Alpes|Human|CNR Virus des Infections Respiratoires - France SUD|CNR Virus des Infections Respiratoires - France SUD|Bal|France
>Spike|hCoV-19/Panama/338657/2020|2020-04-09|EPI_ISL_496875|Original|hCoV-19^^Cocle|Human|Gorgas Memorial Laboratory of Health Studies|Gorgas Memorial Laboratory of Health Studies|Martinez|Panama
>Spike|hCoV-19/Denmark/ALAB-SSI414A/2020|2020-03-23|EPI_ISL_429512|Original|hCoV-19^^Denmark|Human|Department of Virus and Microbiological Special Diagnostics|Albertsen lab|Kirkegaard|Denmark
>Spike|hCoV-19/England/GSTT-265E2C5/2020|2020-03-31|EPI_ISL_484182|Original|hCoV-19^^England|Human|Centre for Clinical Infection and Diagnostics Research and Genomics Innovation Unit|COVID-19 Genomics UK (COG-UK) Consortium|Awan|United Kingdom
>Spike|hCoV-19/Oman/RESP-20-B-16254/2020|2020-05-22|EPI_ISL_518840|Original|hCoV-19^^Muscat|Human|Oman-National Influenza Center|Biotechnology & OMICs Laboratory|Al Mahruqi|Oman
>Spike|hCoV-19/England/EXET-136158/2020|2020-04-15|EPI_ISL_457203|Original|hCoV-19^^England|Human|University of Exeter|COVID-19 Genomics UK (COG-UK) Consortium|Temperton|United Kingdom
>Spike|hCoV-19/Japan/PG-0536/2020|2020-04-00|EPI_ISL_480193|Original|hCoV-19^^Kanto|Human|Ota Health Center Welfare Section|Pathogen Genomics Center|Sekizuka|Japan
>Spike|hCoV-19/Sweden/20-14318/2020|2020-05-05|EPI_ISL_469057|Original|hCoV-19^^Kalmar|Human|Inger Landgren|The Public Health Agency of Sweden|Svartstr\u00f6m|Sweden
>Spike|hCoV-19/USA/TX-HMH-7868/2020|2020-06-30|EPI_ISL_545671|Original|hCoV-19^^Texas|Human|Houston Methodist Hospital|Houston Methodist Hospital|Long|USA
>Spike|hCoV-19/England/BIRM-6B11F/2020|2020-11-14|EPI_ISL_679123|Original|hCoV-19^^England|Human|University of Birmingham|COVID-19 Genomics UK (COG-UK) Consortium|Loman|United Kingdom
>Spike|hCoV-19/Spain/AN-IBV-001956/2020|2020-03-13|EPI_ISL_509602|Original|hCoV-19^^Andalucia|Human|Servicio de Microbiolog\u00eda. HRU de M\u00e1laga. Servicio Andaluz de Salud|SeqCOVID-SPAIN consortium/IBV(CSIC)|Comas|Spain
>Spike|hCoV-19/England/20132100204/2020|2020-03-23|EPI_ISL_423833|Original|hCoV-19^^England|Human|Respiratory Virus Unit|Respiratory Virus Unit|Platt|United Kingdom
>Spike|hCoV-19/USA/NY-NYUMC38/2020|2020-03-18|EPI_ISL_419699|Original|hCoV-19^^New York|Human|NYU Langone Health|Departments of Pathology and Medicine|Maurano|USA
>Spike|hCoV-19/USA/WI-UW-2461/2020|2020-12-21|EPI_ISL_759736|Original|hCoV-19^^Wisconsin|Human|University of Wisconsin-Madison AIDS Vaccine Research Laboratories|University of Wisconsin-Madison AIDS Vaccine Research Laboratories|Moreno|USA
>Spike|hCoV-19/USA/SC-DHEC-0546/2020|2020-06-19|EPI_ISL_529086|Original|hCoV-19^^South Carolina|Human|Microbiology Division|Microbiology Division|Flores,H.|USA
>Spike|hCoV-19/Scotland/QEUH-9657A1/2020|2020-08-14|EPI_ISL_531155|Original|hCoV-19^^Scotland|Human|NHSGGC West of Scotland Specialist Virology Centre / MRC-University of Glasgow Centre for Virus Research|Wellcome Sanger Institute for the COVID-19 Genomics UK (COG-UK) consortium|Davies|United Kingdom

>Spike|hCoV-19/England/BRIS-130D3C/2020|2020-03-24|EPI_ISL_492570|Original|hCoV-19^^England|Human|PHE South West Regional Laboratory|Wellcome Sanger Institute for the COVID-19 Genomics UK (COG-UK) consortium|Davies|United Kingdom
>Spike|hCoV-19/Denmark/DCGC-5952/2020|2020-10-05|EPI_ISL_620217|Original|hCoV-19^^Syddanmark|Human|Department of Virus and Microbiological Special Diagnostics|Albertsen lab|Michaelsen|Denmark
>Spike|hCoV-19/Wales/PHWC-494FA6/2020|2020-11-19|EPI_ISL_726280|Original|hCoV-19^^Wales|Human|Originating lab|COVID-19 Genomics UK (COG-UK) Consortium|Moore|United Kingdom
>Spike|hCoV-19/England/ALDP-953B40/2020|2020-06-08|EPI_ISL_553786|Original|hCoV-19^^England|Human|Lighthouse Lab in Alderley Park|Wellcome Sanger Institute for the COVID-19 Genomics UK (COG-UK) consortium|Davies|United Kingdom
>Spike|hCoV-19/England/QEUH-A364B0/2020|2020-10-03|EPI_ISL_706910|Original|hCoV-19^^England|Human|Oxford Viromics|COVID-19 Genomics UK (COG-UK) Consortium|Golubchik|United Kingdom
>Spike|hCoV-19/South Korea/KCDC2766/2020|2020-07-22|EPI_ISL_526700|Original|hCoV-19^^South Korea|Human|Division of Viral Diseases|Division of Viral Diseases|Kim|South Korea
>Spike|hCoV-19/Japan/PG-3118/2020|2020-05-00|EPI_ISL_691240|Original|hCoV-19^^Japan|Human|Pathogen Genomics Center|Pathogen Genomics Center|Sekizuka|Japan
>Spike|hCoV-19/England/CAMC-A41081/2020|2020-10-06|EPI_ISL_609313|Original|hCoV-19^^England|Human|Lighthouse Lab in Cambridge|Wellcome Sanger Institute for the COVID-19 Genomics UK (COG-UK) consortium|Davies|United Kingdom
>Spike|hCoV-19/Wales/PHWC-49DFF8/2020|2020-12-06|EPI_ISL_742941|Original|hCoV-19^^Wales|Human|Wales Specialist Virology Centre Sequencing lab|COVID-19 Genomics UK (COG-UK) Consortium|Moore|United Kingdom
>Spike|hCoV-19/India/MH-ACTREC-459/2020|2020-08-10|EPI_ISL_700107|Original|hCoV-19^^Maharashtra|Human|Hematopathology Laboratory|Hematopathology Laboratory|Patkar|India
>Spike|hCoV-19/USA/NM-DOH-2020308490/2020|2020-08-24|EPI_ISL_602181|Original|hCoV-19^^New Mexico|Human|New Mexico Department of Health Scientific Laboratory|New Mexico Department of Health Scientific Laboratory|Johnson|USA
>Spike|hCoV-19/England/MILK-9EF1FC/2020|2020-09-24|EPI_ISL_594908|Original|hCoV-19^^England|Human|Oxford Viromics|COVID-19 Genomics UK (COG-UK) Consortium|Golubchik|United Kingdom
>Spike|hCoV-19/England/CAMC-C71E31/2020|2020-12-15|EPI_ISL_768915|Original|hCoV-19^^England|Human|Lighthouse Lab in Cambridge|Wellcome Sanger Institute for the COVID-19 Genomics UK (COG-UK) Consortium|Davies|United Kingdom
>Spike|hCoV-19/Japan/PG-4388/2020|2020-07-00|EPI_ISL_692615|Original|hCoV-19^^Japan|Human|Pathogen Genomics Center|Pathogen Genomics Center|Sekizuka|Japan
>Spike|hCoV-19/England/EXET-139D24/2020|2020-00-00|EPI_ISL_679338|Original|hCoV-19^^England|Human|University of Exeter|COVID-19 Genomics UK (COG-UK) Consortium|Temperton|United Kingdom
>Spike|hCoV-19/England/QEUH-9DE2A2/2020|2020-09-21|EPI_ISL_599568|Original|hCoV-19^^England|Human|Lighthouse Lab in Glasgow|Wellcome Sanger Institute for the COVID-19 Genomics UK (COG-UK) consortium|Davies|United Kingdom
>Spike|hCoV-19/Italy/CAM-INTPas-21/2020|2020-04-22|EPI_ISL_775261|Original|hCoV-19^^Campania|Human|INT Fondazione Pascale|INT Fondazione Pascale|Rachiglio|Italy
>Spike|hCoV-19/England/HECH-259A4F5/2020|2020-07-11|EPI_ISL_529663|Original|hCoV-19^^England|Human|University of Birmingham|COVID-19 Genomics UK (COG-UK) Consortium|Loman|United Kingdom
>Spike|hCoV-19/India/MH-GA46/2020|2020-06-20|EPI_ISL_508240|Original|hCoV-19^^Maharashtra|Human|Government Medical College|National Institute of Biomedical Genomics|Maitra|India
>Spike|hCoV-19/USA/TX-HMH-MCoV-13094/2020|2020-08-26|EPI_ISL_788245|Original|hCoV-19^^Texas|Human|Houston Methodist Hospital|Houston Methodist Hospital|Long|USA
>Spike|hCoV-19/USA/TX-HMH-MCoV-12567/2020|2020-08-31|EPI_ISL_790396|Original|hCoV-19^^Texas|Human|Houston Methodist Hospital|Houston Methodist Hospital|Long|USA

>Spike|hCoV-19/England/QEUH-BA2700/2020|2020-11-18|EPI_ISL_719982|Original|hCoV-19^^England|Human|Lighthouse Lab in Glasgow|Wellcome Sanger Institute for the COVID-19 Genomics UK (COG-UK) Consortium|Davies|United Kingdom
>Spike|hCoV-19/USA/CA-LACPHL-AE00014/2020|2020-07-02|EPI_ISL_707911|Original|hCoV-19^^California|Human|Los Angeles County Public Health Laboratory|Los Angeles County Public Health Laboratory|Hemarajata|USA
>Spike|hCoV-19/Japan/PG-2312/2020|2020-05-00|EPI_ISL_685917|Original|hCoV-19^^Japan|Human|Pathogen Genomics Center|Pathogen Genomics Center|Sekizuka|Japan
>Spike|hCoV-19/India/TG-CCMB-M172/2020|2020-06-06|EPI_ISL_495189|Original|hCoV-19^^Telangana|Human|CSIR-Centre for Cellular and Molecular Biology|CSIR-Centre for Cellular and Molecular Biology|Banu|India
>Spike|hCoV-19/USA/WA-UW-6439/2020|2020-04-03|EPI_ISL_570114|Original|hCoV-19^^Washington|Human|UW Virology Lab|UW Virology Lab|Roychoudhury|USA
>Spike|hCoV-19/Switzerland/AG-ETHZ-130002/2020|2020-05-05|EPI_ISL_466998|Original|hCoV-19^^Aargau|Human|Viollier AG|Department of Biosystems Science and Engineering|Nadeau|Switzerland
>Spike|hCoV-19/Netherlands/UT-RIVM-20114/2020|2020-11-25|EPI_ISL_723290|Orignal|hCoV-19^^Utrecht|Human|Dutch COVID-19 response team|National Institute for Public Health and the Environment (RIVM)|Meijer|Netherlands
>Spike|hCoV-19/USA/UT-0361/2020|2020-03-25|EPI_ISL_420825|Original|hCoV-19^^Utah|Human|Utah Public Health Laboratory|Utah Public Health Laboratory|Young|USA
>Spike|hCoV-19/USA/CA-ALSR-1044-SAN/2020|2020-06-19|EPI_ISL_483480|Original|hCoV-19^^California|Human|UC San Diego Center for Advanced Laboratory Medicine|Andersen lab at Scripps Research|Gangavarapu|USA
>Spike|hCoV-19/Norway/CS-31/2020|2020-04-23|EPI_ISL_666612|Original|hCoV-19^^Viken|Human|Dept. of Microbiology and Infection Control|Dept. of Microbiology and Infection Control|Hesselberg L\u00f8vestad|Norway
>Spike|hCoV-19/Wales/PHWC-4A4459/2020|2020-12-16|EPI_ISL_764694|Original|hCoV-19^^Wales|Human|Wales Specialist Virology Centre Sequencing lab|COVID-19 Genomics UK (COG-UK) Consortium|Moore|United Kingdom
>Spike|hCoV-19/England/QEUH-A8B6D4/2020|2020-10-17|EPI_ISL_643220|Original|hCoV-19^^England|Human|Lighthouse Lab in Glasgow|Wellcome Sanger Institute for the COVID-19 Genomics UK (COG-UK) Consortium|Davies|United Kingdom
>Spike|hCoV-19/England/CAMB-7CA01/2020|2020-04-05|EPI_ISL_442813|Original|hCoV-19^^England|Human|Department of Pathology|Wellcome Sanger Institute for the COVID-19 Genomics UK (COG-UK) consortium|Amato|United Kingdom
>Spike|hCoV-19/England/20129001404/2020|2020-03-20|EPI_ISL_423975|Original|hCoV-19^^England|Human|Respiratory Virus Unit|Respiratory Virus Unit|Platt|United Kingdom
>Spike|hCoV-19/England/ALDP-A6F4AF/2020|2020-10-14|EPI_ISL_607774|Original|hCoV-19^^England|Human|Lighthouse Lab in Alderley Park|Wellcome Sanger Institute for the COVID-19 Genomics UK (COG-UK) consortium|Davies|United Kingdom
>Spike|hCoV-19/United Arab Emirates/0308/2020|2020-05-18|EPI_ISL_698486|Original|hCoV-19^^Abu Dhabi|Human|Group 42 (G42) Healthcare|G42 Healthcare|Ogrodzki|United Arab Emirates
>Spike|hCoV-19/England/ALDP-C8B6B4/2020|2020-12-16|EPI_ISL_756640|Original|hCoV-19^^England|Human|Lighthouse Lab in Alderley Park|Wellcome Sanger Institute for the COVID-19 Genomics UK (COG-UK) Consortium|Davies|United Kingdom
>Spike|hCoV-19/England/ALDP-9BDAB0/2020|2020-09-10|EPI_ISL_568194|Original|hCoV-19^^England|Human|Lighthouse Lab in Alderley Park|Wellcome Sanger Institute for the COVID-19 Genomics UK (COG-UK) consortium|Davies|United Kingdom
>Spike|hCoV-19/USA/MI-MDHHS-SC21171/2020|2020-04-02|EPI_ISL_507428|Original|hCoV-19^^Michigan|Human|Michigan Department of Health and Human Services|Michigan Department of Health and Human Services|Blankenship|USA
>Spike|hCoV-19/USA/UT-UPHL-2012898524/2020|2020-07-26|EPI_ISL_745501|Original|hCoV-19^^Utah|Human|Ginkgo Bioworks Clinical Laboratory|Utah Public Health Laboratory|Young|USA
>Spike|hCoV-19/USA/CA-ALSR-1597/2020|2020-04-22|EPI_ISL_494683|Original|hCoV-19^^California|Human|Scripps Medical Laboratory|Andersen lab at Scripps Research|Gangavarapu|USA

>Spike|hCoV-19/USA/WA-S1504/2020|2020-05-21|EPI_ISL_495930|Original|hCoV-19^^Washington|Human|Washington State Department of Health|Seattle Flu Study|Lee|USA
>Spike|hCoV-19/Netherlands/ZH-EMC-301/2020|2020-07-04|EPI_ISL_523643|Original|hCoV-19^^Zuid Holland|Human|Dutch COVID-19 response team|Erasmus Medical Center|Oude Munnink|Netherlands
>Spike|hCoV-19/USA/WI-UW-301/2020|2020-04-13|EPI_ISL_436595|Original|hCoV-19^^Wisconsin|Human|University of Wisconsin-Madison AIDS Vaccine Research Laboratories|University of Wisconsin-Madison AIDS Vaccine Research Laboratories|Baczenas|USA
>Spike|hCoV-19/England/QEUH-A8C6B5/2020|2020-10-18|EPI_ISL_643093|Original|hCoV-19^^England|Human|Lighthouse Lab in Glasgow|Wellcome Sanger Institute for the COVID-19 Genomics UK (COG-UK) Consortium|Davies|United Kingdom
>Spike|hCoV-19/USA/MI-MDHHS-SC21653/2020|2020-04-01|EPI_ISL_516380|Original|hCoV-19^^Michigan|Human|Michigan Department of Health and Human Services|Michigan Department of Health and Human Services|Blankenship|USA
>Spike|hCoV-19/England/QEUH-98FDEB/2020|2020-08-24|EPI_ISL_539973|Original|hCoV-19^^England|Human|Lighthouse Lab in Glasgow|Wellcome Sanger Institute for the COVID-19 Genomics UK (COG-UK) consortium|Davies|United Kingdom
>Spike|hCoV-19/Brazil/RS-2533/2020|2020-03-18|EPI_ISL_729807|Original|hCoV-19^^Rio Grande do Sul|Human|Laboratorio Central de Saude Publica do Estado do Rio Grande do Sul (LACEN-RS)|Laboratory of Respiratory Viruses and Measles|Silva|Brazil
>Spike|hCoV-19/England/CAMC-B070A4/2020|2020-10-29|EPI_ISL_643676|Original|hCoV-19^^England|Human|Lighthouse Lab in Cambridge|Wellcome Sanger Institute for the COVID-19 Genomics UK (COG-UK) Consortium|Davies|United Kingdom
>Spike|hCoV-19/England/ALDP-94A63C/2020|2020-06-11|EPI_ISL_555795|Original|hCoV-19^^England|Human|Lighthouse Lab in Alderley Park|Wellcome Sanger Institute for the COVID-19 Genomics UK (COG-UK) consortium|Davies|United Kingdom
>Spike|hCoV-19/USA/WI-WSLH-200026/2020|2020-04-17|EPI_ISL_471191|Original|hCoV-19^^Wisconsin|Human|Wisconsin State Laboratory of Hygiene Communicable Disease Division|Wisconsin State Laboratory of Hygiene Communicable Disease Division|Florek|USA
>Spike|hCoV-19/Netherlands/Flevoland_6/2020|2020-04-15|EPI_ISL_460639|Original|hCoV-19^^Flevoland|Human|Dutch COVID-19 response team|Erasmus Medical Center|Oude Munnink|Netherlands
>Spike|hCoV-19/USA/CA-CZB-1034/2020|2020-04-01|EPI_ISL_436675|Original|hCoV-19^^California|Human|County of Santa Clara Public Health Department|Chan-Zuckerberg Biohub|Hao|USA
>Spike|hCoV-19/England/QEUH-A0263F/2020|2020-09-29|EPI_ISL_584917|Original|hCoV-19^^England|Human|Quadram Institute Bioscience|COVID-19 Genomics UK (COG-UK) Consortium|Alikhan|United Kingdom
>Spike|hCoV-19/England/MILK-A7B033/2020|2020-10-14|EPI_ISL_607322|Original|hCoV-19^^England|Human|Lighthouse Lab in Milton Keynes|Wellcome Sanger Institute for the COVID-19 Genomics UK (COG-UK) consortium|Davies|United Kingdom
>Spike|hCoV-19/USA/VA-DCLS-1668/2020|2020-08-29|EPI_ISL_644838|Original|hCoV-19^^Virginia|Human|Virginia DCLS|Virginia DCLS|St. Jacques|USA
>Spike|hCoV-19/England/ALDP-B1CAAE/2020|2020-11-02|EPI_ISL_723526|Original|hCoV-19^^England|Human|Centre for Enzyme Innovation|COVID-19 Genomics UK (COG-UK) Consortium|Robson|United Kingdom
>Spike|hCoV-19/England/QEUH-A0AF87/2020|2020-10-01|EPI_ISL_599263|Original|hCoV-19^^England|Human|Lighthouse Lab in Glasgow|Wellcome Sanger Institute for the COVID-19 Genomics UK (COG-UK) consortium|Davies|United Kingdom
>Spike|hCoV-19/Luxembourg/LNS9623706/2020|2020-10-23|EPI_ISL_744952|Original|hCoV-19^^Luxembourg|Human|Laboratoire national de sant\u00e9|Laboratoire national de sant\u00e9|Wienecke-Baldacchino|Luxembourg
>Spike|hCoV-19/Denmark/DCGC-14890/2020|2020-11-23|EPI_ISL_712049|Original|hCoV-19^^Sjaelland|Human|Department of Virus and Microbiological Special Diagnostics|Albertsen Lab|Michaelsen|Denmark
>Spike|hCoV-19/Luxembourg/LNS7041741/2020|2020-07-06|EPI_ISL_744500|Original|hCoV-19^^Luxembourg|Human|Laboratoire national de sant\u00e9|Laboratoire national de sant\u00e9|Wienecke-Baldacchino|Luxembourg

>Spike|hCoV-19/England/CAMB-1BACD3/2020|2020-11-12|EPI_ISL_664315|Original|hCoV-19^^England|Human|Department of Pathology|COVID-19 Genomics UK (COG-UK) Consortium|Goodfellow|United Kingdom
>Spike|hCoV-19/Japan/NGY-NNH-048/2020|2020-08-25|EPI_ISL_637014|Original|hCoV-19^^Aichi|Human|Department of Infectious Diseases and Immunology|Clinical Research Center|Iwatani|Japan
>Spike|hCoV-19/England/QEUH-B12FE5/2020|2020-11-02|EPI_ISL_641727|Original|hCoV-19^^England|Human|Lighthouse Lab in Glasgow|Wellcome Sanger Institute for the COVID-19 Genomics UK (COG-UK) Consortium|Davies|United Kingdom
>Spike|hCoV-19/England/MILK-956D56/2020|2020-06-08|EPI_ISL_554291|Original|hCoV-19^^England|Human|Lighthouse Lab in Milton Keynes|Wellcome Sanger Institute for the COVID-19 Genomics UK (COG-UK) consortium|Davies|United Kingdom
>Spike|hCoV-19/Northern Ireland/LIVE-1DE0FB/2020|2020-10-09|EPI_ISL_611999|Original|hCoV-19^^Northern Ireland|Human|Liverpool Clinical Laboratories|COVID-19 Genomics UK (COG-UK) Consortium|Haldenby|United Kingdom
>Spike|hCoV-19/USA/CA-CZB-15428/2020|2020-12-02|EPI_ISL_739341|Original|hCoV-19^^California|Human|Orange County Public Health Lab|Chan-Zuckerberg Biohub|Kamm|USA
>Spike|hCoV-19/Switzerland/ZH-ETHZ-270147/2020|2020-08-28|EPI_ISL_541528|Original|hCoV-19^^Zurich|Human|Viollier AG|Department of Biosystems Science and Engineering|Nadeau|Switzerland
>Spike|hCoV-19/England/MILK-A46857/2020|2020-10-06|EPI_ISL_598767|Original|hCoV-19^^England|Human|Lighthouse Lab in Milton Keynes|Wellcome Sanger Institute for the COVID-19 Genomics UK (COG-UK) consortium|Davies|United Kingdom
>Spike|hCoV-19/Scotland/EDB3876/2020|2020-05-01|EPI_ISL_491573|Original|hCoV-19^^Scotland|Human|Virology Department|Wellcome Sanger Institute for the COVID-19 Genomics UK (COG-UK) consortium|Davies|United Kingdom
>Spike|hCoV-19/Switzerland/BE-ETHZ-410563/2020|2020-11-26|EPI_ISL_737735|Original|hCoV-19^^Bern|Human|Viollier AG|Department of Biosystems Science and Engineering|Nadeau|Switzerland
>Spike|hCoV-19/Argentina/argenTAG-12CF5045/2020|2020-07-22|EPI_ISL_648212|Original|hCoV-19^^Buenos Aires|Human|INBIRS-UBA|Laboratorio Mixto de Biotecnolog\u00eda Acu\u00e1tica (LMBA)|Ezpeleta|Argentina
>Spike|hCoV-19/USA/LA-GBCL-26544565/2020|2020-04-18|EPI_ISL_485138|Original|hCoV-19^^Louisiana|Human|River Road Testing Lab|Ginkgo Bioworks Clinical Laboratory|Smith|USA
>Spike|hCoV-19/Denmark/DCGC-5628/2020|2020-09-28|EPI_ISL_619915|Original|hCoV-19^^Hovedstaden|Human|Department of Virus and Microbiological Special Diagnostics|Albertsen lab|Michaelsen|Denmark
>Spike|hCoV-19/USA/MI-MDHHS-SC22358/2020|2020-11-09|EPI_ISL_708338|Original|hCoV-19^^Michigan|Human|Michigan Department of Health and Human Services|Michigan Department of Health and Human Services|Jacob|USA
>Spike|hCoV-19/USA/WI-WSLH-200409/2020|2020-07-28|EPI_ISL_578573|Original|hCoV-19^^Wisconsin|Human|Wisconsin State Laboratory of Hygiene Communicable Disease Division|Wisconsin State Laboratory of Hygiene Communicable Disease Division|Florek|USA
>Spike|hCoV-19/England/QEUH-96B037/2020|2020-08-14|EPI_ISL_531348|Original|hCoV-19^^England|Human|Lighthouse Lab in Glasgow|Wellcome Sanger Institute for the COVID-19 Genomics UK (COG-UK) consortium|Davies|United Kingdom
>Spike|hCoV-19/Wales/QEUH-9CA485/2020|2020-09-10|EPI_ISL_567304|Original|hCoV-19^^Wales|Human|Lighthouse Lab in Glasgow|Wellcome Sanger Institute for the COVID-19 Genomics UK (COG-UK) consortium|Davies|United Kingdom
>Spike|hCoV-19/Netherlands/NoordBrabant_95/2020|2020-03-17|EPI_ISL_422889|Original|hCoV-19^^Noord Brabant|Human|Dutch COVID-19 response team|Erasmus Medical Center|Oude Munnink|Netherlands
>Spike|hCoV-19/USA/NV-NSPHL-A0164/2020|2020-05-15|EPI_ISL_515426|Original|hCoV-19^^Nevada|Human|Nevada State Public Health Laboratory|Nevada State Public Health Laboratory|Sevinsky|USA
>Spike|hCoV-19/Taiwan/CGMH-CGU-18/2020|2020-03-18|EPI_ISL_424974|LLC-MK2|hCoV-19^^Taoyuan|Human|Laboratory Medicine|Department of Laboratory Medicine|Yi Chun|Taiwan

>Spike|hCoV-19/Denmark/DCGC-3003/2020|2020-08-24|EPI_ISL_616779|Original|hCoV-19^^Midtjylland|Human|Department of Virus and Microbiological Special Diagnostics|Albertsen lab|Michaelsen|Denmark
>Spike|hCoV-19/Denmark/DCGC-9459/2020|2020-11-09|EPI_ISL_668982|Original|hCoV-19^^Nordjylland|Human|Department of Virus and Microbiological Special Diagnostics|Albertsen Lab|Michaelsen|Denmark
>Spike|hCoV-19/Wales/PHWC-15CEEC/2020|2020-04-20|EPI_ISL_472637|Original|hCoV-19^^Wales|Human|Wales Specialist Virology Centre Sequencing lab|COVID-19 Genomics UK (COG-UK) Consortium|Moore|United Kingdom
>Spike|hCoV-19/Netherlands/NH-RIVM-20802/2020|2020-12-21|EPI_ISL_790861|Original|hCoV-19^^Noord-Holland|Human|Dutch COVID-19 response team|National Institute for Public Health and the Environment (RIVM)|Meijer|Netherlands
>Spike|hCoV-19/United Arab Emirates/0789/2020|2020-05-19|EPI_ISL_698929|Original|hCoV-19^^Abu Dhabi|Human|Group 42 (G42) Healthcare|G42 Healthcare|Ogrodzki|United Arab Emirates
>Spike|hCoV-19/England/MILK-991F2C/2020|2020-08-27|EPI_ISL_552022|Original|hCoV-19^^England|Human|Lighthouse Lab in Milton Keynes|Wellcome Sanger Institute for the COVID-19 Genomics UK (COG-UK) consortium|Davies|United Kingdom
>Spike|hCoV-19/USA/TX-HMH-MCoV-10048/2020|2020-07-06|EPI_ISL_789304|Original|hCoV-19^^Texas|Human|Houston Methodist Hospital|Houston Methodist Hospital|Long|USA
>Spike|hCoV-19/Denmark/DCGC-971/2020|2020-03-09|EPI_ISL_614575|Original|hCoV-19^^Sjaelland|Human|Department of Virus and Microbiological Special Diagnostics|Albertsen lab|Michaelsen|Denmark
>Spike|hCoV-19/Singapore/597/2020|2020-06-24|EPI_ISL_483620|Original|hCoV-19^^Singapore|Human|National Public Health Laboratory|National Public Health Laboratory|Mak|Singapore
>Spike|hCoV-19/USA/AZ-TG371289/2020|2020-05-19|EPI_ISL_694548|Original|hCoV-19^^Arizona|Human|AZ SPHL|TGen North|French|USA
>Spike|hCoV-19/England/QEUH-9C8F59/2020|2020-09-11|EPI_ISL_567059|Original|hCoV-19^^England|Human|Lighthouse Lab in Glasgow|Wellcome Sanger Institute for the COVID-19 Genomics UK (COG-UK) Consortium|Davies|United Kingdom
>Spike|hCoV-19/Japan/PG-5456/2020|2020-08-00|EPI_ISL_690559|Original|hCoV-19^^Japan|Human|Pathogen Genomics Center|Pathogen Genomics Center|Sekizuka|Japan
>Spike|hCoV-19/USA/TX-HMH-6085/2020|2020-07-02|EPI_ISL_547223|Original|hCoV-19^^Texas|Human|Houston Methodist Hospital|Houston Methodist Hospital|Long|USA
>Spike|hCoV-19/Finland/1LS20C2/2020|2020-10-01|EPI_ISL_757357|Original|hCoV-19^^Uusimaa|Human|Department of Virology and Immunology|Department of Virology|Smura|Finland
>Spike|hCoV-19/USA/NY-NYUMC180/2020|2020-04-01|EPI_ISL_427476|Original|hCoV-19^^New York|Human|NYU Langone Health|Departments of Pathology and Medicine|Maurano|USA
>Spike|hCoV-19/Australia/VIC15430/2020|2020-08-04|EPI_ISL_593303|Original|hCoV-19^^Victoria|Human|Microbiological Diagnostic Unit - Public Health Laboratory (MDU-PHL)|MDU-PHL|Schultz|Australia
>Spike|hCoV-19/Spain/MD-IBV-99007734/2020|2020-08-22|EPI_ISL_582844|Original|hCoV-19^^Madrid|Human|Hospital General Universitario Gregorio Mara\u00f1\u00f3n|SeqCOVID-SPAIN consortium/IBV(CSIC)|Comas|Spain
>Spike|hCoV-19/Australia/VIC565/2020|2020-03-25|EPI_ISL_426825|Original|hCoV-19^^Victoria|Human|Victorian Infectious Diseases Reference Laboratory (VIDRL)|Microbiological Diagnostic Unit Public Health Laboratory and Victorian Infectious Diseases Reference Laboratory|Schultz|Australia
>Spike|hCoV-19/Northern Ireland/QEUH-969E3F/2020|2020-08-14|EPI_ISL_530943|Original|hCoV-19^^Northern Ireland|Human|Lighthouse Lab in Glasgow|Wellcome Sanger Institute for the COVID-19 Genomics UK (COG-UK) consortium|Davies|United Kingdom
>Spike|hCoV-19/Australia/VIC5395/2020|2020-07-19|EPI_ISL_520158|Original|hCoV-19^^Victoria|Human|Microbiological Diagnostic Unit - Public Health Laboratory (MDU-PHL)|MDU-PHL|Schultz|Australia
>Spike|hCoV-19/Japan/PG-6021/2020|2020-07-00|EPI_ISL_689133|Original|hCoV-19^^Japan|Human|Pathogen Genomics Center|Pathogen Genomics Center|Sekizuka|Japan

>Spike|hCoV-19/India/MH-ACTREC-276/2020|2020-07-10|EPI_ISL_699928|Original|hCoV-19^^Maharashtra|Human|Hematopathology Laboratory|Hematopathology Laboratory|Patkar|India
>Spike|hCoV-19/Romania/ROSV_3169/2020|2020-04-18|EPI_ISL_491065|Original|hCoV-19^^Suceava|Human|Suceava County Emergency Hospital|\Stefan cel Mare\ University Metagenomics Lab|Lobiuc|Romania
>Spike|hCoV-19/Wales/MILK-A44C5D/2020|2020-10-05|EPI_ISL_609300|Original|hCoV-19^^Wales|Human|Lighthouse Lab in Milton Keynes|Wellcome Sanger Institute for the COVID-19 Genomics UK (COG-UK) consortium|Davies|United Kingdom
>Spike|hCoV-19/England/ALDP-B5BBEC/2020|2020-11-09|EPI_ISL_655282|Original|hCoV-19^^England|Human|Lighthouse Lab in Alderley Park|Wellcome Sanger Institute for the COVID-19 Genomics UK (COG-UK) Consortium|Davies|United Kingdom
>Spike|hCoV-19/Belgium/UGent-334/2020|2020-12-14|EPI_ISL_745219|Original|hCoV-19^^Ghent|Human|Lab voor klinische biologie|Onderzoeksgroep Virologie|Lambrechts|Belgium
>Spike|hCoV-19/USA/WA-QDX-563/2020|2020-03-18|EPI_ISL_571403|Original|hCoV-19^^Washington|Human|Quest Diagnostics|Quest Diagnostics|Kagan|USA
>Spike|hCoV-19/Wales/MILK-9E04C5/2020|2020-09-20|EPI_ISL_601322|Original|hCoV-19^^Wales|Human|Lighthouse Lab in Milton Keynes|Wellcome Sanger Institute for the COVID-19 Genomics UK (COG-UK) consortium|Davies|United Kingdom
>Spike|hCoV-19/Japan/IC-0171/2020|2020-09-00|EPI_ISL_667600|Original|hCoV-19^^Japan|Human|Pathogen Genomics Center|Pathogen Genomics Center|Sekizuka|Japan
>Spike|hCoV-19/USA/NM-NMDOH-2020321221/2020|2020-09-03|EPI_ISL_569630|Original|hCoV-19^^New Mexico|Human|New Mexico Department of Health Scientific Laboratory|New Mexico Department of Health Scientific Laboratory|Johnson|USA
>Spike|hCoV-19/England/CAMC-B51CC5/2020|2020-11-06|EPI_ISL_657964|Original|hCoV-19^^England|Human|Lighthouse Lab in Cambridge|Wellcome Sanger Institute for the COVID-19 Genomics UK (COG-UK) Consortium|Davies|United Kingdom
>Spike|hCoV-19/Australia/VIC2821/2020|2020-07-02|EPI_ISL_521652|Original|hCoV-19^^Victoria|Human|Microbiological Diagnostic Unit - Public Health Laboratory (MDU-PHL)|MDU-PHL|Schultz|Australia
>Spike|hCoV-19/Netherlands/NB-EMC-381/2020|2020-09-28|EPI_ISL_722835|Original|hCoV-19^^Noord Brabant|Human|Dutch COVID-19 response team|Erasmus Medical Center|Oude Munnink|Netherlands
>Spike|hCoV-19/England/LIVE-AA78B/2020|2020-03-24|EPI_ISL_517499|Original|hCoV-19^^England|Human|Liverpool Clinical Laboratories|COVID-19 Genomics UK (COG-UK) Consortium|Haldenby|United Kingdom
>Spike|hCoV-19/England/CAMB-71F85/2020|2020-03-28|EPI_ISL_439656|Original|hCoV-19^^England|Human|Department of Pathology|Wellcome Sanger Institute for the COVID-19 Genomics UK (COG-UK) consortium|Amato|United Kingdom
>Spike|hCoV-19/USA/CA-CZB-2462/2020|2020-05-21|EPI_ISL_513867|Original|hCoV-19^^California|Human|San Francisco Public Health Laboratory|Chan-Zuckerberg Biohub|Kamm|USA
>Spike|hCoV-19/Senegal/62874/2020|2020-06-11|EPI_ISL_476495|Original|hCoV-19^^Dakar|Human|Institut Pasteur Dakar|Institut Pasteur de Dakar|Dia|Senegal
>Spike|hCoV-19/USA/MI-MDHHS-SC21434/2020|2020-03-24|EPI_ISL_507688|Original|hCoV-19^^Michigan|Human|Michigan Department of Health and Human Services|Michigan Department of Health and Human Services|Blankenship|USA
>Spike|hCoV-19/Scotland/CVR5287/2020|2020-10-27|EPI_ISL_650477|Original|hCoV-19^^Scotland|Human|West of Scotland Specialist Virology Centre|COVID-19 Genomics UK (COG-UK) Consortium|Robertson|United Kingdom
>Spike|hCoV-19/England/QEUH-B4174F/2020|2020-11-08|EPI_ISL_658455|Original|hCoV-19^^England|Human|Lighthouse Lab in Glasgow|Wellcome Sanger Institute for the COVID-19 Genomics UK (COG-UK) Consortium|Davies|United Kingdom
>Spike|hCoV-19/England/MILK-9D50A1/2020|2020-09-17|EPI_ISL_577046|Original|hCoV-19^^England|Human|Centre for Enzyme Innovation|COVID-19 Genomics UK (COG-UK) Consortium|Robson|United Kingdom
>Spike|hCoV-19/Spain/PV-IBV-98007718/2020|2020-09-18|EPI_ISL_582040|Original|hCoV-19^^Basque Country|Human|Servicio de Microbiolog\u00eda. Hospital Universitario

Donostia. OSI Donostialdea. \u00c1rea de Enfermedades Infecciosas|SeqCOVID-SPAIN consortium/IBV(CSIC)|Comas|Spain
>Spike|hCoV-19/Norway/CS-21/2020|2020-03-12|EPI_ISL_666601|Original|hCoV-19^^Viken|Human|Dept. of Microbiology and Infection Control|Dept. of Microbiology and Infection Control|Hesselberg L\u00f8vestad|Norway
>Spike|hCoV-19/England/LIVE-9E966/2020|2020-04-11|EPI_ISL_453347|Original|hCoV-19^^England|Human|Liverpool Clinical Laboratories|COVID-19 Genomics UK (COG-UK) Consortium|Haldenby|United Kingdom
>Spike|hCoV-19/England/MILK-B05DAF/2020|2020-10-23|EPI_ISL_642885|Original|hCoV-19^^England|Human|Lighthouse Lab in Milton Keynes|Wellcome Sanger Institute for the COVID-19 Genomics UK (COG-UK) Consortium|Davies|United Kingdom
>Spike|hCoV-19/England/QEUH-A0C899/2020|2020-10-03|EPI_ISL_599323|Original|hCoV-19^^England|Human|Lighthouse Lab in Glasgow|Wellcome Sanger Institute for the COVID-19 Genomics UK (COG-UK) consortium|Davies|United Kingdom
>Spike|hCoV-19/Japan/PG-5751/2020|2020-08-00|EPI_ISL_688870|Original|hCoV-19^^Japan|Human|Pathogen Genomics Center|Pathogen Genomics Center|Sekizuka|Japan
>Spike|hCoV-19/USA/FL-BPHL-2086/2020|2020-07-16|EPI_ISL_653239|Original|hCoV-19^^Florida|Human|Florida Bureau of Public Health Laboratories|Florida Bureau of Public Health Laboratories|Blanton|USA
>Spike|hCoV-19/England/ALDP-A51B14/2020|2020-10-07|EPI_ISL_598184|Original|hCoV-19^^England|Human|Lighthouse Lab in Alderley Park|Wellcome Sanger Institute for the COVID-19 Genomics UK (COG-UK) consortium|Davies|United Kingdom
>Spike|hCoV-19/Northern Ireland/NIRE-FBE4A/2020|2020-04-10|EPI_ISL_459372|Original|hCoV-19^^Northern Ireland|Human|Regional Virus Laboratory|Wellcome Sanger Institute for the COVID-19 Genomics UK (COG-UK) Consortium|Davies|United Kingdom
>Spike|hCoV-19/England/QEUH-BCC30C/2020|2020-11-26|EPI_ISL_704043|Original|hCoV-19^^England|Human|Lighthouse Lab in Glasgow|Wellcome Sanger Institute for the COVID-19 Genomics UK (COG-UK) Consortium|Davies|United Kingdom
>Spike|hCoV-19/USA/TX-HMH-6099/2020|2020-07-04|EPI_ISL_547236|Original|hCoV-19^^Texas|Human|Houston Methodist Hospital|Houston Methodist Hospital|Long|USA
>Spike|hCoV-19/Spain/PV-IBV-001601/2020|2020-03-12|EPI_ISL_467126|Original|hCoV-19^^Basque Country|Human|Hospital Universitario Araba. Vitoria-Gasteiz|SeqCOVID-SPAIN consortium/IBV(CSIC)|Comas|Spain
>Spike|hCoV-19/England/QEUH-B136FE/2020|2020-11-02|EPI_ISL_641721|Original|hCoV-19^^England|Human|Lighthouse Lab in Glasgow|Wellcome Sanger Institute for the COVID-19 Genomics UK (COG-UK) Consortium|Davies|United Kingdom
>Spike|hCoV-19/Luxembourg/LNS7078792/2020|2020-10-21|EPI_ISL_744506|Original|hCoV-19^^Luxembourg|Human|Laboratoire national de sant\u00e9|Laboratoire national de sant\u00e9|Wienecke-Baldacchino|Luxembourg
>Spike|hCoV-19/USA/WA-S661/2020|2020-04-21|EPI_ISL_438214|Original|hCoV-19^^Washington|Human|Washington State Department of Health|Seattle Flu Study|Bedford|USA
>Spike|hCoV-19/Netherlands/NH-EMC-61/2020|2020-08-20|EPI_ISL_577857|Original|hCoV-19^^Noord Holland|Human|Dutch COVID-19 response team|Erasmus Medical Center|Oude Munnink|Netherlands
>Spike|hCoV-19/Australia/VIC9860/2020|2020-08-09|EPI_ISL_565745|Original|hCoV-19^^Victoria|Human|Microbiological Diagnostic Unit - Public Health Laboratory (MDU-PHL)|MDU-PHL|Schultz|Australia
>Spike|hCoV-19/USA/UT-UPHL-201111665/2020|2020-06-20|EPI_ISL_640298|Original|hCoV-19^^Utah|Human|Utah Public Health Laboratory|Utah Public Health Laboratory|Young|USA
>Spike|hCoV-19/England/MILK-95705C/2020|2020-06-10|EPI_ISL_554297|Original|hCoV-19^^England|Human|Lighthouse Lab in Milton Keynes|Wellcome Sanger Institute for the COVID-19 Genomics UK (COG-UK) consortium|Davies|United Kingdom
>Spike|hCoV-19/Denmark/ALAB-SSI141/2020|2020-03-09|EPI_ISL_429359|Original|hCoV-19^^Denmark|Human|Department of Virus and Microbiological Special Diagnostics|Albertsen lab|Kirkegaard|Denmark
>Spike|hCoV-19/Wales/PHWC-499888/2020|2020-12-03|EPI_ISL_727369|Original|hCoV-19^^Wales|Human|Originating lab|COVID-19 Genomics UK (COG-UK) Consortium|Moore|United Kingdom

>Spike|hCoV-19/Wales/PHWC-484B1E/2020|2020-10-23|EPI_ISL_628080|Original|hCoV-19^^Wales|Human|Wales Specialist Virology Centre Sequencing lab|COVID-19 Genomics UK (COG-UK) Consortium|Moore|United Kingdom
>Spike|hCoV-19/USA/VA-DCLS-0046/2020|2020-03-28|EPI_ISL_420027|Original|hCoV-19^^Virginia|Human|Virginia DCLS|Virginia DCLS|St. Jacques|USA
>Spike|hCoV-19/England/NORW-F59E5/2020|2020-11-13|EPI_ISL_725285|Original|hCoV-19^^England|Human|Quadram Institute Bioscience|COVID-19 Genomics UK (COG-UK) Consortium|Alikhan|United Kingdom
>Spike|hCoV-19/England/ALDP-C498AD/2020|2020-12-12|EPI_ISL_718764|Original|hCoV-19^^England|Human|Lighthouse Lab in Alderley Park|Wellcome Sanger Institute for the COVID-19 Genomics UK (COG-UK) Consortium|Davies|United Kingdom
>Spike|hCoV-19/Wales/PHWC-28EBD/2020|2020-04-04|EPI_ISL_445455|Original|hCoV-19^^Wales|Human|Wales Specialist Virology Centre|Public Health Wales Microbiology Cardiff|Moore|United Kingdom
>Spike|hCoV-19/USA/TX-DSHS-1720/2020|2020-06-20|EPI_ISL_708628|Original|hCoV-19^^Texas|Human|Texas Department of State Health Services|Texas Department of State Health Services|Rahman|USA
>Spike|hCoV-19/South Korea/KCDC2003/2020|2020-02-23|EPI_ISL_426167|Caco-2 P1|hCoV-19^^South Korea|Human|Division of Viral Diseases|Division of Viral Diseases|Kim|South Korea
>Spike|hCoV-19/USA/TX-HMH-MCoV-15293/2020|2020-10-19|EPI_ISL_786777|Original|hCoV-19^^Texas|Human|Houston Methodist Hospital|Houston Methodist Hospital|Long|USA
>Spike|hCoV-19/England/NORT-28993F/2020|2020-04-01|EPI_ISL_458676|Original|hCoV-19^^England|Human|NU-OMICS DNA Sequencing research facility|Wellcome Sanger Institute for the COVID-19 Genomics UK (COG-UK) consortium|Davies|United Kingdom
>Spike|hCoV-19/Denmark/DCGC-21113/2020|2020-12-21|EPI_ISL_758799|Original|hCoV-19^^Hovedstaden|Human|Department of Virus and Microbiological Special Diagnostics|Albertsen Lab|Michaelsen|Denmark
>Spike|hCoV-19/Ireland/D-NVRL-20G35426/2020|2020-03-30|EPI_ISL_605128|Original|hCoV-19^^Dublin|Human|National Virus Reference Laboratory|Irish Coronavirus Sequencing Consortium - Helixworks|Chalapati|Ireland
>Spike|hCoV-19/USA/WI-WSLH-200400/2020|2020-04-23|EPI_ISL_578566|Original|hCoV-19^^Wisconsin|Human|Wisconsin State Laboratory of Hygiene Communicable Disease Division|Wisconsin State Laboratory of Hygiene Communicable Disease Division|Florek|USA
>Spike|hCoV-19/Scotland/EDB8569/2020|2020-10-20|EPI_ISL_651962|Original|hCoV-19^^Scotland|Human|Virology Department|COVID-19 Genomics UK (COG-UK) Consortium|McHugh|United Kingdom
>Spike|hCoV-19/England/ALDP-B23761/2020|2020-11-04|EPI_ISL_647084|Original|hCoV-19^^England|Human|Lighthouse Lab in Alderley Park|Wellcome Sanger Institute for the COVID-19 Genomics UK (COG-UK) Consortium|Davies|United Kingdom
>Spike|hCoV-19/Northern Ireland/NIRE-235237/2020|2020-10-16|EPI_ISL_764421|Original|hCoV-19^^Northern Ireland|Human|Regional Virus Laboratory|COVID-19 Genomics UK (COG-UK) Consortium|Fairley|United Kingdom
>Spike|hCoV-19/Japan/PG-7943/2020|2020-05-00|EPI_ISL_687044|Original|hCoV-19^^Japan|Human|Kanagawa Prefectural Institute of Public Health|Pathogen Genomics Center|Sekizuka|Japan
>Spike|hCoV-19/England/ALDP-94FA77/2020|2020-06-16|EPI_ISL_555176|Original|hCoV-19^^England|Human|Lighthouse Lab in Alderley Park|Wellcome Sanger Institute for the COVID-19 Genomics UK (COG-UK) consortium|Davies|United Kingdom
>Spike|hCoV-19/Sweden/20-53232/2020|2020-10-26|EPI_ISL_648207|Original|hCoV-19^^Sweden|Human|Orebro|The Public Health Agency of Sweden|Svartstr\u00f6m|Sweden
>Spike|hCoV-19/Denmark/DCGC-7623/2020|2020-10-19|EPI_ISL_618553|Original|hCoV-19^^Sjaelland|Human|Department of Virus and Microbiological Special Diagnostics|Albertsen lab|Michaelsen|Denmark
>Spike|hCoV-19/Denmark/DCGC-8967/2020|2020-10-26|EPI_ISL_626202|Original|hCoV-19^^Hovedstaden|Human|Department of Virus and Microbiological Special Diagnostics|Albertsen lab|Michaelsen|Denmark

>Spike|hCoV-19/England/QEUH-CCD7E0/2020|2020-12-24|EPI_ISL_782183|Original|hCoV-19^^England|Human|Lighthouse Lab in Glasgow|Wellcome Sanger Institute for the COVID-19 Genomics UK (COG-UK) Consortium|Davies|United Kingdom
>Spike|hCoV-19/Jordan/SEARCH-5402/2020|2020-11-07|EPI_ISL_755213|Original|hCoV-19^^Amman|Human|Biolab Diagnostic Laboratories|Andersen lab at Scripps Research|Gangavarapu|Jordan
>Spike|hCoV-19/England/ALDP-B75E98/2020|2020-11-12|EPI_ISL_740945|Original|hCoV-19^^England|Human|Oxford Viromics|COVID-19 Genomics UK (COG-UK) Consortium|Golubchik|United Kingdom
>Spike|hCoV-19/England/MILK-B3BFC8/2020|2020-11-01|EPI_ISL_706112|Original|hCoV-19^^England|Human|Oxford Viromics|COVID-19 Genomics UK (COG-UK) Consortium|Golubchik|United Kingdom
>Spike|hCoV-19/England/NORW-EF1D7/2020|2020-09-20|EPI_ISL_573414|Original|hCoV-19^^England|Human|Quadram Institute Bioscience|COVID-19 Genomics UK (COG-UK) Consortium|Alikhan|United Kingdom
>Spike|hCoV-19/Belgium/ULG-10156/2020|2020-04-18|EPI_ISL_447152|Original|hCoV-19^^Liege|Human|Department of Clinical Microbiology|GIGA Medical Genomics|Durkin|Belgium
>Spike|hCoV-19/India/TG-CCMB-AB05/2020|2020-08-13|EPI_ISL_539729|Original|hCoV-19^^Telangana|Human|CSIR-Centre for Cellular and Molecular Biology|CSIR-Centre for Cellular and Molecular Biology|Mukherjee|India
>Spike|hCoV-19/England/CAMC-B7A7A7/2020|2020-11-12|EPI_ISL_741140|Original|hCoV-19^^England|Human|Oxford Viromics|COVID-19 Genomics UK (COG-UK) Consortium|Golubchik|United Kingdom
>Spike|hCoV-19/Denmark/DCGC-20713/2020|2020-12-21|EPI_ISL_759093|Original|hCoV-19^^Hovedstaden|Human|Department of Virus and Microbiological Special Diagnostics|Albertsen Lab|Michaelsen|Denmark
>Spike|hCoV-19/USA/UT-UPHL-2009365/2020|2020-05-14|EPI_ISL_524958|Original|hCoV-19^^Utah|Human|Utah Public Health Laboratory|Utah Public Health Laboratory|Young|USA
>Spike|hCoV-19/England/ALDP-9002A7/2020|2020-06-24|EPI_ISL_551531|Original|hCoV-19^^England|Human|Lighthouse Lab in Alderley Park|Wellcome Sanger Institute for the COVID-19 Genomics UK (COG-UK) consortium|Davies|United Kingdom
>Spike|hCoV-19/Denmark/DCGC-7299/2020|2020-10-12|EPI_ISL_619259|Original|hCoV-19^^Midtjylland|Human|Department of Virus and Microbiological Special Diagnostics|Albertsen lab|Michaelsen|Denmark
>Spike|hCoV-19/England/SHEF-10CE830/2020|2020-12-18|EPI_ISL_742010|Original|hCoV-19^^England|Human|Virology Department|COVID-19 Genomics UK (COG-UK) Consortium|de Silva|United Kingdom
>Spike|hCoV-19/France/BRE-8938/2020|2020-07-29|EPI_ISL_560606|Original|hCoV-19^^Bretagne|Human|Hospital|National Reference Center for Viruses of Respiratory Infections|Enouf|France
>Spike|hCoV-19/England/NORW-EA3F4/2020|2020-05-13|EPI_ISL_457343|Original|hCoV-19^^England|Human|Quadram Institute Bioscience|COVID-19 Genomics UK (COG-UK) Consortium|Alikhan|United Kingdom
>Spike|hCoV-19/England/QEUH-A4B1A2/2020|2020-10-05|EPI_ISL_637816|Original|hCoV-19^^England|Human|Department of Pathology|COVID-19 Genomics UK (COG-UK) Consortium|Goodfellow|United Kingdom
>Spike|hCoV-19/England/NORW-F7D6F/2020|2020-11-13|EPI_ISL_650462|Original|hCoV-19^^England|Human|Quadram Institute Bioscience|COVID-19 Genomics UK (COG-UK) Consortium|Alikhan|United Kingdom
>Spike|hCoV-19/England/NOTT-116BE7/2020|2020-10-18|EPI_ISL_612573|Original|hCoV-19^^England|Human|Queens Medical Centre|COVID-19 Genomics UK (COG-UK) Consortium|Loose|United Kingdom
>Spike|hCoV-19/England/ALDP-B1ABCB/2020|2020-11-01|EPI_ISL_645663|Original|hCoV-19^^England|Human|Lighthouse Lab in Alderley Park|Wellcome Sanger Institute for the COVID-19 Genomics UK (COG-UK) Consortium|Davies|United Kingdom
>Spike|hCoV-19/England/BRIS-12B147/2020|2020-05-08|EPI_ISL_469840|Original|hCoV-19^^England|Human|PHE South West Regional Laboratory|Wellcome Sanger Institute for the COVID-19 Genomics UK (COG-UK) consortium|Davies|United Kingdom

>Spike|hCoV-19/Australia/NSW1304/2020|2020-12-19|EPI_ISL_767870|Original|hCoV-19^^New South Wales|Human|Pathology North - Royal North Shore Hospital - NSW Health Pathology|NSW Health Pathology - Institute of Clinical Pathology and Medical Research; Westmead Hospital; University of Sydney|Gall|Australia
>Spike|hCoV-19/Netherlands/ZH-EMC-1052/2020|2020-12-06|EPI_ISL_722792|Original|hCoV-19^^Zuid Holland|Human|Dutch COVID-19 response team|Erasmus Medical Center|Oude Munnink|Netherlands
>Spike|hCoV-19/Israel/CVL-41-ngs/2020|2020-12-24|EPI_ISL_776632|Original|hCoV-19^^Israel|Human|Israel Central Virology laboratory|Israel Central Virology laboratory|Zuckerman|Israel
>Spike|hCoV-19/Wales/PHWC-47F581/2020|2020-10-06|EPI_ISL_612693|Original|hCoV-19^^Wales|Human|Wales Specialist Virology Centre Sequencing lab|COVID-19 Genomics UK (COG-UK) Consortium|Moore|United Kingdom
>Spike|hCoV-19/USA/WA-UW-2108/2020|2020-03-24|EPI_ISL_426089|Original|hCoV-19^^Washington|Human|UW Virology Lab|UW Virology Lab|Roychoudhury|USA
>Spike|hCoV-19/USA/TX-HMH-MCoV-15215/2020|2020-10-20|EPI_ISL_786699|Original|hCoV-19^^Texas|Human|Houston Methodist Hospital|Houston Methodist Hospital|Long|USA
>Spike|hCoV-19/Denmark/DCGC-14071/2020|2020-11-30|EPI_ISL_711549|Original|hCoV-19^^Syddanmark|Human|Department of Virus and Microbiological Special Diagnostics|Albertsen Lab|Michaelsen|Denmark
>Spike|hCoV-19/England/LOND-126325B/2020|2020-10-28|EPI_ISL_638022|Original|hCoV-19^^England|Human|University College London|COVID-19 Genomics UK (COG-UK) Consortium|Roy|United Kingdom
>Spike|hCoV-19/Australia/VIC6578/2020|2020-07-17|EPI_ISL_521342|Original|hCoV-19^^Victoria|Human|Victorian Infectious Diseases Reference Laboratory (VIDRL)|VIDRL and MDU-PHL|Schultz|Australia
>Spike|hCoV-19/England/BRIS-128A9A/2020|2020-04-13|EPI_ISL_458798|Original|hCoV-19^^England|Human|PHE South West Regional Laboratory|Wellcome Sanger Institute for the COVID-19 Genomics UK (COG-UK) consortium|Davies|United Kingdom
>Spike|hCoV-19/England/CAMC-B336BC/2020|2020-11-05|EPI_ISL_659144|Original|hCoV-19^^England|Human|Lighthouse Lab in Cambridge|Wellcome Sanger Institute for the COVID-19 Genomics UK (COG-UK) Consortium|Davies|United Kingdom
>Spike|hCoV-19/England/MILK-992F2B/2020|2020-08-26|EPI_ISL_551963|Original|hCoV-19^^England|Human|Lighthouse Lab in Milton Keynes|Wellcome Sanger Institute for the COVID-19 Genomics UK (COG-UK) consortium|Davies|United Kingdom
>Spike|hCoV-19/Japan/PG-1439/2020|2020-04-00|EPI_ISL_685046|Original|hCoV-19^^Japan|Human|Saitama Prefectural Institute of Public Health|Pathogen Genomics Center|Sekizuka|Japan
>Spike|hCoV-19/USA/MA-MGH-00307/2020|2020-04-05|EPI_ISL_460431|Original|hCoV-19^^Massachusetts|Human|Massachusetts General Hospital|Infectious Disease Program|Lemieux|USA
>Spike|hCoV-19/England/ALDP-A2EFF6/2020|2020-10-05|EPI_ISL_587045|Original|hCoV-19^^England|Human|Lighthouse Lab in Alderley Park|Wellcome Sanger Institute for the COVID-19 Genomics UK (COG-UK) consortium|Davies|United Kingdom
>Spike|hCoV-19/Netherlands/NH-RIVM-20015/2020|2020-11-20|EPI_ISL_723214|Orignal|hCoV-19^^Noord-Holland|Human|Dutch COVID-19 response team|National Institute for Public Health and the Environment (RIVM)|Meijer|Netherlands
>Spike|hCoV-19/Netherlands/NA_31/2020|2020-03-13|EPI_ISL_415488|Original|hCoV-19^^Netherlands|Human|Dutch COVID-19 response team|Erasmus Medical Center|Oude Munnink|Netherlands
>Spike|hCoV-19/Italy/ABR-IZSGC-TE6580/2020|2020-03-24|EPI_ISL_429235|Original|hCoV-19^^Abruzzo|Human|Ospedale Civile Giuseppe Mazzini|Istituto Zooprofilattico Sperimentale dell'Abruzzo e Molise \G. Caporale\|Lorusso|Italy
>Spike|hCoV-19/England/CAMB-1B2330/2020|2020-05-18|EPI_ISL_456678|Original|hCoV-19^^England|Human|Department of Pathology|COVID-19 Genomics UK (COG-UK) Consortium|Meredith|United Kingdom
>Spike|hCoV-19/England/NORT-290DFD/2020|2020-05-02|EPI_ISL_453535|Original|hCoV-19^^England|Human|Northumbria University / South Tees Hospitals NHS Foundation Trust / North Cumbria Integrated Care NHS Foundation Trust / North Tees and Hartlepool NHS

Foundation Trust / Newcastle Hospitals NHS Foundation Trust|COVID-19 Genomics UK (COG-UK) Consortium|Bashton|United Kingdom
>Spike|hCoV-19/England/QEUH-C31EE2/2020|2020-12-11|EPI_ISL_782557|Original|hCoV-19^^England|Human|Lighthouse Lab in Glasgow|Wellcome Sanger Institute for the COVID-19 Genomics UK (COG-UK) Consortium|Davies|United Kingdom
>Spike|hCoV-19/England/ALDP-B4621C/2020|2020-11-07|EPI_ISL_656018|Original|hCoV-19^^England|Human|Lighthouse Lab in Alderley Park|Wellcome Sanger Institute for the COVID-19 Genomics UK (COG-UK) Consortium|Davies|United Kingdom
>Spike|hCoV-19/Canada/ON-PHL-20-01211/2020|2020-06-00|EPI_ISL_548833|Original|hCoV-19^^Ontario|Human|Public Health Ontario Laboratory|Public Health Ontario Laboratory|Guthrie|Canada
>Spike|hCoV-19/USA/UT-UPHL-2009644/2020|2020-07-19|EPI_ISL_605319|Original|hCoV-19^^Utah|Human|Utah Public Health Laboratory|Utah Public Health Laboratory|Young|USA
>Spike|hCoV-19/England/BRIS-1221D7/2020|2020-03-22|EPI_ISL_440091|Original|hCoV-19^^England|Human|PHE South West Regional Laboratory|Wellcome Sanger Institute for the COVID-19 Genomics UK (COG-UK) consortium|Amato|United Kingdom
>Spike|hCoV-19/USA/NV-NSPHL-A0020/2020|2020-03-16|EPI_ISL_515307|Original|hCoV-19^^Nevada|Human|Nevada State Public Health Laboratory|Nevada State Public Health Laboratory|Sevinsky|USA
>Spike|hCoV-19/Denmark/DCGC-12945/2020|2020-12-07|EPI_ISL_712123|Original|hCoV-19^^Hovedstaden|Human|Department of Virus and Microbiological Special Diagnostics|Albertsen Lab|Michaelsen|Denmark
>Spike|hCoV-19/USA/WA-UW-24937/2020|2020-08-10|EPI_ISL_570775|Original|hCoV-19^^Washington|Human|UW Virology Lab|UW Virology Lab|Roychoudhury|USA
>Spike|hCoV-19/England/QEUH-96A4D2/2020|2020-08-13|EPI_ISL_531469|Original|hCoV-19^^England|Human|Lighthouse Lab in Glasgow|Wellcome Sanger Institute for the COVID-19 Genomics UK (COG-UK) consortium|Davies|United Kingdom
>Spike|hCoV-19/USA/GA-QDX-684/2020|2020-03-17|EPI_ISL_571429|Original|hCoV-19^^Georgia|Human|Quest Diagnostics|Quest Diagnostics|Kagan|USA
>Spike|hCoV-19/France/IDF-8697/2020|2020-06-30|EPI_ISL_560639|Original|hCoV-19^^\u00cele-de-France|Human|Labo Analyses Med|National Reference Center for Viruses of Respiratory Infections|Enouf|France
>Spike|hCoV-19/Netherlands/NoordBrabant_106/2020|2020-04-06|EPI_ISL_455290|Original|hCoV-19^^Noord Brabant|Human|Dutch COVID-19 response team|Erasmus Medical Center|Oude Munnink|Netherlands
>Spike|hCoV-19/SaudiArabia/KAUST-MAKKAH778/2020|2020-04-18|EPI_ISL_513247|Original|hCoV-19^^Makkah|Human|Pathogen Genomics Lab King Abdullah University of Science and Technology(KAUST)|Pathogen Genomics Lab King Abdullah University of Science and Technology(KAUST)|Mohamed Ghazzali|Saudi Arabia
>Spike|hCoV-19/USA/VA-DCLS-0490/2020|2020-05-01|EPI_ISL_614109|Original|hCoV-19^^Virginia|Human|Virginia DCLS|Virginia DCLS|St. Jacques|USA
>Spike|hCoV-19/USA/MI-UM-10035849697/2020|2020-09-08|EPI_ISL_583336|Original|hCoV-19^^Michigan|Human|University of Michigan Clinical Microbiology Laboratory|Lauring Lab|Valesano|USA
>Spike|hCoV-19/USA/NC_200253/2020|2020-03-00|EPI_ISL_454826|Original|hCoV-19^^North Carolina|Human|Dirk Dittmer|Dirk Dittmer|Moorad|USA
>Spike|hCoV-19/Australia/VIC16747/2020|2020-08-08|EPI_ISL_663566|Original|hCoV-19^^Victoria|Human|Microbiological Diagnostic Unit - Public Health Laboratory (MDU-PHL)|MDU-PHL|Schultz|Australia
>Spike|hCoV-19/Australia/NSW568/2020|2020-03-25|EPI_ISL_478680|Original|hCoV-19^^New South Wales|Human|Sydney South West Pathology Service (SSWPS) - Liverpool Hospital - NSW Health Pathology|NSW Health Pathology - Institute of Clinical Pathology and Medical Research; Westmead Hospital; University of Sydney|Gall|Australia
>Spike|hCoV-19/Australia/NSW2145/2020|2020-03-15|EPI_ISL_678300|Original|hCoV-19^^New South Wales|Human|Area of Virology|Virology Research Laboratory; Area of Virology|Foster|Australia
>Spike|hCoV-19/England/ALDP-9C50E0/2020|2020-09-10|EPI_ISL_567877|Original|hCoV-19^^England|Human|Lighthouse Lab in Alderley Park|Wellcome Sanger Institute for the COVID-19 Genomics UK (COG-UK) consortium|Davies|United Kingdom

>Spike|hCoV-19/England/MILK-B89523/2020|2020-11-13|EPI_ISL_675985|Original|hCoV-19^^England|Human|Lighthouse Lab in Milton Keynes|Wellcome Sanger Institute for the COVID-19 Genomics UK (COG-UK) Consortium|Davies|United Kingdom
>Spike|hCoV-19/Canada/QC-LSPQ-L00243675/2020|2020-03-31|EPI_ISL_536370|Original|hCoV-19^^Quebec|Human|Centre hospitalier Anna-Laberge|Laboratoire de sant\u00e9 publique du Qu\u00e9bec|Moreira|Canada
>Spike|hCoV-19/USA/CA-ALSR-4676/2020|2020-11-10|EPI_ISL_667072|Original|hCoV-19^^California|Human|San Diego County Public Health Laboratory|Andersen lab at Scripps Research|Gangavarapu|USA
>Spike|hCoV-19/England/QEUH-9E4B80/2020|2020-09-22|EPI_ISL_579859|Original|hCoV-19^^England|Human|Lighthouse Lab in Glasgow|Wellcome Sanger Institute for the COVID-19 Genomics UK (COG-UK) consortium|Davies|United Kingdom
>Spike|hCoV-19/England/ALDP-A66ED7/2020|2020-10-12|EPI_ISL_630362|Original|hCoV-19^^England|Human|Lighthouse Lab in Alderley Park|Wellcome Sanger Institute for the COVID-19 Genomics UK (COG-UK) consortium|Davies|United Kingdom
>Spike|hCoV-19/England/LIVE-1DE0EC/2020|2020-10-10|EPI_ISL_611741|Original|hCoV-19^^England|Human|Liverpool Clinical Laboratories|COVID-19 Genomics UK (COG-UK) Consortium|Haldenby|United Kingdom
>Spike|hCoV-19/England/20130062504/2020|2020-03-21|EPI_ISL_423643|Original|hCoV-19^^England|Human|Respiratory Virus Unit|Respiratory Virus Unit|Platt|United Kingdom
>Spike|hCoV-19/South Africa/N00141/2020|2020-05-22|EPI_ISL_622975|Original|hCoV-19^^Mpumalanga|Human|National Health Laboratory Service|National Institute for Communicable Diseases of the National Health Laboratory Service|Allam|South Africa
>Spike|hCoV-19/Gibraltar/204641755/2020|2020-00-00|EPI_ISL_637223|Original|hCoV-19^^Gibraltar|Human|Microbiology|Respiratory Virus Unit|Platt|Gibraltar
>Spike|hCoV-19/Australia/VIC5836/2020|2020-07-20|EPI_ISL_518630|Original|hCoV-19^^Victoria|Human|Microbiological Diagnostic Unit - Public Health Laboratory (MDU-PHL)|MDU-PHL|Schultz|Australia
>Spike|hCoV-19/USA/MI-MDHHS-SC21287/2020|2020-04-08|EPI_ISL_507542|Original|hCoV-19^^Michigan|Human|Michigan Department of Health and Human Services|Michigan Department of Health and Human Services|Blankenship|USA
>Spike|hCoV-19/Portugal/PT1773/2020|2020-11-09|EPI_ISL_732004|Original|hCoV-19^^Portugal|Human|Instituto Nacional de Saude (INSA)|Instituto Nacional de Saude (INSA)|Borges|Portugal
>Spike|hCoV-19/England/NOTT-1127D8/2020|2020-07-08|EPI_ISL_493548|Original|hCoV-19^^England|Human|Queens Medical Centre|COVID-19 Genomics UK (COG-UK) Consortium|Loose|United Kingdom
>Spike|hCoV-19/England/ALDP-720CD2/2020|2020-07-01|EPI_ISL_557640|Original|hCoV-19^^England|Human|Lighthouse Lab in Alderley Park|Wellcome Sanger Institute for the COVID-19 Genomics UK (COG-UK) consortium|Davies|United Kingdom
>Spike|hCoV-19/Scotland/CVR5961/2020|2020-11-24|EPI_ISL_705698|Original|hCoV-19^^Scotland|Human|West of Scotland Specialist Virology Centre|COVID-19 Genomics UK (COG-UK) Consortium|Robertson|United Kingdom
>Spike|hCoV-19/Netherlands/OV-RIVM-20674/2020|2020-12-16|EPI_ISL_790744|Original|hCoV-19^^Overijssel|Human|Dutch COVID-19 response team|National Institute for Public Health and the Environment (RIVM)|Meijer|Netherlands
>Spike|hCoV-19/USA/WI-UW-1534/2020|2020-09-14|EPI_ISL_605412|Original|hCoV-19^^Wisconsin|Human|University of Wisconsin-Madison AIDS Vaccine Research Laboratories|University of Wisconsin-Madison AIDS Vaccine Research Laboratories|Baczenas|USA
>Spike|hCoV-19/Denmark/DCGC-2989/2020|2020-08-24|EPI_ISL_616753|Original|hCoV-19^^Sjaelland|Human|Department of Virus and Microbiological Special Diagnostics|Albertsen lab|Michaelsen|Denmark
>Spike|hCoV-19/USA/TX-DSHS-1724/2020|2020-06-19|EPI_ISL_708617|Original|hCoV-19^^Texas|Human|Texas Department of State Health Services|Texas Department of State Health Services|Rahman|USA
>Spike|hCoV-19/England/ALDP-B4811D/2020|2020-11-07|EPI_ISL_649461|Original|hCoV-19^^England|Human|Lighthouse Lab in Alderley Park|Wellcome Sanger Institute for the COVID-19 Genomics UK (COG-UK) Consortium|Davies|United Kingdom

>Spike|hCoV-19/England/MILK-9A6698/2020|2020-09-01|EPI_ISL_549961|Original|hCoV-19^^England|Human|Lighthouse Lab in Milton Keynes|Wellcome Sanger Institute for the COVID-19 Genomics UK (COG-UK) consortium|Davies|United Kingdom
>Spike|hCoV-19/Wales/QEUH-AD5D9A/2020|2020-10-26|EPI_ISL_647951|Original|hCoV-19^^Wales|Human|Lighthouse Lab in Glasgow|Wellcome Sanger Institute for the COVID-19 Genomics UK (COG-UK) Consortium|Davies|United Kingdom
>Spike|hCoV-19/England/CAMC-B98DFC/2020|2020-11-15|EPI_ISL_673624|Original|hCoV-19^^England|Human|Lighthouse Lab in Cambridge|Wellcome Sanger Institute for the COVID-19 Genomics UK (COG-UK) Consortium|Davies|United Kingdom
>Spike|hCoV-19/Switzerland/AG-ETHZ-380242/2020|2020-11-21|EPI_ISL_721713|Original|hCoV-19^^Aargau|Human|Viollier AG|Department of Biosystems Science and Engineering|Nadeau|Switzerland
>Spike|hCoV-19/Luxembourg/LNS3743453/2020|2020-10-21|EPI_ISL_740370|Original|hCoV-19^^Luxembourg|Human|Laboratoire national de sant\u00e9|Laboratoire national de sant\u00e9|Wienecke-Baldacchino|Luxembourg
>Spike|hCoV-19/Scotland/EDB6275/2020|2020-04-06|EPI_ISL_487914|Original|hCoV-19^^Scotland|Human|Virology Department|Wellcome Sanger Institute for the COVID-19 Genomics UK (COG-UK) consortium|Davies|United Kingdom
>Spike|hCoV-19/USA/LA-GBCL-26544528/2020|2020-04-20|EPI_ISL_485112|Original|hCoV-19^^Louisiana|Human|River Road Testing Lab|Ginkgo Bioworks Clinical Laboratory|Smith|USA
>Spike|hCoV-19/England/204200169/2020|2020-10-10|EPI_ISL_609873|Original|hCoV-19^^England|Human|Respiratory Virus Unit|Respiratory Virus Unit|Platt|United Kingdom
>Spike|hCoV-19/England/CAMC-B779FF/2020|2020-11-11|EPI_ISL_661684|Original|hCoV-19^^England|Human|Lighthouse Lab in Cambridge|Wellcome Sanger Institute for the COVID-19 Genomics UK (COG-UK) Consortium|Davies|United Kingdom
>Spike|hCoV-19/Australia/NSW924/2020|2020-08-29|EPI_ISL_545005|Original|hCoV-19^^New South Wales|Human|Sydney South West Pathology Service (SSWPS) - Royal Prince Alfred Hospital - NSW Health Pathology|NSW Health Pathology - Institute of Clinical Pathology and Medical Research; Westmead Hospital; University of Sydney|Gall|Australia
>Spike|hCoV-19/Norway/3208/2020|2020-08-26|EPI_ISL_549036|Original|hCoV-19^^Viken|Human|Hospital of Southern Norway - Kristiansand|Norwegian Institute of Public Health|Kopperud Riis|Norway
>Spike|hCoV-19/England/CAMC-94B02E/2020|2020-07-20|EPI_ISL_556641|Original|hCoV-19^^England|Human|Lighthouse Lab in Cambridge|Wellcome Sanger Institute for the COVID-19 Genomics UK (COG-UK) consortium|Davies|United Kingdom
>Spike|hCoV-19/England/QEUH-CC968D/2020|2020-12-23|EPI_ISL_782101|Original|hCoV-19^^England|Human|Lighthouse Lab in Glasgow|Wellcome Sanger Institute for the COVID-19 Genomics UK (COG-UK) Consortium|Davies|United Kingdom
>Spike|hCoV-19/England/CAMB-77417/2020|2020-03-31|EPI_ISL_439472|Original|hCoV-19^^England|Human|Department of Pathology|Wellcome Sanger Institute for the COVID-19 Genomics UK (COG-UK) consortium|Amato|United Kingdom
>Spike|hCoV-19/USA/TX-HMH-6067/2020|2020-07-02|EPI_ISL_547209|Original|hCoV-19^^Texas|Human|Houston Methodist Hospital|Houston Methodist Hospital|Long|USA
>Spike|hCoV-19/England/ALDP-9EE3AC/2020|2020-05-06|EPI_ISL_589316|Original|hCoV-19^^England|Human|Lighthouse Lab in Alderley Park|Wellcome Sanger Institute for the COVID-19 Genomics UK (COG-UK) consortium|Davies|United Kingdom
>Spike|hCoV-19/USA/FL-BPHL-0370/2020|2020-04-21|EPI_ISL_489779|Original|hCoV-19^^Florida|Human|Florida Bureau of Public Health Laboratories|Florida Bureau of Public Health Laboratories|Blanton|USA
>Spike|hCoV-19/England/QEUH-B80F4C/2020|2020-11-14|EPI_ISL_667876|Original|hCoV-19^^England|Human|Lighthouse Lab in Glasgow|Wellcome Sanger Institute for the COVID-19 Genomics UK (COG-UK) Consortium|Davies|United Kingdom
>Spike|hCoV-19/England/CAMC-9C0768/2020|2020-09-08|EPI_ISL_573193|Original|hCoV-19^^England|Human|Oxford Viromics|COVID-19 Genomics UK (COG-UK) Consortium|Golubchik|United Kingdom
>Spike|hCoV-19/Wales/CAMC-9D6DF4/2020|2020-09-20|EPI_ISL_585620|Original|hCoV-19^^Wales|Human|Virology Department|COVID-19 Genomics UK (COG-UK) Consortium|de Silva|United Kingdom

>Spike|hCoV-19/USA/WI-UW-425/2020|2020-04-27|EPI_ISL_480407|Original|hCoV-19^^Wisconsin|Human|University of Wisconsin-Madison AIDS Vaccine Research Laboratories|University of Wisconsin-Madison AIDS Vaccine Research Laboratories|Baczenas|USA
>Spike|hCoV-19/Russia/MOW-RII-MH4070S/2020|2020-06-09|EPI_ISL_733156|Original|hCoV-19^^Moscow|Human|Pathogenic Microorganisms Variability Laboratory|WHO National Influenza Centre Russian Federation|Komissarov|Russia
>Spike|hCoV-19/USA/MA-MGH-00270/2020|2020-04-01|EPI_ISL_460243|Original|hCoV-19^^Massachusetts|Human|Massachusetts General Hospital|Infectious Disease Program|Lemieux|USA
>Spike|hCoV-19/England/MILK-BACD5F/2020|2020-11-17|EPI_ISL_681625|Original|hCoV-19^^England|Human|Lighthouse Lab in Milton Keynes|Wellcome Sanger Institute for the COVID-19 Genomics UK (COG-UK) Consortium|Davies|United Kingdom
>Spike|hCoV-19/England/ALDP-9C5A4C/2020|2020-09-11|EPI_ISL_567862|Original|hCoV-19^^England|Human|Lighthouse Lab in Alderley Park|Wellcome Sanger Institute for the COVID-19 Genomics UK (COG-UK) consortium|Davies|United Kingdom
>Spike|hCoV-19/Luxembourg/LNS1111678/2020|2020-10-24|EPI_ISL_739889|Original|hCoV-19^^Luxembourg|Human|Laboratoire national de sant\u00e9|Laboratoire national de sant\u00e9|Wienecke-Baldacchino|Luxembourg
>Spike|hCoV-19/England/PORT-2D1D69/2020|2020-04-02|EPI_ISL_499276|Original|hCoV-19^^England|Human|Centre for Enzyme Innovation|COVID-19 Genomics UK (COG-UK) Consortium|Robson|United Kingdom
>Spike|hCoV-19/Australia/VIC13106/2020|2020-07-28|EPI_ISL_640956|Original|hCoV-19^^Victoria|Human|Microbiological Diagnostic Unit - Public Health Laboratory (MDU-PHL)|MDU-PHL|Schultz|Australia
>Spike|hCoV-19/USA/CA-ALSR-4329/2020|2020-10-08|EPI_ISL_648974|Original|hCoV-19^^California|Human|San Diego County Public Health Laboratory|Andersen lab at Scripps Research|Gangavarapu|USA
>Spike|hCoV-19/USA/WI-UW-2088/2020|2020-09-27|EPI_ISL_677493|Original|hCoV-19^^Wisconsin|Human|University of Wisconsin-Madison AIDS Vaccine Research Laboratories|University of Wisconsin-Madison AIDS Vaccine Research Laboratories|Baczenas|USA
>Spike|hCoV-19/England/ALDP-9EA5C8/2020|2020-09-24|EPI_ISL_580193|Original|hCoV-19^^England|Human|Lighthouse Lab in Alderley Park|Wellcome Sanger Institute for the COVID-19 Genomics UK (COG-UK) consortium|Davies|United Kingdom
>Spike|hCoV-19/India/TG-CCMB-M806/2020|2020-06-12|EPI_ISL_495245|Original|hCoV-19^^Telangana|Human|CSIR-Centre for Cellular and Molecular Biology|CSIR-Centre for Cellular and Molecular Biology|Banu|India
>Spike|hCoV-19/Panama/328726/2020|2020-03-08|EPI_ISL_496610|Original|hCoV-19^^Panama Center|Human|Gorgas Memorial Laboratory of Health Studies|Gorgas Memorial Laboratory of Health Studies|Martinez|Panama
>Spike|hCoV-19/England/ALDP-4ACDA1/2020|2020-06-14|EPI_ISL_559539|Original|hCoV-19^^England|Human|Lighthouse Lab in Alderley Park|Wellcome Sanger Institute for the COVID-19 Genomics UK (COG-UK) consortium|Davies|United Kingdom
>Spike|hCoV-19/USA/UT-0289/2020|2020-03-24|EPI_ISL_420809|Original|hCoV-19^^Utah|Human|Utah Public Health Laboratory|Utah Public Health Laboratory|Young|USA
>Spike|hCoV-19/England/20139065204/2020|2020-03-28|EPI_ISL_423353|Original|hCoV-19^^England|Human|Respiratory Virus Unit|Respiratory Virus Unit|Platt|United Kingdom
>Spike|hCoV-19/England/QEUH-AD151E/2020|2020-10-22|EPI_ISL_634583|Original|hCoV-19^^England|Human|Lighthouse Lab in Glasgow|Wellcome Sanger Institute for the COVID-19 Genomics UK (COG-UK) consortium|Davies|United Kingdom
>Spike|hCoV-19/England/MILK-BB1DB1/2020|2020-11-18|EPI_ISL_720509|Original|hCoV-19^^England|Human|Lighthouse Lab in Milton Keynes|Wellcome Sanger Institute for the COVID-19 Genomics UK (COG-UK) Consortium|Davies|United Kingdom
>Spike|hCoV-19/USA/CA-ALSR-4428/2020|2020-10-26|EPI_ISL_649022|Original|hCoV-19^^California|Human|San Diego County Public Health Laboratory|Andersen lab at Scripps Research|Gangavarapu|USA
>Spike|hCoV-19/England/NOTT-12078C/2020|2020-12-10|EPI_ISL_725644|Original|hCoV-19^^England|Human|Queens Medical Centre|COVID-19 Genomics UK (COG-UK) Consortium|Loose|United Kingdom

>Spike|hCoV-19/USA/VA-DCLS-1832/2020|2020-09-23|EPI_ISL_609988|Original|hCoV-19^^Virginia|Human|Virginia DCLS|Virginia DCLS|St. Jacques|USA
>Spike|hCoV-19/Australia/VIC9733/2020|2020-08-01|EPI_ISL_565681|Original|hCoV-19^^Victoria|Human|Microbiological Diagnostic Unit - Public Health Laboratory (MDU-PHL)|MDU-PHL|Schultz|Australia
>Spike|hCoV-19/USA/TX-DSHS-1212/2020|2020-03-19|EPI_ISL_631673|Original|hCoV-19^^Texas|Human|Texas Department of State Health Services|Texas Department of State Health Services|Rahman|USA
>Spike|hCoV-19/England/BRIS-12D8B9/2020|2020-05-06|EPI_ISL_481912|Original|hCoV-19^^England|Human|PHE South West Regional Laboratory|Wellcome Sanger Institute for the COVID-19 Genomics UK (COG-UK) consortium|Davies|United Kingdom
>Spike|hCoV-19/England/20169042204/2020|2020-04-18|EPI_ISL_465253|Original|hCoV-19^^England|Human|Respiratory Virus Unit|Respiratory Virus Unit|Platt|United Kingdom
>Spike|hCoV-19/England/MILK-A7BA9F/2020|2020-10-15|EPI_ISL_607405|Original|hCoV-19^^England|Human|Lighthouse Lab in Milton Keynes|Wellcome Sanger Institute for the COVID-19 Genomics UK (COG-UK) consortium|Davies|United Kingdom
>Spike|hCoV-19/Wales/PHWC-491273/2020|2020-11-17|EPI_ISL_706633|Original|hCoV-19^^Wales|Human|Originating lab|COVID-19 Genomics UK (COG-UK) Consortium|Moore|United Kingdom
>Spike|hCoV-19/Australia/VIC6800/2020|2020-07-21|EPI_ISL_564065|Original|hCoV-19^^Victoria|Human|Victorian Infectious Diseases Reference Laboratory (VIDRL)|VIDRL and MDU-PHL|Schultz|Australia
>Spike|hCoV-19/Wales/ALDP-B1A18D/2020|2020-11-02|EPI_ISL_710930|Original|hCoV-19^^Wales|Human|Lighthouse Lab in Alderley Park|Wellcome Sanger Institute for the COVID-19 Genomics UK (COG-UK) Consortium|Davies|United Kingdom
>Spike|hCoV-19/Japan/PG-5019/2020|2020-08-00|EPI_ISL_690130|Original|hCoV-19^^Japan|Human|Pathogen Genomics Center|Pathogen Genomics Center|Sekizuka|Japan
>Spike|hCoV-19/England/LOND-DA5E2/2020|2020-11-13|EPI_ISL_724645|Original|hCoV-19^^England|Human|University College London|COVID-19 Genomics UK (COG-UK) Consortium|Roy|United Kingdom
>Spike|hCoV-19/USA/WA-S1622/2020|2020-05-27|EPI_ISL_496048|Original|hCoV-19^^Washington|Human|Washington State Department of Health|Seattle Flu Study|Lee|USA
>Spike|hCoV-19/Luxembourg/LNS9348261/2020|2020-11-12|EPI_ISL_744890|Original|hCoV-19^^Luxembourg|Human|Laboratoire national de sant\u00e9|Laboratoire national de sant\u00e9|Wienecke-Baldacchino|Luxembourg
>Spike|hCoV-19/England/BRIS-12C54A/2020|2020-05-03|EPI_ISL_469485|Original|hCoV-19^^England|Human|PHE South West Regional Laboratory|Wellcome Sanger Institute for the COVID-19 Genomics UK (COG-UK) consortium|Davies|United Kingdom
>Spike|hCoV-19/USA/NM-DOH-2020313776/2020|2020-08-26|EPI_ISL_574274|Original|hCoV-19^^New Mexico|Human|New Mexico Department of Health Scientific Laboratory|New Mexico Department of Health Scientific Laboratory|Johnson|USA
>Spike|hCoV-19/England/QEUH-797D6A/2020|2020-07-15|EPI_ISL_533095|Original|hCoV-19^^England|Human|Lighthouse Lab in Glasgow|Wellcome Sanger Institute for the COVID-19 Genomics UK (COG-UK) consortium|Davies|United Kingdom
>Spike|hCoV-19/Australia/VIC11496/2020|2020-08-12|EPI_ISL_563397|Original|hCoV-19^^Victoria|Human|Microbiological Diagnostic Unit - Public Health Laboratory (MDU-PHL)|MDU-PHL|Schultz|Australia
>Spike|hCoV-19/England/CAMC-B0AF1D/2020|2020-10-30|EPI_ISL_643507|Original|hCoV-19^^England|Human|Lighthouse Lab in Cambridge|Wellcome Sanger Institute for the COVID-19 Genomics UK (COG-UK) Consortium|Davies|United Kingdom
>Spike|hCoV-19/Denmark/DCGC-1107/2020|2020-03-16|EPI_ISL_614617|Original|hCoV-19^^Syddanmark|Human|Department of Virus and Microbiological Special Diagnostics|Albertsen lab|Michaelsen|Denmark
>Spike|hCoV-19/England/ALDP-B75AB2/2020|2020-11-12|EPI_ISL_664933|Original|hCoV-19^^England|Human|Quadram Institute Bioscience|COVID-19 Genomics UK (COG-UK) Consortium|Alikhan|United Kingdom
>Spike|hCoV-19/Denmark/DCGC-12018/2020|2020-06-08|EPI_ISL_671265|Original|hCoV-19^^Nordjylland|Human|Department of Virus and Microbiological Special Diagnostics|Albertsen Lab|Michaelsen|Denmark

>Spike|hCoV-19/Chile/RM-170589/2020|2020-09-05|EPI_ISL_746720|Original|hCoV-19^^Region Metropolitana de Santiago|Human|Genetica Molecular and Subdepartamento de Virologia ISP Chile|Instituto de Salud Publica de Chile|Tognarelli|Chile
>Spike|hCoV-19/England/CAMB-81AAF/2020|2020-04-11|EPI_ISL_443087|Original|hCoV-19^^England|Human|Department of Pathology|Wellcome Sanger Institute for the COVID-19 Genomics UK (COG-UK) consortium|Amato|United Kingdom
>Spike|hCoV-19/USA/TX-HMH-2834/2020|2020-06-07|EPI_ISL_543807|Original|hCoV-19^^Texas|Human|Houston Methodist Hospital|Houston Methodist Hospital|Long|USA
>Spike|hCoV-19/USA/WA-UW-10609/2020|2020-06-12|EPI_ISL_570336|Original|hCoV-19^^Washington|Human|UW Virology Lab|UW Virology Lab|Roychoudhury|USA
>Spike|hCoV-19/England/QEUH-B41712/2020|2020-11-08|EPI_ISL_658584|Original|hCoV-19^^England|Human|Lighthouse Lab in Glasgow|Wellcome Sanger Institute for the COVID-19 Genomics UK (COG-UK) Consortium|Davies|United Kingdom
>Spike|hCoV-19/England/BRIS-12E9F1/2020|2020-04-23|EPI_ISL_488430|Original|hCoV-19^^England|Human|PHE South West Regional Laboratory|Wellcome Sanger Institute for the COVID-19 Genomics UK (COG-UK) consortium|Davies|United Kingdom
>Spike|hCoV-19/Switzerland/ZH-ETHZ-190056/2020|2020-07-09|EPI_ISL_500917|Original|hCoV-19^^Zurich|Human|Viollier AG|Department of Biosystems Science and Engineering|Nadeau|Switzerland
>Spike|hCoV-19/Scotland/QEUH-A1E3C0/2020|2020-10-01|EPI_ISL_598844|Original|hCoV-19^^Scotland|Human|Lighthouse Lab in Glasgow|Wellcome Sanger Institute for the COVID-19 Genomics UK (COG-UK) consortium|Davies|United Kingdom
>Spike|hCoV-19/Singapore/966/2020|2020-09-11|EPI_ISL_548975|Original|hCoV-19^^Singapore|Human|National Public Health Laboratory|National Public Health Laboratory|Mak|Singapore
>Spike|hCoV-19/England/QEUH-9AEB73/2020|2020-09-03|EPI_ISL_573483|Original|hCoV-19^^England|Human|University College London|COVID-19 Genomics UK (COG-UK) Consortium|Roy|United Kingdom
>Spike|hCoV-19/Japan/PG-0558/2020|2020-04-00|EPI_ISL_684183|Original|hCoV-19^^Japan|Human|Pathogen Genomics Center|Pathogen Genomics Center|Sekizuka|Japan
>Spike|hCoV-19/USA/MN-MDH-1079/2020|2020-06-03|EPI_ISL_470759|Original|hCoV-19^^Minnesota|Human|Minnesota Department of Health|Minnesota Department of Health|Garfin|USA
>Spike|hCoV-19/England/NORW-F68BA/2020|2020-11-16|EPI_ISL_665560|Original|hCoV-19^^England|Human|Quadram Institute Bioscience|COVID-19 Genomics UK (COG-UK) Consortium|Alikhan|United Kingdom
>Spike|hCoV-19/England/CAMB-1B3092/2020|2020-05-25|EPI_ISL_461549|Original|hCoV-19^^England|Human|Department of Pathology|COVID-19 Genomics UK (COG-UK) Consortium|Meredith|United Kingdom
>Spike|hCoV-19/USA/MN-MDH-1485/2020|2020-03-18|EPI_ISL_518879|Original|hCoV-19^^Minnesota|Human|Mayo Clinic & Mayo Clinic Laboratories|Minnesota Department of Health|Garfin|USA
>Spike|hCoV-19/USA/CA-CZB-1704/2020|2020-04-02|EPI_ISL_468637|Original|hCoV-19^^California|Human|Contra Costa Public Health Lab|Chan-Zuckerberg Biohub|McGeever|USA
>Spike|hCoV-19/Scotland/GCVR-1715BF/2020|2020-03-30|EPI_ISL_469984|Original|hCoV-19^^Scotland|Human|NHSGGC West of Scotland Specialist Virology Centre / MRC-University of Glasgow Centre for Virus Research|Wellcome Sanger Institute for the COVID-19 Genomics UK (COG-UK) consortium|Davies|United Kingdom
>Spike|hCoV-19/Australia/VIC8670/2020|2020-07-24|EPI_ISL_565137|Original|hCoV-19^^Victoria|Human|Victorian Infectious Diseases Reference Laboratory (VIDRL)|VIDRL and MDU-PHL|Schultz|Australia
>Spike|hCoV-19/England/ALDP-AA1E76/2020|2020-10-22|EPI_ISL_625098|Original|hCoV-19^^England|Human|Lighthouse Lab in Alderley Park|Wellcome Sanger Institute for the COVID-19 Genomics UK (COG-UK) consortium|Davies|United Kingdom
>Spike|hCoV-19/Canada/QC-LSPQ-L00239192/2020|2020-03-26|EPI_ISL_536236|Original|hCoV-19^^Quebec|Human|H\u00f4pital Pierre-Boucher|Laboratoire de sant\u00e9 publique du Qu\u00e9bec|Moreira|Canada

>Spike|hCoV-19/England/BRIS-1261A6/2020|2020-04-09|EPI_ISL_459005|Original|hCoV-19^^England|Human|PHE South West Regional Laboratory|Wellcome Sanger Institute for the COVID-19 Genomics UK (COG-UK) consortium|Davies|United Kingdom
>Spike|hCoV-19/USA/WI-UW-2217/2020|2020-09-26|EPI_ISL_677620|Original|hCoV-19^^Wisconsin|Human|University of Wisconsin-Madison AIDS Vaccine Research Laboratories|University of Wisconsin-Madison AIDS Vaccine Research Laboratories|Baczenas|USA
>Spike|hCoV-19/United Arab Emirates/0447/2020|2020-05-18|EPI_ISL_698619|Original|hCoV-19^^Abu Dhabi|Human|Group 42 (G42) Healthcare|G42 Healthcare|Ogrodzki|United Arab Emirates
>Spike|hCoV-19/Switzerland/BE-ETHZ-260021/2020|2020-08-22|EPI_ISL_539355|Original|hCoV-19^^Bern|Human|Viollier AG|Department of Biosystems Science and Engineering|Nadeau|Switzerland
>Spike|hCoV-19/USA/ME-HETL-J0404/2020|2020-12-16|EPI_ISL_755513|Original|hCoV-19^^Maine|Human|Maine Health and Environmental Testing Laboratory|Tewhey Lab|Tewhey|USA
>Spike|hCoV-19/Scotland/QEUH-9F6ABB/2020|2020-09-27|EPI_ISL_600685|Original|hCoV-19^^Scotland|Human|Lighthouse Lab in Glasgow|Wellcome Sanger Institute for the COVID-19 Genomics UK (COG-UK) consortium|Davies|United Kingdom
>Spike|hCoV-19/USA/SC-20-1448-T14/2020|2020-07-08|EPI_ISL_516766|Original|hCoV-19^^South Carolina|Human|van Bakel Laboratory|van Bakel Laboratory|van Bakel|USA
>Spike|hCoV-19/USA/WY-WPHL-20063090/2020|2020-08-17|EPI_ISL_770121|Original|hCoV-19^^Wyoming|Human|Wyoming Public Health Laboratory|Wyoming Public Health Laboratory|Hull|USA
>Spike|hCoV-19/Scotland/EDB929/2020|2020-04-04|EPI_ISL_432888|Original|hCoV-19^^Scotland|Human|Virology Department|COVID-19 Genomics UK (COG-UK) Consortium|Williams|United Kingdom
>Spike|hCoV-19/Russia/SPE-RII-13247V/2020|2020-05-12|EPI_ISL_524004|Vero P1|hCoV-19^^Saint Petersburg|Human|WHO National Influenza Centre Russian Federation|WHO National Influenza Centre Russian Federation|Komissarov|Russia
>Spike|hCoV-19/Australia/VIC8966/2020|2020-07-27|EPI_ISL_565318|Original|hCoV-19^^Victoria|Human|Microbiological Diagnostic Unit - Public Health Laboratory (MDU-PHL)|MDU-PHL|Schultz|Australia
>Spike|hCoV-19/Denmark/DCGC-21812/2020|2020-12-21|EPI_ISL_758379|Original|hCoV-19^^Hovedstaden|Human|Department of Virus and Microbiological Special Diagnostics|Albertsen Lab|Michaelsen|Denmark
>Spike|hCoV-19/Australia/VIC7625/2020|2020-07-23|EPI_ISL_564541|Original|hCoV-19^^Victoria|Human|Microbiological Diagnostic Unit - Public Health Laboratory (MDU-PHL)|MDU-PHL|Schultz|Australia
>Spike|hCoV-19/Netherlands/NA_287/2020|2020-03-31|EPI_ISL_422596|Original|hCoV-19^^Netherlands|Human|Dutch COVID-19 response team|Erasmus Medical Center|Oude Munnink|Netherlands
>Spike|hCoV-19/Switzerland/ZH-ETHZ-280196/2020|2020-09-09|EPI_ISL_560550|Original|hCoV-19^^Zurich|Human|Viollier AG|Department of Biosystems Science and Engineering|Nadeau|Switzerland
>Spike|hCoV-19/England/BRIS-129E7F/2020|2020-04-16|EPI_ISL_458473|Original|hCoV-19^^England|Human|PHE South West Regional Laboratory|Wellcome Sanger Institute for the COVID-19 Genomics UK (COG-UK) consortium|Davies|United Kingdom
>Spike|hCoV-19/England/OXON-B18CA/2020|2020-04-28|EPI_ISL_479050|Original|hCoV-19^^England|Human|Oxford Viromics|COVID-19 Genomics UK (COG-UK) Consortium|Golubchik|United Kingdom
>Spike|hCoV-19/Australia/VIC2197/2020|2020-06-24|EPI_ISL_521943|Original|hCoV-19^^Victoria|Human|Microbiological Diagnostic Unit - Public Health Laboratory (MDU-PHL)|MDU-PHL|Schultz|Australia
>Spike|hCoV-19/England/QEUH-9F4B6F/2020|2020-09-24|EPI_ISL_588428|Original|hCoV-19^^England|Human|Lighthouse Lab in Glasgow|Wellcome Sanger Institute for the COVID-19 Genomics UK (COG-UK) consortium|Davies|United Kingdom
>Spike|hCoV-19/USA/AZ-TG640786/2020|2020-04-27|EPI_ISL_695759|Original|hCoV-19^^Arizona|Human|Sonora Quest Laboratories|TGen North|French|USA

>Spike|hCoV-19/USA/CA-CZB-12385/2020|2020-10-06|EPI_ISL_648507|Original|hCoV-19^^California|Human|Orange County Public Health Lab|Chan-Zuckerberg Biohub|Bhatt|USA
>Spike|hCoV-19/Scotland/CVR2162/2020|2020-04-10|EPI_ISL_477865|Original|hCoV-19^^Scotland|Human|West of Scotland Specialist Virology Centre|COVID-19 Genomics UK (COG-UK) Consortium|Robertson|United Kingdom
>Spike|hCoV-19/England/CAMC-B530C7/2020|2020-11-09|EPI_ISL_657675|Original|hCoV-19^^England|Human|Lighthouse Lab in Cambridge|Wellcome Sanger Institute for the COVID-19 Genomics UK (COG-UK) Consortium|Davies|United Kingdom
>Spike|hCoV-19/USA/MI-MDHHS-SC22129/2020|2020-10-03|EPI_ISL_614180|Original|hCoV-19^^Michigan|Human|Michigan Department of Health and Human Services|Michigan Department of Health and Human Services|Jacob|USA
>Spike|hCoV-19/England/NOTT-11EF7A/2020|2020-12-02|EPI_ISL_725538|Original|hCoV-19^^England|Human|Queens Medical Centre|COVID-19 Genomics UK (COG-UK) Consortium|Loose|United Kingdom
>Spike|hCoV-19/North Macedonia/ChVir7907/2020|2020-06-27|EPI_ISL_492078|Original|hCoV-19^^North Macedonia|Human|Institute for Public Health of the Republic of North Macedonia|Charite Universitatsmedizin Berlin|Muehlemann|North Macedonia
>Spike|hCoV-19/Wales/QEUH-9CA0BE/2020|2020-09-10|EPI_ISL_567411|Original|hCoV-19^^Wales|Human|Lighthouse Lab in Glasgow|Wellcome Sanger Institute for the COVID-19 Genomics UK (COG-UK) consortium|Davies|United Kingdom
>Spike|hCoV-19/England/ALDP-A2D364/2020|2020-10-05|EPI_ISL_586918|Original|hCoV-19^^England|Human|Lighthouse Lab in Alderley Park|Wellcome Sanger Institute for the COVID-19 Genomics UK (COG-UK) consortium|Davies|United Kingdom
>Spike|hCoV-19/USA/NY-PV09482/2020|2020-03-20|EPI_ISL_450154|Original|hCoV-19^^New York|Human|MSHS Clinical Microbiology Laboratories|MSHS Pathogen Surveillance Program|van Bakel|USA
>Spike|hCoV-19/England/QEUH-9DC383/2020|2020-09-21|EPI_ISL_580858|Original|hCoV-19^^England|Human|Lighthouse Lab in Glasgow|Wellcome Sanger Institute for the COVID-19 Genomics UK (COG-UK) consortium|Davies|United Kingdom
>Spike|hCoV-19/USA/CA-CZB-14793/2020|2020-11-13|EPI_ISL_739447|Original|hCoV-19^^California|Human|Humboldt County Public Health Laboratory|Chan-Zuckerberg Biohub|Kamm|USA
>Spike|hCoV-19/India/TG-CCMB-M930/2020|2020-06-13|EPI_ISL_495264|Original|hCoV-19^^Telangana|Human|CSIR-Centre for Cellular and Molecular Biology|CSIR-Centre for Cellular and Molecular Biology|Banu|India
>Spike|hCoV-19/Australia/VIC9413/2020|2020-08-12|EPI_ISL_565540|Original|hCoV-19^^Victoria|Human|Microbiological Diagnostic Unit - Public Health Laboratory (MDU-PHL)|MDU-PHL|Schultz|Australia
>Spike|hCoV-19/USA/WA-UW-1772/2020|2020-03-21|EPI_ISL_424233|Original|hCoV-19^^Washington|Human|UW Virology Lab|UW Virology Lab|Roychoudhury|USA
>Spike|hCoV-19/Japan/PG-0207/2020|2020-03-11|EPI_ISL_479907|Original|hCoV-19^^Kansai|Human|Himeji City Institute of Environment and Health|Pathogen Genomics Center|Sekizuka|Japan
>Spike|hCoV-19/USA/TX-DSHS-0650/2020|2020-07-16|EPI_ISL_576304|Original|hCoV-19^^Texas|Human|Texas Department of State Health Services|Texas Department of State Health Services|Rahman|USA
>Spike|hCoV-19/Switzerland/BE-ETHZ-120068/2020|2020-04-20|EPI_ISL_468240|Original|hCoV-19^^Bern|Human|Viollier AG|Department of Biosystems Science and Engineering|Nadeau|Switzerland
>Spike|hCoV-19/England/CAMB-72625/2020|2020-03-22|EPI_ISL_439648|Original|hCoV-19^^England|Human|Department of Pathology|Wellcome Sanger Institute for the COVID-19 Genomics UK (COG-UK) consortium|Amato|United Kingdom
>Spike|hCoV-19/USA/CA-ALSR-4390/2020|2020-07-31|EPI_ISL_649003|Original|hCoV-19^^California|Human|San Diego County Public Health Laboratory|Andersen lab at Scripps Research|Gangavarapu|USA
>Spike|hCoV-19/Poland/PL_MCB_31/2020|2020-04-19|EPI_ISL_645030|Original|hCoV-19^^Poland|Human|Human Genome Variation Research Group|Human Genome Variation Research Group||Poland

>Spike|hCoV-19/England/MILK-563929/2020|2020-06-10|EPI_ISL_558341|Original|hCoV-19^^England|Human|Lighthouse Lab in Milton Keynes|Wellcome Sanger Institute for the COVID-19 Genomics UK (COG-UK) consortium|Davies|United Kingdom
>Spike|hCoV-19/England/MILK-A48011/2020|2020-10-06|EPI_ISL_598381|Original|hCoV-19^^England|Human|Lighthouse Lab in Milton Keynes|Wellcome Sanger Institute for the COVID-19 Genomics UK (COG-UK) consortium|Davies|United Kingdom
>Spike|hCoV-19/Germany/SH-ChVir20295/2020|2020-11-12|EPI_ISL_729656|Original|hCoV-19^^Schleswig-Holstein|Human|A. Krumbholz|Charit\u00e9 Universit\u00e4tsmedizin Berlin|Muehlemann|Germany
>Spike|hCoV-19/Scotland/EDB2989/2020|2020-04-02|EPI_ISL_489046|Original|hCoV-19^^Scotland|Human|Virology Department|Wellcome Sanger Institute for the COVID-19 Genomics UK (COG-UK) consortium|Davies|United Kingdom
>Spike|hCoV-19/England/QEUH-B819E3/2020|2020-11-14|EPI_ISL_668125|Original|hCoV-19^^England|Human|Lighthouse Lab in Glasgow|Wellcome Sanger Institute for the COVID-19 Genomics UK (COG-UK) Consortium|Davies|United Kingdom
>Spike|hCoV-19/USA/MI-MDHHS-SC20175/2020|2020-03-18|EPI_ISL_436824|Original|hCoV-19^^Michigan|Human|Michigan Department of Health and Human Services|Michigan Department of Health and Human Services|Blankenship|USA
>Spike|hCoV-19/England/QEUH-AF3DC3/2020|2020-10-30|EPI_ISL_662901|Original|hCoV-19^^England|Human|Lighthouse Lab in Glasgow|Wellcome Sanger Institute for the COVID-19 Genomics UK (COG-UK) Consortium|Davies|United Kingdom
>Spike|hCoV-19/Spain/VC-IBV-98000409/2020|2020-03-24|EPI_ISL_539227|Original|hCoV-19^^Comunitat Valenciana|Human|Servicio de Microbiologia. Hospital Clinico Universitario de Valencia|SeqCOVID-SPAIN consortium/IBV(CSIC)|Comas|Spain
>Spike|hCoV-19/Wales/PHWC-49310B/2020|2020-11-28|EPI_ISL_725839|Original|hCoV-19^^Wales|Human|Originating lab|COVID-19 Genomics UK (COG-UK) Consortium|Moore|United Kingdom
>Spike|hCoV-19/England/09c/2020|2020-02-09|EPI_ISL_412116|Original|hCoV-19^^England|Human|Respiratory Virus Unit|Respiratory Virus Unit|Platt|United Kingdom
>Spike|hCoV-19/Japan/PG-6158/2020|2020-08-00|EPI_ISL_689270|Original|hCoV-19^^Japan|Human|Pathogen Genomics Center|Pathogen Genomics Center|Sekizuka|Japan
>Spike|hCoV-19/USA/UT-UPHL-2012209457/2020|2020-08-14|EPI_ISL_746159|Original|hCoV-19^^Utah|Human|Ginkgo Bioworks Clinical Laboratory|Utah Public Health Laboratory|Young|USA
>Spike|hCoV-19/USA/WI-UW-1341/2020|2020-09-08|EPI_ISL_536712|Original|hCoV-19^^Wisconsin|Human|University of Wisconsin-Madison AIDS Vaccine Research Laboratories|University of Wisconsin-Madison AIDS Vaccine Research Laboratories|Baczenas|USA
>Spike|hCoV-19/Brazil/PA-IEC-166687/2020|2020-05-13|EPI_ISL_524800|Original|hCoV-19^^Par\u00e1|Human|\ufeffEvandro Chagas Institute|\ufeffEvandro Chagas Institute|Sousa J\u00fanior|Brazil
>Spike|hCoV-19/Singapore/1358/2020|2020-06-19|EPI_ISL_648709|Original|hCoV-19^^Singapore|Human|Department of Laboratory Medicine|Department of Laboratory Medicine|Chen|Singapore
>Spike|hCoV-19/England/ALDP-5B68B1/2020|2020-06-19|EPI_ISL_558717|Original|hCoV-19^^England|Human|Lighthouse Lab in Alderley Park|Wellcome Sanger Institute for the COVID-19 Genomics UK (COG-UK) consortium|Davies|United Kingdom
>Spike|hCoV-19/Singapore/544/2020|2020-06-02|EPI_ISL_482682|Original|hCoV-19^^Singapore|Human|Singapore General Hospital|Department of Microbiology|Abdul Rahman|Singapore
>Spike|hCoV-19/England/MILK-C60451/2020|2020-12-13|EPI_ISL_769240|Original|hCoV-19^^England|Human|Lighthouse Lab in Milton Keynes|Wellcome Sanger Institute for the COVID-19 Genomics UK (COG-UK) Consortium|Davies|United Kingdom
>Spike|hCoV-19/USA/TX-HMH-MCoV-11626/2020|2020-09-11|EPI_ISL_789910|Original|hCoV-19^^Texas|Human|Houston Methodist Hospital|Houston Methodist Hospital|Long|USA
>Spike|hCoV-19/South Africa/KRISP-K002786/2020|2020-08-23|EPI_ISL_529780|Original|hCoV-19^^KwaZulu-Natal|Human|NHLS-IALCH|KRISP|de Oliveira|South Africa
>Spike|hCoV-19/USA/WI-UW-304/2020|2020-04-13|EPI_ISL_436598|Original|hCoV-19^^Wisconsin|Human|University of Wisconsin-Madison AIDS Vaccine Research

Laboratories|University of Wisconsin-Madison AIDS Vaccine Research Laboratories|Baczenas|USA
>Spike|hCoV-19/USA/TX-HMH-6393/2020|2020-07-05|EPI_ISL_545966|Original|hCoV-19^^Texas|Human|Houston Methodist Hospital|Houston Methodist Hospital|Long|USA
>Spike|hCoV-19/England/CAMC-A57FB8/2020|2020-10-09|EPI_ISL_597051|Original|hCoV-19^^England|Human|Lighthouse Lab in Cambridge|Wellcome Sanger Institute for the COVID-19 Genomics UK (COG-UK) consortium|Davies|United Kingdom
>Spike|hCoV-19/England/CAMC-B98DB0/2020|2020-11-15|EPI_ISL_673610|Original|hCoV-19^^England|Human|Lighthouse Lab in Cambridge|Wellcome Sanger Institute for the COVID-19 Genomics UK (COG-UK) Consortium|Davies|United Kingdom
>Spike|hCoV-19/Argentina/Heritas_HG007/2020|2020-05-09|EPI_ISL_476565|Original|hCoV-19^^Ciudad Autonoma de Buenos Aires|Human|Hospital de Pediatr\u00eda \Prof. Dr. Juan P Garrahan\|H\u00e9ritas|Rohr|Argentina
>Spike|hCoV-19/Spain/MD-IBV-99009637/2020|2020-10-27|EPI_ISL_654373|Original|hCoV-19^^Madrid|Human|Hospital General Universitario Gregorio Mara\u00f1\u00f3n|SeqCOVID-SPAIN consortium/IBV(CSIC)|Comas|Spain
>Spike|hCoV-19/USA/NY-NYCPHL-000165/2020|2020-04-09|EPI_ISL_631701|Original|hCoV-19^^New York|Human|Flushing Hospital Medical Center|New York City Public Health Laboratory|Wang|USA
>Spike|hCoV-19/England/20196060604/2020|2020-05-04|EPI_ISL_465624|Original|hCoV-19^^England|Human|Respiratory Virus Unit|Respiratory Virus Unit|Platt|United Kingdom
>Spike|hCoV-19/Lithuania/C20-05-R8/2020|2020-05-21|EPI_ISL_560403|Original|hCoV-19^^Vilnius r.|Human|Vilnius University Hospital Santaros Klinikos|Institute of Biotechnology|Timinskas|Lithuania
>Spike|hCoV-19/Denmark/DCGC-14272/2020|2020-11-30|EPI_ISL_711114|Original|hCoV-19^^Hovedstaden|Human|Department of Virus and Microbiological Special Diagnostics|Albertsen Lab|Michaelsen|Denmark
>Spike|hCoV-19/Australia/NSW959/2020|2020-09-09|EPI_ISL_545031|Original|hCoV-19^^New South Wales|Human|South Eastern Area Laboratory Services (SEALS)|NSW Health Pathology - Institute of Clinical Pathology and Medical Research; Westmead Hospital; University of Sydney|Gall|Australia
>Spike|hCoV-19/USA/NY-NYUMC585/2020|2020-03-30|EPI_ISL_444702|Original|hCoV-19^^New York|Human|NYU Langone Health|Departments of Pathology and Medicine|Maurano|USA
>Spike|hCoV-19/South Korea/KDCA0399/2020|2020-11-08|EPI_ISL_760183|Original|hCoV-19^^South Korea|Human|Division of Emerging Infectious Diseases|Division of Emerging Infectious Diseases|Park|South Korea
>Spike|hCoV-19/Wales/ALDP-BC640F/2020|2020-11-20|EPI_ISL_767322|Original|hCoV-19^^Wales|Human|Lighthouse Lab in Alderley Park|Wellcome Sanger Institute for the COVID-19 Genomics UK (COG-UK) Consortium|Davies|United Kingdom
>Spike|hCoV-19/Luxembourg/LNS1908675/2020|2020-11-13|EPI_ISL_740019|Original|hCoV-19^^Luxembourg|Human|Laboratoire national de sant\u00e9|Laboratoire national de sant\u00e9|Wienecke-Baldacchino|Luxembourg
>Spike|hCoV-19/Germany/NW-HHU-463/2020|2020-12-11|EPI_ISL_770697|Original|hCoV-19^^North Rhine-Westphalia|Human|ZOTZ KLIMAS MVZ D\u00fcsseldorf-Centrum GbR \u00dcBAG f\u00fcr Labormedizin|Center of Medical Microbiology|Wienemann|Germany
>Spike|hCoV-19/Wales/PHWC-482BB6/2020|2020-10-21|EPI_ISL_613272|Original|hCoV-19^^Wales|Human|Wales Specialist Virology Centre Sequencing lab|COVID-19 Genomics UK (COG-UK) Consortium|Moore|United Kingdom
>Spike|hCoV-19/USA/CA-ALSR-0830-SAN/2020|2020-03-31|EPI_ISL_483386|Original|hCoV-19^^California|Human|UC San Diego Center for Advanced Laboratory Medicine|Andersen lab at Scripps Research|Gangavarapu|USA
>Spike|hCoV-19/Canada/BC_19518330/2020|2020-03-00|EPI_ISL_462765|Original|hCoV-19^^British Columbia|Human|BCCDC Public Health Laboratory|BCCDC Public Health Laboratory|Harrigan|Canada
>Spike|hCoV-19/England/CAMC-C9224D/2020|2020-12-11|EPI_ISL_777133|Original|hCoV-19^^England|Human|Lighthouse Lab in Cambridge|Wellcome Sanger Institute for the COVID-19 Genomics UK (COG-UK) Consortium|Davies|United Kingdom
>Spike|hCoV-19/mink/Netherlands/NB-EMC-1-4/2020|2020-04-28|EPI_ISL_523016|Original|hCoV-19^^Noord Brabant|Neovison vison|Dutch COVID-19 response team|Erasmus Medical Center|Oude Munnink|Netherlands

>Spike|hCoV-19/England/ALDP-9EA3DD/2020|2020-09-24|EPI_ISL_579983|Original|hCoV-19^^England|Human|Lighthouse Lab in Alderley Park|Wellcome Sanger Institute for the COVID-19 Genomics UK (COG-UK) consortium|Davies|United Kingdom
>Spike|hCoV-19/Denmark/DCGC-17951/2020|2020-12-14|EPI_ISL_749662|Original|hCoV-19^^Hovedstaden|Human|Department of Virus and Microbiological Special Diagnostics|Albertsen Lab|Michaelsen|Denmark
>Spike|hCoV-19/England/MILK-975498/2020|2020-08-21|EPI_ISL_552584|Original|hCoV-19^^England|Human|Lighthouse Lab in Milton Keynes|Wellcome Sanger Institute for the COVID-19 Genomics UK (COG-UK) consortium|Davies|United Kingdom
>Spike|hCoV-19/England/LEED-2A81E7/2020|2020-04-26|EPI_ISL_539029|Original|hCoV-19^^England|Human|Leeds Teaching Hospitals NHS Trust and Public Health England|Wellcome Sanger Institute for the COVID-19 Genomics UK (COG-UK) consortium|Davies|United Kingdom
>Spike|hCoV-19/Latvia/051/2020|2020-03-23|EPI_ISL_486435|Original|hCoV-19^^Latvia|Human|Latvijas Infektolo\u0123ijas centrs|Latvian Biomedical Research and Study Centre|Zrelovs|Latvia
>Spike|hCoV-19/England/QEUH-9F50F6/2020|2020-09-27|EPI_ISL_637808|Original|hCoV-19^^England|Human|Department of Pathology|COVID-19 Genomics UK (COG-UK) Consortium|Goodfellow|United Kingdom
>Spike|hCoV-19/USA/MI-MDHHS-SC20080/2020|2020-03-26|EPI_ISL_437167|Original|hCoV-19^^Michigan|Human|Michigan Department of Health and Human Services|Michigan Department of Health and Human Services|Blankenship|USA
>Spike|hCoV-19/USA/TX-HMH-MCoV-12948/2020|2020-08-18|EPI_ISL_788100|Original|hCoV-19^^Texas|Human|Houston Methodist Hospital|Houston Methodist Hospital|Long|USA
>Spike|hCoV-19/United Arab Emirates/0720/2020|2020-05-19|EPI_ISL_698871|Original|hCoV-19^^Abu Dhabi|Human|Group 42 (G42) Healthcare|G42 Healthcare|Ogrodzki|United Arab Emirates
>Spike|hCoV-19/USA/VA-DCLS-1584/2020|2020-08-14|EPI_ISL_610139|Original|hCoV-19^^Virginia|Human|Virginia DCLS|Virginia DCLS|St. Jacques|USA
>Spike|hCoV-19/USA/NC_200256/2020|2020-03-00|EPI_ISL_454807|Original|hCoV-19^^North Carolina|Human|Dirk Dittmer|Dirk Dittmer|Moorad|USA
>Spike|hCoV-19/Wales/PHWC-4A5F1B/2020|2020-12-15|EPI_ISL_764983|Original|hCoV-19^^Wales|Human|Wales Specialist Virology Centre Sequencing lab|COVID-19 Genomics UK (COG-UK) Consortium|Moore|United Kingdom
>Spike|hCoV-19/England/CAMB-1AC454/2020|2020-04-22|EPI_ISL_489556|Original|hCoV-19^^England|Human|Department of Pathology|Wellcome Sanger Institute for the COVID-19 Genomics UK (COG-UK) consortium|Davies|United Kingdom
>Spike|hCoV-19/England/20094061504/2020|2020-02-25|EPI_ISL_464186|Original|hCoV-19^^England|Human|Respiratory Virus Unit|Respiratory Virus Unit|Platt|United Kingdom
>Spike|hCoV-19/Bangladesh/DNAS_DRICM_CPH_isl_441_BGD/2020|2020-04-29|EPI_ISL_774872|Original|hCoV-19^^Dhaka|Human|Designated Reference Institute for Chemical Measurements (DRICM)|DNA SOLUTION LTD.|Rabbi|Bangladesh
>Spike|hCoV-19/USA/LA-QDX-2026/2020|2020-03-17|EPI_ISL_604339|Original|hCoV-19^^Louisiana|Human|Quest Diagnostics|Quest Diagnostics|Kagan|USA
>Spike|hCoV-19/England/QEUH-9F7434/2020|2020-09-25|EPI_ISL_589139|Original|hCoV-19^^England|Human|Lighthouse Lab in Glasgow|Wellcome Sanger Institute for the COVID-19 Genomics UK (COG-UK) consortium|Davies|United Kingdom
>Spike|hCoV-19/England/QEUH-B13685/2020|2020-11-01|EPI_ISL_642284|Original|hCoV-19^^England|Human|Lighthouse Lab in Glasgow|Wellcome Sanger Institute for the COVID-19 Genomics UK (COG-UK) Consortium|Davies|United Kingdom
>Spike|hCoV-19/USA/TX-HMH-MCoV-11979/2020|2020-09-10|EPI_ISL_789856|Original|hCoV-19^^Texas|Human|Houston Methodist Hospital|Houston Methodist Hospital|Long|USA
>Spike|hCoV-19/England/CAMB-78935/2020|2020-04-06|EPI_ISL_433793|Original|hCoV-19^^England|Human|Department of Pathology|COVID-19 Genomics UK (COG-UK) Consortium|Meredith|United Kingdom
>Spike|hCoV-19/England/SHEF-CD236/2020|2020-07-10|EPI_ISL_526445|Original|hCoV-19^^England|Human|Virology Department|COVID-19 Genomics UK (COG-UK) Consortium|de Silva|United Kingdom

>Spike|hCoV-19/England/ALDP-A92A65/2020|2020-10-19|EPI_ISL_611185|Original|hCoV-19^^England|Human|Lighthouse Lab in Alderley Park|Wellcome Sanger Institute for the COVID-19 Genomics UK (COG-UK) consortium|Davies|United Kingdom
>Spike|hCoV-19/England/204260476/2020|2020-10-15|EPI_ISL_609901|Original|hCoV-19^^England|Human|Respiratory Virus Unit|Respiratory Virus Unit|Platt|United Kingdom
>Spike|hCoV-19/Scotland/CVR5662/2020|2020-11-03|EPI_ISL_651892|Original|hCoV-19^^Scotland|Human|West of Scotland Specialist Virology Centre|COVID-19 Genomics UK (COG-UK) Consortium|Robertson|United Kingdom
>Spike|hCoV-19/Poland/PL_MCB_99/2020|2020-05-10|EPI_ISL_645098|Original|hCoV-19^^Poland|Human|Human Genome Variation Research Group|Human Genome Variation Research Group||Poland
>Spike|hCoV-19/USA/OR-OHSU-0332/2020|2020-05-15|EPI_ISL_509104|Original|hCoV-19^^Oregon|Human|OHSU Lab Services Molecular Microbiology Lab|Oregon SARS-CoV-2 Genome Sequencing Center|O'Roak|USA
>Spike|hCoV-19/USA/NY-NYUMC121/2020|2020-03-18|EPI_ISL_421733|Original|hCoV-19^^New York|Human|NYU Langone Health|Departments of Pathology and Medicine|Maurano|USA
>Spike|hCoV-19/USA/TX-HMH-MCoV-14649/2020|2020-10-10|EPI_ISL_787934|Original|hCoV-19^^Texas|Human|Houston Methodist Hospital|Houston Methodist Hospital|Long|USA
>Spike|hCoV-19/England/CAMC-A82CEA/2020|2020-10-13|EPI_ISL_610761|Original|hCoV-19^^England|Human|Lighthouse Lab in Cambridge|Wellcome Sanger Institute for the COVID-19 Genomics UK (COG-UK) consortium|Davies|United Kingdom
>Spike|hCoV-19/Panama/331954/2020|2020-03-20|EPI_ISL_496699|Original|hCoV-19^^Panama Center|Human|Gorgas Memorial Laboratory of Health Studies|Gorgas Memorial Laboratory of Health Studies|Martinez|Panama
>Spike|hCoV-19/Scotland/CAMC-A3F1A1/2020|2020-10-05|EPI_ISL_625447|Original|hCoV-19^^Scotland|Human|Lighthouse Lab in Cambridge|Wellcome Sanger Institute for the COVID-19 Genomics UK (COG-UK) consortium|Davies|United Kingdom
>Spike|hCoV-19/USA/TX-HMH-MCoV-18516/2020|2020-11-19|EPI_ISL_784864|Original|hCoV-19^^Texas|Human|Houston Methodist Hospital|Houston Methodist Hospital|Long|USA
>Spike|hCoV-19/England/NORT-2979A7/2020|2020-04-21|EPI_ISL_488052|Original|hCoV-19^^England|Human|NU-OMICS DNA Sequencing research facility|Wellcome Sanger Institute for the COVID-19 Genomics UK (COG-UK) consortium|Davies|United Kingdom
>Spike|hCoV-19/England/MILK-B01D0D/2020|2020-10-24|EPI_ISL_642833|Original|hCoV-19^^England|Human|Lighthouse Lab in Milton Keynes|Wellcome Sanger Institute for the COVID-19 Genomics UK (COG-UK) Consortium|Davies|United Kingdom
>Spike|hCoV-19/South Africa/NHLS-UCT-GS-0034/2020|2020-08-20|EPI_ISL_640035|Original|hCoV-19^^Western Cape|Human|Riversdale Clinic wc RAV|NHLS/UCT|Iranzadeh|South Africa
>Spike|hCoV-19/England/C200045865/2020|2020-11-10|EPI_ISL_686108|Original|hCoV-19^^England|Human|Respiratory Virus Unit|COVID-19 Genomics UK (COG-UK) Consortium|Platt|United Kingdom
>Spike|hCoV-19/Wales/PHWC-16E694/2020|2020-09-29|EPI_ISL_585752|Original|hCoV-19^^Wales|Human|Wales Specialist Virology Centre Sequencing lab|COVID-19 Genomics UK (COG-UK) Consortium|Moore|United Kingdom
>Spike|hCoV-19/USA/CA-CZB-12394/2020|2020-10-08|EPI_ISL_648512|Original|hCoV-19^^California|Human|Orange County Public Health Lab|Chan-Zuckerberg Biohub|Bhatt|USA
>Spike|hCoV-19/Denmark/DCGC-1065/2020|2020-05-25|EPI_ISL_614490|Original|hCoV-19^^Hovedstaden|Human|Department of Virus and Microbiological Special Diagnostics|Albertsen lab|Michaelsen|Denmark
>Spike|hCoV-19/England/CAMB-769EC/2020|2020-03-30|EPI_ISL_438359|Original|hCoV-19^^England|Human|Department of Pathology|Wellcome Sanger Institute for the COVID-19 Genomics UK (COG-UK) consortium|Amato|United Kingdom
>Spike|hCoV-19/USA/UT-UPHL-2012453480/2020|2020-07-26|EPI_ISL_745717|Original|hCoV-19^^Utah|Human|Ginkgo Bioworks Clinical Laboratory|Utah Public Health Laboratory|Young|USA
>Spike|hCoV-19/Denmark/DCGC-9370/2020|2020-11-16|EPI_ISL_668934|Original|hCoV-19^^Midtjylland|Human|Department of Virus and Microbiological Special Diagnostics|Albertsen Lab|Michaelsen|Denmark

>Spike|hCoV-19/Switzerland/ZH-ETHZ-270183/2020|2020-08-29|EPI_ISL_541530|Original|hCoV-19^^Zurich|Human|Viollier AG|Department of Biosystems Science and Engineering|Nadeau|Switzerland
>Spike|hCoV-19/USA/WA-UW-12832/2020|2020-06-29|EPI_ISL_570908|Original|hCoV-19^^Washington|Human|UW Virology Lab|UW Virology Lab|Roychoudhury|USA
>Spike|hCoV-19/England/MILK-9A7FF6/2020|2020-09-02|EPI_ISL_573252|Original|hCoV-19^^England|Human|Virology Department|COVID-19 Genomics UK (COG-UK) Consortium|Williams|United Kingdom
>Spike|hCoV-19/USA/NY-PV08148/2020|2020-03-14|EPI_ISL_421365|Original|hCoV-19^^New York|Human|MSHS Clinical Microbiology Laboratories|MSHS Pathogen Surveillance Program|van Bakel|USA
>Spike|hCoV-19/Hungary/UD-7417/2020|2020-04-08|EPI_ISL_476075|Original|hCoV-19^^Heves County|Human|University of Debrecen|National Laboratory of Virology|Kemenesi|Hungary
>Spike|hCoV-19/England/MILK-B3DC65/2020|2020-11-01|EPI_ISL_706251|Original|hCoV-19^^England|Human|Oxford Viromics|COVID-19 Genomics UK (COG-UK) Consortium|Golubchik|United Kingdom
>Spike|hCoV-19/Wales/PHWC-168A8F/2020|2020-05-28|EPI_ISL_494323|Original|hCoV-19^^Wales|Human|Originating lab|COVID-19 Genomics UK (COG-UK) Consortium|Moore|United Kingdom
>Spike|hCoV-19/Scotland/EDB11074/2020|2020-12-15|EPI_ISL_742200|Original|hCoV-19^^Scotland|Human|Virology Department|COVID-19 Genomics UK (COG-UK) Consortium|McHugh|United Kingdom
>Spike|hCoV-19/USA/WA-S60/2020|2020-03-02|EPI_ISL_417113|Original|hCoV-19^^Washington|Human|Washington State Department of Health|Seattle Flu Study|Bedford|USA
>Spike|hCoV-19/USA/TX-DSHS-0062/2020|2020-05-09|EPI_ISL_517993|Original|hCoV-19^^Texas|Human|Texas Department of State Health Services|Texas Department of State Health Services|Rahman|USA
>Spike|hCoV-19/England/CAMB-1AA30E/2020|2020-04-16|EPI_ISL_443656|Original|hCoV-19^^England|Human|Department of Pathology|Wellcome Sanger Institute for the COVID-19 Genomics UK (COG-UK) consortium|Amato|United Kingdom
>Spike|hCoV-19/Ireland/D-NVRL-77IRL69365/2020|2020-11-05|EPI_ISL_752514|Original|hCoV-19^^Dublin|Human|National Virus Reference Laboratory|National Virus Reference Laboratory|Gonzalez|Ireland
>Spike|hCoV-19/Netherlands/NH-EMC-53/2020|2020-08-31|EPI_ISL_577849|Original|hCoV-19^^Noord Holland|Human|Dutch COVID-19 response team|Erasmus Medical Center|Oude Munnink|Netherlands
>Spike|hCoV-19/Scotland/EDB8297/2020|2020-10-11|EPI_ISL_594843|Original|hCoV-19^^Scotland|Human|Virology Department|COVID-19 Genomics UK (COG-UK) Consortium|McHugh|United Kingdom
>Spike|hCoV-19/USA/TX-DSHS-1241/2020|2020-07-28|EPI_ISL_676615|Original|hCoV-19^^Texas|Human|Texas Department of State Health Services|Texas Department of State Health Services|Rahman|USA
>Spike|hCoV-19/England/ALDP-95C29D/2020|2020-06-17|EPI_ISL_554883|Original|hCoV-19^^England|Human|Lighthouse Lab in Alderley Park|Wellcome Sanger Institute for the COVID-19 Genomics UK (COG-UK) consortium|Davies|United Kingdom
>Spike|hCoV-19/England/ALDP-A95DB4/2020|2020-10-20|EPI_ISL_624648|Original|hCoV-19^^England|Human|Lighthouse Lab in Alderley Park|Wellcome Sanger Institute for the COVID-19 Genomics UK (COG-UK) consortium|Davies|United Kingdom
>Spike|hCoV-19/Australia/VIC12682/2020|2020-07-28|EPI_ISL_592054|Original|hCoV-19^^Victoria|Human|Microbiological Diagnostic Unit - Public Health Laboratory (MDU-PHL)|MDU-PHL|Schultz|Australia
>Spike|hCoV-19/Scotland/CVR4434/2020|2020-09-15|EPI_ISL_585304|Original|hCoV-19^^Scotland|Human|Lighthouse Lab in Glasgow / MRC-University of Glasgow Centre for Virus Research|COVID-19 Genomics UK (COG-UK) Consortium|Robertson|United Kingdom
>Spike|hCoV-19/Canada/BC_04020783/2020|2020-04-00|EPI_ISL_466761|Original|hCoV-19^^British Columbia|Human|BCCDC Public Health Laboratory|BCCDC Public Health Laboratory|Harrigan|Canada

>Spike|hCoV-19/Peru/LIM-UPCH-0085/2020|2020-06-17|EPI_ISL_540963|Original|hCoV-19^^Lima|Human|Laboratorio de Referencia Nacional de Virus Respiratorios|Laboratorio de Gen\u00f3mica Microbiana|Tsukayama|Peru
>Spike|hCoV-19/India/TN-CCMB-C12/2020|2020-04-26|EPI_ISL_458030|Original|hCoV-19^^Tamil Nadu|Human|King Institute of Preventive Medicine & Research|CSIR-Centre for Cellular and Molecular Biology|Banu|India
>Spike|hCoV-19/Japan/PG-2768/2020|2020-04-00|EPI_ISL_690900|Original|hCoV-19^^Japan|Human|Pathogen Genomics Center|Pathogen Genomics Center|Sekizuka|Japan
>Spike|hCoV-19/Denmark/DCGC-19480/2020|2020-12-07|EPI_ISL_748979|Original|hCoV-19^^Hovedstaden|Human|Department of Virus and Microbiological Special Diagnostics|Albertsen Lab|Michaelsen|Denmark
>Spike|hCoV-19/England/C200044472/2020|2020-11-06|EPI_ISL_666759|Original|hCoV-19^^England|Human|Respiratory Virus Unit|COVID-19 Genomics UK (COG-UK) Consortium|Platt|United Kingdom
>Spike|hCoV-19/Switzerland/BL-UHB-42179281/2020|2020-03-06|EPI_ISL_527945|Original|hCoV-19^^Basel-Land|Human|University Hospital Basel|University Hospital Basel|Roloff Handschin|Switzerland
>Spike|hCoV-19/DRC/10733/2020|2020-05-23|EPI_ISL_513630|Original|hCoV-19^^Kinshasa|Human|Viral Respiratory Lab|Pathogen Sequencing Lab|Lokilo|Democratic Republic of the Congo
>Spike|hCoV-19/Wales/PHWC-28424/2020|2020-03-27|EPI_ISL_432379|Original|hCoV-19^^Wales|Human|Wales Specialist Virology Centre|Public Health Wales Microbiology Cardiff|Moore|United Kingdom
>Spike|hCoV-19/USA/UT-UPHL-201024324/2020|2020-04-12|EPI_ISL_594411|Original|hCoV-19^^Utah|Human|Utah Public Health Laboratory|Utah Public Health Laboratory|Young|USA
>Spike|hCoV-19/Ireland/D-NVRL-20G32319/2020|2020-03-18|EPI_ISL_605136|Original|hCoV-19^^Dublin|Human|National Virus Reference Laboratory|Irish Coronavirus Sequencing Consortium - Helixworks|Chalapati|Ireland
>Spike|hCoV-19/USA/WI-UW-898/2020|2020-07-15|EPI_ISL_509900|Original|hCoV-19^^Wisconsin|Human|University of Wisconsin-Madison AIDS Vaccine Research Laboratories|University of Wisconsin-Madison AIDS Vaccine Research Laboratories|Baczenas|USA
>Spike|hCoV-19/England/20132018004/2020|2020-03-22|EPI_ISL_423731|Original|hCoV-19^^England|Human|Respiratory Virus Unit|Respiratory Virus Unit|Platt|United Kingdom
>Spike|hCoV-19/England/CAMC-A5952D/2020|2020-10-08|EPI_ISL_597044|Original|hCoV-19^^England|Human|Lighthouse Lab in Cambridge|Wellcome Sanger Institute for the COVID-19 Genomics UK (COG-UK) consortium|Davies|United Kingdom
>Spike|hCoV-19/Ireland/MH-NVRL-76IRL30430/2020|2020-10-18|EPI_ISL_732473|Original|hCoV-19^^Meath|Human|National Virus Reference Laboratory|National Virus Reference Laboratory|Gonzalez|Ireland
>Spike|hCoV-19/South Korea/KCDC2728/2020|2020-07-13|EPI_ISL_522507|Original|hCoV-19^^South Korea|Human|Division of Viral Diseases|Division of Viral Diseases|Kim|South Korea
>Spike|hCoV-19/Australia/NSW2252/2020|2020-03-22|EPI_ISL_500691|Original|hCoV-19^^New South Wales|Human|Area of Virology|Area of Virology|Bull|Australia
>Spike|hCoV-19/England/ALDP-B3E657/2020|2020-09-30|EPI_ISL_645473|Original|hCoV-19^^England|Human|Lighthouse Lab in Alderley Park|Wellcome Sanger Institute for the COVID-19 Genomics UK (COG-UK) Consortium|Davies|United Kingdom
>Spike|hCoV-19/England/QEUH-AAA99A/2020|2020-10-21|EPI_ISL_652723|Original|hCoV-19^^England|Human|Oxford Viromics|COVID-19 Genomics UK (COG-UK) Consortium|Golubchik|United Kingdom
>Spike|hCoV-19/USA/WA-UW-3984/2020|2020-03-29|EPI_ISL_427225|Original|hCoV-19^^Washington|Human|UW Virology Lab|UW Virology Lab|Roychoudhury|USA
>Spike|hCoV-19/USA/MD-MDH-0536/2020|2020-11-30|EPI_ISL_778912|Original|hCoV-19^^Maryland|Human|Maryland Public Health Laboratory|Maryland Public Health Laboratory|Keller|USA
>Spike|hCoV-19/England/QEUH-9DABE7/2020|2020-09-22|EPI_ISL_581077|Original|hCoV-19^^England|Human|Lighthouse Lab in Glasgow|Wellcome Sanger Institute for the COVID-19 Genomics UK (COG-UK) consortium|Davies|United Kingdom

>Spike|hCoV-19/England/QEUH-CC9B24/2020|2020-12-23|EPI_ISL_781918|Original|hCoV-19^^England|Human|Lighthouse Lab in Glasgow|Wellcome Sanger Institute for the COVID-19 Genomics UK (COG-UK) Consortium|Davies|United Kingdom
>Spike|hCoV-19/England/ALDP-CECA97/2020|2020-12-21|EPI_ISL_782242|Original|hCoV-19^^England|Human|Lighthouse Lab in Alderley Park|Wellcome Sanger Institute for the COVID-19 Genomics UK (COG-UK) Consortium|Davies|United Kingdom
>Spike|hCoV-19/India/WB-IK65/2020|2020-05-12|EPI_ISL_508389|Original|hCoV-19^^West Bengal|Human|Institute of Post Graduate Medical Education & Research|National Institute of Biomedical Genomics|Maitra|India
>Spike|hCoV-19/USA/SC-DHEC-1129/2020|2020-07-10|EPI_ISL_574574|Original|hCoV-19^^South Carolina|Human|Microbiology Division|Microbiology Division|Flores|USA
>Spike|hCoV-19/USA/CA-CZB-2525/2020|2020-04-29|EPI_ISL_513900|Original|hCoV-19^^California|Human|UCSF Clinical Microbiology Laboratory|Chan-Zuckerberg Biohub|Kamm|USA
>Spike|hCoV-19/England/BRIS-12AB2C/2020|2020-05-01|EPI_ISL_458352|Original|hCoV-19^^England|Human|PHE South West Regional Laboratory|Wellcome Sanger Institute for the COVID-19 Genomics UK (COG-UK) consortium|Davies|United Kingdom
>Spike|hCoV-19/USA/TX-HMH-MCoV-15272/2020|2020-10-19|EPI_ISL_786756|Original|hCoV-19^^Texas|Human|Houston Methodist Hospital|Houston Methodist Hospital|Long|USA
>Spike|hCoV-19/Chile/RM-83540/2020|2020-05-31|EPI_ISL_746796|Original|hCoV-19^^Region Metropolitana de Santiago|Human|Genetica Molecular and Subdepartamento de Virologia ISP Chile|Instituto de Salud Publica de Chile|Tognarelli|Chile
>Spike|hCoV-19/England/MILK-B95CE3/2020|2020-11-14|EPI_ISL_674874|Original|hCoV-19^^England|Human|Lighthouse Lab in Milton Keynes|Wellcome Sanger Institute for the COVID-19 Genomics UK (COG-UK) Consortium|Davies|United Kingdom
>Spike|hCoV-19/USA/MI-UM-2006S000917/2020|2020-03-28|EPI_ISL_484755|Original|hCoV-19^^Michigan|Human|University of Michigan Clinical Microbiology Laboratory|Lauring Lab|Valesano|USA
>Spike|hCoV-19/Scotland/CVR3802/2020|2020-05-21|EPI_ISL_478128|Original|hCoV-19^^Scotland|Human|West of Scotland Specialist Virology Centre|COVID-19 Genomics UK (COG-UK) Consortium|Robertson|United Kingdom
>Spike|hCoV-19/England/NORT-289586/2020|2020-03-30|EPI_ISL_458657|Original|hCoV-19^^England|Human|NU-OMICS DNA Sequencing research facility|Wellcome Sanger Institute for the COVID-19 Genomics UK (COG-UK) consortium|Davies|United Kingdom
>Spike|hCoV-19/USA/AZ-TG377346/2020|2020-06-16|EPI_ISL_694621|Original|hCoV-19^^Arizona|Human|TGen North|TGen North|French|USA
>Spike|hCoV-19/England/QEUH-A0BBEC/2020|2020-10-01|EPI_ISL_599244|Original|hCoV-19^^England|Human|Lighthouse Lab in Glasgow|Wellcome Sanger Institute for the COVID-19 Genomics UK (COG-UK) consortium|Davies|United Kingdom
>Spike|hCoV-19/USA/MN-MDH-1687/2020|2020-04-08|EPI_ISL_560774|Original|hCoV-19^^Minnesota|Human|Minnesota Department of Health|Minnesota Department of Health|Garfin|USA
>Spike|hCoV-19/USA/WI-WSLH-200720/2020|2020-06-29|EPI_ISL_631476|Original|hCoV-19^^Wisconsin|Human|Wisconsin State Laboratory of Hygiene Communicable Disease Division|Wisconsin State Laboratory of Hygiene Communicable Disease Division|Florek|USA
>Spike|hCoV-19/England/LOND-D4A25/2020|2020-04-10|EPI_ISL_444170|Original|hCoV-19^^England|Human|University College London|COVID-19 Genomics UK (COG-UK) Consortium|Roy|United Kingdom
>Spike|hCoV-19/Wales/PHWC-347D1/2020|2020-04-16|EPI_ISL_446976|Original|hCoV-19^^Wales|Human|Wales Specialist Virology Centre|Public Health Wales Microbiology Cardiff|Moore|United Kingdom
>Spike|hCoV-19/England/ALDP-5BD42F/2020|2020-06-21|EPI_ISL_558832|Original|hCoV-19^^England|Human|Lighthouse Lab in Alderley Park|Wellcome Sanger Institute for the COVID-19 Genomics UK (COG-UK) consortium|Davies|United Kingdom
>Spike|hCoV-19/USA/CA-CZB-1011/2020|2020-04-05|EPI_ISL_436652|Original|hCoV-19^^California|Human|County of Santa Clara Public Health Department|Chan-Zuckerberg Biohub|Hao|USA

>Spike|hCoV-19/Singapore/591/2020|2020-06-26|EPI_ISL_483614|Original|hCoV-19^^Singapore|Human|National Public Health Laboratory|National Public Health Laboratory|Mak|Singapore
>Spike|hCoV-19/England/NOTT-113EE1/2020|2020-10-01|EPI_ISL_577121|Original|hCoV-19^^England|Human|Queens Medical Centre|COVID-19 Genomics UK (COG-UK) Consortium|Loose|United Kingdom
>Spike|hCoV-19/England/ALDP-C39CC2/2020|2020-12-09|EPI_ISL_731019|Original|hCoV-19^^England|Human|Lighthouse Lab in Alderley Park|Wellcome Sanger Institute for the COVID-19 Genomics UK (COG-UK) Consortium|Davies|United Kingdom
>Spike|hCoV-19/England/CAMC-B08146/2020|2020-10-29|EPI_ISL_643651|Original|hCoV-19^^England|Human|Lighthouse Lab in Cambridge|Wellcome Sanger Institute for the COVID-19 Genomics UK (COG-UK) Consortium|Davies|United Kingdom
>Spike|hCoV-19/USA/NY-NYCPHL-000999/2020|2020-10-01|EPI_ISL_633022|Original|hCoV-19^^New York|Human|DOHMH Morrisania|New York City Public Health Laboratory|Wang|USA
>Spike|hCoV-19/Spain/AS-HUCA-232017735/2020|2020-03-13|EPI_ISL_710487|Original|hCoV-19^^Asturias|Human|Virology (microbiology)|Virology (microbiology)|Boga|Spain
>Spike|hCoV-19/Australia/VIC8761/2020|2020-07-29|EPI_ISL_565181|Original|hCoV-19^^Victoria|Human|Victorian Infectious Diseases Reference Laboratory (VIDRL)|VIDRL and MDU-PHL|Schultz|Australia
>Spike|hCoV-19/England/ALDP-955847/2020|2020-06-12|EPI_ISL_554453|Original|hCoV-19^^England|Human|Lighthouse Lab in Alderley Park|Wellcome Sanger Institute for the COVID-19 Genomics UK (COG-UK) consortium|Davies|United Kingdom
>Spike|hCoV-19/Canada/NB_247/2020|2020-03-31|EPI_ISL_481745|Original|hCoV-19^^New Brunswick|Human|Dr. Georges-L.-Dumont University Hospital Centre|National Microbiology Laboratory|Grudeski|Canada
>Spike|hCoV-19/USA/CA-CZB-13743/2020|2020-09-10|EPI_ISL_739583|Original|hCoV-19^^California|Human|Alameda County Public Health Lab|Chan-Zuckerberg Biohub|Kamm|USA
>Spike|hCoV-19/Luxembourg/LNS1997895/2020|2020-11-04|EPI_ISL_740033|Original|hCoV-19^^Luxembourg|Human|Laboratoire national de sant\u00e9|Laboratoire national de sant\u00e9|Wienecke-Baldacchino|Luxembourg
>Spike|hCoV-19/England/OXON-B46BE/2020|2020-04-03|EPI_ISL_534966|Original|hCoV-19^^England|Human|Oxford Viromics|COVID-19 Genomics UK (COG-UK) Consortium|Golubchik|United Kingdom
>Spike|hCoV-19/England/QEUH-CAA52B/2020|2020-12-20|EPI_ISL_760843|Original|hCoV-19^^England|Human|Lighthouse Lab in Glasgow|Wellcome Sanger Institute for the COVID-19 Genomics UK (COG-UK) Consortium|Davies|United Kingdom
>Spike|hCoV-19/Australia/VIC6452/2020|2020-07-21|EPI_ISL_521230|Original|hCoV-19^^Victoria|Human|Microbiological Diagnostic Unit - Public Health Laboratory (MDU-PHL)|MDU-PHL|Schultz|Australia
>Spike|hCoV-19/USA/WI-UW-2494/2020|2020-12-30|EPI_ISL_779326|Original|hCoV-19^^Wisconsin|Human|University of Wisconsin-Madison AIDS Vaccine Research Laboratories|University of Wisconsin-Madison AIDS Vaccine Research Laboratories|Moreno|USA
>Spike|hCoV-19/India/TG-NGC-CDFD-13/2020|2020-05-27|EPI_ISL_466851|Original|hCoV-19^^Telangana|Human|National Genomics Core-Center for DNA Fingerprinting and Diagnostics|National Genomics Core- Center for DNA Fingerprinting and Diagnostics (NGC-CDFD)- DBT's PAN-INDIA-1000 Genome consortium|Vashisht|India
>Spike|hCoV-19/USA/MD-MDH-0133/2020|2020-07-10|EPI_ISL_524897|Original|hCoV-19^^Maryland|Human|MD PHL|MD PHL|Keller|USA
>Spike|hCoV-19/England/QEUH-9D0EF7/2020|2020-09-14|EPI_ISL_589902|Original|hCoV-19^^England|Human|Lighthouse Lab in Glasgow|Wellcome Sanger Institute for the COVID-19 Genomics UK (COG-UK) consortium|Davies|United Kingdom
>Spike|hCoV-19/USA/VAN5074/2020|2020-09-14|EPI_ISL_677696|Original|hCoV-19^^New York|Human|Vanda Pharmaceuticals Clinical Site|Vanda Pharmaceuticals|Przychodzen|USA
>Spike|hCoV-19/England/ALDP-8B465C/2020|2020-06-29|EPI_ISL_557006|Original|hCoV-19^^England|Human|Lighthouse Lab in Alderley Park|Wellcome Sanger Institute for the COVID-19 Genomics UK (COG-UK) consortium|Davies|United Kingdom
>Spike|hCoV-19/USA/MA-MASPHL-00411/2020|2020-04-26|EPI_ISL_693138|Original|hCoV-19^^Massachusetts|Human|Massachusetts State Public Health Laboratory|Massachusetts State Public Health Laboratory|Lang|USA

>Spike|hCoV-19/USA/TX-HMH-MCoV-19453/2020|2020-11-30|EPI_ISL_783849|Original|hCoV-19^^Texas|Human|Houston Methodist Hospital|Houston Methodist Hospital|Long|USA
>Spike|hCoV-19/USA/TX-HMH-MCoV-8272/2020|2020-07-05|EPI_ISL_784302|Original|hCoV-19^^Texas|Human|Houston Methodist Hospital|Houston Methodist Hospital|Long|USA
>Spike|hCoV-19/England/ALDP-A928D4/2020|2020-10-20|EPI_ISL_611054|Original|hCoV-19^^England|Human|Lighthouse Lab in Alderley Park|Wellcome Sanger Institute for the COVID-19 Genomics UK (COG-UK) consortium|Davies|United Kingdom
>Spike|hCoV-19/Denmark/DCGC-11173/2020|2020-11-02|EPI_ISL_670437|Original|hCoV-19^^Syddanmark|Human|Department of Virus and Microbiological Special Diagnostics|Albertsen Lab|Michaelsen|Denmark
>Spike|hCoV-19/England/ALDP-C49C56/2020|2020-12-12|EPI_ISL_718919|Original|hCoV-19^^England|Human|Lighthouse Lab in Alderley Park|Wellcome Sanger Institute for the COVID-19 Genomics UK (COG-UK) Consortium|Davies|United Kingdom
>Spike|hCoV-19/United Arab Emirates/1044/2020|2020-06-11|EPI_ISL_698119|Original|hCoV-19^^Abu Dhabi|Human|Group 42 (G42) Healthcare|G42 Healthcare|Ogrodzki|United Arab Emirates
>Spike|hCoV-19/USA/NY-NYUMC216/2020|2020-04-03|EPI_ISL_427511|Original|hCoV-19^^New York|Human|NYU Langone Health|Departments of Pathology and Medicine|Maurano|USA
>Spike|hCoV-19/England/CAMC-B32F1C/2020|2020-11-05|EPI_ISL_659285|Original|hCoV-19^^England|Human|Lighthouse Lab in Cambridge|Wellcome Sanger Institute for the COVID-19 Genomics UK (COG-UK) Consortium|Davies|United Kingdom
>Spike|hCoV-19/Singapore/201/2020|2020-04-24|EPI_ISL_462319|Original|hCoV-19^^Singapore|Human|National Public Health Laboratory|National Public Health Laboratory|Mak|Singapore
>Spike|hCoV-19/Denmark/DCGC-2056/2020|2020-07-20|EPI_ISL_618279|Original|hCoV-19^^Sjaelland|Human|Department of Virus and Microbiological Special Diagnostics|Albertsen lab|Michaelsen|Denmark
>Spike|hCoV-19/Germany/HH-hpi-p1595/2020|2020-03-16|EPI_ISL_775743|Original|hCoV-19^^Hamburg|Human|University Medical Center Hamburg Eppendorf|Heinrich Pette Institute|Robitaille|Germany
>Spike|hCoV-19/Norway/CS-12/2020|2020-04-14|EPI_ISL_666568|Original|hCoV-19^^Viken|Human|Dept. of Microbiology and Infection Control|Dept. of Microbiology and Infection Control|Hesselberg L\u00f8vestad|Norway
>Spike|hCoV-19/England/CAMC-A655F1/2020|2020-10-11|EPI_ISL_608388|Original|hCoV-19^^England|Human|Lighthouse Lab in Cambridge|Wellcome Sanger Institute for the COVID-19 Genomics UK (COG-UK) consortium|Davies|United Kingdom
>Spike|hCoV-19/England/QEUH-B0E4A1/2020|2020-11-02|EPI_ISL_642206|Original|hCoV-19^^England|Human|Lighthouse Lab in Glasgow|Wellcome Sanger Institute for the COVID-19 Genomics UK (COG-UK) Consortium|Davies|United Kingdom
>Spike|hCoV-19/Saudi Arabia/KAUST-Makkah177/2020|2020-04-06|EPI_ISL_437700|Original|hCoV-19^^Makkah|Human|Pathogen Genomics Lab King Abdullah University of Science and Technology(KAUST)|Pathogen Genomics Lab King Abdullah University of Science and Technology(KAUST)|Mohamed Ghazzali|Saudi Arabia
>Spike|hCoV-19/Gambia/NPHL-737/2020|2020-00-00|EPI_ISL_561323|Original|hCoV-19^^Kombo|Human|MRCG at LSHTM Genomics lab|MRCG at LSHTM Genomics lab|Sesay|Gambia
>Spike|hCoV-19/Italy/CAM-INTPas-18/2020|2020-03-19|EPI_ISL_775246|Original|hCoV-19^^Campania|Human|INT Fondazione Pascale|INT Fondazione Pascale|Rachiglio|Italy
>Spike|hCoV-19/Scotland/QEUH-BEAF40/2020|2020-12-04|EPI_ISL_710634|Original|hCoV-19^^Scotland|Human|Lighthouse Lab in Glasgow|Wellcome Sanger Institute for the COVID-19 Genomics UK (COG-UK) Consortium|Davies|United Kingdom
>Spike|hCoV-19/Netherlands/Zeeland_11/2020|2020-04-10|EPI_ISL_461249|Original|hCoV-19^^Zeeland|Human|Dutch COVID-19 response team|Erasmus Medical Center|Oude Munnink|Netherlands
>Spike|hCoV-19/Germany/BY-MVP-0247/2020|2020-04-30|EPI_ISL_466902|Original|hCoV-19^^Bavaria|Human|Max von Pettenkofer Institute|Laboratory for Functional Genome Analysis|Graf|Germany
>Spike|hCoV-19/Netherlands/Flevoland_8/2020|2020-04-18|EPI_ISL_460842|Original|hCoV-19^^Flevoland|Human|Dutch COVID-19 response team|Erasmus Medical Center|Oude Munnink|Netherlands

>Spike|hCoV-19/Brazil/SP-627/2020|2020-03-23|EPI_ISL_515541|Original|hCoV-19^^S\u00e3o Paulo|Human|Hospital Montemagno|Instituto Adolfo Lutz|Sacchi|Brazil
>Spike|hCoV-19/England/20126003902/2020|2020-03-19|EPI_ISL_418744|Original|hCoV-19^^England|Human|Respiratory Virus Unit|Respiratory Virus Unit|Platt|United Kingdom
>Spike|hCoV-19/Spain/VC-IBV-98004423/2020|2020-04-13|EPI_ISL_583922|Original|hCoV-19^^Comunitat_Valenciana|Human|Servicio de Microbiolog\u00eda. Hospital General Universitario de Castell\u00f3n|SeqCOVID-SPAIN consortium/IBV(CSIC)|Comas|Spain
>Spike|hCoV-19/Australia/VIC13601/2020|2020-08-13|EPI_ISL_563777|Original|hCoV-19^^Victoria|Human|Microbiological Diagnostic Unit - Public Health Laboratory (MDU-PHL)|MDU-PHL|Schultz|Australia
>Spike|hCoV-19/Scotland/QEUH-96E4FC/2020|2020-08-20|EPI_ISL_534467|Original|hCoV-19^^Scotland|Human|NHSGGC West of Scotland Specialist Virology Centre / MRC-University of Glasgow Centre for Virus Research|Wellcome Sanger Institute for the COVID-19 Genomics UK (COG-UK) consortium|Davies|United Kingdom
>Spike|hCoV-19/Croatia/Zagreb-11/2020|2020-09-30|EPI_ISL_710569|Original|hCoV-19^^Zagreb|Human|University Hospital Dubrava|Ru\u0111er Bo\u0161kovic Institute; Forensic Science Centre Ivan Vu\u010deti\u0107; University of Zagreb Faculty of Science|Karlic|Croatia
>Spike|hCoV-19/Australia/VIC14387/2020|2020-09-03|EPI_ISL_592480|Original|hCoV-19^^Victoria|Human|Microbiological Diagnostic Unit - Public Health Laboratory (MDU-PHL)|MDU-PHL|Schultz|Australia
>Spike|hCoV-19/England/ALDP-CB6629/2020|2020-12-18|EPI_ISL_761911|Original|hCoV-19^^England|Human|Lighthouse Lab in Alderley Park|Wellcome Sanger Institute for the COVID-19 Genomics UK (COG-UK) Consortium|Davies|United Kingdom
>Spike|hCoV-19/Hong Kong/VM20037427/2020|2020-07-12|EPI_ISL_523838|Original|hCoV-19^^Hong Kong|Human|Princess Margaret Hospital|Hong Kong Department of Health|Mak|China
>Spike|hCoV-19/USA/NY-Wadsworth-292756-01/2020|2020-12-21|EPI_ISL_769933|Original|hCoV-19^^New York|Human|Albany Medical Center Hospital Clinical Laboratories|Wadsworth Center|Lasek-Nesselquist|USA
>Spike|hCoV-19/France/GES-8789/2020|2020-03-28|EPI_ISL_560627|Original|hCoV-19^^Grand-Est|Human|Hospital|National Reference Center for Viruses of Respiratory Infections|Enouf|France
>Spike|hCoV-19/England/EXET-13F465/2020|2020-12-16|EPI_ISL_763412|Original|hCoV-19^^England|Human|University of Exeter|COVID-19 Genomics UK (COG-UK) Consortium|Temperton|United Kingdom
>Spike|hCoV-19/England/ALDP-9EA7D1/2020|2020-09-24|EPI_ISL_580198|Original|hCoV-19^^England|Human|Lighthouse Lab in Alderley Park|Wellcome Sanger Institute for the COVID-19 Genomics UK (COG-UK) consortium|Davies|United Kingdom
>Spike|hCoV-19/France/BRE-5458/2020|2020-03-22|EPI_ISL_443282|Original|hCoV-19^^Bretagne|Human|CHU - H\u00f4pital Cavale Blanche - Labo. de Virologie|National Reference Center for Viruses of Respiratory Infections|Enouf|France
>Spike|hCoV-19/England/QEUH-BEAFE6/2020|2020-12-03|EPI_ISL_708855|Original|hCoV-19^^England|Human|Lighthouse Lab in Glasgow|Wellcome Sanger Institute for the COVID-19 Genomics UK (COG-UK) Consortium|Davies|United Kingdom
>Spike|hCoV-19/England/LOND-12673BE/2020|2020-12-08|EPI_ISL_741281|Original|hCoV-19^^England|Human|University College London|COVID-19 Genomics UK (COG-UK) Consortium|Roy|United Kingdom
>Spike|hCoV-19/England/QEUH-AD35B2/2020|2020-10-25|EPI_ISL_634668|Original|hCoV-19^^England|Human|Lighthouse Lab in Glasgow|Wellcome Sanger Institute for the COVID-19 Genomics UK (COG-UK) consortium|Davies|United Kingdom
>Spike|hCoV-19/Brazil/SP-820/2020|2020-04-24|EPI_ISL_776755|Original|hCoV-19^^S\u00e3o Paulo|Human|Instituto Adolfo Lutz - Central|Instituto Adolfo Lutz|Sacchi|Brazil
>Spike|hCoV-19/England/MILK-957283/2020|2020-06-13|EPI_ISL_554143|Original|hCoV-19^^England|Human|Lighthouse Lab in Milton Keynes|Wellcome Sanger Institute for the COVID-19 Genomics UK (COG-UK) consortium|Davies|United Kingdom
>Spike|hCoV-19/Japan/PG-2466/2020|2020-04-00|EPI_ISL_686071|Original|hCoV-19^^Japan|Human|Pathogen Genomics Center|Pathogen Genomics Center|Sekizuka|Japan

>Spike|hCoV-19/USA/CA-CZB-2760/2020|2020-06-27|EPI_ISL_582865|Original|hCoV-19^^California|Human|County of Santa Clara Public Health Department|Chan-Zuckerberg Biohub|Bhatt|USA
>Spike|hCoV-19/Italy/CAM-IZSM-183/2020|2020-09-12|EPI_ISL_584072|Original|hCoV-19^^Campania|Human|IZSM|IZSM|Viscardi|Italy
>Spike|hCoV-19/Spain/PV-IBV-004302/2020|2020-04-09|EPI_ISL_510417|Original|hCoV-19^^Basque Country|Human|Servicio de Microbiolog\u00eda. Hospital Universitario Donostia. OSI Donostialdea. \u00c1rea de Enfermedades Infecciosas|SeqCOVID-SPAIN consortium/IBV(CSIC)|Comas|Spain
>Spike|hCoV-19/England/LIVE-A60DF/2020|2020-03-21|EPI_ISL_499683|Original|hCoV-19^^England|Human|Liverpool Clinical Laboratories|COVID-19 Genomics UK (COG-UK) Consortium|Haldenby|United Kingdom
>Spike|hCoV-19/Wales/PHWC-31B14/2020|2020-04-11|EPI_ISL_446531|Original|hCoV-19^^Wales|Human|Wales Specialist Virology Centre|Public Health Wales Microbiology Cardiff|Moore|United Kingdom
>Spike|hCoV-19/England/MILK-4673CE/2020|2020-05-21|EPI_ISL_559729|Original|hCoV-19^^England|Human|Lighthouse Lab in Milton Keynes|Wellcome Sanger Institute for the COVID-19 Genomics UK (COG-UK) consortium|Davies|United Kingdom
>Spike|hCoV-19/Denmark/DCGC-3634/2020|2020-08-31|EPI_ISL_616405|Original|hCoV-19^^Hovedstaden|Human|Department of Virus and Microbiological Special Diagnostics|Albertsen lab|Michaelsen|Denmark
>Spike|hCoV-19/England/ALDP-B62AA9/2020|2020-11-09|EPI_ISL_656445|Original|hCoV-19^^England|Human|Lighthouse Lab in Alderley Park|Wellcome Sanger Institute for the COVID-19 Genomics UK (COG-UK) Consortium|Davies|United Kingdom
>Spike|hCoV-19/USA/NY-Wadsworth-200232-01/2020|2020-07-05|EPI_ISL_677066|Original|hCoV-19^^New York|Human|Masonic Medical Research Institute|Wadsworth Center|Lasek-Nesselquist|USA
>Spike|hCoV-19/Italy/APU-IZSPB_313PT/2020|2020-11-02|EPI_ISL_653807|Original|hCoV-19^^Apulia|Human|I.R.C.C.S. \S. De Bellis\ - Ente Ospedaliero|Istituto Zooprofilattico Sperimentale della Puglia e della Basilicata|Parisi|Italy
>Spike|hCoV-19/USA/WA-QDX-917/2020|2020-03-21|EPI_ISL_571691|Original|hCoV-19^^Washington|Human|Quest Diagnostics|Quest Diagnostics|Kagan|USA
>Spike|hCoV-19/Russia/OMS-ORINFI-7121S/2020|2020-05-14|EPI_ISL_569742|Original|hCoV-19^^Omsk|Human|Omsk Research Institute of Natural Focal Infections|WHO National Influenza Centre Russian Federation|Komissarov|Russia
>Spike|hCoV-19/Australia/SAP044/2020|2020-03-22|EPI_ISL_451084|Original|hCoV-19^^South Australia|Human|SA Pathology|SA Pathology|Leong|Australia
>Spike|hCoV-19/Denmark/DCGC-22495/2020|2020-12-14|EPI_ISL_757593|Original|hCoV-19^^Hovedstaden|Human|Department of Virus and Microbiological Special Diagnostics|Albertsen Lab|Michaelsen|Denmark
>Spike|hCoV-19/USA/LA-EVTL1319/2020|2020-12-11|EPI_ISL_778985|Original|hCoV-19^^Louisiana|Human|LSUHS Emerging Viral Threat Laboratory|Microbial Genome Sequencing Center|Kamil|USA
>Spike|hCoV-19/England/QEUH-5EDFDA/2020|2020-06-16|EPI_ISL_533238|Original|hCoV-19^^England|Human|Lighthouse Lab in Glasgow|Wellcome Sanger Institute for the COVID-19 Genomics UK (COG-UK) consortium|Davies|United Kingdom
>Spike|hCoV-19/USA/TX-HMH-5291/2020|2020-06-23|EPI_ISL_544657|Original|hCoV-19^^Texas|Human|Houston Methodist Hospital|Houston Methodist Hospital|Long|USA
>Spike|hCoV-19/Denmark/DCGC-9991/2020|2020-11-09|EPI_ISL_669315|Original|hCoV-19^^Midtjylland|Human|Department of Virus and Microbiological Special Diagnostics|Albertsen Lab|Michaelsen|Denmark
>Spike|hCoV-19/England/CAMC-B53926/2020|2020-11-08|EPI_ISL_656718|Original|hCoV-19^^England|Human|Lighthouse Lab in Cambridge|Wellcome Sanger Institute for the COVID-19 Genomics UK (COG-UK) Consortium|Davies|United Kingdom
>Spike|hCoV-19/England/QEUH-AA04EE/2020|2020-10-19|EPI_ISL_652709|Original|hCoV-19^^England|Human|University of Exeter|COVID-19 Genomics UK (COG-UK) Consortium|Temperton|United Kingdom
>Spike|hCoV-19/Denmark/DCGC-19854/2020|2020-12-07|EPI_ISL_747830|Original|hCoV-19^^Sjaelland|Human|Department of Virus and Microbiological Special Diagnostics|Albertsen Lab|Michaelsen|Denmark

>Spike|hCoV-19/USA/FL-05-UMTL-9TXV/2020|2020-05-21|EPI_ISL_479764|Original|hCoV-19^^Florida|Human|University of Miami Immunology and Histocompatibility Laboratory|University of Miami Immunology and Histocompatibility Laboratory|Margolles-Clark|USA
>Spike|hCoV-19/USA/AZ-TG320609/2020|2020-05-16|EPI_ISL_694331|Original|hCoV-19^^Arizona|Human|TGen North|TGen North|French|USA
>Spike|hCoV-19/USA/MI-UM-10036567026/2020|2020-10-22|EPI_ISL_640211|Original|hCoV-19^^Michigan|Human|University of Michigan Clinical Microbiology Laboratory|Lauring Lab|Valesano|USA
>Spike|hCoV-19/USA/MI-MDHHS-SC22339/2020|2020-11-02|EPI_ISL_708319|Original|hCoV-19^^Michigan|Human|Michigan Department of Health and Human Services|Michigan Department of Health and Human Services|Jacob|USA
>Spike|hCoV-19/Ireland/LS-NVRL-78IRL64932/2020|2020-11-24|EPI_ISL_752583|Original|hCoV-19^^Laois|Human|National Virus Reference Laboratory|National Virus Reference Laboratory|Gonzalez|Ireland
>Spike|hCoV-19/England/EXET-13848A/2020|2020-09-13|EPI_ISL_611619|Original|hCoV-19^^England|Human|University of Exeter|COVID-19 Genomics UK (COG-UK) Consortium|Temperton|United Kingdom
>Spike|hCoV-19/Spain/CT-IBV-97007967/2020|2020-10-06|EPI_ISL_654514|Original|hCoV-19^^Catalunya|Human|Servicio de Microbiolog\u00eda|SeqCOVID-SPAIN consortium/IBV(CSIC)|Comas|Spain
>Spike|hCoV-19/Switzerland/VS-ETHZ-350000/2020|2020-11-03|EPI_ISL_693775|Original|hCoV-19^^Valais|Human|Viollier AG|Department of Biosystems Science and Engineering|Nadeau|Switzerland
>Spike|hCoV-19/Denmark/DCGC-7664/2020|2020-10-19|EPI_ISL_618567|Original|hCoV-19^^Hovedstaden|Human|Department of Virus and Microbiological Special Diagnostics|Albertsen lab|Michaelsen|Denmark
>Spike|hCoV-19/Singapore/578/2020|2020-04-21|EPI_ISL_483601|Original|hCoV-19^^Singapore|Human|National Public Health Laboratory|National Public Health Laboratory|Mak|Singapore
>Spike|hCoV-19/USA/TX-HMH-MCoV-10775/2020|2020-07-05|EPI_ISL_789620|Original|hCoV-19^^Texas|Human|Houston Methodist Hospital|Houston Methodist Hospital|Long|USA
>Spike|hCoV-19/England/CAMC-B538CF/2020|2020-11-08|EPI_ISL_656817|Original|hCoV-19^^England|Human|Lighthouse Lab in Cambridge|Wellcome Sanger Institute for the COVID-19 Genomics UK (COG-UK) Consortium|Davies|United Kingdom
>Spike|hCoV-19/Wales/PHWC-4839E8/2020|2020-10-04|EPI_ISL_626733|Original|hCoV-19^^Wales|Human|Wales Specialist Virology Centre Sequencing lab|COVID-19 Genomics UK (COG-UK) Consortium|Moore|United Kingdom
>Spike|hCoV-19/France/ARA-22088/2020|2020-04-08|EPI_ISL_508996|Original|hCoV-19^^Auvergne-Rh\u00f4ne-Alpes|Human|CNR Virus des Infections Respiratoires - France SUD|CNR Virus des Infections Respiratoires - France SUD|Josset|France
>Spike|hCoV-19/England/ALDP-CC1D9F/2020|2020-12-19|EPI_ISL_760557|Original|hCoV-19^^England|Human|Lighthouse Lab in Alderley Park|Wellcome Sanger Institute for the COVID-19 Genomics UK (COG-UK) Consortium|Davies|United Kingdom
>Spike|hCoV-19/England/NORT-29DD4B/2020|2020-05-07|EPI_ISL_484316|Original|hCoV-19^^England|Human|Northumbria University / South Tees Hospitals NHS Foundation Trust / North Cumbria Integrated Care NHS Foundation Trust / North Tees and Hartlepool NHS Foundation Trust / Newcastle Hospitals NHS Foundation Trust|COVID-19 Genomics UK (COG-UK) Consortium|Bashton|United Kingdom
>Spike|hCoV-19/England/ALDP-B5CA93/2020|2020-11-08|EPI_ISL_655814|Original|hCoV-19^^England|Human|Lighthouse Lab in Alderley Park|Wellcome Sanger Institute for the COVID-19 Genomics UK (COG-UK) Consortium|Davies|United Kingdom
>Spike|hCoV-19/Italy/VEN-IZSVe-20-25/2020|2020-03-24|EPI_ISL_636471|Original|hCoV-19^^Veneto|Human|ULSS6 Piove di Sacco|Istituto Zooprofilattico Sperimentale delle Venezie|Quaranta|Italy
>Spike|hCoV-19/England/CAMB-1AE70E/2020|2020-04-30|EPI_ISL_524583|Original|hCoV-19^^England|Human|Department of Pathology|Wellcome Sanger Institute for the COVID-19 Genomics UK (COG-UK) consortium|Davies|United Kingdom
>Spike|hCoV-19/USA/CA-UCSF-UC44/2020|2020-03-19|EPI_ISL_450236|Original|hCoV-19^^California|Human|UCSF Clinical Microbiology Laboratory|Chiu Laboratory|Chiu|USA

>Spike|hCoV-19/Wales/PHWC-491CC0/2020|2020-11-12|EPI_ISL_705420|Original|hCoV-19^^Wales|Human|Originating lab|COVID-19 Genomics UK (COG-UK) Consortium|Moore|United Kingdom
>Spike|hCoV-19/USA/MI-MDHHS-SC21294/2020|2020-04-11|EPI_ISL_507549|Original|hCoV-19^^Michigan|Human|Michigan Department of Health and Human Services|Michigan Department of Health and Human Services|Blankenship|USA
>Spike|hCoV-19/England/CAMC-B98EAE/2020|2020-11-15|EPI_ISL_673279|Original|hCoV-19^^England|Human|Lighthouse Lab in Cambridge|Wellcome Sanger Institute for the COVID-19 Genomics UK (COG-UK) Consortium|Davies|United Kingdom
>Spike|hCoV-19/England/MILK-B943EF/2020|2020-11-14|EPI_ISL_676134|Original|hCoV-19^^England|Human|Lighthouse Lab in Milton Keynes|Wellcome Sanger Institute for the COVID-19 Genomics UK (COG-UK) Consortium|Davies|United Kingdom
>Spike|hCoV-19/England/PORT-2D0EC1/2020|2020-04-24|EPI_ISL_475301|Original|hCoV-19^^England|Human|Centre for Enzyme Innovation|COVID-19 Genomics UK (COG-UK) Consortium|Robson|United Kingdom
>Spike|hCoV-19/England/QEUH-A8CA40/2020|2020-10-16|EPI_ISL_643212|Original|hCoV-19^^England|Human|Lighthouse Lab in Glasgow|Wellcome Sanger Institute for the COVID-19 Genomics UK (COG-UK) Consortium|Davies|United Kingdom
>Spike|hCoV-19/USA/WA-UW-4917/2020|2020-04-07|EPI_ISL_461404|Original|hCoV-19^^Washington|Human|UW Virology Lab|UW Virology Lab|Roychoudhury|USA
>Spike|hCoV-19/England/ALDP-B83134/2020|2020-11-14|EPI_ISL_675561|Original|hCoV-19^^England|Human|Lighthouse Lab in Alderley Park|Wellcome Sanger Institute for the COVID-19 Genomics UK (COG-UK) Consortium|Davies|United Kingdom
>Spike|hCoV-19/England/LIVE-9A681/2020|2020-03-30|EPI_ISL_449359|Original|hCoV-19^^England|Human|Liverpool Clinical Laboratories|COVID-19 Genomics UK (COG-UK) Consortium|Haldenby|United Kingdom
>Spike|hCoV-19/England/ALDP-95C479/2020|2020-06-20|EPI_ISL_554947|Original|hCoV-19^^England|Human|Lighthouse Lab in Alderley Park|Wellcome Sanger Institute for the COVID-19 Genomics UK (COG-UK) consortium|Davies|United Kingdom
>Spike|hCoV-19/USA/TX-DSHS-0330/2020|2020-06-13|EPI_ISL_534297|Original|hCoV-19^^Texas|Human|Texas Department of State Health Services|Texas Department of State Health Services|Rahman|USA
>Spike|hCoV-19/France/PAC-IHU-1087/2020|2020-08-05|EPI_ISL_569049|Original|hCoV-19^^Provence-Alpes-C\u00f4te-d\u2019Azur|Human|MEPHI|MEPHI|Levasseur|France
>Spike|hCoV-19/Italy/ABR-IZSGC-TE9047/2020|2020-04-01|EPI_ISL_529009|Original|hCoV-19^^Abruzzo|Human|Ospedale Civile S. Liberatore-Atri|Istitituto Zooprofilattico Sperimentale dell'Abruzzo e Molise \G.Caporale\|Lorusso|Italy
>Spike|hCoV-19/England/CAMC-B98020/2020|2020-11-15|EPI_ISL_675064|Original|hCoV-19^^England|Human|Lighthouse Lab in Cambridge|Wellcome Sanger Institute for the COVID-19 Genomics UK (COG-UK) Consortium|Davies|United Kingdom
>Spike|hCoV-19/England/ALDP-A2DCA5/2020|2020-10-05|EPI_ISL_586575|Original|hCoV-19^^England|Human|Lighthouse Lab in Alderley Park|Wellcome Sanger Institute for the COVID-19 Genomics UK (COG-UK) consortium|Davies|United Kingdom
>Spike|hCoV-19/Serbia/Pancevo-210032506/2020|2020-06-24|EPI_ISL_661184|Original|hCoV-19^^Pancevo|Human|Scientific Veterinary Institute Novi Sad|Veterinary Specialized Institute \Kraljevo\|Vidanovic|Serbia
>Spike|hCoV-19/England/PORT-2D3E0A/2020|2020-04-22|EPI_ISL_512415|Original|hCoV-19^^England|Human|Centre for Enzyme Innovation|COVID-19 Genomics UK (COG-UK) Consortium|Robson|United Kingdom
>Spike|hCoV-19/USA/UT-08628/2020|2020-05-20|EPI_ISL_524366|Original|hCoV-19^^Utah|Human|Utah Public Health Laboratory|Utah Public Health Laboratory|Young|USA
>Spike|hCoV-19/England/QEUH-B3F920/2020|2020-11-08|EPI_ISL_658474|Original|hCoV-19^^England|Human|Lighthouse Lab in Glasgow|Wellcome Sanger Institute for the COVID-19 Genomics UK (COG-UK) Consortium|Davies|United Kingdom
>Spike|hCoV-19/India/MH-BJMC-838/2020|2020-04-11|EPI_ISL_496553|Original|hCoV-19^^Maharashtra|Human|B.J. Govt. Medical College|National Centre For Cell Science|Paul|India
>Spike|hCoV-19/Spain/VC-FISABIO-657/2020|2020-03-31|EPI_ISL_455723|Original|hCoV-19^^Comunitat Valenciana|Human|Servicio de Microbiologia. Hospital Clinico

Universitario de Valencia|Sequencing and Bioinformatics Service and Molecular Epidemiology Research Group. FISABIO-Public Health|Gonzalez-Candelas|Spain
>Spike|hCoV-19/USA/VA-DCLS-2093/2020|2020-10-21|EPI_ISL_677135|Original|hCoV-19^^Virginia|Human|Virginia Division of Consolidated Laboratory Services|Virginia Division of Consolidated Laboratory Services|St. Jacques|USA
>Spike|hCoV-19/Wales/PHWC-16E852/2020|2020-10-06|EPI_ISL_585778|Original|hCoV-19^^Wales|Human|Wales Specialist Virology Centre Sequencing lab|COVID-19 Genomics UK (COG-UK) Consortium|Moore|United Kingdom
>Spike|hCoV-19/USA/NY-Wadsworth-290366-01/2020|2020-12-18|EPI_ISL_765547|Original|hCoV-19^^New York|Human|MONTEFIORE MEDICAL CENTER LABORATORIES|Wadsworth Center|Lasek-Nesselquist|USA
>Spike|hCoV-19/Japan/PG-1316/2020|2020-04-00|EPI_ISL_684924|Original|hCoV-19^^Japan|Human|Saitama Prefectural Institute of Public Health|Pathogen Genomics Center|Sekizuka|Japan
>Spike|hCoV-19/USA/LA-GBCL-26684514/2020|2020-04-10|EPI_ISL_485317|Original|hCoV-19^^Louisiana|Human|River Road Testing Lab|Ginkgo Bioworks Clinical Laboratory|Smith|USA
>Spike|hCoV-19/USA/TX-HMH-MCoV-14025/2020|2020-08-14|EPI_ISL_787314|Original|hCoV-19^^Texas|Human|Houston Methodist Hospital|Houston Methodist Hospital|Long|USA
>Spike|hCoV-19/Denmark/DCGC-14933/2020|2020-11-23|EPI_ISL_713491|Original|hCoV-19^^Hovedstaden|Human|Department of Virus and Microbiological Special Diagnostics|Albertsen Lab|Michaelsen|Denmark
>Spike|hCoV-19/Denmark/DCGC-10591/2020|2020-11-02|EPI_ISL_670074|Original|hCoV-19^^Hovedstaden|Human|Department of Virus and Microbiological Special Diagnostics|Albertsen Lab|Michaelsen|Denmark
>Spike|hCoV-19/England/ALDP-A2E2C0/2020|2020-10-06|EPI_ISL_586628|Original|hCoV-19^^England|Human|Lighthouse Lab in Alderley Park|Wellcome Sanger Institute for the COVID-19 Genomics UK (COG-UK) consortium|Davies|United Kingdom
>Spike|hCoV-19/England/ALDP-B8469E/2020|2020-11-15|EPI_ISL_675339|Original|hCoV-19^^England|Human|Lighthouse Lab in Alderley Park|Wellcome Sanger Institute for the COVID-19 Genomics UK (COG-UK) Consortium|Davies|United Kingdom
>Spike|hCoV-19/Canada/ON-PHL-20-00061/2020|2020-00-00|EPI_ISL_548737|Original|hCoV-19^^Ontario|Human|Public Health Ontario Laboratory|Public Health Ontario Laboratory|Guthrie|Canada
>Spike|hCoV-19/Wales/CAMC-A82D05/2020|2020-10-13|EPI_ISL_625288|Original|hCoV-19^^Wales|Human|Lighthouse Lab in Cambridge|Wellcome Sanger Institute for the COVID-19 Genomics UK (COG-UK) consortium|Davies|United Kingdom
>Spike|hCoV-19/USA/CA-CZB-10042/2020|2020-05-19|EPI_ISL_672198|Original|hCoV-19^^California|Human|The Ashley Laboratory|Chan-Zuckerberg Biohub|Bhatt|USA
>Spike|hCoV-19/Belgium/FAE-030948/2020|2020-03-09|EPI_ISL_420445|Original|hCoV-19^^Wandre|Human|KU Leuven|KU Leuven|Maes|Belgium
>Spike|hCoV-19/England/201061456/2020|2020-03-01|EPI_ISL_464414|Original|hCoV-19^^England|Human|Respiratory Virus Unit|Respiratory Virus Unit|Platt|United Kingdom
>Spike|hCoV-19/SaudiArabia/KAUST-MADINAH1107/2020|2020-06-11|EPI_ISL_513111|Original|hCoV-19^^Madinah|Human|Pathogen Genomics Lab King Abdullah University of Science and Technology(KAUST)|Pathogen Genomics Lab King Abdullah University of Science and Technology(KAUST)|Mohamed Ghazzali|Saudi Arabia
>Spike|hCoV-19/Japan/PG-4081/2020|2020-07-00|EPI_ISL_692322|Original|hCoV-19^^Japan|Human|Gunma Prefectural Institute of Public Health and Environmental Sciences|Pathogen Genomics Center|Sekizuka|Japan
>Spike|hCoV-19/Saudi Arabia/KAUST-Madinah258/2020|2020-03-30|EPI_ISL_437742|Original|hCoV-19^^Madinah|Human|Pathogen Genomics Lab King Abdullah University of Science and Technology(KAUST)|Pathogen Genomics Lab King Abdullah University of Science and Technology(KAUST)|Mohamed Ghazzali|Saudi Arabia
>Spike|hCoV-19/England/MILK-AB8DE8/2020|2020-10-21|EPI_ISL_623627|Original|hCoV-19^^England|Human|Lighthouse Lab in Milton Keynes|Wellcome Sanger Institute for the COVID-19 Genomics UK (COG-UK) consortium|Davies|United Kingdom
>Spike|hCoV-19/USA/CA-CZB-10953/2020|2020-09-16|EPI_ISL_583037|Original|hCoV-19^^California|Human|Orange County Public Health Lab|Chan-Zuckerberg Biohub|Bhatt|USA

>Spike|hCoV-19/England/QEUH-AAC78F/2020|2020-10-21|EPI_ISL_651105|Original|hCoV-19^^England|Human|Oxford Viromics|COVID-19 Genomics UK (COG-UK) Consortium|Golubchik|United Kingdom
>Spike|hCoV-19/Iceland/379/2020|2020-03-18|EPI_ISL_424403|Original|hCoV-19^^Reykjavik|Human|The National University Hospital of Iceland|deCODE genetics|J\u00f3nsson|Iceland
>Spike|hCoV-19/England/ALDP-A929A4/2020|2020-10-19|EPI_ISL_611145|Original|hCoV-19^^England|Human|Lighthouse Lab in Alderley Park|Wellcome Sanger Institute for the COVID-19 Genomics UK (COG-UK) consortium|Davies|United Kingdom
>Spike|hCoV-19/USA/TX-HMH-MCoV-13069/2020|2020-08-25|EPI_ISL_788220|Original|hCoV-19^^Texas|Human|Houston Methodist Hospital|Houston Methodist Hospital|Long|USA
>Spike|hCoV-19/England/ALDP-8B602F/2020|2020-06-29|EPI_ISL_557117|Original|hCoV-19^^England|Human|Lighthouse Lab in Alderley Park|Wellcome Sanger Institute for the COVID-19 Genomics UK (COG-UK) consortium|Davies|United Kingdom
>Spike|hCoV-19/Denmark/DCGC-7147/2020|2020-10-12|EPI_ISL_619167|Original|hCoV-19^^Midtjylland|Human|Department of Virus and Microbiological Special Diagnostics|Albertsen lab|Michaelsen|Denmark
>Spike|hCoV-19/England/CAMC-990F2D/2020|2020-08-28|EPI_ISL_551900|Original|hCoV-19^^England|Human|Lighthouse Lab in Cambridge|Wellcome Sanger Institute for the COVID-19 Genomics UK (COG-UK) consortium|Davies|United Kingdom
>Spike|hCoV-19/Scotland/EDB852/2020|2020-04-04|EPI_ISL_433256|Original|hCoV-19^^Scotland|Human|Virology Department|COVID-19 Genomics UK (COG-UK) Consortium|Williams|United Kingdom
>Spike|hCoV-19/USA/MI-MDHHS-SC22075/2020|2020-09-30|EPI_ISL_583532|Original|hCoV-19^^Michigan|Human|Michigan Department of Health and Human Services|Michigan Department of Health and Human Services|Blankenship|USA
>Spike|hCoV-19/England/QEUH-B123CB/2020|2020-11-02|EPI_ISL_642741|Original|hCoV-19^^England|Human|Lighthouse Lab in Glasgow|Wellcome Sanger Institute for the COVID-19 Genomics UK (COG-UK) Consortium|Davies|United Kingdom
>Spike|hCoV-19/England/QEUH-96A0FC/2020|2020-08-12|EPI_ISL_530866|Original|hCoV-19^^England|Human|Lighthouse Lab in Glasgow|Wellcome Sanger Institute for the COVID-19 Genomics UK (COG-UK) consortium|Davies|United Kingdom
>Spike|hCoV-19/England/ALDP-AA3199/2020|2020-10-22|EPI_ISL_624183|Original|hCoV-19^^England|Human|Lighthouse Lab in Alderley Park|Wellcome Sanger Institute for the COVID-19 Genomics UK (COG-UK) consortium|Davies|United Kingdom
>Spike|hCoV-19/England/LIVE-AA42A/2020|2020-03-23|EPI_ISL_517452|Original|hCoV-19^^England|Human|Liverpool Clinical Laboratories|COVID-19 Genomics UK (COG-UK) Consortium|Haldenby|United Kingdom
>Spike|hCoV-19/South Africa/KRISP-K004013/2020|2020-10-10|EPI_ISL_634998|Original|hCoV-19^^KwaZulu-Natal|Human|National Health Laboratory Service - Inkosi Albert Luthuli Central Hospital (NHLS-IALCH)|KRISP|de Oliveira|South Africa
>Spike|hCoV-19/Japan/PG-8130/2020|2020-08-00|EPI_ISL_687229|Original|hCoV-19^^Japan|Human|Pathogen Genomics Center|Pathogen Genomics Center|Sekizuka|Japan
>Spike|hCoV-19/South Africa/KRISP-K000658/2020|2020-07-13|EPI_ISL_509299|Original|hCoV-19^^KwaZulu-Natal|Human|NHLS-IALCH|KRISP|de Oliveira|South Africa
>Spike|hCoV-19/USA/UT-UPHL-2009670/2020|2020-07-25|EPI_ISL_605345|Original|hCoV-19^^Utah|Human|Utah Public Health Laboratory|Utah Public Health Laboratory|Young|USA
>Spike|hCoV-19/Wales/PHWC-2EC35/2020|2020-04-07|EPI_ISL_446130|Original|hCoV-19^^Wales|Human|Wales Specialist Virology Centre|Public Health Wales Microbiology Cardiff|Moore|United Kingdom
>Spike|hCoV-19/Wales/PHWC-36326/2020|2020-04-29|EPI_ISL_474652|Original|hCoV-19^^Wales|Human|Originating lab|COVID-19 Genomics UK (COG-UK) Consortium|Moore|United Kingdom
>Spike|hCoV-19/USA/WA-S557/2020|2020-04-01|EPI_ISL_434317|Original|hCoV-19^^Washington|Human|Washington State Department of Health|Seattle Flu Study|Bedford|USA

>Spike|hCoV-19/Australia/VIC781/2020|2020-03-30|EPI_ISL_427056|Original|hCoV-
19^^Victoria|Human|Microbiological Diagnostic Unit Public Health
Laboratory|Microbiological Diagnostic Unit Public Health Laboratory|Schultz|Australia
>Spike|hCoV-19/India/MH-ACTREC-046/2020|2020-05-13|EPI_ISL_699702|Original|hCoV-
19^^Maharashtra|Human|Hematopathology Laboratory|Hematopathology
Laboratory|Patkar|India
>Spike|hCoV-19/Australia/VIC5730/2020|2020-07-19|EPI_ISL_518531|Original|hCoV-
19^^Victoria|Human|Microbiological Diagnostic Unit - Public Health Laboratory (MDU-
PHL)|MDU-PHL|Schultz|Australia
>Spike|hCoV-19/England/QEUH-961D1C/2020|2020-07-30|EPI_ISL_537070|Original|hCoV-
19^^England|Human|Lighthouse Lab in Glasgow|Wellcome Sanger Institute for the COVID-
19 Genomics UK (COG-UK) consortium|Davies|United Kingdom
>Spike|hCoV-19/USA/CA-ALSR-3392/2020|2020-07-09|EPI_ISL_635999|Original|hCoV-
19^^California|Human|San Diego County Public Health Laboratory|Andersen lab at
Scripps Research|Gangavarapu|USA
>Spike|hCoV-19/Wales/PHWC-16E931/2020|2020-10-03|EPI_ISL_585792|Original|hCoV-
19^^Wales|Human|Wales Specialist Virology Centre Sequencing lab|COVID-19 Genomics UK
(COG-UK) Consortium|Moore|United Kingdom
>Spike|hCoV-19/Wales/ALDP-C47FC8/2020|2020-12-12|EPI_ISL_767144|Original|hCoV-
19^^Wales|Human|Lighthouse Lab in Alderley Park|Wellcome Sanger Institute for the
COVID-19 Genomics UK (COG-UK) Consortium|Davies|United Kingdom
>Spike|hCoV-19/England/QEUH-95FE96/2020|2020-08-07|EPI_ISL_531867|Original|hCoV-
19^^England|Human|Lighthouse Lab in Glasgow|Wellcome Sanger Institute for the COVID-
19 Genomics UK (COG-UK) consortium|Davies|United Kingdom
>Spike|hCoV-19/England/CAMC-C27034/2020|2020-12-07|EPI_ISL_782959|Original|hCoV-
19^^England|Human|Lighthouse Lab in Cambridge|Wellcome Sanger Institute for the
COVID-19 Genomics UK (COG-UK) Consortium|Davies|United Kingdom
>Spike|hCoV-19/Japan/UT-NCGM02/2020|2020-02-01|EPI_ISL_418809|Vero E6 P3|hCoV-
19^^Tokyo|Human|University of Wisconsin - Madison|University of Wisconsin
Madison|Moreno|Japan
>Spike|hCoV-19/Luxembourg/LNS9177314/2020|2020-10-23|EPI_ISL_744864|Original|hCoV-
19^^Luxembourg|Human|Laboratoire national de sant\u00e9|Laboratoire national de
sant\u00e9|Wienecke-Baldacchino|Luxembourg
>Spike|hCoV-19/England/MILK-B3B256/2020|2020-11-01|EPI_ISL_705477|Original|hCoV-
19^^England|Human|Oxford Viromics|COVID-19 Genomics UK (COG-UK)
Consortium|Golubchik|United Kingdom
>Spike|hCoV-19/USA/CA-QDX-2340/2020|2020-03-14|EPI_ISL_604641|Original|hCoV-
19^^California|Human|Quest Diagnostics|Quest Diagnostics|Kagan|USA
>Spike|hCoV-19/Netherlands/NA_13/2020|2020-03-10|EPI_ISL_415469|Original|hCoV-
19^^Netherlands|Human|Dutch COVID-19 response team|Erasmus Medical Center|Oude
Munnink|Netherlands
>Spike|hCoV-19/England/MILK-B97021/2020|2020-11-13|EPI_ISL_674965|Original|hCoV-
19^^England|Human|Lighthouse Lab in Milton Keynes|Wellcome Sanger Institute for the
COVID-19 Genomics UK (COG-UK) Consortium|Davies|United Kingdom
>Spike|hCoV-19/Japan/PG-3055/2020|2020-04-00|EPI_ISL_691178|Original|hCoV-
19^^Japan|Human|Pathogen Genomics Center|Pathogen Genomics Center|Sekizuka|Japan
>Spike|hCoV-19/Denmark/DCGC-6321/2020|2020-09-14|EPI_ISL_615803|Original|hCoV-
19^^NA|Human|Department of Virus and Microbiological Special Diagnostics|Albertsen
lab|Michaelsen|Denmark
>Spike|hCoV-19/Denmark/DCGC-19671/2020|2020-12-07|EPI_ISL_749101|Original|hCoV-
19^^Hovedstaden|Human|Department of Virus and Microbiological Special
Diagnostics|Albertsen Lab|Michaelsen|Denmark
>Spike|hCoV-19/Thailand/Bangkok_29/2020|2020-11-16|EPI_ISL_768522|Vero P1|hCoV-
19^^Bangkok|Human|Synphaet Hospital|National Institute of Health|Phuygun|Thailand
>Spike|hCoV-19/USA/TX-DSHS-1000/2020|2020-07-03|EPI_ISL_614006|Original|hCoV-
19^^Texas|Human|Texas Department of State Health Services|Texas Department of State
Health Services|Rahman|USA
>Spike|hCoV-19/England/CAMB-80D4D/2020|2020-04-10|EPI_ISL_442925|Original|hCoV-
19^^England|Human|Department of Pathology|Wellcome Sanger Institute for the COVID-19
Genomics UK (COG-UK) consortium|Amato|United Kingdom

>Spike|hCoV-19/England/SHEF-CFE03/2020|2020-03-30|EPI_ISL_432601|Original|hCoV-19^^England|Human|Virology Department|COVID-19 Genomics UK (COG-UK) Consortium|de Silva|United Kingdom
>Spike|hCoV-19/Netherlands/ZH-RIVM-20755/2020|2020-12-22|EPI_ISL_790817|Original|hCoV-19^^Zuid-holland|Human|Dutch COVID-19 response team|National Institute for Public Health and the Environment (RIVM)|Meijer|Netherlands
>Spike|hCoV-19/England/LIVE-A2853/2020|2020-05-19|EPI_ISL_500009|Original|hCoV-19^^England|Human|Liverpool Clinical Laboratories|COVID-19 Genomics UK (COG-UK) Consortium|Haldenby|United Kingdom
>Spike|hCoV-19/Denmark/DCGC-8477/2020|2020-06-08|EPI_ISL_618035|Original|hCoV-19^^Nordjylland|Human|Department of Virus and Microbiological Special Diagnostics|Albertsen lab|Michaelsen|Denmark
>Spike|hCoV-19/England/NORW-ECD30/2020|2020-06-02|EPI_ISL_472344|Original|hCoV-19^^England|Human|Quadram Institute Bioscience|COVID-19 Genomics UK (COG-UK) Consortium|Alikhan|United Kingdom
>Spike|hCoV-19/Denmark/DCGC-1476/2020|2020-06-22|EPI_ISL_622270|Original|hCoV-19^^Nordjylland|Human|Department of Virus and Microbiological Special Diagnostics|Albertsen lab|Michaelsen|Denmark
>Spike|hCoV-19/USA/AZ-TG371339/2020|2020-05-22|EPI_ISL_694583|Original|hCoV-19^^Arizona|Human|AZ SPHL|TGen North|French|USA
>Spike|hCoV-19/Canada/ON-S1775/2020|2020-11-08|EPI_ISL_755775|Original|hCoV-19^^Ontario|Human|Toronto Invasive Bacterial Diseases Network|McMaster University|McArthur|Canada
>Spike|hCoV-19/USA/WI-GMF-31258/2020|2020-11-03|EPI_ISL_661080|Original|hCoV-19^^Wisconsin|Human|Gundersen Molecular Diagnostics Laboratory|Kabara Cancer Research Institute|Kenny|USA
>Spike|hCoV-19/USA/CA-IGI-0414/2020|2020-07-13|EPI_ISL_756320|Original|hCoV-19^^California|Human|Innovative Genomics Institute|Innovative Genomics Institute|Wyman|USA
>Spike|hCoV-19/England/ALDP-94E4B6/2020|2020-06-17|EPI_ISL_555410|Original|hCoV-19^^England|Human|Lighthouse Lab in Alderley Park|Wellcome Sanger Institute for the COVID-19 Genomics UK (COG-UK) consortium|Davies|United Kingdom
>Spike|hCoV-19/Netherlands/Gelderland_54/2020|2020-05-18|EPI_ISL_460953|Original|hCoV-19^^Gelderland|Human|Dutch COVID-19 response team|Erasmus Medical Center|Oude Munnink|Netherlands
>Spike|hCoV-19/USA/LA-EVTL553/2020|2020-08-05|EPI_ISL_578957|Original|hCoV-19^^Louisiana|Human|LSUHS Emerging Viral Threat Laboratory|Microbial Genome Sequencing Center|Kamil|USA
>Spike|hCoV-19/USA/GA-CDC-5107/2020|2020-06-30|EPI_ISL_566093|Original|hCoV-19^^Georgia|Human|GA Department of Public Health Laboratory|Pathogen Discovery|Queen|USA
>Spike|hCoV-19/England/LIVE-A5850/2020|2020-03-19|EPI_ISL_499534|Original|hCoV-19^^England|Human|Liverpool Clinical Laboratories|COVID-19 Genomics UK (COG-UK) Consortium|Haldenby|United Kingdom
>Spike|hCoV-19/England/CAMC-BBC82E/2020|2020-11-20|EPI_ISL_705599|Original|hCoV-19^^England|Human|Quadram Institute Bioscience|COVID-19 Genomics UK (COG-UK) Consortium|Alikhan|United Kingdom
>Spike|hCoV-19/Australia/SAP221/2020|2020-03-26|EPI_ISL_468026|Original|hCoV-19^^South Australia|Human|SA Pathology|SA Pathology|Leong|Australia
>Spike|hCoV-19/USA/CA-CZB-10935/2020|2020-09-04|EPI_ISL_583164|Original|hCoV-19^^California|Human|Humboldt County Public Health Laboratory|Chan-Zuckerberg Biohub|Bhatt|USA
>Spike|hCoV-19/Singapore/886/2020|2020-08-31|EPI_ISL_536427|Original|hCoV-19^^Singapore|Human|National Public Health Laboratory|National Public Health Laboratory|Mak|Singapore
>Spike|hCoV-19/USA/OR-OHSU-0648/2020|2020-06-16|EPI_ISL_525869|Original|hCoV-19^^Oregon|Human|OHSU Lab Services Molecular Microbiology Lab|Ginkgo Bioworks Clinical Laboratory|O'Roak|USA

>Spike|hCoV-19/Austria/CeMM0001/2020|2020-03-03|EPI_ISL_419654|Original|hCoV-19^^Austria|Human|Center for Virology|Bergthaler laboratory|Bergthaler|Austria
>Spike|hCoV-19/Scotland/CVR5934/2020|2020-11-21|EPI_ISL_705679|Original|hCoV-19^^Scotland|Human|West of Scotland Specialist Virology Centre|COVID-19 Genomics UK (COG-UK) Consortium|Robertson|United Kingdom
>Spike|hCoV-19/Northern Ireland/QEUH-96996B/2020|2020-08-14|EPI_ISL_531063|Original|hCoV-19^^Northern Ireland|Human|Lighthouse Lab in Glasgow|Wellcome Sanger Institute for the COVID-19 Genomics UK (COG-UK) consortium|Davies|United Kingdom
>Spike|hCoV-19/USA/WA-S217/2020|2020-03-20|EPI_ISL_430255|Original|hCoV-19^^Washington|Human|Washington State Department of Health|Seattle Flu Study|Bedford|USA
>Spike|hCoV-19/Wuhan/HB-WHCM-114/2020|2020-03-02|EPI_ISL_454994|Original|hCoV-19^^Hubei|Human|Wuhan Chain Medical Labs (CMLabs)|State Key Laboratory of Biotherapy of Sichuan University|Tang|China
>Spike|hCoV-19/England/MILK-9C254E/2020|2020-09-07|EPI_ISL_566676|Original|hCoV-19^^England|Human|Lighthouse Lab in Milton Keynes|Wellcome Sanger Institute for the COVID-19 Genomics UK (COG-UK) consortium|Davies|United Kingdom
>Spike|hCoV-19/Denmark/DCGC-4892/2020|2020-09-21|EPI_ISL_621578|Original|hCoV-19^^Midtjylland|Human|Department of Virus and Microbiological Special Diagnostics|Albertsen lab|Michaelsen|Denmark
>Spike|hCoV-19/France/ARA-14443/2020|2020-03-26|EPI_ISL_508887|Original|hCoV-19^^Auvergne-Rh\u00f4ne-Alpes|Human|Institut des Agents Infectieux (IAI)|CNR Virus des Infections Respiratoires - France SUD|Josset|France
>Spike|hCoV-19/England/ALDP-C3AB97/2020|2020-12-09|EPI_ISL_731418|Original|hCoV-19^^England|Human|Lighthouse Lab in Alderley Park|Wellcome Sanger Institute for the COVID-19 Genomics UK (COG-UK) Consortium|Davies|United Kingdom
>Spike|hCoV-19/England/CAMB-75640/2020|2020-03-16|EPI_ISL_425417|Original|hCoV-19^^England|Human|Department of Pathology|COVID-19 Genomics UK (COG-UK) Consortium|Meredith|United Kingdom
>Spike|hCoV-19/Vietnam/HCMC-05003/2020|2020-03-16|EPI_ISL_498176|unknown|hCoV-19^^Ho Chi Minh City|Human|OUCRU|OUCRU|Nguyen|Vietnam
>Spike|hCoV-19/England/MILK-48A34D/2020|2020-05-17|EPI_ISL_559685|Original|hCoV-19^^England|Human|Lighthouse Lab in Milton Keynes|Wellcome Sanger Institute for the COVID-19 Genomics UK (COG-UK) consortium|Davies|United Kingdom
>Spike|hCoV-19/England/OXON-B1D9E/2020|2020-04-13|EPI_ISL_479090|Original|hCoV-19^^England|Human|Oxford Viromics|COVID-19 Genomics UK (COG-UK) Consortium|Golubchik|United Kingdom
>Spike|hCoV-19/Iceland/341/2020|2020-03-15|EPI_ISL_417670|Original|hCoV-19^^Reykjavik|Human|deCODE genetics|deCODE genetics|J\u00f3nsson|Iceland
>Spike|hCoV-19/Belgium/ULG-9536/2020|2020-03-21|EPI_ISL_447124|Original|hCoV-19^^Liege|Human|Department of Clinical Microbiology|GIGA Medical Genomics|Durkin|Belgium
>Spike|hCoV-19/Netherlands/NH-EMC-80/2020|2020-09-02|EPI_ISL_632422|Original|hCoV-19^^Noord Holland|Human|Dutch COVID-19 response team|Erasmus Medical Center|Oude Munnink|Netherlands
>Spike|hCoV-19/Scotland/CVR5574/2020|2020-10-27|EPI_ISL_651847|Original|hCoV-19^^Scotland|Human|West of Scotland Specialist Virology Centre|COVID-19 Genomics UK (COG-UK) Consortium|Robertson|United Kingdom
>Spike|hCoV-19/Denmark/DCGC-19692/2020|2020-12-07|EPI_ISL_749114|Original|hCoV-19^^Syddanmark|Human|Department of Virus and Microbiological Special Diagnostics|Albertsen Lab|Michaelsen|Denmark
>Spike|hCoV-19/USA/UT-03260/2020|2020-05-08|EPI_ISL_493004|Original|hCoV-19^^Utah|Human|Utah Public Health Laboratory|Utah Public Health Laboratory|Butz|USA
>Spike|hCoV-19/USA/NY-NYU-VC-023/2020|2020-04-09|EPI_ISL_479675|Original|hCoV-19^^New York|Human|Center for Genomics and System Biology|Center for Genomics and System Biology|Ghedin|USA
>Spike|hCoV-19/Wales/PHWC-4A26EC/2020|2020-12-16|EPI_ISL_743930|Original|hCoV-19^^Wales|Human|Wales Specialist Virology Centre Sequencing lab|COVID-19 Genomics UK (COG-UK) Consortium|Moore|United Kingdom

>Spike|hCoV-19/USA/CA-ALSR-2800/2020|2020-07-24|EPI_ISL_635683|Original|hCoV-19^^California|Human|San Diego County Public Health Laboratory|Andersen lab at Scripps Research|Gangavarapu|USA
>Spike|hCoV-19/Switzerland/TG-ETHZ-410925/2020|2020-11-30|EPI_ISL_737605|Original|hCoV-19^^Thurgau|Human|Viollier AG|Department of Biosystems Science and Engineering|Nadeau|Switzerland
>Spike|hCoV-19/USA/WI-UW-2306/2020|2020-11-26|EPI_ISL_728072|Original|hCoV-19^^Wisconsin|Human|University of Wisconsin-Madison AIDS Vaccine Research Laboratories|University of Wisconsin-Madison AIDS Vaccine Research Laboratories|Baczenas|USA
>Spike|hCoV-19/Spain/RI-IBV-99005842/2020|2020-04-03|EPI_ISL_537385|Original|hCoV-19^^La Rioja|Human|Centro de Investigaci\u00f3n Biom\u00e9dica de La Rioja - Hospital San Pedro Logro\u00f1o|SeqCOVID-SPAIN consortium/IBV(CSIC)|Comas|Spain
>Spike|hCoV-19/USA/CA-ALSR-3664/2020|2020-09-23|EPI_ISL_636136|Original|hCoV-19^^California|Human|San Diego County Public Health Laboratory|Andersen lab at Scripps Research|Gangavarapu|USA
>Spike|hCoV-19/Wales/ALDP-A85643/2020|2020-10-17|EPI_ISL_625277|Original|hCoV-19^^Wales|Human|Lighthouse Lab in Alderley Park|Wellcome Sanger Institute for the COVID-19 Genomics UK (COG-UK) consortium|Davies|United Kingdom
>Spike|hCoV-19/England/LIVE-1D86D1/2020|2020-09-16|EPI_ISL_576973|Original|hCoV-19^^England|Human|Liverpool Clinical Laboratories|COVID-19 Genomics UK (COG-UK) Consortium|Haldenby|United Kingdom
>Spike|hCoV-19/England/ALDP-B74804/2020|2020-11-12|EPI_ISL_678943|Original|hCoV-19^^England|Human|Queens Medical Centre|COVID-19 Genomics UK (COG-UK) Consortium|Loose|United Kingdom
>Spike|hCoV-19/England/ALDP-90606B/2020|2020-06-24|EPI_ISL_551547|Original|hCoV-19^^England|Human|Lighthouse Lab in Alderley Park|Wellcome Sanger Institute for the COVID-19 Genomics UK (COG-UK) consortium|Davies|United Kingdom
>Spike|hCoV-19/Wales/ALDP-C048EA/2020|2020-12-03|EPI_ISL_720907|Original|hCoV-19^^Wales|Human|Lighthouse Lab in Alderley Park|Wellcome Sanger Institute for the COVID-19 Genomics UK (COG-UK) Consortium|Davies|United Kingdom
>Spike|hCoV-19/Australia/VIC5305/2020|2020-07-18|EPI_ISL_520068|Original|hCoV-19^^Victoria|Human|Microbiological Diagnostic Unit - Public Health Laboratory (MDU-PHL)|MDU-PHL|Schultz|Australia
>Spike|hCoV-19/Japan/PG-6400/2020|2020-08-00|EPI_ISL_689506|Original|hCoV-19^^Japan|Human|Pathogen Genomics Center|Pathogen Genomics Center|Sekizuka|Japan
>Spike|hCoV-19/England/MILK-B05440/2020|2020-10-24|EPI_ISL_638551|Original|hCoV-19^^England|Human|Virology Department|COVID-19 Genomics UK (COG-UK) Consortium|de Silva|United Kingdom
>Spike|hCoV-19/England/CAMB-1AC366/2020|2020-05-01|EPI_ISL_584283|Original|hCoV-19^^England|Human|Department of Pathology|COVID-19 Genomics UK (COG-UK) Consortium|Goodfellow|United Kingdom
>Spike|hCoV-19/England/BRIS-13308B/2020|2020-06-25|EPI_ISL_511955|Original|hCoV-19^^England|Human|PHE South West Regional Laboratory|Wellcome Sanger Institute for the COVID-19 Genomics UK (COG-UK) consortium|Davies|United Kingdom
>Spike|hCoV-19/Spain/MD-IBV-97010045/2020|2020-03-12|EPI_ISL_779727|Original|hCoV-19^^Madrid|Human|IdISSC/Hospital Cl\u00ednico San Carlos de Madrid\u200b|SeqCOVID-SPAIN consortium/IBV(CSIC)|Comas|Spain
>Spike|hCoV-19/Denmark/DCGC-21681/2020|2020-12-21|EPI_ISL_758304|Original|hCoV-19^^Midtjylland|Human|Department of Virus and Microbiological Special Diagnostics|Albertsen Lab|Michaelsen|Denmark
>Spike|hCoV-19/South Korea/KCDC2701/2020|2020-07-05|EPI_ISL_522481|Original|hCoV-19^^South Korea|Human|Center for Laboratory Control of Infectious Diseases|Center for Laboratory Control of Infectious Diseases|Kim|South Korea
>Spike|hCoV-19/England/MILK-A1935C/2020|2020-10-03|EPI_ISL_612505|Original|hCoV-19^^England|Human|Wales Specialist Virology Centre Sequencing lab|COVID-19 Genomics UK (COG-UK) Consortium|Moore|United Kingdom
>Spike|hCoV-19/England/204390345/2020|2020-10-23|EPI_ISL_637137|Original|hCoV-19^^England|Human|Respiratory Virus Unit|COVID-19 Genomics UK (COG-UK) Consortium|Platt|United Kingdom

>Spike|hCoV-19/USA/CA-QDX-443/2020|2020-03-21|EPI_ISL_571165|Original|hCoV-19^^California|Human|Quest Diagnostics|Quest Diagnostics|Kagan|USA
>Spike|hCoV-19/Netherlands/ZH-EMC-544/2020|2020-09-16|EPI_ISL_577972|Original|hCoV-19^^Zuid Holland|Human|Dutch COVID-19 response team|Erasmus Medical Center|Oude Munnink|Netherlands
>Spike|hCoV-19/USA/WI-UW-261/2020|2020-03-26|EPI_ISL_434607|Original|hCoV-19^^Wisconsin|Human|University of Wisconsin-Madison AIDS Vaccine Research Laboratories|University of Wisconsin-Madison AIDS Vaccine Research Laboratories|Baczenas|USA
>Spike|hCoV-19/USA/WI-UW-2067/2020|2020-10-14|EPI_ISL_677472|Original|hCoV-19^^Wisconsin|Human|University of Wisconsin-Madison AIDS Vaccine Research Laboratories|University of Wisconsin-Madison AIDS Vaccine Research Laboratories|Baczenas|USA
>Spike|hCoV-19/England/LOND-D9BC3/2020|2020-10-22|EPI_ISL_612000|Original|hCoV-19^^England|Human|University College London|COVID-19 Genomics UK (COG-UK) Consortium|Roy|United Kingdom
>Spike|hCoV-19/USA/TX-DSHS-0766/2020|2020-05-20|EPI_ISL_584163|Original|hCoV-19^^Texas|Human|Texas Department of State Health Services|Texas Department of State Health Services|Rahman|USA
>Spike|hCoV-19/England/QEUH-AAE143/2020|2020-10-20|EPI_ISL_721554|Original|hCoV-19^^England|Human|Lighthouse Lab in Glasgow|Wellcome Sanger Institute for the COVID-19 Genomics UK (COG-UK) Consortium|Davies|United Kingdom
>Spike|hCoV-19/USA/TX-HMH-5539/2020|2020-06-25|EPI_ISL_544849|Original|hCoV-19^^Texas|Human|Houston Methodist Hospital|Houston Methodist Hospital|Long|USA
>Spike|hCoV-19/Scotland/QEUH-96B27D/2020|2020-08-17|EPI_ISL_531420|Original|hCoV-19^^Scotland|Human|NHSGGC West of Scotland Specialist Virology Centre / MRC-University of Glasgow Centre for Virus Research|Wellcome Sanger Institute for the COVID-19 Genomics UK (COG-UK) consortium|Davies|United Kingdom
>Spike|hCoV-19/USA/AZ-CDC-2-3693500/2020|2020-11-03|EPI_ISL_747125|Original|hCoV-19^^Arizona|Human|Respiratory Viruses Branch|Respiratory Viruses Branch|Queen|USA
>Spike|hCoV-19/Japan/PG-3716/2020|2020-04-00|EPI_ISL_691969|Original|hCoV-19^^Japan|Human|Pathogen Genomics Center|Pathogen Genomics Center|Sekizuka|Japan
>Spike|hCoV-19/USA/WI-UW-1425/2020|2020-09-11|EPI_ISL_541558|Original|hCoV-19^^Wisconsin|Human|University of Wisconsin-Madison Campus AIDS Vaccine Research Laboratories|University of Wisconsin-Madison AIDS Vaccine Research Laboratories|Baczenas|USA
>Spike|hCoV-19/England/ALDP-8E4BDC/2020|2020-06-25|EPI_ISL_556608|Original|hCoV-19^^England|Human|Lighthouse Lab in Alderley Park|Wellcome Sanger Institute for the COVID-19 Genomics UK (COG-UK) consortium|Davies|United Kingdom
>Spike|hCoV-19/Denmark/DCGC-20635/2020|2020-12-21|EPI_ISL_758752|Original|hCoV-19^^Syddanmark|Human|Department of Virus and Microbiological Special Diagnostics|Albertsen Lab|Michaelsen|Denmark
>Spike|hCoV-19/England/ALDP-C49A01/2020|2020-12-12|EPI_ISL_718712|Original|hCoV-19^^England|Human|Lighthouse Lab in Alderley Park|Wellcome Sanger Institute for the COVID-19 Genomics UK (COG-UK) Consortium|Davies|United Kingdom
>Spike|hCoV-19/Morocco/FMP-37/2020|2020-12-00|EPI_ISL_768836|Original|hCoV-19^^Rabat|Human|Laboratoire Biolife|Laboratoire de Biotechnologie|Ouadghiri|Morocco
>Spike|hCoV-19/Austria/Graz-MUG15/2020|2020-04-17|EPI_ISL_437301|Original|hCoV-19^^Styria|Human|Diagnostic- and Research Institute of Pathology|Diagnostic- and Research Institute of Pathology|Kashofer|Austria
>Spike|hCoV-19/USA/CA2/2020|2020-01-22|EPI_ISL_406036|Original|hCoV-19^^California|Human|California Department of Public Health|Pathogen Discovery|Queen|USA
>Spike|hCoV-19/England/QEUH-AE2FA3/2020|2020-10-26|EPI_ISL_633570|Original|hCoV-19^^England|Human|Lighthouse Lab in Glasgow|Wellcome Sanger Institute for the COVID-19 Genomics UK (COG-UK) consortium|Davies|United Kingdom
>Spike|hCoV-19/South Africa/KRISP-102/2020|2020-04-30|EPI_ISL_455632|Original|hCoV-19^^KwaZulu-Natal|Human|KRISP|KRISP|de Oliveira|South Africa

>Spike|hCoV-19/Canada/BC-BCCDC-6406/2020|2020-10-00|EPI_ISL_740561|Original|hCoV-19^^British Columbia|Human|BCCDC Public Health Laboratory|BCCDC Public Health Laboratory|Prystajecky|Canada
>Spike|hCoV-19/England/ALDP-CB3E9D/2020|2020-12-19|EPI_ISL_761142|Original|hCoV-19^^England|Human|Lighthouse Lab in Alderley Park|Wellcome Sanger Institute for the COVID-19 Genomics UK (COG-UK) Consortium|Davies|United Kingdom
>Spike|hCoV-19/Scotland/QEUH-9BA8F5/2020|2020-09-09|EPI_ISL_573830|Original|hCoV-19^^Scotland|Human|Oxford Viromics|COVID-19 Genomics UK (COG-UK) Consortium|Golubchik|United Kingdom
>Spike|hCoV-19/Wales/PHWC-16972D/2020|2020-07-20|EPI_ISL_512504|Original|hCoV-19^^Wales|Human|Wales Specialist Virology Centre Sequencing lab|COVID-19 Genomics UK (COG-UK) Consortium|Moore|United Kingdom
>Spike|hCoV-19/USA/UT-08514/2020|2020-05-18|EPI_ISL_524277|Original|hCoV-19^^Utah|Human|Utah Public Health Laboratory|Utah Public Health Laboratory|Young|USA
>Spike|hCoV-19/England/PORT-2D513C/2020|2020-00-00|EPI_ISL_741680|Original|hCoV-19^^England|Human|Centre for Enzyme Innovation|COVID-19 Genomics UK (COG-UK) Consortium|Robson|United Kingdom
>Spike|hCoV-19/USA/UT-UPHL-2012400168/2020|2020-09-30|EPI_ISL_746124|Original|hCoV-19^^Utah|Human|Ginkgo Bioworks Clinical Laboratory|Utah Public Health Laboratory|Young|USA
>Spike|hCoV-19/England/MILK-B3C89F/2020|2020-11-01|EPI_ISL_706164|Original|hCoV-19^^England|Human|Oxford Viromics|COVID-19 Genomics UK (COG-UK) Consortium|Golubchik|United Kingdom
>Spike|hCoV-19/England/LIVE-9E1F5/2020|2020-04-03|EPI_ISL_453277|Original|hCoV-19^^England|Human|Liverpool Clinical Laboratories|COVID-19 Genomics UK (COG-UK) Consortium|Haldenby|United Kingdom
>Spike|hCoV-19/England/QEUH-BCBD0F/2020|2020-11-26|EPI_ISL_704088|Original|hCoV-19^^England|Human|Lighthouse Lab in Glasgow|Wellcome Sanger Institute for the COVID-19 Genomics UK (COG-UK) Consortium|Davies|United Kingdom
>Spike|hCoV-19/Australia/VIC4095/2020|2020-07-13|EPI_ISL_519476|Original|hCoV-19^^Victoria|Human|Microbiological Diagnostic Unit - Public Health Laboratory (MDU-PHL)|MDU-PHL|Schultz|Australia
>Spike|hCoV-19/Germany/BY-MVP-V2011673/2020|2020-03-16|EPI_ISL_420906|Original|hCoV-19^^Bavaria|Human|Max von Pettenkofer Institute|Laboratory for Functional Genome Analysis|Graf|Germany
>Spike|hCoV-19/Belgium/ULG-8634/2020|2020-03-19|EPI_ISL_418640|Original|hCoV-19^^Li\u00e8ge|Human|Department of Clinical Microbiology|GIGA Medical Genomics|Durkin|Belgium
>Spike|hCoV-19/USA/NV-NSPHL-A0189/2020|2020-05-20|EPI_ISL_515445|Original|hCoV-19^^Nevada|Human|Nevada State Public Health Laboratory|Nevada State Public Health Laboratory|Sevinsky|USA
>Spike|hCoV-19/England/MILK-C7A7F1/2020|2020-12-15|EPI_ISL_762945|Original|hCoV-19^^England|Human|Lighthouse Lab in Milton Keynes|Wellcome Sanger Institute for the COVID-19 Genomics UK (COG-UK) Consortium|Davies|United Kingdom
>Spike|hCoV-19/England/QEUH-9F6365/2020|2020-09-24|EPI_ISL_589002|Original|hCoV-19^^England|Human|Lighthouse Lab in Glasgow|Wellcome Sanger Institute for the COVID-19 Genomics UK (COG-UK) consortium|Davies|United Kingdom
>Spike|hCoV-19/South Africa/LR00010/2020|2020-07-03|EPI_ISL_515140|Original|hCoV-19^^Limpopo|Human|National Institute for Communicable Diseases of the National Health Laboratory Service|National Institute for Communicable Diseases of the National Health Laboratory Service|Allam|South Africa
>Spike|hCoV-19/England/ALDP-498A47/2020|2020-05-25|EPI_ISL_559409|Original|hCoV-19^^England|Human|Lighthouse Lab in Alderley Park|Wellcome Sanger Institute for the COVID-19 Genomics UK (COG-UK) consortium|Davies|United Kingdom
>Spike|hCoV-19/Wales/PHWC-484A99/2020|2020-10-21|EPI_ISL_628074|Original|hCoV-19^^Wales|Human|Wales Specialist Virology Centre Sequencing lab|COVID-19 Genomics UK (COG-UK) Consortium|Moore|United Kingdom
>Spike|hCoV-19/England/QEUH-B7E49D/2020|2020-11-14|EPI_ISL_668034|Original|hCoV-19^^England|Human|Lighthouse Lab in Glasgow|Wellcome Sanger Institute for the COVID-19 Genomics UK (COG-UK) Consortium|Davies|United Kingdom

>Spike|hCoV-19/Germany/BY-MVP-0196/2020|2020-04-16|EPI_ISL_466884|Original|hCoV-19^^Bavaria|Human|Max von Pettenkofer Institute|Laboratory for Functional Genome Analysis|Graf|Germany
>Spike|hCoV-19/England/LEED-2AA701/2020|2020-04-06|EPI_ISL_538980|Original|hCoV-19^^England|Human|Leeds Teaching Hospitals NHS Trust and Public Health England|Wellcome Sanger Institute for the COVID-19 Genomics UK (COG-UK) consortium|Davies|United Kingdom
>Spike|hCoV-19/USA/MI-MDHHS-SC21210/2020|2020-03-30|EPI_ISL_507467|Original|hCoV-19^^Michigan|Human|Michigan Department of Health and Human Services|Michigan Department of Health and Human Services|Blankenship|USA
>Spike|hCoV-19/England/ALDP-9FCB58/2020|2020-10-01|EPI_ISL_587808|Original|hCoV-19^^England|Human|Lighthouse Lab in Alderley Park|Wellcome Sanger Institute for the COVID-19 Genomics UK (COG-UK) consortium|Davies|United Kingdom
>Spike|hCoV-19/England/CAMB-7CF5D/2020|2020-04-06|EPI_ISL_442305|Original|hCoV-19^^England|Human|Department of Pathology|Wellcome Sanger Institute for the COVID-19 Genomics UK (COG-UK) consortium|Amato|United Kingdom
>Spike|hCoV-19/England/NORW-221BAC/2020|2020-12-20|EPI_ISL_764468|Original|hCoV-19^^England|Human|Quadram Institute Bioscience|COVID-19 Genomics UK (COG-UK) Consortium|Alikhan|United Kingdom
>Spike|hCoV-19/Switzerland/BL-ETHZ-250077/2020|2020-08-19|EPI_ISL_535600|Original|hCoV-19^^Basel-Landschaft|Human|Viollier AG|Department of Biosystems Science and Engineering|Nadeau|Switzerland
>Spike|hCoV-19/Japan/PG-0848/2020|2020-04-00|EPI_ISL_684458|Original|hCoV-19^^Japan|Human|Fukuoka Institute of Health and Environmental Sciences|Pathogen Genomics Center|Sekizuka|Japan
>Spike|hCoV-19/England/QEUH-9DD5D7/2020|2020-09-21|EPI_ISL_599729|Original|hCoV-19^^England|Human|Lighthouse Lab in Glasgow|Wellcome Sanger Institute for the COVID-19 Genomics UK (COG-UK) consortium|Davies|United Kingdom
>Spike|hCoV-19/USA/WA-UW-3844/2020|2020-03-25|EPI_ISL_427192|Original|hCoV-19^^Washington|Human|UW Virology Lab|UW Virology Lab|Roychoudhury|USA
>Spike|hCoV-19/England/CAMC-B35456/2020|2020-11-05|EPI_ISL_659606|Original|hCoV-19^^England|Human|Lighthouse Lab in Cambridge|Wellcome Sanger Institute for the COVID-19 Genomics UK (COG-UK) Consortium|Davies|United Kingdom
>Spike|hCoV-19/Switzerland/GR-ETHZ-321464/2020|2020-10-14|EPI_ISL_603679|Original|hCoV-19^^Graubunden|Human|Viollier AG|Department of Biosystems Science and Engineering|Nadeau|Switzerland
>Spike|hCoV-19/England/QEUH-A64361/2020|2020-10-12|EPI_ISL_628691|Original|hCoV-19^^England|Human|Oxford Viromics|COVID-19 Genomics UK (COG-UK) Consortium|Golubchik|United Kingdom
>Spike|hCoV-19/England/ALDP-B1A31E/2020|2020-11-01|EPI_ISL_646071|Original|hCoV-19^^England|Human|Lighthouse Lab in Alderley Park|Wellcome Sanger Institute for the COVID-19 Genomics UK (COG-UK) Consortium|Davies|United Kingdom
>Spike|hCoV-19/Scotland/CVR2716/2020|2020-04-18|EPI_ISL_473537|Original|hCoV-19^^Scotland|Human|West of Scotland Specialist Virology Centre|COVID-19 Genomics UK (COG-UK) Consortium|Robertson|United Kingdom
>Spike|hCoV-19/Netherlands/NH-RIVM-20531/2020|2020-12-08|EPI_ISL_728746|Original|hCoV-19^^Noord-Holland|Human|Dutch COVID-19 response team|National Institute for Public Health and the Environment (RIVM)|Meijer|Netherlands
>Spike|hCoV-19/Wales/PHWC-4A4811/2020|2020-12-17|EPI_ISL_764735|Original|hCoV-19^^Wales|Human|Wales Specialist Virology Centre Sequencing lab|COVID-19 Genomics UK (COG-UK) Consortium|Moore|United Kingdom
>Spike|hCoV-19/England/ALDP-B1E20E/2020|2020-11-01|EPI_ISL_645779|Original|hCoV-19^^England|Human|Lighthouse Lab in Alderley Park|Wellcome Sanger Institute for the COVID-19 Genomics UK (COG-UK) Consortium|Davies|United Kingdom
>Spike|hCoV-19/Australia/VIC5637/2020|2020-07-16|EPI_ISL_518448|Original|hCoV-19^^Victoria|Human|Victorian Infectious Diseases Reference Laboratory (VIDRL)|VIDRL and MDU-PHL|Schultz|Australia

>Spike|hCoV-19/USA/OR-OHSU-0608/2020|2020-06-13|EPI_ISL_525843|Original|hCoV-19^^Oregon|Human|OHSU Lab Services Molecular Microbiology Lab|Ginkgo Bioworks Clinical Laboratory|O'Roak|USA
>Spike|hCoV-19/Wales/PHWC-288DD/2020|2020-04-02|EPI_ISL_432393|Original|hCoV-19^^Wales|Human|Wales Specialist Virology Centre|Public Health Wales Microbiology Cardiff|Moore|United Kingdom
>Spike|hCoV-19/USA/MI-MDHHS-SC20868/2020|2020-04-29|EPI_ISL_471889|Original|hCoV-19^^Michigan|Human|Michigan Department of Health and Human Services|Michigan Department of Health and Human Services|Blankenship|USA
>Spike|hCoV-19/Shaoxing/02/2020|2020-01-30|EPI_ISL_463894|Original|hCoV-19^^Zhejiang|Human|Shaoxing Center for Disease Control and Prevention|Department of Pathology and Laboratory Medicine|Yang|China
>Spike|hCoV-19/England/SHEF-C4E68/2020|2020-09-17|EPI_ISL_573700|Original|hCoV-19^^England|Human|Virology Department|COVID-19 Genomics UK (COG-UK) Consortium|de Silva|United Kingdom
>Spike|hCoV-19/England/ALDP-B5E123/2020|2020-11-06|EPI_ISL_723634|Original|hCoV-19^^England|Human|Oxford Viromics|COVID-19 Genomics UK (COG-UK) Consortium|Golubchik|United Kingdom
>Spike|hCoV-19/South Africa/KRISP-K001343/2020|2020-07-10|EPI_ISL_515828|Original|hCoV-19^^KwaZulu-Natal|Human|Medical Disagnostics Services (MDS)|KRISP|de Oliveira|South Africa
>Spike|hCoV-19/Northern Ireland/NIRE-106B62/2020|2020-05-18|EPI_ISL_501022|Original|hCoV-19^^Northern Ireland|Human|Regional Virus Laboratory|Wellcome Sanger Institute for the COVID-19 Genomics UK (COG-UK) consortium|Davies|United Kingdom
>Spike|hCoV-19/USA/CA-CZB-1578/2020|2020-03-18|EPI_ISL_468509|Original|hCoV-19^^California|Human|San Joaquin County Public Health Lab|Chan-Zuckerberg Biohub|Hao|USA
>Spike|hCoV-19/Belgium/rega-0410574/2020|2020-04-10|EPI_ISL_734564|Original|hCoV-19^^Glabbeek|Human|UZ Leuven|KU Leuven|Maes|Belgium
>Spike|hCoV-19/England/QEUH-A8B7C2/2020|2020-10-17|EPI_ISL_643061|Original|hCoV-19^^England|Human|Lighthouse Lab in Glasgow|Wellcome Sanger Institute for the COVID-19 Genomics UK (COG-UK) Consortium|Davies|United Kingdom
>Spike|hCoV-19/England/MILK-9F232D/2020|2020-09-24|EPI_ISL_589080|Original|hCoV-19^^England|Human|Lighthouse Lab in Milton Keynes|Wellcome Sanger Institute for the COVID-19 Genomics UK (COG-UK) consortium|Davies|United Kingdom
>Spike|hCoV-19/Denmark/DCGC-3020/2020|2020-08-24|EPI_ISL_616798|Original|hCoV-19^^Hovedstaden|Human|Department of Virus and Microbiological Special Diagnostics|Albertsen lab|Michaelsen|Denmark
>Spike|hCoV-19/USA/MO-QDX-2516/2020|2020-03-14|EPI_ISL_604785|Original|hCoV-19^^Missouri|Human|Quest Diagnostics|Quest Diagnostics|Kagan|USA
>Spike|hCoV-19/USA/FL-BPHL-0140/2020|2020-03-09|EPI_ISL_480791|Original|hCoV-19^^Florida|Human|Florida Bureau of Public Health Laboratories|Florida Bureau of Public Health Laboratories|Blanton|USA
>Spike|hCoV-19/USA/WI-GMF-25948/2020|2020-10-15|EPI_ISL_660941|Original|hCoV-19^^Wisconsin|Human|Gundersen Molecular Diagnostics Laboratory|Kabara Cancer Research Institute|Kenny|USA
>Spike|hCoV-19/Australia/NSW2153/2020|2020-01-25|EPI_ISL_509505|Original|hCoV-19^^New South Wales|Human|Area of Virology|Area of Virology|Yeang|Australia
>Spike|hCoV-19/USA/MI-UM-2006S000977/2020|2020-03-29|EPI_ISL_484780|Original|hCoV-19^^Michigan|Human|University of Michigan Clinical Microbiology Laboratory|Lauring Lab|Valesano|USA
>Spike|hCoV-19/Gibraltar/204421205/2020|2020-10-27|EPI_ISL_613666|Original|hCoV-19^^Gibraltar|Human|Microbiology|Respiratory Virus Unit|Platt|Gibraltar
>Spike|hCoV-19/USA/CA-CZB-2339/2020|2020-05-12|EPI_ISL_513789|Original|hCoV-19^^California|Human|Orange County Public Health Laboratory|Chan-Zuckerberg Biohub|Kamm|USA
>Spike|hCoV-19/USA/CA-ALSR-0680-SAN/2020|2020-05-04|EPI_ISL_483297|Original|hCoV-19^^California|Human|UC San Diego Center for Advanced Laboratory Medicine|Andersen lab at Scripps Research|Gangavarapu|USA

>Spike|hCoV-19/Japan/Donner95/2020|2020-08-27|EPI_ISL_644999|Original|hCoV-19^^Kanto|Human|Department of Infectious Diseases|Center for Medical Genetics|Kosaki|Japan
>Spike|hCoV-19/Norway/2800/2020|2020-06-26|EPI_ISL_493366|Original|hCoV-19^^Norway|Human|Furst Medical Laboratory|Norwegian Institute of Public Health|Kopperud Riis|Norway
>Spike|hCoV-19/England/ALDP-951EB2/2020|2020-06-10|EPI_ISL_551320|Original|hCoV-19^^England|Human|Lighthouse Lab in Alderley Park|Wellcome Sanger Institute for the COVID-19 Genomics UK (COG-UK) consortium|Davies|United Kingdom
>Spike|hCoV-19/Wales/PHWC-487FF1/2020|2020-10-26|EPI_ISL_639337|Original|hCoV-19^^Wales|Human|Wales Specialist Virology Centre Sequencing lab|COVID-19 Genomics UK (COG-UK) Consortium|Moore|United Kingdom
>Spike|hCoV-19/England/MILK-A7B561/2020|2020-10-15|EPI_ISL_607466|Original|hCoV-19^^England|Human|Lighthouse Lab in Milton Keynes|Wellcome Sanger Institute for the COVID-19 Genomics UK (COG-UK) consortium|Davies|United Kingdom
>Spike|hCoV-19/Luxembourg/LNS5809570/2020|2020-12-28|EPI_ISL_770889|Original|hCoV-19^^Luxembourg|Human|Laboratoire national de sant\u00e9|Laboratoire national de sant\u00e9|Wienecke-Baldacchino|Luxembourg
>Spike|hCoV-19/Nanchang/JX176/2020|2020-01-29|EPI_ISL_421261|Original|hCoV-19^^Jiangxi|Human|Jiangxi Province Center for Disease Control and Prevention|Jiangxi Province Center for Disease Control and Prevention|Xiong|China
>Spike|hCoV-19/Wales/PHWC-35F8D/2020|2020-04-28|EPI_ISL_474598|Original|hCoV-19^^Wales|Human|Originating lab|COVID-19 Genomics UK (COG-UK) Consortium|Moore|United Kingdom
>Spike|hCoV-19/England/ALDP-B83019/2020|2020-11-14|EPI_ISL_675641|Original|hCoV-19^^England|Human|Lighthouse Lab in Alderley Park|Wellcome Sanger Institute for the COVID-19 Genomics UK (COG-UK) Consortium|Davies|United Kingdom
>Spike|hCoV-19/Denmark/DCGC-3607/2020|2020-08-31|EPI_ISL_616373|Original|hCoV-19^^Syddanmark|Human|Department of Virus and Microbiological Special Diagnostics|Albertsen lab|Michaelsen|Denmark
>Spike|hCoV-19/England/MILK-C30056/2020|2020-12-07|EPI_ISL_783025|Original|hCoV-19^^England|Human|Lighthouse Lab in Milton Keynes|Wellcome Sanger Institute for the COVID-19 Genomics UK (COG-UK) Consortium|Davies|United Kingdom
>Spike|hCoV-19/Kenya/C3143/2020|2020-04-28|EPI_ISL_457900|Original|hCoV-19^^Kenya|Human|KEMRI-CGMR-C|KEMRI-Wellcome Trust Research Programme/KEMRI-CGMR-C Kilifi|Githinji|Kenya
>Spike|hCoV-19/USA/TX-HMH-MCoV-16148/2020|2020-10-30|EPI_ISL_785832|Original|hCoV-19^^Texas|Human|Houston Methodist Hospital|Houston Methodist Hospital|Long|USA
>Spike|hCoV-19/USA/CA-CZB-2043/2020|2020-03-26|EPI_ISL_486287|Original|hCoV-19^^California|Human|San Joaquin County Public Health Lab|Chan-Zuckerberg Biohub|McGeever|USA
>Spike|hCoV-19/Northern Ireland/NIRE-FA6F8/2020|2020-03-31|EPI_ISL_470534|Original|hCoV-19^^Northern Ireland|Human|Regional Virus Laboratory|Wellcome Sanger Institute for the COVID-19 Genomics UK (COG-UK) consortium|Davies|United Kingdom
>Spike|hCoV-19/USA/TX-HMH-2715/2020|2020-06-04|EPI_ISL_545498|Original|hCoV-19^^Texas|Human|Houston Methodist Hospital|Houston Methodist Hospital|Long|USA
>Spike|hCoV-19/USA/CA-LACPHL-AE00040/2020|2020-07-16|EPI_ISL_708732|Original|hCoV-19^^California|Human|Los Angeles County Public Health Laboratory|Los Angeles County Public Health Laboratory|Hemarajata|USA
>Spike|hCoV-19/England/QEUH-96BE79/2020|2020-08-14|EPI_ISL_531247|Original|hCoV-19^^England|Human|Lighthouse Lab in Glasgow|Wellcome Sanger Institute for the COVID-19 Genomics UK (COG-UK) consortium|Davies|United Kingdom
>Spike|hCoV-19/England/ALDP-BC635D/2020|2020-11-20|EPI_ISL_721333|Original|hCoV-19^^England|Human|Lighthouse Lab in Alderley Park|Wellcome Sanger Institute for the COVID-19 Genomics UK (COG-UK) Consortium|Davies|United Kingdom
>Spike|hCoV-19/USA/UT-UPHL-2012392157/2020|2020-08-17|EPI_ISL_746048|Original|hCoV-19^^Utah|Human|Ginkgo Bioworks Clinical Laboratory|Utah Public Health Laboratory|Young|USA

>Spike|hCoV-19/England/ALDP-91F621/2020|2020-06-23|EPI_ISL_553271|Original|hCoV-19^^England|Human|Lighthouse Lab in Alderley Park|Wellcome Sanger Institute for the COVID-19 Genomics UK (COG-UK) consortium|Davies|United Kingdom
>Spike|hCoV-19/USA/CT-Yale-088/2020|2020-04-06|EPI_ISL_428747|Original|hCoV-19^^Connecticut|Human|Yale COVID-19 Biorepository|Grubaugh Lab - Yale School of Public Health|Grubaugh|USA
>Spike|hCoV-19/Denmark/DCGC-15779/2020|2020-11-23|EPI_ISL_714015|Original|hCoV-19^^Nordjylland|Human|Department of Virus and Microbiological Special Diagnostics|Albertsen Lab|Michaelsen|Denmark
>Spike|hCoV-19/USA/OR-OHSU-3183/2020|2020-10-05|EPI_ISL_667501|Original|hCoV-19^^Oregon|Human|OHSU Lab Services Molecular Microbiology Lab|Oregon SARS-CoV-2 Genome Sequencing Center|O'Roak|USA
>Spike|hCoV-19/USA/VA-CDC-8005/2020|2020-05-03|EPI_ISL_540435|original|hCoV-19^^Virginia|Human|VA-Division of Consolidated Laboratory Services|Pathogen Discovery|Queen|USA
>Spike|hCoV-19/USA/WY-WPHL-20161304/2020|2020-12-08|EPI_ISL_765968|Original|hCoV-19^^Wyoming|Human|Wyoming Public Health Laboratory|Wyoming Public Health Laboratory|Hull|USA
>Spike|hCoV-19/England/EXET-13737F/2020|2020-03-27|EPI_ISL_471967|Original|hCoV-19^^England|Human|University of Exeter|COVID-19 Genomics UK (COG-UK) Consortium|Temperton|United Kingdom
>Spike|hCoV-19/USA/WI-GMF-15023/2020|2020-08-12|EPI_ISL_547694|Original|hCoV-19^^Wisconsin|Human|Gundersen Molecular Diagnostics Laboratory|Kabara Cancer Research Institute|Kenny|USA
>Spike|hCoV-19/England/MILK-ABC26D/2020|2020-10-21|EPI_ISL_631048|Original|hCoV-19^^England|Human|Lighthouse Lab in Milton Keynes|Wellcome Sanger Institute for the COVID-19 Genomics UK (COG-UK) consortium|Davies|United Kingdom
>Spike|hCoV-19/India/DL-NCDC4398-CSIR-IGIB/2020|2020-05-21|EPI_ISL_482588|Original|hCoV-19^^Delhi|Human|National Centre for Disease control (NCDC)|NCDC/CSIR-IGIB|Singh|India
>Spike|hCoV-19/Switzerland/SO-ETHZ-420484/2020|2020-12-21|EPI_ISL_768216|Original|hCoV-19^^Solothurn|Human|Viollier AG|Department of Biosystems Science and Engineering|Nadeau|Switzerland
>Spike|hCoV-19/USA/MA-MASPHL-00322/2020|2020-04-18|EPI_ISL_791726|Original|hCoV-19^^Massachusetts|Human|Massachusetts State Public Health Laboratory|Infectious Disease Program|Park|USA
>Spike|hCoV-19/USA/FL-BPHL-1123/2020|2020-08-10|EPI_ISL_526609|Original|hCoV-19^^Florida|Human|Florida Bureau of Public Health Laboratories|Florida Bureau of Public Health Laboratories|Blanton|USA
>Spike|hCoV-19/Northern Ireland/NIRE-1066AD/2020|2020-05-10|EPI_ISL_500961|Original|hCoV-19^^Northern Ireland|Human|Regional Virus Laboratory|Wellcome Sanger Institute for the COVID-19 Genomics UK (COG-UK) consortium|Davies|United Kingdom
>Spike|hCoV-19/England/BRIS-126C3C/2020|2020-04-09|EPI_ISL_458998|Original|hCoV-19^^England|Human|PHE South West Regional Laboratory|Wellcome Sanger Institute for the COVID-19 Genomics UK (COG-UK) consortium|Davies|United Kingdom
>Spike|hCoV-19/USA/WI-WSLH-200528/2020|2020-05-27|EPI_ISL_578667|Original|hCoV-19^^Wisconsin|Human|Wisconsin State Laboratory of Hygiene Communicable Disease Division|Wisconsin State Laboratory of Hygiene Communicable Disease Division|Florek|USA
>Spike|hCoV-19/Belgium/ULG-10639/2020|2020-10-25|EPI_ISL_636617|Original|hCoV-19^^Li\u00e8ge|Human|Department of Clinical Microbiology|GIGA Medical Genomics|Durkin|Belgium
>Spike|hCoV-19/Japan/IC-0102/2020|2020-07-00|EPI_ISL_591443|Original|hCoV-19^^Japan|Human|Pathogen Genomics Center|Pathogen Genomics Center|Sekizuka|Japan
>Spike|hCoV-19/USA/WA-UW170/2020|2020-03-14|EPI_ISL_416708|Original|hCoV-19^^Washington|Human|UW Virology Lab|UW Virology Lab|Roychoudhury|USA
>Spike|hCoV-19/Vietnam/40139/2020|2020-07-00|EPI_ISL_766029|Original|hCoV-19^^Vietnam|Human|National Influenza Center|National Influenza Center|Ung|Vietnam

>Spike|hCoV-19/Wales/PHWC-49D1D4/2020|2020-12-10|EPI_ISL_742747|Original|hCoV-19^^Wales|Human|Wales Specialist Virology Centre Sequencing lab|COVID-19 Genomics UK (COG-UK) Consortium|Moore|United Kingdom
>Spike|hCoV-19/USA/NY-PV09076/2020|2020-03-20|EPI_ISL_421622|Original|hCoV-19^^New York|Human|MSHS Clinical Microbiology Laboratories|MSHS Pathogen Surveillance Program|van Bakel|USA
>Spike|hCoV-19/England/QEUH-B6BDE3/2020|2020-11-11|EPI_ISL_655078|Original|hCoV-19^^England|Human|Lighthouse Lab in Glasgow|Wellcome Sanger Institute for the COVID-19 Genomics UK (COG-UK) Consortium|Davies|United Kingdom
>Spike|hCoV-19/England/ALDP-A51367/2020|2020-10-08|EPI_ISL_597951|Original|hCoV-19^^England|Human|Lighthouse Lab in Alderley Park|Wellcome Sanger Institute for the COVID-19 Genomics UK (COG-UK) consortium|Davies|United Kingdom
>Spike|hCoV-19/Singapore/1114/2020|2020-10-21|EPI_ISL_605823|Original|hCoV-19^^Singapore|Human|National Public Health Laboratory|National Public Health Laboratory|Mak|Singapore
>Spike|hCoV-19/Singapore/877/2020|2020-08-28|EPI_ISL_536418|Original|hCoV-19^^Singapore|Human|National Public Health Laboratory|National Public Health Laboratory|Mak|Singapore
>Spike|hCoV-19/Australia/VIC9791/2020|2020-07-23|EPI_ISL_565719|Original|hCoV-19^^Victoria|Human|Microbiological Diagnostic Unit - Public Health Laboratory (MDU-PHL)|MDU-PHL|Schultz|Australia
>Spike|hCoV-19/Canada/ON-S272/2020|2020-03-28|EPI_ISL_755845|Original|hCoV-19^^Ontario|Human|Toronto Invasive Bacterial Diseases Network|McMaster University|McArthur|Canada
>Spike|hCoV-19/mink/Denmark/mDK-158/2020|2020-11-05|EPI_ISL_683009|Original|hCoV-19^^Denmark|Neovison vison|Department of Virus and Microbiological Special Diagnostics|Albertsen Lab|Michaelsen|Denmark
>Spike|hCoV-19/Switzerland/ZH-ETHZ-250099/2020|2020-08-20|EPI_ISL_535615|Original|hCoV-19^^Zurich|Human|Viollier AG|Department of Biosystems Science and Engineering|Nadeau|Switzerland
>Spike|hCoV-19/Scotland/EDB6005/2020|2020-05-05|EPI_ISL_487977|Original|hCoV-19^^Scotland|Human|Virology Department|Wellcome Sanger Institute for the COVID-19 Genomics UK (COG-UK) consortium|Davies|United Kingdom
>Spike|hCoV-19/England/CAMB-73C9B/2020|2020-04-02|EPI_ISL_433682|Original|hCoV-19^^England|Human|Department of Pathology|COVID-19 Genomics UK (COG-UK) Consortium|Meredith|United Kingdom
>Spike|hCoV-19/England/QEUH-A0131B/2020|2020-10-02|EPI_ISL_595800|Original|hCoV-19^^England|Human|Liverpool Clinical Laboratories|COVID-19 Genomics UK (COG-UK) Consortium|Haldenby|United Kingdom
>Spike|hCoV-19/USA/MI-MDHHS-SC20460/2020|2020-03-30|EPI_ISL_459999|Original|hCoV-19^^Michigan|Human|Michigan Department of Health and Human Services|Michigan Department of Health and Human Services|Blankenship|USA
>Spike|hCoV-19/England/NORT-298ED4/2020|2020-04-22|EPI_ISL_493478|Original|hCoV-19^^England|Human|Northumbria University / South Tees Hospitals NHS Foundation Trust / North Cumbria Integrated Care NHS Foundation Trust / North Tees and Hartlepool NHS Foundation Trust / Newcastle Hospitals NHS Foundation Trust|COVID-19 Genomics UK (COG-UK) Consortium|Bashton|United Kingdom
>Spike|hCoV-19/England/BRIS-12FDD6/2020|2020-04-10|EPI_ISL_492724|Original|hCoV-19^^England|Human|PHE South West Regional Laboratory|Wellcome Sanger Institute for the COVID-19 Genomics UK (COG-UK) consortium|Davies|United Kingdom
>Spike|hCoV-19/USA/MI-MDHHS-SC21215/2020|2020-04-06|EPI_ISL_507472|Original|hCoV-19^^Michigan|Human|Michigan Department of Health and Human Services|Michigan Department of Health and Human Services|Blankenship|USA
>Spike|hCoV-19/USA/OR-OHSU-1863/2020|2020-07-27|EPI_ISL_526051|Original|hCoV-19^^Oregon|Human|OHSU Lab Services Molecular Microbiology Lab|Oregon SARS-CoV-2 Genome Sequencing Center|O'Roak|USA
>Spike|hCoV-19/England/QEUH-A5E5FB/2020|2020-10-12|EPI_ISL_597503|Original|hCoV-19^^England|Human|Lighthouse Lab in Glasgow|Wellcome Sanger Institute for the COVID-19 Genomics UK (COG-UK) consortium|Davies|United Kingdom

>Spike|hCoV-19/Belgium/rega-0411450/2020|2020-04-11|EPI_ISL_476960|Original|hCoV-19^^Ath|Human|KU Leuven|KU Leuven|Maes|Belgium
>Spike|hCoV-19/Scotland/CVR3595/2020|2020-05-19|EPI_ISL_499353|Original|hCoV-19^^Scotland|Human|West of Scotland Specialist Virology Centre|COVID-19 Genomics UK (COG-UK) Consortium|Robertson|United Kingdom
>Spike|hCoV-19/Wales/MILK-9A6B3F/2020|2020-09-02|EPI_ISL_550187|Original|hCoV-19^^Wales|Human|Lighthouse Lab in Milton Keynes|Wellcome Sanger Institute for the COVID-19 Genomics UK (COG-UK) consortium|Davies|United Kingdom
>Spike|hCoV-19/USA/VA-DCLS-1636/2020|2020-08-20|EPI_ISL_614039|Original|hCoV-19^^Virginia|Human|Virginia DCLS|Virginia DCLS|St. Jacques|USA
>Spike|hCoV-19/England/QEUH-9F35CC/2020|2020-09-24|EPI_ISL_588591|Original|hCoV-19^^England|Human|Lighthouse Lab in Glasgow|Wellcome Sanger Institute for the COVID-19 Genomics UK (COG-UK) consortium|Davies|United Kingdom
>Spike|hCoV-19/England/NOTT-10F99C/2020|2020-04-05|EPI_ISL_433067|Original|hCoV-19^^England|Human|Queens Medical Centre|COVID-19 Genomics UK (COG-UK) Consortium|Loose|United Kingdom
>Spike|hCoV-19/USA/WA-S1490/2020|2020-05-04|EPI_ISL_495665|Original|hCoV-19^^Washington|Human|Seattle Flu Study|Seattle Flu Study|Lee|USA
>Spike|hCoV-19/Wales/PHWC-2C1A1/2020|2020-04-04|EPI_ISL_474286|Original|hCoV-19^^Wales|Human|Originating lab|COVID-19 Genomics UK (COG-UK) Consortium|Moore|United Kingdom
>Spike|hCoV-19/Gibraltar/205121255/2020|2020-00-00|EPI_ISL_766374|Original|hCoV-19^^Gibraltar|Human|Microbiology|Respiratory Virus Unit|Platt|Gibraltar
>Spike|hCoV-19/England/QEUH-532765/2020|2020-05-18|EPI_ISL_532251|Original|hCoV-19^^England|Human|Lighthouse Lab in Glasgow|Wellcome Sanger Institute for the COVID-19 Genomics UK (COG-UK) consortium|Davies|United Kingdom
>Spike|hCoV-19/USA/TX-HMH-MCoV-8837/2020|2020-07-01|EPI_ISL_780785|Original|hCoV-19^^Texas|Human|Houston Methodist Hospital|Houston Methodist Hospital|Long|USA
>Spike|hCoV-19/USA/WA-OHSU-0987/2020|2020-07-06|EPI_ISL_526102|Original|hCoV-19^^Washington|Human|OHSU Lab Services Molecular Microbiology Lab|Oregon SARS-CoV-2 Genome Sequencing Center|O'Roak|USA
>Spike|hCoV-19/England/QEUH-AF5769/2020|2020-10-30|EPI_ISL_662956|Original|hCoV-19^^England|Human|Lighthouse Lab in Glasgow|Wellcome Sanger Institute for the COVID-19 Genomics UK (COG-UK) Consortium|Davies|United Kingdom
>Spike|hCoV-19/New Zealand/20VR2103/2020|2020-04-08|EPI_ISL_579230|Original|hCoV-19^^Counties Manukau|Human|Middlemore Hospital|Institute of Environmental Science and Research (ESR)|Wang|New Zealand
>Spike|hCoV-19/USA/CA-CZB-14111/2020|2020-05-26|EPI_ISL_738843|Original|hCoV-19^^California|Human|Alameda County Public Health Lab|Chan-Zuckerberg Biohub|Kamm|USA
>Spike|hCoV-19/England/MILK-9AB01D/2020|2020-08-31|EPI_ISL_551068|Original|hCoV-19^^England|Human|Lighthouse Lab in Milton Keynes|Wellcome Sanger Institute for the COVID-19 Genomics UK (COG-UK) consortium|Davies|United Kingdom
>Spike|hCoV-19/England/CAMC-A6463B/2020|2020-10-09|EPI_ISL_609014|Original|hCoV-19^^England|Human|Lighthouse Lab in Cambridge|Wellcome Sanger Institute for the COVID-19 Genomics UK (COG-UK) consortium|Davies|United Kingdom
>Spike|hCoV-19/England/QEUH-BA273D/2020|2020-11-18|EPI_ISL_719965|Original|hCoV-19^^England|Human|Lighthouse Lab in Glasgow|Wellcome Sanger Institute for the COVID-19 Genomics UK (COG-UK) Consortium|Davies|United Kingdom
>Spike|hCoV-19/Poland/PL_MCB_68/2020|2020-04-16|EPI_ISL_645067|Original|hCoV-19^^Poland|Human|Human Genome Variation Research Group|Human Genome Variation Research Group||Poland
>Spike|hCoV-19/England/CAMC-9497EF/2020|2020-07-27|EPI_ISL_555993|Original|hCoV-19^^England|Human|Lighthouse Lab in Cambridge|Wellcome Sanger Institute for the COVID-19 Genomics UK (COG-UK) consortium|Davies|United Kingdom
>Spike|hCoV-19/Denmark/DCGC-5211/2020|2020-09-21|EPI_ISL_621749|Original|hCoV-19^^Syddanmark|Human|Department of Virus and Microbiological Special Diagnostics|Albertsen lab|Michaelsen|Denmark
>Spike|hCoV-19/Wales/PHWC-162BBF/2020|2020-05-28|EPI_ISL_473174|Original|hCoV-19^^Wales|Human|Wales Specialist Virology Centre Sequencing lab|COVID-19 Genomics UK (COG-UK) Consortium|Moore|United Kingdom

>Spike|hCoV-19/Brazil/RS-00601/2020|2020-11-23|EPI_ISL_779155|Original|hCoV-19^^Rio Grande do Sul|Human|Laborat\u00f3rio de Microbiologia Molecular - Universidade FEEVALE|Bioinformatics Laboratory / LNCC|Almeida|Brazil
>Spike|hCoV-19/India/MH-ACTREC-124/2020|2020-05-25|EPI_ISL_699780|Original|hCoV-19^^Maharashtra|Human|Hematopathology Laboratory|Hematopathology Laboratory|Patkar|India
>Spike|hCoV-19/USA/TX-DSHS-0710/2020|2020-09-08|EPI_ISL_576353|Original|hCoV-19^^Texas|Human|Texas Department of State Health Services|Texas Department of State Health Services|Rahman|USA
>Spike|hCoV-19/Iran/SA-Isf-68/2020|2020-05-13|EPI_ISL_582009|Original|hCoV-19^^Iran|Human|Infectious Diseases and Tropical Medicine Research Center|Infectious Diseases and Tropical Medicine Research Center|Haghjooy Javanmard,S.|Iran
>Spike|hCoV-19/England/CAMC-AAFDF3/2020|2020-10-21|EPI_ISL_623545|Original|hCoV-19^^England|Human|Lighthouse Lab in Cambridge|Wellcome Sanger Institute for the COVID-19 Genomics UK (COG-UK) consortium|Davies|United Kingdom
>Spike|hCoV-19/England/ALDP-A67902/2020|2020-10-12|EPI_ISL_630604|Original|hCoV-19^^England|Human|Lighthouse Lab in Alderley Park|Wellcome Sanger Institute for the COVID-19 Genomics UK (COG-UK) consortium|Davies|United Kingdom
>Spike|hCoV-19/France/ARA-102618/2020|2020-11-06|EPI_ISL_732682|Original|hCoV-19^^Auvergne-Rh\u00f4ne-Alpes|Human|CNR Virus des Infections Respiratoires - France SUD|CNR Virus des Infections Respiratoires - France SUD|Bal|France
>Spike|hCoV-19/England/LOND-D6100/2020|2020-04-20|EPI_ISL_482163|Original|hCoV-19^^England|Human|University College London|Wellcome Sanger Institute for the COVID-19 Genomics UK (COG-UK) consortium|Davies|United Kingdom
>Spike|hCoV-19/England/MILK-A22CFB/2020|2020-10-03|EPI_ISL_612097|Original|hCoV-19^^England|Human|Northumbria University / South Tees Hospitals NHS Foundation Trust / North Cumbria Integrated Care NHS Foundation Trust / North Tees and Hartlepool NHS Foundation Trust / Newcastle Hospitals NHS Foundation Trust|COVID-19 Genomics UK (COG-UK) Consortium|Bashton|United Kingdom
>Spike|hCoV-19/Switzerland/SZ-ETHZ-410171/2020|2020-12-11|EPI_ISL_737572|Original|hCoV-19^^Schwyz|Human|Viollier AG|Department of Biosystems Science and Engineering|Nadeau|Switzerland
>Spike|hCoV-19/USA/TX-HMH-MCoV-13722/2020|2020-08-18|EPI_ISL_788815|Original|hCoV-19^^Texas|Human|Houston Methodist Hospital|Houston Methodist Hospital|Long|USA
>Spike|hCoV-19/England/ALDP-956424/2020|2020-06-08|EPI_ISL_554078|Original|hCoV-19^^England|Human|Lighthouse Lab in Alderley Park|Wellcome Sanger Institute for the COVID-19 Genomics UK (COG-UK) consortium|Davies|United Kingdom
>Spike|hCoV-19/Denmark/DCGC-13137/2020|2020-11-30|EPI_ISL_712140|Original|hCoV-19^^Hovedstaden|Human|Department of Virus and Microbiological Special Diagnostics|Albertsen Lab|Michaelsen|Denmark
>Spike|hCoV-19/USA/TX-HMH-6246/2020|2020-07-04|EPI_ISL_547339|Original|hCoV-19^^Texas|Human|Houston Methodist Hospital|Houston Methodist Hospital|Long|USA
>Spike|hCoV-19/Russia/CRIE168693/2020|2020-03-31|EPI_ISL_486825|Original|hCoV-19^^Moscow|Human|Molecular diagnostic laboratory of Federal Budget Institution of Science \Central Research Institute of Epidemiology\ of The Federal Service on Customers' Rights Protection and Human Well-being Surveillance|Group of Genomics and Postgenomic Technologies of Central Research Institute of Epidemilology|Spera
>nskaya|Russia
>Spike|hCoV-19/England/LOND-DA49A/2020|2020-11-07|EPI_ISL_724633|Original|hCoV-19^^England|Human|University College London|COVID-19 Genomics UK (COG-UK) Consortium|Roy|United Kingdom
>Spike|hCoV-19/Denmark/DCGC-8339/2020|2020-10-26|EPI_ISL_617367|Original|hCoV-19^^Midtjylland|Human|Department of Virus and Microbiological Special Diagnostics|Albertsen lab|Michaelsen|Denmark
>Spike|hCoV-19/England/ALDP-A96269/2020|2020-10-19|EPI_ISL_624759|Original|hCoV-19^^England|Human|Lighthouse Lab in Alderley Park|Wellcome Sanger Institute for the COVID-19 Genomics UK (COG-UK) consortium|Davies|United Kingdom
>Spike|hCoV-19/Australia/VIC13000/2020|2020-08-02|EPI_ISL_592145|Original|hCoV-19^^Victoria|Human|Victorian Infectious Diseases Reference Laboratory (VIDRL)|VIDRL and MDU-PHL|Schultz|Australia

>Spike|hCoV-19/Norway/3596/2020|2020-08-04|EPI_ISL_590943|Original|hCoV-19^^Norway|Human|University Hospital of Northern Norway|Norwegian Institute of Public Health|Vollan|Norway
>Spike|hCoV-19/Scotland/MILK-A6B054/2020|2020-10-08|EPI_ISL_625405|Original|hCoV-19^^Scotland|Human|Lighthouse Lab in Milton Keynes|Wellcome Sanger Institute for the COVID-19 Genomics UK (COG-UK) consortium|Davies|United Kingdom
>Spike|hCoV-19/England/MILK-9955BD/2020|2020-08-25|EPI_ISL_551630|Original|hCoV-19^^England|Human|Lighthouse Lab in Milton Keynes|Wellcome Sanger Institute for the COVID-19 Genomics UK (COG-UK) consortium|Davies|United Kingdom
>Spike|hCoV-19/Russia/MOW-RII-MH2907S/2020|2020-09-27|EPI_ISL_733066|Original|hCoV-19^^Moscow|Human|HELIX LLC|WHO National Influenza Centre Russian Federation|Komissarov|Russia
>Spike|hCoV-19/Scotland/CVR1933/2020|2020-04-07|EPI_ISL_484526|Original|hCoV-19^^Scotland|Human|West of Scotland Specialist Virology Centre|COVID-19 Genomics UK (COG-UK) Consortium|Robertson|United Kingdom
>Spike|hCoV-19/Scotland/EDB2090/2020|2020-04-19|EPI_ISL_433189|Original|hCoV-19^^Scotland|Human|Virology Department|COVID-19 Genomics UK (COG-UK) Consortium|Williams|United Kingdom
>Spike|hCoV-19/USA/TX-HMH-MCoV-12796/2020|2020-08-20|EPI_ISL_790529|Original|hCoV-19^^Texas|Human|Houston Methodist Hospital|Houston Methodist Hospital|Long|USA
>Spike|hCoV-19/Wales/PHWC-483BD3/2020|2020-10-11|EPI_ISL_627906|Original|hCoV-19^^Wales|Human|Wales Specialist Virology Centre Sequencing lab|COVID-19 Genomics UK (COG-UK) Consortium|Moore|United Kingdom
>Spike|hCoV-19/England/MILK-B9424C/2020|2020-11-13|EPI_ISL_676201|Original|hCoV-19^^England|Human|Lighthouse Lab in Milton Keynes|Wellcome Sanger Institute for the COVID-19 Genomics UK (COG-UK) Consortium|Davies|United Kingdom
>Spike|hCoV-19/India/AS-NCDC-2525/2020|2020-03-31|EPI_ISL_436421|Original|hCoV-19^^Assam|Human|National Centre for Disease control (NCDC)|NCDC/CSIR-IGIB|Singh|India
>Spike|hCoV-19/USA/FL-BPHL-1669/2020|2020-07-05|EPI_ISL_594358|Original|hCoV-19^^Florida|Human|Florida Bureau of Public Health Laboratories|Florida Bureau of Public Health Laboratories|Blanton|USA
>Spike|hCoV-19/Denmark/DCGC-17534/2020|2020-12-14|EPI_ISL_751050|Original|hCoV-19^^Hovedstaden|Human|Department of Virus and Microbiological Special Diagnostics|Albertsen Lab|Michaelsen|Denmark
>Spike|hCoV-19/Wales/PHWC-168375/2020|2020-04-20|EPI_ISL_494232|Original|hCoV-19^^Wales|Human|Originating lab|COVID-19 Genomics UK (COG-UK) Consortium|Moore|United Kingdom
>Spike|hCoV-19/Northern Ireland/NIRE-234247/2020|2020-10-15|EPI_ISL_680357|Original|hCoV-19^^Northern Ireland|Human|Regional Virus Laboratory|COVID-19 Genomics UK (COG-UK) Consortium|Fairley|United Kingdom
>Spike|hCoV-19/USA/TX-HMH-MCoV-8761/2020|2020-07-02|EPI_ISL_780738|Original|hCoV-19^^Texas|Human|Houston Methodist Hospital|Houston Methodist Hospital|Long|USA
>Spike|hCoV-19/England/QEUH-CC2D34/2020|2020-12-21|EPI_ISL_778154|Original|hCoV-19^^England|Human|Lighthouse Lab in Glasgow|Wellcome Sanger Institute for the COVID-19 Genomics UK (COG-UK) Consortium|Davies|United Kingdom
>Spike|hCoV-19/Denmark/DCGC-8803/2020|2020-10-26|EPI_ISL_626050|Original|hCoV-19^^Midtjylland|Human|Department of Virus and Microbiological Special Diagnostics|Albertsen lab|Michaelsen|Denmark
>Spike|hCoV-19/England/QEUH-AF1387/2020|2020-10-29|EPI_ISL_666010|Original|hCoV-19^^England|Human|Northumbria University / South Tees Hospitals NHS Foundation Trust / North Cumbria Integrated Care NHS Foundation Trust / North Tees and Hartlepool NHS Foundation Trust / Newcastle Hospitals NHS Foundation Trust|COVID-19 Genomics UK (COG-UK) Consortium|Bashton|United Kingdom
>Spike|hCoV-19/England/CAMC-BC1CDF/2020|2020-11-22|EPI_ISL_703508|Original|hCoV-19^^England|Human|Department of Pathology|COVID-19 Genomics UK (COG-UK) Consortium|Goodfellow|United Kingdom
>Spike|hCoV-19/United Arab Emirates/0625/2020|2020-05-15|EPI_ISL_698781|Original|hCoV-19^^Abu Dhabi|Human|Group 42 (G42) Healthcare|G42 Healthcare|Ogrodzki|United Arab Emirates

>Spike|hCoV-19/USA/WA-UW-12771/2020|2020-06-29|EPI_ISL_570819|Original|hCoV-19^^Washington|Human|UW Virology Lab|UW Virology Lab|Roychoudhury|USA
>Spike|hCoV-19/Denmark/DCGC-12490/2020|2020-11-16|EPI_ISL_682671|Original|hCoV-19^^Hovedstaden|Human|Department of Virus and Microbiological Special Diagnostics|Albertsen Lab|Michaelsen|Denmark
>Spike|hCoV-19/USA/MD-MDH-0148/2020|2020-04-10|EPI_ISL_524876|Original|hCoV-19^^Maryland|Human|MD PHL|MD PHL|Keller|USA
>Spike|hCoV-19/Denmark/DCGC-16488/2020|2020-11-16|EPI_ISL_714797|Original|hCoV-19^^Hovedstaden|Human|Department of Virus and Microbiological Special Diagnostics|Albertsen Lab|Michaelsen|Denmark
>Spike|hCoV-19/Netherlands/NoordBrabant_68/2020|2020-03-12|EPI_ISL_415524|Original|hCoV-19^^Noord Brabant|Human|Dutch COVID-19 response team|Erasmus Medical Center|Oude Munnink|Netherlands
>Spike|hCoV-19/Scotland/QEUH-BC398B/2020|2020-11-25|EPI_ISL_710786|Original|hCoV-19^^Scotland|Human|Lighthouse Lab in Glasgow|Wellcome Sanger Institute for the COVID-19 Genomics UK (COG-UK) Consortium|Davies|United Kingdom
>Spike|hCoV-19/England/QEUH-55484C/2020|2020-05-11|EPI_ISL_532190|Original|hCoV-19^^England|Human|Lighthouse Lab in Glasgow|Wellcome Sanger Institute for the COVID-19 Genomics UK (COG-UK) consortium|Davies|United Kingdom
>Spike|hCoV-19/England/CAMC-B99C67/2020|2020-11-15|EPI_ISL_673363|Original|hCoV-19^^England|Human|Lighthouse Lab in Cambridge|Wellcome Sanger Institute for the COVID-19 Genomics UK (COG-UK) Consortium|Davies|United Kingdom
>Spike|hCoV-19/USA/LA-GBCL-26544579/2020|2020-04-17|EPI_ISL_485150|Original|hCoV-19^^Louisiana|Human|River Road Testing Lab|Ginkgo Bioworks Clinical Laboratory|Smith|USA
>Spike|hCoV-19/Australia/TAS09/2020|2020-04-13|EPI_ISL_522564|Original|hCoV-19^^Tasmania|Human|Royal Hobart Hospital Microbiology Department|MDU-PHL|Schultz|Australia
>Spike|hCoV-19/Denmark/DCGC-7993/2020|2020-10-26|EPI_ISL_617148|Original|hCoV-19^^Midtjylland|Human|Department of Virus and Microbiological Special Diagnostics|Albertsen lab|Michaelsen|Denmark
>Spike|hCoV-19/England/204661790/2020|2020-11-12|EPI_ISL_686580|Original|hCoV-19^^England|Human|Respiratory Virus Unit|COVID-19 Genomics UK (COG-UK) Consortium|Platt|United Kingdom
>Spike|hCoV-19/England/CAMC-B09D50/2020|2020-10-29|EPI_ISL_673083|Original|hCoV-19^^England|Human|Lighthouse Lab in Cambridge|Wellcome Sanger Institute for the COVID-19 Genomics UK (COG-UK) Consortium|Davies|United Kingdom
>Spike|hCoV-19/USA/VA-DCLS-1387/2020|2020-07-07|EPI_ISL_572230|Original|hCoV-19^^Virginia|Human|Virginia DCLS|Virginia DCLS|St. Jacques|USA
>Spike|hCoV-19/Finland/21SS4D3/2020|2020-09-21|EPI_ISL_757382|Original|hCoV-19^^Uusimaa|Human|Department of Virology and Immunology|Department of Virology|Smura|Finland
>Spike|hCoV-19/Colombia/SAN-INS-79752/2020|2020-03-14|EPI_ISL_456131|Original|hCoV-19^^Santander|Human|Instituto Nacional de Salud - Unidad de Secuenciaci\u00f3n y An\u00e1lisis Gen\u00f3mico|Instituto Nacional de Salud|D\u00edaz|Colombia
>Spike|hCoV-19/USA/NY-QDX-2452/2020|2020-03-16|EPI_ISL_604803|Original|hCoV-19^^New York|Human|Quest Diagnostics|Quest Diagnostics|Kagan|USA
>Spike|hCoV-19/England/MILK-A7B938/2020|2020-10-15|EPI_ISL_607359|Original|hCoV-19^^England|Human|Lighthouse Lab in Milton Keynes|Wellcome Sanger Institute for the COVID-19 Genomics UK (COG-UK) consortium|Davies|United Kingdom
>Spike|hCoV-19/England/LIVE-A9CAB/2020|2020-03-24|EPI_ISL_517347|Original|hCoV-19^^England|Human|Liverpool Clinical Laboratories|COVID-19 Genomics UK (COG-UK) Consortium|Haldenby|United Kingdom
>Spike|hCoV-19/USA/FL-BPHL-1142/2020|2020-08-17|EPI_ISL_526628|Original|hCoV-19^^Florida|Human|Florida Bureau of Public Health Laboratories|Florida Bureau of Public Health Laboratories|Blanton|USA
>Spike|hCoV-19/USA/MD-MDH-0417/2020|2020-11-05|EPI_ISL_666787|Original|hCoV-19^^Maryland|Human|Maryland Public Health Laboratory|Maryland Public Health Laboratory|Keller|USA

>Spike|hCoV-19/Japan/PG-8265/2020|2020-08-00|EPI_ISL_687364|Original|hCoV-19^^Japan|Human|Pathogen Genomics Center|Pathogen Genomics Center|Sekizuka|Japan
>Spike|hCoV-19/USA/WI-WSLH-200505/2020|2020-05-22|EPI_ISL_578646|Original|hCoV-19^^Wisconsin|Human|Wisconsin State Laboratory of Hygiene Communicable Disease Division|Wisconsin State Laboratory of Hygiene Communicable Disease Division|Florek|USA
>Spike|hCoV-19/Bangladesh/BCSIR-NILMRC-154/2020|2020-06-18|EPI_ISL_480445|Original|hCoV-19^^Rajshahi|Human|National Institute of Laboratory Medicine and Referral Center|Genomic Research Lab|Mohammad Mahmud|Bangladesh
>Spike|hCoV-19/USA/MI-MDHHS-SC22628/2020|2020-12-07|EPI_ISL_737105|Original|hCoV-19^^Michigan|Human|Michigan Department of Health and Human Services|Michigan Department of Health and Human Services|Blankenship|USA
>Spike|hCoV-19/Wales/PHWC-48C7B8/2020|2020-11-03|EPI_ISL_666467|Original|hCoV-19^^Wales|Human|Wales Specialist Virology Centre Sequencing lab|COVID-19 Genomics UK (COG-UK) Consortium|Moore|United Kingdom
>Spike|hCoV-19/USA/WA-S117/2020|2020-03-02|EPI_ISL_417170|Original|hCoV-19^^Washington|Human|Washington State Department of Health|Seattle Flu Study|Bedford|USA
>Spike|hCoV-19/England/20192018304/2020|2020-05-04|EPI_ISL_528417|Original|hCoV-19^^England|Human|Respiratory Virus Unit|Respiratory Virus Unit|Miah|United Kingdom
>Spike|hCoV-19/England/QEUH-A4D42F/2020|2020-10-06|EPI_ISL_741765|Original|hCoV-19^^England|Human|Oxford Viromics|COVID-19 Genomics UK (COG-UK) Consortium|Golubchik|United Kingdom
>Spike|hCoV-19/Denmark/DCGC-11703/2020|2020-10-26|EPI_ISL_671081|Original|hCoV-19^^Syddanmark|Human|Department of Virus and Microbiological Special Diagnostics|Albertsen Lab|Michaelsen|Denmark
>Spike|hCoV-19/England/NORT-295E31/2020|2020-03-31|EPI_ISL_488534|Original|hCoV-19^^England|Human|NU-OMICS DNA Sequencing research facility|Wellcome Sanger Institute for the COVID-19 Genomics UK (COG-UK) consortium|Davies|United Kingdom
>Spike|hCoV-19/Thailand/SamutSakhon-CONI-0348/2020|2020-12-19|EPI_ISL_733542|Original|hCoV-19^^Samut Sakhon|Human|Institute for Urban Disease Control and Prevention|COVID-19 Network Investigations (CONI) Alliance|Batty|Thailand
>Spike|hCoV-19/USA/WA-S1914/2020|2020-06-02|EPI_ISL_497343|Original|hCoV-19^^Washington|Human|Washington State Department of Health|Seattle Flu Study|Lee|USA
>Spike|hCoV-19/England/CAMB-1B07FA/2020|2020-05-07|EPI_ISL_452924|Original|hCoV-19^^England|Human|Department of Pathology|COVID-19 Genomics UK (COG-UK) Consortium|Meredith|United Kingdom
>Spike|hCoV-19/England/LIVE-DB1159/2020|2020-10-22|EPI_ISL_637439|Original|hCoV-19^^England|Human|Liverpool Clinical Laboratories|COVID-19 Genomics UK (COG-UK) Consortium|Haldenby|United Kingdom
>Spike|hCoV-19/England/CAMC-B37E47/2020|2020-11-06|EPI_ISL_658760|Original|hCoV-19^^England|Human|Lighthouse Lab in Cambridge|Wellcome Sanger Institute for the COVID-19 Genomics UK (COG-UK) Consortium|Davies|United Kingdom
>Spike|hCoV-19/Mali/M002698/2020|2020-04-12|EPI_ISL_487457|Original|hCoV-19^^Bamako|Human|CICM-Mali|Bundeswehr Institut of Microbiology|Antwerpen|Mali
>Spike|hCoV-19/USA/WI-UW-2432/2020|2020-12-14|EPI_ISL_754639|Original|hCoV-19^^Wisconsin|Human|University of Wisconsin-Madison AIDS Vaccine Research Laboratories|University of Wisconsin-Madison AIDS Vaccine Research Laboratories|Moreno|USA
>Spike|hCoV-19/Luxembourg/LNS3035957/2020|2020-03-17|EPI_ISL_421750|Original|hCoV-19^^Luxembourg|Human|Laboratoire National de Sante|Laboratoire National de Sante|Wienecke-Baldacchino|Luxembourg
>Spike|hCoV-19/Spain/CL-IBV-97006264/2020|2020-04-21|EPI_ISL_537636|Original|hCoV-19^^Castilla y Leon|Human|Universidad de Le\u00f3n|SeqCOVID-SPAIN consortium/IBV(CSIC)|Comas|Spain
>Spike|hCoV-19/USA/MN-QDX-1010/2020|2020-03-14|EPI_ISL_571736|Original|hCoV-19^^Minnesota|Human|Quest Diagnostics|Quest Diagnostics|Kagan|USA
>Spike|hCoV-19/England/CAMB-8108F/2020|2020-04-21|EPI_ISL_438562|Original|hCoV-19^^England|Human|Department of Pathology|COVID-19 Genomics UK (COG-UK) Consortium|Meredith|United Kingdom

>Spike|hCoV-19/Denmark/DCGC-19702/2020|2020-12-07|EPI_ISL_748243|Original|hCoV-19^^Hovedstaden|Human|Department of Virus and Microbiological Special Diagnostics|Albertsen Lab|Michaelsen|Denmark
>Spike|hCoV-19/England/MILK-99D37F/2020|2020-08-30|EPI_ISL_549870|Original|hCoV-19^^England|Human|Lighthouse Lab in Milton Keynes|Wellcome Sanger Institute for the COVID-19 Genomics UK (COG-UK) consortium|Davies|United Kingdom
>Spike|hCoV-19/Wales/PHWC-48F481/2020|2020-11-09|EPI_ISL_681097|Original|hCoV-19^^Wales|Human|Wales Specialist Virology Centre Sequencing lab|COVID-19 Genomics UK (COG-UK) Consortium|Moore|United Kingdom
>Spike|hCoV-19/England/ALDP-A6DF91/2020|2020-10-14|EPI_ISL_607800|Original|hCoV-19^^England|Human|Lighthouse Lab in Alderley Park|Wellcome Sanger Institute for the COVID-19 Genomics UK (COG-UK) consortium|Davies|United Kingdom
>Spike|hCoV-19/Australia/VIC15159/2020|2020-08-29|EPI_ISL_593165|Original|hCoV-19^^Victoria|Human|Microbiological Diagnostic Unit - Public Health Laboratory (MDU-PHL)|MDU-PHL|Schultz|Australia
>Spike|hCoV-19/USA/UT-UPHL-2012021960/2020|2020-11-30|EPI_ISL_779294|Original|hCoV-19^^Utah|Human|Utah Public Health Laboratory|Utah Public Health Laboratory|Gallagher|USA
>Spike|hCoV-19/England/ALDP-A93D89/2020|2020-10-19|EPI_ISL_611206|Original|hCoV-19^^England|Human|Lighthouse Lab in Alderley Park|Wellcome Sanger Institute for the COVID-19 Genomics UK (COG-UK) consortium|Davies|United Kingdom
>Spike|hCoV-19/Denmark/DCGC-13500/2020|2020-11-30|EPI_ISL_712836|Original|hCoV-19^^Sjaelland|Human|Department of Virus and Microbiological Special Diagnostics|Albertsen Lab|Michaelsen|Denmark
>Spike|hCoV-19/Denmark/DCGC-4446/2020|2020-09-21|EPI_ISL_621867|Original|hCoV-19^^Nordjylland|Human|Department of Virus and Microbiological Special Diagnostics|Albertsen lab|Michaelsen|Denmark
>Spike|hCoV-19/Northern Ireland/NIRE-108B60/2020|2020-10-09|EPI_ISL_627070|Original|hCoV-19^^Northern Ireland|Human|Regional Virus Laboratory|COVID-19 Genomics UK (COG-UK) Consortium|Fairley|United Kingdom
>Spike|hCoV-19/England/LIVE-A65EF/2020|2020-03-19|EPI_ISL_499724|Original|hCoV-19^^England|Human|Liverpool Clinical Laboratories|COVID-19 Genomics UK (COG-UK) Consortium|Haldenby|United Kingdom
>Spike|hCoV-19/England/ALDP-6BF79C/2020|2020-07-01|EPI_ISL_557751|Original|hCoV-19^^England|Human|Lighthouse Lab in Alderley Park|Wellcome Sanger Institute for the COVID-19 Genomics UK (COG-UK) consortium|Davies|United Kingdom
>Spike|hCoV-19/England/CAMC-B79A27/2020|2020-11-12|EPI_ISL_681672|Original|hCoV-19^^England|Human|Lighthouse Lab in Cambridge|Wellcome Sanger Institute for the COVID-19 Genomics UK (COG-UK) Consortium|Davies|United Kingdom
>Spike|hCoV-19/Spain/RI-IBV-006010/2020|2020-03-28|EPI_ISL_500441|Original|hCoV-19^^La Rioja|Human|Centro de Investigaci\u00f3n Biom\u00e9dica de La Rioja - Hospital San Pedro Logro\u00f1o|SeqCOVID-SPAIN consortium/IBV(CSIC)|Comas|Spain
>Spike|hCoV-19/Switzerland/VD-ETHZ-410088/2020|2020-12-17|EPI_ISL_737901|Original|hCoV-19^^Vaud|Human|Viollier AG|Department of Biosystems Science and Engineering|Nadeau|Switzerland
>Spike|hCoV-19/England/ALDP-B63325/2020|2020-11-08|EPI_ISL_656419|Original|hCoV-19^^England|Human|Lighthouse Lab in Alderley Park|Wellcome Sanger Institute for the COVID-19 Genomics UK (COG-UK) Consortium|Davies|United Kingdom
>Spike|hCoV-19/England/QEUH-7885A0/2020|2020-07-15|EPI_ISL_533184|Original|hCoV-19^^England|Human|Lighthouse Lab in Glasgow|Wellcome Sanger Institute for the COVID-19 Genomics UK (COG-UK) consortium|Davies|United Kingdom
>Spike|hCoV-19/USA/WA-S2272/2020|2020-06-10|EPI_ISL_497700|Original|hCoV-19^^Washington|Human|Washington State Department of Health|Seattle Flu Study|Lee|USA
>Spike|hCoV-19/USA/UT-UPHL-01358/2020|2020-04-01|EPI_ISL_575039|Original|hCoV-19^^Utah|Human|Utah Public Health Laboratory|Utah Public Health Laboratory|Young|USA
>Spike|hCoV-19/mink/Netherlands/NB-EMC-2-3/2020|2020-04-27|EPI_ISL_523075|Original|hCoV-19^^Noord Brabant|Neovison vison|Dutch COVID-19 response team|Erasmus Medical Center|Oude Munnink|Netherlands

>Spike|hCoV-19/India/GJ-GBRC50/2020|2020-04-30|EPI_ISL_444481|Original|hCoV-19^^Gujarat|Human|B.J. Medical College and Civil hospital|Gujarat Biotechnology Research Centre|Joshi|India
>Spike|hCoV-19/Australia/VIC12846/2020|2020-07-24|EPI_ISL_591797|Original|hCoV-19^^Victoria|Human|Microbiological Diagnostic Unit - Public Health Laboratory (MDU-PHL)|MDU-PHL|Schultz|Australia
>Spike|hCoV-19/England/ALDP-A68AC2/2020|2020-10-12|EPI_ISL_608898|Original|hCoV-19^^England|Human|Lighthouse Lab in Alderley Park|Wellcome Sanger Institute for the COVID-19 Genomics UK (COG-UK) consortium|Davies|United Kingdom
>Spike|hCoV-19/USA/TX-HMH-6583/2020|2020-06-24|EPI_ISL_546102|Original|hCoV-19^^Texas|Human|Houston Methodist Hospital|Houston Methodist Hospital|Long|USA
>Spike|hCoV-19/USA/UT-08348/2020|2020-05-14|EPI_ISL_524134|Original|hCoV-19^^Utah|Human|Utah Public Health Laboratory|Utah Public Health Laboratory|Young|USA
>Spike|hCoV-19/England/QEUH-9AE733/2020|2020-09-02|EPI_ISL_577235|Original|hCoV-19^^England|Human|University of Exeter|COVID-19 Genomics UK (COG-UK) Consortium|Temperton|United Kingdom
>Spike|hCoV-19/England/OXON-AEA9D/2020|2020-00-00|EPI_ISL_448752|Original|hCoV-19^^England|Human|Oxford Viromics|COVID-19 Genomics UK (COG-UK) Consortium|Golubchik|United Kingdom
>Spike|hCoV-19/USA/PR-B2K5/2020|2020-08-04|EPI_ISL_527384|Original|hCoV-19^^Puerto Rico|Human|University of Miami Immunology and Histocompatibility Laboratory|University of Miami Immunology and Histocompatibility Laboratory|Margolles-Clark|USA
>Spike|hCoV-19/Netherlands/NA_89/2020|2020-03-16|EPI_ISL_422849|Original|hCoV-19^^Netherlands|Human|Dutch COVID-19 response team|Erasmus Medical Center|Oude Munnink|Netherlands
>Spike|hCoV-19/Netherlands/ZuidHolland_181/2020|2020-04-23|EPI_ISL_461353|Original|hCoV-19^^Zuid Holland|Human|Dutch COVID-19 response team|Erasmus Medical Center|Oude Munnink|Netherlands
>Spike|hCoV-19/USA/DC-CDC-1738/2020|2020-03-28|EPI_ISL_436043|Original|hCoV-19^^District of Columbia|Human|DC Public Health Lab Dept of Forensic Science|Pathogen Discovery|Queen|USA
>Spike|hCoV-19/USA/TX-HMH0146/2020|2020-03-22|EPI_ISL_434845|Original|hCoV-19^^Texas|Human|Houston Methodist Hospital|Houston Methodist Hospital|Long|USA
>Spike|hCoV-19/Denmark/DCGC-5595/2020|2020-09-28|EPI_ISL_620920|Original|hCoV-19^^Syddanmark|Human|Department of Virus and Microbiological Special Diagnostics|Albertsen lab|Michaelsen|Denmark
>Spike|hCoV-19/Serbia/Novi Sad-212182506/2020|2020-06-24|EPI_ISL_676583|Original|hCoV-19^^Novi Sad|Human|Scientific Veterinary Institute Novi Sad|Veterinary Specialized Institute \Kraljevo\|Vidanovic|Serbia
>Spike|hCoV-19/USA/NC-200265/2020|2020-04-08|EPI_ISL_524431|Original|hCoV-19^^North Carolina|Human|Dirk Dittmer|Dirk Dittmer|Dittmer|USA
>Spike|hCoV-19/India/MH-ACTREC-304/2020|2020-07-15|EPI_ISL_699955|Original|hCoV-19^^Maharashtra|Human|Hematopathology Laboratory|Hematopathology Laboratory|Patkar|India
>Spike|hCoV-19/Wales/PHWC-4865E1/2020|2020-10-26|EPI_ISL_637622|Original|hCoV-19^^Wales|Human|Wales Specialist Virology Centre Sequencing lab|COVID-19 Genomics UK (COG-UK) Consortium|Moore|United Kingdom
>Spike|hCoV-19/Scotland/QEUH-A01C98/2020|2020-10-02|EPI_ISL_595834|Original|hCoV-19^^Scotland|Human|Virology Department|COVID-19 Genomics UK (COG-UK) Consortium|de Silva|United Kingdom
>Spike|hCoV-19/Iceland/216/2020|2020-03-16|EPI_ISL_417832|Original|hCoV-19^^Reykjavik|Human|The National University Hospital of Iceland|deCODE genetics|J\u00f3nsson|Iceland
>Spike|hCoV-19/England/BRIS-125D94/2020|2020-03-30|EPI_ISL_443992|Original|hCoV-19^^England|Human|PHE South West Regional Laboratory|Wellcome Sanger Institute for the COVID-19 Genomics UK (COG-UK) consortium|Amato|United Kingdom
>Spike|hCoV-19/England/QEUH-9F6918/2020|2020-09-27|EPI_ISL_588340|Original|hCoV-19^^England|Human|Lighthouse Lab in Glasgow|Wellcome Sanger Institute for the COVID-19 Genomics UK (COG-UK) consortium|Davies|United Kingdom

>Spike|hCoV-19/England/ALDP-9566B5/2020|2020-06-08|EPI_ISL_553909|Original|hCoV-19^^England|Human|Lighthouse Lab in Alderley Park|Wellcome Sanger Institute for the COVID-19 Genomics UK (COG-UK) consortium|Davies|United Kingdom
>Spike|hCoV-19/USA/NY-QDX-863/2020|2020-03-16|EPI_ISL_571670|Original|hCoV-19^^New York|Human|Quest Diagnostics|Quest Diagnostics|Kagan|USA
>Spike|hCoV-19/Australia/VIC11423/2020|2020-08-21|EPI_ISL_563368|Original|hCoV-19^^Victoria|Human|Microbiological Diagnostic Unit - Public Health Laboratory (MDU-PHL)|MDU-PHL|Schultz|Australia
>Spike|hCoV-19/USA/TX-HMH-4181/2020|2020-06-18|EPI_ISL_543417|Original|hCoV-19^^Texas|Human|Houston Methodist Hospital|Houston Methodist Hospital|Long|USA
>Spike|hCoV-19/Scotland/CVR5053/2020|2020-10-10|EPI_ISL_594787|Original|hCoV-19^^Scotland|Human|West of Scotland Specialist Virology Centre|COVID-19 Genomics UK (COG-UK) Consortium|Robertson|United Kingdom
>Spike|hCoV-19/Australia/NSW610/2020|2020-04-14|EPI_ISL_490019|Original|hCoV-19^^New South Wales|Human|South Eastern Area Laboratory Services (SEALS)|NSW Health Pathology - Institute of Clinical Pathology and Medical Research; Westmead Hospital; University of Sydney|Gall|Australia
>Spike|hCoV-19/USA/AZ-TG410668/2020|2020-06-24|EPI_ISL_694967|Original|hCoV-19^^Arizona|Human|TGen North|TGen North|French|USA
>Spike|hCoV-19/England/QEUH-A9F92F/2020|2020-10-20|EPI_ISL_651300|Original|hCoV-19^^England|Human|University of Exeter|COVID-19 Genomics UK (COG-UK) Consortium|Temperton|United Kingdom
>Spike|hCoV-19/England/ALDP-AA31E4/2020|2020-10-22|EPI_ISL_630763|Original|hCoV-19^^England|Human|Lighthouse Lab in Alderley Park|Wellcome Sanger Institute for the COVID-19 Genomics UK (COG-UK) consortium|Davies|United Kingdom
>Spike|hCoV-19/Wales/PHWC-16B07B/2020|2020-09-16|EPI_ISL_560238|Original|hCoV-19^^Wales|Human|Wales Specialist Virology Centre Sequencing lab|COVID-19 Genomics UK (COG-UK) Consortium|Moore|United Kingdom
>Spike|hCoV-19/Denmark/DCGC-6497/2020|2020-10-05|EPI_ISL_620397|Original|hCoV-19^^Midtjylland|Human|Department of Virus and Microbiological Special Diagnostics|Albertsen lab|Michaelsen|Denmark
>Spike|hCoV-19/Ireland/LK-UHL144/2020|2020-04-08|EPI_ISL_681734|Original|hCoV-19^^Limerick|Human|University Hospital Limerick|Irish Coronavirus Sequencing Consortium - Teagasc Moorepark|Walsh|Ireland
>Spike|hCoV-19/Ireland/D-NVRL-75IRL75521/2020|2020-10-13|EPI_ISL_671890|Original|hCoV-19^^Dublin|Human|National Virus Reference Laboratory|National Virus Reference Laboratory|Gonzalez|Ireland
>Spike|hCoV-19/England/CAMB-7283E/2020|2020-03-23|EPI_ISL_439877|Original|hCoV-19^^England|Human|Department of Pathology|Wellcome Sanger Institute for the COVID-19 Genomics UK (COG-UK) consortium|Amato|United Kingdom
>Spike|hCoV-19/England/CAMC-9C0319/2020|2020-09-08|EPI_ISL_573179|Original|hCoV-19^^England|Human|Oxford Viromics|COVID-19 Genomics UK (COG-UK) Consortium|Golubchik|United Kingdom
>Spike|hCoV-19/England/QEUH-A60D2B/2020|2020-10-12|EPI_ISL_665970|Original|hCoV-19^^England|Human|Northumbria University / South Tees Hospitals NHS Foundation Trust / North Cumbria Integrated Care NHS Foundation Trust / North Tees and Hartlepool NHS Foundation Trust / Newcastle Hospitals NHS Foundation Trust|COVID-19 Genomics UK (COG-UK) Consortium|Bashton|United Kingdom
>Spike|hCoV-19/England/QEUH-AF571E/2020|2020-10-30|EPI_ISL_662962|Original|hCoV-19^^England|Human|Lighthouse Lab in Glasgow|Wellcome Sanger Institute for the COVID-19 Genomics UK (COG-UK) Consortium|Davies|United Kingdom
>Spike|hCoV-19/Singapore/472/2020|2020-03-13|EPI_ISL_476807|Original|hCoV-19^^Singapore|Human|Department of Laboratory Medicine\nTan Tock Seng Hospital|Department of Laboratory Medicine\nTan Tock Seng Hospital|Chen|Singapore
>Spike|hCoV-19/Australia/VIC5884/2020|2020-07-20|EPI_ISL_518667|Original|hCoV-19^^Victoria|Human|Microbiological Diagnostic Unit - Public Health Laboratory (MDU-PHL)|MDU-PHL|Schultz|Australia
>Spike|hCoV-19/Scotland/CVR5114/2020|2020-10-08|EPI_ISL_626933|Original|hCoV-19^^Scotland|Human|West of Scotland Specialist Virology Centre|COVID-19 Genomics UK (COG-UK) Consortium|Robertson|United Kingdom

>Spike|hCoV-19/USA/UT-UPHL-2009329/2020|2020-05-17|EPI_ISL_524930|Original|hCoV-19^^Utah|Human|Utah Public Health Laboratory|Utah Public Health Laboratory|Young|USA
>Spike|hCoV-19/England/20166008104/2020|2020-04-14|EPI_ISL_466519|Original|hCoV-19^^England|Human|Respiratory Virus Unit|Respiratory Virus Unit|Platt|United Kingdom
>Spike|hCoV-19/India/KA-nimh-0996/2020|2020-04-14|EPI_ISL_428486|Original|hCoV-19^^Karnataka|Human|District Surveillence Unit|Department of Neurovirology|Pattabiraman|India
>Spike|hCoV-19/Spain/AR-IBV-005037/2020|2020-04-20|EPI_ISL_510340|Original|hCoV-19^^Aragon|Human|Servicio de Microbiolog\u00eda|SeqCOVID-SPAIN consortium/IBV(CSIC)|Comas|Spain
>Spike|hCoV-19/USA/CA-CZB-13013/2020|2020-10-25|EPI_ISL_672299|Original|hCoV-19^^California|Human|Santa Clara County Public Health Laboratory|Chan-Zuckerberg Biohub|Bhatt|USA
>Spike|hCoV-19/Australia/NSW2837/2020|2020-04-09|EPI_ISL_678357|Original|hCoV-19^^New South Wales|Human|Area of Virology|Virology Research Laboratory; Area of Virology|Foster|Australia
>Spike|hCoV-19/England/LIVE-9D0BD/2020|2020-04-06|EPI_ISL_449600|Original|hCoV-19^^England|Human|Liverpool Clinical Laboratories|COVID-19 Genomics UK (COG-UK) Consortium|Haldenby|United Kingdom
>Spike|hCoV-19/Northern Ireland/NIRE-1014A8/2020|2020-04-30|EPI_ISL_482157|Original|hCoV-19^^Northern Ireland|Human|Regional Virus Laboratory|Wellcome Sanger Institute for the COVID-19 Genomics UK (COG-UK) consortium|Davies|United Kingdom
>Spike|hCoV-19/England/MILK-87B6F7/2020|2020-07-19|EPI_ISL_557241|Original|hCoV-19^^England|Human|Lighthouse Lab in Milton Keynes|Wellcome Sanger Institute for the COVID-19 Genomics UK (COG-UK) consortium|Davies|United Kingdom
>Spike|hCoV-19/Japan/PG-7727/2020|2020-08-00|EPI_ISL_686829|Original|hCoV-19^^Japan|Human|Chiba Prefectural Institute of Public Health|Pathogen Genomics Center|Sekizuka|Japan
>Spike|hCoV-19/France/BRE-5400/2020|2020-03-27|EPI_ISL_443276|Original|hCoV-19^^Bretagne|Human|CHU - H\u00f4pital Cavale Blanche - Labo. de Virologie|National Reference Center for Viruses of Respiratory Infections|Enouf|France
>Spike|hCoV-19/USA/WA-S1019/2020|2020-04-30|EPI_ISL_463509|Original|hCoV-19^^Washington|Human|Washington State Department of Health|Seattle Flu Study|Bedford|USA
>Spike|hCoV-19/England/ALDP-C47CEF/2020|2020-12-12|EPI_ISL_718950|Original|hCoV-19^^England|Human|Lighthouse Lab in Alderley Park|Wellcome Sanger Institute for the COVID-19 Genomics UK (COG-UK) Consortium|Davies|United Kingdom
>Spike|hCoV-19/England/QEUH-9DD346/2020|2020-09-21|EPI_ISL_580886|Original|hCoV-19^^England|Human|Lighthouse Lab in Glasgow|Wellcome Sanger Institute for the COVID-19 Genomics UK (COG-UK) consortium|Davies|United Kingdom
>Spike|hCoV-19/USA/CO-QDX-2205/2020|2020-03-16|EPI_ISL_604517|Original|hCoV-19^^Colorado|Human|Quest Diagnostics|Quest Diagnostics|Kagan|USA
>Spike|hCoV-19/Denmark/DCGC-6943/2020|2020-10-12|EPI_ISL_619789|Original|hCoV-19^^Hovedstaden|Human|Department of Virus and Microbiological Special Diagnostics|Albertsen lab|Michaelsen|Denmark
>Spike|hCoV-19/Israel/CVL-n-6105/2020|2020-03-15|EPI_ISL_475000|Original|hCoV-19^^Gedera|Human|Israel Central Virology laboratory|Israel Central Virology laboratory|Zuckerman|Israel
>Spike|hCoV-19/USA/MI-MDHHS-SC21910/2020|2020-03-09|EPI_ISL_565971|Original|hCoV-19^^Michigan|Human|Michigan Department of Health and Human Services|Michigan Department of Health and Human Services|Blankenship|USA
>Spike|hCoV-19/England/MILK-B971C4/2020|2020-11-14|EPI_ISL_675085|Original|hCoV-19^^England|Human|Lighthouse Lab in Milton Keynes|Wellcome Sanger Institute for the COVID-19 Genomics UK (COG-UK) Consortium|Davies|United Kingdom
>Spike|hCoV-19/USA/MI-MDHHS-SC20216/2020|2020-03-13|EPI_ISL_447187|Original|hCoV-19^^Michigan|Human|Michigan Department of Health and Human Services|Michigan Department of Health and Human Services|Blankenship|USA
>Spike|hCoV-19/Japan/PG-0762/2020|2020-03-00|EPI_ISL_684372|Original|hCoV-19^^Japan|Human|Pathogen Genomics Center|Pathogen Genomics Center|Sekizuka|Japan

>Spike|hCoV-19/England/NORT-2926B4/2020|2020-05-13|EPI_ISL_489098|Original|hCoV-19^^England|Human|NU-OMICS DNA Sequencing research facility|Wellcome Sanger Institute for the COVID-19 Genomics UK (COG-UK) consortium|Davies|United Kingdom
>Spike|hCoV-19/England/QEUH-C645A5/2020|2020-12-14|EPI_ISL_778465|Original|hCoV-19^^England|Human|Lighthouse Lab in Glasgow|Wellcome Sanger Institute for the COVID-19 Genomics UK (COG-UK) Consortium|Davies|United Kingdom
>Spike|hCoV-19/England/CAMB-1ABE66/2020|2020-04-28|EPI_ISL_444337|Original|hCoV-19^^England|Human|Department of Pathology|COVID-19 Genomics UK (COG-UK) Consortium|Meredith|United Kingdom
>Spike|hCoV-19/Hong Kong/HKPU-3034/2020|2020-07-30|EPI_ISL_610212|Original|hCoV-19^^Hong Kong|Human|Department of Health Technology and Informatics|Department of Health Technology and Informatics|Leung|China
>Spike|hCoV-19/Wales/PHWC-48CC13/2020|2020-11-09|EPI_ISL_666521|Original|hCoV-19^^Wales|Human|Wales Specialist Virology Centre Sequencing lab|COVID-19 Genomics UK (COG-UK) Consortium|Moore|United Kingdom
>Spike|hCoV-19/England/MILK-A183B7/2020|2020-10-01|EPI_ISL_595030|Original|hCoV-19^^England|Human|Northumbria University / South Tees Hospitals NHS Foundation Trust / North Cumbria Integrated Care NHS Foundation Trust / North Tees and Hartlepool NHS Foundation Trust / Newcastle Hospitals NHS Foundation Trust|COVID-19 Genomics UK (COG-UK) Consortium|Bashton|United Kingdom
>Spike|hCoV-19/Australia/VIC5313/2020|2020-00-00|EPI_ISL_520076|Original|hCoV-19^^Victoria|Human|Microbiological Diagnostic Unit - Public Health Laboratory (MDU-PHL)|MDU-PHL|Schultz|Australia
>Spike|hCoV-19/England/CAMC-B07E31/2020|2020-10-29|EPI_ISL_643416|Original|hCoV-19^^England|Human|Lighthouse Lab in Cambridge|Wellcome Sanger Institute for the COVID-19 Genomics UK (COG-UK) Consortium|Davies|United Kingdom
>Spike|hCoV-19/Japan/PG-2665/2020|2020-06-00|EPI_ISL_690797|Original|hCoV-19^^Japan|Human|Tokyo Metropolitan Institute of Public Health|Pathogen Genomics Center|Sekizuka|Japan
>Spike|hCoV-19/USA/MN-MDH-1591/2020|2020-05-19|EPI_ISL_530187|Original|hCoV-19^^Minnesota|Human|Minnesota Department of Health|Minnesota Department of Health|Garfin|USA
>Spike|hCoV-19/England/ALDP-BB3AA9/2020|2020-11-19|EPI_ISL_701800|Original|hCoV-19^^England|Human|Lighthouse Lab in Alderley Park|Wellcome Sanger Institute for the COVID-19 Genomics UK (COG-UK) Consortium|Davies|United Kingdom
>Spike|hCoV-19/England/ALDP-8E2C26/2020|2020-06-25|EPI_ISL_557123|Original|hCoV-19^^England|Human|Lighthouse Lab in Alderley Park|Wellcome Sanger Institute for the COVID-19 Genomics UK (COG-UK) consortium|Davies|United Kingdom
>Spike|hCoV-19/India/TG-CCMB-L1824-ONT/2020|2020-06-02|EPI_ISL_495165|Original|hCoV-19^^Telangana|Human|CSIR-Centre for Cellular and Molecular Biology|CSIR-Centre for Cellular and Molecular Biology|Banu|India
>Spike|hCoV-19/Japan/PG-1404/2020|2020-04-00|EPI_ISL_685011|Original|hCoV-19^^Japan|Human|Saitama Prefectural Institute of Public Health|Pathogen Genomics Center|Sekizuka|Japan
>Spike|hCoV-19/New Zealand/20VR1936/2020|2020-04-04|EPI_ISL_456315|Original|hCoV-19^^Southern|Human|Southern Community Labs Dunedin|Institute of Environmental Science and Research (ESR)|de Ligt|New Zealand
>Spike|hCoV-19/Denmark/DCGC-19893/2020|2020-12-07|EPI_ISL_747552|Original|hCoV-19^^Syddanmark|Human|Department of Virus and Microbiological Special Diagnostics|Albertsen Lab|Michaelsen|Denmark
>Spike|hCoV-19/USA/ID-UNM-IBL312/2020|2020-03-28|EPI_ISL_632851|Original|hCoV-19^^Idaho|Human|Idaho Bureau of Laboratories|Center for Global Health|Domman|USA
>Spike|hCoV-19/USA/WY-WYPHL-00073/2020|2020-03-28|EPI_ISL_462965|Original|hCoV-19^^Wyoming|Human|Wyoming Public Health Laboratory|Center for Global Health|Domman|USA
>Spike|hCoV-19/Portugal/PT0397a/2020|2020-04-19|EPI_ISL_454119|Original|hCoV-19^^Portugal|Human|unknown|Instituto Nacional de Saude (INSA)|Borges|Portugal
>Spike|hCoV-19/USA/WA-S1941/2020|2020-06-03|EPI_ISL_497369|Original|hCoV-19^^Washington|Human|Washington State Department of Health|Seattle Flu Study|Lee|USA

>Spike|hCoV-19/Japan/IC-0355/2020|2020-12-00|EPI_ISL_721575|Original|hCoV-19^^Japan|Human|Pathogen Genomics Center|Pathogen Genomics Center|Sekizuka|Japan
>Spike|hCoV-19/Scotland/GCVR-16FAA6/2020|2020-03-20|EPI_ISL_489642|Original|hCoV-19^^Scotland|Human|NHSGGC West of Scotland Specialist Virology Centre / MRC-University of Glasgow Centre for Virus Research|Wellcome Sanger Institute for the COVID-19 Genomics UK (COG-UK) consortium|Davies|United Kingdom
>Spike|hCoV-19/England/MILK-A22833/2020|2020-10-02|EPI_ISL_612524|Original|hCoV-19^^England|Human|Northumbria University / South Tees Hospitals NHS Foundation Trust / North Cumbria Integrated Care NHS Foundation Trust / North Tees and Hartlepool NHS Foundation Trust / Newcastle Hospitals NHS Foundation Trust|COVID-19 Genomics UK (COG-UK) Consortium|Bashton|United Kingdom
>Spike|hCoV-19/Wales/PHWC-310CA/2020|2020-04-11|EPI_ISL_446401|Original|hCoV-19^^Wales|Human|Wales Specialist Virology Centre|Public Health Wales Microbiology Cardiff|Moore|United Kingdom
>Spike|hCoV-19/England/CAMC-AEAEDB/2020|2020-10-26|EPI_ISL_633107|Original|hCoV-19^^England|Human|Lighthouse Lab in Cambridge|Wellcome Sanger Institute for the COVID-19 Genomics UK (COG-UK) consortium|Davies|United Kingdom
>Spike|hCoV-19/Wales/PHWC-16D1C1/2020|2020-09-24|EPI_ISL_574209|Original|hCoV-19^^Wales|Human|Wales Specialist Virology Centre Sequencing lab|COVID-19 Genomics UK (COG-UK) Consortium|Moore|United Kingdom
>Spike|hCoV-19/Wales/PHWC-485C0B/2020|2020-10-26|EPI_ISL_628264|Original|hCoV-19^^Wales|Human|Wales Specialist Virology Centre Sequencing lab|COVID-19 Genomics UK (COG-UK) Consortium|Moore|United Kingdom
>Spike|hCoV-19/England/CAMB-1BC48E/2020|2020-11-27|EPI_ISL_723876|Original|hCoV-19^^England|Human|Department of Pathology|COVID-19 Genomics UK (COG-UK) Consortium|Goodfellow|United Kingdom
>Spike|hCoV-19/Jordan/AM-ALSR-4987/2020|2020-10-06|EPI_ISL_730260|Original|hCoV-19^^Amman|Human|Biolab Diagnostic Laboratories|Andersen lab at Scripps Research|Gangavarapu|Jordan
>Spike|hCoV-19/Belgium/ULG-10545/2020|2020-10-23|EPI_ISL_626306|Original|hCoV-19^^Li\u00e8ge|Human|Department of Clinical Microbiology|GIGA Medical Genomics|Durkin|Belgium
>Spike|hCoV-19/USA/FL-UF-14/2020|2020-04-20|EPI_ISL_468719|Original|hCoV-19^^Florida|Human|University of Florida|University of Florida|Elbadry|USA
>Spike|hCoV-19/England/SHEF-C3212/2020|2020-04-03|EPI_ISL_432766|Original|hCoV-19^^England|Human|Virology Department|COVID-19 Genomics UK (COG-UK) Consortium|de Silva|United Kingdom
>Spike|hCoV-19/England/CAMC-B21DCB/2020|2020-11-01|EPI_ISL_647260|Original|hCoV-19^^England|Human|Lighthouse Lab in Cambridge|Wellcome Sanger Institute for the COVID-19 Genomics UK (COG-UK) Consortium|Davies|United Kingdom
>Spike|hCoV-19/England/CAMB-1B872F/2020|2020-10-26|EPI_ISL_650130|Original|hCoV-19^^England|Human|Department of Pathology|COVID-19 Genomics UK (COG-UK) Consortium|Goodfellow|United Kingdom
>Spike|hCoV-19/USA/CA-CZB-9645/2020|2020-04-24|EPI_ISL_672146|Original|hCoV-19^^California|Human|The Ashley Laboratory|Chan-Zuckerberg Biohub|Bhatt|USA
>Spike|hCoV-19/Israel/2046815/2020|2020-04-04|EPI_ISL_447400|Original|hCoV-19^^North District|Human|Clinical Microbiology Laboratory|Stern Lab|Miller|Israel
>Spike|hCoV-19/USA/MI-MDHHS-SC20165/2020|2020-03-27|EPI_ISL_436878|Original|hCoV-19^^Michigan|Human|Michigan Department of Health and Human Services|Michigan Department of Health and Human Services|Blankenship|USA
>Spike|hCoV-19/Portugal/PT0977/2020|2020-03-30|EPI_ISL_511526|Original|hCoV-19^^Portugal|Human|Instituto Nacional de Saude (INSA)|Instituto Nacional de Saude (INSA)|Borges|Portugal
>Spike|hCoV-19/England/ALDP-9CD07F/2020|2020-09-16|EPI_ISL_566149|Original|hCoV-19^^England|Human|Lighthouse Lab in Alderley Park|Wellcome Sanger Institute for the COVID-19 Genomics UK (COG-UK) consortium|Davies|United Kingdom
>Spike|hCoV-19/England/NORW-EA01B/2020|2020-04-30|EPI_ISL_448386|Original|hCoV-19^^England|Human|Quadram Institute Bioscience|COVID-19 Genomics UK (COG-UK) Consortium|Alikhan|United Kingdom

>Spike|hCoV-19/Jordan/AM-ALSR-4052/2020|2020-10-03|EPI_ISL_636412|Original|hCoV-19^^Amman|Human|Biolab Diagnostic Laboratories|Andersen lab at Scripps Research|Gangavarapu|Jordan
>Spike|hCoV-19/Denmark/DCGC-3926/2020|2020-09-14|EPI_ISL_615837|Original|hCoV-19^^Hovedstaden|Human|Department of Virus and Microbiological Special Diagnostics|Albertsen lab|Michaelsen|Denmark
>Spike|hCoV-19/England/ALDP-525422/2020|2020-06-02|EPI_ISL_559041|Original|hCoV-19^^England|Human|Lighthouse Lab in Alderley Park|Wellcome Sanger Institute for the COVID-19 Genomics UK (COG-UK) consortium|Davies|United Kingdom
>Spike|hCoV-19/Australia/VIC185/2020|2020-03-19|EPI_ISL_419881|Original|hCoV-19^^Victoria|Human|Victorian Infectious Diseases Reference Laboratory (VIDRL)|Victorian Infectious Diseases Reference Laboratory and Microbiological Diagnostic Unit Public Health Laboratory|Schultz|Australia
>Spike|hCoV-19/USA/WA-S760/2020|2020-04-14|EPI_ISL_449930|Original|hCoV-19^^Washington|Human|Washington State Department of Health|Seattle Flu Study|Bedford|USA
>Spike|hCoV-19/Scotland/QEUH-95E44A/2020|2020-08-08|EPI_ISL_534411|Original|hCoV-19^^Scotland|Human|NHSGGC West of Scotland Specialist Virology Centre / MRC-University of Glasgow Centre for Virus Research|Wellcome Sanger Institute for the COVID-19 Genomics UK (COG-UK) consortium|Davies|United Kingdom
>Spike|hCoV-19/England/BRIS-12C911/2020|2020-05-13|EPI_ISL_469516|Original|hCoV-19^^England|Human|PHE South West Regional Laboratory|Wellcome Sanger Institute for the COVID-19 Genomics UK (COG-UK) consortium|Davies|United Kingdom
>Spike|hCoV-19/Jordan/SEARCH-5453/2020|2020-09-13|EPI_ISL_755244|Original|hCoV-19^^Amman|Human|Biolab Diagnostic Laboratories|Andersen lab at Scripps Research|Gangavarapu|Jordan
>Spike|hCoV-19/USA/TX-DSHS-1029/2020|2020-03-30|EPI_ISL_644183|Original|hCoV-19^^Texas|Human|Texas Department of State Health Services|Texas Department of State Health Services|Rahman|USA
>Spike|hCoV-19/England/LOND-126367D/2020|2020-11-03|EPI_ISL_679462|Original|hCoV-19^^England|Human|University College London|COVID-19 Genomics UK (COG-UK) Consortium|Roy|United Kingdom
>Spike|hCoV-19/USA/TX-HMH-MCoV-14008/2020|2020-07-05|EPI_ISL_787299|Original|hCoV-19^^Texas|Human|Houston Methodist Hospital|Houston Methodist Hospital|Long|USA
>Spike|hCoV-19/USA/VA-DCLS-1783/2020|2020-09-15|EPI_ISL_644869|Original|hCoV-19^^Virginia|Human|Virginia DCLS|Virginia DCLS|St. Jacques|USA
>Spike|hCoV-19/England/CAMC-B328C6/2020|2020-11-05|EPI_ISL_659618|Original|hCoV-19^^England|Human|Lighthouse Lab in Cambridge|Wellcome Sanger Institute for the COVID-19 Genomics UK (COG-UK) Consortium|Davies|United Kingdom
>Spike|hCoV-19/Canada/ON-S832/2020|2020-04-11|EPI_ISL_755741|Original|hCoV-19^^Ontario|Human|Toronto Invasive Bacterial Diseases Network|McMaster University|McArthur|Canada
>Spike|hCoV-19/India/MH-ACTREC-661/2020|2020-09-15|EPI_ISL_723072|Original|hCoV-19^^Maharashtra|Human|Hematopathology Laboratory|Hematopathology Laboratory|Patkar|India
>Spike|hCoV-19/England/ALDP-A6DEFE/2020|2020-10-14|EPI_ISL_607676|Original|hCoV-19^^England|Human|Lighthouse Lab in Alderley Park|Wellcome Sanger Institute for the COVID-19 Genomics UK (COG-UK) consortium|Davies|United Kingdom
>Spike|hCoV-19/USA/CA-QDX-29/2020|2020-03-15|EPI_ISL_467826|Original|hCoV-19^^California|Human|Quest Diagnostics|Quest Diagnostics|Kagan|USA
>Spike|hCoV-19/England/QEUH-AF4E47/2020|2020-10-30|EPI_ISL_663158|Original|hCoV-19^^England|Human|Lighthouse Lab in Glasgow|Wellcome Sanger Institute for the COVID-19 Genomics UK (COG-UK) Consortium|Davies|United Kingdom
>Spike|hCoV-19/England/QEUH-B402A9/2020|2020-11-08|EPI_ISL_657122|Original|hCoV-19^^England|Human|Lighthouse Lab in Glasgow|Wellcome Sanger Institute for the COVID-19 Genomics UK (COG-UK) Consortium|Davies|United Kingdom
>Spike|hCoV-19/Denmark/DCGC-19887/2020|2020-12-07|EPI_ISL_747853|Original|hCoV-19^^Midtjylland|Human|Department of Virus and Microbiological Special Diagnostics|Albertsen Lab|Michaelsen|Denmark

>Spike|hCoV-19/USA/AZ-TG331778/2020|2020-05-25|EPI_ISL_694352|Original|hCoV-19^^Arizona|Human|TGen North|TGen North|French|USA
>Spike|hCoV-19/England/QEUH-C32CC9/2020|2020-12-11|EPI_ISL_782731|Original|hCoV-19^^England|Human|Lighthouse Lab in Glasgow|Wellcome Sanger Institute for the COVID-19 Genomics UK (COG-UK) Consortium|Davies|United Kingdom
>Spike|hCoV-19/Belgium/DJS-0330307/2020|2020-03-30|EPI_ISL_462161|Original|hCoV-19^^Balen|Human|KU Leuven|KU Leuven|Maes|Belgium
>Spike|hCoV-19/USA/MI-MDHHS-SC20324/2020|2020-03-14|EPI_ISL_450603|Original|hCoV-19^^Michigan|Human|Michigan Department of Health and Human Services|Michigan Department of Health and Human Services|Blankenship|USA
>Spike|hCoV-19/England/MILK-ACC798/2020|2020-10-21|EPI_ISL_629336|Original|hCoV-19^^England|Human|Lighthouse Lab in Milton Keynes|Wellcome Sanger Institute for the COVID-19 Genomics UK (COG-UK) consortium|Davies|United Kingdom
>Spike|hCoV-19/USA/TX-HMH-MCoV-13516/2020|2020-08-23|EPI_ISL_788642|Original|hCoV-19^^Texas|Human|Houston Methodist Hospital|Houston Methodist Hospital|Long|USA
>Spike|hCoV-19/Denmark/DCGC-16318/2020|2020-11-16|EPI_ISL_714700|Original|hCoV-19^^Sjaelland|Human|Department of Virus and Microbiological Special Diagnostics|Albertsen Lab|Michaelsen|Denmark
>Spike|hCoV-19/USA/WI-UW-1447/2020|2020-09-12|EPI_ISL_541579|Original|hCoV-19^^Wisconsin|Human|University of Wisconsin-Madison AIDS Vaccine Research Laboratories|University of Wisconsin-Madison AIDS Vaccine Research Laboratories|Baczenas|USA
>Spike|hCoV-19/Japan/PG-1841/2020|2020-04-00|EPI_ISL_685447|Original|hCoV-19^^Japan|Human|Tokyo Metropolitan Institute of Public Health|Pathogen Genomics Center|Sekizuka|Japan
>Spike|hCoV-19/USA/CA-CZB-4048/2020|2020-08-16|EPI_ISL_548607|Original|hCoV-19^^California|Human|County of Santa Clara Public Health Department|Chan-Zuckerberg Biohub|Bhatt|USA
>Spike|hCoV-19/USA/TX-HMH-4284/2020|2020-06-18|EPI_ISL_543495|Original|hCoV-19^^Texas|Human|Houston Methodist Hospital|Houston Methodist Hospital|Long|USA
>Spike|hCoV-19/Turkey/HSGM-1492/2020|2020-03-19|EPI_ISL_437320|Vero P1|hCoV-19^^Istanbul|Human|Ministry of Health Turkey|Ministry of Health Turkey|Bayrakdar|Turkey
>Spike|hCoV-19/USA/TX-HMH-MCoV-8626/2020|2020-07-01|EPI_ISL_780660|Original|hCoV-19^^Texas|Human|Houston Methodist Hospital|Houston Methodist Hospital|Long|USA
>Spike|hCoV-19/Bahrain/920458273_S24/2020|2020-10-14|EPI_ISL_632902|Original|hCoV-19^^Bahrain|Human|Communicable Disease Laboratory|Communicable Disease Laboratory|Altaif|Bahrain
>Spike|hCoV-19/Switzerland/VD-ETHZ-210061/2020|2020-07-20|EPI_ISL_510794|Original|hCoV-19^^Vaud|Human|Viollier AG|Department of Biosystems Science and Engineering|Nadeau|Switzerland
>Spike|hCoV-19/England/LEED-2A9C8B/2020|2020-04-11|EPI_ISL_538923|Original|hCoV-19^^England|Human|Leeds Teaching Hospitals NHS Trust and Public Health England|Wellcome Sanger Institute for the COVID-19 Genomics UK (COG-UK) consortium|Davies|United Kingdom
>Spike|hCoV-19/USA/CA-SEARCH-5532/2020|2020-11-17|EPI_ISL_755119|Original|hCoV-19^^California|Human|San Diego County Public Health Laboratory|Andersen lab at Scripps Research|Gangavarapu|USA
>Spike|hCoV-19/England/MILK-A7D7D2/2020|2020-10-13|EPI_ISL_606372|Original|hCoV-19^^England|Human|Lighthouse Lab in Milton Keynes|Wellcome Sanger Institute for the COVID-19 Genomics UK (COG-UK) consortium|Davies|United Kingdom
>Spike|hCoV-19/England/ALDP-A2FB79/2020|2020-10-05|EPI_ISL_587141|Original|hCoV-19^^England|Human|Lighthouse Lab in Alderley Park|Wellcome Sanger Institute for the COVID-19 Genomics UK (COG-UK) consortium|Davies|United Kingdom
>Spike|hCoV-19/England/CAMB-78C78/2020|2020-04-03|EPI_ISL_441116|Original|hCoV-19^^England|Human|Department of Pathology|Wellcome Sanger Institute for the COVID-19 Genomics UK (COG-UK) consortium|Amato|United Kingdom
>Spike|hCoV-19/USA/TX-HMH-MCoV-12701/2020|2020-08-31|EPI_ISL_790471|Original|hCoV-19^^Texas|Human|Houston Methodist Hospital|Houston Methodist Hospital|Long|USA

>Spike|hCoV-19/United Arab Emirates/0364/2020|2020-05-15|EPI_ISL_698539|Original|hCoV-19^^Abu Dhabi|Human|Group 42 (G42) Healthcare|G42 Healthcare|Ogrodzki|United Arab Emirates
>Spike|hCoV-19/England/QEUH-9F39FD/2020|2020-09-24|EPI_ISL_588527|Original|hCoV-19^^England|Human|Lighthouse Lab in Glasgow|Wellcome Sanger Institute for the COVID-19 Genomics UK (COG-UK) consortium|Davies|United Kingdom
>Spike|hCoV-19/England/ALDP-954D3A/2020|2020-06-12|EPI_ISL_553381|Original|hCoV-19^^England|Human|Lighthouse Lab in Alderley Park|Wellcome Sanger Institute for the COVID-19 Genomics UK (COG-UK) consortium|Davies|United Kingdom
>Spike|hCoV-19/Denmark/DCGC-21499/2020|2020-12-21|EPI_ISL_758196|Original|hCoV-19^^Hovedstaden|Human|Department of Virus and Microbiological Special Diagnostics|Albertsen Lab|Michaelsen|Denmark
>Spike|hCoV-19/USA/CT-QDX-763/2020|2020-03-18|EPI_ISL_571512|Original|hCoV-19^^Connecticut|Human|Quest Diagnostics|Quest Diagnostics|Kagan|USA
>Spike|hCoV-19/USA/MI-MDHHS-SC22123/2020|2020-10-02|EPI_ISL_614174|Original|hCoV-19^^Michigan|Human|Michigan Department of Health and Human Services|Michigan Department of Health and Human Services|Jacob|USA
>Spike|hCoV-19/USA/FL-BPHL-1329/2020|2020-07-14|EPI_ISL_541267|Original|hCoV-19^^Florida|Human|Florida Bureau of Public Health Laboratories|Florida Bureau of Public Health Laboratories|Blanton|USA
>Spike|hCoV-19/England/QEUH-9AE3F3/2020|2020-09-05|EPI_ISL_638638|Original|hCoV-19^^England|Human|Department of Pathology|COVID-19 Genomics UK (COG-UK) Consortium|Goodfellow|United Kingdom
>Spike|hCoV-19/Japan/PG-1753/2020|2020-04-00|EPI_ISL_685359|Original|hCoV-19^^Japan|Human|Pathogen Genomics Center|Pathogen Genomics Center|Sekizuka|Japan
>Spike|hCoV-19/Spain/AR-IBV-98010673/2020|2020-11-12|EPI_ISL_722268|Original|hCoV-19^^Aragon|Human|Servicio de Microbiolog\u00eda|SeqCOVID-SPAIN consortium/IBV(CSIC)|Comas|Spain
>Spike|hCoV-19/Canada/QC-LSPQ-L00227094/2020|2020-03-18|EPI_ISL_535857|Original|hCoV-19^^Quebec|Human|H\u00f4pital Pierre-Boucher|Laboratoire de sant\u00e9 publique du Qu\u00e9bec|Moreira|Canada
>Spike|hCoV-19/Brazil/SP-122/2020|2020-03-31|EPI_ISL_515522|Original|hCoV-19^^S\u00e3o Paulo|Human|UPA 24HS de Itatiba|Instituto Adolfo Lutz|Sacchi|Brazil
>Spike|hCoV-19/England/ALDP-664A06/2020|2020-07-03|EPI_ISL_555562|Original|hCoV-19^^England|Human|Lighthouse Lab in Alderley Park|Wellcome Sanger Institute for the COVID-19 Genomics UK (COG-UK) consortium|Davies|United Kingdom
>Spike|hCoV-19/Denmark/DCGC-19144/2020|2020-12-07|EPI_ISL_748779|Original|hCoV-19^^Midtjylland|Human|Department of Virus and Microbiological Special Diagnostics|Albertsen Lab|Michaelsen|Denmark
>Spike|hCoV-19/USA/CO-CDPHE-2100005226/2020|2020-11-19|EPI_ISL_771289|Original|hCoV-19^^Colorado|Human|Colorado Department of Public Health and Environment|Colorado Department of Puplic Health and Environment|Bankers|USA
>Spike|hCoV-19/England/QEUH-9B78BC/2020|2020-09-08|EPI_ISL_590652|Original|hCoV-19^^England|Human|Lighthouse Lab in Glasgow|Wellcome Sanger Institute for the COVID-19 Genomics UK (COG-UK) consortium|Davies|United Kingdom
>Spike|hCoV-19/Switzerland/VD-ETHZ-100037/2020|2020-03-11|EPI_ISL_466961|Original|hCoV-19^^Vaud|Human|Viollier AG|Department of Biosystems Science and Engineering|Nadeau|Switzerland
>Spike|hCoV-19/Japan/IC-0533/2020|2020-12-00|EPI_ISL_779216|Original|hCoV-19^^Japan|Human|Pathogen Genomics Center|Pathogen Genomics Center|Sekizuka|Japan
>Spike|hCoV-19/USA/WI-UW-770/2020|2020-07-07|EPI_ISL_495475|Original|hCoV-19^^Wisconsin|Human|University of Wisconsin-Madison AIDS Vaccine Research Laboratories|University of Wisconsin-Madison AIDS Vaccine Research Laboratories|Baczenas|USA
>Spike|hCoV-19/Peru/ICA-INS-039/2020|2020-03-31|EPI_ISL_491464|Original|hCoV-19^^Ica|Human|Laboratorio de Referencia Nacional de Virus Respiratorio. Instituto Nacional de Salud Per\u00fa|Laboratorio de Referencia Nacional de Biotecnolog\u00eda y Biolog\u00eda Molecular. Instituto Nacional de Salud Per\u00fa|Padilla Rojas|Peru

>Spike|hCoV-19/Denmark/DCGC-1489/2020|2020-06-01|EPI_ISL_617973|Original|hCoV-
19^^Hovedstaden|Human|Department of Virus and Microbiological Special
Diagnostics|Albertsen lab|Michaelsen|Denmark
>Spike|hCoV-19/USA/UT-UPHL-2012138984/2020|2020-08-10|EPI_ISL_745844|Original|hCoV-
19^^Utah|Human|Ginkgo Bioworks Clinical Laboratory|Utah Public Health
Laboratory|Young|USA
>Spike|hCoV-19/Japan/PG-6691/2020|2020-07-00|EPI_ISL_687802|Original|hCoV-
19^^Japan|Human|Pathogen Genomics Center|Pathogen Genomics Center|Sekizuka|Japan
>Spike|hCoV-19/England/CAMB-7E61A/2020|2020-04-08|EPI_ISL_524548|Original|hCoV-
19^^England|Human|Department of Pathology|Wellcome Sanger Institute for the COVID-19
Genomics UK (COG-UK) consortium|Davies|United Kingdom
>Spike|hCoV-19/Denmark/DCGC-16930/2020|2020-12-14|EPI_ISL_750312|Original|hCoV-
19^^Midtjylland|Human|Department of Virus and Microbiological Special
Diagnostics|Albertsen Lab|Michaelsen|Denmark
>Spike|hCoV-19/South Korea/KCDC2075/2020|2020-02-21|EPI_ISL_497976|Original|hCoV-
19^^South Korea|Human|Division of Viral Diseases|Division of Viral Diseases|Kim|South
Korea
>Spike|hCoV-19/India/MH-NIV-223537/2020|2020-09-23|EPI_ISL_577649|Original|hCoV-
19^^Maharashtra|Human|NIV Influenza|NIV Influenza|Potdar|India
>Spike|hCoV-19/Brazil/RJ-00417/2020|2020-10-09|EPI_ISL_717819|Original|hCoV-19^^Rio
de Janeiro|Human|Laboratorio de Virologia Molecular / UFRJ|Bioinformatics Laboratory
/ LNCC|Almeida|Brazil
>Spike|hCoV-19/USA/WI-GMF-11002/2020|2020-07-16|EPI_ISL_547622|Original|hCoV-
19^^Wisconsin|Human|Gundersen Molecular Diagnostics Laboratory|Kabara Cancer Research
Institute|Kenny|USA
>Spike|hCoV-19/USA/MI-MDHHS-SC20099/2020|2020-03-30|EPI_ISL_437186|Original|hCoV-
19^^Michigan|Human|Michigan Department of Health and Human Services|Michigan
Department of Health and Human Services|Blankenship|USA
>Spike|hCoV-19/England/EXET-13A075/2020|2020-11-06|EPI_ISL_664202|Original|hCoV-
19^^England|Human|University of Exeter|COVID-19 Genomics UK (COG-UK)
Consortium|Temperton|United Kingdom
>Spike|hCoV-19/France/OCC-108/2020|2020-10-22|EPI_ISL_671954|Original|hCoV-
19^^Occitanie|Human|CHU Purpan - Laboratoire de Virologie - Institut
F\u00e9d\u00e9ratif de Biologie|CHU Purpan - Laboratoire de Virologie - Institut
F\u00e9d\u00e9ratif de Biologie|Latour|France
>Spike|hCoV-19/Scotland/GCVR-170811/2020|2020-03-26|EPI_ISL_489657|Original|hCoV-
19^^Scotland|Human|NHSGGC West of Scotland Specialist Virology Centre / MRC-
University of Glasgow Centre for Virus Research|Wellcome Sanger Institute for the
COVID-19 Genomics UK (COG-UK) consortium|Davies|United Kingdom
>Spike|hCoV-19/USA/VA-DCLS-2109/2020|2020-10-17|EPI_ISL_677143|Original|hCoV-
19^^Virginia|Human|Virginia Division of Consolidated Laboratory Services|Virginia
Division of Consolidated Laboratory Services|St. Jacques|USA
>Spike|hCoV-19/England/ALDP-955DEE/2020|2020-06-12|EPI_ISL_554066|Original|hCoV-
19^^England|Human|Lighthouse Lab in Alderley Park|Wellcome Sanger Institute for the
COVID-19 Genomics UK (COG-UK) consortium|Davies|United Kingdom
>Spike|hCoV-19/England/CAMB-1B73D1/2020|2020-10-15|EPI_ISL_637406|Original|hCoV-
19^^England|Human|Department of Pathology|COVID-19 Genomics UK (COG-UK)
Consortium|Goodfellow|United Kingdom
>Spike|hCoV-19/England/MILK-A06862/2020|2020-10-01|EPI_ISL_600029|Original|hCoV-
19^^England|Human|Lighthouse Lab in Milton Keynes|Wellcome Sanger Institute for the
COVID-19 Genomics UK (COG-UK) consortium|Davies|United Kingdom
>Spike|hCoV-19/England/QEUH-A216B2/2020|2020-10-01|EPI_ISL_587451|Original|hCoV-
19^^England|Human|Lighthouse Lab in Glasgow|Wellcome Sanger Institute for the COVID-
19 Genomics UK (COG-UK) consortium|Davies|United Kingdom
>Spike|hCoV-19/Wales/PHWC-4A2FE1/2020|2020-12-14|EPI_ISL_744041|Original|hCoV-
19^^Wales|Human|Wales Specialist Virology Centre Sequencing lab|COVID-19 Genomics UK
(COG-UK) Consortium|Moore|United Kingdom
>Spike|hCoV-19/England/ALDP-A946AB/2020|2020-10-19|EPI_ISL_610961|Original|hCoV-
19^^England|Human|Lighthouse Lab in Alderley Park|Wellcome Sanger Institute for the
COVID-19 Genomics UK (COG-UK) consortium|Davies|United Kingdom

>Spike|hCoV-19/England/MILK-ACFC1E/2020|2020-10-22|EPI_ISL_652127|Original|hCoV-19^^England|Human|Oxford Viromics|COVID-19 Genomics UK (COG-UK) Consortium|Golubchik|United Kingdom
>Spike|hCoV-19/Scotland/CVR3456/2020|2020-05-10|EPI_ISL_456859|Original|hCoV-19^^Scotland|Human|West of Scotland Specialist Virology Centre|COVID-19 Genomics UK (COG-UK) Consortium|Robertson|United Kingdom
>Spike|hCoV-19/France/ARA-SC345/2020|2020-03-15|EPI_ISL_660340|Original|hCoV-19^^Auvergne-Rh\u00f4ne-Alpes|Human|CHU de Saint-\u00c9tienne H\u00f4pital Nord|CNR Virus des Infections Respiratoires - France SUD|Bal|France
>Spike|hCoV-19/Wales/PHWC-4957CB/2020|2020-11-23|EPI_ISL_726397|Original|hCoV-19^^Wales|Human|Originating lab|COVID-19 Genomics UK (COG-UK) Consortium|Moore|United Kingdom
>Spike|hCoV-19/Gambia/1251/2020|2020-05-07|EPI_ISL_471168|Original|hCoV-19^^Kombo|Human|MRCG at LSHTM Genomics lab|MRCG at LSHTM Genomics lab|Sesay|Gambia
>Spike|hCoV-19/Australia/ACT0047/2020|2020-03-16|EPI_ISL_498514|Original|hCoV-19^^Australian Capital Territory|Human|ACT Pathology|Schwessinger Lab|Hall|Australia
>Spike|hCoV-19/Denmark/DCGC-11812/2020|2020-10-26|EPI_ISL_671150|Original|hCoV-19^^Hovedstaden|Human|Department of Virus and Microbiological Special Diagnostics|Albertsen Lab|Michaelsen|Denmark
>Spike|hCoV-19/Wales/PHWC-16AE90/2020|2020-09-11|EPI_ISL_560212|Original|hCoV-19^^Wales|Human|Wales Specialist Virology Centre Sequencing lab|COVID-19 Genomics UK (COG-UK) Consortium|Moore|United Kingdom
>Spike|hCoV-19/England/QEUH-7A774B/2020|2020-07-08|EPI_ISL_533113|Original|hCoV-19^^England|Human|Lighthouse Lab in Glasgow|Wellcome Sanger Institute for the COVID-19 Genomics UK (COG-UK) consortium|Davies|United Kingdom
>Spike|hCoV-19/USA/CA-CZB-15007/2020|2020-11-18|EPI_ISL_738601|Original|hCoV-19^^California|Human|Santa Clara County Public Health Laboratory|Chan-Zuckerberg Biohub|Kamm|USA
>Spike|hCoV-19/Scotland/QEUH-A2163A/2020|2020-10-01|EPI_ISL_598891|Original|hCoV-19^^Scotland|Human|Lighthouse Lab in Glasgow|Wellcome Sanger Institute for the COVID-19 Genomics UK (COG-UK) consortium|Davies|United Kingdom
>Spike|hCoV-19/USA/WI-WSLH-200646/2020|2020-06-16|EPI_ISL_631415|Original|hCoV-19^^Wisconsin|Human|Wisconsin State Laboratory of Hygiene Communicable Disease Division|Wisconsin State Laboratory of Hygiene Communicable Disease Division|Florek|USA
>Spike|hCoV-19/Luxembourg/LNS7716837/2020|2020-10-27|EPI_ISL_744619|Original|hCoV-19^^Luxembourg|Human|Laboratoire national de sant\u00e9|Laboratoire national de sant\u00e9|Wienecke-Baldacchino|Luxembourg
>Spike|hCoV-19/Wales/PHWC-16C11D/2020|2020-09-22|EPI_ISL_574012|Original|hCoV-19^^Wales|Human|Wales Specialist Virology Centre Sequencing lab|COVID-19 Genomics UK (COG-UK) Consortium|Moore|United Kingdom
>Spike|hCoV-19/England/MILK-A6B31F/2020|2020-10-09|EPI_ISL_608700|Original|hCoV-19^^England|Human|Lighthouse Lab in Milton Keynes|Wellcome Sanger Institute for the COVID-19 Genomics UK (COG-UK) consortium|Davies|United Kingdom
>Spike|hCoV-19/USA/WY-WPHL-20148235/2020|2020-11-23|EPI_ISL_770086|Original|hCoV-19^^Wyoming|Human|Wyoming Public Health Laboratory|Wyoming Public Health Laboratory|Hull|USA
>Spike|hCoV-19/Russia/OMS-ORINFI-7899S/2020|2020-07-21|EPI_ISL_569789|Original|hCoV-19^^Omsk|Human|Omsk Research Institute of Natural Focal Infections|WHO National Influenza Centre Russian Federation|Komissarov|Russia
>Spike|hCoV-19/England/LIVE-A7FA4/2020|2020-03-26|EPI_ISL_517098|Original|hCoV-19^^England|Human|Liverpool Clinical Laboratories|COVID-19 Genomics UK (COG-UK) Consortium|Haldenby|United Kingdom
>Spike|hCoV-19/Iran/K1r-138/2020|2020-05-00|EPI_ISL_759868|Original|hCoV-19^^Iran|Human|Virology|Razi Vaccine and Serum Research Institute|Ranjbar|Iran
>Spike|hCoV-19/England/ALDP-505482/2020|2020-06-24|EPI_ISL_559107|Original|hCoV-19^^England|Human|Lighthouse Lab in Alderley Park|Wellcome Sanger Institute for the COVID-19 Genomics UK (COG-UK) consortium|Davies|United Kingdom

>Spike|hCoV-19/USA/LA-EVTL1125/2020|2020-12-05|EPI_ISL_768525|Original|hCoV-19^^Louisiana|Human|LSUHS Emerging Viral Threat Laboratory|Microbial Genome Sequencing Center|Kamil|USA
>Spike|hCoV-19/Australia/VIC1015/2020|2020-04-01|EPI_ISL_430559|Original|hCoV-19^^Victoria|Human|Victorian Infectious Diseases Reference Laboratory (VIDRL)|Microbiological Diagnostic Unit Public Health Laboratory and Victorian Infectious Diseases Reference Laboratory|Schultz|Australia
>Spike|hCoV-19/Belgium/ULG-7921/2020|2020-03-17|EPI_ISL_447136|Original|hCoV-19^^Liege|Human|Department of Clinical Microbiology|GIGA Medical Genomics|Durkin|Belgium
>Spike|hCoV-19/Scotland/QEUH-95E143/2020|2020-08-07|EPI_ISL_534422|Original|hCoV-19^^Scotland|Human|NHSGGC West of Scotland Specialist Virology Centre / MRC-University of Glasgow Centre for Virus Research|Wellcome Sanger Institute for the COVID-19 Genomics UK (COG-UK) consortium|Davies|United Kingdom
>Spike|hCoV-19/England/MILK-9A90F1/2020|2020-08-31|EPI_ISL_551050|Original|hCoV-19^^England|Human|Lighthouse Lab in Milton Keynes|Wellcome Sanger Institute for the COVID-19 Genomics UK (COG-UK) consortium|Davies|United Kingdom
>Spike|hCoV-19/Chile/RM-157770/2020|2020-08-21|EPI_ISL_746680|Original|hCoV-19^^Region Metropolitana de Santiago|Human|Genetica Molecular and Subdepartamento de Virologia ISP Chile|Instituto de Salud Publica de Chile|Tognarelli|Chile
>Spike|hCoV-19/Portugal/PT1591/2020|2020-07-03|EPI_ISL_693596|Original|hCoV-19^^Portugal|Human|Instituto Nacional de Saude (INSA)|Instituto Nacional de Saude (INSA)|Borges|Portugal
>Spike|hCoV-19/England/QEUH-C31FD0/2020|2020-12-11|EPI_ISL_782651|Original|hCoV-19^^England|Human|Lighthouse Lab in Glasgow|Wellcome Sanger Institute for the COVID-19 Genomics UK (COG-UK) Consortium|Davies|United Kingdom
>Spike|hCoV-19/Spain/AR-IBV-2410/2020|2020-03-28|EPI_ISL_468825|Original|hCoV-19^^Aragon|Human|Servicio de Microbiolog\u00eda|SeqCOVID-SPAIN consortium/IBV(CSIC)|Comas|Spain
>Spike|hCoV-19/Colombia/MET-INS-95637/2020|2020-04-09|EPI_ISL_498164|Original|hCoV-19^^Meta|Human|Instituto Nacional de Salud|Instituto Nacional de Salud|Franco-Mu\u00f1oz|Colombia
>Spike|hCoV-19/Netherlands/un-EMC-737/2020|2020-06-16|EPI_ISL_523492|Original|hCoV-19^^Netherlands|Human|Dutch COVID-19 response team|Erasmus Medical Center|Oude Munnink|Netherlands
>Spike|hCoV-19/Spain/CT-IBV-99009891/2020|2020-08-07|EPI_ISL_660288|Original|hCoV-19^^Catalunya|Human|Servicio de Microbiolog\u00eda|SeqCOVID-SPAIN consortium/IBV(CSIC)|Comas|Spain
>Spike|hCoV-19/England/QEUH-98F4F5/2020|2020-08-25|EPI_ISL_540418|Original|hCoV-19^^England|Human|Lighthouse Lab in Glasgow|Wellcome Sanger Institute for the COVID-19 Genomics UK (COG-UK) consortium|Davies|United Kingdom
>Spike|hCoV-19/England/CAMC-AEAB7A/2020|2020-10-26|EPI_ISL_633189|Original|hCoV-19^^England|Human|Lighthouse Lab in Cambridge|Wellcome Sanger Institute for the COVID-19 Genomics UK (COG-UK) consortium|Davies|United Kingdom
>Spike|hCoV-19/USA/TX-HMH-7973/2020|2020-06-22|EPI_ISL_545755|Original|hCoV-19^^Texas|Human|Houston Methodist Hospital|Houston Methodist Hospital|Long|USA
>Spike|hCoV-19/USA/UT-08585/2020|2020-05-26|EPI_ISL_524336|Original|hCoV-19^^Utah|Human|Utah Public Health Laboratory|Utah Public Health Laboratory|Young|USA
>Spike|hCoV-19/England/OXON-B1C64/2020|2020-04-24|EPI_ISL_479082|Original|hCoV-19^^England|Human|Oxford Viromics|COVID-19 Genomics UK (COG-UK) Consortium|Golubchik|United Kingdom
>Spike|hCoV-19/South Africa/KRISP-K004537/2020|2020-10-18|EPI_ISL_660235|Original|hCoV-19^^KwaZulu-Natal|Human|NHLS-IALCH|KRISP|de Oliveira|South Africa
>Spike|hCoV-19/Spain/AS-MG-100-20/2020|2020-05-30|EPI_ISL_463000|Original|hCoV-19^^Asturias|Human|Molecular Genetics|Molecular Genetics|Submitter|Spain
>Spike|hCoV-19/Thailand/Bangkok-0019/2020|2020-03-20|EPI_ISL_423040|Original|hCoV-19^^Bangkok|Human|Ramathibodi Hospital|COVID-19 Network Investigations (CONI) Alliance|Batty|Thailand

>Spike|hCoV-19/Japan/PG-4370/2020|2020-05-00|EPI_ISL_692597|Original|hCoV-19^^Japan|Human|Pathogen Genomics Center|Pathogen Genomics Center|Sekizuka|Japan
>Spike|hCoV-19/England/ALDP-94E2DA/2020|2020-06-19|EPI_ISL_555619|Original|hCoV-19^^England|Human|Lighthouse Lab in Alderley Park|Wellcome Sanger Institute for the COVID-19 Genomics UK (COG-UK) consortium|Davies|United Kingdom
>Spike|hCoV-19/Wales/PHWC-36353/2020|2020-04-29|EPI_ISL_474655|Original|hCoV-19^^Wales|Human|Originating lab|COVID-19 Genomics UK (COG-UK) Consortium|Moore|United Kingdom
>Spike|hCoV-19/England/QEUH-A625C7/2020|2020-10-12|EPI_ISL_706972|Original|hCoV-19^^England|Human|Oxford Viromics|COVID-19 Genomics UK (COG-UK) Consortium|Golubchik|United Kingdom
>Spike|hCoV-19/Denmark/DCGC-7866/2020|2020-10-26|EPI_ISL_618836|Original|hCoV-19^^Hovedstaden|Human|Department of Virus and Microbiological Special Diagnostics|Albertsen lab|Michaelsen|Denmark
>Spike|hCoV-19/England/ALDP-C48DFA/2020|2020-12-11|EPI_ISL_736616|Original|hCoV-19^^England|Human|Lighthouse Lab in Alderley Park|Wellcome Sanger Institute for the COVID-19 Genomics UK (COG-UK) Consortium|Davies|United Kingdom
>Spike|hCoV-19/England/SHEF-D0300/2020|2020-04-15|EPI_ISL_448902|Original|hCoV-19^^England|Human|Virology Laboratory|COVID-19 Genomics UK (COG-UK) Consortium|de Silva|United Kingdom
>Spike|hCoV-19/Denmark/DCGC-16898/2020|2020-12-14|EPI_ISL_750631|Original|hCoV-19^^Nordjylland|Human|Department of Virus and Microbiological Special Diagnostics|Albertsen Lab|Michaelsen|Denmark
>Spike|hCoV-19/Poland/79288/2020|2020-11-00|EPI_ISL_732908|Original|hCoV-19^^Poland|Human|genXone SA|genXone SA|Kaszuba|Poland
>Spike|hCoV-19/England/MILK-AB828F/2020|2020-10-21|EPI_ISL_623325|Original|hCoV-19^^England|Human|Lighthouse Lab in Milton Keynes|Wellcome Sanger Institute for the COVID-19 Genomics UK (COG-UK) consortium|Davies|United Kingdom
>Spike|hCoV-19/England/MILK-B861AA/2020|2020-11-13|EPI_ISL_674035|Original|hCoV-19^^England|Human|Lighthouse Lab in Milton Keynes|Wellcome Sanger Institute for the COVID-19 Genomics UK (COG-UK) Consortium|Davies|United Kingdom
>Spike|hCoV-19/England/CAMC-A3EC47/2020|2020-10-04|EPI_ISL_609594|Original|hCoV-19^^England|Human|Lighthouse Lab in Cambridge|Wellcome Sanger Institute for the COVID-19 Genomics UK (COG-UK) consortium|Davies|United Kingdom
>Spike|hCoV-19/Wales/PHWC-48EFEB/2020|2020-11-03|EPI_ISL_681033|Original|hCoV-19^^Wales|Human|Wales Specialist Virology Centre Sequencing lab|COVID-19 Genomics UK (COG-UK) Consortium|Moore|United Kingdom
>Spike|hCoV-19/USA/WI-UW-892/2020|2020-07-10|EPI_ISL_509894|Original|hCoV-19^^Wisconsin|Human|University of Wisconsin-Madison AIDS Vaccine Research Laboratories|University of Wisconsin-Madison AIDS Vaccine Research Laboratories|Baczenas|USA
>Spike|hCoV-19/Denmark/DCGC-18266/2020|2020-12-14|EPI_ISL_749851|Original|hCoV-19^^Sjaelland|Human|Department of Virus and Microbiological Special Diagnostics|Albertsen Lab|Michaelsen|Denmark
>Spike|hCoV-19/England/ALDP-9FCEB9/2020|2020-10-01|EPI_ISL_588011|Original|hCoV-19^^England|Human|Lighthouse Lab in Alderley Park|Wellcome Sanger Institute for the COVID-19 Genomics UK (COG-UK) consortium|Davies|United Kingdom
>Spike|hCoV-19/Ecuador/USFQ-344/2020|2020-09-02|EPI_ISL_697789|Original|hCoV-19^^Imbabura|Human|Institute of Microbiology|Institute of Microbiology|Cardenas|Ecuador
>Spike|hCoV-19/Scotland/GCVR-1720CC/2020|2020-03-30|EPI_ISL_469977|Original|hCoV-19^^Scotland|Human|NHSGGC West of Scotland Specialist Virology Centre / MRC-University of Glasgow Centre for Virus Research|Wellcome Sanger Institute for the COVID-19 Genomics UK (COG-UK) consortium|Davies|United Kingdom
>Spike|hCoV-19/England/EXET-13A27F/2020|2020-10-31|EPI_ISL_664592|Original|hCoV-19^^England|Human|University of Exeter|COVID-19 Genomics UK (COG-UK) Consortium|Temperton|United Kingdom
>Spike|hCoV-19/USA/FL-BPHL-1880/2020|2020-04-07|EPI_ISL_613817|Original|hCoV-19^^Florida|Human|Florida Bureau of Public Health Laboratories|Florida Bureau of Public Health Laboratories|Blanton|USA

>Spike|hCoV-19/England/QEUH-B10E35/2020|2020-11-02|EPI_ISL_642310|Original|hCoV-19^^England|Human|Lighthouse Lab in Glasgow|Wellcome Sanger Institute for the COVID-19 Genomics UK (COG-UK) Consortium|Davies|United Kingdom
>Spike|hCoV-19/Japan/PG-6030/2020|2020-07-00|EPI_ISL_689142|Original|hCoV-19^^Japan|Human|Pathogen Genomics Center|Pathogen Genomics Center|Sekizuka|Japan
>Spike|hCoV-19/England/CAMC-A65737/2020|2020-10-11|EPI_ISL_637396|Original|hCoV-19^^England|Human|Department of Pathology|COVID-19 Genomics UK (COG-UK) Consortium|Goodfellow|United Kingdom
>Spike|hCoV-19/Wales/PHWC-16ABE4/2020|2020-09-13|EPI_ISL_560169|Original|hCoV-19^^Wales|Human|Wales Specialist Virology Centre Sequencing lab|COVID-19 Genomics UK (COG-UK) Consortium|Moore|United Kingdom
>Spike|hCoV-19/Spain/VC-IBV-98003111/2020|2020-03-15|EPI_ISL_538047|Original|hCoV-19^^Comunitat Valenciana|Human|Hospital Universitari i Polit\u00e8cnic La Fe de Val\u00e8ncia|SeqCOVID-SPAIN consortium/IBV(CSIC)|Comas|Spain
>Spike|hCoV-19/USA/MD-HP12133/2020|2020-12-28|EPI_ISL_791443|Original|hCoV-19^^Maryland|Human|Johns Hopkins Hospital Department of Pathology|Johns Hopkins Hospital Department of Pathology|Morris|USA
>Spike|hCoV-19/Wales/PHWC-16C2B0/2020|2020-09-27|EPI_ISL_574034|Original|hCoV-19^^Wales|Human|Wales Specialist Virology Centre Sequencing lab|COVID-19 Genomics UK (COG-UK) Consortium|Moore|United Kingdom
>Spike|hCoV-19/USA/WI-UW-2630/2021|2021-01-02|EPI_ISL_791146|Original|hCoV-19^^Wisconsin|Human|University of Wisconsin-Madison AIDS Vaccine Research Laboratories|University of Wisconsin-Madison AIDS Vaccine Research Laboratories|Moreno|USA
>Spike|hCoV-19/England/CAMC-BD1C09/2020|2020-11-27|EPI_ISL_702287|Original|hCoV-19^^England|Human|Lighthouse Lab in Cambridge|Wellcome Sanger Institute for the COVID-19 Genomics UK (COG-UK) Consortium|Davies|United Kingdom
>Spike|hCoV-19/England/LIVE-DCB435/2020|2020-11-22|EPI_ISL_724405|Original|hCoV-19^^England|Human|Liverpool Clinical Laboratories|COVID-19 Genomics UK (COG-UK) Consortium|Haldenby|United Kingdom
>Spike|hCoV-19/Denmark/DCGC-5677/2020|2020-09-28|EPI_ISL_619964|Original|hCoV-19^^Hovedstaden|Human|Department of Virus and Microbiological Special Diagnostics|Albertsen lab|Michaelsen|Denmark
>Spike|hCoV-19/Scotland/QEUH-96CEE1/2020|2020-08-21|EPI_ISL_534571|Original|hCoV-19^^Scotland|Human|NHSGGC West of Scotland Specialist Virology Centre / MRC-University of Glasgow Centre for Virus Research|Wellcome Sanger Institute for the COVID-19 Genomics UK (COG-UK) consortium|Davies|United Kingdom
>Spike|hCoV-19/Denmark/DCGC-14366/2020|2020-11-30|EPI_ISL_711723|Original|hCoV-19^^Nordjylland|Human|Department of Virus and Microbiological Special Diagnostics|Albertsen Lab|Michaelsen|Denmark
>Spike|hCoV-19/England/CAMC-9C01E2/2020|2020-09-09|EPI_ISL_573175|Original|hCoV-19^^England|Human|Oxford Viromics|COVID-19 Genomics UK (COG-UK) Consortium|Golubchik|United Kingdom
>Spike|hCoV-19/England/SHEF-CFDAC/2020|2020-04-22|EPI_ISL_453760|Original|hCoV-19^^England|Human|Virology Department|COVID-19 Genomics UK (COG-UK) Consortium|de Silva|United Kingdom
>Spike|hCoV-19/England/MILK-C5398F/2020|2020-12-12|EPI_ISL_769825|Original|hCoV-19^^England|Human|Lighthouse Lab in Milton Keynes|Wellcome Sanger Institute for the COVID-19 Genomics UK (COG-UK) Consortium|Davies|United Kingdom
>Spike|hCoV-19/USA/UT-UPHL-02828/2020|2020-04-24|EPI_ISL_575088|Original|hCoV-19^^Utah|Human|Utah Public Health Laboratory|Utah Public Health Laboratory|Young|USA
>Spike|hCoV-19/Denmark/DCGC-4473/2020|2020-09-14|EPI_ISL_615658|Original|hCoV-19^^Nordjylland|Human|Department of Virus and Microbiological Special Diagnostics|Albertsen lab|Michaelsen|Denmark
>Spike|hCoV-19/England/20132107304/2020|2020-03-23|EPI_ISL_423844|Original|hCoV-19^^England|Human|Respiratory Virus Unit|Respiratory Virus Unit|Platt|United Kingdom
>Spike|hCoV-19/USA/UT-UPHL-2012263380/2020|2020-10-11|EPI_ISL_746136|Original|hCoV-19^^Utah|Human|Ginkgo Bioworks Clinical Laboratory|Utah Public Health Laboratory|Young|USA

>Spike|hCoV-19/Colombia/QUI-INS-80663/2020|2020-03-18|EPI_ISL_456128|Original|hCoV-19^^Quindio|Human|Instituto Nacional de Salud - Unidad de Secuenciaci\u00f3n y An\u00e1lisis Gen\u00f3mico|Instituto Nacional de Salud|D\u00edaz|Colombia
>Spike|hCoV-19/Singapore/1/2020|2020-01-23|EPI_ISL_406973|Original|hCoV-19^^Singapore|Human|Singapore General Hospital|National Public Health Laboratory|Mak|Singapore
>Spike|hCoV-19/United Arab Emirates/0819/2020|2020-05-19|EPI_ISL_698958|Original|hCoV-19^^Abu Dhabi|Human|Group 42 (G42) Healthcare|G42 Healthcare|Ogrodzki|United Arab Emirates
>Spike|hCoV-19/England/QEUH-9FB4E5/2020|2020-09-28|EPI_ISL_588157|Original|hCoV-19^^England|Human|Lighthouse Lab in Glasgow|Wellcome Sanger Institute for the COVID-19 Genomics UK (COG-UK) consortium|Davies|United Kingdom
>Spike|hCoV-19/USA/VA-DCLS-0469/2020|2020-05-06|EPI_ISL_526777|Original|hCoV-19^^Virginia|Human|Virginia DCLS|Virginia DCLS|St. Jacques|USA
>Spike|hCoV-19/Scotland/CVR5322/2020|2020-10-24|EPI_ISL_650841|Original|hCoV-19^^Scotland|Human|West of Scotland Specialist Virology Centre|COVID-19 Genomics UK (COG-UK) Consortium|Robertson|United Kingdom
>Spike|hCoV-19/Denmark/DCGC-15270/2020|2020-11-23|EPI_ISL_713734|Original|hCoV-19^^Hovedstaden|Human|Department of Virus and Microbiological Special Diagnostics|Albertsen Lab|Michaelsen|Denmark
>Spike|hCoV-19/Wales/PHWC-4A617E/2020|2020-12-16|EPI_ISL_765007|Original|hCoV-19^^Wales|Human|Wales Specialist Virology Centre Sequencing lab|COVID-19 Genomics UK (COG-UK) Consortium|Moore|United Kingdom
>Spike|hCoV-19/Wales/PHWC-485A5C/2020|2020-10-24|EPI_ISL_628242|Original|hCoV-19^^Wales|Human|Wales Specialist Virology Centre Sequencing lab|COVID-19 Genomics UK (COG-UK) Consortium|Moore|United Kingdom
>Spike|hCoV-19/DRC/6313/2020|2020-05-09|EPI_ISL_496516|Original|hCoV-19^^Kinshasa|Human|Viral Respiratory Lab|Pathogen Sequencing Lab|Lokilo|Democratic Republic of the Congo
>Spike|hCoV-19/England/ALDP-955777/2020|2020-06-12|EPI_ISL_554550|Original|hCoV-19^^England|Human|Lighthouse Lab in Alderley Park|Wellcome Sanger Institute for the COVID-19 Genomics UK (COG-UK) consortium|Davies|United Kingdom
>Spike|hCoV-19/India/MH-BJMC-8827/2020|2020-06-03|EPI_ISL_728328|Original|hCoV-19^^Maharashtra|Human|B.J. Govt. Medical College|National Centre For Cell Science|Paul|India
>Spike|hCoV-19/England/ALDP-A865DC/2020|2020-10-17|EPI_ISL_606207|Original|hCoV-19^^England|Human|Lighthouse Lab in Alderley Park|Wellcome Sanger Institute for the COVID-19 Genomics UK (COG-UK) consortium|Davies|United Kingdom
>Spike|hCoV-19/India/TG-CDFD-C1132/2020|2020-05-30|EPI_ISL_528548|Original|hCoV-19^^Telangana|Human|National Genomics Core-Center for DNA Fingerprinting and Diagnostics|National Genomics Core- Center for DNA Fingerprinting and Diagnostics (NGC-CDFD)- DBT's PAN-INDIA-1000 Genome consortium|Vashisht|India
>Spike|hCoV-19/England/205180367/2020|2020-12-16|EPI_ISL_766452|Original|hCoV-19^^England|Human|Respiratory Virus Unit|COVID-19 Genomics UK (COG-UK) Consortium|Platt|United Kingdom
>Spike|hCoV-19/Japan/PG-4128/2020|2020-07-00|EPI_ISL_692367|Original|hCoV-19^^Japan|Human|Pathogen Genomics Center|Pathogen Genomics Center|Sekizuka|Japan
>Spike|hCoV-19/England/CAMC-B778D4/2020|2020-11-11|EPI_ISL_661799|Original|hCoV-19^^England|Human|Lighthouse Lab in Cambridge|Wellcome Sanger Institute for the COVID-19 Genomics UK (COG-UK) Consortium|Davies|United Kingdom
>Spike|hCoV-19/USA/TX-HMH-MCoV-8713/2020|2020-07-01|EPI_ISL_780715|Original|hCoV-19^^Texas|Human|Houston Methodist Hospital|Houston Methodist Hospital|Long|USA
>Spike|hCoV-19/England/QEUH-762111/2020|2020-07-16|EPI_ISL_532998|Original|hCoV-19^^England|Human|Lighthouse Lab in Glasgow|Wellcome Sanger Institute for the COVID-19 Genomics UK (COG-UK) consortium|Davies|United Kingdom
>Spike|hCoV-19/England/ALDP-954B9A/2020|2020-06-13|EPI_ISL_553411|Original|hCoV-19^^England|Human|Lighthouse Lab in Alderley Park|Wellcome Sanger Institute for the COVID-19 Genomics UK (COG-UK) consortium|Davies|United Kingdom

>Spike|hCoV-19/Wales/PHWC-164D5D/2020|2020-04-30|EPI_ISL_479377|Original|hCoV-19^^Wales|Human|Wales Specialist Virology Centre Sequencing lab|COVID-19 Genomics UK (COG-UK) Consortium|Moore|United Kingdom
>Spike|hCoV-19/Netherlands/ZH-EMC-429/2020|2020-08-11|EPI_ISL_523767|Original|hCoV-19^^Zuid Holland|Human|Dutch COVID-19 response team|Erasmus Medical Center|Oude Munnink|Netherlands
>Spike|hCoV-19/Brazil/RJ-00503/2020|2020-11-04|EPI_ISL_717897|Original|hCoV-19^^Rio de Janeiro|Human|Laboratorio de Virologia Molecular / UFRJ|Bioinformatics Laboratory / LNCC|Almeida|Brazil
>Spike|hCoV-19/Spain/CL-IBV-97006337/2020|2020-05-14|EPI_ISL_537687|Original|hCoV-19^^Castilla y Leon|Human|Universidad de Le\u00f3n|SeqCOVID-SPAIN consortium/IBV(CSIC)|Comas|Spain
>Spike|hCoV-19/England/20126036204/2020|2020-03-18|EPI_ISL_423902|Original|hCoV-19^^England|Human|Respiratory Virus Unit|Respiratory Virus Unit|Platt|United Kingdom
>Spike|hCoV-19/USA/FL-BPHL-0675/2020|2020-03-15|EPI_ISL_509733|Original|hCoV-19^^Florida|Human|Florida Bureau of Public Health Laboratories|Florida Bureau of Public Health Laboratories|Blanton|USA
>Spike|hCoV-19/Switzerland/BL-ETHZ-321501/2020|2020-10-15|EPI_ISL_603712|Original|hCoV-19^^Basel-Landschaft|Human|Viollier AG|Department of Biosystems Science and Engineering|Nadeau|Switzerland
>Spike|hCoV-19/Denmark/DCGC-6147/2020|2020-09-14|EPI_ISL_621181|Original|hCoV-19^^Midtjylland|Human|Department of Virus and Microbiological Special Diagnostics|Albertsen lab|Michaelsen|Denmark
>Spike|hCoV-19/England/LIVE-9DB52/2020|2020-04-29|EPI_ISL_449619|Original|hCoV-19^^England|Human|Liverpool Clinical Laboratories|COVID-19 Genomics UK (COG-UK) Consortium|Haldenby|United Kingdom
>Spike|hCoV-19/USA/FL-BPHL-1557/2020|2020-09-09|EPI_ISL_568655|Original|hCoV-19^^Florida|Human|Florida Bureau of Public Health Laboratories|Florida Bureau of Public Health Laboratories|Blanton|USA
>Spike|hCoV-19/Switzerland/BE-UNIBE-156-8498/2020|2020-03-13|EPI_ISL_574797|Original|hCoV-19^^Bern|Human|Institute for Infectious Diseases|Institute for Infectious Diseases|Koch|Switzerland
>Spike|hCoV-19/USA/WA-S2312/2020|2020-06-11|EPI_ISL_496944|Original|hCoV-19^^Washington|Human|Washington State Department of Health|Seattle Flu Study|Lee|USA
>Spike|hCoV-19/England/CAMC-B545F4/2020|2020-11-08|EPI_ISL_658134|Original|hCoV-19^^England|Human|Lighthouse Lab in Cambridge|Wellcome Sanger Institute for the COVID-19 Genomics UK (COG-UK) Consortium|Davies|United Kingdom
>Spike|hCoV-19/England/NORT-28AE3F/2020|2020-04-16|EPI_ISL_492955|Original|hCoV-19^^England|Human|NU-OMICS DNA Sequencing research facility|Wellcome Sanger Institute for the COVID-19 Genomics UK (COG-UK) consortium|Davies|United Kingdom
>Spike|hCoV-19/USA/MD-MDH-0331/2020|2020-10-14|EPI_ISL_648276|Original|hCoV-19^^Maryland|Human|MD PHL|MD PHL|Keller|USA
>Spike|hCoV-19/USA/AZ-TG338539/2020|2020-05-27|EPI_ISL_694374|Original|hCoV-19^^Arizona|Human|TGen North|TGen North|French|USA
>Spike|hCoV-19/USA/CA-CSMC95/2020|2020-04-02|EPI_ISL_475712|Original|hCoV-19^^California|Human|Cedars-Sinai Medical Center|Cedars-Sinai Medical Center|Zhang|USA
>Spike|hCoV-19/USA/UT-UPHL-2009473/2020|2020-07-18|EPI_ISL_605159|Original|hCoV-19^^Utah|Human|Utah Public Health Laboratory|Utah Public Health Laboratory|Young|USA
>Spike|hCoV-19/USA/UT-UPHL-2012053525/2020|2020-08-15|EPI_ISL_745576|Original|hCoV-19^^Utah|Human|Ginkgo Bioworks Clinical Laboratory|Utah Public Health Laboratory|Young|USA
>Spike|hCoV-19/Scotland/EDB4651/2020|2020-05-08|EPI_ISL_478178|Original|hCoV-19^^Scotland|Human|Virology Department|COVID-19 Genomics UK (COG-UK) Consortium|Williams|United Kingdom
>Spike|hCoV-19/Australia/NSW316/2020|2020-03-30|EPI_ISL_451579|Original|hCoV-19^^New South Wales|Human|Pathology West - NSW Health Pathology|NSW Health Pathology - Institute of Clinical Pathology and Medical Research; Westmead Hospital; University of Sydney|Gall|Australia

>Spike|hCoV-19/USA/VA-DCLS-1525/2020|2020-08-08|EPI_ISL_581522|Original|hCoV-
19^^Virginia|Human|Virginia DCLS|Virginia DCLS|St. Jacques|USA
>Spike|hCoV-19/Kenya/C815/2020|2020-04-16|EPI_ISL_568706|Original|hCoV-
19^^Mombasa|Human|KEMRI-Wellcome Trust Research Programme/KEMRI-CGMR-C Kilifi|KEMRI-
Wellcome Trust Research Programme/KEMRI-CGMR-C Kilifi|Githinji|Kenya
>Spike|hCoV-19/England/QEUH-AF291B/2020|2020-10-30|EPI_ISL_662727|Original|hCoV-
19^^England|Human|Lighthouse Lab in Glasgow|Wellcome Sanger Institute for the COVID-
19 Genomics UK (COG-UK) Consortium|Davies|United Kingdom
>Spike|hCoV-19/England/QEUH-A8BD39/2020|2020-10-16|EPI_ISL_643304|Original|hCoV-
19^^England|Human|Lighthouse Lab in Glasgow|Wellcome Sanger Institute for the COVID-
19 Genomics UK (COG-UK) Consortium|Davies|United Kingdom
>Spike|hCoV-19/Norway/3328/2020|2020-09-04|EPI_ISL_590880|original|hCoV-
19^^Viken|Human|Medical Microbiology Unit|Norwegian Institute of Public
Health|Vollan|Norway
>Spike|hCoV-19/USA/TX-HMH-2450/2020|2020-05-29|EPI_ISL_545316|Original|hCoV-
19^^Texas|Human|Houston Methodist Hospital|Houston Methodist Hospital|Long|USA
>Spike|hCoV-19/England/QEUH-A60D0D/2020|2020-10-10|EPI_ISL_665969|Original|hCoV-
19^^England|Human|Northumbria University / South Tees Hospitals NHS Foundation Trust
/ North Cumbria Integrated Care NHS Foundation Trust / North Tees and Hartlepool NHS
Foundation Trust / Newcastle Hospitals NHS Foundation Trust|COVID-19 Genomics UK
(COG-UK) Consortium|Bashton|United Kingdom
>Spike|hCoV-19/England/LIVE-DB60FD/2020|2020-11-06|EPI_ISL_650540|Original|hCoV-
19^^England|Human|Liverpool Clinical Laboratories|COVID-19 Genomics UK (COG-UK)
Consortium|Haldenby|United Kingdom
>Spike|hCoV-19/England/LCST-24D6801/2020|2020-05-30|EPI_ISL_478298|Original|hCoV-
19^^England|Human|University Hospitals Of Leicester NHS Trust and DeepSeq
Nottingham|COVID-19 Genomics UK (COG-UK) Consortium|Loose|United Kingdom
>Spike|hCoV-19/England/QEUH-B414C0/2020|2020-11-08|EPI_ISL_658562|Original|hCoV-
19^^England|Human|Lighthouse Lab in Glasgow|Wellcome Sanger Institute for the COVID-
19 Genomics UK (COG-UK) Consortium|Davies|United Kingdom
>Spike|hCoV-19/Wales/PHWC-49527F/2020|2020-11-21|EPI_ISL_726319|Original|hCoV-
19^^Wales|Human|Originating lab|COVID-19 Genomics UK (COG-UK) Consortium|Moore|United
Kingdom
>Spike|hCoV-19/England/CWAR-24FDE4E/2020|2020-06-04|EPI_ISL_584532|Original|hCoV-
19^^England|Human|UHCW / University of Warwick|COVID-19 Genomics UK (COG-UK)
Consortium|Stark|United Kingdom
>Spike|hCoV-19/USA/MI-MDHHS-SC20367/2020|2020-03-15|EPI_ISL_450578|Original|hCoV-
19^^Michigan|Human|Michigan Department of Health and Human Services|Michigan
Department of Health and Human Services|Blankenship|USA
>Spike|hCoV-19/Denmark/DCGC-16759/2020|2020-12-14|EPI_ISL_750192|Original|hCoV-
19^^Hovedstaden|Human|Department of Virus and Microbiological Special
Diagnostics|Albertsen Lab|Michaelsen|Denmark
>Spike|hCoV-19/England/20129044404/2020|2020-03-11|EPI_ISL_424047|Original|hCoV-
19^^England|Human|Respiratory Virus Unit|Respiratory Virus Unit|Platt|United Kingdom
>Spike|hCoV-19/USA/OR-OHSU-2507/2020|2020-08-17|EPI_ISL_667232|Original|hCoV-
19^^Oregon|Human|OHSU Lab Services Molecular Microbiology Lab|Oregon SARS-CoV-2
Genome Sequencing Center|O'Roak|USA
>Spike|hCoV-19/Japan/PG-1855/2020|2020-05-00|EPI_ISL_685461|Original|hCoV-
19^^Japan|Human|Tokyo Metropolitan Institute of Public Health|Pathogen Genomics
Center|Sekizuka|Japan
>Spike|hCoV-19/USA/TX-HMH-5233/2020|2020-06-26|EPI_ISL_544612|Original|hCoV-
19^^Texas|Human|Houston Methodist Hospital|Houston Methodist Hospital|Long|USA
>Spike|hCoV-19/Canada/QC-LSPQ-L00236430/2020|2020-03-24|EPI_ISL_536130|Original|hCoV-
19^^Quebec|Human|Centre Hospitalier R\u00e9gional de Lanaudi\u00e8re|Laboratoire de
sant\u00e9 publique du Qu\u00e9bec|Moreira|Canada
>Spike|hCoV-19/Australia/SAP293/2020|2020-03-25|EPI_ISL_468034|Original|hCoV-
19^^South Australia|Human|SA Pathology|SA Pathology|Leong|Australia
>Spike|hCoV-19/USA/DE-DHSS-F918613/2020|2020-03-20|EPI_ISL_574599|Original|hCoV-
19^^Delaware|Human|Delaware Public Health Lab|Delaware Public Health Lab|Bajwa|USA

>Spike|hCoV-19/USA/CA-CZB-178/2020|2020-05-08|EPI_ISL_583176|Original|hCoV-19^^California|Human|San Bernardino County Public Health Lab|Chan-Zuckerberg Biohub|Bhatt|USA
>Spike|hCoV-19/South Africa/KRISP-K001325/2020|2020-07-18|EPI_ISL_515810|Original|hCoV-19^^KwaZulu-Natal|Human|Medical Disagnostics Services (MDS)|KRISP|de Oliveira|South Africa
>Spike|hCoV-19/Singapore/894/2020|2020-02-02|EPI_ISL_536435|Original|hCoV-19^^Singapore|Human|National Public Health Laboratory|National Public Health Laboratory|Mak|Singapore
>Spike|hCoV-19/England/MILK-B38875/2020|2020-11-01|EPI_ISL_659066|Original|hCoV-19^^England|Human|Lighthouse Lab in Milton Keynes|Wellcome Sanger Institute for the COVID-19 Genomics UK (COG-UK) Consortium|Davies|United Kingdom
>Spike|hCoV-19/England/205190912/2020|2020-12-18|EPI_ISL_766501|Original|hCoV-19^^England|Human|Respiratory Virus Unit|COVID-19 Genomics UK (COG-UK) Consortium|Platt|United Kingdom
>Spike|hCoV-19/Switzerland/BS-UHB-42192010/2020|2020-03-15|EPI_ISL_528121|Original|hCoV-19^^Basel-Stadt|Human|University Hospital Basel|University Hospital Basel|Roloff Handschin|Switzerland
>Spike|hCoV-19/Wales/PHWC-47E33D/2020|2020-10-11|EPI_ISL_595440|Original|hCoV-19^^Wales|Human|Originating lab|COVID-19 Genomics UK (COG-UK) Consortium|Moore|United Kingdom
>Spike|hCoV-19/DRC/2536/2020|2020-04-15|EPI_ISL_437339|Original|hCoV-19^^Kinshasa|Human|Viral Respiratory Lab|Pathogen Sequencing Lab|Muyembe|Democratic Republic of the Congo
>Spike|hCoV-19/USA/NY-NYCPHL-001121/2020|2020-10-22|EPI_ISL_671704|Original|hCoV-19^^New York|Human|DOHMH Central Harlem|New York City Public Health Laboratory|Wang|USA
>Spike|hCoV-19/France/ARA-1276/2020|2020-03-23|EPI_ISL_509007|Original|hCoV-19^^Auvergne-Rh\u00f4ne-Alpes|Human|Institut des Agents Infectieux (IAI)|CNR Virus des Infections Respiratoires - France SUD|Josset|France
>Spike|hCoV-19/Scotland/QEUH-B7D80B/2020|2020-11-14|EPI_ISL_742250|Original|hCoV-19^^Scotland|Human|Oxford Viromics|COVID-19 Genomics UK (COG-UK) Consortium|Golubchik|United Kingdom
>Spike|hCoV-19/England/20119060904/2020|2020-03-11|EPI_ISL_465084|Original|hCoV-19^^England|Human|Respiratory Virus Unit|Respiratory Virus Unit|Platt|United Kingdom
>Spike|hCoV-19/England/CAMB-1AA74E/2020|2020-04-17|EPI_ISL_470238|Original|hCoV-19^^England|Human|Department of Pathology|Wellcome Sanger Institute for the COVID-19 Genomics UK (COG-UK) consortium|Davies|United Kingdom
>Spike|hCoV-19/Switzerland/BE-ETHZ-230052/2020|2020-08-04|EPI_ISL_516590|Original|hCoV-19^^Bern|Human|Viollier AG|Department of Biosystems Science and Engineering|Nadeau|Switzerland
>Spike|hCoV-19/USA/NY-Wadsworth-251316-01/2020|2020-10-20|EPI_ISL_734386|Original|hCoV-19^^New York|Human|Wadsworth Center|Wadsworth Center|Lasek-Nesselquist|USA
>Spike|hCoV-19/England/ALDP-98AB7D/2020|2020-08-03|EPI_ISL_529498|Original|hCoV-19^^England|Human|Liverpool Clinical Laboratories|COVID-19 Genomics UK (COG-UK) Consortium|Haldenby|United Kingdom
>Spike|hCoV-19/Portugal/PT0035/2020|2020-03-16|EPI_ISL_418020|Original|hCoV-19^^Portugal|Human|H Braga|Instituto Nacional de Saude (INSA)|Borges|Portugal
>Spike|hCoV-19/Denmark/DCGC-18292/2020|2020-12-07|EPI_ISL_749322|Original|hCoV-19^^Midtjylland|Human|Department of Virus and Microbiological Special Diagnostics|Albertsen Lab|Michaelsen|Denmark
>Spike|hCoV-19/England/QEUH-B69ADF/2020|2020-11-11|EPI_ISL_661515|Original|hCoV-19^^England|Human|Lighthouse Lab in Glasgow|Wellcome Sanger Institute for the COVID-19 Genomics UK (COG-UK) Consortium|Davies|United Kingdom
>Spike|hCoV-19/USA/FL-BPHL-2124/2020|2020-03-16|EPI_ISL_653277|Original|hCoV-19^^Florida|Human|Florida Bureau of Public Health Laboratories|Florida Bureau of Public Health Laboratories|Blanton|USA

>Spike|hCoV-19/Kenya/C4822/2020|2020-05-08|EPI_ISL_568739|Original|hCoV-19^^Mombasa|Human|KEMRI-Wellcome Trust Research Programme/KEMRI-CGMR-C Kilifi|KEMRI-Wellcome Trust Research Programme/KEMRI-CGMR-C Kilifi|Githinji|Kenya
>Spike|hCoV-19/USA/CA-SR0249/2020|2020-03-17|EPI_ISL_437561|Original|hCoV-19^^California|Human|Scripps Medical Laboratory|Andersen lab at Scripps Research|Gangavarapu|USA
>Spike|hCoV-19/Malaysia/UNIMAS-15790/2020|2020-11-18|EPI_ISL_718172|Original|hCoV-19^^Sarawak|Human|Ministry of Health Hospitals|Institute of Health and Community Medicine|Jonathan|Malaysia
>Spike|hCoV-19/England/QEUH-AAE0FB/2020|2020-10-22|EPI_ISL_630745|Original|hCoV-19^^England|Human|Lighthouse Lab in Glasgow|Wellcome Sanger Institute for the COVID-19 Genomics UK (COG-UK) consortium|Davies|United Kingdom
>Spike|hCoV-19/USA/CA-ALSR-4139/2020|2020-07-02|EPI_ISL_648843|Original|hCoV-19^^California|Human|San Diego County Public Health Laboratory|Andersen lab at Scripps Research|Gangavarapu|USA
>Spike|hCoV-19/England/QEUH-B66A2D/2020|2020-11-11|EPI_ISL_654981|Original|hCoV-19^^England|Human|Lighthouse Lab in Glasgow|Wellcome Sanger Institute for the COVID-19 Genomics UK (COG-UK) Consortium|Davies|United Kingdom
>Spike|hCoV-19/England/BRIS-12DC71/2020|2020-05-18|EPI_ISL_482022|Original|hCoV-19^^England|Human|PHE South West Regional Laboratory|Wellcome Sanger Institute for the COVID-19 Genomics UK (COG-UK) consortium|Davies|United Kingdom
>Spike|hCoV-19/England/MILK-97738A/2020|2020-07-24|EPI_ISL_550490|Original|hCoV-19^^England|Human|Lighthouse Lab in Milton Keynes|Wellcome Sanger Institute for the COVID-19 Genomics UK (COG-UK) consortium|Davies|United Kingdom
>Spike|hCoV-19/England/20119029504/2020|2020-03-12|EPI_ISL_465039|Original|hCoV-19^^England|Human|Respiratory Virus Unit|Respiratory Virus Unit|Platt|United Kingdom
>Spike|hCoV-19/USA/WA-UW-1954/2020|2020-03-24|EPI_ISL_424325|Original|hCoV-19^^Washington|Human|UW Virology Lab|UW Virology Lab|Roychoudhury|USA
>Spike|hCoV-19/Wales/PHWC-2B6B2/2020|2020-04-12|EPI_ISL_445746|Original|hCoV-19^^Wales|Human|Wales Specialist Virology Centre|Public Health Wales Microbiology Cardiff|Moore|United Kingdom
>Spike|hCoV-19/England/CAMC-B785DC/2020|2020-11-11|EPI_ISL_661947|Original|hCoV-19^^England|Human|Lighthouse Lab in Cambridge|Wellcome Sanger Institute for the COVID-19 Genomics UK (COG-UK) Consortium|Davies|United Kingdom
>Spike|hCoV-19/Wales/PHWC-489DE6/2020|2020-10-24|EPI_ISL_652493|Original|hCoV-19^^Wales|Human|Wales Specialist Virology Centre Sequencing lab|COVID-19 Genomics UK (COG-UK) Consortium|Moore|United Kingdom
>Spike|hCoV-19/England/MILK-9A9000/2020|2020-08-31|EPI_ISL_551129|Original|hCoV-19^^England|Human|Lighthouse Lab in Milton Keynes|Wellcome Sanger Institute for the COVID-19 Genomics UK (COG-UK) consortium|Davies|United Kingdom
>Spike|hCoV-19/USA/WI-WSLH-200358/2020|2020-04-21|EPI_ISL_578528|Original|hCoV-19^^Wisconsin|Human|Wisconsin State Laboratory of Hygiene Communicable Disease Division|Wisconsin State Laboratory of Hygiene Communicable Disease Division|Florek|USA
>Spike|hCoV-19/France/PAC-IHU-0061/2020|2020-03-19|EPI_ISL_569280|Original|hCoV-19^^Provence-Alpes-C\u00f4te-d\u2019Azur|Human|MEPHI|MEPHI|Levasseur|France
>Spike|hCoV-19/USA/MI-MDHHS-SC21203/2020|2020-03-31|EPI_ISL_507460|Original|hCoV-19^^Michigan|Human|Michigan Department of Health and Human Services|Michigan Department of Health and Human Services|Blankenship|USA
>Spike|hCoV-19/Denmark/DCGC-22119/2020|2020-12-14|EPI_ISL_758564|Original|hCoV-19^^Syddanmark|Human|Department of Virus and Microbiological Special Diagnostics|Albertsen Lab|Michaelsen|Denmark
>Spike|hCoV-19/USA/WA-UW-1822/2020|2020-03-22|EPI_ISL_424276|Original|hCoV-19^^Washington|Human|UW Virology Lab|UW Virology Lab|Roychoudhury|USA
>Spike|hCoV-19/Uganda/UG030/2020|2020-05-07|EPI_ISL_737932|Original|hCoV-19^^Uganda|Human|Uganda Central Public Health Lab and Uganda Virus Research Institute|MRC/UVRI & LSHTM Uganda\u00a0Research Unit|Cotten|Uganda
>Spike|hCoV-19/USA/TX-HMH-1975/2020|2020-04-28|EPI_ISL_542931|Original|hCoV-19^^Texas|Human|Houston Methodist Hospital|Houston Methodist Hospital|Long|USA

>Spike|hCoV-19/Canada/ON-S1460/2020|2020-08-00|EPI_ISL_770833|Original|hCoV-19^^Ontario|Human|Toronto Invasive Bacterial Diseases Network|McMaster University|McArthur|Canada
>Spike|hCoV-19/Luxembourg/LNS3120271/2020|2020-11-25|EPI_ISL_740252|Original|hCoV-19^^Luxembourg|Human|Laboratoire national de sant\u00e9|Laboratoire national de sant\u00e9|Wienecke-Baldacchino|Luxembourg
>Spike|hCoV-19/England/CAMC-B09242/2020|2020-10-30|EPI_ISL_643862|Original|hCoV-19^^England|Human|Lighthouse Lab in Cambridge|Wellcome Sanger Institute for the COVID-19 Genomics UK (COG-UK) Consortium|Davies|United Kingdom
>Spike|hCoV-19/England/CAMC-D042DC/2020|2020-12-14|EPI_ISL_781824|Original|hCoV-19^^England|Human|Lighthouse Lab in Cambridge|Wellcome Sanger Institute for the COVID-19 Genomics UK (COG-UK) Consortium|Davies|United Kingdom
>Spike|hCoV-19/Australia/VIC1112/2020|2020-04-07|EPI_ISL_430573|Original|hCoV-19^^Victoria|Human|Victorian Infectious Diseases Reference Laboratory (VIDRL)|Microbiological Diagnostic Unit Public Health Laboratory and Victorian Infectious Diseases Reference Laboratory|Schultz|Australia
>Spike|hCoV-19/Canada/ON-UHTC-0372/2020|2020-10-02|EPI_ISL_671657|Original|hCoV-19^^Ontario|Human|Unity Health Toronto|Ontario Institute for Cancer Research|de Borja|Canada
>Spike|hCoV-19/USA/TX-HMH-MCoV-14679/2020|2020-10-01|EPI_ISL_787964|Original|hCoV-19^^Texas|Human|Houston Methodist Hospital|Houston Methodist Hospital|Long|USA
>Spike|hCoV-19/Switzerland/VD-ETHZ-280165/2020|2020-09-08|EPI_ISL_560515|Original|hCoV-19^^Vaud|Human|Viollier AG|Department of Biosystems Science and Engineering|Nadeau|Switzerland
>Spike|hCoV-19/USA/TX-HMH-MCoV-16204/2020|2020-10-28|EPI_ISL_785888|Original|hCoV-19^^Texas|Human|Houston Methodist Hospital|Houston Methodist Hospital|Long|USA
>Spike|hCoV-19/England/LEED-2AA297/2020|2020-03-30|EPI_ISL_538869|Original|hCoV-19^^England|Human|Leeds Teaching Hospitals NHS Trust and Public Health England|Wellcome Sanger Institute for the COVID-19 Genomics UK (COG-UK) consortium|Davies|United Kingdom
>Spike|hCoV-19/England/MILK-A7B983/2020|2020-10-15|EPI_ISL_607461|Original|hCoV-19^^England|Human|Lighthouse Lab in Milton Keynes|Wellcome Sanger Institute for the COVID-19 Genomics UK (COG-UK) Consortium|Davies|United Kingdom
>Spike|hCoV-19/USA/LA-EVTL423/2020|2020-06-10|EPI_ISL_578829|Original|hCoV-19^^Louisiana|Human|LSUHS Emerging Viral Threat Laboratory|Microbial Genome Sequencing Center|Kamil|USA
>Spike|hCoV-19/England/NORW-F9802/2020|2020-12-02|EPI_ISL_725398|Original|hCoV-19^^England|Human|Quadram Institute Bioscience|COVID-19 Genomics UK (COG-UK) Consortium|Alikhan|United Kingdom
>Spike|hCoV-19/Belgium/VDLH-030548/2020|2020-03-05|EPI_ISL_418796|Original|hCoV-19^^Kalmthout|Human|KU Leuven|KU Leuven|Maes|Belgium
>Spike|hCoV-19/USA/VA-DCLS-0724/2020|2020-07-09|EPI_ISL_526847|Original|hCoV-19^^Virginia|Human|Virginia DCLS|Virginia DCLS|St. Jacques|USA
>Spike|hCoV-19/England/QEUH-9DD012/2020|2020-09-21|EPI_ISL_599520|Original|hCoV-19^^England|Human|Lighthouse Lab in Glasgow|Wellcome Sanger Institute for the COVID-19 Genomics UK (COG-UK) consortium|Davies|United Kingdom
>Spike|hCoV-19/Turkey/IST-GLAB-121/2020|2020-04-27|EPI_ISL_735269|Original|hCoV-19^^Istanbul|Human|Genomic Laboratory (GLAB) (Conjoint lab of Health Directorate of Istanbul and Istanbul Technical University)|Genomic Laboratory (GLAB)|Karacan|Turkey
>Spike|hCoV-19/USA/CA-ALSR-1664/2020|2020-04-01|EPI_ISL_494725|Original|hCoV-19^^California|Human|San Diego County Public Health Laboratory|Andersen lab at Scripps Research|Gangavarapu|USA
>Spike|hCoV-19/India/MH-NIV-18650/2020|2020-04-30|EPI_ISL_479510|Original|hCoV-19^^Maharashtra|Human|NIV Influenza|NIV Influenza|Potdar|India
>Spike|hCoV-19/England/ALDP-95AA2E/2020|2020-06-13|EPI_ISL_554980|Original|hCoV-19^^England|Human|Lighthouse Lab in Alderley Park|Wellcome Sanger Institute for the COVID-19 Genomics UK (COG-UK) consortium|Davies|United Kingdom
>Spike|hCoV-19/Netherlands/ZuidHolland_97/2020|2020-03-31|EPI_ISL_422633|Original|hCoV-19^^Zuid Holland|Human|Dutch COVID-19 response team|Erasmus Medical Center|Oude Munnink|Netherlands

>Spike|hCoV-19/USA/VA-DCLS-2414/2020|2020-11-23|EPI_ISL_723480|Original|hCoV-19^^Virginia|Human|Virginia DCLS|Virginia DCLS|St. Jacques|USA
>Spike|hCoV-19/England/ALDP-BB5263/2020|2020-11-21|EPI_ISL_701716|Original|hCoV-19^^England|Human|Lighthouse Lab in Alderley Park|Wellcome Sanger Institute for the COVID-19 Genomics UK (COG-UK) Consortium|Davies|United Kingdom
>Spike|hCoV-19/England/MILK-995AFA/2020|2020-08-25|EPI_ISL_551622|Original|hCoV-19^^England|Human|Lighthouse Lab in Milton Keynes|Wellcome Sanger Institute for the COVID-19 Genomics UK (COG-UK) consortium|Davies|United Kingdom
>Spike|hCoV-19/Japan/PG-6589/2020|2020-07-00|EPI_ISL_687701|Original|hCoV-19^^Japan|Human|Niigata Prefectural Institute of Public Health and Environmental Sciences|Pathogen Genomics Center|Sekizuka|Japan
>Spike|hCoV-19/USA/VA-DCLS-1131/2020|2020-04-25|EPI_ISL_529924|Original|hCoV-19^^Virginia|Human|Virginia Division of Consolidated Laboratory Services|Virginia Division of Consolidated Laboratory Services|St. Jacques|USA
>Spike|hCoV-19/England/LOND-124F413/2020|2020-08-15|EPI_ISL_665152|Original|hCoV-19^^England|Human|University College London Hospital|COVID-19 Genomics UK (COG-UK) Consortium|Byott|United Kingdom
>Spike|hCoV-19/Scotland/EDB374/2020|2020-03-30|EPI_ISL_425986|Original|hCoV-19^^Scotland|Human|Virology Department|COVID-19 Genomics UK (COG-UK) Consortium|Williams|United Kingdom
>Spike|hCoV-19/Denmark/DCGC-4579/2020|2020-09-14|EPI_ISL_622248|Original|hCoV-19^^Hovedstaden|Human|Department of Virus and Microbiological Special Diagnostics|Albertsen lab|Michaelsen|Denmark
>Spike|hCoV-19/England/MILK-B04739/2020|2020-10-24|EPI_ISL_650985|Original|hCoV-19^^England|Human|Department of Pathology|COVID-19 Genomics UK (COG-UK) Consortium|Goodfellow|United Kingdom
>Spike|hCoV-19/England/ALDP-B23A77/2020|2020-11-04|EPI_ISL_646989|Original|hCoV-19^^England|Human|Lighthouse Lab in Alderley Park|Wellcome Sanger Institute for the COVID-19 Genomics UK (COG-UK) Consortium|Davies|United Kingdom
>Spike|hCoV-19/Wuhan/HB-WH1-134/2020|2020-02-22|EPI_ISL_454911|Original|hCoV-19^^Hubei|Human|Wuhan Chain Medical Labs (CMLabs)|State Key Laboratory of Biotherapy of Sichuan University|Tang|China
>Spike|hCoV-19/England/QEUH-C30AA3/2020|2020-12-10|EPI_ISL_731383|Original|hCoV-19^^England|Human|Lighthouse Lab in Glasgow|Wellcome Sanger Institute for the COVID-19 Genomics UK (COG-UK) Consortium|Davies|United Kingdom
>Spike|hCoV-19/USA/IL-UW-1946/2020|2020-09-27|EPI_ISL_677354|Original|hCoV-19^^Illinois|Human|University of Wisconsin-Madison AIDS Vaccine Research Laboratories|University of Wisconsin-Madison AIDS Vaccine Research Laboratories|Baczenas|USA
>Spike|hCoV-19/Luxembourg/LNS7627646/2020|2020-10-06|EPI_ISL_744600|Original|hCoV-19^^Luxembourg|Human|Laboratoire national de sant\u00e9|Laboratoire national de sant\u00e9|Wienecke-Baldacchino|Luxembourg
>Spike|hCoV-19/Spain/PV-IBV-2084/2020|2020-04-10|EPI_ISL_468949|Original|hCoV-19^^Basque Country|Human|Servicio de Microbiolog\u00eda. Hospital Universitario Donostia. OSI Donostialdea. \u00c1rea de Enfermedades Infecciosas|SeqCOVID-SPAIN consortium/IBV(CSIC)|Comas|Spain
>Spike|hCoV-19/Wales/PHWC-3570D/2020|2020-04-24|EPI_ISL_474464|Original|hCoV-19^^Wales|Human|Originating lab|COVID-19 Genomics UK (COG-UK) Consortium|Moore|United Kingdom
>Spike|hCoV-19/England/MILK-BAB725/2020|2020-11-18|EPI_ISL_783164|Original|hCoV-19^^England|Human|Lighthouse Lab in Milton Keynes|Wellcome Sanger Institute for the COVID-19 Genomics UK (COG-UK) Consortium|Davies|United Kingdom
>Spike|hCoV-19/England/QEUH-CDEAB6/2020|2020-12-26|EPI_ISL_782238|Original|hCoV-19^^England|Human|Lighthouse Lab in Glasgow|Wellcome Sanger Institute for the COVID-19 Genomics UK (COG-UK) Consortium|Davies|United Kingdom
>Spike|hCoV-19/Vietnam/VNHN_1166/2020|2020-03-18|EPI_ISL_511896|Original|hCoV-19^^Hanoi|Human|National Hospital of Tropical Diseases|Oxford University Clinical Research Unit|Nguyen|Vietnam

>Spike|hCoV-19/England/BIRM-65D4E/2020|2020-10-09|EPI_ISL_612223|Original|hCoV-19^^England|Human|University of Birmingham|COVID-19 Genomics UK (COG-UK) Consortium|Loman|United Kingdom
>Spike|hCoV-19/Bangladesh/BCSIR-NILMRC-353/2020|2020-07-15|EPI_ISL_514231|Original|hCoV-19^^Khulna|Human|National Institute of Laboratory Medicine and Referral Center|Genomic Research Lab|Khan|Bangladesh
>Spike|hCoV-19/Japan/PG-2214/2020|2020-04-00|EPI_ISL_685819|Original|hCoV-19^^Japan|Human|Pathogen Genomics Center|Pathogen Genomics Center|Sekizuka|Japan
>Spike|hCoV-19/England/MILK-9A6980/2020|2020-09-01|EPI_ISL_550260|Original|hCoV-19^^England|Human|Lighthouse Lab in Milton Keynes|Wellcome Sanger Institute for the COVID-19 Genomics UK (COG-UK) consortium|Davies|United Kingdom
>Spike|hCoV-19/England/QEUH-B1072B/2020|2020-11-02|EPI_ISL_641875|Original|hCoV-19^^England|Human|Lighthouse Lab in Glasgow|Wellcome Sanger Institute for the COVID-19 Genomics UK (COG-UK) Consortium|Davies|United Kingdom
>Spike|hCoV-19/USA/CT-Yale-306/2020|2020-03-30|EPI_ISL_476535|Original|hCoV-19^^Connecticut|Human|Yale Clinical Virology Laboratory|Grubaugh Lab - Yale School of Public Health|Grubaugh|USA
>Spike|hCoV-19/England/QEUH-B3F401/2020|2020-11-08|EPI_ISL_658615|Original|hCoV-19^^England|Human|Lighthouse Lab in Glasgow|Wellcome Sanger Institute for the COVID-19 Genomics UK (COG-UK) Consortium|Davies|United Kingdom
>Spike|hCoV-19/Wales/PHWC-49D15C/2020|2020-12-10|EPI_ISL_742740|Original|hCoV-19^^Wales|Human|Wales Specialist Virology Centre Sequencing lab|COVID-19 Genomics UK (COG-UK) Consortium|Moore|United Kingdom
>Spike|hCoV-19/Canada/QC_AU6/2020|2020-04-05|EPI_ISL_515230|Original|hCoV-19^^Quebec|Human|Laboratoire de microbiologie|Smith Laboratory|Smith|Canada
>Spike|hCoV-19/USA/TX-HMH-MCoV-14987/2020|2020-10-16|EPI_ISL_786471|Original|hCoV-19^^Texas|Human|Houston Methodist Hospital|Houston Methodist Hospital|Long|USA
>Spike|hCoV-19/Scotland/GCVR-172448/2020|2020-03-30|EPI_ISL_469948|Original|hCoV-19^^Scotland|Human|NHSGGC West of Scotland Specialist Virology Centre / MRC-University of Glasgow Centre for Virus Research|Wellcome Sanger Institute for the COVID-19 Genomics UK (COG-UK) consortium|Davies|United Kingdom
>Spike|hCoV-19/USA/DE-DHSS-F918936/2020|2020-03-17|EPI_ISL_561359|Original|hCoV-19^^Delaware|Human|Delaware Public Health Lab|Delaware Public Health Lab|Bajwa|USA
>Spike|hCoV-19/Australia/VIC6725/2020|2020-00-00|EPI_ISL_564014|Original|hCoV-19^^Victoria|Human|Victorian Infectious Diseases Reference Laboratory (VIDRL)|VIDRL and MDU-PHL|Schultz|Australia
>Spike|hCoV-19/Japan/PG-1912/2020|2020-04-00|EPI_ISL_685518|Original|hCoV-19^^Japan|Human|Pathogen Genomics Center|Pathogen Genomics Center|Sekizuka|Japan
>Spike|hCoV-19/England/CAMC-B080C1/2020|2020-10-30|EPI_ISL_643425|Original|hCoV-19^^England|Human|Lighthouse Lab in Cambridge|Wellcome Sanger Institute for the COVID-19 Genomics UK (COG-UK) Consortium|Davies|United Kingdom
>Spike|hCoV-19/USA/WA-UW-1764/2020|2020-04-14|EPI_ISL_485972|Original|hCoV-19^^Washington|Human|UW Virology Lab|UW Virology Lab|Roychoudhury|USA
>Spike|hCoV-19/England/QEUH-AA0572/2020|2020-10-20|EPI_ISL_650261|Original|hCoV-19^^England|Human|University of Exeter|COVID-19 Genomics UK (COG-UK) Consortium|Temperton|United Kingdom
>Spike|hCoV-19/Spain/MD-IBV-99008759/2020|2020-10-20|EPI_ISL_654270|Original|hCoV-19^^Madrid|Human|Hospital General Universitario Gregorio Mara\u00f1\u00f3n|SeqCOVID-SPAIN consortium/IBV(CSIC)|Comas|Spain
>Spike|hCoV-19/India/TG-CDFD-C1408/2020|2020-06-08|EPI_ISL_528562|Original|hCoV-19^^Telangana|Human|National Genomics Core-Center for DNA Fingerprinting and Diagnostics|National Genomics Core- Center for DNA Fingerprinting and Diagnostics (NGC-CDFD)- DBT's PAN-INDIA-1000 Genome consortium|Vashisht|India
>Spike|hCoV-19/Scotland/QEUH-9CF699/2020|2020-09-17|EPI_ISL_601966|Original|hCoV-19^^Scotland|Human|Lighthouse Lab in Glasgow|Wellcome Sanger Institute for the COVID-19 Genomics UK (COG-UK) consortium|Davies|United Kingdom
>Spike|hCoV-19/England/ALDP-B1B984/2020|2020-11-01|EPI_ISL_645638|Original|hCoV-19^^England|Human|Lighthouse Lab in Alderley Park|Wellcome Sanger Institute for the COVID-19 Genomics UK (COG-UK) Consortium|Davies|United Kingdom

>Spike|hCoV-19/Japan/PG-5861/2020|2020-08-00|EPI_ISL_688978|Original|hCoV-19^^Japan|Human|Pathogen Genomics Center|Pathogen Genomics Center|Sekizuka|Japan
>Spike|hCoV-19/Japan/IC-0332/2020|2020-11-00|EPI_ISL_721579|Original|hCoV-19^^Japan|Human|Pathogen Genomics Center|Pathogen Genomics Center|Sekizuka|Japan
>Spike|hCoV-19/Denmark/DCGC-10561/2020|2020-11-02|EPI_ISL_669471|Original|hCoV-19^^Sjaelland|Human|Department of Virus and Microbiological Special Diagnostics|Albertsen Lab|Michaelsen|Denmark
>Spike|hCoV-19/Denmark/DCGC-20838/2020|2020-12-21|EPI_ISL_759395|Original|hCoV-19^^Syddanmark|Human|Department of Virus and Microbiological Special Diagnostics|Albertsen Lab|Michaelsen|Denmark
>Spike|hCoV-19/England/MILK-807FE6/2020|2020-07-16|EPI_ISL_557272|Original|hCoV-19^^England|Human|Lighthouse Lab in Milton Keynes|Wellcome Sanger Institute for the COVID-19 Genomics UK (COG-UK) consortium|Davies|United Kingdom
>Spike|hCoV-19/Denmark/DCGC-7211/2020|2020-10-12|EPI_ISL_619202|Original|hCoV-19^^Hovedstaden|Human|Department of Virus and Microbiological Special Diagnostics|Albertsen lab|Michaelsen|Denmark
>Spike|hCoV-19/Portugal/PT0720/2020|2020-03-26|EPI_ISL_510979|Original|hCoV-19^^Portugal|Human|Instituto Nacional de Saude (INSA)|Instituto Nacional de Saude (INSA)|Borges|Portugal
>Spike|hCoV-19/England/ALDP-A9250A/2020|2020-10-19|EPI_ISL_611220|Original|hCoV-19^^England|Human|Lighthouse Lab in Alderley Park|Wellcome Sanger Institute for the COVID-19 Genomics UK (COG-UK) consortium|Davies|United Kingdom
>Spike|hCoV-19/England/QEUH-9DE293/2020|2020-09-21|EPI_ISL_599668|Original|hCoV-19^^England|Human|Lighthouse Lab in Glasgow|Wellcome Sanger Institute for the COVID-19 Genomics UK (COG-UK) consortium|Davies|United Kingdom
>Spike|hCoV-19/Australia/VIC7343/2020|2020-08-08|EPI_ISL_564349|Original|hCoV-19^^Victoria|Human|Victorian Infectious Diseases Reference Laboratory (VIDRL)|VIDRL and MDU-PHL|Schultz|Australia
>Spike|hCoV-19/USA/WA-S1281/2020|2020-05-12|EPI_ISL_495731|Original|hCoV-19^^Washington|Human|Washington State Department of Health|Seattle Flu Study|Lee|USA
>Spike|hCoV-19/Belgium/rega-10181081/2020|2020-10-18|EPI_ISL_735121|Original|hCoV-19^^Tervuren|Human|UZ Leuven|KU Leuven|Maes|Belgium
>Spike|hCoV-19/Scotland/EDB1740/2020|2020-04-15|EPI_ISL_433133|Original|hCoV-19^^Scotland|Human|Virology Department|COVID-19 Genomics UK (COG-UK) Consortium|Williams|United Kingdom
>Spike|hCoV-19/Scotland/EDB8486/2020|2020-10-18|EPI_ISL_612370|Original|hCoV-19^^Scotland|Human|Virology Department|COVID-19 Genomics UK (COG-UK) Consortium|McHugh|United Kingdom
>Spike|hCoV-19/England/SHEF-CE1A1/2020|2020-05-19|EPI_ISL_529683|Original|hCoV-19^^England|Human|Virology Department|COVID-19 Genomics UK (COG-UK) Consortium|de Silva|United Kingdom
>Spike|hCoV-19/USA/MN-MDH-1448/2020|2020-03-26|EPI_ISL_514667|Original|hCoV-19^^Minnesota|Human|Minnesota Department of Health|Minnesota Department of Health|Garfin|USA
>Spike|hCoV-19/Denmark/DCGC-877/2020|2020-05-04|EPI_ISL_614788|Original|hCoV-19^^Hovedstaden|Human|Department of Virus and Microbiological Special Diagnostics|Albertsen lab|Michaelsen|Denmark
>Spike|hCoV-19/USA/MN-MDH-1192/2020|2020-05-05|EPI_ISL_477282|Original|hCoV-19^^Minnesota|Human|M Health Fairview|Minnesota Department of Health|Garfin|USA
>Spike|hCoV-19/Germany/HH-hpi-p1537/2020|2020-03-15|EPI_ISL_776031|Original|hCoV-19^^Hamburg|Human|University Medical Center Hamburg Eppendorf|Heinrich Pette Institute|Robitaille|Germany
>Spike|hCoV-19/Australia/VIC2398/2020|2020-06-26|EPI_ISL_522114|Original|hCoV-19^^Victoria|Human|Microbiological Diagnostic Unit - Public Health Laboratory (MDU-PHL)|MDU-PHL|Schultz|Australia
>Spike|hCoV-19/England/SHEF-10B2F0D/2020|2020-05-20|EPI_ISL_537267|Original|hCoV-19^^England|Human|Virology Department|Wellcome Sanger Institute for the COVID-19 Genomics UK (COG-UK) consortium|Davies|United Kingdom

>Spike|hCoV-19/Wales/PHWC-4A4FFE/2020|2020-12-13|EPI_ISL_764809|Original|hCoV-19^^Wales|Human|Wales Specialist Virology Centre Sequencing lab|COVID-19 Genomics UK (COG-UK) Consortium|Moore|United Kingdom
>Spike|hCoV-19/Spain/PV-IBV-98008439/2020|2020-10-22|EPI_ISL_654607|Original|hCoV-19^^Basque Country|Human|Servicio de Microbiolog\u00eda. Hospital Universitario Donostia. OSI Donostialdea. \u00c1rea de Enfermedades Infecciosas|SeqCOVID-SPAIN consortium/IBV(CSIC)|Comas|Spain
>Spike|hCoV-19/England/QEUH-AD0C1A/2020|2020-10-26|EPI_ISL_634138|Original|hCoV-19^^England|Human|Lighthouse Lab in Glasgow|Wellcome Sanger Institute for the COVID-19 Genomics UK (COG-UK) consortium|Davies|United Kingdom
>Spike|hCoV-19/Slovenia/13052/2020|2020-03-25|EPI_ISL_635264|Original|hCoV-19^^Slovenia|Human|Institute of Microbiology and Immunology|Institute of Microbiology and Immunology|Zakotnik|Slovenia
>Spike|hCoV-19/Denmark/DCGC-20846/2020|2020-12-21|EPI_ISL_759402|Original|hCoV-19^^Hovedstaden|Human|Department of Virus and Microbiological Special Diagnostics|Albertsen Lab|Michaelsen|Denmark
>Spike|hCoV-19/USA/WA-UW-24920/2020|2020-08-12|EPI_ISL_570583|Original|hCoV-19^^Washington|Human|UW Virology Lab|UW Virology Lab|Roychoudhury|USA
>Spike|hCoV-19/Scotland/CVR3519/2020|2020-05-12|EPI_ISL_473659|Original|hCoV-19^^Scotland|Human|West of Scotland Specialist Virology Centre|COVID-19 Genomics UK (COG-UK) Consortium|Robertson|United Kingdom
>Spike|hCoV-19/Scotland/QEUH-9D0844/2020|2020-09-16|EPI_ISL_601814|Original|hCoV-19^^Scotland|Human|Lighthouse Lab in Glasgow|Wellcome Sanger Institute for the COVID-19 Genomics UK (COG-UK) consortium|Davies|United Kingdom
>Spike|hCoV-19/England/SHEF-CB7A2/2020|2020-05-12|EPI_ISL_493703|Original|hCoV-19^^England|Human|Virology Department|COVID-19 Genomics UK (COG-UK) Consortium|de Silva|United Kingdom
>Spike|hCoV-19/USA/AZ-TG371267/2020|2020-05-15|EPI_ISL_694527|Original|hCoV-19^^Arizona|Human|AZ SPHL|TGen North|French|USA
>Spike|hCoV-19/Scotland/EDB10915/2020|2020-12-11|EPI_ISL_742184|Original|hCoV-19^^Scotland|Human|Virology Department|COVID-19 Genomics UK (COG-UK) Consortium|McHugh|United Kingdom
>Spike|hCoV-19/Denmark/DCGC-18143/2020|2020-12-14|EPI_ISL_749316|Original|hCoV-19^^Syddanmark|Human|Department of Virus and Microbiological Special Diagnostics|Albertsen Lab|Michaelsen|Denmark
>Spike|hCoV-19/Netherlands/ZH-EMC-783/2020|2020-09-29|EPI_ISL_632620|Original|hCoV-19^^Zuid Holland|Human|Dutch COVID-19 response team|Erasmus Medical Center|Oude Munnink|Netherlands
>Spike|hCoV-19/England/CAMB-80A19/2020|2020-04-08|EPI_ISL_442981|Original|hCoV-19^^England|Human|Department of Pathology|Wellcome Sanger Institute for the COVID-19 Genomics UK (COG-UK) consortium|Amato|United Kingdom
>Spike|hCoV-19/France/ARA-60729/2020|2020-08-13|EPI_ISL_683372|Original|hCoV-19^^Auvergne-Rh\u00f4ne-Alpes|Human|CNR Virus des Infections Respiratoires - France SUD|CNR Virus des Infections Respiratoires - France SUD|Bal|France
>Spike|hCoV-19/USA/MD-MDH-0410/2020|2020-11-04|EPI_ISL_666780|Original|hCoV-19^^Maryland|Human|Maryland Public Health Laboratory|Maryland Public Health Laboratory|Keller|USA
>Spike|hCoV-19/England/BRIS-12E029/2020|2020-04-20|EPI_ISL_482016|Original|hCoV-19^^England|Human|PHE South West Regional Laboratory|Wellcome Sanger Institute for the COVID-19 Genomics UK (COG-UK) consortium|Davies|United Kingdom
>Spike|hCoV-19/USA/VA-DCLS-1739/2020|2020-09-10|EPI_ISL_644844|Original|hCoV-19^^Virginia|Human|Virginia DCLS|Virginia DCLS|St. Jacques|USA
>Spike|hCoV-19/Belgium/ULG-10952/2020|2020-09-06|EPI_ISL_707731|Original|hCoV-19^^Li\u00e8ge|Human|Department of Clinical Microbiology|GIGA Medical Genomics|Durkin|Belgium
>Spike|hCoV-19/India/DL-NCDC1934-CSIR-IGIB/2020|2020-05-18|EPI_ISL_482513|Original|hCoV-19^^Delhi|Human|National Centre for Disease control (NCDC)|NCDC/CSIR-IGIB|Pandey|India
>Spike|hCoV-19/Belgium/rega-10061032/2020|2020-10-06|EPI_ISL_735053|Original|hCoV-19^^Overijse|Human|UZ Leuven|KU Leuven|Maes|Belgium

>Spike|hCoV-19/England/204690838/2020|2020-11-14|EPI_ISL_686587|Original|hCoV-19^^England|Human|Respiratory Virus Unit|COVID-19 Genomics UK (COG-UK) Consortium|Platt|United Kingdom
>Spike|hCoV-19/USA/CA-CZB-1050/2020|2020-04-04|EPI_ISL_437050|Original|hCoV-19^^California|Human|County of Santa Clara Public Health|Chan-Zuckerberg Biohub|Hao|USA
>Spike|hCoV-19/USA/UT-01220/2020|2020-04-08|EPI_ISL_436906|Original|hCoV-19^^Utah|Human|Utah Public Health Laboratory|Utah Public Health Laboratory|Young|USA
>Spike|hCoV-19/England/QEUH-AF472E/2020|2020-10-30|EPI_ISL_662823|Original|hCoV-19^^England|Human|Lighthouse Lab in Glasgow|Wellcome Sanger Institute for the COVID-19 Genomics UK (COG-UK) Consortium|Davies|United Kingdom
>Spike|hCoV-19/England/CAMC-A4127C/2020|2020-10-06|EPI_ISL_609464|Original|hCoV-19^^England|Human|Lighthouse Lab in Cambridge|Wellcome Sanger Institute for the COVID-19 Genomics UK (COG-UK) consortium|Davies|United Kingdom
>Spike|hCoV-19/England/MILK-A46E82/2020|2020-10-06|EPI_ISL_598515|Original|hCoV-19^^England|Human|Lighthouse Lab in Milton Keynes|Wellcome Sanger Institute for the COVID-19 Genomics UK (COG-UK) consortium|Davies|United Kingdom
>Spike|hCoV-19/Spain/AR-IBV-98008097/2020|2020-10-07|EPI_ISL_654409|Original|hCoV-19^^Aragon|Human|Servicio de Microbiolog\u00eda|SeqCOVID-SPAIN consortium/IBV(CSIC)|Comas|Spain
>Spike|hCoV-19/England/QEUH-9B700F/2020|2020-09-04|EPI_ISL_590537|Original|hCoV-19^^England|Human|Lighthouse Lab in Glasgow|Wellcome Sanger Institute for the COVID-19 Genomics UK (COG-UK) consortium|Davies|United Kingdom
>Spike|hCoV-19/USA/UT-02556/2020|2020-04-24|EPI_ISL_487208|Original|hCoV-19^^Utah|Human|Utah Public Health Laboratory|Utah Public Health Laboratory|Butz|USA
>Spike|hCoV-19/Scotland/EDB4658/2020|2020-05-08|EPI_ISL_449317|Original|hCoV-19^^Scotland|Human|Virology Department|COVID-19 Genomics UK (COG-UK) Consortium|Williams|United Kingdom
>Spike|hCoV-19/USA/CA-CZB-14806/2020|2020-11-17|EPI_ISL_739436|Original|hCoV-19^^California|Human|Humboldt County Public Health Laboratory|Chan-Zuckerberg Biohub|Kamm|USA
>Spike|hCoV-19/England/QEUH-9CEE65/2020|2020-09-15|EPI_ISL_590032|Original|hCoV-19^^England|Human|Lighthouse Lab in Glasgow|Wellcome Sanger Institute for the COVID-19 Genomics UK (COG-UK) consortium|Davies|United Kingdom
>Spike|hCoV-19/Denmark/DCGC-15490/2020|2020-11-23|EPI_ISL_713287|Original|hCoV-19^^Syddanmark|Human|Department of Virus and Microbiological Special Diagnostics|Albertsen Lab|Michaelsen|Denmark
>Spike|hCoV-19/Wales/PHWC-49E2B2/2020|2020-12-07|EPI_ISL_742980|Original|hCoV-19^^Wales|Human|Wales Specialist Virology Centre Sequencing lab|COVID-19 Genomics UK (COG-UK) Consortium|Moore|United Kingdom
>Spike|hCoV-19/England/ALDP-5A2CAD/2020|2020-06-14|EPI_ISL_558635|Original|hCoV-19^^England|Human|Lighthouse Lab in Alderley Park|Wellcome Sanger Institute for the COVID-19 Genomics UK (COG-UK) consortium|Davies|United Kingdom
>Spike|hCoV-19/USA/UT-0444/2020|2020-03-30|EPI_ISL_430042|Original|hCoV-19^^Utah|Human|Utah Public Health Laboratory|Utah Public Health Laboratory|Young|USA
>Spike|hCoV-19/Australia/NSW782/2020|2020-07-25|EPI_ISL_526124|Original|hCoV-19^^New South Wales|Human|Sydney South West Pathology Service (SSWPS) - Liverpool Hospital - NSW Health Pathology|NSW Health Pathology - Institute of Clinical Pathology and Medical Research; Westmead Hospital; University of Sydney|Gall|Australia
>Spike|hCoV-19/Canada/BC-BCCDC-8379/2020|2020-11-00|EPI_ISL_740763|Original|hCoV-19^^British Columbia|Human|BCCDC Public Health Laboratory|BCCDC Public Health Laboratory|Prystajecky|Canada
>Spike|hCoV-19/Australia/SAP089/2020|2020-04-01|EPI_ISL_451124|Original|hCoV-19^^South Australia|Human|SA Pathology|SA Pathology|Leong|Australia
>Spike|hCoV-19/Switzerland/BS-42340959/2020|2020-07-06|EPI_ISL_581895|Original|hCoV-19^^Basel-Stadt|Human|University Hospital Basel|University Hospital Basel|Roloff Handschin|Switzerland
>Spike|hCoV-19/Scotland/QEUH-9968C2/2020|2020-08-29|EPI_ISL_559986|Original|hCoV-19^^Scotland|Human|Oxford Viromics|COVID-19 Genomics UK (COG-UK) Consortium|Golubchik|United Kingdom

>Spike|hCoV-19/USA/MI-MDHHS-SC21485/2020|2020-07-26|EPI_ISL_516212|Original|hCoV-19^^Michigan|Human|Michigan Department of Health and Human Services|Michigan Department of Health and Human Services|Blankenship|USA
>Spike|hCoV-19/Northern Ireland/NIRE-234335/2020|2020-10-15|EPI_ISL_680371|Original|hCoV-19^^Northern Ireland|Human|Regional Virus Laboratory|COVID-19 Genomics UK (COG-UK) Consortium|Fairley|United Kingdom
>Spike|hCoV-19/Wales/ALDP-B1AAFB/2020|2020-11-01|EPI_ISL_711036|Original|hCoV-19^^Wales|Human|Lighthouse Lab in Alderley Park|Wellcome Sanger Institute for the COVID-19 Genomics UK (COG-UK) Consortium|Davies|United Kingdom
>Spike|hCoV-19/USA/TX-HMH-MCoV-10412/2020|2020-07-08|EPI_ISL_789460|Original|hCoV-19^^Texas|Human|Houston Methodist Hospital|Houston Methodist Hospital|Long|USA
>Spike|hCoV-19/Wales/PHWC-49CA16/2020|2020-11-19|EPI_ISL_742632|Original|hCoV-19^^Wales|Human|Wales Specialist Virology Centre Sequencing lab|COVID-19 Genomics UK (COG-UK) Consortium|Moore|United Kingdom
>Spike|hCoV-19/England/20169019304/2020|2020-04-18|EPI_ISL_465203|Original|hCoV-19^^England|Human|Respiratory Virus Unit|Respiratory Virus Unit|Platt|United Kingdom
>Spike|hCoV-19/Australia/VIC2490/2020|2020-06-29|EPI_ISL_522188|Original|hCoV-19^^Victoria|Human|Microbiological Diagnostic Unit - Public Health Laboratory (MDU-PHL)|MDU-PHL|Schultz|Australia
>Spike|hCoV-19/Northern Ireland/NIRE-FB536/2020|2020-08-19|EPI_ISL_585233|Original|hCoV-19^^Northern Ireland|Human|Regional Virus Laboratory|COVID-19 Genomics UK (COG-UK) Consortium|Fairley|United Kingdom
>Spike|hCoV-19/United Arab Emirates/1067/2020|2020-06-11|EPI_ISL_698142|Original|hCoV-19^^Abu Dhabi|Human|Group 42 (G42) Healthcare|G42 Healthcare|Ogrodzki|United Arab Emirates
>Spike|hCoV-19/Finland/8CA1/2020|2020-12-04|EPI_ISL_732619|Original|hCoV-19^^Uusimaa|Human|Department of Virology and Immunology|Department of Virology|Smura|Finland
>Spike|hCoV-19/England/QEUH-9DCEEC/2020|2020-09-22|EPI_ISL_580894|Original|hCoV-19^^England|Human|Lighthouse Lab in Glasgow|Wellcome Sanger Institute for the COVID-19 Genomics UK (COG-UK) consortium|Davies|United Kingdom
>Spike|hCoV-19/England/ALDP-AA2F36/2020|2020-10-22|EPI_ISL_623931|Original|hCoV-19^^England|Human|Lighthouse Lab in Alderley Park|Wellcome Sanger Institute for the COVID-19 Genomics UK (COG-UK) consortium|Davies|United Kingdom
>Spike|hCoV-19/England/QEUH-B3F780/2020|2020-11-08|EPI_ISL_658487|Original|hCoV-19^^England|Human|Lighthouse Lab in Glasgow|Wellcome Sanger Institute for the COVID-19 Genomics UK (COG-UK) Consortium|Davies|United Kingdom
>Spike|hCoV-19/Wales/PHWC-16D2EC/2020|2020-09-13|EPI_ISL_574224|Original|hCoV-19^^Wales|Human|Wales Specialist Virology Centre Sequencing lab|COVID-19 Genomics UK (COG-UK) Consortium|Moore|United Kingdom
>Spike|hCoV-19/USA/AZ-TG637306/2020|2020-07-27|EPI_ISL_695728|Original|hCoV-19^^Arizona|Human|AZ SPHL|TGen North|French|USA
>Spike|hCoV-19/England/MILK-B8BF14/2020|2020-11-13|EPI_ISL_674230|Original|hCoV-19^^England|Human|Lighthouse Lab in Milton Keynes|Wellcome Sanger Institute for the COVID-19 Genomics UK (COG-UK) Consortium|Davies|United Kingdom
>Spike|hCoV-19/England/ALDP-B5B487/2020|2020-11-09|EPI_ISL_655613|Original|hCoV-19^^England|Human|Lighthouse Lab in Alderley Park|Wellcome Sanger Institute for the COVID-19 Genomics UK (COG-UK) Consortium|Davies|United Kingdom
>Spike|hCoV-19/Wales/PHWC-49BAEA/2020|2020-12-06|EPI_ISL_742429|Original|hCoV-19^^Wales|Human|Wales Specialist Virology Centre Sequencing lab|COVID-19 Genomics UK (COG-UK) Consortium|Moore|United Kingdom
>Spike|hCoV-19/USA/TX-HMH-5138/2020|2020-06-22|EPI_ISL_544551|Original|hCoV-19^^Texas|Human|Houston Methodist Hospital|Houston Methodist Hospital|Long|USA
>Spike|hCoV-19/England/ALDP-A94AEB/2020|2020-10-20|EPI_ISL_624684|Original|hCoV-19^^England|Human|Lighthouse Lab in Alderley Park|Wellcome Sanger Institute for the COVID-19 Genomics UK (COG-UK) consortium|Davies|United Kingdom
>Spike|hCoV-19/Wuhan/HBCDC-HB-02/2019|2019-12-30|EPI_ISL_412898|Original|hCoV-19^^Hubei|Human|Wuhan Jinyintan Hospital|Hubei Provincial Center for Disease Control and Prevention|Fang|China

>Spike|hCoV-19/England/NORT-288F4D/2020|2020-03-24|EPI_ISL_458619|Original|hCoV-19^^England|Human|NU-OMICS DNA Sequencing research facility|Wellcome Sanger Institute for the COVID-19 Genomics UK (COG-UK) consortium|Davies|United Kingdom
>Spike|hCoV-19/England/CAMC-B7B0D8/2020|2020-11-12|EPI_ISL_668012|Original|hCoV-19^^England|Human|Lighthouse Lab in Cambridge|Wellcome Sanger Institute for the COVID-19 Genomics UK (COG-UK) Consortium|Davies|United Kingdom
>Spike|hCoV-19/USA/TX-HMH-MCoV-14159/2020|2020-10-02|EPI_ISL_787444|Original|hCoV-19^^Texas|Human|Houston Methodist Hospital|Houston Methodist Hospital|Long|USA
>Spike|hCoV-19/USA/TX-HMH-MCoV-15234/2020|2020-10-21|EPI_ISL_786718|Original|hCoV-19^^Texas|Human|Houston Methodist Hospital|Houston Methodist Hospital|Long|USA
>Spike|hCoV-19/USA/ME-HETL-J0381/2020|2020-12-11|EPI_ISL_755499|Original|hCoV-19^^Maine|Human|Maine Health and Environmental Testing Laboratory|Tewhey Lab|Tewhey|USA
>Spike|hCoV-19/Wales/PHWC-1642C7/2020|2020-05-10|EPI_ISL_474155|Original|hCoV-19^^Wales|Human|Originating lab|COVID-19 Genomics UK (COG-UK) Consortium|Moore|United Kingdom
>Spike|hCoV-19/England/204291439/2020|2020-10-16|EPI_ISL_622876|Original|hCoV-19^^England|Human|Respiratory Virus Unit|Respiratory Virus Unit|Platt|United Kingdom
>Spike|hCoV-19/Spain/MD-IBV-99007140/2020|2020-08-04|EPI_ISL_541913|Original|hCoV-19^^Madrid|Human|Hospital General Universitario Gregorio Mara\u00f1\u00f3n|SeqCOVID-SPAIN consortium/IBV(CSIC)|Comas|Spain
>Spike|hCoV-19/England/QEUH-BCCB40/2020|2020-11-25|EPI_ISL_704371|Original|hCoV-19^^England|Human|Lighthouse Lab in Glasgow|Wellcome Sanger Institute for the COVID-19 Genomics UK (COG-UK) Consortium|Davies|United Kingdom
>Spike|hCoV-19/Luxembourg/LNS5966835/2020|2020-11-26|EPI_ISL_744331|Original|hCoV-19^^Luxembourg|Human|Laboratoire national de sant\u00e9|Laboratoire national de sant\u00e9|Wienecke-Baldacchino|Luxembourg
>Spike|hCoV-19/Spain/MD-IBV-005336/2020|2020-03-17|EPI_ISL_510214|Original|hCoV-19^^Madrid|Human|Hospital General Universitario Gregorio Mara\u00f1\u00f3n|SeqCOVID-SPAIN consortium/IBV(CSIC)|Comas|Spain
>Spike|hCoV-19/England/QEUH-9626D4/2020|2020-08-10|EPI_ISL_531637|Original|hCoV-19^^England|Human|Lighthouse Lab in Glasgow|Wellcome Sanger Institute for the COVID-19 Genomics UK (COG-UK) consortium|Davies|United Kingdom
>Spike|hCoV-19/USA/CA-CZB-11795/2020|2020-09-22|EPI_ISL_625638|Original|hCoV-19^^California|Human|Orange County Public Health Lab|Chan-Zuckerberg Biohub|Bhatt|USA
>Spike|hCoV-19/Japan/PG-7110/2020|2020-08-00|EPI_ISL_688215|Original|hCoV-19^^Japan|Human|Pathogen Genomics Center|Pathogen Genomics Center|Sekizuka|Japan
>Spike|hCoV-19/England/QEUH-9B46C4/2020|2020-09-04|EPI_ISL_590229|Original|hCoV-19^^England|Human|Lighthouse Lab in Glasgow|Wellcome Sanger Institute for the COVID-19 Genomics UK (COG-UK) consortium|Davies|United Kingdom
>Spike|hCoV-19/Canada/BC-BCCDC-7530/2020|2020-11-00|EPI_ISL_740698|Original|hCoV-19^^British Columbia|Human|BCCDC Public Health Laboratory|BCCDC Public Health Laboratory|Prystajecky|Canada
>Spike|hCoV-19/Wales/PHWC-4877F9/2020|2020-10-26|EPI_ISL_639242|Original|hCoV-19^^Wales|Human|Wales Specialist Virology Centre Sequencing lab|COVID-19 Genomics UK (COG-UK) Consortium|Moore|United Kingdom
>Spike|hCoV-19/England/QEUH-5FE64A/2020|2020-06-27|EPI_ISL_533263|Original|hCoV-19^^England|Human|Lighthouse Lab in Glasgow|Wellcome Sanger Institute for the COVID-19 Genomics UK (COG-UK) consortium|Davies|United Kingdom
>Spike|hCoV-19/England/MILK-B969FA/2020|2020-11-13|EPI_ISL_676081|Original|hCoV-19^^England|Human|Lighthouse Lab in Milton Keynes|Wellcome Sanger Institute for the COVID-19 Genomics UK (COG-UK) Consortium|Davies|United Kingdom
>Spike|hCoV-19/Netherlands/UT-EMC-113/2020|2020-10-11|EPI_ISL_632635|Original|hCoV-19^^Utrecht|Human|Dutch COVID-19 response team|Erasmus Medical Center|Oude Munnink|Netherlands
>Spike|hCoV-19/England/CAMB-1AEDDF/2020|2020-05-10|EPI_ISL_452865|Original|hCoV-19^^England|Human|Department of Pathology|COVID-19 Genomics UK (COG-UK) Consortium|Meredith|United Kingdom

>Spike|hCoV-19/Iceland/132/2020|2020-03-12|EPI_ISL_417787|Original|hCoV-19^^Reykjavik|Human|The National University Hospital of Iceland|deCODE genetics|J\u00f3nsson|Iceland
>Spike|hCoV-19/Denmark/DCGC-22543/2020|2020-12-14|EPI_ISL_757628|Original|hCoV-19^^Syddanmark|Human|Department of Virus and Microbiological Special Diagnostics|Albertsen Lab|Michaelsen|Denmark
>Spike|hCoV-19/USA/TX-HMH-6975/2020|2020-06-28|EPI_ISL_546400|Original|hCoV-19^^Texas|Human|Houston Methodist Hospital|Houston Methodist Hospital|Long|USA
>Spike|hCoV-19/Japan/PG-5420/2020|2020-08-00|EPI_ISL_690523|Original|hCoV-19^^Japan|Human|Fukuoka Institute of Health and Environmental Sciences|Pathogen Genomics Center|Sekizuka|Japan
>Spike|hCoV-19/Russia/RYA-RII-MH3361S/2020|2020-10-07|EPI_ISL_733106|Original|hCoV-19^^Ryazan|Human|HELIX LLC|WHO National Influenza Centre Russian Federation|Komissarov|Russia
>Spike|hCoV-19/Palestine/48/2020|2020-08-18|EPI_ISL_596531|Original|hCoV-19^^Jericho|Human|Palestinian Ministry of Health|Molecular Genetics Lab|van Dorp|Palestine
>Spike|hCoV-19/England/NORT-284934/2020|2020-03-24|EPI_ISL_488475|Original|hCoV-19^^England|Human|NU-OMICS DNA Sequencing research facility|Wellcome Sanger Institute for the COVID-19 Genomics UK (COG-UK) consortium|Davies|United Kingdom
>Spike|hCoV-19/Wales/PHWC-2671E/2020|2020-03-27|EPI_ISL_422244|Original|hCoV-19^^Wales|Human|Wales Specialist Virology Centre|Public Health Wales Microbiology Cardiff|Moore|United Kingdom
>Spike|hCoV-19/Luxembourg/LNS1355488/2020|2020-10-26|EPI_ISL_739922|Original|hCoV-19^^Luxembourg|Human|Laboratoire national de sant\u00e9|Laboratoire national de sant\u00e9|Wienecke-Baldacchino|Luxembourg
>Spike|hCoV-19/Wales/PHWC-16E10C/2020|2020-10-02|EPI_ISL_585670|Original|hCoV-19^^Wales|Human|Wales Specialist Virology Centre Sequencing lab|COVID-19 Genomics UK (COG-UK) Consortium|Moore|United Kingdom
>Spike|hCoV-19/Colombia/DC-INS-78998/2020|2020-03-11|EPI_ISL_653753|Original|hCoV-19^^Bogota|Human|Instituto Nacional de Salud|Instituto Nacional de Salud|Franco-Mu\u00f1oz|Colombia
>Spike|hCoV-19/England/BRIS-122966/2020|2020-04-03|EPI_ISL_440624|Original|hCoV-19^^England|Human|PHE South West Regional Laboratory|Wellcome Sanger Institute for the COVID-19 Genomics UK (COG-UK) consortium|Amato|United Kingdom
>Spike|hCoV-19/Nanchang/JX149/2020|2020-01-27|EPI_ISL_421247|Original|hCoV-19^^Jiangxi|Human|Jiangxi Province Center for Disease Control and Prevention|Jiangxi Province Center for Disease Control and Prevention|Xiong|China
>Spike|hCoV-19/Wales/PHWC-47E9B3/2020|2020-09-25|EPI_ISL_595532|Original|hCoV-19^^Wales|Human|Originating lab|COVID-19 Genomics UK (COG-UK) Consortium|Moore|United Kingdom
>Spike|hCoV-19/Netherlands/ZH-EMC-1125/2020|2020-12-16|EPI_ISL_763204|Original|hCoV-19^^Zuid Holland|Human|Dutch COVID-19 response team|Erasmus Medical Center|Oude Munnink|Netherlands
>Spike|hCoV-19/England/QEUH-C33C13/2020|2020-12-11|EPI_ISL_734114|Original|hCoV-19^^England|Human|Lighthouse Lab in Glasgow|Wellcome Sanger Institute for the COVID-19 Genomics UK (COG-UK) Consortium|Davies|United Kingdom
>Spike|hCoV-19/Canada/ON-PHL-20-03740/2020|2020-11-00|EPI_ISL_775208|Original|hCoV-19^^Ontario|Human|Public Health Ontario Laboratory|Public Health Ontario Laboratory|Guthrie|Canada
>Spike|hCoV-19/Denmark/DCGC-7764/2020|2020-10-19|EPI_ISL_618732|Original|hCoV-19^^Hovedstaden|Human|Department of Virus and Microbiological Special Diagnostics|Albertsen lab|Michaelsen|Denmark
>Spike|hCoV-19/Scotland/CVR2557/2020|2020-04-15|EPI_ISL_453060|Original|hCoV-19^^Scotland|Human|West of Scotland Specialist Virology Centre|COVID-19 Genomics UK (COG-UK) Consortium|Robertson|United Kingdom
>Spike|hCoV-19/Japan/PG-8703/2020|2020-08-00|EPI_ISL_686299|Original|hCoV-19^^Japan|Human|Pathogen Genomics Center|Pathogen Genomics Center|Sekizuka|Japan

>Spike|hCoV-19/Canada/MB-NML-997/2020|2020-07-19|EPI_ISL_582367|Original|hCoV-19^^Manitoba|Human|Cadham Provincial Laboratory|National Microbiology Laboratory (NML)|Majer|Canada
>Spike|hCoV-19/England/QEUH-9D19F5/2020|2020-09-14|EPI_ISL_573675|Original|hCoV-19^^England|Human|University College London|COVID-19 Genomics UK (COG-UK) Consortium|Roy|United Kingdom
>Spike|hCoV-19/England/ALDP-BB3D0A/2020|2020-11-19|EPI_ISL_701838|Original|hCoV-19^^England|Human|Lighthouse Lab in Alderley Park|Wellcome Sanger Institute for the COVID-19 Genomics UK (COG-UK) Consortium|Davies|United Kingdom
>Spike|hCoV-19/England/NORW-21F88C/2020|2020-12-07|EPI_ISL_725059|Original|hCoV-19^^England|Human|Quadram Institute Bioscience|COVID-19 Genomics UK (COG-UK) Consortium|Alikhan|United Kingdom
>Spike|hCoV-19/Wales/PHWC-4A6299/2020|2020-12-16|EPI_ISL_765019|Original|hCoV-19^^Wales|Human|Wales Specialist Virology Centre Sequencing lab|COVID-19 Genomics UK (COG-UK) Consortium|Moore|United Kingdom
>Spike|hCoV-19/Denmark/DCGC-9743/2020|2020-11-09|EPI_ISL_669140|Original|hCoV-19^^Nordjylland|Human|Department of Virus and Microbiological Special Diagnostics|Albertsen Lab|Michaelsen|Denmark
>Spike|hCoV-19/England/ALDP-CB4281/2020|2020-12-20|EPI_ISL_761008|Original|hCoV-19^^England|Human|Lighthouse Lab in Alderley Park|Wellcome Sanger Institute for the COVID-19 Genomics UK (COG-UK) Consortium|Davies|United Kingdom
>Spike|hCoV-19/England/MILK-9E1673/2020|2020-09-19|EPI_ISL_577081|Original|hCoV-19^^England|Human|Oxford Viromics|COVID-19 Genomics UK (COG-UK) Consortium|Golubchik|United Kingdom
>Spike|hCoV-19/Jordan/AM-ALSR-3906/2020|2020-09-30|EPI_ISL_636272|Original|hCoV-19^^Amman|Human|Biolab Diagnostic Laboratories|Andersen lab at Scripps Research|Gangavarapu|Jordan
>Spike|hCoV-19/South Africa/KRISP-K002560/2020|2020-07-31|EPI_ISL_535428|Original|hCoV-19^^KwaZulu-Natal|Human|NHLS-IALCH|KRISP|de Oliveira|South Africa
>Spike|hCoV-19/England/QEUH-A005CB/2020|2020-10-02|EPI_ISL_584890|Original|hCoV-19^^England|Human|University of Exeter|COVID-19 Genomics UK (COG-UK) Consortium|Temperton|United Kingdom
>Spike|hCoV-19/England/ALDP-CED207/2020|2020-12-18|EPI_ISL_782002|Original|hCoV-19^^England|Human|Lighthouse Lab in Alderley Park|Wellcome Sanger Institute for the COVID-19 Genomics UK (COG-UK) Consortium|Davies|United Kingdom
>Spike|hCoV-19/England/ALDP-656DFF/2020|2020-07-03|EPI_ISL_558078|Original|hCoV-19^^England|Human|Lighthouse Lab in Alderley Park|Wellcome Sanger Institute for the COVID-19 Genomics UK (COG-UK) consortium|Davies|United Kingdom
>Spike|hCoV-19/Brazil/SP-L26-CD548/2020|2020-04-18|EPI_ISL_672726|Original|hCoV-19^^S\u00e3o Paulo|Human|Hospital das Cl\u00ednicas da Faculdade de Medicina da Universidade de S\u00e3o Paulo (HC-FMUSP)|Laborat\u00f3rio de Parasitologia M\u00e9dica - Instituto de Medicina Tropical - Universidade de S\u00e3o Paulo|de Jesus|Brazil
>Spike|hCoV-19/Netherlands/NA_530/2020|2020-03-26|EPI_ISL_460703|Original|hCoV-19^^Netherlands|Human|Dutch COVID-19 response team|Erasmus Medical Center|Oude Munnink|Netherlands
>Spike|hCoV-19/Spain/MD-IBV-99008579/2020|2020-09-21|EPI_ISL_654119|Original|hCoV-19^^Madrid|Human|Hospital General Universitario Gregorio Mara\u00f1\u00f3n|SeqCOVID-SPAIN consortium/IBV(CSIC)|Comas|Spain
>Spike|hCoV-19/England/CAMB-83D95/2020|2020-04-24|EPI_ISL_438690|Original|hCoV-19^^England|Human|Department of Pathology|COVID-19 Genomics UK (COG-UK) Consortium|Meredith|United Kingdom
>Spike|hCoV-19/England/MILK-9C3D45/2020|2020-09-09|EPI_ISL_566794|Original|hCoV-19^^England|Human|Lighthouse Lab in Milton Keynes|Wellcome Sanger Institute for the COVID-19 Genomics UK (COG-UK) consortium|Davies|United Kingdom
>Spike|hCoV-19/Bahrain/341036861/2020|2020-11-16|EPI_ISL_684028|Original|hCoV-19^^Bahrain|Human|Communicable Disease Laboratory|Communicable Disease Laboratory|Altaif|Bahrain

>Spike|hCoV-19/England/QEUH-B2E3F9/2020|2020-11-02|EPI_ISL_646401|Original|hCoV-19^^England|Human|Lighthouse Lab in Glasgow|Wellcome Sanger Institute for the COVID-19 Genomics UK (COG-UK) Consortium|Davies|United Kingdom
>Spike|hCoV-19/USA/CA-CZB-2162/2020|2020-05-22|EPI_ISL_513712|Original|hCoV-19^^California|Human|Orange County Public Health Lab|Chan-Zuckerberg Biohub|Kamm|USA
>Spike|hCoV-19/Netherlands/ZH-EMC-441/2020|2020-08-12|EPI_ISL_523779|Original|hCoV-19^^Zuid Holland|Human|Dutch COVID-19 response team|Erasmus Medical Center|Oude Munnink|Netherlands
>Spike|hCoV-19/England/MILK-99D6D0/2020|2020-08-31|EPI_ISL_549862|Original|hCoV-19^^England|Human|Lighthouse Lab in Milton Keynes|Wellcome Sanger Institute for the COVID-19 Genomics UK (COG-UK) consortium|Davies|United Kingdom
>Spike|hCoV-19/Netherlands/Friesland_17/2020|2020-04-03|EPI_ISL_455120|Original|hCoV-19^^Friesland|Human|Dutch COVID-19 response team|Erasmus Medical Center|Oude Munnink|Netherlands
>Spike|hCoV-19/Burkina Faso/3052/2020|2020-09-25|EPI_ISL_660466|Original|hCoV-19^^Bobo Dioulasso|Human|Laboratoire de Microbiologie CHU Souro Sanou|Centre Muraz|Ouedraogo|Burkina Faso
>Spike|hCoV-19/Wales/PHWC-484D18/2020|2020-10-22|EPI_ISL_628101|Original|hCoV-19^^Wales|Human|Wales Specialist Virology Centre Sequencing lab|COVID-19 Genomics UK (COG-UK) Consortium|Moore|United Kingdom
>Spike|hCoV-19/Denmark/DCGC-19093/2020|2020-12-07|EPI_ISL_748754|Original|hCoV-19^^Hovedstaden|Human|Department of Virus and Microbiological Special Diagnostics|Albertsen Lab|Michaelsen|Denmark
>Spike|hCoV-19/Netherlands/LI-RIVM-20563/2020|2020-12-09|EPI_ISL_790647|Original|hCoV-19^^Limburg|Human|Dutch COVID-19 response team|National Institute for Public Health and the Environment (RIVM)|Meijer|Netherlands
>Spike|hCoV-19/USA/CA-CZB-2391/2020|2020-05-18|EPI_ISL_513822|Original|hCoV-19^^California|Human|Orange County Public Health Laboratory|Chan-Zuckerberg Biohub|Kamm|USA
>Spike|hCoV-19/England/CAMC-9DF274/2020|2020-09-20|EPI_ISL_637749|Original|hCoV-19^^England|Human|Department of Pathology|COVID-19 Genomics UK (COG-UK) Consortium|Goodfellow|United Kingdom
>Spike|hCoV-19/North Macedonia/4637/2020|2020-06-22|EPI_ISL_677709|Original|hCoV-19^^Ohrid|Human|General Hospital - Ohrid|Research Center for Genetic Engineering and Biotechnology \Georgi D. Efremov\u201d|Dimovski|North Macedonia
>Spike|hCoV-19/USA/CA-CSMC48/2020|2020-04-09|EPI_ISL_475660|Original|hCoV-19^^California|Human|Cedars-Sinai Medical Center|Cedars-Sinai Medical Center|Zhang|USA
>Spike|hCoV-19/USA/TX-QDX-2048/2020|2020-03-17|EPI_ISL_604377|Original|hCoV-19^^Texas|Human|Quest Diagnostics|Quest Diagnostics|Kagan|USA
>Spike|hCoV-19/Scotland/QEUH-9F52E1/2020|2020-09-27|EPI_ISL_638925|Original|hCoV-19^^Scotland|Human|Department of Pathology|COVID-19 Genomics UK (COG-UK) Consortium|Goodfellow|United Kingdom
>Spike|hCoV-19/Russia/StPetersburg-RII4328S/2020|2020-03-25|EPI_ISL_427338|Original|hCoV-19^^Saint Petersburg|Human|WHO National Influenza Centre Russian Federation|WHO National Influenza Centre Russian Federation|Komissarov|Russia
>Spike|hCoV-19/Switzerland/VD-ETHZ-270122/2020|2020-09-03|EPI_ISL_541490|Original|hCoV-19^^Vaud|Human|Viollier AG|Department of Biosystems Science and Engineering|Nadeau|Switzerland
>Spike|hCoV-19/England/MILK-A79703/2020|2020-10-15|EPI_ISL_606891|Original|hCoV-19^^England|Human|Lighthouse Lab in Milton Keynes|Wellcome Sanger Institute for the COVID-19 Genomics UK (COG-UK) consortium|Davies|United Kingdom
>Spike|hCoV-19/England/BRIS-12BB3A/2020|2020-05-14|EPI_ISL_469442|Original|hCoV-19^^England|Human|PHE South West Regional Laboratory|Wellcome Sanger Institute for the COVID-19 Genomics UK (COG-UK) consortium|Davies|United Kingdom
>Spike|hCoV-19/Japan/PG-7419/2020|2020-07-00|EPI_ISL_688521|Original|hCoV-19^^Japan|Human|Pathogen Genomics Center|Pathogen Genomics Center|Sekizuka|Japan

>Spike|hCoV-19/Wales/PHWC-2B148/2020|2020-04-07|EPI_ISL_432264|Original|hCoV-19^^Wales|Human|Wales Specialist Virology Centre|Public Health Wales Microbiology Cardiff|Moore|United Kingdom
>Spike|hCoV-19/England/MILK-BDB7E0/2020|2020-11-26|EPI_ISL_709797|Original|hCoV-19^^England|Human|Lighthouse Lab in Milton Keynes|Wellcome Sanger Institute for the COVID-19 Genomics UK (COG-UK) Consortium|Davies|United Kingdom
>Spike|hCoV-19/Chile/RM-109455/2020|2020-06-23|EPI_ISL_746554|Original|hCoV-19^^Region Metropolitana de Santiago|Human|Genetica Molecular and Subdepartamento de Virologia ISP Chile|Instituto de Salud Publica de Chile|Tognarelli|Chile
>Spike|hCoV-19/England/QEUH-B9E154/2020|2020-11-17|EPI_ISL_721077|Original|hCoV-19^^England|Human|Lighthouse Lab in Glasgow|Wellcome Sanger Institute for the COVID-19 Genomics UK (COG-UK) Consortium|Davies|United Kingdom
>Spike|hCoV-19/England/20139010504/2020|2020-03-27|EPI_ISL_421872|Original|hCoV-19^^England|Human|Respiratory Virus Unit|Respiratory Virus Unit|Platt|United Kingdom
>Spike|hCoV-19/England/CAMC-B0A51B/2020|2020-10-30|EPI_ISL_673140|Original|hCoV-19^^England|Human|Lighthouse Lab in Cambridge|Wellcome Sanger Institute for the COVID-19 Genomics UK (COG-UK) Consortium|Davies|United Kingdom
>Spike|hCoV-19/Japan/PG-8550/2020|2020-08-00|EPI_ISL_686146|Original|hCoV-19^^Japan|Human|Pathogen Genomics Center|Pathogen Genomics Center|Sekizuka|Japan
>Spike|hCoV-19/Switzerland/BL-ETHZ-260115/2020|2020-08-26|EPI_ISL_539424|Original|hCoV-19^^Basel-Landschaft|Human|Viollier AG|Department of Biosystems Science and Engineering|Nadeau|Switzerland
>Spike|hCoV-19/Switzerland/TI-ETHZ-100051/2020|2020-03-11|EPI_ISL_466973|Original|hCoV-19^^Ticino|Human|Viollier AG|Department of Biosystems Science and Engineering|Nadeau|Switzerland
>Spike|hCoV-19/England/QEUH-9B6EF7/2020|2020-09-09|EPI_ISL_590640|Original|hCoV-19^^England|Human|Lighthouse Lab in Glasgow|Wellcome Sanger Institute for the COVID-19 Genomics UK (COG-UK) consortium|Davies|United Kingdom
>Spike|hCoV-19/Spain/VC-FISABIO-39/2020|2020-03-09|EPI_ISL_420124|Original|hCoV-19^^Comunitat Valenciana|Human|Servicio de Microbiolog\u00eda. Consorcio Hospital General Universitario de Valencia|Sequencing and Bioinformatics Service and Molecular Epidemiology Research Group. FISABIO-Public Health|Gonzalez-Candelas|Spain
>Spike|hCoV-19/USA/WA-UW119/2020|2020-03-11|EPI_ISL_416657|Original|hCoV-19^^Washington|Human|UW Virology Lab|UW Virology Lab|Roychoudhury|USA
>Spike|hCoV-19/Bangladesh/DNAS_DRCIM_CPH_isl_486_BGD/2020|2020-04-29|EPI_ISL_774881|Original|hCoV-19^^Dhaka|Human|Designated Reference Institute for Chemical Measurements (DRICM)|DNA SOLUTION LTD.|Rabbi|Bangladesh
>Spike|hCoV-19/South Africa/KRISP-K003340/2020|2020-09-07|EPI_ISL_602732|Original|hCoV-19^^KwaZulu-Natal|Human|NHLS-IALCH|KRISP|de Oliveira|South Africa
>Spike|hCoV-19/England/QEUH-96C388/2020|2020-08-13|EPI_ISL_530855|Original|hCoV-19^^England|Human|Lighthouse Lab in Glasgow|Wellcome Sanger Institute for the COVID-19 Genomics UK (COG-UK) consortium|Davies|United Kingdom
>Spike|hCoV-19/Japan/PG-4418/2020|2020-07-00|EPI_ISL_692643|Original|hCoV-19^^Japan|Human|Pathogen Genomics Center|Pathogen Genomics Center|Sekizuka|Japan
>Spike|hCoV-19/New Zealand/20VR2536/2020|2020-00-00|EPI_ISL_579281|Original|hCoV-19^^Auckland|Human|LabPLUS|Institute of Environmental Science and Research (ESR)|Wang|New Zealand
>Spike|hCoV-19/USA/TX-HMH-5416/2020|2020-06-26|EPI_ISL_544760|Original|hCoV-19^^Texas|Human|Houston Methodist Hospital|Houston Methodist Hospital|Long|USA
>Spike|hCoV-19/England/QEUH-BA2D4A/2020|2020-11-18|EPI_ISL_720921|Original|hCoV-19^^England|Human|Lighthouse Lab in Glasgow|Wellcome Sanger Institute for the COVID-19 Genomics UK (COG-UK) Consortium|Davies|United Kingdom
>Spike|hCoV-19/England/BIRM-60754/2020|2020-06-05|EPI_ISL_483884|Original|hCoV-19^^England|Human|University of Birmingham|COVID-19 Genomics UK (COG-UK) Consortium|Loman|United Kingdom
>Spike|hCoV-19/England/ALDP-953E47/2020|2020-06-12|EPI_ISL_554394|Original|hCoV-19^^England|Human|Lighthouse Lab in Alderley Park|Wellcome Sanger Institute for the COVID-19 Genomics UK (COG-UK) consortium|Davies|United Kingdom

>Spike|hCoV-19/England/CAMB-7867A/2020|2020-04-06|EPI_ISL_433760|Original|hCoV-19^^England|Human|Department of Pathology|COVID-19 Genomics UK (COG-UK) Consortium|Meredith|United Kingdom
>Spike|hCoV-19/Denmark/DCGC-21710/2020|2020-12-21|EPI_ISL_758322|Original|hCoV-19^^Sjaelland|Human|Department of Virus and Microbiological Special Diagnostics|Albertsen Lab|Michaelsen|Denmark
>Spike|hCoV-19/USA/UN-UW-7231/2020|2020-04-13|EPI_ISL_576507|Original|hCoV-19^^USA|Human|UW Virology Lab|UW Virology Lab|Roychoudhury|USA
>Spike|hCoV-19/Denmark/DCGC-1944/2020|2020-07-13|EPI_ISL_618208|Original|hCoV-19^^Hovedstaden|Human|Department of Virus and Microbiological Special Diagnostics|Albertsen lab|Michaelsen|Denmark
>Spike|hCoV-19/England/MILK-992E1F/2020|2020-08-26|EPI_ISL_551933|Original|hCoV-19^^England|Human|Lighthouse Lab in Milton Keynes|Wellcome Sanger Institute for the COVID-19 Genomics UK (COG-UK) consortium|Davies|United Kingdom
>Spike|hCoV-19/Wales/PHWC-48E09D/2020|2020-11-09|EPI_ISL_680802|Original|hCoV-19^^Wales|Human|Wales Specialist Virology Centre Sequencing lab|COVID-19 Genomics UK (COG-UK) Consortium|Moore|United Kingdom
>Spike|hCoV-19/India/MH-ACTREC-353/2020|2020-07-22|EPI_ISL_700002|Original|hCoV-19^^Maharashtra|Human|Hematopathology Laboratory|Hematopathology Laboratory|Patkar|India
>Spike|hCoV-19/USA/UT-QDX-1866/2020|2020-03-16|EPI_ISL_604179|Original|hCoV-19^^Utah|Human|Quest Diagnostics|Quest Diagnostics|Kagan|USA
>Spike|hCoV-19/Denmark/DCGC-21987/2020|2020-12-14|EPI_ISL_758494|Original|hCoV-19^^Sjaelland|Human|Department of Virus and Microbiological Special Diagnostics|Albertsen Lab|Michaelsen|Denmark
>Spike|hCoV-19/Japan/PG-4656/2020|2020-07-00|EPI_ISL_689793|Original|hCoV-19^^Japan|Human|Fukuoka Institute of Health and Environmental Sciences|Pathogen Genomics Center|Sekizuka|Japan
>Spike|hCoV-19/South Africa/NHLS-UCT-GS-1695/2020|2020-08-19|EPI_ISL_700507|Original|hCoV-19^^Western Cape|Human|Groote Schuur Hospital wc GSH|NHLS/UCT|Iranzadeh|South Africa
>Spike|hCoV-19/England/CAMC-B796BA/2020|2020-11-13|EPI_ISL_667819|Original|hCoV-19^^England|Human|Lighthouse Lab in Cambridge|Wellcome Sanger Institute for the COVID-19 Genomics UK (COG-UK) Consortium|Davies|United Kingdom
>Spike|hCoV-19/USA/TX-HMH-MCoV-19278/2020|2020-11-24|EPI_ISL_783737|Original|hCoV-19^^Texas|Human|Houston Methodist Hospital|Houston Methodist Hospital|Long|USA
>Spike|hCoV-19/England/CAMC-CB8C61/2020|2020-12-19|EPI_ISL_777882|Original|hCoV-19^^England|Human|Lighthouse Lab in Cambridge|Wellcome Sanger Institute for the COVID-19 Genomics UK (COG-UK) Consortium|Davies|United Kingdom
>Spike|hCoV-19/South Africa/KRISP-K002771/2020|2020-08-21|EPI_ISL_529766|Original|hCoV-19^^KwaZulu-Natal|Human|NHLS-IALCH|KRISP|de Oliveira|South Africa
>Spike|hCoV-19/Japan/PG-6064/2020|2020-07-00|EPI_ISL_689176|Original|hCoV-19^^Japan|Human|Pathogen Genomics Center|Pathogen Genomics Center|Sekizuka|Japan
>Spike|hCoV-19/England/ALDP-C3DCFB/2020|2020-12-10|EPI_ISL_731630|Original|hCoV-19^^England|Human|Lighthouse Lab in Alderley Park|Wellcome Sanger Institute for the COVID-19 Genomics UK (COG-UK) Consortium|Davies|United Kingdom
>Spike|hCoV-19/England/ALDP-BB5148/2020|2020-11-21|EPI_ISL_701954|Original|hCoV-19^^England|Human|Lighthouse Lab in Alderley Park|Wellcome Sanger Institute for the COVID-19 Genomics UK (COG-UK) Consortium|Davies|United Kingdom
>Spike|hCoV-19/England/ALDP-B62C67/2020|2020-11-08|EPI_ISL_655571|Original|hCoV-19^^England|Human|Lighthouse Lab in Alderley Park|Wellcome Sanger Institute for the COVID-19 Genomics UK (COG-UK) Consortium|Davies|United Kingdom
>Spike|hCoV-19/Switzerland/BE-ETHZ-210060/2020|2020-07-20|EPI_ISL_510793|Original|hCoV-19^^Bern|Human|Viollier AG|Department of Biosystems Science and Engineering|Nadeau|Switzerland
>Spike|hCoV-19/Luxembourg/LNS4691488/2020|2020-03-11|EPI_ISL_419587|Original|hCoV-19^^Luxembourg|Human|Laboratoire National de Sant\u00e9|Laboratoire National de Sant\u00e9|Wienecke-Baldacchino|Luxembourg

>Spike|hCoV-19/Denmark/ALAB-HH63/2020|2020-03-18|EPI_ISL_429309|Original|hCoV-19^^Denmark|Human|Department of Clinical Microbiology|Albertsen lab|Kirkegaard|Denmark
>Spike|hCoV-19/USA/DE-DHSS-F918161/2020|2020-03-27|EPI_ISL_576151|Original|hCoV-19^^Delaware|Human|Delaware Public Health Lab|Delaware Public Health Lab|Bajwa|USA
>Spike|hCoV-19/USA/TX-HMH-MCoV-13520/2020|2020-08-22|EPI_ISL_788645|Original|hCoV-19^^Texas|Human|Houston Methodist Hospital|Houston Methodist Hospital|Long|USA
>Spike|hCoV-19/Italy/BAS-IZSPB-393PT/2020|2020-10-25|EPI_ISL_722923|Original|hCoV-19^^Basilicata|Human|Istituto Zooprofilattico Sperimentale della Puglia e della Basilicata|Istituto Zooprofilattico Sperimentale della Puglia e della Basilicata|Parisi|Italy
>Spike|hCoV-19/Denmark/DCGC-14525/2020|2020-11-30|EPI_ISL_711818|Original|hCoV-19^^Midtjylland|Human|Department of Virus and Microbiological Special Diagnostics|Albertsen Lab|Michaelsen|Denmark
>Spike|hCoV-19/USA/TX-HMH-6822/2020|2020-06-25|EPI_ISL_546267|Original|hCoV-19^^Texas|Human|Houston Methodist Hospital|Houston Methodist Hospital|Long|USA
>Spike|hCoV-19/USA/MA-MGH-01169/2020|2020-04-29|EPI_ISL_791898|Original|hCoV-19^^Massachusetts|Human|Massachusetts General Hospital|Infectious Disease Program|Park|USA
>Spike|hCoV-19/Sweden/20-51696/2020|2020-05-24|EPI_ISL_615089|Original|hCoV-19^^Stockholm|Human|Karolinska Universitetslaboratoriet|The Public Health Agency of Sweden|Svartstr\u00f6m|Sweden
>Spike|hCoV-19/Netherlands/ZH-EMC-710/2020|2020-09-29|EPI_ISL_632557|Original|hCoV-19^^Zuid Holland|Human|Dutch COVID-19 response team|Erasmus Medical Center|Oude Munnink|Netherlands
>Spike|hCoV-19/Wales/PHWC-481E09/2020|2020-10-16|EPI_ISL_613174|Original|hCoV-19^^Wales|Human|Wales Specialist Virology Centre Sequencing lab|COVID-19 Genomics UK (COG-UK) Consortium|Moore|United Kingdom
>Spike|hCoV-19/Japan/PG-4684/2020|2020-08-00|EPI_ISL_689821|Original|hCoV-19^^Japan|Human|Fukuoka Institute of Health and Environmental Sciences|Pathogen Genomics Center|Sekizuka|Japan
>Spike|hCoV-19/Japan/PG-7370/2020|2020-06-00|EPI_ISL_688472|Original|hCoV-19^^Japan|Human|Pathogen Genomics Center|Pathogen Genomics Center|Sekizuka|Japan
>Spike|hCoV-19/England/BRIS-12D1DC/2020|2020-05-04|EPI_ISL_481789|Original|hCoV-19^^England|Human|PHE South West Regional Laboratory|Wellcome Sanger Institute for the COVID-19 Genomics UK (COG-UK) consortium|Davies|United Kingdom
>Spike|hCoV-19/Brazil/RS-15280/2020|2020-07-28|EPI_ISL_729851|Original|hCoV-19^^Rio Grande do Sul|Human|Laboratorio Central de Saude Publica do Estado do Rio Grande do Sul (LACEN-RS)|Laboratory of Respiratory Viruses and Measles|Silva|Brazil
>Spike|hCoV-19/USA/RI-CDC-2-3714591/2020|2020-12-04|EPI_ISL_751757|Original|hCoV-19^^Rhode Island|Human|RI State Health Laboratories|Genomics and Discovery|Queen|USA
>Spike|hCoV-19/England/ALDP-5BF047/2020|2020-06-17|EPI_ISL_558843|Original|hCoV-19^^England|Human|Lighthouse Lab in Alderley Park|Wellcome Sanger Institute for the COVID-19 Genomics UK (COG-UK) consortium|Davies|United Kingdom
>Spike|hCoV-19/Wales/PHWC-15D14C/2020|2020-05-04|EPI_ISL_472672|Original|hCoV-19^^Wales|Human|Wales Specialist Virology Centre Sequencing lab|COVID-19 Genomics UK (COG-UK) Consortium|Moore|United Kingdom
>Spike|hCoV-19/USA/TX-DSHS-0888/2020|2020-06-16|EPI_ISL_610024|Original|hCoV-19^^Texas|Human|Texas Department of State Health Services|Texas Department of State Health Services|Rahman|USA
>Spike|hCoV-19/Denmark/DCGC-20641/2020|2020-12-21|EPI_ISL_759270|Original|hCoV-19^^Midtjylland|Human|Department of Virus and Microbiological Special Diagnostics|Albertsen Lab|Michaelsen|Denmark
>Spike|hCoV-19/Netherlands/ZH-RIVM-20764/2020|2020-12-23|EPI_ISL_790824|Original|hCoV-19^^Zuid-holland|Human|Dutch COVID-19 response team|National Institute for Public Health and the Environment (RIVM)|Meijer|Netherlands
>Spike|hCoV-19/England/NORW-EF632/2020|2020-09-13|EPI_ISL_572718|Original|hCoV-19^^England|Human|Quadram Institute Bioscience|COVID-19 Genomics UK (COG-UK) Consortium|Alikhan|United Kingdom

>Spike|hCoV-19/Norway/5764/2020|2020-10-26|EPI_ISL_708166|Original|hCoV-19^^Vestfold og Telemark|Human|Vestfold Hospital|Norwegian Institute of Public Health|Kopperud Riis|Norway
>Spike|hCoV-19/Luxembourg/LNS2353921/2020|2020-11-09|EPI_ISL_740104|Original|hCoV-19^^Luxembourg|Human|Laboratoire national de sant\u00e9|Laboratoire national de sant\u00e9|Wienecke-Baldacchino|Luxembourg
>Spike|hCoV-19/USA/WA-S1601/2020|2020-05-26|EPI_ISL_496027|Original|hCoV-19^^Washington|Human|Washington State Department of Health|Seattle Flu Study|Lee|USA
>Spike|hCoV-19/England/MILK-CA6A20/2020|2020-12-18|EPI_ISL_761527|Original|hCoV-19^^England|Human|Lighthouse Lab in Milton Keynes|Wellcome Sanger Institute for the COVID-19 Genomics UK (COG-UK) Consortium|Davies|United Kingdom
>Spike|hCoV-19/England/MILK-9C3F5E/2020|2020-09-09|EPI_ISL_566703|Original|hCoV-19^^England|Human|Lighthouse Lab in Milton Keynes|Wellcome Sanger Institute for the COVID-19 Genomics UK (COG-UK) consortium|Davies|United Kingdom
>Spike|hCoV-19/USA/MA-MASPHL-00687/2020|2020-04-15|EPI_ISL_692959|Original|hCoV-19^^Massachusetts|Human|Massachusetts State Public Health Laboratory|Massachusetts State Public Health Laboratory|Lang|USA
>Spike|hCoV-19/Denmark/DCGC-7918/2020|2020-10-26|EPI_ISL_618817|Original|hCoV-19^^Hovedstaden|Human|Department of Virus and Microbiological Special Diagnostics|Albertsen lab|Michaelsen|Denmark
>Spike|hCoV-19/England/ALDP-913DA0/2020|2020-06-23|EPI_ISL_552876|Original|hCoV-19^^England|Human|Lighthouse Lab in Alderley Park|Wellcome Sanger Institute for the COVID-19 Genomics UK (COG-UK) consortium|Davies|United Kingdom
>Spike|hCoV-19/Hong Kong/HKPU52-3101/2020|2020-02-13|EPI_ISL_419224|Original|hCoV-19^^Hong Kong|Human|Department of Clinical Pathology|Department of Health Technology and Informatics|Leung|China
>Spike|hCoV-19/Scotland/QEUH-9655E3/2020|2020-08-12|EPI_ISL_531116|Original|hCoV-19^^Scotland|Human|NHSGGC West of Scotland Specialist Virology Centre / MRC-University of Glasgow Centre for Virus Research|Wellcome Sanger Institute for the COVID-19 Genomics UK (COG-UK) consortium|Davies|United Kingdom
>Spike|hCoV-19/England/ALDP-9CD1D6/2020|2020-09-16|EPI_ISL_566206|Original|hCoV-19^^England|Human|Lighthouse Lab in Alderley Park|Wellcome Sanger Institute for the COVID-19 Genomics UK (COG-UK) consortium|Davies|United Kingdom
>Spike|hCoV-19/England/ALDP-95330C/2020|2020-06-11|EPI_ISL_554564|Original|hCoV-19^^England|Human|Lighthouse Lab in Alderley Park|Wellcome Sanger Institute for the COVID-19 Genomics UK (COG-UK) consortium|Davies|United Kingdom
>Spike|hCoV-19/USA/CA-CZB-10359/2020|2020-09-10|EPI_ISL_648549|Original|hCoV-19^^California|Human|Madera County Department of Public Health|Chan-Zuckerberg Biohub|Bhatt|USA
>Spike|hCoV-19/England/NORT-2863F8/2020|2020-10-01|EPI_ISL_638562|Original|hCoV-19^^England|Human|Northumbria University / South Tees Hospitals NHS Foundation Trust / North Cumbria Integrated Care NHS Foundation Trust / North Tees and Hartlepool NHS Foundation Trust / Newcastle Hospitals NHS Foundation Trust|COVID-19 Genomics UK (COG-UK) Consortium|Bashton|United Kingdom
>Spike|hCoV-19/England/204941562/2020|2020-11-30|EPI_ISL_754304|Original|hCoV-19^^England|Human|Respiratory Virus Unit|COVID-19 Genomics UK (COG-UK) Consortium|Platt|United Kingdom
>Spike|hCoV-19/England/ALDP-8FF8C8/2020|2020-06-24|EPI_ISL_557131|Original|hCoV-19^^England|Human|Lighthouse Lab in Alderley Park|Wellcome Sanger Institute for the COVID-19 Genomics UK (COG-UK) consortium|Davies|United Kingdom
>Spike|hCoV-19/Iceland/401/2020|2020-03-19|EPI_ISL_424425|Original|hCoV-19^^Reykjavik|Human|The National University Hospital of Iceland|deCODE genetics|J\u00f3nsson|Iceland
>Spike|hCoV-19/Belgium/rega-12111355/2020|2020-12-11|EPI_ISL_738408|Original|hCoV-19^^Mol|Human|UZ Leuven|KU Leuven|Maes|Belgium
>Spike|hCoV-19/England/QEUH-B66A4B/2020|2020-11-11|EPI_ISL_655132|Original|hCoV-19^^England|Human|Lighthouse Lab in Glasgow|Wellcome Sanger Institute for the COVID-19 Genomics UK (COG-UK) Consortium|Davies|United Kingdom
>Spike|hCoV-19/England/NORT-1B79C79/2020|2020-11-19|EPI_ISL_724925|Original|hCoV-19^^England|Human|Northumbria University / South Tees Hospitals NHS Foundation Trust

/ North Cumbria Integrated Care NHS Foundation Trust / North Tees and Hartlepool NHS Foundation Trust / Newcastle Hospitals NHS Foundation Trust|COVID-19 Genomics UK (COG-UK) Consortium|Bashton|United Kingdom
>Spike|hCoV-19/England/BIRM-61AB4/2020|2020-03-24|EPI_ISL_529325|Original|hCoV-19^^England|Human|University of Birmingham|COVID-19 Genomics UK (COG-UK) Consortium|Loman|United Kingdom
>Spike|hCoV-19/South Africa/KRISP-CH01110222/2020|2020-11-25|EPI_ISL_736985|Original|hCoV-19^^KwaZulu-Natal|Human|NHLS-IALCH|KRISP|de Oliveira|South Africa
>Spike|hCoV-19/Norway/2881/2020|2020-07-13|EPI_ISL_500799|Original|hCoV-19^^Norway|Human|Hospital of Southern Norway - Kristiansand|Norwegian Institute of Public Health|Kopperud Riis|Norway
>Spike|hCoV-19/USA/CA-ALSR-3600/2020|2020-06-24|EPI_ISL_636105|Original|hCoV-19^^California|Human|San Diego County Public Health Laboratory|Andersen lab at Scripps Research|Gangavarapu|USA
>Spike|hCoV-19/USA/TX-HMH-MCoV-15177/2020|2020-10-20|EPI_ISL_786661|Original|hCoV-19^^Texas|Human|Houston Methodist Hospital|Houston Methodist Hospital|Long|USA
>Spike|hCoV-19/Brazil/SP-600/2020|2020-06-30|EPI_ISL_515525|Original|hCoV-19^^S\u00e3o Paulo|Human|National Influenza Center - Instituto Adolfo Lutz|Instituto Adolfo Lutz|Sacchi|Brazil
>Spike|hCoV-19/England/CAMC-A81A0F/2020|2020-10-14|EPI_ISL_610787|Original|hCoV-19^^England|Human|Lighthouse Lab in Cambridge|Wellcome Sanger Institute for the COVID-19 Genomics UK (COG-UK) consortium|Davies|United Kingdom
>Spike|hCoV-19/Scotland/EDB3000/2020|2020-04-03|EPI_ISL_489008|Original|hCoV-19^^Scotland|Human|Virology Department|Wellcome Sanger Institute for the COVID-19 Genomics UK (COG-UK) consortium|Davies|United Kingdom
>Spike|hCoV-19/England/QEUH-9D02EC/2020|2020-09-16|EPI_ISL_589888|Original|hCoV-19^^England|Human|Lighthouse Lab in Glasgow|Wellcome Sanger Institute for the COVID-19 Genomics UK (COG-UK) consortium|Davies|United Kingdom
>Spike|hCoV-19/USA/UT-04138/2020|2020-05-13|EPI_ISL_493037|Original|hCoV-19^^Utah|Human|Utah Public Health Laboratory|Utah Public Health Laboratory|Butz|USA
>Spike|hCoV-19/Japan/PG-8051/2020|2020-08-00|EPI_ISL_687152|Original|hCoV-19^^Japan|Human|Pathogen Genomics Center|Pathogen Genomics Center|Sekizuka|Japan
>Spike|hCoV-19/England/CAMC-B22AA5/2020|2020-11-02|EPI_ISL_647192|Original|hCoV-19^^England|Human|Lighthouse Lab in Cambridge|Wellcome Sanger Institute for the COVID-19 Genomics UK (COG-UK) Consortium|Davies|United Kingdom
>Spike|hCoV-19/Costa Rica/INC-0074/2020|2020-06-30|EPI_ISL_527742|Original|hCoV-19^^San Jose|Human|Centro Nacional De Rehabilitacion Humberto Araya Rojas (Cenare)|Inciensa|Soto-Garita|Costa Rica
>Spike|hCoV-19/England/CAMC-B7A3A3/2020|2020-11-11|EPI_ISL_741105|Original|hCoV-19^^England|Human|Oxford Viromics|COVID-19 Genomics UK (COG-UK) Consortium|Golubchik|United Kingdom
>Spike|hCoV-19/Australia/VIC7355/2020|2020-07-24|EPI_ISL_564357|Original|hCoV-19^^Victoria|Human|Victorian Infectious Diseases Reference Laboratory (VIDRL)|VIDRL and MDU-PHL|Schultz|Australia
>Spike|hCoV-19/Wales/PHWC-4906D2/2020|2020-11-12|EPI_ISL_706501|Original|hCoV-19^^Wales|Human|Originating lab|COVID-19 Genomics UK (COG-UK) Consortium|Moore|United Kingdom
>Spike|hCoV-19/Scotland/QEUH-9FAF9A/2020|2020-09-29|EPI_ISL_600596|Original|hCoV-19^^Scotland|Human|Lighthouse Lab in Glasgow|Wellcome Sanger Institute for the COVID-19 Genomics UK (COG-UK) consortium|Davies|United Kingdom
>Spike|hCoV-19/USA/WI-UW-1049/2020|2020-08-01|EPI_ISL_516475|Original|hCoV-19^^Wisconsin|Human|University of Wisconsin-Madison AIDS Vaccine Research Laboratories|University of Wisconsin-Madison AIDS Vaccine Research Laboratories|Baczenas|USA
>Spike|hCoV-19/Australia/NSW1322/2020|2020-12-16|EPI_ISL_767888|Original|hCoV-19^^New South Wales|Human|South Eastern Area Laboratory Services (SEALS)|NSW Health Pathology - Institute of Clinical Pathology and Medical Research; Westmead Hospital; University of Sydney|Gall|Australia

>Spike|hCoV-19/England/BRIS-12BB85/2020|2020-05-16|EPI_ISL_469457|Original|hCoV-19^^England|Human|PHE South West Regional Laboratory|Wellcome Sanger Institute for the COVID-19 Genomics UK (COG-UK) consortium|Davies|United Kingdom
>Spike|hCoV-19/USA/TX-HMH-MCoV-9009/2020|2020-07-02|EPI_ISL_780893|Original|hCoV-19^^Texas|Human|Houston Methodist Hospital|Houston Methodist Hospital|Long|USA
>Spike|hCoV-19/Denmark/DCGC-15788/2020|2020-11-23|EPI_ISL_714019|Original|hCoV-19^^Nordjylland|Human|Department of Virus and Microbiological Special Diagnostics|Albertsen Lab|Michaelsen|Denmark
>Spike|hCoV-19/USA/WA-UW-10131/2020|2020-05-29|EPI_ISL_461475|Original|hCoV-19^^Washington|Human|UW Virology Lab|UW Virology Lab|Roychoudhury|USA
>Spike|hCoV-19/Wales/PHWC-47DBFA/2020|2020-10-08|EPI_ISL_595340|Original|hCoV-19^^Wales|Human|Originating lab|COVID-19 Genomics UK (COG-UK) Consortium|Moore|United Kingdom
>Spike|hCoV-19/England/BRIS-12EC52/2020|2020-04-26|EPI_ISL_488207|Original|hCoV-19^^England|Human|PHE South West Regional Laboratory|Wellcome Sanger Institute for the COVID-19 Genomics UK (COG-UK) consortium|Davies|United Kingdom
>Spike|hCoV-19/Australia/VIC4752/2020|2020-07-16|EPI_ISL_519140|Original|hCoV-19^^Victoria|Human|Microbiological Diagnostic Unit - Public Health Laboratory (MDU-PHL)|MDU-PHL|Schultz|Australia
>Spike|hCoV-19/Wales/PHWC-2BD62/2020|2020-04-07|EPI_ISL_445842|Original|hCoV-19^^Wales|Human|Wales Specialist Virology Centre|Public Health Wales Microbiology Cardiff|Moore|United Kingdom
>Spike|hCoV-19/Australia/VIC13205/2020|2020-08-16|EPI_ISL_563640|Original|hCoV-19^^Victoria|Human|Microbiological Diagnostic Unit - Public Health Laboratory (MDU-PHL)|MDU-PHL|Schultz|Australia
>Spike|hCoV-19/USA/MI-MDHHS-SC20332/2020|2020-03-19|EPI_ISL_450611|Original|hCoV-19^^Michigan|Human|Michigan Department of Health and Human Services|Michigan Department of Health and Human Services|Blankenship|USA
>Spike|hCoV-19/Netherlands/ZH-EMC-598/2020|2020-09-18|EPI_ISL_578026|Original|hCoV-19^^Zuid Holland|Human|Dutch COVID-19 response team|Erasmus Medical Center|Oude Munnink|Netherlands
>Spike|hCoV-19/Denmark/DCGC-2967/2020|2020-08-24|EPI_ISL_616729|Original|hCoV-19^^Syddanmark|Human|Department of Virus and Microbiological Special Diagnostics|Albertsen lab|Michaelsen|Denmark
>Spike|hCoV-19/Australia/VIC12377/2020|2020-07-29|EPI_ISL_640765|Original|hCoV-19^^Victoria|Human|Microbiological Diagnostic Unit - Public Health Laboratory (MDU-PHL)|MDU-PHL|Schultz|Australia
>Spike|hCoV-19/Kazakhstan/26565/2020|2020-04-26|EPI_ISL_454600|Original|hCoV-19^^Nur-Sultan|Human|Laboratory of virology|Laboratory of molecular-genetic research|Abdaliyev|Kazakhstan
>Spike|hCoV-19/Wales/PHWC-4811F1/2020|2020-10-18|EPI_ISL_611588|Original|hCoV-19^^Wales|Human|Wales Specialist Virology Centre Sequencing lab|COVID-19 Genomics UK (COG-UK) Consortium|Moore|United Kingdom
>Spike|hCoV-19/USA/HI-H200016/2020|2020-03-25|EPI_ISL_752612|Original|hCoV-19^^Hawaii|Human|State Laboratories Division|State Laboratories Division|Sultana|USA
>Spike|hCoV-19/Denmark/DCGC-13208/2020|2020-11-30|EPI_ISL_712652|Original|hCoV-19^^Midtjylland|Human|Department of Virus and Microbiological Special Diagnostics|Albertsen Lab|Michaelsen|Denmark
>Spike|hCoV-19/England/QEUH-9F40E8/2020|2020-09-25|EPI_ISL_588668|Original|hCoV-19^^England|Human|Lighthouse Lab in Glasgow|Wellcome Sanger Institute for the COVID-19 Genomics UK (COG-UK) consortium|Davies|United Kingdom
>Spike|hCoV-19/England/BRIS-123E75/2020|2020-04-08|EPI_ISL_492832|Original|hCoV-19^^England|Human|PHE South West Regional Laboratory|Wellcome Sanger Institute for the COVID-19 Genomics UK (COG-UK) consortium|Davies|United Kingdom
>Spike|hCoV-19/Jordan/SEARCH-5476/2020|2020-09-08|EPI_ISL_755256|Original|hCoV-19^^Amman|Human|Biolab Diagnostic Laboratories|Andersen lab at Scripps Research|Gangavarapu|Jordan
>Spike|hCoV-19/USA/TX-HMH-MCoV-12855/2020|2020-08-19|EPI_ISL_788008|Original|hCoV-19^^Texas|Human|Houston Methodist Hospital|Houston Methodist Hospital|Long|USA

>Spike|hCoV-19/Australia/NSW913/2020|2020-08-31|EPI_ISL_544994|Original|hCoV-19^^New South Wales|Human|St Vincent's Pathology (SydPath)|NSW Health Pathology - Institute of Clinical Pathology and Medical Research; Westmead Hospital; University of Sydney|Gall|Australia
>Spike|hCoV-19/England/NORW-FA36A/2020|2020-12-06|EPI_ISL_725498|Original|hCoV-19^^England|Human|Quadram Institute Bioscience|COVID-19 Genomics UK (COG-UK) Consortium|Alikhan|United Kingdom
>Spike|hCoV-19/Denmark/DCGC-19393/2020|2020-12-07|EPI_ISL_748922|Original|hCoV-19^^Hovedstaden|Human|Department of Virus and Microbiological Special Diagnostics|Albertsen Lab|Michaelsen|Denmark
>Spike|hCoV-19/England/MILK-B8F0D1/2020|2020-11-13|EPI_ISL_675443|Original|hCoV-19^^England|Human|Lighthouse Lab in Milton Keynes|Wellcome Sanger Institute for the COVID-19 Genomics UK (COG-UK) Consortium|Davies|United Kingdom
>Spike|hCoV-19/Switzerland/ZH-ETHZ-939plus/2020|2020-04-17|EPI_ISL_483670|Original|hCoV-19^^Zurich|Human|University Hospital Zurich|Department of Biosystems Science and Engineering|Nadeau|Switzerland
>Spike|hCoV-19/England/CAMB-75130/2020|2020-03-16|EPI_ISL_440400|Original|hCoV-19^^England|Human|Department of Pathology|Wellcome Sanger Institute for the COVID-19 Genomics UK (COG-UK) consortium|Amato|United Kingdom
>Spike|hCoV-19/Germany/NW-HHU-539/2020|2020-12-14|EPI_ISL_779948|Original|hCoV-19^^North Rhine-Westphalia|Human|Center of Medical Microbiology|Center of Medical Microbiology|Wienemann|Germany
>Spike|hCoV-19/USA/NY-NYCPHL-000314/2020|2020-05-01|EPI_ISL_631840|Original|hCoV-19^^New York|Human|OCME Office Of Chief Medical Examiner|New York City Public Health Laboratory|Wang|USA
>Spike|hCoV-19/Scotland/GCVR-17240C/2020|2020-03-30|EPI_ISL_469956|Original|hCoV-19^^Scotland|Human|NHSGGC West of Scotland Specialist Virology Centre / MRC-University of Glasgow Centre for Virus Research|Wellcome Sanger Institute for the COVID-19 Genomics UK (COG-UK) consortium|Davies|United Kingdom
>Spike|hCoV-19/Japan/PG-5406/2020|2020-08-00|EPI_ISL_690509|Original|hCoV-19^^Japan|Human|Fukuoka Institute of Health and Environmental Sciences|Pathogen Genomics Center|Sekizuka|Japan
>Spike|hCoV-19/England/MILK-A7A6D8/2020|2020-10-15|EPI_ISL_606585|Original|hCoV-19^^England|Human|Lighthouse Lab in Milton Keynes|Wellcome Sanger Institute for the COVID-19 Genomics UK (COG-UK) consortium|Davies|United Kingdom
>Spike|hCoV-19/Iceland/369/2020|2020-03-19|EPI_ISL_424393|Original|hCoV-19^^Reykjavik|Human|The National University Hospital of Iceland|deCODE genetics|J\u00f3nsson|Iceland
>Spike|hCoV-19/Wales/PHWC-2DFD3/2020|2020-04-10|EPI_ISL_446045|Original|hCoV-19^^Wales|Human|Wales Specialist Virology Centre|Public Health Wales Microbiology Cardiff|Moore|United Kingdom
>Spike|hCoV-19/Japan/PG-2395/2020|2020-04-00|EPI_ISL_686000|Original|hCoV-19^^Japan|Human|Pathogen Genomics Center|Pathogen Genomics Center|Sekizuka|Japan
>Spike|hCoV-19/Australia/VIC8802/2020|2020-07-31|EPI_ISL_565205|Original|hCoV-19^^Victoria|Human|Victorian Infectious Diseases Reference Laboratory (VIDRL)|VIDRL and MDU-PHL|Schultz|Australia
>Spike|hCoV-19/Denmark/DCGC-17073/2020|2020-12-14|EPI_ISL_750743|Original|hCoV-19^^Nordjylland|Human|Department of Virus and Microbiological Special Diagnostics|Albertsen Lab|Michaelsen|Denmark
>Spike|hCoV-19/Denmark/ALAB-SSI-590/2020|2020-03-28|EPI_ISL_436984|Original|hCoV-19^^Denmark|Human|Department of Virus and Microbiological Special Diagnostics|Albertsen lab|Kirkegaard|Denmark
>Spike|hCoV-19/Netherlands/NH-RIVM-10067/2020|2020-04-21|EPI_ISL_547496|Original|hCoV-19^^Noord Holland|Human|Dutch COVID-19 response team|National Institute for Public Health and the Environment (RIVM)|Meijer|Netherlands
>Spike|hCoV-19/England/CAMC-B09163/2020|2020-10-30|EPI_ISL_672950|Original|hCoV-19^^England|Human|Lighthouse Lab in Cambridge|Wellcome Sanger Institute for the COVID-19 Genomics UK (COG-UK) Consortium|Davies|United Kingdom

>Spike|hCoV-19/England/CAMC-B7A9ED/2020|2020-11-12|EPI_ISL_668085|Original|hCoV-19^^England|Human|Lighthouse Lab in Cambridge|Wellcome Sanger Institute for the COVID-19 Genomics UK (COG-UK) Consortium|Davies|United Kingdom
>Spike|hCoV-19/Germany/HH-hpi-p1679/2020|2020-03-18|EPI_ISL_775674|Original|hCoV-19^^Hamburg|Human|University Medical Center Hamburg Eppendorf|Heinrich Pette Institute|Robitaille|Germany
>Spike|hCoV-19/Scotland/CVR3256/2020|2020-04-24|EPI_ISL_493833|Original|hCoV-19^^Scotland|Human|West of Scotland Specialist Virology Centre|COVID-19 Genomics UK (COG-UK) Consortium|Robertson|United Kingdom
>Spike|hCoV-19/England/ALDP-A51BAB/2020|2020-10-07|EPI_ISL_598221|Original|hCoV-19^^England|Human|Lighthouse Lab in Alderley Park|Wellcome Sanger Institute for the COVID-19 Genomics UK (COG-UK) consortium|Davies|United Kingdom
>Spike|hCoV-19/England/ALDP-B83EE2/2020|2020-11-14|EPI_ISL_674442|Original|hCoV-19^^England|Human|Lighthouse Lab in Alderley Park|Wellcome Sanger Institute for the COVID-19 Genomics UK (COG-UK) Consortium|Davies|United Kingdom
>Spike|hCoV-19/USA/TX-HMH-3774/2020|2020-06-15|EPI_ISL_543182|Original|hCoV-19^^Texas|Human|Houston Methodist Hospital|Houston Methodist Hospital|Long|USA
>Spike|hCoV-19/USA/MA-MGH-00946/2020|2020-05-02|EPI_ISL_791830|Original|hCoV-19^^Massachusetts|Human|Massachusetts General Hospital|Infectious Disease Program|Park|USA
>Spike|hCoV-19/Denmark/DCGC-16899/2020|2020-12-14|EPI_ISL_750211|Original|hCoV-19^^Hovedstaden|Human|Department of Virus and Microbiological Special Diagnostics|Albertsen Lab|Michaelsen|Denmark
>Spike|hCoV-19/England/20139052002/2020|2020-03-27|EPI_ISL_420530|Original|hCoV-19^^England|Human|Respiratory Virus Unit|Respiratory Virus Unit|Platt|United Kingdom
>Spike|hCoV-19/England/MILK-B886D6/2020|2020-11-14|EPI_ISL_674415|Original|hCoV-19^^England|Human|Lighthouse Lab in Milton Keynes|Wellcome Sanger Institute for the COVID-19 Genomics UK (COG-UK) Consortium|Davies|United Kingdom
>Spike|hCoV-19/Wales/PHWC-4A53C4/2020|2020-12-15|EPI_ISL_764861|Original|hCoV-19^^Wales|Human|Wales Specialist Virology Centre Sequencing lab|COVID-19 Genomics UK (COG-UK) Consortium|Moore|United Kingdom
>Spike|hCoV-19/Denmark/DCGC-9375/2020|2020-11-16|EPI_ISL_668938|Original|hCoV-19^^Syddanmark|Human|Department of Virus and Microbiological Special Diagnostics|Albertsen Lab|Michaelsen|Denmark
>Spike|hCoV-19/India/DL-NCDC7084-CSIR-IGIB/2020|2020-05-27|EPI_ISL_482626|Original|hCoV-19^^Delhi|Human|National Centre for Disease control (NCDC)|NCDC/CSIR-IGIB|Singh|India
>Spike|hCoV-19/Bangladesh/DNAS_DRICM_isl_MM-50/2020|2020-06-29|EPI_ISL_774990|Original|hCoV-19^^Dhaka|Human|Designated Reference Institute for Chemical Measurements (DRICM)|DNA SOLUTION LTD.|Rabbi|Bangladesh
>Spike|hCoV-19/England/ALDP-A52885/2020|2020-10-07|EPI_ISL_598196|Original|hCoV-19^^England|Human|Lighthouse Lab in Alderley Park|Wellcome Sanger Institute for the COVID-19 Genomics UK (COG-UK) consortium|Davies|United Kingdom
>Spike|hCoV-19/South Africa/KRISP-0001/2020|2020-03-23|EPI_ISL_467431|Original|hCoV-19^^KwaZulu-Natal|Human|Molecular Diagnostics Services (MDS)|KRISP|de Oliveira|South Africa
>Spike|hCoV-19/Scotland/EDB4300/2020|2020-04-23|EPI_ISL_449291|Original|hCoV-19^^Scotland|Human|Virology Department|COVID-19 Genomics UK (COG-UK) Consortium|Williams|United Kingdom
>Spike|hCoV-19/USA/CA-CHLA-PLM54818837/2020|2020-11-15|EPI_ISL_753341|Original|hCoV-19^^California|Human|Clinical virology Laboratory|Center for Personalized Medicine|Dien Bard|USA
>Spike|hCoV-19/England/OXON-AF4CB/2020|2020-04-08|EPI_ISL_478743|Original|hCoV-19^^England|Human|Oxford Viromics|COVID-19 Genomics UK (COG-UK) Consortium|Golubchik|United Kingdom
>Spike|hCoV-19/Netherlands/NA_517/2020|2020-03-24|EPI_ISL_460690|Original|hCoV-19^^Netherlands|Human|Dutch COVID-19 response team|Erasmus Medical Center|Oude Munnink|Netherlands

>Spike|hCoV-19/USA/FL-BPHL-0345/2020|2020-04-12|EPI_ISL_489754|Original|hCoV-19^^Florida|Human|Florida Bureau of Public Health Laboratories|Florida Bureau of Public Health Laboratories|Blanton|USA
>Spike|hCoV-19/England/20144074804/2020|2020-04-01|EPI_ISL_421963|Original|hCoV-19^^England|Human|Respiratory Virus Unit|Respiratory Virus Unit|Platt|United Kingdom
>Spike|hCoV-19/Switzerland/BL-ETHZ-400102/2020|2020-12-07|EPI_ISL_729144|Original|hCoV-19^^Basel-Landschaft|Human|Viollier AG|Department of Biosystems Science and Engineering|Nadeau|Switzerland
>Spike|hCoV-19/Wales/PHWC-2C0E0/2020|2020-04-04|EPI_ISL_474275|Original|hCoV-19^^Wales|Human|Originating lab|COVID-19 Genomics UK (COG-UK) Consortium|Moore|United Kingdom
>Spike|hCoV-19/USA/WA-S653/2020|2020-04-15|EPI_ISL_438206|Original|hCoV-19^^Washington|Human|Washington State Department of Health|Seattle Flu Study|Bedford|USA
>Spike|hCoV-19/England/PORT-2D5B7A/2020|2020-12-18|EPI_ISL_765137|Original|hCoV-19^^England|Human|Centre for Enzyme Innovation|COVID-19 Genomics UK (COG-UK) Consortium|Robson|United Kingdom
>Spike|hCoV-19/Scotland/EDB6492/2020|2020-06-12|EPI_ISL_478246|Original|hCoV-19^^Scotland|Human|Virology Department|COVID-19 Genomics UK (COG-UK) Consortium|Williams|United Kingdom
>Spike|hCoV-19/England/SHEF-C6516/2020|2020-10-15|EPI_ISL_611682|Original|hCoV-19^^England|Human|Virology Department|COVID-19 Genomics UK (COG-UK) Consortium|de Silva|United Kingdom
>Spike|hCoV-19/England/EKHU-260EFFA/2020|2020-04-12|EPI_ISL_501621|Original|hCoV-19^^England|Human|Lab Microbiology|Wellcome Sanger Institute for the COVID-19 Genomics UK (COG-UK) consortium|Davies|United Kingdom
>Spike|hCoV-19/Serbia/NP-0541007/2020|2020-07-09|EPI_ISL_541661|Original|hCoV-19^^Novi Pazar|Human|Laboratory Diagnostic|Laboratory Diagnostic|Vidanovic|Serbia
>Spike|hCoV-19/Wales/PHWC-492FD5/2020|2020-11-28|EPI_ISL_725820|Original|hCoV-19^^Wales|Human|Originating lab|COVID-19 Genomics UK (COG-UK) Consortium|Moore|United Kingdom
>Spike|hCoV-19/Brazil/SC-L19-CD399/2020|2020-04-01|EPI_ISL_476324|Original|hCoV-19^^Santa Catarina|Human|DB Diagn\u00f3sticos do Brasil|Instituto de Medicina Tropical da Univesidade de S\u00e3o Paulo|Sabino|Brazil
>Spike|hCoV-19/USA/TX-HMH-MCoV-8467/2020|2020-07-01|EPI_ISL_780578|Original|hCoV-19^^Texas|Human|Houston Methodist Hospital|Houston Methodist Hospital|Long|USA
>Spike|hCoV-19/USA/MA-MGH-00665/2020|2020-04-04|EPI_ISL_460328|Original|hCoV-19^^Massachusetts|Human|Massachusetts General Hospital|Infectious Disease Program|Lemieux|USA
>Spike|hCoV-19/England/CAMC-BC0F7D/2020|2020-11-22|EPI_ISL_704906|Original|hCoV-19^^England|Human|Lighthouse Lab in Cambridge|Wellcome Sanger Institute for the COVID-19 Genomics UK (COG-UK) Consortium|Davies|United Kingdom
>Spike|hCoV-19/France/ARA-CDP20536/2020|2020-08-28|EPI_ISL_582114|Original|hCoV-19^^Auvergne-Rh\u00f4ne-Alpes|Human|CNR Virus des Infections Respiratoires - France SUD|CNR Virus des Infections Respiratoires - France SUD|Josset|France
>Spike|hCoV-19/USA/VA-DCLS-1853/2020|2020-09-23|EPI_ISL_614077|Original|hCoV-19^^Virginia|Human|Virginia DCLS|Virginia DCLS|St. Jacques|USA
>Spike|hCoV-19/Northern Ireland/NIRE-10687A/2020|2020-05-10|EPI_ISL_500992|Original|hCoV-19^^Northern Ireland|Human|Regional Virus Laboratory|Wellcome Sanger Institute for the COVID-19 Genomics UK (COG-UK) consortium|Davies|United Kingdom
>Spike|hCoV-19/USA/LA-EVTL782/2020|2020-07-22|EPI_ISL_653473|Original|hCoV-19^^Louisiana|Human|LSUHS Emerging Viral Threat Laboratory|Microbial Genome Sequencing Center|Kamil|USA
>Spike|hCoV-19/Scotland/QEUH-9DD856/2020|2020-09-21|EPI_ISL_599492|Original|hCoV-19^^Scotland|Human|Lighthouse Lab in Glasgow|Wellcome Sanger Institute for the COVID-19 Genomics UK (COG-UK) consortium|Davies|United Kingdom
>Spike|hCoV-19/Spain/VC-FISABIO-589/2020|2020-04-01|EPI_ISL_447516|Original|hCoV-19^^Comunitat Valenciana|Human|Servicio de Microbiologia. Hospital Clinico

Universitario de Valencia|Sequencing and Bioinformatics Service and Molecular Epidemiology Research Group. FISABIO-Public Health|Gonzalez-Candelas|Spain
>Spike|hCoV-19/Denmark/DCGC-18519/2020|2020-12-07|EPI_ISL_749526|Original|hCoV-19^^Hovedstaden|Human|Department of Virus and Microbiological Special Diagnostics|Albertsen Lab|Michaelsen|Denmark
>Spike|hCoV-19/USA/TX-HMH-2061/2020|2020-05-12|EPI_ISL_545095|Original|hCoV-19^^Texas|Human|Houston Methodist Hospital|Houston Methodist Hospital|Long|USA
>Spike|hCoV-19/USA/FL-BPHL-1032/2020|2020-07-23|EPI_ISL_517922|Original|hCoV-19^^Florida|Human|Florida Bureau of Public Health Laboratories|Florida Bureau of Public Health Laboratories|Blanton|USA
>Spike|hCoV-19/Canada/Qc-L00240594/2020|2020-03-27|EPI_ISL_465680|Original|hCoV-19^^Quebec|Human|H\u00f4pital du Suro\u00eet|Laboratoire de sant\u00e9 publique du Qu\u00e9bec|Moreira|Canada
>Spike|hCoV-19/England/BRIS-12BA79/2020|2020-05-17|EPI_ISL_469400|Original|hCoV-19^^England|Human|PHE South West Regional Laboratory|Wellcome Sanger Institute for the COVID-19 Genomics UK (COG-UK) consortium|Davies|United Kingdom
>Spike|hCoV-19/USA/NV-NSPHL-A0129/2020|2020-04-29|EPI_ISL_515397|Original|hCoV-19^^Nevada|Human|Nevada State Public Health Laboratory|Nevada State Public Health Laboratory|Sevinsky|USA
>Spike|hCoV-19/England/QEUH-B2E511/2020|2020-11-03|EPI_ISL_646179|Original|hCoV-19^^England|Human|Lighthouse Lab in Glasgow|Wellcome Sanger Institute for the COVID-19 Genomics UK (COG-UK) Consortium|Davies|United Kingdom
>Spike|hCoV-19/England/ALDP-51D8E5/2020|2020-06-02|EPI_ISL_558952|Original|hCoV-19^^England|Human|Lighthouse Lab in Alderley Park|Wellcome Sanger Institute for the COVID-19 Genomics UK (COG-UK) consortium|Davies|United Kingdom
>Spike|hCoV-19/England/CAMC-B22A5A/2020|2020-11-02|EPI_ISL_647225|Original|hCoV-19^^England|Human|Lighthouse Lab in Cambridge|Wellcome Sanger Institute for the COVID-19 Genomics UK (COG-UK) Consortium|Davies|United Kingdom
>Spike|hCoV-19/Scotland/EDB6410/2020|2020-04-13|EPI_ISL_487765|Original|hCoV-19^^Scotland|Human|Virology Department|Wellcome Sanger Institute for the COVID-19 Genomics UK (COG-UK) consortium|Davies|United Kingdom
>Spike|hCoV-19/North Macedonia/3193/2020|2020-06-12|EPI_ISL_516422|Original|hCoV-19^^Skopje|Human|Center for public health - Skopje|Research Center for Genetic Engineering and Biotechnology \Georgi D. Efremov\u201d|Dimovski|North Macedonia
>Spike|hCoV-19/Wales/PHWC-49C0E7/2020|2020-12-07|EPI_ISL_742507|Original|hCoV-19^^Wales|Human|Wales Specialist Virology Centre Sequencing lab|COVID-19 Genomics UK (COG-UK) Consortium|Moore|United Kingdom
>Spike|hCoV-19/England/LOND-12F1726/2020|2020-12-15|EPI_ISL_764385|Original|hCoV-19^^England|Human|University College London|COVID-19 Genomics UK (COG-UK) Consortium|Roy|United Kingdom
>Spike|hCoV-19/Finland/FIN01032020/2020|2020-03-01|EPI_ISL_413605|Original|hCoV-19^^Helsinki|Human|Department of Virology and Immunology|Department of Virology|Smura|Finland
>Spike|hCoV-19/England/ALDP-C58EF4/2020|2020-12-14|EPI_ISL_735882|Original|hCoV-19^^England|Human|Lighthouse Lab in Alderley Park|Wellcome Sanger Institute for the COVID-19 Genomics UK (COG-UK) Consortium|Davies|United Kingdom
>Spike|hCoV-19/USA/CA-QDX-1518/2020|2020-03-17|EPI_ISL_603771|Original|hCoV-19^^California|Human|Quest Diagnostics|Quest Diagnostics|Kagan|USA
>Spike|hCoV-19/England/NOTT-11B7DF/2020|2020-11-12|EPI_ISL_652379|Original|hCoV-19^^England|Human|Queens Medical Centre|COVID-19 Genomics UK (COG-UK) Consortium|Loose|United Kingdom
>Spike|hCoV-19/Spain/MD-IBV-99008704/2020|2020-10-04|EPI_ISL_654284|Original|hCoV-19^^Madrid|Human|Hospital General Universitario Gregorio Mara\u00f1\u00f3n|SeqCOVID-SPAIN consortium/IBV(CSIC)|Comas|Spain
>Spike|hCoV-19/England/QEUH-BC3BB2/2020|2020-11-25|EPI_ISL_703914|Original|hCoV-19^^England|Human|Lighthouse Lab in Glasgow|Wellcome Sanger Institute for the COVID-19 Genomics UK (COG-UK) Consortium|Davies|United Kingdom
>Spike|hCoV-19/Switzerland/BL-ETHZ-420207/2020|2020-12-19|EPI_ISL_768056|Original|hCoV-19^^Basel-Landschaft|Human|Viollier AG|Department of Biosystems Science and Engineering|Nadeau|Switzerland

>Spike|hCoV-19/Algeria/G37318-8849/2020|2020-06-15|EPI_ISL_766863|Original|hCoV-19^^Tipaza|Human|NIC Viral Respiratory Unit - Institut Pasteur of Algeria|National Reference Center for Viruses of Respiratory Infections|Enouf|Algeria
>Spike|hCoV-19/Austria/CeMM0162/2020|2020-03-05|EPI_ISL_438008|Original|hCoV-19^^Vienna|Human|Center for Virology|Bergthaler laboratory|Bergthaler|Austria
>Spike|hCoV-19/Gambia/NPHL-3055/2020|2020-07-17|EPI_ISL_561310|Original|hCoV-19^^Kombo|Human|MRCG at LSHTM Genomics lab|MRCG at LSHTM Genomics lab|Sesay|Gambia
>Spike|hCoV-19/England/MILK-BAA173/2020|2020-11-18|EPI_ISL_681540|Original|hCoV-19^^England|Human|Lighthouse Lab in Milton Keynes|Wellcome Sanger Institute for the COVID-19 Genomics UK (COG-UK) Consortium|Davies|United Kingdom
>Spike|hCoV-19/USA/WA-QDX-1271/2020|2020-03-16|EPI_ISL_572051|Original|hCoV-19^^Washington|Human|Quest Diagnostics|Quest Diagnostics|Kagan|USA
>Spike|hCoV-19/England/QEUH-969F3C/2020|2020-08-11|EPI_ISL_530817|Original|hCoV-19^^England|Human|Lighthouse Lab in Glasgow|Wellcome Sanger Institute for the COVID-19 Genomics UK (COG-UK) consortium|Davies|United Kingdom
>Spike|hCoV-19/England/BRIS-12277B/2020|2020-04-04|EPI_ISL_440659|Original|hCoV-19^^England|Human|PHE South West Regional Laboratory|Wellcome Sanger Institute for the COVID-19 Genomics UK (COG-UK) consortium|Amato|United Kingdom
>Spike|hCoV-19/England/ALDP-B5B9A6/2020|2020-11-08|EPI_ISL_655460|Original|hCoV-19^^England|Human|Lighthouse Lab in Alderley Park|Wellcome Sanger Institute for the COVID-19 Genomics UK (COG-UK) Consortium|Davies|United Kingdom
>Spike|hCoV-19/England/EXET-138138/2020|2020-10-05|EPI_ISL_612384|Original|hCoV-19^^England|Human|University of Exeter|COVID-19 Genomics UK (COG-UK) Consortium|Temperton|United Kingdom
>Spike|hCoV-19/India/MH-GA30/2020|2020-06-17|EPI_ISL_511931|Original|hCoV-19^^Maharashtra|Human|Government Medical College|National Institute of Biomedical Genomics -\u00a0DBT's PAN-INDIA 1000 SARS--CoV-2 RNA Genome Sequencing Consortium|Maitra|India
>Spike|hCoV-19/England/ALDP-BBB1F7/2020|2020-11-20|EPI_ISL_701845|Original|hCoV-19^^England|Human|Lighthouse Lab in Alderley Park|Wellcome Sanger Institute for the COVID-19 Genomics UK (COG-UK) Consortium|Davies|United Kingdom
>Spike|hCoV-19/USA/AZ-TG668420/2020|2020-05-13|EPI_ISL_696315|Original|hCoV-19^^Arizona|Human|Sonora Quest Laboratories|TGen North|French|USA
>Spike|hCoV-19/England/MILK-B8F53C/2020|2020-11-13|EPI_ISL_674546|Original|hCoV-19^^England|Human|Lighthouse Lab in Milton Keynes|Wellcome Sanger Institute for the COVID-19 Genomics UK (COG-UK) Consortium|Davies|United Kingdom
>Spike|hCoV-19/England/QEUH-BCD5D8/2020|2020-11-26|EPI_ISL_704407|Original|hCoV-19^^England|Human|Lighthouse Lab in Glasgow|Wellcome Sanger Institute for the COVID-19 Genomics UK (COG-UK) Consortium|Davies|United Kingdom
>Spike|hCoV-19/Netherlands/ZH-EMC-1106/2020|2020-12-14|EPI_ISL_722780|Original|hCoV-19^^Zuid Holland|Human|Dutch COVID-19 response team|Erasmus Medical Center|Oude Munnink|Netherlands
>Spike|hCoV-19/Wales/PHWC-2ECF9/2020|2020-04-06|EPI_ISL_446141|Original|hCoV-19^^Wales|Human|Wales Specialist Virology Centre|Public Health Wales Microbiology Cardiff|Moore|United Kingdom
>Spike|hCoV-19/USA/AZ-TG494512/2020|2020-07-13|EPI_ISL_695245|Original|hCoV-19^^Arizona|Human|TGen North|TGen North|French|USA
>Spike|hCoV-19/USA/WA-UW-23213/2020|2020-08-07|EPI_ISL_570758|Original|hCoV-19^^Washington|Human|UW Virology Lab|UW Virology Lab|Roychoudhury|USA
>Spike|hCoV-19/Japan/PG-3132/2020|2020-06-00|EPI_ISL_691254|Original|hCoV-19^^Japan|Human|Pathogen Genomics Center|Pathogen Genomics Center|Sekizuka|Japan
>Spike|hCoV-19/England/MILK-9C32A0/2020|2020-09-10|EPI_ISL_566754|Original|hCoV-19^^England|Human|Lighthouse Lab in Milton Keynes|Wellcome Sanger Institute for the COVID-19 Genomics UK (COG-UK) consortium|Davies|United Kingdom
>Spike|hCoV-19/England/ALDP-9EE139/2020|2020-05-06|EPI_ISL_589393|Original|hCoV-19^^England|Human|Lighthouse Lab in Alderley Park|Wellcome Sanger Institute for the COVID-19 Genomics UK (COG-UK) consortium|Davies|United Kingdom
>Spike|hCoV-19/England/ALDP-BCA76C/2020|2020-11-23|EPI_ISL_703258|Original|hCoV-19^^England|Human|Lighthouse Lab in Alderley Park|Wellcome Sanger Institute for the COVID-19 Genomics UK (COG-UK) Consortium|Davies|United Kingdom

>Spike|hCoV-19/Spain/RI-IBV-99005904/2020|2020-04-02|EPI_ISL_537442|Original|hCoV-19^^La Rioja|Human|Centro de Investigaci\u00f3n Biom\u00e9dica de La Rioja - Hospital San Pedro Logro\u00f1o|SeqCOVID-SPAIN consortium/IBV(CSIC)|Comas|Spain
>Spike|hCoV-19/England/QEUH-9F7CC3/2020|2020-09-27|EPI_ISL_588925|Original|hCoV-19^^England|Human|Lighthouse Lab in Glasgow|Wellcome Sanger Institute for the COVID-19 Genomics UK (COG-UK) consortium|Davies|United Kingdom
>Spike|hCoV-19/England/MILK-B862F2/2020|2020-11-13|EPI_ISL_673913|Original|hCoV-19^^England|Human|Lighthouse Lab in Milton Keynes|Wellcome Sanger Institute for the COVID-19 Genomics UK (COG-UK) Consortium|Davies|United Kingdom
>Spike|hCoV-19/Japan/PG-7322/2020|2020-08-00|EPI_ISL_688425|Original|hCoV-19^^Japan|Human|Pathogen Genomics Center|Pathogen Genomics Center|Sekizuka|Japan
>Spike|hCoV-19/USA/MI-MDHHS-SC2-0008/2020|2020-03-12|EPI_ISL_529169|Original|hCoV-19^^Michigan|Human|Michigan Department of Health and Human Services|Michigan Department of Health and Human Services|Blankenship|USA
>Spike|hCoV-19/England/QEUH-9B89B8/2020|2020-09-07|EPI_ISL_549656|Original|hCoV-19^^England|Human|Lighthouse Lab in Glasgow|Wellcome Sanger Institute for the COVID-19 Genomics UK (COG-UK) consortium|Davies|United Kingdom
>Spike|hCoV-19/India/GJ-GBRC198/2020|2020-06-08|EPI_ISL_469043|Original|hCoV-19^^Gujarat|Human|Dr. N. D. Desai Medical College & Hospital|Gujarat Biotechnology Research Centre|Joshi|India
>Spike|hCoV-19/Luxembourg/LNS6092797/2020|2020-11-03|EPI_ISL_744359|Original|hCoV-19^^Luxembourg|Human|Laboratoire national de sant\u00e9|Laboratoire national de sant\u00e9|Wienecke-Baldacchino|Luxembourg
>Spike|hCoV-19/England/CAMC-BC0344/2020|2020-11-22|EPI_ISL_705045|Original|hCoV-19^^England|Human|Lighthouse Lab in Cambridge|Wellcome Sanger Institute for the COVID-19 Genomics UK (COG-UK) Consortium|Davies|United Kingdom
>Spike|hCoV-19/USA/FL-BPHL-1047/2020|2020-07-31|EPI_ISL_517937|Original|hCoV-19^^Florida|Human|Florida Bureau of Public Health Laboratories|Florida Bureau of Public Health Laboratories|Blanton|USA
>Spike|hCoV-19/United Arab Emirates/0144/2020|2020-05-18|EPI_ISL_698331|Original|hCoV-19^^Abu Dhabi|Human|Group 42 (G42) Healthcare|G42 Healthcare|Ogrodzki|United Arab Emirates
>Spike|hCoV-19/Scotland/EDB8584/2020|2020-10-21|EPI_ISL_651965|Original|hCoV-19^^Scotland|Human|Virology Department|COVID-19 Genomics UK (COG-UK) Consortium|McHugh|United Kingdom
>Spike|hCoV-19/England/QEUH-B4225C/2020|2020-11-08|EPI_ISL_657172|Original|hCoV-19^^England|Human|Lighthouse Lab in Glasgow|Wellcome Sanger Institute for the COVID-19 Genomics UK (COG-UK) Consortium|Davies|United Kingdom
>Spike|hCoV-19/USA/TX-HMH0268/2020|2020-03-26|EPI_ISL_434897|Original|hCoV-19^^Texas|Human|Houston Methodist Hospital|Houston Methodist Hospital|Long|USA
>Spike|hCoV-19/Russia/SVE-RII-MH4681S/2020|2020-09-25|EPI_ISL_733233|Original|hCoV-19^^Sverdlovsk|Human|UMMC-Health|WHO National Influenza Centre Russian Federation|Komissarov|Russia
>Spike|hCoV-19/USA/CO-CDPHE-2008170936/2020|2020-08-17|EPI_ISL_677308|Original|hCoV-19^^Colorado|Human|Colorado Department of Public Health and Environment|Colorado Department of Puplic Health and Environment|Bankers|USA
>Spike|hCoV-19/USA/CA-ALSR-1596/2020|2020-04-27|EPI_ISL_494682|Original|hCoV-19^^California|Human|Scripps Medical Laboratory|Andersen lab at Scripps Research|Gangavarapu|USA
>Spike|hCoV-19/Wales/PHWC-2523C/2020|2020-03-22|EPI_ISL_420977|Original|hCoV-19^^Wales|Human|Wales Specialist Virology Centre|Public Health Wales Microbiology Cardiff|Moore|United Kingdom
>Spike|hCoV-19/USA/TX-HMH-MCoV-15286/2020|2020-10-21|EPI_ISL_786770|Original|hCoV-19^^Texas|Human|Houston Methodist Hospital|Houston Methodist Hospital|Long|USA
>Spike|hCoV-19/Denmark/DCGC-11327/2020|2020-11-02|EPI_ISL_670843|Original|hCoV-19^^Nordjylland|Human|Department of Virus and Microbiological Special Diagnostics|Albertsen Lab|Michaelsen|Denmark
>Spike|hCoV-19/United Arab Emirates/0464/2020|2020-05-18|EPI_ISL_698634|Original|hCoV-19^^Abu Dhabi|Human|Group 42 (G42) Healthcare|G42 Healthcare|Ogrodzki|United Arab Emirates

>Spike|hCoV-19/Guadeloupe/IPG-7326/2020|2020-03-17|EPI_ISL_613426|Original|hCoV-19^^Baie-Mahault|Human|Institut Pasteur de la Guadeloupe|Institut Pasteur de la Guadeloupe|Enouf|Guadeloupe
>Spike|hCoV-19/England/ALDP-943F38/2020|2020-06-07|EPI_ISL_554795|Original|hCoV-19^^England|Human|Lighthouse Lab in Alderley Park|Wellcome Sanger Institute for the COVID-19 Genomics UK (COG-UK) consortium|Davies|United Kingdom
>Spike|hCoV-19/Wales/PHWC-484BC3/2020|2020-10-24|EPI_ISL_628087|Original|hCoV-19^^Wales|Human|Wales Specialist Virology Centre Sequencing lab|COVID-19 Genomics UK (COG-UK) Consortium|Moore|United Kingdom
>Spike|hCoV-19/USA/MN-MDH-70/2020|2020-03-14|EPI_ISL_460060|Original|hCoV-19^^Minnesota|Human|Minnesota Department of Health|Minnesota Department of Health|Garfin|USA
>Spike|hCoV-19/USA/UT-UPHL-2012266699/2020|2020-11-30|EPI_ISL_779300|Original|hCoV-19^^Utah|Human|Utah Public Health Laboratory|Utah Public Health Laboratory|Gallagher|USA
>Spike|hCoV-19/Switzerland/BS-65253015/2020|2020-07-21|EPI_ISL_581906|Original|hCoV-19^^Basel-Stadt|Human|University Hospital Basel|University Hospital Basel|Roloff Handschin|Switzerland
>Spike|hCoV-19/South Africa/KRISP-K002733/2020|2020-08-16|EPI_ISL_529731|Original|hCoV-19^^KwaZulu-Natal|Human|NHLS-IALCH|KRISP|de Oliveira|South Africa
>Spike|hCoV-19/England/MILK-B3866C/2020|2020-11-01|EPI_ISL_659010|Original|hCoV-19^^England|Human|Lighthouse Lab in Milton Keynes|Wellcome Sanger Institute for the COVID-19 Genomics UK (COG-UK) Consortium|Davies|United Kingdom
>Spike|hCoV-19/Scotland/CVR3319/2020|2020-05-05|EPI_ISL_493871|Original|hCoV-19^^Scotland|Human|West of Scotland Specialist Virology Centre|COVID-19 Genomics UK (COG-UK) Consortium|Robertson|United Kingdom
>Spike|hCoV-19/Wales/PHWC-49EF33/2020|2020-12-09|EPI_ISL_743145|Original|hCoV-19^^Wales|Human|Wales Specialist Virology Centre Sequencing lab|COVID-19 Genomics UK (COG-UK) Consortium|Moore|United Kingdom
>Spike|hCoV-19/Australia/NSW1358/2020|2020-12-28|EPI_ISL_767924|Original|hCoV-19^^New South Wales|Human|South Eastern Area Laboratory Services (SEALS)|NSW Health Pathology - Institute of Clinical Pathology and Medical Research; Westmead Hospital; University of Sydney|Gall|Australia
>Spike|hCoV-19/Scotland/EDB1559/2020|2020-04-12|EPI_ISL_433081|Original|hCoV-19^^Scotland|Human|Virology Department|COVID-19 Genomics UK (COG-UK) Consortium|Williams|United Kingdom
>Spike|hCoV-19/England/QEUH-B7E864/2020|2020-11-14|EPI_ISL_668327|Original|hCoV-19^^England|Human|Lighthouse Lab in Glasgow|Wellcome Sanger Institute for the COVID-19 Genomics UK (COG-UK) Consortium|Davies|United Kingdom
>Spike|hCoV-19/Netherlands/NH-EMC-54/2020|2020-08-25|EPI_ISL_577850|Original|hCoV-19^^Noord Holland|Human|Dutch COVID-19 response team|Erasmus Medical Center|Oude Munnink|Netherlands
>Spike|hCoV-19/England/QEUH-C57B85/2020|2020-12-12|EPI_ISL_736223|Original|hCoV-19^^England|Human|Lighthouse Lab in Glasgow|Wellcome Sanger Institute for the COVID-19 Genomics UK (COG-UK) Consortium|Davies|United Kingdom
>Spike|hCoV-19/England/MILK-A480B7/2020|2020-10-06|EPI_ISL_598486|Original|hCoV-19^^England|Human|Lighthouse Lab in Milton Keynes|Wellcome Sanger Institute for the COVID-19 Genomics UK (COG-UK) consortium|Davies|United Kingdom
>Spike|hCoV-19/USA/WI-WSLH-200108/2020|2020-04-03|EPI_ISL_578339|Original|hCoV-19^^Wisconsin|Human|Wisconsin State Laboratory of Hygiene Communicable Disease Division|Wisconsin State Laboratory of Hygiene Communicable Disease Division|Florek|USA
>Spike|hCoV-19/Northern Ireland/QEUH-963D74/2020|2020-08-12|EPI_ISL_531502|Original|hCoV-19^^Northern Ireland|Human|Lighthouse Lab in Glasgow|Wellcome Sanger Institute for the COVID-19 Genomics UK (COG-UK) consortium|Davies|United Kingdom
>Spike|hCoV-19/Australia/VIC6076/2020|2020-07-21|EPI_ISL_518103|Original|hCoV-19^^Victoria|Human|Victorian Infectious Diseases Reference Laboratory (VIDRL)|VIDRL and MDU-PHL|Schultz|Australia

>Spike|hCoV-19/USA/SD-SDPHL-0014/2020|2020-08-05|EPI_ISL_569622|Original|hCoV-19^^South Dakota|Human|Woodrow Wilson Keeble Memorial Healthcare|South Dakota Public Health Laboratory|Carlson|USA
>Spike|hCoV-19/USA/CA-CZB-10793/2020|2020-06-22|EPI_ISL_583053|Original|hCoV-19^^California|Human|Humboldt County Public Health Laboratory|Chan-Zuckerberg Biohub|Bhatt|USA
>Spike|hCoV-19/Wales/PHWC-16869A/2020|2020-04-20|EPI_ISL_494269|Original|hCoV-19^^Wales|Human|Originating lab|COVID-19 Genomics UK (COG-UK) Consortium|Moore|United Kingdom
>Spike|hCoV-19/USA/CA-CZB-1450/2020|2020-04-03|EPI_ISL_468414|Original|hCoV-19^^California|Human|County of San Luis Obispo Public Health Laboratory|Chan-Zuckerberg Biohub|Hao|USA
>Spike|hCoV-19/Denmark/DCGC-17499/2020|2020-12-14|EPI_ISL_750357|Original|hCoV-19^^Midtjylland|Human|Department of Virus and Microbiological Special Diagnostics|Albertsen Lab|Michaelsen|Denmark
>Spike|hCoV-19/England/CAMC-B08261/2020|2020-10-30|EPI_ISL_643479|Original|hCoV-19^^England|Human|Lighthouse Lab in Cambridge|Wellcome Sanger Institute for the COVID-19 Genomics UK (COG-UK) Consortium|Davies|United Kingdom
>Spike|hCoV-19/England/MILK-9AB772/2020|2020-08-31|EPI_ISL_549458|Original|hCoV-19^^England|Human|Oxford Viromics|COVID-19 Genomics UK (COG-UK) Consortium|Golubchik|United Kingdom
>Spike|hCoV-19/England/CAMC-BFB146/2020|2020-12-04|EPI_ISL_760936|Original|hCoV-19^^England|Human|Lighthouse Lab in Cambridge|Wellcome Sanger Institute for the COVID-19 Genomics UK (COG-UK) Consortium|Davies|United Kingdom
>Spike|hCoV-19/USA/TX-DSHS-1052/2020|2020-06-15|EPI_ISL_628731|Original|hCoV-19^^Texas|Human|Texas Department of State Health Services|Texas Department of State Health Services|Rahman|USA
>Spike|hCoV-19/Australia/TAS33/2020|2020-04-12|EPI_ISL_522710|Original|hCoV-19^^Tasmania|Human|Royal Hobart Hospital Microbiology Department|MDU-PHL|Schultz|Australia
>Spike|hCoV-19/England/QEUH-9FB209/2020|2020-09-29|EPI_ISL_588121|Original|hCoV-19^^England|Human|Lighthouse Lab in Glasgow|Wellcome Sanger Institute for the COVID-19 Genomics UK (COG-UK) consortium|Davies|United Kingdom
>Spike|hCoV-19/Japan/PG-4560/2020|2020-07-00|EPI_ISL_689697|Original|hCoV-19^^Japan|Human|Fukuoka Institute of Health and Environmental Sciences|Pathogen Genomics Center|Sekizuka|Japan
>Spike|hCoV-19/Australia/SAP286/2020|2020-04-06|EPI_ISL_467998|Original|hCoV-19^^South Australia|Human|SA Pathology|SA Pathology|Leong|Australia
>Spike|hCoV-19/Australia/VIC12013/2020|2020-07-22|EPI_ISL_640529|Original|hCoV-19^^Victoria|Human|Microbiological Diagnostic Unit - Public Health Laboratory (MDU-PHL)|MDU-PHL|Schultz|Australia
>Spike|hCoV-19/England/201380229/2020|2020-03-25|EPI_ISL_421825|Original|hCoV-19^^England|Human|Respiratory Virus Unit|Respiratory Virus Unit|Platt|United Kingdom
>Spike|hCoV-19/Nigeria/AK-CV271/2020|2020-07-01|EPI_ISL_729955|Original|hCoV-19^^Akwa-Ibom State|Human|Nigeria Centre for Disease Control (NCDC)|African Centre of Excellence for Genomics of Infectious Diseases (ACEGID)|Oluniyi|Nigeria
>Spike|hCoV-19/Russia/KDA-RII-MH2766S/2020|2020-09-22|EPI_ISL_602438|Original|hCoV-19^^Krasnodar|Human|HELIX LLC|WHO National Influenza Centre Russian Federation|Komissarov|Russia
>Spike|hCoV-19/USA/CruiseA-5/2020|2020-02-21|EPI_ISL_413610|Original|hCoV-19^^USA|Human|unknown|Pathogen Discovery|Queen|USA
>Spike|hCoV-19/Jordan/AM-ALSR-4956/2020|2020-10-04|EPI_ISL_730230|Original|hCoV-19^^Amman|Human|Biolab Diagnostic Laboratories|Andersen lab at Scripps Research|Gangavarapu|Jordan
>Spike|hCoV-19/USA/CT-Yale-116/2020|2020-04-10|EPI_ISL_431083|Original|hCoV-19^^Connecticut|Human|Yale COVID-19 Biorepository|Grubaugh Lab - Yale School of Public Health|Grubaugh|USA
>Spike|hCoV-19/Portugal/PT0074/2020|2020-03-21|EPI_ISL_421477|Original|hCoV-19^^Portugal|Human|Instituto Nacional de Saude (INSA)|Instituto Nacional de Saude (INSA)|Borges|Portugal

>Spike|hCoV-19/England/ALDP-C3B60E/2020|2020-12-09|EPI_ISL_730735|Original|hCoV-
19^^England|Human|Lighthouse Lab in Alderley Park|Wellcome Sanger Institute for the
COVID-19 Genomics UK (COG-UK) Consortium|Davies|United Kingdom
>Spike|hCoV-19/England/QEUH-9F92FC/2020|2020-09-26|EPI_ISL_588876|Original|hCoV-
19^^England|Human|Lighthouse Lab in Glasgow|Wellcome Sanger Institute for the COVID-
19 Genomics UK (COG-UK) consortium|Davies|United Kingdom
>Spike|hCoV-19/USA/CA-ALSR-0624-IPL/2020|2020-04-21|EPI_ISL_483264|Original|hCoV-
19^^California|Human|UC San Diego Center for Advanced Laboratory Medicine|Andersen
lab at Scripps Research|Gangavarapu|USA
>Spike|hCoV-19/Denmark/DCGC-13349/2020|2020-11-30|EPI_ISL_712743|Original|hCoV-
19^^Hovedstaden|Human|Department of Virus and Microbiological Special
Diagnostics|Albertsen Lab|Michaelsen|Denmark
>Spike|hCoV-19/England/MILK-A7BE84/2020|2020-10-16|EPI_ISL_607495|Original|hCoV-
19^^England|Human|Lighthouse Lab in Milton Keynes|Wellcome Sanger Institute for the
COVID-19 Genomics UK (COG-UK) consortium|Davies|United Kingdom
>Spike|hCoV-19/USA/TX-HMH-MCoV-15139/2020|2020-10-20|EPI_ISL_786623|Original|hCoV-
19^^Texas|Human|Houston Methodist Hospital|Houston Methodist Hospital|Long|USA
>Spike|hCoV-19/USA/CA-ALSR-3685/2020|2020-09-28|EPI_ISL_636147|Original|hCoV-
19^^California|Human|San Diego County Public Health Laboratory|Andersen lab at
Scripps Research|Gangavarapu|USA
>Spike|hCoV-19/Netherlands/ZH-EMC-371/2020|2020-08-02|EPI_ISL_523711|Original|hCoV-
19^^Zuid Holland|Human|Dutch COVID-19 response team|Erasmus Medical Center|Oude
Munnink|Netherlands
>Spike|hCoV-19/England/NORT-28463D/2020|2020-03-22|EPI_ISL_478558|Original|hCoV-
19^^England|Human|Northumbria University / South Tees Hospitals NHS Foundation Trust
/ North Cumbria Integrated Care NHS Foundation Trust / North Tees and Hartlepool NHS
Foundation Trust / Newcastle Hospitals NHS Foundation Trust|COVID-19 Genomics UK
(COG-UK) Consortium|Bashton|United Kingdom
>Spike|hCoV-19/USA/AZ-TG271591/2020|2020-03-25|EPI_ISL_426536|Original|hCoV-
19^^Arizona|Human|TGen North|TGen North|Lemmer|USA
>Spike|hCoV-19/Netherlands/GE-EMC-434/2020|2020-09-13|EPI_ISL_722818|Original|hCoV-
19^^Gelderland|Human|Dutch COVID-19 response team|Erasmus Medical Center|Oude
Munnink|Netherlands
>Spike|hCoV-19/USA/MI-MDHHS-SC20143/2020|2020-03-16|EPI_ISL_436817|Original|hCoV-
19^^Michigan|Human|Michigan Department of Health and Human Services|Michigan
Department of Health and Human Services|Blankenship|USA
>Spike|hCoV-19/USA/CA-CZB-14773/2020|2020-11-09|EPI_ISL_738827|Original|hCoV-
19^^California|Human|Humboldt County Public Health Laboratory|Chan-Zuckerberg
Biohub|Kamm|USA
>Spike|hCoV-19/England/BRIS-1853C69/2020|2020-04-22|EPI_ISL_492316|Original|hCoV-
19^^England|Human|PHE South West Regional Laboratory|Wellcome Sanger Institute for
the COVID-19 Genomics UK (COG-UK) consortium|Davies|United Kingdom
>Spike|hCoV-19/England/CAMB-773A1/2020|2020-03-31|EPI_ISL_438527|Original|hCoV-
19^^England|Human|Department of Pathology|Wellcome Sanger Institute for the COVID-19
Genomics UK (COG-UK) consortium|Amato|United Kingdom
>Spike|hCoV-19/England/QEUH-94334B/2020|2020-07-29|EPI_ISL_532583|Original|hCoV-
19^^England|Human|Lighthouse Lab in Glasgow|Wellcome Sanger Institute for the COVID-
19 Genomics UK (COG-UK) Consortium|Davies|United Kingdom
>Spike|hCoV-19/Denmark/DCGC-16594/2020|2020-11-16|EPI_ISL_714384|Original|hCoV-
19^^Syddanmark|Human|Department of Virus and Microbiological Special
Diagnostics|Albertsen Lab|Michaelsen|Denmark
>Spike|hCoV-19/Denmark/DCGC-3947/2020|2020-09-14|EPI_ISL_615858|Original|hCoV-
19^^Syddanmark|Human|Department of Virus and Microbiological Special
Diagnostics|Albertsen lab|Michaelsen|Denmark
>Spike|hCoV-19/USA/WA-UW-1723/2020|2020-03-20|EPI_ISL_424200|Original|hCoV-
19^^Washington|Human|UW Virology Lab|UW Virology Lab|Roychoudhury|USA
>Spike|hCoV-19/USA/CA-CZB-2196/2020|2020-05-27|EPI_ISL_513745|Original|hCoV-
19^^California|Human|Orange County Public Health Lab|Chan-Zuckerberg Biohub|Kamm|USA

>Spike|hCoV-19/England/ALDP-AA1D1F/2020|2020-10-22|EPI_ISL_624285|Original|hCoV-
19^^England|Human|Lighthouse Lab in Alderley Park|Wellcome Sanger Institute for the
COVID-19 Genomics UK (COG-UK) consortium|Davies|United Kingdom
>Spike|hCoV-19/Spain/AR-IBV-98010609/2020|2020-11-05|EPI_ISL_722212|Original|hCoV-
19^^Aragon|Human|Servicio de Microbiolog\u00eda|SeqCOVID-SPAIN
consortium/IBV(CSIC)|Comas|Spain
>Spike|hCoV-19/Japan/PG-6198/2020|2020-08-00|EPI_ISL_689310|Original|hCoV-
19^^Japan|Human|Pathogen Genomics Center|Pathogen Genomics Center|Sekizuka|Japan
>Spike|hCoV-19/England/QEUH-B0DEB3/2020|2020-11-02|EPI_ISL_642142|Original|hCoV-
19^^England|Human|Lighthouse Lab in Glasgow|Wellcome Sanger Institute for the COVID-
19 Genomics UK (COG-UK) Consortium|Davies|United Kingdom
>Spike|hCoV-19/USA/CA-QDX-2675/2020|2020-03-16|EPI_ISL_604915|Original|hCoV-
19^^California|Human|Quest Diagnostics|Quest Diagnostics|Kagan|USA
>Spike|hCoV-19/Wales/PHWC-4806C6/2020|2020-10-18|EPI_ISL_612919|Original|hCoV-
19^^Wales|Human|Wales Specialist Virology Centre Sequencing lab|COVID-19 Genomics UK
(COG-UK) Consortium|Moore|United Kingdom
>Spike|hCoV-19/England/LIVE-DC2C36/2020|2020-11-25|EPI_ISL_704195|Original|hCoV-
19^^England|Human|Liverpool Clinical Laboratories|COVID-19 Genomics UK (COG-UK)
Consortium|Haldenby|United Kingdom
>Spike|hCoV-19/USA/CA-CZB-6728/2020|2020-08-19|EPI_ISL_582987|Original|hCoV-
19^^California|Human|Orange County Public Health Lab|Chan-Zuckerberg Biohub|Bhatt|USA
>Spike|hCoV-19/USA/AZ-TG276774/2020|2020-04-16|EPI_ISL_694133|Original|hCoV-
19^^Arizona|Human|AZ SPHL|TGen North|French|USA
>Spike|hCoV-19/Wales/PHWC-4A3CCB/2020|2020-12-12|EPI_ISL_764617|Original|hCoV-
19^^Wales|Human|Wales Specialist Virology Centre Sequencing lab|COVID-19 Genomics UK
(COG-UK) Consortium|Moore|United Kingdom
>Spike|hCoV-19/USA/TX-HMH-5481/2020|2020-06-29|EPI_ISL_544807|Original|hCoV-
19^^Texas|Human|Houston Methodist Hospital|Houston Methodist Hospital|Long|USA
>Spike|hCoV-19/Portugal/PT0452/2020|2020-04-04|EPI_ISL_454176|Original|hCoV-
19^^Portugal|Human|unknown|Instituto Nacional de Saude (INSA)|Borges|Portugal
>Spike|hCoV-19/Denmark/DCGC-21297/2020|2020-12-21|EPI_ISL_758940|Original|hCoV-
19^^Syddanmark|Human|Department of Virus and Microbiological Special
Diagnostics|Albertsen Lab|Michaelsen|Denmark
>Spike|hCoV-19/USA/LA-EVTL660/2020|2020-07-13|EPI_ISL_653352|Original|hCoV-
19^^Louisiana|Human|LSUHS Emerging Viral Threat Laboratory|Microbial Genome
Sequencing Center|Kamil|USA
>Spike|hCoV-19/Australia/VIC7841/2020|2020-07-29|EPI_ISL_564692|Original|hCoV-
19^^Victoria|Human|Microbiological Diagnostic Unit - Public Health Laboratory (MDU-
PHL)|MDU-PHL|Schultz|Australia
>Spike|hCoV-19/England/ALDP-C88444/2020|2020-12-10|EPI_ISL_756470|Original|hCoV-
19^^England|Human|Lighthouse Lab in Alderley Park|Wellcome Sanger Institute for the
COVID-19 Genomics UK (COG-UK) Consortium|Davies|United Kingdom
>Spike|hCoV-19/England/QEUH-9B8D9E/2020|2020-09-07|EPI_ISL_549787|Original|hCoV-
19^^England|Human|Lighthouse Lab in Glasgow|Wellcome Sanger Institute for the COVID-
19 Genomics UK (COG-UK) consortium|Davies|United Kingdom
>Spike|hCoV-19/Scotland/EDB7153/2020|2020-08-05|EPI_ISL_514559|Original|hCoV-
19^^Scotland|Human|Virology Department|COVID-19 Genomics UK (COG-UK)
Consortium|Williams|United Kingdom
>Spike|hCoV-19/Wales/PHWC-16E816/2020|2020-10-04|EPI_ISL_585774|Original|hCoV-
19^^Wales|Human|Wales Specialist Virology Centre Sequencing lab|COVID-19 Genomics UK
(COG-UK) Consortium|Moore|United Kingdom
>Spike|hCoV-19/USA/VA-DCLS-2101/2020|2020-10-21|EPI_ISL_677139|Original|hCoV-
19^^Virginia|Human|Virginia Division of Consolidated Laboratory Services|Virginia
Division of Consolidated Laboratory Services|St. Jacques|USA
>Spike|hCoV-19/Luxembourg/LNS5739154/2020|2020-11-14|EPI_ISL_744288|Original|hCoV-
19^^Luxembourg|Human|Laboratoire national de sant\u00e9|Laboratoire national de
sant\u00e9|Wienecke-Baldacchino|Luxembourg
>Spike|hCoV-19/Iceland/434/2020|2020-03-20|EPI_ISL_424458|Original|hCoV-
19^^Reykjavik|Human|The National University Hospital of Iceland|deCODE
genetics|J\u00f3nsson|Iceland

>Spike|hCoV-19/USA/WI-GMF-07607/2020|2020-06-23|EPI_ISL_489922|Original|hCoV-19^^Wisconsin|Human|Gundersen Molecular Diagnostics Laboratory|Kabara Cancer Research Institute|Kenny|USA
>Spike|hCoV-19/Japan/PG-0712/2020|2020-04-00|EPI_ISL_684322|Original|hCoV-19^^Japan|Human|Pathogen Genomics Center|Pathogen Genomics Center|Sekizuka|Japan
>Spike|hCoV-19/USA/CO-CDPHE-2008281882/2020|2020-08-27|EPI_ISL_710328|Original|hCoV-19^^Colorado|Human|Colorado Department of Public Health and Environment|Colorado Department of Puplic Health and Environment|Bankers|USA
>Spike|hCoV-19/Japan/PG-6445/2020|2020-09-00|EPI_ISL_688614|Original|hCoV-19^^Japan|Human|Pathogen Genomics Center|Pathogen Genomics Center|Sekizuka|Japan
>Spike|hCoV-19/Iceland/235/2020|2020-03-16|EPI_ISL_417563|Original|hCoV-19^^Reykjavik|Human|The National University Hospital of Iceland|deCODE genetics|J\u00f3nsson|Iceland
>Spike|hCoV-19/Denmark/DCGC-10598/2020|2020-11-02|EPI_ISL_670078|Original|hCoV-19^^Midtjylland|Human|Department of Virus and Microbiological Special Diagnostics|Albertsen Lab|Michaelsen|Denmark
>Spike|hCoV-19/Japan/PG-6510/2020|2020-07-00|EPI_ISL_687624|Original|hCoV-19^^Japan|Human|Pathogen Genomics Center|Pathogen Genomics Center|Sekizuka|Japan
>Spike|hCoV-19/Belgium/WCV-0408399/2020|2020-04-08|EPI_ISL_462253|Original|hCoV-19^^Buggenhout|Human|KU Leuven|KU Leuven|Maes|Belgium
>Spike|hCoV-19/Gambia/GC19-3514/2020|2020-07-27|EPI_ISL_561189|Original|hCoV-19^^Kombo|Human|MRCG at LSHTM Genomics lab|MRCG at LSHTM Genomics lab|Sesay|Gambia
>Spike|hCoV-19/England/201420121/2020|2020-03-30|EPI_ISL_465918|Original|hCoV-19^^England|Human|Respiratory Virus Unit|Respiratory Virus Unit|Platt|United Kingdom
>Spike|hCoV-19/France/ARA-116531/2020|2020-12-06|EPI_ISL_745367|Original|hCoV-19^^Auvergne-Rh\u00f4ne-Alpes|Human|CNR Virus des Infections Respiratoires - France SUD|CNR Virus des Infections Respiratoires - France SUD|Bal|France
>Spike|hCoV-19/Scotland/CVR3396/2020|2020-05-09|EPI_ISL_456823|Original|hCoV-19^^Scotland|Human|West of Scotland Specialist Virology Centre|COVID-19 Genomics UK (COG-UK) Consortium|Robertson|United Kingdom
>Spike|hCoV-19/England/QEUH-B67479/2020|2020-11-11|EPI_ISL_662136|Original|hCoV-19^^England|Human|Lighthouse Lab in Glasgow|Wellcome Sanger Institute for the COVID-19 Genomics UK (COG-UK) Consortium|Davies|United Kingdom
>Spike|hCoV-19/England/MILK-A464DB/2020|2020-10-05|EPI_ISL_598759|Original|hCoV-19^^England|Human|Lighthouse Lab in Milton Keynes|Wellcome Sanger Institute for the COVID-19 Genomics UK (COG-UK) consortium|Davies|United Kingdom
>Spike|hCoV-19/Australia/VIC6899/2020|2020-07-21|EPI_ISL_564124|Original|hCoV-19^^Victoria|Human|Microbiological Diagnostic Unit - Public Health Laboratory (MDU-PHL)|MDU-PHL|Schultz|Australia
>Spike|hCoV-19/England/CAMC-94B71A/2020|2020-07-20|EPI_ISL_556646|Original|hCoV-19^^England|Human|Lighthouse Lab in Cambridge|Wellcome Sanger Institute for the COVID-19 Genomics UK (COG-UK) consortium|Davies|United Kingdom
>Spike|hCoV-19/USA/IL-NM-0656/2020|2020-05-07|EPI_ISL_626462|Original|hCoV-19^^Illinois|Human|Northwestern Memorial Hospital|Ozer Lab|Ozer|USA
>Spike|hCoV-19/Slovakia/UKBA-411/2020|2020-11-13|EPI_ISL_718259|Original|hCoV-19^^Bratislava|Human|Institute of Virology|Faculty of Natural Sciences|Vinar|Slovakia
>Spike|hCoV-19/Canada/ON-CVD00784/2020|2020-04-14|EPI_ISL_586272|Original|hCoV-19^^Ontario|Human|Toronto Invasive Bacterial Diseases Network|McMaster University|McArthur|Canada
>Spike|hCoV-19/Australia/NSW560/2020|2020-05-24|EPI_ISL_455099|Original|hCoV-19^^New South Wales|Human|South Eastern Area Laboratory Services|NSW Health Pathology - Institute of Clinical Pathology and Medical Research; Westmead Hospital; University of Sydney|Gall|Australia
>Spike|hCoV-19/England/QEUH-C57B3A/2020|2020-12-12|EPI_ISL_736444|Original|hCoV-19^^England|Human|Lighthouse Lab in Glasgow|Wellcome Sanger Institute for the COVID-19 Genomics UK (COG-UK) Consortium|Davies|United Kingdom
>Spike|hCoV-19/USA/HI-H200497/2020|2020-10-09|EPI_ISL_753036|Original|hCoV-19^^Hawaii|Human|State Laboratories Division|State Laboratories Division|Sultana|USA

>Spike|hCoV-19/India/TG-CCMB-AC437/2020|2020-08-19|EPI_ISL_539642|Original|hCoV-19^^Telangana|Human|CSIR-Centre for Cellular and Molecular Biology|CSIR-Centre for Cellular and Molecular Biology|Mukherjee|India
>Spike|hCoV-19/USA/CA-CHLA-PLM61546012/2020|2020-11-29|EPI_ISL_753533|Original|hCoV-19^^California|Human|Clinical virology Laboratory|Center for Personalized Medicine|Dien Bard|USA
>Spike|hCoV-19/England/MILK-ACE8DF/2020|2020-10-22|EPI_ISL_629359|Original|hCoV-19^^England|Human|Lighthouse Lab in Milton Keynes|Wellcome Sanger Institute for the COVID-19 Genomics UK (COG-UK) consortium|Davies|United Kingdom
>Spike|hCoV-19/England/ALDP-BC73E3/2020|2020-11-22|EPI_ISL_721211|Original|hCoV-19^^England|Human|Lighthouse Lab in Alderley Park|Wellcome Sanger Institute for the COVID-19 Genomics UK (COG-UK) Consortium|Davies|United Kingdom
>Spike|hCoV-19/Switzerland/BL-ETHZ-260009/2020|2020-08-21|EPI_ISL_539347|Original|hCoV-19^^Basel-Landschaft|Human|Viollier AG|Department of Biosystems Science and Engineering|Nadeau|Switzerland
>Spike|hCoV-19/Denmark/DCGC-18937/2020|2020-12-07|EPI_ISL_748650|Original|hCoV-19^^Midtjylland|Human|Department of Virus and Microbiological Special Diagnostics|Albertsen Lab|Michaelsen|Denmark
>Spike|hCoV-19/Australia/VIC9590/2020|2020-08-23|EPI_ISL_562681|Original|hCoV-19^^Victoria|Human|Microbiological Diagnostic Unit - Public Health Laboratory (MDU-PHL)|MDU-PHL|Schultz|Australia
>Spike|hCoV-19/England/204781018/2020|2020-10-18|EPI_ISL_678751|Original|hCoV-19^^England|Human|Respiratory Virus Unit|COVID-19 Genomics UK (COG-UK) Consortium|Platt|United Kingdom
>Spike|hCoV-19/France/PAC-IHU-1325/2020|2020-08-19|EPI_ISL_569116|Original|hCoV-19^^Provence-Alpes-C\u00f4te-d\u2019Azur|Human|MEPHI|MEPHI|Levasseur|France
>Spike|hCoV-19/Wales/PHWC-16B2A2/2020|2020-09-14|EPI_ISL_560268|Original|hCoV-19^^Wales|Human|Wales Specialist Virology Centre Sequencing lab|COVID-19 Genomics UK (COG-UK) Consortium|Moore|United Kingdom
>Spike|hCoV-19/England/204680169/2020|2020-11-12|EPI_ISL_637097|Original|hCoV-19^^England|Human|Respiratory Virus Unit|Respiratory Virus Unit|Platt|United Kingdom
>Spike|hCoV-19/England/QEUH-99C3BC/2020|2020-08-27|EPI_ISL_559798|Original|hCoV-19^^England|Human|Oxford Viromics|COVID-19 Genomics UK (COG-UK) Consortium|Golubchik|United Kingdom
>Spike|hCoV-19/England/ALDP-AA131D/2020|2020-10-22|EPI_ISL_623966|Original|hCoV-19^^England|Human|Lighthouse Lab in Alderley Park|Wellcome Sanger Institute for the COVID-19 Genomics UK (COG-UK) consortium|Davies|United Kingdom
>Spike|hCoV-19/Australia/VIC14280/2020|2020-08-04|EPI_ISL_592431|Original|hCoV-19^^Victoria|Human|Microbiological Diagnostic Unit - Public Health Laboratory (MDU-PHL)|MDU-PHL|Schultz|Australia
>Spike|hCoV-19/USA/TX-DSHS-0736/2020|2020-05-14|EPI_ISL_584138|Original|hCoV-19^^Texas|Human|Texas Department of State Health Services|Texas Department of State Health Services|Rahman|USA
>Spike|hCoV-19/England/CAMC-A579C9/2020|2020-10-08|EPI_ISL_597279|Original|hCoV-19^^England|Human|Lighthouse Lab in Cambridge|Wellcome Sanger Institute for the COVID-19 Genomics UK (COG-UK) consortium|Davies|United Kingdom
>Spike|hCoV-19/England/MILK-A0556C/2020|2020-09-30|EPI_ISL_600046|Original|hCoV-19^^England|Human|Lighthouse Lab in Milton Keynes|Wellcome Sanger Institute for the COVID-19 Genomics UK (COG-UK) consortium|Davies|United Kingdom
>Spike|hCoV-19/England/CAMC-BFBB1B/2020|2020-12-04|EPI_ISL_709252|Original|hCoV-19^^England|Human|Lighthouse Lab in Cambridge|Wellcome Sanger Institute for the COVID-19 Genomics UK (COG-UK) Consortium|Davies|United Kingdom
>Spike|hCoV-19/USA/TX-DSHS-1912/2020|2020-07-11|EPI_ISL_751524|Original|hCoV-19^^Texas|Human|Texas Department of State Health Services|Texas Department of State Health Services|Rahman|USA
>Spike|hCoV-19/Wales/CAMC-A80E13/2020|2020-10-14|EPI_ISL_625291|Original|hCoV-19^^Wales|Human|Lighthouse Lab in Cambridge|Wellcome Sanger Institute for the COVID-19 Genomics UK (COG-UK) consortium|Davies|United Kingdom

>Spike|hCoV-19/Australia/WA98/2020|2020-03-29|EPI_ISL_512751|Original|hCoV-19^^Western Australia|Human|PathWest Laboratory Medicine WA|PathWest Laboratory Medicine WA Microbial Surveillance Unit|Sikazwe|Australia
>Spike|hCoV-19/England/ALDP-A96409/2020|2020-10-20|EPI_ISL_624827|Original|hCoV-19^^England|Human|Lighthouse Lab in Alderley Park|Wellcome Sanger Institute for the COVID-19 Genomics UK (COG-UK) consortium|Davies|United Kingdom
>Spike|hCoV-19/England/MILK-9E2F86/2020|2020-09-21|EPI_ISL_581195|Original|hCoV-19^^England|Human|Lighthouse Lab in Milton Keynes|Wellcome Sanger Institute for the COVID-19 Genomics UK (COG-UK) consortium|Davies|United Kingdom
>Spike|hCoV-19/Denmark/DCGC-5030/2020|2020-09-14|EPI_ISL_621785|Original|hCoV-19^^Hovedstaden|Human|Department of Virus and Microbiological Special Diagnostics|Albertsen lab|Michaelsen|Denmark
>Spike|hCoV-19/England/NORW-E98BA/2020|2020-04-23|EPI_ISL_448341|Original|hCoV-19^^England|Human|Quadram Institute Bioscience|COVID-19 Genomics UK (COG-UK) Consortium|Alikhan|United Kingdom
>Spike|hCoV-19/England/BIRM-6378E/2020|2020-08-16|EPI_ISL_529648|Original|hCoV-19^^England|Human|University of Birmingham|COVID-19 Genomics UK (COG-UK) Consortium|Loman|United Kingdom
>Spike|hCoV-19/USA/MI-QDX-1545/2020|2020-03-17|EPI_ISL_603841|Original|hCoV-19^^Michigan|Human|Quest Diagnostics|Quest Diagnostics|Kagan|USA
>Spike|hCoV-19/Wales/PHWC-2F250/2020|2020-04-06|EPI_ISL_446223|Original|hCoV-19^^Wales|Human|Wales Specialist Virology Centre|Public Health Wales Microbiology Cardiff|Moore|United Kingdom
>Spike|hCoV-19/Netherlands/LI-EMC-91/2020|2020-00-00|EPI_ISL_632479|Original|hCoV-19^^Limburg|Human|Dutch COVID-19 response team|Erasmus Medical Center|Oude Munnink|Netherlands
>Spike|hCoV-19/USA/CA-ALSR-4431/2020|2020-10-26|EPI_ISL_649025|Original|hCoV-19^^California|Human|San Diego County Public Health Laboratory|Andersen lab at Scripps Research|Gangavarapu|USA
>Spike|hCoV-19/Denmark/DCGC-20038/2020|2020-12-07|EPI_ISL_747595|Original|hCoV-19^^Hovedstaden|Human|Department of Virus and Microbiological Special Diagnostics|Albertsen Lab|Michaelsen|Denmark
>Spike|hCoV-19/Austria/CeMM0196/2020|2020-03-14|EPI_ISL_438037|Original|hCoV-19^^Vienna|Human|Center for Virology|Bergthaler laboratory|Bergthaler|Austria
>Spike|hCoV-19/USA/WA-UW-5429/2020|2020-03-26|EPI_ISL_486103|Original|hCoV-19^^Washington|Human|UW Virology Lab|UW Virology Lab|Roychoudhury|USA
>Spike|hCoV-19/England/ALDP-A51ABD/2020|2020-10-07|EPI_ISL_598047|Original|hCoV-19^^England|Human|Lighthouse Lab in Alderley Park|Wellcome Sanger Institute for the COVID-19 Genomics UK (COG-UK) consortium|Davies|United Kingdom
>Spike|hCoV-19/USA/WI-UW-2089/2020|2020-09-27|EPI_ISL_677494|Original|hCoV-19^^Wisconsin|Human|University of Wisconsin-Madison AIDS Vaccine Research Laboratories|University of Wisconsin-Madison AIDS Vaccine Research Laboratories|Baczenas|USA
>Spike|hCoV-19/Australia/WA329/2020|2020-04-07|EPI_ISL_596872|Original|hCoV-19^^Western Australia|Human|PathWest Laboratory Medicine WA|PathWest Laboratory Medicine WA Microbial Surveillance Unit|Sikazwe|Australia
>Spike|hCoV-19/England/MILK-B8C99D/2020|2020-11-13|EPI_ISL_675997|Original|hCoV-19^^England|Human|Lighthouse Lab in Milton Keynes|Wellcome Sanger Institute for the COVID-19 Genomics UK (COG-UK) Consortium|Davies|United Kingdom
>Spike|hCoV-19/Wales/PHWC-49C2D2/2020|2020-12-07|EPI_ISL_742538|Original|hCoV-19^^Wales|Human|Wales Specialist Virology Centre Sequencing lab|COVID-19 Genomics UK (COG-UK) Consortium|Moore|United Kingdom
>Spike|hCoV-19/Switzerland/ZH-ETHZ-120191/2020|2020-04-27|EPI_ISL_468266|Original|hCoV-19^^Zurich|Human|Viollier AG|Department of Biosystems Science and Engineering|Nadeau|Switzerland
>Spike|hCoV-19/USA/UT-02967/2020|2020-05-01|EPI_ISL_522282|Original|hCoV-19^^Utah|Human|Utah Public Health Laboratory|Utah Public Health Laboratory|Young|USA
>Spike|hCoV-19/England/QEUH-A215C4/2020|2020-10-01|EPI_ISL_587630|Original|hCoV-19^^England|Human|Lighthouse Lab in Glasgow|Wellcome Sanger Institute for the COVID-19 Genomics UK (COG-UK) consortium|Davies|United Kingdom

>Spike|hCoV-19/Wales/PHWC-29C0D/2020|2020-04-04|EPI_ISL_432219|Original|hCoV-19^^Wales|Human|Wales Specialist Virology Centre|Public Health Wales Microbiology Cardiff|Moore|United Kingdom
>Spike|hCoV-19/England/CAMC-A65351/2020|2020-10-09|EPI_ISL_637362|Original|hCoV-19^^England|Human|Department of Pathology|COVID-19 Genomics UK (COG-UK) Consortium|Goodfellow|United Kingdom
>Spike|hCoV-19/Denmark/DCGC-2979/2020|2020-08-24|EPI_ISL_616741|Original|hCoV-19^^Midtjylland|Human|Department of Virus and Microbiological Special Diagnostics|Albertsen lab|Michaelsen|Denmark
>Spike|hCoV-19/England/NOTT-10EEE9/2020|2020-03-29|EPI_ISL_432940|Original|hCoV-19^^England|Human|Queens Medical Centre|COVID-19 Genomics UK (COG-UK) Consortium|Loose|United Kingdom
>Spike|hCoV-19/England/QEUH-AA05AF/2020|2020-10-20|EPI_ISL_652710|Original|hCoV-19^^England|Human|University of Exeter|COVID-19 Genomics UK (COG-UK) Consortium|Temperton|United Kingdom
>Spike|hCoV-19/USA/LA-EVTL1255/2020|2020-12-04|EPI_ISL_778921|Original|hCoV-19^^Louisiana|Human|LSUHS Emerging Viral Threat Laboratory|Microbial Genome Sequencing Center|Kamil|USA
>Spike|hCoV-19/USA/MA-MASPHL-00328/2020|2020-04-30|EPI_ISL_693189|Original|hCoV-19^^Massachusetts|Human|Massachusetts State Public Health Laboratory|Massachusetts State Public Health Laboratory|Lang|USA
>Spike|hCoV-19/Wales/QEUH-AD4153/2020|2020-10-26|EPI_ISL_647943|Original|hCoV-19^^Wales|Human|Lighthouse Lab in Glasgow|Wellcome Sanger Institute for the COVID-19 Genomics UK (COG-UK) Consortium|Davies|United Kingdom
>Spike|hCoV-19/England/BRIS-185414B/2020|2020-04-28|EPI_ISL_492250|Original|hCoV-19^^England|Human|PHE South West Regional Laboratory|Wellcome Sanger Institute for the COVID-19 Genomics UK (COG-UK) consortium|Davies|United Kingdom
>Spike|hCoV-19/England/MILK-AC8BAF/2020|2020-10-21|EPI_ISL_629649|Original|hCoV-19^^England|Human|Lighthouse Lab in Milton Keynes|Wellcome Sanger Institute for the COVID-19 Genomics UK (COG-UK) consortium|Davies|United Kingdom
>Spike|hCoV-19/India/DL-NCDC2130-CSIR-IGIB/2020|2020-05-18|EPI_ISL_482544|Original|hCoV-19^^Delhi|Human|National Centre for Disease control (NCDC)|NCDC/CSIR-IGIB|Pandey|India
>Spike|hCoV-19/Australia/VIC6366/2020|2020-07-21|EPI_ISL_521161|Original|hCoV-19^^Victoria|Human|Microbiological Diagnostic Unit - Public Health Laboratory (MDU-PHL)|MDU-PHL|Schultz|Australia
>Spike|hCoV-19/England/NOTT-11D53D/2020|2020-11-22|EPI_ISL_665935|Original|hCoV-19^^England|Human|Queens Medical Centre|COVID-19 Genomics UK (COG-UK) Consortium|Loose|United Kingdom
>Spike|hCoV-19/England/ALDP-9FC87F/2020|2020-10-01|EPI_ISL_587763|Original|hCoV-19^^England|Human|Lighthouse Lab in Alderley Park|Wellcome Sanger Institute for the COVID-19 Genomics UK (COG-UK) consortium|Davies|United Kingdom
>Spike|hCoV-19/Denmark/DCGC-3892/2020|2020-09-07|EPI_ISL_615611|Original|hCoV-19^^Syddanmark|Human|Department of Virus and Microbiological Special Diagnostics|Albertsen lab|Michaelsen|Denmark
>Spike|hCoV-19/England/LIVE-9B93C/2020|2020-04-18|EPI_ISL_440887|Original|hCoV-19^^England|Human|Liverpool Clinical Laboratories|COVID-19 Genomics UK (COG-UK) Consortium|Haldenby|United Kingdom
>Spike|hCoV-19/Scotland/QEUH-9B5177/2020|2020-09-03|EPI_ISL_602044|Original|hCoV-19^^Scotland|Human|Lighthouse Lab in Glasgow|Wellcome Sanger Institute for the COVID-19 Genomics UK (COG-UK) consortium|Davies|United Kingdom
>Spike|hCoV-19/USA/CA-CZB-14649/2020|2020-11-16|EPI_ISL_739532|Original|hCoV-19^^California|Human|Orange County Public Health Lab|Chan-Zuckerberg Biohub|Kamm|USA
>Spike|hCoV-19/England/QEUH-9CED0E/2020|2020-09-17|EPI_ISL_590136|Original|hCoV-19^^England|Human|Lighthouse Lab in Glasgow|Wellcome Sanger Institute for the COVID-19 Genomics UK (COG-UK) consortium|Davies|United Kingdom
>Spike|hCoV-19/Denmark/DCGC-12812/2020|2020-10-26|EPI_ISL_682993|Original|hCoV-19^^Nordjylland|Human|Department of Virus and Microbiological Special Diagnostics|Albertsen Lab|Michaelsen|Denmark

>Spike|hCoV-19/USA/TX-DSHS-0796/2020|2020-06-02|EPI_ISL_590861|Original|hCoV-19^^Texas|Human|Texas Department of State Health Services|Texas Department of State Health Services|Rahman|USA
>Spike|hCoV-19/Switzerland/BL-ETHZ-100086/2020|2020-03-13|EPI_ISL_451678|Original|hCoV-19^^Basel-Landschaft|Human|Viollier AG|Department of Biosystems Science and Engineering|Nadeau|Switzerland
>Spike|hCoV-19/England/QEUH-B80ED6/2020|2020-11-14|EPI_ISL_667864|Original|hCoV-19^^England|Human|Lighthouse Lab in Glasgow|Wellcome Sanger Institute for the COVID-19 Genomics UK (COG-UK) Consortium|Davies|United Kingdom
>Spike|hCoV-19/Scotland/CVR1989/2020|2020-04-08|EPI_ISL_493754|Original|hCoV-19^^Scotland|Human|West of Scotland Specialist Virology Centre|COVID-19 Genomics UK (COG-UK) Consortium|Robertson|United Kingdom
>Spike|hCoV-19/England/BRIS-12ABC2/2020|2020-05-01|EPI_ISL_458506|Original|hCoV-19^^England|Human|PHE South West Regional Laboratory|Wellcome Sanger Institute for the COVID-19 Genomics UK (COG-UK) consortium|Davies|United Kingdom
>Spike|hCoV-19/Colombia/RIS-INS-87267/2020|2020-03-28|EPI_ISL_497745|Original|hCoV-19^^Risaralda|Human|Instituto Nacional de Salud|Instituto Nacional de Salud|Franco-Mu\u00f1oz|Colombia
>Spike|hCoV-19/Spain/RI-IBV-99010989/2020|2020-08-11|EPI_ISL_732812|Original|hCoV-19^^La_Rioja|Human|Centro de Investigaci\u00f3n Biom\u00e9dica de La Rioja - Hospital San Pedro Logro\u00f1o|SeqCOVID-SPAIN consortium/IBV(CSIC)|Comas|Spain
>Spike|hCoV-19/Australia/WA34/2020|2020-02-27|EPI_ISL_470851|Original|hCoV-19^^Western Australia|Human|PathWest Laboratory Medicine WA|PathWest Laboratory Medicine WA|Sikazwe|Australia
>Spike|hCoV-19/USA/TX-HMH-MCoV-19295/2020|2020-11-29|EPI_ISL_783565|Original|hCoV-19^^Texas|Human|Houston Methodist Hospital|Houston Methodist Hospital|Long|USA
>Spike|hCoV-19/USA/NY-NYUMC742/2020|2020-03-21|EPI_ISL_451457|Original|hCoV-19^^New York|Human|NYU Langone Health|Departments of Pathology and Medicine|Maurano|USA
>Spike|hCoV-19/Wales/PHWC-4A6026/2020|2020-12-15|EPI_ISL_764991|Original|hCoV-19^^Wales|Human|Wales Specialist Virology Centre Sequencing lab|COVID-19 Genomics UK (COG-UK) Consortium|Moore|United Kingdom
>Spike|hCoV-19/England/QEUH-9E4CBA/2020|2020-09-22|EPI_ISL_579765|Original|hCoV-19^^England|Human|Lighthouse Lab in Glasgow|Wellcome Sanger Institute for the COVID-19 Genomics UK (COG-UK) consortium|Davies|United Kingdom
>Spike|hCoV-19/Wales/PHWC-35D29/2020|2020-04-26|EPI_ISL_474560|Original|hCoV-19^^Wales|Human|Originating lab|COVID-19 Genomics UK (COG-UK) Consortium|Moore|United Kingdom
>Spike|hCoV-19/USA/WA-S1419/2020|2020-05-19|EPI_ISL_495869|Original|hCoV-19^^Washington|Human|Washington State Department of Health|Seattle Flu Study|Lee|USA
>Spike|hCoV-19/Australia/VIC810/2020|2020-04-01|EPI_ISL_427084|Original|hCoV-19^^Victoria|Human|Victorian Infectious Diseases Reference Laboratory (VIDRL)|Microbiological Diagnostic Unit Public Health Laboratory and Victorian Infectious Diseases Reference Laboratory|Schultz|Australia
>Spike|hCoV-19/England/OXON-ACDB4/2020|2020-04-04|EPI_ISL_448538|Original|hCoV-19^^England|Human|Oxford Viromics|COVID-19 Genomics UK (COG-UK) Consortium|Golubchik|United Kingdom
>Spike|hCoV-19/Singapore/271/2020|2020-03-20|EPI_ISL_462389|Original|hCoV-19^^Singapore|Human|National Public Health Laboratory|National Public Health Laboratory|Mak|Singapore
>Spike|hCoV-19/England/SHEF-10C8678/2020|2020-12-12|EPI_ISL_727881|Original|hCoV-19^^England|Human|Virology Department|COVID-19 Genomics UK (COG-UK) Consortium|de Silva|United Kingdom
>Spike|hCoV-19/Bangladesh/CHRF-0025/2020|2020-06-14|EPI_ISL_477135|Original|hCoV-19^^Bangladesh|Human|Child Health Research Foundation|Child Health Research Foundation|Saha|Bangladesh
>Spike|hCoV-19/Denmark/DCGC-5001/2020|2020-09-21|EPI_ISL_621816|Original|hCoV-19^^Hovedstaden|Human|Department of Virus and Microbiological Special Diagnostics|Albertsen lab|Michaelsen|Denmark

>Spike|hCoV-19/Denmark/DCGC-3993/2020|2020-09-14|EPI_ISL_615763|Original|hCoV-19^^Syddanmark|Human|Department of Virus and Microbiological Special Diagnostics|Albertsen lab|Michaelsen|Denmark
>Spike|hCoV-19/USA/WI-UW-1274/2020|2020-08-25|EPI_ISL_536646|Original|hCoV-19^^Wisconsin|Human|University of Wisconsin-Madison AIDS Vaccine Research Laboratories|University of Wisconsin-Madison AIDS Vaccine Research Laboratories|Baczenas|USA
>Spike|hCoV-19/England/LIVE-9CE78/2020|2020-04-26|EPI_ISL_440948|Original|hCoV-19^^England|Human|Liverpool Clinical Laboratories|COVID-19 Genomics UK (COG-UK) Consortium|Haldenby|United Kingdom
>Spike|hCoV-19/England/CAMB-1B0C64/2020|2020-00-00|EPI_ISL_452955|Original|hCoV-19^^England|Human|Department of Pathology|COVID-19 Genomics UK (COG-UK) Consortium|Meredith|United Kingdom
>Spike|hCoV-19/USA/UN-CDC-7432/2020|2020-04-20|EPI_ISL_514068|Original|hCoV-19^^USA|Human|Navy and Marine Corps Public Health Center|Pathogen Discovery|Queen|USA
>Spike|hCoV-19/USA/MS-CDC-6546/2020|2020-08-25|EPI_ISL_648005|Original|hCoV-19^^Mississippi|Human|MS Public Health Laboratory|Pathogen Discovery|Queen|USA
>Spike|hCoV-19/England/QEUH-C01249/2020|2020-12-04|EPI_ISL_720672|Original|hCoV-19^^England|Human|Lighthouse Lab in Glasgow|Wellcome Sanger Institute for the COVID-19 Genomics UK (COG-UK) Consortium|Davies|United Kingdom
>Spike|hCoV-19/Ireland/D-NVRL-73IRL22431/2020|2020-09-16|EPI_ISL_578261|Original|hCoV-19^^Dublin|Human|National Virus Reference Laboratory|National Virus Reference Laboratory|Gonzalez|Ireland
>Spike|hCoV-19/Scotland/EDB4986/2020|2020-05-14|EPI_ISL_493929|Original|hCoV-19^^Scotland|Human|Virology Department|COVID-19 Genomics UK (COG-UK) Consortium|Williams|United Kingdom
>Spike|hCoV-19/Wales/PHWC-4A5F84/2020|2020-12-15|EPI_ISL_763762|Original|hCoV-19^^Wales|Human|Wales Specialist Virology Centre Sequencing lab|COVID-19 Genomics UK (COG-UK) Consortium|Moore|United Kingdom
>Spike|hCoV-19/Denmark/DCGC-11750/2020|2020-10-26|EPI_ISL_670549|Original|hCoV-19^^Nordjylland|Human|Department of Virus and Microbiological Special Diagnostics|Albertsen Lab|Michaelsen|Denmark
>Spike|hCoV-19/Canada/ON-S1256/2020|2020-05-07|EPI_ISL_586315|Original|hCoV-19^^Ontario|Human|Toronto Invasive Bacterial Diseases Network|McMaster University|McArthur|Canada
>Spike|hCoV-19/Norway/3133/2020|2020-08-07|EPI_ISL_549156|Original|hCoV-19^^Oslo|Human|Ostfold Hospital Trust - Kalnes|Norwegian Institute of Public Health|Kopperud Riis|Norway
>Spike|hCoV-19/USA/CA-CZB-10798/2020|2020-06-26|EPI_ISL_583058|Original|hCoV-19^^California|Human|Humboldt County Public Health Laboratory|Chan-Zuckerberg Biohub|Bhatt|USA
>Spike|hCoV-19/Wales/PHWC-169532/2020|2020-06-25|EPI_ISL_499408|Original|hCoV-19^^Wales|Human|Wales Specialist Virology Centre Sequencing lab|COVID-19 Genomics UK (COG-UK) Consortium|Moore|United Kingdom
>Spike|hCoV-19/England/LIVE-A06C4/2020|2020-05-27|EPI_ISL_472125|Original|hCoV-19^^England|Human|Liverpool Clinical Laboratories|COVID-19 Genomics UK (COG-UK) Consortium|Haldenby|United Kingdom
>Spike|hCoV-19/England/NORT-289920/2020|2020-04-01|EPI_ISL_453506|Original|hCoV-19^^England|Human|Northumbria University / South Tees Hospitals NHS Foundation Trust / North Cumbria Integrated Care NHS Foundation Trust / North Tees and Hartlepool NHS Foundation Trust / Newcastle Hospitals NHS Foundation Trust|COVID-19 Genomics UK (COG-UK) Consortium|Bashton|United Kingdom
>Spike|hCoV-19/England/MILK-B9612F/2020|2020-11-14|EPI_ISL_676302|Original|hCoV-19^^England|Human|Lighthouse Lab in Milton Keynes|Wellcome Sanger Institute for the COVID-19 Genomics UK (COG-UK) Consortium|Davies|United Kingdom
>Spike|hCoV-19/USA/NY-NYCPHL-000283/2020|2020-03-20|EPI_ISL_631809|Original|hCoV-19^^New York|Human|Jamaica Hospital Medical Center|New York City Public Health Laboratory|Wang|USA

>Spike|hCoV-19/England/MILK-BB1EDC/2020|2020-11-17|EPI_ISL_720224|Original|hCoV-19^^England|Human|Lighthouse Lab in Milton Keynes|Wellcome Sanger Institute for the COVID-19 Genomics UK (COG-UK) Consortium|Davies|United Kingdom
>Spike|hCoV-19/Denmark/DCGC-17829/2020|2020-12-14|EPI_ISL_749360|Original|hCoV-19^^Syddanmark|Human|Department of Virus and Microbiological Special Diagnostics|Albertsen Lab|Michaelsen|Denmark
>Spike|hCoV-19/England/QEUH-C159F2/2020|2020-12-05|EPI_ISL_709320|Original|hCoV-19^^England|Human|Lighthouse Lab in Glasgow|Wellcome Sanger Institute for the COVID-19 Genomics UK (COG-UK) Consortium|Davies|United Kingdom
>Spike|hCoV-19/England/MILK-B0206D/2020|2020-10-24|EPI_ISL_642972|Original|hCoV-19^^England|Human|Lighthouse Lab in Milton Keynes|Wellcome Sanger Institute for the COVID-19 Genomics UK (COG-UK) Consortium|Davies|United Kingdom
>Spike|hCoV-19/Chile/RM-84921/2020|2020-05-31|EPI_ISL_746809|Original|hCoV-19^^Region Metropolitana de Santiago|Human|Genetica Molecular and Subdepartamento de Virologia ISP Chile|Instituto de Salud Publica de Chile|Tognarelli|Chile
>Spike|hCoV-19/South Africa/KRISP-K002648/2020|2020-08-04|EPI_ISL_535518|Original|hCoV-19^^KwaZulu-Natal|Human|NHLS-IALCH|KRISP|de Oliveira|South Africa
>Spike|hCoV-19/Chile/RM-169155/2020|2020-09-03|EPI_ISL_746706|Original|hCoV-19^^Region Metropolitana de Santiago|Human|Genetica Molecular and Subdepartamento de Virologia ISP Chile|Instituto de Salud Publica de Chile|Tognarelli|Chile
>Spike|hCoV-19/USA/TX-HMH-MCoV-10583/2020|2020-07-08|EPI_ISL_789545|Original|hCoV-19^^Texas|Human|Houston Methodist Hospital|Houston Methodist Hospital|Long|USA
>Spike|hCoV-19/Denmark/DCGC-8904/2020|2020-10-26|EPI_ISL_626153|Original|hCoV-19^^Hovedstaden|Human|Department of Virus and Microbiological Special Diagnostics|Albertsen lab|Michaelsen|Denmark
>Spike|hCoV-19/England/NORT-29B509/2020|2020-06-01|EPI_ISL_493495|Original|hCoV-19^^England|Human|Northumbria University / South Tees Hospitals NHS Foundation Trust / North Cumbria Integrated Care NHS Foundation Trust / North Tees and Hartlepool NHS Foundation Trust / Newcastle Hospitals NHS Foundation Trust|COVID-19 Genomics UK (COG-UK) Consortium|Bashton|United Kingdom
>Spike|hCoV-19/Denmark/DCGC-10094/2020|2020-11-09|EPI_ISL_669390|Original|hCoV-19^^Sjaelland|Human|Department of Virus and Microbiological Special Diagnostics|Albertsen Lab|Michaelsen|Denmark
>Spike|hCoV-19/Australia/VIC2887/2020|2020-07-05|EPI_ISL_521718|Original|hCoV-19^^Victoria|Human|Microbiological Diagnostic Unit - Public Health Laboratory (MDU-PHL)|MDU-PHL|Schultz|Australia
>Spike|hCoV-19/Jordan/SEARCH-5464/2020|2020-09-14|EPI_ISL_755142|Original|hCoV-19^^Amman|Human|Biolab Diagnostic Laboratories|Andersen lab at Scripps Research|Gangavarapu|Jordan
>Spike|hCoV-19/England/20166000204/2020|2020-04-15|EPI_ISL_466493|Original|hCoV-19^^England|Human|Respiratory Virus Unit|Respiratory Virus Unit|Platt|United Kingdom
>Spike|hCoV-19/USA/FL-BPHL-2015/2020|2020-06-19|EPI_ISL_653168|Original|hCoV-19^^Florida|Human|Florida Bureau of Public Health Laboratories|Florida Bureau of Public Health Laboratories|Blanton|USA
>Spike|hCoV-19/Cote dIvoire/BKE0891/2020|2020-08-18|EPI_ISL_614389|Original|hCoV-19^^Boauk\u00e9|Human|Molecular diagnostic unit for viral haemorrhagic fevers and emerging viruses|Project group Epidemology of Highly Pathogenic Microorganisms|Calvignac-Spencer|C\u00f4te d'Ivoire
>Spike|hCoV-19/England/QEUH-B68CCB/2020|2020-11-11|EPI_ISL_655095|Original|hCoV-19^^England|Human|Lighthouse Lab in Glasgow|Wellcome Sanger Institute for the COVID-19 Genomics UK (COG-UK) Consortium|Davies|United Kingdom
>Spike|hCoV-19/England/LIVE-A0E71/2020|2020-06-09|EPI_ISL_478400|Original|hCoV-19^^England|Human|Liverpool Clinical Laboratories|COVID-19 Genomics UK (COG-UK) Consortium|Haldenby|United Kingdom
>Spike|hCoV-19/USA/MD-MDH-0522/2020|2020-11-30|EPI_ISL_778900|Original|hCoV-19^^Maryland|Human|Maryland Public Health Laboratory|Maryland Public Health Laboratory|Keller|USA

>Spike|hCoV-19/England/ALDP-95A43F/2020|2020-06-12|EPI_ISL_553582|Original|hCoV-19^^England|Human|Lighthouse Lab in Alderley Park|Wellcome Sanger Institute for the COVID-19 Genomics UK (COG-UK) consortium|Davies|United Kingdom
>Spike|hCoV-19/USA/CA-ALSR-1616/2020|2020-04-28|EPI_ISL_494689|Original|hCoV-19^^California|Human|Scripps Medical Laboratory|Andersen lab at Scripps Research|Gangavarapu|USA
>Spike|hCoV-19/England/MILK-99E183/2020|2020-08-31|EPI_ISL_550732|Original|hCoV-19^^England|Human|Lighthouse Lab in Milton Keynes|Wellcome Sanger Institute for the COVID-19 Genomics UK (COG-UK) consortium|Davies|United Kingdom
>Spike|hCoV-19/South Korea/KDCA0354/2020|2020-10-20|EPI_ISL_760139|Original|hCoV-19^^South Korea|Human|Division of Emerging Infectious Diseases|Division of Emerging Infectious Diseases|Park|South Korea
>Spike|hCoV-19/England/CAMC-A4194A/2020|2020-10-06|EPI_ISL_609412|Original|hCoV-19^^England|Human|Lighthouse Lab in Cambridge|Wellcome Sanger Institute for the COVID-19 Genomics UK (COG-UK) consortium|Davies|United Kingdom
>Spike|hCoV-19/USA/LA-CDC-1299/2020|2020-06-10|EPI_ISL_535340|Vero P1|hCoV-19^^Louisiana|Human|LA Office of Public Health Laboratories|Pathogen Discovery|Queen|USA
>Spike|hCoV-19/Finland/13LS5AD3/2020|2020-10-13|EPI_ISL_737229|Original|hCoV-19^^Uusimaa|Human|Department of Virology and Immunology|Department of Virology|Smura|Finland
>Spike|hCoV-19/England/CAMC-B369B0/2020|2020-11-05|EPI_ISL_645284|Original|hCoV-19^^England|Human|Lighthouse Lab in Cambridge|Wellcome Sanger Institute for the COVID-19 Genomics UK (COG-UK) Consortium|Davies|United Kingdom
>Spike|hCoV-19/England/ALDP-A87C5E/2020|2020-10-17|EPI_ISL_606074|Original|hCoV-19^^England|Human|Lighthouse Lab in Alderley Park|Wellcome Sanger Institute for the COVID-19 Genomics UK (COG-UK) consortium|Davies|United Kingdom
>Spike|hCoV-19/England/ALDP-A2F2B0/2020|2020-10-05|EPI_ISL_587247|Original|hCoV-19^^England|Human|Lighthouse Lab in Alderley Park|Wellcome Sanger Institute for the COVID-19 Genomics UK (COG-UK) consortium|Davies|United Kingdom
>Spike|hCoV-19/Netherlands/UT-RIVM-20822/2020|2020-12-15|EPI_ISL_790878|Original|hCoV-19^^Utrecht|Human|Dutch COVID-19 response team|National Institute for Public Health and the Environment (RIVM)|Meijer|Netherlands
>Spike|hCoV-19/England/201100016/2020|2020-03-08|EPI_ISL_464622|Original|hCoV-19^^England|Human|Respiratory Virus Unit|Respiratory Virus Unit|Platt|United Kingdom
>Spike|hCoV-19/England/QEUH-A9EFA6/2020|2020-10-20|EPI_ISL_611324|Original|hCoV-19^^England|Human|Lighthouse Lab in Glasgow|Wellcome Sanger Institute for the COVID-19 Genomics UK (COG-UK) consortium|Davies|United Kingdom
>Spike|hCoV-19/North Macedonia/12026/2020|2020-09-11|EPI_ISL_678260|Original|hCoV-19^^Struga|Human|General Hospital - Struga|Research Center for Genetic Engineering and Biotechnology \Georgi D. Efremov\u201d|Dimovski|North Macedonia
>Spike|hCoV-19/England/ALDP-B5CB36/2020|2020-11-08|EPI_ISL_655670|Original|hCoV-19^^England|Human|Lighthouse Lab in Alderley Park|Wellcome Sanger Institute for the COVID-19 Genomics UK (COG-UK) Consortium|Davies|United Kingdom
>Spike|hCoV-19/England/QEUH-AF5796/2020|2020-10-30|EPI_ISL_663103|Original|hCoV-19^^England|Human|Lighthouse Lab in Glasgow|Wellcome Sanger Institute for the COVID-19 Genomics UK (COG-UK) Consortium|Davies|United Kingdom
>Spike|hCoV-19/Weifang/WF0018/2020|2020-02-00|EPI_ISL_413748|Original|hCoV-19^^Weifang|Human|Weifang Center for Disease Control and Prevention|Weifang Center for Disease Control and Prevention & BGI-Shenzhen|Li|China
>Spike|hCoV-19/England/QEUH-B2B5D8/2020|2020-11-02|EPI_ISL_646531|Original|hCoV-19^^England|Human|Lighthouse Lab in Glasgow|Wellcome Sanger Institute for the COVID-19 Genomics UK (COG-UK) Consortium|Davies|United Kingdom
>Spike|hCoV-19/England/ALDP-953E38/2020|2020-06-13|EPI_ISL_554538|Original|hCoV-19^^England|Human|Lighthouse Lab in Alderley Park|Wellcome Sanger Institute for the COVID-19 Genomics UK (COG-UK) consortium|Davies|United Kingdom
>Spike|hCoV-19/England/ALDP-C8BAF4/2020|2020-12-15|EPI_ISL_777072|Original|hCoV-19^^England|Human|Lighthouse Lab in Alderley Park|Wellcome Sanger Institute for the COVID-19 Genomics UK (COG-UK) Consortium|Davies|United Kingdom

>Spike|hCoV-19/Canada/MB_38/2020|2020-03-18|EPI_ISL_429819|Original|hCoV-19^^Manitoba|Human|Cadham Provincial Laboratory|National Microbiology Laboratory|Majer|Canada
>Spike|hCoV-19/England/OXON-B1763/2020|2020-04-25|EPI_ISL_479042|Original|hCoV-19^^England|Human|Oxford Viromics|COVID-19 Genomics UK (COG-UK) Consortium|Golubchik|United Kingdom
>Spike|hCoV-19/Japan/PG-5303/2020|2020-08-00|EPI_ISL_690406|Original|hCoV-19^^Japan|Human|Pathogen Genomics Center|Pathogen Genomics Center|Sekizuka|Japan
>Spike|hCoV-19/England/MILK-B948C2/2020|2020-11-13|EPI_ISL_676244|Original|hCoV-19^^England|Human|Lighthouse Lab in Milton Keynes|Wellcome Sanger Institute for the COVID-19 Genomics UK (COG-UK) Consortium|Davies|United Kingdom
>Spike|hCoV-19/United Arab Emirates/0242/2020|2020-05-17|EPI_ISL_698424|Original|hCoV-19^^Abu Dhabi|Human|Group 42 (G42) Healthcare|G42 Healthcare|Ogrodzki|United Arab Emirates
>Spike|hCoV-19/France/IDF-10011AS/2020|2020-03-00|EPI_ISL_447713|Original|hCoV-19^^France|Human|H\u00f4pital Henri-Mondor Ap-Hp|H\u00f4pital Henri-Mondor Ap-Hp|Rodriguez|France
>Spike|hCoV-19/USA/WA-UW-23184/2020|2020-08-06|EPI_ISL_570753|Original|hCoV-19^^Washington|Human|UW Virology Lab|UW Virology Lab|Roychoudhury|USA
>Spike|hCoV-19/USA/LA-EVTL365/2020|2020-05-28|EPI_ISL_578771|Original|hCoV-19^^Louisiana|Human|LSUHS Emerging Viral Threat Laboratory|Microbial Genome Sequencing Center|Kamil|USA
>Spike|hCoV-19/USA/NY-NYUMC469/2020|2020-04-20|EPI_ISL_435534|Original|hCoV-19^^New York|Human|NYU Langone Health|Departments of Pathology and Medicine|Maurano|USA
>Spike|hCoV-19/Netherlands/NA_117/2020|2020-03-16|EPI_ISL_422664|Original|hCoV-19^^Netherlands|Human|Dutch COVID-19 response team|Erasmus Medical Center|Oude Munnink|Netherlands
>Spike|hCoV-19/Scotland/CVR5669/2020|2020-11-05|EPI_ISL_650726|Original|hCoV-19^^Scotland|Human|West of Scotland Specialist Virology Centre|COVID-19 Genomics UK (COG-UK) Consortium|Robertson|United Kingdom
>Spike|hCoV-19/Italy/BAS-IZSPB-383PT/2020|2020-10-25|EPI_ISL_722884|Original|hCoV-19^^Basilicata|Human|Istituto Zooprofilattico Sperimentale della Puglia e della Basilicata|Istituto Zooprofilattico Sperimentale della Puglia e della Basilicata|Parisi|Italy
>Spike|hCoV-19/USA/ME-HETL-J0027/2020|2020-03-16|EPI_ISL_513439|Original|hCoV-19^^Maine|Human|Maine HETL|Tewhey Lab|Tewhey|USA
>Spike|hCoV-19/England/EXET-139B93/2020|2020-00-00|EPI_ISL_679327|Original|hCoV-19^^England|Human|University of Exeter|COVID-19 Genomics UK (COG-UK) Consortium|Temperton|United Kingdom
>Spike|hCoV-19/USA/TX-HMH-MCoV-10266/2020|2020-07-07|EPI_ISL_789403|Original|hCoV-19^^Texas|Human|Houston Methodist Hospital|Houston Methodist Hospital|Long|USA
>Spike|hCoV-19/England/QEUH-C329E0/2020|2020-12-10|EPI_ISL_782691|Original|hCoV-19^^England|Human|Lighthouse Lab in Glasgow|Wellcome Sanger Institute for the COVID-19 Genomics UK (COG-UK) Consortium|Davies|United Kingdom
>Spike|hCoV-19/Iceland/456/2020|2020-03-20|EPI_ISL_424479|Original|hCoV-19^^Reykjavik|Human|The National University Hospital of Iceland|deCODE genetics|J\u00f3nsson|Iceland
>Spike|hCoV-19/Denmark/DCGC-21974/2020|2020-12-14|EPI_ISL_757802|Original|hCoV-19^^Hovedstaden|Human|Department of Virus and Microbiological Special Diagnostics|Albertsen Lab|Michaelsen|Denmark
>Spike|hCoV-19/USA/NY-Wadsworth-281400-01/2020|2020-10-27|EPI_ISL_734467|Original|hCoV-19^^New York|Human|Masonic Medical Research Institute|Wadsworth Center|Lasek-Nesselquist|USA
>Spike|hCoV-19/England/LOND-DC568/2020|2020-11-09|EPI_ISL_679622|Original|hCoV-19^^England|Human|University College London|COVID-19 Genomics UK (COG-UK) Consortium|Roy|United Kingdom
>Spike|hCoV-19/England/CAMC-B519CE/2020|2020-11-08|EPI_ISL_658201|Original|hCoV-19^^England|Human|Lighthouse Lab in Cambridge|Wellcome Sanger Institute for the COVID-19 Genomics UK (COG-UK) Consortium|Davies|United Kingdom

>Spike|hCoV-19/Canada/QC_AC9/2020|2020-04-06|EPI_ISL_476499|Original|hCoV-19^^Quebec|Human|Laboratoire de microbiologie|Smith Laboratory|Smith|Canada
>Spike|hCoV-19/England/QEUH-99906D/2020|2020-08-24|EPI_ISL_541836|Original|hCoV-19^^England|Human|Lighthouse Lab in Glasgow|Wellcome Sanger Institute for the COVID-19 Genomics UK (COG-UK) consortium|Davies|United Kingdom
>Spike|hCoV-19/Indonesia/JT-NIHRD-PME3110/2020|2020-07-16|EPI_ISL_791982|Original|hCoV-19^^Center Java|Human|RSUP DR KARIADI Semarang|National Institute of Health Research and Development|Setiawati|Indonesia
>Spike|hCoV-19/England/CAMB-1B82F1/2020|2020-10-19|EPI_ISL_637700|Original|hCoV-19^^England|Human|Department of Pathology|COVID-19 Genomics UK (COG-UK) Consortium|Goodfellow|United Kingdom
>Spike|hCoV-19/England/ALDP-B62AF4/2020|2020-11-08|EPI_ISL_656363|Original|hCoV-19^^England|Human|Lighthouse Lab in Alderley Park|Wellcome Sanger Institute for the COVID-19 Genomics UK (COG-UK) Consortium|Davies|United Kingdom
>Spike|hCoV-19/England/20130072204/2020|2020-03-23|EPI_ISL_423665|Original|hCoV-19^^England|Human|Respiratory Virus Unit|Respiratory Virus Unit|Platt|United Kingdom
>Spike|hCoV-19/England/CAMC-B0703B/2020|2020-10-29|EPI_ISL_643835|Original|hCoV-19^^England|Human|Lighthouse Lab in Cambridge|Wellcome Sanger Institute for the COVID-19 Genomics UK (COG-UK) Consortium|Davies|United Kingdom
>Spike|hCoV-19/England/MILK-A46DEF/2020|2020-10-06|EPI_ISL_598393|Original|hCoV-19^^England|Human|Lighthouse Lab in Milton Keynes|Wellcome Sanger Institute for the COVID-19 Genomics UK (COG-UK) consortium|Davies|United Kingdom
>Spike|hCoV-19/England/ALDP-AA4453/2020|2020-10-22|EPI_ISL_624441|Original|hCoV-19^^England|Human|Lighthouse Lab in Alderley Park|Wellcome Sanger Institute for the COVID-19 Genomics UK (COG-UK) consortium|Davies|United Kingdom
>Spike|hCoV-19/England/MILK-56C4E3/2020|2020-06-09|EPI_ISL_558353|Original|hCoV-19^^England|Human|Lighthouse Lab in Milton Keynes|Wellcome Sanger Institute for the COVID-19 Genomics UK (COG-UK) consortium|Davies|United Kingdom
>Spike|hCoV-19/USA/GA-CDC-5127/2020|2020-07-02|EPI_ISL_566107|Original|hCoV-19^^Georgia|Human|GA Department of Public Health Laboratory|Pathogen Discovery|Queen|USA
>Spike|hCoV-19/Spain/VC-IBV-98000368/2020|2020-03-19|EPI_ISL_539235|Original|hCoV-19^^Comunitat Valenciana|Human|Servicio de Microbiologia. Hospital Clinico Universitario de Valencia|SeqCOVID-SPAIN consortium/IBV(CSIC)|Comas|Spain
>Spike|hCoV-19/Denmark/DCGC-2791/2020|2020-08-24|EPI_ISL_616626|Original|hCoV-19^^Hovedstaden|Human|Department of Virus and Microbiological Special Diagnostics|Albertsen lab|Michaelsen|Denmark
>Spike|hCoV-19/Germany/NW-HHU-396/2020|2020-11-01|EPI_ISL_718076|Orginal|hCoV-19^^North Rhine-Westphalia|Human|ZOTZ KLIMAS MVZ D\u00fcsseldorf-Centrum GbR \u00dcBAG f\u00fcr Labormedizin|Center of Medical Microbiology|Wienemann|Germany
>Spike|hCoV-19/South Africa/KRISP-0581/2020|2020-07-09|EPI_ISL_498119|Original|hCoV-19^^KwaZulu-Natal|Human|NHLS-IALCH|KRISP|de Oliveira|South Africa
>Spike|hCoV-19/England/MILK-50CB95/2020|2020-06-11|EPI_ISL_558959|Original|hCoV-19^^England|Human|Lighthouse Lab in Milton Keynes|Wellcome Sanger Institute for the COVID-19 Genomics UK (COG-UK) consortium|Davies|United Kingdom
>Spike|hCoV-19/England/MILK-957687/2020|2020-06-09|EPI_ISL_554080|Original|hCoV-19^^England|Human|Lighthouse Lab in Milton Keynes|Wellcome Sanger Institute for the COVID-19 Genomics UK (COG-UK) consortium|Davies|United Kingdom
>Spike|hCoV-19/USA/NY-NYUMC491/2020|2020-03-18|EPI_ISL_436739|Original|hCoV-19^^New York|Human|NYU Langone Health|Departments of Pathology and Medicine|Maurano|USA
>Spike|hCoV-19/USA/CA-CZB-1820/2020|2020-04-23|EPI_ISL_486137|Original|hCoV-19^^California|Human|Orange County Public Health Laboratory|Chan-Zuckerberg Biohub|McGeever|USA
>Spike|hCoV-19/Russia/KC-RII-MH2751S/2020|2020-09-21|EPI_ISL_602426|Original|hCoV-19^^Karachay-Cherkess|Human|HELIX LLC|WHO National Influenza Centre Russian Federation|Komissarov|Russia
>Spike|hCoV-19/England/QEUH-B10512/2020|2020-11-02|EPI_ISL_642466|Original|hCoV-19^^England|Human|Lighthouse Lab in Glasgow|Wellcome Sanger Institute for the COVID-19 Genomics UK (COG-UK) Consortium|Davies|United Kingdom

>Spike|hCoV-19/Denmark/DCGC-4004/2020|2020-09-14|EPI_ISL_615776|Original|hCoV-19^^Hovedstaden|Human|Department of Virus and Microbiological Special Diagnostics|Albertsen lab|Michaelsen|Denmark
>Spike|hCoV-19/USA/TX-HMH-MCoV-18083/2020|2020-11-16|EPI_ISL_784431|Original|hCoV-19^^Texas|Human|Houston Methodist Hospital|Houston Methodist Hospital|Long|USA
>Spike|hCoV-19/USA/MN-MDH-2144/2020|2020-11-09|EPI_ISL_708482|Original|hCoV-19^^Minnesota|Human|Minnesota Department of Health|Minnesota Department of Health|Lorentz|USA
>Spike|hCoV-19/England/MILK-BAC6DC/2020|2020-11-18|EPI_ISL_681616|Original|hCoV-19^^England|Human|Lighthouse Lab in Milton Keynes|Wellcome Sanger Institute for the COVID-19 Genomics UK (COG-UK) Consortium|Davies|United Kingdom
>Spike|hCoV-19/Australia/VIC8105/2020|2020-08-16|EPI_ISL_564852|Original|hCoV-19^^Victoria|Human|Microbiological Diagnostic Unit - Public Health Laboratory (MDU-PHL)|MDU-PHL|Schultz|Australia
>Spike|hCoV-19/USA/TX-HMH-MCoV-18334/2020|2020-11-18|EPI_ISL_784682|Original|hCoV-19^^Texas|Human|Houston Methodist Hospital|Houston Methodist Hospital|Long|USA
>Spike|hCoV-19/India/TG-CCMB-GC8/2020|2020-05-23|EPI_ISL_528821|Original|hCoV-19^^Telangana|Human|Department of Medicine|CSIR-Centre for Cellular and Molecular Biology|Banu|India
>Spike|hCoV-19/England/NOTT-1199CC/2020|2020-11-02|EPI_ISL_626811|Original|hCoV-19^^England|Human|Queens Medical Centre|COVID-19 Genomics UK (COG-UK) Consortium|Loose|United Kingdom
>Spike|hCoV-19/Australia/NSW246/2020|2020-03-29|EPI_ISL_451511|Original|hCoV-19^^New South Wales|Human|Pathology West - NSW Health Pathology|NSW Health Pathology - Institute of Clinical Pathology and Medical Research; Westmead Hospital; University of Sydney|Gall|Australia
>Spike|hCoV-19/India/MH-ACTREC-350/2020|2020-07-22|EPI_ISL_699999|Original|hCoV-19^^Maharashtra|Human|Hematopathology Laboratory|Hematopathology Laboratory|Patkar|India
>Spike|hCoV-19/USA/IL-QDX-832/2020|2020-03-14|EPI_ISL_571601|Original|hCoV-19^^Illinois|Human|Quest Diagnostics|Quest Diagnostics|Kagan|USA
>Spike|hCoV-19/Switzerland/AG-ETHZ-390231/2020|2020-11-27|EPI_ISL_721735|Original|hCoV-19^^Aargau|Human|Viollier AG|Department of Biosystems Science and Engineering|Nadeau|Switzerland
>Spike|hCoV-19/Wales/PHWC-160E9A/2020|2020-05-26|EPI_ISL_473062|Original|hCoV-19^^Wales|Human|Wales Specialist Virology Centre Sequencing lab|COVID-19 Genomics UK (COG-UK) Consortium|Moore|United Kingdom
>Spike|hCoV-19/England/NOTT-10F5A7/2020|2020-04-03|EPI_ISL_433022|Original|hCoV-19^^England|Human|Queens Medical Centre|COVID-19 Genomics UK (COG-UK) Consortium|Loose|United Kingdom
>Spike|hCoV-19/USA/CA-CZB-3875/2020|2020-07-21|EPI_ISL_548514|Original|hCoV-19^^California|Human|County of Santa Clara Public Health Department|Chan-Zuckerberg Biohub|Bhatt|USA
>Spike|hCoV-19/USA/UT-UPHL-201016827/2020|2020-04-11|EPI_ISL_594416|Original|hCoV-19^^Utah|Human|Utah Public Health Laboratory|Utah Public Health Laboratory|Young|USA
>Spike|hCoV-19/Austria/CeMM0466/2020|2020-03-25|EPI_ISL_475875|Original|hCoV-19^^Austria|Human|Austrian Agency for Health and Food Safety (AGES)|Bergthaler laboratory|Bergthaler|Austria
>Spike|hCoV-19/England/MILK-A19107/2020|2020-09-30|EPI_ISL_612484|Original|hCoV-19^^England|Human|Wales Specialist Virology Centre Sequencing lab|COVID-19 Genomics UK (COG-UK) Consortium|Moore|United Kingdom
>Spike|hCoV-19/Wales/PHWC-16B14B/2020|2020-09-12|EPI_ISL_560249|Original|hCoV-19^^Wales|Human|Wales Specialist Virology Centre Sequencing lab|COVID-19 Genomics UK (COG-UK) Consortium|Moore|United Kingdom
>Spike|hCoV-19/England/MILK-CA6862/2020|2020-12-16|EPI_ISL_761495|Original|hCoV-19^^England|Human|Lighthouse Lab in Milton Keynes|Wellcome Sanger Institute for the COVID-19 Genomics UK (COG-UK) Consortium|Davies|United Kingdom
>Spike|hCoV-19/England/QEUH-B7CE64/2020|2020-11-14|EPI_ISL_674743|Original|hCoV-19^^England|Human|Lighthouse Lab in Glasgow|Wellcome Sanger Institute for the COVID-19 Genomics UK (COG-UK) Consortium|Davies|United Kingdom

>Spike|hCoV-19/England/QEUH-9CF7C3/2020|2020-09-17|EPI_ISL_590102|Original|hCoV-19^^England|Human|Lighthouse Lab in Glasgow|Wellcome Sanger Institute for the COVID-19 Genomics UK (COG-UK) consortium|Davies|United Kingdom
>Spike|hCoV-19/Denmark/DCGC-15916/2020|2020-11-23|EPI_ISL_714116|Original|hCoV-19^^Midtjylland|Human|Department of Virus and Microbiological Special Diagnostics|Albertsen Lab|Michaelsen|Denmark
>Spike|hCoV-19/England/LIVE-A4B2B/2020|2020-03-12|EPI_ISL_500073|Original|hCoV-19^^England|Human|Liverpool Clinical Laboratories|COVID-19 Genomics UK (COG-UK) Consortium|Haldenby|United Kingdom
>Spike|hCoV-19/USA/WA-UW-2971/2020|2020-03-27|EPI_ISL_486064|Original|hCoV-19^^Washington|Human|UW Virology Lab|UW Virology Lab|Roychoudhury|USA
>Spike|hCoV-19/England/QEUH-A37613/2020|2020-10-05|EPI_ISL_638730|Original|hCoV-19^^England|Human|Wales Specialist Virology Centre Sequencing lab|COVID-19 Genomics UK (COG-UK) Consortium|Moore|United Kingdom
>Spike|hCoV-19/England/ALDP-95C87D/2020|2020-06-16|EPI_ISL_553372|Original|hCoV-19^^England|Human|Lighthouse Lab in Alderley Park|Wellcome Sanger Institute for the COVID-19 Genomics UK (COG-UK) consortium|Davies|United Kingdom
>Spike|hCoV-19/Wales/PHWC-4A4AA2/2020|2020-12-16|EPI_ISL_764765|Original|hCoV-19^^Wales|Human|Wales Specialist Virology Centre Sequencing lab|COVID-19 Genomics UK (COG-UK) Consortium|Moore|United Kingdom
>Spike|hCoV-19/United Arab Emirates/0859/2020|2020-05-19|EPI_ISL_698998|Original|hCoV-19^^Abu Dhabi|Human|Group 42 (G42) Healthcare|G42 Healthcare|Ogrodzki|United Arab Emirates
>Spike|hCoV-19/Wales/PHWC-15D22B/2020|2020-04-22|EPI_ISL_472686|Original|hCoV-19^^Wales|Human|Wales Specialist Virology Centre Sequencing lab|COVID-19 Genomics UK (COG-UK) Consortium|Moore|United Kingdom
>Spike|hCoV-19/USA/TX-HMH-1875/2020|2020-05-02|EPI_ISL_542888|Original|hCoV-19^^Texas|Human|Houston Methodist Hospital|Houston Methodist Hospital|Long|USA
>Spike|hCoV-19/England/SHEF-CA099/2020|2020-04-27|EPI_ISL_475427|Original|hCoV-19^^England|Human|Virology Department|COVID-19 Genomics UK (COG-UK) Consortium|de Silva|United Kingdom
>Spike|hCoV-19/USA/UT-UPHL-2012915706/2020|2020-08-06|EPI_ISL_745643|Original|hCoV-19^^Utah|Human|Ginkgo Bioworks Clinical Laboratory|Utah Public Health Laboratory|Young|USA
>Spike|hCoV-19/Denmark/DCGC-9610/2020|2020-11-09|EPI_ISL_669065|Original|hCoV-19^^Nordjylland|Human|Department of Virus and Microbiological Special Diagnostics|Albertsen Lab|Michaelsen|Denmark
>Spike|hCoV-19/England/LOND-D53A1/2020|2020-04-15|EPI_ISL_444215|Original|hCoV-19^^England|Human|University College London|COVID-19 Genomics UK (COG-UK) Consortium|Roy|United Kingdom
>Spike|hCoV-19/Wuhan/0126-C103/2020|2020-01-26|EPI_ISL_493185|Original|hCoV-19^^Hubei|Human|National Virus Resource Center|Computational Virology Group|Liu|China
>Spike|hCoV-19/Scotland/EDB4745/2020|2020-05-10|EPI_ISL_453145|Original|hCoV-19^^Scotland|Human|Virology Department|COVID-19 Genomics UK (COG-UK) Consortium|Williams|United Kingdom
>Spike|hCoV-19/USA/WA-S61/2020|2020-03-05|EPI_ISL_417114|Original|hCoV-19^^Washington|Human|Washington State Department of Health|Seattle Flu Study|Bedford|USA
>Spike|hCoV-19/England/MILK-69ECA3/2020|2020-07-02|EPI_ISL_551829|Original|hCoV-19^^England|Human|Lighthouse Lab in Milton Keynes|Wellcome Sanger Institute for the COVID-19 Genomics UK (COG-UK) consortium|Davies|United Kingdom
>Spike|hCoV-19/Denmark/DCGC-1977/2020|2020-07-13|EPI_ISL_618312|Original|hCoV-19^^Hovedstaden|Human|Department of Virus and Microbiological Special Diagnostics|Albertsen lab|Michaelsen|Denmark
>Spike|hCoV-19/USA/TX-HMH-2864/2020|2020-06-08|EPI_ISL_543834|Original|hCoV-19^^Texas|Human|Houston Methodist Hospital|Houston Methodist Hospital|Long|USA
>Spike|hCoV-19/England/LIVE-9F24C/2020|2020-05-04|EPI_ISL_453440|Original|hCoV-19^^England|Human|Liverpool Clinical Laboratories|COVID-19 Genomics UK (COG-UK) Consortium|Haldenby|United Kingdom

>Spike|hCoV-19/USA/TX-HMH-MCoV-15879/2020|2020-07-10|EPI_ISL_785563|Original|hCoV-19^^Texas|Human|Houston Methodist Hospital|Houston Methodist Hospital|Long|USA
>Spike|hCoV-19/Switzerland/ZH-ETHZ-270205/2020|2020-08-30|EPI_ISL_541537|Original|hCoV-19^^Zurich|Human|Viollier AG|Department of Biosystems Science and Engineering|Nadeau|Switzerland
>Spike|hCoV-19/Indonesia/JK-EIJK-0317/2020|2020-03-19|EPI_ISL_435282|Original|hCoV-19^^Jakarta|Human|RS Pondok Indah Hospital \u2013 Pondok Indah|Eijkman Institute for Molecular Biology|Soebandrio|Indonesia
>Spike|hCoV-19/England/SHEF-CE2F9/2020|2020-04-20|EPI_ISL_490630|Original|hCoV-19^^England|Human|Virology Department|COVID-19 Genomics UK (COG-UK) Consortium|de Silva|United Kingdom
>Spike|hCoV-19/England/QEUH-7A532B/2020|2020-07-08|EPI_ISL_533215|Original|hCoV-19^^England|Human|Lighthouse Lab in Glasgow|Wellcome Sanger Institute for the COVID-19 Genomics UK (COG-UK) consortium|Davies|United Kingdom
>Spike|hCoV-19/Gibraltar/204462875/2020|2020-10-29|EPI_ISL_615042|Original|hCoV-19^^Gibraltar|Human|Microbiology|Respiratory Virus Unit|Platt|Gibraltar
>Spike|hCoV-19/Japan/PG-8795/2020|2020-08-00|EPI_ISL_686383|Original|hCoV-19^^Japan|Human|Pathogen Genomics Center|Pathogen Genomics Center|Sekizuka|Japan
>Spike|hCoV-19/Denmark/DCGC-9000/2020|2020-10-26|EPI_ISL_626179|Original|hCoV-19^^Nordjylland|Human|Department of Virus and Microbiological Special Diagnostics|Albertsen lab|Michaelsen|Denmark
>Spike|hCoV-19/Hong Kong/VM20095004/2020|2020-08-02|EPI_ISL_602617|Original|hCoV-19^^Hong Kong|Human|Queen Mary Hospital|Hong Kong Department of Health|Mak|China
>Spike|hCoV-19/USA/TX-HMH-2740/2020|2020-06-04|EPI_ISL_545519|Original|hCoV-19^^Texas|Human|Houston Methodist Hospital|Houston Methodist Hospital|Long|USA
>Spike|hCoV-19/Denmark/DCGC-6671/2020|2020-10-12|EPI_ISL_619574|Original|hCoV-19^^Midtjylland|Human|Department of Virus and Microbiological Special Diagnostics|Albertsen lab|Michaelsen|Denmark
>Spike|hCoV-19/Norway/4253/2020|2020-10-07|EPI_ISL_775312|Original|hCoV-19^^Nordland|Human|Nordland Hospital - Bodo|Norwegian Institute of Public Health|Vollan|Norway
>Spike|hCoV-19/England/MILK-9EE870/2020|2020-09-23|EPI_ISL_580694|Original|hCoV-19^^England|Human|Lighthouse Lab in Milton Keynes|Wellcome Sanger Institute for the COVID-19 Genomics UK (COG-UK) consortium|Davies|United Kingdom
>Spike|hCoV-19/USA/WI-QDX-777/2020|2020-03-16|EPI_ISL_571563|Original|hCoV-19^^Wisconsin|Human|Quest Diagnostics|Quest Diagnostics|Kagan|USA
>Spike|hCoV-19/USA/NM-DOH-2020315778/2020|2020-08-27|EPI_ISL_732716|Original|hCoV-19^^New Mexico|Human|New Mexico Department of Health Scientific Laboratory|New Mexico Department of Health Scientific Laboratory|Johnson|USA
>Spike|hCoV-19/Japan/PG-3215/2020|2020-04-00|EPI_ISL_691333|Original|hCoV-19^^Japan|Human|Pathogen Genomics Center|Pathogen Genomics Center|Sekizuka|Japan
>Spike|hCoV-19/Australia/VIC14756/2020|2020-08-12|EPI_ISL_592986|Original|hCoV-19^^Victoria|Human|Microbiological Diagnostic Unit - Public Health Laboratory (MDU-PHL)|MDU-PHL|Schultz|Australia
>Spike|hCoV-19/England/QEUH-A61908/2020|2020-10-12|EPI_ISL_628643|Original|hCoV-19^^England|Human|Oxford Viromics|COVID-19 Genomics UK (COG-UK) Consortium|Golubchik|United Kingdom
>Spike|hCoV-19/Belgium/ULG-10541/2020|2020-10-23|EPI_ISL_626299|Original|hCoV-19^^Li\u00e8ge|Human|Department of Clinical Microbiology|GIGA Medical Genomics|Durkin|Belgium
>Spike|hCoV-19/USA/WI-GMF-31217/2020|2020-11-03|EPI_ISL_661078|Original|hCoV-19^^Wisconsin|Human|Gundersen Molecular Diagnostics Laboratory|Kabara Cancer Research Institute|Kenny|USA
>Spike|hCoV-19/England/EXET-13A6CE/2020|2020-11-07|EPI_ISL_665069|Original|hCoV-19^^England|Human|University of Exeter|COVID-19 Genomics UK (COG-UK) Consortium|Temperton|United Kingdom
>Spike|hCoV-19/USA/CA-CZB-13071/2020|2020-05-03|EPI_ISL_672339|Original|hCoV-19^^California|Human|Alameda County Public Health Lab|Chan-Zuckerberg Biohub|Bhatt|USA

>Spike|hCoV-19/Wales/PHWC-31A9F/2020|2020-04-11|EPI_ISL_446523|Original|hCoV-19^^Wales|Human|Wales Specialist Virology Centre|Public Health Wales Microbiology Cardiff|Moore|United Kingdom
>Spike|hCoV-19/England/CAMB-726F8/2020|2020-03-24|EPI_ISL_439884|Original|hCoV-19^^England|Human|Department of Pathology|Wellcome Sanger Institute for the COVID-19 Genomics UK (COG-UK) consortium|Amato|United Kingdom
>Spike|hCoV-19/Netherlands/Limburg_52/2020|2020-04-14|EPI_ISL_461040|Original|hCoV-19^^Limburg|Human|Dutch COVID-19 response team|Erasmus Medical Center|Oude Munnink|Netherlands
>Spike|hCoV-19/England/CAMC-A659E6/2020|2020-10-11|EPI_ISL_637963|Original|hCoV-19^^England|Human|Department of Pathology|COVID-19 Genomics UK (COG-UK) Consortium|Goodfellow|United Kingdom
>Spike|hCoV-19/Denmark/DCGC-1032/2020|2020-03-09|EPI_ISL_614546|Original|hCoV-19^^Nordjylland|Human|Department of Virus and Microbiological Special Diagnostics|Albertsen lab|Michaelsen|Denmark
>Spike|hCoV-19/mink/Netherlands/NB-EMC-26-1/2020|2020-07-29|EPI_ISL_523086|Original|hCoV-19^^Noord Brabant|Neovison vison|Dutch COVID-19 response team|Erasmus Medical Center|Oude Munnink|Netherlands
>Spike|hCoV-19/England/QEUH-997E38/2020|2020-08-24|EPI_ISL_537166|Original|hCoV-19^^England|Human|Lighthouse Lab in Glasgow|Wellcome Sanger Institute for the COVID-19 Genomics UK (COG-UK) consortium|Davies|United Kingdom
>Spike|hCoV-19/USA/ID-QDX-1292/2020|2020-03-17|EPI_ISL_571931|Original|hCoV-19^^Idaho|Human|Quest Diagnostics|Quest Diagnostics|Kagan|USA
>Spike|hCoV-19/Wales/PHWC-48013E/2020|2020-10-08|EPI_ISL_612854|Original|hCoV-19^^Wales|Human|Wales Specialist Virology Centre Sequencing lab|COVID-19 Genomics UK (COG-UK) Consortium|Moore|United Kingdom
>Spike|hCoV-19/Scotland/QEUH-9F61F2/2020|2020-09-27|EPI_ISL_600849|Original|hCoV-19^^Scotland|Human|Lighthouse Lab in Glasgow|Wellcome Sanger Institute for the COVID-19 Genomics UK (COG-UK) consortium|Davies|United Kingdom
>Spike|hCoV-19/Australia/VIC16479/2020|2020-08-06|EPI_ISL_640809|Original|hCoV-19^^Victoria|Human|Microbiological Diagnostic Unit - Public Health Laboratory (MDU-PHL)|MDU-PHL|Schultz|Australia
>Spike|hCoV-19/Kenya/C5176/2020|2020-05-09|EPI_ISL_568751|Original|hCoV-19^^Mombasa|Human|KEMRI-Wellcome Trust Research Programme/KEMRI-CGMR-C Kilifi|KEMRI-Wellcome Trust Research Programme/KEMRI-CGMR-C Kilifi|Githinji|Kenya
>Spike|hCoV-19/England/MILK-B8EFE4/2020|2020-11-13|EPI_ISL_675420|Original|hCoV-19^^England|Human|Lighthouse Lab in Milton Keynes|Wellcome Sanger Institute for the COVID-19 Genomics UK (COG-UK) Consortium|Davies|United Kingdom
>Spike|hCoV-19/USA/TX-DSHS-1661/2020|2020-06-24|EPI_ISL_738080|Original|hCoV-19^^Texas|Human|Laboratory Services Section|Laboratory Services Section|Rahman|USA
>Spike|hCoV-19/Wales/ALDP-C8C47D/2020|2020-12-14|EPI_ISL_777243|Original|hCoV-19^^Wales|Human|Lighthouse Lab in Alderley Park|Wellcome Sanger Institute for the COVID-19 Genomics UK (COG-UK) Consortium|Davies|United Kingdom
>Spike|hCoV-19/England/QEUH-B6845A/2020|2020-11-11|EPI_ISL_661325|Original|hCoV-19^^England|Human|Lighthouse Lab in Glasgow|Wellcome Sanger Institute for the COVID-19 Genomics UK (COG-UK) Consortium|Davies|United Kingdom
>Spike|hCoV-19/USA/MI-MDHHS-SC20730/2020|2020-04-21|EPI_ISL_471754|Original|hCoV-19^^Michigan|Human|Michigan Department of Health and Human Services|Michigan Department of Health and Human Services|Blankenship|USA
>Spike|hCoV-19/Spain/MD-IBV-99008799/2020|2020-09-21|EPI_ISL_654150|Original|hCoV-19^^Madrid|Human|Hospital General Universitario Gregorio Mara\u00f1\u00f3n|SeqCOVID-SPAIN consortium/IBV(CSIC)|Comas|Spain
>Spike|hCoV-19/England/LOND-1267415/2020|2020-12-08|EPI_ISL_741286|Original|hCoV-19^^England|Human|University College London|COVID-19 Genomics UK (COG-UK) Consortium|Roy|United Kingdom
>Spike|hCoV-19/United Arab Emirates/0698/2020|2020-05-15|EPI_ISL_698849|Original|hCoV-19^^Abu Dhabi|Human|Group 42 (G42) Healthcare|G42 Healthcare|Ogrodzki|United Arab Emirates

>Spike|hCoV-19/England/MILK-B38F07/2020|2020-11-01|EPI_ISL_650141|Original|hCoV-19^^England|Human|University of Exeter|COVID-19 Genomics UK (COG-UK) Consortium|Temperton|United Kingdom
>Spike|hCoV-19/USA/MA-MASPHL-00644/2020|2020-05-14|EPI_ISL_692987|Original|hCoV-19^^Massachusetts|Human|Massachusetts State Public Health Laboratory|Massachusetts State Public Health Laboratory|Lang|USA
>Spike|hCoV-19/England/ALDP-6D6665/2020|2020-07-02|EPI_ISL_556153|Original|hCoV-19^^England|Human|Lighthouse Lab in Alderley Park|Wellcome Sanger Institute for the COVID-19 Genomics UK (COG-UK) consortium|Davies|United Kingdom
>Spike|hCoV-19/USA/WA-UW-31203/2020|2020-10-13|EPI_ISL_735459|Original|hCoV-19^^Washington|Human|UW Virology Lab|UW Virology Lab|Roychoudhury|USA
>Spike|hCoV-19/Spain/AR-IBV-2416/2020|2020-03-21|EPI_ISL_468768|Original|hCoV-19^^Aragon|Human|Servicio de Microbiolog\u00eda|SeqCOVID-SPAIN consortium/IBV(CSIC)|Comas|Spain
>Spike|hCoV-19/USA/TX-HMH-5692/2020|2020-06-24|EPI_ISL_546948|Original|hCoV-19^^Texas|Human|Houston Methodist Hospital|Houston Methodist Hospital|Long|USA
>Spike|hCoV-19/Switzerland/UR-ETHZ-321536/2020|2020-10-15|EPI_ISL_603745|Original|hCoV-19^^Uri|Human|Viollier AG|Department of Biosystems Science and Engineering|Nadeau|Switzerland
>Spike|hCoV-19/England/QEUH-9B87AF/2020|2020-09-10|EPI_ISL_549724|Original|hCoV-19^^England|Human|Lighthouse Lab in Glasgow|Wellcome Sanger Institute for the COVID-19 Genomics UK (COG-UK) consortium|Davies|United Kingdom
>Spike|hCoV-19/USA/TX-HMH-7932/2020|2020-06-30|EPI_ISL_545717|Original|hCoV-19^^Texas|Human|Houston Methodist Hospital|Houston Methodist Hospital|Long|USA
>Spike|hCoV-19/Jordan/AM-ALSR-3920/2020|2020-09-30|EPI_ISL_636286|Original|hCoV-19^^Amman|Human|Biolab Diagnostic Laboratories|Andersen lab at Scripps Research|Gangavarapu|Jordan
>Spike|hCoV-19/USA/TX-DSHS-0676/2020|2020-07-16|EPI_ISL_576329|Original|hCoV-19^^Texas|Human|Texas Department of State Health Services|Texas Department of State Health Services|Rahman|USA
>Spike|hCoV-19/Wales/PHWC-2F2AB/2020|2020-04-06|EPI_ISL_446228|Original|hCoV-19^^Wales|Human|Wales Specialist Virology Centre|Public Health Wales Microbiology Cardiff|Moore|United Kingdom
>Spike|hCoV-19/Australia/VIC4682/2020|2020-07-17|EPI_ISL_519070|Original|hCoV-19^^Victoria|Human|Microbiological Diagnostic Unit - Public Health Laboratory (MDU-PHL)|MDU-PHL|Schultz|Australia
>Spike|hCoV-19/USA/NY-PV09134/2020|2020-03-19|EPI_ISL_422506|Original|hCoV-19^^New York|Human|MSHS Clinical Microbiology Laboratories|MSHS Pathogen Surveillance Program|van Bakel|USA
>Spike|hCoV-19/Denmark/DCGC-12598/2020|2020-11-16|EPI_ISL_682779|Original|hCoV-19^^Hovedstaden|Human|Department of Virus and Microbiological Special Diagnostics|Albertsen Lab|Michaelsen|Denmark
>Spike|hCoV-19/USA/WI-UW-1125/2020|2020-08-05|EPI_ISL_522883|Original|hCoV-19^^Wisconsin|Human|University of Wisconsin-Madison AIDS Vaccine Research Laboratories|University of Wisconsin-Madison AIDS Vaccine Research Laboratories|Baczenas|USA
>Spike|hCoV-19/England/SHEF-C9F9D/2020|2020-05-11|EPI_ISL_462043|Original|hCoV-19^^England|Human|Virology Department|COVID-19 Genomics UK (COG-UK) Consortium|de Silva|United Kingdom
>Spike|hCoV-19/USA/TX-HMH-MCoV-15118/2020|2020-10-17|EPI_ISL_786602|Original|hCoV-19^^Texas|Human|Houston Methodist Hospital|Houston Methodist Hospital|Long|USA
>Spike|hCoV-19/USA/VA-DCLS-0778/2020|2020-06-11|EPI_ISL_526892|Original|hCoV-19^^Virginia|Human|Virginia DCLS|Virginia DCLS|St. Jacques|USA
>Spike|hCoV-19/USA/UT-QDX-2270/2020|2020-03-12|EPI_ISL_604604|Original|hCoV-19^^Utah|Human|Quest Diagnostics|Quest Diagnostics|Kagan|USA
>Spike|hCoV-19/Scotland/QEUH-9610D7/2020|2020-08-05|EPI_ISL_532098|Original|hCoV-19^^Scotland|Human|NHSGGC West of Scotland Specialist Virology Centre / MRC-University of Glasgow Centre for Virus Research|Wellcome Sanger Institute for the COVID-19 Genomics UK (COG-UK) consortium|Davies|United Kingdom

>Spike|hCoV-19/USA/FL-BPHL-0561/2020|2020-03-12|EPI_ISL_508765|Original|hCoV-19^^Florida|Human|Florida Bureau of Public Health Laboratories|Florida Bureau of Public Health Laboratories|Blanton|USA
>Spike|hCoV-19/USA/TX-HMH0366/2020|2020-03-25|EPI_ISL_434958|Original|hCoV-19^^Texas|Human|Houston Methodist Hospital|Houston Methodist Hospital|Long|USA
>Spike|hCoV-19/England/ALDP-9CDA71/2020|2020-09-16|EPI_ISL_566185|Original|hCoV-19^^England|Human|Lighthouse Lab in Alderley Park|Wellcome Sanger Institute for the COVID-19 Genomics UK (COG-UK) consortium|Davies|United Kingdom
>Spike|hCoV-19/England/MILK-BACD8C/2020|2020-11-17|EPI_ISL_681574|Original|hCoV-19^^England|Human|Lighthouse Lab in Milton Keynes|Wellcome Sanger Institute for the COVID-19 Genomics UK (COG-UK) Consortium|Davies|United Kingdom
>Spike|hCoV-19/USA/WI-UW-2410/2020|2020-12-04|EPI_ISL_754662|Original|hCoV-19^^Wisconsin|Human|University of Wisconsin-Madison AIDS Vaccine Research Laboratories|University of Wisconsin-Madison AIDS Vaccine Research Laboratories|Moreno|USA
>Spike|hCoV-19/Gambia/NPHL-748/2020|2020-00-00|EPI_ISL_561324|Original|hCoV-19^^Kombo|Human|MRCG at LSHTM Genomics lab|MRCG at LSHTM Genomics lab|Sesay|Gambia
>Spike|hCoV-19/USA/CA-CZB-14662/2020|2020-11-16|EPI_ISL_738884|Original|hCoV-19^^California|Human|Orange County Public Health Lab|Chan-Zuckerberg Biohub|Kamm|USA
>Spike|hCoV-19/Ireland/D-NVRL-MPV45546/2020|2020-04-09|EPI_ISL_767759|Original|hCoV-19^^Dublin|Human|National Virus Reference Laboratory|Irish Coronavirus Sequencing Consortium - Teagasc Moorepark|Walsh|Ireland
>Spike|hCoV-19/Peru/LIM-INS-136/2020|2020-03-17|EPI_ISL_536531|Original|hCoV-19^^Lima|Human|Instituto Nacional de Salud|Laboratorio de Infecciones Respiratorias Agudas|Lopez|Peru
>Spike|hCoV-19/Scotland/GCVR-16FE13/2020|2020-03-22|EPI_ISL_459702|Original|hCoV-19^^Scotland|Human|NHSGGC West of Scotland Specialist Virology Centre / MRC-University of Glasgow Centre for Virus Research|Wellcome Sanger Institute for the COVID-19 Genomics UK (COG-UK) consortium|Davies|United Kingdom
>Spike|hCoV-19/USA/MI-MDHHS-SC21352/2020|2020-04-03|EPI_ISL_507606|Original|hCoV-19^^Michigan|Human|Michigan Department of Health and Human Services|Michigan Department of Health and Human Services|Blankenship|USA
>Spike|hCoV-19/Luxembourg/LNS4386977/2020|2020-11-07|EPI_ISL_740462|Original|hCoV-19^^Luxembourg|Human|Laboratoire national de sant\u00e9|Laboratoire national de sant\u00e9|Wienecke-Baldacchino|Luxembourg
>Spike|hCoV-19/England/MILK-AC9519/2020|2020-10-21|EPI_ISL_629149|Original|hCoV-19^^England|Human|Lighthouse Lab in Milton Keynes|Wellcome Sanger Institute for the COVID-19 Genomics UK (COG-UK) consortium|Davies|United Kingdom
>Spike|hCoV-19/Denmark/DCGC-21450/2020|2020-12-21|EPI_ISL_757732|Original|hCoV-19^^Hovedstaden|Human|Department of Virus and Microbiological Special Diagnostics|Albertsen Lab|Michaelsen|Denmark
>Spike|hCoV-19/Brazil/PE-IAM1264/2020|2020-05-10|EPI_ISL_572385|Original|hCoV-19^^Pernambuco|Human|LACEN/PE|WallauLab|Wallau|Brazil
>Spike|hCoV-19/England/CAMB-79AB9/2020|2020-03-27|EPI_ISL_433819|Original|hCoV-19^^England|Human|Department of Pathology|COVID-19 Genomics UK (COG-UK) Consortium|Meredith|United Kingdom
>Spike|hCoV-19/England/MILK-489214/2020|2020-05-11|EPI_ISL_559668|Original|hCoV-19^^England|Human|Lighthouse Lab in Milton Keynes|Wellcome Sanger Institute for the COVID-19 Genomics UK (COG-UK) consortium|Davies|United Kingdom
>Spike|hCoV-19/England/EXET-13ABED/2020|2020-11-10|EPI_ISL_664266|Original|hCoV-19^^England|Human|University of Exeter|COVID-19 Genomics UK (COG-UK) Consortium|Temperton|United Kingdom
>Spike|hCoV-19/England/ALDP-BC64C3/2020|2020-11-20|EPI_ISL_721504|Original|hCoV-19^^England|Human|Lighthouse Lab in Alderley Park|Wellcome Sanger Institute for the COVID-19 Genomics UK (COG-UK) Consortium|Davies|United Kingdom
>Spike|hCoV-19/Wales/MILK-9E0823/2020|2020-09-20|EPI_ISL_601311|Original|hCoV-19^^Wales|Human|Lighthouse Lab in Milton Keynes|Wellcome Sanger Institute for the COVID-19 Genomics UK (COG-UK) consortium|Davies|United Kingdom

>Spike|hCoV-19/England/LOND-D35E9/2020|2020-04-02|EPI_ISL_440962|Original|hCoV-19^^England|Human|University College London|COVID-19 Genomics UK (COG-UK) Consortium|Roy|United Kingdom
>Spike|hCoV-19/Northern Ireland/NIRE-FFECE/2020|2020-05-01|EPI_ISL_489336|Original|hCoV-19^^Northern Ireland|Human|Regional Virus Laboratory|Wellcome Sanger Institute for the COVID-19 Genomics UK (COG-UK) consortium|Davies|United Kingdom
>Spike|hCoV-19/England/ALDP-66F1AC/2020|2020-07-03|EPI_ISL_558031|Original|hCoV-19^^England|Human|Lighthouse Lab in Alderley Park|Wellcome Sanger Institute for the COVID-19 Genomics UK (COG-UK) consortium|Davies|United Kingdom
>Spike|hCoV-19/USA/MN-MDH-1679/2020|2020-04-08|EPI_ISL_560766|Original|hCoV-19^^Minnesota|Human|Minnesota Department of Health|Minnesota Department of Health|Garfin|USA
>Spike|hCoV-19/Saudi Arabia/KAUST-MADINAH1689/2020|2020-08-23|EPI_ISL_751231|Original|hCoV-19^^Madinah|Human|Pathogen Genomics Lab King Abdullah University of Science and Technology(KAUST)|Pathogen Genomics Lab King Abdullah University of Science and Technology(KAUST)|Mohamed Ghazzali|Saudi Arabia
>Spike|hCoV-19/England/MILK-4C2C8A/2020|2020-06-06|EPI_ISL_559296|Original|hCoV-19^^England|Human|Lighthouse Lab in Milton Keynes|Wellcome Sanger Institute for the COVID-19 Genomics UK (COG-UK) consortium|Davies|United Kingdom
>Spike|hCoV-19/England/MILK-992B81/2020|2020-08-26|EPI_ISL_552004|Original|hCoV-19^^England|Human|Lighthouse Lab in Milton Keynes|Wellcome Sanger Institute for the COVID-19 Genomics UK (COG-UK) consortium|Davies|United Kingdom
>Spike|hCoV-19/USA/VA-DCLS-1804/2020|2020-09-16|EPI_ISL_614056|Original|hCoV-19^^Virginia|Human|Virginia DCLS|Virginia DCLS|St. Jacques|USA
>Spike|hCoV-19/England/MILK-975B1B/2020|2020-08-20|EPI_ISL_552501|Original|hCoV-19^^England|Human|Lighthouse Lab in Milton Keynes|Wellcome Sanger Institute for the COVID-19 Genomics UK (COG-UK) consortium|Davies|United Kingdom
>Spike|hCoV-19/Australia/SAP487/2020|2020-08-15|EPI_ISL_516550|Original|hCoV-19^^South Australia|Human|SA Pathology|SA Pathology|Leong|Australia
>Spike|hCoV-19/Wales/PHWC-16B248/2020|2020-09-15|EPI_ISL_560263|Original|hCoV-19^^Wales|Human|Wales Specialist Virology Centre Sequencing lab|COVID-19 Genomics UK (COG-UK) Consortium|Moore|United Kingdom
>Spike|hCoV-19/USA/TX-HMH-MCoV-8893/2020|2020-07-02|EPI_ISL_780559|Original|hCoV-19^^Texas|Human|Houston Methodist Hospital|Houston Methodist Hospital|Long|USA
>Spike|hCoV-19/England/ALDP-9E6B06/2020|2020-09-24|EPI_ISL_580339|Original|hCoV-19^^England|Human|Lighthouse Lab in Alderley Park|Wellcome Sanger Institute for the COVID-19 Genomics UK (COG-UK) consortium|Davies|United Kingdom
>Spike|hCoV-19/England/NORT-29A79B/2020|2020-05-03|EPI_ISL_507118|Original|hCoV-19^^England|Human|Northumbria University / South Tees Hospitals NHS Foundation Trust / North Cumbria Integrated Care NHS Foundation Trust / North Tees and Hartlepool NHS Foundation Trust / Newcastle Hospitals NHS Foundation Trust|COVID-19 Genomics UK (COG-UK) Consortium|Bashton|United Kingdom
>Spike|hCoV-19/England/LIVE-DCBC2E/2020|2020-11-27|EPI_ISL_724452|Original|hCoV-19^^England|Human|Liverpool Clinical Laboratories|COVID-19 Genomics UK (COG-UK) Consortium|Haldenby|United Kingdom
>Spike|hCoV-19/Netherlands/ZH-EMC-335/2020|2020-06-26|EPI_ISL_523676|Original|hCoV-19^^Zuid Holland|Human|Dutch COVID-19 response team|Erasmus Medical Center|Oude Munnink|Netherlands
>Spike|hCoV-19/England/BIRM-6096D/2020|2020-06-05|EPI_ISL_483909|Original|hCoV-19^^England|Human|University of Birmingham|COVID-19 Genomics UK (COG-UK) Consortium|Loman|United Kingdom
>Spike|hCoV-19/England/QEUH-B6B1F6/2020|2020-11-11|EPI_ISL_662485|Original|hCoV-19^^England|Human|Lighthouse Lab in Glasgow|Wellcome Sanger Institute for the COVID-19 Genomics UK (COG-UK) Consortium|Davies|United Kingdom
>Spike|hCoV-19/England/ALDP-AA0967/2020|2020-10-22|EPI_ISL_630837|Original|hCoV-19^^England|Human|Lighthouse Lab in Alderley Park|Wellcome Sanger Institute for the COVID-19 Genomics UK (COG-UK) consortium|Davies|United Kingdom

>Spike|hCoV-19/USA/NM-DOH-2020631927/2020|2020-12-20|EPI_ISL_766911|Original|hCoV-19^^New Mexico|Human|New Mexico Department of Health Scientific Laboratory|New Mexico Department of Health Scientific Laboratory|Malone|USA
>Spike|hCoV-19/England/ALDP-9229AA/2020|2020-06-23|EPI_ISL_551451|Original|hCoV-19^^England|Human|Lighthouse Lab in Alderley Park|Wellcome Sanger Institute for the COVID-19 Genomics UK (COG-UK) consortium|Davies|United Kingdom
>Spike|hCoV-19/Bangladesh/CHRF-0008/2020|2020-05-16|EPI_ISL_468076|Original|hCoV-19^^Dhaka|Human|Child Health Research Foundation|Child Health Research Foundation|Saha|Bangladesh
>Spike|hCoV-19/Denmark/DCGC-6658/2020|2020-10-12|EPI_ISL_619561|Original|hCoV-19^^Midtjylland|Human|Department of Virus and Microbiological Special Diagnostics|Albertsen lab|Michaelsen|Denmark
>Spike|hCoV-19/England/MILK-B877D4/2020|2020-11-13|EPI_ISL_724800|Original|hCoV-19^^England|Human|Oxford Viromics|COVID-19 Genomics UK (COG-UK) Consortium|Golubchik|United Kingdom
>Spike|hCoV-19/USA/TX-HMH-MCoV-18267/2020|2020-11-16|EPI_ISL_784615|Original|hCoV-19^^Texas|Human|Houston Methodist Hospital|Houston Methodist Hospital|Long|USA
>Spike|hCoV-19/England/ALDP-B24F2C/2020|2020-11-04|EPI_ISL_646653|Original|hCoV-19^^England|Human|Lighthouse Lab in Alderley Park|Wellcome Sanger Institute for the COVID-19 Genomics UK (COG-UK) Consortium|Davies|United Kingdom
>Spike|hCoV-19/Denmark/DCGC-7118/2020|2020-10-12|EPI_ISL_619064|Original|hCoV-19^^Midtjylland|Human|Department of Virus and Microbiological Special Diagnostics|Albertsen lab|Michaelsen|Denmark
>Spike|hCoV-19/Colombia/DC-INS-4283/2020|2020-11-06|EPI_ISL_739671|Original|hCoV-19^^Bogot\u00e1|Human|Instituto Nacional de Salud|Instituto Nacional de Salud|Franco-Mu\u00f1oz|Colombia
>Spike|hCoV-19/England/LIVE-1D2BAB/2020|2020-03-29|EPI_ISL_612413|Original|hCoV-19^^England|Human|Liverpool Clinical Laboratories|COVID-19 Genomics UK (COG-UK) Consortium|Haldenby|United Kingdom
>Spike|hCoV-19/USA/TX-HMH-MCoV-12606/2020|2020-08-31|EPI_ISL_790415|Original|hCoV-19^^Texas|Human|Houston Methodist Hospital|Houston Methodist Hospital|Long|USA
>Spike|hCoV-19/Australia/VIC2521/2020|2020-06-29|EPI_ISL_563984|Original|hCoV-19^^Victoria|Human|Microbiological Diagnostic Unit - Public Health Laboratory (MDU-PHL)|MDU-PHL|Schultz|Australia
>Spike|hCoV-19/USA/MI-MDHHS-SC20951/2020|2020-03-19|EPI_ISL_507740|Original|hCoV-19^^Michigan|Human|Michigan Department of Health and Human Services|Michigan Department of Health and Human Services|Blankenship|USA
>Spike|hCoV-19/Luxembourg/LNS7202999/2020|2020-11-06|EPI_ISL_744535|Original|hCoV-19^^Luxembourg|Human|Laboratoire national de sant\u00e9|Laboratoire national de sant\u00e9|Wienecke-Baldacchino|Luxembourg
>Spike|hCoV-19/USA/AZ-TG666097/2020|2020-05-02|EPI_ISL_696187|Original|hCoV-19^^Arizona|Human|Sonora Quest Laboratories|TGen North|French|USA
>Spike|hCoV-19/Wales/PHWC-48E464/2020|2020-11-12|EPI_ISL_680861|Original|hCoV-19^^Wales|Human|Wales Specialist Virology Centre Sequencing lab|COVID-19 Genomics UK (COG-UK) Consortium|Moore|United Kingdom
>Spike|hCoV-19/England/LIVE-A8055/2020|2020-03-26|EPI_ISL_517107|Original|hCoV-19^^England|Human|Liverpool Clinical Laboratories|COVID-19 Genomics UK (COG-UK) Consortium|Haldenby|United Kingdom
>Spike|hCoV-19/SaudiArabia/KAUST-MADINAH1112/2020|2020-06-11|EPI_ISL_513116|Original|hCoV-19^^Madinah|Human|Pathogen Genomics Lab King Abdullah University of Science and Technology(KAUST)|Pathogen Genomics Lab King Abdullah University of Science and Technology(KAUST)|Mohamed Ghazzali|Saudi Arabia
>Spike|hCoV-19/Japan/PG-6763/2020|2020-08-00|EPI_ISL_687873|Original|hCoV-19^^Japan|Human|Pathogen Genomics Center|Pathogen Genomics Center|Sekizuka|Japan
>Spike|hCoV-19/Shanghai/SH0022/2020|2020-01-30|EPI_ISL_416331|Original|hCoV-19^^Shanghai|Human|Shanghai Public Health Clinical Center|National Research Center for Translational Medicine (Shanghai)|Zhang|China
>Spike|hCoV-19/England/LIVE-DB7CDA/2020|2020-10-28|EPI_ISL_651155|Original|hCoV-19^^England|Human|Liverpool Clinical Laboratories|COVID-19 Genomics UK (COG-UK) Consortium|Haldenby|United Kingdom

>Spike|hCoV-19/Australia/NSW771/2020|2020-07-30|EPI_ISL_513413|Original|hCoV-19^^New South Wales|Human|4Cyte Pathology|NSW Health Pathology - Institute of Clinical Pathology and Medical Research; Westmead Hospital; University of Sydney|Gall|Australia
>Spike|hCoV-19/Scotland/QEUH-BEAF13/2020|2020-12-04|EPI_ISL_710626|Original|hCoV-19^^Scotland|Human|Lighthouse Lab in Glasgow|Wellcome Sanger Institute for the COVID-19 Genomics UK (COG-UK) Consortium|Davies|United Kingdom
>Spike|hCoV-19/Denmark/DCGC-6532/2020|2020-10-05|EPI_ISL_619432|Original|hCoV-19^^Hovedstaden|Human|Department of Virus and Microbiological Special Diagnostics|Albertsen lab|Michaelsen|Denmark
>Spike|hCoV-19/England/LIVE-DB2DCD/2020|2020-10-31|EPI_ISL_638011|Original|hCoV-19^^England|Human|Liverpool Clinical Laboratories|COVID-19 Genomics UK (COG-UK) Consortium|Haldenby|United Kingdom
>Spike|hCoV-19/Luxembourg/LNS8678541/2020|2020-11-20|EPI_ISL_744786|Original|hCoV-19^^Luxembourg|Human|Laboratoire national de sant\u00e9|Laboratoire national de sant\u00e9|Wienecke-Baldacchino|Luxembourg
>Spike|hCoV-19/Canada/ON-PHL-20-00053/2020|2020-00-00|EPI_ISL_548730|Original|hCoV-19^^Ontario|Human|Public Health Ontario Laboratory|Public Health Ontario Laboratory|Guthrie|Canada
>Spike|hCoV-19/USA/LA-EVTL069/2020|2020-04-30|EPI_ISL_451230|Original|hCoV-19^^Louisiana|Human|LSUHS Emerging Viral Threat Laboratory|Microbial Genome Sequencing Center|Kamil|USA
>Spike|hCoV-19/England/QEUH-A7637B/2020|2020-10-13|EPI_ISL_613319|Original|hCoV-19^^England|Human|Queens Medical Centre|COVID-19 Genomics UK (COG-UK) Consortium|Loose|United Kingdom
>Spike|hCoV-19/England/QEUH-BC44F2/2020|2020-11-25|EPI_ISL_703382|Original|hCoV-19^^England|Human|Lighthouse Lab in Glasgow|Wellcome Sanger Institute for the COVID-19 Genomics UK (COG-UK) Consortium|Davies|United Kingdom
>Spike|hCoV-19/England/MILK-9A6E45/2020|2020-09-01|EPI_ISL_550006|Original|hCoV-19^^England|Human|Lighthouse Lab in Milton Keynes|Wellcome Sanger Institute for the COVID-19 Genomics UK (COG-UK) consortium|Davies|United Kingdom
>Spike|hCoV-19/Australia/TAS49/2020|2020-03-21|EPI_ISL_522724|Original|hCoV-19^^Tasmania|Human|Royal Hobart Hospital Microbiology Department|MDU-PHL|Schultz|Australia
>Spike|hCoV-19/USA/WI-UW-189/2020|2020-03-23|EPI_ISL_428285|Original|hCoV-19^^Wisconsin|Human|University of Wisconsin-Madison AIDS Vaccine Research Laboratories|University of Wisconsin-Madison AIDS Vaccine Research Laboratories|Moreno|USA
>Spike|hCoV-19/Switzerland/BE-ETHZ-240057/2020|2020-08-12|EPI_ISL_523897|Original|hCoV-19^^Bern|Human|Viollier AG|Department of Biosystems Science and Engineering|Nadeau|Switzerland
>Spike|hCoV-19/England/CAMC-9EBDDE/2020|2020-09-23|EPI_ISL_584423|Original|hCoV-19^^England|Human|Queens Medical Centre|COVID-19 Genomics UK (COG-UK) Consortium|Loose|United Kingdom
>Spike|hCoV-19/Spain/AR-IBV-98010607/2020|2020-10-02|EPI_ISL_722219|Original|hCoV-19^^Aragon|Human|Servicio de Microbiolog\u00eda|SeqCOVID-SPAIN consortium/IBV(CSIC)|Comas|Spain
>Spike|hCoV-19/USA/CA-ALSR-1162/2020|2020-04-05|EPI_ISL_494405|Original|hCoV-19^^California|Human|San Diego County Public Health Laboratory|Andersen lab at Scripps Research|Gangavarapu|USA
>Spike|hCoV-19/Wales/PHWC-49C21E/2020|2020-12-07|EPI_ISL_742526|Original|hCoV-19^^Wales|Human|Wales Specialist Virology Centre Sequencing lab|COVID-19 Genomics UK (COG-UK) Consortium|Moore|United Kingdom
>Spike|hCoV-19/Wales/PHWC-490E43/2020|2020-11-18|EPI_ISL_706577|Original|hCoV-19^^Wales|Human|Originating lab|COVID-19 Genomics UK (COG-UK) Consortium|Moore|United Kingdom
>Spike|hCoV-19/Iceland/306/2020|2020-03-18|EPI_ISL_417635|Original|hCoV-19^^Reykjavik|Human|The National University Hospital of Iceland|deCODE genetics|J\u00f3nsson|Iceland

>Spike|hCoV-19/Spain/VC-FISABIO-121/2020|2020-03-13|EPI_ISL_436216|Original|hCoV-19^^Comunitat Valenciana|Human|Servicio de Microbiolog\u00eda. Consorcio Hospital General Universitario de Valencia|Sequencing and Bioinformatics Service and Molecular Epidemiology Research Group. FISABIO-Public Health|Gonzalez-Candelas|Spain
>Spike|hCoV-19/Wales/PHWC-2530C/2020|2020-03-23|EPI_ISL_432207|Original|hCoV-19^^Wales|Human|Wales Specialist Virology Centre|Public Health Wales Microbiology Cardiff|Moore|United Kingdom
>Spike|hCoV-19/Australia/VIC9505/2020|2020-08-14|EPI_ISL_565578|Original|hCoV-19^^Victoria|Human|Microbiological Diagnostic Unit - Public Health Laboratory (MDU-PHL)|MDU-PHL|Schultz|Australia
>Spike|hCoV-19/England/QEUH-9E379C/2020|2020-09-24|EPI_ISL_584871|Original|hCoV-19^^England|Human|Northumbria University / South Tees Hospitals NHS Foundation Trust / North Cumbria Integrated Care NHS Foundation Trust / North Tees and Hartlepool NHS Foundation Trust / Newcastle Hospitals NHS Foundation Trust|COVID-19 Genomics UK (COG-UK) Consortium|Bashton|United Kingdom
>Spike|hCoV-19/Wales/PHWC-15D759/2020|2020-05-13|EPI_ISL_472742|Original|hCoV-19^^Wales|Human|Wales Specialist Virology Centre Sequencing lab|COVID-19 Genomics UK (COG-UK) Consortium|Moore|United Kingdom
>Spike|hCoV-19/Spain/MD-IBV-99008794/2020|2020-09-21|EPI_ISL_654145|Original|hCoV-19^^Madrid|Human|Hospital General Universitario Gregorio Mara\u00f1\u00f3n|SeqCOVID-SPAIN consortium/IBV(CSIC)|Comas|Spain
>Spike|hCoV-19/Denmark/DCGC-5718/2020|2020-09-21|EPI_ISL_621223|Original|hCoV-19^^Sjaelland|Human|Department of Virus and Microbiological Special Diagnostics|Albertsen lab|Michaelsen|Denmark
>Spike|hCoV-19/Wales/PHWC-48D7D5/2020|2020-11-07|EPI_ISL_680680|Original|hCoV-19^^Wales|Human|Wales Specialist Virology Centre Sequencing lab|COVID-19 Genomics UK (COG-UK) Consortium|Moore|United Kingdom
>Spike|hCoV-19/Northern Ireland/NIRE-234414/2020|2020-10-15|EPI_ISL_680385|Original|hCoV-19^^Northern Ireland|Human|Regional Virus Laboratory|COVID-19 Genomics UK (COG-UK) Consortium|Fairley|United Kingdom
>Spike|hCoV-19/Denmark/DCGC-20069/2020|2020-12-07|EPI_ISL_747964|Original|hCoV-19^^Syddanmark|Human|Department of Virus and Microbiological Special Diagnostics|Albertsen Lab|Michaelsen|Denmark
>Spike|hCoV-19/USA/TX-DSHS-1919/2020|2020-10-22|EPI_ISL_751529|Original|hCoV-19^^Texas|Human|Texas Department of State Health Services|Texas Department of State Health Services|Rahman|USA
>Spike|hCoV-19/Wales/PHWC-33B9A/2020|2020-04-20|EPI_ISL_446796|Original|hCoV-19^^Wales|Human|Wales Specialist Virology Centre|Public Health Wales Microbiology Cardiff|Moore|United Kingdom
>Spike|hCoV-19/England/QEUH-AE4B9E/2020|2020-10-26|EPI_ISL_633290|Original|hCoV-19^^England|Human|Lighthouse Lab in Glasgow|Wellcome Sanger Institute for the COVID-19 Genomics UK (COG-UK) consortium|Davies|United Kingdom
>Spike|hCoV-19/Canada/BC-BCCDC-8412/2020|2020-10-00|EPI_ISL_740784|Original|hCoV-19^^British Columbia|Human|BCCDC Public Health Laboratory|BCCDC Public Health Laboratory|Prystajecky|Canada
>Spike|hCoV-19/USA/MA-MGH-00143/2020|2020-03-26|EPI_ISL_460114|Original|hCoV-19^^Massachusetts|Human|Massachusetts General Hospital|Infectious Disease Program|Lemieux|USA
>Spike|hCoV-19/Scotland/EDB612/2020|2020-04-02|EPI_ISL_433215|Original|hCoV-19^^Scotland|Human|Virology Department|COVID-19 Genomics UK (COG-UK) Consortium|Williams|United Kingdom
>Spike|hCoV-19/England/20169043104/2020|2020-04-19|EPI_ISL_465259|Original|hCoV-19^^England|Human|Respiratory Virus Unit|Respiratory Virus Unit|Platt|United Kingdom
>Spike|hCoV-19/Australia/VIC15199/2020|2020-08-11|EPI_ISL_593185|Original|hCoV-19^^Victoria|Human|Microbiological Diagnostic Unit - Public Health Laboratory (MDU-PHL)|MDU-PHL|Schultz|Australia
>Spike|hCoV-19/Iceland/416/2020|2020-03-19|EPI_ISL_424440|Original|hCoV-19^^Reykjavik|Human|The National University Hospital of Iceland|deCODE genetics|J\u00f3nsson|Iceland

>Spike|hCoV-19/Denmark/DCGC-15605/2020|2020-11-23|EPI_ISL_713180|Original|hCoV-19^^Hovedstaden|Human|Department of Virus and Microbiological Special Diagnostics|Albertsen Lab|Michaelsen|Denmark
>Spike|hCoV-19/France/PAC-IHU-0587/2020|2020-04-10|EPI_ISL_569605|Original|hCoV-19^^Provence-Alpes-C\u00f4te-d\u2019Azur|Human|MEPHI|MEPHI|Levasseur|France
>Spike|hCoV-19/Australia/VIC3403/2020|2020-07-11|EPI_ISL_521082|Original|hCoV-19^^Victoria|Human|Microbiological Diagnostic Unit - Public Health Laboratory (MDU-PHL)|MDU-PHL|Schultz|Australia
>Spike|hCoV-19/England/ALDP-9D90DA/2020|2020-09-18|EPI_ISL_576697|Original|hCoV-19^^England|Human|Oxford Viromics|COVID-19 Genomics UK (COG-UK) Consortium|Golubchik|United Kingdom
>Spike|hCoV-19/Australia/VIC2752/2020|2020-07-04|EPI_ISL_521587|Original|hCoV-19^^Victoria|Human|Microbiological Diagnostic Unit - Public Health Laboratory (MDU-PHL)|MDU-PHL|Schultz|Australia
>Spike|hCoV-19/England/ALDP-9CBE68/2020|2020-09-16|EPI_ISL_575427|Original|hCoV-19^^England|Human|Lighthouse Lab in Alderley Park|Wellcome Sanger Institute for the COVID-19 Genomics UK (COG-UK) consortium|Davies|United Kingdom
>Spike|hCoV-19/Thailand/Bangkok-CONI-0203/2020|2020-03-17|EPI_ISL_512853|Original|hCoV-19^^Bangkok|Human|Ramathibodi Hospital|COVID-19 Network Investigations (CONI) Alliance|Batty|Thailand
>Spike|hCoV-19/England/MILK-A17E4E/2020|2020-10-01|EPI_ISL_594993|Original|hCoV-19^^England|Human|Northumbria University / South Tees Hospitals NHS Foundation Trust / North Cumbria Integrated Care NHS Foundation Trust / North Tees and Hartlepool NHS Foundation Trust / Newcastle Hospitals NHS Foundation Trust|COVID-19 Genomics UK (COG-UK) Consortium|Bashton|United Kingdom
>Spike|hCoV-19/Scotland/QEUH-A0C2B9/2020|2020-10-03|EPI_ISL_599316|Original|hCoV-19^^Scotland|Human|Lighthouse Lab in Glasgow|Wellcome Sanger Institute for the COVID-19 Genomics UK (COG-UK) consortium|Davies|United Kingdom
>Spike|hCoV-19/Denmark/DCGC-20768/2020|2020-12-21|EPI_ISL_759353|Original|hCoV-19^^Hovedstaden|Human|Department of Virus and Microbiological Special Diagnostics|Albertsen Lab|Michaelsen|Denmark
>Spike|hCoV-19/England/QEUH-961620/2020|2020-07-31|EPI_ISL_537033|Original|hCoV-19^^England|Human|Lighthouse Lab in Glasgow|Wellcome Sanger Institute for the COVID-19 Genomics UK (COG-UK) consortium|Davies|United Kingdom
>Spike|hCoV-19/USA/MI-MDHHS-SC21683/2020|2020-06-26|EPI_ISL_516410|Original|hCoV-19^^Michigan|Human|Michigan Department of Health and Human Services|Michigan Department of Health and Human Services|Blankenship|USA
>Spike|hCoV-19/England/MILK-ABB85D/2020|2020-10-21|EPI_ISL_631021|Original|hCoV-19^^England|Human|Lighthouse Lab in Milton Keynes|Wellcome Sanger Institute for the COVID-19 Genomics UK (COG-UK) consortium|Davies|United Kingdom
>Spike|hCoV-19/USA/CA-QDX-2013/2020|2020-03-17|EPI_ISL_604307|Original|hCoV-19^^California|Human|Quest Diagnostics|Quest Diagnostics|Kagan|USA
>Spike|hCoV-19/India/TG-CCMB-AB182/2020|2020-08-14|EPI_ISL_539709|Original|hCoV-19^^Telangana|Human|CSIR-Centre for Cellular and Molecular Biology|CSIR-Centre for Cellular and Molecular Biology|Mukherjee|India
>Spike|hCoV-19/Japan/IC-0259/2020|2020-10-00|EPI_ISL_667688|Original|hCoV-19^^Japan|Human|Pathogen Genomics Center|Pathogen Genomics Center|Sekizuka|Japan
>Spike|hCoV-19/USA/CA-ALSR-4435/2020|2020-09-25|EPI_ISL_649028|Original|hCoV-19^^California|Human|San Diego County Public Health Laboratory|Andersen lab at Scripps Research|Gangavarapu|USA
>Spike|hCoV-19/USA/TX-HMH-MCoV-12082/2020|2020-09-20|EPI_ISL_789868|Original|hCoV-19^^Texas|Human|Houston Methodist Hospital|Houston Methodist Hospital|Long|USA
>Spike|hCoV-19/England/CAMC-B9811E/2020|2020-11-15|EPI_ISL_674926|Original|hCoV-19^^England|Human|Lighthouse Lab in Cambridge|Wellcome Sanger Institute for the COVID-19 Genomics UK (COG-UK) Consortium|Davies|United Kingdom
>Spike|hCoV-19/England/NORW-21FB83/2020|2020-12-14|EPI_ISL_741465|Original|hCoV-19^^England|Human|Quadram Institute Bioscience|COVID-19 Genomics UK (COG-UK) Consortium|Alikhan|United Kingdom

>Spike|hCoV-19/Singapore/1253/2020|2020-05-02|EPI_ISL_648671|Original|hCoV-
19^^Singapore|Human|Department of Laboratory Medicine|Department of Laboratory
Medicine|Chen|Singapore
>Spike|hCoV-19/England/ALDP-A2E06C/2020|2020-10-06|EPI_ISL_586681|Original|hCoV-
19^^England|Human|Lighthouse Lab in Alderley Park|Wellcome Sanger Institute for the
COVID-19 Genomics UK (COG-UK) consortium|Davies|United Kingdom
>Spike|hCoV-19/USA/WI-UW-1118/2020|2020-07-28|EPI_ISL_516543|Original|hCoV-
19^^Wisconsin|Human|University of Wisconsin-Madison AIDS Vaccine Research
Laboratories|University of Wisconsin-Madison AIDS Vaccine Research
Laboratories|Baczenas|USA
>Spike|hCoV-19/England/MILK-976567/2020|2020-08-20|EPI_ISL_575926|Original|hCoV-
19^^England|Human|Lighthouse Lab in Milton Keynes|Wellcome Sanger Institute for the
COVID-19 Genomics UK (COG-UK) consortium|Davies|United Kingdom
>Spike|hCoV-19/Wales/PHWC-16E1EE/2020|2020-10-03|EPI_ISL_585682|Original|hCoV-
19^^Wales|Human|Wales Specialist Virology Centre Sequencing lab|COVID-19 Genomics UK
(COG-UK) Consortium|Moore|United Kingdom
>Spike|hCoV-19/Spain/MD-IBV-99012341/2020|2020-11-20|EPI_ISL_779990|Original|hCoV-
19^^Madrid|Human|Hospital General Universitario Gregorio Mara\u00f1\u00f3n|SeqCOVID-
SPAIN consortium/IBV(CSIC)|Comas|Spain
>Spike|hCoV-19/Denmark/DCGC-1382/2020|2020-03-30|EPI_ISL_622233|Original|hCoV-
19^^Sjaelland|Human|Department of Virus and Microbiological Special
Diagnostics|Albertsen lab|Michaelsen|Denmark
>Spike|hCoV-19/Singapore/678/2020|2020-06-23|EPI_ISL_498576|Original|hCoV-
19^^Singapore|Human|National Public Health Laboratory|National Public Health
Laboratory|Mak|Singapore
>Spike|hCoV-19/Wales/PHWC-1667C6/2020|2020-05-27|EPI_ISL_494040|Original|hCoV-
19^^Wales|Human|Originating lab|COVID-19 Genomics UK (COG-UK) Consortium|Moore|United
Kingdom
>Spike|hCoV-19/Spain/RI-IBV-99005930/2020|2020-03-27|EPI_ISL_537465|Original|hCoV-
19^^La Rioja|Human|Centro de Investigaci\u00f3n Biom\u00e9dica de La Rioja - Hospital
San Pedro Logro\u00f1o|SeqCOVID-SPAIN consortium/IBV(CSIC)|Comas|Spain
>Spike|hCoV-19/Wales/PHWC-4800F5/2020|2020-10-22|EPI_ISL_612852|Original|hCoV-
19^^Wales|Human|Wales Specialist Virology Centre Sequencing lab|COVID-19 Genomics UK
(COG-UK) Consortium|Moore|United Kingdom
>Spike|hCoV-19/United Arab Emirates/0439/2020|2020-05-
15|EPI_ISL_698613|Original|hCoV-19^^Abu Dhabi|Human|Group 42 (G42) Healthcare|G42
Healthcare|Ogrodzki|United Arab Emirates
>Spike|hCoV-19/Germany/NW-UBI-38/2020|2020-06-18|EPI_ISL_631292|Original|hCoV-
19^^North Rhine-Westphalia|Human|MVZ DIAMEDIS Diagnostische Medizin Sennestadt
GmbH|Bielefeld University|Brandt|Germany
>Spike|hCoV-19/England/CAMC-BBBD20/2020|2020-11-21|EPI_ISL_705567|Original|hCoV-
19^^England|Human|Quadram Institute Bioscience|COVID-19 Genomics UK (COG-UK)
Consortium|Alikhan|United Kingdom
>Spike|hCoV-19/USA/WI-GMF-00498/2020|2020-03-31|EPI_ISL_422453|Original|hCoV-
19^^Wisconsin|Human|Gundersen Molecular Diagnostics Laboratory|Kabara Cancer Research
Institute|Kenny|USA
>Spike|hCoV-19/England/ALDP-B5C161/2020|2020-11-08|EPI_ISL_655953|Original|hCoV-
19^^England|Human|Lighthouse Lab in Alderley Park|Wellcome Sanger Institute for the
COVID-19 Genomics UK (COG-UK) Consortium|Davies|United Kingdom
>Spike|hCoV-19/Denmark/DCGC-3097/2020|2020-08-31|EPI_ISL_615913|Original|hCoV-
19^^Hovedstaden|Human|Department of Virus and Microbiological Special
Diagnostics|Albertsen lab|Michaelsen|Denmark
>Spike|hCoV-19/England/CAMB-77E82/2020|2020-04-01|EPI_ISL_441836|Original|hCoV-
19^^England|Human|Department of Pathology|Wellcome Sanger Institute for the COVID-19
Genomics UK (COG-UK) consortium|Amato|United Kingdom
>Spike|hCoV-19/USA/WI-WSLH-200377/2020|2020-04-15|EPI_ISL_578546|Original|hCoV-
19^^Wisconsin|Human|Wisconsin State Laboratory of Hygiene Communicable Disease
Division|Wisconsin State Laboratory of Hygiene Communicable Disease
Division|Florek|USA

>Spike|hCoV-19/England/LIVE-A7609/2020|2020-03-28|EPI_ISL_517047|Original|hCoV-19^^England|Human|Liverpool Clinical Laboratories|COVID-19 Genomics UK (COG-UK) Consortium|Haldenby|United Kingdom
>Spike|hCoV-19/Norway/3197/2020|2020-08-19|EPI_ISL_549029|Original|hCoV-19^^Viken|Human|Oslo University Hospital|Norwegian Institute of Public Health|Kopperud Riis|Norway
>Spike|hCoV-19/Canada/UN-CRCHUM-PreFreeze-9/2020|2020-09-04|EPI_ISL_667766|Original|hCoV-19^^Canada|Human|Microbiology|Microbiology|Benoit,P.|Canada
>Spike|hCoV-19/England/MILK-B87929/2020|2020-11-13|EPI_ISL_724813|Original|hCoV-19^^England|Human|Oxford Viromics|COVID-19 Genomics UK (COG-UK) Consortium|Golubchik|United Kingdom
>Spike|hCoV-19/Scotland/EDB6727/2020|2020-04-13|EPI_ISL_491555|Original|hCoV-19^^Scotland|Human|Virology Department|Wellcome Sanger Institute for the COVID-19 Genomics UK (COG-UK) consortium|Davies|United Kingdom
>Spike|hCoV-19/Turkey/IST-GLAB-338/2020|2020-06-23|EPI_ISL_735354|Original|hCoV-19^^Istanbul|Human|Genomic Laboratory (GLAB) (Conjoint lab of Health Directorate of Istanbul and Istanbul Technical University)|Genomic Laboratory (GLAB)|Karacan|Turkey
>Spike|hCoV-19/USA/TX-HMH-MCoV-18258/2020|2020-11-17|EPI_ISL_784606|Original|hCoV-19^^Texas|Human|Houston Methodist Hospital|Houston Methodist Hospital|Long|USA
>Spike|hCoV-19/USA/NM-DOH-2020315506/2020|2020-08-27|EPI_ISL_732710|Original|hCoV-19^^New Mexico|Human|New Mexico Department of Health Scientific Laboratory|New Mexico Department of Health Scientific Laboratory|Johnson|USA
>Spike|hCoV-19/Netherlands/UT-EMC-61/2020|2020-07-31|EPI_ISL_523538|Original|hCoV-19^^Utrecht|Human|Dutch COVID-19 response team|Erasmus Medical Center|Oude Munnink|Netherlands
>Spike|hCoV-19/England/NOTT-1116FA/2020|2020-05-24|EPI_ISL_461911|Original|hCoV-19^^England|Human|Queens Medical Centre|COVID-19 Genomics UK (COG-UK) Consortium|Loose|United Kingdom
>Spike|hCoV-19/England/PORT-2D3471/2020|2020-04-14|EPI_ISL_535031|Original|hCoV-19^^England|Human|Centre for Enzyme Innovation|COVID-19 Genomics UK (COG-UK) Consortium|Robson|United Kingdom
>Spike|hCoV-19/USA/OR-OHSU-2325/2020|2020-08-10|EPI_ISL_667183|Original|hCoV-19^^Oregon|Human|OHSU Lab Services Molecular Microbiology Lab|Oregon SARS-CoV-2 Genome Sequencing Center|O'Roak|USA
>Spike|hCoV-19/Portugal/PT1993/2020|2020-11-14|EPI_ISL_732215|Original|hCoV-19^^Portugal|Human|Instituto Nacional de Saude (INSA) and Instituto Gulbenkian de Ciencia (IGC)|Instituto Nacional de Saude (INSA) and Instituto Gulbenkian de Ciencia (IGC)|Borges|Portugal
>Spike|hCoV-19/Scotland/QEUH-98FD27/2020|2020-08-27|EPI_ISL_568314|Original|hCoV-19^^Scotland|Human|Lighthouse Lab in Glasgow|Wellcome Sanger Institute for the COVID-19 Genomics UK (COG-UK) consortium|Davies|United Kingdom
>Spike|hCoV-19/England/OXON-AC9ED/2020|2020-04-07|EPI_ISL_448501|Original|hCoV-19^^England|Human|Oxford Viromics|COVID-19 Genomics UK (COG-UK) Consortium|Golubchik|United Kingdom
>Spike|hCoV-19/England/ALDP-9BCE00/2020|2020-09-09|EPI_ISL_573077|Original|hCoV-19^^England|Human|Oxford Viromics|COVID-19 Genomics UK (COG-UK) Consortium|Golubchik|United Kingdom
>Spike|hCoV-19/Scotland/EDB1463/2020|2020-04-11|EPI_ISL_433458|Original|hCoV-19^^Scotland|Human|Virology Department|COVID-19 Genomics UK (COG-UK) Consortium|Williams|United Kingdom
>Spike|hCoV-19/Wales/PHWC-33E64/2020|2020-04-15|EPI_ISL_446831|Original|hCoV-19^^Wales|Human|Wales Specialist Virology Centre|Public Health Wales Microbiology Cardiff|Moore|United Kingdom
>Spike|hCoV-19/England/OXON-B2AF0/2020|2020-04-13|EPI_ISL_534793|Original|hCoV-19^^England|Human|Oxford Viromics|COVID-19 Genomics UK (COG-UK) Consortium|Golubchik|United Kingdom
>Spike|hCoV-19/England/QEUH-9BA048/2020|2020-09-05|EPI_ISL_573572|Original|hCoV-19^^England|Human|Oxford Viromics|COVID-19 Genomics UK (COG-UK) Consortium|Golubchik|United Kingdom

>Spike|hCoV-19/Belgium/rega-0508647/2020|2020-05-08|EPI_ISL_734716|Original|hCoV-19^^Houthalen-Helchteren|Human|UZ Leuven|KU Leuven|Maes|Belgium
>Spike|hCoV-19/USA/NY-NYCPHL-001546/2020|2020-12-02|EPI_ISL_745461|Original|hCoV-19^^New York|Human|DOHMH Corona|New York City Public Health Laboratory|Wang|USA
>Spike|hCoV-19/USA/TX-DSHS-1521/2020|2020-06-23|EPI_ISL_683436|Original|hCoV-19^^Texas|Human|Texas Department of State Health Services|Texas Department of State Health Services|Rahman|USA
>Spike|hCoV-19/USA/UT-0342/2020|2020-03-24|EPI_ISL_420816|Original|hCoV-19^^Utah|Human|Utah Public Health Laboratory|Utah Public Health Laboratory|Young|USA
>Spike|hCoV-19/Malaysia/IMR-WC9205/2020|2020-03-04|EPI_ISL_489992|Vero E6|hCoV-19^^Selangor|Human|Institute for Medical Research|Institute for Medical Research|Suppiah|Malaysia
>Spike|hCoV-19/England/MILK-619235/2020|2020-07-06|EPI_ISL_557816|Original|hCoV-19^^England|Human|Lighthouse Lab in Milton Keynes|Wellcome Sanger Institute for the COVID-19 Genomics UK (COG-UK) consortium|Davies|United Kingdom
>Spike|hCoV-19/Luxembourg/LNS2971553/2020|2020-08-12|EPI_ISL_740229|Original|hCoV-19^^Luxembourg|Human|Laboratoire national de sant\u00e9|Laboratoire national de sant\u00e9|Wienecke-Baldacchino|Luxembourg
>Spike|hCoV-19/England/MILK-B54F05/2020|2020-11-06|EPI_ISL_657944|Original|hCoV-19^^England|Human|Lighthouse Lab in Milton Keynes|Wellcome Sanger Institute for the COVID-19 Genomics UK (COG-UK) Consortium|Davies|United Kingdom
>Spike|hCoV-19/Canada/QC-LSPQ-L00219340/2020|2020-03-14|EPI_ISL_535792|Original|hCoV-19^^Quebec|Human|H\u00f4pital Fleurimont|Laboratoire de sant\u00e9 publique du Qu\u00e9bec|Moreira|Canada
>Spike|hCoV-19/England/20122031204/2020|2020-03-15|EPI_ISL_465749|Original|hCoV-19^^England|Human|Respiratory Virus Unit|Respiratory Virus Unit|Platt|United Kingdom
>Spike|hCoV-19/Japan/PG-3626/2020|2020-04-00|EPI_ISL_691879|Original|hCoV-19^^Japan|Human|Pathogen Genomics Center|Pathogen Genomics Center|Sekizuka|Japan
>Spike|hCoV-19/USA/OR-OHSU-2817/2020|2020-09-02|EPI_ISL_667325|Original|hCoV-19^^Oregon|Human|OHSU Lab Services Molecular Microbiology Lab|Oregon SARS-CoV-2 Genome Sequencing Center|O'Roak|USA
>Spike|hCoV-19/USA/CA-CZB-15229/2020|2020-11-27|EPI_ISL_739221|Original|hCoV-19^^California|Human|Santa Clara County Public Health Laboratory|Chan-Zuckerberg Biohub|Kamm|USA
>Spike|hCoV-19/USA/CA-CZB-11250/2020|2020-05-02|EPI_ISL_738897|Original|hCoV-19^^California|Human|Napa-Solano-Yolo- Marin County (NSYM) Public Health Laboratories|Chan-Zuckerberg Biohub|Kamm|USA
>Spike|hCoV-19/England/QEUH-A5E6CB/2020|2020-10-12|EPI_ISL_597484|Original|hCoV-19^^England|Human|Lighthouse Lab in Glasgow|Wellcome Sanger Institute for the COVID-19 Genomics UK (COG-UK) consortium|Davies|United Kingdom
>Spike|hCoV-19/USA/UT-UPHL-2012463625/2020|2020-11-20|EPI_ISL_746963|Original|hCoV-19^^Utah|Human|Utah Public Health Laboratory|Utah Public Health Laboratory|Gallagher|USA
>Spike|hCoV-19/England/CAMC-9492B1/2020|2020-07-19|EPI_ISL_556817|Original|hCoV-19^^England|Human|Lighthouse Lab in Cambridge|Wellcome Sanger Institute for the COVID-19 Genomics UK (COG-UK) consortium|Davies|United Kingdom
>Spike|hCoV-19/Scotland/QEUH-B9E682/2020|2020-11-18|EPI_ISL_757224|Original|hCoV-19^^Scotland|Human|Lighthouse Lab in Glasgow|Wellcome Sanger Institute for the COVID-19 Genomics UK (COG-UK) Consortium|Davies|United Kingdom
>Spike|hCoV-19/England/20144021404/2020|2020-03-29|EPI_ISL_423514|Original|hCoV-19^^England|Human|Respiratory Virus Unit|Respiratory Virus Unit|Platt|United Kingdom
>Spike|hCoV-19/England/CAMB-76B40/2020|2020-03-30|EPI_ISL_438372|Original|hCoV-19^^England|Human|Department of Pathology|Wellcome Sanger Institute for the COVID-19 Genomics UK (COG-UK) consortium|Amato|United Kingdom
>Spike|hCoV-19/England/LEED-2A8CD7/2020|2020-03-12|EPI_ISL_539144|Original|hCoV-19^^England|Human|Leeds Teaching Hospitals NHS Trust and Public Health England|Wellcome Sanger Institute for the COVID-19 Genomics UK (COG-UK) consortium|Davies|United Kingdom
>Spike|hCoV-19/Peru/LAM-HRL-223/2020|2020-04-08|EPI_ISL_593775|Original|hCoV-19^^Lambayeque|Human|HOSPITAL REGIONAL LAMBAYEQUE|GENOMA MAYOR|Mechan Llontop|Peru

>Spike|hCoV-19/India/GJ-GBRC176/2020|2020-06-05|EPI_ISL_467040|Original|hCoV-19^^Gujarat|Human|Government Medical College|Gujarat Biotechnology Research Centre|Joshi|India
>Spike|hCoV-19/DRC/2644/2020|2020-04-15|EPI_ISL_437342|Original|hCoV-19^^Kinshasa|Human|Viral Respiratory Lab|Pathogen Sequencing Lab|Muyembe|Democratic Republic of the Congo
>Spike|hCoV-19/Spain/AN-IBV-002859/2020|2020-03-13|EPI_ISL_474858|Original|hCoV-19^^Andalucia|Human|Hospital Universitario Virgen de las Nieves de Granada-SAS|SeqCOVID-SPAIN consortium/IBV(CSIC)|Comas|Spain
>Spike|hCoV-19/Japan/PG-6305/2020|2020-08-00|EPI_ISL_689416|Original|hCoV-19^^Japan|Human|Pathogen Genomics Center|Pathogen Genomics Center|Sekizuka|Japan
>Spike|hCoV-19/USA/TX-HMH-MCoV-8520/2020|2020-07-01|EPI_ISL_780602|Original|hCoV-19^^Texas|Human|Houston Methodist Hospital|Houston Methodist Hospital|Long|USA
>Spike|hCoV-19/Brazil/RJ-INCA-C38/2020|2020-04-17|EPI_ISL_513546|Original|hCoV-19^^Rio de Janeiro|Human|Programa de Oncovirologia|Programa de Oncovirologia|Ramos Goes|Brazil
>Spike|hCoV-19/England/NORT-281DD1/2020|2020-03-24|EPI_ISL_478484|Original|hCoV-19^^England|Human|Northumbria University / South Tees Hospitals NHS Foundation Trust / North Cumbria Integrated Care NHS Foundation Trust / North Tees and Hartlepool NHS Foundation Trust / Newcastle Hospitals NHS Foundation Trust|COVID-19 Genomics UK (COG-UK) Consortium|Bashton|United Kingdom
>Spike|hCoV-19/England/PORT-2EE7A7/2020|2020-12-21|EPI_ISL_763608|Original|hCoV-19^^England|Human|Centre for Enzyme Innovation|COVID-19 Genomics UK (COG-UK) Consortium|Robson|United Kingdom
>Spike|hCoV-19/Canada/NB_260/2020|2020-04-05|EPI_ISL_481751|Original|hCoV-19^^New Brunswick|Human|Dr. Georges-L.-Dumont University Hospital Centre|National Microbiology Laboratory|Grudeski|Canada
>Spike|hCoV-19/USA/AZ-TG588256/2020|2020-08-06|EPI_ISL_695634|Original|hCoV-19^^Arizona|Human|TGen North|TGen North|French|USA
>Spike|hCoV-19/Australia/TAS243/2020|2020-04-15|EPI_ISL_522671|Original|hCoV-19^^Tasmania|Human|Royal Hobart Hospital Microbiology Department|MDU-PHL|Schultz|Australia
>Spike|hCoV-19/England/CAMB-7A7DF/2020|2020-04-02|EPI_ISL_441294|Original|hCoV-19^^England|Human|Department of Pathology|Wellcome Sanger Institute for the COVID-19 Genomics UK (COG-UK) consortium|Amato|United Kingdom
>Spike|hCoV-19/Spain/MD-IBV-006384/2020|2020-03-26|EPI_ISL_510113|Original|hCoV-19^^Madrid|Human|Hospital General Universitario Gregorio Mara\u00f1\u00f3n|SeqCOVID-SPAIN consortium/IBV(CSIC)|Comas|Spain
>Spike|hCoV-19/England/LOND-1262641/2020|2020-09-28|EPI_ISL_594868|Original|hCoV-19^^England|Human|University College London|COVID-19 Genomics UK (COG-UK) Consortium|Roy|United Kingdom
>Spike|hCoV-19/USA/WI-UW-2158/2020|2020-10-15|EPI_ISL_677562|Original|hCoV-19^^Wisconsin|Human|University of Wisconsin-Madison AIDS Vaccine Research Laboratories|University of Wisconsin-Madison AIDS Vaccine Research Laboratories|Baczenas|USA
>Spike|hCoV-19/USA/TX-HMH-MCoV-11822/2020|2020-09-14|EPI_ISL_790116|Original|hCoV-19^^Texas|Human|Houston Methodist Hospital|Houston Methodist Hospital|Long|USA
>Spike|hCoV-19/Wales/PHWC-48009B/2020|2020-10-17|EPI_ISL_612847|Original|hCoV-19^^Wales|Human|Wales Specialist Virology Centre Sequencing lab|COVID-19 Genomics UK (COG-UK) Consortium|Moore|United Kingdom
>Spike|hCoV-19/England/MILK-A7C7A6/2020|2020-10-16|EPI_ISL_607311|Original|hCoV-19^^England|Human|Lighthouse Lab in Milton Keynes|Wellcome Sanger Institute for the COVID-19 Genomics UK (COG-UK) consortium|Davies|United Kingdom
>Spike|hCoV-19/England/EXET-13EDE3/2020|2020-12-14|EPI_ISL_763555|Original|hCoV-19^^England|Human|University of Exeter|COVID-19 Genomics UK (COG-UK) Consortium|Temperton|United Kingdom
>Spike|hCoV-19/Japan/PG-7825/2020|2020-08-00|EPI_ISL_686927|Original|hCoV-19^^Japan|Human|Pathogen Genomics Center|Pathogen Genomics Center|Sekizuka|Japan

>Spike|hCoV-19/Brazil/RS-00673/2020|2020-11-27|EPI_ISL_770614|Original|hCoV-19^^Rio Grande do Sul|Human|Laborat\u00f3rio de Microbiologia Molecular - Universidade FEEVALE|Bioinformatics Laboratory / LNCC|Almeida|Brazil
>Spike|hCoV-19/England/ALDP-B261E8/2020|2020-11-04|EPI_ISL_646508|Original|hCoV-19^^England|Human|Lighthouse Lab in Alderley Park|Wellcome Sanger Institute for the COVID-19 Genomics UK (COG-UK) Consortium|Davies|United Kingdom
>Spike|hCoV-19/Japan/PG-7314/2020|2020-08-00|EPI_ISL_688417|Original|hCoV-19^^Japan|Human|Pathogen Genomics Center|Pathogen Genomics Center|Sekizuka|Japan
>Spike|hCoV-19/Spain/AN-IBV-001989/2020|2020-03-15|EPI_ISL_509624|Original|hCoV-19^^Andalucia|Human|Servicio de Microbiolog\u00eda. HRU de M\u00e1laga. Servicio Andaluz de Salud|SeqCOVID-SPAIN consortium/IBV(CSIC)|Comas|Spain
>Spike|hCoV-19/England/QEUH-9B855A/2020|2020-09-05|EPI_ISL_549664|Original|hCoV-19^^England|Human|Lighthouse Lab in Glasgow|Wellcome Sanger Institute for the COVID-19 Genomics UK (COG-UK) consortium|Davies|United Kingdom
>Spike|hCoV-19/Spain/MD-IBV-99012332/2020|2020-11-20|EPI_ISL_779985|Original|hCoV-19^^Madrid|Human|Hospital General Universitario Gregorio Mara\u00f1\u00f3n|SeqCOVID-SPAIN consortium/IBV(CSIC)|Comas|Spain
>Spike|hCoV-19/USA/VA-DCLS-0049/2020|2020-03-30|EPI_ISL_426457|Original|hCoV-19^^Virginia|Human|Virginia DCLS|Virginia DCLS|St. Jacques|USA
>Spike|hCoV-19/Australia/VIC7548/2020|2020-00-00|EPI_ISL_564491|Original|hCoV-19^^Victoria|Human|Microbiological Diagnostic Unit - Public Health Laboratory (MDU-PHL)|MDU-PHL|Schultz|Australia
>Spike|hCoV-19/Pakistan/NIBD-03-KHI/2020|2020-06-10|EPI_ISL_709044|Orginal|hCoV-19^^Karachi|Human|National Institute of Blood Diseases (NIBD)|Genomics Lab NIBD|Ahmed|Pakistan
>Spike|hCoV-19/England/QEUH-B82830/2020|2020-11-14|EPI_ISL_661633|Original|hCoV-19^^England|Human|Lighthouse Lab in Glasgow|Wellcome Sanger Institute for the COVID-19 Genomics UK (COG-UK) Consortium|Davies|United Kingdom
>Spike|hCoV-19/England/QEUH-C937A7/2020|2020-12-15|EPI_ISL_777250|Original|hCoV-19^^England|Human|Lighthouse Lab in Glasgow|Wellcome Sanger Institute for the COVID-19 Genomics UK (COG-UK) Consortium|Davies|United Kingdom
>Spike|hCoV-19/England/ALDP-B5D0BE/2020|2020-11-09|EPI_ISL_678853|Original|hCoV-19^^England|Human|Department of Pathology|COVID-19 Genomics UK (COG-UK) Consortium|Goodfellow|United Kingdom
>Spike|hCoV-19/Portugal/PT1458/2020|2020-03-17|EPI_ISL_511417|Original|hCoV-19^^Portugal|Human|Instituto Nacional de Saude (INSA)|Instituto Nacional de Saude (INSA)|Borges|Portugal
>Spike|hCoV-19/England/CAMB-845F6/2020|2020-04-16|EPI_ISL_443520|Original|hCoV-19^^England|Human|Department of Pathology|Wellcome Sanger Institute for the COVID-19 Genomics UK (COG-UK) consortium|Amato|United Kingdom
>Spike|hCoV-19/Denmark/DCGC-13980/2020|2020-11-30|EPI_ISL_711492|Original|hCoV-19^^Syddanmark|Human|Department of Virus and Microbiological Special Diagnostics|Albertsen Lab|Michaelsen|Denmark
>Spike|hCoV-19/USA/TX-HMH-MCoV-15258/2020|2020-10-19|EPI_ISL_786742|Original|hCoV-19^^Texas|Human|Houston Methodist Hospital|Houston Methodist Hospital|Long|USA
>Spike|hCoV-19/Chile/RM-72843/2020|2020-05-22|EPI_ISL_746782|Original|hCoV-19^^Region Metropolitana de Santiago|Human|Genetica Molecular and Subdepartamento de Virologia ISP Chile|Instituto de Salud Publica de Chile|Tognarelli|Chile
>Spike|hCoV-19/Australia/VIC16692/2020|2020-08-04|EPI_ISL_649151|Original|hCoV-19^^Victoria|Human|Microbiological Diagnostic Unit - Public Health Laboratory (MDU-PHL)|Microbiological Diagnostic Unit - Public Health Laboratory (MDU-PHL)|Seemann|Australia
>Spike|hCoV-19/Japan/PG-6079/2020|2020-07-00|EPI_ISL_689191|Original|hCoV-19^^Japan|Human|Pathogen Genomics Center|Pathogen Genomics Center|Sekizuka|Japan
>Spike|hCoV-19/England/MILK-9C1F6F/2020|2020-09-10|EPI_ISL_566542|Original|hCoV-19^^England|Human|Lighthouse Lab in Milton Keynes|Wellcome Sanger Institute for the COVID-19 Genomics UK (COG-UK) consortium|Davies|United Kingdom
>Spike|hCoV-19/England/SHEF-CF340/2020|2020-03-28|EPI_ISL_432588|Original|hCoV-19^^England|Human|Virology Department|COVID-19 Genomics UK (COG-UK) Consortium|de Silva|United Kingdom

>Spike|hCoV-19/Scotland/EDB5500/2020|2020-04-27|EPI_ISL_487921|Original|hCoV-19^^Scotland|Human|Virology Department|Wellcome Sanger Institute for the COVID-19 Genomics UK (COG-UK) consortium|Davies|United Kingdom
>Spike|hCoV-19/India/GJ-GBRC119/2020|2020-04-27|EPI_ISL_455016|Original|hCoV-19^^Gujarat|Human|Pandit Deendayal Upadhyay Government Medical College|Gujarat Biotechnology Research Centre|Joshi|India
>Spike|hCoV-19/Iceland/52/2020|2020-03-05|EPI_ISL_417850|Original|hCoV-19^^Reykjavik|Human|The National University Hospital of Iceland|deCODE genetics|J\u00f3nsson|Iceland
>Spike|hCoV-19/USA/NV-NSPHL-A0182/2020|2020-05-19|EPI_ISL_515439|Original|hCoV-19^^Nevada|Human|Nevada State Public Health Laboratory|Nevada State Public Health Laboratory|Sevinsky|USA
>Spike|hCoV-19/Australia/NSW236/2020|2020-03-26|EPI_ISL_451502|Original|hCoV-19^^New South Wales|Human|Pathology West - NSW Health Pathology|NSW Health Pathology - Institute of Clinical Pathology and Medical Research; Westmead Hospital; University of Sydney|Gall|Australia
>Spike|hCoV-19/Kenya/C21582/2020|2020-06-22|EPI_ISL_568872|Original|hCoV-19^^Mombasa|Human|KEMRI-Wellcome Trust Research Programme/KEMRI-CGMR-C Kilifi|KEMRI-Wellcome Trust Research Programme/KEMRI-CGMR-C Kilifi|Githinji|Kenya
>Spike|hCoV-19/SaudiArabia/KAUST-JEDDAH754/2020|2020-04-02|EPI_ISL_512997|Original|hCoV-19^^Jeddah|Human|Pathogen Genomics Lab King Abdullah University of Science and Technology(KAUST)|Pathogen Genomics Lab King Abdullah University of Science and Technology(KAUST)|Mohamed Ghazzali|Saudi Arabia
>Spike|hCoV-19/USA/TX-HMH-MCoV-15388/2020|2020-10-22|EPI_ISL_786872|Original|hCoV-19^^Texas|Human|Houston Methodist Hospital|Houston Methodist Hospital|Long|USA
>Spike|hCoV-19/England/20139042704/2020|2020-03-28|EPI_ISL_420675|Original|hCoV-19^^England|Human|Respiratory Virus Unit|Respiratory Virus Unit|Platt|United Kingdom
>Spike|hCoV-19/USA/OR-OHSU-0043/2020|2020-04-20|EPI_ISL_437525|Original|hCoV-19^^Oregon|Human|OHSU Lab Services Molecular Microbiology Lab|Oregon SARS-CoV-2 Genome Sequencing Center|O'Roak|USA
>Spike|hCoV-19/England/ALDP-A92607/2020|2020-10-19|EPI_ISL_610994|Original|hCoV-19^^England|Human|Lighthouse Lab in Alderley Park|Wellcome Sanger Institute for the COVID-19 Genomics UK (COG-UK) consortium|Davies|United Kingdom
>Spike|hCoV-19/USA/WA-S995/2020|2020-04-29|EPI_ISL_463485|Original|hCoV-19^^Washington|Human|Washington State Department of Health|Seattle Flu Study|Bedford|USA
>Spike|hCoV-19/USA/TX-HMH-2819/2020|2020-06-08|EPI_ISL_543795|Original|hCoV-19^^Texas|Human|Houston Methodist Hospital|Houston Methodist Hospital|Long|USA
>Spike|hCoV-19/England/QEUH-B68654/2020|2020-11-11|EPI_ISL_662259|Original|hCoV-19^^England|Human|Lighthouse Lab in Glasgow|Wellcome Sanger Institute for the COVID-19 Genomics UK (COG-UK) Consortium|Davies|United Kingdom
>Spike|hCoV-19/USA/AZ-TG670223/2020|2020-04-30|EPI_ISL_696406|Original|hCoV-19^^Arizona|Human|Sonora Quest Laboratories|TGen North|French|USA
>Spike|hCoV-19/USA/VA-DCLS-1307/2020|2020-07-23|EPI_ISL_572247|Original|hCoV-19^^Virginia|Human|Virginia DCLS|Virginia DCLS|St. Jacques|USA
>Spike|hCoV-19/Spain/CT-IBV-97010109/2020|2020-11-10|EPI_ISL_683607|Original|hCoV-19^^Catalunya|Human|Servicio de Microbiolog\u00eda|SeqCOVID-SPAIN consortium/IBV(CSIC)|Comas|Spain
>Spike|hCoV-19/England/NOTT-121590/2020|2020-12-17|EPI_ISL_741641|Original|hCoV-19^^England|Human|Queens Medical Centre|COVID-19 Genomics UK (COG-UK) Consortium|Loose|United Kingdom
>Spike|hCoV-19/England/ALDP-9E69DF/2020|2020-09-24|EPI_ISL_580558|Original|hCoV-19^^England|Human|Lighthouse Lab in Alderley Park|Wellcome Sanger Institute for the COVID-19 Genomics UK (COG-UK) consortium|Davies|United Kingdom
>Spike|hCoV-19/Sweden/20-50304/2020|2020-03-12|EPI_ISL_475131|Original|hCoV-19^^Ostergotland|Human|Orebro klinisk mikrobiologi|The Public Health Agency of Sweden|Svartstr\u00f6m|Sweden
>Spike|hCoV-19/Scotland/QEUH-A0CAA2/2020|2020-10-03|EPI_ISL_585615|Original|hCoV-19^^Scotland|Human|Quadram Institute Bioscience|COVID-19 Genomics UK (COG-UK) Consortium|Alikhan|United Kingdom

>Spike|hCoV-19/England/CAMB-1B86E6/2020|2020-10-27|EPI_ISL_650666|Original|hCoV-19^^England|Human|Department of Pathology|COVID-19 Genomics UK (COG-UK) Consortium|Goodfellow|United Kingdom
>Spike|hCoV-19/England/NORT-29B42A/2020|2020-05-31|EPI_ISL_493493|Original|hCoV-19^^England|Human|Northumbria University / South Tees Hospitals NHS Foundation Trust / North Cumbria Integrated Care NHS Foundation Trust / North Tees and Hartlepool NHS Foundation Trust / Newcastle Hospitals NHS Foundation Trust|COVID-19 Genomics UK (COG-UK) Consortium|Bashton|United Kingdom
>Spike|hCoV-19/USA/DE-DHSS-F1006556/2020|2020-11-12|EPI_ISL_766995|Original|hCoV-19^^Delaware|Human|Delaware Public Health Laboratory|Delaware Public Health Laboratory|Bajwa|USA
>Spike|hCoV-19/USA/MI-UM-MH27501/2020|2020-04-18|EPI_ISL_590749|Original|hCoV-19^^Michigan|Human|University of Michigan Clinical Microbiology Laboratory|Lauring Lab|Valesano|USA
>Spike|hCoV-19/Wales/PHWC-4876A1/2020|2020-10-30|EPI_ISL_639227|Original|hCoV-19^^Wales|Human|Wales Specialist Virology Centre Sequencing lab|COVID-19 Genomics UK (COG-UK) Consortium|Moore|United Kingdom
>Spike|hCoV-19/Wales/PHWC-49CF71/2020|2020-12-10|EPI_ISL_742711|Original|hCoV-19^^Wales|Human|Wales Specialist Virology Centre Sequencing lab|COVID-19 Genomics UK (COG-UK) Consortium|Moore|United Kingdom
>Spike|hCoV-19/USA/VA-DCLS-0422/2020|2020-04-29|EPI_ISL_477638|Original|hCoV-19^^Virginia|Human|Virginia DCLS|Virginia DCLS|St. Jacques|USA
>Spike|hCoV-19/England/C200042081/2020|2020-11-02|EPI_ISL_649806|Original|hCoV-19^^England|Human|Respiratory Virus Unit|COVID-19 Genomics UK (COG-UK) Consortium|Platt|United Kingdom
>Spike|hCoV-19/Wales/PHWC-49AC4F/2020|2020-12-03|EPI_ISL_727666|Original|hCoV-19^^Wales|Human|Originating lab|COVID-19 Genomics UK (COG-UK) Consortium|Moore|United Kingdom
>Spike|hCoV-19/USA/CA-CDPH097/2020|2020-08-17|EPI_ISL_754941|Original|hCoV-19^^California|Human|California Department of Public Health|California Department of Public Health|Bell|USA
>Spike|hCoV-19/South Africa/KRISP-K003684/2020|2020-10-05|EPI_ISL_602867|Original|hCoV-19^^KwaZulu-Natal|Human|NHLS-IALCH|KRISP|de Oliveira|South Africa
>Spike|hCoV-19/England/MILK-A0560F/2020|2020-09-30|EPI_ISL_600061|Original|hCoV-19^^England|Human|Lighthouse Lab in Milton Keynes|Wellcome Sanger Institute for the COVID-19 Genomics UK (COG-UK) consortium|Davies|United Kingdom
>Spike|hCoV-19/Wales/PHWC-49719E/2020|2020-11-26|EPI_ISL_726789|Original|hCoV-19^^Wales|Human|Originating lab|COVID-19 Genomics UK (COG-UK) Consortium|Moore|United Kingdom
>Spike|hCoV-19/Denmark/DCGC-9716/2020|2020-11-09|EPI_ISL_669125|Original|hCoV-19^^Nordjylland|Human|Department of Virus and Microbiological Special Diagnostics|Albertsen Lab|Michaelsen|Denmark
>Spike|hCoV-19/USA/NY-QDX-2292/2020|2020-03-15|EPI_ISL_604575|Original|hCoV-19^^New York|Human|Quest Diagnostics|Quest Diagnostics|Kagan|USA
>Spike|hCoV-19/England/20156047304/2020|2020-04-06|EPI_ISL_466323|Original|hCoV-19^^England|Human|Respiratory Virus Unit|Respiratory Virus Unit|Platt|United Kingdom
>Spike|hCoV-19/Denmark/DCGC-6113/2020|2020-09-21|EPI_ISL_621147|Original|hCoV-19^^Midtjylland|Human|Department of Virus and Microbiological Special Diagnostics|Albertsen lab|Michaelsen|Denmark
>Spike|hCoV-19/USA/UT-UPHL-03913/2020|2020-04-13|EPI_ISL_577601|Original|hCoV-19^^Utah|Human|Utah Public Health Laboratory|Utah Public Health Laboratory|Young|USA
>Spike|hCoV-19/Denmark/DCGC-3665/2020|2020-08-31|EPI_ISL_615438|Original|hCoV-19^^Hovedstaden|Human|Department of Virus and Microbiological Special Diagnostics|Albertsen lab|Michaelsen|Denmark
>Spike|hCoV-19/England/ALDP-B1B623/2020|2020-11-01|EPI_ISL_645589|Original|hCoV-19^^England|Human|Lighthouse Lab in Alderley Park|Wellcome Sanger Institute for the COVID-19 Genomics UK (COG-UK) Consortium|Davies|United Kingdom

>Spike|hCoV-19/Panama/329446/2020|2020-03-09|EPI_ISL_496630|Original|hCoV-19^^Colon|Human|Gorgas Memorial Laboratory of Health Studies|Gorgas Memorial Laboratory of Health Studies|Martinez|Panama

>Spike|hCoV-19/Australia/VIC11127/2020|2020-08-29|EPI_ISL_563259|Original|hCoV-19^^Victoria|Human|Microbiological Diagnostic Unit - Public Health Laboratory (MDU-PHL)|MDU-PHL|Schultz|Australia

>Spike|hCoV-19/USA/CO-CDPHE-2008070206/2020|2020-08-06|EPI_ISL_677256|Original|hCoV-19^^Colorado|Human|Colorado Department of Public Health and Environment|Colorado Department of Puplic Health and Environment|Bankers|USA

>Spike|hCoV-19/USA/TX-HMH-MCoV-14287/2020|2020-10-04|EPI_ISL_787572|Original|hCoV-19^^Texas|Human|Houston Methodist Hospital|Houston Methodist Hospital|Long|USA

>Spike|hCoV-19/England/QEUH-B31135/2020|2020-11-04|EPI_ISL_646281|Original|hCoV-19^^England|Human|Lighthouse Lab in Glasgow|Wellcome Sanger Institute for the COVID-19 Genomics UK (COG-UK) Consortium|Davies|United Kingdom

>Spike|hCoV-19/England/NORW-ECB63/2020|2020-06-18|EPI_ISL_484328|Original|hCoV-19^^England|Human|Quadram Institute Bioscience|COVID-19 Genomics UK (COG-UK) Consortium|Alikhan|United Kingdom

>Spike|hCoV-19/England/CAMB-1B1164/2020|2020-05-07|EPI_ISL_459176|Original|hCoV-19^^England|Human|Department of Pathology|Wellcome Sanger Institute for the COVID-19 Genomics UK (COG-UK) consortium|Davies|United Kingdom

>Spike|hCoV-19/Denmark/DCGC-18310/2020|2020-12-07|EPI_ISL_749882|Original|hCoV-19^^Midtjylland|Human|Department of Virus and Microbiological Special Diagnostics|Albertsen Lab|Michaelsen|Denmark

>Spike|hCoV-19/Belgium/ULG-10164/2020|2020-04-19|EPI_ISL_447158|Original|hCoV-19^^Liege|Human|Department of Clinical Microbiology|GIGA Medical Genomics|Durkin|Belgium

>Spike|hCoV-19/England/CAMC-B08C81/2020|2020-10-30|EPI_ISL_643826|Original|hCoV-19^^England|Human|Lighthouse Lab in Cambridge|Wellcome Sanger Institute for the COVID-19 Genomics UK (COG-UK) Consortium|Davies|United Kingdom

>Spike|hCoV-19/Wales/PHWC-35D83/2020|2020-04-27|EPI_ISL_474566|Original|hCoV-19^^Wales|Human|Originating lab|COVID-19 Genomics UK (COG-UK) Consortium|Moore|United Kingdom

>Spike|hCoV-19/England/ALDP-9E8436/2020|2020-09-24|EPI_ISL_637713|Original|hCoV-19^^England|Human|Department of Pathology|COVID-19 Genomics UK (COG-UK) Consortium|Goodfellow|United Kingdom

>Spike|hCoV-19/England/ALDP-9EA49E/2020|2020-09-24|EPI_ISL_580253|Original|hCoV-19^^England|Human|Lighthouse Lab in Alderley Park|Wellcome Sanger Institute for the COVID-19 Genomics UK (COG-UK) consortium|Davies|United Kingdom

>Spike|hCoV-19/England/SHEF-C1214/2020|2020-10-18|EPI_ISL_611774|Original|hCoV-19^^England|Human|Virology Department|COVID-19 Genomics UK (COG-UK) Consortium|de Silva|United Kingdom

>Spike|hCoV-19/USA/FL-BPHL-0690/2020|2020-03-26|EPI_ISL_509748|Original|hCoV-19^^Florida|Human|Florida Bureau of Public Health Laboratories|Florida Bureau of Public Health Laboratories|Blanton|USA

>Spike|hCoV-19/England/BIRM-6A523/2020|2020-11-09|EPI_ISL_679069|Original|hCoV-19^^England|Human|University of Birmingham|COVID-19 Genomics UK (COG-UK) Consortium|Loman|United Kingdom

>Spike|hCoV-19/England/ALDP-7003D3/2020|2020-06-29|EPI_ISL_557726|Original|hCoV-19^^England|Human|Lighthouse Lab in Alderley Park|Wellcome Sanger Institute for the COVID-19 Genomics UK (COG-UK) consortium|Davies|United Kingdom

>Spike|hCoV-19/England/LOND-D4BF5/2020|2020-04-14|EPI_ISL_449662|Original|hCoV-19^^England|Human|University College London|COVID-19 Genomics UK (COG-UK) Consortium|Roy|United Kingdom

>Spike|hCoV-19/Gambia/GC19-3266/2020|2020-07-27|EPI_ISL_561055|Original|hCoV-19^^Kombo|Human|MRCG at LSHTM Genomics lab|MRCG at LSHTM Genomics lab|Sesay|Gambia

>Spike|hCoV-19/Chile/RM-ISPCH-58/2020|2020-04-02|EPI_ISL_445354|Original|hCoV-19^^Region Metropolitana de Santiago|Human|HOSPITAL DE CARABINEROS|Instituto de Salud Publica de Chile|Fernandez|Chile

>Spike|hCoV-19/Bahrain/920455398_S12_L001/2020|2020-10-13|EPI_ISL_681300|Original|hCoV-19^^Bahrain|Human|Communicable Disease Laboratory|Communicable Disease Laboratory|Altaif|Bahrain
>Spike|hCoV-19/USA/MN-MDH-2204/2020|2020-11-03|EPI_ISL_770778|Original|hCoV-19^^Minnesota|Human|Minnesota Department of Health|Minnesota Department of Health|Lorentz|USA
>Spike|hCoV-19/USA/TX-HMH-MCoV-14111/2020|2020-10-02|EPI_ISL_787397|Original|hCoV-19^^Texas|Human|Houston Methodist Hospital|Houston Methodist Hospital|Long|USA
>Spike|hCoV-19/Canada/BC_20332712/2020|2020-03-00|EPI_ISL_462771|Original|hCoV-19^^British Columbia|Human|BCCDC Public Health Laboratory|BCCDC Public Health Laboratory|Harrigan|Canada
>Spike|hCoV-19/England/EXET-13B144/2020|2020-11-12|EPI_ISL_664609|Original|hCoV-19^^England|Human|University of Exeter|COVID-19 Genomics UK (COG-UK) Consortium|Temperton|United Kingdom
>Spike|hCoV-19/Denmark/DCGC-10461/2020|2020-11-02|EPI_ISL_669989|Original|hCoV-19^^Hovedstaden|Human|Department of Virus and Microbiological Special Diagnostics|Albertsen Lab|Michaelsen|Denmark
>Spike|hCoV-19/Brazil/RS-00611/2020|2020-11-24|EPI_ISL_770563|Original|hCoV-19^^Rio Grande do Sul|Human|Laborat\u00f3rio de Microbiologia Molecular - Universidade FEEVALE|Bioinformatics Laboratory / LNCC|Almeida|Brazil
>Spike|hCoV-19/England/CAMC-BD21DC/2020|2020-11-28|EPI_ISL_702241|Original|hCoV-19^^England|Human|Lighthouse Lab in Cambridge|Wellcome Sanger Institute for the COVID-19 Genomics UK (COG-UK) Consortium|Davies|United Kingdom
>Spike|hCoV-19/USA/TX-HMH-686/2020|2020-04-15|EPI_ISL_546295|Original|hCoV-19^^Texas|Human|Houston Methodist Hospital|Houston Methodist Hospital|Long|USA
>Spike|hCoV-19/England/ALDP-A2D1D3/2020|2020-10-05|EPI_ISL_586750|Original|hCoV-19^^England|Human|Lighthouse Lab in Alderley Park|Wellcome Sanger Institute for the COVID-19 Genomics UK (COG-UK) consortium|Davies|United Kingdom
>Spike|hCoV-19/England/LIVE-9F31C/2020|2020-05-01|EPI_ISL_453446|Original|hCoV-19^^England|Human|Liverpool Clinical Laboratories|COVID-19 Genomics UK (COG-UK) Consortium|Haldenby|United Kingdom
>Spike|hCoV-19/England/ALDP-B4803E/2020|2020-11-07|EPI_ISL_649441|Original|hCoV-19^^England|Human|Lighthouse Lab in Alderley Park|Wellcome Sanger Institute for the COVID-19 Genomics UK (COG-UK) Consortium|Davies|United Kingdom
>Spike|hCoV-19/Japan/PG-3442/2020|2020-06-00|EPI_ISL_691552|Original|hCoV-19^^Japan|Human|Pathogen Genomics Center|Pathogen Genomics Center|Sekizuka|Japan
>Spike|hCoV-19/Denmark/DCGC-14967/2020|2020-11-23|EPI_ISL_713554|Original|hCoV-19^^Hovedstaden|Human|Department of Virus and Microbiological Special Diagnostics|Albertsen Lab|Michaelsen|Denmark
>Spike|hCoV-19/Denmark/DCGC-13138/2020|2020-11-30|EPI_ISL_712608|Original|hCoV-19^^Hovedstaden|Human|Department of Virus and Microbiological Special Diagnostics|Albertsen Lab|Michaelsen|Denmark
>Spike|hCoV-19/USA/MI-MDHHS-SC21174/2020|2020-04-01|EPI_ISL_507431|Original|hCoV-19^^Michigan|Human|Michigan Department of Health and Human Services|Michigan Department of Health and Human Services|Blankenship|USA
>Spike|hCoV-19/Australia/VIC6254/2020|2020-07-19|EPI_ISL_518246|Original|hCoV-19^^Victoria|Human|Victorian Infectious Diseases Reference Laboratory (VIDRL)|VIDRL and MDU-PHL|Schultz|Australia
>Spike|hCoV-19/India/KA-nimh-0999/2020|2020-04-14|EPI_ISL_486382|Original|hCoV-19^^Karnataka|Human|District Surveillence Unit|Department of Neurovirology|Pattabiraman|India
>Spike|hCoV-19/Switzerland/BE-ETHZ-350203/2020|2020-10-30|EPI_ISL_693851|Original|hCoV-19^^Bern|Human|Viollier AG|Department of Biosystems Science and Engineering|Nadeau|Switzerland
>Spike|hCoV-19/USA/TX-HMH-MCoV-12757/2020|2020-08-29|EPI_ISL_789904|Original|hCoV-19^^Texas|Human|Houston Methodist Hospital|Houston Methodist Hospital|Long|USA
>Spike|hCoV-19/England/MILK-C52CC3/2020|2020-12-12|EPI_ISL_736463|Original|hCoV-19^^England|Human|Lighthouse Lab in Milton Keynes|Wellcome Sanger Institute for the COVID-19 Genomics UK (COG-UK) Consortium|Davies|United Kingdom

>Spike|hCoV-19/USA/WA-QDX-1104/2020|2020-03-19|EPI_ISL_571908|Original|hCoV-19^^Washington|Human|Quest Diagnostics|Quest Diagnostics|Kagan|USA
>Spike|hCoV-19/Denmark/DCGC-2748/2020|2020-08-17|EPI_ISL_616493|Original|hCoV-19^^Hovedstaden|Human|Department of Virus and Microbiological Special Diagnostics|Albertsen lab|Michaelsen|Denmark
>Spike|hCoV-19/USA/WA-S1000/2020|2020-04-28|EPI_ISL_463490|Original|hCoV-19^^Washington|Human|Washington State Department of Health|Seattle Flu Study|Bedford|USA
>Spike|hCoV-19/Australia/VIC2582/2020|2020-07-01|EPI_ISL_521456|Original|hCoV-19^^Victoria|Human|Microbiological Diagnostic Unit - Public Health Laboratory (MDU-PHL)|MDU-PHL|Schultz|Australia
>Spike|hCoV-19/England/HECH-2599DEC/2020|2020-05-06|EPI_ISL_482073|Original|hCoV-19^^England|Human|Microbiology Department|Wellcome Sanger Institute for the COVID-19 Genomics UK (COG-UK) consortium|Davies|United Kingdom
>Spike|hCoV-19/Denmark/DCGC-18718/2020|2020-12-07|EPI_ISL_750141|Original|hCoV-19^^Midtjylland|Human|Department of Virus and Microbiological Special Diagnostics|Albertsen Lab|Michaelsen|Denmark
>Spike|hCoV-19/England/CAMC-B330FA/2020|2020-11-05|EPI_ISL_659355|Original|hCoV-19^^England|Human|Lighthouse Lab in Cambridge|Wellcome Sanger Institute for the COVID-19 Genomics UK (COG-UK) Consortium|Davies|United Kingdom
>Spike|hCoV-19/USA/UT-UPHL-201027414/2020|2020-04-18|EPI_ISL_594115|Original|hCoV-19^^Utah|Human|Utah Public Health Laboratory|Utah Public Health Laboratory|Young|USA
>Spike|hCoV-19/England/ALDP-BE9187/2020|2020-12-02|EPI_ISL_708938|Original|hCoV-19^^England|Human|Lighthouse Lab in Alderley Park|Wellcome Sanger Institute for the COVID-19 Genomics UK (COG-UK) Consortium|Davies|United Kingdom
>Spike|hCoV-19/England/QEUH-AF00BE/2020|2020-10-29|EPI_ISL_662749|Original|hCoV-19^^England|Human|Lighthouse Lab in Glasgow|Wellcome Sanger Institute for the COVID-19 Genomics UK (COG-UK) Consortium|Davies|United Kingdom
>Spike|hCoV-19/England/QEUH-A317AD/2020|2020-10-05|EPI_ISL_595910|Original|hCoV-19^^England|Human|Quadram Institute Bioscience|COVID-19 Genomics UK (COG-UK) Consortium|Alikhan|United Kingdom
>Spike|hCoV-19/Indonesia/JI-ITDua-966NTv/2020|2020-03-26|EPI_ISL_759966|Vero P1|hCoV-19^^East Java|Human|RSUD Sidoarjo|Institute of Tropical Disease|Lusida|Indonesia
>Spike|hCoV-19/Scotland/QEUH-9632DF/2020|2020-08-11|EPI_ISL_531714|Original|hCoV-19^^Scotland|Human|NHSGGC West of Scotland Specialist Virology Centre / MRC-University of Glasgow Centre for Virus Research|Wellcome Sanger Institute for the COVID-19 Genomics UK (COG-UK) consortium|Davies|United Kingdom
>Spike|hCoV-19/USA/IL-UW-364/2020|2020-04-16|EPI_ISL_450718|Original|hCoV-19^^Illinois|Human|University of Wisconsin-Madison AIDS Vaccine Research Laboratories|University of Wisconsin-Madison AIDS Vaccine Research Laboratories|Baczenas|USA
>Spike|hCoV-19/England/SHEF-C65F8/2020|2020-03-21|EPI_ISL_432821|Original|hCoV-19^^England|Human|Virology Department|COVID-19 Genomics UK (COG-UK) Consortium|de Silva|United Kingdom
>Spike|hCoV-19/England/LIVE-AA721/2020|2020-03-24|EPI_ISL_517493|Original|hCoV-19^^England|Human|Liverpool Clinical Laboratories|COVID-19 Genomics UK (COG-UK) Consortium|Haldenby|United Kingdom
>Spike|hCoV-19/Denmark/DCGC-21170/2020|2020-12-21|EPI_ISL_759589|Original|hCoV-19^^Nordjylland|Human|Department of Virus and Microbiological Special Diagnostics|Albertsen Lab|Michaelsen|Denmark
>Spike|hCoV-19/England/LEED-2A9A54/2020|2020-04-11|EPI_ISL_538885|Original|hCoV-19^^England|Human|Leeds Teaching Hospitals NHS Trust and Public Health England|Wellcome Sanger Institute for the COVID-19 Genomics UK (COG-UK) consortium|Davies|United Kingdom
>Spike|hCoV-19/USA/WA-S2151/2020|2020-06-08|EPI_ISL_497579|Original|hCoV-19^^Washington|Human|Washington State Department of Health|Seattle Flu Study|Lee|USA
>Spike|hCoV-19/Belgium/ULG-11122/2020|2020-12-17|EPI_ISL_737369|Original|hCoV-19^^Li\u00e8ge|Human|Department of Clinical Microbiology|GIGA Medical Genomics|Durkin|Belgium

>Spike|hCoV-19/Bangladesh/BCSIR-NILMRC-264/2020|2020-07-16|EPI_ISL_504137|Original|hCoV-19^^Chittagong|Human|National Institute of Laboratory Medicine and Referral Center|Genomic Research Lab|Khan|Bangladesh
>Spike|hCoV-19/England/20146060504/2020|2020-03-31|EPI_ISL_423583|Original|hCoV-19^^England|Human|Respiratory Virus Unit|Respiratory Virus Unit|Platt|United Kingdom
>Spike|hCoV-19/England/MILK-A6BFDF/2020|2020-10-09|EPI_ISL_608901|Original|hCoV-19^^England|Human|Lighthouse Lab in Milton Keynes|Wellcome Sanger Institute for the COVID-19 Genomics UK (COG-UK) consortium|Davies|United Kingdom
>Spike|hCoV-19/England/MILK-BAAA4B/2020|2020-11-17|EPI_ISL_681610|Original|hCoV-19^^England|Human|Lighthouse Lab in Milton Keynes|Wellcome Sanger Institute for the COVID-19 Genomics UK (COG-UK) Consortium|Davies|United Kingdom
>Spike|hCoV-19/USA/TX-HMH-MCoV-14402/2020|2020-10-08|EPI_ISL_787687|Original|hCoV-19^^Texas|Human|Houston Methodist Hospital|Houston Methodist Hospital|Long|USA
>Spike|hCoV-19/England/MILK-ACD357/2020|2020-10-21|EPI_ISL_634353|Original|hCoV-19^^England|Human|Lighthouse Lab in Milton Keynes|Wellcome Sanger Institute for the COVID-19 Genomics UK (COG-UK) consortium|Davies|United Kingdom
>Spike|hCoV-19/England/QEUH-A017A6/2020|2020-09-30|EPI_ISL_679996|Original|hCoV-19^^England|Human|Virology Department|COVID-19 Genomics UK (COG-UK) Consortium|de Silva|United Kingdom
>Spike|hCoV-19/USA/WA-S2881/2020|2020-09-28|EPI_ISL_596412|Original|hCoV-19^^Washington|Human|Seattle Flu Study|Seattle Flu Study|Lee|USA
>Spike|hCoV-19/USA/TX-HMH-8016/2020|2020-07-03|EPI_ISL_545793|Original|hCoV-19^^Texas|Human|Houston Methodist Hospital|Houston Methodist Hospital|Long|USA
>Spike|hCoV-19/England/PORT-2E926F/2020|2020-00-00|EPI_ISL_741707|Original|hCoV-19^^England|Human|Centre for Enzyme Innovation|COVID-19 Genomics UK (COG-UK) Consortium|Robson|United Kingdom
>Spike|hCoV-19/England/ALDP-94480F/2020|2020-06-07|EPI_ISL_555662|Original|hCoV-19^^England|Human|Lighthouse Lab in Alderley Park|Wellcome Sanger Institute for the COVID-19 Genomics UK (COG-UK) consortium|Davies|United Kingdom
>Spike|hCoV-19/Denmark/DCGC-9726/2020|2020-11-09|EPI_ISL_669130|Original|hCoV-19^^Hovedstaden|Human|Department of Virus and Microbiological Special Diagnostics|Albertsen Lab|Michaelsen|Denmark
>Spike|hCoV-19/England/ALDP-C3D50F/2020|2020-12-10|EPI_ISL_731823|Original|hCoV-19^^England|Human|Lighthouse Lab in Alderley Park|Wellcome Sanger Institute for the COVID-19 Genomics UK (COG-UK) Consortium|Davies|United Kingdom
>Spike|hCoV-19/Wales/PHWC-168032/2020|2020-04-20|EPI_ISL_494191|Original|hCoV-19^^Wales|Human|Originating lab|COVID-19 Genomics UK (COG-UK) Consortium|Moore|United Kingdom
>Spike|hCoV-19/USA/UT-UPHL-2012804386/2020|2020-11-30|EPI_ISL_746949|Original|hCoV-19^^Utah|Human|Utah Public Health Laboratory|Utah Public Health Laboratory|Gallagher|USA
>Spike|hCoV-19/England/MILK-9C3F30/2020|2020-09-09|EPI_ISL_566759|Original|hCoV-19^^England|Human|Lighthouse Lab in Milton Keynes|Wellcome Sanger Institute for the COVID-19 Genomics UK (COG-UK) consortium|Davies|United Kingdom
>Spike|hCoV-19/USA/NV-NSPHL-A0044/2020|2020-03-24|EPI_ISL_515327|Original|hCoV-19^^Nevada|Human|Nevada State Public Health Laboratory|Nevada State Public Health Laboratory|Sevinsky|USA
>Spike|hCoV-19/Beijing/IVDC-02-06/2020|2020-06-11|EPI_ISL_469255|Original|hCoV-19^^Beijing|Human|National Institute for Viral Disease Control and Prevention|Institute of Viral Disease Control and Prevention|Wu|China
>Spike|hCoV-19/England/OXON-AE259/2020|2020-00-00|EPI_ISL_448676|Original|hCoV-19^^England|Human|Oxford Viromics|COVID-19 Genomics UK (COG-UK) Consortium|Golubchik|United Kingdom
>Spike|hCoV-19/Wales/PHW1/2020|2020-02-27|EPI_ISL_413555|Original|hCoV-19^^Wales|Human|Wales Specialist Virology Centre|Public Health Wales Microbiology Cardiff|Moore|United Kingdom
>Spike|hCoV-19/England/BRIS-127442/2020|2020-04-22|EPI_ISL_469750|Original|hCoV-19^^England|Human|PHE South West Regional Laboratory|Wellcome Sanger Institute for the COVID-19 Genomics UK (COG-UK) consortium|Davies|United Kingdom

>Spike|hCoV-19/Australia/VIC2827/2020|2020-07-02|EPI_ISL_521658|Original|hCoV-19^^Victoria|Human|Microbiological Diagnostic Unit - Public Health Laboratory (MDU-PHL)|MDU-PHL|Schultz|Australia
>Spike|hCoV-19/England/QEUH-B0DB8F/2020|2020-11-02|EPI_ISL_641962|Original|hCoV-19^^England|Human|Lighthouse Lab in Glasgow|Wellcome Sanger Institute for the COVID-19 Genomics UK (COG-UK) Consortium|Davies|United Kingdom
>Spike|hCoV-19/England/QEUH-A8B470/2020|2020-10-16|EPI_ISL_643164|Original|hCoV-19^^England|Human|Lighthouse Lab in Glasgow|Wellcome Sanger Institute for the COVID-19 Genomics UK (COG-UK) Consortium|Davies|United Kingdom
>Spike|hCoV-19/India/MP-NCDC-4878/2020|2020-04-16|EPI_ISL_436462|Original|hCoV-19^^Madhya Pradesh|Human|National Centre for Disease control (NCDC)|NCDC/CSIR-IGIB|Singh|India
>Spike|hCoV-19/England/ALDP-C6B993/2020|2020-12-14|EPI_ISL_778412|Original|hCoV-19^^England|Human|Lighthouse Lab in Alderley Park|Wellcome Sanger Institute for the COVID-19 Genomics UK (COG-UK) Consortium|Davies|United Kingdom
>Spike|hCoV-19/USA/MT-RML-9/2020|2020-07-20|EPI_ISL_591056|Original|hCoV-19^^Montana|Human|Virus Ecology|Virus Ecology|Martens|USA
>Spike|hCoV-19/England/ALDP-A862E4/2020|2020-10-17|EPI_ISL_606269|Original|hCoV-19^^England|Human|Lighthouse Lab in Alderley Park|Wellcome Sanger Institute for the COVID-19 Genomics UK (COG-UK) consortium|Davies|United Kingdom
>Spike|hCoV-19/England/204980818/2020|2020-12-04|EPI_ISL_754291|Original|hCoV-19^^England|Human|Respiratory Virus Unit|COVID-19 Genomics UK (COG-UK) Consortium|Platt|United Kingdom
>Spike|hCoV-19/England/QEUH-B9F144/2020|2020-11-18|EPI_ISL_719854|Original|hCoV-19^^England|Human|Lighthouse Lab in Glasgow|Wellcome Sanger Institute for the COVID-19 Genomics UK (COG-UK) Consortium|Davies|United Kingdom
>Spike|hCoV-19/USA/TX-DSHS-1308/2020|2020-03-31|EPI_ISL_648239|Original|hCoV-19^^Texas|Human|Texas Department of State Health Services|Texas Department of State Health Services|Rahman|USA
>Spike|hCoV-19/England/MILK-A06352/2020|2020-10-01|EPI_ISL_599851|Original|hCoV-19^^England|Human|Lighthouse Lab in Milton Keynes|Wellcome Sanger Institute for the COVID-19 Genomics UK (COG-UK) consortium|Davies|United Kingdom
>Spike|hCoV-19/USA/WI-WSLH-200085/2020|2020-03-16|EPI_ISL_471249|Original|hCoV-19^^Wisconsin|Human|Wisconsin State Laboratory of Hygiene Communicable Disease Division|Wisconsin State Laboratory of Hygiene Communicable Disease Division|Florek|USA
>Spike|hCoV-19/Wales/PHWC-26BC7/2020|2020-03-27|EPI_ISL_422148|Original|hCoV-19^^Wales|Human|Wales Specialist Virology Centre|Public Health Wales Microbiology Cardiff|Moore|United Kingdom
>Spike|hCoV-19/Canada/BC-BCCDC-7307/2020|2020-00-00|EPI_ISL_740674|Original|hCoV-19^^British Columbia|Human|BCCDC Public Health Laboratory|BCCDC Public Health Laboratory|Prystajecky|Canada
>Spike|hCoV-19/Australia/VIC3117/2020|2020-07-05|EPI_ISL_520824|Original|hCoV-19^^Victoria|Human|Victorian Infectious Diseases Reference Laboratory (VIDRL)|VIDRL and MDU-PHL|Schultz|Australia
>Spike|hCoV-19/USA/IL-UW-690/2020|2020-06-28|EPI_ISL_491313|Original|hCoV-19^^Illinois|Human|University of Wisconsin-Madison AIDS Vaccine Research Laboratories|University of Wisconsin-Madison AIDS Vaccine Research Laboratories|Baczenas|USA
>Spike|hCoV-19/Scotland/CVR3170/2020|2020-04-27|EPI_ISL_449200|Original|hCoV-19^^Scotland|Human|West of Scotland Specialist Virology Centre|COVID-19 Genomics UK (COG-UK) Consortium|Robertson|United Kingdom
>Spike|hCoV-19/USA/MD-MDH-0409/2020|2020-11-05|EPI_ISL_666779|Original|hCoV-19^^Maryland|Human|Maryland Public Health Laboratory|Maryland Public Health Laboratory|Keller|USA
>Spike|hCoV-19/USA/AZ-TG670255/2020|2020-03-25|EPI_ISL_696432|Original|hCoV-19^^Arizona|Human|Sonora Quest Laboratories|TGen North|French|USA
>Spike|hCoV-19/USA/TX-HMH-5067/2020|2020-06-21|EPI_ISL_544500|Original|hCoV-19^^Texas|Human|Houston Methodist Hospital|Houston Methodist Hospital|Long|USA

>Spike|hCoV-19/England/NOTT-114642/2020|2020-10-06|EPI_ISL_577183|Original|hCoV-19^^England|Human|Queens Medical Centre|COVID-19 Genomics UK (COG-UK) Consortium|Loose|United Kingdom
>Spike|hCoV-19/Russia/OMS-ORINFI-8499S/2020|2020-07-22|EPI_ISL_569847|Original|hCoV-19^^Omsk|Human|Omsk Research Institute of Natural Focal Infections|WHO National Influenza Centre Russian Federation|Komissarov|Russia
>Spike|hCoV-19/USA/DC-HP00052/2020|2020-03-07|EPI_ISL_457796|Original|hCoV-19^^District of Columbia|Human|Johns Hopkins Hospital Department of Pathology|Johns Hopkins Hospital Department of Pathology|Ramakrishnan|USA
>Spike|hCoV-19/USA/CA-CZB-12810/2020|2020-09-05|EPI_ISL_648461|Original|hCoV-19^^California|Human|Santa Clara County Public Health Laboratory|Chan-Zuckerberg Biohub|Bhatt|USA
>Spike|hCoV-19/Japan/PG-5469/2020|2020-08-00|EPI_ISL_690572|Original|hCoV-19^^Japan|Human|Pathogen Genomics Center|Pathogen Genomics Center|Sekizuka|Japan
>Spike|hCoV-19/Wales/PHWC-4A6114/2020|2020-12-15|EPI_ISL_764247|Original|hCoV-19^^Wales|Human|Wales Specialist Virology Centre Sequencing lab|COVID-19 Genomics UK (COG-UK) Consortium|Moore|United Kingdom
>Spike|hCoV-19/England/QEUH-9AED04/2020|2020-09-03|EPI_ISL_573497|Original|hCoV-19^^England|Human|University College London|COVID-19 Genomics UK (COG-UK) Consortium|Roy|United Kingdom
>Spike|hCoV-19/Wales/PHWC-484EF7/2020|2020-10-24|EPI_ISL_626784|Original|hCoV-19^^Wales|Human|Wales Specialist Virology Centre Sequencing lab|COVID-19 Genomics UK (COG-UK) Consortium|Moore|United Kingdom
>Spike|hCoV-19/DRC/6203/2020|2020-05-09|EPI_ISL_514109|Original|hCoV-19^^Kinshasa|Human|Viral Respiratory Lab|Pathogen Sequencing Lab|Lokilo|Democratic Republic of the Congo
>Spike|hCoV-19/Australia/VIC12476/2020|2020-09-12|EPI_ISL_563566|Original|hCoV-19^^Victoria|Human|Microbiological Diagnostic Unit - Public Health Laboratory (MDU-PHL)|MDU-PHL|Schultz|Australia
>Spike|hCoV-19/USA/NY-NYCPHL-000634/2020|2020-03-10|EPI_ISL_632081|Original|hCoV-19^^New York|Human|New York Presbyterian-Brooklyn Methodist Hospital|New York City Public Health Laboratory|Wang|USA
>Spike|hCoV-19/USA/WA-UW-12909/2020|2020-06-29|EPI_ISL_570928|Original|hCoV-19^^Washington|Human|UW Virology Lab|UW Virology Lab|Roychoudhury|USA
>Spike|hCoV-19/England/OXON-AE5BA/2020|2020-00-00|EPI_ISL_448704|Original|hCoV-19^^England|Human|Oxford Viromics|COVID-19 Genomics UK (COG-UK) Consortium|Golubchik|United Kingdom
>Spike|hCoV-19/USA/WI-GMF-25857/2020|2020-10-14|EPI_ISL_660936|Original|hCoV-19^^Wisconsin|Human|Gundersen Molecular Diagnostics Laboratory|Kabara Cancer Research Institute|Kenny|USA
>Spike|hCoV-19/Denmark/DCGC-1112/2020|2020-03-16|EPI_ISL_614603|Original|hCoV-19^^Hovedstaden|Human|Department of Virus and Microbiological Special Diagnostics|Albertsen lab|Michaelsen|Denmark
>Spike|hCoV-19/Brazil/RJ-00552/2020|2020-10-29|EPI_ISL_717936|Original|hCoV-19^^Rio de Janeiro|Human|Laboratorio de Virologia Molecular / UFRJ|Bioinformatics Laboratory / LNCC|Almeida|Brazil
>Spike|hCoV-19/Jordan/AQ-ALSR-5264/2020|2020-10-15|EPI_ISL_730502|Original|hCoV-19^^Aqaba|Human|Biolab Diagnostic Laboratories|Andersen lab at Scripps Research|Gangavarapu|Jordan
>Spike|hCoV-19/Spain/MD-IBV-99009594/2020|2020-08-26|EPI_ISL_654346|Original|hCoV-19^^Madrid|Human|Hospital General Universitario Gregorio Mara\u00f1\u00f3n|SeqCOVID-SPAIN consortium/IBV(CSIC)|Comas|Spain
>Spike|hCoV-19/England/ALDP-A51D5A/2020|2020-10-07|EPI_ISL_597933|Original|hCoV-19^^England|Human|Lighthouse Lab in Alderley Park|Wellcome Sanger Institute for the COVID-19 Genomics UK (COG-UK) consortium|Davies|United Kingdom
>Spike|hCoV-19/USA/TX-HMH-MCoV-14562/2020|2020-10-08|EPI_ISL_787847|Original|hCoV-19^^Texas|Human|Houston Methodist Hospital|Houston Methodist Hospital|Long|USA
>Spike|hCoV-19/South Africa/NHLS-UCT-GS-0013/2020|2020-08-11|EPI_ISL_640015|Original|hCoV-19^^Western Cape|Human|Conville CDC wc CVC|NHLS/UCT|Iranzadeh|South Africa

>Spike|hCoV-19/England/CAMC-B3673E/2020|2020-11-05|EPI_ISL_645358|Original|hCoV-19^^England|Human|Lighthouse Lab in Cambridge|Wellcome Sanger Institute for the COVID-19 Genomics UK (COG-UK) Consortium|Davies|United Kingdom
>Spike|hCoV-19/USA/NY-PV09489/2020|2020-03-20|EPI_ISL_450161|Original|hCoV-19^^New York|Human|MSHS Clinical Microbiology Laboratories|MSHS Pathogen Surveillance Program|van Bakel|USA
>Spike|hCoV-19/Scotland/CVR4108/2020|2020-09-07|EPI_ISL_540810|Original|hCoV-19^^Scotland|Human|West of Scotland Specialist Virology Centre|COVID-19 Genomics UK (COG-UK) Consortium|Robertson|United Kingdom
>Spike|hCoV-19/England/ALDP-A6C8F1/2020|2020-10-15|EPI_ISL_608077|Original|hCoV-19^^England|Human|Lighthouse Lab in Alderley Park|Wellcome Sanger Institute for the COVID-19 Genomics UK (COG-UK) consortium|Davies|United Kingdom
>Spike|hCoV-19/USA/CA-CZB-13819/2020|2020-08-14|EPI_ISL_738793|Original|hCoV-19^^California|Human|Alameda County Public Health Lab|Chan-Zuckerberg Biohub|Kamm|USA
>Spike|hCoV-19/USA/UT-UPHL-2012720932/2020|2020-11-30|EPI_ISL_746968|Original|hCoV-19^^Utah|Human|Utah Public Health Laboratory|Utah Public Health Laboratory|Gallagher|USA
>Spike|hCoV-19/Switzerland/BS-42208210/2020|2020-03-26|EPI_ISL_581763|Original|hCoV-19^^Basel-Stadt|Human|University Hospital Basel|University Hospital Basel|Roloff Handschin|Switzerland
>Spike|hCoV-19/Scotland/QEUH-9FAD90/2020|2020-09-29|EPI_ISL_600550|Original|hCoV-19^^Scotland|Human|Lighthouse Lab in Glasgow|Wellcome Sanger Institute for the COVID-19 Genomics UK (COG-UK) consortium|Davies|United Kingdom
>Spike|hCoV-19/USA/WA-UW-4032/2020|2020-03-29|EPI_ISL_437810|Original|hCoV-19^^Washington|Human|UW Virology Lab|UW Virology Lab|Roychoudhury|USA
>Spike|hCoV-19/USA/TX-HMH0433/2020|2020-04-02|EPI_ISL_435016|Original|hCoV-19^^Texas|Human|Houston Methodist Hospital|Houston Methodist Hospital|Long|USA
>Spike|hCoV-19/Japan/PG-6660/2020|2020-07-00|EPI_ISL_687771|Original|hCoV-19^^Japan|Human|Pathogen Genomics Center|Pathogen Genomics Center|Sekizuka|Japan
>Spike|hCoV-19/India/MH-GA83/2020|2020-06-18|EPI_ISL_508278|Original|hCoV-19^^Maharashtra|Human|Government Medical College|National Institute of Biomedical Genomics|Maitra|India
>Spike|hCoV-19/Serbia/CA3089-04/2020|2020-04-25|EPI_ISL_454795|Original|hCoV-19^^Cacak|Human|Veterinary Specialized Institute Kraljevo|Veterinary Specialized Institute Kraljevo|Vidanovic|Serbia
>Spike|hCoV-19/Australia/VIC6812/2020|2020-07-21|EPI_ISL_564071|Original|hCoV-19^^Victoria|Human|Victorian Infectious Diseases Reference Laboratory (VIDRL)|VIDRL and MDU-PHL|Schultz|Australia
>Spike|hCoV-19/USA/NY-Wadsworth-63615-01/2020|2020-04-22|EPI_ISL_676869|Original|hCoV-19^^New York|Human|Wadsworth Center|Wadsworth Center|Lasek-Nesselquist|USA
>Spike|hCoV-19/United Arab Emirates/skmc-227217/2020|2020-04-19|EPI_ISL_582633|Original|hCoV-19^^United Arab Emirates|Human|Sheikh Khalifa Medical City|Molecular/Surveillance lab\nSheikh Khalifa Medical City|Amirtharaj|United Arab Emirates
>Spike|hCoV-19/USA/CO-CDPHE-2009121107/2020|2020-09-11|EPI_ISL_677262|Original|hCoV-19^^Colorado|Human|Colorado Department of Public Health and Environment|Colorado Department of Puplic Health and Environment|Bankers|USA
>Spike|hCoV-19/Jordan/SR-048/2020|2020-03-23|EPI_ISL_430006|Original|hCoV-19^^Amman|Human|Biolab Diagnostic Laboratories|Andersen lab at Scripps Research|Gangavarapu|Jordan
>Spike|hCoV-19/England/QEUH-AAD33F/2020|2020-10-20|EPI_ISL_624163|Original|hCoV-19^^England|Human|Lighthouse Lab in Glasgow|Wellcome Sanger Institute for the COVID-19 Genomics UK (COG-UK) consortium|Davies|United Kingdom
>Spike|hCoV-19/USA/UT-UPHL-03126/2020|2020-05-06|EPI_ISL_575261|Original|hCoV-19^^Utah|Human|Utah Public Health Laboratory|Utah Public Health Laboratory|Young|USA
>Spike|hCoV-19/England/ALDP-9CD8FF/2020|2020-09-16|EPI_ISL_566120|Original|hCoV-19^^England|Human|Lighthouse Lab in Alderley Park|Wellcome Sanger Institute for the COVID-19 Genomics UK (COG-UK) consortium|Davies|United Kingdom

>Spike|hCoV-19/England/CAMB-77374/2020|2020-04-01|EPI_ISL_439378|Original|hCoV-19^^England|Human|Department of Pathology|Wellcome Sanger Institute for the COVID-19 Genomics UK (COG-UK) consortium|Amato|United Kingdom
>Spike|hCoV-19/England/QEUH-B9F126/2020|2020-11-18|EPI_ISL_719907|Original|hCoV-19^^England|Human|Lighthouse Lab in Glasgow|Wellcome Sanger Institute for the COVID-19 Genomics UK (COG-UK) Consortium|Davies|United Kingdom
>Spike|hCoV-19/England/CAMB-1AE5E6/2020|2020-04-30|EPI_ISL_524589|Original|hCoV-19^^England|Human|Department of Pathology|Wellcome Sanger Institute for the COVID-19 Genomics UK (COG-UK) consortium|Davies|United Kingdom
>Spike|hCoV-19/India/GJ-GBRC115/2020|2020-05-03|EPI_ISL_451160|Original|hCoV-19^^Gujarat|Human|Government Medical College|Gujarat Biotechnology Research Centre|Joshi|India
>Spike|hCoV-19/England/CAMC-A5800F/2020|2020-10-09|EPI_ISL_597137|Original|hCoV-19^^England|Human|Lighthouse Lab in Cambridge|Wellcome Sanger Institute for the COVID-19 Genomics UK (COG-UK) consortium|Davies|United Kingdom
>Spike|hCoV-19/England/MILK-9C2C85/2020|2020-09-10|EPI_ISL_566425|Original|hCoV-19^^England|Human|Lighthouse Lab in Milton Keynes|Wellcome Sanger Institute for the COVID-19 Genomics UK (COG-UK) consortium|Davies|United Kingdom
>Spike|hCoV-19/Luxembourg/LNS2951750/2020|2020-09-26|EPI_ISL_740222|Original|hCoV-19^^Luxembourg|Human|Laboratoire national de sant\u00e9|Laboratoire national de sant\u00e9|Wienecke-Baldacchino|Luxembourg
>Spike|hCoV-19/Scotland/QEUH-C33CAA/2020|2020-12-11|EPI_ISL_757172|Original|hCoV-19^^Scotland|Human|Lighthouse Lab in Glasgow|Wellcome Sanger Institute for the COVID-19 Genomics UK (COG-UK) Consortium|Davies|United Kingdom
>Spike|hCoV-19/Shanghai/SH0079/2020|2020-02-01|EPI_ISL_416381|Original|hCoV-19^^Shanghai|Human|Shanghai Public Health Clinical Center|National Research Center for Translational Medicine (Shanghai)|Zhang|China
>Spike|hCoV-19/Poland/1107355/2020|2020-03-23|EPI_ISL_428235|Original|hCoV-19^^Pomorskie|Human|Hematology Laboratory|Department of Virology|Smura|Poland
>Spike|hCoV-19/Russia/SVE-RII-MH2066S/2020|2020-09-21|EPI_ISL_602401|Original|hCoV-19^^Sverdlovsk|Human|HELIX LLC|WHO National Influenza Centre Russian Federation|Komissarov|Russia
>Spike|hCoV-19/Denmark/DCGC-1721/2020|2020-03-30|EPI_ISL_621824|Original|hCoV-19^^Sjaelland|Human|Department of Virus and Microbiological Special Diagnostics|Albertsen lab|Michaelsen|Denmark
>Spike|hCoV-19/Netherlands/GE-EMC-422/2020|2020-11-30|EPI_ISL_722711|Original|hCoV-19^^Gelderland|Human|Dutch COVID-19 response team|Erasmus Medical Center|Oude Munnink|Netherlands
>Spike|hCoV-19/Wales/PHWC-496BEC/2020|2020-11-23|EPI_ISL_726700|Original|hCoV-19^^Wales|Human|Originating lab|COVID-19 Genomics UK (COG-UK) Consortium|Moore|United Kingdom
>Spike|hCoV-19/Portugal/IGC4245/2020|2020-06-03|EPI_ISL_491240|Original|hCoV-19^^Portugal|Human|Instituto Gulbenkian de Ci\u00eancia|Instituto Gulbenkian de Ci\u00eancia|Leite|Portugal
>Spike|hCoV-19/Scotland/EDB2754/2020|2020-03-29|EPI_ISL_488925|Original|hCoV-19^^Scotland|Human|Virology Department|Wellcome Sanger Institute for the COVID-19 Genomics UK (COG-UK) consortium|Davies|United Kingdom
>Spike|hCoV-19/England/CAMC-B227CC/2020|2020-11-01|EPI_ISL_647209|Original|hCoV-19^^England|Human|Lighthouse Lab in Cambridge|Wellcome Sanger Institute for the COVID-19 Genomics UK (COG-UK) Consortium|Davies|United Kingdom
>Spike|hCoV-19/Northern Ireland/NIRE-FBDC5/2020|2020-04-10|EPI_ISL_459359|Original|hCoV-19^^Northern Ireland|Human|Regional Virus Laboratory|Wellcome Sanger Institute for the COVID-19 Genomics UK (COG-UK) consortium|Davies|United Kingdom
>Spike|hCoV-19/Hungary/UD-91485/2020|2020-10-05|EPI_ISL_671481|Original|hCoV-19^^Hajd\u00fa-Bihar county|Human|University of Debrecen|National Laboratory of Virology|Kemenesi|Hungary
>Spike|hCoV-19/USA/CA-CHLA-PLM76320099/2020|2020-11-18|EPI_ISL_753378|Original|hCoV-19^^California|Human|Clinical virology Laboratory|Center for Personalized Medicine|Dien Bard|USA

>Spike|hCoV-19/England/ALDP-9C5BA3/2020|2020-09-10|EPI_ISL_567562|Original|hCoV-19^^England|Human|Lighthouse Lab in Alderley Park|Wellcome Sanger Institute for the COVID-19 Genomics UK (COG-UK) consortium|Davies|United Kingdom
>Spike|hCoV-19/South Korea/KDCA0057/2020|2020-09-15|EPI_ISL_747298|Original|hCoV-19^^South Korea|Human|Division of Emerging Infectious Diseases|Division of Emerging Infectious Diseases|Park|South Korea
>Spike|hCoV-19/Canada/ON-S1782/2020|2020-11-08|EPI_ISL_755780|Original|hCoV-19^^Ontario|Human|Toronto Invasive Bacterial Diseases Network|McMaster University|McArthur|Canada
>Spike|hCoV-19/England/ALDP-B1B9C0/2020|2020-11-01|EPI_ISL_645814|Original|hCoV-19^^England|Human|Lighthouse Lab in Alderley Park|Wellcome Sanger Institute for the COVID-19 Genomics UK (COG-UK) Consortium|Davies|United Kingdom
>Spike|hCoV-19/Denmark/DCGC-6236/2020|2020-10-05|EPI_ISL_619996|Original|hCoV-19^^Midtjylland|Human|Department of Virus and Microbiological Special Diagnostics|Albertsen lab|Michaelsen|Denmark
>Spike|hCoV-19/England/MILK-A55F14/2020|2020-10-08|EPI_ISL_595120|Original|hCoV-19^^England|Human|Quadram Institute Bioscience|COVID-19 Genomics UK (COG-UK) Consortium|Alikhan|United Kingdom
>Spike|hCoV-19/Denmark/DCGC-15631/2020|2020-11-23|EPI_ISL_713932|Original|hCoV-19^^Hovedstaden|Human|Department of Virus and Microbiological Special Diagnostics|Albertsen Lab|Michaelsen|Denmark
>Spike|hCoV-19/Australia/NSW939/2020|2020-09-02|EPI_ISL_545011|Original|hCoV-19^^New South Wales|Human|South Eastern Area Laboratory Services (SEALS)|NSW Health Pathology - Institute of Clinical Pathology and Medical Research; Westmead Hospital; University of Sydney|Gall|Australia
>Spike|hCoV-19/England/LIVE-A1317/2020|2020-06-01|EPI_ISL_499993|Original|hCoV-19^^England|Human|Liverpool Clinical Laboratories|COVID-19 Genomics UK (COG-UK) Consortium|Haldenby|United Kingdom
>Spike|hCoV-19/USA/VA-DCLS-0550/2020|2020-03-14|EPI_ISL_485851|Original|hCoV-19^^Virginia|Human|Virginia DCLS|Virginia DCLS|St. Jacques|USA
>Spike|hCoV-19/England/CAMB-8266E/2020|2020-04-13|EPI_ISL_443583|Original|hCoV-19^^England|Human|Department of Pathology|Wellcome Sanger Institute for the COVID-19 Genomics UK (COG-UK) consortium|Amato|United Kingdom
>Spike|hCoV-19/USA/CA-ALSR-1453/2020|2020-03-25|EPI_ISL_494613|Original|hCoV-19^^California|Human|Scripps Medical Laboratory|Andersen lab at Scripps Research|Gangavarapu|USA
>Spike|hCoV-19/Denmark/DCGC-2985/2020|2020-08-24|EPI_ISL_616747|Original|hCoV-19^^Hovedstaden|Human|Department of Virus and Microbiological Special Diagnostics|Albertsen lab|Michaelsen|Denmark
>Spike|hCoV-19/India/TG-CCMB-GC20/2020|2020-07-19|EPI_ISL_528813|Original|hCoV-19^^Telangana|Human|Department of Medicine|CSIR-Centre for Cellular and Molecular Biology|Banu|India
>Spike|hCoV-19/Kenya/C7954/2020|2020-05-18|EPI_ISL_568853|Original|hCoV-19^^Mombasa|Human|KEMRI-Wellcome Trust Research Programme/KEMRI-CGMR-C Kilifi|KEMRI-Wellcome Trust Research Programme/KEMRI-CGMR-C Kilifi|Githinji|Kenya
>Spike|hCoV-19/England/MILK-9E07EA/2020|2020-09-20|EPI_ISL_601417|Original|hCoV-19^^England|Human|Lighthouse Lab in Milton Keynes|Wellcome Sanger Institute for the COVID-19 Genomics UK (COG-UK) consortium|Davies|United Kingdom
>Spike|hCoV-19/England/QEUH-9F6A42/2020|2020-09-27|EPI_ISL_588303|Original|hCoV-19^^England|Human|Lighthouse Lab in Glasgow|Wellcome Sanger Institute for the COVID-19 Genomics UK (COG-UK) consortium|Davies|United Kingdom
>Spike|hCoV-19/Fujian/13/2020|2020-01-22|EPI_ISL_411066|Original|hCoV-19^^Fujian|Human|Fujian Center for Disease Control and Prevention|Fujian Center for Disease Control and Prevention|Jianfeng|China
>Spike|hCoV-19/England/MILK-B38866/2020|2020-11-01|EPI_ISL_659081|Original|hCoV-19^^England|Human|Lighthouse Lab in Milton Keynes|Wellcome Sanger Institute for the COVID-19 Genomics UK (COG-UK) Consortium|Davies|United Kingdom
>Spike|hCoV-19/Guangdong/2020XN4273-P0036/2020|2020-01-30|EPI_ISL_413860|Original|hCoV-19^^Guangdong|Human|Guangdong Provincial Institution of Public Health|Guangdong Provincial Institution of Public Health|Lu|China

>Spike|hCoV-19/Japan/PG-1577/2020|2020-04-00|EPI_ISL_685183|Original|hCoV-19^^Japan|Human|Pathogen Genomics Center|Pathogen Genomics Center|Sekizuka|Japan
>Spike|hCoV-19/Scotland/EDB7814/2020|2020-09-21|EPI_ISL_572483|Original|hCoV-19^^Scotland|Human|Virology Department|COVID-19 Genomics UK (COG-UK) Consortium|Williams|United Kingdom
>Spike|hCoV-19/England/ALDP-BB2DDE/2020|2020-11-19|EPI_ISL_703194|Original|hCoV-19^^England|Human|Lighthouse Lab in Alderley Park|Wellcome Sanger Institute for the COVID-19 Genomics UK (COG-UK) Consortium|Davies|United Kingdom
>Spike|hCoV-19/England/ALDP-A68154/2020|2020-10-12|EPI_ISL_608489|Original|hCoV-19^^England|Human|Lighthouse Lab in Alderley Park|Wellcome Sanger Institute for the COVID-19 Genomics UK (COG-UK) consortium|Davies|United Kingdom
>Spike|hCoV-19/Denmark/DCGC-1719/2020|2020-03-30|EPI_ISL_615617|Original|hCoV-19^^Sjaelland|Human|Department of Virus and Microbiological Special Diagnostics|Albertsen lab|Michaelsen|Denmark
>Spike|hCoV-19/USA/CT-Yale-377/2020|2020-04-21|EPI_ISL_594121|Original|hCoV-19^^Delaware|Human|Yale COVID-19 Biorepository|Grubaugh Lab - Yale School of Public Health|Grubaugh|USA
>Spike|hCoV-19/France/IDF-3276/2020|2020-03-23|EPI_ISL_421508|Original|hCoV-19^^\u00cele-de-France|Human|Le Ch\u00e2teau de Seine-Port|National Reference Center for Viruses of Respiratory Infections|Enouf|France
>Spike|hCoV-19/USA/FL-BPHL-0190/2020|2020-04-03|EPI_ISL_480841|Original|hCoV-19^^Florida|Human|Florida Bureau of Public Health Laboratories|Florida Bureau of Public Health Laboratories|Blanton|USA
>Spike|hCoV-19/USA/AZ-TG664497/2020|2020-04-20|EPI_ISL_696122|Original|hCoV-19^^Arizona|Human|Sonora Quest Laboratories|TGen North|French|USA
>Spike|hCoV-19/England/CAMC-B33A65/2020|2020-11-05|EPI_ISL_659361|Original|hCoV-19^^England|Human|Lighthouse Lab in Cambridge|Wellcome Sanger Institute for the COVID-19 Genomics UK (COG-UK) Consortium|Davies|United Kingdom
>Spike|hCoV-19/Uruguay/CHY-M100/2020|2020-07-16|EPI_ISL_750175|Original|hCoV-19^^Chuy|Human|CENUR Este-Sede Rocha-UdelaR|Institut Pasteur de Montevideo|Spangenberg|Uruguay
>Spike|hCoV-19/Israel/CVL-n14276/2020|2020-03-21|EPI_ISL_514312|Original|hCoV-19^^Israel|Human|Israel Central Virology laboratory|Israel Central Virology laboratory|Zuckerman|Israel
>Spike|hCoV-19/Germany/HH-hpi-p2494/2020|2020-06-17|EPI_ISL_776544|Original|hCoV-19^^Hamburg|Human|University Medical Center Hamburg Eppendorf|Heinrich Pette Institute|Robitaille|Germany
>Spike|hCoV-19/England/MILK-99D069/2020|2020-08-30|EPI_ISL_549826|Original|hCoV-19^^England|Human|Lighthouse Lab in Milton Keynes|Wellcome Sanger Institute for the COVID-19 Genomics UK (COG-UK) consortium|Davies|United Kingdom
>Spike|hCoV-19/Wales/PHWC-49EA6F/2020|2020-12-08|EPI_ISL_743087|Original|hCoV-19^^Wales|Human|Wales Specialist Virology Centre Sequencing lab|COVID-19 Genomics UK (COG-UK) Consortium|Moore|United Kingdom
>Spike|hCoV-19/England/CAMC-B08164/2020|2020-10-29|EPI_ISL_643807|Original|hCoV-19^^England|Human|Lighthouse Lab in Cambridge|Wellcome Sanger Institute for the COVID-19 Genomics UK (COG-UK) Consortium|Davies|United Kingdom
>Spike|hCoV-19/USA/TX-HMH-MCoV-15572/2020|2020-10-26|EPI_ISL_787056|Original|hCoV-19^^Texas|Human|Houston Methodist Hospital|Houston Methodist Hospital|Long|USA
>Spike|hCoV-19/USA/UT-03894/2020|2020-04-13|EPI_ISL_470556|Original|hCoV-19^^Utah|Human|Utah Public Health Laboratory|Utah Public Health Laboratory|Young|USA
>Spike|hCoV-19/Australia/VIC2458/2020|2020-06-26|EPI_ISL_522164|Original|hCoV-19^^Victoria|Human|Victorian Infectious Diseases Reference Laboratory (VIDRL)|VIDRL and MDU-PHL|Schultz|Australia
>Spike|hCoV-19/Scotland/QEUH-B0F297/2020|2020-11-02|EPI_ISL_649526|Original|hCoV-19^^Scotland|Human|Lighthouse Lab in Glasgow|Wellcome Sanger Institute for the COVID-19 Genomics UK (COG-UK) Consortium|Davies|United Kingdom
>Spike|hCoV-19/England/CAMB-729C2/2020|2020-03-23|EPI_ISL_439588|Original|hCoV-19^^England|Human|Department of Pathology|Wellcome Sanger Institute for the COVID-19 Genomics UK (COG-UK) consortium|Amato|United Kingdom

>Spike|hCoV-19/England/LIVE-9EC30/2020|2020-04-04|EPI_ISL_453379|Original|hCoV-19^^England|Human|Liverpool Clinical Laboratories|COVID-19 Genomics UK (COG-UK) Consortium|Haldenby|United Kingdom
>Spike|hCoV-19/England/QEUH-B6C957/2020|2020-11-11|EPI_ISL_655201|Original|hCoV-19^^England|Human|Lighthouse Lab in Glasgow|Wellcome Sanger Institute for the COVID-19 Genomics UK (COG-UK) Consortium|Davies|United Kingdom
>Spike|hCoV-19/USA/WI-UW-528/2020|2020-06-23|EPI_ISL_484903|Original|hCoV-19^^Wisconsin|Human|University of Wisconsin-Madison AIDS Vaccine Research Laboratories|University of Wisconsin-Madison AIDS Vaccine Research Laboratories|Baczenas|USA
>Spike|hCoV-19/Netherlands/NA_482/2020|2020-04-08|EPI_ISL_455284|Original|hCoV-19^^Netherlands|Human|Dutch COVID-19 response team|Erasmus Medical Center|Oude Munnink|Netherlands
>Spike|hCoV-19/Australia/VIC3627/2020|2020-07-10|EPI_ISL_520368|Original|hCoV-19^^Victoria|Human|Victorian Infectious Diseases Reference Laboratory (VIDRL)|VIDRL and MDU-PHL|Schultz|Australia
>Spike|hCoV-19/Portugal/PT1712/2020|2020-08-24|EPI_ISL_693639|Original|hCoV-19^^Portugal|Human|Hospital Vila Franca de Xira|Instituto Nacional de Saude (INSA)|Borges|Portugal
>Spike|hCoV-19/Switzerland/BL-ETHZ-100843/2020|2020-03-25|EPI_ISL_451790|Original|hCoV-19^^Basel-Landschaft|Human|Viollier AG|Department of Biosystems Science and Engineering|Nadeau|Switzerland
>Spike|hCoV-19/Germany/HH-hpi-p1656/2020|2020-03-18|EPI_ISL_775846|Original|hCoV-19^^Hamburg|Human|University Medical Center Hamburg Eppendorf|Heinrich Pette Institute|Robitaille|Germany
>Spike|hCoV-19/Australia/VIC10848/2020|2020-09-03|EPI_ISL_563125|Original|hCoV-19^^Victoria|Human|Microbiological Diagnostic Unit - Public Health Laboratory (MDU-PHL)|MDU-PHL|Schultz|Australia
>Spike|hCoV-19/England/NORW-E9CAF/2020|2020-04-29|EPI_ISL_461849|Original|hCoV-19^^England|Human|Quadram Institute Bioscience|COVID-19 Genomics UK (COG-UK) Consortium|Alikhan|United Kingdom
>Spike|hCoV-19/Wales/PHWC-49C621/2020|2020-12-11|EPI_ISL_742583|Original|hCoV-19^^Wales|Human|Wales Specialist Virology Centre Sequencing lab|COVID-19 Genomics UK (COG-UK) Consortium|Moore|United Kingdom
>Spike|hCoV-19/Denmark/DCGC-18028/2020|2020-12-14|EPI_ISL_749711|Original|hCoV-19^^Nordjylland|Human|Department of Virus and Microbiological Special Diagnostics|Albertsen Lab|Michaelsen|Denmark
>Spike|hCoV-19/Brazil/PE-IAM1315/2020|2020-05-11|EPI_ISL_572388|Original|hCoV-19^^Pernambuco|Human|LACEN/PE|WallauLab|Wallau|Brazil
>Spike|hCoV-19/Japan/IC-0040/2020|2020-05-00|EPI_ISL_591382|Original|hCoV-19^^Japan|Human|Pathogen Genomics Center|Pathogen Genomics Center|Sekizuka|Japan
>Spike|hCoV-19/England/MILK-B385AB/2020|2020-11-01|EPI_ISL_658839|Original|hCoV-19^^England|Human|Lighthouse Lab in Milton Keynes|Wellcome Sanger Institute for the COVID-19 Genomics UK (COG-UK) Consortium|Davies|United Kingdom
>Spike|hCoV-19/Australia/NSW37/2020|2020-03-10|EPI_ISL_417395|Original|hCoV-19^^New South Wales|Human|Centre for Infectious Diseases and Microbiology Public Health|NSW Health Pathology - Institute of Clinical Pathology and Medical Research; Westmead Hospital; University of Sydney|Eden|Australia
>Spike|hCoV-19/Denmark/DCGC-10112/2020|2020-11-09|EPI_ISL_669401|Original|hCoV-19^^Nordjylland|Human|Department of Virus and Microbiological Special Diagnostics|Albertsen Lab|Michaelsen|Denmark
>Spike|hCoV-19/England/CAMC-9485C8/2020|2020-07-15|EPI_ISL_556809|Original|hCoV-19^^England|Human|Lighthouse Lab in Cambridge|Wellcome Sanger Institute for the COVID-19 Genomics UK (COG-UK) consortium|Davies|United Kingdom
>Spike|hCoV-19/Australia/VIC338/2020|2020-03-20|EPI_ISL_426647|Original|hCoV-19^^Victoria|Human|Victorian Infectious Diseases Reference Laboratory (VIDRL)|Microbiological Diagnostic Unit Public Health Laboratory and Victorian Infectious Diseases Reference Laboratory|Schultz|Australia
>Spike|hCoV-19/USA/NY-NYUMC554/2020|2020-03-30|EPI_ISL_444671|Original|hCoV-19^^New York|Human|NYU Langone Health|Departments of Pathology and Medicine|Maurano|USA

>Spike|hCoV-19/England/NORT-289F97/2020|2020-04-07|EPI_ISL_453521|Original|hCoV-19^^England|Human|Northumbria University / South Tees Hospitals NHS Foundation Trust / North Cumbria Integrated Care NHS Foundation Trust / North Tees and Hartlepool NHS Foundation Trust / Newcastle Hospitals NHS Foundation Trust|COVID-19 Genomics UK (COG-UK) Consortium|Bashton|United Kingdom
>Spike|hCoV-19/USA/CA-CDPH-UC4/2020|2020-02-27|EPI_ISL_413561|Original|hCoV-19^^California|Human|California Department of Public Health|Chiu Laboratory|Chiu|USA
>Spike|hCoV-19/Brazil/RJ-DCVN1/2020|2020-03-23|EPI_ISL_509434|Original|hCoV-19^^Rio de Janeiro|Human|Centro de Desenvolvimento Tecnologico em Saude|Centro de Desenvolvimento Tecnologico em Saude|Souza|Brazil
>Spike|hCoV-19/Portugal/PT1551/2020|2020-06-25|EPI_ISL_693564|Original|hCoV-19^^Portugal|Human|Instituto Nacional de Saude (INSA)|Instituto Nacional de Saude (INSA)|Borges|Portugal
>Spike|hCoV-19/Netherlands/ZH-EMC-1165/2020|2020-11-07|EPI_ISL_763240|Original|hCoV-19^^Zuid Holland|Human|Dutch COVID-19 response team|Erasmus Medical Center|Oude Munnink|Netherlands
>Spike|hCoV-19/Netherlands/NH-RIVM-20002/2020|2020-11-20|EPI_ISL_723200|Orignal|hCoV-19^^Noord-Holland|Human|Dutch COVID-19 response team|National Institute for Public Health and the Environment (RIVM)|Meijer|Netherlands
>Spike|hCoV-19/England/QEUH-B6AE1E/2020|2020-11-11|EPI_ISL_662573|Original|hCoV-19^^England|Human|Lighthouse Lab in Glasgow|Wellcome Sanger Institute for the COVID-19 Genomics UK (COG-UK) Consortium|Davies|United Kingdom
>Spike|hCoV-19/USA/NY-NYUMC94/2020|2020-03-18|EPI_ISL_421706|Original|hCoV-19^^New York|Human|NYU Langone Health|Departments of Pathology and Medicine|Maurano|USA
>Spike|hCoV-19/Egypt/CUNCI-HGC3I023/2020|2020-06-02|EPI_ISL_479695|Original|hCoV-19^^Egypt|Human|Egyptian National Cancer Institute (ENCI)|Egyptian National Cancer Institute (ENCI)|Zekri|Egypt
>Spike|hCoV-19/England/MILK-510B52/2020|2020-06-10|EPI_ISL_559080|Original|hCoV-19^^England|Human|Lighthouse Lab in Milton Keynes|Wellcome Sanger Institute for the COVID-19 Genomics UK (COG-UK) consortium|Davies|United Kingdom
>Spike|hCoV-19/England/ALDP-C58595/2020|2020-12-14|EPI_ISL_736083|Original|hCoV-19^^England|Human|Lighthouse Lab in Alderley Park|Wellcome Sanger Institute for the COVID-19 Genomics UK (COG-UK) Consortium|Davies|United Kingdom
>Spike|hCoV-19/England/ALDP-A2DFBB/2020|2020-10-06|EPI_ISL_586608|Original|hCoV-19^^England|Human|Lighthouse Lab in Alderley Park|Wellcome Sanger Institute for the COVID-19 Genomics UK (COG-UK) consortium|Davies|United Kingdom
>Spike|hCoV-19/Spain/RI-ISCIII-201571/2020|2020-03-03|EPI_ISL_578199|Original|hCoV-19^^La Rioja|Human|Hospital San Pedro|Instituto de Salud Carlos III|Caballero|Spain
>Spike|hCoV-19/Russia/StPetersburg-RII5247S/2020|2020-04-10|EPI_ISL_430091|Original|hCoV-19^^Saint Petersburg|Human|WHO National Influenza Centre Russian Federation|WHO National Influenza Centre Russian Federation|Komissarov|Russia
>Spike|hCoV-19/Japan/PG-4380/2020|2020-06-00|EPI_ISL_692607|Original|hCoV-19^^Japan|Human|Pathogen Genomics Center|Pathogen Genomics Center|Sekizuka|Japan
>Spike|hCoV-19/England/ALDP-B1F856/2020|2020-11-01|EPI_ISL_645978|Original|hCoV-19^^England|Human|Lighthouse Lab in Alderley Park|Wellcome Sanger Institute for the COVID-19 Genomics UK (COG-UK) Consortium|Davies|United Kingdom
>Spike|hCoV-19/USA/MA-MGH-01358/2020|2020-05-06|EPI_ISL_791932|Original|hCoV-19^^Massachusetts|Human|Massachusetts General Hospital|Infectious Disease Program|Park|USA
>Spike|hCoV-19/Spain/MD-IBV-97010025/2020|2020-03-24|EPI_ISL_779745|Original|hCoV-19^^Madrid|Human|IdISSC/Hospital Cl\u00ednico San Carlos de Madrid\u200b|SeqCOVID-SPAIN consortium/IBV(CSIC)|Comas|Spain
>Spike|hCoV-19/Saudi Arabia/KAUST-MADINAH344/2020|2020-04-24|EPI_ISL_513131|Original|hCoV-19^^Madinah|Human|Pathogen Genomics Lab King Abdullah University of Science and Technology(KAUST)|Pathogen Genomics Lab King Abdullah University of Science and Technology(KAUST)|Mohamed Ghazzali|Saudi Arabia
>Spike|hCoV-19/United Arab Emirates/0247/2020|2020-05-17|EPI_ISL_698429|Original|hCoV-19^^Abu Dhabi|Human|Group 42 (G42) Healthcare|G42 Healthcare|Ogrodzki|United Arab Emirates

>Spike|hCoV-19/England/CAMC-B08E03/2020|2020-10-29|EPI_ISL_673104|Original|hCoV-19^^England|Human|Lighthouse Lab in Cambridge|Wellcome Sanger Institute for the COVID-19 Genomics UK (COG-UK) Consortium|Davies|United Kingdom
>Spike|hCoV-19/England/ALDP-9E61D7/2020|2020-09-24|EPI_ISL_580415|Original|hCoV-19^^England|Human|Lighthouse Lab in Alderley Park|Wellcome Sanger Institute for the COVID-19 Genomics UK (COG-UK) consortium|Davies|United Kingdom
>Spike|hCoV-19/England/SHEF-C4FCF/2020|2020-04-03|EPI_ISL_432798|Original|hCoV-19^^England|Human|Virology Department|COVID-19 Genomics UK (COG-UK) Consortium|de Silva|United Kingdom
>Spike|hCoV-19/South Korea/KCDC2778/2020|2020-07-24|EPI_ISL_526712|Original|hCoV-19^^South Korea|Human|Division of Viral Diseases|Division of Viral Diseases|Kim|South Korea
>Spike|hCoV-19/England/ALDP-9EA6A7/2020|2020-09-24|EPI_ISL_580110|Original|hCoV-19^^England|Human|Lighthouse Lab in Alderley Park|Wellcome Sanger Institute for the COVID-19 Genomics UK (COG-UK) consortium|Davies|United Kingdom
>Spike|hCoV-19/Bangladesh/BCSIR-NILMRC-320/2020|2020-05-31|EPI_ISL_504181|Original|hCoV-19^^Mymensingh|Human|National Institute of Laboratory Medicine and Referral Center|Genomic Research Lab|Khan|Bangladesh
>Spike|hCoV-19/USA/CA-CZB-1757/2020|2020-03-19|EPI_ISL_476774|Original|hCoV-19^^California|Human|Stanford clinical virology lab|Chan-Zuckerberg Biohub|Kamm|USA
>Spike|hCoV-19/United Arab Emirates/0971/2020|2020-06-29|EPI_ISL_698191|Original|hCoV-19^^Abu Dhabi|Human|Group 42 (G42) Healthcare|G42 Healthcare|Ogrodzki|United Arab Emirates
>Spike|hCoV-19/England/MILK-B3B238/2020|2020-10-30|EPI_ISL_706055|Original|hCoV-19^^England|Human|Oxford Viromics|COVID-19 Genomics UK (COG-UK) Consortium|Golubchik|United Kingdom
>Spike|hCoV-19/Taiwan/NTU32/2020|2020-11-17|EPI_ISL_693303|Vero E6|hCoV-19^^Taipei|Human|Department of Laboratory Medicine|Microbial Genomics Core Lab|Lin|Taiwan
>Spike|hCoV-19/Finland/FIN03032020B/2020|2020-03-03|EPI_ISL_413603|Original|hCoV-19^^Helsinki|Human|Department of Virology and Immunology|Department of Virology|Smura|Finland
>Spike|hCoV-19/England/QEUH-BA0DC4/2020|2020-11-18|EPI_ISL_719622|Original|hCoV-19^^England|Human|Lighthouse Lab in Glasgow|Wellcome Sanger Institute for the COVID-19 Genomics UK (COG-UK) Consortium|Davies|United Kingdom
>Spike|hCoV-19/USA/TX-DSHS-0346/2020|2020-06-13|EPI_ISL_538284|Original|hCoV-19^^Texas|Human|Texas Department of State Health Services|Texas Department of State Health Services|Rahman|USA
>Spike|hCoV-19/Singapore/929/2020|2020-03-28|EPI_ISL_538461|Original|hCoV-19^^Singapore|Human|Department of Laboratory Medicine|Department of Laboratory Medicine|Chen|Singapore
>Spike|hCoV-19/USA/TX-HMH-MCoV-14851/2020|2020-10-13|EPI_ISL_786335|Original|hCoV-19^^Texas|Human|Houston Methodist Hospital|Houston Methodist Hospital|Long|USA
>Spike|hCoV-19/England/BRIS-12F398/2020|2020-04-11|EPI_ISL_488305|Original|hCoV-19^^England|Human|PHE South West Regional Laboratory|Wellcome Sanger Institute for the COVID-19 Genomics UK (COG-UK) consortium|Davies|United Kingdom
>Spike|hCoV-19/England/NORT-281B5F/2020|2020-03-23|EPI_ISL_472180|Original|hCoV-19^^England|Human|Northumbria University / South Tees Hospitals NHS Foundation Trust / North Cumbria Integrated Care NHS Foundation Trust / North Tees and Hartlepool NHS Foundation Trust / Newcastle Hospitals NHS Foundation Trust|COVID-19 Genomics UK (COG-UK) Consortium|Bashton|United Kingdom
>Spike|hCoV-19/Scotland/CVR4546/2020|2020-09-22|EPI_ISL_585405|Original|hCoV-19^^Scotland|Human|Lighthouse Lab in Glasgow / MRC-University of Glasgow Centre for Virus Research|COVID-19 Genomics UK (COG-UK) Consortium|Robertson|United Kingdom
>Spike|hCoV-19/Japan/PG-7333/2020|2020-08-00|EPI_ISL_688436|Original|hCoV-19^^Japan|Human|Pathogen Genomics Center|Pathogen Genomics Center|Sekizuka|Japan
>Spike|hCoV-19/England/MILK-ABBD30/2020|2020-10-21|EPI_ISL_631146|Original|hCoV-19^^England|Human|Lighthouse Lab in Milton Keynes|Wellcome Sanger Institute for the COVID-19 Genomics UK (COG-UK) consortium|Davies|United Kingdom

>Spike|hCoV-19/Wales/PHWC-260E6/2020|2020-03-25|EPI_ISL_422282|Original|hCoV-19^^Wales|Human|Wales Specialist Virology Centre|Public Health Wales Microbiology Cardiff|Moore|United Kingdom
>Spike|hCoV-19/USA/SC-DHEC-1084/2020|2020-07-08|EPI_ISL_541667|Original|hCoV-19^^South Carolina|Human|Microbiology Division|Microbiology Division|Flores|USA
>Spike|hCoV-19/Belgium/20A44/2020|2020-04-09|EPI_ISL_452149|Original|hCoV-19^^Brussels|Human|CUB Hopital Erasme Laboratoire d'Anatomie Pathologique|CUB Hopital Erasme Laboratoire d'Anatomie Pathologique|Van Campenhout|Belgium
>Spike|hCoV-19/Oman/RESP-20-B-2932/2020|2020-04-24|EPI_ISL_518847|Original|hCoV-19^^Muscat|Human|Oman-National Influenza Center|Biotechnology & OMICs Laboratory|Al Mahruqi|Oman
>Spike|hCoV-19/England/20189163501/2020|2020-05-01|EPI_ISL_465566|Original|hCoV-19^^England|Human|Respiratory Virus Unit|Respiratory Virus Unit|Platt|United Kingdom
>Spike|hCoV-19/England/CAMB-1B39D3/2020|2020-05-22|EPI_ISL_492189|Original|hCoV-19^^England|Human|Department of Pathology|Wellcome Sanger Institute for the COVID-19 Genomics UK (COG-UK) consortium|Davies|United Kingdom
>Spike|hCoV-19/Denmark/DCGC-3808/2020|2020-08-31|EPI_ISL_622702|Original|hCoV-19^^Hovedstaden|Human|Department of Virus and Microbiological Special Diagnostics|Albertsen lab|Michaelsen|Denmark
>Spike|hCoV-19/England/QEUH-9DC963/2020|2020-09-21|EPI_ISL_580914|Original|hCoV-19^^England|Human|Lighthouse Lab in Glasgow|Wellcome Sanger Institute for the COVID-19 Genomics UK (COG-UK) consortium|Davies|United Kingdom
>Spike|hCoV-19/USA/TX-HMH-MCoV-18632/2020|2020-11-20|EPI_ISL_784980|Original|hCoV-19^^Texas|Human|Houston Methodist Hospital|Houston Methodist Hospital|Long|USA
>Spike|hCoV-19/USA/WA-S1993/2020|2020-06-04|EPI_ISL_497421|Original|hCoV-19^^Washington|Human|Washington State Department of Health|Seattle Flu Study|Lee|USA
>Spike|hCoV-19/USA/CA-CZB-10003/2020|2020-07-09|EPI_ISL_672056|Original|hCoV-19^^California|Human|The Ashley Laboratory|Chan-Zuckerberg Biohub|Bhatt|USA
>Spike|hCoV-19/England/CAMC-B2260B/2020|2020-11-01|EPI_ISL_645896|Original|hCoV-19^^England|Human|Lighthouse Lab in Cambridge|Wellcome Sanger Institute for the COVID-19 Genomics UK (COG-UK) Consortium|Davies|United Kingdom
>Spike|hCoV-19/USA/FL-BMMG/2020|2020-08-31|EPI_ISL_602320|Original|hCoV-19^^Florida|Human|University of Miami Immunology and Histocompatibility Laboratory|University of Miami Immunology and Histocompatibility Laboratory|Margolles-Clark|USA
>Spike|hCoV-19/USA/FL-BPHL-1759/2020|2020-10-02|EPI_ISL_613867|Original|hCoV-19^^Florida|Human|Florida Bureau of Public Health Laboratories|Florida Bureau of Public Health Laboratories|Blanton|USA
>Spike|hCoV-19/Denmark/DCGC-21560/2020|2020-12-21|EPI_ISL_758235|Original|hCoV-19^^Syddanmark|Human|Department of Virus and Microbiological Special Diagnostics|Albertsen Lab|Michaelsen|Denmark
>Spike|hCoV-19/England/MILK-999CA5/2020|2020-08-29|EPI_ISL_550292|Original|hCoV-19^^England|Human|Lighthouse Lab in Milton Keynes|Wellcome Sanger Institute for the COVID-19 Genomics UK (COG-UK) consortium|Davies|United Kingdom
>Spike|hCoV-19/Wales/PHWC-492E23/2020|2020-11-28|EPI_ISL_725794|Original|hCoV-19^^Wales|Human|Originating lab|COVID-19 Genomics UK (COG-UK) Consortium|Moore|United Kingdom
>Spike|hCoV-19/USA/UT-UPHL-2009497/2020|2020-07-14|EPI_ISL_605181|Original|hCoV-19^^Utah|Human|Utah Public Health Laboratory|Utah Public Health Laboratory|Young|USA
>Spike|hCoV-19/England/QEUH-B2B514/2020|2020-11-03|EPI_ISL_646674|Original|hCoV-19^^England|Human|Lighthouse Lab in Glasgow|Wellcome Sanger Institute for the COVID-19 Genomics UK (COG-UK) Consortium|Davies|United Kingdom
>Spike|hCoV-19/Scotland/GCVR-171397/2020|2020-03-25|EPI_ISL_459583|Original|hCoV-19^^Scotland|Human|NHSGGC West of Scotland Specialist Virology Centre / MRC-University of Glasgow Centre for Virus Research|Wellcome Sanger Institute for the COVID-19 Genomics UK (COG-UK) consortium|Davies|United Kingdom
>Spike|hCoV-19/Scotland/EDB9175/2020|2020-11-02|EPI_ISL_651524|Original|hCoV-19^^Scotland|Human|Virology Department|COVID-19 Genomics UK (COG-UK) Consortium|McHugh|United Kingdom

>Spike|hCoV-19/Netherlands/NA_75/2020|2020-03-10|EPI_ISL_422835|Original|hCoV-19^^Netherlands|Human|Dutch COVID-19 response team|Erasmus Medical Center|Oude Munnink|Netherlands
>Spike|hCoV-19/USA/CA-CZB-4054/2020|2020-07-16|EPI_ISL_548644|Original|hCoV-19^^California|Human|County of San Luis Obispo Public Health Laboratory|Chan-Zuckerberg Biohub|Bhatt|USA
>Spike|hCoV-19/Denmark/DCGC-10092/2020|2020-11-09|EPI_ISL_669388|Original|hCoV-19^^Hovedstaden|Human|Department of Virus and Microbiological Special Diagnostics|Albertsen Lab|Michaelsen|Denmark
>Spike|hCoV-19/England/LIVE-9BE97/2020|2020-03-26|EPI_ISL_449472|Original|hCoV-19^^England|Human|Liverpool Clinical Laboratories|COVID-19 Genomics UK (COG-UK) Consortium|Haldenby|United Kingdom
>Spike|hCoV-19/United Arab Emirates/0856/2020|2020-05-19|EPI_ISL_698995|Original|hCoV-19^^Abu Dhabi|Human|Group 42 (G42) Healthcare|G42 Healthcare|Ogrodzki|United Arab Emirates
>Spike|hCoV-19/England/20142083804/2020|2020-03-30|EPI_ISL_423466|Original|hCoV-19^^England|Human|Respiratory Virus Unit|Respiratory Virus Unit|Platt|United Kingdom
>Spike|hCoV-19/Scotland/EDB6258/2020|2020-06-02|EPI_ISL_478240|Original|hCoV-19^^Scotland|Human|Virology Department|COVID-19 Genomics UK (COG-UK) Consortium|Williams|United Kingdom
>Spike|hCoV-19/USA/WA-UW45/2020|2020-03-08|EPI_ISL_415610|Original|hCoV-19^^Washington|Human|UW Virology Lab|UW Virology Lab|Roychoudhury|USA
>Spike|hCoV-19/Bahrain/110003611/2020|2020-06-25|EPI_ISL_487273|Original|hCoV-19^^Bahrain|Human|unknown|Communicable Disease Laboratory|Submitter|Bahrain
>Spike|hCoV-19/England/CAMB-1AD981/2020|2020-00-00|EPI_ISL_448030|Original|hCoV-19^^England|Human|Department of Pathology|COVID-19 Genomics UK (COG-UK) Consortium|Meredith|United Kingdom
>Spike|hCoV-19/England/20178043904/2020|2020-04-20|EPI_ISL_465420|Original|hCoV-19^^England|Human|Respiratory Virus Unit|Respiratory Virus Unit|Platt|United Kingdom
>Spike|hCoV-19/USA/WA-S1954/2020|2020-06-03|EPI_ISL_497382|Original|hCoV-19^^Washington|Human|Washington State Department of Health|Seattle Flu Study|Lee|USA
>Spike|hCoV-19/England/20110000706/2020|2020-03-05|EPI_ISL_417302|Original|hCoV-19^^England|Human|Respiratory Virus Unit|Respiratory Virus Unit|Platt|United Kingdom
>Spike|hCoV-19/England/MILK-AB7833/2020|2020-10-21|EPI_ISL_623518|Original|hCoV-19^^England|Human|Lighthouse Lab in Milton Keynes|Wellcome Sanger Institute for the COVID-19 Genomics UK (COG-UK) consortium|Davies|United Kingdom
>Spike|hCoV-19/Denmark/DCGC-19237/2020|2020-12-07|EPI_ISL_748835|Original|hCoV-19^^Hovedstaden|Human|Department of Virus and Microbiological Special Diagnostics|Albertsen Lab|Michaelsen|Denmark
>Spike|hCoV-19/USA/WA-S2796/2020|2020-08-19|EPI_ISL_530153|Original|hCoV-19^^Washington|Human|Seattle Flu Study|Seattle Flu Study|Lee|USA
>Spike|hCoV-19/Scotland/EDB4244/2020|2020-04-21|EPI_ISL_487879|Original|hCoV-19^^Scotland|Human|Virology Department|Wellcome Sanger Institute for the COVID-19 Genomics UK (COG-UK) consortium|Davies|United Kingdom
>Spike|hCoV-19/USA/CO-CDPHE-2005220677/2020|2020-05-21|EPI_ISL_710311|Original|hCoV-19^^Colorado|Human|Colorado Department of Public Health and Environment|Colorado Department of Puplic Health and Environment|Bankers|USA
>Spike|hCoV-19/Switzerland/GE-SNRCI-7899/2020|2020-03-00|EPI_ISL_548207|Original|hCoV-19^^Geneve|Human|Laboratoire de Virologie|Swiss National Reference Centre for Influenza|Gon\u00e7alves Cabecinhas|Switzerland
>Spike|hCoV-19/Australia/VIC5097/2020|2020-07-19|EPI_ISL_519900|Original|hCoV-19^^Victoria|Human|Microbiological Diagnostic Unit - Public Health Laboratory (MDU-PHL)|MDU-PHL|Schultz|Australia
>Spike|hCoV-19/Wales/PHWC-4A57C8/2020|2020-12-16|EPI_ISL_764921|Original|hCoV-19^^Wales|Human|Wales Specialist Virology Centre Sequencing lab|COVID-19 Genomics UK (COG-UK) Consortium|Moore|United Kingdom
>Spike|hCoV-19/USA/WA-S895/2020|2020-04-24|EPI_ISL_463385|Original|hCoV-19^^Washington|Human|Washington State Department of Health|Seattle Flu Study|Bedford|USA

>Spike|hCoV-19/England/ALDP-BB4914/2020|2020-11-20|EPI_ISL_702609|Original|hCoV-19^^England|Human|Lighthouse Lab in Alderley Park|Wellcome Sanger Institute for the COVID-19 Genomics UK (COG-UK) Consortium|Davies|United Kingdom
>Spike|hCoV-19/Spain/PV-IBV-000910/2020|2020-03-26|EPI_ISL_452657|Original|hCoV-19^^Basque Country|Human|Servicio de Microbiolog\u00eda. Hospital Universitario Donostia. OSI Donostialdea. \u00c1rea de Enfermedades Infecciosas|SeqCOVID-SPAIN consortium/IBV(CSIC)|Comas|Spain
>Spike|hCoV-19/Portugal/PT1868/2020|2020-11-10|EPI_ISL_732087|Original|hCoV-19^^Portugal|Human|Instituto Nacional de Saude (INSA)|Instituto Nacional de Saude (INSA)|Borges|Portugal
>Spike|hCoV-19/Denmark/DCGC-3661/2020|2020-08-31|EPI_ISL_615433|Original|hCoV-19^^Syddanmark|Human|Department of Virus and Microbiological Special Diagnostics|Albertsen lab|Michaelsen|Denmark
>Spike|hCoV-19/England/BIRM-659D1/2020|2020-10-02|EPI_ISL_594600|Original|hCoV-19^^England|Human|University of Birmingham|COVID-19 Genomics UK (COG-UK) Consortium|Loman|United Kingdom
>Spike|hCoV-19/England/ALDP-645E0F/2020|2020-07-03|EPI_ISL_558016|Original|hCoV-19^^England|Human|Lighthouse Lab in Alderley Park|Wellcome Sanger Institute for the COVID-19 Genomics UK (COG-UK) consortium|Davies|United Kingdom
>Spike|hCoV-19/USA/TX-HMH-MCoV-14032/2020|2020-08-14|EPI_ISL_787321|Original|hCoV-19^^Texas|Human|Houston Methodist Hospital|Houston Methodist Hospital|Long|USA
>Spike|hCoV-19/USA/TX-HMH-MCoV-8091/2020|2020-06-29|EPI_ISL_784191|Original|hCoV-19^^Texas|Human|Houston Methodist Hospital|Houston Methodist Hospital|Long|USA
>Spike|hCoV-19/South Korea/KCDC2773/2020|2020-07-23|EPI_ISL_526707|Original|hCoV-19^^South Korea|Human|Division of Viral Diseases|Division of Viral Diseases|Kim|South Korea
>Spike|hCoV-19/England/ALDP-95412F/2020|2020-06-13|EPI_ISL_553781|Original|hCoV-19^^England|Human|Lighthouse Lab in Alderley Park|Wellcome Sanger Institute for the COVID-19 Genomics UK (COG-UK) consortium|Davies|United Kingdom
>Spike|hCoV-19/New Zealand/20VR1793/2020|2020-03-20|EPI_ISL_579130|Original|hCoV-19^^Counties Manukau|Human|Middlemore Hospital|Institute of Environmental Science and Research (ESR)|Wang|New Zealand
>Spike|hCoV-19/France/ARA-SC141/2020|2020-05-02|EPI_ISL_700405|Original|hCoV-19^^Auvergne-Rh\u00f4ne-Alpes|Human|Centre hospitalier M\u00e9tropole Savoie|CNR Virus des Infections Respiratoires - France SUD|Bal|France
>Spike|hCoV-19/India/MH-NCCS-NR1885/2020|2020-05-19|EPI_ISL_496566|Original|hCoV-19^^Maharashtra|Human|National Centre For Cell Science|National Centre For Cell Science|Paul|India
>Spike|hCoV-19/Spain/VC-FISABIO-204/2020|2020-03-22|EPI_ISL_436299|Original|hCoV-19^^Comunitat Valenciana|Human|Servicio de Microbiologia. Hospital Clinico Universitario de Valencia|Sequencing and Bioinformatics Service and Molecular Epidemiology Research Group. FISABIO-Public Health|Gonzalez-Candelas|Spain
>Spike|hCoV-19/Wales/PHWC-4A684C/2020|2020-12-17|EPI_ISL_765077|Original|hCoV-19^^Wales|Human|Wales Specialist Virology Centre Sequencing lab|COVID-19 Genomics UK (COG-UK) Consortium|Moore|United Kingdom
>Spike|hCoV-19/England/MILK-9E0CFA/2020|2020-09-19|EPI_ISL_601480|Original|hCoV-19^^England|Human|Lighthouse Lab in Milton Keynes|Wellcome Sanger Institute for the COVID-19 Genomics UK (COG-UK) consortium|Davies|United Kingdom
>Spike|hCoV-19/England/NORT-28C8E4/2020|2020-05-16|EPI_ISL_469318|Original|hCoV-19^^England|Human|NU-OMICS DNA Sequencing research facility|Wellcome Sanger Institute for the COVID-19 Genomics UK (COG-UK) consortium|Davies|United Kingdom
>Spike|hCoV-19/South Africa/KRISP-K003674/2020|2020-07-27|EPI_ISL_602630|H1299|hCoV-19^^KwaZulu-Natal|Human|AHRI-Sigal|KRISP|de Oliveira|South Africa
>Spike|hCoV-19/Denmark/DCGC-4358/2020|2020-09-07|EPI_ISL_615560|Original|hCoV-19^^Nordjylland|Human|Department of Virus and Microbiological Special Diagnostics|Albertsen lab|Michaelsen|Denmark
>Spike|hCoV-19/England/QEUH-AD047C/2020|2020-10-23|EPI_ISL_634143|Original|hCoV-19^^England|Human|Lighthouse Lab in Glasgow|Wellcome Sanger Institute for the COVID-19 Genomics UK (COG-UK) consortium|Davies|United Kingdom

>Spike|hCoV-19/England/MILK-A7A0BC/2020|2020-10-15|EPI_ISL_606621|Original|hCoV-19^^England|Human|Lighthouse Lab in Milton Keynes|Wellcome Sanger Institute for the COVID-19 Genomics UK (COG-UK) consortium|Davies|United Kingdom
>Spike|hCoV-19/England/NORT-29DD2D/2020|2020-05-06|EPI_ISL_493498|Original|hCoV-19^^England|Human|Northumbria University / South Tees Hospitals NHS Foundation Trust / North Cumbria Integrated Care NHS Foundation Trust / North Tees and Hartlepool NHS Foundation Trust / Newcastle Hospitals NHS Foundation Trust|COVID-19 Genomics UK (COG-UK) Consortium|Bashton|United Kingdom
>Spike|hCoV-19/England/SHEF-CF920/2020|2020-09-19|EPI_ISL_573748|Original|hCoV-19^^England|Human|Virology Department|COVID-19 Genomics UK (COG-UK) Consortium|de Silva|United Kingdom
>Spike|hCoV-19/England/CAMC-B0986E/2020|2020-10-30|EPI_ISL_673042|Original|hCoV-19^^England|Human|Lighthouse Lab in Cambridge|Wellcome Sanger Institute for the COVID-19 Genomics UK (COG-UK) Consortium|Davies|United Kingdom
>Spike|hCoV-19/Spain/CT-IBV-99009906/2020|2020-08-08|EPI_ISL_660303|Original|hCoV-19^^Catalunya|Human|Servicio de Microbiolog\u00eda|SeqCOVID-SPAIN consortium/IBV(CSIC)|Comas|Spain
>Spike|hCoV-19/USA/WA-UW40/2020|2020-03-05|EPI_ISL_415605|Original|hCoV-19^^Washington|Human|UW Virology Lab|UW Virology Lab|Roychoudhury|USA
>Spike|hCoV-19/England/QEUH-B7C3B0/2020|2020-11-13|EPI_ISL_668321|Original|hCoV-19^^England|Human|Lighthouse Lab in Glasgow|Wellcome Sanger Institute for the COVID-19 Genomics UK (COG-UK) Consortium|Davies|United Kingdom
>Spike|hCoV-19/England/CAMB-1B9E65/2020|2020-11-05|EPI_ISL_650962|Original|hCoV-19^^England|Human|Department of Pathology|COVID-19 Genomics UK (COG-UK) Consortium|Goodfellow|United Kingdom
>Spike|hCoV-19/England/QEUH-A7928A/2020|2020-10-12|EPI_ISL_638795|Original|hCoV-19^^England|Human|Oxford Viromics|COVID-19 Genomics UK (COG-UK) Consortium|Golubchik|United Kingdom
>Spike|hCoV-19/Spain/CM-ISCIII-202018188/2020|2020-03-21|EPI_ISL_539526|Original|hCoV-19^^Castilla La Mancha|Human|Consejeria de Sanidad y Asuntos Sociales|Instituto de Salud Carlos III|Caballero|Spain
>Spike|hCoV-19/England/ALDP-A957F2/2020|2020-10-19|EPI_ISL_624657|Original|hCoV-19^^England|Human|Lighthouse Lab in Alderley Park|Wellcome Sanger Institute for the COVID-19 Genomics UK (COG-UK) consortium|Davies|United Kingdom
>Spike|hCoV-19/Australia/SAP180/2020|2020-03-18|EPI_ISL_492121|Original|hCoV-19^^South Australia|Human|SA Pathology|SA Pathology|Leong|Australia
>Spike|hCoV-19/South Korea/KCDC2458/2020|2020-06-08|EPI_ISL_514861|Original|hCoV-19^^South Korea|Human|Division of Viral Diseases|Division of Viral Diseases|Kim|South Korea
>Spike|hCoV-19/England/LOND-124C5D7/2020|2020-03-30|EPI_ISL_492876|Original|hCoV-19^^England|Human|Royal Free Hospital / Health Services Laboratories|Wellcome Sanger Institute for the COVID-19 Genomics UK (COG-UK) consortium|Davies|United Kingdom
>Spike|hCoV-19/England/CAMC-B0ABFB/2020|2020-10-29|EPI_ISL_643374|Original|hCoV-19^^England|Human|Lighthouse Lab in Cambridge|Wellcome Sanger Institute for the COVID-19 Genomics UK (COG-UK) Consortium|Davies|United Kingdom
>Spike|hCoV-19/Netherlands/UT-RIVM-20819/2020|2020-12-16|EPI_ISL_790875|Original|hCoV-19^^Utrecht|Human|Dutch COVID-19 response team|National Institute for Public Health and the Environment (RIVM)|Meijer|Netherlands
>Spike|hCoV-19/Wales/PHWC-34689/2020|2020-04-20|EPI_ISL_446956|Original|hCoV-19^^Wales|Human|Wales Specialist Virology Centre|Public Health Wales Microbiology Cardiff|Moore|United Kingdom
>Spike|hCoV-19/Bangladesh/BCSIR-NILMRC-192/2020|2020-06-17|EPI_ISL_483634|Original|hCoV-19^^Chittagong|Human|National Institute of Laboratory Medicine and Referral Center|Genomic Research Lab|Khan|Bangladesh
>Spike|hCoV-19/USA/NY-Wadsworth-248789-01/2020|2020-10-19|EPI_ISL_734374|Original|hCoV-19^^New York|Human|Wadsworth Center|Wadsworth Center|Lasek-Nesselquist|USA

>Spike|hCoV-19/Wales/PHWC-28688/2020|2020-03-31|EPI_ISL_432366|Original|hCoV-19^^Wales|Human|Wales Specialist Virology Centre|Public Health Wales Microbiology Cardiff|Moore|United Kingdom
>Spike|hCoV-19/England/QEUH-98FB1E/2020|2020-08-26|EPI_ISL_539974|Original|hCoV-19^^England|Human|Lighthouse Lab in Glasgow|Wellcome Sanger Institute for the COVID-19 Genomics UK (COG-UK) consortium|Davies|United Kingdom
>Spike|hCoV-19/USA/MI-MDHHS-SC20752/2020|2020-04-30|EPI_ISL_471773|Original|hCoV-19^^Michigan|Human|Michigan Department of Health and Human Services|Michigan Department of Health and Human Services|Blankenship|USA
>Spike|hCoV-19/England/PORT-2EA4D1/2020|2020-11-17|EPI_ISL_665616|Original|hCoV-19^^England|Human|Centre for Enzyme Innovation|COVID-19 Genomics UK (COG-UK) Consortium|Robson|United Kingdom
>Spike|hCoV-19/Wales/PHWC-2BAD4/2020|2020-03-28|EPI_ISL_445806|Original|hCoV-19^^Wales|Human|Wales Specialist Virology Centre|Public Health Wales Microbiology Cardiff|Moore|United Kingdom
>Spike|hCoV-19/USA/VA_NIDDL_2996/2020|2020-04-07|EPI_ISL_491926|Original|hCoV-19^^Virginia|Human|Naval Infectious Diseases Diagnostic Laboratory|Naval Medical Research Center Biological Defense Research Directorate|Voegtly|USA
>Spike|hCoV-19/Australia/NSW243/2020|2020-03-30|EPI_ISL_451509|Original|hCoV-19^^New South Wales|Human|Pathology West - NSW Health Pathology|NSW Health Pathology - Institute of Clinical Pathology and Medical Research; Westmead Hospital; University of Sydney|Gall|Australia
>Spike|hCoV-19/England/MILK-BB13BF/2020|2020-11-17|EPI_ISL_720174|Original|hCoV-19^^England|Human|Lighthouse Lab in Milton Keynes|Wellcome Sanger Institute for the COVID-19 Genomics UK (COG-UK) Consortium|Davies|United Kingdom
>Spike|hCoV-19/Denmark/DCGC-9996/2020|2020-11-09|EPI_ISL_669320|Original|hCoV-19^^Syddanmark|Human|Department of Virus and Microbiological Special Diagnostics|Albertsen Lab|Michaelsen|Denmark
>Spike|hCoV-19/England/ALDP-90C79D/2020|2020-06-22|EPI_ISL_551463|Original|hCoV-19^^England|Human|Lighthouse Lab in Alderley Park|Wellcome Sanger Institute for the COVID-19 Genomics UK (COG-UK) consortium|Davies|United Kingdom
>Spike|hCoV-19/Belgium/ULG-8543/2020|2020-03-19|EPI_ISL_418637|Original|hCoV-19^^Li\u00e8ge|Human|Department of Clinical Microbiology|GIGA Medical Genomics|Durkin|Belgium
>Spike|hCoV-19/Portugal/PT1464/2020|2020-03-28|EPI_ISL_511423|Original|hCoV-19^^Portugal|Human|Instituto Nacional de Saude (INSA)|Instituto Nacional de Saude (INSA)|Borges|Portugal
>Spike|hCoV-19/Netherlands/Gelderland_67/2020|2020-05-18|EPI_ISL_460966|Original|hCoV-19^^Gelderland|Human|Dutch COVID-19 response team|Erasmus Medical Center|Oude Munnink|Netherlands
>Spike|hCoV-19/Wales/CAMC-A80E40/2020|2020-10-14|EPI_ISL_625304|Original|hCoV-19^^Wales|Human|Lighthouse Lab in Cambridge|Wellcome Sanger Institute for the COVID-19 Genomics UK (COG-UK) consortium|Davies|United Kingdom
>Spike|hCoV-19/Wales/PHWC-16C05C/2020|2020-09-17|EPI_ISL_574006|Original|hCoV-19^^Wales|Human|Wales Specialist Virology Centre Sequencing lab|COVID-19 Genomics UK (COG-UK) Consortium|Moore|United Kingdom
>Spike|hCoV-19/Scotland/EDB2614/2020|2020-04-25|EPI_ISL_439243|Original|hCoV-19^^Scotland|Human|Virology Department|COVID-19 Genomics UK (COG-UK) Consortium|Williams|United Kingdom
>Spike|hCoV-19/England/MILK-993A83/2020|2020-08-26|EPI_ISL_552381|Original|hCoV-19^^England|Human|Lighthouse Lab in Milton Keynes|Wellcome Sanger Institute for the COVID-19 Genomics UK (COG-UK) consortium|Davies|United Kingdom
>Spike|hCoV-19/Australia/NSW130/2020|2020-03-24|EPI_ISL_427768|Original|hCoV-19^^New South Wales|Human|Centre for Infectious Diseases and Microbiology Public Health|NSW Health Pathology - Institute of Clinical Pathology and Medical Research; Westmead Hospital; University of Sydney|Gall|Australia
>Spike|hCoV-19/England/CAMB-77ECE/2020|2020-04-01|EPI_ISL_441840|Original|hCoV-19^^England|Human|Department of Pathology|Wellcome Sanger Institute for the COVID-19 Genomics UK (COG-UK) consortium|Amato|United Kingdom

>Spike|hCoV-19/England/MILK-99CFB8/2020|2020-08-30|EPI_ISL_549908|Original|hCoV-19^^England|Human|Lighthouse Lab in Milton Keynes|Wellcome Sanger Institute for the COVID-19 Genomics UK (COG-UK) consortium|Davies|United Kingdom
>Spike|hCoV-19/USA/TX-HMH-MCoV-14607/2020|2020-10-09|EPI_ISL_787892|Original|hCoV-19^^Texas|Human|Houston Methodist Hospital|Houston Methodist Hospital|Long|USA
>Spike|hCoV-19/England/ALDP-95C1AF/2020|2020-06-17|EPI_ISL_555120|Original|hCoV-19^^England|Human|Lighthouse Lab in Alderley Park|Wellcome Sanger Institute for the COVID-19 Genomics UK (COG-UK) consortium|Davies|United Kingdom
>Spike|hCoV-19/Wales/PHWC-49003E/2020|2020-11-12|EPI_ISL_706441|Original|hCoV-19^^Wales|Human|Originating lab|COVID-19 Genomics UK (COG-UK) Consortium|Moore|United Kingdom
>Spike|hCoV-19/Canada/BC_21008826/2020|2020-03-00|EPI_ISL_462785|Original|hCoV-19^^British Columbia|Human|BCCDC Public Health Laboratory|BCCDC Public Health Laboratory|Harrigan|Canada
>Spike|hCoV-19/Canada/ON-UHTC_0399/2020|2020-11-24|EPI_ISL_698080|Original|hCoV-19^^Ontario|Human|Unity Health Toronto|Ontario Institute for Cancer Research|de Borja|Canada
>Spike|hCoV-19/England/QEUH-AE286C/2020|2020-10-27|EPI_ISL_633550|Original|hCoV-19^^England|Human|Lighthouse Lab in Glasgow|Wellcome Sanger Institute for the COVID-19 Genomics UK (COG-UK) consortium|Davies|United Kingdom
>Spike|hCoV-19/Australia/VIC14492/2020|2020-08-13|EPI_ISL_592519|Original|hCoV-19^^Victoria|Human|Microbiological Diagnostic Unit - Public Health Laboratory (MDU-PHL)|MDU-PHL|Schultz|Australia
>Spike|hCoV-19/USA/NY-Wadsworth-192873-01/2020|2020-06-24|EPI_ISL_677004|Original|hCoV-19^^New York|Human|Masonic Medical Research Institute|Wadsworth Center|Lasek-Nesselquist|USA
>Spike|hCoV-19/England/CAMC-A3F04A/2020|2020-10-06|EPI_ISL_609715|Original|hCoV-19^^England|Human|Lighthouse Lab in Cambridge|Wellcome Sanger Institute for the COVID-19 Genomics UK (COG-UK) consortium|Davies|United Kingdom
>Spike|hCoV-19/South Africa/KRISP-K002370/2020|2020-07-22|EPI_ISL_515738|Original|hCoV-19^^KwaZulu-Natal|Human|NHLS-IALCH|KRISP|de Oliveira|South Africa
>Spike|hCoV-19/Denmark/DCGC-17620/2020|2020-12-14|EPI_ISL_751109|Original|hCoV-19^^Syddanmark|Human|Department of Virus and Microbiological Special Diagnostics|Albertsen Lab|Michaelsen|Denmark
>Spike|hCoV-19/USA/CA-IGI-0072/2020|2020-05-29|EPI_ISL_511826|Original|hCoV-19^^California|Human|Innovative Genomics Institute|Innovative Genomics Institute|Wyman|USA
>Spike|hCoV-19/USA/UT-UPHL-2012759080/2020|2020-08-11|EPI_ISL_745903|Original|hCoV-19^^Utah|Human|Ginkgo Bioworks Clinical Laboratory|Utah Public Health Laboratory|Young|USA
>Spike|hCoV-19/England/QEUH-B7DC87/2020|2020-11-14|EPI_ISL_741912|Original|hCoV-19^^England|Human|Oxford Viromics|COVID-19 Genomics UK (COG-UK) Consortium|Golubchik|United Kingdom
>Spike|hCoV-19/Scotland/QEUH-9CFDFE/2020|2020-09-16|EPI_ISL_601838|Original|hCoV-19^^Scotland|Human|Lighthouse Lab in Glasgow|Wellcome Sanger Institute for the COVID-19 Genomics UK (COG-UK) consortium|Davies|United Kingdom
>Spike|hCoV-19/Wales/PHWC-328C1/2020|2020-04-14|EPI_ISL_474362|Original|hCoV-19^^Wales|Human|Originating lab|COVID-19 Genomics UK (COG-UK) Consortium|Moore|United Kingdom
>Spike|hCoV-19/Belgium/rega-10191284/2020|2020-10-19|EPI_ISL_735191|Original|hCoV-19^^Blanden|Human|UZ Leuven|KU Leuven|Maes|Belgium
>Spike|hCoV-19/USA/CA-ALSR-3209/2020|2020-07-27|EPI_ISL_635911|Original|hCoV-19^^California|Human|San Diego County Public Health Laboratory|Andersen lab at Scripps Research|Gangavarapu|USA
>Spike|hCoV-19/USA/WI-UW-1803/2020|2020-09-10|EPI_ISL_605675|Original|hCoV-19^^Wisconsin|Human|University of Wisconsin-Madison AIDS Vaccine Research Laboratories|University of Wisconsin-Madison AIDS Vaccine Research Laboratories|Baczenas|USA

>Spike|hCoV-19/Wales/PHWC-16BB2F/2020|2020-09-22|EPI_ISL_573943|Original|hCoV-
19^^Wales|Human|Wales Specialist Virology Centre Sequencing lab|COVID-19 Genomics UK
(COG-UK) Consortium|Moore|United Kingdom
>Spike|hCoV-19/England/CAMC-A829C5/2020|2020-10-10|EPI_ISL_610826|Original|hCoV-
19^^England|Human|Lighthouse Lab in Cambridge|Wellcome Sanger Institute for the
COVID-19 Genomics UK (COG-UK) consortium|Davies|United Kingdom
>Spike|hCoV-19/Singapore/156/2020|2020-03-20|EPI_ISL_443248|Original|hCoV-
19^^Singapore|Human|National Public Health Laboratory|National Public Health
Laboratory|Mak|Singapore
>Spike|hCoV-19/Wales/PHWC-4A351B/2020|2020-12-14|EPI_ISL_744106|Original|hCoV-
19^^Wales|Human|Wales Specialist Virology Centre Sequencing lab|COVID-19 Genomics UK
(COG-UK) Consortium|Moore|United Kingdom
>Spike|hCoV-19/Wales/PHWC-48D66F/2020|2020-11-15|EPI_ISL_680658|Original|hCoV-
19^^Wales|Human|Wales Specialist Virology Centre Sequencing lab|COVID-19 Genomics UK
(COG-UK) Consortium|Moore|United Kingdom
>Spike|hCoV-19/Netherlands/ZuidHolland_56/2020|2020-03-
23|EPI_ISL_422924|Original|hCoV-19^^Zuid Holland|Human|Dutch COVID-19 response
team|Erasmus Medical Center|Oude Munnink|Netherlands
>Spike|hCoV-19/Australia/VIC9654/2020|2020-08-14|EPI_ISL_565640|Original|hCoV-
19^^Victoria|Human|Microbiological Diagnostic Unit - Public Health Laboratory (MDU-
PHL)|MDU-PHL|Schultz|Australia
>Spike|hCoV-19/USA/MA-MGH-00004/2020|2020-03-11|EPI_ISL_460467|Original|hCoV-
19^^Massachusetts|Human|Massachusetts General Hospital|Infectious Disease
Program|Lemieux|USA
>Spike|hCoV-19/USA/WA-S2456/2020|2020-06-16|EPI_ISL_497088|Original|hCoV-
19^^Washington|Human|Washington State Department of Health|Seattle Flu Study|Lee|USA
>Spike|hCoV-19/England/SHEF-C2538/2020|2020-11-03|EPI_ISL_653061|Original|hCoV-
19^^England|Human|Virology Department|COVID-19 Genomics UK (COG-UK) Consortium|de
Silva|United Kingdom
>Spike|hCoV-19/England/NOTT-11235C/2020|2020-04-25|EPI_ISL_484374|Original|hCoV-
19^^England|Human|Queens Medical Centre|COVID-19 Genomics UK (COG-UK)
Consortium|Loose|United Kingdom
>Spike|hCoV-19/Netherlands/Flevoland_12/2020|2020-05-01|EPI_ISL_460835|Original|hCoV-
19^^Flevoland|Human|Dutch COVID-19 response team|Erasmus Medical Center|Oude
Munnink|Netherlands
>Spike|hCoV-19/Japan/PG-7616/2020|2020-08-00|EPI_ISL_686718|Original|hCoV-
19^^Japan|Human|Chiba Prefectural Institute of Public Health|Pathogen Genomics
Center|Sekizuka|Japan
>Spike|hCoV-19/Wales/PHWC-4976CC/2020|2020-11-21|EPI_ISL_726867|Original|hCoV-
19^^Wales|Human|Originating lab|COVID-19 Genomics UK (COG-UK) Consortium|Moore|United
Kingdom
>Spike|hCoV-19/England/ALDP-B74A68/2020|2020-11-12|EPI_ISL_678963|Original|hCoV-
19^^England|Human|Queens Medical Centre|COVID-19 Genomics UK (COG-UK)
Consortium|Loose|United Kingdom
>Spike|hCoV-19/England/20186078004/2020|2020-04-29|EPI_ISL_465477|Original|hCoV-
19^^England|Human|Respiratory Virus Unit|Respiratory Virus Unit|Platt|United Kingdom
>Spike|hCoV-19/South Africa/NHLS-UCT-GS-7030-KRISP/2020|2020-11-
24|EPI_ISL_696509|Original|hCoV-19^^Western Cape|Human|Conville CDC wc CVC &
NHLS/UCT|KRISP|de Oliveira|South Africa
>Spike|hCoV-19/USA/TX-HMH-MCoV-15108/2020|2020-10-18|EPI_ISL_786592|Original|hCoV-
19^^Texas|Human|Houston Methodist Hospital|Houston Methodist Hospital|Long|USA
>Spike|hCoV-19/Spain/AR-IBV-98008080/2020|2020-09-30|EPI_ISL_654425|Original|hCoV-
19^^Aragon|Human|Servicio de Microbiolog\u00eda|SeqCOVID-SPAIN
consortium/IBV(CSIC)|Comas|Spain
>Spike|hCoV-19/Scotland/QEUH-9F705E/2020|2020-09-27|EPI_ISL_595656|Original|hCoV-
19^^Scotland|Human|Oxford Viromics|COVID-19 Genomics UK (COG-UK)
Consortium|Golubchik|United Kingdom
>Spike|hCoV-19/England/MILK-9A863C/2020|2020-09-02|EPI_ISL_550125|Original|hCoV-
19^^England|Human|Lighthouse Lab in Milton Keynes|Wellcome Sanger Institute for the
COVID-19 Genomics UK (COG-UK) consortium|Davies|United Kingdom

>Spike|hCoV-19/SaudiArabia/KAUST-JEDDAH566/2020|2020-04-22|EPI_ISL_512963|Original|hCoV-19^^Jeddah|Human|Pathogen Genomics Lab King Abdullah University of Science and Technology(KAUST)|Pathogen Genomics Lab King Abdullah University of Science and Technology(KAUST)|Mohamed Ghazzali|Saudi Arabia
>Spike|hCoV-19/England/ALDP-B62FC8/2020|2020-11-08|EPI_ISL_655387|Original|hCoV-19^^England|Human|Lighthouse Lab in Alderley Park|Wellcome Sanger Institute for the COVID-19 Genomics UK (COG-UK) Consortium|Davies|United Kingdom
>Spike|hCoV-19/USA/WI-WSLH-200565/2020|2020-05-29|EPI_ISL_578678|Original|hCoV-19^^Wisconsin|Human|Wisconsin State Laboratory of Hygiene Communicable Disease Division|Wisconsin State Laboratory of Hygiene Communicable Disease Division|Florek|USA
>Spike|hCoV-19/England/ALDP-C47EF8/2020|2020-12-12|EPI_ISL_718828|Original|hCoV-19^^England|Human|Lighthouse Lab in Alderley Park|Wellcome Sanger Institute for the COVID-19 Genomics UK (COG-UK) Consortium|Davies|United Kingdom
>Spike|hCoV-19/England/HECH-2599C94/2020|2020-05-05|EPI_ISL_482092|Original|hCoV-19^^England|Human|Microbiology Department|Wellcome Sanger Institute for the COVID-19 Genomics UK (COG-UK) consortium|Davies|United Kingdom
>Spike|hCoV-19/USA/WI-UW-2109/2020|2020-09-29|EPI_ISL_677514|Original|hCoV-19^^Wisconsin|Human|University of Wisconsin-Madison AIDS Vaccine Research Laboratories|University of Wisconsin-Madison AIDS Vaccine Research Laboratories|Baczenas|USA
>Spike|hCoV-19/Canada/BC_22037912/2020|2020-03-00|EPI_ISL_462822|Original|hCoV-19^^British Columbia|Human|BCCDC Public Health Laboratory|BCCDC Public Health Laboratory|Harrigan|Canada
>Spike|hCoV-19/England/NORT-1B72320/2020|2020-11-26|EPI_ISL_724870|Original|hCoV-19^^England|Human|Northumbria University / South Tees Hospitals NHS Foundation Trust / North Cumbria Integrated Care NHS Foundation Trust / North Tees and Hartlepool NHS Foundation Trust / Newcastle Hospitals NHS Foundation Trust|COVID-19 Genomics UK (COG-UK) Consortium|Bashton|United Kingdom
>Spike|hCoV-19/USA/VA-DCLS-0462/2020|2020-05-05|EPI_ISL_477671|Original|hCoV-19^^Virginia|Human|Virginia DCLS|Virginia DCLS|St. Jacques|USA
>Spike|hCoV-19/Switzerland/BS-42386285/2020|2020-08-08|EPI_ISL_581942|Original|hCoV-19^^Basel-Stadt|Human|University Hospital Basel|University Hospital Basel|Roloff Handschin|Switzerland
>Spike|hCoV-19/England/LIVE-A7469/2020|2020-03-28|EPI_ISL_517024|Original|hCoV-19^^England|Human|Liverpool Clinical Laboratories|COVID-19 Genomics UK (COG-UK) Consortium|Haldenby|United Kingdom
>Spike|hCoV-19/Japan/PG-6842/2020|2020-09-00|EPI_ISL_687950|Original|hCoV-19^^Japan|Human|Pathogen Genomics Center|Pathogen Genomics Center|Sekizuka|Japan
>Spike|hCoV-19/England/GSTT-265D581/2020|2020-03-23|EPI_ISL_483979|Original|hCoV-19^^England|Human|Centre for Clinical Infection and Diagnostics Research and Genomics Innovation Unit|COVID-19 Genomics UK (COG-UK) Consortium|Awan|United Kingdom
>Spike|hCoV-19/Wales/PHWC-15CB03/2020|2020-03-29|EPI_ISL_472581|Original|hCoV-19^^Wales|Human|Wales Specialist Virology Centre Sequencing lab|COVID-19 Genomics UK (COG-UK) Consortium|Moore|United Kingdom
>Spike|hCoV-19/England/QEUH-A8A990/2020|2020-10-15|EPI_ISL_638865|Original|hCoV-19^^England|Human|Oxford Viromics|COVID-19 Genomics UK (COG-UK) Consortium|Golubchik|United Kingdom
>Spike|hCoV-19/England/204590493/2020|2020-11-06|EPI_ISL_683513|Original|hCoV-19^^England|Human|Respiratory Virus Unit|COVID-19 Genomics UK (COG-UK) Consortium|Platt|United Kingdom
>Spike|hCoV-19/USA/TX-DSHS-0901/2020|2020-06-16|EPI_ISL_610037|Original|hCoV-19^^Texas|Human|Texas Department of State Health Services|Texas Department of State Health Services|Rahman|USA
>Spike|hCoV-19/England/LIVE-9DE95/2020|2020-04-05|EPI_ISL_453242|Original|hCoV-19^^England|Human|Liverpool Clinical Laboratories|COVID-19 Genomics UK (COG-UK) Consortium|Haldenby|United Kingdom
>Spike|hCoV-19/South Korea/KCDC2109/2020|2020-03-11|EPI_ISL_498010|Original|hCoV-19^^South Korea|Human|Division of Viral Diseases|Division of Viral Diseases|Kim|South Korea

>Spike|hCoV-19/Peru/ANC-UPCH-0105/2020|2020-06-16|EPI_ISL_540983|Original|hCoV-19^^Ancash|Human|Laboratorio de Referencia Nacional de Virus Respiratorios|Laboratorio de Gen\u00f3mica Microbiana|Tsukayama|Peru
>Spike|hCoV-19/USA/UT-UPHL-2012332844/2020|2020-08-14|EPI_ISL_746111|Original|hCoV-19^^Utah|Human|Ginkgo Bioworks Clinical Laboratory|Utah Public Health Laboratory|Young|USA
>Spike|hCoV-19/Japan/PG-0298/2020|2020-03-00|EPI_ISL_684157|Original|hCoV-19^^Japan|Human|Pathogen Genomics Center|Pathogen Genomics Center|Sekizuka|Japan
>Spike|hCoV-19/England/NOTT-113AED/2020|2020-09-26|EPI_ISL_573457|Original|hCoV-19^^England|Human|Queens Medical Centre|COVID-19 Genomics UK (COG-UK) Consortium|Loose|United Kingdom
>Spike|hCoV-19/England/LIVE-A9F75/2020|2020-03-25|EPI_ISL_517379|Original|hCoV-19^^England|Human|Liverpool Clinical Laboratories|COVID-19 Genomics UK (COG-UK) Consortium|Haldenby|United Kingdom
>Spike|hCoV-19/South Korea/KCDC2420/2020|2020-06-03|EPI_ISL_514823|Original|hCoV-19^^South Korea|Human|Division of Viral Diseases|Division of Viral Diseases|Kim|South Korea
>Spike|hCoV-19/Scotland/CVR1624/2020|2020-04-05|EPI_ISL_448121|Original|hCoV-19^^Scotland|Human|West of Scotland Specialist Virology Centre|COVID-19 Genomics UK (COG-UK) Consortium|Robertson|United Kingdom
>Spike|hCoV-19/England/ALDP-C5802B/2020|2020-12-14|EPI_ISL_735980|Original|hCoV-19^^England|Human|Lighthouse Lab in Alderley Park|Wellcome Sanger Institute for the COVID-19 Genomics UK (COG-UK) Consortium|Davies|United Kingdom
>Spike|hCoV-19/Denmark/DCGC-9643/2020|2020-11-09|EPI_ISL_669082|Original|hCoV-19^^Midtjylland|Human|Department of Virus and Microbiological Special Diagnostics|Albertsen Lab|Michaelsen|Denmark
>Spike|hCoV-19/England/ALDP-A6E0CA/2020|2020-10-14|EPI_ISL_607662|Original|hCoV-19^^England|Human|Lighthouse Lab in Alderley Park|Wellcome Sanger Institute for the COVID-19 Genomics UK (COG-UK) consortium|Davies|United Kingdom
>Spike|hCoV-19/England/ALDP-A85704/2020|2020-10-17|EPI_ISL_610532|Original|hCoV-19^^England|Human|Lighthouse Lab in Alderley Park|Wellcome Sanger Institute for the COVID-19 Genomics UK (COG-UK) consortium|Davies|United Kingdom
>Spike|hCoV-19/USA/NY-Wadsworth-280623-01/2020|2020-11-30|EPI_ISL_754409|Original|hCoV-19^^New York|Human|Wadsworth Center|Wadsworth Center|Lasek-Nesselquist|USA
>Spike|hCoV-19/England/NOTT-11416F/2020|2020-10-04|EPI_ISL_577143|Original|hCoV-19^^England|Human|Queens Medical Centre|COVID-19 Genomics UK (COG-UK) Consortium|Loose|United Kingdom
>Spike|hCoV-19/Denmark/DCGC-14341/2020|2020-11-30|EPI_ISL_711704|Original|hCoV-19^^Hovedstaden|Human|Department of Virus and Microbiological Special Diagnostics|Albertsen Lab|Michaelsen|Denmark
>Spike|hCoV-19/Japan/PG-0842/2020|2020-04-00|EPI_ISL_684452|Original|hCoV-19^^Japan|Human|Fukuoka Institute of Health and Environmental Sciences|Pathogen Genomics Center|Sekizuka|Japan
>Spike|hCoV-19/England/ALDP-9BDD3F/2020|2020-09-09|EPI_ISL_568082|Original|hCoV-19^^England|Human|Lighthouse Lab in Alderley Park|Wellcome Sanger Institute for the COVID-19 Genomics UK (COG-UK) consortium|Davies|United Kingdom
>Spike|hCoV-19/Vietnam/HCMC-35020/2020|2020-03-25|EPI_ISL_450744|Original|hCoV-19^^Ho Chi Minh City|Human|OUCRU/HTD|OUCRU/HTD|Tan|Vietnam
>Spike|hCoV-19/England/ALDP-6C3786/2020|2020-07-01|EPI_ISL_557860|Original|hCoV-19^^England|Human|Lighthouse Lab in Alderley Park|Wellcome Sanger Institute for the COVID-19 Genomics UK (COG-UK) consortium|Davies|United Kingdom
>Spike|hCoV-19/Wales/PHWC-2C833/2020|2020-04-01|EPI_ISL_445917|Original|hCoV-19^^Wales|Human|Wales Specialist Virology Centre|Public Health Wales Microbiology Cardiff|Moore|United Kingdom
>Spike|hCoV-19/Scotland/QEUH-A1FDEF/2020|2020-10-01|EPI_ISL_599095|Original|hCoV-19^^Scotland|Human|Lighthouse Lab in Glasgow|Wellcome Sanger Institute for the COVID-19 Genomics UK (COG-UK) consortium|Davies|United Kingdom

>Spike|hCoV-19/England/CAMC-B99EE9/2020|2020-11-15|EPI_ISL_673804|Original|hCoV-19^^England|Human|Lighthouse Lab in Cambridge|Wellcome Sanger Institute for the COVID-19 Genomics UK (COG-UK) Consortium|Davies|United Kingdom
>Spike|hCoV-19/England/MILK-B97531/2020|2020-11-14|EPI_ISL_675013|Original|hCoV-19^^England|Human|Lighthouse Lab in Milton Keynes|Wellcome Sanger Institute for the COVID-19 Genomics UK (COG-UK) Consortium|Davies|United Kingdom
>Spike|hCoV-19/Denmark/DCGC-7536/2020|2020-10-12|EPI_ISL_618443|Original|hCoV-19^^Hovedstaden|Human|Department of Virus and Microbiological Special Diagnostics|Albertsen lab|Michaelsen|Denmark
>Spike|hCoV-19/France/ARA-06813/2020|2020-03-07|EPI_ISL_417340|Original|hCoV-19^^Auvergne-Rh\u00f4ne-Alpes|Human|Centre Hospitalier de Bourg en Bresse|CNR Virus des Infections Respiratoires - France SUD|Josset|France
>Spike|hCoV-19/England/QEUH-B67D32/2020|2020-11-11|EPI_ISL_655269|Original|hCoV-19^^England|Human|Lighthouse Lab in Glasgow|Wellcome Sanger Institute for the COVID-19 Genomics UK (COG-UK) Consortium|Davies|United Kingdom
>Spike|hCoV-19/England/204560323/2020|2020-11-04|EPI_ISL_678679|Original|hCoV-19^^England|Human|Respiratory Virus Unit|COVID-19 Genomics UK (COG-UK) Consortium|Platt|United Kingdom
>Spike|hCoV-19/Denmark/DCGC-1576/2020|2020-06-15|EPI_ISL_618146|Original|hCoV-19^^Sjaelland|Human|Department of Virus and Microbiological Special Diagnostics|Albertsen lab|Michaelsen|Denmark
>Spike|hCoV-19/England/QEUH-9AEDB9/2020|2020-09-03|EPI_ISL_573504|Original|hCoV-19^^England|Human|University College London|COVID-19 Genomics UK (COG-UK) Consortium|Roy|United Kingdom
>Spike|hCoV-19/England/QEUH-9F9511/2020|2020-09-25|EPI_ISL_589239|Original|hCoV-19^^England|Human|Lighthouse Lab in Glasgow|Wellcome Sanger Institute for the COVID-19 Genomics UK (COG-UK) consortium|Davies|United Kingdom
>Spike|hCoV-19/Saudi Arabia/KAUST-Madinah23/2020|2020-03-20|EPI_ISL_437482|Original|hCoV-19^^Madinah|Human|Pathogen Genomics Lab King Abdullah University of Science and Technology(KAUST)|Pathogen Genomics Lab King Abdullah University of Science and Technology(KAUST)|Mohamed Ghazzali|Saudi Arabia
>Spike|hCoV-19/England/LIVE-DB3D08/2020|2020-10-20|EPI_ISL_650199|Original|hCoV-19^^England|Human|Liverpool Clinical Laboratories|COVID-19 Genomics UK (COG-UK) Consortium|Haldenby|United Kingdom
>Spike|hCoV-19/Denmark/DCGC-15602/2020|2020-11-23|EPI_ISL_713203|Original|hCoV-19^^Hovedstaden|Human|Department of Virus and Microbiological Special Diagnostics|Albertsen Lab|Michaelsen|Denmark
>Spike|hCoV-19/USA/WA-S1032/2020|2020-05-01|EPI_ISL_463522|Original|hCoV-19^^Washington|Human|Washington State Department of Health|Seattle Flu Study|Bedford|USA
>Spike|hCoV-19/USA/FL-BPHL-2176/2020|2020-08-18|EPI_ISL_653329|Original|hCoV-19^^Florida|Human|Florida Bureau of Public Health Laboratories|Florida Bureau of Public Health Laboratories|Blanton|USA
>Spike|hCoV-19/England/MILK-ACE550/2020|2020-10-21|EPI_ISL_644079|Original|hCoV-19^^England|Human|Lighthouse Lab in Milton Keynes|Wellcome Sanger Institute for the COVID-19 Genomics UK (COG-UK) Consortium|Davies|United Kingdom
>Spike|hCoV-19/England/EXET-13E563/2020|2020-00-00|EPI_ISL_724329|Original|hCoV-19^^England|Human|University of Exeter|COVID-19 Genomics UK (COG-UK) Consortium|Temperton|United Kingdom
>Spike|hCoV-19/England/ALDP-C3CA7A/2020|2020-12-10|EPI_ISL_731195|Original|hCoV-19^^England|Human|Lighthouse Lab in Alderley Park|Wellcome Sanger Institute for the COVID-19 Genomics UK (COG-UK) Consortium|Davies|United Kingdom
>Spike|hCoV-19/Denmark/DCGC-2545/2020|2020-08-17|EPI_ISL_616521|Original|hCoV-19^^Nordjylland|Human|Department of Virus and Microbiological Special Diagnostics|Albertsen lab|Michaelsen|Denmark
>Spike|hCoV-19/Russia/CRIE7158978/2020|2020-08-24|EPI_ISL_610239|Original|hCoV-19^^Moscow|Human|Molecular diagnostic laboratory of Federal Budget Institution of Science \Central Research Institute of Epidemiology\ of The Federal Service on Customers' Rights Protection and Human Well-being Surveillance|Group of Genomics and Postgenomic Technologies of Central Research Institute of Epidemilology|Samo

>ilov|Russia
>Spike|hCoV-19/USA/VT-CDC-0303/2020|2020-03-05|EPI_ISL_447846|Original|hCoV-19^^Vermont|Human|VT Dept. of Health Laboratory|Pathogen Discovery|Queen|USA
>Spike|hCoV-19/England/ALDP-A86CAA/2020|2020-10-16|EPI_ISL_606235|Original|hCoV-19^^England|Human|Lighthouse Lab in Alderley Park|Wellcome Sanger Institute for the COVID-19 Genomics UK (COG-UK) consortium|Davies|United Kingdom
>Spike|hCoV-19/England/CAMC-B22CFA/2020|2020-11-02|EPI_ISL_647129|Original|hCoV-19^^England|Human|Lighthouse Lab in Cambridge|Wellcome Sanger Institute for the COVID-19 Genomics UK (COG-UK) Consortium|Davies|United Kingdom
>Spike|hCoV-19/Latvia/141/2020|2020-08-31|EPI_ISL_639638|Original|hCoV-19^^Latvia|Human|E. Gulbja Laboratorija|Latvian Biomedical Research and Study Centre|Zrelovs|Latvia
>Spike|hCoV-19/Brazil/RJ-00416/2020|2020-10-08|EPI_ISL_717818|Original|hCoV-19^^Rio de Janeiro|Human|Laboratorio de Virologia Molecular / UFRJ|Bioinformatics Laboratory / LNCC|Almeida|Brazil
>Spike|hCoV-19/Japan/PG-6427/2020|2020-07-00|EPI_ISL_689533|Original|hCoV-19^^Japan|Human|Pathogen Genomics Center|Pathogen Genomics Center|Sekizuka|Japan
>Spike|hCoV-19/South Korea/KDCA0312/2020|2020-10-29|EPI_ISL_760098|Original|hCoV-19^^South Korea|Human|Division of Emerging Infectious Diseases|Division of Emerging Infectious Diseases|Park|South Korea
>Spike|hCoV-19/Denmark/DCGC-10480/2020|2020-11-02|EPI_ISL_670003|Original|hCoV-19^^Hovedstaden|Human|Department of Virus and Microbiological Special Diagnostics|Albertsen Lab|Michaelsen|Denmark
>Spike|hCoV-19/Spain/PV-IBV-98006709/2020|2020-07-13|EPI_ISL_541104|Original|hCoV-19^^Basque Country|Human|Servicio de Microbiolog\u00eda. Hospital Universitario Donostia. OSI Donostialdea. \u00c1rea de Enfermedades Infecciosas|SeqCOVID-SPAIN consortium/Institute of Biomedicine of Valencia|Comas|Spain
>Spike|hCoV-19/Australia/VIC3854/2020|2020-07-12|EPI_ISL_520567|Original|hCoV-19^^Victoria|Human|Microbiological Diagnostic Unit - Public Health Laboratory (MDU-PHL)|MDU-PHL|Schultz|Australia
>Spike|hCoV-19/England/ALDP-AA2437/2020|2020-10-22|EPI_ISL_624499|Original|hCoV-19^^England|Human|Lighthouse Lab in Alderley Park|Wellcome Sanger Institute for the COVID-19 Genomics UK (COG-UK) consortium|Davies|United Kingdom
>Spike|hCoV-19/England/MILK-B39883/2020|2020-11-01|EPI_ISL_679647|Original|hCoV-19^^England|Human|Oxford Viromics|COVID-19 Genomics UK (COG-UK) Consortium|Golubchik|United Kingdom
>Spike|hCoV-19/USA/TX-HMH-2092/2020|2020-05-20|EPI_ISL_545115|Original|hCoV-19^^Texas|Human|Houston Methodist Hospital|Houston Methodist Hospital|Long|USA
>Spike|hCoV-19/Scotland/CVR5812/2020|2020-11-13|EPI_ISL_666030|Original|hCoV-19^^Scotland|Human|West of Scotland Specialist Virology Centre|COVID-19 Genomics UK (COG-UK) Consortium|Robertson|United Kingdom
>Spike|hCoV-19/Wales/PHWC-49153E/2020|2020-11-11|EPI_ISL_706665|Original|hCoV-19^^Wales|Human|Originating lab|COVID-19 Genomics UK (COG-UK) Consortium|Moore|United Kingdom
>Spike|hCoV-19/Chile/RM-131381/2020|2020-07-23|EPI_ISL_746625|Original|hCoV-19^^Region Metropolitana de Santiago|Human|Genetica Molecular and Subdepartamento de Virologia ISP Chile|Instituto de Salud Publica de Chile|Tognarelli|Chile
>Spike|hCoV-19/Denmark/DCGC-16276/2020|2020-11-16|EPI_ISL_714678|Original|hCoV-19^^Hovedstaden|Human|Department of Virus and Microbiological Special Diagnostics|Albertsen Lab|Michaelsen|Denmark
>Spike|hCoV-19/Wales/PHWC-4A4037/2020|2020-12-14|EPI_ISL_764653|Original|hCoV-19^^Wales|Human|Wales Specialist Virology Centre Sequencing lab|COVID-19 Genomics UK (COG-UK) Consortium|Moore|United Kingdom
>Spike|hCoV-19/Wales/PHWC-240BB/2020|2020-03-13|EPI_ISL_418141|Original|hCoV-19^^Wales|Human|Wales Specialist Virology Centre|Public Health Wales Microbiology Cardiff|Moore|United Kingdom
>Spike|hCoV-19/Wales/PHWC-47D00A/2020|2020-10-10|EPI_ISL_586085|Original|hCoV-19^^Wales|Human|Wales Specialist Virology Centre Sequencing lab|COVID-19 Genomics UK (COG-UK) Consortium|Moore|United Kingdom

>Spike|hCoV-19/USA/TX-HMH-MCoV-13742/2020|2020-08-17|EPI_ISL_788835|Original|hCoV-19^^Texas|Human|Houston Methodist Hospital|Houston Methodist Hospital|Long|USA
>Spike|hCoV-19/England/ALDP-A6C387/2020|2020-10-15|EPI_ISL_608035|Original|hCoV-19^^England|Human|Lighthouse Lab in Alderley Park|Wellcome Sanger Institute for the COVID-19 Genomics UK (COG-UK) consortium|Davies|United Kingdom
>Spike|hCoV-19/Scotland/QEUH-AD070A/2020|2020-10-24|EPI_ISL_644046|Original|hCoV-19^^Scotland|Human|Lighthouse Lab in Glasgow|Wellcome Sanger Institute for the COVID-19 Genomics UK (COG-UK) Consortium|Davies|United Kingdom
>Spike|hCoV-19/Portugal/PT0408/2020|2020-03-21|EPI_ISL_454132|Original|hCoV-19^^Portugal|Human|unknown|Instituto Nacional de Saude (INSA)|Borges|Portugal
>Spike|hCoV-19/South Africa/KRISP-K003479/2020|2020-09-14|EPI_ISL_602811|Original|hCoV-19^^KwaZulu-Natal|Human|NHLS-IALCH|KRISP|de Oliveira|South Africa
>Spike|hCoV-19/USA/TX-HMH-MCoV-8897/2020|2020-07-02|EPI_ISL_780817|Original|hCoV-19^^Texas|Human|Houston Methodist Hospital|Houston Methodist Hospital|Long|USA
>Spike|hCoV-19/Denmark/DCGC-21719/2020|2020-12-21|EPI_ISL_757768|Original|hCoV-19^^Hovedstaden|Human|Department of Virus and Microbiological Special Diagnostics|Albertsen Lab|Michaelsen|Denmark
>Spike|hCoV-19/England/NORT-29D17C/2020|2020-05-04|EPI_ISL_499798|Original|hCoV-19^^England|Human|Northumbria University / South Tees Hospitals NHS Foundation Trust / North Cumbria Integrated Care NHS Foundation Trust / North Tees and Hartlepool NHS Foundation Trust / Newcastle Hospitals NHS Foundation Trust|COVID-19 Genomics UK (COG-UK) Consortium|Bashton|United Kingdom
>Spike|hCoV-19/Scotland/CVR378/2020|2020-03-22|EPI_ISL_439067|Original|hCoV-19^^Scotland|Human|West of Scotland Specialist Virology Centre|COVID-19 Genomics UK (COG-UK) Consortium|Robertson|United Kingdom
>Spike|hCoV-19/Sweden/20-08042/2020|2020-03-08|EPI_ISL_454437|Original|hCoV-19^^Halland|Human|Halmstad klinisk mikrobiologi|The Public Health Agency of Sweden|Svartstr\u00f6m|Sweden
>Spike|hCoV-19/Australia/VIC13965/2020|2020-07-25|EPI_ISL_563898|Original|hCoV-19^^Victoria|Human|Microbiological Diagnostic Unit - Public Health Laboratory (MDU-PHL)|MDU-PHL|Schultz|Australia
>Spike|hCoV-19/USA/TX-HMH-MCoV-15021/2020|2020-10-16|EPI_ISL_786505|Original|hCoV-19^^Texas|Human|Houston Methodist Hospital|Houston Methodist Hospital|Long|USA
>Spike|hCoV-19/India/WB-S72/2020|2020-05-20|EPI_ISL_508462|Original|hCoV-19^^West Bengal|Human|ICMR-National Institute of Cholera and Enteric Diseases|National Institute of Biomedical Genomics|Maitra|India
>Spike|hCoV-19/Northern Ireland/NIRE-1027BD/2020|2020-04-18|EPI_ISL_469881|Original|hCoV-19^^Northern Ireland|Human|Regional Virus Laboratory|Wellcome Sanger Institute for the COVID-19 Genomics UK (COG-UK) consortium|Davies|United Kingdom
>Spike|hCoV-19/Egypt/CUNCI-HGC7I025/2020|2020-07-19|EPI_ISL_576373|Original|hCoV-19^^Egypt|Human|Cancer Biology Department|Cancer Biology Department|Zekri|Egypt
>Spike|hCoV-19/Brazil/PE-IAM957/2020|2020-05-03|EPI_ISL_572362|Original|hCoV-19^^Pernambuco|Human|LACEN/PE|WallauLab|Wallau|Brazil
>Spike|hCoV-19/England/QEUH-AE4D4D/2020|2020-10-27|EPI_ISL_633191|Original|hCoV-19^^England|Human|Lighthouse Lab in Glasgow|Wellcome Sanger Institute for the COVID-19 Genomics UK (COG-UK) consortium|Davies|United Kingdom
>Spike|hCoV-19/USA/OR-OHSU-4292/2020|2020-11-03|EPI_ISL_667534|Original|hCoV-19^^Oregon|Human|OHSU Lab Services Molecular Microbiology Lab|Oregon SARS-CoV-2 Genome Sequencing Center|O'Roak|USA
>Spike|hCoV-19/Denmark/DCGC-11983/2020|2020-09-07|EPI_ISL_670664|Original|hCoV-19^^Nordjylland|Human|Department of Virus and Microbiological Special Diagnostics|Albertsen Lab|Michaelsen|Denmark
>Spike|hCoV-19/Australia/WA280/2020|2020-03-07|EPI_ISL_596823|Original|hCoV-19^^Western Australia|Human|PathWest Laboratory Medicine WA|PathWest Laboratory Medicine WA Microbial Surveillance Unit|Sikazwe|Australia
>Spike|hCoV-19/South Korea/KCDC2315/2020|2020-05-26|EPI_ISL_510651|Original|hCoV-19^^South Korea|Human|Division of Viral Diseases|Division of Viral Diseases|Kim|South Korea

>Spike|hCoV-19/England/LIVE-1D88EA/2020|2020-09-24|EPI_ISL_576979|Original|hCoV-19^^England|Human|Liverpool Clinical Laboratories|COVID-19 Genomics UK (COG-UK) Consortium|Haldenby|United Kingdom
>Spike|hCoV-19/USA/TX-HMH-4349/2020|2020-06-17|EPI_ISL_543550|Original|hCoV-19^^Texas|Human|Houston Methodist Hospital|Houston Methodist Hospital|Long|USA
>Spike|hCoV-19/England/MILK-9C371A/2020|2020-09-10|EPI_ISL_566888|Original|hCoV-19^^England|Human|Lighthouse Lab in Milton Keynes|Wellcome Sanger Institute for the COVID-19 Genomics UK (COG-UK) consortium|Davies|United Kingdom
>Spike|hCoV-19/England/CAMC-A65773/2020|2020-10-10|EPI_ISL_637260|Original|hCoV-19^^England|Human|Department of Pathology|COVID-19 Genomics UK (COG-UK) Consortium|Goodfellow|United Kingdom
>Spike|hCoV-19/England/QEUH-C32722/2020|2020-12-11|EPI_ISL_782649|Original|hCoV-19^^England|Human|Lighthouse Lab in Glasgow|Wellcome Sanger Institute for the COVID-19 Genomics UK (COG-UK) Consortium|Davies|United Kingdom
>Spike|hCoV-19/England/ALDP-951113/2020|2020-06-13|EPI_ISL_554305|Original|hCoV-19^^England|Human|Lighthouse Lab in Alderley Park|Wellcome Sanger Institute for the COVID-19 Genomics UK (COG-UK) consortium|Davies|United Kingdom
>Spike|hCoV-19/England/QEUH-9636A6/2020|2020-08-10|EPI_ISL_531777|Original|hCoV-19^^England|Human|Lighthouse Lab in Glasgow|Wellcome Sanger Institute for the COVID-19 Genomics UK (COG-UK) consortium|Davies|United Kingdom
>Spike|hCoV-19/USA/WA-S1150/2020|2020-05-05|EPI_ISL_463640|Original|hCoV-19^^Washington|Human|Washington State Department of Health|Seattle Flu Study|Bedford|USA
>Spike|hCoV-19/Portugal/PT0210/2020|2020-03-30|EPI_ISL_453924|Original|hCoV-19^^Portugal|Human|unknown|Instituto Nacional de Saude (INSA)|Borges|Portugal
>Spike|hCoV-19/Northern Ireland/NIRE-107572/2020|2020-03-30|EPI_ISL_585168|Original|hCoV-19^^Northern Ireland|Human|Regional Virus Laboratory|COVID-19 Genomics UK (COG-UK) Consortium|Fairley|United Kingdom
>Spike|hCoV-19/Japan/PG-1480/2020|2020-04-00|EPI_ISL_685087|Original|hCoV-19^^Japan|Human|Saitama Prefectural Institute of Public Health|Pathogen Genomics Center|Sekizuka|Japan
>Spike|hCoV-19/England/ALDP-AA2938/2020|2020-10-21|EPI_ISL_630777|Original|hCoV-19^^England|Human|Lighthouse Lab in Alderley Park|Wellcome Sanger Institute for the COVID-19 Genomics UK (COG-UK) consortium|Davies|United Kingdom
>Spike|hCoV-19/Wales/PHWC-168F71/2020|2020-07-06|EPI_ISL_499373|Original|hCoV-19^^Wales|Human|Originating lab|COVID-19 Genomics UK (COG-UK) Consortium|Moore|United Kingdom
>Spike|hCoV-19/USA/CA-ALSR-4185/2020|2020-09-02|EPI_ISL_648871|Original|hCoV-19^^California|Human|San Diego County Public Health Laboratory|Andersen lab at Scripps Research|Gangavarapu|USA
>Spike|hCoV-19/England/ALDP-B75B73/2020|2020-11-12|EPI_ISL_740939|Original|hCoV-19^^England|Human|Oxford Viromics|COVID-19 Genomics UK (COG-UK) Consortium|Golubchik|United Kingdom
>Spike|hCoV-19/England/ALDP-9B5A8B/2020|2020-08-28|EPI_ISL_590462|Original|hCoV-19^^England|Human|Lighthouse Lab in Alderley Park|Wellcome Sanger Institute for the COVID-19 Genomics UK (COG-UK) consortium|Davies|United Kingdom
>Spike|hCoV-19/England/LCST-24D67D7/2020|2020-05-30|EPI_ISL_478295|Original|hCoV-19^^England|Human|University Hospitals Of Leicester NHS Trust and DeepSeq Nottingham|COVID-19 Genomics UK (COG-UK) Consortium|Loose|United Kingdom
>Spike|hCoV-19/England/CAMC-A3E45E/2020|2020-10-06|EPI_ISL_609782|Original|hCoV-19^^England|Human|Lighthouse Lab in Cambridge|Wellcome Sanger Institute for the COVID-19 Genomics UK (COG-UK) consortium|Davies|United Kingdom
>Spike|hCoV-19/Luxembourg/LNS5912684/2020|2020-11-25|EPI_ISL_744322|Original|hCoV-19^^Luxembourg|Human|Laboratoire national de sant\u00e9|Laboratoire national de sant\u00e9|Wienecke-Baldacchino|Luxembourg
>Spike|hCoV-19/Denmark/DCGC-12194/2020|2020-11-16|EPI_ISL_682375|Original|hCoV-19^^Denmark|Human|Department of Virus and Microbiological Special Diagnostics|Albertsen Lab|Michaelsen|Denmark

>Spike|hCoV-19/Wales/QEUH-AE4B25/2020|2020-10-26|EPI_ISL_647912|Original|hCoV-19^^Wales|Human|Lighthouse Lab in Glasgow|Wellcome Sanger Institute for the COVID-19 Genomics UK (COG-UK) Consortium|Davies|United Kingdom
>Spike|hCoV-19/England/QEUH-A0173D/2020|2020-10-02|EPI_ISL_595820|Original|hCoV-19^^England|Human|Virology Department|COVID-19 Genomics UK (COG-UK) Consortium|de Silva|United Kingdom
>Spike|hCoV-19/Scotland/CVR5060/2020|2020-10-10|EPI_ISL_594793|Original|hCoV-19^^Scotland|Human|West of Scotland Specialist Virology Centre|COVID-19 Genomics UK (COG-UK) Consortium|Robertson|United Kingdom
>Spike|hCoV-19/England/EXET-13EF47/2020|2020-12-15|EPI_ISL_764158|Original|hCoV-19^^England|Human|University of Exeter|COVID-19 Genomics UK (COG-UK) Consortium|Temperton|United Kingdom
>Spike|hCoV-19/India/TG-CCMB-X103/2020|2020-07-15|EPI_ISL_528844|Original|hCoV-19^^Telangana|Human|CSIR-Centre for Cellular and Molecular Biology|CSIR-Centre for Cellular and Molecular Biology|Banu|India
>Spike|hCoV-19/USA/TX-HMH-MCoV-14172/2020|2020-10-02|EPI_ISL_787457|Original|hCoV-19^^Texas|Human|Houston Methodist Hospital|Houston Methodist Hospital|Long|USA
>Spike|hCoV-19/England/NOTT-10FD09/2020|2020-04-08|EPI_ISL_441438|Original|hCoV-19^^England|Human|Queens Medical Centre|COVID-19 Genomics UK (COG-UK) Consortium|Loose|United Kingdom
>Spike|hCoV-19/Australia/VIC10595/2020|2020-08-03|EPI_ISL_562977|Original|hCoV-19^^Victoria|Human|Microbiological Diagnostic Unit - Public Health Laboratory (MDU-PHL)|MDU-PHL|Schultz|Australia
>Spike|hCoV-19/Denmark/DCGC-13944/2020|2020-11-30|EPI_ISL_711466|Original|hCoV-19^^Sjaelland|Human|Department of Virus and Microbiological Special Diagnostics|Albertsen Lab|Michaelsen|Denmark
>Spike|hCoV-19/Scotland/QEUH-A9B60E/2020|2020-10-20|EPI_ISL_625393|Original|hCoV-19^^Scotland|Human|Lighthouse Lab in Glasgow|Wellcome Sanger Institute for the COVID-19 Genomics UK (COG-UK) consortium|Davies|United Kingdom
>Spike|hCoV-19/England/MILK-A7C08D/2020|2020-10-15|EPI_ISL_606359|Original|hCoV-19^^England|Human|Lighthouse Lab in Milton Keynes|Wellcome Sanger Institute for the COVID-19 Genomics UK (COG-UK) consortium|Davies|United Kingdom
>Spike|hCoV-19/England/CAMC-9B1B22/2020|2020-09-04|EPI_ISL_566589|Original|hCoV-19^^England|Human|Lighthouse Lab in Cambridge|Wellcome Sanger Institute for the COVID-19 Genomics UK (COG-UK) consortium|Davies|United Kingdom
>Spike|hCoV-19/England/NORW-F013D/2020|2020-10-10|EPI_ISL_595177|Original|hCoV-19^^England|Human|Quadram Institute Bioscience|COVID-19 Genomics UK (COG-UK) Consortium|Alikhan|United Kingdom
>Spike|hCoV-19/Denmark/DCGC-15688/2020|2020-11-23|EPI_ISL_713965|Original|hCoV-19^^Nordjylland|Human|Department of Virus and Microbiological Special Diagnostics|Albertsen Lab|Michaelsen|Denmark
>Spike|hCoV-19/Belgium/rega-0409561/2020|2020-04-09|EPI_ISL_734551|Original|hCoV-19^^Braine-Le-Comte|Human|UZ Leuven|KU Leuven|Maes|Belgium
>Spike|hCoV-19/England/ALDP-B5DE69/2020|2020-11-10|EPI_ISL_723601|Original|hCoV-19^^England|Human|Northumbria University / South Tees Hospitals NHS Foundation Trust / North Cumbria Integrated Care NHS Foundation Trust / North Tees and Hartlepool NHS Foundation Trust / Newcastle Hospitals NHS Foundation Trust|COVID-19 Genomics UK (COG-UK) Consortium|Bashton|United Kingdom
>Spike|hCoV-19/England/CAMB-760F6/2020|2020-03-30|EPI_ISL_438268|Original|hCoV-19^^England|Human|Department of Pathology|Wellcome Sanger Institute for the COVID-19 Genomics UK (COG-UK) consortium|Amato|United Kingdom
>Spike|hCoV-19/Ireland/CO-NVRL-78IRL75683/2020|2020-11-22|EPI_ISL_752576|Original|hCoV-19^^Cork|Human|National Virus Reference Laboratory|National Virus Reference Laboratory|Gonzalez|Ireland
>Spike|hCoV-19/England/NOTT-121466/2020|2020-12-16|EPI_ISL_741637|Original|hCoV-19^^England|Human|Queens Medical Centre|COVID-19 Genomics UK (COG-UK) Consortium|Loose|United Kingdom
>Spike|hCoV-19/South Africa/KRISP-108/2020|2020-05-04|EPI_ISL_455638|Original|hCoV-19^^KwaZulu-Natal|Human|KRISP|KRISP|de Oliveira|South Africa

>Spike|hCoV-19/Australia/VIC17297/2020|2020-07-26|EPI_ISL_663824|Original|hCoV-19^^Victoria|Human|Microbiological Diagnostic Unit - Public Health Laboratory (MDU-PHL)|MDU-PHL|Schultz|Australia
>Spike|hCoV-19/Switzerland/BS-ETHZ-190085/2020|2020-07-07|EPI_ISL_500941|Original|hCoV-19^^Basel-Stadt|Human|Viollier AG|Department of Biosystems Science and Engineering|Nadeau|Switzerland
>Spike|hCoV-19/Switzerland/BL-SNRCI-29950902/2020|2020-02-27|EPI_ISL_414021|Original|hCoV-19^^Basel-Landschaft|Human|Laboratoire de Virologie|Swiss National Reference Centre for Influenza|Gon\u00e7alves Cabecinhas|Switzerland
>Spike|hCoV-19/England/NORT-29D4BF/2020|2020-05-08|EPI_ISL_488163|Original|hCoV-19^^England|Human|NU-OMICS DNA Sequencing research facility|Wellcome Sanger Institute for the COVID-19 Genomics UK (COG-UK) consortium|Davies|United Kingdom
>Spike|hCoV-19/England/CAMC-AAF546/2020|2020-10-21|EPI_ISL_644115|Original|hCoV-19^^England|Human|Lighthouse Lab in Cambridge|Wellcome Sanger Institute for the COVID-19 Genomics UK (COG-UK) Consortium|Davies|United Kingdom
>Spike|hCoV-19/Scotland/CVR4503/2020|2020-09-21|EPI_ISL_585366|Original|hCoV-19^^Scotland|Human|Lighthouse Lab in Glasgow / MRC-University of Glasgow Centre for Virus Research|COVID-19 Genomics UK (COG-UK) Consortium|Robertson|United Kingdom
>Spike|hCoV-19/England/QEUH-A5E2D6/2020|2020-10-12|EPI_ISL_597280|Original|hCoV-19^^England|Human|Lighthouse Lab in Glasgow|Wellcome Sanger Institute for the COVID-19 Genomics UK (COG-UK) consortium|Davies|United Kingdom
>Spike|hCoV-19/USA/ME-HETL-J0132/2020|2020-09-08|EPI_ISL_755329|Original|hCoV-19^^Maine|Human|Maine Health and Environmental Testing Laboratory|Tewhey Lab|Tewhey|USA
>Spike|hCoV-19/England/SHEF-10D58CD/2020|2020-12-18|EPI_ISL_742079|Original|hCoV-19^^England|Human|Virology Department|COVID-19 Genomics UK (COG-UK) Consortium|de Silva|United Kingdom
>Spike|hCoV-19/Denmark/DCGC-13478/2020|2020-11-30|EPI_ISL_712822|Original|hCoV-19^^Midtjylland|Human|Department of Virus and Microbiological Special Diagnostics|Albertsen Lab|Michaelsen|Denmark
>Spike|hCoV-19/England/ALDP-9827EA/2020|2020-08-10|EPI_ISL_529551|Original|hCoV-19^^England|Human|Queens Medical Centre|COVID-19 Genomics UK (COG-UK) Consortium|Loose|United Kingdom
>Spike|hCoV-19/Brazil/PE-IAM1093/2020|2020-05-07|EPI_ISL_572377|Original|hCoV-19^^Pernambuco|Human|LACEN/PE|WallauLab|Wallau|Brazil
>Spike|hCoV-19/England/MILK-C2FAA7/2020|2020-12-07|EPI_ISL_733904|Original|hCoV-19^^England|Human|Lighthouse Lab in Milton Keynes|Wellcome Sanger Institute for the COVID-19 Genomics UK (COG-UK) Consortium|Davies|United Kingdom
>Spike|hCoV-19/England/ALDP-A50016/2020|2020-10-07|EPI_ISL_597785|Original|hCoV-19^^England|Human|Lighthouse Lab in Alderley Park|Wellcome Sanger Institute for the COVID-19 Genomics UK (COG-UK) consortium|Davies|United Kingdom
>Spike|hCoV-19/Canada/ON-PHL-20-03667/2020|2020-11-00|EPI_ISL_775153|Original|hCoV-19^^Ontario|Human|Public Health Ontario Laboratory|Public Health Ontario Laboratory|Guthrie|Canada
>Spike|hCoV-19/Scotland/CVR4715/2020|2020-09-21|EPI_ISL_611717|Original|hCoV-19^^Scotland|Human|West of Scotland Specialist Virology Centre|COVID-19 Genomics UK (COG-UK) Consortium|Robertson|United Kingdom
>Spike|hCoV-19/England/QEUH-AD59F0/2020|2020-10-26|EPI_ISL_633761|Original|hCoV-19^^England|Human|Lighthouse Lab in Glasgow|Wellcome Sanger Institute for the COVID-19 Genomics UK (COG-UK) consortium|Davies|United Kingdom
>Spike|hCoV-19/USA/CA-SCCPHD-UC140/2020|2020-03-10|EPI_ISL_435619|Original|hCoV-19^^California|Human|Santa Clara County Public Health Department|Chiu Laboratory|Chiu|USA
>Spike|hCoV-19/Wales/PHWC-49B8B3/2020|2020-12-05|EPI_ISL_742399|Original|hCoV-19^^Wales|Human|Wales Specialist Virology Centre Sequencing lab|COVID-19 Genomics UK (COG-UK) Consortium|Moore|United Kingdom
>Spike|hCoV-19/USA/NY-PV09193/2020|2020-03-20|EPI_ISL_422549|Original|hCoV-19^^New York|Human|MSHS Clinical Microbiology Laboratories|MSHS Pathogen Surveillance Program|van Bakel|USA

>Spike|hCoV-19/England/NORT-28420C/2020|2020-03-15|EPI_ISL_478536|Original|hCoV-19^^England|Human|Northumbria University / South Tees Hospitals NHS Foundation Trust / North Cumbria Integrated Care NHS Foundation Trust / North Tees and Hartlepool NHS Foundation Trust / Newcastle Hospitals NHS Foundation Trust|COVID-19 Genomics UK (COG-UK) Consortium|Bashton|United Kingdom
>Spike|hCoV-19/USA/CA-ALSR-4503/2020|2020-11-02|EPI_ISL_666972|Original|hCoV-19^^California|Human|San Diego County Public Health Laboratory|Andersen lab at Scripps Research|Gangavarapu|USA
>Spike|hCoV-19/USA/AZ-TG547291/2020|2020-07-06|EPI_ISL_695466|Original|hCoV-19^^Arizona|Human|AZ SPHL|TGen North|French|USA
>Spike|hCoV-19/Portugal/PT1831/2020|2020-11-15|EPI_ISL_732052|Original|hCoV-19^^Portugal|Human|Instituto Nacional de Saude (INSA)|Instituto Nacional de Saude (INSA)|Borges|Portugal
>Spike|hCoV-19/England/ALDP-A96287/2020|2020-10-19|EPI_ISL_624863|Original|hCoV-19^^England|Human|Lighthouse Lab in Alderley Park|Wellcome Sanger Institute for the COVID-19 Genomics UK (COG-UK) consortium|Davies|United Kingdom
>Spike|hCoV-19/Ireland/KE-NVRL-20G27987/2020|2020-03-20|EPI_ISL_671375|Original|hCoV-19^^Kerry|Human|National Virus Reference Laboratory|Irish Coronavirus Sequencing Consortium - Teagasc Moorepark|Walsh|Ireland
>Spike|hCoV-19/Wales/PHWC-16817B/2020|2020-05-29|EPI_ISL_494207|Original|hCoV-19^^Wales|Human|Originating lab|COVID-19 Genomics UK (COG-UK) Consortium|Moore|United Kingdom
>Spike|hCoV-19/Luxembourg/LNS0814393/2020|2020-03-23|EPI_ISL_429766|Original|hCoV-19^^Luxembourg|Human|Laboratoire National de Sante|Laboratoire National de Sante|Wienecke-Baldacchino|Luxembourg
>Spike|hCoV-19/England/CAMB-1AB84A/2020|2020-04-26|EPI_ISL_444336|Original|hCoV-19^^England|Human|Department of Pathology|COVID-19 Genomics UK (COG-UK) Consortium|Meredith|United Kingdom
>Spike|hCoV-19/England/ALDP-9CC2F2/2020|2020-09-16|EPI_ISL_573096|Original|hCoV-19^^England|Human|Quadram Institute Bioscience|COVID-19 Genomics UK (COG-UK) Consortium|Alikhan|United Kingdom
>Spike|hCoV-19/England/QEUH-B7E363/2020|2020-11-14|EPI_ISL_727859|Original|hCoV-19^^England|Human|Oxford Viromics|COVID-19 Genomics UK (COG-UK) Consortium|Golubchik|United Kingdom
>Spike|hCoV-19/USA/WA-UW-9016/2020|2020-05-12|EPI_ISL_570226|Original|hCoV-19^^Washington|Human|UW Virology Lab|UW Virology Lab|Roychoudhury|USA
>Spike|hCoV-19/England/ALDP-A2E2A2/2020|2020-10-06|EPI_ISL_586595|Original|hCoV-19^^England|Human|Lighthouse Lab in Alderley Park|Wellcome Sanger Institute for the COVID-19 Genomics UK (COG-UK) consortium|Davies|United Kingdom
>Spike|hCoV-19/Guangzhou/GZMU0081/2020|2020-02-14|EPI_ISL_457692|Original|hCoV-19^^Guangzhou|Human|The First Affiliated Hospital of Guangzhou Medical University|BGI-shenzhen & The First Affiliated Hospital of Guangzhou Medical University|Ji|China
>Spike|hCoV-19/USA/WA-S2224/2020|2020-06-08|EPI_ISL_497652|Original|hCoV-19^^Washington|Human|Washington State Department of Health|Seattle Flu Study|Lee|USA
>Spike|hCoV-19/England/CAMC-B545D6/2020|2020-11-08|EPI_ISL_658191|Original|hCoV-19^^England|Human|Lighthouse Lab in Cambridge|Wellcome Sanger Institute for the COVID-19 Genomics UK (COG-UK) Consortium|Davies|United Kingdom
>Spike|hCoV-19/Denmark/DCGC-20376/2020|2020-12-21|EPI_ISL_759114|Original|hCoV-19^^Syddanmark|Human|Department of Virus and Microbiological Special Diagnostics|Albertsen Lab|Michaelsen|Denmark
>Spike|hCoV-19/USA/CA-CZB-14397/2020|2020-07-02|EPI_ISL_738577|Original|hCoV-19^^California|Human|Alameda County Public Health Lab|Chan-Zuckerberg Biohub|Kamm|USA
>Spike|hCoV-19/Latvia/091/2020|2020-07-07|EPI_ISL_512646|Original|hCoV-19^^Latvia|Human|E. Gulbja Laboratorija|Latvian Biomedical Research and Study Centre|Zrelovs|Latvia
>Spike|hCoV-19/USA/ME-HETL-J0276/2020|2020-10-04|EPI_ISL_755422|Original|hCoV-19^^Maine|Human|Maine Health and Environmental Testing Laboratory|Tewhey Lab|Tewhey|USA

>Spike|hCoV-19/England/MILK-A7B640/2020|2020-10-15|EPI_ISL_607515|Original|hCoV-
19^^England|Human|Lighthouse Lab in Milton Keynes|Wellcome Sanger Institute for the
COVID-19 Genomics UK (COG-UK) consortium|Davies|United Kingdom
>Spike|hCoV-19/USA/TX-HMH-MCoV-18592/2020|2020-11-19|EPI_ISL_784940|Original|hCoV-
19^^Texas|Human|Houston Methodist Hospital|Houston Methodist Hospital|Long|USA
>Spike|hCoV-19/USA/WI-WSLH-200511/2020|2020-05-24|EPI_ISL_578652|Original|hCoV-
19^^Wisconsin|Human|Wisconsin State Laboratory of Hygiene Communicable Disease
Division|Wisconsin State Laboratory of Hygiene Communicable Disease
Division|Florek|USA
>Spike|hCoV-19/USA/MN-MDH-1694/2020|2020-04-08|EPI_ISL_560781|Original|hCoV-
19^^Minnesota|Human|Minnesota Department of Health|Minnesota Department of
Health|Garfin|USA
>Spike|hCoV-19/Germany/TH-IIMK-CaSe-31/2020|2020-11-02|EPI_ISL_768726|Original|hCoV-
19^^Thuringia|Human|Jena University Hospital|Institute of infectious medicine &
hospital hygiene|Brandt|Germany
>Spike|hCoV-19/Netherlands/NA_212/2020|2020-03-24|EPI_ISL_422747|Original|hCoV-
19^^Netherlands|Human|Dutch COVID-19 response team|Erasmus Medical Center|Oude
Munnink|Netherlands
>Spike|hCoV-19/Wales/PHWC-34847/2020|2020-04-14|EPI_ISL_446983|Original|hCoV-
19^^Wales|Human|Wales Specialist Virology Centre|Public Health Wales Microbiology
Cardiff|Moore|United Kingdom
>Spike|hCoV-19/England/20139032404/2020|2020-03-27|EPI_ISL_421918|Original|hCoV-
19^^England|Human|Respiratory Virus Unit|Respiratory Virus Unit|Platt|United Kingdom
>Spike|hCoV-19/USA/MI-MDHHS-SC22633/2020|2020-12-01|EPI_ISL_737111|Original|hCoV-
19^^Michigan|Human|Michigan Department of Health and Human Services|Michigan
Department of Health and Human Services|Blankenship|USA
>Spike|hCoV-19/England/QEUH-9F54EB/2020|2020-09-24|EPI_ISL_588519|Original|hCoV-
19^^England|Human|Lighthouse Lab in Glasgow|Wellcome Sanger Institute for the COVID-
19 Genomics UK (COG-UK) consortium|Davies|United Kingdom
>Spike|hCoV-19/USA/AZ-TG618013/2020|2020-07-17|EPI_ISL_695668|Original|hCoV-
19^^Arizona|Human|AZ SPHL|TGen North|French|USA
>Spike|hCoV-19/Denmark/DCGC-6496/2020|2020-10-05|EPI_ISL_620396|Original|hCoV-
19^^Syddanmark|Human|Department of Virus and Microbiological Special
Diagnostics|Albertsen lab|Michaelsen|Denmark
>Spike|hCoV-19/USA/WA-S1475/2020|2020-05-11|EPI_ISL_495650|Original|hCoV-
19^^Washington|Human|Washington State Department of Health|Seattle Flu Study|Lee|USA
>Spike|hCoV-19/Scotland/EDB10104/2020|2020-10-03|EPI_ISL_680469|Original|hCoV-
19^^Scotland|Human|Virology Department|COVID-19 Genomics UK (COG-UK)
Consortium|McHugh|United Kingdom
>Spike|hCoV-19/Switzerland/VD-ETHZ-321421/2020|2020-10-
14|EPI_ISL_603659|Original|hCoV-19^^Vaud|Human|Viollier AG|Department of Biosystems
Science and Engineering|Nadeau|Switzerland
>Spike|hCoV-19/England/MILK-C5168A/2020|2020-12-11|EPI_ISL_769828|Original|hCoV-
19^^England|Human|Lighthouse Lab in Milton Keynes|Wellcome Sanger Institute for the
COVID-19 Genomics UK (COG-UK) Consortium|Davies|United Kingdom
>Spike|hCoV-19/England/MILK-B8824B/2020|2020-11-13|EPI_ISL_674236|Original|hCoV-
19^^England|Human|Lighthouse Lab in Milton Keynes|Wellcome Sanger Institute for the
COVID-19 Genomics UK (COG-UK) Consortium|Davies|United Kingdom
>Spike|hCoV-19/USA/MA-MGH-00416/2020|2020-03-07|EPI_ISL_460353|Original|hCoV-
19^^Massachusetts|Human|Massachusetts General Hospital|Infectious Disease
Program|Lemieux|USA
>Spike|hCoV-19/England/MILK-A7AA72/2020|2020-10-15|EPI_ISL_606514|Original|hCoV-
19^^England|Human|Lighthouse Lab in Milton Keynes|Wellcome Sanger Institute for the
COVID-19 Genomics UK (COG-UK) consortium|Davies|United Kingdom
>Spike|hCoV-19/England/CAMC-B33519/2020|2020-11-05|EPI_ISL_659323|Original|hCoV-
19^^England|Human|Lighthouse Lab in Cambridge|Wellcome Sanger Institute for the
COVID-19 Genomics UK (COG-UK) Consortium|Davies|United Kingdom
>Spike|hCoV-19/England/ALDP-A95EC0/2020|2020-10-19|EPI_ISL_624682|Original|hCoV-
19^^England|Human|Lighthouse Lab in Alderley Park|Wellcome Sanger Institute for the
COVID-19 Genomics UK (COG-UK) consortium|Davies|United Kingdom

>Spike|hCoV-19/Wales/PHWC-49F837/2020|2020-12-08|EPI_ISL_743276|Original|hCoV-19^^Wales|Human|Wales Specialist Virology Centre Sequencing lab|COVID-19 Genomics UK (COG-UK) Consortium|Moore|United Kingdom
>Spike|hCoV-19/England/MILK-B8E7EC/2020|2020-11-13|EPI_ISL_674733|Original|hCoV-19^^England|Human|Lighthouse Lab in Milton Keynes|Wellcome Sanger Institute for the COVID-19 Genomics UK (COG-UK) Consortium|Davies|United Kingdom
>Spike|hCoV-19/England/20118104004/2020|2020-03-11|EPI_ISL_464948|Original|hCoV-19^^England|Human|Respiratory Virus Unit|Respiratory Virus Unit|Platt|United Kingdom
>Spike|hCoV-19/Canada/BC_24235415/2020|2020-03-00|EPI_ISL_463203|Original|hCoV-19^^British Columbia|Human|BCCDC Public Health Laboratory|BCCDC Public Health Laboratory|Harrigan|Canada
>Spike|hCoV-19/England/MILK-B56440/2020|2020-11-05|EPI_ISL_657419|Original|hCoV-19^^England|Human|Lighthouse Lab in Milton Keynes|Wellcome Sanger Institute for the COVID-19 Genomics UK (COG-UK) Consortium|Davies|United Kingdom
>Spike|hCoV-19/Netherlands/Gelderland_60/2020|2020-05-18|EPI_ISL_460959|Original|hCoV-19^^Gelderland|Human|Dutch COVID-19 response team|Erasmus Medical Center|Oude Munnink|Netherlands
>Spike|hCoV-19/Japan/PG-2543/2020|2020-05-00|EPI_ISL_690678|Original|hCoV-19^^Japan|Human|Saitama Prefectural Institute of Public Health|Pathogen Genomics Center|Sekizuka|Japan
>Spike|hCoV-19/England/QEUH-96B8C6/2020|2020-08-16|EPI_ISL_531334|Original|hCoV-19^^England|Human|Lighthouse Lab in Glasgow|Wellcome Sanger Institute for the COVID-19 Genomics UK (COG-UK) consortium|Davies|United Kingdom
>Spike|hCoV-19/England/ALDP-B47D29/2020|2020-11-07|EPI_ISL_649437|Original|hCoV-19^^England|Human|Lighthouse Lab in Alderley Park|Wellcome Sanger Institute for the COVID-19 Genomics UK (COG-UK) Consortium|Davies|United Kingdom
>Spike|hCoV-19/Portugal/PT0606/2020|2020-03-13|EPI_ISL_454329|Original|hCoV-19^^Portugal|Human|unknown|Instituto Nacional de Saude (INSA)|Borges|Portugal
>Spike|hCoV-19/England/QEUH-AE2A1B/2020|2020-10-27|EPI_ISL_633632|Original|hCoV-19^^England|Human|Lighthouse Lab in Glasgow|Wellcome Sanger Institute for the COVID-19 Genomics UK (COG-UK) consortium|Davies|United Kingdom
>Spike|hCoV-19/Portugal/PT0146/2020|2020-03-27|EPI_ISL_453862|Original|hCoV-19^^Portugal|Human|unknown|Instituto Nacional de Saude (INSA)|Borges|Portugal
>Spike|hCoV-19/Poland/IHG_PAS_1_77/2020|2020-04-11|EPI_ISL_450338|Original|hCoV-19^^Wielkopolskie|Human|Institute of Human Genetics|Institute of Human Genetics|Hryhorowicz|Poland
>Spike|hCoV-19/Scotland/QEUH-C5413B/2020|2020-12-12|EPI_ISL_757076|Original|hCoV-19^^Scotland|Human|Lighthouse Lab in Glasgow|Wellcome Sanger Institute for the COVID-19 Genomics UK (COG-UK) Consortium|Davies|United Kingdom
>Spike|hCoV-19/England/MILK-9A8B1F/2020|2020-09-01|EPI_ISL_550252|Original|hCoV-19^^England|Human|Lighthouse Lab in Milton Keynes|Wellcome Sanger Institute for the COVID-19 Genomics UK (COG-UK) consortium|Davies|United Kingdom
>Spike|hCoV-19/USA/NY-NYCPHL-001457/2020|2020-11-25|EPI_ISL_683819|Original|hCoV-19^^New York|Human|DOHMH Riverside|New York City Public Health Laboratory|Wang|USA
>Spike|hCoV-19/USA/MI-MDHHS-SC21493/2020|2020-07-30|EPI_ISL_516220|Original|hCoV-19^^Michigan|Human|Michigan Department of Health and Human Services|Michigan Department of Health and Human Services|Blankenship|USA
>Spike|hCoV-19/USA/CA-ALSR-4751/2020|2020-10-06|EPI_ISL_730122|Original|hCoV-19^^California|Human|San Diego County Public Health Laboratory|Andersen lab at Scripps Research|Gangavarapu|USA
>Spike|hCoV-19/England/OXON-ADBD7/2020|2020-00-00|EPI_ISL_448621|Original|hCoV-19^^England|Human|Oxford Viromics|COVID-19 Genomics UK (COG-UK) Consortium|Golubchik|United Kingdom
>Spike|hCoV-19/USA/WA-NH23/2020|2020-03-13|EPI_ISL_418790|Original|hCoV-19^^Washington|Human|WA State Department of Health|Pathogen Discovery|Queen|USA
>Spike|hCoV-19/England/QEUH-AD2CBE/2020|2020-10-26|EPI_ISL_634059|Original|hCoV-19^^England|Human|Lighthouse Lab in Glasgow|Wellcome Sanger Institute for the COVID-19 Genomics UK (COG-UK) consortium|Davies|United Kingdom
>Spike|hCoV-19/USA/WI-UW-14/2020|2020-03-16|EPI_ISL_417513|Original|hCoV-19^^Wisconsin|Human|University of Wisconsin-Madison AIDS Vaccine Research

Laboratories|University of Wisconsin-Madison AIDS Vaccine Research Laboratories|Braun|USA
>Spike|hCoV-19/USA/CA-ALSR-0679-IPL/2020|2020-04-24|EPI_ISL_483296|Original|hCoV-19^^California|Human|UC San Diego Center for Advanced Laboratory Medicine|Andersen lab at Scripps Research|Gangavarapu|USA
>Spike|hCoV-19/Denmark/DCGC-17330/2020|2020-12-14|EPI_ISL_750912|Original|hCoV-19^^Midtjylland|Human|Department of Virus and Microbiological Special Diagnostics|Albertsen Lab|Michaelsen|Denmark
>Spike|hCoV-19/Scotland/QEUH-C01D93/2020|2020-12-05|EPI_ISL_757259|Original|hCoV-19^^Scotland|Human|Lighthouse Lab in Glasgow|Wellcome Sanger Institute for the COVID-19 Genomics UK (COG-UK) Consortium|Davies|United Kingdom
>Spike|hCoV-19/USA/WA-S1948/2020|2020-06-03|EPI_ISL_497376|Original|hCoV-19^^Washington|Human|Washington State Department of Health|Seattle Flu Study|Lee|USA
>Spike|hCoV-19/USA/VA-DCLS-1129/2020|2020-04-25|EPI_ISL_529922|Original|hCoV-19^^Virginia|Human|Virginia Division of Consolidated Laboratory Services|Virginia Division of Consolidated Laboratory Services|St. Jacques|USA
>Spike|hCoV-19/USA/FL-BPHL-0909/2020|2020-06-30|EPI_ISL_517799|Original|hCoV-19^^Florida|Human|Florida Bureau of Public Health Laboratories|Florida Bureau of Public Health Laboratories|Blanton|USA
>Spike|hCoV-19/USA/AZ-TG359376/2020|2020-06-08|EPI_ISL_694446|Original|hCoV-19^^Arizona|Human|TGen North|TGen North|French|USA
>Spike|hCoV-19/England/BRIS-129A20/2020|2020-04-01|EPI_ISL_458430|Original|hCoV-19^^England|Human|PHE South West Regional Laboratory|Wellcome Sanger Institute for the COVID-19 Genomics UK (COG-UK) consortium|Davies|United Kingdom
>Spike|hCoV-19/England/LOND-1286DD9/2020|2020-06-14|EPI_ISL_541768|Original|hCoV-19^^England|Human|Barts Health NHS Trust|Wellcome Sanger Institute for the COVID-19 Genomics UK (COG-UK) consortium|Davies|United Kingdom
>Spike|hCoV-19/Uganda/UG023/2020|2020-05-04|EPI_ISL_737934|Original|hCoV-19^^Uganda|Human|Uganda Central Public Health Lab and Uganda Virus Research Institute|MRC/UVRI & LSHTM Uganda\u00a0Research Unit|Cotten|Uganda
>Spike|hCoV-19/USA/TX-DSHS-0704/2020|2020-09-03|EPI_ISL_576347|Original|hCoV-19^^Texas|Human|Texas Department of State Health Services|Texas Department of State Health Services|Rahman|USA
>Spike|hCoV-19/USA/WI-UW-1052/2020|2020-08-03|EPI_ISL_516478|Original|hCoV-19^^Wisconsin|Human|University of Wisconsin-Madison AIDS Vaccine Research Laboratories|University of Wisconsin-Madison AIDS Vaccine Research Laboratories|Baczenas|USA
>Spike|hCoV-19/England/NOTT-117779/2020|2020-10-23|EPI_ISL_612586|Original|hCoV-19^^England|Human|Queens Medical Centre|COVID-19 Genomics UK (COG-UK) Consortium|Loose|United Kingdom
>Spike|hCoV-19/Denmark/DCGC-11031/2020|2020-11-02|EPI_ISL_670355|Original|hCoV-19^^Sjaelland|Human|Department of Virus and Microbiological Special Diagnostics|Albertsen Lab|Michaelsen|Denmark
>Spike|hCoV-19/Denmark/DCGC-13442/2020|2020-11-30|EPI_ISL_712803|Original|hCoV-19^^Syddanmark|Human|Department of Virus and Microbiological Special Diagnostics|Albertsen Lab|Michaelsen|Denmark
>Spike|hCoV-19/England/201140442/2020|2020-03-10|EPI_ISL_464735|Original|hCoV-19^^England|Human|Respiratory Virus Unit|Respiratory Virus Unit|Platt|United Kingdom
>Spike|hCoV-19/Wales/PHWC-15D65C/2020|2020-05-15|EPI_ISL_472739|Original|hCoV-19^^Wales|Human|Wales Specialist Virology Centre Sequencing lab|COVID-19 Genomics UK (COG-UK) Consortium|Moore|United Kingdom
>Spike|hCoV-19/USA/VA-DCLS-1935/2020|2020-09-29|EPI_ISL_644925|Original|hCoV-19^^Virginia|Human|Virginia DCLS|Virginia DCLS|St. Jacques|USA
>Spike|hCoV-19/USA/UN-CDC-7657/2020|2020-04-24|EPI_ISL_514091|Original|hCoV-19^^USA|Human|Navy and Marine Corps Public Health Center|Pathogen Discovery|Queen|USA
>Spike|hCoV-19/England/ALDP-C48659/2020|2020-12-12|EPI_ISL_736687|Original|hCoV-19^^England|Human|Lighthouse Lab in Alderley Park|Wellcome Sanger Institute for the COVID-19 Genomics UK (COG-UK) Consortium|Davies|United Kingdom

>Spike|hCoV-19/Australia/VIC5854/2020|2020-07-19|EPI_ISL_518647|Original|hCoV-
19^^Victoria|Human|Microbiological Diagnostic Unit - Public Health Laboratory (MDU-
PHL)|MDU-PHL|Schultz|Australia
>Spike|hCoV-19/Australia/NSW774/2020|2020-07-28|EPI_ISL_526116|Original|hCoV-19^^New
South Wales|Human|Pathology West - NSW Health Pathology|NSW Health Pathology -
Institute of Clinical Pathology and Medical Research; Westmead Hospital; University
of Sydney|Gall|Australia
>Spike|hCoV-19/England/MILK-A7AB9D/2020|2020-10-16|EPI_ISL_606786|Original|hCoV-
19^^England|Human|Lighthouse Lab in Milton Keynes|Wellcome Sanger Institute for the
COVID-19 Genomics UK (COG-UK) consortium|Davies|United Kingdom
>Spike|hCoV-19/Japan/PG-7604/2020|2020-07-00|EPI_ISL_686706|Original|hCoV-
19^^Japan|Human|Chiba Prefectural Institute of Public Health|Pathogen Genomics
Center|Sekizuka|Japan
>Spike|hCoV-19/Sweden/20-51387/2020|2020-05-12|EPI_ISL_455107|Original|hCoV-
19^^Vastra Gotaland|Human|Narhalsan Mellerud|The Public Health Agency of
Sweden|Svartstr\u00f6m|Sweden
>Spike|hCoV-19/Scotland/EDB5456/2020|2020-05-25|EPI_ISL_529690|Original|hCoV-
19^^Scotland|Human|Virology Department|COVID-19 Genomics UK (COG-UK)
Consortium|Williams|United Kingdom
>Spike|hCoV-19/India/MH-AFCO-8108/2020|2020-06-16|EPI_ISL_721637|Original|hCoV-
19^^Maharashtra|Human|Armed Forces Medical College|National Centre For Cell
Science|Paul|India
>Spike|hCoV-19/USA/PA-MGSC40-05/2020|2020-03-26|EPI_ISL_436488|Original|hCoV-
19^^Pennsylvania|Human|UPMC Clinical Laboratory|Microbial Genome Sequencing
Center|Cooper|USA
>Spike|hCoV-19/England/CAMC-9B1CA7/2020|2020-09-04|EPI_ISL_566453|Original|hCoV-
19^^England|Human|Lighthouse Lab in Cambridge|Wellcome Sanger Institute for the
COVID-19 Genomics UK (COG-UK) consortium|Davies|United Kingdom
>Spike|hCoV-19/England/BRIS-12C920/2020|2020-05-14|EPI_ISL_469509|Original|hCoV-
19^^England|Human|PHE South West Regional Laboratory|Wellcome Sanger Institute for
the COVID-19 Genomics UK (COG-UK) consortium|Davies|United Kingdom
>Spike|hCoV-19/USA/CA-CZB-1734/2020|2020-05-13|EPI_ISL_468655|Original|hCoV-
19^^California|Human|Contra Costa Public Health Lab|Chan-Zuckerberg
Biohub|McGeever|USA
>Spike|hCoV-19/England/MILK-84375F/2020|2020-07-16|EPI_ISL_557332|Original|hCoV-
19^^England|Human|Lighthouse Lab in Milton Keynes|Wellcome Sanger Institute for the
COVID-19 Genomics UK (COG-UK) consortium|Davies|United Kingdom
>Spike|hCoV-19/USA/WI-UW-458/2020|2020-06-18|EPI_ISL_484833|Original|hCoV-
19^^Wisconsin|Human|University of Wisconsin-Madison AIDS Vaccine Research
Laboratories|University of Wisconsin-Madison AIDS Vaccine Research
Laboratories|Baczenas|USA
>Spike|hCoV-19/Switzerland/ZH-UZH-IMV112/2020|2020-12-
19|EPI_ISL_730629|Original|hCoV-19^^Zurich|Human|M\u00e4nnedorf Hospital|Institute of
Medical Virology|Schmutz|Switzerland
>Spike|hCoV-19/England/CAMC-A580B4/2020|2020-10-07|EPI_ISL_596944|Original|hCoV-
19^^England|Human|Lighthouse Lab in Cambridge|Wellcome Sanger Institute for the
COVID-19 Genomics UK (COG-UK) consortium|Davies|United Kingdom
>Spike|hCoV-19/France/PAC-IHU-0337/2020|2020-04-01|EPI_ISL_569463|Original|hCoV-
19^^Provence-Alpes-C\u00f4te-d\u2019Azur|Human|MEPHI|MEPHI|Levasseur|France
>Spike|hCoV-19/Chile/RM-ISPCH-3/2020|2020-03-06|EPI_ISL_445245|Original|hCoV-
19^^Region Metropolitana de Santiago|Human|CLINICA ALEMANA DE SANTIAGO S.A.|Instituto
de Salud Publica de Chile|Fernandez|Chile
>Spike|hCoV-19/Denmark/DCGC-6599/2020|2020-10-12|EPI_ISL_619647|Original|hCoV-
19^^Sjaelland|Human|Department of Virus and Microbiological Special
Diagnostics|Albertsen lab|Michaelsen|Denmark
>Spike|hCoV-19/Australia/VIC7050/2020|2020-07-07|EPI_ISL_564209|Original|hCoV-
19^^Victoria|Human|Victorian Infectious Diseases Reference Laboratory (VIDRL)|VIDRL
and MDU-PHL|Schultz|Australia
>Spike|hCoV-19/USA/TX-HMH-MCoV-13353/2020|2020-08-24|EPI_ISL_788500|Original|hCoV-
19^^Texas|Human|Houston Methodist Hospital|Houston Methodist Hospital|Long|USA

>Spike|hCoV-19/Germany/NW-HHU-154/2020|2020-03-10|EPI_ISL_602524|Orginal|hCoV-19^^North Rhine-Westphalia|Human|Institute for Virology|Center of Medical Microbiology|Wienemann|Germany
>Spike|hCoV-19/Denmark/DCGC-5661/2020|2020-09-28|EPI_ISL_619948|Original|hCoV-19^^Sjaelland|Human|Department of Virus and Microbiological Special Diagnostics|Albertsen lab|Michaelsen|Denmark
>Spike|hCoV-19/Wales/MILK-9575D5/2020|2020-06-10|EPI_ISL_553929|Original|hCoV-19^^Wales|Human|Lighthouse Lab in Milton Keynes|Wellcome Sanger Institute for the COVID-19 Genomics UK (COG-UK) consortium|Davies|United Kingdom
>Spike|hCoV-19/England/QEUH-AF05BF/2020|2020-10-29|EPI_ISL_662811|Original|hCoV-19^^England|Human|Lighthouse Lab in Glasgow|Wellcome Sanger Institute for the COVID-19 Genomics UK (COG-UK) Consortium|Davies|United Kingdom
>Spike|hCoV-19/Netherlands/NB-RIVM-20662/2020|2020-12-18|EPI_ISL_790733|Original|hCoV-19^^Noord-Brabant|Human|Dutch COVID-19 response team|National Institute for Public Health and the Environment (RIVM)|Meijer|Netherlands
>Spike|hCoV-19/England/205090721/2020|2020-12-10|EPI_ISL_766080|Original|hCoV-19^^England|Human|Respiratory Virus Unit|COVID-19 Genomics UK (COG-UK) Consortium|Platt|United Kingdom
>Spike|hCoV-19/USA/LA-EVTL1278/2020|2020-12-05|EPI_ISL_778944|Original|hCoV-19^^Louisiana|Human|LSUHS Emerging Viral Threat Laboratory|Microbial Genome Sequencing Center|Kamil|USA
>Spike|hCoV-19/USA/MD-MDH-0199/2020|2020-06-08|EPI_ISL_560812|Original|hCoV-19^^Maryland|Human|Maryland Public Health Laboratory|Maryland Public Health Laboratory|Keller|USA
>Spike|hCoV-19/USA/ME-HETL-J0250/2020|2020-09-28|EPI_ISL_755403|Original|hCoV-19^^Maine|Human|Maine Health and Environmental Testing Laboratory|Tewhey Lab|Tewhey|USA
>Spike|hCoV-19/USA/MI-UM-10033760952/2020|2020-03-23|EPI_ISL_578096|Original|hCoV-19^^Michigan|Human|University of Michigan Clinical Microbiology Laboratory|Lauring Lab|Valesano|USA
>Spike|hCoV-19/Wales/PHWC-47FC22/2020|2020-10-07|EPI_ISL_612788|Original|hCoV-19^^Wales|Human|Wales Specialist Virology Centre Sequencing lab|COVID-19 Genomics UK (COG-UK) Consortium|Moore|United Kingdom
>Spike|hCoV-19/England/MILK-975841/2020|2020-08-20|EPI_ISL_552627|Original|hCoV-19^^England|Human|Lighthouse Lab in Milton Keynes|Wellcome Sanger Institute for the COVID-19 Genomics UK (COG-UK) consortium|Davies|United Kingdom
>Spike|hCoV-19/Switzerland/BS-UHB-42192489/2020|2020-03-16|EPI_ISL_528127|Original|hCoV-19^^Basel-Stadt|Human|University Hospital Basel|University Hospital Basel|Roloff Handschin|Switzerland
>Spike|hCoV-19/Canada/BC_08940467/2020|2020-04-00|EPI_ISL_466838|Original|hCoV-19^^British Columbia|Human|BCCDC Public Health Laboratory|BCCDC Public Health Laboratory|Harrigan|Canada
>Spike|hCoV-19/USA/CA-IGI-0084/2020|2020-06-08|EPI_ISL_511838|Original|hCoV-19^^California|Human|Innovative Genomics Institute|Innovative Genomics Institute|Wyman|USA
>Spike|hCoV-19/USA/FL-BPHL-2038/2020|2020-07-16|EPI_ISL_653191|Original|hCoV-19^^Florida|Human|Florida Bureau of Public Health Laboratories|Florida Bureau of Public Health Laboratories|Blanton|USA
>Spike|hCoV-19/England/MILK-B55BF1/2020|2020-11-05|EPI_ISL_658303|Original|hCoV-19^^England|Human|Lighthouse Lab in Milton Keynes|Wellcome Sanger Institute for the COVID-19 Genomics UK (COG-UK) Consortium|Davies|United Kingdom
>Spike|hCoV-19/England/QEUH-9FF93D/2020|2020-09-30|EPI_ISL_600290|Original|hCoV-19^^England|Human|Lighthouse Lab in Glasgow|Wellcome Sanger Institute for the COVID-19 Genomics UK (COG-UK) consortium|Davies|United Kingdom
>Spike|hCoV-19/France/BRE-IPP9970/2020|2020-09-14|EPI_ISL_718229|Original|hCoV-19^^Bretagne|Human|Hospital|National Reference Center for Viruses of Respiratory Infections|Enouf|France
>Spike|hCoV-19/USA/IL-NM-0463/2020|2020-04-21|EPI_ISL_626412|Original|hCoV-19^^Illinois|Human|Northwestern Memorial Hospital|Ozer Lab|Ozer|USA

>Spike|hCoV-19/USA/WA-S92/2020|2020-02-29|EPI_ISL_417145|Original|hCoV-19^^Washington|Human|Washington State Department of Health|Seattle Flu Study|Bedford|USA
>Spike|hCoV-19/Wales/MILK-A56256/2020|2020-10-09|EPI_ISL_595143|Original|hCoV-19^^Wales|Human|Quadram Institute Bioscience|COVID-19 Genomics UK (COG-UK) Consortium|Alikhan|United Kingdom
>Spike|hCoV-19/USA/TX-HMH-MCoV-19404/2020|2020-11-29|EPI_ISL_783820|Original|hCoV-19^^Texas|Human|Houston Methodist Hospital|Houston Methodist Hospital|Long|USA
>Spike|hCoV-19/Australia/VIC4419/2020|2020-07-18|EPI_ISL_519715|Original|hCoV-19^^Victoria|Human|Microbiological Diagnostic Unit - Public Health Laboratory (MDU-PHL)|MDU-PHL|Schultz|Australia
>Spike|hCoV-19/England/MILK-A18E5C/2020|2020-10-01|EPI_ISL_612468|Original|hCoV-19^^England|Human|Wales Specialist Virology Centre Sequencing lab|COVID-19 Genomics UK (COG-UK) Consortium|Moore|United Kingdom
>Spike|hCoV-19/Canada/ON-PHL-20-01314/2020|2020-06-00|EPI_ISL_548917|Original|hCoV-19^^Ontario|Human|Public Health Ontario Laboratory|Public Health Ontario Laboratory|Guthrie|Canada
>Spike|hCoV-19/USA/TX-HMH-MCoV-18019/2020|2020-11-16|EPI_ISL_786167|Original|hCoV-19^^Texas|Human|Houston Methodist Hospital|Houston Methodist Hospital|Long|USA
>Spike|hCoV-19/USA/MI-UM-10035952713/2020|2020-09-15|EPI_ISL_583354|Original|hCoV-19^^Michigan|Human|University of Michigan Clinical Microbiology Laboratory|Lauring Lab|Valesano|USA
>Spike|hCoV-19/Scotland/EDB3270/2020|2020-04-07|EPI_ISL_488946|Original|hCoV-19^^Scotland|Human|Virology Department|Wellcome Sanger Institute for the COVID-19 Genomics UK (COG-UK) consortium|Davies|United Kingdom
>Spike|hCoV-19/SaudiArabia/KAUST-JEDDAH1023/2020|2020-06-23|EPI_ISL_512893|Original|hCoV-19^^Jeddah|Human|Pathogen Genomics Lab King Abdullah University of Science and Technology(KAUST)|Pathogen Genomics Lab King Abdullah University of Science and Technology(KAUST)|Mohamed Ghazzali|Saudi Arabia
>Spike|hCoV-19/Denmark/DCGC-13466/2020|2020-11-30|EPI_ISL_712816|Original|hCoV-19^^Nordjylland|Human|Department of Virus and Microbiological Special Diagnostics|Albertsen Lab|Michaelsen|Denmark
>Spike|hCoV-19/England/QEUH-9AE117/2020|2020-09-01|EPI_ISL_638627|Original|hCoV-19^^England|Human|Department of Pathology|COVID-19 Genomics UK (COG-UK) Consortium|Goodfellow|United Kingdom
>Spike|hCoV-19/England/MILK-ACD014/2020|2020-10-21|EPI_ISL_630300|Original|hCoV-19^^England|Human|Lighthouse Lab in Milton Keynes|Wellcome Sanger Institute for the COVID-19 Genomics UK (COG-UK) consortium|Davies|United Kingdom
>Spike|hCoV-19/Germany/NW-HHU-44/2020|2020-03-19|EPI_ISL_425139|Original|hCoV-19^^North Rhine-Westphalia|Human|Center of Medical Microbiology|Center of Medical Microbiology|Wienemann|Germany
>Spike|hCoV-19/France/PAC-IHU-0973/2020|2020-07-22|EPI_ISL_569020|Original|hCoV-19^^Provence-Alpes-C\u00f4te-d\u2019Azur|Human|MEPHI|MEPHI|Levasseur|France
>Spike|hCoV-19/England/CAMC-B345EB/2020|2020-11-05|EPI_ISL_659741|Original|hCoV-19^^England|Human|Lighthouse Lab in Cambridge|Wellcome Sanger Institute for the COVID-19 Genomics UK (COG-UK) Consortium|Davies|United Kingdom
>Spike|hCoV-19/USA/TX-HMH-MCoV-8250/2020|2020-07-07|EPI_ISL_784290|Original|hCoV-19^^Texas|Human|Houston Methodist Hospital|Houston Methodist Hospital|Long|USA
>Spike|hCoV-19/Switzerland/AG-ETHZ-370282/2020|2020-11-06|EPI_ISL_693940|Original|hCoV-19^^Aargau|Human|Viollier AG|Department of Biosystems Science and Engineering|Nadeau|Switzerland
>Spike|hCoV-19/Denmark/DCGC-3572/2020|2020-08-31|EPI_ISL_616367|Original|hCoV-19^^Hovedstaden|Human|Department of Virus and Microbiological Special Diagnostics|Albertsen lab|Michaelsen|Denmark
>Spike|hCoV-19/Scotland/QEUH-AF475B/2020|2020-10-30|EPI_ISL_676458|Original|hCoV-19^^Scotland|Human|Lighthouse Lab in Glasgow|Wellcome Sanger Institute for the COVID-19 Genomics UK (COG-UK) Consortium|Davies|United Kingdom
>Spike|hCoV-19/England/ALDP-B62D46/2020|2020-11-08|EPI_ISL_655492|Original|hCoV-19^^England|Human|Lighthouse Lab in Alderley Park|Wellcome Sanger Institute for the COVID-19 Genomics UK (COG-UK) Consortium|Davies|United Kingdom

>Spike|hCoV-19/England/QEUH-96B18F/2020|2020-08-18|EPI_ISL_531253|Original|hCoV-19^^England|Human|Lighthouse Lab in Glasgow|Wellcome Sanger Institute for the COVID-19 Genomics UK (COG-UK) consortium|Davies|United Kingdom
>Spike|hCoV-19/Denmark/DCGC-2928/2020|2020-08-24|EPI_ISL_616662|Original|hCoV-19^^Midtjylland|Human|Department of Virus and Microbiological Special Diagnostics|Albertsen lab|Michaelsen|Denmark
>Spike|hCoV-19/Australia/VIC5859/2020|2020-07-20|EPI_ISL_518652|Original|hCoV-19^^Victoria|Human|Microbiological Diagnostic Unit - Public Health Laboratory (MDU-PHL)|MDU-PHL|Schultz|Australia
>Spike|hCoV-19/Australia/VIC-CBA5/2020|2020-04-08|EPI_ISL_430065|Original|hCoV-19^^Victoria|Human|Geelong Centre for Emerging Infectious Diseases|Geelong Centre for Emerging Infectious Diseases|Alexandersen|Australia
>Spike|hCoV-19/Denmark/DCGC-9955/2020|2020-11-09|EPI_ISL_669292|Original|hCoV-19^^Hovedstaden|Human|Department of Virus and Microbiological Special Diagnostics|Albertsen Lab|Michaelsen|Denmark
>Spike|hCoV-19/Australia/VIC6721/2020|2020-07-25|EPI_ISL_564012|Original|hCoV-19^^Victoria|Human|Victorian Infectious Diseases Reference Laboratory (VIDRL)|VIDRL and MDU-PHL|Schultz|Australia
>Spike|hCoV-19/Wales/PHWC-48CB9E/2020|2020-11-03|EPI_ISL_666514|Original|hCoV-19^^Wales|Human|Wales Specialist Virology Centre Sequencing lab|COVID-19 Genomics UK (COG-UK) Consortium|Moore|United Kingdom
>Spike|hCoV-19/Russia/CRIE4907775/2020|2020-07-14|EPI_ISL_610227|Original|hCoV-19^^Moscow|Human|Molecular diagnostic laboratory of Federal Budget Institution of Science \Central Research Institute of Epidemiology\ of The Federal Service on Customers' Rights Protection and Human Well-being Surveillance|Group of Genomics and Postgenomic Technologies of Central Research Institute of Epidemilology|Samo
>ilov|Russia
>Spike|hCoV-19/England/QEUH-B6ADF3/2020|2020-11-11|EPI_ISL_662505|Original|hCoV-19^^England|Human|Lighthouse Lab in Glasgow|Wellcome Sanger Institute for the COVID-19 Genomics UK (COG-UK) Consortium|Davies|United Kingdom
>Spike|hCoV-19/Denmark/DCGC-18293/2020|2020-12-07|EPI_ISL_749868|Original|hCoV-19^^Hovedstaden|Human|Department of Virus and Microbiological Special Diagnostics|Albertsen Lab|Michaelsen|Denmark
>Spike|hCoV-19/France/PAC-MEPHI-1913/2020|2020-08-27|EPI_ISL_644387|Original|hCoV-19^^Provence-Alpes-Cote-d\u2019Azur|Human|MEPHI|MEPHI|Levasseur|France
>Spike|hCoV-19/England/MILK-B5A39A/2020|2020-11-06|EPI_ISL_658245|Original|hCoV-19^^England|Human|Lighthouse Lab in Milton Keynes|Wellcome Sanger Institute for the COVID-19 Genomics UK (COG-UK) Consortium|Davies|United Kingdom
>Spike|hCoV-19/USA/CA-ALSR-1900/2020|2020-04-22|EPI_ISL_512224|Original|hCoV-19^^California|Human|San Diego County Public Health Laboratory|Andersen lab at Scripps Research|Gangavarapu|USA
>Spike|hCoV-19/Scotland/CVR315/2020|2020-03-21|EPI_ISL_439046|Original|hCoV-19^^Scotland|Human|West of Scotland Specialist Virology Centre|COVID-19 Genomics UK (COG-UK) Consortium|Robertson|United Kingdom
>Spike|hCoV-19/Russia/MOW-RII-MH2911S/2020|2020-09-21|EPI_ISL_733067|Original|hCoV-19^^Moscow|Human|HELIX LLC|WHO National Influenza Centre Russian Federation|Komissarov|Russia
>Spike|hCoV-19/Austria/Graz-MUG3/2020|2020-03-31|EPI_ISL_437199|Original|hCoV-19^^Styria|Human|Diagnostic- and Research Institute of Pathology|Diagnostic- and Research Institute of Pathology|Karl Kashofer|Austria
>Spike|hCoV-19/Gibraltar/204421211/2020|2020-10-27|EPI_ISL_613672|Original|hCoV-19^^Gibraltar|Human|Microbiology|Respiratory Virus Unit|Platt|Gibraltar
>Spike|hCoV-19/USA/NY-NYUMC311/2020|2020-03-30|EPI_ISL_430338|Original|hCoV-19^^New York|Human|NYU Langone Health|Departments of Pathology and Medicine|Maurano|USA
>Spike|hCoV-19/England/BRIS-1259DC/2020|2020-03-30|EPI_ISL_443746|Original|hCoV-19^^England|Human|PHE South West Regional Laboratory|Wellcome Sanger Institute for the COVID-19 Genomics UK (COG-UK) consortium|Amato|United Kingdom
>Spike|hCoV-19/Wales/PHWC-264EA/2020|2020-03-28|EPI_ISL_422325|Original|hCoV-19^^Wales|Human|Wales Specialist Virology Centre|Public Health Wales Microbiology Cardiff|Moore|United Kingdom

>Spike|hCoV-19/Scotland/QEUH-AAE529/2020|2020-10-22|EPI_ISL_623916|Original|hCoV-19^^Scotland|Human|Lighthouse Lab in Glasgow|Wellcome Sanger Institute for the COVID-19 Genomics UK (COG-UK) consortium|Davies|United Kingdom
>Spike|hCoV-19/USA/CA-CDPH182/2020|2020-10-02|EPI_ISL_754916|Original|hCoV-19^^California|Human|California Department of Public Health|California Department of Public Health|Bell|USA
>Spike|hCoV-19/USA/TX-HMH-4053/2020|2020-06-18|EPI_ISL_543329|Original|hCoV-19^^Texas|Human|Houston Methodist Hospital|Houston Methodist Hospital|Long|USA
>Spike|hCoV-19/Japan/PG-5036/2020|2020-07-00|EPI_ISL_690147|Original|hCoV-19^^Japan|Human|Pathogen Genomics Center|Pathogen Genomics Center|Sekizuka|Japan
>Spike|hCoV-19/England/QEUH-B676EC/2020|2020-11-11|EPI_ISL_661455|Original|hCoV-19^^England|Human|Lighthouse Lab in Glasgow|Wellcome Sanger Institute for the COVID-19 Genomics UK (COG-UK) Consortium|Davies|United Kingdom
>Spike|hCoV-19/England/ALDP-9EA6D4/2020|2020-09-24|EPI_ISL_580159|Original|hCoV-19^^England|Human|Lighthouse Lab in Alderley Park|Wellcome Sanger Institute for the COVID-19 Genomics UK (COG-UK) consortium|Davies|United Kingdom
>Spike|hCoV-19/England/ALDP-49A984/2020|2020-06-04|EPI_ISL_559316|Original|hCoV-19^^England|Human|Lighthouse Lab in Alderley Park|Wellcome Sanger Institute for the COVID-19 Genomics UK (COG-UK) consortium|Davies|United Kingdom
>Spike|hCoV-19/England/SHEF-C9461/2020|2020-10-12|EPI_ISL_613377|Original|hCoV-19^^England|Human|Virology Department|COVID-19 Genomics UK (COG-UK) Consortium|de Silva|United Kingdom
>Spike|hCoV-19/Russia/SVE-RII-MH1928S/2020|2020-09-17|EPI_ISL_602369|Original|hCoV-19^^Sverdlovsk|Human|HELIX LLC|WHO National Influenza Centre Russian Federation|Komissarov|Russia
>Spike|hCoV-19/Turkey/IST-GLAB-196/2020|2020-05-06|EPI_ISL_480280|Original|hCoV-19^^Istanbul|Human|Genomic Laboratory (GLAB) (Conjoint lab of Health Directorate of Istanbul and Istanbul Technical University)|Genomic Laboratory (GLAB)|Karacan|Turkey
>Spike|hCoV-19/England/LOND-D5E55/2020|2020-04-19|EPI_ISL_444251|Original|hCoV-19^^England|Human|University College London|COVID-19 Genomics UK (COG-UK) Consortium|Roy|United Kingdom
>Spike|hCoV-19/Canada/ON-S722/2020|2020-04-00|EPI_ISL_591147|Original|hCoV-19^^Ontario|Human|Toronto Invasive Bacterial Diseases Network|McMaster University|McArthur|Canada
>Spike|hCoV-19/USA/AZ-TG666123/2020|2020-05-01|EPI_ISL_696209|Original|hCoV-19^^Arizona|Human|Sonora Quest Laboratories|TGen North|French|USA
>Spike|hCoV-19/England/MILK-993D6C/2020|2020-08-27|EPI_ISL_552337|Original|hCoV-19^^England|Human|Lighthouse Lab in Milton Keynes|Wellcome Sanger Institute for the COVID-19 Genomics UK (COG-UK) consortium|Davies|United Kingdom
>Spike|hCoV-19/USA/NY-NYUMC637/2020|2020-04-02|EPI_ISL_444754|Original|hCoV-19^^New York|Human|NYU Langone Health|Departments of Pathology and Medicine|Maurano|USA
>Spike|hCoV-19/England/QEUH-B6891F/2020|2020-11-11|EPI_ISL_662153|Original|hCoV-19^^England|Human|Lighthouse Lab in Glasgow|Wellcome Sanger Institute for the COVID-19 Genomics UK (COG-UK) Consortium|Davies|United Kingdom
>Spike|hCoV-19/England/CAMB-1B3047/2020|2020-05-21|EPI_ISL_456749|Original|hCoV-19^^England|Human|Department of Pathology|COVID-19 Genomics UK (COG-UK) Consortium|Meredith|United Kingdom
>Spike|hCoV-19/England/BRIS-122395/2020|2020-04-03|EPI_ISL_440745|Original|hCoV-19^^England|Human|PHE South West Regional Laboratory|Wellcome Sanger Institute for the COVID-19 Genomics UK (COG-UK) consortium|Amato|United Kingdom
>Spike|hCoV-19/Japan/PG-3231/2020|2020-04-00|EPI_ISL_691348|Original|hCoV-19^^Japan|Human|Pathogen Genomics Center|Pathogen Genomics Center|Sekizuka|Japan
>Spike|hCoV-19/England/ALDP-950651/2020|2020-06-17|EPI_ISL_555276|Original|hCoV-19^^England|Human|Lighthouse Lab in Alderley Park|Wellcome Sanger Institute for the COVID-19 Genomics UK (COG-UK) consortium|Davies|United Kingdom
>Spike|hCoV-19/England/EXET-13EACE/2020|2020-12-12|EPI_ISL_764224|Original|hCoV-19^^England|Human|University of Exeter|COVID-19 Genomics UK (COG-UK) Consortium|Temperton|United Kingdom

>Spike|hCoV-19/Denmark/DCGC-17903/2020|2020-12-14|EPI_ISL_749634|Original|hCoV-19^^Hovedstaden|Human|Department of Virus and Microbiological Special Diagnostics|Albertsen Lab|Michaelsen|Denmark
>Spike|hCoV-19/Austria/CeMM0031/2020|2020-03-09|EPI_ISL_437920|Original|hCoV-19^^Ischgl|Human|Institut f\u00fcr Virologie am Department f\u00fcr Hygiene|Bergthaler laboratory|Bergthaler|Austria
>Spike|hCoV-19/Canada/ON-OICR-SLB2789/2020|2020-03-25|EPI_ISL_538323|Original|hCoV-19^^Ontario|Human|Kingston Health Sciences Centre / Queen's University|Ontario Institute for Cancer Research|de Borja|Canada
>Spike|hCoV-19/England/BIRM-67C4F/2020|2020-11-06|EPI_ISL_651630|Original|hCoV-19^^England|Human|University of Birmingham|COVID-19 Genomics UK (COG-UK) Consortium|Loman|United Kingdom
>Spike|hCoV-19/Finland/23LS5G11/2020|2020-10-23|EPI_ISL_759778|Original|hCoV-19^^Uusimaa|Human|Department of Virology and Immunology|Department of Virology|Smura|Finland
>Spike|hCoV-19/Scotland/CVR2440/2020|2020-04-14|EPI_ISL_493799|Original|hCoV-19^^Scotland|Human|West of Scotland Specialist Virology Centre|COVID-19 Genomics UK (COG-UK) Consortium|Robertson|United Kingdom
>Spike|hCoV-19/England/MILK-9D44D3/2020|2020-09-19|EPI_ISL_601781|Original|hCoV-19^^England|Human|Lighthouse Lab in Milton Keynes|Wellcome Sanger Institute for the COVID-19 Genomics UK (COG-UK) consortium|Davies|United Kingdom
>Spike|hCoV-19/USA/MD-MDH-0036/2020|2020-04-07|EPI_ISL_481281|Original|hCoV-19^^Maryland|Human|Maryland Department of Health|Maryland Department of Health|Keller|USA
>Spike|hCoV-19/USA/NY-NYUMC839/2020|2020-04-24|EPI_ISL_456111|Original|hCoV-19^^New York|Human|NYU Langone Health|Departments of Pathology and Medicine|Maurano|USA
>Spike|hCoV-19/USA/HI-H200349/2020|2020-09-15|EPI_ISL_752905|Original|hCoV-19^^Hawaii|Human|State Laboratories Division|State Laboratories Division|Sultana|USA
>Spike|hCoV-19/USA/TX-HMH-3193/2020|2020-06-13|EPI_ISL_544066|Original|hCoV-19^^Texas|Human|Houston Methodist Hospital|Houston Methodist Hospital|Long|USA
>Spike|hCoV-19/England/ALDP-90C6BE/2020|2020-06-22|EPI_ISL_553336|Original|hCoV-19^^England|Human|Lighthouse Lab in Alderley Park|Wellcome Sanger Institute for the COVID-19 Genomics UK (COG-UK) consortium|Davies|United Kingdom
>Spike|hCoV-19/England/BRIS-13030D/2020|2020-04-11|EPI_ISL_492725|Original|hCoV-19^^England|Human|PHE South West Regional Laboratory|Wellcome Sanger Institute for the COVID-19 Genomics UK (COG-UK) consortium|Davies|United Kingdom
>Spike|hCoV-19/USA/MI-MDHHS-SC21959/2020|2020-04-16|EPI_ISL_566010|Original|hCoV-19^^Michigan|Human|Michigan Department of Health and Human Services|Michigan Department of Health and Human Services|Blankenship|USA
>Spike|hCoV-19/England/QEUH-9F341A/2020|2020-09-24|EPI_ISL_588670|Original|hCoV-19^^England|Human|Lighthouse Lab in Glasgow|Wellcome Sanger Institute for the COVID-19 Genomics UK (COG-UK) consortium|Davies|United Kingdom
>Spike|hCoV-19/Netherlands/NoordBrabant_47/2020|2020-03-09|EPI_ISL_415504|Original|hCoV-19^^Noord Brabant|Human|Dutch COVID-19 response team|Erasmus Medical Center|Oude Munnink|Netherlands
>Spike|hCoV-19/USA/WA-S1805/2020|2020-06-02|EPI_ISL_496231|Original|hCoV-19^^Washington|Human|Washington State Department of Health|Seattle Flu Study|Lee|USA
>Spike|hCoV-19/Luxembourg/LNS3459765/2020|2020-11-16|EPI_ISL_740312|Original|hCoV-19^^Luxembourg|Human|Laboratoire national de sant\u00e9|Laboratoire national de sant\u00e9|Wienecke-Baldacchino|Luxembourg
>Spike|hCoV-19/USA/MI-MDHHS-SC22390/2020|2020-11-12|EPI_ISL_708370|Original|hCoV-19^^Michigan|Human|Michigan Department of Health and Human Services|Michigan Department of Health and Human Services|Jacob|USA
>Spike|hCoV-19/USA/NV-NSPHL-A0197/2020|2020-05-27|EPI_ISL_515451|Original|hCoV-19^^Nevada|Human|Nevada State Public Health Laboratory|Nevada State Public Health Laboratory|Sevinsky|USA
>Spike|hCoV-19/England/MILK-A6AE2E/2020|2020-10-09|EPI_ISL_608720|Original|hCoV-19^^England|Human|Lighthouse Lab in Milton Keynes|Wellcome Sanger Institute for the COVID-19 Genomics UK (COG-UK) consortium|Davies|United Kingdom

>Spike|hCoV-19/Denmark/DCGC-9008/2020|2020-10-26|EPI_ISL_626180|Original|hCoV-19^^Hovedstaden|Human|Department of Virus and Microbiological Special Diagnostics|Albertsen lab|Michaelsen|Denmark
>Spike|hCoV-19/England/OXON-B4BCE/2020|2020-04-23|EPI_ISL_534998|Original|hCoV-19^^England|Human|Oxford Viromics|COVID-19 Genomics UK (COG-UK) Consortium|Golubchik|United Kingdom
>Spike|hCoV-19/England/MILK-B96848/2020|2020-11-14|EPI_ISL_676031|Original|hCoV-19^^England|Human|Lighthouse Lab in Milton Keynes|Wellcome Sanger Institute for the COVID-19 Genomics UK (COG-UK) Consortium|Davies|United Kingdom
>Spike|hCoV-19/USA/CA-ALSR-3528/2020|2020-09-08|EPI_ISL_636071|Original|hCoV-19^^California|Human|San Diego County Public Health Laboratory|Andersen lab at Scripps Research|Gangavarapu|USA
>Spike|hCoV-19/England/QEUH-B6983F/2020|2020-11-11|EPI_ISL_661361|Original|hCoV-19^^England|Human|Lighthouse Lab in Glasgow|Wellcome Sanger Institute for the COVID-19 Genomics UK (COG-UK) Consortium|Davies|United Kingdom
>Spike|hCoV-19/USA/WA-UW-4318/2020|2020-03-31|EPI_ISL_427259|Original|hCoV-19^^Washington|Human|UW Virology Lab|UW Virology Lab|Roychoudhury|USA
>Spike|hCoV-19/Spain/VC-IBV-98003105/2020|2020-03-15|EPI_ISL_538041|Original|hCoV-19^^Comunitat Valenciana|Human|Hospital Universitari i Polit\u00e8cnic La Fe de Val\u00e8ncia|SeqCOVID-SPAIN consortium/IBV(CSIC)|Comas|Spain
>Spike|hCoV-19/England/201640112/2020|2020-04-14|EPI_ISL_466440|Original|hCoV-19^^England|Human|Respiratory Virus Unit|Respiratory Virus Unit|Platt|United Kingdom
>Spike|hCoV-19/Australia/VIC3520/2020|2020-07-08|EPI_ISL_520272|Original|hCoV-19^^Victoria|Human|Victorian Infectious Diseases Reference Laboratory (VIDRL)|VIDRL and MDU-PHL|Schultz|Australia
>Spike|hCoV-19/England/BRIS-12414E/2020|2020-04-08|EPI_ISL_492815|Original|hCoV-19^^England|Human|PHE South West Regional Laboratory|Wellcome Sanger Institute for the COVID-19 Genomics UK (COG-UK) consortium|Davies|United Kingdom
>Spike|hCoV-19/England/MILK-B952F0/2020|2020-11-13|EPI_ISL_673599|Original|hCoV-19^^England|Human|Lighthouse Lab in Milton Keynes|Wellcome Sanger Institute for the COVID-19 Genomics UK (COG-UK) Consortium|Davies|United Kingdom
>Spike|hCoV-19/England/QEUH-C5BF94/2020|2020-12-14|EPI_ISL_769471|Original|hCoV-19^^England|Human|Lighthouse Lab in Glasgow|Wellcome Sanger Institute for the COVID-19 Genomics UK (COG-UK) Consortium|Davies|United Kingdom
>Spike|hCoV-19/South Africa/KRISP-0035/2020|2020-03-27|EPI_ISL_467441|Original|hCoV-19^^KwaZulu-Natal|Human|NHLS-IALCH|KRISP|de Oliveira|South Africa
>Spike|hCoV-19/USA/HI-H200444/2020|2020-10-07|EPI_ISL_752987|Original|hCoV-19^^Hawaii|Human|State Laboratories Division|State Laboratories Division|Sultana|USA
>Spike|hCoV-19/England/ALDP-97DD4A/2020|2020-07-22|EPI_ISL_526332|Original|hCoV-19^^England|Human|University of Birmingham|COVID-19 Genomics UK (COG-UK) Consortium|Loman|United Kingdom
>Spike|hCoV-19/Australia/VIC9997/2020|2020-08-24|EPI_ISL_565828|Original|hCoV-19^^Victoria|Human|Microbiological Diagnostic Unit - Public Health Laboratory (MDU-PHL)|MDU-PHL|Schultz|Australia
>Spike|hCoV-19/USA/WA-SFS-S2855/2020|2020-03-03|EPI_ISL_575033|Original|hCoV-19^^Washington|Human|Seattle Flu Study|Seattle Flu Study|Lee|USA
>Spike|hCoV-19/Spain/PV-IBV-001535/2020|2020-03-16|EPI_ISL_467144|Original|hCoV-19^^Basque Country|Human|Hospital Universitario Araba. Vitoria-Gasteiz|SeqCOVID-SPAIN consortium/IBV(CSIC)|Comas|Spain
>Spike|hCoV-19/England/CAMC-9DF669/2020|2020-09-20|EPI_ISL_638416|Original|hCoV-19^^England|Human|Department of Pathology|COVID-19 Genomics UK (COG-UK) Consortium|Goodfellow|United Kingdom
>Spike|hCoV-19/USA/CA-CZB-3996/2020|2020-07-29|EPI_ISL_548632|Original|hCoV-19^^California|Human|County of Santa Clara Public Health Department|Chan-Zuckerberg Biohub|Bhatt|USA
>Spike|hCoV-19/Denmark/DCGC-20583/2020|2020-12-21|EPI_ISL_758746|Original|hCoV-19^^Hovedstaden|Human|Department of Virus and Microbiological Special Diagnostics|Albertsen Lab|Michaelsen|Denmark

>Spike|hCoV-19/England/OXON-AEB21/2020|2020-00-00|EPI_ISL_448758|Original|hCoV-19^^England|Human|Oxford Viromics|COVID-19 Genomics UK (COG-UK) Consortium|Golubchik|United Kingdom
>Spike|hCoV-19/Italy/LOM-ASST-10/2020|2020-03-02|EPI_ISL_486647|Original|hCoV-19^^Lombardy|Human|Microbiology|Microbiology|Comandatore|Italy
>Spike|hCoV-19/Australia/VIC6008/2020|2020-07-23|EPI_ISL_518783|Original|hCoV-19^^Victoria|Human|Microbiological Diagnostic Unit - Public Health Laboratory (MDU-PHL)|MDU-PHL|Schultz|Australia
>Spike|hCoV-19/Japan/PG-4499/2020|2020-07-00|EPI_ISL_692722|Original|hCoV-19^^Japan|Human|Pathogen Genomics Center|Pathogen Genomics Center|Sekizuka|Japan
>Spike|hCoV-19/England/CAMC-B076B1/2020|2020-10-30|EPI_ISL_649632|Original|hCoV-19^^England|Human|Lighthouse Lab in Cambridge|Wellcome Sanger Institute for the COVID-19 Genomics UK (COG-UK) Consortium|Davies|United Kingdom
>Spike|hCoV-19/England/QEUH-B7F05C/2020|2020-11-14|EPI_ISL_661885|Original|hCoV-19^^England|Human|Lighthouse Lab in Glasgow|Wellcome Sanger Institute for the COVID-19 Genomics UK (COG-UK) Consortium|Davies|United Kingdom
>Spike|hCoV-19/England/QEUH-B7E2DF/2020|2020-11-14|EPI_ISL_727852|Original|hCoV-19^^England|Human|Oxford Viromics|COVID-19 Genomics UK (COG-UK) Consortium|Golubchik|United Kingdom
>Spike|hCoV-19/USA/OR-OHSU-2017/2020|2020-07-31|EPI_ISL_667092|Original|hCoV-19^^Oregon|Human|OHSU Lab Services Molecular Microbiology Lab|Oregon SARS-CoV-2 Genome Sequencing Center|O'Roak|USA
>Spike|hCoV-19/USA/LA-GBCL-26791722/2020|2020-04-09|EPI_ISL_485341|Original|hCoV-19^^Louisiana|Human|River Road Testing Lab|Ginkgo Bioworks Clinical Laboratory|Smith|USA
>Spike|hCoV-19/England/CAMB-82103/2020|2020-04-21|EPI_ISL_438620|Original|hCoV-19^^England|Human|Department of Pathology|COVID-19 Genomics UK (COG-UK) Consortium|Meredith|United Kingdom
>Spike|hCoV-19/England/LOND-1262DA6/2020|2020-10-15|EPI_ISL_612014|Original|hCoV-19^^England|Human|University College London|COVID-19 Genomics UK (COG-UK) Consortium|Roy|United Kingdom
>Spike|hCoV-19/Wales/PHWC-4A00B3/2020|2020-12-01|EPI_ISL_743393|Original|hCoV-19^^Wales|Human|Wales Specialist Virology Centre Sequencing lab|COVID-19 Genomics UK (COG-UK) Consortium|Moore|United Kingdom
>Spike|hCoV-19/USA/CA-CZB-5833/2020|2020-07-16|EPI_ISL_582960|Original|hCoV-19^^California|Human|San Luis Obispo Public Health Department|Chan-Zuckerberg Biohub|Bhatt|USA
>Spike|hCoV-19/Australia/NT13/2020|2020-00-00|EPI_ISL_426901|Original|hCoV-19^^Northern territory|Human|Royal Darwin Hospital Pathology|Microbiological Diagnostic Unit Public Health Laboratory and Victorian Infectious Diseases Reference Laboratory|Schultz|Australia
>Spike|hCoV-19/USA/GA-QDX-580/2020|2020-03-18|EPI_ISL_571291|Original|hCoV-19^^Georgia|Human|Quest Diagnostics|Quest Diagnostics|Kagan|USA
>Spike|hCoV-19/Japan/PG-1915/2020|2020-05-00|EPI_ISL_685521|Original|hCoV-19^^Japan|Human|Pathogen Genomics Center|Pathogen Genomics Center|Sekizuka|Japan
>Spike|hCoV-19/England/ALDP-AA1957/2020|2020-10-21|EPI_ISL_624182|Original|hCoV-19^^England|Human|Lighthouse Lab in Alderley Park|Wellcome Sanger Institute for the COVID-19 Genomics UK (COG-UK) consortium|Davies|United Kingdom
>Spike|hCoV-19/USA/WI-UW-2390/2020|2020-10-30|EPI_ISL_754626|Original|hCoV-19^^Wisconsin|Human|University of Wisconsin-Madison AIDS Vaccine Research Laboratories|University of Wisconsin-Madison AIDS Vaccine Research Laboratories|Moreno|USA
>Spike|hCoV-19/USA/WA-UW-2066/2020|2020-03-25|EPI_ISL_477703|Original|hCoV-19^^Washington|Human|UW Virology Lab|UW Virology Lab|Roychoudhury|USA
>Spike|hCoV-19/India/MH-ACTREC-653/2020|2020-09-14|EPI_ISL_700283|Original|hCoV-19^^Maharashtra|Human|Hematopathology Laboratory|Hematopathology Laboratory|Patkar|India
>Spike|hCoV-19/England/CAMB-78C96/2020|2020-04-03|EPI_ISL_441070|Original|hCoV-19^^England|Human|Department of Pathology|Wellcome Sanger Institute for the COVID-19 Genomics UK (COG-UK) consortium|Amato|United Kingdom

>Spike|hCoV-19/South Africa/R12669-20/2020|2020-06-16|EPI_ISL_504217|Original|hCoV-
19^^North West|Human|National Institute for Communicable Diseases of the National
Health Laboratory Service|National Institute for Communicable Diseases of the
National Health Laboratory Service|Allam|South Africa
>Spike|hCoV-19/Scotland/EDB5194/2020|2020-05-18|EPI_ISL_493947|Original|hCoV-
19^^Scotland|Human|Virology Department|COVID-19 Genomics UK (COG-UK)
Consortium|Williams|United Kingdom
>Spike|hCoV-19/Japan/PG-6711/2020|2020-07-00|EPI_ISL_687822|Original|hCoV-
19^^Japan|Human|Pathogen Genomics Center|Pathogen Genomics Center|Sekizuka|Japan
>Spike|hCoV-19/England/ALDP-A4FFE0/2020|2020-10-07|EPI_ISL_597881|Original|hCoV-
19^^England|Human|Lighthouse Lab in Alderley Park|Wellcome Sanger Institute for the
COVID-19 Genomics UK (COG-UK) consortium|Davies|United Kingdom
>Spike|hCoV-19/Netherlands/ZH-EMC-436/2020|2020-08-12|EPI_ISL_523774|Original|hCoV-
19^^Zuid Holland|Human|Dutch COVID-19 response team|Erasmus Medical Center|Oude
Munnink|Netherlands
>Spike|hCoV-19/England/NOTT-121846/2020|2020-12-23|EPI_ISL_764510|Original|hCoV-
19^^England|Human|Queens Medical Centre|COVID-19 Genomics UK (COG-UK)
Consortium|Loose|United Kingdom
>Spike|hCoV-19/Spain/CN-IBV-97006477/2020|2020-03-24|EPI_ISL_537694|Original|hCoV-
19^^Canary Islands|Human|Hospital Universitario de Gran Canaria Dr.
Negr\u00edn|SeqCOVID-SPAIN consortium/IBV(CSIC)|Comas|Spain
>Spike|hCoV-19/England/ALDP-CEC9D6/2020|2020-12-21|EPI_ISL_782352|Original|hCoV-
19^^England|Human|Lighthouse Lab in Alderley Park|Wellcome Sanger Institute for the
COVID-19 Genomics UK (COG-UK) Consortium|Davies|United Kingdom
>Spike|hCoV-19/England/NORT-29E3DF/2020|2020-04-30|EPI_ISL_493515|Original|hCoV-
19^^England|Human|Northumbria University / South Tees Hospitals NHS Foundation Trust
/ North Cumbria Integrated Care NHS Foundation Trust / North Tees and Hartlepool NHS
Foundation Trust / Newcastle Hospitals NHS Foundation Trust|COVID-19 Genomics UK
(COG-UK) Consortium|Bashton|United Kingdom
>Spike|hCoV-19/Belgium/ULG-11063/2020|2020-12-14|EPI_ISL_737314|Original|hCoV-
19^^Li\u00e8ge|Human|Department of Clinical Microbiology|GIGA Medical
Genomics|Durkin|Belgium
>Spike|hCoV-19/USA/IL_0087/2020|2020-03-07|EPI_ISL_424850|Original|hCoV-
19^^Illinois|Human|IL Department of Public Health Chicago Laboratory|Pathogen
Discovery|Queen|USA
>Spike|hCoV-19/Belgium/ULG-10803/2020|2020-11-17|EPI_ISL_661232|Original|hCoV-
19^^Li\u00e8ge|Human|Department of Clinical Microbiology|GIGA Medical
Genomics|Durkin|Belgium
>Spike|hCoV-19/Scotland/QEUH-941116/2020|2020-07-29|EPI_ISL_532639|Original|hCoV-
19^^Scotland|Human|NHSGGC West of Scotland Specialist Virology Centre / MRC-
University of Glasgow Centre for Virus Research|Wellcome Sanger Institute for the
COVID-19 Genomics UK (COG-UK) consortium|Davies|United Kingdom
>Spike|hCoV-19/USA/ME-HETL-J0120/2020|2020-09-01|EPI_ISL_755322|Original|hCoV-
19^^Maine|Human|Maine Health and Environmental Testing Laboratory|Tewhey
Lab|Tewhey|USA
>Spike|hCoV-19/Nigeria/FC-CV245/2020|2020-07-07|EPI_ISL_729930|Original|hCoV-
19^^Abuja|Human|Nigeria Centre for Disease Control (NCDC)|African Centre of
Excellence for Genomics of Infectious Diseases (ACEGID)|Oluniyi|Nigeria
>Spike|hCoV-19/Serbia/Kraljevo-01452507/2020|2020-07-24|EPI_ISL_582532|Original|hCoV-
19^^Kraljevo|Human|Veterinary Specialized Institute \Kraljevo\|Veterinary Specialized
Institute \Kraljevo\|Vidanovic|Serbia
>Spike|hCoV-19/Switzerland/SG-ETHZ-120040/2020|2020-04-
20|EPI_ISL_468229|Original|hCoV-19^^Sankt Gallen|Human|Viollier AG|Department of
Biosystems Science and Engineering|Nadeau|Switzerland
>Spike|hCoV-19/England/ALDP-94ED8E/2020|2020-06-17|EPI_ISL_556412|Original|hCoV-
19^^England|Human|Lighthouse Lab in Alderley Park|Wellcome Sanger Institute for the
COVID-19 Genomics UK (COG-UK) consortium|Davies|United Kingdom
>Spike|hCoV-19/Switzerland/TI-ETHZ-380311/2020|2020-11-
24|EPI_ISL_721912|Original|hCoV-19^^Ticino|Human|Viollier AG|Department of Biosystems
Science and Engineering|Nadeau|Switzerland

>Spike|hCoV-19/Netherlands/OV-EMC-114/2020|2020-11-07|EPI_ISL_763154|Original|hCoV-19^^Overijssel|Human|Dutch COVID-19 response team|Erasmus Medical Center|Oude Munnink|Netherlands
>Spike|hCoV-19/Spain/AR-IBV-98010590/2020|2020-10-03|EPI_ISL_722248|Original|hCoV-19^^Aragon|Human|Servicio de Microbiolog\u00eda|SeqCOVID-SPAIN consortium/IBV(CSIC)|Comas|Spain
>Spike|hCoV-19/USA/TX-HMH-MCoV-14416/2020|2020-10-06|EPI_ISL_787701|Original|hCoV-19^^Texas|Human|Houston Methodist Hospital|Houston Methodist Hospital|Long|USA
>Spike|hCoV-19/Australia/VIC851/2020|2020-04-04|EPI_ISL_427111|Original|hCoV-19^^Victoria|Human|Victorian Infectious Diseases Reference Laboratory (VIDRL)|Microbiological Diagnostic Unit Public Health Laboratory and Victorian Infectious Diseases Reference Laboratory|Schultz|Australia
>Spike|hCoV-19/Austria/CeMM0943/2020|2020-03-26|EPI_ISL_583795|Original|hCoV-19^^Austria|Human|Dr. Gernot Walder GmbH|Bergthaler laboratory|Bergthaler|Austria
>Spike|hCoV-19/Denmark/DCGC-12345/2020|2020-11-16|EPI_ISL_682526|Original|hCoV-19^^Syddanmark|Human|Department of Virus and Microbiological Special Diagnostics|Albertsen Lab|Michaelsen|Denmark
>Spike|hCoV-19/Wales/PHWC-32DFF/2020|2020-04-14|EPI_ISL_446692|Original|hCoV-19^^Wales|Human|Wales Specialist Virology Centre|Public Health Wales Microbiology Cardiff|Moore|United Kingdom
>Spike|hCoV-19/Denmark/DCGC-4779/2020|2020-09-21|EPI_ISL_621389|Original|hCoV-19^^Hovedstaden|Human|Department of Virus and Microbiological Special Diagnostics|Albertsen lab|Michaelsen|Denmark
>Spike|hCoV-19/Australia/SAP214/2020|2020-01-30|EPI_ISL_468032|Original|hCoV-19^^South Australia|Human|SA Pathology|SA Pathology|Leong|Australia
>Spike|hCoV-19/Wales/PHWC-48AEA6/2020|2020-11-03|EPI_ISL_666168|Original|hCoV-19^^Wales|Human|Wales Specialist Virology Centre Sequencing lab|COVID-19 Genomics UK (COG-UK) Consortium|Moore|United Kingdom
>Spike|hCoV-19/England/ALDP-90FCE6/2020|2020-06-24|EPI_ISL_557050|Original|hCoV-19^^England|Human|Lighthouse Lab in Alderley Park|Wellcome Sanger Institute for the COVID-19 Genomics UK (COG-UK) consortium|Davies|United Kingdom
>Spike|hCoV-19/England/MILK-970E71/2020|2020-08-18|EPI_ISL_553041|Original|hCoV-19^^England|Human|Lighthouse Lab in Milton Keynes|Wellcome Sanger Institute for the COVID-19 Genomics UK (COG-UK) consortium|Davies|United Kingdom
>Spike|hCoV-19/Wales/PHWC-48304D/2020|2020-10-21|EPI_ISL_627771|Original|hCoV-19^^Wales|Human|Wales Specialist Virology Centre Sequencing lab|COVID-19 Genomics UK (COG-UK) Consortium|Moore|United Kingdom
>Spike|hCoV-19/USA/TX-HMH-MCoV-8364/2020|2020-07-07|EPI_ISL_784354|Original|hCoV-19^^Texas|Human|Houston Methodist Hospital|Houston Methodist Hospital|Long|USA
>Spike|hCoV-19/England/ALDP-B1F5D7/2020|2020-11-01|EPI_ISL_645848|Original|hCoV-19^^England|Human|Lighthouse Lab in Alderley Park|Wellcome Sanger Institute for the COVID-19 Genomics UK (COG-UK) Consortium|Davies|United Kingdom
>Spike|hCoV-19/Japan/TKY10466-2/2020|2020-04-00|EPI_ISL_462444|unknown|hCoV-19^^Japan|Human|unknown|Ryota Kumagai Tokyo Metropolitan Institute of Public Health|Submitter|Japan
>Spike|hCoV-19/England/MILK-BB2372/2020|2020-11-18|EPI_ISL_720407|Original|hCoV-19^^England|Human|Lighthouse Lab in Milton Keynes|Wellcome Sanger Institute for the COVID-19 Genomics UK (COG-UK) Consortium|Davies|United Kingdom
>Spike|hCoV-19/Denmark/DCGC-1741/2020|2020-03-09|EPI_ISL_619977|Original|hCoV-19^^Syddanmark|Human|Department of Virus and Microbiological Special Diagnostics|Albertsen lab|Michaelsen|Denmark
>Spike|hCoV-19/England/QEUH-C654F2/2020|2020-12-14|EPI_ISL_778594|Original|hCoV-19^^England|Human|Lighthouse Lab in Glasgow|Wellcome Sanger Institute for the COVID-19 Genomics UK (COG-UK) Consortium|Davies|United Kingdom
>Spike|hCoV-19/Netherlands/Gelderland_47/2020|2020-04-22|EPI_ISL_460947|Original|hCoV-19^^Gelderland|Human|Dutch COVID-19 response team|Erasmus Medical Center|Oude Munnink|Netherlands
>Spike|hCoV-19/Denmark/DCGC-4538/2020|2020-09-21|EPI_ISL_621334|Original|hCoV-19^^Syddanmark|Human|Department of Virus and Microbiological Special Diagnostics|Albertsen lab|Michaelsen|Denmark

>Spike|hCoV-19/USA/CA-CZB-1189/2020|2020-04-29|EPI_ISL_454632|Original|hCoV-19^^California|Human|UCSF Clinical Microbiology Laboratory|Chan-Zuckerberg Biohub|Hao|USA
>Spike|hCoV-19/Scotland/CVR5065/2020|2020-10-11|EPI_ISL_594798|Original|hCoV-19^^Scotland|Human|West of Scotland Specialist Virology Centre|COVID-19 Genomics UK (COG-UK) Consortium|Robertson|United Kingdom
>Spike|hCoV-19/USA/AZ-TG382139/2020|2020-06-16|EPI_ISL_694677|Original|hCoV-19^^Arizona|Human|TGen North|TGen North|French|USA
>Spike|hCoV-19/Luxembourg/LNS9475401/2020|2020-11-16|EPI_ISL_744923|Original|hCoV-19^^Luxembourg|Human|Laboratoire national de sant\u00e9|Laboratoire national de sant\u00e9|Wienecke-Baldacchino|Luxembourg
>Spike|hCoV-19/Saudi Arabia/596/2020|2020-02-18|EPI_ISL_490006|Original|hCoV-19^^Saudi Arabia|Human|King Fahad Medical City|King Fahad Medical City|Naeem|Saudi Arabia
>Spike|hCoV-19/USA/CA-ALSR-0771-IPL/2020|2020-04-10|EPI_ISL_483347|Original|hCoV-19^^California|Human|UC San Diego Center for Advanced Laboratory Medicine|Andersen lab at Scripps Research|Gangavarapu|USA
>Spike|hCoV-19/USA/TX-HMH-MCoV-10195/2020|2020-07-07|EPI_ISL_789366|Original|hCoV-19^^Texas|Human|Houston Methodist Hospital|Houston Methodist Hospital|Long|USA
>Spike|hCoV-19/England/MILK-B3DAB6/2020|2020-11-02|EPI_ISL_741384|Original|hCoV-19^^England|Human|Oxford Viromics|COVID-19 Genomics UK (COG-UK) Consortium|Golubchik|United Kingdom
>Spike|hCoV-19/England/20118179304/2020|2020-03-12|EPI_ISL_465000|Original|hCoV-19^^England|Human|Respiratory Virus Unit|Respiratory Virus Unit|Platt|United Kingdom
>Spike|hCoV-19/Wales/PHWC-27BB7/2020|2020-04-01|EPI_ISL_432310|Original|hCoV-19^^Wales|Human|Wales Specialist Virology Centre|Public Health Wales Microbiology Cardiff|Moore|United Kingdom
>Spike|hCoV-19/United Arab Emirates/L6841/2020|2020-03-27|EPI_ISL_469280|Original|hCoV-19^^United Arab Emirates|Human|Mohammed Bin Rashid University of Medicine and Health Sciences|Al Jalila Genomics Center|Ramaswamy|United Arab Emirates
>Spike|hCoV-19/Australia/VIC6833/2020|2020-07-24|EPI_ISL_564085|Original|hCoV-19^^Victoria|Human|Victorian Infectious Diseases Reference Laboratory (VIDRL)|VIDRL and MDU-PHL|Schultz|Australia
>Spike|hCoV-19/Thailand/Samut Sakorn_AS/2020|2020-12-17|EPI_ISL_768536|Original|hCoV-19^^Samut Sakorn|Human|Regional Medical Sciences Center 5 Samut Songkhram|National Institute of Health|Phuygun|Thailand
>Spike|hCoV-19/USA/LA-GBCL-26159759/2020|2020-04-11|EPI_ISL_485009|Original|hCoV-19^^Louisiana|Human|River Road Testing Lab|Ginkgo Bioworks Clinical Laboratory|Smith|USA
>Spike|hCoV-19/USA/WA-S1362/2020|2020-05-18|EPI_ISL_495812|Original|hCoV-19^^Washington|Human|Washington State Department of Health|Seattle Flu Study|Lee|USA
>Spike|hCoV-19/Wales/PHWC-484325/2020|2020-10-21|EPI_ISL_627990|Original|hCoV-19^^Wales|Human|Wales Specialist Virology Centre Sequencing lab|COVID-19 Genomics UK (COG-UK) Consortium|Moore|United Kingdom
>Spike|hCoV-19/Australia/VIC3389/2020|2020-07-06|EPI_ISL_521068|Original|hCoV-19^^Victoria|Human|Microbiological Diagnostic Unit - Public Health Laboratory (MDU-PHL)|MDU-PHL|Schultz|Australia
>Spike|hCoV-19/Canada/QC-LSPQ-L00242878/2020|2020-03-30|EPI_ISL_536338|Original|hCoV-19^^Quebec|Human|H\u00f4pital Charles-LeMoyne|Laboratoire de sant\u00e9 publique du Qu\u00e9bec|Moreira|Canada
>Spike|hCoV-19/Chile/MA-CADIUMAG-12/2020|2020-04-16|EPI_ISL_681678|Original|hCoV-19^^Magallanes|Human|Molecular Medicine Laboratory|Centro Asistencial Docente y de Investigacion|Navarrete|Chile
>Spike|hCoV-19/England/QEUH-BA0AAF/2020|2020-11-18|EPI_ISL_719641|Original|hCoV-19^^England|Human|Lighthouse Lab in Glasgow|Wellcome Sanger Institute for the COVID-19 Genomics UK (COG-UK) Consortium|Davies|United Kingdom
>Spike|hCoV-19/England/CAMB-7CB95/2020|2020-04-05|EPI_ISL_442188|Original|hCoV-19^^England|Human|Department of Pathology|Wellcome Sanger Institute for the COVID-19 Genomics UK (COG-UK) consortium|Amato|United Kingdom

>Spike|hCoV-19/Wales/PHWC-167042/2020|2020-06-21|EPI_ISL_494144|Original|hCoV-19^^Wales|Human|Originating lab|COVID-19 Genomics UK (COG-UK) Consortium|Moore|United Kingdom
>Spike|hCoV-19/USA/CA-ALSR-4273/2020|2020-10-05|EPI_ISL_648929|Original|hCoV-19^^California|Human|San Diego County Public Health Laboratory|Andersen lab at Scripps Research|Gangavarapu|USA
>Spike|hCoV-19/Scotland/CVR4409/2020|2020-09-19|EPI_ISL_585283|Original|hCoV-19^^Scotland|Human|West of Scotland Specialist Virology Centre|COVID-19 Genomics UK (COG-UK) Consortium|Robertson|United Kingdom
>Spike|hCoV-19/USA/CA-CZB-11682/2020|2020-09-24|EPI_ISL_625493|Original|hCoV-19^^California|Human|Santa Clara County Public Health Laboratory|Chan-Zuckerberg Biohub|Bhatt|USA
>Spike|hCoV-19/England/CAMB-7A195/2020|2020-04-02|EPI_ISL_441338|Original|hCoV-19^^England|Human|Department of Pathology|Wellcome Sanger Institute for the COVID-19 Genomics UK (COG-UK) consortium|Amato|United Kingdom
>Spike|hCoV-19/Netherlands/un-EMC-726/2020|2020-04-14|EPI_ISL_523481|Original|hCoV-19^^Netherlands|Human|Dutch COVID-19 response team|Erasmus Medical Center|Oude Munnink|Netherlands
>Spike|hCoV-19/Denmark/DCGC-20691/2020|2020-12-21|EPI_ISL_759304|Original|hCoV-19^^Hovedstaden|Human|Department of Virus and Microbiological Special Diagnostics|Albertsen Lab|Michaelsen|Denmark
>Spike|hCoV-19/Vietnam/HCMC-05006/2020|2020-03-23|EPI_ISL_498177|unknown|hCoV-19^^Ho Chi Minh City|Human|OUCRU|OUCRU|Nguyen|Vietnam
>Spike|hCoV-19/England/ALDP-A94A63/2020|2020-10-20|EPI_ISL_624772|Original|hCoV-19^^England|Human|Lighthouse Lab in Alderley Park|Wellcome Sanger Institute for the COVID-19 Genomics UK (COG-UK) consortium|Davies|United Kingdom
>Spike|hCoV-19/Luxembourg/LNS2529586/2020|2020-10-25|EPI_ISL_740136|Original|hCoV-19^^Luxembourg|Human|Laboratoire national de sant\u00e9|Laboratoire national de sant\u00e9|Wienecke-Baldacchino|Luxembourg
>Spike|hCoV-19/USA/TX-DSHS-1073/2020|2020-06-16|EPI_ISL_628741|Original|hCoV-19^^Texas|Human|Texas Department of State Health Services|Texas Department of State Health Services|Rahman|USA
>Spike|hCoV-19/Japan/PG-1359/2020|2020-04-00|EPI_ISL_684967|Original|hCoV-19^^Japan|Human|Saitama Prefectural Institute of Public Health|Pathogen Genomics Center|Sekizuka|Japan
>Spike|hCoV-19/England/QEUH-A621A5/2020|2020-10-12|EPI_ISL_706961|Original|hCoV-19^^England|Human|Oxford Viromics|COVID-19 Genomics UK (COG-UK) Consortium|Golubchik|United Kingdom
>Spike|hCoV-19/England/QEUH-BCC050/2020|2020-11-27|EPI_ISL_704167|Original|hCoV-19^^England|Human|Lighthouse Lab in Glasgow|Wellcome Sanger Institute for the COVID-19 Genomics UK (COG-UK) Consortium|Davies|United Kingdom
>Spike|hCoV-19/England/ALDP-A67708/2020|2020-10-12|EPI_ISL_630692|Original|hCoV-19^^England|Human|Lighthouse Lab in Alderley Park|Wellcome Sanger Institute for the COVID-19 Genomics UK (COG-UK) consortium|Davies|United Kingdom
>Spike|hCoV-19/England/MILK-A1933E/2020|2020-10-03|EPI_ISL_612504|Original|hCoV-19^^England|Human|Wales Specialist Virology Centre Sequencing lab|COVID-19 Genomics UK (COG-UK) Consortium|Moore|United Kingdom
>Spike|hCoV-19/USA/TX-HMH-MCoV-14367/2020|2020-10-05|EPI_ISL_787652|Original|hCoV-19^^Texas|Human|Houston Methodist Hospital|Houston Methodist Hospital|Long|USA
>Spike|hCoV-19/Bangladesh/G-187/2020|2020-06-15|EPI_ISL_600519|Original|hCoV-19^^Chattogram|Human|Institute of Epidemiology Disease Control And Research|Institute for Developing Science and Health Initiatives|Cowley|Bangladesh
>Spike|hCoV-19/Japan/PG-3940/2020|2020-07-00|EPI_ISL_691734|Original|hCoV-19^^Japan|Human|Pathogen Genomics Center|Pathogen Genomics Center|Sekizuka|Japan
>Spike|hCoV-19/USA/AZ-TG366715/2020|2020-06-02|EPI_ISL_694479|Original|hCoV-19^^Arizona|Human|AZ SPHL|TGen North|French|USA
>Spike|hCoV-19/Oman/6990/2020|2020-03-29|EPI_ISL_491154|Original|hCoV-19^^Dakhiliyah|Human|Oman-National Influenza Center|Biotechnology & OMICs Laboratory|Al Mahruqi|Oman

>Spike|hCoV-19/England/QEUH-9CFC5B/2020|2020-09-15|EPI_ISL_589807|Original|hCoV-19^^England|Human|Lighthouse Lab in Glasgow|Wellcome Sanger Institute for the COVID-19 Genomics UK (COG-UK) consortium|Davies|United Kingdom
>Spike|hCoV-19/USA/CA-IGI-0123/2020|2020-05-20|EPI_ISL_495145|Original|hCoV-19^^California|Human|Innovative Genomics Institute|Innovative Genomics Institute|Shivram|USA
>Spike|hCoV-19/USA/MN-QDX-982/2020|2020-03-15|EPI_ISL_571781|Original|hCoV-19^^Minnesota|Human|Quest Diagnostics|Quest Diagnostics|Kagan|USA
>Spike|hCoV-19/India/MH-ACTREC-670/2020|2020-09-18|EPI_ISL_700296|Original|hCoV-19^^Maharashtra|Human|Hematopathology Laboratory|Hematopathology Laboratory|Patkar|India
>Spike|hCoV-19/USA/CA-CZB-3339/2020|2020-07-12|EPI_ISL_548271|Original|hCoV-19^^California|Human|Orange County Public Health Laboratory|Chan-Zuckerberg Biohub|Bhatt|USA
>Spike|hCoV-19/England/BIRM-61E21/2020|2020-03-26|EPI_ISL_529612|Original|hCoV-19^^England|Human|University of Birmingham|COVID-19 Genomics UK (COG-UK) Consortium|Loman|United Kingdom
>Spike|hCoV-19/Wales/PHWC-4A5B80/2020|2020-12-13|EPI_ISL_764957|Original|hCoV-19^^Wales|Human|Wales Specialist Virology Centre Sequencing lab|COVID-19 Genomics UK (COG-UK) Consortium|Moore|United Kingdom
>Spike|hCoV-19/USA/WA-UW-21695/2020|2020-08-03|EPI_ISL_570832|Original|hCoV-19^^Washington|Human|UW Virology Lab|UW Virology Lab|Roychoudhury|USA
>Spike|hCoV-19/Scotland/CVR5258/2020|2020-10-19|EPI_ISL_626991|Original|hCoV-19^^Scotland|Human|West of Scotland Specialist Virology Centre|COVID-19 Genomics UK (COG-UK) Consortium|Robertson|United Kingdom
>Spike|hCoV-19/England/CAMC-BC09BB/2020|2020-11-22|EPI_ISL_704782|Original|hCoV-19^^England|Human|Lighthouse Lab in Cambridge|Wellcome Sanger Institute for the COVID-19 Genomics UK (COG-UK) Consortium|Davies|United Kingdom
>Spike|hCoV-19/England/CAMB-1ABD78/2020|2020-04-21|EPI_ISL_470258|Original|hCoV-19^^England|Human|Department of Pathology|Wellcome Sanger Institute for the COVID-19 Genomics UK (COG-UK) consortium|Davies|United Kingdom
>Spike|hCoV-19/USA/NY-QDX-1174/2020|2020-03-13|EPI_ISL_571984|Original|hCoV-19^^New York|Human|Quest Diagnostics|Quest Diagnostics|Kagan|USA
>Spike|hCoV-19/Belgium/ULG-10443/2020|2020-08-17|EPI_ISL_581620|Original|hCoV-19^^Li\u00e8ge|Human|Department of Clinical Microbiology|GIGA Medical Genomics|Durkin|Belgium
>Spike|hCoV-19/England/MILK-B3CA7B/2020|2020-11-01|EPI_ISL_706181|Original|hCoV-19^^England|Human|Oxford Viromics|COVID-19 Genomics UK (COG-UK) Consortium|Golubchik|United Kingdom
>Spike|hCoV-19/Wales/MILK-9E1233/2020|2020-09-20|EPI_ISL_580993|Original|hCoV-19^^Wales|Human|Lighthouse Lab in Milton Keynes|Wellcome Sanger Institute for the COVID-19 Genomics UK (COG-UK) consortium|Davies|United Kingdom
>Spike|hCoV-19/Northern Ireland/NIRE-101EE6/2020|2020-03-23|EPI_ISL_453477|Original|hCoV-19^^Northern Ireland|Human|Regional Virus Laboratory|COVID-19 Genomics UK (COG-UK) Consortium|Fairley|United Kingdom
>Spike|hCoV-19/Argentina/PAIS-A0021/2020|2020-04-11|EPI_ISL_430812|Original|hCoV-19^^Ciudad Autonoma de Buenos Aires|Human|Laboratorio de Virolog\u00eda del Hospital de Ni\u00f1os Dr. Ricardo Gutierrez|\u00c1rea de Secuenciaci\u00f3n del Laboratorio de Virolog\u00eda del Hospital de Ni\u00f1os Dr. Ricardo Gutierrez|Viegas|Argentina
>Spike|hCoV-19/England/ALDP-9E72B5/2020|2020-09-24|EPI_ISL_580399|Original|hCoV-19^^England|Human|Lighthouse Lab in Alderley Park|Wellcome Sanger Institute for the COVID-19 Genomics UK (COG-UK) consortium|Davies|United Kingdom
>Spike|hCoV-19/Switzerland/AG-ETHZ-280188/2020|2020-09-10|EPI_ISL_560426|Original|hCoV-19^^Aargau|Human|Viollier AG|Department of Biosystems Science and Engineering|Nadeau|Switzerland
>Spike|hCoV-19/England/BRIS-12CF97/2020|2020-04-25|EPI_ISL_481845|Original|hCoV-19^^England|Human|PHE South West Regional Laboratory|Wellcome Sanger Institute for the COVID-19 Genomics UK (COG-UK) consortium|Davies|United Kingdom

>Spike|hCoV-19/England/LIVE-999C5/2020|2020-04-08|EPI_ISL_439783|Original|hCoV-19^^England|Human|Liverpool Clinical Laboratories|COVID-19 Genomics UK (COG-UK) Consortium|Haldenby|United Kingdom
>Spike|hCoV-19/England/QEUH-96EB60/2020|2020-08-19|EPI_ISL_530597|Original|hCoV-19^^England|Human|Lighthouse Lab in Glasgow|Wellcome Sanger Institute for the COVID-19 Genomics UK (COG-UK) consortium|Davies|United Kingdom
>Spike|hCoV-19/England/ALDP-B1BA36/2020|2020-11-01|EPI_ISL_645811|Original|hCoV-19^^England|Human|Lighthouse Lab in Alderley Park|Wellcome Sanger Institute for the COVID-19 Genomics UK (COG-UK) Consortium|Davies|United Kingdom
>Spike|hCoV-19/England/20132000304/2020|2020-03-23|EPI_ISL_423701|Original|hCoV-19^^England|Human|Respiratory Virus Unit|Respiratory Virus Unit|Platt|United Kingdom
>Spike|hCoV-19/Canada/ON-S1420/2020|2020-08-22|EPI_ISL_591189|Original|hCoV-19^^Ontario|Human|Toronto Invasive Bacterial Diseases Network|McMaster University|McArthur|Canada
>Spike|hCoV-19/USA/MI-MDHHS-SC20419/2020|2020-03-14|EPI_ISL_452293|Original|hCoV-19^^Michigan|Human|Michigan Department of Health and Human Services|Michigan Department of Health and Human Services|Blankenship|USA
>Spike|hCoV-19/Luxembourg/LNS6164249/2020|2020-11-09|EPI_ISL_744370|Original|hCoV-19^^Luxembourg|Human|Laboratoire national de sant\u00e9|Laboratoire national de sant\u00e9|Wienecke-Baldacchino|Luxembourg
>Spike|hCoV-19/Japan/PG-1877/2020|2020-05-00|EPI_ISL_685483|Original|hCoV-19^^Japan|Human|Tokyo Metropolitan Institute of Public Health|Pathogen Genomics Center|Sekizuka|Japan
>Spike|hCoV-19/England/NOTT-10EF22/2020|2020-03-30|EPI_ISL_432943|Original|hCoV-19^^England|Human|Queens Medical Centre|COVID-19 Genomics UK (COG-UK) Consortium|Loose|United Kingdom
>Spike|hCoV-19/Spain/CT-IBV-005598/2020|2020-03-26|EPI_ISL_508683|Original|hCoV-19^^Catalunya|Human|Departamento de Microbiolog\u00eda|SeqCOVID-SPAIN consortium/IBV(CSIC)|Comas|Spain
>Spike|hCoV-19/Belgium/rega-0916958/2020|2020-09-16|EPI_ISL_734951|Original|hCoV-19^^Tervuren|Human|UZ Leuven|KU Leuven|Maes|Belgium
>Spike|hCoV-19/England/ALDP-745C22/2020|2020-06-29|EPI_ISL_557354|Original|hCoV-19^^England|Human|Lighthouse Lab in Alderley Park|Wellcome Sanger Institute for the COVID-19 Genomics UK (COG-UK) consortium|Davies|United Kingdom
>Spike|hCoV-19/England/NOTT-1159C0/2020|2020-10-15|EPI_ISL_595278|Original|hCoV-19^^England|Human|Queens Medical Centre|COVID-19 Genomics UK (COG-UK) Consortium|Loose|United Kingdom
>Spike|hCoV-19/England/ALDP-A2F8DC/2020|2020-10-06|EPI_ISL_587265|Original|hCoV-19^^England|Human|Lighthouse Lab in Alderley Park|Wellcome Sanger Institute for the COVID-19 Genomics UK (COG-UK) Consortium|Davies|United Kingdom
>Spike|hCoV-19/Belgium/ULG-10334/2020|2020-07-30|EPI_ISL_540501|Original|hCoV-19^^Li\u00e8ge|Human|Department of Clinical Microbiology|GIGA Medical Genomics|Durkin|Belgium
>Spike|hCoV-19/USA/CA-CZB-3888/2020|2020-07-21|EPI_ISL_548523|Original|hCoV-19^^California|Human|County of Santa Clara Public Health Department|Chan-Zuckerberg Biohub|Bhatt|USA
>Spike|hCoV-19/USA/TX-DSHS-1168/2020|2020-07-03|EPI_ISL_634968|Original|hCoV-19^^Texas|Human|Texas Department of State Health Services|Texas Department of State Health Services|Rahman|USA
>Spike|hCoV-19/England/MILK-A6C1AB/2020|2020-10-09|EPI_ISL_609066|Original|hCoV-19^^England|Human|Lighthouse Lab in Milton Keynes|Wellcome Sanger Institute for the COVID-19 Genomics UK (COG-UK) consortium|Davies|United Kingdom
>Spike|hCoV-19/Luxembourg/LNS0470029/2020|2020-10-21|EPI_ISL_739782|Original|hCoV-19^^Luxembourg|Human|Laboratoire national de sant\u00e9|Laboratoire national de sant\u00e9|Wienecke-Baldacchino|Luxembourg
>Spike|hCoV-19/Japan/PG-8038/2020|2020-08-00|EPI_ISL_687139|Original|hCoV-19^^Japan|Human|Pathogen Genomics Center|Pathogen Genomics Center|Sekizuka|Japan
>Spike|hCoV-19/Japan/IC-0257/2020|2020-10-00|EPI_ISL_667686|Original|hCoV-19^^Japan|Human|Pathogen Genomics Center|Pathogen Genomics Center|Sekizuka|Japan

>Spike|hCoV-19/England/QEUH-C33C5F/2020|2020-12-11|EPI_ISL_733943|Original|hCoV-19^^England|Human|Lighthouse Lab in Glasgow|Wellcome Sanger Institute for the COVID-19 Genomics UK (COG-UK) Consortium|Davies|United Kingdom
>Spike|hCoV-19/USA/CA-CZB-3719/2020|2020-08-01|EPI_ISL_548470|Original|hCoV-19^^California|Human|County of Santa Clara Public Health Department|Chan-Zuckerberg Biohub|Bhatt|USA
>Spike|hCoV-19/England/PORT-2CF42D/2020|2020-04-28|EPI_ISL_453679|Original|hCoV-19^^England|Human|Centre for Enzyme Innovation|COVID-19 Genomics UK (COG-UK) Consortium|Robson|United Kingdom
>Spike|hCoV-19/USA/VA-DCLS-0488/2020|2020-05-12|EPI_ISL_526795|Original|hCoV-19^^Virginia|Human|Virginia DCLS|Virginia DCLS|St. Jacques|USA
>Spike|hCoV-19/Wales/ALDP-B1F908/2020|2020-11-01|EPI_ISL_710989|Original|hCoV-19^^Wales|Human|Lighthouse Lab in Alderley Park|Wellcome Sanger Institute for the COVID-19 Genomics UK (COG-UK) Consortium|Davies|United Kingdom
>Spike|hCoV-19/England/MILK-C2FDCC/2020|2020-12-07|EPI_ISL_782914|Original|hCoV-19^^England|Human|Lighthouse Lab in Milton Keynes|Wellcome Sanger Institute for the COVID-19 Genomics UK (COG-UK) Consortium|Davies|United Kingdom
>Spike|hCoV-19/Denmark/DCGC-20997/2020|2020-12-21|EPI_ISL_758956|Original|hCoV-19^^Midtjylland|Human|Department of Virus and Microbiological Special Diagnostics|Albertsen Lab|Michaelsen|Denmark
>Spike|hCoV-19/USA/UT-UPHL-2012734844/2020|2020-08-17|EPI_ISL_746092|Original|hCoV-19^^Utah|Human|Ginkgo Bioworks Clinical Laboratory|Utah Public Health Laboratory|Young|USA
>Spike|hCoV-19/USA/CO-CSU-D2680/2020|2020-04-02|EPI_ISL_527474|Original|hCoV-19^^Colorado|Human|Colorado State University - Ebel Lab|Colorado State University - Ebel Lab|Cline|USA
>Spike|hCoV-19/Denmark/DCGC-17427/2020|2020-12-14|EPI_ISL_750974|Original|hCoV-19^^Hovedstaden|Human|Department of Virus and Microbiological Special Diagnostics|Albertsen Lab|Michaelsen|Denmark
>Spike|hCoV-19/Scotland/EDB2711/2020|2020-03-27|EPI_ISL_488988|Original|hCoV-19^^Scotland|Human|Virology Department|Wellcome Sanger Institute for the COVID-19 Genomics UK (COG-UK) consortium|Davies|United Kingdom
>Spike|hCoV-19/Canada/QC-LSPQ-L00229172/2020|2020-03-20|EPI_ISL_535886|Original|hCoV-19^^Quebec|Human|H\u00f4pital de Hull|Laboratoire de sant\u00e9 publique du Qu\u00e9bec|Moreira|Canada
>Spike|hCoV-19/USA/NY-Wadsworth-292843-01/2020|2020-12-28|EPI_ISL_769981|Original|hCoV-19^^New York|Human|Albany Medical Center Hospital Clinical Laboratories|Wadsworth Center|Lasek-Nesselquist|USA
>Spike|hCoV-19/England/QEUH-AD31FA/2020|2020-10-26|EPI_ISL_633894|Original|hCoV-19^^England|Human|Lighthouse Lab in Glasgow|Wellcome Sanger Institute for the COVID-19 Genomics UK (COG-UK) consortium|Davies|United Kingdom
>Spike|hCoV-19/England/NORW-E98D8/2020|2020-04-23|EPI_ISL_448342|Original|hCoV-19^^England|Human|Quadram Institute Bioscience|COVID-19 Genomics UK (COG-UK) Consortium|Alikhan|United Kingdom
>Spike|hCoV-19/USA/FL-BPHL-1256/2020|2020-04-22|EPI_ISL_541156|Original|hCoV-19^^Florida|Human|Florida Bureau of Public Health Laboratories|Florida Bureau of Public Health Laboratories|Blanton|USA
>Spike|hCoV-19/Jordan/IR-ALSR-5203/2020|2020-10-12|EPI_ISL_730441|Original|hCoV-19^^Irbid|Human|Biolab Diagnostic Laboratories|Andersen lab at Scripps Research|Gangavarapu|Jordan
>Spike|hCoV-19/Australia/VIC9651/2020|2020-08-21|EPI_ISL_565638|Original|hCoV-19^^Victoria|Human|Microbiological Diagnostic Unit - Public Health Laboratory (MDU-PHL)|MDU-PHL|Schultz|Australia
>Spike|hCoV-19/USA/TX-HMH-MCoV-14619/2020|2020-10-09|EPI_ISL_787904|Original|hCoV-19^^Texas|Human|Houston Methodist Hospital|Houston Methodist Hospital|Long|USA
>Spike|hCoV-19/England/CAMC-B980E4/2020|2020-11-15|EPI_ISL_674995|Original|hCoV-19^^England|Human|Lighthouse Lab in Cambridge|Wellcome Sanger Institute for the COVID-19 Genomics UK (COG-UK) Consortium|Davies|United Kingdom

>Spike|hCoV-19/Scotland/EDB9219/2020|2020-11-03|EPI_ISL_666062|Original|hCoV-19^^Scotland|Human|Virology Department|COVID-19 Genomics UK (COG-UK) Consortium|McHugh|United Kingdom
>Spike|hCoV-19/England/CAMC-B22A69/2020|2020-11-02|EPI_ISL_647441|Original|hCoV-19^^England|Human|Lighthouse Lab in Cambridge|Wellcome Sanger Institute for the COVID-19 Genomics UK (COG-UK) Consortium|Davies|United Kingdom
>Spike|hCoV-19/England/ALDP-B82CCB/2020|2020-11-14|EPI_ISL_675323|Original|hCoV-19^^England|Human|Lighthouse Lab in Alderley Park|Wellcome Sanger Institute for the COVID-19 Genomics UK (COG-UK) Consortium|Davies|United Kingdom
>Spike|hCoV-19/USA/WI-UW-2552/2020|2020-12-29|EPI_ISL_779381|Original|hCoV-19^^Wisconsin|Human|University of Wisconsin-Madison AIDS Vaccine Research Laboratories|University of Wisconsin-Madison AIDS Vaccine Research Laboratories|Moreno|USA
>Spike|hCoV-19/England/NOTT-10F01F/2020|2020-03-30|EPI_ISL_432956|Original|hCoV-19^^England|Human|Queens Medical Centre|COVID-19 Genomics UK (COG-UK) Consortium|Loose|United Kingdom
>Spike|hCoV-19/England/ALDP-AA1632/2020|2020-10-22|EPI_ISL_624035|Original|hCoV-19^^England|Human|Lighthouse Lab in Alderley Park|Wellcome Sanger Institute for the COVID-19 Genomics UK (COG-UK) consortium|Davies|United Kingdom
>Spike|hCoV-19/England/QEUH-9B448E/2020|2020-09-04|EPI_ISL_559894|Original|hCoV-19^^England|Human|Oxford Viromics|COVID-19 Genomics UK (COG-UK) Consortium|Golubchik|United Kingdom
>Spike|hCoV-19/USA/TX-HMH-5991/2020|2020-06-30|EPI_ISL_547150|Original|hCoV-19^^Texas|Human|Houston Methodist Hospital|Houston Methodist Hospital|Long|USA
>Spike|hCoV-19/England/MILK-9C2344/2020|2020-09-09|EPI_ISL_566573|Original|hCoV-19^^England|Human|Lighthouse Lab in Milton Keynes|Wellcome Sanger Institute for the COVID-19 Genomics UK (COG-UK) consortium|Davies|United Kingdom
>Spike|hCoV-19/Wales/PHWC-4830E3/2020|2020-10-04|EPI_ISL_627780|Original|hCoV-19^^Wales|Human|Wales Specialist Virology Centre Sequencing lab|COVID-19 Genomics UK (COG-UK) Consortium|Moore|United Kingdom
>Spike|hCoV-19/USA/MI-UM-10036832946/2020|2020-11-07|EPI_ISL_728469|Original|hCoV-19^^Michigan|Human|University of Michigan Clinical Microbiology Laboratory|Lauring Lab|Valesano|USA
>Spike|hCoV-19/Denmark/ALAB-SSI-635/2020|2020-03-26|EPI_ISL_437011|Original|hCoV-19^^Denmark|Human|Department of Virus and Microbiological Special Diagnostics|Albertsen lab|Kirkegaard|Denmark
>Spike|hCoV-19/England/CAMB-1B8701/2020|2020-10-26|EPI_ISL_651656|Original|hCoV-19^^England|Human|Department of Pathology|COVID-19 Genomics UK (COG-UK) Consortium|Goodfellow|United Kingdom
>Spike|hCoV-19/Canada/ON-S719/2020|2020-04-00|EPI_ISL_586429|Original|hCoV-19^^Ontario|Human|Toronto Invasive Bacterial Diseases Network|McMaster University|McArthur|Canada
>Spike|hCoV-19/USA/LA-EVTL1395/2020|2020-12-11|EPI_ISL_779061|Original|hCoV-19^^Louisiana|Human|LSUHS Emerging Viral Threat Laboratory|Microbial Genome Sequencing Center|Kamil|USA
>Spike|hCoV-19/Belgium/ULG-10347/2020|2020-08-04|EPI_ISL_540514|Original|hCoV-19^^Li\u00e8ge|Human|Department of Clinical Microbiology|GIGA Medical Genomics|Durkin|Belgium
>Spike|hCoV-19/USA/TX-HMH-MCoV-15635/2020|2020-10-26|EPI_ISL_785319|Original|hCoV-19^^Texas|Human|Houston Methodist Hospital|Houston Methodist Hospital|Long|USA
>Spike|hCoV-19/Australia/VIC9591/2020|2020-08-23|EPI_ISL_562682|Original|hCoV-19^^Victoria|Human|Microbiological Diagnostic Unit - Public Health Laboratory (MDU-PHL)|MDU-PHL|Schultz|Australia
>Spike|hCoV-19/Wales/PHWC-2E2F7/2020|2020-04-15|EPI_ISL_446086|Original|hCoV-19^^Wales|Human|Wales Specialist Virology Centre|Public Health Wales Microbiology Cardiff|Moore|United Kingdom
>Spike|hCoV-19/USA/WA-UW270/2020|2020-03-14|EPI_ISL_418055|Original|hCoV-19^^Washington|Human|UW Virology Lab|UW Virology Lab|Roychoudhury|USA

>Spike|hCoV-19/Wales/PHWC-16DA7B/2020|2020-09-21|EPI_ISL_577483|Original|hCoV-19^^Wales|Human|Wales Specialist Virology Centre Sequencing lab|COVID-19 Genomics UK (COG-UK) Consortium|Moore|United Kingdom
>Spike|hCoV-19/USA/LA-SR0140/2020|2020-03-31|EPI_ISL_435445|Original|hCoV-19^^Louisiana|Human|Robert Garry lab|Andersen lab at Scripps Research|Gangavarapu|USA
>Spike|hCoV-19/England/SHEF-10B2547/2020|2020-04-12|EPI_ISL_469928|Original|hCoV-19^^England|Human|Virology Department|Wellcome Sanger Institute for the COVID-19 Genomics UK (COG-UK) consortium|Davies|United Kingdom
>Spike|hCoV-19/England/ALDP-A5034A/2020|2020-10-07|EPI_ISL_597639|Original|hCoV-19^^England|Human|Lighthouse Lab in Alderley Park|Wellcome Sanger Institute for the COVID-19 Genomics UK (COG-UK) consortium|Davies|United Kingdom
>Spike|hCoV-19/Netherlands/ZuidHolland_21/2020|2020-03-03|EPI_ISL_414563|Original|hCoV-19^^Zuid Holland|Human|Dutch COVID-19 response team|Erasmus Medical Center|Oude Munnink|Netherlands
>Spike|hCoV-19/England/QEUH-B1429F/2020|2020-10-31|EPI_ISL_641815|Original|hCoV-19^^England|Human|Lighthouse Lab in Glasgow|Wellcome Sanger Institute for the COVID-19 Genomics UK (COG-UK) Consortium|Davies|United Kingdom
>Spike|hCoV-19/Denmark/ALAB-SSI-137/2020|2020-03-08|EPI_ISL_437649|Original|hCoV-19^^Denmark|Human|Department of Virus and Microbiological Special Diagnostics|Albertsen lab|Kirkegaard|Denmark
>Spike|hCoV-19/Belgium/ULG-10182/2020|2020-05-10|EPI_ISL_455970|Original|hCoV-19^^Li\u00e8ge|Human|Department of Clinical Microbiology|GIGA Medical Genomics|Durkin|Belgium
>Spike|hCoV-19/Switzerland/BE-ETHZ-280110/2020|2020-09-07|EPI_ISL_560433|Original|hCoV-19^^Bern|Human|Viollier AG|Department of Biosystems Science and Engineering|Nadeau|Switzerland
>Spike|hCoV-19/Netherlands/ZH-EMC-739/2020|2020-09-16|EPI_ISL_632581|Original|hCoV-19^^Zuid Holland|Human|Dutch COVID-19 response team|Erasmus Medical Center|Oude Munnink|Netherlands
>Spike|hCoV-19/England/ALDP-A2E5B8/2020|2020-10-06|EPI_ISL_586774|Original|hCoV-19^^England|Human|Lighthouse Lab in Alderley Park|Wellcome Sanger Institute for the COVID-19 Genomics UK (COG-UK) consortium|Davies|United Kingdom
>Spike|hCoV-19/Denmark/DCGC-20914/2020|2020-12-21|EPI_ISL_759443|Original|hCoV-19^^Hovedstaden|Human|Department of Virus and Microbiological Special Diagnostics|Albertsen Lab|Michaelsen|Denmark
>Spike|hCoV-19/England/MILK-9C4719/2020|2020-09-07|EPI_ISL_566921|Original|hCoV-19^^England|Human|Lighthouse Lab in Milton Keynes|Wellcome Sanger Institute for the COVID-19 Genomics UK (COG-UK) consortium|Davies|United Kingdom
>Spike|hCoV-19/England/ALDP-9EDAD5/2020|2020-05-08|EPI_ISL_580689|Original|hCoV-19^^England|Human|Lighthouse Lab in Alderley Park|Wellcome Sanger Institute for the COVID-19 Genomics UK (COG-UK) consortium|Davies|United Kingdom
>Spike|hCoV-19/USA/CT-Yale-582/2020|2020-05-27|EPI_ISL_738293|Original|hCoV-19^^Connecticut|Human|Yale Pathology Lab|Grubaugh Lab - Yale School of Public Health|Grubaugh|USA
>Spike|hCoV-19/England/OXON-B19C7/2020|2020-05-05|EPI_ISL_479060|Original|hCoV-19^^England|Human|Oxford Viromics|COVID-19 Genomics UK (COG-UK) Consortium|Golubchik|United Kingdom
>Spike|hCoV-19/Norway/4056/2020|2020-10-02|EPI_ISL_635096|Original|hCoV-19^^Tr\u00f8ndelag|Human|Department of Medical Microbiology|Norwegian Institute of Public Health|Vollan|Norway
>Spike|hCoV-19/USA/UT-UPHL-04309/2020|2020-05-15|EPI_ISL_575139|Original|hCoV-19^^Utah|Human|Utah Public Health Laboratory|Utah Public Health Laboratory|Young|USA
>Spike|hCoV-19/India/HR-TF24/2020|2020-06-19|EPI_ISL_508498|Original|hCoV-19^^Haryana|Human|Translational Health Science and Technology Institute|National Institute of Biomedical Genomics|Maitra|India
>Spike|hCoV-19/Russia/Moscow-PMVL-12/2020|2020-05-18|EPI_ISL_572398|Vero E6|hCoV-19^^Moscow|Human|Laboratory of Biology and Identification of Arboviruses|Pathogenic Microorganisms Variability Laboratory|Shchetinin|Russia
>Spike|hCoV-19/England/204290519/2020|2020-10-14|EPI_ISL_613614|Original|hCoV-19^^England|Human|Respiratory Virus Unit|Respiratory Virus Unit|Platt|United Kingdom

>Spike|hCoV-19/USA/CA-ALSR-3822/2020|2020-06-13|EPI_ISL_636226|Original|hCoV-19^^California|Human|San Diego County Public Health Laboratory|Andersen lab at Scripps Research|Gangavarapu|USA
>Spike|hCoV-19/Scotland/QEUH-9FA862/2020|2020-09-28|EPI_ISL_600555|Original|hCoV-19^^Scotland|Human|Lighthouse Lab in Glasgow|Wellcome Sanger Institute for the COVID-19 Genomics UK (COG-UK) consortium|Davies|United Kingdom
>Spike|hCoV-19/England/CAMC-B37D77/2020|2020-11-05|EPI_ISL_658829|Original|hCoV-19^^England|Human|Lighthouse Lab in Cambridge|Wellcome Sanger Institute for the COVID-19 Genomics UK (COG-UK) Consortium|Davies|United Kingdom
>Spike|hCoV-19/Wales/MILK-A05B4C/2020|2020-09-30|EPI_ISL_600050|Original|hCoV-19^^Wales|Human|Lighthouse Lab in Milton Keynes|Wellcome Sanger Institute for the COVID-19 Genomics UK (COG-UK) consortium|Davies|United Kingdom
>Spike|hCoV-19/England/C200036256/2020|2020-10-21|EPI_ISL_636723|Original|hCoV-19^^England|Human|Respiratory Virus Unit|Respiratory Virus Unit|Platt|United Kingdom
>Spike|hCoV-19/Netherlands/Limburg_10/2020|2020-03-20|EPI_ISL_422646|Original|hCoV-19^^Limburg|Human|Dutch COVID-19 response team|Erasmus Medical Center|Oude Munnink|Netherlands
>Spike|hCoV-19/England/CAMB-1B0CDD/2020|2020-05-12|EPI_ISL_452962|Original|hCoV-19^^England|Human|Department of Pathology|COVID-19 Genomics UK (COG-UK) Consortium|Meredith|United Kingdom
>Spike|hCoV-19/England/BRIS-122535/2020|2020-04-04|EPI_ISL_440670|Original|hCoV-19^^England|Human|PHE South West Regional Laboratory|Wellcome Sanger Institute for the COVID-19 Genomics UK (COG-UK) consortium|Amato|United Kingdom
>Spike|hCoV-19/USA/TX-HMH-MCoV-10318/2020|2020-07-08|EPI_ISL_789421|Original|hCoV-19^^Texas|Human|Houston Methodist Hospital|Houston Methodist Hospital|Long|USA
>Spike|hCoV-19/England/ALDP-497DE5/2020|2020-06-03|EPI_ISL_559595|Original|hCoV-19^^England|Human|Lighthouse Lab in Alderley Park|Wellcome Sanger Institute for the COVID-19 Genomics UK (COG-UK) consortium|Davies|United Kingdom
>Spike|hCoV-19/Costa Rica/INC-0122/2020|2020-07-31|EPI_ISL_682261|Original|hCoV-19^^Puntarenas|Human|TAMIZAJE COMUNITARIO- PASO CANOAS|Inciensa|Soto-Garita|Costa Rica
>Spike|hCoV-19/USA/CA-ALSR-4442/2020|2020-07-08|EPI_ISL_649033|Original|hCoV-19^^California|Human|San Diego County Public Health Laboratory|Andersen lab at Scripps Research|Gangavarapu|USA
>Spike|hCoV-19/USA/MN-MDH-1573/2020|2020-08-18|EPI_ISL_530169|Original|hCoV-19^^Minnesota|Human|Hennepin County Medical Center|Minnesota Department of Health|Garfin|USA
>Spike|hCoV-19/England/201161484/2020|2020-03-11|EPI_ISL_464849|Original|hCoV-19^^England|Human|Respiratory Virus Unit|Respiratory Virus Unit|Platt|United Kingdom
>Spike|hCoV-19/England/QEUH-A9D129/2020|2020-10-20|EPI_ISL_652655|Original|hCoV-19^^England|Human|Queens Medical Centre|COVID-19 Genomics UK (COG-UK) Consortium|Loose|United Kingdom
>Spike|hCoV-19/Northern Ireland/QEUH-96C045/2020|2020-08-15|EPI_ISL_530883|Original|hCoV-19^^Northern Ireland|Human|Lighthouse Lab in Glasgow|Wellcome Sanger Institute for the COVID-19 Genomics UK (COG-UK) consortium|Davies|United Kingdom
>Spike|hCoV-19/Denmark/DCGC-20263/2020|2020-11-30|EPI_ISL_747583|Original|hCoV-19^^Sjaelland|Human|Department of Virus and Microbiological Special Diagnostics|Albertsen Lab|Michaelsen|Denmark
>Spike|hCoV-19/England/MILK-B38BF4/2020|2020-11-01|EPI_ISL_658894|Original|hCoV-19^^England|Human|Lighthouse Lab in Milton Keynes|Wellcome Sanger Institute for the COVID-19 Genomics UK (COG-UK) Consortium|Davies|United Kingdom
>Spike|hCoV-19/England/MILK-828A57/2020|2020-07-16|EPI_ISL_557212|Original|hCoV-19^^England|Human|Lighthouse Lab in Milton Keynes|Wellcome Sanger Institute for the COVID-19 Genomics UK (COG-UK) consortium|Davies|United Kingdom
>Spike|hCoV-19/Germany/BY-ChVir-W1068-5746/2020|2020-03-27|EPI_ISL_754027|Original|hCoV-19^^Bavaria|Human|Charit\u00e9 Universit\u00e4tsmedizin Berlin|Charit\u00e9 Universit\u00e4tsmedizin Berlin|Muehlemann|Germany

>Spike|hCoV-19/Spain/AR-IBV-2475/2020|2020-03-24|EPI_ISL_468775|Original|hCoV-19^^Aragon|Human|Servicio de Microbiolog\u00eda|SeqCOVID-SPAIN consortium/IBV(CSIC)|Comas|Spain
>Spike|hCoV-19/England/ALDP-A526C7/2020|2020-10-07|EPI_ISL_598051|Original|hCoV-19^^England|Human|Lighthouse Lab in Alderley Park|Wellcome Sanger Institute for the COVID-19 Genomics UK (COG-UK) consortium|Davies|United Kingdom
>Spike|hCoV-19/Australia/VIC1611/2020|2020-05-09|EPI_ISL_456521|Original|hCoV-19^^Victoria|Human|Victorian Infectious Diseases Reference Laboratory (VIDRL)|Microbiological Diagnostic Unit Public Health Laboratory and Victorian Infectious Diseases Reference Laboratory|Schultz|Australia
>Spike|hCoV-19/England/BIRM-6A453/2020|2020-11-09|EPI_ISL_679058|Original|hCoV-19^^England|Human|University of Birmingham|COVID-19 Genomics UK (COG-UK) Consortium|Loman|United Kingdom
>Spike|hCoV-19/Australia/VIC13878/2020|2020-08-23|EPI_ISL_563862|Original|hCoV-19^^Victoria|Human|Microbiological Diagnostic Unit - Public Health Laboratory (MDU-PHL)|MDU-PHL|Schultz|Australia
>Spike|hCoV-19/England/201160037/2020|2020-03-12|EPI_ISL_464779|Original|hCoV-19^^England|Human|Respiratory Virus Unit|Respiratory Virus Unit|Platt|United Kingdom
>Spike|hCoV-19/USA/FL-BPHL-1522/2020|2020-07-28|EPI_ISL_568620|Original|hCoV-19^^Florida|Human|Florida Bureau of Public Health Laboratories|Florida Bureau of Public Health Laboratories|Blanton|USA
>Spike|hCoV-19/England/MILK-B3AAD7/2020|2020-11-01|EPI_ISL_706045|Original|hCoV-19^^England|Human|Oxford Viromics|COVID-19 Genomics UK (COG-UK) Consortium|Golubchik|United Kingdom
>Spike|hCoV-19/USA/MI-MDHHS-SC20602/2020|2020-04-30|EPI_ISL_462684|Original|hCoV-19^^Michigan|Human|Michigan Department of Health and Human Services|Michigan Department of Health and Human Services|Blankenship|USA
>Spike|hCoV-19/England/20130032604/2020|2020-03-21|EPI_ISL_423598|Original|hCoV-19^^England|Human|Respiratory Virus Unit|Respiratory Virus Unit|Platt|United Kingdom
>Spike|hCoV-19/USA/TX-HMH-MCoV-10747/2020|2020-07-09|EPI_ISL_789246|Original|hCoV-19^^Texas|Human|Houston Methodist Hospital|Houston Methodist Hospital|Long|USA
>Spike|hCoV-19/France/BRE-8934/2020|2020-07-28|EPI_ISL_560602|Original|hCoV-19^^Bretagne|Human|Hospital|National Reference Center for Viruses of Respiratory Infections|Enouf|France
>Spike|hCoV-19/Nigeria/AK-CV272/2020|2020-07-12|EPI_ISL_729956|Original|hCoV-19^^Akwa-Ibom State|Human|Nigeria Centre for Disease Control (NCDC)|African Centre of Excellence for Genomics of Infectious Diseases (ACEGID)|Oluniyi|Nigeria
>Spike|hCoV-19/Switzerland/BS-UHB-42203696/2020|2020-03-23|EPI_ISL_528365|Original|hCoV-19^^Basel-Stadt|Human|University Hospital Basel|University Hospital Basel|Roloff Handschin|Switzerland
>Spike|hCoV-19/USA/TX-HMH-7191/2020|2020-06-29|EPI_ISL_546585|Original|hCoV-19^^Texas|Human|Houston Methodist Hospital|Houston Methodist Hospital|Long|USA
>Spike|hCoV-19/USA/TX-HMH-MCoV-16086/2020|2020-10-30|EPI_ISL_785770|Original|hCoV-19^^Texas|Human|Houston Methodist Hospital|Houston Methodist Hospital|Long|USA
>Spike|hCoV-19/Northern Ireland/NIRE-234CC1/2020|2020-10-16|EPI_ISL_764393|Original|hCoV-19^^Northern Ireland|Human|Regional Virus Laboratory|COVID-19 Genomics UK (COG-UK) Consortium|Fairley|United Kingdom
>Spike|hCoV-19/Wales/PHWC-4A27DA/2020|2020-12-11|EPI_ISL_743942|Original|hCoV-19^^Wales|Human|Wales Specialist Virology Centre Sequencing lab|COVID-19 Genomics UK (COG-UK) Consortium|Moore|United Kingdom
>Spike|hCoV-19/USA/WA-S793/2020|2020-04-15|EPI_ISL_449963|Original|hCoV-19^^Washington|Human|Washington State Department of Health|Seattle Flu Study|Bedford|USA
>Spike|hCoV-19/Denmark/DCGC-20053/2020|2020-12-07|EPI_ISL_747953|Original|hCoV-19^^Midtjylland|Human|Department of Virus and Microbiological Special Diagnostics|Albertsen Lab|Michaelsen|Denmark
>Spike|hCoV-19/Switzerland/BE-ETHZ-360140/2020|2020-11-06|EPI_ISL_693919|Original|hCoV-19^^Bern|Human|Viollier AG|Department of Biosystems Science and Engineering|Nadeau|Switzerland

>Spike|hCoV-19/USA/WI-UW-842/2020|2020-05-07|EPI_ISL_509849|Original|hCoV-19^^Wisconsin|Human|University of Wisconsin-Madison AIDS Vaccine Research Laboratories|University of Wisconsin-Madison AIDS Vaccine Research Laboratories|Baczenas|USA
>Spike|hCoV-19/mink/Netherlands/NB-EMC-54-1/2020|2020-09-22|EPI_ISL_632450|Original|hCoV-19^^Noord Brabant|Neovison vison|Dutch COVID-19 response team|Erasmus Medical Center|Oude Munnink|Netherlands
>Spike|hCoV-19/Wales/PHWC-4992B7/2020|2020-12-03|EPI_ISL_727283|Original|hCoV-19^^Wales|Human|Originating lab|COVID-19 Genomics UK (COG-UK) Consortium|Moore|United Kingdom
>Spike|hCoV-19/England/ALDP-981D8F/2020|2020-08-09|EPI_ISL_552785|Original|hCoV-19^^England|Human|Lighthouse Lab in Alderley Park|Wellcome Sanger Institute for the COVID-19 Genomics UK (COG-UK) consortium|Davies|United Kingdom
>Spike|hCoV-19/Denmark/DCGC-22315/2020|2020-12-14|EPI_ISL_758673|Original|hCoV-19^^Sjaelland|Human|Department of Virus and Microbiological Special Diagnostics|Albertsen Lab|Michaelsen|Denmark
>Spike|hCoV-19/England/QEUH-AA028A/2020|2020-10-20|EPI_ISL_611427|Original|hCoV-19^^England|Human|Lighthouse Lab in Glasgow|Wellcome Sanger Institute for the COVID-19 Genomics UK (COG-UK) consortium|Davies|United Kingdom
>Spike|hCoV-19/England/QEUH-A1E18A/2020|2020-10-01|EPI_ISL_587544|Original|hCoV-19^^England|Human|Lighthouse Lab in Glasgow|Wellcome Sanger Institute for the COVID-19 Genomics UK (COG-UK) consortium|Davies|United Kingdom
>Spike|hCoV-19/Scotland/EDB732/2020|2020-04-02|EPI_ISL_433231|Original|hCoV-19^^Scotland|Human|Virology Department|COVID-19 Genomics UK (COG-UK) Consortium|Williams|United Kingdom
>Spike|hCoV-19/Sweden/20-53248/2020|2020-10-28|EPI_ISL_710611|Original|hCoV-19^^Stockholm|Human|Ektorps Vardcentral|The Public Health Agency of Sweden|Svartstr\u00f6m|Sweden
>Spike|hCoV-19/Australia/VIC6582/2020|2020-07-18|EPI_ISL_521346|Original|hCoV-19^^Victoria|Human|Victorian Infectious Diseases Reference Laboratory (VIDRL)|VIDRL and MDU-PHL|Schultz|Australia
>Spike|hCoV-19/Japan/PG-5956/2020|2020-00-00|EPI_ISL_689071|Original|hCoV-19^^Japan|Human|Gunma Prefectural Institute of Public Health and Environmental Sciences|Pathogen Genomics Center|Sekizuka|Japan
>Spike|hCoV-19/England/MILK-BE7189/2020|2020-11-29|EPI_ISL_709871|Original|hCoV-19^^England|Human|Lighthouse Lab in Milton Keynes|Wellcome Sanger Institute for the COVID-19 Genomics UK (COG-UK) Consortium|Davies|United Kingdom
>Spike|hCoV-19/England/NORW-F204D/2020|2020-10-22|EPI_ISL_627651|Original|hCoV-19^^England|Human|Quadram Institute Bioscience|COVID-19 Genomics UK (COG-UK) Consortium|Alikhan|United Kingdom
>Spike|hCoV-19/Denmark/DCGC-7379/2020|2020-10-12|EPI_ISL_619100|Original|hCoV-19^^Hovedstaden|Human|Department of Virus and Microbiological Special Diagnostics|Albertsen lab|Michaelsen|Denmark
>Spike|hCoV-19/India/MH-NCCS-NR1565/2020|2020-05-17|EPI_ISL_496563|Original|hCoV-19^^Maharashtra|Human|National Centre For Cell Science|National Centre For Cell Science|Paul|India
>Spike|hCoV-19/Australia/VIC2522/2020|2020-06-29|EPI_ISL_522216|Original|hCoV-19^^Victoria|Human|Microbiological Diagnostic Unit - Public Health Laboratory (MDU-PHL)|MDU-PHL|Schultz|Australia
>Spike|hCoV-19/England/MILK-B960E6/2020|2020-11-14|EPI_ISL_676259|Original|hCoV-19^^England|Human|Lighthouse Lab in Milton Keynes|Wellcome Sanger Institute for the COVID-19 Genomics UK (COG-UK) Consortium|Davies|United Kingdom
>Spike|hCoV-19/Australia/VIC754/2020|2020-03-30|EPI_ISL_427031|Original|hCoV-19^^Victoria|Human|Victorian Infectious Diseases Reference Laboratory (VIDRL)|Microbiological Diagnostic Unit Public Health Laboratory and Victorian Infectious Diseases Reference Laboratory|Schultz|Australia
>Spike|hCoV-19/Wales/PHWC-48A222/2020|2020-11-04|EPI_ISL_652537|Original|hCoV-19^^Wales|Human|Wales Specialist Virology Centre Sequencing lab|COVID-19 Genomics UK (COG-UK) Consortium|Moore|United Kingdom

>Spike|hCoV-19/Belgium/Rega-0328209/2020|2020-03-28|EPI_ISL_458179|Original|hCoV-19^^Fl\u00e9nu|Human|KU Leuven|KU Leuven|Maes|Belgium
>Spike|hCoV-19/Israel/CVL-51-ngs/2020|2020-12-24|EPI_ISL_776636|Original|hCoV-19^^Israel|Human|Israel Central Virology laboratory|Israel Central Virology laboratory|Zuckerman|Israel
>Spike|hCoV-19/USA/NY-NYUMC464/2020|2020-04-15|EPI_ISL_435529|Original|hCoV-19^^New York|Human|NYU Langone Health|Departments of Pathology and Medicine|Maurano|USA
>Spike|hCoV-19/USA/WA-S1075/2020|2020-04-30|EPI_ISL_463565|Original|hCoV-19^^Washington|Human|Washington State Department of Health|Seattle Flu Study|Bedford|USA
>Spike|hCoV-19/Northern Ireland/NIRE-103549/2020|2020-04-17|EPI_ISL_489314|Original|hCoV-19^^Northern Ireland|Human|Regional Virus Laboratory|Wellcome Sanger Institute for the COVID-19 Genomics UK (COG-UK) consortium|Davies|United Kingdom
>Spike|hCoV-19/USA/CA-CZB-6974/2020|2020-09-02|EPI_ISL_582906|Original|hCoV-19^^California|Human|County of Santa Clara Public Health Department|Chan-Zuckerberg Biohub|Bhatt|USA
>Spike|hCoV-19/USA/MA-MGH-01089/2020|2020-05-01|EPI_ISL_791871|Original|hCoV-19^^Massachusetts|Human|Massachusetts General Hospital|Infectious Disease Program|Park|USA
>Spike|hCoV-19/USA/CA-CZB-10861/2020|2020-08-02|EPI_ISL_583103|Original|hCoV-19^^California|Human|Humboldt County Public Health Laboratory|Chan-Zuckerberg Biohub|Bhatt|USA
>Spike|hCoV-19/Australia/NSW132/2020|2020-03-23|EPI_ISL_427787|Original|hCoV-19^^New South Wales|Human|Centre for Infectious Diseases and Microbiology Public Health|NSW Health Pathology - Institute of Clinical Pathology and Medical Research; Westmead Hospital; University of Sydney|Gall|Australia
>Spike|hCoV-19/Japan/PG-4000/2020|2020-07-00|EPI_ISL_692241|Original|hCoV-19^^Japan|Human|Pathogen Genomics Center|Pathogen Genomics Center|Sekizuka|Japan
>Spike|hCoV-19/England/BIRM-5F70D/2020|2020-05-02|EPI_ISL_473322|Original|hCoV-19^^England|Human|University of Birmingham|COVID-19 Genomics UK (COG-UK) Consortium|Loman|United Kingdom
>Spike|hCoV-19/England/MILK-A7B6B9/2020|2020-10-15|EPI_ISL_607578|Original|hCoV-19^^England|Human|Lighthouse Lab in Milton Keynes|Wellcome Sanger Institute for the COVID-19 Genomics UK (COG-UK) consortium|Davies|United Kingdom
>Spike|hCoV-19/England/QEUH-A1E005/2020|2020-09-29|EPI_ISL_587417|Original|hCoV-19^^England|Human|Lighthouse Lab in Glasgow|Wellcome Sanger Institute for the COVID-19 Genomics UK (COG-UK) consortium|Davies|United Kingdom
>Spike|hCoV-19/USA/CO-CDPHE-2100015997/2020|2020-11-23|EPI_ISL_771321|Original|hCoV-19^^Colorado|Human|Colorado Department of Public Health and Environment|Colorado Department of Puplic Health and Environment|Bankers|USA
>Spike|hCoV-19/Switzerland/BL-ETHZ-410823/2020|2020-12-14|EPI_ISL_737479|Original|hCoV-19^^Basel-Landschaft|Human|Viollier AG|Department of Biosystems Science and Engineering|Nadeau|Switzerland
>Spike|hCoV-19/USA/TX-HMH-3819/2020|2020-06-16|EPI_ISL_543223|Original|hCoV-19^^Texas|Human|Houston Methodist Hospital|Houston Methodist Hospital|Long|USA
>Spike|hCoV-19/Switzerland/BL-ETHZ-190083/2020|2020-07-07|EPI_ISL_500939|Original|hCoV-19^^Basel-Landschaft|Human|Viollier AG|Department of Biosystems Science and Engineering|Nadeau|Switzerland
>Spike|hCoV-19/USA/NM-DOH-2020318505/2020|2020-08-29|EPI_ISL_732734|Original|hCoV-19^^New Mexico|Human|New Mexico Department of Health Scientific Laboratory|New Mexico Department of Health Scientific Laboratory|Johnson|USA
>Spike|hCoV-19/Portugal/PT0303/2020|2020-03-14|EPI_ISL_454019|Original|hCoV-19^^Portugal|Human|unknown|Instituto Nacional de Saude (INSA)|Borges|Portugal
>Spike|hCoV-19/Norway/5393/2020|2020-10-27|EPI_ISL_708074|Original|hCoV-19^^Vestland|Human|Foerde Hospital|Norwegian Institute of Public Health|Kopperud Riis|Norway
>Spike|hCoV-19/Australia/VIC6071/2020|2020-07-20|EPI_ISL_518100|Original|hCoV-19^^Victoria|Human|Victorian Infectious Diseases Reference Laboratory (VIDRL)|VIDRL and MDU-PHL|Schultz|Australia

>Spike|hCoV-19/USA/CA-CHLA-PLM98478969/2020|2020-11-29|EPI_ISL_753652|Original|hCoV-19^^California|Human|Clinical virology Laboratory|Center for Personalized Medicine|Dien Bard|USA
>Spike|hCoV-19/England/MILK-977F1D/2020|2020-07-29|EPI_ISL_550467|Original|hCoV-19^^England|Human|Lighthouse Lab in Milton Keynes|Wellcome Sanger Institute for the COVID-19 Genomics UK (COG-UK) consortium|Davies|United Kingdom
>Spike|hCoV-19/USA/AZ-TG371304/2020|2020-05-20|EPI_ISL_694559|Original|hCoV-19^^Arizona|Human|AZ SPHL|TGen North|French|USA
>Spike|hCoV-19/Germany/HH-hpi-p1932/2020|2020-03-26|EPI_ISL_776260|Original|hCoV-19^^Hamburg|Human|University Medical Center Hamburg Eppendorf|Heinrich Pette Institute|Robitaille|Germany
>Spike|hCoV-19/USA/AZ-TG569526/2020|2020-07-13|EPI_ISL_695605|Original|hCoV-19^^Arizona|Human|AZ SPHL|TGen North|French|USA
>Spike|hCoV-19/Russia/CRIE166582/2020|2020-03-31|EPI_ISL_534349|Original|hCoV-19^^Moscow|Human|Molecular diagnostic laboratory of Federal Budget Institution of Science \Central Research Institute of Epidemiology\ of The Federal Service on Customers' Rights Protection and Human Well-being Surveillance|Group of Genomics and Postgenomic Technologies of Central Research Institute of Epidemilology|Spera
>nskaya|Russia
>Spike|hCoV-19/Luxembourg/LNS4646138/2020|2020-10-26|EPI_ISL_740513|Original|hCoV-19^^Luxembourg|Human|Laboratoire national de sant\u00e9|Laboratoire national de sant\u00e9|Wienecke-Baldacchino|Luxembourg
>Spike|hCoV-19/Luxembourg/LNS1530163/2020|2020-11-04|EPI_ISL_739949|Original|hCoV-19^^Luxembourg|Human|Laboratoire national de sant\u00e9|Laboratoire national de sant\u00e9|Wienecke-Baldacchino|Luxembourg
>Spike|hCoV-19/Belgium/rega-10081245/2020|2020-10-08|EPI_ISL_735090|Original|hCoV-19^^Hoeilaart|Human|UZ Leuven|KU Leuven|Maes|Belgium
>Spike|hCoV-19/Denmark/DCGC-10465/2020|2020-11-02|EPI_ISL_669992|Original|hCoV-19^^Nordjylland|Human|Department of Virus and Microbiological Special Diagnostics|Albertsen Lab|Michaelsen|Denmark
>Spike|hCoV-19/USA/UT-UPHL-2012644535/2020|2020-06-18|EPI_ISL_746453|Original|hCoV-19^^Utah|Human|Utah Public Health Laboratory|Utah Public Health Laboratory|Gallagher|USA
>Spike|hCoV-19/USA/CA-ALSR-5114/2020|2020-12-03|EPI_ISL_730360|Original|hCoV-19^^California|Human|San Diego County Public Health Laboratory|Andersen lab at Scripps Research|Gangavarapu|USA
>Spike|hCoV-19/Canada/ON-A1/2020|2020-11-24|EPI_ISL_755712|Original|hCoV-19^^Ontario|Human|Toronto Invasive Bacterial Diseases Network|McMaster University|McArthur|Canada
>Spike|hCoV-19/England/ALDP-BE91C3/2020|2020-12-03|EPI_ISL_708923|Original|hCoV-19^^England|Human|Lighthouse Lab in Alderley Park|Wellcome Sanger Institute for the COVID-19 Genomics UK (COG-UK) Consortium|Davies|United Kingdom
>Spike|hCoV-19/England/SHEF-D33C1/2020|2020-04-01|EPI_ISL_432666|Original|hCoV-19^^England|Human|Virology Department|COVID-19 Genomics UK (COG-UK) Consortium|de Silva|United Kingdom
>Spike|hCoV-19/England/CAMB-1B39F1/2020|2020-05-22|EPI_ISL_492199|Original|hCoV-19^^England|Human|Department of Pathology|Wellcome Sanger Institute for the COVID-19 Genomics UK (COG-UK) consortium|Davies|United Kingdom
>Spike|hCoV-19/England/LOND-12607E6/2020|2020-04-05|EPI_ISL_524700|Original|hCoV-19^^England|Human|North West London Pathology|Wellcome Sanger Institute for the COVID-19 Genomics UK (COG-UK) consortium|Davies|United Kingdom
>Spike|hCoV-19/USA/TX-HMH-MCoV-18642/2020|2020-11-20|EPI_ISL_784990|Original|hCoV-19^^Texas|Human|Houston Methodist Hospital|Houston Methodist Hospital|Long|USA
>Spike|hCoV-19/England/WSFT-25C138F/2020|2020-05-05|EPI_ISL_492873|Original|hCoV-19^^England|Human|Department of Medical Microbiology|Wellcome Sanger Institute for the COVID-19 Genomics UK (COG-UK) consortium|Davies|United Kingdom
>Spike|hCoV-19/Luxembourg/LNS6854244/2020|2020-04-01|EPI_ISL_428956|Original|hCoV-19^^Luxembourg|Human|Laboratoire National de Sante|Laboratoire National de Sante|Wienecke-Baldacchino|Luxembourg

>Spike|hCoV-19/England/20174033204/2020|2020-04-21|EPI_ISL_465393|Original|hCoV-19^^England|Human|Respiratory Virus Unit|Respiratory Virus Unit|Platt|United Kingdom
>Spike|hCoV-19/USA/OR-OHSU-1705/2020|2020-07-23|EPI_ISL_526009|Original|hCoV-19^^Oregon|Human|OHSU Lab Services Molecular Microbiology Lab|Oregon SARS-CoV-2 Genome Sequencing Center|O'Roak|USA
>Spike|hCoV-19/Japan/PG-2978/2020|2020-04-00|EPI_ISL_690615|Original|hCoV-19^^Japan|Human|Pathogen Genomics Center|Pathogen Genomics Center|Sekizuka|Japan
>Spike|hCoV-19/Japan/PG-3439/2020|2020-06-00|EPI_ISL_691549|Original|hCoV-19^^Japan|Human|Pathogen Genomics Center|Pathogen Genomics Center|Sekizuka|Japan
>Spike|hCoV-19/England/MILK-C2FC56/2020|2020-12-07|EPI_ISL_733735|Original|hCoV-19^^England|Human|Lighthouse Lab in Milton Keynes|Wellcome Sanger Institute for the COVID-19 Genomics UK (COG-UK) Consortium|Davies|United Kingdom
>Spike|hCoV-19/Northern Ireland/NIRE-2352A0/2020|2020-10-16|EPI_ISL_764425|Original|hCoV-19^^Northern Ireland|Human|Regional Virus Laboratory|COVID-19 Genomics UK (COG-UK) Consortium|Fairley|United Kingdom
>Spike|hCoV-19/England/ALDP-94E322/2020|2020-06-18|EPI_ISL_555172|Original|hCoV-19^^England|Human|Lighthouse Lab in Alderley Park|Wellcome Sanger Institute for the COVID-19 Genomics UK (COG-UK) consortium|Davies|United Kingdom
>Spike|hCoV-19/Australia/VIC11781/2020|2020-07-28|EPI_ISL_591849|Original|hCoV-19^^Victoria|Human|Victorian Infectious Diseases Reference Laboratory (VIDRL)|VIDRL and MDU-PHL|Schultz|Australia
>Spike|hCoV-19/USA/VA-DCLS-0058/2020|2020-03-31|EPI_ISL_426466|Original|hCoV-19^^Virginia|Human|Virginia DCLS|Virginia DCLS|St. Jacques|USA
>Spike|hCoV-19/England/QEUH-9B565A/2020|2020-09-03|EPI_ISL_590304|Original|hCoV-19^^England|Human|Lighthouse Lab in Glasgow|Wellcome Sanger Institute for the COVID-19 Genomics UK (COG-UK) consortium|Davies|United Kingdom
>Spike|hCoV-19/USA/TX-HMH-590/2020|2020-04-08|EPI_ISL_547094|Original|hCoV-19^^Texas|Human|Houston Methodist Hospital|Houston Methodist Hospital|Long|USA
>Spike|hCoV-19/England/MILK-A1D31C/2020|2020-10-03|EPI_ISL_595049|Original|hCoV-19^^England|Human|Queens Medical Centre|COVID-19 Genomics UK (COG-UK) Consortium|Loose|United Kingdom
>Spike|hCoV-19/USA/TX-HMH-MCoV-15204/2020|2020-10-19|EPI_ISL_786688|Original|hCoV-19^^Texas|Human|Houston Methodist Hospital|Houston Methodist Hospital|Long|USA
>Spike|hCoV-19/USA/FL-BPHL-0549/2020|2020-05-28|EPI_ISL_508752|Original|hCoV-19^^Florida|Human|Florida Bureau of Public Health Laboratories|Florida Bureau of Public Health Laboratories|Blanton|USA
>Spike|hCoV-19/Wales/PHWC-16914D/2020|2020-06-25|EPI_ISL_499415|Original|hCoV-19^^Wales|Human|Wales Specialist Virology Centre Sequencing lab|COVID-19 Genomics UK (COG-UK) Consortium|Moore|United Kingdom
>Spike|hCoV-19/South Africa/KRISP-K002463/2020|2020-07-15|EPI_ISL_518046|Original|hCoV-19^^KwaZulu-Natal|Human|NHLS-IALCH|KRISP|de Oliveira|South Africa
>Spike|hCoV-19/England/NOTT-11C0B5/2020|2020-11-15|EPI_ISL_650821|Original|hCoV-19^^England|Human|Queens Medical Centre|COVID-19 Genomics UK (COG-UK) Consortium|Loose|United Kingdom
>Spike|hCoV-19/England/QEUH-BC35C3/2020|2020-11-24|EPI_ISL_721533|Original|hCoV-19^^England|Human|Lighthouse Lab in Glasgow|Wellcome Sanger Institute for the COVID-19 Genomics UK (COG-UK) Consortium|Davies|United Kingdom
>Spike|hCoV-19/England/MILK-B3A5E5/2020|2020-11-02|EPI_ISL_658803|Original|hCoV-19^^England|Human|Lighthouse Lab in Milton Keynes|Wellcome Sanger Institute for the COVID-19 Genomics UK (COG-UK) Consortium|Davies|United Kingdom
>Spike|hCoV-19/Denmark/DCGC-22302/2020|2020-12-14|EPI_ISL_757705|Original|hCoV-19^^Sjaelland|Human|Department of Virus and Microbiological Special Diagnostics|Albertsen Lab|Michaelsen|Denmark
>Spike|hCoV-19/Australia/VIC2508/2020|2020-06-29|EPI_ISL_522203|Original|hCoV-19^^Victoria|Human|Microbiological Diagnostic Unit - Public Health Laboratory (MDU-PHL)|MDU-PHL|Schultz|Australia
>Spike|hCoV-19/Italy/BAS-AMVRC-4162/2020|2020-04-07|EPI_ISL_477195|Original|hCoV-19^^Basilicata|Human|Istituto Zooprofilattico Sperimentale Puglia e Basilicata;|Beaconlab (Bioinformatics|Chiara|Italy

>Spike|hCoV-19/USA/TX-DSHS-1927/2020|2020-06-21|EPI_ISL_765930|Original|hCoV-19^^Texas|Human|TXDSHS|TXDSHS|Rahman|USA
>Spike|hCoV-19/USA/MI-QDX-1125/2020|2020-03-22|EPI_ISL_571855|Original|hCoV-19^^Michigan|Human|Quest Diagnostics|Quest Diagnostics|Kagan|USA
>Spike|hCoV-19/England/LOND-D4D0E/2020|2020-04-16|EPI_ISL_449679|Original|hCoV-19^^England|Human|University College London|COVID-19 Genomics UK (COG-UK) Consortium|Roy|United Kingdom
>Spike|hCoV-19/England/SHEF-C051C/2020|2020-03-22|EPI_ISL_420238|Original|hCoV-19^^England|Human|Virology Department|Department of Infection|de Silva|United Kingdom
>Spike|hCoV-19/Denmark/DCGC-8418/2020|2020-10-26|EPI_ISL_617397|Original|hCoV-19^^Syddanmark|Human|Department of Virus and Microbiological Special Diagnostics|Albertsen lab|Michaelsen|Denmark
>Spike|hCoV-19/Japan/PG-6960/2020|2020-07-00|EPI_ISL_688065|Original|hCoV-19^^Japan|Human|Pathogen Genomics Center|Pathogen Genomics Center|Sekizuka|Japan
>Spike|hCoV-19/Portugal/PT1426/2020|2020-03-16|EPI_ISL_511385|Original|hCoV-19^^Portugal|Human|Instituto Nacional de Saude (INSA)|Instituto Nacional de Saude (INSA)|Borges|Portugal
>Spike|hCoV-19/USA/CA-CZB-15013/2020|2020-11-18|EPI_ISL_738701|Original|hCoV-19^^California|Human|Santa Clara County Public Health Laboratory|Chan-Zuckerberg Biohub|Kamm|USA
>Spike|hCoV-19/England/NOTT-10DF23/2020|2020-03-12|EPI_ISL_425470|Original|hCoV-19^^England|Human|Queens Medical Centre|COVID-19 Genomics UK (COG-UK) Consortium|Loose|United Kingdom
>Spike|hCoV-19/England/NORT-283294/2020|2020-03-28|EPI_ISL_488605|Original|hCoV-19^^England|Human|NU-OMICS DNA Sequencing research facility|Wellcome Sanger Institute for the COVID-19 Genomics UK (COG-UK) consortium|Davies|United Kingdom
>Spike|hCoV-19/Denmark/DCGC-13369/2020|2020-11-30|EPI_ISL_712755|Original|hCoV-19^^Sjaelland|Human|Department of Virus and Microbiological Special Diagnostics|Albertsen Lab|Michaelsen|Denmark
>Spike|hCoV-19/USA/WA-NH5/2020|2020-03-13|EPI_ISL_418774|Original|hCoV-19^^Washington|Human|WA State Department of Health|Pathogen Discovery|Queen|USA
>Spike|hCoV-19/England/MILK-994615/2020|2020-08-25|EPI_ISL_552295|Original|hCoV-19^^England|Human|Lighthouse Lab in Milton Keynes|Wellcome Sanger Institute for the COVID-19 Genomics UK (COG-UK) consortium|Davies|United Kingdom
>Spike|hCoV-19/England/BRIS-12E2C9/2020|2020-04-20|EPI_ISL_481994|Original|hCoV-19^^England|Human|PHE South West Regional Laboratory|Wellcome Sanger Institute for the COVID-19 Genomics UK (COG-UK) consortium|Davies|United Kingdom
>Spike|hCoV-19/Scotland/CVR62/2020|2020-03-12|EPI_ISL_425790|Original|hCoV-19^^Scotland|Human|West of Scotland Specialist Virology Centre|COVID-19 Genomics UK (COG-UK) Consortium|Robertson|United Kingdom
>Spike|hCoV-19/Denmark/DCGC-13610/2020|2020-11-30|EPI_ISL_712909|Original|hCoV-19^^Midtjylland|Human|Department of Virus and Microbiological Special Diagnostics|Albertsen Lab|Michaelsen|Denmark
>Spike|hCoV-19/Denmark/DCGC-1313/2020|2020-06-08|EPI_ISL_618047|Original|hCoV-19^^Syddanmark|Human|Department of Virus and Microbiological Special Diagnostics|Albertsen lab|Michaelsen|Denmark
>Spike|hCoV-19/Japan/PG-3858/2020|2020-07-00|EPI_ISL_692109|Original|hCoV-19^^Japan|Human|Pathogen Genomics Center|Pathogen Genomics Center|Sekizuka|Japan
>Spike|hCoV-19/USA/VA-DCLS-1989/2020|2020-10-09|EPI_ISL_614074|Original|hCoV-19^^Virginia|Human|Virginia DCLS|Virginia DCLS|St. Jacques|USA
>Spike|hCoV-19/Wales/PHWC-25EFC/2020|2020-03-25|EPI_ISL_422287|Original|hCoV-19^^Wales|Human|Wales Specialist Virology Centre|Public Health Wales Microbiology Cardiff|Moore|United Kingdom
>Spike|hCoV-19/England/MILK-B54C4A/2020|2020-11-05|EPI_ISL_657781|Original|hCoV-19^^England|Human|Lighthouse Lab in Milton Keynes|Wellcome Sanger Institute for the COVID-19 Genomics UK (COG-UK) Consortium|Davies|United Kingdom
>Spike|hCoV-19/England/MILK-BDC61F/2020|2020-11-27|EPI_ISL_709685|Original|hCoV-19^^England|Human|Lighthouse Lab in Milton Keynes|Wellcome Sanger Institute for the COVID-19 Genomics UK (COG-UK) Consortium|Davies|United Kingdom

>Spike|hCoV-19/England/ALDP-9CC33B/2020|2020-09-16|EPI_ISL_573100|Original|hCoV-19^^England|Human|Quadram Institute Bioscience|COVID-19 Genomics UK (COG-UK) Consortium|Alikhan|United Kingdom
>Spike|hCoV-19/USA/FL-BPHL-0757/2020|2020-06-27|EPI_ISL_512563|Original|hCoV-19^^Florida|Human|Florida Bureau of Public Health Laboratories|Florida Bureau of Public Health Laboratories|Blanton|USA
>Spike|hCoV-19/England/ALDP-94A9BB/2020|2020-06-11|EPI_ISL_555782|Original|hCoV-19^^England|Human|Lighthouse Lab in Alderley Park|Wellcome Sanger Institute for the COVID-19 Genomics UK (COG-UK) consortium|Davies|United Kingdom
>Spike|hCoV-19/England/NORT-299495/2020|2020-04-24|EPI_ISL_492489|Original|hCoV-19^^England|Human|NU-OMICS DNA Sequencing research facility|Wellcome Sanger Institute for the COVID-19 Genomics UK (COG-UK) consortium|Davies|United Kingdom
>Spike|hCoV-19/Wales/PHWC-48B564/2020|2020-10-31|EPI_ISL_666253|Original|hCoV-19^^Wales|Human|Wales Specialist Virology Centre Sequencing lab|COVID-19 Genomics UK (COG-UK) Consortium|Moore|United Kingdom
>Spike|hCoV-19/Denmark/DCGC-3106/2020|2020-08-31|EPI_ISL_615922|Original|hCoV-19^^Sjaelland|Human|Department of Virus and Microbiological Special Diagnostics|Albertsen lab|Michaelsen|Denmark
>Spike|hCoV-19/England/LIVE-A4806/2020|2020-03-13|EPI_ISL_499914|Original|hCoV-19^^England|Human|Liverpool Clinical Laboratories|COVID-19 Genomics UK (COG-UK) Consortium|Haldenby|United Kingdom
>Spike|hCoV-19/USA/AZ-TG306014/2020|2020-04-30|EPI_ISL_694314|Original|hCoV-19^^Arizona|Human|AZ SPHL|TGen North|French|USA
>Spike|hCoV-19/England/NORT-2812A5/2020|2020-03-09|EPI_ISL_488756|Original|hCoV-19^^England|Human|NU-OMICS DNA Sequencing research facility|Wellcome Sanger Institute for the COVID-19 Genomics UK (COG-UK) consortium|Davies|United Kingdom
>Spike|hCoV-19/Singapore/1362/2020|2020-06-22|EPI_ISL_648713|Original|hCoV-19^^Singapore|Human|Department of Laboratory Medicine|Department of Laboratory Medicine|Chen|Singapore
>Spike|hCoV-19/Gambia/NPHL-1968/2020|2020-07-16|EPI_ISL_561240|Original|hCoV-19^^Kombo|Human|MRCG at LSHTM Genomics lab|MRCG at LSHTM Genomics lab|Sesay|Gambia
>Spike|hCoV-19/Luxembourg/LNS4800679/2020|2020-12-03|EPI_ISL_770982|Original|hCoV-19^^Luxembourg|Human|Laboratoire national de sant\u00e9|Laboratoire national de sant\u00e9|Wienecke-Baldacchino|Luxembourg
>Spike|hCoV-19/Denmark/DCGC-10628/2020|2020-11-02|EPI_ISL_670102|Original|hCoV-19^^Midtjylland|Human|Department of Virus and Microbiological Special Diagnostics|Albertsen Lab|Michaelsen|Denmark
>Spike|hCoV-19/Australia/VIC560/2020|2020-03-25|EPI_ISL_426821|Original|hCoV-19^^Victoria|Human|Victorian Infectious Diseases Reference Laboratory (VIDRL)|Microbiological Diagnostic Unit Public Health Laboratory and Victorian Infectious Diseases Reference Laboratory|Schultz|Australia
>Spike|hCoV-19/England/MILK-99E3C9/2020|2020-08-31|EPI_ISL_550792|Original|hCoV-19^^England|Human|Lighthouse Lab in Milton Keynes|Wellcome Sanger Institute for the COVID-19 Genomics UK (COG-UK) consortium|Davies|United Kingdom
>Spike|hCoV-19/England/NORW-EB95B/2020|2020-05-07|EPI_ISL_457459|Original|hCoV-19^^England|Human|Quadram Institute Bioscience|COVID-19 Genomics UK (COG-UK) Consortium|Alikhan|United Kingdom
>Spike|hCoV-19/India/GJ-GBRC314/2020|2020-06-17|EPI_ISL_495070|Original|hCoV-19^^Gujarat|Human|Department of MicroBiology|Gujarat Biotechnology Research Centre|Joshi|India
>Spike|hCoV-19/England/MILK-677C0A/2020|2020-07-03|EPI_ISL_551821|Original|hCoV-19^^England|Human|Lighthouse Lab in Milton Keynes|Wellcome Sanger Institute for the COVID-19 Genomics UK (COG-UK) consortium|Davies|United Kingdom
>Spike|hCoV-19/Spain/MD-IBV-99009632/2020|2020-08-06|EPI_ISL_654369|Original|hCoV-19^^Madrid|Human|Hospital General Universitario Gregorio Mara\u00f1\u00f3n|SeqCOVID-SPAIN consortium/IBV(CSIC)|Comas|Spain
>Spike|hCoV-19/England/MILK-9569BC/2020|2020-06-06|EPI_ISL_554286|Original|hCoV-19^^England|Human|Lighthouse Lab in Milton Keynes|Wellcome Sanger Institute for the COVID-19 Genomics UK (COG-UK) consortium|Davies|United Kingdom

>Spike|hCoV-19/England/MILK-B046C3/2020|2020-10-24|EPI_ISL_651024|Original|hCoV-19^^England|Human|Department of Pathology|COVID-19 Genomics UK (COG-UK) Consortium|Goodfellow|United Kingdom
>Spike|hCoV-19/England/ALDP-A94887/2020|2020-10-19|EPI_ISL_611064|Original|hCoV-19^^England|Human|Lighthouse Lab in Alderley Park|Wellcome Sanger Institute for the COVID-19 Genomics UK (COG-UK) consortium|Davies|United Kingdom
>Spike|hCoV-19/Scotland/EDB3357/2020|2020-04-09|EPI_ISL_487688|Original|hCoV-19^^Scotland|Human|Virology Department|Wellcome Sanger Institute for the COVID-19 Genomics UK (COG-UK) consortium|Davies|United Kingdom
>Spike|hCoV-19/DRC/11134/2020|2020-05-24|EPI_ISL_527518|Original|hCoV-19^^Kinshasa|Human|Viral Respiratory Lab|Pathogen Sequencing Lab|Lokilo|Democratic Republic of the Congo
>Spike|hCoV-19/Denmark/DCGC-16579/2020|2020-11-16|EPI_ISL_714849|Original|hCoV-19^^Hovedstaden|Human|Department of Virus and Microbiological Special Diagnostics|Albertsen Lab|Michaelsen|Denmark
>Spike|hCoV-19/Australia/TAS226/2020|2020-03-29|EPI_ISL_522655|Original|hCoV-19^^Tasmania|Human|Royal Hobart Hospital Microbiology Department|MDU-PHL|Schultz|Australia
>Spike|hCoV-19/England/SHEF-10CF969/2020|2020-12-20|EPI_ISL_742020|Original|hCoV-19^^England|Human|Virology Department|COVID-19 Genomics UK (COG-UK) Consortium|de Silva|United Kingdom
>Spike|hCoV-19/Singapore/1090/2020|2020-10-02|EPI_ISL_596480|Original|hCoV-19^^Singapore|Human|National Public Health Laboratory|National Public Health Laboratory|Mak|Singapore
>Spike|hCoV-19/Scotland/EDB3512/2020|2020-04-13|EPI_ISL_487747|Original|hCoV-19^^Scotland|Human|Virology Department|Wellcome Sanger Institute for the COVID-19 Genomics UK (COG-UK) consortium|Davies|United Kingdom
>Spike|hCoV-19/Switzerland/BS-UHB-42203366/2020|2020-03-23|EPI_ISL_528351|Original|hCoV-19^^Basel-Stadt|Human|University Hospital Basel|University Hospital Basel|Roloff Handschin|Switzerland
>Spike|hCoV-19/England/CAMC-B20E41/2020|2020-11-02|EPI_ISL_647702|Original|hCoV-19^^England|Human|Lighthouse Lab in Cambridge|Wellcome Sanger Institute for the COVID-19 Genomics UK (COG-UK) Consortium|Davies|United Kingdom
>Spike|hCoV-19/Denmark/DCGC-12967/2020|2020-12-07|EPI_ISL_712494|Original|hCoV-19^^Hovedstaden|Human|Department of Virus and Microbiological Special Diagnostics|Albertsen Lab|Michaelsen|Denmark
>Spike|hCoV-19/Belgium/ULG-10248/2020|2020-07-14|EPI_ISL_515055|Original|hCoV-19^^Liege|Human|Department of Clinical Microbiology|GIGA Medical Genomics|Durkin|Belgium
>Spike|hCoV-19/Northern Ireland/QEUH-96C5DD/2020|2020-08-15|EPI_ISL_530888|Original|hCoV-19^^Northern Ireland|Human|Lighthouse Lab in Glasgow|Wellcome Sanger Institute for the COVID-19 Genomics UK (COG-UK) consortium|Davies|United Kingdom
>Spike|hCoV-19/England/ALDP-4A5E4B/2020|2020-06-09|EPI_ISL_559523|Original|hCoV-19^^England|Human|Lighthouse Lab in Alderley Park|Wellcome Sanger Institute for the COVID-19 Genomics UK (COG-UK) consortium|Davies|United Kingdom
>Spike|hCoV-19/USA/NY-NYCPHL-001550/2020|2020-12-02|EPI_ISL_745464|Original|hCoV-19^^New York|Human|DOHMH Corona|New York City Public Health Laboratory|Wang|USA
>Spike|hCoV-19/Japan/PG-2640/2020|2020-04-00|EPI_ISL_690772|Original|hCoV-19^^Japan|Human|Pathogen Genomics Center|Pathogen Genomics Center|Sekizuka|Japan
>Spike|hCoV-19/England/NORW-E9270/2020|2020-04-27|EPI_ISL_453587|Original|hCoV-19^^England|Human|Quadram Institute Bioscience|COVID-19 Genomics UK (COG-UK) Consortium|Alikhan|United Kingdom
>Spike|hCoV-19/Wales/PHWC-28CD1/2020|2020-04-02|EPI_ISL_432159|Original|hCoV-19^^Wales|Human|Wales Specialist Virology Centre|Public Health Wales Microbiology Cardiff|Moore|United Kingdom
>Spike|hCoV-19/England/ALDP-B5B4C3/2020|2020-11-09|EPI_ISL_655442|Original|hCoV-19^^England|Human|Lighthouse Lab in Alderley Park|Wellcome Sanger Institute for the COVID-19 Genomics UK (COG-UK) Consortium|Davies|United Kingdom

>Spike|hCoV-19/Germany/HH-hpi-p1805/2020|2020-03-19|EPI_ISL_775752|Original|hCoV-19^^Hamburg|Human|University Medical Center Hamburg Eppendorf|Heinrich Pette Institute|Robitaille|Germany
>Spike|hCoV-19/England/CAMC-AB1E19/2020|2020-10-23|EPI_ISL_623674|Original|hCoV-19^^England|Human|Lighthouse Lab in Cambridge|Wellcome Sanger Institute for the COVID-19 Genomics UK (COG-UK) consortium|Davies|United Kingdom
>Spike|hCoV-19/England/ALDP-A66C55/2020|2020-10-12|EPI_ISL_630535|Original|hCoV-19^^England|Human|Lighthouse Lab in Alderley Park|Wellcome Sanger Institute for the COVID-19 Genomics UK (COG-UK) consortium|Davies|United Kingdom
>Spike|hCoV-19/Australia/VIC3418/2020|2020-07-12|EPI_ISL_521092|Original|hCoV-19^^Victoria|Human|Microbiological Diagnostic Unit - Public Health Laboratory (MDU-PHL)|MDU-PHL|Schultz|Australia
>Spike|hCoV-19/Singapore/190/2020|2020-04-20|EPI_ISL_462308|Original|hCoV-19^^Singapore|Human|National Public Health Laboratory|National Public Health Laboratory|Mak|Singapore
>Spike|hCoV-19/Denmark/DCGC-16822/2020|2020-12-14|EPI_ISL_750585|Original|hCoV-19^^Midtjylland|Human|Department of Virus and Microbiological Special Diagnostics|Albertsen Lab|Michaelsen|Denmark
>Spike|hCoV-19/Switzerland/SG-ETHZ-100152/2020|2020-03-16|EPI_ISL_451736|Original|hCoV-19^^Sankt Gallen|Human|Viollier AG|Department of Biosystems Science and Engineering|Nadeau|Switzerland
>Spike|hCoV-19/Japan/PG-0306/2020|2020-03-20|EPI_ISL_479981|Original|hCoV-19^^Kyusyu|Human|Oita Prefectural Institute of Public Health and Environmental Science|Pathogen Genomics Center|Sekizuka|Japan
>Spike|hCoV-19/England/ALDP-9534CD/2020|2020-06-08|EPI_ISL_554511|Original|hCoV-19^^England|Human|Lighthouse Lab in Alderley Park|Wellcome Sanger Institute for the COVID-19 Genomics UK (COG-UK) consortium|Davies|United Kingdom
>Spike|hCoV-19/England/BRIS-1298CC/2020|2020-03-31|EPI_ISL_458448|Original|hCoV-19^^England|Human|PHE South West Regional Laboratory|Wellcome Sanger Institute for the COVID-19 Genomics UK (COG-UK) consortium|Davies|United Kingdom
>Spike|hCoV-19/England/ALDP-A94A36/2020|2020-10-20|EPI_ISL_624735|Original|hCoV-19^^England|Human|Lighthouse Lab in Alderley Park|Wellcome Sanger Institute for the COVID-19 Genomics UK (COG-UK) consortium|Davies|United Kingdom
>Spike|hCoV-19/England/QEUH-96A821/2020|2020-08-13|EPI_ISL_531466|Original|hCoV-19^^England|Human|Lighthouse Lab in Glasgow|Wellcome Sanger Institute for the COVID-19 Genomics UK (COG-UK) consortium|Davies|United Kingdom
>Spike|hCoV-19/Spain/AN-IBV-004077/2020|2020-04-14|EPI_ISL_510045|Original|hCoV-19^^Andalucia|Human|Servicio de Microbiolog\u00eda. HRU de M\u00e1laga. Servicio Andaluz de Salud|SeqCOVID-SPAIN consortium/IBV(CSIC)|Comas|Spain
>Spike|hCoV-19/Ecuador/USFQ-114/2020|2020-06-30|EPI_ISL_486849|Original|hCoV-19^^Imbabura|Human|Institute of Microbiology|Institute of Microbiology|Cardenas|Ecuador
>Spike|hCoV-19/USA/TX-HMH-6456/2020|2020-06-27|EPI_ISL_546009|Original|hCoV-19^^Texas|Human|Houston Methodist Hospital|Houston Methodist Hospital|Long|USA
>Spike|hCoV-19/Northern Ireland/NIRE-FB27B/2020|2020-03-24|EPI_ISL_448956|Original|hCoV-19^^Northern Ireland|Human|Regional Virus Laboratory|COVID-19 Genomics UK (COG-UK) Consortium|Fairley|United Kingdom
>Spike|hCoV-19/Israel/CVL-64-ngs/2020|2020-12-24|EPI_ISL_776602|Original|hCoV-19^^Israel|Human|Israel Central Virology laboratory|Israel Central Virology laboratory|Zuckerman|Israel
>Spike|hCoV-19/England/QEUH-AF0CC9/2020|2020-10-29|EPI_ISL_662688|Original|hCoV-19^^England|Human|Lighthouse Lab in Glasgow|Wellcome Sanger Institute for the COVID-19 Genomics UK (COG-UK) Consortium|Davies|United Kingdom
>Spike|hCoV-19/England/MILK-B9531B/2020|2020-11-13|EPI_ISL_673696|Original|hCoV-19^^England|Human|Lighthouse Lab in Milton Keynes|Wellcome Sanger Institute for the COVID-19 Genomics UK (COG-UK) Consortium|Davies|United Kingdom
>Spike|hCoV-19/England/NORW-E8499/2020|2020-04-16|EPI_ISL_449153|Original|hCoV-19^^England|Human|Quadram Institute Bioscience|COVID-19 Genomics UK (COG-UK) Consortium|Alikhan|United Kingdom

>Spike|hCoV-19/USA/VAN4169/2020|2020-05-11|EPI_ISL_677687|Original|hCoV-19^^New York|Human|Vanda Pharmaceuticals Clinical Site|Vanda Pharmaceuticals|Przychodzen|USA
>Spike|hCoV-19/Japan/PG-5296/2020|2020-08-00|EPI_ISL_690399|Original|hCoV-19^^Japan|Human|Pathogen Genomics Center|Pathogen Genomics Center|Sekizuka|Japan
>Spike|hCoV-19/USA/TX-HMH-MCoV-9842/2020|2020-07-06|EPI_ISL_780302|Original|hCoV-19^^Texas|Human|Houston Methodist Hospital|Houston Methodist Hospital|Long|USA
>Spike|hCoV-19/Spain/CT-IBV-005559/2020|2020-03-18|EPI_ISL_508620|Original|hCoV-19^^Catalunya|Human|Departamento de Microbiolog\u00eda|SeqCOVID-SPAIN consortium/IBV(CSIC)|Comas|Spain
>Spike|hCoV-19/England/CAMC-AAFEA5/2020|2020-10-21|EPI_ISL_623222|Original|hCoV-19^^England|Human|Lighthouse Lab in Cambridge|Wellcome Sanger Institute for the COVID-19 Genomics UK (COG-UK) consortium|Davies|United Kingdom
>Spike|hCoV-19/France/ARA-101325/2020|2020-11-04|EPI_ISL_683338|Original|hCoV-19^^Auvergne-Rh\u00f4ne-Alpes|Human|CNR Virus des Infections Respiratoires - France SUD|CNR Virus des Infections Respiratoires - France SUD|Bal|France
>Spike|hCoV-19/USA/CA-CHLA-PLM04975012/2020|2020-11-17|EPI_ISL_753363|Original|hCoV-19^^California|Human|Clinical virology Laboratory|Center for Personalized Medicine|Dien Bard|USA
>Spike|hCoV-19/England/OXON-AF67A/2020|2020-04-14|EPI_ISL_478761|Original|hCoV-19^^England|Human|Oxford Viromics|COVID-19 Genomics UK (COG-UK) Consortium|Golubchik|United Kingdom
>Spike|hCoV-19/Northern Ireland/NIRE-FC7D5/2020|2020-04-20|EPI_ISL_459342|Original|hCoV-19^^Northern Ireland|Human|Regional Virus Laboratory|Wellcome Sanger Institute for the COVID-19 Genomics UK (COG-UK) consortium|Davies|United Kingdom
>Spike|hCoV-19/Wales/PHWC-48363C/2020|2020-10-16|EPI_ISL_627839|Original|hCoV-19^^Wales|Human|Wales Specialist Virology Centre Sequencing lab|COVID-19 Genomics UK (COG-UK) Consortium|Moore|United Kingdom
>Spike|hCoV-19/Wales/PHWC-4A1C72/2020|2020-12-12|EPI_ISL_743797|Original|hCoV-19^^Wales|Human|Wales Specialist Virology Centre Sequencing lab|COVID-19 Genomics UK (COG-UK) Consortium|Moore|United Kingdom
>Spike|hCoV-19/England/MILK-978AEE/2020|2020-08-01|EPI_ISL_550453|Original|hCoV-19^^England|Human|Lighthouse Lab in Milton Keynes|Wellcome Sanger Institute for the COVID-19 Genomics UK (COG-UK) consortium|Davies|United Kingdom
>Spike|hCoV-19/Portugal/PT1982/2020|2020-11-11|EPI_ISL_732205|Original|hCoV-19^^Portugal|Human|Instituto Nacional de Saude (INSA) and Instituto Gulbenkian de Ciencia (IGC)|Instituto Nacional de Saude (INSA) and Instituto Gulbenkian de Ciencia (IGC)|Borges|Portugal
>Spike|hCoV-19/USA/MN-GMF-15330/2020|2020-08-14|EPI_ISL_547703|Original|hCoV-19^^Minnesota|Human|Gundersen Molecular Diagnostics Laboratory|Kabara Cancer Research Institute|Kenny|USA
>Spike|hCoV-19/India/UN-N5/2020|2020-00-00|EPI_ISL_497758|Original|hCoV-19^^India|Human|CSIR-CDRI/SGPGI|CSIr-CDRI/SGPGI|Singh|India
>Spike|hCoV-19/Ireland/D-NVRL-78IRL78609/2020|2020-11-24|EPI_ISL_752587|Original|hCoV-19^^Dublin|Human|National Virus Reference Laboratory|National Virus Reference Laboratory|Gonzalez|Ireland
>Spike|hCoV-19/England/ALDP-B74C9F/2020|2020-11-12|EPI_ISL_662263|Original|hCoV-19^^England|Human|Lighthouse Lab in Alderley Park|Wellcome Sanger Institute for the COVID-19 Genomics UK (COG-UK) Consortium|Davies|United Kingdom
>Spike|hCoV-19/USA/CA-ALSR-0684-SAN/2020|2020-03-30|EPI_ISL_483300|Original|hCoV-19^^California|Human|UC San Diego Center for Advanced Laboratory Medicine|Andersen lab at Scripps Research|Gangavarapu|USA
>Spike|hCoV-19/New Zealand/20CV0166/2020|2020-08-21|EPI_ISL_548038|Original|hCoV-19^^Auckland|Human|Middlemore Hospital|Institute of Environmental Science and Research (ESR)|Wang|New Zealand
>Spike|hCoV-19/Belgium/ULG-10843/2020|2020-10-27|EPI_ISL_666878|Original|hCoV-19^^Li\u00e8ge|Human|Department of Clinical Microbiology|GIGA Medical Genomics|Durkin|Belgium

>Spike|hCoV-19/Chile/RM-73219/2020|2020-05-22|EPI_ISL_746787|Original|hCoV-19^^Region Metropolitana de Santiago|Human|Genetica Molecular and Subdepartamento de Virologia ISP Chile|Instituto de Salud Publica de Chile|Tognarelli|Chile
>Spike|hCoV-19/USA/MD-HP12099/2020|2020-12-14|EPI_ISL_791417|Original|hCoV-19^^Maryland|Human|Johns Hopkins Hospital Department of Pathology|Johns Hopkins Hospital Department of Pathology|Morris|USA
>Spike|hCoV-19/England/ALDP-B5D0EB/2020|2020-11-09|EPI_ISL_678856|Original|hCoV-19^^England|Human|Department of Pathology|COVID-19 Genomics UK (COG-UK) Consortium|Goodfellow|United Kingdom
>Spike|hCoV-19/USA/LA-EVTL1153/2020|2020-12-10|EPI_ISL_768422|Original|hCoV-19^^Louisiana|Human|LSUHS Emerging Viral Threat Laboratory|Microbial Genome Sequencing Center|Kamil|USA
>Spike|hCoV-19/Turkey/IST-GLAB-149/2020|2020-05-02|EPI_ISL_480250|Original|hCoV-19^^Istanbul|Human|Genomic Laboratory (GLAB) (Conjoint lab of Health Directorate of Istanbul and Istanbul Technical University)|Genomic Laboratory (GLAB)|Karacan|Turkey
>Spike|hCoV-19/USA/FL-BPHL-0083/2020|2020-04-28|EPI_ISL_445026|Original|hCoV-19^^Florida|Human|Florida Bureau of Public Health Laboratories|Florida Bureau of Public Health Laboratories|Blanton|USA
>Spike|hCoV-19/India/MH-GA69/2020|2020-06-18|EPI_ISL_508263|Original|hCoV-19^^Maharashtra|Human|Government Medical College|National Institute of Biomedical Genomics|Maitra|India
>Spike|hCoV-19/England/BIRM-61094/2020|2020-04-23|EPI_ISL_477744|Original|hCoV-19^^England|Human|University of Birmingham|COVID-19 Genomics UK (COG-UK) Consortium|Loman|United Kingdom
>Spike|hCoV-19/Japan/PG-1456/2020|2020-04-00|EPI_ISL_685063|Original|hCoV-19^^Japan|Human|Saitama Prefectural Institute of Public Health|Pathogen Genomics Center|Sekizuka|Japan
>Spike|hCoV-19/Japan/PG-3371/2020|2020-04-00|EPI_ISL_691486|Original|hCoV-19^^Japan|Human|Pathogen Genomics Center|Pathogen Genomics Center|Sekizuka|Japan
>Spike|hCoV-19/England/ALDP-B46AAB/2020|2020-11-07|EPI_ISL_656002|Original|hCoV-19^^England|Human|Lighthouse Lab in Alderley Park|Wellcome Sanger Institute for the COVID-19 Genomics UK (COG-UK) Consortium|Davies|United Kingdom
>Spike|hCoV-19/England/20144009104/2020|2020-03-31|EPI_ISL_423504|Original|hCoV-19^^England|Human|Respiratory Virus Unit|Respiratory Virus Unit|Platt|United Kingdom
>Spike|hCoV-19/Wales/PHWC-2EF2D/2020|2020-04-06|EPI_ISL_446176|Original|hCoV-19^^Wales|Human|Wales Specialist Virology Centre|Public Health Wales Microbiology Cardiff|Moore|United Kingdom
>Spike|hCoV-19/Scotland/EDB8872/2020|2020-10-28|EPI_ISL_652013|Original|hCoV-19^^Scotland|Human|Virology Department|COVID-19 Genomics UK (COG-UK) Consortium|McHugh|United Kingdom
>Spike|hCoV-19/Switzerland/TI-ETHZ-260157/2020|2020-08-26|EPI_ISL_539451|Original|hCoV-19^^Ticino|Human|Viollier AG|Department of Biosystems Science and Engineering|Nadeau|Switzerland
>Spike|hCoV-19/Denmark/DCGC-18638/2020|2020-12-07|EPI_ISL_750095|Original|hCoV-19^^Sjaelland|Human|Department of Virus and Microbiological Special Diagnostics|Albertsen Lab|Michaelsen|Denmark
>Spike|hCoV-19/USA/LA-EVTL065/2020|2020-05-02|EPI_ISL_451226|Original|hCoV-19^^Louisiana|Human|LSUHS Emerging Viral Threat Laboratory|Microbial Genome Sequencing Center|Kamil|USA
>Spike|hCoV-19/England/NORT-28843F/2020|2020-11-21|EPI_ISL_725013|Original|hCoV-19^^England|Human|Northumbria University / South Tees Hospitals NHS Foundation Trust / North Cumbria Integrated Care NHS Foundation Trust / North Tees and Hartlepool NHS Foundation Trust / Newcastle Hospitals NHS Foundation Trust|COVID-19 Genomics UK (COG-UK) Consortium|Bashton|United Kingdom
>Spike|hCoV-19/Scotland/QEUH-AD5B27/2020|2020-10-24|EPI_ISL_649675|Original|hCoV-19^^Scotland|Human|Lighthouse Lab in Glasgow|Wellcome Sanger Institute for the COVID-19 Genomics UK (COG-UK) Consortium|Davies|United Kingdom
>Spike|hCoV-19/New Zealand/20CV0376/2020|2020-09-27|EPI_ISL_579114|Original|hCoV-19^^Auckland|Human|LabPLUS|Institute of Environmental Science and Research (ESR)|Wang|New Zealand

>Spike|hCoV-19/USA/TX-HMH-MCoV-16067/2020|2020-10-27|EPI_ISL_785751|Original|hCoV-19^^Texas|Human|Houston Methodist Hospital|Houston Methodist Hospital|Long|USA
>Spike|hCoV-19/USA/VA-DCLS-0504/2020|2020-05-29|EPI_ISL_614113|Original|hCoV-19^^Virginia|Human|Virginia DCLS|Virginia DCLS|St. Jacques|USA
>Spike|hCoV-19/Portugal/PT0052/2020|2020-03-18|EPI_ISL_421455|Original|hCoV-19^^Portugal|Human|CH Barreiro Montijo|Instituto Nacional de Saude (INSA)|Borges|Portugal
>Spike|hCoV-19/England/QEUH-AAD144/2020|2020-10-20|EPI_ISL_652944|Original|hCoV-19^^England|Human|Oxford Viromics|COVID-19 Genomics UK (COG-UK) Consortium|Golubchik|United Kingdom
>Spike|hCoV-19/Netherlands/ZH-RIVM-20847/2020|2020-12-17|EPI_ISL_790902|Original|hCoV-19^^Zuid-holland|Human|Dutch COVID-19 response team|National Institute for Public Health and the Environment (RIVM)|Meijer|Netherlands
>Spike|hCoV-19/India/DL-60932-CSIR-IGIB/2020|2020-08-06|EPI_ISL_636780|Original|hCoV-19^^Delhi|Human|National Centre for Disease control (NCDC)|NCDC/CSIR-IGIB|Faruq|India
>Spike|hCoV-19/Denmark/DCGC-12694/2020|2020-11-16|EPI_ISL_682875|Original|hCoV-19^^Syddanmark|Human|Department of Virus and Microbiological Special Diagnostics|Albertsen Lab|Michaelsen|Denmark
>Spike|hCoV-19/England/QEUH-96449C/2020|2020-08-12|EPI_ISL_536960|Original|hCoV-19^^England|Human|Lighthouse Lab in Glasgow|Wellcome Sanger Institute for the COVID-19 Genomics UK (COG-UK) consortium|Davies|United Kingdom
>Spike|hCoV-19/Mexico/CMX-INMEGEN-09/2020|2020-08-01|EPI_ISL_522880|Original|hCoV-19^^Mexico City|Human|Instituto Nacional de Medicina Gen\u00f3mica|Instituto Nacional de Medicina Gen\u00f3mica|Hidalgo|Mexico
>Spike|hCoV-19/USA/WI-UW-2252/2020|2020-10-07|EPI_ISL_728021|Original|hCoV-19^^Wisconsin|Human|University of Wisconsin-Madison AIDS Vaccine Research Laboratories|University of Wisconsin-Madison AIDS Vaccine Research Laboratories|Baczenas|USA
>Spike|hCoV-19/Australia/VIC15746/2020|2020-07-31|EPI_ISL_593438|Original|hCoV-19^^Victoria|Human|Microbiological Diagnostic Unit - Public Health Laboratory (MDU-PHL)|MDU-PHL|Schultz|Australia
>Spike|hCoV-19/England/ALDP-C23144/2020|2020-12-06|EPI_ISL_733647|Original|hCoV-19^^England|Human|Lighthouse Lab in Alderley Park|Wellcome Sanger Institute for the COVID-19 Genomics UK (COG-UK) Consortium|Davies|United Kingdom
>Spike|hCoV-19/USA/CA-CDPH240/2020|2020-11-15|EPI_ISL_755085|Original|hCoV-19^^California|Human|California Department of Public Health|California Department of Public Health|Bell|USA
>Spike|hCoV-19/Switzerland/VD-ETHZ-220087/2020|2020-07-28|EPI_ISL_512052|Original|hCoV-19^^Vaud|Human|Viollier AG|Department of Biosystems Science and Engineering|Nadeau|Switzerland
>Spike|hCoV-19/England/NORW-EB162/2020|2020-05-03|EPI_ISL_472302|Original|hCoV-19^^England|Human|Quadram Institute Bioscience|COVID-19 Genomics UK (COG-UK) Consortium|Alikhan|United Kingdom
>Spike|hCoV-19/England/QEUH-AD4E98/2020|2020-10-26|EPI_ISL_633971|Original|hCoV-19^^England|Human|Lighthouse Lab in Glasgow|Wellcome Sanger Institute for the COVID-19 Genomics UK (COG-UK) consortium|Davies|United Kingdom
>Spike|hCoV-19/Scotland/CVR5604/2020|2020-11-01|EPI_ISL_650514|Original|hCoV-19^^Scotland|Human|West of Scotland Specialist Virology Centre|COVID-19 Genomics UK (COG-UK) Consortium|Robertson|United Kingdom
>Spike|hCoV-19/USA/WA-UW-11277/2020|2020-06-20|EPI_ISL_570384|Original|hCoV-19^^Washington|Human|UW Virology Lab|UW Virology Lab|Roychoudhury|USA
>Spike|hCoV-19/Sweden/20-14697/2020|2020-05-15|EPI_ISL_469061|Original|hCoV-19^^Vastra Gotaland|Human|Narhalsan Sjobo vardcentral|The Public Health Agency of Sweden|Svartstr\u00f6m|Sweden
>Spike|hCoV-19/England/ALDP-9E767D/2020|2020-09-24|EPI_ISL_638209|Original|hCoV-19^^England|Human|Department of Pathology|COVID-19 Genomics UK (COG-UK) Consortium|Goodfellow|United Kingdom

>Spike|hCoV-19/England/CAMB-1B5E78/2020|2020-00-00|EPI_ISL_573134|Original|hCoV-19^^England|Human|Department of Pathology|COVID-19 Genomics UK (COG-UK) Consortium|Goodfellow|United Kingdom
>Spike|hCoV-19/USA/CA-CZB-5841/2020|2020-07-22|EPI_ISL_672387|Original|hCoV-19^^California|Human|UCSF Clinical Microbiology Laboratory|Chan-Zuckerberg Biohub|Bhatt|USA
>Spike|hCoV-19/USA/WA-S2483/2020|2020-06-15|EPI_ISL_497115|Original|hCoV-19^^Washington|Human|Washington State Department of Health|Seattle Flu Study|Lee|USA
>Spike|hCoV-19/Netherlands/ZH-EMC-319/2020|2020-07-03|EPI_ISL_523660|Original|hCoV-19^^Zuid Holland|Human|Dutch COVID-19 response team|Erasmus Medical Center|Oude Munnink|Netherlands
>Spike|hCoV-19/Japan/PG-3734/2020|2020-04-00|EPI_ISL_691987|Original|hCoV-19^^Japan|Human|Pathogen Genomics Center|Pathogen Genomics Center|Sekizuka|Japan
>Spike|hCoV-19/Denmark/DCGC-15720/2020|2020-11-23|EPI_ISL_713981|Original|hCoV-19^^Nordjylland|Human|Department of Virus and Microbiological Special Diagnostics|Albertsen Lab|Michaelsen|Denmark
>Spike|hCoV-19/USA/WA-S1982/2020|2020-06-04|EPI_ISL_497410|Original|hCoV-19^^Washington|Human|Washington State Department of Health|Seattle Flu Study|Lee|USA
>Spike|hCoV-19/United Arab Emirates/0442/2020|2020-05-17|EPI_ISL_698616|Original|hCoV-19^^Abu Dhabi|Human|Group 42 (G42) Healthcare|G42 Healthcare|Ogrodzki|United Arab Emirates
>Spike|hCoV-19/Australia/VIC10616/2020|2020-08-02|EPI_ISL_562993|Original|hCoV-19^^Victoria|Human|Microbiological Diagnostic Unit - Public Health Laboratory (MDU-PHL)|MDU-PHL|Schultz|Australia
>Spike|hCoV-19/Denmark/DCGC-21073/2020|2020-12-21|EPI_ISL_758701|Original|hCoV-19^^Sjaelland|Human|Department of Virus and Microbiological Special Diagnostics|Albertsen Lab|Michaelsen|Denmark
>Spike|hCoV-19/Wales/PHWC-493CCE/2020|2020-11-17|EPI_ISL_726010|Original|hCoV-19^^Wales|Human|Originating lab|COVID-19 Genomics UK (COG-UK) Consortium|Moore|United Kingdom
>Spike|hCoV-19/England/QEUH-C31732/2020|2020-12-10|EPI_ISL_731340|Original|hCoV-19^^England|Human|Lighthouse Lab in Glasgow|Wellcome Sanger Institute for the COVID-19 Genomics UK (COG-UK) Consortium|Davies|United Kingdom
>Spike|hCoV-19/England/CAMC-C99501/2020|2020-12-17|EPI_ISL_762041|Original|hCoV-19^^England|Human|Lighthouse Lab in Cambridge|Wellcome Sanger Institute for the COVID-19 Genomics UK (COG-UK) Consortium|Davies|United Kingdom
>Spike|hCoV-19/England/ALDP-A5276A/2020|2020-10-07|EPI_ISL_597928|Original|hCoV-19^^England|Human|Lighthouse Lab in Alderley Park|Wellcome Sanger Institute for the COVID-19 Genomics UK (COG-UK) consortium|Davies|United Kingdom
>Spike|hCoV-19/England/CAMB-1AD189/2020|2020-04-29|EPI_ISL_447973|Original|hCoV-19^^England|Human|Department of Pathology|COVID-19 Genomics UK (COG-UK) Consortium|Meredith|United Kingdom
>Spike|hCoV-19/Brazil/RS-6220/2020|2020-05-27|EPI_ISL_729796|Original|hCoV-19^^Rio Grande do Sul|Human|Laboratorio Central de Saude Publica do Estado do Rio Grande do Sul (LACEN-RS)|Laboratory of Respiratory Viruses and Measles|Silva|Brazil
>Spike|hCoV-19/England/ALDP-C3CB68/2020|2020-12-10|EPI_ISL_731645|Original|hCoV-19^^England|Human|Lighthouse Lab in Alderley Park|Wellcome Sanger Institute for the COVID-19 Genomics UK (COG-UK) Consortium|Davies|United Kingdom
>Spike|hCoV-19/Denmark/DCGC-17123/2020|2020-12-14|EPI_ISL_750226|Original|hCoV-19^^Hovedstaden|Human|Department of Virus and Microbiological Special Diagnostics|Albertsen Lab|Michaelsen|Denmark
>Spike|hCoV-19/USA/AZ-TG577617/2020|2020-08-05|EPI_ISL_695624|Original|hCoV-19^^Arizona|Human|TGen North|TGen North|French|USA
>Spike|hCoV-19/USA/DC-HP00073/2020|2020-03-17|EPI_ISL_438231|Original|hCoV-19^^District of Columbia|Human|Johns Hopkins Hospital Department of Pathology|Johns Hopkins Hospital Department of Pathology|Ramakrishnan|USA
>Spike|hCoV-19/USA/WI-GMF-16060/2020|2020-08-18|EPI_ISL_547716|Original|hCoV-19^^Wisconsin|Human|Gundersen Molecular Diagnostics Laboratory|Kabara Cancer Research Institute|Kenny|USA

>Spike|hCoV-19/USA/FL-BPHL-0354/2020|2020-04-12|EPI_ISL_489763|Original|hCoV-19^^Florida|Human|Florida Bureau of Public Health Laboratories|Florida Bureau of Public Health Laboratories|Blanton|USA
>Spike|hCoV-19/England/CAMB-7B3BC/2020|2020-04-12|EPI_ISL_433949|Original|hCoV-19^^England|Human|Department of Pathology|COVID-19 Genomics UK (COG-UK) Consortium|Meredith|United Kingdom
>Spike|hCoV-19/USA/OR-OHSU-3109/2020|2020-10-02|EPI_ISL_667483|Original|hCoV-19^^Oregon|Human|OHSU Lab Services Molecular Microbiology Lab|Oregon SARS-CoV-2 Genome Sequencing Center|O'Roak|USA
>Spike|hCoV-19/England/CAMB-840C8/2020|2020-04-26|EPI_ISL_438734|Original|hCoV-19^^England|Human|Department of Pathology|COVID-19 Genomics UK (COG-UK) Consortium|Meredith|United Kingdom
>Spike|hCoV-19/England/CAMC-B992B0/2020|2020-11-14|EPI_ISL_673563|Original|hCoV-19^^England|Human|Lighthouse Lab in Cambridge|Wellcome Sanger Institute for the COVID-19 Genomics UK (COG-UK) Consortium|Davies|United Kingdom
>Spike|hCoV-19/England/ALDP-950ADD/2020|2020-06-14|EPI_ISL_554468|Original|hCoV-19^^England|Human|Lighthouse Lab in Alderley Park|Wellcome Sanger Institute for the COVID-19 Genomics UK (COG-UK) consortium|Davies|United Kingdom
>Spike|hCoV-19/Spain/CT-IBV-97010149/2020|2020-11-03|EPI_ISL_683617|Original|hCoV-19^^Catalunya|Human|Servicio de Microbiolog\u00eda|SeqCOVID-SPAIN consortium/IBV(CSIC)|Comas|Spain
>Spike|hCoV-19/Spain/IB-IBV-99011601/2020|2020-12-04|EPI_ISL_779917|Original|hCoV-19^^Balear Islands|Human|Servicio de Microbiolog\u00eda|SeqCOVID-SPAIN consortium/IBV(CSIC)|Comas|Spain
>Spike|hCoV-19/Wales/ALDP-AA2349/2020|2020-10-22|EPI_ISL_624349|Original|hCoV-19^^Wales|Human|Lighthouse Lab in Alderley Park|Wellcome Sanger Institute for the COVID-19 Genomics UK (COG-UK) consortium|Davies|United Kingdom
>Spike|hCoV-19/Denmark/DCGC-2506/2020|2020-08-10|EPI_ISL_617777|Original|hCoV-19^^Midtjylland|Human|Department of Virus and Microbiological Special Diagnostics|Albertsen lab|Michaelsen|Denmark
>Spike|hCoV-19/Denmark/DCGC-8258/2020|2020-10-26|EPI_ISL_617272|Original|hCoV-19^^Hovedstaden|Human|Department of Virus and Microbiological Special Diagnostics|Albertsen lab|Michaelsen|Denmark
>Spike|hCoV-19/Belgium/ULG-10200/2020|2020-05-23|EPI_ISL_471435|Original|hCoV-19^^Liege|Human|Department of Clinical Microbiology|GIGA Medical Genomics|Durkin|Belgium
>Spike|hCoV-19/Netherlands/UT-EMC-117/2020|2020-10-11|EPI_ISL_632640|Original|hCoV-19^^Utrecht|Human|Dutch COVID-19 response team|Erasmus Medical Center|Oude Munnink|Netherlands
>Spike|hCoV-19/USA/WA-S2064/2020|2020-06-05|EPI_ISL_497492|Original|hCoV-19^^Washington|Human|Washington State Department of Health|Seattle Flu Study|Lee|USA
>Spike|hCoV-19/England/CAMC-B084B6/2020|2020-10-30|EPI_ISL_643537|Original|hCoV-19^^England|Human|Lighthouse Lab in Cambridge|Wellcome Sanger Institute for the COVID-19 Genomics UK (COG-UK) Consortium|Davies|United Kingdom
>Spike|hCoV-19/Italy/UMB-IZSUM-48682-14/2020|2020-10-29|EPI_ISL_763322|Original|hCoV-19^^Umbria|Human|Istituto Zooprofilattico Sperimentale dell' Umbria e delle Marche - Togo Rosati|Istituto Superiore di Sanit\u00e0|De Sabato|Italy
>Spike|hCoV-19/England/MILK-974E9B/2020|2020-08-20|EPI_ISL_552675|Original|hCoV-19^^England|Human|Lighthouse Lab in Milton Keynes|Wellcome Sanger Institute for the COVID-19 Genomics UK (COG-UK) consortium|Davies|United Kingdom
>Spike|hCoV-19/USA/TX-HMH-2748/2020|2020-06-04|EPI_ISL_545525|Original|hCoV-19^^Texas|Human|Houston Methodist Hospital|Houston Methodist Hospital|Long|USA
>Spike|hCoV-19/England/ALDP-B1B23E/2020|2020-11-02|EPI_ISL_645825|Original|hCoV-19^^England|Human|Lighthouse Lab in Alderley Park|Wellcome Sanger Institute for the COVID-19 Genomics UK (COG-UK) Consortium|Davies|United Kingdom
>Spike|hCoV-19/USA/TX-HMH-6851/2020|2020-06-25|EPI_ISL_546290|Original|hCoV-19^^Texas|Human|Houston Methodist Hospital|Houston Methodist Hospital|Long|USA
>Spike|hCoV-19/USA/VA-DCLS-0559/2020|2020-03-17|EPI_ISL_485860|Original|hCoV-19^^Virginia|Human|Virginia DCLS|Virginia DCLS|St. Jacques|USA

>Spike|hCoV-19/Germany/MV-Cento-70001814/2020|2020-04-01|EPI_ISL_459962|Original|hCoV-19^^Mecklenburg-Western Pomerania|Human|Centogene AG|Centogene AG|Kandaswamy|Germany
>Spike|hCoV-19/Japan/PG-2926/2020|2020-04-00|EPI_ISL_691052|Original|hCoV-19^^Japan|Human|Pathogen Genomics Center|Pathogen Genomics Center|Sekizuka|Japan
>Spike|hCoV-19/USA/AR-CDC-0843/2020|2020-07-08|EPI_ISL_535653|Original|hCoV-19^^Arkansas|Human|AR Dept. of Health-Public Health Lab|Pathogen Discovery|Queen|USA
>Spike|hCoV-19/Australia/VIC11801/2020|2020-08-10|EPI_ISL_591857|Original|hCoV-19^^Victoria|Human|Victorian Infectious Diseases Reference Laboratory (VIDRL)|VIDRL and MDU-PHL|Schultz|Australia
>Spike|hCoV-19/Spain/PV-IBV-2159/2020|2020-04-12|EPI_ISL_468908|Original|hCoV-19^^Basque Country|Human|Servicio de Microbiolog\u00eda. Hospital Universitario Donostia. OSI Donostialdea. \u00c1rea de Enfermedades Infecciosas|SeqCOVID-SPAIN consortium/IBV(CSIC)|Comas|Spain
>Spike|hCoV-19/Netherlands/LI-RIVM-10130/2020|2020-03-30|EPI_ISL_547474|Original|hCoV-19^^Limburg|Human|Dutch COVID-19 response team|National Institute for Public Health and the Environment (RIVM)|Meijer|Netherlands
>Spike|hCoV-19/England/MILK-C613EA/2020|2020-12-13|EPI_ISL_769251|Original|hCoV-19^^England|Human|Lighthouse Lab in Milton Keynes|Wellcome Sanger Institute for the COVID-19 Genomics UK (COG-UK) Consortium|Davies|United Kingdom
>Spike|hCoV-19/USA/WI-UW-1559/2020|2020-09-15|EPI_ISL_605437|Original|hCoV-19^^Wisconsin|Human|University of Wisconsin-Madison AIDS Vaccine Research Laboratories|University of Wisconsin-Madison AIDS Vaccine Research Laboratories|Baczenas|USA
>Spike|hCoV-19/India/GJ-GBRC134/2020|2020-05-24|EPI_ISL_458089|Original|hCoV-19^^Gujarat|Human|B.J. Medical College and Civil hospital|Gujarat Biotechnology Research Centre|Joshi|India
>Spike|hCoV-19/USA/OR-OHSU-2901/2020|2020-09-04|EPI_ISL_667401|Original|hCoV-19^^Oregon|Human|OHSU Lab Services Molecular Microbiology Lab|Oregon SARS-CoV-2 Genome Sequencing Center|O'Roak|USA
>Spike|hCoV-19/Denmark/DCGC-18722/2020|2020-12-07|EPI_ISL_750144|Original|hCoV-19^^Midtjylland|Human|Department of Virus and Microbiological Special Diagnostics|Albertsen Lab|Michaelsen|Denmark
>Spike|hCoV-19/Denmark/DCGC-8578/2020|2020-10-26|EPI_ISL_625942|Original|hCoV-19^^Hovedstaden|Human|Department of Virus and Microbiological Special Diagnostics|Albertsen lab|Michaelsen|Denmark
>Spike|hCoV-19/England/QEUH-9F9B6A/2020|2020-09-26|EPI_ISL_595749|Original|hCoV-19^^England|Human|Oxford Viromics|COVID-19 Genomics UK (COG-UK) Consortium|Golubchik|United Kingdom
>Spike|hCoV-19/Denmark/ALAB-SSI-670/2020|2020-03-26|EPI_ISL_437030|Original|hCoV-19^^Denmark|Human|Department of Virus and Microbiological Special Diagnostics|Albertsen lab|Kirkegaard|Denmark
>Spike|hCoV-19/USA/CA-CZB-3900/2020|2020-07-24|EPI_ISL_548517|Original|hCoV-19^^California|Human|County of San Luis Obispo Public Health Laboratory|Chan-Zuckerberg Biohub|Bhatt|USA
>Spike|hCoV-19/Scotland/EDB2134/2020|2020-04-20|EPI_ISL_491632|Original|hCoV-19^^Scotland|Human|Virology Department|Wellcome Sanger Institute for the COVID-19 Genomics UK (COG-UK) consortium|Davies|United Kingdom
>Spike|hCoV-19/South Korea/KCDC2301/2020|2020-05-22|EPI_ISL_510637|Original|hCoV-19^^South Korea|Human|Division of Viral Diseases|Division of Viral Diseases|Kim|South Korea
>Spike|hCoV-19/Denmark/DCGC-3717/2020|2020-08-31|EPI_ISL_615495|Original|hCoV-19^^Hovedstaden|Human|Department of Virus and Microbiological Special Diagnostics|Albertsen lab|Michaelsen|Denmark
>Spike|hCoV-19/Australia/VIC4221/2020|2020-07-18|EPI_ISL_519549|Original|hCoV-19^^Victoria|Human|Microbiological Diagnostic Unit - Public Health Laboratory (MDU-PHL)|MDU-PHL|Schultz|Australia

>Spike|hCoV-19/England/QEUH-9FAC2A/2020|2020-09-28|EPI_ISL_588261|Original|hCoV-19^^England|Human|Lighthouse Lab in Glasgow|Wellcome Sanger Institute for the COVID-19 Genomics UK (COG-UK) consortium|Davies|United Kingdom
>Spike|hCoV-19/USA/CA-CDC-4729/2020|2020-06-15|EPI_ISL_535700|Original|hCoV-19^^California|Human|CDPH|Pathogen Discovery|Queen|USA
>Spike|hCoV-19/Japan/PG-4814/2020|2020-08-00|EPI_ISL_689938|Original|hCoV-19^^Japan|Human|Pathogen Genomics Center|Pathogen Genomics Center|Sekizuka|Japan
>Spike|hCoV-19/Australia/VIC13150/2020|2020-07-31|EPI_ISL_641030|Original|hCoV-19^^Victoria|Human|Microbiological Diagnostic Unit - Public Health Laboratory (MDU-PHL)|MDU-PHL|Schultz|Australia
>Spike|hCoV-19/USA/WA-S1136/2020|2020-05-04|EPI_ISL_463626|Original|hCoV-19^^Washington|Human|Washington State Department of Health|Seattle Flu Study|Bedford|USA
>Spike|hCoV-19/England/20128094904/2020|2020-03-20|EPI_ISL_423969|Original|hCoV-19^^England|Human|Respiratory Virus Unit|Respiratory Virus Unit|Platt|United Kingdom
>Spike|hCoV-19/England/20134012504/2020|2020-03-23|EPI_ISL_423083|Original|hCoV-19^^England|Human|Respiratory Virus Unit|Respiratory Virus Unit|Platt|United Kingdom
>Spike|hCoV-19/Australia/VIC7242/2020|2020-07-27|EPI_ISL_564308|Original|hCoV-19^^Victoria|Human|Microbiological Diagnostic Unit - Public Health Laboratory (MDU-PHL)|MDU-PHL|Schultz|Australia
>Spike|hCoV-19/USA/UT-08601/2020|2020-05-18|EPI_ISL_524348|Original|hCoV-19^^Utah|Human|Utah Public Health Laboratory|Utah Public Health Laboratory|Young|USA
>Spike|hCoV-19/Japan/Donner73/2020|2020-07-28|EPI_ISL_644977|Original|hCoV-19^^Kanto|Human|Department of Infectious Diseases|Center for Medical Genetics|Kosaki|Japan
>Spike|hCoV-19/Denmark/DCGC-20797/2020|2020-12-21|EPI_ISL_759369|Original|hCoV-19^^Sjaelland|Human|Department of Virus and Microbiological Special Diagnostics|Albertsen Lab|Michaelsen|Denmark
>Spike|hCoV-19/England/ALDP-A671BF/2020|2020-10-12|EPI_ISL_630642|Original|hCoV-19^^England|Human|Lighthouse Lab in Alderley Park|Wellcome Sanger Institute for the COVID-19 Genomics UK (COG-UK) consortium|Davies|United Kingdom
>Spike|hCoV-19/Scotland/EDB7752/2020|2020-09-11|EPI_ISL_540889|Original|hCoV-19^^Scotland|Human|Virology Department|COVID-19 Genomics UK (COG-UK) Consortium|Williams|United Kingdom
>Spike|hCoV-19/USA/NC_200245/2020|2020-03-00|EPI_ISL_454803|Original|hCoV-19^^North Carolina|Human|Dirk Dittmer|Dirk Dittmer|Moorad|USA
>Spike|hCoV-19/England/ALDP-9EDAF3/2020|2020-05-09|EPI_ISL_580849|Original|hCoV-19^^England|Human|Lighthouse Lab in Alderley Park|Wellcome Sanger Institute for the COVID-19 Genomics UK (COG-UK) consortium|Davies|United Kingdom
>Spike|hCoV-19/England/CAMB-72D7B/2020|2020-03-22|EPI_ISL_440009|Original|hCoV-19^^England|Human|Department of Pathology|Wellcome Sanger Institute for the COVID-19 Genomics UK (COG-UK) consortium|Amato|United Kingdom
>Spike|hCoV-19/USA/UT-07270/2020|2020-06-16|EPI_ISL_487268|Original|hCoV-19^^Utah|Human|Utah Public Health Laboratory|Utah Public Health Laboratory|Butz|USA
>Spike|hCoV-19/Scotland/QEUH-A1FF61/2020|2020-10-01|EPI_ISL_598927|Original|hCoV-19^^Scotland|Human|Lighthouse Lab in Glasgow|Wellcome Sanger Institute for the COVID-19 Genomics UK (COG-UK) consortium|Davies|United Kingdom
>Spike|hCoV-19/England/LOND-DA99B/2020|2020-11-03|EPI_ISL_724675|Original|hCoV-19^^England|Human|University College London|COVID-19 Genomics UK (COG-UK) Consortium|Roy|United Kingdom
>Spike|hCoV-19/USA/NJ-PV09473/2020|2020-03-19|EPI_ISL_450145|Original|hCoV-19^^New Jersey|Human|MSHS Clinical Microbiology Laboratories|MSHS Pathogen Surveillance Program|van Bakel|USA
>Spike|hCoV-19/England/MILK-A7A2F2/2020|2020-10-15|EPI_ISL_606866|Original|hCoV-19^^England|Human|Lighthouse Lab in Milton Keynes|Wellcome Sanger Institute for the COVID-19 Genomics UK (COG-UK) consortium|Davies|United Kingdom
>Spike|hCoV-19/USA/NY-Wadsworth-16600-01/2020|2020-03-15|EPI_ISL_676742|Original|hCoV-19^^New York|Human|Wadsworth Center|Wadsworth Center|Lasek-Nesselquist|USA

>Spike|hCoV-19/Wales/PHWC-485870/2020|2020-10-21|EPI_ISL_628229|Original|hCoV-19^^Wales|Human|Wales Specialist Virology Centre Sequencing lab|COVID-19 Genomics UK (COG-UK) Consortium|Moore|United Kingdom
>Spike|hCoV-19/England/NORT-29596D/2020|2020-04-01|EPI_ISL_488553|Original|hCoV-19^^England|Human|NU-OMICS DNA Sequencing research facility|Wellcome Sanger Institute for the COVID-19 Genomics UK (COG-UK) consortium|Davies|United Kingdom
>Spike|hCoV-19/USA/MN-GMF-07592/2020|2020-06-23|EPI_ISL_489900|Original|hCoV-19^^Minnesota|Human|Gundersen Molecular Diagnostics Laboratory|Kabara Cancer Research Institute|Kenny|USA
>Spike|hCoV-19/USA/PA-MGSC12-04/2020|2020-03-25|EPI_ISL_436467|Original|hCoV-19^^Pennsylvania|Human|UPMC Clinical Laboratory|Microbial Genome Sequencing Center|Cooper|USA
>Spike|hCoV-19/USA/MA-MGH-03040/2020|2020-08-01|EPI_ISL_765689|Original|hCoV-19^^Massachusetts|Human|Massachusetts General Hospital|Infectious Disease Program|Park|USA
>Spike|hCoV-19/USA/NM-UNM-00794/2020|2020-05-05|EPI_ISL_610260|Original|hCoV-19^^New Mexico|Human|TriCore Reference Laboratories|Center for Global Health|Domman|USA
>Spike|hCoV-19/USA/CA-CDPH238/2020|2020-11-15|EPI_ISL_755007|Original|hCoV-19^^California|Human|California Department of Public Health|California Department of Public Health|Bell|USA
>Spike|hCoV-19/Australia/VIC174/2020|2020-03-18|EPI_ISL_419871|Original|hCoV-19^^Victoria|Human|Victorian Infectious Diseases Reference Laboratory (VIDRL)|Victorian Infectious Diseases Reference Laboratory and Microbiological Diagnostic Unit Public Health Laboratory|Schultz|Australia
>Spike|hCoV-19/England/QEUH-B2B49F/2020|2020-11-03|EPI_ISL_646729|Original|hCoV-19^^England|Human|Lighthouse Lab in Glasgow|Wellcome Sanger Institute for the COVID-19 Genomics UK (COG-UK) Consortium|Davies|United Kingdom
>Spike|hCoV-19/USA/CA-ALSR-2127/2020|2020-05-21|EPI_ISL_635357|Original|hCoV-19^^California|Human|San Diego County Public Health Laboratory|Andersen lab at Scripps Research|Gangavarapu|USA
>Spike|hCoV-19/USA/IA_6396/2020|2020-03-08|EPI_ISL_424895|Original|hCoV-19^^Iowa|Human|IA State Hygienic Laboratory|Pathogen Discovery|Queen|USA
>Spike|hCoV-19/England/QEUH-B2FD90/2020|2020-11-03|EPI_ISL_660050|Original|hCoV-19^^England|Human|Lighthouse Lab in Glasgow|Wellcome Sanger Institute for the COVID-19 Genomics UK (COG-UK) Consortium|Davies|United Kingdom
>Spike|hCoV-19/USA/UT-UPHL-2008771/2020|2020-06-01|EPI_ISL_524804|Original|hCoV-19^^Utah|Human|Utah Public Health Laboratory|Utah Public Health Laboratory|Young|USA
>Spike|hCoV-19/Australia/VIC913/2020|2020-03-24|EPI_ISL_426882|Original|hCoV-19^^Victoria|Human|Victorian Infectious Diseases Reference Laboratory (VIDRL)|Microbiological Diagnostic Unit Public Health Laboratory and Victorian Infectious Diseases Reference Laboratory|Schultz|Australia
>Spike|hCoV-19/Luxembourg/LNS7915037/2020|2020-11-09|EPI_ISL_744654|Original|hCoV-19^^Luxembourg|Human|Laboratoire national de sant\u00e9|Laboratoire national de sant\u00e9|Wienecke-Baldacchino|Luxembourg
>Spike|hCoV-19/Bangladesh/DNAS_DRCIM_isl_Hia/2020|2020-07-02|EPI_ISL_774930|Original|hCoV-19^^Dhaka|Human|Designated Reference Institute for Chemical Measurements (DRICM)|DNA SOLUTION LTD.|Rabbi|Bangladesh
>Spike|hCoV-19/England/20132005204/2020|2020-03-23|EPI_ISL_423720|Original|hCoV-19^^England|Human|Respiratory Virus Unit|Respiratory Virus Unit|Platt|United Kingdom
>Spike|hCoV-19/England/CAMB-1B6CAA/2020|2020-10-13|EPI_ISL_612277|Original|hCoV-19^^England|Human|Department of Pathology|COVID-19 Genomics UK (COG-UK) Consortium|Goodfellow|United Kingdom
>Spike|hCoV-19/USA/TX-HMH-MCoV-12258/2020|2020-09-23|EPI_ISL_790271|Original|hCoV-19^^Texas|Human|Houston Methodist Hospital|Houston Methodist Hospital|Long|USA
>Spike|hCoV-19/England/ALDP-68A484/2020|2020-07-02|EPI_ISL_558216|Original|hCoV-19^^England|Human|Lighthouse Lab in Alderley Park|Wellcome Sanger Institute for the COVID-19 Genomics UK (COG-UK) consortium|Davies|United Kingdom
>Spike|hCoV-19/England/QEUH-A4BFF3/2020|2020-10-05|EPI_ISL_628537|Original|hCoV-19^^England|Human|Oxford Viromics|COVID-19 Genomics UK (COG-UK) Consortium|Golubchik|United Kingdom

>Spike|hCoV-19/USA/AZ-TG670219/2020|2020-04-30|EPI_ISL_696403|Original|hCoV-19^^Arizona|Human|Sonora Quest Laboratories|TGen North|French|USA
>Spike|hCoV-19/Belgium/ULG-10353/2020|2020-08-04|EPI_ISL_540520|Original|hCoV-19^^Li\u00e8ge|Human|Department of Clinical Microbiology|GIGA Medical Genomics|Durkin|Belgium
>Spike|hCoV-19/Scotland/CVR5985/2020|2020-10-28|EPI_ISL_703755|Original|hCoV-19^^Scotland|Human|West of Scotland Specialist Virology Centre|COVID-19 Genomics UK (COG-UK) Consortium|Robertson|United Kingdom
>Spike|hCoV-19/Wales/PHWC-4955B2/2020|2020-11-23|EPI_ISL_726367|Original|hCoV-19^^Wales|Human|Originating lab|COVID-19 Genomics UK (COG-UK) Consortium|Moore|United Kingdom
>Spike|hCoV-19/England/BRIS-126416/2020|2020-04-10|EPI_ISL_458976|Original|hCoV-19^^England|Human|PHE South West Regional Laboratory|Wellcome Sanger Institute for the COVID-19 Genomics UK (COG-UK) consortium|Davies|United Kingdom
>Spike|hCoV-19/Japan/PG-1641/2020|2020-04-00|EPI_ISL_685247|Original|hCoV-19^^Japan|Human|Pathogen Genomics Center|Pathogen Genomics Center|Sekizuka|Japan
>Spike|hCoV-19/USA/CA-LACPHL-AA00013/2020|2020-08-05|EPI_ISL_710111|Original|hCoV-19^^California|Human|Los Angeles County PHL|Los Angeles County PHL|Hemarajata|USA
>Spike|hCoV-19/USA/TX-HMH-6164/2020|2020-07-02|EPI_ISL_547284|Original|hCoV-19^^Texas|Human|Houston Methodist Hospital|Houston Methodist Hospital|Long|USA
>Spike|hCoV-19/USA/NM-UNM-00292/2020|2020-05-15|EPI_ISL_508047|Original|hCoV-19^^New Mexico|Human|New Mexico Department of Health Scientific Laboratory Division|Center for Global Health|Domman|USA
>Spike|hCoV-19/USA/UT-QDX-1796/2020|2020-03-15|EPI_ISL_604126|Original|hCoV-19^^Utah|Human|Quest Diagnostics|Quest Diagnostics|Kagan|USA
>Spike|hCoV-19/England/CAMC-A662CC/2020|2020-10-11|EPI_ISL_637518|Original|hCoV-19^^England|Human|Department of Pathology|COVID-19 Genomics UK (COG-UK) Consortium|Goodfellow|United Kingdom
>Spike|hCoV-19/DRC/11191/2020|2020-05-23|EPI_ISL_527506|Original|hCoV-19^^Kongo Central|Human|Viral Respiratory Lab|Pathogen Sequencing Lab|Lokilo|Democratic Republic of the Congo
>Spike|hCoV-19/Wales/ALDP-AA0949/2020|2020-10-22|EPI_ISL_625000|Original|hCoV-19^^Wales|Human|Lighthouse Lab in Alderley Park|Wellcome Sanger Institute for the COVID-19 Genomics UK (COG-UK) consortium|Davies|United Kingdom
>Spike|hCoV-19/Denmark/DCGC-9626/2020|2020-11-09|EPI_ISL_669073|Original|hCoV-19^^Syddanmark|Human|Department of Virus and Microbiological Special Diagnostics|Albertsen Lab|Michaelsen|Denmark
>Spike|hCoV-19/Scotland/EDB1247/2020|2020-04-09|EPI_ISL_439147|Original|hCoV-19^^Scotland|Human|Virology Department|COVID-19 Genomics UK (COG-UK) Consortium|Williams|United Kingdom
>Spike|hCoV-19/England/MILK-AC7988/2020|2020-10-20|EPI_ISL_634448|Original|hCoV-19^^England|Human|Lighthouse Lab in Milton Keynes|Wellcome Sanger Institute for the COVID-19 Genomics UK (COG-UK) consortium|Davies|United Kingdom
>Spike|hCoV-19/Denmark/DCGC-7720/2020|2020-10-19|EPI_ISL_618601|Original|hCoV-19^^Hovedstaden|Human|Department of Virus and Microbiological Special Diagnostics|Albertsen lab|Michaelsen|Denmark
>Spike|hCoV-19/England/ALDP-A2E530/2020|2020-10-05|EPI_ISL_586740|Original|hCoV-19^^England|Human|Lighthouse Lab in Alderley Park|Wellcome Sanger Institute for the COVID-19 Genomics UK (COG-UK) consortium|Davies|United Kingdom
>Spike|hCoV-19/Canada/ON-PHL-20-03705/2020|2020-11-00|EPI_ISL_775188|Original|hCoV-19^^Ontario|Human|Public Health Ontario Laboratory|Public Health Ontario Laboratory|Guthrie|Canada
>Spike|hCoV-19/England/ALDP-C3D790/2020|2020-12-10|EPI_ISL_731833|Original|hCoV-19^^England|Human|Lighthouse Lab in Alderley Park|Wellcome Sanger Institute for the COVID-19 Genomics UK (COG-UK) Consortium|Davies|United Kingdom
>Spike|hCoV-19/Wales/PHWC-2B236/2020|2020-04-08|EPI_ISL_432286|Original|hCoV-19^^Wales|Human|Wales Specialist Virology Centre|Public Health Wales Microbiology Cardiff|Moore|United Kingdom

>Spike|hCoV-19/England/BRIS-1334E9/2020|2020-07-05|EPI_ISL_539894|Original|hCoV-19^^England|Human|PHE South West Regional Laboratory|Wellcome Sanger Institute for the COVID-19 Genomics UK (COG-UK) consortium|Davies|United Kingdom
>Spike|hCoV-19/England/QEUH-C53F50/2020|2020-12-11|EPI_ISL_736378|Original|hCoV-19^^England|Human|Lighthouse Lab in Glasgow|Wellcome Sanger Institute for the COVID-19 Genomics UK (COG-UK) Consortium|Davies|United Kingdom
>Spike|hCoV-19/USA/TX-HMH-MCoV-8968/2020|2020-07-02|EPI_ISL_780864|Original|hCoV-19^^Texas|Human|Houston Methodist Hospital|Houston Methodist Hospital|Long|USA
>Spike|hCoV-19/Malaysia/MKAK-CL-2020-7554/2020|2020-02-06|EPI_ISL_416907|Vero E6|hCoV-19^^Selangor|Human|National Public Health Laboratory|Malaysia Genome Institute|Mat Isa|Malaysia
>Spike|hCoV-19/Scotland/QEUH-AD4B91/2020|2020-10-27|EPI_ISL_644035|Original|hCoV-19^^Scotland|Human|Lighthouse Lab in Glasgow|Wellcome Sanger Institute for the COVID-19 Genomics UK (COG-UK) Consortium|Davies|United Kingdom
>Spike|hCoV-19/Scotland/CVR4123/2020|2020-09-08|EPI_ISL_540821|Original|hCoV-19^^Scotland|Human|West of Scotland Specialist Virology Centre|COVID-19 Genomics UK (COG-UK) Consortium|Robertson|United Kingdom
>Spike|hCoV-19/Japan/PG-1000/2020|2020-04-00|EPI_ISL_684610|Original|hCoV-19^^Japan|Human|Pathogen Genomics Center|Pathogen Genomics Center|Sekizuka|Japan
>Spike|hCoV-19/England/BRIS-122E49/2020|2020-04-04|EPI_ISL_440644|Original|hCoV-19^^England|Human|PHE South West Regional Laboratory|Wellcome Sanger Institute for the COVID-19 Genomics UK (COG-UK) consortium|Amato|United Kingdom
>Spike|hCoV-19/England/NOTT-110488/2020|2020-04-22|EPI_ISL_441518|Original|hCoV-19^^England|Human|Queens Medical Centre|COVID-19 Genomics UK (COG-UK) Consortium|Loose|United Kingdom
>Spike|hCoV-19/England/ALDP-A2E49D/2020|2020-10-06|EPI_ISL_586613|Original|hCoV-19^^England|Human|Lighthouse Lab in Alderley Park|Wellcome Sanger Institute for the COVID-19 Genomics UK (COG-UK) consortium|Davies|United Kingdom
>Spike|hCoV-19/England/ALDP-BBA389/2020|2020-11-20|EPI_ISL_701858|Original|hCoV-19^^England|Human|Lighthouse Lab in Alderley Park|Wellcome Sanger Institute for the COVID-19 Genomics UK (COG-UK) Consortium|Davies|United Kingdom
>Spike|hCoV-19/USA/AZ-TG668405/2020|2020-05-12|EPI_ISL_696308|Original|hCoV-19^^Arizona|Human|Sonora Quest Laboratories|TGen North|French|USA
>Spike|hCoV-19/Belgium/rega-10141390/2020|2020-10-14|EPI_ISL_738442|Original|hCoV-19^^Tervuren|Human|UZ Leuven|KU Leuven|Maes|Belgium
>Spike|hCoV-19/England/QEUH-B4078C/2020|2020-11-08|EPI_ISL_657029|Original|hCoV-19^^England|Human|Lighthouse Lab in Glasgow|Wellcome Sanger Institute for the COVID-19 Genomics UK (COG-UK) Consortium|Davies|United Kingdom
>Spike|hCoV-19/Spain/EX-IBV-005018/2020|2020-03-13|EPI_ISL_510327|Original|hCoV-19^^Extremadura|Human|Hospital San Pedro de Alc\u00e1ntara (C\u00e1ceres)|SeqCOVID-SPAIN consortium/IBV(CSIC)|Comas|Spain
>Spike|hCoV-19/Wales/PHWC-4A285F/2020|2020-12-08|EPI_ISL_743949|Original|hCoV-19^^Wales|Human|Wales Specialist Virology Centre Sequencing lab|COVID-19 Genomics UK (COG-UK) Consortium|Moore|United Kingdom
>Spike|hCoV-19/Gibraltar/204641744/2020|2020-00-00|EPI_ISL_637213|Original|hCoV-19^^Gibraltar|Human|Microbiology|Respiratory Virus Unit|Platt|Gibraltar
>Spike|hCoV-19/Belgium/ULG-11135/2020|2020-12-18|EPI_ISL_737379|Original|hCoV-19^^Li\u00e8ge|Human|Department of Clinical Microbiology|GIGA Medical Genomics|Durkin|Belgium
>Spike|hCoV-19/England/MILK-99C950/2020|2020-08-31|EPI_ISL_550662|Original|hCoV-19^^England|Human|Lighthouse Lab in Milton Keynes|Wellcome Sanger Institute for the COVID-19 Genomics UK (COG-UK) consortium|Davies|United Kingdom
>Spike|hCoV-19/England/20148065004/2020|2020-04-02|EPI_ISL_423593|Original|hCoV-19^^England|Human|Respiratory Virus Unit|Respiratory Virus Unit|Platt|United Kingdom
>Spike|hCoV-19/Denmark/DCGC-1630/2020|2020-04-06|EPI_ISL_619256|Original|hCoV-19^^Sjaelland|Human|Department of Virus and Microbiological Special Diagnostics|Albertsen lab|Michaelsen|Denmark
>Spike|hCoV-19/England/LOND-126042B/2020|2020-03-16|EPI_ISL_524658|Original|hCoV-19^^England|Human|North West London Pathology|Wellcome Sanger Institute for the COVID-19 Genomics UK (COG-UK) consortium|Davies|United Kingdom

>Spike|hCoV-19/England/CAMB-724B2/2020|2020-03-24|EPI_ISL_439943|Original|hCoV-19^^England|Human|Department of Pathology|Wellcome Sanger Institute for the COVID-19 Genomics UK (COG-UK) consortium|Amato|United Kingdom
>Spike|hCoV-19/USA/VA-DCLS-1444/2020|2020-08-18|EPI_ISL_572311|Original|hCoV-19^^Virginia|Human|Virginia DCLS|Virginia DCLS|St. Jacques|USA
>Spike|hCoV-19/Sweden/20-50235/2020|2020-03-10|EPI_ISL_429158|Original|hCoV-19^^Varmland|Human|Klinisk mikrobiologi Orebro|The Public Health Agency of Sweden|Svartstr\u00f6m|Sweden
>Spike|hCoV-19/Russia/SVE-RII-MH3109S/2020|2020-10-03|EPI_ISL_733088|Original|hCoV-19^^Sverdlovsk|Human|HELIX LLC|WHO National Influenza Centre Russian Federation|Komissarov|Russia
>Spike|hCoV-19/Canada/QC-LSPQ-L00242317/2020|2020-03-29|EPI_ISL_536317|Original|hCoV-19^^Quebec|Human|H\u00f4pital Charles-LeMoyne|Laboratoire de sant\u00e9 publique du Qu\u00e9bec|Moreira|Canada
>Spike|hCoV-19/England/ALDP-6BF7AB/2020|2020-07-01|EPI_ISL_557774|Original|hCoV-19^^England|Human|Lighthouse Lab in Alderley Park|Wellcome Sanger Institute for the COVID-19 Genomics UK (COG-UK) consortium|Davies|United Kingdom
>Spike|hCoV-19/USA/WI-UW-1042/2020|2020-08-02|EPI_ISL_516468|Original|hCoV-19^^Wisconsin|Human|University of Wisconsin-Madison AIDS Vaccine Research Laboratories|University of Wisconsin-Madison AIDS Vaccine Research Laboratories|Baczenas|USA
>Spike|hCoV-19/England/QEUH-B2E07A/2020|2020-11-03|EPI_ISL_646436|Original|hCoV-19^^England|Human|Lighthouse Lab in Glasgow|Wellcome Sanger Institute for the COVID-19 Genomics UK (COG-UK) Consortium|Davies|United Kingdom
>Spike|hCoV-19/Scotland/CVR5335/2020|2020-10-24|EPI_ISL_651766|Original|hCoV-19^^Scotland|Human|West of Scotland Specialist Virology Centre|COVID-19 Genomics UK (COG-UK) Consortium|Robertson|United Kingdom
>Spike|hCoV-19/Singapore/436/2020|2020-04-09|EPI_ISL_475967|Original|hCoV-19^^Singapore|Human|National Public Health Laboratory|National Public Health Laboratory|Mak|Singapore
>Spike|hCoV-19/India/GJ-GBRC343a/2020|2020-06-18|EPI_ISL_514584|Original|hCoV-19^^Gujarat|Human|B.J. Medical College and Civil hospital|Gujarat Biotechnology Research Centre|Joshi|India
>Spike|hCoV-19/England/CAMB-7B9AB/2020|2020-04-05|EPI_ISL_441612|Original|hCoV-19^^England|Human|Department of Pathology|Wellcome Sanger Institute for the COVID-19 Genomics UK (COG-UK) consortium|Amato|United Kingdom
>Spike|hCoV-19/Australia/VIC17790/2020|2020-07-27|EPI_ISL_779454|Original|hCoV-19^^Victoria|Human|Microbiological Diagnostic Unit - Public Health Laboratory (MDU-PHL)|MDU-PHL|Beck|Australia
>Spike|hCoV-19/USA/CO-CDPHE-2100010210/2020|2020-11-19|EPI_ISL_771293|Original|hCoV-19^^Colorado|Human|Colorado Department of Public Health and Environment|Colorado Department of Puplic Health and Environment|Bankers|USA
>Spike|hCoV-19/Denmark/DCGC-22428/2020|2020-12-14|EPI_ISL_757411|Original|hCoV-19^^Hovedstaden|Human|Department of Virus and Microbiological Special Diagnostics|Albertsen Lab|Michaelsen|Denmark
>Spike|hCoV-19/England/MILK-B024E9/2020|2020-10-23|EPI_ISL_649555|Original|hCoV-19^^England|Human|Lighthouse Lab in Milton Keynes|Wellcome Sanger Institute for the COVID-19 Genomics UK (COG-UK) Consortium|Davies|United Kingdom
>Spike|hCoV-19/England/ALDP-A6EA80/2020|2020-10-15|EPI_ISL_607825|Original|hCoV-19^^England|Human|Lighthouse Lab in Alderley Park|Wellcome Sanger Institute for the COVID-19 Genomics UK (COG-UK) consortium|Davies|United Kingdom
>Spike|hCoV-19/England/CAMB-7C825/2020|2020-04-05|EPI_ISL_442247|Original|hCoV-19^^England|Human|Department of Pathology|Wellcome Sanger Institute for the COVID-19 Genomics UK (COG-UK) consortium|Amato|United Kingdom
>Spike|hCoV-19/Netherlands/Friesland_14/2020|2020-03-31|EPI_ISL_455117|Original|hCoV-19^^Friesland|Human|Dutch COVID-19 response team|Erasmus Medical Center|Oude Munnink|Netherlands
>Spike|hCoV-19/Denmark/DCGC-19677/2020|2020-12-07|EPI_ISL_748266|Original|hCoV-19^^Hovedstaden|Human|Department of Virus and Microbiological Special Diagnostics|Albertsen Lab|Michaelsen|Denmark

>Spike|hCoV-19/USA/MI-MDHHS-SC22523/2020|2020-11-17|EPI_ISL_708226|Original|hCoV-19^^Michigan|Human|Michigan Department of Health and Human Services|Michigan Department of Health and Human Services|Jacob|USA
>Spike|hCoV-19/USA/CA-QDX-1053/2020|2020-03-17|EPI_ISL_571713|Original|hCoV-19^^California|Human|Quest Diagnostics|Quest Diagnostics|Kagan|USA
>Spike|hCoV-19/Denmark/DCGC-15154/2020|2020-11-23|EPI_ISL_713145|Original|hCoV-19^^Hovedstaden|Human|Department of Virus and Microbiological Special Diagnostics|Albertsen Lab|Michaelsen|Denmark
>Spike|hCoV-19/England/ALDP-A702A1/2020|2020-10-15|EPI_ISL_607898|Original|hCoV-19^^England|Human|Lighthouse Lab in Alderley Park|Wellcome Sanger Institute for the COVID-19 Genomics UK (COG-UK) consortium|Davies|United Kingdom
>Spike|hCoV-19/England/CAMC-BD2BED/2020|2020-11-27|EPI_ISL_702355|Original|hCoV-19^^England|Human|Lighthouse Lab in Cambridge|Wellcome Sanger Institute for the COVID-19 Genomics UK (COG-UK) Consortium|Davies|United Kingdom
>Spike|hCoV-19/USA/TX-HMH-MCoV-19592/2020|2020-11-16|EPI_ISL_783963|Original|hCoV-19^^Texas|Human|Houston Methodist Hospital|Houston Methodist Hospital|Long|USA
>Spike|hCoV-19/Brazil/SP-L8-CD88/2020|2020-03-23|EPI_ISL_476465|Original|hCoV-19^^S\u00e3o Paulo|Human|Hospital da Cl\u00ednicas da Faculdade de Medicina da Universidade de S\u00e3o Paulo|Instituto de Medicina Tropical da Univesidade de S\u00e3o Paulo|Sabino|Brazil
>Spike|hCoV-19/India/TG-CDFD-C1399/2020|2020-06-08|EPI_ISL_528561|Original|hCoV-19^^Telangana|Human|National Genomics Core-Center for DNA Fingerprinting and Diagnostics|National Genomics Core- Center for DNA Fingerprinting and Diagnostics (NGC-CDFD)- DBT's PAN-INDIA-1000 Genome consortium|Vashisht|India
>Spike|hCoV-19/Wales/PHWC-4A5E4B/2020|2020-12-14|EPI_ISL_764978|Original|hCoV-19^^Wales|Human|Wales Specialist Virology Centre Sequencing lab|COVID-19 Genomics UK (COG-UK) Consortium|Moore|United Kingdom
>Spike|hCoV-19/USA/MI-MDHHS-SC22052/2020|2020-09-18|EPI_ISL_583509|Original|hCoV-19^^Michigan|Human|Michigan Department of Health and Human Services|Michigan Department of Health and Human Services|Blankenship|USA
>Spike|hCoV-19/USA/CA-ALSR-0661-IPL/2020|2020-04-18|EPI_ISL_483286|Original|hCoV-19^^California|Human|UC San Diego Center for Advanced Laboratory Medicine|Andersen lab at Scripps Research|Gangavarapu|USA
>Spike|hCoV-19/England/QEUH-B68311/2020|2020-11-11|EPI_ISL_661450|Original|hCoV-19^^England|Human|Lighthouse Lab in Glasgow|Wellcome Sanger Institute for the COVID-19 Genomics UK (COG-UK) Consortium|Davies|United Kingdom
>Spike|hCoV-19/Canada/ON-PHL-20-01232/2020|2020-06-00|EPI_ISL_548854|Original|hCoV-19^^Ontario|Human|Public Health Ontario Laboratory|Public Health Ontario Laboratory|Guthrie|Canada
>Spike|hCoV-19/Northern Ireland/NIRE-108896/2020|2020-09-11|EPI_ISL_585211|Original|hCoV-19^^Northern Ireland|Human|Regional Virus Laboratory|COVID-19 Genomics UK (COG-UK) Consortium|Fairley|United Kingdom
>Spike|hCoV-19/Denmark/DCGC-15305/2020|2020-11-23|EPI_ISL_713218|Original|hCoV-19^^Denmark|Human|Department of Virus and Microbiological Special Diagnostics|Albertsen Lab|Michaelsen|Denmark
>Spike|hCoV-19/Russia/MOS-CRIE-13642350/2020|2020-11-09|EPI_ISL_745254|Original|hCoV-19^^Moscow|Human|Molecular diagnostic laboratory of Federal Budget Institution of Science \Central Research Institute of Epidemiology\ of The Federal Service on Customers' Rights Protection and Human Well-being Surveillance|Group of Genomics and Postgenomic Technologies of Central Research Institute of Epidemilolog
>y|Korneenko|Russia
>Spike|hCoV-19/England/CAMB-720FA/2020|2020-03-28|EPI_ISL_439546|Original|hCoV-19^^England|Human|Department of Pathology|Wellcome Sanger Institute for the COVID-19 Genomics UK (COG-UK) consortium|Amato|United Kingdom
>Spike|hCoV-19/Brazil/SP-L38-CD1014/2020|2020-05-10|EPI_ISL_721995|Original|hCoV-19^^S\u00e3o Paulo|Human|Hospital das Cl\u00ednicas Universidade de S\u00e3o Paulo Medical School|Laborat\u00f3rio de Parasitologia M\u00e9dica - Instituto de Medicina Tropical - Universidade de S\u00e3o Paulo|de Jesus|Brazil

>Spike|hCoV-19/Wales/ALDP-94D2BD/2020|2020-06-15|EPI_ISL_556495|Original|hCoV-19^^Wales|Human|Lighthouse Lab in Alderley Park|Wellcome Sanger Institute for the COVID-19 Genomics UK (COG-UK) consortium|Davies|United Kingdom
>Spike|hCoV-19/India/GJ-GBRC205a/2020|2020-06-11|EPI_ISL_475030|Original|hCoV-19^^Gujarat|Human|Department of MicroBiology|Gujarat Biotechnology Research Centre|Joshi|India
>Spike|hCoV-19/Scotland/CVR3131/2020|2020-04-25|EPI_ISL_439043|Original|hCoV-19^^Scotland|Human|West of Scotland Specialist Virology Centre|COVID-19 Genomics UK (COG-UK) Consortium|Robertson|United Kingdom
>Spike|hCoV-19/England/ALDP-A6F64F/2020|2020-10-15|EPI_ISL_607797|Original|hCoV-19^^England|Human|Lighthouse Lab in Alderley Park|Wellcome Sanger Institute for the COVID-19 Genomics UK (COG-UK) consortium|Davies|United Kingdom
>Spike|hCoV-19/England/ALDP-666266/2020|2020-07-03|EPI_ISL_555625|Original|hCoV-19^^England|Human|Lighthouse Lab in Alderley Park|Wellcome Sanger Institute for the COVID-19 Genomics UK (COG-UK) consortium|Davies|United Kingdom
>Spike|hCoV-19/Chile/RM-192950/2020|2020-09-28|EPI_ISL_746752|Original|hCoV-19^^Region Metropolitana de Santiago|Human|Genetica Molecular and Subdepartamento de Virologia ISP Chile|Instituto de Salud Publica de Chile|Tognarelli|Chile
>Spike|hCoV-19/USA/CA-ALSR-1106-SAN/2020|2020-06-14|EPI_ISL_483530|Original|hCoV-19^^California|Human|UC San Diego Center for Advanced Laboratory Medicine|Andersen lab at Scripps Research|Gangavarapu|USA
>Spike|hCoV-19/Australia/VIC10542/2020|2020-08-03|EPI_ISL_562953|Original|hCoV-19^^Victoria|Human|Microbiological Diagnostic Unit - Public Health Laboratory (MDU-PHL)|MDU-PHL|Schultz|Australia
>Spike|hCoV-19/Switzerland/BE-ETHZ-280239/2020|2020-09-10|EPI_ISL_560450|Original|hCoV-19^^Bern|Human|Viollier AG|Department of Biosystems Science and Engineering|Nadeau|Switzerland
>Spike|hCoV-19/Japan/PG-4758/2020|2020-08-00|EPI_ISL_689894|Original|hCoV-19^^Japan|Human|Pathogen Genomics Center|Pathogen Genomics Center|Sekizuka|Japan
>Spike|hCoV-19/Switzerland/BL-ETHZ-230047/2020|2020-08-04|EPI_ISL_516586|Original|hCoV-19^^Basel-Landschaft|Human|Viollier AG|Department of Biosystems Science and Engineering|Nadeau|Switzerland
>Spike|hCoV-19/USA/MI-QDX-2178/2020|2020-03-17|EPI_ISL_604474|Original|hCoV-19^^Michigan|Human|Quest Diagnostics|Quest Diagnostics|Kagan|USA
>Spike|hCoV-19/Hong Kong/VM20066578/2020|2020-07-21|EPI_ISL_602589|Original|hCoV-19^^Hong Kong|Human|Queen Elizabeth Hospital|Hong Kong Department of Health|Mak|China
>Spike|hCoV-19/France/HDF-6466/2020|2020-04-02|EPI_ISL_443315|Original|hCoV-19^^Hauts-de-France|Human|Ch\u00e2teau de la Source|National Reference Center for Viruses of Respiratory Infections|Enouf|France
>Spike|hCoV-19/Italy/MAR-UnivPM-71205/2020|2020-02-26|EPI_ISL_516083|Original|hCoV-19^^Marche|Human|Biomedical Sciences and Public Health|Biomedical Sciences and Public Health|Bagnarelli|Italy
>Spike|hCoV-19/India/TG-CCMB-L302-P1/2020|2020-04-20|EPI_ISL_458076|Vero P1|hCoV-19^^Telangana|Human|CSIR-Centre for Cellular and Molecular Biology|CSIR-Centre for Cellular and Molecular Biology|Banu|India
>Spike|hCoV-19/USA/TX-HMH-6630/2020|2020-06-24|EPI_ISL_546132|Original|hCoV-19^^Texas|Human|Houston Methodist Hospital|Houston Methodist Hospital|Long|USA
>Spike|hCoV-19/Denmark/DCGC-7234/2020|2020-10-12|EPI_ISL_618959|Original|hCoV-19^^Sjaelland|Human|Department of Virus and Microbiological Special Diagnostics|Albertsen lab|Michaelsen|Denmark
>Spike|hCoV-19/England/ALDP-A6C536/2020|2020-10-15|EPI_ISL_608142|Original|hCoV-19^^England|Human|Lighthouse Lab in Alderley Park|Wellcome Sanger Institute for the COVID-19 Genomics UK (COG-UK) consortium|Davies|United Kingdom
>Spike|hCoV-19/Senegal/5785/2020|2020-04-17|EPI_ISL_481239|Original|hCoV-19^^Dakar|Human|Institut Pasteur Dakar|Institut Pasteur de Dakar|Dia|Senegal
>Spike|hCoV-19/USA/TX-HMH-7577/2020|2020-06-25|EPI_ISL_546865|Original|hCoV-19^^Texas|Human|Houston Methodist Hospital|Houston Methodist Hospital|Long|USA
>Spike|hCoV-19/Wales/PHWC-166799/2020|2020-06-23|EPI_ISL_494038|Original|hCoV-19^^Wales|Human|Originating lab|COVID-19 Genomics UK (COG-UK) Consortium|Moore|United Kingdom

>Spike|hCoV-19/USA/FL-BPHL-0458/2020|2020-03-24|EPI_ISL_495364|Original|hCoV-19^^Florida|Human|Florida Bureau of Public Health Laboratories|Florida Bureau of Public Health Laboratories|Blanton|USA
>Spike|hCoV-19/Scotland/CVR4866/2020|2020-10-05|EPI_ISL_651330|Original|hCoV-19^^Scotland|Human|West of Scotland Specialist Virology Centre|COVID-19 Genomics UK (COG-UK) Consortium|Robertson|United Kingdom
>Spike|hCoV-19/Switzerland/BL-ETHZ-410126/2020|2020-12-17|EPI_ISL_737498|Original|hCoV-19^^Basel-Landschaft|Human|Viollier AG|Department of Biosystems Science and Engineering|Nadeau|Switzerland
>Spike|hCoV-19/USA/MI-MDHHS-SC21938/2020|2020-09-26|EPI_ISL_576204|Original|hCoV-19^^Michigan|Human|Michigan Department of Health and Human Services|Michigan Department of Health and Human Services|Blankenship|USA
>Spike|hCoV-19/Gibraltar/205121248/2020|2020-00-00|EPI_ISL_766370|Original|hCoV-19^^Gibraltar|Human|Microbiology|Respiratory Virus Unit|Platt|Gibraltar
>Spike|hCoV-19/USA/TX-HMH-5157/2020|2020-06-21|EPI_ISL_544566|Original|hCoV-19^^Texas|Human|Houston Methodist Hospital|Houston Methodist Hospital|Long|USA
>Spike|hCoV-19/England/ALDP-953797/2020|2020-06-08|EPI_ISL_554497|Original|hCoV-19^^England|Human|Lighthouse Lab in Alderley Park|Wellcome Sanger Institute for the COVID-19 Genomics UK (COG-UK) Consortium|Davies|United Kingdom
>Spike|hCoV-19/Belgium/DBOM-030566/2020|2020-03-05|EPI_ISL_420322|Original|hCoV-19^^Elsene|Human|KU Leuven|KU Leuven|Maes|Belgium
>Spike|hCoV-19/USA/NY-CDC-8449/2020|2020-03-19|EPI_ISL_594457|original|hCoV-19^^New York|Human|NYS Department of Health|Pathogen Discovery|Queen|USA
>Spike|hCoV-19/Wales/PHWC-2B6FE/2020|2020-04-12|EPI_ISL_445750|Original|hCoV-19^^Wales|Human|Wales Specialist Virology Centre|Public Health Wales Microbiology Cardiff|Moore|United Kingdom
>Spike|hCoV-19/England/MILK-BAC180/2020|2020-11-18|EPI_ISL_783167|Original|hCoV-19^^England|Human|Lighthouse Lab in Milton Keynes|Wellcome Sanger Institute for the COVID-19 Genomics UK (COG-UK) Consortium|Davies|United Kingdom
>Spike|hCoV-19/England/ALDP-B75D13/2020|2020-11-12|EPI_ISL_723698|Original|hCoV-19^^England|Human|Oxford Viromics|COVID-19 Genomics UK (COG-UK) Consortium|Golubchik|United Kingdom
>Spike|hCoV-19/USA/TX-HMH-MCoV-16286/2020|2020-10-26|EPI_ISL_785970|Original|hCoV-19^^Texas|Human|Houston Methodist Hospital|Houston Methodist Hospital|Long|USA
>Spike|hCoV-19/USA/TX-HMH-MCoV-13884/2020|2020-08-17|EPI_ISL_787176|Original|hCoV-19^^Texas|Human|Houston Methodist Hospital|Houston Methodist Hospital|Long|USA
>Spike|hCoV-19/South Africa/UFS-VIRO-NGS-8/2020|2020-10-18|EPI_ISL_682351|Original|hCoV-19^^Free State|Human|NHLS Universitas Academic|UFS Virology|Bester|South Africa
>Spike|hCoV-19/Denmark/DCGC-5671/2020|2020-09-28|EPI_ISL_619958|Original|hCoV-19^^Hovedstaden|Human|Department of Virus and Microbiological Special Diagnostics|Albertsen lab|Michaelsen|Denmark
>Spike|hCoV-19/Japan/PG-8744/2020|2020-09-00|EPI_ISL_686340|Original|hCoV-19^^Japan|Human|Pathogen Genomics Center|Pathogen Genomics Center|Sekizuka|Japan
>Spike|hCoV-19/DRC/7471/2020|2020-05-14|EPI_ISL_513596|Original|hCoV-19^^Kinshasa|Human|Viral Respiratory Lab|Pathogen Sequencing Lab|Lokilo|Democratic Republic of the Congo
>Spike|hCoV-19/England/QEUH-AE4985/2020|2020-10-27|EPI_ISL_633306|Original|hCoV-19^^England|Human|Lighthouse Lab in Glasgow|Wellcome Sanger Institute for the COVID-19 Genomics UK (COG-UK) consortium|Davies|United Kingdom
>Spike|hCoV-19/United Arab Emirates/skmc-358799/2020|2020-04-29|EPI_ISL_582640|Original|hCoV-19^^United Arab Emirates|Human|Sheikh Khalifa Medical City|Molecular/Surveillance lab\nSheikh Khalifa Medical City|Amirtharaj|United Arab Emirates
>Spike|hCoV-19/Germany/BY-MVP-0073/2020|2020-03-28|EPI_ISL_437269|Original|hCoV-19^^Bavaria|Human|Max von Pettenkofer Institute|Laboratory for Functional Genome Analysis|Graf|Germany
>Spike|hCoV-19/Denmark/DCGC-5531/2020|2020-09-28|EPI_ISL_620856|Original|hCoV-19^^Hovedstaden|Human|Department of Virus and Microbiological Special Diagnostics|Albertsen lab|Michaelsen|Denmark

>Spike|hCoV-19/Taiwan/TSGH-11/2020|2020-03-25|EPI_ISL_427398|Original|hCoV-19^^New Taipei City|Human|TSGH-CP molecular lab|TSGH-CP molecular lab|Shang|Taiwan
>Spike|hCoV-19/USA/TX-HMH-2605/2020|2020-06-05|EPI_ISL_545421|Original|hCoV-19^^Texas|Human|Houston Methodist Hospital|Houston Methodist Hospital|Long|USA
>Spike|hCoV-19/Wales/PHWC-49C7E2/2020|2020-11-18|EPI_ISL_742605|Original|hCoV-19^^Wales|Human|Wales Specialist Virology Centre Sequencing lab|COVID-19 Genomics UK (COG-UK) Consortium|Moore|United Kingdom
>Spike|hCoV-19/Denmark/DCGC-2411/2020|2020-08-10|EPI_ISL_617593|Original|hCoV-19^^Midtjylland|Human|Department of Virus and Microbiological Special Diagnostics|Albertsen lab|Michaelsen|Denmark
>Spike|hCoV-19/USA/CA-ALSR-2640/2020|2020-07-11|EPI_ISL_635605|Original|hCoV-19^^California|Human|San Diego County Public Health Laboratory|Andersen lab at Scripps Research|Gangavarapu|USA
>Spike|hCoV-19/Norway/2992/2020|2020-07-31|EPI_ISL_549117|Original|hCoV-19^^Oslo|Human|Ostfold Hospital Trust - Kalnes|Norwegian Institute of Public Health|Kopperud Riis|Norway
>Spike|hCoV-19/Latvia/139/2020|2020-09-04|EPI_ISL_639636|Original|hCoV-19^^Latvia|Human|Centr\u0101l\u0101 Laboratorija|Latvian Biomedical Research and Study Centre|Zrelovs|Latvia
>Spike|hCoV-19/Denmark/DCGC-15406/2020|2020-11-23|EPI_ISL_713807|Original|hCoV-19^^Sjaelland|Human|Department of Virus and Microbiological Special Diagnostics|Albertsen Lab|Michaelsen|Denmark
>Spike|hCoV-19/Australia/VIC51/2020|2020-03-12|EPI_ISL_419826|Original|hCoV-19^^Victoria|Human|Victorian Infectious Diseases Reference Laboratory (VIDRL)|Victorian Infectious Diseases Reference Laboratory and Microbiological Diagnostic Unit Public Health Laboratory|Schultz|Australia
>Spike|hCoV-19/England/MILK-BB221B/2020|2020-11-18|EPI_ISL_720336|Original|hCoV-19^^England|Human|Lighthouse Lab in Milton Keynes|Wellcome Sanger Institute for the COVID-19 Genomics UK (COG-UK) Consortium|Davies|United Kingdom
>Spike|hCoV-19/England/MILK-9F2035/2020|2020-09-24|EPI_ISL_589732|Original|hCoV-19^^England|Human|Lighthouse Lab in Milton Keynes|Wellcome Sanger Institute for the COVID-19 Genomics UK (COG-UK) consortium|Davies|United Kingdom
>Spike|hCoV-19/Finland/S5E7/2020|2020-09-10|EPI_ISL_737066|Original|hCoV-19^^Uusimaa|Human|Department of Virology and Immunology|Department of Virology|Smura|Finland
>Spike|hCoV-19/Luxembourg/LNS6454473/2020|2020-09-02|EPI_ISL_744418|Original|hCoV-19^^Luxembourg|Human|Laboratoire national de sant\u00e9|Laboratoire national de sant\u00e9|Wienecke-Baldacchino|Luxembourg
>Spike|hCoV-19/England/ALDP-94AEBC/2020|2020-06-11|EPI_ISL_556480|Original|hCoV-19^^England|Human|Lighthouse Lab in Alderley Park|Wellcome Sanger Institute for the COVID-19 Genomics UK (COG-UK) consortium|Davies|United Kingdom
>Spike|hCoV-19/England/ALDP-AA3418/2020|2020-10-22|EPI_ISL_625111|Original|hCoV-19^^England|Human|Lighthouse Lab in Alderley Park|Wellcome Sanger Institute for the COVID-19 Genomics UK (COG-UK) consortium|Davies|United Kingdom
>Spike|hCoV-19/USA/NY-NYUMC301/2020|2020-03-30|EPI_ISL_430328|Original|hCoV-19^^New York|Human|NYU Langone Health|Departments of Pathology and Medicine|Maurano|USA
>Spike|hCoV-19/Denmark/DCGC-13216/2020|2020-11-30|EPI_ISL_712151|Original|hCoV-19^^Hovedstaden|Human|Department of Virus and Microbiological Special Diagnostics|Albertsen Lab|Michaelsen|Denmark
>Spike|hCoV-19/Wales/PHWC-4A41BC/2020|2020-12-16|EPI_ISL_764662|Original|hCoV-19^^Wales|Human|Wales Specialist Virology Centre Sequencing lab|COVID-19 Genomics UK (COG-UK) Consortium|Moore|United Kingdom
>Spike|hCoV-19/Denmark/DCGC-10356/2020|2020-11-02|EPI_ISL_669467|Original|hCoV-19^^Syddanmark|Human|Department of Virus and Microbiological Special Diagnostics|Albertsen Lab|Michaelsen|Denmark
>Spike|hCoV-19/England/MILK-A7A720/2020|2020-10-15|EPI_ISL_606831|Original|hCoV-19^^England|Human|Lighthouse Lab in Milton Keynes|Wellcome Sanger Institute for the COVID-19 Genomics UK (COG-UK) consortium|Davies|United Kingdom

>Spike|hCoV-19/England/SHEF-C5605/2020|2020-10-14|EPI_ISL_613367|Original|hCoV-19^^England|Human|Virology Department|COVID-19 Genomics UK (COG-UK) Consortium|de Silva|United Kingdom
>Spike|hCoV-19/USA/WI-UW-2043/2020|2020-10-05|EPI_ISL_677449|Original|hCoV-19^^Wisconsin|Human|University of Wisconsin-Madison AIDS Vaccine Research Laboratories|University of Wisconsin-Madison AIDS Vaccine Research Laboratories|Baczenas|USA
>Spike|hCoV-19/England/QEUH-9D07CF/2020|2020-09-16|EPI_ISL_590005|Original|hCoV-19^^England|Human|Lighthouse Lab in Glasgow|Wellcome Sanger Institute for the COVID-19 Genomics UK (COG-UK) consortium|Davies|United Kingdom
>Spike|hCoV-19/England/ALDP-A51BC9/2020|2020-10-07|EPI_ISL_598027|Original|hCoV-19^^England|Human|Lighthouse Lab in Alderley Park|Wellcome Sanger Institute for the COVID-19 Genomics UK (COG-UK) consortium|Davies|United Kingdom
>Spike|hCoV-19/USA/CA-ALSR-1760/2020|2020-05-13|EPI_ISL_512169|Original|hCoV-19^^California|Human|San Diego County Public Health Laboratory|Andersen lab at Scripps Research|Gangavarapu|USA
>Spike|hCoV-19/England/BIRM-64732/2020|2020-09-23|EPI_ISL_594511|Original|hCoV-19^^England|Human|University of Birmingham|COVID-19 Genomics UK (COG-UK) Consortium|Loman|United Kingdom
>Spike|hCoV-19/England/CAMC-BBDE76/2020|2020-11-22|EPI_ISL_702518|Original|hCoV-19^^England|Human|Virology Department|COVID-19 Genomics UK (COG-UK) Consortium|de Silva|United Kingdom
>Spike|hCoV-19/Australia/VIC8425/2020|2020-07-29|EPI_ISL_565003|Original|hCoV-19^^Victoria|Human|Microbiological Diagnostic Unit - Public Health Laboratory (MDU-PHL)|MDU-PHL|Schultz|Australia
>Spike|hCoV-19/Japan/PG-0256/2020|2020-03-21|EPI_ISL_479941|Original|hCoV-19^^Kanto|Human|Ibaraki Prefectural Institute of Public Health|Pathogen Genomics Center|Sekizuka|Japan
>Spike|hCoV-19/England/MILK-978547/2020|2020-07-30|EPI_ISL_526416|Original|hCoV-19^^England|Human|Queens Medical Centre|COVID-19 Genomics UK (COG-UK) Consortium|Loose|United Kingdom
>Spike|hCoV-19/Wales/PHWC-29F13/2020|2020-04-01|EPI_ISL_432199|Original|hCoV-19^^Wales|Human|Wales Specialist Virology Centre|Public Health Wales Microbiology Cardiff|Moore|United Kingdom
>Spike|hCoV-19/Wales/PHWC-24A62/2020|2020-03-20|EPI_ISL_419474|Original|hCoV-19^^Wales|Human|Wales Specialist Virology Centre|Public Health Wales Microbiology Cardiff|Moore|United Kingdom
>Spike|hCoV-19/Japan/PG-6475/2020|2020-08-00|EPI_ISL_689580|Original|hCoV-19^^Japan|Human|Pathogen Genomics Center|Pathogen Genomics Center|Sekizuka|Japan
>Spike|hCoV-19/Spain/VC-IBV-98003455/2020|2020-04-03|EPI_ISL_539261|Original|hCoV-19^^Comunitat Valenciana|Human|Hospital Universitario de La Ribera (Alzira|SeqCOVID-SPAIN consortium/IBV(CSIC)|Comas|Spain
>Spike|hCoV-19/USA/WA-UW-2072/2020|2020-03-24|EPI_ISL_426079|Original|hCoV-19^^Washington|Human|UW Virology Lab|UW Virology Lab|Roychoudhury|USA
>Spike|hCoV-19/England/CAMC-B7886A/2020|2020-11-12|EPI_ISL_783524|Original|hCoV-19^^England|Human|Lighthouse Lab in Cambridge|Wellcome Sanger Institute for the COVID-19 Genomics UK (COG-UK) Consortium|Davies|United Kingdom
>Spike|hCoV-19/England/LIVE-9A153/2020|2020-04-17|EPI_ISL_439852|Original|hCoV-19^^England|Human|Liverpool Clinical Laboratories|COVID-19 Genomics UK (COG-UK) Consortium|Haldenby|United Kingdom
>Spike|hCoV-19/England/ALDP-954BE5/2020|2020-06-12|EPI_ISL_553623|Original|hCoV-19^^England|Human|Lighthouse Lab in Alderley Park|Wellcome Sanger Institute for the COVID-19 Genomics UK (COG-UK) consortium|Davies|United Kingdom
>Spike|hCoV-19/England/20130054802/2020|2020-03-19|EPI_ISL_420632|Original|hCoV-19^^England|Human|Respiratory Virus Unit|Respiratory Virus Unit|Platt|United Kingdom
>Spike|hCoV-19/England/CAMB-7A56C/2020|2020-04-02|EPI_ISL_441251|Original|hCoV-19^^England|Human|Department of Pathology|Wellcome Sanger Institute for the COVID-19 Genomics UK (COG-UK) consortium|Amato|United Kingdom

>Spike|hCoV-19/Iceland/603/2020|2020-03-28|EPI_ISL_424623|Original|hCoV-19^^Reykjavik|Human|The National University Hospital of Iceland|deCODE genetics|J\u00f3nsson|Iceland
>Spike|hCoV-19/England/LIVE-A3DAE/2020|2020-05-19|EPI_ISL_500027|Original|hCoV-19^^England|Human|Liverpool Clinical Laboratories|COVID-19 Genomics UK (COG-UK) Consortium|Haldenby|United Kingdom
>Spike|hCoV-19/Wales/PHWC-36CFE/2020|2020-05-03|EPI_ISL_474792|Original|hCoV-19^^Wales|Human|Originating lab|COVID-19 Genomics UK (COG-UK) Consortium|Moore|United Kingdom
>Spike|hCoV-19/USA/TX-DSHS-1258/2020|2020-05-31|EPI_ISL_644199|Original|hCoV-19^^Texas|Human|Texas Department of State Health Services|Texas Department of State Health Services|Rahman|USA
>Spike|hCoV-19/England/NOTT-1139C2/2020|2020-09-20|EPI_ISL_572705|Original|hCoV-19^^England|Human|Lincolnshire Hospitals and DeepSeq Nottingham|COVID-19 Genomics UK (COG-UK) Consortium|Loose|United Kingdom
>Spike|hCoV-19/Germany/HH-hpi-p1818/2020|2020-03-22|EPI_ISL_776174|Original|hCoV-19^^Hamburg|Human|University Medical Center Hamburg Eppendorf|Heinrich Pette Institute|Robitaille|Germany
>Spike|hCoV-19/England/CAMC-BBC396/2020|2020-11-18|EPI_ISL_724129|Original|hCoV-19^^England|Human|Quadram Institute Bioscience|COVID-19 Genomics UK (COG-UK) Consortium|Alikhan|United Kingdom
>Spike|hCoV-19/Russia/KDA-RII-MH4875S/2020|2020-10-22|EPI_ISL_733279|Original|hCoV-19^^Krasnodar|Human|WHO National Influenza Centre Russian Federation|WHO National Influenza Centre Russian Federation|Komissarov|Russia
>Spike|hCoV-19/England/ALDP-9C502C/2020|2020-09-10|EPI_ISL_567659|Original|hCoV-19^^England|Human|Lighthouse Lab in Alderley Park|Wellcome Sanger Institute for the COVID-19 Genomics UK (COG-UK) consortium|Davies|United Kingdom
>Spike|hCoV-19/USA/FL-BPHL-1780/2020|2020-07-30|EPI_ISL_613883|Original|hCoV-19^^Florida|Human|Florida Bureau of Public Health Laboratories|Florida Bureau of Public Health Laboratories|Blanton|USA
>Spike|hCoV-19/USA/MA-MGH-01127/2020|2020-05-02|EPI_ISL_791885|Original|hCoV-19^^Massachusetts|Human|Massachusetts General Hospital|Infectious Disease Program|Park|USA
>Spike|hCoV-19/USA/WA-S709/2020|2020-04-09|EPI_ISL_449879|Original|hCoV-19^^Washington|Human|Washington State Department of Health|Seattle Flu Study|Bedford|USA
>Spike|hCoV-19/England/CAMC-A5848B/2020|2020-10-08|EPI_ISL_597017|Original|hCoV-19^^England|Human|Lighthouse Lab in Cambridge|Wellcome Sanger Institute for the COVID-19 Genomics UK (COG-UK) consortium|Davies|United Kingdom
>Spike|hCoV-19/Australia/NSW511/2020|2020-03-28|EPI_ISL_455051|Original|hCoV-19^^New South Wales|Human|Douglas Hanly Moir Pathology|NSW Health Pathology - Institute of Clinical Pathology and Medical Research; Westmead Hospital; University of Sydney|Gall|Australia
>Spike|hCoV-19/USA/CO-CDPHE-2009090330/2020|2020-09-08|EPI_ISL_677647|Original|hCoV-19^^Colorado|Human|Colorado Department of Public Health and Environment|Colorado Department of Puplic Health and Environment|Bankers|USA
>Spike|hCoV-19/England/20206138304/2020|2020-05-14|EPI_ISL_465662|Original|hCoV-19^^England|Human|Respiratory Virus Unit|Respiratory Virus Unit|Platt|United Kingdom
>Spike|hCoV-19/England/NOTT-10E9CA/2020|2020-03-30|EPI_ISL_425622|Original|hCoV-19^^England|Human|Queens Medical Centre|COVID-19 Genomics UK (COG-UK) Consortium|Loose|United Kingdom
>Spike|hCoV-19/Japan/PG-7132/2020|2020-08-00|EPI_ISL_688237|Original|hCoV-19^^Japan|Human|Pathogen Genomics Center|Pathogen Genomics Center|Sekizuka|Japan
>Spike|hCoV-19/USA/WI-WSLH-200451/2020|2020-05-19|EPI_ISL_578596|Original|hCoV-19^^Wisconsin|Human|Wisconsin State Laboratory of Hygiene Communicable Disease Division|Wisconsin State Laboratory of Hygiene Communicable Disease Division|Florek|USA
>Spike|hCoV-19/India/KA-NIV-QC-1801/2020|2020-09-00|EPI_ISL_577715|Original|hCoV-19^^Karnataka|Human|NIV Influenza|NIV Influenza|Potdar|India

>Spike|hCoV-19/USA/TX-HMH0182/2020|2020-03-23|EPI_ISL_434860|Original|hCoV-19^^Texas|Human|Houston Methodist Hospital|Houston Methodist Hospital|Long|USA
>Spike|hCoV-19/USA/WA-UW-5181/2020|2020-04-02|EPI_ISL_430871|Original|hCoV-19^^Washington|Human|UW Virology Lab|UW Virology Lab|Roychoudhury|USA
>Spike|hCoV-19/Italy/APU-PODV-15940334/2020|2020-08-25|EPI_ISL_788957|Original|hCoV-19^^Apulia|Human|Ospedale \Di Venere\|Beaconlab (Bioinformatics|Chiara|Italy
>Spike|hCoV-19/England/ALDP-A6CF29/2020|2020-10-14|EPI_ISL_608157|Original|hCoV-19^^England|Human|Lighthouse Lab in Alderley Park|Wellcome Sanger Institute for the COVID-19 Genomics UK (COG-UK) consortium|Davies|United Kingdom
>Spike|hCoV-19/England/MILK-9C253F/2020|2020-09-07|EPI_ISL_566658|Original|hCoV-19^^England|Human|Lighthouse Lab in Milton Keynes|Wellcome Sanger Institute for the COVID-19 Genomics UK (COG-UK) consortium|Davies|United Kingdom
>Spike|hCoV-19/England/CAMC-B22CCD/2020|2020-11-02|EPI_ISL_647394|Original|hCoV-19^^England|Human|Lighthouse Lab in Cambridge|Wellcome Sanger Institute for the COVID-19 Genomics UK (COG-UK) Consortium|Davies|United Kingdom
>Spike|hCoV-19/Brazil/SE-6567/2020|2020-04-24|EPI_ISL_541386|Original|hCoV-19^^Sergipe|Human|LACEN/SE|Laboratory of Respiratory Viruses and Measles|Silva|Brazil
>Spike|hCoV-19/Netherlands/GE-RIVM-20527/2020|2020-12-04|EPI_ISL_728742|Original|hCoV-19^^Gelderland|Human|Dutch COVID-19 response team|National Institute for Public Health and the Environment (RIVM)|Meijer|Netherlands
>Spike|hCoV-19/South Korea/KDCA0441/2020|2020-10-04|EPI_ISL_760225|Original|hCoV-19^^South Korea|Human|Division of Emerging Infectious Diseases|Division of Emerging Infectious Diseases|Park|South Korea
>Spike|hCoV-19/Denmark/DCGC-9543/2020|2020-11-09|EPI_ISL_669031|Original|hCoV-19^^Nordjylland|Human|Department of Virus and Microbiological Special Diagnostics|Albertsen Lab|Michaelsen|Denmark
>Spike|hCoV-19/USA/CA-CZB-12335/2020|2020-10-08|EPI_ISL_648412|Original|hCoV-19^^California|Human|Santa Clara County Public Health Laboratory|Chan-Zuckerberg Biohub|Bhatt|USA
>Spike|hCoV-19/Australia/VIC14439/2020|2020-08-19|EPI_ISL_592502|Original|hCoV-19^^Victoria|Human|Victorian Infectious Diseases Reference Laboratory (VIDRL)|VIDRL and MDU-PHL|Schultz|Australia
>Spike|hCoV-19/England/NORT-295A3D/2020|2020-04-03|EPI_ISL_488457|Original|hCoV-19^^England|Human|NU-OMICS DNA Sequencing research facility|Wellcome Sanger Institute for the COVID-19 Genomics UK (COG-UK) consortium|Davies|United Kingdom
>Spike|hCoV-19/Belgium/ULG-10474/2020|2020-09-22|EPI_ISL_581643|Original|hCoV-19^^Li\u00e8ge|Human|Department of Clinical Microbiology|GIGA Medical Genomics|Durkin|Belgium
>Spike|hCoV-19/England/CAMC-94275F/2020|2020-07-22|EPI_ISL_556903|Original|hCoV-19^^England|Human|Lighthouse Lab in Cambridge|Wellcome Sanger Institute for the COVID-19 Genomics UK (COG-UK) consortium|Davies|United Kingdom
>Spike|hCoV-19/Denmark/DCGC-20548/2020|2020-12-21|EPI_ISL_758859|Original|hCoV-19^^Sjaelland|Human|Department of Virus and Microbiological Special Diagnostics|Albertsen Lab|Michaelsen|Denmark
>Spike|hCoV-19/England/CAMC-B323C5/2020|2020-11-05|EPI_ISL_659385|Original|hCoV-19^^England|Human|Lighthouse Lab in Cambridge|Wellcome Sanger Institute for the COVID-19 Genomics UK (COG-UK) Consortium|Davies|United Kingdom
>Spike|hCoV-19/England/PORT-2D31A7/2020|2020-04-09|EPI_ISL_628312|Original|hCoV-19^^England|Human|Centre for Enzyme Innovation|COVID-19 Genomics UK (COG-UK) Consortium|Robson|United Kingdom
>Spike|hCoV-19/USA/VA-DCLS-1213/2020|2020-06-23|EPI_ISL_572282|Original|hCoV-19^^Virginia|Human|Virginia DCLS|Virginia DCLS|St. Jacques|USA
>Spike|hCoV-19/USA/CA-CDC-4710/2020|2020-06-04|EPI_ISL_535686|Original|hCoV-19^^California|Human|CDPH|Pathogen Discovery|Queen|USA
>Spike|hCoV-19/England/QEUH-9B0CD5/2020|2020-09-03|EPI_ISL_559860|Original|hCoV-19^^England|Human|Oxford Viromics|COVID-19 Genomics UK (COG-UK) Consortium|Golubchik|United Kingdom

>Spike|hCoV-19/Canada/ON-S83/2020|2020-03-20|EPI_ISL_591168|Original|hCoV-19^^Ontario|Human|Toronto Invasive Bacterial Diseases Network|McMaster University|McArthur|Canada
>Spike|hCoV-19/England/ALDP-BB3BD3/2020|2020-11-19|EPI_ISL_701976|Original|hCoV-19^^England|Human|Lighthouse Lab in Alderley Park|Wellcome Sanger Institute for the COVID-19 Genomics UK (COG-UK) Consortium|Davies|United Kingdom
>Spike|hCoV-19/England/CAMC-94B5E3/2020|2020-07-27|EPI_ISL_556309|Original|hCoV-19^^England|Human|Lighthouse Lab in Cambridge|Wellcome Sanger Institute for the COVID-19 Genomics UK (COG-UK) consortium|Davies|United Kingdom
>Spike|hCoV-19/England/ALDP-B2629A/2020|2020-11-04|EPI_ISL_646612|Original|hCoV-19^^England|Human|Lighthouse Lab in Alderley Park|Wellcome Sanger Institute for the COVID-19 Genomics UK (COG-UK) Consortium|Davies|United Kingdom
>Spike|hCoV-19/England/QEUH-AD294E/2020|2020-10-26|EPI_ISL_634130|Original|hCoV-19^^England|Human|Lighthouse Lab in Glasgow|Wellcome Sanger Institute for the COVID-19 Genomics UK (COG-UK) consortium|Davies|United Kingdom
>Spike|hCoV-19/Australia/VIC6485/2020|2020-07-21|EPI_ISL_521263|Original|hCoV-19^^Victoria|Human|Microbiological Diagnostic Unit - Public Health Laboratory (MDU-PHL)|MDU-PHL|Schultz|Australia
>Spike|hCoV-19/USA/WA-UW68/2020|2020-03-09|EPI_ISL_415596|Original|hCoV-19^^Washington|Human|UW Virology Lab|UW Virology Lab|Roychoudhury|USA
>Spike|hCoV-19/England/ALDP-BCA67E/2020|2020-11-23|EPI_ISL_703475|Original|hCoV-19^^England|Human|Lighthouse Lab in Alderley Park|Wellcome Sanger Institute for the COVID-19 Genomics UK (COG-UK) Consortium|Davies|United Kingdom
>Spike|hCoV-19/England/NORT-29C1C8/2020|2020-06-22|EPI_ISL_499598|Original|hCoV-19^^England|Human|Northumbria University / South Tees Hospitals NHS Foundation Trust / North Cumbria Integrated Care NHS Foundation Trust / North Tees and Hartlepool NHS Foundation Trust / Newcastle Hospitals NHS Foundation Trust|COVID-19 Genomics UK (COG-UK) Consortium|Bashton|United Kingdom
>Spike|hCoV-19/England/204881292/2020|2020-11-00|EPI_ISL_678814|Original|hCoV-19^^England|Human|Respiratory Virus Unit|COVID-19 Genomics UK (COG-UK) Consortium|Platt|United Kingdom
>Spike|hCoV-19/USA/UT-03744/2020|2020-04-05|EPI_ISL_509583|Original|hCoV-19^^Utah|Human|Utah Public Health Laboratory|Utah Public Health Laboratory|Butz|USA
>Spike|hCoV-19/Australia/VIC11441/2020|2020-08-22|EPI_ISL_563372|Original|hCoV-19^^Victoria|Human|Microbiological Diagnostic Unit - Public Health Laboratory (MDU-PHL)|MDU-PHL|Schultz|Australia
>Spike|hCoV-19/England/205080422/2020|2020-12-10|EPI_ISL_747499|Original|hCoV-19^^England|Human|Respiratory Virus Unit|COVID-19 Genomics UK (COG-UK) Consortium|Miah|United Kingdom
>Spike|hCoV-19/USA/UT-08415/2020|2020-05-12|EPI_ISL_524193|Original|hCoV-19^^Utah|Human|Utah Public Health Laboratory|Utah Public Health Laboratory|Young|USA
>Spike|hCoV-19/Denmark/ALAB-SSI195/2020|2020-03-09|EPI_ISL_429396|Original|hCoV-19^^Denmark|Human|Department of Virus and Microbiological Special Diagnostics|Albertsen lab|Kirkegaard|Denmark
>Spike|hCoV-19/Denmark/DCGC-5685/2020|2020-09-28|EPI_ISL_619972|Original|hCoV-19^^Hovedstaden|Human|Department of Virus and Microbiological Special Diagnostics|Albertsen lab|Michaelsen|Denmark
>Spike|hCoV-19/England/MILK-C2FBD1/2020|2020-12-07|EPI_ISL_733743|Original|hCoV-19^^England|Human|Lighthouse Lab in Milton Keynes|Wellcome Sanger Institute for the COVID-19 Genomics UK (COG-UK) Consortium|Davies|United Kingdom
>Spike|hCoV-19/Russia/SPE-RII-MH989S/2020|2020-09-03|EPI_ISL_596374|Original|hCoV-19^^Saint Petersburg|Human|HELIX LCC|WHO National Influenza Centre Russian Federation|Komissarov|Russia
>Spike|hCoV-19/Portugal/PT0609/2020|2020-03-14|EPI_ISL_454332|Original|hCoV-19^^Portugal|Human|unknown|Instituto Nacional de Saude (INSA)|Borges|Portugal
>Spike|hCoV-19/England/BRIS-12F240/2020|2020-04-13|EPI_ISL_488230|Original|hCoV-19^^England|Human|PHE South West Regional Laboratory|Wellcome Sanger Institute for the COVID-19 Genomics UK (COG-UK) consortium|Davies|United Kingdom
>Spike|hCoV-19/Austria/CeMM0977/2020|2020-04-01|EPI_ISL_583822|Original|hCoV-19^^Austria|Human|Dr. Gernot Walder GmbH|Bergthaler laboratory|Bergthaler|Austria

>Spike|hCoV-19/England/MILK-B8E597/2020|2020-11-13|EPI_ISL_673966|Original|hCoV-19^^England|Human|Lighthouse Lab in Milton Keynes|Wellcome Sanger Institute for the COVID-19 Genomics UK (COG-UK) Consortium|Davies|United Kingdom
>Spike|hCoV-19/Scotland/EDB2357/2020|2020-04-22|EPI_ISL_439187|Original|hCoV-19^^Scotland|Human|Virology Department|COVID-19 Genomics UK (COG-UK) Consortium|Williams|United Kingdom
>Spike|hCoV-19/England/NOTT-114536/2020|2020-10-08|EPI_ISL_577172|Original|hCoV-19^^England|Human|Queens Medical Centre|COVID-19 Genomics UK (COG-UK) Consortium|Loose|United Kingdom
>Spike|hCoV-19/Wales/PHWC-49BB32/2020|2020-12-06|EPI_ISL_742434|Original|hCoV-19^^Wales|Human|Wales Specialist Virology Centre Sequencing lab|COVID-19 Genomics UK (COG-UK) Consortium|Moore|United Kingdom
>Spike|hCoV-19/England/LOND-D4454/2020|2020-04-08|EPI_ISL_444123|Original|hCoV-19^^England|Human|University College London|COVID-19 Genomics UK (COG-UK) Consortium|Roy|United Kingdom
>Spike|hCoV-19/Wales/PHWC-49A41A/2020|2020-12-02|EPI_ISL_727543|Original|hCoV-19^^Wales|Human|Originating lab|COVID-19 Genomics UK (COG-UK) Consortium|Moore|United Kingdom
>Spike|hCoV-19/USA/TX-HMH-MCoV-10190/2020|2020-07-07|EPI_ISL_789363|Original|hCoV-19^^Texas|Human|Houston Methodist Hospital|Houston Methodist Hospital|Long|USA
>Spike|hCoV-19/England/QEUH-B2F307/2020|2020-11-03|EPI_ISL_659861|Original|hCoV-19^^England|Human|Lighthouse Lab in Glasgow|Wellcome Sanger Institute for the COVID-19 Genomics UK (COG-UK) Consortium|Davies|United Kingdom
>Spike|hCoV-19/France/OCC-65/2020|2020-08-02|EPI_ISL_593880|Original|hCoV-19^^Occitanie|Human|CHU Purpan - Laboratoire de Virologie - Institut F\u00e9d\u00e9ratif de Biologie|CHU Purpan - Laboratoire de Virologie - Institut F\u00e9d\u00e9ratif de Biologie|Latour|France
>Spike|hCoV-19/England/ALDP-9CAA74/2020|2020-09-16|EPI_ISL_575728|Original|hCoV-19^^England|Human|Lighthouse Lab in Alderley Park|Wellcome Sanger Institute for the COVID-19 Genomics UK (COG-UK) consortium|Davies|United Kingdom
>Spike|hCoV-19/Wales/PHWC-480DFE/2020|2020-10-16|EPI_ISL_613005|Original|hCoV-19^^Wales|Human|Wales Specialist Virology Centre Sequencing lab|COVID-19 Genomics UK (COG-UK) Consortium|Moore|United Kingdom
>Spike|hCoV-19/England/ALDP-B5BFD1/2020|2020-11-10|EPI_ISL_655852|Original|hCoV-19^^England|Human|Lighthouse Lab in Alderley Park|Wellcome Sanger Institute for the COVID-19 Genomics UK (COG-UK) Consortium|Davies|United Kingdom
>Spike|hCoV-19/England/QEUH-CDE403/2020|2020-12-26|EPI_ISL_782342|Original|hCoV-19^^England|Human|Lighthouse Lab in Glasgow|Wellcome Sanger Institute for the COVID-19 Genomics UK (COG-UK) Consortium|Davies|United Kingdom
>Spike|hCoV-19/England/ALDP-9ECEDA/2020|2020-05-04|EPI_ISL_589450|Original|hCoV-19^^England|Human|Lighthouse Lab in Alderley Park|Wellcome Sanger Institute for the COVID-19 Genomics UK (COG-UK) consortium|Davies|United Kingdom
>Spike|hCoV-19/England/201160647/2020|2020-03-12|EPI_ISL_464800|Original|hCoV-19^^England|Human|Respiratory Virus Unit|Respiratory Virus Unit|Platt|United Kingdom
>Spike|hCoV-19/Vietnam/HCMC-2712/2020|2020-03-20|EPI_ISL_498172|unknown|hCoV-19^^Ho Chi Minh City|Human|OUCRU|OUCRU|Nguyen|Vietnam
>Spike|hCoV-19/DRC/10373/2020|2020-05-22|EPI_ISL_527495|Original|hCoV-19^^Kinshasa|Human|Viral Respiratory Lab|Pathogen Sequencing Lab|Lokilo|Democratic Republic of the Congo
>Spike|hCoV-19/England/MILK-9C3598/2020|2020-09-10|EPI_ISL_566789|Original|hCoV-19^^England|Human|Lighthouse Lab in Milton Keynes|Wellcome Sanger Institute for the COVID-19 Genomics UK (COG-UK) consortium|Davies|United Kingdom
>Spike|hCoV-19/Wales/PHWC-167015/2020|2020-06-23|EPI_ISL_494141|Original|hCoV-19^^Wales|Human|Originating lab|COVID-19 Genomics UK (COG-UK) Consortium|Moore|United Kingdom
>Spike|hCoV-19/Portugal/PT0457/2020|2020-04-18|EPI_ISL_454181|Original|hCoV-19^^Portugal|Human|unknown|Instituto Nacional de Saude (INSA)|Borges|Portugal
>Spike|hCoV-19/Scotland/CVR4412/2020|2020-09-20|EPI_ISL_585286|Original|hCoV-19^^Scotland|Human|West of Scotland Specialist Virology Centre|COVID-19 Genomics UK (COG-UK) Consortium|Robertson|United Kingdom

>Spike|hCoV-19/Denmark/DCGC-1768/2020|2020-06-29|EPI_ISL_618177|Original|hCoV-19^^NA|Human|Department of Virus and Microbiological Special Diagnostics|Albertsen lab|Michaelsen|Denmark
>Spike|hCoV-19/England/QEUH-9E4E3C/2020|2020-09-22|EPI_ISL_579814|Original|hCoV-19^^England|Human|Lighthouse Lab in Glasgow|Wellcome Sanger Institute for the COVID-19 Genomics UK (COG-UK) consortium|Davies|United Kingdom
>Spike|hCoV-19/England/MILK-ABBB27/2020|2020-10-21|EPI_ISL_631073|Original|hCoV-19^^England|Human|Lighthouse Lab in Milton Keynes|Wellcome Sanger Institute for the COVID-19 Genomics UK (COG-UK) consortium|Davies|United Kingdom
>Spike|hCoV-19/Japan/PG-7401/2020|2020-07-00|EPI_ISL_688503|Original|hCoV-19^^Japan|Human|Pathogen Genomics Center|Pathogen Genomics Center|Sekizuka|Japan
>Spike|hCoV-19/Netherlands/NoordBrabant_111/2020|2020-03-11|EPI_ISL_461147|Original|hCoV-19^^Noord Brabant|Human|Dutch COVID-19 response team|Erasmus Medical Center|Oude Munnink|Netherlands
>Spike|hCoV-19/England/CAMB-7CD35/2020|2020-04-06|EPI_ISL_441638|Original|hCoV-19^^England|Human|Department of Pathology|Wellcome Sanger Institute for the COVID-19 Genomics UK (COG-UK) consortium|Amato|United Kingdom
>Spike|hCoV-19/England/MILK-484826/2020|2020-06-05|EPI_ISL_559681|Original|hCoV-19^^England|Human|Lighthouse Lab in Milton Keynes|Wellcome Sanger Institute for the COVID-19 Genomics UK (COG-UK) consortium|Davies|United Kingdom
>Spike|hCoV-19/USA/TX-HMH-MCoV-12756/2020|2020-08-29|EPI_ISL_790507|Original|hCoV-19^^Texas|Human|Houston Methodist Hospital|Houston Methodist Hospital|Long|USA
>Spike|hCoV-19/England/ALDP-B1BF28/2020|2020-11-01|EPI_ISL_645611|Original|hCoV-19^^England|Human|Lighthouse Lab in Alderley Park|Wellcome Sanger Institute for the COVID-19 Genomics UK (COG-UK) Consortium|Davies|United Kingdom
>Spike|hCoV-19/England/ALDP-AA4480/2020|2020-10-22|EPI_ISL_624272|Original|hCoV-19^^England|Human|Lighthouse Lab in Alderley Park|Wellcome Sanger Institute for the COVID-19 Genomics UK (COG-UK) consortium|Davies|United Kingdom
>Spike|hCoV-19/England/ALDP-B63B2D/2020|2020-11-08|EPI_ISL_656550|Original|hCoV-19^^England|Human|Lighthouse Lab in Alderley Park|Wellcome Sanger Institute for the COVID-19 Genomics UK (COG-UK) Consortium|Davies|United Kingdom
>Spike|hCoV-19/Japan/PG-3112/2020|2020-05-00|EPI_ISL_691234|Original|hCoV-19^^Japan|Human|Pathogen Genomics Center|Pathogen Genomics Center|Sekizuka|Japan
>Spike|hCoV-19/USA/WA-S1023/2020|2020-05-01|EPI_ISL_463513|Original|hCoV-19^^Washington|Human|Washington State Department of Health|Seattle Flu Study|Bedford|USA
>Spike|hCoV-19/England/MILK-A79985/2020|2020-10-14|EPI_ISL_606701|Original|hCoV-19^^England|Human|Lighthouse Lab in Milton Keynes|Wellcome Sanger Institute for the COVID-19 Genomics UK (COG-UK) consortium|Davies|United Kingdom
>Spike|hCoV-19/England/MILK-9EEC65/2020|2020-09-24|EPI_ISL_580793|Original|hCoV-19^^England|Human|Lighthouse Lab in Milton Keynes|Wellcome Sanger Institute for the COVID-19 Genomics UK (COG-UK) consortium|Davies|United Kingdom
>Spike|hCoV-19/USA/ME-HETL-J0040/2020|2020-03-15|EPI_ISL_513452|Original|hCoV-19^^Maine|Human|Maine HETL|Tewhey Lab|Tewhey|USA
>Spike|hCoV-19/England/ALDP-A86CB9/2020|2020-10-17|EPI_ISL_606204|Original|hCoV-19^^England|Human|Lighthouse Lab in Alderley Park|Wellcome Sanger Institute for the COVID-19 Genomics UK (COG-UK) consortium|Davies|United Kingdom
>Spike|hCoV-19/England/ALDP-9C5C91/2020|2020-09-10|EPI_ISL_567788|Original|hCoV-19^^England|Human|Lighthouse Lab in Alderley Park|Wellcome Sanger Institute for the COVID-19 Genomics UK (COG-UK) consortium|Davies|United Kingdom
>Spike|hCoV-19/India/MH-GA4/2020|2020-06-17|EPI_ISL_508234|Original|hCoV-19^^Maharashtra|Human|Government Medical College|National Institute of Biomedical Genomics|Maitra|India
>Spike|hCoV-19/USA/WI-WSLH-200320/2020|2020-04-20|EPI_ISL_578501|Original|hCoV-19^^Wisconsin|Human|Wisconsin State Laboratory of Hygiene Communicable Disease Division|Wisconsin State Laboratory of Hygiene Communicable Disease Division|Florek|USA
>Spike|hCoV-19/England/QEUH-B7FF04/2020|2020-11-14|EPI_ISL_661661|Original|hCoV-19^^England|Human|Lighthouse Lab in Glasgow|Wellcome Sanger Institute for the COVID-19 Genomics UK (COG-UK) Consortium|Davies|United Kingdom

>Spike|hCoV-19/USA/CT-Yale-494/2020|2020-10-16|EPI_ISL_729769|Original|hCoV-19^^Connecticut|Human|Yale Pathology Lab|Grubaugh Lab - Yale School of Public Health|Grubaugh|USA
>Spike|hCoV-19/USA/AR-CDC-4206/2020|2020-07-11|EPI_ISL_527633|Original|hCoV-19^^Arkansas|Human|AR Dept. of Health-Public Health Lab|Pathogen Discovery|Queen|USA
>Spike|hCoV-19/Portugal/PT0881/2020|2020-04-26|EPI_ISL_511140|Original|hCoV-19^^Portugal|Human|Instituto Nacional de Saude (INSA)|Instituto Nacional de Saude (INSA)|Borges|Portugal
>Spike|hCoV-19/USA/AZ-TG333784/2020|2020-05-27|EPI_ISL_694362|Original|hCoV-19^^Arizona|Human|TGen North|TGen North|French|USA
>Spike|hCoV-19/England/MILK-ACEB12/2020|2020-10-21|EPI_ISL_629603|Original|hCoV-19^^England|Human|Lighthouse Lab in Milton Keynes|Wellcome Sanger Institute for the COVID-19 Genomics UK (COG-UK) consortium|Davies|United Kingdom
>Spike|hCoV-19/England/ALDP-959D26/2020|2020-06-11|EPI_ISL_555111|Original|hCoV-19^^England|Human|Lighthouse Lab in Alderley Park|Wellcome Sanger Institute for the COVID-19 Genomics UK (COG-UK) consortium|Davies|United Kingdom
>Spike|hCoV-19/England/ALDP-C061A1/2020|2020-12-05|EPI_ISL_709513|Original|hCoV-19^^England|Human|Lighthouse Lab in Alderley Park|Wellcome Sanger Institute for the COVID-19 Genomics UK (COG-UK) Consortium|Davies|United Kingdom
>Spike|hCoV-19/USA/NY-NYCPHL-000608/2020|2020-03-11|EPI_ISL_632065|Original|hCoV-19^^New York|Human|Jamaica Hospital Medical Center|New York City Public Health Laboratory|Wang|USA
>Spike|hCoV-19/USA/TX-HMH-MCoV-15226/2020|2020-10-20|EPI_ISL_786710|Original|hCoV-19^^Texas|Human|Houston Methodist Hospital|Houston Methodist Hospital|Long|USA
>Spike|hCoV-19/England/MILK-957E8F/2020|2020-06-13|EPI_ISL_554748|Original|hCoV-19^^England|Human|Lighthouse Lab in Milton Keynes|Wellcome Sanger Institute for the COVID-19 Genomics UK (COG-UK) consortium|Davies|United Kingdom
>Spike|hCoV-19/Wales/PHWC-49793C/2020|2020-11-22|EPI_ISL_726905|Original|hCoV-19^^Wales|Human|Originating lab|COVID-19 Genomics UK (COG-UK) Consortium|Moore|United Kingdom
>Spike|hCoV-19/England/ALDP-AA33C0/2020|2020-10-22|EPI_ISL_624983|Original|hCoV-19^^England|Human|Lighthouse Lab in Alderley Park|Wellcome Sanger Institute for the COVID-19 Genomics UK (COG-UK) consortium|Davies|United Kingdom
>Spike|hCoV-19/Panama/335013/2020|2020-04-01|EPI_ISL_496801|Original|hCoV-19^^Cocle|Human|Gorgas Memorial Laboratory of Health Studies|Gorgas Memorial Laboratory of Health Studies|Martinez|Panama
>Spike|hCoV-19/Denmark/DCGC-18714/2020|2020-12-07|EPI_ISL_750139|Original|hCoV-19^^Syddanmark|Human|Department of Virus and Microbiological Special Diagnostics|Albertsen Lab|Michaelsen|Denmark
>Spike|hCoV-19/Australia/VIC4906/2020|2020-07-14|EPI_ISL_519282|Original|hCoV-19^^Victoria|Human|Victorian Infectious Diseases Reference Laboratory (VIDRL)|VIDRL and MDU-PHL|Schultz|Australia
>Spike|hCoV-19/England/SHEF-CFEE5/2020|2020-03-21|EPI_ISL_432605|Original|hCoV-19^^England|Human|Virology Department|COVID-19 Genomics UK (COG-UK) Consortium|de Silva|United Kingdom
>Spike|hCoV-19/Wales/PHWC-493208/2020|2020-11-28|EPI_ISL_725855|Original|hCoV-19^^Wales|Human|Originating lab|COVID-19 Genomics UK (COG-UK) Consortium|Moore|United Kingdom
>Spike|hCoV-19/Romania/USAFSAM-S577/2020|2020-11-28|EPI_ISL_738321|Original|hCoV-19^^Romania|Human|Landstuhl Regional Medical Center|United States Air Force School of Aerospace Medicine|Fries|Romania
>Spike|hCoV-19/Norway/4040/2020|2020-10-02|EPI_ISL_635124|Original|hCoV-19^^Innlandet|Human|Innlandet Hospital Trust|Norwegian Institute of Public Health|Vollan|Norway
>Spike|hCoV-19/USA/MD-HP00140/2020|2020-03-22|EPI_ISL_457772|Original|hCoV-19^^Maryland|Human|Johns Hopkins Hospital Department of Pathology|Johns Hopkins Hospital Department of Pathology|Ramakrishnan|USA
>Spike|hCoV-19/England/ALDP-AA55C8/2020|2020-10-22|EPI_ISL_625068|Original|hCoV-19^^England|Human|Lighthouse Lab in Alderley Park|Wellcome Sanger Institute for the COVID-19 Genomics UK (COG-UK) consortium|Davies|United Kingdom

>Spike|hCoV-19/Australia/NSW1066/2020|2020-08-02|EPI_ISL_593674|Original|hCoV-19^^New South Wales|Human|Pathology West - NSW Health Pathology|NSW Health Pathology - Institute of Clinical Pathology and Medical Research; Westmead Hospital; University of Sydney|Gall|Australia
>Spike|hCoV-19/England/ALDP-BCA24D/2020|2020-11-23|EPI_ISL_703780|Original|hCoV-19^^England|Human|Lighthouse Lab in Alderley Park|Wellcome Sanger Institute for the COVID-19 Genomics UK (COG-UK) Consortium|Davies|United Kingdom
>Spike|hCoV-19/England/PORT-2D5D0B/2020|2020-12-19|EPI_ISL_765142|Original|hCoV-19^^England|Human|Centre for Enzyme Innovation|COVID-19 Genomics UK (COG-UK) Consortium|Robson|United Kingdom
>Spike|hCoV-19/Scotland/GCVR-17242A/2020|2020-03-30|EPI_ISL_469974|Original|hCoV-19^^Scotland|Human|NHSGGC West of Scotland Specialist Virology Centre / MRC-University of Glasgow Centre for Virus Research|Wellcome Sanger Institute for the COVID-19 Genomics UK (COG-UK) consortium|Davies|United Kingdom
>Spike|hCoV-19/Spain/MD-H12-24/2020|2020-03-22|EPI_ISL_428687|Original|hCoV-19^^Madrid|Human|Hospital Universitario 12 de Octubre|Hospital Universitario 12 de Octubre|Viedma|Spain
>Spike|hCoV-19/England/ALDP-A92EC3/2020|2020-10-20|EPI_ISL_610944|Original|hCoV-19^^England|Human|Lighthouse Lab in Alderley Park|Wellcome Sanger Institute for the COVID-19 Genomics UK (COG-UK) consortium|Davies|United Kingdom
>Spike|hCoV-19/USA/VA-DCLS-0863/2020|2020-04-30|EPI_ISL_516659|Original|hCoV-19^^Virginia|Human|Virginia DCLS|Virginia DCLS|St. Jacques|USA
>Spike|hCoV-19/USA/CA-LACPHL-AF00075/2020|2020-12-21|EPI_ISL_766012|Original|hCoV-19^^California|Human|Los Angeles County PHL|Los Angeles County PHL|Hemarajata|USA
>Spike|hCoV-19/Japan/NF-08/2020|2020-03-00|EPI_ISL_591478|Original|hCoV-19^^Japan|Human|Pathogen Genomics Center|Pathogen Genomics Center|Sekizuka|Japan
>Spike|hCoV-19/Australia/VIC601/2020|2020-03-26|EPI_ISL_426857|Original|hCoV-19^^Victoria|Human|Victorian Infectious Diseases Reference Laboratory (VIDRL)|Microbiological Diagnostic Unit Public Health Laboratory and Victorian Infectious Diseases Reference Laboratory|Schultz|Australia
>Spike|hCoV-19/England/MILK-B3D597/2020|2020-11-01|EPI_ISL_706228|Original|hCoV-19^^England|Human|Oxford Viromics|COVID-19 Genomics UK (COG-UK) Consortium|Golubchik|United Kingdom
>Spike|hCoV-19/Wales/PHWC-48F955/2020|2020-11-10|EPI_ISL_681163|Original|hCoV-19^^Wales|Human|Wales Specialist Virology Centre Sequencing lab|COVID-19 Genomics UK (COG-UK) Consortium|Moore|United Kingdom
>Spike|hCoV-19/England/ALDP-A669A9/2020|2020-10-12|EPI_ISL_630431|Original|hCoV-19^^England|Human|Lighthouse Lab in Alderley Park|Wellcome Sanger Institute for the COVID-19 Genomics UK (COG-UK) consortium|Davies|United Kingdom
>Spike|hCoV-19/England/ALDP-A2F20B/2020|2020-10-05|EPI_ISL_587171|Original|hCoV-19^^England|Human|Lighthouse Lab in Alderley Park|Wellcome Sanger Institute for the COVID-19 Genomics UK (COG-UK) consortium|Davies|United Kingdom
>Spike|hCoV-19/England/20202024904/2020|2020-05-10|EPI_ISL_465648|Original|hCoV-19^^England|Human|Respiratory Virus Unit|Respiratory Virus Unit|Platt|United Kingdom
>Spike|hCoV-19/Wales/PHWC-482F6F/2020|2020-10-23|EPI_ISL_627761|Original|hCoV-19^^Wales|Human|Wales Specialist Virology Centre Sequencing lab|COVID-19 Genomics UK (COG-UK) Consortium|Moore|United Kingdom
>Spike|hCoV-19/USA/WI-UW-2058/2020|2020-10-15|EPI_ISL_677463|Original|hCoV-19^^Wisconsin|Human|University of Wisconsin-Madison AIDS Vaccine Research Laboratories|University of Wisconsin-Madison AIDS Vaccine Research Laboratories|Baczenas|USA
>Spike|hCoV-19/Tunisia/COV1339/2020|2020-03-30|EPI_ISL_463003|Original|hCoV-19^^Tunisia|Human|unknown|Clinical virology|Submitter|Tunisia
>Spike|hCoV-19/England/MILK-9E1822/2020|2020-09-20|EPI_ISL_577089|Original|hCoV-19^^England|Human|Oxford Viromics|COVID-19 Genomics UK (COG-UK) Consortium|Golubchik|United Kingdom
>Spike|hCoV-19/USA/WA-S553/2020|2020-03-31|EPI_ISL_434313|Original|hCoV-19^^Washington|Human|Washington State Department of Health|Seattle Flu Study|Bedford|USA

>Spike|hCoV-19/England/CAMC-B09A3B/2020|2020-10-29|EPI_ISL_673177|Original|hCoV-19^^England|Human|Lighthouse Lab in Cambridge|Wellcome Sanger Institute for the COVID-19 Genomics UK (COG-UK) Consortium|Davies|United Kingdom
>Spike|hCoV-19/England/ALDP-B77124/2020|2020-11-12|EPI_ISL_723846|Original|hCoV-19^^England|Human|Oxford Viromics|COVID-19 Genomics UK (COG-UK) Consortium|Golubchik|United Kingdom
>Spike|hCoV-19/England/LCST-24D709F/2020|2020-06-06|EPI_ISL_478360|Original|hCoV-19^^England|Human|University Hospitals Of Leicester NHS Trust and DeepSeq Nottingham|COVID-19 Genomics UK (COG-UK) Consortium|Loose|United Kingdom
>Spike|hCoV-19/Australia/VIC2786/2020|2020-07-04|EPI_ISL_521620|Original|hCoV-19^^Victoria|Human|Microbiological Diagnostic Unit - Public Health Laboratory (MDU-PHL)|MDU-PHL|Schultz|Australia
>Spike|hCoV-19/USA/VA-DCLS-1696/2020|2020-09-05|EPI_ISL_609978|Original|hCoV-19^^Virginia|Human|Virginia DCLS|Virginia DCLS|St. Jacques|USA
>Spike|hCoV-19/England/QEUH-B10C95/2020|2020-11-02|EPI_ISL_642240|Original|hCoV-19^^England|Human|Lighthouse Lab in Glasgow|Wellcome Sanger Institute for the COVID-19 Genomics UK (COG-UK) Consortium|Davies|United Kingdom
>Spike|hCoV-19/Japan/PG-5897/2020|2020-08-00|EPI_ISL_689012|Original|hCoV-19^^Japan|Human|Pathogen Genomics Center|Pathogen Genomics Center|Sekizuka|Japan
>Spike|hCoV-19/USA/TX-HMH-MCoV-11960/2020|2020-09-15|EPI_ISL_789892|Original|hCoV-19^^Texas|Human|Houston Methodist Hospital|Houston Methodist Hospital|Long|USA
>Spike|hCoV-19/England/204221463/2020|2020-00-00|EPI_ISL_593828|Original|hCoV-19^^England|Human|Respiratory Virus Unit|Respiratory Virus Unit|Platt|United Kingdom
>Spike|hCoV-19/Scotland/EDB4387/2020|2020-05-04|EPI_ISL_526481|Original|hCoV-19^^Scotland|Human|Virology Department|COVID-19 Genomics UK (COG-UK) Consortium|Williams|United Kingdom
>Spike|hCoV-19/USA/HI-H200664/2020|2020-12-01|EPI_ISL_753187|Original|hCoV-19^^Hawaii|Human|State Laboratories Division|State Laboratories Division|Sultana|USA
>Spike|hCoV-19/England/CAMB-7862F/2020|2020-04-05|EPI_ISL_433757|Original|hCoV-19^^England|Human|Department of Pathology|COVID-19 Genomics UK (COG-UK) Consortium|Meredith|United Kingdom
>Spike|hCoV-19/USA/MN-QDX-994/2020|2020-03-14|EPI_ISL_571791|Original|hCoV-19^^Minnesota|Human|Quest Diagnostics|Quest Diagnostics|Kagan|USA
>Spike|hCoV-19/England/NORT-1B61728/2020|2020-11-09|EPI_ISL_703533|Original|hCoV-19^^England|Human|Northumbria University / South Tees Hospitals NHS Foundation Trust / North Cumbria Integrated Care NHS Foundation Trust / North Tees and Hartlepool NHS Foundation Trust / Newcastle Hospitals NHS Foundation Trust|COVID-19 Genomics UK (COG-UK) Consortium|Bashton|United Kingdom
>Spike|hCoV-19/Ireland/WW-NVRL-AIIDV1457v2/2020|2020-12-08|EPI_ISL_778838|Original|hCoV-19^^Wicklow|Human|AIID|Irish Coronavirus Sequencing Consortium-Teagasc Grange|McCabe|Ireland
>Spike|hCoV-19/Wales/PHWC-16A67A/2020|2020-09-09|EPI_ISL_560084|Original|hCoV-19^^Wales|Human|Wales Specialist Virology Centre Sequencing lab|COVID-19 Genomics UK (COG-UK) Consortium|Moore|United Kingdom
>Spike|hCoV-19/Netherlands/GE-EMC-295/2020|2020-00-00|EPI_ISL_523274|Original|hCoV-19^^Gelderland|Human|Dutch COVID-19 response team|Erasmus Medical Center|Oude Munnink|Netherlands
>Spike|hCoV-19/England/ALDP-9FDC63/2020|2020-10-02|EPI_ISL_587975|Original|hCoV-19^^England|Human|Lighthouse Lab in Alderley Park|Wellcome Sanger Institute for the COVID-19 Genomics UK (COG-UK) consortium|Davies|United Kingdom
>Spike|hCoV-19/USA/TX-DSHS-0558/2020|2020-05-14|EPI_ISL_574461|Original|hCoV-19^^Texas|Human|Texas Department of State Health Services|Texas Department of State Health Services|Rahman|USA
>Spike|hCoV-19/USA/WI-UW-372/2020|2020-05-20|EPI_ISL_480354|Original|hCoV-19^^Wisconsin|Human|University of Wisconsin-Madison AIDS Vaccine Research Laboratories|University of Wisconsin-Madison AIDS Vaccine Research Laboratories|Baczenas|USA
>Spike|hCoV-19/England/MILK-A1D77A/2020|2020-10-03|EPI_ISL_595082|Original|hCoV-19^^England|Human|Queens Medical Centre|COVID-19 Genomics UK (COG-UK) Consortium|Loose|United Kingdom

>Spike|hCoV-19/Germany/HH-hpi-p2443/2020|2020-04-19|EPI_ISL_776508|Original|hCoV-19^^Hamburg|Human|University Medical Center Hamburg Eppendorf|Heinrich Pette Institute|Robitaille|Germany
>Spike|hCoV-19/Gambia/GC19-1983/2020|2020-06-07|EPI_ISL_561038|Original|hCoV-19^^Kombo|Human|MRCG at LSHTM Genomics lab|MRCG at LSHTM Genomics lab|Sesay|Gambia
>Spike|hCoV-19/USA/CA-ALSR-1229/2020|2020-05-05|EPI_ISL_494451|Original|hCoV-19^^California|Human|San Diego County Public Health Laboratory|Andersen lab at Scripps Research|Gangavarapu|USA
>Spike|hCoV-19/USA/NY-NYCPHL-001408/2020|2020-11-18|EPI_ISL_683784|Original|hCoV-19^^New York|Human|DOHMH Riverside|New York City Public Health Laboratory|Wang|USA
>Spike|hCoV-19/Wales/PHWC-29B79/2020|2020-04-04|EPI_ISL_432253|Original|hCoV-19^^Wales|Human|Wales Specialist Virology Centre|Public Health Wales Microbiology Cardiff|Moore|United Kingdom
>Spike|hCoV-19/England/MILK-9A9880/2020|2020-08-28|EPI_ISL_550905|Original|hCoV-19^^England|Human|Lighthouse Lab in Milton Keynes|Wellcome Sanger Institute for the COVID-19 Genomics UK (COG-UK) consortium|Davies|United Kingdom
>Spike|hCoV-19/Japan/PG-7724/2020|2020-08-00|EPI_ISL_686826|Original|hCoV-19^^Japan|Human|Chiba Prefectural Institute of Public Health|Pathogen Genomics Center|Sekizuka|Japan
>Spike|hCoV-19/USA/FL-BPHL-1478/2020|2020-06-26|EPI_ISL_549260|Original|hCoV-19^^Florida|Human|Florida Bureau of Public Health Laboratories|Florida Bureau of Public Health Laboratories|Blanton|USA
>Spike|hCoV-19/Netherlands/DR-EMC-13/2020|2020-08-14|EPI_ISL_574781|Original|hCoV-19^^Drenthe|Human|Dutch COVID-19 response team|Erasmus Medical Center|Oude Munnink|Netherlands
>Spike|hCoV-19/England/QEUH-AF280F/2020|2020-10-30|EPI_ISL_662662|Original|hCoV-19^^England|Human|Lighthouse Lab in Glasgow|Wellcome Sanger Institute for the COVID-19 Genomics UK (COG-UK) Consortium|Davies|United Kingdom
>Spike|hCoV-19/Canada/BC_03405412/2020|2020-04-00|EPI_ISL_466739|Original|hCoV-19^^British Columbia|Human|BCCDC Public Health Laboratory|BCCDC Public Health Laboratory|Harrigan|Canada
>Spike|hCoV-19/England/LIVE-A4E04/2020|2020-03-05|EPI_ISL_500091|Original|hCoV-19^^England|Human|Liverpool Clinical Laboratories|COVID-19 Genomics UK (COG-UK) Consortium|Haldenby|United Kingdom
>Spike|hCoV-19/Netherlands/NB-EMC-444/2020|2020-11-24|EPI_ISL_722687|Original|hCoV-19^^Noord Brabant|Human|Dutch COVID-19 response team|Erasmus Medical Center|Oude Munnink|Netherlands
>Spike|hCoV-19/USA/WI-UW-2106/2020|2020-09-29|EPI_ISL_677511|Original|hCoV-19^^Wisconsin|Human|University of Wisconsin-Madison AIDS Vaccine Research Laboratories|University of Wisconsin-Madison AIDS Vaccine Research Laboratories|Baczenas|USA
>Spike|hCoV-19/England/CAMB-77C3D/2020|2020-03-31|EPI_ISL_440851|Original|hCoV-19^^England|Human|Department of Pathology|Wellcome Sanger Institute for the COVID-19 Genomics UK (COG-UK) consortium|Amato|United Kingdom
>Spike|hCoV-19/England/ALDP-722A6D/2020|2020-07-01|EPI_ISL_557684|Original|hCoV-19^^England|Human|Lighthouse Lab in Alderley Park|Wellcome Sanger Institute for the COVID-19 Genomics UK (COG-UK) consortium|Davies|United Kingdom
>Spike|hCoV-19/Denmark/DCGC-21968/2020|2020-12-21|EPI_ISL_758482|Original|hCoV-19^^Hovedstaden|Human|Department of Virus and Microbiological Special Diagnostics|Albertsen Lab|Michaelsen|Denmark
>Spike|hCoV-19/Romania/ROSV_10626/2020|2020-05-26|EPI_ISL_491036|Original|hCoV-19^^Suceava|Human|Suceava County Emergency Hospital|\Stefan cel Mare\ University Metagenomics Lab|Lobiuc|Romania
>Spike|hCoV-19/England/NORT-1B723D5/2020|2020-11-27|EPI_ISL_724875|Original|hCoV-19^^England|Human|Northumbria University / South Tees Hospitals NHS Foundation Trust / North Cumbria Integrated Care NHS Foundation Trust / North Tees and Hartlepool NHS Foundation Trust / Newcastle Hospitals NHS Foundation Trust|COVID-19 Genomics UK (COG-UK) Consortium|Bashton|United Kingdom

>Spike|hCoV-19/Switzerland/BL-ETHZ-260058/2020|2020-08-23|EPI_ISL_539384|Original|hCoV-19^^Basel-Landschaft|Human|Viollier AG|Department of Biosystems Science and Engineering|Nadeau|Switzerland
>Spike|hCoV-19/England/OXON-B3B29/2020|2020-04-05|EPI_ISL_534901|Original|hCoV-19^^England|Human|Oxford Viromics|COVID-19 Genomics UK (COG-UK) Consortium|Golubchik|United Kingdom
>Spike|hCoV-19/Wales/PHWC-28BB6/2020|2020-04-03|EPI_ISL_445413|Original|hCoV-19^^Wales|Human|Wales Specialist Virology Centre|Public Health Wales Microbiology Cardiff|Moore|United Kingdom
>Spike|hCoV-19/England/LIVE-9FFDC/2020|2020-06-01|EPI_ISL_472081|Original|hCoV-19^^England|Human|Liverpool Clinical Laboratories|COVID-19 Genomics UK (COG-UK) Consortium|Haldenby|United Kingdom
>Spike|hCoV-19/England/CAMB-75473/2020|2020-03-12|EPI_ISL_425391|Original|hCoV-19^^England|Human|Department of Pathology|COVID-19 Genomics UK (COG-UK) Consortium|Meredith|United Kingdom
>Spike|hCoV-19/Bangladesh/DNAS_DRICM_isl_SIBL_12/2020|2020-06-28|EPI_ISL_774979|Original|hCoV-19^^Dhaka|Human|Designated Reference Institute for Chemical Measurements (DRICM)|DNA SOLUTION LTD.|Rabbi|Bangladesh
>Spike|hCoV-19/Scotland/CVR3165/2020|2020-04-22|EPI_ISL_449198|Original|hCoV-19^^Scotland|Human|West of Scotland Specialist Virology Centre|COVID-19 Genomics UK (COG-UK) Consortium|Robertson|United Kingdom
>Spike|hCoV-19/USA/TX-HMH-MCoV-12190/2020|2020-09-21|EPI_ISL_790238|Original|hCoV-19^^Texas|Human|Houston Methodist Hospital|Houston Methodist Hospital|Long|USA
>Spike|hCoV-19/Ireland/CE-NVRL-AIIDV1105v4/2020|2020-04-26|EPI_ISL_644272|Original|hCoV-19^^Clare|Human|CEPHR / Vincent's Hospital|Irish Coronavirus Sequencing Consortium - National Virus Reference Laboratory|Gonzalez|Ireland
>Spike|hCoV-19/USA/TX-HMH-MCoV-14597/2020|2020-10-09|EPI_ISL_787882|Original|hCoV-19^^Texas|Human|Houston Methodist Hospital|Houston Methodist Hospital|Long|USA
>Spike|hCoV-19/USA/WI-UW-1509/2020|2020-09-14|EPI_ISL_541635|Original|hCoV-19^^Wisconsin|Human|University of Wisconsin-Madison AIDS Vaccine Research Laboratories|University of Wisconsin-Madison AIDS Vaccine Research Laboratories|Baczenas|USA
>Spike|hCoV-19/Italy/PIE-SLL-NZ142/2020|2020-04-01|EPI_ISL_569882|Original|hCoV-19^^Piedmont|Human|Amedeo di savoia|Crosetto lab|Harbers|Italy
>Spike|hCoV-19/Japan/PG-7362/2020|2020-08-00|EPI_ISL_688464|Original|hCoV-19^^Japan|Human|Pathogen Genomics Center|Pathogen Genomics Center|Sekizuka|Japan
>Spike|hCoV-19/Australia/VIC11931/2020|2020-08-11|EPI_ISL_591704|Original|hCoV-19^^Victoria|Human|Victorian Infectious Diseases Reference Laboratory (VIDRL)|VIDRL and MDU-PHL|Schultz|Australia
>Spike|hCoV-19/USA/VA-DCLS-1795/2020|2020-09-16|EPI_ISL_644878|Original|hCoV-19^^Virginia|Human|Virginia DCLS|Virginia DCLS|St. Jacques|USA
>Spike|hCoV-19/England/CAMC-B0ADD7/2020|2020-10-30|EPI_ISL_643333|Original|hCoV-19^^England|Human|Lighthouse Lab in Cambridge|Wellcome Sanger Institute for the COVID-19 Genomics UK (COG-UK) Consortium|Davies|United Kingdom
>Spike|hCoV-19/Wales/PHWC-495EA8/2020|2020-11-26|EPI_ISL_726498|Original|hCoV-19^^Wales|Human|Originating lab|COVID-19 Genomics UK (COG-UK) Consortium|Moore|United Kingdom
>Spike|hCoV-19/France/ARA-111501/2020|2020-11-24|EPI_ISL_728547|Original|hCoV-19^^Auvergne-Rh\u00f4ne-Alpes|Human|CNR Virus des Infections Respiratoires - France SUD|CNR Virus des Infections Respiratoires - France SUD|Bal|France
>Spike|hCoV-19/USA/LA-GBCL-26492461/2020|2020-04-28|EPI_ISL_485038|Original|hCoV-19^^Louisiana|Human|River Road Testing Lab|Ginkgo Bioworks Clinical Laboratory|Smith|USA
>Spike|hCoV-19/Ireland/D-NVRL-AIIDV1263v1/2020|2020-10-23|EPI_ISL_778819|Original|hCoV-19^^Dublin|Human|AIID|Irish Coronavirus Sequencing Consortium-Teagasc Grange|McCabe|Ireland
>Spike|hCoV-19/Belgium/ULG-11080/2020|2020-12-15|EPI_ISL_737330|Original|hCoV-19^^Li\u00e8ge|Human|Department of Clinical Microbiology|GIGA Medical Genomics|Durkin|Belgium

>Spike|hCoV-19/England/MILK-A7AB42/2020|2020-10-16|EPI_ISL_606887|Original|hCoV-19^^England|Human|Lighthouse Lab in Milton Keynes|Wellcome Sanger Institute for the COVID-19 Genomics UK (COG-UK) consortium|Davies|United Kingdom
>Spike|hCoV-19/India/DL-IGIB11304171/2020|2020-07-00|EPI_ISL_751317|Original|hCoV-19^^Delhi|Human|Devki Devi Foundation|CSIR-IGIB/Max|Pandey|India
>Spike|hCoV-19/England/QEUH-A8B373/2020|2020-10-16|EPI_ISL_643260|Original|hCoV-19^^England|Human|Lighthouse Lab in Glasgow|Wellcome Sanger Institute for the COVID-19 Genomics UK (COG-UK) Consortium|Davies|United Kingdom
>Spike|hCoV-19/Switzerland/ZH-ETHZ-340146/2020|2020-10-27|EPI_ISL_721936|Original|hCoV-19^^Zurich|Human|Viollier AG|Department of Biosystems Science and Engineering|Nadeau|Switzerland
>Spike|hCoV-19/Denmark/DCGC-11584/2020|2020-11-02|EPI_ISL_671007|Original|hCoV-19^^Hovedstaden|Human|Department of Virus and Microbiological Special Diagnostics|Albertsen Lab|Michaelsen|Denmark
>Spike|hCoV-19/USA/TX-HMH-MCoV-16048/2020|2020-10-27|EPI_ISL_785732|Original|hCoV-19^^Texas|Human|Houston Methodist Hospital|Houston Methodist Hospital|Long|USA
>Spike|hCoV-19/England/ALDP-B24029/2020|2020-11-04|EPI_ISL_646428|Original|hCoV-19^^England|Human|Lighthouse Lab in Alderley Park|Wellcome Sanger Institute for the COVID-19 Genomics UK (COG-UK) Consortium|Davies|United Kingdom
>Spike|hCoV-19/Denmark/DCGC-1014/2020|2020-03-30|EPI_ISL_614475|Original|hCoV-19^^Hovedstaden|Human|Department of Virus and Microbiological Special Diagnostics|Albertsen lab|Michaelsen|Denmark
>Spike|hCoV-19/USA/TX-HMH-MCoV-19178/2020|2020-11-23|EPI_ISL_783672|Original|hCoV-19^^Texas|Human|Houston Methodist Hospital|Houston Methodist Hospital|Long|USA
>Spike|hCoV-19/Spain/MD-ISCIII-2014615/2020|2020-08-19|EPI_ISL_691705|Original|hCoV-19^^Madrid|Human|Hospital Cl\u00ednico San Carlos|Instituto de Salud Carlos III|Caballero|Spain
>Spike|hCoV-19/England/QEUH-96992F/2020|2020-08-14|EPI_ISL_531115|Original|hCoV-19^^England|Human|Lighthouse Lab in Glasgow|Wellcome Sanger Institute for the COVID-19 Genomics UK (COG-UK) consortium|Davies|United Kingdom
>Spike|hCoV-19/England/ALDP-95573B/2020|2020-06-12|EPI_ISL_554567|Original|hCoV-19^^England|Human|Lighthouse Lab in Alderley Park|Wellcome Sanger Institute for the COVID-19 Genomics UK (COG-UK) consortium|Davies|United Kingdom
>Spike|hCoV-19/England/ALDP-95C2AC/2020|2020-06-17|EPI_ISL_554988|Original|hCoV-19^^England|Human|Lighthouse Lab in Alderley Park|Wellcome Sanger Institute for the COVID-19 Genomics UK (COG-UK) consortium|Davies|United Kingdom
>Spike|hCoV-19/Vietnam/PIHCM-304/2020|2020-03-16|EPI_ISL_654867|Vero E6|hCoV-19^^Ho Chi Minh city|Human|Pasteur Institute in Ho Chi Minh city|Department of Microbiology and Immunology - Pasteur Institute in Ho Chi Minh city|Vu|Viet Nam
>Spike|hCoV-19/France/OCC-SC528/2020|2020-03-10|EPI_ISL_660712|Original|hCoV-19^^Occitanie|Human|CHU N\u00eemes|CNR Virus des Infections Respiratoires - France SUD|Bal|France
>Spike|hCoV-19/USA/FL-AFFK/2020|2020-06-29|EPI_ISL_513283|Original|hCoV-19^^Florida|Human|University of Miami Immunology and Histocompatibility Laboratory|University of Miami Immunology and Histocompatibility Laboratory|Margolles-Clark|USA
>Spike|hCoV-19/Switzerland/BE-ETHZ-260125/2020|2020-08-26|EPI_ISL_539432|Original|hCoV-19^^Bern|Human|Viollier AG|Department of Biosystems Science and Engineering|Nadeau|Switzerland
>Spike|hCoV-19/India/MH-ACTREC-499/2020|2020-08-14|EPI_ISL_700147|Original|hCoV-19^^Maharashtra|Human|Hematopathology Laboratory|Hematopathology Laboratory|Patkar|India
>Spike|hCoV-19/Scotland/QEUH-BF1A09/2020|2020-12-05|EPI_ISL_710653|Original|hCoV-19^^Scotland|Human|Lighthouse Lab in Glasgow|Wellcome Sanger Institute for the COVID-19 Genomics UK (COG-UK) Consortium|Davies|United Kingdom
>Spike|hCoV-19/Saudi Arabia/KAUST-MADINAH1487/2020|2020-06-19|EPI_ISL_678024|Original|hCoV-19^^Madinah|Human|Pathogen Genomics Lab King Abdullah University of Science and Technology(KAUST)|Pathogen Genomics Lab King Abdullah University of Science and Technology(KAUST)|Mohamed Ghazzali|Saudi Arabia

>Spike|hCoV-19/England/MILK-9581EF/2020|2020-06-14|EPI_ISL_554687|Original|hCoV-19^^England|Human|Lighthouse Lab in Milton Keynes|Wellcome Sanger Institute for the COVID-19 Genomics UK (COG-UK) consortium|Davies|United Kingdom
>Spike|hCoV-19/USA/MA-MGH-00276/2020|2020-04-01|EPI_ISL_460172|Original|hCoV-19^^Massachusetts|Human|Massachusetts General Hospital|Infectious Disease Program|Lemieux|USA
>Spike|hCoV-19/Scotland/CVR2228/2020|2020-04-13|EPI_ISL_433273|Original|hCoV-19^^Scotland|Human|West of Scotland Specialist Virology Centre|COVID-19 Genomics UK (COG-UK) Consortium|Robertson|United Kingdom
>Spike|hCoV-19/England/MILK-A7A27A/2020|2020-10-15|EPI_ISL_606735|Original|hCoV-19^^England|Human|Lighthouse Lab in Milton Keynes|Wellcome Sanger Institute for the COVID-19 Genomics UK (COG-UK) consortium|Davies|United Kingdom
>Spike|hCoV-19/England/QEUH-9E491D/2020|2020-09-22|EPI_ISL_579792|Original|hCoV-19^^England|Human|Lighthouse Lab in Glasgow|Wellcome Sanger Institute for the COVID-19 Genomics UK (COG-UK) consortium|Davies|United Kingdom
>Spike|hCoV-19/England/ALDP-A6DBF7/2020|2020-10-14|EPI_ISL_607886|Original|hCoV-19^^England|Human|Lighthouse Lab in Alderley Park|Wellcome Sanger Institute for the COVID-19 Genomics UK (COG-UK) consortium|Davies|United Kingdom
>Spike|hCoV-19/England/ALDP-B76538/2020|2020-11-12|EPI_ISL_723768|Original|hCoV-19^^England|Human|Oxford Viromics|COVID-19 Genomics UK (COG-UK) Consortium|Golubchik|United Kingdom
>Spike|hCoV-19/USA/TX-HMH-MCoV-13054/2020|2020-08-27|EPI_ISL_788205|Original|hCoV-19^^Texas|Human|Houston Methodist Hospital|Houston Methodist Hospital|Long|USA
>Spike|hCoV-19/England/204742213/2020|2020-00-00|EPI_ISL_649919|Original|hCoV-19^^England|Human|Respiratory Virus Unit|COVID-19 Genomics UK (COG-UK) Consortium|Platt|United Kingdom
>Spike|hCoV-19/England/SHEF-C48F1/2020|2020-09-13|EPI_ISL_559907|Original|hCoV-19^^England|Human|Virology Department|COVID-19 Genomics UK (COG-UK) Consortium|de Silva|United Kingdom
>Spike|hCoV-19/England/ALDP-A87878/2020|2020-10-17|EPI_ISL_606230|Original|hCoV-19^^England|Human|Lighthouse Lab in Alderley Park|Wellcome Sanger Institute for the COVID-19 Genomics UK (COG-UK) consortium|Davies|United Kingdom
>Spike|hCoV-19/Japan/PG-5918/2020|2020-08-00|EPI_ISL_689033|Original|hCoV-19^^Japan|Human|Pathogen Genomics Center|Pathogen Genomics Center|Sekizuka|Japan
>Spike|hCoV-19/England/MILK-BB0E28/2020|2020-11-18|EPI_ISL_720423|Original|hCoV-19^^England|Human|Lighthouse Lab in Milton Keynes|Wellcome Sanger Institute for the COVID-19 Genomics UK (COG-UK) Consortium|Davies|United Kingdom
>Spike|hCoV-19/USA/MI-MDHHS-SC20457/2020|2020-05-07|EPI_ISL_459996|Original|hCoV-19^^Michigan|Human|Michigan Department of Health and Human Services|Michigan Department of Health and Human Services|Blankenship|USA
>Spike|hCoV-19/USA/FL-BPHL-1984/2020|2020-04-17|EPI_ISL_653137|Original|hCoV-19^^Florida|Human|Florida Bureau of Public Health Laboratories|Florida Bureau of Public Health Laboratories|Blanton|USA
>Spike|hCoV-19/England/MILK-994985/2020|2020-08-24|EPI_ISL_552384|Original|hCoV-19^^England|Human|Lighthouse Lab in Milton Keynes|Wellcome Sanger Institute for the COVID-19 Genomics UK (COG-UK) consortium|Davies|United Kingdom
>Spike|hCoV-19/Canada/NS-NML-1285/2020|2020-04-07|EPI_ISL_579669|Original|hCoV-19^^Nova Scotia|Human|QEII Health Sciences Centre|National Microbiology Laboratory (NML)|Majer|Canada
>Spike|hCoV-19/USA/VA-DCLS-0183/2020|2020-03-31|EPI_ISL_437399|Original|hCoV-19^^Virginia|Human|Virginia DCLS|Virginia DCLS|St. Jacques|USA
>Spike|hCoV-19/USA/TX-HMH-1520/2020|2020-04-23|EPI_ISL_542770|Original|hCoV-19^^Texas|Human|Houston Methodist Hospital|Houston Methodist Hospital|Long|USA
>Spike|hCoV-19/Denmark/DCGC-14917/2020|2020-11-23|EPI_ISL_713518|Original|hCoV-19^^Midtjylland|Human|Department of Virus and Microbiological Special Diagnostics|Albertsen Lab|Michaelsen|Denmark
>Spike|hCoV-19/Belgium/rega-0419530/2020|2020-04-19|EPI_ISL_734623|Original|hCoV-19^^Templeuve|Human|UZ Leuven|KU Leuven|Maes|Belgium

>Spike|hCoV-19/England/QEUH-998E64/2020|2020-08-24|EPI_ISL_539926|Original|hCoV-19^^England|Human|Lighthouse Lab in Glasgow|Wellcome Sanger Institute for the COVID-19 Genomics UK (COG-UK) consortium|Davies|United Kingdom
>Spike|hCoV-19/England/QEUH-9B862A/2020|2020-09-08|EPI_ISL_549602|Original|hCoV-19^^England|Human|Lighthouse Lab in Glasgow|Wellcome Sanger Institute for the COVID-19 Genomics UK (COG-UK) consortium|Davies|United Kingdom
>Spike|hCoV-19/England/ALDP-9BDB71/2020|2020-09-10|EPI_ISL_567948|Original|hCoV-19^^England|Human|Lighthouse Lab in Alderley Park|Wellcome Sanger Institute for the COVID-19 Genomics UK (COG-UK) consortium|Davies|United Kingdom
>Spike|hCoV-19/Wales/PHWC-493280/2020|2020-11-28|EPI_ISL_725861|Original|hCoV-19^^Wales|Human|Originating lab|COVID-19 Genomics UK (COG-UK) Consortium|Moore|United Kingdom
>Spike|hCoV-19/Switzerland/UR-ETHZ-240082/2020|2020-08-11|EPI_ISL_523919|Original|hCoV-19^^Uri|Human|Viollier AG|Department of Biosystems Science and Engineering|Nadeau|Switzerland
>Spike|hCoV-19/USA/MI-MDHHS-SC21466/2020|2020-03-14|EPI_ISL_507720|Original|hCoV-19^^Michigan|Human|Michigan Department of Health and Human Services|Michigan Department of Health and Human Services|Blankenship|USA
>Spike|hCoV-19/Spain/RI-IBV-98002325/2020|2020-03-22|EPI_ISL_537844|Original|hCoV-19^^La Rioja|Human|Centro de Investigaci\u00f3n Biom\u00e9dica de La Rioja - Hospital San Pedro Logro\u00f1o|SeqCOVID-SPAIN consortium/IBV(CSIC)|Comas|Spain
>Spike|hCoV-19/USA/NY-NYCPHL-001037/2020|2020-09-30|EPI_ISL_633050|Original|hCoV-19^^New York|Human|DOHMH PHL|New York City Public Health Laboratory|Wang|USA
>Spike|hCoV-19/USA/NY-NYCPHL-000345/2020|2020-04-20|EPI_ISL_631856|Original|hCoV-19^^New York|Human|St Barnabas Hospital|New York City Public Health Laboratory|Wang|USA
>Spike|hCoV-19/Russia/SAR-RARI-3656/2020|2020-07-31|EPI_ISL_678282|Original|hCoV-19^^Saratov|Human|Mikrobiologie|Mikrobiologie|Krasnov|Russia
>Spike|hCoV-19/USA/CA-ALSR-3096/2020|2020-08-20|EPI_ISL_635847|Original|hCoV-19^^California|Human|San Diego County Public Health Laboratory|Andersen lab at Scripps Research|Gangavarapu|USA
>Spike|hCoV-19/Wales/PHWC-298EB/2020|2020-04-01|EPI_ISL_474234|Original|hCoV-19^^Wales|Human|Originating lab|COVID-19 Genomics UK (COG-UK) Consortium|Moore|United Kingdom
>Spike|hCoV-19/Spain/MD-IBV-99012447/2020|2020-11-19|EPI_ISL_780081|Original|hCoV-19^^Madrid|Human|Hospital General Universitario Gregorio Mara\u00f1\u00f3n|SeqCOVID-SPAIN consortium/IBV(CSIC)|Comas|Spain
>Spike|hCoV-19/England/MILK-9A901F/2020|2020-09-02|EPI_ISL_551269|Original|hCoV-19^^England|Human|Lighthouse Lab in Milton Keynes|Wellcome Sanger Institute for the COVID-19 Genomics UK (COG-UK) consortium|Davies|United Kingdom
>Spike|hCoV-19/England/NORW-F7B47/2020|2020-11-18|EPI_ISL_665519|Original|hCoV-19^^England|Human|Quadram Institute Bioscience|COVID-19 Genomics UK (COG-UK) Consortium|Alikhan|United Kingdom
>Spike|hCoV-19/USA/WI-UW-520/2020|2020-06-24|EPI_ISL_484895|Original|hCoV-19^^Wisconsin|Human|University of Wisconsin-Madison AIDS Vaccine Research Laboratories|University of Wisconsin-Madison AIDS Vaccine Research Laboratories|Baczenas|USA
>Spike|hCoV-19/England/QEUH-B1003F/2020|2020-11-02|EPI_ISL_642622|Original|hCoV-19^^England|Human|Lighthouse Lab in Glasgow|Wellcome Sanger Institute for the COVID-19 Genomics UK (COG-UK) Consortium|Davies|United Kingdom
>Spike|hCoV-19/USA/NY-Wadsworth-251941-01/2020|2020-10-22|EPI_ISL_754744|Original|hCoV-19^^New York|Human|Wadsworth Center|Wadsworth Center|Lasek-Nesselquist|USA
>Spike|hCoV-19/Australia/VIC6464/2020|2020-07-23|EPI_ISL_521242|Original|hCoV-19^^Victoria|Human|Microbiological Diagnostic Unit - Public Health Laboratory (MDU-PHL)|MDU-PHL|Schultz|Australia
>Spike|hCoV-19/Japan/PG-6063/2020|2020-07-00|EPI_ISL_689175|Original|hCoV-19^^Japan|Human|Pathogen Genomics Center|Pathogen Genomics Center|Sekizuka|Japan

>Spike|hCoV-19/USA/TX-DSHS-0461/2020|2020-06-21|EPI_ISL_541992|Original|hCoV-
19^^Texas|Human|Texas Department of State Health Services|Texas Department of State
Health Services|Rahman|USA
>Spike|hCoV-19/Japan/PG-1540/2020|2020-04-00|EPI_ISL_685146|Original|hCoV-
19^^Japan|Human|Pathogen Genomics Center|Pathogen Genomics Center|Sekizuka|Japan
>Spike|hCoV-19/England/ALDP-8DF758/2020|2020-06-25|EPI_ISL_557074|Original|hCoV-
19^^England|Human|Lighthouse Lab in Alderley Park|Wellcome Sanger Institute for the
COVID-19 Genomics UK (COG-UK) consortium|Davies|United Kingdom
>Spike|hCoV-19/England/MILK-9A6838/2020|2020-08-31|EPI_ISL_550066|Original|hCoV-
19^^England|Human|Lighthouse Lab in Milton Keynes|Wellcome Sanger Institute for the
COVID-19 Genomics UK (COG-UK) consortium|Davies|United Kingdom
>Spike|hCoV-19/Japan/PG-8549/2020|2020-08-00|EPI_ISL_686145|Original|hCoV-
19^^Japan|Human|Pathogen Genomics Center|Pathogen Genomics Center|Sekizuka|Japan
>Spike|hCoV-19/England/201720576/2020|2020-04-21|EPI_ISL_526755|Original|hCoV-
19^^England|Human|Respiratory Virus Unit|Respiratory Virus Unit|Miah|United Kingdom
>Spike|hCoV-19/England/ALDP-950CE6/2020|2020-06-14|EPI_ISL_554572|Original|hCoV-
19^^England|Human|Lighthouse Lab in Alderley Park|Wellcome Sanger Institute for the
COVID-19 Genomics UK (COG-UK) consortium|Davies|United Kingdom
>Spike|hCoV-19/England/QEUH-9E5035/2020|2020-09-23|EPI_ISL_579888|Original|hCoV-
19^^England|Human|Lighthouse Lab in Glasgow|Wellcome Sanger Institute for the COVID-
19 Genomics UK (COG-UK) consortium|Davies|United Kingdom
>Spike|hCoV-19/England/CAMB-1AFBF2/2020|2020-05-03|EPI_ISL_459192|Original|hCoV-
19^^England|Human|Department of Pathology|Wellcome Sanger Institute for the COVID-19
Genomics UK (COG-UK) consortium|Davies|United Kingdom
>Spike|hCoV-19/Australia/VIC9513/2020|2020-08-19|EPI_ISL_561654|Original|hCoV-
19^^Victoria|Human|Microbiological Diagnostic Unit - Public Health Laboratory (MDU-
PHL)|MDU-PHL|Schultz|Australia
>Spike|hCoV-19/England/QEUH-9CFD67/2020|2020-09-16|EPI_ISL_589823|Original|hCoV-
19^^England|Human|Lighthouse Lab in Glasgow|Wellcome Sanger Institute for the COVID-
19 Genomics UK (COG-UK) consortium|Davies|United Kingdom
>Spike|hCoV-19/Japan/PG-1408/2020|2020-04-00|EPI_ISL_685015|Original|hCoV-
19^^Japan|Human|Saitama Prefectural Institute of Public Health|Pathogen Genomics
Center|Sekizuka|Japan
>Spike|hCoV-19/England/CAMB-1AE66B/2020|2020-04-29|EPI_ISL_459497|Original|hCoV-
19^^England|Human|Department of Pathology|Wellcome Sanger Institute for the COVID-19
Genomics UK (COG-UK) consortium|Davies|United Kingdom
>Spike|hCoV-19/England/BIRM-637F7/2020|2020-08-19|EPI_ISL_529333|Original|hCoV-
19^^England|Human|University of Birmingham|COVID-19 Genomics UK (COG-UK)
Consortium|Loman|United Kingdom
>Spike|hCoV-19/Belgium/ULG-10141/2020|2020-04-08|EPI_ISL_427386|Original|hCoV-
19^^Liege|Human|Department of Clinical Microbiology|GIGA Medical
Genomics|Durkin|Belgium
>Spike|hCoV-19/Denmark/DCGC-1009/2020|2020-03-09|EPI_ISL_614780|Original|hCoV-
19^^Syddanmark|Human|Department of Virus and Microbiological Special
Diagnostics|Albertsen lab|Michaelsen|Denmark
>Spike|hCoV-19/Australia/VIC2025/2020|2020-06-05|EPI_ISL_480665|Original|hCoV-
19^^Victoria|Human|Victorian Infectious Diseases Reference Laboratory (VIDRL)|VIDRL
and MDU-PHL|Schultz|Australia
>Spike|hCoV-19/England/201161203/2020|2020-03-11|EPI_ISL_464821|Original|hCoV-
19^^England|Human|Respiratory Virus Unit|Respiratory Virus Unit|Platt|United Kingdom
>Spike|hCoV-19/Netherlands/ZH-EMC-1215/2020|2020-12-13|EPI_ISL_763285|Original|hCoV-
19^^Zuid Holland|Human|Dutch COVID-19 response team|Erasmus Medical Center|Oude
Munnink|Netherlands
>Spike|hCoV-19/USA/CA-LACPHL-AE00036/2020|2020-09-01|EPI_ISL_708728|Original|hCoV-
19^^California|Human|Los Angeles County Public Health Laboratory|Los Angeles County
Public Health Laboratory|Hemarajata|USA
>Spike|hCoV-19/Japan/PG-2337/2020|2020-05-00|EPI_ISL_685942|Original|hCoV-
19^^Japan|Human|Pathogen Genomics Center|Pathogen Genomics Center|Sekizuka|Japan

>Spike|hCoV-19/Canada/NB-237/2020|2020-03-31|EPI_ISL_479650|Original|hCoV-19^^New Brunswick|Human|Dr. Georges-L.-Dumont University Hospital Centre|National Microbiology Laboratory|Majer|Canada
>Spike|hCoV-19/England/CAMB-84AAC/2020|2020-03-10|EPI_ISL_440545|Original|hCoV-19^^England|Human|Department of Pathology|Wellcome Sanger Institute for the COVID-19 Genomics UK (COG-UK) consortium|Amato|United Kingdom
>Spike|hCoV-19/USA/NY-Wadsworth-292768-01/2020|2020-12-27|EPI_ISL_769941|Original|hCoV-19^^New York|Human|Albany Medical Center Hospital Clinical Laboratories|Wadsworth Center|Lasek-Nesselquist|USA
>Spike|hCoV-19/Iceland/326/2020|2020-03-14|EPI_ISL_417655|Original|hCoV-19^^Reykjavik|Human|deCODE genetics|deCODE genetics|J\u00f3nsson|Iceland
>Spike|hCoV-19/Scotland/QEUH-9FB3E8/2020|2020-09-29|EPI_ISL_600580|Original|hCoV-19^^Scotland|Human|Lighthouse Lab in Glasgow|Wellcome Sanger Institute for the COVID-19 Genomics UK (COG-UK) consortium|Davies|United Kingdom
>Spike|hCoV-19/USA/WY-WPHL-20161708/2020|2020-12-08|EPI_ISL_765972|Original|hCoV-19^^Wyoming|Human|Wyoming Public Health Laboratory|Wyoming Public Health Laboratory|Hull|USA
>Spike|hCoV-19/Hong Kong/VM20002450/2020|2020-02-09|EPI_ISL_426387|Original|hCoV-19^^Hong Kong|Human|Pamela Youde Nethersole Eastern Hospital|Hong Kong Department of Health|Mak|China
>Spike|hCoV-19/USA/TX-HMH-MCoV-15113/2020|2020-10-16|EPI_ISL_786597|Original|hCoV-19^^Texas|Human|Houston Methodist Hospital|Houston Methodist Hospital|Long|USA
>Spike|hCoV-19/Netherlands/NH-RIVM-20950/2020|2020-12-21|EPI_ISL_790974|Original|hCoV-19^^Noord-Holland|Human|Dutch COVID-19 response team|National Institute for Public Health and the Environment (RIVM)|Meijer|Netherlands
>Spike|hCoV-19/Brazil/RJ-MD63-1a/2020|2020-03-16|EPI_ISL_528637|Vero-E6|hCoV-19^^Rio de Janeiro|Human|LVM/UFRJ|Bioinformatics Laboratory / LNCC|Almeida|Brazil
>Spike|hCoV-19/England/QEUH-A204B9/2020|2020-10-01|EPI_ISL_587671|Original|hCoV-19^^England|Human|Lighthouse Lab in Glasgow|Wellcome Sanger Institute for the COVID-19 Genomics UK (COG-UK) consortium|Davies|United Kingdom
>Spike|hCoV-19/USA/CA-ALSR-4585/2020|2020-07-11|EPI_ISL_667021|Original|hCoV-19^^California|Human|San Diego County Public Health Laboratory|Andersen lab at Scripps Research|Gangavarapu|USA
>Spike|hCoV-19/USA/TX-HMH-4882/2020|2020-06-21|EPI_ISL_544401|Original|hCoV-19^^Texas|Human|Houston Methodist Hospital|Houston Methodist Hospital|Long|USA
>Spike|hCoV-19/England/205041753/2020|2020-00-00|EPI_ISL_710524|Original|hCoV-19^^England|Human|Respiratory Virus Unit|COVID-19 Genomics UK (COG-UK) Consortium|Platt|United Kingdom
>Spike|hCoV-19/Canada/QC-LSPQ-L00242505/2020|2020-03-29|EPI_ISL_536323|Original|hCoV-19^^Quebec|Human|H\u00f4pital Pierre-Boucher|Laboratoire de sant\u00e9 publique du Qu\u00e9bec|Moreira|Canada
>Spike|hCoV-19/Switzerland/SG-ETHZ-240025/2020|2020-08-10|EPI_ISL_523875|Original|hCoV-19^^Sankt Gallen|Human|Viollier AG|Department of Biosystems Science and Engineering|Nadeau|Switzerland
>Spike|hCoV-19/USA/UT-UPHL-2012421387/2020|2020-11-30|EPI_ISL_746987|Original|hCoV-19^^Utah|Human|Utah Public Health Laboratory|Utah Public Health Laboratory|Gallagher|USA
>Spike|hCoV-19/Denmark/DCGC-13490/2020|2020-11-30|EPI_ISL_712827|Original|hCoV-19^^Midtjylland|Human|Department of Virus and Microbiological Special Diagnostics|Albertsen Lab|Michaelsen|Denmark
>Spike|hCoV-19/Switzerland/BE-ETHZ-420620/2020|2020-12-22|EPI_ISL_768028|Original|hCoV-19^^Bern|Human|Viollier AG|Department of Biosystems Science and Engineering|Nadeau|Switzerland
>Spike|hCoV-19/England/EXET-138DAD/2020|2020-09-04|EPI_ISL_626937|Original|hCoV-19^^England|Human|University of Exeter|COVID-19 Genomics UK (COG-UK) Consortium|Temperton|United Kingdom
>Spike|hCoV-19/Mexico/BCN-ALSR-1692/2020|2020-04-27|EPI_ISL_496353|Original|hCoV-19^^Baja California|Human|Infectolab|Andersen lab at Scripps Research|Gangavarapu|Mexico

>Spike|hCoV-19/Australia/VIC15179/2020|2020-08-29|EPI_ISL_593174|Original|hCoV-19^^Victoria|Human|Microbiological Diagnostic Unit - Public Health Laboratory (MDU-PHL)|MDU-PHL|Schultz|Australia
>Spike|hCoV-19/England/MILK-9E1F0F/2020|2020-09-20|EPI_ISL_581169|Original|hCoV-19^^England|Human|Lighthouse Lab in Milton Keynes|Wellcome Sanger Institute for the COVID-19 Genomics UK (COG-UK) consortium|Davies|United Kingdom
>Spike|hCoV-19/Senegal/9838/2020|2020-04-25|EPI_ISL_498231|Original|hCoV-19^^Dakar|Human|Institut Pasteur de Dakar|Institut Pasteur de Dakar|Dia|Senegal
>Spike|hCoV-19/USA/NM-NMDOH-2020327831/2020|2020-09-02|EPI_ISL_569649|Original|hCoV-19^^New Mexico|Human|New Mexico Department of Health Scientific Laboratory|New Mexico Department of Health Scientific Laboratory|Johnson|USA
>Spike|hCoV-19/England/MILK-B39A14/2020|2020-11-01|EPI_ISL_679657|Original|hCoV-19^^England|Human|Oxford Viromics|COVID-19 Genomics UK (COG-UK) Consortium|Golubchik|United Kingdom
>Spike|hCoV-19/Australia/VIC3864/2020|2020-07-13|EPI_ISL_520577|Original|hCoV-19^^Victoria|Human|Microbiological Diagnostic Unit - Public Health Laboratory (MDU-PHL)|MDU-PHL|Schultz|Australia
>Spike|hCoV-19/USA/AZ-TG395951/2020|2020-06-08|EPI_ISL_694782|Original|hCoV-19^^Arizona|Human|AZ SPHL|TGen North|French|USA
>Spike|hCoV-19/Australia/VIC1248/2020|2020-04-09|EPI_ISL_436114|Original|hCoV-19^^Victoria|Human|Victorian Infectious Diseases Reference Laboratory (VIDRL)|Microbiological Diagnostic Unit Public Health Laboratory and Victorian Infectious Diseases Reference Laboratory|Schultz|Australia
>Spike|hCoV-19/England/LIVE-A692F/2020|2020-03-19|EPI_ISL_499468|Original|hCoV-19^^England|Human|Liverpool Clinical Laboratories|COVID-19 Genomics UK (COG-UK) Consortium|Haldenby|United Kingdom
>Spike|hCoV-19/England/CAMB-82D78/2020|2020-04-13|EPI_ISL_443497|Original|hCoV-19^^England|Human|Department of Pathology|Wellcome Sanger Institute for the COVID-19 Genomics UK (COG-UK) consortium|Amato|United Kingdom
>Spike|hCoV-19/USA/VA-DCLS-0828/2020|2020-05-06|EPI_ISL_516701|Original|hCoV-19^^Virginia|Human|Virginia DCLS|Virginia DCLS|St. Jacques|USA
>Spike|hCoV-19/Wales/PHWC-165703/2020|2020-06-22|EPI_ISL_490716|Original|hCoV-19^^Wales|Human|Wales Specialist Virology Centre Sequencing lab|COVID-19 Genomics UK (COG-UK) Consortium|Moore|United Kingdom
>Spike|hCoV-19/Scotland/CVR4158/2020|2020-08-29|EPI_ISL_540842|Original|hCoV-19^^Scotland|Human|Lighthouse Lab in Glasgow / MRC-University of Glasgow Centre for Virus Research|COVID-19 Genomics UK (COG-UK) Consortium|Robertson|United Kingdom
>Spike|hCoV-19/Denmark/DCGC-3818/2020|2020-08-31|EPI_ISL_622694|Original|hCoV-19^^Hovedstaden|Human|Department of Virus and Microbiological Special Diagnostics|Albertsen lab|Michaelsen|Denmark
>Spike|hCoV-19/England/NORW-EAEE4/2020|2020-04-29|EPI_ISL_457392|Original|hCoV-19^^England|Human|Quadram Institute Bioscience|COVID-19 Genomics UK (COG-UK) Consortium|Alikhan|United Kingdom
>Spike|hCoV-19/USA/TX-HMH-6522/2020|2020-06-27|EPI_ISL_546055|Original|hCoV-19^^Texas|Human|Houston Methodist Hospital|Houston Methodist Hospital|Long|USA
>Spike|hCoV-19/USA/MA-MASPHL-00666/2020|2020-05-16|EPI_ISL_692970|Original|hCoV-19^^Massachusetts|Human|Massachusetts State Public Health Laboratory|Massachusetts State Public Health Laboratory|Lang|USA
>Spike|hCoV-19/Denmark/DCGC-8378/2020|2020-10-26|EPI_ISL_617357|Original|hCoV-19^^Syddanmark|Human|Department of Virus and Microbiological Special Diagnostics|Albertsen lab|Michaelsen|Denmark
>Spike|hCoV-19/England/QEUH-B423C2/2020|2020-11-08|EPI_ISL_657317|Original|hCoV-19^^England|Human|Lighthouse Lab in Glasgow|Wellcome Sanger Institute for the COVID-19 Genomics UK (COG-UK) Consortium|Davies|United Kingdom
>Spike|hCoV-19/Denmark/DCGC-18667/2020|2020-12-07|EPI_ISL_749253|Original|hCoV-19^^Hovedstaden|Human|Department of Virus and Microbiological Special Diagnostics|Albertsen Lab|Michaelsen|Denmark
>Spike|hCoV-19/England/LOND-1260DB7/2020|2020-04-04|EPI_ISL_524686|Original|hCoV-19^^England|Human|North West London Pathology|Wellcome Sanger Institute for the COVID-19 Genomics UK (COG-UK) consortium|Davies|United Kingdom

>Spike|hCoV-19/Australia/VIC2175/2020|2020-06-14|EPI_ISL_521924|Original|hCoV-19^^Victoria|Human|Victorian Infectious Diseases Reference Laboratory (VIDRL)|VIDRL and MDU-PHL|Schultz|Australia
>Spike|hCoV-19/South Africa/NHLS-UCT-GS-1078/2020|2020-10-29|EPI_ISL_700468|Original|hCoV-19^^Western Cape|Human|Khayelethu Clinic wc KLC|NHLS/UCT|Iranzadeh|South Africa
>Spike|hCoV-19/United Arab Emirates/0270/2020|2020-05-18|EPI_ISL_698451|Original|hCoV-19^^Abu Dhabi|Human|Group 42 (G42) Healthcare|G42 Healthcare|Ogrodzki|United Arab Emirates
>Spike|hCoV-19/Colombia/ANT-GUV-92250/2020|2020-04-01|EPI_ISL_447766|Original|hCoV-19^^Antioquia|Human|Instituto Nacional de Salud|Grupo de Investigaciones Microbiol\u00f3gicas-UR (GIMUR)|Ram\u00edrez|Colombia
>Spike|hCoV-19/Denmark/DCGC-1388/2020|2020-04-20|EPI_ISL_618662|Original|hCoV-19^^Hovedstaden|Human|Department of Virus and Microbiological Special Diagnostics|Albertsen lab|Michaelsen|Denmark
>Spike|hCoV-19/Germany/BY-MVP-0110/2020|2020-03-12|EPI_ISL_451936|Original|hCoV-19^^Bavaria|Human|Max von Pettenkofer Institute|Laboratory for Functional Genome Analysis|Graf|Germany
>Spike|hCoV-19/Scotland/EDB8714/2020|2020-10-24|EPI_ISL_650305|Original|hCoV-19^^Scotland|Human|Virology Department|COVID-19 Genomics UK (COG-UK) Consortium|McHugh|United Kingdom
>Spike|hCoV-19/Brazil/RJ-00580/2020|2020-07-29|EPI_ISL_717964|Original|hCoV-19^^Rio de Janeiro|Human|LACEN Dr. Francisco Rimolo Neto|Bioinformatics Laboratory / LNCC|Almeida|Brazil
>Spike|hCoV-19/Austria/CeMM1068/2020|2020-03-16|EPI_ISL_583881|Original|hCoV-19^^Austria|Human|Center for Virology|Bergthaler laboratory|Bergthaler|Austria
>Spike|hCoV-19/USA/CA-SR-0840/2020|2020-04-22|EPI_ISL_524435|Original|hCoV-19^^California|Human|Department of Immunology|Department of Immunology|Submitter|USA
>Spike|hCoV-19/England/MILK-B3AA40/2020|2020-11-02|EPI_ISL_679704|Original|hCoV-19^^England|Human|Oxford Viromics|COVID-19 Genomics UK (COG-UK) Consortium|Golubchik|United Kingdom
>Spike|hCoV-19/Germany/HH-hpi-p1860/2020|2020-03-23|EPI_ISL_776208|Original|hCoV-19^^Hamburg|Human|University Medical Center Hamburg Eppendorf|Heinrich Pette Institute|Robitaille|Germany
>Spike|hCoV-19/Denmark/DCGC-13010/2020|2020-12-07|EPI_ISL_712526|Original|hCoV-19^^Syddanmark|Human|Department of Virus and Microbiological Special Diagnostics|Albertsen Lab|Michaelsen|Denmark
>Spike|hCoV-19/Greece/56_37161/2020|2020-03-31|EPI_ISL_437911|Original|hCoV-19^^Athens|Human|Laboratory of Microbiology|Laboratory of Biology|Karakasiliotis|Greece
>Spike|hCoV-19/Greece/149_32357/2020|2020-03-12|EPI_ISL_447635|unknown|hCoV-19^^Athens|Human|unknown|Department of Medicine|Submitter|Greece
>Spike|hCoV-19/England/ALDP-AA11B9/2020|2020-10-21|EPI_ISL_625063|Original|hCoV-19^^England|Human|Lighthouse Lab in Alderley Park|Wellcome Sanger Institute for the COVID-19 Genomics UK (COG-UK) consortium|Davies|United Kingdom
>Spike|hCoV-19/Japan/PG-0492/2020|2020-04-06|EPI_ISL_480151|Original|hCoV-19^^HokurikuShinsyu|Human|Fukui Prefectural Institute of Public Health and Environmental Science|Pathogen Genomics Center|Sekizuka|Japan
>Spike|hCoV-19/Wales/PHWC-16A5C8/2020|2020-09-08|EPI_ISL_540911|Original|hCoV-19^^Wales|Human|Wales Specialist Virology Centre Sequencing lab|COVID-19 Genomics UK (COG-UK) Consortium|Moore|United Kingdom
>Spike|hCoV-19/England/ALDP-C04A4E/2020|2020-12-04|EPI_ISL_720741|Original|hCoV-19^^England|Human|Lighthouse Lab in Alderley Park|Wellcome Sanger Institute for the COVID-19 Genomics UK (COG-UK) Consortium|Davies|United Kingdom
>Spike|hCoV-19/Denmark/DCGC-10999/2020|2020-11-02|EPI_ISL_669515|Original|hCoV-19^^Syddanmark|Human|Department of Virus and Microbiological Special Diagnostics|Albertsen Lab|Michaelsen|Denmark
>Spike|hCoV-19/Sweden/20-53086/2020|2020-09-08|EPI_ISL_648136|Original|hCoV-19^^Sweden|Human|The Public Health Agency of Sweden|The Public Health Agency of Sweden|Svartstr\u00f6m|Sweden

>Spike|hCoV-19/Israel/51137031/2020|2020-03-01|EPI_ISL_447330|Original|hCoV-19^^Tel Aviv District|Human|Clinical Virology Laboratory|Stern Lab|Miller|Israel
>Spike|hCoV-19/USA/MI-MDHHS-SC20620/2020|2020-04-16|EPI_ISL_462702|Original|hCoV-19^^Michigan|Human|Michigan Department of Health and Human Services|Michigan Department of Health and Human Services|Blankenship|USA
>Spike|hCoV-19/England/OXON-AF883/2020|2020-04-08|EPI_ISL_478780|Original|hCoV-19^^England|Human|Oxford Viromics|COVID-19 Genomics UK (COG-UK) Consortium|Golubchik|United Kingdom
>Spike|hCoV-19/England/EXET-138C28/2020|2020-09-24|EPI_ISL_627437|Original|hCoV-19^^England|Human|University of Exeter|COVID-19 Genomics UK (COG-UK) Consortium|Temperton|United Kingdom
>Spike|hCoV-19/England/ALDP-9C4F5D/2020|2020-09-10|EPI_ISL_567598|Original|hCoV-19^^England|Human|Lighthouse Lab in Alderley Park|Wellcome Sanger Institute for the COVID-19 Genomics UK (COG-UK) consortium|Davies|United Kingdom
>Spike|hCoV-19/Denmark/DCGC-5423/2020|2020-09-14|EPI_ISL_615355|Original|hCoV-19^^Midtjylland|Human|Department of Virus and Microbiological Special Diagnostics|Albertsen lab|Michaelsen|Denmark
>Spike|hCoV-19/England/MILK-C7B74B/2020|2020-12-01|EPI_ISL_756925|Original|hCoV-19^^England|Human|Lighthouse Lab in Milton Keynes|Wellcome Sanger Institute for the COVID-19 Genomics UK (COG-UK) Consortium|Davies|United Kingdom
>Spike|hCoV-19/USA/MN-CDC-3750/2020|2020-04-25|EPI_ISL_594463|original|hCoV-19^^Minnesota|Human|MN Department of Health|Pathogen Discovery|Queen|USA
>Spike|hCoV-19/USA/WI-UW-238/2020|2020-03-27|EPI_ISL_428334|Original|hCoV-19^^Wisconsin|Human|University of Wisconsin-Madison AIDS Vaccine Research Laboratories|University of Wisconsin-Madison AIDS Vaccine Research Laboratories|Moreno|USA
>Spike|hCoV-19/England/ALDP-7054AE/2020|2020-06-30|EPI_ISL_557613|Original|hCoV-19^^England|Human|Lighthouse Lab in Alderley Park|Wellcome Sanger Institute for the COVID-19 Genomics UK (COG-UK) consortium|Davies|United Kingdom
>Spike|hCoV-19/Denmark/DCGC-10402/2020|2020-11-02|EPI_ISL_669947|Original|hCoV-19^^Hovedstaden|Human|Department of Virus and Microbiological Special Diagnostics|Albertsen Lab|Michaelsen|Denmark
>Spike|hCoV-19/England/MILK-B88A34/2020|2020-11-14|EPI_ISL_675885|Original|hCoV-19^^England|Human|Lighthouse Lab in Milton Keynes|Wellcome Sanger Institute for the COVID-19 Genomics UK (COG-UK) Consortium|Davies|United Kingdom
>Spike|hCoV-19/Germany/SN-RKI-NP-0076/2020|2020-12-21|EPI_ISL_763009|Original|hCoV-19^^Saxony|Human|Unit 17|Project group Epidemiology of Highly Pathogenic Microorganisms|Schubert|Germany
>Spike|hCoV-19/England/CAMB-71FB2/2020|2020-03-28|EPI_ISL_439873|Original|hCoV-19^^England|Human|Department of Pathology|Wellcome Sanger Institute for the COVID-19 Genomics UK (COG-UK) consortium|Amato|United Kingdom
>Spike|hCoV-19/Japan/TKYET29933/2020|2020-08-00|EPI_ISL_649137|Original|hCoV-19^^Japan|Human|Tokyo Metropolitan Institute of Public Health|Tokyo Metropolitan Institute of Public Health|Ryota|Japan
>Spike|hCoV-19/USA/AZ-TG336686/2020|2020-05-25|EPI_ISL_694369|Original|hCoV-19^^Arizona|Human|TGen North|TGen North|French|USA
>Spike|hCoV-19/England/BRIS-126944/2020|2020-04-08|EPI_ISL_459043|Original|hCoV-19^^England|Human|PHE South West Regional Laboratory|Wellcome Sanger Institute for the COVID-19 Genomics UK (COG-UK) consortium|Davies|United Kingdom
>Spike|hCoV-19/Wales/MILK-9A5338/2020|2020-09-02|EPI_ISL_550257|Original|hCoV-19^^Wales|Human|Lighthouse Lab in Milton Keynes|Wellcome Sanger Institute for the COVID-19 Genomics UK (COG-UK) consortium|Davies|United Kingdom
>Spike|hCoV-19/Switzerland/ZH-ETHZ-350124/2020|2020-11-02|EPI_ISL_693822|Original|hCoV-19^^Zurich|Human|Viollier AG|Department of Biosystems Science and Engineering|Nadeau|Switzerland
>Spike|hCoV-19/USA/HI-H200529/2020|2020-11-03|EPI_ISL_753062|Original|hCoV-19^^Hawaii|Human|State Laboratories Division|State Laboratories Division|Sultana|USA
>Spike|hCoV-19/England/BIRM-61665/2020|2020-04-30|EPI_ISL_493448|Original|hCoV-19^^England|Human|University of Birmingham|COVID-19 Genomics UK (COG-UK) Consortium|Loman|United Kingdom

>Spike|hCoV-19/USA/CA-LACPHL-AF00014/2020|2020-12-15|EPI_ISL_737249|Original|hCoV-19^^California|Human|Los Angeles County PHL|Los Angeles County PHL|Hemarajata|USA
>Spike|hCoV-19/USA/LA-EVTL1294/2020|2020-12-10|EPI_ISL_778960|Original|hCoV-19^^Louisiana|Human|LSUHS Emerging Viral Threat Laboratory|Microbial Genome Sequencing Center|Kamil|USA
>Spike|hCoV-19/England/LOND-12623C2/2020|2020-09-25|EPI_ISL_584672|Original|hCoV-19^^England|Human|University College London|COVID-19 Genomics UK (COG-UK) Consortium|Roy|United Kingdom
>Spike|hCoV-19/USA/MI-UM-10036464027/2020|2020-10-15|EPI_ISL_628840|Original|hCoV-19^^Michigan|Human|University of Michigan Clinical Microbiology Laboratory|Lauring Lab|Valesano|USA
>Spike|hCoV-19/England/CAMB-1BB276/2020|2020-11-13|EPI_ISL_664833|Original|hCoV-19^^England|Human|Department of Pathology|COVID-19 Genomics UK (COG-UK) Consortium|Goodfellow|United Kingdom
>Spike|hCoV-19/Denmark/DCGC-8800/2020|2020-10-26|EPI_ISL_626075|Original|hCoV-19^^Hovedstaden|Human|Department of Virus and Microbiological Special Diagnostics|Albertsen lab|Michaelsen|Denmark
>Spike|hCoV-19/England/CAMC-A82AFF/2020|2020-10-12|EPI_ISL_610706|Original|hCoV-19^^England|Human|Lighthouse Lab in Cambridge|Wellcome Sanger Institute for the COVID-19 Genomics UK (COG-UK) consortium|Davies|United Kingdom
>Spike|hCoV-19/USA/TX-HMH0279/2020|2020-03-27|EPI_ISL_434907|Original|hCoV-19^^Texas|Human|Houston Methodist Hospital|Houston Methodist Hospital|Long|USA
>Spike|hCoV-19/England/MILK-9A8809/2020|2020-08-31|EPI_ISL_549426|Original|hCoV-19^^England|Human|Oxford Viromics|COVID-19 Genomics UK (COG-UK) Consortium|Golubchik|United Kingdom
>Spike|hCoV-19/United Arab Emirates/skmc-3819699/2020|2020-09-16|EPI_ISL_582674|Original|hCoV-19^^United Arab Emirates|Human|Sheikh Khalifa Medical City|Molecular/Surveillance lab\nSheikh Khalifa Medical City|Amirtharaj|United Arab Emirates
>Spike|hCoV-19/England/204742222/2020|2020-00-00|EPI_ISL_649926|Original|hCoV-19^^England|Human|Respiratory Virus Unit|COVID-19 Genomics UK (COG-UK) Consortium|Platt|United Kingdom
>Spike|hCoV-19/Russia/SPE-RII-MH1524S/2020|2020-09-12|EPI_ISL_596291|Original|hCoV-19^^Saint Petersburg|Human|HELIX LCC|WHO National Influenza Centre Russian Federation|Komissarov|Russia
>Spike|hCoV-19/USA/MI-MDHHS-SC20226/2020|2020-03-17|EPI_ISL_447196|Original|hCoV-19^^Michigan|Human|Michigan Department of Health and Human Services|Michigan Department of Health and Human Services|Blankenship|USA
>Spike|hCoV-19/Scotland/QEUH-A20440/2020|2020-10-01|EPI_ISL_599012|Original|hCoV-19^^Scotland|Human|Lighthouse Lab in Glasgow|Wellcome Sanger Institute for the COVID-19 Genomics UK (COG-UK) consortium|Davies|United Kingdom
>Spike|hCoV-19/Scotland/QEUH-BA1A62/2020|2020-11-18|EPI_ISL_757188|Original|hCoV-19^^Scotland|Human|Lighthouse Lab in Glasgow|Wellcome Sanger Institute for the COVID-19 Genomics UK (COG-UK) Consortium|Davies|United Kingdom
>Spike|hCoV-19/Scotland/CVR218/2020|2020-03-19|EPI_ISL_425745|Original|hCoV-19^^Scotland|Human|West of Scotland Specialist Virology Centre|COVID-19 Genomics UK (COG-UK) Consortium|Robertson|United Kingdom
>Spike|hCoV-19/England/LOND-124F6D1/2020|2020-03-21|EPI_ISL_665165|Original|hCoV-19^^England|Human|University College London Hospital|COVID-19 Genomics UK (COG-UK) Consortium|Byott|United Kingdom
>Spike|hCoV-19/England/CAMC-B08B66/2020|2020-10-30|EPI_ISL_673169|Original|hCoV-19^^England|Human|Lighthouse Lab in Cambridge|Wellcome Sanger Institute for the COVID-19 Genomics UK (COG-UK) Consortium|Davies|United Kingdom
>Spike|hCoV-19/England/EXET-13E1AB/2020|2020-12-05|EPI_ISL_724313|Original|hCoV-19^^England|Human|University of Exeter|COVID-19 Genomics UK (COG-UK) Consortium|Temperton|United Kingdom
>Spike|hCoV-19/England/OXON-B14E4/2020|2020-04-22|EPI_ISL_479022|Original|hCoV-19^^England|Human|Oxford Viromics|COVID-19 Genomics UK (COG-UK) Consortium|Golubchik|United Kingdom

>Spike|hCoV-19/Denmark/DCGC-8401/2020|2020-10-26|EPI_ISL_617342|Original|hCoV-19^^Hovedstaden|Human|Department of Virus and Microbiological Special Diagnostics|Albertsen lab|Michaelsen|Denmark
>Spike|hCoV-19/Portugal/PT0413/2020|2020-03-23|EPI_ISL_454137|Original|hCoV-19^^Portugal|Human|unknown|Instituto Nacional de Saude (INSA)|Borges|Portugal
>Spike|hCoV-19/England/GSTT-265D74F/2020|2020-03-24|EPI_ISL_484006|Original|hCoV-19^^England|Human|Centre for Clinical Infection and Diagnostics Research and Genomics Innovation Unit|COVID-19 Genomics UK (COG-UK) Consortium|Awan|United Kingdom
>Spike|hCoV-19/Wales/PHWC-48EC02/2020|2020-11-09|EPI_ISL_680978|Original|hCoV-19^^Wales|Human|Wales Specialist Virology Centre Sequencing lab|COVID-19 Genomics UK (COG-UK) Consortium|Moore|United Kingdom
>Spike|hCoV-19/Wales/PHWC-15D382/2020|2020-05-06|EPI_ISL_472708|Original|hCoV-19^^Wales|Human|Wales Specialist Virology Centre Sequencing lab|COVID-19 Genomics UK (COG-UK) Consortium|Moore|United Kingdom
>Spike|hCoV-19/USA/NY-NYUMC791/2020|2020-03-26|EPI_ISL_456066|Original|hCoV-19^^New York|Human|NYU Langone Health|Departments of Pathology and Medicine|Maurano|USA
>Spike|hCoV-19/Australia/WA283/2020|2020-04-03|EPI_ISL_596826|Original|hCoV-19^^Western Australia|Human|PathWest Laboratory Medicine WA|PathWest Laboratory Medicine WA Microbial Surveillance Unit|Sikazwe|Australia
>Spike|hCoV-19/Scotland/EDB3196/2020|2020-04-06|EPI_ISL_488962|Original|hCoV-19^^Scotland|Human|Virology Department|Wellcome Sanger Institute for the COVID-19 Genomics UK (COG-UK) consortium|Davies|United Kingdom
>Spike|hCoV-19/Switzerland/BL-ETHZ-120044/2020|2020-04-20|EPI_ISL_468232|Original|hCoV-19^^Basel-Landschaft|Human|Viollier AG|Department of Biosystems Science and Engineering|Nadeau|Switzerland
>Spike|hCoV-19/England/CAMB-1B7131/2020|2020-10-13|EPI_ISL_638306|Original|hCoV-19^^England|Human|Department of Pathology|COVID-19 Genomics UK (COG-UK) Consortium|Goodfellow|United Kingdom
>Spike|hCoV-19/Spain/VC-IBV-98003560/2020|2020-03-30|EPI_ISL_537980|Original|hCoV-19^^Comunitat Valenciana|Human|Servicio de Microbiolog\u00eda. Hospital General Universitario de Castell\u00f3n|SeqCOVID-SPAIN consortium/IBV(CSIC)|Comas|Spain
>Spike|hCoV-19/England/MILK-ACCCC6/2020|2020-10-21|EPI_ISL_630324|Original|hCoV-19^^England|Human|Lighthouse Lab in Milton Keynes|Wellcome Sanger Institute for the COVID-19 Genomics UK (COG-UK) consortium|Davies|United Kingdom
>Spike|hCoV-19/Wales/PHWC-27BF3/2020|2020-03-31|EPI_ISL_431917|Original|hCoV-19^^Wales|Human|Wales Specialist Virology Centre|Public Health Wales Microbiology Cardiff|Moore|United Kingdom
>Spike|hCoV-19/England/MILK-99D764/2020|2020-08-31|EPI_ISL_549874|Original|hCoV-19^^England|Human|Lighthouse Lab in Milton Keynes|Wellcome Sanger Institute for the COVID-19 Genomics UK (COG-UK) consortium|Davies|United Kingdom
>Spike|hCoV-19/Scotland/QEUH-C30CDA/2020|2020-12-11|EPI_ISL_757135|Original|hCoV-19^^Scotland|Human|Lighthouse Lab in Glasgow|Wellcome Sanger Institute for the COVID-19 Genomics UK (COG-UK) Consortium|Davies|United Kingdom
>Spike|hCoV-19/Luxembourg/LNS3186627/2020|2020-10-28|EPI_ISL_740265|Original|hCoV-19^^Luxembourg|Human|Laboratoire national de sant\u00e9|Laboratoire national de sant\u00e9|Wienecke-Baldacchino|Luxembourg
>Spike|hCoV-19/England/CAMC-B7A798/2020|2020-11-12|EPI_ISL_741139|Original|hCoV-19^^England|Human|Oxford Viromics|COVID-19 Genomics UK (COG-UK) Consortium|Golubchik|United Kingdom
>Spike|hCoV-19/England/ALDP-B628EB/2020|2020-11-09|EPI_ISL_656606|Original|hCoV-19^^England|Human|Lighthouse Lab in Alderley Park|Wellcome Sanger Institute for the COVID-19 Genomics UK (COG-UK) Consortium|Davies|United Kingdom
>Spike|hCoV-19/Japan/PG-0858/2020|2020-04-00|EPI_ISL_684468|Original|hCoV-19^^Japan|Human|Fukuoka Institute of Health and Environmental Sciences|Pathogen Genomics Center|Sekizuka|Japan
>Spike|hCoV-19/England/ALDP-B85035/2020|2020-11-14|EPI_ISL_675890|Original|hCoV-19^^England|Human|Lighthouse Lab in Alderley Park|Wellcome Sanger Institute for the COVID-19 Genomics UK (COG-UK) Consortium|Davies|United Kingdom

>Spike|hCoV-19/Denmark/DCGC-8517/2020|2020-10-26|EPI_ISL_625921|Original|hCoV-19^^Hovedstaden|Human|Department of Virus and Microbiological Special Diagnostics|Albertsen lab|Michaelsen|Denmark
>Spike|hCoV-19/Denmark/DCGC-10830/2020|2020-11-02|EPI_ISL_670237|Original|hCoV-19^^Denmark|Human|Department of Virus and Microbiological Special Diagnostics|Albertsen Lab|Michaelsen|Denmark
>Spike|hCoV-19/England/LCST-24D676E/2020|2020-05-30|EPI_ISL_478290|Original|hCoV-19^^England|Human|University Hospitals Of Leicester NHS Trust and DeepSeq Nottingham|COVID-19 Genomics UK (COG-UK) Consortium|Loose|United Kingdom
>Spike|hCoV-19/Wales/PHWC-1665BD/2020|2020-06-22|EPI_ISL_494019|Original|hCoV-19^^Wales|Human|Originating lab|COVID-19 Genomics UK (COG-UK) Consortium|Moore|United Kingdom
>Spike|hCoV-19/USA/CA-ALSR-4188/2020|2020-06-20|EPI_ISL_648874|Original|hCoV-19^^California|Human|San Diego County Public Health Laboratory|Andersen lab at Scripps Research|Gangavarapu|USA
>Spike|hCoV-19/Australia/NSW709/2020|2020-07-25|EPI_ISL_513352|Original|hCoV-19^^New South Wales|Human|South Eastern Area Laboratory Services (SEALS)|NSW Health Pathology - Institute of Clinical Pathology and Medical Research; Westmead Hospital; University of Sydney|Gall|Australia
>Spike|hCoV-19/Scotland/QEUH-9CE69A/2020|2020-09-17|EPI_ISL_602010|Original|hCoV-19^^Scotland|Human|Lighthouse Lab in Glasgow|Wellcome Sanger Institute for the COVID-19 Genomics UK (COG-UK) consortium|Davies|United Kingdom
>Spike|hCoV-19/Denmark/DCGC-20622/2020|2020-12-21|EPI_ISL_759259|Original|hCoV-19^^Hovedstaden|Human|Department of Virus and Microbiological Special Diagnostics|Albertsen Lab|Michaelsen|Denmark
>Spike|hCoV-19/India/TG-CCMB-X10/2020|2020-07-14|EPI_ISL_528841|Original|hCoV-19^^Telangana|Human|CSIR-Centre for Cellular and Molecular Biology|CSIR-Centre for Cellular and Molecular Biology|Banu|India
>Spike|hCoV-19/England/LIVE-DB2D27/2020|2020-10-31|EPI_ISL_637336|Original|hCoV-19^^England|Human|Liverpool Clinical Laboratories|COVID-19 Genomics UK (COG-UK) Consortium|Haldenby|United Kingdom
>Spike|hCoV-19/New Zealand/20VR2731/2020|2020-00-00|EPI_ISL_579346|Original|hCoV-19^^Auckland|Human|LabPLUS|Institute of Environmental Science and Research (ESR)|Wang|New Zealand
>Spike|hCoV-19/England/CAMC-B08920/2020|2020-10-29|EPI_ISL_643906|Original|hCoV-19^^England|Human|Lighthouse Lab in Cambridge|Wellcome Sanger Institute for the COVID-19 Genomics UK (COG-UK) Consortium|Davies|United Kingdom
>Spike|hCoV-19/Luxembourg/LNS5936689/2020|2020-11-09|EPI_ISL_744327|Original|hCoV-19^^Luxembourg|Human|Laboratoire national de sant\u00e9|Laboratoire national de sant\u00e9|Wienecke-Baldacchino|Luxembourg
>Spike|hCoV-19/USA/FL-BPHL-1059/2020|2020-07-17|EPI_ISL_517949|Original|hCoV-19^^Florida|Human|Florida Bureau of Public Health Laboratories|Florida Bureau of Public Health Laboratories|Blanton|USA
>Spike|hCoV-19/Denmark/DCGC-17654/2020|2020-12-14|EPI_ISL_751126|Original|hCoV-19^^Nordjylland|Human|Department of Virus and Microbiological Special Diagnostics|Albertsen Lab|Michaelsen|Denmark
>Spike|hCoV-19/Wales/PHWC-47DA93/2020|2020-10-08|EPI_ISL_586233|Original|hCoV-19^^Wales|Human|Wales Specialist Virology Centre Sequencing lab|COVID-19 Genomics UK (COG-UK) Consortium|Moore|United Kingdom
>Spike|hCoV-19/USA/ME-HETL-J0290/2020|2020-10-05|EPI_ISL_755432|Original|hCoV-19^^Maine|Human|Maine Health and Environmental Testing Laboratory|Tewhey Lab|Tewhey|USA
>Spike|hCoV-19/Denmark/DCGC-11384/2020|2020-11-02|EPI_ISL_670880|Original|hCoV-19^^Hovedstaden|Human|Department of Virus and Microbiological Special Diagnostics|Albertsen Lab|Michaelsen|Denmark
>Spike|hCoV-19/England/CAMC-9C1DDE/2020|2020-09-08|EPI_ISL_573235|Original|hCoV-19^^England|Human|Oxford Viromics|COVID-19 Genomics UK (COG-UK) Consortium|Golubchik|United Kingdom
>Spike|hCoV-19/USA/UT-02904/2020|2020-04-30|EPI_ISL_509535|Original|hCoV-19^^Utah|Human|Utah Public Health Laboratory|Utah Public Health Laboratory|Butz|USA

>Spike|hCoV-19/Wales/PHWC-47E5FB/2020|2020-10-10|EPI_ISL_595477|Original|hCoV-19^^Wales|Human|Originating lab|COVID-19 Genomics UK (COG-UK) Consortium|Moore|United Kingdom
>Spike|hCoV-19/USA/CA-CZB077/2020|2020-03-28|EPI_ISL_428996|Original|hCoV-19^^California|Human|UCSF Clinical Microbiology Laboratory|Chan-Zuckerberg Biohub|Kamm|USA
>Spike|hCoV-19/Wales/PHWC-48AE88/2020|2020-10-31|EPI_ISL_666166|Original|hCoV-19^^Wales|Human|Wales Specialist Virology Centre Sequencing lab|COVID-19 Genomics UK (COG-UK) Consortium|Moore|United Kingdom
>Spike|hCoV-19/England/MILK-B05EE8/2020|2020-10-24|EPI_ISL_642907|Original|hCoV-19^^England|Human|Lighthouse Lab in Milton Keynes|Wellcome Sanger Institute for the COVID-19 Genomics UK (COG-UK) Consortium|Davies|United Kingdom
>Spike|hCoV-19/Denmark/DCGC-1451/2020|2020-06-08|EPI_ISL_618092|Original|hCoV-19^^Nordjylland|Human|Department of Virus and Microbiological Special Diagnostics|Albertsen lab|Michaelsen|Denmark
>Spike|hCoV-19/England/CAMC-9421AC/2020|2020-07-23|EPI_ISL_556908|Original|hCoV-19^^England|Human|Lighthouse Lab in Cambridge|Wellcome Sanger Institute for the COVID-19 Genomics UK (COG-UK) consortium|Davies|United Kingdom
>Spike|hCoV-19/England/QEUH-9C96EA/2020|2020-09-10|EPI_ISL_567530|Original|hCoV-19^^England|Human|Lighthouse Lab in Glasgow|Wellcome Sanger Institute for the COVID-19 Genomics UK (COG-UK) consortium|Davies|United Kingdom
>Spike|hCoV-19/England/NOTT-1120CE/2020|2020-04-20|EPI_ISL_478728|Original|hCoV-19^^England|Human|Queens Medical Centre|COVID-19 Genomics UK (COG-UK) Consortium|Loose|United Kingdom
>Spike|hCoV-19/England/QEUH-9994CB/2020|2020-08-24|EPI_ISL_559764|Original|hCoV-19^^England|Human|Oxford Viromics|COVID-19 Genomics UK (COG-UK) Consortium|Golubchik|United Kingdom
>Spike|hCoV-19/Wales/PHWC-49BF09/2020|2020-12-06|EPI_ISL_742482|Original|hCoV-19^^Wales|Human|Wales Specialist Virology Centre Sequencing lab|COVID-19 Genomics UK (COG-UK) Consortium|Moore|United Kingdom
>Spike|hCoV-19/Greece/244_32204/2020|2020-03-10|EPI_ISL_437904|Original|hCoV-19^^Athens|Human|Laboratory of Microbiology|Laboratory of Biology|Karakasiliotis|Greece
>Spike|hCoV-19/USA/MI-QDX-295/2020|2020-03-20|EPI_ISL_571039|Original|hCoV-19^^Michigan|Human|Quest Diagnostics|Quest Diagnostics|Kagan|USA
>Spike|hCoV-19/Japan/PG-3921/2020|2020-07-00|EPI_ISL_692172|Original|hCoV-19^^Japan|Human|Pathogen Genomics Center|Pathogen Genomics Center|Sekizuka|Japan
>Spike|hCoV-19/USA/FL-BPHL-0450/2020|2020-03-23|EPI_ISL_495356|Original|hCoV-19^^Florida|Human|Florida Bureau of Public Health Laboratories|Florida Bureau of Public Health Laboratories|Blanton|USA
>Spike|hCoV-19/USA/CA-CZB-6811/2020|2020-07-14|EPI_ISL_582886|Original|hCoV-19^^California|Human|County of Santa Clara Public Health Department|Chan-Zuckerberg Biohub|Bhatt|USA
>Spike|hCoV-19/Netherlands/NB-EMC-424/2020|2020-11-11|EPI_ISL_722616|Original|hCoV-19^^Noord Brabant|Human|Dutch COVID-19 response team|Erasmus Medical Center|Oude Munnink|Netherlands
>Spike|hCoV-19/USA/CA-ALSR-4181/2020|2020-06-20|EPI_ISL_648867|Original|hCoV-19^^California|Human|San Diego County Public Health Laboratory|Andersen lab at Scripps Research|Gangavarapu|USA
>Spike|hCoV-19/England/OXON-AF935/2020|2020-04-15|EPI_ISL_478786|Original|hCoV-19^^England|Human|Oxford Viromics|COVID-19 Genomics UK (COG-UK) Consortium|Golubchik|United Kingdom
>Spike|hCoV-19/Scotland/QEUH-9CF392/2020|2020-09-17|EPI_ISL_602003|Original|hCoV-19^^Scotland|Human|Lighthouse Lab in Glasgow|Wellcome Sanger Institute for the COVID-19 Genomics UK (COG-UK) consortium|Davies|United Kingdom
>Spike|hCoV-19/New Zealand/20CV0213/2020|2020-08-31|EPI_ISL_548062|Original|hCoV-19^^Auckland|Human|North Shore Hospital|Institute of Environmental Science and Research (ESR)|Wang|New Zealand

>Spike|hCoV-19/Wales/PHWC-48E604/2020|2020-11-12|EPI_ISL_680885|Original|hCoV-
19^^Wales|Human|Wales Specialist Virology Centre Sequencing lab|COVID-19 Genomics UK
(COG-UK) Consortium|Moore|United Kingdom
>Spike|hCoV-19/USA/WI-UW-1083/2020|2020-07-24|EPI_ISL_516509|Original|hCoV-
19^^Wisconsin|Human|University of Wisconsin-Madison AIDS Vaccine Research
Laboratories|University of Wisconsin-Madison AIDS Vaccine Research
Laboratories|Baczenas|USA
>Spike|hCoV-19/England/MILK-97528F/2020|2020-08-21|EPI_ISL_552558|Original|hCoV-
19^^England|Human|Lighthouse Lab in Milton Keynes|Wellcome Sanger Institute for the
COVID-19 Genomics UK (COG-UK) consortium|Davies|United Kingdom
>Spike|hCoV-19/England/ALDP-AA1ACD/2020|2020-10-22|EPI_ISL_624384|Original|hCoV-
19^^England|Human|Lighthouse Lab in Alderley Park|Wellcome Sanger Institute for the
COVID-19 Genomics UK (COG-UK) consortium|Davies|United Kingdom
>Spike|hCoV-19/France/PAC-IHU-1365/2020|2020-08-20|EPI_ISL_569144|Original|hCoV-
19^^Provence-Alpes-C\u00f4te-d\u2019Azur|Human|MEPHI|MEPHI|Levasseur|France
>Spike|hCoV-19/USA/AZ-TG668363/2020|2020-05-13|EPI_ISL_696283|Original|hCoV-
19^^Arizona|Human|Sonora Quest Laboratories|TGen North|French|USA
>Spike|hCoV-19/England/CAMC-BD444A/2020|2020-11-27|EPI_ISL_702289|Original|hCoV-
19^^England|Human|Lighthouse Lab in Cambridge|Wellcome Sanger Institute for the
COVID-19 Genomics UK (COG-UK) Consortium|Davies|United Kingdom
>Spike|hCoV-19/USA/WI-UW-2632/2021|2021-01-03|EPI_ISL_791148|Original|hCoV-
19^^Wisconsin|Human|University of Wisconsin-Madison AIDS Vaccine Research
Laboratories|University of Wisconsin-Madison AIDS Vaccine Research
Laboratories|Moreno|USA
>Spike|hCoV-19/Denmark/DCGC-13786/2020|2020-11-30|EPI_ISL_713024|Original|hCoV-
19^^Sjaelland|Human|Department of Virus and Microbiological Special
Diagnostics|Albertsen Lab|Michaelsen|Denmark
>Spike|hCoV-19/Denmark/DCGC-14534/2020|2020-11-30|EPI_ISL_711822|Original|hCoV-
19^^Hovedstaden|Human|Department of Virus and Microbiological Special
Diagnostics|Albertsen Lab|Michaelsen|Denmark
>Spike|hCoV-19/England/ALDP-A2D09A/2020|2020-10-06|EPI_ISL_586798|Original|hCoV-
19^^England|Human|Lighthouse Lab in Alderley Park|Wellcome Sanger Institute for the
COVID-19 Genomics UK (COG-UK) consortium|Davies|United Kingdom
>Spike|hCoV-19/Austria/CeMM0360/2020|2020-04-05|EPI_ISL_438123|Vero P3|hCoV-
19^^Vienna|Human|Center for Virology|Bergthaler laboratory|Bergthaler|Austria
>Spike|hCoV-19/Luxembourg/LNS6035285/2020|2020-12-01|EPI_ISL_770944|Original|hCoV-
19^^Luxembourg|Human|Laboratoire national de sant\u00e9|Laboratoire national de
sant\u00e9|Wienecke-Baldacchino|Luxembourg
>Spike|hCoV-19/Scotland/CVR5051/2020|2020-10-09|EPI_ISL_594785|Original|hCoV-
19^^Scotland|Human|West of Scotland Specialist Virology Centre|COVID-19 Genomics UK
(COG-UK) Consortium|Robertson|United Kingdom
>Spike|hCoV-19/England/MILK-9C3FE5/2020|2020-09-09|EPI_ISL_566970|Original|hCoV-
19^^England|Human|Lighthouse Lab in Milton Keynes|Wellcome Sanger Institute for the
COVID-19 Genomics UK (COG-UK) consortium|Davies|United Kingdom
>Spike|hCoV-19/Northern Ireland/QEUH-5FF88F/2020|2020-06-
27|EPI_ISL_533266|Original|hCoV-19^^Northern Ireland|Human|Lighthouse Lab in
Glasgow|Wellcome Sanger Institute for the COVID-19 Genomics UK (COG-UK)
consortium|Davies|United Kingdom
>Spike|hCoV-19/England/ALDP-AC5D9D/2020|2020-10-14|EPI_ISL_631265|Original|hCoV-
19^^England|Human|Lighthouse Lab in Alderley Park|Wellcome Sanger Institute for the
COVID-19 Genomics UK (COG-UK) consortium|Davies|United Kingdom
>Spike|hCoV-19/France/PAC-IHU-1590/2020|2020-09-08|EPI_ISL_569219|Original|hCoV-
19^^Provence-Alpes-C\u00f4te-d\u2019Azur|Human|MEPHI|MEPHI|Levasseur|France
>Spike|hCoV-19/Sweden/20-13142/2020|2020-12-23|EPI_ISL_789026|Original|hCoV-
19^^Stockholm|Human|ABC|The Public Health Agency of Sweden|Svartstr\u00f6m|Sweden
>Spike|hCoV-19/Germany/BE-ChVir-W1081-8197/2020|2020-04-
06|EPI_ISL_753933|Original|hCoV-19^^Berlin|Human|Charit\u00e9
Universit\u00e4tsmedizin Berlin|Charit\u00e9 Universit\u00e4tsmedizin
Berlin|Muehlemann|Germany

>Spike|hCoV-19/USA/CO-CDPHE-2009151379/2020|2020-09-14|EPI_ISL_710229|Original|hCoV-
19^^Colorado|Human|Colorado Department of Public Health and Environment|Colorado
Department of Puplic Health and Environment|Bankers|USA
>Spike|hCoV-19/USA/FL-UF-4/2020|2020-03-27|EPI_ISL_524433|LLC-MK2|hCoV-
19^^Florida|Human|Environmental and Global Health|University of Florida|Elbadry|USA
>Spike|hCoV-19/Japan/PG-7105/2020|2020-08-00|EPI_ISL_688210|Original|hCoV-
19^^Japan|Human|Pathogen Genomics Center|Pathogen Genomics Center|Sekizuka|Japan
>Spike|hCoV-19/England/ALDP-A8586B/2020|2020-10-17|EPI_ISL_610343|Original|hCoV-
19^^England|Human|Lighthouse Lab in Alderley Park|Wellcome Sanger Institute for the
COVID-19 Genomics UK (COG-UK) consortium|Davies|United Kingdom
>Spike|hCoV-19/Switzerland/BE-ETHZ-360089/2020|2020-11-
08|EPI_ISL_693899|Original|hCoV-19^^Bern|Human|Viollier AG|Department of Biosystems
Science and Engineering|Nadeau|Switzerland
>Spike|hCoV-19/France/ARA-SC409/2020|2020-05-02|EPI_ISL_660368|Original|hCoV-
19^^Auvergne-Rh\u00f4ne-Alpes|Human|CHU Clermont-Ferrand|CNR Virus des Infections
Respiratoires - France SUD|Bal|France
>Spike|hCoV-19/England/LEED-2A81C9/2020|2020-04-26|EPI_ISL_539048|Original|hCoV-
19^^England|Human|Leeds Teaching Hospitals NHS Trust and Public Health
England|Wellcome Sanger Institute for the COVID-19 Genomics UK (COG-UK)
consortium|Davies|United Kingdom
>Spike|hCoV-19/USA/CA-QDX-2185/2020|2020-03-18|EPI_ISL_604446|Original|hCoV-
19^^California|Human|Quest Diagnostics|Quest Diagnostics|Kagan|USA
>Spike|hCoV-19/England/QEUH-960C98/2020|2020-08-02|EPI_ISL_531980|Original|hCoV-
19^^England|Human|Lighthouse Lab in Glasgow|Wellcome Sanger Institute for the COVID-
19 Genomics UK (COG-UK) consortium|Davies|United Kingdom
>Spike|hCoV-19/USA/TX-HMH-4602/2020|2020-06-20|EPI_ISL_543748|Original|hCoV-
19^^Texas|Human|Houston Methodist Hospital|Houston Methodist Hospital|Long|USA
>Spike|hCoV-19/England/MILK-BB1F9D/2020|2020-11-18|EPI_ISL_720242|Original|hCoV-
19^^England|Human|Lighthouse Lab in Milton Keynes|Wellcome Sanger Institute for the
COVID-19 Genomics UK (COG-UK) Consortium|Davies|United Kingdom
>Spike|hCoV-19/Northern Ireland/NIRE-FE427/2020|2020-05-
07|EPI_ISL_489229|Original|hCoV-19^^Northern Ireland|Human|Regional Virus
Laboratory|Wellcome Sanger Institute for the COVID-19 Genomics UK (COG-UK)
consortium|Davies|United Kingdom
>Spike|hCoV-19/Belgium/ULG-10643/2020|2020-10-26|EPI_ISL_636621|Original|hCoV-
19^^Li\u00e8ge|Human|Department of Clinical Microbiology|GIGA Medical
Genomics|Durkin|Belgium
>Spike|hCoV-19/England/NORT-297B92/2020|2020-04-24|EPI_ISL_488175|Original|hCoV-
19^^England|Human|NU-OMICS DNA Sequencing research facility|Wellcome Sanger Institute
for the COVID-19 Genomics UK (COG-UK) consortium|Davies|United Kingdom
>Spike|hCoV-19/Wales/PHWC-4A4635/2020|2020-12-16|EPI_ISL_764716|Original|hCoV-
19^^Wales|Human|Wales Specialist Virology Centre Sequencing lab|COVID-19 Genomics UK
(COG-UK) Consortium|Moore|United Kingdom
>Spike|hCoV-19/Wales/PHWC-4A31F9/2020|2020-12-14|EPI_ISL_744069|Original|hCoV-
19^^Wales|Human|Wales Specialist Virology Centre Sequencing lab|COVID-19 Genomics UK
(COG-UK) Consortium|Moore|United Kingdom
>Spike|hCoV-19/England/QEUH-AF7345/2020|2020-10-29|EPI_ISL_662781|Original|hCoV-
19^^England|Human|Lighthouse Lab in Glasgow|Wellcome Sanger Institute for the COVID-
19 Genomics UK (COG-UK) Consortium|Davies|United Kingdom
>Spike|hCoV-19/Wales/PHWC-48CC6E/2020|2020-11-05|EPI_ISL_666523|Original|hCoV-
19^^Wales|Human|Wales Specialist Virology Centre Sequencing lab|COVID-19 Genomics UK
(COG-UK) Consortium|Moore|United Kingdom
>Spike|hCoV-19/Wales/PHWC-16EB86/2020|2020-10-04|EPI_ISL_585829|Original|hCoV-
19^^Wales|Human|Wales Specialist Virology Centre Sequencing lab|COVID-19 Genomics UK
(COG-UK) Consortium|Moore|United Kingdom
>Spike|hCoV-19/USA/TX-HMH-MCoV-9511/2020|2020-07-03|EPI_ISL_781157|Original|hCoV-
19^^Texas|Human|Houston Methodist Hospital|Houston Methodist Hospital|Long|USA
>Spike|hCoV-19/Sweden/20-53054/2020|2020-09-21|EPI_ISL_661287|Original|hCoV-
19^^Gavleborg|Human|Gavle klinisk mikrobiologi|The Public Health Agency of
Sweden|Svartstr\u00f6m|Sweden

>Spike|hCoV-19/USA/FL-BPHL-0622/2020|2020-04-04|EPI_ISL_508826|Original|hCoV-19^^Florida|Human|Florida Bureau of Public Health Laboratories|Florida Bureau of Public Health Laboratories|Blanton|USA
>Spike|hCoV-19/USA/NY-Wadsworth-13288-01/2020|2020-03-11|EPI_ISL_676732|Original|hCoV-19^^New York|Human|Wadsworth Center|Wadsworth Center|Lasek-Nesselquist|USA
>Spike|hCoV-19/USA/TX-HMH-MCoV-16332/2020|2020-11-02|EPI_ISL_786016|Original|hCoV-19^^Texas|Human|Houston Methodist Hospital|Houston Methodist Hospital|Long|USA
>Spike|hCoV-19/England/NORW-EAF3C/2020|2020-04-30|EPI_ISL_457394|Original|hCoV-19^^England|Human|Quadram Institute Bioscience|COVID-19 Genomics UK (COG-UK) Consortium|Alikhan|United Kingdom
>Spike|hCoV-19/England/20156053104/2020|2020-04-07|EPI_ISL_466343|Original|hCoV-19^^England|Human|Respiratory Virus Unit|Respiratory Virus Unit|Platt|United Kingdom
>Spike|hCoV-19/England/MILK-B562A0/2020|2020-11-05|EPI_ISL_657614|Original|hCoV-19^^England|Human|Lighthouse Lab in Milton Keynes|Wellcome Sanger Institute for the COVID-19 Genomics UK (COG-UK) Consortium|Davies|United Kingdom
>Spike|hCoV-19/England/BRIS-12A12A/2020|2020-04-11|EPI_ISL_458412|Original|hCoV-19^^England|Human|PHE South West Regional Laboratory|Wellcome Sanger Institute for the COVID-19 Genomics UK (COG-UK) consortium|Davies|United Kingdom
>Spike|hCoV-19/England/ALDP-BB3DDD/2020|2020-11-20|EPI_ISL_701732|Original|hCoV-19^^England|Human|Lighthouse Lab in Alderley Park|Wellcome Sanger Institute for the COVID-19 Genomics UK (COG-UK) Consortium|Davies|United Kingdom
>Spike|hCoV-19/USA/OR-PROV-209/2020|2020-04-02|EPI_ISL_482450|Original|hCoV-19^^Oregon|Human|Providence St. Joseph Health Molecular Genomics Laboratory|Providence St. Joseph Health Molecular Genomics Laboratory|Dowdell|USA
>Spike|hCoV-19/Spain/RI-IBV-006026/2020|2020-03-31|EPI_ISL_500456|Original|hCoV-19^^La Rioja|Human|Centro de Investigaci\u00f3n Biom\u00e9dica de La Rioja - Hospital San Pedro Logro\u00f1o|SeqCOVID-SPAIN consortium/IBV(CSIC)|Comas|Spain
>Spike|hCoV-19/Denmark/DCGC-16011/2020|2020-11-23|EPI_ISL_714533|Original|hCoV-19^^Midtjylland|Human|Department of Virus and Microbiological Special Diagnostics|Albertsen Lab|Michaelsen|Denmark
>Spike|hCoV-19/England/QEUH-9617C3/2020|2020-08-02|EPI_ISL_537079|Original|hCoV-19^^England|Human|Lighthouse Lab in Glasgow|Wellcome Sanger Institute for the COVID-19 Genomics UK (COG-UK) consortium|Davies|United Kingdom
>Spike|hCoV-19/Wales/PHWC-160094/2020|2020-05-26|EPI_ISL_473142|Original|hCoV-19^^Wales|Human|Wales Specialist Virology Centre Sequencing lab|COVID-19 Genomics UK (COG-UK) Consortium|Moore|United Kingdom
>Spike|hCoV-19/England/MILK-C517B4/2020|2020-12-07|EPI_ISL_769597|Original|hCoV-19^^England|Human|Lighthouse Lab in Milton Keynes|Wellcome Sanger Institute for the COVID-19 Genomics UK (COG-UK) Consortium|Davies|United Kingdom
>Spike|hCoV-19/England/204500452/2020|2020-10-30|EPI_ISL_660783|Original|hCoV-19^^England|Human|Respiratory Virus Unit|COVID-19 Genomics UK (COG-UK) Consortium|Platt|United Kingdom
>Spike|hCoV-19/South Korea/KDCA0101/2020|2020-09-12|EPI_ISL_747337|Original|hCoV-19^^South Korea|Human|Division of Emerging Infectious Diseases|Division of Emerging Infectious Diseases|Park|South Korea
>Spike|hCoV-19/Denmark/DCGC-5269/2020|2020-09-14|EPI_ISL_615200|Original|hCoV-19^^Hovedstaden|Human|Department of Virus and Microbiological Special Diagnostics|Albertsen lab|Michaelsen|Denmark
>Spike|hCoV-19/Denmark/DCGC-6223/2020|2020-10-05|EPI_ISL_620126|Original|hCoV-19^^Syddanmark|Human|Department of Virus and Microbiological Special Diagnostics|Albertsen lab|Michaelsen|Denmark
>Spike|hCoV-19/Spain/MD-HLP-H10-39/2020|2020-03-12|EPI_ISL_417963|Original|hCoV-19^^Madrid|Human|Hospital Universitario 12 de Octubre|Hospital Universitario La Paz|Viedma|Spain
>Spike|hCoV-19/England/QEUH-A336AE/2020|2020-10-02|EPI_ISL_628401|Original|hCoV-19^^England|Human|Virology Department|COVID-19 Genomics UK (COG-UK) Consortium|de Silva|United Kingdom
>Spike|hCoV-19/USA/CA-CZB-13855/2020|2020-08-14|EPI_ISL_738561|Original|hCoV-19^^California|Human|Alameda County Public Health Lab|Chan-Zuckerberg Biohub|Kamm|USA

>Spike|hCoV-19/England/MILK-ACCC11/2020|2020-10-21|EPI_ISL_634438|Original|hCoV-19^^England|Human|Lighthouse Lab in Milton Keynes|Wellcome Sanger Institute for the COVID-19 Genomics UK (COG-UK) consortium|Davies|United Kingdom
>Spike|hCoV-19/Norway/3024/2020|2020-08-05|EPI_ISL_549076|Original|hCoV-19^^Oslo|Human|Furst Medical Laboratory|Norwegian Institute of Public Health|Kopperud Riis|Norway
>Spike|hCoV-19/England/CAMC-CF9815/2020|2020-12-18|EPI_ISL_781514|Original|hCoV-19^^England|Human|Lighthouse Lab in Cambridge|Wellcome Sanger Institute for the COVID-19 Genomics UK (COG-UK) Consortium|Davies|United Kingdom
>Spike|hCoV-19/USA/TX-HMH-MCoV-18311/2020|2020-11-17|EPI_ISL_784659|Original|hCoV-19^^Texas|Human|Houston Methodist Hospital|Houston Methodist Hospital|Long|USA
>Spike|hCoV-19/Wales/PHWC-1607CC/2020|2020-05-20|EPI_ISL_479297|Original|hCoV-19^^Wales|Human|Wales Specialist Virology Centre Sequencing lab|COVID-19 Genomics UK (COG-UK) Consortium|Moore|United Kingdom
>Spike|hCoV-19/Wales/PHWC-49D2FF/2020|2020-12-10|EPI_ISL_742764|Original|hCoV-19^^Wales|Human|Wales Specialist Virology Centre Sequencing lab|COVID-19 Genomics UK (COG-UK) Consortium|Moore|United Kingdom
>Spike|hCoV-19/Mexico/BCN-ALSR-2452/2020|2020-05-15|EPI_ISL_635484|Original|hCoV-19^^Baja California|Human|Centro de Diagn\u221a\u2265stico COVID-19 UABC Tijuana|Andersen lab at Scripps Research|Gangavarapu|Mexico
>Spike|hCoV-19/Japan/PG-0483/2020|2020-04-03|EPI_ISL_480142|Original|hCoV-19^^HokurikuShinsyu|Human|Fukui Prefectural Institute of Public Health and Environmental Science|Pathogen Genomics Center|Sekizuka|Japan
>Spike|hCoV-19/USA/MN-MDH-1498/2020|2020-03-21|EPI_ISL_518891|Original|hCoV-19^^Minnesota|Human|Mayo Clinic & Mayo Clinic Laboratories|Minnesota Department of Health|Garfin|USA
>Spike|hCoV-19/Portugal/PT0346/2020|2020-03-18|EPI_ISL_454062|Original|hCoV-19^^Portugal|Human|unknown|Instituto Nacional de Saude (INSA)|Borges|Portugal
>Spike|hCoV-19/England/CAMB-1AFCFF/2020|2020-05-14|EPI_ISL_452875|Original|hCoV-19^^England|Human|Department of Pathology|COVID-19 Genomics UK (COG-UK) Consortium|Meredith|United Kingdom
>Spike|hCoV-19/USA/MI-MDHHS-SC21792/2020|2020-04-13|EPI_ISL_565835|Original|hCoV-19^^Michigan|Human|Michigan Department of Health and Human Services|Michigan Department of Health and Human Services|Blankenship|USA
>Spike|hCoV-19/Spain/PV-IBV-001528/2020|2020-03-13|EPI_ISL_467159|Original|hCoV-19^^Basque Country|Human|Hospital Universitario Araba. Vitoria-Gasteiz|SeqCOVID-SPAIN consortium/IBV(CSIC)|Comas|Spain
>Spike|hCoV-19/United Arab Emirates/0335/2020|2020-05-19|EPI_ISL_698511|Original|hCoV-19^^Abu Dhabi|Human|Group 42 (G42) Healthcare|G42 Healthcare|Ogrodzki|United Arab Emirates
>Spike|hCoV-19/Switzerland/BE-42216413/2020|2020-04-01|EPI_ISL_581671|Original|hCoV-19^^Bern|Human|University Hospital Basel|University Hospital Basel|Roloff Handschin|Switzerland
>Spike|hCoV-19/England/ALDP-9FCF7A/2020|2020-10-01|EPI_ISL_587731|Original|hCoV-19^^England|Human|Lighthouse Lab in Alderley Park|Wellcome Sanger Institute for the COVID-19 Genomics UK (COG-UK) consortium|Davies|United Kingdom
>Spike|hCoV-19/USA/TX-HMH-MCoV-15974/2020|2020-07-10|EPI_ISL_785658|Original|hCoV-19^^Texas|Human|Houston Methodist Hospital|Houston Methodist Hospital|Long|USA
>Spike|hCoV-19/England/NOTT-10F747/2020|2020-04-03|EPI_ISL_433044|Original|hCoV-19^^England|Human|Queens Medical Centre|COVID-19 Genomics UK (COG-UK) Consortium|Loose|United Kingdom
>Spike|hCoV-19/England/CAMC-C26448/2020|2020-12-07|EPI_ISL_733841|Original|hCoV-19^^England|Human|Lighthouse Lab in Cambridge|Wellcome Sanger Institute for the COVID-19 Genomics UK (COG-UK) Consortium|Davies|United Kingdom
>Spike|hCoV-19/England/ALDP-6E9C4F/2020|2020-06-29|EPI_ISL_557600|Original|hCoV-19^^England|Human|Lighthouse Lab in Alderley Park|Wellcome Sanger Institute for the COVID-19 Genomics UK (COG-UK) consortium|Davies|United Kingdom
>Spike|hCoV-19/USA/CA-CZB-1353/2020|2020-04-11|EPI_ISL_468367|Original|hCoV-19^^California|Human|Alameda County Public Health Lab|Chan-Zuckerberg Biohub|Hao|USA

>Spike|hCoV-19/Australia/VIC3472/2020|2020-07-05|EPI_ISL_521133|Original|hCoV-19^^Victoria|Human|Victorian Infectious Diseases Reference Laboratory (VIDRL)|VIDRL and MDU-PHL|Schultz|Australia
>Spike|hCoV-19/England/ALDP-B5B539/2020|2020-11-08|EPI_ISL_655306|Original|hCoV-19^^England|Human|Lighthouse Lab in Alderley Park|Wellcome Sanger Institute for the COVID-19 Genomics UK (COG-UK) Consortium|Davies|United Kingdom
>Spike|hCoV-19/Denmark/DCGC-16643/2020|2020-11-16|EPI_ISL_714170|Original|hCoV-19^^Sjaelland|Human|Department of Virus and Microbiological Special Diagnostics|Albertsen Lab|Michaelsen|Denmark
>Spike|hCoV-19/England/C200024019/2020|2020-09-28|EPI_ISL_636689|Original|hCoV-19^^England|Human|Respiratory Virus Unit|Respiratory Virus Unit|Platt|United Kingdom
>Spike|hCoV-19/USA/TX-HMH-7547/2020|2020-06-25|EPI_ISL_546842|Original|hCoV-19^^Texas|Human|Houston Methodist Hospital|Houston Methodist Hospital|Long|USA
>Spike|hCoV-19/England/ALDP-BC6645/2020|2020-11-22|EPI_ISL_721256|Original|hCoV-19^^England|Human|Lighthouse Lab in Alderley Park|Wellcome Sanger Institute for the COVID-19 Genomics UK (COG-UK) Consortium|Davies|United Kingdom
>Spike|hCoV-19/USA/WI-UW-2362/2020|2020-10-04|EPI_ISL_728128|Original|hCoV-19^^Wisconsin|Human|University of Wisconsin-Madison AIDS Vaccine Research Laboratories|University of Wisconsin-Madison AIDS Vaccine Research Laboratories|Baczenas|USA
>Spike|hCoV-19/USA/MD-HP12149/2020|2020-12-20|EPI_ISL_791458|Original|hCoV-19^^Maryland|Human|Johns Hopkins Hospital Department of Pathology|Johns Hopkins Hospital Department of Pathology|Morris|USA
>Spike|hCoV-19/USA/TX-HMH-MCoV-14169/2020|2020-10-02|EPI_ISL_787454|Original|hCoV-19^^Texas|Human|Houston Methodist Hospital|Houston Methodist Hospital|Long|USA
>Spike|hCoV-19/Ireland/LH-NVRL-20ILR08013/2020|2020-11-10|EPI_ISL_732412|Original|hCoV-19^^Louth|Human|National Virus Reference Laboratory|National Virus Reference Laboratory|Gonzalez|Ireland
>Spike|hCoV-19/Australia/VIC5087/2020|2020-07-17|EPI_ISL_519890|Original|hCoV-19^^Victoria|Human|Microbiological Diagnostic Unit - Public Health Laboratory (MDU-PHL)|MDU-PHL|Schultz|Australia
>Spike|hCoV-19/Canada/MB-NML-642/2020|2020-03-20|EPI_ISL_582299|Original|hCoV-19^^Manitoba|Human|Cadham Provincial Laboratory|National Microbiology Laboratory (NML)|Majer|Canada
>Spike|hCoV-19/Singapore/1390/2020|2020-11-04|EPI_ISL_645117|Original|hCoV-19^^Singapore|Human|National Public Health Laboratory|National Public Health Laboratory|Mak|Singapore
>Spike|hCoV-19/England/CAMC-A58658/2020|2020-10-09|EPI_ISL_597025|Original|hCoV-19^^England|Human|Lighthouse Lab in Cambridge|Wellcome Sanger Institute for the COVID-19 Genomics UK (COG-UK) consortium|Davies|United Kingdom
>Spike|hCoV-19/England/ALDP-A67B0C/2020|2020-10-12|EPI_ISL_630627|Original|hCoV-19^^England|Human|Lighthouse Lab in Alderley Park|Wellcome Sanger Institute for the COVID-19 Genomics UK (COG-UK) consortium|Davies|United Kingdom
>Spike|hCoV-19/Denmark/DCGC-7934/2020|2020-10-26|EPI_ISL_618890|Original|hCoV-19^^Syddanmark|Human|Department of Virus and Microbiological Special Diagnostics|Albertsen lab|Michaelsen|Denmark
>Spike|hCoV-19/South Africa/KRISP-K002622/2020|2020-07-31|EPI_ISL_535485|Original|hCoV-19^^KwaZulu-Natal|Human|NHLS-IALCH|KRISP|de Oliveira|South Africa
>Spike|hCoV-19/South Korea/KCDC2157/2020|2020-02-29|EPI_ISL_506951|Original|hCoV-19^^South Korea|Human|Division of Viral Diseases|Division of Viral Diseases|Kim|South Korea
>Spike|hCoV-19/Denmark/DCGC-15793/2020|2020-11-23|EPI_ISL_714023|Original|hCoV-19^^Nordjylland|Human|Department of Virus and Microbiological Special Diagnostics|Albertsen Lab|Michaelsen|Denmark
>Spike|hCoV-19/USA/CA-CZB-13824/2020|2020-08-17|EPI_ISL_739136|Original|hCoV-19^^California|Human|Alameda County Public Health Lab|Chan-Zuckerberg Biohub|Kamm|USA
>Spike|hCoV-19/Jordan/AM-ALSR-4975/2020|2020-10-08|EPI_ISL_730248|Original|hCoV-19^^Amman|Human|Biolab Diagnostic Laboratories|Andersen lab at Scripps Research|Gangavarapu|Jordan

>Spike|hCoV-19/England/CAMC-A64C93/2020|2020-10-11|EPI_ISL_609037|Original|hCoV-19^^England|Human|Lighthouse Lab in Cambridge|Wellcome Sanger Institute for the COVID-19 Genomics UK (COG-UK) consortium|Davies|United Kingdom
>Spike|hCoV-19/Spain/AN-IBV-002028/2020|2020-03-17|EPI_ISL_452391|Original|hCoV-19^^Andalucia|Human|Servicio de Microbiolog\u00eda. HRU de M\u00e1laga. Servicio Andaluz de Salud|SeqCOVID-SPAIN consortium/IBV(CSIC)|Comas|Spain
>Spike|hCoV-19/England/CAMC-A65E50/2020|2020-10-11|EPI_ISL_637766|Original|hCoV-19^^England|Human|Department of Pathology|COVID-19 Genomics UK (COG-UK) Consortium|Goodfellow|United Kingdom
>Spike|hCoV-19/England/LIVE-1D6EAE/2020|2020-09-19|EPI_ISL_572584|Original|hCoV-19^^England|Human|Liverpool Clinical Laboratories|COVID-19 Genomics UK (COG-UK) Consortium|Haldenby|United Kingdom
>Spike|hCoV-19/India/TG-CCMB-AA335/2020|2020-08-11|EPI_ISL_539756|Original|hCoV-19^^Telangana|Human|CSIR-Centre for Cellular and Molecular Biology|CSIR-Centre for Cellular and Molecular Biology|Mukherjee|India
>Spike|hCoV-19/USA/TX-HMH-MCoV-15518/2020|2020-10-24|EPI_ISL_787002|Original|hCoV-19^^Texas|Human|Houston Methodist Hospital|Houston Methodist Hospital|Long|USA
>Spike|hCoV-19/USA/UT-UPHL-2009326/2020|2020-05-16|EPI_ISL_524927|Original|hCoV-19^^Utah|Human|Utah Public Health Laboratory|Utah Public Health Laboratory|Young|USA
>Spike|hCoV-19/England/QEUH-A21DEA/2020|2020-10-01|EPI_ISL_587582|Original|hCoV-19^^England|Human|Lighthouse Lab in Glasgow|Wellcome Sanger Institute for the COVID-19 Genomics UK (COG-UK) consortium|Davies|United Kingdom
>Spike|hCoV-19/USA/FL-BPHL-0577/2020|2020-03-21|EPI_ISL_508781|Original|hCoV-19^^Florida|Human|Florida Bureau of Public Health Laboratories|Florida Bureau of Public Health Laboratories|Blanton|USA
>Spike|hCoV-19/England/QEUH-A33820/2020|2020-10-05|EPI_ISL_626924|Original|hCoV-19^^England|Human|Virology Department|COVID-19 Genomics UK (COG-UK) Consortium|de Silva|United Kingdom
>Spike|hCoV-19/USA/UT-0492/2020|2020-04-02|EPI_ISL_430047|Original|hCoV-19^^Utah|Human|Utah Public Health Laboratory|Utah Public Health Laboratory|Young|USA
>Spike|hCoV-19/Wales/PHWC-16392D/2020|2020-05-06|EPI_ISL_474016|Original|hCoV-19^^Wales|Human|Originating lab|COVID-19 Genomics UK (COG-UK) Consortium|Moore|United Kingdom
>Spike|hCoV-19/USA/WA-S856/2020|2020-04-24|EPI_ISL_463346|Original|hCoV-19^^Washington|Human|Washington State Department of Health|Seattle Flu Study|Bedford|USA
>Spike|hCoV-19/Scotland/GCVR-171B08/2020|2020-03-27|EPI_ISL_489537|Original|hCoV-19^^Scotland|Human|NHSGGC West of Scotland Specialist Virology Centre / MRC-University of Glasgow Centre for Virus Research|Wellcome Sanger Institute for the COVID-19 Genomics UK (COG-UK) consortium|Davies|United Kingdom
>Spike|hCoV-19/South Africa/L00180/2020|2020-07-27|EPI_ISL_622988|Original|hCoV-19^^Gauteng|Human|Lancet Laboratories|National Institute for Communicable Diseases of the National Health Laboratory Service|Allam|South Africa
>Spike|hCoV-19/Scotland/QEUH-9CF81B/2020|2020-09-17|EPI_ISL_601942|Original|hCoV-19^^Scotland|Human|Lighthouse Lab in Glasgow|Wellcome Sanger Institute for the COVID-19 Genomics UK (COG-UK) consortium|Davies|United Kingdom
>Spike|hCoV-19/Iran/K1r-136/2020|2020-04-27|EPI_ISL_568506|Original|hCoV-19^^Iran|Human|Virology|Virology|Ranjbar|Iran
>Spike|hCoV-19/Switzerland/VS-ETHZ-180011/2020|2020-06-30|EPI_ISL_489965|Original|hCoV-19^^Valais|Human|Viollier AG|Department of Biosystems Science and Engineering|Nadeau|Switzerland
>Spike|hCoV-19/England/QEUH-B2D749/2020|2020-11-03|EPI_ISL_647115|Original|hCoV-19^^England|Human|Lighthouse Lab in Glasgow|Wellcome Sanger Institute for the COVID-19 Genomics UK (COG-UK) Consortium|Davies|United Kingdom
>Spike|hCoV-19/Australia/VIC3851/2020|2020-07-12|EPI_ISL_520564|Original|hCoV-19^^Victoria|Human|Microbiological Diagnostic Unit - Public Health Laboratory (MDU-PHL)|MDU-PHL|Schultz|Australia
>Spike|hCoV-19/England/CAMB-1AE2C1/2020|2020-04-28|EPI_ISL_524575|Original|hCoV-19^^England|Human|Department of Pathology|Wellcome Sanger Institute for the COVID-19 Genomics UK (COG-UK) consortium|Davies|United Kingdom

>Spike|hCoV-19/Wales/PHWC-4896BE/2020|2020-10-28|EPI_ISL_639604|Original|hCoV-19^^Wales|Human|Wales Specialist Virology Centre Sequencing lab|COVID-19 Genomics UK (COG-UK) Consortium|Moore|United Kingdom
>Spike|hCoV-19/USA/OR-OHSU-0917/2020|2020-07-02|EPI_ISL_525926|Original|hCoV-19^^Oregon|Human|OHSU Lab Services Molecular Microbiology Lab|Oregon SARS-CoV-2 Genome Sequencing Center|O'Roak|USA
>Spike|hCoV-19/Thailand/SamutSakhon-CONI-0346/2020|2020-12-19|EPI_ISL_733540|Original|hCoV-19^^Samut Sakhon|Human|Institute for Urban Disease Control and Prevention|COVID-19 Network Investigations (CONI) Alliance|Batty|Thailand
>Spike|hCoV-19/Scotland/QEUH-A20422/2020|2020-10-01|EPI_ISL_598942|Original|hCoV-19^^Scotland|Human|Lighthouse Lab in Glasgow|Wellcome Sanger Institute for the COVID-19 Genomics UK (COG-UK) consortium|Davies|United Kingdom
>Spike|hCoV-19/USA/CA-S1920/2020|2020-06-03|EPI_ISL_497341|Original|hCoV-19^^California|Human|Washington State Department of Health|Seattle Flu Study|Lee|USA
>Spike|hCoV-19/Switzerland/GE-SNRCI-7725/2020|2020-03-00|EPI_ISL_548199|Original|hCoV-19^^Geneve|Human|Laboratoire de Virologie|Swiss National Reference Centre for Influenza|Gon\u00e7alves Cabecinhas|Switzerland
>Spike|hCoV-19/Wales/PHWC-49DFCB/2020|2020-12-06|EPI_ISL_742938|Original|hCoV-19^^Wales|Human|Wales Specialist Virology Centre Sequencing lab|COVID-19 Genomics UK (COG-UK) Consortium|Moore|United Kingdom
>Spike|hCoV-19/England/QEUH-A78054/2020|2020-10-14|EPI_ISL_613355|Original|hCoV-19^^England|Human|Queens Medical Centre|COVID-19 Genomics UK (COG-UK) Consortium|Loose|United Kingdom
>Spike|hCoV-19/Wales/PHWC-27847/2020|2020-00-00|EPI_ISL_431993|Original|hCoV-19^^Wales|Human|Wales Specialist Virology Centre|Public Health Wales Microbiology Cardiff|Moore|United Kingdom
>Spike|hCoV-19/mink/Canada/A/2020|2020-12-04|EPI_ISL_717714|Original|hCoV-19^^British Columbia|Mink|Animal Health Centre|National Centre for Foreign Animal Disease|Kruczkiewicz|Canada
>Spike|hCoV-19/USA/WI-UW-1791/2020|2020-09-14|EPI_ISL_605663|Original|hCoV-19^^Wisconsin|Human|University of Wisconsin-Madison AIDS Vaccine Research Laboratories|University of Wisconsin-Madison AIDS Vaccine Research Laboratories|Baczenas|USA
>Spike|hCoV-19/Scotland/GCVR-16FDAD/2020|2020-03-23|EPI_ISL_489655|Original|hCoV-19^^Scotland|Human|NHSGGC West of Scotland Specialist Virology Centre / MRC-University of Glasgow Centre for Virus Research|Wellcome Sanger Institute for the COVID-19 Genomics UK (COG-UK) consortium|Davies|United Kingdom
>Spike|hCoV-19/Japan/PG-3083/2020|2020-04-00|EPI_ISL_691206|Original|hCoV-19^^Japan|Human|Pathogen Genomics Center|Pathogen Genomics Center|Sekizuka|Japan
>Spike|hCoV-19/England/MILK-9A6829/2020|2020-08-31|EPI_ISL_550075|Original|hCoV-19^^England|Human|Lighthouse Lab in Milton Keynes|Wellcome Sanger Institute for the COVID-19 Genomics UK (COG-UK) consortium|Davies|United Kingdom
>Spike|hCoV-19/USA/UT-UPHL-2012091937/2020|2020-11-30|EPI_ISL_746916|Original|hCoV-19^^Utah|Human|Utah Public Health Laboratory|Utah Public Health Laboratory|Gallagher|USA
>Spike|hCoV-19/England/QEUH-C34C30/2020|2020-12-10|EPI_ISL_731493|Original|hCoV-19^^England|Human|Lighthouse Lab in Glasgow|Wellcome Sanger Institute for the COVID-19 Genomics UK (COG-UK) Consortium|Davies|United Kingdom
>Spike|hCoV-19/Brazil/RJ-L10-CD154/2020|2020-03-16|EPI_ISL_476178|Original|hCoV-19^^Rio de Janeiro|Human|DB Diagn\u00f3sticos do Brasil|Instituto de Medicina Tropical da Univesidade de S\u00e3o Paulo|Faria|Brazil
>Spike|hCoV-19/England/MILK-99DEC9/2020|2020-08-31|EPI_ISL_550690|Original|hCoV-19^^England|Human|Lighthouse Lab in Milton Keynes|Wellcome Sanger Institute for the COVID-19 Genomics UK (COG-UK) consortium|Davies|United Kingdom
>Spike|hCoV-19/USA/LA-EVTL421/2020|2020-06-10|EPI_ISL_578827|Original|hCoV-19^^Louisiana|Human|LSUHS Emerging Viral Threat Laboratory|Microbial Genome Sequencing Center|Kamil|USA
>Spike|hCoV-19/USA/TX-HMH-MCoV-15785/2020|2020-07-09|EPI_ISL_785469|Original|hCoV-19^^Texas|Human|Houston Methodist Hospital|Houston Methodist Hospital|Long|USA

>Spike|hCoV-19/England/LEED-2A9AFA/2020|2020-04-12|EPI_ISL_538867|Original|hCoV-19^^England|Human|Leeds Teaching Hospitals NHS Trust and Public Health England|Wellcome Sanger Institute for the COVID-19 Genomics UK (COG-UK) consortium|Davies|United Kingdom
>Spike|hCoV-19/USA/NY-Wadsworth-278092-01/2020|2020-11-22|EPI_ISL_754604|Original|hCoV-19^^New York|Human|Wadsworth Center|Wadsworth Center|Lasek-Nesselquist|USA
>Spike|hCoV-19/Denmark/DCGC-21730/2020|2020-12-21|EPI_ISL_758332|Original|hCoV-19^^Hovedstaden|Human|Department of Virus and Microbiological Special Diagnostics|Albertsen Lab|Michaelsen|Denmark
>Spike|hCoV-19/Luxembourg/LNS0293199/2020|2020-10-28|EPI_ISL_739751|Original|hCoV-19^^Luxembourg|Human|Laboratoire national de sant\u00e9|Laboratoire national de sant\u00e9|Wienecke-Baldacchino|Luxembourg
>Spike|hCoV-19/USA/NY-Wadsworth-17549-01/2020|2020-03-16|EPI_ISL_525616|Original|hCoV-19^^New York|Human|Wadsworth Center|Wadsworth Center|Plitnick|USA
>Spike|hCoV-19/England/CAMC-B21B2B/2020|2020-11-01|EPI_ISL_647197|Original|hCoV-19^^England|Human|Lighthouse Lab in Cambridge|Wellcome Sanger Institute for the COVID-19 Genomics UK (COG-UK) Consortium|Davies|United Kingdom
>Spike|hCoV-19/USA/FL-BPHL-0933/2020|2020-07-15|EPI_ISL_517823|Original|hCoV-19^^Florida|Human|Florida Bureau of Public Health Laboratories|Florida Bureau of Public Health Laboratories|Blanton|USA
>Spike|hCoV-19/England/CAMC-B7A33A/2020|2020-11-12|EPI_ISL_741100|Original|hCoV-19^^England|Human|Oxford Viromics|COVID-19 Genomics UK (COG-UK) Consortium|Golubchik|United Kingdom
>Spike|hCoV-19/Northern Ireland/NIRE-107484/2020|2020-03-30|EPI_ISL_585154|Original|hCoV-19^^Northern Ireland|Human|Regional Virus Laboratory|COVID-19 Genomics UK (COG-UK) Consortium|Fairley|United Kingdom
>Spike|hCoV-19/England/ALDP-AA10DA/2020|2020-10-22|EPI_ISL_624418|Original|hCoV-19^^England|Human|Lighthouse Lab in Alderley Park|Wellcome Sanger Institute for the COVID-19 Genomics UK (COG-UK) consortium|Davies|United Kingdom
>Spike|hCoV-19/England/CAMC-A657DD/2020|2020-10-11|EPI_ISL_637759|Original|hCoV-19^^England|Human|Department of Pathology|COVID-19 Genomics UK (COG-UK) Consortium|Goodfellow|United Kingdom
>Spike|hCoV-19/England/CAMC-B0B41D/2020|2020-10-30|EPI_ISL_643483|Original|hCoV-19^^England|Human|Lighthouse Lab in Cambridge|Wellcome Sanger Institute for the COVID-19 Genomics UK (COG-UK) Consortium|Davies|United Kingdom
>Spike|hCoV-19/USA/TX-DSHS-0505/2020|2020-06-21|EPI_ISL_560949|Original|hCoV-19^^Texas|Human|Texas Department of State Health Services|Texas Department of State Health Services|Rahman|USA
>Spike|hCoV-19/England/MILK-B3C7B0/2020|2020-11-02|EPI_ISL_705454|Original|hCoV-19^^England|Human|Oxford Viromics|COVID-19 Genomics UK (COG-UK) Consortium|Golubchik|United Kingdom
>Spike|hCoV-19/USA/VA-DCLS-2437/2020|2020-11-17|EPI_ISL_734186|Original|hCoV-19^^Virginia|Human|Virginia Division of Consolidated Laboratory Services (DCLS)|Virginia Division of Consolidated Laboratory Services (DCLS)|St. Jacques|USA
>Spike|hCoV-19/England/MILK-B386D5/2020|2020-11-02|EPI_ISL_662645|Original|hCoV-19^^England|Human|Lighthouse Lab in Milton Keynes|Wellcome Sanger Institute for the COVID-19 Genomics UK (COG-UK) Consortium|Davies|United Kingdom
>Spike|hCoV-19/England/GSTT-265DC22/2020|2020-03-21|EPI_ISL_484082|Original|hCoV-19^^England|Human|Centre for Clinical Infection and Diagnostics Research and Genomics Innovation Unit|COVID-19 Genomics UK (COG-UK) Consortium|Awan|United Kingdom
>Spike|hCoV-19/South Korea/KCDC2570/2020|2020-06-25|EPI_ISL_514973|Original|hCoV-19^^South Korea|Human|Division of Viral Diseases|Division of Viral Diseases|Kim|South Korea
>Spike|hCoV-19/Portugal/PT0703/2020|2020-03-17|EPI_ISL_510962|Original|hCoV-19^^Portugal|Human|Instituto Nacional de Saude (INSA)|Instituto Nacional de Saude (INSA)|Borges|Portugal

>Spike|hCoV-19/Denmark/DCGC-1575/2020|2020-06-15|EPI_ISL_618145|Original|hCoV-19^^Sjaelland|Human|Department of Virus and Microbiological Special Diagnostics|Albertsen lab|Michaelsen|Denmark
>Spike|hCoV-19/England/QEUH-CC2745/2020|2020-12-21|EPI_ISL_778215|Original|hCoV-19^^England|Human|Lighthouse Lab in Glasgow|Wellcome Sanger Institute for the COVID-19 Genomics UK (COG-UK) Consortium|Davies|United Kingdom
>Spike|hCoV-19/Bangladesh/BCSIR-NILMRC-059/2020|2020-05-10|EPI_ISL_465164|Original|hCoV-19^^Dhaka|Human|National Institute of Laboratory Medicine and Referral Center|Genomic Research Lab|Khan|Bangladesh
>Spike|hCoV-19/USA/UT-08553/2020|2020-05-07|EPI_ISL_524312|Original|hCoV-19^^Utah|Human|Utah Public Health Laboratory|Utah Public Health Laboratory|Young|USA
>Spike|hCoV-19/USA/VA-DCLS-2316/2020|2020-10-22|EPI_ISL_734266|Original|hCoV-19^^Virginia|Human|Virginia Division of Consolidated Laboratory Services (DCLS)|Virginia Division of Consolidated Laboratory Services (DCLS)|St. Jacques|USA
>Spike|hCoV-19/England/QEUH-AD3DBA/2020|2020-10-26|EPI_ISL_629878|Original|hCoV-19^^England|Human|Lighthouse Lab in Glasgow|Wellcome Sanger Institute for the COVID-19 Genomics UK (COG-UK) consortium|Davies|United Kingdom
>Spike|hCoV-19/England/ALDP-90F211/2020|2020-06-22|EPI_ISL_551422|Original|hCoV-19^^England|Human|Lighthouse Lab in Alderley Park|Wellcome Sanger Institute for the COVID-19 Genomics UK (COG-UK) consortium|Davies|United Kingdom
>Spike|hCoV-19/Scotland/CVR3823/2020|2020-05-19|EPI_ISL_478149|Original|hCoV-19^^Scotland|Human|West of Scotland Specialist Virology Centre|COVID-19 Genomics UK (COG-UK) Consortium|Robertson|United Kingdom
>Spike|hCoV-19/England/204881317/2020|2020-11-00|EPI_ISL_678826|Original|hCoV-19^^England|Human|Respiratory Virus Unit|COVID-19 Genomics UK (COG-UK) Consortium|Platt|United Kingdom
>Spike|hCoV-19/Switzerland/BL-UHB-42195966/2020|2020-03-17|EPI_ISL_528192|Original|hCoV-19^^Basel-Land|Human|University Hospital Basel|University Hospital Basel|Roloff Handschin|Switzerland
>Spike|hCoV-19/Thailand/Bangkok_2854/2020|2020-03-00|EPI_ISL_515473|Vero P2|hCoV-19^^Bangkok|Human|National Institute of Health|National Institute of Health|Phuygun|Thailand
>Spike|hCoV-19/Switzerland/BE-ETHZ-310522/2020|2020-10-08|EPI_ISL_603590|Original|hCoV-19^^Bern|Human|Viollier AG|Department of Biosystems Science and Engineering|Nadeau|Switzerland
>Spike|hCoV-19/England/MILK-97589C/2020|2020-08-20|EPI_ISL_552692|Original|hCoV-19^^England|Human|Lighthouse Lab in Milton Keynes|Wellcome Sanger Institute for the COVID-19 Genomics UK (COG-UK) consortium|Davies|United Kingdom
>Spike|hCoV-19/USA/WI-UW-2351/2020|2020-11-19|EPI_ISL_728117|Original|hCoV-19^^Wisconsin|Human|University of Wisconsin-Madison AIDS Vaccine Research Laboratories|University of Wisconsin-Madison AIDS Vaccine Research Laboratories|Baczenas|USA
>Spike|hCoV-19/USA/GA-CDC-7746/2020|2020-10-27|EPI_ISL_681813|Original|hCoV-19^^Georgia|Human|GA Department of Public Health Laboratory|Pathogen Discovery|Queen|USA
>Spike|hCoV-19/Japan/PG-5251/2020|2020-07-00|EPI_ISL_690354|Original|hCoV-19^^Japan|Human|Pathogen Genomics Center|Pathogen Genomics Center|Sekizuka|Japan
>Spike|hCoV-19/England/LIVE-DCB6F3/2020|2020-11-24|EPI_ISL_724421|Original|hCoV-19^^England|Human|Liverpool Clinical Laboratories|COVID-19 Genomics UK (COG-UK) Consortium|Haldenby|United Kingdom
>Spike|hCoV-19/Scotland/QEUH-9DAD5A/2020|2020-09-22|EPI_ISL_601179|Original|hCoV-19^^Scotland|Human|Lighthouse Lab in Glasgow|Wellcome Sanger Institute for the COVID-19 Genomics UK (COG-UK) consortium|Davies|United Kingdom
>Spike|hCoV-19/Denmark/DCGC-8507/2020|2020-10-26|EPI_ISL_625918|Original|hCoV-19^^Midtjylland|Human|Department of Virus and Microbiological Special Diagnostics|Albertsen lab|Michaelsen|Denmark
>Spike|hCoV-19/England/ALDP-9E9FF8/2020|2020-09-24|EPI_ISL_579975|Original|hCoV-19^^England|Human|Lighthouse Lab in Alderley Park|Wellcome Sanger Institute for the COVID-19 Genomics UK (COG-UK) consortium|Davies|United Kingdom

>Spike|hCoV-19/USA/WI-UW-1098/2020|2020-07-23|EPI_ISL_516523|Original|hCoV-19^^Wisconsin|Human|University of Wisconsin-Madison AIDS Vaccine Research Laboratories|University of Wisconsin-Madison AIDS Vaccine Research Laboratories|Baczenas|USA
>Spike|hCoV-19/Scotland/EDB698/2020|2020-04-01|EPI_ISL_491668|Original|hCoV-19^^Scotland|Human|Virology Department|Wellcome Sanger Institute for the COVID-19 Genomics UK (COG-UK) consortium|Davies|United Kingdom
>Spike|hCoV-19/England/LIVE-DB7E10/2020|2020-10-26|EPI_ISL_651328|Original|hCoV-19^^England|Human|Liverpool Clinical Laboratories|COVID-19 Genomics UK (COG-UK) Consortium|Haldenby|United Kingdom
>Spike|hCoV-19/Wales/PHWC-47FF38/2020|2020-10-16|EPI_ISL_612832|Original|hCoV-19^^Wales|Human|Wales Specialist Virology Centre Sequencing lab|COVID-19 Genomics UK (COG-UK) Consortium|Moore|United Kingdom
>Spike|hCoV-19/USA/WI-UW-339/2020|2020-03-31|EPI_ISL_436633|Original|hCoV-19^^Wisconsin|Human|University of Wisconsin-Madison AIDS Vaccine Research Laboratories|University of Wisconsin-Madison AIDS Vaccine Research Laboratories|Baczenas|USA
>Spike|hCoV-19/England/MILK-9C4333/2020|2020-09-08|EPI_ISL_602029|Original|hCoV-19^^England|Human|Lighthouse Lab in Milton Keynes|Wellcome Sanger Institute for the COVID-19 Genomics UK (COG-UK) consortium|Davies|United Kingdom
>Spike|hCoV-19/England/CAMC-B52699/2020|2020-11-06|EPI_ISL_656863|Original|hCoV-19^^England|Human|Lighthouse Lab in Cambridge|Wellcome Sanger Institute for the COVID-19 Genomics UK (COG-UK) Consortium|Davies|United Kingdom
>Spike|hCoV-19/Denmark/DCGC-13683/2020|2020-11-30|EPI_ISL_712958|Original|hCoV-19^^Midtjylland|Human|Department of Virus and Microbiological Special Diagnostics|Albertsen Lab|Michaelsen|Denmark
>Spike|hCoV-19/England/MILK-956E53/2020|2020-06-09|EPI_ISL_554137|Original|hCoV-19^^England|Human|Lighthouse Lab in Milton Keynes|Wellcome Sanger Institute for the COVID-19 Genomics UK (COG-UK) consortium|Davies|United Kingdom
>Spike|hCoV-19/England/QEUH-A6164D/2020|2020-10-12|EPI_ISL_628625|Original|hCoV-19^^England|Human|Oxford Viromics|COVID-19 Genomics UK (COG-UK) Consortium|Golubchik|United Kingdom
>Spike|hCoV-19/Australia/VIC15549/2020|2020-08-08|EPI_ISL_593356|Original|hCoV-19^^Victoria|Human|Microbiological Diagnostic Unit - Public Health Laboratory (MDU-PHL)|MDU-PHL|Schultz|Australia
>Spike|hCoV-19/England/SHEF-C3D8A/2020|2020-05-28|EPI_ISL_535051|Original|hCoV-19^^England|Human|Virology Department|COVID-19 Genomics UK (COG-UK) Consortium|de Silva|United Kingdom
>Spike|hCoV-19/USA/WA-S238/2020|2020-03-02|EPI_ISL_430276|Original|hCoV-19^^Washington|Human|Washington State Department of Health|Seattle Flu Study|Bedford|USA
>Spike|hCoV-19/Jordan/AM-ALSR-5160/2020|2020-10-21|EPI_ISL_730399|Original|hCoV-19^^Amman|Human|Biolab Diagnostic Laboratories|Andersen lab at Scripps Research|Gangavarapu|Jordan
>Spike|hCoV-19/Ireland/D-NVRL-76IRL28438/2020|2020-10-16|EPI_ISL_732490|Original|hCoV-19^^Dublin|Human|National Virus Reference Laboratory|National Virus Reference Laboratory|Gonzalez|Ireland
>Spike|hCoV-19/England/QEUH-B7DBC6/2020|2020-11-14|EPI_ISL_741904|Original|hCoV-19^^England|Human|Oxford Viromics|COVID-19 Genomics UK (COG-UK) Consortium|Golubchik|United Kingdom
>Spike|hCoV-19/England/205260409/2020|2020-00-00|EPI_ISL_766530|Original|hCoV-19^^England|Human|Respiratory Virus Unit|COVID-19 Genomics UK (COG-UK) Consortium|Platt|United Kingdom
>Spike|hCoV-19/England/20129085704/2020|2020-03-21|EPI_ISL_424076|Original|hCoV-19^^England|Human|Respiratory Virus Unit|Respiratory Virus Unit|Platt|United Kingdom
>Spike|hCoV-19/Belgium/rega-0917990/2020|2020-09-17|EPI_ISL_734982|Original|hCoV-19^^Wilsele|Human|UZ Leuven|KU Leuven|Maes|Belgium
>Spike|hCoV-19/Scotland/CVR2139/2020|2020-04-11|EPI_ISL_490669|Original|hCoV-19^^Scotland|Human|West of Scotland Specialist Virology Centre|COVID-19 Genomics UK (COG-UK) Consortium|Robertson|United Kingdom

>Spike|hCoV-19/South Korea/KDCA0183/2020|2020-07-04|EPI_ISL_747416|Original|hCoV-
19^^South Korea|Human|Division of Emerging Infectious Diseases|Division of Emerging
Infectious Diseases|Park|South Korea
>Spike|hCoV-19/Netherlands/UT-RIVM-20177/2020|2020-11-30|EPI_ISL_723338|Orignal|hCoV-
19^^Utrecht|Human|Dutch COVID-19 response team|National Institute for Public Health
and the Environment (RIVM)|Meijer|Netherlands
>Spike|hCoV-19/Norway/2870/2020|2020-07-09|EPI_ISL_500789|Original|hCoV-
19^^Norway|Human|Furst Medical Laboratory|Norwegian Institute of Public
Health|Kopperud Riis|Norway
>Spike|hCoV-19/Australia/VIC11023/2020|2020-08-03|EPI_ISL_563206|Original|hCoV-
19^^Victoria|Human|Microbiological Diagnostic Unit - Public Health Laboratory (MDU-
PHL)|MDU-PHL|Schultz|Australia
>Spike|hCoV-19/USA/TX-HMH0410/2020|2020-03-30|EPI_ISL_434996|Original|hCoV-
19^^Texas|Human|Houston Methodist Hospital|Houston Methodist Hospital|Long|USA
>Spike|hCoV-19/USA/AZ-TG273304/2020|2020-04-01|EPI_ISL_426564|Original|hCoV-
19^^Arizona|Human|AZ SPHL|TGen North|Lemmer|USA
>Spike|hCoV-19/England/CAMC-C43AAD/2020|2020-12-10|EPI_ISL_719086|Original|hCoV-
19^^England|Human|Lighthouse Lab in Cambridge|Wellcome Sanger Institute for the
COVID-19 Genomics UK (COG-UK) Consortium|Davies|United Kingdom
>Spike|hCoV-19/England/OXON-F40041/2020|2020-12-15|EPI_ISL_764527|Original|hCoV-
19^^England|Human|Oxford Viromics|COVID-19 Genomics UK (COG-UK)
Consortium|Golubchik|United Kingdom
>Spike|hCoV-19/Canada/QC-LSPQ-L00225956/2020|2020-03-18|EPI_ISL_535840|Original|hCoV-
19^^Quebec|Human|H\u00f4pital Pierre-Boucher|Laboratoire de sant\u00e9 publique du
Qu\u00e9bec|Moreira|Canada
>Spike|hCoV-19/England/ALDP-9B65A7/2020|2020-08-28|EPI_ISL_573017|Original|hCoV-
19^^England|Human|Oxford Viromics|COVID-19 Genomics UK (COG-UK)
Consortium|Golubchik|United Kingdom
>Spike|hCoV-19/England/MILK-BAC821/2020|2020-11-18|EPI_ISL_720393|Original|hCoV-
19^^England|Human|Lighthouse Lab in Milton Keynes|Wellcome Sanger Institute for the
COVID-19 Genomics UK (COG-UK) Consortium|Davies|United Kingdom
>Spike|hCoV-19/Scotland/QEUH-A4B5B5/2020|2020-10-08|EPI_ISL_637843|Original|hCoV-
19^^Scotland|Human|Department of Pathology|COVID-19 Genomics UK (COG-UK)
Consortium|Goodfellow|United Kingdom
>Spike|hCoV-19/South Africa/KRISP-K004066/2020|2020-10-
20|EPI_ISL_635045|Original|hCoV-19^^KwaZulu-Natal|Human|National Health Laboratory
Service - Inkosi Albert Luthuli Central Hospital (NHLS-IALCH)|KRISP|de Oliveira|South
Africa
>Spike|hCoV-19/England/MILK-ACDC89/2020|2020-10-22|EPI_ISL_630308|Original|hCoV-
19^^England|Human|Lighthouse Lab in Milton Keynes|Wellcome Sanger Institute for the
COVID-19 Genomics UK (COG-UK) consortium|Davies|United Kingdom
>Spike|hCoV-19/Portugal/PT0718/2020|2020-03-25|EPI_ISL_510977|Original|hCoV-
19^^Portugal|Human|Instituto Nacional de Saude (INSA)|Instituto Nacional de Saude
(INSA)|Borges|Portugal
>Spike|hCoV-19/England/MILK-A91C7F/2020|2020-10-18|EPI_ISL_704812|Original|hCoV-
19^^England|Human|Oxford Viromics|COVID-19 Genomics UK (COG-UK)
Consortium|Golubchik|United Kingdom
>Spike|hCoV-19/England/GSTT-5156/2020|2020-03-18|EPI_ISL_560653|Original|hCoV-
19^^England|Human|Centre for Clinical Infection and Diagnostics Research and Genomics
Innovation Unit|Centre for Clinical Infection and Diagnostics Research and Genomics
Innovation Unit|Awan|United Kingdom
>Spike|hCoV-19/Wales/PHWC-33FBC/2020|2020-04-21|EPI_ISL_446851|Original|hCoV-
19^^Wales|Human|Wales Specialist Virology Centre|Public Health Wales Microbiology
Cardiff|Moore|United Kingdom
>Spike|hCoV-19/England/CAMB-76E74/2020|2020-03-31|EPI_ISL_425434|Original|hCoV-
19^^England|Human|Department of Pathology|COVID-19 Genomics UK (COG-UK)
Consortium|Meredith|United Kingdom
>Spike|hCoV-19/USA/WA-UW-10106/2020|2020-05-29|EPI_ISL_461465|Original|hCoV-
19^^Washington|Human|UW Virology Lab|UW Virology Lab|Roychoudhury|USA

>Spike|hCoV-19/Japan/PG-0418/2020|2020-03-27|EPI_ISL_480086|Original|hCoV-19^^Chubu|Human|Gifu Prefectural Institute of Public Health and Environmental Sciences|Pathogen Genomics Center|Sekizuka|Japan
>Spike|hCoV-19/France/OCC-22/2020|2020-06-08|EPI_ISL_482880|Original|hCoV-19^^Occitanie|Human|CHU Purpan - Laboratoire de Virologie - Institut F\u00e9d\u00e9ratif de Biologie|Laboratoire de virologie - \u00c9cole Nationale V\u00e9t\u00e9rinaire de Toulouse|Croville|France
>Spike|hCoV-19/USA/VA-DCLS-0214/2020|2020-04-04|EPI_ISL_437429|Original|hCoV-19^^Virginia|Human|Virginia DCLS|Virginia DCLS|St. Jacques|USA
>Spike|hCoV-19/Australia/NT31/2020|2020-00-00|EPI_ISL_430635|Original|hCoV-19^^Northern Territory|Human|Royal Darwin Hospital Pathology|Microbiological Diagnostic Unit Public Health Laboratory and Victorian Infectious Diseases Reference Laboratory|Schultz|Australia
>Spike|hCoV-19/USA/TX-DSHS-0738/2020|2020-05-14|EPI_ISL_584140|Original|hCoV-19^^Texas|Human|Texas Department of State Health Services|Texas Department of State Health Services|Rahman|USA
>Spike|hCoV-19/Norway/5794/2020|2020-11-09|EPI_ISL_708176|Original|hCoV-19^^Vestland|Human|Foerde Hospital|Norwegian Institute of Public Health|Kopperud Riis|Norway
>Spike|hCoV-19/India/TG-CCMB-OM2-ONT/2020|2020-05-13|EPI_ISL_495287|Original|hCoV-19^^Telangana|Human|Osmania Medical College|CSIR-Centre for Cellular and Molecular Biology|Banu|India
>Spike|hCoV-19/USA/CA-CZB-11017/2020|2020-08-24|EPI_ISL_582957|Original|hCoV-19^^California|Human|County of Santa Clara Public Health Department|Chan-Zuckerberg Biohub|Bhatt|USA
>Spike|hCoV-19/England/20146005804/2020|2020-04-01|EPI_ISL_423574|Original|hCoV-19^^England|Human|Respiratory Virus Unit|Respiratory Virus Unit|Platt|United Kingdom
>Spike|hCoV-19/Portugal/PT0095/2020|2020-03-24|EPI_ISL_421498|Original|hCoV-19^^Portugal|Human|Instituto Nacional de Saude (INSA)|Instituto Nacional de Saude (INSA)|Borges|Portugal
>Spike|hCoV-19/India/MH-ACTREC-506/2020|2020-08-17|EPI_ISL_700154|Original|hCoV-19^^Maharashtra|Human|Hematopathology Laboratory|Hematopathology Laboratory|Patkar|India
>Spike|hCoV-19/Denmark/DCGC-2903/2020|2020-08-24|EPI_ISL_616537|Original|hCoV-19^^NA|Human|Department of Virus and Microbiological Special Diagnostics|Albertsen lab|Michaelsen|Denmark
>Spike|hCoV-19/England/CWAR-24FD8AA/2020|2020-04-16|EPI_ISL_584445|Original|hCoV-19^^England|Human|UHCW / University of Warwick|COVID-19 Genomics UK (COG-UK) Consortium|Stark|United Kingdom
>Spike|hCoV-19/England/CAMB-1BC460/2020|2020-11-27|EPI_ISL_723874|Original|hCoV-19^^England|Human|Department of Pathology|COVID-19 Genomics UK (COG-UK) Consortium|Goodfellow|United Kingdom
>Spike|hCoV-19/England/205260446/2020|2020-12-21|EPI_ISL_754314|Original|hCoV-19^^England|Human|Respiratory Virus Unit|COVID-19 Genomics UK (COG-UK) Consortium|Platt|United Kingdom
>Spike|hCoV-19/Wales/PHWC-49E777/2020|2020-12-08|EPI_ISL_743044|Original|hCoV-19^^Wales|Human|Wales Specialist Virology Centre Sequencing lab|COVID-19 Genomics UK (COG-UK) Consortium|Moore|United Kingdom
>Spike|hCoV-19/England/QEUH-943E2C/2020|2020-07-28|EPI_ISL_532553|Original|hCoV-19^^England|Human|Lighthouse Lab in Glasgow|Wellcome Sanger Institute for the COVID-19 Genomics UK (COG-UK) consortium|Davies|United Kingdom
>Spike|hCoV-19/Scotland/CVR41/2020|2020-03-13|EPI_ISL_425769|Original|hCoV-19^^Scotland|Human|West of Scotland Specialist Virology Centre|COVID-19 Genomics UK (COG-UK) Consortium|Robertson|United Kingdom
>Spike|hCoV-19/Luxembourg/LNS9744887/2020|2020-12-28|EPI_ISL_770895|Original|hCoV-19^^Luxembourg|Human|Laboratoire national de sant\u00e9|Laboratoire national de sant\u00e9|Wienecke-Baldacchino|Luxembourg
>Spike|hCoV-19/USA/MI-MDHHS-SC21341/2020|2020-03-31|EPI_ISL_507595|Original|hCoV-19^^Michigan|Human|Michigan Department of Health and Human Services|Michigan Department of Health and Human Services|Blankenship|USA

>Spike|hCoV-19/Denmark/DCGC-6417/2020|2020-10-12|EPI_ISL_620317|Original|hCoV-19^^Sjaelland|Human|Department of Virus and Microbiological Special Diagnostics|Albertsen lab|Michaelsen|Denmark
>Spike|hCoV-19/mink/Netherlands/NB-EMC-45-3/2020|2020-09-02|EPI_ISL_577802|Original|hCoV-19^^Noord Brabant|Neovison vison|Dutch COVID-19 response team|Erasmus Medical Center|Oude Munnink|Netherlands
>Spike|hCoV-19/Wales/PHWC-496AEF/2020|2020-11-23|EPI_ISL_726685|Original|hCoV-19^^Wales|Human|Originating lab|COVID-19 Genomics UK (COG-UK) Consortium|Moore|United Kingdom
>Spike|hCoV-19/USA/NY-PV09375/2020|2020-03-19|EPI_ISL_450059|Original|hCoV-19^^New York|Human|MSHS Clinical Microbiology Laboratories|MSHS Pathogen Surveillance Program|van Bakel|USA
>Spike|hCoV-19/Wales/ALDP-A4FEE3/2020|2020-10-07|EPI_ISL_609213|Original|hCoV-19^^Wales|Human|Lighthouse Lab in Alderley Park|Wellcome Sanger Institute for the COVID-19 Genomics UK (COG-UK) consortium|Davies|United Kingdom
>Spike|hCoV-19/Northern Ireland/NIRE-FBF38/2020|2020-04-10|EPI_ISL_459327|Original|hCoV-19^^Northern Ireland|Human|Regional Virus Laboratory|Wellcome Sanger Institute for the COVID-19 Genomics UK (COG-UK) consortium|Davies|United Kingdom
>Spike|hCoV-19/USA/CA-QDX-2620/2020|2020-03-16|EPI_ISL_604896|Original|hCoV-19^^California|Human|Quest Diagnostics|Quest Diagnostics|Kagan|USA
>Spike|hCoV-19/USA/WA-S146/2020|2020-03-14|EPI_ISL_430183|Original|hCoV-19^^Washington|Human|Washington State Department of Health|Seattle Flu Study|Bedford|USA
>Spike|hCoV-19/England/QEUH-96C33D/2020|2020-08-13|EPI_ISL_530903|Original|hCoV-19^^England|Human|Lighthouse Lab in Glasgow|Wellcome Sanger Institute for the COVID-19 Genomics UK (COG-UK) consortium|Davies|United Kingdom
>Spike|hCoV-19/England/LIVE-DC8B33/2020|2020-11-19|EPI_ISL_705940|Original|hCoV-19^^England|Human|Liverpool Clinical Laboratories|COVID-19 Genomics UK (COG-UK) Consortium|Haldenby|United Kingdom
>Spike|hCoV-19/Scotland/CVR2659/2020|2020-04-16|EPI_ISL_493823|Original|hCoV-19^^Scotland|Human|West of Scotland Specialist Virology Centre|COVID-19 Genomics UK (COG-UK) Consortium|Robertson|United Kingdom
>Spike|hCoV-19/Hong Kong/HKU-200723-065/2020|2020-03-19|EPI_ISL_497832|Original|hCoV-19^^Hong Kong|Human|Department of Microbiology|Department of Microbiology|Chan|China
>Spike|hCoV-19/Egypt/MASRI-028/2020|2020-05-13|EPI_ISL_483038|Original|hCoV-19^^Cairo|Human|Medical Ain Shams Research Institute (MASRI)|Medical Ain Shams Research Institute (MASRI)|Moustafa|Egypt
>Spike|hCoV-19/Belgium/rega-0414495/2020|2020-04-14|EPI_ISL_734578|Original|hCoV-19^^Tienen|Human|UZ Leuven|KU Leuven|Maes|Belgium
>Spike|hCoV-19/USA/CA-CZB-15934/2020|2020-12-02|EPI_ISL_739617|Original|hCoV-19^^California|Human|Santa Clara County Public Health Laboratory|Chan-Zuckerberg Biohub|Kamm|USA
>Spike|hCoV-19/England/ALDP-CB65D1/2020|2020-12-19|EPI_ISL_761784|Original|hCoV-19^^England|Human|Lighthouse Lab in Alderley Park|Wellcome Sanger Institute for the COVID-19 Genomics UK (COG-UK) Consortium|Davies|United Kingdom
>Spike|hCoV-19/USA/CA-CZB-10923/2020|2020-08-31|EPI_ISL_583152|Original|hCoV-19^^California|Human|Humboldt County Public Health Laboratory|Chan-Zuckerberg Biohub|Bhatt|USA
>Spike|hCoV-19/USA/LA-GBCL-26629191/2020|2020-04-24|EPI_ISL_485892|Original|hCoV-19^^Louisiana|Human|River Road Testing Lab|Ginkgo Bioworks Clinical Laboratory|Smith|USA
>Spike|hCoV-19/Russia/PRI-RII-MH733S/2020|2020-08-06|EPI_ISL_596353|Original|hCoV-19^^Primorsky|Human|WHO National Influenza Centre Russian Federation|WHO National Influenza Centre Russian Federation|Komissarov|Russia
>Spike|hCoV-19/Wales/PHWC-24EFD/2020|2020-03-20|EPI_ISL_420925|Original|hCoV-19^^Wales|Human|Wales Specialist Virology Centre|Public Health Wales Microbiology Cardiff|Moore|United Kingdom

>Spike|hCoV-19/England/BRIS-130F27/2020|2020-03-24|EPI_ISL_501586|Original|hCoV-19^^England|Human|PHE South West Regional Laboratory|Wellcome Sanger Institute for the COVID-19 Genomics UK (COG-UK) consortium|Davies|United Kingdom
>Spike|hCoV-19/USA/WY-WPHL-20148634/2020|2020-11-23|EPI_ISL_770091|Original|hCoV-19^^Wyoming|Human|Wyoming Public Health Laboratory|Wyoming Public Health Laboratory|Hull|USA
>Spike|hCoV-19/Australia/VIC2132/2020|2020-06-19|EPI_ISL_480730|Original|hCoV-19^^Victoria|Human|Victorian Infectious Diseases Reference Laboratory (VIDRL)|VIDRL and MDU-PHL|Schultz|Australia
>Spike|hCoV-19/Spain/MD-HLP-51/2020|2020-04-20|EPI_ISL_530054|Original|hCoV-19^^Madrid|Human|Hospital Universitario La Paz|Hospital Universitario La Paz|Viedma|Spain
>Spike|hCoV-19/Belgium/ULG-9739/2020|2020-03-22|EPI_ISL_418663|Original|hCoV-19^^Li\u00e8ge|Human|Department of Clinical Microbiology|GIGA Medical Genomics|Durkin|Belgium
>Spike|hCoV-19/United Arab Emirates/0411/2020|2020-05-15|EPI_ISL_698586|Original|hCoV-19^^Abu Dhabi|Human|Group 42 (G42) Healthcare|G42 Healthcare|Ogrodzki|United Arab Emirates
>Spike|hCoV-19/USA/CA-CZB0741/2020|2020-04-03|EPI_ISL_429051|Original|hCoV-19^^California|Human|UCSF Clinical Microbiology Laboratory|Chan-Zuckerberg Biohub|Kamm|USA
>Spike|hCoV-19/Scotland/QEUH-9B8657/2020|2020-09-10|EPI_ISL_568227|Original|hCoV-19^^Scotland|Human|Lighthouse Lab in Glasgow|Wellcome Sanger Institute for the COVID-19 Genomics UK (COG-UK) consortium|Davies|United Kingdom
>Spike|hCoV-19/Wales/PHWC-326B8/2020|2020-04-14|EPI_ISL_474340|Original|hCoV-19^^Wales|Human|Originating lab|COVID-19 Genomics UK (COG-UK) Consortium|Moore|United Kingdom
>Spike|hCoV-19/Switzerland/GE-ETHZ-120219/2020|2020-04-29|EPI_ISL_468282|Original|hCoV-19^^Geneve|Human|Viollier AG|Department of Biosystems Science and Engineering|Nadeau|Switzerland
>Spike|hCoV-19/Belgium/ULG-10764/2020|2020-09-19|EPI_ISL_641606|Original|hCoV-19^^Li\u00e8ge|Human|Department of Clinical Microbiology|GIGA Medical Genomics|Durkin|Belgium
>Spike|hCoV-19/England/ALDP-B62A9A/2020|2020-11-09|EPI_ISL_656556|Original|hCoV-19^^England|Human|Lighthouse Lab in Alderley Park|Wellcome Sanger Institute for the COVID-19 Genomics UK (COG-UK) Consortium|Davies|United Kingdom
>Spike|hCoV-19/Finland/29M18S2/2020|2020-03-29|EPI_ISL_481643|Original|hCoV-19^^Uusimaa|Human|Department of Virology and Immunology|Department of Virology|Smura|Finland
>Spike|hCoV-19/Germany/HH-hpi-p2056/2020|2020-03-21|EPI_ISL_776343|Original|hCoV-19^^Hamburg|Human|University Medical Center Hamburg Eppendorf|Heinrich Pette Institute|Robitaille|Germany
>Spike|hCoV-19/England/BRIS-12B271/2020|2020-05-18|EPI_ISL_469834|Original|hCoV-19^^England|Human|PHE South West Regional Laboratory|Wellcome Sanger Institute for the COVID-19 Genomics UK (COG-UK) consortium|Davies|United Kingdom
>Spike|hCoV-19/Germany/HH-hpi-p1650/2020|2020-03-17|EPI_ISL_775819|Original|hCoV-19^^Hamburg|Human|University Medical Center Hamburg Eppendorf|Heinrich Pette Institute|Robitaille|Germany
>Spike|hCoV-19/Wales/PHWC-4A002C/2020|2020-11-30|EPI_ISL_743385|Original|hCoV-19^^Wales|Human|Wales Specialist Virology Centre Sequencing lab|COVID-19 Genomics UK (COG-UK) Consortium|Moore|United Kingdom
>Spike|hCoV-19/England/LIVE-9CA29/2020|2020-04-24|EPI_ISL_440930|Original|hCoV-19^^England|Human|Liverpool Clinical Laboratories|COVID-19 Genomics UK (COG-UK) Consortium|Haldenby|United Kingdom
>Spike|hCoV-19/England/CAMC-A58148/2020|2020-10-09|EPI_ISL_597220|Original|hCoV-19^^England|Human|Lighthouse Lab in Cambridge|Wellcome Sanger Institute for the COVID-19 Genomics UK (COG-UK) consortium|Davies|United Kingdom
>Spike|hCoV-19/Scotland/EDB9107/2020|2020-10-25|EPI_ISL_652042|Original|hCoV-19^^Scotland|Human|Virology Department|COVID-19 Genomics UK (COG-UK) Consortium|McHugh|United Kingdom

>Spike|hCoV-19/England/CAMC-B375D9/2020|2020-11-05|EPI_ISL_645312|Original|hCoV-19^^England|Human|Lighthouse Lab in Cambridge|Wellcome Sanger Institute for the COVID-19 Genomics UK (COG-UK) Consortium|Davies|United Kingdom
>Spike|hCoV-19/USA/CO-CDPHE-2007234429/2020|2020-07-23|EPI_ISL_677295|Original|hCoV-19^^Colorado|Human|Colorado Department of Public Health and Environment|Colorado Department of Puplic Health and Environment|Bankers|USA
>Spike|hCoV-19/South Africa/KRISP-K003323/2020|2020-09-04|EPI_ISL_602716|Original|hCoV-19^^KwaZulu-Natal|Human|NHLS-IALCH|KRISP|de Oliveira|South Africa
>Spike|hCoV-19/Lithuania/MR-LUHS-Eilnr314/2020|2020-08-17|EPI_ISL_636863|Original|hCoV-19^^Taurages apskritis|Human|Lithuanian University of Health Sciences Hospital|Lithuanian University of Health Sciences|Zemaitis|Lithuania
>Spike|hCoV-19/Denmark/DCGC-1726/2020|2020-05-11|EPI_ISL_617945|Original|hCoV-19^^Syddanmark|Human|Department of Virus and Microbiological Special Diagnostics|Albertsen lab|Michaelsen|Denmark
>Spike|hCoV-19/England/BIRM-61E12/2020|2020-03-25|EPI_ISL_529611|Original|hCoV-19^^England|Human|University of Birmingham|COVID-19 Genomics UK (COG-UK) Consortium|Loman|United Kingdom
>Spike|hCoV-19/South Korea/KCDC2703/2020|2020-07-06|EPI_ISL_522483|Original|hCoV-19^^South Korea|Human|Center for Laboratory Control of Infectious Diseases|Center for Laboratory Control of Infectious Diseases|Kim|South Korea
>Spike|hCoV-19/England/ALDP-9C6331/2020|2020-09-10|EPI_ISL_567733|Original|hCoV-19^^England|Human|Lighthouse Lab in Alderley Park|Wellcome Sanger Institute for the COVID-19 Genomics UK (COG-UK) consortium|Davies|United Kingdom
>Spike|hCoV-19/Luxembourg/LNS5702833/2020|2020-11-27|EPI_ISL_744279|Original|hCoV-19^^Luxembourg|Human|Laboratoire national de sant\u00e9|Laboratoire national de sant\u00e9|Wienecke-Baldacchino|Luxembourg
>Spike|hCoV-19/USA/WA-S44/2020|2020-02-28|EPI_ISL_417097|Original|hCoV-19^^Washington|Human|Washington State Department of Health|Seattle Flu Study|Bedford|USA
>Spike|hCoV-19/USA/WA-S2385/2020|2020-06-13|EPI_ISL_497017|Original|hCoV-19^^Washington|Human|Washington State Department of Health|Seattle Flu Study|Lee|USA
>Spike|hCoV-19/England/PORT-2D12A6/2020|2020-03-24|EPI_ISL_475341|Original|hCoV-19^^England|Human|Centre for Enzyme Innovation|COVID-19 Genomics UK (COG-UK) Consortium|Robson|United Kingdom
>Spike|hCoV-19/Scotland/QEUH-B132BE/2020|2020-11-03|EPI_ISL_649579|Original|hCoV-19^^Scotland|Human|Lighthouse Lab in Glasgow|Wellcome Sanger Institute for the COVID-19 Genomics UK (COG-UK) Consortium|Davies|United Kingdom
>Spike|hCoV-19/England/QEUH-C3224F/2020|2020-12-11|EPI_ISL_730849|Original|hCoV-19^^England|Human|Lighthouse Lab in Glasgow|Wellcome Sanger Institute for the COVID-19 Genomics UK (COG-UK) Consortium|Davies|United Kingdom
>Spike|hCoV-19/USA/CA-ALSR-0551-IPL/2020|2020-05-07|EPI_ISL_483217|Original|hCoV-19^^California|Human|UC San Diego Center for Advanced Laboratory Medicine|Andersen lab at Scripps Research|Gangavarapu|USA
>Spike|hCoV-19/Portugal/PT0463/2020|2020-04-17|EPI_ISL_454187|Original|hCoV-19^^Portugal|Human|unknown|Instituto Nacional de Saude (INSA)|Borges|Portugal
>Spike|hCoV-19/USA/WA-UW-26861/2020|2020-09-01|EPI_ISL_570783|Original|hCoV-19^^Washington|Human|UW Virology Lab|UW Virology Lab|Roychoudhury|USA
>Spike|hCoV-19/USA/NY-NYCPHL-000077/2020|2020-03-12|EPI_ISL_631570|Original|hCoV-19^^New York|Human|OCME Office Of Chief Medical Examiner|New York City Public Health Laboratory|Wang|USA
>Spike|hCoV-19/Scotland/EDB2894/2020|2020-04-01|EPI_ISL_487703|Original|hCoV-19^^Scotland|Human|Virology Department|Wellcome Sanger Institute for the COVID-19 Genomics UK (COG-UK) consortium|Davies|United Kingdom
>Spike|hCoV-19/England/ALDP-BBAD9A/2020|2020-11-19|EPI_ISL_702521|Original|hCoV-19^^England|Human|Lighthouse Lab in Alderley Park|Wellcome Sanger Institute for the COVID-19 Genomics UK (COG-UK) Consortium|Davies|United Kingdom
>Spike|hCoV-19/England/QEUH-AD30C0/2020|2020-10-26|EPI_ISL_633873|Original|hCoV-19^^England|Human|Lighthouse Lab in Glasgow|Wellcome Sanger Institute for the COVID-19 Genomics UK (COG-UK) consortium|Davies|United Kingdom

>Spike|hCoV-19/Belgium/ULG-10533/2020|2020-10-23|EPI_ISL_626325|Original|hCoV-19^^Li\u00e8ge|Human|Department of Clinical Microbiology|GIGA Medical Genomics|Durkin|Belgium
>Spike|hCoV-19/USA/FL-BPHL-0678/2020|2020-03-17|EPI_ISL_509736|Original|hCoV-19^^Florida|Human|Florida Bureau of Public Health Laboratories|Florida Bureau of Public Health Laboratories|Blanton|USA
>Spike|hCoV-19/England/MILK-B04FD7/2020|2020-10-24|EPI_ISL_637862|Original|hCoV-19^^England|Human|Virology Department|COVID-19 Genomics UK (COG-UK) Consortium|de Silva|United Kingdom
>Spike|hCoV-19/England/QEUH-9F7285/2020|2020-09-27|EPI_ISL_595674|Original|hCoV-19^^England|Human|Oxford Viromics|COVID-19 Genomics UK (COG-UK) Consortium|Golubchik|United Kingdom
>Spike|hCoV-19/England/OXON-B0676/2020|2020-04-20|EPI_ISL_478897|Original|hCoV-19^^England|Human|Oxford Viromics|COVID-19 Genomics UK (COG-UK) Consortium|Golubchik|United Kingdom
>Spike|hCoV-19/England/QEUH-A8A866/2020|2020-10-16|EPI_ISL_638852|Original|hCoV-19^^England|Human|Oxford Viromics|COVID-19 Genomics UK (COG-UK) Consortium|Golubchik|United Kingdom
>Spike|hCoV-19/USA/OR-OHSU-1971/2020|2020-07-30|EPI_ISL_526079|Original|hCoV-19^^Oregon|Human|OHSU Lab Services Molecular Microbiology Lab|Oregon SARS-CoV-2 Genome Sequencing Center|O'Roak|USA
>Spike|hCoV-19/Gibraltar/204841717/2020|2020-00-00|EPI_ISL_671286|Original|hCoV-19^^Gibraltar|Human|Microbiology|Respiratory Virus Unit|Platt|Gibraltar
>Spike|hCoV-19/England/CAMC-B7893A/2020|2020-11-12|EPI_ISL_668009|Original|hCoV-19^^England|Human|Lighthouse Lab in Cambridge|Wellcome Sanger Institute for the COVID-19 Genomics UK (COG-UK) Consortium|Davies|United Kingdom
>Spike|hCoV-19/USA/MI-MDHHS-SC20277/2020|2020-04-18|EPI_ISL_447075|Original|hCoV-19^^Michigan|Human|Michigan Department of Health and Human Services|Michigan Department of Health and Human Services|Blankenship|USA
>Spike|hCoV-19/USA/WI-UW-2641/2021|2021-01-01|EPI_ISL_791156|Original|hCoV-19^^Wisconsin|Human|University of Wisconsin-Madison AIDS Vaccine Research Laboratories|University of Wisconsin-Madison AIDS Vaccine Research Laboratories|Moreno|USA
>Spike|hCoV-19/USA/OR-OHSU-0245/2020|2020-04-06|EPI_ISL_509080|Original|hCoV-19^^Oregon|Human|OHSU Lab Services Molecular Microbiology Lab|Oregon SARS-CoV-2 Genome Sequencing Center|O'Roak|USA
>Spike|hCoV-19/USA/UT-08511/2020|2020-05-18|EPI_ISL_524275|Original|hCoV-19^^Utah|Human|Utah Public Health Laboratory|Utah Public Health Laboratory|Young|USA
>Spike|hCoV-19/Canada/MB-NML-1150/2020|2020-07-19|EPI_ISL_582477|Original|hCoV-19^^Manitoba|Human|Cadham Provincial Laboratory|National Microbiology Laboratory (NML)|Majer|Canada
>Spike|hCoV-19/Switzerland/GR-ETHZ-210075/2020|2020-07-22|EPI_ISL_510805|Original|hCoV-19^^Graubunden|Human|Viollier AG|Department of Biosystems Science and Engineering|Nadeau|Switzerland
>Spike|hCoV-19/USA/UT-08712/2020|2020-05-18|EPI_ISL_524770|Original|hCoV-19^^Utah|Human|Utah Public Health Laboratory|Utah Public Health Laboratory|Young|USA
>Spike|hCoV-19/England/SHEF-CB70C/2020|2020-11-22|EPI_ISL_680171|Original|hCoV-19^^England|Human|Virology Department|COVID-19 Genomics UK (COG-UK) Consortium|de Silva|United Kingdom
>Spike|hCoV-19/England/ALDP-94CF7B/2020|2020-06-14|EPI_ISL_556052|Original|hCoV-19^^England|Human|Lighthouse Lab in Alderley Park|Wellcome Sanger Institute for the COVID-19 Genomics UK (COG-UK) consortium|Davies|United Kingdom
>Spike|hCoV-19/Chile/AR-ISPCH-2/2020|2020-03-16|EPI_ISL_445279|Original|hCoV-19^^La Araucania|Human|LABORATORIO INMUNOLAB SPA|Instituto de Salud Publica de Chile|Fernandez|Chile
>Spike|hCoV-19/Japan/PG-4962/2020|2020-07-00|EPI_ISL_690080|Original|hCoV-19^^Japan|Human|Pathogen Genomics Center|Pathogen Genomics Center|Sekizuka|Japan
>Spike|hCoV-19/Spain/MD-IBV-99008492/2020|2020-09-10|EPI_ISL_654051|Original|hCoV-19^^Madrid|Human|Hospital General Universitario Gregorio Mara\u00f1\u00f3n|SeqCOVID-SPAIN consortium/IBV(CSIC)|Comas|Spain

>Spike|hCoV-19/Malaysia/MGI-MAEPS54/2020|2020-06-04|EPI_ISL_528743|Original|hCoV-19^^Selangor|Human|Malaysia Genome Institute|Malaysia Genome Institute|Mat Isa|Malaysia
>Spike|hCoV-19/England/MILK-A6A073/2020|2020-10-08|EPI_ISL_627492|Original|hCoV-19^^England|Human|Virology Department|COVID-19 Genomics UK (COG-UK) Consortium|de Silva|United Kingdom
>Spike|hCoV-19/Norway/2660/2020|2020-05-25|EPI_ISL_481215|Original|hCoV-19^^Norway|Human|Oslo University Hospital|Norwegian Institute of Public Health|Kopperud Riis|Norway
>Spike|hCoV-19/England/NOTT-111979/2020|2020-05-30|EPI_ISL_461945|Original|hCoV-19^^England|Human|Queens Medical Centre|COVID-19 Genomics UK (COG-UK) Consortium|Loose|United Kingdom
>Spike|hCoV-19/Ecuador/USFQ-533/2020|2020-11-30|EPI_ISL_697799|Original|hCoV-19^^Sucumbios|Human|Institute of Microbiology|Institute of Microbiology|Cardenas|Ecuador
>Spike|hCoV-19/Japan/PG-5435/2020|2020-08-00|EPI_ISL_690538|Original|hCoV-19^^Japan|Human|Pathogen Genomics Center|Pathogen Genomics Center|Sekizuka|Japan
>Spike|hCoV-19/England/ALDP-97D384/2020|2020-07-17|EPI_ISL_526324|Original|hCoV-19^^England|Human|University of Birmingham|COVID-19 Genomics UK (COG-UK) Consortium|Loman|United Kingdom
>Spike|hCoV-19/USA/WI-GMF-17647/2020|2020-08-28|EPI_ISL_547747|Original|hCoV-19^^Wisconsin|Human|Gundersen Molecular Diagnostics Laboratory|Kabara Cancer Research Institute|Kenny|USA
>Spike|hCoV-19/Wales/PHWC-168C89/2020|2020-04-20|EPI_ISL_494349|Original|hCoV-19^^Wales|Human|Originating lab|COVID-19 Genomics UK (COG-UK) Consortium|Moore|United Kingdom
>Spike|hCoV-19/USA/NY-NYUMC681/2020|2020-03-30|EPI_ISL_450398|Original|hCoV-19^^New York|Human|NYU Langone Health|Departments of Pathology and Medicine|Maurano|USA
>Spike|hCoV-19/USA/TX-DSHS-0209/2020|2020-03-28|EPI_ISL_525771|Original|hCoV-19^^Texas|Human|Texas Department of State Health Services|Texas Department of State Health Services|Rahman|USA
>Spike|hCoV-19/England/ALDP-A5109D/2020|2020-10-07|EPI_ISL_598210|Original|hCoV-19^^England|Human|Lighthouse Lab in Alderley Park|Wellcome Sanger Institute for the COVID-19 Genomics UK (COG-UK) consortium|Davies|United Kingdom
>Spike|hCoV-19/England/EXET-135B88/2020|2020-03-31|EPI_ISL_457139|Original|hCoV-19^^England|Human|University of Exeter|COVID-19 Genomics UK (COG-UK) Consortium|Temperton|United Kingdom
>Spike|hCoV-19/USA/CA-CDC-4703/2020|2020-06-04|EPI_ISL_535680|Original|hCoV-19^^California|Human|CDPH|Pathogen Discovery|Queen|USA
>Spike|hCoV-19/USA/MA-MGH-00866/2020|2020-04-14|EPI_ISL_791801|Original|hCoV-19^^Massachusetts|Human|Massachusetts General Hospital|Infectious Disease Program|Park|USA
>Spike|hCoV-19/England/MILK-BAAC36/2020|2020-11-18|EPI_ISL_681475|Original|hCoV-19^^England|Human|Lighthouse Lab in Milton Keynes|Wellcome Sanger Institute for the COVID-19 Genomics UK (COG-UK) Consortium|Davies|United Kingdom
>Spike|hCoV-19/Canada/ON-PHL-20-01778/2020|2020-07-00|EPI_ISL_636930|Original|hCoV-19^^Ontario|Human|Public Health Ontario Laboratory|Public Health Ontario Laboratory|Guthrie|Canada
>Spike|hCoV-19/Japan/PG-8374/2020|2020-07-00|EPI_ISL_687473|Original|hCoV-19^^Japan|Human|Pathogen Genomics Center|Pathogen Genomics Center|Sekizuka|Japan
>Spike|hCoV-19/Denmark/DCGC-13317/2020|2020-11-30|EPI_ISL_712725|Original|hCoV-19^^Hovedstaden|Human|Department of Virus and Microbiological Special Diagnostics|Albertsen Lab|Michaelsen|Denmark
>Spike|hCoV-19/Australia/VIC3248/2020|2020-07-06|EPI_ISL_520928|Original|hCoV-19^^Victoria|Human|Victorian Infectious Diseases Reference Laboratory (VIDRL)|VIDRL and MDU-PHL|Schultz|Australia
>Spike|hCoV-19/Australia/VIC11073/2020|2020-07-31|EPI_ISL_563232|Original|hCoV-19^^Victoria|Human|Microbiological Diagnostic Unit - Public Health Laboratory (MDU-PHL)|MDU-PHL|Schultz|Australia

>Spike|hCoV-19/Vietnam/VNHN_0764/2020|2020-03-13|EPI_ISL_435314|Original|hCoV-19^^Hanoi|Human|National Hospital of Tropical Diseases|Oxford University Clinical Research Unit|Nguyen|Vietnam
>Spike|hCoV-19/England/MILK-AB79D6/2020|2020-10-21|EPI_ISL_623698|Original|hCoV-19^^England|Human|Lighthouse Lab in Milton Keynes|Wellcome Sanger Institute for the COVID-19 Genomics UK (COG-UK) consortium|Davies|United Kingdom
>Spike|hCoV-19/Denmark/DCGC-19673/2020|2020-12-07|EPI_ISL_749103|Original|hCoV-19^^Midtjylland|Human|Department of Virus and Microbiological Special Diagnostics|Albertsen Lab|Michaelsen|Denmark
>Spike|hCoV-19/Netherlands/NoordBrabant_119/2020|2020-03-24|EPI_ISL_461154|Original|hCoV-19^^Noord Brabant|Human|Dutch COVID-19 response team|Erasmus Medical Center|Oude Munnink|Netherlands
>Spike|hCoV-19/USA/MA-MGH-00477/2020|2020-04-04|EPI_ISL_460208|Original|hCoV-19^^Massachusetts|Human|Massachusetts General Hospital|Infectious Disease Program|Lemieux|USA
>Spike|hCoV-19/Switzerland/BE-ETHZ-321490/2020|2020-10-15|EPI_ISL_603702|Original|hCoV-19^^Bern|Human|Viollier AG|Department of Biosystems Science and Engineering|Nadeau|Switzerland
>Spike|hCoV-19/England/205041767/2020|2020-00-00|EPI_ISL_710529|Original|hCoV-19^^England|Human|Respiratory Virus Unit|COVID-19 Genomics UK (COG-UK) Consortium|Platt|United Kingdom
>Spike|hCoV-19/England/ALDP-B74EB7/2020|2020-11-12|EPI_ISL_679002|Original|hCoV-19^^England|Human|Queens Medical Centre|COVID-19 Genomics UK (COG-UK) Consortium|Loose|United Kingdom
>Spike|hCoV-19/England/LIVE-9E7E4/2020|2020-04-12|EPI_ISL_453333|Original|hCoV-19^^England|Human|Liverpool Clinical Laboratories|COVID-19 Genomics UK (COG-UK) Consortium|Haldenby|United Kingdom
>Spike|hCoV-19/Japan/PG-6237/2020|2020-08-00|EPI_ISL_689349|Original|hCoV-19^^Japan|Human|Pathogen Genomics Center|Pathogen Genomics Center|Sekizuka|Japan
>Spike|hCoV-19/USA/TX-HMH-3292/2020|2020-06-10|EPI_ISL_544109|Original|hCoV-19^^Texas|Human|Houston Methodist Hospital|Houston Methodist Hospital|Long|USA
>Spike|hCoV-19/England/MILK-B3A363/2020|2020-11-01|EPI_ISL_706020|Original|hCoV-19^^England|Human|Oxford Viromics|COVID-19 Genomics UK (COG-UK) Consortium|Golubchik|United Kingdom
>Spike|hCoV-19/England/NORT-28AE99/2020|2020-04-16|EPI_ISL_492945|Original|hCoV-19^^England|Human|NU-OMICS DNA Sequencing research facility|Wellcome Sanger Institute for the COVID-19 Genomics UK (COG-UK) consortium|Davies|United Kingdom
>Spike|hCoV-19/England/SHEF-10D5089/2020|2020-12-11|EPI_ISL_742071|Original|hCoV-19^^England|Human|Virology Department|COVID-19 Genomics UK (COG-UK) Consortium|de Silva|United Kingdom
>Spike|hCoV-19/England/SHEF-10CAC65/2020|2020-12-05|EPI_ISL_706977|Original|hCoV-19^^England|Human|Virology Department|COVID-19 Genomics UK (COG-UK) Consortium|de Silva|United Kingdom
>Spike|hCoV-19/Denmark/DCGC-7896/2020|2020-10-19|EPI_ISL_618833|Original|hCoV-19^^Syddanmark|Human|Department of Virus and Microbiological Special Diagnostics|Albertsen lab|Michaelsen|Denmark
>Spike|hCoV-19/USA/TX-HMH-MCoV-14054/2020|2020-08-14|EPI_ISL_787341|Original|hCoV-19^^Texas|Human|Houston Methodist Hospital|Houston Methodist Hospital|Long|USA
>Spike|hCoV-19/England/QEUH-A5E91D/2020|2020-10-12|EPI_ISL_597235|Original|hCoV-19^^England|Human|Lighthouse Lab in Glasgow|Wellcome Sanger Institute for the COVID-19 Genomics UK (COG-UK) consortium|Davies|United Kingdom
>Spike|hCoV-19/Germany/TH-IIMK-CaSe-17/2020|2020-12-06|EPI_ISL_728338|Original|hCoV-19^^Thuringia|Human|Jena University Hospital|Institute of infectious medicine & hospital hygiene|Brandt|Germany
>Spike|hCoV-19/Canada/QC-LSPQ-L00244338/2020|2020-03-31|EPI_ISL_536393|Original|hCoV-19^^Quebec|Human|H\u00f4pital de Hull|Laboratoire de sant\u00e9 publique du Qu\u00e9bec|Moreira|Canada
>Spike|hCoV-19/Sweden/20-52918/2020|2020-09-20|EPI_ISL_710594|Original|hCoV-19^^Vasternorrland|Human|Klinisk mikrobiologi Vasternorrland|The Public Health Agency of Sweden|Svartstr\u00f6m|Sweden

>Spike|hCoV-19/England/QEUH-AD1921/2020|2020-10-26|EPI_ISL_634575|Original|hCoV-19^^England|Human|Lighthouse Lab in Glasgow|Wellcome Sanger Institute for the COVID-19 Genomics UK (COG-UK) consortium|Davies|United Kingdom
>Spike|hCoV-19/England/CAMC-BDD010/2020|2020-11-29|EPI_ISL_709814|Original|hCoV-19^^England|Human|Lighthouse Lab in Cambridge|Wellcome Sanger Institute for the COVID-19 Genomics UK (COG-UK) Consortium|Davies|United Kingdom
>Spike|hCoV-19/USA/NM-DOH-2020310691/2020|2020-08-21|EPI_ISL_602206|Original|hCoV-19^^New Mexico|Human|New Mexico Department of Health Scientific Laboratory|New Mexico Department of Health Scientific Laboratory|Johnson|USA
>Spike|hCoV-19/USA/FL-BPHL-1863/2020|2020-04-10|EPI_ISL_613941|Original|hCoV-19^^Florida|Human|Florida Bureau of Public Health Laboratories|Florida Bureau of Public Health Laboratories|Blanton|USA
>Spike|hCoV-19/Brazil/SP-L1-CAMPI18/2020|2020-04-09|EPI_ISL_476159|Original|hCoV-19^^S\u00e3o Paulo|Human|Laborat\u00f3rio de Patologia Cl\u00ednica - UNICAMP|Laborat\u00f3rio de Estudos de V\u00edrus Emergentes - UNICAMP|Faria|Brazil
>Spike|hCoV-19/USA/TX-HMH-MCoV-8693/2020|2020-07-01|EPI_ISL_780705|Original|hCoV-19^^Texas|Human|Houston Methodist Hospital|Houston Methodist Hospital|Long|USA
>Spike|hCoV-19/USA/VA-DCLS-0752/2020|2020-06-23|EPI_ISL_526868|Original|hCoV-19^^Virginia|Human|Virginia DCLS|Virginia DCLS|St. Jacques|USA
>Spike|hCoV-19/USA/WA-UW-12632/2020|2020-06-29|EPI_ISL_570824|Original|hCoV-19^^Washington|Human|UW Virology Lab|UW Virology Lab|Roychoudhury|USA
>Spike|hCoV-19/USA/MD-MDH-0502/2020|2020-11-13|EPI_ISL_778883|Original|hCoV-19^^Maryland|Human|Maryland Public Health Laboratory|Maryland Public Health Laboratory|Keller|USA
>Spike|hCoV-19/Wales/PHWC-4A5986/2020|2020-12-15|EPI_ISL_764941|Original|hCoV-19^^Wales|Human|Wales Specialist Virology Centre Sequencing lab|COVID-19 Genomics UK (COG-UK) Consortium|Moore|United Kingdom
>Spike|hCoV-19/USA/ME-HETL-J0029/2020|2020-03-15|EPI_ISL_513441|Original|hCoV-19^^Maine|Human|Maine HETL|Tewhey Lab|Tewhey|USA
>Spike|hCoV-19/Mexico/CMX-INMEGEN-08/2020|2020-07-31|EPI_ISL_522879|Original|hCoV-19^^Mexico City|Human|Instituto Nacional de Medicina Gen\u00f3mica|Instituto Nacional de Medicina Gen\u00f3mica|Hidalgo|Mexico
>Spike|hCoV-19/Scotland/QEUH-9C8E5C/2020|2020-09-14|EPI_ISL_576033|Original|hCoV-19^^Scotland|Human|Lighthouse Lab in Glasgow|Wellcome Sanger Institute for the COVID-19 Genomics UK (COG-UK) consortium|Davies|United Kingdom
>Spike|hCoV-19/England/SHEF-10CB5C3/2020|2020-12-11|EPI_ISL_727902|Original|hCoV-19^^England|Human|Virology Department|COVID-19 Genomics UK (COG-UK) Consortium|de Silva|United Kingdom
>Spike|hCoV-19/USA/WA-S2281/2020|2020-06-10|EPI_ISL_497709|Original|hCoV-19^^Washington|Human|Washington State Department of Health|Seattle Flu Study|Lee|USA
>Spike|hCoV-19/New Zealand/20CV0630/2020|2020-12-02|EPI_ISL_707797|Original|hCoV-19^^Auckland|Human|LabPLUS|Institute of Environmental Science and Research (ESR)|Wang|New Zealand
>Spike|hCoV-19/England/LEED-2A8F65/2020|2020-04-05|EPI_ISL_539043|Original|hCoV-19^^England|Human|Leeds Teaching Hospitals NHS Trust and Public Health England|Wellcome Sanger Institute for the COVID-19 Genomics UK (COG-UK) consortium|Davies|United Kingdom
>Spike|hCoV-19/England/CAMB-1AA2B6/2020|2020-04-17|EPI_ISL_443629|Original|hCoV-19^^England|Human|Department of Pathology|Wellcome Sanger Institute for the COVID-19 Genomics UK (COG-UK) consortium|Amato|United Kingdom
>Spike|hCoV-19/England/CAMC-94328A/2020|2020-07-25|EPI_ISL_556910|Original|hCoV-19^^England|Human|Lighthouse Lab in Cambridge|Wellcome Sanger Institute for the COVID-19 Genomics UK (COG-UK) consortium|Davies|United Kingdom
>Spike|hCoV-19/England/ALDP-AA0985/2020|2020-10-22|EPI_ISL_644108|Original|hCoV-19^^England|Human|Lighthouse Lab in Alderley Park|Wellcome Sanger Institute for the COVID-19 Genomics UK (COG-UK) Consortium|Davies|United Kingdom
>Spike|hCoV-19/USA/NY-Wadsworth-142749-01/2020|2020-04-20|EPI_ISL_676965|Original|hCoV-19^^New York|Human|Wadsworth Center|Wadsworth Center|Lasek-Nesselquist|USA

>Spike|hCoV-19/England/20129061704/2020|2020-03-12|EPI_ISL_424057|Original|hCoV-19^^England|Human|Respiratory Virus Unit|Respiratory Virus Unit|Platt|United Kingdom
>Spike|hCoV-19/Denmark/DCGC-11409/2020|2020-11-02|EPI_ISL_670898|Original|hCoV-19^^Midtjylland|Human|Department of Virus and Microbiological Special Diagnostics|Albertsen Lab|Michaelsen|Denmark
>Spike|hCoV-19/Scotland/CVR2014/2020|2020-04-08|EPI_ISL_490648|Original|hCoV-19^^Scotland|Human|West of Scotland Specialist Virology Centre|COVID-19 Genomics UK (COG-UK) Consortium|Robertson|United Kingdom
>Spike|hCoV-19/England/QEUH-B11872/2020|2020-11-02|EPI_ISL_642315|Original|hCoV-19^^England|Human|Lighthouse Lab in Glasgow|Wellcome Sanger Institute for the COVID-19 Genomics UK (COG-UK) Consortium|Davies|United Kingdom
>Spike|hCoV-19/USA/TX-HMH-MCoV-13220/2020|2020-08-25|EPI_ISL_788371|Original|hCoV-19^^Texas|Human|Houston Methodist Hospital|Houston Methodist Hospital|Long|USA
>Spike|hCoV-19/Canada/ON-S122/2020|2020-03-23|EPI_ISL_586296|Original|hCoV-19^^Ontario|Human|Toronto Invasive Bacterial Diseases Network|McMaster University|McArthur|Canada
>Spike|hCoV-19/Egypt/CUNCI-HGC5I016/2020|2020-06-02|EPI_ISL_479731|Original|hCoV-19^^Egypt|Human|Egyptian National Cancer Institute (ENCI)|Egyptian National Cancer Institute (ENCI)|Zekri|Egypt
>Spike|hCoV-19/Australia/TAS112/2020|2020-03-27|EPI_ISL_522578|Original|hCoV-19^^Tasmania|Human|Royal Hobart Hospital Microbiology Department|MDU-PHL|Schultz|Australia
>Spike|hCoV-19/Switzerland/BE-ETHZ-410875/2020|2020-12-14|EPI_ISL_737741|Original|hCoV-19^^Bern|Human|Viollier AG|Department of Biosystems Science and Engineering|Nadeau|Switzerland
>Spike|hCoV-19/Netherlands/ZH-EMC-719/2020|2020-10-09|EPI_ISL_632566|Original|hCoV-19^^Zuid Holland|Human|Dutch COVID-19 response team|Erasmus Medical Center|Oude Munnink|Netherlands
>Spike|hCoV-19/Australia/VIC9951/2020|2020-07-23|EPI_ISL_565799|Original|hCoV-19^^Victoria|Human|Microbiological Diagnostic Unit - Public Health Laboratory (MDU-PHL)|MDU-PHL|Schultz|Australia
>Spike|hCoV-19/USA/WI-WSLH-200326/2020|2020-04-19|EPI_ISL_578507|Original|hCoV-19^^Wisconsin|Human|Wisconsin State Laboratory of Hygiene Communicable Disease Division|Wisconsin State Laboratory of Hygiene Communicable Disease Division|Florek|USA
>Spike|hCoV-19/Netherlands/ZH-EMC-574/2020|2020-00-00|EPI_ISL_578002|Original|hCoV-19^^Zuid Holland|Human|Dutch COVID-19 response team|Erasmus Medical Center|Oude Munnink|Netherlands
>Spike|hCoV-19/Denmark/DCGC-11301/2020|2020-11-02|EPI_ISL_670825|Original|hCoV-19^^Midtjylland|Human|Department of Virus and Microbiological Special Diagnostics|Albertsen Lab|Michaelsen|Denmark
>Spike|hCoV-19/Wales/PHWC-32393/2020|2020-04-08|EPI_ISL_446639|Original|hCoV-19^^Wales|Human|Wales Specialist Virology Centre|Public Health Wales Microbiology Cardiff|Moore|United Kingdom
>Spike|hCoV-19/England/CAMC-B21350/2020|2020-11-03|EPI_ISL_647305|Original|hCoV-19^^England|Human|Lighthouse Lab in Cambridge|Wellcome Sanger Institute for the COVID-19 Genomics UK (COG-UK) Consortium|Davies|United Kingdom
>Spike|hCoV-19/England/MILK-A7B419/2020|2020-10-14|EPI_ISL_607345|Original|hCoV-19^^England|Human|Lighthouse Lab in Milton Keynes|Wellcome Sanger Institute for the COVID-19 Genomics UK (COG-UK) consortium|Davies|United Kingdom
>Spike|hCoV-19/England/ALDP-A6F9FB/2020|2020-10-14|EPI_ISL_607640|Original|hCoV-19^^England|Human|Lighthouse Lab in Alderley Park|Wellcome Sanger Institute for the COVID-19 Genomics UK (COG-UK) consortium|Davies|United Kingdom
>Spike|hCoV-19/England/ALDP-AA4CA6/2020|2020-10-21|EPI_ISL_630755|Original|hCoV-19^^England|Human|Lighthouse Lab in Alderley Park|Wellcome Sanger Institute for the COVID-19 Genomics UK (COG-UK) consortium|Davies|United Kingdom
>Spike|hCoV-19/USA/OR-OHSU-2981/2020|2020-09-09|EPI_ISL_667445|Original|hCoV-19^^Oregon|Human|OHSU Lab Services Molecular Microbiology Lab|Oregon SARS-CoV-2 Genome Sequencing Center|O'Roak|USA

>Spike|hCoV-19/Canada/ON-UHTC_0387/2020|2020-11-24|EPI_ISL_698088|Original|hCoV-19^^Ontario|Human|Unity Health Toronto|Ontario Institute for Cancer Research|de Borja|Canada
>Spike|hCoV-19/England/CAMC-B512A5/2020|2020-11-08|EPI_ISL_658048|Original|hCoV-19^^England|Human|Lighthouse Lab in Cambridge|Wellcome Sanger Institute for the COVID-19 Genomics UK (COG-UK) Consortium|Davies|United Kingdom
>Spike|hCoV-19/USA/WI-UW-1376/2020|2020-09-06|EPI_ISL_536747|Original|hCoV-19^^Wisconsin|Human|University of Wisconsin-Madison AIDS Vaccine Research Laboratories|University of Wisconsin-Madison AIDS Vaccine Research Laboratories|Baczenas|USA
>Spike|hCoV-19/England/MILK-9E0850/2020|2020-09-20|EPI_ISL_601287|Original|hCoV-19^^England|Human|Lighthouse Lab in Milton Keynes|Wellcome Sanger Institute for the COVID-19 Genomics UK (COG-UK) consortium|Davies|United Kingdom
>Spike|hCoV-19/Germany/HH-hpi-p1962/2020|2020-03-25|EPI_ISL_776287|Original|hCoV-19^^Hamburg|Human|University Medical Center Hamburg Eppendorf|Heinrich Pette Institute|Robitaille|Germany
>Spike|hCoV-19/Germany/NW-HHU-163/2020|2020-03-15|EPI_ISL_602532|Orginal|hCoV-19^^North Rhine-Westphalia|Human|Institute for Virology|Center of Medical Microbiology|Wienemann|Germany
>Spike|hCoV-19/England/ALDP-B25F0D/2020|2020-11-04|EPI_ISL_646563|Original|hCoV-19^^England|Human|Lighthouse Lab in Alderley Park|Wellcome Sanger Institute for the COVID-19 Genomics UK (COG-UK) Consortium|Davies|United Kingdom
>Spike|hCoV-19/Denmark/DCGC-10791/2020|2020-11-02|EPI_ISL_670210|Original|hCoV-19^^Nordjylland|Human|Department of Virus and Microbiological Special Diagnostics|Albertsen Lab|Michaelsen|Denmark
>Spike|hCoV-19/England/NORT-1B71BA1/2020|2020-11-19|EPI_ISL_703704|Original|hCoV-19^^England|Human|Northumbria University / South Tees Hospitals NHS Foundation Trust / North Cumbria Integrated Care NHS Foundation Trust / North Tees and Hartlepool NHS Foundation Trust / Newcastle Hospitals NHS Foundation Trust|COVID-19 Genomics UK (COG-UK) Consortium|Bashton|United Kingdom
>Spike|hCoV-19/England/QEUH-AD1D9E/2020|2020-10-26|EPI_ISL_629935|Original|hCoV-19^^England|Human|Lighthouse Lab in Glasgow|Wellcome Sanger Institute for the COVID-19 Genomics UK (COG-UK) consortium|Davies|United Kingdom
>Spike|hCoV-19/India/MH-AFMC-5604/2020|2020-05-24|EPI_ISL_497884|Original|hCoV-19^^Maharashtra|Human|Armed Forces Medical College|National Centre For Cell Science|Paul|India
>Spike|hCoV-19/England/MILK-57B0FC/2020|2020-06-12|EPI_ISL_558247|Original|hCoV-19^^England|Human|Lighthouse Lab in Milton Keynes|Wellcome Sanger Institute for the COVID-19 Genomics UK (COG-UK) consortium|Davies|United Kingdom
>Spike|hCoV-19/USA/UT-UPHL-2008836/2020|2020-06-01|EPI_ISL_524855|Original|hCoV-19^^Utah|Human|Utah Public Health Laboratory|Utah Public Health Laboratory|Young|USA
>Spike|hCoV-19/England/ALDP-B2617F/2020|2020-11-04|EPI_ISL_646683|Original|hCoV-19^^England|Human|Lighthouse Lab in Alderley Park|Wellcome Sanger Institute for the COVID-19 Genomics UK (COG-UK) Consortium|Davies|United Kingdom
>Spike|hCoV-19/England/CAMC-B79B42/2020|2020-11-13|EPI_ISL_668023|Original|hCoV-19^^England|Human|Lighthouse Lab in Cambridge|Wellcome Sanger Institute for the COVID-19 Genomics UK (COG-UK) Consortium|Davies|United Kingdom
>Spike|hCoV-19/USA/TX-HMH-2798/2020|2020-06-08|EPI_ISL_545561|Original|hCoV-19^^Texas|Human|Houston Methodist Hospital|Houston Methodist Hospital|Long|USA
>Spike|hCoV-19/USA/WA-S151/2020|2020-03-16|EPI_ISL_430188|Original|hCoV-19^^Washington|Human|Washington State Department of Health|Seattle Flu Study|Bedford|USA
>Spike|hCoV-19/England/MILK-BDC2B1/2020|2020-11-26|EPI_ISL_709597|Original|hCoV-19^^England|Human|Lighthouse Lab in Milton Keynes|Wellcome Sanger Institute for the COVID-19 Genomics UK (COG-UK) Consortium|Davies|United Kingdom
>Spike|hCoV-19/Cote dIvoire/BKE0249/2020|2020-06-20|EPI_ISL_614366|Original|hCoV-19^^Boauk\u00e9|Human|Molecular diagnostic unit for viral haemorrhagic fevers and emerging viruses|Project group Epidemology of Highly Pathogenic Microorganisms|Calvignac-Spencer|C\u00f4te d'Ivoire

>Spike|hCoV-19/Denmark/DCGC-12992/2020|2020-12-07|EPI_ISL_712514|Original|hCoV-19^^Hovedstaden|Human|Department of Virus and Microbiological Special Diagnostics|Albertsen Lab|Michaelsen|Denmark
>Spike|hCoV-19/India/MH-BJMC-22757/2020|2020-07-18|EPI_ISL_728254|Original|hCoV-19^^Maharashtra|Human|B.J. Govt. Medical College|National Centre For Cell Science|Paul|India
>Spike|hCoV-19/England/QEUH-CDE39D/2020|2020-12-26|EPI_ISL_782465|Original|hCoV-19^^England|Human|Lighthouse Lab in Glasgow|Wellcome Sanger Institute for the COVID-19 Genomics UK (COG-UK) Consortium|Davies|United Kingdom
>Spike|hCoV-19/Israel/701002431/2020|2020-03-24|EPI_ISL_447302|Original|hCoV-19^^South Coast District|Human|Microbiology Division|Stern Lab|Miller|Israel
>Spike|hCoV-19/Belgium/rega-10131360/2020|2020-10-13|EPI_ISL_738413|Original|hCoV-19^^Leuven|Human|UZ Leuven|KU Leuven|Maes|Belgium
>Spike|hCoV-19/Denmark/DCGC-3970/2020|2020-09-14|EPI_ISL_615883|Original|hCoV-19^^Sjaelland|Human|Department of Virus and Microbiological Special Diagnostics|Albertsen lab|Michaelsen|Denmark
>Spike|hCoV-19/England/ALDP-B6360E/2020|2020-11-09|EPI_ISL_656399|Original|hCoV-19^^England|Human|Lighthouse Lab in Alderley Park|Wellcome Sanger Institute for the COVID-19 Genomics UK (COG-UK) Consortium|Davies|United Kingdom
>Spike|hCoV-19/Australia/VIC2785/2020|2020-07-05|EPI_ISL_521619|Original|hCoV-19^^Victoria|Human|Microbiological Diagnostic Unit - Public Health Laboratory (MDU-PHL)|MDU-PHL|Schultz|Australia
>Spike|hCoV-19/Switzerland/BL-ETHZ-310386/2020|2020-10-08|EPI_ISL_603460|Original|hCoV-19^^Basel-Landschaft|Human|Viollier AG|Department of Biosystems Science and Engineering|Nadeau|Switzerland
>Spike|hCoV-19/England/NORT-281B13/2020|2020-03-23|EPI_ISL_472177|Original|hCoV-19^^England|Human|Northumbria University / South Tees Hospitals NHS Foundation Trust / North Cumbria Integrated Care NHS Foundation Trust / North Tees and Hartlepool NHS Foundation Trust / Newcastle Hospitals NHS Foundation Trust|COVID-19 Genomics UK (COG-UK) Consortium|Bashton|United Kingdom
>Spike|hCoV-19/Denmark/DCGC-2398/2020|2020-08-10|EPI_ISL_617580|Original|hCoV-19^^Midtjylland|Human|Department of Virus and Microbiological Special Diagnostics|Albertsen lab|Michaelsen|Denmark
>Spike|hCoV-19/Denmark/DCGC-7762/2020|2020-10-19|EPI_ISL_618703|Original|hCoV-19^^Hovedstaden|Human|Department of Virus and Microbiological Special Diagnostics|Albertsen lab|Michaelsen|Denmark
>Spike|hCoV-19/Australia/VIC10376/2020|2020-08-31|EPI_ISL_562709|Original|hCoV-19^^Victoria|Human|Microbiological Diagnostic Unit - Public Health Laboratory (MDU-PHL)|MDU-PHL|Schultz|Australia
>Spike|hCoV-19/England/QEUH-A61F60/2020|2020-10-12|EPI_ISL_706948|Original|hCoV-19^^England|Human|Oxford Viromics|COVID-19 Genomics UK (COG-UK) Consortium|Golubchik|United Kingdom
>Spike|hCoV-19/USA/TX-HMH-5559/2020|2020-06-26|EPI_ISL_544861|Original|hCoV-19^^Texas|Human|Houston Methodist Hospital|Houston Methodist Hospital|Long|USA
>Spike|hCoV-19/Wales/ALDP-B1E295/2020|2020-11-01|EPI_ISL_710944|Original|hCoV-19^^Wales|Human|Lighthouse Lab in Alderley Park|Wellcome Sanger Institute for the COVID-19 Genomics UK (COG-UK) Consortium|Davies|United Kingdom
>Spike|hCoV-19/England/GSTT-265D83D/2020|2020-03-25|EPI_ISL_484021|Original|hCoV-19^^England|Human|Centre for Clinical Infection and Diagnostics Research and Genomics Innovation Unit|COVID-19 Genomics UK (COG-UK) Consortium|Awan|United Kingdom
>Spike|hCoV-19/Singapore/148/2020|2020-03-17|EPI_ISL_443240|Original|hCoV-19^^Singapore|Human|National Public Health Laboratory|National Public Health Laboratory|Mak|Singapore
>Spike|hCoV-19/Netherlands/ZH-EMC-877/2020|2020-10-28|EPI_ISL_632757|Original|hCoV-19^^Zuid Holland|Human|Dutch COVID-19 response team|Erasmus Medical Center|Oude Munnink|Netherlands
>Spike|hCoV-19/England/LOND-12F089D/2020|2020-11-19|EPI_ISL_679585|Original|hCoV-19^^England|Human|University College London|COVID-19 Genomics UK (COG-UK) Consortium|Roy|United Kingdom

>Spike|hCoV-19/Japan/PG-5338/2020|2020-08-00|EPI_ISL_690441|Original|hCoV-19^^Japan|Human|Fukuoka Institute of Health and Environmental Sciences|Pathogen Genomics Center|Sekizuka|Japan
>Spike|hCoV-19/mink/Netherlands/NB-EMC-7-2/2020|2020-05-30|EPI_ISL_523110|Original|hCoV-19^^Noord Brabant|Neovison vison|Dutch COVID-19 response team|Erasmus Medical Center|Oude Munnink|Netherlands
>Spike|hCoV-19/England/MILK-C52C78/2020|2020-12-12|EPI_ISL_736329|Original|hCoV-19^^England|Human|Lighthouse Lab in Milton Keynes|Wellcome Sanger Institute for the COVID-19 Genomics UK (COG-UK) Consortium|Davies|United Kingdom
>Spike|hCoV-19/England/CAMB-7AC94/2020|2020-04-07|EPI_ISL_433861|Original|hCoV-19^^England|Human|Department of Pathology|COVID-19 Genomics UK (COG-UK) Consortium|Meredith|United Kingdom
>Spike|hCoV-19/France/ARA-105851/2020|2020-11-12|EPI_ISL_728307|Original|hCoV-19^^Auvergne-Rh\u00f4ne-Alpes|Human|CNR Virus des Infections Respiratoires - France SUD|CNR Virus des Infections Respiratoires - France SUD|Bal|France
>Spike|hCoV-19/England/20099073004/2020|2020-02-27|EPI_ISL_464226|Original|hCoV-19^^England|Human|Respiratory Virus Unit|Respiratory Virus Unit|Platt|United Kingdom
>Spike|hCoV-19/England/QEUH-C579E5/2020|2020-12-12|EPI_ISL_736394|Original|hCoV-19^^England|Human|Lighthouse Lab in Glasgow|Wellcome Sanger Institute for the COVID-19 Genomics UK (COG-UK) Consortium|Davies|United Kingdom
>Spike|hCoV-19/Brazil/PE-IAM103/2020|2020-04-09|EPI_ISL_500462|Original|hCoV-19^^Pernambuco|Human|LACEN/PE|WallauLab|Wallau|Brazil
>Spike|hCoV-19/Scotland/QEUH-9E4713/2020|2020-09-23|EPI_ISL_600962|Original|hCoV-19^^Scotland|Human|Lighthouse Lab in Glasgow|Wellcome Sanger Institute for the COVID-19 Genomics UK (COG-UK) consortium|Davies|United Kingdom
>Spike|hCoV-19/Australia/VIC8190/2020|2020-08-13|EPI_ISL_561504|Original|hCoV-19^^Victoria|Human|Microbiological Diagnostic Unit - Public Health Laboratory (MDU-PHL)|MDU-PHL|Schultz|Australia
>Spike|hCoV-19/Germany/HE-ChVir-W1339-8077/2020|2020-05-18|EPI_ISL_754042|Original|hCoV-19^^Hesse|Human|Charit\u00e9 Universit\u00e4tsmedizin Berlin|Charit\u00e9 Universit\u00e4tsmedizin Berlin|Muehlemann|Germany
>Spike|hCoV-19/England/MILK-BA9EF5/2020|2020-11-18|EPI_ISL_681354|Original|hCoV-19^^England|Human|Lighthouse Lab in Milton Keynes|Wellcome Sanger Institute for the COVID-19 Genomics UK (COG-UK) Consortium|Davies|United Kingdom
>Spike|hCoV-19/England/QEUH-9AE0CF/2020|2020-09-05|EPI_ISL_638624|Original|hCoV-19^^England|Human|Department of Pathology|COVID-19 Genomics UK (COG-UK) Consortium|Goodfellow|United Kingdom
>Spike|hCoV-19/USA/WA-S2110/2020|2020-06-08|EPI_ISL_497538|Original|hCoV-19^^Washington|Human|Washington State Department of Health|Seattle Flu Study|Lee|USA
>Spike|hCoV-19/USA/TX-DSHS-0514/2020|2020-06-21|EPI_ISL_560957|Original|hCoV-19^^Texas|Human|Texas Department of State Health Services|Texas Department of State Health Services|Rahman|USA
>Spike|hCoV-19/USA/VA-DCLS-2174/2020|2020-11-06|EPI_ISL_677202|Original|hCoV-19^^Virginia|Human|Virginia Division of Consolidated Laboratory Services|Virginia Division of Consolidated Laboratory Services|St. Jacques|USA
>Spike|hCoV-19/England/CAMC-AEAD47/2020|2020-10-26|EPI_ISL_633253|Original|hCoV-19^^England|Human|Lighthouse Lab in Cambridge|Wellcome Sanger Institute for the COVID-19 Genomics UK (COG-UK) consortium|Davies|United Kingdom
>Spike|hCoV-19/England/LIVE-1E18B1/2020|2020-10-11|EPI_ISL_626905|Original|hCoV-19^^England|Human|Liverpool Clinical Laboratories|COVID-19 Genomics UK (COG-UK) Consortium|Haldenby|United Kingdom
>Spike|hCoV-19/England/ALDP-AA12F2/2020|2020-10-22|EPI_ISL_624103|Original|hCoV-19^^England|Human|Lighthouse Lab in Alderley Park|Wellcome Sanger Institute for the COVID-19 Genomics UK (COG-UK) consortium|Davies|United Kingdom
>Spike|hCoV-19/Germany/BY-IMB06494/2020|2020-05-26|EPI_ISL_732560|Original|hCoV-19^^Bavaria|Human|Bundeswehr Institute of Microbiology|Bundeswehr Institute of Microbiology|Antwerpen|Germany
>Spike|hCoV-19/Australia/VIC4607/2020|2020-07-13|EPI_ISL_519008|Original|hCoV-19^^Victoria|Human|Victorian Infectious Diseases Reference Laboratory (VIDRL)|VIDRL and MDU-PHL|Schultz|Australia

>Spike|hCoV-19/England/CAMC-9916CD/2020|2020-08-27|EPI_ISL_551980|Original|hCoV-19^^England|Human|Lighthouse Lab in Cambridge|Wellcome Sanger Institute for the COVID-19 Genomics UK (COG-UK) consortium|Davies|United Kingdom
>Spike|hCoV-19/Czech Republic/2308/2020|2020-04-14|EPI_ISL_434572|Original|hCoV-19^^Vysocina Region|Human|The National Institute of Public Health Center for Epidemiology and Microbiology|The National Institute of Public Health Center for Epidemiology and Microbiology|Nagy|Czech Republic
>Spike|hCoV-19/Japan/PG-4751/2020|2020-07-00|EPI_ISL_689887|Original|hCoV-19^^Japan|Human|Pathogen Genomics Center|Pathogen Genomics Center|Sekizuka|Japan
>Spike|hCoV-19/USA/CA-ALSR-1124/2020|2020-04-27|EPI_ISL_494376|Original|hCoV-19^^California|Human|San Diego County Public Health Laboratory|Andersen lab at Scripps Research|Gangavarapu|USA
>Spike|hCoV-19/Australia/VIC5343/2020|2020-07-19|EPI_ISL_520106|Original|hCoV-19^^Victoria|Human|Microbiological Diagnostic Unit - Public Health Laboratory (MDU-PHL)|MDU-PHL|Schultz|Australia
>Spike|hCoV-19/Brazil/RJ-INCA-C30/2020|2020-04-16|EPI_ISL_513538|Original|hCoV-19^^Rio de Janeiro|Human|Programa de Oncovirologia|Programa de Oncovirologia|Ramos Goes|Brazil
>Spike|hCoV-19/Japan/PG-1097/2020|2020-04-00|EPI_ISL_684707|Original|hCoV-19^^Japan|Human|Pathogen Genomics Center|Pathogen Genomics Center|Sekizuka|Japan
>Spike|hCoV-19/Poland/76112/2020|2020-11-00|EPI_ISL_732874|Original|hCoV-19^^Poland|Human|genXone SA|genXone SA|Kaszuba|Poland
>Spike|hCoV-19/USA/NY-SURV0302/2020|2020-03-09|EPI_ISL_436060|Original|hCoV-19^^New York|Human|NYC Department of Health and Mental Hygiene|Pathogen Discovery|Queen|USA
>Spike|hCoV-19/England/ALDP-B7519F/2020|2020-11-12|EPI_ISL_662145|Original|hCoV-19^^England|Human|Lighthouse Lab in Alderley Park|Wellcome Sanger Institute for the COVID-19 Genomics UK (COG-UK) Consortium|Davies|United Kingdom
>Spike|hCoV-19/Denmark/DCGC-11898/2020|2020-10-26|EPI_ISL_671204|Original|hCoV-19^^Nordjylland|Human|Department of Virus and Microbiological Special Diagnostics|Albertsen Lab|Michaelsen|Denmark
>Spike|hCoV-19/England/LIVE-9F9CF/2020|2020-06-04|EPI_ISL_472031|Original|hCoV-19^^England|Human|Liverpool Clinical Laboratories|COVID-19 Genomics UK (COG-UK) Consortium|Haldenby|United Kingdom
>Spike|hCoV-19/Kenya/C5913/2020|2020-05-12|EPI_ISL_568781|Original|hCoV-19^^Mombasa|Human|KEMRI-Wellcome Trust Research Programme/KEMRI-CGMR-C Kilifi|KEMRI-Wellcome Trust Research Programme/KEMRI-CGMR-C Kilifi|Githinji|Kenya
>Spike|hCoV-19/England/GSTT-265E1D7/2020|2020-03-31|EPI_ISL_484167|Original|hCoV-19^^England|Human|Centre for Clinical Infection and Diagnostics Research and Genomics Innovation Unit|COVID-19 Genomics UK (COG-UK) Consortium|Awan|United Kingdom
>Spike|hCoV-19/Wales/PHWC-1652C6/2020|2020-05-27|EPI_ISL_479446|Original|hCoV-19^^Wales|Human|Wales Specialist Virology Centre Sequencing lab|COVID-19 Genomics UK (COG-UK) Consortium|Moore|United Kingdom
>Spike|hCoV-19/Australia/NSW1096/2020|2020-08-30|EPI_ISL_593658|Original|hCoV-19^^New South Wales|Human|Pathology North - Hunter - NSW Health Pathology|NSW Health Pathology - Institute of Clinical Pathology and Medical Research; Westmead Hospital; University of Sydney|Gall|Australia
>Spike|hCoV-19/USA/WI-WSLH-200148/2020|2020-03-13|EPI_ISL_578375|Original|hCoV-19^^Wisconsin|Human|Wisconsin State Laboratory of Hygiene Communicable Disease Division|Wisconsin State Laboratory of Hygiene Communicable Disease Division|Florek|USA
>Spike|hCoV-19/England/PORT-2D19ED/2020|2020-04-10|EPI_ISL_514479|Original|hCoV-19^^England|Human|Centre for Enzyme Innovation|COVID-19 Genomics UK (COG-UK) Consortium|Robson|United Kingdom
>Spike|hCoV-19/USA/LA-EVTL212/2020|2020-08-15|EPI_ISL_574346|Original|hCoV-19^^Louisiana|Human|LSUHS Emerging Viral Threat Laboratory|Microbial Genome Sequencing Center|Kamil|USA
>Spike|hCoV-19/Japan/PG-4651/2020|2020-07-00|EPI_ISL_689788|Original|hCoV-19^^Japan|Human|Fukuoka Institute of Health and Environmental Sciences|Pathogen Genomics Center|Sekizuka|Japan

>Spike|hCoV-19/Australia/VIC3021/2020|2020-07-03|EPI_ISL_521850|Original|hCoV-19^^Victoria|Human|Victorian Infectious Diseases Reference Laboratory (VIDRL)|VIDRL and MDU-PHL|Schultz|Australia
>Spike|hCoV-19/Denmark/DCGC-7625/2020|2020-10-19|EPI_ISL_618518|Original|hCoV-19^^Hovedstaden|Human|Department of Virus and Microbiological Special Diagnostics|Albertsen lab|Michaelsen|Denmark
>Spike|hCoV-19/India/DL-NCDC2001-CSIR-IGIB/2020|2020-05-18|EPI_ISL_482522|Original|hCoV-19^^Delhi|Human|National Centre for Disease control (NCDC)|NCDC/CSIR-IGIB|Pandey|India
>Spike|hCoV-19/USA/MI-MDHHS-SC21994/2020|2020-09-26|EPI_ISL_576217|Original|hCoV-19^^Michigan|Human|Michigan Department of Health and Human Services|Michigan Department of Health and Human Services|Blankenship|USA
>Spike|hCoV-19/England/ALDP-6EA9A1/2020|2020-06-30|EPI_ISL_557634|Original|hCoV-19^^England|Human|Lighthouse Lab in Alderley Park|Wellcome Sanger Institute for the COVID-19 Genomics UK (COG-UK) consortium|Davies|United Kingdom
>Spike|hCoV-19/Wales/PHWC-4833CC/2020|2020-10-08|EPI_ISL_627115|Original|hCoV-19^^Wales|Human|Wales Specialist Virology Centre Sequencing lab|COVID-19 Genomics UK (COG-UK) Consortium|Moore|United Kingdom
>Spike|hCoV-19/England/MILK-B887B5/2020|2020-11-13|EPI_ISL_674214|Original|hCoV-19^^England|Human|Lighthouse Lab in Milton Keynes|Wellcome Sanger Institute for the COVID-19 Genomics UK (COG-UK) Consortium|Davies|United Kingdom
>Spike|hCoV-19/USA/CO-CDPHE-2010200214/2020|2020-10-19|EPI_ISL_710155|Original|hCoV-19^^Colorado|Human|Colorado Department of Public Health and Environment|Colorado Department of Puplic Health and Environment|Bankers|USA
>Spike|hCoV-19/USA/WI-UW-455/2020|2020-06-16|EPI_ISL_484830|Original|hCoV-19^^Wisconsin|Human|University of Wisconsin-Madison AIDS Vaccine Research Laboratories|University of Wisconsin-Madison AIDS Vaccine Research Laboratories|Baczenas|USA
>Spike|hCoV-19/England/201120049/2020|2020-03-09|EPI_ISL_464665|Original|hCoV-19^^England|Human|Respiratory Virus Unit|Respiratory Virus Unit|Platt|United Kingdom
>Spike|hCoV-19/England/CAMB-1ACDE0/2020|2020-04-23|EPI_ISL_489450|Original|hCoV-19^^England|Human|Department of Pathology|Wellcome Sanger Institute for the COVID-19 Genomics UK (COG-UK) consortium|Davies|United Kingdom
>Spike|hCoV-19/England/QEUH-9F3614/2020|2020-09-24|EPI_ISL_588666|Original|hCoV-19^^England|Human|Lighthouse Lab in Glasgow|Wellcome Sanger Institute for the COVID-19 Genomics UK (COG-UK) consortium|Davies|United Kingdom
>Spike|hCoV-19/Italy/LOM-INMI-5925/2020|2020-02-22|EPI_ISL_460091|Original|hCoV-19^^Lombardy|Human|Molecular Virology Unit|Laboratory of Virology|Gruber|Italy
>Spike|hCoV-19/USA/MI-MDHHS-SC21208/2020|2020-03-29|EPI_ISL_507465|Original|hCoV-19^^Michigan|Human|Michigan Department of Health and Human Services|Michigan Department of Health and Human Services|Blankenship|USA
>Spike|hCoV-19/Australia/VIC8008/2020|2020-08-17|EPI_ISL_564800|Original|hCoV-19^^Victoria|Human|Microbiological Diagnostic Unit - Public Health Laboratory (MDU-PHL)|MDU-PHL|Schultz|Australia
>Spike|hCoV-19/Belgium/rega-0408286/2020|2020-04-08|EPI_ISL_464080|Original|hCoV-19^^Kortenberg|Human|KU Leuven|KU Leuven|Maes|Belgium
>Spike|hCoV-19/Scotland/CVR3329/2020|2020-05-01|EPI_ISL_493880|Original|hCoV-19^^Scotland|Human|West of Scotland Specialist Virology Centre|COVID-19 Genomics UK (COG-UK) Consortium|Robertson|United Kingdom
>Spike|hCoV-19/USA/NY-NYUMC398/2020|2020-04-13|EPI_ISL_430425|Original|hCoV-19^^New York|Human|NYU Langone Health|Departments of Pathology and Medicine|Maurano|USA
>Spike|hCoV-19/England/BRIS-1260E5/2020|2020-04-10|EPI_ISL_459064|Original|hCoV-19^^England|Human|PHE South West Regional Laboratory|Wellcome Sanger Institute for the COVID-19 Genomics UK (COG-UK) consortium|Davies|United Kingdom
>Spike|hCoV-19/Luxembourg/LNS3413350/2020|2020-11-05|EPI_ISL_740304|Original|hCoV-19^^Luxembourg|Human|Laboratoire national de sant\u00e9|Laboratoire national de sant\u00e9|Wienecke-Baldacchino|Luxembourg
>Spike|hCoV-19/Switzerland/BE-ETHZ-380341/2020|2020-11-21|EPI_ISL_721767|Original|hCoV-19^^Bern|Human|Viollier AG|Department of Biosystems Science and Engineering|Nadeau|Switzerland

>Spike|hCoV-19/India/OR-ILSCV32899/2020|2020-06-05|EPI_ISL_481177|Original|hCoV-19^^Odisha|Human|Immunogenomics lab|Immunogenomics lab|Raghav|India
>Spike|hCoV-19/Portugal/IGC4124/2020|2020-06-02|EPI_ISL_491235|Original|hCoV-19^^Portugal|Human|Instituto Gulbenkian de Ci\u00eancia|Instituto Gulbenkian de Ci\u00eancia|Leite|Portugal
>Spike|hCoV-19/Norway/4542/2020|2020-10-14|EPI_ISL_775474|Original|hCoV-19^^Vestfold og Telemark|Human|Vestfold Hospital|Norwegian Institute of Public Health|Vollan|Norway
>Spike|hCoV-19/Denmark/DCGC-3337/2020|2020-08-31|EPI_ISL_616091|Original|hCoV-19^^Hovedstaden|Human|Department of Virus and Microbiological Special Diagnostics|Albertsen lab|Michaelsen|Denmark
>Spike|hCoV-19/Denmark/ALAB-HH-177/2020|2020-04-14|EPI_ISL_452045|Original|hCoV-19^^Denmark|Human|Department of Clinical Microbiology|Albertsen lab|Kirkegaard|Denmark
>Spike|hCoV-19/United Arab Emirates/0671/2020|2020-05-15|EPI_ISL_698824|Original|hCoV-19^^Abu Dhabi|Human|Group 42 (G42) Healthcare|G42 Healthcare|Ogrodzki|United Arab Emirates
>Spike|hCoV-19/North Macedonia/ChVir7915/2020|2020-06-25|EPI_ISL_492085|Original|hCoV-19^^North Macedonia|Human|Institute for Public Health of the Republic of North Macedonia|Charite Universitatsmedizin Berlin|Muehlemann|North Macedonia
>Spike|hCoV-19/USA/WA-UW-1826/2020|2020-03-22|EPI_ISL_424273|Original|hCoV-19^^Washington|Human|UW Virology Lab|UW Virology Lab|Roychoudhury|USA
>Spike|hCoV-19/USA/OR-PROV-003/2020|2020-04-03|EPI_ISL_482294|Original|hCoV-19^^Oregon|Human|Providence St. Joseph Health Molecular Genomics Laboratory|Providence St. Joseph Health Molecular Genomics Laboratory|Dowdell|USA
>Spike|hCoV-19/England/MILK-99CF7C/2020|2020-08-30|EPI_ISL_549846|Original|hCoV-19^^England|Human|Lighthouse Lab in Milton Keynes|Wellcome Sanger Institute for the COVID-19 Genomics UK (COG-UK) consortium|Davies|United Kingdom
>Spike|hCoV-19/USA/CA-QDX-1035/2020|2020-03-17|EPI_ISL_571700|Original|hCoV-19^^California|Human|Quest Diagnostics|Quest Diagnostics|Kagan|USA
>Spike|hCoV-19/Japan/PG-8604/2020|2020-08-00|EPI_ISL_686200|Original|hCoV-19^^Japan|Human|Pathogen Genomics Center|Pathogen Genomics Center|Sekizuka|Japan
>Spike|hCoV-19/Russia/StPetersburg-RII4938S/2020|2020-04-08|EPI_ISL_430073|Original|hCoV-19^^Saint Petersburg|Human|WHO National Influenza Centre Russian Federation|WHO National Influenza Centre Russian Federation|Komissarov|Russia
>Spike|hCoV-19/South Africa/Tygerberg_341_CC2/2020|2020-10-09|EPI_ISL_660147|Original|hCoV-19^^Western Cape|Human|PathCare|National Health Laboratory Service (NHLS)|de Oliveira|South Africa
>Spike|hCoV-19/USA/UT-UPHL-2009307/2020|2020-05-15|EPI_ISL_524913|Original|hCoV-19^^Utah|Human|Utah Public Health Laboratory|Utah Public Health Laboratory|Young|USA
>Spike|hCoV-19/Wales/PHWC-4991E7/2020|2020-12-02|EPI_ISL_727271|Original|hCoV-19^^Wales|Human|Originating lab|COVID-19 Genomics UK (COG-UK) Consortium|Moore|United Kingdom
>Spike|hCoV-19/England/QEUH-960B9B/2020|2020-08-02|EPI_ISL_532048|Original|hCoV-19^^England|Human|Lighthouse Lab in Glasgow|Wellcome Sanger Institute for the COVID-19 Genomics UK (COG-UK) consortium|Davies|United Kingdom
>Spike|hCoV-19/United Arab Emirates/0640/2020|2020-05-15|EPI_ISL_698796|Original|hCoV-19^^Abu Dhabi|Human|Group 42 (G42) Healthcare|G42 Healthcare|Ogrodzki|United Arab Emirates
>Spike|hCoV-19/Australia/WA84/2020|2020-05-11|EPI_ISL_512737|Original|hCoV-19^^Western Australia|Human|PathWest Laboratory Medicine WA|PathWest Laboratory Medicine WA Microbial Surveillance Unit|Sikazwe|Australia
>Spike|hCoV-19/Switzerland/TI-ETHZ-100045/2020|2020-03-11|EPI_ISL_466968|Original|hCoV-19^^Ticino|Human|Viollier AG|Department of Biosystems Science and Engineering|Nadeau|Switzerland
>Spike|hCoV-19/Wales/PHWC-49B1B8/2020|2020-10-27|EPI_ISL_742306|Original|hCoV-19^^Wales|Human|Wales Specialist Virology Centre Sequencing lab|COVID-19 Genomics UK (COG-UK) Consortium|Moore|United Kingdom

>Spike|hCoV-19/Jordan/AM-ALSR-5185/2020|2020-10-15|EPI_ISL_730424|Original|hCoV-19^^Amman|Human|Biolab Diagnostic Laboratories|Andersen lab at Scripps Research|Gangavarapu|Jordan
>Spike|hCoV-19/New Zealand/20VR1924/2020|2020-04-03|EPI_ISL_456309|Original|hCoV-19^^Southern|Human|Southern Community Labs Dunedin|Institute of Environmental Science and Research (ESR)|de Ligt|New Zealand
>Spike|hCoV-19/France/ARA-107506/2020|2020-11-16|EPI_ISL_728312|Original|hCoV-19^^Auvergne-Rh\u00f4ne-Alpes|Human|CNR Virus des Infections Respiratoires - France SUD|CNR Virus des Infections Respiratoires - France SUD|Bal|France
>Spike|hCoV-19/Denmark/DCGC-2553/2020|2020-08-17|EPI_ISL_616529|Original|hCoV-19^^Hovedstaden|Human|Department of Virus and Microbiological Special Diagnostics|Albertsen lab|Michaelsen|Denmark
>Spike|hCoV-19/Wales/PHWC-498C41/2020|2020-11-25|EPI_ISL_727191|Original|hCoV-19^^Wales|Human|Originating lab|COVID-19 Genomics UK (COG-UK) Consortium|Moore|United Kingdom
>Spike|hCoV-19/England/QEUH-B12EBB/2020|2020-11-02|EPI_ISL_642489|Original|hCoV-19^^England|Human|Lighthouse Lab in Glasgow|Wellcome Sanger Institute for the COVID-19 Genomics UK (COG-UK) Consortium|Davies|United Kingdom
>Spike|hCoV-19/Australia/VIC2006/2020|2020-05-02|EPI_ISL_521903|Original|hCoV-19^^Victoria|Human|Victorian Infectious Diseases Reference Laboratory (VIDRL)|VIDRL and MDU-PHL|Schultz|Australia
>Spike|hCoV-19/USA/TX-HMH-MCoV-16098/2020|2020-10-27|EPI_ISL_785782|Original|hCoV-19^^Texas|Human|Houston Methodist Hospital|Houston Methodist Hospital|Long|USA
>Spike|hCoV-19/USA/TX-HMH-5765/2020|2020-06-24|EPI_ISL_546998|Original|hCoV-19^^Texas|Human|Houston Methodist Hospital|Houston Methodist Hospital|Long|USA
>Spike|hCoV-19/England/NORT-297567/2020|2020-04-22|EPI_ISL_488007|Original|hCoV-19^^England|Human|NU-OMICS DNA Sequencing research facility|Wellcome Sanger Institute for the COVID-19 Genomics UK (COG-UK) consortium|Davies|United Kingdom
>Spike|hCoV-19/England/LOND-DA38E/2020|2020-11-15|EPI_ISL_724624|Original|hCoV-19^^England|Human|University College London|COVID-19 Genomics UK (COG-UK) Consortium|Roy|United Kingdom
>Spike|hCoV-19/England/QEUH-A7694C/2020|2020-10-13|EPI_ISL_613336|Original|hCoV-19^^England|Human|Queens Medical Centre|COVID-19 Genomics UK (COG-UK) Consortium|Loose|United Kingdom
>Spike|hCoV-19/USA/WI-UW-2010/2020|2020-09-10|EPI_ISL_677418|Original|hCoV-19^^Wisconsin|Human|University of Wisconsin-Madison AIDS Vaccine Research Laboratories|University of Wisconsin-Madison AIDS Vaccine Research Laboratories|Baczenas|USA
>Spike|hCoV-19/South Korea/KCDC2589/2020|2020-06-29|EPI_ISL_514992|Original|hCoV-19^^South Korea|Human|Division of Viral Diseases|Division of Viral Diseases|Kim|South Korea
>Spike|hCoV-19/England/QEUH-9CA898/2020|2020-09-10|EPI_ISL_567406|Original|hCoV-19^^England|Human|Lighthouse Lab in Glasgow|Wellcome Sanger Institute for the COVID-19 Genomics UK (COG-UK) consortium|Davies|United Kingdom
>Spike|hCoV-19/Senegal/6847/2020|2020-04-02|EPI_ISL_486865|Original|hCoV-19^^Dakar|Human|Institut Pasteur Dakar|Institut Pasteur de Dakar|Dia|Senegal
>Spike|hCoV-19/England/GSTT-265DAEC/2020|2020-03-27|EPI_ISL_484063|Original|hCoV-19^^England|Human|Centre for Clinical Infection and Diagnostics Research and Genomics Innovation Unit|COVID-19 Genomics UK (COG-UK) Consortium|Awan|United Kingdom
>Spike|hCoV-19/England/ALDP-8FC1EE/2020|2020-06-24|EPI_ISL_551375|Original|hCoV-19^^England|Human|Lighthouse Lab in Alderley Park|Wellcome Sanger Institute for the COVID-19 Genomics UK (COG-UK) consortium|Davies|United Kingdom
>Spike|hCoV-19/Denmark/DCGC-19343/2020|2020-12-07|EPI_ISL_748895|Original|hCoV-19^^Syddanmark|Human|Department of Virus and Microbiological Special Diagnostics|Albertsen Lab|Michaelsen|Denmark
>Spike|hCoV-19/USA/MI-MDHHS-SC22146/2020|2020-10-15|EPI_ISL_614197|Original|hCoV-19^^Michigan|Human|Michigan Department of Health and Human Services|Michigan Department of Health and Human Services|Jacob|USA

>Spike|hCoV-19/Wales/PHWC-32551/2020|2020-04-07|EPI_ISL_474328|Original|hCoV-19^^Wales|Human|Originating lab|COVID-19 Genomics UK (COG-UK) Consortium|Moore|United Kingdom
>Spike|hCoV-19/Australia/VIC752/2020|2020-03-30|EPI_ISL_427029|Original|hCoV-19^^Victoria|Human|Victorian Infectious Diseases Reference Laboratory (VIDRL)|Microbiological Diagnostic Unit Public Health Laboratory and Victorian Infectious Diseases Reference Laboratory|Schultz|Australia
>Spike|hCoV-19/USA/MA-MASPHL-00170/2020|2020-04-03|EPI_ISL_791635|Original|hCoV-19^^Massachusetts|Human|Massachusetts State Public Health Laboratory|Infectious Disease Program|Park|USA
>Spike|hCoV-19/Luxembourg/LNS4375344/2020|2020-11-28|EPI_ISL_740458|Original|hCoV-19^^Luxembourg|Human|Laboratoire national de sant\u00e9|Laboratoire national de sant\u00e9|Wienecke-Baldacchino|Luxembourg
>Spike|hCoV-19/Luxembourg/LNS6686752/2020|2020-11-25|EPI_ISL_744449|Original|hCoV-19^^Luxembourg|Human|Laboratoire national de sant\u00e9|Laboratoire national de sant\u00e9|Wienecke-Baldacchino|Luxembourg
>Spike|hCoV-19/USA/TX-HMH-7119/2020|2020-06-29|EPI_ISL_546520|Original|hCoV-19^^Texas|Human|Houston Methodist Hospital|Houston Methodist Hospital|Long|USA
>Spike|hCoV-19/England/NORT-1B70F00/2020|2020-11-12|EPI_ISL_706300|Original|hCoV-19^^England|Human|Northumbria University / South Tees Hospitals NHS Foundation Trust / North Cumbria Integrated Care NHS Foundation Trust / North Tees and Hartlepool NHS Foundation Trust / Newcastle Hospitals NHS Foundation Trust|COVID-19 Genomics UK (COG-UK) Consortium|Bashton|United Kingdom
>Spike|hCoV-19/USA/NM-NMDOH-2020324378/2020|2020-09-03|EPI_ISL_569639|Original|hCoV-19^^New Mexico|Human|New Mexico Department of Health Scientific Laboratory|New Mexico Department of Health Scientific Laboratory|Johnson|USA
>Spike|hCoV-19/England/ALDP-A6794E/2020|2020-10-12|EPI_ISL_630519|Original|hCoV-19^^England|Human|Lighthouse Lab in Alderley Park|Wellcome Sanger Institute for the COVID-19 Genomics UK (COG-UK) consortium|Davies|United Kingdom
>Spike|hCoV-19/Wales/PHWC-4881BE/2020|2020-10-31|EPI_ISL_639362|Original|hCoV-19^^Wales|Human|Wales Specialist Virology Centre Sequencing lab|COVID-19 Genomics UK (COG-UK) Consortium|Moore|United Kingdom
>Spike|hCoV-19/England/QEUH-C14C45/2020|2020-12-06|EPI_ISL_709486|Original|hCoV-19^^England|Human|Lighthouse Lab in Glasgow|Wellcome Sanger Institute for the COVID-19 Genomics UK (COG-UK) Consortium|Davies|United Kingdom
>Spike|hCoV-19/Switzerland/BS-UHB-42187080/2020|2020-03-12|EPI_ISL_528020|Original|hCoV-19^^Basel-Stadt|Human|University Hospital Basel|University Hospital Basel|Roloff Handschin|Switzerland
>Spike|hCoV-19/England/QEUH-C93F54/2020|2020-12-16|EPI_ISL_777397|Original|hCoV-19^^England|Human|Lighthouse Lab in Glasgow|Wellcome Sanger Institute for the COVID-19 Genomics UK (COG-UK) Consortium|Davies|United Kingdom
>Spike|hCoV-19/USA/NY-NYUMC732/2020|2020-03-26|EPI_ISL_451447|Original|hCoV-19^^New York|Human|NYU Langone Health|Departments of Pathology and Medicine|Maurano|USA
>Spike|hCoV-19/England/ALDP-B62520/2020|2020-11-08|EPI_ISL_656663|Original|hCoV-19^^England|Human|Lighthouse Lab in Alderley Park|Wellcome Sanger Institute for the COVID-19 Genomics UK (COG-UK) Consortium|Davies|United Kingdom
>Spike|hCoV-19/Denmark/DCGC-2788/2020|2020-08-24|EPI_ISL_616623|Original|hCoV-19^^Midtjylland|Human|Department of Virus and Microbiological Special Diagnostics|Albertsen lab|Michaelsen|Denmark
>Spike|hCoV-19/Wales/PHWC-24534/2020|2020-03-18|EPI_ISL_419425|Original|hCoV-19^^Wales|Human|Wales Specialist Virology Centre|Public Health Wales Microbiology Cardiff|Moore|United Kingdom
>Spike|hCoV-19/England/MILK-B01DD0/2020|2020-10-24|EPI_ISL_642783|Original|hCoV-19^^England|Human|Lighthouse Lab in Milton Keynes|Wellcome Sanger Institute for the COVID-19 Genomics UK (COG-UK) Consortium|Davies|United Kingdom
>Spike|hCoV-19/Wales/PHWC-485360/2020|2020-10-21|EPI_ISL_628172|Original|hCoV-19^^Wales|Human|Wales Specialist Virology Centre Sequencing lab|COVID-19 Genomics UK (COG-UK) Consortium|Moore|United Kingdom

>Spike|hCoV-19/Denmark/DCGC-19396/2020|2020-12-07|EPI_ISL_748222|Original|hCoV-19^^Sjaelland|Human|Department of Virus and Microbiological Special Diagnostics|Albertsen Lab|Michaelsen|Denmark
>Spike|hCoV-19/USA/MI-MDHHS-SC22429/2020|2020-11-18|EPI_ISL_708262|Original|hCoV-19^^Michigan|Human|Michigan Department of Health and Human Services|Michigan Department of Health and Human Services|Jacob|USA
>Spike|hCoV-19/England/BRIS-18545A9/2020|2020-03-13|EPI_ISL_492332|Original|hCoV-19^^England|Human|PHE South West Regional Laboratory|Wellcome Sanger Institute for the COVID-19 Genomics UK (COG-UK) consortium|Davies|United Kingdom
>Spike|hCoV-19/England/ALDP-A940BC/2020|2020-10-19|EPI_ISL_611187|Original|hCoV-19^^England|Human|Lighthouse Lab in Alderley Park|Wellcome Sanger Institute for the COVID-19 Genomics UK (COG-UK) consortium|Davies|United Kingdom
>Spike|hCoV-19/Wales/PHWC-4925E2/2020|2020-11-26|EPI_ISL_725672|Original|hCoV-19^^Wales|Human|Originating lab|COVID-19 Genomics UK (COG-UK) Consortium|Moore|United Kingdom
>Spike|hCoV-19/Japan/PG-2983/2020|2020-05-00|EPI_ISL_691107|Original|hCoV-19^^Japan|Human|Pathogen Genomics Center|Pathogen Genomics Center|Sekizuka|Japan
>Spike|hCoV-19/USA/WI-UW-1810/2020|2020-09-09|EPI_ISL_605682|Original|hCoV-19^^Wisconsin|Human|University of Wisconsin-Madison AIDS Vaccine Research Laboratories|University of Wisconsin-Madison AIDS Vaccine Research Laboratories|Baczenas|USA
>Spike|hCoV-19/USA/MA-MASPHL-00244/2020|2020-04-16|EPI_ISL_791688|Original|hCoV-19^^Massachusetts|Human|Massachusetts State Public Health Laboratory|Infectious Disease Program|Park|USA
>Spike|hCoV-19/England/20139059904/2020|2020-03-27|EPI_ISL_420730|Original|hCoV-19^^England|Human|Respiratory Virus Unit|Respiratory Virus Unit|Platt|United Kingdom
>Spike|hCoV-19/Australia/WA300/2020|2020-04-04|EPI_ISL_596843|Original|hCoV-19^^Western Australia|Human|PathWest Laboratory Medicine WA|PathWest Laboratory Medicine WA Microbial Surveillance Unit|Sikazwe|Australia
>Spike|hCoV-19/USA/CA-QDX-1674/2020|2020-03-19|EPI_ISL_603923|Original|hCoV-19^^California|Human|Quest Diagnostics|Quest Diagnostics|Kagan|USA
>Spike|hCoV-19/Scotland/EDB6909/2020|2020-05-01|EPI_ISL_526514|Original|hCoV-19^^Scotland|Human|Virology Department|COVID-19 Genomics UK (COG-UK) Consortium|Williams|United Kingdom
>Spike|hCoV-19/Denmark/DCGC-19832/2020|2020-12-07|EPI_ISL_747818|Original|hCoV-19^^Midtjylland|Human|Department of Virus and Microbiological Special Diagnostics|Albertsen Lab|Michaelsen|Denmark
>Spike|hCoV-19/England/BRIS-18531F1/2020|2020-04-02|EPI_ISL_492261|Original|hCoV-19^^England|Human|PHE South West Regional Laboratory|Wellcome Sanger Institute for the COVID-19 Genomics UK (COG-UK) consortium|Davies|United Kingdom
>Spike|hCoV-19/England/CAMC-B51C6B/2020|2020-11-09|EPI_ISL_657883|Original|hCoV-19^^England|Human|Lighthouse Lab in Cambridge|Wellcome Sanger Institute for the COVID-19 Genomics UK (COG-UK) Consortium|Davies|United Kingdom
>Spike|hCoV-19/Scotland/QEUH-96EB24/2020|2020-08-19|EPI_ISL_534509|Original|hCoV-19^^Scotland|Human|NHSGGC West of Scotland Specialist Virology Centre / MRC-University of Glasgow Centre for Virus Research|Wellcome Sanger Institute for the COVID-19 Genomics UK (COG-UK) consortium|Davies|United Kingdom
>Spike|hCoV-19/Canada/BC-BCCDC-7283/2020|2020-11-00|EPI_ISL_740656|Original|hCoV-19^^British Columbia|Human|BCCDC Public Health Laboratory|BCCDC Public Health Laboratory|Prystajecky|Canada
>Spike|hCoV-19/Czech Republic/IAB_16/2020|2020-03-26|EPI_ISL_426891|Original|hCoV-19^^Czech Republic|Human|Motol University Hospital|Institute of Applied Biotechnologies a.s.|Broz|Czech Republic
>Spike|hCoV-19/Denmark/DCGC-7331/2020|2020-10-12|EPI_ISL_619316|Original|hCoV-19^^Syddanmark|Human|Department of Virus and Microbiological Special Diagnostics|Albertsen lab|Michaelsen|Denmark
>Spike|hCoV-19/Australia/VIC7936/2020|2020-08-17|EPI_ISL_564758|Original|hCoV-19^^Victoria|Human|Microbiological Diagnostic Unit - Public Health Laboratory (MDU-PHL)|MDU-PHL|Schultz|Australia

>Spike|hCoV-19/Scotland/QEUH-A0B320/2020|2020-10-03|EPI_ISL_599279|Original|hCoV-19^^Scotland|Human|Lighthouse Lab in Glasgow|Wellcome Sanger Institute for the COVID-19 Genomics UK (COG-UK) consortium|Davies|United Kingdom
>Spike|hCoV-19/USA/UN-UW358/2020|2020-03-00|EPI_ISL_418925|Original|hCoV-19^^USA|Human|UW Virology Lab|UW Virology Lab|Roychoudhury|USA
>Spike|hCoV-19/Wuhan/HB-WH4-206/2020|2020-03-27|EPI_ISL_454956|Original|hCoV-19^^Hubei|Human|Wuhan Chain Medical Labs (CMLabs)|State Key Laboratory of Biotherapy of Sichuan University|Tang|China
>Spike|hCoV-19/England/ALDP-9ECF7D/2020|2020-05-05|EPI_ISL_589388|Original|hCoV-19^^England|Human|Lighthouse Lab in Alderley Park|Wellcome Sanger Institute for the COVID-19 Genomics UK (COG-UK) consortium|Davies|United Kingdom
>Spike|hCoV-19/USA/TX-HMH-MCoV-10734/2020|2020-07-08|EPI_ISL_789608|Original|hCoV-19^^Texas|Human|Houston Methodist Hospital|Houston Methodist Hospital|Long|USA
>Spike|hCoV-19/USA/TX-HMH-MCoV-13416/2020|2020-08-23|EPI_ISL_788554|Original|hCoV-19^^Texas|Human|Houston Methodist Hospital|Houston Methodist Hospital|Long|USA
>Spike|hCoV-19/Canada/ON-PHL-20-03765/2020|2020-11-00|EPI_ISL_775075|Original|hCoV-19^^Ontario|Human|Public Health Ontario Laboratory|Public Health Ontario Laboratory|Guthrie|Canada
>Spike|hCoV-19/USA/AZ-TG670228/2020|2020-05-01|EPI_ISL_696410|Original|hCoV-19^^Arizona|Human|Sonora Quest Laboratories|TGen North|French|USA
>Spike|hCoV-19/England/QEUH-B13D44/2020|2020-11-01|EPI_ISL_641780|Original|hCoV-19^^England|Human|Lighthouse Lab in Glasgow|Wellcome Sanger Institute for the COVID-19 Genomics UK (COG-UK) Consortium|Davies|United Kingdom
>Spike|hCoV-19/England/MILK-B85DF2/2020|2020-11-13|EPI_ISL_673908|Original|hCoV-19^^England|Human|Lighthouse Lab in Milton Keynes|Wellcome Sanger Institute for the COVID-19 Genomics UK (COG-UK) Consortium|Davies|United Kingdom
>Spike|hCoV-19/England/SHEF-10D1894/2020|2020-12-21|EPI_ISL_765184|Original|hCoV-19^^England|Human|Virology Department|COVID-19 Genomics UK (COG-UK) Consortium|de Silva|United Kingdom
>Spike|hCoV-19/England/QEUH-B677F8/2020|2020-11-09|EPI_ISL_655660|Original|hCoV-19^^England|Human|Lighthouse Lab in Glasgow|Wellcome Sanger Institute for the COVID-19 Genomics UK (COG-UK) Consortium|Davies|United Kingdom
>Spike|hCoV-19/USA/CA-CZB-3874/2020|2020-07-21|EPI_ISL_548511|Original|hCoV-19^^California|Human|County of Santa Clara Public Health Department|Chan-Zuckerberg Biohub|Bhatt|USA
>Spike|hCoV-19/Austria/CeMM0887/2020|2020-04-29|EPI_ISL_583743|Original|hCoV-19^^Austria|Human|Dr. Gernot Walder GmbH|Bergthaler laboratory|Bergthaler|Austria
>Spike|hCoV-19/England/MILK-974B1C/2020|2020-08-21|EPI_ISL_552593|Original|hCoV-19^^England|Human|Lighthouse Lab in Milton Keynes|Wellcome Sanger Institute for the COVID-19 Genomics UK (COG-UK) consortium|Davies|United Kingdom
>Spike|hCoV-19/Spain/CT-IBV-97009562/2020|2020-07-29|EPI_ISL_671859|Original|hCoV-19^^Catalunya|Human|Servicio de Microbiolog\u00eda|SeqCOVID-SPAIN consortium/IBV(CSIC)|Comas|Spain
>Spike|hCoV-19/USA/WI-GMF-30626/2020|2020-11-01|EPI_ISL_661042|Original|hCoV-19^^Wisconsin|Human|Gundersen Molecular Diagnostics Laboratory|Kabara Cancer Research Institute|Kenny|USA
>Spike|hCoV-19/Netherlands/NoordBrabant_35/2020|2020-03-09|EPI_ISL_414544|Original|hCoV-19^^Noord Brabant|Human|Dutch COVID-19 response team|Erasmus Medical Center|Oude Munnink|Netherlands
>Spike|hCoV-19/England/QEUH-9FF95B/2020|2020-09-30|EPI_ISL_600421|Original|hCoV-19^^England|Human|Lighthouse Lab in Glasgow|Wellcome Sanger Institute for the COVID-19 Genomics UK (COG-UK) consortium|Davies|United Kingdom
>Spike|hCoV-19/England/QEUH-9C7EA8/2020|2020-09-11|EPI_ISL_567050|Original|hCoV-19^^England|Human|Lighthouse Lab in Glasgow|Wellcome Sanger Institute for the COVID-19 Genomics UK (COG-UK) consortium|Davies|United Kingdom
>Spike|hCoV-19/England/MILK-B049AC/2020|2020-10-24|EPI_ISL_650590|Original|hCoV-19^^England|Human|Department of Pathology|COVID-19 Genomics UK (COG-UK) Consortium|Goodfellow|United Kingdom

>Spike|hCoV-19/USA/WA-S655/2020|2020-04-16|EPI_ISL_438208|Original|hCoV-
19^^Washington|Human|Washington State Department of Health|Seattle Flu
Study|Bedford|USA
>Spike|hCoV-19/England/ALDP-CED252/2020|2020-12-18|EPI_ISL_782122|Original|hCoV-
19^^England|Human|Lighthouse Lab in Alderley Park|Wellcome Sanger Institute for the
COVID-19 Genomics UK (COG-UK) Consortium|Davies|United Kingdom
>Spike|hCoV-19/Wales/PHWC-487744/2020|2020-10-25|EPI_ISL_639231|Original|hCoV-
19^^Wales|Human|Wales Specialist Virology Centre Sequencing lab|COVID-19 Genomics UK
(COG-UK) Consortium|Moore|United Kingdom
>Spike|hCoV-19/England/CAMB-1B2BA1/2020|2020-05-19|EPI_ISL_456708|Original|hCoV-
19^^England|Human|Department of Pathology|COVID-19 Genomics UK (COG-UK)
Consortium|Meredith|United Kingdom
>Spike|hCoV-19/Switzerland/SO-UHB-42195124/2020|2020-03-
17|EPI_ISL_528173|Original|hCoV-19^^Solothurn|Human|University Hospital
Basel|University Hospital Basel|Roloff Handschin|Switzerland
>Spike|hCoV-19/Japan/IC-0061/2020|2020-06-00|EPI_ISL_591403|Original|hCoV-
19^^Japan|Human|Pathogen Genomics Center|Pathogen Genomics Center|Sekizuka|Japan
>Spike|hCoV-19/Wales/PHWC-48F630/2020|2020-11-10|EPI_ISL_681118|Original|hCoV-
19^^Wales|Human|Wales Specialist Virology Centre Sequencing lab|COVID-19 Genomics UK
(COG-UK) Consortium|Moore|United Kingdom
>Spike|hCoV-19/Denmark/DCGC-3170/2020|2020-08-31|EPI_ISL_615992|Original|hCoV-
19^^Hovedstaden|Human|Department of Virus and Microbiological Special
Diagnostics|Albertsen lab|Michaelsen|Denmark
>Spike|hCoV-19/USA/WA-S2559/2020|2020-06-17|EPI_ISL_497191|Original|hCoV-
19^^Washington|Human|Washington State Department of Health|Seattle Flu Study|Lee|USA
>Spike|hCoV-19/Vietnam/19-01S/2020|2020-01-22|EPI_ISL_418269|Vero E6|hCoV-19^^Ho Chi
Minh City|Human|Microbiology and Immunology department|Microbiology and Immunology
department|Submitter|Vietnam
>Spike|hCoV-19/England/MILK-A05BF1/2020|2020-09-30|EPI_ISL_599935|Original|hCoV-
19^^England|Human|Lighthouse Lab in Milton Keynes|Wellcome Sanger Institute for the
COVID-19 Genomics UK (COG-UK) consortium|Davies|United Kingdom
>Spike|hCoV-19/England/QEUH-B11960/2020|2020-11-02|EPI_ISL_642293|Original|hCoV-
19^^England|Human|Lighthouse Lab in Glasgow|Wellcome Sanger Institute for the COVID-
19 Genomics UK (COG-UK) Consortium|Davies|United Kingdom
>Spike|hCoV-19/Switzerland/ZH-ETHZ-220010/2020|2020-07-
24|EPI_ISL_511997|Original|hCoV-19^^Zurich|Human|Viollier AG|Department of Biosystems
Science and Engineering|Nadeau|Switzerland
>Spike|hCoV-19/England/CAMC-A3FB3A/2020|2020-10-05|EPI_ISL_609544|Original|hCoV-
19^^England|Human|Lighthouse Lab in Cambridge|Wellcome Sanger Institute for the
COVID-19 Genomics UK (COG-UK) consortium|Davies|United Kingdom
>Spike|hCoV-19/South Korea/KCDC2287/2020|2020-05-19|EPI_ISL_510624|Original|hCoV-
19^^South Korea|Human|Division of Viral Diseases|Division of Viral Diseases|Kim|South
Korea
>Spike|hCoV-19/Wales/MILK-9D4C80/2020|2020-09-19|EPI_ISL_601566|Original|hCoV-
19^^Wales|Human|Lighthouse Lab in Milton Keynes|Wellcome Sanger Institute for the
COVID-19 Genomics UK (COG-UK) consortium|Davies|United Kingdom
>Spike|hCoV-19/Australia/VIC15700/2020|2020-08-04|EPI_ISL_593414|Original|hCoV-
19^^Victoria|Human|Microbiological Diagnostic Unit - Public Health Laboratory (MDU-
PHL)|MDU-PHL|Schultz|Australia
>Spike|hCoV-19/Italy/ABR-IZSGC-TE26643/2020|2020-04-27|EPI_ISL_436729|Original|hCoV-
19^^Abruzzo|Human|SERVIZIO DI IGIENE E SANIT\u00c0 PUBBLICA ASL Teramo|Istituto
Zooprofilattico Sperimentale dell'Abruzzo e Molise \G.Caporale\|Lorusso|Italy
>Spike|hCoV-19/England/LIVE-A90DC/2020|2020-03-25|EPI_ISL_517260|Original|hCoV-
19^^England|Human|Liverpool Clinical Laboratories|COVID-19 Genomics UK (COG-UK)
Consortium|Haldenby|United Kingdom
>Spike|hCoV-19/Japan/PG-1706/2020|2020-05-00|EPI_ISL_685312|Original|hCoV-
19^^Japan|Human|Pathogen Genomics Center|Pathogen Genomics Center|Sekizuka|Japan
>Spike|hCoV-19/USA/WI-UW-546/2020|2020-06-19|EPI_ISL_484921|Original|hCoV-
19^^Wisconsin|Human|University of Wisconsin-Madison AIDS Vaccine Research

Laboratories|University of Wisconsin-Madison AIDS Vaccine Research Laboratories|Baczenas|USA
>Spike|hCoV-19/England/QEUH-AF53ED/2020|2020-10-30|EPI_ISL_663055|Original|hCoV-19^^England|Human|Lighthouse Lab in Glasgow|Wellcome Sanger Institute for the COVID-19 Genomics UK (COG-UK) Consortium|Davies|United Kingdom
>Spike|hCoV-19/England/QEUH-BA2DB3/2020|2020-11-18|EPI_ISL_721093|Original|hCoV-19^^England|Human|Lighthouse Lab in Glasgow|Wellcome Sanger Institute for the COVID-19 Genomics UK (COG-UK) Consortium|Davies|United Kingdom
>Spike|hCoV-19/Australia/VIC3337/2020|2020-07-08|EPI_ISL_521016|Original|hCoV-19^^Victoria|Human|Microbiological Diagnostic Unit - Public Health Laboratory (MDU-PHL)|MDU-PHL|Schultz|Australia
>Spike|hCoV-19/England/ALDP-B1A4FD/2020|2020-10-31|EPI_ISL_647786|Original|hCoV-19^^England|Human|Lighthouse Lab in Alderley Park|Wellcome Sanger Institute for the COVID-19 Genomics UK (COG-UK) Consortium|Davies|United Kingdom
>Spike|hCoV-19/USA/TX-HMH-4508/2020|2020-06-19|EPI_ISL_543678|Original|hCoV-19^^Texas|Human|Houston Methodist Hospital|Houston Methodist Hospital|Long|USA
>Spike|hCoV-19/France/PAC-MEPHI-2304/2020|2020-10-07|EPI_ISL_644536|Original|hCoV-19^^Provence-Alpes-Cote-d\u2019Azur|Human|MEPHI|MEPHI|Levasseur|France
>Spike|hCoV-19/Wales/PHWC-263A1/2020|2020-03-27|EPI_ISL_422371|Original|hCoV-19^^Wales|Human|Wales Specialist Virology Centre|Public Health Wales Microbiology Cardiff|Moore|United Kingdom
>Spike|hCoV-19/England/BRIS-125374/2020|2020-03-27|EPI_ISL_443712|Original|hCoV-19^^England|Human|PHE South West Regional Laboratory|Wellcome Sanger Institute for the COVID-19 Genomics UK (COG-UK) consortium|Amato|United Kingdom
>Spike|hCoV-19/England/CAMB-1B4B45/2020|2020-06-10|EPI_ISL_473499|Original|hCoV-19^^England|Human|Department of Pathology|COVID-19 Genomics UK (COG-UK) Consortium|Meredith|United Kingdom
>Spike|hCoV-19/England/CAMC-C4325A/2020|2020-12-10|EPI_ISL_719387|Original|hCoV-19^^England|Human|Lighthouse Lab in Cambridge|Wellcome Sanger Institute for the COVID-19 Genomics UK (COG-UK) Consortium|Davies|United Kingdom
>Spike|hCoV-19/England/CAMB-1AF5E5/2020|2020-00-00|EPI_ISL_448085|Original|hCoV-19^^England|Human|Department of Pathology|COVID-19 Genomics UK (COG-UK) Consortium|Meredith|United Kingdom
>Spike|hCoV-19/England/ALDP-B2493D/2020|2020-11-04|EPI_ISL_646835|Original|hCoV-19^^England|Human|Lighthouse Lab in Alderley Park|Wellcome Sanger Institute for the COVID-19 Genomics UK (COG-UK) Consortium|Davies|United Kingdom
>Spike|hCoV-19/Denmark/DCGC-20718/2020|2020-12-21|EPI_ISL_758971|Original|hCoV-19^^Syddanmark|Human|Department of Virus and Microbiological Special Diagnostics|Albertsen Lab|Michaelsen|Denmark
>Spike|hCoV-19/England/ALDP-9EE01E/2020|2020-05-08|EPI_ISL_589496|Original|hCoV-19^^England|Human|Lighthouse Lab in Alderley Park|Wellcome Sanger Institute for the COVID-19 Genomics UK (COG-UK) consortium|Davies|United Kingdom
>Spike|hCoV-19/England/MILK-ABB8F3/2020|2020-10-21|EPI_ISL_623348|Original|hCoV-19^^England|Human|Lighthouse Lab in Milton Keynes|Wellcome Sanger Institute for the COVID-19 Genomics UK (COG-UK) consortium|Davies|United Kingdom
>Spike|hCoV-19/England/BIRM-65A92/2020|2020-10-01|EPI_ISL_594608|Original|hCoV-19^^England|Human|University of Birmingham|COVID-19 Genomics UK (COG-UK) Consortium|Loman|United Kingdom
>Spike|hCoV-19/England/MILK-AC7663/2020|2020-10-21|EPI_ISL_630009|Original|hCoV-19^^England|Human|Lighthouse Lab in Milton Keynes|Wellcome Sanger Institute for the COVID-19 Genomics UK (COG-UK) consortium|Davies|United Kingdom
>Spike|hCoV-19/Wales/CVR4824/2020|2020-09-14|EPI_ISL_627359|Original|hCoV-19^^Wales|Human|West of Scotland Specialist Virology Centre|COVID-19 Genomics UK (COG-UK) Consortium|Robertson|United Kingdom
>Spike|hCoV-19/England/OXON-AE09B/2020|2020-00-00|EPI_ISL_448660|Original|hCoV-19^^England|Human|Oxford Viromics|COVID-19 Genomics UK (COG-UK) Consortium|Golubchik|United Kingdom
>Spike|hCoV-19/New Zealand/20CV0663/2020|2020-12-15|EPI_ISL_732965|Original|hCoV-19^^Auckland|Human|LabPLUS|Institute of Environmental Science and Research (ESR)|Wang|New Zealand

>Spike|hCoV-19/USA/FL-BPHL-1849/2020|2020-04-09|EPI_ISL_613929|Original|hCoV-19^^Florida|Human|Florida Bureau of Public Health Laboratories|Florida Bureau of Public Health Laboratories|Blanton|USA
>Spike|hCoV-19/England/ALDP-955CD2/2020|2020-06-12|EPI_ISL_553886|Original|hCoV-19^^England|Human|Lighthouse Lab in Alderley Park|Wellcome Sanger Institute for the COVID-19 Genomics UK (COG-UK) consortium|Davies|United Kingdom
>Spike|hCoV-19/USA/TX-DSHS-1436/2020|2020-06-24|EPI_ISL_671610|Original|hCoV-19^^Texas|Human|Texas Department of State Health Services|Texas Department of State Health Services|Rahman|USA
>Spike|hCoV-19/Scotland/QEUH-A1FBD6/2020|2020-10-01|EPI_ISL_599062|Original|hCoV-19^^Scotland|Human|Lighthouse Lab in Glasgow|Wellcome Sanger Institute for the COVID-19 Genomics UK (COG-UK) consortium|Davies|United Kingdom
>Spike|hCoV-19/England/ALDP-BB32ED/2020|2020-11-19|EPI_ISL_703058|Original|hCoV-19^^England|Human|Lighthouse Lab in Alderley Park|Wellcome Sanger Institute for the COVID-19 Genomics UK (COG-UK) Consortium|Davies|United Kingdom
>Spike|hCoV-19/USA/WI-UW-2571/2021|2021-01-04|EPI_ISL_788913|Original|hCoV-19^^Wisconsin|Human|University of Wisconsin-Madison AIDS Vaccine Research Laboratories|University of Wisconsin-Madison AIDS Vaccine Research Laboratories|Moreno|USA
>Spike|hCoV-19/Australia/VIC1232/2020|2020-04-02|EPI_ISL_563551|Original|hCoV-19^^Victoria|Human|Victorian Infectious Diseases Reference Laboratory (VIDRL)|VIDRL and MDU-PHL|Schultz|Australia
>Spike|hCoV-19/England/MILK-9927C9/2020|2020-08-27|EPI_ISL_552083|Original|hCoV-19^^England|Human|Lighthouse Lab in Milton Keynes|Wellcome Sanger Institute for the COVID-19 Genomics UK (COG-UK) consortium|Davies|United Kingdom
>Spike|hCoV-19/England/LOND-1263C11/2020|2020-11-10|EPI_ISL_679481|Original|hCoV-19^^England|Human|University College London|COVID-19 Genomics UK (COG-UK) Consortium|Roy|United Kingdom
>Spike|hCoV-19/Portugal/PT0298/2020|2020-03-13|EPI_ISL_454014|Original|hCoV-19^^Portugal|Human|unknown|Instituto Nacional de Saude (INSA)|Borges|Portugal
>Spike|hCoV-19/Switzerland/BS-UHB-42201259/2020|2020-03-21|EPI_ISL_528286|Original|hCoV-19^^Basel-Stadt|Human|University Hospital Basel|University Hospital Basel|Roloff Handschin|Switzerland
>Spike|hCoV-19/Scotland/CVR986/2020|2020-03-27|EPI_ISL_439141|Original|hCoV-19^^Scotland|Human|West of Scotland Specialist Virology Centre|COVID-19 Genomics UK (COG-UK) Consortium|Robertson|United Kingdom
>Spike|hCoV-19/Scotland/EDB6243/2020|2020-05-21|EPI_ISL_473925|Original|hCoV-19^^Scotland|Human|Virology Department|COVID-19 Genomics UK (COG-UK) Consortium|Williams|United Kingdom
>Spike|hCoV-19/England/ALDP-B1CE84/2020|2020-11-01|EPI_ISL_645739|Original|hCoV-19^^England|Human|Lighthouse Lab in Alderley Park|Wellcome Sanger Institute for the COVID-19 Genomics UK (COG-UK) Consortium|Davies|United Kingdom
>Spike|hCoV-19/USA/TX-DSHS-0053/2020|2020-05-09|EPI_ISL_517984|Original|hCoV-19^^Texas|Human|Texas Department of State Health Services|Texas Department of State Health Services|Rahman|USA
>Spike|hCoV-19/Japan/PG-7168/2020|2020-07-00|EPI_ISL_688273|Original|hCoV-19^^Japan|Human|Pathogen Genomics Center|Pathogen Genomics Center|Sekizuka|Japan
>Spike|hCoV-19/England/MILK-B021D3/2020|2020-10-24|EPI_ISL_643021|Original|hCoV-19^^England|Human|Lighthouse Lab in Milton Keynes|Wellcome Sanger Institute for the COVID-19 Genomics UK (COG-UK) Consortium|Davies|United Kingdom
>Spike|hCoV-19/England/LIVE-1D4C88/2020|2020-08-31|EPI_ISL_611579|Original|hCoV-19^^England|Human|Liverpool Clinical Laboratories|COVID-19 Genomics UK (COG-UK) Consortium|Haldenby|United Kingdom
>Spike|hCoV-19/Denmark/DCGC-5616/2020|2020-09-28|EPI_ISL_620941|Original|hCoV-19^^Syddanmark|Human|Department of Virus and Microbiological Special Diagnostics|Albertsen lab|Michaelsen|Denmark
>Spike|hCoV-19/Wales/PHWC-47E0FA/2020|2020-09-29|EPI_ISL_595407|Original|hCoV-19^^Wales|Human|Originating lab|COVID-19 Genomics UK (COG-UK) Consortium|Moore|United Kingdom

>Spike|hCoV-19/Australia/VIC8870/2020|2020-07-23|EPI_ISL_565249|Original|hCoV-19^^Victoria|Human|Microbiological Diagnostic Unit - Public Health Laboratory (MDU-PHL)|MDU-PHL|Schultz|Australia
>Spike|hCoV-19/England/QEUH-9F3E94/2020|2020-09-24|EPI_ISL_588624|Original|hCoV-19^^England|Human|Lighthouse Lab in Glasgow|Wellcome Sanger Institute for the COVID-19 Genomics UK (COG-UK) consortium|Davies|United Kingdom
>Spike|hCoV-19/England/CAMC-AEAD92/2020|2020-10-26|EPI_ISL_633357|Original|hCoV-19^^England|Human|Lighthouse Lab in Cambridge|Wellcome Sanger Institute for the COVID-19 Genomics UK (COG-UK) consortium|Davies|United Kingdom
>Spike|hCoV-19/Denmark/DCGC-18952/2020|2020-12-07|EPI_ISL_748661|Original|hCoV-19^^Hovedstaden|Human|Department of Virus and Microbiological Special Diagnostics|Albertsen Lab|Michaelsen|Denmark
>Spike|hCoV-19/USA/WA-S2507/2020|2020-06-17|EPI_ISL_497139|Original|hCoV-19^^Washington|Human|Washington State Department of Health|Seattle Flu Study|Lee|USA
>Spike|hCoV-19/USA/MA-MGH-03288/2020|2020-10-26|EPI_ISL_765774|Original|hCoV-19^^Massachusetts|Human|Massachusetts General Hospital|Infectious Disease Program|Park|USA
>Spike|hCoV-19/USA/TX-HMH-MCoV-12271/2020|2020-09-23|EPI_ISL_790277|Original|hCoV-19^^Texas|Human|Houston Methodist Hospital|Houston Methodist Hospital|Long|USA
>Spike|hCoV-19/Austria/CeMM0448/2020|2020-04-21|EPI_ISL_475865|Original|hCoV-19^^Austria|Human|Austrian Agency for Health and Food Safety (AGES)|Bergthaler laboratory|Bergthaler|Austria
>Spike|hCoV-19/Gambia/GC19-2427/2020|2020-07-21|EPI_ISL_561105|Original|hCoV-19^^Kombo|Human|MRCG at LSHTM Genomics lab|MRCG at LSHTM Genomics lab|Sesay|Gambia
>Spike|hCoV-19/Japan/PG-0808/2020|2020-04-00|EPI_ISL_684418|Original|hCoV-19^^Japan|Human|Fukuoka Institute of Health and Environmental Sciences|Pathogen Genomics Center|Sekizuka|Japan
>Spike|hCoV-19/England/CAMC-B34BF8/2020|2020-11-05|EPI_ISL_659646|Original|hCoV-19^^England|Human|Lighthouse Lab in Cambridge|Wellcome Sanger Institute for the COVID-19 Genomics UK (COG-UK) Consortium|Davies|United Kingdom
>Spike|hCoV-19/England/LEED-2A8B61/2020|2020-04-03|EPI_ISL_539096|Original|hCoV-19^^England|Human|Leeds Teaching Hospitals NHS Trust and Public Health England|Wellcome Sanger Institute for the COVID-19 Genomics UK (COG-UK) consortium|Davies|United Kingdom
>Spike|hCoV-19/USA/HI-H200406/2020|2020-07-08|EPI_ISL_752951|Original|hCoV-19^^Hawaii|Human|State Laboratories Division|State Laboratories Division|Sultana|USA
>Spike|hCoV-19/USA/WI-UW-630/2020|2020-06-26|EPI_ISL_491324|Original|hCoV-19^^Wisconsin|Human|University of Wisconsin-Madison AIDS Vaccine Research Laboratories|University of Wisconsin-Madison AIDS Vaccine Research Laboratories|Baczenas|USA
>Spike|hCoV-19/USA/TX-HMH-MCoV-10087/2020|2020-07-06|EPI_ISL_789318|Original|hCoV-19^^Texas|Human|Houston Methodist Hospital|Houston Methodist Hospital|Long|USA
>Spike|hCoV-19/England/SHEF-C1593/2020|2020-10-19|EPI_ISL_637973|Original|hCoV-19^^England|Human|Virology Department|COVID-19 Genomics UK (COG-UK) Consortium|de Silva|United Kingdom
>Spike|hCoV-19/England/204960259/2020|2020-12-02|EPI_ISL_723013|Original|hCoV-19^^England|Human|Respiratory Virus Unit|COVID-19 Genomics UK (COG-UK) Consortium|Platt|United Kingdom
>Spike|hCoV-19/England/ALDP-71DF93/2020|2020-07-01|EPI_ISL_557649|Original|hCoV-19^^England|Human|Lighthouse Lab in Alderley Park|Wellcome Sanger Institute for the COVID-19 Genomics UK (COG-UK) consortium|Davies|United Kingdom
>Spike|hCoV-19/Australia/VIC16483/2020|2020-00-00|EPI_ISL_640819|Original|hCoV-19^^Victoria|Human|Microbiological Diagnostic Unit - Public Health Laboratory (MDU-PHL)|MDU-PHL|Schultz|Australia
>Spike|hCoV-19/England/NOTT-11577B/2020|2020-10-14|EPI_ISL_595260|Original|hCoV-19^^England|Human|Queens Medical Centre|COVID-19 Genomics UK (COG-UK) Consortium|Loose|United Kingdom
>Spike|hCoV-19/South Africa/KRISP-K001066/2020|2020-07-01|EPI_ISL_509322|Original|hCoV-19^^KwaZulu-Natal|Human|NHLS-IALCH|KRISP|de Oliveira|South Africa

>Spike|hCoV-19/South Africa/KRISP-0494/2020|2020-07-09|EPI_ISL_498065|Original|hCoV-19^^KwaZulu-Natal|Human|NHLS-IALCH|KRISP|de Oliveira|South Africa
>Spike|hCoV-19/England/LOND-D49FB/2020|2020-04-10|EPI_ISL_444167|Original|hCoV-19^^England|Human|University College London|COVID-19 Genomics UK (COG-UK) Consortium|Roy|United Kingdom
>Spike|hCoV-19/Singapore/530/2020|2020-04-26|EPI_ISL_479486|Original|hCoV-19^^Singapore|Human|Department of Laboratory Medicine Tan Tock Seng Hospital|Department of Laboratory Medicine Tan Tock Seng Hospital|Chen|Singapore
>Spike|hCoV-19/Scotland/EDB10588/2020|2020-12-01|EPI_ISL_742175|Original|hCoV-19^^Scotland|Human|Virology Department|COVID-19 Genomics UK (COG-UK) Consortium|McHugh|United Kingdom
>Spike|hCoV-19/Japan/PG-0101/2020|2020-03-00|EPI_ISL_684122|Original|hCoV-19^^Japan|Human|Pathogen Genomics Center|Pathogen Genomics Center|Sekizuka|Japan
>Spike|hCoV-19/England/ALDP-927FFE/2020|2020-06-22|EPI_ISL_551454|Original|hCoV-19^^England|Human|Lighthouse Lab in Alderley Park|Wellcome Sanger Institute for the COVID-19 Genomics UK (COG-UK) consortium|Davies|United Kingdom
>Spike|hCoV-19/Scotland/EDB2518/2020|2020-04-23|EPI_ISL_439223|Original|hCoV-19^^Scotland|Human|Virology Department|COVID-19 Genomics UK (COG-UK) Consortium|Williams|United Kingdom
>Spike|hCoV-19/England/QEUH-A63D64/2020|2020-10-12|EPI_ISL_627288|Original|hCoV-19^^England|Human|Oxford Viromics|COVID-19 Genomics UK (COG-UK) Consortium|Golubchik|United Kingdom
>Spike|hCoV-19/England/MILK-B5564B/2020|2020-11-05|EPI_ISL_657446|Original|hCoV-19^^England|Human|Lighthouse Lab in Milton Keynes|Wellcome Sanger Institute for the COVID-19 Genomics UK (COG-UK) Consortium|Davies|United Kingdom
>Spike|hCoV-19/Denmark/DCGC-5649/2020|2020-09-28|EPI_ISL_619936|Original|hCoV-19^^Hovedstaden|Human|Department of Virus and Microbiological Special Diagnostics|Albertsen lab|Michaelsen|Denmark
>Spike|hCoV-19/Switzerland/BE-ETHZ-360022/2020|2020-11-10|EPI_ISL_693878|Original|hCoV-19^^Bern|Human|Viollier AG|Department of Biosystems Science and Engineering|Nadeau|Switzerland
>Spike|hCoV-19/Canada/ON-S1422/2020|2020-08-22|EPI_ISL_591191|Original|hCoV-19^^Ontario|Human|Toronto Invasive Bacterial Diseases Network|McMaster University|McArthur|Canada
>Spike|hCoV-19/USA/VA_NIDDL_2686/2020|2020-03-25|EPI_ISL_491911|Original|hCoV-19^^Virginia|Human|Naval Infectious Diseases Diagnostic Laboratory|Naval Medical Research Center Biological Defense Research Directorate|Voegtly|USA
>Spike|hCoV-19/Wales/PHWC-2BCB0/2020|2020-04-11|EPI_ISL_445831|Original|hCoV-19^^Wales|Human|Wales Specialist Virology Centre|Public Health Wales Microbiology Cardiff|Moore|United Kingdom
>Spike|hCoV-19/England/MILK-C4FDD5/2020|2020-12-11|EPI_ISL_769778|Original|hCoV-19^^England|Human|Lighthouse Lab in Milton Keynes|Wellcome Sanger Institute for the COVID-19 Genomics UK (COG-UK) Consortium|Davies|United Kingdom
>Spike|hCoV-19/England/204393937/2020|2020-10-24|EPI_ISL_649887|Original|hCoV-19^^England|Human|Respiratory Virus Unit|COVID-19 Genomics UK (COG-UK) Consortium|Platt|United Kingdom
>Spike|hCoV-19/USA/CA-CZB-14126/2020|2020-05-26|EPI_ISL_738528|Original|hCoV-19^^California|Human|Alameda County Public Health Lab|Chan-Zuckerberg Biohub|Kamm|USA
>Spike|hCoV-19/Italy/APU-IZSPB_311PT/2020|2020-11-03|EPI_ISL_653805|Original|hCoV-19^^Apulia|Human|I.R.C.C.S. \S. De Bellis\ - Ente Ospedaliero|Istituto Zooprofilattico Sperimentale della Puglia e della Basilicata|Parisi|Italy
>Spike|hCoV-19/Japan/PG-8807/2020|2020-08-00|EPI_ISL_686393|Original|hCoV-19^^Japan|Human|Pathogen Genomics Center|Pathogen Genomics Center|Sekizuka|Japan
>Spike|hCoV-19/Wales/PHWC-48F73D/2020|2020-11-10|EPI_ISL_681132|Original|hCoV-19^^Wales|Human|Wales Specialist Virology Centre Sequencing lab|COVID-19 Genomics UK (COG-UK) Consortium|Moore|United Kingdom
>Spike|hCoV-19/USA/NY-MSK-3Ai/2020|2020-03-19|EPI_ISL_583434|Vero e6 P1|hCoV-19^^New York|Human|Garcia-Sastre Laboratory|van Bakel Laboratory|van Bakel|USA

>Spike|hCoV-19/England/ALDP-5A4C23/2020|2020-06-15|EPI_ISL_558797|Original|hCoV-19^^England|Human|Lighthouse Lab in Alderley Park|Wellcome Sanger Institute for the COVID-19 Genomics UK (COG-UK) consortium|Davies|United Kingdom
>Spike|hCoV-19/Australia/NSW321/2020|2020-04-06|EPI_ISL_451583|Original|hCoV-19^^New South Wales|Human|Medlab Pathology|NSW Health Pathology - Institute of Clinical Pathology and Medical Research; Westmead Hospital; University of Sydney|Gall|Australia
>Spike|hCoV-19/USA/AZ-TG517482/2020|2020-06-29|EPI_ISL_695332|Original|hCoV-19^^Arizona|Human|AZ SPHL|TGen North|French|USA
>Spike|hCoV-19/England/PORT-2E9BA0/2020|2020-00-00|EPI_ISL_706873|Original|hCoV-19^^England|Human|Centre for Enzyme Innovation|COVID-19 Genomics UK (COG-UK) Consortium|Robson|United Kingdom
>Spike|hCoV-19/USA/MN-GMF-M00029/2020|2020-08-12|EPI_ISL_547705|Original|hCoV-19^^Minnesota|Human|Gundersen Clinical Microbiology Laboratory|Kabara Cancer Research Institute|Kenny|USA
>Spike|hCoV-19/Denmark/DCGC-19775/2020|2020-12-07|EPI_ISL_747780|Original|hCoV-19^^Hovedstaden|Human|Department of Virus and Microbiological Special Diagnostics|Albertsen Lab|Michaelsen|Denmark
>Spike|hCoV-19/England/MILK-AC93E2/2020|2020-10-21|EPI_ISL_630036|Original|hCoV-19^^England|Human|Lighthouse Lab in Milton Keynes|Wellcome Sanger Institute for the COVID-19 Genomics UK (COG-UK) consortium|Davies|United Kingdom
>Spike|hCoV-19/Australia/VIC8410/2020|2020-07-23|EPI_ISL_564993|Original|hCoV-19^^Victoria|Human|Microbiological Diagnostic Unit - Public Health Laboratory (MDU-PHL)|MDU-PHL|Schultz|Australia
>Spike|hCoV-19/USA/TX-HMH-MCoV-8786/2020|2020-07-02|EPI_ISL_780752|Original|hCoV-19^^Texas|Human|Houston Methodist Hospital|Houston Methodist Hospital|Long|USA
>Spike|hCoV-19/Australia/VIC5263/2020|2020-07-16|EPI_ISL_520028|Original|hCoV-19^^Victoria|Human|Microbiological Diagnostic Unit - Public Health Laboratory (MDU-PHL)|MDU-PHL|Schultz|Australia
>Spike|hCoV-19/USA/SD-SDPHL-0005/2020|2020-07-07|EPI_ISL_569613|Original|hCoV-19^^South Dakota|Human|Cheyenne River Health Center|South Dakota Public Health Laboratory|Carlson|USA
>Spike|hCoV-19/USA/TX-HMH-3034/2020|2020-05-26|EPI_ISL_543948|Original|hCoV-19^^Texas|Human|Houston Methodist Hospital|Houston Methodist Hospital|Long|USA
>Spike|hCoV-19/USA/NY-Wadsworth-281377-01/2020|2020-10-12|EPI_ISL_734448|Original|hCoV-19^^New York|Human|Masonic Medical Research Institute|Wadsworth Center|Lasek-Nesselquist|USA
>Spike|hCoV-19/Jordan/IR-ALSR-5221/2020|2020-10-18|EPI_ISL_730459|Original|hCoV-19^^Irbid|Human|Biolab Diagnostic Laboratories|Andersen lab at Scripps Research|Gangavarapu|Jordan
>Spike|hCoV-19/Ireland/D-NVRL-MPS91730/2020|2020-04-11|EPI_ISL_767749|Original|hCoV-19^^Dublin|Human|National Virus Reference Laboratory|Irish Coronavirus Sequencing Consortium - Teagasc Moorepark|Walsh|Ireland
>Spike|hCoV-19/Australia/VIC2531/2020|2020-07-01|EPI_ISL_522224|Original|hCoV-19^^Victoria|Human|Microbiological Diagnostic Unit - Public Health Laboratory (MDU-PHL)|MDU-PHL|Schultz|Australia
>Spike|hCoV-19/Japan/PG-8879/2020|2020-08-00|EPI_ISL_686425|Original|hCoV-19^^Japan|Human|Pathogen Genomics Center|Pathogen Genomics Center|Sekizuka|Japan
>Spike|hCoV-19/Spain/AN-IBV-001121/2020|2020-03-27|EPI_ISL_452580|Original|hCoV-19^^Andalucia|Human|Servicio de Microbiolog\u00eda y Parasitolog\u00eda cl\u00ednica. UCEIMP. Hospital Universitario Virgen del Roc\u00edo/IBIS/CSIC/US.|SeqCOVID-SPAIN consortium/IBV(CSIC)|Comas|Spain
>Spike|hCoV-19/England/MILK-B8C8AF/2020|2020-11-12|EPI_ISL_675931|Original|hCoV-19^^England|Human|Lighthouse Lab in Milton Keynes|Wellcome Sanger Institute for the COVID-19 Genomics UK (COG-UK) Consortium|Davies|United Kingdom
>Spike|hCoV-19/USA/TX-HMH-MCoV-12813/2020|2020-08-19|EPI_ISL_790543|Original|hCoV-19^^Texas|Human|Houston Methodist Hospital|Houston Methodist Hospital|Long|USA
>Spike|hCoV-19/USA/NY-Wadsworth-11244-01/2020|2020-03-06|EPI_ISL_525580|Original|hCoV-19^^New York|Human|Wadsworth Center|Wadsworth Center|Plitnick|USA

>Spike|hCoV-19/England/CAMC-A82E8A/2020|2020-10-13|EPI_ISL_610846|Original|hCoV-19^^England|Human|Lighthouse Lab in Cambridge|Wellcome Sanger Institute for the COVID-19 Genomics UK (COG-UK) consortium|Davies|United Kingdom
>Spike|hCoV-19/England/MILK-BAB691/2020|2020-11-18|EPI_ISL_783307|Original|hCoV-19^^England|Human|Lighthouse Lab in Milton Keynes|Wellcome Sanger Institute for the COVID-19 Genomics UK (COG-UK) Consortium|Davies|United Kingdom
>Spike|hCoV-19/USA/TX-DSHS-1848/2020|2020-06-22|EPI_ISL_745287|Original|hCoV-19^^Texas|Human|Texas Department of State Health Services|Texas Department of State Health Services|Rahman|USA
>Spike|hCoV-19/USA/FL-BPHL-1449/2020|2020-04-07|EPI_ISL_549231|Original|hCoV-19^^Florida|Human|Florida Bureau of Public Health Laboratories|Florida Bureau of Public Health Laboratories|Blanton|USA
>Spike|hCoV-19/Australia/VIC7743/2020|2020-08-07|EPI_ISL_564621|Original|hCoV-19^^Victoria|Human|Microbiological Diagnostic Unit - Public Health Laboratory (MDU-PHL)|MDU-PHL|Schultz|Australia
>Spike|hCoV-19/USA/CA-ALSR-4319/2020|2020-10-07|EPI_ISL_648968|Original|hCoV-19^^California|Human|San Diego County Public Health Laboratory|Andersen lab at Scripps Research|Gangavarapu|USA
>Spike|hCoV-19/Switzerland/BS-UHB-42203486/2020|2020-03-23|EPI_ISL_528357|Original|hCoV-19^^Basel-Stadt|Human|University Hospital Basel|University Hospital Basel|Roloff Handschin|Switzerland
>Spike|hCoV-19/Wales/PHWC-48BB35/2020|2020-10-30|EPI_ISL_666327|Original|hCoV-19^^Wales|Human|Wales Specialist Virology Centre Sequencing lab|COVID-19 Genomics UK (COG-UK) Consortium|Moore|United Kingdom
>Spike|hCoV-19/Gambia/GC19-3396/2020|2020-07-27|EPI_ISL_561069|Original|hCoV-19^^Kombo|Human|MRCG at LSHTM Genomics lab|MRCG at LSHTM Genomics lab|Sesay|Gambia
>Spike|hCoV-19/England/MILK-9D54E1/2020|2020-09-19|EPI_ISL_577065|Original|hCoV-19^^England|Human|Centre for Enzyme Innovation|COVID-19 Genomics UK (COG-UK) Consortium|Robson|United Kingdom
>Spike|hCoV-19/England/ALDP-6B1D3E/2020|2020-07-01|EPI_ISL_557815|Original|hCoV-19^^England|Human|Lighthouse Lab in Alderley Park|Wellcome Sanger Institute for the COVID-19 Genomics UK (COG-UK) consortium|Davies|United Kingdom
>Spike|hCoV-19/England/ALDP-CBECB6/2020|2020-12-19|EPI_ISL_760555|Original|hCoV-19^^England|Human|Lighthouse Lab in Alderley Park|Wellcome Sanger Institute for the COVID-19 Genomics UK (COG-UK) Consortium|Davies|United Kingdom
>Spike|hCoV-19/USA/WA-S1403/2020|2020-05-19|EPI_ISL_495853|Original|hCoV-19^^Washington|Human|Washington State Department of Health|Seattle Flu Study|Lee|USA
>Spike|hCoV-19/USA/WA-S2423/2020|2020-06-15|EPI_ISL_497055|Original|hCoV-19^^Washington|Human|Washington State Department of Health|Seattle Flu Study|Lee|USA
>Spike|hCoV-19/England/ALDP-A6C78B/2020|2020-10-14|EPI_ISL_608322|Original|hCoV-19^^England|Human|Lighthouse Lab in Alderley Park|Wellcome Sanger Institute for the COVID-19 Genomics UK (COG-UK) consortium|Davies|United Kingdom
>Spike|hCoV-19/USA/LA-GBCL-26492483/2020|2020-04-25|EPI_ISL_485048|Original|hCoV-19^^Louisiana|Human|River Road Testing Lab|Ginkgo Bioworks Clinical Laboratory|Smith|USA
>Spike|hCoV-19/Wales/PHWC-2D261/2020|2020-04-02|EPI_ISL_445962|Original|hCoV-19^^Wales|Human|Wales Specialist Virology Centre|Public Health Wales Microbiology Cardiff|Moore|United Kingdom
>Spike|hCoV-19/India/TG-CCMB-L1542/2020|2020-05-26|EPI_ISL_471617|Original|hCoV-19^^Telangana|Human|CSIR-Centre for Cellular and Molecular Biology|CSIR-Centre for Cellular and Molecular Biology|Banu|India
>Spike|hCoV-19/Wales/PHWC-4972C8/2020|2020-11-26|EPI_ISL_726807|Original|hCoV-19^^Wales|Human|Originating lab|COVID-19 Genomics UK (COG-UK) Consortium|Moore|United Kingdom
>Spike|hCoV-19/England/MILK-AC8635/2020|2020-10-21|EPI_ISL_629204|Original|hCoV-19^^England|Human|Lighthouse Lab in Milton Keynes|Wellcome Sanger Institute for the COVID-19 Genomics UK (COG-UK) consortium|Davies|United Kingdom
>Spike|hCoV-19/Japan/PG-4475/2020|2020-07-00|EPI_ISL_692698|Original|hCoV-19^^Japan|Human|Pathogen Genomics Center|Pathogen Genomics Center|Sekizuka|Japan

>Spike|hCoV-19/India/GJ-GBRC224a/2020|2020-06-08|EPI_ISL_476859|Original|hCoV-19^^Gujarat|Human|GMERS Medical College & Hospital|Gujarat Biotechnology Research Centre|Joshi|India
>Spike|hCoV-19/England/MILK-A6A0CE/2020|2020-10-09|EPI_ISL_626896|Original|hCoV-19^^England|Human|Virology Department|COVID-19 Genomics UK (COG-UK) Consortium|de Silva|United Kingdom
>Spike|hCoV-19/USA/ME-HETL-J0340/2020|2020-11-24|EPI_ISL_755468|Original|hCoV-19^^Maine|Human|Maine Health and Environmental Testing Laboratory|Tewhey Lab|Tewhey|USA
>Spike|hCoV-19/England/QEUH-B2B426/2020|2020-11-02|EPI_ISL_646643|Original|hCoV-19^^England|Human|Lighthouse Lab in Glasgow|Wellcome Sanger Institute for the COVID-19 Genomics UK (COG-UK) Consortium|Davies|United Kingdom
>Spike|hCoV-19/England/MILK-ACDE38/2020|2020-10-21|EPI_ISL_630170|Original|hCoV-19^^England|Human|Lighthouse Lab in Milton Keynes|Wellcome Sanger Institute for the COVID-19 Genomics UK (COG-UK) consortium|Davies|United Kingdom
>Spike|hCoV-19/England/SHEF-C4DA7/2020|2020-10-13|EPI_ISL_611553|Original|hCoV-19^^England|Human|Virology Department|COVID-19 Genomics UK (COG-UK) Consortium|de Silva|United Kingdom
>Spike|hCoV-19/USA/TX-HMH-MCoV-8537/2020|2020-07-01|EPI_ISL_780614|Original|hCoV-19^^Texas|Human|Houston Methodist Hospital|Houston Methodist Hospital|Long|USA
>Spike|hCoV-19/Denmark/DCGC-12511/2020|2020-11-16|EPI_ISL_682692|Original|hCoV-19^^Hovedstaden|Human|Department of Virus and Microbiological Special Diagnostics|Albertsen Lab|Michaelsen|Denmark
>Spike|hCoV-19/USA/TX-HMH-481/2020|2020-04-07|EPI_ISL_544345|Original|hCoV-19^^Texas|Human|Houston Methodist Hospital|Houston Methodist Hospital|Long|USA
>Spike|hCoV-19/Japan/PG-4025/2020|2020-07-00|EPI_ISL_692266|Original|hCoV-19^^Japan|Human|Pathogen Genomics Center|Pathogen Genomics Center|Sekizuka|Japan
>Spike|hCoV-19/India/KA-IB8/2020|2020-06-20|EPI_ISL_508336|Original|hCoV-19^^Karnataka|Human|Indian Institute of Science|National Institute of Biomedical Genomics|Maitra|India
>Spike|hCoV-19/Gibraltar/204462880/2020|2020-10-29|EPI_ISL_615047|Original|hCoV-19^^Gibraltar|Human|Microbiology|Respiratory Virus Unit|Platt|Gibraltar
>Spike|hCoV-19/Denmark/DCGC-18404/2020|2020-12-07|EPI_ISL_749945|Original|hCoV-19^^Hovedstaden|Human|Department of Virus and Microbiological Special Diagnostics|Albertsen Lab|Michaelsen|Denmark
>Spike|hCoV-19/Scotland/CVR2857/2020|2020-04-21|EPI_ISL_473600|Original|hCoV-19^^Scotland|Human|West of Scotland Specialist Virology Centre|COVID-19 Genomics UK (COG-UK) Consortium|Robertson|United Kingdom
>Spike|hCoV-19/USA/MI-MDHHS-SC22464/2020|2020-11-03|EPI_ISL_708556|Original|hCoV-19^^Michigan|Human|Michigan Department of Health and Human Services|Michigan Department of Health and Human Services|Jacob|USA
>Spike|hCoV-19/Canada/QC-LSPQ-L00241444/2020|2020-03-27|EPI_ISL_536282|Original|hCoV-19^^Quebec|Human|H\u00f4pital Charles-LeMoyne|Laboratoire de sant\u00e9 publique du Qu\u00e9bec|Moreira|Canada
>Spike|hCoV-19/Wales/PHWC-2BA3E/2020|2020-03-26|EPI_ISL_445796|Original|hCoV-19^^Wales|Human|Wales Specialist Virology Centre|Public Health Wales Microbiology Cardiff|Moore|United Kingdom
>Spike|hCoV-19/Spain/AR-ISCIII-201597/2020|2020-03-06|EPI_ISL_539504|Original|hCoV-19^^Aragon|Human|Hospital Universitario Miguel Servet|Instituto de Salud Carlos III|Caballero|Spain
>Spike|hCoV-19/USA/VA-DCLS-0255/2020|2020-04-20|EPI_ISL_452829|Original|hCoV-19^^Virginia|Human|Virginia DCLS|Virginia DCLS|St. Jacques|USA
>Spike|hCoV-19/Denmark/DCGC-3360/2020|2020-08-31|EPI_ISL_616117|Original|hCoV-19^^Syddanmark|Human|Department of Virus and Microbiological Special Diagnostics|Albertsen lab|Michaelsen|Denmark
>Spike|hCoV-19/Japan/PG-5731/2020|2020-07-00|EPI_ISL_688853|Original|hCoV-19^^Japan|Human|Pathogen Genomics Center|Pathogen Genomics Center|Sekizuka|Japan
>Spike|hCoV-19/Germany/SH-ChVir8194/2020|2020-07-19|EPI_ISL_729470|Original|hCoV-19^^Schleswig-Holstein|Human|A. Krumbholz|Charit\u00e9 Universit\u00e4tsmedizin Berlin|Muehlemann|Germany

>Spike|hCoV-19/Denmark/DCGC-16746/2020|2020-05-04|EPI_ISL_714191|Original|hCoV-19^^Hovedstaden|Human|Department of Virus and Microbiological Special Diagnostics|Albertsen Lab|Michaelsen|Denmark
>Spike|hCoV-19/England/ALDP-929475/2020|2020-06-22|EPI_ISL_552943|Original|hCoV-19^^England|Human|Lighthouse Lab in Alderley Park|Wellcome Sanger Institute for the COVID-19 Genomics UK (COG-UK) consortium|Davies|United Kingdom
>Spike|hCoV-19/Japan/P5-3/2020|2020-03-13|EPI_ISL_419311|Original|hCoV-19^^Chiba|Human|Chiba Prefectural Institute of Public Health|Pathogen Genomics Center|Sekizuka|Japan
>Spike|hCoV-19/Ghana/TTH6/2020|2020-03-27|EPI_ISL_515248|Original|hCoV-19^^Ghana|Human|Kumasi Centre for Collaborative Research in Tropical Medicine|Institute of Virology|El-Duah|Ghana
>Spike|hCoV-19/India/GJ-GBRC-371/2020|2020-06-18|EPI_ISL_524727|Original|hCoV-19^^Gujarat|Human|B.J. Medical College and Civil hospital|Gujarat Biotechnology Research Centre|Joshi|India
>Spike|hCoV-19/Wales/PHWC-49A210/2020|2020-12-01|EPI_ISL_727512|Original|hCoV-19^^Wales|Human|Originating lab|COVID-19 Genomics UK (COG-UK) Consortium|Moore|United Kingdom
>Spike|hCoV-19/England/C200044483/2020|2020-11-06|EPI_ISL_666764|Original|hCoV-19^^England|Human|Respiratory Virus Unit|COVID-19 Genomics UK (COG-UK) Consortium|Platt|United Kingdom
>Spike|hCoV-19/USA/TX-HMH-MCoV-18210/2020|2020-11-13|EPI_ISL_784558|Original|hCoV-19^^Texas|Human|Houston Methodist Hospital|Houston Methodist Hospital|Long|USA
>Spike|hCoV-19/Wales/PHWC-49AFCE/2020|2020-11-12|EPI_ISL_742280|Original|hCoV-19^^Wales|Human|Wales Specialist Virology Centre Sequencing lab|COVID-19 Genomics UK (COG-UK) Consortium|Moore|United Kingdom
>Spike|hCoV-19/USA/MI-UM-10036775247/2020|2020-11-04|EPI_ISL_707867|Original|hCoV-19^^Michigan|Human|University of Michigan Clinical Microbiology Laboratory|Lauring Lab|Valesano|USA
>Spike|hCoV-19/England/ALDP-A2ECA4/2020|2020-10-05|EPI_ISL_587188|Original|hCoV-19^^England|Human|Lighthouse Lab in Alderley Park|Wellcome Sanger Institute for the COVID-19 Genomics UK (COG-UK) consortium|Davies|United Kingdom
>Spike|hCoV-19/USA/FL-06-UMTL-A81P/2020|2020-04-17|EPI_ISL_479773|Original|hCoV-19^^Florida|Human|University of Miami Immunology and Histocompatibility Laboratory|University of Miami Immunology and Histocompatibility Laboratory|Margolles-Clark|USA
>Spike|hCoV-19/Denmark/DCGC-2453/2020|2020-08-03|EPI_ISL_617413|Original|hCoV-19^^Syddanmark|Human|Department of Virus and Microbiological Special Diagnostics|Albertsen lab|Michaelsen|Denmark
>Spike|hCoV-19/Scotland/QEUH-9F6B03/2020|2020-09-27|EPI_ISL_600743|Original|hCoV-19^^Scotland|Human|Lighthouse Lab in Glasgow|Wellcome Sanger Institute for the COVID-19 Genomics UK (COG-UK) consortium|Davies|United Kingdom
>Spike|hCoV-19/England/SHEF-10CFEC4/2020|2020-12-20|EPI_ISL_764107|Original|hCoV-19^^England|Human|Virology Department|COVID-19 Genomics UK (COG-UK) Consortium|de Silva|United Kingdom
>Spike|hCoV-19/USA/TX-HMH-7368/2020|2020-06-29|EPI_ISL_546711|Original|hCoV-19^^Texas|Human|Houston Methodist Hospital|Houston Methodist Hospital|Long|USA
>Spike|hCoV-19/England/ALDP-9BCBDC/2020|2020-09-09|EPI_ISL_573061|Original|hCoV-19^^England|Human|Oxford Viromics|COVID-19 Genomics UK (COG-UK) Consortium|Golubchik|United Kingdom
>Spike|hCoV-19/Wales/PHWC-2ABE1/2020|2020-04-05|EPI_ISL_445678|Original|hCoV-19^^Wales|Human|Wales Specialist Virology Centre|Public Health Wales Microbiology Cardiff|Moore|United Kingdom
>Spike|hCoV-19/Israel/701002550/2020|2020-03-26|EPI_ISL_447291|Original|hCoV-19^^South Coast District|Human|Microbiology Division|Stern Lab|Miller|Israel
>Spike|hCoV-19/USA/AZ-TG280856/2020|2020-04-21|EPI_ISL_694195|Original|hCoV-19^^Arizona|Human|AZ SPHL|TGen North|French|USA
>Spike|hCoV-19/Canada/ON-PHL-20-02335/2020|2020-09-00|EPI_ISL_671567|Original|hCoV-19^^Ontario|Human|Public Health Ontario Laboratory|Public Health Ontario Laboratory|Guthrie|Canada

>Spike|hCoV-19/USA/CO-CDPHE-2009164054/2020|2020-09-16|EPI_ISL_710333|Original|hCoV-19^^Colorado|Human|Colorado Department of Public Health and Environment|Colorado Department of Puplic Health and Environment|Bankers|USA
>Spike|hCoV-19/England/MILK-9705C7/2020|2020-08-18|EPI_ISL_553146|Original|hCoV-19^^England|Human|Lighthouse Lab in Milton Keynes|Wellcome Sanger Institute for the COVID-19 Genomics UK (COG-UK) consortium|Davies|United Kingdom
>Spike|hCoV-19/Australia/VIC151/2020|2020-03-17|EPI_ISL_419848|Original|hCoV-19^^Victoria|Human|Victorian Infectious Diseases Reference Laboratory (VIDRL)|Victorian Infectious Diseases Reference Laboratory and Microbiological Diagnostic Unit Public Health Laboratory|Schultz|Australia
>Spike|hCoV-19/England/QEUH-9BAF4B/2020|2020-09-07|EPI_ISL_573639|Original|hCoV-19^^England|Human|Oxford Viromics|COVID-19 Genomics UK (COG-UK) Consortium|Golubchik|United Kingdom
>Spike|hCoV-19/Northern Ireland/NIRE-23399E/2020|2020-10-04|EPI_ISL_680328|Original|hCoV-19^^Northern Ireland|Human|Regional Virus Laboratory|COVID-19 Genomics UK (COG-UK) Consortium|Fairley|United Kingdom
>Spike|hCoV-19/USA/CA-CDPH018/2020|2020-02-27|EPI_ISL_515911|Original|hCoV-19^^California|Human|California Department of Public Health|California Department of Public Health|Bell|USA
>Spike|hCoV-19/England/LIVE-DB3931/2020|2020-10-21|EPI_ISL_651145|Original|hCoV-19^^England|Human|Liverpool Clinical Laboratories|COVID-19 Genomics UK (COG-UK) Consortium|Haldenby|United Kingdom
>Spike|hCoV-19/Singapore/1013/2020|2020-03-24|EPI_ISL_574498|Original|hCoV-19^^Singapore|Human|National Public Health Laboratory|National Public Health Laboratory|Mak|Singapore
>Spike|hCoV-19/Wales/PHWC-4A0A97/2020|2020-12-10|EPI_ISL_743537|Original|hCoV-19^^Wales|Human|Wales Specialist Virology Centre Sequencing lab|COVID-19 Genomics UK (COG-UK) Consortium|Moore|United Kingdom
>Spike|hCoV-19/USA/CO-CDPHE-2011199375/2020|2020-11-19|EPI_ISL_771255|Original|hCoV-19^^Colorado|Human|Colorado Department of Public Health and Environment|Colorado Department of Puplic Health and Environment|Bankers|USA
>Spike|hCoV-19/USA/MN-MDH-1066/2020|2020-05-14|EPI_ISL_462911|Original|hCoV-19^^Minnesota|Human|Minnesota Department of Health|Minnesota Department of Health|Garfin|USA
>Spike|hCoV-19/England/NORW-EC5DE/2020|2020-05-10|EPI_ISL_457538|Original|hCoV-19^^England|Human|Quadram Institute Bioscience|COVID-19 Genomics UK (COG-UK) Consortium|Alikhan|United Kingdom
>Spike|hCoV-19/Uruguay/RIV-M10/2020|2020-05-21|EPI_ISL_748140|Original|hCoV-19^^Rivera|Human|Instituto de Investigaciones Biolo\u0301gicas Clemente Estable|Institut Pasteur de Montevideo|Spangenberg|Uruguay
>Spike|hCoV-19/USA/NY-NYUMC276/2020|2020-04-06|EPI_ISL_428790|Original|hCoV-19^^New York|Human|NYU Langone Health|Departments of Pathology and Medicine|Maurano|USA
>Spike|hCoV-19/Northern Ireland/NIRE-104584/2020|2020-04-15|EPI_ISL_489333|Original|hCoV-19^^Northern Ireland|Human|Regional Virus Laboratory|Wellcome Sanger Institute for the COVID-19 Genomics UK (COG-UK) consortium|Davies|United Kingdom
>Spike|hCoV-19/Wales/PHWC-2EEC6/2020|2020-04-06|EPI_ISL_446170|Original|hCoV-19^^Wales|Human|Wales Specialist Virology Centre|Public Health Wales Microbiology Cardiff|Moore|United Kingdom
>Spike|hCoV-19/Scotland/EDB8934/2020|2020-10-29|EPI_ISL_650813|Original|hCoV-19^^Scotland|Human|Virology Department|COVID-19 Genomics UK (COG-UK) Consortium|McHugh|United Kingdom
>Spike|hCoV-19/Japan/PG-5028/2020|2020-07-00|EPI_ISL_690139|Original|hCoV-19^^Japan|Human|Pathogen Genomics Center|Pathogen Genomics Center|Sekizuka|Japan
>Spike|hCoV-19/USA/HI-H200702/2020|2020-12-04|EPI_ISL_753223|Original|hCoV-19^^Hawaii|Human|State Laboratories Division|State Laboratories Division|Sultana|USA
>Spike|hCoV-19/USA/NY-Wadsworth-277726-01/2020|2020-11-20|EPI_ISL_754587|Original|hCoV-19^^New York|Human|Wadsworth Center|Wadsworth Center|Lasek-Nesselquist|USA

>Spike|hCoV-19/England/NOTT-112CD9/2020|2020-07-09|EPI_ISL_493609|Original|hCoV-19^^England|Human|Queens Medical Centre|COVID-19 Genomics UK (COG-UK) Consortium|Loose|United Kingdom
>Spike|hCoV-19/Germany/BE-ChVir-W1588-8297/2020|2020-06-30|EPI_ISL_753726|Original|hCoV-19^^Berlin|Human|Charit\u00e9 Universit\u00e4tsmedizin Berlin|Charit\u00e9 Universit\u00e4tsmedizin Berlin|Muehlemann|Germany
>Spike|hCoV-19/Wales/PHWC-16379C/2020|2020-04-29|EPI_ISL_473993|Original|hCoV-19^^Wales|Human|Originating lab|COVID-19 Genomics UK (COG-UK) Consortium|Moore|United Kingdom
>Spike|hCoV-19/USA/UT-UPHL-2012669491/2020|2020-11-30|EPI_ISL_746455|Original|hCoV-19^^Utah|Human|Utah Public Health Laboratory|Utah Public Health Laboratory|Gallagher|USA
>Spike|hCoV-19/Wales/PHWC-16B178/2020|2020-09-16|EPI_ISL_560252|Original|hCoV-19^^Wales|Human|Wales Specialist Virology Centre Sequencing lab|COVID-19 Genomics UK (COG-UK) Consortium|Moore|United Kingdom
>Spike|hCoV-19/Northern Ireland/NIRE-234520/2020|2020-10-19|EPI_ISL_680402|Original|hCoV-19^^Northern Ireland|Human|Regional Virus Laboratory|COVID-19 Genomics UK (COG-UK) Consortium|Fairley|United Kingdom
>Spike|hCoV-19/Wales/PHWC-26C6A/2020|2020-03-27|EPI_ISL_422294|Original|hCoV-19^^Wales|Human|Wales Specialist Virology Centre|Public Health Wales Microbiology Cardiff|Moore|United Kingdom
>Spike|hCoV-19/England/QEUH-990066/2020|2020-08-26|EPI_ISL_539977|Original|hCoV-19^^England|Human|Lighthouse Lab in Glasgow|Wellcome Sanger Institute for the COVID-19 Genomics UK (COG-UK) consortium|Davies|United Kingdom
>Spike|hCoV-19/USA/TX-HMH-MCoV-15941/2020|2020-07-10|EPI_ISL_785625|Original|hCoV-19^^Texas|Human|Houston Methodist Hospital|Houston Methodist Hospital|Long|USA
>Spike|hCoV-19/Canada/MB-NML-1041/2020|2020-04-28|EPI_ISL_582401|Original|hCoV-19^^Manitoba|Human|Cadham Provincial Laboratory|National Microbiology Laboratory (NML)|Majer|Canada
>Spike|hCoV-19/England/ALDP-955601/2020|2020-06-13|EPI_ISL_553369|Original|hCoV-19^^England|Human|Lighthouse Lab in Alderley Park|Wellcome Sanger Institute for the COVID-19 Genomics UK (COG-UK) consortium|Davies|United Kingdom
>Spike|hCoV-19/England/MILK-AC8F0D/2020|2020-10-22|EPI_ISL_629586|Original|hCoV-19^^England|Human|Lighthouse Lab in Milton Keynes|Wellcome Sanger Institute for the COVID-19 Genomics UK (COG-UK) consortium|Davies|United Kingdom
>Spike|hCoV-19/USA/CA-ALSR-2367/2020|2020-07-08|EPI_ISL_635444|Original|hCoV-19^^California|Human|San Diego County Public Health Laboratory|Andersen lab at Scripps Research|Gangavarapu|USA
>Spike|hCoV-19/Japan/FHU-CS84-0222/2020|2020-02-22|EPI_ISL_728155|Original|hCoV-19^^Aichi|Human|Fujita Health University Okazaki Medical Center|Department of Virology and Parasitology|Komoto|Japan
>Spike|hCoV-19/England/204891510/2020|2020-11-27|EPI_ISL_722971|Original|hCoV-19^^England|Human|Respiratory Virus Unit|COVID-19 Genomics UK (COG-UK) Consortium|Platt|United Kingdom
>Spike|hCoV-19/England/CAMC-A810C1/2020|2020-10-15|EPI_ISL_610693|Original|hCoV-19^^England|Human|Lighthouse Lab in Cambridge|Wellcome Sanger Institute for the COVID-19 Genomics UK (COG-UK) consortium|Davies|United Kingdom
>Spike|hCoV-19/England/ALDP-92AF37/2020|2020-06-22|EPI_ISL_551460|Original|hCoV-19^^England|Human|Lighthouse Lab in Alderley Park|Wellcome Sanger Institute for the COVID-19 Genomics UK (COG-UK) consortium|Davies|United Kingdom
>Spike|hCoV-19/Sweden/20-53005/2020|2020-09-28|EPI_ISL_648205|Original|hCoV-19^^Orebro|Human|Orebro klinisk mikrobiologi|The Public Health Agency of Sweden|Svartstr\u00f6m|Sweden
>Spike|hCoV-19/Denmark/DCGC-16813/2020|2020-12-14|EPI_ISL_750201|Original|hCoV-19^^Hovedstaden|Human|Department of Virus and Microbiological Special Diagnostics|Albertsen Lab|Michaelsen|Denmark
>Spike|hCoV-19/Australia/VIC9862/2020|2020-07-15|EPI_ISL_565747|Original|hCoV-19^^Victoria|Human|Microbiological Diagnostic Unit - Public Health Laboratory (MDU-PHL)|MDU-PHL|Schultz|Australia

>Spike|hCoV-19/England/QEUH-98F291/2020|2020-08-25|EPI_ISL_540375|Original|hCoV-19^^England|Human|Lighthouse Lab in Glasgow|Wellcome Sanger Institute for the COVID-19 Genomics UK (COG-UK) consortium|Davies|United Kingdom
>Spike|hCoV-19/Denmark/DCGC-21544/2020|2020-12-21|EPI_ISL_758223|Original|hCoV-19^^Midtjylland|Human|Department of Virus and Microbiological Special Diagnostics|Albertsen Lab|Michaelsen|Denmark
>Spike|hCoV-19/Japan/PG-1806/2020|2020-04-00|EPI_ISL_685412|Original|hCoV-19^^Japan|Human|Tokyo Metropolitan Institute of Public Health|Pathogen Genomics Center|Sekizuka|Japan
>Spike|hCoV-19/England/20142131604/2020|2020-03-30|EPI_ISL_423470|Original|hCoV-19^^England|Human|Respiratory Virus Unit|Respiratory Virus Unit|Platt|United Kingdom
>Spike|hCoV-19/England/CAMC-A65C83/2020|2020-10-11|EPI_ISL_637515|Original|hCoV-19^^England|Human|Department of Pathology|COVID-19 Genomics UK (COG-UK) Consortium|Goodfellow|United Kingdom
>Spike|hCoV-19/Sichuan/SC-PHCC1-031/2020|2020-02-08|EPI_ISL_451352|Original|hCoV-19^^Sichuan|Human|West China Hospital of Sichuan University|State Key Laboratory of Biotherapy of Sichuan University|Tang|China
>Spike|hCoV-19/England/ALDP-A2DB7B/2020|2020-10-06|EPI_ISL_586842|Original|hCoV-19^^England|Human|Lighthouse Lab in Alderley Park|Wellcome Sanger Institute for the COVID-19 Genomics UK (COG-UK) consortium|Davies|United Kingdom
>Spike|hCoV-19/USA/AZ-TG512977/2020|2020-07-17|EPI_ISL_695306|Original|hCoV-19^^Arizona|Human|TGen North|TGen North|French|USA
>Spike|hCoV-19/England/CAMC-946885/2020|2020-07-14|EPI_ISL_556973|Original|hCoV-19^^England|Human|Lighthouse Lab in Cambridge|Wellcome Sanger Institute for the COVID-19 Genomics UK (COG-UK) consortium|Davies|United Kingdom
>Spike|hCoV-19/Scotland/CVR1429/2020|2020-04-02|EPI_ISL_433581|Original|hCoV-19^^Scotland|Human|West of Scotland Specialist Virology Centre|COVID-19 Genomics UK (COG-UK) Consortium|Robertson|United Kingdom
>Spike|hCoV-19/Spain/MD-IBV-006408/2020|2020-03-23|EPI_ISL_510124|Original|hCoV-19^^Madrid|Human|Hospital General Universitario Gregorio Mara\u00f1\u00f3n|SeqCOVID-SPAIN consortium/IBV(CSIC)|Comas|Spain
>Spike|hCoV-19/USA/VA-DCLS-0379/2020|2020-05-13|EPI_ISL_463135|Original|hCoV-19^^Virginia|Human|Virginia DCLS|Virginia DCLS|St. Jacques|USA
>Spike|hCoV-19/USA/FL-BPHL-0335/2020|2020-04-20|EPI_ISL_489744|Original|hCoV-19^^Florida|Human|Florida Bureau of Public Health Laboratories|Florida Bureau of Public Health Laboratories|Blanton|USA
>Spike|hCoV-19/Belgium/UGent-218/2020|2020-10-19|EPI_ISL_717605|Original|hCoV-19^^Ghent|Human|Lab voor klinische biologie|Onderzoeksgroep Virologie|Lambrechts|Belgium
>Spike|hCoV-19/USA/WA-S336/2020|2020-04-03|EPI_ISL_434096|Original|hCoV-19^^Washington|Human|Washington State Department of Health|Seattle Flu Study|Bedford|USA
>Spike|hCoV-19/England/QEUH-AD25D1/2020|2020-10-24|EPI_ISL_630002|Original|hCoV-19^^England|Human|Lighthouse Lab in Glasgow|Wellcome Sanger Institute for the COVID-19 Genomics UK (COG-UK) consortium|Davies|United Kingdom
>Spike|hCoV-19/Netherlands/NB-EMC-393/2020|2020-00-00|EPI_ISL_722470|Original|hCoV-19^^Noord Brabant|Human|Dutch COVID-19 response team|Erasmus Medical Center|Oude Munnink|Netherlands
>Spike|hCoV-19/Denmark/DCGC-19593/2020|2020-12-07|EPI_ISL_748281|Original|hCoV-19^^Syddanmark|Human|Department of Virus and Microbiological Special Diagnostics|Albertsen Lab|Michaelsen|Denmark
>Spike|hCoV-19/Australia/VIC9585/2020|2020-08-23|EPI_ISL_565614|Original|hCoV-19^^Victoria|Human|Microbiological Diagnostic Unit - Public Health Laboratory (MDU-PHL)|MDU-PHL|Schultz|Australia
>Spike|hCoV-19/England/MILK-ACC516/2020|2020-10-20|EPI_ISL_634516|Original|hCoV-19^^England|Human|Lighthouse Lab in Milton Keynes|Wellcome Sanger Institute for the COVID-19 Genomics UK (COG-UK) consortium|Davies|United Kingdom
>Spike|hCoV-19/England/ALDP-A93F65/2020|2020-10-19|EPI_ISL_611124|Original|hCoV-19^^England|Human|Lighthouse Lab in Alderley Park|Wellcome Sanger Institute for the COVID-19 Genomics UK (COG-UK) consortium|Davies|United Kingdom

>Spike|hCoV-19/USA/NY-WCMP4B06P/2020|2020-03-13|EPI_ISL_427532|Original|hCoV-19^^New York|Human|NewYork-Presbyterian & Mason Lab|Mason Lab|Mason|USA
>Spike|hCoV-19/Scotland/QEUH-AD1D52/2020|2020-10-27|EPI_ISL_644038|Original|hCoV-19^^Scotland|Human|Lighthouse Lab in Glasgow|Wellcome Sanger Institute for the COVID-19 Genomics UK (COG-UK) Consortium|Davies|United Kingdom
>Spike|hCoV-19/Netherlands/UT-EMC-221/2020|2020-12-06|EPI_ISL_722790|Original|hCoV-19^^Utrecht|Human|Dutch COVID-19 response team|Erasmus Medical Center|Oude Munnink|Netherlands
>Spike|hCoV-19/England/ALDP-B4680B/2020|2020-11-07|EPI_ISL_656025|Original|hCoV-19^^England|Human|Lighthouse Lab in Alderley Park|Wellcome Sanger Institute for the COVID-19 Genomics UK (COG-UK) Consortium|Davies|United Kingdom
>Spike|hCoV-19/England/NORT-28C750/2020|2020-05-15|EPI_ISL_478600|Original|hCoV-19^^England|Human|Northumbria University / South Tees Hospitals NHS Foundation Trust / North Cumbria Integrated Care NHS Foundation Trust / North Tees and Hartlepool NHS Foundation Trust / Newcastle Hospitals NHS Foundation Trust|COVID-19 Genomics UK (COG-UK) Consortium|Bashton|United Kingdom
>Spike|hCoV-19/England/CAMB-1B4A66/2020|2020-06-07|EPI_ISL_473492|Original|hCoV-19^^England|Human|Department of Pathology|COVID-19 Genomics UK (COG-UK) Consortium|Meredith|United Kingdom
>Spike|hCoV-19/England/QEUH-9C933E/2020|2020-09-10|EPI_ISL_567418|Original|hCoV-19^^England|Human|Lighthouse Lab in Glasgow|Wellcome Sanger Institute for the COVID-19 Genomics UK (COG-UK) consortium|Davies|United Kingdom
>Spike|hCoV-19/USA/AZ-TG494849/2020|2020-07-10|EPI_ISL_695255|Original|hCoV-19^^Arizona|Human|TGen North|TGen North|French|USA
>Spike|hCoV-19/Denmark/DCGC-12671/2020|2020-11-16|EPI_ISL_682852|Original|hCoV-19^^Syddanmark|Human|Department of Virus and Microbiological Special Diagnostics|Albertsen Lab|Michaelsen|Denmark
>Spike|hCoV-19/USA/CA-CZB-16183/2020|2020-12-02|EPI_ISL_738712|Original|hCoV-19^^California|Human|Renegade|Chan-Zuckerberg Biohub|Kamm|USA
>Spike|hCoV-19/USA/TX-HMH-MCoV-11240/2020|2020-09-02|EPI_ISL_789745|Original|hCoV-19^^Texas|Human|Houston Methodist Hospital|Houston Methodist Hospital|Long|USA
>Spike|hCoV-19/Spain/MD-H12-H12-2107/2020|2020-03-27|EPI_ISL_421177|Original|hCoV-19^^Madrid|Human|Hospital Universitario 12 de Octubre|Hospital Universitario 12 de Octubre|Viedma|Spain
>Spike|hCoV-19/England/QEUH-9E4E69/2020|2020-09-22|EPI_ISL_579864|Original|hCoV-19^^England|Human|Lighthouse Lab in Glasgow|Wellcome Sanger Institute for the COVID-19 Genomics UK (COG-UK) consortium|Davies|United Kingdom
>Spike|hCoV-19/England/CAMC-B205B5/2020|2020-11-02|EPI_ISL_647730|Original|hCoV-19^^England|Human|Lighthouse Lab in Cambridge|Wellcome Sanger Institute for the COVID-19 Genomics UK (COG-UK) Consortium|Davies|United Kingdom
>Spike|hCoV-19/Denmark/DCGC-17908/2020|2020-12-14|EPI_ISL_749160|Original|hCoV-19^^Hovedstaden|Human|Department of Virus and Microbiological Special Diagnostics|Albertsen Lab|Michaelsen|Denmark
>Spike|hCoV-19/England/MILK-B3A13C/2020|2020-11-02|EPI_ISL_679684|Original|hCoV-19^^England|Human|Oxford Viromics|COVID-19 Genomics UK (COG-UK) Consortium|Golubchik|United Kingdom
>Spike|hCoV-19/USA/CA-CZB-13118/2020|2020-10-27|EPI_ISL_672416|Original|hCoV-19^^California|Human|Madera County Department of Public Health|Chan-Zuckerberg Biohub|Bhatt|USA
>Spike|hCoV-19/USA/AZ-TG491474/2020|2020-06-17|EPI_ISL_695108|Original|hCoV-19^^Arizona|Human|AZ SPHL|TGen North|French|USA
>Spike|hCoV-19/England/QEUH-962917/2020|2020-08-10|EPI_ISL_531709|Original|hCoV-19^^England|Human|Lighthouse Lab in Glasgow|Wellcome Sanger Institute for the COVID-19 Genomics UK (COG-UK) consortium|Davies|United Kingdom
>Spike|hCoV-19/Belgium/Rega-0330252/2020|2020-03-30|EPI_ISL_458222|Original|hCoV-19^^Holsbeek|Human|KU Leuven|KU Leuven|Maes|Belgium
>Spike|hCoV-19/USA/CA-CZB-13412/2020|2020-09-02|EPI_ISL_672113|Original|hCoV-19^^California|Human|Alameda County Public Health Lab|Chan-Zuckerberg Biohub|Bhatt|USA

>Spike|hCoV-19/England/LIVE-DB9986/2020|2020-11-09|EPI_ISL_679381|Original|hCoV-19^^England|Human|Liverpool Clinical Laboratories|COVID-19 Genomics UK (COG-UK) Consortium|Haldenby|United Kingdom
>Spike|hCoV-19/England/PORT-2D11A9/2020|2020-04-21|EPI_ISL_475337|Original|hCoV-19^^England|Human|Centre for Enzyme Innovation|COVID-19 Genomics UK (COG-UK) Consortium|Robson|United Kingdom
>Spike|hCoV-19/England/MILK-B39ABA/2020|2020-11-02|EPI_ISL_651527|Original|hCoV-19^^England|Human|University of Exeter|COVID-19 Genomics UK (COG-UK) Consortium|Temperton|United Kingdom
>Spike|hCoV-19/Switzerland/BS-ETHZ-100241/2020|2020-03-18|EPI_ISL_574910|Original|hCoV-19^^Basel-Stadt|Human|Viollier AG|Department of Biosystems Science and Engineering|Nadeau|Switzerland
>Spike|hCoV-19/Denmark/ALAB-SSI-573/2020|2020-03-27|EPI_ISL_437665|Original|hCoV-19^^Denmark|Human|Department of Virus and Microbiological Special Diagnostics|Albertsen lab|Kirkegaard|Denmark
>Spike|hCoV-19/Scotland/CVR3041/2020|2020-04-24|EPI_ISL_461629|Original|hCoV-19^^Scotland|Human|West of Scotland Specialist Virology Centre|COVID-19 Genomics UK (COG-UK) Consortium|Robertson|United Kingdom
>Spike|hCoV-19/USA/TX-HMH-MCoV-18721/2020|2020-11-23|EPI_ISL_785068|Original|hCoV-19^^Texas|Human|Houston Methodist Hospital|Houston Methodist Hospital|Long|USA
>Spike|hCoV-19/Finland/13M82/2020|2020-03-13|EPI_ISL_418399|Original|hCoV-19^^Finland|Human|Department of Virology and Immunology|Department of Virology|Smura|Finland
>Spike|hCoV-19/England/SHEF-10C84D8/2020|2020-12-20|EPI_ISL_741953|Original|hCoV-19^^England|Human|Virology Department|COVID-19 Genomics UK (COG-UK) Consortium|de Silva|United Kingdom
>Spike|hCoV-19/Wales/PHWC-48F5CA/2020|2020-11-09|EPI_ISL_681113|Original|hCoV-19^^Wales|Human|Wales Specialist Virology Centre Sequencing lab|COVID-19 Genomics UK (COG-UK) Consortium|Moore|United Kingdom
>Spike|hCoV-19/England/CAMC-B37FBD/2020|2020-11-05|EPI_ISL_659000|Original|hCoV-19^^England|Human|Lighthouse Lab in Cambridge|Wellcome Sanger Institute for the COVID-19 Genomics UK (COG-UK) Consortium|Davies|United Kingdom
>Spike|hCoV-19/USA/UT-UPHL-01384/2020|2020-04-01|EPI_ISL_575046|Original|hCoV-19^^Utah|Human|Utah Public Health Laboratory|Utah Public Health Laboratory|Young|USA
>Spike|hCoV-19/England/QEUH-B0ED1F/2020|2020-11-02|EPI_ISL_642586|Original|hCoV-19^^England|Human|Lighthouse Lab in Glasgow|Wellcome Sanger Institute for the COVID-19 Genomics UK (COG-UK) Consortium|Davies|United Kingdom
>Spike|hCoV-19/India/WB-S23/2020|2020-04-21|EPI_ISL_455653|Original|hCoV-19^^West Bengal|Human|ICMR-National Institute of Cholera and Enteric Diseases|National Institute of Biomedical Genomics|Maitra|India
>Spike|hCoV-19/England/CAMB-1BB3CE/2020|2020-11-16|EPI_ISL_664304|Original|hCoV-19^^England|Human|Department of Pathology|COVID-19 Genomics UK (COG-UK) Consortium|Goodfellow|United Kingdom
>Spike|hCoV-19/Ireland/WW-NVRL-73IRL23922/2020|2020-09-05|EPI_ISL_578329|Original|hCoV-19^^Wicklow|Human|National Virus Reference Laboratory|National Virus Reference Laboratory|Gonzalez|Ireland
>Spike|hCoV-19/England/QEUH-9FAE06/2020|2020-09-28|EPI_ISL_588062|Original|hCoV-19^^England|Human|Lighthouse Lab in Glasgow|Wellcome Sanger Institute for the COVID-19 Genomics UK (COG-UK) consortium|Davies|United Kingdom
>Spike|hCoV-19/USA/TX-HMH-7627/2020|2020-06-26|EPI_ISL_546897|Original|hCoV-19^^Texas|Human|Houston Methodist Hospital|Houston Methodist Hospital|Long|USA
>Spike|hCoV-19/USA/TX-HMH-1872/2020|2020-05-05|EPI_ISL_542886|Original|hCoV-19^^Texas|Human|Houston Methodist Hospital|Houston Methodist Hospital|Long|USA
>Spike|hCoV-19/England/MILK-B8774D/2020|2020-11-13|EPI_ISL_724796|Original|hCoV-19^^England|Human|Oxford Viromics|COVID-19 Genomics UK (COG-UK) Consortium|Golubchik|United Kingdom
>Spike|hCoV-19/England/MILK-B56AA8/2020|2020-11-05|EPI_ISL_656834|Original|hCoV-19^^England|Human|Lighthouse Lab in Milton Keynes|Wellcome Sanger Institute for the COVID-19 Genomics UK (COG-UK) Consortium|Davies|United Kingdom

>Spike|hCoV-19/USA/WA-S1008/2020|2020-04-29|EPI_ISL_463498|Original|hCoV-19^^Washington|Human|Washington State Department of Health|Seattle Flu Study|Bedford|USA
>Spike|hCoV-19/Brazil/SP-L19-CD393/2020|2020-04-02|EPI_ISL_476321|Original|hCoV-19^^S\u00e3o Paulo|Human|DB Diagn\u00f3sticos do Brasil|Instituto de Medicina Tropical da Univesidade de S\u00e3o Paulo|Faria|Brazil
>Spike|hCoV-19/USA/MI-MDHHS-SC21775/2020|2020-08-13|EPI_ISL_529893|Original|hCoV-19^^Michigan|Human|Michigan Department of Health and Human Services|Michigan Department of Health and Human Services|Blankenship|USA
>Spike|hCoV-19/England/ALDP-94F901/2020|2020-06-15|EPI_ISL_555161|Original|hCoV-19^^England|Human|Lighthouse Lab in Alderley Park|Wellcome Sanger Institute for the COVID-19 Genomics UK (COG-UK) consortium|Davies|United Kingdom
>Spike|hCoV-19/England/CAMC-BC1E8E/2020|2020-11-22|EPI_ISL_704309|Original|hCoV-19^^England|Human|Department of Pathology|COVID-19 Genomics UK (COG-UK) Consortium|Goodfellow|United Kingdom
>Spike|hCoV-19/Japan/PG-5200/2020|2020-08-00|EPI_ISL_690303|Original|hCoV-19^^Japan|Human|Pathogen Genomics Center|Pathogen Genomics Center|Sekizuka|Japan
>Spike|hCoV-19/England/QEUH-A0026A/2020|2020-09-30|EPI_ISL_600272|Original|hCoV-19^^England|Human|Lighthouse Lab in Glasgow|Wellcome Sanger Institute for the COVID-19 Genomics UK (COG-UK) consortium|Davies|United Kingdom
>Spike|hCoV-19/England/QEUH-9D0FC7/2020|2020-09-19|EPI_ISL_589989|Original|hCoV-19^^England|Human|Lighthouse Lab in Glasgow|Wellcome Sanger Institute for the COVID-19 Genomics UK (COG-UK) consortium|Davies|United Kingdom
>Spike|hCoV-19/England/LIVE-A361C/2020|2020-06-11|EPI_ISL_490386|Original|hCoV-19^^England|Human|Liverpool Clinical Laboratories|COVID-19 Genomics UK (COG-UK) Consortium|Haldenby|United Kingdom
>Spike|hCoV-19/United Arab Emirates/0131/2020|2020-05-18|EPI_ISL_698321|Original|hCoV-19^^Abu Dhabi|Human|Group 42 (G42) Healthcare|G42 Healthcare|Ogrodzki|United Arab Emirates
>Spike|hCoV-19/Russia/SVE-RII-MH4633S/2020|2020-08-28|EPI_ISL_733223|Original|hCoV-19^^Sverdlovsk|Human|UMMC-Health|WHO National Influenza Centre Russian Federation|Komissarov|Russia
>Spike|hCoV-19/USA/TX-HMH-4256/2020|2020-06-19|EPI_ISL_543468|Original|hCoV-19^^Texas|Human|Houston Methodist Hospital|Houston Methodist Hospital|Long|USA
>Spike|hCoV-19/England/ALDP-C8B809/2020|2020-12-17|EPI_ISL_756651|Original|hCoV-19^^England|Human|Lighthouse Lab in Alderley Park|Wellcome Sanger Institute for the COVID-19 Genomics UK (COG-UK) Consortium|Davies|United Kingdom
>Spike|hCoV-19/England/QEUH-A60C97/2020|2020-10-11|EPI_ISL_665305|Original|hCoV-19^^England|Human|Northumbria University / South Tees Hospitals NHS Foundation Trust / North Cumbria Integrated Care NHS Foundation Trust / North Tees and Hartlepool NHS Foundation Trust / Newcastle Hospitals NHS Foundation Trust|COVID-19 Genomics UK (COG-UK) Consortium|Bashton|United Kingdom
>Spike|hCoV-19/New Zealand/20VR2666/2020|2020-00-00|EPI_ISL_579328|Original|hCoV-19^^Auckland|Human|LabPLUS|Institute of Environmental Science and Research (ESR)|Wang|New Zealand
>Spike|hCoV-19/USA/MD-HP12116/2021|2021-01-04|EPI_ISL_791430|Original|hCoV-19^^Maryland|Human|Johns Hopkins Hospital Department of Pathology|Johns Hopkins Hospital Department of Pathology|Morris|USA
>Spike|hCoV-19/USA/WI-UW-1452/2020|2020-09-12|EPI_ISL_541584|Original|hCoV-19^^Wisconsin|Human|University of Wisconsin-Madison AIDS Vaccine Research Laboratories|University of Wisconsin-Madison AIDS Vaccine Research Laboratories|Baczenas|USA
>Spike|hCoV-19/USA/MI-UM-10036324614/2020|2020-10-07|EPI_ISL_596614|Original|hCoV-19^^Michigan|Human|University of Michigan Clinical Microbiology Laboratory|Lauring Lab|Valesano|USA
>Spike|hCoV-19/England/QEUH-BA016E/2020|2020-11-18|EPI_ISL_719621|Original|hCoV-19^^England|Human|Lighthouse Lab in Glasgow|Wellcome Sanger Institute for the COVID-19 Genomics UK (COG-UK) Consortium|Davies|United Kingdom

>Spike|hCoV-19/England/MILK-A7C111/2020|2020-10-15|EPI_ISL_606439|Original|hCoV-19^^England|Human|Lighthouse Lab in Milton Keynes|Wellcome Sanger Institute for the COVID-19 Genomics UK (COG-UK) consortium|Davies|United Kingdom
>Spike|hCoV-19/USA/WY-WPHL-20061819/2020|2020-08-14|EPI_ISL_769905|Original|hCoV-19^^Wyoming|Human|Wyoming Public Health Laboratory|Wyoming Public Health Laboratory|Hull|USA
>Spike|hCoV-19/England/ALDP-C5886F/2020|2020-12-14|EPI_ISL_735887|Original|hCoV-19^^England|Human|Lighthouse Lab in Alderley Park|Wellcome Sanger Institute for the COVID-19 Genomics UK (COG-UK) Consortium|Davies|United Kingdom
>Spike|hCoV-19/USA/TX-HMH-1213/2020|2020-03-31|EPI_ISL_542587|Original|hCoV-19^^Texas|Human|Houston Methodist Hospital|Houston Methodist Hospital|Long|USA
>Spike|hCoV-19/Canada/ON-UHTC-0293/2020|2020-07-30|EPI_ISL_569980|Original|hCoV-19^^Ontario|Human|Unity Health Toronto|Ontario Institute for Cancer Research|de Borja|Canada
>Spike|hCoV-19/USA/FL-BPHL-1535/2020|2020-05-14|EPI_ISL_568633|Original|hCoV-19^^Florida|Human|Florida Bureau of Public Health Laboratories|Florida Bureau of Public Health Laboratories|Blanton|USA
>Spike|hCoV-19/Northern Ireland/NIRE-101F5C/2020|2020-03-23|EPI_ISL_441358|Original|hCoV-19^^Northern Ireland|Human|Regional Virus Laboratory|COVID-19 Genomics UK (COG-UK) Consortium|Fairley|United Kingdom
>Spike|hCoV-19/England/SHEF-C15FD/2020|2020-03-30|EPI_ISL_432726|Original|hCoV-19^^England|Human|Virology Department|COVID-19 Genomics UK (COG-UK) Consortium|de Silva|United Kingdom
>Spike|hCoV-19/USA/UT-UPHL-04312_2/2020|2020-05-15|EPI_ISL_591303|Original|hCoV-19^^Utah|Human|Utah Public Health Laboratory|Utah Public Health Laboratory|Young|USA
>Spike|hCoV-19/Wales/PHWC-33CF1/2020|2020-04-18|EPI_ISL_446813|Original|hCoV-19^^Wales|Human|Wales Specialist Virology Centre|Public Health Wales Microbiology Cardiff|Moore|United Kingdom
>Spike|hCoV-19/Norway/7180/2020|2020-12-27|EPI_ISL_775428|Original|hCoV-19^^Viken|Human|Department of Medical Microbiology - section Molde|Norwegian Institute of Public Health|Vollan|Norway
>Spike|hCoV-19/England/SHEF-10CDEC6/2020|2020-12-15|EPI_ISL_742007|Original|hCoV-19^^England|Human|Virology Department|COVID-19 Genomics UK (COG-UK) Consortium|de Silva|United Kingdom
>Spike|hCoV-19/Germany/NW-HHU-290/2020|2020-09-30|EPI_ISL_770765|Original|hCoV-19^^North Rhine-Westphalia|Human|ZOTZ KLIMAS MVZ D\u00fcsseldorf-Centrum GbR \u00dcBAG f\u00fcr Labormedizin|Center of Medical Microbiology|Wienemann|Germany
>Spike|hCoV-19/USA/TX-HMH-5738/2020|2020-06-24|EPI_ISL_546979|Original|hCoV-19^^Texas|Human|Houston Methodist Hospital|Houston Methodist Hospital|Long|USA
>Spike|hCoV-19/Wales/PHWC-16C960/2020|2020-09-22|EPI_ISL_574114|Original|hCoV-19^^Wales|Human|Wales Specialist Virology Centre Sequencing lab|COVID-19 Genomics UK (COG-UK) Consortium|Moore|United Kingdom
>Spike|hCoV-19/England/CAMB-74474/2020|2020-03-19|EPI_ISL_425293|Original|hCoV-19^^England|Human|Department of Pathology|COVID-19 Genomics UK (COG-UK) Consortium|Meredith|United Kingdom
>Spike|hCoV-19/Denmark/DCGC-13367/2020|2020-11-30|EPI_ISL_712753|Original|hCoV-19^^Syddanmark|Human|Department of Virus and Microbiological Special Diagnostics|Albertsen Lab|Michaelsen|Denmark
>Spike|hCoV-19/India/GJ-GBRC236/2020|2020-06-12|EPI_ISL_476878|Original|hCoV-19^^Gujarat|Human|Department of MicroBiology|Gujarat Biotechnology Research Centre|Joshi|India
>Spike|hCoV-19/Canada/ON-PHL-20-03673/2020|2020-11-00|EPI_ISL_778848|Original|hCoV-19^^Ontario|Human|Public Health Ontario Laboratory|Public Health Ontario Laboratory|Guthrie|Canada
>Spike|hCoV-19/England/ALDP-5AB257/2020|2020-06-15|EPI_ISL_558658|Original|hCoV-19^^England|Human|Lighthouse Lab in Alderley Park|Wellcome Sanger Institute for the COVID-19 Genomics UK (COG-UK) consortium|Davies|United Kingdom
>Spike|hCoV-19/Norway/CS-07/2020|2020-04-20|EPI_ISL_665256|Original|hCoV-19^^Viken|Human|Dept. of Microbiology and Infection Control|Dept. of Microbiology and Infection Control|Hesselberg L\u00f8vestad|Norway

>Spike|hCoV-19/Australia/VIC13538/2020|2020-08-03|EPI_ISL_563741|Original|hCoV-19^^Victoria|Human|Microbiological Diagnostic Unit - Public Health Laboratory (MDU-PHL)|MDU-PHL|Schultz|Australia
>Spike|hCoV-19/Scotland/CVR5519/2020|2020-11-01|EPI_ISL_651826|Original|hCoV-19^^Scotland|Human|West of Scotland Specialist Virology Centre|COVID-19 Genomics UK (COG-UK) Consortium|Robertson|United Kingdom
>Spike|hCoV-19/England/BRIS-12E29C/2020|2020-04-19|EPI_ISL_481992|Original|hCoV-19^^England|Human|PHE South West Regional Laboratory|Wellcome Sanger Institute for the COVID-19 Genomics UK (COG-UK) consortium|Davies|United Kingdom
>Spike|hCoV-19/France/OCC-43/2020|2020-06-19|EPI_ISL_593858|Original|hCoV-19^^Occitanie|Human|CHU Purpan - Laboratoire de Virologie - Institut F\u00e9d\u00e9ratif de Biologie|CHU Purpan - Laboratoire de Virologie - Institut F\u00e9d\u00e9ratif de Biologie|Latour|France
>Spike|hCoV-19/Switzerland/GE-SNRCI-7960/2020|2020-03-00|EPI_ISL_548211|Original|hCoV-19^^Geneve|Human|Laboratoire de Virologie|Swiss National Reference Centre for Influenza|Gon\u00e7alves Cabecinhas|Switzerland
>Spike|hCoV-19/USA/MA-MGH-00581/2020|2020-03-28|EPI_ISL_460426|Original|hCoV-19^^Massachusetts|Human|Massachusetts General Hospital|Infectious Disease Program|Lemieux|USA
>Spike|hCoV-19/England/MILK-9713CC/2020|2020-08-18|EPI_ISL_553172|Original|hCoV-19^^England|Human|Lighthouse Lab in Milton Keynes|Wellcome Sanger Institute for the COVID-19 Genomics UK (COG-UK) consortium|Davies|United Kingdom
>Spike|hCoV-19/England/ALDP-94CBA4/2020|2020-06-15|EPI_ISL_555965|Original|hCoV-19^^England|Human|Lighthouse Lab in Alderley Park|Wellcome Sanger Institute for the COVID-19 Genomics UK (COG-UK) consortium|Davies|United Kingdom
>Spike|hCoV-19/USA/TX-HMH-3064/2020|2020-06-10|EPI_ISL_543969|Original|hCoV-19^^Texas|Human|Houston Methodist Hospital|Houston Methodist Hospital|Long|USA
>Spike|hCoV-19/England/MILK-A7BB9C/2020|2020-10-15|EPI_ISL_607438|Original|hCoV-19^^England|Human|Lighthouse Lab in Milton Keynes|Wellcome Sanger Institute for the COVID-19 Genomics UK (COG-UK) consortium|Davies|United Kingdom
>Spike|hCoV-19/Japan/IC-0560/2021|2021-01-00|EPI_ISL_779243|Original|hCoV-19^^Japan|Human|Pathogen Genomics Center|Pathogen Genomics Center|Sekizuka|Japan
>Spike|hCoV-19/Australia/VIC6593/2020|2020-07-17|EPI_ISL_521355|Original|hCoV-19^^Victoria|Human|Victorian Infectious Diseases Reference Laboratory (VIDRL)|VIDRL and MDU-PHL|Schultz|Australia
>Spike|hCoV-19/Scotland/QEUH-9F4A53/2020|2020-09-27|EPI_ISL_600699|Original|hCoV-19^^Scotland|Human|Lighthouse Lab in Glasgow|Wellcome Sanger Institute for the COVID-19 Genomics UK (COG-UK) consortium|Davies|United Kingdom
>Spike|hCoV-19/Japan/PG-0410/2020|2020-03-30|EPI_ISL_480079|Original|hCoV-19^^Kanto|Human|Shizuoka City Institute of Environmental Sciences and Public Health|Pathogen Genomics Center|Sekizuka|Japan
>Spike|hCoV-19/England/QEUH-AE2B54/2020|2020-10-27|EPI_ISL_633442|Original|hCoV-19^^England|Human|Lighthouse Lab in Glasgow|Wellcome Sanger Institute for the COVID-19 Genomics UK (COG-UK) consortium|Davies|United Kingdom
>Spike|hCoV-19/USA/UT-UPHL-2009656/2020|2020-07-26|EPI_ISL_605331|Original|hCoV-19^^Utah|Human|Utah Public Health Laboratory|Utah Public Health Laboratory|Young|USA
>Spike|hCoV-19/England/CAMB-768A3/2020|2020-03-30|EPI_ISL_438350|Original|hCoV-19^^England|Human|Department of Pathology|Wellcome Sanger Institute for the COVID-19 Genomics UK (COG-UK) consortium|Amato|United Kingdom
>Spike|hCoV-19/Finland/26A48S5/2020|2020-04-26|EPI_ISL_481620|Original|hCoV-19^^Uusimaa|Human|Department of Virology and Immunology|Department of Virology|Smura|Finland
>Spike|hCoV-19/France/PAC-SC783/2020|2020-12-29|EPI_ISL_779849|Original|hCoV-19^^Provence-Alpes-C\u00f4te d'Azur|Human|Laboratoire BIOMED 05|CNR Virus des Infections Respiratoires - France SUD|Bal|France
>Spike|hCoV-19/USA/WA-UW-24020/2020|2020-08-13|EPI_ISL_570762|Original|hCoV-19^^Washington|Human|UW Virology Lab|UW Virology Lab|Roychoudhury|USA
>Spike|hCoV-19/Australia/VIC4594/2020|2020-07-12|EPI_ISL_518995|Original|hCoV-19^^Victoria|Human|Victorian Infectious Diseases Reference Laboratory (VIDRL)|VIDRL and MDU-PHL|Schultz|Australia

>Spike|hCoV-19/Bangladesh/CHRF-0058/2020|2020-06-19|EPI_ISL_625471|Original|hCoV-
19^^Bangladesh|Human|Child Health Research Foundation|Child Health Research
Foundation|Saha|Bangladesh
>Spike|hCoV-19/England/CAMB-1B0E8C/2020|2020-05-11|EPI_ISL_452971|Original|hCoV-
19^^England|Human|Department of Pathology|COVID-19 Genomics UK (COG-UK)
Consortium|Meredith|United Kingdom
>Spike|hCoV-19/Switzerland/ZH-UZH-1000482982/2020|2020-03-
15|EPI_ISL_508864|Original|hCoV-19^^Zurich|Human|Division of Infectious Diseases and
Hospital Epidemiology|Institute of Medical Virology|Huber|Switzerland
>Spike|hCoV-19/England/QEUH-BC4191/2020|2020-11-23|EPI_ISL_703399|Original|hCoV-
19^^England|Human|Lighthouse Lab in Glasgow|Wellcome Sanger Institute for the COVID-
19 Genomics UK (COG-UK) Consortium|Davies|United Kingdom
>Spike|hCoV-19/USA/UT-UPHL-2012379451/2020|2020-08-03|EPI_ISL_745721|Original|hCoV-
19^^Utah|Human|Ginkgo Bioworks Clinical Laboratory|Utah Public Health
Laboratory|Young|USA
>Spike|hCoV-19/Australia/VIC7405/2020|2020-07-30|EPI_ISL_564387|Original|hCoV-
19^^Victoria|Human|Victorian Infectious Diseases Reference Laboratory (VIDRL)|VIDRL
and MDU-PHL|Schultz|Australia
>Spike|hCoV-19/Scotland/QEUH-9CEFCC/2020|2020-09-17|EPI_ISL_601915|Original|hCoV-
19^^Scotland|Human|Lighthouse Lab in Glasgow|Wellcome Sanger Institute for the COVID-
19 Genomics UK (COG-UK) consortium|Davies|United Kingdom
>Spike|hCoV-19/USA/MI-UM-10036598511/2020|2020-10-23|EPI_ISL_649977|Original|hCoV-
19^^Michigan|Human|University of Michigan Clinical Microbiology Laboratory|Lauring
Lab|Valesano|USA
>Spike|hCoV-19/Wales/PHWC-49B67D/2020|2020-12-03|EPI_ISL_742365|Original|hCoV-
19^^Wales|Human|Wales Specialist Virology Centre Sequencing lab|COVID-19 Genomics UK
(COG-UK) Consortium|Moore|United Kingdom
>Spike|hCoV-19/England/QEUH-9D01A3/2020|2020-09-16|EPI_ISL_589858|Original|hCoV-
19^^England|Human|Lighthouse Lab in Glasgow|Wellcome Sanger Institute for the COVID-
19 Genomics UK (COG-UK) consortium|Davies|United Kingdom
>Spike|hCoV-19/USA/HI-H200232/2020|2020-08-12|EPI_ISL_752791|Original|hCoV-
19^^Hawaii|Human|State Laboratories Division|State Laboratories Division|Sultana|USA
>Spike|hCoV-19/Denmark/DCGC-8710/2020|2020-10-26|EPI_ISL_626024|Original|hCoV-
19^^Hovedstaden|Human|Department of Virus and Microbiological Special
Diagnostics|Albertsen lab|Michaelsen|Denmark
>Spike|hCoV-19/Switzerland/BL-ETHZ-140062/2020|2020-04-
30|EPI_ISL_476110|Original|hCoV-19^^Basel-Landschaft|Human|Viollier AG|Department of
Biosystems Science and Engineering|Nadeau|Switzerland
>Spike|hCoV-19/USA/CA-CZB-10001/2020|2020-04-01|EPI_ISL_672184|Original|hCoV-
19^^California|Human|The Ashley Laboratory|Chan-Zuckerberg Biohub|Bhatt|USA
>Spike|hCoV-19/England/CAMC-A57307/2020|2020-10-09|EPI_ISL_597424|Original|hCoV-
19^^England|Human|Lighthouse Lab in Cambridge|Wellcome Sanger Institute for the
COVID-19 Genomics UK (COG-UK) consortium|Davies|United Kingdom
>Spike|hCoV-19/USA/AZ-TG491491/2020|2020-06-17|EPI_ISL_695122|Original|hCoV-
19^^Arizona|Human|AZ SPHL|TGen North|French|USA
>Spike|hCoV-19/Canada/QC-LSPQ-L00215586/2020|2020-03-12|EPI_ISL_535732|Original|hCoV-
19^^Quebec|Human|CHU Sainte-Justine|Laboratoire de sant\u00e9 publique du
Qu\u00e9bec|Moreira|Canada
>Spike|hCoV-19/England/MILK-C6C613/2020|2020-12-13|EPI_ISL_778498|Original|hCoV-
19^^England|Human|Lighthouse Lab in Milton Keynes|Wellcome Sanger Institute for the
COVID-19 Genomics UK (COG-UK) Consortium|Davies|United Kingdom
>Spike|hCoV-19/France/PAC-IHU-1131/2020|2020-08-07|EPI_ISL_569055|Original|hCoV-
19^^Provence-Alpes-C\u00f4te-d\u2019Azur|Human|MEPHI|MEPHI|Levasseur|France
>Spike|hCoV-19/England/QEUH-95E671/2020|2020-08-07|EPI_ISL_532376|Original|hCoV-
19^^England|Human|Lighthouse Lab in Glasgow|Wellcome Sanger Institute for the COVID-
19 Genomics UK (COG-UK) consortium|Davies|United Kingdom
>Spike|hCoV-19/Wales/PHWC-493842/2020|2020-11-29|EPI_ISL_725949|Original|hCoV-
19^^Wales|Human|Originating lab|COVID-19 Genomics UK (COG-UK) Consortium|Moore|United
Kingdom

>Spike|hCoV-19/England/CWAR-24FDB56/2020|2020-05-02|EPI_ISL_584485|Original|hCoV-19^^England|Human|UHCW / University of Warwick|COVID-19 Genomics UK (COG-UK) Consortium|Stark|United Kingdom
>Spike|hCoV-19/USA/NY-NYUMC801/2020|2020-03-27|EPI_ISL_456037|Original|hCoV-19^^New York|Human|NYU Langone Health|Departments of Pathology and Medicine|Maurano|USA
>Spike|hCoV-19/Wales/PHWC-48ED0F/2020|2020-11-10|EPI_ISL_680994|Original|hCoV-19^^Wales|Human|Wales Specialist Virology Centre Sequencing lab|COVID-19 Genomics UK (COG-UK) Consortium|Moore|United Kingdom
>Spike|hCoV-19/Denmark/DCGC-14262/2020|2020-11-30|EPI_ISL_711339|Original|hCoV-19^^Sjaelland|Human|Department of Virus and Microbiological Special Diagnostics|Albertsen Lab|Michaelsen|Denmark
>Spike|hCoV-19/England/CAMC-B9C60C/2020|2020-11-15|EPI_ISL_673480|Original|hCoV-19^^England|Human|Lighthouse Lab in Cambridge|Wellcome Sanger Institute for the COVID-19 Genomics UK (COG-UK) Consortium|Davies|United Kingdom
>Spike|hCoV-19/England/LIVE-9A566/2020|2020-03-26|EPI_ISL_449345|Original|hCoV-19^^England|Human|Liverpool Clinical Laboratories|COVID-19 Genomics UK (COG-UK) Consortium|Haldenby|United Kingdom
>Spike|hCoV-19/USA/WI-UW-252/2020|2020-03-24|EPI_ISL_428726|Original|hCoV-19^^Wisconsin|Human|University of Wisconsin-Madison AIDS Vaccine Research Laboratories|University of Wisconsin-Madison AIDS Vaccine Research Laboratories|Baczenas|USA
>Spike|hCoV-19/Netherlands/ZH-EMC-913/2020|2020-11-03|EPI_ISL_722707|Original|hCoV-19^^Zuid Holland|Human|Dutch COVID-19 response team|Erasmus Medical Center|Oude Munnink|Netherlands
>Spike|hCoV-19/USA/WI-UW-1290/2020|2020-09-02|EPI_ISL_536662|Original|hCoV-19^^Wisconsin|Human|University of Wisconsin-Madison AIDS Vaccine Research Laboratories|University of Wisconsin-Madison AIDS Vaccine Research Laboratories|Baczenas|USA
>Spike|hCoV-19/France/PDL-SC600/2020|2020-03-11|EPI_ISL_649173|Original|hCoV-19^^Pays-de-la-Loire|Human|CHU Nantes|CNR Virus des Infections Respiratoires - France SUD|Bal|France
>Spike|hCoV-19/India/WB-IK54/2020|2020-05-06|EPI_ISL_508380|Original|hCoV-19^^West Bengal|Human|Institute of Post Graduate Medical Education & Research|National Institute of Biomedical Genomics|Maitra|India
>Spike|hCoV-19/USA/WI-UW-936/2020|2020-05-05|EPI_ISL_509936|Original|hCoV-19^^Wisconsin|Human|University of Wisconsin-Madison AIDS Vaccine Research Laboratories|University of Wisconsin-Madison AIDS Vaccine Research Laboratories|Baczenas|USA
>Spike|hCoV-19/Luxembourg/LNS8040373/2020|2020-11-05|EPI_ISL_744673|Original|hCoV-19^^Luxembourg|Human|Laboratoire national de sant\u00e9|Laboratoire national de sant\u00e9|Wienecke-Baldacchino|Luxembourg
>Spike|hCoV-19/Scotland/CVR3793/2020|2020-05-16|EPI_ISL_478121|Original|hCoV-19^^Scotland|Human|West of Scotland Specialist Virology Centre|COVID-19 Genomics UK (COG-UK) Consortium|Robertson|United Kingdom
>Spike|hCoV-19/England/MILK-99DF6C/2020|2020-08-31|EPI_ISL_550716|Original|hCoV-19^^England|Human|Lighthouse Lab in Milton Keynes|Wellcome Sanger Institute for the COVID-19 Genomics UK (COG-UK) consortium|Davies|United Kingdom
>Spike|hCoV-19/Germany/NW-HHU-25/2020|2020-03-15|EPI_ISL_419542|Original|hCoV-19^^North Rhine-Westphalia|Human|Center of Medical Microbiology|Center of Medical Microbiology|Wienemann|Germany
>Spike|hCoV-19/Netherlands/ZH-RIVM-20685/2020|2020-12-23|EPI_ISL_790753|Original|hCoV-19^^Zuid-holland|Human|Dutch COVID-19 response team|National Institute for Public Health and the Environment (RIVM)|Meijer|Netherlands
>Spike|hCoV-19/Luxembourg/LNS2264925/2020|2020-11-09|EPI_ISL_740087|Original|hCoV-19^^Luxembourg|Human|Laboratoire national de sant\u00e9|Laboratoire national de sant\u00e9|Wienecke-Baldacchino|Luxembourg
>Spike|hCoV-19/England/EXET-136871/2020|2020-04-28|EPI_ISL_457238|Original|hCoV-19^^England|Human|University of Exeter|COVID-19 Genomics UK (COG-UK) Consortium|Temperton|United Kingdom

>Spike|hCoV-19/USA/TX-HMH-MCoV-19703/2020|2020-11-25|EPI_ISL_784056|Original|hCoV-19^^Texas|Human|Houston Methodist Hospital|Houston Methodist Hospital|Long|USA
>Spike|hCoV-19/England/LIVE-9D74F/2020|2020-04-11|EPI_ISL_453229|Original|hCoV-19^^England|Human|Liverpool Clinical Laboratories|COVID-19 Genomics UK (COG-UK) Consortium|Haldenby|United Kingdom
>Spike|hCoV-19/Wales/PHWC-49F5B8/2020|2020-12-08|EPI_ISL_743237|Original|hCoV-19^^Wales|Human|Wales Specialist Virology Centre Sequencing lab|COVID-19 Genomics UK (COG-UK) Consortium|Moore|United Kingdom
>Spike|hCoV-19/England/ALDP-9D990C/2020|2020-09-18|EPI_ISL_576771|Original|hCoV-19^^England|Human|Oxford Viromics|COVID-19 Genomics UK (COG-UK) Consortium|Golubchik|United Kingdom
>Spike|hCoV-19/England/ALDP-A85537/2020|2020-10-18|EPI_ISL_610559|Original|hCoV-19^^England|Human|Lighthouse Lab in Alderley Park|Wellcome Sanger Institute for the COVID-19 Genomics UK (COG-UK) consortium|Davies|United Kingdom
>Spike|hCoV-19/Canada/BC-BCCDC-6429/2020|2020-10-00|EPI_ISL_740582|Original|hCoV-19^^British Columbia|Human|BCCDC Public Health Laboratory|BCCDC Public Health Laboratory|Prystajecky|Canada
>Spike|hCoV-19/England/QEUH-79F807/2020|2020-07-11|EPI_ISL_533179|Original|hCoV-19^^England|Human|Lighthouse Lab in Glasgow|Wellcome Sanger Institute for the COVID-19 Genomics UK (COG-UK) consortium|Davies|United Kingdom
>Spike|hCoV-19/South Korea/KDCA0456/2020|2020-11-17|EPI_ISL_760240|Original|hCoV-19^^South Korea|Human|Division of Emerging Infectious Diseases|Division of Emerging Infectious Diseases|Park|South Korea
>Spike|hCoV-19/Australia/VIC10758/2020|2020-09-02|EPI_ISL_563075|Original|hCoV-19^^Victoria|Human|Microbiological Diagnostic Unit - Public Health Laboratory (MDU-PHL)|MDU-PHL|Schultz|Australia
>Spike|hCoV-19/England/QEUH-B13E50/2020|2020-11-02|EPI_ISL_641709|Original|hCoV-19^^England|Human|Lighthouse Lab in Glasgow|Wellcome Sanger Institute for the COVID-19 Genomics UK (COG-UK) Consortium|Davies|United Kingdom
>Spike|hCoV-19/USA/MI-UM-10036535538/2020|2020-10-20|EPI_ISL_640150|Original|hCoV-19^^Michigan|Human|University of Michigan Clinical Microbiology Laboratory|Lauring Lab|Valesano|USA
>Spike|hCoV-19/Denmark/DCGC-8165/2020|2020-10-26|EPI_ISL_617240|Original|hCoV-19^^Hovedstaden|Human|Department of Virus and Microbiological Special Diagnostics|Albertsen lab|Michaelsen|Denmark
>Spike|hCoV-19/Belgium/ULG-10691/2020|2020-10-26|EPI_ISL_636663|Original|hCoV-19^^Li\u00e8ge|Human|Department of Clinical Microbiology|GIGA Medical Genomics|Durkin|Belgium
>Spike|hCoV-19/India/DL-IGIB11303760/2020|2020-03-00|EPI_ISL_751297|Original|hCoV-19^^Delhi|Human|Devki Devi Foundation|CSIR-IGIB/Max|Pandey|India
>Spike|hCoV-19/England/NORW-EB979/2020|2020-05-06|EPI_ISL_457460|Original|hCoV-19^^England|Human|Quadram Institute Bioscience|COVID-19 Genomics UK (COG-UK) Consortium|Alikhan|United Kingdom
>Spike|hCoV-19/England/CAMB-74A09/2020|2020-03-18|EPI_ISL_425342|Original|hCoV-19^^England|Human|Department of Pathology|COVID-19 Genomics UK (COG-UK) Consortium|Meredith|United Kingdom
>Spike|hCoV-19/USA/UT-UPHL-2012624197/2020|2020-10-02|EPI_ISL_745592|Original|hCoV-19^^Utah|Human|Ginkgo Bioworks Clinical Laboratory|Utah Public Health Laboratory|Young|USA
>Spike|hCoV-19/Australia/VIC12182/2020|2020-07-27|EPI_ISL_591923|Original|hCoV-19^^Victoria|Human|Microbiological Diagnostic Unit - Public Health Laboratory (MDU-PHL)|MDU-PHL|Schultz|Australia
>Spike|hCoV-19/England/ALDP-9E99DC/2020|2020-09-24|EPI_ISL_584275|Original|hCoV-19^^England|Human|Virology Department|COVID-19 Genomics UK (COG-UK) Consortium|de Silva|United Kingdom
>Spike|hCoV-19/USA/WI-CDC-2-3693678/2020|2020-11-07|EPI_ISL_747080|Original|hCoV-19^^Wisconsin|Human|Respiratory Viruses Branch|Respiratory Viruses Branch|Queen|USA
>Spike|hCoV-19/Oman/RESP-20-2282/2020|2020-03-14|EPI_ISL_457974|Original|hCoV-19^^Muscat|Human|Oman-NIC|Oman-NIC|Al Mahruqi|Oman

>Spike|hCoV-19/USA/HI-H200636/2020|2020-11-23|EPI_ISL_753160|Original|hCoV-
19^^Hawaii|Human|State Laboratories Division|State Laboratories Division|Sultana|USA
>Spike|hCoV-19/Canada/MB-NML-1023/2020|2020-04-18|EPI_ISL_582388|Original|hCoV-
19^^Manitoba|Human|Cadham Provincial Laboratory|National Microbiology Laboratory
(NML)|Majer|Canada
>Spike|hCoV-19/France/PAC-IHU-0653/2020|2020-03-20|EPI_ISL_568916|Original|hCoV-
19^^Provence-Alpes-C\u00f4te-d\u2019Azur|Human|MEPHI|MEPHI|Levasseur|France
>Spike|hCoV-19/USA/FL-BPHL-1286/2020|2020-08-28|EPI_ISL_541218|Original|hCoV-
19^^Florida|Human|Florida Bureau of Public Health Laboratories|Florida Bureau of
Public Health Laboratories|Blanton|USA
>Spike|hCoV-19/Germany/SH-ChVir8325/2020|2020-07-28|EPI_ISL_729580|Original|hCoV-
19^^Schleswig-Holstein|Human|A. Krumbholz|Charit\u00e9 Universit\u00e4tsmedizin
Berlin|Muehlemann|Germany
>Spike|hCoV-19/USA/TX-HMH-5701/2020|2020-06-23|EPI_ISL_546953|Original|hCoV-
19^^Texas|Human|Houston Methodist Hospital|Houston Methodist Hospital|Long|USA
>Spike|hCoV-19/Australia/VIC13167/2020|2020-07-26|EPI_ISL_592199|Original|hCoV-
19^^Victoria|Human|Microbiological Diagnostic Unit - Public Health Laboratory (MDU-
PHL)|MDU-PHL|Schultz|Australia
>Spike|hCoV-19/Denmark/DCGC-3817/2020|2020-08-17|EPI_ISL_622678|Original|hCoV-
19^^Hovedstaden|Human|Department of Virus and Microbiological Special
Diagnostics|Albertsen lab|Michaelsen|Denmark
>Spike|hCoV-19/USA/VA-DCLS-1240/2020|2020-07-30|EPI_ISL_528659|Original|hCoV-
19^^Virginia|Human|Virginia DCLS|Virginia DCLS|St. Jacques|USA
>Spike|hCoV-19/Northern Ireland/NIRE-102067/2020|2020-03-
22|EPI_ISL_441372|Original|hCoV-19^^Northern Ireland|Human|Regional Virus
Laboratory|COVID-19 Genomics UK (COG-UK) Consortium|Fairley|United Kingdom
>Spike|hCoV-19/England/ALDP-B8333E/2020|2020-11-14|EPI_ISL_675568|Original|hCoV-
19^^England|Human|Lighthouse Lab in Alderley Park|Wellcome Sanger Institute for the
COVID-19 Genomics UK (COG-UK) Consortium|Davies|United Kingdom
>Spike|hCoV-19/England/ALDP-B23B74/2020|2020-11-04|EPI_ISL_646156|Original|hCoV-
19^^England|Human|Lighthouse Lab in Alderley Park|Wellcome Sanger Institute for the
COVID-19 Genomics UK (COG-UK) Consortium|Davies|United Kingdom
>Spike|hCoV-19/USA/VA-DCLS-2073/2020|2020-10-21|EPI_ISL_677181|Original|hCoV-
19^^Virginia|Human|Virginia Division of Consolidated Laboratory Services|Virginia
Division of Consolidated Laboratory Services|St. Jacques|USA
>Spike|hCoV-19/Denmark/DCGC-10976/2020|2020-11-02|EPI_ISL_670320|Original|hCoV-
19^^Midtjylland|Human|Department of Virus and Microbiological Special
Diagnostics|Albertsen Lab|Michaelsen|Denmark
>Spike|hCoV-19/Wales/PHWC-162751/2020|2020-05-27|EPI_ISL_473115|Original|hCoV-
19^^Wales|Human|Wales Specialist Virology Centre Sequencing lab|COVID-19 Genomics UK
(COG-UK) Consortium|Moore|United Kingdom
>Spike|hCoV-19/Austria/CeMM0663/2020|2020-05-15|EPI_ISL_583631|Original|hCoV-
19^^Austria|Human|Austrian Agency for Health and Food Safety (AGES)|Bergthaler
laboratory|Bergthaler|Austria
>Spike|hCoV-19/Australia/VIC1619/2020|2020-05-08|EPI_ISL_456527|Original|hCoV-
19^^Victoria|Human|Victorian Infectious Diseases Reference Laboratory
(VIDRL)|Microbiological Diagnostic Unit Public Health Laboratory and Victorian
Infectious Diseases Reference Laboratory|Schultz|Australia
>Spike|hCoV-19/England/QEUH-B11A03/2020|2020-11-02|EPI_ISL_642442|Original|hCoV-
19^^England|Human|Lighthouse Lab in Glasgow|Wellcome Sanger Institute for the COVID-
19 Genomics UK (COG-UK) Consortium|Davies|United Kingdom
>Spike|hCoV-19/Australia/VIC2242/2020|2020-06-24|EPI_ISL_521975|Original|hCoV-
19^^Victoria|Human|Victorian Infectious Diseases Reference Laboratory (VIDRL)|VIDRL
and MDU-PHL|Schultz|Australia
>Spike|hCoV-19/USA/NM-DOH-2020313106/2020|2020-08-26|EPI_ISL_574263|Original|hCoV-
19^^New Mexico|Human|New Mexico Department of Health Scientific Laboratory|New Mexico
Department of Health Scientific Laboratory|Johnson|USA
>Spike|hCoV-19/Denmark/DCGC-9690/2020|2020-11-09|EPI_ISL_669112|Original|hCoV-
19^^Nordjylland|Human|Department of Virus and Microbiological Special
Diagnostics|Albertsen Lab|Michaelsen|Denmark

>Spike|hCoV-19/Denmark/DCGC-4937/2020|2020-09-21|EPI_ISL_621475|Original|hCoV-19^^Nordjylland|Human|Department of Virus and Microbiological Special Diagnostics|Albertsen lab|Michaelsen|Denmark
>Spike|hCoV-19/USA/TX-DSHS-0725/2020|2020-05-13|EPI_ISL_584128|Original|hCoV-19^^Texas|Human|Texas Department of State Health Services|Texas Department of State Health Services|Rahman|USA
>Spike|hCoV-19/USA/NY-PV09490/2020|2020-03-20|EPI_ISL_450162|Original|hCoV-19^^New York|Human|MSHS Clinical Microbiology Laboratories|MSHS Pathogen Surveillance Program|van Bakel|USA
>Spike|hCoV-19/England/ALDP-B248C7/2020|2020-11-04|EPI_ISL_646801|Original|hCoV-19^^England|Human|Lighthouse Lab in Alderley Park|Wellcome Sanger Institute for the COVID-19 Genomics UK (COG-UK) Consortium|Davies|United Kingdom
>Spike|hCoV-19/England/ALDP-9562FD/2020|2020-06-08|EPI_ISL_554019|Original|hCoV-19^^England|Human|Lighthouse Lab in Alderley Park|Wellcome Sanger Institute for the COVID-19 Genomics UK (COG-UK) consortium|Davies|United Kingdom
>Spike|hCoV-19/England/QEUH-AF0A65/2020|2020-10-29|EPI_ISL_662685|Original|hCoV-19^^England|Human|Lighthouse Lab in Glasgow|Wellcome Sanger Institute for the COVID-19 Genomics UK (COG-UK) Consortium|Davies|United Kingdom
>Spike|hCoV-19/England/LOND-DA685/2020|2020-11-09|EPI_ISL_724652|Original|hCoV-19^^England|Human|University College London|COVID-19 Genomics UK (COG-UK) Consortium|Roy|United Kingdom
>Spike|hCoV-19/USA/TX-HMH-6450/2020|2020-06-27|EPI_ISL_546004|Original|hCoV-19^^Texas|Human|Houston Methodist Hospital|Houston Methodist Hospital|Long|USA
>Spike|hCoV-19/England/CAMC-94B237/2020|2020-07-16|EPI_ISL_556710|Original|hCoV-19^^England|Human|Lighthouse Lab in Cambridge|Wellcome Sanger Institute for the COVID-19 Genomics UK (COG-UK) consortium|Davies|United Kingdom
>Spike|hCoV-19/Japan/Donner26/2020|2020-03-25|EPI_ISL_438970|Original|hCoV-19^^Kanto|Human|Keio University School of Medicine|Keio University School of Medicine|Kosaki|Japan
>Spike|hCoV-19/England/NORT-1B72065/2020|2020-11-23|EPI_ISL_705494|Original|hCoV-19^^England|Human|Northumbria University / South Tees Hospitals NHS Foundation Trust / North Cumbria Integrated Care NHS Foundation Trust / North Tees and Hartlepool NHS Foundation Trust / Newcastle Hospitals NHS Foundation Trust|COVID-19 Genomics UK (COG-UK) Consortium|Bashton|United Kingdom
>Spike|hCoV-19/England/QEUH-9A4032/2020|2020-09-01|EPI_ISL_540219|Original|hCoV-19^^England|Human|Lighthouse Lab in Glasgow|Wellcome Sanger Institute for the COVID-19 Genomics UK (COG-UK) consortium|Davies|United Kingdom
>Spike|hCoV-19/England/BRIS-125DFE/2020|2020-03-31|EPI_ISL_443829|Original|hCoV-19^^England|Human|PHE South West Regional Laboratory|Wellcome Sanger Institute for the COVID-19 Genomics UK (COG-UK) consortium|Amato|United Kingdom
>Spike|hCoV-19/USA/SD-SDPHL-0010/2020|2020-07-25|EPI_ISL_569618|Original|hCoV-19^^South Dakota|Human|Bethel Lutheran Home|South Dakota Public Health Laboratory|Carlson|USA
>Spike|hCoV-19/USA/CA-SEARCH-5558/2020|2020-11-25|EPI_ISL_755293|Original|hCoV-19^^California|Human|San Diego County Public Health Laboratory|Andersen lab at Scripps Research|Gangavarapu|USA
>Spike|hCoV-19/England/QEUH-B662DA/2020|2020-11-10|EPI_ISL_661566|Original|hCoV-19^^England|Human|Lighthouse Lab in Glasgow|Wellcome Sanger Institute for the COVID-19 Genomics UK (COG-UK) Consortium|Davies|United Kingdom
>Spike|hCoV-19/England/ALDP-9C63C8/2020|2020-09-10|EPI_ISL_567574|Original|hCoV-19^^England|Human|Lighthouse Lab in Alderley Park|Wellcome Sanger Institute for the COVID-19 Genomics UK (COG-UK) consortium|Davies|United Kingdom
>Spike|hCoV-19/Peru/ANC-UPCH-0051/2020|2020-06-16|EPI_ISL_540940|Original|hCoV-19^^Ancash|Human|Laboratorio de Referencia Nacional de Virus Respiratorios|Laboratorio de Gen\u00f3mica Microbiana|Tsukayama|Peru
>Spike|hCoV-19/England/LIVE-DC4AEF/2020|2020-11-11|EPI_ISL_702869|Original|hCoV-19^^England|Human|Liverpool Clinical Laboratories|COVID-19 Genomics UK (COG-UK) Consortium|Haldenby|United Kingdom

>Spike|hCoV-19/USA/CA-CZB-12811/2020|2020-09-09|EPI_ISL_648462|Original|hCoV-19^^California|Human|Santa Clara County Public Health Laboratory|Chan-Zuckerberg Biohub|Bhatt|USA
>Spike|hCoV-19/England/QEUH-C93877/2020|2020-12-19|EPI_ISL_777105|Original|hCoV-19^^England|Human|Lighthouse Lab in Glasgow|Wellcome Sanger Institute for the COVID-19 Genomics UK (COG-UK) Consortium|Davies|United Kingdom
>Spike|hCoV-19/Scotland/EDB6588/2020|2020-05-14|EPI_ISL_478269|Original|hCoV-19^^Scotland|Human|Virology Department|COVID-19 Genomics UK (COG-UK) Consortium|Williams|United Kingdom
>Spike|hCoV-19/Wales/PHWC-2B9B9/2020|2020-03-28|EPI_ISL_445788|Original|hCoV-19^^Wales|Human|Wales Specialist Virology Centre|Public Health Wales Microbiology Cardiff|Moore|United Kingdom
>Spike|hCoV-19/Australia/VIC2898/2020|2020-07-08|EPI_ISL_521729|Original|hCoV-19^^Victoria|Human|Microbiological Diagnostic Unit - Public Health Laboratory (MDU-PHL)|MDU-PHL|Schultz|Australia
>Spike|hCoV-19/Northern Ireland/NIRE-106DE4/2020|2020-05-29|EPI_ISL_501056|Original|hCoV-19^^Northern Ireland|Human|Regional Virus Laboratory|Wellcome Sanger Institute for the COVID-19 Genomics UK (COG-UK) consortium|Davies|United Kingdom
>Spike|hCoV-19/Denmark/DCGC-2001/2020|2020-07-20|EPI_ISL_618336|Original|hCoV-19^^Midtjylland|Human|Department of Virus and Microbiological Special Diagnostics|Albertsen lab|Michaelsen|Denmark
>Spike|hCoV-19/India/DL-MaxCov0043-CSIR-IGIB/2020|2020-04-29|EPI_ISL_459945|Original|hCoV-19^^Delhi|Human|Devki Devi Foundation|CSIR-IGIB/Max|Pandey|India
>Spike|hCoV-19/Japan/PG-1521/2020|2020-04-00|EPI_ISL_685128|Original|hCoV-19^^Japan|Human|Saitama Prefectural Institute of Public Health|Pathogen Genomics Center|Sekizuka|Japan
>Spike|hCoV-19/England/EXET-135DFB/2020|2020-03-25|EPI_ISL_457170|Original|hCoV-19^^England|Human|University of Exeter|COVID-19 Genomics UK (COG-UK) Consortium|Temperton|United Kingdom
>Spike|hCoV-19/India/KA-IB50/2020|2020-05-09|EPI_ISL_508328|Original|hCoV-19^^Karnataka|Human|Indian Institute of Science|National Institute of Biomedical Genomics|Maitra|India
>Spike|hCoV-19/Canada/ON-CVD01486/2020|2020-05-01|EPI_ISL_586277|Original|hCoV-19^^Ontario|Human|Toronto Invasive Bacterial Diseases Network|McMaster University|McArthur|Canada
>Spike|hCoV-19/Indonesia/NT-EIJK26/2020|2020-04-14|EPI_ISL_766048|Original|hCoV-19^^East Nusa Tenggara|Human|RS Bhayangkara Tk. III|Eijkman Institute for Molecular Biology|Soebandrio|Indonesia
>Spike|hCoV-19/England/SHEF-C8F16/2020|2020-05-07|EPI_ISL_490614|Original|hCoV-19^^England|Human|Virology Department|COVID-19 Genomics UK (COG-UK) Consortium|de Silva|United Kingdom
>Spike|hCoV-19/England/QEUH-A60AD9/2020|2020-10-12|EPI_ISL_665392|Original|hCoV-19^^England|Human|Northumbria University / South Tees Hospitals NHS Foundation Trust / North Cumbria Integrated Care NHS Foundation Trust / North Tees and Hartlepool NHS Foundation Trust / Newcastle Hospitals NHS Foundation Trust|COVID-19 Genomics UK (COG-UK) Consortium|Bashton|United Kingdom
>Spike|hCoV-19/USA/TX-HMH-MCoV-10648/2020|2020-07-06|EPI_ISL_789574|Original|hCoV-19^^Texas|Human|Houston Methodist Hospital|Houston Methodist Hospital|Long|USA
>Spike|hCoV-19/Australia/VIC10499/2020|2020-08-31|EPI_ISL_562936|Original|hCoV-19^^Victoria|Human|Victorian Infectious Diseases Reference Laboratory (VIDRL)|VIDRL and MDU-PHL|Schultz|Australia
>Spike|hCoV-19/England/CAMC-CB8F1D/2020|2020-12-18|EPI_ISL_778066|Original|hCoV-19^^England|Human|Lighthouse Lab in Cambridge|Wellcome Sanger Institute for the COVID-19 Genomics UK (COG-UK) Consortium|Davies|United Kingdom
>Spike|hCoV-19/England/BRIS-122429/2020|2020-04-02|EPI_ISL_440725|Original|hCoV-19^^England|Human|PHE South West Regional Laboratory|Wellcome Sanger Institute for the COVID-19 Genomics UK (COG-UK) consortium|Amato|United Kingdom

>Spike|hCoV-19/England/NOTT-10EFAA/2020|2020-03-31|EPI_ISL_432950|Original|hCoV-19^^England|Human|Queens Medical Centre|COVID-19 Genomics UK (COG-UK) Consortium|Loose|United Kingdom
>Spike|hCoV-19/Canada/BC_27007571/2020|2020-03-00|EPI_ISL_463257|Original|hCoV-19^^British Columbia|Human|BCCDC Public Health Laboratory|BCCDC Public Health Laboratory|Harrigan|Canada
>Spike|hCoV-19/Luxembourg/LNS7990137/2020|2020-10-25|EPI_ISL_744666|Original|hCoV-19^^Luxembourg|Human|Laboratoire national de sant\u00e9|Laboratoire national de sant\u00e9|Wienecke-Baldacchino|Luxembourg
>Spike|hCoV-19/England/QEUH-9F30AD/2020|2020-09-24|EPI_ISL_588610|Original|hCoV-19^^England|Human|Lighthouse Lab in Glasgow|Wellcome Sanger Institute for the COVID-19 Genomics UK (COG-UK) consortium|Davies|United Kingdom
>Spike|hCoV-19/Panama/333706/2020|2020-03-20|EPI_ISL_496754|Original|hCoV-19^^Colon|Human|Gorgas Memorial Laboratory of Health Studies|Gorgas Memorial Laboratory of Health Studies|Martinez|Panama
>Spike|hCoV-19/Denmark/DCGC-15752/2020|2020-11-23|EPI_ISL_713999|Original|hCoV-19^^Nordjylland|Human|Department of Virus and Microbiological Special Diagnostics|Albertsen Lab|Michaelsen|Denmark
>Spike|hCoV-19/Wales/ALDP-AA4866/2020|2020-10-21|EPI_ISL_624536|Original|hCoV-19^^Wales|Human|Lighthouse Lab in Alderley Park|Wellcome Sanger Institute for the COVID-19 Genomics UK (COG-UK) consortium|Davies|United Kingdom
>Spike|hCoV-19/Switzerland/BS-42406439/2020|2020-08-22|EPI_ISL_581981|Original|hCoV-19^^Basel-Stadt|Human|University Hospital Basel|University Hospital Basel|Roloff Handschin|Switzerland
>Spike|hCoV-19/USA/TX-HMH-600/2020|2020-04-07|EPI_ISL_547156|Original|hCoV-19^^Texas|Human|Houston Methodist Hospital|Houston Methodist Hospital|Long|USA
>Spike|hCoV-19/Singapore/585/2020|2020-06-26|EPI_ISL_483608|Original|hCoV-19^^Singapore|Human|National Public Health Laboratory|National Public Health Laboratory|Mak|Singapore
>Spike|hCoV-19/India/DL-NCDC2218-CSIR-IGIB/2020|2020-05-19|EPI_ISL_482554|Original|hCoV-19^^Delhi|Human|National Centre for Disease control (NCDC)|NCDC/CSIR-IGIB|Pandey|India
>Spike|hCoV-19/England/QEUH-AD0ED8/2020|2020-10-24|EPI_ISL_629956|Original|hCoV-19^^England|Human|Lighthouse Lab in Glasgow|Wellcome Sanger Institute for the COVID-19 Genomics UK (COG-UK) consortium|Davies|United Kingdom
>Spike|hCoV-19/England/204891341/2020|2020-11-27|EPI_ISL_723018|Original|hCoV-19^^England|Human|Respiratory Virus Unit|COVID-19 Genomics UK (COG-UK) Consortium|Platt|United Kingdom
>Spike|hCoV-19/England/CAMC-B34985/2020|2020-11-05|EPI_ISL_659672|Original|hCoV-19^^England|Human|Lighthouse Lab in Cambridge|Wellcome Sanger Institute for the COVID-19 Genomics UK (COG-UK) Consortium|Davies|United Kingdom
>Spike|hCoV-19/England/QEUH-532792/2020|2020-05-18|EPI_ISL_532230|Original|hCoV-19^^England|Human|Lighthouse Lab in Glasgow|Wellcome Sanger Institute for the COVID-19 Genomics UK (COG-UK) consortium|Davies|United Kingdom
>Spike|hCoV-19/England/20104004704/2020|2020-03-02|EPI_ISL_464316|Original|hCoV-19^^England|Human|Respiratory Virus Unit|Respiratory Virus Unit|Platt|United Kingdom
>Spike|hCoV-19/England/ALDP-A520D8/2020|2020-10-07|EPI_ISL_598104|Original|hCoV-19^^England|Human|Lighthouse Lab in Alderley Park|Wellcome Sanger Institute for the COVID-19 Genomics UK (COG-UK) consortium|Davies|United Kingdom
>Spike|hCoV-19/England/LOND-125152A/2020|2020-09-06|EPI_ISL_679408|Original|hCoV-19^^England|Human|University College London Hospital|COVID-19 Genomics UK (COG-UK) Consortium|Byott|United Kingdom
>Spike|hCoV-19/Denmark/DCGC-13631/2020|2020-11-30|EPI_ISL_712925|Original|hCoV-19^^Midtjylland|Human|Department of Virus and Microbiological Special Diagnostics|Albertsen Lab|Michaelsen|Denmark
>Spike|hCoV-19/USA/TX-HMH-3120/2020|2020-06-11|EPI_ISL_544006|Original|hCoV-19^^Texas|Human|Houston Methodist Hospital|Houston Methodist Hospital|Long|USA
>Spike|hCoV-19/Japan/PG-3874/2020|2020-06-00|EPI_ISL_692125|Original|hCoV-19^^Japan|Human|Tokyo Metropolitan Institute of Public Health|Pathogen Genomics Center|Sekizuka|Japan

>Spike|hCoV-19/England/SHEF-C3FC0/2020|2020-04-06|EPI_ISL_499332|Original|hCoV-19^^England|Human|Virology Department|COVID-19 Genomics UK (COG-UK) Consortium|de Silva|United Kingdom
>Spike|hCoV-19/New Zealand/20VR0933/2020|2020-03-19|EPI_ISL_456171|Original|hCoV-19^^Bay of Plenty|Human|PathLab Bay of Plenty|Institute of Environmental Science and Research (ESR)|de Ligt|New Zealand
>Spike|hCoV-19/England/MILK-9A6EAF/2020|2020-09-02|EPI_ISL_550093|Original|hCoV-19^^England|Human|Lighthouse Lab in Milton Keynes|Wellcome Sanger Institute for the COVID-19 Genomics UK (COG-UK) consortium|Davies|United Kingdom
>Spike|hCoV-19/England/NORW-EAB92/2020|2020-05-11|EPI_ISL_457358|Original|hCoV-19^^England|Human|Quadram Institute Bioscience|COVID-19 Genomics UK (COG-UK) Consortium|Alikhan|United Kingdom
>Spike|hCoV-19/Netherlands/NA_614/2020|2020-04-23|EPI_ISL_460777|Original|hCoV-19^^Netherlands|Human|Dutch COVID-19 response team|Erasmus Medical Center|Oude Munnink|Netherlands
>Spike|hCoV-19/England/CAMC-B2327F/2020|2020-11-03|EPI_ISL_651725|Original|hCoV-19^^England|Human|Virology Department|COVID-19 Genomics UK (COG-UK) Consortium|de Silva|United Kingdom
>Spike|hCoV-19/England/QEUH-A0164F/2020|2020-09-30|EPI_ISL_679992|Original|hCoV-19^^England|Human|Virology Department|COVID-19 Genomics UK (COG-UK) Consortium|de Silva|United Kingdom
>Spike|hCoV-19/Canada/ON-PHL-20-03613/2020|2020-11-00|EPI_ISL_775121|Original|hCoV-19^^Ontario|Human|Public Health Ontario Laboratory|Public Health Ontario Laboratory|Guthrie|Canada
>Spike|hCoV-19/USA/CA-CZB-2218/2020|2020-05-28|EPI_ISL_513766|Original|hCoV-19^^California|Human|Orange County Public Health Lab|Chan-Zuckerberg Biohub|Kamm|USA
>Spike|hCoV-19/England/ALDP-9052EE/2020|2020-06-24|EPI_ISL_553726|Original|hCoV-19^^England|Human|Lighthouse Lab in Alderley Park|Wellcome Sanger Institute for the COVID-19 Genomics UK (COG-UK) consortium|Davies|United Kingdom
>Spike|hCoV-19/Gibraltar/204421229/2020|2020-10-27|EPI_ISL_613689|Original|hCoV-19^^Gibraltar|Human|Microbiology|Respiratory Virus Unit|Platt|Gibraltar
>Spike|hCoV-19/Denmark/DCGC-6902/2020|2020-10-12|EPI_ISL_619747|Original|hCoV-19^^Hovedstaden|Human|Department of Virus and Microbiological Special Diagnostics|Albertsen lab|Michaelsen|Denmark
>Spike|hCoV-19/Denmark/DCGC-18331/2020|2020-12-07|EPI_ISL_749893|Original|hCoV-19^^Hovedstaden|Human|Department of Virus and Microbiological Special Diagnostics|Albertsen Lab|Michaelsen|Denmark
>Spike|hCoV-19/England/QEUH-96F91A/2020|2020-08-17|EPI_ISL_540282|Original|hCoV-19^^England|Human|Lighthouse Lab in Glasgow|Wellcome Sanger Institute for the COVID-19 Genomics UK (COG-UK) consortium|Davies|United Kingdom
>Spike|hCoV-19/England/ALDP-9E981B/2020|2020-09-24|EPI_ISL_584253|Original|hCoV-19^^England|Human|Virology Department|COVID-19 Genomics UK (COG-UK) Consortium|de Silva|United Kingdom
>Spike|hCoV-19/USA/CT-UW-6379/2020|2020-04-01|EPI_ISL_570109|Original|hCoV-19^^Connecticut|Human|UW Virology Lab|UW Virology Lab|Roychoudhury|USA
>Spike|hCoV-19/Japan/PG-8310/2020|2020-08-00|EPI_ISL_687409|Original|hCoV-19^^Japan|Human|Pathogen Genomics Center|Pathogen Genomics Center|Sekizuka|Japan
>Spike|hCoV-19/USA/CA-CHLA-PLM87793444/2020|2020-11-30|EPI_ISL_753443|Original|hCoV-19^^California|Human|Clinical virology Laboratory|Center for Personalized Medicine|Dien Bard|USA
>Spike|hCoV-19/USA/GA-CDC-5125/2020|2020-06-29|EPI_ISL_566105|Original|hCoV-19^^Georgia|Human|GA Department of Public Health Laboratory|Pathogen Discovery|Queen|USA
>Spike|hCoV-19/England/CAMC-B99DDD/2020|2020-11-15|EPI_ISL_673256|Original|hCoV-19^^England|Human|Lighthouse Lab in Cambridge|Wellcome Sanger Institute for the COVID-19 Genomics UK (COG-UK) Consortium|Davies|United Kingdom
>Spike|hCoV-19/Australia/VIC5820/2020|2020-07-20|EPI_ISL_518614|Original|hCoV-19^^Victoria|Human|Microbiological Diagnostic Unit - Public Health Laboratory (MDU-PHL)|MDU-PHL|Schultz|Australia

>Spike|hCoV-19/England/ALDP-B620C5/2020|2020-11-08|EPI_ISL_656664|Original|hCoV-19^^England|Human|Lighthouse Lab in Alderley Park|Wellcome Sanger Institute for the COVID-19 Genomics UK (COG-UK) Consortium|Davies|United Kingdom
>Spike|hCoV-19/South Africa/L00172/2020|2020-07-27|EPI_ISL_622951|Original|hCoV-19^^Gauteng|Human|Lancet Laboratories|National Institute for Communicable Diseases of the National Health Laboratory Service|Allam|South Africa
>Spike|hCoV-19/Gibraltar/204841740/2020|2020-00-00|EPI_ISL_671307|Original|hCoV-19^^Gibraltar|Human|Microbiology|Respiratory Virus Unit|Platt|Gibraltar
>Spike|hCoV-19/England/ALDP-9BC732/2020|2020-09-09|EPI_ISL_568067|Original|hCoV-19^^England|Human|Lighthouse Lab in Alderley Park|Wellcome Sanger Institute for the COVID-19 Genomics UK (COG-UK) consortium|Davies|United Kingdom
>Spike|hCoV-19/USA/CO-QDX-2314/2020|2020-03-16|EPI_ISL_604684|Original|hCoV-19^^Colorado|Human|Quest Diagnostics|Quest Diagnostics|Kagan|USA
>Spike|hCoV-19/Switzerland/GE-HUG-5708/2020|2020-03-24|EPI_ISL_429211|Original|hCoV-19^^Geneve|Human|University Hospitals of Geneva Laboratory of Virology|University Hospitals of Geneva Laboratory of Virology|Cordey|Switzerland
>Spike|hCoV-19/Japan/PG-7228/2020|2020-07-00|EPI_ISL_688332|Original|hCoV-19^^Japan|Human|Saitama Prefectural Institute of Public Health|Pathogen Genomics Center|Sekizuka|Japan
>Spike|hCoV-19/Scotland/CVR195/2020|2020-03-18|EPI_ISL_425727|Original|hCoV-19^^Scotland|Human|West of Scotland Specialist Virology Centre|COVID-19 Genomics UK (COG-UK) Consortium|Robertson|United Kingdom
>Spike|hCoV-19/Australia/SAP350/2020|2020-03-23|EPI_ISL_483125|Original|hCoV-19^^South Australia|Human|SA Pathology|SA Pathology|Leong|Australia
>Spike|hCoV-19/England/SHEF-CF1BF/2020|2020-10-31|EPI_ISL_653091|Original|hCoV-19^^England|Human|Virology Department|COVID-19 Genomics UK (COG-UK) Consortium|de Silva|United Kingdom
>Spike|hCoV-19/Latvia/163/2020|2020-08-31|EPI_ISL_639660|Original|hCoV-19^^Latvia|Human|E. Gulbja Laboratorija|Latvian Biomedical Research and Study Centre|Zrelovs|Latvia
>Spike|hCoV-19/USA/TX-HMH-1110/2020|2020-04-22|EPI_ISL_542536|Original|hCoV-19^^Texas|Human|Houston Methodist Hospital|Houston Methodist Hospital|Long|USA
>Spike|hCoV-19/USA/TX-HMH-7126/2020|2020-06-29|EPI_ISL_546527|Original|hCoV-19^^Texas|Human|Houston Methodist Hospital|Houston Methodist Hospital|Long|USA
>Spike|hCoV-19/England/QEUH-C318E4/2020|2020-12-11|EPI_ISL_782573|Original|hCoV-19^^England|Human|Lighthouse Lab in Glasgow|Wellcome Sanger Institute for the COVID-19 Genomics UK (COG-UK) Consortium|Davies|United Kingdom
>Spike|hCoV-19/England/BRIS-125523/2020|2020-03-27|EPI_ISL_443700|Original|hCoV-19^^England|Human|PHE South West Regional Laboratory|Wellcome Sanger Institute for the COVID-19 Genomics UK (COG-UK) consortium|Amato|United Kingdom
>Spike|hCoV-19/Belgium/ULG-10855/2020|2020-11-13|EPI_ISL_678399|Original|hCoV-19^^Li\u00e8ge|Human|Department of Clinical Microbiology|GIGA Medical Genomics|Durkin|Belgium
>Spike|hCoV-19/England/NORT-29ECA7/2020|2020-05-10|EPI_ISL_490481|Original|hCoV-19^^England|Human|Northumbria University / South Tees Hospitals NHS Foundation Trust / North Cumbria Integrated Care NHS Foundation Trust / North Tees and Hartlepool NHS Foundation Trust / Newcastle Hospitals NHS Foundation Trust|COVID-19 Genomics UK (COG-UK) Consortium|Bashton|United Kingdom
>Spike|hCoV-19/Hong Kong/HKPU-1319/2020|2020-07-08|EPI_ISL_610181|Original|hCoV-19^^Hong Kong|Human|Department of Health Technology and Informatics|Department of Health Technology and Informatics|Leung|China
>Spike|hCoV-19/Denmark/DCGC-19339/2020|2020-12-07|EPI_ISL_748892|Original|hCoV-19^^Syddanmark|Human|Department of Virus and Microbiological Special Diagnostics|Albertsen Lab|Michaelsen|Denmark
>Spike|hCoV-19/Norway/3262/2020|2020-08-31|EPI_ISL_590904|Original|hCoV-19^^Agder|Human|Hospital of Southern Norway - Kristiansand|Norwegian Institute of Public Health|Vollan|Norway
>Spike|hCoV-19/Belgium/rega-0630826/2020|2020-06-30|EPI_ISL_734785|Original|hCoV-19^^Sint-jans-molenbeek|Human|UZ Leuven|KU Leuven|Maes|Belgium

>Spike|hCoV-19/England/20198018804/2020|2020-05-06|EPI_ISL_465634|Original|hCoV-19^^England|Human|Respiratory Virus Unit|Respiratory Virus Unit|Platt|United Kingdom
>Spike|hCoV-19/England/ALDP-B73656/2020|2020-11-12|EPI_ISL_662410|Original|hCoV-19^^England|Human|Lighthouse Lab in Alderley Park|Wellcome Sanger Institute for the COVID-19 Genomics UK (COG-UK) Consortium|Davies|United Kingdom
>Spike|hCoV-19/Denmark/DCGC-19667/2020|2020-12-07|EPI_ISL_749097|Original|hCoV-19^^Sjaelland|Human|Department of Virus and Microbiological Special Diagnostics|Albertsen Lab|Michaelsen|Denmark
>Spike|hCoV-19/USA/NY-NYCPHL-001323/2020|2020-11-10|EPI_ISL_683919|Original|hCoV-19^^New York|Human|DOHMH Morrisania|New York City Public Health Laboratory|Wang|USA
>Spike|hCoV-19/Wales/PHWC-49F04E/2020|2020-12-08|EPI_ISL_743159|Original|hCoV-19^^Wales|Human|Wales Specialist Virology Centre Sequencing lab|COVID-19 Genomics UK (COG-UK) Consortium|Moore|United Kingdom
>Spike|hCoV-19/South Korea/KDCA0409/2020|2020-11-07|EPI_ISL_760193|Original|hCoV-19^^South Korea|Human|Division of Emerging Infectious Diseases|Division of Emerging Infectious Diseases|Park|South Korea
>Spike|hCoV-19/Spain/MD-IBV-99008535/2020|2020-03-19|EPI_ISL_654083|Original|hCoV-19^^Madrid|Human|Hospital General Universitario Gregorio Mara\u00f1\u00f3n|SeqCOVID-SPAIN consortium/IBV(CSIC)|Comas|Spain
>Spike|hCoV-19/USA/WI-UW-2558/2020|2020-12-29|EPI_ISL_788901|Original|hCoV-19^^Wisconsin|Human|University of Wisconsin-Madison AIDS Vaccine Research Laboratories|University of Wisconsin-Madison AIDS Vaccine Research Laboratories|Moreno|USA
>Spike|hCoV-19/USA/WA-S2644/2020|2020-06-20|EPI_ISL_497276|Original|hCoV-19^^Washington|Human|Washington State Department of Health|Seattle Flu Study|Lee|USA
>Spike|hCoV-19/USA/MI-UM-10036578565/2020|2020-10-22|EPI_ISL_640163|Original|hCoV-19^^Michigan|Human|University of Michigan Clinical Microbiology Laboratory|Lauring Lab|Valesano|USA
>Spike|hCoV-19/Scotland/QEUH-A4CCA0/2020|2020-10-08|EPI_ISL_596040|Original|hCoV-19^^Scotland|Human|Quadram Institute Bioscience|COVID-19 Genomics UK (COG-UK) Consortium|Alikhan|United Kingdom
>Spike|hCoV-19/USA/TX-DSHS-1244/2020|2020-09-17|EPI_ISL_676610|Original|hCoV-19^^Texas|Human|Texas Department of State Health Services|Texas Department of State Health Services|Rahman|USA
>Spike|hCoV-19/USA/VA-DCLS-0236/2020|2020-04-09|EPI_ISL_452811|Original|hCoV-19^^Virginia|Human|Virginia DCLS|Virginia DCLS|St. Jacques|USA
>Spike|hCoV-19/Spain/AN-IBV-006106/2020|2020-05-29|EPI_ISL_510438|Original|hCoV-19^^Andalucia|Human|Hospital Universitario Virgen de las Nieves de Granada-SAS|SeqCOVID-SPAIN consortium/IBV(CSIC)|Comas|Spain
>Spike|hCoV-19/England/CAMC-B08B84/2020|2020-10-30|EPI_ISL_673058|Original|hCoV-19^^England|Human|Lighthouse Lab in Cambridge|Wellcome Sanger Institute for the COVID-19 Genomics UK (COG-UK) Consortium|Davies|United Kingdom
>Spike|hCoV-19/Sweden/20-08801/2020|2020-04-04|EPI_ISL_475120|Original|hCoV-19^^Halland|Human|Halmstad klinisk mikrobiologi|The Public Health Agency of Sweden|Svartstr\u00f6m|Sweden
>Spike|hCoV-19/Bangladesh/BCSIR-NILMRC-180/2020|2020-06-20|EPI_ISL_483693|Original|hCoV-19^^Rajshahi|Human|National Institute of Laboratory Medicine and Referral Center|Genomic Research Lab|Khan|Bangladesh
>Spike|hCoV-19/England/NOTT-11C60E/2020|2020-11-16|EPI_ISL_665903|Original|hCoV-19^^England|Human|Queens Medical Centre|COVID-19 Genomics UK (COG-UK) Consortium|Loose|United Kingdom
>Spike|hCoV-19/Japan/PG-4628/2020|2020-07-00|EPI_ISL_689765|Original|hCoV-19^^Japan|Human|Fukuoka Institute of Health and Environmental Sciences|Pathogen Genomics Center|Sekizuka|Japan
>Spike|hCoV-19/USA/UT-UPHL-2009156/2020|2020-05-22|EPI_ISL_525146|Original|hCoV-19^^Utah|Human|Utah Public Health Laboratory|Utah Public Health Laboratory|Young|USA
>Spike|hCoV-19/Wales/PHWC-4A161A/2020|2020-12-10|EPI_ISL_743701|Original|hCoV-19^^Wales|Human|Wales Specialist Virology Centre Sequencing lab|COVID-19 Genomics UK (COG-UK) Consortium|Moore|United Kingdom

>Spike|hCoV-19/Denmark/DCGC-2752/2020|2020-08-17|EPI_ISL_616497|Original|hCoV-19^^Midtjylland|Human|Department of Virus and Microbiological Special Diagnostics|Albertsen lab|Michaelsen|Denmark
>Spike|hCoV-19/England/CAMB-1B5B53/2020|2020-08-13|EPI_ISL_516997|Original|hCoV-19^^England|Human|Department of Pathology|COVID-19 Genomics UK (COG-UK) Consortium|Meredith|United Kingdom
>Spike|hCoV-19/USA/TX-HMH-MCoV-15379/2020|2020-10-21|EPI_ISL_786863|Original|hCoV-19^^Texas|Human|Houston Methodist Hospital|Houston Methodist Hospital|Long|USA
>Spike|hCoV-19/South Korea/KCDC2147/2020|2020-03-23|EPI_ISL_498048|Original|hCoV-19^^South Korea|Human|Division of Viral Diseases|Division of Viral Diseases|Kim|South Korea
>Spike|hCoV-19/Wales/PHWC-49F381/2020|2020-12-07|EPI_ISL_743204|Original|hCoV-19^^Wales|Human|Wales Specialist Virology Centre Sequencing lab|COVID-19 Genomics UK (COG-UK) Consortium|Moore|United Kingdom
>Spike|hCoV-19/Gibraltar/204462819/2020|2020-10-29|EPI_ISL_614994|Original|hCoV-19^^Gibraltar|Human|Microbiology|Respiratory Virus Unit|Platt|Gibraltar
>Spike|hCoV-19/USA/VA-DCLS-0656/2020|2020-04-09|EPI_ISL_495327|Original|hCoV-19^^Virginia|Human|Virginia DCLS|Virginia DCLS|St. Jacques|USA
>Spike|hCoV-19/USA/UT-UPHL-2012657489/2020|2020-08-14|EPI_ISL_746149|Original|hCoV-19^^Utah|Human|Ginkgo Bioworks Clinical Laboratory|Utah Public Health Laboratory|Young|USA
>Spike|hCoV-19/England/ALDP-A6C9FE/2020|2020-10-15|EPI_ISL_606989|Original|hCoV-19^^England|Human|Lighthouse Lab in Alderley Park|Wellcome Sanger Institute for the COVID-19 Genomics UK (COG-UK) consortium|Davies|United Kingdom
>Spike|hCoV-19/USA/NV-NSPHL-A0167/2020|2020-05-17|EPI_ISL_515428|Original|hCoV-19^^Nevada|Human|Nevada State Public Health Laboratory|Nevada State Public Health Laboratory|Sevinsky|USA
>Spike|hCoV-19/England/ALDP-9BDBF9/2020|2020-09-09|EPI_ISL_568189|Original|hCoV-19^^England|Human|Lighthouse Lab in Alderley Park|Wellcome Sanger Institute for the COVID-19 Genomics UK (COG-UK) consortium|Davies|United Kingdom
>Spike|hCoV-19/England/20129105404/2020|2020-03-13|EPI_ISL_424090|Original|hCoV-19^^England|Human|Respiratory Virus Unit|Respiratory Virus Unit|Platt|United Kingdom
>Spike|hCoV-19/England/CAMC-B78B52/2020|2020-11-12|EPI_ISL_783421|Original|hCoV-19^^England|Human|Lighthouse Lab in Cambridge|Wellcome Sanger Institute for the COVID-19 Genomics UK (COG-UK) Consortium|Davies|United Kingdom
>Spike|hCoV-19/USA/WA-S133/2020|2020-03-13|EPI_ISL_430170|Original|hCoV-19^^Washington|Human|Washington State Department of Health|Seattle Flu Study|Bedford|USA
>Spike|hCoV-19/USA/TX-HMH-6016/2020|2020-06-28|EPI_ISL_547170|Original|hCoV-19^^Texas|Human|Houston Methodist Hospital|Houston Methodist Hospital|Long|USA
>Spike|hCoV-19/Serbia/Sabac-000490807/2020|2020-07-07|EPI_ISL_678492|Original|hCoV-19^^Sabac|Human|Veterinary Specialized Institute \Sabac\|Veterinary Specialized Institute \Kraljevo\|Vidanovic|Serbia
>Spike|hCoV-19/Iceland/26/2020|2020-03-03|EPI_ISL_417841|Original|hCoV-19^^Reykjavik|Human|The National University Hospital of Iceland|deCODE genetics|J\u00f3nsson|Iceland
>Spike|hCoV-19/England/QEUH-9D0668/2020|2020-09-15|EPI_ISL_589985|Original|hCoV-19^^England|Human|Lighthouse Lab in Glasgow|Wellcome Sanger Institute for the COVID-19 Genomics UK (COG-UK) consortium|Davies|United Kingdom
>Spike|hCoV-19/England/QEUH-A02A24/2020|2020-09-28|EPI_ISL_595847|Original|hCoV-19^^England|Human|Quadram Institute Bioscience|COVID-19 Genomics UK (COG-UK) Consortium|Alikhan|United Kingdom
>Spike|hCoV-19/Scotland/CVR5633/2020|2020-10-28|EPI_ISL_651882|Original|hCoV-19^^Scotland|Human|West of Scotland Specialist Virology Centre|COVID-19 Genomics UK (COG-UK) Consortium|Robertson|United Kingdom
>Spike|hCoV-19/USA/AZ-TG668406/2020|2020-04-18|EPI_ISL_696309|Original|hCoV-19^^Arizona|Human|Sonora Quest Laboratories|TGen North|French|USA
>Spike|hCoV-19/Switzerland/VD-ETHZ-270064/2020|2020-09-02|EPI_ISL_541473|Original|hCoV-19^^Vaud|Human|Viollier AG|Department of Biosystems Science and Engineering|Nadeau|Switzerland

>Spike|hCoV-19/England/CAMC-B52893/2020|2020-11-08|EPI_ISL_657590|Original|hCoV-19^^England|Human|Lighthouse Lab in Cambridge|Wellcome Sanger Institute for the COVID-19 Genomics UK (COG-UK) Consortium|Davies|United Kingdom
>Spike|hCoV-19/Portugal/PT1570/2020|2020-06-28|EPI_ISL_693541|Original|hCoV-19^^Portugal|Human|Instituto Nacional de Saude (INSA)|Instituto Nacional de Saude (INSA)|Borges|Portugal
>Spike|hCoV-19/England/ALDP-982058/2020|2020-08-10|EPI_ISL_552732|Original|hCoV-19^^England|Human|Lighthouse Lab in Alderley Park|Wellcome Sanger Institute for the COVID-19 Genomics UK (COG-UK) consortium|Davies|United Kingdom
>Spike|hCoV-19/Sweden/20-05984/2020|2020-03-18|EPI_ISL_475563|Original|hCoV-19^^Vastra Gotaland|Human|Din Klinik|The Public Health Agency of Sweden|Svartstr\u00f6m|Sweden
>Spike|hCoV-19/Northern Ireland/NIRE-FB8E2/2020|2020-06-01|EPI_ISL_585247|Original|hCoV-19^^Northern Ireland|Human|Regional Virus Laboratory|COVID-19 Genomics UK (COG-UK) Consortium|Fairley|United Kingdom
>Spike|hCoV-19/USA/MN-QDX-1732/2020|2020-03-15|EPI_ISL_604074|Original|hCoV-19^^Minnesota|Human|Quest Diagnostics|Quest Diagnostics|Kagan|USA
>Spike|hCoV-19/England/ALDP-9518A5/2020|2020-06-10|EPI_ISL_555376|Original|hCoV-19^^England|Human|Lighthouse Lab in Alderley Park|Wellcome Sanger Institute for the COVID-19 Genomics UK (COG-UK) consortium|Davies|United Kingdom
>Spike|hCoV-19/USA/CA-ALSR-3807/2020|2020-06-05|EPI_ISL_636213|Original|hCoV-19^^California|Human|San Diego County Public Health Laboratory|Andersen lab at Scripps Research|Gangavarapu|USA
>Spike|hCoV-19/England/20169014704/2020|2020-04-17|EPI_ISL_465197|Original|hCoV-19^^England|Human|Respiratory Virus Unit|Respiratory Virus Unit|Platt|United Kingdom
>Spike|hCoV-19/Denmark/DCGC-9709/2020|2020-11-09|EPI_ISL_669121|Original|hCoV-19^^Hovedstaden|Human|Department of Virus and Microbiological Special Diagnostics|Albertsen Lab|Michaelsen|Denmark
>Spike|hCoV-19/Australia/VIC8474/2020|2020-00-00|EPI_ISL_565040|Original|hCoV-19^^Victoria|Human|Microbiological Diagnostic Unit - Public Health Laboratory (MDU-PHL)|MDU-PHL|Schultz|Australia
>Spike|hCoV-19/Wales/PHWC-32A61/2020|2020-04-14|EPI_ISL_474387|Original|hCoV-19^^Wales|Human|Originating lab|COVID-19 Genomics UK (COG-UK) Consortium|Moore|United Kingdom
>Spike|hCoV-19/USA/NY-Wadsworth-264206-01/2020|2020-11-05|EPI_ISL_754517|Original|hCoV-19^^New York|Human|Wadsworth Center|Wadsworth Center|Lasek-Nesselquist|USA
>Spike|hCoV-19/USA/MI-MDHHS-SC22034/2020|2020-09-11|EPI_ISL_577558|Original|hCoV-19^^Michigan|Human|Michigan Department of Health and Human Services|Michigan Department of Health and Human Services|Jacob|USA
>Spike|hCoV-19/USA/TX-HMH-3406/2020|2020-06-11|EPI_ISL_544166|Original|hCoV-19^^Texas|Human|Houston Methodist Hospital|Houston Methodist Hospital|Long|USA
>Spike|hCoV-19/USA/FL-BPHL-1427/2020|2020-09-01|EPI_ISL_549209|Original|hCoV-19^^Florida|Human|Florida Bureau of Public Health Laboratories|Florida Bureau of Public Health Laboratories|Blanton|USA
>Spike|hCoV-19/Netherlands/LI-RIVM-20562/2020|2020-12-09|EPI_ISL_790626|Original|hCoV-19^^Limburg|Human|Dutch COVID-19 response team|National Institute for Public Health and the Environment (RIVM)|Meijer|Netherlands
>Spike|hCoV-19/England/CAMC-A3E62B/2020|2020-10-05|EPI_ISL_609543|Original|hCoV-19^^England|Human|Lighthouse Lab in Cambridge|Wellcome Sanger Institute for the COVID-19 Genomics UK (COG-UK) consortium|Davies|United Kingdom
>Spike|hCoV-19/Wales/PHWC-1647B9/2020|2020-05-25|EPI_ISL_479308|Original|hCoV-19^^Wales|Human|Wales Specialist Virology Centre Sequencing lab|COVID-19 Genomics UK (COG-UK) Consortium|Moore|United Kingdom
>Spike|hCoV-19/USA/MI-QDX-506/2020|2020-03-23|EPI_ISL_571240|Original|hCoV-19^^Michigan|Human|Quest Diagnostics|Quest Diagnostics|Kagan|USA
>Spike|hCoV-19/Netherlands/UT-RIVM-10125/2020|2020-03-27|EPI_ISL_547533|Original|hCoV-19^^Utrecht|Human|Dutch COVID-19 response

team|National Institute for Public Health and the Environment (RIVM)|Meijer|Netherlands
>Spike|hCoV-19/Australia/VIC6316/2020|2020-07-24|EPI_ISL_518304|Original|hCoV-19^^Victoria|Human|Microbiological Diagnostic Unit - Public Health Laboratory (MDU-PHL)|MDU-PHL|Schultz|Australia
>Spike|hCoV-19/USA/TX-HMH-MCoV-15589/2020|2020-10-26|EPI_ISL_787073|Original|hCoV-19^^Texas|Human|Houston Methodist Hospital|Houston Methodist Hospital|Long|USA
>Spike|hCoV-19/England/QEUH-962F7F/2020|2020-08-10|EPI_ISL_531705|Original|hCoV-19^^England|Human|Lighthouse Lab in Glasgow|Wellcome Sanger Institute for the COVID-19 Genomics UK (COG-UK) consortium|Davies|United Kingdom
>Spike|hCoV-19/Luxembourg/LNS5217472/2020|2020-12-18|EPI_ISL_744203|Original|hCoV-19^^Luxembourg|Human|Laboratoire national de sant\u00e9|Laboratoire national de sant\u00e9|Wienecke-Baldacchino|Luxembourg
>Spike|hCoV-19/USA/WI-WSLH-200577/2020|2020-06-07|EPI_ISL_578688|Original|hCoV-19^^Wisconsin|Human|Wisconsin State Laboratory of Hygiene Communicable Disease Division|Wisconsin State Laboratory of Hygiene Communicable Disease Division|Florek|USA
>Spike|hCoV-19/USA/TX-HMH-3159/2020|2020-06-10|EPI_ISL_544035|Original|hCoV-19^^Texas|Human|Houston Methodist Hospital|Houston Methodist Hospital|Long|USA
>Spike|hCoV-19/England/QEUH-AAF300/2020|2020-10-20|EPI_ISL_630705|Original|hCoV-19^^England|Human|Lighthouse Lab in Glasgow|Wellcome Sanger Institute for the COVID-19 Genomics UK (COG-UK) consortium|Davies|United Kingdom
>Spike|hCoV-19/England/ALDP-951F55/2020|2020-06-09|EPI_ISL_551289|Original|hCoV-19^^England|Human|Lighthouse Lab in Alderley Park|Wellcome Sanger Institute for the COVID-19 Genomics UK (COG-UK) consortium|Davies|United Kingdom
>Spike|hCoV-19/England/MILK-A6A90F/2020|2020-10-08|EPI_ISL_626709|Original|hCoV-19^^England|Human|Liverpool Clinical Laboratories|COVID-19 Genomics UK (COG-UK) Consortium|Haldenby|United Kingdom
>Spike|hCoV-19/England/20139038004/2020|2020-03-28|EPI_ISL_420671|Original|hCoV-19^^England|Human|Respiratory Virus Unit|Respiratory Virus Unit|Platt|United Kingdom
>Spike|hCoV-19/Netherlands/NB-EMC-413/2020|2020-00-00|EPI_ISL_722377|Original|hCoV-19^^Noord Brabant|Human|Dutch COVID-19 response team|Erasmus Medical Center|Oude Munnink|Netherlands
>Spike|hCoV-19/Denmark/DCGC-10361/2020|2020-11-02|EPI_ISL_669925|Original|hCoV-19^^Syddanmark|Human|Department of Virus and Microbiological Special Diagnostics|Albertsen Lab|Michaelsen|Denmark
>Spike|hCoV-19/USA/UT-UPHL-2009166/2020|2020-05-27|EPI_ISL_525153|Original|hCoV-19^^Utah|Human|Utah Public Health Laboratory|Utah Public Health Laboratory|Young|USA
>Spike|hCoV-19/USA/MA-MGH-00039/2020|2020-03-24|EPI_ISL_460334|Original|hCoV-19^^Massachusetts|Human|Massachusetts General Hospital|Infectious Disease Program|Lemieux|USA
>Spike|hCoV-19/England/MILK-A7C2F0/2020|2020-10-15|EPI_ISL_606573|Original|hCoV-19^^England|Human|Lighthouse Lab in Milton Keynes|Wellcome Sanger Institute for the COVID-19 Genomics UK (COG-UK) consortium|Davies|United Kingdom
>Spike|hCoV-19/England/CAMB-79846/2020|2020-04-01|EPI_ISL_441818|Original|hCoV-19^^England|Human|Department of Pathology|Wellcome Sanger Institute for the COVID-19 Genomics UK (COG-UK) consortium|Amato|United Kingdom
>Spike|hCoV-19/Russia/SVE-RII-MH1640S/2020|2020-09-09|EPI_ISL_596308|Original|hCoV-19^^Sverdlovsk|Human|HELIX LCC|WHO National Influenza Centre Russian Federation|Komissarov|Russia
>Spike|hCoV-19/Scotland/QEUH-9DAEB1/2020|2020-09-22|EPI_ISL_601137|Original|hCoV-19^^Scotland|Human|Lighthouse Lab in Glasgow|Wellcome Sanger Institute for the COVID-19 Genomics UK (COG-UK) consortium|Davies|United Kingdom
>Spike|hCoV-19/England/ALDP-A6D456/2020|2020-10-15|EPI_ISL_608249|Original|hCoV-19^^England|Human|Lighthouse Lab in Alderley Park|Wellcome Sanger Institute for the COVID-19 Genomics UK (COG-UK) consortium|Davies|United Kingdom
>Spike|hCoV-19/India/MH-ACTREC-676/2020|2020-09-18|EPI_ISL_700301|Original|hCoV-19^^Maharashtra|Human|Hematopathology Laboratory|Hematopathology Laboratory|Patkar|India

>Spike|hCoV-19/South Korea/KCDC2738/2020|2020-07-16|EPI_ISL_522517|Original|hCoV-
19^^South Korea|Human|Division of Viral Diseases|Division of Viral Diseases|Kim|South
Korea
>Spike|hCoV-19/Australia/NSW240/2020|2020-03-25|EPI_ISL_451506|Original|hCoV-19^^New
South Wales|Human|Pathology West - NSW Health Pathology|NSW Health Pathology -
Institute of Clinical Pathology and Medical Research; Westmead Hospital; University
of Sydney|Gall|Australia
>Spike|hCoV-19/Belgium/rega-0916981/2020|2020-09-16|EPI_ISL_734971|Original|hCoV-
19^^Oud-Heverlee|Human|UZ Leuven|KU Leuven|Maes|Belgium
>Spike|hCoV-19/Northern Ireland/QEUH-964E25/2020|2020-08-
13|EPI_ISL_530958|Original|hCoV-19^^Northern Ireland|Human|Lighthouse Lab in
Glasgow|Wellcome Sanger Institute for the COVID-19 Genomics UK (COG-UK)
consortium|Davies|United Kingdom
>Spike|hCoV-19/England/OXON-B0078/2020|2020-04-18|EPI_ISL_478850|Original|hCoV-
19^^England|Human|Oxford Viromics|COVID-19 Genomics UK (COG-UK)
Consortium|Golubchik|United Kingdom
>Spike|hCoV-19/Wales/PHWC-164519/2020|2020-04-29|EPI_ISL_474189|Original|hCoV-
19^^Wales|Human|Originating lab|COVID-19 Genomics UK (COG-UK) Consortium|Moore|United
Kingdom
>Spike|hCoV-19/Australia/VIC10413/2020|2020-08-18|EPI_ISL_562905|Original|hCoV-
19^^Victoria|Human|Microbiological Diagnostic Unit - Public Health Laboratory (MDU-
PHL)|MDU-PHL|Schultz|Australia
>Spike|hCoV-19/Scotland/QEUH-9ADED5/2020|2020-09-05|EPI_ISL_638895|Original|hCoV-
19^^Scotland|Human|Department of Pathology|COVID-19 Genomics UK (COG-UK)
Consortium|Goodfellow|United Kingdom
>Spike|hCoV-19/Wales/PHWC-168B31/2020|2020-05-19|EPI_ISL_494333|Original|hCoV-
19^^Wales|Human|Originating lab|COVID-19 Genomics UK (COG-UK) Consortium|Moore|United
Kingdom
>Spike|hCoV-19/Wales/PHWC-4A40DD/2020|2020-12-16|EPI_ISL_763633|Original|hCoV-
19^^Wales|Human|Wales Specialist Virology Centre Sequencing lab|COVID-19 Genomics UK
(COG-UK) Consortium|Moore|United Kingdom
>Spike|hCoV-19/England/MILK-995210/2020|2020-08-24|EPI_ISL_552364|Original|hCoV-
19^^England|Human|Lighthouse Lab in Milton Keynes|Wellcome Sanger Institute for the
COVID-19 Genomics UK (COG-UK) consortium|Davies|United Kingdom
>Spike|hCoV-19/USA/NY-Wadsworth-292057-01/2020|2020-12-
21|EPI_ISL_767622|Original|hCoV-19^^New York|Human|WHITE PLAINS HOSPITAL CENTER
LABORATORY|Wadsworth Center|Lasek-Nesselquist|USA
>Spike|hCoV-19/Japan/PG-7169/2020|2020-07-00|EPI_ISL_688274|Original|hCoV-
19^^Japan|Human|Pathogen Genomics Center|Pathogen Genomics Center|Sekizuka|Japan
>Spike|hCoV-19/Senegal/64709/2020|2020-06-13|EPI_ISL_476560|Original|hCoV-
19^^Dakar|Human|Institut Pasteur Dakar|Institut Pasteur de Dakar|Dia|Senegal
>Spike|hCoV-19/England/MILK-AC7B37/2020|2020-10-21|EPI_ISL_634449|Original|hCoV-
19^^England|Human|Lighthouse Lab in Milton Keynes|Wellcome Sanger Institute for the
COVID-19 Genomics UK (COG-UK) consortium|Davies|United Kingdom
>Spike|hCoV-19/England/MILK-BDC943/2020|2020-11-27|EPI_ISL_709627|Original|hCoV-
19^^England|Human|Lighthouse Lab in Milton Keynes|Wellcome Sanger Institute for the
COVID-19 Genomics UK (COG-UK) Consortium|Davies|United Kingdom
>Spike|hCoV-19/France/PAC-MEPHI-1929/2020|2020-08-27|EPI_ISL_644462|Original|hCoV-
19^^Provence-Alpes-Cote-d\u2019Azur|Human|MEPHI|MEPHI|Levasseur|France
>Spike|hCoV-19/England/MILK-45ED08/2020|2020-05-14|EPI_ISL_559713|Original|hCoV-
19^^England|Human|Lighthouse Lab in Milton Keynes|Wellcome Sanger Institute for the
COVID-19 Genomics UK (COG-UK) consortium|Davies|United Kingdom
>Spike|hCoV-19/England/ALDP-CBFE46/2020|2020-12-20|EPI_ISL_760266|Original|hCoV-
19^^England|Human|Lighthouse Lab in Alderley Park|Wellcome Sanger Institute for the
COVID-19 Genomics UK (COG-UK) Consortium|Davies|United Kingdom
>Spike|hCoV-19/Wales/PHWC-49A6BA/2020|2020-12-02|EPI_ISL_727582|Original|hCoV-
19^^Wales|Human|Originating lab|COVID-19 Genomics UK (COG-UK) Consortium|Moore|United
Kingdom

>Spike|hCoV-19/England/QEUH-A00983/2020|2020-09-30|EPI_ISL_600136|Original|hCoV-19^^England|Human|Lighthouse Lab in Glasgow|Wellcome Sanger Institute for the COVID-19 Genomics UK (COG-UK) consortium|Davies|United Kingdom
>Spike|hCoV-19/USA/TX-HMH-MCoV-15707/2020|2020-07-09|EPI_ISL_785391|Original|hCoV-19^^Texas|Human|Houston Methodist Hospital|Houston Methodist Hospital|Long|USA
>Spike|hCoV-19/Hong Kong/VM20100570/2020|2020-08-06|EPI_ISL_637023|Original|hCoV-19^^Hong Kong|Human|Queen Elizabeth Hospital|Hong Kong Department of Health|Mak|China
>Spike|hCoV-19/USA/WA-S456/2020|2020-04-06|EPI_ISL_434216|Original|hCoV-19^^Washington|Human|Washington State Department of Health|Seattle Flu Study|Bedford|USA
>Spike|hCoV-19/Scotland/QEUH-AD4A2B/2020|2020-10-26|EPI_ISL_649732|Original|hCoV-19^^Scotland|Human|Lighthouse Lab in Glasgow|Wellcome Sanger Institute for the COVID-19 Genomics UK (COG-UK) Consortium|Davies|United Kingdom
>Spike|hCoV-19/Luxembourg/LNS4695210/2020|2020-10-26|EPI_ISL_740526|Original|hCoV-19^^Luxembourg|Human|Laboratoire national de sant\u00e9|Laboratoire national de sant\u00e9|Wienecke-Baldacchino|Luxembourg
>Spike|hCoV-19/England/QEUH-B423FF/2020|2020-11-08|EPI_ISL_657219|Original|hCoV-19^^England|Human|Lighthouse Lab in Glasgow|Wellcome Sanger Institute for the COVID-19 Genomics UK (COG-UK) Consortium|Davies|United Kingdom
>Spike|hCoV-19/Denmark/DCGC-3351/2020|2020-08-31|EPI_ISL_616105|Original|hCoV-19^^Hovedstaden|Human|Department of Virus and Microbiological Special Diagnostics|Albertsen lab|Michaelsen|Denmark
>Spike|hCoV-19/England/MILK-ACF9DB/2020|2020-10-22|EPI_ISL_629508|Original|hCoV-19^^England|Human|Lighthouse Lab in Milton Keynes|Wellcome Sanger Institute for the COVID-19 Genomics UK (COG-UK) consortium|Davies|United Kingdom
>Spike|hCoV-19/USA/CA-QDX-915/2020|2020-03-23|EPI_ISL_571574|Original|hCoV-19^^California|Human|Quest Diagnostics|Quest Diagnostics|Kagan|USA
>Spike|hCoV-19/England/MILK-BAA331/2020|2020-11-17|EPI_ISL_681430|Original|hCoV-19^^England|Human|Lighthouse Lab in Milton Keynes|Wellcome Sanger Institute for the COVID-19 Genomics UK (COG-UK) Consortium|Davies|United Kingdom
>Spike|hCoV-19/England/MILK-6F4D20/2020|2020-06-30|EPI_ISL_557513|Original|hCoV-19^^England|Human|Lighthouse Lab in Milton Keynes|Wellcome Sanger Institute for the COVID-19 Genomics UK (COG-UK) consortium|Davies|United Kingdom
>Spike|hCoV-19/USA/UT-UPHL-00093/2020|2020-03-15|EPI_ISL_529202|Original|hCoV-19^^Utah|Human|Utah Public Health Laboratory|Utah Public Health Laboratory|Young|USA
>Spike|hCoV-19/England/NORT-28CBEB/2020|2020-08-23|EPI_ISL_572424|Original|hCoV-19^^England|Human|Northumbria University / South Tees Hospitals NHS Foundation Trust / North Cumbria Integrated Care NHS Foundation Trust / North Tees and Hartlepool NHS Foundation Trust / Newcastle Hospitals NHS Foundation Trust|COVID-19 Genomics UK (COG-UK) Consortium|Bashton|United Kingdom
>Spike|hCoV-19/Scotland/CVR4337/2020|2020-09-09|EPI_ISL_573778|Original|hCoV-19^^Scotland|Human|West of Scotland Specialist Virology Centre|COVID-19 Genomics UK (COG-UK) Consortium|Robertson|United Kingdom
>Spike|hCoV-19/England/ALDP-A66DAD/2020|2020-10-12|EPI_ISL_630467|Original|hCoV-19^^England|Human|Lighthouse Lab in Alderley Park|Wellcome Sanger Institute for the COVID-19 Genomics UK (COG-UK) consortium|Davies|United Kingdom
>Spike|hCoV-19/England/QEUH-B1289F/2020|2020-11-02|EPI_ISL_642345|Original|hCoV-19^^England|Human|Lighthouse Lab in Glasgow|Wellcome Sanger Institute for the COVID-19 Genomics UK (COG-UK) Consortium|Davies|United Kingdom
>Spike|hCoV-19/Denmark/DCGC-2616/2020|2020-08-17|EPI_ISL_617853|Original|hCoV-19^^Midtjylland|Human|Department of Virus and Microbiological Special Diagnostics|Albertsen lab|Michaelsen|Denmark
>Spike|hCoV-19/England/MILK-B0598D/2020|2020-10-24|EPI_ISL_652209|Original|hCoV-19^^England|Human|Queens Medical Centre|COVID-19 Genomics UK (COG-UK) Consortium|Loose|United Kingdom
>Spike|hCoV-19/England/QEUH-A60CE2/2020|2020-10-12|EPI_ISL_665626|Original|hCoV-19^^England|Human|Northumbria University / South Tees Hospitals NHS Foundation Trust / North Cumbria Integrated Care NHS Foundation Trust / North Tees and Hartlepool NHS Foundation Trust / Newcastle Hospitals NHS Foundation Trust|COVID-19 Genomics UK (COG-UK) Consortium|Bashton|United Kingdom

>Spike|hCoV-19/Brazil/SP-565/2020|2020-03-20|EPI_ISL_693195|Original|hCoV-19^^S\u00e3o Paulo|Human|Hospital e Pronto Socorro Portinari|Instituto Adolfo Lutz|Sacchi|Brazil
>Spike|hCoV-19/Wales/MILK-9E127F/2020|2020-09-20|EPI_ISL_581067|Original|hCoV-19^^Wales|Human|Lighthouse Lab in Milton Keynes|Wellcome Sanger Institute for the COVID-19 Genomics UK (COG-UK) consortium|Davies|United Kingdom
>Spike|hCoV-19/USA/MI-UM-10036835234/2020|2020-11-07|EPI_ISL_728463|Original|hCoV-19^^Michigan|Human|University of Michigan Clinical Microbiology Laboratory|Lauring Lab|Valesano|USA
>Spike|hCoV-19/USA/MI-UM-10036788362/2020|2020-11-04|EPI_ISL_707875|Original|hCoV-19^^Michigan|Human|University of Michigan Clinical Microbiology Laboratory|Lauring Lab|Valesano|USA
>Spike|hCoV-19/England/LCST-24D6E1E/2020|2020-06-11|EPI_ISL_484240|Original|hCoV-19^^England|Human|University Hospitals Of Leicester NHS Trust and DeepSeq Nottingham|COVID-19 Genomics UK (COG-UK) Consortium|Loose|United Kingdom
>Spike|hCoV-19/France/ARA-SC159/2020|2020-04-08|EPI_ISL_634824|Original|hCoV-19^^Auvergne-Rhone-Alpes|Human|Laboratoire de virologie|CNR Virus des Infections Respiratoires - France SUD|Bal|France
>Spike|hCoV-19/Denmark/DCGC-8475/2020|2020-10-26|EPI_ISL_617401|Original|hCoV-19^^Syddanmark|Human|Department of Virus and Microbiological Special Diagnostics|Albertsen lab|Michaelsen|Denmark
>Spike|hCoV-19/USA/AZ-QDX-2448/2020|2020-03-16|EPI_ISL_604740|Original|hCoV-19^^Arizona|Human|Quest Diagnostics|Quest Diagnostics|Kagan|USA
>Spike|hCoV-19/England/ALDP-C5978F/2020|2020-12-13|EPI_ISL_736148|Original|hCoV-19^^England|Human|Lighthouse Lab in Alderley Park|Wellcome Sanger Institute for the COVID-19 Genomics UK (COG-UK) Consortium|Davies|United Kingdom
>Spike|hCoV-19/Denmark/DCGC-9983/2020|2020-11-09|EPI_ISL_669310|Original|hCoV-19^^Hovedstaden|Human|Department of Virus and Microbiological Special Diagnostics|Albertsen Lab|Michaelsen|Denmark
>Spike|hCoV-19/England/NORT-289726/2020|2020-03-31|EPI_ISL_458647|Original|hCoV-19^^England|Human|NU-OMICS DNA Sequencing research facility|Wellcome Sanger Institute for the COVID-19 Genomics UK (COG-UK) consortium|Davies|United Kingdom
>Spike|hCoV-19/Belgium/ULG-10870/2020|2020-11-13|EPI_ISL_681268|Original|hCoV-19^^Li\u00e8ge|Human|Department of Clinical Microbiology|GIGA Medical Genomics|Durkin|Belgium
>Spike|hCoV-19/Brazil/SP-L24-CD538/2020|2020-04-15|EPI_ISL_672696|Original|hCoV-19^^S\u00e3o Paulo|Human|Hospital das Cl\u00ednicas da Faculdade de Medicina da Universidade de S\u00e3o Paulo (HC-FMUSP)|Laborat\u00f3rio de Parasitologia M\u00e9dica - Instituto de Medicina Tropical - Universidade de S\u00e3o Paulo|de Jesus|Brazil
>Spike|hCoV-19/Wales/PHWC-49A140/2020|2020-11-30|EPI_ISL_727500|Original|hCoV-19^^Wales|Human|Originating lab|COVID-19 Genomics UK (COG-UK) Consortium|Moore|United Kingdom
>Spike|hCoV-19/Denmark/DCGC-14711/2020|2020-11-23|EPI_ISL_711273|Original|hCoV-19^^Nordjylland|Human|Department of Virus and Microbiological Special Diagnostics|Albertsen Lab|Michaelsen|Denmark
>Spike|hCoV-19/Australia/QLD1273/2020|2020-03-20|EPI_ISL_693275|Original|hCoV-19^^Australia|Human|unknown|Public Health Virology Laboratory|Nguyen|Australia
>Spike|hCoV-19/South Africa/KRISP-K001071/2020|2020-07-18|EPI_ISL_509325|Original|hCoV-19^^KwaZulu-Natal|Human|NHLS-IALCH|KRISP|de Oliveira|South Africa
>Spike|hCoV-19/USA/MI-UM-10036575847/2020|2020-10-22|EPI_ISL_650005|Original|hCoV-19^^Michigan|Human|University of Michigan Clinical Microbiology Laboratory|Lauring Lab|Valesano|USA
>Spike|hCoV-19/Wales/PHWC-3472C/2020|2020-04-19|EPI_ISL_446966|Original|hCoV-19^^Wales|Human|Wales Specialist Virology Centre|Public Health Wales Microbiology Cardiff|Moore|United Kingdom
>Spike|hCoV-19/Denmark/DCGC-7681/2020|2020-10-19|EPI_ISL_618562|Original|hCoV-19^^Sjaelland|Human|Department of Virus and Microbiological Special Diagnostics|Albertsen lab|Michaelsen|Denmark

>Spike|hCoV-19/England/QEUH-99C2A0/2020|2020-08-27|EPI_ISL_559791|Original|hCoV-19^^England|Human|Oxford Viromics|COVID-19 Genomics UK (COG-UK) Consortium|Golubchik|United Kingdom
>Spike|hCoV-19/USA/CT-Yale-011/2020|2020-03-11|EPI_ISL_428369|Original|hCoV-19^^Connecticut|Human|Yale Clinical Virology Laboratory|Grubaugh Lab - Yale School of Public Health|Grubaugh|USA
>Spike|hCoV-19/England/20119032104/2020|2020-03-12|EPI_ISL_465046|Original|hCoV-19^^England|Human|Respiratory Virus Unit|Respiratory Virus Unit|Platt|United Kingdom
>Spike|hCoV-19/Australia/VIC1533/2020|2020-05-06|EPI_ISL_456478|Original|hCoV-19^^Victoria|Human|Microbiological Diagnostic Unit Public Health Laboratory|Microbiological Diagnostic Unit Public Health Laboratory|Schultz|Australia
>Spike|hCoV-19/England/QEUH-B80B0C/2020|2020-11-14|EPI_ISL_661938|Original|hCoV-19^^England|Human|Lighthouse Lab in Glasgow|Wellcome Sanger Institute for the COVID-19 Genomics UK (COG-UK) Consortium|Davies|United Kingdom
>Spike|hCoV-19/England/CAMB-84954/2020|2020-03-07|EPI_ISL_440474|Original|hCoV-19^^England|Human|Department of Pathology|Wellcome Sanger Institute for the COVID-19 Genomics UK (COG-UK) consortium|Amato|United Kingdom
>Spike|hCoV-19/England/CAMB-7D54B/2020|2020-04-07|EPI_ISL_441728|Original|hCoV-19^^England|Human|Department of Pathology|Wellcome Sanger Institute for the COVID-19 Genomics UK (COG-UK) consortium|Amato|United Kingdom
>Spike|hCoV-19/USA/CA-CZB-9527/2020|2020-04-07|EPI_ISL_672120|Original|hCoV-19^^California|Human|The Ashley Laboratory|Chan-Zuckerberg Biohub|Bhatt|USA
>Spike|hCoV-19/United Arab Emirates/0593/2020|2020-05-15|EPI_ISL_698751|Original|hCoV-19^^Abu Dhabi|Human|Group 42 (G42) Healthcare|G42 Healthcare|Ogrodzki|United Arab Emirates
>Spike|hCoV-19/Australia/VIC8297/2020|2020-08-15|EPI_ISL_564929|Original|hCoV-19^^Victoria|Human|Microbiological Diagnostic Unit - Public Health Laboratory (MDU-PHL)|MDU-PHL|Schultz|Australia
>Spike|hCoV-19/Scotland/CVR5401/2020|2020-09-22|EPI_ISL_651800|Original|hCoV-19^^Scotland|Human|Lighthouse Lab in Glasgow / MRC-University of Glasgow Centre for Virus Research|COVID-19 Genomics UK (COG-UK) Consortium|Robertson|United Kingdom
>Spike|hCoV-19/England/NORW-F7172/2020|2020-11-25|EPI_ISL_706390|Original|hCoV-19^^England|Human|Quadram Institute Bioscience|COVID-19 Genomics UK (COG-UK) Consortium|Alikhan|United Kingdom
>Spike|hCoV-19/England/NOTT-11773D/2020|2020-10-23|EPI_ISL_611840|Original|hCoV-19^^England|Human|Queens Medical Centre|COVID-19 Genomics UK (COG-UK) Consortium|Loose|United Kingdom
>Spike|hCoV-19/USA/FL-BPHL-1699/2020|2020-08-21|EPI_ISL_594388|Original|hCoV-19^^Florida|Human|Florida Bureau of Public Health Laboratories|Florida Bureau of Public Health Laboratories|Blanton|USA
>Spike|hCoV-19/Scotland/QEUH-96ECC7/2020|2020-08-19|EPI_ISL_534477|Original|hCoV-19^^Scotland|Human|NHSGGC West of Scotland Specialist Virology Centre / MRC-University of Glasgow Centre for Virus Research|Wellcome Sanger Institute for the COVID-19 Genomics UK (COG-UK) consortium|Davies|United Kingdom
>Spike|hCoV-19/Switzerland/BS-ETHZ-110467/2020|2020-04-06|EPI_ISL_451885|Original|hCoV-19^^Basel-Stadt|Human|Viollier AG|Department of Biosystems Science and Engineering|Nadeau|Switzerland
>Spike|hCoV-19/Jordan/AM-ALSR-3926/2020|2020-09-30|EPI_ISL_636291|Original|hCoV-19^^Amman|Human|Biolab Diagnostic Laboratories|Andersen lab at Scripps Research|Gangavarapu|Jordan
>Spike|hCoV-19/England/ALDP-BCA40B/2020|2020-11-23|EPI_ISL_703638|Original|hCoV-19^^England|Human|Lighthouse Lab in Alderley Park|Wellcome Sanger Institute for the COVID-19 Genomics UK (COG-UK) Consortium|Davies|United Kingdom
>Spike|hCoV-19/Netherlands/NA_669/2020|2020-03-09|EPI_ISL_461104|Original|hCoV-19^^Netherlands|Human|Dutch COVID-19 response team|Erasmus Medical Center|Oude Munnink|Netherlands
>Spike|hCoV-19/Switzerland/BS-UHB-42203355/2020|2020-03-23|EPI_ISL_528350|Original|hCoV-19^^Basel-Stadt|Human|University Hospital Basel|University Hospital Basel|Roloff Handschin|Switzerland

>Spike|hCoV-19/England/MILK-ABBB09/2020|2020-10-20|EPI_ISL_631030|Original|hCoV-19^^England|Human|Lighthouse Lab in Milton Keynes|Wellcome Sanger Institute for the COVID-19 Genomics UK (COG-UK) consortium|Davies|United Kingdom
>Spike|hCoV-19/USA/UT-UPHL-2012899488/2020|2020-08-09|EPI_ISL_745785|Original|hCoV-19^^Utah|Human|Ginkgo Bioworks Clinical Laboratory|Utah Public Health Laboratory|Young|USA
>Spike|hCoV-19/England/LIVE-DB7AEF/2020|2020-11-03|EPI_ISL_650453|Original|hCoV-19^^England|Human|Liverpool Clinical Laboratories|COVID-19 Genomics UK (COG-UK) Consortium|Haldenby|United Kingdom
>Spike|hCoV-19/England/QEUH-9BA093/2020|2020-09-05|EPI_ISL_573576|Original|hCoV-19^^England|Human|Oxford Viromics|COVID-19 Genomics UK (COG-UK) Consortium|Golubchik|United Kingdom
>Spike|hCoV-19/France/IDF-8679/2020|2020-06-25|EPI_ISL_560637|Original|hCoV-19^^\u00cele-de-France|Human|Labo Analyses Med|National Reference Center for Viruses of Respiratory Infections|Enouf|France
>Spike|hCoV-19/Iran/K1r-171/2020|2020-05-00|EPI_ISL_591325|Original|hCoV-19^^Iran|Human|Virology|Virology|Ranjbar|Iran
>Spike|hCoV-19/Hong Kong/VM20009614/2020|2020-04-06|EPI_ISL_444033|Original|hCoV-19^^Hong Kong|Human|Queen Mary Hospital|Hong Kong Department of Health|Mak|China
>Spike|hCoV-19/Denmark/DCGC-19193/2020|2020-12-07|EPI_ISL_748805|Original|hCoV-19^^Midtjylland|Human|Department of Virus and Microbiological Special Diagnostics|Albertsen Lab|Michaelsen|Denmark
>Spike|hCoV-19/England/NORW-EAD41/2020|2020-04-29|EPI_ISL_457372|Original|hCoV-19^^England|Human|Quadram Institute Bioscience|COVID-19 Genomics UK (COG-UK) Consortium|Alikhan|United Kingdom
>Spike|hCoV-19/England/CAMC-AAFAB0/2020|2020-10-23|EPI_ISL_623528|Original|hCoV-19^^England|Human|Lighthouse Lab in Cambridge|Wellcome Sanger Institute for the COVID-19 Genomics UK (COG-UK) consortium|Davies|United Kingdom
>Spike|hCoV-19/Wales/PHWC-47DB72/2020|2020-10-07|EPI_ISL_595334|Original|hCoV-19^^Wales|Human|Originating lab|COVID-19 Genomics UK (COG-UK) Consortium|Moore|United Kingdom
>Spike|hCoV-19/USA/AZ-TG280846/2020|2020-04-20|EPI_ISL_694187|Original|hCoV-19^^Arizona|Human|AZ SPHL|TGen North|French|USA
>Spike|hCoV-19/England/MILK-B8EDDB/2020|2020-11-13|EPI_ISL_674638|Original|hCoV-19^^England|Human|Lighthouse Lab in Milton Keynes|Wellcome Sanger Institute for the COVID-19 Genomics UK (COG-UK) Consortium|Davies|United Kingdom
>Spike|hCoV-19/England/CAMC-A811EC/2020|2020-10-13|EPI_ISL_610841|Original|hCoV-19^^England|Human|Lighthouse Lab in Cambridge|Wellcome Sanger Institute for the COVID-19 Genomics UK (COG-UK) consortium|Davies|United Kingdom
>Spike|hCoV-19/USA/TX-HMH-MCoV-11518/2020|2020-09-08|EPI_ISL_790041|Original|hCoV-19^^Texas|Human|Houston Methodist Hospital|Houston Methodist Hospital|Long|USA
>Spike|hCoV-19/England/ALDP-A68BCF/2020|2020-10-12|EPI_ISL_608992|Original|hCoV-19^^England|Human|Lighthouse Lab in Alderley Park|Wellcome Sanger Institute for the COVID-19 Genomics UK (COG-UK) consortium|Davies|United Kingdom
>Spike|hCoV-19/England/CAMC-A57C75/2020|2020-10-09|EPI_ISL_597265|Original|hCoV-19^^England|Human|Lighthouse Lab in Cambridge|Wellcome Sanger Institute for the COVID-19 Genomics UK (COG-UK) consortium|Davies|United Kingdom
>Spike|hCoV-19/Japan/PG-8531/2020|2020-07-00|EPI_ISL_686127|Original|hCoV-19^^Japan|Human|Pathogen Genomics Center|Pathogen Genomics Center|Sekizuka|Japan
>Spike|hCoV-19/Wales/PHWC-49E276/2020|2020-12-07|EPI_ISL_742976|Original|hCoV-19^^Wales|Human|Wales Specialist Virology Centre Sequencing lab|COVID-19 Genomics UK (COG-UK) Consortium|Moore|United Kingdom
>Spike|hCoV-19/USA/MS-CDC-6515/2020|2020-08-22|EPI_ISL_648038|Original|hCoV-19^^Mississippi|Human|MS Public Health Laboratory|Pathogen Discovery|Queen|USA
>Spike|hCoV-19/England/CAMB-7F752/2020|2020-04-09|EPI_ISL_442899|Original|hCoV-19^^England|Human|Department of Pathology|Wellcome Sanger Institute for the COVID-19 Genomics UK (COG-UK) consortium|Amato|United Kingdom
>Spike|hCoV-19/USA/TX-HMH-MCoV-19057/2020|2020-11-23|EPI_ISL_783605|Original|hCoV-19^^Texas|Human|Houston Methodist Hospital|Houston Methodist Hospital|Long|USA

>Spike|hCoV-19/Wales/PHWC-2C2EA/2020|2020-03-30|EPI_ISL_474305|Original|hCoV-19^^Wales|Human|Originating lab|COVID-19 Genomics UK (COG-UK) Consortium|Moore|United Kingdom
>Spike|hCoV-19/England/EXET-13AE7B/2020|2020-11-11|EPI_ISL_664271|Original|hCoV-19^^England|Human|University of Exeter|COVID-19 Genomics UK (COG-UK) Consortium|Temperton|United Kingdom
>Spike|hCoV-19/Wales/PHWC-315F8/2020|2020-04-13|EPI_ISL_446467|Original|hCoV-19^^Wales|Human|Wales Specialist Virology Centre|Public Health Wales Microbiology Cardiff|Moore|United Kingdom
>Spike|hCoV-19/Northern Ireland/NIRE-101684/2020|2020-04-30|EPI_ISL_469871|Original|hCoV-19^^Northern Ireland|Human|Regional Virus Laboratory|Wellcome Sanger Institute for the COVID-19 Genomics UK (COG-UK) consortium|Davies|United Kingdom
>Spike|hCoV-19/South Africa/NHLS-UCT-GS-1025/2020|2020-10-26|EPI_ISL_700435|Original|hCoV-19^^Western Cape|Human|Pacaltsdorp Clinic wc PAC|NHLS/UCT|Iranzadeh|South Africa
>Spike|hCoV-19/Brazil/RJ-DCVN4/2020|2020-07-20|EPI_ISL_510541|Vero E6|hCoV-19^^Rio de Janeiro|Human|Centro de Desenvolvimento Tecnologico em Saude|Centro de Desenvolvimento Tecnologico em Saude|Souza|Brazil
>Spike|hCoV-19/India/UT-AR40/2020|2020-05-28|EPI_ISL_511912|Original|hCoV-19^^Uttarakhand|Human|All india institute of Medical Sciences Rishikesh|National Institute of Biomedical Genomics -\u00a0DBT's PAN-INDIA 1000 SARS--CoV-2 RNA Genome Sequencing Consortium|Maitra|India
>Spike|hCoV-19/Wales/PHWC-495BCF/2020|2020-11-25|EPI_ISL_726452|Original|hCoV-19^^Wales|Human|Originating lab|COVID-19 Genomics UK (COG-UK) Consortium|Moore|United Kingdom
>Spike|hCoV-19/Denmark/DCGC-7093/2020|2020-10-12|EPI_ISL_619135|Original|hCoV-19^^Midtjylland|Human|Department of Virus and Microbiological Special Diagnostics|Albertsen lab|Michaelsen|Denmark
>Spike|hCoV-19/Australia/VIC5567/2020|2020-07-09|EPI_ISL_518386|Original|hCoV-19^^Victoria|Human|Microbiological Diagnostic Unit - Public Health Laboratory (MDU-PHL)|MDU-PHL|Schultz|Australia
>Spike|hCoV-19/Sweden/20-52885/2020|2020-09-13|EPI_ISL_615106|Original|hCoV-19^^Halland|Human|Halmstad klinisk mikrobiologi|The Public Health Agency of Sweden|Svartstr\u00f6m|Sweden
>Spike|hCoV-19/USA/FL-BPHL-1824/2020|2020-04-08|EPI_ISL_613911|Original|hCoV-19^^Florida|Human|Florida Bureau of Public Health Laboratories|Florida Bureau of Public Health Laboratories|Blanton|USA
>Spike|hCoV-19/Denmark/DCGC-14806/2020|2020-11-23|EPI_ISL_711997|Original|hCoV-19^^Nordjylland|Human|Department of Virus and Microbiological Special Diagnostics|Albertsen Lab|Michaelsen|Denmark
>Spike|hCoV-19/England/ALDP-A87A09/2020|2020-10-17|EPI_ISL_606247|Original|hCoV-19^^England|Human|Lighthouse Lab in Alderley Park|Wellcome Sanger Institute for the COVID-19 Genomics UK (COG-UK) consortium|Davies|United Kingdom
>Spike|hCoV-19/England/CAMC-B3576C/2020|2020-11-05|EPI_ISL_659470|Original|hCoV-19^^England|Human|Lighthouse Lab in Cambridge|Wellcome Sanger Institute for the COVID-19 Genomics UK (COG-UK) Consortium|Davies|United Kingdom
>Spike|hCoV-19/Denmark/DCGC-6530/2020|2020-10-05|EPI_ISL_619430|Original|hCoV-19^^Hovedstaden|Human|Department of Virus and Microbiological Special Diagnostics|Albertsen lab|Michaelsen|Denmark
>Spike|hCoV-19/England/ALDP-9B62FB/2020|2020-08-28|EPI_ISL_572998|Original|hCoV-19^^England|Human|Oxford Viromics|COVID-19 Genomics UK (COG-UK) Consortium|Golubchik|United Kingdom
>Spike|hCoV-19/Netherlands/Zeeland_20/2020|2020-03-18|EPI_ISL_461259|Original|hCoV-19^^Zeeland|Human|Dutch COVID-19 response team|Erasmus Medical Center|Oude Munnink|Netherlands
>Spike|hCoV-19/Netherlands/ZuidHolland_75/2020|2020-03-26|EPI_ISL_422943|Original|hCoV-19^^Zuid Holland|Human|Dutch COVID-19 response team|Erasmus Medical Center|Oude Munnink|Netherlands

>Spike|hCoV-19/England/NOTT-11051C/2020|2020-04-23|EPI_ISL_441526|Original|hCoV-19^^England|Human|Queens Medical Centre|COVID-19 Genomics UK (COG-UK) Consortium|Loose|United Kingdom
>Spike|hCoV-19/USA/TX-HMH-7376/2020|2020-06-29|EPI_ISL_546716|Original|hCoV-19^^Texas|Human|Houston Methodist Hospital|Houston Methodist Hospital|Long|USA
>Spike|hCoV-19/India/TG-CCMB-OM21/2020|2020-09-20|EPI_ISL_644715|Original|hCoV-19^^Telangana|Human|Osmania Medical College|CSIR-Centre for Cellular and Molecular Biology|Mukherjee|India
>Spike|hCoV-19/England/QEUH-9FF9A6/2020|2020-09-30|EPI_ISL_600241|Original|hCoV-19^^England|Human|Lighthouse Lab in Glasgow|Wellcome Sanger Institute for the COVID-19 Genomics UK (COG-UK) consortium|Davies|United Kingdom
>Spike|hCoV-19/Denmark/DCGC-18922/2020|2020-12-07|EPI_ISL_748349|Original|hCoV-19^^Hovedstaden|Human|Department of Virus and Microbiological Special Diagnostics|Albertsen Lab|Michaelsen|Denmark
>Spike|hCoV-19/England/SHEF-CF137/2020|2020-05-27|EPI_ISL_535088|Original|hCoV-19^^England|Human|Virology Department|COVID-19 Genomics UK (COG-UK) Consortium|de Silva|United Kingdom
>Spike|hCoV-19/Ireland/G-NVRL-78IRL79027/2020|2020-11-23|EPI_ISL_752593|Original|hCoV-19^^Galway|Human|National Virus Reference Laboratory|National Virus Reference Laboratory|Gonzalez|Ireland
>Spike|hCoV-19/Indonesia/JT-UNS-18_39701/2020|2020-10-14|EPI_ISL_759701|Original|hCoV-19^^Central Java|Human|RS UNS|UNS/RS-UNS|Suryawati|Indonesia
>Spike|hCoV-19/Wales/PHWC-16E227/2020|2020-09-30|EPI_ISL_585686|Original|hCoV-19^^Wales|Human|Wales Specialist Virology Centre Sequencing lab|COVID-19 Genomics UK (COG-UK) Consortium|Moore|United Kingdom
>Spike|hCoV-19/Netherlands/GR-RIVM-20790/2020|2020-12-15|EPI_ISL_790850|Original|hCoV-19^^Groningen|Human|Dutch COVID-19 response team|National Institute for Public Health and the Environment (RIVM)|Meijer|Netherlands
>Spike|hCoV-19/Finland/6LS3H9/2020|2020-10-06|EPI_ISL_756087|Original|hCoV-19^^Uusimaa|Human|Department of Virology and Immunology|Department of Virology|Smura|Finland
>Spike|hCoV-19/Switzerland/BE-ETHZ-300279/2020|2020-10-01|EPI_ISL_603311|Original|hCoV-19^^Bern|Human|Viollier AG|Department of Biosystems Science and Engineering|Nadeau|Switzerland
>Spike|hCoV-19/USA/TX-HMH-MCoV-12917/2020|2020-08-18|EPI_ISL_788069|Original|hCoV-19^^Texas|Human|Houston Methodist Hospital|Houston Methodist Hospital|Long|USA
>Spike|hCoV-19/England/SHEF-C236B/2020|2020-11-22|EPI_ISL_680121|Original|hCoV-19^^England|Human|Virology Department|COVID-19 Genomics UK (COG-UK) Consortium|de Silva|United Kingdom
>Spike|hCoV-19/England/QEUH-9B5001/2020|2020-09-07|EPI_ISL_590182|Original|hCoV-19^^England|Human|Lighthouse Lab in Glasgow|Wellcome Sanger Institute for the COVID-19 Genomics UK (COG-UK) consortium|Davies|United Kingdom
>Spike|hCoV-19/Denmark/DCGC-7176/2020|2020-10-12|EPI_ISL_618925|Original|hCoV-19^^Hovedstaden|Human|Department of Virus and Microbiological Special Diagnostics|Albertsen lab|Michaelsen|Denmark
>Spike|hCoV-19/Wales/PHWC-16912F/2020|2020-05-15|EPI_ISL_499396|Original|hCoV-19^^Wales|Human|Originating lab|COVID-19 Genomics UK (COG-UK) Consortium|Moore|United Kingdom
>Spike|hCoV-19/New Zealand/20VR3894/2020|2020-00-00|EPI_ISL_548091|Original|hCoV-19^^Auckland|Human|LabPLUS|Institute of Environmental Science and Research (ESR)|Wang|New Zealand
>Spike|hCoV-19/Denmark/DCGC-7458/2020|2020-10-12|EPI_ISL_619393|Original|hCoV-19^^Hovedstaden|Human|Department of Virus and Microbiological Special Diagnostics|Albertsen lab|Michaelsen|Denmark
>Spike|hCoV-19/USA/VA-DCLS-0078/2020|2020-03-27|EPI_ISL_429977|Original|hCoV-19^^Virginia|Human|Virginia DCLS|Virginia DCLS|St. Jacques|USA

>Spike|hCoV-19/England/MILK-B561B2/2020|2020-11-06|EPI_ISL_657709|Original|hCoV-19^^England|Human|Lighthouse Lab in Milton Keynes|Wellcome Sanger Institute for the COVID-19 Genomics UK (COG-UK) Consortium|Davies|United Kingdom
>Spike|hCoV-19/Switzerland/BL-ETHZ-100079/2020|2020-03-12|EPI_ISL_451671|Original|hCoV-19^^Basel-Landschaft|Human|Viollier AG|Department of Biosystems Science and Engineering|Nadeau|Switzerland
>Spike|hCoV-19/Australia/TAS114/2020|2020-03-23|EPI_ISL_522580|Original|hCoV-19^^Tasmania|Human|Royal Hobart Hospital Microbiology Department|MDU-PHL|Schultz|Australia
>Spike|hCoV-19/Japan/PG-8793/2020|2020-08-00|EPI_ISL_686381|Original|hCoV-19^^Japan|Human|Pathogen Genomics Center|Pathogen Genomics Center|Sekizuka|Japan
>Spike|hCoV-19/England/MILK-6A7120/2020|2020-07-02|EPI_ISL_551814|Original|hCoV-19^^England|Human|Lighthouse Lab in Milton Keynes|Wellcome Sanger Institute for the COVID-19 Genomics UK (COG-UK) consortium|Davies|United Kingdom
>Spike|hCoV-19/England/LIVE-A5C54/2020|2020-03-18|EPI_ISL_499573|Original|hCoV-19^^England|Human|Liverpool Clinical Laboratories|COVID-19 Genomics UK (COG-UK) Consortium|Haldenby|United Kingdom
>Spike|hCoV-19/USA/MI-MDHHS-SC20505/2020|2020-05-11|EPI_ISL_460043|Original|hCoV-19^^Michigan|Human|Michigan Department of Health and Human Services|Michigan Department of Health and Human Services|Blankenship|USA
>Spike|hCoV-19/England/NOTT-10FFB8/2020|2020-04-06|EPI_ISL_441460|Original|hCoV-19^^England|Human|Queens Medical Centre|COVID-19 Genomics UK (COG-UK) Consortium|Loose|United Kingdom
>Spike|hCoV-19/USA/CA-CZB-4069/2020|2020-08-05|EPI_ISL_548674|Original|hCoV-19^^California|Human|Orange County Public Health Laboratory|Chan-Zuckerberg Biohub|Bhatt|USA
>Spike|hCoV-19/England/MILK-9C6F1F/2020|2020-09-08|EPI_ISL_573268|Original|hCoV-19^^England|Human|Northumbria University / South Tees Hospitals NHS Foundation Trust / North Cumbria Integrated Care NHS Foundation Trust / North Tees and Hartlepool NHS Foundation Trust / Newcastle Hospitals NHS Foundation Trust|COVID-19 Genomics UK (COG-UK) Consortium|Bashton|United Kingdom
>Spike|hCoV-19/Denmark/DCGC-12245/2020|2020-11-16|EPI_ISL_682426|Original|hCoV-19^^Hovedstaden|Human|Department of Virus and Microbiological Special Diagnostics|Albertsen Lab|Michaelsen|Denmark
>Spike|hCoV-19/England/ALDP-B8486B/2020|2020-11-14|EPI_ISL_675923|Original|hCoV-19^^England|Human|Lighthouse Lab in Alderley Park|Wellcome Sanger Institute for the COVID-19 Genomics UK (COG-UK) Consortium|Davies|United Kingdom
>Spike|hCoV-19/England/ALDP-B48FE6/2020|2020-11-07|EPI_ISL_649371|Original|hCoV-19^^England|Human|Lighthouse Lab in Alderley Park|Wellcome Sanger Institute for the COVID-19 Genomics UK (COG-UK) Consortium|Davies|United Kingdom
>Spike|hCoV-19/England/CAMC-B37CF2/2020|2020-11-05|EPI_ISL_658836|Original|hCoV-19^^England|Human|Lighthouse Lab in Cambridge|Wellcome Sanger Institute for the COVID-19 Genomics UK (COG-UK) Consortium|Davies|United Kingdom
>Spike|hCoV-19/England/CAMB-7BB69/2020|2020-04-04|EPI_ISL_442166|Original|hCoV-19^^England|Human|Department of Pathology|Wellcome Sanger Institute for the COVID-19 Genomics UK (COG-UK) consortium|Amato|United Kingdom
>Spike|hCoV-19/South Africa/KRISP-K003355/2020|2020-09-06|EPI_ISL_602746|Original|hCoV-19^^KwaZulu-Natal|Human|NHLS-IALCH|KRISP|de Oliveira|South Africa
>Spike|hCoV-19/Wales/PHWC-4A0A2E/2020|2020-12-10|EPI_ISL_743530|Original|hCoV-19^^Wales|Human|Wales Specialist Virology Centre Sequencing lab|COVID-19 Genomics UK (COG-UK) Consortium|Moore|United Kingdom
>Spike|hCoV-19/England/OXON-B2F79/2020|2020-03-24|EPI_ISL_534830|Original|hCoV-19^^England|Human|Oxford Viromics|COVID-19 Genomics UK (COG-UK) Consortium|Golubchik|United Kingdom
>Spike|hCoV-19/Denmark/DCGC-6525/2020|2020-10-05|EPI_ISL_619425|Original|hCoV-19^^Midtjylland|Human|Department of Virus and Microbiological Special Diagnostics|Albertsen lab|Michaelsen|Denmark

>Spike|hCoV-19/England/MILK-991F1D/2020|2020-08-27|EPI_ISL_551947|Original|hCoV-19^^England|Human|Lighthouse Lab in Milton Keynes|Wellcome Sanger Institute for the COVID-19 Genomics UK (COG-UK) consortium|Davies|United Kingdom
>Spike|hCoV-19/France/ARA-36371/2020|2020-05-11|EPI_ISL_678543|Original|hCoV-19^^Auvergne-Rh\u00f4ne-Alpes|Human|CNR Virus des Infections Respiratoires - France SUD|CNR Virus des Infections Respiratoires - France SUD|Bal|France
>Spike|hCoV-19/USA/CA-CZB-1994/2020|2020-04-30|EPI_ISL_486257|Original|hCoV-19^^California|Human|Orange County Public Health Laboratory|Chan-Zuckerberg Biohub|McGeever|USA
>Spike|hCoV-19/England/NORW-E9A0F/2020|2020-04-22|EPI_ISL_448348|Original|hCoV-19^^England|Human|Quadram Institute Bioscience|COVID-19 Genomics UK (COG-UK) Consortium|Alikhan|United Kingdom
>Spike|hCoV-19/England/ALDP-9C5262/2020|2020-09-10|EPI_ISL_567763|Original|hCoV-19^^England|Human|Lighthouse Lab in Alderley Park|Wellcome Sanger Institute for the COVID-19 Genomics UK (COG-UK) consortium|Davies|United Kingdom
>Spike|hCoV-19/England/ALDP-A7021A/2020|2020-10-15|EPI_ISL_608330|Original|hCoV-19^^England|Human|Lighthouse Lab in Alderley Park|Wellcome Sanger Institute for the COVID-19 Genomics UK (COG-UK) consortium|Davies|United Kingdom
>Spike|hCoV-19/India/MH-ACTREC-648/2020|2020-09-14|EPI_ISL_700278|Original|hCoV-19^^Maharashtra|Human|Hematopathology Laboratory|Hematopathology Laboratory|Patkar|India
>Spike|hCoV-19/Germany/BE-ChVir-D2003-5974/2020|2020-10-15|EPI_ISL_753893|Original|hCoV-19^^Berlin|Human|Charit\u00e9 Universit\u00e4tsmedizin Berlin|Charit\u00e9 Universit\u00e4tsmedizin Berlin|Muehlemann|Germany
>Spike|hCoV-19/England/QEUH-B80577/2020|2020-11-14|EPI_ISL_661771|Original|hCoV-19^^England|Human|Lighthouse Lab in Glasgow|Wellcome Sanger Institute for the COVID-19 Genomics UK (COG-UK) Consortium|Davies|United Kingdom
>Spike|hCoV-19/Australia/VIC16191/2020|2020-08-03|EPI_ISL_640657|Original|hCoV-19^^Victoria|Human|Microbiological Diagnostic Unit - Public Health Laboratory (MDU-PHL)|MDU-PHL|Schultz|Australia
>Spike|hCoV-19/Portugal/PT0645/2020|2020-03-29|EPI_ISL_510906|Original|hCoV-19^^Portugal|Human|Instituto Nacional de Saude (INSA)|Instituto Nacional de Saude (INSA)|Borges|Portugal
>Spike|hCoV-19/Spain/AR-IBV-98008119/2020|2020-10-13|EPI_ISL_654457|Original|hCoV-19^^Aragon|Human|Servicio de Microbiolog\u00eda|SeqCOVID-SPAIN consortium/IBV(CSIC)|Comas|Spain
>Spike|hCoV-19/England/CAMC-B071ED/2020|2020-10-30|EPI_ISL_643707|Original|hCoV-19^^England|Human|Lighthouse Lab in Cambridge|Wellcome Sanger Institute for the COVID-19 Genomics UK (COG-UK) Consortium|Davies|United Kingdom
>Spike|hCoV-19/Wales/PHWC-490CD0/2020|2020-11-16|EPI_ISL_706560|Original|hCoV-19^^Wales|Human|Originating lab|COVID-19 Genomics UK (COG-UK) Consortium|Moore|United Kingdom
>Spike|hCoV-19/Scotland/QEUH-B2D03F/2020|2020-11-04|EPI_ISL_680577|Original|hCoV-19^^Scotland|Human|Department of Pathology|COVID-19 Genomics UK (COG-UK) Consortium|Goodfellow|United Kingdom
>Spike|hCoV-19/England/MILK-976303/2020|2020-08-19|EPI_ISL_575871|Original|hCoV-19^^England|Human|Lighthouse Lab in Milton Keynes|Wellcome Sanger Institute for the COVID-19 Genomics UK (COG-UK) consortium|Davies|United Kingdom
>Spike|hCoV-19/England/MILK-9E15FE/2020|2020-09-19|EPI_ISL_577077|Original|hCoV-19^^England|Human|Oxford Viromics|COVID-19 Genomics UK (COG-UK) Consortium|Golubchik|United Kingdom
>Spike|hCoV-19/England/201060067/2020|2020-03-04|EPI_ISL_464393|Original|hCoV-19^^England|Human|Respiratory Virus Unit|Respiratory Virus Unit|Platt|United Kingdom
>Spike|hCoV-19/Latvia/013/2020|2020-03-20|EPI_ISL_437092|Original|hCoV-19^^Latvia|Human|Latvijas Infektolo\u0123ijas centrs|Latvian Biomedical Research and Study Centre|Zrelovs|Latvia
>Spike|hCoV-19/Poland/Pom9/2020|2020-04-27|EPI_ISL_451650|Original|hCoV-19^^Pomorze|Human|Hematology Laboratory|Laboratory of Recombinant Vaccines|Rabalski|Poland

>Spike|hCoV-19/Spain/IB-IBV-99011585/2020|2020-12-18|EPI_ISL_779908|Original|hCoV-19^^Balear Islands|Human|Servicio de Microbiolog\u00eda|SeqCOVID-SPAIN consortium/IBV(CSIC)|Comas|Spain
>Spike|hCoV-19/England/QEUH-C01337/2020|2020-12-04|EPI_ISL_720728|Original|hCoV-19^^England|Human|Lighthouse Lab in Glasgow|Wellcome Sanger Institute for the COVID-19 Genomics UK (COG-UK) Consortium|Davies|United Kingdom
>Spike|hCoV-19/France/PAC-IHU-0409/2020|2020-04-02|EPI_ISL_569493|Original|hCoV-19^^Provence-Alpes-C\u00f4te-d\u2019Azur|Human|MEPHI|MEPHI|Levasseur|France
>Spike|hCoV-19/Russia/StPetersburg-RII15817S/2020|2020-05-18|EPI_ISL_507273|Original|hCoV-19^^Saint Petersburg|Human|WHO National Influenza Centre Russian Federation|WHO National Influenza Centre Russian Federation|Komissarov|Russia
>Spike|hCoV-19/Denmark/DCGC-3373/2020|2020-08-31|EPI_ISL_616131|Original|hCoV-19^^Hovedstaden|Human|Department of Virus and Microbiological Special Diagnostics|Albertsen lab|Michaelsen|Denmark
>Spike|hCoV-19/USA/TX-HMH-1898/2020|2020-05-06|EPI_ISL_542894|Original|hCoV-19^^Texas|Human|Houston Methodist Hospital|Houston Methodist Hospital|Long|USA
>Spike|hCoV-19/Australia/VIC16180/2020|2020-08-06|EPI_ISL_641221|Original|hCoV-19^^Victoria|Human|Microbiological Diagnostic Unit - Public Health Laboratory (MDU-PHL)|MDU-PHL|Schultz|Australia
>Spike|hCoV-19/USA/TX-HMH-MCoV-13985/2020|2020-08-16|EPI_ISL_787276|Original|hCoV-19^^Texas|Human|Houston Methodist Hospital|Houston Methodist Hospital|Long|USA
>Spike|hCoV-19/Bangladesh/DNAS_DRICM_isl_BRB-13/2020|2020-06-28|EPI_ISL_774987|Original|hCoV-19^^Dhaka|Human|Designated Reference Institute for Chemical Measurements (DRICM)|DNA SOLUTION LTD.|Rabbi|Bangladesh
>Spike|hCoV-19/USA/TX-HMH-MCoV-8572/2020|2020-07-01|EPI_ISL_780632|Original|hCoV-19^^Texas|Human|Houston Methodist Hospital|Houston Methodist Hospital|Long|USA
>Spike|hCoV-19/England/MILK-ACCF90/2020|2020-10-21|EPI_ISL_630156|Original|hCoV-19^^England|Human|Lighthouse Lab in Milton Keynes|Wellcome Sanger Institute for the COVID-19 Genomics UK (COG-UK) consortium|Davies|United Kingdom
>Spike|hCoV-19/Australia/VIC16523/2020|2020-07-25|EPI_ISL_640798|Original|hCoV-19^^Victoria|Human|Microbiological Diagnostic Unit - Public Health Laboratory (MDU-PHL)|MDU-PHL|Schultz|Australia
>Spike|hCoV-19/Italy/LAZ-INMI-99/2020|2020-11-03|EPI_ISL_718264|Original|hCoV-19^^Lazio|Human|National Institute for Infectious Diseases|National Institute for Infectious Diseases|Gruber|Italy
>Spike|hCoV-19/England/ALDP-A2FB4C/2020|2020-10-05|EPI_ISL_587113|Original|hCoV-19^^England|Human|Lighthouse Lab in Alderley Park|Wellcome Sanger Institute for the COVID-19 Genomics UK (COG-UK) consortium|Davies|United Kingdom
>Spike|hCoV-19/Japan/IC-0314/2020|2020-11-00|EPI_ISL_667743|Original|hCoV-19^^Japan|Human|Pathogen Genomics Center|Pathogen Genomics Center|Sekizuka|Japan
>Spike|hCoV-19/SaudiArabia/KAUST-MADINAH1036/2020|2020-06-11|EPI_ISL_513073|Original|hCoV-19^^Madinah|Human|Pathogen Genomics Lab King Abdullah University of Science and Technology(KAUST)|Pathogen Genomics Lab King Abdullah University of Science and Technology(KAUST)|Mohamed Ghazzali|Saudi Arabia
>Spike|hCoV-19/USA/UT-UPHL-2012505034/2020|2020-08-12|EPI_ISL_745950|Original|hCoV-19^^Utah|Human|Ginkgo Bioworks Clinical Laboratory|Utah Public Health Laboratory|Young|USA
>Spike|hCoV-19/USA/CA-CZB-2082/2020|2020-05-04|EPI_ISL_486325|Original|hCoV-19^^California|Human|San Joaquin County Public Health Lab|Chan-Zuckerberg Biohub|McGeever|USA
>Spike|hCoV-19/England/ALDP-94B13A/2020|2020-06-13|EPI_ISL_555824|Original|hCoV-19^^England|Human|Lighthouse Lab in Alderley Park|Wellcome Sanger Institute for the COVID-19 Genomics UK (COG-UK) consortium|Davies|United Kingdom
>Spike|hCoV-19/Australia/SAP195/2020|2020-03-24|EPI_ISL_483068|Original|hCoV-19^^South Australia|Human|SA Pathology|SA Pathology|Leong|Australia
>Spike|hCoV-19/England/BRIS-18541F0/2020|2020-04-28|EPI_ISL_492278|Original|hCoV-19^^England|Human|PHE South West Regional Laboratory|Wellcome Sanger Institute for the COVID-19 Genomics UK (COG-UK) consortium|Davies|United Kingdom

>Spike|hCoV-19/England/BRIS-12A9D7/2020|2020-04-30|EPI_ISL_458376|Original|hCoV-19^^England|Human|PHE South West Regional Laboratory|Wellcome Sanger Institute for the COVID-19 Genomics UK (COG-UK) consortium|Davies|United Kingdom
>Spike|hCoV-19/England/MILK-BAFDF2/2020|2020-11-17|EPI_ISL_720475|Original|hCoV-19^^England|Human|Lighthouse Lab in Milton Keynes|Wellcome Sanger Institute for the COVID-19 Genomics UK (COG-UK) Consortium|Davies|United Kingdom
>Spike|hCoV-19/England/ALDP-94CF11/2020|2020-06-14|EPI_ISL_555899|Original|hCoV-19^^England|Human|Lighthouse Lab in Alderley Park|Wellcome Sanger Institute for the COVID-19 Genomics UK (COG-UK) consortium|Davies|United Kingdom
>Spike|hCoV-19/USA/MI-MDHHS-SC21656/2020|2020-07-04|EPI_ISL_516383|Original|hCoV-19^^Michigan|Human|Michigan Department of Health and Human Services|Michigan Department of Health and Human Services|Blankenship|USA
>Spike|hCoV-19/Wales/PHWC-497D30/2020|2020-11-30|EPI_ISL_726967|Original|hCoV-19^^Wales|Human|Originating lab|COVID-19 Genomics UK (COG-UK) Consortium|Moore|United Kingdom
>Spike|hCoV-19/England/QEUH-896F37/2020|2020-07-25|EPI_ISL_532802|Original|hCoV-19^^England|Human|Lighthouse Lab in Glasgow|Wellcome Sanger Institute for the COVID-19 Genomics UK (COG-UK) consortium|Davies|United Kingdom
>Spike|hCoV-19/Scotland/QEUH-A00AEA/2020|2020-10-02|EPI_ISL_585547|Original|hCoV-19^^Scotland|Human|University of Exeter|COVID-19 Genomics UK (COG-UK) Consortium|Temperton|United Kingdom
>Spike|hCoV-19/Australia/VIC14620/2020|2020-08-05|EPI_ISL_592918|Original|hCoV-19^^Victoria|Human|Microbiological Diagnostic Unit - Public Health Laboratory (MDU-PHL)|MDU-PHL|Schultz|Australia
>Spike|hCoV-19/England/QEUH-C163C6/2020|2020-12-05|EPI_ISL_709394|Original|hCoV-19^^England|Human|Lighthouse Lab in Glasgow|Wellcome Sanger Institute for the COVID-19 Genomics UK (COG-UK) Consortium|Davies|United Kingdom
>Spike|hCoV-19/Wales/PHWC-166984/2020|2020-06-19|EPI_ISL_494065|Original|hCoV-19^^Wales|Human|Originating lab|COVID-19 Genomics UK (COG-UK) Consortium|Moore|United Kingdom
>Spike|hCoV-19/Brazil/SP-699/2020|2020-06-30|EPI_ISL_693241|Original|hCoV-19^^S\u00e3o Paulo|Human|Hospital e Maternidade Sao Lucas|Instituto Adolfo Lutz|Sacchi|Brazil
>Spike|hCoV-19/USA/TX-HMH-7543/2020|2020-06-25|EPI_ISL_546838|Original|hCoV-19^^Texas|Human|Houston Methodist Hospital|Houston Methodist Hospital|Long|USA
>Spike|hCoV-19/Wales/PHWC-27C3C/2020|2020-03-31|EPI_ISL_432362|Original|hCoV-19^^Wales|Human|Wales Specialist Virology Centre|Public Health Wales Microbiology Cardiff|Moore|United Kingdom
>Spike|hCoV-19/Switzerland/VD-ETHZ-321394/2020|2020-10-13|EPI_ISL_603635|Original|hCoV-19^^Vaud|Human|Viollier AG|Department of Biosystems Science and Engineering|Nadeau|Switzerland
>Spike|hCoV-19/England/QEUH-B1210D/2020|2020-11-02|EPI_ISL_642529|Original|hCoV-19^^England|Human|Lighthouse Lab in Glasgow|Wellcome Sanger Institute for the COVID-19 Genomics UK (COG-UK) Consortium|Davies|United Kingdom
>Spike|hCoV-19/England/QEUH-96080D/2020|2020-08-02|EPI_ISL_531952|Original|hCoV-19^^England|Human|Lighthouse Lab in Glasgow|Wellcome Sanger Institute for the COVID-19 Genomics UK (COG-UK) consortium|Davies|United Kingdom
>Spike|hCoV-19/USA/NY-NYCPHL-001533/2020|2020-12-01|EPI_ISL_745451|Original|hCoV-19^^New York|Human|DOHMH Central Harlem|New York City Public Health Laboratory|Wang|USA
>Spike|hCoV-19/New Zealand/20VR2524/2020|2020-00-00|EPI_ISL_579269|Original|hCoV-19^^Auckland|Human|LabPLUS|Institute of Environmental Science and Research (ESR)|Wang|New Zealand
>Spike|hCoV-19/USA/WI-UW-348/2020|2020-04-15|EPI_ISL_450702|Original|hCoV-19^^Wisconsin|Human|University of Wisconsin-Madison AIDS Vaccine Research Laboratories|University of Wisconsin-Madison AIDS Vaccine Research Laboratories|Baczenas|USA
>Spike|hCoV-19/Luxembourg/LNS2924827/2020|2020-10-21|EPI_ISL_740219|Original|hCoV-19^^Luxembourg|Human|Laboratoire national de sant\u00e9|Laboratoire national de sant\u00e9|Wienecke-Baldacchino|Luxembourg

>Spike|hCoV-19/USA/FL-BPHL-0976/2020|2020-07-29|EPI_ISL_517866|Original|hCoV-19^^Florida|Human|Florida Bureau of Public Health Laboratories|Florida Bureau of Public Health Laboratories|Blanton|USA
>Spike|hCoV-19/USA/MO-WUSTL050/2020|2020-06-19|EPI_ISL_493077|Original|hCoV-19^^Missouri|Human|Washington University in St. Louis|Washington University in St. Louis|Tahan|USA
>Spike|hCoV-19/England/CAMB-1AFB6B/2020|2020-05-04|EPI_ISL_459179|Original|hCoV-19^^England|Human|Department of Pathology|Wellcome Sanger Institute for the COVID-19 Genomics UK (COG-UK) consortium|Davies|United Kingdom
>Spike|hCoV-19/USA/WA-S1149/2020|2020-05-05|EPI_ISL_463639|Original|hCoV-19^^Washington|Human|Washington State Department of Health|Seattle Flu Study|Bedford|USA
>Spike|hCoV-19/England/CAMC-B07B76/2020|2020-10-29|EPI_ISL_643829|Original|hCoV-19^^England|Human|Lighthouse Lab in Cambridge|Wellcome Sanger Institute for the COVID-19 Genomics UK (COG-UK) Consortium|Davies|United Kingdom
>Spike|hCoV-19/Australia/VIC13443/2020|2020-09-07|EPI_ISL_592241|Original|hCoV-19^^Victoria|Human|Microbiological Diagnostic Unit - Public Health Laboratory (MDU-PHL)|MDU-PHL|Schultz|Australia
>Spike|hCoV-19/South Africa/KRISP-K004034/2020|2020-10-14|EPI_ISL_635015|Original|hCoV-19^^KwaZulu-Natal|Human|National Health Laboratory Service - Inkosi Albert Luthuli Central Hospital (NHLS-IALCH)|KRISP|de Oliveira|South Africa
>Spike|hCoV-19/England/QEUH-A21EC9/2020|2020-10-01|EPI_ISL_587618|Original|hCoV-19^^England|Human|Lighthouse Lab in Glasgow|Wellcome Sanger Institute for the COVID-19 Genomics UK (COG-UK) consortium|Davies|United Kingdom
>Spike|hCoV-19/Northern Ireland/NIRE-234B79/2020|2020-10-14|EPI_ISL_764392|Original|hCoV-19^^Northern Ireland|Human|Regional Virus Laboratory|COVID-19 Genomics UK (COG-UK) Consortium|Fairley|United Kingdom
>Spike|hCoV-19/Belgium/ULG-10312/2020|2020-07-27|EPI_ISL_540479|Original|hCoV-19^^Li\u00e8ge|Human|Department of Clinical Microbiology|GIGA Medical Genomics|Durkin|Belgium
>Spike|hCoV-19/England/ALDP-982C09/2020|2020-08-10|EPI_ISL_552464|Original|hCoV-19^^England|Human|Lighthouse Lab in Alderley Park|Wellcome Sanger Institute for the COVID-19 Genomics UK (COG-UK) consortium|Davies|United Kingdom
>Spike|hCoV-19/USA/TX-HMH-5593/2020|2020-06-19|EPI_ISL_544887|Original|hCoV-19^^Texas|Human|Houston Methodist Hospital|Houston Methodist Hospital|Long|USA
>Spike|hCoV-19/England/QEUH-96B987/2020|2020-08-14|EPI_ISL_531453|Original|hCoV-19^^England|Human|Lighthouse Lab in Glasgow|Wellcome Sanger Institute for the COVID-19 Genomics UK (COG-UK) consortium|Davies|United Kingdom
>Spike|hCoV-19/England/NORT-292001/2020|2020-05-07|EPI_ISL_478607|Original|hCoV-19^^England|Human|Northumbria University / South Tees Hospitals NHS Foundation Trust / North Cumbria Integrated Care NHS Foundation Trust / North Tees and Hartlepool NHS Foundation Trust / Newcastle Hospitals NHS Foundation Trust|COVID-19 Genomics UK (COG-UK) Consortium|Bashton|United Kingdom
>Spike|hCoV-19/England/MILK-C517A5/2020|2020-12-07|EPI_ISL_769545|Original|hCoV-19^^England|Human|Lighthouse Lab in Milton Keynes|Wellcome Sanger Institute for the COVID-19 Genomics UK (COG-UK) Consortium|Davies|United Kingdom
>Spike|hCoV-19/USA/WI-UW-1993/2020|2020-09-25|EPI_ISL_677401|Original|hCoV-19^^Wisconsin|Human|University of Wisconsin-Madison AIDS Vaccine Research Laboratories|University of Wisconsin-Madison AIDS Vaccine Research Laboratories|Baczenas|USA
>Spike|hCoV-19/USA/TX-HMH-4504/2020|2020-06-19|EPI_ISL_543674|Original|hCoV-19^^Texas|Human|Houston Methodist Hospital|Houston Methodist Hospital|Long|USA
>Spike|hCoV-19/Denmark/DCGC-22157/2020|2020-12-14|EPI_ISL_758592|Original|hCoV-19^^Sjaelland|Human|Department of Virus and Microbiological Special Diagnostics|Albertsen Lab|Michaelsen|Denmark
>Spike|hCoV-19/England/SHEF-CE244/2020|2020-04-03|EPI_ISL_442442|Original|hCoV-19^^England|Human|Virology Department|COVID-19 Genomics UK (COG-UK) Consortium|de Silva|United Kingdom

>Spike|hCoV-19/USA/WA-S2877/2020|2020-09-24|EPI_ISL_596408|Original|hCoV-19^^Washington|Human|Seattle Flu Study|Seattle Flu Study|Lee|USA
>Spike|hCoV-19/England/CAMC-B091EB/2020|2020-10-29|EPI_ISL_673115|Original|hCoV-19^^England|Human|Lighthouse Lab in Cambridge|Wellcome Sanger Institute for the COVID-19 Genomics UK (COG-UK) Consortium|Davies|United Kingdom
>Spike|hCoV-19/Hungary/UD-94339/2020|2020-10-08|EPI_ISL_678387|Original|hCoV-19^^Borsod-Aba\u00faj-Zempl\u00e9n county|Human|University of Debrecen|National Laboratory of Virology|Kemenesi|Hungary
>Spike|hCoV-19/USA/NY-NYUMC333/2020|2020-04-07|EPI_ISL_430360|Original|hCoV-19^^New York|Human|NYU Langone Health|Departments of Pathology and Medicine|Maurano|USA
>Spike|hCoV-19/England/CAMC-B07718/2020|2020-10-30|EPI_ISL_643894|Original|hCoV-19^^England|Human|Lighthouse Lab in Cambridge|Wellcome Sanger Institute for the COVID-19 Genomics UK (COG-UK) Consortium|Davies|United Kingdom
>Spike|hCoV-19/England/QEUH-9D0747/2020|2020-09-16|EPI_ISL_589875|Original|hCoV-19^^England|Human|Lighthouse Lab in Glasgow|Wellcome Sanger Institute for the COVID-19 Genomics UK (COG-UK) consortium|Davies|United Kingdom
>Spike|hCoV-19/Japan/PG-1436/2020|2020-04-00|EPI_ISL_685043|Original|hCoV-19^^Japan|Human|Saitama Prefectural Institute of Public Health|Pathogen Genomics Center|Sekizuka|Japan
>Spike|hCoV-19/India/MH-MW35/2020|2020-06-06|EPI_ISL_511926|Original|hCoV-19^^Maharashtra|Human|Mahatma Gandhi Institute of Medical Sciences|National Institute of Biomedical Genomics -\u00a0DBT's PAN-INDIA 1000 SARS--CoV-2 RNA Genome Sequencing Consortium|Maitra|India
>Spike|hCoV-19/Denmark/DCGC-6304/2020|2020-08-31|EPI_ISL_615437|Original|hCoV-19^^Sjaelland|Human|Department of Virus and Microbiological Special Diagnostics|Albertsen lab|Michaelsen|Denmark
>Spike|hCoV-19/England/SHEF-C08B9/2020|2020-03-24|EPI_ISL_420171|Original|hCoV-19^^England|Human|Virology Department|Department of Infection|de Silva|United Kingdom
>Spike|hCoV-19/England/CAMC-94C360/2020|2020-07-16|EPI_ISL_556728|Original|hCoV-19^^England|Human|Lighthouse Lab in Cambridge|Wellcome Sanger Institute for the COVID-19 Genomics UK (COG-UK) consortium|Davies|United Kingdom
>Spike|hCoV-19/Denmark/DCGC-11345/2020|2020-11-02|EPI_ISL_670854|Original|hCoV-19^^Midtjylland|Human|Department of Virus and Microbiological Special Diagnostics|Albertsen Lab|Michaelsen|Denmark
>Spike|hCoV-19/Spain/MD-H12-82/2020|2020-04-16|EPI_ISL_530011|Original|hCoV-19^^Madrid|Human|Hospital Universitario 12 de Octubre|Hospital Universitario 12 de Octubre|Viedma|Spain
>Spike|hCoV-19/England/LIVE-A1B00/2020|2020-06-16|EPI_ISL_484261|Original|hCoV-19^^England|Human|Liverpool Clinical Laboratories|COVID-19 Genomics UK (COG-UK) Consortium|Haldenby|United Kingdom
>Spike|hCoV-19/Netherlands/ZH-EMC-920/2020|2020-10-13|EPI_ISL_722369|Original|hCoV-19^^Zuid Holland|Human|Dutch COVID-19 response team|Erasmus Medical Center|Oude Munnink|Netherlands
>Spike|hCoV-19/Morocco/RMPS-06/2020|2020-04-01|EPI_ISL_469054|Original|hCoV-19^^Morocco|Human|LNR National Reference Laboratory|Medical Biotechnology Laboratory|Kartti|Morocco
>Spike|hCoV-19/England/NOTT-1126BD/2020|2020-04-15|EPI_ISL_490564|Original|hCoV-19^^England|Human|Lincolnshire Hospitals and DeepSeq Nottingham|COVID-19 Genomics UK (COG-UK) Consortium|Loose|United Kingdom
>Spike|hCoV-19/USA/TX-HMH-7869/2020|2020-06-30|EPI_ISL_545672|Original|hCoV-19^^Texas|Human|Houston Methodist Hospital|Houston Methodist Hospital|Long|USA
>Spike|hCoV-19/England/ALDP-94B880/2020|2020-06-11|EPI_ISL_555783|Original|hCoV-19^^England|Human|Lighthouse Lab in Alderley Park|Wellcome Sanger Institute for the COVID-19 Genomics UK (COG-UK) consortium|Davies|United Kingdom
>Spike|hCoV-19/Wales/MILK-9F1009/2020|2020-09-25|EPI_ISL_589681|Original|hCoV-19^^Wales|Human|Lighthouse Lab in Milton Keynes|Wellcome Sanger Institute for the COVID-19 Genomics UK (COG-UK) consortium|Davies|United Kingdom
>Spike|hCoV-19/England/ALDP-A6E482/2020|2020-10-15|EPI_ISL_607631|Original|hCoV-19^^England|Human|Lighthouse Lab in Alderley Park|Wellcome Sanger Institute for the COVID-19 Genomics UK (COG-UK) consortium|Davies|United Kingdom

>Spike|hCoV-19/England/SHEF-C51B9/2020|2020-04-20|EPI_ISL_453714|Original|hCoV-
19^^England|Human|Virology Department|COVID-19 Genomics UK (COG-UK) Consortium|de
Silva|United Kingdom
>Spike|hCoV-19/England/ALDP-A5218A/2020|2020-10-07|EPI_ISL_598129|Original|hCoV-
19^^England|Human|Lighthouse Lab in Alderley Park|Wellcome Sanger Institute for the
COVID-19 Genomics UK (COG-UK) consortium|Davies|United Kingdom
>Spike|hCoV-19/USA/WI-WSLH-200690/2020|2020-09-14|EPI_ISL_631447|Original|hCoV-
19^^Wisconsin|Human|Wisconsin State Laboratory of Hygiene Communicable Disease
Division|Wisconsin State Laboratory of Hygiene Communicable Disease
Division|Florek|USA
>Spike|hCoV-19/England/CAMC-A65694/2020|2020-10-10|EPI_ISL_637507|Original|hCoV-
19^^England|Human|Department of Pathology|COVID-19 Genomics UK (COG-UK)
Consortium|Goodfellow|United Kingdom
>Spike|hCoV-19/USA/TX-HMH-6088/2020|2020-06-22|EPI_ISL_547226|Original|hCoV-
19^^Texas|Human|Houston Methodist Hospital|Houston Methodist Hospital|Long|USA
>Spike|hCoV-19/Australia/VIC16365/2020|2020-08-04|EPI_ISL_640882|Original|hCoV-
19^^Victoria|Human|Microbiological Diagnostic Unit - Public Health Laboratory (MDU-
PHL)|MDU-PHL|Schultz|Australia
>Spike|hCoV-19/Netherlands/un-EMC-752/2020|2020-06-27|EPI_ISL_523508|Original|hCoV-
19^^Netherlands|Human|Dutch COVID-19 response team|Erasmus Medical Center|Oude
Munnink|Netherlands
>Spike|hCoV-19/USA/MI-MDHHS-SC21710/2020|2020-06-23|EPI_ISL_529829|Original|hCoV-
19^^Michigan|Human|Michigan Department of Health and Human Services|Michigan
Department of Health and Human Services|Blankenship|USA
>Spike|hCoV-19/England/MILK-B889A0/2020|2020-11-14|EPI_ISL_675844|Original|hCoV-
19^^England|Human|Lighthouse Lab in Milton Keynes|Wellcome Sanger Institute for the
COVID-19 Genomics UK (COG-UK) Consortium|Davies|United Kingdom
>Spike|hCoV-19/USA/TX-HMH-5208/2020|2020-06-23|EPI_ISL_544593|Original|hCoV-
19^^Texas|Human|Houston Methodist Hospital|Houston Methodist Hospital|Long|USA
>Spike|hCoV-19/England/201420209/2020|2020-03-29|EPI_ISL_465930|Original|hCoV-
19^^England|Human|Respiratory Virus Unit|Respiratory Virus Unit|Platt|United Kingdom
>Spike|hCoV-19/Ireland/LH-NVRL-73IRL72600/2020|2020-09-
14|EPI_ISL_639837|Original|hCoV-19^^Louth|Human|National Virus Reference
Laboratory|National Virus Reference Laboratory|Gonzalez|Ireland
>Spike|hCoV-19/England/LIVE-A7821/2020|2020-03-27|EPI_ISL_517077|Original|hCoV-
19^^England|Human|Liverpool Clinical Laboratories|COVID-19 Genomics UK (COG-UK)
Consortium|Haldenby|United Kingdom
>Spike|hCoV-19/Germany/HH-hpi-p2400/2020|2020-04-22|EPI_ISL_775621|Original|hCoV-
19^^Hamburg|Human|University Medical Center Hamburg Eppendorf|Heinrich Pette
Institute|Robitaille|Germany
>Spike|hCoV-19/England/ALDP-C3CE32/2020|2020-12-10|EPI_ISL_731670|Original|hCoV-
19^^England|Human|Lighthouse Lab in Alderley Park|Wellcome Sanger Institute for the
COVID-19 Genomics UK (COG-UK) Consortium|Davies|United Kingdom
>Spike|hCoV-19/USA/MA-MASPHL-00451/2020|2020-04-30|EPI_ISL_693109|Original|hCoV-
19^^Massachusetts|Human|Massachusetts State Public Health Laboratory|Massachusetts
State Public Health Laboratory|Lang|USA
>Spike|hCoV-19/USA/TX-HMH-MCoV-9887/2020|2020-07-06|EPI_ISL_780322|Original|hCoV-
19^^Texas|Human|Houston Methodist Hospital|Houston Methodist Hospital|Long|USA
[truncated: 483,257 more chars]
