# Supplementary material for: Epitope Classification and RBD Binding Properties of Neutralizing Antibodies Against SARS-CoV-2 Variants of Concern
Source: Front Immunol. 2021 Jun 4;12:691715. doi: 10.3389/fimmu.2021.691715 (PMC8212047; doi:10.3389/fimmu.2021.691715)
Supplement: Supplementary file 3 [file DataSheet_3.pdf]

Supplementary Table 1. GISAID sequence headers (75,001-150,000)for each SARS-CoV-2 S amino acid sequence used in the frequency analysis.
>Spike|hCoV-19/England/NORW-F98C6/2020|2020-12-03|EPI_ISL_725405|Original|hCoV-19^^England|Human|Quadram Institute Bioscience|COVID-19 Genomics UK (COG-UK) Consortium|Alikhan|United Kingdom
>Spike|hCoV-19/England/QEUH-9628CF/2020|2020-08-10|EPI_ISL_531560|Original|hCoV-19^^England|Human|Lighthouse Lab in Glasgow|Wellcome Sanger Institute for the COVID-19 Genomics UK (COG-UK) consortium|Davies|United Kingdom
>Spike|hCoV-19/USA/CA-CZB-6929/2020|2020-08-19|EPI_ISL_582889|Original|hCoV-19^^California|Human|County of Santa Clara Public Health Department|Chan-Zuckerberg Biohub|Bhatt|USA
>Spike|hCoV-19/South Korea/KCDC2402/2020|2020-05-31|EPI_ISL_514805|Original|hCoV-19^^South Korea|Human|Division of Viral Diseases|Division of Viral Diseases|Kim|South Korea
>Spike|hCoV-19/England/CAMB-834CD/2020|2020-04-15|EPI_ISL_443395|Original|hCoV-19^^England|Human|Department of Pathology|Wellcome Sanger Institute for the COVID-19 Genomics UK (COG-UK) consortium|Amato|United Kingdom
>Spike|hCoV-19/England/CAMC-B516C7/2020|2020-11-08|EPI_ISL_658073|Original|hCoV-19^^England|Human|Lighthouse Lab in Cambridge|Wellcome Sanger Institute for the COVID-19 Genomics UK (COG-UK) Consortium|Davies|United Kingdom
>Spike|hCoV-19/Sichuan/SC-WCH4-286/2020|2020-02-10|EPI_ISL_451388|Original|hCoV-19^^Sichuan|Human|West China Hospital of Sichuan University|State Key Laboratory of Biotherapy of Sichuan University|Tang|China
>Spike|hCoV-19/Australia/VIC4774/2020|2020-07-18|EPI_ISL_519160|Original|hCoV-19^^Victoria|Human|Microbiological Diagnostic Unit - Public Health Laboratory (MDU-PHL)|MDU-PHL|Schultz|Australia
>Spike|hCoV-19/Australia/NSW540/2020|2020-03-22|EPI_ISL_455079|Original|hCoV-19^^New South Wales|Human|South Eastern Area Laboratory Services|NSW Health Pathology - Institute of Clinical Pathology and Medical Research; Westmead Hospital; University of Sydney|Gall|Australia
>Spike|hCoV-19/Denmark/DCGC-19415/2020|2020-12-07|EPI_ISL_748308|Original|hCoV-19^^Midtjylland|Human|Department of Virus and Microbiological Special Diagnostics|Albertsen Lab|Michaelsen|Denmark
>Spike|hCoV-19/South Africa/LR00024/2020|2020-07-05|EPI_ISL_515153|Original|hCoV-19^^Gauteng|Human|National Institute for Communicable Diseases of the National Health Laboratory Service|National Institute for Communicable Diseases of the National Health Laboratory Service|Allam|South Africa
>Spike|hCoV-19/USA/CA-CZB-1163/2020|2020-04-27|EPI_ISL_454620|Original|hCoV-19^^California|Human|UCSF Clinical Microbiology Laboratory|Chan-Zuckerberg Biohub|Hao|USA
>Spike|hCoV-19/USA/WI-UW-1570/2020|2020-09-16|EPI_ISL_605448|Original|hCoV-19^^Wisconsin|Human|University of Wisconsin-Madison AIDS Vaccine Research Laboratories|University of Wisconsin-Madison AIDS Vaccine Research Laboratories|Baczenas|USA
>Spike|hCoV-19/USA/MD-HP12132/2020|2020-12-31|EPI_ISL_791442|Original|hCoV-19^^Maryland|Human|Johns Hopkins Hospital Department of Pathology|Johns Hopkins Hospital Department of Pathology|Morris|USA
>Spike|hCoV-19/Scotland/QEUH-9DA9FC/2020|2020-09-22|EPI_ISL_575369|Original|hCoV-19^^Scotland|Human|Lighthouse Lab in Glasgow|Wellcome Sanger Institute for the COVID-19 Genomics UK (COG-UK) consortium|Davies|United Kingdom
>Spike|hCoV-19/USA/UT-08586/2020|2020-05-07|EPI_ISL_524337|Original|hCoV-19^^Utah|Human|Utah Public Health Laboratory|Utah Public Health Laboratory|Young|USA
>Spike|hCoV-19/Denmark/DCGC-6894/2020|2020-10-12|EPI_ISL_619739|Original|hCoV-19^^Hovedstaden|Human|Department of Virus and Microbiological Special Diagnostics|Albertsen lab|Michaelsen|Denmark
>Spike|hCoV-19/England/CAMC-C859B1/2020|2020-12-17|EPI_ISL_756490|Original|hCoV-19^^England|Human|Lighthouse Lab in Cambridge|Wellcome Sanger Institute for the COVID-19 Genomics UK (COG-UK) Consortium|Davies|United Kingdom
>Spike|hCoV-19/England/20120002404/2020|2020-03-14|EPI_ISL_465141|Original|hCoV-19^^England|Human|Respiratory Virus Unit|Respiratory Virus Unit|Platt|United Kingdom

>Spike|hCoV-19/Belgium/ULG-10567/2020|2020-10-23|EPI_ISL_626334|Original|hCoV-19^^Li\u00e8ge|Human|Department of Clinical Microbiology|GIGA Medical Genomics|Durkin|Belgium
>Spike|hCoV-19/USA/AK-PHL464/2020|2020-06-10|EPI_ISL_512141|Original|hCoV-19^^Alaska|Human|Alaska State Virology Laboratory|Alaska State Virology Laboratory|Drown|USA
>Spike|hCoV-19/Portugal/PT0472/2020|2020-04-18|EPI_ISL_454196|Original|hCoV-19^^Portugal|Human|unknown|Instituto Nacional de Saude (INSA)|Borges|Portugal
>Spike|hCoV-19/Mexico/BCN-ALSR-2569/2020|2020-07-08|EPI_ISL_635570|Original|hCoV-19^^Baja California|Human|Centro de Diagn\u221a\u2265stico COVID-19 UABC Tijuana|Andersen lab at Scripps Research|Gangavarapu|Mexico
>Spike|hCoV-19/England/ALDP-AA2C99/2020|2020-10-22|EPI_ISL_625264|Original|hCoV-19^^England|Human|Lighthouse Lab in Alderley Park|Wellcome Sanger Institute for the COVID-19 Genomics UK (COG-UK) consortium|Davies|United Kingdom
>Spike|hCoV-19/Denmark/DCGC-13307/2020|2020-11-30|EPI_ISL_712721|Original|hCoV-19^^Syddanmark|Human|Department of Virus and Microbiological Special Diagnostics|Albertsen Lab|Michaelsen|Denmark
>Spike|hCoV-19/Spain/AR-IBV-2490/2020|2020-03-24|EPI_ISL_468824|Original|hCoV-19^^Aragon|Human|Servicio de Microbiolog\u00eda|SeqCOVID-SPAIN consortium/IBV(CSIC)|Comas|Spain
>Spike|hCoV-19/Russia/Moscow-62502/2020|2020-03-11|EPI_ISL_428864|Vero|hCoV-19^^Moscow|Human|State Research Center of Virology and Biotechnology VECTOR|State Research Center of Virology and Biotechnology VECTOR|Shvalov|Russia
>Spike|hCoV-19/Denmark/DCGC-4993/2020|2020-09-14|EPI_ISL_621770|Original|hCoV-19^^Hovedstaden|Human|Department of Virus and Microbiological Special Diagnostics|Albertsen lab|Michaelsen|Denmark
>Spike|hCoV-19/USA/CA-SCCPHD-UC136/2020|2020-03-07|EPI_ISL_435615|Original|hCoV-19^^California|Human|Santa Clara County Public Health Department|Chiu Laboratory|Chiu|USA
>Spike|hCoV-19/Denmark/DCGC-15559/2020|2020-11-23|EPI_ISL_713892|Original|hCoV-19^^Sjaelland|Human|Department of Virus and Microbiological Special Diagnostics|Albertsen Lab|Michaelsen|Denmark
>Spike|hCoV-19/United Arab Emirates/0658/2020|2020-05-16|EPI_ISL_698200|Original|hCoV-19^^Abu Dhabi|Human|Group 42 (G42) Healthcare|G42 Healthcare|Ogrodzki|United Arab Emirates
>Spike|hCoV-19/England/CAMB-751E5/2020|2020-03-14|EPI_ISL_440396|Original|hCoV-19^^England|Human|Department of Pathology|Wellcome Sanger Institute for the COVID-19 Genomics UK (COG-UK) consortium|Amato|United Kingdom
>Spike|hCoV-19/Denmark/DCGC-15583/2020|2020-11-23|EPI_ISL_713178|Original|hCoV-19^^Hovedstaden|Human|Department of Virus and Microbiological Special Diagnostics|Albertsen Lab|Michaelsen|Denmark
>Spike|hCoV-19/USA/UT-UPHL-2012724093/2020|2020-08-10|EPI_ISL_745863|Original|hCoV-19^^Utah|Human|Ginkgo Bioworks Clinical Laboratory|Utah Public Health Laboratory|Young|USA
>Spike|hCoV-19/South Africa/R12652-20/2020|2020-06-16|EPI_ISL_504210|Original|hCoV-19^^North West|Human|National Institute for Communicable Diseases of the National Health Laboratory Service|National Institute for Communicable Diseases of the National Health Laboratory Service|Allam|South Africa
>Spike|hCoV-19/England/MILK-B0513A/2020|2020-10-24|EPI_ISL_638542|Original|hCoV-19^^England|Human|Virology Department|COVID-19 Genomics UK (COG-UK) Consortium|de Silva|United Kingdom
>Spike|hCoV-19/USA/TX-HMH-MCoV-9513/2020|2020-07-03|EPI_ISL_781158|Original|hCoV-19^^Texas|Human|Houston Methodist Hospital|Houston Methodist Hospital|Long|USA
>Spike|hCoV-19/Beijing/DT-travelUK04/2020|2020-03-20|EPI_ISL_452336|Original|hCoV-19^^Beijing|Human|Laboratory of Infectious Diseases Center of Beijing Ditan Hospital|Laboratory of Infectious Diseases Center of Beijing Ditan Hospital|Yang|China
>Spike|hCoV-19/Japan/IC-0241/2020|2020-10-00|EPI_ISL_667670|Original|hCoV-19^^Japan|Human|Pathogen Genomics Center|Pathogen Genomics Center|Sekizuka|Japan

>Spike|hCoV-19/Sweden/20-53180/2020|2020-10-21|EPI_ISL_661288|Original|hCoV-19^^Vastmanland|Human|Klinisk mikrobiologi|The Public Health Agency of Sweden|Svartstr\u00f6m|Sweden
>Spike|hCoV-19/Japan/PG-7694/2020|2020-08-00|EPI_ISL_686796|Original|hCoV-19^^Japan|Human|Chiba Prefectural Institute of Public Health|Pathogen Genomics Center|Sekizuka|Japan
>Spike|hCoV-19/England/CAMC-BC22DC/2020|2020-11-22|EPI_ISL_704790|Original|hCoV-19^^England|Human|Lighthouse Lab in Cambridge|Wellcome Sanger Institute for the COVID-19 Genomics UK (COG-UK) Consortium|Davies|United Kingdom
>Spike|hCoV-19/South Africa/KRISP-0165/2020|2020-05-29|EPI_ISL_467515|Original|hCoV-19^^KwaZulu-Natal|Human|NHLS-IALCH|KRISP|de Oliveira|South Africa
>Spike|hCoV-19/Spain/AR-IBV-2403/2020|2020-03-23|EPI_ISL_468796|Original|hCoV-19^^Aragon|Human|Servicio de Microbiolog\u00eda|SeqCOVID-SPAIN consortium/IBV(CSIC)|Comas|Spain
>Spike|hCoV-19/France/OCC-SC565/2020|2020-05-19|EPI_ISL_660731|Original|hCoV-19^^Occitanie|Human|CHU N\u00eemes|CNR Virus des Infections Respiratoires - France SUD|Bal|France
>Spike|hCoV-19/USA/WA-UW-5501/2020|2020-04-30|EPI_ISL_776657|Original|hCoV-19^^Washington|Human|UW Virology Lab|UW Virology Lab|Roychoudhury|USA
>Spike|hCoV-19/England/MILK-C50885/2020|2020-12-11|EPI_ISL_736617|Original|hCoV-19^^England|Human|Lighthouse Lab in Milton Keynes|Wellcome Sanger Institute for the COVID-19 Genomics UK (COG-UK) Consortium|Davies|United Kingdom
>Spike|hCoV-19/South Africa/KRISP-K004536/2020|2020-10-18|EPI_ISL_660234|Original|hCoV-19^^KwaZulu-Natal|Human|NHLS-IALCH|KRISP|de Oliveira|South Africa
>Spike|hCoV-19/USA/DE-DHSS-F1020819/2020|2020-10-29|EPI_ISL_708443|Original|hCoV-19^^Delaware|Human|Delaware Public Health Lab|Delaware Public Health Lab|Bajwa|USA
>Spike|hCoV-19/Wales/PHWC-4A1577/2020|2020-12-10|EPI_ISL_743691|Original|hCoV-19^^Wales|Human|Wales Specialist Virology Centre Sequencing lab|COVID-19 Genomics UK (COG-UK) Consortium|Moore|United Kingdom
>Spike|hCoV-19/England/SHEF-CCD90/2020|2020-03-25|EPI_ISL_442422|Original|hCoV-19^^England|Human|Virology Department|COVID-19 Genomics UK (COG-UK) Consortium|de Silva|United Kingdom
>Spike|hCoV-19/England/NORW-F57CD/2020|2020-11-03|EPI_ISL_665893|Original|hCoV-19^^England|Human|Quadram Institute Bioscience|COVID-19 Genomics UK (COG-UK) Consortium|Alikhan|United Kingdom
>Spike|hCoV-19/England/LCST-24D70BD/2020|2020-06-07|EPI_ISL_478362|Original|hCoV-19^^England|Human|University Hospitals Of Leicester NHS Trust and DeepSeq Nottingham|COVID-19 Genomics UK (COG-UK) Consortium|Loose|United Kingdom
>Spike|hCoV-19/Australia/VIC4920/2020|2020-07-14|EPI_ISL_519295|Original|hCoV-19^^Victoria|Human|Victorian Infectious Diseases Reference Laboratory (VIDRL)|VIDRL and MDU-PHL|Schultz|Australia
>Spike|hCoV-19/Denmark/DCGC-1260/2020|2020-06-01|EPI_ISL_618026|Original|hCoV-19^^Syddanmark|Human|Department of Virus and Microbiological Special Diagnostics|Albertsen lab|Michaelsen|Denmark
>Spike|hCoV-19/USA/AZ-TG666087/2020|2020-05-01|EPI_ISL_696179|Original|hCoV-19^^Arizona|Human|Sonora Quest Laboratories|TGen North|French|USA
>Spike|hCoV-19/England/BRIS-1282A1/2020|2020-04-16|EPI_ISL_469531|Original|hCoV-19^^England|Human|PHE South West Regional Laboratory|Wellcome Sanger Institute for the COVID-19 Genomics UK (COG-UK) consortium|Davies|United Kingdom
>Spike|hCoV-19/Denmark/DCGC-4744/2020|2020-09-21|EPI_ISL_622183|Original|hCoV-19^^Syddanmark|Human|Department of Virus and Microbiological Special Diagnostics|Albertsen lab|Michaelsen|Denmark
>Spike|hCoV-19/Spain/IB-IBV-2505/2020|2020-03-20|EPI_ISL_468962|Original|hCoV-19^^Illes Balears|Human|Servicio de Microbiolog\u00eda|SeqCOVID-SPAIN consortium/IBV(CSIC)|Comas|Spain
>Spike|hCoV-19/England/QEUH-BC3A88/2020|2020-11-25|EPI_ISL_703825|Original|hCoV-19^^England|Human|Lighthouse Lab in Glasgow|Wellcome Sanger Institute for the COVID-19 Genomics UK (COG-UK) Consortium|Davies|United Kingdom

>Spike|hCoV-19/USA/LA-EVTL143/2020|2020-03-25|EPI_ISL_455986|Original|hCoV-
19^^Louisiana|Human|LSUHS Emerging Viral Threat Laboratory|Microbial Genome
Sequencing Center|Kamil|USA
>Spike|hCoV-19/USA/WA-UW-12440/2020|2020-06-27|EPI_ISL_570534|Original|hCoV-
19^^Washington|Human|UW Virology Lab|UW Virology Lab|Roychoudhury|USA
>Spike|hCoV-19/Germany/BW-MVZ-LS-38/2020|2020-04-22|EPI_ISL_640255|Original|hCoV-
19^^Baden-W\u00fcrttemberg|Human|MVZ Labor\u00e4rzte Singen|MVZ Labor\u00e4rzte
Singen|Schmidt|Germany
>Spike|hCoV-19/Brazil/SP-391/2020|2020-06-20|EPI_ISL_574598|Original|hCoV-
19^^S\u00e3o Paulo|Human|Servico de Verificacao de Obito SVO|Instituto Adolfo
Lutz|Sacchi|Brazil
>Spike|hCoV-19/Gibraltar/204462843/2020|2020-10-29|EPI_ISL_615014|Original|hCoV-
19^^Gibraltar|Human|Microbiology|Respiratory Virus Unit|Platt|Gibraltar
>Spike|hCoV-19/England/CAMB-1B7E94/2020|2020-10-14|EPI_ISL_637687|Original|hCoV-
19^^England|Human|Department of Pathology|COVID-19 Genomics UK (COG-UK)
Consortium|Goodfellow|United Kingdom
>Spike|hCoV-19/England/ALDP-4FD776/2020|2020-06-23|EPI_ISL_559188|Original|hCoV-
19^^England|Human|Lighthouse Lab in Alderley Park|Wellcome Sanger Institute for the
COVID-19 Genomics UK (COG-UK) consortium|Davies|United Kingdom
>Spike|hCoV-19/Wales/PHWC-28C86/2020|2020-04-02|EPI_ISL_445425|Original|hCoV-
19^^Wales|Human|Wales Specialist Virology Centre|Public Health Wales Microbiology
Cardiff|Moore|United Kingdom
>Spike|hCoV-19/USA/WI-UW-2548/2020|2020-12-29|EPI_ISL_779377|Original|hCoV-
19^^Wisconsin|Human|University of Wisconsin-Madison AIDS Vaccine Research
Laboratories|University of Wisconsin-Madison AIDS Vaccine Research
Laboratories|Moreno|USA
>Spike|hCoV-19/England/ALDP-A4FB91/2020|2020-10-07|EPI_ISL_597592|Original|hCoV-
19^^England|Human|Lighthouse Lab in Alderley Park|Wellcome Sanger Institute for the
COVID-19 Genomics UK (COG-UK) consortium|Davies|United Kingdom
>Spike|hCoV-19/Scotland/QEUH-B80F00/2020|2020-11-14|EPI_ISL_710839|Original|hCoV-
19^^Scotland|Human|Lighthouse Lab in Glasgow|Wellcome Sanger Institute for the COVID-
19 Genomics UK (COG-UK) Consortium|Davies|United Kingdom
>Spike|hCoV-19/England/CAMC-B37D68/2020|2020-11-05|EPI_ISL_658931|Original|hCoV-
19^^England|Human|Lighthouse Lab in Cambridge|Wellcome Sanger Institute for the
COVID-19 Genomics UK (COG-UK) Consortium|Davies|United Kingdom
>Spike|hCoV-19/USA/WA-UW-22365/2020|2020-08-08|EPI_ISL_570669|Original|hCoV-
19^^Washington|Human|UW Virology Lab|UW Virology Lab|Roychoudhury|USA
>Spike|hCoV-19/England/QEUH-A4D920/2020|2020-10-05|EPI_ISL_628615|Original|hCoV-
19^^England|Human|Oxford Viromics|COVID-19 Genomics UK (COG-UK)
Consortium|Golubchik|United Kingdom
>Spike|hCoV-19/USA/UT-UPHL-2009524/2020|2020-07-18|EPI_ISL_605205|Original|hCoV-
19^^Utah|Human|Utah Public Health Laboratory|Utah Public Health Laboratory|Young|USA
>Spike|hCoV-19/England/QEUH-B7F65A/2020|2020-11-14|EPI_ISL_668350|Original|hCoV-
19^^England|Human|Lighthouse Lab in Glasgow|Wellcome Sanger Institute for the COVID-
19 Genomics UK (COG-UK) Consortium|Davies|United Kingdom
>Spike|hCoV-19/England/CAMB-83445/2020|2020-04-15|EPI_ISL_443638|Original|hCoV-
19^^England|Human|Department of Pathology|Wellcome Sanger Institute for the COVID-19
Genomics UK (COG-UK) consortium|Amato|United Kingdom
>Spike|hCoV-19/Australia/NSW1128/2020|2020-09-12|EPI_ISL_699642|Original|hCoV-19^^New
South Wales|Human|South Eastern Area Laboratory Services (SEALS)|NSW Health Pathology
- Institute of Clinical Pathology and Medical Research; Westmead Hospital; University
of Sydney|Gall|Australia
>Spike|hCoV-19/Germany/BY-MVP-0055/2020|2020-03-17|EPI_ISL_437254|Original|hCoV-
19^^Bavaria|Human|Max von Pettenkofer Institute|Laboratory for Functional Genome
Analysis|Graf|Germany
>Spike|hCoV-19/USA/MI-MDHHS-SC22064/2020|2020-09-28|EPI_ISL_583521|Original|hCoV-
19^^Michigan|Human|Michigan Department of Health and Human Services|Michigan
Department of Health and Human Services|Blankenship|USA

>Spike|hCoV-19/Wales/PHWC-487207/2020|2020-10-29|EPI_ISL_638052|Original|hCoV-19^^Wales|Human|Wales Specialist Virology Centre Sequencing lab|COVID-19 Genomics UK (COG-UK) Consortium|Moore|United Kingdom
>Spike|hCoV-19/England/ALDP-AA31A8/2020|2020-10-22|EPI_ISL_624190|Original|hCoV-19^^England|Human|Lighthouse Lab in Alderley Park|Wellcome Sanger Institute for the COVID-19 Genomics UK (COG-UK) consortium|Davies|United Kingdom
>Spike|hCoV-19/England/CAMC-B9C4A8/2020|2020-11-15|EPI_ISL_673280|Original|hCoV-19^^England|Human|Lighthouse Lab in Cambridge|Wellcome Sanger Institute for the COVID-19 Genomics UK (COG-UK) Consortium|Davies|United Kingdom
>Spike|hCoV-19/England/ALDP-A9408F/2020|2020-10-19|EPI_ISL_611156|Original|hCoV-19^^England|Human|Lighthouse Lab in Alderley Park|Wellcome Sanger Institute for the COVID-19 Genomics UK (COG-UK) consortium|Davies|United Kingdom
>Spike|hCoV-19/England/201080061/2020|2020-03-06|EPI_ISL_464450|Original|hCoV-19^^England|Human|Respiratory Virus Unit|Respiratory Virus Unit|Platt|United Kingdom
>Spike|hCoV-19/Oman/205032386/2020|2020-04-28|EPI_ISL_457984|Original|hCoV-19^^Muscat|Human|Oman-NIC|Department of\u00a0Microbiology\u00a0and Immunology-SQUH|AL Zadjali|Oman
>Spike|hCoV-19/Russia/SVE-RII-MH1618S/2020|2020-09-07|EPI_ISL_596298|Original|hCoV-19^^Sverdlovsk|Human|HELIX LCC|WHO National Influenza Centre Russian Federation|Komissarov|Russia
>Spike|hCoV-19/England/QEUH-B7EFF6/2020|2020-11-14|EPI_ISL_661798|Original|hCoV-19^^England|Human|Lighthouse Lab in Glasgow|Wellcome Sanger Institute for the COVID-19 Genomics UK (COG-UK) Consortium|Davies|United Kingdom
>Spike|hCoV-19/England/MILK-99E10B/2020|2020-08-30|EPI_ISL_550540|Original|hCoV-19^^England|Human|Lighthouse Lab in Milton Keynes|Wellcome Sanger Institute for the COVID-19 Genomics UK (COG-UK) consortium|Davies|United Kingdom
>Spike|hCoV-19/Scotland/QEUH-B108EC/2020|2020-11-02|EPI_ISL_649620|Original|hCoV-19^^Scotland|Human|Lighthouse Lab in Glasgow|Wellcome Sanger Institute for the COVID-19 Genomics UK (COG-UK) Consortium|Davies|United Kingdom
>Spike|hCoV-19/England/MILK-ACCAF9/2020|2020-10-21|EPI_ISL_634381|Original|hCoV-19^^England|Human|Lighthouse Lab in Milton Keynes|Wellcome Sanger Institute for the COVID-19 Genomics UK (COG-UK) consortium|Davies|United Kingdom
>Spike|hCoV-19/England/MILK-9D4567/2020|2020-09-18|EPI_ISL_601642|Original|hCoV-19^^England|Human|Lighthouse Lab in Milton Keynes|Wellcome Sanger Institute for the COVID-19 Genomics UK (COG-UK) consortium|Davies|United Kingdom
>Spike|hCoV-19/England/204390337/2020|2020-10-23|EPI_ISL_637131|Original|hCoV-19^^England|Human|Respiratory Virus Unit|COVID-19 Genomics UK (COG-UK) Consortium|Platt|United Kingdom
>Spike|hCoV-19/USA/WI-UW-1315/2020|2020-08-31|EPI_ISL_536687|Original|hCoV-19^^Wisconsin|Human|University of Wisconsin-Madison AIDS Vaccine Research Laboratories|University of Wisconsin-Madison AIDS Vaccine Research Laboratories|Baczenas|USA
>Spike|hCoV-19/USA/NY-WCMP5F01P/2020|2020-03-13|EPI_ISL_427540|Original|hCoV-19^^New York|Human|NewYork-Presbyterian & Mason Lab|Mason Lab|Mason|USA
>Spike|hCoV-19/Japan/PG-6247/2020|2020-08-00|EPI_ISL_689358|Original|hCoV-19^^Japan|Human|Pathogen Genomics Center|Pathogen Genomics Center|Sekizuka|Japan
>Spike|hCoV-19/USA/CA-CDPH164/2020|2020-06-18|EPI_ISL_754986|Original|hCoV-19^^California|Human|California Department of Public Health|California Department of Public Health|Bell|USA
>Spike|hCoV-19/England/ALDP-A70010/2020|2020-10-15|EPI_ISL_608084|Original|hCoV-19^^England|Human|Lighthouse Lab in Alderley Park|Wellcome Sanger Institute for the COVID-19 Genomics UK (COG-UK) consortium|Davies|United Kingdom
>Spike|hCoV-19/England/ALDP-B852E4/2020|2020-11-15|EPI_ISL_675525|Original|hCoV-19^^England|Human|Lighthouse Lab in Alderley Park|Wellcome Sanger Institute for the COVID-19 Genomics UK (COG-UK) Consortium|Davies|United Kingdom
>Spike|hCoV-19/Denmark/DCGC-11335/2020|2020-11-02|EPI_ISL_670668|Original|hCoV-19^^Hovedstaden|Human|Department of Virus and Microbiological Special Diagnostics|Albertsen Lab|Michaelsen|Denmark

>Spike|hCoV-19/England/ALDP-BB9E10/2020|2020-11-20|EPI_ISL_702020|Original|hCoV-19^^England|Human|Lighthouse Lab in Alderley Park|Wellcome Sanger Institute for the COVID-19 Genomics UK (COG-UK) Consortium|Davies|United Kingdom
>Spike|hCoV-19/Portugal/PT0693/2020|2020-04-25|EPI_ISL_510952|Original|hCoV-19^^Portugal|Human|Instituto Nacional de Saude (INSA)|Instituto Nacional de Saude (INSA)|Borges|Portugal
>Spike|hCoV-19/Belgium/ULG-10527/2020|2020-10-23|EPI_ISL_626321|Original|hCoV-19^^Li\u00e8ge|Human|Department of Clinical Microbiology|GIGA Medical Genomics|Durkin|Belgium
>Spike|hCoV-19/Spain/VC-FISABIO-160/2020|2020-03-19|EPI_ISL_436255|Original|hCoV-19^^Comunitat Valenciana|Human|Servicio de Microbiologia. Hospital Universitario Doctor Peset|Sequencing and Bioinformatics Service and Molecular Epidemiology Research Group. FISABIO-Public Health|Gonzalez-Candelas|Spain
>Spike|hCoV-19/England/QEUH-B415BE/2020|2020-11-08|EPI_ISL_658430|Original|hCoV-19^^England|Human|Lighthouse Lab in Glasgow|Wellcome Sanger Institute for the COVID-19 Genomics UK (COG-UK) Consortium|Davies|United Kingdom
>Spike|hCoV-19/Denmark/DCGC-10317/2020|2020-11-02|EPI_ISL_669808|Original|hCoV-19^^Nordjylland|Human|Department of Virus and Microbiological Special Diagnostics|Albertsen Lab|Michaelsen|Denmark
>Spike|hCoV-19/England/MILK-B94288/2020|2020-11-13|EPI_ISL_676290|Original|hCoV-19^^England|Human|Lighthouse Lab in Milton Keynes|Wellcome Sanger Institute for the COVID-19 Genomics UK (COG-UK) Consortium|Davies|United Kingdom
>Spike|hCoV-19/India/MH-NIV-99083/2020|2020-07-04|EPI_ISL_541750|Original|hCoV-19^^Maharashtra|Human|National Institute of Virology|National Institute of Virology|Potdar|India
>Spike|hCoV-19/Sweden/20-08332/2020|2020-03-20|EPI_ISL_429127|Original|hCoV-19^^Sormland|Human|Unilabs Skovde|The Public Health Agency of Sweden|Svartstr\u00f6m|Sweden
>Spike|hCoV-19/USA/NY-NYCPHL-000158/2020|2020-04-09|EPI_ISL_631694|Original|hCoV-19^^New York|Human|OCME Office Of Chief Medical Examiner|New York City Public Health Laboratory|Wang|USA
>Spike|hCoV-19/England/203290066/2020|2020-08-03|EPI_ISL_514349|Original|hCoV-19^^England|Human|Respiratory Virus Unit|Respiratory Virus Unit|Platt|United Kingdom
>Spike|hCoV-19/England/ALDP-AA232B/2020|2020-10-22|EPI_ISL_624428|Original|hCoV-19^^England|Human|Lighthouse Lab in Alderley Park|Wellcome Sanger Institute for the COVID-19 Genomics UK (COG-UK) consortium|Davies|United Kingdom
>Spike|hCoV-19/England/ALDP-9FD137/2020|2020-10-02|EPI_ISL_587962|Original|hCoV-19^^England|Human|Lighthouse Lab in Alderley Park|Wellcome Sanger Institute for the COVID-19 Genomics UK (COG-UK) consortium|Davies|United Kingdom
>Spike|hCoV-19/England/CAMC-A59675/2020|2020-10-08|EPI_ISL_597179|Original|hCoV-19^^England|Human|Lighthouse Lab in Cambridge|Wellcome Sanger Institute for the COVID-19 Genomics UK (COG-UK) consortium|Davies|United Kingdom
>Spike|hCoV-19/Burkina Faso/2-032-1197/2020|2020-07-25|EPI_ISL_660479|Original|hCoV-19^^Bobo Dioulasso|Human|Laboratoire de Microbiologie CHU Souro Sanou|Centre Muraz|Ouedraogo|Burkina Faso
>Spike|hCoV-19/USA/CA-SCCPHD-UC121/2020|2020-03-05|EPI_ISL_435600|Original|hCoV-19^^California|Human|Santa Clara County Public Health Department|Chiu Laboratory|Chiu|USA
>Spike|hCoV-19/Japan/PG-4828/2020|2020-07-00|EPI_ISL_689952|Original|hCoV-19^^Japan|Human|Pathogen Genomics Center|Pathogen Genomics Center|Sekizuka|Japan
>Spike|hCoV-19/Scotland/QEUH-AF6188/2020|2020-10-30|EPI_ISL_676475|Original|hCoV-19^^Scotland|Human|Lighthouse Lab in Glasgow|Wellcome Sanger Institute for the COVID-19 Genomics UK (COG-UK) Consortium|Davies|United Kingdom
>Spike|hCoV-19/England/ALDP-A683E5/2020|2020-10-12|EPI_ISL_608661|Original|hCoV-19^^England|Human|Lighthouse Lab in Alderley Park|Wellcome Sanger Institute for the COVID-19 Genomics UK (COG-UK) consortium|Davies|United Kingdom
>Spike|hCoV-19/USA/AZ-TG395826/2020|2020-06-22|EPI_ISL_694723|Original|hCoV-19^^Arizona|Human|TGen North|TGen North|French|USA
>Spike|hCoV-19/England/201160755/2020|2020-03-10|EPI_ISL_464802|Original|hCoV-19^^England|Human|Respiratory Virus Unit|Respiratory Virus Unit|Platt|United Kingdom

>Spike|hCoV-19/Israel/13077723/2020|2020-04-03|EPI_ISL_447427|Original|hCoV-19^^Tel Aviv District|Human|Clinical Microbiology Laboratory|Stern Lab|Miller|Israel
>Spike|hCoV-19/England/QEUH-961972/2020|2020-07-31|EPI_ISL_536962|Original|hCoV-19^^England|Human|Lighthouse Lab in Glasgow|Wellcome Sanger Institute for the COVID-19 Genomics UK (COG-UK) consortium|Davies|United Kingdom
>Spike|hCoV-19/Denmark/DCGC-8822/2020|2020-10-26|EPI_ISL_626096|Original|hCoV-19^^Syddanmark|Human|Department of Virus and Microbiological Special Diagnostics|Albertsen lab|Michaelsen|Denmark
>Spike|hCoV-19/England/ALDP-A2E08A/2020|2020-10-06|EPI_ISL_586686|Original|hCoV-19^^England|Human|Lighthouse Lab in Alderley Park|Wellcome Sanger Institute for the COVID-19 Genomics UK (COG-UK) consortium|Davies|United Kingdom
>Spike|hCoV-19/USA/UT-090/2020|2020-03-14|EPI_ISL_420804|Original|hCoV-19^^Utah|Human|Utah Public Health Laboratory|Utah Public Health Laboratory|Young|USA
>Spike|hCoV-19/Scotland/QEUH-A9D059/2020|2020-10-20|EPI_ISL_652649|Original|hCoV-19^^Scotland|Human|Queens Medical Centre|COVID-19 Genomics UK (COG-UK) Consortium|Loose|United Kingdom
>Spike|hCoV-19/Scotland/QEUH-B3179D/2020|2020-11-04|EPI_ISL_676422|Original|hCoV-19^^Scotland|Human|Lighthouse Lab in Glasgow|Wellcome Sanger Institute for the COVID-19 Genomics UK (COG-UK) Consortium|Davies|United Kingdom
>Spike|hCoV-19/England/QEUH-B828B8/2020|2020-11-14|EPI_ISL_661772|Original|hCoV-19^^England|Human|Lighthouse Lab in Glasgow|Wellcome Sanger Institute for the COVID-19 Genomics UK (COG-UK) Consortium|Davies|United Kingdom
>Spike|hCoV-19/England/QEUH-B7C4EA/2020|2020-11-14|EPI_ISL_741782|Original|hCoV-19^^England|Human|Oxford Viromics|COVID-19 Genomics UK (COG-UK) Consortium|Golubchik|United Kingdom
>Spike|hCoV-19/USA/TX-HMH-7931/2020|2020-06-29|EPI_ISL_545716|Original|hCoV-19^^Texas|Human|Houston Methodist Hospital|Houston Methodist Hospital|Long|USA
>Spike|hCoV-19/USA/WA-UW-30946/2020|2020-10-10|EPI_ISL_735458|Original|hCoV-19^^Washington|Human|UW Virology Lab|UW Virology Lab|Roychoudhury|USA
>Spike|hCoV-19/Australia/TAS56/2020|2020-03-22|EPI_ISL_522730|Original|hCoV-19^^Tasmania|Human|Royal Hobart Hospital Microbiology Department|MDU-PHL|Schultz|Australia
>Spike|hCoV-19/USA/WA-S1619/2020|2020-05-27|EPI_ISL_496045|Original|hCoV-19^^Washington|Human|Washington State Department of Health|Seattle Flu Study|Lee|USA
>Spike|hCoV-19/Scotland/EDB3149/2020|2020-04-06|EPI_ISL_488981|Original|hCoV-19^^Scotland|Human|Virology Department|Wellcome Sanger Institute for the COVID-19 Genomics UK (COG-UK) consortium|Davies|United Kingdom
>Spike|hCoV-19/England/LOND-D41C6/2020|2020-04-06|EPI_ISL_444098|Original|hCoV-19^^England|Human|University College London|COVID-19 Genomics UK (COG-UK) Consortium|Roy|United Kingdom
>Spike|hCoV-19/England/QEUH-C4B8D8/2020|2020-12-12|EPI_ISL_769567|Original|hCoV-19^^England|Human|Lighthouse Lab in Glasgow|Wellcome Sanger Institute for the COVID-19 Genomics UK (COG-UK) Consortium|Davies|United Kingdom
>Spike|hCoV-19/England/MILK-9EF684/2020|2020-09-23|EPI_ISL_594929|Original|hCoV-19^^England|Human|Queens Medical Centre|COVID-19 Genomics UK (COG-UK) Consortium|Loose|United Kingdom
>Spike|hCoV-19/USA/CA-CZB-6999/2020|2020-08-04|EPI_ISL_582925|Original|hCoV-19^^California|Human|County of Santa Clara Public Health Department|Chan-Zuckerberg Biohub|Bhatt|USA
>Spike|hCoV-19/England/CAMB-7BA5D/2020|2020-04-04|EPI_ISL_442165|Original|hCoV-19^^England|Human|Department of Pathology|Wellcome Sanger Institute for the COVID-19 Genomics UK (COG-UK) consortium|Amato|United Kingdom
>Spike|hCoV-19/Denmark/DCGC-3288/2020|2020-08-31|EPI_ISL_616040|Original|hCoV-19^^Hovedstaden|Human|Department of Virus and Microbiological Special Diagnostics|Albertsen lab|Michaelsen|Denmark
>Spike|hCoV-19/Wales/PHWC-16BA40/2020|2020-09-14|EPI_ISL_573932|Original|hCoV-19^^Wales|Human|Wales Specialist Virology Centre Sequencing lab|COVID-19 Genomics UK (COG-UK) Consortium|Moore|United Kingdom

>Spike|hCoV-19/Hungary/MH-9653/2020|2020-06-04|EPI_ISL_526224|Original|hCoV-19^^Pest county|Human|Hungarian Defence Forces Military Medical Centre|National Laboratory of Virology|Kemenesi|Hungary
>Spike|hCoV-19/England/CAMC-B09822/2020|2020-10-29|EPI_ISL_673137|Original|hCoV-19^^England|Human|Lighthouse Lab in Cambridge|Wellcome Sanger Institute for the COVID-19 Genomics UK (COG-UK) Consortium|Davies|United Kingdom
>Spike|hCoV-19/Scotland/EDB5797/2020|2020-04-27|EPI_ISL_473858|Original|hCoV-19^^Scotland|Human|Virology Department|COVID-19 Genomics UK (COG-UK) Consortium|Williams|United Kingdom
>Spike|hCoV-19/Spain/AR-IBV-97010741/2020|2020-10-02|EPI_ISL_779776|Original|hCoV-19^^Aragon|Human|Servicio de Microbiolog\u00eda|SeqCOVID-SPAIN consortium/IBV(CSIC)|Comas|Spain
>Spike|hCoV-19/England/QEUH-AD19B8/2020|2020-10-26|EPI_ISL_634533|Original|hCoV-19^^England|Human|Lighthouse Lab in Glasgow|Wellcome Sanger Institute for the COVID-19 Genomics UK (COG-UK) consortium|Davies|United Kingdom
>Spike|hCoV-19/England/BIRM-62AD1/2020|2020-09-02|EPI_ISL_529642|Original|hCoV-19^^England|Human|University of Birmingham|COVID-19 Genomics UK (COG-UK) Consortium|Loman|United Kingdom
>Spike|hCoV-19/Switzerland/GE-SNRCI-8298/2020|2020-03-00|EPI_ISL_548221|Original|hCoV-19^^Geneve|Human|Laboratoire de Virologie|Swiss National Reference Centre for Influenza|Gon\u00e7alves Cabecinhas|Switzerland
>Spike|hCoV-19/England/MILK-A91055/2020|2020-10-16|EPI_ISL_638500|Original|hCoV-19^^England|Human|Oxford Viromics|COVID-19 Genomics UK (COG-UK) Consortium|Golubchik|United Kingdom
>Spike|hCoV-19/England/CAMC-94A1C2/2020|2020-07-24|EPI_ISL_555955|Original|hCoV-19^^England|Human|Lighthouse Lab in Cambridge|Wellcome Sanger Institute for the COVID-19 Genomics UK (COG-UK) consortium|Davies|United Kingdom
>Spike|hCoV-19/USA/TX-HMH0367/2020|2020-03-29|EPI_ISL_434959|Original|hCoV-19^^Texas|Human|Houston Methodist Hospital|Houston Methodist Hospital|Long|USA
>Spike|hCoV-19/Japan/PG-8389/2020|2020-07-00|EPI_ISL_687488|Original|hCoV-19^^Japan|Human|Pathogen Genomics Center|Pathogen Genomics Center|Sekizuka|Japan
>Spike|hCoV-19/Belgium/rega-10191296/2020|2020-10-19|EPI_ISL_735200|Original|hCoV-19^^Leuven|Human|UZ Leuven|KU Leuven|Maes|Belgium
>Spike|hCoV-19/USA/OR-UW-12645/2020|2020-06-26|EPI_ISL_570862|Original|hCoV-19^^Oregon|Human|UW Virology Lab|UW Virology Lab|Roychoudhury|USA
>Spike|hCoV-19/Japan/PG-1028/2020|2020-04-00|EPI_ISL_684638|Original|hCoV-19^^Japan|Human|Fukuoka Institute of Health and Environmental Sciences|Pathogen Genomics Center|Sekizuka|Japan
>Spike|hCoV-19/USA/WI-GMF-15972/2020|2020-08-18|EPI_ISL_547714|Original|hCoV-19^^Wisconsin|Human|Gundersen Molecular Diagnostics Laboratory|Kabara Cancer Research Institute|Kenny|USA
>Spike|hCoV-19/Luxembourg/LNS7132932/2020|2020-09-30|EPI_ISL_744521|Original|hCoV-19^^Luxembourg|Human|Laboratoire national de sant\u00e9|Laboratoire national de sant\u00e9|Wienecke-Baldacchino|Luxembourg
>Spike|hCoV-19/Scotland/GCVR-1709F0/2020|2020-03-26|EPI_ISL_459650|Original|hCoV-19^^Scotland|Human|NHSGGC West of Scotland Specialist Virology Centre / MRC-University of Glasgow Centre for Virus Research|Wellcome Sanger Institute for the COVID-19 Genomics UK (COG-UK) consortium|Davies|United Kingdom
>Spike|hCoV-19/England/CAMB-7E850/2020|2020-04-07|EPI_ISL_442533|Original|hCoV-19^^England|Human|Department of Pathology|Wellcome Sanger Institute for the COVID-19 Genomics UK (COG-UK) consortium|Amato|United Kingdom
>Spike|hCoV-19/Belgium/ULG-10455/2020|2020-08-28|EPI_ISL_581629|Original|hCoV-19^^Li\u00e8ge|Human|Department of Clinical Microbiology|GIGA Medical Genomics|Durkin|Belgium
>Spike|hCoV-19/England/EXET-13A637/2020|2020-11-07|EPI_ISL_665068|Original|hCoV-19^^England|Human|University of Exeter|COVID-19 Genomics UK (COG-UK) Consortium|Temperton|United Kingdom
>Spike|hCoV-19/USA/TX-HMH-MCoV-12572/2020|2020-08-31|EPI_ISL_790401|Original|hCoV-19^^Texas|Human|Houston Methodist Hospital|Houston Methodist Hospital|Long|USA

>Spike|hCoV-19/Hong Kong/VM20031418/2020|2020-07-07|EPI_ISL_510101|Original|hCoV-19^^Hong Kong|Human|Pamela Youde Nethersole Eastern Hospital|Hong Kong Department of Health|Mak|China
>Spike|hCoV-19/England/OXON-AF838/2020|2020-04-15|EPI_ISL_478776|Original|hCoV-19^^England|Human|Oxford Viromics|COVID-19 Genomics UK (COG-UK) Consortium|Golubchik|United Kingdom
>Spike|hCoV-19/USA/TX-HMH-6070/2020|2020-07-05|EPI_ISL_547211|Original|hCoV-19^^Texas|Human|Houston Methodist Hospital|Houston Methodist Hospital|Long|USA
>Spike|hCoV-19/England/ALDP-9CBB70/2020|2020-09-16|EPI_ISL_575573|Original|hCoV-19^^England|Human|Lighthouse Lab in Alderley Park|Wellcome Sanger Institute for the COVID-19 Genomics UK (COG-UK) consortium|Davies|United Kingdom
>Spike|hCoV-19/England/ALDP-9FD1DD/2020|2020-10-02|EPI_ISL_588052|Original|hCoV-19^^England|Human|Lighthouse Lab in Alderley Park|Wellcome Sanger Institute for the COVID-19 Genomics UK (COG-UK) consortium|Davies|United Kingdom
>Spike|hCoV-19/USA/MN-MDH-1074/2020|2020-06-03|EPI_ISL_470754|Original|hCoV-19^^Minnesota|Human|Minnesota Department of Health|Minnesota Department of Health|Garfin|USA
>Spike|hCoV-19/South Africa/KRISP-K003745/2020|2020-09-30|EPI_ISL_602919|Original|hCoV-19^^KwaZulu-Natal|Human|NHLS-IALCH|KRISP|de Oliveira|South Africa
>Spike|hCoV-19/England/SHEF-C9319/2020|2020-04-27|EPI_ISL_475419|Original|hCoV-19^^England|Human|Virology Department|COVID-19 Genomics UK (COG-UK) Consortium|de Silva|United Kingdom
>Spike|hCoV-19/Thailand/NIH-2720/2020|2020-03-16|EPI_ISL_434705|Original|hCoV-19^^Bangkok|Human|Praram 9 Hospital|National Institute of Health. Department of medical Sciences|Okada|Thailand
>Spike|hCoV-19/Switzerland/BS-UHB-42193283/2020|2020-03-16|EPI_ISL_528148|Original|hCoV-19^^Basel-Stadt|Human|University Hospital Basel|University Hospital Basel|Roloff Handschin|Switzerland
>Spike|hCoV-19/USA/CA-ALSR-3570/2020|2020-06-16|EPI_ISL_636089|Original|hCoV-19^^California|Human|San Diego County Public Health Laboratory|Andersen lab at Scripps Research|Gangavarapu|USA
>Spike|hCoV-19/England/ALDP-B84F66/2020|2020-11-15|EPI_ISL_674420|Original|hCoV-19^^England|Human|Lighthouse Lab in Alderley Park|Wellcome Sanger Institute for the COVID-19 Genomics UK (COG-UK) Consortium|Davies|United Kingdom
>Spike|hCoV-19/Japan/PG-8794/2020|2020-08-00|EPI_ISL_686382|Original|hCoV-19^^Japan|Human|Pathogen Genomics Center|Pathogen Genomics Center|Sekizuka|Japan
>Spike|hCoV-19/England/LIVE-A7593/2020|2020-03-28|EPI_ISL_517040|Original|hCoV-19^^England|Human|Liverpool Clinical Laboratories|COVID-19 Genomics UK (COG-UK) Consortium|Haldenby|United Kingdom
>Spike|hCoV-19/England/MILK-C61E25/2020|2020-12-13|EPI_ISL_735656|Original|hCoV-19^^England|Human|Lighthouse Lab in Milton Keynes|Wellcome Sanger Institute for the COVID-19 Genomics UK (COG-UK) Consortium|Davies|United Kingdom
>Spike|hCoV-19/Romania/ROSV_2113/2020|2020-04-11|EPI_ISL_491057|Original|hCoV-19^^Suceava|Human|Suceava County Emergency Hospital|\Stefan cel Mare\ University Metagenomics Lab|Lobiuc|Romania
>Spike|hCoV-19/England/ALDP-A511C7/2020|2020-10-07|EPI_ISL_598243|Original|hCoV-19^^England|Human|Lighthouse Lab in Alderley Park|Wellcome Sanger Institute for the COVID-19 Genomics UK (COG-UK) consortium|Davies|United Kingdom
>Spike|hCoV-19/Portugal/PT0175/2020|2020-03-28|EPI_ISL_453891|Original|hCoV-19^^Portugal|Human|unknown|Instituto Nacional de Saude (INSA)|Borges|Portugal
>Spike|hCoV-19/England/MILK-AC90BE/2020|2020-10-22|EPI_ISL_629643|Original|hCoV-19^^England|Human|Lighthouse Lab in Milton Keynes|Wellcome Sanger Institute for the COVID-19 Genomics UK (COG-UK) consortium|Davies|United Kingdom
>Spike|hCoV-19/USA/TX-DSHS-1289/2020|2020-03-31|EPI_ISL_648220|Original|hCoV-19^^Texas|Human|Texas Department of State Health Services|Texas Department of State Health Services|Rahman|USA
>Spike|hCoV-19/Japan/PG-4720/2020|2020-07-00|EPI_ISL_689856|Original|hCoV-19^^Japan|Human|Pathogen Genomics Center|Pathogen Genomics Center|Sekizuka|Japan

>Spike|hCoV-19/England/QEUH-A002B5/2020|2020-10-02|EPI_ISL_600242|Original|hCoV-19^^England|Human|Lighthouse Lab in Glasgow|Wellcome Sanger Institute for the COVID-19 Genomics UK (COG-UK) consortium|Davies|United Kingdom
>Spike|hCoV-19/England/MILK-BB1C3C/2020|2020-11-18|EPI_ISL_720152|Original|hCoV-19^^England|Human|Lighthouse Lab in Milton Keynes|Wellcome Sanger Institute for the COVID-19 Genomics UK (COG-UK) Consortium|Davies|United Kingdom
>Spike|hCoV-19/Netherlands/ZH-EMC-616/2020|2020-09-16|EPI_ISL_578044|Original|hCoV-19^^Zuid Holland|Human|Dutch COVID-19 response team|Erasmus Medical Center|Oude Munnink|Netherlands
>Spike|hCoV-19/England/CAMB-83612/2020|2020-04-15|EPI_ISL_443532|Original|hCoV-19^^England|Human|Department of Pathology|Wellcome Sanger Institute for the COVID-19 Genomics UK (COG-UK) consortium|Amato|United Kingdom
>Spike|hCoV-19/Australia/VIC10613/2020|2020-08-03|EPI_ISL_562991|Original|hCoV-19^^Victoria|Human|Microbiological Diagnostic Unit - Public Health Laboratory (MDU-PHL)|MDU-PHL|Schultz|Australia
>Spike|hCoV-19/USA/CA-CZB-1242/2020|2020-04-14|EPI_ISL_454662|Original|hCoV-19^^California|Human|County of Santa Clara Public Health Department|Chan-Zuckerberg Biohub|Hao|USA
>Spike|hCoV-19/England/LIVE-9D6CA/2020|2020-04-10|EPI_ISL_453226|Original|hCoV-19^^England|Human|Liverpool Clinical Laboratories|COVID-19 Genomics UK (COG-UK) Consortium|Haldenby|United Kingdom
>Spike|hCoV-19/South Africa/KRISP-0529/2020|2020-07-13|EPI_ISL_498074|Original|hCoV-19^^KwaZulu-Natal|Human|NHLS-IALCH|KRISP|de Oliveira|South Africa
>Spike|hCoV-19/United Arab Emirates/L920647384/2020|2020-04-07|EPI_ISL_520732|Original|hCoV-19^^United Arab Emirates|Human|Mohammed Bin Rashid University of Medicine and Health Sciences|Al Jalila Genomics Center|Ramaswamy|United Arab Emirates
>Spike|hCoV-19/Northern Ireland/NIRE-235923/2020|2020-11-10|EPI_ISL_742164|Original|hCoV-19^^Northern Ireland|Human|Regional Virus Laboratory|COVID-19 Genomics UK (COG-UK) Consortium|Fairley|United Kingdom
>Spike|hCoV-19/Wales/PHWC-16683C/2020|2020-06-27|EPI_ISL_494047|Original|hCoV-19^^Wales|Human|Originating lab|COVID-19 Genomics UK (COG-UK) Consortium|Moore|United Kingdom
>Spike|hCoV-19/India/GJ-GBRC42/2020|2020-04-29|EPI_ISL_444473|Original|hCoV-19^^Gujarat|Human|B.J. Medical College and Civil hospital|Gujarat Biotechnology Research Centre|Joshi|India
>Spike|hCoV-19/Scotland/CVR5935/2020|2020-11-21|EPI_ISL_705680|Original|hCoV-19^^Scotland|Human|West of Scotland Specialist Virology Centre|COVID-19 Genomics UK (COG-UK) Consortium|Robertson|United Kingdom
>Spike|hCoV-19/England/MILK-B85C13/2020|2020-11-13|EPI_ISL_673933|Original|hCoV-19^^England|Human|Lighthouse Lab in Milton Keynes|Wellcome Sanger Institute for the COVID-19 Genomics UK (COG-UK) Consortium|Davies|United Kingdom
>Spike|hCoV-19/Scotland/GCVR-170F0D/2020|2020-03-28|EPI_ISL_489627|Original|hCoV-19^^Scotland|Human|NHSGGC West of Scotland Specialist Virology Centre / MRC-University of Glasgow Centre for Virus Research|Wellcome Sanger Institute for the COVID-19 Genomics UK (COG-UK) consortium|Davies|United Kingdom
>Spike|hCoV-19/Tunisia/61628/2020|2020-11-23|EPI_ISL_707697|Original|hCoV-19^^Nabeul|Human|1-Laboratory of Microbiology|1-Clinical and Experimental Pharmacology Lab|Boutiba-Ben Boubaker|Tunisia
>Spike|hCoV-19/India/MH-ACTREC-097/2020|2020-05-23|EPI_ISL_699753|Original|hCoV-19^^Maharashtra|Human|Hematopathology Laboratory|Hematopathology Laboratory|Patkar|India
>Spike|hCoV-19/USA/UT-UPHL-04289/2020|2020-05-14|EPI_ISL_575130|Original|hCoV-19^^Utah|Human|Utah Public Health Laboratory|Utah Public Health Laboratory|Young|USA
>Spike|hCoV-19/England/CAMB-1BD5A8/2020|2020-00-00|EPI_ISL_764318|Original|hCoV-19^^England|Human|Department of Pathology|COVID-19 Genomics UK (COG-UK) Consortium|Goodfellow|United Kingdom
>Spike|hCoV-19/Australia/VIC15386/2020|2020-08-04|EPI_ISL_593277|Original|hCoV-19^^Victoria|Human|Microbiological Diagnostic Unit - Public Health Laboratory (MDU-PHL)|MDU-PHL|Schultz|Australia

>Spike|hCoV-19/Denmark/DCGC-3303/2020|2020-08-31|EPI_ISL_616055|Original|hCoV-19^^Hovedstaden|Human|Department of Virus and Microbiological Special Diagnostics|Albertsen lab|Michaelsen|Denmark
>Spike|hCoV-19/Japan/PG-3154/2020|2020-05-00|EPI_ISL_691274|Original|hCoV-19^^Japan|Human|Pathogen Genomics Center|Pathogen Genomics Center|Sekizuka|Japan
>Spike|hCoV-19/USA/TX-HMH-6274/2020|2020-06-28|EPI_ISL_547357|Original|hCoV-19^^Texas|Human|Houston Methodist Hospital|Houston Methodist Hospital|Long|USA
>Spike|hCoV-19/England/MILK-AB7EF5/2020|2020-10-21|EPI_ISL_623596|Original|hCoV-19^^England|Human|Lighthouse Lab in Milton Keynes|Wellcome Sanger Institute for the COVID-19 Genomics UK (COG-UK) consortium|Davies|United Kingdom
>Spike|hCoV-19/England/MILK-AC8565/2020|2020-10-21|EPI_ISL_629458|Original|hCoV-19^^England|Human|Lighthouse Lab in Milton Keynes|Wellcome Sanger Institute for the COVID-19 Genomics UK (COG-UK) consortium|Davies|United Kingdom
>Spike|hCoV-19/England/CAMC-C46AB9/2020|2020-12-10|EPI_ISL_718605|Original|hCoV-19^^England|Human|Lighthouse Lab in Cambridge|Wellcome Sanger Institute for the COVID-19 Genomics UK (COG-UK) Consortium|Davies|United Kingdom
>Spike|hCoV-19/Scotland/CVR4836/2020|2020-09-21|EPI_ISL_627362|Original|hCoV-19^^Scotland|Human|West of Scotland Specialist Virology Centre|COVID-19 Genomics UK (COG-UK) Consortium|Robertson|United Kingdom
>Spike|hCoV-19/England/NOTT-11050D/2020|2020-04-23|EPI_ISL_441525|Original|hCoV-19^^England|Human|Queens Medical Centre|COVID-19 Genomics UK (COG-UK) Consortium|Loose|United Kingdom
>Spike|hCoV-19/Portugal/PT0001b/2020|2020-03-03|EPI_ISL_417986|Original|hCoV-19^^Portugal|Human|Centro Hospitalar e Universitario de Sao Joao|Instituto Nacional de Saude (INSA)|Borges|Portugal
>Spike|hCoV-19/England/NOTT-116D96/2020|2020-10-21|EPI_ISL_627667|Original|hCoV-19^^England|Human|Queens Medical Centre|COVID-19 Genomics UK (COG-UK) Consortium|Loose|United Kingdom
>Spike|hCoV-19/England/ALDP-A93ACE/2020|2020-10-19|EPI_ISL_611114|Original|hCoV-19^^England|Human|Lighthouse Lab in Alderley Park|Wellcome Sanger Institute for the COVID-19 Genomics UK (COG-UK) consortium|Davies|United Kingdom
>Spike|hCoV-19/England/MILK-9A9413/2020|2020-09-01|EPI_ISL_551006|Original|hCoV-19^^England|Human|Lighthouse Lab in Milton Keynes|Wellcome Sanger Institute for the COVID-19 Genomics UK (COG-UK) consortium|Davies|United Kingdom
>Spike|hCoV-19/USA/TX-HMH-1308/2020|2020-04-14|EPI_ISL_542642|Original|hCoV-19^^Texas|Human|Houston Methodist Hospital|Houston Methodist Hospital|Long|USA
>Spike|hCoV-19/Singapore/1245/2020|2020-05-01|EPI_ISL_648663|Original|hCoV-19^^Singapore|Human|Department of Laboratory Medicine|Department of Laboratory Medicine|Chen|Singapore
>Spike|hCoV-19/England/NORW-21FD50/2020|2020-12-14|EPI_ISL_741477|Original|hCoV-19^^England|Human|Quadram Institute Bioscience|COVID-19 Genomics UK (COG-UK) Consortium|Alikhan|United Kingdom
>Spike|hCoV-19/Hong Kong/VM20009097/2020|2020-04-06|EPI_ISL_444030|Original|hCoV-19^^Hong Kong|Human|United Christian Hospital|Hong Kong Department of Health|Mak|China
>Spike|hCoV-19/England/BRIS-12232C/2020|2020-03-23|EPI_ISL_440021|Original|hCoV-19^^England|Human|PHE South West Regional Laboratory|Wellcome Sanger Institute for the COVID-19 Genomics UK (COG-UK) consortium|Amato|United Kingdom
>Spike|hCoV-19/England/ALDP-BB9302/2020|2020-11-21|EPI_ISL_702567|Original|hCoV-19^^England|Human|Lighthouse Lab in Alderley Park|Wellcome Sanger Institute for the COVID-19 Genomics UK (COG-UK) Consortium|Davies|United Kingdom
>Spike|hCoV-19/England/MILK-975C72/2020|2020-08-20|EPI_ISL_552453|Original|hCoV-19^^England|Human|Lighthouse Lab in Milton Keynes|Wellcome Sanger Institute for the COVID-19 Genomics UK (COG-UK) consortium|Davies|United Kingdom
>Spike|hCoV-19/Denmark/DCGC-6719/2020|2020-04-20|EPI_ISL_622372|Original|hCoV-19^^Hovedstaden|Human|Department of Virus and Microbiological Special Diagnostics|Albertsen lab|Michaelsen|Denmark
>Spike|hCoV-19/England/CAMB-7DBB2/2020|2020-04-06|EPI_ISL_441678|Original|hCoV-19^^England|Human|Department of Pathology|Wellcome Sanger Institute for the COVID-19 Genomics UK (COG-UK) consortium|Amato|United Kingdom

>Spike|hCoV-19/USA/CA-IGI-0671/2020|2020-07-30|EPI_ISL_677864|Original|hCoV-19^^California|Human|Innovative Genomics Institute|Innovative Genomics Institute|Wyman|USA
>Spike|hCoV-19/Wales/PHWC-163F76/2020|2020-05-06|EPI_ISL_474108|Original|hCoV-19^^Wales|Human|Originating lab|COVID-19 Genomics UK (COG-UK) Consortium|Moore|United Kingdom
>Spike|hCoV-19/USA/NY-NYUMC146/2020|2020-04-01|EPI_ISL_424953|Original|hCoV-19^^New York|Human|NYU Langone Health|Departments of Pathology and Medicine|Maurano|USA
>Spike|hCoV-19/USA/UT-04206/2020|2020-05-14|EPI_ISL_498684|Original|hCoV-19^^Utah|Human|Utah Public Health Laboratory|Utah Public Health Laboratory|Butz|USA
>Spike|hCoV-19/Denmark/DCGC-13161/2020|2020-11-30|EPI_ISL_712625|Original|hCoV-19^^Hovedstaden|Human|Department of Virus and Microbiological Special Diagnostics|Albertsen Lab|Michaelsen|Denmark
>Spike|hCoV-19/Hong Kong/VM20002581/2020|2020-02-13|EPI_ISL_413496|Original|hCoV-19^^Hong Kong|Human|Ruttonjee Hospital|Hong Kong Department of Health|Mak|China
>Spike|hCoV-19/England/CAMC-B9970C/2020|2020-11-16|EPI_ISL_673629|Original|hCoV-19^^England|Human|Lighthouse Lab in Cambridge|Wellcome Sanger Institute for the COVID-19 Genomics UK (COG-UK) Consortium|Davies|United Kingdom
>Spike|hCoV-19/Austria/CeMM1045/2020|2020-04-06|EPI_ISL_583862|Original|hCoV-19^^Austria|Human|Institute for Laboratory Diagnostics and Microbiology|Bergthaler laboratory|Bergthaler|Austria
>Spike|hCoV-19/USA/TX-HMH-6738/2020|2020-06-25|EPI_ISL_546205|Original|hCoV-19^^Texas|Human|Houston Methodist Hospital|Houston Methodist Hospital|Long|USA
>Spike|hCoV-19/England/LIVE-9942E/2020|2020-04-08|EPI_ISL_439722|Original|hCoV-19^^England|Human|Liverpool Clinical Laboratories|COVID-19 Genomics UK (COG-UK) Consortium|Haldenby|United Kingdom
>Spike|hCoV-19/Spain/CT-IBV-003152/2020|2020-03-16|EPI_ISL_510249|Original|hCoV-19^^Catalunya|Human|Hospital de la Santa Creu i Sant Pau. Servicio de Microbiolog\u00eda|SeqCOVID-SPAIN consortium/IBV(CSIC)|Comas|Spain
>Spike|hCoV-19/USA/CA-LACPHL-AA00043/2020|2020-10-14|EPI_ISL_710429|Original|hCoV-19^^California|Human|Los Angeles County PHL|Los Angeles County PHL|Hemarajata|USA
>Spike|hCoV-19/Canada/ON-S566/2020|2020-04-06|EPI_ISL_586420|Original|hCoV-19^^Ontario|Human|Toronto Invasive Bacterial Diseases Network|McMaster University|McArthur|Canada
>Spike|hCoV-19/Denmark/DCGC-15215/2020|2020-11-23|EPI_ISL_713699|Original|hCoV-19^^Hovedstaden|Human|Department of Virus and Microbiological Special Diagnostics|Albertsen Lab|Michaelsen|Denmark
>Spike|hCoV-19/England/20132081804/2020|2020-03-23|EPI_ISL_423812|Original|hCoV-19^^England|Human|Respiratory Virus Unit|Respiratory Virus Unit|Platt|United Kingdom
>Spike|hCoV-19/France/PAC-IHU-0262/2020|2020-03-31|EPI_ISL_569419|Original|hCoV-19^^Provence-Alpes-C\u00f4te-d\u2019Azur|Human|MEPHI|MEPHI|Levasseur|France
>Spike|hCoV-19/England/LIVE-9D7C7/2020|2020-04-15|EPI_ISL_453233|Original|hCoV-19^^England|Human|Liverpool Clinical Laboratories|COVID-19 Genomics UK (COG-UK) Consortium|Haldenby|United Kingdom
>Spike|hCoV-19/Northern Ireland/NIRE-FD631/2020|2020-05-03|EPI_ISL_489271|Original|hCoV-19^^Northern Ireland|Human|Regional Virus Laboratory|Wellcome Sanger Institute for the COVID-19 Genomics UK (COG-UK) consortium|Davies|United Kingdom
>Spike|hCoV-19/Denmark/DCGC-7487/2020|2020-10-12|EPI_ISL_619385|Original|hCoV-19^^Hovedstaden|Human|Department of Virus and Microbiological Special Diagnostics|Albertsen lab|Michaelsen|Denmark
>Spike|hCoV-19/Israel/CVL1133/2020|2020-06-29|EPI_ISL_516903|Original|hCoV-19^^Northern District|Human|Israel Central Virology laboratory|Israel Central Virology laboratory|Zuckerman|Israel
>Spike|hCoV-19/England/ALDP-AA4C4C/2020|2020-10-21|EPI_ISL_630784|Original|hCoV-19^^England|Human|Lighthouse Lab in Alderley Park|Wellcome Sanger Institute for the COVID-19 Genomics UK (COG-UK) consortium|Davies|United Kingdom
>Spike|hCoV-19/England/CAMC-B0901B/2020|2020-10-29|EPI_ISL_673071|Original|hCoV-19^^England|Human|Lighthouse Lab in Cambridge|Wellcome Sanger Institute for the COVID-19 Genomics UK (COG-UK) Consortium|Davies|United Kingdom

>Spike|hCoV-19/USA/CA-ALSR-3067/2020|2020-08-27|EPI_ISL_635826|Original|hCoV-19^^California|Human|San Diego County Public Health Laboratory|Andersen lab at Scripps Research|Gangavarapu|USA
>Spike|hCoV-19/USA/NY-Wadsworth-200226-01/2020|2020-07-01|EPI_ISL_677060|Original|hCoV-19^^New York|Human|Masonic Medical Research Institute|Wadsworth Center|Lasek-Nesselquist|USA
>Spike|hCoV-19/USA/AZ-TG584207/2020|2020-08-05|EPI_ISL_695633|Original|hCoV-19^^Arizona|Human|TGen North|TGen North|French|USA
>Spike|hCoV-19/England/ALDP-BB991F/2020|2020-11-20|EPI_ISL_703130|Original|hCoV-19^^England|Human|Lighthouse Lab in Alderley Park|Wellcome Sanger Institute for the COVID-19 Genomics UK (COG-UK) Consortium|Davies|United Kingdom
>Spike|hCoV-19/Australia/VIC5057/2020|2020-07-19|EPI_ISL_519860|Original|hCoV-19^^Victoria|Human|Microbiological Diagnostic Unit - Public Health Laboratory (MDU-PHL)|MDU-PHL|Schultz|Australia
>Spike|hCoV-19/England/QEUH-B115F3/2020|2020-11-02|EPI_ISL_642346|Original|hCoV-19^^England|Human|Lighthouse Lab in Glasgow|Wellcome Sanger Institute for the COVID-19 Genomics UK (COG-UK) Consortium|Davies|United Kingdom
>Spike|hCoV-19/South Africa/R13168-20/2020|2020-06-30|EPI_ISL_622896|Original|hCoV-19^^Gauteng|Human|National Institute for Communicable Diseases of the National Health Laboratory Service|National Institute for Communicable Diseases of the National Health Laboratory Service|Allam|South Africa
>Spike|hCoV-19/Denmark/DCGC-16436/2020|2020-11-16|EPI_ISL_714765|Original|hCoV-19^^Hovedstaden|Human|Department of Virus and Microbiological Special Diagnostics|Albertsen Lab|Michaelsen|Denmark
>Spike|hCoV-19/England/ALDP-B25EB5/2020|2020-11-04|EPI_ISL_646652|Original|hCoV-19^^England|Human|Lighthouse Lab in Alderley Park|Wellcome Sanger Institute for the COVID-19 Genomics UK (COG-UK) Consortium|Davies|United Kingdom
>Spike|hCoV-19/England/CAMC-B09B74/2020|2020-10-29|EPI_ISL_672927|Original|hCoV-19^^England|Human|Lighthouse Lab in Cambridge|Wellcome Sanger Institute for the COVID-19 Genomics UK (COG-UK) Consortium|Davies|United Kingdom
>Spike|hCoV-19/Wales/PHWC-48203F/2020|2020-10-21|EPI_ISL_613194|Original|hCoV-19^^Wales|Human|Wales Specialist Virology Centre Sequencing lab|COVID-19 Genomics UK (COG-UK) Consortium|Moore|United Kingdom
>Spike|hCoV-19/England/MILK-A4758C/2020|2020-10-06|EPI_ISL_598269|Original|hCoV-19^^England|Human|Lighthouse Lab in Milton Keynes|Wellcome Sanger Institute for the COVID-19 Genomics UK (COG-UK) consortium|Davies|United Kingdom
>Spike|hCoV-19/Spain/AN-IBV-98001775/2020|2020-04-05|EPI_ISL_538596|Original|hCoV-19^^Andalucia|Human|Servicio de Microbiolog\u00eda y Parasitolog\u00eda cl\u00ednica. UCEIMP. Hospital Universitario Virgen del Roc\u00edo/IBIS/CSIC/US|SeqCOVID-SPAIN consortium/IBV(CSIC)|Comas|Spain
>Spike|hCoV-19/England/204661424/2020|2020-11-11|EPI_ISL_693411|Original|hCoV-19^^England|Human|Respiratory Virus Unit|COVID-19 Genomics UK (COG-UK) Consortium|Platt|United Kingdom
>Spike|hCoV-19/Wales/ALDP-BC64B4/2020|2020-11-20|EPI_ISL_767324|Original|hCoV-19^^Wales|Human|Lighthouse Lab in Alderley Park|Wellcome Sanger Institute for the COVID-19 Genomics UK (COG-UK) Consortium|Davies|United Kingdom
>Spike|hCoV-19/England/20118171704/2020|2020-03-12|EPI_ISL_464991|Original|hCoV-19^^England|Human|Respiratory Virus Unit|Respiratory Virus Unit|Platt|United Kingdom
>Spike|hCoV-19/Scotland/EDB656/2020|2020-04-02|EPI_ISL_491578|Original|hCoV-19^^Scotland|Human|Virology Department|Wellcome Sanger Institute for the COVID-19 Genomics UK (COG-UK) consortium|Davies|United Kingdom
>Spike|hCoV-19/Switzerland/ZH-ETHZ-270128/2020|2020-09-03|EPI_ISL_541523|Original|hCoV-19^^Zurich|Human|Viollier AG|Department of Biosystems Science and Engineering|Nadeau|Switzerland
>Spike|hCoV-19/Switzerland/BL-UHB-42194515/2020|2020-03-17|EPI_ISL_528162|Original|hCoV-19^^Basel-Land|Human|University Hospital Basel|University Hospital Basel|Roloff Handschin|Switzerland
>Spike|hCoV-19/USA/UT-QDX-1794/2020|2020-03-16|EPI_ISL_604124|Original|hCoV-19^^Utah|Human|Quest Diagnostics|Quest Diagnostics|Kagan|USA

>Spike|hCoV-19/USA/TX-HMH-MCoV-17974/2020|2020-11-16|EPI_ISL_786122|Original|hCoV-19^^Texas|Human|Houston Methodist Hospital|Houston Methodist Hospital|Long|USA
>Spike|hCoV-19/England/CAMB-83ADA/2020|2020-04-15|EPI_ISL_443340|Original|hCoV-19^^England|Human|Department of Pathology|Wellcome Sanger Institute for the COVID-19 Genomics UK (COG-UK) consortium|Amato|United Kingdom
>Spike|hCoV-19/England/ALDP-B5DD7B/2020|2020-11-09|EPI_ISL_723590|Original|hCoV-19^^England|Human|Northumbria University / South Tees Hospitals NHS Foundation Trust / North Cumbria Integrated Care NHS Foundation Trust / North Tees and Hartlepool NHS Foundation Trust / Newcastle Hospitals NHS Foundation Trust|COVID-19 Genomics UK (COG-UK) Consortium|Bashton|United Kingdom
>Spike|hCoV-19/Australia/VIC12361/2020|2020-09-09|EPI_ISL_563555|Original|hCoV-19^^Victoria|Human|Microbiological Diagnostic Unit - Public Health Laboratory (MDU-PHL)|MDU-PHL|Schultz|Australia
>Spike|hCoV-19/Netherlands/un-EMC-759/2020|2020-07-05|EPI_ISL_523515|Original|hCoV-19^^Netherlands|Human|Dutch COVID-19 response team|Erasmus Medical Center|Oude Munnink|Netherlands
>Spike|hCoV-19/Scotland/EDB6223/2020|2020-05-19|EPI_ISL_473921|Original|hCoV-19^^Scotland|Human|Virology Department|COVID-19 Genomics UK (COG-UK) Consortium|Williams|United Kingdom
>Spike|hCoV-19/England/MILK-9F1986/2020|2020-09-25|EPI_ISL_589621|Original|hCoV-19^^England|Human|Lighthouse Lab in Milton Keynes|Wellcome Sanger Institute for the COVID-19 Genomics UK (COG-UK) consortium|Davies|United Kingdom
>Spike|hCoV-19/England/QEUH-98FB96/2020|2020-08-29|EPI_ISL_530351|Original|hCoV-19^^England|Human|Lighthouse Lab in Glasgow|Wellcome Sanger Institute for the COVID-19 Genomics UK (COG-UK) consortium|Davies|United Kingdom
>Spike|hCoV-19/USA/MA-MGH-00582/2020|2020-03-27|EPI_ISL_460337|Original|hCoV-19^^Massachusetts|Human|Massachusetts General Hospital|Infectious Disease Program|Lemieux|USA
>Spike|hCoV-19/Denmark/DCGC-4124/2020|2020-09-21|EPI_ISL_621944|Original|hCoV-19^^Hovedstaden|Human|Department of Virus and Microbiological Special Diagnostics|Albertsen lab|Michaelsen|Denmark
>Spike|hCoV-19/England/NOTT-10F6B3/2020|2020-04-03|EPI_ISL_433036|Original|hCoV-19^^England|Human|Queens Medical Centre|COVID-19 Genomics UK (COG-UK) Consortium|Loose|United Kingdom
>Spike|hCoV-19/England/LOND-12517CA/2020|2020-11-18|EPI_ISL_679438|Original|hCoV-19^^England|Human|University College London Hospital|COVID-19 Genomics UK (COG-UK) Consortium|Byott|United Kingdom
>Spike|hCoV-19/England/QEUH-99872D/2020|2020-08-24|EPI_ISL_540027|Original|hCoV-19^^England|Human|Lighthouse Lab in Glasgow|Wellcome Sanger Institute for the COVID-19 Genomics UK (COG-UK) consortium|Davies|United Kingdom
>Spike|hCoV-19/Spain/VC-FISABIO-579/2020|2020-03-28|EPI_ISL_447509|Original|hCoV-19^^Comunitat Valenciana|Human|Servicio de Microbiologia. Hospital Clinico Universitario de Valencia|Sequencing and Bioinformatics Service and Molecular Epidemiology Research Group. FISABIO-Public Health|Gonzalez-Candelas|Spain
>Spike|hCoV-19/Australia/VIC8098/2020|2020-08-16|EPI_ISL_564846|Original|hCoV-19^^Victoria|Human|Microbiological Diagnostic Unit - Public Health Laboratory (MDU-PHL)|MDU-PHL|Schultz|Australia
>Spike|hCoV-19/England/MILK-BB0954/2020|2020-11-17|EPI_ISL_720178|Original|hCoV-19^^England|Human|Lighthouse Lab in Milton Keynes|Wellcome Sanger Institute for the COVID-19 Genomics UK (COG-UK) Consortium|Davies|United Kingdom
>Spike|hCoV-19/USA/LA-CDC-1905/2020|2020-05-07|EPI_ISL_535341|Original|hCoV-19^^Louisiana|Human|LA Office of Public Health Laboratories|Pathogen Discovery|Queen|USA
>Spike|hCoV-19/England/ALDP-8E2B65/2020|2020-06-25|EPI_ISL_557097|Original|hCoV-19^^England|Human|Lighthouse Lab in Alderley Park|Wellcome Sanger Institute for the COVID-19 Genomics UK (COG-UK) consortium|Davies|United Kingdom
>Spike|hCoV-19/USA/ID-UW-2425/2020|2020-03-28|EPI_ISL_477682|Original|hCoV-19^^Idaho|Human|UW Virology Lab|UW Virology Lab|Roychoudhury|USA

>Spike|hCoV-19/England/MILK-970284/2020|2020-08-17|EPI_ISL_553235|Original|hCoV-19^^England|Human|Lighthouse Lab in Milton Keynes|Wellcome Sanger Institute for the COVID-19 Genomics UK (COG-UK) consortium|Davies|United Kingdom
>Spike|hCoV-19/USA/TX-DSHS-1968/2020|2020-06-03|EPI_ISL_766773|Original|hCoV-19^^Texas|Human|Texas Department of State Health Services|Texas Department of State Health Services|Rahman|USA
>Spike|hCoV-19/Denmark/DCGC-18493/2020|2020-12-07|EPI_ISL_750000|Original|hCoV-19^^Hovedstaden|Human|Department of Virus and Microbiological Special Diagnostics|Albertsen Lab|Michaelsen|Denmark
>Spike|hCoV-19/England/ALDP-97FC4B/2020|2020-08-04|EPI_ISL_529535|Original|hCoV-19^^England|Human|Queens Medical Centre|COVID-19 Genomics UK (COG-UK) Consortium|Loose|United Kingdom
>Spike|hCoV-19/England/LIVE-DB3F99/2020|2020-10-21|EPI_ISL_651236|Original|hCoV-19^^England|Human|Liverpool Clinical Laboratories|COVID-19 Genomics UK (COG-UK) Consortium|Haldenby|United Kingdom
>Spike|hCoV-19/USA/OR-OHSU-4667/2020|2020-11-09|EPI_ISL_667545|Original|hCoV-19^^Oregon|Human|OHSU Lab Services Molecular Microbiology Lab|Oregon SARS-CoV-2 Genome Sequencing Center|O'Roak|USA
>Spike|hCoV-19/USA/CA-CZB-14057/2020|2020-06-28|EPI_ISL_739144|Original|hCoV-19^^California|Human|Alameda County Public Health Lab|Chan-Zuckerberg Biohub|Kamm|USA
>Spike|hCoV-19/Denmark/DCGC-22050/2020|2020-12-14|EPI_ISL_758527|Original|hCoV-19^^Nordjylland|Human|Department of Virus and Microbiological Special Diagnostics|Albertsen Lab|Michaelsen|Denmark
>Spike|hCoV-19/Russia/KK-RII-MH2759S/2020|2020-09-25|EPI_ISL_602432|Original|hCoV-19^^Khakassia|Human|HELIX LLC|WHO National Influenza Centre Russian Federation|Komissarov|Russia
>Spike|hCoV-19/USA/TX-HMH-MCoV-15586/2020|2020-10-26|EPI_ISL_787070|Original|hCoV-19^^Texas|Human|Houston Methodist Hospital|Houston Methodist Hospital|Long|USA
>Spike|hCoV-19/Portugal/PT0820/2020|2020-03-20|EPI_ISL_511079|Original|hCoV-19^^Portugal|Human|Instituto Nacional de Saude (INSA)|Instituto Nacional de Saude (INSA)|Borges|Portugal
>Spike|hCoV-19/Wales/PHWC-15FF40/2020|2020-05-26|EPI_ISL_472873|Original|hCoV-19^^Wales|Human|Wales Specialist Virology Centre Sequencing lab|COVID-19 Genomics UK (COG-UK) Consortium|Moore|United Kingdom
>Spike|hCoV-19/Finland/16A25S14/2020|2020-04-16|EPI_ISL_481567|Original|hCoV-19^^Uusimaa|Human|Department of Virology and Immunology|Department of Virology|Smura|Finland
>Spike|hCoV-19/Scotland/CVR364/2020|2020-03-22|EPI_ISL_433285|Original|hCoV-19^^Scotland|Human|West of Scotland Specialist Virology Centre|COVID-19 Genomics UK (COG-UK) Consortium|Robertson|United Kingdom
>Spike|hCoV-19/England/CAMC-BC0AC7/2020|2020-11-22|EPI_ISL_704745|Original|hCoV-19^^England|Human|Lighthouse Lab in Cambridge|Wellcome Sanger Institute for the COVID-19 Genomics UK (COG-UK) Consortium|Davies|United Kingdom
>Spike|hCoV-19/England/OXON-B2E6D/2020|2020-04-10|EPI_ISL_534819|Original|hCoV-19^^England|Human|Oxford Viromics|COVID-19 Genomics UK (COG-UK) Consortium|Golubchik|United Kingdom
>Spike|hCoV-19/USA/NY-NYUMC905/2020|2020-04-28|EPI_ISL_467398|Original|hCoV-19^^New York|Human|NYU Langone Health|Departments of Pathology and Medicine|Maurano|USA
>Spike|hCoV-19/USA/WA-UW-2091/2020|2020-03-24|EPI_ISL_426084|Original|hCoV-19^^Washington|Human|UW Virology Lab|UW Virology Lab|Roychoudhury|USA
>Spike|hCoV-19/England/CAMB-7377C/2020|2020-03-29|EPI_ISL_425234|Original|hCoV-19^^England|Human|Department of Pathology|COVID-19 Genomics UK (COG-UK) Consortium|Meredith|United Kingdom
>Spike|hCoV-19/England/CAMC-A820C0/2020|2020-10-13|EPI_ISL_610772|Original|hCoV-19^^England|Human|Lighthouse Lab in Cambridge|Wellcome Sanger Institute for the COVID-19 Genomics UK (COG-UK) consortium|Davies|United Kingdom
>Spike|hCoV-19/France/OCC-33/2020|2020-06-08|EPI_ISL_591541|Original|hCoV-19^^Occitanie|Human|CHU Purpan - Laboratoire de Virologie - Institut F\u00e9d\u00e9ratif de Biologie|CHU Purpan - Laboratoire de Virologie - Institut F\u00e9d\u00e9ratif de Biologie|Latour|France

>Spike|hCoV-19/USA/TX-HMH-MCoV-14907/2020|2020-10-12|EPI_ISL_786391|Original|hCoV-19^^Texas|Human|Houston Methodist Hospital|Houston Methodist Hospital|Long|USA
>Spike|hCoV-19/Scotland/EDB4985/2020|2020-05-14|EPI_ISL_493928|Original|hCoV-19^^Scotland|Human|Virology Department|COVID-19 Genomics UK (COG-UK) Consortium|Williams|United Kingdom
>Spike|hCoV-19/Japan/PG-4871/2020|2020-08-00|EPI_ISL_689992|Original|hCoV-19^^Japan|Human|Pathogen Genomics Center|Pathogen Genomics Center|Sekizuka|Japan
>Spike|hCoV-19/USA/WA-UW-13306/2020|2020-07-03|EPI_ISL_501135|Original|hCoV-19^^Washington|Human|University of Washington Virology Lab|University of Washington Virology Lab|Roychoudhury|USA
>Spike|hCoV-19/USA/IL-NM-1179/2020|2020-06-17|EPI_ISL_626504|Original|hCoV-19^^Illinois|Human|Northwestern Memorial Hospital|Ozer Lab|Ozer|USA
>Spike|hCoV-19/India/MH-ACTREC-209/2020|2020-06-27|EPI_ISL_699863|Original|hCoV-19^^Maharashtra|Human|Hematopathology Laboratory|Hematopathology Laboratory|Patkar|India
>Spike|hCoV-19/USA/NM-UNM-00849/2020|2020-05-04|EPI_ISL_610277|Original|hCoV-19^^New Mexico|Human|New Mexico Department of Health Scientific Laboratory Division|Center for Global Health|Domman|USA
>Spike|hCoV-19/Japan/PG-5963/2020|2020-07-00|EPI_ISL_689078|Original|hCoV-19^^Japan|Human|Tokyo Metropolitan Institute of Public Health|Pathogen Genomics Center|Sekizuka|Japan
>Spike|hCoV-19/USA/TX-HMH-MCoV-19564/2020|2020-11-30|EPI_ISL_783936|Original|hCoV-19^^Texas|Human|Houston Methodist Hospital|Houston Methodist Hospital|Long|USA
>Spike|hCoV-19/South Africa/LR00078/2020|2020-07-13|EPI_ISL_514389|Original|hCoV-19^^Gauteng|Human|National Institute for Communicable Diseases of the National Health Laboratory Service|National Institute for Communicable Diseases of the National Health Laboratory Service|Allam|South Africa
>Spike|hCoV-19/England/PORT-2CFF3B/2020|2020-05-27|EPI_ISL_493627|Original|hCoV-19^^England|Human|Centre for Enzyme Innovation|COVID-19 Genomics UK (COG-UK) Consortium|Robson|United Kingdom
>Spike|hCoV-19/USA/CA-CZB-10308/2020|2020-09-11|EPI_ISL_648501|Original|hCoV-19^^California|Human|Orange County Public Health Lab|Chan-Zuckerberg Biohub|Bhatt|USA
>Spike|hCoV-19/England/MILK-B0209A/2020|2020-10-24|EPI_ISL_642820|Original|hCoV-19^^England|Human|Lighthouse Lab in Milton Keynes|Wellcome Sanger Institute for the COVID-19 Genomics UK (COG-UK) Consortium|Davies|United Kingdom
>Spike|hCoV-19/Canada/ON-PHL-20-01179/2020|2020-06-00|EPI_ISL_548801|Original|hCoV-19^^Ontario|Human|Public Health Ontario Laboratory|Public Health Ontario Laboratory|Guthrie|Canada
>Spike|hCoV-19/England/ALDP-9884B0/2020|2020-08-03|EPI_ISL_529588|Original|hCoV-19^^England|Human|Quadram Institute Bioscience|COVID-19 Genomics UK (COG-UK) Consortium|Alikhan|United Kingdom
>Spike|hCoV-19/USA/CT-Yale-333/2020|2020-05-12|EPI_ISL_452145|Original|hCoV-19^^Connecticut|Human|Yale COVID-19 Biorepository|Grubaugh Lab - Yale School of Public Health|Grubaugh|USA
>Spike|hCoV-19/Canada/BC_09819716/2020|2020-05-00|EPI_ISL_477072|Original|hCoV-19^^British Columbia|Human|BCCDC Public Health Laboratory|BCCDC Public Health Laboratory|Harrigan|Canada
>Spike|hCoV-19/India/MH-ACTREC-041/2020|2020-05-11|EPI_ISL_699697|Original|hCoV-19^^Maharashtra|Human|Hematopathology Laboratory|Hematopathology Laboratory|Patkar|India
>Spike|hCoV-19/England/MILK-A47917/2020|2020-10-05|EPI_ISL_598424|Original|hCoV-19^^England|Human|Lighthouse Lab in Milton Keynes|Wellcome Sanger Institute for the COVID-19 Genomics UK (COG-UK) consortium|Davies|United Kingdom
>Spike|hCoV-19/England/CAMC-B35483/2020|2020-11-05|EPI_ISL_659657|Original|hCoV-19^^England|Human|Lighthouse Lab in Cambridge|Wellcome Sanger Institute for the COVID-19 Genomics UK (COG-UK) Consortium|Davies|United Kingdom
>Spike|hCoV-19/Suriname/SR-16/2020|2020-06-25|EPI_ISL_517627|Original|hCoV-19^^Suriname|Human|Academic Hospital Paramaribo|Erasmus Medical Center|M\u00fcnger|Suriname

>Spike|hCoV-19/Wales/PHWC-4A1629/2020|2020-12-10|EPI_ISL_743702|Original|hCoV-19^^Wales|Human|Wales Specialist Virology Centre Sequencing lab|COVID-19 Genomics UK (COG-UK) Consortium|Moore|United Kingdom
>Spike|hCoV-19/USA/IL-QDX-831/2020|2020-03-14|EPI_ISL_571600|Original|hCoV-19^^Illinois|Human|Quest Diagnostics|Quest Diagnostics|Kagan|USA
>Spike|hCoV-19/USA/PA-MGEL70/2020|2020-03-30|EPI_ISL_454358|Original|hCoV-19^^Pennsylvania|Human|UPMC Clinical Microbiology Laboratory|Microbial Genome Sequencing Center|Mustapha|USA
>Spike|hCoV-19/USA/MD-HP00060/2020|2020-03-10|EPI_ISL_457802|Original|hCoV-19^^Maryland|Human|Johns Hopkins Hospital Department of Pathology|Johns Hopkins Hospital Department of Pathology|Ramakrishnan|USA
>Spike|hCoV-19/South Africa/KRISP-K002882/2020|2020-09-03|EPI_ISL_602694|Original|hCoV-19^^KwaZulu-Natal|Human|NHLS-IALCH|KRISP|de Oliveira|South Africa
>Spike|hCoV-19/England/201360181/2020|2020-03-26|EPI_ISL_423232|Original|hCoV-19^^England|Human|Respiratory Virus Unit|Respiratory Virus Unit|Platt|United Kingdom
>Spike|hCoV-19/USA/TX-DSHS-1717/2020|2020-06-20|EPI_ISL_708630|Original|hCoV-19^^Texas|Human|Texas Department of State Health Services|Texas Department of State Health Services|Rahman|USA
>Spike|hCoV-19/USA/FL-BPHL-2228/2020|2020-09-03|EPI_ISL_666850|Original|hCoV-19^^Florida|Human|Florida Bureau of Public Health Laboratories|Florida Bureau of Public Health Laboratories|Blanton|USA
>Spike|hCoV-19/Denmark/DCGC-4717/2020|2020-09-21|EPI_ISL_622156|Original|hCoV-19^^Syddanmark|Human|Department of Virus and Microbiological Special Diagnostics|Albertsen lab|Michaelsen|Denmark
>Spike|hCoV-19/Switzerland/BL-UHB-42195430/2020|2020-03-17|EPI_ISL_528177|Original|hCoV-19^^Basel-Land|Human|University Hospital Basel|University Hospital Basel|Roloff Handschin|Switzerland
>Spike|hCoV-19/England/CAMC-94AD46/2020|2020-07-16|EPI_ISL_556666|Original|hCoV-19^^England|Human|Lighthouse Lab in Cambridge|Wellcome Sanger Institute for the COVID-19 Genomics UK (COG-UK) consortium|Davies|United Kingdom
>Spike|hCoV-19/England/20149029204/2020|2020-04-03|EPI_ISL_466182|Original|hCoV-19^^England|Human|Respiratory Virus Unit|Respiratory Virus Unit|Platt|United Kingdom
>Spike|hCoV-19/England/NORW-F5EB9/2020|2020-11-08|EPI_ISL_650371|Original|hCoV-19^^England|Human|Quadram Institute Bioscience|COVID-19 Genomics UK (COG-UK) Consortium|Alikhan|United Kingdom
>Spike|hCoV-19/Scotland/CVR3033/2020|2020-04-23|EPI_ISL_461626|Original|hCoV-19^^Scotland|Human|West of Scotland Specialist Virology Centre|COVID-19 Genomics UK (COG-UK) Consortium|Robertson|United Kingdom
>Spike|hCoV-19/England/LIVE-A1696/2020|2020-06-11|EPI_ISL_490334|Original|hCoV-19^^England|Human|Liverpool Clinical Laboratories|COVID-19 Genomics UK (COG-UK) Consortium|Haldenby|United Kingdom
>Spike|hCoV-19/England/ALDP-94EE6D/2020|2020-06-17|EPI_ISL_556363|Original|hCoV-19^^England|Human|Lighthouse Lab in Alderley Park|Wellcome Sanger Institute for the COVID-19 Genomics UK (COG-UK) consortium|Davies|United Kingdom
>Spike|hCoV-19/Bangladesh/BCSIR-NILMRC-135/2020|2020-06-18|EPI_ISL_480420|Original|hCoV-19^^Dhaka|Human|National Institute of Laboratory Medicine and Referral Center|Bangladesh Council of Scientific and Industrial Research|Khan|Bangladesh
>Spike|hCoV-19/England/QEUH-9F3984/2020|2020-09-24|EPI_ISL_588568|Original|hCoV-19^^England|Human|Lighthouse Lab in Glasgow|Wellcome Sanger Institute for the COVID-19 Genomics UK (COG-UK) consortium|Davies|United Kingdom
>Spike|hCoV-19/Switzerland/BS-UHB-42201508/2020|2020-03-21|EPI_ISL_528297|Original|hCoV-19^^Basel-Stadt|Human|University Hospital Basel|University Hospital Basel|Roloff Handschin|Switzerland
>Spike|hCoV-19/England/ALDP-AA1878/2020|2020-10-22|EPI_ISL_625017|Original|hCoV-19^^England|Human|Lighthouse Lab in Alderley Park|Wellcome Sanger Institute for the COVID-19 Genomics UK (COG-UK) consortium|Davies|United Kingdom

>Spike|hCoV-19/Kazakhstan/18243/2020|2020-04-16|EPI_ISL_454500|Original|hCoV-19^^Nur-Sultan|Human|RSE \National Center for Biotechnology\|RSE \National Center for Biotechnology\|Kalendar|Kazakhstan
>Spike|hCoV-19/England/NOTT-111821/2020|2020-05-21|EPI_ISL_461929|Original|hCoV-19^^England|Human|Queens Medical Centre|COVID-19 Genomics UK (COG-UK) Consortium|Loose|United Kingdom
>Spike|hCoV-19/Switzerland/SO-ETHZ-380351/2020|2020-11-21|EPI_ISL_721886|Original|hCoV-19^^Solothurn|Human|Viollier AG|Department of Biosystems Science and Engineering|Nadeau|Switzerland
>Spike|hCoV-19/Scotland/QEUH-B6943B/2020|2020-11-11|EPI_ISL_676316|Original|hCoV-19^^Scotland|Human|Lighthouse Lab in Glasgow|Wellcome Sanger Institute for the COVID-19 Genomics UK (COG-UK) Consortium|Davies|United Kingdom
>Spike|hCoV-19/USA/WA-S1703/2020|2020-05-31|EPI_ISL_496129|Original|hCoV-19^^Washington|Human|Washington State Department of Health|Seattle Flu Study|Lee|USA
>Spike|hCoV-19/USA/MI-UM-10036667418/2020|2020-10-28|EPI_ISL_650091|Original|hCoV-19^^Michigan|Human|University of Michigan Clinical Microbiology Laboratory|Lauring Lab|Valesano|USA
>Spike|hCoV-19/England/CAMC-B381C5/2020|2020-11-05|EPI_ISL_658959|Original|hCoV-19^^England|Human|Lighthouse Lab in Cambridge|Wellcome Sanger Institute for the COVID-19 Genomics UK (COG-UK) Consortium|Davies|United Kingdom
>Spike|hCoV-19/England/QEUH-B42465/2020|2020-11-08|EPI_ISL_657230|Original|hCoV-19^^England|Human|Lighthouse Lab in Glasgow|Wellcome Sanger Institute for the COVID-19 Genomics UK (COG-UK) Consortium|Davies|United Kingdom
>Spike|hCoV-19/USA/GA-EHC-209C/2020|2020-04-23|EPI_ISL_455359|Original|hCoV-19^^Georgia|Human|Emory Molecular Diagnostics Laboratory|Piantadosi Lab|Piantadosi|USA
>Spike|hCoV-19/Netherlands/GE-EMC-349/2020|2020-10-05|EPI_ISL_632642|Original|hCoV-19^^Gelderland|Human|Dutch COVID-19 response team|Erasmus Medical Center|Oude Munnink|Netherlands
>Spike|hCoV-19/Wales/PHWC-15C0B9/2020|2020-03-31|EPI_ISL_472434|Original|hCoV-19^^Wales|Human|Wales Specialist Virology Centre Sequencing lab|COVID-19 Genomics UK (COG-UK) Consortium|Moore|United Kingdom
>Spike|hCoV-19/England/201080039/2020|2020-03-06|EPI_ISL_464438|Original|hCoV-19^^England|Human|Respiratory Virus Unit|Respiratory Virus Unit|Platt|United Kingdom
>Spike|hCoV-19/USA/NM-UNM-00799/2020|2020-05-05|EPI_ISL_610262|Original|hCoV-19^^New Mexico|Human|TriCore Reference Laboratories|Center for Global Health|Domman|USA
>Spike|hCoV-19/Wales/PHWC-168700/2020|2020-04-01|EPI_ISL_494276|Original|hCoV-19^^Wales|Human|Originating lab|COVID-19 Genomics UK (COG-UK) Consortium|Moore|United Kingdom
>Spike|hCoV-19/USA/TX-HMH-2189/2020|2020-05-23|EPI_ISL_545171|Original|hCoV-19^^Texas|Human|Houston Methodist Hospital|Houston Methodist Hospital|Long|USA
>Spike|hCoV-19/England/QEUH-9D31DD/2020|2020-09-18|EPI_ISL_577316|Original|hCoV-19^^England|Human|Centre for Enzyme Innovation|COVID-19 Genomics UK (COG-UK) Consortium|Robson|United Kingdom
>Spike|hCoV-19/England/CAMC-C40105/2020|2020-12-10|EPI_ISL_719396|Original|hCoV-19^^England|Human|Lighthouse Lab in Cambridge|Wellcome Sanger Institute for the COVID-19 Genomics UK (COG-UK) Consortium|Davies|United Kingdom
>Spike|hCoV-19/USA/TX-HMH-MCoV-11616/2020|2020-09-11|EPI_ISL_790068|Original|hCoV-19^^Texas|Human|Houston Methodist Hospital|Houston Methodist Hospital|Long|USA
>Spike|hCoV-19/England/MILK-A063BC/2020|2020-10-01|EPI_ISL_600073|Original|hCoV-19^^England|Human|Lighthouse Lab in Milton Keynes|Wellcome Sanger Institute for the COVID-19 Genomics UK (COG-UK) consortium|Davies|United Kingdom
>Spike|hCoV-19/USA/MN-MDH-2117/2020|2020-10-30|EPI_ISL_683746|Original|hCoV-19^^Minnesota|Human|Minnesota Department of Health|Minnesota Department of Health|Garfin|USA
>Spike|hCoV-19/England/ALDP-A50E0D/2020|2020-10-07|EPI_ISL_597749|Original|hCoV-19^^England|Human|Lighthouse Lab in Alderley Park|Wellcome Sanger Institute for the COVID-19 Genomics UK (COG-UK) consortium|Davies|United Kingdom

>Spike|hCoV-19/Ireland/D-NVRL-73IRL43808/2020|2020-09-09|EPI_ISL_578275|Original|hCoV-19^^Dublin|Human|National Virus Reference Laboratory|National Virus Reference Laboratory|Gonzalez|Ireland
>Spike|hCoV-19/Denmark/DCGC-16021/2020|2020-11-23|EPI_ISL_714541|Original|hCoV-19^^Hovedstaden|Human|Department of Virus and Microbiological Special Diagnostics|Albertsen Lab|Michaelsen|Denmark
>Spike|hCoV-19/Canada/ON-PHL-20-03615/2020|2020-11-00|EPI_ISL_775122|Original|hCoV-19^^Ontario|Human|Public Health Ontario Laboratory|Public Health Ontario Laboratory|Guthrie|Canada
>Spike|hCoV-19/England/ALDP-94A03E/2020|2020-06-11|EPI_ISL_556468|Original|hCoV-19^^England|Human|Lighthouse Lab in Alderley Park|Wellcome Sanger Institute for the COVID-19 Genomics UK (COG-UK) consortium|Davies|United Kingdom
>Spike|hCoV-19/England/ALDP-A2F845/2020|2020-10-06|EPI_ISL_587300|Original|hCoV-19^^England|Human|Lighthouse Lab in Alderley Park|Wellcome Sanger Institute for the COVID-19 Genomics UK (COG-UK) consortium|Davies|United Kingdom
>Spike|hCoV-19/England/CAMB-1B03C9/2020|2020-05-01|EPI_ISL_459284|Original|hCoV-19^^England|Human|Department of Pathology|Wellcome Sanger Institute for the COVID-19 Genomics UK (COG-UK) consortium|Davies|United Kingdom
>Spike|hCoV-19/Denmark/ALAB-HH-137/2020|2020-03-11|EPI_ISL_452016|Original|hCoV-19^^Denmark|Human|Department of Clinical Microbiology|Albertsen lab|Kirkegaard|Denmark
>Spike|hCoV-19/England/ALDP-B23BA1/2020|2020-11-04|EPI_ISL_646173|Original|hCoV-19^^England|Human|Lighthouse Lab in Alderley Park|Wellcome Sanger Institute for the COVID-19 Genomics UK (COG-UK) Consortium|Davies|United Kingdom
>Spike|hCoV-19/Saudi Arabia/KAUST-Makkah10/2020|2020-03-23|EPI_ISL_437464|Original|hCoV-19^^Makkah|Human|Pathogen Genomics Lab King Abdullah University of Science and Technology(KAUST)|Pathogen Genomics Lab King Abdullah University of Science and Technology(KAUST)|Mohamed Ghazzali|Saudi Arabia
>Spike|hCoV-19/England/ALDP-BCAB06/2020|2020-11-23|EPI_ISL_703314|Original|hCoV-19^^England|Human|Lighthouse Lab in Alderley Park|Wellcome Sanger Institute for the COVID-19 Genomics UK (COG-UK) Consortium|Davies|United Kingdom
>Spike|hCoV-19/England/LEED-2AA59E/2020|2020-04-06|EPI_ISL_538882|Original|hCoV-19^^England|Human|Leeds Teaching Hospitals NHS Trust and Public Health England|Wellcome Sanger Institute for the COVID-19 Genomics UK (COG-UK) consortium|Davies|United Kingdom
>Spike|hCoV-19/South Korea/KCDC2299/2020|2020-05-22|EPI_ISL_510635|Original|hCoV-19^^South Korea|Human|Division of Viral Diseases|Division of Viral Diseases|Kim|South Korea
>Spike|hCoV-19/USA/TX-HMH-5468/2020|2020-06-23|EPI_ISL_544795|Original|hCoV-19^^Texas|Human|Houston Methodist Hospital|Houston Methodist Hospital|Long|USA
>Spike|hCoV-19/England/QEUH-A9F1DC/2020|2020-10-19|EPI_ISL_652676|Original|hCoV-19^^England|Human|University of Exeter|COVID-19 Genomics UK (COG-UK) Consortium|Temperton|United Kingdom
>Spike|hCoV-19/Scotland/GCVR-172C5F/2020|2020-04-06|EPI_ISL_459164|Original|hCoV-19^^Scotland|Human|NHSGGC West of Scotland Specialist Virology Centre / MRC-University of Glasgow Centre for Virus Research|Wellcome Sanger Institute for the COVID-19 Genomics UK (COG-UK) consortium|Davies|United Kingdom
>Spike|hCoV-19/South Korea/KCDC2090/2020|2020-02-21|EPI_ISL_497991|Original|hCoV-19^^South Korea|Human|Division of Viral Diseases|Division of Viral Diseases|Kim|South Korea
>Spike|hCoV-19/England/MILK-58FB7C/2020|2020-06-11|EPI_ISL_558352|Original|hCoV-19^^England|Human|Lighthouse Lab in Milton Keynes|Wellcome Sanger Institute for the COVID-19 Genomics UK (COG-UK) consortium|Davies|United Kingdom
>Spike|hCoV-19/England/CAMC-B7A30D/2020|2020-11-12|EPI_ISL_741097|Original|hCoV-19^^England|Human|Oxford Viromics|COVID-19 Genomics UK (COG-UK) Consortium|Golubchik|United Kingdom
>Spike|hCoV-19/USA/TX-HMH-MCoV-14555/2020|2020-10-09|EPI_ISL_787840|Original|hCoV-19^^Texas|Human|Houston Methodist Hospital|Houston Methodist Hospital|Long|USA

>Spike|hCoV-19/England/BIRM-6A5BA/2020|2020-11-09|EPI_ISL_650350|Original|hCoV-19^^England|Human|University of Birmingham|COVID-19 Genomics UK (COG-UK) Consortium|Loman|United Kingdom
>Spike|hCoV-19/England/ALDP-B5E24E/2020|2020-11-09|EPI_ISL_723644|Original|hCoV-19^^England|Human|Oxford Viromics|COVID-19 Genomics UK (COG-UK) Consortium|Golubchik|United Kingdom
>Spike|hCoV-19/USA/NY-Wadsworth-291850-01/2020|2020-12-23|EPI_ISL_767615|Original|hCoV-19^^New York|Human|WHITE PLAINS HOSPITAL CENTER LABORATORY|Wadsworth Center|Lasek-Nesselquist|USA
>Spike|hCoV-19/Spain/CT-IBV-005578/2020|2020-03-26|EPI_ISL_508629|Original|hCoV-19^^Catalunya|Human|Departamento de Microbiolog\u00eda|SeqCOVID-SPAIN consortium/IBV(CSIC)|Comas|Spain
>Spike|hCoV-19/England/MILK-A44DD2/2020|2020-10-06|EPI_ISL_598758|Original|hCoV-19^^England|Human|Lighthouse Lab in Milton Keynes|Wellcome Sanger Institute for the COVID-19 Genomics UK (COG-UK) consortium|Davies|United Kingdom
>Spike|hCoV-19/Japan/PG-1823/2020|2020-04-00|EPI_ISL_685429|Original|hCoV-19^^Japan|Human|Tokyo Metropolitan Institute of Public Health|Pathogen Genomics Center|Sekizuka|Japan
>Spike|hCoV-19/USA/MA-MASPHL-01034/2020|2020-11-17|EPI_ISL_692779|Original|hCoV-19^^Massachusetts|Human|Massachusetts State Public Health Laboratory|Massachusetts State Public Health Laboratory|Lang|USA
>Spike|hCoV-19/England/CAMC-B51A8F/2020|2020-11-08|EPI_ISL_658092|Original|hCoV-19^^England|Human|Lighthouse Lab in Cambridge|Wellcome Sanger Institute for the COVID-19 Genomics UK (COG-UK) Consortium|Davies|United Kingdom
>Spike|hCoV-19/Denmark/DCGC-12806/2020|2020-10-26|EPI_ISL_682987|Original|hCoV-19^^Nordjylland|Human|Department of Virus and Microbiological Special Diagnostics|Albertsen Lab|Michaelsen|Denmark
>Spike|hCoV-19/USA/UT-UPHL-2012692663/2020|2020-08-11|EPI_ISL_745663|Original|hCoV-19^^Utah|Human|Ginkgo Bioworks Clinical Laboratory|Utah Public Health Laboratory|Young|USA
>Spike|hCoV-19/England/QEUH-99045B/2020|2020-08-26|EPI_ISL_530365|Original|hCoV-19^^England|Human|Lighthouse Lab in Glasgow|Wellcome Sanger Institute for the COVID-19 Genomics UK (COG-UK) consortium|Davies|United Kingdom
>Spike|hCoV-19/USA/TX-HMH-7038/2020|2020-06-28|EPI_ISL_546456|Original|hCoV-19^^Texas|Human|Houston Methodist Hospital|Houston Methodist Hospital|Long|USA
>Spike|hCoV-19/USA/TX-HMH-MCoV-10932/2020|2020-07-09|EPI_ISL_789690|Original|hCoV-19^^Texas|Human|Houston Methodist Hospital|Houston Methodist Hospital|Long|USA
>Spike|hCoV-19/England/MILK-AB7E9B/2020|2020-10-21|EPI_ISL_623821|Original|hCoV-19^^England|Human|Lighthouse Lab in Milton Keynes|Wellcome Sanger Institute for the COVID-19 Genomics UK (COG-UK) consortium|Davies|United Kingdom
>Spike|hCoV-19/Palestine/9/2020|2020-07-17|EPI_ISL_596567|Original|hCoV-19^^Jerusalem|Human|Palestinian Ministry of Health|Molecular Genetics Lab|van Dorp|Palestine
>Spike|hCoV-19/England/CAMC-B79D00/2020|2020-11-10|EPI_ISL_667870|Original|hCoV-19^^England|Human|Lighthouse Lab in Cambridge|Wellcome Sanger Institute for the COVID-19 Genomics UK (COG-UK) Consortium|Davies|United Kingdom
>Spike|hCoV-19/England/QEUH-B41879/2020|2020-11-08|EPI_ISL_658597|Original|hCoV-19^^England|Human|Lighthouse Lab in Glasgow|Wellcome Sanger Institute for the COVID-19 Genomics UK (COG-UK) Consortium|Davies|United Kingdom
>Spike|hCoV-19/USA/LA-EVTL255/2020|2020-08-22|EPI_ISL_574389|Original|hCoV-19^^Louisiana|Human|LSUHS Emerging Viral Threat Laboratory|Microbial Genome Sequencing Center|Kamil|USA
>Spike|hCoV-19/England/MILK-A7A84B/2020|2020-10-15|EPI_ISL_606544|Original|hCoV-19^^England|Human|Lighthouse Lab in Milton Keynes|Wellcome Sanger Institute for the COVID-19 Genomics UK (COG-UK) consortium|Davies|United Kingdom
>Spike|hCoV-19/USA/MI-MDHHS-SC20823/2020|2020-05-11|EPI_ISL_471844|Original|hCoV-19^^Michigan|Human|Michigan Department of Health and Human Services|Michigan Department of Health and Human Services|Blankenship|USA

>Spike|hCoV-19/Spain/CE-ISCIII-2016514/2020|2020-10-14|EPI_ISL_691694|Original|hCoV-19^^Ceuta|Human|Hospital Universitario de Ceuta|Instituto de Salud Carlos III|Caballero|Spain
>Spike|hCoV-19/Switzerland/SG-ETHZ-410901/2020|2020-11-30|EPI_ISL_737816|Original|hCoV-19^^St Gallen|Human|Viollier AG|Department of Biosystems Science and Engineering|Nadeau|Switzerland
>Spike|hCoV-19/Singapore/742/2020|2020-07-04|EPI_ISL_500556|Original|hCoV-19^^Singapore|Human|Singapore General Hospital|Department of Microbiology|Abdul Rahman|Singapore
>Spike|hCoV-19/USA/MN-MDH-1301/2020|2020-04-15|EPI_ISL_495600|Original|hCoV-19^^Minnesota|Human|Mayo Clinic & Mayo Clinic Laboratories|Minnesota Department of Health|Garfin|USA
>Spike|hCoV-19/Wales/PHWC-486F2F/2020|2020-10-13|EPI_ISL_639149|Original|hCoV-19^^Wales|Human|Wales Specialist Virology Centre Sequencing lab|COVID-19 Genomics UK (COG-UK) Consortium|Moore|United Kingdom
>Spike|hCoV-19/England/SHEF-C6473/2020|2020-04-04|EPI_ISL_499336|Original|hCoV-19^^England|Human|Virology Department|COVID-19 Genomics UK (COG-UK) Consortium|de Silva|United Kingdom
>Spike|hCoV-19/Scotland/CVR2726/2020|2020-04-18|EPI_ISL_473547|Original|hCoV-19^^Scotland|Human|West of Scotland Specialist Virology Centre|COVID-19 Genomics UK (COG-UK) Consortium|Robertson|United Kingdom
>Spike|hCoV-19/New Zealand/20CV0157/2020|2020-08-20|EPI_ISL_548031|Original|hCoV-19^^Auckland|Human|Middlemore Hospital|Institute of Environmental Science and Research (ESR)|Wang|New Zealand
>Spike|hCoV-19/Scotland/CVR3360/2020|2020-05-02|EPI_ISL_456790|Original|hCoV-19^^Scotland|Human|West of Scotland Specialist Virology Centre|COVID-19 Genomics UK (COG-UK) Consortium|Robertson|United Kingdom
>Spike|hCoV-19/England/LOND-124D98F/2020|2020-00-00|EPI_ISL_584627|Original|hCoV-19^^England|Human|University College London Hospital|COVID-19 Genomics UK (COG-UK) Consortium|Byott|United Kingdom
>Spike|hCoV-19/USA/NM-DOH-2020616835/2020|2020-12-12|EPI_ISL_751831|Original|hCoV-19^^New Mexico|Human|New Mexico Department of Health Scientific Laboratory|New Mexico Department of Health Scientific Laboratory|Malone|USA
>Spike|hCoV-19/England/20132004704/2020|2020-03-23|EPI_ISL_423716|Original|hCoV-19^^England|Human|Respiratory Virus Unit|Respiratory Virus Unit|Platt|United Kingdom
>Spike|hCoV-19/Wales/PHWC-48EC7B/2020|2020-11-09|EPI_ISL_680985|Original|hCoV-19^^Wales|Human|Wales Specialist Virology Centre Sequencing lab|COVID-19 Genomics UK (COG-UK) Consortium|Moore|United Kingdom
>Spike|hCoV-19/Rwanda/NRLNAT1006/2020|2020-10-17|EPI_ISL_735446|Original|hCoV-19^^Kigali|Human|Nucleic Acid Testing - Rwanda National Reference Laboratory|GIGA Medical Genomics|Butera|Rwanda
>Spike|hCoV-19/Japan/PG-3821/2020|2020-07-00|EPI_ISL_692072|Original|hCoV-19^^Japan|Human|Pathogen Genomics Center|Pathogen Genomics Center|Sekizuka|Japan
>Spike|hCoV-19/USA/TX-HMH-5707/2020|2020-06-26|EPI_ISL_546957|Original|hCoV-19^^Texas|Human|Houston Methodist Hospital|Houston Methodist Hospital|Long|USA
>Spike|hCoV-19/England/QEUH-9F4DC3/2020|2020-09-25|EPI_ISL_638643|Original|hCoV-19^^England|Human|Department of Pathology|COVID-19 Genomics UK (COG-UK) Consortium|Goodfellow|United Kingdom
>Spike|hCoV-19/Wales/PHWC-496C52/2020|2020-11-23|EPI_ISL_726707|Original|hCoV-19^^Wales|Human|Originating lab|COVID-19 Genomics UK (COG-UK) Consortium|Moore|United Kingdom
>Spike|hCoV-19/Scotland/QEUH-998DEF/2020|2020-08-26|EPI_ISL_568318|Original|hCoV-19^^Scotland|Human|Lighthouse Lab in Glasgow|Wellcome Sanger Institute for the COVID-19 Genomics UK (COG-UK) consortium|Davies|United Kingdom
>Spike|hCoV-19/Australia/VIC9750/2020|2020-08-24|EPI_ISL_565692|Original|hCoV-19^^Victoria|Human|Microbiological Diagnostic Unit - Public Health Laboratory (MDU-PHL)|MDU-PHL|Schultz|Australia
>Spike|hCoV-19/England/MILK-559D84/2020|2020-06-10|EPI_ISL_558373|Original|hCoV-19^^England|Human|Lighthouse Lab in Milton Keynes|Wellcome Sanger Institute for the COVID-19 Genomics UK (COG-UK) consortium|Davies|United Kingdom

>Spike|hCoV-19/Denmark/DCGC-3271/2020|2020-08-31|EPI_ISL_616303|Original|hCoV-19^^Hovedstaden|Human|Department of Virus and Microbiological Special Diagnostics|Albertsen lab|Michaelsen|Denmark
>Spike|hCoV-19/England/MILK-99CCC0/2020|2020-08-30|EPI_ISL_549834|Original|hCoV-19^^England|Human|Lighthouse Lab in Milton Keynes|Wellcome Sanger Institute for the COVID-19 Genomics UK (COG-UK) consortium|Davies|United Kingdom
>Spike|hCoV-19/England/QEUH-9F8020/2020|2020-09-27|EPI_ISL_588747|Original|hCoV-19^^England|Human|Lighthouse Lab in Glasgow|Wellcome Sanger Institute for the COVID-19 Genomics UK (COG-UK) consortium|Davies|United Kingdom
>Spike|hCoV-19/England/ALDP-92D1F2/2020|2020-06-21|EPI_ISL_551508|Original|hCoV-19^^England|Human|Lighthouse Lab in Alderley Park|Wellcome Sanger Institute for the COVID-19 Genomics UK (COG-UK) consortium|Davies|United Kingdom
>Spike|hCoV-19/USA/UT-UPHL-2009368/2020|2020-05-16|EPI_ISL_524961|Original|hCoV-19^^Utah|Human|Utah Public Health Laboratory|Utah Public Health Laboratory|Young|USA
>Spike|hCoV-19/England/BRIS-123A26/2020|2020-04-06|EPI_ISL_442723|Original|hCoV-19^^England|Human|PHE South West Regional Laboratory|Wellcome Sanger Institute for the COVID-19 Genomics UK (COG-UK) consortium|Amato|United Kingdom
>Spike|hCoV-19/South Africa/Tygerberg_327_CC2/2020|2020-10-09|EPI_ISL_660135|Original|hCoV-19^^Western Cape|Human|Lancet|National Health Laboratory Service (NHLS)|de Oliveira|South Africa
>Spike|hCoV-19/Wales/PHWC-163B90/2020|2020-05-06|EPI_ISL_474050|Original|hCoV-19^^Wales|Human|Originating lab|COVID-19 Genomics UK (COG-UK) Consortium|Moore|United Kingdom
>Spike|hCoV-19/United Arab Emirates/0601/2020|2020-05-15|EPI_ISL_698759|Original|hCoV-19^^Abu Dhabi|Human|Group 42 (G42) Healthcare|G42 Healthcare|Ogrodzki|United Arab Emirates
>Spike|hCoV-19/Northern Ireland/NIRE-FBDD4/2020|2020-04-12|EPI_ISL_470041|Original|hCoV-19^^Northern Ireland|Human|Regional Virus Laboratory|Wellcome Sanger Institute for the COVID-19 Genomics UK (COG-UK) consortium|Davies|United Kingdom
>Spike|hCoV-19/mink/Poland/Pomorskie_67/2020|2020-11-17|EPI_ISL_732955|Original|hCoV-19^^Pomorskie|Neovison vison|Department of Tropical Parasitology|Laboratory of Recombinant Vaccines|Rabalski|Poland
>Spike|hCoV-19/England/CAMB-7568C/2020|2020-03-17|EPI_ISL_440377|Original|hCoV-19^^England|Human|Department of Pathology|Wellcome Sanger Institute for the COVID-19 Genomics UK (COG-UK) consortium|Amato|United Kingdom
>Spike|hCoV-19/Denmark/DCGC-1928/2020|2020-06-22|EPI_ISL_622521|Original|hCoV-19^^Hovedstaden|Human|Department of Virus and Microbiological Special Diagnostics|Albertsen lab|Michaelsen|Denmark
>Spike|hCoV-19/England/EXET-134FD8/2020|2020-00-00|EPI_ISL_457027|Original|hCoV-19^^England|Human|University of Exeter|COVID-19 Genomics UK (COG-UK) Consortium|Temperton|United Kingdom
>Spike|hCoV-19/Australia/VIC155/2020|2020-03-17|EPI_ISL_419852|Original|hCoV-19^^Victoria|Human|Victorian Infectious Diseases Reference Laboratory (VIDRL)|Victorian Infectious Diseases Reference Laboratory and Microbiological Diagnostic Unit Public Health Laboratory|Schultz|Australia
>Spike|hCoV-19/Australia/VIC7683/2020|2020-07-28|EPI_ISL_564573|Original|hCoV-19^^Victoria|Human|Microbiological Diagnostic Unit - Public Health Laboratory (MDU-PHL)|MDU-PHL|Schultz|Australia
>Spike|hCoV-19/England/QEUH-AD27CC/2020|2020-10-26|EPI_ISL_633883|Original|hCoV-19^^England|Human|Lighthouse Lab in Glasgow|Wellcome Sanger Institute for the COVID-19 Genomics UK (COG-UK) consortium|Davies|United Kingdom
>Spike|hCoV-19/USA/NY-NYCPHL-001458/2020|2020-11-25|EPI_ISL_683767|Original|hCoV-19^^New York|Human|DOHMH Riverside|New York City Public Health Laboratory|Wang|USA
>Spike|hCoV-19/England/ALDP-661CB8/2020|2020-07-03|EPI_ISL_555644|Original|hCoV-19^^England|Human|Lighthouse Lab in Alderley Park|Wellcome Sanger Institute for the COVID-19 Genomics UK (COG-UK) consortium|Davies|United Kingdom
>Spike|hCoV-19/Scotland/EDB7152/2020|2020-08-04|EPI_ISL_514558|Original|hCoV-19^^Scotland|Human|Virology Department|COVID-19 Genomics UK (COG-UK) Consortium|Williams|United Kingdom

>Spike|hCoV-19/Scotland/CVR2129/2020|2020-04-10|EPI_ISL_477857|Original|hCoV-19^^Scotland|Human|West of Scotland Specialist Virology Centre|COVID-19 Genomics UK (COG-UK) Consortium|Robertson|United Kingdom
>Spike|hCoV-19/England/QEUH-AEFC81/2020|2020-10-29|EPI_ISL_662681|Original|hCoV-19^^England|Human|Lighthouse Lab in Glasgow|Wellcome Sanger Institute for the COVID-19 Genomics UK (COG-UK) Consortium|Davies|United Kingdom
>Spike|hCoV-19/USA/AZ-TG276772/2020|2020-04-16|EPI_ISL_694132|Original|hCoV-19^^Arizona|Human|AZ SPHL|TGen North|French|USA
>Spike|hCoV-19/USA/MA-MASPHL-00770/2020|2020-09-25|EPI_ISL_692925|Original|hCoV-19^^Massachusetts|Human|Massachusetts State Public Health Laboratory|Massachusetts State Public Health Laboratory|Lang|USA
>Spike|hCoV-19/Canada/ON-PHL-20-01213/2020|2020-06-00|EPI_ISL_548835|Original|hCoV-19^^Ontario|Human|Public Health Ontario Laboratory|Public Health Ontario Laboratory|Guthrie|Canada
>Spike|hCoV-19/Denmark/DCGC-4933/2020|2020-09-21|EPI_ISL_621471|Original|hCoV-19^^Hovedstaden|Human|Department of Virus and Microbiological Special Diagnostics|Albertsen lab|Michaelsen|Denmark
>Spike|hCoV-19/USA/TX-HMH-MCoV-18495/2020|2020-11-19|EPI_ISL_784843|Original|hCoV-19^^Texas|Human|Houston Methodist Hospital|Houston Methodist Hospital|Long|USA
>Spike|hCoV-19/Brazil/SP-601/2020|2020-03-22|EPI_ISL_471545|Original|hCoV-19^^S\u00e3o Paulo|Human|Hospital Sao Paulo de Ensino da Unifesp|Instituto Adolfo Lutz|Sacchi|Brazil
>Spike|hCoV-19/England/204490695/2020|2020-10-30|EPI_ISL_654009|Original|hCoV-19^^England|Human|Respiratory Virus Unit|COVID-19 Genomics UK (COG-UK) Consortium|Platt|United Kingdom
>Spike|hCoV-19/Australia/VIC11118/2020|2020-09-09|EPI_ISL_562695|Original|hCoV-19^^Victoria|Human|Microbiological Diagnostic Unit - Public Health Laboratory (MDU-PHL)|MDU-PHL|Schultz|Australia
>Spike|hCoV-19/USA/CA-CZB-14737/2020|2020-10-12|EPI_ISL_739533|Original|hCoV-19^^California|Human|Humboldt County Public Health Laboratory|Chan-Zuckerberg Biohub|Kamm|USA
>Spike|hCoV-19/USA/MN-MDH-1533/2020|2020-07-22|EPI_ISL_527598|Original|hCoV-19^^Minnesota|Human|Minnesota Department of Health|Minnesota Department of Health|Garfin|USA
>Spike|hCoV-19/USA/NM-DOH-2020641094/2020|2020-12-26|EPI_ISL_767058|Original|hCoV-19^^New Mexico|Human|New Mexico Department of Health Scientific Laboratory|New Mexico Department of Health Scientific Laboratory|Malone|USA
>Spike|hCoV-19/England/CAMC-C44286/2020|2020-12-10|EPI_ISL_718415|Original|hCoV-19^^England|Human|Lighthouse Lab in Cambridge|Wellcome Sanger Institute for the COVID-19 Genomics UK (COG-UK) Consortium|Davies|United Kingdom
>Spike|hCoV-19/Spain/CT-IBV-003161/2020|2020-03-16|EPI_ISL_510257|Original|hCoV-19^^Catalunya|Human|Hospital de la Santa Creu i Sant Pau. Servicio de Microbiolog\u00eda|SeqCOVID-SPAIN consortium/IBV(CSIC)|Comas|Spain
>Spike|hCoV-19/Denmark/DCGC-13855/2020|2020-11-30|EPI_ISL_713062|Original|hCoV-19^^Sjaelland|Human|Department of Virus and Microbiological Special Diagnostics|Albertsen Lab|Michaelsen|Denmark
>Spike|hCoV-19/England/ALDP-9CAB53/2020|2020-09-16|EPI_ISL_575625|Original|hCoV-19^^England|Human|Lighthouse Lab in Alderley Park|Wellcome Sanger Institute for the COVID-19 Genomics UK (COG-UK) consortium|Davies|United Kingdom
>Spike|hCoV-19/Australia/VIC13180/2020|2020-07-27|EPI_ISL_563629|Original|hCoV-19^^Victoria|Human|Microbiological Diagnostic Unit - Public Health Laboratory (MDU-PHL)|MDU-PHL|Schultz|Australia
>Spike|hCoV-19/USA/LA-EVTL1254/2020|2020-12-04|EPI_ISL_778920|Original|hCoV-19^^Louisiana|Human|LSUHS Emerging Viral Threat Laboratory|Microbial Genome Sequencing Center|Kamil|USA
>Spike|hCoV-19/Portugal/PT1934/2020|2020-11-15|EPI_ISL_732144|Original|hCoV-19^^Portugal|Human|Instituto Nacional de Saude (INSA) and Instituto Gulbenkian de Ciencia (IGC)|Instituto Nacional de Saude (INSA) and Instituto Gulbenkian de Ciencia (IGC)|Borges|Portugal

>Spike|hCoV-19/USA/OR-OHSU-1757/2020|2020-07-24|EPI_ISL_526021|Original|hCoV-19^^Oregon|Human|OHSU Lab Services Molecular Microbiology Lab|Oregon SARS-CoV-2 Genome Sequencing Center|O'Roak|USA
>Spike|hCoV-19/Belgium/UGent-168/2020|2020-10-01|EPI_ISL_634884|Original|hCoV-19^^Ghent|Human|Lab voor klinische biologie|Onderzoeksgroep Virologie|Lambrechts|Belgium
>Spike|hCoV-19/USA/UT-UPHL-201109190/2020|2020-10-01|EPI_ISL_628946|Original|hCoV-19^^Utah|Human|Utah Public Health Laboratory|Utah Public Health Laboratory|Young|USA
>Spike|hCoV-19/Spain/AN-IBV-002842/2020|2020-03-09|EPI_ISL_474841|Original|hCoV-19^^Andalucia|Human|Hospital Universitario Virgen de las Nieves de Granada-SAS|SeqCOVID-SPAIN consortium/IBV(CSIC)|Comas|Spain
>Spike|hCoV-19/Wales/PHWC-2C6C0/2020|2020-03-31|EPI_ISL_445896|Original|hCoV-19^^Wales|Human|Wales Specialist Virology Centre|Public Health Wales Microbiology Cardiff|Moore|United Kingdom
>Spike|hCoV-19/Wales/PHWC-168991/2020|2020-05-21|EPI_ISL_494311|Original|hCoV-19^^Wales|Human|Originating lab|COVID-19 Genomics UK (COG-UK) Consortium|Moore|United Kingdom
>Spike|hCoV-19/England/PORT-2EE82C/2020|2020-12-13|EPI_ISL_763611|Original|hCoV-19^^England|Human|Centre for Enzyme Innovation|COVID-19 Genomics UK (COG-UK) Consortium|Robson|United Kingdom
>Spike|hCoV-19/USA/CA-ALSR-2932/2020|2020-06-09|EPI_ISL_635737|Original|hCoV-19^^California|Human|San Diego County Public Health Laboratory|Andersen lab at Scripps Research|Gangavarapu|USA
>Spike|hCoV-19/Wales/PHWC-31AF9/2020|2020-04-11|EPI_ISL_446529|Original|hCoV-19^^Wales|Human|Wales Specialist Virology Centre|Public Health Wales Microbiology Cardiff|Moore|United Kingdom
>Spike|hCoV-19/Japan/PG-0870/2020|2020-00-00|EPI_ISL_684480|Original|hCoV-19^^Japan|Human|Fukuoka Institute of Health and Environmental Sciences|Pathogen Genomics Center|Sekizuka|Japan
>Spike|hCoV-19/USA/UT-UPHL-2012069277/2020|2020-07-27|EPI_ISL_745676|Original|hCoV-19^^Utah|Human|Ginkgo Bioworks Clinical Laboratory|Utah Public Health Laboratory|Young|USA
>Spike|hCoV-19/Australia/VIC10753/2020|2020-09-01|EPI_ISL_563071|Original|hCoV-19^^Victoria|Human|Microbiological Diagnostic Unit - Public Health Laboratory (MDU-PHL)|MDU-PHL|Schultz|Australia
>Spike|hCoV-19/DRC/4020/2020|2020-04-26|EPI_ISL_471403|Original|hCoV-19^^Kinshasa|Human|Viral Respiratory Lab|Pathogen Sequencing Lab|Lusamaki|Democratic Republic of the Congo
>Spike|hCoV-19/Japan/PG-0565/2020|2020-04-00|EPI_ISL_684185|Original|hCoV-19^^Japan|Human|Pathogen Genomics Center|Pathogen Genomics Center|Sekizuka|Japan
>Spike|hCoV-19/USA/WA-UW-22175/2020|2020-08-05|EPI_ISL_570635|Original|hCoV-19^^Washington|Human|UW Virology Lab|UW Virology Lab|Roychoudhury|USA
>Spike|hCoV-19/England/CAMC-A59192/2020|2020-10-09|EPI_ISL_597198|Original|hCoV-19^^England|Human|Lighthouse Lab in Cambridge|Wellcome Sanger Institute for the COVID-19 Genomics UK (COG-UK) consortium|Davies|United Kingdom
>Spike|hCoV-19/Portugal/PT0559/2020|2020-04-22|EPI_ISL_454282|Original|hCoV-19^^Portugal|Human|unknown|Instituto Nacional de Saude (INSA)|Borges|Portugal
>Spike|hCoV-19/Denmark/DCGC-14215/2020|2020-11-30|EPI_ISL_711060|Original|hCoV-19^^Midtjylland|Human|Department of Virus and Microbiological Special Diagnostics|Albertsen Lab|Michaelsen|Denmark
>Spike|hCoV-19/England/LIVE-DBEB21/2020|2020-10-29|EPI_ISL_664489|Original|hCoV-19^^England|Human|Liverpool Clinical Laboratories|COVID-19 Genomics UK (COG-UK) Consortium|Haldenby|United Kingdom
>Spike|hCoV-19/Netherlands/Blaricum_1364780/2020|2020-03-02|EPI_ISL_413566|Original|hCoV-19^^Blaricum|Human|MHC Gooi & Vechtstreek|Erasmus Medical Center|Oude Munnink|Netherlands
>Spike|hCoV-19/Russia/Sverdlovsk-71903/2020|2020-03-19|EPI_ISL_428890|Vero|hCoV-19^^Sverdlovsk|Human|State Research Center of Virology and Biotechnology VECTOR|State Research Center of Virology and Biotechnology VECTOR|Shvalov|Russia

>Spike|hCoV-19/USA/MN-MDH-2021/2020|2020-11-01|EPI_ISL_654702|Original|hCoV-19^^Minnesota|Human|Minnesota Department of Health|Minnesota Department of Health|Lorentz|USA
>Spike|hCoV-19/England/204582194/2020|2020-11-04|EPI_ISL_683492|Original|hCoV-19^^England|Human|Respiratory Virus Unit|COVID-19 Genomics UK (COG-UK) Consortium|Platt|United Kingdom
>Spike|hCoV-19/Netherlands/ZH-RIVM-20451/2020|2020-10-26|EPI_ISL_728670|Original|hCoV-19^^Zuid-holland|Human|Dutch COVID-19 response team|National Institute for Public Health and the Environment (RIVM)|Meijer|Netherlands
>Spike|hCoV-19/England/QEUH-B67EB7/2020|2020-11-11|EPI_ISL_661424|Original|hCoV-19^^England|Human|Lighthouse Lab in Glasgow|Wellcome Sanger Institute for the COVID-19 Genomics UK (COG-UK) Consortium|Davies|United Kingdom
>Spike|hCoV-19/USA/TX-HMH-5490/2020|2020-06-25|EPI_ISL_544813|Original|hCoV-19^^Texas|Human|Houston Methodist Hospital|Houston Methodist Hospital|Long|USA
>Spike|hCoV-19/Denmark/DCGC-10117/2020|2020-11-09|EPI_ISL_669406|Original|hCoV-19^^Nordjylland|Human|Department of Virus and Microbiological Special Diagnostics|Albertsen Lab|Michaelsen|Denmark
>Spike|hCoV-19/Wales/PHWC-49C630/2020|2020-12-11|EPI_ISL_742584|Original|hCoV-19^^Wales|Human|Wales Specialist Virology Centre Sequencing lab|COVID-19 Genomics UK (COG-UK) Consortium|Moore|United Kingdom
>Spike|hCoV-19/England/CAMC-B78DB6/2020|2020-11-12|EPI_ISL_667936|Original|hCoV-19^^England|Human|Lighthouse Lab in Cambridge|Wellcome Sanger Institute for the COVID-19 Genomics UK (COG-UK) Consortium|Davies|United Kingdom
>Spike|hCoV-19/Sweden/20-08950/2020|2020-08-02|EPI_ISL_534232|Original|hCoV-19^^Stockholm|Human|Capio S|The Public Health Agency of Sweden|Svartstr\u00f6m|Sweden
>Spike|hCoV-19/Hong Kong/VM20037347/2020|2020-07-10|EPI_ISL_523835|Original|hCoV-19^^Hong Kong|Human|Queen Elizabeth Hospital|Hong Kong Department of Health|Mak|China
>Spike|hCoV-19/England/QEUH-95E644/2020|2020-08-09|EPI_ISL_532372|Original|hCoV-19^^England|Human|Lighthouse Lab in Glasgow|Wellcome Sanger Institute for the COVID-19 Genomics UK (COG-UK) consortium|Davies|United Kingdom
>Spike|hCoV-19/Chile/MA-194115-B/2020|2020-09-27|EPI_ISL_746528|Original|hCoV-19^^Magallanes|Human|Genetica Molecular and Subdepartamento de Virologia ISP Chile|Instituto de Salud Publica de Chile|Tognarelli|Chile
>Spike|hCoV-19/USA/NH-CDC-2-3714186/2020|2020-11-02|EPI_ISL_751678|Original|hCoV-19^^New Hampshire|Human|NH Dept. of Health and Human Services Public Health Labs|Genomics and Discovery|Queen|USA
>Spike|hCoV-19/Denmark/DCGC-6924/2020|2020-10-12|EPI_ISL_619770|Original|hCoV-19^^Hovedstaden|Human|Department of Virus and Microbiological Special Diagnostics|Albertsen lab|Michaelsen|Denmark
>Spike|hCoV-19/Austria/CeMM0563/2020|2020-03-10|EPI_ISL_475913|Original|hCoV-19^^Losenstein|Human|Klinikum Wels-Grieskirchen|Bergthaler laboratory|Bergthaler|Austria
>Spike|hCoV-19/England/ALDP-A67A87/2020|2020-10-12|EPI_ISL_630371|Original|hCoV-19^^England|Human|Lighthouse Lab in Alderley Park|Wellcome Sanger Institute for the COVID-19 Genomics UK (COG-UK) consortium|Davies|United Kingdom
>Spike|hCoV-19/England/MILK-B5A27F/2020|2020-11-06|EPI_ISL_658169|Original|hCoV-19^^England|Human|Lighthouse Lab in Milton Keynes|Wellcome Sanger Institute for the COVID-19 Genomics UK (COG-UK) Consortium|Davies|United Kingdom
>Spike|hCoV-19/Wales/PHWC-496CBC/2020|2020-11-24|EPI_ISL_726712|Original|hCoV-19^^Wales|Human|Originating lab|COVID-19 Genomics UK (COG-UK) Consortium|Moore|United Kingdom
>Spike|hCoV-19/England/PORT-2D73BC/2020|2020-10-22|EPI_ISL_626678|Original|hCoV-19^^England|Human|Centre for Enzyme Innovation|COVID-19 Genomics UK (COG-UK) Consortium|Robson|United Kingdom
>Spike|hCoV-19/Wales/PHWC-47CEF2/2020|2020-10-09|EPI_ISL_586068|Original|hCoV-19^^Wales|Human|Wales Specialist Virology Centre Sequencing lab|COVID-19 Genomics UK (COG-UK) Consortium|Moore|United Kingdom

>Spike|hCoV-19/England/ALDP-A672F8/2020|2020-10-12|EPI_ISL_630674|Original|hCoV-19^^England|Human|Lighthouse Lab in Alderley Park|Wellcome Sanger Institute for the COVID-19 Genomics UK (COG-UK) consortium|Davies|United Kingdom
>Spike|hCoV-19/USA/CA-CZB-15476/2020|2020-12-05|EPI_ISL_739460|Original|hCoV-19^^California|Human|Orange County Public Health Lab|Chan-Zuckerberg Biohub|Kamm|USA
>Spike|hCoV-19/Northern Ireland/NIRE-FAF66/2020|2020-04-15|EPI_ISL_470054|Original|hCoV-19^^Northern Ireland|Human|Regional Virus Laboratory|Wellcome Sanger Institute for the COVID-19 Genomics UK (COG-UK) consortium|Davies|United Kingdom
>Spike|hCoV-19/England/QEUH-AF5611/2020|2020-10-30|EPI_ISL_662926|Original|hCoV-19^^England|Human|Lighthouse Lab in Glasgow|Wellcome Sanger Institute for the COVID-19 Genomics UK (COG-UK) Consortium|Davies|United Kingdom
>Spike|hCoV-19/England/CAMB-1AE23A/2020|2020-04-26|EPI_ISL_459455|Original|hCoV-19^^England|Human|Department of Pathology|Wellcome Sanger Institute for the COVID-19 Genomics UK (COG-UK) consortium|Davies|United Kingdom
>Spike|hCoV-19/Denmark/DCGC-22391/2020|2020-12-14|EPI_ISL_757465|Original|hCoV-19^^Syddanmark|Human|Department of Virus and Microbiological Special Diagnostics|Albertsen Lab|Michaelsen|Denmark
>Spike|hCoV-19/USA/WI-UW-855/2020|2020-07-09|EPI_ISL_509861|Original|hCoV-19^^Wisconsin|Human|University of Wisconsin-Madison AIDS Vaccine Research Laboratories|University of Wisconsin-Madison AIDS Vaccine Research Laboratories|Baczenas|USA
>Spike|hCoV-19/England/CAMB-1BA68A/2020|2020-11-11|EPI_ISL_664434|Original|hCoV-19^^England|Human|Department of Pathology|COVID-19 Genomics UK (COG-UK) Consortium|Goodfellow|United Kingdom
>Spike|hCoV-19/Equatorial Guinea/43296/2020|2020-07-26|EPI_ISL_648378|Original|hCoV-19^^Bioko Norte|Human|Laboratorio de Investigaciones de Baney|University Hospital Basel|Hosch|Equatorial Guinea
>Spike|hCoV-19/England/BRIS-185322B/2020|2020-04-02|EPI_ISL_492302|Original|hCoV-19^^England|Human|PHE South West Regional Laboratory|Wellcome Sanger Institute for the COVID-19 Genomics UK (COG-UK) consortium|Davies|United Kingdom
>Spike|hCoV-19/USA/IL-UW-360/2020|2020-04-18|EPI_ISL_450714|Original|hCoV-19^^Illinois|Human|University of Wisconsin-Madison AIDS Vaccine Research Laboratories|University of Wisconsin-Madison AIDS Vaccine Research Laboratories|Baczenas|USA
>Spike|hCoV-19/England/ALDP-95B00D/2020|2020-06-15|EPI_ISL_555533|Original|hCoV-19^^England|Human|Lighthouse Lab in Alderley Park|Wellcome Sanger Institute for the COVID-19 Genomics UK (COG-UK) consortium|Davies|United Kingdom
>Spike|hCoV-19/England/CAMB-744B0/2020|2020-03-20|EPI_ISL_425297|Original|hCoV-19^^England|Human|Department of Pathology|COVID-19 Genomics UK (COG-UK) Consortium|Meredith|United Kingdom
>Spike|hCoV-19/Northern Ireland/NIRE-1070DB/2020|2020-03-27|EPI_ISL_585107|Original|hCoV-19^^Northern Ireland|Human|Regional Virus Laboratory|COVID-19 Genomics UK (COG-UK) Consortium|Fairley|United Kingdom
>Spike|hCoV-19/England/QEUH-A9D88E/2020|2020-10-20|EPI_ISL_727791|Original|hCoV-19^^England|Human|Centre for Enzyme Innovation|COVID-19 Genomics UK (COG-UK) Consortium|Robson|United Kingdom
>Spike|hCoV-19/Russia/SPE-RII-35217S/2020|2020-09-25|EPI_ISL_596246|Original|hCoV-19^^Saint Petersburg|Human|WHO National Influenza Centre Russian Federation|WHO National Influenza Centre Russian Federation|Komissarov|Russia
>Spike|hCoV-19/Denmark/DCGC-14682/2020|2020-11-23|EPI_ISL_711908|Original|hCoV-19^^Hovedstaden|Human|Department of Virus and Microbiological Special Diagnostics|Albertsen Lab|Michaelsen|Denmark
>Spike|hCoV-19/Japan/PG-8003/2020|2020-08-00|EPI_ISL_687104|Original|hCoV-19^^Japan|Human|Pathogen Genomics Center|Pathogen Genomics Center|Sekizuka|Japan
>Spike|hCoV-19/Netherlands/ZH-EMC-365/2020|2020-08-02|EPI_ISL_523705|Original|hCoV-19^^Zuid Holland|Human|Dutch COVID-19 response team|Erasmus Medical Center|Oude Munnink|Netherlands

>Spike|hCoV-19/Switzerland/ZH-ETHZ-390225/2020|2020-11-27|EPI_ISL_721963|Original|hCoV-19^^Zurich|Human|Viollier AG|Department of Biosystems Science and Engineering|Nadeau|Switzerland
>Spike|hCoV-19/Denmark/DCGC-9792/2020|2020-11-09|EPI_ISL_669181|Original|hCoV-19^^Midtjylland|Human|Department of Virus and Microbiological Special Diagnostics|Albertsen Lab|Michaelsen|Denmark
>Spike|hCoV-19/Switzerland/BS-UHB-42204477/2020|2020-03-20|EPI_ISL_528372|Original|hCoV-19^^Basel-Stadt|Human|University Hospital Basel|University Hospital Basel|Roloff Handschin|Switzerland
>Spike|hCoV-19/England/ALDP-B46E63/2020|2020-11-07|EPI_ISL_656089|Original|hCoV-19^^England|Human|Lighthouse Lab in Alderley Park|Wellcome Sanger Institute for the COVID-19 Genomics UK (COG-UK) Consortium|Davies|United Kingdom
>Spike|hCoV-19/England/MILK-992A75/2020|2020-08-27|EPI_ISL_552177|Original|hCoV-19^^England|Human|Lighthouse Lab in Milton Keynes|Wellcome Sanger Institute for the COVID-19 Genomics UK (COG-UK) consortium|Davies|United Kingdom
>Spike|hCoV-19/England/204380343/2020|2020-10-23|EPI_ISL_660747|Original|hCoV-19^^England|Human|Respiratory Virus Unit|COVID-19 Genomics UK (COG-UK) Consortium|Platt|United Kingdom
>Spike|hCoV-19/USA/UT-UPHL-2009660/2020|2020-07-25|EPI_ISL_605335|Original|hCoV-19^^Utah|Human|Utah Public Health Laboratory|Utah Public Health Laboratory|Young|USA
>Spike|hCoV-19/Denmark/DCGC-16689/2020|2020-11-09|EPI_ISL_714918|Original|hCoV-19^^Midtjylland|Human|Department of Virus and Microbiological Special Diagnostics|Albertsen Lab|Michaelsen|Denmark
>Spike|hCoV-19/England/ALDP-A870F8/2020|2020-10-17|EPI_ISL_606185|Original|hCoV-19^^England|Human|Lighthouse Lab in Alderley Park|Wellcome Sanger Institute for the COVID-19 Genomics UK (COG-UK) consortium|Davies|United Kingdom
>Spike|hCoV-19/Switzerland/ZH-ETHZ-280172/2020|2020-09-08|EPI_ISL_560548|Original|hCoV-19^^Zurich|Human|Viollier AG|Department of Biosystems Science and Engineering|Nadeau|Switzerland
>Spike|hCoV-19/USA/FL-BPHL-1853/2020|2020-04-09|EPI_ISL_613933|Original|hCoV-19^^Florida|Human|Florida Bureau of Public Health Laboratories|Florida Bureau of Public Health Laboratories|Blanton|USA
>Spike|hCoV-19/England/ALDP-A93651/2020|2020-10-20|EPI_ISL_611135|Original|hCoV-19^^England|Human|Lighthouse Lab in Alderley Park|Wellcome Sanger Institute for the COVID-19 Genomics UK (COG-UK) consortium|Davies|United Kingdom
>Spike|hCoV-19/England/QEUH-B6BE77/2020|2020-11-11|EPI_ISL_655164|Original|hCoV-19^^England|Human|Lighthouse Lab in Glasgow|Wellcome Sanger Institute for the COVID-19 Genomics UK (COG-UK) Consortium|Davies|United Kingdom
>Spike|hCoV-19/USA/WA-S907/2020|2020-04-27|EPI_ISL_463397|Original|hCoV-19^^Washington|Human|Washington State Department of Health|Seattle Flu Study|Bedford|USA
>Spike|hCoV-19/England/NOTT-11036D/2020|2020-04-16|EPI_ISL_441504|Original|hCoV-19^^England|Human|Queens Medical Centre|COVID-19 Genomics UK (COG-UK) Consortium|Loose|United Kingdom
>Spike|hCoV-19/USA/WY-WYPHL-00028/2020|2020-03-23|EPI_ISL_462921|Original|hCoV-19^^Wyoming|Human|Wyoming Public Health Laboratory|Center for Global Health|Domman|USA
>Spike|hCoV-19/Japan/IC-0568/2020|2020-09-00|EPI_ISL_779247|Original|hCoV-19^^Japan|Human|Pathogen Genomics Center|Pathogen Genomics Center|Sekizuka|Japan
>Spike|hCoV-19/England/ALDP-B6397E/2020|2020-11-08|EPI_ISL_656462|Original|hCoV-19^^England|Human|Lighthouse Lab in Alderley Park|Wellcome Sanger Institute for the COVID-19 Genomics UK (COG-UK) Consortium|Davies|United Kingdom
>Spike|hCoV-19/DRC/2133/2020|2020-04-13|EPI_ISL_435168|Original|hCoV-19^^Kinshasa|Human|Viral Respiratory Lab|Pathogen Sequencing Lab|Pratt|Democratic Republic of the Congo
>Spike|hCoV-19/South Africa/NHLS-UCT-GP-5244/2020|2020-04-29|EPI_ISL_700544|Original|hCoV-19^^Western Cape|Human|Masincedane Clinic wc MAS|NHLS/UCT|Iranzadeh|South Africa
>Spike|hCoV-19/South Africa/LR00153/2020|2020-07-23|EPI_ISL_514421|Original|hCoV-19^^Free State|Human|National Institute for Communicable Diseases of the National

Health Laboratory Service|National Institute for Communicable Diseases of the National Health Laboratory Service|Allam|South Africa
>Spike|hCoV-19/Harbin/HRB-25/2020|2020-03-13|EPI_ISL_467430|Vero E6 P1|hCoV-19^^Harbin|Human|Zoonotic and Exotic infection Diseases Division|Zoontic and Exotic infection Diseases Division|Wang|China
>Spike|hCoV-19/England/CAMC-B343A5/2020|2020-11-05|EPI_ISL_659501|Original|hCoV-19^^England|Human|Lighthouse Lab in Cambridge|Wellcome Sanger Institute for the COVID-19 Genomics UK (COG-UK) Consortium|Davies|United Kingdom
>Spike|hCoV-19/England/MILK-CB19DA/2020|2020-12-07|EPI_ISL_760871|Original|hCoV-19^^England|Human|Lighthouse Lab in Milton Keynes|Wellcome Sanger Institute for the COVID-19 Genomics UK (COG-UK) Consortium|Davies|United Kingdom
>Spike|hCoV-19/England/ALDP-BB97E8/2020|2020-11-20|EPI_ISL_702748|Original|hCoV-19^^England|Human|Lighthouse Lab in Alderley Park|Wellcome Sanger Institute for the COVID-19 Genomics UK (COG-UK) Consortium|Davies|United Kingdom
>Spike|hCoV-19/Scotland/EDB10590/2020|2020-11-30|EPI_ISL_703414|Original|hCoV-19^^Scotland|Human|Virology Department|COVID-19 Genomics UK (COG-UK) Consortium|McHugh|United Kingdom
>Spike|hCoV-19/Belgium/BT-0407271/2020|2020-04-07|EPI_ISL_464065|Original|hCoV-19^^Houthalen-Helchteren|Human|KU Leuven|KU Leuven|Maes|Belgium
>Spike|hCoV-19/England/QEUH-9CFA24/2020|2020-09-16|EPI_ISL_589930|Original|hCoV-19^^England|Human|Lighthouse Lab in Glasgow|Wellcome Sanger Institute for the COVID-19 Genomics UK (COG-UK) consortium|Davies|United Kingdom
>Spike|hCoV-19/Spain/PV-IBV-004260/2020|2020-04-07|EPI_ISL_500324|Original|hCoV-19^^Basque Country|Human|Servicio de Microbiolog\u00eda. Hospital Universitario Donostia. OSI Donostialdea. \u00c1rea de Enfermedades Infecciosas|SeqCOVID-SPAIN consortium/IBV(CSIC)|Comas|Spain
>Spike|hCoV-19/Japan/PG-7164/2020|2020-07-00|EPI_ISL_688269|Original|hCoV-19^^Japan|Human|Pathogen Genomics Center|Pathogen Genomics Center|Sekizuka|Japan
>Spike|hCoV-19/Germany/HH-hpi-p2423/2020|2020-04-26|EPI_ISL_776492|Original|hCoV-19^^Hamburg|Human|University Medical Center Hamburg Eppendorf|Heinrich Pette Institute|Robitaille|Germany
>Spike|hCoV-19/England/CAMC-A58C38/2020|2020-10-08|EPI_ISL_597049|Original|hCoV-19^^England|Human|Lighthouse Lab in Cambridge|Wellcome Sanger Institute for the COVID-19 Genomics UK (COG-UK) consortium|Davies|United Kingdom
>Spike|hCoV-19/Spain/RI-IBV-005965/2020|2020-03-08|EPI_ISL_500399|Original|hCoV-19^^La Rioja|Human|Centro de Investigaci\u00f3n Biom\u00e9dica de La Rioja - Hospital San Pedro Logro\u00f1o|SeqCOVID-SPAIN consortium/IBV(CSIC)|Comas|Spain
>Spike|hCoV-19/England/MILK-9718DC/2020|2020-08-18|EPI_ISL_553098|Original|hCoV-19^^England|Human|Lighthouse Lab in Milton Keynes|Wellcome Sanger Institute for the COVID-19 Genomics UK (COG-UK) consortium|Davies|United Kingdom
>Spike|hCoV-19/India/TG-CCMB-AA819/2020|2020-08-13|EPI_ISL_539669|Original|hCoV-19^^Telangana|Human|CSIR-Centre for Cellular and Molecular Biology|CSIR-Centre for Cellular and Molecular Biology|Mukherjee|India
>Spike|hCoV-19/USA/FL-BPHL-1033/2020|2020-07-23|EPI_ISL_517923|Original|hCoV-19^^Florida|Human|Florida Bureau of Public Health Laboratories|Florida Bureau of Public Health Laboratories|Blanton|USA
>Spike|hCoV-19/USA/TX-HMH-3758/2020|2020-06-16|EPI_ISL_543169|Original|hCoV-19^^Texas|Human|Houston Methodist Hospital|Houston Methodist Hospital|Long|USA
>Spike|hCoV-19/England/QEUH-B1411A/2020|2020-11-03|EPI_ISL_641880|Original|hCoV-19^^England|Human|Lighthouse Lab in Glasgow|Wellcome Sanger Institute for the COVID-19 Genomics UK (COG-UK) Consortium|Davies|United Kingdom
>Spike|hCoV-19/Denmark/DCGC-13107/2020|2020-11-30|EPI_ISL_712301|Original|hCoV-19^^Midtjylland|Human|Department of Virus and Microbiological Special Diagnostics|Albertsen Lab|Michaelsen|Denmark
>Spike|hCoV-19/England/CAMB-83296/2020|2020-04-14|EPI_ISL_443506|Original|hCoV-19^^England|Human|Department of Pathology|Wellcome Sanger Institute for the COVID-19 Genomics UK (COG-UK) consortium|Amato|United Kingdom
>Spike|hCoV-19/England/20179042104/2020|2020-04-25|EPI_ISL_527342|Original|hCoV-19^^England|Human|Respiratory Virus Unit|Respiratory Virus Unit|Ledesma|United Kingdom

>Spike|hCoV-19/England/CAMC-B0C83E/2020|2020-10-31|EPI_ISL_643583|Original|hCoV-19^^England|Human|Lighthouse Lab in Cambridge|Wellcome Sanger Institute for the COVID-19 Genomics UK (COG-UK) Consortium|Davies|United Kingdom
>Spike|hCoV-19/USA/WI-WSLH-200687/2020|2020-09-14|EPI_ISL_631444|Original|hCoV-19^^Wisconsin|Human|Wisconsin State Laboratory of Hygiene Communicable Disease Division|Wisconsin State Laboratory of Hygiene Communicable Disease Division|Florek|USA
>Spike|hCoV-19/England/20106005303/2020|2020-03-03|EPI_ISL_417243|Original|hCoV-19^^England|Human|Respiratory Virus Unit|Respiratory Virus Unit|Platt|United Kingdom
>Spike|hCoV-19/Australia/VIC14001/2020|2020-07-17|EPI_ISL_563919|Original|hCoV-19^^Victoria|Human|Microbiological Diagnostic Unit - Public Health Laboratory (MDU-PHL)|MDU-PHL|Schultz|Australia
>Spike|hCoV-19/USA/OH-ODH-SC35001/2020|2020-04-23|EPI_ISL_763076|Original|hCoV-19^^Ohio|Human|Ohio Department Of Health|Ohio Department of Health|Holmes|USA
>Spike|hCoV-19/Wales/PHWC-498106/2020|2020-11-30|EPI_ISL_727027|Original|hCoV-19^^Wales|Human|Originating lab|COVID-19 Genomics UK (COG-UK) Consortium|Moore|United Kingdom
>Spike|hCoV-19/England/201360129/2020|2020-03-24|EPI_ISL_425024|Original|hCoV-19^^England|Human|Respiratory Virus Unit|Respiratory Virus Unit|Platt|United Kingdom
>Spike|hCoV-19/England/MILK-B8E56A/2020|2020-11-13|EPI_ISL_673907|Original|hCoV-19^^England|Human|Lighthouse Lab in Milton Keynes|Wellcome Sanger Institute for the COVID-19 Genomics UK (COG-UK) Consortium|Davies|United Kingdom
>Spike|hCoV-19/England/QEUH-AD08E9/2020|2020-10-23|EPI_ISL_633947|Original|hCoV-19^^England|Human|Lighthouse Lab in Glasgow|Wellcome Sanger Institute for the COVID-19 Genomics UK (COG-UK) consortium|Davies|United Kingdom
>Spike|hCoV-19/Wales/PHWC-4869D6/2020|2020-10-27|EPI_ISL_639098|Original|hCoV-19^^Wales|Human|Wales Specialist Virology Centre Sequencing lab|COVID-19 Genomics UK (COG-UK) Consortium|Moore|United Kingdom
>Spike|hCoV-19/USA/UT-QDX-2192/2020|2020-03-14|EPI_ISL_604579|Original|hCoV-19^^Utah|Human|Quest Diagnostics|Quest Diagnostics|Kagan|USA
>Spike|hCoV-19/England/QEUH-9FAD09/2020|2020-09-28|EPI_ISL_588120|Original|hCoV-19^^England|Human|Lighthouse Lab in Glasgow|Wellcome Sanger Institute for the COVID-19 Genomics UK (COG-UK) consortium|Davies|United Kingdom
>Spike|hCoV-19/Australia/TAS34/2020|2020-04-11|EPI_ISL_522711|Original|hCoV-19^^Tasmania|Human|Royal Hobart Hospital Microbiology Department|MDU-PHL|Schultz|Australia
>Spike|hCoV-19/Norway/3248/2020|2020-08-05|EPI_ISL_590919|original|hCoV-19^^Vestfold og Telemark|Human|Vestfold Hospital|Norwegian Institute of Public Health|Vollan|Norway
>Spike|hCoV-19/England/QEUH-9DA74D/2020|2020-09-22|EPI_ISL_601331|Original|hCoV-19^^England|Human|Lighthouse Lab in Glasgow|Wellcome Sanger Institute for the COVID-19 Genomics UK (COG-UK) consortium|Davies|United Kingdom
>Spike|hCoV-19/Spain/PV-IBV-004274/2020|2020-04-07|EPI_ISL_510393|Original|hCoV-19^^Basque Country|Human|Servicio de Microbiolog\u00eda. Hospital Universitario Donostia. OSI Donostialdea. \u00c1rea de Enfermedades Infecciosas|SeqCOVID-SPAIN consortium/IBV(CSIC)|Comas|Spain
>Spike|hCoV-19/England/QEUH-B7CBE5/2020|2020-11-14|EPI_ISL_741811|Original|hCoV-19^^England|Human|Oxford Viromics|COVID-19 Genomics UK (COG-UK) Consortium|Golubchik|United Kingdom
>Spike|hCoV-19/England/MILK-A468FD/2020|2020-10-06|EPI_ISL_598331|Original|hCoV-19^^England|Human|Lighthouse Lab in Milton Keynes|Wellcome Sanger Institute for the COVID-19 Genomics UK (COG-UK) Consortium|Davies|United Kingdom
>Spike|hCoV-19/USA/MI-MDHHS-SC21153/2020|2020-03-30|EPI_ISL_507929|Original|hCoV-19^^Michigan|Human|Michigan Department of Health and Human Services|Michigan Department of Health and Human Services|Blankenship|USA
>Spike|hCoV-19/Wales/PHWC-4A27BC/2020|2020-12-15|EPI_ISL_743940|Original|hCoV-19^^Wales|Human|Wales Specialist Virology Centre Sequencing lab|COVID-19 Genomics UK (COG-UK) Consortium|Moore|United Kingdom

>Spike|hCoV-19/England/ALDP-6B59DC/2020|2020-07-01|EPI_ISL_557825|Original|hCoV-19^^England|Human|Lighthouse Lab in Alderley Park|Wellcome Sanger Institute for the COVID-19 Genomics UK (COG-UK) consortium|Davies|United Kingdom
>Spike|hCoV-19/USA/WA-UW-11449/2020|2020-06-23|EPI_ISL_570411|Original|hCoV-19^^Washington|Human|UW Virology Lab|UW Virology Lab|Roychoudhury|USA
>Spike|hCoV-19/Denmark/DCGC-19620/2020|2020-12-07|EPI_ISL_748238|Original|hCoV-19^^Hovedstaden|Human|Department of Virus and Microbiological Special Diagnostics|Albertsen Lab|Michaelsen|Denmark
>Spike|hCoV-19/England/MILK-B3A0D5/2020|2020-11-02|EPI_ISL_679680|Original|hCoV-19^^England|Human|Oxford Viromics|COVID-19 Genomics UK (COG-UK) Consortium|Golubchik|United Kingdom
>Spike|hCoV-19/USA/NV-QDX-418/2020|2020-03-18|EPI_ISL_571248|Original|hCoV-19^^Nevada|Human|Quest Diagnostics|Quest Diagnostics|Kagan|USA
>Spike|hCoV-19/England/NORT-1B79A06/2020|2020-11-26|EPI_ISL_741443|Original|hCoV-19^^England|Human|Northumbria University / South Tees Hospitals NHS Foundation Trust / North Cumbria Integrated Care NHS Foundation Trust / North Tees and Hartlepool NHS Foundation Trust / Newcastle Hospitals NHS Foundation Trust|COVID-19 Genomics UK (COG-UK) Consortium|Bashton|United Kingdom
>Spike|hCoV-19/Wales/PHWC-499299/2020|2020-12-03|EPI_ISL_727281|Original|hCoV-19^^Wales|Human|Originating lab|COVID-19 Genomics UK (COG-UK) Consortium|Moore|United Kingdom
>Spike|hCoV-19/England/20148055704/2020|2020-04-01|EPI_ISL_466072|Original|hCoV-19^^England|Human|Respiratory Virus Unit|Respiratory Virus Unit|Platt|United Kingdom
>Spike|hCoV-19/USA/WI-UW-2232/2020|2020-10-25|EPI_ISL_728002|Original|hCoV-19^^Wisconsin|Human|University of Wisconsin-Madison AIDS Vaccine Research Laboratories|University of Wisconsin-Madison AIDS Vaccine Research Laboratories|Baczenas|USA
>Spike|hCoV-19/Scotland/QEUH-AAE2D7/2020|2020-10-22|EPI_ISL_652967|Original|hCoV-19^^Scotland|Human|Oxford Viromics|COVID-19 Genomics UK (COG-UK) Consortium|Golubchik|United Kingdom
>Spike|hCoV-19/USA/WA-UW-25085/2020|2020-08-21|EPI_ISL_776689|Original|hCoV-19^^Washington|Human|UW Virology Lab|UW Virology Lab|Roychoudhury|USA
>Spike|hCoV-19/Mexico/BCN-ALSR-2508/2020|2020-06-08|EPI_ISL_635513|Original|hCoV-19^^Baja California|Human|Centro de Diagn\u221a\u2265stico COVID-19 UABC Tijuana|Andersen lab at Scripps Research|Gangavarapu|Mexico
>Spike|hCoV-19/USA/TX-HMH-5722/2020|2020-06-26|EPI_ISL_546968|Original|hCoV-19^^Texas|Human|Houston Methodist Hospital|Houston Methodist Hospital|Long|USA
>Spike|hCoV-19/Denmark/DCGC-1479/2020|2020-06-15|EPI_ISL_618103|Original|hCoV-19^^Nordjylland|Human|Department of Virus and Microbiological Special Diagnostics|Albertsen lab|Michaelsen|Denmark
>Spike|hCoV-19/Nigeria/AK-CV270/2020|2020-06-29|EPI_ISL_729954|Original|hCoV-19^^Akwa-Ibom State|Human|Nigeria Centre for Disease Control (NCDC)|African Centre of Excellence for Genomics of Infectious Diseases (ACEGID)|Oluniyi|Nigeria
>Spike|hCoV-19/Denmark/DCGC-21315/2020|2020-12-21|EPI_ISL_759670|Original|hCoV-19^^Hovedstaden|Human|Department of Virus and Microbiological Special Diagnostics|Albertsen Lab|Michaelsen|Denmark
>Spike|hCoV-19/Wales/PHWC-490B79/2020|2020-11-16|EPI_ISL_706542|Original|hCoV-19^^Wales|Human|Originating lab|COVID-19 Genomics UK (COG-UK) Consortium|Moore|United Kingdom
>Spike|hCoV-19/Belgium/ULG-7729/2020|2020-03-17|EPI_ISL_418630|Original|hCoV-19^^Li\u00e8ge|Human|Department of Clinical Microbiology|GIGA Medical Genomics|Durkin|Belgium
>Spike|hCoV-19/England/ALDP-904795/2020|2020-06-24|EPI_ISL_551464|Original|hCoV-19^^England|Human|Lighthouse Lab in Alderley Park|Wellcome Sanger Institute for the COVID-19 Genomics UK (COG-UK) consortium|Davies|United Kingdom
>Spike|hCoV-19/Denmark/DCGC-2256/2020|2020-08-10|EPI_ISL_617436|Original|hCoV-19^^Syddanmark|Human|Department of Virus and Microbiological Special Diagnostics|Albertsen lab|Michaelsen|Denmark

>Spike|hCoV-19/New Zealand/20VR2721/2020|2020-00-00|EPI_ISL_579339|Original|hCoV-19^^Auckland|Human|LabPLUS|Institute of Environmental Science and Research (ESR)|Wang|New Zealand
>Spike|hCoV-19/Denmark/DCGC-11326/2020|2020-11-02|EPI_ISL_670842|Original|hCoV-19^^Syddanmark|Human|Department of Virus and Microbiological Special Diagnostics|Albertsen Lab|Michaelsen|Denmark
>Spike|hCoV-19/England/LIVE-A3F02/2020|2020-06-28|EPI_ISL_490397|Original|hCoV-19^^England|Human|Liverpool Clinical Laboratories|COVID-19 Genomics UK (COG-UK) Consortium|Haldenby|United Kingdom
>Spike|hCoV-19/United Arab Emirates/0143/2020|2020-05-18|EPI_ISL_698330|Original|hCoV-19^^Abu Dhabi|Human|Group 42 (G42) Healthcare|G42 Healthcare|Ogrodzki|United Arab Emirates
>Spike|hCoV-19/Wales/PHWC-274F8/2020|2020-03-30|EPI_ISL_432361|Original|hCoV-19^^Wales|Human|Wales Specialist Virology Centre|Public Health Wales Microbiology Cardiff|Moore|United Kingdom
>Spike|hCoV-19/Spain/PV-IBV-004213/2020|2020-04-04|EPI_ISL_510498|Original|hCoV-19^^Basque Country|Human|Servicio de Microbiolog\u00eda. Hospital Universitario Donostia. OSI Donostialdea. \u00c1rea de Enfermedades Infecciosas|SeqCOVID-SPAIN consortium/IBV(CSIC)|Comas|Spain
>Spike|hCoV-19/Romania/USAFSAM-S562/2020|2020-11-28|EPI_ISL_738340|Original|hCoV-19^^Romania|Human|Landstuhl Regional Medical Center|United States Air Force School of Aerospace Medicine|Fries|Romania
>Spike|hCoV-19/England/ALDP-A855CE/2020|2020-10-17|EPI_ISL_610577|Original|hCoV-19^^England|Human|Lighthouse Lab in Alderley Park|Wellcome Sanger Institute for the COVID-19 Genomics UK (COG-UK) consortium|Davies|United Kingdom
>Spike|hCoV-19/England/NORT-1B61272/2020|2020-10-21|EPI_ISL_637943|Original|hCoV-19^^England|Human|Northumbria University / South Tees Hospitals NHS Foundation Trust / North Cumbria Integrated Care NHS Foundation Trust / North Tees and Hartlepool NHS Foundation Trust / Newcastle Hospitals NHS Foundation Trust|COVID-19 Genomics UK (COG-UK) Consortium|Bashton|United Kingdom
>Spike|hCoV-19/England/QEUH-785C71/2020|2020-07-15|EPI_ISL_533157|Original|hCoV-19^^England|Human|Lighthouse Lab in Glasgow|Wellcome Sanger Institute for the COVID-19 Genomics UK (COG-UK) consortium|Davies|United Kingdom
>Spike|hCoV-19/USA/CA-CZB098/2020|2020-03-28|EPI_ISL_429002|Original|hCoV-19^^California|Human|UCSF Clinical Microbiology Laboratory|Chan-Zuckerberg Biohub|Kamm|USA
>Spike|hCoV-19/Australia/VIC17921/2020|2020-08-24|EPI_ISL_779559|Original|hCoV-19^^Victoria|Human|Microbiological Diagnostic Unit - Public Health Laboratory (MDU-PHL)|MDU-PHL|Beck|Australia
>Spike|hCoV-19/Panama/337442/2020|2020-07-04|EPI_ISL_496860|Original|hCoV-19^^Chiriqui|Human|Gorgas Memorial Laboratory of Health Studies|Gorgas Memorial Laboratory of Health Studies|Martinez|Panama
>Spike|hCoV-19/Finland/1LS11G3/2020|2020-10-01|EPI_ISL_757344|Original|hCoV-19^^Uusimaa|Human|Department of Virology and Immunology|Department of Virology|Smura|Finland
>Spike|hCoV-19/England/MILK-A23B39/2020|2020-10-03|EPI_ISL_741348|Original|hCoV-19^^England|Human|Centre for Enzyme Innovation|COVID-19 Genomics UK (COG-UK) Consortium|Robson|United Kingdom
>Spike|hCoV-19/Brazil/RO-01/2020|2020-05-11|EPI_ISL_514131|Original|hCoV-19^^Rond\u00f4nia|Human|Rond\u00f4nia Central Public Health Laboratory (LACEN/RO)|Molecular Virology Laboratory of Oswaldo Cruz Foundation of Rond\u00f4nia|Souza Botelho|Brazil
>Spike|hCoV-19/Japan/PG-3193/2020|2020-04-00|EPI_ISL_691313|Original|hCoV-19^^Japan|Human|Pathogen Genomics Center|Pathogen Genomics Center|Sekizuka|Japan
>Spike|hCoV-19/Australia/VIC16343/2020|2020-08-03|EPI_ISL_641222|Original|hCoV-19^^Victoria|Human|Microbiological Diagnostic Unit - Public Health Laboratory (MDU-PHL)|MDU-PHL|Schultz|Australia
>Spike|hCoV-19/Japan/PG-5757/2020|2020-08-00|EPI_ISL_688876|Original|hCoV-19^^Japan|Human|Pathogen Genomics Center|Pathogen Genomics Center|Sekizuka|Japan

>Spike|hCoV-19/Ireland/WH-NVRL-MPS15031/2020|2020-04-11|EPI_ISL_768749|Original|hCoV-19^^Westmeath|Human|National Virus Reference Laboratory|Irish Coronavirus Sequencing Consortium - Teagasc Moorepark|Walsh|Ireland
>Spike|hCoV-19/Netherlands/NA_193/2020|2020-03-24|EPI_ISL_422728|Original|hCoV-19^^Netherlands|Human|Dutch COVID-19 response team|Erasmus Medical Center|Oude Munnink|Netherlands
>Spike|hCoV-19/USA/WA-S944/2020|2020-04-27|EPI_ISL_463434|Original|hCoV-19^^Washington|Human|Washington State Department of Health|Seattle Flu Study|Bedford|USA
>Spike|hCoV-19/Belgium/ULG-10194/2020|2020-05-20|EPI_ISL_471429|Original|hCoV-19^^Liege|Human|Department of Clinical Microbiology|GIGA Medical Genomics|Durkin|Belgium
>Spike|hCoV-19/USA/TX-HMH-MCoV-13984/2020|2020-08-16|EPI_ISL_787275|Original|hCoV-19^^Texas|Human|Houston Methodist Hospital|Houston Methodist Hospital|Long|USA
>Spike|hCoV-19/Finland/13M29/2020|2020-03-13|EPI_ISL_418388|Original|hCoV-19^^Finland|Human|Department of Virology and Immunology|Department of Virology|Smura|Finland
>Spike|hCoV-19/England/MILK-AB87BD/2020|2020-10-21|EPI_ISL_623711|Original|hCoV-19^^England|Human|Lighthouse Lab in Milton Keynes|Wellcome Sanger Institute for the COVID-19 Genomics UK (COG-UK) consortium|Davies|United Kingdom
>Spike|hCoV-19/Australia/VIC2881/2020|2020-07-06|EPI_ISL_521712|Original|hCoV-19^^Victoria|Human|Microbiological Diagnostic Unit - Public Health Laboratory (MDU-PHL)|MDU-PHL|Schultz|Australia
>Spike|hCoV-19/Wales/PHWC-35846/2020|2020-04-25|EPI_ISL_474484|Original|hCoV-19^^Wales|Human|Originating lab|COVID-19 Genomics UK (COG-UK) Consortium|Moore|United Kingdom
>Spike|hCoV-19/Slovenia/5359/2020|2020-03-09|EPI_ISL_635218|Original|hCoV-19^^Slovenia|Human|Institute of Microbiology and Immunology|Institute of Microbiology and Immunology|Zakotnik|Slovenia
>Spike|hCoV-19/USA/NY-NYCPHL-000881/2020|2020-03-27|EPI_ISL_632953|Original|hCoV-19^^New York|Human|Jamaica Hospital Medical Center|New York City Public Health Laboratory|Wang|USA
>Spike|hCoV-19/Norway/5759/2020|2020-10-23|EPI_ISL_775332|Original|hCoV-19^^Vestfold og Telemark|Human|Vestfold Hospital|Norwegian Institute of Public Health|Vollan|Norway
>Spike|hCoV-19/England/NORT-296191/2020|2020-05-17|EPI_ISL_484264|Original|hCoV-19^^England|Human|Northumbria University / South Tees Hospitals NHS Foundation Trust / North Cumbria Integrated Care NHS Foundation Trust / North Tees and Hartlepool NHS Foundation Trust / Newcastle Hospitals NHS Foundation Trust|COVID-19 Genomics UK (COG-UK) Consortium|Bashton|United Kingdom
>Spike|hCoV-19/Wales/PHWC-49299B/2020|2020-11-20|EPI_ISL_725728|Original|hCoV-19^^Wales|Human|Originating lab|COVID-19 Genomics UK (COG-UK) Consortium|Moore|United Kingdom
>Spike|hCoV-19/Denmark/DCGC-5115/2020|2020-09-21|EPI_ISL_621653|Original|hCoV-19^^Syddanmark|Human|Department of Virus and Microbiological Special Diagnostics|Albertsen lab|Michaelsen|Denmark
>Spike|hCoV-19/USA/OR-PROV-137/2020|2020-05-02|EPI_ISL_482399|Original|hCoV-19^^Oregon|Human|Providence St. Joseph Health Molecular Genomics Laboratory|Providence St. Joseph Health Molecular Genomics Laboratory|Dowdell|USA
>Spike|hCoV-19/Japan/PG-4728/2020|2020-07-00|EPI_ISL_689864|Original|hCoV-19^^Japan|Human|Pathogen Genomics Center|Pathogen Genomics Center|Sekizuka|Japan
>Spike|hCoV-19/England/CAMC-A81AC3/2020|2020-10-14|EPI_ISL_610684|Original|hCoV-19^^England|Human|Lighthouse Lab in Cambridge|Wellcome Sanger Institute for the COVID-19 Genomics UK (COG-UK) consortium|Davies|United Kingdom
>Spike|hCoV-19/England/ALDP-95464E/2020|2020-06-13|EPI_ISL_555466|Original|hCoV-19^^England|Human|Lighthouse Lab in Alderley Park|Wellcome Sanger Institute for the COVID-19 Genomics UK (COG-UK) consortium|Davies|United Kingdom
>Spike|hCoV-19/Australia/VIC2287/2020|2020-06-27|EPI_ISL_522009|Original|hCoV-19^^Victoria|Human|Microbiological Diagnostic Unit - Public Health Laboratory (MDU-PHL)|MDU-PHL|Schultz|Australia

>Spike|hCoV-19/Canada/QC-AK2/2020|2020-04-07|EPI_ISL_450641|Original|hCoV-19^^Quebec|Human|Laboratoire de microbiologie|Smith Laboratory|Smith|Canada
>Spike|hCoV-19/England/CAMB-834AF/2020|2020-04-15|EPI_ISL_443500|Original|hCoV-19^^England|Human|Department of Pathology|Wellcome Sanger Institute for the COVID-19 Genomics UK (COG-UK) consortium|Amato|United Kingdom
>Spike|hCoV-19/England/EXET-13EAA0/2020|2020-12-13|EPI_ISL_763390|Original|hCoV-19^^England|Human|University of Exeter|COVID-19 Genomics UK (COG-UK) Consortium|Temperton|United Kingdom
>Spike|hCoV-19/USA/WA-UW-681/2020|2020-03-13|EPI_ISL_476929|Original|hCoV-19^^Washington|Human|UW Virology Lab|UW Virology Lab|Roychoudhury|USA
>Spike|hCoV-19/USA/NY-Wadsworth-213980-01/2020|2020-09-09|EPI_ISL_765488|Original|hCoV-19^^New York|Human|Wadsworth Center|Wadsworth Center|Lasek-Nesselquist|USA
>Spike|hCoV-19/England/ALDP-9E5967/2020|2020-09-24|EPI_ISL_589381|Original|hCoV-19^^England|Human|Lighthouse Lab in Alderley Park|Wellcome Sanger Institute for the COVID-19 Genomics UK (COG-UK) consortium|Davies|United Kingdom
>Spike|hCoV-19/USA/MN-MDH-1272/2020|2020-03-12|EPI_ISL_482993|Original|hCoV-19^^Minnesota|Human|Minnesota Department of Health|Minnesota Department of Health|Garfin|USA
>Spike|hCoV-19/South Korea/KCDC2472/2020|2020-06-09|EPI_ISL_514875|Original|hCoV-19^^South Korea|Human|Division of Viral Diseases|Division of Viral Diseases|Kim|South Korea
>Spike|hCoV-19/Spain/AN-IBV-001991/2020|2020-03-15|EPI_ISL_509604|Original|hCoV-19^^Andalucia|Human|Servicio de Microbiolog\u00eda. HRU de M\u00e1laga. Servicio Andaluz de Salud|SeqCOVID-SPAIN consortium/IBV(CSIC)|Comas|Spain
>Spike|hCoV-19/Ireland/LK-UHL31/2020|2020-04-10|EPI_ISL_681749|Original|hCoV-19^^Limerick|Human|University Hospital Limerick|Irish Coronavirus Sequencing Consortium - Teagasc Moorepark|Walsh|Ireland
>Spike|hCoV-19/USA/NY-QDX-2125/2020|2020-03-14|EPI_ISL_604484|Original|hCoV-19^^New York|Human|Quest Diagnostics|Quest Diagnostics|Kagan|USA
>Spike|hCoV-19/Switzerland/VD-ETHZ-321486/2020|2020-10-14|EPI_ISL_603698|Original|hCoV-19^^Vaud|Human|Viollier AG|Department of Biosystems Science and Engineering|Nadeau|Switzerland
>Spike|hCoV-19/Scotland/QEUH-AD4065/2020|2020-10-26|EPI_ISL_649716|Original|hCoV-19^^Scotland|Human|Lighthouse Lab in Glasgow|Wellcome Sanger Institute for the COVID-19 Genomics UK (COG-UK) Consortium|Davies|United Kingdom
>Spike|hCoV-19/England/MILK-B59814/2020|2020-11-06|EPI_ISL_679872|Original|hCoV-19^^England|Human|Virology Department|COVID-19 Genomics UK (COG-UK) Consortium|de Silva|United Kingdom
>Spike|hCoV-19/Scotland/EDB6260/2020|2020-06-02|EPI_ISL_478241|Original|hCoV-19^^Scotland|Human|Virology Department|COVID-19 Genomics UK (COG-UK) Consortium|Williams|United Kingdom
>Spike|hCoV-19/England/ALDP-A50CD6/2020|2020-10-07|EPI_ISL_597617|Original|hCoV-19^^England|Human|Lighthouse Lab in Alderley Park|Wellcome Sanger Institute for the COVID-19 Genomics UK (COG-UK) consortium|Davies|United Kingdom
>Spike|hCoV-19/Spain/PV-IBV-001521/2020|2020-03-18|EPI_ISL_467133|Original|hCoV-19^^Basque Country|Human|Hospital Universitario Araba. Vitoria-Gasteiz|SeqCOVID-SPAIN consortium/IBV(CSIC)|Comas|Spain
>Spike|hCoV-19/Wales/PHWC-36C0D/2020|2020-05-01|EPI_ISL_474777|Original|hCoV-19^^Wales|Human|Originating lab|COVID-19 Genomics UK (COG-UK) Consortium|Moore|United Kingdom
>Spike|hCoV-19/England/QEUH-998E46/2020|2020-08-26|EPI_ISL_540082|Original|hCoV-19^^England|Human|Lighthouse Lab in Glasgow|Wellcome Sanger Institute for the COVID-19 Genomics UK (COG-UK) consortium|Davies|United Kingdom
>Spike|hCoV-19/USA/CA-CZB-14996/2020|2020-11-18|EPI_ISL_739352|Original|hCoV-19^^California|Human|Santa Clara County Public Health Laboratory|Chan-Zuckerberg Biohub|Kamm|USA
>Spike|hCoV-19/England/QEUH-A1E357/2020|2020-09-28|EPI_ISL_587406|Original|hCoV-19^^England|Human|Lighthouse Lab in Glasgow|Wellcome Sanger Institute for the COVID-19 Genomics UK (COG-UK) consortium|Davies|United Kingdom

>Spike|hCoV-19/Scotland/CVR5008/2020|2020-10-08|EPI_ISL_594747|Original|hCoV-19^^Scotland|Human|West of Scotland Specialist Virology Centre|COVID-19 Genomics UK (COG-UK) Consortium|Robertson|United Kingdom
>Spike|hCoV-19/England/CAMB-1AB534/2020|2020-04-20|EPI_ISL_470183|Original|hCoV-19^^England|Human|Department of Pathology|Wellcome Sanger Institute for the COVID-19 Genomics UK (COG-UK) consortium|Davies|United Kingdom
>Spike|hCoV-19/Brazil/BA-510/2020|2020-03-06|EPI_ISL_427293|Original|hCoV-19^^Bahia|Human|LACEN-BA - Laborat\u00f3rio Central de Sa\u00fade P\u00fablica Professor Gon\u00e7alo Moniz|Instituto Oswaldo Cruz FIOCRUZ - Laboratory of Respiratory Viruses and Measles (LVRS)|Silva|Brazil
>Spike|hCoV-19/South Korea/KDCA0208/2020|2020-11-25|EPI_ISL_747443|Original|hCoV-19^^South Korea|Human|Division of Emerging Infectious Diseases|Division of Emerging Infectious Diseases|Park|South Korea
>Spike|hCoV-19/Wales/PHWC-4A04B7/2020|2020-12-06|EPI_ISL_743452|Original|hCoV-19^^Wales|Human|Wales Specialist Virology Centre Sequencing lab|COVID-19 Genomics UK (COG-UK) Consortium|Moore|United Kingdom
>Spike|hCoV-19/England/SHEF-10B2A93/2020|2020-05-03|EPI_ISL_537208|Original|hCoV-19^^England|Human|Virology Department|Wellcome Sanger Institute for the COVID-19 Genomics UK (COG-UK) consortium|Davies|United Kingdom
>Spike|hCoV-19/Denmark/DCGC-2657/2020|2020-08-17|EPI_ISL_617877|Original|hCoV-19^^Midtjylland|Human|Department of Virus and Microbiological Special Diagnostics|Albertsen lab|Michaelsen|Denmark
>Spike|hCoV-19/England/CAMB-73071/2020|2020-03-21|EPI_ISL_440208|Original|hCoV-19^^England|Human|Department of Pathology|Wellcome Sanger Institute for the COVID-19 Genomics UK (COG-UK) consortium|Amato|United Kingdom
>Spike|hCoV-19/England/QEUH-9ACD24/2020|2020-09-02|EPI_ISL_559807|Original|hCoV-19^^England|Human|Oxford Viromics|COVID-19 Genomics UK (COG-UK) Consortium|Golubchik|United Kingdom
>Spike|hCoV-19/South Africa/KRISP-K001390/2020|2020-07-20|EPI_ISL_515874|Original|hCoV-19^^KwaZulu-Natal|Human|Medical Disagnostics Services (MDS)|KRISP|de Oliveira|South Africa
>Spike|hCoV-19/England/MILK-BABDD8/2020|2020-11-18|EPI_ISL_783197|Original|hCoV-19^^England|Human|Lighthouse Lab in Milton Keynes|Wellcome Sanger Institute for the COVID-19 Genomics UK (COG-UK) Consortium|Davies|United Kingdom
>Spike|hCoV-19/USA/TX-HMH-MCoV-16296/2020|2020-10-29|EPI_ISL_785980|Original|hCoV-19^^Texas|Human|Houston Methodist Hospital|Houston Methodist Hospital|Long|USA
>Spike|hCoV-19/Northern Ireland/NIRE-FB74F/2020|2020-03-28|EPI_ISL_448973|Original|hCoV-19^^Northern Ireland|Human|Regional Virus Laboratory|COVID-19 Genomics UK (COG-UK) Consortium|Fairley|United Kingdom
>Spike|hCoV-19/Denmark/DCGC-7879/2020|2020-10-19|EPI_ISL_618847|Original|hCoV-19^^Syddanmark|Human|Department of Virus and Microbiological Special Diagnostics|Albertsen lab|Michaelsen|Denmark
>Spike|hCoV-19/USA/TX-HMH-MCoV-15499/2020|2020-10-23|EPI_ISL_786983|Original|hCoV-19^^Texas|Human|Houston Methodist Hospital|Houston Methodist Hospital|Long|USA
>Spike|hCoV-19/Netherlands/NA_405/2020|2020-03-17|EPI_ISL_455207|Original|hCoV-19^^Netherlands|Human|Dutch COVID-19 response team|Erasmus Medical Center|Oude Munnink|Netherlands
>Spike|hCoV-19/USA/LA-EVTL055/2020|2020-04-27|EPI_ISL_451216|Original|hCoV-19^^Louisiana|Human|LSUHS Emerging Viral Threat Laboratory|Microbial Genome Sequencing Center|Kamil|USA
>Spike|hCoV-19/South Africa/KRISP-K002456/2020|2020-07-09|EPI_ISL_518042|Original|hCoV-19^^KwaZulu-Natal|Human|NHLS-IALCH|KRISP|de Oliveira|South Africa
>Spike|hCoV-19/England/QEUH-964DA0/2020|2020-08-13|EPI_ISL_531146|Original|hCoV-19^^England|Human|Lighthouse Lab in Glasgow|Wellcome Sanger Institute for the COVID-19 Genomics UK (COG-UK) consortium|Davies|United Kingdom
>Spike|hCoV-19/England/201080031/2020|2020-03-04|EPI_ISL_464435|Original|hCoV-19^^England|Human|Respiratory Virus Unit|Respiratory Virus Unit|Platt|United Kingdom

>Spike|hCoV-19/USA/PA-MGSC13-04/2020|2020-03-26|EPI_ISL_436468|Original|hCoV-19^^Pennsylvania|Human|UPMC Clinical Laboratory|Microbial Genome Sequencing Center|Cooper|USA
>Spike|hCoV-19/England/MILK-BB1908/2020|2020-11-17|EPI_ISL_719991|Original|hCoV-19^^England|Human|Lighthouse Lab in Milton Keynes|Wellcome Sanger Institute for the COVID-19 Genomics UK (COG-UK) Consortium|Davies|United Kingdom
>Spike|hCoV-19/England/ALDP-B742D9/2020|2020-11-12|EPI_ISL_662048|Original|hCoV-19^^England|Human|Lighthouse Lab in Alderley Park|Wellcome Sanger Institute for the COVID-19 Genomics UK (COG-UK) Consortium|Davies|United Kingdom
>Spike|hCoV-19/Denmark/DCGC-4052/2020|2020-09-14|EPI_ISL_622008|Original|hCoV-19^^Hovedstaden|Human|Department of Virus and Microbiological Special Diagnostics|Albertsen lab|Michaelsen|Denmark
>Spike|hCoV-19/England/QEUH-BC460B/2020|2020-11-25|EPI_ISL_703541|Original|hCoV-19^^England|Human|Lighthouse Lab in Glasgow|Wellcome Sanger Institute for the COVID-19 Genomics UK (COG-UK) Consortium|Davies|United Kingdom
>Spike|hCoV-19/Japan/PG-5559/2020|2020-08-00|EPI_ISL_688684|Original|hCoV-19^^Japan|Human|Pathogen Genomics Center|Pathogen Genomics Center|Sekizuka|Japan
>Spike|hCoV-19/England/ALDP-B74479/2020|2020-11-12|EPI_ISL_678920|Original|hCoV-19^^England|Human|Queens Medical Centre|COVID-19 Genomics UK (COG-UK) Consortium|Loose|United Kingdom
>Spike|hCoV-19/England/ALDP-4A80BB/2020|2020-06-11|EPI_ISL_559506|Original|hCoV-19^^England|Human|Lighthouse Lab in Alderley Park|Wellcome Sanger Institute for the COVID-19 Genomics UK (COG-UK) consortium|Davies|United Kingdom
>Spike|hCoV-19/England/201161648/2020|2020-03-11|EPI_ISL_464867|Original|hCoV-19^^England|Human|Respiratory Virus Unit|Respiratory Virus Unit|Platt|United Kingdom
>Spike|hCoV-19/Switzerland/BL-UHB-42169310/2020|2020-02-27|EPI_ISL_418275|Original|hCoV-19^^Basel|Human|University Hospital Basel|University Hospital Basel|Roloff Handschin|Switzerland
>Spike|hCoV-19/USA/TX-HMH-MCoV-14697/2020|2020-10-12|EPI_ISL_787982|Original|hCoV-19^^Texas|Human|Houston Methodist Hospital|Houston Methodist Hospital|Long|USA
>Spike|hCoV-19/Denmark/DCGC-13054/2020|2020-11-30|EPI_ISL_712254|Original|hCoV-19^^Hovedstaden|Human|Department of Virus and Microbiological Special Diagnostics|Albertsen Lab|Michaelsen|Denmark
>Spike|hCoV-19/Denmark/DCGC-10535/2020|2020-11-02|EPI_ISL_669762|Original|hCoV-19^^Syddanmark|Human|Department of Virus and Microbiological Special Diagnostics|Albertsen Lab|Michaelsen|Denmark
>Spike|hCoV-19/Scotland/EDB3166/2020|2020-04-06|EPI_ISL_489058|Original|hCoV-19^^Scotland|Human|Virology Department|Wellcome Sanger Institute for the COVID-19 Genomics UK (COG-UK) consortium|Davies|United Kingdom
>Spike|hCoV-19/USA/UT-08600/2020|2020-05-19|EPI_ISL_524347|Original|hCoV-19^^Utah|Human|Utah Public Health Laboratory|Utah Public Health Laboratory|Young|USA
>Spike|hCoV-19/Denmark/DCGC-12664/2020|2020-11-16|EPI_ISL_682845|Original|hCoV-19^^Syddanmark|Human|Department of Virus and Microbiological Special Diagnostics|Albertsen Lab|Michaelsen|Denmark
>Spike|hCoV-19/Belgium/rega-0517522/2020|2020-04-17|EPI_ISL_734736|Original|hCoV-19^^Kortenberg|Human|UZ Leuven|KU Leuven|Maes|Belgium
>Spike|hCoV-19/England/QEUH-B687CA/2020|2020-11-11|EPI_ISL_654972|Original|hCoV-19^^England|Human|Lighthouse Lab in Glasgow|Wellcome Sanger Institute for the COVID-19 Genomics UK (COG-UK) Consortium|Davies|United Kingdom
>Spike|hCoV-19/USA/TX-DSHS-2056/2020|2020-12-03|EPI_ISL_766690|Original|hCoV-19^^Texas|Human|Texas Department of State Health Services|Texas Department of State Health Services|Rahman|USA
>Spike|hCoV-19/Spain/MD-IBV-99008557/2020|2020-09-19|EPI_ISL_654098|Original|hCoV-19^^Madrid|Human|Hospital General Universitario Gregorio Mara\u00f1\u00f3n|SeqCOVID-SPAIN consortium/IBV(CSIC)|Comas|Spain
>Spike|hCoV-19/USA/AZ-TG273354/2020|2020-04-03|EPI_ISL_694058|Original|hCoV-19^^Arizona|Human|AZ SPHL|TGen North|French|USA
>Spike|hCoV-19/Australia/SAP169/2020|2020-03-19|EPI_ISL_492120|Original|hCoV-19^^South Australia|Human|SA Pathology|SA Pathology|Leong|Australia

>Spike|hCoV-19/USA/WI-UW-1901/2020|2020-09-18|EPI_ISL_605770|Original|hCoV-19^^Wisconsin|Human|University of Wisconsin-Madison AIDS Vaccine Research Laboratories|University of Wisconsin-Madison AIDS Vaccine Research Laboratories|Baczenas|USA
>Spike|hCoV-19/England/CAMC-C71D52/2020|2020-12-15|EPI_ISL_769116|Original|hCoV-19^^England|Human|Lighthouse Lab in Cambridge|Wellcome Sanger Institute for the COVID-19 Genomics UK (COG-UK) Consortium|Davies|United Kingdom
>Spike|hCoV-19/India/WB-S14/2020|2020-04-02|EPI_ISL_455644|Original|hCoV-19^^West Bengal|Human|ICMR-National Institute of Cholera and Enteric Diseases|National Institute of Biomedical Genomics|Maitra|India
>Spike|hCoV-19/Russia/AST-RII-MH4135S/2020|2020-08-04|EPI_ISL_733169|Original|hCoV-19^^Astrakhan|Human|Pathogenic Microorganisms Variability Laboratory|WHO National Influenza Centre Russian Federation|Komissarov|Russia
>Spike|hCoV-19/England/QEUH-A3285E/2020|2020-10-06|EPI_ISL_628360|Original|hCoV-19^^England|Human|Oxford Viromics|COVID-19 Genomics UK (COG-UK) Consortium|Golubchik|United Kingdom
>Spike|hCoV-19/England/LIVE-DC4CCB/2020|2020-11-11|EPI_ISL_705915|Original|hCoV-19^^England|Human|Liverpool Clinical Laboratories|COVID-19 Genomics UK (COG-UK) Consortium|Haldenby|United Kingdom
>Spike|hCoV-19/USA/UT-UPHL-2012773632/2020|2020-08-13|EPI_ISL_745955|Original|hCoV-19^^Utah|Human|Ginkgo Bioworks Clinical Laboratory|Utah Public Health Laboratory|Young|USA
>Spike|hCoV-19/Wales/PHWC-492D26/2020|2020-11-27|EPI_ISL_725780|Original|hCoV-19^^Wales|Human|Originating lab|COVID-19 Genomics UK (COG-UK) Consortium|Moore|United Kingdom
>Spike|hCoV-19/England/QEUH-9ACE7C/2020|2020-09-02|EPI_ISL_559812|Original|hCoV-19^^England|Human|Oxford Viromics|COVID-19 Genomics UK (COG-UK) Consortium|Golubchik|United Kingdom
>Spike|hCoV-19/USA/CA-CZB-10866/2020|2020-08-04|EPI_ISL_583107|Original|hCoV-19^^California|Human|Humboldt County Public Health Laboratory|Chan-Zuckerberg Biohub|Bhatt|USA
>Spike|hCoV-19/USA/NY-Wadsworth-248637-01/2020|2020-10-19|EPI_ISL_754717|Original|hCoV-19^^New York|Human|Wadsworth Center|Wadsworth Center|Lasek-Nesselquist|USA
>Spike|hCoV-19/England/ALDP-C39A5F/2020|2020-12-09|EPI_ISL_731365|Original|hCoV-19^^England|Human|Lighthouse Lab in Alderley Park|Wellcome Sanger Institute for the COVID-19 Genomics UK (COG-UK) Consortium|Davies|United Kingdom
>Spike|hCoV-19/USA/HI-H200090/2020|2020-06-19|EPI_ISL_752657|Original|hCoV-19^^Hawaii|Human|State Laboratories Division|State Laboratories Division|Sultana|USA
>Spike|hCoV-19/England/ALDP-658944/2020|2020-07-03|EPI_ISL_558219|Original|hCoV-19^^England|Human|Lighthouse Lab in Alderley Park|Wellcome Sanger Institute for the COVID-19 Genomics UK (COG-UK) consortium|Davies|United Kingdom
>Spike|hCoV-19/England/QEUH-B65E8C/2020|2020-11-10|EPI_ISL_655973|Original|hCoV-19^^England|Human|Lighthouse Lab in Glasgow|Wellcome Sanger Institute for the COVID-19 Genomics UK (COG-UK) Consortium|Davies|United Kingdom
>Spike|hCoV-19/England/MILK-A7DAE8/2020|2020-10-13|EPI_ISL_606398|Original|hCoV-19^^England|Human|Lighthouse Lab in Milton Keynes|Wellcome Sanger Institute for the COVID-19 Genomics UK (COG-UK) consortium|Davies|United Kingdom
>Spike|hCoV-19/USA/AZ-TG305977/2020|2020-04-30|EPI_ISL_694305|Original|hCoV-19^^Arizona|Human|AZ SPHL|TGen North|French|USA
>Spike|hCoV-19/England/MILK-61B725/2020|2020-07-01|EPI_ISL_557970|Original|hCoV-19^^England|Human|Lighthouse Lab in Milton Keynes|Wellcome Sanger Institute for the COVID-19 Genomics UK (COG-UK) consortium|Davies|United Kingdom
>Spike|hCoV-19/Japan/PG-0801/2020|2020-04-00|EPI_ISL_684411|Original|hCoV-19^^Japan|Human|Fukuoka Institute of Health and Environmental Sciences|Pathogen Genomics Center|Sekizuka|Japan
>Spike|hCoV-19/Northern Ireland/NIRE-234502/2020|2020-10-19|EPI_ISL_680400|Original|hCoV-19^^Northern Ireland|Human|Regional Virus Laboratory|COVID-19 Genomics UK (COG-UK) Consortium|Fairley|United Kingdom

>Spike|hCoV-19/Norway/4276/2020|2020-10-10|EPI_ISL_635143|Original|hCoV-19^^Agder|Human|Hospital of Southern Norway - Kristiansand|Norwegian Institute of Public Health|Vollan|Norway
>Spike|hCoV-19/Netherlands/NA_631/2020|2020-03-27|EPI_ISL_461066|Original|hCoV-19^^Netherlands|Human|Dutch COVID-19 response team|Erasmus Medical Center|Oude Munnink|Netherlands
>Spike|hCoV-19/Japan/PG-7319/2020|2020-08-00|EPI_ISL_688422|Original|hCoV-19^^Japan|Human|Pathogen Genomics Center|Pathogen Genomics Center|Sekizuka|Japan
>Spike|hCoV-19/Wales/PHWC-16691B/2020|2020-06-27|EPI_ISL_494060|Original|hCoV-19^^Wales|Human|Originating lab|COVID-19 Genomics UK (COG-UK) Consortium|Moore|United Kingdom
>Spike|hCoV-19/England/MILK-9A9F8B/2020|2020-08-31|EPI_ISL_550831|Original|hCoV-19^^England|Human|Lighthouse Lab in Milton Keynes|Wellcome Sanger Institute for the COVID-19 Genomics UK (COG-UK) consortium|Davies|United Kingdom
>Spike|hCoV-19/USA/FL-BPHL-2131/2020|2020-05-07|EPI_ISL_653284|Original|hCoV-19^^Florida|Human|Florida Bureau of Public Health Laboratories|Florida Bureau of Public Health Laboratories|Blanton|USA
>Spike|hCoV-19/England/LIVE-1D15AE/2020|2020-05-15|EPI_ISL_572748|Original|hCoV-19^^England|Human|Liverpool Clinical Laboratories|COVID-19 Genomics UK (COG-UK) Consortium|Haldenby|United Kingdom
>Spike|hCoV-19/Denmark/DCGC-19468/2020|2020-12-07|EPI_ISL_748967|Original|hCoV-19^^Midtjylland|Human|Department of Virus and Microbiological Special Diagnostics|Albertsen Lab|Michaelsen|Denmark
>Spike|hCoV-19/England/ALDP-5B3474/2020|2020-06-18|EPI_ISL_558813|Original|hCoV-19^^England|Human|Lighthouse Lab in Alderley Park|Wellcome Sanger Institute for the COVID-19 Genomics UK (COG-UK) consortium|Davies|United Kingdom
>Spike|hCoV-19/Netherlands/UT-RIVM-20818/2020|2020-12-16|EPI_ISL_790874|Original|hCoV-19^^Utrecht|Human|Dutch COVID-19 response team|National Institute for Public Health and the Environment (RIVM)|Meijer|Netherlands
>Spike|hCoV-19/Wales/PHWC-4802D1/2020|2020-10-18|EPI_ISL_612872|Original|hCoV-19^^Wales|Human|Wales Specialist Virology Centre Sequencing lab|COVID-19 Genomics UK (COG-UK) Consortium|Moore|United Kingdom
>Spike|hCoV-19/Jordan/AM-ALSR-4085/2020|2020-10-03|EPI_ISL_636445|Original|hCoV-19^^Amman|Human|Biolab Diagnostic Laboratories|Andersen lab at Scripps Research|Gangavarapu|Jordan
>Spike|hCoV-19/United Arab Emirates/1007/2020|2020-06-10|EPI_ISL_699139|Original|hCoV-19^^Abu Dhabi|Human|Group 42 (G42) Healthcare|G42 Healthcare|Ogrodzki|United Arab Emirates
>Spike|hCoV-19/Spain/MD-IBV-99008719/2020|2020-10-16|EPI_ISL_654296|Original|hCoV-19^^Madrid|Human|Hospital General Universitario Gregorio Mara\u00f1\u00f3n|SeqCOVID-SPAIN consortium/IBV(CSIC)|Comas|Spain
>Spike|hCoV-19/Australia/VIC13565/2020|2020-08-03|EPI_ISL_563756|Original|hCoV-19^^Victoria|Human|Microbiological Diagnostic Unit - Public Health Laboratory (MDU-PHL)|MDU-PHL|Schultz|Australia
>Spike|hCoV-19/USA/TX-HMH-6935/2020|2020-06-27|EPI_ISL_546364|Original|hCoV-19^^Texas|Human|Houston Methodist Hospital|Houston Methodist Hospital|Long|USA
>Spike|hCoV-19/Scotland/CVR4552/2020|2020-09-22|EPI_ISL_585411|Original|hCoV-19^^Scotland|Human|Lighthouse Lab in Glasgow / MRC-University of Glasgow Centre for Virus Research|COVID-19 Genomics UK (COG-UK) Consortium|Robertson|United Kingdom
>Spike|hCoV-19/Scotland/EDB3868/2020|2020-04-30|EPI_ISL_439346|Original|hCoV-19^^Scotland|Human|Virology Department|COVID-19 Genomics UK (COG-UK) Consortium|Williams|United Kingdom
>Spike|hCoV-19/England/LIVE-DB3D44/2020|2020-10-20|EPI_ISL_650782|Original|hCoV-19^^England|Human|Liverpool Clinical Laboratories|COVID-19 Genomics UK (COG-UK) Consortium|Haldenby|United Kingdom
>Spike|hCoV-19/England/MILK-ACE4DB/2020|2020-10-21|EPI_ISL_629232|Original|hCoV-19^^England|Human|Lighthouse Lab in Milton Keynes|Wellcome Sanger Institute for the COVID-19 Genomics UK (COG-UK) consortium|Davies|United Kingdom

>Spike|hCoV-19/USA/MD-HP12059/2020|2020-12-02|EPI_ISL_791391|Original|hCoV-19^^Maryland|Human|Johns Hopkins Hospital Department of Pathology|Johns Hopkins Hospital Department of Pathology|Morris|USA
>Spike|hCoV-19/Wales/PHWC-33C3D/2020|2020-04-16|EPI_ISL_446804|Original|hCoV-19^^Wales|Human|Wales Specialist Virology Centre|Public Health Wales Microbiology Cardiff|Moore|United Kingdom
>Spike|hCoV-19/Denmark/DCGC-10665/2020|2020-11-02|EPI_ISL_669497|Original|hCoV-19^^Midtjylland|Human|Department of Virus and Microbiological Special Diagnostics|Albertsen Lab|Michaelsen|Denmark
>Spike|hCoV-19/France/ARA-SC140/2020|2020-05-10|EPI_ISL_700404|Original|hCoV-19^^Auvergne-Rh\u00f4ne-Alpes|Human|Centre hospitalier M\u00e9tropole Savoie|CNR Virus des Infections Respiratoires - France SUD|Bal|France
>Spike|hCoV-19/USA/WI-UW-620/2020|2020-06-24|EPI_ISL_484995|Original|hCoV-19^^Wisconsin|Human|University of Wisconsin-Madison AIDS Vaccine Research Laboratories|University of Wisconsin-Madison AIDS Vaccine Research Laboratories|Baczenas|USA
>Spike|hCoV-19/Denmark/DCGC-4910/2020|2020-09-21|EPI_ISL_621448|Original|hCoV-19^^Syddanmark|Human|Department of Virus and Microbiological Special Diagnostics|Albertsen lab|Michaelsen|Denmark
>Spike|hCoV-19/England/C200035466/2020|2020-00-00|EPI_ISL_636718|Original|hCoV-19^^England|Human|Respiratory Virus Unit|Respiratory Virus Unit|Platt|United Kingdom
>Spike|hCoV-19/England/NOTT-11E718/2020|2020-11-28|EPI_ISL_725533|Original|hCoV-19^^England|Human|Queens Medical Centre|COVID-19 Genomics UK (COG-UK) Consortium|Loose|United Kingdom
>Spike|hCoV-19/USA/NM-DOH-2020308907/2020|2020-08-24|EPI_ISL_602187|Original|hCoV-19^^New Mexico|Human|New Mexico Department of Health Scientific Laboratory|New Mexico Department of Health Scientific Laboratory|Johnson|USA
>Spike|hCoV-19/USA/TX-DSHS-1054/2020|2020-06-15|EPI_ISL_628736|Original|hCoV-19^^Texas|Human|Texas Department of State Health Services|Texas Department of State Health Services|Rahman|USA
>Spike|hCoV-19/England/MILK-95755D/2020|2020-06-11|EPI_ISL_554024|Original|hCoV-19^^England|Human|Lighthouse Lab in Milton Keynes|Wellcome Sanger Institute for the COVID-19 Genomics UK (COG-UK) consortium|Davies|United Kingdom
>Spike|hCoV-19/Russia/StPetersburg-RII5029S/2020|2020-04-10|EPI_ISL_430085|Original|hCoV-19^^Saint Petersburg|Human|WHO National Influenza Centre Russian Federation|WHO National Influenza Centre Russian Federation|Komissarov|Russia
>Spike|hCoV-19/England/LIVE-9C3E2/2020|2020-03-29|EPI_ISL_449535|Original|hCoV-19^^England|Human|Liverpool Clinical Laboratories|COVID-19 Genomics UK (COG-UK) Consortium|Haldenby|United Kingdom
>Spike|hCoV-19/England/SHEF-C5E1C/2020|2020-04-29|EPI_ISL_475388|Original|hCoV-19^^England|Human|Virology Department|COVID-19 Genomics UK (COG-UK) Consortium|de Silva|United Kingdom
>Spike|hCoV-19/Belgium/ULG-10750/2020|2020-09-19|EPI_ISL_641592|Original|hCoV-19^^Li\u00e8ge|Human|Department of Clinical Microbiology|GIGA Medical Genomics|Durkin|Belgium
>Spike|hCoV-19/England/20170032604/2020|2020-04-16|EPI_ISL_465279|Original|hCoV-19^^England|Human|Respiratory Virus Unit|Respiratory Virus Unit|Platt|United Kingdom
>Spike|hCoV-19/Scotland/EDB736/2020|2020-04-02|EPI_ISL_433235|Original|hCoV-19^^Scotland|Human|Virology Department|COVID-19 Genomics UK (COG-UK) Consortium|Williams|United Kingdom
>Spike|hCoV-19/USA/TX-HMH-MCoV-13092/2020|2020-08-27|EPI_ISL_788243|Original|hCoV-19^^Texas|Human|Houston Methodist Hospital|Houston Methodist Hospital|Long|USA
>Spike|hCoV-19/Japan/PG-7225/2020|2020-08-00|EPI_ISL_688329|Original|hCoV-19^^Japan|Human|Saitama Prefectural Institute of Public Health|Pathogen Genomics Center|Sekizuka|Japan
>Spike|hCoV-19/Denmark/DCGC-9974/2020|2020-11-09|EPI_ISL_668496|Original|hCoV-19^^Sjaelland|Human|Department of Virus and Microbiological Special Diagnostics|Albertsen Lab|Michaelsen|Denmark

>Spike|hCoV-19/England/MILK-9A93DA/2020|2020-09-01|EPI_ISL_551089|Original|hCoV-19^^England|Human|Lighthouse Lab in Milton Keynes|Wellcome Sanger Institute for the COVID-19 Genomics UK (COG-UK) consortium|Davies|United Kingdom
>Spike|hCoV-19/USA/TX-HMH-2655/2020|2020-06-03|EPI_ISL_545453|Original|hCoV-19^^Texas|Human|Houston Methodist Hospital|Houston Methodist Hospital|Long|USA
>Spike|hCoV-19/USA/OR-UW-4410/2020|2020-03-29|EPI_ISL_430950|Original|hCoV-19^^Oregon|Human|UW Virology Lab|UW Virology Lab|Roychoudhury|USA
>Spike|hCoV-19/Russia/StPetersburg-RII8397S/2020|2020-04-22|EPI_ISL_450280|Original|hCoV-19^^Saint Petersburg|Human|WHO National Influenza Centre Russian Federation|WHO National Influenza Centre Russian Federation|Komissarov|Russia
>Spike|hCoV-19/Switzerland/TI-ETHZ-101173/2020|2020-03-31|EPI_ISL_451836|Original|hCoV-19^^Ticino|Human|Viollier AG|Department of Biosystems Science and Engineering|Nadeau|Switzerland
>Spike|hCoV-19/England/CAMB-1AC014/2020|2020-04-29|EPI_ISL_444360|Original|hCoV-19^^England|Human|Department of Pathology|COVID-19 Genomics UK (COG-UK) Consortium|Meredith|United Kingdom
>Spike|hCoV-19/England/ALDP-A92D7B/2020|2020-10-19|EPI_ISL_611119|Original|hCoV-19^^England|Human|Lighthouse Lab in Alderley Park|Wellcome Sanger Institute for the COVID-19 Genomics UK (COG-UK) consortium|Davies|United Kingdom
>Spike|hCoV-19/Spain/MD-IBV-99008648/2020|2020-09-29|EPI_ISL_654214|Original|hCoV-19^^Madrid|Human|Hospital General Universitario Gregorio Mara\u00f1\u00f3n|SeqCOVID-SPAIN consortium/IBV(CSIC)|Comas|Spain
>Spike|hCoV-19/USA/WI-GMF-07816/2020|2020-06-24|EPI_ISL_489925|Original|hCoV-19^^Wisconsin|Human|Gundersen Molecular Diagnostics Laboratory|Kabara Cancer Research Institute|Kenny|USA
>Spike|hCoV-19/Australia/VIC14262/2020|2020-07-30|EPI_ISL_592423|Original|hCoV-19^^Victoria|Human|Microbiological Diagnostic Unit - Public Health Laboratory (MDU-PHL)|MDU-PHL|Schultz|Australia
>Spike|hCoV-19/England/LIVE-DBB016/2020|2020-11-12|EPI_ISL_665123|Original|hCoV-19^^England|Human|Liverpool Clinical Laboratories|COVID-19 Genomics UK (COG-UK) Consortium|Haldenby|United Kingdom
>Spike|hCoV-19/Iceland/54/2020|2020-03-06|EPI_ISL_417684|Original|hCoV-19^^Reykjavik|Human|The National University Hospital of Iceland|deCODE genetics|J\u00f3nsson|Iceland
>Spike|hCoV-19/USA/TX-HMH-1058/2020|2020-04-09|EPI_ISL_542512|Original|hCoV-19^^Texas|Human|Houston Methodist Hospital|Houston Methodist Hospital|Long|USA
>Spike|hCoV-19/England/CAMC-BFBE7C/2020|2020-12-04|EPI_ISL_709240|Original|hCoV-19^^England|Human|Lighthouse Lab in Cambridge|Wellcome Sanger Institute for the COVID-19 Genomics UK (COG-UK) Consortium|Davies|United Kingdom
>Spike|hCoV-19/Sweden/20-08074/2020|2020-03-04|EPI_ISL_475552|Original|hCoV-19^^Stockholm|Human|Karolinska Universitetslaboratoriet|The Public Health Agency of Sweden|Svartstr\u00f6m|Sweden
>Spike|hCoV-19/USA/CA-UCLA-7/2020|2020-04-14|EPI_ISL_537594|Original|hCoV-19^^California|Human|UCLA Pathology Clinical Microbiology Lab|Kruglyak Lab|Boocock|USA
>Spike|hCoV-19/England/MILK-BAA8D8/2020|2020-11-18|EPI_ISL_681388|Original|hCoV-19^^England|Human|Lighthouse Lab in Milton Keynes|Wellcome Sanger Institute for the COVID-19 Genomics UK (COG-UK) Consortium|Davies|United Kingdom
>Spike|hCoV-19/Australia/VIC6027/2020|2020-07-23|EPI_ISL_518068|Original|hCoV-19^^Victoria|Human|Microbiological Diagnostic Unit - Public Health Laboratory (MDU-PHL)|MDU-PHL|Schultz|Australia
>Spike|hCoV-19/England/QEUH-9F7865/2020|2020-09-27|EPI_ISL_589245|Original|hCoV-19^^England|Human|Lighthouse Lab in Glasgow|Wellcome Sanger Institute for the COVID-19 Genomics UK (COG-UK) consortium|Davies|United Kingdom
>Spike|hCoV-19/USA/CA-CDPH157/2020|2020-09-26|EPI_ISL_754974|Original|hCoV-19^^California|Human|California Department of Public Health|California Department of Public Health|Bell|USA
>Spike|hCoV-19/USA/TX-HMH-5474/2020|2020-06-26|EPI_ISL_544800|Original|hCoV-19^^Texas|Human|Houston Methodist Hospital|Houston Methodist Hospital|Long|USA

>Spike|hCoV-19/Brazil/AP-IEC-162966/2020|2020-04-05|EPI_ISL_458142|Original|hCoV-19^^Amapa|Human|\ufeffEvandro Chagas Institute|\ufeffEvandro Chagas Institute|Sousa J\u00fanior|Brazil
>Spike|hCoV-19/England/ALDP-BCA5AE/2020|2020-11-23|EPI_ISL_703333|Original|hCoV-19^^England|Human|Lighthouse Lab in Alderley Park|Wellcome Sanger Institute for the COVID-19 Genomics UK (COG-UK) Consortium|Davies|United Kingdom
>Spike|hCoV-19/USA/AZ-TG403641/2020|2020-06-23|EPI_ISL_694863|Original|hCoV-19^^Arizona|Human|TGen North|TGen North|French|USA
>Spike|hCoV-19/Denmark/DCGC-13553/2020|2020-11-30|EPI_ISL_712874|Original|hCoV-19^^Midtjylland|Human|Department of Virus and Microbiological Special Diagnostics|Albertsen Lab|Michaelsen|Denmark
>Spike|hCoV-19/England/QEUH-9CF17A/2020|2020-09-14|EPI_ISL_590077|Original|hCoV-19^^England|Human|Lighthouse Lab in Glasgow|Wellcome Sanger Institute for the COVID-19 Genomics UK (COG-UK) consortium|Davies|United Kingdom
>Spike|hCoV-19/USA/TX-HMH-MCoV-19771/2020|2020-11-16|EPI_ISL_784112|Original|hCoV-19^^Texas|Human|Houston Methodist Hospital|Houston Methodist Hospital|Long|USA
>Spike|hCoV-19/USA/CA-QDX-2660/2020|2020-03-16|EPI_ISL_604912|Original|hCoV-19^^California|Human|Quest Diagnostics|Quest Diagnostics|Kagan|USA
>Spike|hCoV-19/Denmark/DCGC-6917/2020|2020-10-12|EPI_ISL_619763|Original|hCoV-19^^Hovedstaden|Human|Department of Virus and Microbiological Special Diagnostics|Albertsen lab|Michaelsen|Denmark
>Spike|hCoV-19/England/CWAR-24FDBCF/2020|2020-05-03|EPI_ISL_584492|Original|hCoV-19^^England|Human|UHCW / University of Warwick|COVID-19 Genomics UK (COG-UK) Consortium|Stark|United Kingdom
>Spike|hCoV-19/Italy/CAM-IZSM-129/2020|2020-09-12|EPI_ISL_572321|Original|hCoV-19^^Campania|Human|IZSM|IZSM|Viscardi|Italy
>Spike|hCoV-19/Belgium/ULG-10276/2020|2020-07-23|EPI_ISL_540443|Original|hCoV-19^^Li\u00e8ge|Human|University of Li\u00e8ge COVID-19 testing center|GIGA Medical Genomics|Durkin|Belgium
>Spike|hCoV-19/USA/MN-MDH-2198/2020|2020-11-24|EPI_ISL_723149|Original|hCoV-19^^Minnesota|Human|Vault Health|Minnesota Department of Health|Lorentz|USA
>Spike|hCoV-19/Wales/PHWC-2ADEB/2020|2020-04-05|EPI_ISL_431951|Original|hCoV-19^^Wales|Human|Wales Specialist Virology Centre|Public Health Wales Microbiology Cardiff|Moore|United Kingdom
>Spike|hCoV-19/England/PORT-2CF2AB/2020|2020-04-24|EPI_ISL_453668|Original|hCoV-19^^England|Human|Centre for Enzyme Innovation|COVID-19 Genomics UK (COG-UK) Consortium|Robson|United Kingdom
>Spike|hCoV-19/USA/CA-IGI-0686/2020|2020-07-30|EPI_ISL_677869|Original|hCoV-19^^California|Human|Innovative Genomics Institute|Innovative Genomics Institute|Wyman|USA
>Spike|hCoV-19/USA/WA-S2131/2020|2020-06-08|EPI_ISL_497559|Original|hCoV-19^^Washington|Human|Washington State Department of Health|Seattle Flu Study|Lee|USA
>Spike|hCoV-19/USA/UT-UPHL-2009176/2020|2020-05-27|EPI_ISL_525162|Original|hCoV-19^^Utah|Human|Utah Public Health Laboratory|Utah Public Health Laboratory|Young|USA
>Spike|hCoV-19/Australia/VIC1993/2020|2020-03-26|EPI_ISL_521899|Original|hCoV-19^^Victoria|Human|Victorian Infectious Diseases Reference Laboratory (VIDRL)|VIDRL and MDU-PHL|Schultz|Australia
>Spike|hCoV-19/USA/TX-HMH-2522/2020|2020-06-02|EPI_ISL_545358|Original|hCoV-19^^Texas|Human|Houston Methodist Hospital|Houston Methodist Hospital|Long|USA
>Spike|hCoV-19/USA/WI-UW-469/2020|2020-06-08|EPI_ISL_484844|Original|hCoV-19^^Wisconsin|Human|University of Wisconsin-Madison AIDS Vaccine Research Laboratories|University of Wisconsin-Madison AIDS Vaccine Research Laboratories|Baczenas|USA
>Spike|hCoV-19/England/CAMC-B33CAB/2020|2020-11-06|EPI_ISL_659375|Original|hCoV-19^^England|Human|Lighthouse Lab in Cambridge|Wellcome Sanger Institute for the COVID-19 Genomics UK (COG-UK) Consortium|Davies|United Kingdom
>Spike|hCoV-19/Denmark/DCGC-11219/2020|2020-11-02|EPI_ISL_670774|Original|hCoV-19^^Hovedstaden|Human|Department of Virus and Microbiological Special Diagnostics|Albertsen Lab|Michaelsen|Denmark

>Spike|hCoV-19/England/BRIS-12DCCC/2020|2020-05-31|EPI_ISL_488877|Original|hCoV-
19^^England|Human|PHE South West Regional Laboratory|Wellcome Sanger Institute for
the COVID-19 Genomics UK (COG-UK) consortium|Davies|United Kingdom
>Spike|hCoV-19/Denmark/DCGC-18946/2020|2020-12-07|EPI_ISL_748657|Original|hCoV-
19^^Hovedstaden|Human|Department of Virus and Microbiological Special
Diagnostics|Albertsen Lab|Michaelsen|Denmark
>Spike|hCoV-19/England/QEUH-9DC013/2020|2020-09-21|EPI_ISL_601403|Original|hCoV-
19^^England|Human|Lighthouse Lab in Glasgow|Wellcome Sanger Institute for the COVID-
19 Genomics UK (COG-UK) consortium|Davies|United Kingdom
>Spike|hCoV-19/Scotland/QEUH-7C5AB7/2020|2020-07-23|EPI_ISL_532838|Original|hCoV-
19^^Scotland|Human|NHSGGC West of Scotland Specialist Virology Centre / MRC-
University of Glasgow Centre for Virus Research|Wellcome Sanger Institute for the
COVID-19 Genomics UK (COG-UK) consortium|Davies|United Kingdom
>Spike|hCoV-19/Portugal/PT1690/2020|2020-11-12|EPI_ISL_732157|Original|hCoV-
19^^Portugal|Human|Instituto Nacional de Saude (INSA) and Instituto Gulbenkian de
Ciencia (IGC)|Instituto Nacional de Saude (INSA) and Instituto Gulbenkian de Ciencia
(IGC)|Borges|Portugal
>Spike|hCoV-19/Luxembourg/LNS4839843/2020|2020-11-30|EPI_ISL_770911|Original|hCoV-
19^^Luxembourg|Human|Laboratoire national de sant\u00e9|Laboratoire national de
sant\u00e9|Wienecke-Baldacchino|Luxembourg
>Spike|hCoV-19/England/CAMC-A41BAE/2020|2020-10-07|EPI_ISL_609421|Original|hCoV-
19^^England|Human|Lighthouse Lab in Cambridge|Wellcome Sanger Institute for the
COVID-19 Genomics UK (COG-UK) consortium|Davies|United Kingdom
>Spike|hCoV-19/Australia/VIC6277/2020|2020-07-25|EPI_ISL_518266|Original|hCoV-
19^^Victoria|Human|Microbiological Diagnostic Unit - Public Health Laboratory (MDU-
PHL)|MDU-PHL|Schultz|Australia
>Spike|hCoV-19/USA/TX-HMH-MCoV-12195/2020|2020-09-21|EPI_ISL_790241|Original|hCoV-
19^^Texas|Human|Houston Methodist Hospital|Houston Methodist Hospital|Long|USA
>Spike|hCoV-19/England/ALDP-951395/2020|2020-06-13|EPI_ISL_554215|Original|hCoV-
19^^England|Human|Lighthouse Lab in Alderley Park|Wellcome Sanger Institute for the
COVID-19 Genomics UK (COG-UK) consortium|Davies|United Kingdom
>Spike|hCoV-19/England/ALDP-A2FCFE/2020|2020-10-06|EPI_ISL_587194|Original|hCoV-
19^^England|Human|Lighthouse Lab in Alderley Park|Wellcome Sanger Institute for the
COVID-19 Genomics UK (COG-UK) consortium|Davies|United Kingdom
>Spike|hCoV-19/Scotland/CVR4329/2020|2020-09-08|EPI_ISL_573776|Original|hCoV-
19^^Scotland|Human|West of Scotland Specialist Virology Centre|COVID-19 Genomics UK
(COG-UK) Consortium|Robertson|United Kingdom
>Spike|hCoV-19/Scotland/QEUH-A1F6F3/2020|2020-10-01|EPI_ISL_599107|Original|hCoV-
19^^Scotland|Human|Lighthouse Lab in Glasgow|Wellcome Sanger Institute for the COVID-
19 Genomics UK (COG-UK) consortium|Davies|United Kingdom
>Spike|hCoV-19/USA/TX-HMH-4829/2020|2020-06-22|EPI_ISL_544359|Original|hCoV-
19^^Texas|Human|Houston Methodist Hospital|Houston Methodist Hospital|Long|USA
>Spike|hCoV-19/Spain/MD-IBV-99009595/2020|2020-10-27|EPI_ISL_654347|Original|hCoV-
19^^Madrid|Human|Hospital General Universitario Gregorio Mara\u00f1\u00f3n|SeqCOVID-
SPAIN consortium/IBV(CSIC)|Comas|Spain
>Spike|hCoV-19/England/LOND-D5550/2020|2020-04-16|EPI_ISL_444233|Original|hCoV-
19^^England|Human|University College London|COVID-19 Genomics UK (COG-UK)
Consortium|Roy|United Kingdom
>Spike|hCoV-19/Australia/WA363/2020|2020-10-18|EPI_ISL_605840|Original|hCoV-
19^^Western Australia|Human|PathWest Laboratory Medicine WA|PathWest Laboratory
Medicine WA Microbial Surveillance Unit|Sikazwe|Australia
>Spike|hCoV-19/England/QEUH-AF1396/2020|2020-10-28|EPI_ISL_665390|Original|hCoV-
19^^England|Human|Northumbria University / South Tees Hospitals NHS Foundation Trust
/ North Cumbria Integrated Care NHS Foundation Trust / North Tees and Hartlepool NHS
Foundation Trust / Newcastle Hospitals NHS Foundation Trust|COVID-19 Genomics UK
(COG-UK) Consortium|Bashton|United Kingdom
>Spike|hCoV-19/England/ALDP-AA30D8/2020|2020-10-22|EPI_ISL_630886|Original|hCoV-
19^^England|Human|Lighthouse Lab in Alderley Park|Wellcome Sanger Institute for the
COVID-19 Genomics UK (COG-UK) consortium|Davies|United Kingdom

>Spike|hCoV-19/USA/WA-UW-12911/2020|2020-06-29|EPI_ISL_570929|Original|hCoV-19^^Washington|Human|UW Virology Lab|UW Virology Lab|Roychoudhury|USA
>Spike|hCoV-19/England/ALDP-B7492F/2020|2020-11-12|EPI_ISL_662131|Original|hCoV-19^^England|Human|Lighthouse Lab in Alderley Park|Wellcome Sanger Institute for the COVID-19 Genomics UK (COG-UK) Consortium|Davies|United Kingdom
>Spike|hCoV-19/Australia/VIC11461/2020|2020-08-21|EPI_ISL_563382|Original|hCoV-19^^Victoria|Human|Microbiological Diagnostic Unit - Public Health Laboratory (MDU-PHL)|MDU-PHL|Schultz|Australia
>Spike|hCoV-19/USA/WI-WSLH-200462/2020|2020-05-18|EPI_ISL_578604|Original|hCoV-19^^Wisconsin|Human|Wisconsin State Laboratory of Hygiene Communicable Disease Division|Wisconsin State Laboratory of Hygiene Communicable Disease Division|Florek|USA
>Spike|hCoV-19/Denmark/DCGC-18740/2020|2020-12-07|EPI_ISL_749353|Original|hCoV-19^^Midtjylland|Human|Department of Virus and Microbiological Special Diagnostics|Albertsen Lab|Michaelsen|Denmark
>Spike|hCoV-19/England/QEUH-A0C5CF/2020|2020-10-01|EPI_ISL_584993|Original|hCoV-19^^England|Human|Quadram Institute Bioscience|COVID-19 Genomics UK (COG-UK) Consortium|Alikhan|United Kingdom
>Spike|hCoV-19/Russia/StPetersburg-RII11271S/2020|2020-05-02|EPI_ISL_507249|Original|hCoV-19^^Saint Petersburg|Human|WHO National Influenza Centre Russian Federation|WHO National Influenza Centre Russian Federation|Komissarov|Russia
>Spike|hCoV-19/DRC/4720/2020|2020-05-01|EPI_ISL_514108|Original|hCoV-19^^Kinshasa|Human|Viral Respiratory Lab|Pathogen Sequencing Lab|Lokilo|Democratic Republic of the Congo
>Spike|hCoV-19/Australia/VIC3543/2020|2020-07-07|EPI_ISL_520292|Original|hCoV-19^^Victoria|Human|Victorian Infectious Diseases Reference Laboratory (VIDRL)|VIDRL and MDU-PHL|Schultz|Australia
>Spike|hCoV-19/Denmark/DCGC-15927/2020|2020-11-23|EPI_ISL_714488|Original|hCoV-19^^Hovedstaden|Human|Department of Virus and Microbiological Special Diagnostics|Albertsen Lab|Michaelsen|Denmark
>Spike|hCoV-19/England/CAMB-7B3F8/2020|2020-04-13|EPI_ISL_433953|Original|hCoV-19^^England|Human|Department of Pathology|COVID-19 Genomics UK (COG-UK) Consortium|Meredith|United Kingdom
>Spike|hCoV-19/Wales/PHWC-49062D/2020|2020-11-11|EPI_ISL_706495|Original|hCoV-19^^Wales|Human|Originating lab|COVID-19 Genomics UK (COG-UK) Consortium|Moore|United Kingdom
>Spike|hCoV-19/England/ALDP-C8B739/2020|2020-12-16|EPI_ISL_756524|Original|hCoV-19^^England|Human|Lighthouse Lab in Alderley Park|Wellcome Sanger Institute for the COVID-19 Genomics UK (COG-UK) Consortium|Davies|United Kingdom
>Spike|hCoV-19/USA/DC-HP00054/2020|2020-03-07|EPI_ISL_457797|Original|hCoV-19^^District of Columbia|Human|Johns Hopkins Hospital Department of Pathology|Johns Hopkins Hospital Department of Pathology|Ramakrishnan|USA
>Spike|hCoV-19/Denmark/DCGC-15099/2020|2020-11-23|EPI_ISL_713629|Original|hCoV-19^^Syddanmark|Human|Department of Virus and Microbiological Special Diagnostics|Albertsen Lab|Michaelsen|Denmark
>Spike|hCoV-19/England/NORT-2A7494/2020|2020-11-01|EPI_ISL_704579|Original|hCoV-19^^England|Human|Northumbria University / South Tees Hospitals NHS Foundation Trust / North Cumbria Integrated Care NHS Foundation Trust / North Tees and Hartlepool NHS Foundation Trust / Newcastle Hospitals NHS Foundation Trust|COVID-19 Genomics UK (COG-UK) Consortium|Bashton|United Kingdom
>Spike|hCoV-19/England/20100005504/2020|2020-02-28|EPI_ISL_464252|Original|hCoV-19^^England|Human|Respiratory Virus Unit|Respiratory Virus Unit|Platt|United Kingdom
>Spike|hCoV-19/India/OR-RMRC46/2020|2020-04-08|EPI_ISL_455780|Original|hCoV-19^^Odisha|Human|REGIONAL VRDL,ICMR-RMRC BBSR|Immunogenomics lab|Raghav|India
>Spike|hCoV-19/Canada/ON-PHL-20-03602/2020|2020-11-00|EPI_ISL_775110|Original|hCoV-19^^Ontario|Human|Public Health Ontario Laboratory|Public Health Ontario Laboratory|Guthrie|Canada

>Spike|hCoV-19/England/MILK-B896F3/2020|2020-11-13|EPI_ISL_675990|Original|hCoV-19^^England|Human|Lighthouse Lab in Milton Keynes|Wellcome Sanger Institute for the COVID-19 Genomics UK (COG-UK) Consortium|Davies|United Kingdom
>Spike|hCoV-19/England/ALDP-9BCC9D/2020|2020-09-09|EPI_ISL_573068|Original|hCoV-19^^England|Human|Oxford Viromics|COVID-19 Genomics UK (COG-UK) Consortium|Golubchik|United Kingdom
>Spike|hCoV-19/USA/TX-HMH-MCoV-9691/2020|2020-07-04|EPI_ISL_780116|Original|hCoV-19^^Texas|Human|Houston Methodist Hospital|Houston Methodist Hospital|Long|USA
>Spike|hCoV-19/USA/TX-HMH-3357/2020|2020-06-13|EPI_ISL_544142|Original|hCoV-19^^Texas|Human|Houston Methodist Hospital|Houston Methodist Hospital|Long|USA
>Spike|hCoV-19/Denmark/DCGC-18536/2020|2020-12-07|EPI_ISL_750027|Original|hCoV-19^^Midtjylland|Human|Department of Virus and Microbiological Special Diagnostics|Albertsen Lab|Michaelsen|Denmark
>Spike|hCoV-19/England/LIVE-A8FC1/2020|2020-03-26|EPI_ISL_517243|Original|hCoV-19^^England|Human|Liverpool Clinical Laboratories|COVID-19 Genomics UK (COG-UK) Consortium|Haldenby|United Kingdom
>Spike|hCoV-19/England/CAMC-C82ED3/2020|2020-12-17|EPI_ISL_762745|Original|hCoV-19^^England|Human|Lighthouse Lab in Cambridge|Wellcome Sanger Institute for the COVID-19 Genomics UK (COG-UK) Consortium|Davies|United Kingdom
>Spike|hCoV-19/Wales/PHWC-16A337/2020|2020-09-06|EPI_ISL_535235|Original|hCoV-19^^Wales|Human|Wales Specialist Virology Centre Sequencing lab|COVID-19 Genomics UK (COG-UK) Consortium|Moore|United Kingdom
>Spike|hCoV-19/England/NORT-2A48F5/2020|2020-09-13|EPI_ISL_573388|Original|hCoV-19^^England|Human|Northumbria University / South Tees Hospitals NHS Foundation Trust / North Cumbria Integrated Care NHS Foundation Trust / North Tees and Hartlepool NHS Foundation Trust / Newcastle Hospitals NHS Foundation Trust|COVID-19 Genomics UK (COG-UK) Consortium|Bashton|United Kingdom
>Spike|hCoV-19/Costa Rica/INC-0131/2020|2020-08-10|EPI_ISL_682270|Original|hCoV-19^^Cartago|Human|HOSPITAL SAN JUAN DE DIOS|Inciensa|Soto-Garita|Costa Rica
>Spike|hCoV-19/Shanghai/SH0104/2020|2020-02-09|EPI_ISL_416395|Original|hCoV-19^^Shanghai|Human|Shanghai Public Health Clinical Center|National Research Center for Translational Medicine (Shanghai)|Zhang|China
>Spike|hCoV-19/USA/HI-H200654/2020|2020-11-30|EPI_ISL_753177|Original|hCoV-19^^Hawaii|Human|State Laboratories Division|State Laboratories Division|Sultana|USA
>Spike|hCoV-19/Denmark/DCGC-15090/2020|2020-11-23|EPI_ISL_713137|Original|hCoV-19^^Midtjylland|Human|Department of Virus and Microbiological Special Diagnostics|Albertsen Lab|Michaelsen|Denmark
>Spike|hCoV-19/USA/MI-MDHHS-SC20207/2020|2020-03-19|EPI_ISL_447178|Original|hCoV-19^^Michigan|Human|Michigan Department of Health and Human Services|Michigan Department of Health and Human Services|Blankenship|USA
>Spike|hCoV-19/USA/UT-UPHL-2012819923/2020|2020-08-12|EPI_ISL_745891|Original|hCoV-19^^Utah|Human|Ginkgo Bioworks Clinical Laboratory|Utah Public Health Laboratory|Young|USA
>Spike|hCoV-19/Scotland/CVR4664/2020|2020-09-28|EPI_ISL_612301|Original|hCoV-19^^Scotland|Human|West of Scotland Specialist Virology Centre|COVID-19 Genomics UK (COG-UK) Consortium|Robertson|United Kingdom
>Spike|hCoV-19/Belgium/UGent-63/2020|2020-04-06|EPI_ISL_451174|Original|hCoV-19^^Ghent|Human|Lab voor klinische biologie|Onderzoeksgroep Virologie|Lambrechts|Belgium
>Spike|hCoV-19/Poland/PL_MCB_12/2020|2020-04-07|EPI_ISL_645011|Original|hCoV-19^^Poland|Human|Human Genome Variation Research Group|Human Genome Variation Research Group||Poland
>Spike|hCoV-19/USA/MI-MDHHS-SC22190/2020|2020-10-16|EPI_ISL_614241|Original|hCoV-19^^Michigan|Human|Michigan Department of Health and Human Services|Michigan Department of Health and Human Services|Jacob|USA
>Spike|hCoV-19/United Arab Emirates/skmc-35676/2020|2020-04-03|EPI_ISL_582627|Original|hCoV-19^^United Arab Emirates|Human|Sheikh Khalifa Medical City|Molecular/Surveillance lab\nSheikh Khalifa Medical City|Amirtharaj|United Arab Emirates

>Spike|hCoV-19/USA/WY-WPHL-20149696/2020|2020-11-24|EPI_ISL_770100|Original|hCoV-19^^Wyoming|Human|Wyoming Public Health Laboratory|Wyoming Public Health Laboratory|Hull|USA
>Spike|hCoV-19/Denmark/DCGC-20714/2020|2020-12-21|EPI_ISL_758952|Original|hCoV-19^^Midtjylland|Human|Department of Virus and Microbiological Special Diagnostics|Albertsen Lab|Michaelsen|Denmark
>Spike|hCoV-19/Wales/PHWC-26259/2020|2020-03-27|EPI_ISL_422352|Original|hCoV-19^^Wales|Human|Wales Specialist Virology Centre|Public Health Wales Microbiology Cardiff|Moore|United Kingdom
>Spike|hCoV-19/USA/CA-IGI-0228/2020|2020-07-01|EPI_ISL_569900|Original|hCoV-19^^California|Human|Innovative Genomics Institute|Innovative Genomics Institute|Wyman|USA
>Spike|hCoV-19/Spain/IB-IBV-2510/2020|2020-03-18|EPI_ISL_468985|Original|hCoV-19^^Illes Balears|Human|Servicio de Microbiolog\u00eda|SeqCOVID-SPAIN consortium/IBV(CSIC)|Comas|Spain
>Spike|hCoV-19/England/NORW-2232B4/2020|2020-12-17|EPI_ISL_741581|Original|hCoV-19^^England|Human|Quadram Institute Bioscience|COVID-19 Genomics UK (COG-UK) Consortium|Alikhan|United Kingdom
>Spike|hCoV-19/England/QEUH-7B73CC/2020|2020-07-22|EPI_ISL_532890|Original|hCoV-19^^England|Human|Lighthouse Lab in Glasgow|Wellcome Sanger Institute for the COVID-19 Genomics UK (COG-UK) consortium|Davies|United Kingdom
>Spike|hCoV-19/Wales/PHWC-48752C/2020|2020-10-25|EPI_ISL_639207|Original|hCoV-19^^Wales|Human|Wales Specialist Virology Centre Sequencing lab|COVID-19 Genomics UK (COG-UK) Consortium|Moore|United Kingdom
>Spike|hCoV-19/Scotland/CVR5832/2020|2020-11-13|EPI_ISL_665385|Original|hCoV-19^^Scotland|Human|West of Scotland Specialist Virology Centre|COVID-19 Genomics UK (COG-UK) Consortium|Robertson|United Kingdom
>Spike|hCoV-19/England/QEUH-A0B3C6/2020|2020-10-01|EPI_ISL_599417|Original|hCoV-19^^England|Human|Lighthouse Lab in Glasgow|Wellcome Sanger Institute for the COVID-19 Genomics UK (COG-UK) consortium|Davies|United Kingdom
>Spike|hCoV-19/England/QEUH-5EF1C3/2020|2020-06-16|EPI_ISL_533226|Original|hCoV-19^^England|Human|Lighthouse Lab in Glasgow|Wellcome Sanger Institute for the COVID-19 Genomics UK (COG-UK) consortium|Davies|United Kingdom
>Spike|hCoV-19/Wales/PHWC-15C8FD/2020|2020-05-04|EPI_ISL_472555|Original|hCoV-19^^Wales|Human|Wales Specialist Virology Centre Sequencing lab|COVID-19 Genomics UK (COG-UK) Consortium|Moore|United Kingdom
>Spike|hCoV-19/England/CAMC-B7A121/2020|2020-11-12|EPI_ISL_741084|Original|hCoV-19^^England|Human|Oxford Viromics|COVID-19 Genomics UK (COG-UK) Consortium|Golubchik|United Kingdom
>Spike|hCoV-19/England/ALDP-B5E40C/2020|2020-11-08|EPI_ISL_723657|Original|hCoV-19^^England|Human|Oxford Viromics|COVID-19 Genomics UK (COG-UK) Consortium|Golubchik|United Kingdom
>Spike|hCoV-19/Denmark/DCGC-21628/2020|2020-12-21|EPI_ISL_757962|Original|hCoV-19^^Hovedstaden|Human|Department of Virus and Microbiological Special Diagnostics|Albertsen Lab|Michaelsen|Denmark
>Spike|hCoV-19/USA/WI-GMF-16207/2020|2020-08-19|EPI_ISL_547719|Original|hCoV-19^^Wisconsin|Human|Gundersen Molecular Diagnostics Laboratory|Kabara Cancer Research Institute|Kenny|USA
>Spike|hCoV-19/Spain/PV-IBV-004293/2020|2020-04-09|EPI_ISL_510409|Original|hCoV-19^^Basque Country|Human|Servicio de Microbiolog\u00eda. Hospital Universitario Donostia. OSI Donostialdea. \u00c1rea de Enfermedades Infecciosas|SeqCOVID-SPAIN consortium/IBV(CSIC)|Comas|Spain
>Spike|hCoV-19/Japan/PG-6522/2020|2020-07-00|EPI_ISL_687636|Original|hCoV-19^^Japan|Human|Pathogen Genomics Center|Pathogen Genomics Center|Sekizuka|Japan
>Spike|hCoV-19/France/PAC-MEPHI-1771/2020|2020-09-19|EPI_ISL_644446|Original|hCoV-19^^Provence-Alpes-Cote-d\u2019Azur|Human|MEPHI|MEPHI|Levasseur|France
>Spike|hCoV-19/Luxembourg/LNS1643579/2020|2020-11-08|EPI_ISL_739967|Original|hCoV-19^^Luxembourg|Human|Laboratoire national de sant\u00e9|Laboratoire national de sant\u00e9|Wienecke-Baldacchino|Luxembourg

>Spike|hCoV-19/England/CAMC-B531A6/2020|2020-11-09|EPI_ISL_657707|Original|hCoV-19^^England|Human|Lighthouse Lab in Cambridge|Wellcome Sanger Institute for the COVID-19 Genomics UK (COG-UK) Consortium|Davies|United Kingdom
>Spike|hCoV-19/England/ALDP-A6D76C/2020|2020-10-15|EPI_ISL_608130|Original|hCoV-19^^England|Human|Lighthouse Lab in Alderley Park|Wellcome Sanger Institute for the COVID-19 Genomics UK (COG-UK) consortium|Davies|United Kingdom
>Spike|hCoV-19/Japan/PG-4944/2020|2020-08-00|EPI_ISL_690063|Original|hCoV-19^^Japan|Human|Pathogen Genomics Center|Pathogen Genomics Center|Sekizuka|Japan
>Spike|hCoV-19/England/QEUH-A4CF6B/2020|2020-10-05|EPI_ISL_596071|Original|hCoV-19^^England|Human|Quadram Institute Bioscience|COVID-19 Genomics UK (COG-UK) Consortium|Alikhan|United Kingdom
>Spike|hCoV-19/USA/NY-NYUMC916/2020|2020-03-20|EPI_ISL_467409|Original|hCoV-19^^New York|Human|NYU Langone Health|Departments of Pathology and Medicine|Maurano|USA
>Spike|hCoV-19/England/CAMC-B5221D/2020|2020-11-08|EPI_ISL_656809|Original|hCoV-19^^England|Human|Lighthouse Lab in Cambridge|Wellcome Sanger Institute for the COVID-19 Genomics UK (COG-UK) Consortium|Davies|United Kingdom
>Spike|hCoV-19/Sweden/20-53777/2020|2020-12-18|EPI_ISL_775546|Original|hCoV-19^^Vastmanland|Human|Klinisk Mikrobiologi|The Public Health Agency of Sweden|Svartstr\u00f6m|Sweden
>Spike|hCoV-19/England/MILK-C60F32/2020|2020-12-13|EPI_ISL_769484|Original|hCoV-19^^England|Human|Lighthouse Lab in Milton Keynes|Wellcome Sanger Institute for the COVID-19 Genomics UK (COG-UK) Consortium|Davies|United Kingdom
>Spike|hCoV-19/Australia/VIC6191/2020|2020-07-22|EPI_ISL_518193|Original|hCoV-19^^Victoria|Human|Victorian Infectious Diseases Reference Laboratory (VIDRL)|VIDRL and MDU-PHL|Schultz|Australia
>Spike|hCoV-19/England/MILK-B3C38F/2020|2020-11-01|EPI_ISL_706138|Original|hCoV-19^^England|Human|Oxford Viromics|COVID-19 Genomics UK (COG-UK) Consortium|Golubchik|United Kingdom
>Spike|hCoV-19/Wales/PHWC-31F81/2020|2020-04-14|EPI_ISL_446592|Original|hCoV-19^^Wales|Human|Wales Specialist Virology Centre|Public Health Wales Microbiology Cardiff|Moore|United Kingdom
>Spike|hCoV-19/USA/LA-GBCL-26544519/2020|2020-04-20|EPI_ISL_485107|Original|hCoV-19^^Louisiana|Human|River Road Testing Lab|Ginkgo Bioworks Clinical Laboratory|Smith|USA
>Spike|hCoV-19/Wales/PHWC-48320B/2020|2020-10-23|EPI_ISL_627791|Original|hCoV-19^^Wales|Human|Wales Specialist Virology Centre Sequencing lab|COVID-19 Genomics UK (COG-UK) Consortium|Moore|United Kingdom
>Spike|hCoV-19/England/QEUH-BCD1D4/2020|2020-11-25|EPI_ISL_704334|Original|hCoV-19^^England|Human|Lighthouse Lab in Glasgow|Wellcome Sanger Institute for the COVID-19 Genomics UK (COG-UK) Consortium|Davies|United Kingdom
>Spike|hCoV-19/Equatorial Guinea/44989/2020|2020-07-29|EPI_ISL_649171|Original|hCoV-19^^Bioko Sur|Human|Laboratorio de Investigaciones de Baney|University Hospital Basel|Hosch|Equatorial Guinea
>Spike|hCoV-19/Canada/NS-NML-1212/2020|2020-04-03|EPI_ISL_579610|Original|hCoV-19^^Nova Scotia|Human|QEII Health Sciences Centre|National Microbiology Laboratory (NML)|Majer|Canada
>Spike|hCoV-19/Denmark/DCGC-14141/2020|2020-11-30|EPI_ISL_711188|Original|hCoV-19^^Syddanmark|Human|Department of Virus and Microbiological Special Diagnostics|Albertsen Lab|Michaelsen|Denmark
>Spike|hCoV-19/England/BIRM-662A8/2020|2020-10-07|EPI_ISL_612252|Original|hCoV-19^^England|Human|University of Birmingham|COVID-19 Genomics UK (COG-UK) Consortium|Loman|United Kingdom
>Spike|hCoV-19/USA/WI-GMF-00557/2020|2020-04-02|EPI_ISL_422459|Original|hCoV-19^^Wisconsin|Human|Gundersen Molecular Diagnostics Laboratory|Kabara Cancer Research Institute|Kenny|USA
>Spike|hCoV-19/Slovenia/V0-4311/2020|2020-04-03|EPI_ISL_635289|Original|hCoV-19^^Slovenia|Human|Institute of Microbiology and Immunology|Institute of Microbiology and Immunology|Zakotnik|Slovenia

>Spike|hCoV-19/England/QEUH-B68D40/2020|2020-11-11|EPI_ISL_662199|Original|hCoV-19^^England|Human|Lighthouse Lab in Glasgow|Wellcome Sanger Institute for the COVID-19 Genomics UK (COG-UK) Consortium|Davies|United Kingdom
>Spike|hCoV-19/USA/NM-DOH-2020631746/2020|2020-12-19|EPI_ISL_766908|Original|hCoV-19^^New Mexico|Human|New Mexico Department of Health Scientific Laboratory|New Mexico Department of Health Scientific Laboratory|Malone|USA
>Spike|hCoV-19/England/MILK-B94CB7/2020|2020-11-14|EPI_ISL_676079|Original|hCoV-19^^England|Human|Lighthouse Lab in Milton Keynes|Wellcome Sanger Institute for the COVID-19 Genomics UK (COG-UK) Consortium|Davies|United Kingdom
>Spike|hCoV-19/Australia/VIC6770/2020|2020-07-29|EPI_ISL_564044|Original|hCoV-19^^Victoria|Human|Victorian Infectious Diseases Reference Laboratory (VIDRL)|VIDRL and MDU-PHL|Schultz|Australia
>Spike|hCoV-19/Wales/MILK-976840/2020|2020-08-21|EPI_ISL_552737|Original|hCoV-19^^Wales|Human|Lighthouse Lab in Milton Keynes|Wellcome Sanger Institute for the COVID-19 Genomics UK (COG-UK) consortium|Davies|United Kingdom
>Spike|hCoV-19/USA/AK-PHL878/2020|2020-07-06|EPI_ISL_528532|Original|hCoV-19^^Alaska|Human|Alaska State Virology Laboratory|Alaska State Virology Laboratory|Drown|USA
>Spike|hCoV-19/Japan/PG-7218/2020|2020-07-00|EPI_ISL_688322|Original|hCoV-19^^Japan|Human|Saitama Prefectural Institute of Public Health|Pathogen Genomics Center|Sekizuka|Japan
>Spike|hCoV-19/Brazil/SP-L7-CD69/2020|2020-03-23|EPI_ISL_476436|Original|hCoV-19^^S\u00e3o Paulo|Human|Hospital da Cl\u00ednicas da Faculdade de Medicina da Universidade de S\u00e3o Paulo|Instituto de Medicina Tropical da Univesidade de S\u00e3o Paulo|Faria|Brazil
>Spike|hCoV-19/USA/TX-HMH-2174/2020|2020-05-22|EPI_ISL_545162|Original|hCoV-19^^Texas|Human|Houston Methodist Hospital|Houston Methodist Hospital|Long|USA
>Spike|hCoV-19/England/LIVE-9F69B/2020|2020-04-22|EPI_ISL_471998|Original|hCoV-19^^England|Human|Liverpool Clinical Laboratories|COVID-19 Genomics UK (COG-UK) Consortium|Haldenby|United Kingdom
>Spike|hCoV-19/Singapore/458/2020|2020-04-20|EPI_ISL_475989|Original|hCoV-19^^Singapore|Human|National Public Health Laboratory|National Public Health Laboratory|Mak|Singapore
>Spike|hCoV-19/USA/UT-UPHL-03760/2020|2020-04-05|EPI_ISL_575109|Original|hCoV-19^^Utah|Human|Utah Public Health Laboratory|Utah Public Health Laboratory|Young|USA
>Spike|hCoV-19/Wales/PHWC-483FAA/2020|2020-10-25|EPI_ISL_627951|Original|hCoV-19^^Wales|Human|Wales Specialist Virology Centre Sequencing lab|COVID-19 Genomics UK (COG-UK) Consortium|Moore|United Kingdom
>Spike|hCoV-19/USA/NY-QDX-2277/2020|2020-03-15|EPI_ISL_604574|Original|hCoV-19^^New York|Human|Quest Diagnostics|Quest Diagnostics|Kagan|USA
>Spike|hCoV-19/Spain/IB-IBV-99011607/2020|2020-12-14|EPI_ISL_779855|Original|hCoV-19^^Balear Islands|Human|Servicio de Microbiolog\u00eda|SeqCOVID-SPAIN consortium/IBV(CSIC)|Comas|Spain
>Spike|hCoV-19/Australia/VIC17477/2020|2020-08-21|EPI_ISL_663900|Original|hCoV-19^^Victoria|Human|Microbiological Diagnostic Unit - Public Health Laboratory (MDU-PHL)|MDU-PHL|Schultz|Australia
>Spike|hCoV-19/Wales/PHWC-26B03/2020|2020-03-29|EPI_ISL_422373|Original|hCoV-19^^Wales|Human|Wales Specialist Virology Centre|Public Health Wales Microbiology Cardiff|Moore|United Kingdom
>Spike|hCoV-19/England/MILK-CA6808/2020|2020-12-16|EPI_ISL_761569|Original|hCoV-19^^England|Human|Lighthouse Lab in Milton Keynes|Wellcome Sanger Institute for the COVID-19 Genomics UK (COG-UK) Consortium|Davies|United Kingdom
>Spike|hCoV-19/England/LOND-12609E0/2020|2020-04-04|EPI_ISL_516869|Original|hCoV-19^^England|Human|North West London Pathology|Wellcome Sanger Institute for the COVID-19 Genomics UK (COG-UK) consortium|Davies|United Kingdom
>Spike|hCoV-19/USA/CA-CDPH122/2020|2020-09-21|EPI_ISL_754940|Original|hCoV-19^^California|Human|California Department of Public Health|California Department of Public Health|Bell|USA

>Spike|hCoV-19/England/CAMC-B21AE2/2020|2020-11-01|EPI_ISL_647281|Original|hCoV-19^^England|Human|Lighthouse Lab in Cambridge|Wellcome Sanger Institute for the COVID-19 Genomics UK (COG-UK) Consortium|Davies|United Kingdom
>Spike|hCoV-19/USA/MA-MASPHL-00779/2020|2020-09-25|EPI_ISL_692917|Original|hCoV-19^^Massachusetts|Human|Massachusetts State Public Health Laboratory|Massachusetts State Public Health Laboratory|Lang|USA
>Spike|hCoV-19/USA/FL-BPHL-0643/2020|2020-04-06|EPI_ISL_508847|Original|hCoV-19^^Florida|Human|Florida Bureau of Public Health Laboratories|Florida Bureau of Public Health Laboratories|Blanton|USA
>Spike|hCoV-19/South Africa/KRISP-K003683/2020|2020-09-26|EPI_ISL_602866|Original|hCoV-19^^KwaZulu-Natal|Human|NHLS-IALCH|KRISP|de Oliveira|South Africa
>Spike|hCoV-19/Norway/2240/2020|2020-03-27|EPI_ISL_590914|original|hCoV-19^^Oslo|Human|Oslo University Hospital|Norwegian Institute of Public Health|Vollan|Norway
>Spike|hCoV-19/England/MILK-A7A4CF/2020|2020-10-16|EPI_ISL_606877|Original|hCoV-19^^England|Human|Lighthouse Lab in Milton Keynes|Wellcome Sanger Institute for the COVID-19 Genomics UK (COG-UK) consortium|Davies|United Kingdom
>Spike|hCoV-19/USA/MN-QDX-1722/2020|2020-03-16|EPI_ISL_604071|Original|hCoV-19^^Minnesota|Human|Quest Diagnostics|Quest Diagnostics|Kagan|USA
>Spike|hCoV-19/England/ALDP-A86211/2020|2020-10-17|EPI_ISL_606120|Original|hCoV-19^^England|Human|Lighthouse Lab in Alderley Park|Wellcome Sanger Institute for the COVID-19 Genomics UK (COG-UK) consortium|Davies|United Kingdom
>Spike|hCoV-19/USA/TX-HMH-MCoV-19788/2020|2020-11-23|EPI_ISL_784126|Original|hCoV-19^^Texas|Human|Houston Methodist Hospital|Houston Methodist Hospital|Long|USA
>Spike|hCoV-19/England/LIVE-9EA63/2020|2020-04-08|EPI_ISL_453359|Original|hCoV-19^^England|Human|Liverpool Clinical Laboratories|COVID-19 Genomics UK (COG-UK) Consortium|Haldenby|United Kingdom
>Spike|hCoV-19/Germany/NW-HHU-388/2020|2020-11-01|EPI_ISL_718064|Orginal|hCoV-19^^North Rhine-Westphalia|Human|ZOTZ KLIMAS MVZ D\u00fcsseldorf-Centrum GbR \u00dcBAG f\u00fcr Labormedizin|Center of Medical Microbiology|Wienemann|Germany
>Spike|hCoV-19/Turkey/DEN-PAU14/2020|2020-08-26|EPI_ISL_708195|Original|hCoV-19^^Denizli|Human|Pamukkale University Hospital|Pamukkale University Department of Medical Genetics|Tokgun|Turkey
>Spike|hCoV-19/England/LIVE-9A7CA/2020|2020-03-30|EPI_ISL_449366|Original|hCoV-19^^England|Human|Liverpool Clinical Laboratories|COVID-19 Genomics UK (COG-UK) Consortium|Haldenby|United Kingdom
>Spike|hCoV-19/Scotland/CVR4867/2020|2020-10-05|EPI_ISL_651735|Original|hCoV-19^^Scotland|Human|West of Scotland Specialist Virology Centre|COVID-19 Genomics UK (COG-UK) Consortium|Robertson|United Kingdom
>Spike|hCoV-19/USA/NY-WC-46317-01/2020|2020-04-11|EPI_ISL_529036|Original|hCoV-19^^New York|Human|Wadsworth Center|Wadsworth Center|Plitnick|USA
>Spike|hCoV-19/Finland/4May88S4/2020|2020-05-04|EPI_ISL_481681|Original|hCoV-19^^Uusimaa|Human|Department of Virology and Immunology|Department of Virology|Smura|Finland
>Spike|hCoV-19/England/204691686/2020|2020-11-14|EPI_ISL_766270|Original|hCoV-19^^England|Human|Respiratory Virus Unit|COVID-19 Genomics UK (COG-UK) Consortium|Platt|United Kingdom
>Spike|hCoV-19/USA/WA-S2436/2020|2020-06-16|EPI_ISL_497068|Original|hCoV-19^^Washington|Human|Washington State Department of Health|Seattle Flu Study|Lee|USA
>Spike|hCoV-19/England/MILK-BB2345/2020|2020-11-18|EPI_ISL_720528|Original|hCoV-19^^England|Human|Lighthouse Lab in Milton Keynes|Wellcome Sanger Institute for the COVID-19 Genomics UK (COG-UK) Consortium|Davies|United Kingdom
>Spike|hCoV-19/USA/TX-HMH-MCoV-15592/2020|2020-10-23|EPI_ISL_787076|Original|hCoV-19^^Texas|Human|Houston Methodist Hospital|Houston Methodist Hospital|Long|USA
>Spike|hCoV-19/India/GJ-GBRC110/2020|2020-05-03|EPI_ISL_451155|Original|hCoV-19^^Gujarat|Human|Government Medical College|Gujarat Biotechnology Research Centre|Joshi|India

>Spike|hCoV-19/England/QEUH-AD593C/2020|2020-10-26|EPI_ISL_633703|Original|hCoV-19^^England|Human|Lighthouse Lab in Glasgow|Wellcome Sanger Institute for the COVID-19 Genomics UK (COG-UK) consortium|Davies|United Kingdom
>Spike|hCoV-19/Scotland/QEUH-9E42F4/2020|2020-09-23|EPI_ISL_601061|Original|hCoV-19^^Scotland|Human|Lighthouse Lab in Glasgow|Wellcome Sanger Institute for the COVID-19 Genomics UK (COG-UK) consortium|Davies|United Kingdom
>Spike|hCoV-19/Denmark/DCGC-3538/2020|2020-08-31|EPI_ISL_616331|Original|hCoV-19^^Nordjylland|Human|Department of Virus and Microbiological Special Diagnostics|Albertsen lab|Michaelsen|Denmark
>Spike|hCoV-19/South Korea/KCDC2081/2020|2020-02-21|EPI_ISL_497982|Original|hCoV-19^^South Korea|Human|Division of Viral Diseases|Division of Viral Diseases|Kim|South Korea
>Spike|hCoV-19/USA/WA-S1147/2020|2020-05-05|EPI_ISL_463637|Original|hCoV-19^^Washington|Human|Washington State Department of Health|Seattle Flu Study|Bedford|USA
>Spike|hCoV-19/England/MILK-AC83A7/2020|2020-10-21|EPI_ISL_629239|Original|hCoV-19^^England|Human|Lighthouse Lab in Milton Keynes|Wellcome Sanger Institute for the COVID-19 Genomics UK (COG-UK) consortium|Davies|United Kingdom
>Spike|hCoV-19/Denmark/DCGC-9932/2020|2020-11-09|EPI_ISL_669279|Original|hCoV-19^^Hovedstaden|Human|Department of Virus and Microbiological Special Diagnostics|Albertsen Lab|Michaelsen|Denmark
>Spike|hCoV-19/Australia/SAP551/2020|2020-11-14|EPI_ISL_654798|Original|hCoV-19^^South Australia|Human|SA Pathology|SA Pathology|Leong|Australia
>Spike|hCoV-19/Australia/VIC3263/2020|2020-07-13|EPI_ISL_520942|Original|hCoV-19^^Victoria|Human|Microbiological Diagnostic Unit - Public Health Laboratory (MDU-PHL)|MDU-PHL|Schultz|Australia
>Spike|hCoV-19/England/ALDP-C58F3D/2020|2020-12-14|EPI_ISL_736021|Original|hCoV-19^^England|Human|Lighthouse Lab in Alderley Park|Wellcome Sanger Institute for the COVID-19 Genomics UK (COG-UK) Consortium|Davies|United Kingdom
>Spike|hCoV-19/Luxembourg/LNS8204416/2020|2020-10-19|EPI_ISL_744703|Original|hCoV-19^^Luxembourg|Human|Laboratoire national de sant\u00e9|Laboratoire national de sant\u00e9|Wienecke-Baldacchino|Luxembourg
>Spike|hCoV-19/Japan/PG-8320/2020|2020-08-00|EPI_ISL_687419|Original|hCoV-19^^Japan|Human|Pathogen Genomics Center|Pathogen Genomics Center|Sekizuka|Japan
>Spike|hCoV-19/USA/MI-MDHHS-SC21321/2020|2020-04-02|EPI_ISL_507576|Original|hCoV-19^^Michigan|Human|Michigan Department of Health and Human Services|Michigan Department of Health and Human Services|Blankenship|USA
>Spike|hCoV-19/USA/CA-CZB-13790/2020|2020-09-24|EPI_ISL_738814|Original|hCoV-19^^California|Human|Alameda County Public Health Lab|Chan-Zuckerberg Biohub|Kamm|USA
>Spike|hCoV-19/England/MILK-A45779/2020|2020-10-06|EPI_ISL_598684|Original|hCoV-19^^England|Human|Lighthouse Lab in Milton Keynes|Wellcome Sanger Institute for the COVID-19 Genomics UK (COG-UK) consortium|Davies|United Kingdom
>Spike|hCoV-19/England/LIVE-DB9A47/2020|2020-11-09|EPI_ISL_679388|Original|hCoV-19^^England|Human|Liverpool Clinical Laboratories|COVID-19 Genomics UK (COG-UK) Consortium|Haldenby|United Kingdom
>Spike|hCoV-19/England/ALDP-AA1483/2020|2020-10-22|EPI_ISL_624156|Original|hCoV-19^^England|Human|Lighthouse Lab in Alderley Park|Wellcome Sanger Institute for the COVID-19 Genomics UK (COG-UK) consortium|Davies|United Kingdom
>Spike|hCoV-19/Australia/VIC1438/2020|2020-04-18|EPI_ISL_456442|Original|hCoV-19^^Victoria|Human|Victorian Infectious Diseases Reference Laboratory (VIDRL)|Microbiological Diagnostic Unit Public Health Laboratory and Victorian Infectious Diseases Reference Laboratory|Schultz|Australia
>Spike|hCoV-19/Belgium/VRJM-0323114/2020|2020-03-23|EPI_ISL_420371|Original|hCoV-19^^Hemisem|Human|KU Leuven|KU Leuven|Maes|Belgium
>Spike|hCoV-19/Scotland/QEUH-9B8208/2020|2020-09-10|EPI_ISL_568279|Original|hCoV-19^^Scotland|Human|Lighthouse Lab in Glasgow|Wellcome Sanger Institute for the COVID-19 Genomics UK (COG-UK) consortium|Davies|United Kingdom
>Spike|hCoV-19/England/ALDP-952DE1/2020|2020-06-10|EPI_ISL_553665|Original|hCoV-19^^England|Human|Lighthouse Lab in Alderley Park|Wellcome Sanger Institute for the COVID-19 Genomics UK (COG-UK) consortium|Davies|United Kingdom

>Spike|hCoV-19/USA/ID-UW-3183/2020|2020-03-26|EPI_ISL_490990|Original|hCoV-19^^Idaho|Human|UW Virology Lab|UW Virology Lab|Roychoudhury|USA
>Spike|hCoV-19/Denmark/DCGC-11624/2020|2020-11-02|EPI_ISL_670740|Original|hCoV-19^^Nordjylland|Human|Department of Virus and Microbiological Special Diagnostics|Albertsen Lab|Michaelsen|Denmark
>Spike|hCoV-19/Australia/VIC14613/2020|2020-08-06|EPI_ISL_592916|Original|hCoV-19^^Victoria|Human|Microbiological Diagnostic Unit - Public Health Laboratory (MDU-PHL)|MDU-PHL|Schultz|Australia
>Spike|hCoV-19/England/CAMC-B542B1/2020|2020-11-08|EPI_ISL_656828|Original|hCoV-19^^England|Human|Lighthouse Lab in Cambridge|Wellcome Sanger Institute for the COVID-19 Genomics UK (COG-UK) Consortium|Davies|United Kingdom
>Spike|hCoV-19/Gibraltar/204462809/2020|2020-10-29|EPI_ISL_614984|Original|hCoV-19^^Gibraltar|Human|Microbiology|Respiratory Virus Unit|Platt|Gibraltar
>Spike|hCoV-19/USA/MN-MDH-1853/2020|2020-10-05|EPI_ISL_602988|Original|hCoV-19^^Minnesota|Human|Minnesota Department of Health|Minnesota Department of Health|Garfin|USA
>Spike|hCoV-19/USA/TX-HMH-7400/2020|2020-06-26|EPI_ISL_546739|Original|hCoV-19^^Texas|Human|Houston Methodist Hospital|Houston Methodist Hospital|Long|USA
>Spike|hCoV-19/England/MILK-9E0BFD/2020|2020-09-21|EPI_ISL_601437|Original|hCoV-19^^England|Human|Lighthouse Lab in Milton Keynes|Wellcome Sanger Institute for the COVID-19 Genomics UK (COG-UK) consortium|Davies|United Kingdom
>Spike|hCoV-19/Japan/PG-2244/2020|2020-04-00|EPI_ISL_685849|Original|hCoV-19^^Japan|Human|Pathogen Genomics Center|Pathogen Genomics Center|Sekizuka|Japan
>Spike|hCoV-19/Denmark/DCGC-14569/2020|2020-11-30|EPI_ISL_711843|Original|hCoV-19^^Hovedstaden|Human|Department of Virus and Microbiological Special Diagnostics|Albertsen Lab|Michaelsen|Denmark
>Spike|hCoV-19/USA/WI-UW-2198/2020|2020-10-25|EPI_ISL_677601|Original|hCoV-19^^Wisconsin|Human|University of Wisconsin-Madison AIDS Vaccine Research Laboratories|University of Wisconsin-Madison AIDS Vaccine Research Laboratories|Baczenas|USA
>Spike|hCoV-19/Scotland/QEUH-9DCEA0/2020|2020-09-22|EPI_ISL_601132|Original|hCoV-19^^Scotland|Human|Lighthouse Lab in Glasgow|Wellcome Sanger Institute for the COVID-19 Genomics UK (COG-UK) consortium|Davies|United Kingdom
>Spike|hCoV-19/Australia/VIC11775/2020|2020-07-24|EPI_ISL_591846|Original|hCoV-19^^Victoria|Human|Microbiological Diagnostic Unit - Public Health Laboratory (MDU-PHL)|MDU-PHL|Schultz|Australia
>Spike|hCoV-19/USA/VA-DCLS-0423/2020|2020-04-29|EPI_ISL_477639|Original|hCoV-19^^Virginia|Human|Virginia DCLS|Virginia DCLS|St. Jacques|USA
>Spike|hCoV-19/USA/TX-HMH-6840/2020|2020-06-25|EPI_ISL_546281|Original|hCoV-19^^Texas|Human|Houston Methodist Hospital|Houston Methodist Hospital|Long|USA
>Spike|hCoV-19/Oman/205038035/2020|2020-05-21|EPI_ISL_491991|Original|hCoV-19^^Muscat|Human|Oman-NIC|Department of\u00a0Microbiology\u00a0and Immunology-SQUH|AL Zadjali|Oman
>Spike|hCoV-19/Jordan/SEARCH-5487/2020|2020-08-22|EPI_ISL_755145|Original|hCoV-19^^Amman|Human|Biolab Diagnostic Laboratories|Andersen lab at Scripps Research|Gangavarapu|Jordan
>Spike|hCoV-19/England/210100346/2021|2021-01-04|EPI_ISL_791194|Original|hCoV-19^^England|Human|Respiratory Virus Unit|COVID-19 Genomics UK (COG-UK) Consortium|Platt|United Kingdom
>Spike|hCoV-19/England/BRIS-1224DE/2020|2020-04-04|EPI_ISL_440713|Original|hCoV-19^^England|Human|PHE South West Regional Laboratory|Wellcome Sanger Institute for the COVID-19 Genomics UK (COG-UK) consortium|Amato|United Kingdom
>Spike|hCoV-19/Brazil/RJ-INCA-I39/2020|2020-05-04|EPI_ISL_513578|Original|hCoV-19^^Rio de Janeiro|Human|Programa de Oncovirologia|Programa de Oncovirologia|Ramos Goes|Brazil
>Spike|hCoV-19/USA/FL-BPHL-2250/2020|2020-11-24|EPI_ISL_763333|Original|hCoV-19^^Florida|Human|Florida Bureau of Public Health Laboratories|Florida Bureau of Public Health Laboratories|Blanton|USA

>Spike|hCoV-19/Wales/PHWC-15D2A3/2020|2020-05-06|EPI_ISL_472694|Original|hCoV-19^^Wales|Human|Wales Specialist Virology Centre Sequencing lab|COVID-19 Genomics UK (COG-UK) Consortium|Moore|United Kingdom
>Spike|hCoV-19/Scotland/CVR4442/2020|2020-09-19|EPI_ISL_585311|Original|hCoV-19^^Scotland|Human|Lighthouse Lab in Glasgow / MRC-University of Glasgow Centre for Virus Research|COVID-19 Genomics UK (COG-UK) Consortium|Robertson|United Kingdom
>Spike|hCoV-19/Canada/BC_04975041/2020|2020-04-00|EPI_ISL_466774|Original|hCoV-19^^British Columbia|Human|BCCDC Public Health Laboratory|BCCDC Public Health Laboratory|Harrigan|Canada
>Spike|hCoV-19/Scotland/CVR3381/2020|2020-05-07|EPI_ISL_456810|Original|hCoV-19^^Scotland|Human|West of Scotland Specialist Virology Centre|COVID-19 Genomics UK (COG-UK) Consortium|Robertson|United Kingdom
>Spike|hCoV-19/Serbia/Pancevo-075362804/2020|2020-04-27|EPI_ISL_676600|Original|hCoV-19^^Pancevo|Human|Scientific Veterinary Institute Novi Sad|Veterinary Specialized Institute \Kraljevo\|Vidanovic|Serbia
>Spike|hCoV-19/England/NOTT-121554/2020|2020-12-16|EPI_ISL_741640|Original|hCoV-19^^England|Human|Queens Medical Centre|COVID-19 Genomics UK (COG-UK) Consortium|Loose|United Kingdom
>Spike|hCoV-19/USA/AZ-TG371301/2020|2020-05-19|EPI_ISL_694556|Original|hCoV-19^^Arizona|Human|AZ SPHL|TGen North|French|USA
>Spike|hCoV-19/Australia/NSW1362/2020|2020-12-30|EPI_ISL_767928|Original|hCoV-19^^New South Wales|Human|South Eastern Area Laboratory Services (SEALS)|NSW Health Pathology - Institute of Clinical Pathology and Medical Research; Westmead Hospital; University of Sydney|Gall|Australia
>Spike|hCoV-19/England/BRIS-12F4FF/2020|2020-04-12|EPI_ISL_488411|Original|hCoV-19^^England|Human|PHE South West Regional Laboratory|Wellcome Sanger Institute for the COVID-19 Genomics UK (COG-UK) Consortium|Davies|United Kingdom
>Spike|hCoV-19/USA/MN-GMF-30816/2020|2020-11-02|EPI_ISL_661055|Original|hCoV-19^^Minnesota|Human|Gundersen Molecular Diagnostics Laboratory|Kabara Cancer Research Institute|Kenny|USA
>Spike|hCoV-19/Switzerland/VD-ETHZ-320578/2020|2020-10-12|EPI_ISL_737899|Original|hCoV-19^^Vaud|Human|Viollier AG|Department of Biosystems Science and Engineering|Nadeau|Switzerland
>Spike|hCoV-19/USA/LA-EVTL1047/2020|2020-08-25|EPI_ISL_653737|Original|hCoV-19^^Louisiana|Human|LSUHS Emerging Viral Threat Laboratory|Microbial Genome Sequencing Center|Kamil|USA
>Spike|hCoV-19/USA/FL-BPHL-1916/2020|2020-07-20|EPI_ISL_613777|Original|hCoV-19^^Florida|Human|Florida Bureau of Public Health Laboratories|Florida Bureau of Public Health Laboratories|Blanton|USA
>Spike|hCoV-19/England/EXET-139234/2020|2020-10-26|EPI_ISL_664318|Original|hCoV-19^^England|Human|University of Exeter|COVID-19 Genomics UK (COG-UK) Consortium|Temperton|United Kingdom
>Spike|hCoV-19/USA/NY-Wadsworth-280806-01/2020|2020-11-30|EPI_ISL_754507|Original|hCoV-19^^New York|Human|Wadsworth Center|Wadsworth Center|Lasek-Nesselquist|USA
>Spike|hCoV-19/Scotland/EDB100/2020|2020-03-20|EPI_ISL_425890|Original|hCoV-19^^Scotland|Human|Virology Department|COVID-19 Genomics UK (COG-UK) Consortium|Williams|United Kingdom
>Spike|hCoV-19/England/ALDP-9C5727/2020|2020-09-10|EPI_ISL_567856|Original|hCoV-19^^England|Human|Lighthouse Lab in Alderley Park|Wellcome Sanger Institute for the COVID-19 Genomics UK (COG-UK) consortium|Davies|United Kingdom
>Spike|hCoV-19/Russia/MOW-RII-MH3352S/2020|2020-10-07|EPI_ISL_733099|Original|hCoV-19^^Moscow|Human|HELIX LLC|WHO National Influenza Centre Russian Federation|Komissarov|Russia
>Spike|hCoV-19/England/LIVE-A686E/2020|2020-03-15|EPI_ISL_499747|Original|hCoV-19^^England|Human|Liverpool Clinical Laboratories|COVID-19 Genomics UK (COG-UK) Consortium|Haldenby|United Kingdom
>Spike|hCoV-19/Scotland/QEUH-AAE7F6/2020|2020-10-22|EPI_ISL_644099|Original|hCoV-19^^Scotland|Human|Lighthouse Lab in Glasgow|Wellcome Sanger Institute for the COVID-19 Genomics UK (COG-UK) Consortium|Davies|United Kingdom

>Spike|hCoV-19/Wales/PHWC-16CE61/2020|2020-09-27|EPI_ISL_572605|Original|hCoV-19^^Wales|Human|Wales Specialist Virology Centre Sequencing lab|COVID-19 Genomics UK (COG-UK) Consortium|Moore|United Kingdom
>Spike|hCoV-19/South Korea/KDCA0107/2020|2020-09-25|EPI_ISL_747343|Original|hCoV-19^^South Korea|Human|Division of Emerging Infectious Diseases|Division of Emerging Infectious Diseases|Park|South Korea
>Spike|hCoV-19/France/BRE-8951/2020|2020-08-07|EPI_ISL_560618|Original|hCoV-19^^Bretagne|Human|Hospital|National Reference Center for Viruses of Respiratory Infections|Enouf|France
>Spike|hCoV-19/USA/TX-HMH-MCoV-19797/2020|2020-11-27|EPI_ISL_784133|Original|hCoV-19^^Texas|Human|Houston Methodist Hospital|Houston Methodist Hospital|Long|USA
>Spike|hCoV-19/England/CAMC-9424A3/2020|2020-07-21|EPI_ISL_556881|Original|hCoV-19^^England|Human|Lighthouse Lab in Cambridge|Wellcome Sanger Institute for the COVID-19 Genomics UK (COG-UK) consortium|Davies|United Kingdom
>Spike|hCoV-19/Japan/IC-0334/2020|2020-11-00|EPI_ISL_721581|Original|hCoV-19^^Japan|Human|Pathogen Genomics Center|Pathogen Genomics Center|Sekizuka|Japan
>Spike|hCoV-19/England/QEUH-AE51B9/2020|2020-10-27|EPI_ISL_633213|Original|hCoV-19^^England|Human|Lighthouse Lab in Glasgow|Wellcome Sanger Institute for the COVID-19 Genomics UK (COG-UK) consortium|Davies|United Kingdom
>Spike|hCoV-19/USA/TX-DSHS-1157/2020|2020-07-03|EPI_ISL_634958|Original|hCoV-19^^Texas|Human|Texas Department of State Health Services|Texas Department of State Health Services|Rahman|USA
>Spike|hCoV-19/England/ALDP-B24812/2020|2020-11-04|EPI_ISL_646790|Original|hCoV-19^^England|Human|Lighthouse Lab in Alderley Park|Wellcome Sanger Institute for the COVID-19 Genomics UK (COG-UK) Consortium|Davies|United Kingdom
>Spike|hCoV-19/England/NORW-E7E9C/2020|2020-05-06|EPI_ISL_449093|Original|hCoV-19^^England|Human|Quadram Institute Bioscience|COVID-19 Genomics UK (COG-UK) Consortium|Alikhan|United Kingdom
>Spike|hCoV-19/Finland/9LS18AH6/2020|2020-10-09|EPI_ISL_755954|Original|hCoV-19^^Uusimaa|Human|Department of Virology and Immunology|Department of Virology|Smura|Finland
>Spike|hCoV-19/USA/CA-LAC-USC23/2020-06-22|2020-06-22|EPI_ISL_569686|Original|hCoV-19^^California|Human|Lee Lab|Lee Lab|Lee|USA
>Spike|hCoV-19/USA/CA-CDC-4735/2020|2020-06-16|EPI_ISL_535704|Original|hCoV-19^^California|Human|CDPH|Pathogen Discovery|Queen|USA
>Spike|hCoV-19/Scotland/GCVR-172DB6/2020|2020-04-06|EPI_ISL_459162|Original|hCoV-19^^Scotland|Human|NHSGGC West of Scotland Specialist Virology Centre / MRC-University of Glasgow Centre for Virus Research|Wellcome Sanger Institute for the COVID-19 Genomics UK (COG-UK) consortium|Davies|United Kingdom
>Spike|hCoV-19/Denmark/DCGC-6301/2020|2020-08-31|EPI_ISL_616399|Original|hCoV-19^^Syddanmark|Human|Department of Virus and Microbiological Special Diagnostics|Albertsen lab|Michaelsen|Denmark
>Spike|hCoV-19/USA/AZ-TG648591/2020|2020-04-27|EPI_ISL_695843|Original|hCoV-19^^Arizona|Human|Sonora Quest Laboratories|TGen North|French|USA
>Spike|hCoV-19/England/QEUH-9BA822/2020|2020-09-09|EPI_ISL_573604|Original|hCoV-19^^England|Human|Oxford Viromics|COVID-19 Genomics UK (COG-UK) Consortium|Golubchik|United Kingdom
>Spike|hCoV-19/Japan/PG-3598/2020|2020-07-00|EPI_ISL_691852|Original|hCoV-19^^Japan|Human|Pathogen Genomics Center|Pathogen Genomics Center|Sekizuka|Japan
>Spike|hCoV-19/England/ALDP-A8730E/2020|2020-10-14|EPI_ISL_605932|Original|hCoV-19^^England|Human|Lighthouse Lab in Alderley Park|Wellcome Sanger Institute for the COVID-19 Genomics UK (COG-UK) consortium|Davies|United Kingdom
>Spike|hCoV-19/England/ALDP-A6DE1C/2020|2020-10-15|EPI_ISL_607134|Original|hCoV-19^^England|Human|Lighthouse Lab in Alderley Park|Wellcome Sanger Institute for the COVID-19 Genomics UK (COG-UK) consortium|Davies|United Kingdom
>Spike|hCoV-19/Japan/Donner8/2020|2020-04-06|EPI_ISL_438953|Original|hCoV-19^^Kanto|Human|Keio University School of Medicine|Keio University School of Medicine|Kosaki|Japan
>Spike|hCoV-19/England/NORT-289C81/2020|2020-04-04|EPI_ISL_453514|Original|hCoV-19^^England|Human|Northumbria University / South Tees Hospitals NHS Foundation Trust

/ North Cumbria Integrated Care NHS Foundation Trust / North Tees and Hartlepool NHS Foundation Trust / Newcastle Hospitals NHS Foundation Trust|COVID-19 Genomics UK (COG-UK) Consortium|Bashton|United Kingdom
>Spike|hCoV-19/England/BRIS-12FC9D/2020|2020-04-07|EPI_ISL_492688|Original|hCoV-19^^England|Human|PHE South West Regional Laboratory|Wellcome Sanger Institute for the COVID-19 Genomics UK (COG-UK) consortium|Davies|United Kingdom
>Spike|hCoV-19/India/DL-59108-CSIR-IGIB/2020|2020-08-04|EPI_ISL_636809|Original|hCoV-19^^Delhi|Human|National Centre for Disease control (NCDC)|NCDC/CSIR-IGIB|Faruq|India
>Spike|hCoV-19/USA/LA-EVTL682/2020|2020-07-13|EPI_ISL_653374|Original|hCoV-19^^Louisiana|Human|LSUHS Emerging Viral Threat Laboratory|Microbial Genome Sequencing Center|Kamil|USA
>Spike|hCoV-19/United Arab Emirates/1051/2020|2020-06-11|EPI_ISL_698126|Original|hCoV-19^^Abu Dhabi|Human|Group 42 (G42) Healthcare|G42 Healthcare|Ogrodzki|United Arab Emirates
>Spike|hCoV-19/Australia/VIC364/2020|2020-03-22|EPI_ISL_426672|Original|hCoV-19^^Victoria|Human|Victorian Infectious Diseases Reference Laboratory (VIDRL)|Microbiological Diagnostic Unit Public Health Laboratory and Victorian Infectious Diseases Reference Laboratory|Schultz|Australia
>Spike|hCoV-19/USA/UT-UPHL-2009663/2020|2020-07-26|EPI_ISL_605338|Original|hCoV-19^^Utah|Human|Utah Public Health Laboratory|Utah Public Health Laboratory|Young|USA
>Spike|hCoV-19/Singapore/108/2020|2020-03-18|EPI_ISL_443200|Original|hCoV-19^^Singapore|Human|National Public Health Laboratory|National Public Health Laboratory|Mak|Singapore
>Spike|hCoV-19/USA/WA-UW-23193/2020|2020-08-06|EPI_ISL_570754|Original|hCoV-19^^Washington|Human|UW Virology Lab|UW Virology Lab|Roychoudhury|USA
>Spike|hCoV-19/England/ALDP-923772/2020|2020-06-23|EPI_ISL_551377|Original|hCoV-19^^England|Human|Lighthouse Lab in Alderley Park|Wellcome Sanger Institute for the COVID-19 Genomics UK (COG-UK) consortium|Davies|United Kingdom
>Spike|hCoV-19/USA/NV-NSPHL-A0043/2020|2020-03-23|EPI_ISL_515326|Original|hCoV-19^^Nevada|Human|Nevada State Public Health Laboratory|Nevada State Public Health Laboratory|Sevinsky|USA
>Spike|hCoV-19/USA/MI-UM-10036689863/2020|2020-10-29|EPI_ISL_682119|Original|hCoV-19^^Michigan|Human|University of Michigan Clinical Microbiology Laboratory|Lauring Lab|Valesano|USA
>Spike|hCoV-19/Germany/HH-hpi-p2027/2020|2020-03-21|EPI_ISL_776329|Original|hCoV-19^^Hamburg|Human|University Medical Center Hamburg Eppendorf|Heinrich Pette Institute|Robitaille|Germany
>Spike|hCoV-19/England/ALDP-9524DD/2020|2020-06-09|EPI_ISL_555663|Original|hCoV-19^^England|Human|Lighthouse Lab in Alderley Park|Wellcome Sanger Institute for the COVID-19 Genomics UK (COG-UK) consortium|Davies|United Kingdom
>Spike|hCoV-19/Denmark/DCGC-9727/2020|2020-11-09|EPI_ISL_669131|Original|hCoV-19^^Midtjylland|Human|Department of Virus and Microbiological Special Diagnostics|Albertsen Lab|Michaelsen|Denmark
>Spike|hCoV-19/USA/OR-OHSU-1013/2020|2020-07-08|EPI_ISL_525947|Original|hCoV-19^^Oregon|Human|OHSU Lab Services Molecular Microbiology Lab|Oregon SARS-CoV-2 Genome Sequencing Center|O'Roak|USA
>Spike|hCoV-19/England/CAMB-A97E64/2020|2020-12-12|EPI_ISL_764334|Original|hCoV-19^^England|Human|Department of Pathology|COVID-19 Genomics UK (COG-UK) Consortium|Goodfellow|United Kingdom
>Spike|hCoV-19/Denmark/DCGC-17026/2020|2020-12-14|EPI_ISL_750718|Original|hCoV-19^^Sjaelland|Human|Department of Virus and Microbiological Special Diagnostics|Albertsen Lab|Michaelsen|Denmark
>Spike|hCoV-19/Denmark/DCGC-7679/2020|2020-10-19|EPI_ISL_618610|Original|hCoV-19^^Syddanmark|Human|Department of Virus and Microbiological Special Diagnostics|Albertsen lab|Michaelsen|Denmark
>Spike|hCoV-19/Portugal/PT0568a/2020|2020-04-09|EPI_ISL_511481|Original|hCoV-19^^Portugal|Human|Instituto Nacional de Saude (INSA)|Instituto Nacional de Saude (INSA)|Borges|Portugal
>Spike|hCoV-19/Morocco/6888/2020|2020-03-15|EPI_ISL_459966|Original|hCoV-19^^Morocco|Human|Institut Pasteur du Maroc|Institut Pasteur du Maroc|Enouf|Morocco

>Spike|hCoV-19/Australia/WA385/2020|2020-05-23|EPI_ISL_605861|Original|hCoV-19^^Western Australia|Human|PathWest Laboratory Medicine WA|PathWest Laboratory Medicine WA Microbial Surveillance Unit|Sikazwe|Australia
>Spike|hCoV-19/England/MILK-956C3B/2020|2020-06-10|EPI_ISL_554289|Original|hCoV-19^^England|Human|Lighthouse Lab in Milton Keynes|Wellcome Sanger Institute for the COVID-19 Genomics UK (COG-UK) consortium|Davies|United Kingdom
>Spike|hCoV-19/USA/CA-ALSR-2838/2020|2020-08-05|EPI_ISL_635705|Original|hCoV-19^^California|Human|San Diego County Public Health Laboratory|Andersen lab at Scripps Research|Gangavarapu|USA
>Spike|hCoV-19/Wales/PHWC-2C138/2020|2020-04-04|EPI_ISL_474279|Original|hCoV-19^^Wales|Human|Originating lab|COVID-19 Genomics UK (COG-UK) Consortium|Moore|United Kingdom
>Spike|hCoV-19/England/CAMC-C721FB/2020|2020-12-15|EPI_ISL_769108|Original|hCoV-19^^England|Human|Lighthouse Lab in Cambridge|Wellcome Sanger Institute for the COVID-19 Genomics UK (COG-UK) Consortium|Davies|United Kingdom
>Spike|hCoV-19/DRC/2122/2020|2020-04-13|EPI_ISL_435165|Original|hCoV-19^^Kinshasa|Human|Viral Respiratory Lab|Pathogen Sequencing Lab|Pratt|Democratic Republic of the Congo
>Spike|hCoV-19/USA/UT-UPHL-2012803086/2020|2020-07-27|EPI_ISL_745644|Original|hCoV-19^^Utah|Human|Ginkgo Bioworks Clinical Laboratory|Utah Public Health Laboratory|Young|USA
>Spike|hCoV-19/Denmark/DCGC-8207/2020|2020-10-26|EPI_ISL_617276|Original|hCoV-19^^Syddanmark|Human|Department of Virus and Microbiological Special Diagnostics|Albertsen lab|Michaelsen|Denmark
>Spike|hCoV-19/England/QEUH-C03EBC/2020|2020-12-04|EPI_ISL_720733|Original|hCoV-19^^England|Human|Lighthouse Lab in Glasgow|Wellcome Sanger Institute for the COVID-19 Genomics UK (COG-UK) Consortium|Davies|United Kingdom
>Spike|hCoV-19/England/GSTT-265DF92/2020|2020-03-29|EPI_ISL_484132|Original|hCoV-19^^England|Human|Centre for Clinical Infection and Diagnostics Research and Genomics Innovation Unit|COVID-19 Genomics UK (COG-UK) Consortium|Awan|United Kingdom
>Spike|hCoV-19/Zimbabwe/ZW-9953/2020|2020-06-01|EPI_ISL_644820|Original|hCoV-19^^Zimbabwe|Human|National Microbiology Reference Laboratory|Quadram Institute Bioscience|Alikhan|Zimbabwe
>Spike|hCoV-19/England/MILK-C2FAB6/2020|2020-12-07|EPI_ISL_733820|Original|hCoV-19^^England|Human|Lighthouse Lab in Milton Keynes|Wellcome Sanger Institute for the COVID-19 Genomics UK (COG-UK) Consortium|Davies|United Kingdom
>Spike|hCoV-19/England/201440136/2020|2020-03-31|EPI_ISL_465983|Original|hCoV-19^^England|Human|Respiratory Virus Unit|Respiratory Virus Unit|Platt|United Kingdom
>Spike|hCoV-19/England/LIVE-99B47/2020|2020-04-13|EPI_ISL_439801|Original|hCoV-19^^England|Human|Liverpool Clinical Laboratories|COVID-19 Genomics UK (COG-UK) Consortium|Haldenby|United Kingdom
>Spike|hCoV-19/Denmark/DCGC-18849/2020|2020-12-07|EPI_ISL_748596|Original|hCoV-19^^Midtjylland|Human|Department of Virus and Microbiological Special Diagnostics|Albertsen Lab|Michaelsen|Denmark
>Spike|hCoV-19/Canada/QC-LSPQ-L00237972/2020|2020-03-25|EPI_ISL_536202|Original|hCoV-19^^Quebec|Human|H\u00f4pital du Suro\u00eet|Laboratoire de sant\u00e9 publique du Qu\u00e9bec|Moreira|Canada
>Spike|hCoV-19/England/MILK-974A1F/2020|2020-08-21|EPI_ISL_575918|Original|hCoV-19^^England|Human|Lighthouse Lab in Milton Keynes|Wellcome Sanger Institute for the COVID-19 Genomics UK (COG-UK) consortium|Davies|United Kingdom
>Spike|hCoV-19/England/CAMC-B77986/2020|2020-11-11|EPI_ISL_661917|Original|hCoV-19^^England|Human|Lighthouse Lab in Cambridge|Wellcome Sanger Institute for the COVID-19 Genomics UK (COG-UK) Consortium|Davies|United Kingdom
>Spike|hCoV-19/USA/MI-QDX-885/2020|2020-03-18|EPI_ISL_571606|Original|hCoV-19^^Michigan|Human|Quest Diagnostics|Quest Diagnostics|Kagan|USA
>Spike|hCoV-19/Wales/PHWC-492940/2020|2020-11-19|EPI_ISL_725723|Original|hCoV-19^^Wales|Human|Originating lab|COVID-19 Genomics UK (COG-UK) Consortium|Moore|United Kingdom

>Spike|hCoV-19/England/CAMB-1ABA35/2020|2020-04-21|EPI_ISL_470135|Original|hCoV-19^^England|Human|Department of Pathology|Wellcome Sanger Institute for the COVID-19 Genomics UK (COG-UK) consortium|Davies|United Kingdom
>Spike|hCoV-19/USA/WA-S138/2020|2020-03-12|EPI_ISL_430175|Original|hCoV-19^^Washington|Human|Washington State Department of Health|Seattle Flu Study|Bedford|USA
>Spike|hCoV-19/Canada/ON-E77/2020|2020-05-13|EPI_ISL_593550|Original|hCoV-19^^Ontario|Human|Eastern Ontario Regional Laboratory Association|McMaster University|McArthur|Canada
>Spike|hCoV-19/England/LIVE-1D7274/2020|2020-09-26|EPI_ISL_584600|Original|hCoV-19^^England|Human|Liverpool Clinical Laboratories|COVID-19 Genomics UK (COG-UK) Consortium|Haldenby|United Kingdom
>Spike|hCoV-19/Netherlands/NH-EMC-79/2020|2020-09-02|EPI_ISL_632526|Original|hCoV-19^^Noord Holland|Human|Dutch COVID-19 response team|Erasmus Medical Center|Oude Munnink|Netherlands
>Spike|hCoV-19/England/ALDP-95018D/2020|2020-06-17|EPI_ISL_555157|Original|hCoV-19^^England|Human|Lighthouse Lab in Alderley Park|Wellcome Sanger Institute for the COVID-19 Genomics UK (COG-UK) consortium|Davies|United Kingdom
>Spike|hCoV-19/USA/MN-CDC-6214/2020|2020-04-24|EPI_ISL_527675|Original|hCoV-19^^Minnesota|Human|MN PHL Division|Pathogen Discovery|Queen|USA
>Spike|hCoV-19/Canada/ON-S1415/2020|2020-08-23|EPI_ISL_591186|Original|hCoV-19^^Ontario|Human|Toronto Invasive Bacterial Diseases Network|McMaster University|McArthur|Canada
>Spike|hCoV-19/Wales/PHWC-48544F/2020|2020-10-21|EPI_ISL_626829|Original|hCoV-19^^Wales|Human|Wales Specialist Virology Centre Sequencing lab|COVID-19 Genomics UK (COG-UK) Consortium|Moore|United Kingdom
>Spike|hCoV-19/England/SHEF-D0EE1/2020|2020-04-25|EPI_ISL_479274|Original|hCoV-19^^England|Human|Virology Department|COVID-19 Genomics UK (COG-UK) Consortium|de Silva|United Kingdom
>Spike|hCoV-19/Belgium/ULG-11038/2020|2020-11-30|EPI_ISL_722952|Original|hCoV-19^^Liege|Human|Department of Clinical Microbiology|GIGA Medical Genomics|Durkin|Belgium
>Spike|hCoV-19/USA/UT-UPHL-2012955167/2020|2020-08-17|EPI_ISL_746173|Original|hCoV-19^^Utah|Human|Ginkgo Bioworks Clinical Laboratory|Utah Public Health Laboratory|Young|USA
>Spike|hCoV-19/Luxembourg/LNS9737544/2020|2020-11-25|EPI_ISL_744975|Original|hCoV-19^^Luxembourg|Human|Laboratoire national de sant\u00e9|Laboratoire national de sant\u00e9|Wienecke-Baldacchino|Luxembourg
>Spike|hCoV-19/Scotland/CVR3502/2020|2020-05-10|EPI_ISL_456887|Original|hCoV-19^^Scotland|Human|West of Scotland Specialist Virology Centre|COVID-19 Genomics UK (COG-UK) Consortium|Robertson|United Kingdom
>Spike|hCoV-19/Slovakia/UKBA-609/2020|2020-11-30|EPI_ISL_788986|Original|hCoV-19^^Bratislava|Human|Institute of Virology|Faculty of Natural Sciences|Vinar|Slovakia
>Spike|hCoV-19/England/QEUH-B0F877/2020|2020-11-02|EPI_ISL_642008|Original|hCoV-19^^England|Human|Lighthouse Lab in Glasgow|Wellcome Sanger Institute for the COVID-19 Genomics UK (COG-UK) Consortium|Davies|United Kingdom
>Spike|hCoV-19/USA/NY-Wadsworth-247373-01/2020|2020-10-16|EPI_ISL_734354|Original|hCoV-19^^New York|Human|Wadsworth Center|Wadsworth Center|Lasek-Nesselquist|USA
>Spike|hCoV-19/USA/DE-CDC-2-3713996/2020|2020-10-21|EPI_ISL_751726|Original|hCoV-19^^Delaware|Human|DE Public Health Laboratory|Genomics and Discovery|Queen|USA
>Spike|hCoV-19/Wales/PHWC-483B00/2020|2020-10-20|EPI_ISL_627895|Original|hCoV-19^^Wales|Human|Wales Specialist Virology Centre Sequencing lab|COVID-19 Genomics UK (COG-UK) Consortium|Moore|United Kingdom
>Spike|hCoV-19/England/NORW-EE69D/2020|2020-04-24|EPI_ISL_490555|Original|hCoV-19^^England|Human|Quadram Institute Bioscience|COVID-19 Genomics UK (COG-UK) Consortium|Alikhan|United Kingdom
>Spike|hCoV-19/Wales/PHWC-492F6C/2020|2020-11-28|EPI_ISL_725813|Original|hCoV-19^^Wales|Human|Originating lab|COVID-19 Genomics UK (COG-UK) Consortium|Moore|United Kingdom

>Spike|hCoV-19/Vietnam/39607/2020|2020-03-07|EPI_ISL_416428|Vero|hCoV-19^^Quangning|Human|National Influenza Center|National Influenza Center|Ung|Vietnam
>Spike|hCoV-19/Wales/PHWC-2559D/2020|2020-03-23|EPI_ISL_421007|Original|hCoV-19^^Wales|Human|Wales Specialist Virology Centre|Public Health Wales Microbiology Cardiff|Moore|United Kingdom
>Spike|hCoV-19/Netherlands/Limburg_61/2020|2020-04-25|EPI_ISL_461047|Original|hCoV-19^^Limburg|Human|Dutch COVID-19 response team|Erasmus Medical Center|Oude Munnink|Netherlands
>Spike|hCoV-19/Spain/NC-IBV-001422/2020|2020-03-22|EPI_ISL_452479|Original|hCoV-19^^Navarra|Human|Cl\u00ednica Universidad de Navarra. Servicio de Enfermedades Infecciosas y Microbiolog\u00eda cl\u00ednica|SeqCOVID-SPAIN consortium/IBV(CSIC)|Comas|Spain
>Spike|hCoV-19/England/20119040106/2020|2020-03-12|EPI_ISL_465056|Original|hCoV-19^^England|Human|Respiratory Virus Unit|Respiratory Virus Unit|Platt|United Kingdom
>Spike|hCoV-19/England/CAMC-B0A44B/2020|2020-10-29|EPI_ISL_673062|Original|hCoV-19^^England|Human|Lighthouse Lab in Cambridge|Wellcome Sanger Institute for the COVID-19 Genomics UK (COG-UK) Consortium|Davies|United Kingdom
>Spike|hCoV-19/England/CAMB-7903F/2020|2020-04-03|EPI_ISL_441785|Original|hCoV-19^^England|Human|Department of Pathology|Wellcome Sanger Institute for the COVID-19 Genomics UK (COG-UK) consortium|Amato|United Kingdom
>Spike|hCoV-19/England/MILK-ACD66D/2020|2020-10-20|EPI_ISL_634283|Original|hCoV-19^^England|Human|Lighthouse Lab in Milton Keynes|Wellcome Sanger Institute for the COVID-19 Genomics UK (COG-UK) consortium|Davies|United Kingdom
>Spike|hCoV-19/Scotland/QEUH-AD13C9/2020|2020-10-27|EPI_ISL_649722|Original|hCoV-19^^Scotland|Human|Lighthouse Lab in Glasgow|Wellcome Sanger Institute for the COVID-19 Genomics UK (COG-UK) Consortium|Davies|United Kingdom
>Spike|hCoV-19/England/QEUH-B7F6A5/2020|2020-11-14|EPI_ISL_668301|Original|hCoV-19^^England|Human|Lighthouse Lab in Glasgow|Wellcome Sanger Institute for the COVID-19 Genomics UK (COG-UK) Consortium|Davies|United Kingdom
>Spike|hCoV-19/USA/MI-MDHHS-SC20964/2020|2020-03-27|EPI_ISL_507752|Original|hCoV-19^^Michigan|Human|Michigan Department of Health and Human Services|Michigan Department of Health and Human Services|Blankenship|USA
>Spike|hCoV-19/England/QEUH-888396/2020|2020-07-24|EPI_ISL_532794|Original|hCoV-19^^England|Human|Lighthouse Lab in Glasgow|Wellcome Sanger Institute for the COVID-19 Genomics UK (COG-UK) consortium|Davies|United Kingdom
>Spike|hCoV-19/USA/TX-HMH-608/2020|2020-04-10|EPI_ISL_547217|Original|hCoV-19^^Texas|Human|Houston Methodist Hospital|Houston Methodist Hospital|Long|USA
>Spike|hCoV-19/Northern Ireland/QEUH-96CC32/2020|2020-08-21|EPI_ISL_530678|Original|hCoV-19^^Northern Ireland|Human|Lighthouse Lab in Glasgow|Wellcome Sanger Institute for the COVID-19 Genomics UK (COG-UK) consortium|Davies|United Kingdom
>Spike|hCoV-19/England/QEUH-A61953/2020|2020-10-12|EPI_ISL_628644|Original|hCoV-19^^England|Human|Oxford Viromics|COVID-19 Genomics UK (COG-UK) Consortium|Golubchik|United Kingdom
>Spike|hCoV-19/England/MILK-CA6A02/2020|2020-12-17|EPI_ISL_761410|Original|hCoV-19^^England|Human|Lighthouse Lab in Milton Keynes|Wellcome Sanger Institute for the COVID-19 Genomics UK (COG-UK) Consortium|Davies|United Kingdom
>Spike|hCoV-19/USA/CA-CZB-2058/2020|2020-04-22|EPI_ISL_486301|Original|hCoV-19^^California|Human|San Joaquin County Public Health Lab|Chan-Zuckerberg Biohub|McGeever|USA
>Spike|hCoV-19/Wales/PHWC-4A243D/2020|2020-12-14|EPI_ISL_743889|Original|hCoV-19^^Wales|Human|Wales Specialist Virology Centre Sequencing lab|COVID-19 Genomics UK (COG-UK) Consortium|Moore|United Kingdom
>Spike|hCoV-19/Germany/HE-FFM5/2020|2020-03-02|EPI_ISL_452221|Caco-2|hCoV-19^^Hesse|Human|Goethe University Hospital Frankfurt|Institute for Medical Virology|Toptan Grabmair|Germany
>Spike|hCoV-19/England/LOND-D3613/2020|2020-04-02|EPI_ISL_440965|Original|hCoV-19^^England|Human|University College London|COVID-19 Genomics UK (COG-UK) Consortium|Roy|United Kingdom

>Spike|hCoV-19/England/MILK-C51936/2020|2020-12-11|EPI_ISL_769561|Original|hCoV-19^^England|Human|Lighthouse Lab in Milton Keynes|Wellcome Sanger Institute for the COVID-19 Genomics UK (COG-UK) Consortium|Davies|United Kingdom
>Spike|hCoV-19/England/QEUH-A5E4EF/2020|2020-10-12|EPI_ISL_597275|Original|hCoV-19^^England|Human|Lighthouse Lab in Glasgow|Wellcome Sanger Institute for the COVID-19 Genomics UK (COG-UK) consortium|Davies|United Kingdom
>Spike|hCoV-19/Switzerland/BL-ETHZ-260162/2020|2020-08-27|EPI_ISL_539455|Original|hCoV-19^^Basel-Landschaft|Human|Viollier AG|Department of Biosystems Science and Engineering|Nadeau|Switzerland
>Spike|hCoV-19/England/CAMB-7F154/2020|2020-04-08|EPI_ISL_442889|Original|hCoV-19^^England|Human|Department of Pathology|Wellcome Sanger Institute for the COVID-19 Genomics UK (COG-UK) consortium|Amato|United Kingdom
>Spike|hCoV-19/England/LIVE-DC1C37/2020|2020-11-20|EPI_ISL_704131|Original|hCoV-19^^England|Human|Liverpool Clinical Laboratories|COVID-19 Genomics UK (COG-UK) Consortium|Haldenby|United Kingdom
>Spike|hCoV-19/USA/TX-HMH-MCoV-19083/2020|2020-11-25|EPI_ISL_783615|Original|hCoV-19^^Texas|Human|Houston Methodist Hospital|Houston Methodist Hospital|Long|USA
>Spike|hCoV-19/England/SHEF-D0124/2020|2020-03-26|EPI_ISL_442466|Original|hCoV-19^^England|Human|Virology Department|COVID-19 Genomics UK (COG-UK) Consortium|de Silva|United Kingdom
>Spike|hCoV-19/Hungary/MH-7051/2020|2020-05-14|EPI_ISL_526222|Original|hCoV-19^^Pest county|Human|Hungarian Defence Forces Military Medical Centre|National Laboratory of Virology|Kemenesi|Hungary
>Spike|hCoV-19/Denmark/DCGC-8549/2020|2020-10-26|EPI_ISL_625897|Original|hCoV-19^^Midtjylland|Human|Department of Virus and Microbiological Special Diagnostics|Albertsen lab|Michaelsen|Denmark
>Spike|hCoV-19/England/BRIS-130B7E/2020|2020-03-13|EPI_ISL_492557|Original|hCoV-19^^England|Human|PHE South West Regional Laboratory|Wellcome Sanger Institute for the COVID-19 Genomics UK (COG-UK) consortium|Davies|United Kingdom
>Spike|hCoV-19/Japan/PG-8886/2020|2020-08-00|EPI_ISL_686432|Original|hCoV-19^^Japan|Human|Pathogen Genomics Center|Pathogen Genomics Center|Sekizuka|Japan
>Spike|hCoV-19/England/ALDP-B84F84/2020|2020-11-15|EPI_ISL_674390|Original|hCoV-19^^England|Human|Lighthouse Lab in Alderley Park|Wellcome Sanger Institute for the COVID-19 Genomics UK (COG-UK) Consortium|Davies|United Kingdom
>Spike|hCoV-19/India/WB-S93/2020|2020-06-02|EPI_ISL_508482|Original|hCoV-19^^West Bengal|Human|ICMR-National Institute of Cholera and Enteric Diseases|National Institute of Biomedical Genomics|Maitra|India
>Spike|hCoV-19/England/CAMB-81E3A/2020|2020-04-21|EPI_ISL_438583|Original|hCoV-19^^England|Human|Department of Pathology|COVID-19 Genomics UK (COG-UK) Consortium|Meredith|United Kingdom
>Spike|hCoV-19/England/201060300/2020|2020-03-03|EPI_ISL_464401|Original|hCoV-19^^England|Human|Respiratory Virus Unit|Respiratory Virus Unit|Platt|United Kingdom
>Spike|hCoV-19/England/CAMB-782A3/2020|2020-04-04|EPI_ISL_433711|Original|hCoV-19^^England|Human|Department of Pathology|COVID-19 Genomics UK (COG-UK) Consortium|Meredith|United Kingdom
>Spike|hCoV-19/USA/AZ-TG491626/2020|2020-06-24|EPI_ISL_695196|Original|hCoV-19^^Arizona|Human|AZ SPHL|TGen North|French|USA
>Spike|hCoV-19/Denmark/DCGC-16967/2020|2020-12-14|EPI_ISL_750674|Original|hCoV-19^^Midtjylland|Human|Department of Virus and Microbiological Special Diagnostics|Albertsen Lab|Michaelsen|Denmark
>Spike|hCoV-19/Canada/BC-BCCDC-8413/2020|2020-10-00|EPI_ISL_740785|Original|hCoV-19^^British Columbia|Human|BCCDC Public Health Laboratory|BCCDC Public Health Laboratory|Prystajecky|Canada
>Spike|hCoV-19/Scotland/QEUH-9FA950/2020|2020-09-29|EPI_ISL_600612|Original|hCoV-19^^Scotland|Human|Lighthouse Lab in Glasgow|Wellcome Sanger Institute for the COVID-19 Genomics UK (COG-UK) consortium|Davies|United Kingdom
>Spike|hCoV-19/Denmark/DCGC-11470/2020|2020-11-02|EPI_ISL_670936|Original|hCoV-19^^Nordjylland|Human|Department of Virus and Microbiological Special Diagnostics|Albertsen Lab|Michaelsen|Denmark

>Spike|hCoV-19/Wales/PHWC-490F04/2020|2020-11-19|EPI_ISL_705435|Original|hCoV-19^^Wales|Human|Originating lab|COVID-19 Genomics UK (COG-UK) Consortium|Moore|United Kingdom
>Spike|hCoV-19/Australia/VIC8323/2020|2020-08-12|EPI_ISL_564939|Original|hCoV-19^^Victoria|Human|Microbiological Diagnostic Unit - Public Health Laboratory (MDU-PHL)|MDU-PHL|Schultz|Australia
>Spike|hCoV-19/India/TG-CCMB-AB966/2020|2020-08-17|EPI_ISL_539708|Original|hCoV-19^^Telangana|Human|CSIR-Centre for Cellular and Molecular Biology|CSIR-Centre for Cellular and Molecular Biology|Mukherjee|India
>Spike|hCoV-19/England/ALDP-9E6799/2020|2020-09-24|EPI_ISL_580278|Original|hCoV-19^^England|Human|Lighthouse Lab in Alderley Park|Wellcome Sanger Institute for the COVID-19 Genomics UK (COG-UK) consortium|Davies|United Kingdom
>Spike|hCoV-19/Netherlands/ZH-EMC-397/2020|2020-07-24|EPI_ISL_523737|Original|hCoV-19^^Zuid Holland|Human|Dutch COVID-19 response team|Erasmus Medical Center|Oude Munnink|Netherlands
>Spike|hCoV-19/USA/CA-ALSR-4436/2020|2020-10-12|EPI_ISL_649029|Original|hCoV-19^^California|Human|San Diego County Public Health Laboratory|Andersen lab at Scripps Research|Gangavarapu|USA
>Spike|hCoV-19/England/MILK-ACF57D/2020|2020-10-22|EPI_ISL_629716|Original|hCoV-19^^England|Human|Lighthouse Lab in Milton Keynes|Wellcome Sanger Institute for the COVID-19 Genomics UK (COG-UK) consortium|Davies|United Kingdom
>Spike|hCoV-19/Luxembourg/LNS1057454/2020|2020-10-26|EPI_ISL_739882|Original|hCoV-19^^Luxembourg|Human|Laboratoire national de sant\u00e9|Laboratoire national de sant\u00e9|Wienecke-Baldacchino|Luxembourg
>Spike|hCoV-19/Germany/NW-HHU-02-1/2020|2020-02-25|EPI_ISL_414497|Original|hCoV-19^^North Rhine-Westphalia|Human|Center of Medical Microbiology|Center of Medical Microbiology|Wienemann|Germany
>Spike|hCoV-19/South Africa/NHLS-UCT-GS-1030/2020|2020-10-27|EPI_ISL_700498|Original|hCoV-19^^Western Cape|Human|Thembalethu CDC wc THC|NHLS/UCT|Iranzadeh|South Africa
>Spike|hCoV-19/Netherlands/NA_679/2020|2020-03-03|EPI_ISL_461114|Original|hCoV-19^^Netherlands|Human|Dutch COVID-19 response team|Erasmus Medical Center|Oude Munnink|Netherlands
>Spike|hCoV-19/Scotland/QEUH-A4B54C/2020|2020-10-08|EPI_ISL_637837|Original|hCoV-19^^Scotland|Human|Department of Pathology|COVID-19 Genomics UK (COG-UK) Consortium|Goodfellow|United Kingdom
>Spike|hCoV-19/England/QEUH-9B8C28/2020|2020-09-07|EPI_ISL_549723|Original|hCoV-19^^England|Human|Lighthouse Lab in Glasgow|Wellcome Sanger Institute for the COVID-19 Genomics UK (COG-UK) consortium|Davies|United Kingdom
>Spike|hCoV-19/Spain/VC-FISABIO-109/2020|2020-03-19|EPI_ISL_436204|Original|hCoV-19^^Comunitat Valenciana|Human|Servicio de Microbiolog\u00eda. Consorcio Hospital General Universitario de Valencia|Sequencing and Bioinformatics Service and Molecular Epidemiology Research Group. FISABIO-Public Health|Gonzalez-Candelas|Spain
>Spike|hCoV-19/England/CAMC-B32617/2020|2020-11-05|EPI_ISL_659238|Original|hCoV-19^^England|Human|Lighthouse Lab in Cambridge|Wellcome Sanger Institute for the COVID-19 Genomics UK (COG-UK) Consortium|Davies|United Kingdom
>Spike|hCoV-19/Thailand/Bangkok-CONI-0257/2020|2020-03-14|EPI_ISL_693336|Original|hCoV-19^^Bangkok|Human|Ramathibodi Hospital|COVID-19 Network Investigations (CONI) Alliance|Batty|Thailand
>Spike|hCoV-19/Switzerland/BL-ETHZ-190047/2020|2020-07-08|EPI_ISL_500909|Original|hCoV-19^^Basel-Landschaft|Human|Viollier AG|Department of Biosystems Science and Engineering|Nadeau|Switzerland
>Spike|hCoV-19/Wales/PHWC-48E4CE/2020|2020-11-12|EPI_ISL_680866|Original|hCoV-19^^Wales|Human|Wales Specialist Virology Centre Sequencing lab|COVID-19 Genomics UK (COG-UK) Consortium|Moore|United Kingdom
>Spike|hCoV-19/Wales/PHWC-16905F/2020|2020-07-12|EPI_ISL_499386|Original|hCoV-19^^Wales|Human|Originating lab|COVID-19 Genomics UK (COG-UK) Consortium|Moore|United Kingdom
>Spike|hCoV-19/USA/AZ-TG666089/2020|2020-05-01|EPI_ISL_696180|Original|hCoV-19^^Arizona|Human|Sonora Quest Laboratories|TGen North|French|USA

>Spike|hCoV-19/mink/Netherlands/NB-EMC-16-1/2020|2020-06-21|EPI_ISL_523029|Original|hCoV-19^^Noord Brabant|Neovison vison|Dutch COVID-19 response team|Erasmus Medical Center|Oude Munnink|Netherlands
>Spike|hCoV-19/England/LEED-2A91D7/2020|2020-04-06|EPI_ISL_539016|Original|hCoV-19^^England|Human|Leeds Teaching Hospitals NHS Trust and Public Health England|Wellcome Sanger Institute for the COVID-19 Genomics UK (COG-UK) consortium|Davies|United Kingdom
>Spike|hCoV-19/England/MILK-9A7EAE/2020|2020-09-02|EPI_ISL_572421|Original|hCoV-19^^England|Human|Virology Department|COVID-19 Genomics UK (COG-UK) Consortium|Williams|United Kingdom
>Spike|hCoV-19/Wales/PHWC-168964/2020|2020-04-20|EPI_ISL_494309|Original|hCoV-19^^Wales|Human|Originating lab|COVID-19 Genomics UK (COG-UK) Consortium|Moore|United Kingdom
>Spike|hCoV-19/Netherlands/NA_54/2020|2020-03-15|EPI_ISL_422815|Original|hCoV-19^^Netherlands|Human|Dutch COVID-19 response team|Erasmus Medical Center|Oude Munnink|Netherlands
>Spike|hCoV-19/Australia/VIC538/2020|2020-03-23|EPI_ISL_426804|Original|hCoV-19^^Victoria|Human|Victorian Infectious Diseases Reference Laboratory (VIDRL)|Microbiological Diagnostic Unit Public Health Laboratory and Victorian Infectious Diseases Reference Laboratory|Schultz|Australia
>Spike|hCoV-19/Scotland/CVR852/2020|2020-03-27|EPI_ISL_433352|Original|hCoV-19^^Scotland|Human|West of Scotland Specialist Virology Centre|COVID-19 Genomics UK (COG-UK) Consortium|Robertson|United Kingdom
>Spike|hCoV-19/England/NORT-2938CC/2020|2020-07-06|EPI_ISL_499563|Original|hCoV-19^^England|Human|Northumbria University / South Tees Hospitals NHS Foundation Trust / North Cumbria Integrated Care NHS Foundation Trust / North Tees and Hartlepool NHS Foundation Trust / Newcastle Hospitals NHS Foundation Trust|COVID-19 Genomics UK (COG-UK) Consortium|Bashton|United Kingdom
>Spike|hCoV-19/England/ALDP-BCA0F8/2020|2020-11-23|EPI_ISL_703433|Original|hCoV-19^^England|Human|Lighthouse Lab in Alderley Park|Wellcome Sanger Institute for the COVID-19 Genomics UK (COG-UK) Consortium|Davies|United Kingdom
>Spike|hCoV-19/England/CAMB-1AD3B0/2020|2020-04-25|EPI_ISL_489492|Original|hCoV-19^^England|Human|Department of Pathology|Wellcome Sanger Institute for the COVID-19 Genomics UK (COG-UK) consortium|Davies|United Kingdom
>Spike|hCoV-19/England/NOTT-119B5D/2020|2020-11-04|EPI_ISL_626881|Original|hCoV-19^^England|Human|Queens Medical Centre|COVID-19 Genomics UK (COG-UK) Consortium|Loose|United Kingdom
>Spike|hCoV-19/England/ALDP-B5D090/2020|2020-11-09|EPI_ISL_678852|Original|hCoV-19^^England|Human|Department of Pathology|COVID-19 Genomics UK (COG-UK) Consortium|Goodfellow|United Kingdom
>Spike|hCoV-19/England/QEUH-9FF6FA/2020|2020-10-02|EPI_ISL_600251|Original|hCoV-19^^England|Human|Lighthouse Lab in Glasgow|Wellcome Sanger Institute for the COVID-19 Genomics UK (COG-UK) consortium|Davies|United Kingdom
>Spike|hCoV-19/USA/MI-MDHHS-SC20828/2020|2020-04-19|EPI_ISL_471849|Original|hCoV-19^^Michigan|Human|Michigan Department of Health and Human Services|Michigan Department of Health and Human Services|Blankenship|USA
>Spike|hCoV-19/Australia/NSW82/2020|2020-03-20|EPI_ISL_427805|Original|hCoV-19^^New South Wales|Human|Centre for Infectious Diseases and Microbiology Public Health|NSW Health Pathology - Institute of Clinical Pathology and Medical Research; Westmead Hospital; University of Sydney|Gall|Australia
>Spike|hCoV-19/Denmark/DCGC-14447/2020|2020-11-30|EPI_ISL_711773|Original|hCoV-19^^Hovedstaden|Human|Department of Virus and Microbiological Special Diagnostics|Albertsen Lab|Michaelsen|Denmark
>Spike|hCoV-19/USA/UT-UPHL-2012749027/2020|2020-11-30|EPI_ISL_746463|Original|hCoV-19^^Utah|Human|Utah Public Health Laboratory|Utah Public Health Laboratory|Gallagher|USA
>Spike|hCoV-19/USA/CA-ALSR-2424/2020|2020-07-06|EPI_ISL_635472|Original|hCoV-19^^California|Human|San Diego County Public Health Laboratory|Andersen lab at Scripps Research|Gangavarapu|USA

>Spike|hCoV-19/Denmark/DCGC-20552/2020|2020-12-21|EPI_ISL_759214|Original|hCoV-19^^Nordjylland|Human|Department of Virus and Microbiological Special Diagnostics|Albertsen Lab|Michaelsen|Denmark
>Spike|hCoV-19/USA/CA-CZB-11513/2020|2020-08-02|EPI_ISL_625583|Original|hCoV-19^^California|Human|NaN|Chan-Zuckerberg Biohub|Bhatt|USA
>Spike|hCoV-19/Brazil/RS-00674/2020|2020-11-27|EPI_ISL_770615|Original|hCoV-19^^Rio Grande do Sul|Human|Laborat\u00f3rio de Microbiologia Molecular - Universidade FEEVALE|Bioinformatics Laboratory / LNCC|Almeida|Brazil
>Spike|hCoV-19/USA/HI-H200645/2020|2020-11-23|EPI_ISL_753169|Original|hCoV-19^^Hawaii|Human|State Laboratories Division|State Laboratories Division|Sultana|USA
>Spike|hCoV-19/England/CAMC-B088F6/2020|2020-10-29|EPI_ISL_643818|Original|hCoV-19^^England|Human|Lighthouse Lab in Cambridge|Wellcome Sanger Institute for the COVID-19 Genomics UK (COG-UK) Consortium|Davies|United Kingdom
>Spike|hCoV-19/England/20166018804/2020|2020-04-15|EPI_ISL_466545|Original|hCoV-19^^England|Human|Respiratory Virus Unit|Respiratory Virus Unit|Platt|United Kingdom
>Spike|hCoV-19/USA/WA-UW-6525/2020|2020-04-04|EPI_ISL_570126|Original|hCoV-19^^Washington|Human|UW Virology Lab|UW Virology Lab|Roychoudhury|USA
>Spike|hCoV-19/Scotland/QEUH-A4B4D6/2020|2020-10-08|EPI_ISL_638941|Original|hCoV-19^^Scotland|Human|Department of Pathology|COVID-19 Genomics UK (COG-UK) Consortium|Goodfellow|United Kingdom
>Spike|hCoV-19/India/GJ-GBRC358/2020|2020-06-25|EPI_ISL_514608|Original|hCoV-19^^Gujarat|Human|B.J. Medical College and Civil hospital|Gujarat Biotechnology Research Centre|Joshi|India
>Spike|hCoV-19/Australia/VIC11141/2020|2020-08-08|EPI_ISL_563267|Original|hCoV-19^^Victoria|Human|Microbiological Diagnostic Unit - Public Health Laboratory (MDU-PHL)|MDU-PHL|Schultz|Australia
>Spike|hCoV-19/Denmark/DCGC-2377/2020|2020-08-10|EPI_ISL_617557|Original|hCoV-19^^Hovedstaden|Human|Department of Virus and Microbiological Special Diagnostics|Albertsen lab|Michaelsen|Denmark
>Spike|hCoV-19/Germany/NW-HHU-497/2020|2020-12-03|EPI_ISL_770746|Original|hCoV-19^^North Rhine-Westphalia|Human|ZOTZ KLIMAS MVZ D\u00fcsseldorf-Centrum GbR \u00dcBAG f\u00fcr Labormedizin|Center of Medical Microbiology|Wienemann|Germany
>Spike|hCoV-19/USA/TX-DSHS-1669/2020|2020-06-24|EPI_ISL_710079|Original|hCoV-19^^Texas|Human|Texas Department of State Health Services|Texas Department of State Health Services|Rahman|USA
>Spike|hCoV-19/Portugal/PT0655/2020|2020-03-28|EPI_ISL_510916|Original|hCoV-19^^Portugal|Human|Instituto Nacional de Saude (INSA)|Instituto Nacional de Saude (INSA)|Borges|Portugal
>Spike|hCoV-19/England/QEUH-B663AA/2020|2020-11-11|EPI_ISL_661481|Original|hCoV-19^^England|Human|Lighthouse Lab in Glasgow|Wellcome Sanger Institute for the COVID-19 Genomics UK (COG-UK) Consortium|Davies|United Kingdom
>Spike|hCoV-19/Denmark/DCGC-12683/2020|2020-11-16|EPI_ISL_682864|Original|hCoV-19^^Sjaelland|Human|Department of Virus and Microbiological Special Diagnostics|Albertsen Lab|Michaelsen|Denmark
>Spike|hCoV-19/England/QEUH-9B9252/2020|2020-09-07|EPI_ISL_549736|Original|hCoV-19^^England|Human|Lighthouse Lab in Glasgow|Wellcome Sanger Institute for the COVID-19 Genomics UK (COG-UK) consortium|Davies|United Kingdom
>Spike|hCoV-19/England/MILK-9D5539/2020|2020-09-19|EPI_ISL_577067|Original|hCoV-19^^England|Human|Centre for Enzyme Innovation|COVID-19 Genomics UK (COG-UK) Consortium|Robson|United Kingdom
>Spike|hCoV-19/Uganda/UG024/2020|2020-05-01|EPI_ISL_737948|Original|hCoV-19^^Uganda|Human|Uganda Central Public Health Lab and Uganda Virus Research Institute|MRC/UVRI & LSHTM Uganda\u00a0Research Unit|Cotten|Uganda
>Spike|hCoV-19/England/ALDP-92C102/2020|2020-06-22|EPI_ISL_553709|Original|hCoV-19^^England|Human|Lighthouse Lab in Alderley Park|Wellcome Sanger Institute for the COVID-19 Genomics UK (COG-UK) consortium|Davies|United Kingdom
>Spike|hCoV-19/Japan/PG-0310/2020|2020-03-24|EPI_ISL_479985|Original|hCoV-19^^Kanto|Human|Ibaraki Prefectural Institute of Public Health|Pathogen Genomics Center|Sekizuka|Japan

>Spike|hCoV-19/England/QEUH-96B468/2020|2020-08-14|EPI_ISL_531462|Original|hCoV-19^^England|Human|Lighthouse Lab in Glasgow|Wellcome Sanger Institute for the COVID-19 Genomics UK (COG-UK) consortium|Davies|United Kingdom
>Spike|hCoV-19/USA/AZ-TG495770/2020|2020-07-13|EPI_ISL_695263|Original|hCoV-19^^Arizona|Human|TGen North|TGen North|French|USA
>Spike|hCoV-19/England/MILK-A79721/2020|2020-10-15|EPI_ISL_606848|Original|hCoV-19^^England|Human|Lighthouse Lab in Milton Keynes|Wellcome Sanger Institute for the COVID-19 Genomics UK (COG-UK) consortium|Davies|United Kingdom
>Spike|hCoV-19/South Africa/KRISP-K003365/2020|2020-09-04|EPI_ISL_602756|Original|hCoV-19^^KwaZulu-Natal|Human|NHLS-IALCH|KRISP|de Oliveira|South Africa
>Spike|hCoV-19/England/MILK-A44CA8/2020|2020-10-06|EPI_ISL_598606|Original|hCoV-19^^England|Human|Lighthouse Lab in Milton Keynes|Wellcome Sanger Institute for the COVID-19 Genomics UK (COG-UK) Consortium|Davies|United Kingdom
>Spike|hCoV-19/England/205260428/2020|2020-00-00|EPI_ISL_766112|Original|hCoV-19^^England|Human|Respiratory Virus Unit|COVID-19 Genomics UK (COG-UK) Consortium|Platt|United Kingdom
>Spike|hCoV-19/USA/PA-MGEL-00256/2020|2020-04-22|EPI_ISL_681989|Original|hCoV-19^^Pennsylvania|Human|UPMC Clinical Microbiology Laboratory|Microbial Genomic Epidemiology Laboratory|Griffith|USA
>Spike|hCoV-19/USA/TX-HMH-MCoV-9753/2020|2020-07-06|EPI_ISL_780255|Original|hCoV-19^^Texas|Human|Houston Methodist Hospital|Houston Methodist Hospital|Long|USA
>Spike|hCoV-19/England/BRIS-12F019/2020|2020-04-24|EPI_ISL_488398|Original|hCoV-19^^England|Human|PHE South West Regional Laboratory|Wellcome Sanger Institute for the COVID-19 Genomics UK (COG-UK) consortium|Davies|United Kingdom
>Spike|hCoV-19/USA/CA-CZB-1473/2020|2020-04-29|EPI_ISL_468427|Original|hCoV-19^^California|Human|County of San Luis Obispo Public Health Laboratory|Chan-Zuckerberg Biohub|Hao|USA
>Spike|hCoV-19/USA/MT-CDC-2-3693496/2020|2020-10-30|EPI_ISL_747034|Original|hCoV-19^^Montana|Human|Respiratory Viruses Branch|Respiratory Viruses Branch|Queen|USA
>Spike|hCoV-19/England/ALDP-C3B01F/2020|2020-12-09|EPI_ISL_731707|Original|hCoV-19^^England|Human|Lighthouse Lab in Alderley Park|Wellcome Sanger Institute for the COVID-19 Genomics UK (COG-UK) Consortium|Davies|United Kingdom
>Spike|hCoV-19/USA/AZ-TG305888/2020|2020-04-29|EPI_ISL_694288|Original|hCoV-19^^Arizona|Human|AZ SPHL|TGen North|French|USA
>Spike|hCoV-19/England/CAMB-79776/2020|2020-04-01|EPI_ISL_441071|Original|hCoV-19^^England|Human|Department of Pathology|Wellcome Sanger Institute for the COVID-19 Genomics UK (COG-UK) consortium|Amato|United Kingdom
>Spike|hCoV-19/Luxembourg/LNS6613781/2020|2020-04-21|EPI_ISL_445060|Original|hCoV-19^^Luxembourg|Human|Laboratoire National de Sante|Laboratoire National de Sante|Wienecke-Baldacchino|Luxembourg
>Spike|hCoV-19/USA/AZ-TG670165/2020|2020-04-04|EPI_ISL_696364|Original|hCoV-19^^Arizona|Human|Sonora Quest Laboratories|TGen North|French|USA
>Spike|hCoV-19/England/CAMC-BC1D90/2020|2020-11-22|EPI_ISL_703032|Original|hCoV-19^^England|Human|Department of Pathology|COVID-19 Genomics UK (COG-UK) Consortium|Goodfellow|United Kingdom
>Spike|hCoV-19/Australia/VIC7613/2020|2020-07-22|EPI_ISL_564529|Original|hCoV-19^^Victoria|Human|Microbiological Diagnostic Unit - Public Health Laboratory (MDU-PHL)|MDU-PHL|Schultz|Australia
>Spike|hCoV-19/Japan/PG-7688/2020|2020-08-00|EPI_ISL_686790|Original|hCoV-19^^Japan|Human|Chiba Prefectural Institute of Public Health|Pathogen Genomics Center|Sekizuka|Japan
>Spike|hCoV-19/Switzerland/BS-ETHZ-100016/2020|2020-03-08|EPI_ISL_466940|Original|hCoV-19^^Basel-Stadt|Human|Viollier AG|Department of Biosystems Science and Engineering|Nadeau|Switzerland
>Spike|hCoV-19/England/LIVE-A67BC/2020|2020-03-16|EPI_ISL_499467|Original|hCoV-19^^England|Human|Liverpool Clinical Laboratories|COVID-19 Genomics UK (COG-UK) Consortium|Haldenby|United Kingdom

>Spike|hCoV-19/England/CAMC-A3F4B7/2020|2020-10-05|EPI_ISL_609593|Original|hCoV-19^^England|Human|Lighthouse Lab in Cambridge|Wellcome Sanger Institute for the COVID-19 Genomics UK (COG-UK) consortium|Davies|United Kingdom
>Spike|hCoV-19/England/MILK-AB7AB5/2020|2020-10-21|EPI_ISL_623322|Original|hCoV-19^^England|Human|Lighthouse Lab in Milton Keynes|Wellcome Sanger Institute for the COVID-19 Genomics UK (COG-UK) consortium|Davies|United Kingdom
>Spike|hCoV-19/Wales/ALDP-C3CC29/2020|2020-12-10|EPI_ISL_767219|Original|hCoV-19^^Wales|Human|Lighthouse Lab in Alderley Park|Wellcome Sanger Institute for the COVID-19 Genomics UK (COG-UK) Consortium|Davies|United Kingdom
>Spike|hCoV-19/Scotland/CVR5218/2020|2020-10-16|EPI_ISL_626985|Original|hCoV-19^^Scotland|Human|West of Scotland Specialist Virology Centre|COVID-19 Genomics UK (COG-UK) Consortium|Robertson|United Kingdom
>Spike|hCoV-19/USA/WI-UW-2391/2020|2020-10-30|EPI_ISL_754627|Original|hCoV-19^^Wisconsin|Human|University of Wisconsin-Madison AIDS Vaccine Research Laboratories|University of Wisconsin-Madison AIDS Vaccine Research Laboratories|Moreno|USA
>Spike|hCoV-19/England/LIVE-DBE893/2020|2020-10-29|EPI_ISL_664726|Original|hCoV-19^^England|Human|Liverpool Clinical Laboratories|COVID-19 Genomics UK (COG-UK) Consortium|Haldenby|United Kingdom
>Spike|hCoV-19/USA/MD-MDH-0476/2020|2020-11-11|EPI_ISL_710093|Original|hCoV-19^^Maryland|Human|Maryland Public Health Laboratory|Maryland Public Health Laboratory|Keller|USA
>Spike|hCoV-19/Scotland/CVR4883/2020|2020-10-05|EPI_ISL_650853|Original|hCoV-19^^Scotland|Human|West of Scotland Specialist Virology Centre|COVID-19 Genomics UK (COG-UK) Consortium|Robertson|United Kingdom
>Spike|hCoV-19/Switzerland/BE-ETHZ-130032/2020|2020-05-13|EPI_ISL_467016|Original|hCoV-19^^Bern|Human|Viollier AG|Department of Biosystems Science and Engineering|Nadeau|Switzerland
>Spike|hCoV-19/Scotland/EDB046/2020|2020-03-15|EPI_ISL_425850|Original|hCoV-19^^Scotland|Human|Virology Department|COVID-19 Genomics UK (COG-UK) Consortium|Williams|United Kingdom
>Spike|hCoV-19/England/CWAR-24FDA86/2020|2020-04-30|EPI_ISL_584473|Original|hCoV-19^^England|Human|UHCW / University of Warwick|COVID-19 Genomics UK (COG-UK) Consortium|Stark|United Kingdom
>Spike|hCoV-19/England/NORT-282BD6/2020|2020-03-28|EPI_ISL_472217|Original|hCoV-19^^England|Human|Northumbria University / South Tees Hospitals NHS Foundation Trust / North Cumbria Integrated Care NHS Foundation Trust / North Tees and Hartlepool NHS Foundation Trust / Newcastle Hospitals NHS Foundation Trust|COVID-19 Genomics UK (COG-UK) Consortium|Bashton|United Kingdom
>Spike|hCoV-19/Wales/PHWC-4842BF/2020|2020-10-22|EPI_ISL_627984|Original|hCoV-19^^Wales|Human|Wales Specialist Virology Centre Sequencing lab|COVID-19 Genomics UK (COG-UK) Consortium|Moore|United Kingdom
>Spike|hCoV-19/Colombia/COR-INS-95319/2020|2020-04-01|EPI_ISL_498163|Original|hCoV-19^^Cordoba|Human|Instituto Nacional de Salud|Instituto Nacional de Salud|Franco-Mu\u00f1oz|Colombia
>Spike|hCoV-19/USA/TX-HMH-MCoV-18906/2020|2020-11-25|EPI_ISL_785212|Original|hCoV-19^^Texas|Human|Houston Methodist Hospital|Houston Methodist Hospital|Long|USA
>Spike|hCoV-19/Singapore/1125/2020|2020-10-29|EPI_ISL_626637|Original|hCoV-19^^Singapore|Human|National Public Health Laboratory|National Public Health Laboratory|Mak|Singapore
>Spike|hCoV-19/South Africa/KRISP-K002629/2020|2020-07-31|EPI_ISL_535499|Original|hCoV-19^^KwaZulu-Natal|Human|NHLS-IALCH|KRISP|de Oliveira|South Africa
>Spike|hCoV-19/England/QEUH-B0E043/2020|2020-11-02|EPI_ISL_642119|Original|hCoV-19^^England|Human|Lighthouse Lab in Glasgow|Wellcome Sanger Institute for the COVID-19 Genomics UK (COG-UK) Consortium|Davies|United Kingdom
>Spike|hCoV-19/England/BRIS-12C5E0/2020|2020-05-03|EPI_ISL_469471|Original|hCoV-19^^England|Human|PHE South West Regional Laboratory|Wellcome Sanger Institute for the COVID-19 Genomics UK (COG-UK) consortium|Davies|United Kingdom

>Spike|hCoV-19/Russia/LEN-RII-MH5616S/2020|2020-11-09|EPI_ISL_733304|Original|hCoV-19^^Leningrad|Human|HELIX LLC|WHO National Influenza Centre Russian Federation|Komissarov|Russia
>Spike|hCoV-19/England/ALDP-C58A0F/2020|2020-12-13|EPI_ISL_736049|Original|hCoV-19^^England|Human|Lighthouse Lab in Alderley Park|Wellcome Sanger Institute for the COVID-19 Genomics UK (COG-UK) Consortium|Davies|United Kingdom
>Spike|hCoV-19/Singapore/1216/2020|2020-04-23|EPI_ISL_648632|Original|hCoV-19^^Singapore|Human|Department of Laboratory Medicine|Department of Laboratory Medicine|Chen|Singapore
>Spike|hCoV-19/USA/NY-NYCPHL-000752/2020|2020-03-21|EPI_ISL_632132|Original|hCoV-19^^New York|Human|Flushing Hospital Medical Center|New York City Public Health Laboratory|Wang|USA
>Spike|hCoV-19/Chile/RM-158239/2020|2020-08-22|EPI_ISL_746687|Original|hCoV-19^^Region Metropolitana de Santiago|Human|Genetica Molecular and Subdepartamento de Virologia ISP Chile|Instituto de Salud Publica de Chile|Tognarelli|Chile
>Spike|hCoV-19/Japan/Donner36/2020|2020-05-11|EPI_ISL_469294|Original|hCoV-19^^Kanto|Human|Keio University Hospital|Keio University Hospital|Kosaki|Japan
>Spike|hCoV-19/USA/NY-NYCPHL-000151/2020|2020-04-05|EPI_ISL_631687|Original|hCoV-19^^New York|Human|OCME Office Of Chief Medical Examiner|New York City Public Health Laboratory|Wang|USA
>Spike|hCoV-19/USA/MA-MASPHL-00469/2020|2020-04-30|EPI_ISL_693094|Original|hCoV-19^^Massachusetts|Human|Massachusetts State Public Health Laboratory|Massachusetts State Public Health Laboratory|Lang|USA
>Spike|hCoV-19/Australia/VIC5538/2020|2020-07-21|EPI_ISL_518369|Original|hCoV-19^^Victoria|Human|Microbiological Diagnostic Unit - Public Health Laboratory (MDU-PHL)|MDU-PHL|Schultz|Australia
>Spike|hCoV-19/Spain/MD-IBV-99008523/2020|2020-09-10|EPI_ISL_654046|Original|hCoV-19^^Madrid|Human|Hospital General Universitario Gregorio Mara\u00f1\u00f3n|SeqCOVID-SPAIN consortium/IBV(CSIC)|Comas|Spain
>Spike|hCoV-19/England/ALDP-B75821/2020|2020-11-12|EPI_ISL_740926|Original|hCoV-19^^England|Human|Oxford Viromics|COVID-19 Genomics UK (COG-UK) Consortium|Golubchik|United Kingdom
>Spike|hCoV-19/England/BRIS-12432A/2020|2020-04-08|EPI_ISL_492814|Original|hCoV-19^^England|Human|PHE South West Regional Laboratory|Wellcome Sanger Institute for the COVID-19 Genomics UK (COG-UK) consortium|Davies|United Kingdom
>Spike|hCoV-19/England/CAMC-9C015B/2020|2020-09-09|EPI_ISL_573172|Original|hCoV-19^^England|Human|Oxford Viromics|COVID-19 Genomics UK (COG-UK) Consortium|Golubchik|United Kingdom
>Spike|hCoV-19/USA/NY-Wadsworth-236443-01/2020|2020-10-03|EPI_ISL_734316|Original|hCoV-19^^New York|Human|Wadsworth Center|Wadsworth Center|Lasek-Nesselquist|USA
>Spike|hCoV-19/USA/AR-CDC-4219/2020|2020-07-12|EPI_ISL_527637|Original|hCoV-19^^Arkansas|Human|AR Dept. of Health-Public Health Lab|Pathogen Discovery|Queen|USA
>Spike|hCoV-19/Ireland/D-NVRL-AIIDV0072v2/2020|2020-04-12|EPI_ISL_644257|Original|hCoV-19^^Dublin|Human|CEPHR / Vincent's Hospital|Irish Coronavirus Sequencing Consortium - National Virus Reference Laboratory|Gonzalez|Ireland
>Spike|hCoV-19/Denmark/DCGC-5087/2020|2020-09-21|EPI_ISL_621625|Original|hCoV-19^^Sjaelland|Human|Department of Virus and Microbiological Special Diagnostics|Albertsen lab|Michaelsen|Denmark
>Spike|hCoV-19/Bosnia and Herzegovina/ChVir7340/2020|2020-03-17|EPI_ISL_462450|Original|hCoV-19^^Bihac|Human|Clinical Center|Charite Universitatsmedizin Berlin|Muehlemann|Bosnia and Herzegovina
>Spike|hCoV-19/Denmark/DCGC-13242/2020|2020-11-30|EPI_ISL_712674|Original|hCoV-19^^Hovedstaden|Human|Department of Virus and Microbiological Special Diagnostics|Albertsen Lab|Michaelsen|Denmark
>Spike|hCoV-19/Wales/PHWC-48DF64/2020|2020-11-08|EPI_ISL_680783|Original|hCoV-19^^Wales|Human|Wales Specialist Virology Centre Sequencing lab|COVID-19 Genomics UK (COG-UK) Consortium|Moore|United Kingdom

>Spike|hCoV-19/England/QEUH-AD0D17/2020|2020-10-26|EPI_ISL_634121|Original|hCoV-19^^England|Human|Lighthouse Lab in Glasgow|Wellcome Sanger Institute for the COVID-19 Genomics UK (COG-UK) consortium|Davies|United Kingdom
>Spike|hCoV-19/South Africa/KRISP-K002484/2020|2020-08-07|EPI_ISL_535392|Original|hCoV-19^^KwaZulu-Natal|Human|NHLS-IALCH|KRISP|de Oliveira|South Africa
>Spike|hCoV-19/Canada/ON-UHTC_0006/2020|2020-03-25|EPI_ISL_464015|unknown|hCoV-19^^Ontario|Human|Unity Health Toronto|Ontario Institute for Cancer Research|de Borja|Canada
>Spike|hCoV-19/USA/ME-HETL-J0285/2020|2020-10-05|EPI_ISL_755428|Original|hCoV-19^^Maine|Human|Maine Health and Environmental Testing Laboratory|Tewhey Lab|Tewhey|USA
>Spike|hCoV-19/Brazil/SP-L34-CD876/2020|2020-04-23|EPI_ISL_722047|Original|hCoV-19^^S\u00e3o Paulo|Human|Hospital das Cl\u00ednicas Universidade de S\u00e3o Paulo Medical School|Laborat\u00f3rio de Parasitologia M\u00e9dica - Instituto de Medicina Tropical - Universidade de S\u00e3o Paulo|de Jesus|Brazil
>Spike|hCoV-19/USA/TX-DSHS-0858/2020|2020-06-16|EPI_ISL_593590|Original|hCoV-19^^Texas|Human|Texas Department of State Health Services|Texas Department of State Health Services|Rahman|USA
>Spike|hCoV-19/Denmark/DCGC-11439/2020|2020-11-02|EPI_ISL_670475|Original|hCoV-19^^Hovedstaden|Human|Department of Virus and Microbiological Special Diagnostics|Albertsen Lab|Michaelsen|Denmark
>Spike|hCoV-19/England/MILK-ACD5D9/2020|2020-10-19|EPI_ISL_634424|Original|hCoV-19^^England|Human|Lighthouse Lab in Milton Keynes|Wellcome Sanger Institute for the COVID-19 Genomics UK (COG-UK) consortium|Davies|United Kingdom
>Spike|hCoV-19/Denmark/DCGC-2531/2020|2020-08-17|EPI_ISL_617801|Original|hCoV-19^^Hovedstaden|Human|Department of Virus and Microbiological Special Diagnostics|Albertsen lab|Michaelsen|Denmark
>Spike|hCoV-19/Northern Ireland/NIRE-107026/2020|2020-03-30|EPI_ISL_585099|Original|hCoV-19^^Northern Ireland|Human|Regional Virus Laboratory|COVID-19 Genomics UK (COG-UK) Consortium|Fairley|United Kingdom
>Spike|hCoV-19/England/CAMB-1B1BC0/2020|2020-05-07|EPI_ISL_458548|Original|hCoV-19^^England|Human|Department of Pathology|Wellcome Sanger Institute for the COVID-19 Genomics UK (COG-UK) consortium|Davies|United Kingdom
>Spike|hCoV-19/Spain/AN-IBV-006110/2020|2020-07-04|EPI_ISL_510440|Original|hCoV-19^^Andalucia|Human|Hospital Universitario Virgen de las Nieves de Granada-SAS|SeqCOVID-SPAIN consortium/IBV(CSIC)|Comas|Spain
>Spike|hCoV-19/Hangzhou/HZ162/2020|2020-01-23|EPI_ISL_418508|Original|hCoV-19^^Zhejiang|Human|Hangzhou Center for Disease Control and Prevention|Inspection Center of Hangzhou Center for Disease Control and Prevention|Renton|China
>Spike|hCoV-19/Australia/VIC1829/2020|2020-05-27|EPI_ISL_521878|Original|hCoV-19^^Victoria|Human|Victorian Infectious Diseases Reference Laboratory (VIDRL)|VIDRL and MDU-PHL|Schultz|Australia
>Spike|hCoV-19/England/20149055204/2020|2020-04-05|EPI_ISL_466219|Original|hCoV-19^^England|Human|Respiratory Virus Unit|Respiratory Virus Unit|Platt|United Kingdom
>Spike|hCoV-19/Wales/PHWC-49C041/2020|2020-12-07|EPI_ISL_742498|Original|hCoV-19^^Wales|Human|Wales Specialist Virology Centre Sequencing lab|COVID-19 Genomics UK (COG-UK) Consortium|Moore|United Kingdom
>Spike|hCoV-19/USA/FL-BPHL-1150/2020|2020-08-18|EPI_ISL_526636|Original|hCoV-19^^Florida|Human|Florida Bureau of Public Health Laboratories|Florida Bureau of Public Health Laboratories|Blanton|USA
>Spike|hCoV-19/Denmark/DCGC-20215/2020|2020-11-30|EPI_ISL_748053|Original|hCoV-19^^Sjaelland|Human|Department of Virus and Microbiological Special Diagnostics|Albertsen Lab|Michaelsen|Denmark
>Spike|hCoV-19/Sri Lanka/CDR-KK57/2020|2020-07-21|EPI_ISL_525486|Original|hCoV-19^^Sri Lanka|Human|Centre for Dengue Research|Centre for Dengue Research|Malavige|Sri Lanka
>Spike|hCoV-19/England/LIVE-DBB702/2020|2020-11-12|EPI_ISL_664675|Original|hCoV-19^^England|Human|Liverpool Clinical Laboratories|COVID-19 Genomics UK (COG-UK) Consortium|Haldenby|United Kingdom

>Spike|hCoV-19/Denmark/ALAB-SSI262/2020|2020-03-10|EPI_ISL_429424|Original|hCoV-19^^Denmark|Human|Department of Virus and Microbiological Special Diagnostics|Albertsen lab|Kirkegaard|Denmark
>Spike|hCoV-19/England/QEUH-A8BF51/2020|2020-10-17|EPI_ISL_643250|Original|hCoV-19^^England|Human|Lighthouse Lab in Glasgow|Wellcome Sanger Institute for the COVID-19 Genomics UK (COG-UK) Consortium|Davies|United Kingdom
>Spike|hCoV-19/England/ALDP-B5C301/2020|2020-11-10|EPI_ISL_655759|Original|hCoV-19^^England|Human|Lighthouse Lab in Alderley Park|Wellcome Sanger Institute for the COVID-19 Genomics UK (COG-UK) Consortium|Davies|United Kingdom
>Spike|hCoV-19/Norway/4673/2020|2020-10-22|EPI_ISL_635066|Original|hCoV-19^^Oslo|Human|Norwegian Institute of Public Health|Norwegian Institute of Public Health|Vollan|Norway
>Spike|hCoV-19/England/LIVE-A552C/2020|2020-03-20|EPI_ISL_499820|Original|hCoV-19^^England|Human|Liverpool Clinical Laboratories|COVID-19 Genomics UK (COG-UK) Consortium|Haldenby|United Kingdom
>Spike|hCoV-19/Portugal/PT0743/2020|2020-03-22|EPI_ISL_511002|Original|hCoV-19^^Portugal|Human|Instituto Nacional de Saude (INSA)|Instituto Nacional de Saude (INSA)|Borges|Portugal
>Spike|hCoV-19/England/CAMB-72731/2020|2020-03-23|EPI_ISL_439635|Original|hCoV-19^^England|Human|Department of Pathology|Wellcome Sanger Institute for the COVID-19 Genomics UK (COG-UK) consortium|Amato|United Kingdom
>Spike|hCoV-19/Scotland/QEUH-95E3F2/2020|2020-08-10|EPI_ISL_534430|Original|hCoV-19^^Scotland|Human|NHSGGC West of Scotland Specialist Virology Centre / MRC-University of Glasgow Centre for Virus Research|Wellcome Sanger Institute for the COVID-19 Genomics UK (COG-UK) consortium|Davies|United Kingdom
>Spike|hCoV-19/Australia/VIC6629/2020|2020-07-18|EPI_ISL_521389|Original|hCoV-19^^Victoria|Human|Victorian Infectious Diseases Reference Laboratory (VIDRL)|VIDRL and MDU-PHL|Schultz|Australia
>Spike|hCoV-19/Denmark/DCGC-14123/2020|2020-11-30|EPI_ISL_711582|Original|hCoV-19^^Hovedstaden|Human|Department of Virus and Microbiological Special Diagnostics|Albertsen Lab|Michaelsen|Denmark
>Spike|hCoV-19/Canada/ON-SK-20H-328MM1080/2020|2020-11-24|EPI_ISL_791502|Original|hCoV-19^^Ontario|Human|The Hosptial for Sick Children|The Hospital for Sick Children|Lau|Canada
>Spike|hCoV-19/Singapore/1211/2020|2020-04-22|EPI_ISL_648627|Original|hCoV-19^^Singapore|Human|Department of Laboratory Medicine|Department of Laboratory Medicine|Chen|Singapore
>Spike|hCoV-19/Wales/PHWC-484E42/2020|2020-10-24|EPI_ISL_628118|Original|hCoV-19^^Wales|Human|Wales Specialist Virology Centre Sequencing lab|COVID-19 Genomics UK (COG-UK) Consortium|Moore|United Kingdom
>Spike|hCoV-19/England/MILK-ACC0E8/2020|2020-10-21|EPI_ISL_629244|Original|hCoV-19^^England|Human|Lighthouse Lab in Milton Keynes|Wellcome Sanger Institute for the COVID-19 Genomics UK (COG-UK) consortium|Davies|United Kingdom
>Spike|hCoV-19/Wales/PHWC-164CD8/2020|2020-05-11|EPI_ISL_479370|Original|hCoV-19^^Wales|Human|Wales Specialist Virology Centre Sequencing lab|COVID-19 Genomics UK (COG-UK) Consortium|Moore|United Kingdom
>Spike|hCoV-19/England/ALDP-9B5CA3/2020|2020-08-28|EPI_ISL_572962|Original|hCoV-19^^England|Human|Oxford Viromics|COVID-19 Genomics UK (COG-UK) Consortium|Golubchik|United Kingdom
>Spike|hCoV-19/Scotland/GCVR-1706F9/2020|2020-03-25|EPI_ISL_489583|Original|hCoV-19^^Scotland|Human|NHSGGC West of Scotland Specialist Virology Centre / MRC-University of Glasgow Centre for Virus Research|Wellcome Sanger Institute for the COVID-19 Genomics UK (COG-UK) consortium|Davies|United Kingdom
>Spike|hCoV-19/Australia/VIC4270/2020|2020-07-13|EPI_ISL_519598|Original|hCoV-19^^Victoria|Human|Microbiological Diagnostic Unit - Public Health Laboratory (MDU-PHL)|MDU-PHL|Schultz|Australia
>Spike|hCoV-19/USA/CO-CDPHE-2010280214/2020|2020-10-26|EPI_ISL_710366|Original|hCoV-19^^Colorado|Human|Colorado Department of Public Health and Environment|Colorado Department of Puplic Health and Environment|Bankers|USA

>Spike|hCoV-19/Japan/PG-6725/2020|2020-08-00|EPI_ISL_687836|Original|hCoV-19^^Japan|Human|Pathogen Genomics Center|Pathogen Genomics Center|Sekizuka|Japan
>Spike|hCoV-19/Netherlands/NB-EMC-333/2020|2020-10-23|EPI_ISL_722404|Original|hCoV-19^^Noord Brabant|Human|Dutch COVID-19 response team|Erasmus Medical Center|Oude Munnink|Netherlands
>Spike|hCoV-19/England/ALDP-8FFEF3/2020|2020-06-24|EPI_ISL_557102|Original|hCoV-19^^England|Human|Lighthouse Lab in Alderley Park|Wellcome Sanger Institute for the COVID-19 Genomics UK (COG-UK) consortium|Davies|United Kingdom
>Spike|hCoV-19/Scotland/CVR2859/2020|2020-04-22|EPI_ISL_453079|Original|hCoV-19^^Scotland|Human|West of Scotland Specialist Virology Centre|COVID-19 Genomics UK (COG-UK) Consortium|Robertson|United Kingdom
>Spike|hCoV-19/USA/MA-MGH-01448/2020|2020-04-30|EPI_ISL_791950|Original|hCoV-19^^Massachusetts|Human|Massachusetts General Hospital|Infectious Disease Program|Park|USA
>Spike|hCoV-19/England/MILK-BE716B/2020|2020-11-29|EPI_ISL_709803|Original|hCoV-19^^England|Human|Lighthouse Lab in Milton Keynes|Wellcome Sanger Institute for the COVID-19 Genomics UK (COG-UK) Consortium|Davies|United Kingdom
>Spike|hCoV-19/USA/CA-CZB-3344/2020|2020-07-13|EPI_ISL_548270|Original|hCoV-19^^California|Human|Orange County Public Health Laboratory|Chan-Zuckerberg Biohub|Bhatt|USA
>Spike|hCoV-19/USA/TX-HMH-MCoV-12747/2020|2020-09-01|EPI_ISL_790503|Original|hCoV-19^^Texas|Human|Houston Methodist Hospital|Houston Methodist Hospital|Long|USA
>Spike|hCoV-19/England/ALDP-AA10BC/2020|2020-10-22|EPI_ISL_624276|Original|hCoV-19^^England|Human|Lighthouse Lab in Alderley Park|Wellcome Sanger Institute for the COVID-19 Genomics UK (COG-UK) consortium|Davies|United Kingdom
>Spike|hCoV-19/Scotland/GCVR-170714/2020|2020-03-25|EPI_ISL_489663|Original|hCoV-19^^Scotland|Human|NHSGGC West of Scotland Specialist Virology Centre / MRC-University of Glasgow Centre for Virus Research|Wellcome Sanger Institute for the COVID-19 Genomics UK (COG-UK) consortium|Davies|United Kingdom
>Spike|hCoV-19/India/OR-RMRC160/2020|2020-05-07|EPI_ISL_455761|Original|hCoV-19^^Odisha|Human|REGIONAL VRDL,ICMR-RMRC BBSR|Immunogenomics lab|Raghav|India
>Spike|hCoV-19/Denmark/DCGC-15269/2020|2020-11-23|EPI_ISL_713733|Original|hCoV-19^^Sjaelland|Human|Department of Virus and Microbiological Special Diagnostics|Albertsen Lab|Michaelsen|Denmark
>Spike|hCoV-19/England/QEUH-B9F2F6/2020|2020-11-18|EPI_ISL_721051|Original|hCoV-19^^England|Human|Lighthouse Lab in Glasgow|Wellcome Sanger Institute for the COVID-19 Genomics UK (COG-UK) Consortium|Davies|United Kingdom
>Spike|hCoV-19/Denmark/DCGC-9766/2020|2020-11-09|EPI_ISL_669159|Original|hCoV-19^^Hovedstaden|Human|Department of Virus and Microbiological Special Diagnostics|Albertsen Lab|Michaelsen|Denmark
>Spike|hCoV-19/USA/TX-DSHS-1071/2020|2020-06-16|EPI_ISL_628740|Original|hCoV-19^^Texas|Human|Texas Department of State Health Services|Texas Department of State Health Services|Rahman|USA
>Spike|hCoV-19/England/CAMC-B381E3/2020|2020-11-05|EPI_ISL_658864|Original|hCoV-19^^England|Human|Lighthouse Lab in Cambridge|Wellcome Sanger Institute for the COVID-19 Genomics UK (COG-UK) Consortium|Davies|United Kingdom
>Spike|hCoV-19/USA/CO-USAFSAM-S040/2020|2020-03-27|EPI_ISL_512805|Original|hCoV-19^^Colorado|Human|Public Health|Public Health|Fries|USA
>Spike|hCoV-19/USA/AZ-TG666139/2020|2020-05-02|EPI_ISL_696222|Original|hCoV-19^^Arizona|Human|Sonora Quest Laboratories|TGen North|French|USA
>Spike|hCoV-19/SaudiArabia/KAUST-JEDDAH757/2020|2020-04-01|EPI_ISL_513000|Original|hCoV-19^^Jeddah|Human|Pathogen Genomics Lab King Abdullah University of Science and Technology(KAUST)|Pathogen Genomics Lab King Abdullah University of Science and Technology(KAUST)|Mohamed Ghazzali|Saudi Arabia
>Spike|hCoV-19/USA/CA-IGI-0051/2020|2020-06-11|EPI_ISL_511806|Original|hCoV-19^^California|Human|Innovative Genomics Institute|Innovative Genomics Institute|Wyman|USA
>Spike|hCoV-19/USA/WA-UW-3158/2020|2020-03-28|EPI_ISL_491006|Original|hCoV-19^^Washington|Human|UW Virology Lab|UW Virology Lab|Roychoudhury|USA

>Spike|hCoV-19/Wales/PHWC-4961F9/2020|2020-11-20|EPI_ISL_726546|Original|hCoV-19^^Wales|Human|Originating lab|COVID-19 Genomics UK (COG-UK) Consortium|Moore|United Kingdom
>Spike|hCoV-19/England/ALDP-B85439/2020|2020-11-15|EPI_ISL_674471|Original|hCoV-19^^England|Human|Lighthouse Lab in Alderley Park|Wellcome Sanger Institute for the COVID-19 Genomics UK (COG-UK) Consortium|Davies|United Kingdom
>Spike|hCoV-19/England/QEUH-B12176/2020|2020-11-02|EPI_ISL_642302|Original|hCoV-19^^England|Human|Lighthouse Lab in Glasgow|Wellcome Sanger Institute for the COVID-19 Genomics UK (COG-UK) Consortium|Davies|United Kingdom
>Spike|hCoV-19/England/CAMC-A6647B/2020|2020-10-11|EPI_ISL_637384|Original|hCoV-19^^England|Human|Department of Pathology|COVID-19 Genomics UK (COG-UK) Consortium|Goodfellow|United Kingdom
>Spike|hCoV-19/Australia/VIC6166/2020|2020-07-23|EPI_ISL_518172|Original|hCoV-19^^Victoria|Human|Victorian Infectious Diseases Reference Laboratory (VIDRL)|VIDRL and MDU-PHL|Schultz|Australia
>Spike|hCoV-19/England/CAMC-B33528/2020|2020-11-05|EPI_ISL_659381|Original|hCoV-19^^England|Human|Lighthouse Lab in Cambridge|Wellcome Sanger Institute for the COVID-19 Genomics UK (COG-UK) Consortium|Davies|United Kingdom
>Spike|hCoV-19/Canada/ON-PHL-20-01326/2020|2020-06-00|EPI_ISL_548929|Original|hCoV-19^^Ontario|Human|Public Health Ontario Laboratory|Public Health Ontario Laboratory|Guthrie|Canada
>Spike|hCoV-19/France/ARA-18440/2020|2020-04-02|EPI_ISL_508969|Original|hCoV-19^^Auvergne-Rh\u00f4ne-Alpes|Human|CNR Virus des Infections Respiratoires - France SUD|CNR Virus des Infections Respiratoires - France SUD|Josset|France
>Spike|hCoV-19/Scotland/EDB6757/2020|2020-04-23|EPI_ISL_507200|Original|hCoV-19^^Scotland|Human|Virology Department|COVID-19 Genomics UK (COG-UK) Consortium|Williams|United Kingdom
>Spike|hCoV-19/New Zealand/20VR1076/2020|2020-03-22|EPI_ISL_456187|Original|hCoV-19^^Wairarapa|Human|Wellington SCL|Institute of Environmental Science and Research (ESR)|de Ligt|New Zealand
>Spike|hCoV-19/USA/TX-HMH-MCoV-19239/2020|2020-11-23|EPI_ISL_783711|Original|hCoV-19^^Texas|Human|Houston Methodist Hospital|Houston Methodist Hospital|Long|USA
>Spike|hCoV-19/USA/WI-WSLH-200118/2020|2020-04-09|EPI_ISL_578348|Original|hCoV-19^^Wisconsin|Human|Wisconsin State Laboratory of Hygiene Communicable Disease Division|Wisconsin State Laboratory of Hygiene Communicable Disease Division|Florek|USA
>Spike|hCoV-19/Denmark/DCGC-7695/2020|2020-10-19|EPI_ISL_618598|Original|hCoV-19^^Midtjylland|Human|Department of Virus and Microbiological Special Diagnostics|Albertsen lab|Michaelsen|Denmark
>Spike|hCoV-19/USA/WI-UW-MAYO8/2020|2020-03-18|EPI_ISL_500519|Original|hCoV-19^^Wisconsin|Human|Mayo Clinic Laboratories|University of Washington Virology Lab|Roychoudhury|USA
>Spike|hCoV-19/Luxembourg/LNS1741668/2020|2020-11-26|EPI_ISL_739986|Original|hCoV-19^^Luxembourg|Human|Laboratoire national de sant\u00e9|Laboratoire national de sant\u00e9|Wienecke-Baldacchino|Luxembourg
>Spike|hCoV-19/Wales/PHWC-486AB5/2020|2020-10-28|EPI_ISL_639106|Original|hCoV-19^^Wales|Human|Wales Specialist Virology Centre Sequencing lab|COVID-19 Genomics UK (COG-UK) Consortium|Moore|United Kingdom
>Spike|hCoV-19/England/PORT-2D5E62/2020|2020-12-19|EPI_ISL_763516|Original|hCoV-19^^England|Human|Centre for Enzyme Innovation|COVID-19 Genomics UK (COG-UK) Consortium|Robson|United Kingdom
>Spike|hCoV-19/England/C200042605/2020|2020-00-00|EPI_ISL_666731|Original|hCoV-19^^England|Human|Respiratory Virus Unit|COVID-19 Genomics UK (COG-UK) Consortium|Platt|United Kingdom
>Spike|hCoV-19/Wales/PHWC-47D583/2020|2020-10-07|EPI_ISL_586161|Original|hCoV-19^^Wales|Human|Wales Specialist Virology Centre Sequencing lab|COVID-19 Genomics UK (COG-UK) Consortium|Moore|United Kingdom
>Spike|hCoV-19/England/ALDP-B83EB5/2020|2020-11-14|EPI_ISL_675295|Original|hCoV-19^^England|Human|Lighthouse Lab in Alderley Park|Wellcome Sanger Institute for the COVID-19 Genomics UK (COG-UK) Consortium|Davies|United Kingdom

>Spike|hCoV-19/Australia/VIC2105/2020|2020-06-15|EPI_ISL_480713|Original|hCoV-19^^Victoria|Human|Victorian Infectious Diseases Reference Laboratory (VIDRL)|VIDRL and MDU-PHL|Schultz|Australia
>Spike|hCoV-19/Wales/QEUH-AE517D/2020|2020-10-27|EPI_ISL_647918|Original|hCoV-19^^Wales|Human|Lighthouse Lab in Glasgow|Wellcome Sanger Institute for the COVID-19 Genomics UK (COG-UK) Consortium|Davies|United Kingdom
>Spike|hCoV-19/USA/TX-HMH-MCoV-14719/2020|2020-10-11|EPI_ISL_786204|Original|hCoV-19^^Texas|Human|Houston Methodist Hospital|Houston Methodist Hospital|Long|USA
>Spike|hCoV-19/USA/MI-MDHHS-SC20680/2020|2020-05-14|EPI_ISL_471704|Original|hCoV-19^^Michigan|Human|Michigan Department of Health and Human Services|Michigan Department of Health and Human Services|Blankenship|USA
>Spike|hCoV-19/England/BRIS-131393/2020|2020-05-23|EPI_ISL_467708|Original|hCoV-19^^England|Human|PHE South West Regional Laboratory|Wellcome Sanger Institute for the COVID-19 Genomics UK (COG-UK) consortium|Davies|United Kingdom
>Spike|hCoV-19/USA/WI-UW-2638/2020|2020-12-31|EPI_ISL_791154|Original|hCoV-19^^Wisconsin|Human|University of Wisconsin-Madison AIDS Vaccine Research Laboratories|University of Wisconsin-Madison AIDS Vaccine Research Laboratories|Moreno|USA
>Spike|hCoV-19/USA/WA-S672/2020|2020-04-08|EPI_ISL_449842|Original|hCoV-19^^Washington|Human|Washington State Department of Health|Seattle Flu Study|Bedford|USA
>Spike|hCoV-19/USA/IL-NM-1021/2020|2020-06-02|EPI_ISL_626491|Original|hCoV-19^^Illinois|Human|Northwestern Memorial Hospital|Ozer Lab|Ozer|USA
>Spike|hCoV-19/England/MILK-9A95C5/2020|2020-09-01|EPI_ISL_551111|Original|hCoV-19^^England|Human|Lighthouse Lab in Milton Keynes|Wellcome Sanger Institute for the COVID-19 Genomics UK (COG-UK) consortium|Davies|United Kingdom
>Spike|hCoV-19/Denmark/DCGC-3877/2020|2020-09-07|EPI_ISL_615595|Original|hCoV-19^^Hovedstaden|Human|Department of Virus and Microbiological Special Diagnostics|Albertsen lab|Michaelsen|Denmark
>Spike|hCoV-19/Denmark/DCGC-10058/2020|2020-11-09|EPI_ISL_669368|Original|hCoV-19^^Nordjylland|Human|Department of Virus and Microbiological Special Diagnostics|Albertsen Lab|Michaelsen|Denmark
>Spike|hCoV-19/Bangladesh/GRMDRC/2020|2020-12-19|EPI_ISL_774976|Original|hCoV-19^^Dhaka|Human|Gonoshasthya-RNA Molecular Diagnostic and Research Center|Gonoshasthya-RNA Molecular Diagnostic and Research Center|Adnan|Bangladesh
>Spike|hCoV-19/Scotland/EDB1096/2020|2020-04-07|EPI_ISL_433392|Original|hCoV-19^^Scotland|Human|Virology Department|COVID-19 Genomics UK (COG-UK) Consortium|Williams|United Kingdom
>Spike|hCoV-19/USA/NY-NYUMC400/2020|2020-04-13|EPI_ISL_430427|Original|hCoV-19^^New York|Human|NYU Langone Health|Departments of Pathology and Medicine|Maurano|USA
>Spike|hCoV-19/Wales/PHWC-487F1F/2020|2020-10-29|EPI_ISL_637603|Original|hCoV-19^^Wales|Human|Wales Specialist Virology Centre Sequencing lab|COVID-19 Genomics UK (COG-UK) Consortium|Moore|United Kingdom
>Spike|hCoV-19/England/BRIS-1853DB1/2020|2020-04-28|EPI_ISL_492234|Original|hCoV-19^^England|Human|PHE South West Regional Laboratory|Wellcome Sanger Institute for the COVID-19 Genomics UK (COG-UK) consortium|Davies|United Kingdom
>Spike|hCoV-19/Scotland/QEUH-C5410E/2020|2020-12-12|EPI_ISL_757056|Original|hCoV-19^^Scotland|Human|Lighthouse Lab in Glasgow|Wellcome Sanger Institute for the COVID-19 Genomics UK (COG-UK) Consortium|Davies|United Kingdom
>Spike|hCoV-19/Canada/QC-LSPQ-L00239612/2020|2020-03-26|EPI_ISL_536255|Original|hCoV-19^^Quebec|Human|Centre hospitalier Anna-Laberge|Laboratoire de sant\u00e9 publique du Qu\u00e9bec|Moreira|Canada
>Spike|hCoV-19/England/PORT-2D76E0/2020|2020-10-22|EPI_ISL_650578|Original|hCoV-19^^England|Human|Centre for Enzyme Innovation|COVID-19 Genomics UK (COG-UK) Consortium|Robson|United Kingdom
>Spike|hCoV-19/Belgium/RS-030677/2020|2020-03-06|EPI_ISL_420333|Original|hCoV-19^^Seraing|Human|KU Leuven|KU Leuven|Maes|Belgium
>Spike|hCoV-19/England/QEUH-9F432B/2020|2020-09-24|EPI_ISL_588584|Original|hCoV-19^^England|Human|Lighthouse Lab in Glasgow|Wellcome Sanger Institute for the COVID-19 Genomics UK (COG-UK) consortium|Davies|United Kingdom

>Spike|hCoV-19/Wales/QEUH-AD3A59/2020|2020-10-26|EPI_ISL_634753|Original|hCoV-19^^Wales|Human|Lighthouse Lab in Glasgow|Wellcome Sanger Institute for the COVID-19 Genomics UK (COG-UK) consortium|Davies|United Kingdom
>Spike|hCoV-19/Brazil/SE-6556/2020|2020-04-27|EPI_ISL_541382|Original|hCoV-19^^Sergipe|Human|LACEN/SE|Laboratory of Respiratory Viruses and Measles|Silva|Brazil
>Spike|hCoV-19/USA/MN-MDH-1842/2020|2020-09-25|EPI_ISL_602977|Original|hCoV-19^^Minnesota|Human|Minnesota Department of Health|Minnesota Department of Health|Garfin|USA
>Spike|hCoV-19/Denmark/DCGC-20537/2020|2020-12-21|EPI_ISL_758862|Original|hCoV-19^^Syddanmark|Human|Department of Virus and Microbiological Special Diagnostics|Albertsen Lab|Michaelsen|Denmark
>Spike|hCoV-19/England/ALDP-A6EA9F/2020|2020-10-15|EPI_ISL_606966|Original|hCoV-19^^England|Human|Lighthouse Lab in Alderley Park|Wellcome Sanger Institute for the COVID-19 Genomics UK (COG-UK) consortium|Davies|United Kingdom
>Spike|hCoV-19/USA/UT-QDX-1844/2020|2020-03-15|EPI_ISL_604160|Original|hCoV-19^^Utah|Human|Quest Diagnostics|Quest Diagnostics|Kagan|USA
>Spike|hCoV-19/Denmark/DCGC-19504/2020|2020-12-07|EPI_ISL_748993|Original|hCoV-19^^Syddanmark|Human|Department of Virus and Microbiological Special Diagnostics|Albertsen Lab|Michaelsen|Denmark
>Spike|hCoV-19/USA/MN-MDH-1251/2020|2020-06-02|EPI_ISL_482972|Original|hCoV-19^^Minnesota|Human|Mayo Clinic & Mayo Clinic Laboratories|Minnesota Department of Health|Garfin|USA
>Spike|hCoV-19/Denmark/DCGC-17227/2020|2020-12-14|EPI_ISL_750840|Original|hCoV-19^^Midtjylland|Human|Department of Virus and Microbiological Special Diagnostics|Albertsen Lab|Michaelsen|Denmark
>Spike|hCoV-19/Germany/HE-FFM4clin/2020|2020-02-00|EPI_ISL_447610|Original|hCoV-19^^Hesse|Human|Goethe University Hospital Frankfurt|Institute for Medical Virology|Toptan Grabmair|Germany
>Spike|hCoV-19/USA/MA-MASPHL-00396/2020|2020-05-07|EPI_ISL_693150|Original|hCoV-19^^Massachusetts|Human|Massachusetts State Public Health Laboratory|Massachusetts State Public Health Laboratory|Lang|USA
>Spike|hCoV-19/USA/CA-CDPH282/2020|2020-11-14|EPI_ISL_755107|Original|hCoV-19^^California|Human|California Department of Public Health|California Department of Public Health|Bell|USA
>Spike|hCoV-19/England/LOND-D5D58/2020|2020-04-18|EPI_ISL_444245|Original|hCoV-19^^England|Human|University College London|COVID-19 Genomics UK (COG-UK) Consortium|Roy|United Kingdom
>Spike|hCoV-19/Wales/PHWC-48DA27/2020|2020-11-01|EPI_ISL_680714|Original|hCoV-19^^Wales|Human|Wales Specialist Virology Centre Sequencing lab|COVID-19 Genomics UK (COG-UK) Consortium|Moore|United Kingdom
>Spike|hCoV-19/USA/LA-EVTL804/2020|2020-07-24|EPI_ISL_653495|Original|hCoV-19^^Louisiana|Human|LSUHS Emerging Viral Threat Laboratory|Microbial Genome Sequencing Center|Kamil|USA
>Spike|hCoV-19/Wales/PHWC-25E1A/2020|2020-03-25|EPI_ISL_422331|Original|hCoV-19^^Wales|Human|Wales Specialist Virology Centre|Public Health Wales Microbiology Cardiff|Moore|United Kingdom
>Spike|hCoV-19/USA/LA-EVTL1080/2020|2020-11-28|EPI_ISL_768350|Original|hCoV-19^^Louisiana|Human|LSUHS Emerging Viral Threat Laboratory|Microbial Genome Sequencing Center|Kamil|USA
>Spike|hCoV-19/USA/CA-QDX-1551/2020|2020-03-18|EPI_ISL_603797|Original|hCoV-19^^California|Human|Quest Diagnostics|Quest Diagnostics|Kagan|USA
>Spike|hCoV-19/Japan/PG-5353/2020|2020-08-00|EPI_ISL_690456|Original|hCoV-19^^Japan|Human|Fukuoka Institute of Health and Environmental Sciences|Pathogen Genomics Center|Sekizuka|Japan
>Spike|hCoV-19/Denmark/DCGC-21004/2020|2020-12-21|EPI_ISL_758931|Original|hCoV-19^^Midtjylland|Human|Department of Virus and Microbiological Special Diagnostics|Albertsen Lab|Michaelsen|Denmark
>Spike|hCoV-19/England/QEUH-AAEC8E/2020|2020-10-22|EPI_ISL_652995|Original|hCoV-19^^England|Human|Oxford Viromics|COVID-19 Genomics UK (COG-UK) Consortium|Golubchik|United Kingdom

>Spike|hCoV-19/Australia/VIC16189/2020|2020-00-00|EPI_ISL_640988|Original|hCoV-19^^Victoria|Human|Microbiological Diagnostic Unit - Public Health Laboratory (MDU-PHL)|MDU-PHL|Schultz|Australia
>Spike|hCoV-19/Scotland/QEUH-C01DC0/2020|2020-12-05|EPI_ISL_757258|Original|hCoV-19^^Scotland|Human|Lighthouse Lab in Glasgow|Wellcome Sanger Institute for the COVID-19 Genomics UK (COG-UK) Consortium|Davies|United Kingdom
>Spike|hCoV-19/Russia/SPE-RII-23386V/2020|2020-06-11|EPI_ISL_524046|Vero P1|hCoV-19^^Saint Petersburg|Human|WHO National Influenza Centre Russian Federation|WHO National Influenza Centre Russian Federation|Komissarov|Russia
>Spike|hCoV-19/Wales/ALDP-BB9469/2020|2020-11-20|EPI_ISL_702641|Original|hCoV-19^^Wales|Human|Lighthouse Lab in Alderley Park|Wellcome Sanger Institute for the COVID-19 Genomics UK (COG-UK) Consortium|Davies|United Kingdom
>Spike|hCoV-19/England/OXON-ADBB9/2020|2020-00-00|EPI_ISL_448620|Original|hCoV-19^^England|Human|Oxford Viromics|COVID-19 Genomics UK (COG-UK) Consortium|Golubchik|United Kingdom
>Spike|hCoV-19/South Korea/KDCA0298/2020|2020-10-27|EPI_ISL_760092|Original|hCoV-19^^South Korea|Human|Division of Emerging Infectious Diseases|Division of Emerging Infectious Diseases|Park|South Korea
>Spike|hCoV-19/France/PAC-IHU-0203/2020|2020-03-27|EPI_ISL_569369|Original|hCoV-19^^Provence-Alpes-C\u00f4te-d\u2019Azur|Human|MEPHI|MEPHI|Levasseur|France
>Spike|hCoV-19/England/MILK-9A8739/2020|2020-08-31|EPI_ISL_549416|Original|hCoV-19^^England|Human|Oxford Viromics|COVID-19 Genomics UK (COG-UK) Consortium|Golubchik|United Kingdom
>Spike|hCoV-19/USA/NY-NYCPHL-001456/2020|2020-11-25|EPI_ISL_683818|Original|hCoV-19^^New York|Human|DOHMH Corona|New York City Public Health Laboratory|Wang|USA
>Spike|hCoV-19/USA/MA-MASPHL-00622/2020|2020-05-11|EPI_ISL_693003|Original|hCoV-19^^Massachusetts|Human|Massachusetts State Public Health Laboratory|Massachusetts State Public Health Laboratory|Lang|USA
>Spike|hCoV-19/Canada/ON-S1313/2020|2020-04-10|EPI_ISL_586350|Original|hCoV-19^^Ontario|Human|Toronto Invasive Bacterial Diseases Network|McMaster University|McArthur|Canada
>Spike|hCoV-19/USA/WA-S1185/2020|2020-05-06|EPI_ISL_463675|Original|hCoV-19^^Washington|Human|Washington State Department of Health|Seattle Flu Study|Bedford|USA
>Spike|hCoV-19/England/MILK-992969/2020|2020-08-27|EPI_ISL_551997|Original|hCoV-19^^England|Human|Lighthouse Lab in Milton Keynes|Wellcome Sanger Institute for the COVID-19 Genomics UK (COG-UK) consortium|Davies|United Kingdom
>Spike|hCoV-19/Canada/NS-NML-1343/2020|2020-04-12|EPI_ISL_579720|Original|hCoV-19^^Nova Scotia|Human|QEII Health Sciences Centre|National Microbiology Laboratory (NML)|Majer|Canada
>Spike|hCoV-19/Scotland/QEUH-C4BFD3/2020|2020-12-14|EPI_ISL_757105|Original|hCoV-19^^Scotland|Human|Lighthouse Lab in Glasgow|Wellcome Sanger Institute for the COVID-19 Genomics UK (COG-UK) Consortium|Davies|United Kingdom
>Spike|hCoV-19/Denmark/DCGC-13831/2020|2020-11-30|EPI_ISL_712208|Original|hCoV-19^^Hovedstaden|Human|Department of Virus and Microbiological Special Diagnostics|Albertsen Lab|Michaelsen|Denmark
>Spike|hCoV-19/England/ALDP-9CD5BC/2020|2020-09-16|EPI_ISL_566157|Original|hCoV-19^^England|Human|Lighthouse Lab in Alderley Park|Wellcome Sanger Institute for the COVID-19 Genomics UK (COG-UK) consortium|Davies|United Kingdom
>Spike|hCoV-19/England/ALDP-A69126/2020|2020-10-13|EPI_ISL_608658|Original|hCoV-19^^England|Human|Lighthouse Lab in Alderley Park|Wellcome Sanger Institute for the COVID-19 Genomics UK (COG-UK) consortium|Davies|United Kingdom
>Spike|hCoV-19/South Korea/KDCA0073/2020|2020-09-23|EPI_ISL_747314|Original|hCoV-19^^South Korea|Human|Division of Emerging Infectious Diseases|Division of Emerging Infectious Diseases|Park|South Korea
>Spike|hCoV-19/USA/WA-S1105/2020|2020-05-04|EPI_ISL_463595|Original|hCoV-19^^Washington|Human|Washington State Department of Health|Seattle Flu Study|Bedford|USA

>Spike|hCoV-19/Rwanda/NRLNAT1001/2020|2020-08-27|EPI_ISL_735436|Original|hCoV-19^^Kigali|Human|Nucleic Acid Testing - Rwanda National Reference Laboratory|GIGA Medical Genomics|Butera|Rwanda
>Spike|hCoV-19/USA/AZ-TG396357/2020|2020-06-10|EPI_ISL_694815|Original|hCoV-19^^Arizona|Human|AZ SPHL|TGen North|French|USA
>Spike|hCoV-19/Australia/VIC13700/2020|2020-09-02|EPI_ISL_563810|Original|hCoV-19^^Victoria|Human|Microbiological Diagnostic Unit - Public Health Laboratory (MDU-PHL)|MDU-PHL|Schultz|Australia
>Spike|hCoV-19/Hong Kong/VM20040267/2020|2020-07-13|EPI_ISL_523850|Original|hCoV-19^^Hong Kong|Human|Tseung Kwan O Hospital|Hong Kong Department of Health|Mak|China
>Spike|hCoV-19/Brazil/PE-IAM238/2020|2020-04-09|EPI_ISL_500475|Original|hCoV-19^^Pernambuco|Human|LACEN/PE|WallauLab|Wallau|Brazil
>Spike|hCoV-19/Denmark/DCGC-6341/2020|2020-09-28|EPI_ISL_622309|Original|hCoV-19^^Hovedstaden|Human|Department of Virus and Microbiological Special Diagnostics|Albertsen lab|Michaelsen|Denmark
>Spike|hCoV-19/India/WB-S67/2020|2020-05-28|EPI_ISL_508457|Original|hCoV-19^^West Bengal|Human|ICMR-National Institute of Cholera and Enteric Diseases|National Institute of Biomedical Genomics|Maitra|India
>Spike|hCoV-19/Wales/PHWC-4A68E2/2020|2020-12-17|EPI_ISL_765082|Original|hCoV-19^^Wales|Human|Wales Specialist Virology Centre Sequencing lab|COVID-19 Genomics UK (COG-UK) Consortium|Moore|United Kingdom
>Spike|hCoV-19/England/MILK-9941BA/2020|2020-08-24|EPI_ISL_551698|Original|hCoV-19^^England|Human|Lighthouse Lab in Milton Keynes|Wellcome Sanger Institute for the COVID-19 Genomics UK (COG-UK) consortium|Davies|United Kingdom
>Spike|hCoV-19/USA/AZ-TG366746/2020|2020-06-02|EPI_ISL_694501|Original|hCoV-19^^Arizona|Human|AZ SPHL|TGen North|French|USA
>Spike|hCoV-19/England/QEUH-CAD740/2020|2020-12-20|EPI_ISL_761168|Original|hCoV-19^^England|Human|Lighthouse Lab in Glasgow|Wellcome Sanger Institute for the COVID-19 Genomics UK (COG-UK) Consortium|Davies|United Kingdom
>Spike|hCoV-19/USA/OR-OHSU-0627/2020|2020-06-13|EPI_ISL_525856|Original|hCoV-19^^Oregon|Human|OHSU Lab Services Molecular Microbiology Lab|Oregon SARS-CoV-2 Genome Sequencing Center|O'Roak|USA
>Spike|hCoV-19/England/NORW-F37FC/2020|2020-12-07|EPI_ISL_725139|Original|hCoV-19^^England|Human|Quadram Institute Bioscience|COVID-19 Genomics UK (COG-UK) Consortium|Alikhan|United Kingdom
>Spike|hCoV-19/India/MH-NIV-124593/2020|2020-07-20|EPI_ISL_541692|Original|hCoV-19^^Maharashtra|Human|National Institute of Virology|National Institute of Virology|Potdar|India
>Spike|hCoV-19/Jordan/AM-ALSR-3948/2020|2020-09-30|EPI_ISL_636312|Original|hCoV-19^^Amman|Human|Biolab Diagnostic Laboratories|Andersen lab at Scripps Research|Gangavarapu|Jordan
>Spike|hCoV-19/USA/NY-NYCPHL-000350/2020|2020-04-25|EPI_ISL_631859|Original|hCoV-19^^New York|Human|St Barnabas Hospital|New York City Public Health Laboratory|Wang|USA
>Spike|hCoV-19/Wales/PHWC-16DF4F/2020|2020-09-29|EPI_ISL_585643|Original|hCoV-19^^Wales|Human|Wales Specialist Virology Centre Sequencing lab|COVID-19 Genomics UK (COG-UK) Consortium|Moore|United Kingdom
>Spike|hCoV-19/England/LOND-DB37E/2020|2020-11-13|EPI_ISL_724760|Original|hCoV-19^^England|Human|University College London|COVID-19 Genomics UK (COG-UK) Consortium|Roy|United Kingdom
>Spike|hCoV-19/Portugal/PT0325/2020|2020-03-16|EPI_ISL_454041|Original|hCoV-19^^Portugal|Human|unknown|Instituto Nacional de Saude (INSA)|Borges|Portugal
>Spike|hCoV-19/England/MILK-A7C7B5/2020|2020-10-15|EPI_ISL_607520|Original|hCoV-19^^England|Human|Lighthouse Lab in Milton Keynes|Wellcome Sanger Institute for the COVID-19 Genomics UK (COG-UK) consortium|Davies|United Kingdom
>Spike|hCoV-19/Portugal/PT1964/2020|2020-11-24|EPI_ISL_732189|Original|hCoV-19^^Portugal|Human|Instituto Nacional de Saude (INSA) and Instituto Gulbenkian de Ciencia (IGC)|Instituto Nacional de Saude (INSA) and Instituto Gulbenkian de Ciencia (IGC)|Borges|Portugal

>Spike|hCoV-19/Denmark/DCGC-20861/2020|2020-12-21|EPI_ISL_758778|Original|hCoV-19^^Hovedstaden|Human|Department of Virus and Microbiological Special Diagnostics|Albertsen Lab|Michaelsen|Denmark
>Spike|hCoV-19/England/BRIS-123EFD/2020|2020-04-08|EPI_ISL_492799|Original|hCoV-19^^England|Human|PHE South West Regional Laboratory|Wellcome Sanger Institute for the COVID-19 Genomics UK (COG-UK) consortium|Davies|United Kingdom
>Spike|hCoV-19/England/NORW-EB29C/2020|2020-05-03|EPI_ISL_457414|Original|hCoV-19^^England|Human|Quadram Institute Bioscience|COVID-19 Genomics UK (COG-UK) Consortium|Alikhan|United Kingdom
>Spike|hCoV-19/Slovenia/7484/2020|2020-03-14|EPI_ISL_635242|Original|hCoV-19^^Slovenia|Human|Institute of Microbiology and Immunology|Institute of Microbiology and Immunology|Zakotnik|Slovenia
>Spike|hCoV-19/Scotland/CVR2232/2020|2020-04-12|EPI_ISL_490675|Original|hCoV-19^^Scotland|Human|West of Scotland Specialist Virology Centre|COVID-19 Genomics UK (COG-UK) Consortium|Robertson|United Kingdom
>Spike|hCoV-19/Cote dIvoire/BKE0247/2020|2020-06-19|EPI_ISL_614364|Original|hCoV-19^^Boauk\u00e9|Human|Molecular diagnostic unit for viral haemorrhagic fevers and emerging viruses|Project group Epidemology of Highly Pathogenic Microorganisms|Calvignac-Spencer|C\u00f4te d'Ivoire
>Spike|hCoV-19/Brazil/SP-437/2020|2020-07-11|EPI_ISL_603034|Original|hCoV-19^^S\u00e3o Paulo|Human|Departamento de Vigil\u00e2ncia \u00e0 Sa\u00fade|Instituto Adolfo Lutz|Sacchi|Brazil
>Spike|hCoV-19/USA/UT-UPHL-2012588723/2020|2020-10-25|EPI_ISL_746253|Original|hCoV-19^^Utah|Human|Ginkgo Bioworks Clinical Laboratory|Utah Public Health Laboratory|Young|USA
>Spike|hCoV-19/USA/UT-UPHL-2012841191/2020|2020-08-13|EPI_ISL_745909|Original|hCoV-19^^Utah|Human|Ginkgo Bioworks Clinical Laboratory|Utah Public Health Laboratory|Young|USA
>Spike|hCoV-19/England/CAMC-9DF863/2020|2020-09-21|EPI_ISL_638428|Original|hCoV-19^^England|Human|Department of Pathology|COVID-19 Genomics UK (COG-UK) Consortium|Goodfellow|United Kingdom
>Spike|hCoV-19/Lithuania/MR-LUHS-Eilnr237654/2020|2020-12-22|EPI_ISL_770520|Original|hCoV-19^^Kauno apskritis|Human|Lithuanian University of Health Sciences Hospital|Lithuanian University of Health Sciences|Zemaitis|Lithuania
>Spike|hCoV-19/Spain/MD-IBV-99007148/2020|2020-08-11|EPI_ISL_541920|Original|hCoV-19^^Madrid|Human|Hospital General Universitario Gregorio Mara\u00f1\u00f3n|SeqCOVID-SPAIN consortium/IBV(CSIC)|Comas|Spain
>Spike|hCoV-19/Denmark/DCGC-2663/2020|2020-08-17|EPI_ISL_617883|Original|hCoV-19^^Hovedstaden|Human|Department of Virus and Microbiological Special Diagnostics|Albertsen lab|Michaelsen|Denmark
>Spike|hCoV-19/Bangladesh/BCSIR-NILMRC-363/2020|2020-07-24|EPI_ISL_514236|Original|hCoV-19^^Chittagong|Human|National Institute of Laboratory Medicine and Referral Center|Genomic Research Lab|Khan|Bangladesh
>Spike|hCoV-19/England/MILK-9570F2/2020|2020-06-10|EPI_ISL_554276|Original|hCoV-19^^England|Human|Lighthouse Lab in Milton Keynes|Wellcome Sanger Institute for the COVID-19 Genomics UK (COG-UK) consortium|Davies|United Kingdom
>Spike|hCoV-19/Japan/PG-7917/2020|2020-07-00|EPI_ISL_687018|Original|hCoV-19^^Japan|Human|Pathogen Genomics Center|Pathogen Genomics Center|Sekizuka|Japan
>Spike|hCoV-19/England/MILK-B06BB3/2020|2020-10-29|EPI_ISL_652264|Original|hCoV-19^^England|Human|Quadram Institute Bioscience|COVID-19 Genomics UK (COG-UK) Consortium|Alikhan|United Kingdom
>Spike|hCoV-19/Northern Ireland/QEUH-96AB19/2020|2020-08-17|EPI_ISL_531301|Original|hCoV-19^^Northern Ireland|Human|Lighthouse Lab in Glasgow|Wellcome Sanger Institute for the COVID-19 Genomics UK (COG-UK) consortium|Davies|United Kingdom
>Spike|hCoV-19/England/204723031/2020|2020-11-17|EPI_ISL_766094|Original|hCoV-19^^England|Human|Respiratory Virus Unit|COVID-19 Genomics UK (COG-UK) Consortium|Platt|United Kingdom

>Spike|hCoV-19/England/QEUH-9CA69E/2020|2020-09-11|EPI_ISL_602035|Original|hCoV-19^^England|Human|Lighthouse Lab in Glasgow|Wellcome Sanger Institute for the COVID-19 Genomics UK (COG-UK) consortium|Davies|United Kingdom
>Spike|hCoV-19/USA/TX-HMH-3194/2020|2020-06-12|EPI_ISL_544067|Original|hCoV-19^^Texas|Human|Houston Methodist Hospital|Houston Methodist Hospital|Long|USA
>Spike|hCoV-19/USA/NY-NYUMC186/2020|2020-04-01|EPI_ISL_427482|Original|hCoV-19^^New York|Human|NYU Langone Health|Departments of Pathology and Medicine|Maurano|USA
>Spike|hCoV-19/Wales/PHWC-48FC3E/2020|2020-11-11|EPI_ISL_681205|Original|hCoV-19^^Wales|Human|Wales Specialist Virology Centre Sequencing lab|COVID-19 Genomics UK (COG-UK) Consortium|Moore|United Kingdom
>Spike|hCoV-19/Wales/PHWC-16D8BD/2020|2020-09-25|EPI_ISL_577459|Original|hCoV-19^^Wales|Human|Wales Specialist Virology Centre Sequencing lab|COVID-19 Genomics UK (COG-UK) Consortium|Moore|United Kingdom
>Spike|hCoV-19/Canada/BC_23252816/2020|2020-04-00|EPI_ISL_477036|Original|hCoV-19^^British Columbia|Human|BCCDC Public Health Laboratory|BCCDC Public Health Laboratory|Harrigan|Canada
>Spike|hCoV-19/USA/CA-CZB0114/2020|2020-03-28|EPI_ISL_429058|Original|hCoV-19^^California|Human|UCSF Clinical Microbiology Laboratory|Chan-Zuckerberg Biohub|Kamm|USA
>Spike|hCoV-19/Denmark/DCGC-19750/2020|2020-12-07|EPI_ISL_747757|Original|hCoV-19^^Midtjylland|Human|Department of Virus and Microbiological Special Diagnostics|Albertsen Lab|Michaelsen|Denmark
>Spike|hCoV-19/England/ALDP-952D96/2020|2020-06-11|EPI_ISL_553632|Original|hCoV-19^^England|Human|Lighthouse Lab in Alderley Park|Wellcome Sanger Institute for the COVID-19 Genomics UK (COG-UK) consortium|Davies|United Kingdom
>Spike|hCoV-19/Australia/VIC2397/2020|2020-06-30|EPI_ISL_522113|Original|hCoV-19^^Victoria|Human|Microbiological Diagnostic Unit - Public Health Laboratory (MDU-PHL)|MDU-PHL|Schultz|Australia
>Spike|hCoV-19/Japan/PG-7610/2020|2020-07-00|EPI_ISL_686712|Original|hCoV-19^^Japan|Human|Chiba Prefectural Institute of Public Health|Pathogen Genomics Center|Sekizuka|Japan
>Spike|hCoV-19/Switzerland/BE-ETHZ-420316/2020|2020-12-20|EPI_ISL_768018|Original|hCoV-19^^Bern|Human|Viollier AG|Department of Biosystems Science and Engineering|Nadeau|Switzerland
>Spike|hCoV-19/England/NORT-295C64/2020|2020-04-04|EPI_ISL_488648|Original|hCoV-19^^England|Human|NU-OMICS DNA Sequencing research facility|Wellcome Sanger Institute for the COVID-19 Genomics UK (COG-UK) consortium|Davies|United Kingdom
>Spike|hCoV-19/USA/MI-MDHHS-SC22281/2020|2020-10-20|EPI_ISL_666947|Original|hCoV-19^^Michigan|Human|Michigan Department of Health and Human Services|Michigan Department of Health and Human Services|Jacob|USA
>Spike|hCoV-19/England/ALDP-955689/2020|2020-06-15|EPI_ISL_554325|Original|hCoV-19^^England|Human|Lighthouse Lab in Alderley Park|Wellcome Sanger Institute for the COVID-19 Genomics UK (COG-UK) consortium|Davies|United Kingdom
>Spike|hCoV-19/England/CAMC-B98E08/2020|2020-11-15|EPI_ISL_673714|Original|hCoV-19^^England|Human|Lighthouse Lab in Cambridge|Wellcome Sanger Institute for the COVID-19 Genomics UK (COG-UK) Consortium|Davies|United Kingdom
>Spike|hCoV-19/Jordan/AM-ALSR-4059/2020|2020-10-03|EPI_ISL_636419|Original|hCoV-19^^Amman|Human|Biolab Diagnostic Laboratories|Andersen lab at Scripps Research|Gangavarapu|Jordan
>Spike|hCoV-19/Scotland/EDB3268/2020|2020-04-07|EPI_ISL_489011|Original|hCoV-19^^Scotland|Human|Virology Department|Wellcome Sanger Institute for the COVID-19 Genomics UK (COG-UK) consortium|Davies|United Kingdom
>Spike|hCoV-19/Netherlands/Flevoland_4/2020|2020-04-01|EPI_ISL_455113|Original|hCoV-19^^Flevoland|Human|Dutch COVID-19 response team|Erasmus Medical Center|Oude Munnink|Netherlands
>Spike|hCoV-19/Wales/PHWC-2B0D2/2020|2020-04-08|EPI_ISL_432268|Original|hCoV-19^^Wales|Human|Wales Specialist Virology Centre|Public Health Wales Microbiology Cardiff|Moore|United Kingdom
>Spike|hCoV-19/USA/CA-CZB-13807/2020|2020-08-17|EPI_ISL_739115|Original|hCoV-19^^California|Human|Alameda County Public Health Lab|Chan-Zuckerberg Biohub|Kamm|USA

>Spike|hCoV-19/France/PAC-IHU-0974/2020|2020-07-22|EPI_ISL_569021|Original|hCoV-19^^Provence-Alpes-C\u00f4te-d\u2019Azur|Human|MEPHI|MEPHI|Levasseur|France
>Spike|hCoV-19/England/QEUH-B7F97F/2020|2020-11-14|EPI_ISL_668292|Original|hCoV-19^^England|Human|Lighthouse Lab in Glasgow|Wellcome Sanger Institute for the COVID-19 Genomics UK (COG-UK) Consortium|Davies|United Kingdom
>Spike|hCoV-19/England/ALDP-B1BFBF/2020|2020-11-01|EPI_ISL_645682|Original|hCoV-19^^England|Human|Lighthouse Lab in Alderley Park|Wellcome Sanger Institute for the COVID-19 Genomics UK (COG-UK) Consortium|Davies|United Kingdom
>Spike|hCoV-19/Scotland/QEUH-962AF6/2020|2020-08-11|EPI_ISL_531858|Original|hCoV-19^^Scotland|Human|NHSGGC West of Scotland Specialist Virology Centre / MRC-University of Glasgow Centre for Virus Research|Wellcome Sanger Institute for the COVID-19 Genomics UK (COG-UK) consortium|Davies|United Kingdom
>Spike|hCoV-19/Wales/PHWC-490872/2020|2020-11-14|EPI_ISL_706518|Original|hCoV-19^^Wales|Human|Originating lab|COVID-19 Genomics UK (COG-UK) Consortium|Moore|United Kingdom
>Spike|hCoV-19/Netherlands/NB-EMC-430/2020|2020-11-16|EPI_ISL_722276|Original|hCoV-19^^Noord Brabant|Human|Dutch COVID-19 response team|Erasmus Medical Center|Oude Munnink|Netherlands
>Spike|hCoV-19/England/QEUH-B0E2C5/2020|2020-11-02|EPI_ISL_642126|Original|hCoV-19^^England|Human|Lighthouse Lab in Glasgow|Wellcome Sanger Institute for the COVID-19 Genomics UK (COG-UK) Consortium|Davies|United Kingdom
>Spike|hCoV-19/England/ALDP-B1DAAD/2020|2020-11-01|EPI_ISL_646137|Original|hCoV-19^^England|Human|Lighthouse Lab in Alderley Park|Wellcome Sanger Institute for the COVID-19 Genomics UK (COG-UK) Consortium|Davies|United Kingdom
>Spike|hCoV-19/England/ALDP-6C9A0F/2020|2020-07-02|EPI_ISL_556129|Original|hCoV-19^^England|Human|Lighthouse Lab in Alderley Park|Wellcome Sanger Institute for the COVID-19 Genomics UK (COG-UK) consortium|Davies|United Kingdom
>Spike|hCoV-19/Russia/MOS-CRIE-13668879/2020|2020-11-09|EPI_ISL_745250|Original|hCoV-19^^Moscow|Human|Molecular diagnostic laboratory of Federal Budget Institution of Science \Central Research Institute of Epidemiology\ of The Federal Service on Customers' Rights Protection and Human Well-being Surveillance|Group of Genomics and Postgenomic Technologies of Central Research Institute of Epidemilolog
>y|Korneenko|Russia
>Spike|hCoV-19/Portugal/PT1416/2020|2020-03-30|EPI_ISL_511375|Original|hCoV-19^^Portugal|Human|Instituto Nacional de Saude (INSA)|Instituto Nacional de Saude (INSA)|Borges|Portugal
>Spike|hCoV-19/USA/NE-10939/2020|2020-05-04|EPI_ISL_466670|Original|hCoV-19^^Nebraska|Human|Nebraska Public Health Laboratory|UNMC COVID-19 Response Team|Pratt|USA
>Spike|hCoV-19/Netherlands/ZH-EMC-874/2020|2020-10-05|EPI_ISL_632755|Original|hCoV-19^^Zuid Holland|Human|Dutch COVID-19 response team|Erasmus Medical Center|Oude Munnink|Netherlands
>Spike|hCoV-19/England/CAMC-A6599B/2020|2020-10-11|EPI_ISL_608510|Original|hCoV-19^^England|Human|Lighthouse Lab in Cambridge|Wellcome Sanger Institute for the COVID-19 Genomics UK (COG-UK) consortium|Davies|United Kingdom
>Spike|hCoV-19/India/GJ-GBRC207b/2020|2020-06-11|EPI_ISL_475034|Original|hCoV-19^^Gujarat|Human|Department of MicroBiology|Gujarat Biotechnology Research Centre|Joshi|India
>Spike|hCoV-19/USA/TX-HMH-6958/2020|2020-06-29|EPI_ISL_546385|Original|hCoV-19^^Texas|Human|Houston Methodist Hospital|Houston Methodist Hospital|Long|USA
>Spike|hCoV-19/USA/WA-UW-2022/2020|2020-03-23|EPI_ISL_485975|Original|hCoV-19^^Washington|Human|UW Virology Lab|UW Virology Lab|Roychoudhury|USA
>Spike|hCoV-19/South Africa/R12695-20/2020|2020-06-16|EPI_ISL_504229|Original|hCoV-19^^North West|Human|National Institute for Communicable Diseases of the National Health Laboratory Service|National Institute for Communicable Diseases of the National Health Laboratory Service|Allam|South Africa
>Spike|hCoV-19/USA/MA-MGH-00471/2020|2020-04-04|EPI_ISL_460168|Original|hCoV-19^^Massachusetts|Human|Massachusetts General Hospital|Infectious Disease Program|Lemieux|USA

>Spike|hCoV-19/England/CAMC-9B1F26/2020|2020-09-03|EPI_ISL_549403|Original|hCoV-19^^England|Human|Oxford Viromics|COVID-19 Genomics UK (COG-UK) Consortium|Golubchik|United Kingdom
>Spike|hCoV-19/England/CAMC-B0AE01/2020|2020-10-30|EPI_ISL_643422|Original|hCoV-19^^England|Human|Lighthouse Lab in Cambridge|Wellcome Sanger Institute for the COVID-19 Genomics UK (COG-UK) Consortium|Davies|United Kingdom
>Spike|hCoV-19/Scotland/CVR5327/2020|2020-10-24|EPI_ISL_650763|Original|hCoV-19^^Scotland|Human|West of Scotland Specialist Virology Centre|COVID-19 Genomics UK (COG-UK) Consortium|Robertson|United Kingdom
>Spike|hCoV-19/USA/WI-GMF-01455/2020|2020-04-21|EPI_ISL_435394|Original|hCoV-19^^Iowa|Human|Gundersen Molecular Diagnostics Laboratory|Kabara Cancer Research Institute|Kenny|USA
>Spike|hCoV-19/Spain/AN-IBV-98001177/2020|2020-03-26|EPI_ISL_538191|Original|hCoV-19^^Andalucia|Human|Servicio de Microbiolog\u00eda y Parasitolog\u00eda cl\u00ednica. UCEIMP. Hospital Universitario Virgen del Roc\u00edo/IBIS/CSIC/US|SeqCOVID-SPAIN consortium/IBV(CSIC)|Comas|Spain
>Spike|hCoV-19/England/MILK-9C37CF/2020|2020-09-10|EPI_ISL_566815|Original|hCoV-19^^England|Human|Lighthouse Lab in Milton Keynes|Wellcome Sanger Institute for the COVID-19 Genomics UK (COG-UK) consortium|Davies|United Kingdom
>Spike|hCoV-19/DRC/D09-0678/2020|2020-05-12|EPI_ISL_636984|Original|hCoV-19^^Kinshasa|Human|Virology Lab|Project group Epidemology of Highly Pathogenic Microorganisms|Calvignac-Spencer|Democratic Republic of the Congo
>Spike|hCoV-19/USA/MI-MDHHS-SC20350/2020|2020-03-30|EPI_ISL_450629|Original|hCoV-19^^Michigan|Human|Michigan Department of Health and Human Services|Michigan Department of Health and Human Services|Blankenship|USA
>Spike|hCoV-19/England/MILK-9A69BD/2020|2020-09-01|EPI_ISL_550246|Original|hCoV-19^^England|Human|Lighthouse Lab in Milton Keynes|Wellcome Sanger Institute for the COVID-19 Genomics UK (COG-UK) consortium|Davies|United Kingdom
>Spike|hCoV-19/France/ARA-SC108/2020|2020-04-07|EPI_ISL_700372|Original|hCoV-19^^Auvergne-Rh\u00f4ne-Alpes|Human|Centre hospitalier M\u00e9tropole Savoie|CNR Virus des Infections Respiratoires - France SUD|Bal|France
>Spike|hCoV-19/England/QEUH-A4C138/2020|2020-10-05|EPI_ISL_628545|Original|hCoV-19^^England|Human|Oxford Viromics|COVID-19 Genomics UK (COG-UK) Consortium|Golubchik|United Kingdom
>Spike|hCoV-19/Australia/VIC13331/2020|2020-07-28|EPI_ISL_563678|Original|hCoV-19^^Victoria|Human|Microbiological Diagnostic Unit - Public Health Laboratory (MDU-PHL)|MDU-PHL|Schultz|Australia
>Spike|hCoV-19/Denmark/DCGC-16269/2020|2020-11-16|EPI_ISL_714292|Original|hCoV-19^^Syddanmark|Human|Department of Virus and Microbiological Special Diagnostics|Albertsen Lab|Michaelsen|Denmark
>Spike|hCoV-19/England/LCST-24D67C8/2020|2020-05-30|EPI_ISL_478294|Original|hCoV-19^^England|Human|University Hospitals Of Leicester NHS Trust and DeepSeq Nottingham|COVID-19 Genomics UK (COG-UK) Consortium|Loose|United Kingdom
>Spike|hCoV-19/Denmark/DCGC-4456/2020|2020-08-31|EPI_ISL_615641|Original|hCoV-19^^Midtjylland|Human|Department of Virus and Microbiological Special Diagnostics|Albertsen lab|Michaelsen|Denmark
>Spike|hCoV-19/Belgium/rega-10061033/2020|2020-10-06|EPI_ISL_735054|Original|hCoV-19^^Leuven|Human|UZ Leuven|KU Leuven|Maes|Belgium
>Spike|hCoV-19/USA/WA-S1469/2020|2020-05-07|EPI_ISL_495644|Original|hCoV-19^^Washington|Human|Washington State Department of Health|Seattle Flu Study|Lee|USA
>Spike|hCoV-19/USA/MI-MDHHS-SC21998/2020|2020-09-26|EPI_ISL_576221|Original|hCoV-19^^Michigan|Human|Michigan Department of Health and Human Services|Michigan Department of Health and Human Services|Blankenship|USA
>Spike|hCoV-19/Wales/MILK-A46392/2020|2020-10-04|EPI_ISL_609286|Original|hCoV-19^^Wales|Human|Lighthouse Lab in Milton Keynes|Wellcome Sanger Institute for the COVID-19 Genomics UK (COG-UK) consortium|Davies|United Kingdom
>Spike|hCoV-19/England/CAMC-A581FD/2020|2020-10-09|EPI_ISL_596971|Original|hCoV-19^^England|Human|Lighthouse Lab in Cambridge|Wellcome Sanger Institute for the COVID-19 Genomics UK (COG-UK) consortium|Davies|United Kingdom

>Spike|hCoV-19/England/ALDP-A2EB4D/2020|2020-10-05|EPI_ISL_587236|Original|hCoV-19^^England|Human|Lighthouse Lab in Alderley Park|Wellcome Sanger Institute for the COVID-19 Genomics UK (COG-UK) consortium|Davies|United Kingdom
>Spike|hCoV-19/Chile/AR-ISPCH-1/2020|2020-03-14|EPI_ISL_445270|Original|hCoV-19^^La Araucania|Human|HOSPITAL DR.HERNAN HENRIQUEZ ARAVENA|Instituto de Salud Publica de Chile|Fernandez|Chile
>Spike|hCoV-19/England/ALDP-A86DA7/2020|2020-10-16|EPI_ISL_606005|Original|hCoV-19^^England|Human|Lighthouse Lab in Alderley Park|Wellcome Sanger Institute for the COVID-19 Genomics UK (COG-UK) consortium|Davies|United Kingdom
>Spike|hCoV-19/England/CAMC-B22D60/2020|2020-11-02|EPI_ISL_647319|Original|hCoV-19^^England|Human|Lighthouse Lab in Cambridge|Wellcome Sanger Institute for the COVID-19 Genomics UK (COG-UK) Consortium|Davies|United Kingdom
>Spike|hCoV-19/USA/TX-HMH-1803/2020|2020-05-16|EPI_ISL_542851|Original|hCoV-19^^Texas|Human|Houston Methodist Hospital|Houston Methodist Hospital|Long|USA
>Spike|hCoV-19/Japan/PG-0444/2020|2020-03-00|EPI_ISL_684169|Original|hCoV-19^^Japan|Human|Pathogen Genomics Center|Pathogen Genomics Center|Sekizuka|Japan
>Spike|hCoV-19/England/ALDP-68BD3D/2020|2020-07-02|EPI_ISL_558184|Original|hCoV-19^^England|Human|Lighthouse Lab in Alderley Park|Wellcome Sanger Institute for the COVID-19 Genomics UK (COG-UK) consortium|Davies|United Kingdom
>Spike|hCoV-19/Italy/APU-PODV-15732017/2020|2020-05-06|EPI_ISL_788967|Original|hCoV-19^^Apulia|Human|Ospedale \Di Venere\|Beaconlab (Bioinformatics|Chiara|Italy
>Spike|hCoV-19/Japan/PG-2433/2020|2020-03-00|EPI_ISL_686038|Original|hCoV-19^^Japan|Human|Pathogen Genomics Center|Pathogen Genomics Center|Sekizuka|Japan
>Spike|hCoV-19/Canada/ON-PHL-20-01319/2020|2020-06-00|EPI_ISL_548922|Original|hCoV-19^^Ontario|Human|Public Health Ontario Laboratory|Public Health Ontario Laboratory|Guthrie|Canada
>Spike|hCoV-19/Scotland/CVR5819/2020|2020-11-13|EPI_ISL_665537|Original|hCoV-19^^Scotland|Human|West of Scotland Specialist Virology Centre|COVID-19 Genomics UK (COG-UK) Consortium|Robertson|United Kingdom
>Spike|hCoV-19/Australia/QLD1285/2020|2020-11-11|EPI_ISL_693284|Original|hCoV-19^^Australia|Human|unknown|Public Health Virology Laboratory|Nguyen|Australia
>Spike|hCoV-19/USA/LA-EVTL236/2020|2020-08-22|EPI_ISL_574370|Original|hCoV-19^^Louisiana|Human|LSUHS Emerging Viral Threat Laboratory|Microbial Genome Sequencing Center|Kamil|USA
>Spike|hCoV-19/USA/WA-S2287/2020|2020-06-10|EPI_ISL_497715|Original|hCoV-19^^Washington|Human|Washington State Department of Health|Seattle Flu Study|Lee|USA
>Spike|hCoV-19/England/CAMC-B23321/2020|2020-11-02|EPI_ISL_665032|Original|hCoV-19^^England|Human|Virology Department|COVID-19 Genomics UK (COG-UK) Consortium|de Silva|United Kingdom
>Spike|hCoV-19/USA/WI-GMF-M00007/2020|2020-06-01|EPI_ISL_471157|Original|hCoV-19^^Wisconsin|Human|Gundersen Clinical Microbiology Laboratory|Kabara Cancer Research Institute|Kenny|USA
>Spike|hCoV-19/Singapore/330/2020|2020-04-17|EPI_ISL_469084|Original|hCoV-19^^Singapore|Human|National Public Health Laboratory|National Public Health Laboratory|Mak|Singapore
>Spike|hCoV-19/England/LIVE-A1A30/2020|2020-06-16|EPI_ISL_490351|Original|hCoV-19^^England|Human|Liverpool Clinical Laboratories|COVID-19 Genomics UK (COG-UK) Consortium|Haldenby|United Kingdom
>Spike|hCoV-19/USA/AZ-TG666148/2020|2020-05-01|EPI_ISL_696229|Original|hCoV-19^^Arizona|Human|Sonora Quest Laboratories|TGen North|French|USA
>Spike|hCoV-19/USA/WI-GMF-27309/2020|2020-10-23|EPI_ISL_660975|Original|hCoV-19^^Wisconsin|Human|Gundersen Molecular Diagnostics Laboratory|Kabara Cancer Research Institute|Kenny|USA
>Spike|hCoV-19/USA/WA-S636/2020|2020-04-17|EPI_ISL_438189|Original|hCoV-19^^Washington|Human|Washington State Department of Health|Seattle Flu Study|Bedford|USA
>Spike|hCoV-19/Wales/PHWC-28196/2020|2020-03-27|EPI_ISL_432047|Original|hCoV-19^^Wales|Human|Wales Specialist Virology Centre|Public Health Wales Microbiology Cardiff|Moore|United Kingdom

>Spike|hCoV-19/USA/CA-ALSR-1530/2020|2020-05-01|EPI_ISL_494645|Original|hCoV-19^^California|Human|Scripps Medical Laboratory|Andersen lab at Scripps Research|Gangavarapu|USA
>Spike|hCoV-19/England/ALDP-4A6C9B/2020|2020-06-08|EPI_ISL_559480|Original|hCoV-19^^England|Human|Lighthouse Lab in Alderley Park|Wellcome Sanger Institute for the COVID-19 Genomics UK (COG-UK) consortium|Davies|United Kingdom
>Spike|hCoV-19/Portugal/PT1104/2020|2020-03-27|EPI_ISL_511651|Original|hCoV-19^^Portugal|Human|Instituto Nacional de Saude (INSA)|Instituto Nacional de Saude (INSA)|Borges|Portugal
>Spike|hCoV-19/Canada/ON-PHL-20-02481/2020|2020-09-00|EPI_ISL_671584|Original|hCoV-19^^Ontario|Human|Public Health Ontario Laboratory|Public Health Ontario Laboratory|Guthrie|Canada
>Spike|hCoV-19/Wales/PHWC-47CFFF/2020|2020-10-08|EPI_ISL_586084|Original|hCoV-19^^Wales|Human|Wales Specialist Virology Centre Sequencing lab|COVID-19 Genomics UK (COG-UK) Consortium|Moore|United Kingdom
>Spike|hCoV-19/Denmark/DCGC-17352/2020|2020-12-14|EPI_ISL_750927|Original|hCoV-19^^Midtjylland|Human|Department of Virus and Microbiological Special Diagnostics|Albertsen Lab|Michaelsen|Denmark
>Spike|hCoV-19/England/MILK-AC92E5/2020|2020-10-21|EPI_ISL_629417|Original|hCoV-19^^England|Human|Lighthouse Lab in Milton Keynes|Wellcome Sanger Institute for the COVID-19 Genomics UK (COG-UK) consortium|Davies|United Kingdom
>Spike|hCoV-19/United Arab Emirates/1071/2020|2020-06-11|EPI_ISL_698145|Original|hCoV-19^^Abu Dhabi|Human|Group 42 (G42) Healthcare|G42 Healthcare|Ogrodzki|United Arab Emirates
>Spike|hCoV-19/USA/MN-QDX-1691/2020|2020-03-16|EPI_ISL_604007|Original|hCoV-19^^Minnesota|Human|Quest Diagnostics|Quest Diagnostics|Kagan|USA
>Spike|hCoV-19/India/DL-60949-CSIR-IGIB/2020|2020-08-06|EPI_ISL_636765|Original|hCoV-19^^Delhi|Human|National Centre for Disease control (NCDC)|NCDC/CSIR-IGIB|Faruq|India
>Spike|hCoV-19/USA/UT-UPHL-2008884/2020|2020-05-28|EPI_ISL_524998|Original|hCoV-19^^Utah|Human|Utah Public Health Laboratory|Utah Public Health Laboratory|Young|USA
>Spike|hCoV-19/Denmark/DCGC-5541/2020|2020-09-28|EPI_ISL_620866|Original|hCoV-19^^Syddanmark|Human|Department of Virus and Microbiological Special Diagnostics|Albertsen lab|Michaelsen|Denmark
>Spike|hCoV-19/Czech Republic/NRL_8779/2020|2020-09-10|EPI_ISL_626574|Original|hCoV-19^^South Bohemian Region|Human|The National Institute of Public Health|State Veterinary Institute Prague|Nagy|Czech Republic
>Spike|hCoV-19/England/ALDP-8E2FF0/2020|2020-06-25|EPI_ISL_557109|Original|hCoV-19^^England|Human|Lighthouse Lab in Alderley Park|Wellcome Sanger Institute for the COVID-19 Genomics UK (COG-UK) consortium|Davies|United Kingdom
>Spike|hCoV-19/Australia/VIC46/2020|2020-03-11|EPI_ISL_419816|Original|hCoV-19^^Victoria|Human|Victorian Infectious Diseases Reference Laboratory (VIDRL)|Victorian Infectious Diseases Reference Laboratory and Microbiological Diagnostic Unit Public Health Laboratory|Schultz|Australia
>Spike|hCoV-19/Denmark/DCGC-4979/2020|2020-09-14|EPI_ISL_621768|Original|hCoV-19^^Hovedstaden|Human|Department of Virus and Microbiological Special Diagnostics|Albertsen lab|Michaelsen|Denmark
>Spike|hCoV-19/Australia/VIC12342/2020|2020-07-23|EPI_ISL_591963|Original|hCoV-19^^Victoria|Human|Microbiological Diagnostic Unit - Public Health Laboratory (MDU-PHL)|MDU-PHL|Schultz|Australia
>Spike|hCoV-19/England/QEUH-AD3567/2020|2020-10-26|EPI_ISL_633775|Original|hCoV-19^^England|Human|Lighthouse Lab in Glasgow|Wellcome Sanger Institute for the COVID-19 Genomics UK (COG-UK) consortium|Davies|United Kingdom
>Spike|hCoV-19/England/ALDP-9B5E16/2020|2020-08-28|EPI_ISL_572969|Original|hCoV-19^^England|Human|Oxford Viromics|COVID-19 Genomics UK (COG-UK) Consortium|Golubchik|United Kingdom
>Spike|hCoV-19/USA/TX-DSHS-1821/2020|2020-06-22|EPI_ISL_738188|Original|hCoV-19^^Texas|Human|Texas Department of State Health Services|Texas Department of State Health Services|Rahman|USA
>Spike|hCoV-19/USA/MN-MDH-2226/2020|2020-12-06|EPI_ISL_770800|Original|hCoV-19^^Minnesota|Human|Vault Health|Minnesota Department of Health|Lorentz|USA

>Spike|hCoV-19/Canada/BC-BCCDC-8380/2020|2020-11-00|EPI_ISL_740764|Original|hCoV-
19^^British Columbia|Human|BCCDC Public Health Laboratory|BCCDC Public Health
Laboratory|Prystajecky|Canada
>Spike|hCoV-19/Wales/PHWC-48A90F/2020|2020-10-29|EPI_ISL_652613|Original|hCoV-
19^^Wales|Human|Wales Specialist Virology Centre Sequencing lab|COVID-19 Genomics UK
(COG-UK) Consortium|Moore|United Kingdom
>Spike|hCoV-19/England/MILK-9C4281/2020|2020-09-09|EPI_ISL_566889|Original|hCoV-
19^^England|Human|Lighthouse Lab in Milton Keynes|Wellcome Sanger Institute for the
COVID-19 Genomics UK (COG-UK) consortium|Davies|United Kingdom
>Spike|hCoV-19/England/ALDP-5A9B7C/2020|2020-06-15|EPI_ISL_558632|Original|hCoV-
19^^England|Human|Lighthouse Lab in Alderley Park|Wellcome Sanger Institute for the
COVID-19 Genomics UK (COG-UK) consortium|Davies|United Kingdom
>Spike|hCoV-19/England/QEUH-9AE2C9/2020|2020-09-02|EPI_ISL_637261|Original|hCoV-
19^^England|Human|Department of Pathology|COVID-19 Genomics UK (COG-UK)
Consortium|Goodfellow|United Kingdom
>Spike|hCoV-19/Denmark/DCGC-15126/2020|2020-11-23|EPI_ISL_713280|Original|hCoV-
19^^Hovedstaden|Human|Department of Virus and Microbiological Special
Diagnostics|Albertsen Lab|Michaelsen|Denmark
>Spike|hCoV-19/England/BIRM-612AD/2020|2020-06-08|EPI_ISL_473450|Original|hCoV-
19^^England|Human|University of Birmingham|COVID-19 Genomics UK (COG-UK)
Consortium|Loman|United Kingdom
>Spike|hCoV-19/USA/AZ-TG507341/2020|2020-07-16|EPI_ISL_695297|Original|hCoV-
19^^Arizona|Human|TGen North|TGen North|French|USA
>Spike|hCoV-19/Australia/VIC10121/2020|2020-08-28|EPI_ISL_562797|Original|hCoV-
19^^Victoria|Human|Microbiological Diagnostic Unit - Public Health Laboratory (MDU-
PHL)|MDU-PHL|Schultz|Australia
>Spike|hCoV-19/USA/TX-HMH-1919/2020|2020-04-15|EPI_ISL_542902|Original|hCoV-
19^^Texas|Human|Houston Methodist Hospital|Houston Methodist Hospital|Long|USA
>Spike|hCoV-19/Australia/ACT0011/2020|2020-03-21|EPI_ISL_498478|Original|hCoV-
19^^Australian Capital Territory|Human|ACT Pathology|Schwessinger Lab|Hall|Australia
>Spike|hCoV-19/Egypt/CUNCI-HGC7I023/2020|2020-07-19|EPI_ISL_576372|Original|hCoV-
19^^Egypt|Human|Cancer Biology Department|Cancer Biology Department|Zekri|Egypt
>Spike|hCoV-19/England/MILK-A47522/2020|2020-10-06|EPI_ISL_598512|Original|hCoV-
19^^England|Human|Lighthouse Lab in Milton Keynes|Wellcome Sanger Institute for the
COVID-19 Genomics UK (COG-UK) consortium|Davies|United Kingdom
>Spike|hCoV-19/England/ALDP-9C4DBD/2020|2020-09-10|EPI_ISL_567835|Original|hCoV-
19^^England|Human|Lighthouse Lab in Alderley Park|Wellcome Sanger Institute for the
COVID-19 Genomics UK (COG-UK) consortium|Davies|United Kingdom
>Spike|hCoV-19/Australia/VIC6407/2020|2020-07-23|EPI_ISL_521199|Original|hCoV-
19^^Victoria|Human|Microbiological Diagnostic Unit - Public Health Laboratory (MDU-
PHL)|MDU-PHL|Schultz|Australia
>Spike|hCoV-19/USA/CA-CZB-12774/2020|2020-10-12|EPI_ISL_648437|Original|hCoV-
19^^California|Human|Santa Clara County Public Health Laboratory|Chan-Zuckerberg
Biohub|Bhatt|USA
>Spike|hCoV-19/Norway/4555/2020|2020-10-12|EPI_ISL_635196|Original|hCoV-
19^^Rogaland|Human|Dept. of Medical Microbiology|Norwegian Institute of Public
Health|Vollan|Norway
>Spike|hCoV-19/England/ALDP-C8B7DF/2020|2020-12-17|EPI_ISL_756573|Original|hCoV-
19^^England|Human|Lighthouse Lab in Alderley Park|Wellcome Sanger Institute for the
COVID-19 Genomics UK (COG-UK) Consortium|Davies|United Kingdom
>Spike|hCoV-19/Australia/VIC9979/2020|2020-07-23|EPI_ISL_565822|Original|hCoV-
19^^Victoria|Human|Microbiological Diagnostic Unit - Public Health Laboratory (MDU-
PHL)|MDU-PHL|Schultz|Australia
>Spike|hCoV-19/Israel/2115990/2020|2020-04-16|EPI_ISL_447334|Original|hCoV-
19^^Jerusalem District|Human|Clinical Virology Unit|Stern Lab|Miller|Israel
>Spike|hCoV-19/USA/MN-MDH-1693/2020|2020-04-08|EPI_ISL_560780|Original|hCoV-
19^^Minnesota|Human|Minnesota Department of Health|Minnesota Department of
Health|Garfin|USA

>Spike|hCoV-19/Wales/PHWC-165448/2020|2020-06-10|EPI_ISL_479465|Original|hCoV-19^^Wales|Human|Wales Specialist Virology Centre Sequencing lab|COVID-19 Genomics UK (COG-UK) Consortium|Moore|United Kingdom
>Spike|hCoV-19/England/NOTT-113625/2020|2020-09-09|EPI_ISL_549381|Original|hCoV-19^^England|Human|Lincolnshire Hospitals and DeepSeq Nottingham|COVID-19 Genomics UK (COG-UK) Consortium|Loose|United Kingdom
>Spike|hCoV-19/Wales/PHWC-4993F0/2020|2020-12-04|EPI_ISL_727301|Original|hCoV-19^^Wales|Human|Originating lab|COVID-19 Genomics UK (COG-UK) Consortium|Moore|United Kingdom
>Spike|hCoV-19/USA/TX-HMH-MCoV-10530/2020|2020-07-08|EPI_ISL_789521|Original|hCoV-19^^Texas|Human|Houston Methodist Hospital|Houston Methodist Hospital|Long|USA
>Spike|hCoV-19/Denmark/DCGC-9383/2020|2020-11-16|EPI_ISL_668942|Original|hCoV-19^^Syddanmark|Human|Department of Virus and Microbiological Special Diagnostics|Albertsen Lab|Michaelsen|Denmark
>Spike|hCoV-19/England/QEUH-C331C9/2020|2020-12-11|EPI_ISL_734040|Original|hCoV-19^^England|Human|Lighthouse Lab in Glasgow|Wellcome Sanger Institute for the COVID-19 Genomics UK (COG-UK) Consortium|Davies|United Kingdom
>Spike|hCoV-19/Gambia/NPHL-1921/2020|2020-00-00|EPI_ISL_561239|Original|hCoV-19^^Kombo|Human|MRCG at LSHTM Genomics lab|MRCG at LSHTM Genomics lab|Sesay|Gambia
>Spike|hCoV-19/England/CAMC-BBC32D/2020|2020-11-22|EPI_ISL_705592|Original|hCoV-19^^England|Human|Quadram Institute Bioscience|COVID-19 Genomics UK (COG-UK) Consortium|Alikhan|United Kingdom
>Spike|hCoV-19/Denmark/DCGC-1179/2020|2020-03-16|EPI_ISL_614655|Original|hCoV-19^^Hovedstaden|Human|Department of Virus and Microbiological Special Diagnostics|Albertsen lab|Michaelsen|Denmark
>Spike|hCoV-19/England/BIRM-63D9B/2020|2020-09-04|EPI_ISL_576883|Original|hCoV-19^^England|Human|University of Birmingham|COVID-19 Genomics UK (COG-UK) Consortium|Loman|United Kingdom
>Spike|hCoV-19/England/ALDP-B74637/2020|2020-11-12|EPI_ISL_662166|Original|hCoV-19^^England|Human|Lighthouse Lab in Alderley Park|Wellcome Sanger Institute for the COVID-19 Genomics UK (COG-UK) Consortium|Davies|United Kingdom
>Spike|hCoV-19/Belgium/rega-0408893/2020|2020-04-08|EPI_ISL_734545|Original|hCoV-19^^Gembloux|Human|UZ Leuven|KU Leuven|Maes|Belgium
>Spike|hCoV-19/USA/WA-UW-4892/2020|2020-04-07|EPI_ISL_429634|Original|hCoV-19^^Washington|Human|UW Virology Lab|UW Virology Lab|Roychoudhury|USA
>Spike|hCoV-19/Northern Ireland/NIRE-232E63/2020|2020-09-24|EPI_ISL_680230|Original|hCoV-19^^Northern Ireland|Human|Regional Virus Laboratory|COVID-19 Genomics UK (COG-UK) Consortium|Fairley|United Kingdom
>Spike|hCoV-19/England/SHEF-C97C2/2020|2020-09-18|EPI_ISL_573721|Original|hCoV-19^^England|Human|Virology Department|COVID-19 Genomics UK (COG-UK) Consortium|de Silva|United Kingdom
>Spike|hCoV-19/USA/CA-ALSR-2985/2020|2020-08-27|EPI_ISL_635773|Original|hCoV-19^^California|Human|San Diego County Public Health Laboratory|Andersen lab at Scripps Research|Gangavarapu|USA
>Spike|hCoV-19/Austria/CeMM0994/2020|2020-04-12|EPI_ISL_583838|Original|hCoV-19^^Austria|Human|Dr. Gernot Walder GmbH|Bergthaler laboratory|Bergthaler|Austria
>Spike|hCoV-19/Kenya/C2239/2020|2020-04-23|EPI_ISL_457872|Original|hCoV-19^^Kenya|Human|KEMRI-CGMR-C|KEMRI-Wellcome Trust Research Programme/KEMRI-CGMR-C Kilifi|Githinji|Kenya
>Spike|hCoV-19/USA/MI-QDX-1612/2020|2020-03-16|EPI_ISL_603957|Original|hCoV-19^^Michigan|Human|Quest Diagnostics|Quest Diagnostics|Kagan|USA
>Spike|hCoV-19/USA/UT-UPHL-2012725139/2020|2020-08-08|EPI_ISL_745980|Original|hCoV-19^^Utah|Human|Ginkgo Bioworks Clinical Laboratory|Utah Public Health Laboratory|Young|USA
>Spike|hCoV-19/USA/TX-HMH-MCoV-12137/2020|2020-09-21|EPI_ISL_790216|Original|hCoV-19^^Texas|Human|Houston Methodist Hospital|Houston Methodist Hospital|Long|USA
>Spike|hCoV-19/France/ARA-SC193/2020|2020-04-28|EPI_ISL_700367|Original|hCoV-19^^Auvergne-Rh\u00f4ne-Alpes|Human|Laboratoire de virologie|CNR Virus des Infections Respiratoires - France SUD|Bal|France

>Spike|hCoV-19/Australia/VIC17184/2020|2020-00-00|EPI_ISL_663782|Original|hCoV-
19^^Victoria|Human|Microbiological Diagnostic Unit - Public Health Laboratory (MDU-
PHL)|MDU-PHL|Schultz|Australia
>Spike|hCoV-19/England/LIVE-DCB541/2020|2020-11-24|EPI_ISL_724409|Original|hCoV-
19^^England|Human|Liverpool Clinical Laboratories|COVID-19 Genomics UK (COG-UK)
Consortium|Haldenby|United Kingdom
>Spike|hCoV-19/Wales/PHWC-48DFBF/2020|2020-11-09|EPI_ISL_680788|Original|hCoV-
19^^Wales|Human|Wales Specialist Virology Centre Sequencing lab|COVID-19 Genomics UK
(COG-UK) Consortium|Moore|United Kingdom
>Spike|hCoV-19/England/QEUH-9D1500/2020|2020-09-14|EPI_ISL_577288|Original|hCoV-
19^^England|Human|University of Exeter|COVID-19 Genomics UK (COG-UK)
Consortium|Temperton|United Kingdom
>Spike|hCoV-19/England/MILK-B566C2/2020|2020-11-05|EPI_ISL_657418|Original|hCoV-
19^^England|Human|Lighthouse Lab in Milton Keynes|Wellcome Sanger Institute for the
COVID-19 Genomics UK (COG-UK) Consortium|Davies|United Kingdom
>Spike|hCoV-19/Luxembourg/LNS7802129/2020|2020-10-09|EPI_ISL_744633|Original|hCoV-
19^^Luxembourg|Human|Laboratoire national de sant\u00e9|Laboratoire national de
sant\u00e9|Wienecke-Baldacchino|Luxembourg
>Spike|hCoV-19/Wales/PHWC-49A526/2020|2020-12-02|EPI_ISL_727559|Original|hCoV-
19^^Wales|Human|Originating lab|COVID-19 Genomics UK (COG-UK) Consortium|Moore|United
Kingdom
>Spike|hCoV-19/England/ALDP-A95ADB/2020|2020-10-19|EPI_ISL_624683|Original|hCoV-
19^^England|Human|Lighthouse Lab in Alderley Park|Wellcome Sanger Institute for the
COVID-19 Genomics UK (COG-UK) consortium|Davies|United Kingdom
>Spike|hCoV-19/England/MILK-855C79/2020|2020-07-17|EPI_ISL_557312|Original|hCoV-
19^^England|Human|Lighthouse Lab in Milton Keynes|Wellcome Sanger Institute for the
COVID-19 Genomics UK (COG-UK) consortium|Davies|United Kingdom
>Spike|hCoV-19/Belgium/rega-0417687/2020|2020-04-17|EPI_ISL_734618|Original|hCoV-
19^^Tervuren|Human|UZ Leuven|KU Leuven|Maes|Belgium
>Spike|hCoV-19/Canada/BC_15710616/2020|2020-04-00|EPI_ISL_468675|Original|hCoV-
19^^British Columbia|Human|BCCDC Public Health Laboratory|BCCDC Public Health
Laboratory|Harrigan|Canada
>Spike|hCoV-19/Spain/MD-IBV-99012364/2020|2020-11-22|EPI_ISL_780007|Original|hCoV-
19^^Madrid|Human|Hospital General Universitario Gregorio Mara\u00f1\u00f3n|SeqCOVID-
SPAIN consortium/IBV(CSIC)|Comas|Spain
>Spike|hCoV-19/Belgium/ULG-10380/2020|2020-08-07|EPI_ISL_540547|Original|hCoV-
19^^Li\u00e8ge|Human|Department of Clinical Microbiology|GIGA Medical
Genomics|Durkin|Belgium
>Spike|hCoV-19/Wales/PHWC-160AF0/2020|2020-05-20|EPI_ISL_473013|Original|hCoV-
19^^Wales|Human|Wales Specialist Virology Centre Sequencing lab|COVID-19 Genomics UK
(COG-UK) Consortium|Moore|United Kingdom
>Spike|hCoV-19/England/CAMC-B21314/2020|2020-11-02|EPI_ISL_647609|Original|hCoV-
19^^England|Human|Lighthouse Lab in Cambridge|Wellcome Sanger Institute for the
COVID-19 Genomics UK (COG-UK) Consortium|Davies|United Kingdom
>Spike|hCoV-19/England/EXET-13602E/2020|2020-04-19|EPI_ISL_478281|Original|hCoV-
19^^England|Human|University of Exeter|COVID-19 Genomics UK (COG-UK)
Consortium|Temperton|United Kingdom
>Spike|hCoV-19/England/BRIS-123C6C/2020|2020-04-07|EPI_ISL_442772|Original|hCoV-
19^^England|Human|PHE South West Regional Laboratory|Wellcome Sanger Institute for
the COVID-19 Genomics UK (COG-UK) consortium|Amato|United Kingdom
>Spike|hCoV-19/Denmark/DCGC-6221/2020|2020-10-05|EPI_ISL_620124|Original|hCoV-
19^^Syddanmark|Human|Department of Virus and Microbiological Special
Diagnostics|Albertsen lab|Michaelsen|Denmark
>Spike|hCoV-19/Austria/CeMM0495/2020|2020-03-26|EPI_ISL_583584|Original|hCoV-
19^^Linz|Human|Institute for Medical and Chemical Laboratory Diagnostics|Bergthaler
laboratory|Bergthaler|Austria
>Spike|hCoV-19/Japan/PG-3361/2020|2020-04-00|EPI_ISL_691476|Original|hCoV-
19^^Japan|Human|Pathogen Genomics Center|Pathogen Genomics Center|Sekizuka|Japan

>Spike|hCoV-19/USA/NY-PV09192/2020|2020-03-20|EPI_ISL_422548|Original|hCoV-19^^New York|Human|MSHS Clinical Microbiology Laboratories|MSHS Pathogen Surveillance Program|van Bakel|USA
>Spike|hCoV-19/England/NOTT-12031F/2020|2020-12-07|EPI_ISL_725606|Original|hCoV-19^^England|Human|Queens Medical Centre|COVID-19 Genomics UK (COG-UK) Consortium|Loose|United Kingdom
>Spike|hCoV-19/England/LIVE-DC41DB/2020|2020-11-14|EPI_ISL_705179|Original|hCoV-19^^England|Human|Liverpool Clinical Laboratories|COVID-19 Genomics UK (COG-UK) Consortium|Haldenby|United Kingdom
>Spike|hCoV-19/USA/CA-CZB-2165/2020|2020-05-22|EPI_ISL_513715|Original|hCoV-19^^California|Human|Orange County Public Health Lab|Chan-Zuckerberg Biohub|Kamm|USA
>Spike|hCoV-19/England/MILK-A7D24A/2020|2020-10-13|EPI_ISL_606446|Original|hCoV-19^^England|Human|Lighthouse Lab in Milton Keynes|Wellcome Sanger Institute for the COVID-19 Genomics UK (COG-UK) consortium|Davies|United Kingdom
>Spike|hCoV-19/Netherlands/NB-EMC-422/2020|2020-11-08|EPI_ISL_722747|Original|hCoV-19^^Noord Brabant|Human|Dutch COVID-19 response team|Erasmus Medical Center|Oude Munnink|Netherlands
>Spike|hCoV-19/England/MILK-9D51DB/2020|2020-09-18|EPI_ISL_637491|Original|hCoV-19^^England|Human|Centre for Enzyme Innovation|COVID-19 Genomics UK (COG-UK) Consortium|Robson|United Kingdom
>Spike|hCoV-19/England/CAMB-1BAC88/2020|2020-11-12|EPI_ISL_664790|Original|hCoV-19^^England|Human|Department of Pathology|COVID-19 Genomics UK (COG-UK) Consortium|Goodfellow|United Kingdom
>Spike|hCoV-19/Wales/PHWC-496F86/2020|2020-11-25|EPI_ISL_726756|Original|hCoV-19^^Wales|Human|Originating lab|COVID-19 Genomics UK (COG-UK) Consortium|Moore|United Kingdom
>Spike|hCoV-19/England/MILK-9C3F21/2020|2020-09-09|EPI_ISL_566793|Original|hCoV-19^^England|Human|Lighthouse Lab in Milton Keynes|Wellcome Sanger Institute for the COVID-19 Genomics UK (COG-UK) consortium|Davies|United Kingdom
>Spike|hCoV-19/Australia/VIC4558/2020|2020-07-18|EPI_ISL_518968|Original|hCoV-19^^Victoria|Human|Microbiological Diagnostic Unit - Public Health Laboratory (MDU-PHL)|MDU-PHL|Schultz|Australia
>Spike|hCoV-19/England/QEUH-B402D6/2020|2020-11-08|EPI_ISL_657313|Original|hCoV-19^^England|Human|Lighthouse Lab in Glasgow|Wellcome Sanger Institute for the COVID-19 Genomics UK (COG-UK) Consortium|Davies|United Kingdom
>Spike|hCoV-19/England/MILK-B0638E/2020|2020-10-24|EPI_ISL_642845|Original|hCoV-19^^England|Human|Lighthouse Lab in Milton Keynes|Wellcome Sanger Institute for the COVID-19 Genomics UK (COG-UK) Consortium|Davies|United Kingdom
>Spike|hCoV-19/USA/CA-CZB-14856/2020|2020-11-23|EPI_ISL_739499|Original|hCoV-19^^California|Human|Humboldt County Public Health Laboratory|Chan-Zuckerberg Biohub|Kamm|USA
>Spike|hCoV-19/Japan/PG-3568/2020|2020-06-00|EPI_ISL_691824|Original|hCoV-19^^Japan|Human|Pathogen Genomics Center|Pathogen Genomics Center|Sekizuka|Japan
>Spike|hCoV-19/Wales/PHWC-49E9EA/2020|2020-12-08|EPI_ISL_743079|Original|hCoV-19^^Wales|Human|Wales Specialist Virology Centre Sequencing lab|COVID-19 Genomics UK (COG-UK) Consortium|Moore|United Kingdom
>Spike|hCoV-19/USA/WI-UW-896/2020|2020-07-11|EPI_ISL_509898|Original|hCoV-19^^Wisconsin|Human|University of Wisconsin-Madison AIDS Vaccine Research Laboratories|University of Wisconsin-Madison AIDS Vaccine Research Laboratories|Baczenas|USA
>Spike|hCoV-19/England/204742482/2020|2020-11-17|EPI_ISL_649937|Original|hCoV-19^^England|Human|Respiratory Virus Unit|COVID-19 Genomics UK (COG-UK) Consortium|Platt|United Kingdom
>Spike|hCoV-19/Belgium/VLG-0325162/2020|2020-03-25|EPI_ISL_420419|Original|hCoV-19^^Winksele|Human|KU Leuven|KU Leuven|Maes|Belgium
>Spike|hCoV-19/USA/TX-HMH-MCoV-9504/2020|2020-07-03|EPI_ISL_781155|Original|hCoV-19^^Texas|Human|Houston Methodist Hospital|Houston Methodist Hospital|Long|USA
>Spike|hCoV-19/USA/AZ-TG305865/2020|2020-04-27|EPI_ISL_694283|Original|hCoV-19^^Arizona|Human|AZ SPHL|TGen North|French|USA

>Spike|hCoV-19/Switzerland/BL-ETHZ-420387/2020|2020-12-21|EPI_ISL_768091|Original|hCoV-19^^Basel-Landschaft|Human|Viollier AG|Department of Biosystems Science and Engineering|Nadeau|Switzerland
>Spike|hCoV-19/England/CAMC-B37ECF/2020|2020-11-05|EPI_ISL_658775|Original|hCoV-19^^England|Human|Lighthouse Lab in Cambridge|Wellcome Sanger Institute for the COVID-19 Genomics UK (COG-UK) Consortium|Davies|United Kingdom
>Spike|hCoV-19/USA/CA-ALSR-4718/2020|2020-10-09|EPI_ISL_730102|Original|hCoV-19^^California|Human|San Diego County Public Health Laboratory|Andersen lab at Scripps Research|Gangavarapu|USA
>Spike|hCoV-19/Wales/PHWC-4A5A1A/2020|2020-12-17|EPI_ISL_764948|Original|hCoV-19^^Wales|Human|Wales Specialist Virology Centre Sequencing lab|COVID-19 Genomics UK (COG-UK) Consortium|Moore|United Kingdom
>Spike|hCoV-19/England/CAMB-1AFF6F/2020|2020-05-04|EPI_ISL_459196|Original|hCoV-19^^England|Human|Department of Pathology|Wellcome Sanger Institute for the COVID-19 Genomics UK (COG-UK) consortium|Davies|United Kingdom
>Spike|hCoV-19/England/QEUH-A9F8B9/2020|2020-10-20|EPI_ISL_652684|Original|hCoV-19^^England|Human|University of Exeter|COVID-19 Genomics UK (COG-UK) Consortium|Temperton|United Kingdom
>Spike|hCoV-19/Wales/PHWC-2A2EC/2020|2020-04-06|EPI_ISL_445551|Original|hCoV-19^^Wales|Human|Wales Specialist Virology Centre|Public Health Wales Microbiology Cardiff|Moore|United Kingdom
>Spike|hCoV-19/Australia/VIC638/2020|2020-03-29|EPI_ISL_426934|Original|hCoV-19^^Victoria|Human|Victorian Infectious Diseases Reference Laboratory (VIDRL)|Microbiological Diagnostic Unit Public Health Laboratory and Victorian Infectious Diseases Reference Laboratory|Schultz|Australia
>Spike|hCoV-19/England/MILK-AB7E22/2020|2020-10-21|EPI_ISL_623316|Original|hCoV-19^^England|Human|Lighthouse Lab in Milton Keynes|Wellcome Sanger Institute for the COVID-19 Genomics UK (COG-UK) consortium|Davies|United Kingdom
>Spike|hCoV-19/England/QEUH-AD3CAE/2020|2020-10-26|EPI_ISL_634046|Original|hCoV-19^^England|Human|Lighthouse Lab in Glasgow|Wellcome Sanger Institute for the COVID-19 Genomics UK (COG-UK) consortium|Davies|United Kingdom
>Spike|hCoV-19/Denmark/ALAB-SSI-780/2020|2020-03-19|EPI_ISL_444902|Original|hCoV-19^^Denmark|Human|Department of Virus and Microbiological Special Diagnostics|Albertsen lab|Kirkegaard|Denmark
>Spike|hCoV-19/USA/UT-UPHL-2009697/2020|2020-07-27|EPI_ISL_605371|Original|hCoV-19^^Utah|Human|Utah Public Health Laboratory|Utah Public Health Laboratory|Young|USA
>Spike|hCoV-19/Belgium/ULG-10329/2020|2020-07-30|EPI_ISL_540496|Original|hCoV-19^^Li\u00e8ge|Human|Department of Clinical Microbiology|GIGA Medical Genomics|Durkin|Belgium
>Spike|hCoV-19/Wales/MILK-9D450D/2020|2020-09-19|EPI_ISL_601665|Original|hCoV-19^^Wales|Human|Lighthouse Lab in Milton Keynes|Wellcome Sanger Institute for the COVID-19 Genomics UK (COG-UK) consortium|Davies|United Kingdom
>Spike|hCoV-19/England/BRIS-12B03B/2020|2020-05-04|EPI_ISL_469817|Original|hCoV-19^^England|Human|PHE South West Regional Laboratory|Wellcome Sanger Institute for the COVID-19 Genomics UK (COG-UK) consortium|Davies|United Kingdom
>Spike|hCoV-19/Spain/MD-ISCIII-2014631/2020|2020-08-18|EPI_ISL_691714|Original|hCoV-19^^Madrid|Human|Hospital Cl\u00ednico San Carlos|Instituto de Salud Carlos III|Caballero|Spain
>Spike|hCoV-19/Canada/QC-LSPQ-L00226142/2020|2020-03-17|EPI_ISL_535842|Original|hCoV-19^^Quebec|Human|Centre Hospitalier R\u00e9gional de Lanaudi\u00e8re|Laboratoire de sant\u00e9 publique du Qu\u00e9bec|Moreira|Canada
>Spike|hCoV-19/Germany/NW-HHU-356/2020|2020-09-10|EPI_ISL_666643|Orginal|hCoV-19^^North Rhine-Westphalia|Human|ZOTZ KLIMAS MVZ D\u00fcsseldorf-Centrum GbR \u00dcBAG f\u00fcr Labormedizin|Center of Medical Microbiology|Wienemann|Germany
>Spike|hCoV-19/Denmark/DCGC-8310/2020|2020-10-26|EPI_ISL_616967|Original|hCoV-19^^Syddanmark|Human|Department of Virus and Microbiological Special Diagnostics|Albertsen lab|Michaelsen|Denmark
>Spike|hCoV-19/Netherlands/Flevoland_11/2020|2020-04-23|EPI_ISL_460834|Original|hCoV-19^^Flevoland|Human|Dutch COVID-19 response team|Erasmus Medical Center|Oude Munnink|Netherlands

>Spike|hCoV-19/Japan/PG-7617/2020|2020-08-00|EPI_ISL_686719|Original|hCoV-19^^Japan|Human|Chiba Prefectural Institute of Public Health|Pathogen Genomics Center|Sekizuka|Japan
>Spike|hCoV-19/South Africa/KRISP-K003400/2020|2020-09-11|EPI_ISL_602788|Original|hCoV-19^^KwaZulu-Natal|Human|NHLS-IALCH|KRISP|de Oliveira|South Africa
>Spike|hCoV-19/Wales/PHWC-495E11/2020|2020-11-26|EPI_ISL_726489|Original|hCoV-19^^Wales|Human|Originating lab|COVID-19 Genomics UK (COG-UK) Consortium|Moore|United Kingdom
>Spike|hCoV-19/USA/AZ-TG280867/2020|2020-04-21|EPI_ISL_694201|Original|hCoV-19^^Arizona|Human|AZ SPHL|TGen North|French|USA
>Spike|hCoV-19/England/ALDP-6B8EAD/2020|2020-07-02|EPI_ISL_557874|Original|hCoV-19^^England|Human|Lighthouse Lab in Alderley Park|Wellcome Sanger Institute for the COVID-19 Genomics UK (COG-UK) consortium|Davies|United Kingdom
>Spike|hCoV-19/England/CAMB-72A83/2020|2020-03-23|EPI_ISL_439614|Original|hCoV-19^^England|Human|Department of Pathology|Wellcome Sanger Institute for the COVID-19 Genomics UK (COG-UK) consortium|Amato|United Kingdom
>Spike|hCoV-19/Denmark/DCGC-19971/2020|2020-12-07|EPI_ISL_747900|Original|hCoV-19^^Hovedstaden|Human|Department of Virus and Microbiological Special Diagnostics|Albertsen Lab|Michaelsen|Denmark
>Spike|hCoV-19/England/QEUH-9F6101/2020|2020-09-25|EPI_ISL_588975|Original|hCoV-19^^England|Human|Lighthouse Lab in Glasgow|Wellcome Sanger Institute for the COVID-19 Genomics UK (COG-UK) consortium|Davies|United Kingdom
>Spike|hCoV-19/USA/VA-DCLS-1629/2020|2020-08-17|EPI_ISL_614032|Original|hCoV-19^^Virginia|Human|Virginia DCLS|Virginia DCLS|St. Jacques|USA
>Spike|hCoV-19/Denmark/DCGC-19406/2020|2020-12-07|EPI_ISL_748930|Original|hCoV-19^^Hovedstaden|Human|Department of Virus and Microbiological Special Diagnostics|Albertsen Lab|Michaelsen|Denmark
>Spike|hCoV-19/USA/CA-SR0222/2020|2020-04-15|EPI_ISL_437578|Original|hCoV-19^^California|Human|Scripps Medical Laboratory|Andersen lab at Scripps Research|Gangavarapu|USA
>Spike|hCoV-19/USA/NY-QDX-2535/2020|2020-03-14|EPI_ISL_604829|Original|hCoV-19^^New York|Human|Quest Diagnostics|Quest Diagnostics|Kagan|USA
>Spike|hCoV-19/England/NOTT-110E99/2020|2020-04-29|EPI_ISL_448430|Original|hCoV-19^^England|Human|Queens Medical Centre|COVID-19 Genomics UK (COG-UK) Consortium|Loose|United Kingdom
>Spike|hCoV-19/Scotland/CVR6020/2020|2020-11-29|EPI_ISL_680457|Original|hCoV-19^^Scotland|Human|West of Scotland Specialist Virology Centre|COVID-19 Genomics UK (COG-UK) Consortium|Robertson|United Kingdom
>Spike|hCoV-19/Switzerland/UR-ETHZ-321442/2020|2020-10-14|EPI_ISL_603667|Original|hCoV-19^^Uri|Human|Viollier AG|Department of Biosystems Science and Engineering|Nadeau|Switzerland
>Spike|hCoV-19/Australia/VIC4764/2020|2020-07-17|EPI_ISL_519152|Original|hCoV-19^^Victoria|Human|Microbiological Diagnostic Unit - Public Health Laboratory (MDU-PHL)|MDU-PHL|Schultz|Australia
>Spike|hCoV-19/Netherlands/LI-RIVM-10331/2020|2020-03-23|EPI_ISL_636541|Original|hCoV-19^^Limburg|Human|Dutch COVID-19 response team|National Institute for Public Health and the Environment (RIVM)|Meijer|Netherlands
>Spike|hCoV-19/England/QEUH-A00473/2020|2020-09-30|EPI_ISL_600232|Original|hCoV-19^^England|Human|Lighthouse Lab in Glasgow|Wellcome Sanger Institute for the COVID-19 Genomics UK (COG-UK) consortium|Davies|United Kingdom
>Spike|hCoV-19/England/ALDP-9560F3/2020|2020-06-08|EPI_ISL_553936|Original|hCoV-19^^England|Human|Lighthouse Lab in Alderley Park|Wellcome Sanger Institute for the COVID-19 Genomics UK (COG-UK) consortium|Davies|United Kingdom
>Spike|hCoV-19/England/SHEF-C2468/2020|2020-06-15|EPI_ISL_479202|Original|hCoV-19^^England|Human|Virology Department|COVID-19 Genomics UK (COG-UK) Consortium|de Silva|United Kingdom

>Spike|hCoV-19/England/MILK-975559/2020|2020-08-21|EPI_ISL_552638|Original|hCoV-19^^England|Human|Lighthouse Lab in Milton Keynes|Wellcome Sanger Institute for the COVID-19 Genomics UK (COG-UK) consortium|Davies|United Kingdom
>Spike|hCoV-19/Senegal/315/2020|2020-03-17|EPI_ISL_420070|Original|hCoV-19^^Dakar|Human|Institut Pasteur Dakar|Institut Pasteur de Dakar|Dia|Senegal
>Spike|hCoV-19/England/CAMC-9DF793/2020|2020-09-21|EPI_ISL_638423|Original|hCoV-19^^England|Human|Department of Pathology|COVID-19 Genomics UK (COG-UK) Consortium|Goodfellow|United Kingdom
>Spike|hCoV-19/USA/VA-DCLS-0399/2020|2020-04-21|EPI_ISL_467794|Original|hCoV-19^^Virginia|Human|Virginia DCLS|Virginia DCLS|St. Jacques|USA
>Spike|hCoV-19/England/NORW-E8A5B/2020|2020-04-15|EPI_ISL_448268|Original|hCoV-19^^England|Human|Quadram Institute Bioscience|COVID-19 Genomics UK (COG-UK) Consortium|Alikhan|United Kingdom
>Spike|hCoV-19/England/205260464/2020|2020-12-20|EPI_ISL_754245|Original|hCoV-19^^England|Human|Respiratory Virus Unit|COVID-19 Genomics UK (COG-UK) Consortium|Platt|United Kingdom
>Spike|hCoV-19/USA/NY-PV09125/2020|2020-03-19|EPI_ISL_422499|Original|hCoV-19^^New York|Human|MSHS Clinical Microbiology Laboratories|MSHS Pathogen Surveillance Program|van Bakel|USA
>Spike|hCoV-19/England/ALDP-94C588/2020|2020-06-15|EPI_ISL_555876|Original|hCoV-19^^England|Human|Lighthouse Lab in Alderley Park|Wellcome Sanger Institute for the COVID-19 Genomics UK (COG-UK) consortium|Davies|United Kingdom
>Spike|hCoV-19/Denmark/DCGC-11184/2020|2020-11-02|EPI_ISL_670441|Original|hCoV-19^^Hovedstaden|Human|Department of Virus and Microbiological Special Diagnostics|Albertsen Lab|Michaelsen|Denmark
>Spike|hCoV-19/Wales/PHWC-16F314/2020|2020-10-04|EPI_ISL_585947|Original|hCoV-19^^Wales|Human|Wales Specialist Virology Centre Sequencing lab|COVID-19 Genomics UK (COG-UK) Consortium|Moore|United Kingdom
>Spike|hCoV-19/England/ALDP-C48B0F/2020|2020-12-12|EPI_ISL_736496|Original|hCoV-19^^England|Human|Lighthouse Lab in Alderley Park|Wellcome Sanger Institute for the COVID-19 Genomics UK (COG-UK) Consortium|Davies|United Kingdom
>Spike|hCoV-19/South Africa/KRISP-K002765/2020|2020-08-12|EPI_ISL_529761|Original|hCoV-19^^KwaZulu-Natal|Human|NHLS-IALCH|KRISP|de Oliveira|South Africa
>Spike|hCoV-19/England/ALDP-B848F2/2020|2020-11-14|EPI_ISL_675803|Original|hCoV-19^^England|Human|Lighthouse Lab in Alderley Park|Wellcome Sanger Institute for the COVID-19 Genomics UK (COG-UK) Consortium|Davies|United Kingdom
>Spike|hCoV-19/Palestine/22/2020|2020-03-21|EPI_ISL_596509|Original|hCoV-19^^Bethlehem|Human|Palestinian Ministry of Health|Molecular Genetics Lab|van Dorp|Palestine
>Spike|hCoV-19/USA/DC-HP00090/2020|2020-03-27|EPI_ISL_438243|Original|hCoV-19^^District of Columbia|Human|Johns Hopkins Hospital Department of Pathology|Johns Hopkins Hospital Department of Pathology|Mehoke|USA
>Spike|hCoV-19/England/ALDP-9CD1A9/2020|2020-09-16|EPI_ISL_566347|Original|hCoV-19^^England|Human|Lighthouse Lab in Alderley Park|Wellcome Sanger Institute for the COVID-19 Genomics UK (COG-UK) consortium|Davies|United Kingdom
>Spike|hCoV-19/Denmark/DCGC-15119/2020|2020-11-23|EPI_ISL_713641|Original|hCoV-19^^Hovedstaden|Human|Department of Virus and Microbiological Special Diagnostics|Albertsen Lab|Michaelsen|Denmark
>Spike|hCoV-19/England/ALDP-9558A1/2020|2020-06-12|EPI_ISL_554425|Original|hCoV-19^^England|Human|Lighthouse Lab in Alderley Park|Wellcome Sanger Institute for the COVID-19 Genomics UK (COG-UK) consortium|Davies|United Kingdom
>Spike|hCoV-19/Switzerland/VD-ETHZ-100328/2020|2020-03-18|EPI_ISL_575006|Original|hCoV-19^^Vaud|Human|Viollier AG|Department of Biosystems Science and Engineering|Nadeau|Switzerland
>Spike|hCoV-19/Scotland/QEUH-98F6D1/2020|2020-08-27|EPI_ISL_568298|Original|hCoV-19^^Scotland|Human|Lighthouse Lab in Glasgow|Wellcome Sanger Institute for the COVID-19 Genomics UK (COG-UK) consortium|Davies|United Kingdom

>Spike|hCoV-19/Germany/HH-hpi-p1452/2020|2020-03-11|EPI_ISL_775969|Original|hCoV-
19^^Hamburg|Human|University Medical Center Hamburg Eppendorf|Heinrich Pette
Institute|Robitaille|Germany
>Spike|hCoV-19/England/ALDP-A9409E/2020|2020-10-19|EPI_ISL_610974|Original|hCoV-
19^^England|Human|Lighthouse Lab in Alderley Park|Wellcome Sanger Institute for the
COVID-19 Genomics UK (COG-UK) consortium|Davies|United Kingdom
>Spike|hCoV-19/England/NOTT-10FB3C/2020|2020-04-07|EPI_ISL_432688|Original|hCoV-
19^^England|Human|Queens Medical Centre|COVID-19 Genomics UK (COG-UK)
Consortium|Loose|United Kingdom
>Spike|hCoV-19/England/ALDP-A6CC6E/2020|2020-10-14|EPI_ISL_607235|Original|hCoV-
19^^England|Human|Lighthouse Lab in Alderley Park|Wellcome Sanger Institute for the
COVID-19 Genomics UK (COG-UK) consortium|Davies|United Kingdom
>Spike|hCoV-19/England/MILK-B5A43D/2020|2020-11-06|EPI_ISL_658107|Original|hCoV-
19^^England|Human|Lighthouse Lab in Milton Keynes|Wellcome Sanger Institute for the
COVID-19 Genomics UK (COG-UK) Consortium|Davies|United Kingdom
>Spike|hCoV-19/Portugal/PT0701/2020|2020-03-17|EPI_ISL_510960|Original|hCoV-
19^^Portugal|Human|Instituto Nacional de Saude (INSA)|Instituto Nacional de Saude
(INSA)|Borges|Portugal
>Spike|hCoV-19/Denmark/DCGC-21251/2020|2020-12-21|EPI_ISL_759634|Original|hCoV-
19^^Nordjylland|Human|Department of Virus and Microbiological Special
Diagnostics|Albertsen Lab|Michaelsen|Denmark
>Spike|hCoV-19/Japan/PG-0890/2020|2020-03-00|EPI_ISL_684500|Original|hCoV-
19^^Japan|Human|Pathogen Genomics Center|Pathogen Genomics Center|Sekizuka|Japan
>Spike|hCoV-19/USA/UT-QDX-2194/2020|2020-03-12|EPI_ISL_604580|Original|hCoV-
19^^Utah|Human|Quest Diagnostics|Quest Diagnostics|Kagan|USA
>Spike|hCoV-19/Scotland/QEUH-9FB3AC/2020|2020-09-29|EPI_ISL_600591|Original|hCoV-
19^^Scotland|Human|Lighthouse Lab in Glasgow|Wellcome Sanger Institute for the COVID-
19 Genomics UK (COG-UK) consortium|Davies|United Kingdom
>Spike|hCoV-19/Canada/NB-225/2020|2020-03-26|EPI_ISL_479641|Original|hCoV-19^^New
Brunswick|Human|Dr. Georges-L.-Dumont University Hospital Centre|National
Microbiology Laboratory|Majer|Canada
>Spike|hCoV-19/England/CAMC-B7A358/2020|2020-11-12|EPI_ISL_741102|Original|hCoV-
19^^England|Human|Oxford Viromics|COVID-19 Genomics UK (COG-UK)
Consortium|Golubchik|United Kingdom
>Spike|hCoV-19/England/QEUH-BF0D7A/2020|2020-12-03|EPI_ISL_709160|Original|hCoV-
19^^England|Human|Lighthouse Lab in Glasgow|Wellcome Sanger Institute for the COVID-
19 Genomics UK (COG-UK) Consortium|Davies|United Kingdom
>Spike|hCoV-19/Finland/10A57S10/2020|2020-04-10|EPI_ISL_481528|Original|hCoV-
19^^Uusimaa|Human|Department of Virology and Immunology|Department of
Virology|Smura|Finland
>Spike|hCoV-19/Spain/AR-IBV-2446/2020|2020-03-17|EPI_ISL_468803|Original|hCoV-
19^^Aragon|Human|Servicio de Microbiolog\u00eda|SeqCOVID-SPAIN
consortium/IBV(CSIC)|Comas|Spain
>Spike|hCoV-19/USA/WI-UW-2279/2020|2020-11-15|EPI_ISL_728046|Original|hCoV-
19^^Wisconsin|Human|University of Wisconsin-Madison AIDS Vaccine Research
Laboratories|University of Wisconsin-Madison AIDS Vaccine Research
Laboratories|Baczenas|USA
>Spike|hCoV-19/USA/VA-DCLS-2413/2020|2020-11-18|EPI_ISL_734184|Original|hCoV-
19^^Virginia|Human|Virginia Division of Consolidated Laboratory Services
(DCLS)|Virginia Division of Consolidated Laboratory Services (DCLS)|St. Jacques|USA
>Spike|hCoV-19/England/ALDP-AA5768/2020|2020-10-22|EPI_ISL_625243|Original|hCoV-
19^^England|Human|Lighthouse Lab in Alderley Park|Wellcome Sanger Institute for the
COVID-19 Genomics UK (COG-UK) consortium|Davies|United Kingdom
>Spike|hCoV-19/Vietnam/HCMC-35021/2020|2020-03-26|EPI_ISL_450745|Original|hCoV-19^^Ho
Chi Minh City|Human|OUCRU/HTD|OUCRU/HTD|Tan|Vietnam
>Spike|hCoV-19/England/NORW-EC76F/2020|2020-05-11|EPI_ISL_457559|Original|hCoV-
19^^England|Human|Quadram Institute Bioscience|COVID-19 Genomics UK (COG-UK)
Consortium|Alikhan|United Kingdom

>Spike|hCoV-19/Lithuania/MR-LUHS-Eilnr258/2020|2020-08-07|EPI_ISL_636855|Original|hCoV-19^^Kauno apskritis|Human|Lithuanian University of Health Sciences Hospital|Lithuanian University of Health Sciences|Zemaitis|Lithuania
>Spike|hCoV-19/England/CAMB-7CA5C/2020|2020-04-05|EPI_ISL_441555|Original|hCoV-19^^England|Human|Department of Pathology|Wellcome Sanger Institute for the COVID-19 Genomics UK (COG-UK) consortium|Amato|United Kingdom
>Spike|hCoV-19/Australia/VIC490/2020|2020-03-30|EPI_ISL_426921|Original|hCoV-19^^Victoria|Human|Microbiological Diagnostic Unit Public Health Laboratory|Microbiological Diagnostic Unit Public Health Laboratory|Schultz|Australia
>Spike|hCoV-19/Australia/VIC6530/2020|2020-07-24|EPI_ISL_521300|Original|hCoV-19^^Victoria|Human|Microbiological Diagnostic Unit - Public Health Laboratory (MDU-PHL)|MDU-PHL|Schultz|Australia
>Spike|hCoV-19/Wales/PHWC-4A0BC1/2020|2020-12-11|EPI_ISL_743556|Original|hCoV-19^^Wales|Human|Wales Specialist Virology Centre Sequencing lab|COVID-19 Genomics UK (COG-UK) Consortium|Moore|United Kingdom
>Spike|hCoV-19/Canada/QC-LSPQ-L00234302/2020|2020-03-23|EPI_ISL_536050|Original|hCoV-19^^Quebec|Human|Centre Hospitalier R\u00e9gional de Lanaudi\u00e8re|Laboratoire de sant\u00e9 publique du Qu\u00e9bec|Moreira|Canada
>Spike|hCoV-19/England/ALDP-C3A4D8/2020|2020-12-07|EPI_ISL_731274|Original|hCoV-19^^England|Human|Lighthouse Lab in Alderley Park|Wellcome Sanger Institute for the COVID-19 Genomics UK (COG-UK) Consortium|Davies|United Kingdom
>Spike|hCoV-19/Denmark/DCGC-6543/2020|2020-10-05|EPI_ISL_619443|Original|hCoV-19^^Midtjylland|Human|Department of Virus and Microbiological Special Diagnostics|Albertsen lab|Michaelsen|Denmark
>Spike|hCoV-19/England/CAMB-1B08AC/2020|2020-05-04|EPI_ISL_459183|Original|hCoV-19^^England|Human|Department of Pathology|Wellcome Sanger Institute for the COVID-19 Genomics UK (COG-UK) consortium|Davies|United Kingdom
>Spike|hCoV-19/Brazil/SP-RP7/2020|2020-03-24|EPI_ISL_613962|Original|hCoV-19^^S\u00e3o Paulo|Human|Laboratory of Molecular Biology|Laboratory of Molecular Biology|Slavov|Brazil
>Spike|hCoV-19/England/QEUH-9D1791/2020|2020-09-16|EPI_ISL_573658|Original|hCoV-19^^England|Human|University College London|COVID-19 Genomics UK (COG-UK) Consortium|Roy|United Kingdom
>Spike|hCoV-19/Australia/VIC7576/2020|2020-07-26|EPI_ISL_564508|Original|hCoV-19^^Victoria|Human|Microbiological Diagnostic Unit - Public Health Laboratory (MDU-PHL)|MDU-PHL|Schultz|Australia
>Spike|hCoV-19/Germany/BE-ChVir-W1446-8293/2020|2020-06-24|EPI_ISL_753747|Original|hCoV-19^^Berlin|Human|Charit\u00e9 Universit\u00e4tsmedizin Berlin|Charit\u00e9 Universit\u00e4tsmedizin Berlin|Muehlemann|Germany
>Spike|hCoV-19/Denmark/DCGC-15225/2020|2020-11-23|EPI_ISL_713707|Original|hCoV-19^^Hovedstaden|Human|Department of Virus and Microbiological Special Diagnostics|Albertsen Lab|Michaelsen|Denmark
>Spike|hCoV-19/USA/AZ-TG630778/2020|2020-08-21|EPI_ISL_695710|Original|hCoV-19^^Arizona|Human|TGen North|TGen North|French|USA
>Spike|hCoV-19/Australia/NSW1050/2020|2020-07-08|EPI_ISL_593702|Original|hCoV-19^^New South Wales|Human|South Eastern Area Laboratory Services (SEALS)|NSW Health Pathology - Institute of Clinical Pathology and Medical Research; Westmead Hospital; University of Sydney|Gall|Australia
>Spike|hCoV-19/USA/NY-NYCPHL-000440/2020|2020-03-12|EPI_ISL_631914|Original|hCoV-19^^New York|Human|Mount Sinai Hospital|New York City Public Health Laboratory|Wang|USA
>Spike|hCoV-19/England/205041306/2020|2020-12-04|EPI_ISL_766284|Original|hCoV-19^^England|Human|Respiratory Virus Unit|COVID-19 Genomics UK (COG-UK) Consortium|Platt|United Kingdom
>Spike|hCoV-19/USA/MI-QDX-1703/2020|2020-03-18|EPI_ISL_604038|Original|hCoV-19^^Michigan|Human|Quest Diagnostics|Quest Diagnostics|Kagan|USA
>Spike|hCoV-19/USA/MI-MDHHS-SC22103/2020|2020-05-01|EPI_ISL_594215|Original|hCoV-19^^Michigan|Human|Michigan Department of Health and Human Services|Michigan Department of Health and Human Services|Jacob|USA

>Spike|hCoV-19/Wales/PHWC-16F36F/2020|2020-10-03|EPI_ISL_585952|Original|hCoV-19^^Wales|Human|Wales Specialist Virology Centre Sequencing lab|COVID-19 Genomics UK (COG-UK) Consortium|Moore|United Kingdom
>Spike|hCoV-19/England/CAMC-B0788E/2020|2020-10-31|EPI_ISL_643980|Original|hCoV-19^^England|Human|Lighthouse Lab in Cambridge|Wellcome Sanger Institute for the COVID-19 Genomics UK (COG-UK) Consortium|Davies|United Kingdom
>Spike|hCoV-19/USA/NY-NYCPHL-001232/2020|2020-11-05|EPI_ISL_683940|Original|hCoV-19^^New York|Human|DOHMH Morrisania|New York City Public Health Laboratory|Wang|USA
>Spike|hCoV-19/Australia/VIC617/2020|2020-03-26|EPI_ISL_426872|Original|hCoV-19^^Victoria|Human|Victorian Infectious Diseases Reference Laboratory (VIDRL)|Microbiological Diagnostic Unit Public Health Laboratory and Victorian Infectious Diseases Reference Laboratory|Schultz|Australia
>Spike|hCoV-19/Spain/CT-IBV-005607/2020|2020-03-26|EPI_ISL_508652|Original|hCoV-19^^Catalunya|Human|Departamento de Microbiolog\u00eda|SeqCOVID-SPAIN consortium/IBV(CSIC)|Comas|Spain
>Spike|hCoV-19/England/LIVE-ABD88/2020|2020-08-07|EPI_ISL_540630|Original|hCoV-19^^England|Human|Liverpool Clinical Laboratories|COVID-19 Genomics UK (COG-UK) Consortium|Haldenby|United Kingdom
>Spike|hCoV-19/England/ALDP-9CABBD/2020|2020-09-16|EPI_ISL_575516|Original|hCoV-19^^England|Human|Lighthouse Lab in Alderley Park|Wellcome Sanger Institute for the COVID-19 Genomics UK (COG-UK) consortium|Davies|United Kingdom
>Spike|hCoV-19/England/CAMC-B35EC1/2020|2020-11-05|EPI_ISL_645277|Original|hCoV-19^^England|Human|Lighthouse Lab in Cambridge|Wellcome Sanger Institute for the COVID-19 Genomics UK (COG-UK) Consortium|Davies|United Kingdom
>Spike|hCoV-19/USA/UT-UPHL-2009558/2020|2020-07-18|EPI_ISL_605237|Original|hCoV-19^^Utah|Human|Utah Public Health Laboratory|Utah Public Health Laboratory|Young|USA
>Spike|hCoV-19/Switzerland/ZH-ETHZ-100194/2020|2020-03-17|EPI_ISL_575013|Original|hCoV-19^^Zurich|Human|Viollier AG|Department of Biosystems Science and Engineering|Nadeau|Switzerland
>Spike|hCoV-19/Australia/VIC3030/2020|2020-07-01|EPI_ISL_521859|Original|hCoV-19^^Victoria|Human|Victorian Infectious Diseases Reference Laboratory (VIDRL)|VIDRL and MDU-PHL|Schultz|Australia
>Spike|hCoV-19/Denmark/DCGC-15141/2020|2020-11-23|EPI_ISL_713654|Original|hCoV-19^^Hovedstaden|Human|Department of Virus and Microbiological Special Diagnostics|Albertsen Lab|Michaelsen|Denmark
>Spike|hCoV-19/England/QEUH-AD33B8/2020|2020-10-26|EPI_ISL_629994|Original|hCoV-19^^England|Human|Lighthouse Lab in Glasgow|Wellcome Sanger Institute for the COVID-19 Genomics UK (COG-UK) consortium|Davies|United Kingdom
>Spike|hCoV-19/Wales/PHWC-27B6C/2020|2020-04-01|EPI_ISL_432325|Original|hCoV-19^^Wales|Human|Wales Specialist Virology Centre|Public Health Wales Microbiology Cardiff|Moore|United Kingdom
>Spike|hCoV-19/England/20119025604/2020|2020-03-13|EPI_ISL_465035|Original|hCoV-19^^England|Human|Respiratory Virus Unit|Respiratory Virus Unit|Platt|United Kingdom
>Spike|hCoV-19/England/201340854/2020|2020-03-24|EPI_ISL_423188|Original|hCoV-19^^England|Human|Respiratory Virus Unit|Respiratory Virus Unit|Platt|United Kingdom
>Spike|hCoV-19/USA/NC-QDX-180/2020|2020-04-28|EPI_ISL_494527|Original|hCoV-19^^North Carolina|Human|Quest Diagnostics|Quest Diagnostics|Kagan|USA
>Spike|hCoV-19/England/ALDP-BBA6BD/2020|2020-11-20|EPI_ISL_702444|Original|hCoV-19^^England|Human|Lighthouse Lab in Alderley Park|Wellcome Sanger Institute for the COVID-19 Genomics UK (COG-UK) Consortium|Davies|United Kingdom
>Spike|hCoV-19/Wales/PHWC-486D9E/2020|2020-10-27|EPI_ISL_639132|Original|hCoV-19^^Wales|Human|Wales Specialist Virology Centre Sequencing lab|COVID-19 Genomics UK (COG-UK) Consortium|Moore|United Kingdom
>Spike|hCoV-19/England/SHEF-D0D99/2020|2020-03-28|EPI_ISL_432620|Original|hCoV-19^^England|Human|Virology Department|COVID-19 Genomics UK (COG-UK) Consortium|de Silva|United Kingdom
>Spike|hCoV-19/USA/CA-CZB-5667/2020|2020-08-08|EPI_ISL_583218|Original|hCoV-19^^California|Human|UCSF Clinical Microbiology Laboratory|Chan-Zuckerberg Biohub|Bhatt|USA

>Spike|hCoV-19/USA/VA-DCLS-1620/2020|2020-08-20|EPI_ISL_614027|Original|hCoV-19^^Virginia|Human|Virginia DCLS|Virginia DCLS|St. Jacques|USA
>Spike|hCoV-19/Denmark/DCGC-12760/2020|2020-11-09|EPI_ISL_682941|Original|hCoV-19^^Syddanmark|Human|Department of Virus and Microbiological Special Diagnostics|Albertsen Lab|Michaelsen|Denmark
>Spike|hCoV-19/Portugal/PT1501/2020|2020-03-15|EPI_ISL_511460|Original|hCoV-19^^Portugal|Human|Instituto Nacional de Saude (INSA)|Instituto Nacional de Saude (INSA)|Borges|Portugal
>Spike|hCoV-19/England/QEUH-7B2B32/2020|2020-07-18|EPI_ISL_532845|Original|hCoV-19^^England|Human|Lighthouse Lab in Glasgow|Wellcome Sanger Institute for the COVID-19 Genomics UK (COG-UK) consortium|Davies|United Kingdom
>Spike|hCoV-19/England/BRIS-13106F/2020|2020-03-26|EPI_ISL_501591|Original|hCoV-19^^England|Human|PHE South West Regional Laboratory|Wellcome Sanger Institute for the COVID-19 Genomics UK (COG-UK) consortium|Davies|United Kingdom
>Spike|hCoV-19/England/MILK-B9720D/2020|2020-11-14|EPI_ISL_674817|Original|hCoV-19^^England|Human|Lighthouse Lab in Milton Keynes|Wellcome Sanger Institute for the COVID-19 Genomics UK (COG-UK) Consortium|Davies|United Kingdom
>Spike|hCoV-19/USA/MN-MDH-1983/2020|2020-10-21|EPI_ISL_634857|Original|hCoV-19^^Minnesota|Human|Minneapolis VA Health Care System|Minnesota Department of Health|Lorentz|USA
>Spike|hCoV-19/England/CAMB-1B9D2C/2020|2020-11-06|EPI_ISL_650396|Original|hCoV-19^^England|Human|Department of Pathology|COVID-19 Genomics UK (COG-UK) Consortium|Goodfellow|United Kingdom
>Spike|hCoV-19/USA/CO-CDPHE-2009163250/2020|2020-09-16|EPI_ISL_677648|Original|hCoV-19^^Colorado|Human|Colorado Department of Public Health and Environment|Colorado Department of Puplic Health and Environment|Bankers|USA
>Spike|hCoV-19/USA/HI_4970/2020|2020-03-05|EPI_ISL_426420|Original|hCoV-19^^Hawaii|Human|HI Dept. of Health|Pathogen Discovery|Queen|USA
>Spike|hCoV-19/India/TG-CCMB-AA622/2020|2020-08-12|EPI_ISL_539638|Original|hCoV-19^^Telangana|Human|CSIR-Centre for Cellular and Molecular Biology|CSIR-Centre for Cellular and Molecular Biology|Mukherjee|India
>Spike|hCoV-19/Italy/TUS-DBM-23/2020|2020-03-29|EPI_ISL_583967|Original|hCoV-19^^Tuscany|Human|UOC Microbiologia e Virologia|Dipartimento di Biotecnologie Mediche|Pinzauti|Italy
>Spike|hCoV-19/Wales/QEUH-A8B59B/2020|2020-10-16|EPI_ISL_647859|Original|hCoV-19^^Wales|Human|Lighthouse Lab in Glasgow|Wellcome Sanger Institute for the COVID-19 Genomics UK (COG-UK) Consortium|Davies|United Kingdom
>Spike|hCoV-19/England/QEUH-AF630A/2020|2020-10-29|EPI_ISL_653053|Original|hCoV-19^^England|Human|Quadram Institute Bioscience|COVID-19 Genomics UK (COG-UK) Consortium|Alikhan|United Kingdom
>Spike|hCoV-19/USA/TX-HMH-MCoV-12461/2020|2020-09-28|EPI_ISL_790349|Original|hCoV-19^^Texas|Human|Houston Methodist Hospital|Houston Methodist Hospital|Long|USA
>Spike|hCoV-19/USA/CT-Yale-346/2020|2020-08-17|EPI_ISL_527763|Original|hCoV-19^^Connecticut|Human|Connecticut State Department of Public Health|Grubaugh Lab - Yale School of Public Health|Fauver|USA
>Spike|hCoV-19/England/MILK-A05B1F/2020|2020-09-30|EPI_ISL_599958|Original|hCoV-19^^England|Human|Lighthouse Lab in Milton Keynes|Wellcome Sanger Institute for the COVID-19 Genomics UK (COG-UK) consortium|Davies|United Kingdom
>Spike|hCoV-19/USA/NY-Wadsworth-11666-01/2020|2020-03-08|EPI_ISL_426050|Original|hCoV-19^^New York|Human|Wadsworth Center|Wadsworth Center|Lasek-Nesselquist|USA
>Spike|hCoV-19/England/CAMC-B09B65/2020|2020-10-31|EPI_ISL_673229|Original|hCoV-19^^England|Human|Lighthouse Lab in Cambridge|Wellcome Sanger Institute for the COVID-19 Genomics UK (COG-UK) Consortium|Davies|United Kingdom
>Spike|hCoV-19/Wales/PHWC-25C3E/2020|2020-03-25|EPI_ISL_422041|Original|hCoV-19^^Wales|Human|Wales Specialist Virology Centre|Public Health Wales Microbiology Cardiff|Moore|United Kingdom
>Spike|hCoV-19/England/SHEF-C119F/2020|2020-04-28|EPI_ISL_475351|Original|hCoV-19^^England|Human|Virology Department|COVID-19 Genomics UK (COG-UK) Consortium|de Silva|United Kingdom

>Spike|hCoV-19/Denmark/DCGC-10625/2020|2020-11-02|EPI_ISL_670099|Original|hCoV-19^^Nordjylland|Human|Department of Virus and Microbiological Special Diagnostics|Albertsen Lab|Michaelsen|Denmark
>Spike|hCoV-19/England/NORW-EFB60/2020|2020-00-00|EPI_ISL_549486|Original|hCoV-19^^England|Human|Quadram Institute Bioscience|COVID-19 Genomics UK (COG-UK) Consortium|Alikhan|United Kingdom
>Spike|hCoV-19/England/QEUH-B686EB/2020|2020-11-11|EPI_ISL_662084|Original|hCoV-19^^England|Human|Lighthouse Lab in Glasgow|Wellcome Sanger Institute for the COVID-19 Genomics UK (COG-UK) Consortium|Davies|United Kingdom
>Spike|hCoV-19/England/20139063804/2020|2020-03-28|EPI_ISL_420748|Original|hCoV-19^^England|Human|Respiratory Virus Unit|Respiratory Virus Unit|Platt|United Kingdom
>Spike|hCoV-19/Scotland/EDB9463/2020|2020-11-01|EPI_ISL_680537|Original|hCoV-19^^Scotland|Human|Virology Department|COVID-19 Genomics UK (COG-UK) Consortium|McHugh|United Kingdom
>Spike|hCoV-19/Norway/4814/2020|2020-10-26|EPI_ISL_635074|Original|hCoV-19^^Oslo|Human|Norwegian Institute of Public Health|Norwegian Institute of Public Health|Vollan|Norway
>Spike|hCoV-19/England/20136016004/2020|2020-03-25|EPI_ISL_465817|Original|hCoV-19^^England|Human|Respiratory Virus Unit|Respiratory Virus Unit|Platt|United Kingdom
>Spike|hCoV-19/USA/AZ-TG637315/2020|2020-07-30|EPI_ISL_695736|Original|hCoV-19^^Arizona|Human|AZ SPHL|TGen North|French|USA
>Spike|hCoV-19/USA/TX-HMH-1142/2020|2020-04-23|EPI_ISL_542549|Original|hCoV-19^^Texas|Human|Houston Methodist Hospital|Houston Methodist Hospital|Long|USA
>Spike|hCoV-19/England/CAMC-A3F98B/2020|2020-10-06|EPI_ISL_609674|Original|hCoV-19^^England|Human|Lighthouse Lab in Cambridge|Wellcome Sanger Institute for the COVID-19 Genomics UK (COG-UK) consortium|Davies|United Kingdom
>Spike|hCoV-19/England/ALDP-B770FA/2020|2020-11-12|EPI_ISL_723845|Original|hCoV-19^^England|Human|Oxford Viromics|COVID-19 Genomics UK (COG-UK) Consortium|Golubchik|United Kingdom
>Spike|hCoV-19/England/CAMB-76551/2020|2020-03-29|EPI_ISL_438323|Original|hCoV-19^^England|Human|Department of Pathology|Wellcome Sanger Institute for the COVID-19 Genomics UK (COG-UK) consortium|Amato|United Kingdom
>Spike|hCoV-19/England/ALDP-B46C2D/2020|2020-11-06|EPI_ISL_656043|Original|hCoV-19^^England|Human|Lighthouse Lab in Alderley Park|Wellcome Sanger Institute for the COVID-19 Genomics UK (COG-UK) Consortium|Davies|United Kingdom
>Spike|hCoV-19/Denmark/DCGC-5445/2020|2020-09-21|EPI_ISL_620770|Original|hCoV-19^^Syddanmark|Human|Department of Virus and Microbiological Special Diagnostics|Albertsen lab|Michaelsen|Denmark
>Spike|hCoV-19/USA/TX-DSHS-1843/2020|2020-06-23|EPI_ISL_745268|Original|hCoV-19^^Texas|Human|Texas Department of State Health Services|Texas Department of State Health Services|Rahman|USA
>Spike|hCoV-19/Wales/PHWC-28A13/2020|2020-04-01|EPI_ISL_445387|Original|hCoV-19^^Wales|Human|Wales Specialist Virology Centre|Public Health Wales Microbiology Cardiff|Moore|United Kingdom
>Spike|hCoV-19/England/CAMC-A40FA3/2020|2020-10-05|EPI_ISL_609371|Original|hCoV-19^^England|Human|Lighthouse Lab in Cambridge|Wellcome Sanger Institute for the COVID-19 Genomics UK (COG-UK) consortium|Davies|United Kingdom
>Spike|hCoV-19/Wales/PHWC-48EB23/2020|2020-11-03|EPI_ISL_680965|Original|hCoV-19^^Wales|Human|Wales Specialist Virology Centre Sequencing lab|COVID-19 Genomics UK (COG-UK) Consortium|Moore|United Kingdom
>Spike|hCoV-19/Australia/VIC10384/2020|2020-08-31|EPI_ISL_562733|Original|hCoV-19^^Victoria|Human|Microbiological Diagnostic Unit - Public Health Laboratory (MDU-PHL)|MDU-PHL|Schultz|Australia
>Spike|hCoV-19/Scotland/QEUH-B9FCAD/2020|2020-11-18|EPI_ISL_757275|Original|hCoV-19^^Scotland|Human|Lighthouse Lab in Glasgow|Wellcome Sanger Institute for the COVID-19 Genomics UK (COG-UK) Consortium|Davies|United Kingdom
>Spike|hCoV-19/Denmark/DCGC-5930/2020|2020-10-05|EPI_ISL_620195|Original|hCoV-19^^Hovedstaden|Human|Department of Virus and Microbiological Special Diagnostics|Albertsen lab|Michaelsen|Denmark

>Spike|hCoV-19/France/HDF-rega-0417506/2020|2020-04-17|EPI_ISL_735243|Original|hCoV-19^^Hauts-de-France|Human|UZ Leuven|KU Leuven|Maes|France
>Spike|hCoV-19/England/ALDP-746052/2020|2020-06-29|EPI_ISL_557415|Original|hCoV-19^^England|Human|Lighthouse Lab in Alderley Park|Wellcome Sanger Institute for the COVID-19 Genomics UK (COG-UK) consortium|Davies|United Kingdom
>Spike|hCoV-19/England/ALDP-9E634A/2020|2020-09-24|EPI_ISL_580280|Original|hCoV-19^^England|Human|Lighthouse Lab in Alderley Park|Wellcome Sanger Institute for the COVID-19 Genomics UK (COG-UK) consortium|Davies|United Kingdom
>Spike|hCoV-19/England/LOND-1266D0C/2020|2020-12-10|EPI_ISL_741199|Original|hCoV-19^^England|Human|University College London|COVID-19 Genomics UK (COG-UK) Consortium|Roy|United Kingdom
>Spike|hCoV-19/USA/FL-BPHL-1866/2020|2020-04-09|EPI_ISL_613944|Original|hCoV-19^^Florida|Human|Florida Bureau of Public Health Laboratories|Florida Bureau of Public Health Laboratories|Blanton|USA
>Spike|hCoV-19/Denmark/DCGC-3391/2020|2020-08-31|EPI_ISL_616150|Original|hCoV-19^^Hovedstaden|Human|Department of Virus and Microbiological Special Diagnostics|Albertsen lab|Michaelsen|Denmark
>Spike|hCoV-19/Denmark/DCGC-17675/2020|2020-12-14|EPI_ISL_750187|Original|hCoV-19^^Sjaelland|Human|Department of Virus and Microbiological Special Diagnostics|Albertsen Lab|Michaelsen|Denmark
>Spike|hCoV-19/Wales/MILK-A46C00/2020|2020-10-06|EPI_ISL_609222|Original|hCoV-19^^Wales|Human|Lighthouse Lab in Milton Keynes|Wellcome Sanger Institute for the COVID-19 Genomics UK (COG-UK) consortium|Davies|United Kingdom
>Spike|hCoV-19/USA/WA-S1729/2020|2020-06-01|EPI_ISL_496155|Original|hCoV-19^^Washington|Human|Washington State Department of Health|Seattle Flu Study|Lee|USA
>Spike|hCoV-19/Australia/SAP379/2020|2020-03-22|EPI_ISL_492144|Original|hCoV-19^^South Australia|Human|SA Pathology|SA Pathology|Leong|Australia
>Spike|hCoV-19/England/CAMB-1AF952/2020|2020-05-01|EPI_ISL_459316|Original|hCoV-19^^England|Human|Department of Pathology|Wellcome Sanger Institute for the COVID-19 Genomics UK (COG-UK) consortium|Davies|United Kingdom
>Spike|hCoV-19/Spain/PV-IBV-001594/2020|2020-03-13|EPI_ISL_467142|Original|hCoV-19^^Basque Country|Human|Hospital Universitario Araba. Vitoria-Gasteiz|SeqCOVID-SPAIN consortium/IBV(CSIC)|Comas|Spain
>Spike|hCoV-19/USA/CA-QDX-291/2020|2020-03-20|EPI_ISL_571024|Original|hCoV-19^^California|Human|Quest Diagnostics|Quest Diagnostics|Kagan|USA
>Spike|hCoV-19/Denmark/DCGC-15170/2020|2020-11-23|EPI_ISL_713672|Original|hCoV-19^^Midtjylland|Human|Department of Virus and Microbiological Special Diagnostics|Albertsen Lab|Michaelsen|Denmark
>Spike|hCoV-19/USA/TX-HMH-4748/2020|2020-06-22|EPI_ISL_544302|Original|hCoV-19^^Texas|Human|Houston Methodist Hospital|Houston Methodist Hospital|Long|USA
>Spike|hCoV-19/Scotland/CVR2543/2020|2020-04-15|EPI_ISL_493812|Original|hCoV-19^^Scotland|Human|West of Scotland Specialist Virology Centre|COVID-19 Genomics UK (COG-UK) Consortium|Robertson|United Kingdom
>Spike|hCoV-19/England/CAMC-A57C57/2020|2020-10-08|EPI_ISL_597083|Original|hCoV-19^^England|Human|Lighthouse Lab in Cambridge|Wellcome Sanger Institute for the COVID-19 Genomics UK (COG-UK) consortium|Davies|United Kingdom
>Spike|hCoV-19/England/ALDP-BB9DF5/2020|2020-11-20|EPI_ISL_701960|Original|hCoV-19^^England|Human|Lighthouse Lab in Alderley Park|Wellcome Sanger Institute for the COVID-19 Genomics UK (COG-UK) Consortium|Davies|United Kingdom
>Spike|hCoV-19/England/ALDP-A2EFD8/2020|2020-10-05|EPI_ISL_587172|Original|hCoV-19^^England|Human|Lighthouse Lab in Alderley Park|Wellcome Sanger Institute for the COVID-19 Genomics UK (COG-UK) consortium|Davies|United Kingdom
>Spike|hCoV-19/Japan/IC-0580/2020|2020-10-00|EPI_ISL_779666|Original|hCoV-19^^Japan|Human|Pathogen Genomics Center|Pathogen Genomics Center|Sekizuka|Japan
>Spike|hCoV-19/USA/AZ-TG486013/2020|2020-07-09|EPI_ISL_695080|Original|hCoV-19^^Arizona|Human|TGen North|TGen North|French|USA
>Spike|hCoV-19/Luxembourg/LNS3959009/2020|2020-10-07|EPI_ISL_740396|Original|hCoV-19^^Luxembourg|Human|Laboratoire national de sant\u00e9|Laboratoire national de sant\u00e9|Wienecke-Baldacchino|Luxembourg

>Spike|hCoV-19/India/WB-IK44/2020|2020-05-04|EPI_ISL_508371|Original|hCoV-19^^West Bengal|Human|Institute of Post Graduate Medical Education & Research|National Institute of Biomedical Genomics|Maitra|India
>Spike|hCoV-19/Scotland/EDB2280/2020|2020-04-21|EPI_ISL_491511|Original|hCoV-19^^Scotland|Human|Virology Department|Wellcome Sanger Institute for the COVID-19 Genomics UK (COG-UK) consortium|Davies|United Kingdom
>Spike|hCoV-19/Australia/QLD1078/2020|2020-03-19|EPI_ISL_593597|Original|hCoV-19^^Australia|Human|unknown|Public Health Virology Laboratory|Nguyen|Australia
>Spike|hCoV-19/Denmark/DCGC-10744/2020|2020-11-02|EPI_ISL_670177|Original|hCoV-19^^Hovedstaden|Human|Department of Virus and Microbiological Special Diagnostics|Albertsen Lab|Michaelsen|Denmark
>Spike|hCoV-19/mink/Denmark/mDK-25/2020|2020-10-16|EPI_ISL_641418|unknown|hCoV-19^^Denmark|Neovison vison|Department of Virus and Microbiological Special Diagnostics|Albertsen lab|Michaelsen|Denmark
>Spike|hCoV-19/England/ALDP-4A30B0/2020|2020-05-23|EPI_ISL_559424|Original|hCoV-19^^England|Human|Lighthouse Lab in Alderley Park|Wellcome Sanger Institute for the COVID-19 Genomics UK (COG-UK) consortium|Davies|United Kingdom
>Spike|hCoV-19/Egypt/EGY-028/2020|2020-08-14|EPI_ISL_526983|Original|hCoV-19^^Egypt|Human|Biological prevention|Biological prevention|Seadawy|Egypt
>Spike|hCoV-19/Japan/PG-1185/2020|2020-04-00|EPI_ISL_684795|Original|hCoV-19^^Japan|Human|Pathogen Genomics Center|Pathogen Genomics Center|Sekizuka|Japan
>Spike|hCoV-19/Japan/PG-7577/2020|2020-07-00|EPI_ISL_686679|Original|hCoV-19^^Japan|Human|Chiba Prefectural Institute of Public Health|Pathogen Genomics Center|Sekizuka|Japan
>Spike|hCoV-19/Taiwan/NTU15/2020|2020-03-18|EPI_ISL_422417|Vero E6|hCoV-19^^Taipei|Human|Department of Laboratory Medicine|Microbial Genomics Core Lab|Lin|Taiwan
>Spike|hCoV-19/England/ALDP-B5CAEE/2020|2020-11-08|EPI_ISL_655917|Original|hCoV-19^^England|Human|Lighthouse Lab in Alderley Park|Wellcome Sanger Institute for the COVID-19 Genomics UK (COG-UK) Consortium|Davies|United Kingdom
>Spike|hCoV-19/England/QEUH-B2D3CD/2020|2020-11-04|EPI_ISL_659924|Original|hCoV-19^^England|Human|Lighthouse Lab in Glasgow|Wellcome Sanger Institute for the COVID-19 Genomics UK (COG-UK) Consortium|Davies|United Kingdom
>Spike|hCoV-19/USA/TX-DSHS-1277/2020|2020-03-31|EPI_ISL_648263|Original|hCoV-19^^Texas|Human|Texas Department of State Health Services|Texas Department of State Health Services|Rahman|USA
>Spike|hCoV-19/Spain/MD-IBV-99007146/2020|2020-08-07|EPI_ISL_541918|Original|hCoV-19^^Madrid|Human|Hospital General Universitario Gregorio Mara\u00f1\u00f3n|SeqCOVID-SPAIN consortium/IBV(CSIC)|Comas|Spain
>Spike|hCoV-19/USA/CO-CDC-2-3713975/2020|2020-12-02|EPI_ISL_751763|Original|hCoV-19^^Colorado|Human|CO Dept. of Public Health and Environment|Genomics and Discovery|Queen|USA
>Spike|hCoV-19/Switzerland/BE-ETHZ-150006plus/2020|2020-06-11|EPI_ISL_483651|Original|hCoV-19^^Bern|Human|Viollier AG|Department of Biosystems Science and Engineering|Nadeau|Switzerland
>Spike|hCoV-19/Belgium/UGent-9/2020|2020-03-25|EPI_ISL_425053|Original|hCoV-19^^Ghent|Human|Lab voor klinische biologie|Onderzoeksgroep Virologie|Lambrechts|Belgium
>Spike|hCoV-19/USA/DC-HP00139/2020|2020-03-21|EPI_ISL_457771|Original|hCoV-19^^District of Columbia|Human|Johns Hopkins Hospital Department of Pathology|Johns Hopkins Hospital Department of Pathology|Ramakrishnan|USA
>Spike|hCoV-19/USA/FL-BPHL-0452/2020|2020-03-24|EPI_ISL_495358|Original|hCoV-19^^Florida|Human|Florida Bureau of Public Health Laboratories|Florida Bureau of Public Health Laboratories|Blanton|USA
>Spike|hCoV-19/England/ALDP-B831F8/2020|2020-11-14|EPI_ISL_675599|Original|hCoV-19^^England|Human|Lighthouse Lab in Alderley Park|Wellcome Sanger Institute for the COVID-19 Genomics UK (COG-UK) Consortium|Davies|United Kingdom
>Spike|hCoV-19/England/CAMC-A81E12/2020|2020-10-12|EPI_ISL_610801|Original|hCoV-19^^England|Human|Lighthouse Lab in Cambridge|Wellcome Sanger Institute for the COVID-19 Genomics UK (COG-UK) consortium|Davies|United Kingdom

>Spike|hCoV-19/Denmark/DCGC-13576/2020|2020-11-30|EPI_ISL_712887|Original|hCoV-19^^Hovedstaden|Human|Department of Virus and Microbiological Special Diagnostics|Albertsen Lab|Michaelsen|Denmark
>Spike|hCoV-19/Canada/ON-UHTC_0132/2020|2020-05-22|EPI_ISL_526272|Original|hCoV-19^^Ontario|Human|Unity Health Toronto|Ontario Institute for Cancer Research|de Borja|Canada
>Spike|hCoV-19/England/QEUH-AF5848/2020|2020-10-30|EPI_ISL_663090|Original|hCoV-19^^England|Human|Lighthouse Lab in Glasgow|Wellcome Sanger Institute for the COVID-19 Genomics UK (COG-UK) Consortium|Davies|United Kingdom
>Spike|hCoV-19/England/BRIS-12B0FF/2020|2020-05-04|EPI_ISL_469831|Original|hCoV-19^^England|Human|PHE South West Regional Laboratory|Wellcome Sanger Institute for the COVID-19 Genomics UK (COG-UK) consortium|Davies|United Kingdom
>Spike|hCoV-19/Wales/PHWC-48A301/2020|2020-11-03|EPI_ISL_652542|Original|hCoV-19^^Wales|Human|Wales Specialist Virology Centre Sequencing lab|COVID-19 Genomics UK (COG-UK) Consortium|Moore|United Kingdom
>Spike|hCoV-19/USA/NY-NYCPHL-000453/2020|2020-04-04|EPI_ISL_631927|Original|hCoV-19^^New York|Human|Jamaica Hospital Medical Center|New York City Public Health Laboratory|Wang|USA
>Spike|hCoV-19/Israel/CVL-n-5991/2020|2020-03-15|EPI_ISL_475019|Original|hCoV-19^^Israel|Human|Israel Central Virology laboratory|Israel Central Virology laboratory|Zuckerman|Israel
>Spike|hCoV-19/Finland/8LS2H5/2020|2020-10-08|EPI_ISL_755986|Original|hCoV-19^^Uusimaa|Human|Department of Virology and Immunology|Department of Virology|Smura|Finland
>Spike|hCoV-19/USA/UN-UW-2849/2020|2020-04-15|EPI_ISL_485958|Original|hCoV-19^^USA|Human|UW Virology Lab|UW Virology Lab|Roychoudhury|USA
>Spike|hCoV-19/England/CAMC-B334A3/2020|2020-11-03|EPI_ISL_659135|Original|hCoV-19^^England|Human|Lighthouse Lab in Cambridge|Wellcome Sanger Institute for the COVID-19 Genomics UK (COG-UK) Consortium|Davies|United Kingdom
>Spike|hCoV-19/England/ALDP-AA1F64/2020|2020-10-22|EPI_ISL_624494|Original|hCoV-19^^England|Human|Lighthouse Lab in Alderley Park|Wellcome Sanger Institute for the COVID-19 Genomics UK (COG-UK) consortium|Davies|United Kingdom
>Spike|hCoV-19/Portugal/PT0280/2020|2020-04-05|EPI_ISL_453994|Original|hCoV-19^^Portugal|Human|unknown|Instituto Nacional de Saude (INSA)|Borges|Portugal
>Spike|hCoV-19/Netherlands/NoordHolland_29/2020|2020-05-02|EPI_ISL_461219|Original|hCoV-19^^Noord Holland|Human|Dutch COVID-19 response team|Erasmus Medical Center|Oude Munnink|Netherlands
>Spike|hCoV-19/Denmark/DCGC-12612/2020|2020-11-16|EPI_ISL_682793|Original|hCoV-19^^Nordjylland|Human|Department of Virus and Microbiological Special Diagnostics|Albertsen Lab|Michaelsen|Denmark
>Spike|hCoV-19/Denmark/DCGC-10520/2020|2020-11-02|EPI_ISL_670031|Original|hCoV-19^^Nordjylland|Human|Department of Virus and Microbiological Special Diagnostics|Albertsen Lab|Michaelsen|Denmark
>Spike|hCoV-19/Australia/SAP599/2020|2020-12-29|EPI_ISL_755569|Original|hCoV-19^^South Australia|Human|SA Pathology|SA Pathology|Leong|Australia
>Spike|hCoV-19/USA/UT-UPHL-2009016/2020|2020-05-11|EPI_ISL_525322|Original|hCoV-19^^Utah|Human|Utah Public Health Laboratory|Utah Public Health Laboratory|Young|USA
>Spike|hCoV-19/England/QEUH-9604FA/2020|2020-08-07|EPI_ISL_532072|Original|hCoV-19^^England|Human|Lighthouse Lab in Glasgow|Wellcome Sanger Institute for the COVID-19 Genomics UK (COG-UK) consortium|Davies|United Kingdom
>Spike|hCoV-19/England/NOTT-114F56/2020|2020-10-12|EPI_ISL_584781|Original|hCoV-19^^England|Human|Queens Medical Centre|COVID-19 Genomics UK (COG-UK) Consortium|Loose|United Kingdom
>Spike|hCoV-19/England/SHEF-C41F6/2020|2020-04-19|EPI_ISL_462014|Original|hCoV-19^^England|Human|Virology Department|COVID-19 Genomics UK (COG-UK) Consortium|de Silva|United Kingdom
>Spike|hCoV-19/Denmark/DCGC-5756/2020|2020-09-28|EPI_ISL_621261|Original|hCoV-19^^Hovedstaden|Human|Department of Virus and Microbiological Special Diagnostics|Albertsen lab|Michaelsen|Denmark

>Spike|hCoV-19/USA/TX-HMH-MCoV-14224/2020|2020-10-03|EPI_ISL_787509|Original|hCoV-19^^Texas|Human|Houston Methodist Hospital|Houston Methodist Hospital|Long|USA
>Spike|hCoV-19/USA/CT-Yale-360/2020|2020-08-17|EPI_ISL_527776|Original|hCoV-19^^Connecticut|Human|Connecticut State Department of Public Health|Grubaugh Lab - Yale School of Public Health|Fauver|USA
>Spike|hCoV-19/DRC/16145/2020|2020-06-01|EPI_ISL_591085|Original|hCoV-19^^Kinshasa|Human|Viral Respiratory Lab|Pathogen Sequencing Lab|Lokilo|Democratic Republic of the Congo
>Spike|hCoV-19/England/CAMB-79943/2020|2020-04-20|EPI_ISL_444393|Original|hCoV-19^^England|Human|Department of Pathology|COVID-19 Genomics UK (COG-UK) Consortium|Meredith|United Kingdom
>Spike|hCoV-19/Israel/CVL-n3304/2020|2020-03-09|EPI_ISL_649079|Original|hCoV-19^^Maale-Edumim|Human|Israel Central Virology laboratory|Israel Central Virology laboratory|Zuckerman|Israel
>Spike|hCoV-19/Equatorial Guinea/3280/2020|2020-04-24|EPI_ISL_648325|Original|hCoV-19^^Litoral|Human|Laboratorio de Investigaciones de Baney|University Hospital Basel|Hosch|Equatorial Guinea
>Spike|hCoV-19/USA/MN-QDX-2059/2020|2020-03-14|EPI_ISL_604356|Original|hCoV-19^^Minnesota|Human|Quest Diagnostics|Quest Diagnostics|Kagan|USA
>Spike|hCoV-19/Denmark/DCGC-21193/2020|2020-12-21|EPI_ISL_759607|Original|hCoV-19^^Nordjylland|Human|Department of Virus and Microbiological Special Diagnostics|Albertsen Lab|Michaelsen|Denmark
>Spike|hCoV-19/India/GJ-GBRC-407b/2020|2020-06-25|EPI_ISL_586530|Original|hCoV-19^^Gujarat|Human|B.J. Medical College and Civil hospital|Gujarat Biotechnology Research Centre|Joshi|India
>Spike|hCoV-19/Scotland/EDB9419/2020|2020-10-29|EPI_ISL_680522|Original|hCoV-19^^Scotland|Human|Virology Department|COVID-19 Genomics UK (COG-UK) Consortium|McHugh|United Kingdom
>Spike|hCoV-19/USA/CA-ALSR-1223/2020|2020-04-10|EPI_ISL_494447|Original|hCoV-19^^California|Human|San Diego County Public Health Laboratory|Andersen lab at Scripps Research|Gangavarapu|USA
>Spike|hCoV-19/England/CAMC-B22692/2020|2020-11-02|EPI_ISL_647246|Original|hCoV-19^^England|Human|Lighthouse Lab in Cambridge|Wellcome Sanger Institute for the COVID-19 Genomics UK (COG-UK) Consortium|Davies|United Kingdom
>Spike|hCoV-19/Japan/IC-0020/2020|2020-04-00|EPI_ISL_591365|Original|hCoV-19^^Japan|Human|Pathogen Genomics Center|Pathogen Genomics Center|Sekizuka|Japan
>Spike|hCoV-19/England/20182013104/2020|2020-04-23|EPI_ISL_465450|Original|hCoV-19^^England|Human|Respiratory Virus Unit|Respiratory Virus Unit|Platt|United Kingdom
>Spike|hCoV-19/USA/NY-NYCPHL-000082/2020|2020-03-18|EPI_ISL_631575|Original|hCoV-19^^New York|Human|Jamaica Hospital Medical Center|New York City Public Health Laboratory|Wang|USA
>Spike|hCoV-19/Italy/VEN-UniVR-119/2020|2020-03-29|EPI_ISL_751320|Original|hCoV-19^^Veneto|Human|IRCCS Sacro Cuore Don Calabria Hospital|University of Verona|Mori|Italy
>Spike|hCoV-19/Egypt/CUNCI-HGC5I030/2020|2020-06-02|EPI_ISL_479721|Original|hCoV-19^^Egypt|Human|Egyptian National Cancer Institute (ENCI)|Egyptian National Cancer Institute (ENCI)|Zekri|Egypt
>Spike|hCoV-19/USA/WA-UW-12443/2020|2020-06-27|EPI_ISL_570535|Original|hCoV-19^^Washington|Human|UW Virology Lab|UW Virology Lab|Roychoudhury|USA
>Spike|hCoV-19/England/QEUH-96458A/2020|2020-08-11|EPI_ISL_536867|Original|hCoV-19^^England|Human|Lighthouse Lab in Glasgow|Wellcome Sanger Institute for the COVID-19 Genomics UK (COG-UK) consortium|Davies|United Kingdom
>Spike|hCoV-19/USA/WI-WSLH-200041/2020|2020-04-02|EPI_ISL_471206|Original|hCoV-19^^Wisconsin|Human|Wisconsin State Laboratory of Hygiene Communicable Disease Division|Wisconsin State Laboratory of Hygiene Communicable Disease Division|Florek|USA
>Spike|hCoV-19/England/MILK-B94D3C/2020|2020-11-13|EPI_ISL_676022|Original|hCoV-19^^England|Human|Lighthouse Lab in Milton Keynes|Wellcome Sanger Institute for the COVID-19 Genomics UK (COG-UK) Consortium|Davies|United Kingdom

>Spike|hCoV-19/USA/TX-HMH-MCoV-10671/2020|2020-07-08|EPI_ISL_789585|Original|hCoV-19^^Texas|Human|Houston Methodist Hospital|Houston Methodist Hospital|Long|USA
>Spike|hCoV-19/USA/CA-CZB-14706/2020|2020-11-17|EPI_ISL_738632|Original|hCoV-19^^California|Human|Orange County Public Health Lab|Chan-Zuckerberg Biohub|Kamm|USA
>Spike|hCoV-19/England/MILK-A7B112/2020|2020-10-15|EPI_ISL_607355|Original|hCoV-19^^England|Human|Lighthouse Lab in Milton Keynes|Wellcome Sanger Institute for the COVID-19 Genomics UK (COG-UK) consortium|Davies|United Kingdom
>Spike|hCoV-19/England/QEUH-B686AF/2020|2020-11-11|EPI_ISL_662222|Original|hCoV-19^^England|Human|Lighthouse Lab in Glasgow|Wellcome Sanger Institute for the COVID-19 Genomics UK (COG-UK) Consortium|Davies|United Kingdom
>Spike|hCoV-19/Japan/PG-7351/2020|2020-08-00|EPI_ISL_688453|Original|hCoV-19^^Japan|Human|Pathogen Genomics Center|Pathogen Genomics Center|Sekizuka|Japan
>Spike|hCoV-19/USA/WA-S1473/2020|2020-05-12|EPI_ISL_495648|Original|hCoV-19^^Washington|Human|Washington State Department of Health|Seattle Flu Study|Lee|USA
>Spike|hCoV-19/Spain/RI-IBV-98002370/2020|2020-03-20|EPI_ISL_537866|Original|hCoV-19^^La Rioja|Human|Centro de Investigaci\u00f3n Biom\u00e9dica de La Rioja - Hospital San Pedro Logro\u00f1o|SeqCOVID-SPAIN consortium/IBV(CSIC)|Comas|Spain
>Spike|hCoV-19/Wales/PHWC-486147/2020|2020-10-25|EPI_ISL_628311|Original|hCoV-19^^Wales|Human|Wales Specialist Virology Centre Sequencing lab|COVID-19 Genomics UK (COG-UK) Consortium|Moore|United Kingdom
>Spike|hCoV-19/England/NORT-29AB71/2020|2020-05-21|EPI_ISL_492454|Original|hCoV-19^^England|Human|NU-OMICS DNA Sequencing research facility|Wellcome Sanger Institute for the COVID-19 Genomics UK (COG-UK) consortium|Davies|United Kingdom
>Spike|hCoV-19/Canada/QC_AA9/2020|2020-04-06|EPI_ISL_444498|Original|hCoV-19^^Quebec|Human|Laboratoire de microbiologie|Smith Laboratory|Smith|Canada
>Spike|hCoV-19/Spain/NC-IBV-001407/2020|2020-03-20|EPI_ISL_452494|Original|hCoV-19^^Navarra|Human|Cl\u00ednica Universidad de Navarra. Servicio de Enfermedades Infecciosas y Microbiolog\u00eda cl\u00ednica|SeqCOVID-SPAIN consortium/IBV(CSIC)|Comas|Spain
>Spike|hCoV-19/England/ALDP-A50CB8/2020|2020-10-07|EPI_ISL_597673|Original|hCoV-19^^England|Human|Lighthouse Lab in Alderley Park|Wellcome Sanger Institute for the COVID-19 Genomics UK (COG-UK) consortium|Davies|United Kingdom
>Spike|hCoV-19/Wales/PHWC-492281/2020|2020-11-17|EPI_ISL_706834|Original|hCoV-19^^Wales|Human|Originating lab|COVID-19 Genomics UK (COG-UK) Consortium|Moore|United Kingdom
>Spike|hCoV-19/England/ALDP-9FCBD0/2020|2020-10-01|EPI_ISL_588004|Original|hCoV-19^^England|Human|Lighthouse Lab in Alderley Park|Wellcome Sanger Institute for the COVID-19 Genomics UK (COG-UK) consortium|Davies|United Kingdom
>Spike|hCoV-19/Luxembourg/LNS2122915/2020|2020-08-16|EPI_ISL_740063|Original|hCoV-19^^Luxembourg|Human|Laboratoire national de sant\u00e9|Laboratoire national de sant\u00e9|Wienecke-Baldacchino|Luxembourg
>Spike|hCoV-19/Canada/NS-NML-12/2020|2020-03-13|EPI_ISL_579516|Original|hCoV-19^^Nova Scotia|Human|QEII Health Sciences Centre|National Microbiology Laboratory (NML)|Majer|Canada
>Spike|hCoV-19/Australia/VIC4845/2020|2020-00-00|EPI_ISL_519229|Original|hCoV-19^^Victoria|Human|Microbiological Diagnostic Unit - Public Health Laboratory (MDU-PHL)|MDU-PHL|Schultz|Australia
>Spike|hCoV-19/USA/HI-H200451/2020|2020-10-09|EPI_ISL_752994|Original|hCoV-19^^Hawaii|Human|State Laboratories Division|State Laboratories Division|Sultana|USA
>Spike|hCoV-19/Australia/VIC16065/2020|2020-08-02|EPI_ISL_641255|Original|hCoV-19^^Victoria|Human|Microbiological Diagnostic Unit - Public Health Laboratory (MDU-PHL)|MDU-PHL|Schultz|Australia
>Spike|hCoV-19/Denmark/DCGC-3386/2020|2020-08-31|EPI_ISL_616145|Original|hCoV-19^^Hovedstaden|Human|Department of Virus and Microbiological Special Diagnostics|Albertsen lab|Michaelsen|Denmark
>Spike|hCoV-19/Denmark/DCGC-20853/2020|2020-12-21|EPI_ISL_759409|Original|hCoV-19^^Hovedstaden|Human|Department of Virus and Microbiological Special Diagnostics|Albertsen Lab|Michaelsen|Denmark

>Spike|hCoV-19/England/QEUH-A1E4BE/2020|2020-09-29|EPI_ISL_587462|Original|hCoV-19^^England|Human|Lighthouse Lab in Glasgow|Wellcome Sanger Institute for the COVID-19 Genomics UK (COG-UK) consortium|Davies|United Kingdom
>Spike|hCoV-19/Canada/BC-BCCDC-7515/2020|2020-11-00|EPI_ISL_740686|Original|hCoV-19^^British Columbia|Human|BCCDC Public Health Laboratory|BCCDC Public Health Laboratory|Prystajecky|Canada
>Spike|hCoV-19/Northern Ireland/NIRE-FB4FD/2020|2020-03-17|EPI_ISL_441435|Original|hCoV-19^^Northern Ireland|Human|Regional Virus Laboratory|COVID-19 Genomics UK (COG-UK) Consortium|Fairley|United Kingdom
>Spike|hCoV-19/England/BRIS-122A36/2020|2020-04-03|EPI_ISL_440769|Original|hCoV-19^^England|Human|PHE South West Regional Laboratory|Wellcome Sanger Institute for the COVID-19 Genomics UK (COG-UK) consortium|Amato|United Kingdom
>Spike|hCoV-19/USA/MN-QDX-1372/2020|2020-03-15|EPI_ISL_572161|Original|hCoV-19^^Minnesota|Human|Quest Diagnostics|Quest Diagnostics|Kagan|USA
>Spike|hCoV-19/Wales/PHWC-499527/2020|2020-12-03|EPI_ISL_727318|Original|hCoV-19^^Wales|Human|Originating lab|COVID-19 Genomics UK (COG-UK) Consortium|Moore|United Kingdom
>Spike|hCoV-19/Denmark/DCGC-16366/2020|2020-11-16|EPI_ISL_714726|Original|hCoV-19^^Midtjylland|Human|Department of Virus and Microbiological Special Diagnostics|Albertsen Lab|Michaelsen|Denmark
>Spike|hCoV-19/England/ALDP-B1A5FA/2020|2020-11-01|EPI_ISL_647572|Original|hCoV-19^^England|Human|Lighthouse Lab in Alderley Park|Wellcome Sanger Institute for the COVID-19 Genomics UK (COG-UK) Consortium|Davies|United Kingdom
>Spike|hCoV-19/USA/WA-UW-5182/2020|2020-04-02|EPI_ISL_430872|Original|hCoV-19^^Washington|Human|UW Virology Lab|UW Virology Lab|Roychoudhury|USA
>Spike|hCoV-19/England/EXET-13F3B3/2020|2020-12-16|EPI_ISL_763376|Original|hCoV-19^^England|Human|University of Exeter|COVID-19 Genomics UK (COG-UK) Consortium|Temperton|United Kingdom
>Spike|hCoV-19/England/QEUH-96C591/2020|2020-08-13|EPI_ISL_530918|Original|hCoV-19^^England|Human|Lighthouse Lab in Glasgow|Wellcome Sanger Institute for the COVID-19 Genomics UK (COG-UK) consortium|Davies|United Kingdom
>Spike|hCoV-19/England/CAMB-1BBD0C/2020|2020-11-14|EPI_ISL_679223|Original|hCoV-19^^England|Human|Department of Pathology|COVID-19 Genomics UK (COG-UK) Consortium|Goodfellow|United Kingdom
>Spike|hCoV-19/Wales/PHWC-482E44/2020|2020-09-19|EPI_ISL_627748|Original|hCoV-19^^Wales|Human|Wales Specialist Virology Centre Sequencing lab|COVID-19 Genomics UK (COG-UK) Consortium|Moore|United Kingdom
>Spike|hCoV-19/Denmark/DCGC-18379/2020|2020-12-07|EPI_ISL_749928|Original|hCoV-19^^Midtjylland|Human|Department of Virus and Microbiological Special Diagnostics|Albertsen Lab|Michaelsen|Denmark
>Spike|hCoV-19/England/BRIS-121C7D/2020|2020-03-18|EPI_ISL_440253|Original|hCoV-19^^England|Human|PHE South West Regional Laboratory|Wellcome Sanger Institute for the COVID-19 Genomics UK (COG-UK) consortium|Amato|United Kingdom
>Spike|hCoV-19/USA/MA-MASPHL-00387/2020|2020-05-12|EPI_ISL_693157|Original|hCoV-19^^Massachusetts|Human|Massachusetts State Public Health Laboratory|Massachusetts State Public Health Laboratory|Lang|USA
>Spike|hCoV-19/England/MILK-9F1E1E/2020|2020-09-24|EPI_ISL_589662|Original|hCoV-19^^England|Human|Lighthouse Lab in Milton Keynes|Wellcome Sanger Institute for the COVID-19 Genomics UK (COG-UK) consortium|Davies|United Kingdom
>Spike|hCoV-19/USA/TX-HMH-MCoV-14980/2020|2020-10-15|EPI_ISL_786464|Original|hCoV-19^^Texas|Human|Houston Methodist Hospital|Houston Methodist Hospital|Long|USA
>Spike|hCoV-19/Czech Republic/NRL_5556/2020|2020-03-22|EPI_ISL_489708|Original|hCoV-19^^Brno|Human|The National Institute of Public Health|The National Institute of Public Health and State Veterinary Institute Prague|Nagy|Czech Republic
>Spike|hCoV-19/England/CAMC-C738AA/2020|2020-12-15|EPI_ISL_769158|Original|hCoV-19^^England|Human|Lighthouse Lab in Cambridge|Wellcome Sanger Institute for the COVID-19 Genomics UK (COG-UK) Consortium|Davies|United Kingdom
>Spike|hCoV-19/Denmark/DCGC-16527/2020|2020-11-16|EPI_ISL_714816|Original|hCoV-19^^Syddanmark|Human|Department of Virus and Microbiological Special Diagnostics|Albertsen Lab|Michaelsen|Denmark

>Spike|hCoV-19/France/ARA-103983/2020|2020-11-09|EPI_ISL_683346|Original|hCoV-
19^^Auvergne-Rh\u00f4ne-Alpes|Human|CNR Virus des Infections Respiratoires - France
SUD|CNR Virus des Infections Respiratoires - France SUD|Bal|France
>Spike|hCoV-19/Northern Ireland/QEUH-96EAAF/2020|2020-08-
19|EPI_ISL_530617|Original|hCoV-19^^Northern Ireland|Human|Lighthouse Lab in
Glasgow|Wellcome Sanger Institute for the COVID-19 Genomics UK (COG-UK)
consortium|Davies|United Kingdom
>Spike|hCoV-19/England/MILK-B02A50/2020|2020-10-25|EPI_ISL_642930|Original|hCoV-
19^^England|Human|Lighthouse Lab in Milton Keynes|Wellcome Sanger Institute for the
COVID-19 Genomics UK (COG-UK) Consortium|Davies|United Kingdom
>Spike|hCoV-19/Hungary/SRC-03670w/2020|2020-03-29|EPI_ISL_435430|Original|hCoV-
19^^Kecskemet|Human|Virological Research Group|Bioinformatics Research
Group|Herczeg|Hungary
>Spike|hCoV-19/Canada/ON-PHL-20-01308/2020|2020-06-00|EPI_ISL_548911|Original|hCoV-
19^^Ontario|Human|Public Health Ontario Laboratory|Public Health Ontario
Laboratory|Guthrie|Canada
>Spike|hCoV-19/England/CAMB-78DA2/2020|2020-04-03|EPI_ISL_441252|Original|hCoV-
19^^England|Human|Department of Pathology|Wellcome Sanger Institute for the COVID-19
Genomics UK (COG-UK) consortium|Amato|United Kingdom
>Spike|hCoV-19/England/CAMC-AAFD5D/2020|2020-10-21|EPI_ISL_623404|Original|hCoV-
19^^England|Human|Lighthouse Lab in Cambridge|Wellcome Sanger Institute for the
COVID-19 Genomics UK (COG-UK) consortium|Davies|United Kingdom
>Spike|hCoV-19/USA/AZ-TG408205/2020|2020-06-17|EPI_ISL_694941|Original|hCoV-
19^^Arizona|Human|AZ SPHL|TGen North|French|USA
>Spike|hCoV-19/Wales/PHWC-36CEF/2020|2020-05-03|EPI_ISL_474791|Original|hCoV-
19^^Wales|Human|Originating lab|COVID-19 Genomics UK (COG-UK) Consortium|Moore|United
Kingdom
>Spike|hCoV-19/England/MILK-992BDC/2020|2020-08-26|EPI_ISL_552079|Original|hCoV-
19^^England|Human|Lighthouse Lab in Milton Keynes|Wellcome Sanger Institute for the
COVID-19 Genomics UK (COG-UK) consortium|Davies|United Kingdom
>Spike|hCoV-19/Scotland/GCVR-1713F1/2020|2020-03-26|EPI_ISL_459684|Original|hCoV-
19^^Scotland|Human|NHSGGC West of Scotland Specialist Virology Centre / MRC-
University of Glasgow Centre for Virus Research|Wellcome Sanger Institute for the
COVID-19 Genomics UK (COG-UK) consortium|Davies|United Kingdom
>Spike|hCoV-19/Luxembourg/LNS4702094/2020|2020-12-02|EPI_ISL_771036|Original|hCoV-
19^^Luxembourg|Human|Laboratoire national de sant\u00e9|Laboratoire national de
sant\u00e9|Wienecke-Baldacchino|Luxembourg
>Spike|hCoV-19/Japan/PG-4215/2020|2020-07-00|EPI_ISL_692453|Original|hCoV-
19^^Japan|Human|Fukuoka Institute of Health and Environmental Sciences|Pathogen
Genomics Center|Sekizuka|Japan
>Spike|hCoV-19/Saudi Arabia/KAUST-RIYADH1264/2020|2020-07-
18|EPI_ISL_678222|Original|hCoV-19^^Riyadh|Human|Pathogen Genomics Lab King Abdullah
University of Science and Technology(KAUST)|Pathogen Genomics Lab King Abdullah
University of Science and Technology(KAUST)|Mohamed Ghazzali|Saudi Arabia
>Spike|hCoV-19/USA/WA-UW-3858/2020|2020-03-26|EPI_ISL_427195|Original|hCoV-
19^^Washington|Human|UW Virology Lab|UW Virology Lab|Roychoudhury|USA
>Spike|hCoV-19/USA/MI-QDX-1304/2020|2020-03-17|EPI_ISL_571959|Original|hCoV-
19^^Michigan|Human|Quest Diagnostics|Quest Diagnostics|Kagan|USA
>Spike|hCoV-19/Canada/ON-S1637/2020|2020-09-15|EPI_ISL_755758|Original|hCoV-
19^^Ontario|Human|Toronto Invasive Bacterial Diseases Network|McMaster
University|McArthur|Canada
>Spike|hCoV-19/USA/AZ-TG670267/2020|2020-03-25|EPI_ISL_696442|Original|hCoV-
19^^Arizona|Human|Sonora Quest Laboratories|TGen North|French|USA
>Spike|hCoV-19/USA/CA-CZB-1324/2020|2020-05-21|EPI_ISL_468356|Original|hCoV-
19^^California|Human|County of Santa Clara Public Health Department|Chan-Zuckerberg
Biohub|Hao|USA
>Spike|hCoV-19/England/20196050704/2020|2020-05-07|EPI_ISL_526546|Original|hCoV-
19^^England|Human|Respiratory Virus Unit|Respiratory Virus Unit|Ledesma|United
Kingdom

>Spike|hCoV-19/Switzerland/BE-ETHZ-380284/2020|2020-11-25|EPI_ISL_721762|Original|hCoV-19^^Bern|Human|Viollier AG|Department of Biosystems Science and Engineering|Nadeau|Switzerland
>Spike|hCoV-19/Canada/ON-S294/2020|2020-03-28|EPI_ISL_755857|Original|hCoV-19^^Ontario|Human|Toronto Invasive Bacterial Diseases Network|McMaster University|McArthur|Canada
>Spike|hCoV-19/England/QEUH-9F48B3/2020|2020-09-25|EPI_ISL_588366|Original|hCoV-19^^England|Human|Lighthouse Lab in Glasgow|Wellcome Sanger Institute for the COVID-19 Genomics UK (COG-UK) consortium|Davies|United Kingdom
>Spike|hCoV-19/England/BIRM-66114/2020|2020-10-07|EPI_ISL_612236|Original|hCoV-19^^England|Human|University of Birmingham|COVID-19 Genomics UK (COG-UK) Consortium|Loman|United Kingdom
>Spike|hCoV-19/England/LIVE-A7F59/2020|2020-03-26|EPI_ISL_517094|Original|hCoV-19^^England|Human|Liverpool Clinical Laboratories|COVID-19 Genomics UK (COG-UK) Consortium|Haldenby|United Kingdom
>Spike|hCoV-19/USA/MI-MDHHS-SC22383/2020|2020-11-09|EPI_ISL_708363|Original|hCoV-19^^Michigan|Human|Michigan Department of Health and Human Services|Michigan Department of Health and Human Services|Jacob|USA
>Spike|hCoV-19/Belgium/VJ-0409291/2020|2020-04-09|EPI_ISL_464085|Original|hCoV-19^^Houthalen-Helchteren|Human|KU Leuven|KU Leuven|Maes|Belgium
>Spike|hCoV-19/Netherlands/NoordBrabant_61/2020|2020-00-00|EPI_ISL_415517|Original|hCoV-19^^Noord Brabant|Human|Dutch COVID-19 response team|Erasmus Medical Center|Oude Munnink|Netherlands
>Spike|hCoV-19/England/CAMC-B543BE/2020|2020-11-08|EPI_ISL_656762|Original|hCoV-19^^England|Human|Lighthouse Lab in Cambridge|Wellcome Sanger Institute for the COVID-19 Genomics UK (COG-UK) Consortium|Davies|United Kingdom
>Spike|hCoV-19/Wales/PHWC-169B12/2020|2020-07-21|EPI_ISL_517591|Original|hCoV-19^^Wales|Human|Wales Specialist Virology Centre Sequencing lab|COVID-19 Genomics UK (COG-UK) Consortium|Moore|United Kingdom
>Spike|hCoV-19/Canada/BC_25416870/2020|2020-03-00|EPI_ISL_463224|Original|hCoV-19^^British Columbia|Human|BCCDC Public Health Laboratory|BCCDC Public Health Laboratory|Harrigan|Canada
>Spike|hCoV-19/USA/MD-MDH-0439/2020|2020-11-10|EPI_ISL_666806|Original|hCoV-19^^Maryland|Human|Maryland Public Health Laboratory|Maryland Public Health Laboratory|Keller|USA
>Spike|hCoV-19/USA/LA-EVTL1276/2020|2020-12-04|EPI_ISL_778942|Original|hCoV-19^^Louisiana|Human|LSUHS Emerging Viral Threat Laboratory|Microbial Genome Sequencing Center|Kamil|USA
>Spike|hCoV-19/England/CAMC-B77D20/2020|2020-11-12|EPI_ISL_668280|Original|hCoV-19^^England|Human|Lighthouse Lab in Cambridge|Wellcome Sanger Institute for the COVID-19 Genomics UK (COG-UK) Consortium|Davies|United Kingdom
>Spike|hCoV-19/England/NOTT-111047/2020|2020-05-05|EPI_ISL_453619|Original|hCoV-19^^England|Human|Queens Medical Centre|COVID-19 Genomics UK (COG-UK) Consortium|Loose|United Kingdom
>Spike|hCoV-19/Brazil/PE-IAM220/2020|2020-04-09|EPI_ISL_500472|Original|hCoV-19^^Pernambuco|Human|LACEN/PE|WallauLab|Wallau|Brazil
>Spike|hCoV-19/Brazil/PE-IAM707/2020|2020-04-26|EPI_ISL_572350|Original|hCoV-19^^Pernambuco|Human|LACEN/PE|WallauLab|Wallau|Brazil
>Spike|hCoV-19/Wales/ALDP-C3B3DA/2020|2020-12-08|EPI_ISL_767169|Original|hCoV-19^^Wales|Human|Lighthouse Lab in Alderley Park|Wellcome Sanger Institute for the COVID-19 Genomics UK (COG-UK) Consortium|Davies|United Kingdom
>Spike|hCoV-19/Australia/NSW2939/2020|2020-10-16|EPI_ISL_678371|Original|hCoV-19^^New South Wales|Human|Area of Virology|Virology Research Laboratory; Area of Virology|Foster|Australia
>Spike|hCoV-19/USA/NM-DOH-2020308244/2020|2020-08-24|EPI_ISL_602174|Original|hCoV-19^^New Mexico|Human|New Mexico Department of Health Scientific Laboratory|New Mexico Department of Health Scientific Laboratory|Johnson|USA
>Spike|hCoV-19/England/ALDP-A51A35/2020|2020-10-07|EPI_ISL_598024|Original|hCoV-19^^England|Human|Lighthouse Lab in Alderley Park|Wellcome Sanger Institute for the COVID-19 Genomics UK (COG-UK) consortium|Davies|United Kingdom

>Spike|hCoV-19/Canada/BC_4078583/2020|2020-03-03|EPI_ISL_418824|Original|hCoV-19^^British Columbia|Human|BCCDC Public Health Laboratory|BCCDC Public Health Laboratory|Harrigan|Canada
>Spike|hCoV-19/England/NORT-29EE29/2020|2020-05-11|EPI_ISL_493539|Original|hCoV-19^^England|Human|Northumbria University / South Tees Hospitals NHS Foundation Trust / North Cumbria Integrated Care NHS Foundation Trust / North Tees and Hartlepool NHS Foundation Trust / Newcastle Hospitals NHS Foundation Trust|COVID-19 Genomics UK (COG-UK) Consortium|Bashton|United Kingdom
>Spike|hCoV-19/Portugal/PT1856/2020|2020-11-13|EPI_ISL_732075|Original|hCoV-19^^Portugal|Human|Instituto Nacional de Saude (INSA)|Instituto Nacional de Saude (INSA)|Borges|Portugal
>Spike|hCoV-19/India/MH-NCCS-NR-5693/2020|2020-06-11|EPI_ISL_722184|Original|hCoV-19^^Maharashtra|Human|National Centre For Cell Science|National Centre For Cell Science|Paul|India
>Spike|hCoV-19/Thailand/Phuket-SQ-JS21/2020|2020-03-23|EPI_ISL_708735|Vero 1|hCoV-19^^Phuket|Human|Regional Medical Sciences Center 11/1 phuket|National Institute of Health|Phuygun|Thailand
>Spike|hCoV-19/USA/TX-HMH-MCoV-12412/2020|2020-09-27|EPI_ISL_789806|Original|hCoV-19^^Texas|Human|Houston Methodist Hospital|Houston Methodist Hospital|Long|USA
>Spike|hCoV-19/England/QEUH-A34EF0/2020|2020-10-05|EPI_ISL_628425|Original|hCoV-19^^England|Human|Oxford Viromics|COVID-19 Genomics UK (COG-UK) Consortium|Golubchik|United Kingdom
>Spike|hCoV-19/England/ALDP-955470/2020|2020-06-12|EPI_ISL_554337|Original|hCoV-19^^England|Human|Lighthouse Lab in Alderley Park|Wellcome Sanger Institute for the COVID-19 Genomics UK (COG-UK) consortium|Davies|United Kingdom
>Spike|hCoV-19/USA/NM-UNM-00168/2020|2020-04-04|EPI_ISL_467663|Original|hCoV-19^^New Mexico|Human|New Mexico Department of Health Scientific Laboratory Division|Center for Global Health|Domman|USA
>Spike|hCoV-19/Peru/LIM-UPCH-0143/2020|2020-08-27|EPI_ISL_568537|Original|hCoV-19^^Lima|Human|Laboratorio de Referencia Nacional de Virus Respiratorios|Laboratorio de Gen\u00f3mica Microbiana|Tsukayama|Peru
>Spike|hCoV-19/India/MH-GA12/2020|2020-06-17|EPI_ISL_508208|Original|hCoV-19^^Maharashtra|Human|Government Medical College|National Institute of Biomedical Genomics|Maitra|India
>Spike|hCoV-19/USA/NY-NYCPHL-000126/2020|2020-04-01|EPI_ISL_631617|Original|hCoV-19^^New York|Human|OCME Office Of Chief Medical Examiner|New York City Public Health Laboratory|Wang|USA
>Spike|hCoV-19/Australia/VIC16234/2020|2020-08-11|EPI_ISL_640385|Original|hCoV-19^^Victoria|Human|Microbiological Diagnostic Unit - Public Health Laboratory (MDU-PHL)|MDU-PHL|Schultz|Australia
>Spike|hCoV-19/Australia/NSW989/2020|2020-09-15|EPI_ISL_591502|Original|hCoV-19^^New South Wales|Human|South Eastern Area Laboratory Services (SEALS)|NSW Health Pathology - Institute of Clinical Pathology and Medical Research; Westmead Hospital; University of Sydney|Gall|Australia
>Spike|hCoV-19/Wales/PHWC-2A2BF/2020|2020-04-06|EPI_ISL_445548|Original|hCoV-19^^Wales|Human|Wales Specialist Virology Centre|Public Health Wales Microbiology Cardiff|Moore|United Kingdom
>Spike|hCoV-19/Wales/PHWC-48D535/2020|2020-11-12|EPI_ISL_680640|Original|hCoV-19^^Wales|Human|Wales Specialist Virology Centre Sequencing lab|COVID-19 Genomics UK (COG-UK) Consortium|Moore|United Kingdom
>Spike|hCoV-19/Netherlands/ZH-EMC-1149/2020|2020-12-19|EPI_ISL_763225|Original|hCoV-19^^Zuid Holland|Human|Dutch COVID-19 response team|Erasmus Medical Center|Oude Munnink|Netherlands
>Spike|hCoV-19/Denmark/DCGC-20813/2020|2020-12-21|EPI_ISL_759380|Original|hCoV-19^^Sjaelland|Human|Department of Virus and Microbiological Special Diagnostics|Albertsen Lab|Michaelsen|Denmark
>Spike|hCoV-19/Norway/2795/2020|2020-06-25|EPI_ISL_493361|Original|hCoV-19^^Norway|Human|Furst Medical Laboratory|Norwegian Institute of Public Health|Kopperud Riis|Norway

>Spike|hCoV-19/USA/NMDOH-2020320943/2020|2020-09-01|EPI_ISL_542066|Original|hCoV-19^^Texas|Human|New Mexico Department of Health Scientific Laboratory|New Mexico Department of Health Scientific Laboratory|Johnson|USA
>Spike|hCoV-19/England/LIVE-DB40B3/2020|2020-10-19|EPI_ISL_652078|Original|hCoV-19^^England|Human|Liverpool Clinical Laboratories|COVID-19 Genomics UK (COG-UK) Consortium|Haldenby|United Kingdom
>Spike|hCoV-19/Germany/HH-hpi-p1638/2020|2020-03-17|EPI_ISL_775823|Original|hCoV-19^^Hamburg|Human|University Medical Center Hamburg Eppendorf|Heinrich Pette Institute|Robitaille|Germany
>Spike|hCoV-19/USA/MI-MDHHS-SC21896/2020|2020-04-03|EPI_ISL_565957|Original|hCoV-19^^Michigan|Human|Michigan Department of Health and Human Services|Michigan Department of Health and Human Services|Blankenship|USA
>Spike|hCoV-19/India/OR-RMRC158/2020|2020-05-07|EPI_ISL_455759|Original|hCoV-19^^Odisha|Human|REGIONAL VRDL,ICMR-RMRC BBSR|Immunogenomics lab|Raghav|India
>Spike|hCoV-19/Australia/VIC12849/2020|2020-07-29|EPI_ISL_640560|Original|hCoV-19^^Victoria|Human|Microbiological Diagnostic Unit - Public Health Laboratory (MDU-PHL)|MDU-PHL|Schultz|Australia
>Spike|hCoV-19/USA/LA-EVTL1087/2020|2020-11-30|EPI_ISL_768357|Original|hCoV-19^^Louisiana|Human|LSUHS Emerging Viral Threat Laboratory|Microbial Genome Sequencing Center|Kamil|USA
>Spike|hCoV-19/Wales/PHWC-483C58/2020|2020-10-24|EPI_ISL_627914|Original|hCoV-19^^Wales|Human|Wales Specialist Virology Centre Sequencing lab|COVID-19 Genomics UK (COG-UK) Consortium|Moore|United Kingdom
>Spike|hCoV-19/England/ALDP-95365E/2020|2020-06-08|EPI_ISL_554494|Original|hCoV-19^^England|Human|Lighthouse Lab in Alderley Park|Wellcome Sanger Institute for the COVID-19 Genomics UK (COG-UK) consortium|Davies|United Kingdom
>Spike|hCoV-19/Japan/PG-7848/2020|2020-08-00|EPI_ISL_686949|Original|hCoV-19^^Japan|Human|Pathogen Genomics Center|Pathogen Genomics Center|Sekizuka|Japan
>Spike|hCoV-19/England/LIVE-A9DA8/2020|2020-03-24|EPI_ISL_517361|Original|hCoV-19^^England|Human|Liverpool Clinical Laboratories|COVID-19 Genomics UK (COG-UK) Consortium|Haldenby|United Kingdom
>Spike|hCoV-19/England/MILK-9E0401/2020|2020-09-19|EPI_ISL_601282|Original|hCoV-19^^England|Human|Lighthouse Lab in Milton Keynes|Wellcome Sanger Institute for the COVID-19 Genomics UK (COG-UK) consortium|Davies|United Kingdom
>Spike|hCoV-19/Northern Ireland/NIRE-FC6D8/2020|2020-04-21|EPI_ISL_459394|Original|hCoV-19^^Northern Ireland|Human|Regional Virus Laboratory|Wellcome Sanger Institute for the COVID-19 Genomics UK (COG-UK) consortium|Davies|United Kingdom
>Spike|hCoV-19/England/QEUH-A9E8C9/2020|2020-10-20|EPI_ISL_611373|Original|hCoV-19^^England|Human|Lighthouse Lab in Glasgow|Wellcome Sanger Institute for the COVID-19 Genomics UK (COG-UK) consortium|Davies|United Kingdom
>Spike|hCoV-19/Wales/PHWC-485852/2020|2020-10-25|EPI_ISL_626853|Original|hCoV-19^^Wales|Human|Wales Specialist Virology Centre Sequencing lab|COVID-19 Genomics UK (COG-UK) Consortium|Moore|United Kingdom
>Spike|hCoV-19/England/QEUH-AE53E0/2020|2020-10-27|EPI_ISL_633305|Original|hCoV-19^^England|Human|Lighthouse Lab in Glasgow|Wellcome Sanger Institute for the COVID-19 Genomics UK (COG-UK) consortium|Davies|United Kingdom
>Spike|hCoV-19/Denmark/DCGC-18908/2020|2020-12-07|EPI_ISL_748633|Original|hCoV-19^^Hovedstaden|Human|Department of Virus and Microbiological Special Diagnostics|Albertsen Lab|Michaelsen|Denmark
>Spike|hCoV-19/USA/WA-S361/2020|2020-03-26|EPI_ISL_434121|Original|hCoV-19^^Washington|Human|Washington State Department of Health|Seattle Flu Study|Bedford|USA
>Spike|hCoV-19/USA/CA-CHLA-PLM76481572/2020|2020-11-30|EPI_ISL_753550|Original|hCoV-19^^California|Human|Clinical virology Laboratory|Center for Personalized Medicine|Dien Bard|USA
>Spike|hCoV-19/USA/FL-BPHL-1796/2020|2020-07-31|EPI_ISL_613895|Original|hCoV-19^^Florida|Human|Florida Bureau of Public Health Laboratories|Florida Bureau of Public Health Laboratories|Blanton|USA

>Spike|hCoV-19/USA/NJ-QDX-852/2020|2020-03-16|EPI_ISL_571643|Original|hCoV-19^^New Jersey|Human|Quest Diagnostics|Quest Diagnostics|Kagan|USA
>Spike|hCoV-19/Denmark/DCGC-13772/2020|2020-11-30|EPI_ISL_713015|Original|hCoV-19^^Midtjylland|Human|Department of Virus and Microbiological Special Diagnostics|Albertsen Lab|Michaelsen|Denmark
>Spike|hCoV-19/USA/NY-PV09120/2020|2020-03-19|EPI_ISL_422495|Original|hCoV-19^^New York|Human|MSHS Clinical Microbiology Laboratories|MSHS Pathogen Surveillance Program|van Bakel|USA
>Spike|hCoV-19/Wales/PHWC-49ED84/2020|2020-12-03|EPI_ISL_743119|Original|hCoV-19^^Wales|Human|Wales Specialist Virology Centre Sequencing lab|COVID-19 Genomics UK (COG-UK) Consortium|Moore|United Kingdom
>Spike|hCoV-19/Japan/PG-0602/2020|2020-04-00|EPI_ISL_684212|Original|hCoV-19^^Japan|Human|Pathogen Genomics Center|Pathogen Genomics Center|Sekizuka|Japan
>Spike|hCoV-19/Singapore/1115/2020|2020-10-20|EPI_ISL_605824|Original|hCoV-19^^Singapore|Human|National Public Health Laboratory|National Public Health Laboratory|Mak|Singapore
>Spike|hCoV-19/England/QEUH-9AF529/2020|2020-09-01|EPI_ISL_559849|Original|hCoV-19^^England|Human|Oxford Viromics|COVID-19 Genomics UK (COG-UK) Consortium|Golubchik|United Kingdom
>Spike|hCoV-19/Wales/PHWC-48C563/2020|2020-11-07|EPI_ISL_666441|Original|hCoV-19^^Wales|Human|Wales Specialist Virology Centre Sequencing lab|COVID-19 Genomics UK (COG-UK) Consortium|Moore|United Kingdom
>Spike|hCoV-19/Scotland/QEUH-A0B7F7/2020|2020-10-03|EPI_ISL_599466|Original|hCoV-19^^Scotland|Human|Lighthouse Lab in Glasgow|Wellcome Sanger Institute for the COVID-19 Genomics UK (COG-UK) consortium|Davies|United Kingdom
>Spike|hCoV-19/Wales/PHWC-497E4C/2020|2020-11-30|EPI_ISL_726984|Original|hCoV-19^^Wales|Human|Originating lab|COVID-19 Genomics UK (COG-UK) Consortium|Moore|United Kingdom
>Spike|hCoV-19/England/CAMC-AAF7C8/2020|2020-10-23|EPI_ISL_631254|Original|hCoV-19^^England|Human|Lighthouse Lab in Cambridge|Wellcome Sanger Institute for the COVID-19 Genomics UK (COG-UK) consortium|Davies|United Kingdom
>Spike|hCoV-19/England/CAMB-1B95F7/2020|2020-11-06|EPI_ISL_664942|Original|hCoV-19^^England|Human|Department of Pathology|COVID-19 Genomics UK (COG-UK) Consortium|Goodfellow|United Kingdom
>Spike|hCoV-19/England/QEUH-B40115/2020|2020-11-08|EPI_ISL_657273|Original|hCoV-19^^England|Human|Lighthouse Lab in Glasgow|Wellcome Sanger Institute for the COVID-19 Genomics UK (COG-UK) Consortium|Davies|United Kingdom
>Spike|hCoV-19/England/NOTT-10FAE4/2020|2020-04-07|EPI_ISL_432684|Original|hCoV-19^^England|Human|Queens Medical Centre|COVID-19 Genomics UK (COG-UK) Consortium|Loose|United Kingdom
>Spike|hCoV-19/DRC/11024/2020|2020-05-24|EPI_ISL_527552|Original|hCoV-19^^Kinshasa|Human|Viral Respiratory Lab|Pathogen Sequencing Lab|Lokilo|Democratic Republic of the Congo
>Spike|hCoV-19/England/QEUH-96E6E7/2020|2020-08-17|EPI_ISL_530602|Original|hCoV-19^^England|Human|Lighthouse Lab in Glasgow|Wellcome Sanger Institute for the COVID-19 Genomics UK (COG-UK) consortium|Davies|United Kingdom
>Spike|hCoV-19/Denmark/DCGC-19485/2020|2020-12-07|EPI_ISL_748981|Original|hCoV-19^^Sjaelland|Human|Department of Virus and Microbiological Special Diagnostics|Albertsen Lab|Michaelsen|Denmark
>Spike|hCoV-19/USA/VA-DCLS-1862/2020|2020-09-23|EPI_ISL_614083|Original|hCoV-19^^Virginia|Human|Virginia DCLS|Virginia DCLS|St. Jacques|USA
>Spike|hCoV-19/Ireland/D-NVRL-71IRL07248/2020|2020-07-07|EPI_ISL_500589|Original|hCoV-19^^Dublin|Human|National Virus Reference Laboratory|National Virus Reference Laboratory|Gonzalez|Ireland
>Spike|hCoV-19/India/MH-ACTREC-013/2020|2020-05-02|EPI_ISL_699669|Original|hCoV-19^^Maharashtra|Human|Hematopathology Laboratory|Hematopathology Laboratory|Patkar|India
>Spike|hCoV-19/USA/SC-DHEC-0182/2020|2020-05-06|EPI_ISL_529188|Original|hCoV-19^^South Carolina|Human|South Carolina Department of Health and Environmental Control|South Carolina Department of Health and Environmental Control|Flores|USA

>Spike|hCoV-19/USA/MD-MDH-0287/2020|2020-10-13|EPI_ISL_629067|Original|hCoV-19^^Maryland|Human|Maryland Public Health Laboratory|Maryland Public Health Laboratory|Keller|USA
>Spike|hCoV-19/England/CAMC-94BC39/2020|2020-07-16|EPI_ISL_556776|Original|hCoV-19^^England|Human|Lighthouse Lab in Cambridge|Wellcome Sanger Institute for the COVID-19 Genomics UK (COG-UK) consortium|Davies|United Kingdom
>Spike|hCoV-19/USA/TX-HMH-1398/2020|2020-04-13|EPI_ISL_542696|Original|hCoV-19^^Texas|Human|Houston Methodist Hospital|Houston Methodist Hospital|Long|USA
>Spike|hCoV-19/Netherlands/Utrecht_35/2020|2020-00-00|EPI_ISL_461233|Original|hCoV-19^^Utrecht|Human|Dutch COVID-19 response team|Erasmus Medical Center|Oude Munnink|Netherlands
>Spike|hCoV-19/Australia/NSW990/2020|2020-09-25|EPI_ISL_591517|Original|hCoV-19^^New South Wales|Human|Sydney South West Pathology Service (SSWPS) - Royal Prince Alfred Hospital - NSW Health Pathology|NSW Health Pathology - Institute of Clinical Pathology and Medical Research; Westmead Hospital; University of Sydney|Gall|Australia
>Spike|hCoV-19/England/QEUH-CC2402/2020|2020-12-21|EPI_ISL_778153|Original|hCoV-19^^England|Human|Lighthouse Lab in Glasgow|Wellcome Sanger Institute for the COVID-19 Genomics UK (COG-UK) Consortium|Davies|United Kingdom
>Spike|hCoV-19/England/CAMC-A81CFA/2020|2020-10-12|EPI_ISL_610724|Original|hCoV-19^^England|Human|Lighthouse Lab in Cambridge|Wellcome Sanger Institute for the COVID-19 Genomics UK (COG-UK) consortium|Davies|United Kingdom
>Spike|hCoV-19/United Arab Emirates/0091/2020|2020-05-15|EPI_ISL_698283|Original|hCoV-19^^Abu Dhabi|Human|Group 42 (G42) Healthcare|G42 Healthcare|Ogrodzki|United Arab Emirates
>Spike|hCoV-19/Singapore/959/2020|2020-04-09|EPI_ISL_538493|Original|hCoV-19^^Singapore|Human|Department of Laboratory Medicine|Department of Laboratory Medicine|Chen|Singapore
>Spike|hCoV-19/USA/MI-MDHHS-SC22179/2020|2020-10-19|EPI_ISL_614230|Original|hCoV-19^^Michigan|Human|Michigan Department of Health and Human Services|Michigan Department of Health and Human Services|Jacob|USA
>Spike|hCoV-19/USA/TX-HMH-MCoV-19589/2020|2020-11-21|EPI_ISL_783960|Original|hCoV-19^^Texas|Human|Houston Methodist Hospital|Houston Methodist Hospital|Long|USA
>Spike|hCoV-19/England/ALDP-4A9454/2020|2020-06-09|EPI_ISL_559615|Original|hCoV-19^^England|Human|Lighthouse Lab in Alderley Park|Wellcome Sanger Institute for the COVID-19 Genomics UK (COG-UK) consortium|Davies|United Kingdom
>Spike|hCoV-19/England/MILK-974DAD/2020|2020-08-22|EPI_ISL_552487|Original|hCoV-19^^England|Human|Lighthouse Lab in Milton Keynes|Wellcome Sanger Institute for the COVID-19 Genomics UK (COG-UK) consortium|Davies|United Kingdom
>Spike|hCoV-19/Denmark/DCGC-18110/2020|2020-12-14|EPI_ISL_749761|Original|hCoV-19^^Syddanmark|Human|Department of Virus and Microbiological Special Diagnostics|Albertsen Lab|Michaelsen|Denmark
>Spike|hCoV-19/England/MILK-B93D3D/2020|2020-11-13|EPI_ISL_676206|Original|hCoV-19^^England|Human|Lighthouse Lab in Milton Keynes|Wellcome Sanger Institute for the COVID-19 Genomics UK (COG-UK) Consortium|Davies|United Kingdom
>Spike|hCoV-19/England/QEUH-969567/2020|2020-08-12|EPI_ISL_531108|Original|hCoV-19^^England|Human|Lighthouse Lab in Glasgow|Wellcome Sanger Institute for the COVID-19 Genomics UK (COG-UK) consortium|Davies|United Kingdom
>Spike|hCoV-19/Northern Ireland/NIRE-1088A5/2020|2020-09-02|EPI_ISL_585212|Original|hCoV-19^^Northern Ireland|Human|Regional Virus Laboratory|COVID-19 Genomics UK (COG-UK) Consortium|Fairley|United Kingdom
>Spike|hCoV-19/England/ALDP-A2E925/2020|2020-10-05|EPI_ISL_586948|Original|hCoV-19^^England|Human|Lighthouse Lab in Alderley Park|Wellcome Sanger Institute for the COVID-19 Genomics UK (COG-UK) consortium|Davies|United Kingdom
>Spike|hCoV-19/England/NOTT-11F7BD/2020|2020-11-26|EPI_ISL_725568|Original|hCoV-19^^England|Human|Lincolnshire Hospitals and DeepSeq Nottingham|COVID-19 Genomics UK (COG-UK) Consortium|Loose|United Kingdom
>Spike|hCoV-19/England/CAMC-B32F67/2020|2020-11-05|EPI_ISL_659242|Original|hCoV-19^^England|Human|Lighthouse Lab in Cambridge|Wellcome Sanger Institute for the COVID-19 Genomics UK (COG-UK) Consortium|Davies|United Kingdom

>Spike|hCoV-19/England/CAMC-A81E21/2020|2020-10-12|EPI_ISL_610896|Original|hCoV-19^^England|Human|Lighthouse Lab in Cambridge|Wellcome Sanger Institute for the COVID-19 Genomics UK (COG-UK) consortium|Davies|United Kingdom
>Spike|hCoV-19/USA/TX-HMH-MCoV-15486/2020|2020-10-23|EPI_ISL_786970|Original|hCoV-19^^Texas|Human|Houston Methodist Hospital|Houston Methodist Hospital|Long|USA
>Spike|hCoV-19/Scotland/EDB6168/2020|2020-05-15|EPI_ISL_473899|Original|hCoV-19^^Scotland|Human|Virology Department|COVID-19 Genomics UK (COG-UK) Consortium|Williams|United Kingdom
>Spike|hCoV-19/USA/UT-08549/2020|2020-05-18|EPI_ISL_524309|Original|hCoV-19^^Utah|Human|Utah Public Health Laboratory|Utah Public Health Laboratory|Young|USA
>Spike|hCoV-19/Denmark/DCGC-10770/2020|2020-11-02|EPI_ISL_669823|Original|hCoV-19^^Hovedstaden|Human|Department of Virus and Microbiological Special Diagnostics|Albertsen Lab|Michaelsen|Denmark
>Spike|hCoV-19/Denmark/DCGC-2889/2020|2020-08-24|EPI_ISL_616584|Original|hCoV-19^^Nordjylland|Human|Department of Virus and Microbiological Special Diagnostics|Albertsen lab|Michaelsen|Denmark
>Spike|hCoV-19/England/BRIS-130C7B/2020|2020-03-22|EPI_ISL_492590|Original|hCoV-19^^England|Human|PHE South West Regional Laboratory|Wellcome Sanger Institute for the COVID-19 Genomics UK (COG-UK) consortium|Davies|United Kingdom
>Spike|hCoV-19/England/PORT-2D71C1/2020|2020-10-26|EPI_ISL_627056|Original|hCoV-19^^England|Human|Centre for Enzyme Innovation|COVID-19 Genomics UK (COG-UK) Consortium|Robson|United Kingdom
>Spike|hCoV-19/Denmark/DCGC-905/2020|2020-05-11|EPI_ISL_614468|Original|hCoV-19^^Hovedstaden|Human|Department of Virus and Microbiological Special Diagnostics|Albertsen lab|Michaelsen|Denmark
>Spike|hCoV-19/England/CAMB-7CCED/2020|2020-04-06|EPI_ISL_441611|Original|hCoV-19^^England|Human|Department of Pathology|Wellcome Sanger Institute for the COVID-19 Genomics UK (COG-UK) consortium|Amato|United Kingdom
>Spike|hCoV-19/Australia/VIC15113/2020|2020-08-13|EPI_ISL_593143|Original|hCoV-19^^Victoria|Human|Microbiological Diagnostic Unit - Public Health Laboratory (MDU-PHL)|MDU-PHL|Schultz|Australia
>Spike|hCoV-19/Burkina Faso/2-035-1347/2020|2020-11-04|EPI_ISL_660452|Original|hCoV-19^^Bobo Dioulasso|Human|Laboratoire de Microbiologie CHU Souro Sanou|Centre Muraz|Ouedraogo|Burkina Faso
>Spike|hCoV-19/USA/MI-MDHHS-SC22522/2020|2020-11-17|EPI_ISL_708225|Original|hCoV-19^^Michigan|Human|Michigan Department of Health and Human Services|Michigan Department of Health and Human Services|Jacob|USA
>Spike|hCoV-19/USA/WA-S1177/2020|2020-05-05|EPI_ISL_463667|Original|hCoV-19^^Washington|Human|Washington State Department of Health|Seattle Flu Study|Bedford|USA
>Spike|hCoV-19/England/CAMB-1B833A/2020|2020-10-19|EPI_ISL_637742|Original|hCoV-19^^England|Human|Department of Pathology|COVID-19 Genomics UK (COG-UK) Consortium|Goodfellow|United Kingdom
>Spike|hCoV-19/Japan/PG-3016/2020|2020-04-00|EPI_ISL_691140|Original|hCoV-19^^Japan|Human|Pathogen Genomics Center|Pathogen Genomics Center|Sekizuka|Japan
>Spike|hCoV-19/Denmark/DCGC-6962/2020|2020-10-12|EPI_ISL_619808|Original|hCoV-19^^Nordjylland|Human|Department of Virus and Microbiological Special Diagnostics|Albertsen lab|Michaelsen|Denmark
>Spike|hCoV-19/USA/WI-UW-1651/2020|2020-09-10|EPI_ISL_605525|Original|hCoV-19^^Wisconsin|Human|University of Wisconsin-Madison AIDS Vaccine Research Laboratories|University of Wisconsin-Madison AIDS Vaccine Research Laboratories|Baczenas|USA
>Spike|hCoV-19/England/ALDP-C05C65/2020|2020-12-04|EPI_ISL_720858|Original|hCoV-19^^England|Human|Lighthouse Lab in Alderley Park|Wellcome Sanger Institute for the COVID-19 Genomics UK (COG-UK) Consortium|Davies|United Kingdom
>Spike|hCoV-19/USA/VA-DCLS-1035/2020|2020-06-03|EPI_ISL_526920|Original|hCoV-19^^Virginia|Human|Virginia DCLS|Virginia DCLS|St. Jacques|USA
>Spike|hCoV-19/England/QEUH-9B86C0/2020|2020-09-10|EPI_ISL_549576|Original|hCoV-19^^England|Human|Lighthouse Lab in Glasgow|Wellcome Sanger Institute for the COVID-19 Genomics UK (COG-UK) consortium|Davies|United Kingdom

>Spike|hCoV-19/Wales/PHWC-48589E/2020|2020-10-24|EPI_ISL_627279|Original|hCoV-19^^Wales|Human|Wales Specialist Virology Centre Sequencing lab|COVID-19 Genomics UK (COG-UK) Consortium|Moore|United Kingdom
>Spike|hCoV-19/USA/WA-UW-6190/2020|2020-04-01|EPI_ISL_570086|Original|hCoV-19^^Washington|Human|UW Virology Lab|UW Virology Lab|Roychoudhury|USA
>Spike|hCoV-19/England/BRIS-12D2BB/2020|2020-05-04|EPI_ISL_481811|Original|hCoV-19^^England|Human|PHE South West Regional Laboratory|Wellcome Sanger Institute for the COVID-19 Genomics UK (COG-UK) consortium|Davies|United Kingdom
>Spike|hCoV-19/Denmark/DCGC-16312/2020|2020-11-16|EPI_ISL_714228|Original|hCoV-19^^Hovedstaden|Human|Department of Virus and Microbiological Special Diagnostics|Albertsen Lab|Michaelsen|Denmark
>Spike|hCoV-19/USA/CA-CZB-14050/2020|2020-07-10|EPI_ISL_739638|Original|hCoV-19^^California|Human|Alameda County Public Health Lab|Chan-Zuckerberg Biohub|Kamm|USA
>Spike|hCoV-19/England/CWAR-24FDEB7/2020|2020-06-07|EPI_ISL_584539|Original|hCoV-19^^England|Human|UHCW / University of Warwick|COVID-19 Genomics UK (COG-UK) Consortium|Stark|United Kingdom
>Spike|hCoV-19/USA/WI-GMF-30052/2020|2020-10-29|EPI_ISL_661024|Original|hCoV-19^^Wisconsin|Human|Gundersen Molecular Diagnostics Laboratory|Kabara Cancer Research Institute|Kenny|USA
>Spike|hCoV-19/Australia/VIC16691/2020|2020-00-00|EPI_ISL_640407|Original|hCoV-19^^Victoria|Human|Microbiological Diagnostic Unit - Public Health Laboratory (MDU-PHL)|MDU-PHL|Schultz|Australia
>Spike|hCoV-19/USA/MI-MDHHS-SC21724/2020|2020-07-31|EPI_ISL_529843|Original|hCoV-19^^Michigan|Human|Michigan Department of Health and Human Services|Michigan Department of Health and Human Services|Blankenship|USA
>Spike|hCoV-19/USA/MI-MDHHS-SC22629/2020|2020-12-01|EPI_ISL_737102|Original|hCoV-19^^Michigan|Human|Michigan Department of Health and Human Services|Michigan Department of Health and Human Services|Blankenship|USA
>Spike|hCoV-19/South Africa/KRISP-K002341/2020|2020-07-22|EPI_ISL_515714|Original|hCoV-19^^KwaZulu-Natal|Human|NHLS-IALCH|KRISP|de Oliveira|South Africa
>Spike|hCoV-19/Mexico/BCN-ALSR-2447/2020|2020-05-14|EPI_ISL_635481|Original|hCoV-19^^Baja California|Human|Centro de Diagn\u221a\u2265stico COVID-19 UABC Tijuana|Andersen lab at Scripps Research|Gangavarapu|Mexico
>Spike|hCoV-19/England/LIVE-A2880/2020|2020-06-08|EPI_ISL_500010|Original|hCoV-19^^England|Human|Liverpool Clinical Laboratories|COVID-19 Genomics UK (COG-UK) Consortium|Haldenby|United Kingdom
>Spike|hCoV-19/USA/FL-BPHL-0440/2020|2020-03-21|EPI_ISL_495346|Original|hCoV-19^^Florida|Human|Florida Bureau of Public Health Laboratories|Florida Bureau of Public Health Laboratories|Blanton|USA
>Spike|hCoV-19/Scotland/QEUH-9CE3C0/2020|2020-09-16|EPI_ISL_602013|Original|hCoV-19^^Scotland|Human|Lighthouse Lab in Glasgow|Wellcome Sanger Institute for the COVID-19 Genomics UK (COG-UK) consortium|Davies|United Kingdom
>Spike|hCoV-19/France/PAC-IHU-0319/2020|2020-04-01|EPI_ISL_569451|Original|hCoV-19^^Provence-Alpes-C\u00f4te-d\u2019Azur|Human|MEPHI|MEPHI|Levasseur|France
>Spike|hCoV-19/Wales/PHWC-48E6B9/2020|2020-11-12|EPI_ISL_680895|Original|hCoV-19^^Wales|Human|Wales Specialist Virology Centre Sequencing lab|COVID-19 Genomics UK (COG-UK) Consortium|Moore|United Kingdom
>Spike|hCoV-19/Wales/PHWC-493192/2020|2020-11-29|EPI_ISL_725848|Original|hCoV-19^^Wales|Human|Originating lab|COVID-19 Genomics UK (COG-UK) Consortium|Moore|United Kingdom
>Spike|hCoV-19/Denmark/DCGC-6626/2020|2020-10-12|EPI_ISL_619675|Original|hCoV-19^^Sjaelland|Human|Department of Virus and Microbiological Special Diagnostics|Albertsen lab|Michaelsen|Denmark
>Spike|hCoV-19/Ireland/LK-UHL126/2020|2020-04-07|EPI_ISL_681726|Original|hCoV-19^^Limerick|Human|University Hospital Limerick|Irish Coronavirus Sequencing Consortium - Teagasc Moorepark|Walsh|Ireland
>Spike|hCoV-19/England/NOTT-11DFF3/2020|2020-11-25|EPI_ISL_703720|Original|hCoV-19^^England|Human|Queens Medical Centre|COVID-19 Genomics UK (COG-UK) Consortium|Loose|United Kingdom

>Spike|hCoV-19/USA/CA-CZB-1346/2020|2020-04-09|EPI_ISL_468362|Original|hCoV-19^^California|Human|Alameda County Public Health Lab|Chan-Zuckerberg Biohub|Hao|USA
>Spike|hCoV-19/England/MILK-A22D25/2020|2020-10-03|EPI_ISL_611657|Original|hCoV-19^^England|Human|Northumbria University / South Tees Hospitals NHS Foundation Trust / North Cumbria Integrated Care NHS Foundation Trust / North Tees and Hartlepool NHS Foundation Trust / Newcastle Hospitals NHS Foundation Trust|COVID-19 Genomics UK (COG-UK) Consortium|Bashton|United Kingdom
>Spike|hCoV-19/USA/OR-OHSU-2269/2020|2020-08-08|EPI_ISL_667164|Original|hCoV-19^^Oregon|Human|OHSU Lab Services Molecular Microbiology Lab|Oregon SARS-CoV-2 Genome Sequencing Center|O'Roak|USA
>Spike|hCoV-19/England/QEUH-B2BA33/2020|2020-11-02|EPI_ISL_646547|Original|hCoV-19^^England|Human|Lighthouse Lab in Glasgow|Wellcome Sanger Institute for the COVID-19 Genomics UK (COG-UK) Consortium|Davies|United Kingdom
>Spike|hCoV-19/Denmark/DCGC-3130/2020|2020-08-31|EPI_ISL_615949|Original|hCoV-19^^Hovedstaden|Human|Department of Virus and Microbiological Special Diagnostics|Albertsen lab|Michaelsen|Denmark
>Spike|hCoV-19/Indonesia/BT-SHSIU-01-4920/2020|2020-10-18|EPI_ISL_708652|Original|hCoV-19^^Banten|Human|Medical Research Center|Medical Research Center|Adhiyanto|Indonesia
>Spike|hCoV-19/USA/LA-CDC-1295/2020|2020-06-11|EPI_ISL_535336|Vero P1|hCoV-19^^Louisiana|Human|LA Office of Public Health Laboratories|Pathogen Discovery|Queen|USA
>Spike|hCoV-19/Denmark/DCGC-4750/2020|2020-09-14|EPI_ISL_622189|Original|hCoV-19^^Syddanmark|Human|Department of Virus and Microbiological Special Diagnostics|Albertsen lab|Michaelsen|Denmark
>Spike|hCoV-19/Australia/VIC2345/2020|2020-06-24|EPI_ISL_522066|Original|hCoV-19^^Victoria|Human|Victorian Infectious Diseases Reference Laboratory (VIDRL)|VIDRL and MDU-PHL|Schultz|Australia
>Spike|hCoV-19/Spain/CT-IBV-99009899/2020|2020-08-08|EPI_ISL_660296|Original|hCoV-19^^Catalunya|Human|Servicio de Microbiolog\u00eda|SeqCOVID-SPAIN consortium/IBV(CSIC)|Comas|Spain
>Spike|hCoV-19/England/CAMC-C720FE/2020|2020-12-15|EPI_ISL_769082|Original|hCoV-19^^England|Human|Lighthouse Lab in Cambridge|Wellcome Sanger Institute for the COVID-19 Genomics UK (COG-UK) Consortium|Davies|United Kingdom
>Spike|hCoV-19/England/204221380/2020|2020-00-00|EPI_ISL_593787|Original|hCoV-19^^England|Human|Respiratory Virus Unit|Respiratory Virus Unit|Platt|United Kingdom
>Spike|hCoV-19/Brazil/RJ-00481/2020|2020-10-09|EPI_ISL_717793|Original|hCoV-19^^Rio de Janeiro|Human|Laboratorio de Virologia Molecular / UFRJ|Bioinformatics Laboratory / LNCC|Almeida|Brazil
>Spike|hCoV-19/Netherlands/GE-EMC-207/2020|2020-05-27|EPI_ISL_523189|Original|hCoV-19^^Gelderland|Human|Dutch COVID-19 response team|Erasmus Medical Center|Oude Munnink|Netherlands
>Spike|hCoV-19/England/QEUH-B825C0/2020|2020-11-14|EPI_ISL_661820|Original|hCoV-19^^England|Human|Lighthouse Lab in Glasgow|Wellcome Sanger Institute for the COVID-19 Genomics UK (COG-UK) Consortium|Davies|United Kingdom
>Spike|hCoV-19/Australia/VIC1659/2020|2020-05-12|EPI_ISL_456560|Original|hCoV-19^^Victoria|Human|Victorian Infectious Diseases Reference Laboratory (VIDRL)|Microbiological Diagnostic Unit Public Health Laboratory and Victorian Infectious Diseases Reference Laboratory|Schultz|Australia
>Spike|hCoV-19/Japan/PG-6677/2020|2020-07-00|EPI_ISL_687788|Original|hCoV-19^^Japan|Human|Pathogen Genomics Center|Pathogen Genomics Center|Sekizuka|Japan
>Spike|hCoV-19/Denmark/DCGC-7244/2020|2020-10-12|EPI_ISL_619223|Original|hCoV-19^^Sjaelland|Human|Department of Virus and Microbiological Special Diagnostics|Albertsen lab|Michaelsen|Denmark
>Spike|hCoV-19/Australia/WA282/2020|2020-04-01|EPI_ISL_596825|Original|hCoV-19^^Western Australia|Human|PathWest Laboratory Medicine WA|PathWest Laboratory Medicine WA Microbial Surveillance Unit|Sikazwe|Australia
>Spike|hCoV-19/USA/MI-MDHHS-SC22295/2020|2020-10-23|EPI_ISL_666961|Original|hCoV-19^^Michigan|Human|Michigan Department of Health and Human Services|Michigan Department of Health and Human Services|Jacob|USA

>Spike|hCoV-19/England/ALDP-9CD7A7/2020|2020-09-15|EPI_ISL_566164|Original|hCoV-
19^^England|Human|Lighthouse Lab in Alderley Park|Wellcome Sanger Institute for the
COVID-19 Genomics UK (COG-UK) consortium|Davies|United Kingdom
>Spike|hCoV-19/Belgium/rega-0801793/2020|2020-08-01|EPI_ISL_734856|Original|hCoV-
19^^Sint-Niklaas|Human|UZ Leuven|KU Leuven|Maes|Belgium
>Spike|hCoV-19/Luxembourg/LNS3192468/2020|2020-10-30|EPI_ISL_740266|Original|hCoV-
19^^Luxembourg|Human|Laboratoire national de sant\u00e9|Laboratoire national de
sant\u00e9|Wienecke-Baldacchino|Luxembourg
>Spike|hCoV-19/USA/TX-HMH-MCoV-10157/2020|2020-07-06|EPI_ISL_789165|Original|hCoV-
19^^Texas|Human|Houston Methodist Hospital|Houston Methodist Hospital|Long|USA
>Spike|hCoV-19/England/ALDP-B76301/2020|2020-11-12|EPI_ISL_723754|Original|hCoV-
19^^England|Human|Oxford Viromics|COVID-19 Genomics UK (COG-UK)
Consortium|Golubchik|United Kingdom
>Spike|hCoV-19/Shanghai/SH0075/2020|2020-01-30|EPI_ISL_416378|Original|hCoV-
19^^Shanghai|Human|Shanghai Public Health Clinical Center|National Research Center
for Translational Medicine (Shanghai)|Zhang|China
>Spike|hCoV-19/USA/NY-NYCPHL-000132/2020|2020-04-02|EPI_ISL_631623|Original|hCoV-
19^^New York|Human|Richmond University Medical Center|New York City Public Health
Laboratory|Wang|USA
>Spike|hCoV-19/USA/UT-08269/2020|2020-05-20|EPI_ISL_524075|Original|hCoV-
19^^Utah|Human|Utah Public Health Laboratory|Utah Public Health Laboratory|Young|USA
>Spike|hCoV-19/USA/CA-ALSR-1646/2020|2020-05-18|EPI_ISL_494711|Original|hCoV-
19^^California|Human|Scripps Medical Laboratory|Andersen lab at Scripps
Research|Gangavarapu|USA
>Spike|hCoV-19/Denmark/ALAB-HH11/2020|2020-03-10|EPI_ISL_429270|Original|hCoV-
19^^Denmark|Human|Department of Clinical Microbiology|Albertsen
lab|Kirkegaard|Denmark
>Spike|hCoV-19/England/MILK-9A87FD/2020|2020-08-31|EPI_ISL_549425|Original|hCoV-
19^^England|Human|Oxford Viromics|COVID-19 Genomics UK (COG-UK)
Consortium|Golubchik|United Kingdom
>Spike|hCoV-19/England/QEUH-A77FEF/2020|2020-10-14|EPI_ISL_613350|Original|hCoV-
19^^England|Human|Queens Medical Centre|COVID-19 Genomics UK (COG-UK)
Consortium|Loose|United Kingdom
>Spike|hCoV-19/USA/MD-HP12014/2020|2020-11-05|EPI_ISL_791356|Original|hCoV-
19^^Maryland|Human|Johns Hopkins Hospital Department of Pathology|Johns Hopkins
Hospital Department of Pathology|Morris|USA
>Spike|hCoV-19/Canada/ON-PHL-20-01261/2020|2020-06-00|EPI_ISL_548876|Original|hCoV-
19^^Ontario|Human|Public Health Ontario Laboratory|Public Health Ontario
Laboratory|Guthrie|Canada
>Spike|hCoV-19/England/CAMC-B52857/2020|2020-11-08|EPI_ISL_657604|Original|hCoV-
19^^England|Human|Lighthouse Lab in Cambridge|Wellcome Sanger Institute for the
COVID-19 Genomics UK (COG-UK) Consortium|Davies|United Kingdom
>Spike|hCoV-19/England/BRIS-124A25/2020|2020-03-25|EPI_ISL_443761|Original|hCoV-
19^^England|Human|PHE South West Regional Laboratory|Wellcome Sanger Institute for
the COVID-19 Genomics UK (COG-UK) consortium|Amato|United Kingdom
>Spike|hCoV-19/USA/FL-BPHL-0727/2020|2020-07-14|EPI_ISL_509785|Original|hCoV-
19^^Florida|Human|Florida Bureau of Public Health Laboratories|Florida Bureau of
Public Health Laboratories|Blanton|USA
>Spike|hCoV-19/England/MILK-ACC5DA/2020|2020-10-21|EPI_ISL_634270|Original|hCoV-
19^^England|Human|Lighthouse Lab in Milton Keynes|Wellcome Sanger Institute for the
COVID-19 Genomics UK (COG-UK) consortium|Davies|United Kingdom
>Spike|hCoV-19/England/MILK-ACDB31/2020|2020-10-21|EPI_ISL_630261|Original|hCoV-
19^^England|Human|Lighthouse Lab in Milton Keynes|Wellcome Sanger Institute for the
COVID-19 Genomics UK (COG-UK) consortium|Davies|United Kingdom
>Spike|hCoV-19/USA/UT-UPHL-2012142641/2020|2020-07-25|EPI_ISL_745688|Original|hCoV-
19^^Utah|Human|Ginkgo Bioworks Clinical Laboratory|Utah Public Health
Laboratory|Young|USA
>Spike|hCoV-19/USA/CA-CZB-13970/2020|2020-06-18|EPI_ISL_739075|Original|hCoV-
19^^California|Human|Alameda County Public Health Lab|Chan-Zuckerberg Biohub|Kamm|USA

>Spike|hCoV-19/Wales/PHWC-4A1AE1/2020|2020-12-11|EPI_ISL_743775|Original|hCoV-19^^Wales|Human|Wales Specialist Virology Centre Sequencing lab|COVID-19 Genomics UK (COG-UK) Consortium|Moore|United Kingdom
>Spike|hCoV-19/Wales/PHWC-495163/2020|2020-11-21|EPI_ISL_726307|Original|hCoV-19^^Wales|Human|Originating lab|COVID-19 Genomics UK (COG-UK) Consortium|Moore|United Kingdom
>Spike|hCoV-19/USA/VA-DCLS-1750/2020|2020-09-10|EPI_ISL_644851|Original|hCoV-19^^Virginia|Human|Virginia DCLS|Virginia DCLS|St. Jacques|USA
>Spike|hCoV-19/Canada/ON-S787/2020|2020-04-00|EPI_ISL_591228|Original|hCoV-19^^Ontario|Human|Toronto Invasive Bacterial Diseases Network|McMaster University|McArthur|Canada
>Spike|hCoV-19/England/QEUH-BC3B94/2020|2020-11-25|EPI_ISL_704091|Original|hCoV-19^^England|Human|Lighthouse Lab in Glasgow|Wellcome Sanger Institute for the COVID-19 Genomics UK (COG-UK) Consortium|Davies|United Kingdom
>Spike|hCoV-19/Japan/PG-2844/2020|2020-04-00|EPI_ISL_690971|Original|hCoV-19^^Japan|Human|Pathogen Genomics Center|Pathogen Genomics Center|Sekizuka|Japan
>Spike|hCoV-19/England/QEUH-9B9F88/2020|2020-09-07|EPI_ISL_572727|Original|hCoV-19^^England|Human|Oxford Viromics|COVID-19 Genomics UK (COG-UK) Consortium|Golubchik|United Kingdom
>Spike|hCoV-19/England/ALDP-9DA042/2020|2020-09-18|EPI_ISL_576839|Original|hCoV-19^^England|Human|Oxford Viromics|COVID-19 Genomics UK (COG-UK) Consortium|Golubchik|United Kingdom
>Spike|hCoV-19/Panama/332252/2020|2020-03-23|EPI_ISL_496706|Original|hCoV-19^^Guna Yala|Human|Gorgas Memorial Laboratory of Health Studies|Gorgas Memorial Laboratory of Health Studies|Martinez|Panama
>Spike|hCoV-19/England/NOTT-1102E8/2020|2020-04-11|EPI_ISL_441496|Original|hCoV-19^^England|Human|Queens Medical Centre|COVID-19 Genomics UK (COG-UK) Consortium|Loose|United Kingdom
>Spike|hCoV-19/Scotland/QEUH-BCCCB6/2020|2020-11-26|EPI_ISL_710794|Original|hCoV-19^^Scotland|Human|Lighthouse Lab in Glasgow|Wellcome Sanger Institute for the COVID-19 Genomics UK (COG-UK) Consortium|Davies|United Kingdom
>Spike|hCoV-19/USA/WI-GMF-03823/2020|2020-05-19|EPI_ISL_455576|Original|hCoV-19^^Wisconsin|Human|Gundersen Molecular Diagnostics Laboratory|Kabara Cancer Research Institute|Kenny|USA
>Spike|hCoV-19/England/QEUH-9DA81D/2020|2020-09-22|EPI_ISL_601453|Original|hCoV-19^^England|Human|Lighthouse Lab in Glasgow|Wellcome Sanger Institute for the COVID-19 Genomics UK (COG-UK) consortium|Davies|United Kingdom
>Spike|hCoV-19/USA/LA-EVTL156/2020|2020-04-01|EPI_ISL_455999|Original|hCoV-19^^Louisiana|Human|LSUHS Emerging Viral Threat Laboratory|Microbial Genome Sequencing Center|Kamil|USA
>Spike|hCoV-19/England/MILK-9C2292/2020|2020-09-11|EPI_ISL_566527|Original|hCoV-19^^England|Human|Lighthouse Lab in Milton Keynes|Wellcome Sanger Institute for the COVID-19 Genomics UK (COG-UK) consortium|Davies|United Kingdom
>Spike|hCoV-19/Singapore/21/2020|2020-02-13|EPI_ISL_419000|Original|hCoV-19^^Singapore|Human|National Public Health Laboratory|National Public Health Laboratory|Mak|Singapore
>Spike|hCoV-19/Spain/AR-IBV-97009510/2020|2020-07-24|EPI_ISL_671798|Original|hCoV-19^^Aragon|Human|Hospital Cl\u00ednico Universitario Lozano Blesa de Zaragoza (Espa\u00f1a)|SeqCOVID-SPAIN consortium/IBV(CSIC)|Comas|Spain
>Spike|hCoV-19/Netherlands/UT-RIVM-10377/2020|2020-09-29|EPI_ISL_636579|Original|hCoV-19^^Utrecht|Human|Dutch COVID-19 response team|National Institute for Public Health and the Environment (RIVM)|Meijer|Netherlands
>Spike|hCoV-19/United Arab Emirates/0866/2020|2020-05-19|EPI_ISL_699005|Original|hCoV-19^^Abu Dhabi|Human|Group 42 (G42) Healthcare|G42 Healthcare|Ogrodzki|United Arab Emirates
>Spike|hCoV-19/USA/IL-NM-0500/2020|2020-04-24|EPI_ISL_626435|Original|hCoV-19^^Illinois|Human|Northwestern Memorial Hospital|Ozer Lab|Ozer|USA
>Spike|hCoV-19/Australia/VIC264/2020|2020-03-22|EPI_ISL_419957|Original|hCoV-19^^Victoria|Human|Victorian Infectious Diseases Reference Laboratory

(VIDRL)|Victorian Infectious Diseases Reference Laboratory and Microbiological Diagnostic Unit Public Health Laboratory|Schultz|Australia
>Spike|hCoV-19/England/MILK-B3AB5C/2020|2020-11-02|EPI_ISL_679707|Original|hCoV-19^^England|Human|Oxford Viromics|COVID-19 Genomics UK (COG-UK) Consortium|Golubchik|United Kingdom
>Spike|hCoV-19/USA/TX-HMH-1990/2020|2020-05-19|EPI_ISL_545055|Original|hCoV-19^^Texas|Human|Houston Methodist Hospital|Houston Methodist Hospital|Long|USA
>Spike|hCoV-19/USA/NY-NYUMC649/2020|2020-04-03|EPI_ISL_444766|Original|hCoV-19^^New York|Human|NYU Langone Health|Departments of Pathology and Medicine|Maurano|USA
>Spike|hCoV-19/USA/WI-UW-56/2020|2020-03-18|EPI_ISL_421314|Original|hCoV-19^^Wisconsin|Human|University of Wisconsin-Madison AIDS Vaccine Research Laboratories|University of Wisconsin-Madison AIDS Vaccine Research Laboratories|Baczenas|USA
>Spike|hCoV-19/England/QEUH-9AE690/2020|2020-09-05|EPI_ISL_577232|Original|hCoV-19^^England|Human|University of Exeter|COVID-19 Genomics UK (COG-UK) Consortium|Temperton|United Kingdom
>Spike|hCoV-19/Wales/PHWC-34865/2020|2020-04-13|EPI_ISL_446985|Original|hCoV-19^^Wales|Human|Wales Specialist Virology Centre|Public Health Wales Microbiology Cardiff|Moore|United Kingdom
>Spike|hCoV-19/Russia/Novy_Urengoy-RII24776S/2020|2020-05-00|EPI_ISL_507219|Original|hCoV-19^^Yamalo-Nenets|Human|WHO National Influenza Centre Russian Federation|WHO National Influenza Centre Russian Federation|Komissarov|Russia
>Spike|hCoV-19/England/205201136/2020|2020-12-18|EPI_ISL_791210|Original|hCoV-19^^England|Human|Respiratory Virus Unit|COVID-19 Genomics UK (COG-UK) Consortium|Platt|United Kingdom
>Spike|hCoV-19/Luxembourg/LNS9795610/2020|2020-11-25|EPI_ISL_744986|Original|hCoV-19^^Luxembourg|Human|Laboratoire national de sant\u00e9|Laboratoire national de sant\u00e9|Wienecke-Baldacchino|Luxembourg
>Spike|hCoV-19/Netherlands/NA_206/2020|2020-03-25|EPI_ISL_422741|Original|hCoV-19^^Netherlands|Human|Dutch COVID-19 response team|Erasmus Medical Center|Oude Munnink|Netherlands
>Spike|hCoV-19/USA/WY-WPHL-20062464/2020|2020-08-17|EPI_ISL_769893|Original|hCoV-19^^Wyoming|Human|Wyoming Public Health Laboratory|Wyoming Public Health Laboratory|Hull|USA
>Spike|hCoV-19/England/QEUH-9B8648/2020|2020-09-08|EPI_ISL_549683|Original|hCoV-19^^England|Human|Lighthouse Lab in Glasgow|Wellcome Sanger Institute for the COVID-19 Genomics UK (COG-UK) consortium|Davies|United Kingdom
>Spike|hCoV-19/Ireland/LH-NVRL-73IRL41660/2020|2020-09-09|EPI_ISL_639848|Original|hCoV-19^^Louth|Human|National Virus Reference Laboratory|National Virus Reference Laboratory|Gonzalez|Ireland
>Spike|hCoV-19/Scotland/CVR5926/2020|2020-11-21|EPI_ISL_705675|Original|hCoV-19^^Scotland|Human|West of Scotland Specialist Virology Centre|COVID-19 Genomics UK (COG-UK) Consortium|Robertson|United Kingdom
>Spike|hCoV-19/USA/WA-UW-11078/2020|2020-06-17|EPI_ISL_570373|Original|hCoV-19^^Washington|Human|UW Virology Lab|UW Virology Lab|Roychoudhury|USA
>Spike|hCoV-19/USA/WI-GMF-30272/2020|2020-10-30|EPI_ISL_661031|Original|hCoV-19^^Wisconsin|Human|Gundersen Molecular Diagnostics Laboratory|Kabara Cancer Research Institute|Kenny|USA
>Spike|hCoV-19/United Arab Emirates/0325/2020|2020-05-18|EPI_ISL_698501|Original|hCoV-19^^Abu Dhabi|Human|Group 42 (G42) Healthcare|G42 Healthcare|Ogrodzki|United Arab Emirates
>Spike|hCoV-19/New Zealand/20CV0635/2020|2020-12-03|EPI_ISL_707799|Original|hCoV-19^^Auckland|Human|LabPLUS|Institute of Environmental Science and Research (ESR)|Wang|New Zealand
>Spike|hCoV-19/USA/TX-DSHS-1368/2020|2020-05-07|EPI_ISL_654741|Original|hCoV-19^^Texas|Human|Texas Department of State Health Services|Texas Department of State Health Services|Rahman|USA
>Spike|hCoV-19/USA/MN-MDH-1584/2020|2020-05-22|EPI_ISL_530180|Original|hCoV-19^^Minnesota|Human|Minnesota Department of Health|Minnesota Department of Health|Garfin|USA

>Spike|hCoV-19/USA/CA-ALSR-3773/2020|2020-05-23|EPI_ISL_636192|Original|hCoV-19^^California|Human|San Diego County Public Health Laboratory|Andersen lab at Scripps Research|Gangavarapu|USA
>Spike|hCoV-19/Wales/ALDP-C39758/2020|2020-12-07|EPI_ISL_767215|Original|hCoV-19^^Wales|Human|Lighthouse Lab in Alderley Park|Wellcome Sanger Institute for the COVID-19 Genomics UK (COG-UK) Consortium|Davies|United Kingdom
>Spike|hCoV-19/England/ALDP-9C56DF/2020|2020-09-10|EPI_ISL_567795|Original|hCoV-19^^England|Human|Lighthouse Lab in Alderley Park|Wellcome Sanger Institute for the COVID-19 Genomics UK (COG-UK) consortium|Davies|United Kingdom
>Spike|hCoV-19/USA/NY-NYCPHL-001082/2020|2020-10-13|EPI_ISL_671676|Original|hCoV-19^^New York|Human|DOHMH PHL|New York City Public Health Laboratory|Wang|USA
>Spike|hCoV-19/USA/AZ-TG645817/2020|2020-08-06|EPI_ISL_695832|Original|hCoV-19^^Arizona|Human|AZ SPHL|TGen North|French|USA
>Spike|hCoV-19/USA/NY-NYCPHL-000469/2020|2020-05-01|EPI_ISL_631939|Original|hCoV-19^^New York|Human|St Barnabas Hospital|New York City Public Health Laboratory|Wang|USA
>Spike|hCoV-19/USA/UT-08647/2020|2020-05-07|EPI_ISL_524380|Original|hCoV-19^^Utah|Human|Utah Public Health Laboratory|Utah Public Health Laboratory|Young|USA
>Spike|hCoV-19/Denmark/DCGC-2314/2020|2020-08-10|EPI_ISL_617651|Original|hCoV-19^^Syddanmark|Human|Department of Virus and Microbiological Special Diagnostics|Albertsen lab|Michaelsen|Denmark
>Spike|hCoV-19/Singapore/239/2020|2020-04-06|EPI_ISL_462357|Original|hCoV-19^^Singapore|Human|National Public Health Laboratory|National Public Health Laboratory|Mak|Singapore
>Spike|hCoV-19/England/CAMB-1B4F3A/2020|2020-06-18|EPI_ISL_477801|Original|hCoV-19^^England|Human|Department of Pathology|COVID-19 Genomics UK (COG-UK) Consortium|Meredith|United Kingdom
>Spike|hCoV-19/Kazakhstan/NCB-3/2020|2020-03-25|EPI_ISL_435047|Original|hCoV-19^^Nur-Sultan|Human|Laboratory of Applied Genetics|RSE \National Center for Biotechnology\|Kalendar|Kazakhstan
>Spike|hCoV-19/England/ALDP-9520E8/2020|2020-06-09|EPI_ISL_551306|Original|hCoV-19^^England|Human|Lighthouse Lab in Alderley Park|Wellcome Sanger Institute for the COVID-19 Genomics UK (COG-UK) consortium|Davies|United Kingdom
>Spike|hCoV-19/South Africa/NHLS-UCT-GS-0081/2020|2020-04-01|EPI_ISL_640044|Original|hCoV-19^^Western Cape|Human|District 6 CDC wc DSI|NHLS/UCT|Iranzadeh|South Africa
>Spike|hCoV-19/USA/MI-MDHHS-SC22028/2020|2020-09-09|EPI_ISL_577584|Original|hCoV-19^^Michigan|Human|Michigan Department of Health and Human Services|Michigan Department of Health and Human Services|Jacob|USA
>Spike|hCoV-19/Denmark/DCGC-20771/2020|2020-12-21|EPI_ISL_758917|Original|hCoV-19^^Midtjylland|Human|Department of Virus and Microbiological Special Diagnostics|Albertsen Lab|Michaelsen|Denmark
>Spike|hCoV-19/England/QEUH-A8BC4B/2020|2020-10-16|EPI_ISL_643249|Original|hCoV-19^^England|Human|Lighthouse Lab in Glasgow|Wellcome Sanger Institute for the COVID-19 Genomics UK (COG-UK) Consortium|Davies|United Kingdom
>Spike|hCoV-19/England/QEUH-9ADF2D/2020|2020-09-05|EPI_ISL_638615|Original|hCoV-19^^England|Human|Department of Pathology|COVID-19 Genomics UK (COG-UK) Consortium|Goodfellow|United Kingdom
>Spike|hCoV-19/England/QEUH-B11D73/2020|2020-11-02|EPI_ISL_642410|Original|hCoV-19^^England|Human|Lighthouse Lab in Glasgow|Wellcome Sanger Institute for the COVID-19 Genomics UK (COG-UK) Consortium|Davies|United Kingdom
>Spike|hCoV-19/Denmark/DCGC-4263/2020|2020-09-07|EPI_ISL_615700|Original|hCoV-19^^Hovedstaden|Human|Department of Virus and Microbiological Special Diagnostics|Albertsen lab|Michaelsen|Denmark
>Spike|hCoV-19/England/CAMB-1B722F/2020|2020-10-13|EPI_ISL_638310|Original|hCoV-19^^England|Human|Department of Pathology|COVID-19 Genomics UK (COG-UK) Consortium|Goodfellow|United Kingdom
>Spike|hCoV-19/USA/CA-ALSR-3386/2020|2020-07-09|EPI_ISL_635995|Original|hCoV-19^^California|Human|San Diego County Public Health Laboratory|Andersen lab at Scripps Research|Gangavarapu|USA

>Spike|hCoV-19/Northern Ireland/QEUH-96ECE5/2020|2020-08-19|EPI_ISL_530438|Original|hCoV-19^^Northern Ireland|Human|Lighthouse Lab in Glasgow|Wellcome Sanger Institute for the COVID-19 Genomics UK (COG-UK) consortium|Davies|United Kingdom
>Spike|hCoV-19/England/ALDP-9C5B0D/2020|2020-09-10|EPI_ISL_567728|Original|hCoV-19^^England|Human|Lighthouse Lab in Alderley Park|Wellcome Sanger Institute for the COVID-19 Genomics UK (COG-UK) consortium|Davies|United Kingdom
>Spike|hCoV-19/England/MILK-956FBA/2020|2020-06-10|EPI_ISL_554316|Original|hCoV-19^^England|Human|Lighthouse Lab in Milton Keynes|Wellcome Sanger Institute for the COVID-19 Genomics UK (COG-UK) consortium|Davies|United Kingdom
>Spike|hCoV-19/England/CAMB-80123/2020|2020-04-09|EPI_ISL_470512|Original|hCoV-19^^England|Human|Department of Pathology|Wellcome Sanger Institute for the COVID-19 Genomics UK (COG-UK) consortium|Davies|United Kingdom
>Spike|hCoV-19/Australia/SAP111/2020|2020-03-15|EPI_ISL_451131|Original|hCoV-19^^South Australia|Human|SA Pathology|SA Pathology|Leong|Australia
>Spike|hCoV-19/England/ALDP-51D6FA/2020|2020-06-02|EPI_ISL_559079|Original|hCoV-19^^England|Human|Lighthouse Lab in Alderley Park|Wellcome Sanger Institute for the COVID-19 Genomics UK (COG-UK) consortium|Davies|United Kingdom
>Spike|hCoV-19/Spain/CN-IBV-97006484/2020|2020-03-25|EPI_ISL_537699|Original|hCoV-19^^Canary Islands|Human|Hospital Universitario de Gran Canaria Dr. Negr\u00edn|SeqCOVID-SPAIN consortium/IBV(CSIC)|Comas|Spain
>Spike|hCoV-19/England/ALDP-9EA188/2020|2020-09-24|EPI_ISL_580236|Original|hCoV-19^^England|Human|Lighthouse Lab in Alderley Park|Wellcome Sanger Institute for the COVID-19 Genomics UK (COG-UK) consortium|Davies|United Kingdom
>Spike|hCoV-19/Scotland/QEUH-9E5439/2020|2020-09-24|EPI_ISL_601005|Original|hCoV-19^^Scotland|Human|Lighthouse Lab in Glasgow|Wellcome Sanger Institute for the COVID-19 Genomics UK (COG-UK) consortium|Davies|United Kingdom
>Spike|hCoV-19/France/GES-8541/2020|2020-05-22|EPI_ISL_560589|Original|hCoV-19^^Grand-Est|Human|Hopital|National Reference Center for Viruses of Respiratory Infections|Enouf|France
>Spike|hCoV-19/USA/CA-CHLA-PLM40173004/2020|2020-11-30|EPI_ISL_753437|Original|hCoV-19^^California|Human|Clinical virology Laboratory|Center for Personalized Medicine|Dien Bard|USA
>Spike|hCoV-19/Denmark/DCGC-10618/2020|2020-11-02|EPI_ISL_670092|Original|hCoV-19^^Hovedstaden|Human|Department of Virus and Microbiological Special Diagnostics|Albertsen Lab|Michaelsen|Denmark
>Spike|hCoV-19/USA/WI-UW-1637/2020|2020-09-11|EPI_ISL_605511|Original|hCoV-19^^Wisconsin|Human|University of Wisconsin-Madison AIDS Vaccine Research Laboratories|University of Wisconsin-Madison AIDS Vaccine Research Laboratories|Baczenas|USA
>Spike|hCoV-19/Japan/PG-5983/2020|2020-08-00|EPI_ISL_689098|Original|hCoV-19^^Japan|Human|Pathogen Genomics Center|Pathogen Genomics Center|Sekizuka|Japan
>Spike|hCoV-19/England/QEUH-A8B3BF/2020|2020-10-15|EPI_ISL_643196|Original|hCoV-19^^England|Human|Lighthouse Lab in Glasgow|Wellcome Sanger Institute for the COVID-19 Genomics UK (COG-UK) Consortium|Davies|United Kingdom
>Spike|hCoV-19/USA/TX-HMH-5858/2020|2020-06-26|EPI_ISL_547068|Original|hCoV-19^^Texas|Human|Houston Methodist Hospital|Houston Methodist Hospital|Long|USA
>Spike|hCoV-19/Australia/VIC190/2020|2020-03-19|EPI_ISL_419886|Original|hCoV-19^^Victoria|Human|Victorian Infectious Diseases Reference Laboratory (VIDRL)|Victorian Infectious Diseases Reference Laboratory and Microbiological Diagnostic Unit Public Health Laboratory|Schultz|Australia
>Spike|hCoV-19/England/QEUH-7B810D/2020|2020-07-22|EPI_ISL_532904|Original|hCoV-19^^England|Human|Lighthouse Lab in Glasgow|Wellcome Sanger Institute for the COVID-19 Genomics UK (COG-UK) consortium|Davies|United Kingdom
>Spike|hCoV-19/England/ALDP-91B713/2020|2020-06-22|EPI_ISL_551561|Original|hCoV-19^^England|Human|Lighthouse Lab in Alderley Park|Wellcome Sanger Institute for the COVID-19 Genomics UK (COG-UK) consortium|Davies|United Kingdom
>Spike|hCoV-19/Wales/PHWC-48845B/2020|2020-10-28|EPI_ISL_639393|Original|hCoV-19^^Wales|Human|Wales Specialist Virology Centre Sequencing lab|COVID-19 Genomics UK (COG-UK) Consortium|Moore|United Kingdom

>Spike|hCoV-19/Wales/ALDP-C04E06/2020|2020-12-03|EPI_ISL_767297|Original|hCoV-19^^Wales|Human|Lighthouse Lab in Alderley Park|Wellcome Sanger Institute for the COVID-19 Genomics UK (COG-UK) Consortium|Davies|United Kingdom
>Spike|hCoV-19/USA/MA-MASPHL-00018/2020|2020-04-01|EPI_ISL_791524|Original|hCoV-19^^Massachusetts|Human|Massachusetts State Public Health Laboratory|Infectious Disease Program|Park|USA
>Spike|hCoV-19/England/ALDP-910D0D/2020|2020-06-22|EPI_ISL_551384|Original|hCoV-19^^England|Human|Lighthouse Lab in Alderley Park|Wellcome Sanger Institute for the COVID-19 Genomics UK (COG-UK) consortium|Davies|United Kingdom
>Spike|hCoV-19/England/ALDP-94E049/2020|2020-06-17|EPI_ISL_555395|Original|hCoV-19^^England|Human|Lighthouse Lab in Alderley Park|Wellcome Sanger Institute for the COVID-19 Genomics UK (COG-UK) consortium|Davies|United Kingdom
>Spike|hCoV-19/Japan/PG-6267/2020|2020-08-00|EPI_ISL_689378|Original|hCoV-19^^Japan|Human|Pathogen Genomics Center|Pathogen Genomics Center|Sekizuka|Japan
>Spike|hCoV-19/USA/UT-UPHL-2012754321/2020|2020-08-17|EPI_ISL_746051|Original|hCoV-19^^Utah|Human|Ginkgo Bioworks Clinical Laboratory|Utah Public Health Laboratory|Young|USA
>Spike|hCoV-19/Spain/AN-ISCIII-202657/2020|2020-03-12|EPI_ISL_539499|Original|hCoV-19^^Andalucia|Human|Hospital Costa del Sol|Instituto de Salud Carlos III|Caballero|Spain
>Spike|hCoV-19/USA/MI-MDHHS-SC20042/2020|2020-03-19|EPI_ISL_437129|Original|hCoV-19^^Michigan|Human|Michigan Department of Health and Human Services|Michigan Department of Health and Human Services|Blankenship|USA
>Spike|hCoV-19/USA/MA-MGH-00437/2020|2020-03-07|EPI_ISL_460239|Original|hCoV-19^^Massachusetts|Human|Massachusetts General Hospital|Infectious Disease Program|Lemieux|USA
>Spike|hCoV-19/Canada/MB-NML-1186/2020|2020-08-10|EPI_ISL_582500|Original|hCoV-19^^Manitoba|Human|Cadham Provincial Laboratory|National Microbiology Laboratory (NML)|Majer|Canada
>Spike|hCoV-19/USA/RI-Yale-412/2020|2020-04-17|EPI_ISL_738245|Original|hCoV-19^^Rhode Island|Human|Connecticut Veterans' Affairs Hospital|Grubaugh Lab - Yale School of Public Health|Grubaugh|USA
>Spike|hCoV-19/England/ALDP-9B64E6/2020|2020-08-27|EPI_ISL_573012|Original|hCoV-19^^England|Human|Oxford Viromics|COVID-19 Genomics UK (COG-UK) Consortium|Golubchik|United Kingdom
>Spike|hCoV-19/Japan/PG-0902/2020|2020-03-00|EPI_ISL_684512|Original|hCoV-19^^Japan|Human|Tokyo Metropolitan Institute of Public Health|Pathogen Genomics Center|Sekizuka|Japan
>Spike|hCoV-19/USA/MA-MGH-00453/2020|2020-04-04|EPI_ISL_460284|Original|hCoV-19^^Massachusetts|Human|Massachusetts General Hospital|Infectious Disease Program|Lemieux|USA
>Spike|hCoV-19/Finland/3A47S9/2020|2020-04-03|EPI_ISL_481667|Original|hCoV-19^^Uusimaa|Human|Department of Virology and Immunology|Department of Virology|Smura|Finland
>Spike|hCoV-19/England/BRIS-12A4F4/2020|2020-04-29|EPI_ISL_458428|Original|hCoV-19^^England|Human|PHE South West Regional Laboratory|Wellcome Sanger Institute for the COVID-19 Genomics UK (COG-UK) consortium|Davies|United Kingdom
>Spike|hCoV-19/Scotland/GCVR-170EF1/2020|2020-03-29|EPI_ISL_489645|Original|hCoV-19^^Scotland|Human|NHSGGC West of Scotland Specialist Virology Centre / MRC-University of Glasgow Centre for Virus Research|Wellcome Sanger Institute for the COVID-19 Genomics UK (COG-UK) consortium|Davies|United Kingdom
>Spike|hCoV-19/USA/MN-MDH-2138/2020|2020-11-06|EPI_ISL_708476|Original|hCoV-19^^Minnesota|Human|Minnesota Department of Health|Minnesota Department of Health|Lorentz|USA
>Spike|hCoV-19/England/QEUH-C3485A/2020|2020-12-11|EPI_ISL_733980|Original|hCoV-19^^England|Human|Lighthouse Lab in Glasgow|Wellcome Sanger Institute for the COVID-19 Genomics UK (COG-UK) Consortium|Davies|United Kingdom
>Spike|hCoV-19/Japan/IC-0369/2020|2020-11-00|EPI_ISL_721572|Original|hCoV-19^^Japan|Human|Pathogen Genomics Center|Pathogen Genomics Center|Sekizuka|Japan

>Spike|hCoV-19/Luxembourg/LNS2388068/2020|2020-12-17|EPI_ISL_740111|Original|hCoV-19^^Luxembourg|Human|Laboratoire national de sant\u00e9|Laboratoire national de sant\u00e9|Wienecke-Baldacchino|Luxembourg
>Spike|hCoV-19/Singapore/1150/2020|2020-04-17|EPI_ISL_648059|Original|hCoV-19^^Singapore|Human|Department of Laboratory Medicine|Department of Laboratory Medicine|Chen|Singapore
>Spike|hCoV-19/England/ALDP-A52621/2020|2020-10-07|EPI_ISL_598087|Original|hCoV-19^^England|Human|Lighthouse Lab in Alderley Park|Wellcome Sanger Institute for the COVID-19 Genomics UK (COG-UK) consortium|Davies|United Kingdom
>Spike|hCoV-19/England/LIVE-A927C/2020|2020-03-25|EPI_ISL_517284|Original|hCoV-19^^England|Human|Liverpool Clinical Laboratories|COVID-19 Genomics UK (COG-UK) Consortium|Haldenby|United Kingdom
>Spike|hCoV-19/England/NORW-F9574/2020|2020-12-02|EPI_ISL_725386|Original|hCoV-19^^England|Human|Quadram Institute Bioscience|COVID-19 Genomics UK (COG-UK) Consortium|Alikhan|United Kingdom
>Spike|hCoV-19/England/CAMC-CB6E7C/2020|2020-12-19|EPI_ISL_778158|Original|hCoV-19^^England|Human|Lighthouse Lab in Cambridge|Wellcome Sanger Institute for the COVID-19 Genomics UK (COG-UK) Consortium|Davies|United Kingdom
>Spike|hCoV-19/England/QEUH-AA02A8/2020|2020-10-20|EPI_ISL_611473|Original|hCoV-19^^England|Human|Lighthouse Lab in Glasgow|Wellcome Sanger Institute for the COVID-19 Genomics UK (COG-UK) consortium|Davies|United Kingdom
>Spike|hCoV-19/USA/TX-HMH-MCoV-8751/2020|2020-07-01|EPI_ISL_780734|Original|hCoV-19^^Texas|Human|Houston Methodist Hospital|Houston Methodist Hospital|Long|USA
>Spike|hCoV-19/England/ALDP-BCA9A2/2020|2020-11-23|EPI_ISL_703504|Original|hCoV-19^^England|Human|Lighthouse Lab in Alderley Park|Wellcome Sanger Institute for the COVID-19 Genomics UK (COG-UK) Consortium|Davies|United Kingdom
>Spike|hCoV-19/Denmark/DCGC-1540/2020|2020-05-25|EPI_ISL_617961|Original|hCoV-19^^Hovedstaden|Human|Department of Virus and Microbiological Special Diagnostics|Albertsen lab|Michaelsen|Denmark
>Spike|hCoV-19/England/QEUH-B69BCD/2020|2020-11-11|EPI_ISL_655127|Original|hCoV-19^^England|Human|Lighthouse Lab in Glasgow|Wellcome Sanger Institute for the COVID-19 Genomics UK (COG-UK) Consortium|Davies|United Kingdom
>Spike|hCoV-19/England/MILK-B55292/2020|2020-11-05|EPI_ISL_657921|Original|hCoV-19^^England|Human|Lighthouse Lab in Milton Keynes|Wellcome Sanger Institute for the COVID-19 Genomics UK (COG-UK) Consortium|Davies|United Kingdom
>Spike|hCoV-19/England/QEUH-96F622/2020|2020-08-17|EPI_ISL_540274|Original|hCoV-19^^England|Human|Lighthouse Lab in Glasgow|Wellcome Sanger Institute for the COVID-19 Genomics UK (COG-UK) consortium|Davies|United Kingdom
>Spike|hCoV-19/Wales/MILK-9C2335/2020|2020-09-09|EPI_ISL_566645|Original|hCoV-19^^Wales|Human|Lighthouse Lab in Milton Keynes|Wellcome Sanger Institute for the COVID-19 Genomics UK (COG-UK) consortium|Davies|United Kingdom
>Spike|hCoV-19/South Africa/KRISP-0121/2020|2020-04-01|EPI_ISL_467473|Original|hCoV-19^^KwaZulu-Natal|Human|AMPATH-DBN|KRISP|de Oliveira|South Africa
>Spike|hCoV-19/Denmark/DCGC-15130/2020|2020-11-23|EPI_ISL_713646|Original|hCoV-19^^Hovedstaden|Human|Department of Virus and Microbiological Special Diagnostics|Albertsen Lab|Michaelsen|Denmark
>Spike|hCoV-19/England/SHEF-10D00F7/2020|2020-12-16|EPI_ISL_765175|Original|hCoV-19^^England|Human|Virology Department|COVID-19 Genomics UK (COG-UK) Consortium|de Silva|United Kingdom
>Spike|hCoV-19/England/ALDP-AA1A72/2020|2020-10-22|EPI_ISL_624303|Original|hCoV-19^^England|Human|Lighthouse Lab in Alderley Park|Wellcome Sanger Institute for the COVID-19 Genomics UK (COG-UK) consortium|Davies|United Kingdom
>Spike|hCoV-19/USA/NY-Wadsworth-252108-01/2020|2020-10-22|EPI_ISL_734398|Original|hCoV-19^^New York|Human|Wadsworth Center|Wadsworth Center|Lasek-Nesselquist|USA
>Spike|hCoV-19/USA/TX-HMH-MCoV-19432/2020|2020-11-30|EPI_ISL_783838|Original|hCoV-19^^Texas|Human|Houston Methodist Hospital|Houston Methodist Hospital|Long|USA
>Spike|hCoV-19/England/QEUH-C4BD33/2020|2020-12-14|EPI_ISL_736491|Original|hCoV-19^^England|Human|Lighthouse Lab in Glasgow|Wellcome Sanger Institute for the COVID-19 Genomics UK (COG-UK) Consortium|Davies|United Kingdom

>Spike|hCoV-19/Japan/PG-0609/2020|2020-03-00|EPI_ISL_684219|Original|hCoV-19^^Japan|Human|Pathogen Genomics Center|Pathogen Genomics Center|Sekizuka|Japan
>Spike|hCoV-19/USA/OR-PROV-090/2020|2020-04-20|EPI_ISL_482361|Original|hCoV-19^^Oregon|Human|Providence St. Joseph Health Molecular Genomics Laboratory|Providence St. Joseph Health Molecular Genomics Laboratory|Dowdell|USA
>Spike|hCoV-19/USA/NY-Wadsworth-116460-01/2020|2020-05-24|EPI_ISL_676954|Original|hCoV-19^^New York|Human|Wadsworth Center|Wadsworth Center|Lasek-Nesselquist|USA
>Spike|hCoV-19/England/ALDP-AA367C/2020|2020-10-22|EPI_ISL_624909|Original|hCoV-19^^England|Human|Lighthouse Lab in Alderley Park|Wellcome Sanger Institute for the COVID-19 Genomics UK (COG-UK) Consortium|Davies|United Kingdom
>Spike|hCoV-19/England/LIVE-9B35C/2020|2020-03-26|EPI_ISL_449436|Original|hCoV-19^^England|Human|Liverpool Clinical Laboratories|COVID-19 Genomics UK (COG-UK) Consortium|Haldenby|United Kingdom
>Spike|hCoV-19/Japan/PG-4487/2020|2020-07-00|EPI_ISL_692710|Original|hCoV-19^^Japan|Human|Pathogen Genomics Center|Pathogen Genomics Center|Sekizuka|Japan
>Spike|hCoV-19/Singapore/1268/2020|2020-05-05|EPI_ISL_648752|Original|hCoV-19^^Singapore|Human|Department of Laboratory Medicine|Department of Laboratory Medicine|Chen|Singapore
>Spike|hCoV-19/England/NORT-298D22/2020|2020-04-21|EPI_ISL_493474|Original|hCoV-19^^England|Human|Northumbria University / South Tees Hospitals NHS Foundation Trust / North Cumbria Integrated Care NHS Foundation Trust / North Tees and Hartlepool NHS Foundation Trust / Newcastle Hospitals NHS Foundation Trust|COVID-19 Genomics UK (COG-UK) Consortium|Bashton|United Kingdom
>Spike|hCoV-19/England/QEUH-9CA93B/2020|2020-09-10|EPI_ISL_567341|Original|hCoV-19^^England|Human|Lighthouse Lab in Glasgow|Wellcome Sanger Institute for the COVID-19 Genomics UK (COG-UK) consortium|Davies|United Kingdom
>Spike|hCoV-19/India/WB-IICB-028/2020|2020-08-12|EPI_ISL_581503|Original|hCoV-19^^West Bengal|Human|CSIR-Indian Institute of Chemical Biology|CSIR-Indian Institute of Chemical Biology|Bhowmik|India
>Spike|hCoV-19/USA/CA-CZB-1150/2020|2020-04-26|EPI_ISL_454615|Original|hCoV-19^^California|Human|UCSF Clinical Microbiology Laboratory|Chan-Zuckerberg Biohub|Hao|USA
>Spike|hCoV-19/Australia/NSW294/2020|2020-04-08|EPI_ISL_451558|Original|hCoV-19^^New South Wales|Human|Medlab Pathology|NSW Health Pathology - Institute of Clinical Pathology and Medical Research; Westmead Hospital; University of Sydney|Gall|Australia
>Spike|hCoV-19/USA/CA-CZB-11931/2020|2020-10-06|EPI_ISL_625607|Original|hCoV-19^^California|Human|Santa Clara County Public Health Laboratory|Chan-Zuckerberg Biohub|Bhatt|USA
>Spike|hCoV-19/England/ALDP-4F4C25/2020|2020-06-24|EPI_ISL_559115|Original|hCoV-19^^England|Human|Lighthouse Lab in Alderley Park|Wellcome Sanger Institute for the COVID-19 Genomics UK (COG-UK) consortium|Davies|United Kingdom
>Spike|hCoV-19/Wales/PHWC-47F563/2020|2020-10-15|EPI_ISL_611972|Original|hCoV-19^^Wales|Human|Wales Specialist Virology Centre Sequencing lab|COVID-19 Genomics UK (COG-UK) Consortium|Moore|United Kingdom
>Spike|hCoV-19/England/CAMB-1BB66B/2020|2020-11-21|EPI_ISL_679160|Original|hCoV-19^^England|Human|Department of Pathology|COVID-19 Genomics UK (COG-UK) Consortium|Goodfellow|United Kingdom
>Spike|hCoV-19/England/CAMC-948FCA/2020|2020-07-21|EPI_ISL_556573|Original|hCoV-19^^England|Human|Lighthouse Lab in Cambridge|Wellcome Sanger Institute for the COVID-19 Genomics UK (COG-UK) consortium|Davies|United Kingdom
>Spike|hCoV-19/Denmark/DCGC-17989/2020|2020-12-14|EPI_ISL_749684|Original|hCoV-19^^Midtjylland|Human|Department of Virus and Microbiological Special Diagnostics|Albertsen Lab|Michaelsen|Denmark
>Spike|hCoV-19/Peru/LIM-UPCH-0113/2020|2020-06-18|EPI_ISL_540990|Original|hCoV-19^^Lima|Human|Laboratorio de Referencia Nacional de Virus Respiratorios|Laboratorio de Gen\u00f3mica Microbiana|Tsukayama|Peru

>Spike|hCoV-19/England/MILK-A7BCF3/2020|2020-10-15|EPI_ISL_607374|Original|hCoV-19^^England|Human|Lighthouse Lab in Milton Keynes|Wellcome Sanger Institute for the COVID-19 Genomics UK (COG-UK) consortium|Davies|United Kingdom
>Spike|hCoV-19/England/SHEF-10D82EA/2020|2020-12-18|EPI_ISL_742113|Original|hCoV-19^^England|Human|Virology Department|COVID-19 Genomics UK (COG-UK) Consortium|de Silva|United Kingdom
>Spike|hCoV-19/South Africa/NHLS-UCT-GS-0808/2020|2020-10-15|EPI_ISL_700441|Original|hCoV-19^^Western Cape|Human|Conville CDC wc CVC|NHLS/UCT|Iranzadeh|South Africa
>Spike|hCoV-19/England/CAMC-A3ECDE/2020|2020-10-05|EPI_ISL_609655|Original|hCoV-19^^England|Human|Lighthouse Lab in Cambridge|Wellcome Sanger Institute for the COVID-19 Genomics UK (COG-UK) consortium|Davies|United Kingdom
>Spike|hCoV-19/Denmark/DCGC-17533/2020|2020-12-14|EPI_ISL_751049|Original|hCoV-19^^Sjaelland|Human|Department of Virus and Microbiological Special Diagnostics|Albertsen Lab|Michaelsen|Denmark
>Spike|hCoV-19/SaudiArabia/KAUST-MADINAH450/2020|2020-04-21|EPI_ISL_513160|Original|hCoV-19^^Madinah|Human|Pathogen Genomics Lab King Abdullah University of Science and Technology(KAUST)|Pathogen Genomics Lab King Abdullah University of Science and Technology(KAUST)|Mohamed Ghazzali|Saudi Arabia
>Spike|hCoV-19/USA/UT-UPHL-2012742341/2020|2020-08-09|EPI_ISL_745843|Original|hCoV-19^^Utah|Human|Ginkgo Bioworks Clinical Laboratory|Utah Public Health Laboratory|Young|USA
>Spike|hCoV-19/England/BRIS-132DA3/2020|2020-06-16|EPI_ISL_511966|Original|hCoV-19^^England|Human|PHE South West Regional Laboratory|Wellcome Sanger Institute for the COVID-19 Genomics UK (COG-UK) consortium|Davies|United Kingdom
>Spike|hCoV-19/USA/CA-CDPH151/2020|2020-09-27|EPI_ISL_755058|Original|hCoV-19^^California|Human|California Department of Public Health|California Department of Public Health|Bell|USA
>Spike|hCoV-19/England/ALDP-A87ABE/2020|2020-10-17|EPI_ISL_605969|Original|hCoV-19^^England|Human|Lighthouse Lab in Alderley Park|Wellcome Sanger Institute for the COVID-19 Genomics UK (COG-UK) consortium|Davies|United Kingdom
>Spike|hCoV-19/England/ALDP-A2EA7D/2020|2020-10-05|EPI_ISL_587000|Original|hCoV-19^^England|Human|Lighthouse Lab in Alderley Park|Wellcome Sanger Institute for the COVID-19 Genomics UK (COG-UK) consortium|Davies|United Kingdom
>Spike|hCoV-19/Japan/PG-5915/2020|2020-08-00|EPI_ISL_689030|Original|hCoV-19^^Japan|Human|Pathogen Genomics Center|Pathogen Genomics Center|Sekizuka|Japan
>Spike|hCoV-19/Denmark/DCGC-18297/2020|2020-12-07|EPI_ISL_749870|Original|hCoV-19^^Hovedstaden|Human|Department of Virus and Microbiological Special Diagnostics|Albertsen Lab|Michaelsen|Denmark
>Spike|hCoV-19/England/QEUH-B9F8E5/2020|2020-11-18|EPI_ISL_720934|Original|hCoV-19^^England|Human|Lighthouse Lab in Glasgow|Wellcome Sanger Institute for the COVID-19 Genomics UK (COG-UK) Consortium|Davies|United Kingdom
>Spike|hCoV-19/USA/NY-Wadsworth-278033-01/2020|2020-11-22|EPI_ISL_754601|Original|hCoV-19^^New York|Human|Wadsworth Center|Wadsworth Center|Lasek-Nesselquist|USA
>Spike|hCoV-19/Denmark/DCGC-19852/2020|2020-12-07|EPI_ISL_747540|Original|hCoV-19^^Sjaelland|Human|Department of Virus and Microbiological Special Diagnostics|Albertsen Lab|Michaelsen|Denmark
>Spike|hCoV-19/Saudi Arabia/KAUST-MADINAH250/2020|2020-04-02|EPI_ISL_678027|Original|hCoV-19^^Madinah|Human|Pathogen Genomics Lab King Abdullah University of Science and Technology(KAUST)|Pathogen Genomics Lab King Abdullah University of Science and Technology(KAUST)|Mohamed Ghazzali|Saudi Arabia
>Spike|hCoV-19/England/QEUH-9F6295/2020|2020-09-25|EPI_ISL_588786|Original|hCoV-19^^England|Human|Lighthouse Lab in Glasgow|Wellcome Sanger Institute for the COVID-19 Genomics UK (COG-UK) consortium|Davies|United Kingdom
>Spike|hCoV-19/England/MILK-99C5D4/2020|2020-08-30|EPI_ISL_550695|Original|hCoV-19^^England|Human|Lighthouse Lab in Milton Keynes|Wellcome Sanger Institute for the COVID-19 Genomics UK (COG-UK) consortium|Davies|United Kingdom
>Spike|hCoV-19/Australia/NSW983/2020|2020-08-02|EPI_ISL_591509|Original|hCoV-19^^New South Wales|Human|Sydney South West Pathology Service (SSWPS) - Liverpool Hospital -

NSW Health Pathology|NSW Health Pathology - Institute of Clinical Pathology and Medical Research; Westmead Hospital; University of Sydney|Gall|Australia
>Spike|hCoV-19/Belgium/rega-0801927/2020|2020-08-01|EPI_ISL_734869|Original|hCoV-19^^Hasselt|Human|UZ Leuven|KU Leuven|Maes|Belgium
>Spike|hCoV-19/Scotland/CVR3868/2020|2020-05-25|EPI_ISL_484662|Original|hCoV-19^^Scotland|Human|West of Scotland Specialist Virology Centre|COVID-19 Genomics UK (COG-UK) Consortium|Robertson|United Kingdom
>Spike|hCoV-19/England/CAMB-81D3D/2020|2020-04-13|EPI_ISL_443106|Original|hCoV-19^^England|Human|Department of Pathology|Wellcome Sanger Institute for the COVID-19 Genomics UK (COG-UK) consortium|Amato|United Kingdom
>Spike|hCoV-19/England/20129040602/2020|2020-03-20|EPI_ISL_420489|Original|hCoV-19^^England|Human|Respiratory Virus Unit|Respiratory Virus Unit|Platt|United Kingdom
>Spike|hCoV-19/Wales/ALDP-C39794/2020|2020-12-08|EPI_ISL_767162|Original|hCoV-19^^Wales|Human|Lighthouse Lab in Alderley Park|Wellcome Sanger Institute for the COVID-19 Genomics UK (COG-UK) Consortium|Davies|United Kingdom
>Spike|hCoV-19/England/QEUH-962504/2020|2020-08-10|EPI_ISL_531841|Original|hCoV-19^^England|Human|Lighthouse Lab in Glasgow|Wellcome Sanger Institute for the COVID-19 Genomics UK (COG-UK) consortium|Davies|United Kingdom
>Spike|hCoV-19/Sweden/20-50341/2020|2020-03-13|EPI_ISL_475090|Original|hCoV-19^^Stockholm|Human|Karolinska Universitetslaboratoriet|The Public Health Agency of Sweden|Svartstr\u00f6m|Sweden
>Spike|hCoV-19/USA/MA-Yale-448/2020|2020-04-16|EPI_ISL_738250|Original|hCoV-19^^Massachusetts|Human|Connecticut Veterans' Affairs Hospital|Grubaugh Lab - Yale School of Public Health|Grubaugh|USA
>Spike|hCoV-19/England/20120037404/2020|2020-03-15|EPI_ISL_465161|Original|hCoV-19^^England|Human|Respiratory Virus Unit|Respiratory Virus Unit|Platt|United Kingdom
>Spike|hCoV-19/Wales/MILK-9D56EB/2020|2020-09-19|EPI_ISL_637853|Original|hCoV-19^^Wales|Human|Centre for Enzyme Innovation|COVID-19 Genomics UK (COG-UK) Consortium|Robson|United Kingdom
>Spike|hCoV-19/England/ALDP-9E56E8/2020|2020-09-24|EPI_ISL_589282|Original|hCoV-19^^England|Human|Lighthouse Lab in Alderley Park|Wellcome Sanger Institute for the COVID-19 Genomics UK (COG-UK) consortium|Davies|United Kingdom
>Spike|hCoV-19/Wales/PHWC-16F675/2020|2020-10-04|EPI_ISL_586001|Original|hCoV-19^^Wales|Human|Wales Specialist Virology Centre Sequencing lab|COVID-19 Genomics UK (COG-UK) Consortium|Moore|United Kingdom
>Spike|hCoV-19/Hong Kong/VM20009034/2020|2020-04-04|EPI_ISL_444047|Original|hCoV-19^^Hong Kong|Human|United Christian Hospital|Hong Kong Department of Health|Mak|China
>Spike|hCoV-19/Belgium/Rega-0327215/2020|2020-03-27|EPI_ISL_458185|Original|hCoV-19^^Wilrijk|Human|KU Leuven|KU Leuven|Maes|Belgium
>Spike|hCoV-19/USA/VA-DCLS-1941/2020|2020-09-30|EPI_ISL_644928|Original|hCoV-19^^Virginia|Human|Virginia DCLS|Virginia DCLS|St. Jacques|USA
>Spike|hCoV-19/England/ALDP-524942/2020|2020-06-02|EPI_ISL_558905|Original|hCoV-19^^England|Human|Lighthouse Lab in Alderley Park|Wellcome Sanger Institute for the COVID-19 Genomics UK (COG-UK) consortium|Davies|United Kingdom
>Spike|hCoV-19/Russia/MOW-RII-MH1999S/2020|2020-06-11|EPI_ISL_596343|Original|hCoV-19^^Moscow|Human|Pathogenic Microorganisms Variability Laboratory|WHO National Influenza Centre Russian Federation|Komissarov|Russia
>Spike|hCoV-19/England/CAMB-1B6D7A/2020|2020-10-12|EPI_ISL_611967|Original|hCoV-19^^England|Human|Department of Pathology|COVID-19 Genomics UK (COG-UK) Consortium|Goodfellow|United Kingdom
>Spike|hCoV-19/Scotland/QEUH-9CF514/2020|2020-09-17|EPI_ISL_601896|Original|hCoV-19^^Scotland|Human|Lighthouse Lab in Glasgow|Wellcome Sanger Institute for the COVID-19 Genomics UK (COG-UK) consortium|Davies|United Kingdom
>Spike|hCoV-19/England/CAMC-B79E85/2020|2020-11-12|EPI_ISL_741056|Original|hCoV-19^^England|Human|Oxford Viromics|COVID-19 Genomics UK (COG-UK) Consortium|Golubchik|United Kingdom
>Spike|hCoV-19/England/ALDP-5AE96E/2020|2020-06-17|EPI_ISL_558759|Original|hCoV-19^^England|Human|Lighthouse Lab in Alderley Park|Wellcome Sanger Institute for the COVID-19 Genomics UK (COG-UK) consortium|Davies|United Kingdom

>Spike|hCoV-19/Spain/MD-HLP-40/2020|2020-04-12|EPI_ISL_530044|Original|hCoV-19^^Madrid|Human|Hospital Universitario La Paz|Hospital Universitario La Paz|Viedma|Spain
>Spike|hCoV-19/Australia/VIC11364/2020|2020-08-02|EPI_ISL_563345|Original|hCoV-19^^Victoria|Human|Microbiological Diagnostic Unit - Public Health Laboratory (MDU-PHL)|MDU-PHL|Schultz|Australia
>Spike|hCoV-19/Spain/AN-IBV-003023/2020|2020-04-03|EPI_ISL_474895|Original|hCoV-19^^Andalucia|Human|Hospital Universitario Virgen de las Nieves de Granada-SAS|SeqCOVID-SPAIN consortium/IBV(CSIC)|Comas|Spain
>Spike|hCoV-19/India/GJ-GBRC-381/2020|2020-06-18|EPI_ISL_524741|Original|hCoV-19^^Gujarat|Human|GMERS Medical College and Hospital|Gujarat Biotechnology Research Centre|Joshi|India
>Spike|hCoV-19/England/QEUH-5472C6/2020|2020-05-14|EPI_ISL_533411|Original|hCoV-19^^England|Human|Lighthouse Lab in Glasgow|Wellcome Sanger Institute for the COVID-19 Genomics UK (COG-UK) consortium|Davies|United Kingdom
>Spike|hCoV-19/Norway/5422/2020|2020-11-03|EPI_ISL_708088|Original|hCoV-19^^Troms og Finnmark|Human|University Hospital of Northern Norway|Norwegian Institute of Public Health|Kopperud Riis|Norway
>Spike|hCoV-19/England/CAMC-B21E13/2020|2020-11-01|EPI_ISL_647321|Original|hCoV-19^^England|Human|Lighthouse Lab in Cambridge|Wellcome Sanger Institute for the COVID-19 Genomics UK (COG-UK) Consortium|Davies|United Kingdom
>Spike|hCoV-19/England/LIVE-1E0339/2020|2020-10-11|EPI_ISL_626742|Original|hCoV-19^^England|Human|Liverpool Clinical Laboratories|COVID-19 Genomics UK (COG-UK) Consortium|Haldenby|United Kingdom
>Spike|hCoV-19/Chile/RM-ISPCH-5/2020|2020-03-09|EPI_ISL_445248|Original|hCoV-19^^Region Metropolitana de Santiago|Human|CLINICA ALEMANA DE SANTIAGO S.A.|Instituto de Salud Publica de Chile|Fernandez|Chile
>Spike|hCoV-19/England/BIRM-5FDCF/2020|2020-00-00|EPI_ISL_461526|Original|hCoV-19^^England|Human|University of Birmingham|COVID-19 Genomics UK (COG-UK) Consortium|Loman|United Kingdom
>Spike|hCoV-19/Spain/PV-IBV-98008313/2020|2020-10-07|EPI_ISL_654565|Original|hCoV-19^^Basque Country|Human|Servicio de Microbiolog\u00eda. Hospital Universitario Donostia. OSI Donostialdea. \u00c1rea de Enfermedades Infecciosas|SeqCOVID-SPAIN consortium/IBV(CSIC)|Comas|Spain
>Spike|hCoV-19/Norway/3384/2020|2020-08-18|EPI_ISL_635106|Original|hCoV-19^^Vestfold og Telemark|Human|Vestfold Hospital|Norwegian Institute of Public Health|Vollan|Norway
>Spike|hCoV-19/England/ALDP-C58C09/2020|2020-12-14|EPI_ISL_735986|Original|hCoV-19^^England|Human|Lighthouse Lab in Alderley Park|Wellcome Sanger Institute for the COVID-19 Genomics UK (COG-UK) Consortium|Davies|United Kingdom
>Spike|hCoV-19/England/CAMB-1BD5B7/2020|2020-12-02|EPI_ISL_764281|Original|hCoV-19^^England|Human|Department of Pathology|COVID-19 Genomics UK (COG-UK) Consortium|Goodfellow|United Kingdom
>Spike|hCoV-19/Austria/CeMM0058/2020|2020-04-03|EPI_ISL_437938|Original|hCoV-19^^Innsbruck|Human|Universitaetsklinik f\u00fcr Innere Medizin II Innsbruck|Bergthaler laboratory|Bergthaler|Austria
>Spike|hCoV-19/Australia/NSW1070/2020|2020-08-04|EPI_ISL_593656|Original|hCoV-19^^New South Wales|Human|Pathology North - Hunter - NSW Health Pathology|NSW Health Pathology - Institute of Clinical Pathology and Medical Research; Westmead Hospital; University of Sydney|Gall|Australia
>Spike|hCoV-19/England/ALDP-95306F/2020|2020-06-11|EPI_ISL_553696|Original|hCoV-19^^England|Human|Lighthouse Lab in Alderley Park|Wellcome Sanger Institute for the COVID-19 Genomics UK (COG-UK) consortium|Davies|United Kingdom
>Spike|hCoV-19/Canada/BC-BCCDC-6395/2020|2020-10-00|EPI_ISL_740552|Original|hCoV-19^^British Columbia|Human|BCCDC Public Health Laboratory|BCCDC Public Health Laboratory|Prystajecky|Canada
>Spike|hCoV-19/England/SHEF-C4A64/2020|2020-05-28|EPI_ISL_535054|Original|hCoV-19^^England|Human|Virology Department|COVID-19 Genomics UK (COG-UK) Consortium|de Silva|United Kingdom

>Spike|hCoV-19/England/CAMB-A97936/2020|2020-12-10|EPI_ISL_723937|Original|hCoV-19^^England|Human|Department of Pathology|COVID-19 Genomics UK (COG-UK) Consortium|Goodfellow|United Kingdom
>Spike|hCoV-19/England/CAMB-1B8F81/2020|2020-11-02|EPI_ISL_651695|Original|hCoV-19^^England|Human|Department of Pathology|COVID-19 Genomics UK (COG-UK) Consortium|Goodfellow|United Kingdom
>Spike|hCoV-19/England/LIVE-DB3C65/2020|2020-10-21|EPI_ISL_650926|Original|hCoV-19^^England|Human|Liverpool Clinical Laboratories|COVID-19 Genomics UK (COG-UK) Consortium|Haldenby|United Kingdom
>Spike|hCoV-19/England/MILK-956FC9/2020|2020-06-08|EPI_ISL_554132|Original|hCoV-19^^England|Human|Lighthouse Lab in Milton Keynes|Wellcome Sanger Institute for the COVID-19 Genomics UK (COG-UK) consortium|Davies|United Kingdom
>Spike|hCoV-19/Wales/PHWC-48E18B/2020|2020-11-09|EPI_ISL_680817|Original|hCoV-19^^Wales|Human|Wales Specialist Virology Centre Sequencing lab|COVID-19 Genomics UK (COG-UK) Consortium|Moore|United Kingdom
>Spike|hCoV-19/Wales/PHWC-4A51E8/2020|2020-12-15|EPI_ISL_764836|Original|hCoV-19^^Wales|Human|Wales Specialist Virology Centre Sequencing lab|COVID-19 Genomics UK (COG-UK) Consortium|Moore|United Kingdom
>Spike|hCoV-19/England/QEUH-C01276/2020|2020-12-03|EPI_ISL_720867|Original|hCoV-19^^England|Human|Lighthouse Lab in Glasgow|Wellcome Sanger Institute for the COVID-19 Genomics UK (COG-UK) Consortium|Davies|United Kingdom
>Spike|hCoV-19/Denmark/DCGC-3016/2020|2020-08-24|EPI_ISL_616794|Original|hCoV-19^^Hovedstaden|Human|Department of Virus and Microbiological Special Diagnostics|Albertsen lab|Michaelsen|Denmark
>Spike|hCoV-19/Portugal/PT1965/2020|2020-11-24|EPI_ISL_732190|Original|hCoV-19^^Portugal|Human|Instituto Nacional de Saude (INSA) and Instituto Gulbenkian de Ciencia (IGC)|Instituto Nacional de Saude (INSA) and Instituto Gulbenkian de Ciencia (IGC)|Borges|Portugal
>Spike|hCoV-19/England/BRIS-12415D/2020|2020-04-08|EPI_ISL_492780|Original|hCoV-19^^England|Human|PHE South West Regional Laboratory|Wellcome Sanger Institute for the COVID-19 Genomics UK (COG-UK) consortium|Davies|United Kingdom
>Spike|hCoV-19/England/CAMB-1B31DB/2020|2020-05-22|EPI_ISL_456751|Original|hCoV-19^^England|Human|Department of Pathology|COVID-19 Genomics UK (COG-UK) Consortium|Meredith|United Kingdom
>Spike|hCoV-19/Switzerland/BE-ETHZ-350243/2020|2020-10-30|EPI_ISL_728845|Original|hCoV-19^^Bern|Human|Viollier AG|Department of Biosystems Science and Engineering|Nadeau|Switzerland
>Spike|hCoV-19/USA/AZ-TG666125/2020|2020-05-02|EPI_ISL_696211|Original|hCoV-19^^Arizona|Human|Sonora Quest Laboratories|TGen North|French|USA
>Spike|hCoV-19/Wales/PHWC-4963E4/2020|2020-11-20|EPI_ISL_726575|Original|hCoV-19^^Wales|Human|Originating lab|COVID-19 Genomics UK (COG-UK) Consortium|Moore|United Kingdom
>Spike|hCoV-19/England/QEUH-B68B91/2020|2020-11-11|EPI_ISL_655061|Original|hCoV-19^^England|Human|Lighthouse Lab in Glasgow|Wellcome Sanger Institute for the COVID-19 Genomics UK (COG-UK) Consortium|Davies|United Kingdom
>Spike|hCoV-19/England/QEUH-9B0C6C/2020|2020-09-03|EPI_ISL_559857|Original|hCoV-19^^England|Human|Oxford Viromics|COVID-19 Genomics UK (COG-UK) Consortium|Golubchik|United Kingdom
>Spike|hCoV-19/USA/CA-CZB-1623/2020|2020-05-07|EPI_ISL_468552|Original|hCoV-19^^California|Human|San Joaquin County Public Health Lab|Chan-Zuckerberg Biohub|Hao|USA
>Spike|hCoV-19/Germany/HH-hpi-p2316/2020|2020-03-23|EPI_ISL_775623|Original|hCoV-19^^Hamburg|Human|University Medical Center Hamburg Eppendorf|Heinrich Pette Institute|Robitaille|Germany
>Spike|hCoV-19/SaudiArabia/KAUST-JEDDAH990/2020|2020-06-23|EPI_ISL_513064|Original|hCoV-19^^Jeddah|Human|Pathogen Genomics Lab King Abdullah University of Science and Technology(KAUST)|Pathogen Genomics Lab King Abdullah University of Science and Technology(KAUST)|Mohamed Ghazzali|Saudi Arabia

>Spike|hCoV-19/England/MILK-A7D356/2020|2020-10-13|EPI_ISL_606337|Original|hCoV-19^^England|Human|Lighthouse Lab in Milton Keynes|Wellcome Sanger Institute for the COVID-19 Genomics UK (COG-UK) consortium|Davies|United Kingdom
>Spike|hCoV-19/Japan/FHU-CS24-0221/2020|2020-02-21|EPI_ISL_718273|Original|hCoV-19^^Aichi|Human|Fujita Health University Okazaki Medical Center|Department of Virology and Parasitology|Komoto|Japan
>Spike|hCoV-19/Wales/PHWC-4A04E4/2020|2020-12-07|EPI_ISL_743455|Original|hCoV-19^^Wales|Human|Wales Specialist Virology Centre Sequencing lab|COVID-19 Genomics UK (COG-UK) Consortium|Moore|United Kingdom
>Spike|hCoV-19/Denmark/DCGC-16402/2020|2020-11-16|EPI_ISL_714745|Original|hCoV-19^^Hovedstaden|Human|Department of Virus and Microbiological Special Diagnostics|Albertsen Lab|Michaelsen|Denmark
>Spike|hCoV-19/Australia/VIC561/2020|2020-03-25|EPI_ISL_426822|Original|hCoV-19^^Victoria|Human|Victorian Infectious Diseases Reference Laboratory (VIDRL)|Microbiological Diagnostic Unit Public Health Laboratory and Victorian Infectious Diseases Reference Laboratory|Schultz|Australia
>Spike|hCoV-19/Denmark/DCGC-10627/2020|2020-11-02|EPI_ISL_670101|Original|hCoV-19^^Nordjylland|Human|Department of Virus and Microbiological Special Diagnostics|Albertsen Lab|Michaelsen|Denmark
>Spike|hCoV-19/Spain/VC-IBV-98006468/2020|2020-06-27|EPI_ISL_538669|Original|hCoV-19^^Comunitat Valenciana|Human|Servicio de Microbiolog\u00eda. Hospital General Universitario de Castell\u00f3n|SeqCOVID-SPAIN consortium/IBV(CSIC)|Comas|Spain
>Spike|hCoV-19/Denmark/DCGC-21056/2020|2020-12-21|EPI_ISL_758839|Original|hCoV-19^^Hovedstaden|Human|Department of Virus and Microbiological Special Diagnostics|Albertsen Lab|Michaelsen|Denmark
>Spike|hCoV-19/England/MILK-B04AB8/2020|2020-10-24|EPI_ISL_650971|Original|hCoV-19^^England|Human|Department of Pathology|COVID-19 Genomics UK (COG-UK) Consortium|Goodfellow|United Kingdom
>Spike|hCoV-19/Japan/PG-7083/2020|2020-08-00|EPI_ISL_688188|Original|hCoV-19^^Japan|Human|Pathogen Genomics Center|Pathogen Genomics Center|Sekizuka|Japan
>Spike|hCoV-19/Scotland/GCVR-170A0C/2020|2020-03-26|EPI_ISL_459665|Original|hCoV-19^^Scotland|Human|NHSGGC West of Scotland Specialist Virology Centre / MRC-University of Glasgow Centre for Virus Research|Wellcome Sanger Institute for the COVID-19 Genomics UK (COG-UK) consortium|Davies|United Kingdom
>Spike|hCoV-19/England/QEUH-B3F8C9/2020|2020-11-08|EPI_ISL_658501|Original|hCoV-19^^England|Human|Lighthouse Lab in Glasgow|Wellcome Sanger Institute for the COVID-19 Genomics UK (COG-UK) Consortium|Davies|United Kingdom
>Spike|hCoV-19/Wales/PHWC-1636EA/2020|2020-05-11|EPI_ISL_473983|Original|hCoV-19^^Wales|Human|Originating lab|COVID-19 Genomics UK (COG-UK) Consortium|Moore|United Kingdom
>Spike|hCoV-19/Scotland/QEUH-9B8ED7/2020|2020-09-07|EPI_ISL_568260|Original|hCoV-19^^Scotland|Human|Lighthouse Lab in Glasgow|Wellcome Sanger Institute for the COVID-19 Genomics UK (COG-UK) consortium|Davies|United Kingdom
>Spike|hCoV-19/England/SHEF-C576C/2020|2020-04-22|EPI_ISL_453716|Original|hCoV-19^^England|Human|Virology Department|COVID-19 Genomics UK (COG-UK) Consortium|de Silva|United Kingdom
>Spike|hCoV-19/Denmark/DCGC-14446/2020|2020-11-30|EPI_ISL_711250|Original|hCoV-19^^Midtjylland|Human|Department of Virus and Microbiological Special Diagnostics|Albertsen Lab|Michaelsen|Denmark
>Spike|hCoV-19/Denmark/DCGC-8482/2020|2020-06-08|EPI_ISL_618044|Original|hCoV-19^^Nordjylland|Human|Department of Virus and Microbiological Special Diagnostics|Albertsen lab|Michaelsen|Denmark
>Spike|hCoV-19/England/LOND-D4621/2020|2020-04-08|EPI_ISL_444143|Original|hCoV-19^^England|Human|University College London|COVID-19 Genomics UK (COG-UK) Consortium|Roy|United Kingdom
>Spike|hCoV-19/England/QEUH-AAE6AE/2020|2020-10-22|EPI_ISL_630719|Original|hCoV-19^^England|Human|Lighthouse Lab in Glasgow|Wellcome Sanger Institute for the COVID-19 Genomics UK (COG-UK) consortium|Davies|United Kingdom

>Spike|hCoV-19/England/C200044442/2020|2020-11-06|EPI_ISL_666755|Original|hCoV-19^^England|Human|Respiratory Virus Unit|COVID-19 Genomics UK (COG-UK) Consortium|Platt|United Kingdom
>Spike|hCoV-19/Canada/ON-S888/2020|2020-04-12|EPI_ISL_591255|Original|hCoV-19^^Ontario|Human|Toronto Invasive Bacterial Diseases Network|McMaster University|McArthur|Canada
>Spike|hCoV-19/England/MILK-A7AFFB/2020|2020-10-14|EPI_ISL_607336|Original|hCoV-19^^England|Human|Lighthouse Lab in Milton Keynes|Wellcome Sanger Institute for the COVID-19 Genomics UK (COG-UK) consortium|Davies|United Kingdom
>Spike|hCoV-19/USA/CA-ALSR-2387/2020|2020-06-28|EPI_ISL_635454|Original|hCoV-19^^California|Human|San Diego County Public Health Laboratory|Andersen lab at Scripps Research|Gangavarapu|USA
>Spike|hCoV-19/Australia/VIC4026/2020|2020-07-10|EPI_ISL_519412|Original|hCoV-19^^Victoria|Human|Microbiological Diagnostic Unit - Public Health Laboratory (MDU-PHL)|MDU-PHL|Schultz|Australia
>Spike|hCoV-19/Spain/VC-IBV-98003095/2020|2020-03-14|EPI_ISL_538031|Original|hCoV-19^^Comunitat Valenciana|Human|Hospital Universitari i Polit\u00e8cnic La Fe de Val\u00e8ncia|SeqCOVID-SPAIN consortium/IBV(CSIC)|Comas|Spain
>Spike|hCoV-19/Taiwan/CGMH-CGU-17/2020|2020-03-17|EPI_ISL_424973|LLC-MK2|hCoV-19^^Taoyuan|Human|Laboratory Medicine|Department of Laboratory Medicine|Yi Chun|Taiwan
>Spike|hCoV-19/Denmark/DCGC-4554/2020|2020-09-21|EPI_ISL_621351|Original|hCoV-19^^Sjaelland|Human|Department of Virus and Microbiological Special Diagnostics|Albertsen lab|Michaelsen|Denmark
>Spike|hCoV-19/England/ALDP-9E667E/2020|2020-09-23|EPI_ISL_580318|Original|hCoV-19^^England|Human|Lighthouse Lab in Alderley Park|Wellcome Sanger Institute for the COVID-19 Genomics UK (COG-UK) consortium|Davies|United Kingdom
>Spike|hCoV-19/USA/MD-HP00067/2020|2020-03-11|EPI_ISL_457808|Original|hCoV-19^^Maryland|Human|Johns Hopkins Hospital Department of Pathology|Johns Hopkins Hospital Department of Pathology|Ramakrishnan|USA
>Spike|hCoV-19/USA/WI-UW-2613/2021|2021-01-02|EPI_ISL_791130|Original|hCoV-19^^Wisconsin|Human|University of Wisconsin-Madison AIDS Vaccine Research Laboratories|University of Wisconsin-Madison AIDS Vaccine Research Laboratories|Moreno|USA
>Spike|hCoV-19/Bangladesh/BARJ-CVASU-CTG-511/2020|2020-05-09|EPI_ISL_450343|Original|hCoV-19^^Chittagong|Human|Bangladesh Institute of Tropical & Infectious Diseases|Basic and Applied Research on Jute Project|Ahmed|Bangladesh
>Spike|hCoV-19/Ireland/D-NVRL-AIIDV0192v1/2020|2020-04-14|EPI_ISL_644226|Original|hCoV-19^^Dublin|Human|CEPHR / Vincent's Hospital|Irish Coronavirus Sequencing Consortium - National Virus Reference Laboratory|Gonzalez|Ireland
>Spike|hCoV-19/Wales/PHWC-166E58/2020|2020-06-27|EPI_ISL_494121|Original|hCoV-19^^Wales|Human|Originating lab|COVID-19 Genomics UK (COG-UK) Consortium|Moore|United Kingdom
>Spike|hCoV-19/United Arab Emirates/0560/2020|2020-05-19|EPI_ISL_698197|Original|hCoV-19^^Abu Dhabi|Human|Group 42 (G42) Healthcare|G42 Healthcare|Ogrodzki|United Arab Emirates
>Spike|hCoV-19/USA/WA-S1963/2020|2020-06-03|EPI_ISL_497391|Original|hCoV-19^^Washington|Human|Washington State Department of Health|Seattle Flu Study|Lee|USA
>Spike|hCoV-19/Denmark/DCGC-11109/2020|2020-11-02|EPI_ISL_669485|Original|hCoV-19^^Syddanmark|Human|Department of Virus and Microbiological Special Diagnostics|Albertsen Lab|Michaelsen|Denmark
>Spike|hCoV-19/England/20180026004/2020|2020-04-27|EPI_ISL_465433|Original|hCoV-19^^England|Human|Respiratory Virus Unit|Respiratory Virus Unit|Platt|United Kingdom
>Spike|hCoV-19/USA/MA-MASPHL-00241/2020|2020-04-16|EPI_ISL_791686|Original|hCoV-19^^Massachusetts|Human|Massachusetts State Public Health Laboratory|Infectious Disease Program|Park|USA
>Spike|hCoV-19/India/TN-NCDC-02333/2020|2020-03-29|EPI_ISL_435087|Original|hCoV-19^^Tamil Nadu|Human|National Centre for Disease control (NCDC)|NCDC/CSIR-IGIB|Faruq|India

>Spike|hCoV-19/Singapore/350/2020|2020-04-06|EPI_ISL_469105|Original|hCoV-19^^Singapore|Human|National Public Health Laboratory|National Public Health Laboratory|Mak|Singapore
>Spike|hCoV-19/Australia/VIC1588/2020|2020-05-05|EPI_ISL_456508|Original|hCoV-19^^Victoria|Human|Victorian Infectious Diseases Reference Laboratory (VIDRL)|Microbiological Diagnostic Unit Public Health Laboratory and Victorian Infectious Diseases Reference Laboratory|Schultz|Australia
>Spike|hCoV-19/USA/AZ-TG269358/2020|2020-03-20|EPI_ISL_426523|Original|hCoV-19^^Arizona|Human|TGen North|TGen North|Lemmer|USA
>Spike|hCoV-19/England/QEUH-A00130/2020|2020-10-02|EPI_ISL_600324|Original|hCoV-19^^England|Human|Lighthouse Lab in Glasgow|Wellcome Sanger Institute for the COVID-19 Genomics UK (COG-UK) consortium|Davies|United Kingdom
>Spike|hCoV-19/England/ALDP-B240FC/2020|2020-11-04|EPI_ISL_646299|Original|hCoV-19^^England|Human|Lighthouse Lab in Alderley Park|Wellcome Sanger Institute for the COVID-19 Genomics UK (COG-UK) Consortium|Davies|United Kingdom
>Spike|hCoV-19/England/201360506/2020|2020-03-25|EPI_ISL_421782|Original|hCoV-19^^England|Human|Respiratory Virus Unit|Respiratory Virus Unit|Platt|United Kingdom
>Spike|hCoV-19/Denmark/DCGC-5697/2020|2020-09-21|EPI_ISL_621202|Original|hCoV-19^^Syddanmark|Human|Department of Virus and Microbiological Special Diagnostics|Albertsen lab|Michaelsen|Denmark
>Spike|hCoV-19/USA/CA-CZB-12932/2020|2020-10-18|EPI_ISL_672103|Original|hCoV-19^^California|Human|Orange County Public Health Lab|Chan-Zuckerberg Biohub|Bhatt|USA
>Spike|hCoV-19/England/MILK-A6A626/2020|2020-10-09|EPI_ISL_626949|Original|hCoV-19^^England|Human|Virology Department|COVID-19 Genomics UK (COG-UK) Consortium|de Silva|United Kingdom
>Spike|hCoV-19/Denmark/DCGC-19175/2020|2020-12-07|EPI_ISL_748794|Original|hCoV-19^^Hovedstaden|Human|Department of Virus and Microbiological Special Diagnostics|Albertsen Lab|Michaelsen|Denmark
>Spike|hCoV-19/Germany/BY-MVP-0037/2020|2020-03-22|EPI_ISL_437236|Original|hCoV-19^^Bavaria|Human|Max von Pettenkofer Institute|Laboratory for Functional Genome Analysis|Graf|Germany
>Spike|hCoV-19/Denmark/DCGC-3648/2020|2020-08-31|EPI_ISL_615420|Original|hCoV-19^^Midtjylland|Human|Department of Virus and Microbiological Special Diagnostics|Albertsen lab|Michaelsen|Denmark
>Spike|hCoV-19/England/MILK-BB0514/2020|2020-11-17|EPI_ISL_720089|Original|hCoV-19^^England|Human|Lighthouse Lab in Milton Keynes|Wellcome Sanger Institute for the COVID-19 Genomics UK (COG-UK) Consortium|Davies|United Kingdom
>Spike|hCoV-19/Scotland/QEUH-A1FFBC/2020|2020-10-01|EPI_ISL_599079|Original|hCoV-19^^Scotland|Human|Lighthouse Lab in Glasgow|Wellcome Sanger Institute for the COVID-19 Genomics UK (COG-UK) consortium|Davies|United Kingdom
>Spike|hCoV-19/USA/MA-MASPHL-00519/2020|2020-05-05|EPI_ISL_693058|Original|hCoV-19^^Massachusetts|Human|Massachusetts State Public Health Laboratory|Massachusetts State Public Health Laboratory|Lang|USA
>Spike|hCoV-19/Latvia/172/2020|2020-10-05|EPI_ISL_639669|Original|hCoV-19^^Latvia|Human|E. Gulbja Laboratorija|Latvian Biomedical Research and Study Centre|Zrelovs|Latvia
>Spike|hCoV-19/Wales/PHWC-49E452/2020|2020-12-07|EPI_ISL_743003|Original|hCoV-19^^Wales|Human|Wales Specialist Virology Centre Sequencing lab|COVID-19 Genomics UK (COG-UK) Consortium|Moore|United Kingdom
>Spike|hCoV-19/Brazil/RJ-00454/2020|2020-06-01|EPI_ISL_717849|Original|hCoV-19^^Rio de Janeiro|Human|Laboratorio de Virologia Molecular / UFRJ|Bioinformatics Laboratory / LNCC|Almeida|Brazil
>Spike|hCoV-19/USA/MA-MGH-00565/2020|2020-03-22|EPI_ISL_460423|Original|hCoV-19^^Massachusetts|Human|Massachusetts General Hospital|Infectious Disease Program|Lemieux|USA
>Spike|hCoV-19/England/QEUH-AD2E4F/2020|2020-10-26|EPI_ISL_629798|Original|hCoV-19^^England|Human|Lighthouse Lab in Glasgow|Wellcome Sanger Institute for the COVID-19 Genomics UK (COG-UK) consortium|Davies|United Kingdom
>Spike|hCoV-19/England/LEED-2A8E68/2020|2020-03-31|EPI_ISL_539163|Original|hCoV-19^^England|Human|Leeds Teaching Hospitals NHS Trust and Public Health

England|Wellcome Sanger Institute for the COVID-19 Genomics UK (COG-UK) consortium|Davies|United Kingdom
>Spike|hCoV-19/Scotland/EDB015/2020|2020-03-11|EPI_ISL_425821|Original|hCoV-19^^Scotland|Human|Virology Department|COVID-19 Genomics UK (COG-UK) Consortium|Williams|United Kingdom
>Spike|hCoV-19/England/BRIS-124B8C/2020|2020-03-24|EPI_ISL_443709|Original|hCoV-19^^England|Human|PHE South West Regional Laboratory|Wellcome Sanger Institute for the COVID-19 Genomics UK (COG-UK) consortium|Amato|United Kingdom
>Spike|hCoV-19/India/OR-ILSCV20408/2020|2020-05-15|EPI_ISL_463076|Original|hCoV-19^^Odisha|Human|Institute of Life Sciences|Immunogenomics lab|Raghav|India
>Spike|hCoV-19/Russia/ROS-RII-MH2821S/2020|2020-09-24|EPI_ISL_639966|Original|hCoV-19^^Rostov|Human|HELIX LLC|WHO National Influenza Centre Russian Federation|Komissarov|Russia
>Spike|hCoV-19/England/204561548/2020|2020-11-05|EPI_ISL_678695|Original|hCoV-19^^England|Human|Respiratory Virus Unit|COVID-19 Genomics UK (COG-UK) Consortium|Platt|United Kingdom
>Spike|hCoV-19/England/MILK-C3001A/2020|2020-12-07|EPI_ISL_782995|Original|hCoV-19^^England|Human|Lighthouse Lab in Milton Keynes|Wellcome Sanger Institute for the COVID-19 Genomics UK (COG-UK) Consortium|Davies|United Kingdom
>Spike|hCoV-19/England/CAMB-80A37/2020|2020-04-07|EPI_ISL_442941|Original|hCoV-19^^England|Human|Department of Pathology|Wellcome Sanger Institute for the COVID-19 Genomics UK (COG-UK) consortium|Amato|United Kingdom
>Spike|hCoV-19/England/NOTT-1155CC/2020|2020-10-14|EPI_ISL_595251|Original|hCoV-19^^England|Human|Queens Medical Centre|COVID-19 Genomics UK (COG-UK) Consortium|Loose|United Kingdom
>Spike|hCoV-19/Wales/PHWC-3462F/2020|2020-04-20|EPI_ISL_446950|Original|hCoV-19^^Wales|Human|Wales Specialist Virology Centre|Public Health Wales Microbiology Cardiff|Moore|United Kingdom
>Spike|hCoV-19/England/ALDP-5C43A0/2020|2020-06-19|EPI_ISL_558814|Original|hCoV-19^^England|Human|Lighthouse Lab in Alderley Park|Wellcome Sanger Institute for the COVID-19 Genomics UK (COG-UK) consortium|Davies|United Kingdom
>Spike|hCoV-19/USA/VA-DCLS-1671/2020|2020-08-29|EPI_ISL_644839|Original|hCoV-19^^Virginia|Human|Virginia DCLS|Virginia DCLS|St. Jacques|USA
>Spike|hCoV-19/England/LIVE-9D4EE/2020|2020-04-16|EPI_ISL_453206|Original|hCoV-19^^England|Human|Liverpool Clinical Laboratories|COVID-19 Genomics UK (COG-UK) Consortium|Haldenby|United Kingdom
>Spike|hCoV-19/Jordan/SEARCH-5403/2020|2020-09-15|EPI_ISL_755214|Original|hCoV-19^^Amman|Human|Biolab Diagnostic Laboratories|Andersen lab at Scripps Research|Gangavarapu|Jordan
>Spike|hCoV-19/Japan/PG-0079/2020|2020-02-00|EPI_ISL_684113|Original|hCoV-19^^Japan|Human|Pathogen Genomics Center|Pathogen Genomics Center|Sekizuka|Japan
>Spike|hCoV-19/Japan/PG-0132/2020|2020-03-12|EPI_ISL_479858|Original|hCoV-19^^Kansai|Human|Department of Infectious Diseases|Pathogen Genomics Center|Sekizuka|Japan
>Spike|hCoV-19/Italy/APU-IZSPB_267PT/2020|2020-09-30|EPI_ISL_653786|Original|hCoV-19^^Apulia|Human|Istituto Zooprofilattico Sperimentale della Puglia e della Basilicata|Istituto Zooprofilattico Sperimentale della Puglia e della Basilicata|Parisi|Italy
>Spike|hCoV-19/England/ALDP-B238B9/2020|2020-11-04|EPI_ISL_647083|Original|hCoV-19^^England|Human|Lighthouse Lab in Alderley Park|Wellcome Sanger Institute for the COVID-19 Genomics UK (COG-UK) Consortium|Davies|United Kingdom
>Spike|hCoV-19/Scotland/QEUH-96EBD9/2020|2020-08-19|EPI_ISL_534512|Original|hCoV-19^^Scotland|Human|NHSGGC West of Scotland Specialist Virology Centre / MRC-University of Glasgow Centre for Virus Research|Wellcome Sanger Institute for the COVID-19 Genomics UK (COG-UK) consortium|Davies|United Kingdom
>Spike|hCoV-19/Australia/VIC13724/2020|2020-09-16|EPI_ISL_592310|Original|hCoV-19^^Victoria|Human|Microbiological Diagnostic Unit - Public Health Laboratory (MDU-PHL)|MDU-PHL|Schultz|Australia

>Spike|hCoV-19/England/ALDP-C8CC2A/2020|2020-12-15|EPI_ISL_777262|Original|hCoV-19^^England|Human|Lighthouse Lab in Alderley Park|Wellcome Sanger Institute for the COVID-19 Genomics UK (COG-UK) Consortium|Davies|United Kingdom
>Spike|hCoV-19/South Korea/KDCA0346/2020|2020-08-09|EPI_ISL_760132|Original|hCoV-19^^South Korea|Human|Division of Emerging Infectious Diseases|Division of Emerging Infectious Diseases|Park|South Korea
>Spike|hCoV-19/Denmark/DCGC-4547/2020|2020-09-21|EPI_ISL_621344|Original|hCoV-19^^Hovedstaden|Human|Department of Virus and Microbiological Special Diagnostics|Albertsen lab|Michaelsen|Denmark
>Spike|hCoV-19/England/ALDP-7240E1/2020|2020-07-02|EPI_ISL_557598|Original|hCoV-19^^England|Human|Lighthouse Lab in Alderley Park|Wellcome Sanger Institute for the COVID-19 Genomics UK (COG-UK) consortium|Davies|United Kingdom
>Spike|hCoV-19/Wales/PHWC-487CDC/2020|2020-10-27|EPI_ISL_639297|Original|hCoV-19^^Wales|Human|Wales Specialist Virology Centre Sequencing lab|COVID-19 Genomics UK (COG-UK) Consortium|Moore|United Kingdom
>Spike|hCoV-19/Poland/79330/2020|2020-11-00|EPI_ISL_732885|Original|hCoV-19^^Poland|Human|genXone SA|genXone SA|Kaszuba|Poland
>Spike|hCoV-19/USA/NY-NYCPHL-000545/2020|2020-03-22|EPI_ISL_632005|Original|hCoV-19^^New York|Human|Jamaica Hospital Medical Center|New York City Public Health Laboratory|Wang|USA
>Spike|hCoV-19/USA/CA-CZB-14394/2020|2020-07-02|EPI_ISL_739340|Original|hCoV-19^^California|Human|Alameda County Public Health Lab|Chan-Zuckerberg Biohub|Kamm|USA
>Spike|hCoV-19/Spain/PV-IBV-000845/2020|2020-03-11|EPI_ISL_452702|Original|hCoV-19^^Basque Country|Human|Hospital Universitario Araba. Vitoria-Gasteiz,|SeqCOVID-SPAIN consortium/IBV(CSIC)|Comas|Spain
>Spike|hCoV-19/England/205100231/2020|2020-12-14|EPI_ISL_766072|Original|hCoV-19^^England|Human|Respiratory Virus Unit|COVID-19 Genomics UK (COG-UK) Consortium|Platt|United Kingdom
>Spike|hCoV-19/USA/WI-UW-1623/2020|2020-09-24|EPI_ISL_605497|Original|hCoV-19^^Wisconsin|Human|University of Wisconsin-Madison AIDS Vaccine Research Laboratories|University of Wisconsin-Madison AIDS Vaccine Research Laboratories|Baczenas|USA
>Spike|hCoV-19/Scotland/EDB6701/2020|2020-05-06|EPI_ISL_507191|Original|hCoV-19^^Scotland|Human|Virology Department|COVID-19 Genomics UK (COG-UK) Consortium|Williams|United Kingdom
>Spike|hCoV-19/India/MH-NIV-7439/2020|2020-04-05|EPI_ISL_452207|Original|hCoV-19^^Maharashtra|Human|NIV Influenza|NIV Influenza|Potdar|India
>Spike|hCoV-19/Scotland/CVR2907/2020|2020-04-22|EPI_ISL_473624|Original|hCoV-19^^Scotland|Human|West of Scotland Specialist Virology Centre|COVID-19 Genomics UK (COG-UK) Consortium|Robertson|United Kingdom
>Spike|hCoV-19/Scotland/CVR3051/2020|2020-04-24|EPI_ISL_461639|Original|hCoV-19^^Scotland|Human|West of Scotland Specialist Virology Centre|COVID-19 Genomics UK (COG-UK) Consortium|Robertson|United Kingdom
>Spike|hCoV-19/England/CAMB-78003/2020|2020-04-01|EPI_ISL_441146|Original|hCoV-19^^England|Human|Department of Pathology|Wellcome Sanger Institute for the COVID-19 Genomics UK (COG-UK) consortium|Amato|United Kingdom
>Spike|hCoV-19/Australia/VIC9851/2020|2020-08-24|EPI_ISL_565742|Original|hCoV-19^^Victoria|Human|Victorian Infectious Diseases Reference Laboratory (VIDRL)|VIDRL and MDU-PHL|Schultz|Australia
>Spike|hCoV-19/England/ALDP-C3ADBF/2020|2020-12-09|EPI_ISL_731667|Original|hCoV-19^^England|Human|Lighthouse Lab in Alderley Park|Wellcome Sanger Institute for the COVID-19 Genomics UK (COG-UK) Consortium|Davies|United Kingdom
>Spike|hCoV-19/Belgium/ULG-10891/2020|2020-11-14|EPI_ISL_678433|Original|hCoV-19^^Li\u00e8ge|Human|Department of Clinical Microbiology|GIGA Medical Genomics|Durkin|Belgium
>Spike|hCoV-19/Germany/HH-hpi-p1817/2020|2020-03-22|EPI_ISL_775636|Original|hCoV-19^^Hamburg|Human|University Medical Center Hamburg Eppendorf|Heinrich Pette Institute|Robitaille|Germany

>Spike|hCoV-19/England/ALDP-94B99C/2020|2020-06-11|EPI_ISL_555826|Original|hCoV-19^^England|Human|Lighthouse Lab in Alderley Park|Wellcome Sanger Institute for the COVID-19 Genomics UK (COG-UK) consortium|Davies|United Kingdom
>Spike|hCoV-19/USA/WA-S660/2020|2020-04-21|EPI_ISL_438213|Original|hCoV-19^^Washington|Human|Washington State Department of Health|Seattle Flu Study|Bedford|USA
>Spike|hCoV-19/Spain/CB-IBV-98002761/2020|2020-03-22|EPI_ISL_537810|Original|hCoV-19^^Cantabria|Human|Hospital Universitario Marqu\u00e9s de Valdecilla (Santander)|SeqCOVID-SPAIN consortium/IBV(CSIC)|Comas|Spain
>Spike|hCoV-19/Luxembourg/LNS4614580/2020|2020-03-22|EPI_ISL_740510|Original|hCoV-19^^Luxembourg|Human|Laboratoire national de sant\u00e9|Laboratoire national de sant\u00e9|Wienecke-Baldacchino|Luxembourg
>Spike|hCoV-19/South Africa/NHLS-UCT-GS-1362/2020|2020-11-12|EPI_ISL_700550|Original|hCoV-19^^Western Cape|Human|Great Brak River Clinic wc GBC|NHLS/UCT|Iranzadeh|South Africa
>Spike|hCoV-19/England/CAMB-1ABC3F/2020|2020-04-20|EPI_ISL_470271|Original|hCoV-19^^England|Human|Department of Pathology|Wellcome Sanger Institute for the COVID-19 Genomics UK (COG-UK) consortium|Davies|United Kingdom
>Spike|hCoV-19/England/ALDP-B624E7/2020|2020-11-08|EPI_ISL_656476|Original|hCoV-19^^England|Human|Lighthouse Lab in Alderley Park|Wellcome Sanger Institute for the COVID-19 Genomics UK (COG-UK) Consortium|Davies|United Kingdom
>Spike|hCoV-19/Wales/QEUH-A63AA9/2020|2020-10-12|EPI_ISL_628662|Original|hCoV-19^^Wales|Human|Oxford Viromics|COVID-19 Genomics UK (COG-UK) Consortium|Golubchik|United Kingdom
>Spike|hCoV-19/England/QEUH-A9EE5E/2020|2020-10-20|EPI_ISL_611392|Original|hCoV-19^^England|Human|Lighthouse Lab in Glasgow|Wellcome Sanger Institute for the COVID-19 Genomics UK (COG-UK) consortium|Davies|United Kingdom
>Spike|hCoV-19/England/SHEF-CB65A/2020|2020-03-21|EPI_ISL_475439|Original|hCoV-19^^England|Human|Virology Department|COVID-19 Genomics UK (COG-UK) Consortium|de Silva|United Kingdom
>Spike|hCoV-19/England/MILK-BAAF1F/2020|2020-11-18|EPI_ISL_783265|Original|hCoV-19^^England|Human|Lighthouse Lab in Milton Keynes|Wellcome Sanger Institute for the COVID-19 Genomics UK (COG-UK) Consortium|Davies|United Kingdom
>Spike|hCoV-19/England/CAMB-1B74DE/2020|2020-10-19|EPI_ISL_637510|Original|hCoV-19^^England|Human|Department of Pathology|COVID-19 Genomics UK (COG-UK) Consortium|Goodfellow|United Kingdom
>Spike|hCoV-19/USA/WI-UW-722/2020|2020-06-30|EPI_ISL_491397|Original|hCoV-19^^Wisconsin|Human|University of Wisconsin-Madison AIDS Vaccine Research Laboratories|University of Wisconsin-Madison AIDS Vaccine Research Laboratories|Baczenas|USA
>Spike|hCoV-19/England/LOND-D3983/2020|2020-04-03|EPI_ISL_441015|Original|hCoV-19^^England|Human|University College London|COVID-19 Genomics UK (COG-UK) Consortium|Roy|United Kingdom
>Spike|hCoV-19/USA/TX-HMH-5879/2020|2020-06-30|EPI_ISL_547082|Original|hCoV-19^^Texas|Human|Houston Methodist Hospital|Houston Methodist Hospital|Long|USA
>Spike|hCoV-19/Netherlands/NB-EMC-398/2020|2020-00-00|EPI_ISL_722475|Original|hCoV-19^^Noord Brabant|Human|Dutch COVID-19 response team|Erasmus Medical Center|Oude Munnink|Netherlands
>Spike|hCoV-19/England/204960393/2020|2020-12-01|EPI_ISL_754346|Original|hCoV-19^^England|Human|Respiratory Virus Unit|COVID-19 Genomics UK (COG-UK) Consortium|Platt|United Kingdom
>Spike|hCoV-19/England/205160918/2020|2020-12-16|EPI_ISL_766424|Original|hCoV-19^^England|Human|Respiratory Virus Unit|COVID-19 Genomics UK (COG-UK) Consortium|Platt|United Kingdom
>Spike|hCoV-19/Belgium/rega-0803751/2020|2020-08-03|EPI_ISL_734896|Original|hCoV-19^^Merksem|Human|UZ Leuven|KU Leuven|Maes|Belgium
>Spike|hCoV-19/Canada/ON-PHL-20-00023/2020|2020-00-00|EPI_ISL_548704|Original|hCoV-19^^Ontario|Human|Public Health Ontario Laboratory|Public Health Ontario Laboratory|Guthrie|Canada

>Spike|hCoV-19/Morocco/FMP-59/2020|2020-12-21|EPI_ISL_775260|Original|hCoV-
19^^Temara|Human|Laboratoire Biolife|Laboratoire de Biotechnologie|Ouadghiri|Morocco
>Spike|hCoV-19/Japan/PG-8515/2020|2020-07-00|EPI_ISL_686111|Original|hCoV-
19^^Japan|Human|Pathogen Genomics Center|Pathogen Genomics Center|Sekizuka|Japan
>Spike|hCoV-19/England/MILK-9C3E60/2020|2020-09-09|EPI_ISL_566741|Original|hCoV-
19^^England|Human|Lighthouse Lab in Milton Keynes|Wellcome Sanger Institute for the
COVID-19 Genomics UK (COG-UK) consortium|Davies|United Kingdom
>Spike|hCoV-19/South Africa/KRISP-K002467/2020|2020-07-
01|EPI_ISL_518050|Original|hCoV-19^^KwaZulu-Natal|Human|NHLS-IALCH|KRISP|de
Oliveira|South Africa
>Spike|hCoV-19/Scotland/EDB6133/2020|2020-05-14|EPI_ISL_473878|Original|hCoV-
19^^Scotland|Human|Virology Department|COVID-19 Genomics UK (COG-UK)
Consortium|Williams|United Kingdom
>Spike|hCoV-19/Wales/PHWC-494FB5/2020|2020-11-25|EPI_ISL_726281|Original|hCoV-
19^^Wales|Human|Originating lab|COVID-19 Genomics UK (COG-UK) Consortium|Moore|United
Kingdom
>Spike|hCoV-19/England/20119089004/2020|2020-03-14|EPI_ISL_465123|Original|hCoV-
19^^England|Human|Respiratory Virus Unit|Respiratory Virus Unit|Platt|United Kingdom
>Spike|hCoV-19/USA/TX-HMH-MCoV-16089/2020|2020-10-27|EPI_ISL_785773|Original|hCoV-
19^^Texas|Human|Houston Methodist Hospital|Houston Methodist Hospital|Long|USA
>Spike|hCoV-19/India/MH-NIV-57774/2020|2020-06-10|EPI_ISL_541727|Original|hCoV-
19^^Maharashtra|Human|National Institute of Virology|National Institute of
Virology|Potdar|India
>Spike|hCoV-19/England/CAMB-7D0A4/2020|2020-04-07|EPI_ISL_442812|Original|hCoV-
19^^England|Human|Department of Pathology|Wellcome Sanger Institute for the COVID-19
Genomics UK (COG-UK) consortium|Amato|United Kingdom
>Spike|hCoV-19/England/CAMC-B53452/2020|2020-11-08|EPI_ISL_656977|Original|hCoV-
19^^England|Human|Lighthouse Lab in Cambridge|Wellcome Sanger Institute for the
COVID-19 Genomics UK (COG-UK) Consortium|Davies|United Kingdom
>Spike|hCoV-19/Norway/7016/2020|2020-12-17|EPI_ISL_775438|Original|hCoV-
19^^Nordland|Human|Nordland Hospital - Bodo|Norwegian Institute of Public
Health|Vollan|Norway
>Spike|hCoV-19/England/QEUH-964BA6/2020|2020-08-11|EPI_ISL_531209|Original|hCoV-
19^^England|Human|Lighthouse Lab in Glasgow|Wellcome Sanger Institute for the COVID-
19 Genomics UK (COG-UK) consortium|Davies|United Kingdom
>Spike|hCoV-19/Japan/PG-3304/2020|2020-04-00|EPI_ISL_691419|Original|hCoV-
19^^Japan|Human|Pathogen Genomics Center|Pathogen Genomics Center|Sekizuka|Japan
>Spike|hCoV-19/Singapore/1239/2020|2020-04-28|EPI_ISL_648656|Original|hCoV-
19^^Singapore|Human|Department of Laboratory Medicine|Department of Laboratory
Medicine|Chen|Singapore
>Spike|hCoV-19/England/ALDP-AA4DFE/2020|2020-10-21|EPI_ISL_630950|Original|hCoV-
19^^England|Human|Lighthouse Lab in Alderley Park|Wellcome Sanger Institute for the
COVID-19 Genomics UK (COG-UK) consortium|Davies|United Kingdom
>Spike|hCoV-19/Israel/13075703/2020|2020-03-29|EPI_ISL_447450|Original|hCoV-19^^Tel
Aviv District|Human|Clinical Microbiology Laboratory|Stern Lab|Miller|Israel
>Spike|hCoV-19/England/MILK-B97355/2020|2020-11-14|EPI_ISL_674901|Original|hCoV-
19^^England|Human|Lighthouse Lab in Milton Keynes|Wellcome Sanger Institute for the
COVID-19 Genomics UK (COG-UK) Consortium|Davies|United Kingdom
>Spike|hCoV-19/Scotland/QEUH-9F6778/2020|2020-09-27|EPI_ISL_600634|Original|hCoV-
19^^Scotland|Human|Lighthouse Lab in Glasgow|Wellcome Sanger Institute for the COVID-
19 Genomics UK (COG-UK) consortium|Davies|United Kingdom
>Spike|hCoV-19/Ireland/MH-NVRL-20G49920/2020|2020-04-07|EPI_ISL_681907|Original|hCoV-
19^^Meath|Human|National Virus Reference Laboratory|Irish Coronavirus Sequencing
Consortium - Teagasc Moorepark|Walsh|Ireland
>Spike|hCoV-19/Equatorial Guinea/9357/2020|2020-05-19|EPI_ISL_648353|Original|hCoV-
19^^Bioko Norte|Human|Laboratorio de Investigaciones de Baney|University Hospital
Basel|Hosch|Equatorial Guinea
>Spike|hCoV-19/Denmark/DCGC-13887/2020|2020-11-30|EPI_ISL_712246|Original|hCoV-
19^^Syddanmark|Human|Department of Virus and Microbiological Special
Diagnostics|Albertsen Lab|Michaelsen|Denmark

>Spike|hCoV-19/USA/TX-HMH-MCoV-14705/2020|2020-10-01|EPI_ISL_787990|Original|hCoV-19^^Texas|Human|Houston Methodist Hospital|Houston Methodist Hospital|Long|USA
>Spike|hCoV-19/England/CAMB-72740/2020|2020-03-23|EPI_ISL_439644|Original|hCoV-19^^England|Human|Department of Pathology|Wellcome Sanger Institute for the COVID-19 Genomics UK (COG-UK) consortium|Amato|United Kingdom
>Spike|hCoV-19/Malaysia/1121/2020|2020-03-24|EPI_ISL_501182|Original|hCoV-19^^Kuala Lumpur|Human|Department of Medical Microbiology|Department of Medical Microbiology|Chan|Malaysia
>Spike|hCoV-19/Netherlands/NA_343/2020|2020-04-04|EPI_ISL_455148|Original|hCoV-19^^Netherlands|Human|Dutch COVID-19 response team|Erasmus Medical Center|Oude Munnink|Netherlands
>Spike|hCoV-19/England/ALDP-B5C78D/2020|2020-11-08|EPI_ISL_655827|Original|hCoV-19^^England|Human|Lighthouse Lab in Alderley Park|Wellcome Sanger Institute for the COVID-19 Genomics UK (COG-UK) Consortium|Davies|United Kingdom
>Spike|hCoV-19/USA/NY-PV09163/2020|2020-03-21|EPI_ISL_422527|Original|hCoV-19^^New York|Human|MSHS Clinical Microbiology Laboratories|MSHS Pathogen Surveillance Program|van Bakel|USA
>Spike|hCoV-19/USA/MI-MDHHS-SC22212/2020|2020-10-26|EPI_ISL_644360|Original|hCoV-19^^Michigan|Human|Michigan Department of Health and Human Services|Michigan Department of Health and Human Services|Jacob|USA
>Spike|hCoV-19/England/QEUH-CADF0C/2020|2020-12-20|EPI_ISL_761302|Original|hCoV-19^^England|Human|Lighthouse Lab in Glasgow|Wellcome Sanger Institute for the COVID-19 Genomics UK (COG-UK) Consortium|Davies|United Kingdom
>Spike|hCoV-19/Portugal/PT1001/2020|2020-03-22|EPI_ISL_511549|Original|hCoV-19^^Portugal|Human|Instituto Nacional de Saude (INSA)|Instituto Nacional de Saude (INSA)|Borges|Portugal
>Spike|hCoV-19/Denmark/DCGC-14710/2020|2020-11-23|EPI_ISL_711923|Original|hCoV-19^^Hovedstaden|Human|Department of Virus and Microbiological Special Diagnostics|Albertsen Lab|Michaelsen|Denmark
>Spike|hCoV-19/England/CAMC-B7B533/2020|2020-11-12|EPI_ISL_741178|Original|hCoV-19^^England|Human|Oxford Viromics|COVID-19 Genomics UK (COG-UK) Consortium|Golubchik|United Kingdom
>Spike|hCoV-19/England/CAMB-7A5E4/2020|2020-04-02|EPI_ISL_441097|Original|hCoV-19^^England|Human|Department of Pathology|Wellcome Sanger Institute for the COVID-19 Genomics UK (COG-UK) consortium|Amato|United Kingdom
>Spike|hCoV-19/Denmark/DCGC-22015/2020|2020-12-14|EPI_ISL_758512|Original|hCoV-19^^Hovedstaden|Human|Department of Virus and Microbiological Special Diagnostics|Albertsen Lab|Michaelsen|Denmark
>Spike|hCoV-19/Norway/6731/2020|2020-12-06|EPI_ISL_775407|Original|hCoV-19^^Vestland|Human|Haukeland University Hospital|Norwegian Institute of Public Health|Vollan|Norway
>Spike|hCoV-19/England/MILK-9709DA/2020|2020-08-18|EPI_ISL_553200|Original|hCoV-19^^England|Human|Lighthouse Lab in Milton Keynes|Wellcome Sanger Institute for the COVID-19 Genomics UK (COG-UK) consortium|Davies|United Kingdom
>Spike|hCoV-19/England/MILK-9F2B07/2020|2020-09-25|EPI_ISL_589242|Original|hCoV-19^^England|Human|Lighthouse Lab in Milton Keynes|Wellcome Sanger Institute for the COVID-19 Genomics UK (COG-UK) consortium|Davies|United Kingdom
>Spike|hCoV-19/Wales/PHWC-34197/2020|2020-04-17|EPI_ISL_446881|Original|hCoV-19^^Wales|Human|Wales Specialist Virology Centre|Public Health Wales Microbiology Cardiff|Moore|United Kingdom
>Spike|hCoV-19/Sweden/20-05856/2020|2020-03-17|EPI_ISL_434649|Original|hCoV-19^^Dalarna|Human|Svardsjo VC|The Public Health Agency of Sweden|Svartstr\u00f6m|Sweden
>Spike|hCoV-19/DRC/3688/2020|2020-04-24|EPI_ISL_447237|Original|hCoV-19^^Kinshasa|Human|Viral Respiratory Lab|Pathogen Sequencing Lab|Amuri|Democratic Republic of the Congo
>Spike|hCoV-19/England/CAMC-A3E6B2/2020|2020-10-05|EPI_ISL_609628|Original|hCoV-19^^England|Human|Lighthouse Lab in Cambridge|Wellcome Sanger Institute for the COVID-19 Genomics UK (COG-UK) consortium|Davies|United Kingdom

>Spike|hCoV-19/England/MILK-AC9616/2020|2020-10-21|EPI_ISL_629157|Original|hCoV-19^^England|Human|Lighthouse Lab in Milton Keynes|Wellcome Sanger Institute for the COVID-19 Genomics UK (COG-UK) consortium|Davies|United Kingdom
>Spike|hCoV-19/England/ALDP-AA3BD7/2020|2020-10-22|EPI_ISL_624238|Original|hCoV-19^^England|Human|Lighthouse Lab in Alderley Park|Wellcome Sanger Institute for the COVID-19 Genomics UK (COG-UK) consortium|Davies|United Kingdom
>Spike|hCoV-19/Scotland/QEUH-9FB421/2020|2020-09-29|EPI_ISL_600556|Original|hCoV-19^^Scotland|Human|Lighthouse Lab in Glasgow|Wellcome Sanger Institute for the COVID-19 Genomics UK (COG-UK) consortium|Davies|United Kingdom
>Spike|hCoV-19/England/204490335/2020|2020-10-29|EPI_ISL_637179|Original|hCoV-19^^England|Human|Respiratory Virus Unit|COVID-19 Genomics UK (COG-UK) Consortium|Platt|United Kingdom
>Spike|hCoV-19/Scotland/CVR2263/2020|2020-04-11|EPI_ISL_453037|Original|hCoV-19^^Scotland|Human|West of Scotland Specialist Virology Centre|COVID-19 Genomics UK (COG-UK) Consortium|Robertson|United Kingdom
>Spike|hCoV-19/Australia/VIC5136/2020|2020-07-19|EPI_ISL_519939|Original|hCoV-19^^Victoria|Human|Microbiological Diagnostic Unit - Public Health Laboratory (MDU-PHL)|MDU-PHL|Schultz|Australia
>Spike|hCoV-19/England/SHEF-C8963/2020|2020-04-01|EPI_ISL_432454|Original|hCoV-19^^England|Human|Virology Department|COVID-19 Genomics UK (COG-UK) Consortium|de Silva|United Kingdom
>Spike|hCoV-19/England/ALDP-A4FA76/2020|2020-10-07|EPI_ISL_597866|Original|hCoV-19^^England|Human|Lighthouse Lab in Alderley Park|Wellcome Sanger Institute for the COVID-19 Genomics UK (COG-UK) consortium|Davies|United Kingdom
>Spike|hCoV-19/India/DL-NCDC-3982/2020|2020-04-09|EPI_ISL_436445|Original|hCoV-19^^Delhi|Human|National Centre for Disease control (NCDC)|NCDC/CSIR-IGIB|Singh|India
>Spike|hCoV-19/India/KA-nimh-4376/2020|2020-04-24|EPI_ISL_436140|Original|hCoV-19^^Karnataka|Human|District Surveillance Unit|Department of Neurovirology|Pattabiraman|India
>Spike|hCoV-19/Japan/PG-6442/2020|2020-08-00|EPI_ISL_689548|Original|hCoV-19^^Japan|Human|Pathogen Genomics Center|Pathogen Genomics Center|Sekizuka|Japan
>Spike|hCoV-19/England/ALDP-9E8102/2020|2020-09-24|EPI_ISL_637299|Original|hCoV-19^^England|Human|Department of Pathology|COVID-19 Genomics UK (COG-UK) Consortium|Goodfellow|United Kingdom
>Spike|hCoV-19/USA/TX-HMH-5352/2020|2020-06-25|EPI_ISL_544708|Original|hCoV-19^^Texas|Human|Houston Methodist Hospital|Houston Methodist Hospital|Long|USA
>Spike|hCoV-19/USA/ME-HETL-J0005/2020|2020-03-13|EPI_ISL_513418|Original|hCoV-19^^Maine|Human|Maine HETL|Tewhey Lab|Tewhey|USA
>Spike|hCoV-19/mink/Netherlands/NB-EMC-15-3/2020|2020-06-21|EPI_ISL_523025|Original|hCoV-19^^Noord Brabant|Neovison vison|Dutch COVID-19 response team|Erasmus Medical Center|Oude Munnink|Netherlands
>Spike|hCoV-19/England/20144049604/2020|2020-03-31|EPI_ISL_421949|Original|hCoV-19^^England|Human|Respiratory Virus Unit|Respiratory Virus Unit|Platt|United Kingdom
>Spike|hCoV-19/England/CAMC-B3821D/2020|2020-11-05|EPI_ISL_658979|Original|hCoV-19^^England|Human|Lighthouse Lab in Cambridge|Wellcome Sanger Institute for the COVID-19 Genomics UK (COG-UK) Consortium|Davies|United Kingdom
>Spike|hCoV-19/Australia/VIC14786/2020|2020-08-17|EPI_ISL_592998|Original|hCoV-19^^Victoria|Human|Microbiological Diagnostic Unit - Public Health Laboratory (MDU-PHL)|MDU-PHL|Schultz|Australia
>Spike|hCoV-19/USA/NM-DOH-2020315376/2020|2020-08-27|EPI_ISL_732708|Original|hCoV-19^^New Mexico|Human|New Mexico Department of Health Scientific Laboratory|New Mexico Department of Health Scientific Laboratory|Johnson|USA
>Spike|hCoV-19/South Africa/N00360/2020|2020-09-29|EPI_ISL_712077|Original|hCoV-19^^Eastern Cape|Human|East London NHLS\u00a0Laboratory|National Institute for Communicable Diseases of the National Health Laboratory Service|Bhiman|South Africa
>Spike|hCoV-19/USA/TX-HMH-MCoV-18563/2020|2020-11-19|EPI_ISL_784911|Original|hCoV-19^^Texas|Human|Houston Methodist Hospital|Houston Methodist Hospital|Long|USA
>Spike|hCoV-19/Scotland/CVR4676/2020|2020-09-29|EPI_ISL_611548|Original|hCoV-19^^Scotland|Human|West of Scotland Specialist Virology Centre|COVID-19 Genomics UK (COG-UK) Consortium|Robertson|United Kingdom

>Spike|hCoV-19/England/ALDP-A2E6F1/2020|2020-10-05|EPI_ISL_586777|Original|hCoV-19^^England|Human|Lighthouse Lab in Alderley Park|Wellcome Sanger Institute for the COVID-19 Genomics UK (COG-UK) consortium|Davies|United Kingdom
>Spike|hCoV-19/Australia/VIC11467/2020|2020-08-30|EPI_ISL_563385|Original|hCoV-19^^Victoria|Human|Microbiological Diagnostic Unit - Public Health Laboratory (MDU-PHL)|MDU-PHL|Schultz|Australia
>Spike|hCoV-19/USA/TX-HMH-2724/2020|2020-06-06|EPI_ISL_545507|Original|hCoV-19^^Texas|Human|Houston Methodist Hospital|Houston Methodist Hospital|Long|USA
>Spike|hCoV-19/Australia/VIC4105/2020|2020-07-14|EPI_ISL_519485|Original|hCoV-19^^Victoria|Human|Microbiological Diagnostic Unit - Public Health Laboratory (MDU-PHL)|MDU-PHL|Schultz|Australia
>Spike|hCoV-19/Wales/PHWC-4A245B/2020|2020-12-12|EPI_ISL_743890|Original|hCoV-19^^Wales|Human|Wales Specialist Virology Centre Sequencing lab|COVID-19 Genomics UK (COG-UK) Consortium|Moore|United Kingdom
>Spike|hCoV-19/Northern Ireland/NIRE-233178/2020|2020-09-26|EPI_ISL_680260|Original|hCoV-19^^Northern Ireland|Human|Regional Virus Laboratory|COVID-19 Genomics UK (COG-UK) Consortium|Fairley|United Kingdom
>Spike|hCoV-19/USA/TX-HMH-MCoV-13177/2020|2020-08-25|EPI_ISL_788328|Original|hCoV-19^^Texas|Human|Houston Methodist Hospital|Houston Methodist Hospital|Long|USA
>Spike|hCoV-19/Wales/PHWC-49AB8E/2020|2020-12-03|EPI_ISL_727654|Original|hCoV-19^^Wales|Human|Originating lab|COVID-19 Genomics UK (COG-UK) Consortium|Moore|United Kingdom
>Spike|hCoV-19/Luxembourg/LNS3825568/2020|2020-11-26|EPI_ISL_740377|Original|hCoV-19^^Luxembourg|Human|Laboratoire national de sant\u00e9|Laboratoire national de sant\u00e9|Wienecke-Baldacchino|Luxembourg
>Spike|hCoV-19/Denmark/DCGC-9501/2020|2020-11-09|EPI_ISL_668540|Original|hCoV-19^^Midtjylland|Human|Department of Virus and Microbiological Special Diagnostics|Albertsen Lab|Michaelsen|Denmark
>Spike|hCoV-19/South Africa/UFS-VIRO-NGS-25/2020|2020-10-16|EPI_ISL_682331|Original|hCoV-19^^Free State|Human|NHLS Universitas Academic|UFS Virology|Bester|South Africa
>Spike|hCoV-19/South Korea/KCDC2742/2020|2020-07-18|EPI_ISL_522521|Original|hCoV-19^^South Korea|Human|Division of Viral Diseases|Division of Viral Diseases|Kim|South Korea
>Spike|hCoV-19/New Zealand/20VR1887/2020|2020-04-02|EPI_ISL_456294|Original|hCoV-19^^Waitemata|Human|North Shore Hospital|Institute of Environmental Science and Research (ESR)|de Ligt|New Zealand
>Spike|hCoV-19/England/NORW-F637D/2020|2020-11-23|EPI_ISL_703187|Original|hCoV-19^^England|Human|Quadram Institute Bioscience|COVID-19 Genomics UK (COG-UK) Consortium|Alikhan|United Kingdom
>Spike|hCoV-19/United Arab Emirates/0914/2020|2020-06-26|EPI_ISL_699052|Original|hCoV-19^^Abu Dhabi|Human|Group 42 (G42) Healthcare|G42 Healthcare|Ogrodzki|United Arab Emirates
>Spike|hCoV-19/India/GJ-GBRC64/2020|2020-05-03|EPI_ISL_447039|Original|hCoV-19^^Gujarat|Human|B.J. Medical College and Civil hospital|Gujarat Biotechnology Research Centre|Joshi|India
>Spike|hCoV-19/USA/TX-HMH-MCoV-16226/2020|2020-10-27|EPI_ISL_785910|Original|hCoV-19^^Texas|Human|Houston Methodist Hospital|Houston Methodist Hospital|Long|USA
>Spike|hCoV-19/Netherlands/NB-EMC-255/2020|2020-06-16|EPI_ISL_523378|Original|hCoV-19^^Noord Brabant|Human|Dutch COVID-19 response team|Erasmus Medical Center|Oude Munnink|Netherlands
>Spike|hCoV-19/Belgium/rega-0915957/2020|2020-09-15|EPI_ISL_734947|Original|hCoV-19^^Overijse|Human|UZ Leuven|KU Leuven|Maes|Belgium
>Spike|hCoV-19/Australia/VIC7026/2020|2020-07-24|EPI_ISL_564199|Original|hCoV-19^^Victoria|Human|Victorian Infectious Diseases Reference Laboratory (VIDRL)|VIDRL and MDU-PHL|Schultz|Australia
>Spike|hCoV-19/USA/UT-QDX-2454/2020|2020-03-13|EPI_ISL_604846|Original|hCoV-19^^Utah|Human|Quest Diagnostics|Quest Diagnostics|Kagan|USA

>Spike|hCoV-19/Spain/MD-IBV-99012477/2020|2020-11-14|EPI_ISL_780057|Original|hCoV-19^^Madrid|Human|Hospital General Universitario Gregorio Mara\u00f1\u00f3n|SeqCOVID-SPAIN consortium/IBV(CSIC)|Comas|Spain
>Spike|hCoV-19/England/QEUH-C30B46/2020|2020-12-11|EPI_ISL_731505|Original|hCoV-19^^England|Human|Lighthouse Lab in Glasgow|Wellcome Sanger Institute for the COVID-19 Genomics UK (COG-UK) Consortium|Davies|United Kingdom
>Spike|hCoV-19/England/LIVE-A48CA/2020|2020-03-09|EPI_ISL_500052|Original|hCoV-19^^England|Human|Liverpool Clinical Laboratories|COVID-19 Genomics UK (COG-UK) Consortium|Haldenby|United Kingdom
>Spike|hCoV-19/Spain/CE-ISCIII-2016516/2020|2020-10-14|EPI_ISL_691695|Original|hCoV-19^^Ceuta|Human|Hospital Universitario de Ceuta|Instituto de Salud Carlos III|Caballero|Spain
>Spike|hCoV-19/Wales/PHWC-169C3D/2020|2020-08-13|EPI_ISL_517607|Original|hCoV-19^^Wales|Human|Wales Specialist Virology Centre Sequencing lab|COVID-19 Genomics UK (COG-UK) Consortium|Moore|United Kingdom
>Spike|hCoV-19/England/ALDP-9E773E/2020|2020-09-24|EPI_ISL_638214|Original|hCoV-19^^England|Human|Department of Pathology|COVID-19 Genomics UK (COG-UK) Consortium|Goodfellow|United Kingdom
>Spike|hCoV-19/USA/CT-Yale-261/2020|2020-04-01|EPI_ISL_469187|Original|hCoV-19^^Connecticut|Human|Yale Clinical Virology Laboratory|Grubaugh Lab - Yale School of Public Health|Grubaugh|USA
>Spike|hCoV-19/England/CAMC-C737E9/2020|2020-12-15|EPI_ISL_769068|Original|hCoV-19^^England|Human|Lighthouse Lab in Cambridge|Wellcome Sanger Institute for the COVID-19 Genomics UK (COG-UK) Consortium|Davies|United Kingdom
>Spike|hCoV-19/Denmark/DCGC-15333/2020|2020-11-23|EPI_ISL_713768|Original|hCoV-19^^Hovedstaden|Human|Department of Virus and Microbiological Special Diagnostics|Albertsen Lab|Michaelsen|Denmark
>Spike|hCoV-19/England/MILK-ABBC9D/2020|2020-10-20|EPI_ISL_631091|Original|hCoV-19^^England|Human|Lighthouse Lab in Milton Keynes|Wellcome Sanger Institute for the COVID-19 Genomics UK (COG-UK) consortium|Davies|United Kingdom
>Spike|hCoV-19/USA/CT-Yale-591/2020|2020-04-02|EPI_ISL_738285|Original|hCoV-19^^Connecticut|Human|Connecticut Veterans' Affairs Hospital|Grubaugh Lab - Yale School of Public Health|Grubaugh|USA
>Spike|hCoV-19/Luxembourg/LNS9385975/2020|2020-10-24|EPI_ISL_744897|Original|hCoV-19^^Luxembourg|Human|Laboratoire national de sant\u00e9|Laboratoire national de sant\u00e9|Wienecke-Baldacchino|Luxembourg
>Spike|hCoV-19/England/MILK-A7A08F/2020|2020-10-15|EPI_ISL_606851|Original|hCoV-19^^England|Human|Lighthouse Lab in Milton Keynes|Wellcome Sanger Institute for the COVID-19 Genomics UK (COG-UK) consortium|Davies|United Kingdom
>Spike|hCoV-19/England/SHEF-10D597F/2020|2020-12-18|EPI_ISL_742080|Original|hCoV-19^^England|Human|Virology Department|COVID-19 Genomics UK (COG-UK) Consortium|de Silva|United Kingdom
>Spike|hCoV-19/England/MILK-B060D2/2020|2020-10-24|EPI_ISL_642800|Original|hCoV-19^^England|Human|Lighthouse Lab in Milton Keynes|Wellcome Sanger Institute for the COVID-19 Genomics UK (COG-UK) Consortium|Davies|United Kingdom
>Spike|hCoV-19/England/ALDP-A700F2/2020|2020-10-15|EPI_ISL_608064|Original|hCoV-19^^England|Human|Lighthouse Lab in Alderley Park|Wellcome Sanger Institute for the COVID-19 Genomics UK (COG-UK) consortium|Davies|United Kingdom
>Spike|hCoV-19/Wales/PHWC-498E3C/2020|2020-11-28|EPI_ISL_727217|Original|hCoV-19^^Wales|Human|Originating lab|COVID-19 Genomics UK (COG-UK) Consortium|Moore|United Kingdom
>Spike|hCoV-19/Chile/RM-157308/2020|2020-08-20|EPI_ISL_746674|Original|hCoV-19^^Region Metropolitana de Santiago|Human|Genetica Molecular and Subdepartamento de Virologia ISP Chile|Instituto de Salud Publica de Chile|Tognarelli|Chile
>Spike|hCoV-19/Australia/VIC5268/2020|2020-00-00|EPI_ISL_520033|Original|hCoV-19^^Victoria|Human|Microbiological Diagnostic Unit - Public Health Laboratory (MDU-PHL)|MDU-PHL|Schultz|Australia
>Spike|hCoV-19/USA/TX-HMH-MCoV-15720/2020|2020-07-09|EPI_ISL_785404|Original|hCoV-19^^Texas|Human|Houston Methodist Hospital|Houston Methodist Hospital|Long|USA

>Spike|hCoV-19/USA/TX-DSHS-1790/2020|2020-06-13|EPI_ISL_738165|Original|hCoV-19^^Texas|Human|Texas Department of State Health Services|Texas Department of State Health Services|Rahman|USA
>Spike|hCoV-19/England/CAMC-A650D2/2020|2020-10-11|EPI_ISL_608681|Original|hCoV-19^^England|Human|Lighthouse Lab in Cambridge|Wellcome Sanger Institute for the COVID-19 Genomics UK (COG-UK) Consortium|Davies|United Kingdom
>Spike|hCoV-19/USA/LA-GBCL-26684504/2020|2020-04-10|EPI_ISL_485904|Original|hCoV-19^^Louisiana|Human|River Road Testing Lab|Ginkgo Bioworks Clinical Laboratory|Smith|USA
>Spike|hCoV-19/England/ALDP-A6889B/2020|2020-10-12|EPI_ISL_608384|Original|hCoV-19^^England|Human|Lighthouse Lab in Alderley Park|Wellcome Sanger Institute for the COVID-19 Genomics UK (COG-UK) consortium|Davies|United Kingdom
>Spike|hCoV-19/India/MH-ACTREC-189/2020|2020-06-22|EPI_ISL_699843|Original|hCoV-19^^Maharashtra|Human|Hematopathology Laboratory|Hematopathology Laboratory|Patkar|India
>Spike|hCoV-19/Wales/PHWC-35BE3/2020|2020-04-27|EPI_ISL_474541|Original|hCoV-19^^Wales|Human|Originating lab|COVID-19 Genomics UK (COG-UK) Consortium|Moore|United Kingdom
>Spike|hCoV-19/Poland/PL_MCB_17/2020|2020-03-28|EPI_ISL_645016|Original|hCoV-19^^Poland|Human|Human Genome Variation Research Group|Human Genome Variation Research Group||Poland
>Spike|hCoV-19/India/TG-CCMB-AC329/2020|2020-08-19|EPI_ISL_539704|Original|hCoV-19^^Telangana|Human|CSIR-Centre for Cellular and Molecular Biology|CSIR-Centre for Cellular and Molecular Biology|Mukherjee|India
>Spike|hCoV-19/USA/MA-MGH-02730/2020|2020-09-29|EPI_ISL_765603|Original|hCoV-19^^Massachusetts|Human|Brigham and Womens Hospital|Infectious Disease Program|Park|USA
>Spike|hCoV-19/England/QEUH-A9CE8D/2020|2020-10-20|EPI_ISL_652643|Original|hCoV-19^^England|Human|Queens Medical Centre|COVID-19 Genomics UK (COG-UK) Consortium|Loose|United Kingdom
>Spike|hCoV-19/England/OXON-B3734/2020|2020-04-01|EPI_ISL_534884|Original|hCoV-19^^England|Human|Oxford Viromics|COVID-19 Genomics UK (COG-UK) Consortium|Golubchik|United Kingdom
>Spike|hCoV-19/England/CAMC-9DFC85/2020|2020-09-21|EPI_ISL_576928|Original|hCoV-19^^England|Human|Oxford Viromics|COVID-19 Genomics UK (COG-UK) Consortium|Golubchik|United Kingdom
>Spike|hCoV-19/England/ALDP-CB356B/2020|2020-12-20|EPI_ISL_761317|Original|hCoV-19^^England|Human|Lighthouse Lab in Alderley Park|Wellcome Sanger Institute for the COVID-19 Genomics UK (COG-UK) Consortium|Davies|United Kingdom
>Spike|hCoV-19/Denmark/DCGC-13539/2020|2020-11-30|EPI_ISL_712866|Original|hCoV-19^^Sjaelland|Human|Department of Virus and Microbiological Special Diagnostics|Albertsen Lab|Michaelsen|Denmark
>Spike|hCoV-19/Japan/PG-3815/2020|2020-06-00|EPI_ISL_692066|Original|hCoV-19^^Japan|Human|Pathogen Genomics Center|Pathogen Genomics Center|Sekizuka|Japan
>Spike|hCoV-19/USA/TX-HMH-MCoV-12982/2020|2020-08-28|EPI_ISL_788133|Original|hCoV-19^^Texas|Human|Houston Methodist Hospital|Houston Methodist Hospital|Long|USA
>Spike|hCoV-19/USA/WI-UW-1295/2020|2020-09-01|EPI_ISL_536667|Original|hCoV-19^^Wisconsin|Human|University of Wisconsin-Madison AIDS Vaccine Research Laboratories|University of Wisconsin-Madison AIDS Vaccine Research Laboratories|Baczenas|USA
>Spike|hCoV-19/France/PAC-IHU-0500/2020|2020-04-03|EPI_ISL_569555|Original|hCoV-19^^Provence-Alpes-C\u00f4te-d\u2019Azur|Human|MEPHI|MEPHI|Levasseur|France
>Spike|hCoV-19/Denmark/DCGC-10245/2020|2020-11-09|EPI_ISL_669855|Original|hCoV-19^^Hovedstaden|Human|Department of Virus and Microbiological Special Diagnostics|Albertsen Lab|Michaelsen|Denmark
>Spike|hCoV-19/England/OXON-ACC11/2020|2020-04-05|EPI_ISL_448519|Original|hCoV-19^^England|Human|Oxford Viromics|COVID-19 Genomics UK (COG-UK) Consortium|Golubchik|United Kingdom
>Spike|hCoV-19/Spain/RI-IBV-001375/2020|2020-03-13|EPI_ISL_452526|Original|hCoV-19^^La Rioja|Human|Cl\u00ednica Universidad de Navarra. Servicio de Enfermedades

Infecciosas y Microbiolog\u00eda cl\u00ednica|SeqCOVID-SPAIN
consortium/IBV(CSIC)|Comas|Spain
>Spike|hCoV-19/England/NORT-283FAC/2020|2020-04-27|EPI_ISL_472259|Original|hCoV-19^^England|Human|Northumbria University / South Tees Hospitals NHS Foundation Trust / North Cumbria Integrated Care NHS Foundation Trust / North Tees and Hartlepool NHS Foundation Trust / Newcastle Hospitals NHS Foundation Trust|COVID-19 Genomics UK (COG-UK) Consortium|Bashton|United Kingdom
>Spike|hCoV-19/USA/WA-S397/2020|2020-03-25|EPI_ISL_434157|Original|hCoV-19^^Washington|Human|Washington State Department of Health|Seattle Flu Study|Bedford|USA
>Spike|hCoV-19/Belgium/rega-0916959/2020|2020-09-16|EPI_ISL_734952|Original|hCoV-19^^Bost|Human|UZ Leuven|KU Leuven|Maes|Belgium
>Spike|hCoV-19/England/MILK-BB04EA/2020|2020-11-17|EPI_ISL_720158|Original|hCoV-19^^England|Human|Lighthouse Lab in Milton Keynes|Wellcome Sanger Institute for the COVID-19 Genomics UK (COG-UK) Consortium|Davies|United Kingdom
>Spike|hCoV-19/Scotland/EDB5811/2020|2020-04-27|EPI_ISL_487906|Original|hCoV-19^^Scotland|Human|Virology Department|Wellcome Sanger Institute for the COVID-19 Genomics UK (COG-UK) consortium|Davies|United Kingdom
>Spike|hCoV-19/USA/AZ-TG482920/2020|2020-07-08|EPI_ISL_695074|Original|hCoV-19^^Arizona|Human|TGen North|TGen North|French|USA
>Spike|hCoV-19/England/LIVE-A5AE1/2020|2020-03-17|EPI_ISL_499627|Original|hCoV-19^^England|Human|Liverpool Clinical Laboratories|COVID-19 Genomics UK (COG-UK) Consortium|Haldenby|United Kingdom
>Spike|hCoV-19/Netherlands/NB-EMC-427/2020|2020-11-16|EPI_ISL_722651|Original|hCoV-19^^Noord Brabant|Human|Dutch COVID-19 response team|Erasmus Medical Center|Oude Munnink|Netherlands
>Spike|hCoV-19/England/QEUH-98FEF7/2020|2020-08-26|EPI_ISL_540007|Original|hCoV-19^^England|Human|Lighthouse Lab in Glasgow|Wellcome Sanger Institute for the COVID-19 Genomics UK (COG-UK) consortium|Davies|United Kingdom
>Spike|hCoV-19/Scotland/QEUH-961FF8/2020|2020-08-10|EPI_ISL_531595|Original|hCoV-19^^Scotland|Human|NHSGGC West of Scotland Specialist Virology Centre / MRC-University of Glasgow Centre for Virus Research|Wellcome Sanger Institute for the COVID-19 Genomics UK (COG-UK) consortium|Davies|United Kingdom
>Spike|hCoV-19/Netherlands/NA_168/2020|2020-03-16|EPI_ISL_422703|Original|hCoV-19^^Netherlands|Human|Dutch COVID-19 response team|Erasmus Medical Center|Oude Munnink|Netherlands
>Spike|hCoV-19/Wales/PHWC-482952/2020|2020-10-19|EPI_ISL_613254|Original|hCoV-19^^Wales|Human|Wales Specialist Virology Centre Sequencing lab|COVID-19 Genomics UK (COG-UK) Consortium|Moore|United Kingdom
>Spike|hCoV-19/Canada/BC_03489456/2020|2020-04-00|EPI_ISL_466752|Original|hCoV-19^^British Columbia|Human|BCCDC Public Health Laboratory|BCCDC Public Health Laboratory|Harrigan|Canada
>Spike|hCoV-19/England/CAMC-9B1B31/2020|2020-09-04|EPI_ISL_566638|Original|hCoV-19^^England|Human|Lighthouse Lab in Cambridge|Wellcome Sanger Institute for the COVID-19 Genomics UK (COG-UK) consortium|Davies|United Kingdom
>Spike|hCoV-19/England/QEUH-BA35C9/2020|2020-11-18|EPI_ISL_721159|Original|hCoV-19^^England|Human|Lighthouse Lab in Glasgow|Wellcome Sanger Institute for the COVID-19 Genomics UK (COG-UK) Consortium|Davies|United Kingdom
>Spike|hCoV-19/England/CAMC-B5176A/2020|2020-11-08|EPI_ISL_658366|Original|hCoV-19^^England|Human|Lighthouse Lab in Cambridge|Wellcome Sanger Institute for the COVID-19 Genomics UK (COG-UK) Consortium|Davies|United Kingdom
>Spike|hCoV-19/England/ALDP-94A71B/2020|2020-06-11|EPI_ISL_556356|Original|hCoV-19^^England|Human|Lighthouse Lab in Alderley Park|Wellcome Sanger Institute for the COVID-19 Genomics UK (COG-UK) consortium|Davies|United Kingdom
>Spike|hCoV-19/USA/WA-S67/2020|2020-03-04|EPI_ISL_417120|Original|hCoV-19^^Washington|Human|Washington State Department of Health|Seattle Flu Study|Bedford|USA
>Spike|hCoV-19/England/SHEF-CC9C9/2020|2020-11-27|EPI_ISL_702776|Original|hCoV-19^^England|Human|Virology Department|COVID-19 Genomics UK (COG-UK) Consortium|de Silva|United Kingdom

>Spike|hCoV-19/Wales/ALDP-C04DCD/2020|2020-12-03|EPI_ISL_767304|Original|hCoV-19^^Wales|Human|Lighthouse Lab in Alderley Park|Wellcome Sanger Institute for the COVID-19 Genomics UK (COG-UK) Consortium|Davies|United Kingdom
>Spike|hCoV-19/England/MILK-A7B525/2020|2020-10-15|EPI_ISL_607551|Original|hCoV-19^^England|Human|Lighthouse Lab in Milton Keynes|Wellcome Sanger Institute for the COVID-19 Genomics UK (COG-UK) consortium|Davies|United Kingdom
>Spike|hCoV-19/Wales/PHWC-163C9D/2020|2020-04-23|EPI_ISL_474065|Original|hCoV-19^^Wales|Human|Originating lab|COVID-19 Genomics UK (COG-UK) Consortium|Moore|United Kingdom
>Spike|hCoV-19/England/LIVE-9C82F/2020|2020-03-21|EPI_ISL_449584|Original|hCoV-19^^England|Human|Liverpool Clinical Laboratories|COVID-19 Genomics UK (COG-UK) Consortium|Haldenby|United Kingdom
>Spike|hCoV-19/England/BRIS-123A53/2020|2020-04-07|EPI_ISL_442716|Original|hCoV-19^^England|Human|PHE South West Regional Laboratory|Wellcome Sanger Institute for the COVID-19 Genomics UK (COG-UK) consortium|Amato|United Kingdom
>Spike|hCoV-19/Australia/VIC5808/2020|2020-07-19|EPI_ISL_518603|Original|hCoV-19^^Victoria|Human|Microbiological Diagnostic Unit - Public Health Laboratory (MDU-PHL)|MDU-PHL|Schultz|Australia
>Spike|hCoV-19/England/ALDP-CB463A/2020|2020-12-19|EPI_ISL_761336|Original|hCoV-19^^England|Human|Lighthouse Lab in Alderley Park|Wellcome Sanger Institute for the COVID-19 Genomics UK (COG-UK) Consortium|Davies|United Kingdom
>Spike|hCoV-19/England/NORW-E78BC/2020|2020-05-05|EPI_ISL_449081|Original|hCoV-19^^England|Human|Quadram Institute Bioscience|COVID-19 Genomics UK (COG-UK) Consortium|Alikhan|United Kingdom
>Spike|hCoV-19/USA/MN-QDX-1021/2020|2020-03-16|EPI_ISL_571747|Original|hCoV-19^^Minnesota|Human|Quest Diagnostics|Quest Diagnostics|Kagan|USA
>Spike|hCoV-19/Wales/PHWC-494C63/2020|2020-11-24|EPI_ISL_726237|Original|hCoV-19^^Wales|Human|Originating lab|COVID-19 Genomics UK (COG-UK) Consortium|Moore|United Kingdom
>Spike|hCoV-19/England/QEUH-9B749A/2020|2020-09-07|EPI_ISL_590541|Original|hCoV-19^^England|Human|Lighthouse Lab in Glasgow|Wellcome Sanger Institute for the COVID-19 Genomics UK (COG-UK) consortium|Davies|United Kingdom
>Spike|hCoV-19/USA/WI-UW-111/2020|2020-03-31|EPI_ISL_425168|Original|hCoV-19^^Wisconsin|Human|University of Wisconsin-Madison AIDS Vaccine Research Laboratories|University of Wisconsin-Madison AIDS Vaccine Research Laboratories|Baczenas|USA
>Spike|hCoV-19/India/WB-IK96/2020|2020-05-24|EPI_ISL_508412|Original|hCoV-19^^West Bengal|Human|Institute of Post Graduate Medical Education & Research|National Institute of Biomedical Genomics|Maitra|India
>Spike|hCoV-19/Netherlands/ZH-EMC-917/2020|2020-11-04|EPI_ISL_722723|Original|hCoV-19^^Zuid Holland|Human|Dutch COVID-19 response team|Erasmus Medical Center|Oude Munnink|Netherlands
>Spike|hCoV-19/South Korea/KDCA0116/2020|2020-09-29|EPI_ISL_747351|Original|hCoV-19^^South Korea|Human|Division of Emerging Infectious Diseases|Division of Emerging Infectious Diseases|Park|South Korea
>Spike|hCoV-19/USA/TX-DSHS-1995/2020|2020-06-21|EPI_ISL_766772|Original|hCoV-19^^Texas|Human|Texas Department of State Health Services|Texas Department of State Health Services|Rahman|USA
>Spike|hCoV-19/Wales/PHWC-496DF5/2020|2020-11-25|EPI_ISL_726732|Original|hCoV-19^^Wales|Human|Originating lab|COVID-19 Genomics UK (COG-UK) Consortium|Moore|United Kingdom
>Spike|hCoV-19/England/CAMC-B336CB/2020|2020-11-05|EPI_ISL_659215|Original|hCoV-19^^England|Human|Lighthouse Lab in Cambridge|Wellcome Sanger Institute for the COVID-19 Genomics UK (COG-UK) Consortium|Davies|United Kingdom
>Spike|hCoV-19/USA/FL-BPHL-1530/2020|2020-07-29|EPI_ISL_568628|Original|hCoV-19^^Florida|Human|Florida Bureau of Public Health Laboratories|Florida Bureau of Public Health Laboratories|Blanton|USA
>Spike|hCoV-19/England/ALDP-92AACD/2020|2020-06-22|EPI_ISL_552882|Original|hCoV-19^^England|Human|Lighthouse Lab in Alderley Park|Wellcome Sanger Institute for the COVID-19 Genomics UK (COG-UK) consortium|Davies|United Kingdom

>Spike|hCoV-19/Wales/MILK-A069F6/2020|2020-10-01|EPI_ISL_600067|Original|hCoV-19^^Wales|Human|Lighthouse Lab in Milton Keynes|Wellcome Sanger Institute for the COVID-19 Genomics UK (COG-UK) consortium|Davies|United Kingdom
>Spike|hCoV-19/India/GJ-GBRC153a/2020|2020-04-27|EPI_ISL_461481|Original|hCoV-19^^Gujarat|Human|Pandit Deendayal Upadhyay Government Medical College|Gujarat Biotechnology Research Centre|Joshi|India
>Spike|hCoV-19/Denmark/DCGC-13817/2020|2020-11-30|EPI_ISL_712110|Original|hCoV-19^^Sjaelland|Human|Department of Virus and Microbiological Special Diagnostics|Albertsen Lab|Michaelsen|Denmark
>Spike|hCoV-19/England/CAMB-7AA8B/2020|2020-04-08|EPI_ISL_433833|Original|hCoV-19^^England|Human|Department of Pathology|COVID-19 Genomics UK (COG-UK) Consortium|Meredith|United Kingdom
>Spike|hCoV-19/England/QEUH-AD1CEC/2020|2020-10-26|EPI_ISL_629889|Original|hCoV-19^^England|Human|Lighthouse Lab in Glasgow|Wellcome Sanger Institute for the COVID-19 Genomics UK (COG-UK) consortium|Davies|United Kingdom
>Spike|hCoV-19/England/20169014104/2020|2020-04-16|EPI_ISL_465195|Original|hCoV-19^^England|Human|Respiratory Virus Unit|Respiratory Virus Unit|Platt|United Kingdom
>Spike|hCoV-19/USA/WA-S2209/2020|2020-06-08|EPI_ISL_497637|Original|hCoV-19^^Washington|Human|Washington State Department of Health|Seattle Flu Study|Lee|USA
>Spike|hCoV-19/USA/CA-CZB-10299/2020|2020-09-08|EPI_ISL_648500|Original|hCoV-19^^California|Human|Orange County Public Health Lab|Chan-Zuckerberg Biohub|Bhatt|USA
>Spike|hCoV-19/USA/AZ-TG648601/2020|2020-04-27|EPI_ISL_695851|Original|hCoV-19^^Arizona|Human|Sonora Quest Laboratories|TGen North|French|USA
>Spike|hCoV-19/USA/FL-BPHL-1267/2020|2020-06-17|EPI_ISL_541174|Original|hCoV-19^^Florida|Human|Florida Bureau of Public Health Laboratories|Florida Bureau of Public Health Laboratories|Blanton|USA
>Spike|hCoV-19/England/NOTT-112556/2020|2020-04-27|EPI_ISL_484393|Original|hCoV-19^^England|Human|Lincolnshire Hospitals and DeepSeq Nottingham|COVID-19 Genomics UK (COG-UK) Consortium|Loose|United Kingdom
>Spike|hCoV-19/England/CAMB-1B2093/2020|2020-05-18|EPI_ISL_453003|Original|hCoV-19^^England|Human|Department of Pathology|COVID-19 Genomics UK (COG-UK) Consortium|Meredith|United Kingdom
>Spike|hCoV-19/England/LOND-126509B/2020|2020-11-27|EPI_ISL_724583|Original|hCoV-19^^England|Human|University College London|COVID-19 Genomics UK (COG-UK) Consortium|Roy|United Kingdom
>Spike|hCoV-19/England/ALDP-9529CF/2020|2020-06-13|EPI_ISL_553883|Original|hCoV-19^^England|Human|Lighthouse Lab in Alderley Park|Wellcome Sanger Institute for the COVID-19 Genomics UK (COG-UK) consortium|Davies|United Kingdom
>Spike|hCoV-19/England/QEUH-B0FC20/2020|2020-11-02|EPI_ISL_641971|Original|hCoV-19^^England|Human|Lighthouse Lab in Glasgow|Wellcome Sanger Institute for the COVID-19 Genomics UK (COG-UK) Consortium|Davies|United Kingdom
>Spike|hCoV-19/USA/AK-PHL914/2020|2020-07-07|EPI_ISL_528535|Original|hCoV-19^^Alaska|Human|Alaska State Virology Laboratory|Alaska State Virology Laboratory|Drown|USA
>Spike|hCoV-19/Wales/PHWC-2617A/2020|2020-03-25|EPI_ISL_422094|Original|hCoV-19^^Wales|Human|Wales Specialist Virology Centre|Public Health Wales Microbiology Cardiff|Moore|United Kingdom
>Spike|hCoV-19/USA/MI-UM-10036456089/2020|2020-10-15|EPI_ISL_628835|Original|hCoV-19^^Michigan|Human|University of Michigan Clinical Microbiology Laboratory|Lauring Lab|Valesano|USA
>Spike|hCoV-19/England/QEUH-9DDC96/2020|2020-09-21|EPI_ISL_599562|Original|hCoV-19^^England|Human|Lighthouse Lab in Glasgow|Wellcome Sanger Institute for the COVID-19 Genomics UK (COG-UK) consortium|Davies|United Kingdom
>Spike|hCoV-19/Scotland/QEUH-BEB1D0/2020|2020-12-04|EPI_ISL_710619|Original|hCoV-19^^Scotland|Human|Lighthouse Lab in Glasgow|Wellcome Sanger Institute for the COVID-19 Genomics UK (COG-UK) Consortium|Davies|United Kingdom
>Spike|hCoV-19/Germany/NW-MPP-4/2020|2020-05-25|EPI_ISL_487426|Original|hCoV-19^^North Rhine-Westphalia|Human|Labor Knei\u00dfler GmbH & Co. KG|Heinrich Pette Institute|G\u00fcnther|Germany

>Spike|hCoV-19/England/20170031904/2020|2020-04-07|EPI_ISL_465275|Original|hCoV-19^^England|Human|Respiratory Virus Unit|Respiratory Virus Unit|Platt|United Kingdom
>Spike|hCoV-19/Wales/PHWC-27FBB/2020|2020-03-24|EPI_ISL_432165|Original|hCoV-19^^Wales|Human|Wales Specialist Virology Centre|Public Health Wales Microbiology Cardiff|Moore|United Kingdom
>Spike|hCoV-19/Sweden/20-50370/2020|2020-03-16|EPI_ISL_475137|Original|hCoV-19^^Kalmar|Human|Kalmar klinisk mikrobiologi|The Public Health Agency of Sweden|Svartstr\u00f6m|Sweden
>Spike|hCoV-19/USA/CA-ALSR-3054/2020|2020-09-04|EPI_ISL_635818|Original|hCoV-19^^California|Human|San Diego County Public Health Laboratory|Andersen lab at Scripps Research|Gangavarapu|USA
>Spike|hCoV-19/England/CAMB-1AA3B3/2020|2020-04-15|EPI_ISL_443644|Original|hCoV-19^^England|Human|Department of Pathology|Wellcome Sanger Institute for the COVID-19 Genomics UK (COG-UK) consortium|Amato|United Kingdom
>Spike|hCoV-19/United Arab Emirates/0387/2020|2020-05-15|EPI_ISL_698562|Original|hCoV-19^^Abu Dhabi|Human|Group 42 (G42) Healthcare|G42 Healthcare|Ogrodzki|United Arab Emirates
>Spike|hCoV-19/Scotland/EDB10781/2020|2020-12-08|EPI_ISL_724235|Original|hCoV-19^^Scotland|Human|Virology Department|COVID-19 Genomics UK (COG-UK) Consortium|McHugh|United Kingdom
>Spike|hCoV-19/USA/UT-QDX-1795/2020|2020-03-16|EPI_ISL_604125|Original|hCoV-19^^Utah|Human|Quest Diagnostics|Quest Diagnostics|Kagan|USA
>Spike|hCoV-19/Denmark/DCGC-3379/2020|2020-08-31|EPI_ISL_616138|Original|hCoV-19^^Hovedstaden|Human|Department of Virus and Microbiological Special Diagnostics|Albertsen lab|Michaelsen|Denmark
>Spike|hCoV-19/USA/CA-ALSR-0821-SAN/2020|2020-04-02|EPI_ISL_483381|Original|hCoV-19^^California|Human|UC San Diego Center for Advanced Laboratory Medicine|Andersen lab at Scripps Research|Gangavarapu|USA
>Spike|hCoV-19/Australia/VIC12117/2020|2020-07-24|EPI_ISL_641228|Original|hCoV-19^^Victoria|Human|Microbiological Diagnostic Unit - Public Health Laboratory (MDU-PHL)|MDU-PHL|Schultz|Australia
>Spike|hCoV-19/England/QEUH-9F93DB/2020|2020-09-25|EPI_ISL_588993|Original|hCoV-19^^England|Human|Lighthouse Lab in Glasgow|Wellcome Sanger Institute for the COVID-19 Genomics UK (COG-UK) consortium|Davies|United Kingdom
>Spike|hCoV-19/Scotland/QEUH-A21816/2020|2020-10-01|EPI_ISL_598862|Original|hCoV-19^^Scotland|Human|Lighthouse Lab in Glasgow|Wellcome Sanger Institute for the COVID-19 Genomics UK (COG-UK) consortium|Davies|United Kingdom
>Spike|hCoV-19/Australia/NSW879/2020|2020-08-10|EPI_ISL_544960|Original|hCoV-19^^New South Wales|Human|Histopath|NSW Health Pathology - Institute of Clinical Pathology and Medical Research; Westmead Hospital; University of Sydney|Gall|Australia
>Spike|hCoV-19/Switzerland/BE-ETHZ-300429/2020|2020-09-29|EPI_ISL_603384|Original|hCoV-19^^Bern|Human|Viollier AG|Department of Biosystems Science and Engineering|Nadeau|Switzerland
>Spike|hCoV-19/Wales/PHWC-487629/2020|2020-10-28|EPI_ISL_639221|Original|hCoV-19^^Wales|Human|Wales Specialist Virology Centre Sequencing lab|COVID-19 Genomics UK (COG-UK) Consortium|Moore|United Kingdom
>Spike|hCoV-19/USA/TX-HMH-2758/2020|2020-06-04|EPI_ISL_545533|Original|hCoV-19^^Texas|Human|Houston Methodist Hospital|Houston Methodist Hospital|Long|USA
>Spike|hCoV-19/England/LIVE-1D1EEF/2020|2020-05-13|EPI_ISL_573240|Original|hCoV-19^^England|Human|Liverpool Clinical Laboratories|COVID-19 Genomics UK (COG-UK) Consortium|Haldenby|United Kingdom
>Spike|hCoV-19/Ukraine/203100344/2020|2020-05-27|EPI_ISL_512623|Original|hCoV-19^^Ukraine|Human|National Laboratory for Influenza/Virology reference laboratory|Respiratory Virus Unit|Platt|Ukraine
>Spike|hCoV-19/USA/UT-UPHL-201112213/2020|2020-05-13|EPI_ISL_628966|Original|hCoV-19^^Utah|Human|Utah Public Health Laboratory|Utah Public Health Laboratory|Young|USA
>Spike|hCoV-19/Germany/BE-ChVir-D672-4/2020|2020-03-07|EPI_ISL_516632|Original|hCoV-19^^Berlin|Human|Charite\u0301 Universita\u0308tsmedizin Berlin|Charite\u0301 Universita\u0308tsmedizin Berlin|Muehlemann|Germany

>Spike|hCoV-19/Spain/CN-IBV-97006506/2020|2020-03-29|EPI_ISL_537714|Original|hCoV-19^^Canary Islands|Human|Hospital Universitario de Gran Canaria Dr. Negr\u00edn|SeqCOVID-SPAIN consortium/IBV(CSIC)|Comas|Spain
>Spike|hCoV-19/England/BRIS-12CD33/2020|2020-05-14|EPI_ISL_481867|Original|hCoV-19^^England|Human|PHE South West Regional Laboratory|Wellcome Sanger Institute for the COVID-19 Genomics UK (COG-UK) consortium|Davies|United Kingdom
>Spike|hCoV-19/England/CAMC-9DF784/2020|2020-09-21|EPI_ISL_637646|Original|hCoV-19^^England|Human|Department of Pathology|COVID-19 Genomics UK (COG-UK) Consortium|Goodfellow|United Kingdom
>Spike|hCoV-19/Canada/NS-NML-1244/2020|2020-04-05|EPI_ISL_579636|Original|hCoV-19^^Nova Scotia|Human|QEII Health Sciences Centre|National Microbiology Laboratory (NML)|Majer|Canada
>Spike|hCoV-19/Australia/VIC14892/2020|2020-08-21|EPI_ISL_593047|Original|hCoV-19^^Victoria|Human|Microbiological Diagnostic Unit - Public Health Laboratory (MDU-PHL)|MDU-PHL|Schultz|Australia
>Spike|hCoV-19/England/MILK-4BC4F4/2020|2020-06-04|EPI_ISL_559149|Original|hCoV-19^^England|Human|Lighthouse Lab in Milton Keynes|Wellcome Sanger Institute for the COVID-19 Genomics UK (COG-UK) consortium|Davies|United Kingdom
>Spike|hCoV-19/USA/TX-HMH-MCoV-13829/2020|2020-08-17|EPI_ISL_787122|Original|hCoV-19^^Texas|Human|Houston Methodist Hospital|Houston Methodist Hospital|Long|USA
>Spike|hCoV-19/USA/HI-H200459/2020|2020-10-06|EPI_ISL_753002|Original|hCoV-19^^Hawaii|Human|State Laboratories Division|State Laboratories Division|Sultana|USA
>Spike|hCoV-19/England/CAMB-1AAADC/2020|2020-04-17|EPI_ISL_470127|Original|hCoV-19^^England|Human|Department of Pathology|Wellcome Sanger Institute for the COVID-19 Genomics UK (COG-UK) consortium|Davies|United Kingdom
>Spike|hCoV-19/Luxembourg/LNS0641910/2020|2020-03-05|EPI_ISL_419566|Original|hCoV-19^^Luxembourg|Human|Laboratoire National de Sant\u00e9|Laboratoire National de Sant\u00e9|Wienecke-Baldacchino|Luxembourg
>Spike|hCoV-19/England/ALDP-BC6575/2020|2020-11-20|EPI_ISL_721225|Original|hCoV-19^^England|Human|Lighthouse Lab in Alderley Park|Wellcome Sanger Institute for the COVID-19 Genomics UK (COG-UK) Consortium|Davies|United Kingdom
>Spike|hCoV-19/England/ALDP-983831/2020|2020-07-09|EPI_ISL_529282|Original|hCoV-19^^England|Human|Quadram Institute Bioscience|COVID-19 Genomics UK (COG-UK) Consortium|Alikhan|United Kingdom
>Spike|hCoV-19/Wales/PHWC-49696A/2020|2020-11-22|EPI_ISL_726661|Original|hCoV-19^^Wales|Human|Originating lab|COVID-19 Genomics UK (COG-UK) Consortium|Moore|United Kingdom
>Spike|hCoV-19/USA/WA-UW-45075/2020|2020-12-08|EPI_ISL_747202|Original|hCoV-19^^Washington|Human|UW Virology Lab|UW Virology Lab|Roychoudhury|USA
>Spike|hCoV-19/Denmark/DCGC-3423/2020|2020-08-31|EPI_ISL_616185|Original|hCoV-19^^Hovedstaden|Human|Department of Virus and Microbiological Special Diagnostics|Albertsen lab|Michaelsen|Denmark
>Spike|hCoV-19/England/MILK-CA9CEB/2020|2020-12-18|EPI_ISL_762335|Original|hCoV-19^^England|Human|Lighthouse Lab in Milton Keynes|Wellcome Sanger Institute for the COVID-19 Genomics UK (COG-UK) Consortium|Davies|United Kingdom
>Spike|hCoV-19/Scotland/CVR5997/2020|2020-11-18|EPI_ISL_703756|Original|hCoV-19^^Scotland|Human|West of Scotland Specialist Virology Centre|COVID-19 Genomics UK (COG-UK) Consortium|Robertson|United Kingdom
>Spike|hCoV-19/USA/AZ-TG670211/2020|2020-04-30|EPI_ISL_696400|Original|hCoV-19^^Arizona|Human|Sonora Quest Laboratories|TGen North|French|USA
>Spike|hCoV-19/Australia/VIC8619/2020|2020-07-29|EPI_ISL_565111|Original|hCoV-19^^Victoria|Human|Microbiological Diagnostic Unit - Public Health Laboratory (MDU-PHL)|MDU-PHL|Schultz|Australia
>Spike|hCoV-19/Iceland/166/2020|2020-03-13|EPI_ISL_417759|Original|hCoV-19^^Reykjavik|Human|The National University Hospital of Iceland|deCODE genetics|J\u00f3nsson|Iceland
>Spike|hCoV-19/USA/AK-PHL634/2020|2020-06-25|EPI_ISL_602560|Original|hCoV-19^^Alaska|Human|Department of Biology and Wildlife|Department of Biology and Wildlife|Chen|USA

>Spike|hCoV-19/Australia/VIC1463/2020|2020-04-21|EPI_ISL_456445|Original|hCoV-
19^^Victoria|Human|Victorian Infectious Diseases Reference Laboratory
(VIDRL)|Microbiological Diagnostic Unit Public Health Laboratory and Victorian
Infectious Diseases Reference Laboratory|Schultz|Australia
>Spike|hCoV-19/England/20104012804/2020|2020-03-03|EPI_ISL_464327|Original|hCoV-
19^^England|Human|Respiratory Virus Unit|Respiratory Virus Unit|Platt|United Kingdom
>Spike|hCoV-19/England/NORT-29951A/2020|2020-04-24|EPI_ISL_492494|Original|hCoV-
19^^England|Human|NU-OMICS DNA Sequencing research facility|Wellcome Sanger Institute
for the COVID-19 Genomics UK (COG-UK) consortium|Davies|United Kingdom
>Spike|hCoV-19/England/LIVE-1D2E84/2020|2020-04-03|EPI_ISL_611621|Original|hCoV-
19^^England|Human|Liverpool Clinical Laboratories|COVID-19 Genomics UK (COG-UK)
Consortium|Haldenby|United Kingdom
>Spike|hCoV-19/South Africa/KRISP-K002228/2020|2020-07-
20|EPI_ISL_515624|Original|hCoV-19^^KwaZulu-Natal|Human|NHLS-IALCH|KRISP|de
Oliveira|South Africa
>Spike|hCoV-19/Australia/VIC10832/2020|2020-08-02|EPI_ISL_563117|Original|hCoV-
19^^Victoria|Human|Microbiological Diagnostic Unit - Public Health Laboratory (MDU-
PHL)|MDU-PHL|Schultz|Australia
>Spike|hCoV-19/USA/TX-HMH-1108/2020|2020-04-25|EPI_ISL_542534|Original|hCoV-
19^^Texas|Human|Houston Methodist Hospital|Houston Methodist Hospital|Long|USA
>Spike|hCoV-19/South Korea/KCDC2231/2020|2020-05-08|EPI_ISL_510569|Original|hCoV-
19^^South Korea|Human|Division of Viral Diseases|Division of Viral Diseases|Kim|South
Korea
>Spike|hCoV-19/USA/NY-NYCPHL-000838/2020|2020-03-29|EPI_ISL_632215|Original|hCoV-
19^^New York|Human|Richmond University Medical Center|New York City Public Health
Laboratory|Wang|USA
>Spike|hCoV-19/Netherlands/LI-RIVM-20959/2020|2020-12-
24|EPI_ISL_790983|Original|hCoV-19^^Limburg|Human|Dutch COVID-19 response
team|National Institute for Public Health and the Environment
(RIVM)|Meijer|Netherlands
>Spike|hCoV-19/France/PAC-IHU-0153/2020|2020-03-23|EPI_ISL_569339|Original|hCoV-
19^^Provence-Alpes-C\u00f4te-d\u2019Azur|Human|MEPHI|MEPHI|Levasseur|France
>Spike|hCoV-19/Switzerland/VD-ETHZ-310353/2020|2020-10-
07|EPI_ISL_603427|Original|hCoV-19^^Vaud|Human|Viollier AG|Department of Biosystems
Science and Engineering|Nadeau|Switzerland
>Spike|hCoV-19/USA/TX-HMH-5377/2020|2020-06-25|EPI_ISL_544729|Original|hCoV-
19^^Texas|Human|Houston Methodist Hospital|Houston Methodist Hospital|Long|USA
>Spike|hCoV-19/USA/MI-UM-10036483429/2020|2020-10-16|EPI_ISL_628820|Original|hCoV-
19^^Michigan|Human|University of Michigan Clinical Microbiology Laboratory|Lauring
Lab|Valesano|USA
>Spike|hCoV-19/Canada/ON-PHL-20-02344/2020|2020-09-00|EPI_ISL_671600|Original|hCoV-
19^^Ontario|Human|Public Health Ontario Laboratory|Public Health Ontario
Laboratory|Guthrie|Canada
>Spike|hCoV-19/New Zealand/20CV0067/2020|2020-08-13|EPI_ISL_547976|Original|hCoV-
19^^Combined Auckland|Human|LabPLUS|Institute of Environmental Science and Research
(ESR)|Wang|New Zealand
>Spike|hCoV-19/USA/TX-HMH-3570/2020|2020-06-15|EPI_ISL_543030|Original|hCoV-
19^^Texas|Human|Houston Methodist Hospital|Houston Methodist Hospital|Long|USA
>Spike|hCoV-19/USA/CA-SR0353/2020|2020-04-08|EPI_ISL_458239|Original|hCoV-
19^^California|Human|Scripps Medical Laboratory|Andersen lab at Scripps
Research|Gangavarapu|USA
>Spike|hCoV-19/England/MILK-99D139/2020|2020-08-30|EPI_ISL_549814|Original|hCoV-
19^^England|Human|Lighthouse Lab in Milton Keynes|Wellcome Sanger Institute for the
COVID-19 Genomics UK (COG-UK) consortium|Davies|United Kingdom
>Spike|hCoV-19/USA/AZ-TG269856/2020|2020-03-14|EPI_ISL_426528|Original|hCoV-
19^^Arizona|Human|AZ SPHL|TGen North|Lemmer|USA
>Spike|hCoV-19/England/CAMC-BBFFDB/2020|2020-11-22|EPI_ISL_704751|Original|hCoV-
19^^England|Human|Lighthouse Lab in Cambridge|Wellcome Sanger Institute for the
COVID-19 Genomics UK (COG-UK) Consortium|Davies|United Kingdom

>Spike|hCoV-19/Scotland/CVR4549/2020|2020-09-22|EPI_ISL_585408|Original|hCoV-19^^Scotland|Human|Lighthouse Lab in Glasgow / MRC-University of Glasgow Centre for Virus Research|COVID-19 Genomics UK (COG-UK) Consortium|Robertson|United Kingdom
>Spike|hCoV-19/Belgium/ULG-10831/2020|2020-11-13|EPI_ISL_661249|Original|hCoV-19^^Li\u00e8ge|Human|Department of Clinical Microbiology|GIGA Medical Genomics|Durkin|Belgium
>Spike|hCoV-19/Australia/VIC17736/2020|2020-08-30|EPI_ISL_779415|Original|hCoV-19^^Victoria|Human|Microbiological Diagnostic Unit - Public Health Laboratory (MDU-PHL)|MDU-PHL|Beck|Australia
>Spike|hCoV-19/India/TG-CCMB-OM10/2020|2020-05-15|EPI_ISL_495276|Original|hCoV-19^^Telangana|Human|Osmania Medical College|CSIR-Centre for Cellular and Molecular Biology|Banu|India
>Spike|hCoV-19/Australia/VIC2657/2020|2020-06-30|EPI_ISL_521503|Original|hCoV-19^^Victoria|Human|Victorian Infectious Diseases Reference Laboratory (VIDRL)|VIDRL and MDU-PHL|Schultz|Australia
>Spike|hCoV-19/USA/TX-DSHS-0662/2020|2020-07-19|EPI_ISL_576316|Original|hCoV-19^^Texas|Human|Texas Department of State Health Services|Texas Department of State Health Services|Rahman|USA
>Spike|hCoV-19/England/201340119/2020|2020-03-23|EPI_ISL_423080|Original|hCoV-19^^England|Human|Respiratory Virus Unit|Respiratory Virus Unit|Platt|United Kingdom
>Spike|hCoV-19/Wales/PHWC-25111/2020|2020-03-22|EPI_ISL_420969|Original|hCoV-19^^Wales|Human|Wales Specialist Virology Centre|Public Health Wales Microbiology Cardiff|Moore|United Kingdom
>Spike|hCoV-19/Singapore/542/2020|2020-06-02|EPI_ISL_482690|Original|hCoV-19^^Singapore|Human|Singapore General Hospital|Department of Microbiology|Abdul Rahman|Singapore
>Spike|hCoV-19/England/QEUH-9F3289/2020|2020-09-24|EPI_ISL_600779|Original|hCoV-19^^England|Human|Lighthouse Lab in Glasgow|Wellcome Sanger Institute for the COVID-19 Genomics UK (COG-UK) consortium|Davies|United Kingdom
>Spike|hCoV-19/England/QEUH-AF28C3/2020|2020-10-30|EPI_ISL_662840|Original|hCoV-19^^England|Human|Lighthouse Lab in Glasgow|Wellcome Sanger Institute for the COVID-19 Genomics UK (COG-UK) Consortium|Davies|United Kingdom
>Spike|hCoV-19/Luxembourg/LNS6058776/2020|2020-11-03|EPI_ISL_744347|Original|hCoV-19^^Luxembourg|Human|Laboratoire national de sant\u00e9|Laboratoire national de sant\u00e9|Wienecke-Baldacchino|Luxembourg
>Spike|hCoV-19/USA/WA-S2497/2020|2020-06-17|EPI_ISL_497129|Original|hCoV-19^^Washington|Human|Washington State Department of Health|Seattle Flu Study|Lee|USA
>Spike|hCoV-19/England/QEUH-CC5D5F/2020|2020-12-22|EPI_ISL_760429|Original|hCoV-19^^England|Human|Lighthouse Lab in Glasgow|Wellcome Sanger Institute for the COVID-19 Genomics UK (COG-UK) Consortium|Davies|United Kingdom
>Spike|hCoV-19/India/MH-ACTREC-691/2020|2020-09-24|EPI_ISL_700316|Original|hCoV-19^^Maharashtra|Human|Hematopathology Laboratory|Hematopathology Laboratory|Patkar|India
>Spike|hCoV-19/Ireland/D-NVRL-20G32315/2020|2020-03-18|EPI_ISL_605131|Original|hCoV-19^^Dublin|Human|National Virus Reference Laboratory|Irish Coronavirus Sequencing Consortium - Helixworks|Chalapati|Ireland
>Spike|hCoV-19/England/NORT-299486/2020|2020-04-24|EPI_ISL_492481|Original|hCoV-19^^England|Human|NU-OMICS DNA Sequencing research facility|Wellcome Sanger Institute for the COVID-19 Genomics UK (COG-UK) consortium|Davies|United Kingdom
>Spike|hCoV-19/Brazil/RJ-00353/2020|2020-04-13|EPI_ISL_623156|Original|hCoV-19^^Rio de Janeiro|Human|Laboratorio de Virologia Molecular / UFRJ|Bioinformatics Laboratory / LNCC|Almeida|Brazil
>Spike|hCoV-19/England/ALDP-B62F7D/2020|2020-11-08|EPI_ISL_655360|Original|hCoV-19^^England|Human|Lighthouse Lab in Alderley Park|Wellcome Sanger Institute for the COVID-19 Genomics UK (COG-UK) Consortium|Davies|United Kingdom
>Spike|hCoV-19/Denmark/DCGC-20589/2020|2020-12-21|EPI_ISL_759239|Original|hCoV-19^^Sjaelland|Human|Department of Virus and Microbiological Special Diagnostics|Albertsen Lab|Michaelsen|Denmark
>Spike|hCoV-19/USA/CA-CZB-13980/2020|2020-06-18|EPI_ISL_739506|Original|hCoV-19^^California|Human|Alameda County Public Health Lab|Chan-Zuckerberg Biohub|Kamm|USA

>Spike|hCoV-19/England/QEUH-A024BD/2020|2020-09-29|EPI_ISL_584908|Original|hCoV-19^^England|Human|Quadram Institute Bioscience|COVID-19 Genomics UK (COG-UK) Consortium|Alikhan|United Kingdom
>Spike|hCoV-19/England/BRIS-126EAF/2020|2020-04-23|EPI_ISL_469558|Original|hCoV-19^^England|Human|PHE South West Regional Laboratory|Wellcome Sanger Institute for the COVID-19 Genomics UK (COG-UK) consortium|Davies|United Kingdom
>Spike|hCoV-19/Scotland/CVR3208/2020|2020-04-16|EPI_ISL_449227|Original|hCoV-19^^Scotland|Human|West of Scotland Specialist Virology Centre|COVID-19 Genomics UK (COG-UK) Consortium|Robertson|United Kingdom
>Spike|hCoV-19/Denmark/ALAB-SSI420/2020|2020-03-11|EPI_ISL_429518|Original|hCoV-19^^Denmark|Human|Department of Virus and Microbiological Special Diagnostics|Albertsen lab|Kirkegaard|Denmark
>Spike|hCoV-19/England/ALDP-6FC9D9/2020|2020-06-30|EPI_ISL_557363|Original|hCoV-19^^England|Human|Lighthouse Lab in Alderley Park|Wellcome Sanger Institute for the COVID-19 Genomics UK (COG-UK) consortium|Davies|United Kingdom
>Spike|hCoV-19/Australia/VIC9127/2020|2020-08-07|EPI_ISL_565401|Original|hCoV-19^^Victoria|Human|Victorian Infectious Diseases Reference Laboratory (VIDRL)|VIDRL and MDU-PHL|Schultz|Australia
>Spike|hCoV-19/Australia/NSW689/2020|2020-07-18|EPI_ISL_513332|Original|hCoV-19^^New South Wales|Human|Pathology West - NSW Health Pathology|NSW Health Pathology - Institute of Clinical Pathology and Medical Research; Westmead Hospital; University of Sydney|Gall|Australia
>Spike|hCoV-19/USA/NY-NYUMC86/2020|2020-03-18|EPI_ISL_421587|Original|hCoV-19^^New York|Human|NYU Langone Health|Departments of Pathology and Medicine|Maurano|USA
>Spike|hCoV-19/Switzerland/SO-ETHZ-410347/2020|2020-12-17|EPI_ISL_737464|Original|hCoV-19^^Solothurn|Human|Viollier AG|Department of Biosystems Science and Engineering|Nadeau|Switzerland
>Spike|hCoV-19/England/NORW-FA1BB/2020|2020-12-08|EPI_ISL_725479|Original|hCoV-19^^England|Human|Quadram Institute Bioscience|COVID-19 Genomics UK (COG-UK) Consortium|Alikhan|United Kingdom
>Spike|hCoV-19/England/CAMB-83A52/2020|2020-04-16|EPI_ISL_443354|Original|hCoV-19^^England|Human|Department of Pathology|Wellcome Sanger Institute for the COVID-19 Genomics UK (COG-UK) consortium|Amato|United Kingdom
>Spike|hCoV-19/USA/WI-WSLH-200481/2020|2020-05-20|EPI_ISL_578622|Original|hCoV-19^^Wisconsin|Human|Wisconsin State Laboratory of Hygiene Communicable Disease Division|Wisconsin State Laboratory of Hygiene Communicable Disease Division|Florek|USA
>Spike|hCoV-19/England/QEUH-B10BC5/2020|2020-11-02|EPI_ISL_642588|Original|hCoV-19^^England|Human|Lighthouse Lab in Glasgow|Wellcome Sanger Institute for the COVID-19 Genomics UK (COG-UK) Consortium|Davies|United Kingdom
>Spike|hCoV-19/England/ALDP-664954/2020|2020-07-03|EPI_ISL_555737|Original|hCoV-19^^England|Human|Lighthouse Lab in Alderley Park|Wellcome Sanger Institute for the COVID-19 Genomics UK (COG-UK) consortium|Davies|United Kingdom
>Spike|hCoV-19/Austria/CeMM0103/2020|2020-03-19|EPI_ISL_437964|Original|hCoV-19^^Innsbruck|Human|Universitaetsklinik f\u00fcr Innere Medizin II Innsbruck|Bergthaler laboratory|Bergthaler|Austria
>Spike|hCoV-19/Belgium/VLS-0507418/2020|2020-05-07|EPI_ISL_462272|Original|hCoV-19^^Turnhout|Human|KU Leuven|KU Leuven|Maes|Belgium
>Spike|hCoV-19/Scotland/CVR133/2020|2020-03-16|EPI_ISL_425676|Original|hCoV-19^^Scotland|Human|West of Scotland Specialist Virology Centre|COVID-19 Genomics UK (COG-UK) Consortium|Robertson|United Kingdom
>Spike|hCoV-19/New Zealand/20VR1283/2020|2020-03-20|EPI_ISL_456208|Original|hCoV-19^^Auckland|Human|LabPLUS|Institute of Environmental Science and Research (ESR)|de Ligt|New Zealand
>Spike|hCoV-19/England/201060024/2020|2020-03-02|EPI_ISL_459961|Original|hCoV-19^^England|Human|Respiratory Virus Unit|Respiratory Virus Unit|Platt|United Kingdom
>Spike|hCoV-19/Australia/NSW1163/2020|2020-10-07|EPI_ISL_639731|Original|hCoV-19^^New South Wales|Human|Laverty Pathology|NSW Health Pathology - Institute of Clinical Pathology and Medical Research; Westmead Hospital; University of Sydney|Gall|Australia

>Spike|hCoV-19/USA/CA-CZB-1632/2020|2020-03-28|EPI_ISL_468559|Original|hCoV-19^^California|Human|San Joaquin County Public Health Lab|Chan-Zuckerberg Biohub|Hao|USA
>Spike|hCoV-19/Denmark/DCGC-1122/2020|2020-03-16|EPI_ISL_614624|Original|hCoV-19^^Hovedstaden|Human|Department of Virus and Microbiological Special Diagnostics|Albertsen lab|Michaelsen|Denmark
>Spike|hCoV-19/England/ALDP-B74118/2020|2020-11-12|EPI_ISL_664900|Original|hCoV-19^^England|Human|Quadram Institute Bioscience|COVID-19 Genomics UK (COG-UK) Consortium|Alikhan|United Kingdom
>Spike|hCoV-19/Australia/VIC569/2020|2020-03-25|EPI_ISL_426829|Original|hCoV-19^^Victoria|Human|Victorian Infectious Diseases Reference Laboratory (VIDRL)|Microbiological Diagnostic Unit Public Health Laboratory and Victorian Infectious Diseases Reference Laboratory|Schultz|Australia
>Spike|hCoV-19/England/QEUH-997E65/2020|2020-08-23|EPI_ISL_537204|Original|hCoV-19^^England|Human|Lighthouse Lab in Glasgow|Wellcome Sanger Institute for the COVID-19 Genomics UK (COG-UK) consortium|Davies|United Kingdom
>Spike|hCoV-19/Denmark/ALAB-SSI-1427/2020|2020-04-04|EPI_ISL_437654|Original|hCoV-19^^Denmark|Human|Department of Virus and Microbiological Special Diagnostics|Albertsen lab|Kirkegaard|Denmark
>Spike|hCoV-19/Scotland/CVR102/2020|2020-03-13|EPI_ISL_425649|Original|hCoV-19^^Scotland|Human|West of Scotland Specialist Virology Centre|COVID-19 Genomics UK (COG-UK) Consortium|Robertson|United Kingdom
>Spike|hCoV-19/USA/TX-HMH-MCoV-18711/2020|2020-11-20|EPI_ISL_785059|Original|hCoV-19^^Texas|Human|Houston Methodist Hospital|Houston Methodist Hospital|Long|USA
>Spike|hCoV-19/SaudiArabia/KAUST-JEDDAH919/2020|2020-04-06|EPI_ISL_513050|Original|hCoV-19^^Jeddah|Human|Pathogen Genomics Lab King Abdullah University of Science and Technology(KAUST)|Pathogen Genomics Lab King Abdullah University of Science and Technology(KAUST)|Mohamed Ghazzali|Saudi Arabia
>Spike|hCoV-19/Denmark/DCGC-17116/2020|2020-12-14|EPI_ISL_750225|Original|hCoV-19^^Hovedstaden|Human|Department of Virus and Microbiological Special Diagnostics|Albertsen Lab|Michaelsen|Denmark
>Spike|hCoV-19/USA/TX-HMH-5275/2020|2020-06-23|EPI_ISL_544645|Original|hCoV-19^^Texas|Human|Houston Methodist Hospital|Houston Methodist Hospital|Long|USA
>Spike|hCoV-19/England/ALDP-95A3F6/2020|2020-06-16|EPI_ISL_553515|Original|hCoV-19^^England|Human|Lighthouse Lab in Alderley Park|Wellcome Sanger Institute for the COVID-19 Genomics UK (COG-UK) consortium|Davies|United Kingdom
>Spike|hCoV-19/USA/WA-UW263/2020|2020-03-16|EPI_ISL_418048|Original|hCoV-19^^Washington|Human|UW Virology Lab|UW Virology Lab|Roychoudhury|USA
>Spike|hCoV-19/England/MILK-9A5444/2020|2020-09-03|EPI_ISL_550140|Original|hCoV-19^^England|Human|Lighthouse Lab in Milton Keynes|Wellcome Sanger Institute for the COVID-19 Genomics UK (COG-UK) consortium|Davies|United Kingdom
>Spike|hCoV-19/USA/OR-OHSU-3095/2020|2020-10-01|EPI_ISL_667480|Original|hCoV-19^^Oregon|Human|OHSU Lab Services Molecular Microbiology Lab|Oregon SARS-CoV-2 Genome Sequencing Center|O'Roak|USA
>Spike|hCoV-19/USA/CA-SEARCH-5551/2020|2020-11-29|EPI_ISL_755288|Original|hCoV-19^^California|Human|San Diego County Public Health Laboratory|Andersen lab at Scripps Research|Gangavarapu|USA
>Spike|hCoV-19/Spain/VC-FISABIO-255/2020|2020-03-26|EPI_ISL_436350|Original|hCoV-19^^Comunitat Valenciana|Human|Servicio de Microbiologia. Hospital Clinico Universitario de Valencia|Sequencing and Bioinformatics Service and Molecular Epidemiology Research Group. FISABIO-Public Health|Gonzalez-Candelas|Spain
>Spike|hCoV-19/Switzerland/BS-42227651/2020|2020-04-10|EPI_ISL_581695|Original|hCoV-19^^Basel-Stadt|Human|University Hospital Basel|University Hospital Basel|Roloff Handschin|Switzerland
>Spike|hCoV-19/England/EKHU-260F14E/2020|2020-03-24|EPI_ISL_501615|Original|hCoV-19^^England|Human|Lab Microbiology|Wellcome Sanger Institute for the COVID-19 Genomics UK (COG-UK) consortium|Davies|United Kingdom
>Spike|hCoV-19/England/QEUH-960EDE/2020|2020-08-04|EPI_ISL_531971|Original|hCoV-19^^England|Human|Lighthouse Lab in Glasgow|Wellcome Sanger Institute for the COVID-19 Genomics UK (COG-UK) consortium|Davies|United Kingdom

>Spike|hCoV-19/USA/UT-UPHL-2012863648/2020|2020-06-18|EPI_ISL_746471|Original|hCoV-19^^Utah|Human|Utah Public Health Laboratory|Utah Public Health Laboratory|Gallagher|USA
>Spike|hCoV-19/France/GES-IPP9562/2020|2020-11-03|EPI_ISL_693767|Original|hCoV-19^^Grand-Est|Human|General practitioner|National Reference Center for Viruses of Respiratory Infections|Enouf|France
>Spike|hCoV-19/USA/MN-MDH-1481/2020|2020-03-18|EPI_ISL_518875|Original|hCoV-19^^Minnesota|Human|Mayo Clinic & Mayo Clinic Laboratories|Minnesota Department of Health|Garfin|USA
>Spike|hCoV-19/Scotland/QEUH-A0C0BF/2020|2020-10-01|EPI_ISL_599243|Original|hCoV-19^^Scotland|Human|Lighthouse Lab in Glasgow|Wellcome Sanger Institute for the COVID-19 Genomics UK (COG-UK) consortium|Davies|United Kingdom
>Spike|hCoV-19/USA/CA-ALSR-0707-SAN/2020|2020-03-30|EPI_ISL_483316|Original|hCoV-19^^California|Human|UC San Diego Center for Advanced Laboratory Medicine|Andersen lab at Scripps Research|Gangavarapu|USA
>Spike|hCoV-19/England/BRIS-122EA3/2020|2020-04-03|EPI_ISL_440783|Original|hCoV-19^^England|Human|PHE South West Regional Laboratory|Wellcome Sanger Institute for the COVID-19 Genomics UK (COG-UK) consortium|Amato|United Kingdom
>Spike|hCoV-19/Denmark/DCGC-11355/2020|2020-11-02|EPI_ISL_670862|Original|hCoV-19^^Midtjylland|Human|Department of Virus and Microbiological Special Diagnostics|Albertsen Lab|Michaelsen|Denmark
>Spike|hCoV-19/England/QEUH-9B43AF/2020|2020-09-04|EPI_ISL_559889|Original|hCoV-19^^England|Human|Oxford Viromics|COVID-19 Genomics UK (COG-UK) Consortium|Golubchik|United Kingdom
>Spike|hCoV-19/England/BIRM-5E601/2020|2020-03-24|EPI_ISL_444297|Original|hCoV-19^^England|Human|University of Birmingham|COVID-19 Genomics UK (COG-UK) Consortium|Loman|United Kingdom
>Spike|hCoV-19/Denmark/DCGC-5122/2020|2020-09-21|EPI_ISL_621660|Original|hCoV-19^^Syddanmark|Human|Department of Virus and Microbiological Special Diagnostics|Albertsen lab|Michaelsen|Denmark
>Spike|hCoV-19/England/CAMC-C3F321/2020|2020-12-10|EPI_ISL_719315|Original|hCoV-19^^England|Human|Lighthouse Lab in Cambridge|Wellcome Sanger Institute for the COVID-19 Genomics UK (COG-UK) Consortium|Davies|United Kingdom
>Spike|hCoV-19/USA/TX-HMH-MCoV-12987/2020|2020-08-28|EPI_ISL_788138|Original|hCoV-19^^Texas|Human|Houston Methodist Hospital|Houston Methodist Hospital|Long|USA
>Spike|hCoV-19/Wales/PHWC-166DD3/2020|2020-06-27|EPI_ISL_494115|Original|hCoV-19^^Wales|Human|Originating lab|COVID-19 Genomics UK (COG-UK) Consortium|Moore|United Kingdom
>Spike|hCoV-19/Israel/701002504/2020|2020-03-25|EPI_ISL_447294|Original|hCoV-19^^South Coast District|Human|Microbiology Division|Stern Lab|Miller|Israel
>Spike|hCoV-19/England/MILK-A7C339/2020|2020-10-15|EPI_ISL_606408|Original|hCoV-19^^England|Human|Lighthouse Lab in Milton Keynes|Wellcome Sanger Institute for the COVID-19 Genomics UK (COG-UK) consortium|Davies|United Kingdom
>Spike|hCoV-19/England/ALDP-A84A1B/2020|2020-10-18|EPI_ISL_610404|Original|hCoV-19^^England|Human|Lighthouse Lab in Alderley Park|Wellcome Sanger Institute for the COVID-19 Genomics UK (COG-UK) consortium|Davies|United Kingdom
>Spike|hCoV-19/Denmark/DCGC-21275/2020|2020-12-21|EPI_ISL_759650|Original|hCoV-19^^Nordjylland|Human|Department of Virus and Microbiological Special Diagnostics|Albertsen Lab|Michaelsen|Denmark
>Spike|hCoV-19/USA/MA-JLL-154/2020|2020-00-00|EPI_ISL_632297|Original|hCoV-19^^Massachusetts|Human|Brigham and Women's Hospital|Jonathan Li laboratory|Choudhary|USA
>Spike|hCoV-19/Wales/PHWC-490D0A/2020|2020-11-16|EPI_ISL_706562|Original|hCoV-19^^Wales|Human|Originating lab|COVID-19 Genomics UK (COG-UK) Consortium|Moore|United Kingdom
>Spike|hCoV-19/Australia/VIC3623/2020|2020-07-10|EPI_ISL_520365|Original|hCoV-19^^Victoria|Human|Victorian Infectious Diseases Reference Laboratory (VIDRL)|VIDRL and MDU-PHL|Schultz|Australia
>Spike|hCoV-19/USA/WA-UW-47141/2020|2020-12-16|EPI_ISL_756236|Original|hCoV-19^^Washington|Human|UW Virology Lab|UW Virology Lab|Roychoudhury|USA

>Spike|hCoV-19/USA/AZ-TG268913/2020|2020-03-14|EPI_ISL_426517|Original|hCoV-19^^Arizona|Human|AZ SPHL|TGen North|Lemmer|USA
>Spike|hCoV-19/USA/MA-MASPHL-00052/2020|2020-04-03|EPI_ISL_791552|Original|hCoV-19^^Massachusetts|Human|Massachusetts State Public Health Laboratory|Infectious Disease Program|Park|USA
>Spike|hCoV-19/Gambia/3668/2020|2020-07-30|EPI_ISL_560992|Original|hCoV-19^^Kombo|Human|MRCG at LSHTM Genomics lab|MRCG at LSHTM Genomics lab|Sesay|Gambia
>Spike|hCoV-19/England/QEUH-B12EAC/2020|2020-11-02|EPI_ISL_642704|Original|hCoV-19^^England|Human|Lighthouse Lab in Glasgow|Wellcome Sanger Institute for the COVID-19 Genomics UK (COG-UK) Consortium|Davies|United Kingdom
>Spike|hCoV-19/England/LIVE-AA1D8/2020|2020-03-23|EPI_ISL_517417|Original|hCoV-19^^England|Human|Liverpool Clinical Laboratories|COVID-19 Genomics UK (COG-UK) Consortium|Haldenby|United Kingdom
>Spike|hCoV-19/Wales/PHWC-4A0A4C/2020|2020-12-10|EPI_ISL_743532|Original|hCoV-19^^Wales|Human|Wales Specialist Virology Centre Sequencing lab|COVID-19 Genomics UK (COG-UK) Consortium|Moore|United Kingdom
>Spike|hCoV-19/England/QEUH-AF5D85/2020|2020-10-30|EPI_ISL_662991|Original|hCoV-19^^England|Human|Lighthouse Lab in Glasgow|Wellcome Sanger Institute for the COVID-19 Genomics UK (COG-UK) Consortium|Davies|United Kingdom
>Spike|hCoV-19/Japan/PG-1029/2020|2020-04-00|EPI_ISL_684639|Original|hCoV-19^^Japan|Human|Fukuoka Institute of Health and Environmental Sciences|Pathogen Genomics Center|Sekizuka|Japan
>Spike|hCoV-19/England/205260370/2020|2020-12-18|EPI_ISL_766100|Original|hCoV-19^^England|Human|Respiratory Virus Unit|COVID-19 Genomics UK (COG-UK) Consortium|Platt|United Kingdom
>Spike|hCoV-19/England/ALDP-9551D3/2020|2020-06-12|EPI_ISL_553446|Original|hCoV-19^^England|Human|Lighthouse Lab in Alderley Park|Wellcome Sanger Institute for the COVID-19 Genomics UK (COG-UK) consortium|Davies|United Kingdom
>Spike|hCoV-19/Australia/VIC8985/2020|2020-07-28|EPI_ISL_565335|Original|hCoV-19^^Victoria|Human|Microbiological Diagnostic Unit - Public Health Laboratory (MDU-PHL)|MDU-PHL|Schultz|Australia
>Spike|hCoV-19/England/QEUH-9B5283/2020|2020-09-03|EPI_ISL_590333|Original|hCoV-19^^England|Human|Lighthouse Lab in Glasgow|Wellcome Sanger Institute for the COVID-19 Genomics UK (COG-UK) consortium|Davies|United Kingdom
>Spike|hCoV-19/USA/AZ-TG491607/2020|2020-06-24|EPI_ISL_695191|Original|hCoV-19^^Arizona|Human|AZ SPHL|TGen North|French|USA
>Spike|hCoV-19/England/QEUH-AD3F5A/2020|2020-10-25|EPI_ISL_634532|Original|hCoV-19^^England|Human|Lighthouse Lab in Glasgow|Wellcome Sanger Institute for the COVID-19 Genomics UK (COG-UK) consortium|Davies|United Kingdom
>Spike|hCoV-19/Wales/PHWC-30939/2020|2020-04-08|EPI_ISL_446295|Original|hCoV-19^^Wales|Human|Wales Specialist Virology Centre|Public Health Wales Microbiology Cardiff|Moore|United Kingdom
>Spike|hCoV-19/Germany/HH-hpi-p1548/2020|2020-03-15|EPI_ISL_775744|Original|hCoV-19^^Hamburg|Human|University Medical Center Hamburg Eppendorf|Heinrich Pette Institute|Robitaille|Germany
>Spike|hCoV-19/USA/TX-HMH-MCoV-10167/2020|2020-07-06|EPI_ISL_789139|Original|hCoV-19^^Texas|Human|Houston Methodist Hospital|Houston Methodist Hospital|Long|USA
>Spike|hCoV-19/England/MILK-A23A87/2020|2020-10-03|EPI_ISL_637782|Original|hCoV-19^^England|Human|Centre for Enzyme Innovation|COVID-19 Genomics UK (COG-UK) Consortium|Robson|United Kingdom
>Spike|hCoV-19/Australia/VIC2547/2020|2020-06-29|EPI_ISL_522234|Original|hCoV-19^^Victoria|Human|Victorian Infectious Diseases Reference Laboratory (VIDRL)|VIDRL and MDU-PHL|Schultz|Australia
>Spike|hCoV-19/England/QEUH-7A5B7E/2020|2020-07-09|EPI_ISL_533099|Original|hCoV-19^^England|Human|Lighthouse Lab in Glasgow|Wellcome Sanger Institute for the COVID-19 Genomics UK (COG-UK) consortium|Davies|United Kingdom
>Spike|hCoV-19/England/204481940/2020|2020-10-22|EPI_ISL_637167|Original|hCoV-19^^England|Human|Respiratory Virus Unit|COVID-19 Genomics UK (COG-UK) Consortium|Platt|United Kingdom

>Spike|hCoV-19/Spain/CT-IBV-99009880/2020|2020-08-03|EPI_ISL_660277|Original|hCoV-19^^Catalunya|Human|Servicio de Microbiolog\u00eda|SeqCOVID-SPAIN consortium/IBV(CSIC)|Comas|Spain
>Spike|hCoV-19/USA/TX-HMH-MCoV-19076/2020|2020-11-24|EPI_ISL_783612|Original|hCoV-19^^Texas|Human|Houston Methodist Hospital|Houston Methodist Hospital|Long|USA
>Spike|hCoV-19/England/ALDP-956099/2020|2020-06-08|EPI_ISL_554002|Original|hCoV-19^^England|Human|Lighthouse Lab in Alderley Park|Wellcome Sanger Institute for the COVID-19 Genomics UK (COG-UK) consortium|Davies|United Kingdom
>Spike|hCoV-19/Singapore/146/2020|2020-03-17|EPI_ISL_443238|Original|hCoV-19^^Singapore|Human|National Public Health Laboratory|National Public Health Laboratory|Mak|Singapore
>Spike|hCoV-19/USA/FL-BPHL-0208/2020|2020-04-16|EPI_ISL_480859|Original|hCoV-19^^Florida|Human|Florida Bureau of Public Health Laboratories|Florida Bureau of Public Health Laboratories|Blanton|USA
>Spike|hCoV-19/Brazil/SP-L24-CD534/2020|2020-04-14|EPI_ISL_672694|Original|hCoV-19^^S\u00e3o Paulo|Human|Hospital das Cl\u00ednicas da Faculdade de Medicina da Universidade de S\u00e3o Paulo (HC-FMUSP)|Laborat\u00f3rio de Parasitologia M\u00e9dica - Instituto de Medicina Tropical - Universidade de S\u00e3o Paulo|de Jesus|Brazil
>Spike|hCoV-19/England/ALDP-BE93AF/2020|2020-12-03|EPI_ISL_708914|Original|hCoV-19^^England|Human|Lighthouse Lab in Alderley Park|Wellcome Sanger Institute for the COVID-19 Genomics UK (COG-UK) Consortium|Davies|United Kingdom
>Spike|hCoV-19/USA/CA-ALSR-2429/2020|2020-07-02|EPI_ISL_635475|Original|hCoV-19^^California|Human|San Diego County Public Health Laboratory|Andersen lab at Scripps Research|Gangavarapu|USA
>Spike|hCoV-19/USA/TX-HMH-MCoV-10786/2020|2020-07-05|EPI_ISL_789627|Original|hCoV-19^^Texas|Human|Houston Methodist Hospital|Houston Methodist Hospital|Long|USA
>Spike|hCoV-19/England/QEUH-997ECF/2020|2020-08-24|EPI_ISL_537173|Original|hCoV-19^^England|Human|Lighthouse Lab in Glasgow|Wellcome Sanger Institute for the COVID-19 Genomics UK (COG-UK) consortium|Davies|United Kingdom
>Spike|hCoV-19/Kazakhstan/26577/2020|2020-04-26|EPI_ISL_454596|Original|hCoV-19^^Nur-Sultan|Human|Laboratory of virology|Laboratory of molecular-genetic research|Abdaliyev|Kazakhstan
>Spike|hCoV-19/England/MILK-ABB86C/2020|2020-10-22|EPI_ISL_631161|Original|hCoV-19^^England|Human|Lighthouse Lab in Milton Keynes|Wellcome Sanger Institute for the COVID-19 Genomics UK (COG-UK) consortium|Davies|United Kingdom
>Spike|hCoV-19/England/QEUH-AD11DE/2020|2020-10-24|EPI_ISL_633967|Original|hCoV-19^^England|Human|Lighthouse Lab in Glasgow|Wellcome Sanger Institute for the COVID-19 Genomics UK (COG-UK) consortium|Davies|United Kingdom
>Spike|hCoV-19/England/ALDP-BBB2C7/2020|2020-11-21|EPI_ISL_701685|Original|hCoV-19^^England|Human|Lighthouse Lab in Alderley Park|Wellcome Sanger Institute for the COVID-19 Genomics UK (COG-UK) Consortium|Davies|United Kingdom
>Spike|hCoV-19/England/QEUH-9F9D19/2020|2020-09-26|EPI_ISL_595760|Original|hCoV-19^^England|Human|Oxford Viromics|COVID-19 Genomics UK (COG-UK) Consortium|Golubchik|United Kingdom
>Spike|hCoV-19/Australia/NSW1209/2020|2020-12-02|EPI_ISL_672574|Original|hCoV-19^^New South Wales|Human|Histopath|NSW Health Pathology - Institute of Clinical Pathology and Medical Research; Westmead Hospital; University of Sydney|Gall|Australia
>Spike|hCoV-19/Japan/PG-4705/2020|2020-07-00|EPI_ISL_689842|Original|hCoV-19^^Japan|Human|Pathogen Genomics Center|Pathogen Genomics Center|Sekizuka|Japan
>Spike|hCoV-19/England/ALDP-B5BCBC/2020|2020-11-09|EPI_ISL_655940|Original|hCoV-19^^England|Human|Lighthouse Lab in Alderley Park|Wellcome Sanger Institute for the COVID-19 Genomics UK (COG-UK) Consortium|Davies|United Kingdom
>Spike|hCoV-19/USA/WA-UW-26495/2020|2020-09-01|EPI_ISL_570577|Original|hCoV-19^^Washington|Human|UW Virology Lab|UW Virology Lab|Roychoudhury|USA
>Spike|hCoV-19/USA/CA-CZB-15047/2020|2020-11-18|EPI_ISL_738590|Original|hCoV-19^^California|Human|Santa Clara County Public Health Laboratory|Chan-Zuckerberg Biohub|Kamm|USA

>Spike|hCoV-19/Wales/PHWC-16ACC3/2020|2020-09-13|EPI_ISL_560183|Original|hCoV-19^^Wales|Human|Wales Specialist Virology Centre Sequencing lab|COVID-19 Genomics UK (COG-UK) Consortium|Moore|United Kingdom
>Spike|hCoV-19/Netherlands/ZH-EMC-622/2020|2020-09-24|EPI_ISL_578050|Original|hCoV-19^^Zuid Holland|Human|Dutch COVID-19 response team|Erasmus Medical Center|Oude Munnink|Netherlands
>Spike|hCoV-19/USA/TX-HMH-MCoV-14133/2020|2020-10-02|EPI_ISL_787418|Original|hCoV-19^^Texas|Human|Houston Methodist Hospital|Houston Methodist Hospital|Long|USA
>Spike|hCoV-19/USA/FL_5125/2020|2020-02-28|EPI_ISL_419559|Original|hCoV-19^^Florida|Human|FL Bureau of Public Health Laboratories-Tampa|Pathogen Discovery|Queen|USA
>Spike|hCoV-19/Indonesia/BT-MRINUPH-200624-219/2020|2020-06-18|EPI_ISL_779389|Original|hCoV-19^^Banten|Human|Siloam Hospital Lippo Village|Mochtar Riady Institute for Nanotechnology-Universitas Pelita Harapan|Suriapranata|Indonesia
>Spike|hCoV-19/England/OXON-B47D9/2020|2020-04-24|EPI_ISL_534974|Original|hCoV-19^^England|Human|Oxford Viromics|COVID-19 Genomics UK (COG-UK) Consortium|Golubchik|United Kingdom
>Spike|hCoV-19/Denmark/DCGC-9343/2020|2020-11-16|EPI_ISL_668919|Original|hCoV-19^^Nordjylland|Human|Department of Virus and Microbiological Special Diagnostics|Albertsen Lab|Michaelsen|Denmark
>Spike|hCoV-19/USA/MN-MDH-1766/2020|2020-04-11|EPI_ISL_590770|Original|hCoV-19^^Minnesota|Human|Minnesota Department of Health|Minnesota Department of Health|Lorentz|USA
>Spike|hCoV-19/England/20172002204/2020|2020-04-20|EPI_ISL_465319|Original|hCoV-19^^England|Human|Respiratory Virus Unit|Respiratory Virus Unit|Platt|United Kingdom
>Spike|hCoV-19/England/MILK-A478A1/2020|2020-10-05|EPI_ISL_598257|Original|hCoV-19^^England|Human|Lighthouse Lab in Milton Keynes|Wellcome Sanger Institute for the COVID-19 Genomics UK (COG-UK) consortium|Davies|United Kingdom
>Spike|hCoV-19/USA/IL-NM-0683/2020|2020-05-09|EPI_ISL_626469|Original|hCoV-19^^Illinois|Human|Northwestern Memorial Hospital|Ozer Lab|Ozer|USA
>Spike|hCoV-19/England/QEUH-9F332C/2020|2020-09-24|EPI_ISL_588543|Original|hCoV-19^^England|Human|Lighthouse Lab in Glasgow|Wellcome Sanger Institute for the COVID-19 Genomics UK (COG-UK) consortium|Davies|United Kingdom
>Spike|hCoV-19/England/ALDP-953472/2020|2020-06-08|EPI_ISL_554441|Original|hCoV-19^^England|Human|Lighthouse Lab in Alderley Park|Wellcome Sanger Institute for the COVID-19 Genomics UK (COG-UK) consortium|Davies|United Kingdom
>Spike|hCoV-19/Australia/VIC16368/2020|2020-08-03|EPI_ISL_641217|Original|hCoV-19^^Victoria|Human|Microbiological Diagnostic Unit - Public Health Laboratory (MDU-PHL)|MDU-PHL|Schultz|Australia
>Spike|hCoV-19/USA/UT-03180/2020|2020-04-22|EPI_ISL_470740|Original|hCoV-19^^Utah|Human|Utah Public Health Laboratory|Utah Public Health Laboratory|Butz|USA
>Spike|hCoV-19/Finland/20SS11F9/2020|2020-09-20|EPI_ISL_757371|Original|hCoV-19^^Uusimaa|Human|Department of Virology and Immunology|Department of Virology|Smura|Finland
>Spike|hCoV-19/France/ARA-98766/2020|2020-11-01|EPI_ISL_732703|Original|hCoV-19^^Auvergne-Rh\u00f4ne-Alpes|Human|CNR Virus des Infections Respiratoires - France SUD|CNR Virus des Infections Respiratoires - France SUD|Bal|France
>Spike|hCoV-19/Japan/IC-0197/2020|2020-07-00|EPI_ISL_667626|Original|hCoV-19^^Japan|Human|Pathogen Genomics Center|Pathogen Genomics Center|Sekizuka|Japan
>Spike|hCoV-19/England/MILK-A45955/2020|2020-10-06|EPI_ISL_598601|Original|hCoV-19^^England|Human|Lighthouse Lab in Milton Keynes|Wellcome Sanger Institute for the COVID-19 Genomics UK (COG-UK) consortium|Davies|United Kingdom
>Spike|hCoV-19/Germany/BY-MVP-0268/2020|2020-05-17|EPI_ISL_466910|Original|hCoV-19^^Bavaria|Human|Max von Pettenkofer Institute|Laboratory for Functional Genome Analysis|Graf|Germany
>Spike|hCoV-19/England/ALDP-9CD9DE/2020|2020-09-16|EPI_ISL_566190|Original|hCoV-19^^England|Human|Lighthouse Lab in Alderley Park|Wellcome Sanger Institute for the COVID-19 Genomics UK (COG-UK) consortium|Davies|United Kingdom

>Spike|hCoV-19/USA/WI-GMF-23240/2020|2020-09-30|EPI_ISL_660868|Original|hCoV-19^^Wisconsin|Human|Gundersen Molecular Diagnostics Laboratory|Kabara Cancer Research Institute|Kenny|USA
>Spike|hCoV-19/Japan/PG-5791/2020|2020-08-00|EPI_ISL_688910|Original|hCoV-19^^Japan|Human|Fukuoka Institute of Health and Environmental Sciences|Pathogen Genomics Center|Sekizuka|Japan
>Spike|hCoV-19/Singapore/1414/2020|2020-11-12|EPI_ISL_654812|Original|hCoV-19^^Singapore|Human|National Public Health Laboratory|National Public Health Laboratory|Mak|Singapore
>Spike|hCoV-19/USA/HI-H200712/2020|2020-12-09|EPI_ISL_753233|Original|hCoV-19^^Hawaii|Human|State Laboratories Division|State Laboratories Division|Sultana|USA
>Spike|hCoV-19/Luxembourg/LNS2699765/2020|2020-10-22|EPI_ISL_740172|Original|hCoV-19^^Luxembourg|Human|Laboratoire national de sant\u00e9|Laboratoire national de sant\u00e9|Wienecke-Baldacchino|Luxembourg
>Spike|hCoV-19/Spain/VC-FISABIO-618/2020|2020-03-31|EPI_ISL_455724|Original|hCoV-19^^Comunitat Valenciana|Human|Servicio de Microbiologia. Hospital Clinico Universitario de Valencia|Sequencing and Bioinformatics Service and Molecular Epidemiology Research Group. FISABIO-Public Health|Gonzalez-Candelas|Spain
>Spike|hCoV-19/USA/TX-HMH-4465/2020|2020-06-17|EPI_ISL_543642|Original|hCoV-19^^Texas|Human|Houston Methodist Hospital|Houston Methodist Hospital|Long|USA
>Spike|hCoV-19/England/LCST-24D6B62/2020|2020-06-08|EPI_ISL_478334|Original|hCoV-19^^England|Human|University Hospitals Of Leicester NHS Trust and DeepSeq Nottingham|COVID-19 Genomics UK (COG-UK) Consortium|Loose|United Kingdom
>Spike|hCoV-19/Russia/PRI-RII-MH760S/2020|2020-08-10|EPI_ISL_733009|Original|hCoV-19^^Primorsky|Human|WHO National Influenza Centre Russian Federation|WHO National Influenza Centre Russian Federation|Komissarov|Russia
>Spike|hCoV-19/USA/MI-QDX-1147/2020|2020-03-23|EPI_ISL_571877|Original|hCoV-19^^Michigan|Human|Quest Diagnostics|Quest Diagnostics|Kagan|USA
>Spike|hCoV-19/Japan/PG-1722/2020|2020-04-00|EPI_ISL_685328|Original|hCoV-19^^Japan|Human|Pathogen Genomics Center|Pathogen Genomics Center|Sekizuka|Japan
>Spike|hCoV-19/Switzerland/GE-SNRCI-3496/2020|2020-03-00|EPI_ISL_548168|Original|hCoV-19^^Geneve|Human|Laboratoire de Virologie|Swiss National Reference Centre for Influenza|Gon\u00e7alves Cabecinhas|Switzerland
>Spike|hCoV-19/England/ALDP-C063E7/2020|2020-12-06|EPI_ISL_709414|Original|hCoV-19^^England|Human|Lighthouse Lab in Alderley Park|Wellcome Sanger Institute for the COVID-19 Genomics UK (COG-UK) Consortium|Davies|United Kingdom
>Spike|hCoV-19/England/MILK-B95DFF/2020|2020-11-13|EPI_ISL_676133|Original|hCoV-19^^England|Human|Lighthouse Lab in Milton Keynes|Wellcome Sanger Institute for the COVID-19 Genomics UK (COG-UK) Consortium|Davies|United Kingdom
>Spike|hCoV-19/England/MILK-BB017A/2020|2020-11-18|EPI_ISL_720015|Original|hCoV-19^^England|Human|Lighthouse Lab in Milton Keynes|Wellcome Sanger Institute for the COVID-19 Genomics UK (COG-UK) Consortium|Davies|United Kingdom
>Spike|hCoV-19/England/BRIS-12EEB6/2020|2020-04-22|EPI_ISL_488322|Original|hCoV-19^^England|Human|PHE South West Regional Laboratory|Wellcome Sanger Institute for the COVID-19 Genomics UK (COG-UK) consortium|Davies|United Kingdom
>Spike|hCoV-19/Italy/LAZ-INMI-83/2020|2020-08-20|EPI_ISL_609991|Original|hCoV-19^^Lazio|Human|INMI Lazzaro Spallanzani IRCCS|INMI Lazzaro Spallanzani IRCCS|Gruber|Italy
>Spike|hCoV-19/Denmark/DCGC-3518/2020|2020-08-31|EPI_ISL_616309|Original|hCoV-19^^Midtjylland|Human|Department of Virus and Microbiological Special Diagnostics|Albertsen lab|Michaelsen|Denmark
>Spike|hCoV-19/United Arab Emirates/0048/2020|2020-05-18|EPI_ISL_698256|Original|hCoV-19^^Abu Dhabi|Human|Group 42 (G42) Healthcare|G42 Healthcare|Ogrodzki|United Arab Emirates
>Spike|hCoV-19/England/ALDP-A70845/2020|2020-10-14|EPI_ISL_608327|Original|hCoV-19^^England|Human|Lighthouse Lab in Alderley Park|Wellcome Sanger Institute for the COVID-19 Genomics UK (COG-UK) consortium|Davies|United Kingdom
>Spike|hCoV-19/Spain/PV-ISCIII-201382/2020|2020-03-02|EPI_ISL_418253|Original|hCoV-19^^Basque Country|Human|HOSPITAL TXAGORRITXU|Instituto de Salud Carlos III|Caballero|Spain

>Spike|hCoV-19/Canada/NB-214/2020|2020-03-27|EPI_ISL_479630|Original|hCoV-19^^New Brunswick|Human|Dr. Georges-L.-Dumont University Hospital Centre|National Microbiology Laboratory|Majer|Canada
>Spike|hCoV-19/USA/TX-HMH-6926/2020|2020-06-25|EPI_ISL_546357|Original|hCoV-19^^Texas|Human|Houston Methodist Hospital|Houston Methodist Hospital|Long|USA
>Spike|hCoV-19/Ireland/D-NVRL-71IRL96183/2020|2020-08-01|EPI_ISL_525373|Original|hCoV-19^^Dublin|Human|National Virus Reference Laboratory|National Virus Reference Laboratory|Gonzalez|Ireland
>Spike|hCoV-19/Switzerland/ZH-ETHZ-140070/2020|2020-04-28|EPI_ISL_476118|Original|hCoV-19^^Zurich|Human|Viollier AG|Department of Biosystems Science and Engineering|Nadeau|Switzerland
>Spike|hCoV-19/England/NOTT-11060A/2020|2020-04-27|EPI_ISL_441537|Original|hCoV-19^^England|Human|Queens Medical Centre|COVID-19 Genomics UK (COG-UK) Consortium|Loose|United Kingdom
>Spike|hCoV-19/England/MILK-A79A55/2020|2020-10-15|EPI_ISL_606753|Original|hCoV-19^^England|Human|Lighthouse Lab in Milton Keynes|Wellcome Sanger Institute for the COVID-19 Genomics UK (COG-UK) consortium|Davies|United Kingdom
>Spike|hCoV-19/USA/TX-DSHS-1794/2020|2020-06-13|EPI_ISL_738168|Original|hCoV-19^^Texas|Human|Texas Department of State Health Services|Texas Department of State Health Services|Rahman|USA
>Spike|hCoV-19/Australia/VIC5579/2020|2020-07-16|EPI_ISL_518397|Original|hCoV-19^^Victoria|Human|Victorian Infectious Diseases Reference Laboratory (VIDRL)|VIDRL and MDU-PHL|Schultz|Australia
>Spike|hCoV-19/England/CAMC-A59402/2020|2020-10-09|EPI_ISL_597194|Original|hCoV-19^^England|Human|Lighthouse Lab in Cambridge|Wellcome Sanger Institute for the COVID-19 Genomics UK (COG-UK) consortium|Davies|United Kingdom
>Spike|hCoV-19/Denmark/DCGC-14793/2020|2020-11-23|EPI_ISL_711986|Original|hCoV-19^^Hovedstaden|Human|Department of Virus and Microbiological Special Diagnostics|Albertsen Lab|Michaelsen|Denmark
>Spike|hCoV-19/Switzerland/GE-SNRCI-8431/2020|2020-03-00|EPI_ISL_548226|Original|hCoV-19^^Geneve|Human|Laboratoire de Virologie|Swiss National Reference Centre for Influenza|Gon\u00e7alves Cabecinhas|Switzerland
>Spike|hCoV-19/Denmark/DCGC-8450/2020|2020-10-26|EPI_ISL_617911|Original|hCoV-19^^Hovedstaden|Human|Department of Virus and Microbiological Special Diagnostics|Albertsen lab|Michaelsen|Denmark
>Spike|hCoV-19/Australia/VIC16887/2020|2020-08-06|EPI_ISL_663624|Original|hCoV-19^^Victoria|Human|Microbiological Diagnostic Unit - Public Health Laboratory (MDU-PHL)|MDU-PHL|Schultz|Australia
>Spike|hCoV-19/USA/NY-NYUMC318/2020|2020-03-30|EPI_ISL_430345|Original|hCoV-19^^New York|Human|NYU Langone Health|Departments of Pathology and Medicine|Maurano|USA
>Spike|hCoV-19/Japan/PG-0316/2020|2020-03-25|EPI_ISL_479991|Original|hCoV-19^^Kyusyu|Human|Kumamoto City Public Health Research Institute|Pathogen Genomics Center|Sekizuka|Japan
>Spike|hCoV-19/Switzerland/BE-ETHZ-350250/2020|2020-10-30|EPI_ISL_729025|Original|hCoV-19^^Bern|Human|Viollier AG|Department of Biosystems Science and Engineering|Nadeau|Switzerland
>Spike|hCoV-19/Austria/CeMM0758/2020|2020-03-09|EPI_ISL_583691|Original|hCoV-19^^Ischgl|Human|Institut f\u00fcr Virologie am Department f\u00fcr Hygiene|Bergthaler laboratory|Bergthaler|Austria
>Spike|hCoV-19/England/ALDP-A2DE09/2020|2020-10-06|EPI_ISL_586933|Original|hCoV-19^^England|Human|Lighthouse Lab in Alderley Park|Wellcome Sanger Institute for the COVID-19 Genomics UK (COG-UK) consortium|Davies|United Kingdom
>Spike|hCoV-19/England/CAMB-7EC90/2020|2020-04-07|EPI_ISL_442888|Original|hCoV-19^^England|Human|Department of Pathology|Wellcome Sanger Institute for the COVID-19 Genomics UK (COG-UK) consortium|Amato|United Kingdom
>Spike|hCoV-19/England/ALDP-929D4D/2020|2020-06-22|EPI_ISL_552813|Original|hCoV-19^^England|Human|Lighthouse Lab in Alderley Park|Wellcome Sanger Institute for the COVID-19 Genomics UK (COG-UK) consortium|Davies|United Kingdom

>Spike|hCoV-19/England/NORW-F1178/2020|2020-10-15|EPI_ISL_627595|Original|hCoV-
19^^England|Human|Quadram Institute Bioscience|COVID-19 Genomics UK (COG-UK)
Consortium|Alikhan|United Kingdom
>Spike|hCoV-19/Australia/VIC11778/2020|2020-07-25|EPI_ISL_563496|Original|hCoV-
19^^Victoria|Human|Microbiological Diagnostic Unit - Public Health Laboratory (MDU-
PHL)|MDU-PHL|Schultz|Australia
>Spike|hCoV-19/England/MILK-B56C48/2020|2020-11-04|EPI_ISL_656950|Original|hCoV-
19^^England|Human|Lighthouse Lab in Milton Keynes|Wellcome Sanger Institute for the
COVID-19 Genomics UK (COG-UK) Consortium|Davies|United Kingdom
>Spike|hCoV-19/England/CAMB-7F3B8/2020|2020-04-07|EPI_ISL_442835|Original|hCoV-
19^^England|Human|Department of Pathology|Wellcome Sanger Institute for the COVID-19
Genomics UK (COG-UK) consortium|Amato|United Kingdom
>Spike|hCoV-19/South Africa/KRISP-K000615/2020|2020-07-
02|EPI_ISL_509267|Original|hCoV-19^^KwaZulu-Natal|Human|NHLS-IALCH|KRISP|de
Oliveira|South Africa
>Spike|hCoV-19/Japan/PG-5077/2020|2020-08-00|EPI_ISL_690187|Original|hCoV-
19^^Japan|Human|Pathogen Genomics Center|Pathogen Genomics Center|Sekizuka|Japan
>Spike|hCoV-19/USA/TX-HMH-MCoV-18965/2020|2020-11-25|EPI_ISL_785251|Original|hCoV-
19^^Texas|Human|Houston Methodist Hospital|Houston Methodist Hospital|Long|USA
>Spike|hCoV-19/England/LIVE-9EB9D/2020|2020-04-04|EPI_ISL_453372|Original|hCoV-
19^^England|Human|Liverpool Clinical Laboratories|COVID-19 Genomics UK (COG-UK)
Consortium|Haldenby|United Kingdom
>Spike|hCoV-19/India/MH-NIV-13868/2020|2020-04-23|EPI_ISL_541700|Original|hCoV-
19^^Maharashtra|Human|National Institute of Virology|National Institute of
Virology|Potdar|India
>Spike|hCoV-19/USA/NY-QDX-75/2020|2020-03-13|EPI_ISL_467902|Original|hCoV-19^^New
York|Human|Quest Diagnostics|Quest Diagnostics|Kagan|USA
>Spike|hCoV-19/Iceland/297/2020|2020-03-18|EPI_ISL_417626|Original|hCoV-
19^^Reykjavik|Human|The National University Hospital of Iceland|deCODE
genetics|J\u00f3nsson|Iceland
>Spike|hCoV-19/Australia/VIC4688/2020|2020-07-15|EPI_ISL_519076|Original|hCoV-
19^^Victoria|Human|Microbiological Diagnostic Unit - Public Health Laboratory (MDU-
PHL)|MDU-PHL|Schultz|Australia
>Spike|hCoV-19/England/CAMB-1AEAE7/2020|2020-04-30|EPI_ISL_524597|Original|hCoV-
19^^England|Human|Department of Pathology|Wellcome Sanger Institute for the COVID-19
Genomics UK (COG-UK) consortium|Davies|United Kingdom
>Spike|hCoV-19/USA/MI-MDHHS-SC22223/2020|2020-10-27|EPI_ISL_644347|Original|hCoV-
19^^Michigan|Human|Michigan Department of Health and Human Services|Michigan
Department of Health and Human Services|Jacob|USA
>Spike|hCoV-19/Japan/PG-8036/2020|2020-08-00|EPI_ISL_687137|Original|hCoV-
19^^Japan|Human|Pathogen Genomics Center|Pathogen Genomics Center|Sekizuka|Japan
>Spike|hCoV-19/USA/TX-HMH-3546/2020|2020-06-14|EPI_ISL_543009|Original|hCoV-
19^^Texas|Human|Houston Methodist Hospital|Houston Methodist Hospital|Long|USA
>Spike|hCoV-19/USA/FL-BPHL-0953/2020|2020-07-07|EPI_ISL_517843|Original|hCoV-
19^^Florida|Human|Florida Bureau of Public Health Laboratories|Florida Bureau of
Public Health Laboratories|Blanton|USA
>Spike|hCoV-19/England/MILK-B3A56D/2020|2020-11-02|EPI_ISL_658780|Original|hCoV-
19^^England|Human|Lighthouse Lab in Milton Keynes|Wellcome Sanger Institute for the
COVID-19 Genomics UK (COG-UK) Consortium|Davies|United Kingdom
>Spike|hCoV-19/England/ALDP-8FE771/2020|2020-06-23|EPI_ISL_551511|Original|hCoV-
19^^England|Human|Lighthouse Lab in Alderley Park|Wellcome Sanger Institute for the
COVID-19 Genomics UK (COG-UK) consortium|Davies|United Kingdom
>Spike|hCoV-19/Australia/VIC8322/2020|2020-08-13|EPI_ISL_564938|Original|hCoV-
19^^Victoria|Human|Microbiological Diagnostic Unit - Public Health Laboratory (MDU-
PHL)|MDU-PHL|Schultz|Australia
>Spike|hCoV-19/England/QEUH-9C895B/2020|2020-09-10|EPI_ISL_567262|Original|hCoV-
19^^England|Human|Lighthouse Lab in Glasgow|Wellcome Sanger Institute for the COVID-
19 Genomics UK (COG-UK) consortium|Davies|United Kingdom

>Spike|hCoV-19/Canada/ON-PHL-20-03788/2020|2020-11-00|EPI_ISL_775049|Original|hCoV-19^^Ontario|Human|Public Health Ontario Laboratory|Public Health Ontario Laboratory|Guthrie|Canada
>Spike|hCoV-19/England/QEUH-960BE6/2020|2020-07-27|EPI_ISL_531977|Original|hCoV-19^^England|Human|Lighthouse Lab in Glasgow|Wellcome Sanger Institute for the COVID-19 Genomics UK (COG-UK) consortium|Davies|United Kingdom
>Spike|hCoV-19/Saudi Arabia/KAUST-JEDDAH1296/2020|2020-06-30|EPI_ISL_677925|Original|hCoV-19^^Jeddah|Human|Pathogen Genomics Lab King Abdullah University of Science and Technology(KAUST)|Pathogen Genomics Lab King Abdullah University of Science and Technology(KAUST)|Mohamed Ghazzali|Saudi Arabia
>Spike|hCoV-19/USA/MN-MDH-2061/2020|2020-11-07|EPI_ISL_683690|Original|hCoV-19^^Minnesota|Human|Essentia Health-St. Mary's Medical Center|Minnesota Department of Health|Garfin|USA
>Spike|hCoV-19/Ireland/LS-NVRL-78IRL80633/2020|2020-11-24|EPI_ISL_752597|Original|hCoV-19^^Laois|Human|National Virus Reference Laboratory|National Virus Reference Laboratory|Gonzalez|Ireland
>Spike|hCoV-19/Luxembourg/LNS9732596/2020|2020-10-27|EPI_ISL_744974|Original|hCoV-19^^Luxembourg|Human|Laboratoire national de sant\u00e9|Laboratoire national de sant\u00e9|Wienecke-Baldacchino|Luxembourg
>Spike|hCoV-19/England/NOTT-10E20B/2020|2020-03-19|EPI_ISL_425512|Original|hCoV-19^^England|Human|Queens Medical Centre|COVID-19 Genomics UK (COG-UK) Consortium|Loose|United Kingdom
>Spike|hCoV-19/USA/WI-WSLH-200721/2020|2020-10-03|EPI_ISL_631477|Original|hCoV-19^^Wisconsin|Human|Wisconsin State Laboratory of Hygiene Communicable Disease Division|Wisconsin State Laboratory of Hygiene Communicable Disease Division|Florek|USA
>Spike|hCoV-19/Wales/PHWC-15CC88/2020|2020-05-07|EPI_ISL_472602|Original|hCoV-19^^Wales|Human|Wales Specialist Virology Centre Sequencing lab|COVID-19 Genomics UK (COG-UK) Consortium|Moore|United Kingdom
>Spike|hCoV-19/Wales/PHWC-497BAF/2020|2020-11-23|EPI_ISL_726942|Original|hCoV-19^^Wales|Human|Originating lab|COVID-19 Genomics UK (COG-UK) Consortium|Moore|United Kingdom
>Spike|hCoV-19/Serbia/Pancevo-007910104/2020|2020-03-31|EPI_ISL_676576|Original|hCoV-19^^Pancevo|Human|Scientific Veterinary Institute Novi Sad|Veterinary Specialized Institute \Kraljevo\|Vidanovic|Serbia
>Spike|hCoV-19/Denmark/DCGC-3462/2020|2020-08-31|EPI_ISL_616249|Original|hCoV-19^^Sjaelland|Human|Department of Virus and Microbiological Special Diagnostics|Albertsen lab|Michaelsen|Denmark
>Spike|hCoV-19/England/QEUH-CC9377/2020|2020-12-21|EPI_ISL_781919|Original|hCoV-19^^England|Human|Lighthouse Lab in Glasgow|Wellcome Sanger Institute for the COVID-19 Genomics UK (COG-UK) Consortium|Davies|United Kingdom
>Spike|hCoV-19/South Africa/UFS-VIRO-NGS-20/2020|2020-10-09|EPI_ISL_682329|Original|hCoV-19^^Free State|Human|NHLS Universitas Academic|UFS Virology|Bester|South Africa
>Spike|hCoV-19/England/ALDP-A511D6/2020|2020-10-07|EPI_ISL_597990|Original|hCoV-19^^England|Human|Lighthouse Lab in Alderley Park|Wellcome Sanger Institute for the COVID-19 Genomics UK (COG-UK) consortium|Davies|United Kingdom
>Spike|hCoV-19/USA/AZ-TG668352/2020|2020-04-19|EPI_ISL_696274|Original|hCoV-19^^Arizona|Human|Sonora Quest Laboratories|TGen North|French|USA
>Spike|hCoV-19/Romania/ROSV_1296/2020|2020-04-09|EPI_ISL_491050|Original|hCoV-19^^Suceava|Human|Suceava County Emergency Hospital|\Stefan cel Mare\ University Metagenomics Lab|Lobiuc|Romania
>Spike|hCoV-19/Denmark/DCGC-2605/2020|2020-08-17|EPI_ISL_617842|Original|hCoV-19^^Midtjylland|Human|Department of Virus and Microbiological Special Diagnostics|Albertsen lab|Michaelsen|Denmark
>Spike|hCoV-19/England/ALDP-A2FB97/2020|2020-10-05|EPI_ISL_587136|Original|hCoV-19^^England|Human|Lighthouse Lab in Alderley Park|Wellcome Sanger Institute for the COVID-19 Genomics UK (COG-UK) consortium|Davies|United Kingdom

>Spike|hCoV-19/England/CAMB-1AD1C5/2020|2020-04-29|EPI_ISL_447976|Original|hCoV-19^^England|Human|Department of Pathology|COVID-19 Genomics UK (COG-UK) Consortium|Meredith|United Kingdom
>Spike|hCoV-19/England/ALDP-AA4EBF/2020|2020-10-22|EPI_ISL_630899|Original|hCoV-19^^England|Human|Lighthouse Lab in Alderley Park|Wellcome Sanger Institute for the COVID-19 Genomics UK (COG-UK) consortium|Davies|United Kingdom
>Spike|hCoV-19/USA/CA-CZB-4028/2020|2020-08-06|EPI_ISL_548670|Original|hCoV-19^^California|Human|County of Santa Clara Public Health Department|Chan-Zuckerberg Biohub|Bhatt|USA
>Spike|hCoV-19/Denmark/DCGC-12510/2020|2020-11-16|EPI_ISL_682691|Original|hCoV-19^^Hovedstaden|Human|Department of Virus and Microbiological Special Diagnostics|Albertsen Lab|Michaelsen|Denmark
>Spike|hCoV-19/USA/NY-Wadsworth-97721-01/2020|2020-05-13|EPI_ISL_676924|Original|hCoV-19^^New York|Human|Wadsworth Center|Wadsworth Center|Lasek-Nesselquist|USA
>Spike|hCoV-19/Australia/VIC3201/2020|2020-07-02|EPI_ISL_520887|Original|hCoV-19^^Victoria|Human|Victorian Infectious Diseases Reference Laboratory (VIDRL)|VIDRL and MDU-PHL|Schultz|Australia
>Spike|hCoV-19/Australia/VIC1297/2020|2020-04-09|EPI_ISL_430713|Original|hCoV-19^^Victoria|Human|Victorian Infectious Diseases Reference Laboratory (VIDRL)|Microbiological Diagnostic Unit Public Health Laboratory and Victorian Infectious Diseases Reference Laboratory|Schultz|Australia
>Spike|hCoV-19/USA/TX-HMH-MCoV-9047/2020|2020-07-03|EPI_ISL_780918|Original|hCoV-19^^Texas|Human|Houston Methodist Hospital|Houston Methodist Hospital|Long|USA
>Spike|hCoV-19/DRC/11310/2020|2020-05-25|EPI_ISL_527529|Original|hCoV-19^^Kinshasa|Human|Viral Respiratory Lab|Pathogen Sequencing Lab|Lokilo|Democratic Republic of the Congo
>Spike|hCoV-19/Spain/MD-IBV-006363/2020|2020-05-04|EPI_ISL_510106|Original|hCoV-19^^Madrid|Human|Hospital General Universitario Gregorio Mara\u00f1\u00f3n|SeqCOVID-SPAIN consortium/IBV(CSIC)|Comas|Spain
>Spike|hCoV-19/USA/TX-HMH-3803/2020|2020-06-15|EPI_ISL_543207|Original|hCoV-19^^Texas|Human|Houston Methodist Hospital|Houston Methodist Hospital|Long|USA
>Spike|hCoV-19/USA/WA-S1685/2020|2020-05-29|EPI_ISL_496111|Original|hCoV-19^^Washington|Human|Washington State Department of Health|Seattle Flu Study|Lee|USA
>Spike|hCoV-19/India/WB-S42/2020|2020-05-03|EPI_ISL_455664|Original|hCoV-19^^West Bengal|Human|ICMR-National Institute of Cholera and Enteric Diseases|National Institute of Biomedical Genomics|Maitra|India
>Spike|hCoV-19/USA/WA-S447/2020|2020-04-01|EPI_ISL_434207|Original|hCoV-19^^Washington|Human|Washington State Department of Health|Seattle Flu Study|Bedford|USA
>Spike|hCoV-19/Gambia/GC19-3529/2020|2020-07-27|EPI_ISL_561191|Original|hCoV-19^^Kombo|Human|MRCG at LSHTM Genomics lab|MRCG at LSHTM Genomics lab|Sesay|Gambia
>Spike|hCoV-19/Denmark/DCGC-19800/2020|2020-12-07|EPI_ISL_747796|Original|hCoV-19^^Midtjylland|Human|Department of Virus and Microbiological Special Diagnostics|Albertsen Lab|Michaelsen|Denmark
>Spike|hCoV-19/England/ALDP-B1AB9E/2020|2020-11-01|EPI_ISL_645710|Original|hCoV-19^^England|Human|Lighthouse Lab in Alderley Park|Wellcome Sanger Institute for the COVID-19 Genomics UK (COG-UK) Consortium|Davies|United Kingdom
>Spike|hCoV-19/England/MILK-BB1FBB/2020|2020-11-18|EPI_ISL_720208|Original|hCoV-19^^England|Human|Lighthouse Lab in Milton Keynes|Wellcome Sanger Institute for the COVID-19 Genomics UK (COG-UK) Consortium|Davies|United Kingdom
>Spike|hCoV-19/USA/MN-GMF-15923/2020|2020-08-18|EPI_ISL_547713|Original|hCoV-19^^Minnesota|Human|Gundersen Molecular Diagnostics Laboratory|Kabara Cancer Research Institute|Kenny|USA
>Spike|hCoV-19/Belgium/rega-0617717/2020|2020-06-17|EPI_ISL_738220|Original|hCoV-19^^Brussel|Human|UZ Leuven|KU Leuven|Maes|Belgium
>Spike|hCoV-19/USA/TX-DSHS-1950/2020|2020-06-21|EPI_ISL_765914|Original|hCoV-19^^Texas|Human|TXDSHS|TXDSHS|Rahman|USA

>Spike|hCoV-19/England/CAMC-B08E8B/2020|2020-10-29|EPI_ISL_673198|Original|hCoV-
19^^England|Human|Lighthouse Lab in Cambridge|Wellcome Sanger Institute for the
COVID-19 Genomics UK (COG-UK) Consortium|Davies|United Kingdom
>Spike|hCoV-19/England/CAMC-B347A9/2020|2020-11-05|EPI_ISL_659729|Original|hCoV-
19^^England|Human|Lighthouse Lab in Cambridge|Wellcome Sanger Institute for the
COVID-19 Genomics UK (COG-UK) Consortium|Davies|United Kingdom
>Spike|hCoV-19/England/QEUH-9E394B/2020|2020-09-22|EPI_ISL_584877|Original|hCoV-
19^^England|Human|Northumbria University / South Tees Hospitals NHS Foundation Trust
/ North Cumbria Integrated Care NHS Foundation Trust / North Tees and Hartlepool NHS
Foundation Trust / Newcastle Hospitals NHS Foundation Trust|COVID-19 Genomics UK
(COG-UK) Consortium|Bashton|United Kingdom
>Spike|hCoV-19/England/NORW-EADAB/2020|2020-04-29|EPI_ISL_457376|Original|hCoV-
19^^England|Human|Quadram Institute Bioscience|COVID-19 Genomics UK (COG-UK)
Consortium|Alikhan|United Kingdom
>Spike|hCoV-19/Wales/PHWC-488EC6/2020|2020-10-27|EPI_ISL_639513|Original|hCoV-
19^^Wales|Human|Wales Specialist Virology Centre Sequencing lab|COVID-19 Genomics UK
(COG-UK) Consortium|Moore|United Kingdom
>Spike|hCoV-19/England/ALDP-A2D4F8/2020|2020-10-05|EPI_ISL_586694|Original|hCoV-
19^^England|Human|Lighthouse Lab in Alderley Park|Wellcome Sanger Institute for the
COVID-19 Genomics UK (COG-UK) consortium|Davies|United Kingdom
>Spike|hCoV-19/USA/OR-OHSU-2898/2020|2020-09-04|EPI_ISL_667398|Original|hCoV-
19^^Oregon|Human|OHSU Lab Services Molecular Microbiology Lab|Oregon SARS-CoV-2
Genome Sequencing Center|O'Roak|USA
>Spike|hCoV-19/Singapore/1246/2020|2020-05-01|EPI_ISL_648664|Original|hCoV-
19^^Singapore|Human|Department of Laboratory Medicine|Department of Laboratory
Medicine|Chen|Singapore
>Spike|hCoV-19/Australia/VIC4675/2020|2020-07-16|EPI_ISL_519063|Original|hCoV-
19^^Victoria|Human|Microbiological Diagnostic Unit - Public Health Laboratory (MDU-
PHL)|MDU-PHL|Schultz|Australia
>Spike|hCoV-19/USA/WA-UW-46926/2020|2020-12-08|EPI_ISL_756298|Original|hCoV-
19^^Washington|Human|UW Virology Lab|UW Virology Lab|Roychoudhury|USA
>Spike|hCoV-19/USA/NY-NYCPHL-001273/2020|2020-11-09|EPI_ISL_683864|Original|hCoV-
19^^New York|Human|DOHMH Jamaica|New York City Public Health Laboratory|Wang|USA
>Spike|hCoV-19/Denmark/DCGC-22260/2020|2020-12-14|EPI_ISL_758642|Original|hCoV-
19^^Hovedstaden|Human|Department of Virus and Microbiological Special
Diagnostics|Albertsen Lab|Michaelsen|Denmark
>Spike|hCoV-19/USA/CO-CDPHE-2011020331/2020|2020-11-01|EPI_ISL_771243|Original|hCoV-
19^^Colorado|Human|Colorado Department of Public Health and Environment|Colorado
Department of Puplic Health and Environment|Bankers|USA
>Spike|hCoV-19/Wales/PHWC-4A576E/2020|2020-12-16|EPI_ISL_764915|Original|hCoV-
19^^Wales|Human|Wales Specialist Virology Centre Sequencing lab|COVID-19 Genomics UK
(COG-UK) Consortium|Moore|United Kingdom
>Spike|hCoV-19/USA/CA-QDX-706/2020|2020-03-20|EPI_ISL_571423|Original|hCoV-
19^^California|Human|Quest Diagnostics|Quest Diagnostics|Kagan|USA
>Spike|hCoV-19/Japan/PG-3153/2020|2020-05-00|EPI_ISL_691273|Original|hCoV-
19^^Japan|Human|Pathogen Genomics Center|Pathogen Genomics Center|Sekizuka|Japan
>Spike|hCoV-19/Denmark/DCGC-4660/2020|2020-09-21|EPI_ISL_621892|Original|hCoV-
19^^Sjaelland|Human|Department of Virus and Microbiological Special
Diagnostics|Albertsen lab|Michaelsen|Denmark
>Spike|hCoV-19/Germany/HH-hpi-p1984/2020|2020-03-26|EPI_ISL_776302|Original|hCoV-
19^^Hamburg|Human|University Medical Center Hamburg Eppendorf|Heinrich Pette
Institute|Robitaille|Germany
>Spike|hCoV-19/USA/UT-UPHL-04307/2020|2020-05-15|EPI_ISL_575137|Original|hCoV-
19^^Utah|Human|Utah Public Health Laboratory|Utah Public Health Laboratory|Young|USA
>Spike|hCoV-19/England/NOTT-1100FD/2020|2020-04-10|EPI_ISL_441471|Original|hCoV-
19^^England|Human|Queens Medical Centre|COVID-19 Genomics UK (COG-UK)
Consortium|Loose|United Kingdom
>Spike|hCoV-19/England/ALDP-A859FF/2020|2020-10-18|EPI_ISL_610619|Original|hCoV-
19^^England|Human|Lighthouse Lab in Alderley Park|Wellcome Sanger Institute for the
COVID-19 Genomics UK (COG-UK) consortium|Davies|United Kingdom

>Spike|hCoV-19/Scotland/QEUH-AAAA3D/2020|2020-10-21|EPI_ISL_651505|Original|hCoV-19^^Scotland|Human|Oxford Viromics|COVID-19 Genomics UK (COG-UK) Consortium|Golubchik|United Kingdom
>Spike|hCoV-19/Australia/NSW2032/2020|2020-03-21|EPI_ISL_500607|Original|hCoV-19^^New South Wales|Human|Area of Virology|Area of Virology|Bull|Australia
>Spike|hCoV-19/USA/CO-CDPHE-2100015096/2020|2020-11-21|EPI_ISL_771310|Original|hCoV-19^^Colorado|Human|Colorado Department of Public Health and Environment|Colorado Department of Puplic Health and Environment|Bankers|USA
>Spike|hCoV-19/Australia/VIC5064/2020|2020-07-19|EPI_ISL_519867|Original|hCoV-19^^Victoria|Human|Microbiological Diagnostic Unit - Public Health Laboratory (MDU-PHL)|MDU-PHL|Schultz|Australia
>Spike|hCoV-19/England/CAMC-A57738/2020|2020-10-08|EPI_ISL_609193|Original|hCoV-19^^England|Human|Lighthouse Lab in Cambridge|Wellcome Sanger Institute for the COVID-19 Genomics UK (COG-UK) consortium|Davies|United Kingdom
>Spike|hCoV-19/England/ALDP-A95E48/2020|2020-10-20|EPI_ISL_624583|Original|hCoV-19^^England|Human|Lighthouse Lab in Alderley Park|Wellcome Sanger Institute for the COVID-19 Genomics UK (COG-UK) consortium|Davies|United Kingdom
>Spike|hCoV-19/Australia/SAP046/2020|2020-03-22|EPI_ISL_451085|Original|hCoV-19^^South Australia|Human|SA Pathology|SA Pathology|Leong|Australia
>Spike|hCoV-19/USA/WA-UW-23355/2020|2020-08-08|EPI_ISL_576431|Original|hCoV-19^^Washington|Human|UW Virology Lab|UW Virology Lab|Roychoudhury|USA
>Spike|hCoV-19/USA/WI-GMF-15609/2020|2020-08-15|EPI_ISL_547706|Original|hCoV-19^^Wisconsin|Human|Gundersen Molecular Diagnostics Laboratory|Kabara Cancer Research Institute|Kenny|USA
>Spike|hCoV-19/England/QEUH-BA2D0E/2020|2020-11-18|EPI_ISL_721141|Original|hCoV-19^^England|Human|Lighthouse Lab in Glasgow|Wellcome Sanger Institute for the COVID-19 Genomics UK (COG-UK) Consortium|Davies|United Kingdom
>Spike|hCoV-19/Scotland/EDB6215/2020|2020-05-19|EPI_ISL_473919|Original|hCoV-19^^Scotland|Human|Virology Department|COVID-19 Genomics UK (COG-UK) Consortium|Williams|United Kingdom
>Spike|hCoV-19/USA/UT-08526/2020|2020-05-22|EPI_ISL_524289|Original|hCoV-19^^Utah|Human|Utah Public Health Laboratory|Utah Public Health Laboratory|Young|USA
>Spike|hCoV-19/USA/CA-CZB-1415/2020|2020-03-15|EPI_ISL_468390|Original|hCoV-19^^California|Human|County of San Luis Obispo Public Health Laboratory|Chan-Zuckerberg Biohub|Hao|USA
>Spike|hCoV-19/England/OXON-AEF9E/2020|2020-04-07|EPI_ISL_448800|Original|hCoV-19^^England|Human|Oxford Viromics|COVID-19 Genomics UK (COG-UK) Consortium|Golubchik|United Kingdom
>Spike|hCoV-19/England/NORW-F8F95/2020|2020-11-25|EPI_ISL_702861|Original|hCoV-19^^England|Human|Quadram Institute Bioscience|COVID-19 Genomics UK (COG-UK) Consortium|Alikhan|United Kingdom
>Spike|hCoV-19/Northern Ireland/NIRE-FC8D2/2020|2020-04-22|EPI_ISL_459347|Original|hCoV-19^^Northern Ireland|Human|Regional Virus Laboratory|Wellcome Sanger Institute for the COVID-19 Genomics UK (COG-UK) consortium|Davies|United Kingdom
>Spike|hCoV-19/England/LIVE-DBE981/2020|2020-10-30|EPI_ISL_664623|Original|hCoV-19^^England|Human|Liverpool Clinical Laboratories|COVID-19 Genomics UK (COG-UK) Consortium|Haldenby|United Kingdom
>Spike|hCoV-19/USA/UT-03851/2020|2020-04-07|EPI_ISL_470746|Original|hCoV-19^^Utah|Human|Utah Public Health Laboratory|Utah Public Health Laboratory|Butz|USA
>Spike|hCoV-19/Denmark/DCGC-3076/2020|2020-08-31|EPI_ISL_616856|Original|hCoV-19^^Hovedstaden|Human|Department of Virus and Microbiological Special Diagnostics|Albertsen lab|Michaelsen|Denmark
>Spike|hCoV-19/Sweden/20-07666/2020|2020-04-20|EPI_ISL_445238|Original|hCoV-19^^Skane|Human|\u00c3\u2013resundslakarna|The Public Health Agency of Sweden|Svartstr\u00f6m|Sweden
>Spike|hCoV-19/USA/TX-DSHS-0905/2020|2020-06-23|EPI_ISL_610041|Original|hCoV-19^^Texas|Human|Texas Department of State Health Services|Texas Department of State Health Services|Rahman|USA

>Spike|hCoV-19/Netherlands/UT-RIVM-20873/2020|2020-12-
21|EPI_ISL_790841|Original|hCoV-19^^Utrecht|Human|Dutch COVID-19 response
team|National Institute for Public Health and the Environment
(RIVM)|Meijer|Netherlands
>Spike|hCoV-19/USA/VA-DCLS-0637/2020|2020-04-10|EPI_ISL_495309|Original|hCoV-
19^^Virginia|Human|Virginia DCLS|Virginia DCLS|St. Jacques|USA
>Spike|hCoV-19/Japan/PG-0096/2020|2020-03-00|EPI_ISL_684121|Original|hCoV-
19^^Japan|Human|Pathogen Genomics Center|Pathogen Genomics Center|Sekizuka|Japan
>Spike|hCoV-19/England/ALDP-AA34CD/2020|2020-10-22|EPI_ISL_623890|Original|hCoV-
19^^England|Human|Lighthouse Lab in Alderley Park|Wellcome Sanger Institute for the
COVID-19 Genomics UK (COG-UK) consortium|Davies|United Kingdom
>Spike|hCoV-19/England/ALDP-CB5BA3/2020|2020-12-20|EPI_ISL_761856|Original|hCoV-
19^^England|Human|Lighthouse Lab in Alderley Park|Wellcome Sanger Institute for the
COVID-19 Genomics UK (COG-UK) Consortium|Davies|United Kingdom
>Spike|hCoV-19/Japan/PG-2272/2020|2020-04-00|EPI_ISL_685877|Original|hCoV-
19^^Japan|Human|Pathogen Genomics Center|Pathogen Genomics Center|Sekizuka|Japan
>Spike|hCoV-19/USA/UT-UPHL-2012739315/2020|2020-08-15|EPI_ISL_746287|Original|hCoV-
19^^Utah|Human|Ginkgo Bioworks Clinical Laboratory|Utah Public Health
Laboratory|Young|USA
>Spike|hCoV-19/Belgium/ULG-11028/2020|2020-11-30|EPI_ISL_722946|Original|hCoV-
19^^Liege|Human|Department of Clinical Microbiology|GIGA Medical
Genomics|Durkin|Belgium
>Spike|hCoV-19/England/QEUH-9B9225/2020|2020-09-07|EPI_ISL_549584|Original|hCoV-
19^^England|Human|Lighthouse Lab in Glasgow|Wellcome Sanger Institute for the COVID-
19 Genomics UK (COG-UK) consortium|Davies|United Kingdom
>Spike|hCoV-19/England/NORT-289771/2020|2020-04-01|EPI_ISL_458643|Original|hCoV-
19^^England|Human|NU-OMICS DNA Sequencing research facility|Wellcome Sanger Institute
for the COVID-19 Genomics UK (COG-UK) consortium|Davies|United Kingdom
>Spike|hCoV-19/England/SHEF-10D510E/2020|2020-12-20|EPI_ISL_764111|Original|hCoV-
19^^England|Human|Virology Department|COVID-19 Genomics UK (COG-UK) Consortium|de
Silva|United Kingdom
>Spike|hCoV-19/France/ARA-71481/2020|2020-09-12|EPI_ISL_693494|Original|hCoV-
19^^Auvergne-Rh\u00f4ne-Alpes|Human|CNR Virus des Infections Respiratoires - France
SUD|CNR Virus des Infections Respiratoires - France SUD|Bal|France
>Spike|hCoV-19/Luxembourg/LNS3640938/2020|2020-12-15|EPI_ISL_740345|Original|hCoV-
19^^Luxembourg|Human|Laboratoire national de sant\u00e9|Laboratoire national de
sant\u00e9|Wienecke-Baldacchino|Luxembourg
>Spike|hCoV-19/USA/MI-QDX-891/2020|2020-03-19|EPI_ISL_571612|Original|hCoV-
19^^Michigan|Human|Quest Diagnostics|Quest Diagnostics|Kagan|USA
>Spike|hCoV-19/England/ALDP-8D1E9D/2020|2020-06-26|EPI_ISL_557090|Original|hCoV-
19^^England|Human|Lighthouse Lab in Alderley Park|Wellcome Sanger Institute for the
COVID-19 Genomics UK (COG-UK) consortium|Davies|United Kingdom
>Spike|hCoV-19/England/20132005904/2020|2020-03-23|EPI_ISL_423725|Original|hCoV-
19^^England|Human|Respiratory Virus Unit|Respiratory Virus Unit|Platt|United Kingdom
>Spike|hCoV-19/England/CAMC-A57E33/2020|2020-10-09|EPI_ISL_597050|Original|hCoV-
19^^England|Human|Lighthouse Lab in Cambridge|Wellcome Sanger Institute for the
COVID-19 Genomics UK (COG-UK) consortium|Davies|United Kingdom
>Spike|hCoV-19/England/CAMC-B98336/2020|2020-11-15|EPI_ISL_673611|Original|hCoV-
19^^England|Human|Lighthouse Lab in Cambridge|Wellcome Sanger Institute for the
COVID-19 Genomics UK (COG-UK) Consortium|Davies|United Kingdom
>Spike|hCoV-19/Canada/NS-NML-1236/2020|2020-04-05|EPI_ISL_579630|Original|hCoV-
19^^Nova Scotia|Human|QEII Health Sciences Centre|National Microbiology Laboratory
(NML)|Majer|Canada
>Spike|hCoV-19/USA/MI-MDHHS-SC21167/2020|2020-04-03|EPI_ISL_507424|Original|hCoV-
19^^Michigan|Human|Michigan Department of Health and Human Services|Michigan
Department of Health and Human Services|Blankenship|USA
>Spike|hCoV-19/Portugal/PT1469/2020|2020-03-17|EPI_ISL_511428|Original|hCoV-
19^^Portugal|Human|Instituto Nacional de Saude (INSA)|Instituto Nacional de Saude
(INSA)|Borges|Portugal

>Spike|hCoV-19/Bangladesh/DNAS_DRCIM_isl_B-1/2020|2020-07-05|EPI_ISL_774935|Original|hCoV-19^^Dhaka|Human|Designated Reference Institute for Chemical Measurements (DRICM)|DNA SOLUTION LTD.|Rabbi|Bangladesh
>Spike|hCoV-19/Singapore/318/2020|2020-06-02|EPI_ISL_482683|Original|hCoV-19^^Singapore|Human|Singapore General Hospital|Department of Microbiology|Abdul Rahman|Singapore
>Spike|hCoV-19/Denmark/DCGC-6255/2020|2020-10-05|EPI_ISL_620015|Original|hCoV-19^^Hovedstaden|Human|Department of Virus and Microbiological Special Diagnostics|Albertsen lab|Michaelsen|Denmark
>Spike|hCoV-19/England/QEUH-AD1FA7/2020|2020-10-26|EPI_ISL_634710|Original|hCoV-19^^England|Human|Lighthouse Lab in Glasgow|Wellcome Sanger Institute for the COVID-19 Genomics UK (COG-UK) consortium|Davies|United Kingdom
>Spike|hCoV-19/England/MILK-6F6DB5/2020|2020-06-30|EPI_ISL_557370|Original|hCoV-19^^England|Human|Lighthouse Lab in Milton Keynes|Wellcome Sanger Institute for the COVID-19 Genomics UK (COG-UK) consortium|Davies|United Kingdom
>Spike|hCoV-19/England/CAMB-1B88FF/2020|2020-10-30|EPI_ISL_651667|Original|hCoV-19^^England|Human|Department of Pathology|COVID-19 Genomics UK (COG-UK) Consortium|Goodfellow|United Kingdom
>Spike|hCoV-19/England/PORT-2CFCCB/2020|2020-05-07|EPI_ISL_461986|Original|hCoV-19^^England|Human|Centre for Enzyme Innovation|COVID-19 Genomics UK (COG-UK) Consortium|Robson|United Kingdom
>Spike|hCoV-19/Scotland/CVR5927/2020|2020-11-21|EPI_ISL_703750|Original|hCoV-19^^Scotland|Human|West of Scotland Specialist Virology Centre|COVID-19 Genomics UK (COG-UK) Consortium|Robertson|United Kingdom
>Spike|hCoV-19/England/LEED-2A878B/2020|2020-04-01|EPI_ISL_539095|Original|hCoV-19^^England|Human|Leeds Teaching Hospitals NHS Trust and Public Health England|Wellcome Sanger Institute for the COVID-19 Genomics UK (COG-UK) consortium|Davies|United Kingdom
>Spike|hCoV-19/England/LIVE-99D14/2020|2020-04-14|EPI_ISL_439815|Original|hCoV-19^^England|Human|Liverpool Clinical Laboratories|COVID-19 Genomics UK (COG-UK) Consortium|Haldenby|United Kingdom
>Spike|hCoV-19/England/ALDP-A70739/2020|2020-10-15|EPI_ISL_607696|Original|hCoV-19^^England|Human|Lighthouse Lab in Alderley Park|Wellcome Sanger Institute for the COVID-19 Genomics UK (COG-UK) consortium|Davies|United Kingdom
>Spike|hCoV-19/Egypt/EGY-050/2020|2020-08-22|EPI_ISL_527006|Original|hCoV-19^^Egypt|Human|Biological prevention|Biological prevention|Seadawy|Egypt
>Spike|hCoV-19/Italy/PIE-SLL-MS57/2020|2020-03-31|EPI_ISL_569873|Original|hCoV-19^^Piedmont|Human|Amedeo di savoia|Crosetto lab|Harbers|Italy
>Spike|hCoV-19/USA/NY-Wadsworth-200223-01/2020|2020-06-28|EPI_ISL_677058|Original|hCoV-19^^New York|Human|Masonic Medical Research Institute|Wadsworth Center|Lasek-Nesselquist|USA
>Spike|hCoV-19/United Arab Emirates/0389/2020|2020-05-15|EPI_ISL_698564|Original|hCoV-19^^Abu Dhabi|Human|Group 42 (G42) Healthcare|G42 Healthcare|Ogrodzki|United Arab Emirates
>Spike|hCoV-19/New Zealand/20CV0276/2020|2020-03-24|EPI_ISL_579068|Original|hCoV-19^^Hawkes Bay|Human|Canterbury Health Laboratories|Institute of Environmental Science and Research (ESR)|Wang|New Zealand
>Spike|hCoV-19/Australia/NSW885/2020|2020-08-19|EPI_ISL_544966|Original|hCoV-19^^New South Wales|Human|Australian Clinical Labs|NSW Health Pathology - Institute of Clinical Pathology and Medical Research; Westmead Hospital; University of Sydney|Gall|Australia
>Spike|hCoV-19/USA/AZ-TG271575/2020|2020-03-17|EPI_ISL_694052|Original|hCoV-19^^Arizona|Human|TGen North|TGen North|French|USA
>Spike|hCoV-19/England/LOND-1263C20/2020|2020-11-10|EPI_ISL_679482|Original|hCoV-19^^England|Human|University College London|COVID-19 Genomics UK (COG-UK) Consortium|Roy|United Kingdom
>Spike|hCoV-19/England/20144001904/2020|2020-03-30|EPI_ISL_423478|Original|hCoV-19^^England|Human|Respiratory Virus Unit|Respiratory Virus Unit|Platt|United Kingdom
>Spike|hCoV-19/USA/NY-NYUMC586/2020|2020-03-30|EPI_ISL_444703|Original|hCoV-19^^New York|Human|NYU Langone Health|Departments of Pathology and Medicine|Maurano|USA

>Spike|hCoV-19/Japan/IC-0073/2020|2020-07-00|EPI_ISL_591415|Original|hCoV-19^^Japan|Human|Pathogen Genomics Center|Pathogen Genomics Center|Sekizuka|Japan
>Spike|hCoV-19/England/ALDP-B835ED/2020|2020-11-15|EPI_ISL_675254|Original|hCoV-19^^England|Human|Lighthouse Lab in Alderley Park|Wellcome Sanger Institute for the COVID-19 Genomics UK (COG-UK) Consortium|Davies|United Kingdom
>Spike|hCoV-19/Wales/PHWC-48DDB5/2020|2020-11-07|EPI_ISL_680757|Original|hCoV-19^^Wales|Human|Wales Specialist Virology Centre Sequencing lab|COVID-19 Genomics UK (COG-UK) Consortium|Moore|United Kingdom
>Spike|hCoV-19/Mexico/CMX-INMEGEN-11/2020|2020-07-31|EPI_ISL_522941|Original|hCoV-19^^Mexico City|Human|Instituto Nacional de Medicina Gen\u00f3mica|Instituto Nacional de Medicina Gen\u00f3mica|Hidalgo|Mexico
>Spike|hCoV-19/England/QEUH-9AD0DF/2020|2020-09-05|EPI_ISL_559818|Original|hCoV-19^^England|Human|Oxford Viromics|COVID-19 Genomics UK (COG-UK) Consortium|Golubchik|United Kingdom
>Spike|hCoV-19/USA/TX-HMH-MCoV-18730/2020|2020-11-20|EPI_ISL_785075|Original|hCoV-19^^Texas|Human|Houston Methodist Hospital|Houston Methodist Hospital|Long|USA
>Spike|hCoV-19/England/ALDP-B758A9/2020|2020-11-12|EPI_ISL_723677|Original|hCoV-19^^England|Human|Oxford Viromics|COVID-19 Genomics UK (COG-UK) Consortium|Golubchik|United Kingdom
>Spike|hCoV-19/England/ALDP-94E638/2020|2020-06-17|EPI_ISL_555372|Original|hCoV-19^^England|Human|Lighthouse Lab in Alderley Park|Wellcome Sanger Institute for the COVID-19 Genomics UK (COG-UK) consortium|Davies|United Kingdom
>Spike|hCoV-19/Denmark/DCGC-17390/2020|2020-12-14|EPI_ISL_750950|Original|hCoV-19^^Midtjylland|Human|Department of Virus and Microbiological Special Diagnostics|Albertsen Lab|Michaelsen|Denmark
>Spike|hCoV-19/England/C200032845/2020|2020-10-15|EPI_ISL_636712|Original|hCoV-19^^England|Human|Respiratory Virus Unit|Respiratory Virus Unit|Platt|United Kingdom
>Spike|hCoV-19/England/MILK-A7DEFB/2020|2020-10-13|EPI_ISL_606499|Original|hCoV-19^^England|Human|Lighthouse Lab in Milton Keynes|Wellcome Sanger Institute for the COVID-19 Genomics UK (COG-UK) consortium|Davies|United Kingdom
>Spike|hCoV-19/Wales/PHWC-2B05A/2020|2020-04-08|EPI_ISL_432283|Original|hCoV-19^^Wales|Human|Wales Specialist Virology Centre|Public Health Wales Microbiology Cardiff|Moore|United Kingdom
>Spike|hCoV-19/Scotland/CVR3021/2020|2020-04-24|EPI_ISL_439011|Original|hCoV-19^^Scotland|Human|West of Scotland Specialist Virology Centre|COVID-19 Genomics UK (COG-UK) Consortium|Robertson|United Kingdom
>Spike|hCoV-19/England/MILK-BB0927/2020|2020-11-17|EPI_ISL_720371|Original|hCoV-19^^England|Human|Lighthouse Lab in Milton Keynes|Wellcome Sanger Institute for the COVID-19 Genomics UK (COG-UK) Consortium|Davies|United Kingdom
>Spike|hCoV-19/England/ALDP-BBAC51/2020|2020-11-21|EPI_ISL_701752|Original|hCoV-19^^England|Human|Lighthouse Lab in Alderley Park|Wellcome Sanger Institute for the COVID-19 Genomics UK (COG-UK) Consortium|Davies|United Kingdom
>Spike|hCoV-19/Thailand/Bangkok-CONI-0163/2020|2020-03-24|EPI_ISL_498257|Original|hCoV-19^^Bangkok|Human|Hospital for Tropical Diseases|COVID-19 Network Investigations (CONI) Alliance|Batty|Thailand
>Spike|hCoV-19/Scotland/QEUH-9CE551/2020|2020-09-16|EPI_ISL_601937|Original|hCoV-19^^Scotland|Human|Lighthouse Lab in Glasgow|Wellcome Sanger Institute for the COVID-19 Genomics UK (COG-UK) consortium|Davies|United Kingdom
>Spike|hCoV-19/Wales/PHWC-495CDB/2020|2020-11-26|EPI_ISL_726469|Original|hCoV-19^^Wales|Human|Originating lab|COVID-19 Genomics UK (COG-UK) Consortium|Moore|United Kingdom
>Spike|hCoV-19/USA/TX-HMH-MCoV-13098/2020|2020-08-27|EPI_ISL_788249|Original|hCoV-19^^Texas|Human|Houston Methodist Hospital|Houston Methodist Hospital|Long|USA
>Spike|hCoV-19/England/BRIS-12CBC0/2020|2020-04-18|EPI_ISL_481932|Original|hCoV-19^^England|Human|PHE South West Regional Laboratory|Wellcome Sanger Institute for the COVID-19 Genomics UK (COG-UK) consortium|Davies|United Kingdom
>Spike|hCoV-19/Canada/ON-PHL-20-00028/2020|2020-00-00|EPI_ISL_548709|Original|hCoV-19^^Ontario|Human|Public Health Ontario Laboratory|Public Health Ontario Laboratory|Guthrie|Canada

>Spike|hCoV-19/USA/CT-UW-6314/2020|2020-04-01|EPI_ISL_430965|Original|hCoV-
19^^Connecticut|Human|UW Virology Lab|UW Virology Lab|Roychoudhury|USA
>Spike|hCoV-19/Belgium/ULG-10756/2020|2020-09-20|EPI_ISL_641598|Original|hCoV-
19^^Li\u00e8ge|Human|Department of Clinical Microbiology|GIGA Medical
Genomics|Durkin|Belgium
>Spike|hCoV-19/Scotland/QEUH-545F2B/2020|2020-05-14|EPI_ISL_533355|Original|hCoV-
19^^Scotland|Human|NHSGGC West of Scotland Specialist Virology Centre / MRC-
University of Glasgow Centre for Virus Research|Wellcome Sanger Institute for the
COVID-19 Genomics UK (COG-UK) consortium|Davies|United Kingdom
>Spike|hCoV-19/USA/WA-UW-2087/2020|2020-03-25|EPI_ISL_426083|Original|hCoV-
19^^Washington|Human|UW Virology Lab|UW Virology Lab|Roychoudhury|USA
>Spike|hCoV-19/Wales/PHWC-47F080/2020|2020-10-10|EPI_ISL_612624|Original|hCoV-
19^^Wales|Human|Wales Specialist Virology Centre Sequencing lab|COVID-19 Genomics UK
(COG-UK) Consortium|Moore|United Kingdom
>Spike|hCoV-19/Scotland/EDB5993/2020|2020-05-05|EPI_ISL_487872|Original|hCoV-
19^^Scotland|Human|Virology Department|Wellcome Sanger Institute for the COVID-19
Genomics UK (COG-UK) consortium|Davies|United Kingdom
>Spike|hCoV-19/Uruguay/UY-651/2020|2020-05-25|EPI_ISL_480339|Original|hCoV-
19^^Rivera|Human|Microbial Genomics Laboratory|Microbial Genomics
Laboratory|Iraola|Uruguay
>Spike|hCoV-19/England/QEUH-B9EDC9/2020|2020-11-18|EPI_ISL_719933|Original|hCoV-
19^^England|Human|Lighthouse Lab in Glasgow|Wellcome Sanger Institute for the COVID-
19 Genomics UK (COG-UK) Consortium|Davies|United Kingdom
>Spike|hCoV-19/England/ALDP-B63A02/2020|2020-11-09|EPI_ISL_656370|Original|hCoV-
19^^England|Human|Lighthouse Lab in Alderley Park|Wellcome Sanger Institute for the
COVID-19 Genomics UK (COG-UK) Consortium|Davies|United Kingdom
>Spike|hCoV-19/England/20129009304/2020|2020-03-20|EPI_ISL_423979|Original|hCoV-
19^^England|Human|Respiratory Virus Unit|Respiratory Virus Unit|Platt|United Kingdom
>Spike|hCoV-19/USA/MA-MGH-00190/2020|2020-03-26|EPI_ISL_460263|Original|hCoV-
19^^Massachusetts|Human|Massachusetts General Hospital|Infectious Disease
Program|Lemieux|USA
>Spike|hCoV-19/Russia/ALT-RII-MH2762S/2020|2020-09-26|EPI_ISL_602435|Original|hCoV-
19^^Altai K|Human|HELIX LLC|WHO National Influenza Centre Russian
Federation|Komissarov|Russia
>Spike|hCoV-19/England/LIVE-9EA54/2020|2020-04-12|EPI_ISL_453358|Original|hCoV-
19^^England|Human|Liverpool Clinical Laboratories|COVID-19 Genomics UK (COG-UK)
Consortium|Haldenby|United Kingdom
>Spike|hCoV-19/USA/AZ-TG402310/2020|2020-06-23|EPI_ISL_694856|Original|hCoV-
19^^Arizona|Human|TGen North|TGen North|French|USA
>Spike|hCoV-19/England/ALDP-C3A687/2020|2020-12-09|EPI_ISL_731464|Original|hCoV-
19^^England|Human|Lighthouse Lab in Alderley Park|Wellcome Sanger Institute for the
COVID-19 Genomics UK (COG-UK) Consortium|Davies|United Kingdom
>Spike|hCoV-19/England/QEUH-9F53B1/2020|2020-09-25|EPI_ISL_638670|Original|hCoV-
19^^England|Human|Department of Pathology|COVID-19 Genomics UK (COG-UK)
Consortium|Goodfellow|United Kingdom
>Spike|hCoV-19/Northern Ireland/QEUH-998602/2020|2020-08-
24|EPI_ISL_540088|Original|hCoV-19^^Northern Ireland|Human|Lighthouse Lab in
Glasgow|Wellcome Sanger Institute for the COVID-19 Genomics UK (COG-UK)
consortium|Davies|United Kingdom
>Spike|hCoV-19/England/BRIS-1228E1/2020|2020-04-03|EPI_ISL_440808|Original|hCoV-
19^^England|Human|PHE South West Regional Laboratory|Wellcome Sanger Institute for
the COVID-19 Genomics UK (COG-UK) consortium|Amato|United Kingdom
>Spike|hCoV-19/England/LOND-1263E66/2020|2020-11-11|EPI_ISL_679497|Original|hCoV-
19^^England|Human|University College London|COVID-19 Genomics UK (COG-UK)
Consortium|Roy|United Kingdom
>Spike|hCoV-19/USA/LA-EVTL1033/2020|2020-08-25|EPI_ISL_653723|Original|hCoV-
19^^Louisiana|Human|LSUHS Emerging Viral Threat Laboratory|Microbial Genome
Sequencing Center|Kamil|USA

>Spike|hCoV-19/England/ALDP-9EADA2/2020|2020-09-24|EPI_ISL_579997|Original|hCoV-19^^England|Human|Lighthouse Lab in Alderley Park|Wellcome Sanger Institute for the COVID-19 Genomics UK (COG-UK) consortium|Davies|United Kingdom
>Spike|hCoV-19/England/CAMC-A65175/2020|2020-10-08|EPI_ISL_608400|Original|hCoV-19^^England|Human|Lighthouse Lab in Cambridge|Wellcome Sanger Institute for the COVID-19 Genomics UK (COG-UK) consortium|Davies|United Kingdom
>Spike|hCoV-19/Brazil/SP-L3-CAMPI44/2020|2020-04-18|EPI_ISL_476394|Original|hCoV-19^^S\u00e3o Paulo|Human|Laborat\u00f3rio de Patologia Cl\u00ednica - UNICAMP|Laborat\u00f3rio de Estudos de V\u00edrus Emergentes - UNICAMP|Sabino|Brazil
>Spike|hCoV-19/England/SHEF-D3112/2020|2020-09-19|EPI_ISL_573756|Original|hCoV-19^^England|Human|Virology Department|COVID-19 Genomics UK (COG-UK) Consortium|de Silva|United Kingdom
>Spike|hCoV-19/Wales/MILK-9712B0/2020|2020-08-18|EPI_ISL_553029|Original|hCoV-19^^Wales|Human|Lighthouse Lab in Milton Keynes|Wellcome Sanger Institute for the COVID-19 Genomics UK (COG-UK) consortium|Davies|United Kingdom
>Spike|hCoV-19/USA/NY-NYCPHL-001175/2020|2020-10-29|EPI_ISL_671755|Original|hCoV-19^^New York|Human|DOHMH Morrisania|New York City Public Health Laboratory|Wang|USA
>Spike|hCoV-19/USA/AK-PHL683/2020|2020-06-29|EPI_ISL_586255|Original|hCoV-19^^Alaska|Human|Alaska State Virology Laboratory|Alaska State Virology Laboratory|Chen|USA
>Spike|hCoV-19/Finland/13M57/2020|2020-03-13|EPI_ISL_418391|Original|hCoV-19^^Finland|Human|Department of Virology and Immunology|Department of Virology|Smura|Finland
>Spike|hCoV-19/USA/UT-UPHL-201024487/2020|2020-05-28|EPI_ISL_594248|Original|hCoV-19^^Utah|Human|Utah Public Health Laboratory|Utah Public Health Laboratory|Young|USA
>Spike|hCoV-19/England/BRIS-125189/2020|2020-03-26|EPI_ISL_443958|Original|hCoV-19^^England|Human|PHE South West Regional Laboratory|Wellcome Sanger Institute for the COVID-19 Genomics UK (COG-UK) consortium|Amato|United Kingdom
>Spike|hCoV-19/Denmark/DCGC-9371/2020|2020-11-16|EPI_ISL_668935|Original|hCoV-19^^Midtjylland|Human|Department of Virus and Microbiological Special Diagnostics|Albertsen Lab|Michaelsen|Denmark
>Spike|hCoV-19/Nigeria/FC-CV363/2020|2020-07-07|EPI_ISL_730026|Original|hCoV-19^^Abuja|Human|Nigeria Centre for Disease Control (NCDC)|African Centre of Excellence for Genomics of Infectious Diseases (ACEGID)|Oluniyi|Nigeria
>Spike|hCoV-19/England/CAMC-B21138/2020|2020-11-02|EPI_ISL_647523|Original|hCoV-19^^England|Human|Lighthouse Lab in Cambridge|Wellcome Sanger Institute for the COVID-19 Genomics UK (COG-UK) Consortium|Davies|United Kingdom
>Spike|hCoV-19/England/MILK-A6A310/2020|2020-10-09|EPI_ISL_626817|Original|hCoV-19^^England|Human|Virology Department|COVID-19 Genomics UK (COG-UK) Consortium|de Silva|United Kingdom
>Spike|hCoV-19/England/CAMC-B99511/2020|2020-11-15|EPI_ISL_673301|Original|hCoV-19^^England|Human|Lighthouse Lab in Cambridge|Wellcome Sanger Institute for the COVID-19 Genomics UK (COG-UK) Consortium|Davies|United Kingdom
>Spike|hCoV-19/England/LIVE-1D7292/2020|2020-09-28|EPI_ISL_584601|Original|hCoV-19^^England|Human|Liverpool Clinical Laboratories|COVID-19 Genomics UK (COG-UK) Consortium|Haldenby|United Kingdom
>Spike|hCoV-19/England/204621524/2020|2020-11-10|EPI_ISL_700615|Original|hCoV-19^^England|Human|Respiratory Virus Unit|COVID-19 Genomics UK (COG-UK) Consortium|Platt|United Kingdom
>Spike|hCoV-19/USA/WA-S603/2020|2020-04-15|EPI_ISL_438156|Original|hCoV-19^^Washington|Human|Seattle Flu Study|Seattle Flu Study|Bedford|USA
>Spike|hCoV-19/Australia/VIC11787/2020|2020-08-08|EPI_ISL_591852|Original|hCoV-19^^Victoria|Human|Victorian Infectious Diseases Reference Laboratory (VIDRL)|VIDRL and MDU-PHL|Schultz|Australia
>Spike|hCoV-19/England/MILK-9C2C2B/2020|2020-09-10|EPI_ISL_566557|Original|hCoV-19^^England|Human|Lighthouse Lab in Milton Keynes|Wellcome Sanger Institute for the COVID-19 Genomics UK (COG-UK) consortium|Davies|United Kingdom
>Spike|hCoV-19/England/ALDP-CB448B/2020|2020-12-18|EPI_ISL_761773|Original|hCoV-19^^England|Human|Lighthouse Lab in Alderley Park|Wellcome Sanger Institute for the COVID-19 Genomics UK (COG-UK) Consortium|Davies|United Kingdom

>Spike|hCoV-19/Denmark/DCGC-14424/2020|2020-11-30|EPI_ISL_711758|Original|hCoV-19^^Midtjylland|Human|Department of Virus and Microbiological Special Diagnostics|Albertsen Lab|Michaelsen|Denmark
>Spike|hCoV-19/Denmark/DCGC-16666/2020|2020-11-16|EPI_ISL_714415|Original|hCoV-19^^Hovedstaden|Human|Department of Virus and Microbiological Special Diagnostics|Albertsen Lab|Michaelsen|Denmark
>Spike|hCoV-19/England/201040152/2020|2020-03-01|EPI_ISL_464334|Original|hCoV-19^^England|Human|Respiratory Virus Unit|Respiratory Virus Unit|Platt|United Kingdom
>Spike|hCoV-19/Denmark/DCGC-3434/2020|2020-08-31|EPI_ISL_616196|Original|hCoV-19^^Hovedstaden|Human|Department of Virus and Microbiological Special Diagnostics|Albertsen lab|Michaelsen|Denmark
>Spike|hCoV-19/England/CAMB-792EE/2020|2020-04-03|EPI_ISL_441203|Original|hCoV-19^^England|Human|Department of Pathology|Wellcome Sanger Institute for the COVID-19 Genomics UK (COG-UK) consortium|Amato|United Kingdom
>Spike|hCoV-19/USA/AR-UAMS002/2020|2020-07-02|EPI_ISL_492182|Original|hCoV-19^^Arkansas|Human|University of Arkansas for Medical Sciences (UAMS)|Department of Biomedical Informatics|Jenjaroenpun|USA
>Spike|hCoV-19/USA/TX-HMH-MCoV-15383/2020|2020-10-22|EPI_ISL_786867|Original|hCoV-19^^Texas|Human|Houston Methodist Hospital|Houston Methodist Hospital|Long|USA
>Spike|hCoV-19/England/MILK-BAA0EF/2020|2020-11-18|EPI_ISL_783073|Original|hCoV-19^^England|Human|Lighthouse Lab in Milton Keynes|Wellcome Sanger Institute for the COVID-19 Genomics UK (COG-UK) Consortium|Davies|United Kingdom
>Spike|hCoV-19/India/TG-CCMB-AA620/2020|2020-08-12|EPI_ISL_539736|Original|hCoV-19^^Telangana|Human|CSIR-Centre for Cellular and Molecular Biology|CSIR-Centre for Cellular and Molecular Biology|Mukherjee|India
>Spike|hCoV-19/England/QEUH-AD138D/2020|2020-10-27|EPI_ISL_633717|Original|hCoV-19^^England|Human|Lighthouse Lab in Glasgow|Wellcome Sanger Institute for the COVID-19 Genomics UK (COG-UK) consortium|Davies|United Kingdom
>Spike|hCoV-19/England/BRIS-1232F1/2020|2020-04-05|EPI_ISL_442705|Original|hCoV-19^^England|Human|PHE South West Regional Laboratory|Wellcome Sanger Institute for the COVID-19 Genomics UK (COG-UK) consortium|Amato|United Kingdom
>Spike|hCoV-19/USA/UT-UPHL-2009350/2020|2020-05-15|EPI_ISL_524947|Original|hCoV-19^^Utah|Human|Utah Public Health Laboratory|Utah Public Health Laboratory|Young|USA
>Spike|hCoV-19/USA/UT-UPHL-03079/2020|2020-05-04|EPI_ISL_560916|Original|hCoV-19^^Utah|Human|Utah Public Health Laboratory|Utah Public Health Laboratory|Young|USA
>Spike|hCoV-19/Germany/BE-ChVir-W1713-6783/2020|2020-08-05|EPI_ISL_754003|Original|hCoV-19^^Berlin|Human|Charit\u00e9 Universit\u00e4tsmedizin Berlin|Charit\u00e9 Universit\u00e4tsmedizin Berlin|Muehlemann|Germany
>Spike|hCoV-19/England/ALDP-A6FD77/2020|2020-10-14|EPI_ISL_607268|Original|hCoV-19^^England|Human|Lighthouse Lab in Alderley Park|Wellcome Sanger Institute for the COVID-19 Genomics UK (COG-UK) consortium|Davies|United Kingdom
>Spike|hCoV-19/Scotland/QEUH-9B6F12/2020|2020-09-09|EPI_ISL_602125|Original|hCoV-19^^Scotland|Human|Lighthouse Lab in Glasgow|Wellcome Sanger Institute for the COVID-19 Genomics UK (COG-UK) consortium|Davies|United Kingdom
>Spike|hCoV-19/Australia/WA297/2020|2020-04-09|EPI_ISL_596840|Original|hCoV-19^^Western Australia|Human|PathWest Laboratory Medicine WA|PathWest Laboratory Medicine WA Microbial Surveillance Unit|Sikazwe|Australia
>Spike|hCoV-19/Uganda/UG043/2020|2020-05-04|EPI_ISL_737963|Original|hCoV-19^^Uganda|Human|Uganda Central Public Health Lab and Uganda Virus Research Institute|MRC/UVRI & LSHTM Uganda\u00a0Research Unit|Cotten|Uganda
>Spike|hCoV-19/Latvia/050/2020|2020-03-16|EPI_ISL_486434|Original|hCoV-19^^Latvia|Human|Latvijas Infektolo\u0123ijas centrs|Latvian Biomedical Research and Study Centre|Zrelovs|Latvia
>Spike|hCoV-19/Germany/HH-hpi-p2165/2020|2020-03-28|EPI_ISL_776395|Original|hCoV-19^^Hamburg|Human|University Medical Center Hamburg Eppendorf|Heinrich Pette Institute|Robitaille|Germany
>Spike|hCoV-19/England/MILK-99E46C/2020|2020-08-30|EPI_ISL_550592|Original|hCoV-19^^England|Human|Lighthouse Lab in Milton Keynes|Wellcome Sanger Institute for the COVID-19 Genomics UK (COG-UK) consortium|Davies|United Kingdom

>Spike|hCoV-19/England/QEUH-9B762B/2020|2020-09-04|EPI_ISL_590552|Original|hCoV-19^^England|Human|Lighthouse Lab in Glasgow|Wellcome Sanger Institute for the COVID-19 Genomics UK (COG-UK) consortium|Davies|United Kingdom
>Spike|hCoV-19/England/QEUH-B9E075/2020|2020-11-18|EPI_ISL_721038|Original|hCoV-19^^England|Human|Lighthouse Lab in Glasgow|Wellcome Sanger Institute for the COVID-19 Genomics UK (COG-UK) Consortium|Davies|United Kingdom
>Spike|hCoV-19/USA/UT-UPHL-2012070308/2020|2020-07-26|EPI_ISL_745716|Original|hCoV-19^^Utah|Human|Ginkgo Bioworks Clinical Laboratory|Utah Public Health Laboratory|Young|USA
>Spike|hCoV-19/USA/NY-Wadsworth-288367-01/2020|2020-12-16|EPI_ISL_767480|Original|hCoV-19^^New York|Human|Wadsworth Center|Wadsworth Center|Lasek-Nesselquist|USA
>Spike|hCoV-19/Northern Ireland/QEUH-96AFD1/2020|2020-08-15|EPI_ISL_531449|Original|hCoV-19^^Northern Ireland|Human|Lighthouse Lab in Glasgow|Wellcome Sanger Institute for the COVID-19 Genomics UK (COG-UK) consortium|Davies|United Kingdom
>Spike|hCoV-19/England/ALDP-90218A/2020|2020-06-24|EPI_ISL_551376|Original|hCoV-19^^England|Human|Lighthouse Lab in Alderley Park|Wellcome Sanger Institute for the COVID-19 Genomics UK (COG-UK) consortium|Davies|United Kingdom
>Spike|hCoV-19/England/QEUH-A364A1/2020|2020-10-05|EPI_ISL_706909|Original|hCoV-19^^England|Human|Oxford Viromics|COVID-19 Genomics UK (COG-UK) Consortium|Golubchik|United Kingdom
>Spike|hCoV-19/Hong Kong/VM20017766/2020|2020-05-20|EPI_ISL_482778|Original|hCoV-19^^Hong Kong|Human|Tuen Mun Hospital|Hong Kong Department of Health|Mak|China
>Spike|hCoV-19/USA/TX-HMH-MCoV-15752/2020|2020-07-09|EPI_ISL_785436|Original|hCoV-19^^Texas|Human|Houston Methodist Hospital|Houston Methodist Hospital|Long|USA
>Spike|hCoV-19/England/MILK-A7BD2D/2020|2020-10-15|EPI_ISL_607430|Original|hCoV-19^^England|Human|Lighthouse Lab in Milton Keynes|Wellcome Sanger Institute for the COVID-19 Genomics UK (COG-UK) consortium|Davies|United Kingdom
>Spike|hCoV-19/USA/NY-Wadsworth-46884-01/2020|2020-04-14|EPI_ISL_676848|Original|hCoV-19^^New York|Human|Wadsworth Center|Wadsworth Center|Lasek-Nesselquist|USA
>Spike|hCoV-19/England/ALDP-95C52B/2020|2020-06-17|EPI_ISL_555087|Original|hCoV-19^^England|Human|Lighthouse Lab in Alderley Park|Wellcome Sanger Institute for the COVID-19 Genomics UK (COG-UK) consortium|Davies|United Kingdom
>Spike|hCoV-19/USA/NY-NYUMC120/2020|2020-03-18|EPI_ISL_421732|Original|hCoV-19^^New York|Human|NYU Langone Health|Departments of Pathology and Medicine|Maurano|USA
>Spike|hCoV-19/England/20122071604/2020|2020-03-16|EPI_ISL_465763|Original|hCoV-19^^England|Human|Respiratory Virus Unit|Respiratory Virus Unit|Platt|United Kingdom
>Spike|hCoV-19/England/CAMC-B07815/2020|2020-10-31|EPI_ISL_643941|Original|hCoV-19^^England|Human|Lighthouse Lab in Cambridge|Wellcome Sanger Institute for the COVID-19 Genomics UK (COG-UK) Consortium|Davies|United Kingdom
>Spike|hCoV-19/England/ALDP-9E6E3A/2020|2020-09-24|EPI_ISL_580334|Original|hCoV-19^^England|Human|Lighthouse Lab in Alderley Park|Wellcome Sanger Institute for the COVID-19 Genomics UK (COG-UK) consortium|Davies|United Kingdom
>Spike|hCoV-19/USA/WA-UW-29527/2020|2020-09-29|EPI_ISL_576487|Original|hCoV-19^^Washington|Human|UW Virology Lab|UW Virology Lab|Roychoudhury|USA
>Spike|hCoV-19/England/QEUH-AF1581/2020|2020-10-28|EPI_ISL_665629|Original|hCoV-19^^England|Human|Northumbria University / South Tees Hospitals NHS Foundation Trust / North Cumbria Integrated Care NHS Foundation Trust / North Tees and Hartlepool NHS Foundation Trust / Newcastle Hospitals NHS Foundation Trust|COVID-19 Genomics UK (COG-UK) Consortium|Bashton|United Kingdom
>Spike|hCoV-19/Netherlands/ZH-EMC-683/2020|2020-10-02|EPI_ISL_632539|Original|hCoV-19^^Zuid Holland|Human|Dutch COVID-19 response team|Erasmus Medical Center|Oude Munnink|Netherlands
>Spike|hCoV-19/Wales/PHWC-32C10/2020|2020-04-15|EPI_ISL_446666|Original|hCoV-19^^Wales|Human|Wales Specialist Virology Centre|Public Health Wales Microbiology Cardiff|Moore|United Kingdom

>Spike|hCoV-19/England/NOTT-10EFD7/2020|2020-03-30|EPI_ISL_432953|Original|hCoV-19^^England|Human|Queens Medical Centre|COVID-19 Genomics UK (COG-UK) Consortium|Loose|United Kingdom
>Spike|hCoV-19/England/BRIS-12319A/2020|2020-04-05|EPI_ISL_442677|Original|hCoV-19^^England|Human|PHE South West Regional Laboratory|Wellcome Sanger Institute for the COVID-19 Genomics UK (COG-UK) consortium|Amato|United Kingdom
>Spike|hCoV-19/Malaysia/IMR-WC253038/2020|2020-11-02|EPI_ISL_728169|Original|hCoV-19^^Malaysia|Human|Institute for Medical Research|Institute for Medical Research|Suppiah|Malaysia
>Spike|hCoV-19/Denmark/DCGC-18945/2020|2020-12-07|EPI_ISL_748656|Original|hCoV-19^^Sjaelland|Human|Department of Virus and Microbiological Special Diagnostics|Albertsen Lab|Michaelsen|Denmark
>Spike|hCoV-19/USA/CA-CDC-2602/2020|2020-02-26|EPI_ISL_419554|Original|hCoV-19^^California|Human|California Department of Public Health|Pathogen Discovery|Queen|USA
>Spike|hCoV-19/Denmark/DCGC-18746/2020|2020-12-07|EPI_ISL_750160|Original|hCoV-19^^Midtjylland|Human|Department of Virus and Microbiological Special Diagnostics|Albertsen Lab|Michaelsen|Denmark
>Spike|hCoV-19/Switzerland/ZH-ETHZ-220081/2020|2020-07-27|EPI_ISL_512049|Original|hCoV-19^^Zurich|Human|Viollier AG|Department of Biosystems Science and Engineering|Nadeau|Switzerland
>Spike|hCoV-19/England/QEUH-A335DE/2020|2020-10-05|EPI_ISL_626739|Original|hCoV-19^^England|Human|Virology Department|COVID-19 Genomics UK (COG-UK) Consortium|de Silva|United Kingdom
>Spike|hCoV-19/Denmark/DCGC-5376/2020|2020-09-14|EPI_ISL_615307|Original|hCoV-19^^Syddanmark|Human|Department of Virus and Microbiological Special Diagnostics|Albertsen lab|Michaelsen|Denmark
>Spike|hCoV-19/India/DL-NCDC2301-CSIR-IGIB/2020|2020-05-19|EPI_ISL_482557|Original|hCoV-19^^Delhi|Human|National Centre for Disease control (NCDC)|NCDC/CSIR-IGIB|Pandey|India
>Spike|hCoV-19/England/ALDP-BBA43B/2020|2020-11-21|EPI_ISL_701688|Original|hCoV-19^^England|Human|Lighthouse Lab in Alderley Park|Wellcome Sanger Institute for the COVID-19 Genomics UK (COG-UK) Consortium|Davies|United Kingdom
>Spike|hCoV-19/Scotland/GCVR-172026/2020|2020-03-30|EPI_ISL_469990|Original|hCoV-19^^Scotland|Human|NHSGGC West of Scotland Specialist Virology Centre / MRC-University of Glasgow Centre for Virus Research|Wellcome Sanger Institute for the COVID-19 Genomics UK (COG-UK) consortium|Davies|United Kingdom
>Spike|hCoV-19/England/CAMB-75B32/2020|2020-03-29|EPI_ISL_438297|Original|hCoV-19^^England|Human|Department of Pathology|Wellcome Sanger Institute for the COVID-19 Genomics UK (COG-UK) consortium|Amato|United Kingdom
>Spike|hCoV-19/Denmark/DCGC-8619/2020|2020-10-26|EPI_ISL_625966|Original|hCoV-19^^Nordjylland|Human|Department of Virus and Microbiological Special Diagnostics|Albertsen lab|Michaelsen|Denmark
>Spike|hCoV-19/England/QEUH-CCDDC0/2020|2020-12-24|EPI_ISL_782405|Original|hCoV-19^^England|Human|Lighthouse Lab in Glasgow|Wellcome Sanger Institute for the COVID-19 Genomics UK (COG-UK) Consortium|Davies|United Kingdom
>Spike|hCoV-19/Denmark/DCGC-19788/2020|2020-12-07|EPI_ISL_747788|Original|hCoV-19^^Syddanmark|Human|Department of Virus and Microbiological Special Diagnostics|Albertsen Lab|Michaelsen|Denmark
>Spike|hCoV-19/USA/UT-CDC-9755/2020|2020-04-27|EPI_ISL_509692|Original|hCoV-19^^Utah|Human|Utah Public Health Laboratory|Pathogen Discovery|Queen|USA
>Spike|hCoV-19/Canada/MB-NML-1154/2020|2020-07-31|EPI_ISL_582480|Original|hCoV-19^^Manitoba|Human|Cadham Provincial Laboratory|National Microbiology Laboratory (NML)|Majer|Canada
>Spike|hCoV-19/USA/CA-CZB-14670/2020|2020-11-16|EPI_ISL_739099|Original|hCoV-19^^California|Human|Orange County Public Health Lab|Chan-Zuckerberg Biohub|Kamm|USA
>Spike|hCoV-19/England/QEUH-AE2547/2020|2020-10-27|EPI_ISL_633538|Original|hCoV-19^^England|Human|Lighthouse Lab in Glasgow|Wellcome Sanger Institute for the COVID-19 Genomics UK (COG-UK) consortium|Davies|United Kingdom

>Spike|hCoV-19/DRC/04186/2020|2020-04-25|EPI_ISL_471411|Original|hCoV-19^^Haut-Katanga|Human|Viral Respiratory Lab|Pathogen Sequencing Lab|Lusamaki|Democratic Republic of the Congo
>Spike|hCoV-19/USA/MI-UM-10036540781/2020|2020-10-20|EPI_ISL_640146|Original|hCoV-19^^Michigan|Human|University of Michigan Clinical Microbiology Laboratory|Lauring Lab|Valesano|USA
>Spike|hCoV-19/Canada/ON-UHTC_0062/2020|2020-04-01|EPI_ISL_464048|unknown|hCoV-19^^Ontario|Human|Unity Health Toronto|Ontario Institute for Cancer Research|de Borja|Canada
>Spike|hCoV-19/USA/TX-HMH0111/2020|2020-03-20|EPI_ISL_434813|Original|hCoV-19^^Texas|Human|Houston Methodist Hospital|Houston Methodist Hospital|Long|USA
>Spike|hCoV-19/Hong Kong/HKU-200723-092/2020|2020-07-10|EPI_ISL_497859|Original|hCoV-19^^Hong Kong|Human|Department of Microbiology|Department of Microbiology|Chan|China
>Spike|hCoV-19/England/LIVE-A76EB/2020|2020-03-28|EPI_ISL_517059|Original|hCoV-19^^England|Human|Liverpool Clinical Laboratories|COVID-19 Genomics UK (COG-UK) Consortium|Haldenby|United Kingdom
>Spike|hCoV-19/Spain/PV-IBV-004245/2020|2020-04-06|EPI_ISL_500309|Original|hCoV-19^^Basque Country|Human|Servicio de Microbiolog\u00eda. Hospital Universitario Donostia. OSI Donostialdea. \u00c1rea de Enfermedades Infecciosas|SeqCOVID-SPAIN consortium/IBV(CSIC)|Comas|Spain
>Spike|hCoV-19/England/MILK-993801/2020|2020-08-26|EPI_ISL_552354|Original|hCoV-19^^England|Human|Lighthouse Lab in Milton Keynes|Wellcome Sanger Institute for the COVID-19 Genomics UK (COG-UK) consortium|Davies|United Kingdom
>Spike|hCoV-19/USA/TX-HMH-MCoV-18693/2020|2020-11-22|EPI_ISL_785041|Original|hCoV-19^^Texas|Human|Houston Methodist Hospital|Houston Methodist Hospital|Long|USA
>Spike|hCoV-19/Portugal/PT0446/2020|2020-03-27|EPI_ISL_454170|Original|hCoV-19^^Portugal|Human|unknown|Instituto Nacional de Saude (INSA)|Borges|Portugal
>Spike|hCoV-19/USA/NY-NYUMC446/2020|2020-04-14|EPI_ISL_435513|Original|hCoV-19^^New York|Human|NYU Langone Health|Departments of Pathology and Medicine|Maurano|USA
>Spike|hCoV-19/USA/MA-MASPHL-00773/2020|2020-09-25|EPI_ISL_692922|Original|hCoV-19^^Massachusetts|Human|Massachusetts State Public Health Laboratory|Massachusetts State Public Health Laboratory|Lang|USA
>Spike|hCoV-19/USA/NY-NYCPHL-000094/2020|2020-03-23|EPI_ISL_631587|Original|hCoV-19^^New York|Human|NYC HH Lincoln Medical And Mental Health Center|New York City Public Health Laboratory|Wang|USA
>Spike|hCoV-19/England/20156043304/2020|2020-04-09|EPI_ISL_466317|Original|hCoV-19^^England|Human|Respiratory Virus Unit|Respiratory Virus Unit|Platt|United Kingdom
>Spike|hCoV-19/USA/LA-EVTL960/2020|2020-08-13|EPI_ISL_653651|Original|hCoV-19^^Louisiana|Human|LSUHS Emerging Viral Threat Laboratory|Microbial Genome Sequencing Center|Kamil|USA
>Spike|hCoV-19/Australia/VIC14258/2020|2020-08-03|EPI_ISL_592422|Original|hCoV-19^^Victoria|Human|Microbiological Diagnostic Unit - Public Health Laboratory (MDU-PHL)|MDU-PHL|Schultz|Australia
>Spike|hCoV-19/USA/VA-DCLS-1264/2020|2020-08-12|EPI_ISL_529954|Original|hCoV-19^^Virginia|Human|Virginia DCLS|Virginia DCLS|St. Jacques|USA
>Spike|hCoV-19/England/BRIS-124A61/2020|2020-03-25|EPI_ISL_443725|Original|hCoV-19^^England|Human|PHE South West Regional Laboratory|Wellcome Sanger Institute for the COVID-19 Genomics UK (COG-UK) consortium|Amato|United Kingdom
>Spike|hCoV-19/England/QEUH-C32AED/2020|2020-12-11|EPI_ISL_782556|Original|hCoV-19^^England|Human|Lighthouse Lab in Glasgow|Wellcome Sanger Institute for the COVID-19 Genomics UK (COG-UK) Consortium|Davies|United Kingdom
>Spike|hCoV-19/Northern Ireland/NIRE-108711/2020|2020-09-13|EPI_ISL_585197|Original|hCoV-19^^Northern Ireland|Human|Regional Virus Laboratory|COVID-19 Genomics UK (COG-UK) Consortium|Fairley|United Kingdom
>Spike|hCoV-19/Japan/PG-0714/2020|2020-04-00|EPI_ISL_684324|Original|hCoV-19^^Japan|Human|Pathogen Genomics Center|Pathogen Genomics Center|Sekizuka|Japan
>Spike|hCoV-19/Brazil/RJ-00470/2020|2020-08-03|EPI_ISL_717865|Original|hCoV-19^^Rio de Janeiro|Human|Laboratorio de Virologia Molecular / UFRJ|Bioinformatics Laboratory / LNCC|Almeida|Brazil

>Spike|hCoV-19/England/BRIS-12FB09/2020|2020-04-03|EPI_ISL_488392|Original|hCoV-19^^England|Human|PHE South West Regional Laboratory|Wellcome Sanger Institute for the COVID-19 Genomics UK (COG-UK) consortium|Davies|United Kingdom
>Spike|hCoV-19/England/NOTT-111E2F/2020|2020-06-10|EPI_ISL_472412|Original|hCoV-19^^England|Human|Queens Medical Centre|COVID-19 Genomics UK (COG-UK) Consortium|Loose|United Kingdom
>Spike|hCoV-19/Belgium/rega-12111421/2020|2020-12-11|EPI_ISL_738473|Original|hCoV-19^^Mol|Human|UZ Leuven|KU Leuven|Maes|Belgium
>Spike|hCoV-19/Scotland/EDB816/2020|2020-04-03|EPI_ISL_457020|Original|hCoV-19^^Scotland|Human|Virology Department|COVID-19 Genomics UK (COG-UK) Consortium|Williams|United Kingdom
>Spike|hCoV-19/Scotland/QEUH-9CE287/2020|2020-09-16|EPI_ISL_601903|Original|hCoV-19^^Scotland|Human|Lighthouse Lab in Glasgow|Wellcome Sanger Institute for the COVID-19 Genomics UK (COG-UK) consortium|Davies|United Kingdom
>Spike|hCoV-19/England/QEUH-B6B9D0/2020|2020-11-11|EPI_ISL_655149|Original|hCoV-19^^England|Human|Lighthouse Lab in Glasgow|Wellcome Sanger Institute for the COVID-19 Genomics UK (COG-UK) Consortium|Davies|United Kingdom
>Spike|hCoV-19/England/QEUH-AF568A/2020|2020-10-30|EPI_ISL_662989|Original|hCoV-19^^England|Human|Lighthouse Lab in Glasgow|Wellcome Sanger Institute for the COVID-19 Genomics UK (COG-UK) Consortium|Davies|United Kingdom
>Spike|hCoV-19/England/NORT-286273/2020|2020-09-02|EPI_ISL_573321|Original|hCoV-19^^England|Human|Northumbria University / South Tees Hospitals NHS Foundation Trust / North Cumbria Integrated Care NHS Foundation Trust / North Tees and Hartlepool NHS Foundation Trust / Newcastle Hospitals NHS Foundation Trust|COVID-19 Genomics UK (COG-UK) Consortium|Bashton|United Kingdom
>Spike|hCoV-19/England/QEUH-9DCFBC/2020|2020-09-21|EPI_ISL_599550|Original|hCoV-19^^England|Human|Lighthouse Lab in Glasgow|Wellcome Sanger Institute for the COVID-19 Genomics UK (COG-UK) consortium|Davies|United Kingdom
>Spike|hCoV-19/England/QEUH-5460BE/2020|2020-05-14|EPI_ISL_533361|Original|hCoV-19^^England|Human|Lighthouse Lab in Glasgow|Wellcome Sanger Institute for the COVID-19 Genomics UK (COG-UK) consortium|Davies|United Kingdom
>Spike|hCoV-19/France/PAC-IHU-0651/2020|2020-03-20|EPI_ISL_568915|Original|hCoV-19^^Provence-Alpes-C\u00f4te-d\u2019Azur|Human|MEPHI|MEPHI|Levasseur|France
>Spike|hCoV-19/England/LEED-2A926B/2020|2020-04-05|EPI_ISL_539212|Original|hCoV-19^^England|Human|Leeds Teaching Hospitals NHS Trust and Public Health England|Wellcome Sanger Institute for the COVID-19 Genomics UK (COG-UK) consortium|Davies|United Kingdom
>Spike|hCoV-19/Wales/PHWC-485CED/2020|2020-10-22|EPI_ISL_626965|Original|hCoV-19^^Wales|Human|Wales Specialist Virology Centre Sequencing lab|COVID-19 Genomics UK (COG-UK) Consortium|Moore|United Kingdom
>Spike|hCoV-19/USA/OH-ODH-SC35176 /2020|2020-04-23|EPI_ISL_778861|Original|hCoV-19^^Ohio|Human|Ohio Department of Health Laboratory|Ohio Department of Health Laboratory|Holmes|USA
>Spike|hCoV-19/USA/AZ-TG547220/2020|2020-07-02|EPI_ISL_695431|Original|hCoV-19^^Arizona|Human|AZ SPHL|TGen North|French|USA
>Spike|hCoV-19/Wales/PHWC-485139/2020|2020-10-21|EPI_ISL_628150|Original|hCoV-19^^Wales|Human|Wales Specialist Virology Centre Sequencing lab|COVID-19 Genomics UK (COG-UK) Consortium|Moore|United Kingdom
>Spike|hCoV-19/United Arab Emirates/0972/2020|2020-06-29|EPI_ISL_699107|Original|hCoV-19^^Abu Dhabi|Human|Group 42 (G42) Healthcare|G42 Healthcare|Ogrodzki|United Arab Emirates
>Spike|hCoV-19/Canada/NS-NML-1382/2020|2020-04-16|EPI_ISL_579757|Original|hCoV-19^^Nova Scotia|Human|QEII Health Sciences Centre|National Microbiology Laboratory (NML)|Majer|Canada
>Spike|hCoV-19/Australia/VIC16359/2020|2020-08-04|EPI_ISL_641014|Original|hCoV-19^^Victoria|Human|Microbiological Diagnostic Unit - Public Health Laboratory (MDU-PHL)|MDU-PHL|Schultz|Australia
>Spike|hCoV-19/Japan/IC-0267/2020|2020-11-00|EPI_ISL_667696|Original|hCoV-19^^Japan|Human|Pathogen Genomics Center|Pathogen Genomics Center|Sekizuka|Japan

>Spike|hCoV-19/USA/LA-GBCL-26492465/2020|2020-04-27|EPI_ISL_485040|Original|hCoV-19^^Louisiana|Human|River Road Testing Lab|Ginkgo Bioworks Clinical Laboratory|Smith|USA
>Spike|hCoV-19/Australia/VIC5217/2020|2020-00-00|EPI_ISL_519982|Original|hCoV-19^^Victoria|Human|Microbiological Diagnostic Unit - Public Health Laboratory (MDU-PHL)|MDU-PHL|Schultz|Australia
>Spike|hCoV-19/England/MILK-ACF3DD/2020|2020-10-21|EPI_ISL_629709|Original|hCoV-19^^England|Human|Lighthouse Lab in Milton Keynes|Wellcome Sanger Institute for the COVID-19 Genomics UK (COG-UK) consortium|Davies|United Kingdom
>Spike|hCoV-19/Netherlands/LI-EMC-140/2020|2020-00-00|EPI_ISL_763108|Original|hCoV-19^^Limburg|Human|Dutch COVID-19 response team|Erasmus Medical Center|Oude Munnink|Netherlands
>Spike|hCoV-19/Scotland/EDB1897/2020|2020-04-17|EPI_ISL_433156|Original|hCoV-19^^Scotland|Human|Virology Department|COVID-19 Genomics UK (COG-UK) Consortium|Williams|United Kingdom
>Spike|hCoV-19/Wales/PHWC-16BF9C/2020|2020-09-15|EPI_ISL_573996|Original|hCoV-19^^Wales|Human|Wales Specialist Virology Centre Sequencing lab|COVID-19 Genomics UK (COG-UK) Consortium|Moore|United Kingdom
>Spike|hCoV-19/Canada/BC_09021025/2020|2020-04-00|EPI_ISL_477107|Original|hCoV-19^^British Columbia|Human|BCCDC Public Health Laboratory|BCCDC Public Health Laboratory|Harrigan|Canada
>Spike|hCoV-19/India/TG-CCMB-M830/2020|2020-06-12|EPI_ISL_495251|Original|hCoV-19^^Telangana|Human|CSIR-Centre for Cellular and Molecular Biology|CSIR-Centre for Cellular and Molecular Biology|Banu|India
>Spike|hCoV-19/England/CAMC-B9A05B/2020|2020-11-15|EPI_ISL_673496|Original|hCoV-19^^England|Human|Lighthouse Lab in Cambridge|Wellcome Sanger Institute for the COVID-19 Genomics UK (COG-UK) Consortium|Davies|United Kingdom
>Spike|hCoV-19/India/MH-ACTREC-536/2020|2020-08-25|EPI_ISL_700182|Original|hCoV-19^^Maharashtra|Human|Hematopathology Laboratory|Hematopathology Laboratory|Patkar|India
>Spike|hCoV-19/France/PAC-IHU-0490/2020|2020-04-03|EPI_ISL_569546|Original|hCoV-19^^Provence-Alpes-C\u00f4te-d\u2019Azur|Human|MEPHI|MEPHI|Levasseur|France
>Spike|hCoV-19/England/QEUH-CC2217/2020|2020-12-21|EPI_ISL_760746|Original|hCoV-19^^England|Human|Lighthouse Lab in Glasgow|Wellcome Sanger Institute for the COVID-19 Genomics UK (COG-UK) Consortium|Davies|United Kingdom
>Spike|hCoV-19/Belgium/ULG-10688/2020|2020-10-26|EPI_ISL_636660|Original|hCoV-19^^Li\u00e8ge|Human|Department of Clinical Microbiology|GIGA Medical Genomics|Durkin|Belgium
>Spike|hCoV-19/England/MILK-BAC2AB/2020|2020-11-18|EPI_ISL_783249|Original|hCoV-19^^England|Human|Lighthouse Lab in Milton Keynes|Wellcome Sanger Institute for the COVID-19 Genomics UK (COG-UK) Consortium|Davies|United Kingdom
>Spike|hCoV-19/Wales/PHWC-36A4F/2020|2020-05-02|EPI_ISL_474749|Original|hCoV-19^^Wales|Human|Originating lab|COVID-19 Genomics UK (COG-UK) Consortium|Moore|United Kingdom
>Spike|hCoV-19/Scotland/CVR4467/2020|2020-09-20|EPI_ISL_585331|Original|hCoV-19^^Scotland|Human|Lighthouse Lab in Glasgow / MRC-University of Glasgow Centre for Virus Research|COVID-19 Genomics UK (COG-UK) Consortium|Robertson|United Kingdom
>Spike|hCoV-19/England/CAMC-A81216/2020|2020-10-13|EPI_ISL_610908|Original|hCoV-19^^England|Human|Lighthouse Lab in Cambridge|Wellcome Sanger Institute for the COVID-19 Genomics UK (COG-UK) consortium|Davies|United Kingdom
>Spike|hCoV-19/Brazil/AP-IEC-162620/2020|2020-04-03|EPI_ISL_524792|Original|hCoV-19^^Amapa|Human|\ufeffEvandro Chagas Institute|\ufeffEvandro Chagas Institute|Sousa J\u00fanior|Brazil
>Spike|hCoV-19/Spain/VC-IBV-98004313/2020|2020-03-24|EPI_ISL_538650|Original|hCoV-19^^Comunitat Valenciana|Human|Servicio de Microbiolog\u00eda. Hospital General Universitario de Castell\u00f3n|SeqCOVID-SPAIN consortium/IBV(CSIC)|Comas|Spain
>Spike|hCoV-19/Saudi Arabia/KAUST-MADINAH1141/2020|2020-06-27|EPI_ISL_677993|Original|hCoV-19^^Madinah|Human|Pathogen Genomics Lab King Abdullah University of Science and Technology(KAUST)|Pathogen Genomics Lab King Abdullah University of Science and Technology(KAUST)|Mohamed Ghazzali|Saudi Arabia

>Spike|hCoV-19/USA/AL-CDC-2-3714495/2020|2020-11-09|EPI_ISL_751791|Original|hCoV-19^^Alabama|Human|AL Dept. of Public Health Bureau of Clinical Laboratories|Genomics and Discovery|Queen|USA
>Spike|hCoV-19/Denmark/DCGC-2478/2020|2020-08-10|EPI_ISL_617746|Original|hCoV-19^^Hovedstaden|Human|Department of Virus and Microbiological Special Diagnostics|Albertsen lab|Michaelsen|Denmark
>Spike|hCoV-19/England/QEUH-9AF3B6/2020|2020-09-03|EPI_ISL_559839|Original|hCoV-19^^England|Human|Oxford Viromics|COVID-19 Genomics UK (COG-UK) Consortium|Golubchik|United Kingdom
>Spike|hCoV-19/England/MILK-B026D4/2020|2020-10-24|EPI_ISL_643003|Original|hCoV-19^^England|Human|Lighthouse Lab in Milton Keynes|Wellcome Sanger Institute for the COVID-19 Genomics UK (COG-UK) Consortium|Davies|United Kingdom
>Spike|hCoV-19/Netherlands/ZH-EMC-750/2020|2020-09-24|EPI_ISL_632591|Original|hCoV-19^^Zuid Holland|Human|Dutch COVID-19 response team|Erasmus Medical Center|Oude Munnink|Netherlands
>Spike|hCoV-19/England/BRIS-127248/2020|2020-04-23|EPI_ISL_469637|Original|hCoV-19^^England|Human|PHE South West Regional Laboratory|Wellcome Sanger Institute for the COVID-19 Genomics UK (COG-UK) consortium|Davies|United Kingdom
>Spike|hCoV-19/USA/TX-HMH0101/2020|2020-03-19|EPI_ISL_434806|Original|hCoV-19^^Texas|Human|Houston Methodist Hospital|Houston Methodist Hospital|Long|USA
>Spike|hCoV-19/England/NORT-28CB54/2020|2020-05-18|EPI_ISL_469332|Original|hCoV-19^^England|Human|NU-OMICS DNA Sequencing research facility|Wellcome Sanger Institute for the COVID-19 Genomics UK (COG-UK) consortium|Davies|United Kingdom
>Spike|hCoV-19/Wales/PHWC-48DEFE/2020|2020-11-07|EPI_ISL_680776|Original|hCoV-19^^Wales|Human|Wales Specialist Virology Centre Sequencing lab|COVID-19 Genomics UK (COG-UK) Consortium|Moore|United Kingdom
>Spike|hCoV-19/Denmark/DCGC-9309/2020|2020-11-16|EPI_ISL_668901|Original|hCoV-19^^Nordjylland|Human|Department of Virus and Microbiological Special Diagnostics|Albertsen Lab|Michaelsen|Denmark
>Spike|hCoV-19/Denmark/DCGC-6676/2020|2020-10-12|EPI_ISL_619579|Original|hCoV-19^^Midtjylland|Human|Department of Virus and Microbiological Special Diagnostics|Albertsen lab|Michaelsen|Denmark
>Spike|hCoV-19/England/QEUH-B7EB98/2020|2020-11-14|EPI_ISL_668056|Original|hCoV-19^^England|Human|Lighthouse Lab in Glasgow|Wellcome Sanger Institute for the COVID-19 Genomics UK (COG-UK) Consortium|Davies|United Kingdom
>Spike|hCoV-19/Spain/PV-IBV-2134/2020|2020-04-12|EPI_ISL_468939|Original|hCoV-19^^Basque Country|Human|Servicio de Microbiolog\u00eda. Hospital Universitario Donostia. OSI Donostialdea. \u00c1rea de Enfermedades Infecciosas|SeqCOVID-SPAIN consortium/IBV(CSIC)|Comas|Spain
>Spike|hCoV-19/Bangladesh/DNAS_DRCIM_isl_DSIMCH-3/2020|2020-07-03|EPI_ISL_774914|Original|hCoV-19^^Dhaka|Human|Designated Reference Institute for Chemical Measurements (DRICM)|DNA SOLUTION LTD.|Rabbi|Bangladesh
>Spike|hCoV-19/Denmark/DCGC-14982/2020|2020-11-23|EPI_ISL_713560|Original|hCoV-19^^Hovedstaden|Human|Department of Virus and Microbiological Special Diagnostics|Albertsen Lab|Michaelsen|Denmark
>Spike|hCoV-19/Australia/VIC6515/2020|2020-07-28|EPI_ISL_521287|Original|hCoV-19^^Victoria|Human|Microbiological Diagnostic Unit - Public Health Laboratory (MDU-PHL)|MDU-PHL|Schultz|Australia
>Spike|hCoV-19/Australia/VIC6002/2020|2020-07-23|EPI_ISL_518777|Original|hCoV-19^^Victoria|Human|Microbiological Diagnostic Unit - Public Health Laboratory (MDU-PHL)|MDU-PHL|Schultz|Australia
>Spike|hCoV-19/England/MILK-A06765/2020|2020-10-01|EPI_ISL_599844|Original|hCoV-19^^England|Human|Lighthouse Lab in Milton Keynes|Wellcome Sanger Institute for the COVID-19 Genomics UK (COG-UK) consortium|Davies|United Kingdom
>Spike|hCoV-19/Spain/CT-IBV-003153/2020|2020-03-16|EPI_ISL_510250|Original|hCoV-19^^Catalunya|Human|Hospital de la Santa Creu i Sant Pau. Servicio de Microbiolog\u00eda|SeqCOVID-SPAIN consortium/IBV(CSIC)|Comas|Spain
>Spike|hCoV-19/Bangladesh/BCSIR-NILMRC-370/2020|2020-07-21|EPI_ISL_514241|Original|hCoV-19^^Chittagong|Human|National Institute of Laboratory Medicine and Referral Center|Genomic Research Lab|Khan|Bangladesh

>Spike|hCoV-19/Switzerland/BS-42418743/2020|2020-08-31|EPI_ISL_581997|Original|hCoV-19^^Basel-Stadt|Human|University Hospital Basel|University Hospital Basel|Roloff Handschin|Switzerland
>Spike|hCoV-19/Luxembourg/LNS7580348/2020|2020-11-14|EPI_ISL_744590|Original|hCoV-19^^Luxembourg|Human|Laboratoire national de sant\u00e9|Laboratoire national de sant\u00e9|Wienecke-Baldacchino|Luxembourg
>Spike|hCoV-19/England/NOTT-10E283/2020|2020-03-20|EPI_ISL_425519|Original|hCoV-19^^England|Human|Queens Medical Centre|COVID-19 Genomics UK (COG-UK) Consortium|Loose|United Kingdom
>Spike|hCoV-19/USA/CA-CZB-13995/2020|2020-06-14|EPI_ISL_738668|Original|hCoV-19^^California|Human|Alameda County Public Health Lab|Chan-Zuckerberg Biohub|Kamm|USA
>Spike|hCoV-19/Switzerland/AG-ETHZ-410268/2020|2020-12-14|EPI_ISL_737658|Original|hCoV-19^^Aargau|Human|Viollier AG|Department of Biosystems Science and Engineering|Nadeau|Switzerland
>Spike|hCoV-19/Luxembourg/LNS0490194/2020|2020-10-22|EPI_ISL_739787|Original|hCoV-19^^Luxembourg|Human|Laboratoire national de sant\u00e9|Laboratoire national de sant\u00e9|Wienecke-Baldacchino|Luxembourg
>Spike|hCoV-19/USA/UT-QDX-1462/2020|2020-03-16|EPI_ISL_603847|Original|hCoV-19^^Utah|Human|Quest Diagnostics|Quest Diagnostics|Kagan|USA
>Spike|hCoV-19/Finland/26SS7D3/2020|2020-09-26|EPI_ISL_759952|Original|hCoV-19^^Uusimaa|Human|Department of Virology and Immunology|Department of Virology|Smura|Finland
>Spike|hCoV-19/England/CAMC-B9A228/2020|2020-11-15|EPI_ISL_673763|Original|hCoV-19^^England|Human|Lighthouse Lab in Cambridge|Wellcome Sanger Institute for the COVID-19 Genomics UK (COG-UK) Consortium|Davies|United Kingdom
>Spike|hCoV-19/USA/MD-MDH-0085/2020|2020-05-26|EPI_ISL_509489|Original|hCoV-19^^Maryland|Human|Maryland Department of Health|Maryland Department of Health|Keller|USA
>Spike|hCoV-19/England/CAMB-7DA1F/2020|2020-04-05|EPI_ISL_441581|Original|hCoV-19^^England|Human|Department of Pathology|Wellcome Sanger Institute for the COVID-19 Genomics UK (COG-UK) consortium|Amato|United Kingdom
>Spike|hCoV-19/Canada/BC_09320741/2020|2020-04-00|EPI_ISL_467303|Original|hCoV-19^^British Columbia|Human|BCCDC Public Health Laboratory|BCCDC Public Health Laboratory|Harrigan|Canada
>Spike|hCoV-19/Scotland/QEUH-A9CEF6/2020|2020-10-20|EPI_ISL_652645|Original|hCoV-19^^Scotland|Human|Queens Medical Centre|COVID-19 Genomics UK (COG-UK) Consortium|Loose|United Kingdom
>Spike|hCoV-19/Denmark/DCGC-20781/2020|2020-12-21|EPI_ISL_759358|Original|hCoV-19^^Hovedstaden|Human|Department of Virus and Microbiological Special Diagnostics|Albertsen Lab|Michaelsen|Denmark
>Spike|hCoV-19/England/QEUH-9CE31B/2020|2020-09-14|EPI_ISL_590063|Original|hCoV-19^^England|Human|Lighthouse Lab in Glasgow|Wellcome Sanger Institute for the COVID-19 Genomics UK (COG-UK) consortium|Davies|United Kingdom
>Spike|hCoV-19/India/MH-ACTREC-552/2020|2020-08-28|EPI_ISL_700197|Original|hCoV-19^^Maharashtra|Human|Hematopathology Laboratory|Hematopathology Laboratory|Patkar|India
>Spike|hCoV-19/Japan/PG-1724/2020|2020-04-00|EPI_ISL_685330|Original|hCoV-19^^Japan|Human|Pathogen Genomics Center|Pathogen Genomics Center|Sekizuka|Japan
>Spike|hCoV-19/USA/CA-ALSR-4330/2020|2020-10-16|EPI_ISL_648975|Original|hCoV-19^^California|Human|San Diego County Public Health Laboratory|Andersen lab at Scripps Research|Gangavarapu|USA
>Spike|hCoV-19/USA/WI-UW-251/2020|2020-03-14|EPI_ISL_428725|Original|hCoV-19^^Wisconsin|Human|University of Wisconsin-Madison AIDS Vaccine Research Laboratories|University of Wisconsin-Madison AIDS Vaccine Research Laboratories|Baczenas|USA
>Spike|hCoV-19/England/QEUH-B7EC0E/2020|2020-11-14|EPI_ISL_661871|Original|hCoV-19^^England|Human|Lighthouse Lab in Glasgow|Wellcome Sanger Institute for the COVID-19 Genomics UK (COG-UK) Consortium|Davies|United Kingdom

>Spike|hCoV-19/Austria/CeMM0657/2020|2020-03-27|EPI_ISL_583627|Original|hCoV-19^^T\u00f6sens|Human|Pathologie-Labor Dr. Obrist- Dr. Brunhuber|Bergthaler laboratory|Bergthaler|Austria
>Spike|hCoV-19/Wales/PHWC-16E324/2020|2020-09-29|EPI_ISL_627026|Original|hCoV-19^^Wales|Human|Wales Specialist Virology Centre Sequencing lab|COVID-19 Genomics UK (COG-UK) Consortium|Moore|United Kingdom
>Spike|hCoV-19/England/PORT-2D4A05/2020|2020-10-17|EPI_ISL_611755|Original|hCoV-19^^England|Human|Centre for Enzyme Innovation|COVID-19 Genomics UK (COG-UK) Consortium|Robson|United Kingdom
>Spike|hCoV-19/USA/TX-HMH-MCoV-13282/2020|2020-08-24|EPI_ISL_788430|Original|hCoV-19^^Texas|Human|Houston Methodist Hospital|Houston Methodist Hospital|Long|USA
>Spike|hCoV-19/USA/AZ-TG640824/2020|2020-04-27|EPI_ISL_695779|Original|hCoV-19^^Arizona|Human|Sonora Quest Laboratories|TGen North|French|USA
>Spike|hCoV-19/Iceland/125/2020|2020-03-11|EPI_ISL_417690|Original|hCoV-19^^Reykjavik|Human|The National University Hospital of Iceland|deCODE genetics|J\u00f3nsson|Iceland
>Spike|hCoV-19/Denmark/DCGC-2440/2020|2020-08-10|EPI_ISL_617615|Original|hCoV-19^^Nordjylland|Human|Department of Virus and Microbiological Special Diagnostics|Albertsen lab|Michaelsen|Denmark
>Spike|hCoV-19/Denmark/DCGC-1606/2020|2020-04-13|EPI_ISL_618496|Original|hCoV-19^^Syddanmark|Human|Department of Virus and Microbiological Special Diagnostics|Albertsen lab|Michaelsen|Denmark
>Spike|hCoV-19/Finland/S3CE11/2020|2020-10-30|EPI_ISL_732648|Original|hCoV-19^^Uusimaa|Human|Department of Virology and Immunology|Department of Virology|Smura|Finland
>Spike|hCoV-19/USA/NY-Wadsworth-290379-01/2020|2020-12-14|EPI_ISL_765558|Original|hCoV-19^^New York|Human|MONTEFIORE MEDICAL CENTER LABORATORIES|Wadsworth Center|Lasek-Nesselquist|USA
>Spike|hCoV-19/England/ALDP-49B74D/2020|2020-06-11|EPI_ISL_559360|Original|hCoV-19^^England|Human|Lighthouse Lab in Alderley Park|Wellcome Sanger Institute for the COVID-19 Genomics UK (COG-UK) consortium|Davies|United Kingdom
>Spike|hCoV-19/Ireland/un-NVRL-AIIDB0132v1/2020|2020-12-11|EPI_ISL_791307|Original|hCoV-19^^Ireland|Human|National Virus Reference Laboratory|Irish Coronavirus Sequencing Consortium - Teagasc Moorepark|Walsh|Ireland
>Spike|hCoV-19/England/PORT-2F57DA/2020|2020-00-00|EPI_ISL_613301|Original|hCoV-19^^England|Human|Centre for Enzyme Innovation|COVID-19 Genomics UK (COG-UK) Consortium|Robson|United Kingdom
>Spike|hCoV-19/Japan/PG-5789/2020|2020-08-00|EPI_ISL_688908|Original|hCoV-19^^Japan|Human|Fukuoka Institute of Health and Environmental Sciences|Pathogen Genomics Center|Sekizuka|Japan
>Spike|hCoV-19/Germany/NW-MPP-13/2020|2020-05-25|EPI_ISL_487401|Original|hCoV-19^^North Rhine-Westphalia|Human|Labor Knei\u00dfler GmbH & Co. KG|Heinrich Pette Institute|G\u00fcnther|Germany
>Spike|hCoV-19/Wales/PHWC-29C2B/2020|2020-04-05|EPI_ISL_432447|Original|hCoV-19^^Wales|Human|Wales Specialist Virology Centre|Public Health Wales Microbiology Cardiff|Moore|United Kingdom
>Spike|hCoV-19/USA/WA-UW-12612/2020|2020-06-28|EPI_ISL_501113|Original|hCoV-19^^Washington|Human|University of Washington Virology Lab|University of Washington Virology Lab|Roychoudhury|USA
>Spike|hCoV-19/Wales/PHWC-1631E9/2020|2020-05-28|EPI_ISL_473261|Original|hCoV-19^^Wales|Human|Wales Specialist Virology Centre Sequencing lab|COVID-19 Genomics UK (COG-UK) Consortium|Moore|United Kingdom
>Spike|hCoV-19/USA/TX-HMH-MCoV-13650/2020|2020-08-20|EPI_ISL_788752|Original|hCoV-19^^Texas|Human|Houston Methodist Hospital|Houston Methodist Hospital|Long|USA
>Spike|hCoV-19/Egypt/MASRI-027/2020|2020-05-08|EPI_ISL_482773|Original|hCoV-19^^Egypt|Human|Medical Ain Shams Research Institute (MASRI)|Medical Ain Shams Research Institute (MASRI)|Moustafa|Egypt
>Spike|hCoV-19/USA/CO-CDPHE-2011201244/2020|2020-11-19|EPI_ISL_771262|Original|hCoV-19^^Colorado|Human|Colorado Department of Public Health and Environment|Colorado Department of Puplic Health and Environment|Bankers|USA

>Spike|hCoV-19/Belgium/rega-12141407/2020|2020-12-14|EPI_ISL_738459|Original|hCoV-19^^Mol|Human|UZ Leuven|KU Leuven|Maes|Belgium
>Spike|hCoV-19/Japan/PG-5946/2020|2020-00-00|EPI_ISL_689061|Original|hCoV-19^^Japan|Human|Gunma Prefectural Institute of Public Health and Environmental Sciences|Pathogen Genomics Center|Sekizuka|Japan
>Spike|hCoV-19/England/BRIS-1255AB/2020|2020-03-27|EPI_ISL_443800|Original|hCoV-19^^England|Human|PHE South West Regional Laboratory|Wellcome Sanger Institute for the COVID-19 Genomics UK (COG-UK) consortium|Amato|United Kingdom
>Spike|hCoV-19/England/LOND-D38FF/2020|2020-04-03|EPI_ISL_441006|Original|hCoV-19^^England|Human|University College London|COVID-19 Genomics UK (COG-UK) Consortium|Roy|United Kingdom
>Spike|hCoV-19/Switzerland/GE-SNRCI-7986/2020|2020-03-00|EPI_ISL_548212|Original|hCoV-19^^Geneve|Human|Laboratoire de Virologie|Swiss National Reference Centre for Influenza|Gon\u00e7alves Cabecinhas|Switzerland
>Spike|hCoV-19/England/MILK-A479AE/2020|2020-10-05|EPI_ISL_598323|Original|hCoV-19^^England|Human|Lighthouse Lab in Milton Keynes|Wellcome Sanger Institute for the COVID-19 Genomics UK (COG-UK) consortium|Davies|United Kingdom
>Spike|hCoV-19/USA/TX-HMH-7127/2020|2020-06-29|EPI_ISL_546528|Original|hCoV-19^^Texas|Human|Houston Methodist Hospital|Houston Methodist Hospital|Long|USA
>Spike|hCoV-19/Hungary/MH-732/2020|2020-04-15|EPI_ISL_526217|Original|hCoV-19^^Pest county|Human|Hungarian Defence Forces Military Medical Centre|National Laboratory of Virology|Kemenesi|Hungary
>Spike|hCoV-19/England/SHEF-C06A0/2020|2020-03-21|EPI_ISL_420280|Original|hCoV-19^^England|Human|Virology Department|Department of Infection|de Silva|United Kingdom
>Spike|hCoV-19/England/CAMC-A64D54/2020|2020-10-11|EPI_ISL_608558|Original|hCoV-19^^England|Human|Lighthouse Lab in Cambridge|Wellcome Sanger Institute for the COVID-19 Genomics UK (COG-UK) consortium|Davies|United Kingdom
>Spike|hCoV-19/USA/NY-Wadsworth-226483-01/2020|2020-07-28|EPI_ISL_677115|Original|hCoV-19^^New York|Human|Masonic Medical Research Institute|Wadsworth Center|Lasek-Nesselquist|USA
>Spike|hCoV-19/India/DL-NCDC1542-CSIR-IGIB/2020|2020-05-17|EPI_ISL_482493|Original|hCoV-19^^Delhi|Human|National Centre for Disease control (NCDC)|NCDC/CSIR-IGIB|Pandey|India
>Spike|hCoV-19/USA/TX-HMH-4090/2020|2020-06-17|EPI_ISL_543360|Original|hCoV-19^^Texas|Human|Houston Methodist Hospital|Houston Methodist Hospital|Long|USA
>Spike|hCoV-19/England/ALDP-9562C0/2020|2020-06-08|EPI_ISL_554030|Original|hCoV-19^^England|Human|Lighthouse Lab in Alderley Park|Wellcome Sanger Institute for the COVID-19 Genomics UK (COG-UK) consortium|Davies|United Kingdom
>Spike|hCoV-19/Brazil/RJ-0268/2020|2020-04-15|EPI_ISL_470634|Original|hCoV-19^^Rio de Janeiro|Human|Laboratorio de Virologia Molecular / UFRJ|Bioinformatics Laboratory / LNCC|Almeida|Brazil
>Spike|hCoV-19/England/BRIS-128BB5/2020|2020-04-14|EPI_ISL_458883|Original|hCoV-19^^England|Human|PHE South West Regional Laboratory|Wellcome Sanger Institute for the COVID-19 Genomics UK (COG-UK) consortium|Davies|United Kingdom
>Spike|hCoV-19/USA/VA-DCLS-1071/2020|2020-06-10|EPI_ISL_528670|Original|hCoV-19^^Virginia|Human|Virginia Division of Consolidated Laboratory Services (DCLS)|Virginia Division of Consolidated Laboratory Services (DCLS)|St. Jacques|USA
>Spike|hCoV-19/England/CAMB-734EE/2020|2020-03-21|EPI_ISL_440007|Original|hCoV-19^^England|Human|Department of Pathology|Wellcome Sanger Institute for the COVID-19 Genomics UK (COG-UK) consortium|Amato|United Kingdom
>Spike|hCoV-19/England/BRIS-124D2C/2020|2020-03-26|EPI_ISL_444016|Original|hCoV-19^^England|Human|PHE South West Regional Laboratory|Wellcome Sanger Institute for the COVID-19 Genomics UK (COG-UK) consortium|Amato|United Kingdom
>Spike|hCoV-19/Denmark/DCGC-8417/2020|2020-10-26|EPI_ISL_617392|Original|hCoV-19^^NA|Human|Department of Virus and Microbiological Special Diagnostics|Albertsen lab|Michaelsen|Denmark
>Spike|hCoV-19/England/ALDP-BCAA36/2020|2020-11-23|EPI_ISL_703318|Original|hCoV-19^^England|Human|Lighthouse Lab in Alderley Park|Wellcome Sanger Institute for the COVID-19 Genomics UK (COG-UK) Consortium|Davies|United Kingdom

>Spike|hCoV-19/Vietnam/PIHCM-320/2020|2020-03-18|EPI_ISL_654888|Vero E6|hCoV-19^^Ho Chi Minh city|Human|Pasteur Institute in Ho Chi Minh city|Department of Microbiology and Immunology - Pasteur Institute in Ho Chi Minh city|Vu|Viet Nam
>Spike|hCoV-19/Denmark/DCGC-15832/2020|2020-11-23|EPI_ISL_714048|Original|hCoV-19^^Nordjylland|Human|Department of Virus and Microbiological Special Diagnostics|Albertsen Lab|Michaelsen|Denmark
>Spike|hCoV-19/England/ALDP-B851E7/2020|2020-11-15|EPI_ISL_674318|Original|hCoV-19^^England|Human|Lighthouse Lab in Alderley Park|Wellcome Sanger Institute for the COVID-19 Genomics UK (COG-UK) Consortium|Davies|United Kingdom
>Spike|hCoV-19/USA/MT-CDC-2-3714260/2020|2020-12-02|EPI_ISL_751640|Original|hCoV-19^^Montana|Human|MT Public Health Laboratory|Genomics and Discovery|Queen|USA
>Spike|hCoV-19/Wales/PHWC-498AFC/2020|2020-12-04|EPI_ISL_727173|Original|hCoV-19^^Wales|Human|Originating lab|COVID-19 Genomics UK (COG-UK) Consortium|Moore|United Kingdom
>Spike|hCoV-19/USA/MI-QDX-1120/2020|2020-03-21|EPI_ISL_571850|Original|hCoV-19^^Michigan|Human|Quest Diagnostics|Quest Diagnostics|Kagan|USA
>Spike|hCoV-19/USA/TX-HMH-6941/2020|2020-06-27|EPI_ISL_546370|Original|hCoV-19^^Texas|Human|Houston Methodist Hospital|Houston Methodist Hospital|Long|USA
>Spike|hCoV-19/Switzerland/SZ-ETHZ-360101/2020|2020-11-08|EPI_ISL_693909|Original|hCoV-19^^Schwyz|Human|Viollier AG|Department of Biosystems Science and Engineering|Nadeau|Switzerland
>Spike|hCoV-19/Belgium/ULG-10264/2020|2020-07-11|EPI_ISL_515071|Original|hCoV-19^^Liege|Human|Department of Clinical Microbiology|GIGA Medical Genomics|Durkin|Belgium
>Spike|hCoV-19/USA/WA-S803/2020|2020-04-16|EPI_ISL_449973|Original|hCoV-19^^Washington|Human|Washington State Department of Health|Seattle Flu Study|Bedford|USA
>Spike|hCoV-19/Denmark/DCGC-7974/2020|2020-10-26|EPI_ISL_617072|Original|hCoV-19^^Nordjylland|Human|Department of Virus and Microbiological Special Diagnostics|Albertsen lab|Michaelsen|Denmark
>Spike|hCoV-19/England/QEUH-9C826F/2020|2020-09-11|EPI_ISL_567256|Original|hCoV-19^^England|Human|Lighthouse Lab in Glasgow|Wellcome Sanger Institute for the COVID-19 Genomics UK (COG-UK) consortium|Davies|United Kingdom
>Spike|hCoV-19/Algeria/G38218-8852/2020|2020-06-19|EPI_ISL_766865|Original|hCoV-19^^S\u00e9tif|Human|NIC Viral Respiratory Unit - Institut Pasteur of Algeria|National Reference Center for Viruses of Respiratory Infections|Enouf|Algeria
>Spike|hCoV-19/France/NAQ-SC283/2020|2020-04-05|EPI_ISL_644676|Original|hCoV-19^^Nouvelle-Aquitaine|Human|CHU de Limoges|CNR Virus des Infections Respiratoires - France SUD|Bal|France
>Spike|hCoV-19/USA/TX-HMH-MCoV-15146/2020|2020-10-21|EPI_ISL_786630|Original|hCoV-19^^Texas|Human|Houston Methodist Hospital|Houston Methodist Hospital|Long|USA
>Spike|hCoV-19/United Arab Emirates/0618/2020|2020-05-15|EPI_ISL_698774|Original|hCoV-19^^Abu Dhabi|Human|Group 42 (G42) Healthcare|G42 Healthcare|Ogrodzki|United Arab Emirates
>Spike|hCoV-19/England/ALDP-94B12B/2020|2020-06-13|EPI_ISL_555787|Original|hCoV-19^^England|Human|Lighthouse Lab in Alderley Park|Wellcome Sanger Institute for the COVID-19 Genomics UK (COG-UK) consortium|Davies|United Kingdom
>Spike|hCoV-19/Austria/CeMM0473/2020|2020-03-07|EPI_ISL_475878|Original|hCoV-19^^Austria|Human|Austrian Agency for Health and Food Safety (AGES)|Bergthaler laboratory|Bergthaler|Austria
>Spike|hCoV-19/Iceland/145/2020|2020-03-13|EPI_ISL_417741|Original|hCoV-19^^Reykjavik|Human|The National University Hospital of Iceland|deCODE genetics|J\u00f3nsson|Iceland
>Spike|hCoV-19/England/ALDP-A84CF7/2020|2020-10-17|EPI_ISL_610565|Original|hCoV-19^^England|Human|Lighthouse Lab in Alderley Park|Wellcome Sanger Institute for the COVID-19 Genomics UK (COG-UK) consortium|Davies|United Kingdom
>Spike|hCoV-19/England/NORT-2896ED/2020|2020-03-31|EPI_ISL_458582|Original|hCoV-19^^England|Human|NU-OMICS DNA Sequencing research facility|Wellcome Sanger Institute for the COVID-19 Genomics UK (COG-UK) consortium|Davies|United Kingdom

>Spike|hCoV-19/USA/MI-MDHHS-SC20848/2020|2020-04-29|EPI_ISL_471869|Original|hCoV-19^^Michigan|Human|Michigan Department of Health and Human Services|Michigan Department of Health and Human Services|Blankenship|USA
>Spike|hCoV-19/Switzerland/NE-ETHZ-390325/2020|2020-11-29|EPI_ISL_721837|Original|hCoV-19^^Neuch\u00e2tel|Human|Viollier AG|Department of Biosystems Science and Engineering|Nadeau|Switzerland
>Spike|hCoV-19/Switzerland/ZH-ETHZ-230037/2020|2020-08-04|EPI_ISL_516577|Original|hCoV-19^^Zurich|Human|Viollier AG|Department of Biosystems Science and Engineering|Nadeau|Switzerland
>Spike|hCoV-19/Japan/PG-0795/2020|2020-04-00|EPI_ISL_684405|Original|hCoV-19^^Japan|Human|Pathogen Genomics Center|Pathogen Genomics Center|Sekizuka|Japan
>Spike|hCoV-19/Finland/13LS7AH2/2020|2020-10-13|EPI_ISL_737231|Original|hCoV-19^^Uusimaa|Human|Department of Virology and Immunology|Department of Virology|Smura|Finland
>Spike|hCoV-19/United Arab Emirates/0457/2020|2020-05-18|EPI_ISL_698627|Original|hCoV-19^^Abu Dhabi|Human|Group 42 (G42) Healthcare|G42 Healthcare|Ogrodzki|United Arab Emirates
>Spike|hCoV-19/Scotland/QEUH-AAAA4C/2020|2020-10-21|EPI_ISL_652729|Original|hCoV-19^^Scotland|Human|Oxford Viromics|COVID-19 Genomics UK (COG-UK) Consortium|Golubchik|United Kingdom
>Spike|hCoV-19/Wales/PHWC-487BC0/2020|2020-10-28|EPI_ISL_639284|Original|hCoV-19^^Wales|Human|Wales Specialist Virology Centre Sequencing lab|COVID-19 Genomics UK (COG-UK) Consortium|Moore|United Kingdom
>Spike|hCoV-19/USA/CA-ALSR-4702/2020|2020-10-05|EPI_ISL_730090|Original|hCoV-19^^California|Human|San Diego County Public Health Laboratory|Andersen lab at Scripps Research|Gangavarapu|USA
>Spike|hCoV-19/England/MILK-B96B12/2020|2020-11-13|EPI_ISL_676047|Original|hCoV-19^^England|Human|Lighthouse Lab in Milton Keynes|Wellcome Sanger Institute for the COVID-19 Genomics UK (COG-UK) Consortium|Davies|United Kingdom
>Spike|hCoV-19/Scotland/QEUH-9F3131/2020|2020-09-24|EPI_ISL_600774|Original|hCoV-19^^Scotland|Human|Lighthouse Lab in Glasgow|Wellcome Sanger Institute for the COVID-19 Genomics UK (COG-UK) consortium|Davies|United Kingdom
>Spike|hCoV-19/England/MILK-BB19EA/2020|2020-11-18|EPI_ISL_720464|Original|hCoV-19^^England|Human|Lighthouse Lab in Milton Keynes|Wellcome Sanger Institute for the COVID-19 Genomics UK (COG-UK) Consortium|Davies|United Kingdom
>Spike|hCoV-19/England/LIVE-DC2F3D/2020|2020-11-24|EPI_ISL_705056|Original|hCoV-19^^England|Human|Liverpool Clinical Laboratories|COVID-19 Genomics UK (COG-UK) Consortium|Haldenby|United Kingdom
>Spike|hCoV-19/England/ALDP-A84978/2020|2020-10-18|EPI_ISL_610380|Original|hCoV-19^^England|Human|Lighthouse Lab in Alderley Park|Wellcome Sanger Institute for the COVID-19 Genomics UK (COG-UK) consortium|Davies|United Kingdom
>Spike|hCoV-19/USA/PA-MGEL63/2020|2020-03-30|EPI_ISL_454354|Original|hCoV-19^^Pennsylvania|Human|UPMC Clinical Microbiology Laboratory|Microbial Genome Sequencing Center; Microbial Genomic Epidemiology Laboratory|Mustapha|USA
>Spike|hCoV-19/USA/NY-QDX-1935/2020|2020-03-16|EPI_ISL_604260|Original|hCoV-19^^New York|Human|Quest Diagnostics|Quest Diagnostics|Kagan|USA
>Spike|hCoV-19/England/CAMC-990804/2020|2020-08-27|EPI_ISL_551899|Original|hCoV-19^^England|Human|Lighthouse Lab in Cambridge|Wellcome Sanger Institute for the COVID-19 Genomics UK (COG-UK) consortium|Davies|United Kingdom
>Spike|hCoV-19/England/GSTT-265D660/2020|2020-03-24|EPI_ISL_483993|Original|hCoV-19^^England|Human|Centre for Clinical Infection and Diagnostics Research and Genomics Innovation Unit|COVID-19 Genomics UK (COG-UK) Consortium|Awan|United Kingdom
>Spike|hCoV-19/Germany/HH-hpi-p2426/2020|2020-04-29|EPI_ISL_776495|Original|hCoV-19^^Hamburg|Human|University Medical Center Hamburg Eppendorf|Heinrich Pette Institute|Robitaille|Germany
>Spike|hCoV-19/England/20109047304/2020|2020-03-07|EPI_ISL_464567|Original|hCoV-19^^England|Human|Respiratory Virus Unit|Respiratory Virus Unit|Platt|United Kingdom
>Spike|hCoV-19/India/MH-NCCS-NR2255/2020|2020-05-21|EPI_ISL_497873|Original|hCoV-19^^Maharashtra|Human|National Centre For Cell Science|National Centre For Cell Science|Paul|India

>Spike|hCoV-19/India/GJ-GBRC318a/2020|2020-06-17|EPI_ISL_495075|Original|hCoV-19^^Gujarat|Human|Department of MicroBiology|Gujarat Biotechnology Research Centre|Joshi|India
>Spike|hCoV-19/Romania/ROSV_3019/2020|2020-04-17|EPI_ISL_491064|Original|hCoV-19^^Suceava|Human|Suceava County Emergency Hospital|\Stefan cel Mare\ University Metagenomics Lab|Lobiuc|Romania
>Spike|hCoV-19/England/MILK-A799A3/2020|2020-10-15|EPI_ISL_606634|Original|hCoV-19^^England|Human|Lighthouse Lab in Milton Keynes|Wellcome Sanger Institute for the COVID-19 Genomics UK (COG-UK) consortium|Davies|United Kingdom
>Spike|hCoV-19/France/OCC-SC569/2020|2020-05-23|EPI_ISL_663246|Original|hCoV-19^^Occitanie|Human|CHU N\u00eemes|CNR Virus des Infections Respiratoires - France SUD|Bal|France
>Spike|hCoV-19/England/CAMB-7717A/2020|2020-04-01|EPI_ISL_439459|Original|hCoV-19^^England|Human|Department of Pathology|Wellcome Sanger Institute for the COVID-19 Genomics UK (COG-UK) consortium|Amato|United Kingdom
>Spike|hCoV-19/USA/VA-DCLS-1982/2020|2020-10-07|EPI_ISL_644897|Original|hCoV-19^^Virginia|Human|Virginia DCLS|Virginia DCLS|St. Jacques|USA
>Spike|hCoV-19/USA/TX-HMH-6084/2020|2020-06-27|EPI_ISL_547222|Original|hCoV-19^^Texas|Human|Houston Methodist Hospital|Houston Methodist Hospital|Long|USA
>Spike|hCoV-19/England/GSTT-265DFFC/2020|2020-03-29|EPI_ISL_484138|Original|hCoV-19^^England|Human|Centre for Clinical Infection and Diagnostics Research and Genomics Innovation Unit|COVID-19 Genomics UK (COG-UK) Consortium|Awan|United Kingdom
>Spike|hCoV-19/Japan/PG-3181/2020|2020-04-00|EPI_ISL_691301|Original|hCoV-19^^Japan|Human|Pathogen Genomics Center|Pathogen Genomics Center|Sekizuka|Japan
>Spike|hCoV-19/England/OXON-AF294/2020|2020-03-23|EPI_ISL_448821|Original|hCoV-19^^England|Human|Oxford Viromics|COVID-19 Genomics UK (COG-UK) Consortium|Golubchik|United Kingdom
>Spike|hCoV-19/England/CAMC-B9AC1B/2020|2020-11-15|EPI_ISL_673552|Original|hCoV-19^^England|Human|Lighthouse Lab in Cambridge|Wellcome Sanger Institute for the COVID-19 Genomics UK (COG-UK) Consortium|Davies|United Kingdom
>Spike|hCoV-19/USA/NY-NYUMC181/2020|2020-04-01|EPI_ISL_427477|Original|hCoV-19^^New York|Human|NYU Langone Health|Departments of Pathology and Medicine|Maurano|USA
>Spike|hCoV-19/USA/TX-HMH-MCoV-11931/2020|2020-09-10|EPI_ISL_790137|Original|hCoV-19^^Texas|Human|Houston Methodist Hospital|Houston Methodist Hospital|Long|USA
>Spike|hCoV-19/England/CAMB-83982/2020|2020-04-15|EPI_ISL_443359|Original|hCoV-19^^England|Human|Department of Pathology|Wellcome Sanger Institute for the COVID-19 Genomics UK (COG-UK) consortium|Amato|United Kingdom
>Spike|hCoV-19/USA/TX-HMH-1865/2020|2020-05-07|EPI_ISL_542885|Original|hCoV-19^^Texas|Human|Houston Methodist Hospital|Houston Methodist Hospital|Long|USA
>Spike|hCoV-19/Portugal/PT2000/2020|2020-11-13|EPI_ISL_732221|Original|hCoV-19^^Portugal|Human|Instituto Nacional de Saude (INSA) and Instituto Gulbenkian de Ciencia (IGC)|Instituto Nacional de Saude (INSA) and Instituto Gulbenkian de Ciencia (IGC)|Borges|Portugal
>Spike|hCoV-19/England/LOND-124C70E/2020|2020-00-00|EPI_ISL_490426|Original|hCoV-19^^England|Human|University College London Hospital|COVID-19 Genomics UK (COG-UK) Consortium|Byott|United Kingdom
>Spike|hCoV-19/England/201620049/2020|2020-04-13|EPI_ISL_466395|Original|hCoV-19^^England|Human|Respiratory Virus Unit|Respiratory Virus Unit|Platt|United Kingdom
>Spike|hCoV-19/Spain/MD-H12-1804/2020|2020-03-29|EPI_ISL_421174|Original|hCoV-19^^Madrid|Human|Hospital Universitario 12 de Octubre|Hospital Universitario 12 de Octubre|Viedma|Spain
>Spike|hCoV-19/Portugal/PT0664/2020|2020-03-23|EPI_ISL_510923|Original|hCoV-19^^Portugal|Human|Instituto Nacional de Saude (INSA)|Instituto Nacional de Saude (INSA)|Borges|Portugal
>Spike|hCoV-19/USA/NY-Wadsworth-290715-01/2020|2020-12-21|EPI_ISL_767423|Original|hCoV-19^^New York|Human|URMC LABS|Wadsworth Center|Lasek-Nesselquist|USA
>Spike|hCoV-19/England/QEUH-9F9335/2020|2020-09-25|EPI_ISL_588870|Original|hCoV-19^^England|Human|Lighthouse Lab in Glasgow|Wellcome Sanger Institute for the COVID-19 Genomics UK (COG-UK) consortium|Davies|United Kingdom

>Spike|hCoV-19/England/LIVE-A5692/2020|2020-03-19|EPI_ISL_500138|Original|hCoV-19^^England|Human|Liverpool Clinical Laboratories|COVID-19 Genomics UK (COG-UK) Consortium|Haldenby|United Kingdom
>Spike|hCoV-19/USA/TX-HMH-MCoV-18992/2020|2020-11-25|EPI_ISL_785270|Original|hCoV-19^^Texas|Human|Houston Methodist Hospital|Houston Methodist Hospital|Long|USA
>Spike|hCoV-19/USA/UT-UPHL-04341_2/2020|2020-05-18|EPI_ISL_591315|Original|hCoV-19^^Utah|Human|Utah Public Health Laboratory|Utah Public Health Laboratory|Young|USA
>Spike|hCoV-19/Spain/PV-IBV-98007584/2020|2020-09-01|EPI_ISL_582054|Original|hCoV-19^^Basque Country|Human|Servicio de Microbiolog\u00eda. Hospital Universitario Donostia. OSI Donostialdea. \u00c1rea de Enfermedades Infecciosas|SeqCOVID-SPAIN consortium/IBV(CSIC)|Comas|Spain
>Spike|hCoV-19/England/201161700/2020|2020-03-11|EPI_ISL_464873|Original|hCoV-19^^England|Human|Respiratory Virus Unit|Respiratory Virus Unit|Platt|United Kingdom
>Spike|hCoV-19/USA/TX-HMH-MCoV-15811/2020|2020-07-09|EPI_ISL_785495|Original|hCoV-19^^Texas|Human|Houston Methodist Hospital|Houston Methodist Hospital|Long|USA
>Spike|hCoV-19/Australia/VIC13466/2020|2020-08-04|EPI_ISL_592245|Original|hCoV-19^^Victoria|Human|Microbiological Diagnostic Unit - Public Health Laboratory (MDU-PHL)|MDU-PHL|Schultz|Australia
>Spike|hCoV-19/USA/TX-DSHS-0588/2020|2020-06-03|EPI_ISL_569699|Original|hCoV-19^^Texas|Human|Texas Department of State Health Services|Texas Department of State Health Services|Rahman|USA
>Spike|hCoV-19/Japan/PG-5139/2020|2020-08-00|EPI_ISL_690246|Original|hCoV-19^^Japan|Human|Gunma Prefectural Institute of Public Health and Environmental Sciences|Pathogen Genomics Center|Sekizuka|Japan
>Spike|hCoV-19/Guangdong/20SF190/2020|2020-01-23|EPI_ISL_428452|Original|hCoV-19^^Guangdong|Human|Guangdong Provincial Center for Diseases Control and Prevention;Guangdong Provincial Institute of Public Health|School of Public Health|Gu|China
>Spike|hCoV-19/Denmark/DCGC-3618/2020|2020-08-31|EPI_ISL_616386|Original|hCoV-19^^Syddanmark|Human|Department of Virus and Microbiological Special Diagnostics|Albertsen lab|Michaelsen|Denmark
>Spike|hCoV-19/USA/WY-WYPHL-20008859/2020|2020-04-13|EPI_ISL_539795|Original|hCoV-19^^Wyoming|Human|Wyoming Public Health Laboratory|Wyoming Public Health Laboratory|Sevinsky|USA
>Spike|hCoV-19/England/MILK-A4714C/2020|2020-10-06|EPI_ISL_598336|Original|hCoV-19^^England|Human|Lighthouse Lab in Milton Keynes|Wellcome Sanger Institute for the COVID-19 Genomics UK (COG-UK) consortium|Davies|United Kingdom
>Spike|hCoV-19/USA/MA-MGH-00304/2020|2020-04-01|EPI_ISL_460430|Original|hCoV-19^^Massachusetts|Human|Massachusetts General Hospital|Infectious Disease Program|Lemieux|USA
>Spike|hCoV-19/USA/MA-MGH-03047/2020|2020-08-02|EPI_ISL_765692|Original|hCoV-19^^Massachusetts|Human|Massachusetts General Hospital|Infectious Disease Program|Park|USA
>Spike|hCoV-19/England/BRIS-1853D57/2020|2020-04-27|EPI_ISL_492392|Original|hCoV-19^^England|Human|PHE South West Regional Laboratory|Wellcome Sanger Institute for the COVID-19 Genomics UK (COG-UK) consortium|Davies|United Kingdom
>Spike|hCoV-19/USA/WA-UW-2105/2020|2020-03-25|EPI_ISL_426088|Original|hCoV-19^^Washington|Human|UW Virology Lab|UW Virology Lab|Roychoudhury|USA
>Spike|hCoV-19/Denmark/DCGC-7459/2020|2020-10-12|EPI_ISL_619400|Original|hCoV-19^^Hovedstaden|Human|Department of Virus and Microbiological Special Diagnostics|Albertsen lab|Michaelsen|Denmark
>Spike|hCoV-19/Wales/PHWC-497125/2020|2020-11-26|EPI_ISL_726782|Original|hCoV-19^^Wales|Human|Originating lab|COVID-19 Genomics UK (COG-UK) Consortium|Moore|United Kingdom
>Spike|hCoV-19/USA/TX-HMH-MCoV-13755/2020|2020-08-18|EPI_ISL_788848|Original|hCoV-19^^Texas|Human|Houston Methodist Hospital|Houston Methodist Hospital|Long|USA
>Spike|hCoV-19/England/ALDP-9CBB8F/2020|2020-09-16|EPI_ISL_575406|Original|hCoV-19^^England|Human|Lighthouse Lab in Alderley Park|Wellcome Sanger Institute for the COVID-19 Genomics UK (COG-UK) consortium|Davies|United Kingdom

>Spike|hCoV-19/England/LOND-126090E/2020|2020-03-16|EPI_ISL_516863|Original|hCoV-19^^England|Human|North West London Pathology|Wellcome Sanger Institute for the COVID-19 Genomics UK (COG-UK) consortium|Davies|United Kingdom
>Spike|hCoV-19/England/NOTT-11690B/2020|2020-10-13|EPI_ISL_612572|Original|hCoV-19^^England|Human|Queens Medical Centre|COVID-19 Genomics UK (COG-UK) Consortium|Loose|United Kingdom
>Spike|hCoV-19/England/ALDP-B1C5AD/2020|2020-11-01|EPI_ISL_645662|Original|hCoV-19^^England|Human|Lighthouse Lab in Alderley Park|Wellcome Sanger Institute for the COVID-19 Genomics UK (COG-UK) Consortium|Davies|United Kingdom
>Spike|hCoV-19/Australia/VIC5755/2020|2020-07-21|EPI_ISL_518553|Original|hCoV-19^^Victoria|Human|Microbiological Diagnostic Unit - Public Health Laboratory (MDU-PHL)|MDU-PHL|Schultz|Australia
>Spike|hCoV-19/USA/TX-HMH-MCoV-9130/2020|2020-07-02|EPI_ISL_780485|Original|hCoV-19^^Texas|Human|Houston Methodist Hospital|Houston Methodist Hospital|Long|USA
>Spike|hCoV-19/England/MILK-99E077/2020|2020-08-30|EPI_ISL_550531|Original|hCoV-19^^England|Human|Lighthouse Lab in Milton Keynes|Wellcome Sanger Institute for the COVID-19 Genomics UK (COG-UK) consortium|Davies|United Kingdom
>Spike|hCoV-19/England/QEUH-BCD620/2020|2020-11-26|EPI_ISL_704414|Original|hCoV-19^^England|Human|Lighthouse Lab in Glasgow|Wellcome Sanger Institute for the COVID-19 Genomics UK (COG-UK) Consortium|Davies|United Kingdom
>Spike|hCoV-19/Australia/NSW860/2020|2020-08-02|EPI_ISL_526202|Original|hCoV-19^^New South Wales|Human|Laverty Pathology|NSW Health Pathology - Institute of Clinical Pathology and Medical Research; Westmead Hospital; University of Sydney|Gall|Australia
>Spike|hCoV-19/England/MILK-BA9B1C/2020|2020-11-17|EPI_ISL_681400|Original|hCoV-19^^England|Human|Lighthouse Lab in Milton Keynes|Wellcome Sanger Institute for the COVID-19 Genomics UK (COG-UK) Consortium|Davies|United Kingdom
>Spike|hCoV-19/USA/CA-ALSR-1517/2020|2020-05-04|EPI_ISL_494639|Original|hCoV-19^^California|Human|Scripps Medical Laboratory|Andersen lab at Scripps Research|Gangavarapu|USA
>Spike|hCoV-19/Netherlands/NH-RIVM-20229/2020|2020-12-06|EPI_ISL_723379|Orignal|hCoV-19^^Noord-Holland|Human|Dutch COVID-19 response team|National Institute for Public Health and the Environment (RIVM)|Meijer|Netherlands
>Spike|hCoV-19/Brazil/RJ-INCA-C95/2020|2020-04-24|EPI_ISL_513572|Original|hCoV-19^^Rio de Janeiro|Human|Programa de Oncovirologia|Programa de Oncovirologia|Ramos Goes|Brazil
>Spike|hCoV-19/England/ALDP-BC7C33/2020|2020-11-23|EPI_ISL_721295|Original|hCoV-19^^England|Human|Lighthouse Lab in Alderley Park|Wellcome Sanger Institute for the COVID-19 Genomics UK (COG-UK) Consortium|Davies|United Kingdom
>Spike|hCoV-19/USA/MN-MDH-1015/2020|2020-04-30|EPI_ISL_462860|Original|hCoV-19^^Minnesota|Human|Minnesota Department of Health|Minnesota Department of Health|Garfin|USA
>Spike|hCoV-19/Australia/NSW1161/2020|2020-08-02|EPI_ISL_639729|Original|hCoV-19^^New South Wales|Human|Sydney South West Pathology Service (SSWPS) - Liverpool Hospital - NSW Health Pathology|NSW Health Pathology - Institute of Clinical Pathology and Medical Research; Westmead Hospital; University of Sydney|Gall|Australia
>Spike|hCoV-19/Netherlands/ZH-EMC-932/2020|2020-10-20|EPI_ISL_722520|Original|hCoV-19^^Zuid Holland|Human|Dutch COVID-19 response team|Erasmus Medical Center|Oude Munnink|Netherlands
>Spike|hCoV-19/Wales/PHWC-48F515/2020|2020-11-10|EPI_ISL_681105|Original|hCoV-19^^Wales|Human|Wales Specialist Virology Centre Sequencing lab|COVID-19 Genomics UK (COG-UK) Consortium|Moore|United Kingdom
>Spike|hCoV-19/Wales/PHWC-3191A/2020|2020-04-11|EPI_ISL_446504|Original|hCoV-19^^Wales|Human|Wales Specialist Virology Centre|Public Health Wales Microbiology Cardiff|Moore|United Kingdom
>Spike|hCoV-19/Wales/PHWC-498528/2020|2020-12-01|EPI_ISL_727088|Original|hCoV-19^^Wales|Human|Originating lab|COVID-19 Genomics UK (COG-UK) Consortium|Moore|United Kingdom
>Spike|hCoV-19/Belgium/rega-10151114/2020|2020-10-15|EPI_ISL_735095|Original|hCoV-19^^Hoeilaart|Human|UZ Leuven|KU Leuven|Maes|Belgium

>Spike|hCoV-19/Netherlands/ZuidHolland_17/2020|2020-03-07|EPI_ISL_414559|Original|hCoV-19^^Zuid Holland|Human|Dutch COVID-19 response team|Erasmus Medical Center|Oude Munnink|Netherlands
>Spike|hCoV-19/England/ALDP-9EA7E0/2020|2020-09-24|EPI_ISL_579989|Original|hCoV-19^^England|Human|Lighthouse Lab in Alderley Park|Wellcome Sanger Institute for the COVID-19 Genomics UK (COG-UK) consortium|Davies|United Kingdom
>Spike|hCoV-19/England/ALDP-A2E4D9/2020|2020-10-06|EPI_ISL_586841|Original|hCoV-19^^England|Human|Lighthouse Lab in Alderley Park|Wellcome Sanger Institute for the COVID-19 Genomics UK (COG-UK) consortium|Davies|United Kingdom
>Spike|hCoV-19/Luxembourg/LNS1895902/2020|2020-10-22|EPI_ISL_740013|Original|hCoV-19^^Luxembourg|Human|Laboratoire national de sant\u00e9|Laboratoire national de sant\u00e9|Wienecke-Baldacchino|Luxembourg
>Spike|hCoV-19/Wales/PHWC-4A2CAE/2020|2020-12-14|EPI_ISL_744002|Original|hCoV-19^^Wales|Human|Wales Specialist Virology Centre Sequencing lab|COVID-19 Genomics UK (COG-UK) Consortium|Moore|United Kingdom
>Spike|hCoV-19/England/MILK-A7B7C5/2020|2020-10-15|EPI_ISL_607398|Original|hCoV-19^^England|Human|Lighthouse Lab in Milton Keynes|Wellcome Sanger Institute for the COVID-19 Genomics UK (COG-UK) consortium|Davies|United Kingdom
>Spike|hCoV-19/Jordan/SEARCH-5401/2020|2020-10-07|EPI_ISL_755212|Original|hCoV-19^^Aqaba|Human|Biolab Diagnostic Laboratories|Andersen lab at Scripps Research|Gangavarapu|Jordan
>Spike|hCoV-19/Australia/VIC6692/2020|2020-07-20|EPI_ISL_521448|Original|hCoV-19^^Victoria|Human|Victorian Infectious Diseases Reference Laboratory (VIDRL)|VIDRL and MDU-PHL|Schultz|Australia
>Spike|hCoV-19/USA/TX-HMH-MCoV-10062/2020|2020-07-06|EPI_ISL_789310|Original|hCoV-19^^Texas|Human|Houston Methodist Hospital|Houston Methodist Hospital|Long|USA
>Spike|hCoV-19/USA/HI-H200415/2020|2020-08-09|EPI_ISL_752959|Original|hCoV-19^^Hawaii|Human|State Laboratories Division|State Laboratories Division|Sultana|USA
>Spike|hCoV-19/Denmark/DCGC-14303/2020|2020-11-30|EPI_ISL_711243|Original|hCoV-19^^Midtjylland|Human|Department of Virus and Microbiological Special Diagnostics|Albertsen Lab|Michaelsen|Denmark
>Spike|hCoV-19/Wales/PHWC-49A33B/2020|2020-12-01|EPI_ISL_727530|Original|hCoV-19^^Wales|Human|Originating lab|COVID-19 Genomics UK (COG-UK) Consortium|Moore|United Kingdom
>Spike|hCoV-19/Japan/PG-7811/2020|2020-07-00|EPI_ISL_686913|Original|hCoV-19^^Japan|Human|Pathogen Genomics Center|Pathogen Genomics Center|Sekizuka|Japan
>Spike|hCoV-19/USA/VA-DCLS-1917/2020|2020-10-05|EPI_ISL_614064|Original|hCoV-19^^Virginia|Human|Virginia DCLS|Virginia DCLS|St. Jacques|USA
>Spike|hCoV-19/England/CAMC-B21D70/2020|2020-11-03|EPI_ISL_647365|Original|hCoV-19^^England|Human|Lighthouse Lab in Cambridge|Wellcome Sanger Institute for the COVID-19 Genomics UK (COG-UK) Consortium|Davies|United Kingdom
>Spike|hCoV-19/Wales/PHWC-48030B/2020|2020-10-18|EPI_ISL_612873|Original|hCoV-19^^Wales|Human|Wales Specialist Virology Centre Sequencing lab|COVID-19 Genomics UK (COG-UK) Consortium|Moore|United Kingdom
>Spike|hCoV-19/Italy/APU-IZSPB_289PT/2020|2020-11-02|EPI_ISL_653780|Original|hCoV-19^^Apulia|Human|I.R.C.C.S. \S. De Bellis\ - Ente Ospedaliero|Istituto Zooprofilattico Sperimentale della Puglia e della Basilicata|Parisi|Italy
>Spike|hCoV-19/England/LOND-D7EEA/2020|2020-04-16|EPI_ISL_492499|Original|hCoV-19^^England|Human|University College London|Wellcome Sanger Institute for the COVID-19 Genomics UK (COG-UK) consortium|Davies|United Kingdom
>Spike|hCoV-19/Australia/VIC1459/2020|2020-04-21|EPI_ISL_456448|Original|hCoV-19^^Victoria|Human|Victorian Infectious Diseases Reference Laboratory (VIDRL)|Microbiological Diagnostic Unit Public Health Laboratory and Victorian Infectious Diseases Reference Laboratory|Schultz|Australia
>Spike|hCoV-19/Belgium/rega-0416499/2020|2020-04-16|EPI_ISL_734591|Original|hCoV-19^^Heverlee|Human|UZ Leuven|KU Leuven|Maes|Belgium
>Spike|hCoV-19/England/CAMB-A97DC1/2020|2020-12-13|EPI_ISL_741012|Original|hCoV-19^^England|Human|Department of Pathology|COVID-19 Genomics UK (COG-UK) Consortium|Goodfellow|United Kingdom

>Spike|hCoV-19/Singapore/540/2020|2020-06-02|EPI_ISL_482688|Original|hCoV-
19^^Singapore|Human|Singapore General Hospital|Department of Microbiology|Abdul
Rahman|Singapore
>Spike|hCoV-19/Japan/FHU-CS108-0222/2020|2020-02-22|EPI_ISL_728156|Original|hCoV-
19^^Aichi|Human|Fujita Health University Okazaki Medical Center|Department of
Virology and Parasitology|Komoto|Japan
>Spike|hCoV-19/Scotland/QEUH-A61346/2020|2020-10-13|EPI_ISL_665313|Original|hCoV-
19^^Scotland|Human|Northumbria University / South Tees Hospitals NHS Foundation Trust
/ North Cumbria Integrated Care NHS Foundation Trust / North Tees and Hartlepool NHS
Foundation Trust / Newcastle Hospitals NHS Foundation Trust|COVID-19 Genomics UK
(COG-UK) Consortium|Bashton|United Kingdom
>Spike|hCoV-19/Australia/VIC2149/2020|2020-06-17|EPI_ISL_480764|Original|hCoV-
19^^Victoria|Human|Victorian Infectious Diseases Reference Laboratory (VIDRL)|VIDRL
and MDU-PHL|Schultz|Australia
>Spike|hCoV-19/England/ALDP-C58C45/2020|2020-12-14|EPI_ISL_736178|Original|hCoV-
19^^England|Human|Lighthouse Lab in Alderley Park|Wellcome Sanger Institute for the
COVID-19 Genomics UK (COG-UK) Consortium|Davies|United Kingdom
>Spike|hCoV-19/Wales/QEUH-9C98D5/2020|2020-09-10|EPI_ISL_567310|Original|hCoV-
19^^Wales|Human|Lighthouse Lab in Glasgow|Wellcome Sanger Institute for the COVID-19
Genomics UK (COG-UK) consortium|Davies|United Kingdom
>Spike|hCoV-19/Denmark/DCGC-6289/2020|2020-10-05|EPI_ISL_620049|Original|hCoV-
19^^Hovedstaden|Human|Department of Virus and Microbiological Special
Diagnostics|Albertsen lab|Michaelsen|Denmark
>Spike|hCoV-19/Belgium/DWW-0330306/2020|2020-03-30|EPI_ISL_462160|Original|hCoV-
19^^Oevel|Human|KU Leuven|KU Leuven|Maes|Belgium
>Spike|hCoV-19/England/CAMB-836B8/2020|2020-04-15|EPI_ISL_443543|Original|hCoV-
19^^England|Human|Department of Pathology|Wellcome Sanger Institute for the COVID-19
Genomics UK (COG-UK) consortium|Amato|United Kingdom
>Spike|hCoV-19/Denmark/DCGC-2167/2020|2020-08-10|EPI_ISL_617466|Original|hCoV-
19^^Midtjylland|Human|Department of Virus and Microbiological Special
Diagnostics|Albertsen lab|Michaelsen|Denmark
>Spike|hCoV-19/USA/TX-HMH-5330/2020|2020-06-26|EPI_ISL_544688|Original|hCoV-
19^^Texas|Human|Houston Methodist Hospital|Houston Methodist Hospital|Long|USA
>Spike|hCoV-19/Australia/VIC4074/2020|2020-07-11|EPI_ISL_519456|Original|hCoV-
19^^Victoria|Human|Microbiological Diagnostic Unit - Public Health Laboratory (MDU-
PHL)|MDU-PHL|Schultz|Australia
>Spike|hCoV-19/USA/MI-MDHHS-SC20323/2020|2020-03-15|EPI_ISL_450602|Original|hCoV-
19^^Michigan|Human|Michigan Department of Health and Human Services|Michigan
Department of Health and Human Services|Blankenship|USA
>Spike|hCoV-19/USA/WA-S2035/2020|2020-06-06|EPI_ISL_497463|Original|hCoV-
19^^Washington|Human|Washington State Department of Health|Seattle Flu Study|Lee|USA
>Spike|hCoV-19/Italy/BAS-IZSPB-397PT/2020|2020-10-21|EPI_ISL_722925|Original|hCoV-
19^^Basilicata|Human|Istituto Zooprofilattico Sperimentale della Puglia e della
Basilicata|Istituto Zooprofilattico Sperimentale della Puglia e della
Basilicata|Parisi|Italy
>Spike|hCoV-19/Wales/PHWC-2BDF9/2020|2020-04-09|EPI_ISL_445851|Original|hCoV-
19^^Wales|Human|Wales Specialist Virology Centre|Public Health Wales Microbiology
Cardiff|Moore|United Kingdom
>Spike|hCoV-19/USA/MI-MDHHS-SC20454/2020|2020-05-06|EPI_ISL_459993|Original|hCoV-
19^^Michigan|Human|Michigan Department of Health and Human Services|Michigan
Department of Health and Human Services|Blankenship|USA
>Spike|hCoV-19/Brazil/RS-00652/2020|2020-11-25|EPI_ISL_770597|Original|hCoV-19^^Rio
Grande do Sul|Human|Laborat\u00f3rio de Microbiologia Molecular - Universidade
FEEVALE|Bioinformatics Laboratory / LNCC|Almeida|Brazil
>Spike|hCoV-19/England/20132035904/2020|2020-03-23|EPI_ISL_423779|Original|hCoV-
19^^England|Human|Respiratory Virus Unit|Respiratory Virus Unit|Platt|United Kingdom
>Spike|hCoV-19/Scotland/CVR3144/2020|2020-04-19|EPI_ISL_449188|Original|hCoV-
19^^Scotland|Human|West of Scotland Specialist Virology Centre|COVID-19 Genomics UK
(COG-UK) Consortium|Robertson|United Kingdom

>Spike|hCoV-19/USA/UT-UPHL-2012762024/2020|2020-06-19|EPI_ISL_746401|Original|hCoV-19^^Utah|Human|Utah Public Health Laboratory|Utah Public Health Laboratory|Gallagher|USA
>Spike|hCoV-19/USA/LA-QDX-1195/2020|2020-03-16|EPI_ISL_571941|Original|hCoV-19^^Louisiana|Human|Quest Diagnostics|Quest Diagnostics|Kagan|USA
>Spike|hCoV-19/Spain/IB-IBV-2526/2020|2020-03-20|EPI_ISL_469012|Original|hCoV-19^^Illes Balears|Human|Servicio de Microbiolog\u00eda|SeqCOVID-SPAIN consortium/IBV(CSIC)|Comas|Spain
>Spike|hCoV-19/Austria/CeMM0148/2020|2020-03-02|EPI_ISL_437996|Original|hCoV-19^^Vienna|Human|Center for Virology|Bergthaler laboratory|Bergthaler|Austria
>Spike|hCoV-19/England/CAMC-B0BA93/2020|2020-10-29|EPI_ISL_643626|Original|hCoV-19^^England|Human|Lighthouse Lab in Cambridge|Wellcome Sanger Institute for the COVID-19 Genomics UK (COG-UK) Consortium|Davies|United Kingdom
>Spike|hCoV-19/Japan/PG-7372/2020|2020-06-00|EPI_ISL_688474|Original|hCoV-19^^Japan|Human|Pathogen Genomics Center|Pathogen Genomics Center|Sekizuka|Japan
>Spike|hCoV-19/England/ALDP-B8579A/2020|2020-11-14|EPI_ISL_675155|Original|hCoV-19^^England|Human|Lighthouse Lab in Alderley Park|Wellcome Sanger Institute for the COVID-19 Genomics UK (COG-UK) Consortium|Davies|United Kingdom
>Spike|hCoV-19/Hong Kong/HKU-200723-016/2020|2020-02-03|EPI_ISL_497783|Original|hCoV-19^^Hong Kong|Human|Department of Microbiology|Department of Microbiology|Chan|China
>Spike|hCoV-19/Denmark/DCGC-8254/2020|2020-10-26|EPI_ISL_617283|Original|hCoV-19^^Hovedstaden|Human|Department of Virus and Microbiological Special Diagnostics|Albertsen lab|Michaelsen|Denmark
>Spike|hCoV-19/England/ALDP-B24171/2020|2020-11-04|EPI_ISL_646984|Original|hCoV-19^^England|Human|Lighthouse Lab in Alderley Park|Wellcome Sanger Institute for the COVID-19 Genomics UK (COG-UK) Consortium|Davies|United Kingdom
>Spike|hCoV-19/USA/WA-UW-4614/2020|2020-03-26|EPI_ISL_515280|Original|hCoV-19^^Washington|Human|University of Washington Virology Lab|University of Washington Virology Lab|Roychoudhury|USA
>Spike|hCoV-19/USA/VA-DCLS-1630/2020|2020-08-20|EPI_ISL_614033|Original|hCoV-19^^Virginia|Human|Virginia DCLS|Virginia DCLS|St. Jacques|USA
>Spike|hCoV-19/Wales/PHWC-47FEB3/2020|2020-10-07|EPI_ISL_612824|Original|hCoV-19^^Wales|Human|Wales Specialist Virology Centre Sequencing lab|COVID-19 Genomics UK (COG-UK) Consortium|Moore|United Kingdom
>Spike|hCoV-19/Canada/ON-S743/2020|2020-04-10|EPI_ISL_591211|Original|hCoV-19^^Ontario|Human|Toronto Invasive Bacterial Diseases Network|McMaster University|McArthur|Canada
>Spike|hCoV-19/Scotland/CVR2686/2020|2020-04-17|EPI_ISL_448189|Original|hCoV-19^^Scotland|Human|West of Scotland Specialist Virology Centre|COVID-19 Genomics UK (COG-UK) Consortium|Robertson|United Kingdom
>Spike|hCoV-19/England/QEUH-C5BDF4/2020|2020-12-14|EPI_ISL_769445|Original|hCoV-19^^England|Human|Lighthouse Lab in Glasgow|Wellcome Sanger Institute for the COVID-19 Genomics UK (COG-UK) Consortium|Davies|United Kingdom
>Spike|hCoV-19/Sweden/20-53825/2020|2020-12-14|EPI_ISL_775587|Original|hCoV-19^^Stockholm|Human|Synlab Medilab|The Public Health Agency of Sweden|Svartstr\u00f6m|Sweden
>Spike|hCoV-19/Scotland/QEUH-9DE8A0/2020|2020-09-21|EPI_ISL_601232|Original|hCoV-19^^Scotland|Human|Lighthouse Lab in Glasgow|Wellcome Sanger Institute for the COVID-19 Genomics UK (COG-UK) consortium|Davies|United Kingdom
>Spike|hCoV-19/Denmark/DCGC-15062/2020|2020-11-23|EPI_ISL_713345|Original|hCoV-19^^Hovedstaden|Human|Department of Virus and Microbiological Special Diagnostics|Albertsen Lab|Michaelsen|Denmark
>Spike|hCoV-19/England/CAMB-8467B/2020|2020-04-27|EPI_ISL_438747|Original|hCoV-19^^England|Human|Department of Pathology|COVID-19 Genomics UK (COG-UK) Consortium|Meredith|United Kingdom
>Spike|hCoV-19/England/ALDP-B1BA18/2020|2020-11-01|EPI_ISL_645766|Original|hCoV-19^^England|Human|Lighthouse Lab in Alderley Park|Wellcome Sanger Institute for the COVID-19 Genomics UK (COG-UK) Consortium|Davies|United Kingdom

>Spike|hCoV-19/England/ALDP-94F8D7/2020|2020-06-16|EPI_ISL_555778|Original|hCoV-19^^England|Human|Lighthouse Lab in Alderley Park|Wellcome Sanger Institute for the COVID-19 Genomics UK (COG-UK) consortium|Davies|United Kingdom
>Spike|hCoV-19/Australia/VIC9784/2020|2020-07-28|EPI_ISL_565713|Original|hCoV-19^^Victoria|Human|Microbiological Diagnostic Unit - Public Health Laboratory (MDU-PHL)|MDU-PHL|Schultz|Australia
>Spike|hCoV-19/England/MILK-B3D0C3/2020|2020-11-01|EPI_ISL_706219|Original|hCoV-19^^England|Human|Oxford Viromics|COVID-19 Genomics UK (COG-UK) Consortium|Golubchik|United Kingdom
>Spike|hCoV-19/Netherlands/LI-EMC-107/2020|2020-10-30|EPI_ISL_722577|Original|hCoV-19^^Limburg|Human|Dutch COVID-19 response team|Erasmus Medical Center|Oude Munnink|Netherlands
>Spike|hCoV-19/England/ALDP-94CD9F/2020|2020-06-15|EPI_ISL_555877|Original|hCoV-19^^England|Human|Lighthouse Lab in Alderley Park|Wellcome Sanger Institute for the COVID-19 Genomics UK (COG-UK) consortium|Davies|United Kingdom
>Spike|hCoV-19/England/BRIS-185348F/2020|2020-04-07|EPI_ISL_492296|Original|hCoV-19^^England|Human|PHE South West Regional Laboratory|Wellcome Sanger Institute for the COVID-19 Genomics UK (COG-UK) consortium|Davies|United Kingdom
>Spike|hCoV-19/USA/MA-MGH-03346/2020|2020-11-05|EPI_ISL_765796|Original|hCoV-19^^Massachusetts|Human|Massachusetts General Hospital|Infectious Disease Program|Park|USA
>Spike|hCoV-19/England/ALDP-CB5BB2/2020|2020-12-20|EPI_ISL_761787|Original|hCoV-19^^England|Human|Lighthouse Lab in Alderley Park|Wellcome Sanger Institute for the COVID-19 Genomics UK (COG-UK) Consortium|Davies|United Kingdom
>Spike|hCoV-19/South Africa/KRISP-0407/2020|2020-06-24|EPI_ISL_495532|Original|hCoV-19^^KwaZulu-Natal|Human|NHLS-IALCH|KRISP|de Oliveira|South Africa
>Spike|hCoV-19/Denmark/DCGC-16320/2020|2020-11-16|EPI_ISL_714701|Original|hCoV-19^^Sjaelland|Human|Department of Virus and Microbiological Special Diagnostics|Albertsen Lab|Michaelsen|Denmark
>Spike|hCoV-19/USA/WA-UW164/2020|2020-03-13|EPI_ISL_416702|Original|hCoV-19^^Washington|Human|UW Virology Lab|UW Virology Lab|Roychoudhury|USA
>Spike|hCoV-19/England/MILK-ABBC60/2020|2020-10-21|EPI_ISL_631259|Original|hCoV-19^^England|Human|Lighthouse Lab in Milton Keynes|Wellcome Sanger Institute for the COVID-19 Genomics UK (COG-UK) consortium|Davies|United Kingdom
>Spike|hCoV-19/England/LEED-2A9B15/2020|2020-04-08|EPI_ISL_538922|Original|hCoV-19^^England|Human|Leeds Teaching Hospitals NHS Trust and Public Health England|Wellcome Sanger Institute for the COVID-19 Genomics UK (COG-UK) consortium|Davies|United Kingdom
>Spike|hCoV-19/USA/FL-BPHL-0897/2020|2020-04-16|EPI_ISL_517787|Original|hCoV-19^^Florida|Human|Florida Bureau of Public Health Laboratories|Florida Bureau of Public Health Laboratories|Blanton|USA
>Spike|hCoV-19/Scotland/EDB5671/2020|2020-04-12|EPI_ISL_473829|Original|hCoV-19^^Scotland|Human|Virology Department|COVID-19 Genomics UK (COG-UK) Consortium|Williams|United Kingdom
>Spike|hCoV-19/Denmark/DCGC-15234/2020|2020-11-23|EPI_ISL_713713|Original|hCoV-19^^Hovedstaden|Human|Department of Virus and Microbiological Special Diagnostics|Albertsen Lab|Michaelsen|Denmark
>Spike|hCoV-19/England/BRIS-1301B8/2020|2020-04-11|EPI_ISL_492520|Original|hCoV-19^^England|Human|PHE South West Regional Laboratory|Wellcome Sanger Institute for the COVID-19 Genomics UK (COG-UK) consortium|Davies|United Kingdom
>Spike|hCoV-19/Switzerland/BL-ETHZ-260113/2020|2020-08-26|EPI_ISL_539422|Original|hCoV-19^^Basel-Landschaft|Human|Viollier AG|Department of Biosystems Science and Engineering|Nadeau|Switzerland
>Spike|hCoV-19/Germany/BE-ChVir7622/2020|2020-06-05|EPI_ISL_729556|Original|hCoV-19^^Berlin|Human|Charit\u00e9 Universit\u00e4tsmedizin Berlin|Charit\u00e9 Universit\u00e4tsmedizin Berlin|Muehlemann|Germany
>Spike|hCoV-19/England/ALDP-955D2A/2020|2020-06-12|EPI_ISL_554072|Original|hCoV-19^^England|Human|Lighthouse Lab in Alderley Park|Wellcome Sanger Institute for the COVID-19 Genomics UK (COG-UK) consortium|Davies|United Kingdom

>Spike|hCoV-19/England/MILK-BB1D57/2020|2020-11-18|EPI_ISL_720628|Original|hCoV-19^^England|Human|Lighthouse Lab in Milton Keynes|Wellcome Sanger Institute for the COVID-19 Genomics UK (COG-UK) Consortium|Davies|United Kingdom
>Spike|hCoV-19/England/QEUH-9DCD85/2020|2020-09-21|EPI_ISL_599538|Original|hCoV-19^^England|Human|Lighthouse Lab in Glasgow|Wellcome Sanger Institute for the COVID-19 Genomics UK (COG-UK) consortium|Davies|United Kingdom
>Spike|hCoV-19/Spain/VC-FISABIO-37/2020|2020-03-09|EPI_ISL_420122|Original|hCoV-19^^Comunitat Valenciana|Human|Servicio de Microbiolog\u00eda. Consorcio Hospital General Universitario de Valencia|Sequencing and Bioinformatics Service and Molecular Epidemiology Research Group. FISABIO-Public Health|Gonzalez-Candelas|Spain
>Spike|hCoV-19/England/MILK-CA97BD/2020|2020-12-17|EPI_ISL_762464|Original|hCoV-19^^England|Human|Lighthouse Lab in Milton Keynes|Wellcome Sanger Institute for the COVID-19 Genomics UK (COG-UK) Consortium|Davies|United Kingdom
>Spike|hCoV-19/USA/AZ-TG670146/2020|2020-04-30|EPI_ISL_696351|Original|hCoV-19^^Arizona|Human|Sonora Quest Laboratories|TGen North|French|USA
>Spike|hCoV-19/USA/CT-Yale-117/2020|2020-04-11|EPI_ISL_428405|Original|hCoV-19^^Connecticut|Human|Yale COVID-19 Biorepository|Grubaugh Lab - Yale School of Public Health|Grubaugh|USA
>Spike|hCoV-19/USA/TX-HMH-3007/2020|2020-05-29|EPI_ISL_543928|Original|hCoV-19^^Texas|Human|Houston Methodist Hospital|Houston Methodist Hospital|Long|USA
>Spike|hCoV-19/USA/AK-CDC-2-3693795/2020|2020-11-09|EPI_ISL_747056|Original|hCoV-19^^Alaska|Human|Respiratory Viruses Branch|Respiratory Viruses Branch|Queen|USA
>Spike|hCoV-19/Denmark/DCGC-7119/2020|2020-10-12|EPI_ISL_619152|Original|hCoV-19^^Hovedstaden|Human|Department of Virus and Microbiological Special Diagnostics|Albertsen lab|Michaelsen|Denmark
>Spike|hCoV-19/Belgium/STM-0324170/2020|2020-03-24|EPI_ISL_420427|Original|hCoV-19^^Boussu|Human|KU Leuven|KU Leuven|Maes|Belgium
>Spike|hCoV-19/Switzerland/BS-42209704/2020|2020-03-27|EPI_ISL_581804|Original|hCoV-19^^Basel-Stadt|Human|University Hospital Basel|University Hospital Basel|Roloff Handschin|Switzerland
>Spike|hCoV-19/Spain/MD-IBV-004953/2020|2020-04-22|EPI_ISL_481084|Original|hCoV-19^^Madrid|Human|Hospital General Universitario Gregorio Mara\u00f1\u00f3n|SeqCOVID-SPAIN consortium/IBV(CSIC)|Comas|Spain
>Spike|hCoV-19/England/BIRM-5E883/2020|2020-03-23|EPI_ISL_444309|Original|hCoV-19^^England|Human|University of Birmingham|COVID-19 Genomics UK (COG-UK) Consortium|Loman|United Kingdom
>Spike|hCoV-19/United Arab Emirates/0023/2020|2020-05-12|EPI_ISL_698233|Original|hCoV-19^^Abu Dhabi|Human|Group 42 (G42) Healthcare|G42 Healthcare|Ogrodzki|United Arab Emirates
>Spike|hCoV-19/England/ALDP-C48D18/2020|2020-12-12|EPI_ISL_736579|Original|hCoV-19^^England|Human|Lighthouse Lab in Alderley Park|Wellcome Sanger Institute for the COVID-19 Genomics UK (COG-UK) Consortium|Davies|United Kingdom
>Spike|hCoV-19/England/NORT-28373B/2020|2020-03-31|EPI_ISL_478509|Original|hCoV-19^^England|Human|Northumbria University / South Tees Hospitals NHS Foundation Trust / North Cumbria Integrated Care NHS Foundation Trust / North Tees and Hartlepool NHS Foundation Trust / Newcastle Hospitals NHS Foundation Trust|COVID-19 Genomics UK (COG-UK) Consortium|Bashton|United Kingdom
>Spike|hCoV-19/England/ALDP-B63871/2020|2020-11-09|EPI_ISL_656629|Original|hCoV-19^^England|Human|Lighthouse Lab in Alderley Park|Wellcome Sanger Institute for the COVID-19 Genomics UK (COG-UK) Consortium|Davies|United Kingdom
>Spike|hCoV-19/England/SHEF-C25FC/2020|2020-04-27|EPI_ISL_475363|Original|hCoV-19^^England|Human|Virology Department|COVID-19 Genomics UK (COG-UK) Consortium|de Silva|United Kingdom
>Spike|hCoV-19/USA/NY-NYCPHL-001554/2020|2020-12-02|EPI_ISL_745402|Original|hCoV-19^^New York|Human|DOHMH Jamaica|New York City Public Health Laboratory|Wang|USA
>Spike|hCoV-19/England/CAMB-1AE285/2020|2020-04-28|EPI_ISL_524601|Original|hCoV-19^^England|Human|Department of Pathology|Wellcome Sanger Institute for the COVID-19 Genomics UK (COG-UK) consortium|Davies|United Kingdom

>Spike|hCoV-19/France/ARA-108984/2020|2020-11-18|EPI_ISL_732688|Original|hCoV-
19^^Auvergne-Rh\u00f4ne-Alpes|Human|CNR Virus des Infections Respiratoires - France
SUD|CNR Virus des Infections Respiratoires - France SUD|Bal|France
>Spike|hCoV-19/England/QEUH-A8BE72/2020|2020-10-15|EPI_ISL_643270|Original|hCoV-
19^^England|Human|Lighthouse Lab in Glasgow|Wellcome Sanger Institute for the COVID-
19 Genomics UK (COG-UK) Consortium|Davies|United Kingdom
>Spike|hCoV-19/Scotland/CVR3957/2020|2020-07-25|EPI_ISL_512477|Original|hCoV-
19^^Scotland|Human|West of Scotland Specialist Virology Centre|COVID-19 Genomics UK
(COG-UK) Consortium|Robertson|United Kingdom
>Spike|hCoV-19/England/QEUH-B41563/2020|2020-11-08|EPI_ISL_658416|Original|hCoV-
19^^England|Human|Lighthouse Lab in Glasgow|Wellcome Sanger Institute for the COVID-
19 Genomics UK (COG-UK) Consortium|Davies|United Kingdom
>Spike|hCoV-19/Jordan/AM-ALSR-4988/2020|2020-10-08|EPI_ISL_730261|Original|hCoV-
19^^Amman|Human|Biolab Diagnostic Laboratories|Andersen lab at Scripps
Research|Gangavarapu|Jordan
>Spike|hCoV-19/England/LIVE-1DD7F7/2020|2020-09-29|EPI_ISL_611728|Original|hCoV-
19^^England|Human|Liverpool Clinical Laboratories|COVID-19 Genomics UK (COG-UK)
Consortium|Haldenby|United Kingdom
>Spike|hCoV-19/USA/CA-SR0450/2020|2020-03-12|EPI_ISL_467974|Original|hCoV-
19^^California|Human|San Diego County Public Health Laboratory|Andersen lab at
Scripps Research|Gangavarapu|USA
>Spike|hCoV-19/USA/WI-GMF-18594/2020|2020-09-03|EPI_ISL_547770|Original|hCoV-
19^^Wisconsin|Human|Gundersen Molecular Diagnostics Laboratory|Kabara Cancer Research
Institute|Kenny|USA
>Spike|hCoV-19/England/CAMB-1AAA90/2020|2020-04-18|EPI_ISL_470235|Original|hCoV-
19^^England|Human|Department of Pathology|Wellcome Sanger Institute for the COVID-19
Genomics UK (COG-UK) consortium|Davies|United Kingdom
>Spike|hCoV-19/England/ALDP-A87D6A/2020|2020-10-17|EPI_ISL_606086|Original|hCoV-
19^^England|Human|Lighthouse Lab in Alderley Park|Wellcome Sanger Institute for the
COVID-19 Genomics UK (COG-UK) consortium|Davies|United Kingdom
>Spike|hCoV-19/England/ALDP-920D28/2020|2020-06-23|EPI_ISL_552923|Original|hCoV-
19^^England|Human|Lighthouse Lab in Alderley Park|Wellcome Sanger Institute for the
COVID-19 Genomics UK (COG-UK) consortium|Davies|United Kingdom
>Spike|hCoV-19/England/WSFT-25C12FB/2020|2020-05-04|EPI_ISL_492889|Original|hCoV-
19^^England|Human|Department of Medical Microbiology|Wellcome Sanger Institute for
the COVID-19 Genomics UK (COG-UK) consortium|Davies|United Kingdom
>Spike|hCoV-19/Netherlands/NB-EMC-442/2020|2020-11-20|EPI_ISL_722682|Original|hCoV-
19^^Noord Brabant|Human|Dutch COVID-19 response team|Erasmus Medical Center|Oude
Munnink|Netherlands
>Spike|hCoV-19/USA/CA-CZB-14233/2020|2020-11-15|EPI_ISL_739304|Original|hCoV-
19^^California|Human|Orange County Public Health Lab|Chan-Zuckerberg Biohub|Kamm|USA
>Spike|hCoV-19/England/LEED-2A9B60/2020|2020-04-12|EPI_ISL_538937|Original|hCoV-
19^^England|Human|Leeds Teaching Hospitals NHS Trust and Public Health
England|Wellcome Sanger Institute for the COVID-19 Genomics UK (COG-UK)
consortium|Davies|United Kingdom
>Spike|hCoV-19/USA/MI-MDHHS-SC20891/2020|2020-05-20|EPI_ISL_476028|Original|hCoV-
19^^Michigan|Human|Michigan Department of Health and Human Services|Michigan
Department of Health and Human Services|Blankenship|USA
>Spike|hCoV-19/Denmark/DCGC-13928/2020|2020-11-30|EPI_ISL_711459|Original|hCoV-
19^^Midtjylland|Human|Department of Virus and Microbiological Special
Diagnostics|Albertsen Lab|Michaelsen|Denmark
>Spike|hCoV-19/Russia/OMS-ORINFI-5710S/2020|2020-05-08|EPI_ISL_569735|Original|hCoV-
19^^Omsk|Human|Omsk Research Institute of Natural Focal Infections|WHO National
Influenza Centre Russian Federation|Komissarov|Russia
>Spike|hCoV-19/England/ALDP-94CBC2/2020|2020-06-15|EPI_ISL_555862|Original|hCoV-
19^^England|Human|Lighthouse Lab in Alderley Park|Wellcome Sanger Institute for the
COVID-19 Genomics UK (COG-UK) consortium|Davies|United Kingdom
>Spike|hCoV-19/USA/TX-HMH-MCoV-13202/2020|2020-08-25|EPI_ISL_788353|Original|hCoV-
19^^Texas|Human|Houston Methodist Hospital|Houston Methodist Hospital|Long|USA

>Spike|hCoV-19/Denmark/DCGC-17549/2020|2020-12-14|EPI_ISL_750240|Original|hCoV-19^^Hovedstaden|Human|Department of Virus and Microbiological Special Diagnostics|Albertsen Lab|Michaelsen|Denmark
>Spike|hCoV-19/Scotland/CVR149/2020|2020-03-15|EPI_ISL_425691|Original|hCoV-19^^Scotland|Human|West of Scotland Specialist Virology Centre|COVID-19 Genomics UK (COG-UK) Consortium|Robertson|United Kingdom
>Spike|hCoV-19/England/MILK-99A4BB/2020|2020-08-29|EPI_ISL_550278|Original|hCoV-19^^England|Human|Lighthouse Lab in Milton Keynes|Wellcome Sanger Institute for the COVID-19 Genomics UK (COG-UK) consortium|Davies|United Kingdom
>Spike|hCoV-19/England/QEUH-A0CA2A/2020|2020-10-03|EPI_ISL_585004|Original|hCoV-19^^England|Human|Quadram Institute Bioscience|COVID-19 Genomics UK (COG-UK) Consortium|Alikhan|United Kingdom
>Spike|hCoV-19/England/CAMC-9D729A/2020|2020-09-20|EPI_ISL_584358|Original|hCoV-19^^England|Human|Virology Department|COVID-19 Genomics UK (COG-UK) Consortium|de Silva|United Kingdom
>Spike|hCoV-19/Japan/PG-2516/2020|2020-04-00|EPI_ISL_690652|Original|hCoV-19^^Japan|Human|Saitama Prefectural Institute of Public Health|Pathogen Genomics Center|Sekizuka|Japan
>Spike|hCoV-19/Australia/VIC3153/2020|2020-07-10|EPI_ISL_520859|Original|hCoV-19^^Victoria|Human|Microbiological Diagnostic Unit - Public Health Laboratory (MDU-PHL)|MDU-PHL|Schultz|Australia
>Spike|hCoV-19/England/QEUH-9646A5/2020|2020-08-12|EPI_ISL_536855|Original|hCoV-19^^England|Human|Lighthouse Lab in Glasgow|Wellcome Sanger Institute for the COVID-19 Genomics UK (COG-UK) consortium|Davies|United Kingdom
>Spike|hCoV-19/Scotland/EDB8276/2020|2020-10-11|EPI_ISL_585501|Original|hCoV-19^^Scotland|Human|Virology Department|COVID-19 Genomics UK (COG-UK) Consortium|McHugh|United Kingdom
>Spike|hCoV-19/England/ALDP-AA4E19/2020|2020-10-22|EPI_ISL_630847|Original|hCoV-19^^England|Human|Lighthouse Lab in Alderley Park|Wellcome Sanger Institute for the COVID-19 Genomics UK (COG-UK) consortium|Davies|United Kingdom
>Spike|hCoV-19/Spain/MD-IBV-99008495/2020|2020-09-10|EPI_ISL_654066|Original|hCoV-19^^Madrid|Human|Hospital General Universitario Gregorio Mara\u00f1\u00f3n|SeqCOVID-SPAIN consortium/IBV(CSIC)|Comas|Spain
>Spike|hCoV-19/Australia/NSW725/2020|2020-07-27|EPI_ISL_513368|Original|hCoV-19^^New South Wales|Human|Pathology North - Hunter - NSW Health Pathology|NSW Health Pathology - Institute of Clinical Pathology and Medical Research; Westmead Hospital; University of Sydney|Gall|Australia
>Spike|hCoV-19/Canada/NS-NML-1198/2020|2020-04-02|EPI_ISL_579599|Original|hCoV-19^^Nova Scotia|Human|QEII Health Sciences Centre|National Microbiology Laboratory (NML)|Majer|Canada
>Spike|hCoV-19/USA/MI-MDHHS-SC22613/2020|2020-11-10|EPI_ISL_737186|Original|hCoV-19^^Michigan|Human|Michigan Department of Health and Human Services|Michigan Department of Health and Human Services|Blankenship|USA
>Spike|hCoV-19/Iceland/174/2020|2020-03-16|EPI_ISL_417716|Original|hCoV-19^^Reykjavik|Human|The National University Hospital of Iceland|deCODE genetics|J\u00f3nsson|Iceland
>Spike|hCoV-19/England/SHEF-CCDBE/2020|2020-04-22|EPI_ISL_453747|Original|hCoV-19^^England|Human|Virology Department|COVID-19 Genomics UK (COG-UK) Consortium|de Silva|United Kingdom
>Spike|hCoV-19/England/QEUH-AD5D30/2020|2020-10-26|EPI_ISL_633917|Original|hCoV-19^^England|Human|Lighthouse Lab in Glasgow|Wellcome Sanger Institute for the COVID-19 Genomics UK (COG-UK) consortium|Davies|United Kingdom
>Spike|hCoV-19/USA/WA-UW-1963/2020|2020-03-24|EPI_ISL_424328|Original|hCoV-19^^Washington|Human|UW Virology Lab|UW Virology Lab|Roychoudhury|USA
>Spike|hCoV-19/England/201060025/2020|2020-03-02|EPI_ISL_464368|Original|hCoV-19^^England|Human|Respiratory Virus Unit|Respiratory Virus Unit|Platt|United Kingdom
>Spike|hCoV-19/USA/TX-HMH-MCoV-19370/2020|2020-11-29|EPI_ISL_783799|Original|hCoV-19^^Texas|Human|Houston Methodist Hospital|Houston Methodist Hospital|Long|USA
>Spike|hCoV-19/England/20170036904/2020|2020-04-10|EPI_ISL_465287|Original|hCoV-19^^England|Human|Respiratory Virus Unit|Respiratory Virus Unit|Platt|United Kingdom

>Spike|hCoV-19/England/ALDP-B74B56/2020|2020-11-12|EPI_ISL_678976|Original|hCoV-19^^England|Human|Queens Medical Centre|COVID-19 Genomics UK (COG-UK) Consortium|Loose|United Kingdom
>Spike|hCoV-19/England/QEUH-96EFBF/2020|2020-08-15|EPI_ISL_530414|Original|hCoV-19^^England|Human|Lighthouse Lab in Glasgow|Wellcome Sanger Institute for the COVID-19 Genomics UK (COG-UK) consortium|Davies|United Kingdom
>Spike|hCoV-19/France/HDF-2060/2020|2020-03-05|EPI_ISL_418223|Original|hCoV-19^^Hauts-de-France|Human|Centre Hospitalier Compi\u00e8gne Laboratoire de Biologie|National Reference Center for Viruses of Respiratory Infections|Enouf|France
>Spike|hCoV-19/England/QEUH-A8BC96/2020|2020-10-16|EPI_ISL_643185|Original|hCoV-19^^England|Human|Lighthouse Lab in Glasgow|Wellcome Sanger Institute for the COVID-19 Genomics UK (COG-UK) Consortium|Davies|United Kingdom
>Spike|hCoV-19/United Arab Emirates/0016/2020|2020-05-07|EPI_ISL_698226|Original|hCoV-19^^Abu Dhabi|Human|Group 42 (G42) Healthcare|G42 Healthcare|Ogrodzki|United Arab Emirates
>Spike|hCoV-19/Wales/PHWC-2A7B0/2020|2020-04-07|EPI_ISL_445618|Original|hCoV-19^^Wales|Human|Wales Specialist Virology Centre|Public Health Wales Microbiology Cardiff|Moore|United Kingdom
>Spike|hCoV-19/Denmark/DCGC-11576/2020|2020-11-02|EPI_ISL_671002|Original|hCoV-19^^Hovedstaden|Human|Department of Virus and Microbiological Special Diagnostics|Albertsen Lab|Michaelsen|Denmark
>Spike|hCoV-19/England/NOTT-110FF0/2020|2020-05-04|EPI_ISL_453614|Original|hCoV-19^^England|Human|Queens Medical Centre|COVID-19 Genomics UK (COG-UK) Consortium|Loose|United Kingdom
>Spike|hCoV-19/South Africa/KRISP-K004077/2020|2020-10-17|EPI_ISL_635053|Original|hCoV-19^^KwaZulu-Natal|Human|National Health Laboratory Service - Inkosi Albert Luthuli Central Hospital (NHLS-IALCH)|KRISP|de Oliveira|South Africa
>Spike|hCoV-19/England/QEUH-B7EB3E/2020|2020-11-14|EPI_ISL_661819|Original|hCoV-19^^England|Human|Lighthouse Lab in Glasgow|Wellcome Sanger Institute for the COVID-19 Genomics UK (COG-UK) Consortium|Davies|United Kingdom
>Spike|hCoV-19/USA/CA-ALSR-1277/2020|2020-04-30|EPI_ISL_494475|Original|hCoV-19^^California|Human|San Diego County Public Health Laboratory|Andersen lab at Scripps Research|Gangavarapu|USA
>Spike|hCoV-19/Czech Republic/NRL_9888/2020|2020-10-06|EPI_ISL_626617|Original|hCoV-19^^Prague|Human|The National Institute of Public Health|State Veterinary Institute Prague|Nagy|Czech Republic
>Spike|hCoV-19/USA/AZ-TG654719/2020|2020-04-01|EPI_ISL_696019|Original|hCoV-19^^Arizona|Human|Sonora Quest Laboratories|TGen North|French|USA
>Spike|hCoV-19/England/SHEF-10D3012/2020|2020-12-08|EPI_ISL_742053|Original|hCoV-19^^England|Human|Virology Department|COVID-19 Genomics UK (COG-UK) Consortium|de Silva|United Kingdom
>Spike|hCoV-19/England/MILK-BAAFF1/2020|2020-11-17|EPI_ISL_681349|Original|hCoV-19^^England|Human|Lighthouse Lab in Milton Keynes|Wellcome Sanger Institute for the COVID-19 Genomics UK (COG-UK) Consortium|Davies|United Kingdom
>Spike|hCoV-19/England/QEUH-A01BE6/2020|2020-10-01|EPI_ISL_680015|Original|hCoV-19^^England|Human|Virology Department|COVID-19 Genomics UK (COG-UK) Consortium|de Silva|United Kingdom
>Spike|hCoV-19/India/MH-ACTREC-494/2020|2020-08-14|EPI_ISL_700142|Original|hCoV-19^^Maharashtra|Human|Hematopathology Laboratory|Hematopathology Laboratory|Patkar|India
>Spike|hCoV-19/Poland/Masovia-3342/2020|2020-05-20|EPI_ISL_754908|Original|hCoV-19^^Masovia|Human|Laboratory Diagnostics and Clinical Immunology of Developmental Age|genXone SA|Kaszuba|Poland
>Spike|hCoV-19/Bangladesh/CHRF-0019/2020|2020-05-14|EPI_ISL_492030|Original|hCoV-19^^Bangladesh|Human|Child Health Research Foundation|Child Health Research Foundation|Saha|Bangladesh
>Spike|hCoV-19/England/LIVE-DC294E/2020|2020-11-23|EPI_ISL_704153|Original|hCoV-19^^England|Human|Liverpool Clinical Laboratories|COVID-19 Genomics UK (COG-UK) Consortium|Haldenby|United Kingdom

>Spike|hCoV-19/Japan/PG-8559/2020|2020-08-00|EPI_ISL_686155|Original|hCoV-19^^Japan|Human|Pathogen Genomics Center|Pathogen Genomics Center|Sekizuka|Japan
>Spike|hCoV-19/England/CAMB-7BF30/2020|2020-04-04|EPI_ISL_442101|Original|hCoV-19^^England|Human|Department of Pathology|Wellcome Sanger Institute for the COVID-19 Genomics UK (COG-UK) consortium|Amato|United Kingdom
>Spike|hCoV-19/England/LIVE-A9968/2020|2020-03-23|EPI_ISL_517297|Original|hCoV-19^^England|Human|Liverpool Clinical Laboratories|COVID-19 Genomics UK (COG-UK) Consortium|Haldenby|United Kingdom
>Spike|hCoV-19/Denmark/DCGC-20337/2020|2020-11-09|EPI_ISL_748120|Original|hCoV-19^^Hovedstaden|Human|Department of Virus and Microbiological Special Diagnostics|Albertsen Lab|Michaelsen|Denmark
>Spike|hCoV-19/France/OCC-SC541/2020|2020-03-30|EPI_ISL_660717|Original|hCoV-19^^Occitanie|Human|CHU N\u00eemes|CNR Virus des Infections Respiratoires - France SUD|Bal|France
>Spike|hCoV-19/USA/TX-HMH-MCoV-11502/2020|2020-09-09|EPI_ISL_790033|Original|hCoV-19^^Texas|Human|Houston Methodist Hospital|Houston Methodist Hospital|Long|USA
>Spike|hCoV-19/England/LIVE-DB3C74/2020|2020-10-20|EPI_ISL_652075|Original|hCoV-19^^England|Human|Liverpool Clinical Laboratories|COVID-19 Genomics UK (COG-UK) Consortium|Haldenby|United Kingdom
>Spike|hCoV-19/England/201380099/2020|2020-03-21|EPI_ISL_465830|Original|hCoV-19^^England|Human|Respiratory Virus Unit|Respiratory Virus Unit|Platt|United Kingdom
>Spike|hCoV-19/Scotland/EDB073/2020|2020-03-20|EPI_ISL_425870|Original|hCoV-19^^Scotland|Human|Virology Department|COVID-19 Genomics UK (COG-UK) Consortium|Williams|United Kingdom
>Spike|hCoV-19/Wales/PHWC-48A592/2020|2020-11-03|EPI_ISL_652570|Original|hCoV-19^^Wales|Human|Wales Specialist Virology Centre Sequencing lab|COVID-19 Genomics UK (COG-UK) Consortium|Moore|United Kingdom
>Spike|hCoV-19/Netherlands/Limburg_5/2020|2020-03-04|EPI_ISL_414448|Original|hCoV-19^^Limburg|Human|Dutch COVID-19 response team|Erasmus Medical Center|Oude Munnink|Netherlands
>Spike|hCoV-19/USA/CA-ALSR-3186/2020|2020-06-02|EPI_ISL_635904|Original|hCoV-19^^California|Human|San Diego County Public Health Laboratory|Andersen lab at Scripps Research|Gangavarapu|USA
>Spike|hCoV-19/England/ALDP-A93E86/2020|2020-10-19|EPI_ISL_611165|Original|hCoV-19^^England|Human|Lighthouse Lab in Alderley Park|Wellcome Sanger Institute for the COVID-19 Genomics UK (COG-UK) consortium|Davies|United Kingdom
>Spike|hCoV-19/England/CAMB-787C2/2020|2020-04-06|EPI_ISL_433773|Original|hCoV-19^^England|Human|Department of Pathology|COVID-19 Genomics UK (COG-UK) Consortium|Meredith|United Kingdom
>Spike|hCoV-19/England/QEUH-B129AB/2020|2020-11-02|EPI_ISL_642264|Original|hCoV-19^^England|Human|Lighthouse Lab in Glasgow|Wellcome Sanger Institute for the COVID-19 Genomics UK (COG-UK) Consortium|Davies|United Kingdom
>Spike|hCoV-19/USA/UT-UPHL-2012380154/2020|2020-08-10|EPI_ISL_745845|Original|hCoV-19^^Utah|Human|Ginkgo Bioworks Clinical Laboratory|Utah Public Health Laboratory|Young|USA
>Spike|hCoV-19/England/MILK-B3A0B7/2020|2020-11-02|EPI_ISL_679678|Original|hCoV-19^^England|Human|Oxford Viromics|COVID-19 Genomics UK (COG-UK) Consortium|Golubchik|United Kingdom
>Spike|hCoV-19/South Africa/R12709-20/2020|2020-06-16|EPI_ISL_504236|Original|hCoV-19^^North West|Human|National Institute for Communicable Diseases of the National Health Laboratory Service|National Institute for Communicable Diseases of the National Health Laboratory Service|Allam|South Africa
>Spike|hCoV-19/England/ALDP-BB33F9/2020|2020-11-19|EPI_ISL_703012|Original|hCoV-19^^England|Human|Lighthouse Lab in Alderley Park|Wellcome Sanger Institute for the COVID-19 Genomics UK (COG-UK) Consortium|Davies|United Kingdom
>Spike|hCoV-19/Spain/VC-IBV-98000317/2020|2020-03-15|EPI_ISL_537608|Original|hCoV-19^^Comunitat Valenciana|Human|Servicio de Microbiolog\u00eda. Consorcio Hospital General Universitario de Valencia|SeqCOVID-SPAIN consortium/IBV(CSIC)|Comas|Spain

>Spike|hCoV-19/Australia/VIC13654/2020|2020-08-14|EPI_ISL_563797|Original|hCoV-19^^Victoria|Human|Microbiological Diagnostic Unit - Public Health Laboratory (MDU-PHL)|MDU-PHL|Schultz|Australia
>Spike|hCoV-19/Canada/ON-S1530/2020|2020-08-31|EPI_ISL_586365|Original|hCoV-19^^Ontario|Human|Toronto Invasive Bacterial Diseases Network|McMaster University|McArthur|Canada
>Spike|hCoV-19/England/CAMB-1AE6F2/2020|2020-04-29|EPI_ISL_524576|Original|hCoV-19^^England|Human|Department of Pathology|Wellcome Sanger Institute for the COVID-19 Genomics UK (COG-UK) consortium|Davies|United Kingdom
>Spike|hCoV-19/USA/TX-HMH0274/2020|2020-03-27|EPI_ISL_434902|Original|hCoV-19^^Texas|Human|Houston Methodist Hospital|Houston Methodist Hospital|Long|USA
>Spike|hCoV-19/Denmark/DCGC-20187/2020|2020-11-30|EPI_ISL_747579|Original|hCoV-19^^Hovedstaden|Human|Department of Virus and Microbiological Special Diagnostics|Albertsen Lab|Michaelsen|Denmark
>Spike|hCoV-19/England/QEUH-CC27AF/2020|2020-12-21|EPI_ISL_760642|Original|hCoV-19^^England|Human|Lighthouse Lab in Glasgow|Wellcome Sanger Institute for the COVID-19 Genomics UK (COG-UK) Consortium|Davies|United Kingdom
>Spike|hCoV-19/Denmark/DCGC-12922/2020|2020-12-07|EPI_ISL_712465|Original|hCoV-19^^Hovedstaden|Human|Department of Virus and Microbiological Special Diagnostics|Albertsen Lab|Michaelsen|Denmark
>Spike|hCoV-19/England/OXON-B15C3/2020|2020-04-23|EPI_ISL_479027|Original|hCoV-19^^England|Human|Oxford Viromics|COVID-19 Genomics UK (COG-UK) Consortium|Golubchik|United Kingdom
>Spike|hCoV-19/England/ALDP-4AD915/2020|2020-06-09|EPI_ISL_559632|Original|hCoV-19^^England|Human|Lighthouse Lab in Alderley Park|Wellcome Sanger Institute for the COVID-19 Genomics UK (COG-UK) consortium|Davies|United Kingdom
>Spike|hCoV-19/Japan/PG-4512/2020|2020-07-00|EPI_ISL_689651|Original|hCoV-19^^Japan|Human|Pathogen Genomics Center|Pathogen Genomics Center|Sekizuka|Japan
>Spike|hCoV-19/Wales/PHWC-29D73/2020|2020-04-05|EPI_ISL_432248|Original|hCoV-19^^Wales|Human|Wales Specialist Virology Centre|Public Health Wales Microbiology Cardiff|Moore|United Kingdom
>Spike|hCoV-19/USA/TX-HMH-MCoV-16024/2020|2020-10-29|EPI_ISL_785708|Original|hCoV-19^^Texas|Human|Houston Methodist Hospital|Houston Methodist Hospital|Long|USA
>Spike|hCoV-19/Wales/PHWC-494D42/2020|2020-11-23|EPI_ISL_726247|Original|hCoV-19^^Wales|Human|Originating lab|COVID-19 Genomics UK (COG-UK) Consortium|Moore|United Kingdom
>Spike|hCoV-19/USA/NY-NYCPHL-000537/2020|2020-03-18|EPI_ISL_631997|Original|hCoV-19^^New York|Human|Jamaica Hospital Medical Center|New York City Public Health Laboratory|Wang|USA
>Spike|hCoV-19/England/CAMB-1B70E9/2020|2020-10-13|EPI_ISL_638303|Original|hCoV-19^^England|Human|Department of Pathology|COVID-19 Genomics UK (COG-UK) Consortium|Goodfellow|United Kingdom
>Spike|hCoV-19/Australia/NSW2760/2020|2020-09-15|EPI_ISL_678343|Original|hCoV-19^^New South Wales|Human|Area of Virology|Virology Research Laboratory; Area of Virology|Foster|Australia
>Spike|hCoV-19/Australia/VIC8462/2020|2020-07-28|EPI_ISL_565032|Original|hCoV-19^^Victoria|Human|Microbiological Diagnostic Unit - Public Health Laboratory (MDU-PHL)|MDU-PHL|Schultz|Australia
>Spike|hCoV-19/England/CAMB-1B3EB6/2020|2020-06-09|EPI_ISL_473451|Original|hCoV-19^^England|Human|Department of Pathology|COVID-19 Genomics UK (COG-UK) Consortium|Meredith|United Kingdom
>Spike|hCoV-19/England/20140038604/2020|2020-03-23|EPI_ISL_420750|Original|hCoV-19^^England|Human|Respiratory Virus Unit|Respiratory Virus Unit|Platt|United Kingdom
>Spike|hCoV-19/Brazil/SP-743/2020|2020-07-04|EPI_ISL_735403|Original|hCoV-19^^S\u00e3o Paulo|Human|Instituto Adolfo Lutz - Regional de Rio Claro|Instituto Adolfo Lutz|Sacchi|Brazil
>Spike|hCoV-19/England/MILK-99E192/2020|2020-08-31|EPI_ISL_550611|Original|hCoV-19^^England|Human|Lighthouse Lab in Milton Keynes|Wellcome Sanger Institute for the COVID-19 Genomics UK (COG-UK) consortium|Davies|United Kingdom

>Spike|hCoV-19/USA/HI-H200219/2020|2020-08-10|EPI_ISL_752778|Original|hCoV-19^^Hawaii|Human|State Laboratories Division|State Laboratories Division|Sultana|USA
>Spike|hCoV-19/Portugal/PT1825/2020|2020-11-15|EPI_ISL_732047|Original|hCoV-19^^Portugal|Human|Instituto Nacional de Saude (INSA)|Instituto Nacional de Saude (INSA)|Borges|Portugal
>Spike|hCoV-19/Australia/VIC3255/2020|2020-07-12|EPI_ISL_520935|Original|hCoV-19^^Victoria|Human|Microbiological Diagnostic Unit - Public Health Laboratory (MDU-PHL)|MDU-PHL|Schultz|Australia
>Spike|hCoV-19/Denmark/DCGC-16990/2020|2020-12-14|EPI_ISL_750694|Original|hCoV-19^^Midtjylland|Human|Department of Virus and Microbiological Special Diagnostics|Albertsen Lab|Michaelsen|Denmark
>Spike|hCoV-19/South Africa/R13159-20/2020|2020-06-30|EPI_ISL_622936|Original|hCoV-19^^Gauteng|Human|National Institute for Communicable Diseases of the National Health Laboratory Service|National Institute for Communicable Diseases of the National Health Laboratory Service|Allam|South Africa
>Spike|hCoV-19/England/NORT-283EBE/2020|2020-04-04|EPI_ISL_478523|Original|hCoV-19^^England|Human|Northumbria University / South Tees Hospitals NHS Foundation Trust / North Cumbria Integrated Care NHS Foundation Trust / North Tees and Hartlepool NHS Foundation Trust / Newcastle Hospitals NHS Foundation Trust|COVID-19 Genomics UK (COG-UK) Consortium|Bashton|United Kingdom
>Spike|hCoV-19/England/MILK-99CBC3/2020|2020-08-30|EPI_ISL_549871|Original|hCoV-19^^England|Human|Lighthouse Lab in Milton Keynes|Wellcome Sanger Institute for the COVID-19 Genomics UK (COG-UK) consortium|Davies|United Kingdom
>Spike|hCoV-19/USA/CA-CZB-14298/2020|2020-08-20|EPI_ISL_739563|Original|hCoV-19^^California|Human|Alameda County Public Health Lab|Chan-Zuckerberg Biohub|Kamm|USA
>Spike|hCoV-19/USA/VI-CDC-3661/2020|2020-03-23|EPI_ISL_452121|Original|hCoV-19^^Virgin Islands|Human|VI-US Virgin Islands Department of Health|Pathogen Discovery|Queen|USA
>Spike|hCoV-19/USA/CA-CZB-6932/2020|2020-08-22|EPI_ISL_582890|Original|hCoV-19^^California|Human|County of Santa Clara Public Health Department|Chan-Zuckerberg Biohub|Bhatt|USA
>Spike|hCoV-19/England/ALDP-9D93FF/2020|2020-09-18|EPI_ISL_576725|Original|hCoV-19^^England|Human|Oxford Viromics|COVID-19 Genomics UK (COG-UK) Consortium|Golubchik|United Kingdom
>Spike|hCoV-19/Japan/PG-1628/2020|2020-04-00|EPI_ISL_685234|Original|hCoV-19^^Japan|Human|Tokyo Metropolitan Institute of Public Health|Pathogen Genomics Center|Sekizuka|Japan
>Spike|hCoV-19/England/ALDP-64675E/2020|2020-07-03|EPI_ISL_558185|Original|hCoV-19^^England|Human|Lighthouse Lab in Alderley Park|Wellcome Sanger Institute for the COVID-19 Genomics UK (COG-UK) Consortium|Davies|United Kingdom
>Spike|hCoV-19/England/SHEF-C3476/2020|2020-10-18|EPI_ISL_611651|Original|hCoV-19^^England|Human|Virology Department|COVID-19 Genomics UK (COG-UK) Consortium|de Silva|United Kingdom
>Spike|hCoV-19/Wales/PHWC-16B6B5/2020|2020-09-13|EPI_ISL_573887|Original|hCoV-19^^Wales|Human|Wales Specialist Virology Centre Sequencing lab|COVID-19 Genomics UK (COG-UK) Consortium|Moore|United Kingdom
>Spike|hCoV-19/Ecuador/USFQ-097/2020|2020-06-17|EPI_ISL_477016|Original|hCoV-19^^Quito|Human|Institute of Microbiology|Institute of Microbiology|Cardenas|Ecuador
>Spike|hCoV-19/England/MILK-A46CF1/2020|2020-10-02|EPI_ISL_598440|Original|hCoV-19^^England|Human|Lighthouse Lab in Milton Keynes|Wellcome Sanger Institute for the COVID-19 Genomics UK (COG-UK) consortium|Davies|United Kingdom
>Spike|hCoV-19/USA/NY-NYUMC385/2020|2020-04-10|EPI_ISL_430412|Original|hCoV-19^^New York|Human|NYU Langone Health|Departments of Pathology and Medicine|Maurano|USA
>Spike|hCoV-19/England/MILK-BAC7AC/2020|2020-11-18|EPI_ISL_783143|Original|hCoV-19^^England|Human|Lighthouse Lab in Milton Keynes|Wellcome Sanger Institute for the COVID-19 Genomics UK (COG-UK) Consortium|Davies|United Kingdom
>Spike|hCoV-19/Scotland/QEUH-9DC1D4/2020|2020-09-22|EPI_ISL_575370|Original|hCoV-19^^Scotland|Human|Lighthouse Lab in Glasgow|Wellcome Sanger Institute for the COVID-19 Genomics UK (COG-UK) consortium|Davies|United Kingdom

>Spike|hCoV-19/Scotland/GCVR-171388/2020|2020-03-26|EPI_ISL_459556|Original|hCoV-19^^Scotland|Human|NHSGGC West of Scotland Specialist Virology Centre / MRC-University of Glasgow Centre for Virus Research|Wellcome Sanger Institute for the COVID-19 Genomics UK (COG-UK) consortium|Davies|United Kingdom
>Spike|hCoV-19/England/ALDP-B84959/2020|2020-11-14|EPI_ISL_675222|Original|hCoV-19^^England|Human|Lighthouse Lab in Alderley Park|Wellcome Sanger Institute for the COVID-19 Genomics UK (COG-UK) Consortium|Davies|United Kingdom
>Spike|hCoV-19/England/MILK-A7B5E9/2020|2020-10-15|EPI_ISL_607500|Original|hCoV-19^^England|Human|Lighthouse Lab in Milton Keynes|Wellcome Sanger Institute for the COVID-19 Genomics UK (COG-UK) consortium|Davies|United Kingdom
>Spike|hCoV-19/Denmark/DCGC-4772/2020|2020-09-21|EPI_ISL_621382|Original|hCoV-19^^Hovedstaden|Human|Department of Virus and Microbiological Special Diagnostics|Albertsen lab|Michaelsen|Denmark
>Spike|hCoV-19/Netherlands/ZH-EMC-343/2020|2020-06-26|EPI_ISL_523684|Original|hCoV-19^^Zuid Holland|Human|Dutch COVID-19 response team|Erasmus Medical Center|Oude Munnink|Netherlands
>Spike|hCoV-19/USA/TX-HMH-MCoV-19394/2020|2020-11-29|EPI_ISL_783814|Original|hCoV-19^^Texas|Human|Houston Methodist Hospital|Houston Methodist Hospital|Long|USA
>Spike|hCoV-19/Japan/PG-7101/2020|2020-08-00|EPI_ISL_688206|Original|hCoV-19^^Japan|Human|Pathogen Genomics Center|Pathogen Genomics Center|Sekizuka|Japan
>Spike|hCoV-19/USA/AZ-TG666146/2020|2020-05-02|EPI_ISL_696228|Original|hCoV-19^^Arizona|Human|Sonora Quest Laboratories|TGen North|French|USA
>Spike|hCoV-19/England/CAMB-1B1331/2020|2020-05-07|EPI_ISL_458557|Original|hCoV-19^^England|Human|Department of Pathology|Wellcome Sanger Institute for the COVID-19 Genomics UK (COG-UK) consortium|Davies|United Kingdom
>Spike|hCoV-19/Scotland/QEUH-B68117/2020|2020-11-11|EPI_ISL_676372|Original|hCoV-19^^Scotland|Human|Lighthouse Lab in Glasgow|Wellcome Sanger Institute for the COVID-19 Genomics UK (COG-UK) Consortium|Davies|United Kingdom
>Spike|hCoV-19/Finland/5LS5E7/2020|2020-10-05|EPI_ISL_756062|Original|hCoV-19^^Uusimaa|Human|Department of Virology and Immunology|Department of Virology|Smura|Finland
>Spike|hCoV-19/England/LIVE-DC4C25/2020|2020-11-11|EPI_ISL_705363|Original|hCoV-19^^England|Human|Liverpool Clinical Laboratories|COVID-19 Genomics UK (COG-UK) Consortium|Haldenby|United Kingdom
>Spike|hCoV-19/USA/WI-WSLH-200741/2020|2020-09-17|EPI_ISL_631496|Original|hCoV-19^^Wisconsin|Human|Wisconsin State Laboratory of Hygiene Communicable Disease Division|Wisconsin State Laboratory of Hygiene Communicable Disease Division|Florek|USA
>Spike|hCoV-19/Scotland/EDB8045/2020|2020-10-01|EPI_ISL_594821|Original|hCoV-19^^Scotland|Human|Virology Department|COVID-19 Genomics UK (COG-UK) Consortium|McHugh|United Kingdom
>Spike|hCoV-19/England/BRIS-127187/2020|2020-04-24|EPI_ISL_469580|Original|hCoV-19^^England|Human|PHE South West Regional Laboratory|Wellcome Sanger Institute for the COVID-19 Genomics UK (COG-UK) consortium|Davies|United Kingdom
>Spike|hCoV-19/England/QEUH-A77FD0/2020|2020-10-14|EPI_ISL_612159|Original|hCoV-19^^England|Human|Queens Medical Centre|COVID-19 Genomics UK (COG-UK) Consortium|Loose|United Kingdom
>Spike|hCoV-19/USA/TX-HMH-7641/2020|2020-06-26|EPI_ISL_546907|Original|hCoV-19^^Texas|Human|Houston Methodist Hospital|Houston Methodist Hospital|Long|USA
>Spike|hCoV-19/SaudiArabia/KAUST-MADINAH961/2020|2020-05-12|EPI_ISL_513211|Original|hCoV-19^^Madinah|Human|Pathogen Genomics Lab King Abdullah University of Science and Technology(KAUST)|Pathogen Genomics Lab King Abdullah University of Science and Technology(KAUST)|Mohamed Ghazzali|Saudi Arabia
>Spike|hCoV-19/New Zealand/20VR3755/2020|2020-06-21|EPI_ISL_548132|Original|hCoV-19^^Auckland|Human|Middlemore Hospital|Institute of Environmental Science and Research (ESR)|Wang|New Zealand
>Spike|hCoV-19/England/20139063504/2020|2020-03-28|EPI_ISL_420745|Original|hCoV-19^^England|Human|Respiratory Virus Unit|Respiratory Virus Unit|Platt|United Kingdom
>Spike|hCoV-19/USA/TX-HMH-MCoV-19813/2020|2020-11-21|EPI_ISL_784144|Original|hCoV-19^^Texas|Human|Houston Methodist Hospital|Houston Methodist Hospital|Long|USA

>Spike|hCoV-19/Japan/PG-3172/2020|2020-03-00|EPI_ISL_691292|Original|hCoV-19^^Japan|Human|Pathogen Genomics Center|Pathogen Genomics Center|Sekizuka|Japan
>Spike|hCoV-19/Wales/MILK-99530E/2020|2020-08-24|EPI_ISL_552408|Original|hCoV-19^^Wales|Human|Lighthouse Lab in Milton Keynes|Wellcome Sanger Institute for the COVID-19 Genomics UK (COG-UK) consortium|Davies|United Kingdom
>Spike|hCoV-19/Denmark/DCGC-7586/2020|2020-10-12|EPI_ISL_618469|Original|hCoV-19^^Hovedstaden|Human|Department of Virus and Microbiological Special Diagnostics|Albertsen lab|Michaelsen|Denmark
>Spike|hCoV-19/Switzerland/VS-ETHZ-340153/2020|2020-10-29|EPI_ISL_721919|Original|hCoV-19^^Valais|Human|Viollier AG|Department of Biosystems Science and Engineering|Nadeau|Switzerland
>Spike|hCoV-19/USA/CA-ALSR-3382/2020|2020-05-29|EPI_ISL_635993|Original|hCoV-19^^California|Human|San Diego County Public Health Laboratory|Andersen lab at Scripps Research|Gangavarapu|USA
>Spike|hCoV-19/Wales/PHWC-16291F/2020|2020-05-28|EPI_ISL_473141|Original|hCoV-19^^Wales|Human|Wales Specialist Virology Centre Sequencing lab|COVID-19 Genomics UK (COG-UK) Consortium|Moore|United Kingdom
>Spike|hCoV-19/Scotland/QEUH-998B03/2020|2020-08-27|EPI_ISL_568348|Original|hCoV-19^^Scotland|Human|Lighthouse Lab in Glasgow|Wellcome Sanger Institute for the COVID-19 Genomics UK (COG-UK) consortium|Davies|United Kingdom
>Spike|hCoV-19/Japan/PG-5161/2020|2020-08-00|EPI_ISL_689644|Original|hCoV-19^^Japan|Human|Pathogen Genomics Center|Pathogen Genomics Center|Sekizuka|Japan
>Spike|hCoV-19/England/ALDP-B7388D/2020|2020-11-12|EPI_ISL_662369|Original|hCoV-19^^England|Human|Lighthouse Lab in Alderley Park|Wellcome Sanger Institute for the COVID-19 Genomics UK (COG-UK) Consortium|Davies|United Kingdom
>Spike|hCoV-19/USA/TX-HMH-MCoV-15530/2020|2020-10-23|EPI_ISL_787014|Original|hCoV-19^^Texas|Human|Houston Methodist Hospital|Houston Methodist Hospital|Long|USA
>Spike|hCoV-19/USA/WA-S1165/2020|2020-05-06|EPI_ISL_463655|Original|hCoV-19^^Washington|Human|Washington State Department of Health|Seattle Flu Study|Bedford|USA
>Spike|hCoV-19/England/ALDP-B5CC60/2020|2020-11-08|EPI_ISL_655849|Original|hCoV-19^^England|Human|Lighthouse Lab in Alderley Park|Wellcome Sanger Institute for the COVID-19 Genomics UK (COG-UK) Consortium|Davies|United Kingdom
>Spike|hCoV-19/Japan/PG-3049/2020|2020-04-00|EPI_ISL_691172|Original|hCoV-19^^Japan|Human|Pathogen Genomics Center|Pathogen Genomics Center|Sekizuka|Japan
>Spike|hCoV-19/England/ALDP-BCA016/2020|2020-11-23|EPI_ISL_703580|Original|hCoV-19^^England|Human|Lighthouse Lab in Alderley Park|Wellcome Sanger Institute for the COVID-19 Genomics UK (COG-UK) Consortium|Davies|United Kingdom
>Spike|hCoV-19/Denmark/DCGC-2830/2020|2020-08-24|EPI_ISL_616656|Original|hCoV-19^^Sjaelland|Human|Department of Virus and Microbiological Special Diagnostics|Albertsen lab|Michaelsen|Denmark
>Spike|hCoV-19/England/CAMB-7E10A/2020|2020-04-15|EPI_ISL_434040|Original|hCoV-19^^England|Human|Department of Pathology|COVID-19 Genomics UK (COG-UK) Consortium|Meredith|United Kingdom
>Spike|hCoV-19/England/ALDP-943FCF/2020|2020-06-07|EPI_ISL_554615|Original|hCoV-19^^England|Human|Lighthouse Lab in Alderley Park|Wellcome Sanger Institute for the COVID-19 Genomics UK (COG-UK) consortium|Davies|United Kingdom
>Spike|hCoV-19/England/MILK-B8E61C/2020|2020-11-13|EPI_ISL_674085|Original|hCoV-19^^England|Human|Lighthouse Lab in Milton Keynes|Wellcome Sanger Institute for the COVID-19 Genomics UK (COG-UK) Consortium|Davies|United Kingdom
>Spike|hCoV-19/Australia/VIC2415/2020|2020-06-28|EPI_ISL_522126|Original|hCoV-19^^Victoria|Human|Victorian Infectious Diseases Reference Laboratory (VIDRL)|VIDRL and MDU-PHL|Schultz|Australia
>Spike|hCoV-19/Germany/HH-hpi-p1497/2020|2020-03-14|EPI_ISL_776003|Original|hCoV-19^^Hamburg|Human|University Medical Center Hamburg Eppendorf|Heinrich Pette Institute|Robitaille|Germany
>Spike|hCoV-19/England/LOND-12F0D61/2020|2020-12-01|EPI_ISL_702661|Original|hCoV-19^^England|Human|University College London|COVID-19 Genomics UK (COG-UK) Consortium|Roy|United Kingdom

>Spike|hCoV-19/Wales/PHWC-49909F/2020|2020-12-02|EPI_ISL_727253|Original|hCoV-19^^Wales|Human|Originating lab|COVID-19 Genomics UK (COG-UK) Consortium|Moore|United Kingdom
>Spike|hCoV-19/England/MILK-994E2C/2020|2020-08-25|EPI_ISL_552250|Original|hCoV-19^^England|Human|Lighthouse Lab in Milton Keynes|Wellcome Sanger Institute for the COVID-19 Genomics UK (COG-UK) consortium|Davies|United Kingdom
>Spike|hCoV-19/Hong Kong/HKPU-4424/2020|2020-08-16|EPI_ISL_610213|Original|hCoV-19^^Hong Kong|Human|Department of Health Technology and Informatics|Department of Health Technology and Informatics|Leung|China
>Spike|hCoV-19/Scotland/CVR2651/2020|2020-04-16|EPI_ISL_453066|Original|hCoV-19^^Scotland|Human|West of Scotland Specialist Virology Centre|COVID-19 Genomics UK (COG-UK) Consortium|Robertson|United Kingdom
>Spike|hCoV-19/Wales/PHWC-24859/2020|2020-03-00|EPI_ISL_432405|Original|hCoV-19^^Wales|Human|Wales Specialist Virology Centre|Public Health Wales Microbiology Cardiff|Moore|United Kingdom
>Spike|hCoV-19/England/ALDP-AA15DB/2020|2020-10-22|EPI_ISL_624269|Original|hCoV-19^^England|Human|Lighthouse Lab in Alderley Park|Wellcome Sanger Institute for the COVID-19 Genomics UK (COG-UK) consortium|Davies|United Kingdom
>Spike|hCoV-19/Singapore/233/2020|2020-03-26|EPI_ISL_462351|Original|hCoV-19^^Singapore|Human|National Public Health Laboratory|National Public Health Laboratory|Mak|Singapore
>Spike|hCoV-19/England/MILK-9F18F2/2020|2020-09-25|EPI_ISL_589716|Original|hCoV-19^^England|Human|Lighthouse Lab in Milton Keynes|Wellcome Sanger Institute for the COVID-19 Genomics UK (COG-UK) consortium|Davies|United Kingdom
>Spike|hCoV-19/England/NORT-296B75/2020|2020-04-28|EPI_ISL_488025|Original|hCoV-19^^England|Human|NU-OMICS DNA Sequencing research facility|Wellcome Sanger Institute for the COVID-19 Genomics UK (COG-UK) consortium|Davies|United Kingdom
>Spike|hCoV-19/England/PORT-2D1E84/2020|2020-04-05|EPI_ISL_499288|Original|hCoV-19^^England|Human|Centre for Enzyme Innovation|COVID-19 Genomics UK (COG-UK) Consortium|Robson|United Kingdom
>Spike|hCoV-19/Denmark/DCGC-14974/2020|2020-11-23|EPI_ISL_713229|Original|hCoV-19^^Midtjylland|Human|Department of Virus and Microbiological Special Diagnostics|Albertsen Lab|Michaelsen|Denmark
>Spike|hCoV-19/England/LIVE-A1915/2020|2020-06-09|EPI_ISL_490345|Original|hCoV-19^^England|Human|Liverpool Clinical Laboratories|COVID-19 Genomics UK (COG-UK) Consortium|Haldenby|United Kingdom
>Spike|hCoV-19/Scotland/EDB8643/2020|2020-10-23|EPI_ISL_650300|Original|hCoV-19^^Scotland|Human|Virology Department|COVID-19 Genomics UK (COG-UK) Consortium|McHugh|United Kingdom
>Spike|hCoV-19/England/MILK-9C3D81/2020|2020-09-09|EPI_ISL_566973|Original|hCoV-19^^England|Human|Lighthouse Lab in Milton Keynes|Wellcome Sanger Institute for the COVID-19 Genomics UK (COG-UK) consortium|Davies|United Kingdom
>Spike|hCoV-19/England/MILK-B95C98/2020|2020-11-14|EPI_ISL_674881|Original|hCoV-19^^England|Human|Lighthouse Lab in Milton Keynes|Wellcome Sanger Institute for the COVID-19 Genomics UK (COG-UK) Consortium|Davies|United Kingdom
>Spike|hCoV-19/England/SHEF-D2A81/2020|2020-05-22|EPI_ISL_479281|Original|hCoV-19^^England|Human|Virology Department|COVID-19 Genomics UK (COG-UK) Consortium|de Silva|United Kingdom
>Spike|hCoV-19/England/CAMC-BBC35A/2020|2020-11-19|EPI_ISL_705593|Original|hCoV-19^^England|Human|Quadram Institute Bioscience|COVID-19 Genomics UK (COG-UK) Consortium|Alikhan|United Kingdom
>Spike|hCoV-19/USA/TX-HMH-MCoV-15829/2020|2020-07-10|EPI_ISL_785513|Original|hCoV-19^^Texas|Human|Houston Methodist Hospital|Houston Methodist Hospital|Long|USA
>Spike|hCoV-19/Japan/PG-3490/2020|2020-07-00|EPI_ISL_691599|Original|hCoV-19^^Japan|Human|Pathogen Genomics Center|Pathogen Genomics Center|Sekizuka|Japan
>Spike|hCoV-19/England/LIVE-9F0E8/2020|2020-04-05|EPI_ISL_453430|Original|hCoV-19^^England|Human|Liverpool Clinical Laboratories|COVID-19 Genomics UK (COG-UK) Consortium|Haldenby|United Kingdom

>Spike|hCoV-19/Slovenia/V0-17818/2020|2020-04-28|EPI_ISL_635277|Original|hCoV-19^^Slovenia|Human|Institute of Microbiology and Immunology|Institute of Microbiology and Immunology|Zakotnik|Slovenia
>Spike|hCoV-19/USA/MI-MDHHS-SC20625/2020|2020-04-17|EPI_ISL_462707|Original|hCoV-19^^Michigan|Human|Michigan Department of Health and Human Services|Michigan Department of Health and Human Services|Blankenship|USA
>Spike|hCoV-19/England/QEUH-AF24FC/2020|2020-10-30|EPI_ISL_662782|Original|hCoV-19^^England|Human|Lighthouse Lab in Glasgow|Wellcome Sanger Institute for the COVID-19 Genomics UK (COG-UK) Consortium|Davies|United Kingdom
>Spike|hCoV-19/USA/CA-IGI-0337/2020|2020-07-17|EPI_ISL_754871|Original|hCoV-19^^California|Human|Innovative Genomics Institute|Innovative Genomics Institute|Wyman|USA
>Spike|hCoV-19/India/TG-GMC-KN443/2020|2020-03-16|EPI_ISL_431103|Original|hCoV-19^^Telangana|Human|Department of Microbiology|Department of Microbiology|Muttineni|India
>Spike|hCoV-19/Norway/4693/2020|2020-10-06|EPI_ISL_635197|Original|hCoV-19^^Oslo|Human|Oslo University Hospital|Norwegian Institute of Public Health|Vollan|Norway
>Spike|hCoV-19/USA/UT-UPHL-01354/2020|2020-04-01|EPI_ISL_575038|Original|hCoV-19^^Utah|Human|Utah Public Health Laboratory|Utah Public Health Laboratory|Young|USA
>Spike|hCoV-19/Hangzhou/HZ60/2020|2020-01-22|EPI_ISL_418502|Original|hCoV-19^^Hangzhou|Human|Hangzhou Center for Disease Control and Prevention|Inspection Center of Hangzhou Center for Disease Control and Prevention|Renton|China
>Spike|hCoV-19/USA/UT-UPHL-2012467786/2020|2020-07-25|EPI_ISL_745633|Original|hCoV-19^^Utah|Human|Ginkgo Bioworks Clinical Laboratory|Utah Public Health Laboratory|Young|USA
>Spike|hCoV-19/Wales/PHWC-16B08A/2020|2020-09-14|EPI_ISL_560239|Original|hCoV-19^^Wales|Human|Wales Specialist Virology Centre Sequencing lab|COVID-19 Genomics UK (COG-UK) Consortium|Moore|United Kingdom
>Spike|hCoV-19/USA/VA-DCLS-1371/2020|2020-07-07|EPI_ISL_572224|Original|hCoV-19^^Virginia|Human|Virginia DCLS|Virginia DCLS|St. Jacques|USA
>Spike|hCoV-19/Peru/LIM-UPCH-0238/2020|2020-10-15|EPI_ISL_729868|Original|hCoV-19^^Lima|Human|Laboratorio de Referencia Nacional de Virus Respiratorios|Laboratorio de Gen\u00f3mica Microbiana|Tsukayama|Peru
>Spike|hCoV-19/England/QEUH-B1006C/2020|2020-11-02|EPI_ISL_642545|Original|hCoV-19^^England|Human|Lighthouse Lab in Glasgow|Wellcome Sanger Institute for the COVID-19 Genomics UK (COG-UK) Consortium|Davies|United Kingdom
>Spike|hCoV-19/England/MILK-AC7E4D/2020|2020-10-21|EPI_ISL_634276|Original|hCoV-19^^England|Human|Lighthouse Lab in Milton Keynes|Wellcome Sanger Institute for the COVID-19 Genomics UK (COG-UK) consortium|Davies|United Kingdom
>Spike|hCoV-19/USA/WA-UW-1878/2020|2020-03-24|EPI_ISL_424302|Original|hCoV-19^^Washington|Human|UW Virology Lab|UW Virology Lab|Roychoudhury|USA
>Spike|hCoV-19/Wales/MILK-958668/2020|2020-06-09|EPI_ISL_554849|Original|hCoV-19^^Wales|Human|Lighthouse Lab in Milton Keynes|Wellcome Sanger Institute for the COVID-19 Genomics UK (COG-UK) consortium|Davies|United Kingdom
>Spike|hCoV-19/USA/NY-QDX-2498/2020|2020-03-15|EPI_ISL_604817|Original|hCoV-19^^New York|Human|Quest Diagnostics|Quest Diagnostics|Kagan|USA
>Spike|hCoV-19/England/SHEF-10C7521/2020|2020-11-23|EPI_ISL_702880|Original|hCoV-19^^England|Human|Virology Department|COVID-19 Genomics UK (COG-UK) Consortium|de Silva|United Kingdom
>Spike|hCoV-19/USA/MA-MASPHL-01014/2020|2020-11-11|EPI_ISL_692794|Original|hCoV-19^^Massachusetts|Human|Massachusetts State Public Health Laboratory|Massachusetts State Public Health Laboratory|Lang|USA
>Spike|hCoV-19/Russia/StPetersburg-RII6039S/2020|2020-04-14|EPI_ISL_430102|Original|hCoV-19^^Saint Petersburg|Human|WHO National Influenza Centre Russian Federation|WHO National Influenza Centre Russian Federation|Komissarov|Russia
>Spike|hCoV-19/England/C200044528/2020|2020-11-06|EPI_ISL_664027|Original|hCoV-19^^England|Human|Respiratory Virus Unit|COVID-19 Genomics UK (COG-UK) Consortium|Platt|United Kingdom

>Spike|hCoV-19/USA/LA-EVTL1299/2020|2020-12-11|EPI_ISL_778965|Original|hCoV-19^^Louisiana|Human|LSUHS Emerging Viral Threat Laboratory|Microbial Genome Sequencing Center|Kamil|USA
>Spike|hCoV-19/USA/WI-UW-1958/2020|2020-09-29|EPI_ISL_677366|Original|hCoV-19^^Wisconsin|Human|University of Wisconsin-Madison AIDS Vaccine Research Laboratories|University of Wisconsin-Madison AIDS Vaccine Research Laboratories|Baczenas|USA
>Spike|hCoV-19/Denmark/DCGC-19378/2020|2020-12-07|EPI_ISL_748304|Original|hCoV-19^^Midtjylland|Human|Department of Virus and Microbiological Special Diagnostics|Albertsen Lab|Michaelsen|Denmark
>Spike|hCoV-19/England/CAMB-80A73/2020|2020-04-09|EPI_ISL_470447|Original|hCoV-19^^England|Human|Department of Pathology|Wellcome Sanger Institute for the COVID-19 Genomics UK (COG-UK) consortium|Davies|United Kingdom
>Spike|hCoV-19/Canada/NS-NML-1265/2020|2020-04-07|EPI_ISL_579653|Original|hCoV-19^^Nova Scotia|Human|QEII Health Sciences Centre|National Microbiology Laboratory (NML)|Majer|Canada
>Spike|hCoV-19/England/BRIS-125DB2/2020|2020-03-30|EPI_ISL_443993|Original|hCoV-19^^England|Human|PHE South West Regional Laboratory|Wellcome Sanger Institute for the COVID-19 Genomics UK (COG-UK) consortium|Amato|United Kingdom
>Spike|hCoV-19/England/CAMB-7673D/2020|2020-03-30|EPI_ISL_438425|Original|hCoV-19^^England|Human|Department of Pathology|Wellcome Sanger Institute for the COVID-19 Genomics UK (COG-UK) consortium|Amato|United Kingdom
>Spike|hCoV-19/Scotland/QEUH-961FDA/2020|2020-08-10|EPI_ISL_531631|Original|hCoV-19^^Scotland|Human|NHSGGC West of Scotland Specialist Virology Centre / MRC-University of Glasgow Centre for Virus Research|Wellcome Sanger Institute for the COVID-19 Genomics UK (COG-UK) consortium|Davies|United Kingdom
>Spike|hCoV-19/England/CAMC-C2CF9C/2020|2020-12-08|EPI_ISL_733758|Original|hCoV-19^^England|Human|Lighthouse Lab in Cambridge|Wellcome Sanger Institute for the COVID-19 Genomics UK (COG-UK) Consortium|Davies|United Kingdom
>Spike|hCoV-19/England/ALDP-A6F427/2020|2020-10-15|EPI_ISL_607888|Original|hCoV-19^^England|Human|Lighthouse Lab in Alderley Park|Wellcome Sanger Institute for the COVID-19 Genomics UK (COG-UK) consortium|Davies|United Kingdom
>Spike|hCoV-19/Japan/PG-3548/2020|2020-06-00|EPI_ISL_691804|Original|hCoV-19^^Japan|Human|Pathogen Genomics Center|Pathogen Genomics Center|Sekizuka|Japan
>Spike|hCoV-19/USA/MA-MASPHL-00623/2020|2020-05-11|EPI_ISL_693002|Original|hCoV-19^^Massachusetts|Human|Massachusetts State Public Health Laboratory|Massachusetts State Public Health Laboratory|Lang|USA
>Spike|hCoV-19/USA/CO-CDPHE-2010201090/2020|2020-10-19|EPI_ISL_710358|Original|hCoV-19^^Colorado|Human|Colorado Department of Public Health and Environment|Colorado Department of Puplic Health and Environment|Bankers|USA
>Spike|hCoV-19/United Arab Emirates/0099/2020|2020-05-19|EPI_ISL_698290|Original|hCoV-19^^Abu Dhabi|Human|Group 42 (G42) Healthcare|G42 Healthcare|Ogrodzki|United Arab Emirates
>Spike|hCoV-19/Peru/LIM-UPCH-0135/2020|2020-08-27|EPI_ISL_568531|Original|hCoV-19^^Lima|Human|Laboratorio de Referencia Nacional de Virus Respiratorios|Laboratorio de Gen\u00f3mica Microbiana|Tsukayama|Peru
>Spike|hCoV-19/England/ALDP-A6F20F/2020|2020-10-15|EPI_ISL_607162|Original|hCoV-19^^England|Human|Lighthouse Lab in Alderley Park|Wellcome Sanger Institute for the COVID-19 Genomics UK (COG-UK) consortium|Davies|United Kingdom
>Spike|hCoV-19/England/20118012704/2020|2020-03-11|EPI_ISL_464887|Original|hCoV-19^^England|Human|Respiratory Virus Unit|Respiratory Virus Unit|Platt|United Kingdom
>Spike|hCoV-19/England/QEUH-9DCE46/2020|2020-09-21|EPI_ISL_599526|Original|hCoV-19^^England|Human|Lighthouse Lab in Glasgow|Wellcome Sanger Institute for the COVID-19 Genomics UK (COG-UK) consortium|Davies|United Kingdom
>Spike|hCoV-19/USA/CT-Yale-279/2020|2020-04-02|EPI_ISL_469202|Original|hCoV-19^^Connecticut|Human|Yale Clinical Virology Laboratory|Grubaugh Lab - Yale School of Public Health|Grubaugh|USA
>Spike|hCoV-19/Scotland/QEUH-A78643/2020|2020-10-14|EPI_ISL_638980|Original|hCoV-19^^Scotland|Human|Oxford Viromics|COVID-19 Genomics UK (COG-UK) Consortium|Golubchik|United Kingdom

>Spike|hCoV-19/England/LEED-2A97F3/2020|2020-04-10|EPI_ISL_538760|Original|hCoV-
19^^England|Human|Leeds Teaching Hospitals NHS Trust and Public Health
England|Wellcome Sanger Institute for the COVID-19 Genomics UK (COG-UK)
consortium|Davies|United Kingdom
>Spike|hCoV-19/Northern Ireland/NIRE-10877B/2020|2020-09-
09|EPI_ISL_585201|Original|hCoV-19^^Northern Ireland|Human|Regional Virus
Laboratory|COVID-19 Genomics UK (COG-UK) Consortium|Fairley|United Kingdom
>Spike|hCoV-19/USA/NY-NYUMC169/2020|2020-04-01|EPI_ISL_426624|Original|hCoV-19^^New
York|Human|NYU Langone Health|Departments of Pathology and Medicine|Maurano|USA
>Spike|hCoV-19/Sweden/20-19194/2020|2020-05-28|EPI_ISL_475525|Original|hCoV-
19^^Stockholm|Human|Huddinge VC|The Public Health Agency of
Sweden|Svartstr\u00f6m|Sweden
>Spike|hCoV-19/USA/WA-S1039/2020|2020-05-03|EPI_ISL_463529|Original|hCoV-
19^^Washington|Human|Washington State Department of Health|Seattle Flu
Study|Bedford|USA
>Spike|hCoV-19/Finland/3LS3AA4/2020|2020-10-03|EPI_ISL_756105|Original|hCoV-
19^^Uusimaa|Human|Department of Virology and Immunology|Department of
Virology|Smura|Finland
>Spike|hCoV-19/Spain/MD-IBV-99008646/2020|2020-09-29|EPI_ISL_654183|Original|hCoV-
19^^Madrid|Human|Hospital General Universitario Gregorio Mara\u00f1\u00f3n|SeqCOVID-
SPAIN consortium/IBV(CSIC)|Comas|Spain
>Spike|hCoV-19/England/MILK-9F1CD8/2020|2020-09-24|EPI_ISL_589737|Original|hCoV-
19^^England|Human|Lighthouse Lab in Milton Keynes|Wellcome Sanger Institute for the
COVID-19 Genomics UK (COG-UK) consortium|Davies|United Kingdom
>Spike|hCoV-19/Switzerland/AG-ETHZ-120002/2020|2020-04-
17|EPI_ISL_468203|Original|hCoV-19^^Aargau|Human|Viollier AG|Department of Biosystems
Science and Engineering|Nadeau|Switzerland
>Spike|hCoV-19/Belgium/rega-10181111/2020|2020-10-18|EPI_ISL_735132|Original|hCoV-
19^^Overijse|Human|UZ Leuven|KU Leuven|Maes|Belgium
>Spike|hCoV-19/Denmark/DCGC-2763/2020|2020-08-24|EPI_ISL_616597|Original|hCoV-
19^^Hovedstaden|Human|Department of Virus and Microbiological Special
Diagnostics|Albertsen lab|Michaelsen|Denmark
>Spike|hCoV-19/England/QEUH-C53EBD/2020|2020-12-11|EPI_ISL_736382|Original|hCoV-
19^^England|Human|Lighthouse Lab in Glasgow|Wellcome Sanger Institute for the COVID-
19 Genomics UK (COG-UK) Consortium|Davies|United Kingdom
>Spike|hCoV-19/South Africa/KRISP-K004082/2020|2020-10-
19|EPI_ISL_635058|Original|hCoV-19^^KwaZulu-Natal|Human|National Health Laboratory
Service - Inkosi Albert Luthuli Central Hospital (NHLS-IALCH)|KRISP|de Oliveira|South
Africa
>Spike|hCoV-19/England/LIVE-1DD8F4/2020|2020-09-28|EPI_ISL_611736|Original|hCoV-
19^^England|Human|Liverpool Clinical Laboratories|COVID-19 Genomics UK (COG-UK)
Consortium|Haldenby|United Kingdom
>Spike|hCoV-19/England/ALDP-A93BDA/2020|2020-10-19|EPI_ISL_611233|Original|hCoV-
19^^England|Human|Lighthouse Lab in Alderley Park|Wellcome Sanger Institute for the
COVID-19 Genomics UK (COG-UK) consortium|Davies|United Kingdom
>Spike|hCoV-19/Germany/NW-HHU-342/2020|2020-10-13|EPI_ISL_718027|Orginal|hCoV-
19^^North Rhine-Westphalia|Human|ZOTZ KLIMAS MVZ D\u00fcsseldorf-Centrum GbR
\u00dcBAG f\u00fcr Labormedizin|Center of Medical Microbiology|Wienemann|Germany
>Spike|hCoV-19/USA/OR-OHSU-3179/2020|2020-10-05|EPI_ISL_667500|Original|hCoV-
19^^Oregon|Human|OHSU Lab Services Molecular Microbiology Lab|Oregon SARS-CoV-2
Genome Sequencing Center|O'Roak|USA
>Spike|hCoV-19/USA/NY-NYUMC698/2020|2020-03-26|EPI_ISL_451413|Original|hCoV-19^^New
York|Human|NYU Langone Health|Departments of Pathology and Medicine|Maurano|USA
>Spike|hCoV-19/Wales/PHWC-49496C/2020|2020-11-21|EPI_ISL_726193|Original|hCoV-
19^^Wales|Human|Originating lab|COVID-19 Genomics UK (COG-UK) Consortium|Moore|United
Kingdom
>Spike|hCoV-19/Belgium/ULG-10781/2020|2020-11-16|EPI_ISL_661218|Original|hCoV-
19^^Li\u00e8ge|Human|Department of Clinical Microbiology|GIGA Medical
Genomics|Durkin|Belgium

>Spike|hCoV-19/Wales/MILK-9D4CEA/2020|2020-09-19|EPI_ISL_601527|Original|hCoV-19^^Wales|Human|Lighthouse Lab in Milton Keynes|Wellcome Sanger Institute for the COVID-19 Genomics UK (COG-UK) consortium|Davies|United Kingdom
>Spike|hCoV-19/Panama/330771/2020|2020-03-12|EPI_ISL_496672|Original|hCoV-19^^Colon|Human|Gorgas Memorial Laboratory of Health Studies|Gorgas Memorial Laboratory of Health Studies|Martinez|Panama
>Spike|hCoV-19/England/ALDP-A68E20/2020|2020-10-12|EPI_ISL_608659|Original|hCoV-19^^England|Human|Lighthouse Lab in Alderley Park|Wellcome Sanger Institute for the COVID-19 Genomics UK (COG-UK) consortium|Davies|United Kingdom
>Spike|hCoV-19/England/QEUH-9CFDB2/2020|2020-09-16|EPI_ISL_589885|Original|hCoV-19^^England|Human|Lighthouse Lab in Glasgow|Wellcome Sanger Institute for the COVID-19 Genomics UK (COG-UK) consortium|Davies|United Kingdom
>Spike|hCoV-19/Denmark/DCGC-15318/2020|2020-11-23|EPI_ISL_713155|Original|hCoV-19^^Hovedstaden|Human|Department of Virus and Microbiological Special Diagnostics|Albertsen Lab|Michaelsen|Denmark
>Spike|hCoV-19/Denmark/DCGC-18487/2020|2020-12-07|EPI_ISL_749399|Original|hCoV-19^^Syddanmark|Human|Department of Virus and Microbiological Special Diagnostics|Albertsen Lab|Michaelsen|Denmark
>Spike|hCoV-19/Australia/NSW720/2020|2020-07-27|EPI_ISL_513363|Original|hCoV-19^^New South Wales|Human|St Vincent's Pathology (SydPath)|NSW Health Pathology - Institute of Clinical Pathology and Medical Research; Westmead Hospital; University of Sydney|Gall|Australia
>Spike|hCoV-19/England/NORT-281041/2020|2020-03-02|EPI_ISL_488800|Original|hCoV-19^^England|Human|NU-OMICS DNA Sequencing research facility|Wellcome Sanger Institute for the COVID-19 Genomics UK (COG-UK) consortium|Davies|United Kingdom
>Spike|hCoV-19/USA/LA-GBCL-26684465/2020|2020-04-11|EPI_ISL_485285|Original|hCoV-19^^Louisiana|Human|River Road Testing Lab|Ginkgo Bioworks Clinical Laboratory|Smith|USA
>Spike|hCoV-19/Wales/PHWC-49529D/2020|2020-11-21|EPI_ISL_726320|Original|hCoV-19^^Wales|Human|Originating lab|COVID-19 Genomics UK (COG-UK) Consortium|Moore|United Kingdom
>Spike|hCoV-19/Japan/PG-0054/2020|2020-03-07|EPI_ISL_479817|Original|hCoV-19^^Hokkaido|Human|Hokkaido Institute of Public Health|Pathogen Genomics Center|Sekizuka|Japan
>Spike|hCoV-19/USA/TX-HMH-5176/2020|2020-06-22|EPI_ISL_544576|Original|hCoV-19^^Texas|Human|Houston Methodist Hospital|Houston Methodist Hospital|Long|USA
>Spike|hCoV-19/Denmark/DCGC-10428/2020|2020-11-02|EPI_ISL_669963|Original|hCoV-19^^Sjaelland|Human|Department of Virus and Microbiological Special Diagnostics|Albertsen Lab|Michaelsen|Denmark
>Spike|hCoV-19/England/20134082004/2020|2020-03-20|EPI_ISL_423182|Original|hCoV-19^^England|Human|Respiratory Virus Unit|Respiratory Virus Unit|Platt|United Kingdom
>Spike|hCoV-19/USA/MI-MDHHS-SC22479/2020|2020-11-08|EPI_ISL_708580|Original|hCoV-19^^Michigan|Human|Michigan Department of Health and Human Services|Michigan Department of Health and Human Services|Jacob|USA
>Spike|hCoV-19/USA/CA-CZB-14760/2020|2020-11-04|EPI_ISL_739052|Original|hCoV-19^^California|Human|Humboldt County Public Health Laboratory|Chan-Zuckerberg Biohub|Kamm|USA
>Spike|hCoV-19/USA/MI-MDHHS-SC20814/2020|2020-05-01|EPI_ISL_471835|Original|hCoV-19^^Michigan|Human|Michigan Department of Health and Human Services|Michigan Department of Health and Human Services|Blankenship|USA
>Spike|hCoV-19/Denmark/DCGC-8527/2020|2020-10-26|EPI_ISL_625910|Original|hCoV-19^^Hovedstaden|Human|Department of Virus and Microbiological Special Diagnostics|Albertsen lab|Michaelsen|Denmark
>Spike|hCoV-19/Spain/PV-IBV-98008335/2020|2020-10-09|EPI_ISL_654568|Original|hCoV-19^^Basque Country|Human|Servicio de Microbiolog\u00eda. Hospital Universitario Donostia. OSI Donostialdea. \u00c1rea de Enfermedades Infecciosas|SeqCOVID-SPAIN consortium/IBV(CSIC)|Comas|Spain
>Spike|hCoV-19/England/BRIS-1268B0/2020|2020-04-09|EPI_ISL_459067|Original|hCoV-19^^England|Human|PHE South West Regional Laboratory|Wellcome Sanger Institute for the COVID-19 Genomics UK (COG-UK) consortium|Davies|United Kingdom

>Spike|hCoV-19/USA/MN-MDH-585/2020|2020-04-14|EPI_ISL_437382|Original|hCoV-19^^Minnesota|Human|Minnesota Department of Health|Minnesota Department of Health|Garfin|USA
>Spike|hCoV-19/England/PORT-2D48FC/2020|2020-10-13|EPI_ISL_611585|Original|hCoV-19^^England|Human|Centre for Enzyme Innovation|COVID-19 Genomics UK (COG-UK) Consortium|Robson|United Kingdom
>Spike|hCoV-19/England/MILK-B04D82/2020|2020-10-24|EPI_ISL_650699|Original|hCoV-19^^England|Human|Department of Pathology|COVID-19 Genomics UK (COG-UK) Consortium|Goodfellow|United Kingdom
>Spike|hCoV-19/Japan/PG-7553/2020|2020-09-00|EPI_ISL_686655|Original|hCoV-19^^Japan|Human|Pathogen Genomics Center|Pathogen Genomics Center|Sekizuka|Japan
>Spike|hCoV-19/England/ALDP-C4820A/2020|2020-12-12|EPI_ISL_718791|Original|hCoV-19^^England|Human|Lighthouse Lab in Alderley Park|Wellcome Sanger Institute for the COVID-19 Genomics UK (COG-UK) Consortium|Davies|United Kingdom
>Spike|hCoV-19/USA/OR-OHSU-1843/2020|2020-07-27|EPI_ISL_526045|Original|hCoV-19^^Oregon|Human|OHSU Lab Services Molecular Microbiology Lab|Oregon SARS-CoV-2 Genome Sequencing Center|O'Roak|USA
>Spike|hCoV-19/England/QEUH-9F2C7D/2020|2020-09-24|EPI_ISL_600795|Original|hCoV-19^^England|Human|Lighthouse Lab in Glasgow|Wellcome Sanger Institute for the COVID-19 Genomics UK (COG-UK) consortium|Davies|United Kingdom
>Spike|hCoV-19/USA/CA-ALSR-1582/2020|2020-05-03|EPI_ISL_494670|Original|hCoV-19^^California|Human|Scripps Medical Laboratory|Andersen lab at Scripps Research|Gangavarapu|USA
>Spike|hCoV-19/England/ALDP-B73FA6/2020|2020-11-12|EPI_ISL_662146|Original|hCoV-19^^England|Human|Lighthouse Lab in Alderley Park|Wellcome Sanger Institute for the COVID-19 Genomics UK (COG-UK) Consortium|Davies|United Kingdom
>Spike|hCoV-19/Denmark/DCGC-1044/2020|2020-03-16|EPI_ISL_614675|Original|hCoV-19^^Syddanmark|Human|Department of Virus and Microbiological Special Diagnostics|Albertsen lab|Michaelsen|Denmark
>Spike|hCoV-19/Wales/PHWC-497F1C/2020|2020-11-30|EPI_ISL_726997|Original|hCoV-19^^Wales|Human|Originating lab|COVID-19 Genomics UK (COG-UK) Consortium|Moore|United Kingdom
>Spike|hCoV-19/Denmark/DCGC-14673/2020|2020-11-23|EPI_ISL_711157|Original|hCoV-19^^Nordjylland|Human|Department of Virus and Microbiological Special Diagnostics|Albertsen Lab|Michaelsen|Denmark
>Spike|hCoV-19/India/MH-NIV-229297/2020|2020-09-28|EPI_ISL_577710|Original|hCoV-19^^Maharashtra|Human|NIV Influenza|NIV Influenza|Potdar|India
>Spike|hCoV-19/USA/CA-CZB-1577/2020|2020-03-14|EPI_ISL_468508|Original|hCoV-19^^California|Human|San Joaquin County Public Health Lab|Chan-Zuckerberg Biohub|Hao|USA
>Spike|hCoV-19/USA/TX-HMH-MCoV-18689/2020|2020-11-20|EPI_ISL_785037|Original|hCoV-19^^Texas|Human|Houston Methodist Hospital|Houston Methodist Hospital|Long|USA
>Spike|hCoV-19/Scotland/EDB4233/2020|2020-04-21|EPI_ISL_453114|Original|hCoV-19^^Scotland|Human|Virology Department|COVID-19 Genomics UK (COG-UK) Consortium|Williams|United Kingdom
>Spike|hCoV-19/England/QEUH-9FF557/2020|2020-10-02|EPI_ISL_600170|Original|hCoV-19^^England|Human|Lighthouse Lab in Glasgow|Wellcome Sanger Institute for the COVID-19 Genomics UK (COG-UK) consortium|Davies|United Kingdom
>Spike|hCoV-19/Netherlands/ZH-RIVM-20532/2020|2020-12-15|EPI_ISL_728747|Original|hCoV-19^^Zuid-holland|Human|Dutch COVID-19 response team|National Institute for Public Health and the Environment (RIVM)|Meijer|Netherlands
>Spike|hCoV-19/England/MILK-B0223A/2020|2020-10-24|EPI_ISL_642936|Original|hCoV-19^^England|Human|Lighthouse Lab in Milton Keynes|Wellcome Sanger Institute for the COVID-19 Genomics UK (COG-UK) Consortium|Davies|United Kingdom
>Spike|hCoV-19/South Africa/NHLS-UCT-GS-2089/2020|2020-09-23|EPI_ISL_640130|Original|hCoV-19^^Western Cape|Human|Groote Schuur Hospital wc GSH|NHLS/UCT|Iranzadeh|South Africa
>Spike|hCoV-19/USA/WA-S1753/2020|2020-06-01|EPI_ISL_496179|Original|hCoV-19^^Washington|Human|Washington State Department of Health|Seattle Flu Study|Lee|USA

>Spike|hCoV-19/USA/VA-DCLS-1902/2020|2020-10-05|EPI_ISL_614090|Original|hCoV-
19^^Virginia|Human|Virginia DCLS|Virginia DCLS|St. Jacques|USA
>Spike|hCoV-19/Netherlands/ZH-RIVM-20825/2020|2020-12-
24|EPI_ISL_790881|Original|hCoV-19^^Zuid-holland|Human|Dutch COVID-19 response
team|National Institute for Public Health and the Environment
(RIVM)|Meijer|Netherlands
>Spike|hCoV-19/USA/VA-DCLS-1948/2020|2020-09-29|EPI_ISL_644930|Original|hCoV-
19^^Virginia|Human|Virginia DCLS|Virginia DCLS|St. Jacques|USA
>Spike|hCoV-19/USA/LA-BIE-023/2020|2020-04-06|EPI_ISL_468713|Original|hCoV-
19^^Louisiana|Human|Ochsner Health|Bioinfoexperts|Cross|USA
>Spike|hCoV-19/Denmark/DCGC-1690/2020|2020-06-29|EPI_ISL_618158|Original|hCoV-
19^^Nordjylland|Human|Department of Virus and Microbiological Special
Diagnostics|Albertsen lab|Michaelsen|Denmark
>Spike|hCoV-19/Ireland/D-NVRL-20ILR10854/2020|2020-11-
26|EPI_ISL_732386|Original|hCoV-19^^Dublin|Human|National Virus Reference
Laboratory|National Virus Reference Laboratory|Gonzalez|Ireland
>Spike|hCoV-19/USA/UN-UW-1834/2020|2020-03-22|EPI_ISL_424265|Original|hCoV-
19^^USA|Human|UW Virology Lab|UW Virology Lab|Roychoudhury|USA
>Spike|hCoV-19/Denmark/DCGC-11007/2020|2020-11-02|EPI_ISL_670340|Original|hCoV-
19^^Sjaelland|Human|Department of Virus and Microbiological Special
Diagnostics|Albertsen Lab|Michaelsen|Denmark
>Spike|hCoV-19/Hong Kong/VM20002454/2020|2020-02-09|EPI_ISL_426389|Original|hCoV-
19^^Hong Kong|Human|Ruttonjee Hospital|Hong Kong Department of Health|Mak|China
>Spike|hCoV-19/Wales/PHWC-494EA9/2020|2020-11-26|EPI_ISL_726266|Original|hCoV-
19^^Wales|Human|Originating lab|COVID-19 Genomics UK (COG-UK) Consortium|Moore|United
Kingdom
>Spike|hCoV-19/England/NORT-28367A/2020|2020-03-31|EPI_ISL_478502|Original|hCoV-
19^^England|Human|Northumbria University / South Tees Hospitals NHS Foundation Trust
/ North Cumbria Integrated Care NHS Foundation Trust / North Tees and Hartlepool NHS
Foundation Trust / Newcastle Hospitals NHS Foundation Trust|COVID-19 Genomics UK
(COG-UK) Consortium|Bashton|United Kingdom
>Spike|hCoV-19/England/MILK-B54FF6/2020|2020-11-03|EPI_ISL_657991|Original|hCoV-
19^^England|Human|Lighthouse Lab in Milton Keynes|Wellcome Sanger Institute for the
COVID-19 Genomics UK (COG-UK) Consortium|Davies|United Kingdom
>Spike|hCoV-19/England/CAMB-7D578/2020|2020-04-02|EPI_ISL_442242|Original|hCoV-
19^^England|Human|Department of Pathology|Wellcome Sanger Institute for the COVID-19
Genomics UK (COG-UK) consortium|Amato|United Kingdom
>Spike|hCoV-19/USA/WA-S1171/2020|2020-05-05|EPI_ISL_463661|Original|hCoV-
19^^Washington|Human|Washington State Department of Health|Seattle Flu
Study|Bedford|USA
>Spike|hCoV-19/USA/MI-MDHHS-SC21561/2020|2020-03-21|EPI_ISL_516288|Original|hCoV-
19^^Michigan|Human|Michigan Department of Health and Human Services|Michigan
Department of Health and Human Services|Blankenship|USA
>Spike|hCoV-19/England/CAMC-B07A5B/2020|2020-10-29|EPI_ISL_643871|Original|hCoV-
19^^England|Human|Lighthouse Lab in Cambridge|Wellcome Sanger Institute for the
COVID-19 Genomics UK (COG-UK) Consortium|Davies|United Kingdom
>Spike|hCoV-19/USA/TX-HMH-4374/2020|2020-06-17|EPI_ISL_543571|Original|hCoV-
19^^Texas|Human|Houston Methodist Hospital|Houston Methodist Hospital|Long|USA
>Spike|hCoV-19/Wales/PHWC-29C76/2020|2020-04-05|EPI_ISL_432189|Original|hCoV-
19^^Wales|Human|Wales Specialist Virology Centre|Public Health Wales Microbiology
Cardiff|Moore|United Kingdom
>Spike|hCoV-19/USA/LA-EVTL483/2020|2020-06-23|EPI_ISL_578887|Original|hCoV-
19^^Louisiana|Human|LSUHS Emerging Viral Threat Laboratory|Microbial Genome
Sequencing Center|Kamil|USA
>Spike|hCoV-19/Luxembourg/LNS8527149/2020|2020-10-21|EPI_ISL_744755|Original|hCoV-
19^^Luxembourg|Human|Laboratoire national de sant\u00e9|Laboratoire national de
sant\u00e9|Wienecke-Baldacchino|Luxembourg
>Spike|hCoV-19/England/20169030004/2020|2020-04-10|EPI_ISL_465224|Original|hCoV-
19^^England|Human|Respiratory Virus Unit|Respiratory Virus Unit|Platt|United Kingdom

>Spike|hCoV-19/Wales/PHWC-28F8D/2020|2020-03-31|EPI_ISL_432134|Original|hCoV-19^^Wales|Human|Wales Specialist Virology Centre|Public Health Wales Microbiology Cardiff|Moore|United Kingdom
>Spike|hCoV-19/USA/CA-CZB-3655/2020|2020-07-18|EPI_ISL_516730|Original|hCoV-19^^California|Human|UCSF Clinical Microbiology Laboratory|Chan-Zuckerberg Biohub|Kamm|USA
>Spike|hCoV-19/Portugal/PT0835/2020|2020-03-22|EPI_ISL_511094|Original|hCoV-19^^Portugal|Human|Instituto Nacional de Saude (INSA)|Instituto Nacional de Saude (INSA)|Borges|Portugal
>Spike|hCoV-19/England/20120039604/2020|2020-03-16|EPI_ISL_465721|Original|hCoV-19^^England|Human|Respiratory Virus Unit|Respiratory Virus Unit|Platt|United Kingdom
>Spike|hCoV-19/USA/CA-CZB-7131/2020|2020-08-31|EPI_ISL_583009|Original|hCoV-19^^California|Human|Orange County Public Health Lab|Chan-Zuckerberg Biohub|Bhatt|USA
>Spike|hCoV-19/England/MILK-970D47/2020|2020-08-18|EPI_ISL_553138|Original|hCoV-19^^England|Human|Lighthouse Lab in Milton Keynes|Wellcome Sanger Institute for the COVID-19 Genomics UK (COG-UK) consortium|Davies|United Kingdom
>Spike|hCoV-19/England/QEUH-9BB056/2020|2020-09-07|EPI_ISL_573647|Original|hCoV-19^^England|Human|Oxford Viromics|COVID-19 Genomics UK (COG-UK) Consortium|Golubchik|United Kingdom
>Spike|hCoV-19/England/BRIS-127C2C/2020|2020-04-17|EPI_ISL_469712|Original|hCoV-19^^England|Human|PHE South West Regional Laboratory|Wellcome Sanger Institute for the COVID-19 Genomics UK (COG-UK) consortium|Davies|United Kingdom
>Spike|hCoV-19/Singapore/85/2020|2020-04-19|EPI_ISL_435690|Original|hCoV-19^^Singapore|Human|National Public Health Laboratory|National Public Health Laboratory|Mak|Singapore
>Spike|hCoV-19/Wales/PHWC-16E3D9/2020|2020-09-30|EPI_ISL_585711|Original|hCoV-19^^Wales|Human|Wales Specialist Virology Centre Sequencing lab|COVID-19 Genomics UK (COG-UK) Consortium|Moore|United Kingdom
>Spike|hCoV-19/England/QEUH-B80FA6/2020|2020-11-14|EPI_ISL_667947|Original|hCoV-19^^England|Human|Lighthouse Lab in Glasgow|Wellcome Sanger Institute for the COVID-19 Genomics UK (COG-UK) Consortium|Davies|United Kingdom
>Spike|hCoV-19/USA/VA-DCLS-1244/2020|2020-07-30|EPI_ISL_529937|Original|hCoV-19^^Virginia|Human|Virginia DCLS|Virginia DCLS|St. Jacques|USA
>Spike|hCoV-19/India/TN-NCDC-02331/2020|2020-03-29|EPI_ISL_435075|Original|hCoV-19^^Tamil Nadu|Human|National Centre for Disease control (NCDC)|NCDC/CSIR-IGIB|Faruq|India
>Spike|hCoV-19/England/MILK-9F2062/2020|2020-09-24|EPI_ISL_589227|Original|hCoV-19^^England|Human|Lighthouse Lab in Milton Keynes|Wellcome Sanger Institute for the COVID-19 Genomics UK (COG-UK) consortium|Davies|United Kingdom
>Spike|hCoV-19/Cote dIvoire/BKE0248/2020|2020-06-20|EPI_ISL_614365|Original|hCoV-19^^Boauk\u00e9|Human|Molecular diagnostic unit for viral haemorrhagic fevers and emerging viruses|Project group Epidemology of Highly Pathogenic Microorganisms|Calvignac-Spencer|C\u00f4te d'Ivoire
>Spike|hCoV-19/Brazil/SP-438/2020|2020-07-10|EPI_ISL_603035|Original|hCoV-19^^S\u00e3o Paulo|Human|Secretaria Municipal de Sa\u00fade|Instituto Adolfo Lutz|Sacchi|Brazil
>Spike|hCoV-19/England/NORT-29CB06/2020|2020-05-01|EPI_ISL_484291|Original|hCoV-19^^England|Human|Northumbria University / South Tees Hospitals NHS Foundation Trust / North Cumbria Integrated Care NHS Foundation Trust / North Tees and Hartlepool NHS Foundation Trust / Newcastle Hospitals NHS Foundation Trust|COVID-19 Genomics UK (COG-UK) Consortium|Bashton|United Kingdom
>Spike|hCoV-19/England/ALDP-B5DFB1/2020|2020-11-09|EPI_ISL_723620|Original|hCoV-19^^England|Human|Northumbria University / South Tees Hospitals NHS Foundation Trust / North Cumbria Integrated Care NHS Foundation Trust / North Tees and Hartlepool NHS Foundation Trust / Newcastle Hospitals NHS Foundation Trust|COVID-19 Genomics UK (COG-UK) Consortium|Bashton|United Kingdom
>Spike|hCoV-19/England/LIVE-A1FAA/2020|2020-05-18|EPI_ISL_490371|Original|hCoV-19^^England|Human|Liverpool Clinical Laboratories|COVID-19 Genomics UK (COG-UK) Consortium|Haldenby|United Kingdom

>Spike|hCoV-19/England/ALDP-92650C/2020|2020-06-23|EPI_ISL_551482|Original|hCoV-19^^England|Human|Lighthouse Lab in Alderley Park|Wellcome Sanger Institute for the COVID-19 Genomics UK (COG-UK) consortium|Davies|United Kingdom
>Spike|hCoV-19/USA/WI-UW-68/2020|2020-03-24|EPI_ISL_421326|Original|hCoV-19^^Wisconsin|Human|University of Wisconsin-Madison AIDS Vaccine Research Laboratories|University of Wisconsin-Madison AIDS Vaccine Research Laboratories|Baczenas|USA
>Spike|hCoV-19/USA/ME-HETL-J0270/2020|2020-10-01|EPI_ISL_755416|Original|hCoV-19^^Maine|Human|Maine Health and Environmental Testing Laboratory|Tewhey Lab|Tewhey|USA
>Spike|hCoV-19/USA/TX-HMH-MCoV-10520/2020|2020-07-08|EPI_ISL_789514|Original|hCoV-19^^Texas|Human|Houston Methodist Hospital|Houston Methodist Hospital|Long|USA
>Spike|hCoV-19/England/MILK-B55B88/2020|2020-11-05|EPI_ISL_657490|Original|hCoV-19^^England|Human|Lighthouse Lab in Milton Keynes|Wellcome Sanger Institute for the COVID-19 Genomics UK (COG-UK) Consortium|Davies|United Kingdom
>Spike|hCoV-19/Wales/PHWC-25418/2020|2020-03-23|EPI_ISL_420990|Original|hCoV-19^^Wales|Human|Wales Specialist Virology Centre|Public Health Wales Microbiology Cardiff|Moore|United Kingdom
>Spike|hCoV-19/USA/CT-QDX-769/2020|2020-03-18|EPI_ISL_571518|Original|hCoV-19^^Connecticut|Human|Quest Diagnostics|Quest Diagnostics|Kagan|USA
>Spike|hCoV-19/England/MILK-9C27FD/2020|2020-09-10|EPI_ISL_566648|Original|hCoV-19^^England|Human|Lighthouse Lab in Milton Keynes|Wellcome Sanger Institute for the COVID-19 Genomics UK (COG-UK) consortium|Davies|United Kingdom
>Spike|hCoV-19/Australia/VIC12010/2020|2020-09-10|EPI_ISL_563505|Original|hCoV-19^^Victoria|Human|Microbiological Diagnostic Unit - Public Health Laboratory (MDU-PHL)|MDU-PHL|Schultz|Australia
>Spike|hCoV-19/USA/CA-CZB-11573/2020|2020-07-16|EPI_ISL_625557|Original|hCoV-19^^California|Human|UCSF Clinical Microbiology Laboratory|Chan-Zuckerberg Biohub|Bhatt|USA
>Spike|hCoV-19/Belgium/rega-0501633/2020|2020-05-01|EPI_ISL_734690|Original|hCoV-19^^Mouscron|Human|UZ Leuven|KU Leuven|Maes|Belgium
>Spike|hCoV-19/Scotland/CVR5887/2020|2020-11-17|EPI_ISL_705647|Original|hCoV-19^^Scotland|Human|West of Scotland Specialist Virology Centre|COVID-19 Genomics UK (COG-UK) Consortium|Robertson|United Kingdom
>Spike|hCoV-19/England/OXON-AC83B/2020|2020-04-05|EPI_ISL_448487|Original|hCoV-19^^England|Human|Oxford Viromics|COVID-19 Genomics UK (COG-UK) Consortium|Golubchik|United Kingdom
>Spike|hCoV-19/Wales/PHWC-163608/2020|2020-05-10|EPI_ISL_473971|Original|hCoV-19^^Wales|Human|Originating lab|COVID-19 Genomics UK (COG-UK) Consortium|Moore|United Kingdom
>Spike|hCoV-19/Germany/HH-hpi-p1959/2020|2020-03-25|EPI_ISL_776284|Original|hCoV-19^^Hamburg|Human|University Medical Center Hamburg Eppendorf|Heinrich Pette Institute|Robitaille|Germany
>Spike|hCoV-19/USA/LA-EVTL1341/2020|2020-12-11|EPI_ISL_779007|Original|hCoV-19^^Louisiana|Human|LSUHS Emerging Viral Threat Laboratory|Microbial Genome Sequencing Center|Kamil|USA
>Spike|hCoV-19/USA/TX-HMH-MCoV-18178/2020|2020-11-15|EPI_ISL_784526|Original|hCoV-19^^Texas|Human|Houston Methodist Hospital|Houston Methodist Hospital|Long|USA
>Spike|hCoV-19/England/SHEF-CF8BA/2020|2020-04-21|EPI_ISL_453759|Original|hCoV-19^^England|Human|Virology Department|COVID-19 Genomics UK (COG-UK) Consortium|de Silva|United Kingdom
>Spike|hCoV-19/USA/OR-OHSU-2195/2020|2020-08-06|EPI_ISL_667143|Original|hCoV-19^^Oregon|Human|OHSU Lab Services Molecular Microbiology Lab|Oregon SARS-CoV-2 Genome Sequencing Center|O'Roak|USA
>Spike|hCoV-19/Canada/QC-LSPQ-L00239255/2020|2020-03-26|EPI_ISL_536241|Original|hCoV-19^^Quebec|Human|Centre hospitalier Anna-Laberge|Laboratoire de sant\u00e9 publique du Qu\u00e9bec|Moreira|Canada
>Spike|hCoV-19/Gibraltar/205121256/2020|2020-00-00|EPI_ISL_766375|Original|hCoV-19^^Gibraltar|Human|Microbiology|Respiratory Virus Unit|Platt|Gibraltar

>Spike|hCoV-19/England/MILK-AB8313/2020|2020-10-21|EPI_ISL_623417|Original|hCoV-19^^England|Human|Lighthouse Lab in Milton Keynes|Wellcome Sanger Institute for the COVID-19 Genomics UK (COG-UK) consortium|Davies|United Kingdom
>Spike|hCoV-19/England/QEUH-B7CECE/2020|2020-11-14|EPI_ISL_674707|Original|hCoV-19^^England|Human|Lighthouse Lab in Glasgow|Wellcome Sanger Institute for the COVID-19 Genomics UK (COG-UK) Consortium|Davies|United Kingdom
>Spike|hCoV-19/Denmark/DCGC-18653/2020|2020-12-07|EPI_ISL_750103|Original|hCoV-19^^Nordjylland|Human|Department of Virus and Microbiological Special Diagnostics|Albertsen Lab|Michaelsen|Denmark
>Spike|hCoV-19/Australia/VIC3140/2020|2020-07-11|EPI_ISL_520847|Original|hCoV-19^^Victoria|Human|Microbiological Diagnostic Unit - Public Health Laboratory (MDU-PHL)|MDU-PHL|Schultz|Australia
>Spike|hCoV-19/England/QEUH-B0F1F4/2020|2020-11-02|EPI_ISL_642691|Original|hCoV-19^^England|Human|Lighthouse Lab in Glasgow|Wellcome Sanger Institute for the COVID-19 Genomics UK (COG-UK) Consortium|Davies|United Kingdom
>Spike|hCoV-19/Wales/PHWC-4A55FB/2020|2020-12-16|EPI_ISL_764893|Original|hCoV-19^^Wales|Human|Wales Specialist Virology Centre Sequencing lab|COVID-19 Genomics UK (COG-UK) Consortium|Moore|United Kingdom
>Spike|hCoV-19/England/CAMC-B9A932/2020|2020-11-15|EPI_ISL_673343|Original|hCoV-19^^England|Human|Lighthouse Lab in Cambridge|Wellcome Sanger Institute for the COVID-19 Genomics UK (COG-UK) Consortium|Davies|United Kingdom
>Spike|hCoV-19/New Zealand/20VR2940/2020|2020-00-00|EPI_ISL_579362|Original|hCoV-19^^Auckland|Human|LabPLUS|Institute of Environmental Science and Research (ESR)|Wang|New Zealand
>Spike|hCoV-19/England/CAMB-1B80D9/2020|2020-10-16|EPI_ISL_638191|Original|hCoV-19^^England|Human|Department of Pathology|COVID-19 Genomics UK (COG-UK) Consortium|Goodfellow|United Kingdom
>Spike|hCoV-19/Sweden/20-22145/2020|2020-05-22|EPI_ISL_615084|Original|hCoV-19^^Skane|Human|Klinisk mikrobiologi|The Public Health Agency of Sweden|Svartstr\u00f6m|Sweden
>Spike|hCoV-19/England/ALDP-9E6FBF/2020|2020-09-24|EPI_ISL_580357|Original|hCoV-19^^England|Human|Lighthouse Lab in Alderley Park|Wellcome Sanger Institute for the COVID-19 Genomics UK (COG-UK) consortium|Davies|United Kingdom
>Spike|hCoV-19/Denmark/DCGC-3591/2020|2020-08-31|EPI_ISL_616234|Original|hCoV-19^^Syddanmark|Human|Department of Virus and Microbiological Special Diagnostics|Albertsen lab|Michaelsen|Denmark
>Spike|hCoV-19/Northern Ireland/QEUH-964502/2020|2020-08-11|EPI_ISL_536874|Original|hCoV-19^^Northern Ireland|Human|Lighthouse Lab in Glasgow|Wellcome Sanger Institute for the COVID-19 Genomics UK (COG-UK) consortium|Davies|United Kingdom
>Spike|hCoV-19/England/NORW-ECDC7/2020|2020-06-17|EPI_ISL_499588|Original|hCoV-19^^England|Human|Quadram Institute Bioscience|COVID-19 Genomics UK (COG-UK) Consortium|Alikhan|United Kingdom
>Spike|hCoV-19/USA/NY-NYCPHL-000602/2020|2020-03-10|EPI_ISL_632060|Original|hCoV-19^^New York|Human|NYC HH Queens Hospital Center|New York City Public Health Laboratory|Wang|USA
>Spike|hCoV-19/England/CAMB-1AA9EE/2020|2020-04-18|EPI_ISL_470149|Original|hCoV-19^^England|Human|Department of Pathology|Wellcome Sanger Institute for the COVID-19 Genomics UK (COG-UK) consortium|Davies|United Kingdom
>Spike|hCoV-19/England/NORW-F7C08/2020|2020-11-17|EPI_ISL_665370|Original|hCoV-19^^England|Human|Quadram Institute Bioscience|COVID-19 Genomics UK (COG-UK) Consortium|Alikhan|United Kingdom
>Spike|hCoV-19/Iceland/223/2020|2020-03-16|EPI_ISL_417838|Original|hCoV-19^^Reykjavik|Human|The National University Hospital of Iceland|deCODE genetics|J\u00f3nsson|Iceland
>Spike|hCoV-19/Northern Ireland/NIRE-2333CD/2020|2020-04-06|EPI_ISL_680287|Original|hCoV-19^^Northern Ireland|Human|Regional Virus Laboratory|COVID-19 Genomics UK (COG-UK) Consortium|Fairley|United Kingdom

>Spike|hCoV-19/England/QEUH-9B4B89/2020|2020-09-03|EPI_ISL_590179|Original|hCoV-19^^England|Human|Lighthouse Lab in Glasgow|Wellcome Sanger Institute for the COVID-19 Genomics UK (COG-UK) consortium|Davies|United Kingdom
>Spike|hCoV-19/Canada/ON-S778/2020|2020-04-10|EPI_ISL_591225|Original|hCoV-19^^Ontario|Human|Toronto Invasive Bacterial Diseases Network|McMaster University|McArthur|Canada
>Spike|hCoV-19/England/204881362/2020|2020-00-00|EPI_ISL_666725|Original|hCoV-19^^England|Human|Respiratory Virus Unit|COVID-19 Genomics UK (COG-UK) Consortium|Platt|United Kingdom
>Spike|hCoV-19/Hungary/UD-90554/2020|2020-10-04|EPI_ISL_671475|Original|hCoV-19^^Borsod-Aba\u00faj-Zempl\u00e9n county|Human|University of Debrecen|National Laboratory of Virology|Kemenesi|Hungary
>Spike|hCoV-19/France/ARA-1186-49/2020|2020-12-11|EPI_ISL_745380|Original|hCoV-19^^Auvergne-Rh\u00f4ne-Alpes|Human|CNR Virus des Infections Respiratoires - France SUD|CNR Virus des Infections Respiratoires - France SUD|Bal|France
>Spike|hCoV-19/USA/FL-BPHL-1377/2020|2020-07-21|EPI_ISL_541314|Original|hCoV-19^^Florida|Human|Florida Bureau of Public Health Laboratories|Florida Bureau of Public Health Laboratories|Blanton|USA
>Spike|hCoV-19/England/QEUH-A0B584/2020|2020-10-03|EPI_ISL_599207|Original|hCoV-19^^England|Human|Lighthouse Lab in Glasgow|Wellcome Sanger Institute for the COVID-19 Genomics UK (COG-UK) consortium|Davies|United Kingdom
>Spike|hCoV-19/Spain/RI-IBV-99010906/2020|2020-11-06|EPI_ISL_732752|Original|hCoV-19^^La_Rioja|Human|Centro de Investigaci\u00f3n Biom\u00e9dica de La Rioja - Hospital San Pedro Logro\u00f1o|SeqCOVID-SPAIN consortium/IBV(CSIC)|Comas|Spain
>Spike|hCoV-19/USA/UT-UPHL-2009537/2020|2020-07-18|EPI_ISL_605217|Original|hCoV-19^^Utah|Human|Utah Public Health Laboratory|Utah Public Health Laboratory|Young|USA
>Spike|hCoV-19/Scotland/GCVR-171ED2/2020|2020-03-30|EPI_ISL_469971|Original|hCoV-19^^Scotland|Human|NHSGGC West of Scotland Specialist Virology Centre / MRC-University of Glasgow Centre for Virus Research|Wellcome Sanger Institute for the COVID-19 Genomics UK (COG-UK) consortium|Davies|United Kingdom
>Spike|hCoV-19/Germany/HH-hpi-p1857/2020|2020-03-23|EPI_ISL_776206|Original|hCoV-19^^Hamburg|Human|University Medical Center Hamburg Eppendorf|Heinrich Pette Institute|Robitaille|Germany
>Spike|hCoV-19/Netherlands/LI-EMC-89/2020|2020-09-25|EPI_ISL_577835|Original|hCoV-19^^Limburg|Human|Dutch COVID-19 response team|Erasmus Medical Center|Oude Munnink|Netherlands
>Spike|hCoV-19/Scotland/QEUH-AD01FD/2020|2020-10-26|EPI_ISL_649761|Original|hCoV-19^^Scotland|Human|Lighthouse Lab in Glasgow|Wellcome Sanger Institute for the COVID-19 Genomics UK (COG-UK) Consortium|Davies|United Kingdom
>Spike|hCoV-19/USA/WI-UW-1840/2020|2020-09-11|EPI_ISL_605712|Original|hCoV-19^^Wisconsin|Human|University of Wisconsin-Madison AIDS Vaccine Research Laboratories|University of Wisconsin-Madison AIDS Vaccine Research Laboratories|Baczenas|USA
>Spike|hCoV-19/England/MILK-B5514A/2020|2020-11-06|EPI_ISL_657840|Original|hCoV-19^^England|Human|Lighthouse Lab in Milton Keynes|Wellcome Sanger Institute for the COVID-19 Genomics UK (COG-UK) Consortium|Davies|United Kingdom
>Spike|hCoV-19/England/MILK-B56079/2020|2020-11-06|EPI_ISL_658329|Original|hCoV-19^^England|Human|Lighthouse Lab in Milton Keynes|Wellcome Sanger Institute for the COVID-19 Genomics UK (COG-UK) Consortium|Davies|United Kingdom
>Spike|hCoV-19/Belgium/UGent-232/2020|2020-11-12|EPI_ISL_717585|Original|hCoV-19^^Ghent|Human|Lab voor klinische biologie|Onderzoeksgroep Virologie|Lambrechts|Belgium
>Spike|hCoV-19/USA/CO-CDPHE-2100037717/2020|2020-11-25|EPI_ISL_771347|Original|hCoV-19^^Colorado|Human|Colorado Department of Public Health and Environment|Colorado Department of Puplic Health and Environment|Bankers|USA
>Spike|hCoV-19/Denmark/DCGC-17114/2020|2020-12-14|EPI_ISL_750764|Original|hCoV-19^^Midtjylland|Human|Department of Virus and Microbiological Special Diagnostics|Albertsen Lab|Michaelsen|Denmark

>Spike|hCoV-19/Italy/VEN-UniVR-141/2020|2020-04-05|EPI_ISL_751438|Original|hCoV-19^^Veneto|Human|IRCCS Sacro Cuore Don Calabria Hospital|University of Verona|Mori|Italy
>Spike|hCoV-19/Scotland/CVR4021/2020|2020-08-29|EPI_ISL_535120|Original|hCoV-19^^Scotland|Human|West of Scotland Specialist Virology Centre|COVID-19 Genomics UK (COG-UK) Consortium|Robertson|United Kingdom
>Spike|hCoV-19/England/CAMB-7D5B4/2020|2020-04-05|EPI_ISL_441695|Original|hCoV-19^^England|Human|Department of Pathology|Wellcome Sanger Institute for the COVID-19 Genomics UK (COG-UK) consortium|Amato|United Kingdom
>Spike|hCoV-19/England/ALDP-9E72C4/2020|2020-09-24|EPI_ISL_580463|Original|hCoV-19^^England|Human|Lighthouse Lab in Alderley Park|Wellcome Sanger Institute for the COVID-19 Genomics UK (COG-UK) consortium|Davies|United Kingdom
>Spike|hCoV-19/England/ALDP-B850AE/2020|2020-11-13|EPI_ISL_675965|Original|hCoV-19^^England|Human|Lighthouse Lab in Alderley Park|Wellcome Sanger Institute for the COVID-19 Genomics UK (COG-UK) Consortium|Davies|United Kingdom
>Spike|hCoV-19/Netherlands/ZuidHolland_11/2020|2020-03-04|EPI_ISL_414469|Original|hCoV-19^^Zuid Holland|Human|Dutch COVID-19 response team|Erasmus Medical Center|Oude Munnink|Netherlands
>Spike|hCoV-19/England/CAMC-B09172/2020|2020-10-29|EPI_ISL_673172|Original|hCoV-19^^England|Human|Lighthouse Lab in Cambridge|Wellcome Sanger Institute for the COVID-19 Genomics UK (COG-UK) Consortium|Davies|United Kingdom
>Spike|hCoV-19/England/QEUH-AE4A28/2020|2020-10-27|EPI_ISL_633132|Original|hCoV-19^^England|Human|Lighthouse Lab in Glasgow|Wellcome Sanger Institute for the COVID-19 Genomics UK (COG-UK) consortium|Davies|United Kingdom
>Spike|hCoV-19/England/QEUH-96B486/2020|2020-08-17|EPI_ISL_531434|Original|hCoV-19^^England|Human|Lighthouse Lab in Glasgow|Wellcome Sanger Institute for the COVID-19 Genomics UK (COG-UK) consortium|Davies|United Kingdom
>Spike|hCoV-19/England/OXON-B28BA/2020|2020-04-15|EPI_ISL_534779|Original|hCoV-19^^England|Human|Oxford Viromics|COVID-19 Genomics UK (COG-UK) Consortium|Golubchik|United Kingdom
>Spike|hCoV-19/USA/NY-WCMP9G04P/2020|2020-03-14|EPI_ISL_427552|Original|hCoV-19^^New York|Human|NewYork-Presbyterian & Mason Lab|Mason Lab|Mason|USA
>Spike|hCoV-19/Wales/PHWC-2A3DA/2020|2020-04-06|EPI_ISL_445564|Original|hCoV-19^^Wales|Human|Wales Specialist Virology Centre|Public Health Wales Microbiology Cardiff|Moore|United Kingdom
>Spike|hCoV-19/Japan/IC-0553/2020|2020-12-00|EPI_ISL_779236|Original|hCoV-19^^Japan|Human|Pathogen Genomics Center|Pathogen Genomics Center|Sekizuka|Japan
>Spike|hCoV-19/England/NORW-E8F6B/2020|2020-04-25|EPI_ISL_453568|Original|hCoV-19^^England|Human|Quadram Institute Bioscience|COVID-19 Genomics UK (COG-UK) Consortium|Alikhan|United Kingdom
>Spike|hCoV-19/England/CAMB-1BB20D/2020|2020-11-10|EPI_ISL_664828|Original|hCoV-19^^England|Human|Department of Pathology|COVID-19 Genomics UK (COG-UK) Consortium|Goodfellow|United Kingdom
>Spike|hCoV-19/USA/WI-GMF-21773/2020|2020-09-21|EPI_ISL_660839|Original|hCoV-19^^Wisconsin|Human|Gundersen Molecular Diagnostics Laboratory|Kabara Cancer Research Institute|Kenny|USA
>Spike|hCoV-19/USA/UT-UPHL-2009025/2020|2020-05-27|EPI_ISL_525331|Original|hCoV-19^^Utah|Human|Utah Public Health Laboratory|Utah Public Health Laboratory|Young|USA
>Spike|hCoV-19/New Zealand/20VR1950/2020|2020-03-24|EPI_ISL_456320|Original|hCoV-19^^Combined Wellington|Human|Wellington SCL|Institute of Environmental Science and Research (ESR)|de Ligt|New Zealand
>Spike|hCoV-19/Saudi Arabia/KAUST-Madinah260/2020|2020-03-30|EPI_ISL_437743|Original|hCoV-19^^Madinah|Human|Pathogen Genomics Lab King Abdullah University of Science and Technology(KAUST)|Pathogen Genomics Lab King Abdullah University of Science and Technology(KAUST)|Mohamed Ghazzali|Saudi Arabia
>Spike|hCoV-19/England/MILK-B8F51E/2020|2020-11-13|EPI_ISL_674536|Original|hCoV-19^^England|Human|Lighthouse Lab in Milton Keynes|Wellcome Sanger Institute for the COVID-19 Genomics UK (COG-UK) Consortium|Davies|United Kingdom
>Spike|hCoV-19/USA/TX-HMH-MCoV-11719/2020|2020-09-08|EPI_ISL_789897|Original|hCoV-19^^Texas|Human|Houston Methodist Hospital|Houston Methodist Hospital|Long|USA

>Spike|hCoV-19/England/QEUH-9B50C5/2020|2020-09-07|EPI_ISL_590377|Original|hCoV-19^^England|Human|Lighthouse Lab in Glasgow|Wellcome Sanger Institute for the COVID-19 Genomics UK (COG-UK) consortium|Davies|United Kingdom
>Spike|hCoV-19/USA/TX-HMH-MCoV-8121/2020|2020-07-01|EPI_ISL_784212|Original|hCoV-19^^Texas|Human|Houston Methodist Hospital|Houston Methodist Hospital|Long|USA
>Spike|hCoV-19/England/ALDP-A2ED92/2020|2020-10-05|EPI_ISL_587027|Original|hCoV-19^^England|Human|Lighthouse Lab in Alderley Park|Wellcome Sanger Institute for the COVID-19 Genomics UK (COG-UK) consortium|Davies|United Kingdom
>Spike|hCoV-19/Australia/VIC2792/2020|2020-07-07|EPI_ISL_521625|Original|hCoV-19^^Victoria|Human|Microbiological Diagnostic Unit - Public Health Laboratory (MDU-PHL)|MDU-PHL|Schultz|Australia
>Spike|hCoV-19/Sri Lanka/C28324/2020|2020-11-04|EPI_ISL_668449|Original|hCoV-19^^Colombo|Human|Centre for Dengue Research|Centre for Dengue Research|Malavige|Sri Lanka
>Spike|hCoV-19/USA/CA-ALSR-2822/2020|2020-07-24|EPI_ISL_635697|Original|hCoV-19^^California|Human|San Diego County Public Health Laboratory|Andersen lab at Scripps Research|Gangavarapu|USA
>Spike|hCoV-19/USA/TX-DSHS-0562/2020|2020-05-14|EPI_ISL_574464|Original|hCoV-19^^Texas|Human|Texas Department of State Health Services|Texas Department of State Health Services|Rahman|USA
>Spike|hCoV-19/Japan/PG-3640/2020|2020-04-00|EPI_ISL_691893|Original|hCoV-19^^Japan|Human|Pathogen Genomics Center|Pathogen Genomics Center|Sekizuka|Japan
>Spike|hCoV-19/Scotland/QEUH-9F4F27/2020|2020-09-27|EPI_ISL_638917|Original|hCoV-19^^Scotland|Human|Department of Pathology|COVID-19 Genomics UK (COG-UK) Consortium|Goodfellow|United Kingdom
>Spike|hCoV-19/USA/NY-Wadsworth-71356-01/2020|2020-04-28|EPI_ISL_676888|Original|hCoV-19^^New York|Human|Wadsworth Center|Wadsworth Center|Lasek-Nesselquist|USA
>Spike|hCoV-19/Singapore/503/2020|2020-04-06|EPI_ISL_479581|Original|hCoV-19^^Singapore|Human|National Public Health Laboratory|National Public Health Laboratory|Mak|Singapore
>Spike|hCoV-19/England/BRIS-128784/2020|2020-04-14|EPI_ISL_458848|Original|hCoV-19^^England|Human|PHE South West Regional Laboratory|Wellcome Sanger Institute for the COVID-19 Genomics UK (COG-UK) consortium|Davies|United Kingdom
>Spike|hCoV-19/England/MILK-B3A3CD/2020|2020-10-31|EPI_ISL_706023|Original|hCoV-19^^England|Human|Oxford Viromics|COVID-19 Genomics UK (COG-UK) Consortium|Golubchik|United Kingdom
>Spike|hCoV-19/England/QEUH-9ADFC3/2020|2020-09-02|EPI_ISL_638618|Original|hCoV-19^^England|Human|Department of Pathology|COVID-19 Genomics UK (COG-UK) Consortium|Goodfellow|United Kingdom
>Spike|hCoV-19/England/ALDP-BBB4C1/2020|2020-11-21|EPI_ISL_701787|Original|hCoV-19^^England|Human|Lighthouse Lab in Alderley Park|Wellcome Sanger Institute for the COVID-19 Genomics UK (COG-UK) Consortium|Davies|United Kingdom
>Spike|hCoV-19/USA/OR-OHSU-1599/2020|2020-07-21|EPI_ISL_525986|Original|hCoV-19^^Oregon|Human|OHSU Lab Services Molecular Microbiology Lab|Oregon SARS-CoV-2 Genome Sequencing Center|O'Roak|USA
>Spike|hCoV-19/Spain/IB-IBV-99011527/2020|2020-12-01|EPI_ISL_779881|Original|hCoV-19^^Balear Islands|Human|Servicio de Microbiolog\u00eda|SeqCOVID-SPAIN consortium/IBV(CSIC)|Comas|Spain
>Spike|hCoV-19/Scotland/CVR1559/2020|2020-04-04|EPI_ISL_512443|Original|hCoV-19^^Scotland|Human|West of Scotland Specialist Virology Centre|COVID-19 Genomics UK (COG-UK) Consortium|Robertson|United Kingdom
>Spike|hCoV-19/Denmark/DCGC-18765/2020|2020-12-07|EPI_ISL_748542|Original|hCoV-19^^Nordjylland|Human|Department of Virus and Microbiological Special Diagnostics|Albertsen Lab|Michaelsen|Denmark
>Spike|hCoV-19/USA/MI-MDHHS-SC20637/2020|2020-04-26|EPI_ISL_462719|Original|hCoV-19^^Michigan|Human|Michigan Department of Health and Human Services|Michigan Department of Health and Human Services|Blankenship|USA

>Spike|hCoV-19/Scotland/EDB11006/2020|2020-12-13|EPI_ISL_742189|Original|hCoV-19^^Scotland|Human|Virology Department|COVID-19 Genomics UK (COG-UK) Consortium|McHugh|United Kingdom
>Spike|hCoV-19/Scotland/CVR1113/2020|2020-03-29|EPI_ISL_438766|Original|hCoV-19^^Scotland|Human|West of Scotland Specialist Virology Centre|COVID-19 Genomics UK (COG-UK) Consortium|Robertson|United Kingdom
>Spike|hCoV-19/Australia/VIC6021/2020|2020-07-25|EPI_ISL_518796|Original|hCoV-19^^Victoria|Human|Microbiological Diagnostic Unit - Public Health Laboratory (MDU-PHL)|MDU-PHL|Schultz|Australia
>Spike|hCoV-19/England/QEUH-B3FBA2/2020|2020-11-08|EPI_ISL_658639|Original|hCoV-19^^England|Human|Lighthouse Lab in Glasgow|Wellcome Sanger Institute for the COVID-19 Genomics UK (COG-UK) Consortium|Davies|United Kingdom
>Spike|hCoV-19/England/QEUH-AE530E/2020|2020-10-27|EPI_ISL_633394|Original|hCoV-19^^England|Human|Lighthouse Lab in Glasgow|Wellcome Sanger Institute for the COVID-19 Genomics UK (COG-UK) consortium|Davies|United Kingdom
>Spike|hCoV-19/England/LIVE-9B107/2020|2020-03-25|EPI_ISL_449411|Original|hCoV-19^^England|Human|Liverpool Clinical Laboratories|COVID-19 Genomics UK (COG-UK) Consortium|Haldenby|United Kingdom
>Spike|hCoV-19/England/MILK-BAB46A/2020|2020-11-18|EPI_ISL_681476|Original|hCoV-19^^England|Human|Lighthouse Lab in Milton Keynes|Wellcome Sanger Institute for the COVID-19 Genomics UK (COG-UK) Consortium|Davies|United Kingdom
>Spike|hCoV-19/England/ALDP-A2E336/2020|2020-10-05|EPI_ISL_586738|Original|hCoV-19^^England|Human|Lighthouse Lab in Alderley Park|Wellcome Sanger Institute for the COVID-19 Genomics UK (COG-UK) consortium|Davies|United Kingdom
>Spike|hCoV-19/USA/AZ-TG569514/2020|2020-07-11|EPI_ISL_695596|Original|hCoV-19^^Arizona|Human|AZ SPHL|TGen North|French|USA
>Spike|hCoV-19/Wales/PHWC-48FA07/2020|2020-11-10|EPI_ISL_681173|Original|hCoV-19^^Wales|Human|Wales Specialist Virology Centre Sequencing lab|COVID-19 Genomics UK (COG-UK) Consortium|Moore|United Kingdom
>Spike|hCoV-19/Belgium/ULG-10901/2020|2020-11-14|EPI_ISL_678443|Original|hCoV-19^^Li\u00e8ge|Human|Department of Clinical Microbiology|GIGA Medical Genomics|Durkin|Belgium
>Spike|hCoV-19/Russia/SVE-RII-MH1697S/2020|2020-09-20|EPI_ISL_602343|Original|hCoV-19^^Sverdlovsk|Human|HELIX LLC|WHO National Influenza Centre Russian Federation|Komissarov|Russia
>Spike|hCoV-19/Denmark/DCGC-3126/2020|2020-08-31|EPI_ISL_615944|Original|hCoV-19^^Hovedstaden|Human|Department of Virus and Microbiological Special Diagnostics|Albertsen lab|Michaelsen|Denmark
>Spike|hCoV-19/USA/MI-MDHHS-SC20278/2020|2020-03-28|EPI_ISL_447076|Original|hCoV-19^^Michigan|Human|Michigan Department of Health and Human Services|Michigan Department of Health and Human Services|Blankenship|USA
>Spike|hCoV-19/USA/LA-EVTL138/2020|2020-03-28|EPI_ISL_455981|Original|hCoV-19^^Louisiana|Human|LSUHS Emerging Viral Threat Laboratory|Microbial Genome Sequencing Center|Kamil|USA
>Spike|hCoV-19/England/EXET-135FD7/2020|2020-04-17|EPI_ISL_457187|Original|hCoV-19^^England|Human|University of Exeter|COVID-19 Genomics UK (COG-UK) Consortium|Temperton|United Kingdom
>Spike|hCoV-19/Japan/PG-0576/2020|2020-04-06|EPI_ISL_480217|Original|hCoV-19^^Kansai|Human|Department of Infectious Diseases|Pathogen Genomics Center|Sekizuka|Japan
>Spike|hCoV-19/England/ALDP-A6D7D5/2020|2020-10-15|EPI_ISL_608230|Original|hCoV-19^^England|Human|Lighthouse Lab in Alderley Park|Wellcome Sanger Institute for the COVID-19 Genomics UK (COG-UK) consortium|Davies|United Kingdom
>Spike|hCoV-19/USA/WA-S2515/2020|2020-06-15|EPI_ISL_497147|Original|hCoV-19^^Washington|Human|Washington State Department of Health|Seattle Flu Study|Lee|USA
>Spike|hCoV-19/USA/CA-CZB-12989/2020|2020-10-21|EPI_ISL_672035|Original|hCoV-19^^California|Human|Santa Clara County Public Health Laboratory|Chan-Zuckerberg Biohub|Bhatt|USA
>Spike|hCoV-19/England/MILK-9C71AF/2020|2020-09-08|EPI_ISL_573286|Original|hCoV-19^^England|Human|Northumbria University / South Tees Hospitals NHS Foundation Trust

/ North Cumbria Integrated Care NHS Foundation Trust / North Tees and Hartlepool NHS
Foundation Trust / Newcastle Hospitals NHS Foundation Trust|COVID-19 Genomics UK
(COG-UK) Consortium|Bashton|United Kingdom
>Spike|hCoV-19/England/QEUH-C14C27/2020|2020-12-06|EPI_ISL_709503|Original|hCoV-
19^^England|Human|Lighthouse Lab in Glasgow|Wellcome Sanger Institute for the COVID-
19 Genomics UK (COG-UK) Consortium|Davies|United Kingdom
>Spike|hCoV-19/Russia/KDA-RII-MH4710S/2020|2020-10-05|EPI_ISL_733243|Original|hCoV-
19^^Krasnodar|Human|WHO National Influenza Centre Russian Federation|WHO National
Influenza Centre Russian Federation|Komissarov|Russia
>Spike|hCoV-19/England/ALDP-AA324B/2020|2020-10-22|EPI_ISL_624993|Original|hCoV-
19^^England|Human|Lighthouse Lab in Alderley Park|Wellcome Sanger Institute for the
COVID-19 Genomics UK (COG-UK) consortium|Davies|United Kingdom
>Spike|hCoV-19/Australia/VIC7894/2020|2020-08-06|EPI_ISL_564733|Original|hCoV-
19^^Victoria|Human|Microbiological Diagnostic Unit - Public Health Laboratory (MDU-
PHL)|MDU-PHL|Schultz|Australia
>Spike|hCoV-19/England/BRIS-127020/2020|2020-04-23|EPI_ISL_469536|Original|hCoV-
19^^England|Human|PHE South West Regional Laboratory|Wellcome Sanger Institute for
the COVID-19 Genomics UK (COG-UK) consortium|Davies|United Kingdom
>Spike|hCoV-19/Spain/RI-IBV-99005890/2020|2020-03-21|EPI_ISL_537432|Original|hCoV-
19^^La Rioja|Human|Centro de Investigaci\u00f3n Biom\u00e9dica de La Rioja - Hospital
San Pedro Logro\u00f1o|SeqCOVID-SPAIN consortium/IBV(CSIC)|Comas|Spain
>Spike|hCoV-19/South Korea/KCDC2123/2020|2020-03-15|EPI_ISL_498024|Original|hCoV-
19^^South Korea|Human|Division of Viral Diseases|Division of Viral Diseases|Kim|South
Korea
>Spike|hCoV-19/England/QEUH-9616E4/2020|2020-08-01|EPI_ISL_537137|Original|hCoV-
19^^England|Human|Lighthouse Lab in Glasgow|Wellcome Sanger Institute for the COVID-
19 Genomics UK (COG-UK) consortium|Davies|United Kingdom
>Spike|hCoV-19/Bangladesh/BCSIR-NILMRC-198/2020|2020-06-
20|EPI_ISL_483642|Original|hCoV-19^^Chittagong|Human|National Institute of Laboratory
Medicine and Referral Center|Genomic Research Lab|Khan|Bangladesh
>Spike|hCoV-19/England/CWAR-24FE232/2020|2020-04-16|EPI_ISL_584589|Original|hCoV-
19^^England|Human|UHCW / University of Warwick|COVID-19 Genomics UK (COG-UK)
Consortium|Stark|United Kingdom
>Spike|hCoV-19/USA/AZ-TG371439/2020|2020-05-29|EPI_ISL_694597|Original|hCoV-
19^^Arizona|Human|AZ SPHL|TGen North|French|USA
>Spike|hCoV-19/England/NOTT-1132F4/2020|2020-09-15|EPI_ISL_549495|Original|hCoV-
19^^England|Human|Queens Medical Centre|COVID-19 Genomics UK (COG-UK)
Consortium|Loose|United Kingdom
>Spike|hCoV-19/USA/NM-NMDOH-2020099258/2020|2020-06-01|EPI_ISL_535268|Original|hCoV-
19^^New Mexico|Human|New Mexico Department of Health Scientific Laboratory|New Mexico
Department of Health Scientific Laboratory|Johnson|USA
>Spike|hCoV-19/England/20124034902/2020|2020-03-16|EPI_ISL_418712|Original|hCoV-
19^^England|Human|Respiratory Virus Unit|Respiratory Virus Unit|Platt|United Kingdom
>Spike|hCoV-19/USA/TX-HMH-MCoV-13138/2020|2020-08-27|EPI_ISL_788289|Original|hCoV-
19^^Texas|Human|Houston Methodist Hospital|Houston Methodist Hospital|Long|USA
>Spike|hCoV-19/Portugal/PT0540/2020|2020-04-08|EPI_ISL_454264|Original|hCoV-
19^^Portugal|Human|unknown|Instituto Nacional de Saude (INSA)|Borges|Portugal
>Spike|hCoV-19/England/NORT-283F60/2020|2020-04-05|EPI_ISL_478528|Original|hCoV-
19^^England|Human|Northumbria University / South Tees Hospitals NHS Foundation Trust
/ North Cumbria Integrated Care NHS Foundation Trust / North Tees and Hartlepool NHS
Foundation Trust / Newcastle Hospitals NHS Foundation Trust|COVID-19 Genomics UK
(COG-UK) Consortium|Bashton|United Kingdom
>Spike|hCoV-19/USA/FL-BPHL-1408/2020|2020-05-27|EPI_ISL_549190|Original|hCoV-
19^^Florida|Human|Florida Bureau of Public Health Laboratories|Florida Bureau of
Public Health Laboratories|Blanton|USA
>Spike|hCoV-19/Uruguay/UY-82/2020|2020-04-07|EPI_ISL_480344|Original|hCoV-
19^^Montevideo|Human|Microbial Genomics Laboratory|Microbial Genomics
Laboratory|Iraola|Uruguay

>Spike|hCoV-19/Scotland/EDB10585/2020|2020-12-01|EPI_ISL_705783|Original|hCoV-19^^Scotland|Human|Virology Department|COVID-19 Genomics UK (COG-UK) Consortium|McHugh|United Kingdom
>Spike|hCoV-19/USA/FL-BPHL-1314/2020|2020-07-13|EPI_ISL_541252|Original|hCoV-19^^Florida|Human|Florida Bureau of Public Health Laboratories|Florida Bureau of Public Health Laboratories|Blanton|USA
>Spike|hCoV-19/Singapore/516/2020|2020-05-27|EPI_ISL_479594|Original|hCoV-19^^Singapore|Human|National Public Health Laboratory|National Public Health Laboratory|Mak|Singapore
>Spike|hCoV-19/England/201861227/2020|2020-04-29|EPI_ISL_526771|Original|hCoV-19^^England|Human|Respiratory Virus Unit|Respiratory Virus Unit|Miah|United Kingdom
>Spike|hCoV-19/Australia/NSW1123/2020|2020-09-13|EPI_ISL_593737|Original|hCoV-19^^New South Wales|Human|South Eastern Area Laboratory Services (SEALS)|NSW Health Pathology - Institute of Clinical Pathology and Medical Research; Westmead Hospital; University of Sydney|Gall|Australia
>Spike|hCoV-19/Australia/QLD1024/2020|2020-08-27|EPI_ISL_530278|Original|hCoV-19^^Queensland|Human|Queensland Health Forensic and Scientific Services|Public Health Virology Laboratory|Nguyen|Australia
>Spike|hCoV-19/England/QEUH-B7EFE7/2020|2020-11-14|EPI_ISL_661890|Original|hCoV-19^^England|Human|Lighthouse Lab in Glasgow|Wellcome Sanger Institute for the COVID-19 Genomics UK (COG-UK) Consortium|Davies|United Kingdom
>Spike|hCoV-19/Germany/HH-hpi-p1683/2020|2020-03-15|EPI_ISL_775715|Original|hCoV-19^^Hamburg|Human|University Medical Center Hamburg Eppendorf|Heinrich Pette Institute|Robitaille|Germany
>Spike|hCoV-19/England/NORW-F1BE3/2020|2020-10-22|EPI_ISL_627622|Original|hCoV-19^^England|Human|Quadram Institute Bioscience|COVID-19 Genomics UK (COG-UK) Consortium|Alikhan|United Kingdom
>Spike|hCoV-19/England/OXON-B350D/2020|2020-04-04|EPI_ISL_534863|Original|hCoV-19^^England|Human|Oxford Viromics|COVID-19 Genomics UK (COG-UK) Consortium|Golubchik|United Kingdom
>Spike|hCoV-19/Australia/VIC6569/2020|2020-07-17|EPI_ISL_521335|Original|hCoV-19^^Victoria|Human|Victorian Infectious Diseases Reference Laboratory (VIDRL)|VIDRL and MDU-PHL|Schultz|Australia
>Spike|hCoV-19/Australia/VIC5306/2020|2020-07-17|EPI_ISL_520069|Original|hCoV-19^^Victoria|Human|Microbiological Diagnostic Unit - Public Health Laboratory (MDU-PHL)|MDU-PHL|Schultz|Australia
>Spike|hCoV-19/South Africa/KRISP-K003322/2020|2020-09-04|EPI_ISL_602715|Original|hCoV-19^^KwaZulu-Natal|Human|NHLS-IALCH|KRISP|de Oliveira|South Africa
>Spike|hCoV-19/Wales/PHWC-2A49B/2020|2020-04-05|EPI_ISL_445571|Original|hCoV-19^^Wales|Human|Wales Specialist Virology Centre|Public Health Wales Microbiology Cardiff|Moore|United Kingdom
>Spike|hCoV-19/USA/WA-S449/2020|2020-03-31|EPI_ISL_434209|Original|hCoV-19^^Washington|Human|Washington State Department of Health|Seattle Flu Study|Bedford|USA
>Spike|hCoV-19/USA/WI-GMF-31071/2020|2020-11-03|EPI_ISL_661070|Original|hCoV-19^^Wisconsin|Human|Gundersen Molecular Diagnostics Laboratory|Kabara Cancer Research Institute|Kenny|USA
>Spike|hCoV-19/Australia/NSW2486/2020|2020-06-30|EPI_ISL_527022|Original|hCoV-19^^New South Wales|Human|Area of Virology|Area of Virology|Yeang|Australia
>Spike|hCoV-19/Wales/CAMC-9C00B8/2020|2020-09-09|EPI_ISL_573868|Original|hCoV-19^^Wales|Human|Oxford Viromics|COVID-19 Genomics UK (COG-UK) Consortium|Golubchik|United Kingdom
>Spike|hCoV-19/South Africa/KRISP-K002372/2020|2020-07-20|EPI_ISL_515740|Original|hCoV-19^^KwaZulu-Natal|Human|NHLS-IALCH|KRISP|de Oliveira|South Africa
>Spike|hCoV-19/USA/MI-UM-10036537564/2020|2020-10-20|EPI_ISL_640156|Original|hCoV-19^^Michigan|Human|University of Michigan Clinical Microbiology Laboratory|Lauring Lab|Valesano|USA

>Spike|hCoV-19/USA/FL-BPHL-2064/2020|2020-06-22|EPI_ISL_653217|Original|hCoV-19^^Florida|Human|Florida Bureau of Public Health Laboratories|Florida Bureau of Public Health Laboratories|Blanton|USA
>Spike|hCoV-19/Japan/PG-7880/2020|2020-07-00|EPI_ISL_686981|Original|hCoV-19^^Japan|Human|Pathogen Genomics Center|Pathogen Genomics Center|Sekizuka|Japan
>Spike|hCoV-19/USA/TX-HMH-MCoV-18559/2020|2020-11-20|EPI_ISL_784907|Original|hCoV-19^^Texas|Human|Houston Methodist Hospital|Houston Methodist Hospital|Long|USA
>Spike|hCoV-19/Switzerland/SO-ETHZ-410845/2020|2020-12-12|EPI_ISL_737453|Original|hCoV-19^^Solothurn|Human|Viollier AG|Department of Biosystems Science and Engineering|Nadeau|Switzerland
>Spike|hCoV-19/Spain/RI-IBV-99010988/2020|2020-08-07|EPI_ISL_732811|Original|hCoV-19^^La_Rioja|Human|Centro de Investigaci\u00f3n Biom\u00e9dica de La Rioja - Hospital San Pedro Logro\u00f1o|SeqCOVID-SPAIN consortium/IBV(CSIC)|Comas|Spain
>Spike|hCoV-19/Denmark/DCGC-17730/2020|2020-12-14|EPI_ISL_751171|Original|hCoV-19^^Nordjylland|Human|Department of Virus and Microbiological Special Diagnostics|Albertsen Lab|Michaelsen|Denmark
>Spike|hCoV-19/Denmark/DCGC-1982/2020|2020-07-20|EPI_ISL_618325|Original|hCoV-19^^Hovedstaden|Human|Department of Virus and Microbiological Special Diagnostics|Albertsen lab|Michaelsen|Denmark
>Spike|hCoV-19/USA/UT-UPHL-201224344/2020|2020-05-12|EPI_ISL_672569|Original|hCoV-19^^Utah|Human|Utah Public Health Laboratory|Utah Public Health Laboratory|Young|USA
>Spike|hCoV-19/USA/MI-MDHHS-SC22327/2020|2020-11-09|EPI_ISL_708303|Original|hCoV-19^^Michigan|Human|Michigan Department of Health and Human Services|Michigan Department of Health and Human Services|Jacob|USA
>Spike|hCoV-19/England/NORT-291E08/2020|2020-05-05|EPI_ISL_489130|Original|hCoV-19^^England|Human|NU-OMICS DNA Sequencing research facility|Wellcome Sanger Institute for the COVID-19 Genomics UK (COG-UK) consortium|Davies|United Kingdom
>Spike|hCoV-19/England/CAMB-1B51D9/2020|2020-06-23|EPI_ISL_483914|Original|hCoV-19^^England|Human|Department of Pathology|COVID-19 Genomics UK (COG-UK) Consortium|Meredith|United Kingdom
>Spike|hCoV-19/England/ALDP-A4FFA4/2020|2020-10-07|EPI_ISL_597571|Original|hCoV-19^^England|Human|Lighthouse Lab in Alderley Park|Wellcome Sanger Institute for the COVID-19 Genomics UK (COG-UK) consortium|Davies|United Kingdom
>Spike|hCoV-19/USA/VA-DCLS-0218/2020|2020-04-09|EPI_ISL_452796|Original|hCoV-19^^Virginia|Human|Virginia DCLS|Virginia DCLS|St. Jacques|USA
>Spike|hCoV-19/USA/FL-BPHL-0193/2020|2020-04-04|EPI_ISL_480844|Original|hCoV-19^^Florida|Human|Florida Bureau of Public Health Laboratories|Florida Bureau of Public Health Laboratories|Blanton|USA
>Spike|hCoV-19/Switzerland/BS-ETHZ-250061/2020|2020-08-18|EPI_ISL_535587|Original|hCoV-19^^Basel-Stadt|Human|Viollier AG|Department of Biosystems Science and Engineering|Nadeau|Switzerland
>Spike|hCoV-19/New Zealand/20CV0150/2020|2020-08-24|EPI_ISL_548026|Original|hCoV-19^^Auckland|Human|LabPLUS|Institute of Environmental Science and Research (ESR)|Wang|New Zealand
>Spike|hCoV-19/Wales/MILK-9C2F9B/2020|2020-09-10|EPI_ISL_566686|Original|hCoV-19^^Wales|Human|Lighthouse Lab in Milton Keynes|Wellcome Sanger Institute for the COVID-19 Genomics UK (COG-UK) consortium|Davies|United Kingdom
>Spike|hCoV-19/England/201090724/2020|2020-03-08|EPI_ISL_464600|Original|hCoV-19^^England|Human|Respiratory Virus Unit|Respiratory Virus Unit|Platt|United Kingdom
>Spike|hCoV-19/Australia/VIC5686/2020|2020-07-20|EPI_ISL_518492|Original|hCoV-19^^Victoria|Human|Microbiological Diagnostic Unit - Public Health Laboratory (MDU-PHL)|MDU-PHL|Schultz|Australia
>Spike|hCoV-19/Denmark/DCGC-10875/2020|2020-11-02|EPI_ISL_670260|Original|hCoV-19^^Hovedstaden|Human|Department of Virus and Microbiological Special Diagnostics|Albertsen Lab|Michaelsen|Denmark
>Spike|hCoV-19/Luxembourg/LNS6642658/2020|2020-12-24|EPI_ISL_770853|Original|hCoV-19^^Luxembourg|Human|Laboratoire national de sant\u00e9|Laboratoire national de sant\u00e9|Wienecke-Baldacchino|Luxembourg

>Spike|hCoV-19/England/MILK-957D55/2020|2020-06-12|EPI_ISL_554652|Original|hCoV-19^^England|Human|Lighthouse Lab in Milton Keynes|Wellcome Sanger Institute for the COVID-19 Genomics UK (COG-UK) consortium|Davies|United Kingdom
>Spike|hCoV-19/Denmark/DCGC-7200/2020|2020-10-12|EPI_ISL_619197|Original|hCoV-19^^Midtjylland|Human|Department of Virus and Microbiological Special Diagnostics|Albertsen lab|Michaelsen|Denmark
>Spike|hCoV-19/USA/MA-MGH-02725/2020|2020-09-26|EPI_ISL_765600|Original|hCoV-19^^Massachusetts|Human|Brigham and Womens Hospital|Infectious Disease Program|Park|USA
>Spike|hCoV-19/Denmark/DCGC-14726/2020|2020-11-23|EPI_ISL_711935|Original|hCoV-19^^Midtjylland|Human|Department of Virus and Microbiological Special Diagnostics|Albertsen Lab|Michaelsen|Denmark
>Spike|hCoV-19/USA/TX-HMH-MCoV-11332/2020|2020-09-03|EPI_ISL_789769|Original|hCoV-19^^Texas|Human|Houston Methodist Hospital|Houston Methodist Hospital|Long|USA
>Spike|hCoV-19/USA/WA-S1294/2020|2020-05-14|EPI_ISL_495744|Original|hCoV-19^^Washington|Human|Washington State Department of Health|Seattle Flu Study|Lee|USA
>Spike|hCoV-19/England/QEUH-9B796E/2020|2020-09-08|EPI_ISL_590563|Original|hCoV-19^^England|Human|Lighthouse Lab in Glasgow|Wellcome Sanger Institute for the COVID-19 Genomics UK (COG-UK) consortium|Davies|United Kingdom
>Spike|hCoV-19/England/CAMC-C3F6A0/2020|2020-12-10|EPI_ISL_718544|Original|hCoV-19^^England|Human|Lighthouse Lab in Cambridge|Wellcome Sanger Institute for the COVID-19 Genomics UK (COG-UK) Consortium|Davies|United Kingdom
>Spike|hCoV-19/Denmark/DCGC-21296/2020|2020-12-21|EPI_ISL_759658|Original|hCoV-19^^Hovedstaden|Human|Department of Virus and Microbiological Special Diagnostics|Albertsen Lab|Michaelsen|Denmark
>Spike|hCoV-19/Japan/PG-2731/2020|2020-03-00|EPI_ISL_690863|Original|hCoV-19^^Japan|Human|Pathogen Genomics Center|Pathogen Genomics Center|Sekizuka|Japan
>Spike|hCoV-19/USA/TX-HMH-MCoV-12979/2020|2020-08-29|EPI_ISL_788130|Original|hCoV-19^^Texas|Human|Houston Methodist Hospital|Houston Methodist Hospital|Long|USA
>Spike|hCoV-19/England/HECH-2599D91/2020|2020-05-06|EPI_ISL_482111|Original|hCoV-19^^England|Human|Microbiology Department|Wellcome Sanger Institute for the COVID-19 Genomics UK (COG-UK) consortium|Davies|United Kingdom
>Spike|hCoV-19/USA/CA-CZB-15432/2020|2020-12-02|EPI_ISL_739462|Original|hCoV-19^^California|Human|Orange County Public Health Lab|Chan-Zuckerberg Biohub|Kamm|USA
>Spike|hCoV-19/Denmark/ALAB-HH-141/2020|2020-03-11|EPI_ISL_452020|Original|hCoV-19^^Denmark|Human|Department of Clinical Microbiology|Albertsen lab|Kirkegaard|Denmark
>Spike|hCoV-19/England/QEUH-9DA4BF/2020|2020-09-21|EPI_ISL_601319|Original|hCoV-19^^England|Human|Lighthouse Lab in Glasgow|Wellcome Sanger Institute for the COVID-19 Genomics UK (COG-UK) consortium|Davies|United Kingdom
>Spike|hCoV-19/Canada/ON-PHL-20-01200/2020|2020-06-00|EPI_ISL_548822|Original|hCoV-19^^Ontario|Human|Public Health Ontario Laboratory|Public Health Ontario Laboratory|Guthrie|Canada
>Spike|hCoV-19/Denmark/DCGC-13042/2020|2020-11-30|EPI_ISL_712132|Original|hCoV-19^^Hovedstaden|Human|Department of Virus and Microbiological Special Diagnostics|Albertsen Lab|Michaelsen|Denmark
>Spike|hCoV-19/England/ALDP-A701D1/2020|2020-10-15|EPI_ISL_608067|Original|hCoV-19^^England|Human|Lighthouse Lab in Alderley Park|Wellcome Sanger Institute for the COVID-19 Genomics UK (COG-UK) consortium|Davies|United Kingdom
>Spike|hCoV-19/Botswana/BOT2254/2020|2020-04-08|EPI_ISL_560390|Original|hCoV-19^^Gaborone|Human|National Health Laboratory|Botswana Institute for Technology Research and Innovation|Tumedi|Botswana
>Spike|hCoV-19/USA/MI-UM-10036815482/2020|2020-11-06|EPI_ISL_728377|Original|hCoV-19^^Michigan|Human|University of Michigan Clinical Microbiology Laboratory|Lauring Lab|Valesano|USA
>Spike|hCoV-19/Denmark/DCGC-14311/2020|2020-11-30|EPI_ISL_711687|Original|hCoV-19^^Midtjylland|Human|Department of Virus and Microbiological Special Diagnostics|Albertsen Lab|Michaelsen|Denmark

>Spike|hCoV-19/USA/AK-PHL040/2020|2020-03-24|EPI_ISL_431017|Original|hCoV-19^^Alaska|Human|Alaska State Virology Laboratory|Alaska State Virology Laboratory|Chen|USA
>Spike|hCoV-19/England/ALDP-A68CCC/2020|2020-10-12|EPI_ISL_608562|Original|hCoV-19^^England|Human|Lighthouse Lab in Alderley Park|Wellcome Sanger Institute for the COVID-19 Genomics UK (COG-UK) consortium|Davies|United Kingdom
>Spike|hCoV-19/England/QEUH-9B85A5/2020|2020-09-10|EPI_ISL_549791|Original|hCoV-19^^England|Human|Lighthouse Lab in Glasgow|Wellcome Sanger Institute for the COVID-19 Genomics UK (COG-UK) consortium|Davies|United Kingdom
>Spike|hCoV-19/Denmark/DCGC-1848/2020|2020-05-04|EPI_ISL_622536|Original|hCoV-19^^Hovedstaden|Human|Department of Virus and Microbiological Special Diagnostics|Albertsen lab|Michaelsen|Denmark
>Spike|hCoV-19/Singapore/1010/2020|2020-03-23|EPI_ISL_574495|Original|hCoV-19^^Singapore|Human|National Public Health Laboratory|National Public Health Laboratory|Mak|Singapore
>Spike|hCoV-19/England/QEUH-B68575/2020|2020-11-11|EPI_ISL_655159|Original|hCoV-19^^England|Human|Lighthouse Lab in Glasgow|Wellcome Sanger Institute for the COVID-19 Genomics UK (COG-UK) Consortium|Davies|United Kingdom
>Spike|hCoV-19/Belgium/rega-12111411/2020|2020-12-11|EPI_ISL_738463|Original|hCoV-19^^Dessel|Human|UZ Leuven|KU Leuven|Maes|Belgium
>Spike|hCoV-19/Canada/ON-S1290/2020|2020-05-17|EPI_ISL_586337|Original|hCoV-19^^Ontario|Human|Toronto Invasive Bacterial Diseases Network|McMaster University|McArthur|Canada
>Spike|hCoV-19/Germany/NW-HHU-239/2020|2020-09-15|EPI_ISL_631372|Orginal|hCoV-19^^North Rhine-Westphalia|Human|ZOTZ KLIMAS MVZ D\u00fcsseldorf-Centrum GbR \u00dcBAG f\u00fcr Labormedizin|Center of Medical Microbiology|Wienemann|Germany
>Spike|hCoV-19/England/MILK-A7C120/2020|2020-10-15|EPI_ISL_607401|Original|hCoV-19^^England|Human|Lighthouse Lab in Milton Keynes|Wellcome Sanger Institute for the COVID-19 Genomics UK (COG-UK) consortium|Davies|United Kingdom
>Spike|hCoV-19/England/ALDP-B4938F/2020|2020-11-07|EPI_ISL_649465|Original|hCoV-19^^England|Human|Lighthouse Lab in Alderley Park|Wellcome Sanger Institute for the COVID-19 Genomics UK (COG-UK) Consortium|Davies|United Kingdom
>Spike|hCoV-19/United Arab Emirates/0434/2020|2020-05-15|EPI_ISL_698608|Original|hCoV-19^^Abu Dhabi|Human|Group 42 (G42) Healthcare|G42 Healthcare|Ogrodzki|United Arab Emirates
>Spike|hCoV-19/England/CAMC-B35F91/2020|2020-11-05|EPI_ISL_645456|Original|hCoV-19^^England|Human|Lighthouse Lab in Cambridge|Wellcome Sanger Institute for the COVID-19 Genomics UK (COG-UK) Consortium|Davies|United Kingdom
>Spike|hCoV-19/Switzerland/BE-ETHZ-360017/2020|2020-11-10|EPI_ISL_693875|Original|hCoV-19^^Bern|Human|Viollier AG|Department of Biosystems Science and Engineering|Nadeau|Switzerland
>Spike|hCoV-19/England/BRIS-1853B6C/2020|2020-04-26|EPI_ISL_492246|Original|hCoV-19^^England|Human|PHE South West Regional Laboratory|Wellcome Sanger Institute for the COVID-19 Genomics UK (COG-UK) consortium|Davies|United Kingdom
>Spike|hCoV-19/USA/VA-DCLS-0051/2020|2020-03-28|EPI_ISL_426459|Original|hCoV-19^^Virginia|Human|Virginia DCLS|Virginia DCLS|St. Jacques|USA
>Spike|hCoV-19/Finland/24ES21A11/2020|2020-08-24|EPI_ISL_759811|Original|hCoV-19^^Uusimaa|Human|Department of Virology and Immunology|Department of Virology|Smura|Finland
>Spike|hCoV-19/Scotland/QEUH-9B82CC/2020|2020-09-10|EPI_ISL_568249|Original|hCoV-19^^Scotland|Human|Lighthouse Lab in Glasgow|Wellcome Sanger Institute for the COVID-19 Genomics UK (COG-UK) consortium|Davies|United Kingdom
>Spike|hCoV-19/Belgium/LM-030685/2020|2020-03-06|EPI_ISL_420341|Original|hCoV-19^^Theux|Human|KU Leuven|KU Leuven|Maes|Belgium
>Spike|hCoV-19/England/CAMC-949EBD/2020|2020-07-21|EPI_ISL_556518|Original|hCoV-19^^England|Human|Lighthouse Lab in Cambridge|Wellcome Sanger Institute for the COVID-19 Genomics UK (COG-UK) consortium|Davies|United Kingdom
>Spike|hCoV-19/USA/UT-UPHL-2009183/2020|2020-05-30|EPI_ISL_525166|Original|hCoV-19^^Utah|Human|Utah Public Health Laboratory|Utah Public Health Laboratory|Young|USA

>Spike|hCoV-19/England/ALDP-B47F6F/2020|2020-11-07|EPI_ISL_649280|Original|hCoV-19^^England|Human|Lighthouse Lab in Alderley Park|Wellcome Sanger Institute for the COVID-19 Genomics UK (COG-UK) Consortium|Davies|United Kingdom
>Spike|hCoV-19/USA/TX-HMH-1757/2020|2020-05-12|EPI_ISL_542817|Original|hCoV-19^^Texas|Human|Houston Methodist Hospital|Houston Methodist Hospital|Long|USA
>Spike|hCoV-19/Hungary/UD-93992/2020|2020-10-08|EPI_ISL_671491|Original|hCoV-19^^Hajd\u00fa-Bihar county|Human|University of Debrecen|National Laboratory of Virology|Kemenesi|Hungary
>Spike|hCoV-19/USA/WA-S2624/2020|2020-06-19|EPI_ISL_497256|Original|hCoV-19^^Washington|Human|Washington State Department of Health|Seattle Flu Study|Lee|USA
>Spike|hCoV-19/Wales/PHWC-4827A3/2020|2020-10-20|EPI_ISL_613242|Original|hCoV-19^^Wales|Human|Wales Specialist Virology Centre Sequencing lab|COVID-19 Genomics UK (COG-UK) Consortium|Moore|United Kingdom
>Spike|hCoV-19/Japan/PG-0575/2020|2020-04-00|EPI_ISL_684192|Original|hCoV-19^^Japan|Human|Pathogen Genomics Center|Pathogen Genomics Center|Sekizuka|Japan
>Spike|hCoV-19/England/MILK-A79B16/2020|2020-10-15|EPI_ISL_606914|Original|hCoV-19^^England|Human|Lighthouse Lab in Milton Keynes|Wellcome Sanger Institute for the COVID-19 Genomics UK (COG-UK) consortium|Davies|United Kingdom
>Spike|hCoV-19/USA/AZ-TG426799/2020|2020-06-25|EPI_ISL_694989|Original|hCoV-19^^Arizona|Human|TGen North|TGen North|French|USA
>Spike|hCoV-19/USA/TX-DSHS-0415/2020|2020-06-16|EPI_ISL_542445|Original|hCoV-19^^Texas|Human|Texas Department of State Health Services|Texas Department of State Health Services|Rahman|USA
>Spike|hCoV-19/England/MILK-993DE4/2020|2020-08-22|EPI_ISL_551704|Original|hCoV-19^^England|Human|Lighthouse Lab in Milton Keynes|Wellcome Sanger Institute for the COVID-19 Genomics UK (COG-UK) consortium|Davies|United Kingdom
>Spike|hCoV-19/South Africa/KRISP-K002808/2020|2020-08-25|EPI_ISL_529802|Original|hCoV-19^^KwaZulu-Natal|Human|NHLS-IALCH|KRISP|de Oliveira|South Africa
>Spike|hCoV-19/Canada/ON-CVD00726/2020|2020-04-09|EPI_ISL_586271|Original|hCoV-19^^Ontario|Human|Toronto Invasive Bacterial Diseases Network|McMaster University|McArthur|Canada
>Spike|hCoV-19/Italy/APU-IZSPB_314PT/2020|2020-11-02|EPI_ISL_653808|Original|hCoV-19^^Apulia|Human|I.R.C.C.S. \S. De Bellis\ - Ente Ospedaliero|Istituto Zooprofilattico Sperimentale della Puglia e della Basilicata|Parisi|Italy
>Spike|hCoV-19/USA/IL-NM-0642/2020|2020-05-06|EPI_ISL_626461|Original|hCoV-19^^Illinois|Human|Northwestern Memorial Hospital|Ozer Lab|Ozer|USA
>Spike|hCoV-19/Japan/PG-3671/2020|2020-04-00|EPI_ISL_691924|Original|hCoV-19^^Japan|Human|Pathogen Genomics Center|Pathogen Genomics Center|Sekizuka|Japan
>Spike|hCoV-19/Scotland/EDB3583/2020|2020-04-27|EPI_ISL_439293|Original|hCoV-19^^Scotland|Human|Virology Department|COVID-19 Genomics UK (COG-UK) Consortium|Williams|United Kingdom
>Spike|hCoV-19/USA/NY-MSK-4A/2020|2020-04-13|EPI_ISL_583439|Original|hCoV-19^^New York|Human|Memorial Sloan Kettering Cancer Center|van Bakel Laboratory|van Bakel|USA
>Spike|hCoV-19/England/QEUH-A0BA94/2020|2020-09-30|EPI_ISL_599406|Original|hCoV-19^^England|Human|Lighthouse Lab in Glasgow|Wellcome Sanger Institute for the COVID-19 Genomics UK (COG-UK) consortium|Davies|United Kingdom
>Spike|hCoV-19/Israel/CVL-248-ngs/2020|2020-12-27|EPI_ISL_776855|Original|hCoV-19^^Israel|Human|Israel Central Virology laboratory|Israel Central Virology laboratory|Zuckerman|Israel
>Spike|hCoV-19/Denmark/DCGC-9330/2020|2020-11-16|EPI_ISL_668911|Original|hCoV-19^^Nordjylland|Human|Department of Virus and Microbiological Special Diagnostics|Albertsen Lab|Michaelsen|Denmark
>Spike|hCoV-19/Scotland/EDB10480/2020|2020-11-02|EPI_ISL_705772|Original|hCoV-19^^Scotland|Human|Virology Department|COVID-19 Genomics UK (COG-UK) Consortium|McHugh|United Kingdom
>Spike|hCoV-19/England/QEUH-9C8AEF/2020|2020-09-10|EPI_ISL_567124|Original|hCoV-19^^England|Human|Lighthouse Lab in Glasgow|Wellcome Sanger Institute for the COVID-19 Genomics UK (COG-UK) consortium|Davies|United Kingdom

>Spike|hCoV-19/USA/NMDOH-2020117134/2020|2020-06-09|EPI_ISL_542013|Original|hCoV-19^^New Mexico|Human|New Mexico Department of Health Scientific Laboratory|New Mexico Department of Health Scientific Laboratory|Johnson|USA
>Spike|hCoV-19/USA/WA-UW-24906/2020|2020-08-11|EPI_ISL_570771|Original|hCoV-19^^Washington|Human|UW Virology Lab|UW Virology Lab|Roychoudhury|USA
>Spike|hCoV-19/USA/NY-Wadsworth-290367-01/2020|2020-12-14|EPI_ISL_765548|Original|hCoV-19^^New York|Human|MONTEFIORE MEDICAL CENTER LABORATORIES|Wadsworth Center|Lasek-Nesselquist|USA
>Spike|hCoV-19/Brazil/RJ-UFRJ-58271/2020|2020-06-01|EPI_ISL_492048|Original|hCoV-19^^Rio de Janeiro|Human|Instituto de Biologia do Ex\u00e9rcito|Laborat\u00f3rio Metabolismo Macromolecular FirminoTorres de Castro|Moura-Neto|Brazil
>Spike|hCoV-19/USA/NY-Wadsworth-29242-01/2020|2020-03-25|EPI_ISL_676763|Original|hCoV-19^^New York|Human|Wadsworth Center|Wadsworth Center|Lasek-Nesselquist|USA
>Spike|hCoV-19/England/ALDP-4AB258/2020|2020-06-17|EPI_ISL_559370|Original|hCoV-19^^England|Human|Lighthouse Lab in Alderley Park|Wellcome Sanger Institute for the COVID-19 Genomics UK (COG-UK) consortium|Davies|United Kingdom
>Spike|hCoV-19/Australia/VIC348/2020|2020-03-20|EPI_ISL_426657|Original|hCoV-19^^Victoria|Human|Victorian Infectious Diseases Reference Laboratory (VIDRL)|Microbiological Diagnostic Unit Public Health Laboratory and Victorian Infectious Diseases Reference Laboratory|Schultz|Australia
>Spike|hCoV-19/England/ALDP-A2D81A/2020|2020-10-06|EPI_ISL_586928|Original|hCoV-19^^England|Human|Lighthouse Lab in Alderley Park|Wellcome Sanger Institute for the COVID-19 Genomics UK (COG-UK) consortium|Davies|United Kingdom
>Spike|hCoV-19/USA/UT-UPHL-2012972277/2020|2020-08-08|EPI_ISL_746087|Original|hCoV-19^^Utah|Human|Ginkgo Bioworks Clinical Laboratory|Utah Public Health Laboratory|Young|USA
>Spike|hCoV-19/England/MILK-9C47BF/2020|2020-09-05|EPI_ISL_549464|Original|hCoV-19^^England|Human|Centre for Enzyme Innovation|COVID-19 Genomics UK (COG-UK) Consortium|Robson|United Kingdom
>Spike|hCoV-19/England/CAMC-A81BFD/2020|2020-10-14|EPI_ISL_610744|Original|hCoV-19^^England|Human|Lighthouse Lab in Cambridge|Wellcome Sanger Institute for the COVID-19 Genomics UK (COG-UK) consortium|Davies|United Kingdom
>Spike|hCoV-19/Australia/NSW644/2020|2020-07-10|EPI_ISL_498763|Original|hCoV-19^^New South Wales|Human|Sydney South West Pathology Service (SSWPS) - Liverpool Hospital - NSW Health Pathology|NSW Health Pathology - Institute of Clinical Pathology and Medical Research; Westmead Hospital; University of Sydney|Gall|Australia
>Spike|hCoV-19/Japan/PG-1972/2020|2020-05-00|EPI_ISL_685578|Original|hCoV-19^^Japan|Human|Pathogen Genomics Center|Pathogen Genomics Center|Sekizuka|Japan
>Spike|hCoV-19/USA/WA-UW-46847/2020|2020-12-11|EPI_ISL_756296|Original|hCoV-19^^Washington|Human|UW Virology Lab|UW Virology Lab|Roychoudhury|USA
>Spike|hCoV-19/England/MILK-95D9D3/2020|2020-06-19|EPI_ISL_556213|Original|hCoV-19^^England|Human|Lighthouse Lab in Milton Keynes|Wellcome Sanger Institute for the COVID-19 Genomics UK (COG-UK) consortium|Davies|United Kingdom
>Spike|hCoV-19/USA/CA-CZB-2430/2020|2020-05-21|EPI_ISL_513849|Original|hCoV-19^^California|Human|Humboldt County Public Health Laboratory|Chan-Zuckerberg Biohub|Kamm|USA
>Spike|hCoV-19/Japan/IC-0264/2020|2020-10-00|EPI_ISL_667693|Original|hCoV-19^^Japan|Human|Pathogen Genomics Center|Pathogen Genomics Center|Sekizuka|Japan
>Spike|hCoV-19/Japan/PG-3310/2020|2020-04-00|EPI_ISL_691425|Original|hCoV-19^^Japan|Human|Pathogen Genomics Center|Pathogen Genomics Center|Sekizuka|Japan
>Spike|hCoV-19/USA/TX-HMH-MCoV-14258/2020|2020-10-05|EPI_ISL_787543|Original|hCoV-19^^Texas|Human|Houston Methodist Hospital|Houston Methodist Hospital|Long|USA
>Spike|hCoV-19/England/MILK-B8F912/2020|2020-11-13|EPI_ISL_674632|Original|hCoV-19^^England|Human|Lighthouse Lab in Milton Keynes|Wellcome Sanger Institute for the COVID-19 Genomics UK (COG-UK) Consortium|Davies|United Kingdom
>Spike|hCoV-19/England/ALDP-BB28EC/2020|2020-11-19|EPI_ISL_702981|Original|hCoV-19^^England|Human|Lighthouse Lab in Alderley Park|Wellcome Sanger Institute for the COVID-19 Genomics UK (COG-UK) Consortium|Davies|United Kingdom

>Spike|hCoV-19/India/GJ-GBRC18/2020|2020-04-26|EPI_ISL_437447|Original|hCoV-19^^Gujarat|Human|B.J. Medical College and Civil hospital|Gujarat Biotechnology Research Centre|Joshi|India
>Spike|hCoV-19/England/BRIS-128F5F/2020|2020-04-18|EPI_ISL_458811|Original|hCoV-19^^England|Human|PHE South West Regional Laboratory|Wellcome Sanger Institute for the COVID-19 Genomics UK (COG-UK) consortium|Davies|United Kingdom
>Spike|hCoV-19/Japan/PG-1714/2020|2020-04-00|EPI_ISL_685320|Original|hCoV-19^^Japan|Human|Pathogen Genomics Center|Pathogen Genomics Center|Sekizuka|Japan
>Spike|hCoV-19/Scotland/EDB1841/2020|2020-04-17|EPI_ISL_433153|Original|hCoV-19^^Scotland|Human|Virology Department|COVID-19 Genomics UK (COG-UK) Consortium|Williams|United Kingdom
>Spike|hCoV-19/England/NORT-28CD8B/2020|2020-10-02|EPI_ISL_665870|Original|hCoV-19^^England|Human|Northumbria University / South Tees Hospitals NHS Foundation Trust / North Cumbria Integrated Care NHS Foundation Trust / North Tees and Hartlepool NHS Foundation Trust / Newcastle Hospitals NHS Foundation Trust|COVID-19 Genomics UK (COG-UK) Consortium|Bashton|United Kingdom
>Spike|hCoV-19/Wales/PHWC-162733/2020|2020-05-28|EPI_ISL_473113|Original|hCoV-19^^Wales|Human|Wales Specialist Virology Centre Sequencing lab|COVID-19 Genomics UK (COG-UK) Consortium|Moore|United Kingdom
>Spike|hCoV-19/England/QEUH-95F29A/2020|2020-08-06|EPI_ISL_532365|Original|hCoV-19^^England|Human|Lighthouse Lab in Glasgow|Wellcome Sanger Institute for the COVID-19 Genomics UK (COG-UK) consortium|Davies|United Kingdom
>Spike|hCoV-19/USA/OR-PROV-143/2020|2020-05-02|EPI_ISL_482401|Original|hCoV-19^^Oregon|Human|Providence St. Joseph Health Molecular Genomics Laboratory|Providence St. Joseph Health Molecular Genomics Laboratory|Dowdell|USA
>Spike|hCoV-19/USA/WA-S1301/2020|2020-05-14|EPI_ISL_495751|Original|hCoV-19^^Washington|Human|Washington State Department of Health|Seattle Flu Study|Lee|USA
>Spike|hCoV-19/Portugal/PT1431/2020|2020-03-24|EPI_ISL_511390|Original|hCoV-19^^Portugal|Human|Instituto Nacional de Saude (INSA)|Instituto Nacional de Saude (INSA)|Borges|Portugal
>Spike|hCoV-19/England/CAMB-7E304/2020|2020-04-17|EPI_ISL_433469|Original|hCoV-19^^England|Human|Department of Pathology|COVID-19 Genomics UK (COG-UK) Consortium|Meredith|United Kingdom
>Spike|hCoV-19/England/MILK-9EEE32/2020|2020-09-24|EPI_ISL_580857|Original|hCoV-19^^England|Human|Lighthouse Lab in Milton Keynes|Wellcome Sanger Institute for the COVID-19 Genomics UK (COG-UK) consortium|Davies|United Kingdom
>Spike|hCoV-19/USA/UT-UPHL-2012347682/2020|2020-08-07|EPI_ISL_746005|Original|hCoV-19^^Utah|Human|Ginkgo Bioworks Clinical Laboratory|Utah Public Health Laboratory|Young|USA
>Spike|hCoV-19/Sweden/20-22284/2020|2020-09-17|EPI_ISL_654898|Original|hCoV-19^^Skane|Human|Klinisk mikrobiologi|The Public Health Agency of Sweden|Svartstr\u00f6m|Sweden
>Spike|hCoV-19/England/EXET-13F4B0/2020|2020-12-16|EPI_ISL_763437|Original|hCoV-19^^England|Human|University of Exeter|COVID-19 Genomics UK (COG-UK) Consortium|Temperton|United Kingdom
>Spike|hCoV-19/England/CAMC-B077DC/2020|2020-10-31|EPI_ISL_643727|Original|hCoV-19^^England|Human|Lighthouse Lab in Cambridge|Wellcome Sanger Institute for the COVID-19 Genomics UK (COG-UK) Consortium|Davies|United Kingdom
>Spike|hCoV-19/USA/MI-MDHHS-SC21445/2020|2020-03-22|EPI_ISL_507699|Original|hCoV-19^^Michigan|Human|Michigan Department of Health and Human Services|Michigan Department of Health and Human Services|Blankenship|USA
>Spike|hCoV-19/England/ALDP-A85C32/2020|2020-10-17|EPI_ISL_610426|Original|hCoV-19^^England|Human|Lighthouse Lab in Alderley Park|Wellcome Sanger Institute for the COVID-19 Genomics UK (COG-UK) consortium|Davies|United Kingdom
>Spike|hCoV-19/Belgium/ULG-10311/2020|2020-07-27|EPI_ISL_540478|Original|hCoV-19^^Li\u00e8ge|Human|Department of Clinical Microbiology|GIGA Medical Genomics|Durkin|Belgium
>Spike|hCoV-19/Chile/AR-178998/2020|2020-09-15|EPI_ISL_746500|Original|hCoV-19^^La Araucania|Human|Genetica Molecular and Subdepartamento de Virologia ISP Chile|Instituto de Salud Publica de Chile|Tognarelli|Chile

>Spike|hCoV-19/England/CAMB-1B1261/2020|2020-05-06|EPI_ISL_459178|Original|hCoV-19^^England|Human|Department of Pathology|Wellcome Sanger Institute for the COVID-19 Genomics UK (COG-UK) consortium|Davies|United Kingdom
>Spike|hCoV-19/England/MILK-A05441/2020|2020-09-30|EPI_ISL_599938|Original|hCoV-19^^England|Human|Lighthouse Lab in Milton Keynes|Wellcome Sanger Institute for the COVID-19 Genomics UK (COG-UK) consortium|Davies|United Kingdom
>Spike|hCoV-19/USA/WA-UW-2414/2020|2020-04-18|EPI_ISL_485990|Original|hCoV-19^^Washington|Human|UW Virology Lab|UW Virology Lab|Roychoudhury|USA
>Spike|hCoV-19/USA/WA-S2134/2020|2020-06-08|EPI_ISL_497562|Original|hCoV-19^^Washington|Human|Washington State Department of Health|Seattle Flu Study|Lee|USA
>Spike|hCoV-19/USA/TX-HMH-MCoV-15136/2020|2020-10-16|EPI_ISL_786620|Original|hCoV-19^^Texas|Human|Houston Methodist Hospital|Houston Methodist Hospital|Long|USA
>Spike|hCoV-19/Switzerland/BL-UHB-42188802/2020|2020-03-12|EPI_ISL_528038|Original|hCoV-19^^Basel-Land|Human|University Hospital Basel|University Hospital Basel|Roloff Handschin|Switzerland
>Spike|hCoV-19/USA/TX-HMH-MCoV-13636/2020|2020-08-20|EPI_ISL_788742|Original|hCoV-19^^Texas|Human|Houston Methodist Hospital|Houston Methodist Hospital|Long|USA
>Spike|hCoV-19/England/LOND-D3992/2020|2020-04-06|EPI_ISL_441016|Original|hCoV-19^^England|Human|University College London|COVID-19 Genomics UK (COG-UK) Consortium|Roy|United Kingdom
>Spike|hCoV-19/Japan/IC-0182/2020|2020-10-00|EPI_ISL_667611|Original|hCoV-19^^Japan|Human|Pathogen Genomics Center|Pathogen Genomics Center|Sekizuka|Japan
>Spike|hCoV-19/England/SHEF-C9294/2020|2020-04-02|EPI_ISL_432468|Original|hCoV-19^^England|Human|Virology Department|COVID-19 Genomics UK (COG-UK) Consortium|de Silva|United Kingdom
>Spike|hCoV-19/England/BRIS-1854521/2020|2020-03-03|EPI_ISL_492397|Original|hCoV-19^^England|Human|PHE South West Regional Laboratory|Wellcome Sanger Institute for the COVID-19 Genomics UK (COG-UK) consortium|Davies|United Kingdom
>Spike|hCoV-19/Spain/MD-IBV-97010058/2020|2020-03-12|EPI_ISL_779755|Original|hCoV-19^^Madrid|Human|IdISSC/Hospital Cl\u00ednico San Carlos de Madrid\u200b|SeqCOVID-SPAIN consortium/IBV(CSIC)|Comas|Spain
>Spike|hCoV-19/England/ALDP-A2FAF4/2020|2020-10-05|EPI_ISL_587041|Original|hCoV-19^^England|Human|Lighthouse Lab in Alderley Park|Wellcome Sanger Institute for the COVID-19 Genomics UK (COG-UK) consortium|Davies|United Kingdom
>Spike|hCoV-19/England/ALDP-BB3A30/2020|2020-11-19|EPI_ISL_701853|Original|hCoV-19^^England|Human|Lighthouse Lab in Alderley Park|Wellcome Sanger Institute for the COVID-19 Genomics UK (COG-UK) Consortium|Davies|United Kingdom
>Spike|hCoV-19/England/QEUH-B7D944/2020|2020-11-14|EPI_ISL_727834|Original|hCoV-19^^England|Human|Oxford Viromics|COVID-19 Genomics UK (COG-UK) Consortium|Golubchik|United Kingdom
>Spike|hCoV-19/England/204390353/2020|2020-10-23|EPI_ISL_637145|Original|hCoV-19^^England|Human|Respiratory Virus Unit|COVID-19 Genomics UK (COG-UK) Consortium|Platt|United Kingdom
>Spike|hCoV-19/Denmark/DCGC-6115/2020|2020-09-21|EPI_ISL_621149|Original|hCoV-19^^Midtjylland|Human|Department of Virus and Microbiological Special Diagnostics|Albertsen lab|Michaelsen|Denmark
>Spike|hCoV-19/Spain/AN-IBV-001897/2020|2020-03-15|EPI_ISL_467071|Original|hCoV-19^^Andalucia|Human|Hospital Universitario Puerta del Mar de C\u00e1diz - INIBICA|SeqCOVID-SPAIN consortium/IBV(CSIC)|Comas|Spain
>Spike|hCoV-19/USA/NY-NYUMC334/2020|2020-04-07|EPI_ISL_430361|Original|hCoV-19^^New York|Human|NYU Langone Health|Departments of Pathology and Medicine|Maurano|USA
>Spike|hCoV-19/England/BIRM-67A90/2020|2020-11-02|EPI_ISL_651282|Original|hCoV-19^^England|Human|University of Birmingham|COVID-19 Genomics UK (COG-UK) Consortium|Loman|United Kingdom
>Spike|hCoV-19/England/CAMC-941583/2020|2020-07-20|EPI_ISL_556826|Original|hCoV-19^^England|Human|Lighthouse Lab in Cambridge|Wellcome Sanger Institute for the COVID-19 Genomics UK (COG-UK) consortium|Davies|United Kingdom
>Spike|hCoV-19/USA/TX-HMH-MCoV-14035/2020|2020-08-14|EPI_ISL_787324|Original|hCoV-19^^Texas|Human|Houston Methodist Hospital|Houston Methodist Hospital|Long|USA

>Spike|hCoV-19/South Africa/R12566-20/2020|2020-06-16|EPI_ISL_504191|Original|hCoV-19^^North West|Human|National Institute for Communicable Diseases of the National Health Laboratory Service|National Institute for Communicable Diseases of the National Health Laboratory Service|Allam|South Africa
>Spike|hCoV-19/Wales/PHWC-16F0FF/2020|2020-10-05|EPI_ISL_585914|Original|hCoV-19^^Wales|Human|Wales Specialist Virology Centre Sequencing lab|COVID-19 Genomics UK (COG-UK) Consortium|Moore|United Kingdom
>Spike|hCoV-19/Scotland/CVR2914/2020|2020-04-22|EPI_ISL_473627|Original|hCoV-19^^Scotland|Human|West of Scotland Specialist Virology Centre|COVID-19 Genomics UK (COG-UK) Consortium|Robertson|United Kingdom
>Spike|hCoV-19/England/MILK-B8C57B/2020|2020-11-13|EPI_ISL_674403|Original|hCoV-19^^England|Human|Lighthouse Lab in Milton Keynes|Wellcome Sanger Institute for the COVID-19 Genomics UK (COG-UK) Consortium|Davies|United Kingdom
>Spike|hCoV-19/USA/VA-DCLS-0420/2020|2020-04-28|EPI_ISL_477636|Original|hCoV-19^^Virginia|Human|Virginia DCLS|Virginia DCLS|St. Jacques|USA
>Spike|hCoV-19/Scotland/EDB7844/2020|2020-09-22|EPI_ISL_572411|Original|hCoV-19^^Scotland|Human|Virology Department|COVID-19 Genomics UK (COG-UK) Consortium|Williams|United Kingdom
>Spike|hCoV-19/Japan/PG-1504/2020|2020-04-00|EPI_ISL_685111|Original|hCoV-19^^Japan|Human|Saitama Prefectural Institute of Public Health|Pathogen Genomics Center|Sekizuka|Japan
>Spike|hCoV-19/Denmark/DCGC-19656/2020|2020-12-07|EPI_ISL_749088|Original|hCoV-19^^Sjaelland|Human|Department of Virus and Microbiological Special Diagnostics|Albertsen Lab|Michaelsen|Denmark
>Spike|hCoV-19/England/CAMB-1B2BDE/2020|2020-05-18|EPI_ISL_456710|Original|hCoV-19^^England|Human|Department of Pathology|COVID-19 Genomics UK (COG-UK) Consortium|Meredith|United Kingdom
>Spike|hCoV-19/USA/MI-UM-10036102177/2020|2020-09-23|EPI_ISL_583406|Original|hCoV-19^^Michigan|Human|University of Michigan Clinical Microbiology Laboratory|Lauring Lab|Valesano|USA
>Spike|hCoV-19/England/ALDP-B62D55/2020|2020-11-08|EPI_ISL_655525|Original|hCoV-19^^England|Human|Lighthouse Lab in Alderley Park|Wellcome Sanger Institute for the COVID-19 Genomics UK (COG-UK) Consortium|Davies|United Kingdom
>Spike|hCoV-19/England/ALDP-B263A6/2020|2020-11-04|EPI_ISL_646664|Original|hCoV-19^^England|Human|Lighthouse Lab in Alderley Park|Wellcome Sanger Institute for the COVID-19 Genomics UK (COG-UK) Consortium|Davies|United Kingdom
>Spike|hCoV-19/England/20129036004/2020|2020-03-20|EPI_ISL_424032|Original|hCoV-19^^England|Human|Respiratory Virus Unit|Respiratory Virus Unit|Platt|United Kingdom
>Spike|hCoV-19/England/MILK-A22DCB/2020|2020-10-03|EPI_ISL_611574|Original|hCoV-19^^England|Human|Northumbria University / South Tees Hospitals NHS Foundation Trust / North Cumbria Integrated Care NHS Foundation Trust / North Tees and Hartlepool NHS Foundation Trust / Newcastle Hospitals NHS Foundation Trust|COVID-19 Genomics UK (COG-UK) Consortium|Bashton|United Kingdom
>Spike|hCoV-19/Wales/PHWC-160920/2020|2020-04-18|EPI_ISL_472994|Original|hCoV-19^^Wales|Human|Wales Specialist Virology Centre Sequencing lab|COVID-19 Genomics UK (COG-UK) Consortium|Moore|United Kingdom
>Spike|hCoV-19/England/QEUH-A63A9A/2020|2020-10-12|EPI_ISL_628661|Original|hCoV-19^^England|Human|Oxford Viromics|COVID-19 Genomics UK (COG-UK) Consortium|Golubchik|United Kingdom
>Spike|hCoV-19/Japan/PG-0480/2020|2020-04-02|EPI_ISL_480139|Original|hCoV-19^^HokurikuShinsyu|Human|Fukui Prefectural Institute of Public Health and Environmental Science|Pathogen Genomics Center|Sekizuka|Japan
>Spike|hCoV-19/Denmark/DCGC-20685/2020|2020-12-21|EPI_ISL_759299|Original|hCoV-19^^Midtjylland|Human|Department of Virus and Microbiological Special Diagnostics|Albertsen Lab|Michaelsen|Denmark
>Spike|hCoV-19/England/CAMC-9B1C3E/2020|2020-09-04|EPI_ISL_566611|Original|hCoV-19^^England|Human|Lighthouse Lab in Cambridge|Wellcome Sanger Institute for the COVID-19 Genomics UK (COG-UK) consortium|Davies|United Kingdom

>Spike|hCoV-19/England/EXET-139146/2020|2020-10-27|EPI_ISL_664612|Original|hCoV-19^^England|Human|University of Exeter|COVID-19 Genomics UK (COG-UK) Consortium|Temperton|United Kingdom
>Spike|hCoV-19/USA/UT-UPHL-2009593/2020|2020-07-18|EPI_ISL_605271|Original|hCoV-19^^Utah|Human|Utah Public Health Laboratory|Utah Public Health Laboratory|Young|USA
>Spike|hCoV-19/England/ALDP-9CDD1E/2020|2020-09-16|EPI_ISL_566314|Original|hCoV-19^^England|Human|Lighthouse Lab in Alderley Park|Wellcome Sanger Institute for the COVID-19 Genomics UK (COG-UK) consortium|Davies|United Kingdom
>Spike|hCoV-19/Spain/AN-IBV-006091/2020|2020-05-08|EPI_ISL_510427|Original|hCoV-19^^Andalucia|Human|Hospital Universitario Virgen de las Nieves de Granada-SAS|SeqCOVID-SPAIN consortium/IBV(CSIC)|Comas|Spain
>Spike|hCoV-19/England/QEUH-BA3C97/2020|2020-11-17|EPI_ISL_721170|Original|hCoV-19^^England|Human|Lighthouse Lab in Glasgow|Wellcome Sanger Institute for the COVID-19 Genomics UK (COG-UK) Consortium|Davies|United Kingdom
>Spike|hCoV-19/England/CAMC-C6FEBE/2020|2020-12-15|EPI_ISL_769112|Original|hCoV-19^^England|Human|Lighthouse Lab in Cambridge|Wellcome Sanger Institute for the COVID-19 Genomics UK (COG-UK) Consortium|Davies|United Kingdom
>Spike|hCoV-19/Norway/4761/2020|2020-10-21|EPI_ISL_668399|Original|hCoV-19^^Nordland|Human|Nordland Hospital - Bodo|Norwegian Institute of Public Health|Kopperud Riis|Norway
>Spike|hCoV-19/Denmark/DCGC-4453/2020|2020-08-31|EPI_ISL_615638|Original|hCoV-19^^Syddanmark|Human|Department of Virus and Microbiological Special Diagnostics|Albertsen lab|Michaelsen|Denmark
>Spike|hCoV-19/Italy/APU-IZSPB_266PT/2020|2020-09-29|EPI_ISL_653785|Original|hCoV-19^^Apulia|Human|Istituto Zooprofilattico Sperimentale della Puglia e della Basilicata|Istituto Zooprofilattico Sperimentale della Puglia e della Basilicata|Parisi|Italy
>Spike|hCoV-19/USA/SC-DHEC-0942/2020|2020-06-26|EPI_ISL_541677|Original|hCoV-19^^South Carolina|Human|Microbiology Division|Microbiology Division|Flores|USA
>Spike|hCoV-19/South Korea/KCDC2461/2020|2020-06-08|EPI_ISL_514864|Original|hCoV-19^^South Korea|Human|Division of Viral Diseases|Division of Viral Diseases|Kim|South Korea
>Spike|hCoV-19/England/ALDP-C39354/2020|2020-12-08|EPI_ISL_731539|Original|hCoV-19^^England|Human|Lighthouse Lab in Alderley Park|Wellcome Sanger Institute for the COVID-19 Genomics UK (COG-UK) Consortium|Davies|United Kingdom
>Spike|hCoV-19/Singapore/1355/2020|2020-06-18|EPI_ISL_648706|Original|hCoV-19^^Singapore|Human|Department of Laboratory Medicine|Department of Laboratory Medicine|Chen|Singapore
>Spike|hCoV-19/Denmark/DCGC-3212/2020|2020-08-24|EPI_ISL_616768|Original|hCoV-19^^Syddanmark|Human|Department of Virus and Microbiological Special Diagnostics|Albertsen lab|Michaelsen|Denmark
>Spike|hCoV-19/Denmark/DCGC-17987/2020|2020-12-14|EPI_ISL_749530|Original|hCoV-19^^Hovedstaden|Human|Department of Virus and Microbiological Special Diagnostics|Albertsen Lab|Michaelsen|Denmark
>Spike|hCoV-19/England/PORT-2D0BD9/2020|2020-04-30|EPI_ISL_475262|Original|hCoV-19^^England|Human|Centre for Enzyme Innovation|COVID-19 Genomics UK (COG-UK) Consortium|Robson|United Kingdom
>Spike|hCoV-19/England/MILK-9E0595/2020|2020-09-20|EPI_ISL_601438|Original|hCoV-19^^England|Human|Lighthouse Lab in Milton Keynes|Wellcome Sanger Institute for the COVID-19 Genomics UK (COG-UK) consortium|Davies|United Kingdom
>Spike|hCoV-19/Wales/PHWC-1603E6/2020|2020-05-24|EPI_ISL_472929|Original|hCoV-19^^Wales|Human|Wales Specialist Virology Centre Sequencing lab|COVID-19 Genomics UK (COG-UK) Consortium|Moore|United Kingdom
>Spike|hCoV-19/England/QEUH-96E140/2020|2020-08-18|EPI_ISL_530500|Original|hCoV-19^^England|Human|Lighthouse Lab in Glasgow|Wellcome Sanger Institute for the COVID-19 Genomics UK (COG-UK) consortium|Davies|United Kingdom
>Spike|hCoV-19/USA/NC-SLPH-0018/2020|2020-08-08|EPI_ISL_538522|Original|hCoV-19^^North Carolina|Human|Infectious Diseases|North Carolina State Laboratory of Public Health|Chase|USA

>Spike|hCoV-19/Wales/PHWC-4964C3/2020|2020-11-20|EPI_ISL_726589|Original|hCoV-19^^Wales|Human|Originating lab|COVID-19 Genomics UK (COG-UK) Consortium|Moore|United Kingdom
>Spike|hCoV-19/India/GJ-GBRC121/2020|2020-05-02|EPI_ISL_455018|Original|hCoV-19^^Gujarat|Human|Government Medical College|Gujarat Biotechnology Research Centre|Joshi|India
>Spike|hCoV-19/England/ALDP-C4857A/2020|2020-12-12|EPI_ISL_736583|Original|hCoV-19^^England|Human|Lighthouse Lab in Alderley Park|Wellcome Sanger Institute for the COVID-19 Genomics UK (COG-UK) Consortium|Davies|United Kingdom
>Spike|hCoV-19/England/205091071/2020|2020-12-11|EPI_ISL_747514|Original|hCoV-19^^England|Human|Respiratory Virus Unit|COVID-19 Genomics UK (COG-UK) Consortium|Miah|United Kingdom
>Spike|hCoV-19/USA/WA-UW-2144/2020|2020-03-25|EPI_ISL_426098|Original|hCoV-19^^Washington|Human|UW Virology Lab|UW Virology Lab|Roychoudhury|USA
>Spike|hCoV-19/USA/TX-HMH-MCoV-8822/2020|2020-07-02|EPI_ISL_780775|Original|hCoV-19^^Texas|Human|Houston Methodist Hospital|Houston Methodist Hospital|Long|USA
>Spike|hCoV-19/England/BIRM-65F57/2020|2020-10-08|EPI_ISL_594610|Original|hCoV-19^^England|Human|University of Birmingham|COVID-19 Genomics UK (COG-UK) Consortium|Loman|United Kingdom
>Spike|hCoV-19/Denmark/DCGC-10557/2020|2020-11-02|EPI_ISL_670051|Original|hCoV-19^^Midtjylland|Human|Department of Virus and Microbiological Special Diagnostics|Albertsen Lab|Michaelsen|Denmark
>Spike|hCoV-19/England/CAMC-B3624C/2020|2020-11-05|EPI_ISL_645377|Original|hCoV-19^^England|Human|Lighthouse Lab in Cambridge|Wellcome Sanger Institute for the COVID-19 Genomics UK (COG-UK) Consortium|Davies|United Kingdom
>Spike|hCoV-19/USA/CA-ALSR-3310/2020|2020-09-22|EPI_ISL_635956|Original|hCoV-19^^California|Human|San Diego County Public Health Laboratory|Andersen lab at Scripps Research|Gangavarapu|USA
>Spike|hCoV-19/Wales/PHWC-26550/2020|2020-03-26|EPI_ISL_422369|Original|hCoV-19^^Wales|Human|Wales Specialist Virology Centre|Public Health Wales Microbiology Cardiff|Moore|United Kingdom
>Spike|hCoV-19/Finland/28A50S11/2020|2020-04-28|EPI_ISL_481626|Original|hCoV-19^^Uusimaa|Human|Department of Virology and Immunology|Department of Virology|Smura|Finland
>Spike|hCoV-19/Northern Ireland/QEUH-79CD65/2020|2020-07-15|EPI_ISL_533013|Original|hCoV-19^^Northern Ireland|Human|Lighthouse Lab in Glasgow|Wellcome Sanger Institute for the COVID-19 Genomics UK (COG-UK) consortium|Davies|United Kingdom
>Spike|hCoV-19/England/MILK-9D5047/2020|2020-09-18|EPI_ISL_577042|Original|hCoV-19^^England|Human|Centre for Enzyme Innovation|COVID-19 Genomics UK (COG-UK) Consortium|Robson|United Kingdom
>Spike|hCoV-19/Denmark/DCGC-10272/2020|2020-11-09|EPI_ISL_669869|Original|hCoV-19^^Hovedstaden|Human|Department of Virus and Microbiological Special Diagnostics|Albertsen Lab|Michaelsen|Denmark
>Spike|hCoV-19/England/20134016404/2020|2020-03-24|EPI_ISL_423088|Original|hCoV-19^^England|Human|Respiratory Virus Unit|Respiratory Virus Unit|Platt|United Kingdom
>Spike|hCoV-19/USA/VA-DCLS-0322/2020|2020-05-04|EPI_ISL_467933|Original|hCoV-19^^Virginia|Human|Virginia DCLS|Virginia DCLS|St. Jacques|USA
>Spike|hCoV-19/England/QEUH-AD1569/2020|2020-10-24|EPI_ISL_633816|Original|hCoV-19^^England|Human|Lighthouse Lab in Glasgow|Wellcome Sanger Institute for the COVID-19 Genomics UK (COG-UK) consortium|Davies|United Kingdom
>Spike|hCoV-19/England/QEUH-BA09A2/2020|2020-11-18|EPI_ISL_719627|Original|hCoV-19^^England|Human|Lighthouse Lab in Glasgow|Wellcome Sanger Institute for the COVID-19 Genomics UK (COG-UK) Consortium|Davies|United Kingdom
>Spike|hCoV-19/England/SHEF-CC9E7/2020|2020-05-02|EPI_ISL_457657|Original|hCoV-19^^England|Human|Virology Department|COVID-19 Genomics UK (COG-UK) Consortium|de Silva|United Kingdom
>Spike|hCoV-19/England/CAMB-84839/2020|2020-03-04|EPI_ISL_440507|Original|hCoV-19^^England|Human|Department of Pathology|Wellcome Sanger Institute for the COVID-19 Genomics UK (COG-UK) consortium|Amato|United Kingdom

>Spike|hCoV-19/England/SHEF-10CD6A0/2020|2020-12-08|EPI_ISL_742001|Original|hCoV-19^^England|Human|Virology Department|COVID-19 Genomics UK (COG-UK) Consortium|de Silva|United Kingdom
>Spike|hCoV-19/England/ALDP-B244E1/2020|2020-11-04|EPI_ISL_646890|Original|hCoV-19^^England|Human|Lighthouse Lab in Alderley Park|Wellcome Sanger Institute for the COVID-19 Genomics UK (COG-UK) Consortium|Davies|United Kingdom
>Spike|hCoV-19/Taiwan/NTU35/2020|2020-11-20|EPI_ISL_693306|Vero|hCoV-19^^Taipei|Human|Department of Laboratory Medicine|Microbial Genomics Core Lab|Lin|Taiwan
>Spike|hCoV-19/England/NOTT-1162B5/2020|2020-10-19|EPI_ISL_595314|Original|hCoV-19^^England|Human|Queens Medical Centre|COVID-19 Genomics UK (COG-UK) Consortium|Loose|United Kingdom
>Spike|hCoV-19/England/QEUH-C32A0B/2020|2020-12-09|EPI_ISL_782624|Original|hCoV-19^^England|Human|Lighthouse Lab in Glasgow|Wellcome Sanger Institute for the COVID-19 Genomics UK (COG-UK) Consortium|Davies|United Kingdom
>Spike|hCoV-19/England/ALDP-95574A/2020|2020-06-12|EPI_ISL_581411|Original|hCoV-19^^England|Human|Lighthouse Lab in Alderley Park|Wellcome Sanger Institute for the COVID-19 Genomics UK (COG-UK) consortium|Davies|United Kingdom
>Spike|hCoV-19/England/MILK-B556A5/2020|2020-11-04|EPI_ISL_657532|Original|hCoV-19^^England|Human|Lighthouse Lab in Milton Keynes|Wellcome Sanger Institute for the COVID-19 Genomics UK (COG-UK) Consortium|Davies|United Kingdom
>Spike|hCoV-19/Northern Ireland/NIRE-FA740/2020|2020-04-06|EPI_ISL_470069|Original|hCoV-19^^Northern Ireland|Human|Regional Virus Laboratory|Wellcome Sanger Institute for the COVID-19 Genomics UK (COG-UK) consortium|Davies|United Kingdom
>Spike|hCoV-19/USA/NY-Wadsworth-10496-01/2020|2020-03-03|EPI_ISL_426025|Original|hCoV-19^^New York|Human|Wadsworth Center|Wadsworth Center|Lasek-Nesselquist|USA
>Spike|hCoV-19/Denmark/DCGC-9903/2020|2020-11-09|EPI_ISL_669263|Original|hCoV-19^^Hovedstaden|Human|Department of Virus and Microbiological Special Diagnostics|Albertsen Lab|Michaelsen|Denmark
>Spike|hCoV-19/England/ALDP-936BAD/2020|2020-06-21|EPI_ISL_551520|Original|hCoV-19^^England|Human|Lighthouse Lab in Alderley Park|Wellcome Sanger Institute for the COVID-19 Genomics UK (COG-UK) consortium|Davies|United Kingdom
>Spike|hCoV-19/England/LIVE-A2B5A/2020|2020-05-18|EPI_ISL_499847|Original|hCoV-19^^England|Human|Liverpool Clinical Laboratories|COVID-19 Genomics UK (COG-UK) Consortium|Haldenby|United Kingdom
>Spike|hCoV-19/England/LIVE-A86AE/2020|2020-03-26|EPI_ISL_517198|Original|hCoV-19^^England|Human|Liverpool Clinical Laboratories|COVID-19 Genomics UK (COG-UK) Consortium|Haldenby|United Kingdom
>Spike|hCoV-19/India/DL-ILBS-1921M/2020|2020-04-06|EPI_ISL_578697|Original|hCoV-19^^Delhi|Human|Clinical Virology Laboratory|ILBS - IGIB|Rooge|India
>Spike|hCoV-19/England/ALDP-A516D7/2020|2020-10-07|EPI_ISL_598128|Original|hCoV-19^^England|Human|Lighthouse Lab in Alderley Park|Wellcome Sanger Institute for the COVID-19 Genomics UK (COG-UK) consortium|Davies|United Kingdom
>Spike|hCoV-19/USA/TX-HMH-MCoV-9197/2020|2020-07-02|EPI_ISL_780994|Original|hCoV-19^^Texas|Human|Houston Methodist Hospital|Houston Methodist Hospital|Long|USA
>Spike|hCoV-19/Netherlands/NA_248/2020|2020-03-28|EPI_ISL_422782|Original|hCoV-19^^Netherlands|Human|Dutch COVID-19 response team|Erasmus Medical Center|Oude Munnink|Netherlands
>Spike|hCoV-19/England/ALDP-9522E2/2020|2020-06-09|EPI_ISL_551296|Original|hCoV-19^^England|Human|Lighthouse Lab in Alderley Park|Wellcome Sanger Institute for the COVID-19 Genomics UK (COG-UK) consortium|Davies|United Kingdom
>Spike|hCoV-19/England/QEUH-AABCEB/2020|2020-10-21|EPI_ISL_652823|Original|hCoV-19^^England|Human|Oxford Viromics|COVID-19 Genomics UK (COG-UK) Consortium|Golubchik|United Kingdom
>Spike|hCoV-19/Wales/PHWC-345D7/2020|2020-04-20|EPI_ISL_446946|Original|hCoV-19^^Wales|Human|Wales Specialist Virology Centre|Public Health Wales Microbiology Cardiff|Moore|United Kingdom

>Spike|hCoV-19/England/NOTT-115580/2020|2020-10-14|EPI_ISL_595247|Original|hCoV-19^^England|Human|Queens Medical Centre|COVID-19 Genomics UK (COG-UK) Consortium|Loose|United Kingdom
>Spike|hCoV-19/England/HECH-259A2FB/2020|2020-06-04|EPI_ISL_488859|Original|hCoV-19^^England|Human|Microbiology Department|Wellcome Sanger Institute for the COVID-19 Genomics UK (COG-UK) consortium|Davies|United Kingdom
>Spike|hCoV-19/Canada/MB-NML-1053/2020|2020-07-29|EPI_ISL_582412|Original|hCoV-19^^Manitoba|Human|Cadham Provincial Laboratory|National Microbiology Laboratory (NML)|Majer|Canada
>Spike|hCoV-19/England/NORT-289832/2020|2020-04-01|EPI_ISL_458587|Original|hCoV-19^^England|Human|NU-OMICS DNA Sequencing research facility|Wellcome Sanger Institute for the COVID-19 Genomics UK (COG-UK) consortium|Davies|United Kingdom
>Spike|hCoV-19/England/SHEF-C5614/2020|2020-04-22|EPI_ISL_453715|Original|hCoV-19^^England|Human|Virology Department|COVID-19 Genomics UK (COG-UK) Consortium|de Silva|United Kingdom
>Spike|hCoV-19/Australia/VIC303/2020|2020-03-23|EPI_ISL_419994|Original|hCoV-19^^Victoria|Human|Victorian Infectious Diseases Reference Laboratory (VIDRL)|Victorian Infectious Diseases Reference Laboratory and Microbiological Diagnostic Unit Public Health Laboratory|Schultz|Australia
>Spike|hCoV-19/Denmark/DCGC-14588/2020|2020-11-23|EPI_ISL_711859|Original|hCoV-19^^Syddanmark|Human|Department of Virus and Microbiological Special Diagnostics|Albertsen Lab|Michaelsen|Denmark
>Spike|hCoV-19/USA/TX-HMH-7705/2020|2020-06-30|EPI_ISL_545593|Original|hCoV-19^^Texas|Human|Houston Methodist Hospital|Houston Methodist Hospital|Long|USA
>Spike|hCoV-19/USA/TX-HMH-MCoV-15282/2020|2020-10-19|EPI_ISL_786766|Original|hCoV-19^^Texas|Human|Houston Methodist Hospital|Houston Methodist Hospital|Long|USA
>Spike|hCoV-19/USA/CA-CZB-1234/2020|2020-04-10|EPI_ISL_454654|Original|hCoV-19^^California|Human|County of Santa Clara Public Health Department|Chan-Zuckerberg Biohub|Hao|USA
>Spike|hCoV-19/Scotland/EDB2416/2020|2020-04-22|EPI_ISL_439204|Original|hCoV-19^^Scotland|Human|Virology Department|COVID-19 Genomics UK (COG-UK) Consortium|Williams|United Kingdom
>Spike|hCoV-19/England/MILK-BB14E9/2020|2020-11-18|EPI_ISL_720164|Original|hCoV-19^^England|Human|Lighthouse Lab in Milton Keynes|Wellcome Sanger Institute for the COVID-19 Genomics UK (COG-UK) Consortium|Davies|United Kingdom
>Spike|hCoV-19/USA/TX-HMH-1454/2020|2020-04-13|EPI_ISL_542732|Original|hCoV-19^^Texas|Human|Houston Methodist Hospital|Houston Methodist Hospital|Long|USA
>Spike|hCoV-19/Germany/HH-hpi-p1753/2020|2020-03-14|EPI_ISL_776128|Original|hCoV-19^^Hamburg|Human|University Medical Center Hamburg Eppendorf|Heinrich Pette Institute|Robitaille|Germany
>Spike|hCoV-19/Saudi Arabia/KAUST-MAKKAH1595/2020|2020-04-06|EPI_ISL_678120|Original|hCoV-19^^Makkah|Human|Pathogen Genomics Lab King Abdullah University of Science and Technology(KAUST)|Pathogen Genomics Lab King Abdullah University of Science and Technology(KAUST)|Mohamed Ghazzali|Saudi Arabia
>Spike|hCoV-19/Norway/5390/2020|2020-10-19|EPI_ISL_775325|Original|hCoV-19^^Norway|Human|Foerde Hospital|Norwegian Institute of Public Health|Vollan|Norway
>Spike|hCoV-19/England/CAMC-B78642/2020|2020-11-12|EPI_ISL_783496|Original|hCoV-19^^England|Human|Lighthouse Lab in Cambridge|Wellcome Sanger Institute for the COVID-19 Genomics UK (COG-UK) Consortium|Davies|United Kingdom
>Spike|hCoV-19/Denmark/DCGC-20491/2020|2020-12-21|EPI_ISL_758735|Original|hCoV-19^^Hovedstaden|Human|Department of Virus and Microbiological Special Diagnostics|Albertsen Lab|Michaelsen|Denmark
>Spike|hCoV-19/USA/WA-UW-23277/2020|2020-08-07|EPI_ISL_576415|Original|hCoV-19^^Washington|Human|UW Virology Lab|UW Virology Lab|Roychoudhury|USA
>Spike|hCoV-19/England/QEUH-A4D313/2020|2020-10-04|EPI_ISL_628594|Original|hCoV-19^^England|Human|Oxford Viromics|COVID-19 Genomics UK (COG-UK) Consortium|Golubchik|United Kingdom
>Spike|hCoV-19/USA/TX-HMH-MCoV-19496/2020|2020-11-17|EPI_ISL_783879|Original|hCoV-19^^Texas|Human|Houston Methodist Hospital|Houston Methodist Hospital|Long|USA

>Spike|hCoV-19/England/MILK-AB9BED/2020|2020-10-22|EPI_ISL_623706|Original|hCoV-19^^England|Human|Lighthouse Lab in Milton Keynes|Wellcome Sanger Institute for the COVID-19 Genomics UK (COG-UK) consortium|Davies|United Kingdom
>Spike|hCoV-19/USA/TX-HMH-MCoV-19343/2020|2020-11-29|EPI_ISL_783776|Original|hCoV-19^^Texas|Human|Houston Methodist Hospital|Houston Methodist Hospital|Long|USA
>Spike|hCoV-19/Luxembourg/LNS4605001/2020|2020-11-02|EPI_ISL_740506|Original|hCoV-19^^Luxembourg|Human|Laboratoire national de sant\u00e9|Laboratoire national de sant\u00e9|Wienecke-Baldacchino|Luxembourg
>Spike|hCoV-19/USA/CA-CHLA-PLM90356598/2020|2020-11-29|EPI_ISL_753647|Original|hCoV-19^^California|Human|Clinical virology Laboratory|Center for Personalized Medicine|Dien Bard|USA
>Spike|hCoV-19/Canada/NS-NML-946/2020|2020-03-26|EPI_ISL_579576|Original|hCoV-19^^Nova Scotia|Human|QEII Health Sciences Centre|National Microbiology Laboratory (NML)|Majer|Canada
>Spike|hCoV-19/Sichuan/SC-NJ-092/2020|2020-02-03|EPI_ISL_451341|Original|hCoV-19^^Sichuan|Human|West China Hospital of Sichuan University|State Key Laboratory of Biotherapy of Sichuan University|Tang|China
>Spike|hCoV-19/England/201121119/2020|2020-03-10|EPI_ISL_464682|Original|hCoV-19^^England|Human|Respiratory Virus Unit|Respiratory Virus Unit|Platt|United Kingdom
>Spike|hCoV-19/USA/MI-UM-10036669656/2020|2020-10-28|EPI_ISL_650098|Original|hCoV-19^^Michigan|Human|University of Michigan Clinical Microbiology Laboratory|Lauring Lab|Valesano|USA
>Spike|hCoV-19/England/ALDP-920450/2020|2020-06-23|EPI_ISL_552971|Original|hCoV-19^^England|Human|Lighthouse Lab in Alderley Park|Wellcome Sanger Institute for the COVID-19 Genomics UK (COG-UK) consortium|Davies|United Kingdom
>Spike|hCoV-19/England/LOND-1251C52/2020|2020-11-26|EPI_ISL_704052|Original|hCoV-19^^England|Human|University College London Hospital|COVID-19 Genomics UK (COG-UK) Consortium|Byott|United Kingdom
>Spike|hCoV-19/France/PAC-IHU-0396/2020|2020-03-29|EPI_ISL_569487|Original|hCoV-19^^Provence-Alpes-C\u00f4te-d\u2019Azur|Human|MEPHI|MEPHI|Levasseur|France
>Spike|hCoV-19/England/ALDP-9EE324/2020|2020-05-06|EPI_ISL_589338|Original|hCoV-19^^England|Human|Lighthouse Lab in Alderley Park|Wellcome Sanger Institute for the COVID-19 Genomics UK (COG-UK) consortium|Davies|United Kingdom
>Spike|hCoV-19/USA/UT-UPHL-2012198597/2020|2020-08-04|EPI_ISL_746031|Original|hCoV-19^^Utah|Human|Ginkgo Bioworks Clinical Laboratory|Utah Public Health Laboratory|Young|USA
>Spike|hCoV-19/USA/TX-DSHS-1836/2020|2020-06-23|EPI_ISL_745281|Original|hCoV-19^^Texas|Human|Texas Department of State Health Services|Texas Department of State Health Services|Rahman|USA
>Spike|hCoV-19/England/QEUH-A8B4CB/2020|2020-10-17|EPI_ISL_643216|Original|hCoV-19^^England|Human|Lighthouse Lab in Glasgow|Wellcome Sanger Institute for the COVID-19 Genomics UK (COG-UK) Consortium|Davies|United Kingdom
>Spike|hCoV-19/USA/AZ-TG280845/2020|2020-04-21|EPI_ISL_694186|Original|hCoV-19^^Arizona|Human|AZ SPHL|TGen North|French|USA
>Spike|hCoV-19/England/QEUH-9DCBB8/2020|2020-09-21|EPI_ISL_580863|Original|hCoV-19^^England|Human|Lighthouse Lab in Glasgow|Wellcome Sanger Institute for the COVID-19 Genomics UK (COG-UK) consortium|Davies|United Kingdom
>Spike|hCoV-19/England/ALDP-CB5E40/2020|2020-12-18|EPI_ISL_761900|Original|hCoV-19^^England|Human|Lighthouse Lab in Alderley Park|Wellcome Sanger Institute for the COVID-19 Genomics UK (COG-UK) Consortium|Davies|United Kingdom
>Spike|hCoV-19/England/QEUH-AD2207/2020|2020-10-26|EPI_ISL_629808|Original|hCoV-19^^England|Human|Lighthouse Lab in Glasgow|Wellcome Sanger Institute for the COVID-19 Genomics UK (COG-UK) consortium|Davies|United Kingdom
>Spike|hCoV-19/England/SHEF-C037C/2020|2020-03-28|EPI_ISL_420219|Original|hCoV-19^^England|Human|Virology Department|Department of Infection|de Silva|United Kingdom
>Spike|hCoV-19/Denmark/DCGC-11690/2020|2020-10-26|EPI_ISL_671071|Original|hCoV-19^^Syddanmark|Human|Department of Virus and Microbiological Special Diagnostics|Albertsen Lab|Michaelsen|Denmark
>Spike|hCoV-19/Hong Kong/VM20106598/2020|2020-08-12|EPI_ISL_539816|Original|hCoV-19^^Hong Kong|Human|Queen Mary Hospital|Hong Kong Department of Health|Tsang|China

>Spike|hCoV-19/Scotland/EDB1927/2020|2020-04-17|EPI_ISL_433158|Original|hCoV-19^^Scotland|Human|Virology Department|COVID-19 Genomics UK (COG-UK) Consortium|Williams|United Kingdom
>Spike|hCoV-19/Northern Ireland/NIRE-234423/2020|2020-10-18|EPI_ISL_680386|Original|hCoV-19^^Northern Ireland|Human|Regional Virus Laboratory|COVID-19 Genomics UK (COG-UK) Consortium|Fairley|United Kingdom
>Spike|hCoV-19/Australia/VIC15076/2020|2020-09-24|EPI_ISL_593128|Original|hCoV-19^^Victoria|Human|Victorian Infectious Diseases Reference Laboratory (VIDRL)|VIDRL and MDU-PHL|Schultz|Australia
>Spike|hCoV-19/USA/PA-MGEL-00442/2020|2020-07-22|EPI_ISL_681930|Original|hCoV-19^^Pennsylvania|Human|UPMC Clinical Microbiology Laboratory|Microbial Genomic Epidemiology Laboratory|Griffith|USA
>Spike|hCoV-19/Belgium/Rega-0329240/2020|2020-03-29|EPI_ISL_458210|Original|hCoV-19^^Ghlin|Human|KU Leuven|KU Leuven|Maes|Belgium
>Spike|hCoV-19/USA/TX-HMH-MCoV-14263/2020|2020-10-04|EPI_ISL_787548|Original|hCoV-19^^Texas|Human|Houston Methodist Hospital|Houston Methodist Hospital|Long|USA
>Spike|hCoV-19/Scotland/GCVR-170820/2020|2020-03-26|EPI_ISL_489578|Original|hCoV-19^^Scotland|Human|NHSGGC West of Scotland Specialist Virology Centre / MRC-University of Glasgow Centre for Virus Research|Wellcome Sanger Institute for the COVID-19 Genomics UK (COG-UK) consortium|Davies|United Kingdom
>Spike|hCoV-19/USA/TX-HMH-MCoV-8177/2020|2020-06-29|EPI_ISL_783559|Original|hCoV-19^^Texas|Human|Houston Methodist Hospital|Houston Methodist Hospital|Long|USA
>Spike|hCoV-19/England/NORT-2892AD/2020|2020-03-28|EPI_ISL_458715|Original|hCoV-19^^England|Human|NU-OMICS DNA Sequencing research facility|Wellcome Sanger Institute for the COVID-19 Genomics UK (COG-UK) consortium|Davies|United Kingdom
>Spike|hCoV-19/England/QEUH-A5E6AD/2020|2020-10-12|EPI_ISL_597481|Original|hCoV-19^^England|Human|Lighthouse Lab in Glasgow|Wellcome Sanger Institute for the COVID-19 Genomics UK (COG-UK) consortium|Davies|United Kingdom
>Spike|hCoV-19/England/OXON-B2ED6/2020|2020-03-31|EPI_ISL_534824|Original|hCoV-19^^England|Human|Oxford Viromics|COVID-19 Genomics UK (COG-UK) Consortium|Golubchik|United Kingdom
>Spike|hCoV-19/England/NORT-295921/2020|2020-04-02|EPI_ISL_488699|Original|hCoV-19^^England|Human|NU-OMICS DNA Sequencing research facility|Wellcome Sanger Institute for the COVID-19 Genomics UK (COG-UK) consortium|Davies|United Kingdom
>Spike|hCoV-19/England/QEUH-B12BA5/2020|2020-11-02|EPI_ISL_642712|Original|hCoV-19^^England|Human|Lighthouse Lab in Glasgow|Wellcome Sanger Institute for the COVID-19 Genomics UK (COG-UK) Consortium|Davies|United Kingdom
>Spike|hCoV-19/USA/MI-MDHHS-SC21531/2020|2020-04-04|EPI_ISL_516258|Original|hCoV-19^^Michigan|Human|Michigan Department of Health and Human Services|Michigan Department of Health and Human Services|Blankenship|USA
>Spike|hCoV-19/Switzerland/BS-42221337/2020|2020-03-05|EPI_ISL_581862|Original|hCoV-19^^Basel-Stadt|Human|University Hospital Basel|University Hospital Basel|Roloff Handschin|Switzerland
>Spike|hCoV-19/Australia/VIC5128/2020|2020-07-18|EPI_ISL_519931|Original|hCoV-19^^Victoria|Human|Microbiological Diagnostic Unit - Public Health Laboratory (MDU-PHL)|MDU-PHL|Schultz|Australia
>Spike|hCoV-19/England/201390302/2020|2020-03-26|EPI_ISL_465843|Original|hCoV-19^^England|Human|Respiratory Virus Unit|Respiratory Virus Unit|Platt|United Kingdom
>Spike|hCoV-19/Czech Republic/Seq6/2020|2020-03-17|EPI_ISL_426359|Original|hCoV-19^^Central Czechia and Prague|Human|Laboratory of Molecular Genetics|Laboratory of Molecular Genetics|Kramna|Czech Republic
>Spike|hCoV-19/Brazil/PE-IAM1440/2020|2020-05-13|EPI_ISL_572393|Original|hCoV-19^^Pernambuco|Human|LACEN/PE|WallauLab|Wallau|Brazil
>Spike|hCoV-19/India/HR-TF14/2020|2020-06-17|EPI_ISL_508493|Original|hCoV-19^^Haryana|Human|Translational Health Science and Technology Institute|National Institute of Biomedical Genomics|Maitra|India
>Spike|hCoV-19/England/ALDP-658FAC/2020|2020-07-03|EPI_ISL_558027|Original|hCoV-19^^England|Human|Lighthouse Lab in Alderley Park|Wellcome Sanger Institute for the COVID-19 Genomics UK (COG-UK) consortium|Davies|United Kingdom

>Spike|hCoV-19/Japan/IC-0057/2020|2020-06-00|EPI_ISL_591399|Original|hCoV-19^^Japan|Human|Pathogen Genomics Center|Pathogen Genomics Center|Sekizuka|Japan
>Spike|hCoV-19/Australia/TAS52/2020|2020-03-23|EPI_ISL_522727|Original|hCoV-19^^Tasmania|Human|Royal Hobart Hospital Microbiology Department|MDU-PHL|Schultz|Australia
>Spike|hCoV-19/England/ALDP-C3BFF4/2020|2020-12-10|EPI_ISL_731650|Original|hCoV-19^^England|Human|Lighthouse Lab in Alderley Park|Wellcome Sanger Institute for the COVID-19 Genomics UK (COG-UK) Consortium|Davies|United Kingdom
>Spike|hCoV-19/Japan/PG-1518/2020|2020-04-00|EPI_ISL_685125|Original|hCoV-19^^Japan|Human|Saitama Prefectural Institute of Public Health|Pathogen Genomics Center|Sekizuka|Japan
>Spike|hCoV-19/England/ALDP-9C5AF1/2020|2020-09-11|EPI_ISL_567699|Original|hCoV-19^^England|Human|Lighthouse Lab in Alderley Park|Wellcome Sanger Institute for the COVID-19 Genomics UK (COG-UK) consortium|Davies|United Kingdom
>Spike|hCoV-19/Canada/QC-LSPQ-L00237287/2020|2020-03-25|EPI_ISL_536171|Original|hCoV-19^^Quebec|Human|H\u00f4pital de Papineau|Laboratoire de sant\u00e9 publique du Qu\u00e9bec|Moreira|Canada
>Spike|hCoV-19/Japan/PG-6302/2020|2020-08-00|EPI_ISL_689413|Original|hCoV-19^^Japan|Human|Pathogen Genomics Center|Pathogen Genomics Center|Sekizuka|Japan
>Spike|hCoV-19/USA/CA-CHLA-PLM40259119/2020|2020-12-02|EPI_ISL_753586|Original|hCoV-19^^California|Human|Clinical virology Laboratory|Center for Personalized Medicine|Dien Bard|USA
>Spike|hCoV-19/England/CAMC-A5752F/2020|2020-10-09|EPI_ISL_597337|Original|hCoV-19^^England|Human|Lighthouse Lab in Cambridge|Wellcome Sanger Institute for the COVID-19 Genomics UK (COG-UK) consortium|Davies|United Kingdom
>Spike|hCoV-19/USA/UT-UPHL-2009566/2020|2020-07-19|EPI_ISL_605245|Original|hCoV-19^^Utah|Human|Utah Public Health Laboratory|Utah Public Health Laboratory|Young|USA
>Spike|hCoV-19/USA/WA-S1675/2020|2020-05-29|EPI_ISL_496101|Original|hCoV-19^^Washington|Human|Washington State Department of Health|Seattle Flu Study|Lee|USA
>Spike|hCoV-19/England/PORT-2D5460/2020|2020-12-20|EPI_ISL_765115|Original|hCoV-19^^England|Human|Centre for Enzyme Innovation|COVID-19 Genomics UK (COG-UK) Consortium|Robson|United Kingdom
>Spike|hCoV-19/USA/MO-CDC-2-3714191/2020|2020-11-07|EPI_ISL_751737|Original|hCoV-19^^Missouri|Human|MO State Public Health Laboratory|Genomics and Discovery|Queen|USA
>Spike|hCoV-19/Canada/ON-A28/2020|2020-11-24|EPI_ISL_755726|Original|hCoV-19^^Ontario|Human|Toronto Invasive Bacterial Diseases Network|McMaster University|McArthur|Canada
>Spike|hCoV-19/Wales/PHWC-285A9/2020|2020-03-30|EPI_ISL_432357|Original|hCoV-19^^Wales|Human|Wales Specialist Virology Centre|Public Health Wales Microbiology Cardiff|Moore|United Kingdom
>Spike|hCoV-19/France/ARA-21163/2020|2020-04-07|EPI_ISL_508992|Original|hCoV-19^^Auvergne-Rh\u00f4ne-Alpes|Human|CNR Virus des Infections Respiratoires - France SUD|CNR Virus des Infections Respiratoires - France SUD|Josset|France
>Spike|hCoV-19/England/204491014/2020|2020-10-31|EPI_ISL_654015|Original|hCoV-19^^England|Human|Respiratory Virus Unit|COVID-19 Genomics UK (COG-UK) Consortium|Platt|United Kingdom
>Spike|hCoV-19/Australia/VIC8284/2020|2020-08-20|EPI_ISL_562689|Original|hCoV-19^^Victoria|Human|Microbiological Diagnostic Unit - Public Health Laboratory (MDU-PHL)|MDU-PHL|Schultz|Australia
>Spike|hCoV-19/Wales/PHWC-26D2B/2020|2020-03-30|EPI_ISL_422146|Original|hCoV-19^^Wales|Human|Wales Specialist Virology Centre|Public Health Wales Microbiology Cardiff|Moore|United Kingdom
>Spike|hCoV-19/India/MP-NIHSAD-2104-06/2020|2020-04-19|EPI_ISL_452788|Original|hCoV-19^^Madhya Pradesh|Human|ICAR-National Institute of High Security Animal Diseases|ICAR-National Institute of High Security Animal Diseases|Raut|India
>Spike|hCoV-19/Shanghai/SH0014/2020|2020-01-28|EPI_ISL_416327|Original|hCoV-19^^Shanghai|Human|Shanghai Public Health Clinical Center|National Research Center for Translational Medicine (Shanghai)|Zhang|China

>Spike|hCoV-19/USA/UT-UPHL-2012162581/2020|2020-12-02|EPI_ISL_747025|Original|hCoV-19^^Utah|Human|Utah Public Health Laboratory|Utah Public Health Laboratory|Gallagher|USA
>Spike|hCoV-19/Japan/PG-3616/2020|2020-07-00|EPI_ISL_691869|Original|hCoV-19^^Japan|Human|Pathogen Genomics Center|Pathogen Genomics Center|Sekizuka|Japan
>Spike|hCoV-19/Denmark/DCGC-17505/2020|2020-12-14|EPI_ISL_751029|Original|hCoV-19^^Syddanmark|Human|Department of Virus and Microbiological Special Diagnostics|Albertsen Lab|Michaelsen|Denmark
>Spike|hCoV-19/Indonesia/EJ-ITD-3601NT/2020|2020-04-14|EPI_ISL_560991|Original|hCoV-19^^East Java|Human|RSUD Dr. Soetomo|Institute of Tropical Disease|Lusida|Indonesia
>Spike|hCoV-19/France/PAC-IHU-0094/2020|2020-03-22|EPI_ISL_569305|Original|hCoV-19^^Provence-Alpes-C\u00f4te-d\u2019Azur|Human|MEPHI|MEPHI|Levasseur|France
>Spike|hCoV-19/USA/TX-HMH-2644/2020|2020-06-04|EPI_ISL_545449|Original|hCoV-19^^Texas|Human|Houston Methodist Hospital|Houston Methodist Hospital|Long|USA
>Spike|hCoV-19/USA/UT-UPHL-2012828939/2020|2020-10-02|EPI_ISL_746099|Original|hCoV-19^^Utah|Human|Ginkgo Bioworks Clinical Laboratory|Utah Public Health Laboratory|Young|USA
>Spike|hCoV-19/Denmark/DCGC-21444/2020|2020-12-21|EPI_ISL_757731|Original|hCoV-19^^Hovedstaden|Human|Department of Virus and Microbiological Special Diagnostics|Albertsen Lab|Michaelsen|Denmark
>Spike|hCoV-19/Spain/VC-IBV-98003528/2020|2020-03-27|EPI_ISL_537961|Original|hCoV-19^^Comunitat Valenciana|Human|Servicio de Microbiolog\u00eda. Hospital General Universitario de Castell\u00f3n|SeqCOVID-SPAIN consortium/IBV(CSIC)|Comas|Spain
>Spike|hCoV-19/Ireland/CE-NVRL-73IRL26235/2020|2020-09-06|EPI_ISL_578248|Original|hCoV-19^^Clare|Human|National Virus Reference Laboratory|National Virus Reference Laboratory|Gonzalez|Ireland
>Spike|hCoV-19/Nigeria/ON752-CV144/2020|2020-06-11|EPI_ISL_527890|Original|hCoV-19^^Ondo|Human|Nigeria Centre for Disease Control (NCDC)|African Centre of Excellence for Genomics of Infectious Diseases (ACEGID)|Oluniyi|Nigeria
>Spike|hCoV-19/England/LIVE-DBEABB/2020|2020-10-27|EPI_ISL_665139|Original|hCoV-19^^England|Human|Liverpool Clinical Laboratories|COVID-19 Genomics UK (COG-UK) Consortium|Haldenby|United Kingdom
>Spike|hCoV-19/England/201380050/2020|2020-03-25|EPI_ISL_421807|Original|hCoV-19^^England|Human|Respiratory Virus Unit|Respiratory Virus Unit|Platt|United Kingdom
>Spike|hCoV-19/England/PORT-2CF3A8/2020|2020-04-28|EPI_ISL_453672|Original|hCoV-19^^England|Human|Centre for Enzyme Innovation|COVID-19 Genomics UK (COG-UK) Consortium|Robson|United Kingdom
>Spike|hCoV-19/England/QEUH-9C8BA0/2020|2020-09-11|EPI_ISL_567187|Original|hCoV-19^^England|Human|Lighthouse Lab in Glasgow|Wellcome Sanger Institute for the COVID-19 Genomics UK (COG-UK) consortium|Davies|United Kingdom
>Spike|hCoV-19/France/PAC-IHU-1138/2020|2020-08-07|EPI_ISL_569058|Original|hCoV-19^^Provence-Alpes-C\u00f4te-d\u2019Azur|Human|MEPHI|MEPHI|Levasseur|France
>Spike|hCoV-19/USA/TX-DSHS-0319/2020|2020-06-13|EPI_ISL_534286|Original|hCoV-19^^Texas|Human|Texas Department of State Health Services|Texas Department of State Health Services|Rahman|USA
>Spike|hCoV-19/Iceland/294/2020|2020-03-18|EPI_ISL_417623|Original|hCoV-19^^Reykjavik|Human|The National University Hospital of Iceland|deCODE genetics|J\u00f3nsson|Iceland
>Spike|hCoV-19/England/QEUH-C347D5/2020|2020-12-11|EPI_ISL_733948|Original|hCoV-19^^England|Human|Lighthouse Lab in Glasgow|Wellcome Sanger Institute for the COVID-19 Genomics UK (COG-UK) Consortium|Davies|United Kingdom
>Spike|hCoV-19/Jordan/AM-ALSR-3919/2020|2020-09-30|EPI_ISL_636285|Original|hCoV-19^^Amman|Human|Biolab Diagnostic Laboratories|Andersen lab at Scripps Research|Gangavarapu|Jordan
>Spike|hCoV-19/Scotland/QEUH-9F94F6/2020|2020-09-27|EPI_ISL_600919|Original|hCoV-19^^Scotland|Human|Lighthouse Lab in Glasgow|Wellcome Sanger Institute for the COVID-19 Genomics UK (COG-UK) consortium|Davies|United Kingdom
>Spike|hCoV-19/England/MILK-D069D5/2020|2020-12-19|EPI_ISL_781245|Original|hCoV-19^^England|Human|Lighthouse Lab in Milton Keynes|Wellcome Sanger Institute for the COVID-19 Genomics UK (COG-UK) Consortium|Davies|United Kingdom

>Spike|hCoV-19/Scotland/QEUH-A642FB/2020|2020-10-11|EPI_ISL_596190|Original|hCoV-19^^Scotland|Human|Quadram Institute Bioscience|COVID-19 Genomics UK (COG-UK) Consortium|Alikhan|United Kingdom
>Spike|hCoV-19/Canada/BC-BCCDC-8586/2020|2020-11-00|EPI_ISL_740833|Original|hCoV-19^^British Columbia|Human|BCCDC Public Health Laboratory|BCCDC Public Health Laboratory|Prystajecky|Canada
>Spike|hCoV-19/England/CAMC-B5472B/2020|2020-11-08|EPI_ISL_658160|Original|hCoV-19^^England|Human|Lighthouse Lab in Cambridge|Wellcome Sanger Institute for the COVID-19 Genomics UK (COG-UK) Consortium|Davies|United Kingdom
>Spike|hCoV-19/England/MILK-958774/2020|2020-06-09|EPI_ISL_554659|Original|hCoV-19^^England|Human|Lighthouse Lab in Milton Keynes|Wellcome Sanger Institute for the COVID-19 Genomics UK (COG-UK) consortium|Davies|United Kingdom
>Spike|hCoV-19/England/MILK-C906E9/2020|2020-12-09|EPI_ISL_777313|Original|hCoV-19^^England|Human|Lighthouse Lab in Milton Keynes|Wellcome Sanger Institute for the COVID-19 Genomics UK (COG-UK) Consortium|Davies|United Kingdom
>Spike|hCoV-19/England/NORT-288F5C/2020|2020-03-24|EPI_ISL_458700|Original|hCoV-19^^England|Human|NU-OMICS DNA Sequencing research facility|Wellcome Sanger Institute for the COVID-19 Genomics UK (COG-UK) consortium|Davies|United Kingdom
>Spike|hCoV-19/Denmark/ALAB-HH36/2020|2020-03-12|EPI_ISL_429292|Original|hCoV-19^^Denmark|Human|Department of Clinical Microbiology|Albertsen lab|Kirkegaard|Denmark
>Spike|hCoV-19/Japan/PG-1077/2020|2020-04-00|EPI_ISL_684687|Original|hCoV-19^^Japan|Human|Fukuoka Institute of Health and Environmental Sciences|Pathogen Genomics Center|Sekizuka|Japan
>Spike|hCoV-19/USA/TX-HMH-MCoV-15117/2020|2020-10-17|EPI_ISL_786601|Original|hCoV-19^^Texas|Human|Houston Methodist Hospital|Houston Methodist Hospital|Long|USA
>Spike|hCoV-19/USA/AZ-TG305772/2020|2020-04-27|EPI_ISL_694273|Original|hCoV-19^^Arizona|Human|AZ SPHL|TGen North|French|USA
>Spike|hCoV-19/Scotland/QEUH-C33976/2020|2020-12-11|EPI_ISL_757169|Original|hCoV-19^^Scotland|Human|Lighthouse Lab in Glasgow|Wellcome Sanger Institute for the COVID-19 Genomics UK (COG-UK) Consortium|Davies|United Kingdom
>Spike|hCoV-19/Canada/MB-NML-1025/2020|2020-07-17|EPI_ISL_582390|Original|hCoV-19^^Manitoba|Human|Cadham Provincial Laboratory|National Microbiology Laboratory (NML)|Majer|Canada
>Spike|hCoV-19/USA/NY-PV09132/2020|2020-03-19|EPI_ISL_422505|Original|hCoV-19^^New York|Human|MSHS Clinical Microbiology Laboratories|MSHS Pathogen Surveillance Program|van Bakel|USA
>Spike|hCoV-19/Wales/PHWC-47D36B/2020|2020-10-07|EPI_ISL_586132|Original|hCoV-19^^Wales|Human|Wales Specialist Virology Centre Sequencing lab|COVID-19 Genomics UK (COG-UK) Consortium|Moore|United Kingdom
>Spike|hCoV-19/Denmark/DCGC-3066/2020|2020-08-31|EPI_ISL_616846|Original|hCoV-19^^Hovedstaden|Human|Department of Virus and Microbiological Special Diagnostics|Albertsen lab|Michaelsen|Denmark
>Spike|hCoV-19/Spain/RI-IBV-99005923/2020|2020-03-28|EPI_ISL_537460|Original|hCoV-19^^La Rioja|Human|Centro de Investigaci\u00f3n Biom\u00e9dica de La Rioja - Hospital San Pedro Logro\u00f1o|SeqCOVID-SPAIN consortium/IBV(CSIC)|Comas|Spain
>Spike|hCoV-19/Brazil/PR-L17-CD367/2020|2020-03-31|EPI_ISL_476313|Original|hCoV-19^^Paran\u00e1|Human|DB Diagn\u00f3sticos do Brasil|Instituto de Medicina Tropical da Univesidade de S\u00e3o Paulo|Sabino|Brazil
>Spike|hCoV-19/England/SHEF-CF79F/2020|2020-08-24|EPI_ISL_535089|Original|hCoV-19^^England|Human|Virology Department|COVID-19 Genomics UK (COG-UK) Consortium|de Silva|United Kingdom
>Spike|hCoV-19/England/ALDP-A51859/2020|2020-10-07|EPI_ISL_597995|Original|hCoV-19^^England|Human|Lighthouse Lab in Alderley Park|Wellcome Sanger Institute for the COVID-19 Genomics UK (COG-UK) consortium|Davies|United Kingdom
>Spike|hCoV-19/England/QEUH-C5465A/2020|2020-12-10|EPI_ISL_736459|Original|hCoV-19^^England|Human|Lighthouse Lab in Glasgow|Wellcome Sanger Institute for the COVID-19 Genomics UK (COG-UK) Consortium|Davies|United Kingdom

>Spike|hCoV-19/England/ALDP-95259E/2020|2020-06-09|EPI_ISL_553944|Original|hCoV-19^^England|Human|Lighthouse Lab in Alderley Park|Wellcome Sanger Institute for the COVID-19 Genomics UK (COG-UK) consortium|Davies|United Kingdom
>Spike|hCoV-19/Japan/PG-5228/2020|2020-08-00|EPI_ISL_690331|Original|hCoV-19^^Japan|Human|Pathogen Genomics Center|Pathogen Genomics Center|Sekizuka|Japan
>Spike|hCoV-19/USA/TX-HMH-MCoV-13238/2020|2020-08-25|EPI_ISL_788388|Original|hCoV-19^^Texas|Human|Houston Methodist Hospital|Houston Methodist Hospital|Long|USA
>Spike|hCoV-19/England/QEUH-B6796B/2020|2020-11-11|EPI_ISL_661623|Original|hCoV-19^^England|Human|Lighthouse Lab in Glasgow|Wellcome Sanger Institute for the COVID-19 Genomics UK (COG-UK) Consortium|Davies|United Kingdom
>Spike|hCoV-19/USA/PA-MGEL80/2020|2020-03-30|EPI_ISL_454365|Original|hCoV-19^^Pennsylvania|Human|UPMC Clinical Microbiology Laboratory|Microbial Genome Sequencing Center|Mustapha|USA
>Spike|hCoV-19/Netherlands/NH-EMC-74/2020|2020-09-02|EPI_ISL_632522|Original|hCoV-19^^Noord Holland|Human|Dutch COVID-19 response team|Erasmus Medical Center|Oude Munnink|Netherlands
>Spike|hCoV-19/Denmark/DCGC-14134/2020|2020-11-30|EPI_ISL_711187|Original|hCoV-19^^Midtjylland|Human|Department of Virus and Microbiological Special Diagnostics|Albertsen Lab|Michaelsen|Denmark
>Spike|hCoV-19/USA/TX-HMH-7378/2020|2020-06-29|EPI_ISL_546717|Original|hCoV-19^^Texas|Human|Houston Methodist Hospital|Houston Methodist Hospital|Long|USA
>Spike|hCoV-19/Northern Ireland/QEUH-96A9F1/2020|2020-08-17|EPI_ISL_531378|Original|hCoV-19^^Northern Ireland|Human|Lighthouse Lab in Glasgow|Wellcome Sanger Institute for the COVID-19 Genomics UK (COG-UK) consortium|Davies|United Kingdom
>Spike|hCoV-19/USA/CA-QDX-2485/2020|2020-03-15|EPI_ISL_604754|Original|hCoV-19^^California|Human|Quest Diagnostics|Quest Diagnostics|Kagan|USA
>Spike|hCoV-19/USA/UT-UPHL-2008976/2020|2020-05-15|EPI_ISL_525298|Original|hCoV-19^^Utah|Human|Utah Public Health Laboratory|Utah Public Health Laboratory|Young|USA
>Spike|hCoV-19/USA/MI-MDHHS-SC21931/2020|2020-09-26|EPI_ISL_576199|Original|hCoV-19^^Michigan|Human|Michigan Department of Health and Human Services|Michigan Department of Health and Human Services|Blankenship|USA
>Spike|hCoV-19/England/MILK-BAAC27/2020|2020-11-18|EPI_ISL_681499|Original|hCoV-19^^England|Human|Lighthouse Lab in Milton Keynes|Wellcome Sanger Institute for the COVID-19 Genomics UK (COG-UK) Consortium|Davies|United Kingdom
>Spike|hCoV-19/USA/MO-QDX-534/2020|2020-03-18|EPI_ISL_571385|Original|hCoV-19^^Missouri|Human|Quest Diagnostics|Quest Diagnostics|Kagan|USA
>Spike|hCoV-19/Scotland/QEUH-965774/2020|2020-08-14|EPI_ISL_530973|Original|hCoV-19^^Scotland|Human|NHSGGC West of Scotland Specialist Virology Centre / MRC-University of Glasgow Centre for Virus Research|Wellcome Sanger Institute for the COVID-19 Genomics UK (COG-UK) consortium|Davies|United Kingdom
>Spike|hCoV-19/France/ARA-67686/2020|2020-09-04|EPI_ISL_692765|Original|hCoV-19^^Auvergne-Rh\u00f4ne-Alpes|Human|CNR Virus des Infections Respiratoires - France SUD|CNR Virus des Infections Respiratoires - France SUD|Bal|France
>Spike|hCoV-19/Wales/PHWC-486A3D/2020|2020-10-21|EPI_ISL_638154|Original|hCoV-19^^Wales|Human|Wales Specialist Virology Centre Sequencing lab|COVID-19 Genomics UK (COG-UK) Consortium|Moore|United Kingdom
>Spike|hCoV-19/Egypt/CUNCI-HGC014/2020|2020-05-02|EPI_ISL_468060|Original|hCoV-19^^Egypt|Human|Egyptian National Cancer Institute (ENCI)|Egyptian National Cancer Institute (ENCI)|Zekri|Egypt
>Spike|hCoV-19/England/MILK-B38D94/2020|2020-11-01|EPI_ISL_709903|Original|hCoV-19^^England|Human|Lighthouse Lab in Milton Keynes|Wellcome Sanger Institute for the COVID-19 Genomics UK (COG-UK) Consortium|Davies|United Kingdom
>Spike|hCoV-19/Italy/APU-IZSPB_323PT/2020|2020-04-11|EPI_ISL_653816|Original|hCoV-19^^Apulia|Human|I.R.C.C.S. \S. De Bellis\ - Ente Ospedaliero|Istituto Zooprofilattico Sperimentale della Puglia e della Basilicata|Parisi|Italy
>Spike|hCoV-19/USA/NY-NYCPHL-001363/2020|2020-11-16|EPI_ISL_683927|Original|hCoV-19^^New York|Human|DOHMH Central Harlem|New York City Public Health Laboratory|Wang|USA

>Spike|hCoV-19/England/QEUH-BA3338/2020|2020-11-18|EPI_ISL_721151|Original|hCoV-19^^England|Human|Lighthouse Lab in Glasgow|Wellcome Sanger Institute for the COVID-19 Genomics UK (COG-UK) Consortium|Davies|United Kingdom
>Spike|hCoV-19/Japan/PG-6525/2020|2020-07-00|EPI_ISL_687639|Original|hCoV-19^^Japan|Human|Pathogen Genomics Center|Pathogen Genomics Center|Sekizuka|Japan
>Spike|hCoV-19/USA/CA-CHLA-PLM58754054/2020|2020-12-15|EPI_ISL_753673|Original|hCoV-19^^California|Human|Clinical virology Laboratory|Center for Personalized Medicine|Dien Bard|USA
>Spike|hCoV-19/Wales/PHWC-4A1B57/2020|2020-12-14|EPI_ISL_743782|Original|hCoV-19^^Wales|Human|Wales Specialist Virology Centre Sequencing lab|COVID-19 Genomics UK (COG-UK) Consortium|Moore|United Kingdom
>Spike|hCoV-19/USA/WI-UW-1886/2020|2020-09-16|EPI_ISL_605755|Original|hCoV-19^^Wisconsin|Human|University of Wisconsin-Madison AIDS Vaccine Research Laboratories|University of Wisconsin-Madison AIDS Vaccine Research Laboratories|Baczenas|USA
>Spike|hCoV-19/England/MILK-9C2D82/2020|2020-09-10|EPI_ISL_566630|Original|hCoV-19^^England|Human|Lighthouse Lab in Milton Keynes|Wellcome Sanger Institute for the COVID-19 Genomics UK (COG-UK) consortium|Davies|United Kingdom
>Spike|hCoV-19/Ecuador/USFQ-026/2020|2020-04-17|EPI_ISL_481244|Original|hCoV-19^^Los Rios|Human|Hospital IESS Babahoyo|Institute of Microbiology|Cardenas|Ecuador
>Spike|hCoV-19/England/LIVE-DBB31D/2020|2020-11-14|EPI_ISL_664633|Original|hCoV-19^^England|Human|Liverpool Clinical Laboratories|COVID-19 Genomics UK (COG-UK) Consortium|Haldenby|United Kingdom
>Spike|hCoV-19/Wales/PHWC-2DDF7/2020|2020-04-14|EPI_ISL_446025|Original|hCoV-19^^Wales|Human|Wales Specialist Virology Centre|Public Health Wales Microbiology Cardiff|Moore|United Kingdom
>Spike|hCoV-19/USA/MA-MGH-00774/2020|2020-04-12|EPI_ISL_791760|Original|hCoV-19^^Massachusetts|Human|Massachusetts General Hospital|Infectious Disease Program|Park|USA
>Spike|hCoV-19/England/EXET-139164/2020|2020-10-27|EPI_ISL_665055|Original|hCoV-19^^England|Human|University of Exeter|COVID-19 Genomics UK (COG-UK) Consortium|Temperton|United Kingdom
>Spike|hCoV-19/Denmark/DCGC-5817/2020|2020-09-28|EPI_ISL_621004|Original|hCoV-19^^Hovedstaden|Human|Department of Virus and Microbiological Special Diagnostics|Albertsen lab|Michaelsen|Denmark
>Spike|hCoV-19/Switzerland/GE-ETHZ-300439/2020|2020-09-29|EPI_ISL_603392|Original|hCoV-19^^Geneva|Human|Viollier AG|Department of Biosystems Science and Engineering|Nadeau|Switzerland
>Spike|hCoV-19/England/201121198/2020|2020-03-08|EPI_ISL_464689|Original|hCoV-19^^England|Human|Respiratory Virus Unit|Respiratory Virus Unit|Platt|United Kingdom
>Spike|hCoV-19/England/LOND-DAED8/2020|2020-11-14|EPI_ISL_724726|Original|hCoV-19^^England|Human|University College London|COVID-19 Genomics UK (COG-UK) Consortium|Roy|United Kingdom
>Spike|hCoV-19/England/NOTT-11C8F9/2020|2020-11-17|EPI_ISL_665550|Original|hCoV-19^^England|Human|Queens Medical Centre|COVID-19 Genomics UK (COG-UK) Consortium|Loose|United Kingdom
>Spike|hCoV-19/Denmark/DCGC-4715/2020|2020-09-21|EPI_ISL_622154|Original|hCoV-19^^Syddanmark|Human|Department of Virus and Microbiological Special Diagnostics|Albertsen lab|Michaelsen|Denmark
>Spike|hCoV-19/USA/TX-HMH-MCoV-10596/2020|2020-07-08|EPI_ISL_789553|Original|hCoV-19^^Texas|Human|Houston Methodist Hospital|Houston Methodist Hospital|Long|USA
>Spike|hCoV-19/USA/TX-DSHS-0124/2020|2020-06-11|EPI_ISL_522951|Original|hCoV-19^^Texas|Human|Texas Department of State Health Services|Texas Department of State Health Services|Rahman|USA
>Spike|hCoV-19/Denmark/ALAB-SSI111/2020|2020-03-09|EPI_ISL_429343|Original|hCoV-19^^Denmark|Human|Department of Virus and Microbiological Special Diagnostics|Albertsen lab|Kirkegaard|Denmark
>Spike|hCoV-19/England/SHEF-CFC54/2020|2020-04-27|EPI_ISL_490633|Original|hCoV-19^^England|Human|Virology Department|COVID-19 Genomics UK (COG-UK) Consortium|de Silva|United Kingdom

>Spike|hCoV-19/Denmark/DCGC-6646/2020|2020-10-12|EPI_ISL_619548|Original|hCoV-19^^Midtjylland|Human|Department of Virus and Microbiological Special Diagnostics|Albertsen lab|Michaelsen|Denmark
>Spike|hCoV-19/Scotland/EDB4746/2020|2020-05-11|EPI_ISL_453146|Original|hCoV-19^^Scotland|Human|Virology Department|COVID-19 Genomics UK (COG-UK) Consortium|Williams|United Kingdom
>Spike|hCoV-19/Denmark/DCGC-15574/2020|2020-11-23|EPI_ISL_713903|Original|hCoV-19^^Hovedstaden|Human|Department of Virus and Microbiological Special Diagnostics|Albertsen Lab|Michaelsen|Denmark
>Spike|hCoV-19/Luxembourg/LNS9953002/2020|2020-11-05|EPI_ISL_745018|Original|hCoV-19^^Luxembourg|Human|Laboratoire national de sant\u00e9|Laboratoire national de sant\u00e9|Wienecke-Baldacchino|Luxembourg
>Spike|hCoV-19/England/BRIS-125FF8/2020|2020-04-09|EPI_ISL_459035|Original|hCoV-19^^England|Human|PHE South West Regional Laboratory|Wellcome Sanger Institute for the COVID-19 Genomics UK (COG-UK) consortium|Davies|United Kingdom
>Spike|hCoV-19/Switzerland/GE-ETHZ-350206/2020|2020-10-30|EPI_ISL_693854|Original|hCoV-19^^Geneva|Human|Viollier AG|Department of Biosystems Science and Engineering|Nadeau|Switzerland
>Spike|hCoV-19/Australia/VIC991/2020|2020-03-31|EPI_ISL_430474|Original|hCoV-19^^Victoria|Human|Victorian Infectious Diseases Reference Laboratory (VIDRL)|Microbiological Diagnostic Unit Public Health Laboratory and Victorian Infectious Diseases Reference Laboratory|Schultz|Australia
>Spike|hCoV-19/France/NAQ-SC231/2020|2020-05-01|EPI_ISL_645172|Original|hCoV-19^^Nouvelle-Aquitaine|Human|CHU Bordeaux|CNR Virus des Infections Respiratoires - France SUD|Bal|France
>Spike|hCoV-19/England/ALDP-B5C9A5/2020|2020-11-08|EPI_ISL_655969|Original|hCoV-19^^England|Human|Lighthouse Lab in Alderley Park|Wellcome Sanger Institute for the COVID-19 Genomics UK (COG-UK) Consortium|Davies|United Kingdom
>Spike|hCoV-19/England/ALDP-9BC6F9/2020|2020-09-09|EPI_ISL_568188|Original|hCoV-19^^England|Human|Lighthouse Lab in Alderley Park|Wellcome Sanger Institute for the COVID-19 Genomics UK (COG-UK) consortium|Davies|United Kingdom
>Spike|hCoV-19/USA/NV-NSHL-00317251/2020|2020-12-28|EPI_ISL_766938|Original|hCoV-19^^Nevada|Human|Nevada State Health Lab|Nevada State Health Lab|Gorzalski|USA
>Spike|hCoV-19/USA/UN-UW-672/2020|2020-03-12|EPI_ISL_476926|Original|hCoV-19^^USA|Human|UW Virology Lab|UW Virology Lab|Roychoudhury|USA
>Spike|hCoV-19/England/CAMC-AEA64C/2020|2020-10-25|EPI_ISL_633344|Original|hCoV-19^^England|Human|Lighthouse Lab in Cambridge|Wellcome Sanger Institute for the COVID-19 Genomics UK (COG-UK) consortium|Davies|United Kingdom
>Spike|hCoV-19/USA/FL-BPHL-0844/2020|2020-06-22|EPI_ISL_514190|Original|hCoV-19^^Florida|Human|Florida Bureau of Public Health Laboratories|Florida Bureau of Public Health Laboratories|Blanton|USA
>Spike|hCoV-19/Scotland/QEUH-9B4E44/2020|2020-09-07|EPI_ISL_602073|Original|hCoV-19^^Scotland|Human|Lighthouse Lab in Glasgow|Wellcome Sanger Institute for the COVID-19 Genomics UK (COG-UK) consortium|Davies|United Kingdom
>Spike|hCoV-19/Australia/VIC11599/2020|2020-07-18|EPI_ISL_640835|Original|hCoV-19^^Victoria|Human|Microbiological Diagnostic Unit - Public Health Laboratory (MDU-PHL)|MDU-PHL|Schultz|Australia
>Spike|hCoV-19/USA/CA-CZB-14381/2020|2020-07-07|EPI_ISL_738659|Original|hCoV-19^^California|Human|Alameda County Public Health Lab|Chan-Zuckerberg Biohub|Kamm|USA
>Spike|hCoV-19/Wales/PHWC-15CEFB/2020|2020-05-09|EPI_ISL_472638|Original|hCoV-19^^Wales|Human|Wales Specialist Virology Centre Sequencing lab|COVID-19 Genomics UK (COG-UK) Consortium|Moore|United Kingdom
>Spike|hCoV-19/England/QEUH-9B456D/2020|2020-09-04|EPI_ISL_590404|Original|hCoV-19^^England|Human|Lighthouse Lab in Glasgow|Wellcome Sanger Institute for the COVID-19 Genomics UK (COG-UK) consortium|Davies|United Kingdom
>Spike|hCoV-19/England/NORT-2938DB/2020|2020-07-06|EPI_ISL_499610|Original|hCoV-19^^England|Human|Northumbria University / South Tees Hospitals NHS Foundation Trust / North Cumbria Integrated Care NHS Foundation Trust / North Tees and Hartlepool NHS Foundation Trust / Newcastle Hospitals NHS Foundation Trust|COVID-19 Genomics UK (COG-UK) Consortium|Bashton|United Kingdom

>Spike|hCoV-19/Wales/PHWC-48015C/2020|2020-10-17|EPI_ISL_611637|Original|hCoV-
19^^Wales|Human|Wales Specialist Virology Centre Sequencing lab|COVID-19 Genomics UK
(COG-UK) Consortium|Moore|United Kingdom
>Spike|hCoV-19/USA/TX-HMH-4659/2020|2020-06-22|EPI_ISL_543785|Original|hCoV-
19^^Texas|Human|Houston Methodist Hospital|Houston Methodist Hospital|Long|USA
>Spike|hCoV-19/Japan/PG-2152/2020|2020-04-00|EPI_ISL_685757|Original|hCoV-
19^^Japan|Human|Pathogen Genomics Center|Pathogen Genomics Center|Sekizuka|Japan
>Spike|hCoV-19/England/BRIS-1309A1/2020|2020-03-12|EPI_ISL_492631|Original|hCoV-
19^^England|Human|PHE South West Regional Laboratory|Wellcome Sanger Institute for
the COVID-19 Genomics UK (COG-UK) consortium|Davies|United Kingdom
>Spike|hCoV-19/Wales/PHWC-16C9F7/2020|2020-09-24|EPI_ISL_574119|Original|hCoV-
19^^Wales|Human|Wales Specialist Virology Centre Sequencing lab|COVID-19 Genomics UK
(COG-UK) Consortium|Moore|United Kingdom
>Spike|hCoV-19/Wales/PHWC-16A13D/2020|2020-09-06|EPI_ISL_535205|Original|hCoV-
19^^Wales|Human|Wales Specialist Virology Centre Sequencing lab|COVID-19 Genomics UK
(COG-UK) Consortium|Moore|United Kingdom
>Spike|hCoV-19/Wales/PHWC-48538E/2020|2020-10-24|EPI_ISL_628174|Original|hCoV-
19^^Wales|Human|Wales Specialist Virology Centre Sequencing lab|COVID-19 Genomics UK
(COG-UK) Consortium|Moore|United Kingdom
>Spike|hCoV-19/Netherlands/NH-RIVM-20628/2020|2020-12-
18|EPI_ISL_790632|Original|hCoV-19^^Noord-Holland|Human|Dutch COVID-19 response
team|National Institute for Public Health and the Environment
(RIVM)|Meijer|Netherlands
>Spike|hCoV-19/England/MILK-A44DF0/2020|2020-10-06|EPI_ISL_598695|Original|hCoV-
19^^England|Human|Lighthouse Lab in Milton Keynes|Wellcome Sanger Institute for the
COVID-19 Genomics UK (COG-UK) consortium|Davies|United Kingdom
>Spike|hCoV-19/USA/MI-MDHHS-SC22235/2020|2020-10-29|EPI_ISL_666901|Original|hCoV-
19^^Michigan|Human|Michigan Department of Health and Human Services|Michigan
Department of Health and Human Services|Jacob|USA
>Spike|hCoV-19/England/QEUH-9993CE/2020|2020-08-24|EPI_ISL_541835|Original|hCoV-
19^^England|Human|Lighthouse Lab in Glasgow|Wellcome Sanger Institute for the COVID-
19 Genomics UK (COG-UK) consortium|Davies|United Kingdom
>Spike|hCoV-19/England/QEUH-B2F5B6/2020|2020-11-04|EPI_ISL_659809|Original|hCoV-
19^^England|Human|Lighthouse Lab in Glasgow|Wellcome Sanger Institute for the COVID-
19 Genomics UK (COG-UK) Consortium|Davies|United Kingdom
>Spike|hCoV-19/Australia/VIC15012/2020|2020-07-29|EPI_ISL_593102|Original|hCoV-
19^^Victoria|Human|Microbiological Diagnostic Unit - Public Health Laboratory (MDU-
PHL)|MDU-PHL|Schultz|Australia
>Spike|hCoV-19/England/ALDP-C3D781/2020|2020-12-10|EPI_ISL_731794|Original|hCoV-
19^^England|Human|Lighthouse Lab in Alderley Park|Wellcome Sanger Institute for the
COVID-19 Genomics UK (COG-UK) Consortium|Davies|United Kingdom
>Spike|hCoV-19/Scotland/QEUH-A9E137/2020|2020-10-20|EPI_ISL_727798|Original|hCoV-
19^^Scotland|Human|Centre for Enzyme Innovation|COVID-19 Genomics UK (COG-UK)
Consortium|Robson|United Kingdom
>Spike|hCoV-19/England/20102000704/2020|2020-03-02|EPI_ISL_464262|Original|hCoV-
19^^England|Human|Respiratory Virus Unit|Respiratory Virus Unit|Platt|United Kingdom
>Spike|hCoV-19/Spain/AR-IBV-98010561/2020|2020-10-04|EPI_ISL_722230|Original|hCoV-
19^^Aragon|Human|Servicio de Microbiolog\u00eda|SeqCOVID-SPAIN
consortium/IBV(CSIC)|Comas|Spain
>Spike|hCoV-19/England/CAMC-B34017/2020|2020-11-05|EPI_ISL_659706|Original|hCoV-
19^^England|Human|Lighthouse Lab in Cambridge|Wellcome Sanger Institute for the
COVID-19 Genomics UK (COG-UK) Consortium|Davies|United Kingdom
>Spike|hCoV-19/USA/UT-UPHL-201000304/2020|2020-07-06|EPI_ISL_594043|Original|hCoV-
19^^Utah|Human|Utah Public Health Laboratory|Utah Public Health Laboratory|Young|USA
>Spike|hCoV-19/England/20186120704/2020|2020-04-28|EPI_ISL_465486|Original|hCoV-
19^^England|Human|Respiratory Virus Unit|Respiratory Virus Unit|Platt|United Kingdom
>Spike|hCoV-19/Poland/PL_P44/2020|2020-04-02|EPI_ISL_492069|Original|hCoV-
19^^Mazowieckie|Human|1. ViroGenetics - BSL3 Laboratory of Virology|1. ViroGenetics -
BSL3 Laboratory of Virology|Pyrc|Poland

>Spike|hCoV-19/USA/MS-CDC-6561/2020|2020-09-03|EPI_ISL_648013|Original|hCoV-19^^Mississippi|Human|MS Public Health Laboratory|Pathogen Discovery|Queen|USA
>Spike|hCoV-19/Brazil/PE-IAM355/2020|2020-04-13|EPI_ISL_500874|Original|hCoV-19^^Pernambuco|Human|LACEN/PE|WallauLab|Wallau|Brazil
>Spike|hCoV-19/Denmark/DCGC-16681/2020|2020-11-09|EPI_ISL_714911|Original|hCoV-19^^Denmark|Human|Department of Virus and Microbiological Special Diagnostics|Albertsen Lab|Michaelsen|Denmark
>Spike|hCoV-19/Northern Ireland/NIRE-FB949/2020|2020-09-01|EPI_ISL_585253|Original|hCoV-19^^Northern Ireland|Human|Regional Virus Laboratory|COVID-19 Genomics UK (COG-UK) Consortium|Fairley|United Kingdom
>Spike|hCoV-19/Australia/VIC4937/2020|2020-07-13|EPI_ISL_519311|Original|hCoV-19^^Victoria|Human|Victorian Infectious Diseases Reference Laboratory (VIDRL)|VIDRL and MDU-PHL|Schultz|Australia
>Spike|hCoV-19/Denmark/DCGC-8296/2020|2020-10-26|EPI_ISL_617321|Original|hCoV-19^^Syddanmark|Human|Department of Virus and Microbiological Special Diagnostics|Albertsen lab|Michaelsen|Denmark
>Spike|hCoV-19/Wales/PHWC-4A39A6/2020|2020-12-16|EPI_ISL_763494|Original|hCoV-19^^Wales|Human|Wales Specialist Virology Centre Sequencing lab|COVID-19 Genomics UK (COG-UK) Consortium|Moore|United Kingdom
>Spike|hCoV-19/Denmark/DCGC-4914/2020|2020-09-21|EPI_ISL_621452|Original|hCoV-19^^Hovedstaden|Human|Department of Virus and Microbiological Special Diagnostics|Albertsen lab|Michaelsen|Denmark
>Spike|hCoV-19/USA/NY-NYUMC472/2020|2020-04-20|EPI_ISL_435537|Original|hCoV-19^^New York|Human|NYU Langone Health|Departments of Pathology and Medicine|Maurano|USA
>Spike|hCoV-19/Spain/VC-FISABIO-187/2020|2020-03-25|EPI_ISL_436282|Original|hCoV-19^^Comunitat Valenciana|Human|Servicio de Microbiologia. Hospital Clinico Universitario de Valencia|Sequencing and Bioinformatics Service and Molecular Epidemiology Research Group. FISABIO-Public Health|Gonzalez-Candelas|Spain
>Spike|hCoV-19/USA/TX-HMH-1111/2020|2020-04-19|EPI_ISL_542537|Original|hCoV-19^^Texas|Human|Houston Methodist Hospital|Houston Methodist Hospital|Long|USA
>Spike|hCoV-19/England/ALDP-AA5689/2020|2020-10-22|EPI_ISL_623951|Original|hCoV-19^^England|Human|Lighthouse Lab in Alderley Park|Wellcome Sanger Institute for the COVID-19 Genomics UK (COG-UK) consortium|Davies|United Kingdom
>Spike|hCoV-19/USA/WA-S2886/2020|2020-09-29|EPI_ISL_596417|Original|hCoV-19^^Washington|Human|Seattle Flu Study|Seattle Flu Study|Lee|USA
>Spike|hCoV-19/England/NORT-29EC89/2020|2020-05-10|EPI_ISL_490480|Original|hCoV-19^^England|Human|Northumbria University / South Tees Hospitals NHS Foundation Trust / North Cumbria Integrated Care NHS Foundation Trust / North Tees and Hartlepool NHS Foundation Trust / Newcastle Hospitals NHS Foundation Trust|COVID-19 Genomics UK (COG-UK) Consortium|Bashton|United Kingdom
>Spike|hCoV-19/France/IDF-10017GS/2020|2020-03-00|EPI_ISL_447710|Original|hCoV-19^^France|Human|H\u00f4pital Henri-Mondor Ap-Hp|H\u00f4pital Henri-Mondor Ap-Hp|Rodriguez|France
>Spike|hCoV-19/England/20140002504/2020|2020-03-26|EPI_ISL_465869|Original|hCoV-19^^England|Human|Respiratory Virus Unit|Respiratory Virus Unit|Platt|United Kingdom
>Spike|hCoV-19/England/NORT-2A7FEE/2020|2020-10-10|EPI_ISL_612569|Original|hCoV-19^^England|Human|Northumbria University / South Tees Hospitals NHS Foundation Trust / North Cumbria Integrated Care NHS Foundation Trust / North Tees and Hartlepool NHS Foundation Trust / Newcastle Hospitals NHS Foundation Trust|COVID-19 Genomics UK (COG-UK) Consortium|Bashton|United Kingdom
>Spike|hCoV-19/England/CAMB-75859/2020|2020-03-19|EPI_ISL_440354|Original|hCoV-19^^England|Human|Department of Pathology|Wellcome Sanger Institute for the COVID-19 Genomics UK (COG-UK) consortium|Amato|United Kingdom
>Spike|hCoV-19/Brazil/SP-L5-CD25/2020|2020-03-08|EPI_ISL_476427|Original|hCoV-19^^S\u00e3o Paulo|Human|Laboratory Fleury|Instituto de Medicina Tropical da Univesidade de S\u00e3o Paulo|Sabino|Brazil
>Spike|hCoV-19/Switzerland/ZH-ETHZ-280045/2020|2020-09-05|EPI_ISL_560541|Original|hCoV-19^^Zurich|Human|Viollier AG|Department of Biosystems Science and Engineering|Nadeau|Switzerland

>Spike|hCoV-19/Denmark/DCGC-21962/2020|2020-12-21|EPI_ISL_757801|Original|hCoV-19^^Hovedstaden|Human|Department of Virus and Microbiological Special Diagnostics|Albertsen Lab|Michaelsen|Denmark
>Spike|hCoV-19/Germany/BE-ChVir-W1718-6763/2020|2020-08-19|EPI_ISL_753810|Original|hCoV-19^^Berlin|Human|Charit\u00e9 Universit\u00e4tsmedizin Berlin|Charit\u00e9 Universit\u00e4tsmedizin Berlin|Muehlemann|Germany
>Spike|hCoV-19/New Zealand/20CV0061/2020|2020-08-13|EPI_ISL_547975|Original|hCoV-19^^Combined Auckland|Human|LabTests|Institute of Environmental Science and Research (ESR)|Wang|New Zealand
>Spike|hCoV-19/Scotland/CVR5318/2020|2020-10-23|EPI_ISL_650725|Original|hCoV-19^^Scotland|Human|West of Scotland Specialist Virology Centre|COVID-19 Genomics UK (COG-UK) Consortium|Robertson|United Kingdom
>Spike|hCoV-19/USA/MI-UM-10036463292/2020|2020-10-15|EPI_ISL_628823|Original|hCoV-19^^Michigan|Human|University of Michigan Clinical Microbiology Laboratory|Lauring Lab|Valesano|USA
>Spike|hCoV-19/USA/WA-UW-687/2020|2020-03-13|EPI_ISL_476933|Original|hCoV-19^^Washington|Human|UW Virology Lab|UW Virology Lab|Roychoudhury|USA
>Spike|hCoV-19/England/CAMB-1B68F1/2020|2020-10-07|EPI_ISL_584314|Original|hCoV-19^^England|Human|Department of Pathology|COVID-19 Genomics UK (COG-UK) Consortium|Goodfellow|United Kingdom
>Spike|hCoV-19/England/CAMB-80A64/2020|2020-04-10|EPI_ISL_470421|Original|hCoV-19^^England|Human|Department of Pathology|Wellcome Sanger Institute for the COVID-19 Genomics UK (COG-UK) consortium|Davies|United Kingdom
>Spike|hCoV-19/India/OR-ILSCV29960/2020|2020-05-23|EPI_ISL_481133|Original|hCoV-19^^Odisha|Human|Immunogenomics lab|Immunogenomics lab|Raghav|India
>Spike|hCoV-19/India/DL-NCDC3721-CSIR-IGIB/2020|2020-05-20|EPI_ISL_482566|Original|hCoV-19^^Delhi|Human|National Centre for Disease control (NCDC)|NCDC/CSIR-IGIB|Pandey|India
>Spike|hCoV-19/USA/TX-HMH-2814/2020|2020-06-08|EPI_ISL_543790|Original|hCoV-19^^Texas|Human|Houston Methodist Hospital|Houston Methodist Hospital|Long|USA
>Spike|hCoV-19/England/CAMC-C45B8A/2020|2020-12-10|EPI_ISL_718436|Original|hCoV-19^^England|Human|Lighthouse Lab in Cambridge|Wellcome Sanger Institute for the COVID-19 Genomics UK (COG-UK) Consortium|Davies|United Kingdom
>Spike|hCoV-19/Ireland/D-NVRL-73IRL37614/2020|2020-09-08|EPI_ISL_596903|Original|hCoV-19^^Dublin|Human|National Virus Reference Laboratory|National Virus Reference Laboratory|Gonzalez|Ireland
>Spike|hCoV-19/Spain/CM-ISCIII-202018271/2020|2020-03-22|EPI_ISL_539528|Original|hCoV-19^^Castilla La Mancha|Human|Consejeria de Sanidad y Asuntos Sociales|Instituto de Salud Carlos III|Caballero|Spain
>Spike|hCoV-19/Australia/NSW757/2020|2020-07-19|EPI_ISL_513399|Original|hCoV-19^^New South Wales|Human|St Vincent's Pathology (SydPath)|NSW Health Pathology - Institute of Clinical Pathology and Medical Research; Westmead Hospital; University of Sydney|Gall|Australia
>Spike|hCoV-19/England/QEUH-B40203/2020|2020-11-08|EPI_ISL_657227|Original|hCoV-19^^England|Human|Lighthouse Lab in Glasgow|Wellcome Sanger Institute for the COVID-19 Genomics UK (COG-UK) Consortium|Davies|United Kingdom
>Spike|hCoV-19/England/LIVE-AA008/2020|2020-03-23|EPI_ISL_517388|Original|hCoV-19^^England|Human|Liverpool Clinical Laboratories|COVID-19 Genomics UK (COG-UK) Consortium|Haldenby|United Kingdom
>Spike|hCoV-19/Belgium/KWA-0323410/2020|2020-03-23|EPI_ISL_462264|Original|hCoV-19^^Sint-Niklaas|Human|KU Leuven|KU Leuven|Maes|Belgium
>Spike|hCoV-19/Wales/PHWC-2C04A/2020|2020-04-04|EPI_ISL_474268|Original|hCoV-19^^Wales|Human|Originating lab|COVID-19 Genomics UK (COG-UK) Consortium|Moore|United Kingdom
>Spike|hCoV-19/USA/WA-S468/2020|2020-04-05|EPI_ISL_434228|Original|hCoV-19^^Washington|Human|Washington State Department of Health|Seattle Flu Study|Bedford|USA

>Spike|hCoV-19/Spain/IB-IBV-99011574/2020|2020-12-23|EPI_ISL_779902|Original|hCoV-19^^Balear Islands|Human|Servicio de Microbiolog\u00eda|SeqCOVID-SPAIN consortium/IBV(CSIC)|Comas|Spain
>Spike|hCoV-19/Japan/PG-5440/2020|2020-08-00|EPI_ISL_690543|Original|hCoV-19^^Japan|Human|Pathogen Genomics Center|Pathogen Genomics Center|Sekizuka|Japan
>Spike|hCoV-19/Sweden/20-53867/2020|2020-12-14|EPI_ISL_789043|Original|hCoV-19^^Uppsala|Human|Klinisk mikrobiologi|The Public Health Agency of Sweden|Svartstr\u00f6m|Sweden
>Spike|hCoV-19/Denmark/DCGC-17693/2020|2020-12-14|EPI_ISL_751150|Original|hCoV-19^^Hovedstaden|Human|Department of Virus and Microbiological Special Diagnostics|Albertsen Lab|Michaelsen|Denmark
>Spike|hCoV-19/USA/UT-UPHL-201014927/2020|2020-06-22|EPI_ISL_594258|Original|hCoV-19^^Utah|Human|Utah Public Health Laboratory|Utah Public Health Laboratory|Young|USA
>Spike|hCoV-19/Scotland/CVR1442/2020|2020-04-02|EPI_ISL_438811|Original|hCoV-19^^Scotland|Human|West of Scotland Specialist Virology Centre|COVID-19 Genomics UK (COG-UK) Consortium|Robertson|United Kingdom
>Spike|hCoV-19/USA/TX-HMH-MCoV-14214/2020|2020-10-04|EPI_ISL_787499|Original|hCoV-19^^Texas|Human|Houston Methodist Hospital|Houston Methodist Hospital|Long|USA
>Spike|hCoV-19/England/20138020504/2020|2020-03-25|EPI_ISL_423298|Original|hCoV-19^^England|Human|Respiratory Virus Unit|Respiratory Virus Unit|Platt|United Kingdom
>Spike|hCoV-19/England/SHEF-10B2A39/2020|2020-05-03|EPI_ISL_537212|Original|hCoV-19^^England|Human|Virology Department|Wellcome Sanger Institute for the COVID-19 Genomics UK (COG-UK) consortium|Davies|United Kingdom
>Spike|hCoV-19/USA/CA-LACPHL-AF00046/2020|2020-12-20|EPI_ISL_765993|Original|hCoV-19^^California|Human|UCLA Clinical Micro Lab|Los Angeles County PHL|Hemarajata|USA
>Spike|hCoV-19/Australia/VIC16369/2020|2020-08-03|EPI_ISL_640521|Original|hCoV-19^^Victoria|Human|Microbiological Diagnostic Unit - Public Health Laboratory (MDU-PHL)|MDU-PHL|Schultz|Australia
>Spike|hCoV-19/England/BRIS-130112/2020|2020-04-11|EPI_ISL_492579|Original|hCoV-19^^England|Human|PHE South West Regional Laboratory|Wellcome Sanger Institute for the COVID-19 Genomics UK (COG-UK) consortium|Davies|United Kingdom
>Spike|hCoV-19/USA/WA-UW-9255/2020|2020-05-15|EPI_ISL_570240|Original|hCoV-19^^Washington|Human|UW Virology Lab|UW Virology Lab|Roychoudhury|USA
>Spike|hCoV-19/USA/WA-S2355/2020|2020-06-12|EPI_ISL_496987|Original|hCoV-19^^Washington|Human|Washington State Department of Health|Seattle Flu Study|Lee|USA
>Spike|hCoV-19/England/NORT-2964F2/2020|2020-04-12|EPI_ISL_488043|Original|hCoV-19^^England|Human|NU-OMICS DNA Sequencing research facility|Wellcome Sanger Institute for the COVID-19 Genomics UK (COG-UK) consortium|Davies|United Kingdom
>Spike|hCoV-19/England/QEUH-A4D2CB/2020|2020-10-05|EPI_ISL_596107|Original|hCoV-19^^England|Human|Quadram Institute Bioscience|COVID-19 Genomics UK (COG-UK) Consortium|Alikhan|United Kingdom
>Spike|hCoV-19/South Africa/NHLS-UCT-GS-0023/2020|2020-08-13|EPI_ISL_640024|Original|hCoV-19^^Western Cape|Human|Dysselsdorp Clinic wc DDC|NHLS/UCT|Iranzadeh|South Africa
>Spike|hCoV-19/Scotland/QEUH-A0BC61/2020|2020-10-03|EPI_ISL_599384|Original|hCoV-19^^Scotland|Human|Lighthouse Lab in Glasgow|Wellcome Sanger Institute for the COVID-19 Genomics UK (COG-UK) consortium|Davies|United Kingdom
>Spike|hCoV-19/Kenya/C247/2020|2020-04-04|EPI_ISL_568696|Original|hCoV-19^^Mombasa|Human|KEMRI-Wellcome Trust Research Programme/KEMRI-CGMR-C Kilifi|KEMRI-Wellcome Trust Research Programme/KEMRI-CGMR-C Kilifi|Githinji|Kenya
>Spike|hCoV-19/England/ALDP-A6C572/2020|2020-10-15|EPI_ISL_608046|Original|hCoV-19^^England|Human|Lighthouse Lab in Alderley Park|Wellcome Sanger Institute for the COVID-19 Genomics UK (COG-UK) consortium|Davies|United Kingdom
>Spike|hCoV-19/Wales/ALDP-B1A27B/2020|2020-11-01|EPI_ISL_710915|Original|hCoV-19^^Wales|Human|Lighthouse Lab in Alderley Park|Wellcome Sanger Institute for the COVID-19 Genomics UK (COG-UK) Consortium|Davies|United Kingdom
>Spike|hCoV-19/Japan/PG-3127/2020|2020-04-00|EPI_ISL_691249|Original|hCoV-19^^Japan|Human|Pathogen Genomics Center|Pathogen Genomics Center|Sekizuka|Japan

>Spike|hCoV-19/USA/VA-DCLS-2403/2020|2020-11-16|EPI_ISL_734174|Original|hCoV-19^^Virginia|Human|Virginia Division of Consolidated Laboratory Services (DCLS)|Virginia Division of Consolidated Laboratory Services (DCLS)|St. Jacques|USA
>Spike|hCoV-19/USA/CO-QDX-2401/2020|2020-03-16|EPI_ISL_604690|Original|hCoV-19^^Colorado|Human|Quest Diagnostics|Quest Diagnostics|Kagan|USA
>Spike|hCoV-19/Peru/LIM-INS-120/2020|2020-05-05|EPI_ISL_536518|Original|hCoV-19^^Lima|Human|Instituto Nacional de Salud|Laboratorio de Infecciones Respiratorias Agudas|Lopez|Peru
>Spike|hCoV-19/Australia/VIC2817/2020|2020-07-01|EPI_ISL_521648|Original|hCoV-19^^Victoria|Human|Microbiological Diagnostic Unit - Public Health Laboratory (MDU-PHL)|MDU-PHL|Schultz|Australia
>Spike|hCoV-19/England/BRIS-1281A4/2020|2020-04-16|EPI_ISL_469740|Original|hCoV-19^^England|Human|PHE South West Regional Laboratory|Wellcome Sanger Institute for the COVID-19 Genomics UK (COG-UK) consortium|Davies|United Kingdom
>Spike|hCoV-19/England/QEUH-B31B46/2020|2020-11-04|EPI_ISL_646239|Original|hCoV-19^^England|Human|Lighthouse Lab in Glasgow|Wellcome Sanger Institute for the COVID-19 Genomics UK (COG-UK) Consortium|Davies|United Kingdom
>Spike|hCoV-19/England/QEUH-B0DEE0/2020|2020-11-02|EPI_ISL_641972|Original|hCoV-19^^England|Human|Lighthouse Lab in Glasgow|Wellcome Sanger Institute for the COVID-19 Genomics UK (COG-UK) Consortium|Davies|United Kingdom
>Spike|hCoV-19/England/ALDP-59A8C2/2020|2020-06-15|EPI_ISL_558345|Original|hCoV-19^^England|Human|Lighthouse Lab in Alderley Park|Wellcome Sanger Institute for the COVID-19 Genomics UK (COG-UK) consortium|Davies|United Kingdom
>Spike|hCoV-19/England/QEUH-9E4F75/2020|2020-09-22|EPI_ISL_579766|Original|hCoV-19^^England|Human|Lighthouse Lab in Glasgow|Wellcome Sanger Institute for the COVID-19 Genomics UK (COG-UK) consortium|Davies|United Kingdom
>Spike|hCoV-19/Luxembourg/LNS5507695/2020|2020-05-15|EPI_ISL_459899|Original|hCoV-19^^Luxembourg|Human|Laboratoire National de Sante|Laboratoire National de Sante|Wienecke-Baldacchino|Luxembourg
>Spike|hCoV-19/USA/WI-UW-835/2020|2020-05-22|EPI_ISL_509842|Original|hCoV-19^^Wisconsin|Human|University of Wisconsin-Madison AIDS Vaccine Research Laboratories|University of Wisconsin-Madison AIDS Vaccine Research Laboratories|Baczenas|USA
>Spike|hCoV-19/USA/MN-CDC-6361/2020|2020-05-21|EPI_ISL_527708|Original|hCoV-19^^Minnesota|Human|MN PHL Division|Pathogen Discovery|Queen|USA
>Spike|hCoV-19/England/QEUH-B2DDA1/2020|2020-11-03|EPI_ISL_646284|Original|hCoV-19^^England|Human|Lighthouse Lab in Glasgow|Wellcome Sanger Institute for the COVID-19 Genomics UK (COG-UK) Consortium|Davies|United Kingdom
>Spike|hCoV-19/England/ALDP-A92ED2/2020|2020-10-20|EPI_ISL_611194|Original|hCoV-19^^England|Human|Lighthouse Lab in Alderley Park|Wellcome Sanger Institute for the COVID-19 Genomics UK (COG-UK) consortium|Davies|United Kingdom
>Spike|hCoV-19/USA/CA-ALSR-4399/2020|2020-10-26|EPI_ISL_649007|Original|hCoV-19^^California|Human|San Diego County Public Health Laboratory|Andersen lab at Scripps Research|Gangavarapu|USA
>Spike|hCoV-19/USA/LA-EVTL275/2020|2020-08-26|EPI_ISL_574409|Original|hCoV-19^^Louisiana|Human|LSUHS Emerging Viral Threat Laboratory|Microbial Genome Sequencing Center|Kamil|USA
>Spike|hCoV-19/USA/TX-HMH-MCoV-19755/2020|2020-11-20|EPI_ISL_783566|Original|hCoV-19^^Texas|Human|Houston Methodist Hospital|Houston Methodist Hospital|Long|USA
>Spike|hCoV-19/Canada/BC_11656063/2020|2020-04-00|EPI_ISL_467327|Original|hCoV-19^^British Columbia|Human|BCCDC Public Health Laboratory|BCCDC Public Health Laboratory|Harrigan|Canada
>Spike|hCoV-19/England/ALDP-B1AF65/2020|2020-11-01|EPI_ISL_645531|Original|hCoV-19^^England|Human|Lighthouse Lab in Alderley Park|Wellcome Sanger Institute for the COVID-19 Genomics UK (COG-UK) Consortium|Davies|United Kingdom
>Spike|hCoV-19/France/PAC-IHU-0229/2020|2020-03-29|EPI_ISL_569392|Original|hCoV-19^^Provence-Alpes-C\u00f4te-d\u2019Azur|Human|MEPHI|MEPHI|Levasseur|France
>Spike|hCoV-19/Wales/PHWC-16A197/2020|2020-09-05|EPI_ISL_535210|Original|hCoV-19^^Wales|Human|Wales Specialist Virology Centre Sequencing lab|COVID-19 Genomics UK (COG-UK) Consortium|Moore|United Kingdom

>Spike|hCoV-19/USA/MI-QDX-507/2020|2020-03-24|EPI_ISL_571241|Original|hCoV-
19^^Michigan|Human|Quest Diagnostics|Quest Diagnostics|Kagan|USA
>Spike|hCoV-19/South Korea/KDCA0117/2020|2020-10-01|EPI_ISL_747352|Original|hCoV-
19^^South Korea|Human|Division of Emerging Infectious Diseases|Division of Emerging
Infectious Diseases|Park|South Korea
>Spike|hCoV-19/Saudi Arabia/KAUST-Madinah44/2020|2020-03-
29|EPI_ISL_437473|Original|hCoV-19^^Madinah|Human|Pathogen Genomics Lab King Abdullah
University of Science and Technology(KAUST)|Pathogen Genomics Lab King Abdullah
University of Science and Technology(KAUST)|Mohamed Ghazzali|Saudi Arabia
>Spike|hCoV-19/England/QEUH-A8C503/2020|2020-10-18|EPI_ISL_643229|Original|hCoV-
19^^England|Human|Lighthouse Lab in Glasgow|Wellcome Sanger Institute for the COVID-
19 Genomics UK (COG-UK) Consortium|Davies|United Kingdom
>Spike|hCoV-19/Switzerland/GE-SNRCI-2482/2020|2020-03-
00|EPI_ISL_548154|Original|hCoV-19^^Geneve|Human|Laboratoire de Virologie|Swiss
National Reference Centre for Influenza|Gon\u00e7alves Cabecinhas|Switzerland
>Spike|hCoV-19/Sweden/20-06097/2020|2020-03-20|EPI_ISL_450807|Original|hCoV-
19^^Skane|Human|Victoria Vard och Halsa|The Public Health Agency of
Sweden|Svartstr\u00f6m|Sweden
>Spike|hCoV-19/Scotland/QEUH-95FA92/2020|2020-08-06|EPI_ISL_532351|Original|hCoV-
19^^Scotland|Human|NHSGGC West of Scotland Specialist Virology Centre / MRC-
University of Glasgow Centre for Virus Research|Wellcome Sanger Institute for the
COVID-19 Genomics UK (COG-UK) consortium|Davies|United Kingdom
>Spike|hCoV-19/Croatia/Zagreb-27/2020|2020-09-03|EPI_ISL_710547|Original|hCoV-
19^^Zagreb|Human|Sestre Milosrdnice University Hospital Center|Ru\u0111er
Bo\u0161kovic Institute; Forensic Science Centre Ivan Vu\u010deti\u0107; University
of Zagreb Faculty of Science|Karlic|Croatia
>Spike|hCoV-19/Scotland/EDB5521/2020|2020-04-22|EPI_ISL_487778|Original|hCoV-
19^^Scotland|Human|Virology Department|Wellcome Sanger Institute for the COVID-19
Genomics UK (COG-UK) consortium|Davies|United Kingdom
>Spike|hCoV-19/Belgium/ULG-10306/2020|2020-07-24|EPI_ISL_540473|Original|hCoV-
19^^Li\u00e8ge|Human|Department of Clinical Microbiology|GIGA Medical
Genomics|Durkin|Belgium
>Spike|hCoV-19/USA/MI-MDHHS-SC21438/2020|2020-03-20|EPI_ISL_507692|Original|hCoV-
19^^Michigan|Human|Michigan Department of Health and Human Services|Michigan
Department of Health and Human Services|Blankenship|USA
>Spike|hCoV-19/England/QEUH-95F018/2020|2020-08-07|EPI_ISL_532306|Original|hCoV-
19^^England|Human|Lighthouse Lab in Glasgow|Wellcome Sanger Institute for the COVID-
19 Genomics UK (COG-UK) consortium|Davies|United Kingdom
>Spike|hCoV-19/England/QEUH-B114BA/2020|2020-11-02|EPI_ISL_642427|Original|hCoV-
19^^England|Human|Lighthouse Lab in Glasgow|Wellcome Sanger Institute for the COVID-
19 Genomics UK (COG-UK) Consortium|Davies|United Kingdom
>Spike|hCoV-19/Israel/CVL-n17372/2020|2020-06-02|EPI_ISL_516887|Original|hCoV-19^^Tel
Aviv|Human|Israel Central Virology laboratory|Israel Central Virology
laboratory|Zuckerman|Israel
>Spike|hCoV-19/USA/UN-CDC-7529/2020|2020-04-22|EPI_ISL_514081|Original|hCoV-
19^^USA|Human|Navy and Marine Corps Public Health Center|Pathogen Discovery|Queen|USA
>Spike|hCoV-19/Norway/2861/2020|2020-07-06|EPI_ISL_500784|Original|hCoV-
19^^Norway|Human|Furst Medical Laboratory|Norwegian Institute of Public
Health|Kopperud Riis|Norway
>Spike|hCoV-19/Denmark/DCGC-5743/2020|2020-09-28|EPI_ISL_621248|Original|hCoV-
19^^Hovedstaden|Human|Department of Virus and Microbiological Special
Diagnostics|Albertsen lab|Michaelsen|Denmark
>Spike|hCoV-19/France/PAC-IHU-0810/2020|2020-05-06|EPI_ISL_568973|Original|hCoV-
19^^Provence-Alpes-C\u00f4te-d\u2019Azur|Human|MEPHI|MEPHI|Levasseur|France
>Spike|hCoV-19/England/ALDP-B1B5BD/2020|2020-11-01|EPI_ISL_645566|Original|hCoV-
19^^England|Human|Lighthouse Lab in Alderley Park|Wellcome Sanger Institute for the
COVID-19 Genomics UK (COG-UK) Consortium|Davies|United Kingdom
>Spike|hCoV-19/Spain/VC-IBV-98003476/2020|2020-04-07|EPI_ISL_539274|Original|hCoV-
19^^Comunitat Valenciana|Human|Hospital Universitario de La Ribera (Alzira|SeqCOVID-
SPAIN consortium/IBV(CSIC)|Comas|Spain

>Spike|hCoV-19/Russia/StPetersburg-RII4711S/2020|2020-04-07|EPI_ISL_427330|Original|hCoV-19^^Saint Petersburg|Human|WHO National Influenza Centre Russian Federation|WHO National Influenza Centre Russian Federation|Komissarov|Russia
>Spike|hCoV-19/Wales/PHWC-4948C9/2020|2020-11-20|EPI_ISL_726183|Original|hCoV-19^^Wales|Human|Originating lab|COVID-19 Genomics UK (COG-UK) Consortium|Moore|United Kingdom
>Spike|hCoV-19/England/QEUH-9F87FE/2020|2020-09-27|EPI_ISL_588726|Original|hCoV-19^^England|Human|Lighthouse Lab in Glasgow|Wellcome Sanger Institute for the COVID-19 Genomics UK (COG-UK) consortium|Davies|United Kingdom
>Spike|hCoV-19/Canada/ON-E72b/2020|2020-04-03|EPI_ISL_770825|Original|hCoV-19^^Ontario|Human|Eastern Ontario Regional Laboratory Association|McMaster University|McArthur|Canada
>Spike|hCoV-19/Finland/25SS22H2/2020|2020-09-25|EPI_ISL_759921|Original|hCoV-19^^Uusimaa|Human|Department of Virology and Immunology|Department of Virology|Smura|Finland
>Spike|hCoV-19/Australia/SAP068/2020|2020-03-30|EPI_ISL_451106|Original|hCoV-19^^South Australia|Human|SA Pathology|SA Pathology|Leong|Australia
>Spike|hCoV-19/Belgium/BC-03016/2020|2020-03-01|EPI_ISL_415157|Original|hCoV-19^^Sint-Niklaas|Human|KU Leuven|KU Leuven|Maes|Belgium
>Spike|hCoV-19/United Arab Emirates/0072/2020|2020-05-18|EPI_ISL_698280|Original|hCoV-19^^Abu Dhabi|Human|Group 42 (G42) Healthcare|G42 Healthcare|Ogrodzki|United Arab Emirates
>Spike|hCoV-19/USA/TX-HMH-MCoV-18858/2020|2020-11-24|EPI_ISL_785174|Original|hCoV-19^^Texas|Human|Houston Methodist Hospital|Houston Methodist Hospital|Long|USA
>Spike|hCoV-19/England/ALDP-95A648/2020|2020-06-12|EPI_ISL_553438|Original|hCoV-19^^England|Human|Lighthouse Lab in Alderley Park|Wellcome Sanger Institute for the COVID-19 Genomics UK (COG-UK) consortium|Davies|United Kingdom
>Spike|hCoV-19/USA/MD-MDH-0043/2020|2020-04-04|EPI_ISL_509447|Original|hCoV-19^^Maryland|Human|Maryland Department of Health|Maryland Department of Health|Keller|USA
>Spike|hCoV-19/England/MILK-B86580/2020|2020-11-14|EPI_ISL_674009|Original|hCoV-19^^England|Human|Lighthouse Lab in Milton Keynes|Wellcome Sanger Institute for the COVID-19 Genomics UK (COG-UK) Consortium|Davies|United Kingdom
>Spike|hCoV-19/England/BRIS-133CB4/2020|2020-08-20|EPI_ISL_529338|Original|hCoV-19^^England|Human|University of Birmingham|COVID-19 Genomics UK (COG-UK) Consortium|Loman|United Kingdom
>Spike|hCoV-19/USA/WA-UW-6178/2020|2020-04-01|EPI_ISL_430937|Original|hCoV-19^^Washington|Human|UW Virology Lab|UW Virology Lab|Roychoudhury|USA
>Spike|hCoV-19/Norway/3564/2020|2020-07-31|EPI_ISL_590947|Original|hCoV-19^^Norway|Human|University Hospital of Northern Norway|Norwegian Institute of Public Health|Vollan|Norway
>Spike|hCoV-19/Norway/2812/2020|2020-06-29|EPI_ISL_493377|Original|hCoV-19^^Norway|Human|Furst Medical Laboratory|Norwegian Institute of Public Health|Kopperud Riis|Norway
>Spike|hCoV-19/Japan/PG-8113/2020|2020-08-00|EPI_ISL_687212|Original|hCoV-19^^Japan|Human|Pathogen Genomics Center|Pathogen Genomics Center|Sekizuka|Japan
>Spike|hCoV-19/Luxembourg/LNS0889216/2020|2020-11-22|EPI_ISL_739846|Original|hCoV-19^^Luxembourg|Human|Laboratoire national de sant\u00e9|Laboratoire national de sant\u00e9|Wienecke-Baldacchino|Luxembourg
>Spike|hCoV-19/USA/TX-HMH-MCoV-15037/2020|2020-10-16|EPI_ISL_786521|Original|hCoV-19^^Texas|Human|Houston Methodist Hospital|Houston Methodist Hospital|Long|USA
>Spike|hCoV-19/Japan/PG-5344/2020|2020-08-00|EPI_ISL_690447|Original|hCoV-19^^Japan|Human|Fukuoka Institute of Health and Environmental Sciences|Pathogen Genomics Center|Sekizuka|Japan
>Spike|hCoV-19/USA/TX-HMH-MCoV-16107/2020|2020-10-29|EPI_ISL_785791|Original|hCoV-19^^Texas|Human|Houston Methodist Hospital|Houston Methodist Hospital|Long|USA
>Spike|hCoV-19/England/ALDP-C8F2E9/2020|2020-12-10|EPI_ISL_777033|Original|hCoV-19^^England|Human|Lighthouse Lab in Alderley Park|Wellcome Sanger Institute for the COVID-19 Genomics UK (COG-UK) Consortium|Davies|United Kingdom

>Spike|hCoV-19/USA/VA-DCLS-1006/2020|2020-06-03|EPI_ISL_526902|Original|hCoV-19^^Virginia|Human|Virginia DCLS|Virginia DCLS|St. Jacques|USA
>Spike|hCoV-19/USA/AZ-TG654300/2020|2020-04-10|EPI_ISL_695956|Original|hCoV-19^^Arizona|Human|Sonora Quest Laboratories|TGen North|French|USA
>Spike|hCoV-19/USA/TX-HMH-MCoV-14812/2020|2020-10-12|EPI_ISL_786297|Original|hCoV-19^^Texas|Human|Houston Methodist Hospital|Houston Methodist Hospital|Long|USA
>Spike|hCoV-19/England/QEUH-B2DD47/2020|2020-11-03|EPI_ISL_647061|Original|hCoV-19^^England|Human|Lighthouse Lab in Glasgow|Wellcome Sanger Institute for the COVID-19 Genomics UK (COG-UK) Consortium|Davies|United Kingdom
>Spike|hCoV-19/England/QEUH-969B83/2020|2020-08-12|EPI_ISL_531058|Original|hCoV-19^^England|Human|Lighthouse Lab in Glasgow|Wellcome Sanger Institute for the COVID-19 Genomics UK (COG-UK) consortium|Davies|United Kingdom
>Spike|hCoV-19/England/QEUH-B40959/2020|2020-11-08|EPI_ISL_657270|Original|hCoV-19^^England|Human|Lighthouse Lab in Glasgow|Wellcome Sanger Institute for the COVID-19 Genomics UK (COG-UK) Consortium|Davies|United Kingdom
>Spike|hCoV-19/Scotland/CVR2433/2020|2020-04-14|EPI_ISL_448187|Original|hCoV-19^^Scotland|Human|West of Scotland Specialist Virology Centre|COVID-19 Genomics UK (COG-UK) Consortium|Robertson|United Kingdom
>Spike|hCoV-19/Japan/PG-4890/2020|2020-08-00|EPI_ISL_690011|Original|hCoV-19^^Japan|Human|Sakai City Institute of Public Health|Pathogen Genomics Center|Sekizuka|Japan
>Spike|hCoV-19/USA/TX-DSHS-2063/2020|2020-12-11|EPI_ISL_766675|Original|hCoV-19^^Texas|Human|Texas Department of State Health Services|Texas Department of State Health Services|Rahman|USA
>Spike|hCoV-19/USA/TX-HMH-1837/2020|2020-05-12|EPI_ISL_542874|Original|hCoV-19^^Texas|Human|Houston Methodist Hospital|Houston Methodist Hospital|Long|USA
>Spike|hCoV-19/England/ALDP-A873E0/2020|2020-10-17|EPI_ISL_606020|Original|hCoV-19^^England|Human|Lighthouse Lab in Alderley Park|Wellcome Sanger Institute for the COVID-19 Genomics UK (COG-UK) consortium|Davies|United Kingdom
>Spike|hCoV-19/USA/CA-CZB-169/2020|2020-08-31|EPI_ISL_583202|Original|hCoV-19^^California|Human|UCSF Clinical Microbiology Laboratory|Chan-Zuckerberg Biohub|Bhatt|USA
>Spike|hCoV-19/India/DL-NCDC2037-CSIR-IGIB/2020|2020-05-18|EPI_ISL_482531|Original|hCoV-19^^Delhi|Human|National Centre for Disease control (NCDC)|NCDC/CSIR-IGIB|Pandey|India
>Spike|hCoV-19/Canada/ON-PHL-20-03821/2020|2020-11-00|EPI_ISL_775031|Original|hCoV-19^^Ontario|Human|Public Health Ontario Laboratory|Public Health Ontario Laboratory|Guthrie|Canada
>Spike|hCoV-19/England/QEUH-52E78A/2020|2020-05-18|EPI_ISL_532255|Original|hCoV-19^^England|Human|Lighthouse Lab in Glasgow|Wellcome Sanger Institute for the COVID-19 Genomics UK (COG-UK) consortium|Davies|United Kingdom
>Spike|hCoV-19/Spain/PV-IBV-005168/2020|2020-06-24|EPI_ISL_509649|Original|hCoV-19^^Basque Country|Human|Servicio de Microbiolog\u00eda. Hospital Universitario Donostia. OSI Donostialdea. \u00c1rea de Enfermedades Infecciosas|SeqCOVID-SPAIN consortium/IBV(CSIC)|Comas|Spain
>Spike|hCoV-19/Denmark/DCGC-777/2020|2020-03-30|EPI_ISL_614538|Original|hCoV-19^^Nordjylland|Human|Department of Virus and Microbiological Special Diagnostics|Albertsen lab|Michaelsen|Denmark
>Spike|hCoV-19/Spain/PV-IBV-98008371/2020|2020-10-14|EPI_ISL_654578|Original|hCoV-19^^Basque Country|Human|Servicio de Microbiolog\u00eda. Hospital Universitario Donostia. OSI Donostialdea. \u00c1rea de Enfermedades Infecciosas|SeqCOVID-SPAIN consortium/IBV(CSIC)|Comas|Spain
>Spike|hCoV-19/Portugal/PT0595/2020|2020-03-19|EPI_ISL_454318|Original|hCoV-19^^Portugal|Human|unknown|Instituto Nacional de Saude (INSA)|Borges|Portugal
>Spike|hCoV-19/England/20132035604/2020|2020-03-24|EPI_ISL_423777|Original|hCoV-19^^England|Human|Respiratory Virus Unit|Respiratory Virus Unit|Platt|United Kingdom
>Spike|hCoV-19/England/ALDP-B46373/2020|2020-11-07|EPI_ISL_656271|Original|hCoV-19^^England|Human|Lighthouse Lab in Alderley Park|Wellcome Sanger Institute for the COVID-19 Genomics UK (COG-UK) Consortium|Davies|United Kingdom

>Spike|hCoV-19/USA/MI-MDHHS-SC21123/2020|2020-03-30|EPI_ISL_507899|Original|hCoV-19^^Michigan|Human|Michigan Department of Health and Human Services|Michigan Department of Health and Human Services|Blankenship|USA
>Spike|hCoV-19/USA/AZ-TG445190/2020|2020-06-26|EPI_ISL_695001|Original|hCoV-19^^Arizona|Human|TGen North|TGen North|French|USA
>Spike|hCoV-19/Denmark/DCGC-18463/2020|2020-12-07|EPI_ISL_749983|Original|hCoV-19^^Hovedstaden|Human|Department of Virus and Microbiological Special Diagnostics|Albertsen Lab|Michaelsen|Denmark
>Spike|hCoV-19/USA/MN-MDH-1947/2020|2020-09-19|EPI_ISL_613479|Original|hCoV-19^^Minnesota|Human|Public Health Laboratory - Infectious Disease Lab|Minnesota Department of Health|Lorentz|USA
>Spike|hCoV-19/England/QEUH-C31231/2020|2020-12-10|EPI_ISL_731380|Original|hCoV-19^^England|Human|Lighthouse Lab in Glasgow|Wellcome Sanger Institute for the COVID-19 Genomics UK (COG-UK) Consortium|Davies|United Kingdom
>Spike|hCoV-19/England/CAMC-A57783/2020|2020-10-08|EPI_ISL_597507|Original|hCoV-19^^England|Human|Lighthouse Lab in Cambridge|Wellcome Sanger Institute for the COVID-19 Genomics UK (COG-UK) consortium|Davies|United Kingdom
>Spike|hCoV-19/England/CAMC-B78BBC/2020|2020-11-09|EPI_ISL_783462|Original|hCoV-19^^England|Human|Lighthouse Lab in Cambridge|Wellcome Sanger Institute for the COVID-19 Genomics UK (COG-UK) Consortium|Davies|United Kingdom
>Spike|hCoV-19/Japan/PG-2512/2020|2020-04-00|EPI_ISL_690649|Original|hCoV-19^^Japan|Human|Saitama Prefectural Institute of Public Health|Pathogen Genomics Center|Sekizuka|Japan
>Spike|hCoV-19/England/MILK-A7B0BB/2020|2020-10-14|EPI_ISL_607599|Original|hCoV-19^^England|Human|Lighthouse Lab in Milton Keynes|Wellcome Sanger Institute for the COVID-19 Genomics UK (COG-UK) consortium|Davies|United Kingdom
>Spike|hCoV-19/Wales/PHWC-47E84D/2020|2020-10-14|EPI_ISL_595510|Original|hCoV-19^^Wales|Human|Originating lab|COVID-19 Genomics UK (COG-UK) Consortium|Moore|United Kingdom
>Spike|hCoV-19/Wales/PHWC-49925D/2020|2020-12-02|EPI_ISL_727277|Original|hCoV-19^^Wales|Human|Originating lab|COVID-19 Genomics UK (COG-UK) Consortium|Moore|United Kingdom
>Spike|hCoV-19/USA/WA-UW-19547/2020|2020-08-01|EPI_ISL_570808|Original|hCoV-19^^Washington|Human|UW Virology Lab|UW Virology Lab|Roychoudhury|USA
>Spike|hCoV-19/Australia/WA78/2020|2020-07-13|EPI_ISL_512731|Original|hCoV-19^^Western Australia|Human|PathWest Laboratory Medicine WA|PathWest Laboratory Medicine WA Microbial Surveillance Unit|Sikazwe|Australia
>Spike|hCoV-19/England/ALDP-950730/2020|2020-06-13|EPI_ISL_554389|Original|hCoV-19^^England|Human|Lighthouse Lab in Alderley Park|Wellcome Sanger Institute for the COVID-19 Genomics UK (COG-UK) consortium|Davies|United Kingdom
>Spike|hCoV-19/Australia/VIC3843/2020|2020-07-13|EPI_ISL_520556|Original|hCoV-19^^Victoria|Human|Microbiological Diagnostic Unit - Public Health Laboratory (MDU-PHL)|MDU-PHL|Schultz|Australia
>Spike|hCoV-19/England/MILK-995984/2020|2020-08-23|EPI_ISL_551621|Original|hCoV-19^^England|Human|Lighthouse Lab in Milton Keynes|Wellcome Sanger Institute for the COVID-19 Genomics UK (COG-UK) consortium|Davies|United Kingdom
>Spike|hCoV-19/Scotland/CVR1517/2020|2020-04-03|EPI_ISL_477823|Original|hCoV-19^^Scotland|Human|West of Scotland Specialist Virology Centre|COVID-19 Genomics UK (COG-UK) Consortium|Robertson|United Kingdom
>Spike|hCoV-19/France/ARA-NRC2905/2020|2020-12-08|EPI_ISL_779813|Original|hCoV-19^^Auvergne-Rh\u00f4ne-Alpes|Human|CNR Virus des Infections Respiratoires - France SUD|CNR Virus des Infections Respiratoires - France SUD|Bal|France
>Spike|hCoV-19/England/NORT-1B71F0F/2020|2020-11-23|EPI_ISL_705201|Original|hCoV-19^^England|Human|Northumbria University / South Tees Hospitals NHS Foundation Trust / North Cumbria Integrated Care NHS Foundation Trust / North Tees and Hartlepool NHS Foundation Trust / Newcastle Hospitals NHS Foundation Trust|COVID-19 Genomics UK (COG-UK) Consortium|Bashton|United Kingdom
>Spike|hCoV-19/United Arab Emirates/306/2020|2020-06-02|EPI_ISL_528694|Original|hCoV-19^^Dubai|Human|Alsafar - Khalifa University Abu Dhabi|Alsafar - Khalifa University Abu Dhabi|Daw Elbait|United Arab Emirates

>Spike|hCoV-19/England/ALDP-956354/2020|2020-06-08|EPI_ISL_554069|Original|hCoV-19^^England|Human|Lighthouse Lab in Alderley Park|Wellcome Sanger Institute for the COVID-19 Genomics UK (COG-UK) consortium|Davies|United Kingdom
>Spike|hCoV-19/South Korea/KDCA0069/2020|2020-09-18|EPI_ISL_747310|Original|hCoV-19^^South Korea|Human|Division of Emerging Infectious Diseases|Division of Emerging Infectious Diseases|Park|South Korea
>Spike|hCoV-19/India/PB-IMT-I8/2020|2020-04-27|EPI_ISL_539484|Original|hCoV-19^^Punjab|Human|Civil Hospital|CSIR-Institute of Microbial Technology|Patil|India
>Spike|hCoV-19/India/TG-CCMB-M664/2020|2020-06-11|EPI_ISL_495222|Original|hCoV-19^^Telangana|Human|CSIR-Centre for Cellular and Molecular Biology|CSIR-Centre for Cellular and Molecular Biology|Banu|India
>Spike|hCoV-19/South Africa/N00056/2020|2020-08-12|EPI_ISL_623051|Original|hCoV-19^^Eastern Cape|Human|National Health Laboratory Service|National Institute for Communicable Diseases of the National Health Laboratory Service|Allam|South Africa
>Spike|hCoV-19/Singapore/441/2020|2020-04-08|EPI_ISL_475972|Original|hCoV-19^^Singapore|Human|National Public Health Laboratory|National Public Health Laboratory|Mak|Singapore
>Spike|hCoV-19/England/MILK-A7C357/2020|2020-10-15|EPI_ISL_606463|Original|hCoV-19^^England|Human|Lighthouse Lab in Milton Keynes|Wellcome Sanger Institute for the COVID-19 Genomics UK (COG-UK) consortium|Davies|United Kingdom
>Spike|hCoV-19/USA/TX-HMH-MCoV-15105/2020|2020-10-17|EPI_ISL_786589|Original|hCoV-19^^Texas|Human|Houston Methodist Hospital|Houston Methodist Hospital|Long|USA
>Spike|hCoV-19/England/QEUH-95F5BF/2020|2020-08-06|EPI_ISL_532313|Original|hCoV-19^^England|Human|Lighthouse Lab in Glasgow|Wellcome Sanger Institute for the COVID-19 Genomics UK (COG-UK) consortium|Davies|United Kingdom
>Spike|hCoV-19/England/ALDP-9CAE4B/2020|2020-09-16|EPI_ISL_575717|Original|hCoV-19^^England|Human|Lighthouse Lab in Alderley Park|Wellcome Sanger Institute for the COVID-19 Genomics UK (COG-UK) consortium|Davies|United Kingdom
>Spike|hCoV-19/Scotland/GCVR-173E2B/2020|2020-04-05|EPI_ISL_459131|Original|hCoV-19^^Scotland|Human|NHSGGC West of Scotland Specialist Virology Centre / MRC-University of Glasgow Centre for Virus Research|Wellcome Sanger Institute for the COVID-19 Genomics UK (COG-UK) consortium|Davies|United Kingdom
>Spike|hCoV-19/England/NOTT-12050A/2020|2020-12-08|EPI_ISL_725623|Original|hCoV-19^^England|Human|Queens Medical Centre|COVID-19 Genomics UK (COG-UK) Consortium|Loose|United Kingdom
>Spike|hCoV-19/USA/TX-HMH-MCoV-8849/2020|2020-07-02|EPI_ISL_780794|Original|hCoV-19^^Texas|Human|Houston Methodist Hospital|Houston Methodist Hospital|Long|USA
>Spike|hCoV-19/Netherlands/ZuidHolland_125/2020|2020-04-24|EPI_ISL_461298|Original|hCoV-19^^Zuid Holland|Human|Dutch COVID-19 response team|Erasmus Medical Center|Oude Munnink|Netherlands
>Spike|hCoV-19/Australia/VIC15772/2020|2020-08-07|EPI_ISL_593450|Original|hCoV-19^^Victoria|Human|Microbiological Diagnostic Unit - Public Health Laboratory (MDU-PHL)|MDU-PHL|Schultz|Australia
>Spike|hCoV-19/USA/WI-UW-2282/2020|2020-11-18|EPI_ISL_728049|Original|hCoV-19^^Wisconsin|Human|University of Wisconsin-Madison AIDS Vaccine Research Laboratories|University of Wisconsin-Madison AIDS Vaccine Research Laboratories|Baczenas|USA
>Spike|hCoV-19/England/BRIS-123042/2020|2020-04-05|EPI_ISL_442757|Original|hCoV-19^^England|Human|PHE South West Regional Laboratory|Wellcome Sanger Institute for the COVID-19 Genomics UK (COG-UK) consortium|Amato|United Kingdom
>Spike|hCoV-19/Wales/PHWC-2B6A3/2020|2020-04-12|EPI_ISL_445745|Original|hCoV-19^^Wales|Human|Wales Specialist Virology Centre|Public Health Wales Microbiology Cardiff|Moore|United Kingdom
>Spike|hCoV-19/England/SHEF-C82E0/2020|2020-11-01|EPI_ISL_727993|Original|hCoV-19^^England|Human|Virology Department|COVID-19 Genomics UK (COG-UK) Consortium|de Silva|United Kingdom
>Spike|hCoV-19/Denmark/DCGC-10781/2020|2020-11-02|EPI_ISL_670203|Original|hCoV-19^^Syddanmark|Human|Department of Virus and Microbiological Special Diagnostics|Albertsen Lab|Michaelsen|Denmark

>Spike|hCoV-19/Denmark/DCGC-8545/2020|2020-10-26|EPI_ISL_625915|Original|hCoV-19^^Hovedstaden|Human|Department of Virus and Microbiological Special Diagnostics|Albertsen lab|Michaelsen|Denmark
>Spike|hCoV-19/Denmark/DCGC-4672/2020|2020-09-14|EPI_ISL_621904|Original|hCoV-19^^Hovedstaden|Human|Department of Virus and Microbiological Special Diagnostics|Albertsen lab|Michaelsen|Denmark
>Spike|hCoV-19/England/ALDP-9E8876/2020|2020-09-24|EPI_ISL_638261|Original|hCoV-19^^England|Human|Department of Pathology|COVID-19 Genomics UK (COG-UK) Consortium|Goodfellow|United Kingdom
>Spike|hCoV-19/England/CAMB-1AA7E4/2020|2020-04-17|EPI_ISL_470286|Original|hCoV-19^^England|Human|Department of Pathology|Wellcome Sanger Institute for the COVID-19 Genomics UK (COG-UK) consortium|Davies|United Kingdom
>Spike|hCoV-19/England/QEUH-AD0324/2020|2020-10-26|EPI_ISL_629926|Original|hCoV-19^^England|Human|Lighthouse Lab in Glasgow|Wellcome Sanger Institute for the COVID-19 Genomics UK (COG-UK) consortium|Davies|United Kingdom
>Spike|hCoV-19/Australia/VIC14056/2020|2020-09-18|EPI_ISL_563947|Original|hCoV-19^^Victoria|Human|Microbiological Diagnostic Unit - Public Health Laboratory (MDU-PHL)|MDU-PHL|Schultz|Australia
>Spike|hCoV-19/England/MILK-ABB3F2/2020|2020-10-21|EPI_ISL_623407|Original|hCoV-19^^England|Human|Lighthouse Lab in Milton Keynes|Wellcome Sanger Institute for the COVID-19 Genomics UK (COG-UK) consortium|Davies|United Kingdom
>Spike|hCoV-19/USA/NY-QDX-2545/2020|2020-03-15|EPI_ISL_604833|Original|hCoV-19^^New York|Human|Quest Diagnostics|Quest Diagnostics|Kagan|USA
>Spike|hCoV-19/Russia/StPetersburg-RII18090S/2020|2020-05-27|EPI_ISL_507286|Original|hCoV-19^^Saint Petersburg|Human|WHO National Influenza Centre Russian Federation|WHO National Influenza Centre Russian Federation|Komissarov|Russia
>Spike|hCoV-19/USA/TX-HMH-MCoV-15351/2020|2020-10-21|EPI_ISL_786835|Original|hCoV-19^^Texas|Human|Houston Methodist Hospital|Houston Methodist Hospital|Long|USA
>Spike|hCoV-19/Wales/MILK-848FF8/2020|2020-07-16|EPI_ISL_557337|Original|hCoV-19^^Wales|Human|Lighthouse Lab in Milton Keynes|Wellcome Sanger Institute for the COVID-19 Genomics UK (COG-UK) consortium|Davies|United Kingdom
>Spike|hCoV-19/Japan/PG-2826/2020|2020-04-00|EPI_ISL_690953|Original|hCoV-19^^Japan|Human|Pathogen Genomics Center|Pathogen Genomics Center|Sekizuka|Japan
>Spike|hCoV-19/England/ALDP-C57F6B/2020|2020-12-13|EPI_ISL_736134|Original|hCoV-19^^England|Human|Lighthouse Lab in Alderley Park|Wellcome Sanger Institute for the COVID-19 Genomics UK (COG-UK) Consortium|Davies|United Kingdom
>Spike|hCoV-19/England/20136100204/2020|2020-03-26|EPI_ISL_423267|Original|hCoV-19^^England|Human|Respiratory Virus Unit|Respiratory Virus Unit|Platt|United Kingdom
>Spike|hCoV-19/England/QEUH-9DBE29/2020|2020-09-21|EPI_ISL_601471|Original|hCoV-19^^England|Human|Lighthouse Lab in Glasgow|Wellcome Sanger Institute for the COVID-19 Genomics UK (COG-UK) consortium|Davies|United Kingdom
>Spike|hCoV-19/England/MILK-B06D80/2020|2020-10-29|EPI_ISL_652275|Original|hCoV-19^^England|Human|Quadram Institute Bioscience|COVID-19 Genomics UK (COG-UK) Consortium|Alikhan|United Kingdom
>Spike|hCoV-19/USA/MI-MDHHS-SC22380/2020|2020-11-09|EPI_ISL_708360|Original|hCoV-19^^Michigan|Human|Michigan Department of Health and Human Services|Michigan Department of Health and Human Services|Jacob|USA
>Spike|hCoV-19/Denmark/DCGC-19732/2020|2020-12-07|EPI_ISL_748320|Original|hCoV-19^^Midtjylland|Human|Department of Virus and Microbiological Special Diagnostics|Albertsen Lab|Michaelsen|Denmark
>Spike|hCoV-19/England/LIVE-DCC179/2020|2020-11-23|EPI_ISL_724491|Original|hCoV-19^^England|Human|Liverpool Clinical Laboratories|COVID-19 Genomics UK (COG-UK) Consortium|Haldenby|United Kingdom
>Spike|hCoV-19/USA/WA-S2758/2020|2020-07-28|EPI_ISL_525748|Original|hCoV-19^^Washington|Human|Seattle Flu Study|Seattle Flu Study|Lee|USA
>Spike|hCoV-19/Japan/PG-3699/2020|2020-04-00|EPI_ISL_691952|Original|hCoV-19^^Japan|Human|Pathogen Genomics Center|Pathogen Genomics Center|Sekizuka|Japan

>Spike|hCoV-19/Wales/PHWC-168E29/2020|2020-04-18|EPI_ISL_494370|Original|hCoV-19^^Wales|Human|Originating lab|COVID-19 Genomics UK (COG-UK) Consortium|Moore|United Kingdom
>Spike|hCoV-19/Canada/MB-NML-633/2020|2020-03-21|EPI_ISL_632918|Original|hCoV-19^^Manitoba|Human|Cadham Provincial laboratory|Cadham Provincial laboratory|Majer|Canada
>Spike|hCoV-19/USA/WI-UW-1430/2020|2020-09-11|EPI_ISL_541563|Original|hCoV-19^^Wisconsin|Human|University of Wisconsin-Madison Campus AIDS Vaccine Research Laboratories|University of Wisconsin-Madison AIDS Vaccine Research Laboratories|Baczenas|USA
>Spike|hCoV-19/England/OXON-AFC1E/2020|2020-04-15|EPI_ISL_478813|Original|hCoV-19^^England|Human|Oxford Viromics|COVID-19 Genomics UK (COG-UK) Consortium|Golubchik|United Kingdom
>Spike|hCoV-19/England/CAMB-76E29/2020|2020-04-01|EPI_ISL_439400|Original|hCoV-19^^England|Human|Department of Pathology|Wellcome Sanger Institute for the COVID-19 Genomics UK (COG-UK) consortium|Amato|United Kingdom
>Spike|hCoV-19/Wales/PHWC-166BBB/2020|2020-06-22|EPI_ISL_494090|Original|hCoV-19^^Wales|Human|Originating lab|COVID-19 Genomics UK (COG-UK) Consortium|Moore|United Kingdom
>Spike|hCoV-19/England/MILK-B028B0/2020|2020-10-24|EPI_ISL_642933|Original|hCoV-19^^England|Human|Lighthouse Lab in Milton Keynes|Wellcome Sanger Institute for the COVID-19 Genomics UK (COG-UK) Consortium|Davies|United Kingdom
>Spike|hCoV-19/Wales/PHWC-4807D2/2020|2020-10-11|EPI_ISL_612168|Original|hCoV-19^^Wales|Human|Wales Specialist Virology Centre Sequencing lab|COVID-19 Genomics UK (COG-UK) Consortium|Moore|United Kingdom
>Spike|hCoV-19/England/CAMC-B3675C/2020|2020-11-05|EPI_ISL_645278|Original|hCoV-19^^England|Human|Lighthouse Lab in Cambridge|Wellcome Sanger Institute for the COVID-19 Genomics UK (COG-UK) Consortium|Davies|United Kingdom
>Spike|hCoV-19/England/ALDP-B24153/2020|2020-11-04|EPI_ISL_646922|Original|hCoV-19^^England|Human|Lighthouse Lab in Alderley Park|Wellcome Sanger Institute for the COVID-19 Genomics UK (COG-UK) Consortium|Davies|United Kingdom
>Spike|hCoV-19/Scotland/CVR5898/2020|2020-11-17|EPI_ISL_705657|Original|hCoV-19^^Scotland|Human|West of Scotland Specialist Virology Centre|COVID-19 Genomics UK (COG-UK) Consortium|Robertson|United Kingdom
>Spike|hCoV-19/England/SHEF-10CC3B9/2020|2020-12-19|EPI_ISL_765168|Original|hCoV-19^^England|Human|Virology Department|COVID-19 Genomics UK (COG-UK) Consortium|de Silva|United Kingdom
>Spike|hCoV-19/England/ALDP-AA559B/2020|2020-10-22|EPI_ISL_625042|Original|hCoV-19^^England|Human|Lighthouse Lab in Alderley Park|Wellcome Sanger Institute for the COVID-19 Genomics UK (COG-UK) consortium|Davies|United Kingdom
>Spike|hCoV-19/England/20152055004/2020|2020-04-06|EPI_ISL_466237|Original|hCoV-19^^England|Human|Respiratory Virus Unit|Respiratory Virus Unit|Platt|United Kingdom
>Spike|hCoV-19/Austria/CeMM0042/2020|2020-03-09|EPI_ISL_437930|Original|hCoV-19^^Ischgl|Human|Institut f\u00fcr Virologie am Department f\u00fcr Hygiene|Bergthaler laboratory|Bergthaler|Austria
>Spike|hCoV-19/USA/MI-QDX-820/2020|2020-03-23|EPI_ISL_571537|Original|hCoV-19^^Michigan|Human|Quest Diagnostics|Quest Diagnostics|Kagan|USA
>Spike|hCoV-19/Scotland/QEUH-9633CD/2020|2020-08-11|EPI_ISL_531577|Original|hCoV-19^^Scotland|Human|NHSGGC West of Scotland Specialist Virology Centre / MRC-University of Glasgow Centre for Virus Research|Wellcome Sanger Institute for the COVID-19 Genomics UK (COG-UK) consortium|Davies|United Kingdom
>Spike|hCoV-19/USA/LA-GBCL-26556575/2020|2020-04-14|EPI_ISL_485202|Original|hCoV-19^^Louisiana|Human|River Road Testing Lab|Ginkgo Bioworks Clinical Laboratory|Smith|USA
>Spike|hCoV-19/Sichuan/SC-MY-056/2020|2020-02-01|EPI_ISL_451322|Original|hCoV-19^^Sichuan|Human|West China Hospital of Sichuan University|State Key Laboratory of Biotherapy of Sichuan University|Tang|China
>Spike|hCoV-19/Scotland/GCVR-172387/2020|2020-03-30|EPI_ISL_469961|Original|hCoV-19^^Scotland|Human|NHSGGC West of Scotland Specialist Virology Centre / MRC-

University of Glasgow Centre for Virus Research|Wellcome Sanger Institute for the COVID-19 Genomics UK (COG-UK) consortium|Davies|United Kingdom
>Spike|hCoV-19/England/SHEF-C73EE/2020|2020-05-27|EPI_ISL_535063|Original|hCoV-19^^England|Human|Virology Department|COVID-19 Genomics UK (COG-UK) Consortium|de Silva|United Kingdom
>Spike|hCoV-19/Spain/AN-IBV-006122/2020|2020-04-10|EPI_ISL_510451|Original|hCoV-19^^Andalucia|Human|Hospital Universitario Virgen de las Nieves de Granada-SAS|SeqCOVID-SPAIN consortium/IBV(CSIC)|Comas|Spain
>Spike|hCoV-19/England/CAMB-8191B/2020|2020-04-12|EPI_ISL_442566|Original|hCoV-19^^England|Human|Department of Pathology|Wellcome Sanger Institute for the COVID-19 Genomics UK (COG-UK) consortium|Amato|United Kingdom
>Spike|hCoV-19/England/ALDP-AA10F8/2020|2020-10-22|EPI_ISL_624321|Original|hCoV-19^^England|Human|Lighthouse Lab in Alderley Park|Wellcome Sanger Institute for the COVID-19 Genomics UK (COG-UK) consortium|Davies|United Kingdom
>Spike|hCoV-19/England/ALDP-5D05D8/2020|2020-06-19|EPI_ISL_558433|Original|hCoV-19^^England|Human|Lighthouse Lab in Alderley Park|Wellcome Sanger Institute for the COVID-19 Genomics UK (COG-UK) consortium|Davies|United Kingdom
>Spike|hCoV-19/Switzerland/BL-42402436/2020|2020-08-19|EPI_ISL_581973|Original|hCoV-19^^Basel-Land|Human|University Hospital Basel|University Hospital Basel|Roloff Handschin|Switzerland
>Spike|hCoV-19/USA/CA-CZB-3294/2020|2020-07-20|EPI_ISL_548327|Original|hCoV-19^^California|Human|County of Santa Clara Public Health Department|Chan-Zuckerberg Biohub|Bhatt|USA
>Spike|hCoV-19/India/WB-IK39/2020|2020-05-03|EPI_ISL_508367|Original|hCoV-19^^West Bengal|Human|Institute of Post Graduate Medical Education & Research|National Institute of Biomedical Genomics|Maitra|India
>Spike|hCoV-19/Thailand/Bangkok-CONI-0145/2020|2020-03-15|EPI_ISL_455939|Original|hCoV-19^^Bangkok|Human|Ramathibodi Hospital|COVID-19 Network Investigations (CONI) Alliance|Batty|Thailand
>Spike|hCoV-19/Japan/IC-0584/2020|2020-11-00|EPI_ISL_779670|Original|hCoV-19^^Japan|Human|Pathogen Genomics Center|Pathogen Genomics Center|Sekizuka|Japan
>Spike|hCoV-19/Japan/PG-2961/2020|2020-04-00|EPI_ISL_691087|Original|hCoV-19^^Japan|Human|Pathogen Genomics Center|Pathogen Genomics Center|Sekizuka|Japan
>Spike|hCoV-19/England/CAMC-A58CED/2020|2020-10-09|EPI_ISL_597095|Original|hCoV-19^^England|Human|Lighthouse Lab in Cambridge|Wellcome Sanger Institute for the COVID-19 Genomics UK (COG-UK) consortium|Davies|United Kingdom
>Spike|hCoV-19/England/SHEF-D314F/2020|2020-09-21|EPI_ISL_585088|Original|hCoV-19^^England|Human|Virology Department|COVID-19 Genomics UK (COG-UK) Consortium|de Silva|United Kingdom
>Spike|hCoV-19/SaudiArabia/KAUST-JEDDAH830/2020|2020-05-24|EPI_ISL_513004|Original|hCoV-19^^Jeddah|Human|Pathogen Genomics Lab King Abdullah University of Science and Technology(KAUST)|Pathogen Genomics Lab King Abdullah University of Science and Technology(KAUST)|Mohamed Ghazzali|Saudi Arabia
>Spike|hCoV-19/Japan/PG-1561/2020|2020-04-00|EPI_ISL_685167|Original|hCoV-19^^Japan|Human|Pathogen Genomics Center|Pathogen Genomics Center|Sekizuka|Japan
>Spike|hCoV-19/Australia/VIC6308/2020|2020-07-23|EPI_ISL_518296|Original|hCoV-19^^Victoria|Human|Microbiological Diagnostic Unit - Public Health Laboratory (MDU-PHL)|MDU-PHL|Schultz|Australia
>Spike|hCoV-19/USA/CA-CZB-14354/2020|2020-07-15|EPI_ISL_738983|Original|hCoV-19^^California|Human|Alameda County Public Health Lab|Chan-Zuckerberg Biohub|Kamm|USA
>Spike|hCoV-19/Spain/MD-HLP-74/2020|2020-04-26|EPI_ISL_530073|Original|hCoV-19^^Madrid|Human|Hospital Universitario La Paz|Hospital Universitario La Paz|Viedma|Spain
>Spike|hCoV-19/England/ALDP-AA3EED/2020|2020-10-22|EPI_ISL_624996|Original|hCoV-19^^England|Human|Lighthouse Lab in Alderley Park|Wellcome Sanger Institute for the COVID-19 Genomics UK (COG-UK) consortium|Davies|United Kingdom
>Spike|hCoV-19/USA/TX-HMH-7401/2020|2020-06-29|EPI_ISL_546740|Original|hCoV-19^^Texas|Human|Houston Methodist Hospital|Houston Methodist Hospital|Long|USA

>Spike|hCoV-19/Denmark/DCGC-22448/2020|2020-12-14|EPI_ISL_757568|Original|hCoV-19^^Nordjylland|Human|Department of Virus and Microbiological Special Diagnostics|Albertsen Lab|Michaelsen|Denmark
>Spike|hCoV-19/USA/CA-CZB-9641/2020|2020-04-23|EPI_ISL_672030|Original|hCoV-19^^California|Human|The Ashley Laboratory|Chan-Zuckerberg Biohub|Bhatt|USA
>Spike|hCoV-19/Scotland/QEUH-BCC3B1/2020|2020-11-27|EPI_ISL_710789|Original|hCoV-19^^Scotland|Human|Lighthouse Lab in Glasgow|Wellcome Sanger Institute for the COVID-19 Genomics UK (COG-UK) Consortium|Davies|United Kingdom
>Spike|hCoV-19/USA/TX-HMH-MCoV-10681/2020|2020-07-09|EPI_ISL_789586|Original|hCoV-19^^Texas|Human|Houston Methodist Hospital|Houston Methodist Hospital|Long|USA
>Spike|hCoV-19/USA/GA-CDC-7459/2020|2020-10-23|EPI_ISL_681821|Original|hCoV-19^^Georgia|Human|GA Department of Public Health Laboratory|Pathogen Discovery|Queen|USA
>Spike|hCoV-19/England/LIVE-DC6F2A/2020|2020-11-24|EPI_ISL_705030|Original|hCoV-19^^England|Human|Liverpool Clinical Laboratories|COVID-19 Genomics UK (COG-UK) Consortium|Haldenby|United Kingdom
>Spike|hCoV-19/England/ALDP-A67067/2020|2020-10-10|EPI_ISL_630499|Original|hCoV-19^^England|Human|Lighthouse Lab in Alderley Park|Wellcome Sanger Institute for the COVID-19 Genomics UK (COG-UK) consortium|Davies|United Kingdom
>Spike|hCoV-19/England/QEUH-B807F9/2020|2020-11-14|EPI_ISL_661674|Original|hCoV-19^^England|Human|Lighthouse Lab in Glasgow|Wellcome Sanger Institute for the COVID-19 Genomics UK (COG-UK) Consortium|Davies|United Kingdom
>Spike|hCoV-19/Norway/4045/2020|2020-09-24|EPI_ISL_635131|Original|hCoV-19^^Innlandet|Human|Innlandet Hospital Trust|Norwegian Institute of Public Health|Vollan|Norway
>Spike|hCoV-19/Denmark/DCGC-22649/2020|2020-12-07|EPI_ISL_757688|Original|hCoV-19^^Hovedstaden|Human|Department of Virus and Microbiological Special Diagnostics|Albertsen Lab|Michaelsen|Denmark
>Spike|hCoV-19/USA/UT-UPHL-2012227413/2020|2020-07-27|EPI_ISL_745695|Original|hCoV-19^^Utah|Human|Ginkgo Bioworks Clinical Laboratory|Utah Public Health Laboratory|Young|USA
>Spike|hCoV-19/England/BRIS-12563F/2020|2020-03-28|EPI_ISL_443687|Original|hCoV-19^^England|Human|PHE South West Regional Laboratory|Wellcome Sanger Institute for the COVID-19 Genomics UK (COG-UK) consortium|Amato|United Kingdom
>Spike|hCoV-19/Wales/PHWC-16AF33/2020|2020-09-11|EPI_ISL_560222|Original|hCoV-19^^Wales|Human|Wales Specialist Virology Centre Sequencing lab|COVID-19 Genomics UK (COG-UK) Consortium|Moore|United Kingdom
>Spike|hCoV-19/England/MILK-A7BA71/2020|2020-10-15|EPI_ISL_607356|Original|hCoV-19^^England|Human|Lighthouse Lab in Milton Keynes|Wellcome Sanger Institute for the COVID-19 Genomics UK (COG-UK) consortium|Davies|United Kingdom
>Spike|hCoV-19/Japan/PG-6097/2020|2020-08-00|EPI_ISL_689209|Original|hCoV-19^^Japan|Human|Pathogen Genomics Center|Pathogen Genomics Center|Sekizuka|Japan
>Spike|hCoV-19/Japan/PG-7050/2020|2020-08-00|EPI_ISL_688155|Original|hCoV-19^^Japan|Human|Pathogen Genomics Center|Pathogen Genomics Center|Sekizuka|Japan
>Spike|hCoV-19/Japan/PG-0428/2020|2020-04-00|EPI_ISL_684166|Original|hCoV-19^^Japan|Human|Pathogen Genomics Center|Pathogen Genomics Center|Sekizuka|Japan
>Spike|hCoV-19/Denmark/DCGC-12790/2020|2020-11-02|EPI_ISL_682971|Original|hCoV-19^^Nordjylland|Human|Department of Virus and Microbiological Special Diagnostics|Albertsen Lab|Michaelsen|Denmark
>Spike|hCoV-19/USA/NY-NYU-VC-017/2020|2020-03-25|EPI_ISL_479671|Original|hCoV-19^^New York|Human|Center for Genomics and System Biology|Center for Genomics and System Biology|Ghedin|USA
>Spike|hCoV-19/Canada/ON-S1598/2020|2020-09-04|EPI_ISL_755938|Original|hCoV-19^^Ontario|Human|Toronto Invasive Bacterial Diseases Network|McMaster University|McArthur|Canada
>Spike|hCoV-19/Scotland/CVR5568/2020|2020-10-28|EPI_ISL_651843|Original|hCoV-19^^Scotland|Human|West of Scotland Specialist Virology Centre|COVID-19 Genomics UK (COG-UK) Consortium|Robertson|United Kingdom

>Spike|hCoV-19/USA/CA-CSMC31/2020|2020-04-01|EPI_ISL_475642|Original|hCoV-19^^California|Human|Cedars-Sinai Medical Center|Cedars-Sinai Medical Center|Zhang|USA
>Spike|hCoV-19/Scotland/QEUH-7B166F/2020|2020-07-19|EPI_ISL_532926|Original|hCoV-19^^Scotland|Human|Virology Department|Wellcome Sanger Institute for the COVID-19 Genomics UK (COG-UK) consortium|Davies|United Kingdom
>Spike|hCoV-19/USA/TX-DSHS-1130/2020|2020-06-16|EPI_ISL_634934|Original|hCoV-19^^Texas|Human|Texas Department of State Health Services|Texas Department of State Health Services|Rahman|USA
>Spike|hCoV-19/England/QEUH-B69C42/2020|2020-11-11|EPI_ISL_655258|Original|hCoV-19^^England|Human|Lighthouse Lab in Glasgow|Wellcome Sanger Institute for the COVID-19 Genomics UK (COG-UK) Consortium|Davies|United Kingdom
>Spike|hCoV-19/Wales/PHWC-47DED3/2020|2020-09-30|EPI_ISL_595377|Original|hCoV-19^^Wales|Human|Originating lab|COVID-19 Genomics UK (COG-UK) Consortium|Moore|United Kingdom
>Spike|hCoV-19/England/LIVE-DB2B5A/2020|2020-11-03|EPI_ISL_650115|Original|hCoV-19^^England|Human|Liverpool Clinical Laboratories|COVID-19 Genomics UK (COG-UK) Consortium|Haldenby|United Kingdom
>Spike|hCoV-19/Netherlands/LI-RIVM-20478/2020|2020-12-03|EPI_ISL_728693|Original|hCoV-19^^Limburg|Human|Dutch COVID-19 response team|National Institute for Public Health and the Environment (RIVM)|Meijer|Netherlands
>Spike|hCoV-19/England/BRIS-12C216/2020|2020-05-11|EPI_ISL_469460|Original|hCoV-19^^England|Human|PHE South West Regional Laboratory|Wellcome Sanger Institute for the COVID-19 Genomics UK (COG-UK) consortium|Davies|United Kingdom
>Spike|hCoV-19/England/QEUH-94368E/2020|2020-07-30|EPI_ISL_532629|Original|hCoV-19^^England|Human|Lighthouse Lab in Glasgow|Wellcome Sanger Institute for the COVID-19 Genomics UK (COG-UK) consortium|Davies|United Kingdom
>Spike|hCoV-19/England/MILK-B3A767/2020|2020-11-02|EPI_ISL_706033|Original|hCoV-19^^England|Human|Oxford Viromics|COVID-19 Genomics UK (COG-UK) Consortium|Golubchik|United Kingdom
>Spike|hCoV-19/Australia/VIC9409/2020|2020-08-19|EPI_ISL_565539|Original|hCoV-19^^Victoria|Human|Microbiological Diagnostic Unit - Public Health Laboratory (MDU-PHL)|MDU-PHL|Schultz|Australia
>Spike|hCoV-19/England/LOND-1251232/2020|2020-11-16|EPI_ISL_665839|Original|hCoV-19^^England|Human|University College London Hospital|COVID-19 Genomics UK (COG-UK) Consortium|Byott|United Kingdom
>Spike|hCoV-19/USA/IL-NM084/2020|2020-03-19|EPI_ISL_444575|Original|hCoV-19^^Illinois|Human|Northwestern Memorial Hospital|Ozer Lab|Ozer|USA
>Spike|hCoV-19/USA/NY-NYCPHL-001420/2020|2020-11-19|EPI_ISL_683791|Original|hCoV-19^^New York|Human|DOHMH Jamaica|New York City Public Health Laboratory|Wang|USA
>Spike|hCoV-19/Wales/PHWC-48438F/2020|2020-10-21|EPI_ISL_627995|Original|hCoV-19^^Wales|Human|Wales Specialist Virology Centre Sequencing lab|COVID-19 Genomics UK (COG-UK) Consortium|Moore|United Kingdom
>Spike|hCoV-19/England/PORT-2D1C3F/2020|2020-04-03|EPI_ISL_493658|Original|hCoV-19^^England|Human|Centre for Enzyme Innovation|COVID-19 Genomics UK (COG-UK) Consortium|Robson|United Kingdom
>Spike|hCoV-19/England/ALDP-9531D5/2020|2020-06-12|EPI_ISL_554553|Original|hCoV-19^^England|Human|Lighthouse Lab in Alderley Park|Wellcome Sanger Institute for the COVID-19 Genomics UK (COG-UK) consortium|Davies|United Kingdom
>Spike|hCoV-19/England/QEUH-AF3E66/2020|2020-10-30|EPI_ISL_663093|Original|hCoV-19^^England|Human|Lighthouse Lab in Glasgow|Wellcome Sanger Institute for the COVID-19 Genomics UK (COG-UK) Consortium|Davies|United Kingdom
>Spike|hCoV-19/England/20129003804/2020|2020-03-11|EPI_ISL_465801|Original|hCoV-19^^England|Human|Respiratory Virus Unit|Respiratory Virus Unit|Platt|United Kingdom
>Spike|hCoV-19/Japan/PG-4353/2020|2020-07-00|EPI_ISL_692581|Original|hCoV-19^^Japan|Human|Pathogen Genomics Center|Pathogen Genomics Center|Sekizuka|Japan
>Spike|hCoV-19/Canada/QC-LSPQ-L00243862/2020|2020-03-31|EPI_ISL_536380|Original|hCoV-19^^Quebec|Human|H\u00f4pital Pierre-Boucher|Laboratoire de sant\u00e9 publique du Qu\u00e9bec|Moreira|Canada

>Spike|hCoV-19/Belgium/ULG-10758/2020|2020-09-20|EPI_ISL_641600|Original|hCoV-19^^Li\u00e8ge|Human|Department of Clinical Microbiology|GIGA Medical Genomics|Durkin|Belgium
>Spike|hCoV-19/USA/CA-CZB-5703/2020|2020-07-23|EPI_ISL_582970|Original|hCoV-19^^California|Human|San Luis Obispo Public Health Department|Chan-Zuckerberg Biohub|Bhatt|USA
>Spike|hCoV-19/Denmark/DCGC-13573/2020|2020-11-30|EPI_ISL_712884|Original|hCoV-19^^Syddanmark|Human|Department of Virus and Microbiological Special Diagnostics|Albertsen Lab|Michaelsen|Denmark
>Spike|hCoV-19/England/MILK-B85DB6/2020|2020-11-13|EPI_ISL_674023|Original|hCoV-19^^England|Human|Lighthouse Lab in Milton Keynes|Wellcome Sanger Institute for the COVID-19 Genomics UK (COG-UK) Consortium|Davies|United Kingdom
>Spike|hCoV-19/USA/CA-CZB-14919/2020|2020-11-27|EPI_ISL_739266|Original|hCoV-19^^California|Human|Orange County Public Health Lab|Chan-Zuckerberg Biohub|Kamm|USA
>Spike|hCoV-19/South Africa/Tygerberg_48/2020|2020-04-08|EPI_ISL_464157|Original|hCoV-19^^Western Cape|Human|National Health Laboratory Service (NHLS)|Division of Medical Virology|Engelbrecht|South Africa
>Spike|hCoV-19/USA/TX-HMH-MCoV-18359/2020|2020-11-20|EPI_ISL_784707|Original|hCoV-19^^Texas|Human|Houston Methodist Hospital|Houston Methodist Hospital|Long|USA
>Spike|hCoV-19/USA/OR-OHSU-2859/2020|2020-09-03|EPI_ISL_667362|Original|hCoV-19^^Oregon|Human|OHSU Lab Services Molecular Microbiology Lab|Oregon SARS-CoV-2 Genome Sequencing Center|O'Roak|USA
>Spike|hCoV-19/Spain/VC-FISABIO-566/2020|2020-03-27|EPI_ISL_447498|Original|hCoV-19^^Comunitat Valenciana|Human|Servicio de Microbiologia. Hospital Clinico Universitario de Valencia|Sequencing and Bioinformatics Service and Molecular Epidemiology Research Group. FISABIO-Public Health|Gonzalez-Candelas|Spain
>Spike|hCoV-19/England/QEUH-96EE3A/2020|2020-08-19|EPI_ISL_530498|Original|hCoV-19^^England|Human|Lighthouse Lab in Glasgow|Wellcome Sanger Institute for the COVID-19 Genomics UK (COG-UK) consortium|Davies|United Kingdom
>Spike|hCoV-19/Netherlands/NA_378/2020|2020-04-09|EPI_ISL_455180|Original|hCoV-19^^Netherlands|Human|Dutch COVID-19 response team|Erasmus Medical Center|Oude Munnink|Netherlands
>Spike|hCoV-19/England/ALDP-A6E90B/2020|2020-10-15|EPI_ISL_607949|Original|hCoV-19^^England|Human|Lighthouse Lab in Alderley Park|Wellcome Sanger Institute for the COVID-19 Genomics UK (COG-UK) consortium|Davies|United Kingdom
>Spike|hCoV-19/England/NORT-1B70A4B/2020|2020-11-07|EPI_ISL_706295|Original|hCoV-19^^England|Human|Northumbria University / South Tees Hospitals NHS Foundation Trust / North Cumbria Integrated Care NHS Foundation Trust / North Tees and Hartlepool NHS Foundation Trust / Newcastle Hospitals NHS Foundation Trust|COVID-19 Genomics UK (COG-UK) Consortium|Bashton|United Kingdom
>Spike|hCoV-19/Wales/PHWC-2BE8D/2020|2020-04-10|EPI_ISL_445860|Original|hCoV-19^^Wales|Human|Wales Specialist Virology Centre|Public Health Wales Microbiology Cardiff|Moore|United Kingdom
>Spike|hCoV-19/Denmark/DCGC-5083/2020|2020-09-21|EPI_ISL_621621|Original|hCoV-19^^Sjaelland|Human|Department of Virus and Microbiological Special Diagnostics|Albertsen lab|Michaelsen|Denmark
>Spike|hCoV-19/England/QEUH-9DC3B0/2020|2020-09-22|EPI_ISL_581031|Original|hCoV-19^^England|Human|Lighthouse Lab in Glasgow|Wellcome Sanger Institute for the COVID-19 Genomics UK (COG-UK) consortium|Davies|United Kingdom
>Spike|hCoV-19/Luxembourg/LNS0025790/2020|2020-10-22|EPI_ISL_739698|Original|hCoV-19^^Luxembourg|Human|Laboratoire national de sant\u00e9|Laboratoire national de sant\u00e9|Wienecke-Baldacchino|Luxembourg
>Spike|hCoV-19/England/CAMC-B79FEC/2020|2020-11-12|EPI_ISL_741074|Original|hCoV-19^^England|Human|Oxford Viromics|COVID-19 Genomics UK (COG-UK) Consortium|Golubchik|United Kingdom
>Spike|hCoV-19/Luxembourg/LNS3529687/2020|2020-11-07|EPI_ISL_740328|Original|hCoV-19^^Luxembourg|Human|Laboratoire national de sant\u00e9|Laboratoire national de sant\u00e9|Wienecke-Baldacchino|Luxembourg
>Spike|hCoV-19/Cote dIvoire/BKE0366/2020|2020-06-18|EPI_ISL_681834|Original|hCoV-19^^S\u00e9gu\u00e9la|Human|Molecular diagnostic unit for viral haemorrhagic fevers

and emerging viruses|Project group Epidemology of Highly Pathogenic Microorganisms|Schubert|C\u00f4te d'Ivoire
>Spike|hCoV-19/England/MILK-B9758C/2020|2020-11-11|EPI_ISL_674832|Original|hCoV-19^^England|Human|Lighthouse Lab in Milton Keynes|Wellcome Sanger Institute for the COVID-19 Genomics UK (COG-UK) Consortium|Davies|United Kingdom
>Spike|hCoV-19/England/ALDP-BB5773/2020|2020-11-20|EPI_ISL_702781|Original|hCoV-19^^England|Human|Lighthouse Lab in Alderley Park|Wellcome Sanger Institute for the COVID-19 Genomics UK (COG-UK) Consortium|Davies|United Kingdom
>Spike|hCoV-19/Japan/PG-1186/2020|2020-04-00|EPI_ISL_684796|Original|hCoV-19^^Japan|Human|Pathogen Genomics Center|Pathogen Genomics Center|Sekizuka|Japan
>Spike|hCoV-19/England/ALDP-AA0D7A/2020|2020-10-22|EPI_ISL_624069|Original|hCoV-19^^England|Human|Lighthouse Lab in Alderley Park|Wellcome Sanger Institute for the COVID-19 Genomics UK (COG-UK) consortium|Davies|United Kingdom
>Spike|hCoV-19/Ireland/WX-NVRL-AIIDW001v1/2020|2020-11-09|EPI_ISL_768795|Original|hCoV-19^^Wexford|Human|AIID|Irish Coronavirus Sequencing Consortium - National Virus Reference Laboratory|Gonzalez|Ireland
>Spike|hCoV-19/Singapore/977/2020|2020-03-22|EPI_ISL_548985|Original|hCoV-19^^Singapore|Human|National Public Health Laboratory|National Public Health Laboratory|Mak|Singapore
>Spike|hCoV-19/Wales/PHWC-1664ED/2020|2020-06-19|EPI_ISL_494007|Original|hCoV-19^^Wales|Human|Originating lab|COVID-19 Genomics UK (COG-UK) Consortium|Moore|United Kingdom
>Spike|hCoV-19/England/QEUH-AF2562/2020|2020-10-30|EPI_ISL_662731|Original|hCoV-19^^England|Human|Lighthouse Lab in Glasgow|Wellcome Sanger Institute for the COVID-19 Genomics UK (COG-UK) Consortium|Davies|United Kingdom
>Spike|hCoV-19/USA/TX-HMH-MCoV-19660/2020|2020-11-19|EPI_ISL_784019|Original|hCoV-19^^Texas|Human|Houston Methodist Hospital|Houston Methodist Hospital|Long|USA
>Spike|hCoV-19/Japan/PG-0092/2020|2020-03-06|EPI_ISL_479838|Original|hCoV-19^^Hokkaido|Human|Sapporo City Institute of Public Health|Pathogen Genomics Center|Sekizuka|Japan
>Spike|hCoV-19/England/CAMB-1BA5D8/2020|2020-11-13|EPI_ISL_679157|Original|hCoV-19^^England|Human|Department of Pathology|COVID-19 Genomics UK (COG-UK) Consortium|Goodfellow|United Kingdom
>Spike|hCoV-19/Japan/PG-4582/2020|2020-07-00|EPI_ISL_689719|Original|hCoV-19^^Japan|Human|Pathogen Genomics Center|Pathogen Genomics Center|Sekizuka|Japan
>Spike|hCoV-19/Australia/NSW371/2020|2020-03-27|EPI_ISL_451632|Original|hCoV-19^^New South Wales|Human|South Eastern Area Laboratory Services|NSW Health Pathology - Institute of Clinical Pathology and Medical Research; Westmead Hospital; University of Sydney|Gall|Australia
>Spike|hCoV-19/England/QEUH-B9F690/2020|2020-11-18|EPI_ISL_721113|Original|hCoV-19^^England|Human|Lighthouse Lab in Glasgow|Wellcome Sanger Institute for the COVID-19 Genomics UK (COG-UK) Consortium|Davies|United Kingdom
>Spike|hCoV-19/England/LIVE-DB652B/2020|2020-11-06|EPI_ISL_652102|Original|hCoV-19^^England|Human|Liverpool Clinical Laboratories|COVID-19 Genomics UK (COG-UK) Consortium|Haldenby|United Kingdom
>Spike|hCoV-19/USA/HI-H200439/2020|2020-10-06|EPI_ISL_752982|Original|hCoV-19^^Hawaii|Human|State Laboratories Division|State Laboratories Division|Sultana|USA
>Spike|hCoV-19/New Zealand/20VR3482/2020|2020-04-03|EPI_ISL_579500|Original|hCoV-19^^Canterbury|Human|Canterbury Health Laboratories|Institute of Environmental Science and Research (ESR)|Wang|New Zealand
>Spike|hCoV-19/England/MILK-A18CE9/2020|2020-10-03|EPI_ISL_627481|Original|hCoV-19^^England|Human|University College London|COVID-19 Genomics UK (COG-UK) Consortium|Roy|United Kingdom
>Spike|hCoV-19/Australia/SAP263/2020|2020-03-25|EPI_ISL_468022|Original|hCoV-19^^South Australia|Human|SA Pathology|SA Pathology|Leong|Australia
>Spike|hCoV-19/England/LIVE-9AF4A/2020|2020-03-28|EPI_ISL_449401|Original|hCoV-19^^England|Human|Liverpool Clinical Laboratories|COVID-19 Genomics UK (COG-UK) Consortium|Haldenby|United Kingdom

>Spike|hCoV-19/USA/CA-CZB-15338/2020|2020-11-25|EPI_ISL_738996|Original|hCoV-19^^California|Human|Santa Clara County Public Health Laboratory|Chan-Zuckerberg Biohub|Kamm|USA
>Spike|hCoV-19/England/ALDP-B1C543/2020|2020-11-01|EPI_ISL_649484|Original|hCoV-19^^England|Human|Lighthouse Lab in Alderley Park|Wellcome Sanger Institute for the COVID-19 Genomics UK (COG-UK) Consortium|Davies|United Kingdom
>Spike|hCoV-19/England/MILK-97106B/2020|2020-08-16|EPI_ISL_553051|Original|hCoV-19^^England|Human|Lighthouse Lab in Milton Keynes|Wellcome Sanger Institute for the COVID-19 Genomics UK (COG-UK) consortium|Davies|United Kingdom
>Spike|hCoV-19/USA/OR-PROV-085/2020|2020-04-19|EPI_ISL_482356|Original|hCoV-19^^Oregon|Human|Providence St. Joseph Health Molecular Genomics Laboratory|Providence St. Joseph Health Molecular Genomics Laboratory|Dowdell|USA
>Spike|hCoV-19/Iceland/534/2020|2020-03-28|EPI_ISL_424554|Original|hCoV-19^^Reykjavik|Human|The National University Hospital of Iceland|deCODE genetics|J\u00f3nsson|Iceland
>Spike|hCoV-19/Denmark/DCGC-13903/2020|2020-11-30|EPI_ISL_713094|Original|hCoV-19^^Hovedstaden|Human|Department of Virus and Microbiological Special Diagnostics|Albertsen Lab|Michaelsen|Denmark
>Spike|hCoV-19/England/CAMC-B512D2/2020|2020-11-08|EPI_ISL_657845|Original|hCoV-19^^England|Human|Lighthouse Lab in Cambridge|Wellcome Sanger Institute for the COVID-19 Genomics UK (COG-UK) Consortium|Davies|United Kingdom
>Spike|hCoV-19/USA/NY-WCMP10B03P/2020|2020-03-15|EPI_ISL_427557|Original|hCoV-19^^New York|Human|NewYork-Presbyterian & Mason Lab|Mason Lab|Mason|USA
>Spike|hCoV-19/England/CAMB-7E094/2020|2020-04-06|EPI_ISL_442125|Original|hCoV-19^^England|Human|Department of Pathology|Wellcome Sanger Institute for the COVID-19 Genomics UK (COG-UK) consortium|Amato|United Kingdom
>Spike|hCoV-19/Denmark/DCGC-1001/2020|2020-05-11|EPI_ISL_614806|Original|hCoV-19^^Hovedstaden|Human|Department of Virus and Microbiological Special Diagnostics|Albertsen lab|Michaelsen|Denmark
>Spike|hCoV-19/Japan/PG-0336/2020|2020-03-31|EPI_ISL_480009|Original|hCoV-19^^Kanto|Human|Chiba Prefectural Institute of Public Health|Pathogen Genomics Center|Sekizuka|Japan
>Spike|hCoV-19/Scotland/GCVR-170802/2020|2020-03-26|EPI_ISL_489672|Original|hCoV-19^^Scotland|Human|NHSGGC West of Scotland Specialist Virology Centre / MRC-University of Glasgow Centre for Virus Research|Wellcome Sanger Institute for the COVID-19 Genomics UK (COG-UK) consortium|Davies|United Kingdom
>Spike|hCoV-19/USA/TX-DSHS-0312/2020|2020-06-13|EPI_ISL_534279|Original|hCoV-19^^Texas|Human|Texas Department of State Health Services|Texas Department of State Health Services|Rahman|USA
>Spike|hCoV-19/Luxembourg/LNS6572463/2020|2020-11-25|EPI_ISL_744432|Original|hCoV-19^^Luxembourg|Human|Laboratoire national de sant\u00e9|Laboratoire national de sant\u00e9|Wienecke-Baldacchino|Luxembourg
>Spike|hCoV-19/Uganda/UG002/2020|2020-03-26|EPI_ISL_451184|Original|hCoV-19^^Uganda|Human|Uganda Virus Research Institute|MRC/UVRI & LSHTM Uganda\u00a0Research Unit|Cotten|Uganda
>Spike|hCoV-19/England/ALDP-C3CBA4/2020|2020-12-10|EPI_ISL_731754|Original|hCoV-19^^England|Human|Lighthouse Lab in Alderley Park|Wellcome Sanger Institute for the COVID-19 Genomics UK (COG-UK) Consortium|Davies|United Kingdom
>Spike|hCoV-19/USA/TX-DSHS-1113/2020|2020-06-21|EPI_ISL_615150|Original|hCoV-19^^Texas|Human|Texas Department of State Health Services|Texas Department of State Health Services|Rahman|USA
>Spike|hCoV-19/England/NORW-F1D0B/2020|2020-10-19|EPI_ISL_627632|Original|hCoV-19^^England|Human|Quadram Institute Bioscience|COVID-19 Genomics UK (COG-UK) Consortium|Alikhan|United Kingdom
>Spike|hCoV-19/Australia/VIC6557/2020|2020-07-17|EPI_ISL_521325|Original|hCoV-19^^Victoria|Human|Victorian Infectious Diseases Reference Laboratory (VIDRL)|VIDRL and MDU-PHL|Schultz|Australia
>Spike|hCoV-19/USA/GA-QDX-585/2020|2020-03-18|EPI_ISL_571294|Original|hCoV-19^^Georgia|Human|Quest Diagnostics|Quest Diagnostics|Kagan|USA

>Spike|hCoV-19/England/CAMC-A81A4B/2020|2020-10-12|EPI_ISL_610718|Original|hCoV-19^^England|Human|Lighthouse Lab in Cambridge|Wellcome Sanger Institute for the COVID-19 Genomics UK (COG-UK) consortium|Davies|United Kingdom
>Spike|hCoV-19/England/LEED-2AA4DD/2020|2020-04-02|EPI_ISL_538840|Original|hCoV-19^^England|Human|Leeds Teaching Hospitals NHS Trust and Public Health England|Wellcome Sanger Institute for the COVID-19 Genomics UK (COG-UK) consortium|Davies|United Kingdom
>Spike|hCoV-19/England/LIVE-A5C72/2020|2020-03-17|EPI_ISL_499463|Original|hCoV-19^^England|Human|Liverpool Clinical Laboratories|COVID-19 Genomics UK (COG-UK) Consortium|Haldenby|United Kingdom
>Spike|hCoV-19/USA/WA-UW122/2020|2020-03-10|EPI_ISL_416660|Original|hCoV-19^^Washington|Human|UW Virology Lab|UW Virology Lab|Roychoudhury|USA
>Spike|hCoV-19/Australia/VIC6850/2020|2020-07-25|EPI_ISL_564095|Original|hCoV-19^^Victoria|Human|Victorian Infectious Diseases Reference Laboratory (VIDRL)|VIDRL and MDU-PHL|Schultz|Australia
>Spike|hCoV-19/Portugal/PT1590/2020|2020-07-02|EPI_ISL_693595|Original|hCoV-19^^Portugal|Human|Instituto Nacional de Saude (INSA)|Instituto Nacional de Saude (INSA)|Borges|Portugal
>Spike|hCoV-19/USA/MN-MDH-1911/2020|2020-10-18|EPI_ISL_613515|Original|hCoV-19^^Minnesota|Human|Public Health Laboratory - Infectious Disease Lab|Minnesota Department of Health|Lorentz|USA
>Spike|hCoV-19/USA/CT-QDX-1403/2020|2020-03-16|EPI_ISL_572099|Original|hCoV-19^^Connecticut|Human|Quest Diagnostics|Quest Diagnostics|Kagan|USA
>Spike|hCoV-19/England/MILK-B94EDF/2020|2020-11-13|EPI_ISL_676167|Original|hCoV-19^^England|Human|Lighthouse Lab in Milton Keynes|Wellcome Sanger Institute for the COVID-19 Genomics UK (COG-UK) Consortium|Davies|United Kingdom
>Spike|hCoV-19/Portugal/PT0967/2020|2020-03-29|EPI_ISL_511516|Original|hCoV-19^^Portugal|Human|Instituto Nacional de Saude (INSA)|Instituto Nacional de Saude (INSA)|Borges|Portugal
>Spike|hCoV-19/England/BRIS-1295C5/2020|2020-04-20|EPI_ISL_458742|Original|hCoV-19^^England|Human|PHE South West Regional Laboratory|Wellcome Sanger Institute for the COVID-19 Genomics UK (COG-UK) consortium|Davies|United Kingdom
>Spike|hCoV-19/England/ALDP-9ED079/2020|2020-05-05|EPI_ISL_589445|Original|hCoV-19^^England|Human|Lighthouse Lab in Alderley Park|Wellcome Sanger Institute for the COVID-19 Genomics UK (COG-UK) consortium|Davies|United Kingdom
>Spike|hCoV-19/USA/FL-BPHL-0595/2020|2020-03-24|EPI_ISL_508799|Original|hCoV-19^^Florida|Human|Florida Bureau of Public Health Laboratories|Florida Bureau of Public Health Laboratories|Blanton|USA
>Spike|hCoV-19/England/MILK-BB0462/2020|2020-11-17|EPI_ISL_720041|Original|hCoV-19^^England|Human|Lighthouse Lab in Milton Keynes|Wellcome Sanger Institute for the COVID-19 Genomics UK (COG-UK) Consortium|Davies|United Kingdom
>Spike|hCoV-19/Australia/VIC12408/2020|2020-07-29|EPI_ISL_591979|Original|hCoV-19^^Victoria|Human|Microbiological Diagnostic Unit - Public Health Laboratory (MDU-PHL)|MDU-PHL|Schultz|Australia
>Spike|hCoV-19/Mexico/BCN-ALSR-1697/2020|2020-04-24|EPI_ISL_496358|Original|hCoV-19^^Baja California|Human|Infectolab|Andersen lab at Scripps Research|Gangavarapu|Mexico
>Spike|hCoV-19/England/CAMB-74F37/2020|2020-03-18|EPI_ISL_440394|Original|hCoV-19^^England|Human|Department of Pathology|Wellcome Sanger Institute for the COVID-19 Genomics UK (COG-UK) consortium|Amato|United Kingdom
>Spike|hCoV-19/England/NOTT-121859/2020|2020-12-22|EPI_ISL_764515|Original|hCoV-19^^England|Human|Queens Medical Centre|COVID-19 Genomics UK (COG-UK) Consortium|Loose|United Kingdom
>Spike|hCoV-19/England/ALDP-954611/2020|2020-06-14|EPI_ISL_553855|Original|hCoV-19^^England|Human|Lighthouse Lab in Alderley Park|Wellcome Sanger Institute for the COVID-19 Genomics UK (COG-UK) consortium|Davies|United Kingdom
>Spike|hCoV-19/Wales/PHWC-28442/2020|2020-03-27|EPI_ISL_431909|Original|hCoV-19^^Wales|Human|Wales Specialist Virology Centre|Public Health Wales Microbiology Cardiff|Moore|United Kingdom

>Spike|hCoV-19/USA/WA-UW-10317/2020|2020-06-03|EPI_ISL_570295|Original|hCoV-19^^Washington|Human|UW Virology Lab|UW Virology Lab|Roychoudhury|USA
>Spike|hCoV-19/England/ALDP-A85713/2020|2020-10-17|EPI_ISL_610445|Original|hCoV-19^^England|Human|Lighthouse Lab in Alderley Park|Wellcome Sanger Institute for the COVID-19 Genomics UK (COG-UK) consortium|Davies|United Kingdom
>Spike|hCoV-19/Wales/PHWC-496FB3/2020|2020-11-25|EPI_ISL_726759|Original|hCoV-19^^Wales|Human|Originating lab|COVID-19 Genomics UK (COG-UK) Consortium|Moore|United Kingdom
>Spike|hCoV-19/England/LOND-126109F/2020|2020-04-03|EPI_ISL_524630|Original|hCoV-19^^England|Human|North West London Pathology|Wellcome Sanger Institute for the COVID-19 Genomics UK (COG-UK) consortium|Davies|United Kingdom
>Spike|hCoV-19/USA/NY-PV08474/2020|2020-03-17|EPI_ISL_421417|Original|hCoV-19^^New York|Human|MSHS Clinical Microbiology Laboratories|MSHS Pathogen Surveillance Program|van Bakel|USA
>Spike|hCoV-19/USA/MN-MDH-1122/2020|2020-04-23|EPI_ISL_476707|Original|hCoV-19^^Minnesota|Human|Minnesota Department of Health|Minnesota Department of Health|Garfin|USA
>Spike|hCoV-19/Japan/IC-0500/2020|2020-12-00|EPI_ISL_768709|Original|hCoV-19^^Japan|Human|Pathogen Genomics Center|Pathogen Genomics Center|Sekizuka|Japan
>Spike|hCoV-19/USA/TX-HMH055/2020|2020-03-17|EPI_ISL_434764|Original|hCoV-19^^Texas|Human|Houston Methodist Hospital|Houston Methodist Hospital|Long|USA
>Spike|hCoV-19/England/ALDP-A2F098/2020|2020-10-05|EPI_ISL_598828|Original|hCoV-19^^England|Human|Lighthouse Lab in Alderley Park|Wellcome Sanger Institute for the COVID-19 Genomics UK (COG-UK) consortium|Davies|United Kingdom
>Spike|hCoV-19/Japan/PG-1195/2020|2020-04-00|EPI_ISL_684805|Original|hCoV-19^^Japan|Human|Yamagata Prefectural Institute of Public Health|Pathogen Genomics Center|Sekizuka|Japan
>Spike|hCoV-19/India/TG-CCMB-O3/2020|2020-04-14|EPI_ISL_458068|Original|hCoV-19^^Telangana|Human|Osmania Medical College|CSIR-Centre for Cellular and Molecular Biology|Banu|India
>Spike|hCoV-19/Japan/PG-6168/2020|2020-08-00|EPI_ISL_689280|Original|hCoV-19^^Japan|Human|Pathogen Genomics Center|Pathogen Genomics Center|Sekizuka|Japan
>Spike|hCoV-19/England/ALDP-9E6702/2020|2020-09-25|EPI_ISL_580514|Original|hCoV-19^^England|Human|Lighthouse Lab in Alderley Park|Wellcome Sanger Institute for the COVID-19 Genomics UK (COG-UK) consortium|Davies|United Kingdom
>Spike|hCoV-19/Wales/PHWC-48992E/2020|2020-10-26|EPI_ISL_652426|Original|hCoV-19^^Wales|Human|Wales Specialist Virology Centre Sequencing lab|COVID-19 Genomics UK (COG-UK) Consortium|Moore|United Kingdom
>Spike|hCoV-19/Spain/AR-IBV-97009524/2020|2020-10-05|EPI_ISL_671803|Original|hCoV-19^^Aragon|Human|Hospital Cl\u00ednico Universitario Lozano Blesa de Zaragoza (Espa\u00f1a)|SeqCOVID-SPAIN consortium/IBV(CSIC)|Comas|Spain
>Spike|hCoV-19/South Africa/KRISP-K002357/2020|2020-07-21|EPI_ISL_515728|Original|hCoV-19^^KwaZulu-Natal|Human|NHLS-IALCH|KRISP|de Oliveira|South Africa
>Spike|hCoV-19/mink/Netherlands/NB-EMC-47-3/2020|2020-09-04|EPI_ISL_577811|Original|hCoV-19^^Noord Brabant|Neovison vison|Dutch COVID-19 response team|Erasmus Medical Center|Oude Munnink|Netherlands
>Spike|hCoV-19/Denmark/DCGC-22507/2020|2020-12-14|EPI_ISL_757601|Original|hCoV-19^^Midtjylland|Human|Department of Virus and Microbiological Special Diagnostics|Albertsen Lab|Michaelsen|Denmark
>Spike|hCoV-19/England/205260416/2020|2020-00-00|EPI_ISL_766536|Original|hCoV-19^^England|Human|Respiratory Virus Unit|COVID-19 Genomics UK (COG-UK) Consortium|Platt|United Kingdom
>Spike|hCoV-19/England/CAMB-7816A/2020|2020-04-01|EPI_ISL_441107|Original|hCoV-19^^England|Human|Department of Pathology|Wellcome Sanger Institute for the COVID-19 Genomics UK (COG-UK) consortium|Amato|United Kingdom
>Spike|hCoV-19/Wales/PHWC-15C7C3/2020|2020-04-01|EPI_ISL_472539|Original|hCoV-19^^Wales|Human|Wales Specialist Virology Centre Sequencing lab|COVID-19 Genomics UK (COG-UK) Consortium|Moore|United Kingdom

>Spike|hCoV-19/Australia/WA362/2020|2020-10-18|EPI_ISL_605839|Original|hCoV-19^^Western Australia|Human|PathWest Laboratory Medicine WA|PathWest Laboratory Medicine WA Microbial Surveillance Unit|Sikazwe|Australia
>Spike|hCoV-19/Japan/PG-1850/2020|2020-05-00|EPI_ISL_685456|Original|hCoV-19^^Japan|Human|Tokyo Metropolitan Institute of Public Health|Pathogen Genomics Center|Sekizuka|Japan
>Spike|hCoV-19/USA/WA-S153/2020|2020-03-16|EPI_ISL_430190|Original|hCoV-19^^Washington|Human|Washington State Department of Health|Seattle Flu Study|Bedford|USA
>Spike|hCoV-19/England/205090691/2020|2020-12-12|EPI_ISL_768806|Original|hCoV-19^^England|Human|Respiratory Virus Unit|COVID-19 Genomics UK (COG-UK) Consortium|Platt|United Kingdom
>Spike|hCoV-19/England/MILK-992538/2020|2020-08-27|EPI_ISL_540689|Original|hCoV-19^^England|Human|Queens Medical Centre|COVID-19 Genomics UK (COG-UK) Consortium|Loose|United Kingdom
>Spike|hCoV-19/England/MILK-B9392A/2020|2020-11-13|EPI_ISL_676015|Original|hCoV-19^^England|Human|Lighthouse Lab in Milton Keynes|Wellcome Sanger Institute for the COVID-19 Genomics UK (COG-UK) Consortium|Davies|United Kingdom
>Spike|hCoV-19/Wales/PHWC-24C99/2020|2020-03-21|EPI_ISL_420936|Original|hCoV-19^^Wales|Human|Wales Specialist Virology Centre|Public Health Wales Microbiology Cardiff|Moore|United Kingdom
>Spike|hCoV-19/Denmark/DCGC-2177/2020|2020-08-10|EPI_ISL_617476|Original|hCoV-19^^Midtjylland|Human|Department of Virus and Microbiological Special Diagnostics|Albertsen lab|Michaelsen|Denmark
>Spike|hCoV-19/England/QEUH-C93631/2020|2020-12-16|EPI_ISL_777223|Original|hCoV-19^^England|Human|Lighthouse Lab in Glasgow|Wellcome Sanger Institute for the COVID-19 Genomics UK (COG-UK) Consortium|Davies|United Kingdom
>Spike|hCoV-19/Netherlands/NB-EMC-183/2020|2020-05-08|EPI_ISL_523306|Original|hCoV-19^^Noord Brabant|Human|Dutch COVID-19 response team|Erasmus Medical Center|Oude Munnink|Netherlands
>Spike|hCoV-19/USA/AZ-TG380688/2020|2020-06-16|EPI_ISL_694646|Original|hCoV-19^^Arizona|Human|TGen North|TGen North|French|USA
>Spike|hCoV-19/England/NORW-222F27/2020|2020-12-19|EPI_ISL_741550|Original|hCoV-19^^England|Human|Quadram Institute Bioscience|COVID-19 Genomics UK (COG-UK) Consortium|Alikhan|United Kingdom
>Spike|hCoV-19/Wales/QEUH-96A2BA/2020|2020-08-13|EPI_ISL_536850|Original|hCoV-19^^Wales|Human|Lighthouse Lab in Glasgow|Wellcome Sanger Institute for the COVID-19 Genomics UK (COG-UK) consortium|Davies|United Kingdom
>Spike|hCoV-19/England/BRIS-12F310/2020|2020-04-13|EPI_ISL_488204|Original|hCoV-19^^England|Human|PHE South West Regional Laboratory|Wellcome Sanger Institute for the COVID-19 Genomics UK (COG-UK) consortium|Davies|United Kingdom
>Spike|hCoV-19/USA/CA-CZB-13468/2020|2020-10-22|EPI_ISL_672501|Original|hCoV-19^^California|Human|UCSF Clinical Microbiology Laboratory|Chan-Zuckerberg Biohub|Bhatt|USA
>Spike|hCoV-19/England/ALDP-4FB550/2020|2020-06-23|EPI_ISL_559103|Original|hCoV-19^^England|Human|Lighthouse Lab in Alderley Park|Wellcome Sanger Institute for the COVID-19 Genomics UK (COG-UK) consortium|Davies|United Kingdom
>Spike|hCoV-19/USA/MI-MDHHS-SC20710/2020|2020-04-27|EPI_ISL_471734|Original|hCoV-19^^Michigan|Human|Michigan Department of Health and Human Services|Michigan Department of Health and Human Services|Blankenship|USA
>Spike|hCoV-19/Denmark/DCGC-20498/2020|2020-12-21|EPI_ISL_759186|Original|hCoV-19^^Syddanmark|Human|Department of Virus and Microbiological Special Diagnostics|Albertsen Lab|Michaelsen|Denmark
>Spike|hCoV-19/Denmark/DCGC-18001/2020|2020-12-14|EPI_ISL_749692|Original|hCoV-19^^Midtjylland|Human|Department of Virus and Microbiological Special Diagnostics|Albertsen Lab|Michaelsen|Denmark
>Spike|hCoV-19/USA/TX-HMH-7766/2020|2020-06-26|EPI_ISL_545624|Original|hCoV-19^^Texas|Human|Houston Methodist Hospital|Houston Methodist Hospital|Long|USA

>Spike|hCoV-19/England/ALDP-CBE023/2020|2020-12-20|EPI_ISL_760293|Original|hCoV-19^^England|Human|Lighthouse Lab in Alderley Park|Wellcome Sanger Institute for the COVID-19 Genomics UK (COG-UK) Consortium|Davies|United Kingdom
>Spike|hCoV-19/England/CAMC-A66648/2020|2020-10-11|EPI_ISL_627118|Original|hCoV-19^^England|Human|University of Exeter|COVID-19 Genomics UK (COG-UK) Consortium|Temperton|United Kingdom
>Spike|hCoV-19/Denmark/DCGC-10789/2020|2020-11-02|EPI_ISL_670208|Original|hCoV-19^^Nordjylland|Human|Department of Virus and Microbiological Special Diagnostics|Albertsen Lab|Michaelsen|Denmark
>Spike|hCoV-19/England/CAMB-7FD9C/2020|2020-04-19|EPI_ISL_444412|Original|hCoV-19^^England|Human|Department of Pathology|COVID-19 Genomics UK (COG-UK) Consortium|Meredith|United Kingdom
>Spike|hCoV-19/Ireland/D-NVRL-AIIDV1366v1s2/2020|2020-11-13|EPI_ISL_791284|Original|hCoV-19^^Dublin|Human|National Virus Reference Laboratory|Irish Coronavirus Sequencing Consortium - Teagasc Moorepark|Walsh|Ireland
>Spike|hCoV-19/Spain/CM-IBV-003751/2020|2020-03-31|EPI_ISL_474799|Original|hCoV-19^^Castilla La Mancha|Human|Complejo Hospitalario Universitario de Albacete|SeqCOVID-SPAIN consortium/IBV(CSIC)|Comas|Spain
>Spike|hCoV-19/Scotland/QEUH-9F84AC/2020|2020-09-27|EPI_ISL_600829|Original|hCoV-19^^Scotland|Human|Lighthouse Lab in Glasgow|Wellcome Sanger Institute for the COVID-19 Genomics UK (COG-UK) consortium|Davies|United Kingdom
>Spike|hCoV-19/USA/WI-UW-341/2020|2020-04-02|EPI_ISL_436635|Original|hCoV-19^^Wisconsin|Human|University of Wisconsin-Madison AIDS Vaccine Research Laboratories|University of Wisconsin-Madison AIDS Vaccine Research Laboratories|Baczenas|USA
>Spike|hCoV-19/Australia/SAP082/2020|2020-03-31|EPI_ISL_451118|Original|hCoV-19^^South Australia|Human|SA Pathology|SA Pathology|Leong|Australia
>Spike|hCoV-19/England/ALDP-9FDBEE/2020|2020-10-02|EPI_ISL_587954|Original|hCoV-19^^England|Human|Lighthouse Lab in Alderley Park|Wellcome Sanger Institute for the COVID-19 Genomics UK (COG-UK) consortium|Davies|United Kingdom
>Spike|hCoV-19/USA/CA-CSMC62/2020|2020-04-01|EPI_ISL_475676|Original|hCoV-19^^California|Human|Cedars-Sinai Medical Center|Cedars-Sinai Medical Center|Zhang|USA
>Spike|hCoV-19/England/20102068506/2020|2020-03-01|EPI_ISL_415149|Original|hCoV-19^^England|Human|Respiratory Virus Unit|Respiratory Virus Unit|Platt|United Kingdom
>Spike|hCoV-19/USA/CA-CZB-9714/2020|2020-03-20|EPI_ISL_672157|Original|hCoV-19^^California|Human|The Ashley Laboratory|Chan-Zuckerberg Biohub|Bhatt|USA
>Spike|hCoV-19/USA/TX-HMH-1299/2020|2020-04-25|EPI_ISL_542636|Original|hCoV-19^^Texas|Human|Houston Methodist Hospital|Houston Methodist Hospital|Long|USA
>Spike|hCoV-19/Singapore/68/2020|2020-04-15|EPI_ISL_428847|Original|hCoV-19^^Singapore|Human|National Public Health Laboratory|National Public Health Laboratory|Mak|Singapore
>Spike|hCoV-19/Scotland/CVR5752/2020|2020-09-29|EPI_ISL_651498|Original|hCoV-19^^Scotland|Human|West of Scotland Specialist Virology Centre|COVID-19 Genomics UK (COG-UK) Consortium|Robertson|United Kingdom
>Spike|hCoV-19/USA/HI-H200182/2020|2020-07-30|EPI_ISL_752742|Original|hCoV-19^^Hawaii|Human|State Laboratories Division|State Laboratories Division|Sultana|USA
>Spike|hCoV-19/Denmark/DCGC-14500/2020|2020-11-30|EPI_ISL_711357|Original|hCoV-19^^Hovedstaden|Human|Department of Virus and Microbiological Special Diagnostics|Albertsen Lab|Michaelsen|Denmark
>Spike|hCoV-19/USA/CA-CZB-1863/2020|2020-04-28|EPI_ISL_486168|Original|hCoV-19^^California|Human|Orange County Public Health Laboratory|Chan-Zuckerberg Biohub|McGeever|USA
>Spike|hCoV-19/USA/LA-EVTL387/2020|2020-06-04|EPI_ISL_578793|Original|hCoV-19^^Louisiana|Human|LSUHS Emerging Viral Threat Laboratory|Microbial Genome Sequencing Center|Kamil|USA
>Spike|hCoV-19/Germany/NW-HHU-327/2020|2020-10-12|EPI_ISL_770734|Original|hCoV-19^^North Rhine-Westphalia|Human|ZOTZ KLIMAS MVZ D\u00fcsseldorf-Centrum GbR \u00dcBAG f\u00fcr Labormedizin|Center of Medical Microbiology|Wienemann|Germany

>Spike|hCoV-19/England/ALDP-C3A757/2020|2020-12-09|EPI_ISL_731428|Original|hCoV-19^^England|Human|Lighthouse Lab in Alderley Park|Wellcome Sanger Institute for the COVID-19 Genomics UK (COG-UK) Consortium|Davies|United Kingdom
>Spike|hCoV-19/Portugal/PT0705/2020|2020-03-21|EPI_ISL_510964|Original|hCoV-19^^Portugal|Human|Instituto Nacional de Saude (INSA)|Instituto Nacional de Saude (INSA)|Borges|Portugal
>Spike|hCoV-19/USA/TX-DSHS-1996/2020|2020-06-21|EPI_ISL_766738|Original|hCoV-19^^Texas|Human|Texas Department of State Health Services|Texas Department of State Health Services|Rahman|USA
>Spike|hCoV-19/Japan/PG-0427/2020|2020-04-05|EPI_ISL_480095|Original|hCoV-19^^Kansai|Human|Department of Infectious Diseases|Pathogen Genomics Center|Sekizuka|Japan
>Spike|hCoV-19/Belgium/ULG-10240/2020|2020-06-30|EPI_ISL_498146|Original|hCoV-19^^Li\u00e8ge|Human|Department of Clinical Microbiology|GIGA Medical Genomics|Durkin|Belgium
>Spike|hCoV-19/USA/CA-CZB-14749/2020|2020-10-21|EPI_ISL_738756|Original|hCoV-19^^California|Human|Humboldt County Public Health Laboratory|Chan-Zuckerberg Biohub|Kamm|USA
>Spike|hCoV-19/USA/MN-MDH-2179/2020|2020-11-26|EPI_ISL_723130|Original|hCoV-19^^Minnesota|Human|Minnesota Department of Health|Minnesota Department of Health|Lorentz|USA
>Spike|hCoV-19/Scotland/QEUH-9FB5B5/2020|2020-09-29|EPI_ISL_600595|Original|hCoV-19^^Scotland|Human|Lighthouse Lab in Glasgow|Wellcome Sanger Institute for the COVID-19 Genomics UK (COG-UK) consortium|Davies|United Kingdom
>Spike|hCoV-19/England/ALDP-A8584D/2020|2020-10-17|EPI_ISL_610464|Original|hCoV-19^^England|Human|Lighthouse Lab in Alderley Park|Wellcome Sanger Institute for the COVID-19 Genomics UK (COG-UK) consortium|Davies|United Kingdom
>Spike|hCoV-19/USA/CA-CZB-3728/2020|2020-07-21|EPI_ISL_548418|Original|hCoV-19^^California|Human|Orange County Public Health Laboratory|Chan-Zuckerberg Biohub|Bhatt|USA
>Spike|hCoV-19/England/QEUH-B137FB/2020|2020-11-02|EPI_ISL_641634|Original|hCoV-19^^England|Human|Lighthouse Lab in Glasgow|Wellcome Sanger Institute for the COVID-19 Genomics UK (COG-UK) Consortium|Davies|United Kingdom
>Spike|hCoV-19/Denmark/DCGC-15220/2020|2020-11-23|EPI_ISL_713703|Original|hCoV-19^^Sjaelland|Human|Department of Virus and Microbiological Special Diagnostics|Albertsen Lab|Michaelsen|Denmark
>Spike|hCoV-19/Finland/4A70S1/2020|2020-04-04|EPI_ISL_481671|Original|hCoV-19^^Uusimaa|Human|Department of Virology and Immunology|Department of Virology|Smura|Finland
>Spike|hCoV-19/England/20144075204/2020|2020-04-01|EPI_ISL_423567|Original|hCoV-19^^England|Human|Respiratory Virus Unit|Respiratory Virus Unit|Platt|United Kingdom
>Spike|hCoV-19/England/ALDP-9EAD48/2020|2020-09-24|EPI_ISL_580142|Original|hCoV-19^^England|Human|Lighthouse Lab in Alderley Park|Wellcome Sanger Institute for the COVID-19 Genomics UK (COG-UK) consortium|Davies|United Kingdom
>Spike|hCoV-19/Portugal/PT0390a/2020|2020-04-14|EPI_ISL_454108|Original|hCoV-19^^Portugal|Human|unknown|Instituto Nacional de Saude (INSA)|Borges|Portugal
>Spike|hCoV-19/Singapore/847/2020|2020-07-12|EPI_ISL_518011|Original|hCoV-19^^Singapore|Human|Singapore General Hospital|Department of Microbiology|Abdul Rahman|Singapore
>Spike|hCoV-19/England/QEUH-9FB360/2020|2020-09-28|EPI_ISL_588160|Original|hCoV-19^^England|Human|Lighthouse Lab in Glasgow|Wellcome Sanger Institute for the COVID-19 Genomics UK (COG-UK) consortium|Davies|United Kingdom
>Spike|hCoV-19/USA/MI-QDX-717/2020|2020-03-20|EPI_ISL_571450|Original|hCoV-19^^Michigan|Human|Quest Diagnostics|Quest Diagnostics|Kagan|USA
>Spike|hCoV-19/Japan/PG-4431/2020|2020-07-00|EPI_ISL_692655|Original|hCoV-19^^Japan|Human|Sakai City Institute of Public Health|Pathogen Genomics Center|Sekizuka|Japan
>Spike|hCoV-19/Denmark/DCGC-6547/2020|2020-10-05|EPI_ISL_619447|Original|hCoV-19^^Midtjylland|Human|Department of Virus and Microbiological Special Diagnostics|Albertsen lab|Michaelsen|Denmark

>Spike|hCoV-19/Wales/PHWC-489830/2020|2020-11-02|EPI_ISL_638064|Original|hCoV-
19^^Wales|Human|Wales Specialist Virology Centre Sequencing lab|COVID-19 Genomics UK
(COG-UK) Consortium|Moore|United Kingdom
>Spike|hCoV-19/Australia/VIC6718/2020|2020-07-25|EPI_ISL_564009|Original|hCoV-
19^^Victoria|Human|Victorian Infectious Diseases Reference Laboratory (VIDRL)|VIDRL
and MDU-PHL|Schultz|Australia
>Spike|hCoV-19/Denmark/DCGC-19785/2020|2020-12-07|EPI_ISL_747543|Original|hCoV-
19^^Hovedstaden|Human|Department of Virus and Microbiological Special
Diagnostics|Albertsen Lab|Michaelsen|Denmark
>Spike|hCoV-19/England/QEUH-AD50FB/2020|2020-10-26|EPI_ISL_634057|Original|hCoV-
19^^England|Human|Lighthouse Lab in Glasgow|Wellcome Sanger Institute for the COVID-
19 Genomics UK (COG-UK) consortium|Davies|United Kingdom
>Spike|hCoV-19/Germany/HH-hpi-p2446/2020|2020-04-21|EPI_ISL_775724|Original|hCoV-
19^^Hamburg|Human|University Medical Center Hamburg Eppendorf|Heinrich Pette
Institute|Robitaille|Germany
>Spike|hCoV-19/England/MILK-D0B475/2020|2020-12-20|EPI_ISL_781891|Original|hCoV-
19^^England|Human|Lighthouse Lab in Milton Keynes|Wellcome Sanger Institute for the
COVID-19 Genomics UK (COG-UK) Consortium|Davies|United Kingdom
>Spike|hCoV-19/England/NORW-E7852/2020|2020-05-06|EPI_ISL_449076|Original|hCoV-
19^^England|Human|Quadram Institute Bioscience|COVID-19 Genomics UK (COG-UK)
Consortium|Alikhan|United Kingdom
>Spike|hCoV-19/USA/MA-MGH-01502/2020|2020-05-07|EPI_ISL_791960|Original|hCoV-
19^^Massachusetts|Human|Massachusetts General Hospital|Infectious Disease
Program|Park|USA
>Spike|hCoV-19/Switzerland/BE-ETHZ-320581/2020|2020-10-
12|EPI_ISL_737688|Original|hCoV-19^^Bern|Human|Viollier AG|Department of Biosystems
Science and Engineering|Nadeau|Switzerland
>Spike|hCoV-19/USA/WA-UW-12367/2020|2020-06-27|EPI_ISL_570517|Original|hCoV-
19^^Washington|Human|UW Virology Lab|UW Virology Lab|Roychoudhury|USA
>Spike|hCoV-19/South Africa/LR00038/2020|2020-07-04|EPI_ISL_515165|Original|hCoV-
19^^Limpopo|Human|National Institute for Communicable Diseases of the National Health
Laboratory Service|National Institute for Communicable Diseases of the National
Health Laboratory Service|Allam|South Africa
>Spike|hCoV-19/Trinidad/TT47007/2020|2020-12-17|EPI_ISL_756310|Original|hCoV-
19^^Trinidad|Human|Caribbean Regional Health Authority|Carrington
Lab|Sahadeo|Trinidad
>Spike|hCoV-19/Switzerland/SH-ETHZ-410531/2020|2020-11-
26|EPI_ISL_737773|Original|hCoV-19^^Schaffhausen|Human|Viollier AG|Department of
Biosystems Science and Engineering|Nadeau|Switzerland
>Spike|hCoV-19/Denmark/DCGC-8085/2020|2020-10-26|EPI_ISL_617166|Original|hCoV-
19^^Hovedstaden|Human|Department of Virus and Microbiological Special
Diagnostics|Albertsen lab|Michaelsen|Denmark
>Spike|hCoV-19/USA/UN-UW-13444/2020|2020-06-30|EPI_ISL_501085|Original|hCoV-
19^^USA|Human|University of Washington Virology Lab|University of Washington Virology
Lab|Roychoudhury|USA
>Spike|hCoV-19/Crimea/SRC-80603/2020|2020-03-23|EPI_ISL_428901|Vero|hCoV-
19^^Crimea|Human|State Research Center of Virology and Biotechnology VECTOR|State
Research Center of Virology and Biotechnology VECTOR|Shvalov|Crimea
>Spike|hCoV-19/Spain/MD-HLP-49/2020|2020-04-20|EPI_ISL_530052|Original|hCoV-
19^^Madrid|Human|Hospital Universitario La Paz|Hospital Universitario La
Paz|Viedma|Spain
>Spike|hCoV-19/USA/DE-DHSS-F1045879/2020|2020-12-21|EPI_ISL_766971|Original|hCoV-
19^^Delaware|Human|Delaware Public Health Laboratory|Delaware Public Health
Laboratory|Bajwa|USA
>Spike|hCoV-19/Luxembourg/LNS7658808/2020|2020-11-21|EPI_ISL_744608|Original|hCoV-
19^^Luxembourg|Human|Laboratoire national de sant\u00e9|Laboratoire national de
sant\u00e9|Wienecke-Baldacchino|Luxembourg
>Spike|hCoV-19/USA/WA-S369/2020|2020-03-26|EPI_ISL_434129|Original|hCoV-
19^^Washington|Human|Washington State Department of Health|Seattle Flu
Study|Bedford|USA

>Spike|hCoV-19/USA/TX-HMH-MCoV-15240/2020|2020-10-19|EPI_ISL_786724|Original|hCoV-19^^Texas|Human|Houston Methodist Hospital|Houston Methodist Hospital|Long|USA
>Spike|hCoV-19/Denmark/DCGC-3050/2020|2020-08-31|EPI_ISL_616830|Original|hCoV-19^^Midtjylland|Human|Department of Virus and Microbiological Special Diagnostics|Albertsen lab|Michaelsen|Denmark
>Spike|hCoV-19/Wales/PHWC-47FE77/2020|2020-10-07|EPI_ISL_612821|Original|hCoV-19^^Wales|Human|Wales Specialist Virology Centre Sequencing lab|COVID-19 Genomics UK (COG-UK) Consortium|Moore|United Kingdom
>Spike|hCoV-19/Wales/PHWC-4A4DC7/2020|2020-12-09|EPI_ISL_764792|Original|hCoV-19^^Wales|Human|Wales Specialist Virology Centre Sequencing lab|COVID-19 Genomics UK (COG-UK) Consortium|Moore|United Kingdom
>Spike|hCoV-19/Canada/ON-UHTC_0407/2020|2020-11-24|EPI_ISL_698085|Original|hCoV-19^^Ontario|Human|Unity Health Toronto|Ontario Institute for Cancer Research|de Borja|Canada
>Spike|hCoV-19/England/CAMC-B33C6F/2020|2020-11-05|EPI_ISL_659319|Original|hCoV-19^^England|Human|Lighthouse Lab in Cambridge|Wellcome Sanger Institute for the COVID-19 Genomics UK (COG-UK) Consortium|Davies|United Kingdom
>Spike|hCoV-19/Russia/AST-RII-MH4438S/2020|2020-08-15|EPI_ISL_733187|Original|hCoV-19^^Astrakhan Region|Human|Pathogenic Microorganisms Variability Laboratory|WHO National Influenza Centre Russian Federation|Komissarov|Russia
>Spike|hCoV-19/England/ALDP-9EA443/2020|2020-09-24|EPI_ISL_579966|Original|hCoV-19^^England|Human|Lighthouse Lab in Alderley Park|Wellcome Sanger Institute for the COVID-19 Genomics UK (COG-UK) consortium|Davies|United Kingdom
>Spike|hCoV-19/Belgium/UGent-25/2020|2020-03-25|EPI_ISL_425064|Original|hCoV-19^^Ghent|Human|Lab voor klinische biologie|Onderzoeksgroep Virologie|Lambrechts|Belgium
>Spike|hCoV-19/Austria/CeMM0011/2020|2020-03-23|EPI_ISL_419664|Original|hCoV-19^^Austria|Human|Center for Virology|Bergthaler laboratory|Bergthaler|Austria
>Spike|hCoV-19/England/ALDP-952F27/2020|2020-06-11|EPI_ISL_553680|Original|hCoV-19^^England|Human|Lighthouse Lab in Alderley Park|Wellcome Sanger Institute for the COVID-19 Genomics UK (COG-UK) consortium|Davies|United Kingdom
>Spike|hCoV-19/Belgium/LMA-0330384/2020|2020-03-30|EPI_ISL_462238|Original|hCoV-19^^Mariakerke|Human|KU Leuven|KU Leuven|Maes|Belgium
>Spike|hCoV-19/Ireland/MH-NVRL-20G47269/2020|2020-04-03|EPI_ISL_708526|Original|hCoV-19^^Meath|Human|National Virus Reference Laboratory|Irish Coronavirus Sequencing Consortium - Teagasc Oakpark|Della Bartola|Ireland
>Spike|hCoV-19/England/QEUH-CDEB4A/2020|2020-12-26|EPI_ISL_782468|Original|hCoV-19^^England|Human|Lighthouse Lab in Glasgow|Wellcome Sanger Institute for the COVID-19 Genomics UK (COG-UK) Consortium|Davies|United Kingdom
>Spike|hCoV-19/USA/WA-UW-5488/2020|2020-04-30|EPI_ISL_776732|Original|hCoV-19^^Washington|Human|UW Virology Lab|UW Virology Lab|Roychoudhury|USA
>Spike|hCoV-19/Australia/VIC14065/2020|2020-08-22|EPI_ISL_562657|Original|hCoV-19^^Victoria|Human|Microbiological Diagnostic Unit - Public Health Laboratory (MDU-PHL)|MDU-PHL|Schultz|Australia
>Spike|hCoV-19/India/UP-NBRI-N33-TCX148/2020|2020-04-16|EPI_ISL_516962|Original|hCoV-19^^Uttar Pradesh|Human|King Georges Medical University|CSIR-National Botanical Research Institute|Bag|India
>Spike|hCoV-19/USA/CA-CHLA-PLM31514880/2020|2020-11-10|EPI_ISL_753300|Original|hCoV-19^^California|Human|Clinical virology Laboratory|Center for Personalized Medicine|Dien Bard|USA
>Spike|hCoV-19/England/NORW-F9BEB/2020|2020-12-11|EPI_ISL_725433|Original|hCoV-19^^England|Human|Quadram Institute Bioscience|COVID-19 Genomics UK (COG-UK) Consortium|Alikhan|United Kingdom
>Spike|hCoV-19/England/QEUH-B682F6/2020|2020-11-11|EPI_ISL_661508|Original|hCoV-19^^England|Human|Lighthouse Lab in Glasgow|Wellcome Sanger Institute for the COVID-19 Genomics UK (COG-UK) Consortium|Davies|United Kingdom
>Spike|hCoV-19/England/MILK-874069/2020|2020-07-20|EPI_ISL_557394|Original|hCoV-19^^England|Human|Lighthouse Lab in Milton Keynes|Wellcome Sanger Institute for the COVID-19 Genomics UK (COG-UK) consortium|Davies|United Kingdom

>Spike|hCoV-19/Belgium/ULG-10640/2020|2020-10-27|EPI_ISL_636618|Original|hCoV-
19^^Li\u00e8ge|Human|Department of Clinical Microbiology|GIGA Medical
Genomics|Durkin|Belgium
>Spike|hCoV-19/USA/WI-UW-2099/2020|2020-10-30|EPI_ISL_677504|Original|hCoV-
19^^Wisconsin|Human|University of Wisconsin-Madison AIDS Vaccine Research
Laboratories|University of Wisconsin-Madison AIDS Vaccine Research
Laboratories|Baczenas|USA
>Spike|hCoV-19/England/BRIS-126D75/2020|2020-04-09|EPI_ISL_458997|Original|hCoV-
19^^England|Human|PHE South West Regional Laboratory|Wellcome Sanger Institute for
the COVID-19 Genomics UK (COG-UK) consortium|Davies|United Kingdom
>Spike|hCoV-19/Northern Ireland/NIRE-1091E5/2020|2020-10-
20|EPI_ISL_650930|Original|hCoV-19^^Northern Ireland|Human|Regional Virus
Laboratory|COVID-19 Genomics UK (COG-UK) Consortium|Fairley|United Kingdom
>Spike|hCoV-19/Switzerland/NE-ETHZ-350428/2020|2020-11-
04|EPI_ISL_728853|Original|hCoV-19^^Neuch\u00e2tel|Human|Viollier AG|Department of
Biosystems Science and Engineering|Nadeau|Switzerland
>Spike|hCoV-19/USA/UT-03428/2020|2020-04-20|EPI_ISL_498658|Original|hCoV-
19^^Utah|Human|Utah Public Health Laboratory|Utah Public Health Laboratory|Butz|USA
>Spike|hCoV-19/South Africa/KRISP-0412/2020|2020-07-02|EPI_ISL_495537|Original|hCoV-
19^^KwaZulu-Natal|Human|Medical Disagnostics Services (MDS)|KRISP|de Oliveira|South
Africa
>Spike|hCoV-19/England/OXON-B3E8A/2020|2020-04-02|EPI_ISL_534914|Original|hCoV-
19^^England|Human|Oxford Viromics|COVID-19 Genomics UK (COG-UK)
Consortium|Golubchik|United Kingdom
>Spike|hCoV-19/Norway/6249/2020|2020-11-18|EPI_ISL_775367|Original|hCoV-
19^^Vestland|Human|Foerde Hospital|Norwegian Institute of Public Health|Vollan|Norway
>Spike|hCoV-19/Denmark/DCGC-19740/2020|2020-12-07|EPI_ISL_748383|Original|hCoV-
19^^Sjaelland|Human|Department of Virus and Microbiological Special
Diagnostics|Albertsen Lab|Michaelsen|Denmark
>Spike|hCoV-19/Hong Kong/HKU-200723-067/2020|2020-03-20|EPI_ISL_497834|Original|hCoV-
19^^Hong Kong|Human|Department of Microbiology|Department of Microbiology|Chan|China
>Spike|hCoV-19/England/CAMB-7D03B/2020|2020-04-06|EPI_ISL_442111|Original|hCoV-
19^^England|Human|Department of Pathology|Wellcome Sanger Institute for the COVID-19
Genomics UK (COG-UK) consortium|Amato|United Kingdom
>Spike|hCoV-19/England/LIVE-9AB46/2020|2020-04-01|EPI_ISL_449383|Original|hCoV-
19^^England|Human|Liverpool Clinical Laboratories|COVID-19 Genomics UK (COG-UK)
Consortium|Haldenby|United Kingdom
>Spike|hCoV-19/Australia/NSW1098/2020|2020-08-23|EPI_ISL_593713|Original|hCoV-19^^New
South Wales|Human|South Eastern Area Laboratory Services (SEALS)|NSW Health Pathology
- Institute of Clinical Pathology and Medical Research; Westmead Hospital; University
of Sydney|Gall|Australia
>Spike|hCoV-19/England/ALDP-B1B0E9/2020|2020-11-01|EPI_ISL_645630|Original|hCoV-
19^^England|Human|Lighthouse Lab in Alderley Park|Wellcome Sanger Institute for the
COVID-19 Genomics UK (COG-UK) Consortium|Davies|United Kingdom
>Spike|hCoV-19/England/QEUH-B69B81/2020|2020-11-11|EPI_ISL_655124|Original|hCoV-
19^^England|Human|Lighthouse Lab in Glasgow|Wellcome Sanger Institute for the COVID-
19 Genomics UK (COG-UK) Consortium|Davies|United Kingdom
>Spike|hCoV-19/England/ALDP-A68664/2020|2020-10-12|EPI_ISL_630556|Original|hCoV-
19^^England|Human|Lighthouse Lab in Alderley Park|Wellcome Sanger Institute for the
COVID-19 Genomics UK (COG-UK) consortium|Davies|United Kingdom
>Spike|hCoV-19/England/CAMC-B22902/2020|2020-11-01|EPI_ISL_647139|Original|hCoV-
19^^England|Human|Lighthouse Lab in Cambridge|Wellcome Sanger Institute for the
COVID-19 Genomics UK (COG-UK) Consortium|Davies|United Kingdom
>Spike|hCoV-19/England/NORW-E76B2/2020|2020-05-04|EPI_ISL_449063|Original|hCoV-
19^^England|Human|Quadram Institute Bioscience|COVID-19 Genomics UK (COG-UK)
Consortium|Alikhan|United Kingdom
>Spike|hCoV-19/Australia/VIC2780/2020|2020-07-06|EPI_ISL_521614|Original|hCoV-
19^^Victoria|Human|Microbiological Diagnostic Unit - Public Health Laboratory (MDU-
PHL)|MDU-PHL|Schultz|Australia

>Spike|hCoV-19/Scotland/QEUH-A225C3/2020|2020-10-01|EPI_ISL_599038|Original|hCoV-
19^^Scotland|Human|Lighthouse Lab in Glasgow|Wellcome Sanger Institute for the COVID-
19 Genomics UK (COG-UK) consortium|Davies|United Kingdom
>Spike|hCoV-19/Brazil/MG-0288/2020|2020-04-09|EPI_ISL_470593|Original|hCoV-19^^Minas
Gerais|Human|Simile|Bioinformatics Laboratory / LNCC|Almeida|Brazil
>Spike|hCoV-19/Denmark/DCGC-21152/2020|2020-12-21|EPI_ISL_759578|Original|hCoV-
19^^Nordjylland|Human|Department of Virus and Microbiological Special
Diagnostics|Albertsen Lab|Michaelsen|Denmark
>Spike|hCoV-19/Denmark/DCGC-22353/2020|2020-12-14|EPI_ISL_758699|Original|hCoV-
19^^Hovedstaden|Human|Department of Virus and Microbiological Special
Diagnostics|Albertsen Lab|Michaelsen|Denmark
>Spike|hCoV-19/Japan/PG-8342/2020|2020-08-00|EPI_ISL_687441|Original|hCoV-
19^^Japan|Human|Pathogen Genomics Center|Pathogen Genomics Center|Sekizuka|Japan
>Spike|hCoV-19/England/QEUH-9BA2AC/2020|2020-09-07|EPI_ISL_572947|Original|hCoV-
19^^England|Human|Oxford Viromics|COVID-19 Genomics UK (COG-UK)
Consortium|Golubchik|United Kingdom
>Spike|hCoV-19/Scotland/EDB3740/2020|2020-04-28|EPI_ISL_439315|Original|hCoV-
19^^Scotland|Human|Virology Department|COVID-19 Genomics UK (COG-UK)
Consortium|Williams|United Kingdom
>Spike|hCoV-19/Scotland/EDB7892/2020|2020-09-23|EPI_ISL_585442|Original|hCoV-
19^^Scotland|Human|Virology Department|COVID-19 Genomics UK (COG-UK)
Consortium|McHugh|United Kingdom
>Spike|hCoV-19/Wales/PHWC-33866/2020|2020-04-20|EPI_ISL_446758|Original|hCoV-
19^^Wales|Human|Wales Specialist Virology Centre|Public Health Wales Microbiology
Cardiff|Moore|United Kingdom
>Spike|hCoV-19/Germany/NW-UBI-8/2020|2020-04-03|EPI_ISL_602289|original|hCoV-
19^^North Rhine-Westphalia|Human|Evangelisches Klinikum Bethel|Bielefeld
University|Brandt|Germany
>Spike|hCoV-19/Wales/PHWC-4913AD/2020|2020-11-18|EPI_ISL_703955|Original|hCoV-
19^^Wales|Human|Originating lab|COVID-19 Genomics UK (COG-UK) Consortium|Moore|United
Kingdom
>Spike|hCoV-19/USA/IN-QDX-951/2020|2020-03-16|EPI_ISL_571718|Original|hCoV-
19^^Indiana|Human|Quest Diagnostics|Quest Diagnostics|Kagan|USA
>Spike|hCoV-19/England/ALDP-A85063/2020|2020-10-16|EPI_ISL_610471|Original|hCoV-
19^^England|Human|Lighthouse Lab in Alderley Park|Wellcome Sanger Institute for the
COVID-19 Genomics UK (COG-UK) consortium|Davies|United Kingdom
>Spike|hCoV-19/Scotland/CVR3929/2020|2020-06-05|EPI_ISL_512472|Original|hCoV-
19^^Scotland|Human|West of Scotland Specialist Virology Centre|COVID-19 Genomics UK
(COG-UK) Consortium|Robertson|United Kingdom
>Spike|hCoV-19/England/202881394/2020|2020-07-03|EPI_ISL_491728|Original|hCoV-
19^^England|Human|Respiratory Virus Unit|Respiratory Virus Unit|Platt|United Kingdom
>Spike|hCoV-19/Wales/PHWC-493727/2020|2020-11-29|EPI_ISL_725932|Original|hCoV-
19^^Wales|Human|Originating lab|COVID-19 Genomics UK (COG-UK) Consortium|Moore|United
Kingdom
>Spike|hCoV-19/Germany/NW-HHU-360/2020|2020-09-11|EPI_ISL_666647|Orginal|hCoV-
19^^North Rhine-Westphalia|Human|ZOTZ KLIMAS MVZ D\u00fcsseldorf-Centrum GbR
\u00dcBAG f\u00fcr Labormedizin|Center of Medical Microbiology|Wienemann|Germany
>Spike|hCoV-19/USA/MN-MDH-1148/2020|2020-04-30|EPI_ISL_476733|Original|hCoV-
19^^Minnesota|Human|Minnesota Department of Health|Minnesota Department of
Health|Garfin|USA
>Spike|hCoV-19/England/ALDP-C26008/2020|2020-12-06|EPI_ISL_782969|Original|hCoV-
19^^England|Human|Lighthouse Lab in Alderley Park|Wellcome Sanger Institute for the
COVID-19 Genomics UK (COG-UK) Consortium|Davies|United Kingdom
>Spike|hCoV-19/USA/TX-HMH-MCoV-17944/2020|2020-11-16|EPI_ISL_786092|Original|hCoV-
19^^Texas|Human|Houston Methodist Hospital|Houston Methodist Hospital|Long|USA
>Spike|hCoV-19/England/204541203/2020|2020-11-03|EPI_ISL_678669|Original|hCoV-
19^^England|Human|Respiratory Virus Unit|COVID-19 Genomics UK (COG-UK)
Consortium|Platt|United Kingdom

>Spike|hCoV-19/England/ALDP-A51877/2020|2020-10-07|EPI_ISL_598039|Original|hCoV-19^^England|Human|Lighthouse Lab in Alderley Park|Wellcome Sanger Institute for the COVID-19 Genomics UK (COG-UK) consortium|Davies|United Kingdom
>Spike|hCoV-19/mink/Denmark/mDK-5/2020|2020-10-29|EPI_ISL_641399|unknown|hCoV-19^^Denmark|Neovison vison|Department of Virus and Microbiological Special Diagnostics|Albertsen lab|Michaelsen|Denmark
>Spike|hCoV-19/England/CAMB-1B2242/2020|2020-05-11|EPI_ISL_469891|Original|hCoV-19^^England|Human|Department of Pathology|Wellcome Sanger Institute for the COVID-19 Genomics UK (COG-UK) consortium|Davies|United Kingdom
>Spike|hCoV-19/England/CAMB-1B7298/2020|2020-10-19|EPI_ISL_638313|Original|hCoV-19^^England|Human|Department of Pathology|COVID-19 Genomics UK (COG-UK) Consortium|Goodfellow|United Kingdom
>Spike|hCoV-19/England/MILK-973676/2020|2020-08-20|EPI_ISL_575839|Original|hCoV-19^^England|Human|Lighthouse Lab in Milton Keynes|Wellcome Sanger Institute for the COVID-19 Genomics UK (COG-UK) consortium|Davies|United Kingdom
>Spike|hCoV-19/Canada/ON-PHL-20-03670/2020|2020-11-00|EPI_ISL_775156|Original|hCoV-19^^Ontario|Human|Public Health Ontario Laboratory|Public Health Ontario Laboratory|Guthrie|Canada
>Spike|hCoV-19/England/CAMC-AB1286/2020|2020-10-23|EPI_ISL_623575|Original|hCoV-19^^England|Human|Lighthouse Lab in Cambridge|Wellcome Sanger Institute for the COVID-19 Genomics UK (COG-UK) consortium|Davies|United Kingdom
>Spike|hCoV-19/USA/MI-UM-10035986324/2020|2020-09-16|EPI_ISL_583365|Original|hCoV-19^^Michigan|Human|University of Michigan Clinical Microbiology Laboratory|Lauring Lab|Valesano|USA
>Spike|hCoV-19/England/MILK-BAA216/2020|2020-11-18|EPI_ISL_681366|Original|hCoV-19^^England|Human|Lighthouse Lab in Milton Keynes|Wellcome Sanger Institute for the COVID-19 Genomics UK (COG-UK) Consortium|Davies|United Kingdom
>Spike|hCoV-19/USA/TX-HMH-MCoV-13021/2020|2020-08-28|EPI_ISL_788172|Original|hCoV-19^^Texas|Human|Houston Methodist Hospital|Houston Methodist Hospital|Long|USA
>Spike|hCoV-19/Japan/PG-3775/2020|2020-04-00|EPI_ISL_692027|Original|hCoV-19^^Japan|Human|Pathogen Genomics Center|Pathogen Genomics Center|Sekizuka|Japan
>Spike|hCoV-19/USA/AK-PHL575/2020|2020-06-22|EPI_ISL_512148|Original|hCoV-19^^Alaska|Human|Alaska State Virology Laboratory|Alaska State Virology Laboratory|Drown|USA
>Spike|hCoV-19/England/ALDP-C58BEE/2020|2020-12-14|EPI_ISL_736046|Original|hCoV-19^^England|Human|Lighthouse Lab in Alderley Park|Wellcome Sanger Institute for the COVID-19 Genomics UK (COG-UK) Consortium|Davies|United Kingdom
>Spike|hCoV-19/Australia/NSW790/2020|2020-08-04|EPI_ISL_526132|Original|hCoV-19^^New South Wales|Human|Pathology North - Royal North Shore Hospital - NSW Health Pathology|NSW Health Pathology - Institute of Clinical Pathology and Medical Research; Westmead Hospital; University of Sydney|Gall|Australia
>Spike|hCoV-19/Netherlands/NA_10/2020|2020-03-09|EPI_ISL_415466|Original|hCoV-19^^Netherlands|Human|Dutch COVID-19 response team|Erasmus Medical Center|Oude Munnink|Netherlands
>Spike|hCoV-19/England/CAMC-A57185/2020|2020-10-09|EPI_ISL_597460|Original|hCoV-19^^England|Human|Lighthouse Lab in Cambridge|Wellcome Sanger Institute for the COVID-19 Genomics UK (COG-UK) consortium|Davies|United Kingdom
>Spike|hCoV-19/Wales/PHWC-15E3DC/2020|2020-05-12|EPI_ISL_472812|Original|hCoV-19^^Wales|Human|Wales Specialist Virology Centre Sequencing lab|COVID-19 Genomics UK (COG-UK) Consortium|Moore|United Kingdom
>Spike|hCoV-19/Wales/PHWC-48F3C0/2020|2020-11-09|EPI_ISL_681086|Original|hCoV-19^^Wales|Human|Wales Specialist Virology Centre Sequencing lab|COVID-19 Genomics UK (COG-UK) Consortium|Moore|United Kingdom
>Spike|hCoV-19/Belgium/rega-12111368/2020|2020-12-11|EPI_ISL_738421|Original|hCoV-19^^Mol|Human|UZ Leuven|KU Leuven|Maes|Belgium
>Spike|hCoV-19/Spain/MD-IBV-99008694/2020|2020-10-08|EPI_ISL_654247|Original|hCoV-19^^Madrid|Human|Hospital General Universitario Gregorio Mara\u00f1\u00f3n|SeqCOVID-SPAIN consortium/IBV(CSIC)|Comas|Spain
>Spike|hCoV-19/USA/CA-CZB-10955/2020|2020-09-16|EPI_ISL_583038|Original|hCoV-19^^California|Human|Orange County Public Health Lab|Chan-Zuckerberg Biohub|Bhatt|USA

>Spike|hCoV-19/USA/VA-DCLS-1683/2020|2020-09-03|EPI_ISL_609973|Original|hCoV-19^^Virginia|Human|Virginia DCLS|Virginia DCLS|St. Jacques|USA
>Spike|hCoV-19/Wales/PHWC-47E9A4/2020|2020-10-11|EPI_ISL_595531|Original|hCoV-19^^Wales|Human|Originating lab|COVID-19 Genomics UK (COG-UK) Consortium|Moore|United Kingdom
>Spike|hCoV-19/Japan/PG-5515/2020|2020-08-00|EPI_ISL_688640|Original|hCoV-19^^Japan|Human|Pathogen Genomics Center|Pathogen Genomics Center|Sekizuka|Japan
>Spike|hCoV-19/England/ALDP-9BE985/2020|2020-09-09|EPI_ISL_568010|Original|hCoV-19^^England|Human|Lighthouse Lab in Alderley Park|Wellcome Sanger Institute for the COVID-19 Genomics UK (COG-UK) consortium|Davies|United Kingdom
>Spike|hCoV-19/USA/MI-UM-10036496560/2020|2020-10-17|EPI_ISL_628855|Original|hCoV-19^^Michigan|Human|University of Michigan Clinical Microbiology Laboratory|Lauring Lab|Valesano|USA
>Spike|hCoV-19/Wales/ALDP-C3BA6C/2020|2020-12-10|EPI_ISL_767216|Original|hCoV-19^^Wales|Human|Lighthouse Lab in Alderley Park|Wellcome Sanger Institute for the COVID-19 Genomics UK (COG-UK) Consortium|Davies|United Kingdom
>Spike|hCoV-19/England/MILK-BB19DB/2020|2020-11-18|EPI_ISL_720612|Original|hCoV-19^^England|Human|Lighthouse Lab in Milton Keynes|Wellcome Sanger Institute for the COVID-19 Genomics UK (COG-UK) Consortium|Davies|United Kingdom
>Spike|hCoV-19/Mexico/NAY-InDRE_28/2020|2020-05-29|EPI_ISL_493343|Original|hCoV-19^^Nayarit|Human|Instituto de Diagnostico y Referencia Epidemiologicos (INDRE)|Instituto de Diagnostico y Referencia Epidemiologicos (INDRE)|Rodriguez|Mexico
>Spike|hCoV-19/Denmark/DCGC-5453/2020|2020-09-21|EPI_ISL_620778|Original|hCoV-19^^Syddanmark|Human|Department of Virus and Microbiological Special Diagnostics|Albertsen lab|Michaelsen|Denmark
>Spike|hCoV-19/Australia/VIC3975/2020|2020-07-10|EPI_ISL_519367|Original|hCoV-19^^Victoria|Human|Microbiological Diagnostic Unit - Public Health Laboratory (MDU-PHL)|MDU-PHL|Schultz|Australia
>Spike|hCoV-19/USA/TX-HMH-7153/2020|2020-06-29|EPI_ISL_546550|Original|hCoV-19^^Texas|Human|Houston Methodist Hospital|Houston Methodist Hospital|Long|USA
>Spike|hCoV-19/Denmark/DCGC-11715/2020|2020-10-26|EPI_ISL_671090|Original|hCoV-19^^Hovedstaden|Human|Department of Virus and Microbiological Special Diagnostics|Albertsen Lab|Michaelsen|Denmark
>Spike|hCoV-19/Portugal/PT1093/2020|2020-03-28|EPI_ISL_511640|Original|hCoV-19^^Portugal|Human|Instituto Nacional de Saude (INSA)|Instituto Nacional de Saude (INSA)|Borges|Portugal
>Spike|hCoV-19/USA/UT-08651/2020|2020-05-24|EPI_ISL_524383|Original|hCoV-19^^Utah|Human|Utah Public Health Laboratory|Utah Public Health Laboratory|Young|USA
>Spike|hCoV-19/England/LEED-2A9869/2020|2020-04-12|EPI_ISL_538918|Original|hCoV-19^^England|Human|Leeds Teaching Hospitals NHS Trust and Public Health England|Wellcome Sanger Institute for the COVID-19 Genomics UK (COG-UK) consortium|Davies|United Kingdom
>Spike|hCoV-19/USA/TX-HMH0142/2020|2020-03-21|EPI_ISL_434841|Original|hCoV-19^^Texas|Human|Houston Methodist Hospital|Houston Methodist Hospital|Long|USA
>Spike|hCoV-19/Japan/PG-7111/2020|2020-08-00|EPI_ISL_688216|Original|hCoV-19^^Japan|Human|Pathogen Genomics Center|Pathogen Genomics Center|Sekizuka|Japan
>Spike|hCoV-19/Scotland/QEUH-9D046E/2020|2020-09-16|EPI_ISL_601802|Original|hCoV-19^^Scotland|Human|Lighthouse Lab in Glasgow|Wellcome Sanger Institute for the COVID-19 Genomics UK (COG-UK) consortium|Davies|United Kingdom
>Spike|hCoV-19/USA/CA-CDC-4727/2020|2020-06-15|EPI_ISL_535698|Original|hCoV-19^^California|Human|CDPH|Pathogen Discovery|Queen|USA
>Spike|hCoV-19/Denmark/DCGC-17861/2020|2020-12-14|EPI_ISL_749605|Original|hCoV-19^^Hovedstaden|Human|Department of Virus and Microbiological Special Diagnostics|Albertsen Lab|Michaelsen|Denmark
>Spike|hCoV-19/Palestine/49/2020|2020-08-18|EPI_ISL_596532|Original|hCoV-19^^Jericho|Human|Palestinian Ministry of Health|Molecular Genetics Lab|van Dorp|Palestine

>Spike|hCoV-19/Netherlands/ZH-EMC-684/2020|2020-10-05|EPI_ISL_632333|Original|hCoV-
19^^Zuid Holland|Human|Dutch COVID-19 response team|Erasmus Medical Center|Oude
Munnink|Netherlands
>Spike|hCoV-19/Denmark/DCGC-14392/2020|2020-11-30|EPI_ISL_711293|Original|hCoV-
19^^Syddanmark|Human|Department of Virus and Microbiological Special
Diagnostics|Albertsen Lab|Michaelsen|Denmark
>Spike|hCoV-19/England/LOND-126471F/2020|2020-11-15|EPI_ISL_704092|Original|hCoV-
19^^England|Human|University College London|COVID-19 Genomics UK (COG-UK)
Consortium|Roy|United Kingdom
>Spike|hCoV-19/England/QEUH-AA0590/2020|2020-10-20|EPI_ISL_611344|Original|hCoV-
19^^England|Human|Lighthouse Lab in Glasgow|Wellcome Sanger Institute for the COVID-
19 Genomics UK (COG-UK) consortium|Davies|United Kingdom
>Spike|hCoV-19/USA/TX-DSHS-0970/2020|2020-06-15|EPI_ISL_614009|Original|hCoV-
19^^Texas|Human|Texas Department of State Health Services|Texas Department of State
Health Services|Rahman|USA
>Spike|hCoV-19/Australia/WA250/2020|2020-08-20|EPI_ISL_594166|Original|hCoV-
19^^Western Australia|Human|PathWest Laboratory Medicine WA|PathWest Laboratory
Medicine WA Microbial Surveillance Unit|Sikazwe|Australia
>Spike|hCoV-19/Sweden/20-53801/2020|2020-12-14|EPI_ISL_766641|Original|hCoV-
19^^Gavleborg|Human|Laboratoriemedicin|The Public Health Agency of
Sweden|Svartstr\u00f6m|Sweden
>Spike|hCoV-19/England/SHEF-10D472B/2020|2020-12-06|EPI_ISL_704978|Original|hCoV-
19^^England|Human|Virology Department|COVID-19 Genomics UK (COG-UK) Consortium|de
Silva|United Kingdom
>Spike|hCoV-19/USA/TX-HMH-7559/2020|2020-06-25|EPI_ISL_546851|Original|hCoV-
19^^Texas|Human|Houston Methodist Hospital|Houston Methodist Hospital|Long|USA
>Spike|hCoV-19/Wales/PHWC-16B767/2020|2020-09-19|EPI_ISL_573897|Original|hCoV-
19^^Wales|Human|Wales Specialist Virology Centre Sequencing lab|COVID-19 Genomics UK
(COG-UK) Consortium|Moore|United Kingdom
>Spike|hCoV-19/USA/UT-UPHL-2009136/2020|2020-05-23|EPI_ISL_525131|Original|hCoV-
19^^Utah|Human|Utah Public Health Laboratory|Utah Public Health Laboratory|Young|USA
>Spike|hCoV-19/Scotland/QEUH-96D92B/2020|2020-08-20|EPI_ISL_534579|Original|hCoV-
19^^Scotland|Human|NHSGGC West of Scotland Specialist Virology Centre / MRC-
University of Glasgow Centre for Virus Research|Wellcome Sanger Institute for the
COVID-19 Genomics UK (COG-UK) consortium|Davies|United Kingdom
>Spike|hCoV-19/Wales/PHWC-4A238B/2020|2020-12-13|EPI_ISL_743879|Original|hCoV-
19^^Wales|Human|Wales Specialist Virology Centre Sequencing lab|COVID-19 Genomics UK
(COG-UK) Consortium|Moore|United Kingdom
>Spike|hCoV-19/Spain/MD-IBV-99007137/2020|2020-08-04|EPI_ISL_541910|Original|hCoV-
19^^Madrid|Human|Hospital General Universitario Gregorio Mara\u00f1\u00f3n|SeqCOVID-
SPAIN consortium/IBV(CSIC)|Comas|Spain
>Spike|hCoV-19/Spain/MD-IBV-005339/2020|2020-03-18|EPI_ISL_510217|Original|hCoV-
19^^Madrid|Human|Hospital General Universitario Gregorio Mara\u00f1\u00f3n|SeqCOVID-
SPAIN consortium/IBV(CSIC)|Comas|Spain
>Spike|hCoV-19/USA/MA-MGH-03124/2020|2020-11-15|EPI_ISL_765711|Original|hCoV-
19^^Massachusetts|Human|Massachusetts General Hospital|Infectious Disease
Program|Park|USA
>Spike|hCoV-19/England/CAMB-75D4B/2020|2020-03-29|EPI_ISL_438420|Original|hCoV-
19^^England|Human|Department of Pathology|Wellcome Sanger Institute for the COVID-19
Genomics UK (COG-UK) consortium|Amato|United Kingdom
>Spike|hCoV-19/Denmark/DCGC-19287/2020|2020-12-07|EPI_ISL_748301|Original|hCoV-
19^^Syddanmark|Human|Department of Virus and Microbiological Special
Diagnostics|Albertsen Lab|Michaelsen|Denmark
>Spike|hCoV-19/England/MILK-BAA6B0/2020|2020-11-17|EPI_ISL_783153|Original|hCoV-
19^^England|Human|Lighthouse Lab in Milton Keynes|Wellcome Sanger Institute for the
COVID-19 Genomics UK (COG-UK) Consortium|Davies|United Kingdom
>Spike|hCoV-19/Australia/VIC4783/2020|2020-07-14|EPI_ISL_519169|Original|hCoV-
19^^Victoria|Human|Microbiological Diagnostic Unit - Public Health Laboratory (MDU-
PHL)|MDU-PHL|Schultz|Australia

>Spike|hCoV-19/USA/OR-OHSU-1893/2020|2020-07-28|EPI_ISL_526061|Original|hCoV-19^^Oregon|Human|OHSU Lab Services Molecular Microbiology Lab|Oregon SARS-CoV-2 Genome Sequencing Center|O'Roak|USA
>Spike|hCoV-19/England/QEUH-9F3A45/2020|2020-09-24|EPI_ISL_588641|Original|hCoV-19^^England|Human|Lighthouse Lab in Glasgow|Wellcome Sanger Institute for the COVID-19 Genomics UK (COG-UK) consortium|Davies|United Kingdom
>Spike|hCoV-19/USA/TX-HMH-4176/2020|2020-06-18|EPI_ISL_543413|Original|hCoV-19^^Texas|Human|Houston Methodist Hospital|Houston Methodist Hospital|Long|USA
>Spike|hCoV-19/USA/WA-S2031/2020|2020-06-06|EPI_ISL_497459|Original|hCoV-19^^Washington|Human|Washington State Department of Health|Seattle Flu Study|Lee|USA
>Spike|hCoV-19/India/TG-CCMB-X42/2020|2020-07-14|EPI_ISL_528854|Original|hCoV-19^^Telangana|Human|CSIR-Centre for Cellular and Molecular Biology|CSIR-Centre for Cellular and Molecular Biology|Banu|India
>Spike|hCoV-19/England/CAMB-1ACCA7/2020|2020-04-22|EPI_ISL_489477|Original|hCoV-19^^England|Human|Department of Pathology|Wellcome Sanger Institute for the COVID-19 Genomics UK (COG-UK) consortium|Davies|United Kingdom
>Spike|hCoV-19/England/ALDP-981ED7/2020|2020-08-09|EPI_ISL_552745|Original|hCoV-19^^England|Human|Lighthouse Lab in Alderley Park|Wellcome Sanger Institute for the COVID-19 Genomics UK (COG-UK) consortium|Davies|United Kingdom
>Spike|hCoV-19/Denmark/ALAB-HH15/2020|2020-03-10|EPI_ISL_429273|Original|hCoV-19^^Denmark|Human|Department of Clinical Microbiology|Albertsen lab|Kirkegaard|Denmark
>Spike|hCoV-19/Scotland/CVR5823/2020|2020-11-13|EPI_ISL_666035|Original|hCoV-19^^Scotland|Human|West of Scotland Specialist Virology Centre|COVID-19 Genomics UK (COG-UK) Consortium|Robertson|United Kingdom
>Spike|hCoV-19/USA/AZ-TG396023/2020|2020-06-22|EPI_ISL_694797|Original|hCoV-19^^Arizona|Human|TGen North|TGen North|French|USA
>Spike|hCoV-19/England/CAMC-A3F4C6/2020|2020-10-06|EPI_ISL_609589|Original|hCoV-19^^England|Human|Lighthouse Lab in Cambridge|Wellcome Sanger Institute for the COVID-19 Genomics UK (COG-UK) consortium|Davies|United Kingdom
>Spike|hCoV-19/England/EXET-13F4CF/2020|2020-12-16|EPI_ISL_763717|Original|hCoV-19^^England|Human|University of Exeter|COVID-19 Genomics UK (COG-UK) Consortium|Temperton|United Kingdom
>Spike|hCoV-19/Japan/PG-0753/2020|2020-04-00|EPI_ISL_684363|Original|hCoV-19^^Japan|Human|Pathogen Genomics Center|Pathogen Genomics Center|Sekizuka|Japan
>Spike|hCoV-19/Wales/ALDP-B1AB07/2020|2020-11-01|EPI_ISL_711035|Original|hCoV-19^^Wales|Human|Lighthouse Lab in Alderley Park|Wellcome Sanger Institute for the COVID-19 Genomics UK (COG-UK) Consortium|Davies|United Kingdom
>Spike|hCoV-19/England/MILK-B5A37C/2020|2020-11-02|EPI_ISL_658261|Original|hCoV-19^^England|Human|Lighthouse Lab in Milton Keynes|Wellcome Sanger Institute for the COVID-19 Genomics UK (COG-UK) Consortium|Davies|United Kingdom
>Spike|hCoV-19/England/ALDP-9C594F/2020|2020-09-10|EPI_ISL_567783|Original|hCoV-19^^England|Human|Lighthouse Lab in Alderley Park|Wellcome Sanger Institute for the COVID-19 Genomics UK (COG-UK) consortium|Davies|United Kingdom
>Spike|hCoV-19/USA/TX-HMH-MCoV-14668/2020|2020-10-09|EPI_ISL_787953|Original|hCoV-19^^Texas|Human|Houston Methodist Hospital|Houston Methodist Hospital|Long|USA
>Spike|hCoV-19/Scotland/CVR2795/2020|2020-04-20|EPI_ISL_453076|Original|hCoV-19^^Scotland|Human|West of Scotland Specialist Virology Centre|COVID-19 Genomics UK (COG-UK) Consortium|Robertson|United Kingdom
>Spike|hCoV-19/England/MILK-9F0AFD/2020|2020-09-24|EPI_ISL_589706|Original|hCoV-19^^England|Human|Lighthouse Lab in Milton Keynes|Wellcome Sanger Institute for the COVID-19 Genomics UK (COG-UK) consortium|Davies|United Kingdom
>Spike|hCoV-19/Australia/VIC2185/2020|2020-06-23|EPI_ISL_521931|Original|hCoV-19^^Victoria|Human|Microbiological Diagnostic Unit - Public Health Laboratory (MDU-PHL)|MDU-PHL|Schultz|Australia
>Spike|hCoV-19/Netherlands/FL-EMC-28/2020|2020-07-29|EPI_ISL_523137|Original|hCoV-19^^Flevoland|Human|Dutch COVID-19 response team|Erasmus Medical Center|Oude Munnink|Netherlands

>Spike|hCoV-19/England/CAMC-B080B2/2020|2020-10-30|EPI_ISL_649629|Original|hCoV-19^^England|Human|Lighthouse Lab in Cambridge|Wellcome Sanger Institute for the COVID-19 Genomics UK (COG-UK) Consortium|Davies|United Kingdom
>Spike|hCoV-19/Japan/PG-5363/2020|2020-08-00|EPI_ISL_690466|Original|hCoV-19^^Japan|Human|Fukuoka Institute of Health and Environmental Sciences|Pathogen Genomics Center|Sekizuka|Japan
>Spike|hCoV-19/USA/NY-WCMP9B09P/2020|2020-03-14|EPI_ISL_427549|Original|hCoV-19^^New York|Human|NewYork-Presbyterian & Mason Lab|Mason Lab|Mason|USA
>Spike|hCoV-19/USA/TX-HMH-MCoV-10014/2020|2020-07-06|EPI_ISL_789286|Original|hCoV-19^^Texas|Human|Houston Methodist Hospital|Houston Methodist Hospital|Long|USA
>Spike|hCoV-19/England/LOND-DA2EB/2020|2020-11-16|EPI_ISL_724616|Original|hCoV-19^^England|Human|University College London|COVID-19 Genomics UK (COG-UK) Consortium|Roy|United Kingdom
>Spike|hCoV-19/England/ALDP-B858F1/2020|2020-11-14|EPI_ISL_675591|Original|hCoV-19^^England|Human|Lighthouse Lab in Alderley Park|Wellcome Sanger Institute for the COVID-19 Genomics UK (COG-UK) Consortium|Davies|United Kingdom
>Spike|hCoV-19/USA/NMDOH-2020305762/2020|2020-08-21|EPI_ISL_542051|Original|hCoV-19^^New Mexico|Human|New Mexico Department of Health Scientific Laboratory|New Mexico Department of Health Scientific Laboratory|Johnson|USA
>Spike|hCoV-19/England/MILK-C51990/2020|2020-12-08|EPI_ISL_769584|Original|hCoV-19^^England|Human|Lighthouse Lab in Milton Keynes|Wellcome Sanger Institute for the COVID-19 Genomics UK (COG-UK) Consortium|Davies|United Kingdom
>Spike|hCoV-19/Japan/PG-3162/2020|2020-06-00|EPI_ISL_691282|Original|hCoV-19^^Japan|Human|Pathogen Genomics Center|Pathogen Genomics Center|Sekizuka|Japan
>Spike|hCoV-19/Australia/VIC10115/2020|2020-08-26|EPI_ISL_562793|Original|hCoV-19^^Victoria|Human|Microbiological Diagnostic Unit - Public Health Laboratory (MDU-PHL)|MDU-PHL|Schultz|Australia
>Spike|hCoV-19/USA/VA-DCLS-0189/2020|2020-04-15|EPI_ISL_437405|Original|hCoV-19^^Virginia|Human|Virginia DCLS|Virginia DCLS|St. Jacques|USA
>Spike|hCoV-19/England/ALDP-9539EC/2020|2020-06-08|EPI_ISL_553744|Original|hCoV-19^^England|Human|Lighthouse Lab in Alderley Park|Wellcome Sanger Institute for the COVID-19 Genomics UK (COG-UK) consortium|Davies|United Kingdom
>Spike|hCoV-19/England/MILK-B96295/2020|2020-11-13|EPI_ISL_675029|Original|hCoV-19^^England|Human|Lighthouse Lab in Milton Keynes|Wellcome Sanger Institute for the COVID-19 Genomics UK (COG-UK) Consortium|Davies|United Kingdom
>Spike|hCoV-19/England/CAMC-B08735/2020|2020-10-29|EPI_ISL_643855|Original|hCoV-19^^England|Human|Lighthouse Lab in Cambridge|Wellcome Sanger Institute for the COVID-19 Genomics UK (COG-UK) Consortium|Davies|United Kingdom
>Spike|hCoV-19/Australia/SAP092/2020|2020-04-01|EPI_ISL_451127|Original|hCoV-19^^South Australia|Human|SA Pathology|SA Pathology|Leong|Australia
>Spike|hCoV-19/India/TG-CCMB-AA590/2020|2020-08-12|EPI_ISL_539750|Original|hCoV-19^^Telangana|Human|CSIR-Centre for Cellular and Molecular Biology|CSIR-Centre for Cellular and Molecular Biology|Mukherjee|India
>Spike|hCoV-19/USA/TX-HMH-MCoV-14874/2020|2020-10-14|EPI_ISL_786358|Original|hCoV-19^^Texas|Human|Houston Methodist Hospital|Houston Methodist Hospital|Long|USA
>Spike|hCoV-19/India/MH-GA87/2020|2020-06-15|EPI_ISL_508282|Original|hCoV-19^^Maharashtra|Human|Government Medical College|National Institute of Biomedical Genomics|Maitra|India
>Spike|hCoV-19/England/CAMB-810CB/2020|2020-04-20|EPI_ISL_438566|Original|hCoV-19^^England|Human|Department of Pathology|COVID-19 Genomics UK (COG-UK) Consortium|Meredith|United Kingdom
>Spike|hCoV-19/Spain/AR-IBV-005051/2020|2020-04-21|EPI_ISL_510351|Original|hCoV-19^^Aragon|Human|Servicio de Microbiolog\u00eda|SeqCOVID-SPAIN consortium/IBV(CSIC)|Comas|Spain
>Spike|hCoV-19/England/BRIS-12FC7F/2020|2020-04-07|EPI_ISL_492652|Original|hCoV-19^^England|Human|PHE South West Regional Laboratory|Wellcome Sanger Institute for the COVID-19 Genomics UK (COG-UK) consortium|Davies|United Kingdom
>Spike|hCoV-19/Australia/NSW785/2020|2020-07-30|EPI_ISL_526127|Original|hCoV-19^^New South Wales|Human|Sydney South West Pathology Service (SSWPS) - Liverpool Hospital -

NSW Health Pathology|NSW Health Pathology - Institute of Clinical Pathology and Medical Research; Westmead Hospital; University of Sydney|Gall|Australia
>Spike|hCoV-19/Panama/329694/2020|2020-03-10|EPI_ISL_496643|Original|hCoV-19^^Panama West|Human|Gorgas Memorial Laboratory of Health Studies|Gorgas Memorial Laboratory of Health Studies|Martinez|Panama
>Spike|hCoV-19/USA/TX-DSHS-1586/2020|2020-06-22|EPI_ISL_700720|Original|hCoV-19^^Texas|Human|Texas Department of State Health Services|Texas Department of State Health Services|Rahman|USA
>Spike|hCoV-19/Wales/ALDP-AA480C/2020|2020-10-21|EPI_ISL_624397|Original|hCoV-19^^Wales|Human|Lighthouse Lab in Alderley Park|Wellcome Sanger Institute for the COVID-19 Genomics UK (COG-UK) consortium|Davies|United Kingdom
>Spike|hCoV-19/England/ALDP-C3C3D9/2020|2020-12-10|EPI_ISL_731041|Original|hCoV-19^^England|Human|Lighthouse Lab in Alderley Park|Wellcome Sanger Institute for the COVID-19 Genomics UK (COG-UK) Consortium|Davies|United Kingdom
>Spike|hCoV-19/Denmark/DCGC-19767/2020|2020-12-07|EPI_ISL_747772|Original|hCoV-19^^Hovedstaden|Human|Department of Virus and Microbiological Special Diagnostics|Albertsen Lab|Michaelsen|Denmark
>Spike|hCoV-19/Australia/VIC14114/2020|2020-09-14|EPI_ISL_563973|Original|hCoV-19^^Victoria|Human|Victorian Infectious Diseases Reference Laboratory (VIDRL)|VIDRL and MDU-PHL|Schultz|Australia
>Spike|hCoV-19/USA/OR-OHSU-2275/2020|2020-08-08|EPI_ISL_667167|Original|hCoV-19^^Oregon|Human|OHSU Lab Services Molecular Microbiology Lab|Oregon SARS-CoV-2 Genome Sequencing Center|O'Roak|USA
>Spike|hCoV-19/Northern Ireland/NIRE-FEABC/2020|2020-05-01|EPI_ISL_489287|Original|hCoV-19^^Northern Ireland|Human|Regional Virus Laboratory|Wellcome Sanger Institute for the COVID-19 Genomics UK (COG-UK) consortium|Davies|United Kingdom
>Spike|hCoV-19/Wales/PHWC-49F794/2020|2020-12-08|EPI_ISL_743266|Original|hCoV-19^^Wales|Human|Wales Specialist Virology Centre Sequencing lab|COVID-19 Genomics UK (COG-UK) Consortium|Moore|United Kingdom
>Spike|hCoV-19/USA/TX-HMH-MCoV-10198/2020|2020-07-07|EPI_ISL_789368|Original|hCoV-19^^Texas|Human|Houston Methodist Hospital|Houston Methodist Hospital|Long|USA
>Spike|hCoV-19/Denmark/DCGC-16336/2020|2020-11-16|EPI_ISL_714176|Original|hCoV-19^^Syddanmark|Human|Department of Virus and Microbiological Special Diagnostics|Albertsen Lab|Michaelsen|Denmark
>Spike|hCoV-19/England/BRIS-12DE6C/2020|2020-05-31|EPI_ISL_482015|Original|hCoV-19^^England|Human|PHE South West Regional Laboratory|Wellcome Sanger Institute for the COVID-19 Genomics UK (COG-UK) consortium|Davies|United Kingdom
>Spike|hCoV-19/USA/WA-UW-2606/2020|2020-03-30|EPI_ISL_486004|Original|hCoV-19^^Washington|Human|UW Virology Lab|UW Virology Lab|Roychoudhury|USA
>Spike|hCoV-19/Denmark/DCGC-5408/2020|2020-09-14|EPI_ISL_615340|Original|hCoV-19^^Nordjylland|Human|Department of Virus and Microbiological Special Diagnostics|Albertsen lab|Michaelsen|Denmark
>Spike|hCoV-19/Wales/PHWC-48E6C8/2020|2020-11-12|EPI_ISL_680896|Original|hCoV-19^^Wales|Human|Wales Specialist Virology Centre Sequencing lab|COVID-19 Genomics UK (COG-UK) Consortium|Moore|United Kingdom
>Spike|hCoV-19/France/PAC-IHU-0320/2020|2020-04-01|EPI_ISL_569452|Original|hCoV-19^^Provence-Alpes-C\u00f4te-d\u2019Azur|Human|MEPHI|MEPHI|Levasseur|France
>Spike|hCoV-19/England/QEUH-B7F58A/2020|2020-11-14|EPI_ISL_674723|Original|hCoV-19^^England|Human|Lighthouse Lab in Glasgow|Wellcome Sanger Institute for the COVID-19 Genomics UK (COG-UK) Consortium|Davies|United Kingdom
>Spike|hCoV-19/England/CAMB-1B4D03/2020|2020-06-12|EPI_ISL_473507|Original|hCoV-19^^England|Human|Department of Pathology|COVID-19 Genomics UK (COG-UK) Consortium|Meredith|United Kingdom
>Spike|hCoV-19/England/CAMC-B77C14/2020|2020-11-12|EPI_ISL_783443|Original|hCoV-19^^England|Human|Lighthouse Lab in Cambridge|Wellcome Sanger Institute for the COVID-19 Genomics UK (COG-UK) Consortium|Davies|United Kingdom
>Spike|hCoV-19/USA/FL-BPHL-2209/2020|2020-07-29|EPI_ISL_666831|Original|hCoV-19^^Florida|Human|Florida Bureau of Public Health Laboratories|Florida Bureau of Public Health Laboratories|Blanton|USA

>Spike|hCoV-19/Australia/VIC3157/2020|2020-07-11|EPI_ISL_520863|Original|hCoV-19^^Victoria|Human|Microbiological Diagnostic Unit - Public Health Laboratory (MDU-PHL)|MDU-PHL|Schultz|Australia
>Spike|hCoV-19/Portugal/PT1897/2020|2020-11-23|EPI_ISL_732111|Original|hCoV-19^^Portugal|Human|Instituto Nacional de Saude (INSA)|Instituto Nacional de Saude (INSA)|Borges|Portugal
>Spike|hCoV-19/England/20132024204/2020|2020-03-24|EPI_ISL_423756|Original|hCoV-19^^England|Human|Respiratory Virus Unit|Respiratory Virus Unit|Platt|United Kingdom
>Spike|hCoV-19/England/SHEF-10CB99A/2020|2020-12-01|EPI_ISL_741980|Original|hCoV-19^^England|Human|Virology Department|COVID-19 Genomics UK (COG-UK) Consortium|de Silva|United Kingdom
>Spike|hCoV-19/England/MILK-C300ED/2020|2020-12-07|EPI_ISL_733748|Original|hCoV-19^^England|Human|Lighthouse Lab in Milton Keynes|Wellcome Sanger Institute for the COVID-19 Genomics UK (COG-UK) Consortium|Davies|United Kingdom
>Spike|hCoV-19/England/MILK-AC793D/2020|2020-10-21|EPI_ISL_634483|Original|hCoV-19^^England|Human|Lighthouse Lab in Milton Keynes|Wellcome Sanger Institute for the COVID-19 Genomics UK (COG-UK) Consortium|Davies|United Kingdom
>Spike|hCoV-19/Spain/MD-IBV-99007132/2020|2020-08-01|EPI_ISL_541905|Original|hCoV-19^^Madrid|Human|Hospital General Universitario Gregorio Mara\u00f1\u00f3n|SeqCOVID-SPAIN consortium/IBV(CSIC)|Comas|Spain
>Spike|hCoV-19/England/QEUH-B13B77/2020|2020-10-31|EPI_ISL_641857|Original|hCoV-19^^England|Human|Lighthouse Lab in Glasgow|Wellcome Sanger Institute for the COVID-19 Genomics UK (COG-UK) Consortium|Davies|United Kingdom
>Spike|hCoV-19/USA/WI-UW-1424/2020|2020-09-11|EPI_ISL_541557|Original|hCoV-19^^Wisconsin|Human|University of Wisconsin-Madison Campus AIDS Vaccine Research Laboratories|University of Wisconsin-Madison AIDS Vaccine Research Laboratories|Baczenas|USA
>Spike|hCoV-19/England/MILK-97547A/2020|2020-08-21|EPI_ISL_552616|Original|hCoV-19^^England|Human|Lighthouse Lab in Milton Keynes|Wellcome Sanger Institute for the COVID-19 Genomics UK (COG-UK) consortium|Davies|United Kingdom
>Spike|hCoV-19/Wales/ALDP-A87D3D/2020|2020-10-17|EPI_ISL_625396|Original|hCoV-19^^Wales|Human|Lighthouse Lab in Alderley Park|Wellcome Sanger Institute for the COVID-19 Genomics UK (COG-UK) consortium|Davies|United Kingdom
>Spike|hCoV-19/Denmark/DCGC-13933/2020|2020-11-30|EPI_ISL_711463|Original|hCoV-19^^Syddanmark|Human|Department of Virus and Microbiological Special Diagnostics|Albertsen Lab|Michaelsen|Denmark
>Spike|hCoV-19/England/MILK-9C3E33/2020|2020-09-09|EPI_ISL_566963|Original|hCoV-19^^England|Human|Lighthouse Lab in Milton Keynes|Wellcome Sanger Institute for the COVID-19 Genomics UK (COG-UK) consortium|Davies|United Kingdom
>Spike|hCoV-19/England/CAMC-B07E22/2020|2020-10-29|EPI_ISL_643554|Original|hCoV-19^^England|Human|Lighthouse Lab in Cambridge|Wellcome Sanger Institute for the COVID-19 Genomics UK (COG-UK) Consortium|Davies|United Kingdom
>Spike|hCoV-19/Spain/MD-H12-36/2020|2020-03-09|EPI_ISL_428699|Original|hCoV-19^^Madrid|Human|Hospital Universitario 12 de Octubre|Hospital Universitario 12 de Octubre|Viedma|Spain
>Spike|hCoV-19/England/MILK-9D4725/2020|2020-09-19|EPI_ISL_581372|Original|hCoV-19^^England|Human|Lighthouse Lab in Milton Keynes|Wellcome Sanger Institute for the COVID-19 Genomics UK (COG-UK) consortium|Davies|United Kingdom
>Spike|hCoV-19/England/MILK-BFF582/2020|2020-12-02|EPI_ISL_720754|Original|hCoV-19^^England|Human|Lighthouse Lab in Milton Keynes|Wellcome Sanger Institute for the COVID-19 Genomics UK (COG-UK) Consortium|Davies|United Kingdom
>Spike|hCoV-19/India/MH-NIV-12019/2020|2020-04-20|EPI_ISL_452199|Original|hCoV-19^^Maharashtra|Human|NIV Influenza|NIV Influenza|Potdar|India
>Spike|hCoV-19/USA/VA-DCLS-1298/2020|2020-07-23|EPI_ISL_572289|Original|hCoV-19^^Virginia|Human|Virginia DCLS|Virginia DCLS|St. Jacques|USA
>Spike|hCoV-19/USA/GA-CDC-7595/2020|2020-10-25|EPI_ISL_681798|Original|hCoV-19^^Georgia|Human|GA Department of Public Health Laboratory|Pathogen Discovery|Queen|USA
>Spike|hCoV-19/USA/WA-UW-11036/2020|2020-06-16|EPI_ISL_570365|Original|hCoV-19^^Washington|Human|UW Virology Lab|UW Virology Lab|Roychoudhury|USA

>Spike|hCoV-19/Hangzhou/HZ481/2020|2020-01-25|EPI_ISL_418512|Original|hCoV-19^^Zhejiang|Human|Hangzhou Center for Disease Control and Prevention|Inspection Center of Hangzhou Center for Disease Control and Prevention|Renton|China
>Spike|hCoV-19/USA/AZ-TG664546/2020|2020-04-20|EPI_ISL_696163|Original|hCoV-19^^Arizona|Human|Sonora Quest Laboratories|TGen North|French|USA
>Spike|hCoV-19/England/LIVE-DC80BC/2020|2020-11-17|EPI_ISL_703235|Original|hCoV-19^^England|Human|Liverpool Clinical Laboratories|COVID-19 Genomics UK (COG-UK) Consortium|Haldenby|United Kingdom
>Spike|hCoV-19/USA/NY-QDX-2483/2020|2020-03-13|EPI_ISL_604814|Original|hCoV-19^^New York|Human|Quest Diagnostics|Quest Diagnostics|Kagan|USA
>Spike|hCoV-19/Wales/PHWC-47FE4A/2020|2020-10-06|EPI_ISL_612818|Original|hCoV-19^^Wales|Human|Wales Specialist Virology Centre Sequencing lab|COVID-19 Genomics UK (COG-UK) Consortium|Moore|United Kingdom
>Spike|hCoV-19/India/MH-ACTREC-436/2020|2020-08-05|EPI_ISL_700085|Original|hCoV-19^^Maharashtra|Human|Hematopathology Laboratory|Hematopathology Laboratory|Patkar|India
>Spike|hCoV-19/USA/MN-MDH-1727/2020|2020-09-16|EPI_ISL_576236|Original|hCoV-19^^Minnesota|Human|Minnesota Department of Health|Minnesota Department of Health|Garfin|USA
>Spike|hCoV-19/USA/LA-EVTL1364/2020|2020-12-11|EPI_ISL_779030|Original|hCoV-19^^Louisiana|Human|LSUHS Emerging Viral Threat Laboratory|Microbial Genome Sequencing Center|Kamil|USA
>Spike|hCoV-19/USA/CT-Yale-523/2020|2020-11-04|EPI_ISL_729764|Original|hCoV-19^^Connecticut|Human|Yale Pathology Lab|Grubaugh Lab - Yale School of Public Health|Grubaugh|USA
>Spike|hCoV-19/Uruguay/RIV-M155/2020|2020-07-21|EPI_ISL_751188|Original|hCoV-19^^Rivera|Human|CENUR Litoral Norte \u2013 UdelaR|Institut Pasteur de Montevideo|Spangenberg|Uruguay
>Spike|hCoV-19/Hong Kong/VM20003767/2020|2020-03-18|EPI_ISL_426408|Original|hCoV-19^^Hong Kong|Human|Ruttonjee Hospital|Hong Kong Department of Health|Mak|China
>Spike|hCoV-19/England/QEUH-C53DFC/2020|2020-12-12|EPI_ISL_736416|Original|hCoV-19^^England|Human|Lighthouse Lab in Glasgow|Wellcome Sanger Institute for the COVID-19 Genomics UK (COG-UK) Consortium|Davies|United Kingdom
>Spike|hCoV-19/Australia/NSW1190/2020|2020-10-25|EPI_ISL_667791|Original|hCoV-19^^New South Wales|Human|South Eastern Area Laboratory Services (SEALS)|NSW Health Pathology - Institute of Clinical Pathology and Medical Research; Westmead Hospital; University of Sydney|Gall|Australia
>Spike|hCoV-19/England/PORT-2D23FD/2020|2020-03-24|EPI_ISL_507141|Original|hCoV-19^^England|Human|Centre for Enzyme Innovation|COVID-19 Genomics UK (COG-UK) Consortium|Robson|United Kingdom
>Spike|hCoV-19/Wales/PHWC-497969/2020|2020-11-23|EPI_ISL_726908|Original|hCoV-19^^Wales|Human|Originating lab|COVID-19 Genomics UK (COG-UK) Consortium|Moore|United Kingdom
>Spike|hCoV-19/Wales/PHWC-24C11/2020|2020-03-20|EPI_ISL_419500|Original|hCoV-19^^Wales|Human|Wales Specialist Virology Centre|Public Health Wales Microbiology Cardiff|Moore|United Kingdom
>Spike|hCoV-19/USA/MN-MDH-1792/2020|2020-04-14|EPI_ISL_590794|Original|hCoV-19^^Minnesota|Human|Minnesota Department of Health|Minnesota Department of Health|Lorentz|USA
>Spike|hCoV-19/England/LIVE-A5BC0/2020|2020-03-18|EPI_ISL_499580|Original|hCoV-19^^England|Human|Liverpool Clinical Laboratories|COVID-19 Genomics UK (COG-UK) Consortium|Haldenby|United Kingdom
>Spike|hCoV-19/England/NORT-1B721EA/2020|2020-11-25|EPI_ISL_724859|Original|hCoV-19^^England|Human|Northumbria University / South Tees Hospitals NHS Foundation Trust / North Cumbria Integrated Care NHS Foundation Trust / North Tees and Hartlepool NHS Foundation Trust / Newcastle Hospitals NHS Foundation Trust|COVID-19 Genomics UK (COG-UK) Consortium|Bashton|United Kingdom
>Spike|hCoV-19/England/QEUH-A4B05A/2020|2020-10-05|EPI_ISL_637398|Original|hCoV-19^^England|Human|Department of Pathology|COVID-19 Genomics UK (COG-UK) Consortium|Goodfellow|United Kingdom

>Spike|hCoV-19/Canada/ON-UHTC_0322/2020|2020-09-06|EPI_ISL_609826|Original|hCoV-19^^Ontario|Human|Unity Health Toronto|Ontario Institute for Cancer Research|de Borja|Canada
>Spike|hCoV-19/England/204381401/2020|2020-10-22|EPI_ISL_649866|Original|hCoV-19^^England|Human|Respiratory Virus Unit|COVID-19 Genomics UK (COG-UK) Consortium|Platt|United Kingdom
>Spike|hCoV-19/Australia/NSW978/2020|2020-09-18|EPI_ISL_545050|Original|hCoV-19^^New South Wales|Human|Pathology West - NSW Health Pathology|NSW Health Pathology - Institute of Clinical Pathology and Medical Research; Westmead Hospital; University of Sydney|Gall|Australia
>Spike|hCoV-19/USA/WA-UW-22809/2020|2020-08-04|EPI_ISL_570593|Original|hCoV-19^^Washington|Human|UW Virology Lab|UW Virology Lab|Roychoudhury|USA
>Spike|hCoV-19/England/CAMB-8316C/2020|2020-04-14|EPI_ISL_443622|Original|hCoV-19^^England|Human|Department of Pathology|Wellcome Sanger Institute for the COVID-19 Genomics UK (COG-UK) consortium|Amato|United Kingdom
>Spike|hCoV-19/Australia/VIC4441/2020|2020-07-16|EPI_ISL_519737|Original|hCoV-19^^Victoria|Human|Microbiological Diagnostic Unit - Public Health Laboratory (MDU-PHL)|MDU-PHL|Schultz|Australia
>Spike|hCoV-19/England/QEUH-79B407/2020|2020-07-15|EPI_ISL_533186|Original|hCoV-19^^England|Human|Lighthouse Lab in Glasgow|Wellcome Sanger Institute for the COVID-19 Genomics UK (COG-UK) consortium|Davies|United Kingdom
>Spike|hCoV-19/Japan/PG-5423/2020|2020-08-00|EPI_ISL_690526|Original|hCoV-19^^Japan|Human|Fukuoka Institute of Health and Environmental Sciences|Pathogen Genomics Center|Sekizuka|Japan
>Spike|hCoV-19/Denmark/DCGC-13001/2020|2020-12-07|EPI_ISL_712520|Original|hCoV-19^^Midtjylland|Human|Department of Virus and Microbiological Special Diagnostics|Albertsen Lab|Michaelsen|Denmark
>Spike|hCoV-19/England/OXON-B2647/2020|2020-04-02|EPI_ISL_479162|Original|hCoV-19^^England|Human|Oxford Viromics|COVID-19 Genomics UK (COG-UK) Consortium|Golubchik|United Kingdom
>Spike|hCoV-19/Denmark/DCGC-1298/2020|2020-06-08|EPI_ISL_618081|Original|hCoV-19^^Hovedstaden|Human|Department of Virus and Microbiological Special Diagnostics|Albertsen lab|Michaelsen|Denmark
>Spike|hCoV-19/South Africa/NHLS-UCT-GS-1779/2020|2020-09-04|EPI_ISL_640112|Original|hCoV-19^^Western Cape|Human|Groote Schuur Hospital wc GSH|NHLS/UCT|Iranzadeh|South Africa
>Spike|hCoV-19/England/CAMB-1B0402/2020|2020-05-01|EPI_ISL_459318|Original|hCoV-19^^England|Human|Department of Pathology|Wellcome Sanger Institute for the COVID-19 Genomics UK (COG-UK) consortium|Davies|United Kingdom
>Spike|hCoV-19/England/LOND-12E8555/2020|2020-12-14|EPI_ISL_741324|Original|hCoV-19^^England|Human|University College London|COVID-19 Genomics UK (COG-UK) Consortium|Roy|United Kingdom
>Spike|hCoV-19/England/NORW-F6744/2020|2020-12-01|EPI_ISL_706376|Original|hCoV-19^^England|Human|Quadram Institute Bioscience|COVID-19 Genomics UK (COG-UK) Consortium|Alikhan|United Kingdom
>Spike|hCoV-19/India/MH-NIV-207705/2020|2020-09-11|EPI_ISL_577661|Original|hCoV-19^^Maharashtra|Human|NIV Influenza|NIV Influenza|Potdar|India
>Spike|hCoV-19/England/204221460/2020|2020-09-19|EPI_ISL_593825|Original|hCoV-19^^England|Human|Respiratory Virus Unit|Respiratory Virus Unit|Platt|United Kingdom
>Spike|hCoV-19/Canada/ON-S18/2020|2020-03-16|EPI_ISL_586373|Original|hCoV-19^^Ontario|Human|Toronto Invasive Bacterial Diseases Network|McMaster University|McArthur|Canada
>Spike|hCoV-19/USA/MI-MDHHS-SC22150/2020|2020-10-20|EPI_ISL_614201|Original|hCoV-19^^Michigan|Human|Michigan Department of Health and Human Services|Michigan Department of Health and Human Services|Jacob|USA
>Spike|hCoV-19/USA/TX-HMH-MCoV-10425/2020|2020-07-08|EPI_ISL_789470|Original|hCoV-19^^Texas|Human|Houston Methodist Hospital|Houston Methodist Hospital|Long|USA
>Spike|hCoV-19/Switzerland/BL-UHB-42189907/2020|2020-03-13|EPI_ISL_528056|Original|hCoV-19^^Basel-Land|Human|University Hospital Basel|University Hospital Basel|Roloff Handschin|Switzerland

>Spike|hCoV-19/England/QEUH-961C2E/2020|2020-07-28|EPI_ISL_536992|Original|hCoV-19^^England|Human|Lighthouse Lab in Glasgow|Wellcome Sanger Institute for the COVID-19 Genomics UK (COG-UK) consortium|Davies|United Kingdom
>Spike|hCoV-19/Netherlands/ZuidHolland_47/2020|2020-03-19|EPI_ISL_422918|Original|hCoV-19^^Zuid Holland|Human|Dutch COVID-19 response team|Erasmus Medical Center|Oude Munnink|Netherlands
>Spike|hCoV-19/Switzerland/VS-ETHZ-310380/2020|2020-10-08|EPI_ISL_603454|Original|hCoV-19^^Valais|Human|Viollier AG|Department of Biosystems Science and Engineering|Nadeau|Switzerland
>Spike|hCoV-19/Japan/PG-7794/2020|2020-07-00|EPI_ISL_686896|Original|hCoV-19^^Japan|Human|Pathogen Genomics Center|Pathogen Genomics Center|Sekizuka|Japan
>Spike|hCoV-19/USA/LA-GBCL-26791731/2020|2020-04-09|EPI_ISL_485349|Original|hCoV-19^^Louisiana|Human|River Road Testing Lab|Ginkgo Bioworks Clinical Laboratory|Smith|USA
>Spike|hCoV-19/Thailand/Bangkok-0044/2020|2020-03-28|EPI_ISL_429180|Original|hCoV-19^^Bangkok|Human|Ramathibodi Hospital|COVID-19 Network Investigations (CONI) Alliance|Batty|Thailand
>Spike|hCoV-19/USA/UT-02956/2020|2020-05-01|EPI_ISL_522278|Original|hCoV-19^^Utah|Human|Utah Public Health Laboratory|Utah Public Health Laboratory|Young|USA
>Spike|hCoV-19/Wales/PHWC-486499/2020|2020-10-20|EPI_ISL_639049|Original|hCoV-19^^Wales|Human|Wales Specialist Virology Centre Sequencing lab|COVID-19 Genomics UK (COG-UK) Consortium|Moore|United Kingdom
>Spike|hCoV-19/England/LOND-1250E4E/2020|2020-11-14|EPI_ISL_665821|Original|hCoV-19^^England|Human|University College London Hospital|COVID-19 Genomics UK (COG-UK) Consortium|Byott|United Kingdom
>Spike|hCoV-19/Wales/PHWC-2B175/2020|2020-04-07|EPI_ISL_431904|Original|hCoV-19^^Wales|Human|Wales Specialist Virology Centre|Public Health Wales Microbiology Cardiff|Moore|United Kingdom
>Spike|hCoV-19/England/20099068604/2020|2020-02-29|EPI_ISL_464212|Original|hCoV-19^^England|Human|Respiratory Virus Unit|Respiratory Virus Unit|Platt|United Kingdom
>Spike|hCoV-19/Denmark/DCGC-11069/2020|2020-11-02|EPI_ISL_670377|Original|hCoV-19^^Hovedstaden|Human|Department of Virus and Microbiological Special Diagnostics|Albertsen Lab|Michaelsen|Denmark
>Spike|hCoV-19/USA/NM-DOH-2020315508/2020|2020-08-27|EPI_ISL_574289|Original|hCoV-19^^New Mexico|Human|New Mexico Department of Health Scientific Laboratory|New Mexico Department of Health Scientific Laboratory|Johnson|USA
>Spike|hCoV-19/Spain/CT-HUVH-VH5325/2020|2020-03-21|EPI_ISL_444982|Original|hCoV-19^^Catalunya|Human|Hospital Universitari Vall d'Hebron - Vall d\hebron Institut de Recerca|Hospital Universitari Vall d'Hebron|Anton|Spain
>Spike|hCoV-19/USA/NY-Wadsworth-229107-01/2020|2020-09-26|EPI_ISL_767448|Original|hCoV-19^^New York|Human|Wadsworth Center|Wadsworth Center|Lasek-Nesselquist|USA
>Spike|hCoV-19/England/ALDP-9CCA72/2020|2020-09-16|EPI_ISL_572671|Original|hCoV-19^^England|Human|Quadram Institute Bioscience|COVID-19 Genomics UK (COG-UK) Consortium|Alikhan|United Kingdom
>Spike|hCoV-19/Sweden/20-50090/2020|2020-03-03|EPI_ISL_454873|Original|hCoV-19^^Stockholm|Human|Karolinska Universitetslaboratoriet|The Public Health Agency of Sweden|Svartstr\u00f6m|Sweden
>Spike|hCoV-19/Wales/PHWC-16383F/2020|2020-05-09|EPI_ISL_474003|Original|hCoV-19^^Wales|Human|Originating lab|COVID-19 Genomics UK (COG-UK) Consortium|Moore|United Kingdom
>Spike|hCoV-19/Australia/VIC8375/2020|2020-08-12|EPI_ISL_564972|Original|hCoV-19^^Victoria|Human|Microbiological Diagnostic Unit - Public Health Laboratory (MDU-PHL)|MDU-PHL|Schultz|Australia
>Spike|hCoV-19/Germany/SH-ChVir20299/2020|2020-11-13|EPI_ISL_729637|Original|hCoV-19^^Schleswig-Holstein|Human|A. Krumbholz|Charit\u00e9 Universit\u00e4tsmedizin Berlin|Muehlemann|Germany
>Spike|hCoV-19/Japan/PG-3048/2020|2020-04-00|EPI_ISL_691171|Original|hCoV-19^^Japan|Human|Pathogen Genomics Center|Pathogen Genomics Center|Sekizuka|Japan

>Spike|hCoV-19/Denmark/DCGC-6993/2020|2020-10-12|EPI_ISL_619839|Original|hCoV-19^^Hovedstaden|Human|Department of Virus and Microbiological Special Diagnostics|Albertsen lab|Michaelsen|Denmark
>Spike|hCoV-19/USA/FL-BPHL-0919/2020|2020-07-02|EPI_ISL_517809|Original|hCoV-19^^Florida|Human|Florida Bureau of Public Health Laboratories|Florida Bureau of Public Health Laboratories|Blanton|USA
>Spike|hCoV-19/Norway/4592/2020|2020-10-15|EPI_ISL_668441|Original|hCoV-19^^Trondelag|Human|Department of Medical Microbiology|Norwegian Institute of Public Health|Kopperud Riis|Norway
>Spike|hCoV-19/Wales/PHWC-49A8C3/2020|2020-12-02|EPI_ISL_727613|Original|hCoV-19^^Wales|Human|Originating lab|COVID-19 Genomics UK (COG-UK) Consortium|Moore|United Kingdom
>Spike|hCoV-19/USA/WI-GMF-19743/2020|2020-09-11|EPI_ISL_547843|Original|hCoV-19^^Wisconsin|Human|Gundersen Molecular Diagnostics Laboratory|Kabara Cancer Research Institute|Kenny|USA
>Spike|hCoV-19/Philippines/RITM-05/2020|2020-03-22|EPI_ISL_430843|Original|hCoV-19^^Philippines|Human|Bethany Hospital|Research Institute for Tropical Medicine|Polotan|Philippines
>Spike|hCoV-19/Switzerland/VD-ETHZ-100167/2020|2020-03-16|EPI_ISL_574978|Original|hCoV-19^^Vaud|Human|Viollier AG|Department of Biosystems Science and Engineering|Nadeau|Switzerland
>Spike|hCoV-19/USA/MI-MDHHS-SC22707/2020|2020-11-17|EPI_ISL_767356|Original|hCoV-19^^Michigan|Human|Michigan Department of Health and Human Services|Michigan Department of Health and Human Services|Jacob|USA
>Spike|hCoV-19/England/QEUH-5E819D/2020|2020-06-16|EPI_ISL_533273|Original|hCoV-19^^England|Human|Lighthouse Lab in Glasgow|Wellcome Sanger Institute for the COVID-19 Genomics UK (COG-UK) consortium|Davies|United Kingdom
>Spike|hCoV-19/England/CAMC-9B1E38/2020|2020-09-04|EPI_ISL_549396|Original|hCoV-19^^England|Human|Oxford Viromics|COVID-19 Genomics UK (COG-UK) Consortium|Golubchik|United Kingdom
>Spike|hCoV-19/USA/WI-WSLH-200739/2020|2020-09-14|EPI_ISL_631495|Original|hCoV-19^^Wisconsin|Human|Wisconsin State Laboratory of Hygiene Communicable Disease Division|Wisconsin State Laboratory of Hygiene Communicable Disease Division|Florek|USA
>Spike|hCoV-19/Scotland/QEUH-A006C8/2020|2020-09-30|EPI_ISL_600147|Original|hCoV-19^^Scotland|Human|Lighthouse Lab in Glasgow|Wellcome Sanger Institute for the COVID-19 Genomics UK (COG-UK) consortium|Davies|United Kingdom
>Spike|hCoV-19/Luxembourg/LNS9837378/2020|2020-11-26|EPI_ISL_744996|Original|hCoV-19^^Luxembourg|Human|Laboratoire national de sant\u00e9|Laboratoire national de sant\u00e9|Wienecke-Baldacchino|Luxembourg
>Spike|hCoV-19/Australia/VIC12603/2020|2020-07-28|EPI_ISL_592035|Original|hCoV-19^^Victoria|Human|Microbiological Diagnostic Unit - Public Health Laboratory (MDU-PHL)|MDU-PHL|Schultz|Australia
>Spike|hCoV-19/England/MILK-ABC546/2020|2020-10-21|EPI_ISL_644116|Original|hCoV-19^^England|Human|Lighthouse Lab in Milton Keynes|Wellcome Sanger Institute for the COVID-19 Genomics UK (COG-UK) Consortium|Davies|United Kingdom
>Spike|hCoV-19/Belgium/UGent-1/2020|2020-03-24|EPI_ISL_425048|Original|hCoV-19^^Ghent|Human|Lab voor klinische biologie|Onderzoeksgroep Virologie|Lambrechts|Belgium
>Spike|hCoV-19/England/LIVE-A977D/2020|2020-07-17|EPI_ISL_526392|Original|hCoV-19^^England|Human|Liverpool Clinical Laboratories|COVID-19 Genomics UK (COG-UK) Consortium|Haldenby|United Kingdom
>Spike|hCoV-19/England/ALDP-9E8542/2020|2020-09-24|EPI_ISL_638253|Original|hCoV-19^^England|Human|Centre for Enzyme Innovation|COVID-19 Genomics UK (COG-UK) Consortium|Robson|United Kingdom
>Spike|hCoV-19/Wales/PHWC-49046F/2020|2020-10-30|EPI_ISL_702896|Original|hCoV-19^^Wales|Human|Originating lab|COVID-19 Genomics UK (COG-UK) Consortium|Moore|United Kingdom

>Spike|hCoV-19/United Arab Emirates/0804/2020|2020-05-19|EPI_ISL_698943|Original|hCoV-19^^Abu Dhabi|Human|Group 42 (G42) Healthcare|G42 Healthcare|Ogrodzki|United Arab Emirates
>Spike|hCoV-19/Japan/PG-2482/2020|2020-04-00|EPI_ISL_686087|Original|hCoV-19^^Japan|Human|Saitama Prefectural Institute of Public Health|Pathogen Genomics Center|Sekizuka|Japan
>Spike|hCoV-19/England/LIVE-A2A7B/2020|2020-05-19|EPI_ISL_500015|Original|hCoV-19^^England|Human|Liverpool Clinical Laboratories|COVID-19 Genomics UK (COG-UK) Consortium|Haldenby|United Kingdom
>Spike|hCoV-19/England/QEUH-A78485/2020|2020-10-12|EPI_ISL_638756|Original|hCoV-19^^England|Human|Oxford Viromics|COVID-19 Genomics UK (COG-UK) Consortium|Golubchik|United Kingdom
>Spike|hCoV-19/England/MILK-993072/2020|2020-08-26|EPI_ISL_552048|Original|hCoV-19^^England|Human|Lighthouse Lab in Milton Keynes|Wellcome Sanger Institute for the COVID-19 Genomics UK (COG-UK) consortium|Davies|United Kingdom
>Spike|hCoV-19/Germany/HH-hpi-p1772/2020|2020-03-19|EPI_ISL_776146|Original|hCoV-19^^Hamburg|Human|University Medical Center Hamburg Eppendorf|Heinrich Pette Institute|Robitaille|Germany
>Spike|hCoV-19/England/QEUH-A8A8FD/2020|2020-10-16|EPI_ISL_638859|Original|hCoV-19^^England|Human|Oxford Viromics|COVID-19 Genomics UK (COG-UK) Consortium|Golubchik|United Kingdom
>Spike|hCoV-19/England/ALDP-AA4198/2020|2020-10-22|EPI_ISL_624071|Original|hCoV-19^^England|Human|Lighthouse Lab in Alderley Park|Wellcome Sanger Institute for the COVID-19 Genomics UK (COG-UK) consortium|Davies|United Kingdom
>Spike|hCoV-19/Australia/VIC17208/2020|2020-08-14|EPI_ISL_663795|Original|hCoV-19^^Victoria|Human|Microbiological Diagnostic Unit - Public Health Laboratory (MDU-PHL)|MDU-PHL|Schultz|Australia
>Spike|hCoV-19/England/ALDP-A6D4B0/2020|2020-10-15|EPI_ISL_608238|Original|hCoV-19^^England|Human|Lighthouse Lab in Alderley Park|Wellcome Sanger Institute for the COVID-19 Genomics UK (COG-UK) consortium|Davies|United Kingdom
>Spike|hCoV-19/England/CAMB-1BA66C/2020|2020-11-11|EPI_ISL_664534|Original|hCoV-19^^England|Human|Department of Pathology|COVID-19 Genomics UK (COG-UK) Consortium|Goodfellow|United Kingdom
>Spike|hCoV-19/USA/WI-WSLH-200345/2020|2020-04-20|EPI_ISL_578519|Original|hCoV-19^^Wisconsin|Human|Wisconsin State Laboratory of Hygiene Communicable Disease Division|Wisconsin State Laboratory of Hygiene Communicable Disease Division|Florek|USA
>Spike|hCoV-19/England/MILK-B5A6CE/2020|2020-11-06|EPI_ISL_658389|Original|hCoV-19^^England|Human|Lighthouse Lab in Milton Keynes|Wellcome Sanger Institute for the COVID-19 Genomics UK (COG-UK) Consortium|Davies|United Kingdom
>Spike|hCoV-19/Scotland/QEUH-9DCEBF/2020|2020-09-22|EPI_ISL_601146|Original|hCoV-19^^Scotland|Human|Lighthouse Lab in Glasgow|Wellcome Sanger Institute for the COVID-19 Genomics UK (COG-UK) consortium|Davies|United Kingdom
>Spike|hCoV-19/England/ALDP-A93FED/2020|2020-10-19|EPI_ISL_611204|Original|hCoV-19^^England|Human|Lighthouse Lab in Alderley Park|Wellcome Sanger Institute for the COVID-19 Genomics UK (COG-UK) consortium|Davies|United Kingdom
>Spike|hCoV-19/USA/TX-HMH-MCoV-9744/2020|2020-07-06|EPI_ISL_780143|Original|hCoV-19^^Texas|Human|Houston Methodist Hospital|Houston Methodist Hospital|Long|USA
>Spike|hCoV-19/England/QEUH-A0AD9C/2020|2020-10-01|EPI_ISL_599349|Original|hCoV-19^^England|Human|Lighthouse Lab in Glasgow|Wellcome Sanger Institute for the COVID-19 Genomics UK (COG-UK) consortium|Davies|United Kingdom
>Spike|hCoV-19/Hong Kong/HKPU36-0702/2020|2020-02-09|EPI_ISL_418815|Original|hCoV-19^^Hong Kong|Human|Department of Clinical Pathology|Department of Health Technology and Informatics|Leung|China
>Spike|hCoV-19/Japan/PG-3525/2020|2020-06-00|EPI_ISL_691781|Original|hCoV-19^^Japan|Human|Pathogen Genomics Center|Pathogen Genomics Center|Sekizuka|Japan
>Spike|hCoV-19/Mexico/NLE-UANL-009/2020|2020-05-19|EPI_ISL_779176|Original|hCoV-19^^Nuevo Leon|Human|Laboratorio de Infectolog\u00eda|Laboratorio de Infectolog\u00eda Molecular|Gal\u00e1n|Mexico

>Spike|hCoV-19/Denmark/DCGC-10304/2020|2020-11-02|EPI_ISL_669807|Original|hCoV-19^^Nordjylland|Human|Department of Virus and Microbiological Special Diagnostics|Albertsen Lab|Michaelsen|Denmark
>Spike|hCoV-19/Switzerland/UR-ETHZ-110014/2020|2020-04-01|EPI_ISL_486454|Original|hCoV-19^^Uri|Human|Viollier AG|Department of Biosystems Science and Engineering|Nadeau|Switzerland
>Spike|hCoV-19/USA/TX-HMH-MCoV-12081/2020|2020-09-20|EPI_ISL_789837|Original|hCoV-19^^Texas|Human|Houston Methodist Hospital|Houston Methodist Hospital|Long|USA
>Spike|hCoV-19/USA/CA-CZB-10851/2020|2020-07-28|EPI_ISL_583096|Original|hCoV-19^^California|Human|Humboldt County Public Health Laboratory|Chan-Zuckerberg Biohub|Bhatt|USA
>Spike|hCoV-19/USA/MI-UM-10036767342/2020|2020-11-03|EPI_ISL_682225|Original|hCoV-19^^Michigan|Human|University of Michigan Clinical Microbiology Laboratory|Lauring Lab|Valesano|USA
>Spike|hCoV-19/USA/TX-HMH-3600/2020|2020-06-15|EPI_ISL_543056|Original|hCoV-19^^Texas|Human|Houston Methodist Hospital|Houston Methodist Hospital|Long|USA
>Spike|hCoV-19/Germany/SH-ChVir9946/2020|2020-11-02|EPI_ISL_729622|Original|hCoV-19^^Schleswig-Holstein|Human|A. Krumbholz|Charit\u00e9 Universit\u00e4tsmedizin Berlin|Muehlemann|Germany
>Spike|hCoV-19/Denmark/DCGC-19666/2020|2020-12-07|EPI_ISL_748241|Original|hCoV-19^^Hovedstaden|Human|Department of Virus and Microbiological Special Diagnostics|Albertsen Lab|Michaelsen|Denmark
>Spike|hCoV-19/Canada/NS-NML-1338/2020|2020-04-11|EPI_ISL_579716|Original|hCoV-19^^Nova Scotia|Human|QEII Health Sciences Centre|National Microbiology Laboratory (NML)|Majer|Canada
>Spike|hCoV-19/Denmark/DCGC-15089/2020|2020-11-23|EPI_ISL_713467|Original|hCoV-19^^Syddanmark|Human|Department of Virus and Microbiological Special Diagnostics|Albertsen Lab|Michaelsen|Denmark
>Spike|hCoV-19/Portugal/PT1822/2020|2020-11-15|EPI_ISL_732044|Original|hCoV-19^^Portugal|Human|Instituto Nacional de Saude (INSA)|Instituto Nacional de Saude (INSA)|Borges|Portugal
>Spike|hCoV-19/South Korea/KCDC2316/2020|2020-05-25|EPI_ISL_510652|Original|hCoV-19^^South Korea|Human|Division of Viral Diseases|Division of Viral Diseases|Kim|South Korea
>Spike|hCoV-19/Scotland/CVR2708/2020|2020-04-17|EPI_ISL_478025|Original|hCoV-19^^Scotland|Human|West of Scotland Specialist Virology Centre|COVID-19 Genomics UK (COG-UK) Consortium|Robertson|United Kingdom
>Spike|hCoV-19/USA/MN-MDH-320/2020|2020-03-30|EPI_ISL_450778|Original|hCoV-19^^Minnesota|Human|Minnesota Department of Health|Minnesota Department of Health|Garfin|USA
>Spike|hCoV-19/USA/UT-UPHL-2012167424/2020|2020-08-03|EPI_ISL_745496|Original|hCoV-19^^Utah|Human|Ginkgo Bioworks Clinical Laboratory|Utah Public Health Laboratory|Young|USA
>Spike|hCoV-19/Denmark/DCGC-21098/2020|2020-12-21|EPI_ISL_759554|Original|hCoV-19^^Hovedstaden|Human|Department of Virus and Microbiological Special Diagnostics|Albertsen Lab|Michaelsen|Denmark
>Spike|hCoV-19/Denmark/DCGC-13709/2020|2020-11-30|EPI_ISL_712283|Original|hCoV-19^^Syddanmark|Human|Department of Virus and Microbiological Special Diagnostics|Albertsen Lab|Michaelsen|Denmark
>Spike|hCoV-19/USA/TX-HMH-MCoV-10625/2020|2020-07-08|EPI_ISL_789160|Original|hCoV-19^^Texas|Human|Houston Methodist Hospital|Houston Methodist Hospital|Long|USA
>Spike|hCoV-19/Wales/PHWC-2D568/2020|2020-04-04|EPI_ISL_445996|Original|hCoV-19^^Wales|Human|Wales Specialist Virology Centre|Public Health Wales Microbiology Cardiff|Moore|United Kingdom
>Spike|hCoV-19/England/LIVE-DC2BB1/2020|2020-11-25|EPI_ISL_705839|Original|hCoV-19^^England|Human|Liverpool Clinical Laboratories|COVID-19 Genomics UK (COG-UK) Consortium|Haldenby|United Kingdom
>Spike|hCoV-19/USA/UT-UPHL-2012972043/2020|2020-08-13|EPI_ISL_745879|Original|hCoV-19^^Utah|Human|Ginkgo Bioworks Clinical Laboratory|Utah Public Health Laboratory|Young|USA

>Spike|hCoV-19/Australia/QLD1010/2020|2020-03-22|EPI_ISL_530270|Original|hCoV-19^^Queensland|Human|Queensland Health Forensic and Scientific Services|Public Health Virology Laboratory|Nguyen|Australia
>Spike|hCoV-19/England/NORW-F5675/2020|2020-10-31|EPI_ISL_665889|Original|hCoV-19^^England|Human|Quadram Institute Bioscience|COVID-19 Genomics UK (COG-UK) Consortium|Alikhan|United Kingdom
>Spike|hCoV-19/England/LIVE-DC4997/2020|2020-11-10|EPI_ISL_702597|Original|hCoV-19^^England|Human|Liverpool Clinical Laboratories|COVID-19 Genomics UK (COG-UK) Consortium|Haldenby|United Kingdom
>Spike|hCoV-19/USA/MI-QDX-1156/2020|2020-03-24|EPI_ISL_571886|Original|hCoV-19^^Michigan|Human|Quest Diagnostics|Quest Diagnostics|Kagan|USA
>Spike|hCoV-19/England/ALDP-951BE8/2020|2020-06-10|EPI_ISL_551316|Original|hCoV-19^^England|Human|Lighthouse Lab in Alderley Park|Wellcome Sanger Institute for the COVID-19 Genomics UK (COG-UK) consortium|Davies|United Kingdom
>Spike|hCoV-19/USA/AZ-TG491725/2020|2020-06-26|EPI_ISL_695228|Original|hCoV-19^^Arizona|Human|AZ SPHL|TGen North|French|USA
>Spike|hCoV-19/England/LOND-1260FA2/2020|2020-04-02|EPI_ISL_524695|Original|hCoV-19^^England|Human|North West London Pathology|Wellcome Sanger Institute for the COVID-19 Genomics UK (COG-UK) consortium|Davies|United Kingdom
>Spike|hCoV-19/Scotland/EDB4219/2020|2020-04-21|EPI_ISL_456937|Original|hCoV-19^^Scotland|Human|Virology Department|COVID-19 Genomics UK (COG-UK) Consortium|Williams|United Kingdom
>Spike|hCoV-19/England/CAMC-A3F842/2020|2020-10-06|EPI_ISL_609650|Original|hCoV-19^^England|Human|Lighthouse Lab in Cambridge|Wellcome Sanger Institute for the COVID-19 Genomics UK (COG-UK) consortium|Davies|United Kingdom
>Spike|hCoV-19/Denmark/DCGC-4020/2020|2020-09-14|EPI_ISL_615792|Original|hCoV-19^^Syddanmark|Human|Department of Virus and Microbiological Special Diagnostics|Albertsen lab|Michaelsen|Denmark
>Spike|hCoV-19/India/MH-ACTREC-065/2020|2020-05-17|EPI_ISL_699721|Original|hCoV-19^^Maharashtra|Human|Hematopathology Laboratory|Hematopathology Laboratory|Patkar|India
>Spike|hCoV-19/England/LIVE-DC7D2F/2020|2020-11-16|EPI_ISL_702947|Original|hCoV-19^^England|Human|Liverpool Clinical Laboratories|COVID-19 Genomics UK (COG-UK) Consortium|Haldenby|United Kingdom
>Spike|hCoV-19/India/MH-ACTREC-493/2020|2020-08-14|EPI_ISL_700141|Original|hCoV-19^^Maharashtra|Human|Hematopathology Laboratory|Hematopathology Laboratory|Patkar|India
>Spike|hCoV-19/Scotland/QEUH-96B28C/2020|2020-08-17|EPI_ISL_531416|Original|hCoV-19^^Scotland|Human|NHSGGC West of Scotland Specialist Virology Centre / MRC-University of Glasgow Centre for Virus Research|Wellcome Sanger Institute for the COVID-19 Genomics UK (COG-UK) consortium|Davies|United Kingdom
>Spike|hCoV-19/USA/NY-Wadsworth-141055-01/2020|2020-06-16|EPI_ISL_676718|Original|hCoV-19^^New York|Human|Wadsworth Center|Wadsworth Center|Lasek-Nesselquist|USA
>Spike|hCoV-19/England/ALDP-A947A8/2020|2020-10-19|EPI_ISL_624745|Original|hCoV-19^^England|Human|Lighthouse Lab in Alderley Park|Wellcome Sanger Institute for the COVID-19 Genomics UK (COG-UK) consortium|Davies|United Kingdom
>Spike|hCoV-19/USA/UT-UPHL-2009692/2020|2020-07-25|EPI_ISL_605366|Original|hCoV-19^^Utah|Human|Utah Public Health Laboratory|Utah Public Health Laboratory|Young|USA
>Spike|hCoV-19/England/ALDP-A2EE35/2020|2020-10-05|EPI_ISL_586953|Original|hCoV-19^^England|Human|Lighthouse Lab in Alderley Park|Wellcome Sanger Institute for the COVID-19 Genomics UK (COG-UK) consortium|Davies|United Kingdom
>Spike|hCoV-19/England/MILK-994DF2/2020|2020-08-25|EPI_ISL_552199|Original|hCoV-19^^England|Human|Lighthouse Lab in Milton Keynes|Wellcome Sanger Institute for the COVID-19 Genomics UK (COG-UK) consortium|Davies|United Kingdom
>Spike|hCoV-19/Denmark/DCGC-22254/2020|2020-12-14|EPI_ISL_758637|Original|hCoV-19^^Hovedstaden|Human|Department of Virus and Microbiological Special Diagnostics|Albertsen Lab|Michaelsen|Denmark
>Spike|hCoV-19/USA/UT-0887/2020|2020-04-05|EPI_ISL_435388|Original|hCoV-19^^Utah|Human|Utah Public Health Laboratory|Utah Public Health Laboratory|Young|USA

>Spike|hCoV-19/Denmark/DCGC-19837/2020|2020-12-07|EPI_ISL_747545|Original|hCoV-19^^Hovedstaden|Human|Department of Virus and Microbiological Special Diagnostics|Albertsen Lab|Michaelsen|Denmark
>Spike|hCoV-19/USA/UT-UPHL-2009003/2020|2020-05-27|EPI_ISL_525313|Original|hCoV-19^^Utah|Human|Utah Public Health Laboratory|Utah Public Health Laboratory|Young|USA
>Spike|hCoV-19/England/CAMC-B337D7/2020|2020-11-05|EPI_ISL_659104|Original|hCoV-19^^England|Human|Lighthouse Lab in Cambridge|Wellcome Sanger Institute for the COVID-19 Genomics UK (COG-UK) Consortium|Davies|United Kingdom
>Spike|hCoV-19/Wales/PHWC-35F14/2020|2020-04-27|EPI_ISL_474591|Original|hCoV-19^^Wales|Human|Originating lab|COVID-19 Genomics UK (COG-UK) Consortium|Moore|United Kingdom
>Spike|hCoV-19/England/MILK-B954EB/2020|2020-11-13|EPI_ISL_673708|Original|hCoV-19^^England|Human|Lighthouse Lab in Milton Keynes|Wellcome Sanger Institute for the COVID-19 Genomics UK (COG-UK) Consortium|Davies|United Kingdom
>Spike|hCoV-19/Netherlands/ZH-EMC-680/2020|2020-10-02|EPI_ISL_632414|Original|hCoV-19^^Zuid Holland|Human|Dutch COVID-19 response team|Erasmus Medical Center|Oude Munnink|Netherlands
>Spike|hCoV-19/England/EXET-139331/2020|2020-10-25|EPI_ISL_664458|Original|hCoV-19^^England|Human|University of Exeter|COVID-19 Genomics UK (COG-UK) Consortium|Temperton|United Kingdom
>Spike|hCoV-19/Portugal/PT0529/2020|2020-04-06|EPI_ISL_454253|Original|hCoV-19^^Portugal|Human|unknown|Instituto Nacional de Saude (INSA)|Borges|Portugal
>Spike|hCoV-19/England/QEUH-CCCF52/2020|2020-12-23|EPI_ISL_782370|Original|hCoV-19^^England|Human|Lighthouse Lab in Glasgow|Wellcome Sanger Institute for the COVID-19 Genomics UK (COG-UK) Consortium|Davies|United Kingdom
>Spike|hCoV-19/Wales/PHWC-497705/2020|2020-11-21|EPI_ISL_726871|Original|hCoV-19^^Wales|Human|Originating lab|COVID-19 Genomics UK (COG-UK) Consortium|Moore|United Kingdom
>Spike|hCoV-19/England/CAMB-8459C/2020|2020-04-16|EPI_ISL_443406|Original|hCoV-19^^England|Human|Department of Pathology|Wellcome Sanger Institute for the COVID-19 Genomics UK (COG-UK) consortium|Amato|United Kingdom
>Spike|hCoV-19/Wales/PHWC-165C9B/2020|2020-06-07|EPI_ISL_490787|Original|hCoV-19^^Wales|Human|Wales Specialist Virology Centre Sequencing lab|COVID-19 Genomics UK (COG-UK) Consortium|Moore|United Kingdom
>Spike|hCoV-19/England/CAMC-B989CB/2020|2020-11-15|EPI_ISL_673807|Original|hCoV-19^^England|Human|Lighthouse Lab in Cambridge|Wellcome Sanger Institute for the COVID-19 Genomics UK (COG-UK) Consortium|Davies|United Kingdom
>Spike|hCoV-19/USA/TX-HMH-MCoV-9477/2020|2020-07-03|EPI_ISL_780479|Original|hCoV-19^^Texas|Human|Houston Methodist Hospital|Houston Methodist Hospital|Long|USA
>Spike|hCoV-19/England/NORT-28903A/2020|2020-03-26|EPI_ISL_458636|Original|hCoV-19^^England|Human|NU-OMICS DNA Sequencing research facility|Wellcome Sanger Institute for the COVID-19 Genomics UK (COG-UK) consortium|Davies|United Kingdom
>Spike|hCoV-19/USA/TX-HMH-MCoV-15903/2020|2020-07-10|EPI_ISL_785587|Original|hCoV-19^^Texas|Human|Houston Methodist Hospital|Houston Methodist Hospital|Long|USA
>Spike|hCoV-19/England/ALDP-BC8546/2020|2020-11-23|EPI_ISL_721269|Original|hCoV-19^^England|Human|Lighthouse Lab in Alderley Park|Wellcome Sanger Institute for the COVID-19 Genomics UK (COG-UK) Consortium|Davies|United Kingdom
>Spike|hCoV-19/Denmark/DCGC-17797/2020|2020-12-14|EPI_ISL_749574|Original|hCoV-19^^Midtjylland|Human|Department of Virus and Microbiological Special Diagnostics|Albertsen Lab|Michaelsen|Denmark
>Spike|hCoV-19/Japan/PG-2197/2020|2020-04-00|EPI_ISL_685802|Original|hCoV-19^^Japan|Human|Pathogen Genomics Center|Pathogen Genomics Center|Sekizuka|Japan
>Spike|hCoV-19/England/BRIS-12DA3B/2020|2020-05-07|EPI_ISL_481896|Original|hCoV-19^^England|Human|PHE South West Regional Laboratory|Wellcome Sanger Institute for the COVID-19 Genomics UK (COG-UK) consortium|Davies|United Kingdom
>Spike|hCoV-19/England/MILK-B3BC0D/2020|2020-10-31|EPI_ISL_679795|Original|hCoV-19^^England|Human|Oxford Viromics|COVID-19 Genomics UK (COG-UK) Consortium|Golubchik|United Kingdom

>Spike|hCoV-19/Brazil/RS-00633/2020|2020-11-24|EPI_ISL_770581|Original|hCoV-19^^Rio Grande do Sul|Human|Laborat\u00f3rio de Microbiologia Molecular - Universidade FEEVALE|Bioinformatics Laboratory / LNCC|Almeida|Brazil
>Spike|hCoV-19/USA/LA-GBCL-26544554/2020|2020-04-18|EPI_ISL_485887|Original|hCoV-19^^Louisiana|Human|River Road Testing Lab|Ginkgo Bioworks Clinical Laboratory|Smith|USA
>Spike|hCoV-19/Spain/VC-FISABIO-241/2020|2020-03-26|EPI_ISL_436336|Original|hCoV-19^^Comunitat Valenciana|Human|Servicio de Microbiologia. Hospital Clinico Universitario de Valencia|Sequencing and Bioinformatics Service and Molecular Epidemiology Research Group. FISABIO-Public Health|Gonzalez-Candelas|Spain
>Spike|hCoV-19/England/MILK-B8691B/2020|2020-11-14|EPI_ISL_673954|Original|hCoV-19^^England|Human|Lighthouse Lab in Milton Keynes|Wellcome Sanger Institute for the COVID-19 Genomics UK (COG-UK) Consortium|Davies|United Kingdom
>Spike|hCoV-19/Australia/VIC7029/2020|2020-07-24|EPI_ISL_564201|Original|hCoV-19^^Victoria|Human|Victorian Infectious Diseases Reference Laboratory (VIDRL)|VIDRL and MDU-PHL|Schultz|Australia
>Spike|hCoV-19/England/ALDP-A87720/2020|2020-10-17|EPI_ISL_606038|Original|hCoV-19^^England|Human|Lighthouse Lab in Alderley Park|Wellcome Sanger Institute for the COVID-19 Genomics UK (COG-UK) consortium|Davies|United Kingdom
>Spike|hCoV-19/England/CAMC-B21DF8/2020|2020-11-01|EPI_ISL_647324|Original|hCoV-19^^England|Human|Lighthouse Lab in Cambridge|Wellcome Sanger Institute for the COVID-19 Genomics UK (COG-UK) Consortium|Davies|United Kingdom
>Spike|hCoV-19/England/MILK-C30830/2020|2020-12-07|EPI_ISL_782829|Original|hCoV-19^^England|Human|Lighthouse Lab in Milton Keynes|Wellcome Sanger Institute for the COVID-19 Genomics UK (COG-UK) Consortium|Davies|United Kingdom
>Spike|hCoV-19/Switzerland/AG-UZH-IMV122/2020|2020-12-20|EPI_ISL_730621|Original|hCoV-19^^Aargau|Human|Limmattal Hospital|Institute of Medical Virology|Schmutz|Switzerland
>Spike|hCoV-19/SaudiArabia/KAUST-JEDDAH559/2020|2020-04-21|EPI_ISL_512960|Original|hCoV-19^^Jeddah|Human|Pathogen Genomics Lab King Abdullah University of Science and Technology(KAUST)|Pathogen Genomics Lab King Abdullah University of Science and Technology(KAUST)|Mohamed Ghazzali|Saudi Arabia
>Spike|hCoV-19/Russia/StPetersburg-RII11151S/2020|2020-04-30|EPI_ISL_507236|Original|hCoV-19^^Saint Petersburg|Human|WHO National Influenza Centre Russian Federation|WHO National Influenza Centre Russian Federation|Komissarov|Russia
>Spike|hCoV-19/Scotland/QEUH-96C7AA/2020|2020-08-21|EPI_ISL_534608|Original|hCoV-19^^Scotland|Human|NHSGGC West of Scotland Specialist Virology Centre / MRC-University of Glasgow Centre for Virus Research|Wellcome Sanger Institute for the COVID-19 Genomics UK (COG-UK) consortium|Davies|United Kingdom
>Spike|hCoV-19/Wales/PHWC-49D972/2020|2020-12-07|EPI_ISL_742854|Original|hCoV-19^^Wales|Human|Wales Specialist Virology Centre Sequencing lab|COVID-19 Genomics UK (COG-UK) Consortium|Moore|United Kingdom
>Spike|hCoV-19/USA/LA-EVTL618/2020|2020-07-02|EPI_ISL_579022|Original|hCoV-19^^Louisiana|Human|LSUHS Emerging Viral Threat Laboratory|Microbial Genome Sequencing Center|Kamil|USA
>Spike|hCoV-19/England/CAMC-B09F69/2020|2020-10-30|EPI_ISL_673003|Original|hCoV-19^^England|Human|Lighthouse Lab in Cambridge|Wellcome Sanger Institute for the COVID-19 Genomics UK (COG-UK) Consortium|Davies|United Kingdom
>Spike|hCoV-19/Cote dIvoire/BKE0629/2020|2020-07-17|EPI_ISL_614378|Original|hCoV-19^^Yamoussoukro|Human|Molecular diagnostic unit for viral haemorrhagic fevers and emerging viruses|Project group Epidemology of Highly Pathogenic Microorganisms|Calvignac-Spencer|C\u00f4te d'Ivoire
>Spike|hCoV-19/USA/CA-CZB-15294/2020|2020-11-20|EPI_ISL_739602|Original|hCoV-19^^California|Human|Santa Clara County Public Health Laboratory|Chan-Zuckerberg Biohub|Kamm|USA
>Spike|hCoV-19/England/MILK-971775/2020|2020-08-18|EPI_ISL_553076|Original|hCoV-19^^England|Human|Lighthouse Lab in Milton Keynes|Wellcome Sanger Institute for the COVID-19 Genomics UK (COG-UK) consortium|Davies|United Kingdom

>Spike|hCoV-19/England/LOND-1251CBC/2020|2020-11-26|EPI_ISL_705955|Original|hCoV-19^^England|Human|University College London Hospital|COVID-19 Genomics UK (COG-UK) Consortium|Byott|United Kingdom
>Spike|hCoV-19/USA/TX-HMH-MCoV-12289/2020|2020-09-24|EPI_ISL_790283|Original|hCoV-19^^Texas|Human|Houston Methodist Hospital|Houston Methodist Hospital|Long|USA
>Spike|hCoV-19/Japan/PG-0599/2020|2020-04-00|EPI_ISL_684209|Original|hCoV-19^^Japan|Human|Pathogen Genomics Center|Pathogen Genomics Center|Sekizuka|Japan
>Spike|hCoV-19/Canada/ON-UHTC_0050/2020|2020-04-08|EPI_ISL_464036|unknown|hCoV-19^^Ontario|Human|Unity Health Toronto|Ontario Institute for Cancer Research|de Borja|Canada
>Spike|hCoV-19/South Africa/KRISP-K002856/2020|2020-08-31|EPI_ISL_602671|Original|hCoV-19^^KwaZulu-Natal|Human|NHLS-IALCH|KRISP|de Oliveira|South Africa
>Spike|hCoV-19/England/CAMC-B78A91/2020|2020-11-11|EPI_ISL_668080|Original|hCoV-19^^England|Human|Lighthouse Lab in Cambridge|Wellcome Sanger Institute for the COVID-19 Genomics UK (COG-UK) Consortium|Davies|United Kingdom
>Spike|hCoV-19/Norway/6276/2020|2020-11-22|EPI_ISL_775374|Original|hCoV-19^^Nordland|Human|Nordland Hospital - Bodo|Norwegian Institute of Public Health|Vollan|Norway
>Spike|hCoV-19/England/ALDP-4A7456/2020|2020-06-13|EPI_ISL_559546|Original|hCoV-19^^England|Human|Lighthouse Lab in Alderley Park|Wellcome Sanger Institute for the COVID-19 Genomics UK (COG-UK) consortium|Davies|United Kingdom
>Spike|hCoV-19/Denmark/DCGC-2231/2020|2020-08-10|EPI_ISL_617536|Original|hCoV-19^^Hovedstaden|Human|Department of Virus and Microbiological Special Diagnostics|Albertsen lab|Michaelsen|Denmark
>Spike|hCoV-19/USA/NY-NYCPHL-001495/2020|2020-11-30|EPI_ISL_745401|Original|hCoV-19^^New York|Human|DOHMH Corona|New York City Public Health Laboratory|Wang|USA
>Spike|hCoV-19/England/CAMC-A64F6D/2020|2020-10-11|EPI_ISL_608644|Original|hCoV-19^^England|Human|Lighthouse Lab in Cambridge|Wellcome Sanger Institute for the COVID-19 Genomics UK (COG-UK) consortium|Davies|United Kingdom
>Spike|hCoV-19/Wales/PHWC-1687B5/2020|2020-03-30|EPI_ISL_494286|Original|hCoV-19^^Wales|Human|Originating lab|COVID-19 Genomics UK (COG-UK) Consortium|Moore|United Kingdom
>Spike|hCoV-19/England/SHEF-C6279/2020|2020-10-05|EPI_ISL_596200|Original|hCoV-19^^England|Human|Virology Department|COVID-19 Genomics UK (COG-UK) Consortium|de Silva|United Kingdom
>Spike|hCoV-19/USA/TX-HMH-3107/2020|2020-05-25|EPI_ISL_543996|Original|hCoV-19^^Texas|Human|Houston Methodist Hospital|Houston Methodist Hospital|Long|USA
>Spike|hCoV-19/USA/MI-MDHHS-SC20310/2020|2020-03-14|EPI_ISL_447107|Original|hCoV-19^^Michigan|Human|Michigan Department of Health and Human Services|Michigan Department of Health and Human Services|Blankenship|USA
>Spike|hCoV-19/USA/SC-DHEC-0929/2020|2020-06-26|EPI_ISL_538420|Original|hCoV-19^^South Carolina|Human|Microbiology Division|Microbiology Division|Flores|USA
>Spike|hCoV-19/USA/TX-HMH-2204/2020|2020-05-22|EPI_ISL_545181|Original|hCoV-19^^Texas|Human|Houston Methodist Hospital|Houston Methodist Hospital|Long|USA
>Spike|hCoV-19/England/CAMB-80105/2020|2020-04-09|EPI_ISL_470528|Original|hCoV-19^^England|Human|Department of Pathology|Wellcome Sanger Institute for the COVID-19 Genomics UK (COG-UK) consortium|Davies|United Kingdom
>Spike|hCoV-19/Australia/VIC13379/2020|2020-07-27|EPI_ISL_563703|Original|hCoV-19^^Victoria|Human|Microbiological Diagnostic Unit - Public Health Laboratory (MDU-PHL)|MDU-PHL|Schultz|Australia
>Spike|hCoV-19/Iceland/465/2020|2020-03-20|EPI_ISL_424488|Original|hCoV-19^^Reykjavik|Human|The National University Hospital of Iceland|deCODE genetics|J\u00f3nsson|Iceland
>Spike|hCoV-19/Denmark/DCGC-7555/2020|2020-10-12|EPI_ISL_618455|Original|hCoV-19^^Sjaelland|Human|Department of Virus and Microbiological Special Diagnostics|Albertsen lab|Michaelsen|Denmark
>Spike|hCoV-19/England/MILK-ACC33A/2020|2020-10-21|EPI_ISL_629187|Original|hCoV-19^^England|Human|Lighthouse Lab in Milton Keynes|Wellcome Sanger Institute for the COVID-19 Genomics UK (COG-UK) consortium|Davies|United Kingdom

>Spike|hCoV-19/England/QEUH-9E48C5/2020|2020-09-22|EPI_ISL_579833|Original|hCoV-19^^England|Human|Lighthouse Lab in Glasgow|Wellcome Sanger Institute for the COVID-19 Genomics UK (COG-UK) consortium|Davies|United Kingdom
>Spike|hCoV-19/Japan/PG-2686/2020|2020-03-00|EPI_ISL_690818|Original|hCoV-19^^Japan|Human|Kanagawa Prefectural Institute of Public Health|Pathogen Genomics Center|Sekizuka|Japan
>Spike|hCoV-19/Germany/NW-HHU-511/2020|2020-09-18|EPI_ISL_770727|Original|hCoV-19^^North Rhine-Westphalia|Human|ZOTZ KLIMAS MVZ D\u00fcsseldorf-Centrum GbR \u00dcBAG f\u00fcr Labormedizin|Center of Medical Microbiology|Wienemann|Germany
>Spike|hCoV-19/USA/CA-CSMC11/2020|2020-04-01|EPI_ISL_475585|Original|hCoV-19^^California|Human|Cedars-Sinai Medical Center|Cedars-Sinai Medical Center|Zhang|USA
>Spike|hCoV-19/USA/UT-QDX-2644/2020|2020-03-12|EPI_ISL_604978|Original|hCoV-19^^Utah|Human|Quest Diagnostics|Quest Diagnostics|Kagan|USA
>Spike|hCoV-19/England/CAMC-B53F60/2020|2020-11-06|EPI_ISL_656925|Original|hCoV-19^^England|Human|Lighthouse Lab in Cambridge|Wellcome Sanger Institute for the COVID-19 Genomics UK (COG-UK) Consortium|Davies|United Kingdom
>Spike|hCoV-19/England/QEUH-C35F63/2020|2020-12-11|EPI_ISL_731486|Original|hCoV-19^^England|Human|Lighthouse Lab in Glasgow|Wellcome Sanger Institute for the COVID-19 Genomics UK (COG-UK) Consortium|Davies|United Kingdom
>Spike|hCoV-19/England/CAMC-A80DDA/2020|2020-10-14|EPI_ISL_610825|Original|hCoV-19^^England|Human|Lighthouse Lab in Cambridge|Wellcome Sanger Institute for the COVID-19 Genomics UK (COG-UK) consortium|Davies|United Kingdom
>Spike|hCoV-19/France/OCC-9/2020|2020-03-00|EPI_ISL_434624|Original|hCoV-19^^Occitanie|Human|CHU Purpan - Laboratoire de Virologie - Institut F\u00e9d\u00e9ratif de Biologie|Laboratoire de virologie - \u00c9cole Nationale V\u00e9t\u00e9rinaire de Toulouse|Croville|France
>Spike|hCoV-19/England/MILK-B395AA/2020|2020-11-02|EPI_ISL_658847|Original|hCoV-19^^England|Human|Lighthouse Lab in Milton Keynes|Wellcome Sanger Institute for the COVID-19 Genomics UK (COG-UK) Consortium|Davies|United Kingdom
>Spike|hCoV-19/England/CAMB-1AEF51/2020|2020-04-28|EPI_ISL_459274|Original|hCoV-19^^England|Human|Department of Pathology|Wellcome Sanger Institute for the COVID-19 Genomics UK (COG-UK) consortium|Davies|United Kingdom
>Spike|hCoV-19/England/ALDP-CEC165/2020|2020-12-21|EPI_ISL_781288|Original|hCoV-19^^England|Human|Lighthouse Lab in Alderley Park|Wellcome Sanger Institute for the COVID-19 Genomics UK (COG-UK) Consortium|Davies|United Kingdom
>Spike|hCoV-19/USA/MI-UM-10036721022/2020|2020-10-31|EPI_ISL_682152|Original|hCoV-19^^Michigan|Human|University of Michigan Clinical Microbiology Laboratory|Lauring Lab|Valesano|USA
>Spike|hCoV-19/England/204191376/2020|2020-10-10|EPI_ISL_637114|Original|hCoV-19^^England|Human|Respiratory Virus Unit|COVID-19 Genomics UK (COG-UK) Consortium|Platt|United Kingdom
>Spike|hCoV-19/Denmark/DCGC-12276/2020|2020-11-16|EPI_ISL_682457|Original|hCoV-19^^Hovedstaden|Human|Department of Virus and Microbiological Special Diagnostics|Albertsen Lab|Michaelsen|Denmark
>Spike|hCoV-19/USA/TX-HMH-7173/2020|2020-06-29|EPI_ISL_546569|Original|hCoV-19^^Texas|Human|Houston Methodist Hospital|Houston Methodist Hospital|Long|USA
>Spike|hCoV-19/Japan/PG-4102/2020|2020-06-00|EPI_ISL_692343|Original|hCoV-19^^Japan|Human|Pathogen Genomics Center|Pathogen Genomics Center|Sekizuka|Japan
>Spike|hCoV-19/Ireland/D-NVRL-20G44442/2020|2020-03-29|EPI_ISL_605093|Original|hCoV-19^^Dublin|Human|National Virus Reference Laboratory|Irish Coronavirus Sequencing Consortium - Helixworks|Chalapati|Ireland
>Spike|hCoV-19/England/BIRM-605D2/2020|2020-05-28|EPI_ISL_477734|Original|hCoV-19^^England|Human|University of Birmingham|COVID-19 Genomics UK (COG-UK) Consortium|Loman|United Kingdom
>Spike|hCoV-19/England/BIRM-606EE/2020|2020-06-02|EPI_ISL_483881|Original|hCoV-19^^England|Human|University of Birmingham|COVID-19 Genomics UK (COG-UK) Consortium|Loman|United Kingdom

>Spike|hCoV-19/England/QEUH-B31E7A/2020|2020-11-04|EPI_ISL_646263|Original|hCoV-19^^England|Human|Lighthouse Lab in Glasgow|Wellcome Sanger Institute for the COVID-19 Genomics UK (COG-UK) Consortium|Davies|United Kingdom
>Spike|hCoV-19/England/QEUH-A326DC/2020|2020-10-05|EPI_ISL_627305|Original|hCoV-19^^England|Human|Oxford Viromics|COVID-19 Genomics UK (COG-UK) Consortium|Golubchik|United Kingdom
>Spike|hCoV-19/England/MILK-A18F0E/2020|2020-10-01|EPI_ISL_612475|Original|hCoV-19^^England|Human|Wales Specialist Virology Centre Sequencing lab|COVID-19 Genomics UK (COG-UK) Consortium|Moore|United Kingdom
>Spike|hCoV-19/England/CAMC-B09479/2020|2020-10-29|EPI_ISL_673016|Original|hCoV-19^^England|Human|Lighthouse Lab in Cambridge|Wellcome Sanger Institute for the COVID-19 Genomics UK (COG-UK) Consortium|Davies|United Kingdom
>Spike|hCoV-19/USA/CA-ALSR-3089/2020|2020-09-03|EPI_ISL_635841|Original|hCoV-19^^California|Human|San Diego County Public Health Laboratory|Andersen lab at Scripps Research|Gangavarapu|USA
>Spike|hCoV-19/England/QEUH-9C7B83/2020|2020-09-11|EPI_ISL_567019|Original|hCoV-19^^England|Human|Lighthouse Lab in Glasgow|Wellcome Sanger Institute for the COVID-19 Genomics UK (COG-UK) consortium|Davies|United Kingdom
>Spike|hCoV-19/England/MILK-9F0FFE/2020|2020-09-25|EPI_ISL_589679|Original|hCoV-19^^England|Human|Lighthouse Lab in Milton Keynes|Wellcome Sanger Institute for the COVID-19 Genomics UK (COG-UK) consortium|Davies|United Kingdom
>Spike|hCoV-19/USA/UT-03043/2020|2020-05-01|EPI_ISL_522314|Original|hCoV-19^^Utah|Human|Utah Public Health Laboratory|Utah Public Health Laboratory|Young|USA
>Spike|hCoV-19/Denmark/DCGC-3402/2020|2020-08-31|EPI_ISL_616161|Original|hCoV-19^^Sjaelland|Human|Department of Virus and Microbiological Special Diagnostics|Albertsen lab|Michaelsen|Denmark
>Spike|hCoV-19/England/MILK-C30038/2020|2020-12-07|EPI_ISL_782881|Original|hCoV-19^^England|Human|Lighthouse Lab in Milton Keynes|Wellcome Sanger Institute for the COVID-19 Genomics UK (COG-UK) Consortium|Davies|United Kingdom
>Spike|hCoV-19/England/QEUH-963C3B/2020|2020-08-11|EPI_ISL_531550|Original|hCoV-19^^England|Human|Lighthouse Lab in Glasgow|Wellcome Sanger Institute for the COVID-19 Genomics UK (COG-UK) consortium|Davies|United Kingdom
>Spike|hCoV-19/USA/CT-QDX-761/2020|2020-03-18|EPI_ISL_571510|Original|hCoV-19^^Connecticut|Human|Quest Diagnostics|Quest Diagnostics|Kagan|USA
>Spike|hCoV-19/Australia/VIC8818/2020|2020-08-02|EPI_ISL_565215|Original|hCoV-19^^Victoria|Human|Victorian Infectious Diseases Reference Laboratory (VIDRL)|VIDRL and MDU-PHL|Schultz|Australia
>Spike|hCoV-19/Belgium/rega-09181055/2020|2020-09-18|EPI_ISL_734997|Original|hCoV-19^^Steenokkerzeel|Human|UZ Leuven|KU Leuven|Maes|Belgium
>Spike|hCoV-19/Spain/CT-IBV-005597/2020|2020-03-18|EPI_ISL_508645|Original|hCoV-19^^Catalunya|Human|Departamento de Microbiolog\u00eda|SeqCOVID-SPAIN consortium/IBV(CSIC)|Comas|Spain
>Spike|hCoV-19/England/ALDP-A5181D/2020|2020-10-07|EPI_ISL_597958|Original|hCoV-19^^England|Human|Lighthouse Lab in Alderley Park|Wellcome Sanger Institute for the COVID-19 Genomics UK (COG-UK) consortium|Davies|United Kingdom
>Spike|hCoV-19/Canada/Qc-L00240652/2020|2020-03-27|EPI_ISL_450304|Original|hCoV-19^^Quebec|Human|H\u00f4pital Charles-LeMoyne|Laboratoire de sant\u00e9 publique du Qu\u00e9bec|Moreira|Canada
>Spike|hCoV-19/USA/LA-EVTL1178/2020|2020-12-11|EPI_ISL_768447|Original|hCoV-19^^Louisiana|Human|LSUHS Emerging Viral Threat Laboratory|Microbial Genome Sequencing Center|Kamil|USA
>Spike|hCoV-19/Denmark/DCGC-19619/2020|2020-12-07|EPI_ISL_749064|Original|hCoV-19^^Hovedstaden|Human|Department of Virus and Microbiological Special Diagnostics|Albertsen Lab|Michaelsen|Denmark
>Spike|hCoV-19/Wales/PHWC-492C56/2020|2020-11-27|EPI_ISL_725768|Original|hCoV-19^^Wales|Human|Originating lab|COVID-19 Genomics UK (COG-UK) Consortium|Moore|United Kingdom
>Spike|hCoV-19/Germany/HH-hpi-p2394/2020|2020-05-04|EPI_ISL_776469|Original|hCoV-19^^Hamburg|Human|University Medical Center Hamburg Eppendorf|Heinrich Pette Institute|Robitaille|Germany

>Spike|hCoV-19/Wales/PHWC-4A1FD3/2020|2020-12-14|EPI_ISL_743840|Original|hCoV-19^^Wales|Human|Wales Specialist Virology Centre Sequencing lab|COVID-19 Genomics UK (COG-UK) Consortium|Moore|United Kingdom
>Spike|hCoV-19/Scotland/QEUH-9E384E/2020|2020-09-24|EPI_ISL_585517|Original|hCoV-19^^Scotland|Human|Northumbria University / South Tees Hospitals NHS Foundation Trust / North Cumbria Integrated Care NHS Foundation Trust / North Tees and Hartlepool NHS Foundation Trust / Newcastle Hospitals NHS Foundation Trust|COVID-19 Genomics UK (COG-UK) Consortium|Bashton|United Kingdom
>Spike|hCoV-19/USA/WI-UW-1601/2020|2020-09-23|EPI_ISL_605478|Original|hCoV-19^^Wisconsin|Human|University of Wisconsin-Madison AIDS Vaccine Research Laboratories|University of Wisconsin-Madison AIDS Vaccine Research Laboratories|Baczenas|USA
>Spike|hCoV-19/Denmark/DCGC-10066/2020|2020-11-09|EPI_ISL_669374|Original|hCoV-19^^Midtjylland|Human|Department of Virus and Microbiological Special Diagnostics|Albertsen Lab|Michaelsen|Denmark
>Spike|hCoV-19/Japan/PG-2506/2020|2020-04-00|EPI_ISL_690644|Original|hCoV-19^^Japan|Human|Saitama Prefectural Institute of Public Health|Pathogen Genomics Center|Sekizuka|Japan
>Spike|hCoV-19/USA/MI-UM-2006S000867/2020|2020-03-27|EPI_ISL_484721|Original|hCoV-19^^Michigan|Human|University of Michigan Clinical Microbiology Laboratory|Lauring Lab|Valesano|USA
>Spike|hCoV-19/USA/CA-ALSR-4157/2020|2020-06-26|EPI_ISL_648858|Original|hCoV-19^^California|Human|San Diego County Public Health Laboratory|Andersen lab at Scripps Research|Gangavarapu|USA
>Spike|hCoV-19/England/ALDP-BE910F/2020|2020-12-06|EPI_ISL_708972|Original|hCoV-19^^England|Human|Lighthouse Lab in Alderley Park|Wellcome Sanger Institute for the COVID-19 Genomics UK (COG-UK) Consortium|Davies|United Kingdom
>Spike|hCoV-19/Switzerland/SO-ETHZ-410284/2020|2020-12-15|EPI_ISL_737495|Original|hCoV-19^^Solothurn|Human|Viollier AG|Department of Biosystems Science and Engineering|Nadeau|Switzerland
>Spike|hCoV-19/England/QEUH-9DC9CD/2020|2020-09-21|EPI_ISL_580955|Original|hCoV-19^^England|Human|Lighthouse Lab in Glasgow|Wellcome Sanger Institute for the COVID-19 Genomics UK (COG-UK) consortium|Davies|United Kingdom
>Spike|hCoV-19/England/20189042401/2020|2020-05-01|EPI_ISL_465527|Original|hCoV-19^^England|Human|Respiratory Virus Unit|Respiratory Virus Unit|Platt|United Kingdom
>Spike|hCoV-19/England/MILK-B8676C/2020|2020-11-13|EPI_ISL_673941|Original|hCoV-19^^England|Human|Lighthouse Lab in Milton Keynes|Wellcome Sanger Institute for the COVID-19 Genomics UK (COG-UK) Consortium|Davies|United Kingdom
>Spike|hCoV-19/England/ALDP-AC5D60/2020|2020-10-14|EPI_ISL_631107|Original|hCoV-19^^England|Human|Lighthouse Lab in Alderley Park|Wellcome Sanger Institute for the COVID-19 Genomics UK (COG-UK) consortium|Davies|United Kingdom
>Spike|hCoV-19/England/ALDP-B1A509/2020|2020-10-31|EPI_ISL_647634|Original|hCoV-19^^England|Human|Lighthouse Lab in Alderley Park|Wellcome Sanger Institute for the COVID-19 Genomics UK (COG-UK) Consortium|Davies|United Kingdom
>Spike|hCoV-19/USA/CA-CZB-3948/2020|2020-08-03|EPI_ISL_548558|Original|hCoV-19^^California|Human|Contra Costa Public Health Lab|Chan-Zuckerberg Biohub|Bhatt|USA
>Spike|hCoV-19/Switzerland/BS-ETHZ-130005/2020|2020-05-06|EPI_ISL_467001|Original|hCoV-19^^Basel-Stadt|Human|Viollier AG|Department of Biosystems Science and Engineering|Nadeau|Switzerland
>Spike|hCoV-19/Wales/PHWC-48A398/2020|2020-11-03|EPI_ISL_652547|Original|hCoV-19^^Wales|Human|Wales Specialist Virology Centre Sequencing lab|COVID-19 Genomics UK (COG-UK) Consortium|Moore|United Kingdom
>Spike|hCoV-19/England/ALDP-C3CBFF/2020|2020-12-10|EPI_ISL_731848|Original|hCoV-19^^England|Human|Lighthouse Lab in Alderley Park|Wellcome Sanger Institute for the COVID-19 Genomics UK (COG-UK) Consortium|Davies|United Kingdom
>Spike|hCoV-19/Canada/ON-UHTC_0143/2020|2020-05-30|EPI_ISL_526277|Original|hCoV-19^^Ontario|Human|Unity Health Toronto|Ontario Institute for Cancer Research|de Borja|Canada
>Spike|hCoV-19/Spain/PV-IBV-004220/2020|2020-04-04|EPI_ISL_510504|Original|hCoV-19^^Basque Country|Human|Servicio de Microbiolog\u00eda. Hospital Universitario

Donostia. OSI Donostialdea. \u00c1rea de Enfermedades Infecciosas|SeqCOVID-SPAIN consortium/IBV(CSIC)|Comas|Spain
>Spike|hCoV-19/Portugal/PT1111/2020|2020-03-27|EPI_ISL_511658|Original|hCoV-19^^Portugal|Human|Instituto Nacional de Saude (INSA)|Instituto Nacional de Saude (INSA)|Borges|Portugal
>Spike|hCoV-19/England/QEUH-BCDD2B/2020|2020-11-26|EPI_ISL_704461|Original|hCoV-19^^England|Human|Lighthouse Lab in Glasgow|Wellcome Sanger Institute for the COVID-19 Genomics UK (COG-UK) Consortium|Davies|United Kingdom
>Spike|hCoV-19/England/QEUH-B813D6/2020|2020-11-14|EPI_ISL_661893|Original|hCoV-19^^England|Human|Lighthouse Lab in Glasgow|Wellcome Sanger Institute for the COVID-19 Genomics UK (COG-UK) Consortium|Davies|United Kingdom
>Spike|hCoV-19/England/CAMB-7D98B/2020|2020-04-02|EPI_ISL_441683|Original|hCoV-19^^England|Human|Department of Pathology|Wellcome Sanger Institute for the COVID-19 Genomics UK (COG-UK) consortium|Amato|United Kingdom
>Spike|hCoV-19/Denmark/DCGC-3190/2020|2020-08-31|EPI_ISL_616013|Original|hCoV-19^^Hovedstaden|Human|Department of Virus and Microbiological Special Diagnostics|Albertsen lab|Michaelsen|Denmark
>Spike|hCoV-19/USA/ME-HETL-J0232/2020|2020-09-25|EPI_ISL_755389|Original|hCoV-19^^Maine|Human|Maine Health and Environmental Testing Laboratory|Tewhey Lab|Tewhey|USA
>Spike|hCoV-19/USA/LA-EVTL116/2020|2020-05-06|EPI_ISL_451277|Original|hCoV-19^^Louisiana|Human|LSUHS Emerging Viral Threat Laboratory|Microbial Genome Sequencing Center|Kamil|USA
>Spike|hCoV-19/England/LIVE-DC3910/2020|2020-11-11|EPI_ISL_705238|Original|hCoV-19^^England|Human|Liverpool Clinical Laboratories|COVID-19 Genomics UK (COG-UK) Consortium|Haldenby|United Kingdom
>Spike|hCoV-19/Japan/IC-0399/2020|2020-12-00|EPI_ISL_736909|Original|hCoV-19^^Japan|Human|Pathogen Genomics Center|Pathogen Genomics Center|Sekizuka|Japan
>Spike|hCoV-19/USA/MD-MDH-0515/2020|2020-11-30|EPI_ISL_778896|Original|hCoV-19^^Maryland|Human|Maryland Public Health Laboratory|Maryland Public Health Laboratory|Keller|USA
>Spike|hCoV-19/USA/NY-Wadsworth-41424-01/2020|2020-04-08|EPI_ISL_676823|Original|hCoV-19^^New York|Human|Wadsworth Center|Wadsworth Center|Lasek-Nesselquist|USA
>Spike|hCoV-19/England/CAMC-A666FD/2020|2020-10-11|EPI_ISL_627324|Original|hCoV-19^^England|Human|University of Exeter|COVID-19 Genomics UK (COG-UK) Consortium|Temperton|United Kingdom
>Spike|hCoV-19/England/CAMC-BC0C58/2020|2020-11-22|EPI_ISL_704781|Original|hCoV-19^^England|Human|Lighthouse Lab in Cambridge|Wellcome Sanger Institute for the COVID-19 Genomics UK (COG-UK) Consortium|Davies|United Kingdom
>Spike|hCoV-19/Japan/PG-1425/2020|2020-04-00|EPI_ISL_685032|Original|hCoV-19^^Japan|Human|Saitama Prefectural Institute of Public Health|Pathogen Genomics Center|Sekizuka|Japan
>Spike|hCoV-19/USA/UT-UPHL-2012685247/2020|2020-08-10|EPI_ISL_745872|Original|hCoV-19^^Utah|Human|Ginkgo Bioworks Clinical Laboratory|Utah Public Health Laboratory|Young|USA
>Spike|hCoV-19/England/QEUH-AD5F3A/2020|2020-10-26|EPI_ISL_634560|Original|hCoV-19^^England|Human|Lighthouse Lab in Glasgow|Wellcome Sanger Institute for the COVID-19 Genomics UK (COG-UK) consortium|Davies|United Kingdom
>Spike|hCoV-19/Finland/8LS19AG8/2020|2020-10-08|EPI_ISL_755983|Original|hCoV-19^^Uusimaa|Human|Department of Virology and Immunology|Department of Virology|Smura|Finland
>Spike|hCoV-19/Australia/VIC13349/2020|2020-07-29|EPI_ISL_592230|Original|hCoV-19^^Victoria|Human|Microbiological Diagnostic Unit - Public Health Laboratory (MDU-PHL)|MDU-PHL|Schultz|Australia
>Spike|hCoV-19/Australia/VIC4629/2020|2020-07-12|EPI_ISL_519022|Original|hCoV-19^^Victoria|Human|Victorian Infectious Diseases Reference Laboratory (VIDRL)|VIDRL and MDU-PHL|Schultz|Australia
>Spike|hCoV-19/USA/CA-QDX-2362/2020|2020-03-16|EPI_ISL_604656|Original|hCoV-19^^California|Human|Quest Diagnostics|Quest Diagnostics|Kagan|USA

>Spike|hCoV-19/Denmark/DCGC-7815/2020|2020-10-19|EPI_ISL_618794|Original|hCoV-19^^Hovedstaden|Human|Department of Virus and Microbiological Special Diagnostics|Albertsen lab|Michaelsen|Denmark
>Spike|hCoV-19/South Korea/KDCA0247/2020|2020-08-20|EPI_ISL_760012|Original|hCoV-19^^South Korea|Human|Division of Emerging Infectious Diseases|Division of Emerging Infectious Diseases|Park|South Korea
>Spike|hCoV-19/USA/WI-WSLH-200593/2020|2020-04-17|EPI_ISL_631395|Original|hCoV-19^^Wisconsin|Human|Wisconsin State Laboratory of Hygiene Communicable Disease Division|Wisconsin State Laboratory of Hygiene Communicable Disease Division|Florek|USA
>Spike|hCoV-19/England/ALDP-B747DA/2020|2020-11-12|EPI_ISL_678941|Original|hCoV-19^^England|Human|Queens Medical Centre|COVID-19 Genomics UK (COG-UK) Consortium|Loose|United Kingdom
>Spike|hCoV-19/France/ARA-14350/2020|2020-03-26|EPI_ISL_508885|Original|hCoV-19^^Auvergne-Rh\u00f4ne-Alpes|Human|Institut des Agents Infectieux (IAI)|CNR Virus des Infections Respiratoires - France SUD|Josset|France
>Spike|hCoV-19/England/QEUH-BA2EA1/2020|2020-11-18|EPI_ISL_672780|Original|hCoV-19^^England|Human|Lighthouse Lab in Glasgow|Wellcome Sanger Institute for the COVID-19 Genomics UK (COG-UK) Consortium|Davies|United Kingdom
>Spike|hCoV-19/USA/VA-DCLS-0193/2020|2020-04-16|EPI_ISL_437408|Original|hCoV-19^^Virginia|Human|Virginia DCLS|Virginia DCLS|St. Jacques|USA
>Spike|hCoV-19/Switzerland/BE-UNIBE-151-6539/2020|2020-03-10|EPI_ISL_574792|Original|hCoV-19^^Bern|Human|Institute for Infectious Diseases|Institute for Infectious Diseases|Koch|Switzerland
>Spike|hCoV-19/Czech Republic/NRL_9893/2020|2020-10-12|EPI_ISL_626620|Original|hCoV-19^^Czech Republic|Human|The National Institute of Public Health|State Veterinary Institute Prague|Nagy|Czech Republic
>Spike|hCoV-19/Denmark/DCGC-17452/2020|2020-12-14|EPI_ISL_750993|Original|hCoV-19^^Nordjylland|Human|Department of Virus and Microbiological Special Diagnostics|Albertsen Lab|Michaelsen|Denmark
>Spike|hCoV-19/England/QEUH-B2D49D/2020|2020-11-04|EPI_ISL_659768|Original|hCoV-19^^England|Human|Lighthouse Lab in Glasgow|Wellcome Sanger Institute for the COVID-19 Genomics UK (COG-UK) Consortium|Davies|United Kingdom
>Spike|hCoV-19/England/ALDP-AA0B70/2020|2020-10-22|EPI_ISL_625195|Original|hCoV-19^^England|Human|Lighthouse Lab in Alderley Park|Wellcome Sanger Institute for the COVID-19 Genomics UK (COG-UK) consortium|Davies|United Kingdom
>Spike|hCoV-19/England/QEUH-95DF3B/2020|2020-08-07|EPI_ISL_537067|Original|hCoV-19^^England|Human|Lighthouse Lab in Glasgow|Wellcome Sanger Institute for the COVID-19 Genomics UK (COG-UK) consortium|Davies|United Kingdom
>Spike|hCoV-19/England/MILK-991B82/2020|2020-08-27|EPI_ISL_577027|Original|hCoV-19^^England|Human|Northumbria University / South Tees Hospitals NHS Foundation Trust / North Cumbria Integrated Care NHS Foundation Trust / North Tees and Hartlepool NHS Foundation Trust / Newcastle Hospitals NHS Foundation Trust|COVID-19 Genomics UK (COG-UK) Consortium|Bashton|United Kingdom
>Spike|hCoV-19/Sweden/20-08169/2020|2020-03-12|EPI_ISL_429123|Original|hCoV-19^^Halland|Human|Klinisk mikrobiologi och vardhygien Halmstad|The Public Health Agency of Sweden|Svartstr\u00f6m|Sweden
>Spike|hCoV-19/England/20120009504/2020|2020-03-14|EPI_ISL_465150|Original|hCoV-19^^England|Human|Respiratory Virus Unit|Respiratory Virus Unit|Platt|United Kingdom
>Spike|hCoV-19/USA/CA-ALSR-4613/2020|2020-06-19|EPI_ISL_667039|Original|hCoV-19^^California|Human|San Diego County Public Health Laboratory|Andersen lab at Scripps Research|Gangavarapu|USA
>Spike|hCoV-19/Nigeria/NA-CV240/2020|2020-05-31|EPI_ISL_729926|Original|hCoV-19^^Nasarawa State|Human|Nigeria Centre for Disease Control (NCDC)|African Centre of Excellence for Genomics of Infectious Diseases (ACEGID)|Oluniyi|Nigeria
>Spike|hCoV-19/USA/WA-UW-2212/2020|2020-03-25|EPI_ISL_426113|Original|hCoV-19^^Washington|Human|UW Virology Lab|UW Virology Lab|Roychoudhury|USA
>Spike|hCoV-19/USA/CA-CZB-14828/2020|2020-11-19|EPI_ISL_738938|Original|hCoV-19^^California|Human|Humboldt County Public Health Laboratory|Chan-Zuckerberg Biohub|Kamm|USA

>Spike|hCoV-19/Switzerland/SH-ETHZ-420382/2020|2020-12-21|EPI_ISL_768163|Original|hCoV-19^^Schaffhausen|Human|Viollier AG|Department of Biosystems Science and Engineering|Nadeau|Switzerland
>Spike|hCoV-19/Canada/QC-LSPQ-L00237598/2020|2020-03-25|EPI_ISL_536188|Original|hCoV-19^^Quebec|Human|H\u00f4pital Pierre-Boucher|Laboratoire de sant\u00e9 publique du Qu\u00e9bec|Moreira|Canada
>Spike|hCoV-19/England/ALDP-C8F6B0/2020|2020-12-11|EPI_ISL_777211|Original|hCoV-19^^England|Human|Lighthouse Lab in Alderley Park|Wellcome Sanger Institute for the COVID-19 Genomics UK (COG-UK) Consortium|Davies|United Kingdom
>Spike|hCoV-19/Australia/VIC13320/2020|2020-07-27|EPI_ISL_563671|Original|hCoV-19^^Victoria|Human|Microbiological Diagnostic Unit - Public Health Laboratory (MDU-PHL)|MDU-PHL|Schultz|Australia
>Spike|hCoV-19/USA/TX-HMH-920/2020|2020-04-17|EPI_ISL_545919|Original|hCoV-19^^Texas|Human|Houston Methodist Hospital|Houston Methodist Hospital|Long|USA
>Spike|hCoV-19/Australia/VIC17450/2020|2020-08-19|EPI_ISL_663891|Original|hCoV-19^^Victoria|Human|Microbiological Diagnostic Unit - Public Health Laboratory (MDU-PHL)|MDU-PHL|Schultz|Australia
>Spike|hCoV-19/England/CAMB-74F64/2020|2020-03-17|EPI_ISL_440431|Original|hCoV-19^^England|Human|Department of Pathology|Wellcome Sanger Institute for the COVID-19 Genomics UK (COG-UK) consortium|Amato|United Kingdom
>Spike|hCoV-19/England/ALDP-B1A536/2020|2020-10-31|EPI_ISL_647615|Original|hCoV-19^^England|Human|Lighthouse Lab in Alderley Park|Wellcome Sanger Institute for the COVID-19 Genomics UK (COG-UK) Consortium|Davies|United Kingdom
>Spike|hCoV-19/England/MILK-9EF02C/2020|2020-09-24|EPI_ISL_594894|Original|hCoV-19^^England|Human|Oxford Viromics|COVID-19 Genomics UK (COG-UK) Consortium|Golubchik|United Kingdom
>Spike|hCoV-19/Wales/PHWC-47FD7A/2020|2020-10-14|EPI_ISL_612806|Original|hCoV-19^^Wales|Human|Wales Specialist Virology Centre Sequencing lab|COVID-19 Genomics UK (COG-UK) Consortium|Moore|United Kingdom
>Spike|hCoV-19/England/QEUH-AAC1BE/2020|2020-10-21|EPI_ISL_652846|Original|hCoV-19^^England|Human|Oxford Viromics|COVID-19 Genomics UK (COG-UK) Consortium|Golubchik|United Kingdom
>Spike|hCoV-19/Switzerland/BL-ETHZ-300309/2020|2020-09-25|EPI_ISL_603341|Original|hCoV-19^^Basel-Landschaft|Human|Viollier AG|Department of Biosystems Science and Engineering|Nadeau|Switzerland
>Spike|hCoV-19/Japan/PG-1675/2020|2020-05-00|EPI_ISL_685281|Original|hCoV-19^^Japan|Human|Gunma Prefectural Institute of Public Health and Environmental Sciences|Pathogen Genomics Center|Sekizuka|Japan
>Spike|hCoV-19/South Africa/KRISP-0088/2020|2020-04-07|EPI_ISL_467457|Original|hCoV-19^^KwaZulu-Natal|Human|AMPATH-DBN|KRISP|de Oliveira|South Africa
>Spike|hCoV-19/Portugal/PT0353/2020|2020-03-18|EPI_ISL_454069|Original|hCoV-19^^Portugal|Human|unknown|Instituto Nacional de Saude (INSA)|Borges|Portugal
>Spike|hCoV-19/Australia/VIC12421/2020|2020-07-30|EPI_ISL_641250|Original|hCoV-19^^Victoria|Human|Microbiological Diagnostic Unit - Public Health Laboratory (MDU-PHL)|MDU-PHL|Schultz|Australia
>Spike|hCoV-19/Scotland/CVR1094/2020|2020-03-29|EPI_ISL_438765|Original|hCoV-19^^Scotland|Human|West of Scotland Specialist Virology Centre|COVID-19 Genomics UK (COG-UK) Consortium|Robertson|United Kingdom
>Spike|hCoV-19/England/LOND-D508C/2020|2020-04-15|EPI_ISL_444198|Original|hCoV-19^^England|Human|University College London|COVID-19 Genomics UK (COG-UK) Consortium|Roy|United Kingdom
>Spike|hCoV-19/England/ALDP-903505/2020|2020-06-22|EPI_ISL_552914|Original|hCoV-19^^England|Human|Lighthouse Lab in Alderley Park|Wellcome Sanger Institute for the COVID-19 Genomics UK (COG-UK) consortium|Davies|United Kingdom
>Spike|hCoV-19/USA/TX-HMH-3851/2020|2020-06-15|EPI_ISL_543253|Original|hCoV-19^^Texas|Human|Houston Methodist Hospital|Houston Methodist Hospital|Long|USA
>Spike|hCoV-19/Finland/18MR9cC4/2020|2020-11-18|EPI_ISL_757336|Original|hCoV-19^^Uusimaa|Human|Department of Virology and Immunology|Department of Virology|Smura|Finland

>Spike|hCoV-19/Wales/PHWC-16B503/2020|2020-09-14|EPI_ISL_560299|Original|hCoV-19^^Wales|Human|Wales Specialist Virology Centre Sequencing lab|COVID-19 Genomics UK (COG-UK) Consortium|Moore|United Kingdom
>Spike|hCoV-19/Denmark/DCGC-9506/2020|2020-11-09|EPI_ISL_669008|Original|hCoV-19^^Sjaelland|Human|Department of Virus and Microbiological Special Diagnostics|Albertsen Lab|Michaelsen|Denmark
>Spike|hCoV-19/Germany/HH-hpi-p1564/2020|2020-03-15|EPI_ISL_775645|Original|hCoV-19^^Hamburg|Human|University Medical Center Hamburg Eppendorf|Heinrich Pette Institute|Robitaille|Germany
>Spike|hCoV-19/Scotland/QEUH-A4B46D/2020|2020-10-08|EPI_ISL_637683|Original|hCoV-19^^Scotland|Human|Department of Pathology|COVID-19 Genomics UK (COG-UK) Consortium|Goodfellow|United Kingdom
>Spike|hCoV-19/England/204391017/2020|2020-10-23|EPI_ISL_649878|Original|hCoV-19^^England|Human|Respiratory Virus Unit|COVID-19 Genomics UK (COG-UK) Consortium|Platt|United Kingdom
>Spike|hCoV-19/USA/MI-MDHHS-SC21032/2020|2020-03-28|EPI_ISL_507808|Original|hCoV-19^^Michigan|Human|Michigan Department of Health and Human Services|Michigan Department of Health and Human Services|Blankenship|USA
>Spike|hCoV-19/USA/TX-HMH-4760/2020|2020-06-22|EPI_ISL_544312|Original|hCoV-19^^Texas|Human|Houston Methodist Hospital|Houston Methodist Hospital|Long|USA
>Spike|hCoV-19/England/CAMC-990B92/2020|2020-08-26|EPI_ISL_551844|Original|hCoV-19^^England|Human|Lighthouse Lab in Cambridge|Wellcome Sanger Institute for the COVID-19 Genomics UK (COG-UK) consortium|Davies|United Kingdom
>Spike|hCoV-19/USA/CA-LACPHL-AF00064/2020|2020-12-21|EPI_ISL_766001|Original|hCoV-19^^California|Human|Los Angeles County PHL|Los Angeles County PHL|Hemarajata|USA
>Spike|hCoV-19/Canada/ON-UHTC_0008/2020|2020-03-27|EPI_ISL_464002|unknown|hCoV-19^^Ontario|Human|Unity Health Toronto|Ontario Institute for Cancer Research|de Borja|Canada
>Spike|hCoV-19/Denmark/DCGC-10730/2020|2020-11-02|EPI_ISL_670167|Original|hCoV-19^^Syddanmark|Human|Department of Virus and Microbiological Special Diagnostics|Albertsen Lab|Michaelsen|Denmark
>Spike|hCoV-19/Sweden/20-08144/2020|2020-03-11|EPI_ISL_430848|Original|hCoV-19^^Halland|Human|Klinisk mikrobiologi och vardhygien Halmstad|The Public Health Agency of Sweden|Svartstr\u00f6m|Sweden
>Spike|hCoV-19/England/LIVE-A6127/2020|2020-03-21|EPI_ISL_499523|Original|hCoV-19^^England|Human|Liverpool Clinical Laboratories|COVID-19 Genomics UK (COG-UK) Consortium|Haldenby|United Kingdom
>Spike|hCoV-19/Northern Ireland/NIRE-FB4A2/2020|2020-03-18|EPI_ISL_441430|Original|hCoV-19^^Northern Ireland|Human|Regional Virus Laboratory|COVID-19 Genomics UK (COG-UK) Consortium|Fairley|United Kingdom
>Spike|hCoV-19/USA/TX-HMH0160/2020|2020-03-23|EPI_ISL_434859|Original|hCoV-19^^Texas|Human|Houston Methodist Hospital|Houston Methodist Hospital|Long|USA
>Spike|hCoV-19/France/NAQ-SC287/2020|2020-04-10|EPI_ISL_663226|Original|hCoV-19^^Nouvelle-Aquitaine|Human|CHU de Limoges|CNR Virus des Infections Respiratoires - France SUD|Bal|France
>Spike|hCoV-19/USA/NY-NYUMC569/2020|2020-03-30|EPI_ISL_444686|Original|hCoV-19^^New York|Human|NYU Langone Health|Departments of Pathology and Medicine|Maurano|USA
>Spike|hCoV-19/England/ALDP-9B5F04/2020|2020-08-28|EPI_ISL_572975|Original|hCoV-19^^England|Human|Oxford Viromics|COVID-19 Genomics UK (COG-UK) Consortium|Golubchik|United Kingdom
>Spike|hCoV-19/USA/WA-UW-7164/2020|2020-04-11|EPI_ISL_570173|Original|hCoV-19^^Washington|Human|UW Virology Lab|UW Virology Lab|Roychoudhury|USA
>Spike|hCoV-19/England/MILK-A6BE1E/2020|2020-10-09|EPI_ISL_609029|Original|hCoV-19^^England|Human|Lighthouse Lab in Milton Keynes|Wellcome Sanger Institute for the COVID-19 Genomics UK (COG-UK) consortium|Davies|United Kingdom
>Spike|hCoV-19/USA/FL-BPHL-0912/2020|2020-06-30|EPI_ISL_517802|Original|hCoV-19^^Florida|Human|Florida Bureau of Public Health Laboratories|Florida Bureau of Public Health Laboratories|Blanton|USA
>Spike|hCoV-19/USA/WI-WSLH-200234/2020|2020-04-14|EPI_ISL_578454|Original|hCoV-19^^Wisconsin|Human|Wisconsin State Laboratory of Hygiene Communicable Disease

Division|Wisconsin State Laboratory of Hygiene Communicable Disease Division|Florek|USA
>Spike|hCoV-19/Portugal/PT0225/2020|2020-03-31|EPI_ISL_453939|Original|hCoV-19^^Portugal|Human|unknown|Instituto Nacional de Saude (INSA)|Borges|Portugal
>Spike|hCoV-19/Canada/NS-NML-1328/2020|2020-04-10|EPI_ISL_579708|Original|hCoV-19^^Nova Scotia|Human|QEII Health Sciences Centre|National Microbiology Laboratory (NML)|Majer|Canada
>Spike|hCoV-19/Denmark/DCGC-888/2020|2020-05-11|EPI_ISL_614452|Original|hCoV-19^^Hovedstaden|Human|Department of Virus and Microbiological Special Diagnostics|Albertsen lab|Michaelsen|Denmark
>Spike|hCoV-19/Wales/PHWC-49C59D/2020|2020-12-12|EPI_ISL_742574|Original|hCoV-19^^Wales|Human|Wales Specialist Virology Centre Sequencing lab|COVID-19 Genomics UK (COG-UK) Consortium|Moore|United Kingdom
>Spike|hCoV-19/England/SHEF-C2B45/2020|2020-04-02|EPI_ISL_432750|Original|hCoV-19^^England|Human|Virology Department|COVID-19 Genomics UK (COG-UK) Consortium|de Silva|United Kingdom
>Spike|hCoV-19/Scotland/QEUH-AACB65/2020|2020-10-21|EPI_ISL_652900|Original|hCoV-19^^Scotland|Human|Oxford Viromics|COVID-19 Genomics UK (COG-UK) Consortium|Golubchik|United Kingdom
>Spike|hCoV-19/England/CAMB-75507/2020|2020-03-13|EPI_ISL_425400|Original|hCoV-19^^England|Human|Department of Pathology|COVID-19 Genomics UK (COG-UK) Consortium|Meredith|United Kingdom
>Spike|hCoV-19/USA/CA-CZB-1105/2020|2020-04-30|EPI_ISL_444063|Original|hCoV-19^^California|Human|UCSF Clinical Microbiology Laboratory|Chan-Zuckerberg Biohub|Hao|USA
>Spike|hCoV-19/Denmark/DCGC-19263/2020|2020-12-07|EPI_ISL_748855|Original|hCoV-19^^Hovedstaden|Human|Department of Virus and Microbiological Special Diagnostics|Albertsen Lab|Michaelsen|Denmark
>Spike|hCoV-19/England/CAMB-7ED8E/2020|2020-04-07|EPI_ISL_442874|Original|hCoV-19^^England|Human|Department of Pathology|Wellcome Sanger Institute for the COVID-19 Genomics UK (COG-UK) consortium|Amato|United Kingdom
>Spike|hCoV-19/England/QEUH-AD1693/2020|2020-10-26|EPI_ISL_634628|Original|hCoV-19^^England|Human|Lighthouse Lab in Glasgow|Wellcome Sanger Institute for the COVID-19 Genomics UK (COG-UK) consortium|Davies|United Kingdom
>Spike|hCoV-19/USA/TX-HMH-MCoV-11390/2020|2020-09-06|EPI_ISL_790011|Original|hCoV-19^^Texas|Human|Houston Methodist Hospital|Houston Methodist Hospital|Long|USA
>Spike|hCoV-19/England/CAMC-B369FC/2020|2020-11-05|EPI_ISL_645307|Original|hCoV-19^^England|Human|Lighthouse Lab in Cambridge|Wellcome Sanger Institute for the COVID-19 Genomics UK (COG-UK) Consortium|Davies|United Kingdom
>Spike|hCoV-19/Denmark/DCGC-7369/2020|2020-10-12|EPI_ISL_619285|Original|hCoV-19^^Hovedstaden|Human|Department of Virus and Microbiological Special Diagnostics|Albertsen lab|Michaelsen|Denmark
>Spike|hCoV-19/England/QEUH-C57A3D/2020|2020-12-12|EPI_ISL_736316|Original|hCoV-19^^England|Human|Lighthouse Lab in Glasgow|Wellcome Sanger Institute for the COVID-19 Genomics UK (COG-UK) Consortium|Davies|United Kingdom
>Spike|hCoV-19/England/ALDP-B77054/2020|2020-11-12|EPI_ISL_723840|Original|hCoV-19^^England|Human|Oxford Viromics|COVID-19 Genomics UK (COG-UK) Consortium|Golubchik|United Kingdom
>Spike|hCoV-19/Scotland/CVR6069/2020|2020-11-30|EPI_ISL_705728|Original|hCoV-19^^Scotland|Human|West of Scotland Specialist Virology Centre|COVID-19 Genomics UK (COG-UK) Consortium|Robertson|United Kingdom
>Spike|hCoV-19/USA/NY-NYUMC44/2020|2020-03-18|EPI_ISL_419705|Original|hCoV-19^^New York|Human|NYU Langone Health|Departments of Pathology and Medicine|Maurano|USA
>Spike|hCoV-19/England/20134000804/2020|2020-03-24|EPI_ISL_423045|Original|hCoV-19^^England|Human|Respiratory Virus Unit|Respiratory Virus Unit|Platt|United Kingdom
>Spike|hCoV-19/England/20138087204/2020|2020-03-24|EPI_ISL_421843|Original|hCoV-19^^England|Human|Respiratory Virus Unit|Respiratory Virus Unit|Platt|United Kingdom
>Spike|hCoV-19/England/ALDP-9BDC32/2020|2020-09-09|EPI_ISL_567894|Original|hCoV-19^^England|Human|Lighthouse Lab in Alderley Park|Wellcome Sanger Institute for the COVID-19 Genomics UK (COG-UK) consortium|Davies|United Kingdom

>Spike|hCoV-19/Sweden/20-50229/2020|2020-03-07|EPI_ISL_455879|Original|hCoV-
19^^Stockholm|Human|Karolinska Universitetslaboratoriet|The Public Health Agency of
Sweden|Svartstr\u00f6m|Sweden
>Spike|hCoV-19/USA/NY-NYCPHL-000147/2020|2020-04-04|EPI_ISL_631638|Original|hCoV-
19^^New York|Human|Flushing Hospital Medical Center|New York City Public Health
Laboratory|Wang|USA
>Spike|hCoV-19/USA/CA-IGI-0132/2020|2020-06-18|EPI_ISL_495153|Original|hCoV-
19^^California|Human|Innovative Genomics Institute|Innovative Genomics
Institute|Shivram|USA
>Spike|hCoV-19/South Korea/KCDC2034/2020|2020-02-20|EPI_ISL_497955|Original|hCoV-
19^^South Korea|Human|Division of Viral Diseases|Division of Viral Diseases|Kim|South
Korea
>Spike|hCoV-19/England/CAMC-B325A1/2020|2020-11-05|EPI_ISL_659125|Original|hCoV-
19^^England|Human|Lighthouse Lab in Cambridge|Wellcome Sanger Institute for the
COVID-19 Genomics UK (COG-UK) Consortium|Davies|United Kingdom
>Spike|hCoV-19/Wales/PHWC-287D0/2020|2020-04-01|EPI_ISL_432108|Original|hCoV-
19^^Wales|Human|Wales Specialist Virology Centre|Public Health Wales Microbiology
Cardiff|Moore|United Kingdom
>Spike|hCoV-19/USA/LA-EVTL400/2020|2020-06-01|EPI_ISL_578806|Original|hCoV-
19^^Louisiana|Human|LSUHS Emerging Viral Threat Laboratory|Microbial Genome
Sequencing Center|Kamil|USA
>Spike|hCoV-19/South Africa/Tygerberg_33/2020|2020-04-
02|EPI_ISL_464144|Original|hCoV-19^^Western Cape|Human|National Health Laboratory
Service (NHLS)|Division of Medical Virology|Engelbrecht|South Africa
>Spike|hCoV-19/England/QEUH-AD3312/2020|2020-10-26|EPI_ISL_633988|Original|hCoV-
19^^England|Human|Lighthouse Lab in Glasgow|Wellcome Sanger Institute for the COVID-
19 Genomics UK (COG-UK) consortium|Davies|United Kingdom
>Spike|hCoV-19/England/OXON-B2708/2020|2020-04-02|EPI_ISL_479169|Original|hCoV-
19^^England|Human|Oxford Viromics|COVID-19 Genomics UK (COG-UK)
Consortium|Golubchik|United Kingdom
>Spike|hCoV-19/Denmark/DCGC-7480/2020|2020-10-12|EPI_ISL_618960|Original|hCoV-
19^^Hovedstaden|Human|Department of Virus and Microbiological Special
Diagnostics|Albertsen lab|Michaelsen|Denmark
>Spike|hCoV-19/England/ALDP-9303D8/2020|2020-06-20|EPI_ISL_552901|Original|hCoV-
19^^England|Human|Lighthouse Lab in Alderley Park|Wellcome Sanger Institute for the
COVID-19 Genomics UK (COG-UK) consortium|Davies|United Kingdom
>Spike|hCoV-19/Denmark/DCGC-20210/2020|2020-11-30|EPI_ISL_748051|Original|hCoV-
19^^Hovedstaden|Human|Department of Virus and Microbiological Special
Diagnostics|Albertsen Lab|Michaelsen|Denmark
>Spike|hCoV-19/England/ALDP-B5B083/2020|2020-11-09|EPI_ISL_655540|Original|hCoV-
19^^England|Human|Lighthouse Lab in Alderley Park|Wellcome Sanger Institute for the
COVID-19 Genomics UK (COG-UK) Consortium|Davies|United Kingdom
>Spike|hCoV-19/USA/LA-EVTL551/2020|2020-08-05|EPI_ISL_578955|Original|hCoV-
19^^Louisiana|Human|LSUHS Emerging Viral Threat Laboratory|Microbial Genome
Sequencing Center|Kamil|USA
>Spike|hCoV-19/Gambia/NPHL-1446/2020|2020-07-04|EPI_ISL_561230|Original|hCoV-
19^^Kombo|Human|MRCG at LSHTM Genomics lab|MRCG at LSHTM Genomics lab|Sesay|Gambia
>Spike|hCoV-19/Australia/VIC6005/2020|2020-07-23|EPI_ISL_518780|Original|hCoV-
19^^Victoria|Human|Microbiological Diagnostic Unit - Public Health Laboratory (MDU-
PHL)|MDU-PHL|Schultz|Australia
>Spike|hCoV-19/Denmark/DCGC-15716/2020|2020-11-23|EPI_ISL_713978|Original|hCoV-
19^^Hovedstaden|Human|Department of Virus and Microbiological Special
Diagnostics|Albertsen Lab|Michaelsen|Denmark
>Spike|hCoV-19/USA/CT-CDC-2-3693584/2020|2020-10-25|EPI_ISL_747132|Original|hCoV-
19^^Connecticut|Human|Respiratory Viruses Branch|Respiratory Viruses Branch|Queen|USA
>Spike|hCoV-19/England/QEUH-97008A/2020|2020-08-17|EPI_ISL_540316|Original|hCoV-
19^^England|Human|Lighthouse Lab in Glasgow|Wellcome Sanger Institute for the COVID-
19 Genomics UK (COG-UK) consortium|Davies|United Kingdom

>Spike|hCoV-19/England/LIVE-A734E/2020|2020-03-28|EPI_ISL_517006|Original|hCoV-19^^England|Human|Liverpool Clinical Laboratories|COVID-19 Genomics UK (COG-UK) Consortium|Haldenby|United Kingdom
>Spike|hCoV-19/England/QEUH-B7DEEB/2020|2020-11-13|EPI_ISL_741925|Original|hCoV-19^^England|Human|Oxford Viromics|COVID-19 Genomics UK (COG-UK) Consortium|Golubchik|United Kingdom
>Spike|hCoV-19/England/ALDP-955AF6/2020|2020-06-12|EPI_ISL_553900|Original|hCoV-19^^England|Human|Lighthouse Lab in Alderley Park|Wellcome Sanger Institute for the COVID-19 Genomics UK (COG-UK) consortium|Davies|United Kingdom
>Spike|hCoV-19/England/QEUH-B2E915/2020|2020-11-03|EPI_ISL_659921|Original|hCoV-19^^England|Human|Lighthouse Lab in Glasgow|Wellcome Sanger Institute for the COVID-19 Genomics UK (COG-UK) Consortium|Davies|United Kingdom
>Spike|hCoV-19/USA/WA-S685/2020|2020-04-11|EPI_ISL_449855|Original|hCoV-19^^Washington|Human|Washington State Department of Health|Seattle Flu Study|Bedford|USA
>Spike|hCoV-19/England/QEUH-B6785F/2020|2020-11-10|EPI_ISL_655912|Original|hCoV-19^^England|Human|Lighthouse Lab in Glasgow|Wellcome Sanger Institute for the COVID-19 Genomics UK (COG-UK) Consortium|Davies|United Kingdom
>Spike|hCoV-19/USA/MI-UM-10036817055/2020|2020-11-06|EPI_ISL_728433|Original|hCoV-19^^Michigan|Human|University of Michigan Clinical Microbiology Laboratory|Lauring Lab|Valesano|USA
>Spike|hCoV-19/Australia/VIC1026/2020|2020-04-02|EPI_ISL_561788|Original|hCoV-19^^Victoria|Human|Victorian Infectious Diseases Reference Laboratory (VIDRL)|VIDRL and MDU-PHL|Schultz|Australia
>Spike|hCoV-19/England/ALDP-9ED000/2020|2020-05-05|EPI_ISL_589510|Original|hCoV-19^^England|Human|Lighthouse Lab in Alderley Park|Wellcome Sanger Institute for the COVID-19 Genomics UK (COG-UK) consortium|Davies|United Kingdom
>Spike|hCoV-19/England/ALDP-94E37D/2020|2020-06-17|EPI_ISL_555412|Original|hCoV-19^^England|Human|Lighthouse Lab in Alderley Park|Wellcome Sanger Institute for the COVID-19 Genomics UK (COG-UK) consortium|Davies|United Kingdom
>Spike|hCoV-19/USA/WA-UW-2180/2020|2020-03-24|EPI_ISL_426106|Original|hCoV-19^^Washington|Human|UW Virology Lab|UW Virology Lab|Roychoudhury|USA
>Spike|hCoV-19/Wales/PHWC-166474/2020|2020-06-27|EPI_ISL_494001|Original|hCoV-19^^Wales|Human|Originating lab|COVID-19 Genomics UK (COG-UK) Consortium|Moore|United Kingdom
>Spike|hCoV-19/England/ALDP-9D9562/2020|2020-09-18|EPI_ISL_576738|Original|hCoV-19^^England|Human|Oxford Viromics|COVID-19 Genomics UK (COG-UK) Consortium|Golubchik|United Kingdom
>Spike|hCoV-19/Belgium/rega-0417519/2020|2020-04-17|EPI_ISL_734611|Original|hCoV-19^^Tournai|Human|UZ Leuven|KU Leuven|Maes|Belgium
>Spike|hCoV-19/England/QEUH-B679B6/2020|2020-11-11|EPI_ISL_661587|Original|hCoV-19^^England|Human|Lighthouse Lab in Glasgow|Wellcome Sanger Institute for the COVID-19 Genomics UK (COG-UK) Consortium|Davies|United Kingdom
>Spike|hCoV-19/USA/HI-H200392/2020|2020-09-29|EPI_ISL_752939|Original|hCoV-19^^Hawaii|Human|State Laboratories Division|State Laboratories Division|Sultana|USA
>Spike|hCoV-19/Australia/VIC4908/2020|2020-07-14|EPI_ISL_519284|Original|hCoV-19^^Victoria|Human|Victorian Infectious Diseases Reference Laboratory (VIDRL)|VIDRL and MDU-PHL|Schultz|Australia
>Spike|hCoV-19/England/ALDP-690FA8/2020|2020-07-02|EPI_ISL_558198|Original|hCoV-19^^England|Human|Lighthouse Lab in Alderley Park|Wellcome Sanger Institute for the COVID-19 Genomics UK (COG-UK) consortium|Davies|United Kingdom
>Spike|hCoV-19/England/CAMB-1B9AF8/2020|2020-11-06|EPI_ISL_679151|Original|hCoV-19^^England|Human|Department of Pathology|COVID-19 Genomics UK (COG-UK) Consortium|Goodfellow|United Kingdom
>Spike|hCoV-19/USA/AL-QDX-207/2020|2020-05-01|EPI_ISL_494504|Original|hCoV-19^^Alabama|Human|Quest Diagnostics|Quest Diagnostics|Kagan|USA
>Spike|hCoV-19/Denmark/DCGC-22039/2020|2020-12-14|EPI_ISL_758122|Original|hCoV-19^^Hovedstaden|Human|Department of Virus and Microbiological Special Diagnostics|Albertsen Lab|Michaelsen|Denmark

>Spike|hCoV-19/Chile/RM-ISPCH-10/2020|2020-03-10|EPI_ISL_445253|Original|hCoV-19^^Region Metropolitana de Santiago|Human|CLINICA ALEMANA DE SANTIAGO S.A.|Instituto de Salud Publica de Chile|Fernandez|Chile
>Spike|hCoV-19/England/LIVE-DB5F88/2020|2020-11-06|EPI_ISL_651336|Original|hCoV-19^^England|Human|Liverpool Clinical Laboratories|COVID-19 Genomics UK (COG-UK) Consortium|Haldenby|United Kingdom
>Spike|hCoV-19/Portugal/PT1340/2020|2020-03-19|EPI_ISL_511299|Original|hCoV-19^^Portugal|Human|Instituto Nacional de Saude (INSA) and Instituto Gulbenkian de Ciencia (IGC)|Instituto Nacional de Saude (INSA) and Instituto Gulbenkian de Ciencia (IGC)|Borges|Portugal
>Spike|hCoV-19/England/BRIS-12D6CE/2020|2020-05-08|EPI_ISL_481787|Original|hCoV-19^^England|Human|PHE South West Regional Laboratory|Wellcome Sanger Institute for the COVID-19 Genomics UK (COG-UK) consortium|Davies|United Kingdom
>Spike|hCoV-19/Japan/PG-4583/2020|2020-08-00|EPI_ISL_689720|Original|hCoV-19^^Japan|Human|Pathogen Genomics Center|Pathogen Genomics Center|Sekizuka|Japan
>Spike|hCoV-19/Denmark/DCGC-8505/2020|2020-10-26|EPI_ISL_625906|Original|hCoV-19^^Syddanmark|Human|Department of Virus and Microbiological Special Diagnostics|Albertsen lab|Michaelsen|Denmark
>Spike|hCoV-19/Denmark/DCGC-6603/2020|2020-10-12|EPI_ISL_619651|Original|hCoV-19^^Hovedstaden|Human|Department of Virus and Microbiological Special Diagnostics|Albertsen lab|Michaelsen|Denmark
>Spike|hCoV-19/USA/LA-QDX-1241/2020|2020-03-15|EPI_ISL_571949|Original|hCoV-19^^Louisiana|Human|Quest Diagnostics|Quest Diagnostics|Kagan|USA
>Spike|hCoV-19/USA/TX-DSHS-1273/2020|2020-03-31|EPI_ISL_648259|Original|hCoV-19^^Texas|Human|Texas Department of State Health Services|Texas Department of State Health Services|Rahman|USA
>Spike|hCoV-19/South Africa/LR00041/2020|2020-07-06|EPI_ISL_515168|Original|hCoV-19^^Gauteng|Human|National Institute for Communicable Diseases of the National Health Laboratory Service|National Institute for Communicable Diseases of the National Health Laboratory Service|Allam|South Africa
>Spike|hCoV-19/Reunion/RUN-PIMIT1/2020|2020-03-24|EPI_ISL_522547|Original|hCoV-19^^Reunion|Human|Platforme CYROI|UMR PIMIT Universit\u00e9 de La R\u00e9union|Wilkinson|Reunion
>Spike|hCoV-19/England/MILK-99A15A/2020|2020-08-30|EPI_ISL_550265|Original|hCoV-19^^England|Human|Lighthouse Lab in Milton Keynes|Wellcome Sanger Institute for the COVID-19 Genomics UK (COG-UK) consortium|Davies|United Kingdom
>Spike|hCoV-19/Iceland/157/2020|2020-03-13|EPI_ISL_417804|Original|hCoV-19^^Reykjavik|Human|The National University Hospital of Iceland|deCODE genetics|J\u00f3nsson|Iceland
>Spike|hCoV-19/Wales/PHWC-48497E/2020|2020-10-24|EPI_ISL_628063|Original|hCoV-19^^Wales|Human|Wales Specialist Virology Centre Sequencing lab|COVID-19 Genomics UK (COG-UK) Consortium|Moore|United Kingdom
>Spike|hCoV-19/USA/WI-WSLH-200064/2020|2020-04-24|EPI_ISL_471229|Original|hCoV-19^^Wisconsin|Human|Wisconsin State Laboratory of Hygiene Communicable Disease Division|Wisconsin State Laboratory of Hygiene Communicable Disease Division|Florek|USA
>Spike|hCoV-19/England/CAMC-B535AA/2020|2020-11-08|EPI_ISL_656772|Original|hCoV-19^^England|Human|Lighthouse Lab in Cambridge|Wellcome Sanger Institute for the COVID-19 Genomics UK (COG-UK) Consortium|Davies|United Kingdom
>Spike|hCoV-19/Denmark/DCGC-8867/2020|2020-10-26|EPI_ISL_626101|Original|hCoV-19^^Hovedstaden|Human|Department of Virus and Microbiological Special Diagnostics|Albertsen lab|Michaelsen|Denmark
>Spike|hCoV-19/Chile/RM-193465/2020|2020-09-29|EPI_ISL_746754|Original|hCoV-19^^Region Metropolitana de Santiago|Human|Genetica Molecular and Subdepartamento de Virologia ISP Chile|Instituto de Salud Publica de Chile|Tognarelli|Chile
>Spike|hCoV-19/England/MILK-A56414/2020|2020-10-08|EPI_ISL_595155|Original|hCoV-19^^England|Human|Quadram Institute Bioscience|COVID-19 Genomics UK (COG-UK) Consortium|Alikhan|United Kingdom

>Spike|hCoV-19/USA/NY-PV09073/2020|2020-03-20|EPI_ISL_421620|Original|hCoV-19^^New York|Human|MSHS Clinical Microbiology Laboratories|MSHS Pathogen Surveillance Program|van Bakel|USA
>Spike|hCoV-19/England/20208072004/2020|2020-05-15|EPI_ISL_465671|Original|hCoV-19^^England|Human|Respiratory Virus Unit|Respiratory Virus Unit|Platt|United Kingdom
>Spike|hCoV-19/England/204381148/2020|2020-10-21|EPI_ISL_649852|Original|hCoV-19^^England|Human|Respiratory Virus Unit|COVID-19 Genomics UK (COG-UK) Consortium|Platt|United Kingdom
>Spike|hCoV-19/mink/Netherlands/NB-EMC-47-1/2020|2020-09-04|EPI_ISL_577809|Original|hCoV-19^^Noord Brabant|Neovison vison|Dutch COVID-19 response team|Erasmus Medical Center|Oude Munnink|Netherlands
>Spike|hCoV-19/England/ALDP-C8CC66/2020|2020-12-15|EPI_ISL_777166|Original|hCoV-19^^England|Human|Lighthouse Lab in Alderley Park|Wellcome Sanger Institute for the COVID-19 Genomics UK (COG-UK) Consortium|Davies|United Kingdom
>Spike|hCoV-19/USA/TX-HMH-MCoV-18250/2020|2020-07-05|EPI_ISL_784598|Original|hCoV-19^^Texas|Human|Houston Methodist Hospital|Houston Methodist Hospital|Long|USA
>Spike|hCoV-19/USA/MI-MDHHS-SC21749/2020|2020-08-02|EPI_ISL_529867|Original|hCoV-19^^Michigan|Human|Michigan Department of Health and Human Services|Michigan Department of Health and Human Services|Blankenship|USA
>Spike|hCoV-19/USA/TX-HMH-MCoV-12715/2020|2020-08-30|EPI_ISL_790478|Original|hCoV-19^^Texas|Human|Houston Methodist Hospital|Houston Methodist Hospital|Long|USA
>Spike|hCoV-19/USA/TX-HMH-MCoV-14443/2020|2020-10-07|EPI_ISL_787728|Original|hCoV-19^^Texas|Human|Houston Methodist Hospital|Houston Methodist Hospital|Long|USA
>Spike|hCoV-19/England/GSTT-265E22F/2020|2020-03-31|EPI_ISL_484172|Original|hCoV-19^^England|Human|Centre for Clinical Infection and Diagnostics Research and Genomics Innovation Unit|COVID-19 Genomics UK (COG-UK) Consortium|Awan|United Kingdom
>Spike|hCoV-19/England/MILK-9ECC3A/2020|2020-04-29|EPI_ISL_589377|Original|hCoV-19^^England|Human|Lighthouse Lab in Milton Keynes|Wellcome Sanger Institute for the COVID-19 Genomics UK (COG-UK) consortium|Davies|United Kingdom
>Spike|hCoV-19/England/MILK-A69AB2/2020|2020-10-08|EPI_ISL_665514|Original|hCoV-19^^England|Human|University of Exeter|COVID-19 Genomics UK (COG-UK) Consortium|Temperton|United Kingdom
>Spike|hCoV-19/England/QEUH-A5DF94/2020|2020-10-09|EPI_ISL_597359|Original|hCoV-19^^England|Human|Lighthouse Lab in Glasgow|Wellcome Sanger Institute for the COVID-19 Genomics UK (COG-UK) consortium|Davies|United Kingdom
>Spike|hCoV-19/Italy/MAR-UnivPM-73702/2020|2020-02-28|EPI_ISL_516085|Original|hCoV-19^^Marche|Human|Biomedical Sciences and Public Health|Biomedical Sciences and Public Health|Bagnarelli|Italy
>Spike|hCoV-19/Belgium/ULG-10637/2020|2020-10-25|EPI_ISL_636615|Original|hCoV-19^^Li\u00e8ge|Human|Department of Clinical Microbiology|GIGA Medical Genomics|Durkin|Belgium
>Spike|hCoV-19/Panama/338777/2020|2020-04-09|EPI_ISL_496885|Original|hCoV-19^^Panama Center|Human|Gorgas Memorial Laboratory of Health Studies|Gorgas Memorial Laboratory of Health Studies|Martinez|Panama
>Spike|hCoV-19/USA/TX-DSHS-1417/2020|2020-06-22|EPI_ISL_671647|Original|hCoV-19^^Texas|Human|Texas Department of State Health Services|Texas Department of State Health Services|Rahman|USA
>Spike|hCoV-19/England/ALDP-A877A8/2020|2020-10-17|EPI_ISL_605972|Original|hCoV-19^^England|Human|Lighthouse Lab in Alderley Park|Wellcome Sanger Institute for the COVID-19 Genomics UK (COG-UK) consortium|Davies|United Kingdom
>Spike|hCoV-19/England/ALDP-A8790C/2020|2020-10-17|EPI_ISL_606228|Original|hCoV-19^^England|Human|Lighthouse Lab in Alderley Park|Wellcome Sanger Institute for the COVID-19 Genomics UK (COG-UK) consortium|Davies|United Kingdom
>Spike|hCoV-19/India/TG-CDFD-C1509/2020|2020-06-10|EPI_ISL_528567|Original|hCoV-19^^Telangana|Human|National Genomics Core-Center for DNA Fingerprinting and Diagnostics|National Genomics Core- Center for DNA Fingerprinting and Diagnostics (NGC-CDFD)- DBT's PAN-INDIA-1000 Genome consortium|Vashisht|India
>Spike|hCoV-19/England/QEUH-B0F3D0/2020|2020-11-02|EPI_ISL_641923|Original|hCoV-19^^England|Human|Lighthouse Lab in Glasgow|Wellcome Sanger Institute for the COVID-19 Genomics UK (COG-UK) Consortium|Davies|United Kingdom

>Spike|hCoV-19/Scotland/QEUH-A1FB9A/2020|2020-10-01|EPI_ISL_599035|Original|hCoV-19^^Scotland|Human|Lighthouse Lab in Glasgow|Wellcome Sanger Institute for the COVID-19 Genomics UK (COG-UK) consortium|Davies|United Kingdom
>Spike|hCoV-19/USA/MI-MDHHS-SC21509/2020|2020-04-01|EPI_ISL_516236|Original|hCoV-19^^Michigan|Human|Michigan Department of Health and Human Services|Michigan Department of Health and Human Services|Blankenship|USA
>Spike|hCoV-19/Italy/CAM-INTPas-14/2020|2020-04-02|EPI_ISL_775239|Original|hCoV-19^^Campania|Human|INT Fondazione Pascale|INT Fondazione Pascale|Rachiglio|Italy
>Spike|hCoV-19/Scotland/EDB7781/2020|2020-09-12|EPI_ISL_577350|Original|hCoV-19^^Scotland|Human|Virology Department|COVID-19 Genomics UK (COG-UK) Consortium|Williams|United Kingdom
>Spike|hCoV-19/India/UN-15337/2020|2020-00-00|EPI_ISL_491478|Original|hCoV-19^^India|Human|CSIR-CDRI/SGPGI|CSIR-CDRI/SGPGI|Singh|India
>Spike|hCoV-19/Japan/PG-8552/2020|2020-08-00|EPI_ISL_686148|Original|hCoV-19^^Japan|Human|Pathogen Genomics Center|Pathogen Genomics Center|Sekizuka|Japan
>Spike|hCoV-19/England/QEUH-96BB36/2020|2020-08-14|EPI_ISL_531408|Original|hCoV-19^^England|Human|Lighthouse Lab in Glasgow|Wellcome Sanger Institute for the COVID-19 Genomics UK (COG-UK) consortium|Davies|United Kingdom
>Spike|hCoV-19/Denmark/DCGC-8390/2020|2020-10-26|EPI_ISL_617365|Original|hCoV-19^^Nordjylland|Human|Department of Virus and Microbiological Special Diagnostics|Albertsen lab|Michaelsen|Denmark
>Spike|hCoV-19/England/CAMB-1B06A2/2020|2020-00-00|EPI_ISL_452907|Original|hCoV-19^^England|Human|Department of Pathology|COVID-19 Genomics UK (COG-UK) Consortium|Meredith|United Kingdom
>Spike|hCoV-19/Portugal/PT1125/2020|2020-03-26|EPI_ISL_511672|Original|hCoV-19^^Portugal|Human|Instituto Nacional de Saude (INSA)|Instituto Nacional de Saude (INSA)|Borges|Portugal
>Spike|hCoV-19/Scotland/CVR6051/2020|2020-11-30|EPI_ISL_705717|Original|hCoV-19^^Scotland|Human|West of Scotland Specialist Virology Centre|COVID-19 Genomics UK (COG-UK) Consortium|Robertson|United Kingdom
>Spike|hCoV-19/Scotland/EDB2743/2020|2020-03-27|EPI_ISL_487794|Original|hCoV-19^^Scotland|Human|Virology Department|Wellcome Sanger Institute for the COVID-19 Genomics UK (COG-UK) consortium|Davies|United Kingdom
>Spike|hCoV-19/Colombia/CUN-GUR-1215/2020|2020-03-31|EPI_ISL_447754|Original|hCoV-19^^Cundinamarca|Human|Grupo de Investigaciones Microbiol\u00f3gicas-UR (GIMUR)|Grupo de Investigaciones Microbiol\u00f3gicas-UR (GIMUR)|Ram\u00edrez|Colombia
>Spike|hCoV-19/Australia/VIC12343/2020|2020-07-30|EPI_ISL_640570|Original|hCoV-19^^Victoria|Human|Microbiological Diagnostic Unit - Public Health Laboratory (MDU-PHL)|MDU-PHL|Schultz|Australia
>Spike|hCoV-19/France/PAC-NRC02800/2020|2020-10-19|EPI_ISL_639997|Original|hCoV-19^^Provence-Alpes-Cote-d\u2019Azur|Human|CNR Virus des Infections Respiratoires - France SUD|CNR Virus des Infections Respiratoires - France SUD|Josset|France
>Spike|hCoV-19/Australia/VIC1641/2020|2020-05-02|EPI_ISL_456544|Original|hCoV-19^^Victoria|Human|Victorian Infectious Diseases Reference Laboratory (VIDRL)|Microbiological Diagnostic Unit Public Health Laboratory and Victorian Infectious Diseases Reference Laboratory|Schultz|Australia
>Spike|hCoV-19/Jordan/AM-ALSR-4996/2020|2020-10-10|EPI_ISL_730269|Original|hCoV-19^^Amman|Human|Biolab Diagnostic Laboratories|Andersen lab at Scripps Research|Gangavarapu|Jordan
>Spike|hCoV-19/Denmark/DCGC-2886/2020|2020-08-24|EPI_ISL_616581|Original|hCoV-19^^Hovedstaden|Human|Department of Virus and Microbiological Special Diagnostics|Albertsen lab|Michaelsen|Denmark
>Spike|hCoV-19/England/CAMC-AB1E55/2020|2020-10-23|EPI_ISL_623547|Original|hCoV-19^^England|Human|Lighthouse Lab in Cambridge|Wellcome Sanger Institute for the COVID-19 Genomics UK (COG-UK) consortium|Davies|United Kingdom
>Spike|hCoV-19/England/CAMB-1B44B3/2020|2020-06-02|EPI_ISL_473473|Original|hCoV-19^^England|Human|Department of Pathology|COVID-19 Genomics UK (COG-UK) Consortium|Meredith|United Kingdom

>Spike|hCoV-19/Denmark/DCGC-3330/2020|2020-08-31|EPI_ISL_616084|Original|hCoV-
19^^Hovedstaden|Human|Department of Virus and Microbiological Special
Diagnostics|Albertsen lab|Michaelsen|Denmark
>Spike|hCoV-19/England/QEUH-943651/2020|2020-07-30|EPI_ISL_532605|Original|hCoV-
19^^England|Human|Lighthouse Lab in Glasgow|Wellcome Sanger Institute for the COVID-
19 Genomics UK (COG-UK) consortium|Davies|United Kingdom
>Spike|hCoV-19/Denmark/DCGC-22346/2020|2020-12-14|EPI_ISL_758694|Original|hCoV-
19^^Hovedstaden|Human|Department of Virus and Microbiological Special
Diagnostics|Albertsen Lab|Michaelsen|Denmark
>Spike|hCoV-19/USA/MI-QDX-476/2020|2020-03-23|EPI_ISL_571210|Original|hCoV-
19^^Michigan|Human|Quest Diagnostics|Quest Diagnostics|Kagan|USA
>Spike|hCoV-19/Portugal/PT1845/2020|2020-11-11|EPI_ISL_732065|Original|hCoV-
19^^Portugal|Human|Instituto Nacional de Saude (INSA)|Instituto Nacional de Saude
(INSA)|Borges|Portugal
>Spike|hCoV-19/Switzerland/GE-HUG-1422/2020|2020-02-28|EPI_ISL_415454|Original|hCoV-
19^^Geneve|Human|University Hospitals of Geneva Laboratory of Virology|University
Hospitals of Geneva Laboratory of Virology|Cordey|Switzerland
>Spike|hCoV-19/USA/MI-MDHHS-SC22279/2020|2020-11-02|EPI_ISL_666945|Original|hCoV-
19^^Michigan|Human|Michigan Department of Health and Human Services|Michigan
Department of Health and Human Services|Jacob|USA
>Spike|hCoV-19/England/MILK-99A56D/2020|2020-08-29|EPI_ISL_550336|Original|hCoV-
19^^England|Human|Lighthouse Lab in Milton Keynes|Wellcome Sanger Institute for the
COVID-19 Genomics UK (COG-UK) consortium|Davies|United Kingdom
>Spike|hCoV-19/India/GJ-GBRC252/2020|2020-06-12|EPI_ISL_483837|Original|hCoV-
19^^Gujarat|Human|Pandit Deendayal Upadhyay Government Medical College|Gujarat
Biotechnology Research Centre|Joshi|India
>Spike|hCoV-19/USA/MI-UM-10036747334/2020|2020-11-02|EPI_ISL_682204|Original|hCoV-
19^^Michigan|Human|University of Michigan Clinical Microbiology Laboratory|Lauring
Lab|Valesano|USA
>Spike|hCoV-19/Luxembourg/LNS3063798/2020|2020-03-13|EPI_ISL_740242|Original|hCoV-
19^^Luxembourg|Human|Laboratoire national de sant\u00e9|Laboratoire national de
sant\u00e9|Wienecke-Baldacchino|Luxembourg
>Spike|hCoV-19/England/MILK-565AE8/2020|2020-06-10|EPI_ISL_558314|Original|hCoV-
19^^England|Human|Lighthouse Lab in Milton Keynes|Wellcome Sanger Institute for the
COVID-19 Genomics UK (COG-UK) consortium|Davies|United Kingdom
>Spike|hCoV-19/Spain/MD-HLP-H7-36/2020|2020-03-12|EPI_ISL_417957|Original|hCoV-
19^^Madrid|Human|Hospital Universitario 12 de Octubre|Hospital Universitario La
Paz|Viedma|Spain
>Spike|hCoV-19/England/QEUH-B437B6/2020|2020-11-08|EPI_ISL_657078|Original|hCoV-
19^^England|Human|Lighthouse Lab in Glasgow|Wellcome Sanger Institute for the COVID-
19 Genomics UK (COG-UK) Consortium|Davies|United Kingdom
>Spike|hCoV-19/Germany/BE-ChVir-D2034-8234/2020|2020-10-
13|EPI_ISL_753912|Original|hCoV-19^^Berlin|Human|Charit\u00e9
Universit\u00e4tsmedizin Berlin|Charit\u00e9 Universit\u00e4tsmedizin
Berlin|Muehlemann|Germany
>Spike|hCoV-19/USA/VA-DCLS-0529/2020|2020-03-11|EPI_ISL_485831|Original|hCoV-
19^^Virginia|Human|Virginia DCLS|Virginia DCLS|St. Jacques|USA
>Spike|hCoV-19/England/BRIS-1271A5/2020|2020-04-24|EPI_ISL_469657|Original|hCoV-
19^^England|Human|PHE South West Regional Laboratory|Wellcome Sanger Institute for
the COVID-19 Genomics UK (COG-UK) consortium|Davies|United Kingdom
>Spike|hCoV-19/England/ALDP-B1BCD6/2020|2020-11-01|EPI_ISL_645874|Original|hCoV-
19^^England|Human|Lighthouse Lab in Alderley Park|Wellcome Sanger Institute for the
COVID-19 Genomics UK (COG-UK) Consortium|Davies|United Kingdom
>Spike|hCoV-19/Sweden/01/2020|2020-02-07|EPI_ISL_411951|unknown|hCoV-
19^^Sweden|Human|Unit for Laboratory Development and Technology Transfer|Unit for
Laboratory Development and Technology Transfer|Submitter|Sweden
>Spike|hCoV-19/France/ARA-1125/2020|2020-03-19|EPI_ISL_509002|Original|hCoV-
19^^Auvergne-Rh\u00f4ne-Alpes|Human|CNR Virus des Infections Respiratoires - France
SUD|CNR Virus des Infections Respiratoires - France SUD|Josset|France

>Spike|hCoV-19/Scotland/QEUH-9F3D1F/2020|2020-09-27|EPI_ISL_600796|Original|hCoV-19^^Scotland|Human|Lighthouse Lab in Glasgow|Wellcome Sanger Institute for the COVID-19 Genomics UK (COG-UK) consortium|Davies|United Kingdom
>Spike|hCoV-19/Wales/PHWC-491871/2020|2020-11-06|EPI_ISL_706701|Original|hCoV-19^^Wales|Human|Originating lab|COVID-19 Genomics UK (COG-UK) Consortium|Moore|United Kingdom
>Spike|hCoV-19/England/QEUH-B41800/2020|2020-11-08|EPI_ISL_658605|Original|hCoV-19^^England|Human|Lighthouse Lab in Glasgow|Wellcome Sanger Institute for the COVID-19 Genomics UK (COG-UK) Consortium|Davies|United Kingdom
>Spike|hCoV-19/Wales/PHWC-487A69/2020|2020-10-26|EPI_ISL_639266|Original|hCoV-19^^Wales|Human|Wales Specialist Virology Centre Sequencing lab|COVID-19 Genomics UK (COG-UK) Consortium|Moore|United Kingdom
>Spike|hCoV-19/England/CAMC-C92483/2020|2020-12-11|EPI_ISL_777293|Original|hCoV-19^^England|Human|Lighthouse Lab in Cambridge|Wellcome Sanger Institute for the COVID-19 Genomics UK (COG-UK) Consortium|Davies|United Kingdom
>Spike|hCoV-19/USA/WA-UW-524/2020|2020-03-12|EPI_ISL_430877|Original|hCoV-19^^Washington|Human|UW Virology Lab|UW Virology Lab|Roychoudhury|USA
>Spike|hCoV-19/Scotland/QEUH-A0AE5D/2020|2020-10-03|EPI_ISL_599293|Original|hCoV-19^^Scotland|Human|Lighthouse Lab in Glasgow|Wellcome Sanger Institute for the COVID-19 Genomics UK (COG-UK) consortium|Davies|United Kingdom
>Spike|hCoV-19/Wales/ALDP-A2D57D/2020|2020-10-06|EPI_ISL_598825|Original|hCoV-19^^Wales|Human|Lighthouse Lab in Alderley Park|Wellcome Sanger Institute for the COVID-19 Genomics UK (COG-UK) consortium|Davies|United Kingdom
>Spike|hCoV-19/England/QEUH-96F67D/2020|2020-08-17|EPI_ISL_530612|Original|hCoV-19^^England|Human|Lighthouse Lab in Glasgow|Wellcome Sanger Institute for the COVID-19 Genomics UK (COG-UK) consortium|Davies|United Kingdom
>Spike|hCoV-19/Denmark/DCGC-2301/2020|2020-08-10|EPI_ISL_617726|Original|hCoV-19^^Midtjylland|Human|Department of Virus and Microbiological Special Diagnostics|Albertsen lab|Michaelsen|Denmark
>Spike|hCoV-19/England/MILK-9F0C7F/2020|2020-09-24|EPI_ISL_589667|Original|hCoV-19^^England|Human|Lighthouse Lab in Milton Keynes|Wellcome Sanger Institute for the COVID-19 Genomics UK (COG-UK) consortium|Davies|United Kingdom
>Spike|hCoV-19/USA/TX-HMH-4631/2020|2020-06-22|EPI_ISL_543769|Original|hCoV-19^^Texas|Human|Houston Methodist Hospital|Houston Methodist Hospital|Long|USA
>Spike|hCoV-19/England/QEUH-54C777/2020|2020-05-12|EPI_ISL_532246|Original|hCoV-19^^England|Human|Lighthouse Lab in Glasgow|Wellcome Sanger Institute for the COVID-19 Genomics UK (COG-UK) consortium|Davies|United Kingdom
>Spike|hCoV-19/Switzerland/BE-ETHZ-240061/2020|2020-08-13|EPI_ISL_523901|Original|hCoV-19^^Bern|Human|Viollier AG|Department of Biosystems Science and Engineering|Nadeau|Switzerland
>Spike|hCoV-19/USA/TX-HMH-MCoV-19362/2020|2020-11-27|EPI_ISL_783791|Original|hCoV-19^^Texas|Human|Houston Methodist Hospital|Houston Methodist Hospital|Long|USA
>Spike|hCoV-19/England/LIVE-DC0A98/2020|2020-11-04|EPI_ISL_703711|Original|hCoV-19^^England|Human|Liverpool Clinical Laboratories|COVID-19 Genomics UK (COG-UK) Consortium|Haldenby|United Kingdom
>Spike|hCoV-19/USA/ID-UW-1939/2020|2020-03-21|EPI_ISL_424320|Original|hCoV-19^^Idaho|Human|UW Virology Lab|UW Virology Lab|Roychoudhury|USA
>Spike|hCoV-19/England/CAMC-C4672B/2020|2020-12-10|EPI_ISL_718472|Original|hCoV-19^^England|Human|Lighthouse Lab in Cambridge|Wellcome Sanger Institute for the COVID-19 Genomics UK (COG-UK) Consortium|Davies|United Kingdom
>Spike|hCoV-19/USA/WA-UW86/2020|2020-03-10|EPI_ISL_416442|Original|hCoV-19^^Washington|Human|UW Virology Lab|UW Virology Lab|Roychoudhury|USA
>Spike|hCoV-19/Wales/MILK-9E0841/2020|2020-09-20|EPI_ISL_601292|Original|hCoV-19^^Wales|Human|Lighthouse Lab in Milton Keynes|Wellcome Sanger Institute for the COVID-19 Genomics UK (COG-UK) consortium|Davies|United Kingdom
>Spike|hCoV-19/USA/NY-NYUMC26/2020|2020-03-17|EPI_ISL_418972|Original|hCoV-19^^New York|Human|NYU Langone Health|Department of Pathology and Medicine|Maurano|USA
>Spike|hCoV-19/Wales/PHWC-496715/2020|2020-11-20|EPI_ISL_726626|Original|hCoV-19^^Wales|Human|Originating lab|COVID-19 Genomics UK (COG-UK) Consortium|Moore|United Kingdom

>Spike|hCoV-19/Denmark/DCGC-21383/2020|2020-12-21|EPI_ISL_758137|Original|hCoV-19^^Nordjylland|Human|Department of Virus and Microbiological Special Diagnostics|Albertsen Lab|Michaelsen|Denmark
>Spike|hCoV-19/Denmark/DCGC-18893/2020|2020-12-07|EPI_ISL_748623|Original|hCoV-19^^Sjaelland|Human|Department of Virus and Microbiological Special Diagnostics|Albertsen Lab|Michaelsen|Denmark
>Spike|hCoV-19/Hunan/HN-CIDC-P5/2020|2020-01-25|EPI_ISL_539334|Original|hCoV-19^^Hunan|Human|Institute of Disease Control and Prevention|Institute of Disease Control and Prevention|Qiu|China
>Spike|hCoV-19/Scotland/QEUH-A9E98A/2020|2020-10-20|EPI_ISL_625319|Original|hCoV-19^^Scotland|Human|Lighthouse Lab in Glasgow|Wellcome Sanger Institute for the COVID-19 Genomics UK (COG-UK) consortium|Davies|United Kingdom
>Spike|hCoV-19/Austria/CeMM0275/2020|2020-03-24|EPI_ISL_438102|Original|hCoV-19^^Vienna|Human|Center for Virology|Bergthaler laboratory|Bergthaler|Austria
>Spike|hCoV-19/England/BIRM-65E4B/2020|2020-10-08|EPI_ISL_612233|Original|hCoV-19^^England|Human|University of Birmingham|COVID-19 Genomics UK (COG-UK) Consortium|Loman|United Kingdom
>Spike|hCoV-19/England/LOND-124FF21/2020|2020-10-12|EPI_ISL_664500|Original|hCoV-19^^England|Human|University College London Hospital|COVID-19 Genomics UK (COG-UK) Consortium|Byott|United Kingdom
>Spike|hCoV-19/England/20132027404/2020|2020-03-23|EPI_ISL_423764|Original|hCoV-19^^England|Human|Respiratory Virus Unit|Respiratory Virus Unit|Platt|United Kingdom
>Spike|hCoV-19/Spain/MD-IBV-005353/2020|2020-03-19|EPI_ISL_510230|Original|hCoV-19^^Madrid|Human|Hospital General Universitario Gregorio Mara\u00f1\u00f3n|SeqCOVID-SPAIN consortium/IBV(CSIC)|Comas|Spain
>Spike|hCoV-19/Sweden/20-52111/2020|2020-05-22|EPI_ISL_615092|Original|hCoV-19^^Vasterbotten|Human|Umea klinisk mikrobiologi|The Public Health Agency of Sweden|Svartstr\u00f6m|Sweden
>Spike|hCoV-19/Denmark/DCGC-2571/2020|2020-08-17|EPI_ISL_617894|Original|hCoV-19^^Midtjylland|Human|Department of Virus and Microbiological Special Diagnostics|Albertsen lab|Michaelsen|Denmark
>Spike|hCoV-19/England/204761422/2020|2020-11-17|EPI_ISL_649110|Original|hCoV-19^^England|Human|Respiratory Virus Unit|COVID-19 Genomics UK (COG-UK) Consortium|Platt|United Kingdom
>Spike|hCoV-19/Denmark/DCGC-18477/2020|2020-12-07|EPI_ISL_749990|Original|hCoV-19^^Hovedstaden|Human|Department of Virus and Microbiological Special Diagnostics|Albertsen Lab|Michaelsen|Denmark
>Spike|hCoV-19/England/ALDP-9D824E/2020|2020-09-18|EPI_ISL_576641|Original|hCoV-19^^England|Human|Oxford Viromics|COVID-19 Genomics UK (COG-UK) Consortium|Golubchik|United Kingdom
>Spike|hCoV-19/USA/UT-QDX-2596/2020|2020-03-13|EPI_ISL_604966|Original|hCoV-19^^Utah|Human|Quest Diagnostics|Quest Diagnostics|Kagan|USA
>Spike|hCoV-19/Wales/PHWC-4A0F98/2020|2020-12-13|EPI_ISL_743602|Original|hCoV-19^^Wales|Human|Wales Specialist Virology Centre Sequencing lab|COVID-19 Genomics UK (COG-UK) Consortium|Moore|United Kingdom
>Spike|hCoV-19/Australia/VIC3559/2020|2020-00-00|EPI_ISL_520308|Original|hCoV-19^^Victoria|Human|Victorian Infectious Diseases Reference Laboratory (VIDRL)|VIDRL and MDU-PHL|Schultz|Australia
>Spike|hCoV-19/Scotland/EDB3563/2020|2020-04-14|EPI_ISL_487833|Original|hCoV-19^^Scotland|Human|Virology Department|Wellcome Sanger Institute for the COVID-19 Genomics UK (COG-UK) consortium|Davies|United Kingdom
>Spike|hCoV-19/Denmark/DCGC-21818/2020|2020-12-21|EPI_ISL_758384|Original|hCoV-19^^Midtjylland|Human|Department of Virus and Microbiological Special Diagnostics|Albertsen Lab|Michaelsen|Denmark
>Spike|hCoV-19/Luxembourg/LNS5523733/2020|2020-10-19|EPI_ISL_744246|Original|hCoV-19^^Luxembourg|Human|Laboratoire national de sant\u00e9|Laboratoire national de sant\u00e9|Wienecke-Baldacchino|Luxembourg
>Spike|hCoV-19/England/CAMB-80FFC/2020|2020-04-20|EPI_ISL_438554|Original|hCoV-19^^England|Human|Department of Pathology|COVID-19 Genomics UK (COG-UK) Consortium|Meredith|United Kingdom

>Spike|hCoV-19/USA/CA-CZB-14850/2020|2020-11-22|EPI_ISL_738854|Original|hCoV-19^^California|Human|Humboldt County Public Health Laboratory|Chan-Zuckerberg Biohub|Kamm|USA
>Spike|hCoV-19/England/ALDP-B1BF55/2020|2020-11-01|EPI_ISL_645575|Original|hCoV-19^^England|Human|Lighthouse Lab in Alderley Park|Wellcome Sanger Institute for the COVID-19 Genomics UK (COG-UK) Consortium|Davies|United Kingdom
>Spike|hCoV-19/USA/MI-MDHHS-SC22284/2020|2020-10-28|EPI_ISL_666950|Original|hCoV-19^^Michigan|Human|Michigan Department of Health and Human Services|Michigan Department of Health and Human Services|Jacob|USA
>Spike|hCoV-19/USA/VA-DCLS-2043/2020|2020-10-26|EPI_ISL_677217|Original|hCoV-19^^Virginia|Human|Virginia Division of Consolidated Laboratory Services|Virginia Division of Consolidated Laboratory Services|St. Jacques|USA
>Spike|hCoV-19/USA/WA-UW-1728/2020|2020-03-21|EPI_ISL_424193|Original|hCoV-19^^Washington|Human|UW Virology Lab|UW Virology Lab|Roychoudhury|USA
>Spike|hCoV-19/Austria/CeMM0217/2020|2020-03-16|EPI_ISL_438051|Original|hCoV-19^^Vienna|Human|Center for Virology|Bergthaler laboratory|Bergthaler|Austria
>Spike|hCoV-19/England/CAMC-A81717/2020|2020-10-13|EPI_ISL_610789|Original|hCoV-19^^England|Human|Lighthouse Lab in Cambridge|Wellcome Sanger Institute for the COVID-19 Genomics UK (COG-UK) consortium|Davies|United Kingdom
>Spike|hCoV-19/USA/TX-DSHS-1594/2020|2020-06-22|EPI_ISL_700712|Original|hCoV-19^^Texas|Human|Texas Department of State Health Services|Texas Department of State Health Services|Rahman|USA
>Spike|hCoV-19/USA/TX-HMH-2612/2020|2020-06-05|EPI_ISL_545427|Original|hCoV-19^^Texas|Human|Houston Methodist Hospital|Houston Methodist Hospital|Long|USA
>Spike|hCoV-19/Belgium/SS-031047/2020|2020-03-10|EPI_ISL_418793|Original|hCoV-19^^Zonhoven|Human|KU Leuven|KU Leuven|Maes|Belgium
>Spike|hCoV-19/Canada/ON-PHL-20-00073/2020|2020-00-00|EPI_ISL_548748|Original|hCoV-19^^Ontario|Human|Public Health Ontario Laboratory|Public Health Ontario Laboratory|Guthrie|Canada
>Spike|hCoV-19/Wales/MILK-A18056/2020|2020-10-01|EPI_ISL_595013|Original|hCoV-19^^Wales|Human|Northumbria University / South Tees Hospitals NHS Foundation Trust / North Cumbria Integrated Care NHS Foundation Trust / North Tees and Hartlepool NHS Foundation Trust / Newcastle Hospitals NHS Foundation Trust|COVID-19 Genomics UK (COG-UK) Consortium|Bashton|United Kingdom
>Spike|hCoV-19/England/CAMB-1B83FE/2020|2020-10-15|EPI_ISL_638389|Original|hCoV-19^^England|Human|Department of Pathology|COVID-19 Genomics UK (COG-UK) Consortium|Goodfellow|United Kingdom
>Spike|hCoV-19/USA/TX-HMH-1176/2020|2020-04-17|EPI_ISL_542566|Original|hCoV-19^^Texas|Human|Houston Methodist Hospital|Houston Methodist Hospital|Long|USA
>Spike|hCoV-19/England/QEUH-9E478C/2020|2020-09-23|EPI_ISL_579890|Original|hCoV-19^^England|Human|Lighthouse Lab in Glasgow|Wellcome Sanger Institute for the COVID-19 Genomics UK (COG-UK) consortium|Davies|United Kingdom
>Spike|hCoV-19/Portugal/PT1120/2020|2020-03-30|EPI_ISL_511667|Original|hCoV-19^^Portugal|Human|Instituto Nacional de Saude (INSA)|Instituto Nacional de Saude (INSA)|Borges|Portugal
>Spike|hCoV-19/Scotland/CVR5965/2020|2020-11-24|EPI_ISL_705702|Original|hCoV-19^^Scotland|Human|West of Scotland Specialist Virology Centre|COVID-19 Genomics UK (COG-UK) Consortium|Robertson|United Kingdom
>Spike|hCoV-19/England/MILK-ACE24A/2020|2020-10-21|EPI_ISL_644071|Original|hCoV-19^^England|Human|Lighthouse Lab in Milton Keynes|Wellcome Sanger Institute for the COVID-19 Genomics UK (COG-UK) Consortium|Davies|United Kingdom
>Spike|hCoV-19/Japan/PG-5040/2020|2020-07-00|EPI_ISL_690151|Original|hCoV-19^^Japan|Human|Pathogen Genomics Center|Pathogen Genomics Center|Sekizuka|Japan
>Spike|hCoV-19/Germany/NW-HHU-453/2020|2020-10-26|EPI_ISL_733523|Original|hCoV-19^^North Rhine-Westphalia|Human|ZOTZ KLIMAS MVZ D\u00fcsseldorf-Centrum GbR \u00dcBAG f\u00fcr Labormedizin|Center of Medical Microbiology|Wienemann|Germany
>Spike|hCoV-19/India/MH-NIV-38793/2020|2020-05-22|EPI_ISL_479542|Original|hCoV-19^^Maharashtra|Human|NIV Influenza|NIV Influenza|Potdar|India
>Spike|hCoV-19/USA/UN-CDC-7498/2020|2020-04-22|EPI_ISL_514078|Original|hCoV-19^^USA|Human|Navy and Marine Corps Public Health Center|Pathogen Discovery|Queen|USA

>Spike|hCoV-19/India/GJ-GBRC160a/2020|2020-05-27|EPI_ISL_461491|Original|hCoV-19^^Gujarat|Human|B.J. Medical College and Civil hospital|Gujarat Biotechnology Research Centre|Joshi|India
>Spike|hCoV-19/USA/WA-UW-1904/2020|2020-03-23|EPI_ISL_424299|Original|hCoV-19^^Washington|Human|UW Virology Lab|UW Virology Lab|Roychoudhury|USA
>Spike|hCoV-19/England/LIVE-DCA6C7/2020|2020-12-07|EPI_ISL_724376|Original|hCoV-19^^England|Human|Liverpool Clinical Laboratories|COVID-19 Genomics UK (COG-UK) Consortium|Haldenby|United Kingdom
>Spike|hCoV-19/Japan/PG-6641/2020|2020-06-00|EPI_ISL_687753|Original|hCoV-19^^Japan|Human|Pathogen Genomics Center|Pathogen Genomics Center|Sekizuka|Japan
>Spike|hCoV-19/USA/TX-HMH-MCoV-14896/2020|2020-10-13|EPI_ISL_786380|Original|hCoV-19^^Texas|Human|Houston Methodist Hospital|Houston Methodist Hospital|Long|USA
>Spike|hCoV-19/Ireland/D-NVRL-MPW29674/2020|2020-04-07|EPI_ISL_767763|Original|hCoV-19^^Dublin|Human|National Virus Reference Laboratory|Irish Coronavirus Sequencing Consortium - Teagasc Moorepark|Walsh|Ireland
>Spike|hCoV-19/England/QEUH-A8B461/2020|2020-10-16|EPI_ISL_643167|Original|hCoV-19^^England|Human|Lighthouse Lab in Glasgow|Wellcome Sanger Institute for the COVID-19 Genomics UK (COG-UK) Consortium|Davies|United Kingdom
>Spike|hCoV-19/England/CAMC-BDD089/2020|2020-11-29|EPI_ISL_709869|Original|hCoV-19^^England|Human|Lighthouse Lab in Cambridge|Wellcome Sanger Institute for the COVID-19 Genomics UK (COG-UK) Consortium|Davies|United Kingdom
>Spike|hCoV-19/England/LOND-D6AE7/2020|2020-05-14|EPI_ISL_461771|Original|hCoV-19^^England|Human|University College London|COVID-19 Genomics UK (COG-UK) Consortium|Roy|United Kingdom
>Spike|hCoV-19/England/OXON-B07FB/2020|2020-04-21|EPI_ISL_478911|Original|hCoV-19^^England|Human|Oxford Viromics|COVID-19 Genomics UK (COG-UK) Consortium|Golubchik|United Kingdom
>Spike|hCoV-19/USA/TX-HMH-MCoV-15201/2020|2020-10-20|EPI_ISL_786685|Original|hCoV-19^^Texas|Human|Houston Methodist Hospital|Houston Methodist Hospital|Long|USA
>Spike|hCoV-19/env/Beijing/IVDC-03-06/2020|2020-06-11|EPI_ISL_469256|Original|hCoV-19^^Beijing|Environment|National Institute for Viral Disease Control and Prevention|National Institute for Viral Disease Control and Prevention|Tan|China
>Spike|hCoV-19/England/BRIS-1292DD/2020|2020-04-19|EPI_ISL_458784|Original|hCoV-19^^England|Human|PHE South West Regional Laboratory|Wellcome Sanger Institute for the COVID-19 Genomics UK (COG-UK) consortium|Davies|United Kingdom
>Spike|hCoV-19/England/CAMC-A82D8D/2020|2020-10-14|EPI_ISL_610763|Original|hCoV-19^^England|Human|Lighthouse Lab in Cambridge|Wellcome Sanger Institute for the COVID-19 Genomics UK (COG-UK) consortium|Davies|United Kingdom
>Spike|hCoV-19/England/ALDP-9BE581/2020|2020-09-10|EPI_ISL_568060|Original|hCoV-19^^England|Human|Lighthouse Lab in Alderley Park|Wellcome Sanger Institute for the COVID-19 Genomics UK (COG-UK) consortium|Davies|United Kingdom
>Spike|hCoV-19/Switzerland/SO-ETHZ-140079/2020|2020-04-22|EPI_ISL_476124|Original|hCoV-19^^Solothurn|Human|Viollier AG|Department of Biosystems Science and Engineering|Nadeau|Switzerland
>Spike|hCoV-19/Wales/PHWC-47F123/2020|2020-10-12|EPI_ISL_612632|Original|hCoV-19^^Wales|Human|Wales Specialist Virology Centre Sequencing lab|COVID-19 Genomics UK (COG-UK) Consortium|Moore|United Kingdom
>Spike|hCoV-19/Canada/BC_12251717/2020|2020-04-00|EPI_ISL_467334|Original|hCoV-19^^British Columbia|Human|BCCDC Public Health Laboratory|BCCDC Public Health Laboratory|Harrigan|Canada
>Spike|hCoV-19/South Africa/L00193/2020|2020-00-00|EPI_ISL_622956|Original|hCoV-19^^South Africa|Human|Lancet Laboratories|National Institute for Communicable Diseases of the National Health Laboratory Service|Allam|South Africa
>Spike|hCoV-19/Australia/VIC2447/2020|2020-06-24|EPI_ISL_522153|Original|hCoV-19^^Victoria|Human|Victorian Infectious Diseases Reference Laboratory (VIDRL)|VIDRL and MDU-PHL|Schultz|Australia
>Spike|hCoV-19/Belgium/rega-0731779/2020|2020-07-31|EPI_ISL_734830|Original|hCoV-19^^Deurne|Human|UZ Leuven|KU Leuven|Maes|Belgium

>Spike|hCoV-19/USA/WA-S1130/2020|2020-05-04|EPI_ISL_463620|Original|hCoV-19^^Washington|Human|Washington State Department of Health|Seattle Flu Study|Bedford|USA
>Spike|hCoV-19/USA/LA-ALSR-0076-NBG/2020|2020-03-31|EPI_ISL_434530|Original|hCoV-19^^Louisiana|Human|Robert Garry lab|Andersen lab at Scripps Research|Gangavarapu|USA
>Spike|hCoV-19/Canada/ON-S1938/2020|2020-11-24|EPI_ISL_755659|Original|hCoV-19^^Ontario|Human|Toronto Invasive Bacterial Diseases Network|McMaster University|McArthur|Canada
>Spike|hCoV-19/Australia/NSW2626/2020|2020-04-05|EPI_ISL_678323|Original|hCoV-19^^New South Wales|Human|Area of Virology|Virology Research Laboratory; Area of Virology|Foster|Australia
>Spike|hCoV-19/Wales/PHWC-490CB2/2020|2020-11-16|EPI_ISL_706558|Original|hCoV-19^^Wales|Human|Originating lab|COVID-19 Genomics UK (COG-UK) Consortium|Moore|United Kingdom
>Spike|hCoV-19/Bangladesh/CHRF-0012/2020|2020-05-05|EPI_ISL_477125|Original|hCoV-19^^Bangladesh|Human|Child Health Research Foundation|Child Health Research Foundation|Saha|Bangladesh
>Spike|hCoV-19/USA/TX-HMH-MCoV-18086/2020|2020-11-13|EPI_ISL_784434|Original|hCoV-19^^Texas|Human|Houston Methodist Hospital|Houston Methodist Hospital|Long|USA
>Spike|hCoV-19/USA/LA-EVTL1449/2020|2020-12-17|EPI_ISL_779115|Original|hCoV-19^^Louisiana|Human|LSUHS Emerging Viral Threat Laboratory|Microbial Genome Sequencing Center|Kamil|USA
>Spike|hCoV-19/Japan/PG-1701/2020|2020-04-00|EPI_ISL_685307|Original|hCoV-19^^Japan|Human|Niigata Prefectural Institute of Public Health and Environmental Sciences|Pathogen Genomics Center|Sekizuka|Japan
>Spike|hCoV-19/Vietnam/PIHCM-507-4H/2020|2020-03-25|EPI_ISL_654880|Vero E6|hCoV-19^^Ho Chi Minh city|Human|Pasteur Institute in Ho Chi Minh city|Department of Microbiology and Immunology - Pasteur Institute in Ho Chi Minh city|Vu|Viet Nam
>Spike|hCoV-19/USA/TX-HMH-6577/2020|2020-06-24|EPI_ISL_546098|Original|hCoV-19^^Texas|Human|Houston Methodist Hospital|Houston Methodist Hospital|Long|USA
>Spike|hCoV-19/Guangdong/2020XN4448-P0002/2020|2020-01-31|EPI_ISL_413857|Original|hCoV-19^^Guangdong|Human|Guangdong Provincial Institution of Public Health|Guangdong Provincial Institution of Public Health|Lu|China
>Spike|hCoV-19/Scotland/QEUH-9DC4F9/2020|2020-09-22|EPI_ISL_601125|Original|hCoV-19^^Scotland|Human|Lighthouse Lab in Glasgow|Wellcome Sanger Institute for the COVID-19 Genomics UK (COG-UK) consortium|Davies|United Kingdom
>Spike|hCoV-19/Luxembourg/LNS6034314/2020|2020-11-23|EPI_ISL_744342|Original|hCoV-19^^Luxembourg|Human|Laboratoire national de sant\u00e9|Laboratoire national de sant\u00e9|Wienecke-Baldacchino|Luxembourg
>Spike|hCoV-19/Netherlands/ZH-EMC-317/2020|2020-07-01|EPI_ISL_523659|Original|hCoV-19^^Zuid Holland|Human|Dutch COVID-19 response team|Erasmus Medical Center|Oude Munnink|Netherlands
>Spike|hCoV-19/USA/AZ-TG456496/2020|2020-06-26|EPI_ISL_695045|Original|hCoV-19^^Arizona|Human|TGen North|TGen North|French|USA
>Spike|hCoV-19/England/MILK-AC88F3/2020|2020-10-21|EPI_ISL_629678|Original|hCoV-19^^England|Human|Lighthouse Lab in Milton Keynes|Wellcome Sanger Institute for the COVID-19 Genomics UK (COG-UK) consortium|Davies|United Kingdom
>Spike|hCoV-19/Wales/PHWC-160410/2020|2020-05-21|EPI_ISL_472931|Original|hCoV-19^^Wales|Human|Wales Specialist Virology Centre Sequencing lab|COVID-19 Genomics UK (COG-UK) Consortium|Moore|United Kingdom
>Spike|hCoV-19/England/ALDP-CB3ECA/2020|2020-12-19|EPI_ISL_761275|Original|hCoV-19^^England|Human|Lighthouse Lab in Alderley Park|Wellcome Sanger Institute for the COVID-19 Genomics UK (COG-UK) Consortium|Davies|United Kingdom
>Spike|hCoV-19/England/204621947/2020|2020-11-10|EPI_ISL_700616|Original|hCoV-19^^England|Human|Respiratory Virus Unit|COVID-19 Genomics UK (COG-UK) Consortium|Platt|United Kingdom
>Spike|hCoV-19/England/ALDP-BC7D30/2020|2020-11-23|EPI_ISL_721235|Original|hCoV-19^^England|Human|Lighthouse Lab in Alderley Park|Wellcome Sanger Institute for the COVID-19 Genomics UK (COG-UK) Consortium|Davies|United Kingdom

>Spike|hCoV-19/Wales/PHWC-4A4B27/2020|2020-12-09|EPI_ISL_764138|Original|hCoV-19^^Wales|Human|Wales Specialist Virology Centre Sequencing lab|COVID-19 Genomics UK (COG-UK) Consortium|Moore|United Kingdom
>Spike|hCoV-19/Denmark/DCGC-20928/2020|2020-12-21|EPI_ISL_758785|Original|hCoV-19^^Hovedstaden|Human|Department of Virus and Microbiological Special Diagnostics|Albertsen Lab|Michaelsen|Denmark
>Spike|hCoV-19/USA/WA-UW-2961/2020|2020-03-26|EPI_ISL_486061|Original|hCoV-19^^Washington|Human|UW Virology Lab|UW Virology Lab|Roychoudhury|USA
>Spike|hCoV-19/England/205020748/2020|2020-12-07|EPI_ISL_723039|Original|hCoV-19^^England|Human|Respiratory Virus Unit|COVID-19 Genomics UK (COG-UK) Consortium|Platt|United Kingdom
>Spike|hCoV-19/Wales/PHWC-48065D/2020|2020-09-27|EPI_ISL_611631|Original|hCoV-19^^Wales|Human|Wales Specialist Virology Centre Sequencing lab|COVID-19 Genomics UK (COG-UK) Consortium|Moore|United Kingdom
>Spike|hCoV-19/England/ALDP-B83A1B/2020|2020-11-14|EPI_ISL_674243|Original|hCoV-19^^England|Human|Lighthouse Lab in Alderley Park|Wellcome Sanger Institute for the COVID-19 Genomics UK (COG-UK) Consortium|Davies|United Kingdom
>Spike|hCoV-19/England/PORT-2D26C7/2020|2020-03-27|EPI_ISL_514493|Original|hCoV-19^^England|Human|Centre for Enzyme Innovation|COVID-19 Genomics UK (COG-UK) Consortium|Robson|United Kingdom
>Spike|hCoV-19/Australia/VIC1486/2020|2020-04-25|EPI_ISL_456455|Original|hCoV-19^^Victoria|Human|Victorian Infectious Diseases Reference Laboratory (VIDRL)|Microbiological Diagnostic Unit Public Health Laboratory and Victorian Infectious Diseases Reference Laboratory|Schultz|Australia
>Spike|hCoV-19/Scotland/EDB5641/2020|2020-03-25|EPI_ISL_487685|Original|hCoV-19^^Scotland|Human|Virology Department|Wellcome Sanger Institute for the COVID-19 Genomics UK (COG-UK) consortium|Davies|United Kingdom
>Spike|hCoV-19/Australia/VIC16055/2020|2020-08-04|EPI_ISL_640461|Original|hCoV-19^^Victoria|Human|Microbiological Diagnostic Unit - Public Health Laboratory (MDU-PHL)|MDU-PHL|Schultz|Australia
>Spike|hCoV-19/Canada/BC_20363111/2020|2020-03-00|EPI_ISL_462772|Original|hCoV-19^^British Columbia|Human|BCCDC Public Health Laboratory|BCCDC Public Health Laboratory|Harrigan|Canada
>Spike|hCoV-19/France/ARA-15296/2020|2020-03-27|EPI_ISL_508903|Original|hCoV-19^^Auvergne-Rh\u00f4ne-Alpes|Human|Institut des Agents Infectieux (IAI)|CNR Virus des Infections Respiratoires - France SUD|Josset|France
>Spike|hCoV-19/England/SHEF-C96F2/2020|2020-05-07|EPI_ISL_475422|Original|hCoV-19^^England|Human|Virology Department|COVID-19 Genomics UK (COG-UK) Consortium|de Silva|United Kingdom
>Spike|hCoV-19/USA/CA-CZB-11125/2020|2020-05-30|EPI_ISL_625516|Original|hCoV-19^^California|Human|Alameda County Public Health Lab|Chan-Zuckerberg Biohub|Bhatt|USA
>Spike|hCoV-19/Peru/LIM-UPCH-0083/2020|2020-06-18|EPI_ISL_540961|Original|hCoV-19^^Lima|Human|Laboratorio de Referencia Nacional de Virus Respiratorios|Laboratorio de Gen\u00f3mica Microbiana|Tsukayama|Peru
>Spike|hCoV-19/Denmark/DCGC-16571/2020|2020-11-16|EPI_ISL_714844|Original|hCoV-19^^Hovedstaden|Human|Department of Virus and Microbiological Special Diagnostics|Albertsen Lab|Michaelsen|Denmark
>Spike|hCoV-19/Mexico/SON-InDRE_35/2020|2020-05-15|EPI_ISL_516622|Original|hCoV-19^^Sonora|Human|Instituto de Diagnostico y Referencia Epidemiologicos (INDRE)|Instituto de Diagnostico y Referencia Epidemiologicos (INDRE)|Rodriguez|Mexico
>Spike|hCoV-19/Netherlands/ZuidHolland_154/2020|2020-04-28|EPI_ISL_461327|Original|hCoV-19^^Zuid Holland|Human|Dutch COVID-19 response team|Erasmus Medical Center|Oude Munnink|Netherlands
>Spike|hCoV-19/Egypt/NRC-01/2020|2020-03-18|EPI_ISL_430820|Original|hCoV-19^^Egypt|Human|Center of Scientific Excellence for Influenza Viruses|Center of Scientific Excellence for Influenza Viruses|Kandeil|Egypt

>Spike|hCoV-19/England/EXET-13F447/2020|2020-12-17|EPI_ISL_764185|Original|hCoV-19^^England|Human|University of Exeter|COVID-19 Genomics UK (COG-UK) Consortium|Temperton|United Kingdom
>Spike|hCoV-19/USA/TX-HMH-MCoV-16305/2020|2020-10-31|EPI_ISL_785989|Original|hCoV-19^^Texas|Human|Houston Methodist Hospital|Houston Methodist Hospital|Long|USA
>Spike|hCoV-19/England/ALDP-A84B45/2020|2020-10-18|EPI_ISL_610335|Original|hCoV-19^^England|Human|Lighthouse Lab in Alderley Park|Wellcome Sanger Institute for the COVID-19 Genomics UK (COG-UK) consortium|Davies|United Kingdom
>Spike|hCoV-19/India/MH-ACTREC-269/2020|2020-07-09|EPI_ISL_699921|Original|hCoV-19^^Maharashtra|Human|Hematopathology Laboratory|Hematopathology Laboratory|Patkar|India
>Spike|hCoV-19/Scotland/CVR58/2020|2020-03-12|EPI_ISL_425788|Original|hCoV-19^^Scotland|Human|West of Scotland Specialist Virology Centre|COVID-19 Genomics UK (COG-UK) Consortium|Robertson|United Kingdom
>Spike|hCoV-19/England/ALDP-9CDDB4/2020|2020-09-16|EPI_ISL_566412|Original|hCoV-19^^England|Human|Lighthouse Lab in Alderley Park|Wellcome Sanger Institute for the COVID-19 Genomics UK (COG-UK) consortium|Davies|United Kingdom
>Spike|hCoV-19/Germany/BY-IMB07597/2020|2020-06-16|EPI_ISL_732537|Original|hCoV-19^^Bavaria|Human|Bundeswehr Institute of Microbiology|Bundeswehr Institute of Microbiology|Antwerpen|Germany
>Spike|hCoV-19/USA/NM-UNM-00043/2020|2020-03-18|EPI_ISL_467624|Original|hCoV-19^^New Mexico|Human|New Mexico Department of Health Scientific Laboratory Division|Center for Global Health|Domman|USA
>Spike|hCoV-19/England/QEUH-96FABD/2020|2020-08-17|EPI_ISL_540290|Original|hCoV-19^^England|Human|Lighthouse Lab in Glasgow|Wellcome Sanger Institute for the COVID-19 Genomics UK (COG-UK) consortium|Davies|United Kingdom
>Spike|hCoV-19/Sweden/20-22304/2020|2020-04-02|EPI_ISL_660400|Original|hCoV-19^^Ostergotland|Human|Klinsisk mikrobiologi Linkoping|The Public Health Agency of Sweden|Svartstr\u00f6m|Sweden
>Spike|hCoV-19/England/QEUH-9F624A/2020|2020-09-25|EPI_ISL_588777|Original|hCoV-19^^England|Human|Lighthouse Lab in Glasgow|Wellcome Sanger Institute for the COVID-19 Genomics UK (COG-UK) consortium|Davies|United Kingdom
>Spike|hCoV-19/USA/MD-MDH-0387/2020|2020-10-26|EPI_ISL_660105|Original|hCoV-19^^Maryland|Human|MD PHL|MD PHL|Keller|USA
>Spike|hCoV-19/USA/WA-UW-5714/2020|2020-04-06|EPI_ISL_461439|Original|hCoV-19^^Washington|Human|UW Virology Lab|UW Virology Lab|Roychoudhury|USA
>Spike|hCoV-19/England/MILK-C2FDAE/2020|2020-12-07|EPI_ISL_782933|Original|hCoV-19^^England|Human|Lighthouse Lab in Milton Keynes|Wellcome Sanger Institute for the COVID-19 Genomics UK (COG-UK) Consortium|Davies|United Kingdom
>Spike|hCoV-19/England/QEUH-9CEC2F/2020|2020-09-17|EPI_ISL_590041|Original|hCoV-19^^England|Human|Lighthouse Lab in Glasgow|Wellcome Sanger Institute for the COVID-19 Genomics UK (COG-UK) consortium|Davies|United Kingdom
>Spike|hCoV-19/Australia/VIC2671/2020|2020-07-02|EPI_ISL_521513|Original|hCoV-19^^Victoria|Human|Victorian Infectious Diseases Reference Laboratory (VIDRL)|VIDRL and MDU-PHL|Schultz|Australia
>Spike|hCoV-19/England/LIVE-DB41CF/2020|2020-10-19|EPI_ISL_652082|Original|hCoV-19^^England|Human|Liverpool Clinical Laboratories|COVID-19 Genomics UK (COG-UK) Consortium|Haldenby|United Kingdom
>Spike|hCoV-19/USA/TX-HMH-MCoV-14406/2020|2020-10-07|EPI_ISL_787691|Original|hCoV-19^^Texas|Human|Houston Methodist Hospital|Houston Methodist Hospital|Long|USA
>Spike|hCoV-19/England/ALDP-982A78/2020|2020-08-09|EPI_ISL_552468|Original|hCoV-19^^England|Human|Lighthouse Lab in Alderley Park|Wellcome Sanger Institute for the COVID-19 Genomics UK (COG-UK) consortium|Davies|United Kingdom
>Spike|hCoV-19/Wales/PHWC-4A1225/2020|2020-12-09|EPI_ISL_743640|Original|hCoV-19^^Wales|Human|Wales Specialist Virology Centre Sequencing lab|COVID-19 Genomics UK (COG-UK) Consortium|Moore|United Kingdom
>Spike|hCoV-19/USA/MN-MDH-1629/2020|2020-08-19|EPI_ISL_534217|Original|hCoV-19^^Minnesota|Human|M Health Fairview|Minnesota Department of Health|Garfin|USA

>Spike|hCoV-19/South Africa/KRISP-K002243/2020|2020-07-21|EPI_ISL_515634|Original|hCoV-19^^KwaZulu-Natal|Human|NHLS-IALCH|KRISP|de Oliveira|South Africa
>Spike|hCoV-19/Northern Ireland/NIRE-FF31A/2020|2020-04-25|EPI_ISL_469849|Original|hCoV-19^^Northern Ireland|Human|Regional Virus Laboratory|Wellcome Sanger Institute for the COVID-19 Genomics UK (COG-UK) consortium|Davies|United Kingdom
>Spike|hCoV-19/England/NORT-29F67B/2020|2020-04-29|EPI_ISL_490493|Original|hCoV-19^^England|Human|Northumbria University / South Tees Hospitals NHS Foundation Trust / North Cumbria Integrated Care NHS Foundation Trust / North Tees and Hartlepool NHS Foundation Trust / Newcastle Hospitals NHS Foundation Trust|COVID-19 Genomics UK (COG-UK) Consortium|Bashton|United Kingdom
>Spike|hCoV-19/Luxembourg/LNS0694152/2020|2020-11-02|EPI_ISL_739817|Original|hCoV-19^^Luxembourg|Human|Laboratoire national de sant\u00e9|Laboratoire national de sant\u00e9|Wienecke-Baldacchino|Luxembourg
>Spike|hCoV-19/Indonesia/JI-ITD-17398NT/2020|2020-07-18|EPI_ISL_529963|Original|hCoV-19^^East Java|Human|Universitas Airlangga Hospital|Institute of Tropical Disease|Lusida|Indonesia
>Spike|hCoV-19/Luxembourg/LNS2589258/2020|2020-11-28|EPI_ISL_740148|Original|hCoV-19^^Luxembourg|Human|Laboratoire national de sant\u00e9|Laboratoire national de sant\u00e9|Wienecke-Baldacchino|Luxembourg
>Spike|hCoV-19/Portugal/PT1361/2020|2020-03-24|EPI_ISL_511320|Original|hCoV-19^^Portugal|Human|Instituto Nacional de Saude (INSA)|Instituto Nacional de Saude (INSA)|Borges|Portugal
>Spike|hCoV-19/Denmark/DCGC-18437/2020|2020-12-07|EPI_ISL_749236|Original|hCoV-19^^Hovedstaden|Human|Department of Virus and Microbiological Special Diagnostics|Albertsen Lab|Michaelsen|Denmark
>Spike|hCoV-19/Denmark/DCGC-4903/2020|2020-09-21|EPI_ISL_621441|Original|hCoV-19^^Syddanmark|Human|Department of Virus and Microbiological Special Diagnostics|Albertsen lab|Michaelsen|Denmark
>Spike|hCoV-19/England/20144023804/2020|2020-03-26|EPI_ISL_423528|Original|hCoV-19^^England|Human|Respiratory Virus Unit|Respiratory Virus Unit|Platt|United Kingdom
>Spike|hCoV-19/England/MILK-6F98DF/2020|2020-06-30|EPI_ISL_557478|Original|hCoV-19^^England|Human|Lighthouse Lab in Milton Keynes|Wellcome Sanger Institute for the COVID-19 Genomics UK (COG-UK) consortium|Davies|United Kingdom
>Spike|hCoV-19/Scotland/QEUH-9F4464/2020|2020-09-27|EPI_ISL_600687|Original|hCoV-19^^Scotland|Human|Lighthouse Lab in Glasgow|Wellcome Sanger Institute for the COVID-19 Genomics UK (COG-UK) consortium|Davies|United Kingdom
>Spike|hCoV-19/England/EXET-139D15/2020|2020-00-00|EPI_ISL_679337|Original|hCoV-19^^England|Human|University of Exeter|COVID-19 Genomics UK (COG-UK) Consortium|Temperton|United Kingdom
>Spike|hCoV-19/USA/TX-HMH-MCoV-19560/2020|2020-11-17|EPI_ISL_783932|Original|hCoV-19^^Texas|Human|Houston Methodist Hospital|Houston Methodist Hospital|Long|USA
>Spike|hCoV-19/Denmark/DCGC-17474/2020|2020-12-14|EPI_ISL_751005|Original|hCoV-19^^Nordjylland|Human|Department of Virus and Microbiological Special Diagnostics|Albertsen Lab|Michaelsen|Denmark
>Spike|hCoV-19/Scotland/CVR5643/2020|2020-11-08|EPI_ISL_651885|Original|hCoV-19^^Scotland|Human|West of Scotland Specialist Virology Centre|COVID-19 Genomics UK (COG-UK) Consortium|Robertson|United Kingdom
>Spike|hCoV-19/Japan/PG-4875/2020|2020-08-00|EPI_ISL_689996|Original|hCoV-19^^Japan|Human|Pathogen Genomics Center|Pathogen Genomics Center|Sekizuka|Japan
>Spike|hCoV-19/England/CAMC-B37E38/2020|2020-11-05|EPI_ISL_658948|Original|hCoV-19^^England|Human|Lighthouse Lab in Cambridge|Wellcome Sanger Institute for the COVID-19 Genomics UK (COG-UK) Consortium|Davies|United Kingdom
>Spike|hCoV-19/England/CAMC-B524CC/2020|2020-11-08|EPI_ISL_656897|Original|hCoV-19^^England|Human|Lighthouse Lab in Cambridge|Wellcome Sanger Institute for the COVID-19 Genomics UK (COG-UK) Consortium|Davies|United Kingdom
>Spike|hCoV-19/England/ALDP-9EE02D/2020|2020-05-08|EPI_ISL_589320|Original|hCoV-19^^England|Human|Lighthouse Lab in Alderley Park|Wellcome Sanger Institute for the COVID-19 Genomics UK (COG-UK) consortium|Davies|United Kingdom

>Spike|hCoV-19/England/MILK-9AAC65/2020|2020-09-01|EPI_ISL_550885|Original|hCoV-19^^England|Human|Lighthouse Lab in Milton Keynes|Wellcome Sanger Institute for the COVID-19 Genomics UK (COG-UK) consortium|Davies|United Kingdom
>Spike|hCoV-19/England/20126026506/2020|2020-03-18|EPI_ISL_423894|Original|hCoV-19^^England|Human|Respiratory Virus Unit|Respiratory Virus Unit|Platt|United Kingdom
>Spike|hCoV-19/Switzerland/VS-ETHZ-420692/2020|2020-12-24|EPI_ISL_768243|Original|hCoV-19^^Valais|Human|Viollier AG|Department of Biosystems Science and Engineering|Nadeau|Switzerland
>Spike|hCoV-19/United Arab Emirates/0392/2020|2020-05-15|EPI_ISL_698567|Original|hCoV-19^^Abu Dhabi|Human|Group 42 (G42) Healthcare|G42 Healthcare|Ogrodzki|United Arab Emirates
>Spike|hCoV-19/England/CAMC-BD1F5B/2020|2020-11-28|EPI_ISL_702257|Original|hCoV-19^^England|Human|Lighthouse Lab in Cambridge|Wellcome Sanger Institute for the COVID-19 Genomics UK (COG-UK) Consortium|Davies|United Kingdom
>Spike|hCoV-19/Lithuania/C20-05-R5/2020|2020-05-18|EPI_ISL_560401|Original|hCoV-19^^Vilnius r.|Human|Vilnius University Hospital Santaros Klinikos|Institute of Biotechnology|Timinskas|Lithuania
>Spike|hCoV-19/Spain/CT-IBV-005558/2020|2020-03-18|EPI_ISL_508619|Original|hCoV-19^^Catalunya|Human|Departamento de Microbiolog\u00eda|SeqCOVID-SPAIN consortium/IBV(CSIC)|Comas|Spain
>Spike|hCoV-19/Denmark/DCGC-7661/2020|2020-10-19|EPI_ISL_618607|Original|hCoV-19^^Hovedstaden|Human|Department of Virus and Microbiological Special Diagnostics|Albertsen lab|Michaelsen|Denmark
>Spike|hCoV-19/England/MILK-A46329/2020|2020-10-04|EPI_ISL_598768|Original|hCoV-19^^England|Human|Lighthouse Lab in Milton Keynes|Wellcome Sanger Institute for the COVID-19 Genomics UK (COG-UK) consortium|Davies|United Kingdom
>Spike|hCoV-19/Bahrein/BAH-20/2020|2020-04-08|EPI_ISL_483561|Original|hCoV-19^^Bahrain|Human|Kingdom of Bahrein Ministry of Health|Erasmus Medical Center|Oude Munnink|Bahrain
>Spike|hCoV-19/Australia/VIC4023/2020|2020-07-10|EPI_ISL_519409|Original|hCoV-19^^Victoria|Human|Microbiological Diagnostic Unit - Public Health Laboratory (MDU-PHL)|MDU-PHL|Schultz|Australia
>Spike|hCoV-19/USA/FL-BPHL-1049/2020|2020-07-31|EPI_ISL_517939|Original|hCoV-19^^Florida|Human|Florida Bureau of Public Health Laboratories|Florida Bureau of Public Health Laboratories|Blanton|USA
>Spike|hCoV-19/England/MILK-A7D453/2020|2020-10-13|EPI_ISL_606391|Original|hCoV-19^^England|Human|Lighthouse Lab in Milton Keynes|Wellcome Sanger Institute for the COVID-19 Genomics UK (COG-UK) consortium|Davies|United Kingdom
>Spike|hCoV-19/Luxembourg/LNS6090235/2020|2020-10-14|EPI_ISL_744357|Original|hCoV-19^^Luxembourg|Human|Laboratoire national de sant\u00e9|Laboratoire national de sant\u00e9|Wienecke-Baldacchino|Luxembourg
>Spike|hCoV-19/Denmark/DCGC-1035/2020|2020-03-09|EPI_ISL_614569|Original|hCoV-19^^Sjaelland|Human|Department of Virus and Microbiological Special Diagnostics|Albertsen lab|Michaelsen|Denmark
>Spike|hCoV-19/USA/CA-CZB-14899/2020|2020-11-25|EPI_ISL_739013|Original|hCoV-19^^California|Human|Orange County Public Health Lab|Chan-Zuckerberg Biohub|Kamm|USA
>Spike|hCoV-19/England/ALDP-9FD28F/2020|2020-10-02|EPI_ISL_587747|Original|hCoV-19^^England|Human|Lighthouse Lab in Alderley Park|Wellcome Sanger Institute for the COVID-19 Genomics UK (COG-UK) consortium|Davies|United Kingdom
>Spike|hCoV-19/England/QEUH-B2CA23/2020|2020-11-01|EPI_ISL_646534|Original|hCoV-19^^England|Human|Lighthouse Lab in Glasgow|Wellcome Sanger Institute for the COVID-19 Genomics UK (COG-UK) Consortium|Davies|United Kingdom
>Spike|hCoV-19/Brazil/SP-146/2020|2020-03-26|EPI_ISL_468311|Original|hCoV-19^^S\u00e3o Paulo|Human|Hospital Municipal Dr Ignacio Proenca de Gouvea|Instituto Adolfo Lutz|Sacchi|Brazil
>Spike|hCoV-19/England/QEUH-79AFE9/2020|2020-07-15|EPI_ISL_533158|Original|hCoV-19^^England|Human|Lighthouse Lab in Glasgow|Wellcome Sanger Institute for the COVID-19 Genomics UK (COG-UK) consortium|Davies|United Kingdom

>Spike|hCoV-19/Belgium/ULG-10963/2020|2020-09-08|EPI_ISL_707742|Original|hCoV-19^^Li\u00e8ge|Human|Department of Clinical Microbiology|GIGA Medical Genomics|Durkin|Belgium
>Spike|hCoV-19/Malaysia/UNIMAS-C121/2020|2020-05-29|EPI_ISL_718135|Original|hCoV-19^^Sarawak|Human|Ministry of Health Hospitals|Institute of Health and Community Medicine|Jonathan|Malaysia
>Spike|hCoV-19/USA/WA-UW-3003/2020|2020-03-27|EPI_ISL_486074|Original|hCoV-19^^Washington|Human|UW Virology Lab|UW Virology Lab|Roychoudhury|USA
>Spike|hCoV-19/USA/WI-UW-1339/2020|2020-09-07|EPI_ISL_536710|Original|hCoV-19^^Wisconsin|Human|University of Wisconsin-Madison AIDS Vaccine Research Laboratories|University of Wisconsin-Madison AIDS Vaccine Research Laboratories|Baczenas|USA
>Spike|hCoV-19/Japan/PG-4611/2020|2020-08-00|EPI_ISL_689748|Original|hCoV-19^^Japan|Human|Pathogen Genomics Center|Pathogen Genomics Center|Sekizuka|Japan
>Spike|hCoV-19/England/NORW-EE439/2020|2020-04-24|EPI_ISL_490542|Original|hCoV-19^^England|Human|Quadram Institute Bioscience|COVID-19 Genomics UK (COG-UK) Consortium|Alikhan|United Kingdom
>Spike|hCoV-19/USA/UT-08466/2020|2020-05-19|EPI_ISL_524237|Original|hCoV-19^^Utah|Human|Utah Public Health Laboratory|Utah Public Health Laboratory|Young|USA
>Spike|hCoV-19/United Arab Emirates/44/2020|2020-05-11|EPI_ISL_528701|Original|hCoV-19^^Abu Dhabi|Human|Alsafar - Khalifa University Abu Dhabi|Alsafar - Khalifa University Abu Dhabi|Daw Elbait|United Arab Emirates
>Spike|hCoV-19/Wales/PHWC-4A3320/2020|2020-12-14|EPI_ISL_744088|Original|hCoV-19^^Wales|Human|Wales Specialist Virology Centre Sequencing lab|COVID-19 Genomics UK (COG-UK) Consortium|Moore|United Kingdom
>Spike|hCoV-19/Scotland/CVR3184/2020|2020-04-28|EPI_ISL_449208|Original|hCoV-19^^Scotland|Human|West of Scotland Specialist Virology Centre|COVID-19 Genomics UK (COG-UK) Consortium|Robertson|United Kingdom
>Spike|hCoV-19/Wales/PHWC-49A1C8/2020|2020-12-01|EPI_ISL_727507|Original|hCoV-19^^Wales|Human|Originating lab|COVID-19 Genomics UK (COG-UK) Consortium|Moore|United Kingdom
>Spike|hCoV-19/England/MILK-9AA46D/2020|2020-09-01|EPI_ISL_550807|Original|hCoV-19^^England|Human|Lighthouse Lab in Milton Keynes|Wellcome Sanger Institute for the COVID-19 Genomics UK (COG-UK) consortium|Davies|United Kingdom
>Spike|hCoV-19/USA/WA-S1975/2020|2020-06-03|EPI_ISL_497403|Original|hCoV-19^^Washington|Human|Washington State Department of Health|Seattle Flu Study|Lee|USA
>Spike|hCoV-19/South Korea/KCDC2356/2020|2020-05-28|EPI_ISL_514759|Original|hCoV-19^^South Korea|Human|Division of Viral Diseases|Division of Viral Diseases|Kim|South Korea
>Spike|hCoV-19/USA/AZ-TG379342/2020|2020-06-16|EPI_ISL_694636|Original|hCoV-19^^Arizona|Human|TGen North|TGen North|French|USA
>Spike|hCoV-19/USA/TX-HMH-1424/2020|2020-04-29|EPI_ISL_542715|Original|hCoV-19^^Texas|Human|Houston Methodist Hospital|Houston Methodist Hospital|Long|USA
>Spike|hCoV-19/England/CAMC-BBC545/2020|2020-11-21|EPI_ISL_724152|Original|hCoV-19^^England|Human|Quadram Institute Bioscience|COVID-19 Genomics UK (COG-UK) Consortium|Alikhan|United Kingdom
>Spike|hCoV-19/Denmark/DCGC-13156/2020|2020-11-30|EPI_ISL_712621|Original|hCoV-19^^Syddanmark|Human|Department of Virus and Microbiological Special Diagnostics|Albertsen Lab|Michaelsen|Denmark
>Spike|hCoV-19/Denmark/DCGC-13878/2020|2020-11-30|EPI_ISL_713078|Original|hCoV-19^^Hovedstaden|Human|Department of Virus and Microbiological Special Diagnostics|Albertsen Lab|Michaelsen|Denmark
>Spike|hCoV-19/USA/GA-CDC-6427/2020|2020-07-29|EPI_ISL_527662|Original|hCoV-19^^Georgia|Human|GA Department of Public Health Laboratory|Pathogen Discovery|Queen|USA
>Spike|hCoV-19/England/ALDP-9C536F/2020|2020-09-10|EPI_ISL_567622|Original|hCoV-19^^England|Human|Lighthouse Lab in Alderley Park|Wellcome Sanger Institute for the COVID-19 Genomics UK (COG-UK) consortium|Davies|United Kingdom

>Spike|hCoV-19/Wales/PHWC-48C9C1/2020|2020-11-04|EPI_ISL_666491|Original|hCoV-19^^Wales|Human|Wales Specialist Virology Centre Sequencing lab|COVID-19 Genomics UK (COG-UK) Consortium|Moore|United Kingdom
>Spike|hCoV-19/USA/WI-WSLH-200383/2020|2020-04-22|EPI_ISL_578550|Original|hCoV-19^^Wisconsin|Human|Wisconsin State Laboratory of Hygiene Communicable Disease Division|Wisconsin State Laboratory of Hygiene Communicable Disease Division|Florek|USA
>Spike|hCoV-19/Japan/PG-3676/2020|2020-04-00|EPI_ISL_691929|Original|hCoV-19^^Japan|Human|Pathogen Genomics Center|Pathogen Genomics Center|Sekizuka|Japan
>Spike|hCoV-19/England/ALDP-A957A7/2020|2020-10-20|EPI_ISL_624580|Original|hCoV-19^^England|Human|Lighthouse Lab in Alderley Park|Wellcome Sanger Institute for the COVID-19 Genomics UK (COG-UK) consortium|Davies|United Kingdom
>Spike|hCoV-19/Denmark/DCGC-14413/2020|2020-11-30|EPI_ISL_711129|Original|hCoV-19^^Hovedstaden|Human|Department of Virus and Microbiological Special Diagnostics|Albertsen Lab|Michaelsen|Denmark
>Spike|hCoV-19/England/ALDP-94A99D/2020|2020-06-11|EPI_ISL_555817|Original|hCoV-19^^England|Human|Lighthouse Lab in Alderley Park|Wellcome Sanger Institute for the COVID-19 Genomics UK (COG-UK) consortium|Davies|United Kingdom
>Spike|hCoV-19/Denmark/DCGC-12506/2020|2020-11-16|EPI_ISL_682687|Original|hCoV-19^^Hovedstaden|Human|Department of Virus and Microbiological Special Diagnostics|Albertsen Lab|Michaelsen|Denmark
>Spike|hCoV-19/England/NOTT-110109/2020|2020-04-10|EPI_ISL_441472|Original|hCoV-19^^England|Human|Queens Medical Centre|COVID-19 Genomics UK (COG-UK) Consortium|Loose|United Kingdom
>Spike|hCoV-19/USA/TX-HMH-MCoV-18262/2020|2020-11-17|EPI_ISL_784610|Original|hCoV-19^^Texas|Human|Houston Methodist Hospital|Houston Methodist Hospital|Long|USA
>Spike|hCoV-19/Denmark/DCGC-10671/2020|2020-11-02|EPI_ISL_670125|Original|hCoV-19^^Midtjylland|Human|Department of Virus and Microbiological Special Diagnostics|Albertsen Lab|Michaelsen|Denmark
>Spike|hCoV-19/England/204441569/2020|2020-10-00|EPI_ISL_622892|Original|hCoV-19^^England|Human|Respiratory Virus Unit|Respiratory Virus Unit|Platt|United Kingdom
>Spike|hCoV-19/England/QEUH-B3FB84/2020|2020-11-08|EPI_ISL_658678|Original|hCoV-19^^England|Human|Lighthouse Lab in Glasgow|Wellcome Sanger Institute for the COVID-19 Genomics UK (COG-UK) Consortium|Davies|United Kingdom
>Spike|hCoV-19/USA/NY-NYCPHL-000671/2020|2020-03-18|EPI_ISL_632089|Original|hCoV-19^^New York|Human|Brookdale University Hospital Medical Center|New York City Public Health Laboratory|Wang|USA
>Spike|hCoV-19/England/CAMC-9450AC/2020|2020-07-16|EPI_ISL_556947|Original|hCoV-19^^England|Human|Lighthouse Lab in Cambridge|Wellcome Sanger Institute for the COVID-19 Genomics UK (COG-UK) consortium|Davies|United Kingdom
>Spike|hCoV-19/USA/CA-ALSR-4206/2020|2020-08-06|EPI_ISL_648886|Original|hCoV-19^^California|Human|San Diego County Public Health Laboratory|Andersen lab at Scripps Research|Gangavarapu|USA
>Spike|hCoV-19/South Africa/KRISP-K004602/2020|2020-11-06|EPI_ISL_660631|Original|hCoV-19^^KwaZulu-Natal|Human|NHLS-IALCH|KRISP|de Oliveira|South Africa
>Spike|hCoV-19/USA/AZ-TG666092/2020|2020-05-02|EPI_ISL_696182|Original|hCoV-19^^Arizona|Human|Sonora Quest Laboratories|TGen North|French|USA
>Spike|hCoV-19/England/MILK-B02197/2020|2020-10-24|EPI_ISL_642948|Original|hCoV-19^^England|Human|Lighthouse Lab in Milton Keynes|Wellcome Sanger Institute for the COVID-19 Genomics UK (COG-UK) Consortium|Davies|United Kingdom
>Spike|hCoV-19/Japan/PG-7898/2020|2020-07-00|EPI_ISL_686999|Original|hCoV-19^^Japan|Human|Pathogen Genomics Center|Pathogen Genomics Center|Sekizuka|Japan
>Spike|hCoV-19/Denmark/ALAB-SSI-1256/2020|2020-03-11|EPI_ISL_444830|Original|hCoV-19^^Denmark|Human|Department of Virus and Microbiological Special Diagnostics|Albertsen lab|Kirkegaard|Denmark
>Spike|hCoV-19/Australia/VIC6113/2020|2020-07-22|EPI_ISL_518132|Original|hCoV-19^^Victoria|Human|Victorian Infectious Diseases Reference Laboratory (VIDRL)|VIDRL and MDU-PHL|Schultz|Australia

>Spike|hCoV-19/England/CAMC-B219F4/2020|2020-11-02|EPI_ISL_647459|Original|hCoV-19^^England|Human|Lighthouse Lab in Cambridge|Wellcome Sanger Institute for the COVID-19 Genomics UK (COG-UK) Consortium|Davies|United Kingdom
>Spike|hCoV-19/USA/LA-EVTL447/2020|2020-06-19|EPI_ISL_578851|Original|hCoV-19^^Louisiana|Human|LSUHS Emerging Viral Threat Laboratory|Microbial Genome Sequencing Center|Kamil|USA
>Spike|hCoV-19/Netherlands/ZuidHolland_76/2020|2020-03-26|EPI_ISL_422944|Original|hCoV-19^^Zuid Holland|Human|Dutch COVID-19 response team|Erasmus Medical Center|Oude Munnink|Netherlands
>Spike|hCoV-19/France/PAC-IHU-0116/2020|2020-03-24|EPI_ISL_569316|Original|hCoV-19^^Provence-Alpes-C\u00f4te-d\u2019Azur|Human|MEPHI|MEPHI|Levasseur|France
>Spike|hCoV-19/Northern Ireland/QEUH-96C4B2/2020|2020-08-13|EPI_ISL_530833|Original|hCoV-19^^Northern Ireland|Human|Lighthouse Lab in Glasgow|Wellcome Sanger Institute for the COVID-19 Genomics UK (COG-UK) consortium|Davies|United Kingdom
>Spike|hCoV-19/England/SHEF-C1144/2020|2020-10-28|EPI_ISL_637297|Original|hCoV-19^^England|Human|Virology Department|COVID-19 Genomics UK (COG-UK) Consortium|de Silva|United Kingdom
>Spike|hCoV-19/England/BIRM-638F4/2020|2020-08-21|EPI_ISL_529653|Original|hCoV-19^^England|Human|University of Birmingham|COVID-19 Genomics UK (COG-UK) Consortium|Loman|United Kingdom
>Spike|hCoV-19/England/BRIS-12564E/2020|2020-03-27|EPI_ISL_443822|Original|hCoV-19^^England|Human|PHE South West Regional Laboratory|Wellcome Sanger Institute for the COVID-19 Genomics UK (COG-UK) consortium|Amato|United Kingdom
>Spike|hCoV-19/Japan/IC-0194/2020|2020-07-00|EPI_ISL_667623|Original|hCoV-19^^Japan|Human|Pathogen Genomics Center|Pathogen Genomics Center|Sekizuka|Japan
>Spike|hCoV-19/England/CAMC-A58CDE/2020|2020-10-08|EPI_ISL_597182|Original|hCoV-19^^England|Human|Lighthouse Lab in Cambridge|Wellcome Sanger Institute for the COVID-19 Genomics UK (COG-UK) consortium|Davies|United Kingdom
>Spike|hCoV-19/USA/AZ-TG670113/2020|2020-05-02|EPI_ISL_696331|Original|hCoV-19^^Arizona|Human|Sonora Quest Laboratories|TGen North|French|USA
>Spike|hCoV-19/USA/TX-HMH-3500/2020|2020-06-15|EPI_ISL_544203|Original|hCoV-19^^Texas|Human|Houston Methodist Hospital|Houston Methodist Hospital|Long|USA
>Spike|hCoV-19/England/PORT-2E952A/2020|2020-10-14|EPI_ISL_727709|Original|hCoV-19^^England|Human|Centre for Enzyme Innovation|COVID-19 Genomics UK (COG-UK) Consortium|Robson|United Kingdom
>Spike|hCoV-19/USA/NY-NYCPHL-001474/2020|2020-11-27|EPI_ISL_683768|Original|hCoV-19^^New York|Human|DOHMH Riverside|New York City Public Health Laboratory|Wang|USA
>Spike|hCoV-19/USA/MI-MDHHS-SC21362/2020|2020-04-03|EPI_ISL_507616|Original|hCoV-19^^Michigan|Human|Michigan Department of Health and Human Services|Michigan Department of Health and Human Services|Blankenship|USA
>Spike|hCoV-19/Spain/CT-IBV-99009870/2020|2020-07-29|EPI_ISL_660267|Original|hCoV-19^^Catalunya|Human|Servicio de Microbiolog\u00eda|SeqCOVID-SPAIN consortium/IBV(CSIC)|Comas|Spain
>Spike|hCoV-19/England/CAMB-1BAA24/2020|2020-11-13|EPI_ISL_664773|Original|hCoV-19^^England|Human|Department of Pathology|COVID-19 Genomics UK (COG-UK) Consortium|Goodfellow|United Kingdom
>Spike|hCoV-19/England/CAMB-81289/2020|2020-04-11|EPI_ISL_442823|Original|hCoV-19^^England|Human|Department of Pathology|Wellcome Sanger Institute for the COVID-19 Genomics UK (COG-UK) consortium|Amato|United Kingdom
>Spike|hCoV-19/Northern Ireland/NIRE-1020D0/2020|2020-03-21|EPI_ISL_441379|Original|hCoV-19^^Northern Ireland|Human|Regional Virus Laboratory|COVID-19 Genomics UK (COG-UK) Consortium|Fairley|United Kingdom
>Spike|hCoV-19/USA/LA-EVTL127/2020|2020-05-07|EPI_ISL_451288|Original|hCoV-19^^Louisiana|Human|LSUHS Emerging Viral Threat Laboratory|Microbial Genome Sequencing Center|Kamil|USA
>Spike|hCoV-19/France/IDF-5847/2020|2020-03-19|EPI_ISL_443295|Original|hCoV-19^^\u00cele-de-France|Human|H\u00f4pital Necker - Enfants - Malades\nLaboratoire de Virologie|National Reference Center for Viruses of Respiratory Infections|Enouf|France

>Spike|hCoV-19/England/CAMB-1AD7A5/2020|2020-05-01|EPI_ISL_448007|Original|hCoV-19^^England|Human|Department of Pathology|COVID-19 Genomics UK (COG-UK) Consortium|Meredith|United Kingdom
>Spike|hCoV-19/Australia/VIC10989/2020|2020-08-01|EPI_ISL_563185|Original|hCoV-19^^Victoria|Human|Microbiological Diagnostic Unit - Public Health Laboratory (MDU-PHL)|MDU-PHL|Schultz|Australia
>Spike|hCoV-19/England/NOTT-11140F/2020|2020-05-16|EPI_ISL_453661|Original|hCoV-19^^England|Human|Queens Medical Centre|COVID-19 Genomics UK (COG-UK) Consortium|Loose|United Kingdom
>Spike|hCoV-19/Wales/PHWC-481258/2020|2020-10-18|EPI_ISL_611748|Original|hCoV-19^^Wales|Human|Wales Specialist Virology Centre Sequencing lab|COVID-19 Genomics UK (COG-UK) Consortium|Moore|United Kingdom
>Spike|hCoV-19/England/CAMB-1B9CF2/2020|2020-11-06|EPI_ISL_651708|Original|hCoV-19^^England|Human|Department of Pathology|COVID-19 Genomics UK (COG-UK) Consortium|Goodfellow|United Kingdom
>Spike|hCoV-19/England/QEUH-AD3594/2020|2020-10-26|EPI_ISL_633831|Original|hCoV-19^^England|Human|Lighthouse Lab in Glasgow|Wellcome Sanger Institute for the COVID-19 Genomics UK (COG-UK) consortium|Davies|United Kingdom
>Spike|hCoV-19/Canada/QC-LSPQ-L00224795/2020|2020-03-11|EPI_ISL_535823|Original|hCoV-19^^Quebec|Human|CHUL-LABO MULTI / MICRO|Laboratoire de sant\u00e9 publique du Qu\u00e9bec|Moreira|Canada
>Spike|hCoV-19/Australia/VIC7272/2020|2020-07-23|EPI_ISL_564324|Original|hCoV-19^^Victoria|Human|Victorian Infectious Diseases Reference Laboratory (VIDRL)|VIDRL and MDU-PHL|Schultz|Australia
>Spike|hCoV-19/USA/LA-EVTL296/2020|2020-08-26|EPI_ISL_574430|Original|hCoV-19^^Louisiana|Human|LSUHS Emerging Viral Threat Laboratory|Microbial Genome Sequencing Center|Kamil|USA
>Spike|hCoV-19/Scotland/QEUH-96E975/2020|2020-08-19|EPI_ISL_534470|Original|hCoV-19^^Scotland|Human|NHSGGC West of Scotland Specialist Virology Centre / MRC-University of Glasgow Centre for Virus Research|Wellcome Sanger Institute for the COVID-19 Genomics UK (COG-UK) consortium|Davies|United Kingdom
>Spike|hCoV-19/Iran/KHGRC-3-2178/2020|2020-03-26|EPI_ISL_445088|unknown|hCoV-19^^Iran|Human|Human Genetic Research Center|Human Genetic Research Center|Submitter|Iran
>Spike|hCoV-19/England/ALDP-9C5280/2020|2020-09-10|EPI_ISL_567771|Original|hCoV-19^^England|Human|Lighthouse Lab in Alderley Park|Wellcome Sanger Institute for the COVID-19 Genomics UK (COG-UK) consortium|Davies|United Kingdom
>Spike|hCoV-19/USA/CA-CSMC129/2020|2020-04-05|EPI_ISL_475606|Original|hCoV-19^^California|Human|Cedars-Sinai Medical Center|Cedars-Sinai Medical Center|Zhang|USA
>Spike|hCoV-19/Wales/QEUH-9C9EA6/2020|2020-09-09|EPI_ISL_567274|Original|hCoV-19^^Wales|Human|Lighthouse Lab in Glasgow|Wellcome Sanger Institute for the COVID-19 Genomics UK (COG-UK) consortium|Davies|United Kingdom
>Spike|hCoV-19/England/ALDP-B5D4D0/2020|2020-11-09|EPI_ISL_678903|Original|hCoV-19^^England|Human|Department of Pathology|COVID-19 Genomics UK (COG-UK) Consortium|Goodfellow|United Kingdom
>Spike|hCoV-19/United Arab Emirates/skmc-3939189/2020|2020-09-24|EPI_ISL_582686|Original|hCoV-19^^United Arab Emirates|Human|Sheikh Khalifa Medical City|Molecular/Surveillance lab\nSheikh Khalifa Medical City|Amirtharaj|United Arab Emirates
>Spike|hCoV-19/England/ALDP-4AA16B/2020|2020-06-10|EPI_ISL_559525|Original|hCoV-19^^England|Human|Lighthouse Lab in Alderley Park|Wellcome Sanger Institute for the COVID-19 Genomics UK (COG-UK) consortium|Davies|United Kingdom
>Spike|hCoV-19/England/201161466/2020|2020-03-11|EPI_ISL_464844|Original|hCoV-19^^England|Human|Respiratory Virus Unit|Respiratory Virus Unit|Platt|United Kingdom
>Spike|hCoV-19/USA/CA-ALSR-3738/2020|2020-06-05|EPI_ISL_636176|Original|hCoV-19^^California|Human|San Diego County Public Health Laboratory|Andersen lab at Scripps Research|Gangavarapu|USA
>Spike|hCoV-19/USA/MN-QDX-1344/2020|2020-03-15|EPI_ISL_572133|Original|hCoV-19^^Minnesota|Human|Quest Diagnostics|Quest Diagnostics|Kagan|USA

>Spike|hCoV-19/England/MILK-A4615C/2020|2020-10-06|EPI_ISL_598742|Original|hCoV-19^^England|Human|Lighthouse Lab in Milton Keynes|Wellcome Sanger Institute for the COVID-19 Genomics UK (COG-UK) consortium|Davies|United Kingdom
>Spike|hCoV-19/Spain/PV-IBV-000779/2020|2020-03-09|EPI_ISL_452726|Original|hCoV-19^^Basque Country|Human|Hospital Universitario Araba. Vitoria-Gasteiz,|SeqCOVID-SPAIN consortium/IBV(CSIC)|Comas|Spain
>Spike|hCoV-19/USA/NY-NYCPHL-000921/2020|2020-09-15|EPI_ISL_632976|Original|hCoV-19^^New York|Human|Empire City Laboratories|New York City Public Health Laboratory|Wang|USA
>Spike|hCoV-19/England/QEUH-AAB6DE/2020|2020-10-21|EPI_ISL_651513|Original|hCoV-19^^England|Human|Oxford Viromics|COVID-19 Genomics UK (COG-UK) Consortium|Golubchik|United Kingdom
>Spike|hCoV-19/Switzerland/BE-ETHZ-350375/2020|2020-10-31|EPI_ISL_728823|Original|hCoV-19^^Bern|Human|Viollier AG|Department of Biosystems Science and Engineering|Nadeau|Switzerland
>Spike|hCoV-19/France/PAC-IHU-0021/2020|2020-03-10|EPI_ISL_569250|Original|hCoV-19^^Provence-Alpes-C\u00f4te-d\u2019Azur|Human|MEPHI|MEPHI|Levasseur|France
>Spike|hCoV-19/USA/AZ-TG668356/2020|2020-05-13|EPI_ISL_696277|Original|hCoV-19^^Arizona|Human|Sonora Quest Laboratories|TGen North|French|USA
>Spike|hCoV-19/USA/OR-CDC-2-3693481/2020|2020-11-23|EPI_ISL_747170|Original|hCoV-19^^Oregon|Human|Respiratory Viruses Branch|Respiratory Viruses Branch|Queen|USA
>Spike|hCoV-19/England/MILK-B3830B/2020|2020-11-01|EPI_ISL_709905|Original|hCoV-19^^England|Human|Lighthouse Lab in Milton Keynes|Wellcome Sanger Institute for the COVID-19 Genomics UK (COG-UK) Consortium|Davies|United Kingdom
>Spike|hCoV-19/Wales/PHWC-48C536/2020|2020-11-08|EPI_ISL_666439|Original|hCoV-19^^Wales|Human|Wales Specialist Virology Centre Sequencing lab|COVID-19 Genomics UK (COG-UK) Consortium|Moore|United Kingdom
>Spike|hCoV-19/USA/FL-BPHL-1110/2020|2020-08-03|EPI_ISL_526596|Original|hCoV-19^^Florida|Human|Florida Bureau of Public Health Laboratories|Florida Bureau of Public Health Laboratories|Blanton|USA
>Spike|hCoV-19/England/ALDP-B63853/2020|2020-11-09|EPI_ISL_656452|Original|hCoV-19^^England|Human|Lighthouse Lab in Alderley Park|Wellcome Sanger Institute for the COVID-19 Genomics UK (COG-UK) Consortium|Davies|United Kingdom
>Spike|hCoV-19/Australia/QLD1113/2020|2020-03-26|EPI_ISL_593630|Original|hCoV-19^^Australia|Human|unknown|Public Health Virology Laboratory|Nguyen|Australia
>Spike|hCoV-19/England/PORT-2D349F/2020|2020-04-14|EPI_ISL_535032|Original|hCoV-19^^England|Human|Centre for Enzyme Innovation|COVID-19 Genomics UK (COG-UK) Consortium|Robson|United Kingdom
>Spike|hCoV-19/England/LIVE-A941C/2020|2020-07-13|EPI_ISL_526373|Original|hCoV-19^^England|Human|Liverpool Clinical Laboratories|COVID-19 Genomics UK (COG-UK) Consortium|Haldenby|United Kingdom
>Spike|hCoV-19/Denmark/ALAB-SSI-1246/2020|2020-03-11|EPI_ISL_444825|Original|hCoV-19^^Denmark|Human|Department of Virus and Microbiological Special Diagnostics|Albertsen lab|Kirkegaard|Denmark
>Spike|hCoV-19/Canada/NS-NML-895/2020|2020-03-20|EPI_ISL_579544|Original|hCoV-19^^Nova Scotia|Human|QEII Health Sciences Centre|National Microbiology Laboratory (NML)|Majer|Canada
>Spike|hCoV-19/USA/CA-CZB-11172/2020|2020-09-15|EPI_ISL_625529|Original|hCoV-19^^California|Human|Alameda County Public Health Lab|Chan-Zuckerberg Biohub|Bhatt|USA
>Spike|hCoV-19/USA/WA-S921/2020|2020-04-25|EPI_ISL_463411|Original|hCoV-19^^Washington|Human|Washington State Department of Health|Seattle Flu Study|Bedford|USA
>Spike|hCoV-19/Northern Ireland/NIRE-FEBC8/2020|2020-05-01|EPI_ISL_489260|Original|hCoV-19^^Northern Ireland|Human|Regional Virus Laboratory|Wellcome Sanger Institute for the COVID-19 Genomics UK (COG-UK) consortium|Davies|United Kingdom
>Spike|hCoV-19/England/ALDP-AA273E/2020|2020-10-22|EPI_ISL_630795|Original|hCoV-19^^England|Human|Lighthouse Lab in Alderley Park|Wellcome Sanger Institute for the COVID-19 Genomics UK (COG-UK) consortium|Davies|United Kingdom

>Spike|hCoV-19/USA/WA-OHSU-3111/2020|2020-10-02|EPI_ISL_667485|Original|hCoV-19^^Washington|Human|OHSU Lab Services Molecular Microbiology Lab|Oregon SARS-CoV-2 Genome Sequencing Center|O'Roak|USA
>Spike|hCoV-19/England/ALDP-B83310/2020|2020-11-14|EPI_ISL_675498|Original|hCoV-19^^England|Human|Lighthouse Lab in Alderley Park|Wellcome Sanger Institute for the COVID-19 Genomics UK (COG-UK) Consortium|Davies|United Kingdom
>Spike|hCoV-19/England/ALDP-A707A2/2020|2020-10-15|EPI_ISL_608139|Original|hCoV-19^^England|Human|Lighthouse Lab in Alderley Park|Wellcome Sanger Institute for the COVID-19 Genomics UK (COG-UK) consortium|Davies|United Kingdom
>Spike|hCoV-19/USA/MN-MDH-1689/2020|2020-04-08|EPI_ISL_560776|Original|hCoV-19^^Minnesota|Human|Minnesota Department of Health|Minnesota Department of Health|Garfin|USA
>Spike|hCoV-19/England/LOND-D574B/2020|2020-04-17|EPI_ISL_449718|Original|hCoV-19^^England|Human|University College London|COVID-19 Genomics UK (COG-UK) Consortium|Roy|United Kingdom
>Spike|hCoV-19/Canada/MB-NML-1065/2020|2020-08-05|EPI_ISL_582420|Original|hCoV-19^^Manitoba|Human|Cadham Provincial Laboratory|National Microbiology Laboratory (NML)|Majer|Canada
>Spike|hCoV-19/Belgium/UM-0318103/2020|2020-03-18|EPI_ISL_420359|Original|hCoV-19^^Honnelles|Human|KU Leuven|KU Leuven|Maes|Belgium
>Spike|hCoV-19/USA/LA-EVTL1413/2020|2020-12-11|EPI_ISL_779079|Original|hCoV-19^^Louisiana|Human|LSUHS Emerging Viral Threat Laboratory|Microbial Genome Sequencing Center|Kamil|USA
>Spike|hCoV-19/Japan/PG-6570/2020|2020-09-00|EPI_ISL_687682|Original|hCoV-19^^Japan|Human|Pathogen Genomics Center|Pathogen Genomics Center|Sekizuka|Japan
>Spike|hCoV-19/England/QEUH-BA00F8/2020|2020-11-18|EPI_ISL_719746|Original|hCoV-19^^England|Human|Lighthouse Lab in Glasgow|Wellcome Sanger Institute for the COVID-19 Genomics UK (COG-UK) Consortium|Davies|United Kingdom
>Spike|hCoV-19/USA/MI-UM-10036558247/2020|2020-10-21|EPI_ISL_640206|Original|hCoV-19^^Michigan|Human|University of Michigan Clinical Microbiology Laboratory|Lauring Lab|Valesano|USA
>Spike|hCoV-19/Australia/VIC13865/2020|2020-09-03|EPI_ISL_592334|Original|hCoV-19^^Victoria|Human|Microbiological Diagnostic Unit - Public Health Laboratory (MDU-PHL)|MDU-PHL|Schultz|Australia
>Spike|hCoV-19/England/ALDP-AA482A/2020|2020-10-22|EPI_ISL_624528|Original|hCoV-19^^England|Human|Lighthouse Lab in Alderley Park|Wellcome Sanger Institute for the COVID-19 Genomics UK (COG-UK) consortium|Davies|United Kingdom
>Spike|hCoV-19/England/SHEF-CE01D/2020|2020-09-26|EPI_ISL_585064|Original|hCoV-19^^England|Human|Virology Department|COVID-19 Genomics UK (COG-UK) Consortium|de Silva|United Kingdom
>Spike|hCoV-19/Wales/PHWC-4826B5/2020|2020-10-16|EPI_ISL_611901|Original|hCoV-19^^Wales|Human|Wales Specialist Virology Centre Sequencing lab|COVID-19 Genomics UK (COG-UK) Consortium|Moore|United Kingdom
>Spike|hCoV-19/Japan/PG-5234/2020|2020-08-00|EPI_ISL_690337|Original|hCoV-19^^Japan|Human|Pathogen Genomics Center|Pathogen Genomics Center|Sekizuka|Japan
>Spike|hCoV-19/Denmark/DCGC-17168/2020|2020-12-14|EPI_ISL_750798|Original|hCoV-19^^Sjaelland|Human|Department of Virus and Microbiological Special Diagnostics|Albertsen Lab|Michaelsen|Denmark
>Spike|hCoV-19/Canada/BC_26649386/2020|2020-03-00|EPI_ISL_463249|Original|hCoV-19^^British Columbia|Human|BCCDC Public Health Laboratory|BCCDC Public Health Laboratory|Harrigan|Canada
>Spike|hCoV-19/England/LIVE-A40B3/2020|2020-06-26|EPI_ISL_490404|Original|hCoV-19^^England|Human|Liverpool Clinical Laboratories|COVID-19 Genomics UK (COG-UK) Consortium|Haldenby|United Kingdom
>Spike|hCoV-19/Ecuador/UEES-4040/2020|2020-00-00|EPI_ISL_671988|Original|hCoV-19^^Ecuador|Human|UEES BioLab|Omics Sciences Laboratory|Fernandez Cadena|Ecuador
>Spike|hCoV-19/England/LIVE-A0E53/2020|2020-06-09|EPI_ISL_478399|Original|hCoV-19^^England|Human|Liverpool Clinical Laboratories|COVID-19 Genomics UK (COG-UK) Consortium|Haldenby|United Kingdom

>Spike|hCoV-19/England/CAMC-B08C63/2020|2020-10-29|EPI_ISL_643700|Original|hCoV-19^^England|Human|Lighthouse Lab in Cambridge|Wellcome Sanger Institute for the COVID-19 Genomics UK (COG-UK) Consortium|Davies|United Kingdom
>Spike|hCoV-19/USA/CA-UCLA-23/2020|2020-05-21|EPI_ISL_537535|Original|hCoV-19^^California|Human|UCLA Pathology Clinical Microbiology Lab|Kruglyak Lab|Boocock|USA
>Spike|hCoV-19/Denmark/DCGC-12692/2020|2020-11-16|EPI_ISL_682873|Original|hCoV-19^^Nordjylland|Human|Department of Virus and Microbiological Special Diagnostics|Albertsen Lab|Michaelsen|Denmark
>Spike|hCoV-19/USA/VA-DCLS-1222/2020|2020-07-27|EPI_ISL_528647|Original|hCoV-19^^Virginia|Human|Virginia DCLS|Virginia DCLS|St. Jacques|USA
>Spike|hCoV-19/USA/NY-NYUMC764/2020|2020-03-20|EPI_ISL_451478|Original|hCoV-19^^New York|Human|NYU Langone Health|Departments of Pathology and Medicine|Maurano|USA
>Spike|hCoV-19/USA/VA-DCLS-0793/2020|2020-07-07|EPI_ISL_526897|Original|hCoV-19^^Virginia|Human|Virginia DCLS|Virginia DCLS|St. Jacques|USA
>Spike|hCoV-19/USA/MA-MASPHL-00058/2020|2020-04-03|EPI_ISL_791557|Original|hCoV-19^^Massachusetts|Human|Massachusetts State Public Health Laboratory|Infectious Disease Program|Park|USA
>Spike|hCoV-19/United Arab Emirates/0585/2020|2020-05-16|EPI_ISL_698743|Original|hCoV-19^^Abu Dhabi|Human|Group 42 (G42) Healthcare|G42 Healthcare|Ogrodzki|United Arab Emirates
>Spike|hCoV-19/Singapore/43/2020|2020-02-16|EPI_ISL_428822|Original|hCoV-19^^Singapore|Human|National Public Health Laboratory|National Public Health Laboratory|Mak|Singapore
>Spike|hCoV-19/Denmark/DCGC-9881/2020|2020-11-09|EPI_ISL_669251|Original|hCoV-19^^Nordjylland|Human|Department of Virus and Microbiological Special Diagnostics|Albertsen Lab|Michaelsen|Denmark
>Spike|hCoV-19/Wales/PHWC-16494A/2020|2020-06-03|EPI_ISL_479325|Original|hCoV-19^^Wales|Human|Wales Specialist Virology Centre Sequencing lab|COVID-19 Genomics UK (COG-UK) Consortium|Moore|United Kingdom
>Spike|hCoV-19/Northern Ireland/NIRE-10731E/2020|2020-03-30|EPI_ISL_585137|Original|hCoV-19^^Northern Ireland|Human|Regional Virus Laboratory|COVID-19 Genomics UK (COG-UK) Consortium|Fairley|United Kingdom
>Spike|hCoV-19/Australia/VIC6349/2020|2020-07-21|EPI_ISL_518329|Original|hCoV-19^^Victoria|Human|Microbiological Diagnostic Unit - Public Health Laboratory (MDU-PHL)|MDU-PHL|Schultz|Australia
>Spike|hCoV-19/England/ALDP-C23C70/2020|2020-12-06|EPI_ISL_733641|Original|hCoV-19^^England|Human|Lighthouse Lab in Alderley Park|Wellcome Sanger Institute for the COVID-19 Genomics UK (COG-UK) Consortium|Davies|United Kingdom
>Spike|hCoV-19/Canada/QC-LSPQ-L00215817/2020|2020-03-12|EPI_ISL_535739|Original|hCoV-19^^Quebec|Human|H\u00f4pital de l'Enfant-J\u00e9sus|Laboratoire de sant\u00e9 publique du Qu\u00e9bec|Moreira|Canada
>Spike|hCoV-19/England/QEUH-CC3D42/2020|2020-12-21|EPI_ISL_760540|Original|hCoV-19^^England|Human|Lighthouse Lab in Glasgow|Wellcome Sanger Institute for the COVID-19 Genomics UK (COG-UK) Consortium|Davies|United Kingdom
>Spike|hCoV-19/SaudiArabia/KAUST-MADINAH1099/2020|2020-06-13|EPI_ISL_513106|Original|hCoV-19^^Madinah|Human|Pathogen Genomics Lab King Abdullah University of Science and Technology(KAUST)|Pathogen Genomics Lab King Abdullah University of Science and Technology(KAUST)|Mohamed Ghazzali|Saudi Arabia
>Spike|hCoV-19/Northern Ireland/QEUH-962434/2020|2020-08-10|EPI_ISL_531795|Original|hCoV-19^^Northern Ireland|Human|Lighthouse Lab in Glasgow|Wellcome Sanger Institute for the COVID-19 Genomics UK (COG-UK) consortium|Davies|United Kingdom
>Spike|hCoV-19/Canada/ON-E3/2020|2020-04-23|EPI_ISL_593483|Original|hCoV-19^^Ontario|Human|Eastern Ontario Regional Laboratory Association|McMaster University|McArthur|Canada
>Spike|hCoV-19/Denmark/DCGC-21022/2020|2020-12-21|EPI_ISL_759508|Original|hCoV-19^^Hovedstaden|Human|Department of Virus and Microbiological Special Diagnostics|Albertsen Lab|Michaelsen|Denmark

>Spike|hCoV-19/England/CAMC-C3F3A9/2020|2020-12-10|EPI_ISL_719548|Original|hCoV-19^^England|Human|Lighthouse Lab in Cambridge|Wellcome Sanger Institute for the COVID-19 Genomics UK (COG-UK) Consortium|Davies|United Kingdom
>Spike|hCoV-19/England/QEUH-96A681/2020|2020-08-12|EPI_ISL_531475|Original|hCoV-19^^England|Human|Lighthouse Lab in Glasgow|Wellcome Sanger Institute for the COVID-19 Genomics UK (COG-UK) consortium|Davies|United Kingdom
>Spike|hCoV-19/England/CAMC-B08D9D/2020|2020-10-29|EPI_ISL_673133|Original|hCoV-19^^England|Human|Lighthouse Lab in Cambridge|Wellcome Sanger Institute for the COVID-19 Genomics UK (COG-UK) Consortium|Davies|United Kingdom
>Spike|hCoV-19/England/ALDP-AA207F/2020|2020-10-22|EPI_ISL_624223|Original|hCoV-19^^England|Human|Lighthouse Lab in Alderley Park|Wellcome Sanger Institute for the COVID-19 Genomics UK (COG-UK) consortium|Davies|United Kingdom
>Spike|hCoV-19/Spain/CT-IBV-99009875/2020|2020-08-01|EPI_ISL_660272|Original|hCoV-19^^Catalunya|Human|Servicio de Microbiolog\u00eda|SeqCOVID-SPAIN consortium/IBV(CSIC)|Comas|Spain
>Spike|hCoV-19/USA/DE-DHSS-F1009549/2020|2020-10-04|EPI_ISL_708405|Original|hCoV-19^^Delaware|Human|Delaware Public Health Lab|Delaware Public Health Lab|Bajwa|USA
>Spike|hCoV-19/England/MILK-B59BEE/2020|2020-11-06|EPI_ISL_679917|Original|hCoV-19^^England|Human|Virology Department|COVID-19 Genomics UK (COG-UK) Consortium|de Silva|United Kingdom
>Spike|hCoV-19/Switzerland/BS-UHB-42181950/2020|2020-03-09|EPI_ISL_527959|Original|hCoV-19^^Basel-Stadt|Human|University Hospital Basel|University Hospital Basel|Roloff Handschin|Switzerland
>Spike|hCoV-19/Tunisia/7899/2020|2020-04-06|EPI_ISL_763067|Original|hCoV-19^^Tunis|Human|1-Laboratory of Microbiology|1-Clinical and Experimental Pharmacology Lab|Boutiba-Ben Boubaker|Tunisia
>Spike|hCoV-19/England/LIVE-A5BFD/2020|2020-03-17|EPI_ISL_499608|Original|hCoV-19^^England|Human|Liverpool Clinical Laboratories|COVID-19 Genomics UK (COG-UK) Consortium|Haldenby|United Kingdom
>Spike|hCoV-19/USA/MN-MDH-1846/2020|2020-09-30|EPI_ISL_602981|Original|hCoV-19^^Minnesota|Human|Minnesota Department of Health|Minnesota Department of Health|Garfin|USA
>Spike|hCoV-19/Wales/PHWC-488BCF/2020|2020-11-02|EPI_ISL_639477|Original|hCoV-19^^Wales|Human|Wales Specialist Virology Centre Sequencing lab|COVID-19 Genomics UK (COG-UK) Consortium|Moore|United Kingdom
>Spike|hCoV-19/Sweden/20-53401/2020|2020-11-04|EPI_ISL_661301|Original|hCoV-19^^Vastra Gotaland|Human|Klinisk Mikrobiologi|The Public Health Agency of Sweden|Svartstr\u00f6m|Sweden
>Spike|hCoV-19/Netherlands/ZH-EMC-633/2020|2020-09-21|EPI_ISL_578061|Original|hCoV-19^^Zuid Holland|Human|Dutch COVID-19 response team|Erasmus Medical Center|Oude Munnink|Netherlands
>Spike|hCoV-19/Netherlands/NH-RIVM-20713/2020|2020-12-21|EPI_ISL_790779|Original|hCoV-19^^Noord-Holland|Human|Dutch COVID-19 response team|National Institute for Public Health and the Environment (RIVM)|Meijer|Netherlands
>Spike|hCoV-19/Spain/PV-IBV-2086/2020|2020-04-10|EPI_ISL_468910|Original|hCoV-19^^Basque Country|Human|Servicio de Microbiolog\u00eda. Hospital Universitario Donostia. OSI Donostialdea. \u00c1rea de Enfermedades Infecciosas|SeqCOVID-SPAIN consortium/IBV(CSIC)|Comas|Spain
>Spike|hCoV-19/USA/WA-S2009/2020|2020-06-04|EPI_ISL_497437|Original|hCoV-19^^Washington|Human|Washington State Department of Health|Seattle Flu Study|Lee|USA
>Spike|hCoV-19/Japan/PG-8444/2020|2020-09-00|EPI_ISL_687543|Original|hCoV-19^^Japan|Human|Fukuoka Institute of Health and Environmental Sciences|Pathogen Genomics Center|Sekizuka|Japan
>Spike|hCoV-19/United Arab Emirates/0147/2020|2020-05-18|EPI_ISL_698334|Original|hCoV-19^^Abu Dhabi|Human|Group 42 (G42) Healthcare|G42 Healthcare|Ogrodzki|United Arab Emirates
>Spike|hCoV-19/England/LIVE-A4B3A/2020|2020-03-10|EPI_ISL_499907|Original|hCoV-19^^England|Human|Liverpool Clinical Laboratories|COVID-19 Genomics UK (COG-UK) Consortium|Haldenby|United Kingdom

>Spike|hCoV-19/England/QEUH-B4091D/2020|2020-11-08|EPI_ISL_657177|Original|hCoV-19^^England|Human|Lighthouse Lab in Glasgow|Wellcome Sanger Institute for the COVID-19 Genomics UK (COG-UK) Consortium|Davies|United Kingdom
>Spike|hCoV-19/Aruba/AW-RIVM-10388/2020|2020-08-04|EPI_ISL_636514|Original|hCoV-19^^Aruba|Human|Dutch COVID-19 response team|National Institute for Public Health and the Environment (RIVM)|Meijer|Aruba
>Spike|hCoV-19/USA/VA_NIDDL_2951/2020|2020-04-06|EPI_ISL_491925|Original|hCoV-19^^Virginia|Human|Naval Infectious Diseases Diagnostic Laboratory|Naval Medical Research Center Biological Defense Research Directorate|Voegtly|USA
>Spike|hCoV-19/England/ALDP-B23734/2020|2020-11-04|EPI_ISL_647086|Original|hCoV-19^^England|Human|Lighthouse Lab in Alderley Park|Wellcome Sanger Institute for the COVID-19 Genomics UK (COG-UK) Consortium|Davies|United Kingdom
>Spike|hCoV-19/USA/TX-HMH-MCoV-12761/2020|2020-08-19|EPI_ISL_789919|Original|hCoV-19^^Texas|Human|Houston Methodist Hospital|Houston Methodist Hospital|Long|USA
>Spike|hCoV-19/England/ALDP-BB504B/2020|2020-11-20|EPI_ISL_701906|Original|hCoV-19^^England|Human|Lighthouse Lab in Alderley Park|Wellcome Sanger Institute for the COVID-19 Genomics UK (COG-UK) Consortium|Davies|United Kingdom
>Spike|hCoV-19/USA/TX-HMH-685/2020|2020-04-15|EPI_ISL_546288|Original|hCoV-19^^Texas|Human|Houston Methodist Hospital|Houston Methodist Hospital|Long|USA
>Spike|hCoV-19/USA/CA-CZB-14132/2020|2020-05-25|EPI_ISL_739640|Original|hCoV-19^^California|Human|Alameda County Public Health Lab|Chan-Zuckerberg Biohub|Kamm|USA
>Spike|hCoV-19/England/MILK-870D93/2020|2020-07-16|EPI_ISL_557520|Original|hCoV-19^^England|Human|Lighthouse Lab in Milton Keynes|Wellcome Sanger Institute for the COVID-19 Genomics UK (COG-UK) consortium|Davies|United Kingdom
>Spike|hCoV-19/USA/MN-QDX-1323/2020|2020-03-15|EPI_ISL_572112|Original|hCoV-19^^Minnesota|Human|Quest Diagnostics|Quest Diagnostics|Kagan|USA
>Spike|hCoV-19/USA/MI-UM-10036255029/2020|2020-10-02|EPI_ISL_590741|Original|hCoV-19^^Michigan|Human|University of Michigan Clinical Microbiology Laboratory|Lauring Lab|Valesano|USA
>Spike|hCoV-19/England/QEUH-B699D2/2020|2020-11-11|EPI_ISL_661559|Original|hCoV-19^^England|Human|Lighthouse Lab in Glasgow|Wellcome Sanger Institute for the COVID-19 Genomics UK (COG-UK) Consortium|Davies|United Kingdom
>Spike|hCoV-19/USA/WA-S940/2020|2020-04-23|EPI_ISL_463430|Original|hCoV-19^^Washington|Human|Washington State Department of Health|Seattle Flu Study|Bedford|USA
>Spike|hCoV-19/USA/CA-CHLA-PLM02957000/2020|2020-11-16|EPI_ISL_753351|Original|hCoV-19^^California|Human|Clinical virology Laboratory|Center for Personalized Medicine|Dien Bard|USA
>Spike|hCoV-19/England/ALDP-928CF6/2020|2020-06-22|EPI_ISL_552962|Original|hCoV-19^^England|Human|Lighthouse Lab in Alderley Park|Wellcome Sanger Institute for the COVID-19 Genomics UK (COG-UK) consortium|Davies|United Kingdom
>Spike|hCoV-19/England/20146007404/2020|2020-04-01|EPI_ISL_421973|Original|hCoV-19^^England|Human|Respiratory Virus Unit|Respiratory Virus Unit|Platt|United Kingdom
>Spike|hCoV-19/Luxembourg/LNS7064269/2020|2020-11-23|EPI_ISL_744504|Original|hCoV-19^^Luxembourg|Human|Laboratoire national de sant\u00e9|Laboratoire national de sant\u00e9|Wienecke-Baldacchino|Luxembourg
>Spike|hCoV-19/Netherlands/NH-EMC-59/2020|2020-08-27|EPI_ISL_577855|Original|hCoV-19^^Noord Holland|Human|Dutch COVID-19 response team|Erasmus Medical Center|Oude Munnink|Netherlands
>Spike|hCoV-19/Wales/ALDP-C3AC2B/2020|2020-12-09|EPI_ISL_767278|Original|hCoV-19^^Wales|Human|Lighthouse Lab in Alderley Park|Wellcome Sanger Institute for the COVID-19 Genomics UK (COG-UK) Consortium|Davies|United Kingdom
>Spike|hCoV-19/Morocco/FMP-63/2020|2020-11-00|EPI_ISL_775265|Original|hCoV-19^^Morocco|Human|Laboratoire Biolife|Laboratoire de Biotechnologie|Ouadghiri|Morocco
>Spike|hCoV-19/Switzerland/BE-UNIBE-220-1253/2020|2020-04-09|EPI_ISL_574856|Original|hCoV-19^^Bern|Human|Institute for Infectious Diseases|Institute for Infectious Diseases|Koch|Switzerland
>Spike|hCoV-19/England/ALDP-A9581D/2020|2020-10-20|EPI_ISL_624808|Original|hCoV-19^^England|Human|Lighthouse Lab in Alderley Park|Wellcome Sanger Institute for the COVID-19 Genomics UK (COG-UK) consortium|Davies|United Kingdom

>Spike|hCoV-19/Wales/PHWC-24B23/2020|2020-03-20|EPI_ISL_419486|Original|hCoV-19^^Wales|Human|Wales Specialist Virology Centre|Public Health Wales Microbiology Cardiff|Moore|United Kingdom
>Spike|hCoV-19/England/ALDP-C49843/2020|2020-12-12|EPI_ISL_718635|Original|hCoV-19^^England|Human|Lighthouse Lab in Alderley Park|Wellcome Sanger Institute for the COVID-19 Genomics UK (COG-UK) Consortium|Davies|United Kingdom
>Spike|hCoV-19/Scotland/EDB4984/2020|2020-05-14|EPI_ISL_493927|Original|hCoV-19^^Scotland|Human|Virology Department|COVID-19 Genomics UK (COG-UK) Consortium|Williams|United Kingdom
>Spike|hCoV-19/England/CAMB-801F6/2020|2020-04-09|EPI_ISL_524508|Original|hCoV-19^^England|Human|Department of Pathology|Wellcome Sanger Institute for the COVID-19 Genomics UK (COG-UK) consortium|Davies|United Kingdom
>Spike|hCoV-19/Saudi Arabia/KAUST-Makkah227/2020|2020-04-06|EPI_ISL_437723|Original|hCoV-19^^Makkah|Human|Pathogen Genomics Lab King Abdullah University of Science and Technology(KAUST)|Pathogen Genomics Lab King Abdullah University of Science and Technology(KAUST)|Mohamed Ghazzali|Saudi Arabia
>Spike|hCoV-19/USA/TX-HMH-5886/2020|2020-06-29|EPI_ISL_547087|Original|hCoV-19^^Texas|Human|Houston Methodist Hospital|Houston Methodist Hospital|Long|USA
>Spike|hCoV-19/England/SHEF-D1EFF/2020|2020-09-11|EPI_ISL_559943|Original|hCoV-19^^England|Human|Virology Department|COVID-19 Genomics UK (COG-UK) Consortium|de Silva|United Kingdom
>Spike|hCoV-19/Finland/28A90S17/2020|2020-04-28|EPI_ISL_481635|Original|hCoV-19^^Uusimaa|Human|Department of Virology and Immunology|Department of Virology|Smura|Finland
>Spike|hCoV-19/Scotland/EDB9018/2020|2020-10-31|EPI_ISL_652034|Original|hCoV-19^^Scotland|Human|Virology Department|COVID-19 Genomics UK (COG-UK) Consortium|McHugh|United Kingdom
>Spike|hCoV-19/England/202960225/2020|2020-07-15|EPI_ISL_534719|Original|hCoV-19^^England|Human|Respiratory Virus Unit|Respiratory Virus Unit|Platt|United Kingdom
>Spike|hCoV-19/USA/AZ-TG276548/2020|2020-04-15|EPI_ISL_694109|Original|hCoV-19^^Arizona|Human|AZ SPHL|TGen North|French|USA
>Spike|hCoV-19/England/ALDP-B2444B/2020|2020-11-04|EPI_ISL_646883|Original|hCoV-19^^England|Human|Lighthouse Lab in Alderley Park|Wellcome Sanger Institute for the COVID-19 Genomics UK (COG-UK) Consortium|Davies|United Kingdom
>Spike|hCoV-19/England/LOND-12601E8/2020|2020-04-05|EPI_ISL_524655|Original|hCoV-19^^England|Human|North West London Pathology|Wellcome Sanger Institute for the COVID-19 Genomics UK (COG-UK) consortium|Davies|United Kingdom
>Spike|hCoV-19/England/LIVE-DB601B/2020|2020-11-07|EPI_ISL_652089|Original|hCoV-19^^England|Human|Liverpool Clinical Laboratories|COVID-19 Genomics UK (COG-UK) Consortium|Haldenby|United Kingdom
>Spike|hCoV-19/Wales/PHWC-15DD2A/2020|2020-05-14|EPI_ISL_472773|Original|hCoV-19^^Wales|Human|Wales Specialist Virology Centre Sequencing lab|COVID-19 Genomics UK (COG-UK) Consortium|Moore|United Kingdom
>Spike|hCoV-19/England/QEUH-9FAC93/2020|2020-09-28|EPI_ISL_588199|Original|hCoV-19^^England|Human|Lighthouse Lab in Glasgow|Wellcome Sanger Institute for the COVID-19 Genomics UK (COG-UK) consortium|Davies|United Kingdom
>Spike|hCoV-19/Denmark/DCGC-5337/2020|2020-09-14|EPI_ISL_615268|Original|hCoV-19^^Syddanmark|Human|Department of Virus and Microbiological Special Diagnostics|Albertsen lab|Michaelsen|Denmark
>Spike|hCoV-19/USA/WI-UW-1006/2020|2020-07-17|EPI_ISL_516432|Original|hCoV-19^^Wisconsin|Human|University of Wisconsin-Madison AIDS Vaccine Research Laboratories|University of Wisconsin-Madison AIDS Vaccine Research Laboratories|Baczenas|USA
>Spike|hCoV-19/Wales/PHWC-490BE2/2020|2020-11-15|EPI_ISL_706546|Original|hCoV-19^^Wales|Human|Originating lab|COVID-19 Genomics UK (COG-UK) Consortium|Moore|United Kingdom
>Spike|hCoV-19/Spain/VC-IBV-98004326/2020|2020-03-24|EPI_ISL_654471|Original|hCoV-19^^Comunitat_Valenciana|Human|Servicio de Microbiolog\u00eda. Hospital General Universitario de Castell\u00f3n|SeqCOVID-SPAIN consortium/IBV(CSIC)|Comas|Spain

>Spike|hCoV-19/USA/WA-UW-32886/2020|2020-10-17|EPI_ISL_737094|Original|hCoV-
19^^Washington|Human|UW Virology Lab|UW Virology Lab|Roychoudhury|USA
>Spike|hCoV-19/USA/TX-HMH-MCoV-16092/2020|2020-10-29|EPI_ISL_785776|Original|hCoV-
19^^Texas|Human|Houston Methodist Hospital|Houston Methodist Hospital|Long|USA
>Spike|hCoV-19/Singapore/943/2020|2020-04-04|EPI_ISL_538477|Original|hCoV-
19^^Singapore|Human|Department of Laboratory Medicine|Department of Laboratory
Medicine|Chen|Singapore
>Spike|hCoV-19/USA/WA-UW-2537/2020|2020-03-30|EPI_ISL_485996|Original|hCoV-
19^^Washington|Human|UW Virology Lab|UW Virology Lab|Roychoudhury|USA
>Spike|hCoV-19/Wales/PHWC-4814AD/2020|2020-10-12|EPI_ISL_613075|Original|hCoV-
19^^Wales|Human|Wales Specialist Virology Centre Sequencing lab|COVID-19 Genomics UK
(COG-UK) Consortium|Moore|United Kingdom
>Spike|hCoV-19/Wales/PHWC-15C4AE/2020|2020-03-29|EPI_ISL_472493|Original|hCoV-
19^^Wales|Human|Wales Specialist Virology Centre Sequencing lab|COVID-19 Genomics UK
(COG-UK) Consortium|Moore|United Kingdom
>Spike|hCoV-19/England/LIVE-DCCECD/2020|2020-12-12|EPI_ISL_724530|Original|hCoV-
19^^England|Human|Liverpool Clinical Laboratories|COVID-19 Genomics UK (COG-UK)
Consortium|Haldenby|United Kingdom
>Spike|hCoV-19/USA/MI-QDX-387/2020|2020-03-24|EPI_ISL_661252|Original|hCoV-
19^^Michigan|Human|Quest Diagnostics|Quest Diagnostics|Kagan|USA
>Spike|hCoV-19/Scotland/QEUH-9F5E29/2020|2020-09-27|EPI_ISL_600631|Original|hCoV-
19^^Scotland|Human|Lighthouse Lab in Glasgow|Wellcome Sanger Institute for the COVID-
19 Genomics UK (COG-UK) consortium|Davies|United Kingdom
>Spike|hCoV-19/USA/WA-S2793/2020|2020-08-14|EPI_ISL_530150|Original|hCoV-
19^^Washington|Human|Seattle Flu Study|Seattle Flu Study|Lee|USA
>Spike|hCoV-19/Finland/16A68S11/2020|2020-04-16|EPI_ISL_481568|Original|hCoV-
19^^Uusimaa|Human|Department of Virology and Immunology|Department of
Virology|Smura|Finland
>Spike|hCoV-19/Denmark/DCGC-20478/2020|2020-12-21|EPI_ISL_758733|Original|hCoV-
19^^Syddanmark|Human|Department of Virus and Microbiological Special
Diagnostics|Albertsen Lab|Michaelsen|Denmark
>Spike|hCoV-19/Spain/PV-IBV-000816/2020|2020-03-10|EPI_ISL_452712|Original|hCoV-
19^^Basque Country|Human|Hospital Universitario Araba. Vitoria-Gasteiz,|SeqCOVID-
SPAIN consortium/IBV(CSIC)|Comas|Spain
>Spike|hCoV-19/USA/MI-MDHHS-SC21228/2020|2020-04-09|EPI_ISL_507484|Original|hCoV-
19^^Michigan|Human|Michigan Department of Health and Human Services|Michigan
Department of Health and Human Services|Blankenship|USA
>Spike|hCoV-19/Wales/PHWC-493D8F/2020|2020-11-20|EPI_ISL_726022|Original|hCoV-
19^^Wales|Human|Originating lab|COVID-19 Genomics UK (COG-UK) Consortium|Moore|United
Kingdom
>Spike|hCoV-19/Scotland/QEUH-AAB559/2020|2020-10-20|EPI_ISL_652797|Original|hCoV-
19^^Scotland|Human|Oxford Viromics|COVID-19 Genomics UK (COG-UK)
Consortium|Golubchik|United Kingdom
>Spike|hCoV-19/USA/WI-UW-1610/2020|2020-09-17|EPI_ISL_605487|Original|hCoV-
19^^Wisconsin|Human|University of Wisconsin-Madison AIDS Vaccine Research
Laboratories|University of Wisconsin-Madison AIDS Vaccine Research
Laboratories|Baczenas|USA
>Spike|hCoV-19/England/CAMC-94C2CD/2020|2020-07-15|EPI_ISL_556669|Original|hCoV-
19^^England|Human|Lighthouse Lab in Cambridge|Wellcome Sanger Institute for the
COVID-19 Genomics UK (COG-UK) consortium|Davies|United Kingdom
>Spike|hCoV-19/England/MILK-BFCC08/2020|2020-12-02|EPI_ISL_720835|Original|hCoV-
19^^England|Human|Lighthouse Lab in Milton Keynes|Wellcome Sanger Institute for the
COVID-19 Genomics UK (COG-UK) Consortium|Davies|United Kingdom
>Spike|hCoV-19/Wales/PHWC-164528/2020|2020-05-09|EPI_ISL_474190|Original|hCoV-
19^^Wales|Human|Originating lab|COVID-19 Genomics UK (COG-UK) Consortium|Moore|United
Kingdom
>Spike|hCoV-19/England/MILK-A7DA42/2020|2020-10-16|EPI_ISL_638463|Original|hCoV-
19^^England|Human|Oxford Viromics|COVID-19 Genomics UK (COG-UK)
Consortium|Golubchik|United Kingdom

>Spike|hCoV-19/Wales/PHWC-4960A1/2020|2020-11-25|EPI_ISL_726527|Original|hCoV-19^^Wales|Human|Originating lab|COVID-19 Genomics UK (COG-UK) Consortium|Moore|United Kingdom
>Spike|hCoV-19/England/MILK-B3DD53/2020|2020-11-01|EPI_ISL_706258|Original|hCoV-19^^England|Human|Oxford Viromics|COVID-19 Genomics UK (COG-UK) Consortium|Golubchik|United Kingdom
>Spike|hCoV-19/Luxembourg/LNS4577509/2020|2020-10-21|EPI_ISL_740500|Original|hCoV-19^^Luxembourg|Human|Laboratoire national de sant\u00e9|Laboratoire national de sant\u00e9|Wienecke-Baldacchino|Luxembourg
>Spike|hCoV-19/England/MILK-B597DB/2020|2020-11-06|EPI_ISL_679870|Original|hCoV-19^^England|Human|Virology Department|COVID-19 Genomics UK (COG-UK) Consortium|de Silva|United Kingdom
>Spike|hCoV-19/mink/Netherlands/NB-EMC-64-2/2020|2020-10-14|EPI_ISL_632348|Original|hCoV-19^^Noord Brabant|Neovison vison|Dutch COVID-19 response team|Erasmus Medical Center|Oude Munnink|Netherlands
>Spike|hCoV-19/England/CAMB-1ABC6C/2020|2020-04-19|EPI_ISL_470261|Original|hCoV-19^^England|Human|Department of Pathology|Wellcome Sanger Institute for the COVID-19 Genomics UK (COG-UK) consortium|Davies|United Kingdom
>Spike|hCoV-19/England/LEED-2A8660/2020|2020-03-26|EPI_ISL_539035|Original|hCoV-19^^England|Human|Leeds Teaching Hospitals NHS Trust and Public Health England|Wellcome Sanger Institute for the COVID-19 Genomics UK (COG-UK) consortium|Davies|United Kingdom
>Spike|hCoV-19/Australia/VIC5600/2020|2020-07-19|EPI_ISL_518416|Original|hCoV-19^^Victoria|Human|Microbiological Diagnostic Unit - Public Health Laboratory (MDU-PHL)|MDU-PHL|Schultz|Australia
>Spike|hCoV-19/USA/CA-CHLA-PLM72377620/2020|2020-11-15|EPI_ISL_753344|Original|hCoV-19^^California|Human|Clinical virology Laboratory|Center for Personalized Medicine|Dien Bard|USA
>Spike|hCoV-19/USA/LA-GBCL-26684442/2020|2020-04-11|EPI_ISL_485267|Original|hCoV-19^^Louisiana|Human|River Road Testing Lab|Ginkgo Bioworks Clinical Laboratory|Smith|USA
>Spike|hCoV-19/India/TN-CCMB-C16/2020|2020-05-06|EPI_ISL_458039|Original|hCoV-19^^Tamil Nadu|Human|King Institute of Preventive Medicine & Research|CSIR-Centre for Cellular and Molecular Biology|Banu|India
>Spike|hCoV-19/USA/TX-HMH-MCoV-17990/2020|2020-11-16|EPI_ISL_786138|Original|hCoV-19^^Texas|Human|Houston Methodist Hospital|Houston Methodist Hospital|Long|USA
>Spike|hCoV-19/England/CAMC-A57B2D/2020|2020-10-08|EPI_ISL_597310|Original|hCoV-19^^England|Human|Lighthouse Lab in Cambridge|Wellcome Sanger Institute for the COVID-19 Genomics UK (COG-UK) consortium|Davies|United Kingdom
>Spike|hCoV-19/Ireland/D-NVRL-AIIDV0073v1/2020|2020-04-10|EPI_ISL_768758|Original|hCoV-19^^Dublin|Human|AIID|Irish Coronavirus Sequencing Consortium - National Virus Reference Laboratory|Gonzalez|Ireland
>Spike|hCoV-19/India/WB-S10/2020|2020-04-05|EPI_ISL_437538|Original|hCoV-19^^West Bengal|Human|ICMR-National Institute of Cholera and Enteric Diseases|National Institute of Biomedical Genomics|Maitra|India
>Spike|hCoV-19/USA/WI-WSLH-200580/2020|2020-06-05|EPI_ISL_578691|Original|hCoV-19^^Wisconsin|Human|Wisconsin State Laboratory of Hygiene Communicable Disease Division|Wisconsin State Laboratory of Hygiene Communicable Disease Division|Florek|USA
>Spike|hCoV-19/England/QEUH-9F86B5/2020|2020-09-27|EPI_ISL_588935|Original|hCoV-19^^England|Human|Lighthouse Lab in Glasgow|Wellcome Sanger Institute for the COVID-19 Genomics UK (COG-UK) consortium|Davies|United Kingdom
>Spike|hCoV-19/England/SHEF-D1062/2020|2020-04-29|EPI_ISL_475494|Original|hCoV-19^^England|Human|Virology Department|COVID-19 Genomics UK (COG-UK) Consortium|de Silva|United Kingdom
>Spike|hCoV-19/Japan/PG-4632/2020|2020-07-00|EPI_ISL_689769|Original|hCoV-19^^Japan|Human|Fukuoka Institute of Health and Environmental Sciences|Pathogen Genomics Center|Sekizuka|Japan

>Spike|hCoV-19/Wales/PHWC-28776/2020|2020-03-31|EPI_ISL_432370|Original|hCoV-19^^Wales|Human|Wales Specialist Virology Centre|Public Health Wales Microbiology Cardiff|Moore|United Kingdom
>Spike|hCoV-19/USA/NY-Wadsworth-247837-01/2020|2020-10-17|EPI_ISL_734362|Original|hCoV-19^^New York|Human|Wadsworth Center|Wadsworth Center|Lasek-Nesselquist|USA
>Spike|hCoV-19/USA/CA-CZB080/2020|2020-03-28|EPI_ISL_429013|Original|hCoV-19^^California|Human|UCSF Clinical Microbiology Laboratory|Chan-Zuckerberg Biohub|Kamm|USA
>Spike|hCoV-19/Scotland/CVR5947/2020|2020-11-23|EPI_ISL_703842|Original|hCoV-19^^Scotland|Human|West of Scotland Specialist Virology Centre|COVID-19 Genomics UK (COG-UK) Consortium|Robertson|United Kingdom
>Spike|hCoV-19/USA/TX-HMH-6755/2020|2020-06-24|EPI_ISL_546220|Original|hCoV-19^^Texas|Human|Houston Methodist Hospital|Houston Methodist Hospital|Long|USA
>Spike|hCoV-19/England/NORW-E8C82/2020|2020-04-15|EPI_ISL_448295|Original|hCoV-19^^England|Human|Quadram Institute Bioscience|COVID-19 Genomics UK (COG-UK) Consortium|Alikhan|United Kingdom
>Spike|hCoV-19/USA/LA-EVTL1136/2020|2020-12-07|EPI_ISL_768405|Original|hCoV-19^^Louisiana|Human|LSUHS Emerging Viral Threat Laboratory|Microbial Genome Sequencing Center|Kamil|USA
>Spike|hCoV-19/England/QEUH-B67716/2020|2020-11-11|EPI_ISL_661611|Original|hCoV-19^^England|Human|Lighthouse Lab in Glasgow|Wellcome Sanger Institute for the COVID-19 Genomics UK (COG-UK) Consortium|Davies|United Kingdom
>Spike|hCoV-19/Russia/KK-RII-MH1680S/2020|2020-09-03|EPI_ISL_596311|Original|hCoV-19^^Khakassia|Human|HELIX LCC|WHO National Influenza Centre Russian Federation|Komissarov|Russia
>Spike|hCoV-19/USA/HI-H200055/2020|2020-04-15|EPI_ISL_752640|Original|hCoV-19^^Hawaii|Human|State Laboratories Division|State Laboratories Division|Sultana|USA
>Spike|hCoV-19/England/204760990/2020|2020-11-18|EPI_ISL_683547|Original|hCoV-19^^England|Human|Respiratory Virus Unit|COVID-19 Genomics UK (COG-UK) Consortium|Platt|United Kingdom
>Spike|hCoV-19/England/MILK-ACBD4C/2020|2020-10-21|EPI_ISL_629376|Original|hCoV-19^^England|Human|Lighthouse Lab in Milton Keynes|Wellcome Sanger Institute for the COVID-19 Genomics UK (COG-UK) consortium|Davies|United Kingdom
>Spike|hCoV-19/Mexico/BCN-ALSR-4935/2020|2020-08-26|EPI_ISL_730220|Original|hCoV-19^^Baja California|Human|Genomica Lab Molecular|Andersen lab at Scripps Research|Gangavarapu|Mexico
>Spike|hCoV-19/England/OXON-ACC99/2020|2020-04-05|EPI_ISL_448523|Original|hCoV-19^^England|Human|Oxford Viromics|COVID-19 Genomics UK (COG-UK) Consortium|Golubchik|United Kingdom
>Spike|hCoV-19/USA/VA-DCLS-0520/2020|2020-04-08|EPI_ISL_614121|Original|hCoV-19^^Virginia|Human|Virginia DCLS|Virginia DCLS|St. Jacques|USA
>Spike|hCoV-19/England/CAMB-73660/2020|2020-03-21|EPI_ISL_440004|Original|hCoV-19^^England|Human|Department of Pathology|Wellcome Sanger Institute for the COVID-19 Genomics UK (COG-UK) consortium|Amato|United Kingdom
>Spike|hCoV-19/USA/TX-HMH-6570/2020|2020-06-24|EPI_ISL_546093|Original|hCoV-19^^Texas|Human|Houston Methodist Hospital|Houston Methodist Hospital|Long|USA
>Spike|hCoV-19/USA/MN-MDH-1336/2020|2020-03-24|EPI_ISL_496922|Original|hCoV-19^^Minnesota|Human|Minnesota Department of Health|Minnesota Department of Health|Garfin|USA
>Spike|hCoV-19/Japan/PG-8306/2020|2020-08-00|EPI_ISL_687405|Original|hCoV-19^^Japan|Human|Pathogen Genomics Center|Pathogen Genomics Center|Sekizuka|Japan
>Spike|hCoV-19/Singapore/930/2020|2020-03-29|EPI_ISL_538462|Original|hCoV-19^^Singapore|Human|Department of Laboratory Medicine|Department of Laboratory Medicine|Chen|Singapore
>Spike|hCoV-19/USA/TX-HMH-877/2020|2020-04-20|EPI_ISL_545894|Original|hCoV-19^^Texas|Human|Houston Methodist Hospital|Houston Methodist Hospital|Long|USA
>Spike|hCoV-19/Beijing/DT-travelES02/2020|2020-03-12|EPI_ISL_452344|Original|hCoV-19^^Beijing|Human|Laboratory of Infectious Diseases Center of Beijing Ditan

Hospital|Laboratory of Infectious Diseases Center of Beijing Ditan Hospital|Yang|China
>Spike|hCoV-19/England/PORT-2D38FD/2020|2020-04-17|EPI_ISL_529672|Original|hCoV-19^^England|Human|Centre for Enzyme Innovation|COVID-19 Genomics UK (COG-UK) Consortium|Robson|United Kingdom
>Spike|hCoV-19/England/ALDP-9E583D/2020|2020-09-24|EPI_ISL_589488|Original|hCoV-19^^England|Human|Lighthouse Lab in Alderley Park|Wellcome Sanger Institute for the COVID-19 Genomics UK (COG-UK) consortium|Davies|United Kingdom
>Spike|hCoV-19/Spain/AN-IBV-002943/2020|2020-03-27|EPI_ISL_474929|Original|hCoV-19^^Andalucia|Human|Hospital Universitario Virgen de las Nieves de Granada-SAS|SeqCOVID-SPAIN consortium/IBV(CSIC)|Comas|Spain
>Spike|hCoV-19/Scotland/CVR4776/2020|2020-09-20|EPI_ISL_627347|Original|hCoV-19^^Scotland|Human|West of Scotland Specialist Virology Centre|COVID-19 Genomics UK (COG-UK) Consortium|Robertson|United Kingdom
>Spike|hCoV-19/South Korea/KCDC2351/2020|2020-05-28|EPI_ISL_510687|Original|hCoV-19^^South Korea|Human|Division of Viral Diseases|Division of Viral Diseases|Kim|South Korea
>Spike|hCoV-19/Peru/ARE-INS-026/2020|2020-03-06|EPI_ISL_491431|Original|hCoV-19^^Arequipa|Human|Laboratorio de Referencia Nacional de Virus Respiratorio. Instituto Nacional de Salud. Per\u00fa|Laboratorio de Referencia Nacional de Biotecnolog\u00eda y Biolog\u00eda Molecular. Instituto Nacional de Salud.Per\u00fa|Padilla Rojas|Peru
>Spike|hCoV-19/England/QEUH-9DE363/2020|2020-09-21|EPI_ISL_599579|Original|hCoV-19^^England|Human|Lighthouse Lab in Glasgow|Wellcome Sanger Institute for the COVID-19 Genomics UK (COG-UK) consortium|Davies|United Kingdom
>Spike|hCoV-19/USA/ME-HETL-J0025/2020|2020-03-16|EPI_ISL_513437|Original|hCoV-19^^Maine|Human|Maine HETL|Tewhey Lab|Tewhey|USA
>Spike|hCoV-19/USA/TX-HMH-6298/2020|2020-06-30|EPI_ISL_547372|Original|hCoV-19^^Texas|Human|Houston Methodist Hospital|Houston Methodist Hospital|Long|USA
>Spike|hCoV-19/England/NORT-28852D/2020|2020-12-05|EPI_ISL_725021|Original|hCoV-19^^England|Human|Northumbria University / South Tees Hospitals NHS Foundation Trust / North Cumbria Integrated Care NHS Foundation Trust / North Tees and Hartlepool NHS Foundation Trust / Newcastle Hospitals NHS Foundation Trust|COVID-19 Genomics UK (COG-UK) Consortium|Bashton|United Kingdom
>Spike|hCoV-19/Denmark/DCGC-22140/2020|2020-12-14|EPI_ISL_758580|Original|hCoV-19^^Hovedstaden|Human|Department of Virus and Microbiological Special Diagnostics|Albertsen Lab|Michaelsen|Denmark
>Spike|hCoV-19/Scotland/QEUH-A1FE55/2020|2020-10-01|EPI_ISL_598915|Original|hCoV-19^^Scotland|Human|Lighthouse Lab in Glasgow|Wellcome Sanger Institute for the COVID-19 Genomics UK (COG-UK) consortium|Davies|United Kingdom
>Spike|hCoV-19/England/QEUH-BC3DF8/2020|2020-11-25|EPI_ISL_704138|Original|hCoV-19^^England|Human|Lighthouse Lab in Glasgow|Wellcome Sanger Institute for the COVID-19 Genomics UK (COG-UK) Consortium|Davies|United Kingdom
>Spike|hCoV-19/USA/AZ-TG276750/2020|2020-04-16|EPI_ISL_694123|Original|hCoV-19^^Arizona|Human|AZ SPHL|TGen North|French|USA
>Spike|hCoV-19/Wales/PHWC-47DFB2/2020|2020-10-12|EPI_ISL_595390|Original|hCoV-19^^Wales|Human|Originating lab|COVID-19 Genomics UK (COG-UK) Consortium|Moore|United Kingdom
>Spike|hCoV-19/England/NORT-1B61412/2020|2020-10-24|EPI_ISL_637883|Original|hCoV-19^^England|Human|Northumbria University / South Tees Hospitals NHS Foundation Trust / North Cumbria Integrated Care NHS Foundation Trust / North Tees and Hartlepool NHS Foundation Trust / Newcastle Hospitals NHS Foundation Trust|COVID-19 Genomics UK (COG-UK) Consortium|Bashton|United Kingdom
>Spike|hCoV-19/England/ALDP-B76D6D/2020|2020-11-12|EPI_ISL_723809|Original|hCoV-19^^England|Human|Oxford Viromics|COVID-19 Genomics UK (COG-UK) Consortium|Golubchik|United Kingdom
>Spike|hCoV-19/England/BRIS-129C57/2020|2020-04-15|EPI_ISL_458450|Original|hCoV-19^^England|Human|PHE South West Regional Laboratory|Wellcome Sanger Institute for the COVID-19 Genomics UK (COG-UK) consortium|Davies|United Kingdom

>Spike|hCoV-19/USA/WA-UW-4015/2020|2020-03-28|EPI_ISL_437806|Original|hCoV-19^^Washington|Human|UW Virology Lab|UW Virology Lab|Roychoudhury|USA
>Spike|hCoV-19/Norway/2904/2020|2020-07-13|EPI_ISL_549172|Original|hCoV-19^^Viken|Human|Unilabs Laboratory Medicine|Norwegian Institute of Public Health|Kopperud Riis|Norway
>Spike|hCoV-19/England/MILK-9E22BA/2020|2020-09-20|EPI_ISL_581190|Original|hCoV-19^^England|Human|Lighthouse Lab in Milton Keynes|Wellcome Sanger Institute for the COVID-19 Genomics UK (COG-UK) consortium|Davies|United Kingdom
>Spike|hCoV-19/England/BRIS-12CFF1/2020|2020-04-25|EPI_ISL_481910|Original|hCoV-19^^England|Human|PHE South West Regional Laboratory|Wellcome Sanger Institute for the COVID-19 Genomics UK (COG-UK) consortium|Davies|United Kingdom
>Spike|hCoV-19/France/CVL-8271/2020|2020-03-27|EPI_ISL_560573|Original|hCoV-19^^Centre-Val de Loire|Human|h\u00f4pital|National Reference Center for Viruses of Respiratory Infections|Enouf|France
>Spike|hCoV-19/Wales/PHWC-47EC9C/2020|2020-10-06|EPI_ISL_595575|Original|hCoV-19^^Wales|Human|Originating lab|COVID-19 Genomics UK (COG-UK) Consortium|Moore|United Kingdom
>Spike|hCoV-19/Northern Ireland/QEUH-96C31F/2020|2020-08-13|EPI_ISL_530890|Original|hCoV-19^^Northern Ireland|Human|Lighthouse Lab in Glasgow|Wellcome Sanger Institute for the COVID-19 Genomics UK (COG-UK) consortium|Davies|United Kingdom
>Spike|hCoV-19/Luxembourg/LNS3019031/2020|2020-11-10|EPI_ISL_740234|Original|hCoV-19^^Luxembourg|Human|Laboratoire national de sant\u00e9|Laboratoire national de sant\u00e9|Wienecke-Baldacchino|Luxembourg
>Spike|hCoV-19/Japan/IC-0251/2020|2020-10-00|EPI_ISL_667680|Original|hCoV-19^^Japan|Human|Pathogen Genomics Center|Pathogen Genomics Center|Sekizuka|Japan
>Spike|hCoV-19/England/MILK-A05906/2020|2020-09-30|EPI_ISL_599914|Original|hCoV-19^^England|Human|Lighthouse Lab in Milton Keynes|Wellcome Sanger Institute for the COVID-19 Genomics UK (COG-UK) consortium|Davies|United Kingdom
>Spike|hCoV-19/USA/CA-CSMC14/2020|2020-03-24|EPI_ISL_475618|Original|hCoV-19^^California|Human|Cedars-Sinai Medical Center|Cedars-Sinai Medical Center|Zhang|USA
>Spike|hCoV-19/Netherlands/NA_363/2020|2020-04-08|EPI_ISL_455167|Original|hCoV-19^^Netherlands|Human|Dutch COVID-19 response team|Erasmus Medical Center|Oude Munnink|Netherlands
>Spike|hCoV-19/England/CAMB-7C843/2020|2020-04-06|EPI_ISL_442237|Original|hCoV-19^^England|Human|Department of Pathology|Wellcome Sanger Institute for the COVID-19 Genomics UK (COG-UK) consortium|Amato|United Kingdom
>Spike|hCoV-19/Australia/VIC12254/2020|2020-07-27|EPI_ISL_591936|Original|hCoV-19^^Victoria|Human|Microbiological Diagnostic Unit - Public Health Laboratory (MDU-PHL)|MDU-PHL|Schultz|Australia
>Spike|hCoV-19/England/QEUH-943509/2020|2020-07-31|EPI_ISL_532593|Original|hCoV-19^^England|Human|Lighthouse Lab in Glasgow|Wellcome Sanger Institute for the COVID-19 Genomics UK (COG-UK) consortium|Davies|United Kingdom
>Spike|hCoV-19/USA/OK-CDC-2-3693683/2020|2020-11-05|EPI_ISL_747095|Original|hCoV-19^^Oklahoma|Human|Respiratory Viruses Branch|Respiratory Viruses Branch|Queen|USA
>Spike|hCoV-19/Japan/PG-8940/2020|2020-08-00|EPI_ISL_686486|Original|hCoV-19^^Japan|Human|Pathogen Genomics Center|Pathogen Genomics Center|Sekizuka|Japan
>Spike|hCoV-19/England/QEUH-C34508/2020|2020-12-11|EPI_ISL_733945|Original|hCoV-19^^England|Human|Lighthouse Lab in Glasgow|Wellcome Sanger Institute for the COVID-19 Genomics UK (COG-UK) Consortium|Davies|United Kingdom
>Spike|hCoV-19/England/CAMC-B32B27/2020|2020-11-05|EPI_ISL_659734|Original|hCoV-19^^England|Human|Lighthouse Lab in Cambridge|Wellcome Sanger Institute for the COVID-19 Genomics UK (COG-UK) Consortium|Davies|United Kingdom
>Spike|hCoV-19/Scotland/EDB1432/2020|2020-04-12|EPI_ISL_433445|Original|hCoV-19^^Scotland|Human|Virology Department|COVID-19 Genomics UK (COG-UK) Consortium|Williams|United Kingdom
>Spike|hCoV-19/Spain/AR-IBV-2468/2020|2020-03-27|EPI_ISL_468766|Original|hCoV-19^^Aragon|Human|Servicio de Microbiolog\u00eda|SeqCOVID-SPAIN consortium/IBV(CSIC)|Comas|Spain

>Spike|hCoV-19/Northern Ireland/NIRE-2350B5/2020|2020-10-15|EPI_ISL_764409|Original|hCoV-19^^Northern Ireland|Human|Regional Virus Laboratory|COVID-19 Genomics UK (COG-UK) Consortium|Fairley|United Kingdom
>Spike|hCoV-19/Wales/MILK-9C2423/2020|2020-09-09|EPI_ISL_566560|Original|hCoV-19^^Wales|Human|Lighthouse Lab in Milton Keynes|Wellcome Sanger Institute for the COVID-19 Genomics UK (COG-UK) consortium|Davies|United Kingdom
>Spike|hCoV-19/Denmark/DCGC-9585/2020|2020-11-09|EPI_ISL_668550|Original|hCoV-19^^Nordjylland|Human|Department of Virus and Microbiological Special Diagnostics|Albertsen Lab|Michaelsen|Denmark
>Spike|hCoV-19/Wales/QEUH-96B820/2020|2020-08-14|EPI_ISL_531375|Original|hCoV-19^^Wales|Human|Lighthouse Lab in Glasgow|Wellcome Sanger Institute for the COVID-19 Genomics UK (COG-UK) consortium|Davies|United Kingdom
>Spike|hCoV-19/England/CAMC-B093C7/2020|2020-10-29|EPI_ISL_672980|Original|hCoV-19^^England|Human|Lighthouse Lab in Cambridge|Wellcome Sanger Institute for the COVID-19 Genomics UK (COG-UK) Consortium|Davies|United Kingdom
>Spike|hCoV-19/Spain/CL-IBV-97006140/2020|2020-03-31|EPI_ISL_537288|Original|hCoV-19^^Castilla y Leon|Human|Universidad de Le\u00f3n|SeqCOVID-SPAIN consortium/IBV(CSIC)|Comas|Spain
>Spike|hCoV-19/England/CAMB-1AA8F0/2020|2020-04-18|EPI_ISL_470103|Original|hCoV-19^^England|Human|Department of Pathology|Wellcome Sanger Institute for the COVID-19 Genomics UK (COG-UK) consortium|Davies|United Kingdom
>Spike|hCoV-19/Turkey/IMU-SP-02/2020|2020-04-16|EPI_ISL_460617|Original|hCoV-19^^Turkey|Human|unknown|Physiology|Submitter|Turkey
>Spike|hCoV-19/England/ALDP-94423E/2020|2020-06-07|EPI_ISL_554808|Original|hCoV-19^^England|Human|Lighthouse Lab in Alderley Park|Wellcome Sanger Institute for the COVID-19 Genomics UK (COG-UK) consortium|Davies|United Kingdom
>Spike|hCoV-19/France/OCC-SC498/2020|2020-03-30|EPI_ISL_660692|Original|hCoV-19^^Occitanie|Human|CHU Montpellier|CNR Virus des Infections Respiratoires - France SUD|Bal|France
>Spike|hCoV-19/Australia/VIC2588/2020|2020-06-30|EPI_ISL_521461|Original|hCoV-19^^Victoria|Human|Microbiological Diagnostic Unit - Public Health Laboratory (MDU-PHL)|MDU-PHL|Schultz|Australia
>Spike|hCoV-19/Japan/PG-6577/2020|2020-09-00|EPI_ISL_687689|Original|hCoV-19^^Japan|Human|Pathogen Genomics Center|Pathogen Genomics Center|Sekizuka|Japan
>Spike|hCoV-19/Belgium/ULG-10996/2020|2020-11-14|EPI_ISL_707763|Original|hCoV-19^^Li\u00e8ge|Human|Department of Clinical Microbiology|GIGA Medical Genomics|Durkin|Belgium
>Spike|hCoV-19/Belgium/rega-09191058/2020|2020-09-19|EPI_ISL_735001|Original|hCoV-19^^Bost|Human|UZ Leuven|KU Leuven|Maes|Belgium
>Spike|hCoV-19/New Zealand/20CV0532/2020|2020-11-08|EPI_ISL_637087|Original|hCoV-19^^Auckland|Human|Middlemore Hospital|Institute of Environmental Science and Research (ESR)|Wang|New Zealand
>Spike|hCoV-19/England/MILK-C6F65C/2020|2020-12-13|EPI_ISL_778393|Original|hCoV-19^^England|Human|Lighthouse Lab in Milton Keynes|Wellcome Sanger Institute for the COVID-19 Genomics UK (COG-UK) Consortium|Davies|United Kingdom
>Spike|hCoV-19/Scotland/QEUH-964335/2020|2020-08-12|EPI_ISL_534365|Original|hCoV-19^^Scotland|Human|NHSGGC West of Scotland Specialist Virology Centre / MRC-University of Glasgow Centre for Virus Research|Wellcome Sanger Institute for the COVID-19 Genomics UK (COG-UK) consortium|Davies|United Kingdom
>Spike|hCoV-19/USA/TX-DSHS-0084/2020|2020-05-11|EPI_ISL_522369|Original|hCoV-19^^Texas|Human|Texas Department of State Health Services|Texas Department of State Health Services|Rahman|USA
>Spike|hCoV-19/USA/AL-CDC-2-3714557/2020|2020-11-04|EPI_ISL_751792|Original|hCoV-19^^Alabama|Human|AL Dept. of Public Health Bureau of Clinical Laboratories|Genomics and Discovery|Queen|USA
>Spike|hCoV-19/Guangzhou/GZMU0041/2020|2020-02-08|EPI_ISL_429091|Original|hCoV-19^^Guangzhou|Human|The First Affiliated Hospital of Guangzhou Medical University|BGI-shenzhen & The First Affiliated Hospital of Guangzhou Medical University|Ji|China

>Spike|hCoV-19/USA/CA-CDC-9/2020|2020-02-23|EPI_ISL_412862|Original|hCoV-19^^California|Human|California Department of Public Health|Pathogen Discovery|Queen|USA
>Spike|hCoV-19/Singapore/1310/2020|2020-05-17|EPI_ISL_648797|Original|hCoV-19^^Singapore|Human|Department of Laboratory Medicine|Department of Laboratory Medicine|Chen|Singapore
>Spike|hCoV-19/England/CAMC-B08595/2020|2020-10-29|EPI_ISL_643505|Original|hCoV-19^^England|Human|Lighthouse Lab in Cambridge|Wellcome Sanger Institute for the COVID-19 Genomics UK (COG-UK) Consortium|Davies|United Kingdom
>Spike|hCoV-19/Spain/AN-IBV-002921/2020|2020-03-19|EPI_ISL_474845|Original|hCoV-19^^Andalucia|Human|Hospital Universitario Virgen de las Nieves de Granada-SAS|SeqCOVID-SPAIN consortium/IBV(CSIC)|Comas|Spain
>Spike|hCoV-19/England/ALDP-B2519E/2020|2020-11-04|EPI_ISL_646648|Original|hCoV-19^^England|Human|Lighthouse Lab in Alderley Park|Wellcome Sanger Institute for the COVID-19 Genomics UK (COG-UK) Consortium|Davies|United Kingdom
>Spike|hCoV-19/Wales/ALDP-C04BB4/2020|2020-12-03|EPI_ISL_767301|Original|hCoV-19^^Wales|Human|Lighthouse Lab in Alderley Park|Wellcome Sanger Institute for the COVID-19 Genomics UK (COG-UK) Consortium|Davies|United Kingdom
>Spike|hCoV-19/Wales/PHWC-48793F/2020|2020-10-30|EPI_ISL_638159|Original|hCoV-19^^Wales|Human|Wales Specialist Virology Centre Sequencing lab|COVID-19 Genomics UK (COG-UK) Consortium|Moore|United Kingdom
>Spike|hCoV-19/England/LIVE-DB4D9E/2020|2020-10-23|EPI_ISL_650341|Original|hCoV-19^^England|Human|Liverpool Clinical Laboratories|COVID-19 Genomics UK (COG-UK) Consortium|Haldenby|United Kingdom
>Spike|hCoV-19/England/MILK-C6C67D/2020|2020-12-13|EPI_ISL_778576|Original|hCoV-19^^England|Human|Lighthouse Lab in Milton Keynes|Wellcome Sanger Institute for the COVID-19 Genomics UK (COG-UK) Consortium|Davies|United Kingdom
>Spike|hCoV-19/USA/TX-HMH-MCoV-19440/2020|2020-11-29|EPI_ISL_783840|Original|hCoV-19^^Texas|Human|Houston Methodist Hospital|Houston Methodist Hospital|Long|USA
>Spike|hCoV-19/Denmark/DCGC-1980/2020|2020-07-20|EPI_ISL_618323|Original|hCoV-19^^Hovedstaden|Human|Department of Virus and Microbiological Special Diagnostics|Albertsen lab|Michaelsen|Denmark
>Spike|hCoV-19/Northern Ireland/NIRE-10725D/2020|2020-03-27|EPI_ISL_585129|Original|hCoV-19^^Northern Ireland|Human|Regional Virus Laboratory|COVID-19 Genomics UK (COG-UK) Consortium|Fairley|United Kingdom
>Spike|hCoV-19/England/CAMB-1B74CF/2020|2020-10-19|EPI_ISL_637724|Original|hCoV-19^^England|Human|Department of Pathology|COVID-19 Genomics UK (COG-UK) Consortium|Goodfellow|United Kingdom
>Spike|hCoV-19/England/LOND-124CDFD/2020|2020-04-17|EPI_ISL_507045|Original|hCoV-19^^England|Human|University College London Hospital|COVID-19 Genomics UK (COG-UK) Consortium|Byott|United Kingdom
>Spike|hCoV-19/USA/TX-HMH-5773/2020|2020-06-24|EPI_ISL_547005|Original|hCoV-19^^Texas|Human|Houston Methodist Hospital|Houston Methodist Hospital|Long|USA
>Spike|hCoV-19/Panama/328719/2020|2020-02-22|EPI_ISL_496607|Original|hCoV-19^^Panama Center|Human|Gorgas Memorial Laboratory of Health Studies|Gorgas Memorial Laboratory of Health Studies|Martinez|Panama
>Spike|hCoV-19/Finland/15A41S5/2020|2020-04-15|EPI_ISL_481557|Original|hCoV-19^^Uusimaa|Human|Department of Virology and Immunology|Department of Virology|Smura|Finland
>Spike|hCoV-19/England/ALDP-B5D528/2020|2020-11-09|EPI_ISL_678908|Original|hCoV-19^^England|Human|Department of Pathology|COVID-19 Genomics UK (COG-UK) Consortium|Goodfellow|United Kingdom
>Spike|hCoV-19/Norway/6604/2020|2020-12-01|EPI_ISL_775402|Original|hCoV-19^^Nordland|Human|Nordland Hospital - Bodo|Norwegian Institute of Public Health|Vollan|Norway
>Spike|hCoV-19/USA/AZ-TG664266/2020|2020-04-28|EPI_ISL_696067|Original|hCoV-19^^Arizona|Human|Sonora Quest Laboratories|TGen North|French|USA
>Spike|hCoV-19/England/CAMC-A645C5/2020|2020-10-10|EPI_ISL_608923|Original|hCoV-19^^England|Human|Lighthouse Lab in Cambridge|Wellcome Sanger Institute for the COVID-19 Genomics UK (COG-UK) consortium|Davies|United Kingdom

>Spike|hCoV-19/Mexico/BCN-ALSR-2524/2020|2020-06-16|EPI_ISL_635528|Original|hCoV-19^^Baja California|Human|Centro de Diagn\u221a\u2265stico COVID-19 UABC Tijuana|Andersen lab at Scripps Research|Gangavarapu|Mexico
>Spike|hCoV-19/USA/CA-QDX-803/2020|2020-03-21|EPI_ISL_571496|Original|hCoV-19^^California|Human|Quest Diagnostics|Quest Diagnostics|Kagan|USA
>Spike|hCoV-19/Denmark/DCGC-14214/2020|2020-11-30|EPI_ISL_711629|Original|hCoV-19^^Syddanmark|Human|Department of Virus and Microbiological Special Diagnostics|Albertsen Lab|Michaelsen|Denmark
>Spike|hCoV-19/Canada/ON-PHL-20-03748/2020|2020-11-00|EPI_ISL_775107|Original|hCoV-19^^Ontario|Human|Public Health Ontario Laboratory|Public Health Ontario Laboratory|Guthrie|Canada
>Spike|hCoV-19/Canada/ON-S1962/2020|2020-11-24|EPI_ISL_755678|Original|hCoV-19^^Ontario|Human|Toronto Invasive Bacterial Diseases Network|McMaster University|McArthur|Canada
>Spike|hCoV-19/USA/MI-QDX-2251/2020|2020-03-15|EPI_ISL_604542|Original|hCoV-19^^Michigan|Human|Quest Diagnostics|Quest Diagnostics|Kagan|USA
>Spike|hCoV-19/Denmark/DCGC-16826/2020|2020-12-14|EPI_ISL_750588|Original|hCoV-19^^Midtjylland|Human|Department of Virus and Microbiological Special Diagnostics|Albertsen Lab|Michaelsen|Denmark
>Spike|hCoV-19/USA/TX-HMH-MCoV-14333/2020|2020-10-05|EPI_ISL_787618|Original|hCoV-19^^Texas|Human|Houston Methodist Hospital|Houston Methodist Hospital|Long|USA
>Spike|hCoV-19/Luxembourg/LNS0730142/2020|2020-11-26|EPI_ISL_739823|Original|hCoV-19^^Luxembourg|Human|Laboratoire national de sant\u00e9|Laboratoire national de sant\u00e9|Wienecke-Baldacchino|Luxembourg
>Spike|hCoV-19/Wales/PHWC-16E7EC/2020|2020-10-02|EPI_ISL_585771|Original|hCoV-19^^Wales|Human|Wales Specialist Virology Centre Sequencing lab|COVID-19 Genomics UK (COG-UK) Consortium|Moore|United Kingdom
>Spike|hCoV-19/Tunisia/61627/2020|2020-11-23|EPI_ISL_707698|Original|hCoV-19^^Soliman|Human|1-Laboratory of Microbiology|1-Clinical and Experimental Pharmacology Lab|Boutiba-Ben Boubaker|Tunisia
>Spike|hCoV-19/India/GJ-GBRC218b/2020|2020-06-08|EPI_ISL_475055|Original|hCoV-19^^Gujarat|Human|GMERS Medical College & Hospital|Gujarat Biotechnology Research Centre|Joshi|India
>Spike|hCoV-19/USA/OR-OHSU-1075/2020|2020-07-09|EPI_ISL_525964|Original|hCoV-19^^Oregon|Human|OHSU Lab Services Molecular Microbiology Lab|Oregon SARS-CoV-2 Genome Sequencing Center|O'Roak|USA
>Spike|hCoV-19/England/MILK-B59B2A/2020|2020-11-04|EPI_ISL_679909|Original|hCoV-19^^England|Human|Virology Department|COVID-19 Genomics UK (COG-UK) Consortium|de Silva|United Kingdom
>Spike|hCoV-19/Denmark/DCGC-15351/2020|2020-11-23|EPI_ISL_713778|Original|hCoV-19^^Midtjylland|Human|Department of Virus and Microbiological Special Diagnostics|Albertsen Lab|Michaelsen|Denmark
>Spike|hCoV-19/England/MILK-993EF0/2020|2020-08-25|EPI_ISL_551651|Original|hCoV-19^^England|Human|Lighthouse Lab in Milton Keynes|Wellcome Sanger Institute for the COVID-19 Genomics UK (COG-UK) consortium|Davies|United Kingdom
>Spike|hCoV-19/England/CAMC-A57097/2020|2020-10-09|EPI_ISL_597269|Original|hCoV-19^^England|Human|Lighthouse Lab in Cambridge|Wellcome Sanger Institute for the COVID-19 Genomics UK (COG-UK) consortium|Davies|United Kingdom
>Spike|hCoV-19/USA/NMDOH-2020117409/2020|2020-06-09|EPI_ISL_542015|Original|hCoV-19^^New Mexico|Human|New Mexico Department of Health Scientific Laboratory|New Mexico Department of Health Scientific Laboratory|Johnson|USA
>Spike|hCoV-19/England/QEUH-96D141/2020|2020-08-18|EPI_ISL_530737|Original|hCoV-19^^England|Human|Lighthouse Lab in Glasgow|Wellcome Sanger Institute for the COVID-19 Genomics UK (COG-UK) consortium|Davies|United Kingdom
>Spike|hCoV-19/England/ALDP-BB356C/2020|2020-11-19|EPI_ISL_702635|Original|hCoV-19^^England|Human|Lighthouse Lab in Alderley Park|Wellcome Sanger Institute for the COVID-19 Genomics UK (COG-UK) Consortium|Davies|United Kingdom
>Spike|hCoV-19/USA/TX-HMH-MCoV-15730/2020|2020-07-09|EPI_ISL_785414|Original|hCoV-19^^Texas|Human|Houston Methodist Hospital|Houston Methodist Hospital|Long|USA

>Spike|hCoV-19/South Africa/Tygerberg_07/2020|2020-04-08|EPI_ISL_464118|Original|hCoV-19^^Western Cape|Human|National Health Laboratory Service (NHLS)|Division of Medical Virology|Engelbrecht|South Africa
>Spike|hCoV-19/USA/TX-HMH-2164/2020|2020-05-21|EPI_ISL_545154|Original|hCoV-19^^Texas|Human|Houston Methodist Hospital|Houston Methodist Hospital|Long|USA
>Spike|hCoV-19/Denmark/DCGC-22180/2020|2020-12-14|EPI_ISL_757826|Original|hCoV-19^^Hovedstaden|Human|Department of Virus and Microbiological Special Diagnostics|Albertsen Lab|Michaelsen|Denmark
>Spike|hCoV-19/England/QEUH-A4CEF5/2020|2020-10-05|EPI_ISL_596066|Original|hCoV-19^^England|Human|Quadram Institute Bioscience|COVID-19 Genomics UK (COG-UK) Consortium|Alikhan|United Kingdom
>Spike|hCoV-19/USA/TX-DSHS-1481/2020|2020-06-22|EPI_ISL_676636|Original|hCoV-19^^Texas|Human|Texas Department of State Health Services|Texas Department of State Health Services|Rahman|USA
>Spike|hCoV-19/England/CAMB-83B40/2020|2020-04-16|EPI_ISL_443370|Original|hCoV-19^^England|Human|Department of Pathology|Wellcome Sanger Institute for the COVID-19 Genomics UK (COG-UK) consortium|Amato|United Kingdom
>Spike|hCoV-19/USA/FL-BPHL-1182/2020|2020-08-20|EPI_ISL_526668|Original|hCoV-19^^Florida|Human|Florida Bureau of Public Health Laboratories|Florida Bureau of Public Health Laboratories|Blanton|USA
>Spike|hCoV-19/Denmark/DCGC-17217/2020|2020-12-14|EPI_ISL_750834|Original|hCoV-19^^Hovedstaden|Human|Department of Virus and Microbiological Special Diagnostics|Albertsen Lab|Michaelsen|Denmark
>Spike|hCoV-19/England/QEUH-A61EBE/2020|2020-10-12|EPI_ISL_628658|Original|hCoV-19^^England|Human|Oxford Viromics|COVID-19 Genomics UK (COG-UK) Consortium|Golubchik|United Kingdom
>Spike|hCoV-19/USA/UT-CDC-9750/2020|2020-04-25|EPI_ISL_509691|Original|hCoV-19^^Utah|Human|Utah Public Health Laboratory|Pathogen Discovery|Queen|USA
>Spike|hCoV-19/USA/MD-HP00051/2020|2020-03-19|EPI_ISL_457795|Original|hCoV-19^^Maryland|Human|Johns Hopkins Hospital Department of Pathology|Johns Hopkins Hospital Department of Pathology|Ramakrishnan|USA
>Spike|hCoV-19/Scotland/EDB2243/2020|2020-04-21|EPI_ISL_439175|Original|hCoV-19^^Scotland|Human|Virology Department|COVID-19 Genomics UK (COG-UK) Consortium|Williams|United Kingdom
>Spike|hCoV-19/USA/MI-MDHHS-SC21834/2020|2020-03-26|EPI_ISL_565877|Original|hCoV-19^^Michigan|Human|Michigan Department of Health and Human Services|Michigan Department of Health and Human Services|Blankenship|USA
>Spike|hCoV-19/Wales/PHWC-1688B2/2020|2020-05-19|EPI_ISL_494300|Original|hCoV-19^^Wales|Human|Originating lab|COVID-19 Genomics UK (COG-UK) Consortium|Moore|United Kingdom
>Spike|hCoV-19/England/MILK-59718E/2020|2020-06-20|EPI_ISL_558299|Original|hCoV-19^^England|Human|Lighthouse Lab in Milton Keynes|Wellcome Sanger Institute for the COVID-19 Genomics UK (COG-UK) consortium|Davies|United Kingdom
>Spike|hCoV-19/Denmark/DCGC-15616/2020|2020-11-23|EPI_ISL_713924|Original|hCoV-19^^Nordjylland|Human|Department of Virus and Microbiological Special Diagnostics|Albertsen Lab|Michaelsen|Denmark
>Spike|hCoV-19/USA/WA-S77/2020|2020-03-05|EPI_ISL_417130|Original|hCoV-19^^Washington|Human|Washington State Department of Health|Seattle Flu Study|Bedford|USA
>Spike|hCoV-19/Ireland/MH-NVRL-72IRL08381/2020|2020-08-11|EPI_ISL_528457|Original|hCoV-19^^Meath|Human|National Virus Reference Laboratory|National Virus Reference Laboratory|Gonzalez|Ireland
>Spike|hCoV-19/USA/CA-ALSR-2896/2020|2020-07-11|EPI_ISL_635726|Original|hCoV-19^^California|Human|San Diego County Public Health Laboratory|Andersen lab at Scripps Research|Gangavarapu|USA
>Spike|hCoV-19/Scotland/EDB4835/2020|2020-04-02|EPI_ISL_453161|Original|hCoV-19^^Scotland|Human|Virology Department|COVID-19 Genomics UK (COG-UK) Consortium|Williams|United Kingdom

>Spike|hCoV-19/England/SHEF-CB0F2/2020|2020-03-25|EPI_ISL_432502|Original|hCoV-19^^England|Human|Virology Department|COVID-19 Genomics UK (COG-UK) Consortium|de Silva|United Kingdom
>Spike|hCoV-19/Australia/VIC4099/2020|2020-07-14|EPI_ISL_519480|Original|hCoV-19^^Victoria|Human|Microbiological Diagnostic Unit - Public Health Laboratory (MDU-PHL)|MDU-PHL|Schultz|Australia
>Spike|hCoV-19/Denmark/DCGC-14478/2020|2020-11-30|EPI_ISL_711427|Original|hCoV-19^^Hovedstaden|Human|Department of Virus and Microbiological Special Diagnostics|Albertsen Lab|Michaelsen|Denmark
>Spike|hCoV-19/Norway/4062/2020|2020-10-03|EPI_ISL_775309|Original|hCoV-19^^Viken|Human|Medical Microbiology Unit|Norwegian Institute of Public Health|Vollan|Norway
>Spike|hCoV-19/South Africa/R13196-20/2020|2020-06-30|EPI_ISL_622899|Original|hCoV-19^^Gauteng|Human|National Institute for Communicable Diseases of the National Health Laboratory Service|National Institute for Communicable Diseases of the National Health Laboratory Service|Allam|South Africa
>Spike|hCoV-19/Denmark/DCGC-8879/2020|2020-11-02|EPI_ISL_626162|Original|hCoV-19^^Midtjylland|Human|Department of Virus and Microbiological Special Diagnostics|Albertsen lab|Michaelsen|Denmark
>Spike|hCoV-19/USA/TX-HMH-MCoV-15863/2020|2020-07-10|EPI_ISL_785547|Original|hCoV-19^^Texas|Human|Houston Methodist Hospital|Houston Methodist Hospital|Long|USA
>Spike|hCoV-19/USA/TX-HMH-MCoV-14131/2020|2020-10-02|EPI_ISL_787416|Original|hCoV-19^^Texas|Human|Houston Methodist Hospital|Houston Methodist Hospital|Long|USA
>Spike|hCoV-19/USA/WA-S1980/2020|2020-06-03|EPI_ISL_497408|Original|hCoV-19^^Washington|Human|Washington State Department of Health|Seattle Flu Study|Lee|USA
>Spike|hCoV-19/Scotland/QEUH-9DAF18/2020|2020-09-22|EPI_ISL_601104|Original|hCoV-19^^Scotland|Human|Lighthouse Lab in Glasgow|Wellcome Sanger Institute for the COVID-19 Genomics UK (COG-UK) consortium|Davies|United Kingdom
>Spike|hCoV-19/USA/CA-CZB-6113/2020|2020-05-01|EPI_ISL_583187|Original|hCoV-19^^California|Human|San Bernardino County Public Health Lab|Chan-Zuckerberg Biohub|Bhatt|USA
>Spike|hCoV-19/England/CAMB-813C2/2020|2020-04-10|EPI_ISL_442594|Original|hCoV-19^^England|Human|Department of Pathology|Wellcome Sanger Institute for the COVID-19 Genomics UK (COG-UK) consortium|Amato|United Kingdom
>Spike|hCoV-19/France/PAC-IHU-1307/2020|2020-08-18|EPI_ISL_569113|Original|hCoV-19^^Provence-Alpes-C\u00f4te-d\u2019Azur|Human|MEPHI|MEPHI|Levasseur|France
>Spike|hCoV-19/Australia/VIC7970/2020|2020-08-06|EPI_ISL_564779|Original|hCoV-19^^Victoria|Human|Microbiological Diagnostic Unit - Public Health Laboratory (MDU-PHL)|MDU-PHL|Schultz|Australia
>Spike|hCoV-19/USA/MN-MDH-1681/2020|2020-04-07|EPI_ISL_560768|Original|hCoV-19^^Minnesota|Human|Minnesota Department of Health|Minnesota Department of Health|Garfin|USA
>Spike|hCoV-19/Scotland/CVR2722/2020|2020-04-18|EPI_ISL_473543|Original|hCoV-19^^Scotland|Human|West of Scotland Specialist Virology Centre|COVID-19 Genomics UK (COG-UK) Consortium|Robertson|United Kingdom
>Spike|hCoV-19/New Zealand/20CV0162/2020|2020-08-23|EPI_ISL_548035|Original|hCoV-19^^Auckland|Human|Middlemore Hospital|Institute of Environmental Science and Research (ESR)|Wang|New Zealand
>Spike|hCoV-19/Japan/PG-6134/2020|2020-08-00|EPI_ISL_689246|Original|hCoV-19^^Japan|Human|Pathogen Genomics Center|Pathogen Genomics Center|Sekizuka|Japan
>Spike|hCoV-19/England/ALDP-B23ED5/2020|2020-11-04|EPI_ISL_646170|Original|hCoV-19^^England|Human|Lighthouse Lab in Alderley Park|Wellcome Sanger Institute for the COVID-19 Genomics UK (COG-UK) Consortium|Davies|United Kingdom
>Spike|hCoV-19/USA/CA-CZB-15320/2020|2020-11-25|EPI_ISL_739457|Original|hCoV-19^^California|Human|Santa Clara County Public Health Laboratory|Chan-Zuckerberg Biohub|Kamm|USA
>Spike|hCoV-19/Peru/LIM-INS-002/2020|2020-03-06|EPI_ISL_487269|Original|hCoV-19^^Lima|Human|Laboratorio de Referencia Nacional de Virus Respiratorio. Instituto Nacional de Salud.|Laboratorio de Referencia Nacional de Biotecnolog\u00eda y Biolog\u00eda Molecular. Instituto Nacional de Salud.|Padilla Rojas|Peru

>Spike|hCoV-19/Wales/PHWC-496D8C/2020|2020-11-24|EPI_ISL_726725|Original|hCoV-19^^Wales|Human|Originating lab|COVID-19 Genomics UK (COG-UK) Consortium|Moore|United Kingdom
>Spike|hCoV-19/England/ALDP-9CCACD/2020|2020-09-16|EPI_ISL_572600|Original|hCoV-19^^England|Human|Quadram Institute Bioscience|COVID-19 Genomics UK (COG-UK) Consortium|Alikhan|United Kingdom
>Spike|hCoV-19/Wales/PHWC-2A950/2020|2020-04-08|EPI_ISL_445640|Original|hCoV-19^^Wales|Human|Wales Specialist Virology Centre|Public Health Wales Microbiology Cardiff|Moore|United Kingdom
>Spike|hCoV-19/England/QEUH-A215A6/2020|2020-10-01|EPI_ISL_587606|Original|hCoV-19^^England|Human|Lighthouse Lab in Glasgow|Wellcome Sanger Institute for the COVID-19 Genomics UK (COG-UK) consortium|Davies|United Kingdom
>Spike|hCoV-19/USA/MI-MDHHS-SC21432/2020|2020-03-25|EPI_ISL_507686|Original|hCoV-19^^Michigan|Human|Michigan Department of Health and Human Services|Michigan Department of Health and Human Services|Blankenship|USA
>Spike|hCoV-19/USA/WA-S1321/2020|2020-05-15|EPI_ISL_495771|Original|hCoV-19^^Washington|Human|Washington State Department of Health|Seattle Flu Study|Lee|USA
>Spike|hCoV-19/Australia/VIC3615/2020|2020-07-10|EPI_ISL_520358|Original|hCoV-19^^Victoria|Human|Victorian Infectious Diseases Reference Laboratory (VIDRL)|VIDRL and MDU-PHL|Schultz|Australia
>Spike|hCoV-19/England/ALDP-9BCBBE/2020|2020-09-08|EPI_ISL_573059|Original|hCoV-19^^England|Human|Oxford Viromics|COVID-19 Genomics UK (COG-UK) Consortium|Golubchik|United Kingdom
>Spike|hCoV-19/USA/WA-S1898/2020|2020-05-28|EPI_ISL_496324|Original|hCoV-19^^Washington|Human|Washington State Department of Health|Seattle Flu Study|Lee|USA
>Spike|hCoV-19/USA/CA-ALSR-0519-IPL/2020|2020-04-02|EPI_ISL_483198|Original|hCoV-19^^California|Human|UC San Diego Center for Advanced Laboratory Medicine|Andersen lab at Scripps Research|Gangavarapu|USA
>Spike|hCoV-19/USA/WA-S1137/2020|2020-05-04|EPI_ISL_463627|Original|hCoV-19^^Washington|Human|Washington State Department of Health|Seattle Flu Study|Bedford|USA
>Spike|hCoV-19/Australia/SAP147/2020|2020-04-11|EPI_ISL_451145|Original|hCoV-19^^South Australia|Human|SA Pathology|SA Pathology|Leong|Australia
>Spike|hCoV-19/Belgium/ULG-10153/2020|2020-04-18|EPI_ISL_447149|Original|hCoV-19^^Liege|Human|Department of Clinical Microbiology|GIGA Medical Genomics|Durkin|Belgium
>Spike|hCoV-19/Switzerland/BS-ETHZ-100063/2020|2020-03-12|EPI_ISL_466985|Original|hCoV-19^^Basel-Stadt|Human|Viollier AG|Department of Biosystems Science and Engineering|Nadeau|Switzerland
>Spike|hCoV-19/Belgium/ULG-10127/2020|2020-04-07|EPI_ISL_427373|Original|hCoV-19^^Liege|Human|Department of Clinical Microbiology|GIGA Medical Genomics|Durkin|Belgium
>Spike|hCoV-19/Scotland/EDB6604/2020|2020-05-12|EPI_ISL_484681|Original|hCoV-19^^Scotland|Human|Virology Department|COVID-19 Genomics UK (COG-UK) Consortium|Williams|United Kingdom
>Spike|hCoV-19/Switzerland/GR-ETHZ-100013/2020|2020-03-05|EPI_ISL_466938|Original|hCoV-19^^Graubunden|Human|Viollier AG|Department of Biosystems Science and Engineering|Nadeau|Switzerland
>Spike|hCoV-19/USA/CA-ALSR-2121/2020|2020-05-29|EPI_ISL_635356|Original|hCoV-19^^California|Human|San Diego County Public Health Laboratory|Andersen lab at Scripps Research|Gangavarapu|USA
>Spike|hCoV-19/Australia/VIC6786/2020|2020-07-30|EPI_ISL_564054|Original|hCoV-19^^Victoria|Human|Microbiological Diagnostic Unit - Public Health Laboratory (MDU-PHL)|MDU-PHL|Schultz|Australia
>Spike|hCoV-19/USA/VA-DCLS-0942/2020|2020-05-21|EPI_ISL_522803|Original|hCoV-19^^Virginia|Human|Virginia DCLS|Virginia DCLS|St. Jacques|USA
>Spike|hCoV-19/Norway/3118/2020|2020-08-06|EPI_ISL_549141|Original|hCoV-19^^Viken|Human|Ostfold Hospital Trust - Kalnes|Norwegian Institute of Public Health|Kopperud Riis|Norway

>Spike|hCoV-19/Tunisia/MHT_2/2020|2020-03-24|EPI_ISL_458286|Original|hCoV-19^^Ben Arous|Human|unknown|Bundeswehr Institute of Microbiology|Walter|Tunisia
>Spike|hCoV-19/Beijing/DT-travelHU02/2020|2020-03-18|EPI_ISL_452348|Original|hCoV-19^^Beijing|Human|Laboratory of Infectious Diseases Center of Beijing Ditan Hospital|Laboratory of Infectious Diseases Center of Beijing Ditan Hospital|Yang|China
>Spike|hCoV-19/USA/CA-IGI-0107/2020|2020-05-22|EPI_ISL_495129|Original|hCoV-19^^California|Human|Innovative Genomics Institute|Innovative Genomics Institute|Shivram|USA
>Spike|hCoV-19/Wales/PHWC-49B5BC/2020|2020-12-07|EPI_ISL_742356|Original|hCoV-19^^Wales|Human|Wales Specialist Virology Centre Sequencing lab|COVID-19 Genomics UK (COG-UK) Consortium|Moore|United Kingdom
>Spike|hCoV-19/USA/WA-UW-13233/2020|2020-06-29|EPI_ISL_501123|Original|hCoV-19^^Washington|Human|University of Washington Virology Lab|University of Washington Virology Lab|Roychoudhury|USA
>Spike|hCoV-19/Australia/VIC7650/2020|2020-08-06|EPI_ISL_564551|Original|hCoV-19^^Victoria|Human|Victorian Infectious Diseases Reference Laboratory (VIDRL)|VIDRL and MDU-PHL|Schultz|Australia
>Spike|hCoV-19/Japan/PG-6642/2020|2020-07-00|EPI_ISL_687754|Original|hCoV-19^^Japan|Human|Pathogen Genomics Center|Pathogen Genomics Center|Sekizuka|Japan
>Spike|hCoV-19/England/QEUH-B1401D/2020|2020-11-02|EPI_ISL_641669|Original|hCoV-19^^England|Human|Lighthouse Lab in Glasgow|Wellcome Sanger Institute for the COVID-19 Genomics UK (COG-UK) Consortium|Davies|United Kingdom
>Spike|hCoV-19/Switzerland/BL-42206306/2020|2020-03-25|EPI_ISL_581746|Original|hCoV-19^^Basel-Land|Human|University Hospital Basel|University Hospital Basel|Roloff Handschin|Switzerland
>Spike|hCoV-19/England/NORT-294E14/2020|2020-03-29|EPI_ISL_478619|Original|hCoV-19^^England|Human|Northumbria University / South Tees Hospitals NHS Foundation Trust / North Cumbria Integrated Care NHS Foundation Trust / North Tees and Hartlepool NHS Foundation Trust / Newcastle Hospitals NHS Foundation Trust|COVID-19 Genomics UK (COG-UK) Consortium|Bashton|United Kingdom
>Spike|hCoV-19/France/ARA-100644/2020|2020-11-03|EPI_ISL_732678|Original|hCoV-19^^Auvergne-Rh\u00f4ne-Alpes|Human|CNR Virus des Infections Respiratoires - France SUD|CNR Virus des Infections Respiratoires - France SUD|Bal|France
>Spike|hCoV-19/Netherlands/GR-RIVM-20427/2020|2020-12-01|EPI_ISL_728647|Original|hCoV-19^^Groningen|Human|Dutch COVID-19 response team|National Institute for Public Health and the Environment (RIVM)|Meijer|Netherlands
>Spike|hCoV-19/USA/IL-NM-0521/2020|2020-04-27|EPI_ISL_626441|Original|hCoV-19^^Illinois|Human|Northwestern Memorial Hospital|Ozer Lab|Ozer|USA
>Spike|hCoV-19/England/ALDP-C22EB7/2020|2020-12-06|EPI_ISL_733797|Original|hCoV-19^^England|Human|Lighthouse Lab in Alderley Park|Wellcome Sanger Institute for the COVID-19 Genomics UK (COG-UK) Consortium|Davies|United Kingdom
>Spike|hCoV-19/England/ALDP-A84969/2020|2020-10-18|EPI_ISL_610332|Original|hCoV-19^^England|Human|Lighthouse Lab in Alderley Park|Wellcome Sanger Institute for the COVID-19 Genomics UK (COG-UK) Consortium|Davies|United Kingdom
>Spike|hCoV-19/England/MILK-A23C45/2020|2020-10-03|EPI_ISL_595110|Original|hCoV-19^^England|Human|Centre for Enzyme Innovation|COVID-19 Genomics UK (COG-UK) Consortium|Robson|United Kingdom
>Spike|hCoV-19/USA/MI-MDHHS-SC20531/2020|2020-05-14|EPI_ISL_460579|Original|hCoV-19^^Michigan|Human|Michigan Department of Health and Human Services|Michigan Department of Health and Human Services|Blankenship|USA
>Spike|hCoV-19/Japan/PG-0642/2020|2020-04-00|EPI_ISL_684252|Original|hCoV-19^^Japan|Human|Gunma Prefectural Institute of Public Health and Environmental Sciences|Pathogen Genomics Center|Sekizuka|Japan
>Spike|hCoV-19/Sweden/20-53905/2020|2020-12-17|EPI_ISL_789057|Original|hCoV-19^^Stockholm|Human|Klinisk mikrobiologi|The Public Health Agency of Sweden|Svartstr\u00f6m|Sweden

>Spike|hCoV-19/USA/MI-MDHHS-SC22669/2020|2020-11-29|EPI_ISL_737283|Original|hCoV-19^^Michigan|Human|Michigan Department of Health and Human Services|Michigan Department of Health and Human Services|Blankenship|USA
>Spike|hCoV-19/USA/TX-HMH-1545/2020|2020-04-13|EPI_ISL_542785|Original|hCoV-19^^Texas|Human|Houston Methodist Hospital|Houston Methodist Hospital|Long|USA
>Spike|hCoV-19/Denmark/DCGC-20944/2020|2020-12-21|EPI_ISL_759463|Original|hCoV-19^^Hovedstaden|Human|Department of Virus and Microbiological Special Diagnostics|Albertsen Lab|Michaelsen|Denmark
>Spike|hCoV-19/England/LOND-D63EC/2020|2020-04-20|EPI_ISL_487562|Original|hCoV-19^^England|Human|University College London|Wellcome Sanger Institute for the COVID-19 Genomics UK (COG-UK) consortium|Davies|United Kingdom
>Spike|hCoV-19/England/QEUH-B41994/2020|2020-11-08|EPI_ISL_658655|Original|hCoV-19^^England|Human|Lighthouse Lab in Glasgow|Wellcome Sanger Institute for the COVID-19 Genomics UK (COG-UK) Consortium|Davies|United Kingdom
>Spike|hCoV-19/USA/TX-HMH-2747/2020|2020-06-04|EPI_ISL_545524|Original|hCoV-19^^Texas|Human|Houston Methodist Hospital|Houston Methodist Hospital|Long|USA
>Spike|hCoV-19/Switzerland/VS-ETHZ-300440/2020|2020-09-29|EPI_ISL_603393|Original|hCoV-19^^Valais|Human|Viollier AG|Department of Biosystems Science and Engineering|Nadeau|Switzerland
>Spike|hCoV-19/Peru/LIM-UPCH-0088/2020|2020-06-17|EPI_ISL_540966|Original|hCoV-19^^Lima|Human|Laboratorio de Referencia Nacional de Virus Respiratorios|Laboratorio de Gen\u00f3mica Microbiana|Tsukayama|Peru
>Spike|hCoV-19/England/BHRT-24B3A13/2020|2020-05-28|EPI_ISL_528791|Original|hCoV-19^^England|Human|Microbiology Department|Wellcome Sanger Institute for the COVID-19 Genomics UK (COG-UK) consortium|Davies|United Kingdom
>Spike|hCoV-19/France/ARA-113416/2020|2020-11-28|EPI_ISL_745344|Original|hCoV-19^^Auvergne-Rh\u00f4ne-Alpes|Human|CNR Virus des Infections Respiratoires - France SUD|CNR Virus des Infections Respiratoires - France SUD|Bal|France
>Spike|hCoV-19/USA/MI-UM-10036671254/2020|2020-10-28|EPI_ISL_650092|Original|hCoV-19^^Michigan|Human|University of Michigan Clinical Microbiology Laboratory|Lauring Lab|Valesano|USA
>Spike|hCoV-19/Netherlands/NA_200/2020|2020-03-25|EPI_ISL_422735|Original|hCoV-19^^Netherlands|Human|Dutch COVID-19 response team|Erasmus Medical Center|Oude Munnink|Netherlands
>Spike|hCoV-19/England/210100387/2021|2021-01-04|EPI_ISL_791264|Original|hCoV-19^^England|Human|Respiratory Virus Unit|COVID-19 Genomics UK (COG-UK) Consortium|Platt|United Kingdom
>Spike|hCoV-19/Scotland/CVR2075/2020|2020-04-09|EPI_ISL_493762|Original|hCoV-19^^Scotland|Human|West of Scotland Specialist Virology Centre|COVID-19 Genomics UK (COG-UK) Consortium|Robertson|United Kingdom
>Spike|hCoV-19/USA/MN-GMF-16735/2020|2020-08-23|EPI_ISL_547728|Original|hCoV-19^^Minnesota|Human|Gundersen Molecular Diagnostics Laboratory|Kabara Cancer Research Institute|Kenny|USA
>Spike|hCoV-19/Scotland/QEUH-AD13AB/2020|2020-10-27|EPI_ISL_649718|Original|hCoV-19^^Scotland|Human|Lighthouse Lab in Glasgow|Wellcome Sanger Institute for the COVID-19 Genomics UK (COG-UK) Consortium|Davies|United Kingdom
>Spike|hCoV-19/Netherlands/NoordBrabant_34/2020|2020-03-00|EPI_ISL_414543|Original|hCoV-19^^Noord Brabant|Human|Dutch COVID-19 response team|Erasmus Medical Center|Oude Munnink|Netherlands
>Spike|hCoV-19/USA/CA-CZB-14817/2020|2020-11-18|EPI_ISL_739001|Original|hCoV-19^^California|Human|Humboldt County Public Health Laboratory|Chan-Zuckerberg Biohub|Kamm|USA
>Spike|hCoV-19/Germany/HH-hpi-p1489/2020|2020-03-13|EPI_ISL_775998|Original|hCoV-19^^Hamburg|Human|University Medical Center Hamburg Eppendorf|Heinrich Pette Institute|Robitaille|Germany
>Spike|hCoV-19/USA/SC-DHEC-0064/2020|2020-03-30|EPI_ISL_529175|Original|hCoV-19^^South Carolina|Human|South Carolina Department of Health and Environmental Control|South Carolina Department of Health and Environmental Control|Flores|USA

>Spike|hCoV-19/India/MH-ACTREC-038/2020|2020-05-09|EPI_ISL_699694|Original|hCoV-19^^Maharashtra|Human|Hematopathology Laboratory|Hematopathology Laboratory|Patkar|India
>Spike|hCoV-19/England/MILK-A19152/2020|2020-09-30|EPI_ISL_612486|Original|hCoV-19^^England|Human|Wales Specialist Virology Centre Sequencing lab|COVID-19 Genomics UK (COG-UK) Consortium|Moore|United Kingdom
>Spike|hCoV-19/England/EKHU-260EFCD/2020|2020-03-30|EPI_ISL_501628|Original|hCoV-19^^England|Human|Lab Microbiology|Wellcome Sanger Institute for the COVID-19 Genomics UK (COG-UK) consortium|Davies|United Kingdom
>Spike|hCoV-19/Netherlands/GE-EMC-405/2020|2020-08-11|EPI_ISL_722362|Original|hCoV-19^^Gelderland|Human|Dutch COVID-19 response team|Erasmus Medical Center|Oude Munnink|Netherlands
>Spike|hCoV-19/England/NORT-294588/2020|2020-08-03|EPI_ISL_572753|Original|hCoV-19^^England|Human|Northumbria University / South Tees Hospitals NHS Foundation Trust / North Cumbria Integrated Care NHS Foundation Trust / North Tees and Hartlepool NHS Foundation Trust / Newcastle Hospitals NHS Foundation Trust|COVID-19 Genomics UK (COG-UK) Consortium|Bashton|United Kingdom
>Spike|hCoV-19/USA/CA-LACPHL-AA00007/2020|2020-08-03|EPI_ISL_710105|Original|hCoV-19^^California|Human|Los Angeles County PHL|Los Angeles County PHL|Hemarajata|USA
>Spike|hCoV-19/England/CAMC-A41889/2020|2020-10-06|EPI_ISL_609405|Original|hCoV-19^^England|Human|Lighthouse Lab in Cambridge|Wellcome Sanger Institute for the COVID-19 Genomics UK (COG-UK) consortium|Davies|United Kingdom
>Spike|hCoV-19/England/ALDP-C3D208/2020|2020-12-10|EPI_ISL_730975|Original|hCoV-19^^England|Human|Lighthouse Lab in Alderley Park|Wellcome Sanger Institute for the COVID-19 Genomics UK (COG-UK) Consortium|Davies|United Kingdom
>Spike|hCoV-19/Switzerland/GR-ETHZ-350476/2020|2020-11-03|EPI_ISL_729210|Original|hCoV-19^^Graub\u00fcnden|Human|Viollier AG|Department of Biosystems Science and Engineering|Nadeau|Switzerland
>Spike|hCoV-19/England/ALDP-AA2F81/2020|2020-10-22|EPI_ISL_624015|Original|hCoV-19^^England|Human|Lighthouse Lab in Alderley Park|Wellcome Sanger Institute for the COVID-19 Genomics UK (COG-UK) consortium|Davies|United Kingdom
>Spike|hCoV-19/Japan/PG-8707/2020|2020-08-00|EPI_ISL_686303|Original|hCoV-19^^Japan|Human|Pathogen Genomics Center|Pathogen Genomics Center|Sekizuka|Japan
>Spike|hCoV-19/Wales/MILK-9C26C3/2020|2020-09-10|EPI_ISL_566553|Original|hCoV-19^^Wales|Human|Lighthouse Lab in Milton Keynes|Wellcome Sanger Institute for the COVID-19 Genomics UK (COG-UK) consortium|Davies|United Kingdom
>Spike|hCoV-19/USA/LA-GBCL-26544561/2020|2020-04-18|EPI_ISL_485136|Original|hCoV-19^^Louisiana|Human|River Road Testing Lab|Ginkgo Bioworks Clinical Laboratory|Smith|USA
>Spike|hCoV-19/England/CAMC-990F5A/2020|2020-08-29|EPI_ISL_551896|Original|hCoV-19^^England|Human|Lighthouse Lab in Cambridge|Wellcome Sanger Institute for the COVID-19 Genomics UK (COG-UK) consortium|Davies|United Kingdom
>Spike|hCoV-19/England/ALDP-9E7446/2020|2020-09-24|EPI_ISL_580311|Original|hCoV-19^^England|Human|Lighthouse Lab in Alderley Park|Wellcome Sanger Institute for the COVID-19 Genomics UK (COG-UK) consortium|Davies|United Kingdom
>Spike|hCoV-19/USA/CA-QDX-1675/2020|2020-03-18|EPI_ISL_603924|Original|hCoV-19^^California|Human|Quest Diagnostics|Quest Diagnostics|Kagan|USA
>Spike|hCoV-19/USA/LA-EVTL1140/2020|2020-12-08|EPI_ISL_768409|Original|hCoV-19^^Louisiana|Human|LSUHS Emerging Viral Threat Laboratory|Microbial Genome Sequencing Center|Kamil|USA
>Spike|hCoV-19/Denmark/DCGC-19163/2020|2020-12-07|EPI_ISL_748788|Original|hCoV-19^^Hovedstaden|Human|Department of Virus and Microbiological Special Diagnostics|Albertsen Lab|Michaelsen|Denmark
>Spike|hCoV-19/Italy/APU-IZSPB-332PT/2020|2020-10-10|EPI_ISL_722893|Vero|hCoV-19^^Apulia|Human|Istituto Zooprofilattico Sperimentale della Puglia e della Basilicata|Istituto Zooprofilattico Sperimentale della Puglia e della Basilicata|Parisi|Italy
>Spike|hCoV-19/England/ALDP-B1CA80/2020|2020-11-02|EPI_ISL_664844|Original|hCoV-19^^England|Human|Centre for Enzyme Innovation|COVID-19 Genomics UK (COG-UK) Consortium|Robson|United Kingdom

>Spike|hCoV-19/USA/MD-MDH-0178/2020|2020-06-04|EPI_ISL_534715|Original|hCoV-19^^Maryland|Human|MD PHL|MD PHL|Keller|USA
>Spike|hCoV-19/Denmark/DCGC-19331/2020|2020-12-07|EPI_ISL_748887|Original|hCoV-19^^Syddanmark|Human|Department of Virus and Microbiological Special Diagnostics|Albertsen Lab|Michaelsen|Denmark
>Spike|hCoV-19/England/GSTT-265DB61/2020|2020-03-27|EPI_ISL_484071|Original|hCoV-19^^England|Human|Centre for Clinical Infection and Diagnostics Research and Genomics Innovation Unit|COVID-19 Genomics UK (COG-UK) Consortium|Awan|United Kingdom
>Spike|hCoV-19/Spain/AN-IBV-98001286/2020|2020-03-24|EPI_ISL_538223|Original|hCoV-19^^Andalucia|Human|Servicio de Microbiolog\u00eda y Parasitolog\u00eda cl\u00ednica. UCEIMP. Hospital Universitario Virgen del Roc\u00edo/IBIS/CSIC/US|SeqCOVID-SPAIN consortium/IBV(CSIC)|Comas|Spain
>Spike|hCoV-19/Switzerland/JU-ETHZ-100124/2020|2020-03-16|EPI_ISL_451708|Original|hCoV-19^^Jura|Human|Viollier AG|Department of Biosystems Science and Engineering|Nadeau|Switzerland
>Spike|hCoV-19/England/ALDP-4A6E86/2020|2020-06-08|EPI_ISL_559640|Original|hCoV-19^^England|Human|Lighthouse Lab in Alderley Park|Wellcome Sanger Institute for the COVID-19 Genomics UK (COG-UK) consortium|Davies|United Kingdom
>Spike|hCoV-19/Denmark/DCGC-22174/2020|2020-12-14|EPI_ISL_758599|Original|hCoV-19^^Hovedstaden|Human|Department of Virus and Microbiological Special Diagnostics|Albertsen Lab|Michaelsen|Denmark
>Spike|hCoV-19/USA/TX-HMH-1080/2020|2020-04-12|EPI_ISL_542523|Original|hCoV-19^^Texas|Human|Houston Methodist Hospital|Houston Methodist Hospital|Long|USA
>Spike|hCoV-19/England/20139024204/2020|2020-03-27|EPI_ISL_421891|Original|hCoV-19^^England|Human|Respiratory Virus Unit|Respiratory Virus Unit|Platt|United Kingdom
>Spike|hCoV-19/USA/TX-HMH-MCoV-19202/2020|2020-11-28|EPI_ISL_783687|Original|hCoV-19^^Texas|Human|Houston Methodist Hospital|Houston Methodist Hospital|Long|USA
>Spike|hCoV-19/Denmark/DCGC-18164/2020|2020-12-14|EPI_ISL_749789|Original|hCoV-19^^Syddanmark|Human|Department of Virus and Microbiological Special Diagnostics|Albertsen Lab|Michaelsen|Denmark
>Spike|hCoV-19/England/CAMB-1BC767/2020|2020-11-29|EPI_ISL_723892|Original|hCoV-19^^England|Human|Department of Pathology|COVID-19 Genomics UK (COG-UK) Consortium|Goodfellow|United Kingdom
>Spike|hCoV-19/India/WB-IK92/2020|2020-05-20|EPI_ISL_508410|Original|hCoV-19^^West Bengal|Human|Institute of Post Graduate Medical Education & Research|National Institute of Biomedical Genomics|Maitra|India
>Spike|hCoV-19/England/LIVE-A5683/2020|2020-03-21|EPI_ISL_500137|Original|hCoV-19^^England|Human|Liverpool Clinical Laboratories|COVID-19 Genomics UK (COG-UK) Consortium|Haldenby|United Kingdom
>Spike|hCoV-19/USA/UT-UPHL-2009723/2020|2020-07-26|EPI_ISL_605395|Original|hCoV-19^^Utah|Human|Utah Public Health Laboratory|Utah Public Health Laboratory|Young|USA
>Spike|hCoV-19/England/CAMB-1AC7E2/2020|2020-04-22|EPI_ISL_489546|Original|hCoV-19^^England|Human|Department of Pathology|Wellcome Sanger Institute for the COVID-19 Genomics UK (COG-UK) consortium|Davies|United Kingdom
>Spike|hCoV-19/USA/CA-ALSR-1057-SAN/2020|2020-06-20|EPI_ISL_483488|Original|hCoV-19^^California|Human|UC San Diego Center for Advanced Laboratory Medicine|Andersen lab at Scripps Research|Gangavarapu|USA
>Spike|hCoV-19/England/LIVE-DBAC7D/2020|2020-11-14|EPI_ISL_664113|Original|hCoV-19^^England|Human|Liverpool Clinical Laboratories|COVID-19 Genomics UK (COG-UK) Consortium|Haldenby|United Kingdom
>Spike|hCoV-19/Scotland/QEUH-B13FC6/2020|2020-11-01|EPI_ISL_649503|Original|hCoV-19^^Scotland|Human|Lighthouse Lab in Glasgow|Wellcome Sanger Institute for the COVID-19 Genomics UK (COG-UK) Consortium|Davies|United Kingdom
>Spike|hCoV-19/Germany/BY-MVP-0107/2020|2020-04-13|EPI_ISL_437297|Original|hCoV-19^^Bavaria|Human|Max von Pettenkofer Institute|Laboratory for Functional Genome Analysis|Graf|Germany
>Spike|hCoV-19/USA/CA-CHLA-PLM29218152/2020|2020-11-30|EPI_ISL_753456|Original|hCoV-19^^California|Human|Clinical virology Laboratory|Center for Personalized Medicine|Dien Bard|USA

>Spike|hCoV-19/USA/TX-HMH-MCoV-16191/2020|2020-10-28|EPI_ISL_785875|Original|hCoV-19^^Texas|Human|Houston Methodist Hospital|Houston Methodist Hospital|Long|USA
>Spike|hCoV-19/USA/HI-H200628/2020|2020-11-13|EPI_ISL_753153|Original|hCoV-19^^Hawaii|Human|State Laboratories Division|State Laboratories Division|Sultana|USA
>Spike|hCoV-19/Switzerland/SZ-ETHZ-360118/2020|2020-11-08|EPI_ISL_693913|Original|hCoV-19^^Schwyz|Human|Viollier AG|Department of Biosystems Science and Engineering|Nadeau|Switzerland
>Spike|hCoV-19/England/CAMB-1AFB98/2020|2020-05-02|EPI_ISL_459172|Original|hCoV-19^^England|Human|Department of Pathology|Wellcome Sanger Institute for the COVID-19 Genomics UK (COG-UK) consortium|Davies|United Kingdom
>Spike|hCoV-19/England/204191453/2020|2020-10-10|EPI_ISL_603188|Original|hCoV-19^^England|Human|Respiratory Virus Unit|Respiratory Virus Unit|Platt|United Kingdom
>Spike|hCoV-19/Netherlands/ZH-EMC-378/2020|2020-08-02|EPI_ISL_523718|Original|hCoV-19^^Zuid Holland|Human|Dutch COVID-19 response team|Erasmus Medical Center|Oude Munnink|Netherlands
>Spike|hCoV-19/Finland/9LS11AC1/2020|2020-10-09|EPI_ISL_755944|Original|hCoV-19^^Uusimaa|Human|Department of Virology and Immunology|Department of Virology|Smura|Finland
>Spike|hCoV-19/India/GJ-GBRC270/2020|2020-06-13|EPI_ISL_483867|Original|hCoV-19^^Gujarat|Human|Department of Microbiology|Gujarat Biotechnology Research Centre|Joshi|India
>Spike|hCoV-19/Denmark/DCGC-1498/2020|2020-06-01|EPI_ISL_617982|Original|hCoV-19^^Hovedstaden|Human|Department of Virus and Microbiological Special Diagnostics|Albertsen lab|Michaelsen|Denmark
>Spike|hCoV-19/USA/WA-S991/2020|2020-04-27|EPI_ISL_463481|Original|hCoV-19^^Washington|Human|Washington State Department of Health|Seattle Flu Study|Bedford|USA
>Spike|hCoV-19/Singapore/1316/2020|2020-05-19|EPI_ISL_648803|Original|hCoV-19^^Singapore|Human|Department of Laboratory Medicine|Department of Laboratory Medicine|Chen|Singapore
>Spike|hCoV-19/England/QEUH-99000C/2020|2020-08-26|EPI_ISL_540024|Original|hCoV-19^^England|Human|Lighthouse Lab in Glasgow|Wellcome Sanger Institute for the COVID-19 Genomics UK (COG-UK) consortium|Davies|United Kingdom
>Spike|hCoV-19/Australia/VIC6691/2020|2020-07-20|EPI_ISL_521447|Original|hCoV-19^^Victoria|Human|Victorian Infectious Diseases Reference Laboratory (VIDRL)|VIDRL and MDU-PHL|Schultz|Australia
>Spike|hCoV-19/USA/CA-CZB-14098/2020|2020-05-27|EPI_ISL_739511|Original|hCoV-19^^California|Human|Alameda County Public Health Lab|Chan-Zuckerberg Biohub|Kamm|USA
>Spike|hCoV-19/England/PORT-2D3A8E/2020|2020-04-17|EPI_ISL_529465|Original|hCoV-19^^England|Human|Centre for Enzyme Innovation|COVID-19 Genomics UK (COG-UK) Consortium|Robson|United Kingdom
>Spike|hCoV-19/USA/MN-MDH-1267/2020|2020-03-09|EPI_ISL_482988|Original|hCoV-19^^Minnesota|Human|Minnesota Department of Health|Minnesota Department of Health|Garfin|USA
>Spike|hCoV-19/Canada/QC-LSPQ-L00231249/2020|2020-03-22|EPI_ISL_535948|Original|hCoV-19^^Quebec|Human|H\u00f4pital Pierre-Boucher|Laboratoire de sant\u00e9 publique du Qu\u00e9bec|Moreira|Canada
>Spike|hCoV-19/USA/WI-WSLH-200034/2020|2020-03-28|EPI_ISL_471199|Original|hCoV-19^^Wisconsin|Human|Wisconsin State Laboratory of Hygiene Communicable Disease Division|Wisconsin State Laboratory of Hygiene Communicable Disease Division|Florek|USA
>Spike|hCoV-19/England/QEUH-B8237B/2020|2020-11-14|EPI_ISL_661907|Original|hCoV-19^^England|Human|Lighthouse Lab in Glasgow|Wellcome Sanger Institute for the COVID-19 Genomics UK (COG-UK) Consortium|Davies|United Kingdom
>Spike|hCoV-19/Denmark/DCGC-8562/2020|2020-10-26|EPI_ISL_625956|Original|hCoV-19^^Midtjylland|Human|Department of Virus and Microbiological Special Diagnostics|Albertsen lab|Michaelsen|Denmark
>Spike|hCoV-19/USA/ID-UW-2067/2020|2020-03-22|EPI_ISL_477681|Original|hCoV-19^^Idaho|Human|UW Virology Lab|UW Virology Lab|Roychoudhury|USA

>Spike|hCoV-19/Luxembourg/LNS6071962/2020|2020-11-09|EPI_ISL_744350|Original|hCoV-19^^Luxembourg|Human|Laboratoire national de sant\u00e9|Laboratoire national de sant\u00e9|Wienecke-Baldacchino|Luxembourg
>Spike|hCoV-19/Iceland/560/2020|2020-03-27|EPI_ISL_424580|Original|hCoV-19^^Reykjavik|Human|The National University Hospital of Iceland|deCODE genetics|J\u00f3nsson|Iceland
>Spike|hCoV-19/England/QEUH-BCCF80/2020|2020-11-26|EPI_ISL_704310|Original|hCoV-19^^England|Human|Lighthouse Lab in Glasgow|Wellcome Sanger Institute for the COVID-19 Genomics UK (COG-UK) Consortium|Davies|United Kingdom
>Spike|hCoV-19/USA/WA-UW-4035/2020|2020-03-28|EPI_ISL_427235|Original|hCoV-19^^Washington|Human|UW Virology Lab|UW Virology Lab|Roychoudhury|USA
>Spike|hCoV-19/England/QEUH-9AE241/2020|2020-09-01|EPI_ISL_637341|Original|hCoV-19^^England|Human|Department of Pathology|COVID-19 Genomics UK (COG-UK) Consortium|Goodfellow|United Kingdom
>Spike|hCoV-19/England/ALDP-B5D1CA/2020|2020-11-09|EPI_ISL_678868|Original|hCoV-19^^England|Human|Department of Pathology|COVID-19 Genomics UK (COG-UK) Consortium|Goodfellow|United Kingdom
>Spike|hCoV-19/Belgium/ULG-8578/2020|2020-03-19|EPI_ISL_418638|Original|hCoV-19^^Li\u00e8ge|Human|Department of Clinical Microbiology|GIGA Medical Genomics|Durkin|Belgium
>Spike|hCoV-19/England/20129099504/2020|2020-03-13|EPI_ISL_424085|Original|hCoV-19^^England|Human|Respiratory Virus Unit|Respiratory Virus Unit|Platt|United Kingdom
>Spike|hCoV-19/England/QEUH-9F3562/2020|2020-09-23|EPI_ISL_588623|Original|hCoV-19^^England|Human|Lighthouse Lab in Glasgow|Wellcome Sanger Institute for the COVID-19 Genomics UK (COG-UK) consortium|Davies|United Kingdom
>Spike|hCoV-19/England/ALDP-B5B5C0/2020|2020-11-08|EPI_ISL_655592|Original|hCoV-19^^England|Human|Lighthouse Lab in Alderley Park|Wellcome Sanger Institute for the COVID-19 Genomics UK (COG-UK) Consortium|Davies|United Kingdom
>Spike|hCoV-19/USA/MN-MDH-1934/2020|2020-10-01|EPI_ISL_613492|Original|hCoV-19^^Minnesota|Human|Public Health Laboratory - Infectious Disease Lab|Minnesota Department of Health|Lorentz|USA
>Spike|hCoV-19/USA/WA-UW-12876/2020|2020-06-29|EPI_ISL_570918|Original|hCoV-19^^Washington|Human|UW Virology Lab|UW Virology Lab|Roychoudhury|USA
>Spike|hCoV-19/USA/OH-OSUP0016/2020|2020-03-31|EPI_ISL_427288|Original|hCoV-19^^Ohio|Human|The Ohio State University|The Ohio State University-James Molecular Lab at Polaris|Jones|USA
>Spike|hCoV-19/Wales/ALDP-BC887A/2020|2020-11-22|EPI_ISL_767327|Original|hCoV-19^^Wales|Human|Lighthouse Lab in Alderley Park|Wellcome Sanger Institute for the COVID-19 Genomics UK (COG-UK) Consortium|Davies|United Kingdom
>Spike|hCoV-19/Scotland/QEUH-BC4656/2020|2020-11-25|EPI_ISL_710748|Original|hCoV-19^^Scotland|Human|Lighthouse Lab in Glasgow|Wellcome Sanger Institute for the COVID-19 Genomics UK (COG-UK) Consortium|Davies|United Kingdom
>Spike|hCoV-19/England/204661580/2020|2020-11-12|EPI_ISL_693412|Original|hCoV-19^^England|Human|Respiratory Virus Unit|COVID-19 Genomics UK (COG-UK) Consortium|Platt|United Kingdom
>Spike|hCoV-19/Denmark/DCGC-17826/2020|2020-12-14|EPI_ISL_749587|Original|hCoV-19^^Hovedstaden|Human|Department of Virus and Microbiological Special Diagnostics|Albertsen Lab|Michaelsen|Denmark
>Spike|hCoV-19/Northern Ireland/NIRE-106D02/2020|2020-05-19|EPI_ISL_501065|Original|hCoV-19^^Northern Ireland|Human|Regional Virus Laboratory|Wellcome Sanger Institute for the COVID-19 Genomics UK (COG-UK) consortium|Davies|United Kingdom
>Spike|hCoV-19/Poland/IHG_PAS_3_57/2020|2020-05-13|EPI_ISL_462480|Original|hCoV-19^^Wielkopolskie|Human|Institute of Human Genetics|Institute of Human Genetics|Hryhorowicz|Poland
>Spike|hCoV-19/England/C200042062/2020|2020-11-02|EPI_ISL_649802|Original|hCoV-19^^England|Human|Respiratory Virus Unit|COVID-19 Genomics UK (COG-UK) Consortium|Platt|United Kingdom
>Spike|hCoV-19/USA/IL-NM-0547/2020|2020-04-29|EPI_ISL_626454|Original|hCoV-19^^Illinois|Human|Northwestern Memorial Hospital|Ozer Lab|Ozer|USA

>Spike|hCoV-19/USA/AK-PHL592/2020|2020-06-23|EPI_ISL_586244|Original|hCoV-19^^Alaska|Human|Alaska State Virology Laboratory|Alaska State Virology Laboratory|Chen|USA
>Spike|hCoV-19/England/CAMC-B520F5/2020|2020-11-08|EPI_ISL_657723|Original|hCoV-19^^England|Human|Lighthouse Lab in Cambridge|Wellcome Sanger Institute for the COVID-19 Genomics UK (COG-UK) Consortium|Davies|United Kingdom
>Spike|hCoV-19/Switzerland/SO-ETHZ-400300/2020|2020-12-07|EPI_ISL_729158|Original|hCoV-19^^Solothurn|Human|Viollier AG|Department of Biosystems Science and Engineering|Nadeau|Switzerland
>Spike|hCoV-19/Wales/PHWC-4933E7/2020|2020-11-28|EPI_ISL_725881|Original|hCoV-19^^Wales|Human|Originating lab|COVID-19 Genomics UK (COG-UK) Consortium|Moore|United Kingdom
>Spike|hCoV-19/Ireland/WW-NVRL-73IRL23933/2020|2020-09-05|EPI_ISL_578330|Original|hCoV-19^^Wicklow|Human|National Virus Reference Laboratory|National Virus Reference Laboratory|Gonzalez|Ireland
>Spike|hCoV-19/USA/TX-HMH-MCoV-14940/2020|2020-10-15|EPI_ISL_786424|Original|hCoV-19^^Texas|Human|Houston Methodist Hospital|Houston Methodist Hospital|Long|USA
>Spike|hCoV-19/South Africa/KRISP-K002395/2020|2020-07-28|EPI_ISL_515760|Original|hCoV-19^^KwaZulu-Natal|Human|NHLS-IALCH|KRISP|de Oliveira|South Africa
>Spike|hCoV-19/Germany/HH-hpi-p1655/2020|2020-03-17|EPI_ISL_775930|Original|hCoV-19^^Hamburg|Human|University Medical Center Hamburg Eppendorf|Heinrich Pette Institute|Robitaille|Germany
>Spike|hCoV-19/USA/TX-DSHS-0411/2020|2020-06-01|EPI_ISL_542470|Original|hCoV-19^^Texas|Human|Texas Department of State Health Services|Texas Department of State Health Services|Rahman|USA
>Spike|hCoV-19/Netherlands/UT-EMC-122/2020|2020-10-06|EPI_ISL_632654|Original|hCoV-19^^Utrecht|Human|Dutch COVID-19 response team|Erasmus Medical Center|Oude Munnink|Netherlands
>Spike|hCoV-19/England/CWAR-24FE0ED/2020|2020-05-01|EPI_ISL_584572|Original|hCoV-19^^England|Human|UHCW / University of Warwick|COVID-19 Genomics UK (COG-UK) Consortium|Stark|United Kingdom
>Spike|hCoV-19/Wales/PHWC-29F22/2020|2020-04-02|EPI_ISL_474256|Original|hCoV-19^^Wales|Human|Originating lab|COVID-19 Genomics UK (COG-UK) Consortium|Moore|United Kingdom
>Spike|hCoV-19/Australia/VIC3010/2020|2020-07-02|EPI_ISL_563986|Original|hCoV-19^^Victoria|Human|Microbiological Diagnostic Unit - Public Health Laboratory (MDU-PHL)|MDU-PHL|Schultz|Australia
>Spike|hCoV-19/USA/MI-MDHHS-SC20953/2020|2020-03-20|EPI_ISL_507742|Original|hCoV-19^^Michigan|Human|Michigan Department of Health and Human Services|Michigan Department of Health and Human Services|Blankenship|USA
>Spike|hCoV-19/Denmark/ALAB-SSI-1265/2020|2020-03-09|EPI_ISL_444837|Original|hCoV-19^^Denmark|Human|Department of Virus and Microbiological Special Diagnostics|Albertsen lab|Kirkegaard|Denmark
>Spike|hCoV-19/Czech Republic/NRL-7322-4/2020|2020-06-21|EPI_ISL_584077|Original|hCoV-19^^Northern Bohemia Region|Human|The National Institute of Public Health|State Veterinary Institute Prague|Nagy|Czech Republic
>Spike|hCoV-19/USA/WI-UW-145/2020|2020-04-10|EPI_ISL_427452|Original|hCoV-19^^Wisconsin|Human|University of Wisconsin-Madison AIDS Vaccine Research Laboratories|University of Wisconsin-Madison AIDS Vaccine Research Laboratories|Baczenas|USA
>Spike|hCoV-19/France/ARA-101389/2020|2020-11-04|EPI_ISL_683339|Original|hCoV-19^^Auvergne-Rh\u00f4ne-Alpes|Human|CNR Virus des Infections Respiratoires - France SUD|CNR Virus des Infections Respiratoires - France SUD|Bal|France
>Spike|hCoV-19/Denmark/DCGC-15470/2020|2020-11-23|EPI_ISL_713322|Original|hCoV-19^^Syddanmark|Human|Department of Virus and Microbiological Special Diagnostics|Albertsen Lab|Michaelsen|Denmark
>Spike|hCoV-19/Chile/BI-ISPCH-1-2/2020|2020-03-17|EPI_ISL_445281|Original|hCoV-19^^Biobio|Human|HOSPITAL CLINICO DEL SUR|Instituto de Salud Publica de Chile|Fernandez|Chile

>Spike|hCoV-19/Australia/NSW322/2020|2020-03-28|EPI_ISL_451584|Original|hCoV-19^^New South Wales|Human|Pathology West - NSW Health Pathology|NSW Health Pathology - Institute of Clinical Pathology and Medical Research; Westmead Hospital; University of Sydney|Gall|Australia
>Spike|hCoV-19/Australia/VIC3210/2020|2020-07-03|EPI_ISL_520896|Original|hCoV-19^^Victoria|Human|Victorian Infectious Diseases Reference Laboratory (VIDRL)|VIDRL and MDU-PHL|Schultz|Australia
>Spike|hCoV-19/USA/LA-EVTL501/2020|2020-07-30|EPI_ISL_578905|Original|hCoV-19^^Louisiana|Human|LSUHS Emerging Viral Threat Laboratory|Microbial Genome Sequencing Center|Kamil|USA
>Spike|hCoV-19/USA/AZ-TG668340/2020|2020-05-13|EPI_ISL_696265|Original|hCoV-19^^Arizona|Human|Sonora Quest Laboratories|TGen North|French|USA
>Spike|hCoV-19/England/MILK-C63A7A/2020|2020-12-13|EPI_ISL_735640|Original|hCoV-19^^England|Human|Lighthouse Lab in Milton Keynes|Wellcome Sanger Institute for the COVID-19 Genomics UK (COG-UK) Consortium|Davies|United Kingdom
>Spike|hCoV-19/USA/UT-02524/2020|2020-04-23|EPI_ISL_493110|Original|hCoV-19^^Utah|Human|Utah Public Health Laboratory|Utah Public Health Laboratory|Butz|USA
>Spike|hCoV-19/Wales/PHWC-48CA28/2020|2020-11-08|EPI_ISL_666496|Original|hCoV-19^^Wales|Human|Wales Specialist Virology Centre Sequencing lab|COVID-19 Genomics UK (COG-UK) Consortium|Moore|United Kingdom
>Spike|hCoV-19/England/ALDP-A6D2A7/2020|2020-10-14|EPI_ISL_608276|Original|hCoV-19^^England|Human|Lighthouse Lab in Alderley Park|Wellcome Sanger Institute for the COVID-19 Genomics UK (COG-UK) consortium|Davies|United Kingdom
>Spike|hCoV-19/England/BRIS-121C40/2020|2020-03-18|EPI_ISL_440291|Original|hCoV-19^^England|Human|PHE South West Regional Laboratory|Wellcome Sanger Institute for the COVID-19 Genomics UK (COG-UK) consortium|Amato|United Kingdom
>Spike|hCoV-19/France/HDF-1986/2020|2020-03-04|EPI_ISL_414634|Original|hCoV-19^^Hauts-de-France|Human|Centre Hospitalier Compi\u00e8gne Laboratoire de Biologie|National Reference Center for Viruses of Respiratory Infections|Enouf|France
>Spike|hCoV-19/Wales/PHWC-2706D/2020|2020-03-28|EPI_ISL_422147|Original|hCoV-19^^Wales|Human|Wales Specialist Virology Centre|Public Health Wales Microbiology Cardiff|Moore|United Kingdom
>Spike|hCoV-19/France/OCC-SC531/2020|2020-03-12|EPI_ISL_644700|Original|hCoV-19^^Occitanie|Human|CHU N\u00eemes|CNR Virus des Infections Respiratoires - France SUD|Bal|France
>Spike|hCoV-19/USA/MD-HP12098/2020|2020-12-18|EPI_ISL_791416|Original|hCoV-19^^Maryland|Human|Johns Hopkins Hospital Department of Pathology|Johns Hopkins Hospital Department of Pathology|Morris|USA
>Spike|hCoV-19/Wales/PHWC-16C4E7/2020|2020-09-25|EPI_ISL_574061|Original|hCoV-19^^Wales|Human|Wales Specialist Virology Centre Sequencing lab|COVID-19 Genomics UK (COG-UK) Consortium|Moore|United Kingdom
>Spike|hCoV-19/USA/VA-DCLS-2267/2020|2020-11-13|EPI_ISL_723511|Original|hCoV-19^^Virginia|Human|Virginia Division of Consolidated Laboratory Services (DCLS)|Virginia Division of Consolidated Laboratory Services (DCLS)|St. Jacques|USA
>Spike|hCoV-19/United Arab Emirates/L920633058/2020|2020-03-30|EPI_ISL_520724|Original|hCoV-19^^United Arab Emirates|Human|Mohammed Bin Rashid University of Medicine and Health Sciences|Al Jalila Genomics Center|Ramaswamy|United Arab Emirates
>Spike|hCoV-19/USA/WA-UW-22476/2020|2020-08-05|EPI_ISL_570695|Original|hCoV-19^^Washington|Human|UW Virology Lab|UW Virology Lab|Roychoudhury|USA
>Spike|hCoV-19/Wales/PHWC-47D2F5/2020|2020-10-10|EPI_ISL_586126|Original|hCoV-19^^Wales|Human|Wales Specialist Virology Centre Sequencing lab|COVID-19 Genomics UK (COG-UK) Consortium|Moore|United Kingdom
>Spike|hCoV-19/Denmark/DCGC-20547/2020|2020-12-21|EPI_ISL_759211|Original|hCoV-19^^Sjaelland|Human|Department of Virus and Microbiological Special Diagnostics|Albertsen Lab|Michaelsen|Denmark
>Spike|hCoV-19/USA/MN-MDH-1520/2020|2020-07-14|EPI_ISL_527585|Original|hCoV-19^^Minnesota|Human|Minnesota Department of Health|Minnesota Department of Health|Garfin|USA

>Spike|hCoV-19/England/CAMC-B220C1/2020|2020-11-02|EPI_ISL_646009|Original|hCoV-19^^England|Human|Lighthouse Lab in Cambridge|Wellcome Sanger Institute for the COVID-19 Genomics UK (COG-UK) Consortium|Davies|United Kingdom
>Spike|hCoV-19/France/IDF-IPP9725/2020|2020-11-16|EPI_ISL_693770|Original|hCoV-19^^\u00cele-de-France|Human|General practitioner|National Reference Center for Viruses of Respiratory Infections|Enouf|France
>Spike|hCoV-19/Australia/VIC5472/2020|2020-07-20|EPI_ISL_520221|Original|hCoV-19^^Victoria|Human|Microbiological Diagnostic Unit - Public Health Laboratory (MDU-PHL)|MDU-PHL|Schultz|Australia
>Spike|hCoV-19/England/QEUH-B66067/2020|2020-11-10|EPI_ISL_676382|Original|hCoV-19^^England|Human|Lighthouse Lab in Glasgow|Wellcome Sanger Institute for the COVID-19 Genomics UK (COG-UK) Consortium|Davies|United Kingdom
>Spike|hCoV-19/England/ALDP-94A39F/2020|2020-06-11|EPI_ISL_556512|Original|hCoV-19^^England|Human|Lighthouse Lab in Alderley Park|Wellcome Sanger Institute for the COVID-19 Genomics UK (COG-UK) consortium|Davies|United Kingdom
>Spike|hCoV-19/Japan/PG-6578/2020|2020-09-00|EPI_ISL_687690|Original|hCoV-19^^Japan|Human|Pathogen Genomics Center|Pathogen Genomics Center|Sekizuka|Japan
>Spike|hCoV-19/Spain/VC-FISABIO-252/2020|2020-03-29|EPI_ISL_436347|Original|hCoV-19^^Comunitat Valenciana|Human|Servicio de Microbiologia. Hospital Clinico Universitario de Valencia|Sequencing and Bioinformatics Service and Molecular Epidemiology Research Group. FISABIO-Public Health|Gonzalez-Candelas|Spain
>Spike|hCoV-19/Spain/AN-IBV-001981/2020|2020-03-15|EPI_ISL_509620|Original|hCoV-19^^Andalucia|Human|Servicio de Microbiolog\u00eda. HRU de M\u00e1laga. Servicio Andaluz de Salud|SeqCOVID-SPAIN consortium/IBV(CSIC)|Comas|Spain
>Spike|hCoV-19/Scotland/QEUH-9B9B93/2020|2020-09-09|EPI_ISL_568243|Original|hCoV-19^^Scotland|Human|Lighthouse Lab in Glasgow|Wellcome Sanger Institute for the COVID-19 Genomics UK (COG-UK) consortium|Davies|United Kingdom
>Spike|hCoV-19/Belgium/ITM_C376/2020|2020-04-08|EPI_ISL_450736|Original|hCoV-19^^Antwerp|Human|Hospital AZ Rivierenland|Institute of Tropical Medicine|Selhorst|Belgium
>Spike|hCoV-19/England/OXON-B3A4A/2020|2020-03-22|EPI_ISL_534898|Original|hCoV-19^^England|Human|Oxford Viromics|COVID-19 Genomics UK (COG-UK) Consortium|Golubchik|United Kingdom
>Spike|hCoV-19/England/CAMC-B7B3A2/2020|2020-11-12|EPI_ISL_724006|Original|hCoV-19^^England|Human|Oxford Viromics|COVID-19 Genomics UK (COG-UK) Consortium|Golubchik|United Kingdom
>Spike|hCoV-19/England/CAMC-BC0C67/2020|2020-11-20|EPI_ISL_704779|Original|hCoV-19^^England|Human|Lighthouse Lab in Cambridge|Wellcome Sanger Institute for the COVID-19 Genomics UK (COG-UK) Consortium|Davies|United Kingdom
>Spike|hCoV-19/Denmark/DCGC-8846/2020|2020-10-26|EPI_ISL_626080|Original|hCoV-19^^Syddanmark|Human|Department of Virus and Microbiological Special Diagnostics|Albertsen lab|Michaelsen|Denmark
>Spike|hCoV-19/England/SHEF-C3063/2020|2020-04-23|EPI_ISL_457597|Original|hCoV-19^^England|Human|Virology Department|COVID-19 Genomics UK (COG-UK) Consortium|de Silva|United Kingdom
>Spike|hCoV-19/Denmark/DCGC-17579/2020|2020-12-14|EPI_ISL_751080|Original|hCoV-19^^Sjaelland|Human|Department of Virus and Microbiological Special Diagnostics|Albertsen Lab|Michaelsen|Denmark
>Spike|hCoV-19/Bulgaria/21/2020|2020-05-07|EPI_ISL_480302|Original|hCoV-19^^Dupnitza|Human|National Reference Laboratory \Influenza and acute respiratory diseases\|NRL-HIV|Ivanov|Bulgaria
>Spike|hCoV-19/England/LIVE-DC827A/2020|2020-11-17|EPI_ISL_703937|Original|hCoV-19^^England|Human|Liverpool Clinical Laboratories|COVID-19 Genomics UK (COG-UK) Consortium|Haldenby|United Kingdom
>Spike|hCoV-19/England/ALDP-9B5B88/2020|2020-08-28|EPI_ISL_590399|Original|hCoV-19^^England|Human|Lighthouse Lab in Alderley Park|Wellcome Sanger Institute for the COVID-19 Genomics UK (COG-UK) consortium|Davies|United Kingdom
>Spike|hCoV-19/Czech Republic/NRL_9075/2020|2020-09-18|EPI_ISL_626585|Original|hCoV-19^^Prague|Human|The National Institute of Public Health|State Veterinary Institute Prague|Nagy|Czech Republic

>Spike|hCoV-19/USA/MI-MDHHS-SC21674/2020|2020-07-06|EPI_ISL_516401|Original|hCoV-19^^Michigan|Human|Michigan Department of Health and Human Services|Michigan Department of Health and Human Services|Blankenship|USA
>Spike|hCoV-19/Portugal/PT1622/2020|2020-07-10|EPI_ISL_693623|Original|hCoV-19^^Portugal|Human|Instituto Nacional de Saude (INSA)|Instituto Nacional de Saude (INSA)|Borges|Portugal
>Spike|hCoV-19/England/20128015104/2020|2020-03-19|EPI_ISL_423942|Original|hCoV-19^^England|Human|Respiratory Virus Unit|Respiratory Virus Unit|Platt|United Kingdom
>Spike|hCoV-19/England/SHEF-C02D9/2020|2020-03-27|EPI_ISL_420218|Original|hCoV-19^^England|Human|Virology Department|Department of Infection|de Silva|United Kingdom
>Spike|hCoV-19/England/MILK-B55D37/2020|2020-11-06|EPI_ISL_658174|Original|hCoV-19^^England|Human|Lighthouse Lab in Milton Keynes|Wellcome Sanger Institute for the COVID-19 Genomics UK (COG-UK) Consortium|Davies|United Kingdom
>Spike|hCoV-19/USA/TX-HMH-3399/2020|2020-06-12|EPI_ISL_544159|Original|hCoV-19^^Texas|Human|Houston Methodist Hospital|Houston Methodist Hospital|Long|USA
>Spike|hCoV-19/Wales/PHWC-499E4A/2020|2020-11-24|EPI_ISL_727453|Original|hCoV-19^^Wales|Human|Originating lab|COVID-19 Genomics UK (COG-UK) Consortium|Moore|United Kingdom
>Spike|hCoV-19/Japan/PG-6940/2020|2020-09-00|EPI_ISL_688045|Original|hCoV-19^^Japan|Human|Pathogen Genomics Center|Pathogen Genomics Center|Sekizuka|Japan
>Spike|hCoV-19/USA/HI-H200329/2020|2020-08-20|EPI_ISL_752887|Original|hCoV-19^^Hawaii|Human|State Laboratories Division|State Laboratories Division|Sultana|USA
>Spike|hCoV-19/Brazil/PA-0237/2020|2020-04-03|EPI_ISL_470611|Original|hCoV-19^^Par\u00e1|Human|Hermes Pardini|Bioinformatics Laboratory / LNCC|Almeida|Brazil
>Spike|hCoV-19/England/ALDP-9EA8ED/2020|2020-09-24|EPI_ISL_580135|Original|hCoV-19^^England|Human|Lighthouse Lab in Alderley Park|Wellcome Sanger Institute for the COVID-19 Genomics UK (COG-UK) consortium|Davies|United Kingdom
>Spike|hCoV-19/England/ALDP-B45CA6/2020|2020-11-07|EPI_ISL_656016|Original|hCoV-19^^England|Human|Lighthouse Lab in Alderley Park|Wellcome Sanger Institute for the COVID-19 Genomics UK (COG-UK) Consortium|Davies|United Kingdom
>Spike|hCoV-19/USA/CO-QDX-673/2020|2020-03-17|EPI_ISL_571427|Original|hCoV-19^^Colorado|Human|Quest Diagnostics|Quest Diagnostics|Kagan|USA
>Spike|hCoV-19/Wales/PHWC-16618C/2020|2020-05-15|EPI_ISL_490844|Original|hCoV-19^^Wales|Human|Wales Specialist Virology Centre Sequencing lab|COVID-19 Genomics UK (COG-UK) Consortium|Moore|United Kingdom
>Spike|hCoV-19/Luxembourg/LNS2691541/2020|2020-10-26|EPI_ISL_740170|Original|hCoV-19^^Luxembourg|Human|Laboratoire national de sant\u00e9|Laboratoire national de sant\u00e9|Wienecke-Baldacchino|Luxembourg
>Spike|hCoV-19/Wales/PHWC-48E8FF/2020|2020-11-13|EPI_ISL_680930|Original|hCoV-19^^Wales|Human|Wales Specialist Virology Centre Sequencing lab|COVID-19 Genomics UK (COG-UK) Consortium|Moore|United Kingdom
>Spike|hCoV-19/USA/TX-HMH-MCoV-13785/2020|2020-08-17|EPI_ISL_788878|Original|hCoV-19^^Texas|Human|Houston Methodist Hospital|Houston Methodist Hospital|Long|USA
>Spike|hCoV-19/England/QEUH-AF0F75/2020|2020-10-29|EPI_ISL_662689|Original|hCoV-19^^England|Human|Lighthouse Lab in Glasgow|Wellcome Sanger Institute for the COVID-19 Genomics UK (COG-UK) Consortium|Davies|United Kingdom
>Spike|hCoV-19/England/CAMC-C71CDD/2020|2020-12-15|EPI_ISL_769074|Original|hCoV-19^^England|Human|Lighthouse Lab in Cambridge|Wellcome Sanger Institute for the COVID-19 Genomics UK (COG-UK) Consortium|Davies|United Kingdom
>Spike|hCoV-19/Japan/PG-0304/2020|2020-03-02|EPI_ISL_479980|Original|hCoV-19^^Kyusyu|Human|Oita Prefectural Institute of Public Health and Environmental Science|Pathogen Genomics Center|Sekizuka|Japan
>Spike|hCoV-19/England/201390301/2020|2020-03-26|EPI_ISL_465842|Original|hCoV-19^^England|Human|Respiratory Virus Unit|Respiratory Virus Unit|Platt|United Kingdom
>Spike|hCoV-19/Timor-Leste/TL26/2020|2020-00-00|EPI_ISL_456596|Original|hCoV-19^^Timor-Leste|Human|National Health Laboratory|Microbiological Diagnostic Unit Public Health Laboratory|Schultz|Timor-Leste
>Spike|hCoV-19/Australia/ACT0022/2020|2020-03-20|EPI_ISL_498489|Original|hCoV-19^^Australian Capital Territory|Human|ACT Pathology|Schwessinger Lab|Hall|Australia

>Spike|hCoV-19/England/QEUH-BCB76B/2020|2020-11-27|EPI_ISL_704424|Original|hCoV-19^^England|Human|Lighthouse Lab in Glasgow|Wellcome Sanger Institute for the COVID-19 Genomics UK (COG-UK) Consortium|Davies|United Kingdom
>Spike|hCoV-19/USA/UT-UPHL-2012772719/2020|2020-08-03|EPI_ISL_745738|Original|hCoV-19^^Utah|Human|Ginkgo Bioworks Clinical Laboratory|Utah Public Health Laboratory|Young|USA
>Spike|hCoV-19/England/LIVE-A8037/2020|2020-03-26|EPI_ISL_517105|Original|hCoV-19^^England|Human|Liverpool Clinical Laboratories|COVID-19 Genomics UK (COG-UK) Consortium|Haldenby|United Kingdom
>Spike|hCoV-19/Wales/PHWC-307E4/2020|2020-04-08|EPI_ISL_446282|Original|hCoV-19^^Wales|Human|Wales Specialist Virology Centre|Public Health Wales Microbiology Cardiff|Moore|United Kingdom
>Spike|hCoV-19/Australia/VIC12451/2020|2020-07-24|EPI_ISL_591992|Original|hCoV-19^^Victoria|Human|Microbiological Diagnostic Unit - Public Health Laboratory (MDU-PHL)|MDU-PHL|Schultz|Australia
>Spike|hCoV-19/USA/FL-BPHL-0089/2020|2020-04-27|EPI_ISL_445032|Original|hCoV-19^^Florida|Human|Florida Bureau of Public Health Laboratories|Florida Bureau of Public Health Laboratories|Blanton|USA
>Spike|hCoV-19/Denmark/DCGC-20208/2020|2020-11-30|EPI_ISL_747534|Original|hCoV-19^^Syddanmark|Human|Department of Virus and Microbiological Special Diagnostics|Albertsen Lab|Michaelsen|Denmark
>Spike|hCoV-19/England/ALDP-954FCB/2020|2020-06-11|EPI_ISL_553451|Original|hCoV-19^^England|Human|Lighthouse Lab in Alderley Park|Wellcome Sanger Institute for the COVID-19 Genomics UK (COG-UK) consortium|Davies|United Kingdom
>Spike|hCoV-19/USA/FL-BPHL-0500/2020|2020-06-03|EPI_ISL_495406|Original|hCoV-19^^Florida|Human|Florida Bureau of Public Health Laboratories|Florida Bureau of Public Health Laboratories|Blanton|USA
>Spike|hCoV-19/Singapore/640/2020|2020-05-05|EPI_ISL_493400|Original|hCoV-19^^Singapore|Human|National Public Health Laboratory|National Public Health Laboratory|Mak|Singapore
>Spike|hCoV-19/USA/TX-HMH-MCoV-15269/2020|2020-10-19|EPI_ISL_786753|Original|hCoV-19^^Texas|Human|Houston Methodist Hospital|Houston Methodist Hospital|Long|USA
>Spike|hCoV-19/Germany/HH-hpi-p2190/2020|2020-03-18|EPI_ISL_776418|Original|hCoV-19^^Hamburg|Human|University Medical Center Hamburg Eppendorf|Heinrich Pette Institute|Robitaille|Germany
>Spike|hCoV-19/England/CAMB-76858/2020|2020-03-29|EPI_ISL_438468|Original|hCoV-19^^England|Human|Department of Pathology|Wellcome Sanger Institute for the COVID-19 Genomics UK (COG-UK) consortium|Amato|United Kingdom
>Spike|hCoV-19/England/QEUH-9DCE37/2020|2020-09-21|EPI_ISL_581072|Original|hCoV-19^^England|Human|Lighthouse Lab in Glasgow|Wellcome Sanger Institute for the COVID-19 Genomics UK (COG-UK) consortium|Davies|United Kingdom
>Spike|hCoV-19/Australia/VIC4733/2020|2020-07-15|EPI_ISL_519121|Original|hCoV-19^^Victoria|Human|Microbiological Diagnostic Unit - Public Health Laboratory (MDU-PHL)|MDU-PHL|Schultz|Australia
>Spike|hCoV-19/USA/NY-QDX-1912/2020|2020-03-15|EPI_ISL_604250|Original|hCoV-19^^New York|Human|Quest Diagnostics|Quest Diagnostics|Kagan|USA
>Spike|hCoV-19/Switzerland/BL-42227720/2020|2020-04-10|EPI_ISL_581697|Original|hCoV-19^^Basel-Land|Human|University Hospital Basel|University Hospital Basel|Roloff Handschin|Switzerland
>Spike|hCoV-19/England/ALDP-982A3C/2020|2020-08-09|EPI_ISL_552516|Original|hCoV-19^^England|Human|Lighthouse Lab in Alderley Park|Wellcome Sanger Institute for the COVID-19 Genomics UK (COG-UK) consortium|Davies|United Kingdom
>Spike|hCoV-19/USA/FL-BPHL-0750/2020|2020-06-22|EPI_ISL_512556|Original|hCoV-19^^Florida|Human|Florida Bureau of Public Health Laboratories|Florida Bureau of Public Health Laboratories|Blanton|USA
>Spike|hCoV-19/USA/MI-UM-10036399786/2020|2020-10-12|EPI_ISL_610050|Original|hCoV-19^^Michigan|Human|University of Michigan Clinical Microbiology Laboratory|Lauring Lab|Valesano|USA

>Spike|hCoV-19/England/CAMB-7EB84/2020|2020-04-07|EPI_ISL_442962|Original|hCoV-19^^England|Human|Department of Pathology|Wellcome Sanger Institute for the COVID-19 Genomics UK (COG-UK) consortium|Amato|United Kingdom
>Spike|hCoV-19/Japan/PG-1257/2020|2020-00-00|EPI_ISL_684867|Original|hCoV-19^^Japan|Human|Pathogen Genomics Center|Pathogen Genomics Center|Sekizuka|Japan
>Spike|hCoV-19/Wales/MILK-A46BA9/2020|2020-10-06|EPI_ISL_609262|Original|hCoV-19^^Wales|Human|Lighthouse Lab in Milton Keynes|Wellcome Sanger Institute for the COVID-19 Genomics UK (COG-UK) consortium|Davies|United Kingdom
>Spike|hCoV-19/Portugal/PT0323/2020|2020-03-15|EPI_ISL_454039|Original|hCoV-19^^Portugal|Human|unknown|Instituto Nacional de Saude (INSA)|Borges|Portugal
>Spike|hCoV-19/Northern Ireland/NIRE-FE6A9/2020|2020-05-01|EPI_ISL_489267|Original|hCoV-19^^Northern Ireland|Human|Regional Virus Laboratory|Wellcome Sanger Institute for the COVID-19 Genomics UK (COG-UK) consortium|Davies|United Kingdom
>Spike|hCoV-19/USA/AL-CDC-0731/2020|2020-05-19|EPI_ISL_509688|Original|hCoV-19^^Alabama|Human|Alabama Department of Public Health Bureau of Clinical Laboratories|Pathogen Discovery|Queen|USA
>Spike|hCoV-19/England/MILK-ACDB40/2020|2020-10-21|EPI_ISL_630297|Original|hCoV-19^^England|Human|Lighthouse Lab in Milton Keynes|Wellcome Sanger Institute for the COVID-19 Genomics UK (COG-UK) consortium|Davies|United Kingdom
>Spike|hCoV-19/Scotland/EDB6192/2020|2020-05-18|EPI_ISL_473908|Original|hCoV-19^^Scotland|Human|Virology Department|COVID-19 Genomics UK (COG-UK) Consortium|Williams|United Kingdom
>Spike|hCoV-19/England/CAMB-73017/2020|2020-03-21|EPI_ISL_440239|Original|hCoV-19^^England|Human|Department of Pathology|Wellcome Sanger Institute for the COVID-19 Genomics UK (COG-UK) consortium|Amato|United Kingdom
>Spike|hCoV-19/USA/DE-DHSS-F918037/2020|2020-03-22|EPI_ISL_593938|Original|hCoV-19^^Delaware|Human|Delaware Public Health Lab|Delaware Public Health Lab|Bajwa|USA
>Spike|hCoV-19/England/LIVE-A75C0/2020|2020-03-28|EPI_ISL_517043|Original|hCoV-19^^England|Human|Liverpool Clinical Laboratories|COVID-19 Genomics UK (COG-UK) Consortium|Haldenby|United Kingdom
>Spike|hCoV-19/England/CAMC-B230A2/2020|2020-11-02|EPI_ISL_651514|Original|hCoV-19^^England|Human|Virology Department|COVID-19 Genomics UK (COG-UK) Consortium|de Silva|United Kingdom
>Spike|hCoV-19/Denmark/DCGC-12514/2020|2020-11-16|EPI_ISL_682695|Original|hCoV-19^^Nordjylland|Human|Department of Virus and Microbiological Special Diagnostics|Albertsen Lab|Michaelsen|Denmark
>Spike|hCoV-19/USA/UT-UPHL-2009225/2020|2020-05-14|EPI_ISL_525224|Original|hCoV-19^^Utah|Human|Utah Public Health Laboratory|Utah Public Health Laboratory|Young|USA
>Spike|hCoV-19/Japan/PG-8891/2020|2020-08-00|EPI_ISL_686437|Original|hCoV-19^^Japan|Human|Pathogen Genomics Center|Pathogen Genomics Center|Sekizuka|Japan
>Spike|hCoV-19/Wales/PHWC-4A65FA/2020|2020-12-17|EPI_ISL_765052|Original|hCoV-19^^Wales|Human|Wales Specialist Virology Centre Sequencing lab|COVID-19 Genomics UK (COG-UK) Consortium|Moore|United Kingdom
>Spike|hCoV-19/England/QEUH-96561D/2020|2020-08-12|EPI_ISL_531177|Original|hCoV-19^^England|Human|Lighthouse Lab in Glasgow|Wellcome Sanger Institute for the COVID-19 Genomics UK (COG-UK) consortium|Davies|United Kingdom
>Spike|hCoV-19/England/ALDP-A4FDE6/2020|2020-10-07|EPI_ISL_597775|Original|hCoV-19^^England|Human|Lighthouse Lab in Alderley Park|Wellcome Sanger Institute for the COVID-19 Genomics UK (COG-UK) consortium|Davies|United Kingdom
>Spike|hCoV-19/England/ALDP-BB3353/2020|2020-11-19|EPI_ISL_702923|Original|hCoV-19^^England|Human|Lighthouse Lab in Alderley Park|Wellcome Sanger Institute for the COVID-19 Genomics UK (COG-UK) Consortium|Davies|United Kingdom
>Spike|hCoV-19/Australia/TAS60/2020|2020-04-07|EPI_ISL_522733|Original|hCoV-19^^Tasmania|Human|Royal Hobart Hospital Microbiology Department|MDU-PHL|Schultz|Australia
>Spike|hCoV-19/Northern Ireland/NIRE-FB16F/2020|2020-03-24|EPI_ISL_448942|Original|hCoV-19^^Northern Ireland|Human|Regional Virus Laboratory|COVID-19 Genomics UK (COG-UK) Consortium|Fairley|United Kingdom

>Spike|hCoV-19/Denmark/DCGC-13115/2020|2020-11-30|EPI_ISL_712138|Original|hCoV-19^^Sjaelland|Human|Department of Virus and Microbiological Special Diagnostics|Albertsen Lab|Michaelsen|Denmark
>Spike|hCoV-19/USA/TX-HMH-6252/2020|2020-07-02|EPI_ISL_547341|Original|hCoV-19^^Texas|Human|Houston Methodist Hospital|Houston Methodist Hospital|Long|USA
>Spike|hCoV-19/Netherlands/NA_434/2020|2020-04-07|EPI_ISL_455236|Original|hCoV-19^^Netherlands|Human|Dutch COVID-19 response team|Erasmus Medical Center|Oude Munnink|Netherlands
>Spike|hCoV-19/England/ALDP-A2E20C/2020|2020-10-05|EPI_ISL_586937|Original|hCoV-19^^England|Human|Lighthouse Lab in Alderley Park|Wellcome Sanger Institute for the COVID-19 Genomics UK (COG-UK) consortium|Davies|United Kingdom
>Spike|hCoV-19/United Arab Emirates/skmc-207823c/2020|2020-04-13|EPI_ISL_582629|Original|hCoV-19^^United Arab Emirates|Human|Sheikh Khalifa Medical City|Molecular/Surveillance lab\nSheikh Khalifa Medical City|Amirtharaj|United Arab Emirates
>Spike|hCoV-19/USA/FL-BPHL-0563/2020|2020-03-11|EPI_ISL_508767|Original|hCoV-19^^Florida|Human|Florida Bureau of Public Health Laboratories|Florida Bureau of Public Health Laboratories|Blanton|USA
>Spike|hCoV-19/England/MILK-99D44F/2020|2020-08-30|EPI_ISL_549894|Original|hCoV-19^^England|Human|Lighthouse Lab in Milton Keynes|Wellcome Sanger Institute for the COVID-19 Genomics UK (COG-UK) consortium|Davies|United Kingdom
>Spike|hCoV-19/Denmark/DCGC-17598/2020|2020-12-14|EPI_ISL_751095|Original|hCoV-19^^Sjaelland|Human|Department of Virus and Microbiological Special Diagnostics|Albertsen Lab|Michaelsen|Denmark
>Spike|hCoV-19/England/MILK-B5A5D0/2020|2020-11-06|EPI_ISL_658281|Original|hCoV-19^^England|Human|Lighthouse Lab in Milton Keynes|Wellcome Sanger Institute for the COVID-19 Genomics UK (COG-UK) Consortium|Davies|United Kingdom
>Spike|hCoV-19/England/MILK-9E1D41/2020|2020-09-20|EPI_ISL_581121|Original|hCoV-19^^England|Human|Lighthouse Lab in Milton Keynes|Wellcome Sanger Institute for the COVID-19 Genomics UK (COG-UK) consortium|Davies|United Kingdom
>Spike|hCoV-19/Netherlands/GE-EMC-258/2020|2020-04-24|EPI_ISL_523237|Original|hCoV-19^^Gelderland|Human|Dutch COVID-19 response team|Erasmus Medical Center|Oude Munnink|Netherlands
>Spike|hCoV-19/England/ALDP-A95613/2020|2020-10-20|EPI_ISL_624893|Original|hCoV-19^^England|Human|Lighthouse Lab in Alderley Park|Wellcome Sanger Institute for the COVID-19 Genomics UK (COG-UK) consortium|Davies|United Kingdom
>Spike|hCoV-19/Denmark/ALAB-HH37/2020|2020-03-13|EPI_ISL_429293|Original|hCoV-19^^Denmark|Human|Department of Clinical Microbiology|Albertsen lab|Kirkegaard|Denmark
>Spike|hCoV-19/United Arab Emirates/0586/2020|2020-05-16|EPI_ISL_698744|Original|hCoV-19^^Abu Dhabi|Human|Group 42 (G42) Healthcare|G42 Healthcare|Ogrodzki|United Arab Emirates
>Spike|hCoV-19/New Zealand/20VR2491/2020|2020-00-00|EPI_ISL_579248|Original|hCoV-19^^Auckland|Human|LabPLUS|Institute of Environmental Science and Research (ESR)|Wang|New Zealand
>Spike|hCoV-19/England/NORW-EAC80/2020|2020-05-11|EPI_ISL_457367|Original|hCoV-19^^England|Human|Quadram Institute Bioscience|COVID-19 Genomics UK (COG-UK) Consortium|Alikhan|United Kingdom
>Spike|hCoV-19/Finland/12MR1cG4/2020|2020-11-12|EPI_ISL_757288|Original|hCoV-19^^Uusimaa|Human|Department of Virology and Immunology|Department of Virology|Smura|Finland
>Spike|hCoV-19/Netherlands/NA_584/2020|2020-00-00|EPI_ISL_460748|Original|hCoV-19^^Netherlands|Human|Dutch COVID-19 response team|Erasmus Medical Center|Oude Munnink|Netherlands
>Spike|hCoV-19/Spain/MD-IBV-99008801/2020|2020-09-21|EPI_ISL_654152|Original|hCoV-19^^Madrid|Human|Hospital General Universitario Gregorio Mara\u00f1\u00f3n|SeqCOVID-SPAIN consortium/IBV(CSIC)|Comas|Spain
>Spike|hCoV-19/England/ALDP-A6F700/2020|2020-10-14|EPI_ISL_607253|Original|hCoV-19^^England|Human|Lighthouse Lab in Alderley Park|Wellcome Sanger Institute for the COVID-19 Genomics UK (COG-UK) consortium|Davies|United Kingdom

>Spike|hCoV-19/Scotland/EDB1444/2020|2020-04-13|EPI_ISL_433449|Original|hCoV-
19^^Scotland|Human|Virology Department|COVID-19 Genomics UK (COG-UK)
Consortium|Williams|United Kingdom
>Spike|hCoV-19/USA/TX-DSHS-1859/2020|2020-06-24|EPI_ISL_745295|Original|hCoV-
19^^Texas|Human|Texas Department of State Health Services|Texas Department of State
Health Services|Rahman|USA
>Spike|hCoV-19/Denmark/DCGC-15874/2020|2020-11-23|EPI_ISL_714078|Original|hCoV-
19^^Nordjylland|Human|Department of Virus and Microbiological Special
Diagnostics|Albertsen Lab|Michaelsen|Denmark
>Spike|hCoV-19/USA/FL-BPHL-1269/2020|2020-08-04|EPI_ISL_541201|Original|hCoV-
19^^Florida|Human|Florida Bureau of Public Health Laboratories|Florida Bureau of
Public Health Laboratories|Blanton|USA
>Spike|hCoV-19/USA/TX-QDX-25/2020|2020-03-15|EPI_ISL_467906|Original|hCoV-
19^^Texas|Human|Quest Diagnostics|Quest Diagnostics|Kagan|USA
>Spike|hCoV-19/Italy/ABR-IZSGC-TE27016/2020|2020-04-27|EPI_ISL_529019|Original|hCoV-
19^^Abruzzo|Human|RSA/RP Villa San Giovanni - Gruppo Edos|Istitituto Zooprofilattico
Sperimentale dell'Abruzzo e Molise \G.Caporale\|Lorusso|Italy
>Spike|hCoV-19/England/QEUH-9B9243/2020|2020-09-07|EPI_ISL_549726|Original|hCoV-
19^^England|Human|Lighthouse Lab in Glasgow|Wellcome Sanger Institute for the COVID-
19 Genomics UK (COG-UK) consortium|Davies|United Kingdom
>Spike|hCoV-19/England/SHEF-CDC92/2020|2020-04-29|EPI_ISL_457662|Original|hCoV-
19^^England|Human|Virology Department|COVID-19 Genomics UK (COG-UK) Consortium|de
Silva|United Kingdom
>Spike|hCoV-19/England/MILK-9751FB/2020|2020-08-21|EPI_ISL_552445|Original|hCoV-
19^^England|Human|Lighthouse Lab in Milton Keynes|Wellcome Sanger Institute for the
COVID-19 Genomics UK (COG-UK) consortium|Davies|United Kingdom
>Spike|hCoV-19/Russia/CRIE166064/2020|2020-03-31|EPI_ISL_534348|Original|hCoV-
19^^Moscow|Human|Molecular diagnostic laboratory of Federal Budget Institution of
Science \Central Research Institute of Epidemiology\ of The Federal Service on
Customers' Rights Protection and Human Well-being Surveillance|Group of Genomics and
Postgenomic Technologies of Central Research Institute of Epidemilology|Spera
>nskaya|Russia
>Spike|hCoV-19/France/PAC-MEPHI-1989/2020|2020-08-28|EPI_ISL_644469|Original|hCoV-
19^^Provence-Alpes-Cote-d\u2019Azur|Human|MEPHI|MEPHI|Levasseur|France
>Spike|hCoV-19/USA/TX-HMH-3818/2020|2020-06-11|EPI_ISL_543222|Original|hCoV-
19^^Texas|Human|Houston Methodist Hospital|Houston Methodist Hospital|Long|USA
>Spike|hCoV-19/Peru/LIM-UPCH-0100/2020|2020-06-16|EPI_ISL_540978|Original|hCoV-
19^^Lima|Human|Laboratorio de Referencia Nacional de Virus Respiratorios|Laboratorio
de Gen\u00f3mica Microbiana|Tsukayama|Peru
>Spike|hCoV-19/England/ALDP-929369/2020|2020-06-22|EPI_ISL_552965|Original|hCoV-
19^^England|Human|Lighthouse Lab in Alderley Park|Wellcome Sanger Institute for the
COVID-19 Genomics UK (COG-UK) consortium|Davies|United Kingdom
>Spike|hCoV-19/Denmark/DCGC-20183/2020|2020-11-30|EPI_ISL_748035|Original|hCoV-
19^^Sjaelland|Human|Department of Virus and Microbiological Special
Diagnostics|Albertsen Lab|Michaelsen|Denmark
>Spike|hCoV-19/USA/TX-HMH-7603/2020|2020-06-26|EPI_ISL_546885|Original|hCoV-
19^^Texas|Human|Houston Methodist Hospital|Houston Methodist Hospital|Long|USA
>Spike|hCoV-19/Gambia/NPHL-2463/2020|2020-00-00|EPI_ISL_561254|Original|hCoV-
19^^Kombo|Human|MRCG at LSHTM Genomics lab|MRCG at LSHTM Genomics lab|Sesay|Gambia
>Spike|hCoV-19/England/ALDP-BCA1B9/2020|2020-11-23|EPI_ISL_703402|Original|hCoV-
19^^England|Human|Lighthouse Lab in Alderley Park|Wellcome Sanger Institute for the
COVID-19 Genomics UK (COG-UK) Consortium|Davies|United Kingdom
>Spike|hCoV-19/USA/CO-CDPHE-2100015982/2020|2020-11-20|EPI_ISL_771320|Original|hCoV-
19^^Colorado|Human|Colorado Department of Public Health and Environment|Colorado
Department of Puplic Health and Environment|Bankers|USA
>Spike|hCoV-19/England/CAMB-1ACFDB/2020|2020-04-27|EPI_ISL_447953|Original|hCoV-
19^^England|Human|Department of Pathology|COVID-19 Genomics UK (COG-UK)
Consortium|Meredith|United Kingdom

>Spike|hCoV-19/Australia/VIC17992/2020|2020-00-00|EPI_ISL_779607|Original|hCoV-19^^Victoria|Human|Victorian Infectious Diseases Reference Laboratory (VIDRL)|VIDRL and MDU-PHL|Beck|Australia
>Spike|hCoV-19/Switzerland/BL-ETHZ-380353/2020|2020-11-21|EPI_ISL_721794|Original|hCoV-19^^Basel-Landschaft|Human|Viollier AG|Department of Biosystems Science and Engineering|Nadeau|Switzerland
>Spike|hCoV-19/Australia/VIC17131/2020|2020-08-11|EPI_ISL_663752|Original|hCoV-19^^Victoria|Human|Microbiological Diagnostic Unit - Public Health Laboratory (MDU-PHL)|MDU-PHL|Schultz|Australia
>Spike|hCoV-19/England/ALDP-B45FAD/2020|2020-11-07|EPI_ISL_656291|Original|hCoV-19^^England|Human|Lighthouse Lab in Alderley Park|Wellcome Sanger Institute for the COVID-19 Genomics UK (COG-UK) Consortium|Davies|United Kingdom
>Spike|hCoV-19/USA/VA-DCLS-0059/2020|2020-03-31|EPI_ISL_426467|Original|hCoV-19^^Virginia|Human|Virginia DCLS|Virginia DCLS|St. Jacques|USA
>Spike|hCoV-19/England/ALDP-9FDD51/2020|2020-10-01|EPI_ISL_587844|Original|hCoV-19^^England|Human|Lighthouse Lab in Alderley Park|Wellcome Sanger Institute for the COVID-19 Genomics UK (COG-UK) consortium|Davies|United Kingdom
>Spike|hCoV-19/Denmark/DCGC-19202/2020|2020-12-07|EPI_ISL_748368|Original|hCoV-19^^Syddanmark|Human|Department of Virus and Microbiological Special Diagnostics|Albertsen Lab|Michaelsen|Denmark
>Spike|hCoV-19/England/CAMB-80BAD/2020|2020-04-10|EPI_ISL_443015|Original|hCoV-19^^England|Human|Department of Pathology|Wellcome Sanger Institute for the COVID-19 Genomics UK (COG-UK) consortium|Amato|United Kingdom
>Spike|hCoV-19/England/NORW-222CB7/2020|2020-12-19|EPI_ISL_741513|Original|hCoV-19^^England|Human|Quadram Institute Bioscience|COVID-19 Genomics UK (COG-UK) Consortium|Alikhan|United Kingdom
>Spike|hCoV-19/England/201090129/2020|2020-03-07|EPI_ISL_566048|Original|hCoV-19^^England|Human|Respiratory Virus Unit|Respiratory Virus Unit|Platt|United Kingdom
>Spike|hCoV-19/England/QEUH-9DE0B7/2020|2020-09-21|EPI_ISL_599485|Original|hCoV-19^^England|Human|Lighthouse Lab in Glasgow|Wellcome Sanger Institute for the COVID-19 Genomics UK (COG-UK) consortium|Davies|United Kingdom
>Spike|hCoV-19/USA/UT-01525/2020|2020-04-10|EPI_ISL_452158|Original|hCoV-19^^Utah|Human|Utah Public Health Laboratory|Utah Public Health Laboratory|Young|USA
>Spike|hCoV-19/England/ALDP-94C658/2020|2020-06-14|EPI_ISL_556023|Original|hCoV-19^^England|Human|Lighthouse Lab in Alderley Park|Wellcome Sanger Institute for the COVID-19 Genomics UK (COG-UK) consortium|Davies|United Kingdom
>Spike|hCoV-19/England/LOND-1251742/2020|2020-11-17|EPI_ISL_679430|Original|hCoV-19^^England|Human|University College London Hospital|COVID-19 Genomics UK (COG-UK) Consortium|Byott|United Kingdom
>Spike|hCoV-19/England/NORT-2895D1/2020|2020-03-30|EPI_ISL_458593|Original|hCoV-19^^England|Human|NU-OMICS DNA Sequencing research facility|Wellcome Sanger Institute for the COVID-19 Genomics UK (COG-UK) consortium|Davies|United Kingdom
>Spike|hCoV-19/England/CAMC-B20913/2020|2020-11-02|EPI_ISL_647664|Original|hCoV-19^^England|Human|Lighthouse Lab in Cambridge|Wellcome Sanger Institute for the COVID-19 Genomics UK (COG-UK) Consortium|Davies|United Kingdom
>Spike|hCoV-19/USA/CA-IGI-0816/2020|2020-07-13|EPI_ISL_677900|Original|hCoV-19^^California|Human|Innovative Genomics Institute|Innovative Genomics Institute|Wyman|USA
>Spike|hCoV-19/Wales/PHWC-26990/2020|2020-03-29|EPI_ISL_422293|Original|hCoV-19^^Wales|Human|Wales Specialist Virology Centre|Public Health Wales Microbiology Cardiff|Moore|United Kingdom
>Spike|hCoV-19/Australia/VIC3489/2020|2020-07-08|EPI_ISL_521149|Original|hCoV-19^^Victoria|Human|Victorian Infectious Diseases Reference Laboratory (VIDRL)|VIDRL and MDU-PHL|Schultz|Australia
>Spike|hCoV-19/England/QEUH-9C89B5/2020|2020-09-10|EPI_ISL_567247|Original|hCoV-19^^England|Human|Lighthouse Lab in Glasgow|Wellcome Sanger Institute for the COVID-19 Genomics UK (COG-UK) consortium|Davies|United Kingdom
>Spike|hCoV-19/Denmark/DCGC-21175/2020|2020-12-21|EPI_ISL_759593|Original|hCoV-19^^Syddanmark|Human|Department of Virus and Microbiological Special Diagnostics|Albertsen Lab|Michaelsen|Denmark

>Spike|hCoV-19/USA/TX-HMH-MCoV-15137/2020|2020-10-16|EPI_ISL_786621|Original|hCoV-19^^Texas|Human|Houston Methodist Hospital|Houston Methodist Hospital|Long|USA
>Spike|hCoV-19/England/CAMC-B9939F/2020|2020-11-15|EPI_ISL_673248|Original|hCoV-19^^England|Human|Lighthouse Lab in Cambridge|Wellcome Sanger Institute for the COVID-19 Genomics UK (COG-UK) Consortium|Davies|United Kingdom
>Spike|hCoV-19/Scotland/CVR4094/2020|2020-09-04|EPI_ISL_540799|Original|hCoV-19^^Scotland|Human|West of Scotland Specialist Virology Centre|COVID-19 Genomics UK (COG-UK) Consortium|Robertson|United Kingdom
>Spike|hCoV-19/Wales/PHWC-4A27E9/2020|2020-12-09|EPI_ISL_743943|Original|hCoV-19^^Wales|Human|Wales Specialist Virology Centre Sequencing lab|COVID-19 Genomics UK (COG-UK) Consortium|Moore|United Kingdom
>Spike|hCoV-19/Netherlands/ZH-EMC-373/2020|2020-08-02|EPI_ISL_523713|Original|hCoV-19^^Zuid Holland|Human|Dutch COVID-19 response team|Erasmus Medical Center|Oude Munnink|Netherlands
>Spike|hCoV-19/Uruguay/UY-59/2020|2020-04-25|EPI_ISL_480336|Original|hCoV-19^^Montevideo|Human|Microbial Genomics Laboratory|Microbial Genomics Laboratory|Iraola|Uruguay
>Spike|hCoV-19/Scotland/QEUH-C339C1/2020|2020-12-11|EPI_ISL_757149|Original|hCoV-19^^Scotland|Human|Lighthouse Lab in Glasgow|Wellcome Sanger Institute for the COVID-19 Genomics UK (COG-UK) Consortium|Davies|United Kingdom
>Spike|hCoV-19/USA/MN-CDC-6326/2020|2020-05-19|EPI_ISL_527702|Original|hCoV-19^^Minnesota|Human|MN PHL Division|Pathogen Discovery|Queen|USA
>Spike|hCoV-19/Scotland/EDB3175/2020|2020-04-06|EPI_ISL_489048|Original|hCoV-19^^Scotland|Human|Virology Department|Wellcome Sanger Institute for the COVID-19 Genomics UK (COG-UK) consortium|Davies|United Kingdom
>Spike|hCoV-19/Scotland/QEUH-A003D0/2020|2020-10-02|EPI_ISL_600333|Original|hCoV-19^^Scotland|Human|Lighthouse Lab in Glasgow|Wellcome Sanger Institute for the COVID-19 Genomics UK (COG-UK) consortium|Davies|United Kingdom
>Spike|hCoV-19/Russia/SPE-RII-MH1007S/2020|2020-09-04|EPI_ISL_596250|Original|hCoV-19^^Saint Petersburg|Human|HELIX LCC|WHO National Influenza Centre Russian Federation|Komissarov|Russia
>Spike|hCoV-19/England/ALDP-9FCF01/2020|2020-10-01|EPI_ISL_587917|Original|hCoV-19^^England|Human|Lighthouse Lab in Alderley Park|Wellcome Sanger Institute for the COVID-19 Genomics UK (COG-UK) consortium|Davies|United Kingdom
>Spike|hCoV-19/Nigeria/AK-CV273/2020|2020-07-20|EPI_ISL_729957|Original|hCoV-19^^Akwa-Ibom State|Human|Nigeria Centre for Disease Control (NCDC)|African Centre of Excellence for Genomics of Infectious Diseases (ACEGID)|Oluniyi|Nigeria
>Spike|hCoV-19/England/SHEF-CAA5F/2020|2020-04-02|EPI_ISL_457643|Original|hCoV-19^^England|Human|Virology Department|COVID-19 Genomics UK (COG-UK) Consortium|de Silva|United Kingdom
>Spike|hCoV-19/Switzerland/BS-UHB-42203665/2020|2020-03-23|EPI_ISL_528364|Original|hCoV-19^^Basel-Stadt|Human|University Hospital Basel|University Hospital Basel|Roloff Handschin|Switzerland
>Spike|hCoV-19/England/QEUH-A4B139/2020|2020-10-05|EPI_ISL_637813|Original|hCoV-19^^England|Human|Department of Pathology|COVID-19 Genomics UK (COG-UK) Consortium|Goodfellow|United Kingdom
>Spike|hCoV-19/England/ALDP-A85C14/2020|2020-10-17|EPI_ISL_610427|Original|hCoV-19^^England|Human|Lighthouse Lab in Alderley Park|Wellcome Sanger Institute for the COVID-19 Genomics UK (COG-UK) consortium|Davies|United Kingdom
>Spike|hCoV-19/England/OXON-ACD2D/2020|2020-04-06|EPI_ISL_448531|Original|hCoV-19^^England|Human|Oxford Viromics|COVID-19 Genomics UK (COG-UK) Consortium|Golubchik|United Kingdom
>Spike|hCoV-19/Scotland/QEUH-A1FC3D/2020|2020-10-01|EPI_ISL_598985|Original|hCoV-19^^Scotland|Human|Lighthouse Lab in Glasgow|Wellcome Sanger Institute for the COVID-19 Genomics UK (COG-UK) consortium|Davies|United Kingdom
>Spike|hCoV-19/England/QEUH-B6B053/2020|2020-11-11|EPI_ISL_662600|Original|hCoV-19^^England|Human|Lighthouse Lab in Glasgow|Wellcome Sanger Institute for the COVID-19 Genomics UK (COG-UK) Consortium|Davies|United Kingdom

>Spike|hCoV-19/South Korea/KCDC2533/2020|2020-06-19|EPI_ISL_514936|Original|hCoV-19^^South Korea|Human|Division of Viral Diseases|Division of Viral Diseases|Kim|South Korea
>Spike|hCoV-19/USA/ME-HETL-J0162/2020|2020-09-14|EPI_ISL_755345|Original|hCoV-19^^Maine|Human|Maine Health and Environmental Testing Laboratory|Tewhey Lab|Tewhey|USA
>Spike|hCoV-19/Wales/PHWC-48E74D/2020|2020-11-13|EPI_ISL_680904|Original|hCoV-19^^Wales|Human|Wales Specialist Virology Centre Sequencing lab|COVID-19 Genomics UK (COG-UK) Consortium|Moore|United Kingdom
>Spike|hCoV-19/USA/CA-CZB-12390/2020|2020-10-08|EPI_ISL_648509|Original|hCoV-19^^California|Human|Orange County Public Health Lab|Chan-Zuckerberg Biohub|Bhatt|USA
>Spike|hCoV-19/England/QEUH-9F4E84/2020|2020-09-25|EPI_ISL_588426|Original|hCoV-19^^England|Human|Lighthouse Lab in Glasgow|Wellcome Sanger Institute for the COVID-19 Genomics UK (COG-UK) consortium|Davies|United Kingdom
>Spike|hCoV-19/England/NORW-21F5FE/2020|2020-12-11|EPI_ISL_725033|Original|hCoV-19^^England|Human|Quadram Institute Bioscience|COVID-19 Genomics UK (COG-UK) Consortium|Alikhan|United Kingdom
>Spike|hCoV-19/England/MILK-B949B0/2020|2020-11-14|EPI_ISL_676258|Original|hCoV-19^^England|Human|Lighthouse Lab in Milton Keynes|Wellcome Sanger Institute for the COVID-19 Genomics UK (COG-UK) Consortium|Davies|United Kingdom
>Spike|hCoV-19/USA/TX-HMH-MCoV-19111/2020|2020-11-23|EPI_ISL_783631|Original|hCoV-19^^Texas|Human|Houston Methodist Hospital|Houston Methodist Hospital|Long|USA
>Spike|hCoV-19/USA/TX-DSHS-1093/2020|2020-05-21|EPI_ISL_615132|Original|hCoV-19^^Texas|Human|Texas Department of State Health Services|Texas Department of State Health Services|Rahman|USA
>Spike|hCoV-19/Australia/VIC5101/2020|2020-07-19|EPI_ISL_519904|Original|hCoV-19^^Victoria|Human|Microbiological Diagnostic Unit - Public Health Laboratory (MDU-PHL)|MDU-PHL|Schultz|Australia
>Spike|hCoV-19/Guangdong/20SF243/2020|2020-01-22|EPI_ISL_428456|Original|hCoV-19^^Guangdong|Human|Guangdong Provincial Center for Diseases Control and Prevention;Guangdong Provincial Institute of Public Health|School of Public Health|Gu|China
>Spike|hCoV-19/USA/CA-IGI-0115/2020|2020-06-03|EPI_ISL_495137|Original|hCoV-19^^California|Human|Innovative Genomics Institute|Innovative Genomics Institute|Shivram|USA
>Spike|hCoV-19/USA/TX-HMH-MCoV-13095/2020|2020-08-28|EPI_ISL_788246|Original|hCoV-19^^Texas|Human|Houston Methodist Hospital|Houston Methodist Hospital|Long|USA
>Spike|hCoV-19/England/20129177304/2020|2020-03-22|EPI_ISL_424160|Original|hCoV-19^^England|Human|Respiratory Virus Unit|Respiratory Virus Unit|Platt|United Kingdom
>Spike|hCoV-19/Denmark/DCGC-22314/2020|2020-12-14|EPI_ISL_758672|Original|hCoV-19^^Midtjylland|Human|Department of Virus and Microbiological Special Diagnostics|Albertsen Lab|Michaelsen|Denmark
>Spike|hCoV-19/Japan/PG-6487/2020|2020-08-00|EPI_ISL_688629|Original|hCoV-19^^Japan|Human|Pathogen Genomics Center|Pathogen Genomics Center|Sekizuka|Japan
>Spike|hCoV-19/Singapore/552/2020|2020-06-06|EPI_ISL_482699|Original|hCoV-19^^Singapore|Human|Singapore General Hospital|Department of Microbiology|Abdul Rahman|Singapore
>Spike|hCoV-19/England/QEUH-88CEDD/2020|2020-07-27|EPI_ISL_532817|Original|hCoV-19^^England|Human|Lighthouse Lab in Glasgow|Wellcome Sanger Institute for the COVID-19 Genomics UK (COG-UK) consortium|Davies|United Kingdom
>Spike|hCoV-19/England/ALDP-508179/2020|2020-06-25|EPI_ISL_559155|Original|hCoV-19^^England|Human|Lighthouse Lab in Alderley Park|Wellcome Sanger Institute for the COVID-19 Genomics UK (COG-UK) consortium|Davies|United Kingdom
>Spike|hCoV-19/Wales/PHWC-48C16F/2020|2020-11-04|EPI_ISL_665607|Original|hCoV-19^^Wales|Human|Wales Specialist Virology Centre Sequencing lab|COVID-19 Genomics UK (COG-UK) Consortium|Moore|United Kingdom
>Spike|hCoV-19/South Korea/KCDC2730/2020|2020-07-14|EPI_ISL_522509|Original|hCoV-19^^South Korea|Human|Division of Viral Diseases|Division of Viral Diseases|Kim|South Korea

>Spike|hCoV-19/England/QEUH-B69F67/2020|2020-11-11|EPI_ISL_655089|Original|hCoV-19^^England|Human|Lighthouse Lab in Glasgow|Wellcome Sanger Institute for the COVID-19 Genomics UK (COG-UK) Consortium|Davies|United Kingdom
>Spike|hCoV-19/USA/SC-DHEC-1153/2020|2020-07-10|EPI_ISL_574545|Original|hCoV-19^^South Carolina|Human|Microbiology Division|Microbiology Division|Flores|USA
>Spike|hCoV-19/Japan/PG-8712/2020|2020-08-00|EPI_ISL_686308|Original|hCoV-19^^Japan|Human|Pathogen Genomics Center|Pathogen Genomics Center|Sekizuka|Japan
>Spike|hCoV-19/England/ALDP-BBAA2A/2020|2020-11-20|EPI_ISL_702416|Original|hCoV-19^^England|Human|Lighthouse Lab in Alderley Park|Wellcome Sanger Institute for the COVID-19 Genomics UK (COG-UK) Consortium|Davies|United Kingdom
>Spike|hCoV-19/USA/CA-ALSR-1384/2020|2020-03-15|EPI_ISL_494575|Original|hCoV-19^^California|Human|San Diego County Public Health Laboratory|Andersen lab at Scripps Research|Gangavarapu|USA
>Spike|hCoV-19/England/ALDP-CB5147/2020|2020-12-18|EPI_ISL_761873|Original|hCoV-19^^England|Human|Lighthouse Lab in Alderley Park|Wellcome Sanger Institute for the COVID-19 Genomics UK (COG-UK) Consortium|Davies|United Kingdom
>Spike|hCoV-19/USA/WA-QDX-1085/2020|2020-03-19|EPI_ISL_571907|Original|hCoV-19^^Washington|Human|Quest Diagnostics|Quest Diagnostics|Kagan|USA
>Spike|hCoV-19/Australia/VIC1495/2020|2020-04-29|EPI_ISL_456459|Original|hCoV-19^^Victoria|Human|Victorian Infectious Diseases Reference Laboratory (VIDRL)|Microbiological Diagnostic Unit Public Health Laboratory and Victorian Infectious Diseases Reference Laboratory|Schultz|Australia
>Spike|hCoV-19/England/QEUH-AD11B0/2020|2020-10-26|EPI_ISL_633944|Original|hCoV-19^^England|Human|Lighthouse Lab in Glasgow|Wellcome Sanger Institute for the COVID-19 Genomics UK (COG-UK) consortium|Davies|United Kingdom
>Spike|hCoV-19/England/QEUH-A77BDC/2020|2020-10-14|EPI_ISL_638739|Original|hCoV-19^^England|Human|Oxford Viromics|COVID-19 Genomics UK (COG-UK) Consortium|Golubchik|United Kingdom
>Spike|hCoV-19/England/ALDP-A6F22D/2020|2020-10-15|EPI_ISL_606982|Original|hCoV-19^^England|Human|Lighthouse Lab in Alderley Park|Wellcome Sanger Institute for the COVID-19 Genomics UK (COG-UK) consortium|Davies|United Kingdom
>Spike|hCoV-19/England/QEUH-A00E39/2020|2020-09-30|EPI_ISL_584901|Original|hCoV-19^^England|Human|University of Exeter|COVID-19 Genomics UK (COG-UK) Consortium|Temperton|United Kingdom
>Spike|hCoV-19/Beijing/BJ416/2020|2020-02-01|EPI_ISL_430723|Original|hCoV-19^^Beijing|Human|Chinese PLA Institute for Disease Control and Prevention|Chinese PLA Institute for Disease Control and Prevention|Li|China
>Spike|hCoV-19/England/MILK-BAAEA9/2020|2020-11-18|EPI_ISL_783272|Original|hCoV-19^^England|Human|Lighthouse Lab in Milton Keynes|Wellcome Sanger Institute for the COVID-19 Genomics UK (COG-UK) Consortium|Davies|United Kingdom
>Spike|hCoV-19/England/QEUH-A63EE9/2020|2020-10-12|EPI_ISL_628675|Original|hCoV-19^^England|Human|Oxford Viromics|COVID-19 Genomics UK (COG-UK) Consortium|Golubchik|United Kingdom
>Spike|hCoV-19/England/CWAR-24FDF96/2020|2020-06-12|EPI_ISL_584553|Original|hCoV-19^^England|Human|UHCW / University of Warwick|COVID-19 Genomics UK (COG-UK) Consortium|Stark|United Kingdom
>Spike|hCoV-19/England/MILK-A4805D/2020|2020-10-06|EPI_ISL_598491|Original|hCoV-19^^England|Human|Lighthouse Lab in Milton Keynes|Wellcome Sanger Institute for the COVID-19 Genomics UK (COG-UK) consortium|Davies|United Kingdom
>Spike|hCoV-19/England/QEUH-B9FDB9/2020|2020-11-18|EPI_ISL_721164|Original|hCoV-19^^England|Human|Lighthouse Lab in Glasgow|Wellcome Sanger Institute for the COVID-19 Genomics UK (COG-UK) Consortium|Davies|United Kingdom
>Spike|hCoV-19/England/ALDP-9EA30A/2020|2020-09-24|EPI_ISL_579998|Original|hCoV-19^^England|Human|Lighthouse Lab in Alderley Park|Wellcome Sanger Institute for the COVID-19 Genomics UK (COG-UK) consortium|Davies|United Kingdom
>Spike|hCoV-19/Scotland/EDB1632/2020|2020-04-14|EPI_ISL_433096|Original|hCoV-19^^Scotland|Human|Virology Department|COVID-19 Genomics UK (COG-UK) Consortium|Williams|United Kingdom

>Spike|hCoV-19/England/SHEF-10C9AF3/2020|2020-12-20|EPI_ISL_764269|Original|hCoV-19^^England|Human|Virology Department|COVID-19 Genomics UK (COG-UK) Consortium|de Silva|United Kingdom
>Spike|hCoV-19/England/QEUH-9658CC/2020|2020-08-12|EPI_ISL_531010|Original|hCoV-19^^England|Human|Lighthouse Lab in Glasgow|Wellcome Sanger Institute for the COVID-19 Genomics UK (COG-UK) consortium|Davies|United Kingdom
>Spike|hCoV-19/Singapore/892_2/2020|2020-09-05|EPI_ISL_536454|Original|hCoV-19^^Singapore|Human|National Public Health Laboratory|National Public Health Laboratory|Mak|Singapore
>Spike|hCoV-19/England/SHEF-10C8872/2020|2020-12-18|EPI_ISL_741954|Original|hCoV-19^^England|Human|Virology Department|COVID-19 Genomics UK (COG-UK) Consortium|de Silva|United Kingdom
>Spike|hCoV-19/England/MILK-971915/2020|2020-08-18|EPI_ISL_553026|Original|hCoV-19^^England|Human|Lighthouse Lab in Milton Keynes|Wellcome Sanger Institute for the COVID-19 Genomics UK (COG-UK) consortium|Davies|United Kingdom
>Spike|hCoV-19/England/MILK-957B4C/2020|2020-06-09|EPI_ISL_554287|Original|hCoV-19^^England|Human|Lighthouse Lab in Milton Keynes|Wellcome Sanger Institute for the COVID-19 Genomics UK (COG-UK) consortium|Davies|United Kingdom
>Spike|hCoV-19/USA/TX-HMH-MCoV-14584/2020|2020-10-09|EPI_ISL_787869|Original|hCoV-19^^Texas|Human|Houston Methodist Hospital|Houston Methodist Hospital|Long|USA
>Spike|hCoV-19/Scotland/EDB5782/2020|2020-04-27|EPI_ISL_487986|Original|hCoV-19^^Scotland|Human|Virology Department|Wellcome Sanger Institute for the COVID-19 Genomics UK (COG-UK) consortium|Davies|United Kingdom
>Spike|hCoV-19/England/ALDP-C3A766/2020|2020-12-09|EPI_ISL_731375|Original|hCoV-19^^England|Human|Lighthouse Lab in Alderley Park|Wellcome Sanger Institute for the COVID-19 Genomics UK (COG-UK) Consortium|Davies|United Kingdom
>Spike|hCoV-19/India/DL-NCDC7077-CSIR-IGIB/2020|2020-05-27|EPI_ISL_482624|Original|hCoV-19^^Delhi|Human|National Centre for Disease control (NCDC)|NCDC/CSIR-IGIB|Singh|India
>Spike|hCoV-19/England/OXON-B44C3/2020|2020-03-27|EPI_ISL_534957|Original|hCoV-19^^England|Human|Oxford Viromics|COVID-19 Genomics UK (COG-UK) Consortium|Golubchik|United Kingdom
>Spike|hCoV-19/England/ALDP-9445EA/2020|2020-06-07|EPI_ISL_554782|Original|hCoV-19^^England|Human|Lighthouse Lab in Alderley Park|Wellcome Sanger Institute for the COVID-19 Genomics UK (COG-UK) consortium|Davies|United Kingdom
>Spike|hCoV-19/Netherlands/Drente_1/2020|2020-03-25|EPI_ISL_423034|Original|hCoV-19^^Drente|Human|Dutch COVID-19 response team|Erasmus Medical Center|Oude Munnink|Netherlands
>Spike|hCoV-19/England/QEUH-AD3E3F/2020|2020-10-26|EPI_ISL_629895|Original|hCoV-19^^England|Human|Lighthouse Lab in Glasgow|Wellcome Sanger Institute for the COVID-19 Genomics UK (COG-UK) consortium|Davies|United Kingdom
>Spike|hCoV-19/India/DL-NCDC1566-CSIR-IGIB/2020|2020-05-16|EPI_ISL_482497|Original|hCoV-19^^Delhi|Human|National Centre for Disease control (NCDC)|NCDC/CSIR-IGIB|Pandey|India
>Spike|hCoV-19/Vietnam/VNHN_4189/2020|2020-04-02|EPI_ISL_455707|Original|hCoV-19^^Hanoi|Human|National Hospital of Tropical Diseases|Oxford University Clinical Research Unit|Nguyen|Vietnam
>Spike|hCoV-19/USA/UT-UPHL-03764/2020|2020-04-04|EPI_ISL_575112|Original|hCoV-19^^Utah|Human|Utah Public Health Laboratory|Utah Public Health Laboratory|Young|USA
>Spike|hCoV-19/England/CAMC-A3F00E/2020|2020-10-06|EPI_ISL_609600|Original|hCoV-19^^England|Human|Lighthouse Lab in Cambridge|Wellcome Sanger Institute for the COVID-19 Genomics UK (COG-UK) Consortium|Davies|United Kingdom
>Spike|hCoV-19/Japan/PG-4542/2020|2020-00-00|EPI_ISL_689680|Original|hCoV-19^^Japan|Human|Pathogen Genomics Center|Pathogen Genomics Center|Sekizuka|Japan
>Spike|hCoV-19/Sweden/20-07010/2020|2020-04-07|EPI_ISL_434661|Original|hCoV-19^^Vastra Gotaland|Human|Omtanken Grimmered|The Public Health Agency of Sweden|Svartstr\u00f6m|Sweden
>Spike|hCoV-19/New Zealand/20VR3117/2020|2020-04-29|EPI_ISL_579424|Original|hCoV-19^^Combined Auckland|Human|LabTests|Institute of Environmental Science and Research (ESR)|Wang|New Zealand

>Spike|hCoV-19/USA/TX-HMH-2418/2020|2020-04-20|EPI_ISL_545299|Original|hCoV-19^^Texas|Human|Houston Methodist Hospital|Houston Methodist Hospital|Long|USA
>Spike|hCoV-19/Denmark/DCGC-2864/2020|2020-08-17|EPI_ISL_616692|Original|hCoV-19^^Midtjylland|Human|Department of Virus and Microbiological Special Diagnostics|Albertsen lab|Michaelsen|Denmark
>Spike|hCoV-19/England/MILK-6F100C/2020|2020-06-30|EPI_ISL_557461|Original|hCoV-19^^England|Human|Lighthouse Lab in Milton Keynes|Wellcome Sanger Institute for the COVID-19 Genomics UK (COG-UK) consortium|Davies|United Kingdom
>Spike|hCoV-19/England/ALDP-B74E7B/2020|2020-11-12|EPI_ISL_678998|Original|hCoV-19^^England|Human|Queens Medical Centre|COVID-19 Genomics UK (COG-UK) Consortium|Loose|United Kingdom
>Spike|hCoV-19/Australia/VIC9570/2020|2020-08-06|EPI_ISL_565606|Original|hCoV-19^^Victoria|Human|Microbiological Diagnostic Unit - Public Health Laboratory (MDU-PHL)|MDU-PHL|Schultz|Australia
>Spike|hCoV-19/USA/CA-ALSR-1971/2020|2020-06-26|EPI_ISL_512269|Original|hCoV-19^^California|Human|San Diego County Public Health Laboratory|Andersen lab at Scripps Research|Gangavarapu|USA
>Spike|hCoV-19/Japan/PG-6740/2020|2020-08-00|EPI_ISL_687850|Original|hCoV-19^^Japan|Human|Pathogen Genomics Center|Pathogen Genomics Center|Sekizuka|Japan
>Spike|hCoV-19/USA/WA-S1201/2020|2020-05-07|EPI_ISL_463691|Original|hCoV-19^^Washington|Human|Washington State Department of Health|Seattle Flu Study|Bedford|USA
>Spike|hCoV-19/England/CAMB-78346/2020|2020-04-03|EPI_ISL_433720|Original|hCoV-19^^England|Human|Department of Pathology|COVID-19 Genomics UK (COG-UK) Consortium|Meredith|United Kingdom
>Spike|hCoV-19/England/MILK-A1D30D/2020|2020-10-03|EPI_ISL_595048|Original|hCoV-19^^England|Human|Queens Medical Centre|COVID-19 Genomics UK (COG-UK) Consortium|Loose|United Kingdom
>Spike|hCoV-19/Australia/VIC4657/2020|2020-07-13|EPI_ISL_519046|Original|hCoV-19^^Victoria|Human|Victorian Infectious Diseases Reference Laboratory (VIDRL)|VIDRL and MDU-PHL|Schultz|Australia
>Spike|hCoV-19/USA/WY-WYPHL-20006987/2020|2020-03-31|EPI_ISL_493062|Original|hCoV-19^^Wyoming|Human|Wyoming Public Health Laboratory|Wyoming Public Health Laboratory|Sevinsky|USA
>Spike|hCoV-19/Switzerland/BL-ETHZ-410056/2020|2020-12-17|EPI_ISL_737663|Original|hCoV-19^^Basel-Landschaft|Human|Viollier AG|Department of Biosystems Science and Engineering|Nadeau|Switzerland
>Spike|hCoV-19/England/CAMB-1ACF08/2020|2020-04-23|EPI_ISL_489558|Original|hCoV-19^^England|Human|Department of Pathology|Wellcome Sanger Institute for the COVID-19 Genomics UK (COG-UK) consortium|Davies|United Kingdom
>Spike|hCoV-19/USA/CA-ALSR-3691/2020|2020-09-28|EPI_ISL_636150|Original|hCoV-19^^California|Human|San Diego County Public Health Laboratory|Andersen lab at Scripps Research|Gangavarapu|USA
>Spike|hCoV-19/Denmark/DCGC-13368/2020|2020-11-30|EPI_ISL_712754|Original|hCoV-19^^Hovedstaden|Human|Department of Virus and Microbiological Special Diagnostics|Albertsen Lab|Michaelsen|Denmark
>Spike|hCoV-19/USA/WA-UW-6353/2020|2020-04-02|EPI_ISL_570102|Original|hCoV-19^^Washington|Human|UW Virology Lab|UW Virology Lab|Roychoudhury|USA
>Spike|hCoV-19/England/CAMC-BD2BDE/2020|2020-11-27|EPI_ISL_702040|Original|hCoV-19^^England|Human|Lighthouse Lab in Cambridge|Wellcome Sanger Institute for the COVID-19 Genomics UK (COG-UK) Consortium|Davies|United Kingdom
>Spike|hCoV-19/Belgium/rega-0514670/2020|2020-05-14|EPI_ISL_734733|Original|hCoV-19^^Heverlee|Human|UZ Leuven|KU Leuven|Maes|Belgium
>Spike|hCoV-19/England/BRIS-123BAB/2020|2020-04-06|EPI_ISL_442805|Original|hCoV-19^^England|Human|PHE South West Regional Laboratory|Wellcome Sanger Institute for the COVID-19 Genomics UK (COG-UK) consortium|Amato|United Kingdom
>Spike|hCoV-19/Italy/ABR-IZSGC-TE26969/2020|2020-04-27|EPI_ISL_436730|Original|hCoV-19^^Abruzzo|Human|Servizio di igiene epidemiologia e sanit\u00e0 pubblica (Siesp) Chieti|Istituto Zooprofilattico Sperimentale dell'Abruzzo e Molise \G.Caporale\|Lorusso|Italy

>Spike|hCoV-19/Iceland/288/2020|2020-03-18|EPI_ISL_417616|Original|hCoV-19^^Reykjavik|Human|The National University Hospital of Iceland|deCODE genetics|J\u00f3nsson|Iceland
>Spike|hCoV-19/Canada/BC_7515663/2020|2020-03-11|EPI_ISL_418845|Original|hCoV-19^^British Columbia|Human|BCCDC Public Health Laboratory|BCCDC Public Health Laboratory|Harrigan|Canada
>Spike|hCoV-19/Luxembourg/LNS8965091/2020|2020-10-22|EPI_ISL_744828|Original|hCoV-19^^Luxembourg|Human|Laboratoire national de sant\u00e9|Laboratoire national de sant\u00e9|Wienecke-Baldacchino|Luxembourg
>Spike|hCoV-19/USA/MN-MDH-2011/2020|2020-10-23|EPI_ISL_644942|Original|hCoV-19^^Minnesota|Human|Hennepin County Medical Center|Minnesota Department of Health|Lorentz|USA
>Spike|hCoV-19/India/GJ-GBRC24a/2020|2020-04-26|EPI_ISL_437453|Original|hCoV-19^^Gujarat|Human|B.J. Medical College and Civil hospital|Gujarat Biotechnology Research Centre|Joshi|India
>Spike|hCoV-19/England/QEUH-A8BAAB/2020|2020-10-15|EPI_ISL_643209|Original|hCoV-19^^England|Human|Lighthouse Lab in Glasgow|Wellcome Sanger Institute for the COVID-19 Genomics UK (COG-UK) Consortium|Davies|United Kingdom
>Spike|hCoV-19/USA/VA-DCLS-0913/2020|2020-05-13|EPI_ISL_522785|Original|hCoV-19^^Virginia|Human|Virginia DCLS|Virginia DCLS|St. Jacques|USA
>Spike|hCoV-19/England/ALDP-AA4B7C/2020|2020-10-22|EPI_ISL_624292|Original|hCoV-19^^England|Human|Lighthouse Lab in Alderley Park|Wellcome Sanger Institute for the COVID-19 Genomics UK (COG-UK) consortium|Davies|United Kingdom
>Spike|hCoV-19/England/CWAR-24FDB29/2020|2020-05-02|EPI_ISL_584482|Original|hCoV-19^^England|Human|UHCW / University of Warwick|COVID-19 Genomics UK (COG-UK) Consortium|Stark|United Kingdom
>Spike|hCoV-19/Japan/DP0121/2020|2020-02-15|EPI_ISL_416575|Original|hCoV-19^^Japan|Human|Japanese Quarantine Stations|Pathogen Genomics Center|Sekizuka|Japan
>Spike|hCoV-19/England/MILK-AD001E/2020|2020-10-22|EPI_ISL_634363|Original|hCoV-19^^England|Human|Lighthouse Lab in Milton Keynes|Wellcome Sanger Institute for the COVID-19 Genomics UK (COG-UK) consortium|Davies|United Kingdom
>Spike|hCoV-19/England/QEUH-AD3682/2020|2020-10-25|EPI_ISL_633828|Original|hCoV-19^^England|Human|Lighthouse Lab in Glasgow|Wellcome Sanger Institute for the COVID-19 Genomics UK (COG-UK) consortium|Davies|United Kingdom
>Spike|hCoV-19/England/ALDP-9E6359/2020|2020-09-24|EPI_ISL_580276|Original|hCoV-19^^England|Human|Lighthouse Lab in Alderley Park|Wellcome Sanger Institute for the COVID-19 Genomics UK (COG-UK) consortium|Davies|United Kingdom
>Spike|hCoV-19/USA/OH-QDX-1501/2020|2020-03-17|EPI_ISL_603843|Original|hCoV-19^^Ohio|Human|Quest Diagnostics|Quest Diagnostics|Kagan|USA
>Spike|hCoV-19/England/CAMB-76D86/2020|2020-03-31|EPI_ISL_438530|Original|hCoV-19^^England|Human|Department of Pathology|Wellcome Sanger Institute for the COVID-19 Genomics UK (COG-UK) consortium|Amato|United Kingdom
>Spike|hCoV-19/Scotland/EDB3829/2020|2020-04-30|EPI_ISL_526474|Original|hCoV-19^^Scotland|Human|Virology Department|COVID-19 Genomics UK (COG-UK) Consortium|Williams|United Kingdom
>Spike|hCoV-19/USA/HI-H200648/2020|2020-11-23|EPI_ISL_753172|Original|hCoV-19^^Hawaii|Human|State Laboratories Division|State Laboratories Division|Sultana|USA
>Spike|hCoV-19/USA/WI-UW-1677/2020|2020-09-11|EPI_ISL_605551|Original|hCoV-19^^Wisconsin|Human|University of Wisconsin-Madison AIDS Vaccine Research Laboratories|University of Wisconsin-Madison AIDS Vaccine Research Laboratories|Baczenas|USA
>Spike|hCoV-19/England/LEED-2A84C0/2020|2020-04-01|EPI_ISL_539203|Original|hCoV-19^^England|Human|Leeds Teaching Hospitals NHS Trust and Public Health England|Wellcome Sanger Institute for the COVID-19 Genomics UK (COG-UK) consortium|Davies|United Kingdom
>Spike|hCoV-19/USA/CO-CDPHE-2008170651/2020|2020-08-17|EPI_ISL_677273|Original|hCoV-19^^Colorado|Human|Colorado Department of Public Health and Environment|Colorado Department of Puplic Health and Environment|Bankers|USA

>Spike|hCoV-19/Australia/VIC8315/2020|2020-08-06|EPI_ISL_564937|Original|hCoV-19^^Victoria|Human|Microbiological Diagnostic Unit - Public Health Laboratory (MDU-PHL)|MDU-PHL|Schultz|Australia
>Spike|hCoV-19/England/QEUH-9D3270/2020|2020-09-18|EPI_ISL_577321|Original|hCoV-19^^England|Human|Centre for Enzyme Innovation|COVID-19 Genomics UK (COG-UK) Consortium|Robson|United Kingdom
>Spike|hCoV-19/England/BRIS-12333A/2020|2020-04-05|EPI_ISL_442788|Original|hCoV-19^^England|Human|PHE South West Regional Laboratory|Wellcome Sanger Institute for the COVID-19 Genomics UK (COG-UK) consortium|Amato|United Kingdom
>Spike|hCoV-19/Denmark/DCGC-5509/2020|2020-09-21|EPI_ISL_620834|Original|hCoV-19^^Hovedstaden|Human|Department of Virus and Microbiological Special Diagnostics|Albertsen lab|Michaelsen|Denmark
>Spike|hCoV-19/Brazil/RJ-00479/2020|2020-10-09|EPI_ISL_717874|Original|hCoV-19^^Rio de Janeiro|Human|Laboratorio de Virologia Molecular / UFRJ|Bioinformatics Laboratory / LNCC|Almeida|Brazil
>Spike|hCoV-19/England/CAMB-7E726/2020|2020-04-07|EPI_ISL_524525|Original|hCoV-19^^England|Human|Department of Pathology|Wellcome Sanger Institute for the COVID-19 Genomics UK (COG-UK) consortium|Davies|United Kingdom
>Spike|hCoV-19/England/QEUH-A8C3AF/2020|2020-10-18|EPI_ISL_643154|Original|hCoV-19^^England|Human|Lighthouse Lab in Glasgow|Wellcome Sanger Institute for the COVID-19 Genomics UK (COG-UK) Consortium|Davies|United Kingdom
>Spike|hCoV-19/USA/OR-OHSU-0423/2020|2020-05-25|EPI_ISL_509135|Original|hCoV-19^^Oregon|Human|OHSU Lab Services Molecular Microbiology Lab|Oregon SARS-CoV-2 Genome Sequencing Center|O'Roak|USA
>Spike|hCoV-19/Singapore/199/2020|2020-04-23|EPI_ISL_462317|Original|hCoV-19^^Singapore|Human|National Public Health Laboratory|National Public Health Laboratory|Mak|Singapore
>Spike|hCoV-19/Scotland/EDB5733/2020|2020-04-08|EPI_ISL_473850|Original|hCoV-19^^Scotland|Human|Virology Department|COVID-19 Genomics UK (COG-UK) Consortium|Williams|United Kingdom
>Spike|hCoV-19/Denmark/DCGC-8543/2020|2020-10-26|EPI_ISL_625899|Original|hCoV-19^^Hovedstaden|Human|Department of Virus and Microbiological Special Diagnostics|Albertsen lab|Michaelsen|Denmark
>Spike|hCoV-19/Spain/AN-IBV-001087/2020|2020-03-27|EPI_ISL_452599|Original|hCoV-19^^Andalucia|Human|Servicio de Microbiolog\u00eda y Parasitolog\u00eda cl\u00ednica. UCEIMP. Hospital Universitario Virgen del Roc\u00edo/IBIS/CSIC/US.|SeqCOVID-SPAIN consortium/IBV(CSIC)|Comas|Spain
>Spike|hCoV-19/Scotland/EDB5094/2020|2020-03-29|EPI_ISL_456967|Original|hCoV-19^^Scotland|Human|Virology Department|COVID-19 Genomics UK (COG-UK) Consortium|Williams|United Kingdom
>Spike|hCoV-19/Wales/ALDP-B1B6AB/2020|2020-11-01|EPI_ISL_710935|Original|hCoV-19^^Wales|Human|Lighthouse Lab in Alderley Park|Wellcome Sanger Institute for the COVID-19 Genomics UK (COG-UK) Consortium|Davies|United Kingdom
>Spike|hCoV-19/Japan/PG-8166/2020|2020-07-00|EPI_ISL_687265|Original|hCoV-19^^Japan|Human|Pathogen Genomics Center|Pathogen Genomics Center|Sekizuka|Japan
>Spike|hCoV-19/South Africa/KRISP-K002604/2020|2020-07-31|EPI_ISL_535468|Original|hCoV-19^^KwaZulu-Natal|Human|NHLS-IALCH|KRISP|de Oliveira|South Africa
>Spike|hCoV-19/Jordan/AQ-ALSR-5275/2020|2020-10-08|EPI_ISL_730513|Original|hCoV-19^^Aqaba|Human|Biolab Diagnostic Laboratories|Andersen lab at Scripps Research|Gangavarapu|Jordan
>Spike|hCoV-19/England/ALDP-9C551E/2020|2020-09-10|EPI_ISL_567686|Original|hCoV-19^^England|Human|Lighthouse Lab in Alderley Park|Wellcome Sanger Institute for the COVID-19 Genomics UK (COG-UK) consortium|Davies|United Kingdom
>Spike|hCoV-19/Australia/VIC13371/2020|2020-07-27|EPI_ISL_563697|Original|hCoV-19^^Victoria|Human|Microbiological Diagnostic Unit - Public Health Laboratory (MDU-PHL)|MDU-PHL|Schultz|Australia
>Spike|hCoV-19/Wales/PHWC-16A8ED/2020|2020-09-09|EPI_ISL_560122|Original|hCoV-19^^Wales|Human|Wales Specialist Virology Centre Sequencing lab|COVID-19 Genomics UK (COG-UK) Consortium|Moore|United Kingdom

>Spike|hCoV-19/DRC/1151/2020|2020-04-05|EPI_ISL_447596|Original|hCoV-19^^Kinshasa|Human|Viral Respiratory Lab|Pathogen Sequencing Lab|Lusamaki|Democratic Republic of the Congo
>Spike|hCoV-19/Japan/PG-1153/2020|2020-03-00|EPI_ISL_684763|Original|hCoV-19^^Japan|Human|Pathogen Genomics Center|Pathogen Genomics Center|Sekizuka|Japan
>Spike|hCoV-19/England/QEUH-9962B5/2020|2020-08-28|EPI_ISL_549523|Original|hCoV-19^^England|Human|Oxford Viromics|COVID-19 Genomics UK (COG-UK) Consortium|Golubchik|United Kingdom
>Spike|hCoV-19/USA/WI-UW-2496/2020|2020-12-30|EPI_ISL_779328|Original|hCoV-19^^Wisconsin|Human|University of Wisconsin-Madison AIDS Vaccine Research Laboratories|University of Wisconsin-Madison AIDS Vaccine Research Laboratories|Moreno|USA
>Spike|hCoV-19/Netherlands/OV-RIVM-20505/2020|2020-11-17|EPI_ISL_728720|Original|hCoV-19^^Overijssel|Human|Dutch COVID-19 response team|National Institute for Public Health and the Environment (RIVM)|Meijer|Netherlands
>Spike|hCoV-19/USA/LA-SR0148/2020|2020-03-27|EPI_ISL_435451|Original|hCoV-19^^Louisiana|Human|Robert Garry lab|Andersen lab at Scripps Research|Gangavarapu|USA
>Spike|hCoV-19/Wales/PHWC-4829CB/2020|2020-10-19|EPI_ISL_613256|Original|hCoV-19^^Wales|Human|Wales Specialist Virology Centre Sequencing lab|COVID-19 Genomics UK (COG-UK) Consortium|Moore|United Kingdom
>Spike|hCoV-19/Australia/VIC4901/2020|2020-07-13|EPI_ISL_519277|Original|hCoV-19^^Victoria|Human|Victorian Infectious Diseases Reference Laboratory (VIDRL)|VIDRL and MDU-PHL|Schultz|Australia
>Spike|hCoV-19/England/MILK-A7D0AA/2020|2020-10-11|EPI_ISL_606900|Original|hCoV-19^^England|Human|Lighthouse Lab in Milton Keynes|Wellcome Sanger Institute for the COVID-19 Genomics UK (COG-UK) consortium|Davies|United Kingdom
>Spike|hCoV-19/Wales/PHWC-4834D8/2020|2020-10-13|EPI_ISL_627826|Original|hCoV-19^^Wales|Human|Wales Specialist Virology Centre Sequencing lab|COVID-19 Genomics UK (COG-UK) Consortium|Moore|United Kingdom
>Spike|hCoV-19/England/MILK-45DD72/2020|2020-05-05|EPI_ISL_559732|Original|hCoV-19^^England|Human|Lighthouse Lab in Milton Keynes|Wellcome Sanger Institute for the COVID-19 Genomics UK (COG-UK) consortium|Davies|United Kingdom
>Spike|hCoV-19/Ireland/LK-NVRL-72IRL77937/2020|2020-09-16|EPI_ISL_578311|Original|hCoV-19^^Limerick|Human|National Virus Reference Laboratory|National Virus Reference Laboratory|Gonzalez|Ireland
>Spike|hCoV-19/England/CAMB-7B334/2020|2020-04-12|EPI_ISL_433943|Original|hCoV-19^^England|Human|Department of Pathology|COVID-19 Genomics UK (COG-UK) Consortium|Meredith|United Kingdom
>Spike|hCoV-19/USA/LA-EVTL1022/2020|2020-08-25|EPI_ISL_653713|Original|hCoV-19^^Louisiana|Human|LSUHS Emerging Viral Threat Laboratory|Microbial Genome Sequencing Center|Kamil|USA
>Spike|hCoV-19/Netherlands/ZH-EMC-766/2020|2020-10-08|EPI_ISL_632370|Original|hCoV-19^^Zuid Holland|Human|Dutch COVID-19 response team|Erasmus Medical Center|Oude Munnink|Netherlands
>Spike|hCoV-19/USA/TX-HMH-MCoV-19302/2020|2020-11-27|EPI_ISL_783748|Original|hCoV-19^^Texas|Human|Houston Methodist Hospital|Houston Methodist Hospital|Long|USA
>Spike|hCoV-19/England/CAMB-84392/2020|2020-04-16|EPI_ISL_443667|Original|hCoV-19^^England|Human|Department of Pathology|Wellcome Sanger Institute for the COVID-19 Genomics UK (COG-UK) consortium|Amato|United Kingdom
>Spike|hCoV-19/Germany/HH-hpi-p1451/2020|2020-03-10|EPI_ISL_775911|Original|hCoV-19^^Hamburg|Human|University Medical Center Hamburg Eppendorf|Heinrich Pette Institute|Robitaille|Germany
>Spike|hCoV-19/Wales/PHWC-49BDF0/2020|2020-12-06|EPI_ISL_742469|Original|hCoV-19^^Wales|Human|Wales Specialist Virology Centre Sequencing lab|COVID-19 Genomics UK (COG-UK) Consortium|Moore|United Kingdom
>Spike|hCoV-19/Scotland/QEUH-9F84CA/2020|2020-09-27|EPI_ISL_600840|Original|hCoV-19^^Scotland|Human|Lighthouse Lab in Glasgow|Wellcome Sanger Institute for the COVID-19 Genomics UK (COG-UK) consortium|Davies|United Kingdom

>Spike|hCoV-19/Denmark/DCGC-5226/2020|2020-09-14|EPI_ISL_615316|Original|hCoV-19^^Syddanmark|Human|Department of Virus and Microbiological Special Diagnostics|Albertsen lab|Michaelsen|Denmark
>Spike|hCoV-19/England/QEUH-AD1A97/2020|2020-10-26|EPI_ISL_634735|Original|hCoV-19^^England|Human|Lighthouse Lab in Glasgow|Wellcome Sanger Institute for the COVID-19 Genomics UK (COG-UK) consortium|Davies|United Kingdom
>Spike|hCoV-19/England/QEUH-AF0133/2020|2020-10-29|EPI_ISL_662876|Original|hCoV-19^^England|Human|Lighthouse Lab in Glasgow|Wellcome Sanger Institute for the COVID-19 Genomics UK (COG-UK) Consortium|Davies|United Kingdom
>Spike|hCoV-19/Brazil/SP-459/2020|2020-04-15|EPI_ISL_583490|Original|hCoV-19^^S\u00e3o Paulo|Human|Hospital Estadual Sumare|Instituto Adolfo Lutz|Sacchi|Brazil
>Spike|hCoV-19/Luxembourg/LNS8043806/2020|2020-10-23|EPI_ISL_744674|Original|hCoV-19^^Luxembourg|Human|Laboratoire national de sant\u00e9|Laboratoire national de sant\u00e9|Wienecke-Baldacchino|Luxembourg
>Spike|hCoV-19/USA/TX-HMH-MCoV-13232/2020|2020-08-25|EPI_ISL_788382|Original|hCoV-19^^Texas|Human|Houston Methodist Hospital|Houston Methodist Hospital|Long|USA
>Spike|hCoV-19/Portugal/PT0849/2020|2020-03-27|EPI_ISL_511108|Original|hCoV-19^^Portugal|Human|Instituto Nacional de Saude (INSA)|Instituto Nacional de Saude (INSA)|Borges|Portugal
>Spike|hCoV-19/England/MILK-A1D4BF/2020|2020-10-02|EPI_ISL_595062|Original|hCoV-19^^England|Human|Queens Medical Centre|COVID-19 Genomics UK (COG-UK) Consortium|Loose|United Kingdom
>Spike|hCoV-19/Denmark/DCGC-2078/2020|2020-07-20|EPI_ISL_618262|Original|hCoV-19^^Sjaelland|Human|Department of Virus and Microbiological Special Diagnostics|Albertsen lab|Michaelsen|Denmark
>Spike|hCoV-19/USA/WA-UW-11636/2020|2020-06-18|EPI_ISL_501102|Original|hCoV-19^^Washington|Human|University of Washington Virology Lab|University of Washington Virology Lab|Roychoudhury|USA
>Spike|hCoV-19/Denmark/DCGC-4071/2020|2020-09-14|EPI_ISL_622027|Original|hCoV-19^^Sjaelland|Human|Department of Virus and Microbiological Special Diagnostics|Albertsen lab|Michaelsen|Denmark
>Spike|hCoV-19/England/CAMC-B22D33/2020|2020-11-02|EPI_ISL_647455|Original|hCoV-19^^England|Human|Lighthouse Lab in Cambridge|Wellcome Sanger Institute for the COVID-19 Genomics UK (COG-UK) Consortium|Davies|United Kingdom
>Spike|hCoV-19/Denmark/DCGC-7844/2020|2020-10-19|EPI_ISL_618767|Original|hCoV-19^^Hovedstaden|Human|Department of Virus and Microbiological Special Diagnostics|Albertsen lab|Michaelsen|Denmark
>Spike|hCoV-19/Germany/HH-hpi-p1275/2020|2020-03-12|EPI_ISL_775857|Original|hCoV-19^^Hamburg|Human|University Medical Center Hamburg Eppendorf|Heinrich Pette Institute|Robitaille|Germany
>Spike|hCoV-19/Australia/VIC10871/2020|2020-08-04|EPI_ISL_563134|Original|hCoV-19^^Victoria|Human|Microbiological Diagnostic Unit - Public Health Laboratory (MDU-PHL)|MDU-PHL|Schultz|Australia
>Spike|hCoV-19/England/CAMC-BBBCAB/2020|2020-11-22|EPI_ISL_704492|Original|hCoV-19^^England|Human|Department of Pathology|COVID-19 Genomics UK (COG-UK) Consortium|Goodfellow|United Kingdom
>Spike|hCoV-19/England/QEUH-B1157B/2020|2020-11-02|EPI_ISL_642445|Original|hCoV-19^^England|Human|Lighthouse Lab in Glasgow|Wellcome Sanger Institute for the COVID-19 Genomics UK (COG-UK) Consortium|Davies|United Kingdom
>Spike|hCoV-19/USA/FL-BPHL-0720/2020|2020-07-21|EPI_ISL_509778|Original|hCoV-19^^Florida|Human|Florida Bureau of Public Health Laboratories|Florida Bureau of Public Health Laboratories|Blanton|USA
>Spike|hCoV-19/Scotland/GCVR-16F9E5/2020|2020-03-17|EPI_ISL_489692|Original|hCoV-19^^Scotland|Human|NHSGGC West of Scotland Specialist Virology Centre / MRC-University of Glasgow Centre for Virus Research|Wellcome Sanger Institute for the COVID-19 Genomics UK (COG-UK) consortium|Davies|United Kingdom
>Spike|hCoV-19/England/ALDP-A92555/2020|2020-10-19|EPI_ISL_611241|Original|hCoV-19^^England|Human|Lighthouse Lab in Alderley Park|Wellcome Sanger Institute for the COVID-19 Genomics UK (COG-UK) consortium|Davies|United Kingdom

>Spike|hCoV-19/USA/FL-BPHL-0252/2020|2020-05-04|EPI_ISL_480903|Original|hCoV-19^^Florida|Human|Florida Bureau of Public Health Laboratories|Florida Bureau of Public Health Laboratories|Blanton|USA
>Spike|hCoV-19/England/MILK-ACDDA4/2020|2020-10-21|EPI_ISL_630087|Original|hCoV-19^^England|Human|Lighthouse Lab in Milton Keynes|Wellcome Sanger Institute for the COVID-19 Genomics UK (COG-UK) consortium|Davies|United Kingdom
>Spike|hCoV-19/England/MILK-99E217/2020|2020-08-31|EPI_ISL_550657|Original|hCoV-19^^England|Human|Lighthouse Lab in Milton Keynes|Wellcome Sanger Institute for the COVID-19 Genomics UK (COG-UK) consortium|Davies|United Kingdom
>Spike|hCoV-19/Gibraltar/204641758/2020|2020-00-00|EPI_ISL_637226|Original|hCoV-19^^Gibraltar|Human|Microbiology|Respiratory Virus Unit|Platt|Gibraltar
>Spike|hCoV-19/USA/MI-MDHHS-SC22255/2020|2020-11-04|EPI_ISL_666921|Original|hCoV-19^^Michigan|Human|Michigan Department of Health and Human Services|Michigan Department of Health and Human Services|Jacob|USA
>Spike|hCoV-19/England/BRIS-1854178/2020|2020-04-28|EPI_ISL_492314|Original|hCoV-19^^England|Human|PHE South West Regional Laboratory|Wellcome Sanger Institute for the COVID-19 Genomics UK (COG-UK) consortium|Davies|United Kingdom
>Spike|hCoV-19/England/ALDP-9532A5/2020|2020-06-11|EPI_ISL_554343|Original|hCoV-19^^England|Human|Lighthouse Lab in Alderley Park|Wellcome Sanger Institute for the COVID-19 Genomics UK (COG-UK) consortium|Davies|United Kingdom
>Spike|hCoV-19/Japan/PG-7826/2020|2020-08-00|EPI_ISL_686928|Original|hCoV-19^^Japan|Human|Pathogen Genomics Center|Pathogen Genomics Center|Sekizuka|Japan
>Spike|hCoV-19/England/ALDP-9D9869/2020|2020-09-18|EPI_ISL_576763|Original|hCoV-19^^England|Human|Oxford Viromics|COVID-19 Genomics UK (COG-UK) Consortium|Golubchik|United Kingdom
>Spike|hCoV-19/England/CAMB-7BC75/2020|2020-04-05|EPI_ISL_442096|Original|hCoV-19^^England|Human|Department of Pathology|Wellcome Sanger Institute for the COVID-19 Genomics UK (COG-UK) consortium|Amato|United Kingdom
>Spike|hCoV-19/England/ALDP-B5C26E/2020|2020-11-10|EPI_ISL_655646|Original|hCoV-19^^England|Human|Lighthouse Lab in Alderley Park|Wellcome Sanger Institute for the COVID-19 Genomics UK (COG-UK) Consortium|Davies|United Kingdom
>Spike|hCoV-19/Denmark/DCGC-1378/2020|2020-03-30|EPI_ISL_622229|Original|hCoV-19^^Sjaelland|Human|Department of Virus and Microbiological Special Diagnostics|Albertsen lab|Michaelsen|Denmark
>Spike|hCoV-19/Gambia/GC19-2730/2020|2020-07-21|EPI_ISL_561136|Original|hCoV-19^^Kombo|Human|MRCG at LSHTM Genomics lab|MRCG at LSHTM Genomics lab|Sesay|Gambia
>Spike|hCoV-19/USA/MD-MDH-0302/2020|2020-10-22|EPI_ISL_633084|Original|hCoV-19^^Maryland|Human|MD PHL|MD PHL|Keller|USA
>Spike|hCoV-19/England/CAMC-B356D8/2020|2020-11-05|EPI_ISL_659675|Original|hCoV-19^^England|Human|Lighthouse Lab in Cambridge|Wellcome Sanger Institute for the COVID-19 Genomics UK (COG-UK) Consortium|Davies|United Kingdom
>Spike|hCoV-19/Switzerland/BS-UHB-42182218/2020|2020-03-09|EPI_ISL_527961|Original|hCoV-19^^Basel-Stadt|Human|University Hospital Basel|University Hospital Basel|Roloff Handschin|Switzerland
>Spike|hCoV-19/USA/TX-HMH-7839/2020|2020-07-02|EPI_ISL_545658|Original|hCoV-19^^Texas|Human|Houston Methodist Hospital|Houston Methodist Hospital|Long|USA
>Spike|hCoV-19/Czech Republic/NRL-7944/2020|2020-07-28|EPI_ISL_545957|Original|hCoV-19^^Olomouc Region|Human|The National Institute of Public Health|State Veterinary Institute Prague|Nagy|Czech Republic
>Spike|hCoV-19/USA/WI-UW-2551/2020|2020-12-29|EPI_ISL_779380|Original|hCoV-19^^Wisconsin|Human|University of Wisconsin-Madison AIDS Vaccine Research Laboratories|University of Wisconsin-Madison AIDS Vaccine Research Laboratories|Moreno|USA
>Spike|hCoV-19/Spain/PV-IBV-2147/2020|2020-04-12|EPI_ISL_468928|Original|hCoV-19^^Basque Country|Human|Servicio de Microbiolog\u00eda. Hospital Universitario Donostia. OSI Donostialdea. \u00c1rea de Enfermedades Infecciosas|SeqCOVID-SPAIN consortium/IBV(CSIC)|Comas|Spain
>Spike|hCoV-19/England/CAMC-B99C2B/2020|2020-11-15|EPI_ISL_673324|Original|hCoV-19^^England|Human|Lighthouse Lab in Cambridge|Wellcome Sanger Institute for the COVID-19 Genomics UK (COG-UK) Consortium|Davies|United Kingdom

>Spike|hCoV-19/England/QEUH-AE4FCF/2020|2020-10-27|EPI_ISL_633364|Original|hCoV-19^^England|Human|Lighthouse Lab in Glasgow|Wellcome Sanger Institute for the COVID-19 Genomics UK (COG-UK) consortium|Davies|United Kingdom
>Spike|hCoV-19/USA/UT-UPHL-2008960/2020|2020-05-13|EPI_ISL_525287|Original|hCoV-19^^Utah|Human|Utah Public Health Laboratory|Utah Public Health Laboratory|Young|USA
>Spike|hCoV-19/England/BHRT-24B365B/2020|2020-05-12|EPI_ISL_528802|Original|hCoV-19^^England|Human|Microbiology Department|Wellcome Sanger Institute for the COVID-19 Genomics UK (COG-UK) consortium|Davies|United Kingdom
>Spike|hCoV-19/England/ALDP-9ED792/2020|2020-05-09|EPI_ISL_580635|Original|hCoV-19^^England|Human|Lighthouse Lab in Alderley Park|Wellcome Sanger Institute for the COVID-19 Genomics UK (COG-UK) consortium|Davies|United Kingdom
>Spike|hCoV-19/Wales/PHWC-47E2D6/2020|2020-10-08|EPI_ISL_595434|Original|hCoV-19^^Wales|Human|Originating lab|COVID-19 Genomics UK (COG-UK) Consortium|Moore|United Kingdom
>Spike|hCoV-19/France/PAC-IHU-0020/2020|2020-03-11|EPI_ISL_569249|Original|hCoV-19^^Provence-Alpes-C\u00f4te-d\u2019Azur|Human|MEPHI|MEPHI|Levasseur|France
>Spike|hCoV-19/Wales/PHWC-49978B/2020|2020-12-03|EPI_ISL_727353|Original|hCoV-19^^Wales|Human|Originating lab|COVID-19 Genomics UK (COG-UK) Consortium|Moore|United Kingdom
>Spike|hCoV-19/England/20134081702/2020|2020-03-23|EPI_ISL_420518|Original|hCoV-19^^England|Human|Respiratory Virus Unit|Respiratory Virus Unit|Platt|United Kingdom
>Spike|hCoV-19/USA/TX-HMH-MCoV-9312/2020|2020-07-03|EPI_ISL_781054|Original|hCoV-19^^Texas|Human|Houston Methodist Hospital|Houston Methodist Hospital|Long|USA
>Spike|hCoV-19/Scotland/QEUH-9CFEA0/2020|2020-09-16|EPI_ISL_601854|Original|hCoV-19^^Scotland|Human|Lighthouse Lab in Glasgow|Wellcome Sanger Institute for the COVID-19 Genomics UK (COG-UK) consortium|Davies|United Kingdom
>Spike|hCoV-19/Northern Ireland/NIRE-106397/2020|2020-05-02|EPI_ISL_501051|Original|hCoV-19^^Northern Ireland|Human|Regional Virus Laboratory|Wellcome Sanger Institute for the COVID-19 Genomics UK (COG-UK) consortium|Davies|United Kingdom
>Spike|hCoV-19/Australia/VIC7452/2020|2020-07-23|EPI_ISL_564423|Original|hCoV-19^^Victoria|Human|Microbiological Diagnostic Unit - Public Health Laboratory (MDU-PHL)|MDU-PHL|Schultz|Australia
>Spike|hCoV-19/England/LCST-24D7387/2020|2020-06-30|EPI_ISL_499982|Original|hCoV-19^^England|Human|University Hospitals Of Leicester NHS Trust and DeepSeq Nottingham|COVID-19 Genomics UK (COG-UK) Consortium|Loose|United Kingdom
>Spike|hCoV-19/USA/MD-HP00048/2020|2020-03-15|EPI_ISL_457792|Original|hCoV-19^^Maryland|Human|Johns Hopkins Hospital Department of Pathology|Johns Hopkins Hospital Department of Pathology|Ramakrishnan|USA
>Spike|hCoV-19/England/ALDP-95092B/2020|2020-06-14|EPI_ISL_554448|Original|hCoV-19^^England|Human|Lighthouse Lab in Alderley Park|Wellcome Sanger Institute for the COVID-19 Genomics UK (COG-UK) consortium|Davies|United Kingdom
>Spike|hCoV-19/England/OXON-B2261/2020|2020-04-29|EPI_ISL_479132|Original|hCoV-19^^England|Human|Oxford Viromics|COVID-19 Genomics UK (COG-UK) Consortium|Golubchik|United Kingdom
>Spike|hCoV-19/USA/WI-GMF-30893/2020|2020-11-02|EPI_ISL_661064|Original|hCoV-19^^Wisconsin|Human|Gundersen Molecular Diagnostics Laboratory|Kabara Cancer Research Institute|Kenny|USA
>Spike|hCoV-19/England/ALDP-950369/2020|2020-06-18|EPI_ISL_555211|Original|hCoV-19^^England|Human|Lighthouse Lab in Alderley Park|Wellcome Sanger Institute for the COVID-19 Genomics UK (COG-UK) consortium|Davies|United Kingdom
>Spike|hCoV-19/DRC/4250/2020|2020-04-28|EPI_ISL_471415|Original|hCoV-19^^Kinshasa|Human|Viral Respiratory Lab|Pathogen Sequencing Lab|Lusamaki|Democratic Republic of the Congo
>Spike|hCoV-19/England/CAMC-BD2118/2020|2020-11-27|EPI_ISL_702184|Original|hCoV-19^^England|Human|Lighthouse Lab in Cambridge|Wellcome Sanger Institute for the COVID-19 Genomics UK (COG-UK) Consortium|Davies|United Kingdom
>Spike|hCoV-19/Switzerland/BE-ETHZ-330421/2020|2020-10-18|EPI_ISL_614908|Original|hCoV-19^^Bern|Human|Viollier AG|Department of Biosystems Science and Engineering|Nadeau|Switzerland

>Spike|hCoV-19/USA/CA-CZB-14635/2020|2020-11-16|EPI_ISL_738693|Original|hCoV-19^^California|Human|Orange County Public Health Lab|Chan-Zuckerberg Biohub|Kamm|USA
>Spike|hCoV-19/USA/WA-UW-22398/2020|2020-08-07|EPI_ISL_570674|Original|hCoV-19^^Washington|Human|UW Virology Lab|UW Virology Lab|Roychoudhury|USA
>Spike|hCoV-19/England/CAMB-80DD4/2020|2020-04-10|EPI_ISL_442996|Original|hCoV-19^^England|Human|Department of Pathology|Wellcome Sanger Institute for the COVID-19 Genomics UK (COG-UK) consortium|Amato|United Kingdom
>Spike|hCoV-19/Denmark/DCGC-10917/2020|2020-11-02|EPI_ISL_669514|Original|hCoV-19^^Midtjylland|Human|Department of Virus and Microbiological Special Diagnostics|Albertsen Lab|Michaelsen|Denmark
>Spike|hCoV-19/England/ALDP-A6F393/2020|2020-10-15|EPI_ISL_606942|Original|hCoV-19^^England|Human|Lighthouse Lab in Alderley Park|Wellcome Sanger Institute for the COVID-19 Genomics UK (COG-UK) consortium|Davies|United Kingdom
>Spike|hCoV-19/USA/NY-PV09378/2020|2020-03-19|EPI_ISL_450061|Original|hCoV-19^^New York|Human|MSHS Clinical Microbiology Laboratories|MSHS Pathogen Surveillance Program|van Bakel|USA
>Spike|hCoV-19/England/CAMC-B367C5/2020|2020-11-05|EPI_ISL_645258|Original|hCoV-19^^England|Human|Lighthouse Lab in Cambridge|Wellcome Sanger Institute for the COVID-19 Genomics UK (COG-UK) Consortium|Davies|United Kingdom
>Spike|hCoV-19/Scotland/EDB8116/2020|2020-10-05|EPI_ISL_594830|Original|hCoV-19^^Scotland|Human|Virology Department|COVID-19 Genomics UK (COG-UK) Consortium|McHugh|United Kingdom
>Spike|hCoV-19/Australia/VIC16539/2020|2020-07-23|EPI_ISL_641249|Original|hCoV-19^^Victoria|Human|Microbiological Diagnostic Unit - Public Health Laboratory (MDU-PHL)|MDU-PHL|Schultz|Australia
>Spike|hCoV-19/Denmark/DCGC-9510/2020|2020-11-09|EPI_ISL_669011|Original|hCoV-19^^Hovedstaden|Human|Department of Virus and Microbiological Special Diagnostics|Albertsen Lab|Michaelsen|Denmark
>Spike|hCoV-19/England/MILK-9F0E5B/2020|2020-09-24|EPI_ISL_589641|Original|hCoV-19^^England|Human|Lighthouse Lab in Milton Keynes|Wellcome Sanger Institute for the COVID-19 Genomics UK (COG-UK) consortium|Davies|United Kingdom
>Spike|hCoV-19/Australia/VIC12142/2020|2020-08-13|EPI_ISL_640410|Original|hCoV-19^^Victoria|Human|Microbiological Diagnostic Unit - Public Health Laboratory (MDU-PHL)|MDU-PHL|Schultz|Australia
>Spike|hCoV-19/Australia/VIC14496/2020|2020-08-09|EPI_ISL_592522|Original|hCoV-19^^Victoria|Human|Microbiological Diagnostic Unit - Public Health Laboratory (MDU-PHL)|MDU-PHL|Schultz|Australia
>Spike|hCoV-19/England/QEUH-B11748/2020|2020-11-02|EPI_ISL_642319|Original|hCoV-19^^England|Human|Lighthouse Lab in Glasgow|Wellcome Sanger Institute for the COVID-19 Genomics UK (COG-UK) Consortium|Davies|United Kingdom
>Spike|hCoV-19/England/ALDP-A520C9/2020|2020-10-07|EPI_ISL_597960|Original|hCoV-19^^England|Human|Lighthouse Lab in Alderley Park|Wellcome Sanger Institute for the COVID-19 Genomics UK (COG-UK) consortium|Davies|United Kingdom
>Spike|hCoV-19/England/NORW-E894F/2020|2020-04-11|EPI_ISL_448252|Original|hCoV-19^^England|Human|Quadram Institute Bioscience|COVID-19 Genomics UK (COG-UK) Consortium|Alikhan|United Kingdom
>Spike|hCoV-19/Northern Ireland/NIRE-232EEB/2020|2020-09-25|EPI_ISL_680235|Original|hCoV-19^^Northern Ireland|Human|Regional Virus Laboratory|COVID-19 Genomics UK (COG-UK) Consortium|Fairley|United Kingdom
>Spike|hCoV-19/USA/MI-MDHHS-SC21629/2020|2020-03-29|EPI_ISL_516356|Original|hCoV-19^^Michigan|Human|Michigan Department of Health and Human Services|Michigan Department of Health and Human Services|Blankenship|USA
>Spike|hCoV-19/England/ALDP-498230/2020|2020-06-03|EPI_ISL_559490|Original|hCoV-19^^England|Human|Lighthouse Lab in Alderley Park|Wellcome Sanger Institute for the COVID-19 Genomics UK (COG-UK) consortium|Davies|United Kingdom
>Spike|hCoV-19/Wales/PHWC-4877DB/2020|2020-10-31|EPI_ISL_639240|Original|hCoV-19^^Wales|Human|Wales Specialist Virology Centre Sequencing lab|COVID-19 Genomics UK (COG-UK) Consortium|Moore|United Kingdom

>Spike|hCoV-19/England/MILK-BB0A7F/2020|2020-11-18|EPI_ISL_720118|Original|hCoV-19^^England|Human|Lighthouse Lab in Milton Keynes|Wellcome Sanger Institute for the COVID-19 Genomics UK (COG-UK) Consortium|Davies|United Kingdom
>Spike|hCoV-19/England/ALDP-B485A8/2020|2020-11-07|EPI_ISL_649433|Original|hCoV-19^^England|Human|Lighthouse Lab in Alderley Park|Wellcome Sanger Institute for the COVID-19 Genomics UK (COG-UK) Consortium|Davies|United Kingdom
>Spike|hCoV-19/USA/LA-EVTL1295/2020|2020-12-11|EPI_ISL_778961|Original|hCoV-19^^Louisiana|Human|LSUHS Emerging Viral Threat Laboratory|Microbial Genome Sequencing Center|Kamil|USA
>Spike|hCoV-19/England/BRIS-1238FF/2020|2020-04-06|EPI_ISL_442624|Original|hCoV-19^^England|Human|PHE South West Regional Laboratory|Wellcome Sanger Institute for the COVID-19 Genomics UK (COG-UK) consortium|Amato|United Kingdom
>Spike|hCoV-19/England/MILK-956CC2/2020|2020-06-08|EPI_ISL_554247|Original|hCoV-19^^England|Human|Lighthouse Lab in Milton Keynes|Wellcome Sanger Institute for the COVID-19 Genomics UK (COG-UK) consortium|Davies|United Kingdom
>Spike|hCoV-19/England/CAMB-1AD2B3/2020|2020-00-00|EPI_ISL_447986|Original|hCoV-19^^England|Human|Department of Pathology|COVID-19 Genomics UK (COG-UK) Consortium|Meredith|United Kingdom
>Spike|hCoV-19/England/PORT-2D2481/2020|2020-03-25|EPI_ISL_507146|Original|hCoV-19^^England|Human|Centre for Enzyme Innovation|COVID-19 Genomics UK (COG-UK) Consortium|Robson|United Kingdom
>Spike|hCoV-19/USA/HI-H200530/2020|2020-10-24|EPI_ISL_753063|Original|hCoV-19^^Hawaii|Human|State Laboratories Division|State Laboratories Division|Sultana|USA
>Spike|hCoV-19/Scotland/EDB5515/2020|2020-04-21|EPI_ISL_487946|Original|hCoV-19^^Scotland|Human|Virology Department|Wellcome Sanger Institute for the COVID-19 Genomics UK (COG-UK) consortium|Davies|United Kingdom
>Spike|hCoV-19/India/TG-CCMB-AB465/2020|2020-08-14|EPI_ISL_539617|Original|hCoV-19^^Telangana|Human|CSIR-Centre for Cellular and Molecular Biology|CSIR-Centre for Cellular and Molecular Biology|Mukherjee|India
>Spike|hCoV-19/USA/TX-DSHS-0108/2020|2020-05-28|EPI_ISL_522391|Original|hCoV-19^^Texas|Human|Texas Department of State Health Services|Texas Department of State Health Services|Rahman|USA
>Spike|hCoV-19/England/CAMB-7532B/2020|2020-03-15|EPI_ISL_440428|Original|hCoV-19^^England|Human|Department of Pathology|Wellcome Sanger Institute for the COVID-19 Genomics UK (COG-UK) consortium|Amato|United Kingdom
>Spike|hCoV-19/England/ALDP-9EA030/2020|2020-09-24|EPI_ISL_579958|Original|hCoV-19^^England|Human|Lighthouse Lab in Alderley Park|Wellcome Sanger Institute for the COVID-19 Genomics UK (COG-UK) consortium|Davies|United Kingdom
>Spike|hCoV-19/USA/LA-EVTL1371/2020|2020-12-11|EPI_ISL_779037|Original|hCoV-19^^Louisiana|Human|LSUHS Emerging Viral Threat Laboratory|Microbial Genome Sequencing Center|Kamil|USA
>Spike|hCoV-19/England/CAMB-80E95/2020|2020-04-16|EPI_ISL_444439|Original|hCoV-19^^England|Human|Department of Pathology|COVID-19 Genomics UK (COG-UK) Consortium|Meredith|United Kingdom
>Spike|hCoV-19/Germany/NW-HHU-56/2020|2020-03-07|EPI_ISL_523927|Original|hCoV-19^^North Rhine-Westphalia|Human|Center of Medical Microbiology|Center of Medical Microbiology|Wienemann|Germany
>Spike|hCoV-19/USA/NY-Wadsworth-237026-01/2020|2020-10-05|EPI_ISL_734318|Original|hCoV-19^^New York|Human|Wadsworth Center|Wadsworth Center|Lasek-Nesselquist|USA
>Spike|hCoV-19/India/GJ-GBRC187a/2020|2020-06-05|EPI_ISL_469029|Original|hCoV-19^^Gujarat|Human|Government Medical College|Gujarat Biotechnology Research Centre|Joshi|India
>Spike|hCoV-19/Saudi Arabia/KAUST-RIYADH1195/2020|2020-06-15|EPI_ISL_678201|Original|hCoV-19^^Riyadh|Human|Pathogen Genomics Lab King Abdullah University of Science and Technology(KAUST)|Pathogen Genomics Lab King Abdullah University of Science and Technology(KAUST)|Mohamed Ghazzali|Saudi Arabia
>Spike|hCoV-19/Belgium/ULG-10994/2020|2020-11-14|EPI_ISL_707726|Original|hCoV-19^^Li\u00e8ge|Human|Department of Clinical Microbiology|GIGA Medical Genomics|Durkin|Belgium

>Spike|hCoV-19/Scotland/QEUH-96DDD4/2020|2020-08-21|EPI_ISL_530689|Original|hCoV-19^^Scotland|Human|NHSGGC West of Scotland Specialist Virology Centre / MRC-University of Glasgow Centre for Virus Research|Wellcome Sanger Institute for the COVID-19 Genomics UK (COG-UK) consortium|Davies|United Kingdom
>Spike|hCoV-19/USA/TX-HMH-MCoV-19653/2020|2020-11-16|EPI_ISL_784013|Original|hCoV-19^^Texas|Human|Houston Methodist Hospital|Houston Methodist Hospital|Long|USA
>Spike|hCoV-19/Spain/MD-IBV-99008481/2020|2020-08-11|EPI_ISL_654048|Original|hCoV-19^^Madrid|Human|Hospital General Universitario Gregorio Mara\u00f1\u00f3n|SeqCOVID-SPAIN consortium/IBV(CSIC)|Comas|Spain
>Spike|hCoV-19/England/CAMB-7A793/2020|2020-04-02|EPI_ISL_441271|Original|hCoV-19^^England|Human|Department of Pathology|Wellcome Sanger Institute for the COVID-19 Genomics UK (COG-UK) consortium|Amato|United Kingdom
>Spike|hCoV-19/Australia/VIC6668/2020|2020-07-20|EPI_ISL_521424|Original|hCoV-19^^Victoria|Human|Victorian Infectious Diseases Reference Laboratory (VIDRL)|VIDRL and MDU-PHL|Schultz|Australia
>Spike|hCoV-19/USA/NY-WCMP12C01P/2020|2020-03-16|EPI_ISL_427600|Original|hCoV-19^^New York|Human|NewYork-Presbyterian & Mason Lab|Mason Lab|Mason|USA
>Spike|hCoV-19/USA/TX-HMH-4334/2020|2020-06-18|EPI_ISL_543541|Original|hCoV-19^^Texas|Human|Houston Methodist Hospital|Houston Methodist Hospital|Long|USA
>Spike|hCoV-19/Austria/CeMM0247/2020|2020-03-21|EPI_ISL_438077|Original|hCoV-19^^Vienna|Human|Center for Virology|Bergthaler laboratory|Bergthaler|Austria
>Spike|hCoV-19/England/QEUH-C327D7/2020|2020-12-11|EPI_ISL_782658|Original|hCoV-19^^England|Human|Lighthouse Lab in Glasgow|Wellcome Sanger Institute for the COVID-19 Genomics UK (COG-UK) Consortium|Davies|United Kingdom
>Spike|hCoV-19/Australia/VIC5876/2020|2020-07-20|EPI_ISL_518662|Original|hCoV-19^^Victoria|Human|Microbiological Diagnostic Unit - Public Health Laboratory (MDU-PHL)|MDU-PHL|Schultz|Australia
>Spike|hCoV-19/Wales/QEUH-96B1DA/2020|2020-08-16|EPI_ISL_602157|Original|hCoV-19^^Wales|Human|Lighthouse Lab in Glasgow|Wellcome Sanger Institute for the COVID-19 Genomics UK (COG-UK) consortium|Davies|United Kingdom
>Spike|hCoV-19/India/UT-AR29/2020|2020-05-26|EPI_ISL_508176|Original|hCoV-19^^Uttarakhand|Human|All india institute of Medical Sciences Rishikesh|National Institute of Biomedical Genomics|Maitra|India
>Spike|hCoV-19/France/PAC-IHU-0447/2020|2020-04-04|EPI_ISL_569515|Original|hCoV-19^^Provence-Alpes-C\u00f4te-d\u2019Azur|Human|MEPHI|MEPHI|Levasseur|France
>Spike|hCoV-19/Australia/VIC17798/2020|2020-07-22|EPI_ISL_779461|Original|hCoV-19^^Victoria|Human|Microbiological Diagnostic Unit - Public Health Laboratory (MDU-PHL)|MDU-PHL|Beck|Australia
>Spike|hCoV-19/Denmark/DCGC-4960/2020|2020-09-21|EPI_ISL_621498|Original|hCoV-19^^Hovedstaden|Human|Department of Virus and Microbiological Special Diagnostics|Albertsen lab|Michaelsen|Denmark
>Spike|hCoV-19/Scotland/EDB9268/2020|2020-11-03|EPI_ISL_705799|Original|hCoV-19^^Scotland|Human|Virology Department|COVID-19 Genomics UK (COG-UK) Consortium|McHugh|United Kingdom
>Spike|hCoV-19/England/MILK-A7AF46/2020|2020-10-15|EPI_ISL_606443|Original|hCoV-19^^England|Human|Lighthouse Lab in Milton Keynes|Wellcome Sanger Institute for the COVID-19 Genomics UK (COG-UK) consortium|Davies|United Kingdom
>Spike|hCoV-19/England/QEUH-98EFAA/2020|2020-08-28|EPI_ISL_540411|Original|hCoV-19^^England|Human|Lighthouse Lab in Glasgow|Wellcome Sanger Institute for the COVID-19 Genomics UK (COG-UK) consortium|Davies|United Kingdom
>Spike|hCoV-19/USA/WI-UW-170/2020|2020-03-19|EPI_ISL_428266|Original|hCoV-19^^Wisconsin|Human|University of Wisconsin-Madison AIDS Vaccine Research Laboratories|University of Wisconsin-Madison AIDS Vaccine Research Laboratories|Moreno|USA
>Spike|hCoV-19/England/OXON-B16C0/2020|2020-04-30|EPI_ISL_479036|Original|hCoV-19^^England|Human|Oxford Viromics|COVID-19 Genomics UK (COG-UK) Consortium|Golubchik|United Kingdom
>Spike|hCoV-19/USA/NY-Wadsworth-27162-01/2020|2020-03-23|EPI_ISL_426304|Original|hCoV-19^^New York|Human|Wadsworth Center|Wadsworth Center|Lasek-Nesselquist|USA

>Spike|hCoV-19/Kazakhstan/NCB-1/2020|2020-03-22|EPI_ISL_435045|Original|hCoV-19^^Nur-Sultan|Human|Laboratory of Applied Genetics|RSE \National Center for Biotechnology\|Kalendar|Kazakhstan
>Spike|hCoV-19/Singapore/139nan/2020|2020-03-19|EPI_ISL_648064|Original|hCoV-19^^Singapore|Human|Department of Laboratory Medicine|Department of Laboratory Medicine|Chen|Singapore
>Spike|hCoV-19/USA/NV-NSPHL-A0078/2020|2020-04-13|EPI_ISL_515355|Original|hCoV-19^^Nevada|Human|Nevada State Public Health Laboratory|Nevada State Public Health Laboratory|Sevinsky|USA
>Spike|hCoV-19/Wales/PHWC-4A3F0E/2020|2020-12-13|EPI_ISL_764642|Original|hCoV-19^^Wales|Human|Wales Specialist Virology Centre Sequencing lab|COVID-19 Genomics UK (COG-UK) Consortium|Moore|United Kingdom
>Spike|hCoV-19/Wales/PHWC-165F56/2020|2020-06-13|EPI_ISL_490818|Original|hCoV-19^^Wales|Human|Wales Specialist Virology Centre Sequencing lab|COVID-19 Genomics UK (COG-UK) Consortium|Moore|United Kingdom
>Spike|hCoV-19/Denmark/DCGC-12699/2020|2020-11-16|EPI_ISL_682880|Original|hCoV-19^^Syddanmark|Human|Department of Virus and Microbiological Special Diagnostics|Albertsen Lab|Michaelsen|Denmark
>Spike|hCoV-19/England/SHEF-D1F1A/2020|2020-03-24|EPI_ISL_475500|Original|hCoV-19^^England|Human|Virology Department|COVID-19 Genomics UK (COG-UK) Consortium|de Silva|United Kingdom
>Spike|hCoV-19/England/MILK-A797A9/2020|2020-10-15|EPI_ISL_606811|Original|hCoV-19^^England|Human|Lighthouse Lab in Milton Keynes|Wellcome Sanger Institute for the COVID-19 Genomics UK (COG-UK) consortium|Davies|United Kingdom
>Spike|hCoV-19/USA/MA-MGH-00467/2020|2020-04-04|EPI_ISL_460159|Original|hCoV-19^^Massachusetts|Human|Massachusetts General Hospital|Infectious Disease Program|Lemieux|USA
>Spike|hCoV-19/England/ALDP-A2DF60/2020|2020-10-06|EPI_ISL_586671|Original|hCoV-19^^England|Human|Lighthouse Lab in Alderley Park|Wellcome Sanger Institute for the COVID-19 Genomics UK (COG-UK) consortium|Davies|United Kingdom
>Spike|hCoV-19/England/EXET-139F3D/2020|2020-00-00|EPI_ISL_679353|Original|hCoV-19^^England|Human|University of Exeter|COVID-19 Genomics UK (COG-UK) Consortium|Temperton|United Kingdom
>Spike|hCoV-19/Denmark/DCGC-7036/2020|2020-10-12|EPI_ISL_619883|Original|hCoV-19^^Hovedstaden|Human|Department of Virus and Microbiological Special Diagnostics|Albertsen lab|Michaelsen|Denmark
>Spike|hCoV-19/Scotland/CVR3138/2020|2020-04-26|EPI_ISL_461700|Original|hCoV-19^^Scotland|Human|West of Scotland Specialist Virology Centre|COVID-19 Genomics UK (COG-UK) Consortium|Robertson|United Kingdom
>Spike|hCoV-19/USA/NM-NMDOH-2020327510/2020|2020-09-03|EPI_ISL_569648|Original|hCoV-19^^New Mexico|Human|New Mexico Department of Health Scientific Laboratory|New Mexico Department of Health Scientific Laboratory|Johnson|USA
>Spike|hCoV-19/Sweden/20-50218/2020|2020-03-06|EPI_ISL_455875|Original|hCoV-19^^Stockholm|Human|Karolinska Universitetslaboratoriet|The Public Health Agency of Sweden|Svartstr\u00f6m|Sweden
>Spike|hCoV-19/USA/VA-DCLS-1682/2020|2020-09-03|EPI_ISL_609972|Original|hCoV-19^^Virginia|Human|Virginia DCLS|Virginia DCLS|St. Jacques|USA
>Spike|hCoV-19/Scotland/QEUH-96FC6C/2020|2020-08-17|EPI_ISL_540249|Original|hCoV-19^^Scotland|Human|Lighthouse Lab in Glasgow|Wellcome Sanger Institute for the COVID-19 Genomics UK (COG-UK) consortium|Davies|United Kingdom
>Spike|hCoV-19/England/QEUH-AE29B4/2020|2020-10-27|EPI_ISL_633623|Original|hCoV-19^^England|Human|Lighthouse Lab in Glasgow|Wellcome Sanger Institute for the COVID-19 Genomics UK (COG-UK) consortium|Davies|United Kingdom
>Spike|hCoV-19/England/LIVE-DCB6C6/2020|2020-11-24|EPI_ISL_724419|Original|hCoV-19^^England|Human|Liverpool Clinical Laboratories|COVID-19 Genomics UK (COG-UK) Consortium|Haldenby|United Kingdom
>Spike|hCoV-19/England/MILK-C2FC38/2020|2020-12-07|EPI_ISL_733749|Original|hCoV-19^^England|Human|Lighthouse Lab in Milton Keynes|Wellcome Sanger Institute for the COVID-19 Genomics UK (COG-UK) Consortium|Davies|United Kingdom

>Spike|hCoV-19/England/ALDP-CB4ED8/2020|2020-12-17|EPI_ISL_761714|Original|hCoV-19^^England|Human|Lighthouse Lab in Alderley Park|Wellcome Sanger Institute for the COVID-19 Genomics UK (COG-UK) Consortium|Davies|United Kingdom
>Spike|hCoV-19/USA/AZ-TG548494/2020|2020-07-27|EPI_ISL_695524|Original|hCoV-19^^Arizona|Human|TGen North|TGen North|French|USA
>Spike|hCoV-19/USA/NY-NYCPHL-000953/2020|2020-09-30|EPI_ISL_632990|Original|hCoV-19^^New York|Human|DOHMH Corona|New York City Public Health Laboratory|Wang|USA
>Spike|hCoV-19/USA/FL-BPHL-0161/2020|2020-03-31|EPI_ISL_480812|Original|hCoV-19^^Florida|Human|Florida Bureau of Public Health Laboratories|Florida Bureau of Public Health Laboratories|Blanton|USA
>Spike|hCoV-19/Finland/22SS11D1/2020|2020-09-22|EPI_ISL_757387|Original|hCoV-19^^Uusimaa|Human|Department of Virology and Immunology|Department of Virology|Smura|Finland
>Spike|hCoV-19/England/LIVE-9F5F8/2020|2020-05-15|EPI_ISL_457268|Original|hCoV-19^^England|Human|Liverpool Clinical Laboratories|COVID-19 Genomics UK (COG-UK) Consortium|Haldenby|United Kingdom
>Spike|hCoV-19/England/PORT-2E9D31/2020|2020-00-00|EPI_ISL_706880|Original|hCoV-19^^England|Human|Centre for Enzyme Innovation|COVID-19 Genomics UK (COG-UK) Consortium|Robson|United Kingdom
>Spike|hCoV-19/Denmark/DCGC-19768/2020|2020-12-07|EPI_ISL_747773|Original|hCoV-19^^Syddanmark|Human|Department of Virus and Microbiological Special Diagnostics|Albertsen Lab|Michaelsen|Denmark
>Spike|hCoV-19/Scotland/EDB1233/2020|2020-04-08|EPI_ISL_491650|Original|hCoV-19^^Scotland|Human|Virology Department|Wellcome Sanger Institute for the COVID-19 Genomics UK (COG-UK) consortium|Davies|United Kingdom
>Spike|hCoV-19/Belgium/ULG-10954/2020|2020-09-07|EPI_ISL_707733|Original|hCoV-19^^Li\u00e8ge|Human|Department of Clinical Microbiology|GIGA Medical Genomics|Durkin|Belgium
>Spike|hCoV-19/USA/VA-DCLS-1394/2020|2020-07-08|EPI_ISL_572235|Original|hCoV-19^^Virginia|Human|Virginia DCLS|Virginia DCLS|St. Jacques|USA
>Spike|hCoV-19/India/DL-NCDC1892-CSIR-IGIB/2020|2020-05-18|EPI_ISL_482511|Original|hCoV-19^^Delhi|Human|National Centre for Disease control (NCDC)|NCDC/CSIR-IGIB|Pandey|India
>Spike|hCoV-19/England/LCST-24D677D/2020|2020-05-30|EPI_ISL_478291|Original|hCoV-19^^England|Human|University Hospitals Of Leicester NHS Trust and DeepSeq Nottingham|COVID-19 Genomics UK (COG-UK) Consortium|Loose|United Kingdom
>Spike|hCoV-19/England/QEUH-997E0B/2020|2020-08-24|EPI_ISL_537185|Original|hCoV-19^^England|Human|Lighthouse Lab in Glasgow|Wellcome Sanger Institute for the COVID-19 Genomics UK (COG-UK) consortium|Davies|United Kingdom
>Spike|hCoV-19/Wales/PHWC-26B7C/2020|2020-03-28|EPI_ISL_422253|Original|hCoV-19^^Wales|Human|Wales Specialist Virology Centre|Public Health Wales Microbiology Cardiff|Moore|United Kingdom
>Spike|hCoV-19/England/MILK-B54DFC/2020|2020-11-06|EPI_ISL_657755|Original|hCoV-19^^England|Human|Lighthouse Lab in Milton Keynes|Wellcome Sanger Institute for the COVID-19 Genomics UK (COG-UK) Consortium|Davies|United Kingdom
>Spike|hCoV-19/Ecuador/USFQ-524/2020|2020-09-29|EPI_ISL_697795|Original|hCoV-19^^Morona Santiago|Human|Institute of Microbiology|Institute of Microbiology|Cardenas|Ecuador
>Spike|hCoV-19/Japan/PG-5431/2020|2020-07-00|EPI_ISL_690534|Original|hCoV-19^^Japan|Human|Pathogen Genomics Center|Pathogen Genomics Center|Sekizuka|Japan
>Spike|hCoV-19/USA/MD-MDH-0263/2020|2020-10-13|EPI_ISL_629044|Original|hCoV-19^^Maryland|Human|Maryland Public Health Laboratory|Maryland Public Health Laboratory|Keller|USA
>Spike|hCoV-19/England/QEUH-A4CB49/2020|2020-10-05|EPI_ISL_596023|Original|hCoV-19^^England|Human|Quadram Institute Bioscience|COVID-19 Genomics UK (COG-UK) Consortium|Alikhan|United Kingdom
>Spike|hCoV-19/Scotland/EDB10938/2020|2020-12-12|EPI_ISL_742186|Original|hCoV-19^^Scotland|Human|Virology Department|COVID-19 Genomics UK (COG-UK) Consortium|McHugh|United Kingdom

>Spike|hCoV-19/USA/MD-MDH-0412/2020|2020-11-04|EPI_ISL_666782|Original|hCoV-19^^Maryland|Human|Maryland Public Health Laboratory|Maryland Public Health Laboratory|Keller|USA
>Spike|hCoV-19/Russia/SVE-RII-MH9206S/2020|2020-10-03|EPI_ISL_733414|Original|hCoV-19^^Sverdlovsk|Human|UMMC-Health|WHO National Influenza Centre Russian Federation|Komissarov|Russia
>Spike|hCoV-19/USA/LA-GBCL-26556586/2020|2020-04-14|EPI_ISL_485208|Original|hCoV-19^^Louisiana|Human|River Road Testing Lab|Ginkgo Bioworks Clinical Laboratory|Smith|USA
>Spike|hCoV-19/England/OXON-AF337/2020|2020-04-12|EPI_ISL_478729|Original|hCoV-19^^England|Human|Oxford Viromics|COVID-19 Genomics UK (COG-UK) Consortium|Golubchik|United Kingdom
>Spike|hCoV-19/England/MILK-BAB998/2020|2020-11-18|EPI_ISL_681479|Original|hCoV-19^^England|Human|Lighthouse Lab in Milton Keynes|Wellcome Sanger Institute for the COVID-19 Genomics UK (COG-UK) Consortium|Davies|United Kingdom
>Spike|hCoV-19/USA/WI-CDC-6230/2020|2020-03-24|EPI_ISL_576179|Original|hCoV-19^^Wisconsin|Human|WI State Laboratory of Hygiene|Pathogen Discovery|Queen|USA
>Spike|hCoV-19/USA/ME-HETL-J0291/2020|2020-10-06|EPI_ISL_755433|Original|hCoV-19^^Maine|Human|Maine Health and Environmental Testing Laboratory|Tewhey Lab|Tewhey|USA
>Spike|hCoV-19/Northern Ireland/QEUH-960DB3/2020|2020-08-04|EPI_ISL_532128|Original|hCoV-19^^Northern Ireland|Human|Lighthouse Lab in Glasgow|Wellcome Sanger Institute for the COVID-19 Genomics UK (COG-UK) consortium|Davies|United Kingdom
>Spike|hCoV-19/England/ALDP-B82D04/2020|2020-11-14|EPI_ISL_675226|Original|hCoV-19^^England|Human|Lighthouse Lab in Alderley Park|Wellcome Sanger Institute for the COVID-19 Genomics UK (COG-UK) Consortium|Davies|United Kingdom
>Spike|hCoV-19/Scotland/QEUH-A3152B/2020|2020-10-05|EPI_ISL_595899|Original|hCoV-19^^Scotland|Human|Quadram Institute Bioscience|COVID-19 Genomics UK (COG-UK) Consortium|Alikhan|United Kingdom
>Spike|hCoV-19/Russia/MOS-CRIE-13379339/2020|2020-11-06|EPI_ISL_717970|Original|hCoV-19^^Moscow|Human|Molecular diagnostic laboratory of Federal Budget Institution of Science \Central Research Institute of Epidemiology\ of The Federal Service on Customers' Rights Protection and Human Well-being Surveillance|Group of Genomics and Postgenomic Technologies of Central Research Institute of Epidemilolog
>y|Korneenko|Russia
>Spike|hCoV-19/England/CAMC-BC0159/2020|2020-11-22|EPI_ISL_705087|Original|hCoV-19^^England|Human|Lighthouse Lab in Cambridge|Wellcome Sanger Institute for the COVID-19 Genomics UK (COG-UK) Consortium|Davies|United Kingdom
>Spike|hCoV-19/England/QEUH-B6A7AA/2020|2020-11-11|EPI_ISL_662473|Original|hCoV-19^^England|Human|Lighthouse Lab in Glasgow|Wellcome Sanger Institute for the COVID-19 Genomics UK (COG-UK) Consortium|Davies|United Kingdom
>Spike|hCoV-19/Israel/990059217/2020|2020-03-25|EPI_ISL_447277|Original|hCoV-19^^South Coast District|Human|Microbiology laboratory|Stern Lab|Miller|Israel
>Spike|hCoV-19/England/MILK-B3D5C4/2020|2020-11-01|EPI_ISL_704882|Original|hCoV-19^^England|Human|Oxford Viromics|COVID-19 Genomics UK (COG-UK) Consortium|Golubchik|United Kingdom
>Spike|hCoV-19/England/QEUH-B697BA/2020|2020-11-11|EPI_ISL_661470|Original|hCoV-19^^England|Human|Lighthouse Lab in Glasgow|Wellcome Sanger Institute for the COVID-19 Genomics UK (COG-UK) Consortium|Davies|United Kingdom
>Spike|hCoV-19/USA/LA-EVTL017/2020|2020-04-24|EPI_ISL_435555|Original|hCoV-19^^Louisiana|Human|LSUHS Emerging Viral Threat Laboratory|Microbial Genome Sequencing Center|Kamil|USA
>Spike|hCoV-19/USA/CA-CZB-14721/2020|2020-09-25|EPI_ISL_739489|Original|hCoV-19^^California|Human|Humboldt County Public Health Laboratory|Chan-Zuckerberg Biohub|Kamm|USA
>Spike|hCoV-19/USA/CO-CSU-D0769-2/2020|2020-04-15|EPI_ISL_527422|Original|hCoV-19^^Colorado|Human|Colorado State University - Ebel Lab|Colorado State University - Ebel Lab|Cline|USA

>Spike|hCoV-19/USA/UT-UPHL-201117199/2020|2020-04-06|EPI_ISL_648574|Original|hCoV-19^^Utah|Human|Utah Public Health Laboratory|Utah Public Health Laboratory|Young|USA
>Spike|hCoV-19/USA/IL-NM-0413/2020|2020-04-13|EPI_ISL_626394|Original|hCoV-19^^Illinois|Human|Northwestern Memorial Hospital|Ozer Lab|Ozer|USA
>Spike|hCoV-19/England/204742224/2020|2020-00-00|EPI_ISL_649927|Original|hCoV-19^^England|Human|Respiratory Virus Unit|COVID-19 Genomics UK (COG-UK) Consortium|Platt|United Kingdom
>Spike|hCoV-19/England/BRIS-127A6E/2020|2020-04-17|EPI_ISL_469737|Original|hCoV-19^^England|Human|PHE South West Regional Laboratory|Wellcome Sanger Institute for the COVID-19 Genomics UK (COG-UK) consortium|Davies|United Kingdom
>Spike|hCoV-19/Italy/LAZ-INMI-36/2020|2020-06-12|EPI_ISL_603143|Original|hCoV-19^^Lazio|Human|INMI Lazzaro Spallanzani IRCCS|INMI Lazzaro Spallanzani IRCCS|Gruber|Italy
>Spike|hCoV-19/England/ALDP-A6DD3D/2020|2020-10-15|EPI_ISL_607152|Original|hCoV-19^^England|Human|Lighthouse Lab in Alderley Park|Wellcome Sanger Institute for the COVID-19 Genomics UK (COG-UK) consortium|Davies|United Kingdom
>Spike|hCoV-19/Sweden/20-53291/2020|2020-10-26|EPI_ISL_661295|Original|hCoV-19^^Sormland|Human|Unilabs|The Public Health Agency of Sweden|Svartstr\u00f6m|Sweden
>Spike|hCoV-19/Australia/VIC10191/2020|2020-08-26|EPI_ISL_562820|Original|hCoV-19^^Victoria|Human|Microbiological Diagnostic Unit - Public Health Laboratory (MDU-PHL)|MDU-PHL|Schultz|Australia
>Spike|hCoV-19/USA/WA-UW-12627/2020|2020-06-28|EPI_ISL_570880|Original|hCoV-19^^Washington|Human|UW Virology Lab|UW Virology Lab|Roychoudhury|USA
>Spike|hCoV-19/England/ALDP-B639AB/2020|2020-11-09|EPI_ISL_656607|Original|hCoV-19^^England|Human|Lighthouse Lab in Alderley Park|Wellcome Sanger Institute for the COVID-19 Genomics UK (COG-UK) Consortium|Davies|United Kingdom
>Spike|hCoV-19/Belgium/ULG-10794/2020|2020-11-16|EPI_ISL_661228|Original|hCoV-19^^Li\u00e8ge|Human|Department of Clinical Microbiology|GIGA Medical Genomics|Durkin|Belgium
>Spike|hCoV-19/Japan/PG-7634/2020|2020-08-00|EPI_ISL_686736|Original|hCoV-19^^Japan|Human|Chiba Prefectural Institute of Public Health|Pathogen Genomics Center|Sekizuka|Japan
>Spike|hCoV-19/USA/WI-UW-828/2020|2020-05-13|EPI_ISL_509835|Original|hCoV-19^^Wisconsin|Human|University of Wisconsin-Madison AIDS Vaccine Research Laboratories|University of Wisconsin-Madison AIDS Vaccine Research Laboratories|Baczenas|USA
>Spike|hCoV-19/USA/TX-HMH-MCoV-12473/2020|2020-09-28|EPI_ISL_790355|Original|hCoV-19^^Texas|Human|Houston Methodist Hospital|Houston Methodist Hospital|Long|USA
>Spike|hCoV-19/Wales/PHWC-47FDB6/2020|2020-10-13|EPI_ISL_611809|Original|hCoV-19^^Wales|Human|Wales Specialist Virology Centre Sequencing lab|COVID-19 Genomics UK (COG-UK) Consortium|Moore|United Kingdom
>Spike|hCoV-19/Germany/BY-IMB02204/2020|2020-03-18|EPI_ISL_732546|Original|hCoV-19^^Bavaria|Human|Bundeswehr Institute of Microbiology|Bundeswehr Institute of Microbiology|Antwerpen|Germany
>Spike|hCoV-19/Japan/PG-8637/2020|2020-09-00|EPI_ISL_686233|Original|hCoV-19^^Japan|Human|Pathogen Genomics Center|Pathogen Genomics Center|Sekizuka|Japan
>Spike|hCoV-19/England/MILK-A47C96/2020|2020-10-05|EPI_ISL_598517|Original|hCoV-19^^England|Human|Lighthouse Lab in Milton Keynes|Wellcome Sanger Institute for the COVID-19 Genomics UK (COG-UK) consortium|Davies|United Kingdom
>Spike|hCoV-19/England/MILK-994AFB/2020|2020-08-25|EPI_ISL_552419|Original|hCoV-19^^England|Human|Lighthouse Lab in Milton Keynes|Wellcome Sanger Institute for the COVID-19 Genomics UK (COG-UK) consortium|Davies|United Kingdom
>Spike|hCoV-19/Scotland/QEUH-AD5B36/2020|2020-10-26|EPI_ISL_649695|Original|hCoV-19^^Scotland|Human|Lighthouse Lab in Glasgow|Wellcome Sanger Institute for the COVID-19 Genomics UK (COG-UK) Consortium|Davies|United Kingdom
>Spike|hCoV-19/England/QEUH-AF40C9/2020|2020-10-30|EPI_ISL_663024|Original|hCoV-19^^England|Human|Lighthouse Lab in Glasgow|Wellcome Sanger Institute for the COVID-19 Genomics UK (COG-UK) Consortium|Davies|United Kingdom

>Spike|hCoV-19/USA/CA-CZB-14865/2020|2020-11-23|EPI_ISL_739131|Original|hCoV-19^^California|Human|Humboldt County Public Health Laboratory|Chan-Zuckerberg Biohub|Kamm|USA
>Spike|hCoV-19/England/MILK-ABBEC4/2020|2020-10-22|EPI_ISL_631079|Original|hCoV-19^^England|Human|Lighthouse Lab in Milton Keynes|Wellcome Sanger Institute for the COVID-19 Genomics UK (COG-UK) consortium|Davies|United Kingdom
>Spike|hCoV-19/England/ALDP-A52849/2020|2020-10-07|EPI_ISL_598012|Original|hCoV-19^^England|Human|Lighthouse Lab in Alderley Park|Wellcome Sanger Institute for the COVID-19 Genomics UK (COG-UK) consortium|Davies|United Kingdom
>Spike|hCoV-19/England/QEUH-B0E465/2020|2020-11-02|EPI_ISL_642102|Original|hCoV-19^^England|Human|Lighthouse Lab in Glasgow|Wellcome Sanger Institute for the COVID-19 Genomics UK (COG-UK) Consortium|Davies|United Kingdom
>Spike|hCoV-19/Hong Kong/VM20002529/2020|2020-02-11|EPI_ISL_426393|Original|hCoV-19^^Hong Kong|Human|Kwong Wah Hospital|Hong Kong Department of Health|Mak|China
>Spike|hCoV-19/Australia/NSW2949/2020|2020-10-24|EPI_ISL_678378|Original|hCoV-19^^New South Wales|Human|Area of Virology|Virology Research Laboratory; Area of Virology|Foster|Australia
>Spike|hCoV-19/England/QEUH-B41897/2020|2020-11-08|EPI_ISL_658407|Original|hCoV-19^^England|Human|Lighthouse Lab in Glasgow|Wellcome Sanger Institute for the COVID-19 Genomics UK (COG-UK) Consortium|Davies|United Kingdom
>Spike|hCoV-19/Scotland/EDB9403/2020|2020-10-27|EPI_ISL_680514|Original|hCoV-19^^Scotland|Human|Virology Department|COVID-19 Genomics UK (COG-UK) Consortium|McHugh|United Kingdom
>Spike|hCoV-19/Wales/PHWC-2B218/2020|2020-04-08|EPI_ISL_432273|Original|hCoV-19^^Wales|Human|Wales Specialist Virology Centre|Public Health Wales Microbiology Cardiff|Moore|United Kingdom
>Spike|hCoV-19/USA/WI-CDC-0946/2020|2020-04-21|EPI_ISL_509705|Original|hCoV-19^^Wisconsin|Human|Wisconsin Department of Health Services|Pathogen Discovery|Queen|USA
>Spike|hCoV-19/Switzerland/BS-UHB-42202238/2020|2020-03-21|EPI_ISL_528326|Original|hCoV-19^^Basel-Stadt|Human|University Hospital Basel|University Hospital Basel|Roloff Handschin|Switzerland
>Spike|hCoV-19/USA/TX-HMH-7671/2020|2020-06-26|EPI_ISL_546929|Original|hCoV-19^^Texas|Human|Houston Methodist Hospital|Houston Methodist Hospital|Long|USA
>Spike|hCoV-19/England/CAMC-BC049C/2020|2020-11-22|EPI_ISL_704800|Original|hCoV-19^^England|Human|Lighthouse Lab in Cambridge|Wellcome Sanger Institute for the COVID-19 Genomics UK (COG-UK) Consortium|Davies|United Kingdom
>Spike|hCoV-19/England/NORT-28315B/2020|2020-03-31|EPI_ISL_488653|Original|hCoV-19^^England|Human|NU-OMICS DNA Sequencing research facility|Wellcome Sanger Institute for the COVID-19 Genomics UK (COG-UK) consortium|Davies|United Kingdom
>Spike|hCoV-19/USA/CA-CZB-14136/2020|2020-05-24|EPI_ISL_738858|Original|hCoV-19^^California|Human|Alameda County Public Health Lab|Chan-Zuckerberg Biohub|Kamm|USA
>Spike|hCoV-19/Russia/SPE-RII-MH5889S/2020|2020-11-06|EPI_ISL_733320|Original|hCoV-19^^Saint Petersburg|Human|HELIX LLC|WHO National Influenza Centre Russian Federation|Komissarov|Russia
>Spike|hCoV-19/Australia/VIC12324/2020|2020-08-10|EPI_ISL_591959|Original|hCoV-19^^Victoria|Human|Victorian Infectious Diseases Reference Laboratory (VIDRL)|VIDRL and MDU-PHL|Schultz|Australia
>Spike|hCoV-19/Australia/VIC12789/2020|2020-07-28|EPI_ISL_640331|Original|hCoV-19^^Victoria|Human|Microbiological Diagnostic Unit - Public Health Laboratory (MDU-PHL)|MDU-PHL|Schultz|Australia
>Spike|hCoV-19/England/CAMB-726CB/2020|2020-03-24|EPI_ISL_439604|Original|hCoV-19^^England|Human|Department of Pathology|Wellcome Sanger Institute for the COVID-19 Genomics UK (COG-UK) consortium|Amato|United Kingdom
>Spike|hCoV-19/France/HDF-2393/2020|2020-03-12|EPI_ISL_418227|Original|hCoV-19^^Hauts-de-France|Human|Centre Hospitalier Compi\u00e8gne Laboratoire de Biologie|National Reference Center for Viruses of Respiratory Infections|Enouf|France
>Spike|hCoV-19/USA/TX-HMH-MCoV-15603/2020|2020-10-23|EPI_ISL_787087|Original|hCoV-19^^Texas|Human|Houston Methodist Hospital|Houston Methodist Hospital|Long|USA

>Spike|hCoV-19/USA/CO-CDPHE-2010200815/2020|2020-10-19|EPI_ISL_710357|Original|hCoV-19^^Colorado|Human|Colorado Department of Public Health and Environment|Colorado Department of Puplic Health and Environment|Bankers|USA
>Spike|hCoV-19/England/CAMC-B32495/2020|2020-11-05|EPI_ISL_659103|Original|hCoV-19^^England|Human|Lighthouse Lab in Cambridge|Wellcome Sanger Institute for the COVID-19 Genomics UK (COG-UK) Consortium|Davies|United Kingdom
>Spike|hCoV-19/India/TG-CCMB-L1963/2020|2020-06-04|EPI_ISL_495175|Original|hCoV-19^^Telangana|Human|CSIR-Centre for Cellular and Molecular Biology|CSIR-Centre for Cellular and Molecular Biology|Banu|India
>Spike|hCoV-19/England/MILK-BABCEA/2020|2020-11-18|EPI_ISL_783096|Original|hCoV-19^^England|Human|Lighthouse Lab in Milton Keynes|Wellcome Sanger Institute for the COVID-19 Genomics UK (COG-UK) Consortium|Davies|United Kingdom
>Spike|hCoV-19/Scotland/CVR2409/2020|2020-04-14|EPI_ISL_453048|Original|hCoV-19^^Scotland|Human|West of Scotland Specialist Virology Centre|COVID-19 Genomics UK (COG-UK) Consortium|Robertson|United Kingdom
>Spike|hCoV-19/England/MILK-AB8814/2020|2020-10-21|EPI_ISL_623603|Original|hCoV-19^^England|Human|Lighthouse Lab in Milton Keynes|Wellcome Sanger Institute for the COVID-19 Genomics UK (COG-UK) consortium|Davies|United Kingdom
>Spike|hCoV-19/Australia/VIC5296/2020|2020-07-16|EPI_ISL_520059|Original|hCoV-19^^Victoria|Human|Microbiological Diagnostic Unit - Public Health Laboratory (MDU-PHL)|MDU-PHL|Schultz|Australia
>Spike|hCoV-19/India/MP-DRDE 4661/2020|2020-05-27|EPI_ISL_476895|Original|hCoV-19^^Madhya Pradesh|Human|Defence Research & Development Establishment (DRDE)|Defence Research & Development Establishment (DRDE)|Dash|India
>Spike|hCoV-19/England/ALDP-6FCD55/2020|2020-06-29|EPI_ISL_557436|Original|hCoV-19^^England|Human|Lighthouse Lab in Alderley Park|Wellcome Sanger Institute for the COVID-19 Genomics UK (COG-UK) consortium|Davies|United Kingdom
>Spike|hCoV-19/England/CAMC-B32486/2020|2020-11-05|EPI_ISL_659406|Original|hCoV-19^^England|Human|Lighthouse Lab in Cambridge|Wellcome Sanger Institute for the COVID-19 Genomics UK (COG-UK) Consortium|Davies|United Kingdom
>Spike|hCoV-19/Germany/BY-MVP-0064/2020|2020-03-24|EPI_ISL_437263|Original|hCoV-19^^Bavaria|Human|Max von Pettenkofer Institute|Laboratory for Functional Genome Analysis|Graf|Germany
>Spike|hCoV-19/Denmark/DCGC-9947/2020|2020-11-09|EPI_ISL_669288|Original|hCoV-19^^Midtjylland|Human|Department of Virus and Microbiological Special Diagnostics|Albertsen Lab|Michaelsen|Denmark
>Spike|hCoV-19/USA/MI-MDHHS-SC21332/2020|2020-04-03|EPI_ISL_507587|Original|hCoV-19^^Michigan|Human|Michigan Department of Health and Human Services|Michigan Department of Health and Human Services|Blankenship|USA
>Spike|hCoV-19/England/ALDP-B45BF4/2020|2020-11-07|EPI_ISL_656060|Original|hCoV-19^^England|Human|Lighthouse Lab in Alderley Park|Wellcome Sanger Institute for the COVID-19 Genomics UK (COG-UK) Consortium|Davies|United Kingdom
>Spike|hCoV-19/Northern Ireland/NIRE-103646/2020|2020-04-17|EPI_ISL_489357|Original|hCoV-19^^Northern Ireland|Human|Regional Virus Laboratory|Wellcome Sanger Institute for the COVID-19 Genomics UK (COG-UK) consortium|Davies|United Kingdom
>Spike|hCoV-19/England/CAMB-7BBC3/2020|2020-04-05|EPI_ISL_442105|Original|hCoV-19^^England|Human|Department of Pathology|Wellcome Sanger Institute for the COVID-19 Genomics UK (COG-UK) consortium|Amato|United Kingdom
>Spike|hCoV-19/Wales/PHWC-364F6/2020|2020-05-01|EPI_ISL_474676|Original|hCoV-19^^Wales|Human|Originating lab|COVID-19 Genomics UK (COG-UK) Consortium|Moore|United Kingdom
>Spike|hCoV-19/England/CAMC-A57DFA/2020|2020-10-08|EPI_ISL_597213|Original|hCoV-19^^England|Human|Lighthouse Lab in Cambridge|Wellcome Sanger Institute for the COVID-19 Genomics UK (COG-UK) consortium|Davies|United Kingdom
>Spike|hCoV-19/South Africa/KRISP-K002504/2020|2020-08-06|EPI_ISL_535400|Original|hCoV-19^^KwaZulu-Natal|Human|NHLS-IALCH|KRISP|de Oliveira|South Africa

>Spike|hCoV-19/Singapore/798/2020|2020-07-17|EPI_ISL_512834|Original|hCoV-19^^Singapore|Human|National Public Health Laboratory|National Public Health Laboratory|Mak|Singapore
>Spike|hCoV-19/Denmark/DCGC-7905/2020|2020-10-26|EPI_ISL_618815|Original|hCoV-19^^Hovedstaden|Human|Department of Virus and Microbiological Special Diagnostics|Albertsen lab|Michaelsen|Denmark
>Spike|hCoV-19/India/OR-ILSCV13744/2020|2020-05-09|EPI_ISL_463023|Original|hCoV-19^^Odisha|Human|Institute of Life Sciences|Immunogenomics lab|Raghav|India
>Spike|hCoV-19/England/BRIS-12B1B0/2020|2020-05-11|EPI_ISL_469807|Original|hCoV-19^^England|Human|PHE South West Regional Laboratory|Wellcome Sanger Institute for the COVID-19 Genomics UK (COG-UK) consortium|Davies|United Kingdom
>Spike|hCoV-19/Denmark/DCGC-1603/2020|2020-04-13|EPI_ISL_618649|Original|hCoV-19^^Syddanmark|Human|Department of Virus and Microbiological Special Diagnostics|Albertsen lab|Michaelsen|Denmark
>Spike|hCoV-19/Gambia/NPHL-1216/2020|2020-06-30|EPI_ISL_561216|Original|hCoV-19^^Kombo|Human|MRCG at LSHTM Genomics lab|MRCG at LSHTM Genomics lab|Sesay|Gambia
>Spike|hCoV-19/Canada/ON-UHTC_0002/2020|2020-03-19|EPI_ISL_464031|unknown|hCoV-19^^Ontario|Human|Unity Health Toronto|Ontario Institute for Cancer Research|de Borja|Canada
>Spike|hCoV-19/USA/CA-CZB-13768/2020|2020-09-18|EPI_ISL_739300|Original|hCoV-19^^California|Human|Alameda County Public Health Lab|Chan-Zuckerberg Biohub|Kamm|USA
>Spike|hCoV-19/Scotland/CVR663/2020|2020-03-25|EPI_ISL_433321|Original|hCoV-19^^Scotland|Human|West of Scotland Specialist Virology Centre|COVID-19 Genomics UK (COG-UK) Consortium|Robertson|United Kingdom
>Spike|hCoV-19/Greece/150/2020|2020-03-12|EPI_ISL_434485|Original|hCoV-19^^Athens|Human|Laboratory of Microbiology|Laboratory of Biology|Karakasiliotis|Greece
>Spike|hCoV-19/England/ALDP-94C3E8/2020|2020-06-16|EPI_ISL_555866|Original|hCoV-19^^England|Human|Lighthouse Lab in Alderley Park|Wellcome Sanger Institute for the COVID-19 Genomics UK (COG-UK) consortium|Davies|United Kingdom
>Spike|hCoV-19/USA/TX-HMH-4774/2020|2020-06-22|EPI_ISL_544321|Original|hCoV-19^^Texas|Human|Houston Methodist Hospital|Houston Methodist Hospital|Long|USA
>Spike|hCoV-19/England/MILK-B3CF4F/2020|2020-11-02|EPI_ISL_706208|Original|hCoV-19^^England|Human|Oxford Viromics|COVID-19 Genomics UK (COG-UK) Consortium|Golubchik|United Kingdom
>Spike|hCoV-19/England/ALDP-A679A8/2020|2020-10-12|EPI_ISL_630411|Original|hCoV-19^^England|Human|Lighthouse Lab in Alderley Park|Wellcome Sanger Institute for the COVID-19 Genomics UK (COG-UK) consortium|Davies|United Kingdom
>Spike|hCoV-19/England/20144014704/2020|2020-03-31|EPI_ISL_421936|Original|hCoV-19^^England|Human|Respiratory Virus Unit|Respiratory Virus Unit|Platt|United Kingdom
>Spike|hCoV-19/England/ALDP-A50650/2020|2020-10-07|EPI_ISL_597645|Original|hCoV-19^^England|Human|Lighthouse Lab in Alderley Park|Wellcome Sanger Institute for the COVID-19 Genomics UK (COG-UK) consortium|Davies|United Kingdom
>Spike|hCoV-19/England/ALDP-AA1F28/2020|2020-10-22|EPI_ISL_624314|Original|hCoV-19^^England|Human|Lighthouse Lab in Alderley Park|Wellcome Sanger Institute for the COVID-19 Genomics UK (COG-UK) consortium|Davies|United Kingdom
>Spike|hCoV-19/USA/MI-MDHHS-SC20866/2020|2020-05-05|EPI_ISL_471887|Original|hCoV-19^^Michigan|Human|Michigan Department of Health and Human Services|Michigan Department of Health and Human Services|Blankenship|USA
>Spike|hCoV-19/USA/UT-UPHL-2009553/2020|2020-07-18|EPI_ISL_605232|Original|hCoV-19^^Utah|Human|Utah Public Health Laboratory|Utah Public Health Laboratory|Young|USA
>Spike|hCoV-19/USA/TX-HMH-MCoV-18020/2020|2020-11-16|EPI_ISL_786168|Original|hCoV-19^^Texas|Human|Houston Methodist Hospital|Houston Methodist Hospital|Long|USA
>Spike|hCoV-19/Russia/StPetersburg-RII11126S/2020|2020-05-02|EPI_ISL_507231|Original|hCoV-19^^Saint Petersburg|Human|WHO National Influenza Centre Russian Federation|WHO National Influenza Centre Russian Federation|Komissarov|Russia
>Spike|hCoV-19/England/ALDP-9CAEA5/2020|2020-09-16|EPI_ISL_575513|Original|hCoV-19^^England|Human|Lighthouse Lab in Alderley Park|Wellcome Sanger Institute for the COVID-19 Genomics UK (COG-UK) consortium|Davies|United Kingdom

>Spike|hCoV-19/Switzerland/ZH-UZH-IMV126/2020|2020-11-21|EPI_ISL_733496|Original|hCoV-19^^Zurich|Human|University Hospital Z\u00fcrich|Institute of Medical Virology|Huber|Switzerland
>Spike|hCoV-19/South Korea/KCDC2113/2020|2020-03-12|EPI_ISL_498014|Original|hCoV-19^^South Korea|Human|Division of Viral Diseases|Division of Viral Diseases|Kim|South Korea
>Spike|hCoV-19/England/CAMB-7F0C0/2020|2020-04-07|EPI_ISL_442901|Original|hCoV-19^^England|Human|Department of Pathology|Wellcome Sanger Institute for the COVID-19 Genomics UK (COG-UK) consortium|Amato|United Kingdom
>Spike|hCoV-19/Spain/MD-IBV-99012395/2020|2020-10-12|EPI_ISL_780028|Original|hCoV-19^^Madrid|Human|Hospital General Universitario Gregorio Mara\u00f1\u00f3n|SeqCOVID-SPAIN consortium/IBV(CSIC)|Comas|Spain
>Spike|hCoV-19/Spain/IB-IBV-2532/2020|2020-03-27|EPI_ISL_469003|Original|hCoV-19^^Illes Balears|Human|Servicio de Microbiolog\u00eda|SeqCOVID-SPAIN consortium/IBV(CSIC)|Comas|Spain
>Spike|hCoV-19/USA/TX-HMH-MCoV-10839/2020|2020-07-05|EPI_ISL_789653|Original|hCoV-19^^Texas|Human|Houston Methodist Hospital|Houston Methodist Hospital|Long|USA
>Spike|hCoV-19/England/ALDP-C23117/2020|2020-12-06|EPI_ISL_733879|Original|hCoV-19^^England|Human|Lighthouse Lab in Alderley Park|Wellcome Sanger Institute for the COVID-19 Genomics UK (COG-UK) Consortium|Davies|United Kingdom
>Spike|hCoV-19/England/20126001904/2020|2020-03-18|EPI_ISL_423867|Original|hCoV-19^^England|Human|Respiratory Virus Unit|Respiratory Virus Unit|Platt|United Kingdom
>Spike|hCoV-19/Switzerland/BS-UHB-42200536/2020|2020-03-20|EPI_ISL_528275|Original|hCoV-19^^Basel-Stadt|Human|University Hospital Basel|University Hospital Basel|Roloff Handschin|Switzerland
>Spike|hCoV-19/England/NORW-ED31F/2020|2020-06-02|EPI_ISL_472360|Original|hCoV-19^^England|Human|Quadram Institute Bioscience|COVID-19 Genomics UK (COG-UK) Consortium|Alikhan|United Kingdom
>Spike|hCoV-19/Scotland/CVR2188/2020|2020-04-11|EPI_ISL_493783|Original|hCoV-19^^Scotland|Human|West of Scotland Specialist Virology Centre|COVID-19 Genomics UK (COG-UK) Consortium|Robertson|United Kingdom
>Spike|hCoV-19/England/MILK-BA9D9E/2020|2020-11-17|EPI_ISL_783178|Original|hCoV-19^^England|Human|Lighthouse Lab in Milton Keynes|Wellcome Sanger Institute for the COVID-19 Genomics UK (COG-UK) Consortium|Davies|United Kingdom
>Spike|hCoV-19/USA/FL-BPHL-1970/2020|2020-04-16|EPI_ISL_653123|Original|hCoV-19^^Florida|Human|Florida Bureau of Public Health Laboratories|Florida Bureau of Public Health Laboratories|Blanton|USA
>Spike|hCoV-19/USA/MI-MDHHS-SC21110/2020|2020-03-25|EPI_ISL_507886|Original|hCoV-19^^Michigan|Human|Michigan Department of Health and Human Services|Michigan Department of Health and Human Services|Blankenship|USA
>Spike|hCoV-19/Brazil/RS-00638/2020|2020-11-25|EPI_ISL_770586|Original|hCoV-19^^Rio Grande do Sul|Human|Laborat\u00f3rio de Microbiologia Molecular - Universidade FEEVALE|Bioinformatics Laboratory / LNCC|Almeida|Brazil
>Spike|hCoV-19/France/ARA-SC148/2020|2020-03-09|EPI_ISL_700348|Original|hCoV-19^^Auvergne-Rh\u00f4ne-Alpes|Human|Laboratoire de virologie|CNR Virus des Infections Respiratoires - France SUD|Bal|France
>Spike|hCoV-19/USA/TX-HMH-5343/2020|2020-06-25|EPI_ISL_544699|Original|hCoV-19^^Texas|Human|Houston Methodist Hospital|Houston Methodist Hospital|Long|USA
>Spike|hCoV-19/New Zealand/20VR2959/2020|2020-00-00|EPI_ISL_579378|Original|hCoV-19^^Auckland|Human|LabPLUS|Institute of Environmental Science and Research (ESR)|Wang|New Zealand
>Spike|hCoV-19/England/CAMB-82CF3/2020|2020-04-12|EPI_ISL_443552|Original|hCoV-19^^England|Human|Department of Pathology|Wellcome Sanger Institute for the COVID-19 Genomics UK (COG-UK) consortium|Amato|United Kingdom
>Spike|hCoV-19/England/MILK-AC9379/2020|2020-10-21|EPI_ISL_630052|Original|hCoV-19^^England|Human|Lighthouse Lab in Milton Keynes|Wellcome Sanger Institute for the COVID-19 Genomics UK (COG-UK) consortium|Davies|United Kingdom
>Spike|hCoV-19/Denmark/DCGC-16862/2020|2020-12-14|EPI_ISL_750607|Original|hCoV-19^^Midtjylland|Human|Department of Virus and Microbiological Special Diagnostics|Albertsen Lab|Michaelsen|Denmark

>Spike|hCoV-19/England/SHEF-D2E85/2020|2020-09-10|EPI_ISL_540773|Original|hCoV-19^^England|Human|Virology Department|COVID-19 Genomics UK (COG-UK) Consortium|de Silva|United Kingdom
>Spike|hCoV-19/Australia/WA500/2020|2020-11-29|EPI_ISL_708794|Original|hCoV-19^^Western Australia|Human|PathWest Laboratory Medicine WA|PathWest Laboratory Medicine WA Microbial Surveillance Unit|Sikazwe|Australia
>Spike|hCoV-19/New Zealand/20VR1925/2020|2020-04-02|EPI_ISL_456310|Original|hCoV-19^^Southern|Human|Southern Community Labs Dunedin|Institute of Environmental Science and Research (ESR)|de Ligt|New Zealand
>Spike|hCoV-19/USA/TX-DSHS-0973/2020|2020-06-16|EPI_ISL_613972|Original|hCoV-19^^Texas|Human|Texas Department of State Health Services|Texas Department of State Health Services|Rahman|USA
>Spike|hCoV-19/USA/LA-GBCL-26513055/2020|2020-04-21|EPI_ISL_485091|Original|hCoV-19^^Louisiana|Human|River Road Testing Lab|Ginkgo Bioworks Clinical Laboratory|Smith|USA
>Spike|hCoV-19/USA/TX-HMH-MCoV-8132/2020|2020-06-30|EPI_ISL_784222|Original|hCoV-19^^Texas|Human|Houston Methodist Hospital|Houston Methodist Hospital|Long|USA
>Spike|hCoV-19/England/ALDP-BBB0EB/2020|2020-11-20|EPI_ISL_701805|Original|hCoV-19^^England|Human|Lighthouse Lab in Alderley Park|Wellcome Sanger Institute for the COVID-19 Genomics UK (COG-UK) Consortium|Davies|United Kingdom
>Spike|hCoV-19/USA/WA-S2241/2020|2020-06-09|EPI_ISL_497669|Original|hCoV-19^^Washington|Human|Washington State Department of Health|Seattle Flu Study|Lee|USA
>Spike|hCoV-19/England/EXET-13A4A6/2020|2020-11-06|EPI_ISL_664212|Original|hCoV-19^^England|Human|University of Exeter|COVID-19 Genomics UK (COG-UK) Consortium|Temperton|United Kingdom
>Spike|hCoV-19/USA/WA-UW-22784/2020|2020-08-06|EPI_ISL_570718|Original|hCoV-19^^Washington|Human|UW Virology Lab|UW Virology Lab|Roychoudhury|USA
>Spike|hCoV-19/England/201690346/2020|2020-04-16|EPI_ISL_526754|Original|hCoV-19^^England|Human|Respiratory Virus Unit|Respiratory Virus Unit|Miah|United Kingdom
>Spike|hCoV-19/Japan/IC-0381/2020|2020-11-00|EPI_ISL_721570|Original|hCoV-19^^Japan|Human|Pathogen Genomics Center|Pathogen Genomics Center|Sekizuka|Japan
>Spike|hCoV-19/South Africa/KRISP-0172/2020|2020-06-02|EPI_ISL_467522|Original|hCoV-19^^KwaZulu-Natal|Human|NHLS-IALCH|KRISP|de Oliveira|South Africa
>Spike|hCoV-19/Wales/PHWC-2EA2C/2020|2020-04-07|EPI_ISL_446101|Original|hCoV-19^^Wales|Human|Wales Specialist Virology Centre|Public Health Wales Microbiology Cardiff|Moore|United Kingdom
>Spike|hCoV-19/England/ALDP-A2E970/2020|2020-10-05|EPI_ISL_586941|Original|hCoV-19^^England|Human|Lighthouse Lab in Alderley Park|Wellcome Sanger Institute for the COVID-19 Genomics UK (COG-UK) consortium|Davies|United Kingdom
>Spike|hCoV-19/Australia/VIC15245/2020|2020-08-21|EPI_ISL_593217|Original|hCoV-19^^Victoria|Human|Microbiological Diagnostic Unit - Public Health Laboratory (MDU-PHL)|MDU-PHL|Schultz|Australia
>Spike|hCoV-19/USA/WI-UW-2051/2020|2020-10-10|EPI_ISL_677456|Original|hCoV-19^^Wisconsin|Human|University of Wisconsin-Madison AIDS Vaccine Research Laboratories|University of Wisconsin-Madison AIDS Vaccine Research Laboratories|Baczenas|USA
>Spike|hCoV-19/Wales/PHWC-15D48F/2020|2020-05-07|EPI_ISL_472723|Original|hCoV-19^^Wales|Human|Wales Specialist Virology Centre Sequencing lab|COVID-19 Genomics UK (COG-UK) Consortium|Moore|United Kingdom
>Spike|hCoV-19/Denmark/DCGC-20715/2020|2020-12-21|EPI_ISL_759321|Original|hCoV-19^^Hovedstaden|Human|Department of Virus and Microbiological Special Diagnostics|Albertsen Lab|Michaelsen|Denmark
>Spike|hCoV-19/England/MILK-A05942/2020|2020-09-29|EPI_ISL_600084|Original|hCoV-19^^England|Human|Lighthouse Lab in Milton Keynes|Wellcome Sanger Institute for the COVID-19 Genomics UK (COG-UK) consortium|Davies|United Kingdom
>Spike|hCoV-19/USA/CA-CSMC107/2020|2020-04-02|EPI_ISL_475582|Original|hCoV-19^^California|Human|Cedars-Sinai Medical Center|Cedars-Sinai Medical Center|Zhang|USA
>Spike|hCoV-19/England/20128001806/2020|2020-03-19|EPI_ISL_423921|Original|hCoV-19^^England|Human|Respiratory Virus Unit|Respiratory Virus Unit|Platt|United Kingdom

>Spike|hCoV-19/England/QEUH-AD1314/2020|2020-10-27|EPI_ISL_633855|Original|hCoV-19^^England|Human|Lighthouse Lab in Glasgow|Wellcome Sanger Institute for the COVID-19 Genomics UK (COG-UK) consortium|Davies|United Kingdom
>Spike|hCoV-19/England/ALDP-CC096F/2020|2020-12-20|EPI_ISL_778207|Original|hCoV-19^^England|Human|Lighthouse Lab in Alderley Park|Wellcome Sanger Institute for the COVID-19 Genomics UK (COG-UK) Consortium|Davies|United Kingdom
>Spike|hCoV-19/Belgium/ULG-10544/2020|2020-10-23|EPI_ISL_626303|Original|hCoV-19^^Li\u00e8ge|Human|Department of Clinical Microbiology|GIGA Medical Genomics|Durkin|Belgium
>Spike|hCoV-19/USA/VA-DCLS-2326/2020|2020-10-27|EPI_ISL_734274|Original|hCoV-19^^Virginia|Human|Virginia Division of Consolidated Laboratory Services (DCLS)|Virginia Division of Consolidated Laboratory Services (DCLS)|St. Jacques|USA
>Spike|hCoV-19/England/OXON-AD13F/2020|2020-00-00|EPI_ISL_448566|Original|hCoV-19^^England|Human|Oxford Viromics|COVID-19 Genomics UK (COG-UK) Consortium|Golubchik|United Kingdom
>Spike|hCoV-19/England/QEUH-9F7452/2020|2020-09-25|EPI_ISL_589175|Original|hCoV-19^^England|Human|Lighthouse Lab in Glasgow|Wellcome Sanger Institute for the COVID-19 Genomics UK (COG-UK) consortium|Davies|United Kingdom
>Spike|hCoV-19/USA/WI-WSLH-200251/2020|2020-04-14|EPI_ISL_578467|Original|hCoV-19^^Wisconsin|Human|Wisconsin State Laboratory of Hygiene Communicable Disease Division|Wisconsin State Laboratory of Hygiene Communicable Disease Division|Florek|USA
>Spike|hCoV-19/USA/AZ-TG273302/2020|2020-03-31|EPI_ISL_426563|Original|hCoV-19^^Arizona|Human|AZ SPHL|TGen North|Lemmer|USA
>Spike|hCoV-19/Japan/PG-8341/2020|2020-08-00|EPI_ISL_687440|Original|hCoV-19^^Japan|Human|Pathogen Genomics Center|Pathogen Genomics Center|Sekizuka|Japan
>Spike|hCoV-19/Spain/VC-FISABIO-102/2020|2020-03-17|EPI_ISL_436197|Original|hCoV-19^^Comunitat Valenciana|Human|Servicio de Microbiolog\u00eda. Consorcio Hospital General Universitario de Valencia|Sequencing and Bioinformatics Service and Molecular Epidemiology Research Group. FISABIO-Public Health|Gonzalez-Candelas|Spain
>Spike|hCoV-19/USA/MA-MASPHL-00020/2020|2020-04-01|EPI_ISL_791526|Original|hCoV-19^^Massachusetts|Human|Massachusetts State Public Health Laboratory|Infectious Disease Program|Park|USA
>Spike|hCoV-19/Scotland/EDB7895/2020|2020-09-23|EPI_ISL_585443|Original|hCoV-19^^Scotland|Human|Virology Department|COVID-19 Genomics UK (COG-UK) Consortium|McHugh|United Kingdom
>Spike|hCoV-19/USA/TX-HMH-2695/2020|2020-06-04|EPI_ISL_545483|Original|hCoV-19^^Texas|Human|Houston Methodist Hospital|Houston Methodist Hospital|Long|USA
>Spike|hCoV-19/England/MILK-B95CB6/2020|2020-11-14|EPI_ISL_674954|Original|hCoV-19^^England|Human|Lighthouse Lab in Milton Keynes|Wellcome Sanger Institute for the COVID-19 Genomics UK (COG-UK) Consortium|Davies|United Kingdom
>Spike|hCoV-19/Scotland/QEUH-9F43FE/2020|2020-09-27|EPI_ISL_600661|Original|hCoV-19^^Scotland|Human|Lighthouse Lab in Glasgow|Wellcome Sanger Institute for the COVID-19 Genomics UK (COG-UK) consortium|Davies|United Kingdom
>Spike|hCoV-19/Belgium/ULG-10829/2020|2020-11-13|EPI_ISL_661202|Original|hCoV-19^^Li\u00e8ge|Human|Department of Clinical Microbiology|GIGA Medical Genomics|Durkin|Belgium
>Spike|hCoV-19/England/ALDP-9BDF2A/2020|2020-09-10|EPI_ISL_568220|Original|hCoV-19^^England|Human|Lighthouse Lab in Alderley Park|Wellcome Sanger Institute for the COVID-19 Genomics UK (COG-UK) consortium|Davies|United Kingdom
>Spike|hCoV-19/Spain/PV-IBV-98008353/2020|2020-10-27|EPI_ISL_654572|Original|hCoV-19^^Basque Country|Human|Servicio de Microbiolog\u00eda. Hospital Universitario Donostia. OSI Donostialdea. \u00c1rea de Enfermedades Infecciosas|SeqCOVID-SPAIN consortium/IBV(CSIC)|Comas|Spain
>Spike|hCoV-19/Scotland/QEUH-960032/2020|2020-08-06|EPI_ISL_531905|Original|hCoV-19^^Scotland|Human|NHSGGC West of Scotland Specialist Virology Centre / MRC-University of Glasgow Centre for Virus Research|Wellcome Sanger Institute for the COVID-19 Genomics UK (COG-UK) consortium|Davies|United Kingdom

>Spike|hCoV-19/Wales/PHWC-169839/2020|2020-07-25|EPI_ISL_512520|Original|hCoV-19^^Wales|Human|Wales Specialist Virology Centre Sequencing lab|COVID-19 Genomics UK (COG-UK) Consortium|Moore|United Kingdom
>Spike|hCoV-19/England/QEUH-9DCBE5/2020|2020-09-21|EPI_ISL_599732|Original|hCoV-19^^England|Human|Lighthouse Lab in Glasgow|Wellcome Sanger Institute for the COVID-19 Genomics UK (COG-UK) consortium|Davies|United Kingdom
>Spike|hCoV-19/Singapore/166/2020|2020-03-18|EPI_ISL_462284|Original|hCoV-19^^Singapore|Human|National Public Health Laboratory|National Public Health Laboratory|Mak|Singapore
>Spike|hCoV-19/Russia/BA-RII-5-5798V/2020|2020-00-00|EPI_ISL_507299|Vero P1|hCoV-19^^Bashkortostan|Human|Republican Medical Genetics Center|WHO National Influenza Centre Russian Federation|Komissarov|Russia
>Spike|hCoV-19/USA/TX-HMH-MCoV-15686/2020|2020-07-09|EPI_ISL_785370|Original|hCoV-19^^Texas|Human|Houston Methodist Hospital|Houston Methodist Hospital|Long|USA
>Spike|hCoV-19/Wales/PHWC-166711/2020|2020-06-23|EPI_ISL_494032|Original|hCoV-19^^Wales|Human|Originating lab|COVID-19 Genomics UK (COG-UK) Consortium|Moore|United Kingdom
>Spike|hCoV-19/Panama/336023/2020|2020-04-03|EPI_ISL_496834|Original|hCoV-19^^Panama Center|Human|Gorgas Memorial Laboratory of Health Studies|Gorgas Memorial Laboratory of Health Studies|Martinez|Panama
>Spike|hCoV-19/Jordan/SEARCH-5445/2020|2020-09-09|EPI_ISL_755241|Original|hCoV-19^^Amman|Human|Biolab Diagnostic Laboratories|Andersen lab at Scripps Research|Gangavarapu|Jordan
>Spike|hCoV-19/Australia/VIC3033/2020|2020-07-01|EPI_ISL_520747|Original|hCoV-19^^Victoria|Human|Victorian Infectious Diseases Reference Laboratory (VIDRL)|VIDRL and MDU-PHL|Schultz|Australia
>Spike|hCoV-19/USA/TX-HMH-1521/2020|2020-05-01|EPI_ISL_542771|Original|hCoV-19^^Texas|Human|Houston Methodist Hospital|Houston Methodist Hospital|Long|USA
>Spike|hCoV-19/England/CAMB-1BCB7A/2020|2020-12-01|EPI_ISL_764176|Original|hCoV-19^^England|Human|Department of Pathology|COVID-19 Genomics UK (COG-UK) Consortium|Goodfellow|United Kingdom
>Spike|hCoV-19/England/CAMC-C70C92/2020|2020-12-16|EPI_ISL_769219|Original|hCoV-19^^England|Human|Lighthouse Lab in Cambridge|Wellcome Sanger Institute for the COVID-19 Genomics UK (COG-UK) Consortium|Davies|United Kingdom
>Spike|hCoV-19/England/CAMC-B781D8/2020|2020-11-12|EPI_ISL_783468|Original|hCoV-19^^England|Human|Lighthouse Lab in Cambridge|Wellcome Sanger Institute for the COVID-19 Genomics UK (COG-UK) Consortium|Davies|United Kingdom
>Spike|hCoV-19/England/CAMB-1AAD88/2020|2020-04-19|EPI_ISL_470179|Original|hCoV-19^^England|Human|Department of Pathology|Wellcome Sanger Institute for the COVID-19 Genomics UK (COG-UK) consortium|Davies|United Kingdom
>Spike|hCoV-19/USA/TX-HMH-6820/2020|2020-06-25|EPI_ISL_546265|Original|hCoV-19^^Texas|Human|Houston Methodist Hospital|Houston Methodist Hospital|Long|USA
>Spike|hCoV-19/Wales/PHWC-4A20FD/2020|2020-12-14|EPI_ISL_743847|Original|hCoV-19^^Wales|Human|Wales Specialist Virology Centre Sequencing lab|COVID-19 Genomics UK (COG-UK) Consortium|Moore|United Kingdom
>Spike|hCoV-19/Australia/VIC4769/2020|2020-07-19|EPI_ISL_519157|Original|hCoV-19^^Victoria|Human|Microbiological Diagnostic Unit - Public Health Laboratory (MDU-PHL)|MDU-PHL|Schultz|Australia
>Spike|hCoV-19/Wales/PHWC-486DDA/2020|2020-10-16|EPI_ISL_637618|Original|hCoV-19^^Wales|Human|Wales Specialist Virology Centre Sequencing lab|COVID-19 Genomics UK (COG-UK) Consortium|Moore|United Kingdom
>Spike|hCoV-19/England/QEUH-9AF3D4/2020|2020-08-31|EPI_ISL_559840|Original|hCoV-19^^England|Human|Oxford Viromics|COVID-19 Genomics UK (COG-UK) Consortium|Golubchik|United Kingdom
>Spike|hCoV-19/USA/CA-CZB-10926/2020|2020-08-31|EPI_ISL_583155|Original|hCoV-19^^California|Human|Humboldt County Public Health Laboratory|Chan-Zuckerberg Biohub|Bhatt|USA
>Spike|hCoV-19/England/ALDP-9504A2/2020|2020-06-16|EPI_ISL_555199|Original|hCoV-19^^England|Human|Lighthouse Lab in Alderley Park|Wellcome Sanger Institute for the COVID-19 Genomics UK (COG-UK) consortium|Davies|United Kingdom

>Spike|hCoV-19/England/MILK-97165A/2020|2020-08-18|EPI_ISL_553064|Original|hCoV-19^^England|Human|Lighthouse Lab in Milton Keynes|Wellcome Sanger Institute for the COVID-19 Genomics UK (COG-UK) consortium|Davies|United Kingdom
>Spike|hCoV-19/Wales/PHWC-48F8EF/2020|2020-11-10|EPI_ISL_681156|Original|hCoV-19^^Wales|Human|Wales Specialist Virology Centre Sequencing lab|COVID-19 Genomics UK (COG-UK) Consortium|Moore|United Kingdom
>Spike|hCoV-19/Panama/334610/2020|2020-03-31|EPI_ISL_496786|Original|hCoV-19^^Panama Center|Human|Gorgas Memorial Laboratory of Health Studies|Gorgas Memorial Laboratory of Health Studies|Martinez|Panama
>Spike|hCoV-19/England/NORT-2893AA/2020|2020-03-29|EPI_ISL_458699|Original|hCoV-19^^England|Human|NU-OMICS DNA Sequencing research facility|Wellcome Sanger Institute for the COVID-19 Genomics UK (COG-UK) consortium|Davies|United Kingdom
>Spike|hCoV-19/England/QEUH-B2B9BE/2020|2020-11-01|EPI_ISL_646632|Original|hCoV-19^^England|Human|Lighthouse Lab in Glasgow|Wellcome Sanger Institute for the COVID-19 Genomics UK (COG-UK) Consortium|Davies|United Kingdom
>Spike|hCoV-19/USA/TX-HMH-MCoV-15314/2020|2020-10-19|EPI_ISL_786798|Original|hCoV-19^^Texas|Human|Houston Methodist Hospital|Houston Methodist Hospital|Long|USA
>Spike|hCoV-19/USA/AZ-TG519144/2020|2020-07-01|EPI_ISL_695356|Original|hCoV-19^^Arizona|Human|AZ SPHL|TGen North|French|USA
>Spike|hCoV-19/England/ALDP-9500AE/2020|2020-06-15|EPI_ISL_555279|Original|hCoV-19^^England|Human|Lighthouse Lab in Alderley Park|Wellcome Sanger Institute for the COVID-19 Genomics UK (COG-UK) consortium|Davies|United Kingdom
>Spike|hCoV-19/Germany/SH-ChVir9980/2020|2020-11-02|EPI_ISL_729401|Original|hCoV-19^^Schleswig-Holstein|Human|A. Krumbholz|Charit\u00e9 Universit\u00e4tsmedizin Berlin|Muehlemann|Germany
>Spike|hCoV-19/USA/TX-HMH061/2020|2020-03-18|EPI_ISL_434770|Original|hCoV-19^^Texas|Human|Houston Methodist Hospital|Houston Methodist Hospital|Long|USA
>Spike|hCoV-19/England/ALDP-9CDEFD/2020|2020-09-15|EPI_ISL_566342|Original|hCoV-19^^England|Human|Lighthouse Lab in Alderley Park|Wellcome Sanger Institute for the COVID-19 Genomics UK (COG-UK) consortium|Davies|United Kingdom
>Spike|hCoV-19/Scotland/EDB10631/2020|2020-12-02|EPI_ISL_705787|Original|hCoV-19^^Scotland|Human|Virology Department|COVID-19 Genomics UK (COG-UK) Consortium|McHugh|United Kingdom
>Spike|hCoV-19/Japan/PG-6670/2020|2020-07-00|EPI_ISL_687781|Original|hCoV-19^^Japan|Human|Pathogen Genomics Center|Pathogen Genomics Center|Sekizuka|Japan
>Spike|hCoV-19/England/CAMC-B0800D/2020|2020-10-29|EPI_ISL_643750|Original|hCoV-19^^England|Human|Lighthouse Lab in Cambridge|Wellcome Sanger Institute for the COVID-19 Genomics UK (COG-UK) Consortium|Davies|United Kingdom
>Spike|hCoV-19/Netherlands/NoordBrabant_110/2020|2020-03-04|EPI_ISL_461146|Original|hCoV-19^^Noord Brabant|Human|Dutch COVID-19 response team|Erasmus Medical Center|Oude Munnink|Netherlands
>Spike|hCoV-19/Australia/VIC3582/2020|2020-00-00|EPI_ISL_520330|Original|hCoV-19^^Victoria|Human|Victorian Infectious Diseases Reference Laboratory (VIDRL)|VIDRL and MDU-PHL|Schultz|Australia
>Spike|hCoV-19/Switzerland/BE-ETHZ-120064/2020|2020-04-20|EPI_ISL_468238|Original|hCoV-19^^Bern|Human|Viollier AG|Department of Biosystems Science and Engineering|Nadeau|Switzerland
>Spike|hCoV-19/England/QEUH-96A715/2020|2020-08-13|EPI_ISL_531498|Original|hCoV-19^^England|Human|Lighthouse Lab in Glasgow|Wellcome Sanger Institute for the COVID-19 Genomics UK (COG-UK) consortium|Davies|United Kingdom
>Spike|hCoV-19/DRC/10508/2020|2020-05-20|EPI_ISL_527494|Original|hCoV-19^^Sud Kivu|Human|Viral Respiratory Lab|Pathogen Sequencing Lab|Lokilo|Democratic Republic of the Congo
>Spike|hCoV-19/Scotland/QEUH-A0C0FB/2020|2020-10-03|EPI_ISL_599345|Original|hCoV-19^^Scotland|Human|Lighthouse Lab in Glasgow|Wellcome Sanger Institute for the COVID-19 Genomics UK (COG-UK) consortium|Davies|United Kingdom
>Spike|hCoV-19/England/QEUH-BA13EF/2020|2020-11-18|EPI_ISL_672787|Original|hCoV-19^^England|Human|Lighthouse Lab in Glasgow|Wellcome Sanger Institute for the COVID-19 Genomics UK (COG-UK) Consortium|Davies|United Kingdom

>Spike|hCoV-19/USA/MI-MDHHS-SC22691/2020|2020-11-25|EPI_ISL_737305|Original|hCoV-19^^Michigan|Human|Michigan Department of Health and Human Services|Michigan Department of Health and Human Services|Blankenship|USA
>Spike|hCoV-19/USA/NY-NYCPHL-001502/2020|2020-11-30|EPI_ISL_745427|Original|hCoV-19^^New York|Human|DOHMH Corona|New York City Public Health Laboratory|Wang|USA
>Spike|hCoV-19/USA/WY-WYPHL-20007938/2020|2020-04-06|EPI_ISL_493001|Original|hCoV-19^^Wyoming|Human|Wyoming Public Health Laboratory|Wyoming Public Health Laboratory|Sevinsky|USA
>Spike|hCoV-19/England/ALDP-9C5745/2020|2020-09-10|EPI_ISL_567734|Original|hCoV-19^^England|Human|Lighthouse Lab in Alderley Park|Wellcome Sanger Institute for the COVID-19 Genomics UK (COG-UK) consortium|Davies|United Kingdom
>Spike|hCoV-19/England/NOTT-112F49/2020|2020-08-13|EPI_ISL_526428|Original|hCoV-19^^England|Human|Queens Medical Centre|COVID-19 Genomics UK (COG-UK) Consortium|Loose|United Kingdom
>Spike|hCoV-19/Japan/PG-1481/2020|2020-04-00|EPI_ISL_685088|Original|hCoV-19^^Japan|Human|Saitama Prefectural Institute of Public Health|Pathogen Genomics Center|Sekizuka|Japan
>Spike|hCoV-19/Wales/PHWC-2C97C/2020|2020-03-31|EPI_ISL_445927|Original|hCoV-19^^Wales|Human|Wales Specialist Virology Centre|Public Health Wales Microbiology Cardiff|Moore|United Kingdom
>Spike|hCoV-19/Denmark/DCGC-8592/2020|2020-10-26|EPI_ISL_625777|Original|hCoV-19^^Hovedstaden|Human|Department of Virus and Microbiological Special Diagnostics|Albertsen lab|Michaelsen|Denmark
>Spike|hCoV-19/Denmark/DCGC-15549/2020|2020-11-23|EPI_ISL_713884|Original|hCoV-19^^Midtjylland|Human|Department of Virus and Microbiological Special Diagnostics|Albertsen Lab|Michaelsen|Denmark
>Spike|hCoV-19/England/ALDP-B24250/2020|2020-11-04|EPI_ISL_647119|Original|hCoV-19^^England|Human|Lighthouse Lab in Alderley Park|Wellcome Sanger Institute for the COVID-19 Genomics UK (COG-UK) Consortium|Davies|United Kingdom
>Spike|hCoV-19/Denmark/DCGC-20127/2020|2020-12-07|EPI_ISL_747999|Original|hCoV-19^^Syddanmark|Human|Department of Virus and Microbiological Special Diagnostics|Albertsen Lab|Michaelsen|Denmark
>Spike|hCoV-19/USA/TX-HMH-MCoV-19576/2020|2020-11-28|EPI_ISL_783948|Original|hCoV-19^^Texas|Human|Houston Methodist Hospital|Houston Methodist Hospital|Long|USA
>Spike|hCoV-19/Wales/PHWC-4A2BA1/2020|2020-12-13|EPI_ISL_743988|Original|hCoV-19^^Wales|Human|Wales Specialist Virology Centre Sequencing lab|COVID-19 Genomics UK (COG-UK) Consortium|Moore|United Kingdom
>Spike|hCoV-19/USA/CA-CZB-1513/2020|2020-04-13|EPI_ISL_468460|Original|hCoV-19^^California|Human|Humboldt County Public Health Laboratory|Chan-Zuckerberg Biohub|Hao|USA
>Spike|hCoV-19/England/BRIS-122CF4/2020|2020-04-02|EPI_ISL_440638|Original|hCoV-19^^England|Human|PHE South West Regional Laboratory|Wellcome Sanger Institute for the COVID-19 Genomics UK (COG-UK) consortium|Amato|United Kingdom
>Spike|hCoV-19/USA/NY-NYUMC512/2020|2020-03-30|EPI_ISL_444629|Original|hCoV-19^^New York|Human|NYU Langone Health|Departments of Pathology and Medicine|Maurano|USA
>Spike|hCoV-19/USA/CO-CDPHE-2005300277/2020|2020-05-29|EPI_ISL_677292|Original|hCoV-19^^Colorado|Human|Colorado Department of Public Health and Environment|Colorado Department of Puplic Health and Environment|Bankers|USA
>Spike|hCoV-19/Ireland/LK-NVRL-73IRL35422/2020|2020-09-08|EPI_ISL_596891|Original|hCoV-19^^Limerick|Human|National Virus Reference Laboratory|National Virus Reference Laboratory|Gonzalez|Ireland
>Spike|hCoV-19/Scotland/QEUH-C014BC/2020|2020-12-05|EPI_ISL_757262|Original|hCoV-19^^Scotland|Human|Lighthouse Lab in Glasgow|Wellcome Sanger Institute for the COVID-19 Genomics UK (COG-UK) Consortium|Davies|United Kingdom
>Spike|hCoV-19/England/QEUH-AD1E8C/2020|2020-10-26|EPI_ISL_634687|Original|hCoV-19^^England|Human|Lighthouse Lab in Glasgow|Wellcome Sanger Institute for the COVID-19 Genomics UK (COG-UK) consortium|Davies|United Kingdom
>Spike|hCoV-19/England/ALDP-9CB67F/2020|2020-09-16|EPI_ISL_575474|Original|hCoV-19^^England|Human|Lighthouse Lab in Alderley Park|Wellcome Sanger Institute for the COVID-19 Genomics UK (COG-UK) consortium|Davies|United Kingdom

>Spike|hCoV-19/USA/CA-CZB-14685/2020|2020-11-17|EPI_ISL_739482|Original|hCoV-19^^California|Human|Orange County Public Health Lab|Chan-Zuckerberg Biohub|Kamm|USA
>Spike|hCoV-19/Japan/PG-8140/2020|2020-08-00|EPI_ISL_687239|Original|hCoV-19^^Japan|Human|Pathogen Genomics Center|Pathogen Genomics Center|Sekizuka|Japan
>Spike|hCoV-19/USA/CA-ALSR-4364/2020|2020-08-04|EPI_ISL_648990|Original|hCoV-19^^California|Human|San Diego County Public Health Laboratory|Andersen lab at Scripps Research|Gangavarapu|USA
>Spike|hCoV-19/England/CAMC-B98FC9/2020|2020-11-15|EPI_ISL_673466|Original|hCoV-19^^England|Human|Lighthouse Lab in Cambridge|Wellcome Sanger Institute for the COVID-19 Genomics UK (COG-UK) Consortium|Davies|United Kingdom
>Spike|hCoV-19/England/BRIS-12B1CF/2020|2020-05-15|EPI_ISL_469833|Original|hCoV-19^^England|Human|PHE South West Regional Laboratory|Wellcome Sanger Institute for the COVID-19 Genomics UK (COG-UK) consortium|Davies|United Kingdom
>Spike|hCoV-19/England/CAMC-B0A539/2020|2020-10-29|EPI_ISL_673163|Original|hCoV-19^^England|Human|Lighthouse Lab in Cambridge|Wellcome Sanger Institute for the COVID-19 Genomics UK (COG-UK) Consortium|Davies|United Kingdom
>Spike|hCoV-19/Denmark/DCGC-18089/2020|2020-12-14|EPI_ISL_749749|Original|hCoV-19^^Sjaelland|Human|Department of Virus and Microbiological Special Diagnostics|Albertsen Lab|Michaelsen|Denmark
>Spike|hCoV-19/England/CAMC-C470F2/2020|2020-12-10|EPI_ISL_718717|Original|hCoV-19^^England|Human|Lighthouse Lab in Cambridge|Wellcome Sanger Institute for the COVID-19 Genomics UK (COG-UK) Consortium|Davies|United Kingdom
>Spike|hCoV-19/Luxembourg/LNS8175845/2020|2020-11-06|EPI_ISL_744695|Original|hCoV-19^^Luxembourg|Human|Laboratoire national de sant\u00e9|Laboratoire national de sant\u00e9|Wienecke-Baldacchino|Luxembourg
>Spike|hCoV-19/Canada/ON-UHTC_0025/2020|2020-04-01|EPI_ISL_464005|unknown|hCoV-19^^Ontario|Human|Unity Health Toronto|Ontario Institute for Cancer Research|de Borja|Canada
>Spike|hCoV-19/USA/CO-CDPHE-2100042725/2020|2020-11-27|EPI_ISL_771356|Original|hCoV-19^^Colorado|Human|Colorado Department of Public Health and Environment|Colorado Department of Puplic Health and Environment|Bankers|USA
>Spike|hCoV-19/England/QEUH-C935CB/2020|2020-12-16|EPI_ISL_762017|Original|hCoV-19^^England|Human|Lighthouse Lab in Glasgow|Wellcome Sanger Institute for the COVID-19 Genomics UK (COG-UK) Consortium|Davies|United Kingdom
>Spike|hCoV-19/Gibraltar/204462847/2020|2020-10-29|EPI_ISL_615017|Original|hCoV-19^^Gibraltar|Human|Microbiology|Respiratory Virus Unit|Platt|Gibraltar
>Spike|hCoV-19/South Africa/KRISP-K001327/2020|2020-07-18|EPI_ISL_515812|Original|hCoV-19^^KwaZulu-Natal|Human|Medical Disagnostics Services (MDS)|KRISP|de Oliveira|South Africa
>Spike|hCoV-19/England/ALDP-8DBA9F/2020|2020-06-24|EPI_ISL_557054|Original|hCoV-19^^England|Human|Lighthouse Lab in Alderley Park|Wellcome Sanger Institute for the COVID-19 Genomics UK (COG-UK) consortium|Davies|United Kingdom
>Spike|hCoV-19/Australia/VIC2936/2020|2020-07-08|EPI_ISL_521767|Original|hCoV-19^^Victoria|Human|Microbiological Diagnostic Unit - Public Health Laboratory (MDU-PHL)|MDU-PHL|Schultz|Australia
>Spike|hCoV-19/SaudiArabia/KAUST-JEDDAH831/2020|2020-05-24|EPI_ISL_513005|Original|hCoV-19^^Jeddah|Human|Pathogen Genomics Lab King Abdullah University of Science and Technology(KAUST)|Pathogen Genomics Lab King Abdullah University of Science and Technology(KAUST)|Mohamed Ghazzali|Saudi Arabia
>Spike|hCoV-19/Switzerland/BE-ETHZ-400074/2020|2020-12-06|EPI_ISL_729125|Original|hCoV-19^^Bern|Human|Viollier AG|Department of Biosystems Science and Engineering|Nadeau|Switzerland
>Spike|hCoV-19/USA/MD-MDH-0421/2020|2020-11-05|EPI_ISL_666789|Original|hCoV-19^^Maryland|Human|Maryland Public Health Laboratory|Maryland Public Health Laboratory|Keller|USA
>Spike|hCoV-19/England/MILK-A6A222/2020|2020-10-08|EPI_ISL_627495|Original|hCoV-19^^England|Human|Virology Department|COVID-19 Genomics UK (COG-UK) Consortium|de Silva|United Kingdom

>Spike|hCoV-19/Scotland/EDB2917/2020|2020-04-01|EPI_ISL_488886|Original|hCoV-19^^Scotland|Human|Virology Department|Wellcome Sanger Institute for the COVID-19 Genomics UK (COG-UK) consortium|Davies|United Kingdom

>Spike|hCoV-19/USA/WA-S1716/2020|2020-06-01|EPI_ISL_496142|Original|hCoV-19^^Washington|Human|Washington State Department of Health|Seattle Flu Study|Lee|USA

>Spike|hCoV-19/England/MILK-ABB76F/2020|2020-10-21|EPI_ISL_623484|Original|hCoV-19^^England|Human|Lighthouse Lab in Milton Keynes|Wellcome Sanger Institute for the COVID-19 Genomics UK (COG-UK) Consortium|Davies|United Kingdom

>Spike|hCoV-19/Australia/VIC2641/2020|2020-06-29|EPI_ISL_521487|Original|hCoV-19^^Victoria|Human|Victorian Infectious Diseases Reference Laboratory (VIDRL)|VIDRL and MDU-PHL|Schultz|Australia

>Spike|hCoV-19/France/OCC-SC470/2020|2020-03-09|EPI_ISL_660674|Original|hCoV-19^^Occitanie|Human|CHU Montpellier|CNR Virus des Infections Respiratoires - France SUD|Bal|France

>Spike|hCoV-19/USA/WI-WSLH-200658/2020|2020-06-19|EPI_ISL_631424|Original|hCoV-19^^Wisconsin|Human|Wisconsin State Laboratory of Hygiene Communicable Disease Division|Wisconsin State Laboratory of Hygiene Communicable Disease Division|Florek|USA

>Spike|hCoV-19/Russia/SVE-RII-MH8647S/2020|2020-09-29|EPI_ISL_770652|Original|hCoV-19^^Sverdlovsk|Human|UMMC-Health|WHO National Influenza Centre Russian Federation|Komissarov|Russia

>Spike|hCoV-19/England/NOTT-113BF9/2020|2020-09-28|EPI_ISL_573467|Original|hCoV-19^^England|Human|Queens Medical Centre|COVID-19 Genomics UK (COG-UK) Consortium|Loose|United Kingdom

>Spike|hCoV-19/Netherlands/ZH-EMC-818/2020|2020-10-15|EPI_ISL_632694|Original|hCoV-19^^Zuid Holland|Human|Dutch COVID-19 response team|Erasmus Medical Center|Oude Munnink|Netherlands

>Spike|hCoV-19/Belgium/rega-0417511/2020|2020-04-17|EPI_ISL_734603|Original|hCoV-19^^Bertem|Human|UZ Leuven|KU Leuven|Maes|Belgium

>Spike|hCoV-19/England/ALDP-9EAB4E/2020|2020-09-24|EPI_ISL_579946|Original|hCoV-19^^England|Human|Lighthouse Lab in Alderley Park|Wellcome Sanger Institute for the COVID-19 Genomics UK (COG-UK) consortium|Davies|United Kingdom

>Spike|hCoV-19/Japan/PG-5479/2020|2020-08-00|EPI_ISL_690582|Original|hCoV-19^^Japan|Human|Pathogen Genomics Center|Pathogen Genomics Center|Sekizuka|Japan

>Spike|hCoV-19/USA/WI-GMF-31430/2020|2020-11-04|EPI_ISL_661090|Original|hCoV-19^^Wisconsin|Human|Gundersen Molecular Diagnostics Laboratory|Kabara Cancer Research Institute|Kenny|USA

>Spike|hCoV-19/Australia/VIC3508/2020|2020-07-06|EPI_ISL_520263|Original|hCoV-19^^Victoria|Human|Victorian Infectious Diseases Reference Laboratory (VIDRL)|VIDRL and MDU-PHL|Schultz|Australia

>Spike|hCoV-19/USA/UT-UPHL-2012438158/2020|2020-06-18|EPI_ISL_746424|Original|hCoV-19^^Utah|Human|Utah Public Health Laboratory|Utah Public Health Laboratory|Gallagher|USA

>Spike|hCoV-19/Denmark/DCGC-5799/2020|2020-09-28|EPI_ISL_620986|Original|hCoV-19^^Nordjylland|Human|Department of Virus and Microbiological Special Diagnostics|Albertsen lab|Michaelsen|Denmark

>Spike|hCoV-19/USA/NY-NYCPHL-000614/2020|2020-03-11|EPI_ISL_632071|Original|hCoV-19^^New York|Human|New York Presbyterian Queens|New York City Public Health Laboratory|Wang|USA

>Spike|hCoV-19/Wales/PHWC-4A0754/2020|2020-12-08|EPI_ISL_743489|Original|hCoV-19^^Wales|Human|Wales Specialist Virology Centre Sequencing lab|COVID-19 Genomics UK (COG-UK) Consortium|Moore|United Kingdom

>Spike|hCoV-19/USA/MD-MDH-0503/2020|2020-11-13|EPI_ISL_778884|Original|hCoV-19^^Maryland|Human|Maryland Public Health Laboratory|Maryland Public Health Laboratory|Keller|USA

>Spike|hCoV-19/Scotland/CVR5210/2020|2020-10-18|EPI_ISL_626980|Original|hCoV-19^^Scotland|Human|West of Scotland Specialist Virology Centre|COVID-19 Genomics UK (COG-UK) Consortium|Robertson|United Kingdom

>Spike|hCoV-19/England/ALDP-BCA957/2020|2020-11-23|EPI_ISL_703532|Original|hCoV-19^^England|Human|Lighthouse Lab in Alderley Park|Wellcome Sanger Institute for the COVID-19 Genomics UK (COG-UK) Consortium|Davies|United Kingdom
>Spike|hCoV-19/USA/LA-EVTL395/2020|2020-06-01|EPI_ISL_578801|Original|hCoV-19^^Louisiana|Human|LSUHS Emerging Viral Threat Laboratory|Microbial Genome Sequencing Center|Kamil|USA
>Spike|hCoV-19/Italy/APU-UniMI-854/2020|2020-03-20|EPI_ISL_477202|Original|hCoV-19^^Apulia|Human|Istituto Zooprofilattico Sperimentale Puglia e Basilicata;|Beaconlab (Bioinformatics|Chiara|Italy
>Spike|hCoV-19/USA/VA-DCLS-1698/2020|2020-09-04|EPI_ISL_609979|Original|hCoV-19^^Virginia|Human|Virginia DCLS|Virginia DCLS|St. Jacques|USA
>Spike|hCoV-19/Japan/PG-4131/2020|2020-07-00|EPI_ISL_692370|Original|hCoV-19^^Japan|Human|Pathogen Genomics Center|Pathogen Genomics Center|Sekizuka|Japan
>Spike|hCoV-19/India/KA-IB49/2020|2020-05-25|EPI_ISL_511945|Original|hCoV-19^^Karnataka|Human|Indian Institute of Science|National Institute of Biomedical Genomics -\u00a0DBT's PAN-INDIA 1000 SARS--CoV-2 RNA Genome Sequencing Consortium|Maitra|India
>Spike|hCoV-19/SaudiArabia/KAUST-MADINAH378/2020|2020-04-24|EPI_ISL_513143|Original|hCoV-19^^Madinah|Human|Pathogen Genomics Lab King Abdullah University of Science and Technology(KAUST)|Pathogen Genomics Lab King Abdullah University of Science and Technology(KAUST)|Mohamed Ghazzali|Saudi Arabia
>Spike|hCoV-19/England/MILK-B8DC66/2020|2020-11-13|EPI_ISL_674532|Original|hCoV-19^^England|Human|Lighthouse Lab in Milton Keynes|Wellcome Sanger Institute for the COVID-19 Genomics UK (COG-UK) Consortium|Davies|United Kingdom
>Spike|hCoV-19/England/BIRM-61C45/2020|2020-03-25|EPI_ISL_529603|Original|hCoV-19^^England|Human|University of Birmingham|COVID-19 Genomics UK (COG-UK) Consortium|Loman|United Kingdom
>Spike|hCoV-19/Denmark/DCGC-7040/2020|2020-10-12|EPI_ISL_619888|Original|hCoV-19^^Hovedstaden|Human|Department of Virus and Microbiological Special Diagnostics|Albertsen lab|Michaelsen|Denmark
>Spike|hCoV-19/Iceland/382/2020|2020-03-19|EPI_ISL_424406|Original|hCoV-19^^Reykjavik|Human|The National University Hospital of Iceland|deCODE genetics|J\u00f3nsson|Iceland
>Spike|hCoV-19/South Africa/KRISP-K004539/2020|2020-10-18|EPI_ISL_660237|Original|hCoV-19^^KwaZulu-Natal|Human|NHLS-IALCH|KRISP|de Oliveira|South Africa
>Spike|hCoV-19/England/CAMB-1AB464/2020|2020-04-21|EPI_ISL_470343|Original|hCoV-19^^England|Human|Department of Pathology|Wellcome Sanger Institute for the COVID-19 Genomics UK (COG-UK) consortium|Davies|United Kingdom
>Spike|hCoV-19/New Zealand/20VR1889/2020|2020-04-01|EPI_ISL_579157|Original|hCoV-19^^Southern|Human|Southern Community Labs Dunedin|Institute of Environmental Science and Research (ESR)|Wang|New Zealand
>Spike|hCoV-19/England/QEUH-B824F0/2020|2020-11-14|EPI_ISL_668215|Original|hCoV-19^^England|Human|Lighthouse Lab in Glasgow|Wellcome Sanger Institute for the COVID-19 Genomics UK (COG-UK) Consortium|Davies|United Kingdom
>Spike|hCoV-19/Wales/PHWC-16BDB0/2020|2020-09-14|EPI_ISL_573973|Original|hCoV-19^^Wales|Human|Wales Specialist Virology Centre Sequencing lab|COVID-19 Genomics UK (COG-UK) Consortium|Moore|United Kingdom
>Spike|hCoV-19/England/ALDP-A944DE/2020|2020-10-20|EPI_ISL_624763|Original|hCoV-19^^England|Human|Lighthouse Lab in Alderley Park|Wellcome Sanger Institute for the COVID-19 Genomics UK (COG-UK) consortium|Davies|United Kingdom
>Spike|hCoV-19/England/MILK-B3D500/2020|2020-11-01|EPI_ISL_645300|Original|hCoV-19^^England|Human|Lighthouse Lab in Milton Keynes|Wellcome Sanger Institute for the COVID-19 Genomics UK (COG-UK) Consortium|Davies|United Kingdom
>Spike|hCoV-19/New Zealand/20VR3162/2020|2020-03-23|EPI_ISL_579452|Original|hCoV-19^^Canterbury|Human|Canterbury Health Laboratories|Institute of Environmental Science and Research (ESR)|Wang|New Zealand
>Spike|hCoV-19/England/ALDP-A2F6F0/2020|2020-10-05|EPI_ISL_586975|Original|hCoV-19^^England|Human|Lighthouse Lab in Alderley Park|Wellcome Sanger Institute for the COVID-19 Genomics UK (COG-UK) consortium|Davies|United Kingdom

>Spike|hCoV-19/Germany/SH-ChVir8183/2020|2020-07-11|EPI_ISL_729566|Original|hCoV-19^^Schleswig-Holstein|Human|A. Krumbholz|Charit\u00e9 Universit\u00e4tsmedizin Berlin|Muehlemann|Germany
>Spike|hCoV-19/USA/TX-HMH-6193/2020|2020-07-04|EPI_ISL_547303|Original|hCoV-19^^Texas|Human|Houston Methodist Hospital|Houston Methodist Hospital|Long|USA
>Spike|hCoV-19/England/QEUH-CAF632/2020|2020-12-15|EPI_ISL_760658|Original|hCoV-19^^England|Human|Lighthouse Lab in Glasgow|Wellcome Sanger Institute for the COVID-19 Genomics UK (COG-UK) Consortium|Davies|United Kingdom
>Spike|hCoV-19/Wales/PHWC-489153/2020|2020-10-30|EPI_ISL_639540|Original|hCoV-19^^Wales|Human|Wales Specialist Virology Centre Sequencing lab|COVID-19 Genomics UK (COG-UK) Consortium|Moore|United Kingdom
>Spike|hCoV-19/Denmark/DCGC-20704/2020|2020-12-21|EPI_ISL_759315|Original|hCoV-19^^Midtjylland|Human|Department of Virus and Microbiological Special Diagnostics|Albertsen Lab|Michaelsen|Denmark
>Spike|hCoV-19/England/QEUH-9DECFF/2020|2020-09-21|EPI_ISL_584824|Original|hCoV-19^^England|Human|University College London|COVID-19 Genomics UK (COG-UK) Consortium|Roy|United Kingdom
>Spike|hCoV-19/USA/WA-UW-10040/2020|2020-05-29|EPI_ISL_461456|Original|hCoV-19^^Washington|Human|UW Virology Lab|UW Virology Lab|Roychoudhury|USA
>Spike|hCoV-19/England/20126104404/2020|2020-03-18|EPI_ISL_423915|Original|hCoV-19^^England|Human|Respiratory Virus Unit|Respiratory Virus Unit|Platt|United Kingdom
>Spike|hCoV-19/England/20120003004/2020|2020-03-15|EPI_ISL_465142|Original|hCoV-19^^England|Human|Respiratory Virus Unit|Respiratory Virus Unit|Platt|United Kingdom
>Spike|hCoV-19/England/CAMB-7219D/2020|2020-03-28|EPI_ISL_439625|Original|hCoV-19^^England|Human|Department of Pathology|Wellcome Sanger Institute for the COVID-19 Genomics UK (COG-UK) consortium|Amato|United Kingdom
>Spike|hCoV-19/England/NOTT-112125/2020|2020-06-18|EPI_ISL_484344|Original|hCoV-19^^England|Human|Queens Medical Centre|COVID-19 Genomics UK (COG-UK) Consortium|Loose|United Kingdom
>Spike|hCoV-19/Australia/VIC4207/2020|2020-07-14|EPI_ISL_519535|Original|hCoV-19^^Victoria|Human|Microbiological Diagnostic Unit - Public Health Laboratory (MDU-PHL)|MDU-PHL|Schultz|Australia
>Spike|hCoV-19/USA/MN-MDH-292/2020|2020-03-30|EPI_ISL_450761|Original|hCoV-19^^Minnesota|Human|Minnesota Department of Health|Minnesota Department of Health|Garfin|USA
>Spike|hCoV-19/Scotland/EDB8091/2020|2020-10-02|EPI_ISL_585477|Original|hCoV-19^^Scotland|Human|Virology Department|COVID-19 Genomics UK (COG-UK) Consortium|McHugh|United Kingdom
>Spike|hCoV-19/Denmark/DCGC-13312/2020|2020-11-30|EPI_ISL_712722|Original|hCoV-19^^Hovedstaden|Human|Department of Virus and Microbiological Special Diagnostics|Albertsen Lab|Michaelsen|Denmark
>Spike|hCoV-19/England/ALDP-9B5ECB/2020|2020-08-28|EPI_ISL_572972|Original|hCoV-19^^England|Human|Oxford Viromics|COVID-19 Genomics UK (COG-UK) Consortium|Golubchik|United Kingdom
>Spike|hCoV-19/Wales/PHWC-484D81/2020|2020-10-23|EPI_ISL_628108|Original|hCoV-19^^Wales|Human|Wales Specialist Virology Centre Sequencing lab|COVID-19 Genomics UK (COG-UK) Consortium|Moore|United Kingdom
>Spike|hCoV-19/Wales/PHWC-4982D6/2020|2020-12-01|EPI_ISL_727052|Original|hCoV-19^^Wales|Human|Originating lab|COVID-19 Genomics UK (COG-UK) Consortium|Moore|United Kingdom
>Spike|hCoV-19/France/IDF-10071BR/2020|2020-03-00|EPI_ISL_447682|Original|hCoV-19^^France|Human|H\u00f4pital Henri-Mondor Ap-Hp|H\u00f4pital Henri-Mondor Ap-Hp|Rodriguez|France
>Spike|hCoV-19/England/ALDP-AA1F0A/2020|2020-10-22|EPI_ISL_624388|Original|hCoV-19^^England|Human|Lighthouse Lab in Alderley Park|Wellcome Sanger Institute for the COVID-19 Genomics UK (COG-UK) consortium|Davies|United Kingdom
>Spike|hCoV-19/Switzerland/SG-ETHZ-380398/2020|2020-11-23|EPI_ISL_721845|Original|hCoV-19^^St Gallen|Human|Viollier AG|Department of Biosystems Science and Engineering|Nadeau|Switzerland

>Spike|hCoV-19/Japan/PG-7873/2020|2020-07-00|EPI_ISL_686974|Original|hCoV-19^^Japan|Human|Pathogen Genomics Center|Pathogen Genomics Center|Sekizuka|Japan
>Spike|hCoV-19/England/QEUH-A0C32F/2020|2020-10-03|EPI_ISL_599203|Original|hCoV-19^^England|Human|Lighthouse Lab in Glasgow|Wellcome Sanger Institute for the COVID-19 Genomics UK (COG-UK) consortium|Davies|United Kingdom
>Spike|hCoV-19/Switzerland/LU-ETHZ-220028/2020|2020-07-27|EPI_ISL_512011|Original|hCoV-19^^Luzern|Human|Viollier AG|Department of Biosystems Science and Engineering|Nadeau|Switzerland
>Spike|hCoV-19/India/GJ-GBRC168a/2020|2020-05-27|EPI_ISL_461505|Original|hCoV-19^^Gujarat|Human|B.J. Medical College and Civil hospital|Gujarat Biotechnology Research Centre|Joshi|India
>Spike|hCoV-19/Sweden/20-08976/2020|2020-08-13|EPI_ISL_648181|Original|hCoV-19^^Skane|Human|The Public Health Agency of Sweden|The Public Health Agency of Sweden|Svartstr\u00f6m|Sweden
>Spike|hCoV-19/USA/TX-HMH-MCoV-15660/2020|2020-07-09|EPI_ISL_785344|Original|hCoV-19^^Texas|Human|Houston Methodist Hospital|Houston Methodist Hospital|Long|USA
>Spike|hCoV-19/England/MILK-99C45F/2020|2020-08-30|EPI_ISL_550758|Original|hCoV-19^^England|Human|Lighthouse Lab in Milton Keynes|Wellcome Sanger Institute for the COVID-19 Genomics UK (COG-UK) consortium|Davies|United Kingdom
>Spike|hCoV-19/Sweden/20-21513/2020|2020-08-03|EPI_ISL_676530|Original|hCoV-19^^Uppsala|Human|Uppsala klinisk mikrobiologi|The Public Health Agency of Sweden|Svartstr\u00f6m|Sweden
>Spike|hCoV-19/Denmark/DCGC-3136/2020|2020-08-31|EPI_ISL_615955|Original|hCoV-19^^Hovedstaden|Human|Department of Virus and Microbiological Special Diagnostics|Albertsen lab|Michaelsen|Denmark
>Spike|hCoV-19/USA/FL-BPHL-1066/2020|2020-07-20|EPI_ISL_517956|Original|hCoV-19^^Florida|Human|Florida Bureau of Public Health Laboratories|Florida Bureau of Public Health Laboratories|Blanton|USA
>Spike|hCoV-19/USA/CA-IGI-0061/2020|2020-05-22|EPI_ISL_511816|Original|hCoV-19^^California|Human|Innovative Genomics Institute|Innovative Genomics Institute|Wyman|USA
>Spike|hCoV-19/Singapore/881/2020|2020-04-19|EPI_ISL_536422|Original|hCoV-19^^Singapore|Human|National Public Health Laboratory|National Public Health Laboratory|Mak|Singapore
>Spike|hCoV-19/Wales/PHWC-49EDDF/2020|2020-12-01|EPI_ISL_743124|Original|hCoV-19^^Wales|Human|Wales Specialist Virology Centre Sequencing lab|COVID-19 Genomics UK (COG-UK) Consortium|Moore|United Kingdom
>Spike|hCoV-19/England/MILK-9A6E90/2020|2020-09-02|EPI_ISL_549985|Original|hCoV-19^^England|Human|Lighthouse Lab in Milton Keynes|Wellcome Sanger Institute for the COVID-19 Genomics UK (COG-UK) consortium|Davies|United Kingdom
>Spike|hCoV-19/England/QEUH-96FA80/2020|2020-08-14|EPI_ISL_540311|Original|hCoV-19^^England|Human|Lighthouse Lab in Glasgow|Wellcome Sanger Institute for the COVID-19 Genomics UK (COG-UK) consortium|Davies|United Kingdom
>Spike|hCoV-19/England/NORW-E80C2/2020|2020-04-08|EPI_ISL_449105|Original|hCoV-19^^England|Human|Quadram Institute Bioscience|COVID-19 Genomics UK (COG-UK) Consortium|Alikhan|United Kingdom
>Spike|hCoV-19/England/ALDP-A66D34/2020|2020-10-12|EPI_ISL_630430|Original|hCoV-19^^England|Human|Lighthouse Lab in Alderley Park|Wellcome Sanger Institute for the COVID-19 Genomics UK (COG-UK) consortium|Davies|United Kingdom
>Spike|hCoV-19/Wales/PHWC-482F50/2020|2020-10-14|EPI_ISL_627760|Original|hCoV-19^^Wales|Human|Wales Specialist Virology Centre Sequencing lab|COVID-19 Genomics UK (COG-UK) Consortium|Moore|United Kingdom
>Spike|hCoV-19/England/QEUH-B684C3/2020|2020-11-11|EPI_ISL_655042|Original|hCoV-19^^England|Human|Lighthouse Lab in Glasgow|Wellcome Sanger Institute for the COVID-19 Genomics UK (COG-UK) Consortium|Davies|United Kingdom
>Spike|hCoV-19/USA/TX-HMH-MCoV-9226/2020|2020-07-02|EPI_ISL_781008|Original|hCoV-19^^Texas|Human|Houston Methodist Hospital|Houston Methodist Hospital|Long|USA
>Spike|hCoV-19/USA/TX-HMH-MCoV-9955/2020|2020-07-06|EPI_ISL_780354|Original|hCoV-19^^Texas|Human|Houston Methodist Hospital|Houston Methodist Hospital|Long|USA

>Spike|hCoV-19/Denmark/DCGC-17105/2020|2020-12-14|EPI_ISL_750760|Original|hCoV-
19^^Sjaelland|Human|Department of Virus and Microbiological Special
Diagnostics|Albertsen Lab|Michaelsen|Denmark
>Spike|hCoV-19/England/MILK-B8EDBD/2020|2020-11-13|EPI_ISL_675238|Original|hCoV-
19^^England|Human|Lighthouse Lab in Milton Keynes|Wellcome Sanger Institute for the
COVID-19 Genomics UK (COG-UK) Consortium|Davies|United Kingdom
>Spike|hCoV-19/England/ALDP-9EA2D0/2020|2020-09-24|EPI_ISL_580031|Original|hCoV-
19^^England|Human|Lighthouse Lab in Alderley Park|Wellcome Sanger Institute for the
COVID-19 Genomics UK (COG-UK) consortium|Davies|United Kingdom
>Spike|hCoV-19/England/LIVE-9B705/2020|2020-04-20|EPI_ISL_440871|Original|hCoV-
19^^England|Human|Liverpool Clinical Laboratories|COVID-19 Genomics UK (COG-UK)
Consortium|Haldenby|United Kingdom
>Spike|hCoV-19/Hungary/SRC-01136/2020|2020-04-02|EPI_ISL_435418|Original|hCoV-
19^^Baranya|Human|Virological Research Group|Bioinformatics Research
Group|Herczeg|Hungary
>Spike|hCoV-19/Denmark/DCGC-20218/2020|2020-11-30|EPI_ISL_748056|Original|hCoV-
19^^Syddanmark|Human|Department of Virus and Microbiological Special
Diagnostics|Albertsen Lab|Michaelsen|Denmark
>Spike|hCoV-19/England/SHEF-CD63A/2020|2020-05-05|EPI_ISL_475458|Original|hCoV-
19^^England|Human|Virology Department|COVID-19 Genomics UK (COG-UK) Consortium|de
Silva|United Kingdom
>Spike|hCoV-19/Spain/MD-H12-23/2020|2020-03-22|EPI_ISL_428686|Original|hCoV-
19^^Madrid|Human|Hospital Universitario 12 de Octubre|Hospital Universitario 12 de
Octubre|Viedma|Spain
>Spike|hCoV-19/Thailand/UDC300674-3/2020|2020-03-24|EPI_ISL_450980|Original|hCoV-
19^^Thailand|Human|Center of Excellence in Clinical Virology|Center of Excellence in
Clinical Virology|Poovorawan|Thailand
>Spike|hCoV-19/Denmark/DCGC-6424/2020|2020-10-12|EPI_ISL_620324|Original|hCoV-
19^^Hovedstaden|Human|Department of Virus and Microbiological Special
Diagnostics|Albertsen lab|Michaelsen|Denmark
>Spike|hCoV-19/Wales/MILK-9D3B2A/2020|2020-09-18|EPI_ISL_601707|Original|hCoV-
19^^Wales|Human|Lighthouse Lab in Milton Keynes|Wellcome Sanger Institute for the
COVID-19 Genomics UK (COG-UK) consortium|Davies|United Kingdom
>Spike|hCoV-19/England/QEUH-9F36AB/2020|2020-09-24|EPI_ISL_588516|Original|hCoV-
19^^England|Human|Lighthouse Lab in Glasgow|Wellcome Sanger Institute for the COVID-
19 Genomics UK (COG-UK) consortium|Davies|United Kingdom
>Spike|hCoV-19/South Africa/KRISP-K002333/2020|2020-07-
22|EPI_ISL_515708|Original|hCoV-19^^KwaZulu-Natal|Human|NHLS-IALCH|KRISP|de
Oliveira|South Africa
>Spike|hCoV-19/Belgium/rega-0501666/2020|2020-05-01|EPI_ISL_734694|Original|hCoV-
19^^Hertain|Human|UZ Leuven|KU Leuven|Maes|Belgium
>Spike|hCoV-19/Brazil/SE-6601/2020|2020-04-20|EPI_ISL_541392|Original|hCoV-
19^^Sergipe|Human|LACEN/SE|Laboratory of Respiratory Viruses and Measles|Silva|Brazil
>Spike|hCoV-19/Denmark/DCGC-21501/2020|2020-12-21|EPI_ISL_758198|Original|hCoV-
19^^Midtjylland|Human|Department of Virus and Microbiological Special
Diagnostics|Albertsen Lab|Michaelsen|Denmark
>Spike|hCoV-19/England/QEUH-9C9ED3/2020|2020-09-10|EPI_ISL_567510|Original|hCoV-
19^^England|Human|Lighthouse Lab in Glasgow|Wellcome Sanger Institute for the COVID-
19 Genomics UK (COG-UK) consortium|Davies|United Kingdom
>Spike|hCoV-19/USA/WI-UW-1857/2020|2020-09-17|EPI_ISL_605729|Original|hCoV-
19^^Wisconsin|Human|University of Wisconsin-Madison AIDS Vaccine Research
Laboratories|University of Wisconsin-Madison AIDS Vaccine Research
Laboratories|Baczenas|USA
>Spike|hCoV-19/mink/Netherlands/NB-EMC-18-20/2020|2020-07-
04|EPI_ISL_523044|Original|hCoV-19^^Noord Brabant|Neovison vison|Dutch COVID-19
response team|Erasmus Medical Center|Oude Munnink|Netherlands
>Spike|hCoV-19/Denmark/DCGC-960/2020|2020-03-09|EPI_ISL_614584|Original|hCoV-
19^^Sjaelland|Human|Department of Virus and Microbiological Special
Diagnostics|Albertsen lab|Michaelsen|Denmark

>Spike|hCoV-19/Wales/PHWC-29C67/2020|2020-04-05|EPI_ISL_432383|Original|hCoV-19^^Wales|Human|Wales Specialist Virology Centre|Public Health Wales Microbiology Cardiff|Moore|United Kingdom
>Spike|hCoV-19/USA/MI-MDHHS-SC20878/2020|2020-05-15|EPI_ISL_471899|Original|hCoV-19^^Michigan|Human|Michigan Department of Health and Human Services|Michigan Department of Health and Human Services|Blankenship|USA
>Spike|hCoV-19/Japan/PG-5412/2020|2020-08-00|EPI_ISL_690515|Original|hCoV-19^^Japan|Human|Fukuoka Institute of Health and Environmental Sciences|Pathogen Genomics Center|Sekizuka|Japan
>Spike|hCoV-19/Canada/ON-UHTC_0339/2020|2020-09-10|EPI_ISL_609815|Original|hCoV-19^^Ontario|Human|Unity Health Toronto|Ontario Institute for Cancer Research|de Borja|Canada
>Spike|hCoV-19/England/ALDP-A5231B/2020|2020-10-07|EPI_ISL_597954|Original|hCoV-19^^England|Human|Lighthouse Lab in Alderley Park|Wellcome Sanger Institute for the COVID-19 Genomics UK (COG-UK) consortium|Davies|United Kingdom
>Spike|hCoV-19/Switzerland/VS-ETHZ-110492/2020|2020-04-07|EPI_ISL_451903|Original|hCoV-19^^Valais|Human|Viollier AG|Department of Biosystems Science and Engineering|Nadeau|Switzerland
>Spike|hCoV-19/USA/VA-DCLS-1009/2020|2020-06-03|EPI_ISL_526903|Original|hCoV-19^^Virginia|Human|Virginia DCLS|Virginia DCLS|St. Jacques|USA
>Spike|hCoV-19/Australia/VIC5351/2020|2020-07-17|EPI_ISL_520114|Original|hCoV-19^^Victoria|Human|Microbiological Diagnostic Unit - Public Health Laboratory (MDU-PHL)|MDU-PHL|Schultz|Australia
>Spike|hCoV-19/Wales/PHWC-48ACD9/2020|2020-11-07|EPI_ISL_666146|Original|hCoV-19^^Wales|Human|Wales Specialist Virology Centre Sequencing lab|COVID-19 Genomics UK (COG-UK) Consortium|Moore|United Kingdom
>Spike|hCoV-19/Switzerland/LU-ETHZ-360257/2020|2020-11-09|EPI_ISL_728859|Original|hCoV-19^^Lucerne|Human|Viollier AG|Department of Biosystems Science and Engineering|Nadeau|Switzerland
>Spike|hCoV-19/Scotland/EDB1922/2020|2020-04-17|EPI_ISL_491591|Original|hCoV-19^^Scotland|Human|Virology Department|Wellcome Sanger Institute for the COVID-19 Genomics UK (COG-UK) consortium|Davies|United Kingdom
>Spike|hCoV-19/England/CAMB-1B819A/2020|2020-10-19|EPI_ISL_638195|Original|hCoV-19^^England|Human|Department of Pathology|COVID-19 Genomics UK (COG-UK) Consortium|Goodfellow|United Kingdom
>Spike|hCoV-19/Netherlands/Gelderland_70/2020|2020-05-18|EPI_ISL_460969|Original|hCoV-19^^Gelderland|Human|Dutch COVID-19 response team|Erasmus Medical Center|Oude Munnink|Netherlands
>Spike|hCoV-19/Gibraltar/203360721/2020|2020-07-22|EPI_ISL_547437|Original|hCoV-19^^Gibraltar|Human|Microbiology|Respiratory Virus Unit|Platt|Gibraltar
>Spike|hCoV-19/USA/MN-MDH-1040/2020|2020-05-07|EPI_ISL_462885|Original|hCoV-19^^Minnesota|Human|Minnesota Department of Health|Minnesota Department of Health|Garfin|USA
>Spike|hCoV-19/Scotland/QEUH-9DC3DE/2020|2020-09-22|EPI_ISL_601160|Original|hCoV-19^^Scotland|Human|Lighthouse Lab in Glasgow|Wellcome Sanger Institute for the COVID-19 Genomics UK (COG-UK) consortium|Davies|United Kingdom
>Spike|hCoV-19/Burkina Faso/2828/2020|2020-10-23|EPI_ISL_660503|Original|hCoV-19^^Hound\u00e9|Human|Laboratoire de Microbiologie CHU Souro Sanou|Centre Muraz|Ouedraogo|Burkina Faso
>Spike|hCoV-19/England/ALDP-B75D22/2020|2020-11-12|EPI_ISL_723699|Original|hCoV-19^^England|Human|Oxford Viromics|COVID-19 Genomics UK (COG-UK) Consortium|Golubchik|United Kingdom
>Spike|hCoV-19/England/CAMB-8075E/2020|2020-04-10|EPI_ISL_470477|Original|hCoV-19^^England|Human|Department of Pathology|Wellcome Sanger Institute for the COVID-19 Genomics UK (COG-UK) consortium|Davies|United Kingdom
>Spike|hCoV-19/Canada/ON-S261/2020|2020-03-28|EPI_ISL_755840|Original|hCoV-19^^Ontario|Human|Toronto Invasive Bacterial Diseases Network|McMaster University|McArthur|Canada

>Spike|hCoV-19/Spain/MD-IBV-99007153/2020|2020-08-14|EPI_ISL_541925|Original|hCoV-19^^Madrid|Human|Hospital General Universitario Gregorio Mara\u00f1\u00f3n|SeqCOVID-SPAIN consortium/IBV(CSIC)|Comas|Spain
>Spike|hCoV-19/Turkey/IST-GLAB-063/2020|2020-04-22|EPI_ISL_480239|Original|hCoV-19^^Istanbul|Human|Genomic Laboratory (GLAB) (Conjoint lab of Health Directorate of Istanbul and Istanbul Technical University)|Genomic Laboratory (GLAB)|Karacan|Turkey
>Spike|hCoV-19/Denmark/DCGC-2666/2020|2020-08-17|EPI_ISL_617886|Original|hCoV-19^^Hovedstaden|Human|Department of Virus and Microbiological Special Diagnostics|Albertsen lab|Michaelsen|Denmark
>Spike|hCoV-19/Denmark/DCGC-14514/2020|2020-11-30|EPI_ISL_711811|Original|hCoV-19^^Sjaelland|Human|Department of Virus and Microbiological Special Diagnostics|Albertsen Lab|Michaelsen|Denmark
>Spike|hCoV-19/USA/TX-HMH-597/2020|2020-04-08|EPI_ISL_547132|Original|hCoV-19^^Texas|Human|Houston Methodist Hospital|Houston Methodist Hospital|Long|USA
>Spike|hCoV-19/Australia/VIC3493/2020|2020-07-05|EPI_ISL_520249|Original|hCoV-19^^Victoria|Human|Victorian Infectious Diseases Reference Laboratory (VIDRL)|VIDRL and MDU-PHL|Schultz|Australia
>Spike|hCoV-19/Switzerland/ZH-ETHZ-240075/2020|2020-08-10|EPI_ISL_523913|Original|hCoV-19^^Zurich|Human|Viollier AG|Department of Biosystems Science and Engineering|Nadeau|Switzerland
>Spike|hCoV-19/USA/UT-08490/2020|2020-05-21|EPI_ISL_524258|Original|hCoV-19^^Utah|Human|Utah Public Health Laboratory|Utah Public Health Laboratory|Young|USA
>Spike|hCoV-19/England/LIVE-1E0BB9/2020|2020-10-13|EPI_ISL_627107|Original|hCoV-19^^England|Human|Liverpool Clinical Laboratories|COVID-19 Genomics UK (COG-UK) Consortium|Haldenby|United Kingdom
>Spike|hCoV-19/USA/TX-HMH-3188/2020|2020-06-13|EPI_ISL_544062|Original|hCoV-19^^Texas|Human|Houston Methodist Hospital|Houston Methodist Hospital|Long|USA
>Spike|hCoV-19/Wales/QEUH-AD344C/2020|2020-10-26|EPI_ISL_634743|Original|hCoV-19^^Wales|Human|Lighthouse Lab in Glasgow|Wellcome Sanger Institute for the COVID-19 Genomics UK (COG-UK) consortium|Davies|United Kingdom
>Spike|hCoV-19/England/ALDP-CEBAD4/2020|2020-12-21|EPI_ISL_782369|Original|hCoV-19^^England|Human|Lighthouse Lab in Alderley Park|Wellcome Sanger Institute for the COVID-19 Genomics UK (COG-UK) Consortium|Davies|United Kingdom
>Spike|hCoV-19/Scotland/QEUH-9E542A/2020|2020-09-24|EPI_ISL_601033|Original|hCoV-19^^Scotland|Human|Lighthouse Lab in Glasgow|Wellcome Sanger Institute for the COVID-19 Genomics UK (COG-UK) consortium|Davies|United Kingdom
>Spike|hCoV-19/USA/LA-GBCL-26492444/2020|2020-04-28|EPI_ISL_485027|Original|hCoV-19^^Louisiana|Human|River Road Testing Lab|Ginkgo Bioworks Clinical Laboratory|Smith|USA
>Spike|hCoV-19/Spain/AN-IBV-98001690/2020|2020-04-13|EPI_ISL_538129|Original|hCoV-19^^Andalucia|Human|Servicio de Microbiolog\u00eda y Parasitolog\u00eda cl\u00ednica. UCEIMP. Hospital Universitario Virgen del Roc\u00edo/IBIS/CSIC/US|SeqCOVID-SPAIN consortium/IBV(CSIC)|Comas|Spain
>Spike|hCoV-19/Australia/VIC695/2020|2020-03-28|EPI_ISL_426987|Original|hCoV-19^^Victoria|Human|Victorian Infectious Diseases Reference Laboratory (VIDRL)|Microbiological Diagnostic Unit Public Health Laboratory and Victorian Infectious Diseases Reference Laboratory|Schultz|Australia
>Spike|hCoV-19/Japan/PG-3759/2020|2020-04-00|EPI_ISL_692012|Original|hCoV-19^^Japan|Human|Pathogen Genomics Center|Pathogen Genomics Center|Sekizuka|Japan
>Spike|hCoV-19/Belgium/Rega-0329229/2020|2020-03-29|EPI_ISL_458199|Original|hCoV-19^^Saint-Ghislain|Human|KU Leuven|KU Leuven|Maes|Belgium
>Spike|hCoV-19/USA/CA-ALSR-4574/2020|2020-07-14|EPI_ISL_667014|Original|hCoV-19^^California|Human|San Diego County Public Health Laboratory|Andersen lab at Scripps Research|Gangavarapu|USA
>Spike|hCoV-19/England/QEUH-B31A1C/2020|2020-11-04|EPI_ISL_646437|Original|hCoV-19^^England|Human|Lighthouse Lab in Glasgow|Wellcome Sanger Institute for the COVID-19 Genomics UK (COG-UK) Consortium|Davies|United Kingdom
>Spike|hCoV-19/USA/WI-GMF-M00028/2020|2020-08-09|EPI_ISL_547684|Original|hCoV-19^^Wisconsin|Human|Gundersen Clinical Microbiology Laboratory|Kabara Cancer Research Institute|Kenny|USA

>Spike|hCoV-19/USA/WA-UW-1784/2020|2020-03-21|EPI_ISL_424261|Original|hCoV-19^^Washington|Human|UW Virology Lab|UW Virology Lab|Roychoudhury|USA
>Spike|hCoV-19/USA/CA-CZB-13749/2020|2020-09-14|EPI_ISL_739110|Original|hCoV-19^^California|Human|Alameda County Public Health Lab|Chan-Zuckerberg Biohub|Kamm|USA
>Spike|hCoV-19/Germany/NW-HHU-192/2020|2020-03-30|EPI_ISL_602466|Orginal|hCoV-19^^North Rhine-Westphalia|Human|Institute for Virology|Center of Medical Microbiology|Wienemann|Germany
>Spike|hCoV-19/England/ALDP-B7393F/2020|2020-11-12|EPI_ISL_662359|Original|hCoV-19^^England|Human|Lighthouse Lab in Alderley Park|Wellcome Sanger Institute for the COVID-19 Genomics UK (COG-UK) Consortium|Davies|United Kingdom
>Spike|hCoV-19/South Africa/KRISP-K004071/2020|2020-10-20|EPI_ISL_635049|Original|hCoV-19^^KwaZulu-Natal|Human|National Health Laboratory Service - Inkosi Albert Luthuli Central Hospital (NHLS-IALCH)|KRISP|de Oliveira|South Africa
>Spike|hCoV-19/France/ARA-124840/2020|2020-12-25|EPI_ISL_779807|Original|hCoV-19^^Auvergne-Rh\u00f4ne-Alpes|Human|CNR Virus des Infections Respiratoires - France SUD|CNR Virus des Infections Respiratoires - France SUD|Bal|France
>Spike|hCoV-19/England/LEED-2A8C04/2020|2020-04-05|EPI_ISL_539128|Original|hCoV-19^^England|Human|Leeds Teaching Hospitals NHS Trust and Public Health England|Wellcome Sanger Institute for the COVID-19 Genomics UK (COG-UK) consortium|Davies|United Kingdom
>Spike|hCoV-19/Netherlands/NoordBrabant_12/2020|2020-03-06|EPI_ISL_414452|Original|hCoV-19^^Noord Brabant|Human|Dutch COVID-19 response team|Erasmus Medical Center|Oude Munnink|Netherlands
>Spike|hCoV-19/Scotland/EDB7120/2020|2020-08-03|EPI_ISL_514538|Original|hCoV-19^^Scotland|Human|Virology Department|COVID-19 Genomics UK (COG-UK) Consortium|Williams|United Kingdom
>Spike|hCoV-19/Japan/PG-7493/2020|2020-08-00|EPI_ISL_688595|Original|hCoV-19^^Japan|Human|Tokyo Metropolitan Institute of Public Health|Pathogen Genomics Center|Sekizuka|Japan
>Spike|hCoV-19/England/QEUH-B67B1A/2020|2020-11-11|EPI_ISL_661451|Original|hCoV-19^^England|Human|Lighthouse Lab in Glasgow|Wellcome Sanger Institute for the COVID-19 Genomics UK (COG-UK) Consortium|Davies|United Kingdom
>Spike|hCoV-19/USA/TX-HMH-MCoV-8921/2020|2020-07-02|EPI_ISL_780832|Original|hCoV-19^^Texas|Human|Houston Methodist Hospital|Houston Methodist Hospital|Long|USA
>Spike|hCoV-19/England/CAMC-A8229D/2020|2020-10-12|EPI_ISL_610829|Original|hCoV-19^^England|Human|Lighthouse Lab in Cambridge|Wellcome Sanger Institute for the COVID-19 Genomics UK (COG-UK) consortium|Davies|United Kingdom
>Spike|hCoV-19/USA/NE-11308/2020|2020-05-04|EPI_ISL_466675|Original|hCoV-19^^Nebraska|Human|Nebraska Public Health Laboratory|UNMC COVID-19 Response Team|Pratt|USA
>Spike|hCoV-19/USA/NMDOH-2020321813/2020|2020-09-01|EPI_ISL_542071|Original|hCoV-19^^New Mexico|Human|New Mexico Department of Health Scientific Laboratory|New Mexico Department of Health Scientific Laboratory|Johnson|USA
>Spike|hCoV-19/Wales/PHWC-15E10F/2020|2020-05-12|EPI_ISL_472795|Original|hCoV-19^^Wales|Human|Wales Specialist Virology Centre Sequencing lab|COVID-19 Genomics UK (COG-UK) Consortium|Moore|United Kingdom
>Spike|hCoV-19/Scotland/CVR249/2020|2020-03-19|EPI_ISL_438914|Original|hCoV-19^^Scotland|Human|West of Scotland Specialist Virology Centre|COVID-19 Genomics UK (COG-UK) Consortium|Robertson|United Kingdom
>Spike|hCoV-19/USA/NM-DOH-2020632199/2020|2020-12-20|EPI_ISL_766895|Original|hCoV-19^^New Mexico|Human|New Mexico Department of Health Scientific Laboratory|New Mexico Department of Health Scientific Laboratory|Malone|USA
>Spike|hCoV-19/England/QEUH-AF15DC/2020|2020-10-28|EPI_ISL_666015|Original|hCoV-19^^England|Human|Northumbria University / South Tees Hospitals NHS Foundation Trust / North Cumbria Integrated Care NHS Foundation Trust / North Tees and Hartlepool NHS Foundation Trust / Newcastle Hospitals NHS Foundation Trust|COVID-19 Genomics UK (COG-UK) Consortium|Bashton|United Kingdom

>Spike|hCoV-19/Wales/PHWC-4815F5/2020|2020-10-17|EPI_ISL_613093|Original|hCoV-19^^Wales|Human|Wales Specialist Virology Centre Sequencing lab|COVID-19 Genomics UK (COG-UK) Consortium|Moore|United Kingdom
>Spike|hCoV-19/Canada/ON_QGLO-012/2020|2020-03-26|EPI_ISL_459872|Original|hCoV-19^^Ontario|Human|Kingston Health Sciences Center|Queen's Genomics Lab at Ongwanada (Q-GLO)|Sjaarda|Canada
>Spike|hCoV-19/Wales/PHWC-35EEA/2020|2020-04-28|EPI_ISL_474588|Original|hCoV-19^^Wales|Human|Originating lab|COVID-19 Genomics UK (COG-UK) Consortium|Moore|United Kingdom
>Spike|hCoV-19/Luxembourg/LNS4946091/2020|2020-03-27|EPI_ISL_429756|Original|hCoV-19^^Luxembourg|Human|Laboratoire National de Sante|Laboratoire National de Sante|Wienecke-Baldacchino|Luxembourg
>Spike|hCoV-19/England/ALDP-C6B869/2020|2020-12-14|EPI_ISL_778454|Original|hCoV-19^^England|Human|Lighthouse Lab in Alderley Park|Wellcome Sanger Institute for the COVID-19 Genomics UK (COG-UK) Consortium|Davies|United Kingdom
>Spike|hCoV-19/Denmark/DCGC-14506/2020|2020-11-30|EPI_ISL_711804|Original|hCoV-19^^Sjaelland|Human|Department of Virus and Microbiological Special Diagnostics|Albertsen Lab|Michaelsen|Denmark
>Spike|hCoV-19/Scotland/EDB3439/2020|2020-04-10|EPI_ISL_487967|Original|hCoV-19^^Scotland|Human|Virology Department|Wellcome Sanger Institute for the COVID-19 Genomics UK (COG-UK) consortium|Davies|United Kingdom
>Spike|hCoV-19/England/QEUH-AE2820/2020|2020-10-27|EPI_ISL_633617|Original|hCoV-19^^England|Human|Lighthouse Lab in Glasgow|Wellcome Sanger Institute for the COVID-19 Genomics UK (COG-UK) consortium|Davies|United Kingdom
>Spike|hCoV-19/Australia/NSW510/2020|2020-03-26|EPI_ISL_455050|Original|hCoV-19^^New South Wales|Human|ACT Pathology|NSW Health Pathology - Institute of Clinical Pathology and Medical Research; Westmead Hospital; University of Sydney|Gall|Australia
>Spike|hCoV-19/Scotland/QEUH-98F158/2020|2020-08-28|EPI_ISL_540386|Original|hCoV-19^^Scotland|Human|Lighthouse Lab in Glasgow|Wellcome Sanger Institute for the COVID-19 Genomics UK (COG-UK) consortium|Davies|United Kingdom
>Spike|hCoV-19/England/ALDP-9E6D3D/2020|2020-09-24|EPI_ISL_580346|Original|hCoV-19^^England|Human|Lighthouse Lab in Alderley Park|Wellcome Sanger Institute for the COVID-19 Genomics UK (COG-UK) consortium|Davies|United Kingdom
>Spike|hCoV-19/Wales/PHWC-48738C/2020|2020-10-13|EPI_ISL_637606|Original|hCoV-19^^Wales|Human|Wales Specialist Virology Centre Sequencing lab|COVID-19 Genomics UK (COG-UK) Consortium|Moore|United Kingdom
>Spike|hCoV-19/USA/WA-S2051/2020|2020-06-05|EPI_ISL_497479|Original|hCoV-19^^Washington|Human|Washington State Department of Health|Seattle Flu Study|Lee|USA
>Spike|hCoV-19/England/QEUH-A5E458/2020|2020-10-12|EPI_ISL_597513|Original|hCoV-19^^England|Human|Lighthouse Lab in Glasgow|Wellcome Sanger Institute for the COVID-19 Genomics UK (COG-UK) consortium|Davies|United Kingdom
>Spike|hCoV-19/Wales/PHWC-4A5546/2020|2020-12-16|EPI_ISL_764882|Original|hCoV-19^^Wales|Human|Wales Specialist Virology Centre Sequencing lab|COVID-19 Genomics UK (COG-UK) Consortium|Moore|United Kingdom
>Spike|hCoV-19/Denmark/DCGC-10992/2020|2020-11-02|EPI_ISL_669588|Original|hCoV-19^^Midtjylland|Human|Department of Virus and Microbiological Special Diagnostics|Albertsen Lab|Michaelsen|Denmark
>Spike|hCoV-19/England/CAMB-79E35/2020|2020-04-01|EPI_ISL_441250|Original|hCoV-19^^England|Human|Department of Pathology|Wellcome Sanger Institute for the COVID-19 Genomics UK (COG-UK) consortium|Amato|United Kingdom
>Spike|hCoV-19/Netherlands/UT-RIVM-20870/2020|2020-12-16|EPI_ISL_790838|Original|hCoV-19^^Utrecht|Human|Dutch COVID-19 response team|National Institute for Public Health and the Environment (RIVM)|Meijer|Netherlands
>Spike|hCoV-19/Scotland/EDB3986/2020|2020-05-03|EPI_ISL_449272|Original|hCoV-19^^Scotland|Human|Virology Department|COVID-19 Genomics UK (COG-UK) Consortium|Williams|United Kingdom
>Spike|hCoV-19/Hong Kong/HKU-200723-032/2020|2020-03-17|EPI_ISL_497799|Original|hCoV-19^^Hong Kong|Human|Department of Microbiology|Department of Microbiology|Chan|China

>Spike|hCoV-19/USA/TX-HMH-MCoV-14502/2020|2020-10-08|EPI_ISL_787787|Original|hCoV-19^^Texas|Human|Houston Methodist Hospital|Houston Methodist Hospital|Long|USA
>Spike|hCoV-19/Australia/VIC9961/2020|2020-07-23|EPI_ISL_565806|Original|hCoV-19^^Victoria|Human|Microbiological Diagnostic Unit - Public Health Laboratory (MDU-PHL)|MDU-PHL|Schultz|Australia
>Spike|hCoV-19/Scotland/QEUH-9B8D25/2020|2020-09-07|EPI_ISL_568283|Original|hCoV-19^^Scotland|Human|Lighthouse Lab in Glasgow|Wellcome Sanger Institute for the COVID-19 Genomics UK (COG-UK) consortium|Davies|United Kingdom
>Spike|hCoV-19/USA/CA-CZB-12336/2020|2020-10-10|EPI_ISL_648413|Original|hCoV-19^^California|Human|Santa Clara County Public Health Laboratory|Chan-Zuckerberg Biohub|Bhatt|USA
>Spike|hCoV-19/England/CAMB-7B11C/2020|2020-04-11|EPI_ISL_433920|Original|hCoV-19^^England|Human|Department of Pathology|COVID-19 Genomics UK (COG-UK) Consortium|Meredith|United Kingdom
>Spike|hCoV-19/Jordan/AM-ALSR-5156/2020|2020-10-15|EPI_ISL_730395|Original|hCoV-19^^Amman|Human|Biolab Diagnostic Laboratories|Andersen lab at Scripps Research|Gangavarapu|Jordan
>Spike|hCoV-19/Denmark/DCGC-5865/2020|2020-09-28|EPI_ISL_621052|Original|hCoV-19^^Midtjylland|Human|Department of Virus and Microbiological Special Diagnostics|Albertsen lab|Michaelsen|Denmark
>Spike|hCoV-19/England/ALDP-B84CF6/2020|2020-11-15|EPI_ISL_675818|Original|hCoV-19^^England|Human|Lighthouse Lab in Alderley Park|Wellcome Sanger Institute for the COVID-19 Genomics UK (COG-UK) Consortium|Davies|United Kingdom
>Spike|hCoV-19/England/ALDP-A6CB16/2020|2020-10-14|EPI_ISL_608156|Original|hCoV-19^^England|Human|Lighthouse Lab in Alderley Park|Wellcome Sanger Institute for the COVID-19 Genomics UK (COG-UK) consortium|Davies|United Kingdom
>Spike|hCoV-19/England/CAMC-B0842F/2020|2020-10-30|EPI_ISL_643384|Original|hCoV-19^^England|Human|Lighthouse Lab in Cambridge|Wellcome Sanger Institute for the COVID-19 Genomics UK (COG-UK) Consortium|Davies|United Kingdom
>Spike|hCoV-19/England/QEUH-96E82D/2020|2020-08-19|EPI_ISL_530615|Original|hCoV-19^^England|Human|Lighthouse Lab in Glasgow|Wellcome Sanger Institute for the COVID-19 Genomics UK (COG-UK) consortium|Davies|United Kingdom
>Spike|hCoV-19/Japan/PG-3243/2020|2020-04-00|EPI_ISL_691359|Original|hCoV-19^^Japan|Human|Pathogen Genomics Center|Pathogen Genomics Center|Sekizuka|Japan
>Spike|hCoV-19/England/QEUH-A60C1F/2020|2020-10-12|EPI_ISL_665506|Original|hCoV-19^^England|Human|Northumbria University / South Tees Hospitals NHS Foundation Trust / North Cumbria Integrated Care NHS Foundation Trust / North Tees and Hartlepool NHS Foundation Trust / Newcastle Hospitals NHS Foundation Trust|COVID-19 Genomics UK (COG-UK) Consortium|Bashton|United Kingdom
>Spike|hCoV-19/England/MILK-4BF558/2020|2020-06-05|EPI_ISL_559254|Original|hCoV-19^^England|Human|Lighthouse Lab in Milton Keynes|Wellcome Sanger Institute for the COVID-19 Genomics UK (COG-UK) consortium|Davies|United Kingdom
>Spike|hCoV-19/USA/CA-CZB-11694/2020|2020-09-28|EPI_ISL_625546|Original|hCoV-19^^California|Human|Alameda County Public Health Lab|Chan-Zuckerberg Biohub|Bhatt|USA
>Spike|hCoV-19/Australia/SAP027/2020|2020-04-06|EPI_ISL_468009|Original|hCoV-19^^South Australia|Human|SA Pathology|SA Pathology|Leong|Australia
>Spike|hCoV-19/USA/CA-ALSR-1097-SAN/2020|2020-06-18|EPI_ISL_483524|Original|hCoV-19^^California|Human|UC San Diego Center for Advanced Laboratory Medicine|Andersen lab at Scripps Research|Gangavarapu|USA
>Spike|hCoV-19/England/BRIS-12B6DF/2020|2020-05-20|EPI_ISL_487535|Original|hCoV-19^^England|Human|PHE South West Regional Laboratory|Wellcome Sanger Institute for the COVID-19 Genomics UK (COG-UK) consortium|Davies|United Kingdom
>Spike|hCoV-19/USA/MI-MDHHS-SC20003/2020|2020-03-11|EPI_ISL_424990|unknown|hCoV-19^^Michigan|Human|Bureau of Laboratories|Michigan Department of Health and Human Services|Blankenship|USA
>Spike|hCoV-19/England/BRIS-12A4C7/2020|2020-04-30|EPI_ISL_458382|Original|hCoV-19^^England|Human|PHE South West Regional Laboratory|Wellcome Sanger Institute for the COVID-19 Genomics UK (COG-UK) consortium|Davies|United Kingdom

>Spike|hCoV-19/USA/NY-NYCPHL-000214/2020|2020-04-21|EPI_ISL_631746|Original|hCoV-19^^New York|Human|Jamaica Hospital Medical Center|New York City Public Health Laboratory|Wang|USA
>Spike|hCoV-19/England/ALDP-94D9A9/2020|2020-06-20|EPI_ISL_555187|Original|hCoV-19^^England|Human|Lighthouse Lab in Alderley Park|Wellcome Sanger Institute for the COVID-19 Genomics UK (COG-UK) consortium|Davies|United Kingdom
>Spike|hCoV-19/Norway/3145/2020|2020-08-09|EPI_ISL_549045|Original|hCoV-19^^Viken|Human|Ostfold Hospital Trust - Kalnes|Norwegian Institute of Public Health|Kopperud Riis|Norway
>Spike|hCoV-19/England/QEUH-9F97B1/2020|2020-09-25|EPI_ISL_589085|Original|hCoV-19^^England|Human|Lighthouse Lab in Glasgow|Wellcome Sanger Institute for the COVID-19 Genomics UK (COG-UK) consortium|Davies|United Kingdom
>Spike|hCoV-19/USA/UT-UPHL-2012288941/2020|2020-08-10|EPI_ISL_745919|Original|hCoV-19^^Utah|Human|Ginkgo Bioworks Clinical Laboratory|Utah Public Health Laboratory|Young|USA
>Spike|hCoV-19/USA/WI-WSLH-200452/2020|2020-05-19|EPI_ISL_578597|Original|hCoV-19^^Wisconsin|Human|Wisconsin State Laboratory of Hygiene Communicable Disease Division|Wisconsin State Laboratory of Hygiene Communicable Disease Division|Florek|USA
>Spike|hCoV-19/Wales/PHWC-15DE90/2020|2020-05-14|EPI_ISL_472780|Original|hCoV-19^^Wales|Human|Wales Specialist Virology Centre Sequencing lab|COVID-19 Genomics UK (COG-UK) Consortium|Moore|United Kingdom
>Spike|hCoV-19/USA/AZ-TG280193/2020|2020-04-29|EPI_ISL_694147|Original|hCoV-19^^Arizona|Human|TGen North|TGen North|French|USA
>Spike|hCoV-19/USA/TX-HMH-4065/2020|2020-06-18|EPI_ISL_543338|Original|hCoV-19^^Texas|Human|Houston Methodist Hospital|Houston Methodist Hospital|Long|USA
>Spike|hCoV-19/USA/TX-HMH-MCoV-18488/2020|2020-11-18|EPI_ISL_784836|Original|hCoV-19^^Texas|Human|Houston Methodist Hospital|Houston Methodist Hospital|Long|USA
>Spike|hCoV-19/USA/LA-EVTL317/2020|2020-05-15|EPI_ISL_578724|Original|hCoV-19^^Louisiana|Human|LSUHS Emerging Viral Threat Laboratory|Microbial Genome Sequencing Center|Kamil|USA
>Spike|hCoV-19/Brazil/SP-L25-CD635/2020|2020-04-08|EPI_ISL_672705|Original|hCoV-19^^S\u00e3o Paulo|Human|Institute of Tropical Medicine at the University of S\u00e3o Paulo (IMT-USP)|Laborat\u00f3rio de Parasitologia M\u00e9dica - Instituto de Medicina Tropical - Universidade de S\u00e3o Paulo|de Jesus|Brazil
>Spike|hCoV-19/Denmark/DCGC-4794/2020|2020-09-21|EPI_ISL_621404|Original|hCoV-19^^Sjaelland|Human|Department of Virus and Microbiological Special Diagnostics|Albertsen lab|Michaelsen|Denmark
>Spike|hCoV-19/USA/NY-Wadsworth-105920-01/2020|2020-05-17|EPI_ISL_676714|Original|hCoV-19^^New York|Human|Wadsworth Center|Wadsworth Center|Lasek-Nesselquist|USA
>Spike|hCoV-19/Luxembourg/LNS6456581/2020|2020-11-02|EPI_ISL_744419|Original|hCoV-19^^Luxembourg|Human|Laboratoire national de sant\u00e9|Laboratoire national de sant\u00e9|Wienecke-Baldacchino|Luxembourg
>Spike|hCoV-19/Japan/PG-5350/2020|2020-08-00|EPI_ISL_690453|Original|hCoV-19^^Japan|Human|Fukuoka Institute of Health and Environmental Sciences|Pathogen Genomics Center|Sekizuka|Japan
>Spike|hCoV-19/Switzerland/BE-ETHZ-320191/2020|2020-10-10|EPI_ISL_737682|Original|hCoV-19^^Bern|Human|Viollier AG|Department of Biosystems Science and Engineering|Nadeau|Switzerland
>Spike|hCoV-19/USA/CA-LAC-USC6/2020-05-09|2020-05-09|EPI_ISL_569669|Original|hCoV-19^^California|Human|Lee Lab|Lee Lab|Lee|USA
>Spike|hCoV-19/Scotland/CVR2300/2020|2020-04-12|EPI_ISL_453043|Original|hCoV-19^^Scotland|Human|West of Scotland Specialist Virology Centre|COVID-19 Genomics UK (COG-UK) Consortium|Robertson|United Kingdom
>Spike|hCoV-19/USA/TX-HMH-3012/2020|2020-05-21|EPI_ISL_543932|Original|hCoV-19^^Texas|Human|Houston Methodist Hospital|Houston Methodist Hospital|Long|USA
>Spike|hCoV-19/USA/AZ-TG640907/2020|2020-04-27|EPI_ISL_695811|Original|hCoV-19^^Arizona|Human|Sonora Quest Laboratories|TGen North|French|USA

>Spike|hCoV-19/Wales/PHWC-1682C3/2020|2020-04-19|EPI_ISL_494222|Original|hCoV-19^^Wales|Human|Originating lab|COVID-19 Genomics UK (COG-UK) Consortium|Moore|United Kingdom
>Spike|hCoV-19/Denmark/DCGC-17523/2020|2020-12-14|EPI_ISL_751040|Original|hCoV-19^^Midtjylland|Human|Department of Virus and Microbiological Special Diagnostics|Albertsen Lab|Michaelsen|Denmark
>Spike|hCoV-19/England/CAMC-B22BC0/2020|2020-11-02|EPI_ISL_647423|Original|hCoV-19^^England|Human|Lighthouse Lab in Cambridge|Wellcome Sanger Institute for the COVID-19 Genomics UK (COG-UK) Consortium|Davies|United Kingdom
>Spike|hCoV-19/England/CAMC-B0B32F/2020|2020-10-29|EPI_ISL_643432|Original|hCoV-19^^England|Human|Lighthouse Lab in Cambridge|Wellcome Sanger Institute for the COVID-19 Genomics UK (COG-UK) Consortium|Davies|United Kingdom
>Spike|hCoV-19/England/20140009802/2020|2020-03-30|EPI_ISL_423388|Original|hCoV-19^^England|Human|Respiratory Virus Unit|Respiratory Virus Unit|Platt|United Kingdom
>Spike|hCoV-19/Panama/330817/2020|2020-03-10|EPI_ISL_496676|Original|hCoV-19^^Panama Center|Human|Gorgas Memorial Laboratory of Health Studies|Gorgas Memorial Laboratory of Health Studies|Martinez|Panama
>Spike|hCoV-19/Scotland/EDB899/2020|2020-04-05|EPI_ISL_432872|Original|hCoV-19^^Scotland|Human|Virology Department|COVID-19 Genomics UK (COG-UK) Consortium|Williams|United Kingdom
>Spike|hCoV-19/England/LIVE-DBAE59/2020|2020-11-13|EPI_ISL_664151|Original|hCoV-19^^England|Human|Liverpool Clinical Laboratories|COVID-19 Genomics UK (COG-UK) Consortium|Haldenby|United Kingdom
>Spike|hCoV-19/England/QEUH-B9E34F/2020|2020-11-17|EPI_ISL_720980|Original|hCoV-19^^England|Human|Lighthouse Lab in Glasgow|Wellcome Sanger Institute for the COVID-19 Genomics UK (COG-UK) Consortium|Davies|United Kingdom
>Spike|hCoV-19/Scotland/EDB7373/2020|2020-08-19|EPI_ISL_529694|Original|hCoV-19^^Scotland|Human|Virology Department|COVID-19 Genomics UK (COG-UK) Consortium|Williams|United Kingdom
>Spike|hCoV-19/England/ALDP-A6937B/2020|2020-10-09|EPI_ISL_609004|Original|hCoV-19^^England|Human|Lighthouse Lab in Alderley Park|Wellcome Sanger Institute for the COVID-19 Genomics UK (COG-UK) consortium|Davies|United Kingdom
>Spike|hCoV-19/England/MILK-B94E93/2020|2020-11-14|EPI_ISL_709884|Original|hCoV-19^^England|Human|Lighthouse Lab in Milton Keynes|Wellcome Sanger Institute for the COVID-19 Genomics UK (COG-UK) Consortium|Davies|United Kingdom
>Spike|hCoV-19/England/MILK-9A93CB/2020|2020-09-01|EPI_ISL_551078|Original|hCoV-19^^England|Human|Lighthouse Lab in Milton Keynes|Wellcome Sanger Institute for the COVID-19 Genomics UK (COG-UK) consortium|Davies|United Kingdom
>Spike|hCoV-19/Germany/HH-hpi-p1329/2020|2020-03-23|EPI_ISL_775793|Original|hCoV-19^^Hamburg|Human|University Medical Center Hamburg Eppendorf|Heinrich Pette Institute|Robitaille|Germany
>Spike|hCoV-19/England/QEUH-BA23A2/2020|2020-11-18|EPI_ISL_719975|Original|hCoV-19^^England|Human|Lighthouse Lab in Glasgow|Wellcome Sanger Institute for the COVID-19 Genomics UK (COG-UK) Consortium|Davies|United Kingdom
>Spike|hCoV-19/England/SHEF-C078F/2020|2020-03-23|EPI_ISL_420265|Original|hCoV-19^^England|Human|Virology Department|Department of Infection|de Silva|United Kingdom
>Spike|hCoV-19/England/ALDP-B8524E/2020|2020-11-15|EPI_ISL_674340|Original|hCoV-19^^England|Human|Lighthouse Lab in Alderley Park|Wellcome Sanger Institute for the COVID-19 Genomics UK (COG-UK) Consortium|Davies|United Kingdom
>Spike|hCoV-19/Germany/NW-HHU-42-3/2020|2020-03-20|EPI_ISL_425128|Original|hCoV-19^^North Rhine-Westphalia|Human|Center of Medical Microbiology|Center of Medical Microbiology|Wienemann|Germany
>Spike|hCoV-19/USA/MA-Yale-437/2020|2020-04-10|EPI_ISL_738259|Original|hCoV-19^^Massachusetts|Human|Connecticut Veterans' Affairs Hospital|Grubaugh Lab - Yale School of Public Health|Grubaugh|USA
>Spike|hCoV-19/Australia/VIC13717/2020|2020-08-17|EPI_ISL_563815|Original|hCoV-19^^Victoria|Human|Microbiological Diagnostic Unit - Public Health Laboratory (MDU-PHL)|MDU-PHL|Schultz|Australia

>Spike|hCoV-19/Wales/PHWC-4A0FD4/2020|2020-12-14|EPI_ISL_743605|Original|hCoV-19^^Wales|Human|Wales Specialist Virology Centre Sequencing lab|COVID-19 Genomics UK (COG-UK) Consortium|Moore|United Kingdom
>Spike|hCoV-19/Northern Ireland/QEUH-962443/2020|2020-08-10|EPI_ISL_531848|Original|hCoV-19^^Northern Ireland|Human|Lighthouse Lab in Glasgow|Wellcome Sanger Institute for the COVID-19 Genomics UK (COG-UK) consortium|Davies|United Kingdom
>Spike|hCoV-19/USA/TX-HMH-MCoV-11667/2020|2020-09-11|EPI_ISL_790081|Original|hCoV-19^^Texas|Human|Houston Methodist Hospital|Houston Methodist Hospital|Long|USA
>Spike|hCoV-19/Spain/CT-IBV-003738/2020|2020-04-07|EPI_ISL_480975|Original|hCoV-19^^Catalunya|Human|ISGlobal|SeqCOVID-SPAIN consortium/IBV(CSIC)|Comas|Spain
>Spike|hCoV-19/USA/CO-QDX-2254/2020|2020-03-16|EPI_ISL_604520|Original|hCoV-19^^Colorado|Human|Quest Diagnostics|Quest Diagnostics|Kagan|USA
>Spike|hCoV-19/France/PAC-MEPHI-2411/2020|2020-10-13|EPI_ISL_644560|Original|hCoV-19^^Provence-Alpes-Cote-d\u2019Azur|Human|MEPHI|MEPHI|Levasseur|France
>Spike|hCoV-19/Luxembourg/LNS0849119/2020|2020-10-20|EPI_ISL_739841|Original|hCoV-19^^Luxembourg|Human|Laboratoire national de sant\u00e9|Laboratoire national de sant\u00e9|Wienecke-Baldacchino|Luxembourg
>Spike|hCoV-19/Wales/PHWC-493AC4/2020|2020-11-20|EPI_ISL_725983|Original|hCoV-19^^Wales|Human|Originating lab|COVID-19 Genomics UK (COG-UK) Consortium|Moore|United Kingdom
>Spike|hCoV-19/Switzerland/VD-ETHZ-300252/2020|2020-09-30|EPI_ISL_603284|Original|hCoV-19^^Vaud|Human|Viollier AG|Department of Biosystems Science and Engineering|Nadeau|Switzerland
>Spike|hCoV-19/USA/FL-BPHL-0609/2020|2020-04-03|EPI_ISL_508813|Original|hCoV-19^^Florida|Human|Florida Bureau of Public Health Laboratories|Florida Bureau of Public Health Laboratories|Blanton|USA
>Spike|hCoV-19/England/CAMC-C47854/2020|2020-12-10|EPI_ISL_718668|Original|hCoV-19^^England|Human|Lighthouse Lab in Cambridge|Wellcome Sanger Institute for the COVID-19 Genomics UK (COG-UK) Consortium|Davies|United Kingdom
>Spike|hCoV-19/USA/WI-UW-763/2020|2020-07-04|EPI_ISL_495468|Original|hCoV-19^^Wisconsin|Human|University of Wisconsin-Madison AIDS Vaccine Research Laboratories|University of Wisconsin-Madison AIDS Vaccine Research Laboratories|Baczenas|USA
>Spike|hCoV-19/England/MILK-B38909/2020|2020-11-01|EPI_ISL_658742|Original|hCoV-19^^England|Human|Lighthouse Lab in Milton Keynes|Wellcome Sanger Institute for the COVID-19 Genomics UK (COG-UK) Consortium|Davies|United Kingdom
>Spike|hCoV-19/Wales/PHWC-1657B8/2020|2020-05-15|EPI_ISL_490725|Original|hCoV-19^^Wales|Human|Wales Specialist Virology Centre Sequencing lab|COVID-19 Genomics UK (COG-UK) Consortium|Moore|United Kingdom
>Spike|hCoV-19/Scotland/CVR34/2020|2020-03-12|EPI_ISL_425763|Original|hCoV-19^^Scotland|Human|West of Scotland Specialist Virology Centre|COVID-19 Genomics UK (COG-UK) Consortium|Robertson|United Kingdom
>Spike|hCoV-19/Wales/PHWC-48CBBC/2020|2020-11-02|EPI_ISL_666516|Original|hCoV-19^^Wales|Human|Wales Specialist Virology Centre Sequencing lab|COVID-19 Genomics UK (COG-UK) Consortium|Moore|United Kingdom
>Spike|hCoV-19/England/ALDP-B23CF9/2020|2020-11-04|EPI_ISL_646269|Original|hCoV-19^^England|Human|Lighthouse Lab in Alderley Park|Wellcome Sanger Institute for the COVID-19 Genomics UK (COG-UK) Consortium|Davies|United Kingdom
>Spike|hCoV-19/USA/TX-HMH-MCoV-14690/2020|2020-10-12|EPI_ISL_787975|Original|hCoV-19^^Texas|Human|Houston Methodist Hospital|Houston Methodist Hospital|Long|USA
>Spike|hCoV-19/England/QEUH-B0DB61/2020|2020-11-02|EPI_ISL_641922|Original|hCoV-19^^England|Human|Lighthouse Lab in Glasgow|Wellcome Sanger Institute for the COVID-19 Genomics UK (COG-UK) Consortium|Davies|United Kingdom
>Spike|hCoV-19/Colombia/NAR-INS-IP004/2020|2020-12-08|EPI_ISL_791093|Original|hCoV-19^^Narino|Human|Instituto Nacional de Salud - Unidad de Secuenciaci\u00f3n y An\u00e1lisis Gen\u00f3mico|Instituto Nacional de Salud - Direcci\u00f3n de Investigaci\u00f3n en Salud P\u00fablica|D\u00edaz|Colombia

>Spike|hCoV-19/Ireland/MH-NVRL-MPB44944/2020|2020-04-16|EPI_ISL_767720|Original|hCoV-19^^Meath|Human|National Virus Reference Laboratory|Irish Coronavirus Sequencing Consortium - Teagasc Moorepark|Walsh|Ireland
>Spike|hCoV-19/France/ARA-31289/2020|2020-04-27|EPI_ISL_678538|Original|hCoV-19^^Auvergne-Rh\u00f4ne-Alpes|Human|CNR Virus des Infections Respiratoires - France SUD|CNR Virus des Infections Respiratoires - France SUD|Bal|France
>Spike|hCoV-19/Portugal/PT0191/2020|2020-03-29|EPI_ISL_453907|Original|hCoV-19^^Portugal|Human|unknown|Instituto Nacional de Saude (INSA)|Borges|Portugal
>Spike|hCoV-19/Wales/PHWC-484F9A/2020|2020-10-24|EPI_ISL_628131|Original|hCoV-19^^Wales|Human|Wales Specialist Virology Centre Sequencing lab|COVID-19 Genomics UK (COG-UK) Consortium|Moore|United Kingdom
>Spike|hCoV-19/Panama/330490/2020|2020-03-19|EPI_ISL_496665|Original|hCoV-19^^Panama Center|Human|Gorgas Memorial Laboratory of Health Studies|Gorgas Memorial Laboratory of Health Studies|Martinez|Panama
>Spike|hCoV-19/Belgium/rega-0802763/2020|2020-08-02|EPI_ISL_734873|Original|hCoV-19^^Deurne|Human|UZ Leuven|KU Leuven|Maes|Belgium
>Spike|hCoV-19/England/CAMB-1AE2D0/2020|2020-04-27|EPI_ISL_459426|Original|hCoV-19^^England|Human|Department of Pathology|Wellcome Sanger Institute for the COVID-19 Genomics UK (COG-UK) consortium|Davies|United Kingdom
>Spike|hCoV-19/England/ALDP-746BAC/2020|2020-06-29|EPI_ISL_557493|Original|hCoV-19^^England|Human|Lighthouse Lab in Alderley Park|Wellcome Sanger Institute for the COVID-19 Genomics UK (COG-UK) consortium|Davies|United Kingdom
>Spike|hCoV-19/England/ALDP-B1CE0C/2020|2020-11-01|EPI_ISL_645561|Original|hCoV-19^^England|Human|Lighthouse Lab in Alderley Park|Wellcome Sanger Institute for the COVID-19 Genomics UK (COG-UK) Consortium|Davies|United Kingdom
>Spike|hCoV-19/USA/MN-MDH-289/2020|2020-03-31|EPI_ISL_450758|Original|hCoV-19^^Minnesota|Human|Minnesota Department of Health|Minnesota Department of Health|Garfin|USA
>Spike|hCoV-19/Netherlands/LI-RIVM-20954/2020|2020-12-22|EPI_ISL_790978|Original|hCoV-19^^Limburg|Human|Dutch COVID-19 response team|National Institute for Public Health and the Environment (RIVM)|Meijer|Netherlands
>Spike|hCoV-19/Gibraltar/205000688/2020|2020-12-00|EPI_ISL_710012|Original|hCoV-19^^Gibraltar|Human|Microbiology|Respiratory Virus Unit|Platt|Gibraltar
>Spike|hCoV-19/South Korea/KDCA0061/2020|2020-09-23|EPI_ISL_747302|Original|hCoV-19^^South Korea|Human|Division of Emerging Infectious Diseases|Division of Emerging Infectious Diseases|Park|South Korea
>Spike|hCoV-19/England/CAMC-B98907/2020|2020-11-15|EPI_ISL_673818|Original|hCoV-19^^England|Human|Lighthouse Lab in Cambridge|Wellcome Sanger Institute for the COVID-19 Genomics UK (COG-UK) Consortium|Davies|United Kingdom
>Spike|hCoV-19/India/MH-NCCS-NR2527/2020|2020-05-24|EPI_ISL_496585|Original|hCoV-19^^Maharashtra|Human|National Centre For Cell Science|National Centre For Cell Science|Paul|India
>Spike|hCoV-19/India/WB-S100/2020|2020-06-09|EPI_ISL_508441|Original|hCoV-19^^West Bengal|Human|ICMR-National Institute of Cholera and Enteric Diseases|National Institute of Biomedical Genomics|Maitra|India
>Spike|hCoV-19/Japan/PG-4282/2020|2020-07-00|EPI_ISL_692511|Original|hCoV-19^^Japan|Human|Pathogen Genomics Center|Pathogen Genomics Center|Sekizuka|Japan
>Spike|hCoV-19/USA/CA-ALSR-1269/2020|2020-05-04|EPI_ISL_494469|Original|hCoV-19^^California|Human|San Diego County Public Health Laboratory|Andersen lab at Scripps Research|Gangavarapu|USA
>Spike|hCoV-19/USA/TX-HMH-MCoV-13296/2020|2020-08-24|EPI_ISL_788444|Original|hCoV-19^^Texas|Human|Houston Methodist Hospital|Houston Methodist Hospital|Long|USA
>Spike|hCoV-19/England/CAMB-1AB90B/2020|2020-04-21|EPI_ISL_470174|Original|hCoV-19^^England|Human|Department of Pathology|Wellcome Sanger Institute for the COVID-19 Genomics UK (COG-UK) consortium|Davies|United Kingdom
>Spike|hCoV-19/England/QEUH-B2E827/2020|2020-11-02|EPI_ISL_660000|Original|hCoV-19^^England|Human|Lighthouse Lab in Glasgow|Wellcome Sanger Institute for the COVID-19 Genomics UK (COG-UK) Consortium|Davies|United Kingdom

>Spike|hCoV-19/Germany/HH-hpi-p2385/2020|2020-06-10|EPI_ISL_776463|Original|hCoV-19^^Hamburg|Human|University Medical Center Hamburg Eppendorf|Heinrich Pette Institute|Robitaille|Germany
>Spike|hCoV-19/India/OR-ILSCV32814/2020|2020-06-04|EPI_ISL_481163|Original|hCoV-19^^Odisha|Human|Immunogenomics lab|Immunogenomics lab|Raghav|India
>Spike|hCoV-19/USA/AZ-TG446877/2020|2020-06-26|EPI_ISL_695006|Original|hCoV-19^^Arizona|Human|TGen North|TGen North|French|USA
>Spike|hCoV-19/England/QEUH-B128CC/2020|2020-11-02|EPI_ISL_642396|Original|hCoV-19^^England|Human|Lighthouse Lab in Glasgow|Wellcome Sanger Institute for the COVID-19 Genomics UK (COG-UK) Consortium|Davies|United Kingdom
>Spike|hCoV-19/Australia/VIC14128/2020|2020-09-16|EPI_ISL_592348|Original|hCoV-19^^Victoria|Human|Microbiological Diagnostic Unit - Public Health Laboratory (MDU-PHL)|MDU-PHL|Schultz|Australia
>Spike|hCoV-19/USA/TX-HMH-MCoV-9168/2020|2020-07-02|EPI_ISL_780535|Original|hCoV-19^^Texas|Human|Houston Methodist Hospital|Houston Methodist Hospital|Long|USA
>Spike|hCoV-19/Australia/VIC1375/2020|2020-04-14|EPI_ISL_456416|Original|hCoV-19^^Victoria|Human|Victorian Infectious Diseases Reference Laboratory (VIDRL)|Microbiological Diagnostic Unit Public Health Laboratory and Victorian Infectious Diseases Reference Laboratory|Schultz|Australia
>Spike|hCoV-19/England/ALDP-71CF0D/2020|2020-07-01|EPI_ISL_557620|Original|hCoV-19^^England|Human|Lighthouse Lab in Alderley Park|Wellcome Sanger Institute for the COVID-19 Genomics UK (COG-UK) consortium|Davies|United Kingdom
>Spike|hCoV-19/USA/TX-HMH-5152/2020|2020-06-29|EPI_ISL_544561|Original|hCoV-19^^Texas|Human|Houston Methodist Hospital|Houston Methodist Hospital|Long|USA
>Spike|hCoV-19/England/QEUH-95FBDB/2020|2020-08-05|EPI_ISL_531908|Original|hCoV-19^^England|Human|Lighthouse Lab in Glasgow|Wellcome Sanger Institute for the COVID-19 Genomics UK (COG-UK) consortium|Davies|United Kingdom
>Spike|hCoV-19/USA/WA-UW-3829/2020|2020-03-25|EPI_ISL_427184|Original|hCoV-19^^Washington|Human|UW Virology Lab|UW Virology Lab|Roychoudhury|USA
>Spike|hCoV-19/Australia/VIC17850/2020|2020-08-31|EPI_ISL_779500|Original|hCoV-19^^Victoria|Human|Microbiological Diagnostic Unit - Public Health Laboratory (MDU-PHL)|MDU-PHL|Beck|Australia
>Spike|hCoV-19/England/ALDP-B844D0/2020|2020-11-14|EPI_ISL_675459|Original|hCoV-19^^England|Human|Lighthouse Lab in Alderley Park|Wellcome Sanger Institute for the COVID-19 Genomics UK (COG-UK) Consortium|Davies|United Kingdom
>Spike|hCoV-19/Denmark/DCGC-19429/2020|2020-12-07|EPI_ISL_748938|Original|hCoV-19^^Hovedstaden|Human|Department of Virus and Microbiological Special Diagnostics|Albertsen Lab|Michaelsen|Denmark
>Spike|hCoV-19/USA/TX-DSHS-2035/2020|2020-08-09|EPI_ISL_766672|Original|hCoV-19^^Texas|Human|Texas Department of State Health Services|Texas Department of State Health Services|Rahman|USA
>Spike|hCoV-19/England/QEUH-9DA613/2020|2020-09-22|EPI_ISL_601286|Original|hCoV-19^^England|Human|Lighthouse Lab in Glasgow|Wellcome Sanger Institute for the COVID-19 Genomics UK (COG-UK) consortium|Davies|United Kingdom
>Spike|hCoV-19/Denmark/DCGC-19490/2020|2020-12-07|EPI_ISL_748985|Original|hCoV-19^^Hovedstaden|Human|Department of Virus and Microbiological Special Diagnostics|Albertsen Lab|Michaelsen|Denmark
>Spike|hCoV-19/Australia/VIC4822/2020|2020-07-17|EPI_ISL_519207|Original|hCoV-19^^Victoria|Human|Microbiological Diagnostic Unit - Public Health Laboratory (MDU-PHL)|MDU-PHL|Schultz|Australia
>Spike|hCoV-19/USA/FL-BPHL-2113/2020|2020-03-12|EPI_ISL_653266|Original|hCoV-19^^Florida|Human|Florida Bureau of Public Health Laboratories|Florida Bureau of Public Health Laboratories|Blanton|USA
>Spike|hCoV-19/USA/NY-Wadsworth-99944-01/2020|2020-05-14|EPI_ISL_676654|Original|hCoV-19^^New York|Human|Wadsworth Center|Wadsworth Center|Lasek-Nesselquist|USA
>Spike|hCoV-19/Luxembourg/LNS7385935/2020|2020-10-31|EPI_ISL_744559|Original|hCoV-19^^Luxembourg|Human|Laboratoire national de sant\u00e9|Laboratoire national de sant\u00e9|Wienecke-Baldacchino|Luxembourg

>Spike|hCoV-19/Denmark/DCGC-17905/2020|2020-12-14|EPI_ISL_749636|Original|hCoV-19^^Hovedstaden|Human|Department of Virus and Microbiological Special Diagnostics|Albertsen Lab|Michaelsen|Denmark
>Spike|hCoV-19/Wales/PHWC-487410/2020|2020-10-27|EPI_ISL_639192|Original|hCoV-19^^Wales|Human|Wales Specialist Virology Centre Sequencing lab|COVID-19 Genomics UK (COG-UK) Consortium|Moore|United Kingdom
>Spike|hCoV-19/Wales/PHWC-163626/2020|2020-05-07|EPI_ISL_473973|Original|hCoV-19^^Wales|Human|Originating lab|COVID-19 Genomics UK (COG-UK) Consortium|Moore|United Kingdom
>Spike|hCoV-19/Wales/PHWC-4837B1/2020|2020-10-22|EPI_ISL_627856|Original|hCoV-19^^Wales|Human|Wales Specialist Virology Centre Sequencing lab|COVID-19 Genomics UK (COG-UK) Consortium|Moore|United Kingdom
>Spike|hCoV-19/England/ALDP-AA178A/2020|2020-10-22|EPI_ISL_625050|Original|hCoV-19^^England|Human|Lighthouse Lab in Alderley Park|Wellcome Sanger Institute for the COVID-19 Genomics UK (COG-UK) consortium|Davies|United Kingdom
>Spike|hCoV-19/USA/MI-QDX-2172/2020|2020-03-17|EPI_ISL_604473|Original|hCoV-19^^Michigan|Human|Quest Diagnostics|Quest Diagnostics|Kagan|USA
>Spike|hCoV-19/Gibraltar/205000644/2020|2020-12-00|EPI_ISL_709979|Original|hCoV-19^^Gibraltar|Human|Microbiology|Respiratory Virus Unit|Platt|Gibraltar
>Spike|hCoV-19/USA/DE-DHSS-F918126/2020|2020-03-28|EPI_ISL_593943|Original|hCoV-19^^Delaware|Human|Delaware Public Health Lab|Delaware Public Health Lab|Bajwa|USA
>Spike|hCoV-19/England/MILK-B06FA8/2020|2020-10-29|EPI_ISL_650633|Original|hCoV-19^^England|Human|Department of Pathology|COVID-19 Genomics UK (COG-UK) Consortium|Goodfellow|United Kingdom
>Spike|hCoV-19/USA/NY-NYUMC639/2020|2020-04-02|EPI_ISL_444756|Original|hCoV-19^^New York|Human|NYU Langone Health|Departments of Pathology and Medicine|Maurano|USA
>Spike|hCoV-19/England/20172061704/2020|2020-04-20|EPI_ISL_465375|Original|hCoV-19^^England|Human|Respiratory Virus Unit|Respiratory Virus Unit|Platt|United Kingdom
>Spike|hCoV-19/England/LIVE-9EEC1/2020|2020-04-09|EPI_ISL_453406|Original|hCoV-19^^England|Human|Liverpool Clinical Laboratories|COVID-19 Genomics UK (COG-UK) Consortium|Haldenby|United Kingdom
>Spike|hCoV-19/Japan/IC-0099/2020|2020-07-00|EPI_ISL_591440|Original|hCoV-19^^Japan|Human|Pathogen Genomics Center|Pathogen Genomics Center|Sekizuka|Japan
>Spike|hCoV-19/USA/WI-UW-66/2020|2020-03-24|EPI_ISL_421324|Original|hCoV-19^^Wisconsin|Human|University of Wisconsin-Madison AIDS Vaccine Research Laboratories|University of Wisconsin-Madison AIDS Vaccine Research Laboratories|Baczenas|USA
>Spike|hCoV-19/USA/TX-HMH-MCoV-10522/2020|2020-07-08|EPI_ISL_789516|Original|hCoV-19^^Texas|Human|Houston Methodist Hospital|Houston Methodist Hospital|Long|USA
>Spike|hCoV-19/Denmark/DCGC-3279/2020|2020-08-31|EPI_ISL_616370|Original|hCoV-19^^Hovedstaden|Human|Department of Virus and Microbiological Special Diagnostics|Albertsen lab|Michaelsen|Denmark
>Spike|hCoV-19/North Macedonia/5312/2020|2020-06-28|EPI_ISL_677713|Original|hCoV-19^^Prilep|Human|General Hospital - Prilep|Research Center for Genetic Engineering and Biotechnology \Georgi D. Efremov\u201d|Dimovski|North Macedonia
>Spike|hCoV-19/Switzerland/VS-ETHZ-411035/2020|2020-12-04|EPI_ISL_737639|Original|hCoV-19^^Valais|Human|Viollier AG|Department of Biosystems Science and Engineering|Nadeau|Switzerland
>Spike|hCoV-19/England/QEUH-B68A85/2020|2020-11-11|EPI_ISL_655180|Original|hCoV-19^^England|Human|Lighthouse Lab in Glasgow|Wellcome Sanger Institute for the COVID-19 Genomics UK (COG-UK) Consortium|Davies|United Kingdom
>Spike|hCoV-19/England/NOTT-1115C0/2020|2020-05-18|EPI_ISL_461895|Original|hCoV-19^^England|Human|Queens Medical Centre|COVID-19 Genomics UK (COG-UK) Consortium|Loose|United Kingdom
>Spike|hCoV-19/Hong Kong/VM20040270/2020|2020-07-13|EPI_ISL_523853|Original|hCoV-19^^Hong Kong|Human|Hong Kong Baptist Hospital|Hong Kong Department of Health|Mak|China
>Spike|hCoV-19/England/LIVE-9DD01/2020|2020-04-30|EPI_ISL_449622|Original|hCoV-19^^England|Human|Liverpool Clinical Laboratories|COVID-19 Genomics UK (COG-UK) Consortium|Haldenby|United Kingdom

>Spike|hCoV-19/USA/UT-08560/2020|2020-05-18|EPI_ISL_524318|Original|hCoV-19^^Utah|Human|Utah Public Health Laboratory|Utah Public Health Laboratory|Young|USA
>Spike|hCoV-19/USA/MO-WUSTL-C1/2020|2020-08-14|EPI_ISL_693658|Original|hCoV-19^^Missouri|Human|Washington University in St. Louis|Washington University in St. Louis|Tahan|USA
>Spike|hCoV-19/USA/WI-GMF-15072/2020|2020-08-12|EPI_ISL_547697|Original|hCoV-19^^Wisconsin|Human|Gundersen Molecular Diagnostics Laboratory|Kabara Cancer Research Institute|Kenny|USA
>Spike|hCoV-19/England/CAMC-B32F2B/2020|2020-11-05|EPI_ISL_659253|Original|hCoV-19^^England|Human|Lighthouse Lab in Cambridge|Wellcome Sanger Institute for the COVID-19 Genomics UK (COG-UK) Consortium|Davies|United Kingdom
>Spike|hCoV-19/Germany/NW-HHU-489/2020|2020-12-03|EPI_ISL_770738|Original|hCoV-19^^North Rhine-Westphalia|Human|ZOTZ KLIMAS MVZ D\u00fcsseldorf-Centrum GbR \u00dcBAG f\u00fcr Labormedizin|Center of Medical Microbiology|Wienemann|Germany
>Spike|hCoV-19/England/QEUH-C30D5F/2020|2020-12-11|EPI_ISL_730778|Original|hCoV-19^^England|Human|Lighthouse Lab in Glasgow|Wellcome Sanger Institute for the COVID-19 Genomics UK (COG-UK) Consortium|Davies|United Kingdom
>Spike|hCoV-19/Wales/PHWC-49FA13/2020|2020-12-09|EPI_ISL_743306|Original|hCoV-19^^Wales|Human|Wales Specialist Virology Centre Sequencing lab|COVID-19 Genomics UK (COG-UK) Consortium|Moore|United Kingdom
>Spike|hCoV-19/North Macedonia/ChVir7914/2020|2020-06-25|EPI_ISL_492084|Original|hCoV-19^^North Macedonia|Human|Institute for Public Health of the Republic of North Macedonia|Charite Universitatsmedizin Berlin|Muehlemann|North Macedonia
>Spike|hCoV-19/Australia/VIC16558/2020|2020-08-08|EPI_ISL_640936|Original|hCoV-19^^Victoria|Human|Microbiological Diagnostic Unit - Public Health Laboratory (MDU-PHL)|MDU-PHL|Schultz|Australia
>Spike|hCoV-19/Northern Ireland/NIRE-1087A8/2020|2020-09-03|EPI_ISL_585203|Original|hCoV-19^^Northern Ireland|Human|Regional Virus Laboratory|COVID-19 Genomics UK (COG-UK) Consortium|Fairley|United Kingdom
>Spike|hCoV-19/England/LOND-DA3BB/2020|2020-11-04|EPI_ISL_724625|Original|hCoV-19^^England|Human|University College London|COVID-19 Genomics UK (COG-UK) Consortium|Roy|United Kingdom
>Spike|hCoV-19/France/ARA-107560/2020|2020-11-16|EPI_ISL_728313|Original|hCoV-19^^Auvergne-Rh\u00f4ne-Alpes|Human|CNR Virus des Infections Respiratoires - France SUD|CNR Virus des Infections Respiratoires - France SUD|Bal|France
>Spike|hCoV-19/Denmark/DCGC-5998/2020|2020-09-28|EPI_ISL_621107|Original|hCoV-19^^Midtjylland|Human|Department of Virus and Microbiological Special Diagnostics|Albertsen lab|Michaelsen|Denmark
>Spike|hCoV-19/USA/AZ-TG359374/2020|2020-06-08|EPI_ISL_694444|Original|hCoV-19^^Arizona|Human|TGen North|TGen North|French|USA
>Spike|hCoV-19/Denmark/DCGC-13622/2020|2020-11-30|EPI_ISL_712453|Original|hCoV-19^^Hovedstaden|Human|Department of Virus and Microbiological Special Diagnostics|Albertsen Lab|Michaelsen|Denmark
>Spike|hCoV-19/England/BRIS-133030/2020|2020-06-24|EPI_ISL_511969|Original|hCoV-19^^England|Human|PHE South West Regional Laboratory|Wellcome Sanger Institute for the COVID-19 Genomics UK (COG-UK) consortium|Davies|United Kingdom
>Spike|hCoV-19/USA/CA-CZB-1967/2020|2020-05-06|EPI_ISL_486239|Original|hCoV-19^^California|Human|Orange County Public Health Laboratory|Chan-Zuckerberg Biohub|McGeever|USA
>Spike|hCoV-19/Australia/VIC7562/2020|2020-00-00|EPI_ISL_564500|Original|hCoV-19^^Victoria|Human|Microbiological Diagnostic Unit - Public Health Laboratory (MDU-PHL)|MDU-PHL|Schultz|Australia
>Spike|hCoV-19/USA/UN-UW-2859/2020|2020-04-15|EPI_ISL_485963|Original|hCoV-19^^USA|Human|UW Virology Lab|UW Virology Lab|Roychoudhury|USA
>Spike|hCoV-19/England/QEUH-B0F956/2020|2020-11-02|EPI_ISL_641937|Original|hCoV-19^^England|Human|Lighthouse Lab in Glasgow|Wellcome Sanger Institute for the COVID-19 Genomics UK (COG-UK) Consortium|Davies|United Kingdom
>Spike|hCoV-19/USA/NY-QDX-1233/2020|2020-03-17|EPI_ISL_572009|Original|hCoV-19^^New York|Human|Quest Diagnostics|Quest Diagnostics|Kagan|USA

>Spike|hCoV-19/England/QEUH-9DCDC1/2020|2020-09-21|EPI_ISL_599524|Original|hCoV-19^^England|Human|Lighthouse Lab in Glasgow|Wellcome Sanger Institute for the COVID-19 Genomics UK (COG-UK) consortium|Davies|United Kingdom
>Spike|hCoV-19/Canada/QC-LSPQ-L00234540/2020|2020-03-23|EPI_ISL_536058|Original|hCoV-19^^Quebec|Human|H\u00f4pital de Saint-Eustache|Laboratoire de sant\u00e9 publique du Qu\u00e9bec|Moreira|Canada
>Spike|hCoV-19/England/QEUH-B2BAAC/2020|2020-11-01|EPI_ISL_646779|Original|hCoV-19^^England|Human|Lighthouse Lab in Glasgow|Wellcome Sanger Institute for the COVID-19 Genomics UK (COG-UK) Consortium|Davies|United Kingdom
>Spike|hCoV-19/England/SHEF-C1A85/2020|2020-05-05|EPI_ISL_490583|Original|hCoV-19^^England|Human|Virology Department|COVID-19 Genomics UK (COG-UK) Consortium|de Silva|United Kingdom
>Spike|hCoV-19/England/20150040104/2020|2020-03-31|EPI_ISL_466230|Original|hCoV-19^^England|Human|Respiratory Virus Unit|Respiratory Virus Unit|Platt|United Kingdom
>Spike|hCoV-19/USA/CA-CZB-5773/2020|2020-07-12|EPI_ISL_672355|Original|hCoV-19^^California|Human|County of San Luis Obispo Public Health Laboratory|Chan-Zuckerberg Biohub|Bhatt|USA
>Spike|hCoV-19/England/QEUH-9DDF9D/2020|2020-09-21|EPI_ISL_599797|Original|hCoV-19^^England|Human|Lighthouse Lab in Glasgow|Wellcome Sanger Institute for the COVID-19 Genomics UK (COG-UK) consortium|Davies|United Kingdom
>Spike|hCoV-19/USA/WI-WSLH-200147/2020|2020-03-13|EPI_ISL_578374|Original|hCoV-19^^Wisconsin|Human|Wisconsin State Laboratory of Hygiene Communicable Disease Division|Wisconsin State Laboratory of Hygiene Communicable Disease Division|Florek|USA
>Spike|hCoV-19/Switzerland/ZH-ETHZ-230058/2020|2020-08-05|EPI_ISL_516594|Original|hCoV-19^^Zurich|Human|Viollier AG|Department of Biosystems Science and Engineering|Nadeau|Switzerland
>Spike|hCoV-19/USA/WI-GMF-18731/2020|2020-09-04|EPI_ISL_547779|Original|hCoV-19^^Wisconsin|Human|Gundersen Molecular Diagnostics Laboratory|Kabara Cancer Research Institute|Kenny|USA
>Spike|hCoV-19/USA/MN-MDH-1804/2020|2020-04-14|EPI_ISL_590806|Original|hCoV-19^^Minnesota|Human|Minnesota Department of Health|Minnesota Department of Health|Lorentz|USA
>Spike|hCoV-19/England/ALDP-A92801/2020|2020-10-19|EPI_ISL_610969|Original|hCoV-19^^England|Human|Lighthouse Lab in Alderley Park|Wellcome Sanger Institute for the COVID-19 Genomics UK (COG-UK) consortium|Davies|United Kingdom
>Spike|hCoV-19/USA/FL-BPHL-2120/2020|2020-03-12|EPI_ISL_653273|Original|hCoV-19^^Florida|Human|Florida Bureau of Public Health Laboratories|Florida Bureau of Public Health Laboratories|Blanton|USA
>Spike|hCoV-19/England/ALDP-94FF2D/2020|2020-06-15|EPI_ISL_555261|Original|hCoV-19^^England|Human|Lighthouse Lab in Alderley Park|Wellcome Sanger Institute for the COVID-19 Genomics UK (COG-UK) consortium|Davies|United Kingdom
>Spike|hCoV-19/England/CAMC-B21842/2020|2020-11-03|EPI_ISL_647393|Original|hCoV-19^^England|Human|Lighthouse Lab in Cambridge|Wellcome Sanger Institute for the COVID-19 Genomics UK (COG-UK) Consortium|Davies|United Kingdom
>Spike|hCoV-19/Brazil/RJ-INCA-C31/2020|2020-04-16|EPI_ISL_513539|Original|hCoV-19^^Rio de Janeiro|Human|Programa de Oncovirologia|Programa de Oncovirologia|Ramos Goes|Brazil
>Spike|hCoV-19/England/MILK-974AA6/2020|2020-08-21|EPI_ISL_552625|Original|hCoV-19^^England|Human|Lighthouse Lab in Milton Keynes|Wellcome Sanger Institute for the COVID-19 Genomics UK (COG-UK) consortium|Davies|United Kingdom
>Spike|hCoV-19/England/ALDP-9BE976/2020|2020-09-09|EPI_ISL_568165|Original|hCoV-19^^England|Human|Lighthouse Lab in Alderley Park|Wellcome Sanger Institute for the COVID-19 Genomics UK (COG-UK) consortium|Davies|United Kingdom
>Spike|hCoV-19/England/ALDP-B5D379/2020|2020-11-09|EPI_ISL_678891|Original|hCoV-19^^England|Human|Department of Pathology|COVID-19 Genomics UK (COG-UK) Consortium|Goodfellow|United Kingdom
>Spike|hCoV-19/Japan/PG-3702/2020|2020-04-00|EPI_ISL_691955|Original|hCoV-19^^Japan|Human|Pathogen Genomics Center|Pathogen Genomics Center|Sekizuka|Japan

>Spike|hCoV-19/Denmark/DCGC-21465/2020|2020-12-21|EPI_ISL_757902|Original|hCoV-
19^^Syddanmark|Human|Department of Virus and Microbiological Special
Diagnostics|Albertsen Lab|Michaelsen|Denmark
>Spike|hCoV-19/England/204380844/2020|2020-10-22|EPI_ISL_649846|Original|hCoV-
19^^England|Human|Respiratory Virus Unit|COVID-19 Genomics UK (COG-UK)
Consortium|Platt|United Kingdom
>Spike|hCoV-19/Scotland/CVR3531/2020|2020-05-14|EPI_ISL_473669|Original|hCoV-
19^^Scotland|Human|West of Scotland Specialist Virology Centre|COVID-19 Genomics UK
(COG-UK) Consortium|Robertson|United Kingdom
>Spike|hCoV-19/England/NOTT-10E9F7/2020|2020-03-28|EPI_ISL_425625|Original|hCoV-
19^^England|Human|Queens Medical Centre|COVID-19 Genomics UK (COG-UK)
Consortium|Loose|United Kingdom
>Spike|hCoV-19/England/QEUH-A8BD57/2020|2020-10-16|EPI_ISL_643062|Original|hCoV-
19^^England|Human|Lighthouse Lab in Glasgow|Wellcome Sanger Institute for the COVID-
19 Genomics UK (COG-UK) Consortium|Davies|United Kingdom
>Spike|hCoV-19/England/ALDP-B47011/2020|2020-11-07|EPI_ISL_656263|Original|hCoV-
19^^England|Human|Lighthouse Lab in Alderley Park|Wellcome Sanger Institute for the
COVID-19 Genomics UK (COG-UK) Consortium|Davies|United Kingdom
>Spike|hCoV-19/USA/NM-DOH-2020617083/2020|2020-12-12|EPI_ISL_751842|Original|hCoV-
19^^New Mexico|Human|New Mexico Department of Health Scientific Laboratory|New Mexico
Department of Health Scientific Laboratory|Malone|USA
>Spike|hCoV-19/England/MILK-A6BF2A/2020|2020-10-09|EPI_ISL_609151|Original|hCoV-
19^^England|Human|Lighthouse Lab in Milton Keynes|Wellcome Sanger Institute for the
COVID-19 Genomics UK (COG-UK) consortium|Davies|United Kingdom
>Spike|hCoV-19/England/QEUH-9F5E1A/2020|2020-09-25|EPI_ISL_588362|Original|hCoV-
19^^England|Human|Lighthouse Lab in Glasgow|Wellcome Sanger Institute for the COVID-
19 Genomics UK (COG-UK) consortium|Davies|United Kingdom
>Spike|hCoV-19/USA/TX-HMH-MCoV-14176/2020|2020-10-01|EPI_ISL_787461|Original|hCoV-
19^^Texas|Human|Houston Methodist Hospital|Houston Methodist Hospital|Long|USA
>Spike|hCoV-19/Wales/PHWC-484AD5/2020|2020-10-24|EPI_ISL_628077|Original|hCoV-
19^^Wales|Human|Wales Specialist Virology Centre Sequencing lab|COVID-19 Genomics UK
(COG-UK) Consortium|Moore|United Kingdom
>Spike|hCoV-19/England/ALDP-9D9289/2020|2020-09-18|EPI_ISL_576713|Original|hCoV-
19^^England|Human|Oxford Viromics|COVID-19 Genomics UK (COG-UK)
Consortium|Golubchik|United Kingdom
>Spike|hCoV-19/USA/UT-UPHL-2009604/2020|2020-07-18|EPI_ISL_605282|Original|hCoV-
19^^Utah|Human|Utah Public Health Laboratory|Utah Public Health Laboratory|Young|USA
>Spike|hCoV-19/England/ALDP-A2EF6F/2020|2020-10-05|EPI_ISL_587201|Original|hCoV-
19^^England|Human|Lighthouse Lab in Alderley Park|Wellcome Sanger Institute for the
COVID-19 Genomics UK (COG-UK) consortium|Davies|United Kingdom
>Spike|hCoV-19/Scotland/CVR5249/2020|2020-10-14|EPI_ISL_627411|Original|hCoV-
19^^Scotland|Human|West of Scotland Specialist Virology Centre|COVID-19 Genomics UK
(COG-UK) Consortium|Robertson|United Kingdom
>Spike|hCoV-19/South Korea/KCDC2118/2020|2020-03-15|EPI_ISL_498019|Original|hCoV-
19^^South Korea|Human|Division of Viral Diseases|Division of Viral Diseases|Kim|South
Korea
>Spike|hCoV-19/England/CAMC-B35368/2020|2020-11-05|EPI_ISL_659605|Original|hCoV-
19^^England|Human|Lighthouse Lab in Cambridge|Wellcome Sanger Institute for the
COVID-19 Genomics UK (COG-UK) Consortium|Davies|United Kingdom
>Spike|hCoV-19/USA/UT-02856/2020|2020-04-29|EPI_ISL_513664|Original|hCoV-
19^^Utah|Human|Utah Public Health Laboratory|Utah Public Health Laboratory|Butz|USA
>Spike|hCoV-19/England/CAMC-9473DE/2020|2020-07-21|EPI_ISL_556569|Original|hCoV-
19^^England|Human|Lighthouse Lab in Cambridge|Wellcome Sanger Institute for the
COVID-19 Genomics UK (COG-UK) consortium|Davies|United Kingdom
>Spike|hCoV-19/New Zealand/20VR3064/2020|2020-04-28|EPI_ISL_456403|Original|hCoV-
19^^Counties Manukau|Human|Middlemore Hospital|Institute of Environmental Science and
Research (ESR)|de Ligt|New Zealand
>Spike|hCoV-19/Netherlands/ZH-EMC-815/2020|2020-10-15|EPI_ISL_632692|Original|hCoV-
19^^Zuid Holland|Human|Dutch COVID-19 response team|Erasmus Medical Center|Oude
Munnink|Netherlands

>Spike|hCoV-19/England/QEUH-C31028/2020|2020-12-10|EPI_ISL_731548|Original|hCoV-19^^England|Human|Lighthouse Lab in Glasgow|Wellcome Sanger Institute for the COVID-19 Genomics UK (COG-UK) Consortium|Davies|United Kingdom
>Spike|hCoV-19/Netherlands/NB-EMC-419/2020|2020-11-10|EPI_ISL_722609|Original|hCoV-19^^Noord Brabant|Human|Dutch COVID-19 response team|Erasmus Medical Center|Oude Munnink|Netherlands
>Spike|hCoV-19/England/ALDP-94C7A0/2020|2020-06-15|EPI_ISL_556053|Original|hCoV-19^^England|Human|Lighthouse Lab in Alderley Park|Wellcome Sanger Institute for the COVID-19 Genomics UK (COG-UK) consortium|Davies|United Kingdom
>Spike|hCoV-19/England/NOTT-10E450/2020|2020-03-22|EPI_ISL_425547|Original|hCoV-19^^England|Human|Queens Medical Centre|COVID-19 Genomics UK (COG-UK) Consortium|Loose|United Kingdom
>Spike|hCoV-19/England/CAMB-773DE/2020|2020-03-31|EPI_ISL_439485|Original|hCoV-19^^England|Human|Department of Pathology|Wellcome Sanger Institute for the COVID-19 Genomics UK (COG-UK) consortium|Amato|United Kingdom
>Spike|hCoV-19/England/LIVE-9E1B9/2020|2020-04-03|EPI_ISL_453274|Original|hCoV-19^^England|Human|Liverpool Clinical Laboratories|COVID-19 Genomics UK (COG-UK) Consortium|Haldenby|United Kingdom
>Spike|hCoV-19/England/CAMB-7A809/2020|2020-04-02|EPI_ISL_441269|Original|hCoV-19^^England|Human|Department of Pathology|Wellcome Sanger Institute for the COVID-19 Genomics UK (COG-UK) consortium|Amato|United Kingdom
>Spike|hCoV-19/USA/MN-MDH-1720/2020|2020-09-16|EPI_ISL_576229|Original|hCoV-19^^Minnesota|Human|Minnesota Department of Health|Minnesota Department of Health|Garfin|USA
>Spike|hCoV-19/Wales/PHWC-49BB23/2020|2020-12-06|EPI_ISL_742433|Original|hCoV-19^^Wales|Human|Wales Specialist Virology Centre Sequencing lab|COVID-19 Genomics UK (COG-UK) Consortium|Moore|United Kingdom
>Spike|hCoV-19/England/QEUH-9DDE81/2020|2020-09-21|EPI_ISL_599715|Original|hCoV-19^^England|Human|Lighthouse Lab in Glasgow|Wellcome Sanger Institute for the COVID-19 Genomics UK (COG-UK) consortium|Davies|United Kingdom
>Spike|hCoV-19/Austria/CeMM0375/2020|2020-03-28|EPI_ISL_475819|Original|hCoV-19^^Mayrhofen|Human|Institut f\u00fcr Virologie am Department f\u00fcr Hygiene|Bergthaler laboratory|Bergthaler|Austria
>Spike|hCoV-19/Spain/PV-IBV-98008273/2020|2020-10-02|EPI_ISL_654555|Original|hCoV-19^^Basque Country|Human|Servicio de Microbiolog\u00eda. Hospital Universitario Donostia. OSI Donostialdea. \u00c1rea de Enfermedades Infecciosas|SeqCOVID-SPAIN consortium/IBV(CSIC)|Comas|Spain
>Spike|hCoV-19/England/CAMB-7FE20/2020|2020-04-19|EPI_ISL_444421|Original|hCoV-19^^England|Human|Department of Pathology|COVID-19 Genomics UK (COG-UK) Consortium|Meredith|United Kingdom
>Spike|hCoV-19/USA/LA-EVTL1198/2020|2020-12-11|EPI_ISL_768467|Original|hCoV-19^^Louisiana|Human|LSUHS Emerging Viral Threat Laboratory|Microbial Genome Sequencing Center|Kamil|USA
>Spike|hCoV-19/Canada/ON-S104/2020|2020-03-21|EPI_ISL_586282|Original|hCoV-19^^Ontario|Human|Toronto Invasive Bacterial Diseases Network|McMaster University|McArthur|Canada
>Spike|hCoV-19/England/CAMC-BC0C3A/2020|2020-11-22|EPI_ISL_705037|Original|hCoV-19^^England|Human|Lighthouse Lab in Cambridge|Wellcome Sanger Institute for the COVID-19 Genomics UK (COG-UK) Consortium|Davies|United Kingdom
>Spike|hCoV-19/USA/TX-HMH-6786/2020|2020-06-25|EPI_ISL_546242|Original|hCoV-19^^Texas|Human|Houston Methodist Hospital|Houston Methodist Hospital|Long|USA
>Spike|hCoV-19/Ireland/LH-NVRL-20ILR08028/2020|2020-11-09|EPI_ISL_732418|Original|hCoV-19^^Louth|Human|National Virus Reference Laboratory|National Virus Reference Laboratory|Gonzalez|Ireland
>Spike|hCoV-19/USA/MI-MDHHS-SC20342/2020|2020-03-26|EPI_ISL_450621|Original|hCoV-19^^Michigan|Human|Michigan Department of Health and Human Services|Michigan Department of Health and Human Services|Blankenship|USA
>Spike|hCoV-19/England/QEUH-53E4AE/2020|2020-05-18|EPI_ISL_532198|Original|hCoV-19^^England|Human|Lighthouse Lab in Glasgow|Wellcome Sanger Institute for the COVID-19 Genomics UK (COG-UK) consortium|Davies|United Kingdom

>Spike|hCoV-19/USA/NE-11445/2020|2020-05-05|EPI_ISL_475213|Original|hCoV-19^^Nebraska|Human|Nebraska Public Health Laboratory|UNMC COVID-19 Response Team|Pratt|USA
>Spike|hCoV-19/Australia/VIC5434/2020|2020-07-15|EPI_ISL_520185|Original|hCoV-19^^Victoria|Human|Victorian Infectious Diseases Reference Laboratory (VIDRL)|VIDRL and MDU-PHL|Schultz|Australia
>Spike|hCoV-19/Belgium/rega-0409497/2020|2020-04-09|EPI_ISL_476949|Original|hCoV-19^^Steenokkerzeel|Human|KU Leuven|KU Leuven|Maes|Belgium
>Spike|hCoV-19/Denmark/DCGC-8961/2020|2020-10-26|EPI_ISL_626197|Original|hCoV-19^^Hovedstaden|Human|Department of Virus and Microbiological Special Diagnostics|Albertsen lab|Michaelsen|Denmark
>Spike|hCoV-19/England/QEUH-96AC43/2020|2020-08-17|EPI_ISL_531328|Original|hCoV-19^^England|Human|Lighthouse Lab in Glasgow|Wellcome Sanger Institute for the COVID-19 Genomics UK (COG-UK) consortium|Davies|United Kingdom
>Spike|hCoV-19/Australia/VIC2349/2020|2020-06-25|EPI_ISL_522069|Original|hCoV-19^^Victoria|Human|Victorian Infectious Diseases Reference Laboratory (VIDRL)|VIDRL and MDU-PHL|Schultz|Australia
>Spike|hCoV-19/England/MILK-A45D77/2020|2020-10-06|EPI_ISL_598714|Original|hCoV-19^^England|Human|Lighthouse Lab in Milton Keynes|Wellcome Sanger Institute for the COVID-19 Genomics UK (COG-UK) consortium|Davies|United Kingdom
>Spike|hCoV-19/Wales/PHWC-32DC2/2020|2020-04-14|EPI_ISL_446690|Original|hCoV-19^^Wales|Human|Wales Specialist Virology Centre|Public Health Wales Microbiology Cardiff|Moore|United Kingdom
>Spike|hCoV-19/England/204740531/2020|2020-11-14|EPI_ISL_693469|Original|hCoV-19^^England|Human|Respiratory Virus Unit|COVID-19 Genomics UK (COG-UK) Consortium|Platt|United Kingdom
>Spike|hCoV-19/England/LEED-2AA613/2020|2020-03-31|EPI_ISL_538901|Original|hCoV-19^^England|Human|Leeds Teaching Hospitals NHS Trust and Public Health England|Wellcome Sanger Institute for the COVID-19 Genomics UK (COG-UK) consortium|Davies|United Kingdom
>Spike|hCoV-19/Scotland/QEUH-A4B2BE/2020|2020-10-08|EPI_ISL_637682|Original|hCoV-19^^Scotland|Human|Department of Pathology|COVID-19 Genomics UK (COG-UK) Consortium|Goodfellow|United Kingdom
>Spike|hCoV-19/England/MILK-B38523/2020|2020-11-01|EPI_ISL_659062|Original|hCoV-19^^England|Human|Lighthouse Lab in Milton Keynes|Wellcome Sanger Institute for the COVID-19 Genomics UK (COG-UK) Consortium|Davies|United Kingdom
>Spike|hCoV-19/USA/NJ-NYUMC682/2020|2020-03-30|EPI_ISL_450399|Original|hCoV-19^^New Jersey|Human|NYU Langone Health|Departments of Pathology and Medicine|Maurano|USA
>Spike|hCoV-19/Australia/VIC6392/2020|2020-07-24|EPI_ISL_521184|Original|hCoV-19^^Victoria|Human|Microbiological Diagnostic Unit - Public Health Laboratory (MDU-PHL)|MDU-PHL|Schultz|Australia
>Spike|hCoV-19/USA/WA-S492/2020|2020-03-26|EPI_ISL_434252|Original|hCoV-19^^Washington|Human|Washington State Department of Health|Seattle Flu Study|Bedford|USA
>Spike|hCoV-19/England/LOND-D9C66/2020|2020-08-02|EPI_ISL_612455|Original|hCoV-19^^England|Human|University College London|COVID-19 Genomics UK (COG-UK) Consortium|Roy|United Kingdom
>Spike|hCoV-19/Scotland/QEUH-AD0579/2020|2020-10-26|EPI_ISL_649776|Original|hCoV-19^^Scotland|Human|Lighthouse Lab in Glasgow|Wellcome Sanger Institute for the COVID-19 Genomics UK (COG-UK) Consortium|Davies|United Kingdom
>Spike|hCoV-19/Canada/BC-BCCDC-6407/2020|2020-10-00|EPI_ISL_740562|Original|hCoV-19^^British Columbia|Human|BCCDC Public Health Laboratory|BCCDC Public Health Laboratory|Prystajecky|Canada
>Spike|hCoV-19/England/SHEF-C8B7C/2020|2020-05-28|EPI_ISL_535064|Original|hCoV-19^^England|Human|Virology Department|COVID-19 Genomics UK (COG-UK) Consortium|de Silva|United Kingdom
>Spike|hCoV-19/England/SHEF-10CD761/2020|2020-12-13|EPI_ISL_727916|Original|hCoV-19^^England|Human|Virology Department|COVID-19 Genomics UK (COG-UK) Consortium|de Silva|United Kingdom

>Spike|hCoV-19/England/ALDP-9CDD3C/2020|2020-09-16|EPI_ISL_566365|Original|hCoV-19^^England|Human|Lighthouse Lab in Alderley Park|Wellcome Sanger Institute for the COVID-19 Genomics UK (COG-UK) consortium|Davies|United Kingdom
>Spike|hCoV-19/USA/WI-GMF-17570/2020|2020-08-28|EPI_ISL_547746|Original|hCoV-19^^Wisconsin|Human|Gundersen Molecular Diagnostics Laboratory|Kabara Cancer Research Institute|Kenny|USA
>Spike|hCoV-19/England/ALDP-A96490/2020|2020-10-20|EPI_ISL_624623|Original|hCoV-19^^England|Human|Lighthouse Lab in Alderley Park|Wellcome Sanger Institute for the COVID-19 Genomics UK (COG-UK) consortium|Davies|United Kingdom
>Spike|hCoV-19/Wales/PHWC-2E233/2020|2020-04-07|EPI_ISL_446075|Original|hCoV-19^^Wales|Human|Wales Specialist Virology Centre|Public Health Wales Microbiology Cardiff|Moore|United Kingdom
>Spike|hCoV-19/USA/TX-HMH-MCoV-12933/2020|2020-08-18|EPI_ISL_788085|Original|hCoV-19^^Texas|Human|Houston Methodist Hospital|Houston Methodist Hospital|Long|USA
>Spike|hCoV-19/England/GSTT-265E15F/2020|2020-03-30|EPI_ISL_484159|Original|hCoV-19^^England|Human|Centre for Clinical Infection and Diagnostics Research and Genomics Innovation Unit|COVID-19 Genomics UK (COG-UK) Consortium|Awan|United Kingdom
>Spike|hCoV-19/England/MILK-B389EB/2020|2020-10-31|EPI_ISL_658934|Original|hCoV-19^^England|Human|Lighthouse Lab in Milton Keynes|Wellcome Sanger Institute for the COVID-19 Genomics UK (COG-UK) Consortium|Davies|United Kingdom
>Spike|hCoV-19/England/ALDP-927028/2020|2020-06-22|EPI_ISL_552915|Original|hCoV-19^^England|Human|Lighthouse Lab in Alderley Park|Wellcome Sanger Institute for the COVID-19 Genomics UK (COG-UK) consortium|Davies|United Kingdom
>Spike|hCoV-19/Luxembourg/LNS2148417/2020|2020-11-14|EPI_ISL_740067|Original|hCoV-19^^Luxembourg|Human|Laboratoire national de sant\u00e9|Laboratoire national de sant\u00e9|Wienecke-Baldacchino|Luxembourg
>Spike|hCoV-19/USA/UT-UPHL-2012810942/2020|2020-08-10|EPI_ISL_745873|Original|hCoV-19^^Utah|Human|Ginkgo Bioworks Clinical Laboratory|Utah Public Health Laboratory|Young|USA
>Spike|hCoV-19/Denmark/DCGC-11798/2020|2020-10-26|EPI_ISL_670530|Original|hCoV-19^^Hovedstaden|Human|Department of Virus and Microbiological Special Diagnostics|Albertsen Lab|Michaelsen|Denmark
>Spike|hCoV-19/England/QEUH-9B50E3/2020|2020-09-07|EPI_ISL_590360|Original|hCoV-19^^England|Human|Lighthouse Lab in Glasgow|Wellcome Sanger Institute for the COVID-19 Genomics UK (COG-UK) consortium|Davies|United Kingdom
>Spike|hCoV-19/Denmark/DCGC-13821/2020|2020-11-30|EPI_ISL_712289|Original|hCoV-19^^Midtjylland|Human|Department of Virus and Microbiological Special Diagnostics|Albertsen Lab|Michaelsen|Denmark
>Spike|hCoV-19/USA/HI-H200672/2020|2020-09-21|EPI_ISL_753195|Original|hCoV-19^^Hawaii|Human|State Laboratories Division|State Laboratories Division|Sultana|USA
>Spike|hCoV-19/England/ALDP-A86F74/2020|2020-10-17|EPI_ISL_606203|Original|hCoV-19^^England|Human|Lighthouse Lab in Alderley Park|Wellcome Sanger Institute for the COVID-19 Genomics UK (COG-UK) consortium|Davies|United Kingdom
>Spike|hCoV-19/USA/TX-DSHS-0914/2020|2020-06-04|EPI_ISL_602212|Original|hCoV-19^^Texas|Human|Texas Department of State Health Services|Texas Department of State Health Services|Rahman|USA
>Spike|hCoV-19/England/SHEF-CB6A5/2020|2020-08-30|EPI_ISL_540754|Original|hCoV-19^^England|Human|Virology Department|COVID-19 Genomics UK (COG-UK) Consortium|de Silva|United Kingdom
>Spike|hCoV-19/Turkey/KU-032/2020|2020-05-02|EPI_ISL_495442|Original|hCoV-19^^Kars Province|Human|Kafkas University|Kafkas University|Karamese|Turkey
>Spike|hCoV-19/USA/WA-S1024/2020|2020-05-01|EPI_ISL_463514|Original|hCoV-19^^Washington|Human|Washington State Department of Health|Seattle Flu Study|Bedford|USA
>Spike|hCoV-19/England/QEUH-CC9B7F/2020|2020-12-23|EPI_ISL_782018|Original|hCoV-19^^England|Human|Lighthouse Lab in Glasgow|Wellcome Sanger Institute for the COVID-19 Genomics UK (COG-UK) Consortium|Davies|United Kingdom
>Spike|hCoV-19/England/CAMC-A58DCC/2020|2020-10-08|EPI_ISL_597221|Original|hCoV-19^^England|Human|Lighthouse Lab in Cambridge|Wellcome Sanger Institute for the COVID-19 Genomics UK (COG-UK) consortium|Davies|United Kingdom

>Spike|hCoV-19/USA/MD-HP12017/2020|2020-11-04|EPI_ISL_791359|Original|hCoV-19^^Maryland|Human|Johns Hopkins Hospital Department of Pathology|Johns Hopkins Hospital Department of Pathology|Morris|USA
>Spike|hCoV-19/England/QEUH-BC3666/2020|2020-11-25|EPI_ISL_704165|Original|hCoV-19^^England|Human|Lighthouse Lab in Glasgow|Wellcome Sanger Institute for the COVID-19 Genomics UK (COG-UK) Consortium|Davies|United Kingdom
>Spike|hCoV-19/Wales/PHWC-165DA7/2020|2020-05-17|EPI_ISL_490800|Original|hCoV-19^^Wales|Human|Wales Specialist Virology Centre Sequencing lab|COVID-19 Genomics UK (COG-UK) Consortium|Moore|United Kingdom
>Spike|hCoV-19/Australia/VIC5726/2020|2020-07-19|EPI_ISL_518527|Original|hCoV-19^^Victoria|Human|Microbiological Diagnostic Unit - Public Health Laboratory (MDU-PHL)|MDU-PHL|Schultz|Australia
>Spike|hCoV-19/England/LEED-2A9043/2020|2020-04-04|EPI_ISL_539104|Original|hCoV-19^^England|Human|Leeds Teaching Hospitals NHS Trust and Public Health England|Wellcome Sanger Institute for the COVID-19 Genomics UK (COG-UK) consortium|Davies|United Kingdom
>Spike|hCoV-19/England/ALDP-5C8791/2020|2020-06-19|EPI_ISL_558567|Original|hCoV-19^^England|Human|Lighthouse Lab in Alderley Park|Wellcome Sanger Institute for the COVID-19 Genomics UK (COG-UK) consortium|Davies|United Kingdom
>Spike|hCoV-19/Australia/VIC764/2020|2020-03-29|EPI_ISL_427040|Original|hCoV-19^^Victoria|Human|Victorian Infectious Diseases Reference Laboratory (VIDRL)|Microbiological Diagnostic Unit Public Health Laboratory and Victorian Infectious Diseases Reference Laboratory|Schultz|Australia
>Spike|hCoV-19/Denmark/DCGC-6029/2020|2020-09-21|EPI_ISL_621138|Original|hCoV-19^^Midtjylland|Human|Department of Virus and Microbiological Special Diagnostics|Albertsen lab|Michaelsen|Denmark
>Spike|hCoV-19/USA/CA-CZB-3889/2020|2020-07-21|EPI_ISL_548496|Original|hCoV-19^^California|Human|County of Santa Clara Public Health Department|Chan-Zuckerberg Biohub|Bhatt|USA
>Spike|hCoV-19/USA/CA-CZB-11898/2020|2020-10-02|EPI_ISL_672284|Original|hCoV-19^^California|Human|Santa Clara County Public Health Laboratory|Chan-Zuckerberg Biohub|Bhatt|USA
>Spike|hCoV-19/England/QEUH-9B7F7B/2020|2020-09-08|EPI_ISL_590538|Original|hCoV-19^^England|Human|Lighthouse Lab in Glasgow|Wellcome Sanger Institute for the COVID-19 Genomics UK (COG-UK) consortium|Davies|United Kingdom
>Spike|hCoV-19/Spain/AR-IBV-98008084/2020|2020-10-01|EPI_ISL_654406|Original|hCoV-19^^Aragon|Human|Servicio de Microbiolog\u00eda|SeqCOVID-SPAIN consortium/IBV(CSIC)|Comas|Spain
>Spike|hCoV-19/France/PAC-IHU-0956/2020|2020-07-13|EPI_ISL_569008|Original|hCoV-19^^Provence-Alpes-C\u00f4te-d\u2019Azur|Human|MEPHI|MEPHI|Levasseur|France
>Spike|hCoV-19/Wales/PHWC-489241/2020|2020-11-03|EPI_ISL_639553|Original|hCoV-19^^Wales|Human|Wales Specialist Virology Centre Sequencing lab|COVID-19 Genomics UK (COG-UK) Consortium|Moore|United Kingdom
>Spike|hCoV-19/USA/UT-02555/2020|2020-04-24|EPI_ISL_487207|Original|hCoV-19^^Utah|Human|Utah Public Health Laboratory|Utah Public Health Laboratory|Butz|USA
>Spike|hCoV-19/Japan/PG-2706/2020|2020-04-00|EPI_ISL_690838|Original|hCoV-19^^Japan|Human|Sakai City Institute of Public Health|Pathogen Genomics Center|Sekizuka|Japan
>Spike|hCoV-19/Scotland/QEUH-9E51C9/2020|2020-09-24|EPI_ISL_600949|Original|hCoV-19^^Scotland|Human|Lighthouse Lab in Glasgow|Wellcome Sanger Institute for the COVID-19 Genomics UK (COG-UK) consortium|Davies|United Kingdom
>Spike|hCoV-19/USA/WI-UW-2305/2020|2020-11-25|EPI_ISL_728071|Original|hCoV-19^^Wisconsin|Human|University of Wisconsin-Madison AIDS Vaccine Research Laboratories|University of Wisconsin-Madison AIDS Vaccine Research Laboratories|Baczenas|USA
>Spike|hCoV-19/Scotland/CVR2908/2020|2020-04-22|EPI_ISL_484652|Original|hCoV-19^^Scotland|Human|West of Scotland Specialist Virology Centre|COVID-19 Genomics UK (COG-UK) Consortium|Robertson|United Kingdom

>Spike|hCoV-19/United Arab Emirates/0046/2020|2020-05-18|EPI_ISL_698210|Original|hCoV-19^^Abu Dhabi|Human|Group 42 (G42) Healthcare|G42 Healthcare|Ogrodzki|United Arab Emirates
>Spike|hCoV-19/USA/WY-WPHL-20156911/2020|2020-12-02|EPI_ISL_754389|Original|hCoV-19^^Wyoming|Human|Wyoming Public Health Laboratory|Wyoming Public Health Laboratory|Hull|USA
>Spike|hCoV-19/USA/NY-PV09200/2020|2020-03-22|EPI_ISL_422555|Original|hCoV-19^^New York|Human|MSHS Clinical Microbiology Laboratories|MSHS Pathogen Surveillance Program|van Bakel|USA
>Spike|hCoV-19/Luxembourg/LNS5702473/2020|2020-10-27|EPI_ISL_744278|Original|hCoV-19^^Luxembourg|Human|Laboratoire national de sant\u00e9|Laboratoire national de sant\u00e9|Wienecke-Baldacchino|Luxembourg
>Spike|hCoV-19/England/QEUH-96F1B8/2020|2020-08-17|EPI_ISL_530422|Original|hCoV-19^^England|Human|Lighthouse Lab in Glasgow|Wellcome Sanger Institute for the COVID-19 Genomics UK (COG-UK) consortium|Davies|United Kingdom
>Spike|hCoV-19/USA/UT-03267/2020|2020-05-08|EPI_ISL_493007|Original|hCoV-19^^Utah|Human|Utah Public Health Laboratory|Utah Public Health Laboratory|Butz|USA
>Spike|hCoV-19/USA/WA-S43/2020|2020-02-27|EPI_ISL_417096|Original|hCoV-19^^Washington|Human|Washington State Department of Health|Seattle Flu Study|Bedford|USA
>Spike|hCoV-19/England/ALDP-A68761/2020|2020-10-12|EPI_ISL_630436|Original|hCoV-19^^England|Human|Lighthouse Lab in Alderley Park|Wellcome Sanger Institute for the COVID-19 Genomics UK (COG-UK) consortium|Davies|United Kingdom
>Spike|hCoV-19/Brazil/SP-492/2020|2020-04-27|EPI_ISL_574588|Original|hCoV-19^^S\u00e3o Paulo|Human|Hospital Estadual Sumare|Instituto Adolfo Lutz|Sacchi|Brazil
>Spike|hCoV-19/USA/MA-MASPHL-00353/2020|2020-04-29|EPI_ISL_693179|Original|hCoV-19^^Massachusetts|Human|Massachusetts State Public Health Laboratory|Massachusetts State Public Health Laboratory|Lang|USA
>Spike|hCoV-19/Denmark/DCGC-15717/2020|2020-11-23|EPI_ISL_713979|Original|hCoV-19^^Hovedstaden|Human|Department of Virus and Microbiological Special Diagnostics|Albertsen Lab|Michaelsen|Denmark
>Spike|hCoV-19/SaudiArabia/KAUST-JEDDAH844/2020|2020-05-24|EPI_ISL_513016|Original|hCoV-19^^Jeddah|Human|Pathogen Genomics Lab King Abdullah University of Science and Technology(KAUST)|Pathogen Genomics Lab King Abdullah University of Science and Technology(KAUST)|Mohamed Ghazzali|Saudi Arabia
>Spike|hCoV-19/South Africa/KRISP-K001122/2020|2020-07-16|EPI_ISL_515801|Original|hCoV-19^^KwaZulu-Natal|Human|Medical Disagnostics Services (MDS)|KRISP|de Oliveira|South Africa
>Spike|hCoV-19/England/MILK-9A51F2/2020|2020-09-02|EPI_ISL_549983|Original|hCoV-19^^England|Human|Lighthouse Lab in Milton Keynes|Wellcome Sanger Institute for the COVID-19 Genomics UK (COG-UK) consortium|Davies|United Kingdom
>Spike|hCoV-19/France/NAQ-SC581/2020|2020-03-07|EPI_ISL_663232|Original|hCoV-19^^Nouvelle-Aquitaine|Human|CHU Poitiers|CNR Virus des Infections Respiratoires - France SUD|Bal|France
>Spike|hCoV-19/England/QEUH-96C072/2020|2020-08-15|EPI_ISL_530936|Original|hCoV-19^^England|Human|Lighthouse Lab in Glasgow|Wellcome Sanger Institute for the COVID-19 Genomics UK (COG-UK) consortium|Davies|United Kingdom
>Spike|hCoV-19/England/ALDP-9FDAB4/2020|2020-10-01|EPI_ISL_588015|Original|hCoV-19^^England|Human|Lighthouse Lab in Alderley Park|Wellcome Sanger Institute for the COVID-19 Genomics UK (COG-UK) consortium|Davies|United Kingdom
>Spike|hCoV-19/Denmark/DCGC-11256/2020|2020-11-02|EPI_ISL_670796|Original|hCoV-19^^Hovedstaden|Human|Department of Virus and Microbiological Special Diagnostics|Albertsen Lab|Michaelsen|Denmark
>Spike|hCoV-19/England/CAMC-A57440/2020|2020-10-07|EPI_ISL_597367|Original|hCoV-19^^England|Human|Lighthouse Lab in Cambridge|Wellcome Sanger Institute for the COVID-19 Genomics UK (COG-UK) consortium|Davies|United Kingdom
>Spike|hCoV-19/England/CAMB-1BA82A/2020|2020-11-12|EPI_ISL_664676|Original|hCoV-19^^England|Human|Department of Pathology|COVID-19 Genomics UK (COG-UK) Consortium|Goodfellow|United Kingdom

>Spike|hCoV-19/England/ALDP-97DCC5/2020|2020-07-22|EPI_ISL_526330|Original|hCoV-19^^England|Human|University of Birmingham|COVID-19 Genomics UK (COG-UK) Consortium|Loman|United Kingdom
>Spike|hCoV-19/England/LIVE-1E1453/2020|2020-10-11|EPI_ISL_627183|Original|hCoV-19^^England|Human|Liverpool Clinical Laboratories|COVID-19 Genomics UK (COG-UK) Consortium|Haldenby|United Kingdom
>Spike|hCoV-19/South Korea/KDCA0424/2020|2020-11-14|EPI_ISL_760208|Original|hCoV-19^^South Korea|Human|Division of Emerging Infectious Diseases|Division of Emerging Infectious Diseases|Park|South Korea
>Spike|hCoV-19/Austria/CeMM0930/2020|2020-03-20|EPI_ISL_583782|Original|hCoV-19^^Austria|Human|Dr. Gernot Walder GmbH|Bergthaler laboratory|Bergthaler|Austria
>Spike|hCoV-19/USA/UT-UPHL-201111436/2020|2020-04-09|EPI_ISL_623192|Original|hCoV-19^^Utah|Human|Utah Public Health Laboratory|Utah Public Health Laboratory|Young|USA
>Spike|hCoV-19/England/CAMC-B79535/2020|2020-11-10|EPI_ISL_667829|Original|hCoV-19^^England|Human|Lighthouse Lab in Cambridge|Wellcome Sanger Institute for the COVID-19 Genomics UK (COG-UK) Consortium|Davies|United Kingdom
>Spike|hCoV-19/England/ALDP-9E9F43/2020|2020-09-24|EPI_ISL_579955|Original|hCoV-19^^England|Human|Lighthouse Lab in Alderley Park|Wellcome Sanger Institute for the COVID-19 Genomics UK (COG-UK) consortium|Davies|United Kingdom
>Spike|hCoV-19/England/QEUH-B68733/2020|2020-11-11|EPI_ISL_661969|Original|hCoV-19^^England|Human|Lighthouse Lab in Glasgow|Wellcome Sanger Institute for the COVID-19 Genomics UK (COG-UK) Consortium|Davies|United Kingdom
>Spike|hCoV-19/Belgium/SN-03031/2020|2020-03-03|EPI_ISL_416469|Original|hCoV-19^^Kessel-Lo|Human|KU Leuven|KU Leuven|Maes|Belgium
>Spike|hCoV-19/USA/WY-WYPHL006/2020|2020-03-15|EPI_ISL_421543|Original|hCoV-19^^Wyoming|Human|Wyoming Public Health Laboratory|Center for Global Health|Domman|USA
>Spike|hCoV-19/Australia/TAS270/2020|2020-04-13|EPI_ISL_522699|Original|hCoV-19^^Tasmania|Human|Royal Hobart Hospital Microbiology Department|MDU-PHL|Schultz|Australia
>Spike|hCoV-19/Denmark/DCGC-16636/2020|2020-11-16|EPI_ISL_714885|Original|hCoV-19^^Hovedstaden|Human|Department of Virus and Microbiological Special Diagnostics|Albertsen Lab|Michaelsen|Denmark
>Spike|hCoV-19/England/ALDP-BE9248/2020|2020-12-03|EPI_ISL_708947|Original|hCoV-19^^England|Human|Lighthouse Lab in Alderley Park|Wellcome Sanger Institute for the COVID-19 Genomics UK (COG-UK) Consortium|Davies|United Kingdom
>Spike|hCoV-19/USA/AZ-TG664553/2020|2020-04-20|EPI_ISL_696169|Original|hCoV-19^^Arizona|Human|Sonora Quest Laboratories|TGen North|French|USA
>Spike|hCoV-19/Poland/PL_P9/2020|2020-03-28|EPI_ISL_428932|Original|hCoV-19^^Malopolskie|Human|ViroGenetics - BSL3 Laboratory of Virology; Human Genome Variation Research Group & Genomics Centre MCB; Bioinformatics Research Group; Wojew\u00f3dzka Stacja Sanitarno-Epidemiologiczna w Krakowie|ViroGenetics - BSL3 Laboratory of Virology; Human Genome Variation Research Group & Genomics Centre MCB; Bioi
>nformatics Research Group|Kopera|Poland
>Spike|hCoV-19/USA/ME-HETL-J0375/2020|2020-12-09|EPI_ISL_755496|Original|hCoV-19^^Maine|Human|Maine Health and Environmental Testing Laboratory|Tewhey Lab|Tewhey|USA
>Spike|hCoV-19/mink/Netherlands/NB-EMC-11-2/2020|2020-06-07|EPI_ISL_522996|Original|hCoV-19^^Noord Brabant|Neovison vison|Dutch COVID-19 response team|Erasmus Medical Center|Oude Munnink|Netherlands
>Spike|hCoV-19/USA/VA-DCLS-1538/2020|2020-08-07|EPI_ISL_581533|Original|hCoV-19^^Virginia|Human|Virginia DCLS|Virginia DCLS|St. Jacques|USA
>Spike|hCoV-19/USA/MI-MDHHS-SC20355/2020|2020-03-30|EPI_ISL_450634|Original|hCoV-19^^Michigan|Human|Michigan Department of Health and Human Services|Michigan Department of Health and Human Services|Blankenship|USA
>Spike|hCoV-19/Scotland/QEUH-96363D/2020|2020-08-10|EPI_ISL_531638|Original|hCoV-19^^Scotland|Human|NHSGGC West of Scotland Specialist Virology Centre / MRC-University of Glasgow Centre for Virus Research|Wellcome Sanger Institute for the COVID-19 Genomics UK (COG-UK) consortium|Davies|United Kingdom

>Spike|hCoV-19/England/MILK-ACEEBF/2020|2020-10-22|EPI_ISL_629604|Original|hCoV-19^^England|Human|Lighthouse Lab in Milton Keynes|Wellcome Sanger Institute for the COVID-19 Genomics UK (COG-UK) consortium|Davies|United Kingdom
>Spike|hCoV-19/France/ARA-SC062/2020|2020-04-15|EPI_ISL_666674|Original|hCoV-19^^Auvergne-Rh\u00f4ne-Alpes|Human|Laboratoire du Centre Hospitalier Annecy Genevois|CNR Virus des Infections Respiratoires - France SUD|Bal|France
>Spike|hCoV-19/USA/NY-NYCPHL-000071/2020|2020-03-12|EPI_ISL_631564|Original|hCoV-19^^New York|Human|NYC HH Elmhurst Hospital Medical Center|New York City Public Health Laboratory|Wang|USA
>Spike|hCoV-19/England/LIVE-A878D/2020|2020-03-26|EPI_ISL_517209|Original|hCoV-19^^England|Human|Liverpool Clinical Laboratories|COVID-19 Genomics UK (COG-UK) Consortium|Haldenby|United Kingdom
>Spike|hCoV-19/USA/WA-UW-10298/2020|2020-06-03|EPI_ISL_570292|Original|hCoV-19^^Washington|Human|UW Virology Lab|UW Virology Lab|Roychoudhury|USA
>Spike|hCoV-19/England/QEUH-95DD7D/2020|2020-08-06|EPI_ISL_537073|Original|hCoV-19^^England|Human|Lighthouse Lab in Glasgow|Wellcome Sanger Institute for the COVID-19 Genomics UK (COG-UK) consortium|Davies|United Kingdom
>Spike|hCoV-19/Wales/PHWC-48643F/2020|2020-10-27|EPI_ISL_639043|Original|hCoV-19^^Wales|Human|Wales Specialist Virology Centre Sequencing lab|COVID-19 Genomics UK (COG-UK) Consortium|Moore|United Kingdom
>Spike|hCoV-19/USA/CA-CZB-10844/2020|2020-07-23|EPI_ISL_583089|Original|hCoV-19^^California|Human|Humboldt County Public Health Laboratory|Chan-Zuckerberg Biohub|Bhatt|USA
>Spike|hCoV-19/USA/PA-MGEL-00204/2020|2020-04-10|EPI_ISL_681960|Original|hCoV-19^^Pennsylvania|Human|UPMC Clinical Microbiology Laboratory|Microbial Genomic Epidemiology Laboratory|Griffith|USA
>Spike|hCoV-19/England/MILK-99A327/2020|2020-08-30|EPI_ISL_550308|Original|hCoV-19^^England|Human|Lighthouse Lab in Milton Keynes|Wellcome Sanger Institute for the COVID-19 Genomics UK (COG-UK) consortium|Davies|United Kingdom
>Spike|hCoV-19/Spain/CN-IBV-97006481/2020|2020-03-24|EPI_ISL_537696|Original|hCoV-19^^Canary Islands|Human|Hospital Universitario de Gran Canaria Dr. Negr\u00edn|SeqCOVID-SPAIN consortium/IBV(CSIC)|Comas|Spain
>Spike|hCoV-19/Russia/StPetersburg-RII4307V/2020|2020-03-24|EPI_ISL_427308|Original|hCoV-19^^Saint Petersburg|Human|WHO National Influenza Centre Russian Federation|WHO National Influenza Centre Russian Federation|Komissarov|Russia
>Spike|hCoV-19/England/ALDP-AA2507/2020|2020-10-22|EPI_ISL_630937|Original|hCoV-19^^England|Human|Lighthouse Lab in Alderley Park|Wellcome Sanger Institute for the COVID-19 Genomics UK (COG-UK) consortium|Davies|United Kingdom
>Spike|hCoV-19/Denmark/DCGC-7825/2020|2020-10-19|EPI_ISL_618780|Original|hCoV-19^^Hovedstaden|Human|Department of Virus and Microbiological Special Diagnostics|Albertsen lab|Michaelsen|Denmark
>Spike|hCoV-19/Wales/PHWC-49CE38/2020|2020-12-09|EPI_ISL_742695|Original|hCoV-19^^Wales|Human|Wales Specialist Virology Centre Sequencing lab|COVID-19 Genomics UK (COG-UK) Consortium|Moore|United Kingdom
>Spike|hCoV-19/England/SHEF-C2459/2020|2020-05-20|EPI_ISL_462005|Original|hCoV-19^^England|Human|Virology Department|COVID-19 Genomics UK (COG-UK) Consortium|de Silva|United Kingdom
>Spike|hCoV-19/Bangladesh/BCSIR-NILMRC-095/2020|2020-05-23|EPI_ISL_469297|Original|hCoV-19^^Rangpur|Human|National Institute of Laboratory Medicine and Referral Center|Genomic Research Lab|Khan|Bangladesh
>Spike|hCoV-19/Denmark/DCGC-22212/2020|2020-12-14|EPI_ISL_758617|Original|hCoV-19^^Nordjylland|Human|Department of Virus and Microbiological Special Diagnostics|Albertsen Lab|Michaelsen|Denmark
>Spike|hCoV-19/England/QEUH-A1DDC3/2020|2020-09-29|EPI_ISL_587520|Original|hCoV-19^^England|Human|Lighthouse Lab in Glasgow|Wellcome Sanger Institute for the COVID-19 Genomics UK (COG-UK) consortium|Davies|United Kingdom
>Spike|hCoV-19/England/ALDP-CECF5C/2020|2020-12-21|EPI_ISL_781458|Original|hCoV-19^^England|Human|Lighthouse Lab in Alderley Park|Wellcome Sanger Institute for the COVID-19 Genomics UK (COG-UK) Consortium|Davies|United Kingdom

>Spike|hCoV-19/USA/MA-MGH-00013/2020|2020-03-23|EPI_ISL_460154|Original|hCoV-19^^Massachusetts|Human|Massachusetts General Hospital|Infectious Disease Program|Lemieux|USA
>Spike|hCoV-19/England/ALDP-747C3F/2020|2020-06-29|EPI_ISL_557411|Original|hCoV-19^^England|Human|Lighthouse Lab in Alderley Park|Wellcome Sanger Institute for the COVID-19 Genomics UK (COG-UK) consortium|Davies|United Kingdom
>Spike|hCoV-19/Switzerland/BE-UNIBE-195-4728/2020|2020-03-24|EPI_ISL_574834|Original|hCoV-19^^Bern|Human|Institute for Infectious Diseases|Institute for Infectious Diseases|Koch|Switzerland
>Spike|hCoV-19/USA/NM-NMDOH-2020327107/2020|2020-09-03|EPI_ISL_569645|Original|hCoV-19^^New Mexico|Human|New Mexico Department of Health Scientific Laboratory|New Mexico Department of Health Scientific Laboratory|Johnson|USA
>Spike|hCoV-19/Portugal/PT0271/2020|2020-04-05|EPI_ISL_453985|Original|hCoV-19^^Portugal|Human|unknown|Instituto Nacional de Saude (INSA)|Borges|Portugal
>Spike|hCoV-19/Wales/PHWC-4A15A4/2020|2020-12-10|EPI_ISL_743694|Original|hCoV-19^^Wales|Human|Wales Specialist Virology Centre Sequencing lab|COVID-19 Genomics UK (COG-UK) Consortium|Moore|United Kingdom
>Spike|hCoV-19/England/20138086904/2020|2020-03-23|EPI_ISL_421842|Original|hCoV-19^^England|Human|Respiratory Virus Unit|Respiratory Virus Unit|Platt|United Kingdom
>Spike|hCoV-19/England/ALDP-B1A026/2020|2020-11-01|EPI_ISL_645603|Original|hCoV-19^^England|Human|Lighthouse Lab in Alderley Park|Wellcome Sanger Institute for the COVID-19 Genomics UK (COG-UK) Consortium|Davies|United Kingdom
>Spike|hCoV-19/France/OCC-99/2020|2020-03-24|EPI_ISL_671976|Original|hCoV-19^^Occitanie|Human|CHU Purpan - Laboratoire de Virologie - Institut F\u00e9d\u00e9ratif de Biologie|CHU Purpan - Laboratoire de Virologie - Institut F\u00e9d\u00e9ratif de Biologie|Latour|France
>Spike|hCoV-19/Wales/PHWC-49DEDD/2020|2020-12-05|EPI_ISL_742927|Original|hCoV-19^^Wales|Human|Wales Specialist Virology Centre Sequencing lab|COVID-19 Genomics UK (COG-UK) Consortium|Moore|United Kingdom
>Spike|hCoV-19/USA/TX-HMH-4034/2020|2020-06-16|EPI_ISL_543314|Original|hCoV-19^^Texas|Human|Houston Methodist Hospital|Houston Methodist Hospital|Long|USA
>Spike|hCoV-19/USA/NMDOH-2020305749/2020|2020-08-21|EPI_ISL_542048|Original|hCoV-19^^New Mexico|Human|New Mexico Department of Health Scientific Laboratory|New Mexico Department of Health Scientific Laboratory|Johnson|USA
>Spike|hCoV-19/South Africa/KRISP-0160/2020|2020-06-01|EPI_ISL_467510|Original|hCoV-19^^KwaZulu-Natal|Human|NHLS-IALCH|KRISP|de Oliveira|South Africa
>Spike|hCoV-19/Ireland/DL-NVRL-20G45120/2020|2020-04-02|EPI_ISL_666706|Original|hCoV-19^^Donegal|Human|National Virus Reference Lab (NVRL)|Irish Coronavirus Sequencing Consortium-Teagasc Grange|McCabe|Ireland
>Spike|hCoV-19/England/LIVE-9DE68/2020|2020-04-04|EPI_ISL_453240|Original|hCoV-19^^England|Human|Liverpool Clinical Laboratories|COVID-19 Genomics UK (COG-UK) Consortium|Haldenby|United Kingdom
>Spike|hCoV-19/USA/NM-UNM-00319/2020|2020-05-01|EPI_ISL_508009|Original|hCoV-19^^New Mexico|Human|New Mexico Department of Health Scientific Laboratory Division|Center for Global Health|Domman|USA
>Spike|hCoV-19/England/ALDP-B5B33F/2020|2020-11-09|EPI_ISL_655338|Original|hCoV-19^^England|Human|Lighthouse Lab in Alderley Park|Wellcome Sanger Institute for the COVID-19 Genomics UK (COG-UK) Consortium|Davies|United Kingdom
>Spike|hCoV-19/USA/TX-DSHS-1254/2020|2020-05-30|EPI_ISL_644195|Original|hCoV-19^^Texas|Human|Texas Department of State Health Services|Texas Department of State Health Services|Rahman|USA
>Spike|hCoV-19/USA/UT-UPHL-2009611/2020|2020-07-19|EPI_ISL_605289|Original|hCoV-19^^Utah|Human|Utah Public Health Laboratory|Utah Public Health Laboratory|Young|USA
>Spike|hCoV-19/Scotland/QEUH-9AEEA7/2020|2020-09-03|EPI_ISL_572709|Original|hCoV-19^^Scotland|Human|University College London|COVID-19 Genomics UK (COG-UK) Consortium|Roy|United Kingdom
>Spike|hCoV-19/Wales/MILK-9AA685/2020|2020-08-30|EPI_ISL_550870|Original|hCoV-19^^Wales|Human|Lighthouse Lab in Milton Keynes|Wellcome Sanger Institute for the COVID-19 Genomics UK (COG-UK) consortium|Davies|United Kingdom

>Spike|hCoV-19/England/CAMB-751C7/2020|2020-03-13|EPI_ISL_440404|Original|hCoV-19^^England|Human|Department of Pathology|Wellcome Sanger Institute for the COVID-19 Genomics UK (COG-UK) consortium|Amato|United Kingdom
>Spike|hCoV-19/England/MILK-C5157E/2020|2020-12-11|EPI_ISL_769800|Original|hCoV-19^^England|Human|Lighthouse Lab in Milton Keynes|Wellcome Sanger Institute for the COVID-19 Genomics UK (COG-UK) Consortium|Davies|United Kingdom
>Spike|hCoV-19/England/CAMC-B07E9B/2020|2020-10-29|EPI_ISL_643631|Original|hCoV-19^^England|Human|Lighthouse Lab in Cambridge|Wellcome Sanger Institute for the COVID-19 Genomics UK (COG-UK) Consortium|Davies|United Kingdom
>Spike|hCoV-19/USA/TX-HMH-MCoV-10844/2020|2020-07-05|EPI_ISL_789655|Original|hCoV-19^^Texas|Human|Houston Methodist Hospital|Houston Methodist Hospital|Long|USA
>Spike|hCoV-19/England/QEUH-95DD22/2020|2020-08-07|EPI_ISL_537101|Original|hCoV-19^^England|Human|Lighthouse Lab in Glasgow|Wellcome Sanger Institute for the COVID-19 Genomics UK (COG-UK) consortium|Davies|United Kingdom
>Spike|hCoV-19/Wales/PHWC-169909/2020|2020-07-15|EPI_ISL_512533|Original|hCoV-19^^Wales|Human|Wales Specialist Virology Centre Sequencing lab|COVID-19 Genomics UK (COG-UK) Consortium|Moore|United Kingdom
>Spike|hCoV-19/Denmark/DCGC-9453/2020|2020-11-09|EPI_ISL_668539|Original|hCoV-19^^Hovedstaden|Human|Department of Virus and Microbiological Special Diagnostics|Albertsen Lab|Michaelsen|Denmark
>Spike|hCoV-19/England/ALDP-A6D878/2020|2020-10-14|EPI_ISL_608206|Original|hCoV-19^^England|Human|Lighthouse Lab in Alderley Park|Wellcome Sanger Institute for the COVID-19 Genomics UK (COG-UK) consortium|Davies|United Kingdom
>Spike|hCoV-19/England/QEUH-A4C930/2020|2020-10-05|EPI_ISL_628579|Original|hCoV-19^^England|Human|Oxford Viromics|COVID-19 Genomics UK (COG-UK) Consortium|Golubchik|United Kingdom
>Spike|hCoV-19/England/CAMC-BBC34B/2020|2020-11-17|EPI_ISL_724125|Original|hCoV-19^^England|Human|Quadram Institute Bioscience|COVID-19 Genomics UK (COG-UK) Consortium|Alikhan|United Kingdom
>Spike|hCoV-19/Scotland/EDB2662/2020|2020-04-26|EPI_ISL_439254|Original|hCoV-19^^Scotland|Human|Virology Department|COVID-19 Genomics UK (COG-UK) Consortium|Williams|United Kingdom
>Spike|hCoV-19/England/LOND-D648F/2020|2020-04-22|EPI_ISL_487585|Original|hCoV-19^^England|Human|University College London|Wellcome Sanger Institute for the COVID-19 Genomics UK (COG-UK) consortium|Davies|United Kingdom
>Spike|hCoV-19/USA/WY-WPHL-20095809/2020|2020-10-12|EPI_ISL_770085|Original|hCoV-19^^Wyoming|Human|Wyoming Public Health Laboratory|Wyoming Public Health Laboratory|Hull|USA
>Spike|hCoV-19/USA/TX-HMH-MCoV-8649/2020|2020-07-01|EPI_ISL_780676|Original|hCoV-19^^Texas|Human|Houston Methodist Hospital|Houston Methodist Hospital|Long|USA
>Spike|hCoV-19/Northern Ireland/NIRE-10236E/2020|2020-03-21|EPI_ISL_441405|Original|hCoV-19^^Northern Ireland|Human|Regional Virus Laboratory|COVID-19 Genomics UK (COG-UK) Consortium|Fairley|United Kingdom
>Spike|hCoV-19/Latvia/202/2020|2020-09-18|EPI_ISL_770038|Original|hCoV-19^^Latvia|Human|Centr\u0101l\u0101 Laboratorija|Latvian Biomedical Research and Study Centre|Zrelovs|Latvia
>Spike|hCoV-19/USA/WA-UW-13201/2020|2020-07-01|EPI_ISL_570957|Original|hCoV-19^^Washington|Human|UW Virology Lab|UW Virology Lab|Roychoudhury|USA
>Spike|hCoV-19/Denmark/DCGC-16349/2020|2020-11-16|EPI_ISL_714716|Original|hCoV-19^^Hovedstaden|Human|Department of Virus and Microbiological Special Diagnostics|Albertsen Lab|Michaelsen|Denmark
>Spike|hCoV-19/Gibraltar/204462857/2020|2020-10-29|EPI_ISL_615025|Original|hCoV-19^^Gibraltar|Human|Microbiology|Respiratory Virus Unit|Platt|Gibraltar
>Spike|hCoV-19/England/QEUH-9AF617/2020|2020-09-01|EPI_ISL_559853|Original|hCoV-19^^England|Human|Oxford Viromics|COVID-19 Genomics UK (COG-UK) Consortium|Golubchik|United Kingdom
>Spike|hCoV-19/Japan/PG-1548/2020|2020-04-00|EPI_ISL_685154|Original|hCoV-19^^Japan|Human|Pathogen Genomics Center|Pathogen Genomics Center|Sekizuka|Japan

>Spike|hCoV-19/Australia/VIC9770/2020|2020-07-24|EPI_ISL_565704|Original|hCoV-19^^Victoria|Human|Microbiological Diagnostic Unit - Public Health Laboratory (MDU-PHL)|MDU-PHL|Schultz|Australia
>Spike|hCoV-19/Japan/PG-4645/2020|2020-07-00|EPI_ISL_689782|Original|hCoV-19^^Japan|Human|Fukuoka Institute of Health and Environmental Sciences|Pathogen Genomics Center|Sekizuka|Japan
>Spike|hCoV-19/England/CAMB-71DF4/2020|2020-03-30|EPI_ISL_438255|Original|hCoV-19^^England|Human|Department of Pathology|Wellcome Sanger Institute for the COVID-19 Genomics UK (COG-UK) consortium|Amato|United Kingdom
>Spike|hCoV-19/England/CAMC-CB89A6/2020|2020-12-19|EPI_ISL_777893|Original|hCoV-19^^England|Human|Lighthouse Lab in Cambridge|Wellcome Sanger Institute for the COVID-19 Genomics UK (COG-UK) Consortium|Davies|United Kingdom
>Spike|hCoV-19/Australia/NSW353/2020|2020-04-17|EPI_ISL_451614|Original|hCoV-19^^New South Wales|Human|Pathology West - NSW Health Pathology|NSW Health Pathology - Institute of Clinical Pathology and Medical Research; Westmead Hospital; University of Sydney|Gall|Australia
>Spike|hCoV-19/Wales/PHWC-31C02/2020|2020-04-09|EPI_ISL_446544|Original|hCoV-19^^Wales|Human|Wales Specialist Virology Centre|Public Health Wales Microbiology Cardiff|Moore|United Kingdom
>Spike|hCoV-19/USA/FL-BPHL-0830/2020|2020-06-19|EPI_ISL_514176|Original|hCoV-19^^Florida|Human|Florida Bureau of Public Health Laboratories|Florida Bureau of Public Health Laboratories|Blanton|USA
>Spike|hCoV-19/England/LOND-124D318/2020|2020-03-19|EPI_ISL_507084|Original|hCoV-19^^England|Human|University College London Hospital|COVID-19 Genomics UK (COG-UK) Consortium|Byott|United Kingdom
>Spike|hCoV-19/USA/IL-NM-1722/2020|2020-07-26|EPI_ISL_626548|Original|hCoV-19^^Illinois|Human|Northwestern Memorial Hospital|Ozer Lab|Ozer|USA
>Spike|hCoV-19/USA/FL-BPHL-0099/2020|2020-04-28|EPI_ISL_445042|Original|hCoV-19^^Florida|Human|Florida Bureau of Public Health Laboratories|Florida Bureau of Public Health Laboratories|Blanton|USA
>Spike|hCoV-19/Wales/PHWC-4959B6/2020|2020-11-25|EPI_ISL_726422|Original|hCoV-19^^Wales|Human|Originating lab|COVID-19 Genomics UK (COG-UK) Consortium|Moore|United Kingdom
>Spike|hCoV-19/Denmark/DCGC-6308/2020|2020-08-31|EPI_ISL_615473|Original|hCoV-19^^Syddanmark|Human|Department of Virus and Microbiological Special Diagnostics|Albertsen lab|Michaelsen|Denmark
>Spike|hCoV-19/England/QEUH-A1DF90/2020|2020-10-01|EPI_ISL_587501|Original|hCoV-19^^England|Human|Lighthouse Lab in Glasgow|Wellcome Sanger Institute for the COVID-19 Genomics UK (COG-UK) consortium|Davies|United Kingdom
>Spike|hCoV-19/England/ALDP-74920E/2020|2020-06-29|EPI_ISL_557430|Original|hCoV-19^^England|Human|Lighthouse Lab in Alderley Park|Wellcome Sanger Institute for the COVID-19 Genomics UK (COG-UK) consortium|Davies|United Kingdom
>Spike|hCoV-19/England/MILK-BAA3E6/2020|2020-11-17|EPI_ISL_681513|Original|hCoV-19^^England|Human|Lighthouse Lab in Milton Keynes|Wellcome Sanger Institute for the COVID-19 Genomics UK (COG-UK) Consortium|Davies|United Kingdom
>Spike|hCoV-19/Morocco/FMP-50/2020|2020-12-00|EPI_ISL_775226|Original|hCoV-19^^Rabat|Human|Laboratoire Biolife|Laboratoire de Biotechnologie|Ouadghiri|Morocco
>Spike|hCoV-19/Denmark/DCGC-14001/2020|2020-11-30|EPI_ISL_711508|Original|hCoV-19^^Midtjylland|Human|Department of Virus and Microbiological Special Diagnostics|Albertsen Lab|Michaelsen|Denmark
>Spike|hCoV-19/Australia/VIC16362/2020|2020-08-04|EPI_ISL_640980|Original|hCoV-19^^Victoria|Human|Microbiological Diagnostic Unit - Public Health Laboratory (MDU-PHL)|MDU-PHL|Schultz|Australia
>Spike|hCoV-19/England/ALDP-9CB897/2020|2020-09-16|EPI_ISL_575446|Original|hCoV-19^^England|Human|Lighthouse Lab in Alderley Park|Wellcome Sanger Institute for the COVID-19 Genomics UK (COG-UK) consortium|Davies|United Kingdom
>Spike|hCoV-19/Denmark/DCGC-21632/2020|2020-12-21|EPI_ISL_757755|Original|hCoV-19^^Hovedstaden|Human|Department of Virus and Microbiological Special Diagnostics|Albertsen Lab|Michaelsen|Denmark

>Spike|hCoV-19/England/205041401/2020|2020-12-04|EPI_ISL_766287|Original|hCoV-
19^^England|Human|Respiratory Virus Unit|COVID-19 Genomics UK (COG-UK)
Consortium|Platt|United Kingdom
>Spike|hCoV-19/England/ALDP-9E6E67/2020|2020-09-24|EPI_ISL_580440|Original|hCoV-
19^^England|Human|Lighthouse Lab in Alderley Park|Wellcome Sanger Institute for the
COVID-19 Genomics UK (COG-UK) consortium|Davies|United Kingdom
>Spike|hCoV-19/Scotland/GCVR-16FE22/2020|2020-03-22|EPI_ISL_489654|Original|hCoV-
19^^Scotland|Human|NHSGGC West of Scotland Specialist Virology Centre / MRC-
University of Glasgow Centre for Virus Research|Wellcome Sanger Institute for the
COVID-19 Genomics UK (COG-UK) consortium|Davies|United Kingdom
>Spike|hCoV-19/England/MILK-994FCF/2020|2020-08-25|EPI_ISL_552420|Original|hCoV-
19^^England|Human|Lighthouse Lab in Milton Keynes|Wellcome Sanger Institute for the
COVID-19 Genomics UK (COG-UK) consortium|Davies|United Kingdom
>Spike|hCoV-19/England/NORT-294B77/2020|2020-03-28|EPI_ISL_490466|Original|hCoV-
19^^England|Human|Northumbria University / South Tees Hospitals NHS Foundation Trust
/ North Cumbria Integrated Care NHS Foundation Trust / North Tees and Hartlepool NHS
Foundation Trust / Newcastle Hospitals NHS Foundation Trust|COVID-19 Genomics UK
(COG-UK) Consortium|Bashton|United Kingdom
>Spike|hCoV-19/Mexico/BCN-ALSR-2551/2020|2020-07-01|EPI_ISL_635554|Original|hCoV-
19^^Baja California|Human|Centro de Diagn\u221a\u2265stico COVID-19 UABC
Tijuana|Andersen lab at Scripps Research|Gangavarapu|Mexico
>Spike|hCoV-19/England/ALDP-519D53/2020|2020-06-02|EPI_ISL_558901|Original|hCoV-
19^^England|Human|Lighthouse Lab in Alderley Park|Wellcome Sanger Institute for the
COVID-19 Genomics UK (COG-UK) consortium|Davies|United Kingdom
>Spike|hCoV-19/South Korea/KCDC2569/2020|2020-06-25|EPI_ISL_514972|Original|hCoV-
19^^South Korea|Human|Division of Viral Diseases|Division of Viral Diseases|Kim|South
Korea
>Spike|hCoV-19/England/MILK-A06325/2020|2020-10-01|EPI_ISL_600097|Original|hCoV-
19^^England|Human|Lighthouse Lab in Milton Keynes|Wellcome Sanger Institute for the
COVID-19 Genomics UK (COG-UK) consortium|Davies|United Kingdom
>Spike|hCoV-19/USA/CA-CSMC115/2020|2020-04-11|EPI_ISL_475591|Original|hCoV-
19^^California|Human|Cedars-Sinai Medical Center|Cedars-Sinai Medical
Center|Zhang|USA
>Spike|hCoV-19/England/MILK-A919C3/2020|2020-10-19|EPI_ISL_706000|Original|hCoV-
19^^England|Human|Oxford Viromics|COVID-19 Genomics UK (COG-UK)
Consortium|Golubchik|United Kingdom
>Spike|hCoV-19/USA/CA-ALSR-1751/2020|2020-05-13|EPI_ISL_512165|Original|hCoV-
19^^California|Human|San Diego County Public Health Laboratory|Andersen lab at
Scripps Research|Gangavarapu|USA
>Spike|hCoV-19/Wales/PHWC-48935D/2020|2020-11-03|EPI_ISL_639567|Original|hCoV-
19^^Wales|Human|Wales Specialist Virology Centre Sequencing lab|COVID-19 Genomics UK
(COG-UK) Consortium|Moore|United Kingdom
>Spike|hCoV-19/England/QEUH-9C7B65/2020|2020-09-12|EPI_ISL_567203|Original|hCoV-
19^^England|Human|Lighthouse Lab in Glasgow|Wellcome Sanger Institute for the COVID-
19 Genomics UK (COG-UK) consortium|Davies|United Kingdom
>Spike|hCoV-19/Wales/PHWC-1633C5/2020|2020-05-07|EPI_ISL_473289|Original|hCoV-
19^^Wales|Human|Wales Specialist Virology Centre Sequencing lab|COVID-19 Genomics UK
(COG-UK) Consortium|Moore|United Kingdom
>Spike|hCoV-19/England/SHEF-C3C14/2020|2020-05-11|EPI_ISL_475374|Original|hCoV-
19^^England|Human|Virology Department|COVID-19 Genomics UK (COG-UK) Consortium|de
Silva|United Kingdom
>Spike|hCoV-19/England/NORT-283267/2020|2020-03-30|EPI_ISL_488655|Original|hCoV-
19^^England|Human|NU-OMICS DNA Sequencing research facility|Wellcome Sanger Institute
for the COVID-19 Genomics UK (COG-UK) consortium|Davies|United Kingdom
>Spike|hCoV-19/USA/TX-HMH-MCoV-14254/2020|2020-10-05|EPI_ISL_787539|Original|hCoV-
19^^Texas|Human|Houston Methodist Hospital|Houston Methodist Hospital|Long|USA
>Spike|hCoV-19/USA/NC_0031/2020|2020-03-07|EPI_ISL_424873|Original|hCoV-19^^North
Carolina|Human|NC State Laboratory of Public Health|Pathogen Discovery|Queen|USA

>Spike|hCoV-19/USA/CA-CZB-2409/2020|2020-05-21|EPI_ISL_513833|Original|hCoV-19^^California|Human|Orange County Public Health Laboratory|Chan-Zuckerberg Biohub|Kamm|USA
>Spike|hCoV-19/Wales/ALDP-95AAE2/2020|2020-06-11|EPI_ISL_555064|Original|hCoV-19^^Wales|Human|Lighthouse Lab in Alderley Park|Wellcome Sanger Institute for the COVID-19 Genomics UK (COG-UK) consortium|Davies|United Kingdom
>Spike|hCoV-19/Latvia/190/2020|2020-10-07|EPI_ISL_639687|Original|hCoV-19^^Latvia|Human|E. Gulbja Laboratorija|Latvian Biomedical Research and Study Centre|Zrelovs|Latvia
>Spike|hCoV-19/USA/CA-CSMC58/2020|2020-04-01|EPI_ISL_475671|Original|hCoV-19^^California|Human|Cedars-Sinai Medical Center|Cedars-Sinai Medical Center|Zhang|USA
>Spike|hCoV-19/Australia/NSW2208/2020|2020-03-21|EPI_ISL_500666|Original|hCoV-19^^New South Wales|Human|Area of Virology|Area of Virology|Bull|Australia
>Spike|hCoV-19/England/LIVE-ABB24/2020|2020-08-12|EPI_ISL_540626|Original|hCoV-19^^England|Human|Liverpool Clinical Laboratories|COVID-19 Genomics UK (COG-UK) Consortium|Haldenby|United Kingdom
>Spike|hCoV-19/USA/LA-BIE-035/2020|2020-04-04|EPI_ISL_463283|Original|hCoV-19^^Louisiana|Human|Ochsner Health|Bioinfoexperts|Cross|USA
>Spike|hCoV-19/England/PORT-2D36D5/2020|2020-04-15|EPI_ISL_517536|Original|hCoV-19^^England|Human|Centre for Enzyme Innovation|COVID-19 Genomics UK (COG-UK) Consortium|Robson|United Kingdom
>Spike|hCoV-19/Scotland/CVR715/2020|2020-03-26|EPI_ISL_433332|Original|hCoV-19^^Scotland|Human|West of Scotland Specialist Virology Centre|COVID-19 Genomics UK (COG-UK) Consortium|Robertson|United Kingdom
>Spike|hCoV-19/England/ALDP-C3A353/2020|2020-12-09|EPI_ISL_730987|Original|hCoV-19^^England|Human|Lighthouse Lab in Alderley Park|Wellcome Sanger Institute for the COVID-19 Genomics UK (COG-UK) Consortium|Davies|United Kingdom
>Spike|hCoV-19/Canada/ON-UHTC_0070/2020|2020-04-12|EPI_ISL_464022|unknown|hCoV-19^^Ontario|Human|Unity Health Toronto|Ontario Institute for Cancer Research|de Borja|Canada
>Spike|hCoV-19/Denmark/DCGC-20769/2020|2020-12-21|EPI_ISL_758770|Original|hCoV-19^^Hovedstaden|Human|Department of Virus and Microbiological Special Diagnostics|Albertsen Lab|Michaelsen|Denmark
>Spike|hCoV-19/Portugal/PT0333/2020|2020-03-18|EPI_ISL_454049|Original|hCoV-19^^Portugal|Human|unknown|Instituto Nacional de Saude (INSA)|Borges|Portugal
>Spike|hCoV-19/England/MILK-9572CF/2020|2020-06-10|EPI_ISL_554123|Original|hCoV-19^^England|Human|Lighthouse Lab in Milton Keynes|Wellcome Sanger Institute for the COVID-19 Genomics UK (COG-UK) consortium|Davies|United Kingdom
>Spike|hCoV-19/England/ALDP-BC74C2/2020|2020-11-22|EPI_ISL_721425|Original|hCoV-19^^England|Human|Lighthouse Lab in Alderley Park|Wellcome Sanger Institute for the COVID-19 Genomics UK (COG-UK) Consortium|Davies|United Kingdom
>Spike|hCoV-19/USA/TX-HMH-3904/2020|2020-06-16|EPI_ISL_543273|Original|hCoV-19^^Texas|Human|Houston Methodist Hospital|Houston Methodist Hospital|Long|USA
>Spike|hCoV-19/USA/LA-GBCL-26629220/2020|2020-04-23|EPI_ISL_485224|Original|hCoV-19^^Louisiana|Human|River Road Testing Lab|Ginkgo Bioworks Clinical Laboratory|Smith|USA
>Spike|hCoV-19/Japan/PG-3224/2020|2020-04-00|EPI_ISL_691341|Original|hCoV-19^^Japan|Human|Pathogen Genomics Center|Pathogen Genomics Center|Sekizuka|Japan
>Spike|hCoV-19/England/ALDP-B1ABDA/2020|2020-11-01|EPI_ISL_645764|Original|hCoV-19^^England|Human|Lighthouse Lab in Alderley Park|Wellcome Sanger Institute for the COVID-19 Genomics UK (COG-UK) Consortium|Davies|United Kingdom
>Spike|hCoV-19/Japan/PG-5415/2020|2020-08-00|EPI_ISL_690518|Original|hCoV-19^^Japan|Human|Fukuoka Institute of Health and Environmental Sciences|Pathogen Genomics Center|Sekizuka|Japan
>Spike|hCoV-19/Hong Kong/VM20063289/2020|2020-07-20|EPI_ISL_602585|Original|hCoV-19^^Hong Kong|Human|Private medical practitioner|Hong Kong Department of Health|Mak|China

>Spike|hCoV-19/England/MILK-A47962/2020|2020-10-05|EPI_ISL_598468|Original|hCoV-19^^England|Human|Lighthouse Lab in Milton Keynes|Wellcome Sanger Institute for the COVID-19 Genomics UK (COG-UK) consortium|Davies|United Kingdom
>Spike|hCoV-19/Scotland/EDB3386/2020|2020-04-09|EPI_ISL_487670|Original|hCoV-19^^Scotland|Human|Virology Department|Wellcome Sanger Institute for the COVID-19 Genomics UK (COG-UK) consortium|Davies|United Kingdom
>Spike|hCoV-19/Canada/QC-LSPQ-L00239397/2020|2020-03-24|EPI_ISL_536248|Original|hCoV-19^^Quebec|Human|H\u00f4pital du Centre-de-la-Mauricie|Laboratoire de sant\u00e9 publique du Qu\u00e9bec|Moreira|Canada
>Spike|hCoV-19/Ireland/CE-UHL135/2020|2020-04-07|EPI_ISL_681708|Original|hCoV-19^^Clare|Human|University Hospital Limerick|Irish Coronavirus Sequencing Consortium - Teagasc Moorepark|Walsh|Ireland
>Spike|hCoV-19/Denmark/DCGC-17983/2020|2020-12-14|EPI_ISL_749680|Original|hCoV-19^^Hovedstaden|Human|Department of Virus and Microbiological Special Diagnostics|Albertsen Lab|Michaelsen|Denmark
>Spike|hCoV-19/USA/CA-ALSR-0797-SAN/2020|2020-04-02|EPI_ISL_483364|Original|hCoV-19^^California|Human|UC San Diego Center for Advanced Laboratory Medicine|Andersen lab at Scripps Research|Gangavarapu|USA
>Spike|hCoV-19/USA/NY-NYUMC430/2020|2020-04-21|EPI_ISL_435497|Original|hCoV-19^^New York|Human|NYU Langone Health|Departments of Pathology and Medicine|Maurano|USA
>Spike|hCoV-19/Suriname/SR-58/2020|2020-07-16|EPI_ISL_518807|Original|hCoV-19^^Suriname|Human|Academic Hospital Paramaribo|Erasmus Medical Center|M\u00fcnger|Suriname
>Spike|hCoV-19/USA/LA-EVTL1292/2020|2020-12-10|EPI_ISL_778958|Original|hCoV-19^^Louisiana|Human|LSUHS Emerging Viral Threat Laboratory|Microbial Genome Sequencing Center|Kamil|USA
>Spike|hCoV-19/Denmark/DCGC-10067/2020|2020-11-09|EPI_ISL_669375|Original|hCoV-19^^Hovedstaden|Human|Department of Virus and Microbiological Special Diagnostics|Albertsen Lab|Michaelsen|Denmark
>Spike|hCoV-19/Japan/PG-5672/2020|2020-04-00|EPI_ISL_688796|Original|hCoV-19^^Japan|Human|Pathogen Genomics Center|Pathogen Genomics Center|Sekizuka|Japan
>Spike|hCoV-19/Switzerland/TI-ETHZ-310516/2020|2020-10-08|EPI_ISL_603584|Original|hCoV-19^^Ticino|Human|Viollier AG|Department of Biosystems Science and Engineering|Nadeau|Switzerland
>Spike|hCoV-19/USA/MI-MDHHS-SC21119/2020|2020-03-27|EPI_ISL_507895|Original|hCoV-19^^Michigan|Human|Michigan Department of Health and Human Services|Michigan Department of Health and Human Services|Blankenship|USA
>Spike|hCoV-19/Scotland/CVR142/2020|2020-03-16|EPI_ISL_425686|Original|hCoV-19^^Scotland|Human|West of Scotland Specialist Virology Centre|COVID-19 Genomics UK (COG-UK) Consortium|Robertson|United Kingdom
>Spike|hCoV-19/Singapore/1016/2020|2020-03-22|EPI_ISL_574501|Original|hCoV-19^^Singapore|Human|National Public Health Laboratory|National Public Health Laboratory|Mak|Singapore
>Spike|hCoV-19/England/MILK-992C7F/2020|2020-08-26|EPI_ISL_552130|Original|hCoV-19^^England|Human|Lighthouse Lab in Milton Keynes|Wellcome Sanger Institute for the COVID-19 Genomics UK (COG-UK) consortium|Davies|United Kingdom
>Spike|hCoV-19/England/MILK-A7CCE3/2020|2020-10-13|EPI_ISL_606895|Original|hCoV-19^^England|Human|Lighthouse Lab in Milton Keynes|Wellcome Sanger Institute for the COVID-19 Genomics UK (COG-UK) consortium|Davies|United Kingdom
>Spike|hCoV-19/England/QEUH-AAD056/2020|2020-10-21|EPI_ISL_652935|Original|hCoV-19^^England|Human|Oxford Viromics|COVID-19 Genomics UK (COG-UK) Consortium|Golubchik|United Kingdom
>Spike|hCoV-19/Wales/PHWC-33FAD/2020|2020-04-18|EPI_ISL_446850|Original|hCoV-19^^Wales|Human|Wales Specialist Virology Centre|Public Health Wales Microbiology Cardiff|Moore|United Kingdom
>Spike|hCoV-19/Japan/PG-6912/2020|2020-09-00|EPI_ISL_688020|Original|hCoV-19^^Japan|Human|Pathogen Genomics Center|Pathogen Genomics Center|Sekizuka|Japan
>Spike|hCoV-19/USA/MN-MDH-2171/2020|2020-11-15|EPI_ISL_723122|Original|hCoV-19^^Minnesota|Human|Minnesota Department of Health|Minnesota Department of Health|Lorentz|USA

>Spike|hCoV-19/India/GJ-GBRC346/2020|2020-06-18|EPI_ISL_514588|Original|hCoV-19^^Gujarat|Human|B.J. Medical College and Civil hospital|Gujarat Biotechnology Research Centre|Joshi|India
>Spike|hCoV-19/England/QEUH-9B9E8B/2020|2020-09-07|EPI_ISL_549756|Original|hCoV-19^^England|Human|Lighthouse Lab in Glasgow|Wellcome Sanger Institute for the COVID-19 Genomics UK (COG-UK) consortium|Davies|United Kingdom
>Spike|hCoV-19/Denmark/DCGC-11023/2020|2020-11-02|EPI_ISL_670349|Original|hCoV-19^^Sjaelland|Human|Department of Virus and Microbiological Special Diagnostics|Albertsen Lab|Michaelsen|Denmark
>Spike|hCoV-19/Belgium/rega-10021225/2020|2020-10-02|EPI_ISL_734497|Original|hCoV-19^^Winksele|Human|UZ Leuven|KU Leuven|Maes|Belgium
>Spike|hCoV-19/England/SHEF-C6BAB/2020|2020-09-29|EPI_ISL_596201|Original|hCoV-19^^England|Human|Virology Department|COVID-19 Genomics UK (COG-UK) Consortium|de Silva|United Kingdom
>Spike|hCoV-19/Scotland/CVR5610/2020|2020-11-01|EPI_ISL_651865|Original|hCoV-19^^Scotland|Human|West of Scotland Specialist Virology Centre|COVID-19 Genomics UK (COG-UK) Consortium|Robertson|United Kingdom
>Spike|hCoV-19/England/QEUH-CC2903/2020|2020-12-21|EPI_ISL_760537|Original|hCoV-19^^England|Human|Lighthouse Lab in Glasgow|Wellcome Sanger Institute for the COVID-19 Genomics UK (COG-UK) Consortium|Davies|United Kingdom
>Spike|hCoV-19/Netherlands/ZH-EMC-402/2020|2020-07-23|EPI_ISL_523742|Original|hCoV-19^^Zuid Holland|Human|Dutch COVID-19 response team|Erasmus Medical Center|Oude Munnink|Netherlands
>Spike|hCoV-19/Japan/PG-2340/2020|2020-05-00|EPI_ISL_685945|Original|hCoV-19^^Japan|Human|Pathogen Genomics Center|Pathogen Genomics Center|Sekizuka|Japan
>Spike|hCoV-19/USA/TX-HMH-MCoV-15186/2020|2020-10-21|EPI_ISL_786670|Original|hCoV-19^^Texas|Human|Houston Methodist Hospital|Houston Methodist Hospital|Long|USA
>Spike|hCoV-19/England/QEUH-9DD100/2020|2020-09-21|EPI_ISL_599587|Original|hCoV-19^^England|Human|Lighthouse Lab in Glasgow|Wellcome Sanger Institute for the COVID-19 Genomics UK (COG-UK) consortium|Davies|United Kingdom
>Spike|hCoV-19/USA/WI-UW-2283/2020|2020-10-13|EPI_ISL_728050|Original|hCoV-19^^Wisconsin|Human|University of Wisconsin-Madison AIDS Vaccine Research Laboratories|University of Wisconsin-Madison AIDS Vaccine Research Laboratories|Baczenas|USA
>Spike|hCoV-19/USA/VA-DCLS-2443/2020|2020-11-17|EPI_ISL_734192|Original|hCoV-19^^Virginia|Human|Virginia Division of Consolidated Laboratory Services (DCLS)|Virginia Division of Consolidated Laboratory Services (DCLS)|St. Jacques|USA
>Spike|hCoV-19/Norway/2868/2020|2020-07-08|EPI_ISL_500788|Original|hCoV-19^^Norway|Human|Furst Medical Laboratory|Norwegian Institute of Public Health|Kopperud Riis|Norway
>Spike|hCoV-19/England/ALDP-985996/2020|2020-07-15|EPI_ISL_529389|Original|hCoV-19^^England|Human|Quadram Institute Bioscience|COVID-19 Genomics UK (COG-UK) Consortium|Alikhan|United Kingdom
>Spike|hCoV-19/England/CAMC-C83731/2020|2020-12-17|EPI_ISL_762834|Original|hCoV-19^^England|Human|Lighthouse Lab in Cambridge|Wellcome Sanger Institute for the COVID-19 Genomics UK (COG-UK) Consortium|Davies|United Kingdom
>Spike|hCoV-19/England/MILK-A4630B/2020|2020-10-04|EPI_ISL_598666|Original|hCoV-19^^England|Human|Lighthouse Lab in Milton Keynes|Wellcome Sanger Institute for the COVID-19 Genomics UK (COG-UK) consortium|Davies|United Kingdom
>Spike|hCoV-19/Australia/WA498/2020|2020-11-26|EPI_ISL_708792|Original|hCoV-19^^Western Australia|Human|PathWest Laboratory Medicine WA|PathWest Laboratory Medicine WA Microbial Surveillance Unit|Sikazwe|Australia
>Spike|hCoV-19/USA/NM-DOH-2020631873/2020|2020-12-18|EPI_ISL_766898|Original|hCoV-19^^New Mexico|Human|New Mexico Department of Health Scientific Laboratory|New Mexico Department of Health Scientific Laboratory|Malone|USA
>Spike|hCoV-19/England/20079000204/2020|2020-02-14|EPI_ISL_464171|Original|hCoV-19^^England|Human|Respiratory Virus Unit|Respiratory Virus Unit|Platt|United Kingdom
>Spike|hCoV-19/Australia/NSW984/2020|2020-07-27|EPI_ISL_591518|Original|hCoV-19^^New South Wales|Human|The Children's Hospital at Westmead|NSW Health Pathology -

Institute of Clinical Pathology and Medical Research; Westmead Hospital; University of Sydney|Gall|Australia
>Spike|hCoV-19/Germany/BY-MVP-0075/2020|2020-03-29|EPI_ISL_437270|Original|hCoV-19^^Bavaria|Human|Max von Pettenkofer Institute|Laboratory for Functional Genome Analysis|Graf|Germany
>Spike|hCoV-19/Japan/PG-6418/2020|2020-07-00|EPI_ISL_689524|Original|hCoV-19^^Japan|Human|Pathogen Genomics Center|Pathogen Genomics Center|Sekizuka|Japan
>Spike|hCoV-19/Japan/PG-5553/2020|2020-08-00|EPI_ISL_688678|Original|hCoV-19^^Japan|Human|Pathogen Genomics Center|Pathogen Genomics Center|Sekizuka|Japan
>Spike|hCoV-19/Wales/PHWC-48ED96/2020|2020-11-10|EPI_ISL_681003|Original|hCoV-19^^Wales|Human|Wales Specialist Virology Centre Sequencing lab|COVID-19 Genomics UK (COG-UK) Consortium|Moore|United Kingdom
>Spike|hCoV-19/England/ALDP-B61FF6/2020|2020-11-08|EPI_ISL_656393|Original|hCoV-19^^England|Human|Lighthouse Lab in Alderley Park|Wellcome Sanger Institute for the COVID-19 Genomics UK (COG-UK) Consortium|Davies|United Kingdom
>Spike|hCoV-19/England/QEUH-AD16C0/2020|2020-10-26|EPI_ISL_634540|Original|hCoV-19^^England|Human|Lighthouse Lab in Glasgow|Wellcome Sanger Institute for the COVID-19 Genomics UK (COG-UK) consortium|Davies|United Kingdom
>Spike|hCoV-19/England/ALDP-A68DC9/2020|2020-10-12|EPI_ISL_630551|Original|hCoV-19^^England|Human|Lighthouse Lab in Alderley Park|Wellcome Sanger Institute for the COVID-19 Genomics UK (COG-UK) consortium|Davies|United Kingdom
>Spike|hCoV-19/Spain/VC-FISABIO-654/2020|2020-03-30|EPI_ISL_455743|Original|hCoV-19^^Comunitat Valenciana|Human|Servicio de Microbiologia. Hospital Clinico Universitario de Valencia|Sequencing and Bioinformatics Service and Molecular Epidemiology Research Group. FISABIO-Public Health|Gonzalez-Candelas|Spain
>Spike|hCoV-19/USA/CA-CZB-10110/2020|2020-06-04|EPI_ISL_672218|Original|hCoV-19^^California|Human|The Ashley Laboratory|Chan-Zuckerberg Biohub|Bhatt|USA
>Spike|hCoV-19/USA/NY-NYCPHL-000419/2020|2020-03-30|EPI_ISL_631902|Original|hCoV-19^^New York|Human|Jamaica Hospital Medical Center|New York City Public Health Laboratory|Wang|USA
>Spike|hCoV-19/Wales/PHWC-485C38/2020|2020-10-21|EPI_ISL_628267|Original|hCoV-19^^Wales|Human|Wales Specialist Virology Centre Sequencing lab|COVID-19 Genomics UK (COG-UK) Consortium|Moore|United Kingdom
>Spike|hCoV-19/Luxembourg/LNS6356413/2020|2020-11-01|EPI_ISL_744401|Original|hCoV-19^^Luxembourg|Human|Laboratoire national de sant\u00e9|Laboratoire national de sant\u00e9|Wienecke-Baldacchino|Luxembourg
>Spike|hCoV-19/USA/TX-HMH-MCoV-14088/2020|2020-08-14|EPI_ISL_787374|Original|hCoV-19^^Texas|Human|Houston Methodist Hospital|Houston Methodist Hospital|Long|USA
>Spike|hCoV-19/USA/FL-BPHL-0614/2020|2020-04-03|EPI_ISL_508818|Original|hCoV-19^^Florida|Human|Florida Bureau of Public Health Laboratories|Florida Bureau of Public Health Laboratories|Blanton|USA
>Spike|hCoV-19/England/MILK-9764E2/2020|2020-08-20|EPI_ISL_575883|Original|hCoV-19^^England|Human|Lighthouse Lab in Milton Keynes|Wellcome Sanger Institute for the COVID-19 Genomics UK (COG-UK) consortium|Davies|United Kingdom
>Spike|hCoV-19/England/MILK-B94E0C/2020|2020-11-14|EPI_ISL_676236|Original|hCoV-19^^England|Human|Lighthouse Lab in Milton Keynes|Wellcome Sanger Institute for the COVID-19 Genomics UK (COG-UK) Consortium|Davies|United Kingdom
>Spike|hCoV-19/England/MILK-BDC749/2020|2020-11-27|EPI_ISL_709711|Original|hCoV-19^^England|Human|Lighthouse Lab in Milton Keynes|Wellcome Sanger Institute for the COVID-19 Genomics UK (COG-UK) Consortium|Davies|United Kingdom
>Spike|hCoV-19/Japan/PG-5371/2020|2020-08-00|EPI_ISL_690474|Original|hCoV-19^^Japan|Human|Fukuoka Institute of Health and Environmental Sciences|Pathogen Genomics Center|Sekizuka|Japan
>Spike|hCoV-19/USA/MI-MDHHS-SC21911/2020|2020-03-27|EPI_ISL_565972|Original|hCoV-19^^Michigan|Human|Michigan Department of Health and Human Services|Michigan Department of Health and Human Services|Blankenship|USA
>Spike|hCoV-19/Japan/IC-0468/2020|2020-12-00|EPI_ISL_768677|Original|hCoV-19^^Japan|Human|Pathogen Genomics Center|Pathogen Genomics Center|Sekizuka|Japan

>Spike|hCoV-19/England/CAMC-941671/2020|2020-07-24|EPI_ISL_556849|Original|hCoV-19^^England|Human|Lighthouse Lab in Cambridge|Wellcome Sanger Institute for the COVID-19 Genomics UK (COG-UK) consortium|Davies|United Kingdom
>Spike|hCoV-19/USA/MA-MASPHL-00656/2020|2020-05-16|EPI_ISL_692977|Original|hCoV-19^^Massachusetts|Human|Massachusetts State Public Health Laboratory|Massachusetts State Public Health Laboratory|Lang|USA
>Spike|hCoV-19/Denmark/DCGC-794/2020|2020-04-20|EPI_ISL_614705|Original|hCoV-19^^Hovedstaden|Human|Department of Virus and Microbiological Special Diagnostics|Albertsen lab|Michaelsen|Denmark
>Spike|hCoV-19/USA/NC-QDX-254/2020|2020-04-30|EPI_ISL_498733|Original|hCoV-19^^North Carolina|Human|Quest Diagnostics|Quest Diagnostics|Kagan|USA
>Spike|hCoV-19/Australia/VIC13629/2020|2020-08-01|EPI_ISL_592292|Original|hCoV-19^^Victoria|Human|Microbiological Diagnostic Unit - Public Health Laboratory (MDU-PHL)|MDU-PHL|Schultz|Australia
>Spike|hCoV-19/Oman/20751/2020|2020-04-13|EPI_ISL_491148|Original|hCoV-19^^South Batinah|Human|Oman-National Influenza Center|Biotechnology & OMICs Laboratory|Al Mahruqi|Oman
>Spike|hCoV-19/Germany/HH-hpi-p1562/2020|2020-03-15|EPI_ISL_775709|Original|hCoV-19^^Hamburg|Human|University Medical Center Hamburg Eppendorf|Heinrich Pette Institute|Robitaille|Germany
>Spike|hCoV-19/England/QEUH-A8C239/2020|2020-10-17|EPI_ISL_643110|Original|hCoV-19^^England|Human|Lighthouse Lab in Glasgow|Wellcome Sanger Institute for the COVID-19 Genomics UK (COG-UK) Consortium|Davies|United Kingdom
>Spike|hCoV-19/USA/TX-HMH-5839/2020|2020-06-30|EPI_ISL_547053|Original|hCoV-19^^Texas|Human|Houston Methodist Hospital|Houston Methodist Hospital|Long|USA
>Spike|hCoV-19/USA/WA-S35/2020|2020-03-02|EPI_ISL_417088|Original|hCoV-19^^Washington|Human|Washington State Department of Health|Seattle Flu Study|Bedford|USA
>Spike|hCoV-19/England/QEUH-998EDD/2020|2020-08-24|EPI_ISL_541844|Original|hCoV-19^^England|Human|Lighthouse Lab in Glasgow|Wellcome Sanger Institute for the COVID-19 Genomics UK (COG-UK) consortium|Davies|United Kingdom
>Spike|hCoV-19/Wales/PHWC-16A6B6/2020|2020-09-09|EPI_ISL_560088|Original|hCoV-19^^Wales|Human|Wales Specialist Virology Centre Sequencing lab|COVID-19 Genomics UK (COG-UK) Consortium|Moore|United Kingdom
>Spike|hCoV-19/Scotland/QEUH-9F6550/2020|2020-09-27|EPI_ISL_600658|Original|hCoV-19^^Scotland|Human|Lighthouse Lab in Glasgow|Wellcome Sanger Institute for the COVID-19 Genomics UK (COG-UK) consortium|Davies|United Kingdom
>Spike|hCoV-19/Switzerland/AG-ETHZ-100381/2020|2020-03-20|EPI_ISL_574882|Original|hCoV-19^^Aargau|Human|Viollier AG|Department of Biosystems Science and Engineering|Nadeau|Switzerland
>Spike|hCoV-19/Denmark/DCGC-16979/2020|2020-12-14|EPI_ISL_750683|Original|hCoV-19^^Midtjylland|Human|Department of Virus and Microbiological Special Diagnostics|Albertsen Lab|Michaelsen|Denmark
>Spike|hCoV-19/Wales/PHWC-15E415/2020|2020-05-13|EPI_ISL_472815|Original|hCoV-19^^Wales|Human|Wales Specialist Virology Centre Sequencing lab|COVID-19 Genomics UK (COG-UK) Consortium|Moore|United Kingdom
>Spike|hCoV-19/USA/NY-Wadsworth-65860-01/2020|2020-04-22|EPI_ISL_676881|Original|hCoV-19^^New York|Human|Wadsworth Center|Wadsworth Center|Lasek-Nesselquist|USA
>Spike|hCoV-19/USA/MI-QDX-491/2020|2020-03-23|EPI_ISL_571225|Original|hCoV-19^^Michigan|Human|Quest Diagnostics|Quest Diagnostics|Kagan|USA
>Spike|hCoV-19/Japan/PG-7623/2020|2020-08-00|EPI_ISL_686725|Original|hCoV-19^^Japan|Human|Chiba Prefectural Institute of Public Health|Pathogen Genomics Center|Sekizuka|Japan
>Spike|hCoV-19/England/LIVE-DC43B7/2020|2020-11-14|EPI_ISL_703367|Original|hCoV-19^^England|Human|Liverpool Clinical Laboratories|COVID-19 Genomics UK (COG-UK) Consortium|Haldenby|United Kingdom
>Spike|hCoV-19/Belgium/rega-0603698/2020|2020-06-03|EPI_ISL_734748|Original|hCoV-19^^Tervuren|Human|UZ Leuven|KU Leuven|Maes|Belgium

>Spike|hCoV-19/Australia/VIC2736/2020|2020-07-06|EPI_ISL_521571|Original|hCoV-19^^Victoria|Human|Microbiological Diagnostic Unit - Public Health Laboratory (MDU-PHL)|MDU-PHL|Schultz|Australia
>Spike|hCoV-19/Japan/PG-3767/2020|2020-04-00|EPI_ISL_692020|Original|hCoV-19^^Japan|Human|Pathogen Genomics Center|Pathogen Genomics Center|Sekizuka|Japan
>Spike|hCoV-19/Australia/VIC10065/2020|2020-08-21|EPI_ISL_562781|Original|hCoV-19^^Victoria|Human|Microbiological Diagnostic Unit - Public Health Laboratory (MDU-PHL)|MDU-PHL|Schultz|Australia
>Spike|hCoV-19/Scotland/QEUH-96A751/2020|2020-08-16|EPI_ISL_531480|Original|hCoV-19^^Scotland|Human|NHSGGC West of Scotland Specialist Virology Centre / MRC-University of Glasgow Centre for Virus Research|Wellcome Sanger Institute for the COVID-19 Genomics UK (COG-UK) consortium|Davies|United Kingdom
>Spike|hCoV-19/Denmark/DCGC-7945/2020|2020-10-26|EPI_ISL_618884|Original|hCoV-19^^Sjaelland|Human|Department of Virus and Microbiological Special Diagnostics|Albertsen lab|Michaelsen|Denmark
>Spike|hCoV-19/Australia/VIC3580/2020|2020-07-08|EPI_ISL_520328|Original|hCoV-19^^Victoria|Human|Victorian Infectious Diseases Reference Laboratory (VIDRL)|VIDRL and MDU-PHL|Schultz|Australia
>Spike|hCoV-19/USA/OR-PROV-216/2020|2020-03-09|EPI_ISL_482451|Original|hCoV-19^^Oregon|Human|Providence St. Joseph Health Molecular Genomics Laboratory|Providence St. Joseph Health Molecular Genomics Laboratory|Dowdell|USA
>Spike|hCoV-19/England/ALDP-9CDF72/2020|2020-09-16|EPI_ISL_566295|Original|hCoV-19^^England|Human|Lighthouse Lab in Alderley Park|Wellcome Sanger Institute for the COVID-19 Genomics UK (COG-UK) consortium|Davies|United Kingdom
>Spike|hCoV-19/Denmark/DCGC-11067/2020|2020-11-02|EPI_ISL_670376|Original|hCoV-19^^Hovedstaden|Human|Department of Virus and Microbiological Special Diagnostics|Albertsen Lab|Michaelsen|Denmark
>Spike|hCoV-19/England/MILK-ACD07E/2020|2020-10-21|EPI_ISL_630336|Original|hCoV-19^^England|Human|Lighthouse Lab in Milton Keynes|Wellcome Sanger Institute for the COVID-19 Genomics UK (COG-UK) consortium|Davies|United Kingdom
>Spike|hCoV-19/Wales/PHWC-483F5F/2020|2020-10-23|EPI_ISL_626863|Original|hCoV-19^^Wales|Human|Wales Specialist Virology Centre Sequencing lab|COVID-19 Genomics UK (COG-UK) Consortium|Moore|United Kingdom
>Spike|hCoV-19/USA/VA-DCLS-1547/2020|2020-08-07|EPI_ISL_581538|Original|hCoV-19^^Virginia|Human|Virginia DCLS|Virginia DCLS|St. Jacques|USA
>Spike|hCoV-19/England/QEUH-BCCAAD/2020|2020-11-25|EPI_ISL_704670|Original|hCoV-19^^England|Human|Lighthouse Lab in Glasgow|Wellcome Sanger Institute for the COVID-19 Genomics UK (COG-UK) Consortium|Davies|United Kingdom
>Spike|hCoV-19/Portugal/PT1888/2020|2020-11-12|EPI_ISL_732103|Original|hCoV-19^^Portugal|Human|Instituto Nacional de Saude (INSA)|Instituto Nacional de Saude (INSA)|Borges|Portugal
>Spike|hCoV-19/Italy/APU-IZSPB-377PT/2020|2020-10-14|EPI_ISL_722918|Original|hCoV-19^^Apulia|Human|Istituto Zooprofilattico Sperimentale della Puglia e della Basilicata|Istituto Zooprofilattico Sperimentale della Puglia e della Basilicata|Parisi|Italy
>Spike|hCoV-19/Denmark/DCGC-16260/2020|2020-11-16|EPI_ISL_714284|Original|hCoV-19^^Syddanmark|Human|Department of Virus and Microbiological Special Diagnostics|Albertsen Lab|Michaelsen|Denmark
>Spike|hCoV-19/Peru/LIM-INS-093/2020|2020-07-04|EPI_ISL_536492|Original|hCoV-19^^Lima|Human|Instituto Nacional de Salud|Laboratorio de Infecciones Respiratorias Agudas|Lopez|Peru
>Spike|hCoV-19/USA/WI-UW-2044/2020|2020-10-07|EPI_ISL_677661|Original|hCoV-19^^Wisconsin|Human|University of Wisconsin-Madison AIDS Vaccine Research Laboratories|University of Wisconsin-Madison AIDS Vaccine Research Laboratories|Baczenas|USA
>Spike|hCoV-19/England/ALDP-9E6A27/2020|2020-09-24|EPI_ISL_580361|Original|hCoV-19^^England|Human|Lighthouse Lab in Alderley Park|Wellcome Sanger Institute for the COVID-19 Genomics UK (COG-UK) consortium|Davies|United Kingdom

>Spike|hCoV-19/England/ALDP-B73D7F/2020|2020-11-12|EPI_ISL_664520|Original|hCoV-19^^England|Human|Quadram Institute Bioscience|COVID-19 Genomics UK (COG-UK) Consortium|Alikhan|United Kingdom
>Spike|hCoV-19/England/CAMB-7955E/2020|2020-04-03|EPI_ISL_441197|Original|hCoV-19^^England|Human|Department of Pathology|Wellcome Sanger Institute for the COVID-19 Genomics UK (COG-UK) consortium|Amato|United Kingdom
>Spike|hCoV-19/Israel/CVL-46324/2020|2020-12-13|EPI_ISL_745069|Original|hCoV-19^^Tel Aviv|Human|Israel Central Virology laboratory|Israel Central Virology laboratory|Zuckerman|Israel
>Spike|hCoV-19/England/QEUH-AD0F20/2020|2020-10-25|EPI_ISL_629900|Original|hCoV-19^^England|Human|Lighthouse Lab in Glasgow|Wellcome Sanger Institute for the COVID-19 Genomics UK (COG-UK) consortium|Davies|United Kingdom
>Spike|hCoV-19/Switzerland/SG-ETHZ-390217/2020|2020-11-27|EPI_ISL_721848|Original|hCoV-19^^St Gallen|Human|Viollier AG|Department of Biosystems Science and Engineering|Nadeau|Switzerland
>Spike|hCoV-19/Suriname/SR-43/2020|2020-06-27|EPI_ISL_517654|Original|hCoV-19^^Suriname|Human|Academic Hospital Paramaribo|Erasmus Medical Center|M\u00fcnger|Suriname
>Spike|hCoV-19/Wales/PHWC-493253/2020|2020-11-28|EPI_ISL_725859|Original|hCoV-19^^Wales|Human|Originating lab|COVID-19 Genomics UK (COG-UK) Consortium|Moore|United Kingdom
>Spike|hCoV-19/England/ALDP-C3C825/2020|2020-12-10|EPI_ISL_731053|Original|hCoV-19^^England|Human|Lighthouse Lab in Alderley Park|Wellcome Sanger Institute for the COVID-19 Genomics UK (COG-UK) Consortium|Davies|United Kingdom
>Spike|hCoV-19/England/QEUH-A77E88/2020|2020-10-12|EPI_ISL_638742|Original|hCoV-19^^England|Human|Oxford Viromics|COVID-19 Genomics UK (COG-UK) Consortium|Golubchik|United Kingdom
>Spike|hCoV-19/Wales/PHWC-48FDFF/2020|2020-11-11|EPI_ISL_681232|Original|hCoV-19^^Wales|Human|Wales Specialist Virology Centre Sequencing lab|COVID-19 Genomics UK (COG-UK) Consortium|Moore|United Kingdom
>Spike|hCoV-19/USA/TX-HMH-MCoV-15665/2020|2020-07-09|EPI_ISL_785349|Original|hCoV-19^^Texas|Human|Houston Methodist Hospital|Houston Methodist Hospital|Long|USA
>Spike|hCoV-19/England/MILK-C2F412/2020|2020-12-07|EPI_ISL_782802|Original|hCoV-19^^England|Human|Lighthouse Lab in Milton Keynes|Wellcome Sanger Institute for the COVID-19 Genomics UK (COG-UK) Consortium|Davies|United Kingdom
>Spike|hCoV-19/Scotland/CVR5856/2020|2020-11-16|EPI_ISL_665521|Original|hCoV-19^^Scotland|Human|West of Scotland Specialist Virology Centre|COVID-19 Genomics UK (COG-UK) Consortium|Robertson|United Kingdom
>Spike|hCoV-19/USA/CA-UCLA-154/2020|2020-03-29|EPI_ISL_537493|Original|hCoV-19^^California|Human|UCLA Pathology Clinical Microbiology Lab|Kruglyak Lab|Boocock|USA
>Spike|hCoV-19/Denmark/DCGC-8167/2020|2020-10-26|EPI_ISL_617203|Original|hCoV-19^^Midtjylland|Human|Department of Virus and Microbiological Special Diagnostics|Albertsen lab|Michaelsen|Denmark
>Spike|hCoV-19/USA/AZ-TG333625/2020|2020-05-27|EPI_ISL_694033|Original|hCoV-19^^Arizona|Human|TGen North|TGen North|French|USA
>Spike|hCoV-19/Scotland/CVR2495/2020|2020-04-15|EPI_ISL_477956|Original|hCoV-19^^Scotland|Human|West of Scotland Specialist Virology Centre|COVID-19 Genomics UK (COG-UK) Consortium|Robertson|United Kingdom
>Spike|hCoV-19/Switzerland/BE-ETHZ-350097/2020|2020-11-02|EPI_ISL_693797|Original|hCoV-19^^Bern|Human|Viollier AG|Department of Biosystems Science and Engineering|Nadeau|Switzerland
>Spike|hCoV-19/Wales/MILK-9F1616/2020|2020-09-25|EPI_ISL_589647|Original|hCoV-19^^Wales|Human|Lighthouse Lab in Milton Keynes|Wellcome Sanger Institute for the COVID-19 Genomics UK (COG-UK) consortium|Davies|United Kingdom
>Spike|hCoV-19/England/ALDP-B7435E/2020|2020-11-12|EPI_ISL_662285|Original|hCoV-19^^England|Human|Lighthouse Lab in Alderley Park|Wellcome Sanger Institute for the COVID-19 Genomics UK (COG-UK) Consortium|Davies|United Kingdom

>Spike|hCoV-19/England/ALDP-950B70/2020|2020-06-14|EPI_ISL_554419|Original|hCoV-19^^England|Human|Lighthouse Lab in Alderley Park|Wellcome Sanger Institute for the COVID-19 Genomics UK (COG-UK) consortium|Davies|United Kingdom
>Spike|hCoV-19/USA/MD-MDH-0276/2020|2020-10-13|EPI_ISL_629057|Original|hCoV-19^^Maryland|Human|Maryland Public Health Laboratory|Maryland Public Health Laboratory|Keller|USA
>Spike|hCoV-19/England/SHEF-10CCA78/2020|2020-11-22|EPI_ISL_727908|Original|hCoV-19^^England|Human|Virology Department|COVID-19 Genomics UK (COG-UK) Consortium|de Silva|United Kingdom
>Spike|hCoV-19/Wales/PHWC-49EE27/2020|2020-11-29|EPI_ISL_743129|Original|hCoV-19^^Wales|Human|Wales Specialist Virology Centre Sequencing lab|COVID-19 Genomics UK (COG-UK) Consortium|Moore|United Kingdom
>Spike|hCoV-19/England/BRIS-12D7E9/2020|2020-05-05|EPI_ISL_481800|Original|hCoV-19^^England|Human|PHE South West Regional Laboratory|Wellcome Sanger Institute for the COVID-19 Genomics UK (COG-UK) consortium|Davies|United Kingdom
>Spike|hCoV-19/USA/WA-UW-4925/2020|2020-04-07|EPI_ISL_429637|Original|hCoV-19^^Washington|Human|UW Virology Lab|UW Virology Lab|Roychoudhury|USA
>Spike|hCoV-19/England/ALDP-A5130D/2020|2020-10-08|EPI_ISL_597966|Original|hCoV-19^^England|Human|Lighthouse Lab in Alderley Park|Wellcome Sanger Institute for the COVID-19 Genomics UK (COG-UK) consortium|Davies|United Kingdom
>Spike|hCoV-19/Japan/PG-7991/2020|2020-08-00|EPI_ISL_687092|Original|hCoV-19^^Japan|Human|Pathogen Genomics Center|Pathogen Genomics Center|Sekizuka|Japan
>Spike|hCoV-19/USA/MD-MDH-0395/2020|2020-11-02|EPI_ISL_660089|Original|hCoV-19^^Maryland|Human|MD PHL|MD PHL|Keller|USA
>Spike|hCoV-19/England/ALDP-A4FC34/2020|2020-10-07|EPI_ISL_597589|Original|hCoV-19^^England|Human|Lighthouse Lab in Alderley Park|Wellcome Sanger Institute for the COVID-19 Genomics UK (COG-UK) consortium|Davies|United Kingdom
>Spike|hCoV-19/England/MILK-ABC124/2020|2020-10-22|EPI_ISL_631264|Original|hCoV-19^^England|Human|Lighthouse Lab in Milton Keynes|Wellcome Sanger Institute for the COVID-19 Genomics UK (COG-UK) consortium|Davies|United Kingdom
>Spike|hCoV-19/Japan/PG-3345/2020|2020-04-00|EPI_ISL_691460|Original|hCoV-19^^Japan|Human|Pathogen Genomics Center|Pathogen Genomics Center|Sekizuka|Japan
>Spike|hCoV-19/Japan/PG-7628/2020|2020-08-00|EPI_ISL_686730|Original|hCoV-19^^Japan|Human|Chiba Prefectural Institute of Public Health|Pathogen Genomics Center|Sekizuka|Japan
>Spike|hCoV-19/England/ALDP-95079A/2020|2020-06-14|EPI_ISL_554602|Original|hCoV-19^^England|Human|Lighthouse Lab in Alderley Park|Wellcome Sanger Institute for the COVID-19 Genomics UK (COG-UK) consortium|Davies|United Kingdom
>Spike|hCoV-19/Panama/329700/2020|2020-03-16|EPI_ISL_496644|Original|hCoV-19^^Panama Center|Human|Gorgas Memorial Laboratory of Health Studies|Gorgas Memorial Laboratory of Health Studies|Martinez|Panama
>Spike|hCoV-19/France/OCC-SC415/2020|2020-03-14|EPI_ISL_641551|Original|hCoV-19^^Occitanie|Human|CHU Toulouse|CNR Virus des Infections Respiratoires - France SUD|Bal|France
>Spike|hCoV-19/England/BRIS-185420C/2020|2020-04-28|EPI_ISL_492350|Original|hCoV-19^^England|Human|PHE South West Regional Laboratory|Wellcome Sanger Institute for the COVID-19 Genomics UK (COG-UK) consortium|Davies|United Kingdom
>Spike|hCoV-19/Scotland/CVR4091/2020|2020-08-31|EPI_ISL_535181|Original|hCoV-19^^Scotland|Human|West of Scotland Specialist Virology Centre|COVID-19 Genomics UK (COG-UK) Consortium|Robertson|United Kingdom
>Spike|hCoV-19/England/ALDP-B5BFE0/2020|2020-11-10|EPI_ISL_655751|Original|hCoV-19^^England|Human|Lighthouse Lab in Alderley Park|Wellcome Sanger Institute for the COVID-19 Genomics UK (COG-UK) Consortium|Davies|United Kingdom
>Spike|hCoV-19/Scotland/EDB1681/2020|2020-04-15|EPI_ISL_433116|Original|hCoV-19^^Scotland|Human|Virology Department|COVID-19 Genomics UK (COG-UK) Consortium|Williams|United Kingdom
>Spike|hCoV-19/USA/WA-S1264/2020|2020-05-13|EPI_ISL_495714|Original|hCoV-19^^Washington|Human|Washington State Department of Health|Seattle Flu Study|Lee|USA
>Spike|hCoV-19/USA/VA-DCLS-0186/2020|2020-04-07|EPI_ISL_437402|Original|hCoV-19^^Virginia|Human|Virginia DCLS|Virginia DCLS|St. Jacques|USA

>Spike|hCoV-19/USA/TX-HMH-7645/2020|2020-06-26|EPI_ISL_546910|Original|hCoV-19^^Texas|Human|Houston Methodist Hospital|Houston Methodist Hospital|Long|USA
>Spike|hCoV-19/India/TG-CCMB-M471/2020|2020-06-09|EPI_ISL_495211|Original|hCoV-19^^Telangana|Human|CSIR-Centre for Cellular and Molecular Biology|CSIR-Centre for Cellular and Molecular Biology|Banu|India
>Spike|hCoV-19/England/MILK-BA9F98/2020|2020-11-17|EPI_ISL_681374|Original|hCoV-19^^England|Human|Lighthouse Lab in Milton Keynes|Wellcome Sanger Institute for the COVID-19 Genomics UK (COG-UK) Consortium|Davies|United Kingdom
>Spike|hCoV-19/England/QEUH-96DADD/2020|2020-08-20|EPI_ISL_530774|Original|hCoV-19^^England|Human|Lighthouse Lab in Glasgow|Wellcome Sanger Institute for the COVID-19 Genomics UK (COG-UK) consortium|Davies|United Kingdom
>Spike|hCoV-19/Canada/ON-PHL-20-02475/2020|2020-09-00|EPI_ISL_671587|Original|hCoV-19^^Ontario|Human|Public Health Ontario Laboratory|Public Health Ontario Laboratory|Guthrie|Canada
>Spike|hCoV-19/Australia/VIC9526/2020|2020-08-20|EPI_ISL_565588|Original|hCoV-19^^Victoria|Human|Microbiological Diagnostic Unit - Public Health Laboratory (MDU-PHL)|MDU-PHL|Schultz|Australia
>Spike|hCoV-19/Wales/MILK-9EFA2E/2020|2020-09-23|EPI_ISL_594970|Original|hCoV-19^^Wales|Human|Queens Medical Centre|COVID-19 Genomics UK (COG-UK) Consortium|Loose|United Kingdom
>Spike|hCoV-19/England/CAMB-76797/2020|2020-03-30|EPI_ISL_438339|Original|hCoV-19^^England|Human|Department of Pathology|Wellcome Sanger Institute for the COVID-19 Genomics UK (COG-UK) consortium|Amato|United Kingdom
>Spike|hCoV-19/England/SHEF-CD98C/2020|2020-05-19|EPI_ISL_484504|Original|hCoV-19^^England|Human|Virology Department|COVID-19 Genomics UK (COG-UK) Consortium|de Silva|United Kingdom
>Spike|hCoV-19/Bangladesh/G-182/2020|2020-06-16|EPI_ISL_600510|Original|hCoV-19^^Chattogram|Human|Institute of Epidemiology Disease Control And Research|Institute for Developing Science and Health Initiatives|Cowley|Bangladesh
>Spike|hCoV-19/England/QEUH-C327C8/2020|2020-12-11|EPI_ISL_782516|Original|hCoV-19^^England|Human|Lighthouse Lab in Glasgow|Wellcome Sanger Institute for the COVID-19 Genomics UK (COG-UK) Consortium|Davies|United Kingdom
>Spike|hCoV-19/Denmark/DCGC-6024/2020|2020-09-21|EPI_ISL_621133|Original|hCoV-19^^Midtjylland|Human|Department of Virus and Microbiological Special Diagnostics|Albertsen lab|Michaelsen|Denmark
>Spike|hCoV-19/Japan/PG-0971/2020|2020-04-00|EPI_ISL_684581|Original|hCoV-19^^Japan|Human|Pathogen Genomics Center|Pathogen Genomics Center|Sekizuka|Japan
>Spike|hCoV-19/USA/UT-08665/2020|2020-05-08|EPI_ISL_524391|Original|hCoV-19^^Utah|Human|Utah Public Health Laboratory|Utah Public Health Laboratory|Young|USA
>Spike|hCoV-19/Switzerland/BE-ETHZ-110458/2020|2020-04-07|EPI_ISL_451877|Original|hCoV-19^^Bern|Human|Viollier AG|Department of Biosystems Science and Engineering|Nadeau|Switzerland
>Spike|hCoV-19/England/ALDP-B1D533/2020|2020-11-01|EPI_ISL_645912|Original|hCoV-19^^England|Human|Lighthouse Lab in Alderley Park|Wellcome Sanger Institute for the COVID-19 Genomics UK (COG-UK) Consortium|Davies|United Kingdom
>Spike|hCoV-19/Wales/ALDP-B1AB16/2020|2020-11-01|EPI_ISL_711032|Original|hCoV-19^^Wales|Human|Lighthouse Lab in Alderley Park|Wellcome Sanger Institute for the COVID-19 Genomics UK (COG-UK) Consortium|Davies|United Kingdom
>Spike|hCoV-19/England/20169020204/2020|2020-04-17|EPI_ISL_465207|Original|hCoV-19^^England|Human|Respiratory Virus Unit|Respiratory Virus Unit|Platt|United Kingdom
>Spike|hCoV-19/Singapore/1343/2020|2020-06-09|EPI_ISL_648692|Original|hCoV-19^^Singapore|Human|Department of Laboratory Medicine|Department of Laboratory Medicine|Chen|Singapore
>Spike|hCoV-19/Wales/PHWC-487D24/2020|2020-10-29|EPI_ISL_639300|Original|hCoV-19^^Wales|Human|Wales Specialist Virology Centre Sequencing lab|COVID-19 Genomics UK (COG-UK) Consortium|Moore|United Kingdom
>Spike|hCoV-19/England/PORT-2D12C4/2020|2020-03-24|EPI_ISL_479176|Original|hCoV-19^^England|Human|Centre for Enzyme Innovation|COVID-19 Genomics UK (COG-UK) Consortium|Robson|United Kingdom

>Spike|hCoV-19/USA/TX-HMH-MCoV-14423/2020|2020-10-07|EPI_ISL_787708|Original|hCoV-19^^Texas|Human|Houston Methodist Hospital|Houston Methodist Hospital|Long|USA
>Spike|hCoV-19/USA/LA-EVTL1052/2020|2020-08-28|EPI_ISL_653742|Original|hCoV-19^^Louisiana|Human|LSUHS Emerging Viral Threat Laboratory|Microbial Genome Sequencing Center|Kamil|USA
>Spike|hCoV-19/Australia/VIC17300/2020|2020-00-00|EPI_ISL_663826|Original|hCoV-19^^Victoria|Human|Microbiological Diagnostic Unit - Public Health Laboratory (MDU-PHL)|MDU-PHL|Schultz|Australia
>Spike|hCoV-19/England/20109097506/2020|2020-03-08|EPI_ISL_417293|Original|hCoV-19^^England|Human|Respiratory Virus Unit|Respiratory Virus Unit|Platt|United Kingdom
>Spike|hCoV-19/England/LOND-DB1A1/2020|2020-11-12|EPI_ISL_724742|Original|hCoV-19^^England|Human|University College London|COVID-19 Genomics UK (COG-UK) Consortium|Roy|United Kingdom
>Spike|hCoV-19/England/MILK-9A855D/2020|2020-09-02|EPI_ISL_550118|Original|hCoV-19^^England|Human|Lighthouse Lab in Milton Keynes|Wellcome Sanger Institute for the COVID-19 Genomics UK (COG-UK) consortium|Davies|United Kingdom
>Spike|hCoV-19/England/MILK-9C3413/2020|2020-09-10|EPI_ISL_581375|Original|hCoV-19^^England|Human|Lighthouse Lab in Milton Keynes|Wellcome Sanger Institute for the COVID-19 Genomics UK (COG-UK) consortium|Davies|United Kingdom
>Spike|hCoV-19/Canada/NS-NML-142/2020|2020-04-13|EPI_ISL_579519|Original|hCoV-19^^Nova Scotia|Human|QEII Health Sciences Centre|National Microbiology Laboratory (NML)|Majer|Canada
>Spike|hCoV-19/England/ALDP-A6FBD7/2020|2020-10-15|EPI_ISL_606951|Original|hCoV-19^^England|Human|Lighthouse Lab in Alderley Park|Wellcome Sanger Institute for the COVID-19 Genomics UK (COG-UK) consortium|Davies|United Kingdom
>Spike|hCoV-19/England/LIVE-A63D6/2020|2020-03-18|EPI_ISL_499529|Original|hCoV-19^^England|Human|Liverpool Clinical Laboratories|COVID-19 Genomics UK (COG-UK) Consortium|Haldenby|United Kingdom
>Spike|hCoV-19/England/LIVE-9F6F5/2020|2020-04-21|EPI_ISL_472004|Original|hCoV-19^^England|Human|Liverpool Clinical Laboratories|COVID-19 Genomics UK (COG-UK) Consortium|Haldenby|United Kingdom
>Spike|hCoV-19/Denmark/ALAB-HH87/2020|2020-03-15|EPI_ISL_429327|Original|hCoV-19^^Denmark|Human|Department of Clinical Microbiology|Albertsen lab|Kirkegaard|Denmark
>Spike|hCoV-19/Wales/PHWC-49D31A/2020|2020-12-10|EPI_ISL_742765|Original|hCoV-19^^Wales|Human|Wales Specialist Virology Centre Sequencing lab|COVID-19 Genomics UK (COG-UK) Consortium|Moore|United Kingdom
>Spike|hCoV-19/Japan/PG-2276/2020|2020-04-00|EPI_ISL_685881|Original|hCoV-19^^Japan|Human|Pathogen Genomics Center|Pathogen Genomics Center|Sekizuka|Japan
>Spike|hCoV-19/Canada/QC_AP8/2020|2020-04-07|EPI_ISL_476510|Original|hCoV-19^^Quebec|Human|Laboratoire de microbiologie|Smith Laboratory|Smith|Canada
>Spike|hCoV-19/England/ALDP-A51534/2020|2020-10-07|EPI_ISL_597973|Original|hCoV-19^^England|Human|Lighthouse Lab in Alderley Park|Wellcome Sanger Institute for the COVID-19 Genomics UK (COG-UK) consortium|Davies|United Kingdom
>Spike|hCoV-19/Denmark/DCGC-18360/2020|2020-12-07|EPI_ISL_749918|Original|hCoV-19^^Midtjylland|Human|Department of Virus and Microbiological Special Diagnostics|Albertsen Lab|Michaelsen|Denmark
>Spike|hCoV-19/Denmark/DCGC-14554/2020|2020-11-30|EPI_ISL_711836|Original|hCoV-19^^Sjaelland|Human|Department of Virus and Microbiological Special Diagnostics|Albertsen Lab|Michaelsen|Denmark
>Spike|hCoV-19/Australia/VIC17206/2020|2020-08-14|EPI_ISL_663794|Original|hCoV-19^^Victoria|Human|Microbiological Diagnostic Unit - Public Health Laboratory (MDU-PHL)|MDU-PHL|Schultz|Australia
>Spike|hCoV-19/USA/FL-BPHL-1604/2020|2020-07-05|EPI_ISL_594294|Original|hCoV-19^^Florida|Human|Florida Bureau of Public Health Laboratories|Florida Bureau of Public Health Laboratories|Blanton|USA
>Spike|hCoV-19/Canada/MB-NML-641/2020|2020-03-24|EPI_ISL_582298|Original|hCoV-19^^Manitoba|Human|Cadham Provincial Laboratory|National Microbiology Laboratory (NML)|Majer|Canada

>Spike|hCoV-19/England/MILK-57AEA8/2020|2020-06-12|EPI_ISL_558267|Original|hCoV-19^^England|Human|Lighthouse Lab in Milton Keynes|Wellcome Sanger Institute for the COVID-19 Genomics UK (COG-UK) consortium|Davies|United Kingdom
>Spike|hCoV-19/USA/WI-UW-962/2020|2020-06-04|EPI_ISL_509960|Original|hCoV-19^^Wisconsin|Human|University of Wisconsin-Madison AIDS Vaccine Research Laboratories|University of Wisconsin-Madison AIDS Vaccine Research Laboratories|Baczenas|USA
>Spike|hCoV-19/England/BRIS-123B14/2020|2020-04-07|EPI_ISL_442637|Original|hCoV-19^^England|Human|PHE South West Regional Laboratory|Wellcome Sanger Institute for the COVID-19 Genomics UK (COG-UK) consortium|Amato|United Kingdom
>Spike|hCoV-19/mink/Netherlands/NB-EMC-10-2/2020|2020-06-07|EPI_ISL_522988|Original|hCoV-19^^Noord Brabant|Neovison vison|Dutch COVID-19 response team|Erasmus Medical Center|Oude Munnink|Netherlands
>Spike|hCoV-19/USA/VA-DCLS-1672/2020|2020-08-29|EPI_ISL_644840|Original|hCoV-19^^Virginia|Human|Virginia DCLS|Virginia DCLS|St. Jacques|USA
>Spike|hCoV-19/England/ALDP-A87993/2020|2020-10-16|EPI_ISL_606006|Original|hCoV-19^^England|Human|Lighthouse Lab in Alderley Park|Wellcome Sanger Institute for the COVID-19 Genomics UK (COG-UK) consortium|Davies|United Kingdom
>Spike|hCoV-19/Wuhan/HB-WH3-187/2020|2020-03-03|EPI_ISL_455382|Original|hCoV-19^^Hubei|Human|Wuhan Chain Medical Labs (CMLabs)|State Key Laboratory of Biotherapy of Sichuan University|Tang|China
>Spike|hCoV-19/Denmark/DCGC-13699/2020|2020-11-30|EPI_ISL_712966|Original|hCoV-19^^Hovedstaden|Human|Department of Virus and Microbiological Special Diagnostics|Albertsen Lab|Michaelsen|Denmark
>Spike|hCoV-19/USA/WA-UW-2599/2020|2020-03-29|EPI_ISL_486003|Original|hCoV-19^^Washington|Human|UW Virology Lab|UW Virology Lab|Roychoudhury|USA
>Spike|hCoV-19/England/QEUH-A60B03/2020|2020-10-12|EPI_ISL_665966|Original|hCoV-19^^England|Human|Northumbria University / South Tees Hospitals NHS Foundation Trust / North Cumbria Integrated Care NHS Foundation Trust / North Tees and Hartlepool NHS Foundation Trust / Newcastle Hospitals NHS Foundation Trust|COVID-19 Genomics UK (COG-UK) Consortium|Bashton|United Kingdom
>Spike|hCoV-19/Denmark/DCGC-12411/2020|2020-11-16|EPI_ISL_682592|Original|hCoV-19^^Hovedstaden|Human|Department of Virus and Microbiological Special Diagnostics|Albertsen Lab|Michaelsen|Denmark
>Spike|hCoV-19/England/ALDP-B62D73/2020|2020-11-08|EPI_ISL_655602|Original|hCoV-19^^England|Human|Lighthouse Lab in Alderley Park|Wellcome Sanger Institute for the COVID-19 Genomics UK (COG-UK) Consortium|Davies|United Kingdom
>Spike|hCoV-19/England/QEUH-AD2DCA/2020|2020-10-26|EPI_ISL_629915|Original|hCoV-19^^England|Human|Lighthouse Lab in Glasgow|Wellcome Sanger Institute for the COVID-19 Genomics UK (COG-UK) consortium|Davies|United Kingdom
>Spike|hCoV-19/Denmark/DCGC-15187/2020|2020-11-23|EPI_ISL_713682|Original|hCoV-19^^Hovedstaden|Human|Department of Virus and Microbiological Special Diagnostics|Albertsen Lab|Michaelsen|Denmark
>Spike|hCoV-19/England/LIVE-DA7B49/2020|2020-10-04|EPI_ISL_627177|Original|hCoV-19^^England|Human|Liverpool Clinical Laboratories|COVID-19 Genomics UK (COG-UK) Consortium|Haldenby|United Kingdom
>Spike|hCoV-19/Denmark/ALAB-SSI-623/2020|2020-03-24|EPI_ISL_437003|Original|hCoV-19^^Denmark|Human|Department of Virus and Microbiological Special Diagnostics|Albertsen lab|Kirkegaard|Denmark
>Spike|hCoV-19/Spain/PV-IBV-005148/2020|2020-06-19|EPI_ISL_509638|Original|hCoV-19^^Basque Country|Human|Servicio de Microbiolog\u00eda. Hospital Universitario Donostia. OSI Donostialdea. \u00c1rea de Enfermedades Infecciosas|SeqCOVID-SPAIN consortium/IBV(CSIC)|Comas|Spain
>Spike|hCoV-19/Wales/PHWC-48703A/2020|2020-10-27|EPI_ISL_639159|Original|hCoV-19^^Wales|Human|Wales Specialist Virology Centre Sequencing lab|COVID-19 Genomics UK (COG-UK) Consortium|Moore|United Kingdom
>Spike|hCoV-19/Japan/PG-0166/2020|2020-03-12|EPI_ISL_479876|Original|hCoV-19^^Hokkaido|Human|Sapporo City Institute of Public Health|Pathogen Genomics Center|Sekizuka|Japan

>Spike|hCoV-19/England/ALDP-B4813B/2020|2020-11-07|EPI_ISL_649292|Original|hCoV-19^^England|Human|Lighthouse Lab in Alderley Park|Wellcome Sanger Institute for the COVID-19 Genomics UK (COG-UK) Consortium|Davies|United Kingdom
>Spike|hCoV-19/England/ALDP-B5B4B4/2020|2020-11-09|EPI_ISL_655350|Original|hCoV-19^^England|Human|Lighthouse Lab in Alderley Park|Wellcome Sanger Institute for the COVID-19 Genomics UK (COG-UK) Consortium|Davies|United Kingdom
>Spike|hCoV-19/Wales/PHWC-49684F/2020|2020-11-20|EPI_ISL_726644|Original|hCoV-19^^Wales|Human|Originating lab|COVID-19 Genomics UK (COG-UK) Consortium|Moore|United Kingdom
>Spike|hCoV-19/USA/TX-HMH-7514/2020|2020-06-25|EPI_ISL_546814|Original|hCoV-19^^Texas|Human|Houston Methodist Hospital|Houston Methodist Hospital|Long|USA
>Spike|hCoV-19/USA/AZ-TG378552/2020|2020-06-16|EPI_ISL_694629|Original|hCoV-19^^Arizona|Human|TGen North|TGen North|French|USA
>Spike|hCoV-19/Scotland/QEUH-5E32CC/2020|2020-06-17|EPI_ISL_533298|Original|hCoV-19^^Scotland|Human|NHSGGC West of Scotland Specialist Virology Centre / MRC-University of Glasgow Centre for Virus Research|Wellcome Sanger Institute for the COVID-19 Genomics UK (COG-UK) consortium|Davies|United Kingdom
>Spike|hCoV-19/Japan/PG-8735/2020|2020-08-00|EPI_ISL_686331|Original|hCoV-19^^Japan|Human|Pathogen Genomics Center|Pathogen Genomics Center|Sekizuka|Japan
>Spike|hCoV-19/USA/TX-HMH-979/2020|2020-04-09|EPI_ISL_545944|Original|hCoV-19^^Texas|Human|Houston Methodist Hospital|Houston Methodist Hospital|Long|USA
>Spike|hCoV-19/USA/FL-BPHL-1029/2020|2020-07-22|EPI_ISL_517919|Original|hCoV-19^^Florida|Human|Florida Bureau of Public Health Laboratories|Florida Bureau of Public Health Laboratories|Blanton|USA
>Spike|hCoV-19/Finland/23SS15H5/2020|2020-09-23|EPI_ISL_759803|Original|hCoV-19^^Uusimaa|Human|Department of Virology and Immunology|Department of Virology|Smura|Finland
>Spike|hCoV-19/England/ALDP-B2726C/2020|2020-11-04|EPI_ISL_664562|Original|hCoV-19^^England|Human|Northumbria University / South Tees Hospitals NHS Foundation Trust / North Cumbria Integrated Care NHS Foundation Trust / North Tees and Hartlepool NHS Foundation Trust / Newcastle Hospitals NHS Foundation Trust|COVID-19 Genomics UK (COG-UK) Consortium|Bashton|United Kingdom
>Spike|hCoV-19/Wales/QEUH-AD3330/2020|2020-10-26|EPI_ISL_647947|Original|hCoV-19^^Wales|Human|Lighthouse Lab in Glasgow|Wellcome Sanger Institute for the COVID-19 Genomics UK (COG-UK) Consortium|Davies|United Kingdom
>Spike|hCoV-19/Denmark/DCGC-19547/2020|2020-12-07|EPI_ISL_749018|Original|hCoV-19^^Hovedstaden|Human|Department of Virus and Microbiological Special Diagnostics|Albertsen Lab|Michaelsen|Denmark
>Spike|hCoV-19/England/NORT-28358C/2020|2020-03-31|EPI_ISL_478498|Original|hCoV-19^^England|Human|Northumbria University / South Tees Hospitals NHS Foundation Trust / North Cumbria Integrated Care NHS Foundation Trust / North Tees and Hartlepool NHS Foundation Trust / Newcastle Hospitals NHS Foundation Trust|COVID-19 Genomics UK (COG-UK) Consortium|Bashton|United Kingdom
>Spike|hCoV-19/USA/NY-NYCPHL-001184/2020|2020-10-30|EPI_ISL_671763|Original|hCoV-19^^New York|Human|DOHMH Chelsea|New York City Public Health Laboratory|Wang|USA
>Spike|hCoV-19/England/CAMB-79266/2020|2020-04-03|EPI_ISL_441168|Original|hCoV-19^^England|Human|Department of Pathology|Wellcome Sanger Institute for the COVID-19 Genomics UK (COG-UK) consortium|Amato|United Kingdom
>Spike|hCoV-19/England/CAMC-B515BB/2020|2020-11-08|EPI_ISL_658362|Original|hCoV-19^^England|Human|Lighthouse Lab in Cambridge|Wellcome Sanger Institute for the COVID-19 Genomics UK (COG-UK) Consortium|Davies|United Kingdom
>Spike|hCoV-19/England/MILK-B3E226/2020|2020-11-01|EPI_ISL_706266|Original|hCoV-19^^England|Human|Oxford Viromics|COVID-19 Genomics UK (COG-UK) Consortium|Golubchik|United Kingdom
>Spike|hCoV-19/Switzerland/AG-ETHZ-280134/2020|2020-09-07|EPI_ISL_560421|Original|hCoV-19^^Aargau|Human|Viollier AG|Department of Biosystems Science and Engineering|Nadeau|Switzerland
>Spike|hCoV-19/England/ALDP-9BE1AB/2020|2020-09-09|EPI_ISL_568106|Original|hCoV-19^^England|Human|Lighthouse Lab in Alderley Park|Wellcome Sanger Institute for the COVID-19 Genomics UK (COG-UK) consortium|Davies|United Kingdom

>Spike|hCoV-19/England/QEUH-AA0536/2020|2020-10-20|EPI_ISL_650645|Original|hCoV-19^^England|Human|University of Exeter|COVID-19 Genomics UK (COG-UK) Consortium|Temperton|United Kingdom
>Spike|hCoV-19/USA/WA-OHSU-0821/2020|2020-06-23|EPI_ISL_526099|Original|hCoV-19^^Washington|Human|OHSU Lab Services Molecular Microbiology Lab|Oregon SARS-CoV-2 Genome Sequencing Center|O'Roak|USA
>Spike|hCoV-19/England/QEUH-9C978D/2020|2020-09-10|EPI_ISL_567385|Original|hCoV-19^^England|Human|Lighthouse Lab in Glasgow|Wellcome Sanger Institute for the COVID-19 Genomics UK (COG-UK) consortium|Davies|United Kingdom
>Spike|hCoV-19/England/ALDP-5A3CF7/2020|2020-06-14|EPI_ISL_558788|Original|hCoV-19^^England|Human|Lighthouse Lab in Alderley Park|Wellcome Sanger Institute for the COVID-19 Genomics UK (COG-UK) consortium|Davies|United Kingdom
>Spike|hCoV-19/England/MILK-B3CB4B/2020|2020-11-01|EPI_ISL_706186|Original|hCoV-19^^England|Human|Oxford Viromics|COVID-19 Genomics UK (COG-UK) Consortium|Golubchik|United Kingdom
>Spike|hCoV-19/USA/FL-BPHL-2057/2020|2020-05-18|EPI_ISL_653210|Original|hCoV-19^^Florida|Human|Florida Bureau of Public Health Laboratories|Florida Bureau of Public Health Laboratories|Blanton|USA
>Spike|hCoV-19/England/CAMC-A655D3/2020|2020-10-10|EPI_ISL_608436|Original|hCoV-19^^England|Human|Lighthouse Lab in Cambridge|Wellcome Sanger Institute for the COVID-19 Genomics UK (COG-UK) consortium|Davies|United Kingdom
>Spike|hCoV-19/Denmark/DCGC-9595/2020|2020-11-09|EPI_ISL_669055|Original|hCoV-19^^Midtjylland|Human|Department of Virus and Microbiological Special Diagnostics|Albertsen Lab|Michaelsen|Denmark
>Spike|hCoV-19/Netherlands/GE-EMC-336/2020|2020-10-14|EPI_ISL_632376|Original|hCoV-19^^Gelderland|Human|Dutch COVID-19 response team|Erasmus Medical Center|Oude Munnink|Netherlands
>Spike|hCoV-19/Wales/PHWC-483441/2020|2020-10-13|EPI_ISL_627820|Original|hCoV-19^^Wales|Human|Wales Specialist Virology Centre Sequencing lab|COVID-19 Genomics UK (COG-UK) Consortium|Moore|United Kingdom
>Spike|hCoV-19/Wales/PHWC-164EA5/2020|2020-05-27|EPI_ISL_479392|Original|hCoV-19^^Wales|Human|Wales Specialist Virology Centre Sequencing lab|COVID-19 Genomics UK (COG-UK) Consortium|Moore|United Kingdom
>Spike|hCoV-19/Wales/PHWC-47EC8D/2020|2020-10-09|EPI_ISL_595574|Original|hCoV-19^^Wales|Human|Originating lab|COVID-19 Genomics UK (COG-UK) Consortium|Moore|United Kingdom
>Spike|hCoV-19/England/EXET-139A4B/2020|2020-00-00|EPI_ISL_679317|Original|hCoV-19^^England|Human|University of Exeter|COVID-19 Genomics UK (COG-UK) Consortium|Temperton|United Kingdom
>Spike|hCoV-19/England/QEUH-A5E5CE/2020|2020-10-12|EPI_ISL_597498|Original|hCoV-19^^England|Human|Lighthouse Lab in Glasgow|Wellcome Sanger Institute for the COVID-19 Genomics UK (COG-UK) consortium|Davies|United Kingdom
>Spike|hCoV-19/USA/PA-MGEL-00264/2020|2020-04-23|EPI_ISL_681994|Original|hCoV-19^^Pennsylvania|Human|UPMC Clinical Microbiology Laboratory|Microbial Genomic Epidemiology Laboratory|Griffith|USA
>Spike|hCoV-19/Japan/PG-2380/2020|2020-05-00|EPI_ISL_685985|Original|hCoV-19^^Japan|Human|Tokyo Metropolitan Institute of Public Health|Pathogen Genomics Center|Sekizuka|Japan
>Spike|hCoV-19/England/BRIS-1279F8/2020|2020-04-17|EPI_ISL_469766|Original|hCoV-19^^England|Human|PHE South West Regional Laboratory|Wellcome Sanger Institute for the COVID-19 Genomics UK (COG-UK) consortium|Davies|United Kingdom
>Spike|hCoV-19/USA/AZ-TG352081/2020|2020-05-29|EPI_ISL_694427|Original|hCoV-19^^Arizona|Human|AZ SPHL|TGen North|French|USA
>Spike|hCoV-19/USA/NY-NYCPHL-001203/2020|2020-11-02|EPI_ISL_683777|Original|hCoV-19^^New York|Human|DOHMH Corona|New York City Public Health Laboratory|Wang|USA
>Spike|hCoV-19/Denmark/DCGC-9679/2020|2020-11-09|EPI_ISL_669106|Original|hCoV-19^^Sjaelland|Human|Department of Virus and Microbiological Special Diagnostics|Albertsen Lab|Michaelsen|Denmark

>Spike|hCoV-19/USA/NY-Wadsworth-226518-01/2020|2020-08-04|EPI_ISL_677128|Original|hCoV-19^^New York|Human|Masonic Medical Research Institute|Wadsworth Center|Lasek-Nesselquist|USA
>Spike|hCoV-19/USA/MA-MASPHL-00121/2020|2020-04-03|EPI_ISL_791600|Original|hCoV-19^^Massachusetts|Human|Massachusetts State Public Health Laboratory|Infectious Disease Program|Park|USA
>Spike|hCoV-19/USA/NY-NYCPHL-000098/2020|2020-03-24|EPI_ISL_631590|Original|hCoV-19^^New York|Human|Jamaica Hospital Medical Center|New York City Public Health Laboratory|Wang|USA
>Spike|hCoV-19/Denmark/DCGC-16693/2020|2020-11-09|EPI_ISL_714922|Original|hCoV-19^^Midtjylland|Human|Department of Virus and Microbiological Special Diagnostics|Albertsen Lab|Michaelsen|Denmark
>Spike|hCoV-19/USA/UT-UPHL-2012753967/2020|2020-10-02|EPI_ISL_746095|Original|hCoV-19^^Utah|Human|Ginkgo Bioworks Clinical Laboratory|Utah Public Health Laboratory|Young|USA
>Spike|hCoV-19/England/CAMC-A82181/2020|2020-10-14|EPI_ISL_610859|Original|hCoV-19^^England|Human|Lighthouse Lab in Cambridge|Wellcome Sanger Institute for the COVID-19 Genomics UK (COG-UK) consortium|Davies|United Kingdom
>Spike|hCoV-19/USA/TX-HMH-MCoV-18505/2020|2020-11-19|EPI_ISL_784853|Original|hCoV-19^^Texas|Human|Houston Methodist Hospital|Houston Methodist Hospital|Long|USA
>Spike|hCoV-19/Czech Republic/NRL_5184/2020|2020-03-17|EPI_ISL_471555|Original|hCoV-19^^Prague|Human|The National Institute of Public Health|State Veterinary Institute Prague and The National Institute of Public Health|Nagy|Czech Republic
>Spike|hCoV-19/England/QEUH-B43507/2020|2020-11-08|EPI_ISL_657352|Original|hCoV-19^^England|Human|Lighthouse Lab in Glasgow|Wellcome Sanger Institute for the COVID-19 Genomics UK (COG-UK) Consortium|Davies|United Kingdom
>Spike|hCoV-19/England/20144013704/2020|2020-03-31|EPI_ISL_423508|Original|hCoV-19^^England|Human|Respiratory Virus Unit|Respiratory Virus Unit|Platt|United Kingdom
>Spike|hCoV-19/England/C200040575/2020|2020-00-00|EPI_ISL_636731|Original|hCoV-19^^England|Human|Respiratory Virus Unit|Respiratory Virus Unit|Platt|United Kingdom
>Spike|hCoV-19/Sweden/20-07860/2020|2020-04-23|EPI_ISL_450821|Original|hCoV-19^^Jamtland|Human|Jarpens HC|The Public Health Agency of Sweden|Svartstr\u00f6m|Sweden
>Spike|hCoV-19/Australia/SAP441/2020|2020-04-05|EPI_ISL_508132|Original|hCoV-19^^South Australia|Human|SA Pathology|SA Pathology|Leong|Australia
>Spike|hCoV-19/England/MILK-B8EBC2/2020|2020-11-13|EPI_ISL_674620|Original|hCoV-19^^England|Human|Lighthouse Lab in Milton Keynes|Wellcome Sanger Institute for the COVID-19 Genomics UK (COG-UK) Consortium|Davies|United Kingdom
>Spike|hCoV-19/England/CAMC-B77B71/2020|2020-11-12|EPI_ISL_661873|Original|hCoV-19^^England|Human|Lighthouse Lab in Cambridge|Wellcome Sanger Institute for the COVID-19 Genomics UK (COG-UK) Consortium|Davies|United Kingdom
>Spike|hCoV-19/England/ALDP-BCA92A/2020|2020-11-23|EPI_ISL_703620|Original|hCoV-19^^England|Human|Lighthouse Lab in Alderley Park|Wellcome Sanger Institute for the COVID-19 Genomics UK (COG-UK) Consortium|Davies|United Kingdom
>Spike|hCoV-19/England/ALDP-9C58CA/2020|2020-09-11|EPI_ISL_567861|Original|hCoV-19^^England|Human|Lighthouse Lab in Alderley Park|Wellcome Sanger Institute for the COVID-19 Genomics UK (COG-UK) consortium|Davies|United Kingdom
>Spike|hCoV-19/Scotland/QEUH-C3377C/2020|2020-12-11|EPI_ISL_757165|Original|hCoV-19^^Scotland|Human|Lighthouse Lab in Glasgow|Wellcome Sanger Institute for the COVID-19 Genomics UK (COG-UK) Consortium|Davies|United Kingdom
>Spike|hCoV-19/England/QEUH-999197/2020|2020-08-24|EPI_ISL_541806|Original|hCoV-19^^England|Human|Lighthouse Lab in Glasgow|Wellcome Sanger Institute for the COVID-19 Genomics UK (COG-UK) consortium|Davies|United Kingdom
>Spike|hCoV-19/Scotland/EDB3052/2020|2020-04-03|EPI_ISL_487851|Original|hCoV-19^^Scotland|Human|Virology Department|Wellcome Sanger Institute for the COVID-19 Genomics UK (COG-UK) consortium|Davies|United Kingdom
>Spike|hCoV-19/India/TG-CCMB-AC568/2020|2020-08-19|EPI_ISL_539700|Original|hCoV-19^^Telangana|Human|CSIR-Centre for Cellular and Molecular Biology|CSIR-Centre for Cellular and Molecular Biology|Mukherjee|India

>Spike|hCoV-19/USA/NM-UNM-00023/2020|2020-03-16|EPI_ISL_467604|Original|hCoV-19^^New Mexico|Human|New Mexico Department of Health Scientific Laboratory Division|Center for Global Health|Domman|USA
>Spike|hCoV-19/Japan/PG-7665/2020|2020-08-00|EPI_ISL_686767|Original|hCoV-19^^Japan|Human|Chiba Prefectural Institute of Public Health|Pathogen Genomics Center|Sekizuka|Japan
>Spike|hCoV-19/Austria/CeMM0192/2020|2020-03-13|EPI_ISL_438033|Original|hCoV-19^^Vienna|Human|Center for Virology|Bergthaler laboratory|Bergthaler|Austria
>Spike|hCoV-19/Japan/PG-3322/2020|2020-04-00|EPI_ISL_691437|Original|hCoV-19^^Japan|Human|Pathogen Genomics Center|Pathogen Genomics Center|Sekizuka|Japan
>Spike|hCoV-19/England/SHEF-C9328/2020|2020-04-26|EPI_ISL_490615|Original|hCoV-19^^England|Human|Virology Department|COVID-19 Genomics UK (COG-UK) Consortium|de Silva|United Kingdom
>Spike|hCoV-19/England/CAMC-9462B4/2020|2020-07-14|EPI_ISL_556642|Original|hCoV-19^^England|Human|Lighthouse Lab in Cambridge|Wellcome Sanger Institute for the COVID-19 Genomics UK (COG-UK) consortium|Davies|United Kingdom
>Spike|hCoV-19/USA/UT-01819/2020|2020-04-09|EPI_ISL_509570|Original|hCoV-19^^Utah|Human|Utah Public Health Laboratory|Utah Public Health Laboratory|Butz|USA
>Spike|hCoV-19/England/NORW-EBAC1/2020|2020-05-08|EPI_ISL_457474|Original|hCoV-19^^England|Human|Quadram Institute Bioscience|COVID-19 Genomics UK (COG-UK) Consortium|Alikhan|United Kingdom
>Spike|hCoV-19/USA/MI-MDHHS-SC20921/2020|2020-05-26|EPI_ISL_476057|Original|hCoV-19^^Michigan|Human|Michigan Department of Health and Human Services|Michigan Department of Health and Human Services|Blankenship|USA
>Spike|hCoV-19/England/MILK-B3B326/2020|2020-11-01|EPI_ISL_679754|Original|hCoV-19^^England|Human|Oxford Viromics|COVID-19 Genomics UK (COG-UK) Consortium|Golubchik|United Kingdom
>Spike|hCoV-19/Norway/3207/2020|2020-08-15|EPI_ISL_549035|Original|hCoV-19^^Viken|Human|Oslo University Hospital|Norwegian Institute of Public Health|Kopperud Riis|Norway
>Spike|hCoV-19/USA/FL-BPHL-1103/2020|2020-08-03|EPI_ISL_526589|Original|hCoV-19^^Florida|Human|Florida Bureau of Public Health Laboratories|Florida Bureau of Public Health Laboratories|Blanton|USA
>Spike|hCoV-19/USA/CA-CZB-1428/2020|2020-03-23|EPI_ISL_468399|Original|hCoV-19^^California|Human|County of San Luis Obispo Public Health Laboratory|Chan-Zuckerberg Biohub|Hao|USA
>Spike|hCoV-19/Wales/PHWC-4A6035/2020|2020-12-15|EPI_ISL_764245|Original|hCoV-19^^Wales|Human|Wales Specialist Virology Centre Sequencing lab|COVID-19 Genomics UK (COG-UK) Consortium|Moore|United Kingdom
>Spike|hCoV-19/USA/OR-OHSU-0538/2020|2020-06-15|EPI_ISL_525836|Original|hCoV-19^^Oregon|Human|OHSU Lab Services Molecular Microbiology Lab|Ginkgo Bioworks Clinical Laboratory|O'Roak|USA
>Spike|hCoV-19/USA/TX-HMH-7035/2020|2020-06-28|EPI_ISL_546453|Original|hCoV-19^^Texas|Human|Houston Methodist Hospital|Houston Methodist Hospital|Long|USA
>Spike|hCoV-19/Australia/VIC400/2020|2020-03-24|EPI_ISL_426703|Original|hCoV-19^^Victoria|Human|Victorian Infectious Diseases Reference Laboratory (VIDRL)|Microbiological Diagnostic Unit Public Health Laboratory and Victorian Infectious Diseases Reference Laboratory|Schultz|Australia
>Spike|hCoV-19/Israel/2089863/2020|2020-04-01|EPI_ISL_447360|Original|hCoV-19^^Jerusalem District|Human|Clinical Virology Unit|Stern Lab|Miller|Israel
>Spike|hCoV-19/France/ARA-23001/2020|2020-04-10|EPI_ISL_508923|Original|hCoV-19^^Auvergne-Rh\u00f4ne-Alpes|Human|CNR Virus des Infections Respiratoires - France SUD|CNR Virus des Infections Respiratoires - France SUD|Josset|France
>Spike|hCoV-19/England/QEUH-9DE7FE/2020|2020-09-21|EPI_ISL_581337|Original|hCoV-19^^England|Human|Lighthouse Lab in Glasgow|Wellcome Sanger Institute for the COVID-19 Genomics UK (COG-UK) consortium|Davies|United Kingdom
>Spike|hCoV-19/Netherlands/NA_177/2020|2020-03-22|EPI_ISL_422712|Original|hCoV-19^^Netherlands|Human|Dutch COVID-19 response team|Erasmus Medical Center|Oude Munnink|Netherlands

>Spike|hCoV-19/USA/TX-DSHS-1473/2020|2020-06-22|EPI_ISL_676637|Original|hCoV-19^^Texas|Human|Texas Department of State Health Services|Texas Department of State Health Services|Rahman|USA
>Spike|hCoV-19/England/QEUH-9CEC10/2020|2020-09-17|EPI_ISL_590127|Original|hCoV-19^^England|Human|Lighthouse Lab in Glasgow|Wellcome Sanger Institute for the COVID-19 Genomics UK (COG-UK) consortium|Davies|United Kingdom
>Spike|hCoV-19/England/MILK-A7B0D9/2020|2020-10-14|EPI_ISL_607318|Original|hCoV-19^^England|Human|Lighthouse Lab in Milton Keynes|Wellcome Sanger Institute for the COVID-19 Genomics UK (COG-UK) consortium|Davies|United Kingdom
>Spike|hCoV-19/England/LOND-DC322/2020|2020-08-30|EPI_ISL_627467|Original|hCoV-19^^England|Human|University College London|COVID-19 Genomics UK (COG-UK) Consortium|Roy|United Kingdom
>Spike|hCoV-19/France/IDF-3831/2020|2020-03-23|EPI_ISL_428364|Original|hCoV-19^^\u00cele-de-France|Human|Cabinet M\u00e9dical|National Reference Center for Viruses of Respiratory Infections|Enouf|France
>Spike|hCoV-19/Kazakhstan/26562/2020|2020-04-26|EPI_ISL_454599|Original|hCoV-19^^Nur-Sultan|Human|Laboratory of virology|Laboratory of molecular-genetic research|Abdaliyev|Kazakhstan
>Spike|hCoV-19/USA/TX-HMH-MCoV-13439/2020|2020-08-21|EPI_ISL_788574|Original|hCoV-19^^Texas|Human|Houston Methodist Hospital|Houston Methodist Hospital|Long|USA
>Spike|hCoV-19/Australia/NSW1040/2020|2020-06-29|EPI_ISL_593692|Original|hCoV-19^^New South Wales|Human|South Eastern Area Laboratory Services (SEALS)|NSW Health Pathology - Institute of Clinical Pathology and Medical Research; Westmead Hospital; University of Sydney|Gall|Australia
>Spike|hCoV-19/England/QEUH-B12598/2020|2020-11-02|EPI_ISL_642510|Original|hCoV-19^^England|Human|Lighthouse Lab in Glasgow|Wellcome Sanger Institute for the COVID-19 Genomics UK (COG-UK) Consortium|Davies|United Kingdom
>Spike|hCoV-19/Canada/BC-BCCDC-6446/2020|2020-10-00|EPI_ISL_740596|Original|hCoV-19^^British Columbia|Human|BCCDC Public Health Laboratory|BCCDC Public Health Laboratory|Prystajecky|Canada
>Spike|hCoV-19/England/ALDP-9FC912/2020|2020-10-01|EPI_ISL_587772|Original|hCoV-19^^England|Human|Lighthouse Lab in Alderley Park|Wellcome Sanger Institute for the COVID-19 Genomics UK (COG-UK) consortium|Davies|United Kingdom
>Spike|hCoV-19/USA/NY-NYCPHL-001362/2020|2020-11-16|EPI_ISL_683983|Original|hCoV-19^^New York|Human|DOHMH Central Harlem|New York City Public Health Laboratory|Wang|USA
>Spike|hCoV-19/Denmark/DCGC-17310/2020|2020-12-14|EPI_ISL_750282|Original|hCoV-19^^Nordjylland|Human|Department of Virus and Microbiological Special Diagnostics|Albertsen Lab|Michaelsen|Denmark
>Spike|hCoV-19/USA/TX-DSHS-0225/2020|2020-04-07|EPI_ISL_525784|Original|hCoV-19^^Texas|Human|Texas Department of State Health Services|Texas Department of State Health Services|Rahman|USA
>Spike|hCoV-19/Belgium/ULG-10839/2020|2020-10-27|EPI_ISL_666874|Original|hCoV-19^^Li\u00e8ge|Human|Department of Clinical Microbiology|GIGA Medical Genomics|Durkin|Belgium
>Spike|hCoV-19/USA/AZ-TG566375/2020|2020-07-31|EPI_ISL_695560|Original|hCoV-19^^Arizona|Human|TGen North|TGen North|French|USA
>Spike|hCoV-19/Slovakia/UKBA-315/2020|2020-09-10|EPI_ISL_583483|Original|hCoV-19^^Kamenicna|Human|Institute of Virology|Faculty of Natural Sciences|Vinar|Slovakia
>Spike|hCoV-19/Denmark/DCGC-6914/2020|2020-10-12|EPI_ISL_619760|Original|hCoV-19^^Hovedstaden|Human|Department of Virus and Microbiological Special Diagnostics|Albertsen lab|Michaelsen|Denmark
>Spike|hCoV-19/Denmark/DCGC-14213/2020|2020-11-30|EPI_ISL_711628|Original|hCoV-19^^Hovedstaden|Human|Department of Virus and Microbiological Special Diagnostics|Albertsen Lab|Michaelsen|Denmark
>Spike|hCoV-19/Latvia/034/2020|2020-05-08|EPI_ISL_486417|Original|hCoV-19^^Riga|Human|Centrala laboratorija|Latvian Biomedical Research and Study Centre|Zrelovs|Latvia

>Spike|hCoV-19/Luxembourg/LNS4335636/2020|2020-12-27|EPI_ISL_771117|Original|hCoV-19^^Luxembourg|Human|Laboratoire national de sant\u00e9|Laboratoire national de sant\u00e9|Wienecke-Baldacchino|Luxembourg
>Spike|hCoV-19/England/20119086104/2020|2020-03-15|EPI_ISL_465118|Original|hCoV-19^^England|Human|Respiratory Virus Unit|Respiratory Virus Unit|Platt|United Kingdom
>Spike|hCoV-19/Wales/PHWC-480D67/2020|2020-10-18|EPI_ISL_611742|Original|hCoV-19^^Wales|Human|Wales Specialist Virology Centre Sequencing lab|COVID-19 Genomics UK (COG-UK) Consortium|Moore|United Kingdom
>Spike|hCoV-19/Austria/CeMM0662/2020|2020-05-14|EPI_ISL_583630|Original|hCoV-19^^Austria|Human|Austrian Agency for Health and Food Safety (AGES)|Bergthaler laboratory|Bergthaler|Austria
>Spike|hCoV-19/Netherlands/ZH-EMC-898/2020|2020-10-21|EPI_ISL_632788|Original|hCoV-19^^Zuid Holland|Human|Dutch COVID-19 response team|Erasmus Medical Center|Oude Munnink|Netherlands
>Spike|hCoV-19/England/MILK-992B54/2020|2020-08-27|EPI_ISL_552158|Original|hCoV-19^^England|Human|Lighthouse Lab in Milton Keynes|Wellcome Sanger Institute for the COVID-19 Genomics UK (COG-UK) consortium|Davies|United Kingdom
>Spike|hCoV-19/Australia/WA320/2020|2020-03-07|EPI_ISL_596863|Original|hCoV-19^^Western Australia|Human|PathWest Laboratory Medicine WA|PathWest Laboratory Medicine WA Microbial Surveillance Unit|Sikazwe|Australia
>Spike|hCoV-19/Japan/PG-6202/2020|2020-08-00|EPI_ISL_689314|Original|hCoV-19^^Japan|Human|Pathogen Genomics Center|Pathogen Genomics Center|Sekizuka|Japan
>Spike|hCoV-19/USA/NC_200252/2020|2020-03-00|EPI_ISL_454825|Original|hCoV-19^^North Carolina|Human|Dirk Dittmer|Dirk Dittmer|Moorad|USA
>Spike|hCoV-19/USA/CA-CZB-13865/2020|2020-08-12|EPI_ISL_739470|Original|hCoV-19^^California|Human|Alameda County Public Health Lab|Chan-Zuckerberg Biohub|Kamm|USA
>Spike|hCoV-19/Wales/PHWC-265E7/2020|2020-03-27|EPI_ISL_422019|Original|hCoV-19^^Wales|Human|Wales Specialist Virology Centre|Public Health Wales Microbiology Cardiff|Moore|United Kingdom
>Spike|hCoV-19/USA/TX-HMH-MCoV-8182/2020|2020-07-07|EPI_ISL_783555|Original|hCoV-19^^Texas|Human|Houston Methodist Hospital|Houston Methodist Hospital|Long|USA
>Spike|hCoV-19/Denmark/DCGC-19027/2020|2020-12-07|EPI_ISL_748707|Original|hCoV-19^^Syddanmark|Human|Department of Virus and Microbiological Special Diagnostics|Albertsen Lab|Michaelsen|Denmark
>Spike|hCoV-19/England/ALDP-5B475C/2020|2020-06-17|EPI_ISL_558673|Original|hCoV-19^^England|Human|Lighthouse Lab in Alderley Park|Wellcome Sanger Institute for the COVID-19 Genomics UK (COG-UK) consortium|Davies|United Kingdom
>Spike|hCoV-19/USA/WI-UW-1668/2020|2020-09-17|EPI_ISL_605542|Original|hCoV-19^^Wisconsin|Human|University of Wisconsin-Madison AIDS Vaccine Research Laboratories|University of Wisconsin-Madison AIDS Vaccine Research Laboratories|Baczenas|USA
>Spike|hCoV-19/Netherlands/NH-RIVM-10103/2020|2020-03-10|EPI_ISL_547501|Original|hCoV-19^^Noord Holland|Human|Dutch COVID-19 response team|National Institute for Public Health and the Environment (RIVM)|Meijer|Netherlands
>Spike|hCoV-19/Singapore/129nan/2020|2020-03-20|EPI_ISL_538449|Original|hCoV-19^^Singapore|Human|Department of Laboratory Medicine|Department of Laboratory Medicine|Chen|Singapore
>Spike|hCoV-19/Denmark/DCGC-3515/2020|2020-08-31|EPI_ISL_616306|Original|hCoV-19^^Syddanmark|Human|Department of Virus and Microbiological Special Diagnostics|Albertsen lab|Michaelsen|Denmark
>Spike|hCoV-19/USA/MI-MDHHS-SC21137/2020|2020-04-01|EPI_ISL_507913|Original|hCoV-19^^Michigan|Human|Michigan Department of Health and Human Services|Michigan Department of Health and Human Services|Blankenship|USA
>Spike|hCoV-19/England/ALDP-B75E5C/2020|2020-11-12|EPI_ISL_723712|Original|hCoV-19^^England|Human|Oxford Viromics|COVID-19 Genomics UK (COG-UK) Consortium|Golubchik|United Kingdom
>Spike|hCoV-19/USA/NY-Wadsworth-269648-01/2020|2020-11-10|EPI_ISL_734442|Original|hCoV-19^^New York|Human|Wadsworth Center|Wadsworth Center|Lasek-Nesselquist|USA

>Spike|hCoV-19/Peru/LIM-UPCH-0151/2020|2020-08-28|EPI_ISL_568545|Original|hCoV-19^^Lima|Human|Laboratorio de Referencia Nacional de Virus Respiratorios|Laboratorio de Gen\u00f3mica Microbiana|Tsukayama|Peru
>Spike|hCoV-19/England/MILK-975823/2020|2020-08-20|EPI_ISL_552700|Original|hCoV-19^^England|Human|Lighthouse Lab in Milton Keynes|Wellcome Sanger Institute for the COVID-19 Genomics UK (COG-UK) consortium|Davies|United Kingdom
>Spike|hCoV-19/Scotland/EDB8239/2020|2020-10-03|EPI_ISL_585490|Original|hCoV-19^^Scotland|Human|Virology Department|COVID-19 Genomics UK (COG-UK) Consortium|McHugh|United Kingdom
>Spike|hCoV-19/Denmark/DCGC-11561/2020|2020-11-02|EPI_ISL_670996|Original|hCoV-19^^Nordjylland|Human|Department of Virus and Microbiological Special Diagnostics|Albertsen Lab|Michaelsen|Denmark
>Spike|hCoV-19/England/OXON-B0A6B/2020|2020-04-14|EPI_ISL_478931|Original|hCoV-19^^England|Human|Oxford Viromics|COVID-19 Genomics UK (COG-UK) Consortium|Golubchik|United Kingdom
>Spike|hCoV-19/USA/TX-HMH-MCoV-8792/2020|2020-07-01|EPI_ISL_780758|Original|hCoV-19^^Texas|Human|Houston Methodist Hospital|Houston Methodist Hospital|Long|USA
>Spike|hCoV-19/Germany/BY-MVP-0017/2020|2020-03-12|EPI_ISL_437218|Original|hCoV-19^^Bavaria|Human|Max von Pettenkofer Institute|Laboratory for Functional Genome Analysis|Graf|Germany
>Spike|hCoV-19/Australia/VIC8419/2020|2020-07-27|EPI_ISL_564999|Original|hCoV-19^^Victoria|Human|Microbiological Diagnostic Unit - Public Health Laboratory (MDU-PHL)|MDU-PHL|Schultz|Australia
>Spike|hCoV-19/Netherlands/Loon_op_zand_1363512/2020|2020-02-29|EPI_ISL_413576|Original|hCoV-19^^Loon op zand|Human|RIVM|Erasmus Medical Center|Oude Munnink|Netherlands
>Spike|hCoV-19/England/GSTT-5561/2020|2020-03-22|EPI_ISL_560687|Original|hCoV-19^^England|Human|Centre for Clinical Infection and Diagnostics Research and Genomics Innovation Unit|Centre for Clinical Infection and Diagnostics Research and Genomics Innovation Unit|Awan|United Kingdom
>Spike|hCoV-19/Czech Republic/NRL_2307/2020|2020-03-10|EPI_ISL_471540|Original|hCoV-19^^Vysocina|Human|The National Institute of Public Health|State Veterinary Institute Prague and The National Institute of Public Health|Nagy|Czech Republic
>Spike|hCoV-19/USA/TX-HMH-MCoV-13782/2020|2020-08-18|EPI_ISL_788875|Original|hCoV-19^^Texas|Human|Houston Methodist Hospital|Houston Methodist Hospital|Long|USA
>Spike|hCoV-19/Wales/PHWC-23CB8/2020|2020-03-15|EPI_ISL_418101|Original|hCoV-19^^Wales|Human|Wales Specialist Virology Centre|Public Health Wales Microbiology Cardiff|Moore|United Kingdom
>Spike|hCoV-19/USA/CO-CSU-D2399-2/2020|2020-04-08|EPI_ISL_527466|Original|hCoV-19^^Colorado|Human|Colorado State University - Ebel Lab|Colorado State University - Ebel Lab|Cline|USA
>Spike|hCoV-19/Denmark/DCGC-17415/2020|2020-12-14|EPI_ISL_750966|Original|hCoV-19^^Nordjylland|Human|Department of Virus and Microbiological Special Diagnostics|Albertsen Lab|Michaelsen|Denmark
>Spike|hCoV-19/USA/DE-DHSS-F970840/2020|2020-06-15|EPI_ISL_693693|Original|hCoV-19^^Delaware|Human|Delaware Public Health Laboratory|Delaware Public Health Laboratory|Bajwa|USA
>Spike|hCoV-19/Wales/PHWC-4840F1/2020|2020-10-23|EPI_ISL_627966|Original|hCoV-19^^Wales|Human|Wales Specialist Virology Centre Sequencing lab|COVID-19 Genomics UK (COG-UK) Consortium|Moore|United Kingdom
>Spike|hCoV-19/England/NORT-2822C2/2020|2020-03-25|EPI_ISL_488575|Original|hCoV-19^^England|Human|NU-OMICS DNA Sequencing research facility|Wellcome Sanger Institute for the COVID-19 Genomics UK (COG-UK) consortium|Davies|United Kingdom
>Spike|hCoV-19/Japan/PG-5320/2020|2020-08-00|EPI_ISL_690423|Original|hCoV-19^^Japan|Human|Pathogen Genomics Center|Pathogen Genomics Center|Sekizuka|Japan
>Spike|hCoV-19/Australia/VIC2237/2020|2020-06-22|EPI_ISL_521974|Original|hCoV-19^^Victoria|Human|Microbiological Diagnostic Unit - Public Health Laboratory (MDU-PHL)|MDU-PHL|Schultz|Australia

>Spike|hCoV-19/England/QEUH-9F7373/2020|2020-09-27|EPI_ISL_595681|Original|hCoV-19^^England|Human|Oxford Viromics|COVID-19 Genomics UK (COG-UK) Consortium|Golubchik|United Kingdom
>Spike|hCoV-19/Wales/PHWC-361C2/2020|2020-04-29|EPI_ISL_474632|Original|hCoV-19^^Wales|Human|Originating lab|COVID-19 Genomics UK (COG-UK) Consortium|Moore|United Kingdom
>Spike|hCoV-19/India/MH-ACTREC-454/2020|2020-08-09|EPI_ISL_700102|Original|hCoV-19^^Maharashtra|Human|Hematopathology Laboratory|Hematopathology Laboratory|Patkar|India
>Spike|hCoV-19/England/ALDP-95656D/2020|2020-06-08|EPI_ISL_554027|Original|hCoV-19^^England|Human|Lighthouse Lab in Alderley Park|Wellcome Sanger Institute for the COVID-19 Genomics UK (COG-UK) consortium|Davies|United Kingdom
>Spike|hCoV-19/Australia/NSW2955/2020|2020-10-22|EPI_ISL_678381|Original|hCoV-19^^New South Wales|Human|Area of Virology|Virology Research Laboratory; Area of Virology|Foster|Australia
>Spike|hCoV-19/England/ALDP-9531B7/2020|2020-06-12|EPI_ISL_554522|Original|hCoV-19^^England|Human|Lighthouse Lab in Alderley Park|Wellcome Sanger Institute for the COVID-19 Genomics UK (COG-UK) consortium|Davies|United Kingdom
>Spike|hCoV-19/USA/SC-DHEC-0535/2020|2020-06-04|EPI_ISL_529083|Original|hCoV-19^^South Carolina|Human|Microbiology Division|Microbiology Division|Flores,H.|USA
>Spike|hCoV-19/USA/TX-HMH-MCoV-13089/2020|2020-08-27|EPI_ISL_788240|Original|hCoV-19^^Texas|Human|Houston Methodist Hospital|Houston Methodist Hospital|Long|USA
>Spike|hCoV-19/England/MILK-992A39/2020|2020-08-27|EPI_ISL_551960|Original|hCoV-19^^England|Human|Lighthouse Lab in Milton Keynes|Wellcome Sanger Institute for the COVID-19 Genomics UK (COG-UK) consortium|Davies|United Kingdom
>Spike|hCoV-19/USA/UT-UPHL-2012706876/2020|2020-08-09|EPI_ISL_745805|Original|hCoV-19^^Utah|Human|Ginkgo Bioworks Clinical Laboratory|Utah Public Health Laboratory|Young|USA
>Spike|hCoV-19/England/QEUH-95FAED/2020|2020-08-02|EPI_ISL_532505|Original|hCoV-19^^England|Human|Lighthouse Lab in Glasgow|Wellcome Sanger Institute for the COVID-19 Genomics UK (COG-UK) consortium|Davies|United Kingdom
>Spike|hCoV-19/Wales/QEUH-AE2E1F/2020|2020-10-27|EPI_ISL_647883|Original|hCoV-19^^Wales|Human|Lighthouse Lab in Glasgow|Wellcome Sanger Institute for the COVID-19 Genomics UK (COG-UK) Consortium|Davies|United Kingdom
>Spike|hCoV-19/Japan/PG-5961/2020|2020-00-00|EPI_ISL_689076|Original|hCoV-19^^Japan|Human|Gunma Prefectural Institute of Public Health and Environmental Sciences|Pathogen Genomics Center|Sekizuka|Japan
>Spike|hCoV-19/England/ALDP-A87A45/2020|2020-10-17|EPI_ISL_606275|Original|hCoV-19^^England|Human|Lighthouse Lab in Alderley Park|Wellcome Sanger Institute for the COVID-19 Genomics UK (COG-UK) consortium|Davies|United Kingdom
>Spike|hCoV-19/Denmark/DCGC-16941/2020|2020-12-14|EPI_ISL_750656|Original|hCoV-19^^Midtjylland|Human|Department of Virus and Microbiological Special Diagnostics|Albertsen Lab|Michaelsen|Denmark
>Spike|hCoV-19/Japan/PG-1934/2020|2020-04-00|EPI_ISL_685540|Original|hCoV-19^^Japan|Human|Pathogen Genomics Center|Pathogen Genomics Center|Sekizuka|Japan
>Spike|hCoV-19/England/QEUH-96C537/2020|2020-08-13|EPI_ISL_530806|Original|hCoV-19^^England|Human|Lighthouse Lab in Glasgow|Wellcome Sanger Institute for the COVID-19 Genomics UK (COG-UK) consortium|Davies|United Kingdom
>Spike|hCoV-19/England/CAMC-BF612D/2020|2020-12-04|EPI_ISL_760932|Original|hCoV-19^^England|Human|Lighthouse Lab in Cambridge|Wellcome Sanger Institute for the COVID-19 Genomics UK (COG-UK) Consortium|Davies|United Kingdom
>Spike|hCoV-19/Gibraltar/204462907/2020|2020-10-29|EPI_ISL_632805|Original|hCoV-19^^Gibraltar|Human|Microbiology|Respiratory Virus Unit|Platt|Gibraltar
>Spike|hCoV-19/Australia/VIC1965/2020|2020-06-12|EPI_ISL_480688|Original|hCoV-19^^Victoria|Human|Victorian Infectious Diseases Reference Laboratory (VIDRL)|VIDRL and MDU-PHL|Schultz|Australia
>Spike|hCoV-19/USA/MA-MGH-00628/2020|2020-03-28|EPI_ISL_460137|Original|hCoV-19^^Massachusetts|Human|Massachusetts General Hospital|Infectious Disease Program|Lemieux|USA

>Spike|hCoV-19/England/MILK-BB0981/2020|2020-11-17|EPI_ISL_720098|Original|hCoV-19^^England|Human|Lighthouse Lab in Milton Keynes|Wellcome Sanger Institute for the COVID-19 Genomics UK (COG-UK) Consortium|Davies|United Kingdom
>Spike|hCoV-19/Denmark/DCGC-19074/2020|2020-12-07|EPI_ISL_748738|Original|hCoV-19^^Hovedstaden|Human|Department of Virus and Microbiological Special Diagnostics|Albertsen Lab|Michaelsen|Denmark
>Spike|hCoV-19/Wales/PHWC-16C836/2020|2020-09-16|EPI_ISL_574100|Original|hCoV-19^^Wales|Human|Wales Specialist Virology Centre Sequencing lab|COVID-19 Genomics UK (COG-UK) Consortium|Moore|United Kingdom
>Spike|hCoV-19/England/BRIS-12F978/2020|2020-03-30|EPI_ISL_488312|Original|hCoV-19^^England|Human|PHE South West Regional Laboratory|Wellcome Sanger Institute for the COVID-19 Genomics UK (COG-UK) consortium|Davies|United Kingdom
>Spike|hCoV-19/Jordan/AM-ALSR-4086/2020|2020-10-03|EPI_ISL_636446|Original|hCoV-19^^Amman|Human|Biolab Diagnostic Laboratories|Andersen lab at Scripps Research|Gangavarapu|Jordan
>Spike|hCoV-19/England/QEUH-9F74CB/2020|2020-09-27|EPI_ISL_589124|Original|hCoV-19^^England|Human|Lighthouse Lab in Glasgow|Wellcome Sanger Institute for the COVID-19 Genomics UK (COG-UK) consortium|Davies|United Kingdom
>Spike|hCoV-19/Switzerland/BL-ETHZ-410252/2020|2020-12-14|EPI_ISL_737670|Original|hCoV-19^^Basel-Landschaft|Human|Viollier AG|Department of Biosystems Science and Engineering|Nadeau|Switzerland
>Spike|hCoV-19/England/ALDP-9C5A2E/2020|2020-09-11|EPI_ISL_567744|Original|hCoV-19^^England|Human|Lighthouse Lab in Alderley Park|Wellcome Sanger Institute for the COVID-19 Genomics UK (COG-UK) consortium|Davies|United Kingdom
>Spike|hCoV-19/USA/WA-S1319/2020|2020-05-15|EPI_ISL_495769|Original|hCoV-19^^Washington|Human|Washington State Department of Health|Seattle Flu Study|Lee|USA
>Spike|hCoV-19/Brazil/SP-880/2020|2020-12-07|EPI_ISL_755646|Original|hCoV-19^^S\u00e3o Paulo|Human|Instituto Adolfo Lutz - Regional de Santo Andre|Instituto Adolfo Lutz|Sacchi|Brazil
>Spike|hCoV-19/Denmark/DCGC-18153/2020|2020-12-14|EPI_ISL_749784|Original|hCoV-19^^Hovedstaden|Human|Department of Virus and Microbiological Special Diagnostics|Albertsen Lab|Michaelsen|Denmark
>Spike|hCoV-19/England/ALDP-9BCA2A/2020|2020-09-09|EPI_ISL_573041|Original|hCoV-19^^England|Human|Oxford Viromics|COVID-19 Genomics UK (COG-UK) Consortium|Golubchik|United Kingdom
>Spike|hCoV-19/England/20162005204/2020|2020-04-13|EPI_ISL_466397|Original|hCoV-19^^England|Human|Respiratory Virus Unit|Respiratory Virus Unit|Platt|United Kingdom
>Spike|hCoV-19/England/SHEF-CA406/2020|2020-05-12|EPI_ISL_493694|Original|hCoV-19^^England|Human|Virology Department|COVID-19 Genomics UK (COG-UK) Consortium|de Silva|United Kingdom
>Spike|hCoV-19/England/MILK-B01EBF/2020|2020-10-24|EPI_ISL_642893|Original|hCoV-19^^England|Human|Lighthouse Lab in Milton Keynes|Wellcome Sanger Institute for the COVID-19 Genomics UK (COG-UK) Consortium|Davies|United Kingdom
>Spike|hCoV-19/Denmark/DCGC-12672/2020|2020-11-16|EPI_ISL_682853|Original|hCoV-19^^Syddanmark|Human|Department of Virus and Microbiological Special Diagnostics|Albertsen Lab|Michaelsen|Denmark
>Spike|hCoV-19/USA/FL-BPHL-2014/2020|2020-06-19|EPI_ISL_653167|Original|hCoV-19^^Florida|Human|Florida Bureau of Public Health Laboratories|Florida Bureau of Public Health Laboratories|Blanton|USA
>Spike|hCoV-19/England/BRIS-12F7C9/2020|2020-03-10|EPI_ISL_488241|Original|hCoV-19^^England|Human|PHE South West Regional Laboratory|Wellcome Sanger Institute for the COVID-19 Genomics UK (COG-UK) consortium|Davies|United Kingdom
>Spike|hCoV-19/USA/MD-HP00131/2020|2020-03-18|EPI_ISL_457819|Original|hCoV-19^^Maryland|Human|Johns Hopkins Hospital Department of Pathology|Johns Hopkins Hospital Department of Pathology|Ramakrishnan|USA
>Spike|hCoV-19/Japan/PG-2236/2020|2020-04-00|EPI_ISL_685841|Original|hCoV-19^^Japan|Human|Pathogen Genomics Center|Pathogen Genomics Center|Sekizuka|Japan
>Spike|hCoV-19/England/CAMC-C2D3F9/2020|2020-12-07|EPI_ISL_733732|Original|hCoV-19^^England|Human|Lighthouse Lab in Cambridge|Wellcome Sanger Institute for the COVID-19 Genomics UK (COG-UK) Consortium|Davies|United Kingdom

>Spike|hCoV-19/USA/TX-CDC-0406/2020|2020-03-30|EPI_ISL_454646|Original|hCoV-19^^Texas|Human|City of El Paso Department of Public Health Laboratory|Pathogen Discovery|Queen|USA
>Spike|hCoV-19/USA/LA-EVTL771/2020|2020-07-21|EPI_ISL_653462|Original|hCoV-19^^Louisiana|Human|LSUHS Emerging Viral Threat Laboratory|Microbial Genome Sequencing Center|Kamil|USA
>Spike|hCoV-19/USA/WA-S2008/2020|2020-06-04|EPI_ISL_497436|Original|hCoV-19^^Washington|Human|Washington State Department of Health|Seattle Flu Study|Lee|USA
>Spike|hCoV-19/Wales/PHWC-2855E/2020|2020-03-30|EPI_ISL_432084|Original|hCoV-19^^Wales|Human|Wales Specialist Virology Centre|Public Health Wales Microbiology Cardiff|Moore|United Kingdom
>Spike|hCoV-19/England/LIVE-A4AC4/2020|2020-03-11|EPI_ISL_500069|Original|hCoV-19^^England|Human|Liverpool Clinical Laboratories|COVID-19 Genomics UK (COG-UK) Consortium|Haldenby|United Kingdom
>Spike|hCoV-19/Wales/PHWC-4871FB/2020|2020-10-31|EPI_ISL_639173|Original|hCoV-19^^Wales|Human|Wales Specialist Virology Centre Sequencing lab|COVID-19 Genomics UK (COG-UK) Consortium|Moore|United Kingdom
>Spike|hCoV-19/England/ALDP-9CC508/2020|2020-09-14|EPI_ISL_573112|Original|hCoV-19^^England|Human|Quadram Institute Bioscience|COVID-19 Genomics UK (COG-UK) Consortium|Alikhan|United Kingdom
>Spike|hCoV-19/USA/TX-HMH-MCoV-10765/2020|2020-07-09|EPI_ISL_789617|Original|hCoV-19^^Texas|Human|Houston Methodist Hospital|Houston Methodist Hospital|Long|USA
>Spike|hCoV-19/USA/TX-HMH-MCoV-15940/2020|2020-07-10|EPI_ISL_785624|Original|hCoV-19^^Texas|Human|Houston Methodist Hospital|Houston Methodist Hospital|Long|USA
>Spike|hCoV-19/Iceland/344/2020|2020-03-00|EPI_ISL_424368|Original|hCoV-19^^Reykjavik|Human|The National University Hospital of Iceland|deCODE genetics|J\u00f3nsson|Iceland
>Spike|hCoV-19/England/MILK-B95E29/2020|2020-11-14|EPI_ISL_675100|Original|hCoV-19^^England|Human|Lighthouse Lab in Milton Keynes|Wellcome Sanger Institute for the COVID-19 Genomics UK (COG-UK) Consortium|Davies|United Kingdom
>Spike|hCoV-19/England/QEUH-B7C24A/2020|2020-11-14|EPI_ISL_668323|Original|hCoV-19^^England|Human|Lighthouse Lab in Glasgow|Wellcome Sanger Institute for the COVID-19 Genomics UK (COG-UK) Consortium|Davies|United Kingdom
>Spike|hCoV-19/Scotland/CVR1426/2020|2020-04-02|EPI_ISL_433580|Original|hCoV-19^^Scotland|Human|West of Scotland Specialist Virology Centre|COVID-19 Genomics UK (COG-UK) Consortium|Robertson|United Kingdom
>Spike|hCoV-19/USA/CA-LACPHL-AE00046/2020|2020-07-09|EPI_ISL_735377|Original|hCoV-19^^California|Human|Los Angeles County Public Health Laboratory|Los Angeles County Public Health Laboratory|Hemarajata|USA
>Spike|hCoV-19/England/20149044504/2020|2020-04-03|EPI_ISL_466199|Original|hCoV-19^^England|Human|Respiratory Virus Unit|Respiratory Virus Unit|Platt|United Kingdom
>Spike|hCoV-19/Uganda/UG111/2020|2020-08-17|EPI_ISL_738018|Original|hCoV-19^^Uganda|Human|Uganda Central Public Health Lab and Uganda Virus Research Institute|MRC/UVRI & LSHTM Uganda\u00a0Research Unit|Cotten|Uganda
>Spike|hCoV-19/USA/WA-S2716/2020|2020-06-26|EPI_ISL_525706|Original|hCoV-19^^Washington|Human|Seattle Flu Study|Seattle Flu Study|Lee|USA
>Spike|hCoV-19/USA/MN-QDX-1601/2020|2020-03-16|EPI_ISL_603981|Original|hCoV-19^^Minnesota|Human|Quest Diagnostics|Quest Diagnostics|Kagan|USA
>Spike|hCoV-19/USA/AZ-TG668330/2020|2020-05-12|EPI_ISL_696257|Original|hCoV-19^^Arizona|Human|Sonora Quest Laboratories|TGen North|French|USA
>Spike|hCoV-19/Scotland/EDB6316/2020|2020-04-07|EPI_ISL_487995|Original|hCoV-19^^Scotland|Human|Virology Department|Wellcome Sanger Institute for the COVID-19 Genomics UK (COG-UK) consortium|Davies|United Kingdom
>Spike|hCoV-19/Italy/LAZ-INMI-97/2020|2020-11-02|EPI_ISL_718262|Original|hCoV-19^^Lazio|Human|National Institute for Infectious Diseases|National Institute for Infectious Diseases|Gruber|Italy
>Spike|hCoV-19/Germany/BY-IMB11046/2020|2020-09-24|EPI_ISL_732528|Original|hCoV-19^^Bavaria|Human|Bundeswehr Institute of Microbiology|Bundeswehr Institute of Microbiology|Antwerpen|Germany

>Spike|hCoV-19/USA/MN-CDC-6498/2020|2020-04-09|EPI_ISL_527711|Original|hCoV-19^^Minnesota|Human|MN PHL Division|Pathogen Discovery|Queen|USA
>Spike|hCoV-19/England/ALDP-C486A4/2020|2020-12-12|EPI_ISL_736539|Original|hCoV-19^^England|Human|Lighthouse Lab in Alderley Park|Wellcome Sanger Institute for the COVID-19 Genomics UK (COG-UK) Consortium|Davies|United Kingdom
>Spike|hCoV-19/England/MILK-BB21C3/2020|2020-11-18|EPI_ISL_720535|Original|hCoV-19^^England|Human|Lighthouse Lab in Milton Keynes|Wellcome Sanger Institute for the COVID-19 Genomics UK (COG-UK) Consortium|Davies|United Kingdom
>Spike|hCoV-19/England/QEUH-9F7F9D/2020|2020-09-27|EPI_ISL_588768|Original|hCoV-19^^England|Human|Lighthouse Lab in Glasgow|Wellcome Sanger Institute for the COVID-19 Genomics UK (COG-UK) consortium|Davies|United Kingdom
>Spike|hCoV-19/England/MILK-A7B798/2020|2020-10-15|EPI_ISL_607484|Original|hCoV-19^^England|Human|Lighthouse Lab in Milton Keynes|Wellcome Sanger Institute for the COVID-19 Genomics UK (COG-UK) consortium|Davies|United Kingdom
>Spike|hCoV-19/England/SHEF-D06DA/2020|2020-04-19|EPI_ISL_462073|Original|hCoV-19^^England|Human|Virology Department|COVID-19 Genomics UK (COG-UK) Consortium|de Silva|United Kingdom
>Spike|hCoV-19/England/CAMC-9D236F/2020|2020-09-19|EPI_ISL_601579|Original|hCoV-19^^England|Human|Lighthouse Lab in Cambridge|Wellcome Sanger Institute for the COVID-19 Genomics UK (COG-UK) consortium|Davies|United Kingdom
>Spike|hCoV-19/United Arab Emirates/0620/2020|2020-05-15|EPI_ISL_698776|Original|hCoV-19^^Abu Dhabi|Human|Group 42 (G42) Healthcare|G42 Healthcare|Ogrodzki|United Arab Emirates
>Spike|hCoV-19/Denmark/DCGC-11412/2020|2020-11-02|EPI_ISL_670901|Original|hCoV-19^^Syddanmark|Human|Department of Virus and Microbiological Special Diagnostics|Albertsen Lab|Michaelsen|Denmark
>Spike|hCoV-19/England/QEUH-A8B5AA/2020|2020-10-16|EPI_ISL_643230|Original|hCoV-19^^England|Human|Lighthouse Lab in Glasgow|Wellcome Sanger Institute for the COVID-19 Genomics UK (COG-UK) Consortium|Davies|United Kingdom
>Spike|hCoV-19/Wales/PHWC-166EA3/2020|2020-06-15|EPI_ISL_494125|Original|hCoV-19^^Wales|Human|Originating lab|COVID-19 Genomics UK (COG-UK) Consortium|Moore|United Kingdom
>Spike|hCoV-19/England/MILK-9A9835/2020|2020-08-23|EPI_ISL_550974|Original|hCoV-19^^England|Human|Lighthouse Lab in Milton Keynes|Wellcome Sanger Institute for the COVID-19 Genomics UK (COG-UK) consortium|Davies|United Kingdom
>Spike|hCoV-19/Belgium/rega-03061153/2020|2020-03-06|EPI_ISL_738203|Original|hCoV-19^^Sint-Lambrechts-Woluwe|Human|UZ Leuven|KU Leuven|Maes|Belgium
>Spike|hCoV-19/England/ALDP-A85ACF/2020|2020-10-17|EPI_ISL_610434|Original|hCoV-19^^England|Human|Lighthouse Lab in Alderley Park|Wellcome Sanger Institute for the COVID-19 Genomics UK (COG-UK) consortium|Davies|United Kingdom
>Spike|hCoV-19/England/ALDP-CBE1D5/2020|2020-12-19|EPI_ISL_760390|Original|hCoV-19^^England|Human|Lighthouse Lab in Alderley Park|Wellcome Sanger Institute for the COVID-19 Genomics UK (COG-UK) Consortium|Davies|United Kingdom
>Spike|hCoV-19/England/20128090404/2020|2020-03-20|EPI_ISL_423965|Original|hCoV-19^^England|Human|Respiratory Virus Unit|Respiratory Virus Unit|Platt|United Kingdom
>Spike|hCoV-19/USA/TX-DSHS-0665/2020|2020-07-19|EPI_ISL_576319|Original|hCoV-19^^Texas|Human|Texas Department of State Health Services|Texas Department of State Health Services|Rahman|USA
>Spike|hCoV-19/USA/TX-HMH-MCoV-18135/2020|2020-11-13|EPI_ISL_784483|Original|hCoV-19^^Texas|Human|Houston Methodist Hospital|Houston Methodist Hospital|Long|USA
>Spike|hCoV-19/Germany/NW-HHU-172/2020|2020-03-25|EPI_ISL_602494|Orginal|hCoV-19^^North Rhine-Westphalia|Human|Institute for Virology|Center of Medical Microbiology|Wienemann|Germany
>Spike|hCoV-19/Luxembourg/LNS4352628/2020|2020-11-24|EPI_ISL_740452|Original|hCoV-19^^Luxembourg|Human|Laboratoire national de sant\u00e9|Laboratoire national de sant\u00e9|Wienecke-Baldacchino|Luxembourg
>Spike|hCoV-19/USA/WI-UW-1762/2020|2020-09-14|EPI_ISL_605635|Original|hCoV-19^^Wisconsin|Human|University of Wisconsin-Madison AIDS Vaccine Research Laboratories|University of Wisconsin-Madison AIDS Vaccine Research Laboratories|Baczenas|USA

>Spike|hCoV-19/USA/TX-HMH-MCoV-12841/2020|2020-08-19|EPI_ISL_790563|Original|hCoV-19^^Texas|Human|Houston Methodist Hospital|Houston Methodist Hospital|Long|USA
>Spike|hCoV-19/England/ALDP-954198/2020|2020-06-12|EPI_ISL_553796|Original|hCoV-19^^England|Human|Lighthouse Lab in Alderley Park|Wellcome Sanger Institute for the COVID-19 Genomics UK (COG-UK) consortium|Davies|United Kingdom
>Spike|hCoV-19/England/QEUH-B2BC88/2020|2020-11-02|EPI_ISL_721534|Original|hCoV-19^^England|Human|Lighthouse Lab in Glasgow|Wellcome Sanger Institute for the COVID-19 Genomics UK (COG-UK) Consortium|Davies|United Kingdom
>Spike|hCoV-19/USA/TX-DSHS-1386/2020|2020-05-15|EPI_ISL_654738|Original|hCoV-19^^Texas|Human|Texas Department of State Health Services|Texas Department of State Health Services|Rahman|USA
>Spike|hCoV-19/England/QEUH-AA02B7/2020|2020-10-19|EPI_ISL_611435|Original|hCoV-19^^England|Human|Lighthouse Lab in Glasgow|Wellcome Sanger Institute for the COVID-19 Genomics UK (COG-UK) consortium|Davies|United Kingdom
>Spike|hCoV-19/Wales/PHWC-48D3C2/2020|2020-10-25|EPI_ISL_680618|Original|hCoV-19^^Wales|Human|Wales Specialist Virology Centre Sequencing lab|COVID-19 Genomics UK (COG-UK) Consortium|Moore|United Kingdom
>Spike|hCoV-19/Scotland/QEUH-9C983F/2020|2020-09-10|EPI_ISL_576044|Original|hCoV-19^^Scotland|Human|Lighthouse Lab in Glasgow|Wellcome Sanger Institute for the COVID-19 Genomics UK (COG-UK) consortium|Davies|United Kingdom
>Spike|hCoV-19/Netherlands/UT-RIVM-20600/2020|2020-12-16|EPI_ISL_790683|Original|hCoV-19^^Utrecht|Human|Dutch COVID-19 response team|National Institute for Public Health and the Environment (RIVM)|Meijer|Netherlands
>Spike|hCoV-19/USA/FL-BPHL-0072/2020|2020-04-20|EPI_ISL_445015|Original|hCoV-19^^Florida|Human|Florida Bureau of Public Health Laboratories|Florida Bureau of Public Health Laboratories|Blanton|USA
>Spike|hCoV-19/India/MH-GA55/2020|2020-06-20|EPI_ISL_508250|Original|hCoV-19^^Maharashtra|Human|Government Medical College|National Institute of Biomedical Genomics|Maitra|India
>Spike|hCoV-19/Poland/PL_MCB_91/2020|2020-05-10|EPI_ISL_645090|Original|hCoV-19^^Poland|Human|Human Genome Variation Research Group|Human Genome Variation Research Group||Poland
>Spike|hCoV-19/Australia/TAS223/2020|2020-03-27|EPI_ISL_522652|Original|hCoV-19^^Tasmania|Human|Royal Hobart Hospital Microbiology Department|MDU-PHL|Schultz|Australia
>Spike|hCoV-19/South Africa/KRISP-K002632/2020|2020-07-31|EPI_ISL_535502|Original|hCoV-19^^KwaZulu-Natal|Human|NHLS-IALCH|KRISP|de Oliveira|South Africa
>Spike|hCoV-19/USA/FL-BPHL-0491/2020|2020-03-26|EPI_ISL_495397|Original|hCoV-19^^Florida|Human|Florida Bureau of Public Health Laboratories|Florida Bureau of Public Health Laboratories|Blanton|USA
>Spike|hCoV-19/USA/MI-QDX-312/2020|2020-03-22|EPI_ISL_571056|Original|hCoV-19^^Michigan|Human|Quest Diagnostics|Quest Diagnostics|Kagan|USA
>Spike|hCoV-19/Scotland/QEUH-A0CA39/2020|2020-10-03|EPI_ISL_585609|Original|hCoV-19^^Scotland|Human|Quadram Institute Bioscience|COVID-19 Genomics UK (COG-UK) Consortium|Alikhan|United Kingdom
>Spike|hCoV-19/India/OR-ILSCV35457/2020|2020-06-10|EPI_ISL_481197|Original|hCoV-19^^Odisha|Human|Immunogenomics lab|Immunogenomics lab|Raghav|India
>Spike|hCoV-19/England/QEUH-B131EE/2020|2020-11-02|EPI_ISL_642362|Original|hCoV-19^^England|Human|Lighthouse Lab in Glasgow|Wellcome Sanger Institute for the COVID-19 Genomics UK (COG-UK) Consortium|Davies|United Kingdom
>Spike|hCoV-19/Switzerland/BL-ETHZ-380283/2020|2020-11-25|EPI_ISL_721787|Original|hCoV-19^^Basel-Landschaft|Human|Viollier AG|Department of Biosystems Science and Engineering|Nadeau|Switzerland
>Spike|hCoV-19/USA/LA-SR0291/2020|2020-04-02|EPI_ISL_445146|Original|hCoV-19^^Louisiana|Human|Robert Garry lab|Andersen lab at Scripps Research|Gangavarapu|USA
>Spike|hCoV-19/England/NORW-F5A97/2020|2020-11-12|EPI_ISL_725293|Original|hCoV-19^^England|Human|Quadram Institute Bioscience|COVID-19 Genomics UK (COG-UK) Consortium|Alikhan|United Kingdom

>Spike|hCoV-19/Wales/PHWC-484CB1/2020|2020-10-23|EPI_ISL_628096|Original|hCoV-19^^Wales|Human|Wales Specialist Virology Centre Sequencing lab|COVID-19 Genomics UK (COG-UK) Consortium|Moore|United Kingdom
>Spike|hCoV-19/South Korea/KDCA0205/2020|2020-11-26|EPI_ISL_747440|Original|hCoV-19^^South Korea|Human|Division of Emerging Infectious Diseases|Division of Emerging Infectious Diseases|Park|South Korea
>Spike|hCoV-19/USA/WA-UW-12763/2020|2020-06-30|EPI_ISL_570900|Original|hCoV-19^^Washington|Human|UW Virology Lab|UW Virology Lab|Roychoudhury|USA
>Spike|hCoV-19/USA/MN-QDX-1317/2020|2020-03-14|EPI_ISL_572106|Original|hCoV-19^^Minnesota|Human|Quest Diagnostics|Quest Diagnostics|Kagan|USA
>Spike|hCoV-19/USA/UN-UW-5199/2020|2020-04-02|EPI_ISL_430897|Original|hCoV-19^^USA|Human|UW Virology Lab|UW Virology Lab|Roychoudhury|USA
>Spike|hCoV-19/USA/UT-UPHL-2012091701/2020|2020-08-04|EPI_ISL_745511|Original|hCoV-19^^Utah|Human|Ginkgo Bioworks Clinical Laboratory|Utah Public Health Laboratory|Young|USA
>Spike|hCoV-19/Singapore/1363/2020|2020-06-24|EPI_ISL_648714|Original|hCoV-19^^Singapore|Human|Department of Laboratory Medicine|Department of Laboratory Medicine|Chen|Singapore
>Spike|hCoV-19/England/CAMC-A416CB/2020|2020-10-07|EPI_ISL_609408|Original|hCoV-19^^England|Human|Lighthouse Lab in Cambridge|Wellcome Sanger Institute for the COVID-19 Genomics UK (COG-UK) consortium|Davies|United Kingdom
>Spike|hCoV-19/England/ALDP-95562F/2020|2020-06-13|EPI_ISL_553598|Original|hCoV-19^^England|Human|Lighthouse Lab in Alderley Park|Wellcome Sanger Institute for the COVID-19 Genomics UK (COG-UK) consortium|Davies|United Kingdom
>Spike|hCoV-19/Argentina/argenTAG-1283F02A/2020|2020-07-22|EPI_ISL_648211|Original|hCoV-19^^Buenos Aires|Human|INBIRS-UBA|Laboratorio Mixto de Biotecnolog\u00eda Acu\u00e1tica (LMBA)|Ezpeleta|Argentina
>Spike|hCoV-19/England/QEUH-9F98CD/2020|2020-09-26|EPI_ISL_589062|Original|hCoV-19^^England|Human|Lighthouse Lab in Glasgow|Wellcome Sanger Institute for the COVID-19 Genomics UK (COG-UK) consortium|Davies|United Kingdom
>Spike|hCoV-19/England/QEUH-C4B683/2020|2020-12-13|EPI_ISL_769758|Original|hCoV-19^^England|Human|Lighthouse Lab in Glasgow|Wellcome Sanger Institute for the COVID-19 Genomics UK (COG-UK) Consortium|Davies|United Kingdom
>Spike|hCoV-19/England/QEUH-A0AC26/2020|2020-10-01|EPI_ISL_599193|Original|hCoV-19^^England|Human|Lighthouse Lab in Glasgow|Wellcome Sanger Institute for the COVID-19 Genomics UK (COG-UK) consortium|Davies|United Kingdom
>Spike|hCoV-19/Ireland/LH-NVRL-20Z06929/2020|2020-04-17|EPI_ISL_671401|Original|hCoV-19^^Louth|Human|National Virus Reference Laboratory|Irish Coronavirus Sequencing Consortium - Teagasc Moorepark|Walsh|Ireland
>Spike|hCoV-19/England/LIVE-9D615/2020|2020-04-10|EPI_ISL_453217|Original|hCoV-19^^England|Human|Liverpool Clinical Laboratories|COVID-19 Genomics UK (COG-UK) Consortium|Haldenby|United Kingdom
>Spike|hCoV-19/USA/CA-CZB-1024/2020|2020-04-10|EPI_ISL_436665|Original|hCoV-19^^California|Human|County of Santa Clara Public Health Department|Chan-Zuckerberg Biohub|Hao|USA
>Spike|hCoV-19/USA/TX-HMH-5483/2020|2020-06-29|EPI_ISL_544808|Original|hCoV-19^^Texas|Human|Houston Methodist Hospital|Houston Methodist Hospital|Long|USA
>Spike|hCoV-19/USA/AZ-TG495452/2020|2020-07-13|EPI_ISL_695260|Original|hCoV-19^^Arizona|Human|TGen North|TGen North|French|USA
>Spike|hCoV-19/England/ALDP-9CB387/2020|2020-09-16|EPI_ISL_575430|Original|hCoV-19^^England|Human|Lighthouse Lab in Alderley Park|Wellcome Sanger Institute for the COVID-19 Genomics UK (COG-UK) consortium|Davies|United Kingdom
>Spike|hCoV-19/Denmark/DCGC-18086/2020|2020-12-14|EPI_ISL_749208|Original|hCoV-19^^Sjaelland|Human|Department of Virus and Microbiological Special Diagnostics|Albertsen Lab|Michaelsen|Denmark
>Spike|hCoV-19/England/MILK-B884DC/2020|2020-11-13|EPI_ISL_674386|Original|hCoV-19^^England|Human|Lighthouse Lab in Milton Keynes|Wellcome Sanger Institute for the COVID-19 Genomics UK (COG-UK) Consortium|Davies|United Kingdom

>Spike|hCoV-19/Hungary/MH-726/2020|2020-04-15|EPI_ISL_526234|Original|hCoV-19^^Pest county|Human|Hungarian Defence Forces Military Medical Centre|National Laboratory of Virology|Kemenesi|Hungary
>Spike|hCoV-19/Australia/VIC15213/2020|2020-08-03|EPI_ISL_593192|Original|hCoV-19^^Victoria|Human|Microbiological Diagnostic Unit - Public Health Laboratory (MDU-PHL)|MDU-PHL|Schultz|Australia
>Spike|hCoV-19/England/MILK-A911E9/2020|2020-10-16|EPI_ISL_638510|Original|hCoV-19^^England|Human|Oxford Viromics|COVID-19 Genomics UK (COG-UK) Consortium|Golubchik|United Kingdom
>Spike|hCoV-19/USA/LA-EVTL446/2020|2020-07-30|EPI_ISL_578850|Original|hCoV-19^^Louisiana|Human|LSUHS Emerging Viral Threat Laboratory|Microbial Genome Sequencing Center|Kamil|USA
>Spike|hCoV-19/Japan/PG-1968/2020|2020-05-00|EPI_ISL_685574|Original|hCoV-19^^Japan|Human|Pathogen Genomics Center|Pathogen Genomics Center|Sekizuka|Japan
>Spike|hCoV-19/Japan/PG-4398/2020|2020-07-00|EPI_ISL_692624|Original|hCoV-19^^Japan|Human|Pathogen Genomics Center|Pathogen Genomics Center|Sekizuka|Japan
>Spike|hCoV-19/USA/WI-UW-2409/2020|2020-12-07|EPI_ISL_754673|Original|hCoV-19^^Wisconsin|Human|University of Wisconsin-Madison AIDS Vaccine Research Laboratories|University of Wisconsin-Madison AIDS Vaccine Research Laboratories|Moreno|USA
>Spike|hCoV-19/England/ALDP-9CCD79/2020|2020-09-16|EPI_ISL_566129|Original|hCoV-19^^England|Human|Lighthouse Lab in Alderley Park|Wellcome Sanger Institute for the COVID-19 Genomics UK (COG-UK) consortium|Davies|United Kingdom
>Spike|hCoV-19/USA/LA-EVTL814/2020|2020-07-27|EPI_ISL_653505|Original|hCoV-19^^Louisiana|Human|LSUHS Emerging Viral Threat Laboratory|Microbial Genome Sequencing Center|Kamil|USA
>Spike|hCoV-19/England/20146057204/2020|2020-04-01|EPI_ISL_466043|Original|hCoV-19^^England|Human|Respiratory Virus Unit|Respiratory Virus Unit|Platt|United Kingdom
>Spike|hCoV-19/USA/UT-0301/2020|2020-03-27|EPI_ISL_420814|Original|hCoV-19^^Utah|Human|Utah Public Health Laboratory|Utah Public Health Laboratory|Young|USA
>Spike|hCoV-19/England/CAMC-B7B524/2020|2020-11-12|EPI_ISL_741177|Original|hCoV-19^^England|Human|Oxford Viromics|COVID-19 Genomics UK (COG-UK) Consortium|Golubchik|United Kingdom
>Spike|hCoV-19/England/NORT-2847C1/2020|2020-03-24|EPI_ISL_478568|Original|hCoV-19^^England|Human|Northumbria University / South Tees Hospitals NHS Foundation Trust / North Cumbria Integrated Care NHS Foundation Trust / North Tees and Hartlepool NHS Foundation Trust / Newcastle Hospitals NHS Foundation Trust|COVID-19 Genomics UK (COG-UK) Consortium|Bashton|United Kingdom
>Spike|hCoV-19/England/QEUH-AAED03/2020|2020-10-20|EPI_ISL_653000|Original|hCoV-19^^England|Human|Oxford Viromics|COVID-19 Genomics UK (COG-UK) Consortium|Golubchik|United Kingdom
>Spike|hCoV-19/Belgium/UGent-58/2020|2020-04-05|EPI_ISL_451169|Original|hCoV-19^^Ghent|Human|Lab voor klinische biologie|Onderzoeksgroep Virologie|Lambrechts|Belgium
>Spike|hCoV-19/Australia/VIC17662/2020|2020-08-05|EPI_ISL_663981|Original|hCoV-19^^Victoria|Human|Microbiological Diagnostic Unit - Public Health Laboratory (MDU-PHL)|MDU-PHL|Schultz|Australia
>Spike|hCoV-19/England/QEUH-AADE5C/2020|2020-10-22|EPI_ISL_652956|Original|hCoV-19^^England|Human|Oxford Viromics|COVID-19 Genomics UK (COG-UK) Consortium|Golubchik|United Kingdom
>Spike|hCoV-19/USA/CA-CZB-16172/2020|2020-11-25|EPI_ISL_738727|Original|hCoV-19^^California|Human|Renegade|Chan-Zuckerberg Biohub|Kamm|USA
>Spike|hCoV-19/USA/FL-BPHL-1448/2020|2020-04-08|EPI_ISL_549230|Original|hCoV-19^^Florida|Human|Florida Bureau of Public Health Laboratories|Florida Bureau of Public Health Laboratories|Blanton|USA
>Spike|hCoV-19/USA/ME-HETL-J0214/2020|2020-09-22|EPI_ISL_755377|Original|hCoV-19^^Maine|Human|Maine Health and Environmental Testing Laboratory|Tewhey Lab|Tewhey|USA

>Spike|hCoV-19/England/HECH-2599DCE/2020|2020-05-06|EPI_ISL_482108|Original|hCoV-19^^England|Human|Microbiology Department|Wellcome Sanger Institute for the COVID-19 Genomics UK (COG-UK) consortium|Davies|United Kingdom
>Spike|hCoV-19/Spain/AR-IBV-2472/2020|2020-03-24|EPI_ISL_468779|Original|hCoV-19^^Aragon|Human|Servicio de Microbiolog\u00eda|SeqCOVID-SPAIN consortium/IBV(CSIC)|Comas|Spain
>Spike|hCoV-19/Denmark/DCGC-13879/2020|2020-11-30|EPI_ISL_713079|Original|hCoV-19^^Midtjylland|Human|Department of Virus and Microbiological Special Diagnostics|Albertsen Lab|Michaelsen|Denmark
>Spike|hCoV-19/Japan/PG-7972/2020|2020-05-00|EPI_ISL_687073|Original|hCoV-19^^Japan|Human|Kanagawa Prefectural Institute of Public Health|Pathogen Genomics Center|Sekizuka|Japan
>Spike|hCoV-19/USA/WA-S1451/2020|2020-05-21|EPI_ISL_495901|Original|hCoV-19^^Washington|Human|Washington State Department of Health|Seattle Flu Study|Lee|USA
>Spike|hCoV-19/England/SHEF-CDEAB/2020|2020-06-03|EPI_ISL_479262|Original|hCoV-19^^England|Human|Virology Department|COVID-19 Genomics UK (COG-UK) Consortium|de Silva|United Kingdom
>Spike|hCoV-19/England/NORW-EB6CD/2020|2020-05-04|EPI_ISL_472310|Original|hCoV-19^^England|Human|Quadram Institute Bioscience|COVID-19 Genomics UK (COG-UK) Consortium|Alikhan|United Kingdom
>Spike|hCoV-19/Wales/PHWC-255F7/2020|2020-03-24|EPI_ISL_421011|Original|hCoV-19^^Wales|Human|Wales Specialist Virology Centre|Public Health Wales Microbiology Cardiff|Moore|United Kingdom
>Spike|hCoV-19/Japan/PG-7312/2020|2020-08-00|EPI_ISL_688415|Original|hCoV-19^^Japan|Human|Pathogen Genomics Center|Pathogen Genomics Center|Sekizuka|Japan
>Spike|hCoV-19/Portugal/PT1979/2020|2020-11-10|EPI_ISL_732202|Original|hCoV-19^^Portugal|Human|Instituto Nacional de Saude (INSA) and Instituto Gulbenkian de Ciencia (IGC)|Instituto Nacional de Saude (INSA) and Instituto Gulbenkian de Ciencia (IGC)|Borges|Portugal
>Spike|hCoV-19/England/MILK-993E1E/2020|2020-08-22|EPI_ISL_551708|Original|hCoV-19^^England|Human|Lighthouse Lab in Milton Keynes|Wellcome Sanger Institute for the COVID-19 Genomics UK (COG-UK) consortium|Davies|United Kingdom
>Spike|hCoV-19/England/BRIS-1853A41/2020|2020-04-16|EPI_ISL_492412|Original|hCoV-19^^England|Human|PHE South West Regional Laboratory|Wellcome Sanger Institute for the COVID-19 Genomics UK (COG-UK) consortium|Davies|United Kingdom
>Spike|hCoV-19/Denmark/DCGC-22611/2020|2020-12-14|EPI_ISL_757670|Original|hCoV-19^^Syddanmark|Human|Department of Virus and Microbiological Special Diagnostics|Albertsen Lab|Michaelsen|Denmark
>Spike|hCoV-19/Canada/QC-LSPQ-L00241924/2020|2020-03-28|EPI_ISL_536296|Original|hCoV-19^^Quebec|Human|H\u00f4pital Charles-LeMoyne|Laboratoire de sant\u00e9 publique du Qu\u00e9bec|Moreira|Canada
>Spike|hCoV-19/England/CAMC-A82AA4/2020|2020-10-09|EPI_ISL_610914|Original|hCoV-19^^England|Human|Lighthouse Lab in Cambridge|Wellcome Sanger Institute for the COVID-19 Genomics UK (COG-UK) consortium|Davies|United Kingdom
>Spike|hCoV-19/England/LIVE-1DD803/2020|2020-10-02|EPI_ISL_612112|Original|hCoV-19^^England|Human|Liverpool Clinical Laboratories|COVID-19 Genomics UK (COG-UK) Consortium|Haldenby|United Kingdom
>Spike|hCoV-19/Spain/AR-ISCIII-201688/2020|2020-03-06|EPI_ISL_539510|Original|hCoV-19^^Aragon|Human|Hospital Cl\u00ednico Universitario Lozano Blesa|Instituto de Salud Carlos III|Caballero|Spain
>Spike|hCoV-19/England/ALDP-B24135/2020|2020-11-04|EPI_ISL_646958|Original|hCoV-19^^England|Human|Lighthouse Lab in Alderley Park|Wellcome Sanger Institute for the COVID-19 Genomics UK (COG-UK) Consortium|Davies|United Kingdom
>Spike|hCoV-19/USA/MN-MDH-1211/2020|2020-05-20|EPI_ISL_477300|Original|hCoV-19^^Minnesota|Human|Mayo Clinic & Mayo Clinic Laboratories|Minnesota Department of Health|Garfin|USA
>Spike|hCoV-19/Slovenia/6455/2020|2020-03-12|EPI_ISL_635229|Original|hCoV-19^^Slovenia|Human|Institute of Microbiology and Immunology|Institute of Microbiology and Immunology|Zakotnik|Slovenia

>Spike|hCoV-19/India/DL-NCDC4764-CSIR-IGIB/2020|2020-05-24|EPI_ISL_482603|Original|hCoV-19^^Delhi|Human|National Centre for Disease control (NCDC)|NCDC/CSIR-IGIB|Singh|India
>Spike|hCoV-19/Scotland/CVR2878/2020|2020-04-21|EPI_ISL_473614|Original|hCoV-19^^Scotland|Human|West of Scotland Specialist Virology Centre|COVID-19 Genomics UK (COG-UK) Consortium|Robertson|United Kingdom
>Spike|hCoV-19/USA/TX-HMH-MCoV-9853/2020|2020-07-06|EPI_ISL_780305|Original|hCoV-19^^Texas|Human|Houston Methodist Hospital|Houston Methodist Hospital|Long|USA
>Spike|hCoV-19/England/CAMC-BD48E4/2020|2020-11-27|EPI_ISL_702269|Original|hCoV-19^^England|Human|Lighthouse Lab in Cambridge|Wellcome Sanger Institute for the COVID-19 Genomics UK (COG-UK) Consortium|Davies|United Kingdom
>Spike|hCoV-19/USA/MA-MASPHL-00057/2020|2020-04-03|EPI_ISL_791556|Original|hCoV-19^^Massachusetts|Human|Massachusetts State Public Health Laboratory|Infectious Disease Program|Park|USA
>Spike|hCoV-19/Russia/PSK-RII-MH6094S/2020|2020-11-14|EPI_ISL_733345|Original|hCoV-19^^Pskov|Human|HELIX LLC|WHO National Influenza Centre Russian Federation|Komissarov|Russia
>Spike|hCoV-19/Portugal/PT0639/2020|2020-03-28|EPI_ISL_510900|Original|hCoV-19^^Portugal|Human|Instituto Nacional de Saude (INSA)|Instituto Nacional de Saude (INSA)|Borges|Portugal
>Spike|hCoV-19/Singapore/1168/2020|2020-04-09|EPI_ISL_648096|Original|hCoV-19^^Singapore|Human|Department of Laboratory Medicine|Department of Laboratory Medicine|Chen|Singapore
>Spike|hCoV-19/Scotland/EDB2955/2020|2020-04-02|EPI_ISL_488939|Original|hCoV-19^^Scotland|Human|Virology Department|Wellcome Sanger Institute for the COVID-19 Genomics UK (COG-UK) consortium|Davies|United Kingdom
>Spike|hCoV-19/USA/FL-BPHL-1508/2020|2020-07-24|EPI_ISL_568606|Original|hCoV-19^^Florida|Human|Florida Bureau of Public Health Laboratories|Florida Bureau of Public Health Laboratories|Blanton|USA
>Spike|hCoV-19/England/OXON-B29A8/2020|2020-04-09|EPI_ISL_534784|Original|hCoV-19^^England|Human|Oxford Viromics|COVID-19 Genomics UK (COG-UK) Consortium|Golubchik|United Kingdom
>Spike|hCoV-19/England/QEUH-B0EA72/2020|2020-11-02|EPI_ISL_642158|Original|hCoV-19^^England|Human|Lighthouse Lab in Glasgow|Wellcome Sanger Institute for the COVID-19 Genomics UK (COG-UK) Consortium|Davies|United Kingdom
>Spike|hCoV-19/Scotland/QEUH-9B73CA/2020|2020-09-09|EPI_ISL_602118|Original|hCoV-19^^Scotland|Human|Lighthouse Lab in Glasgow|Wellcome Sanger Institute for the COVID-19 Genomics UK (COG-UK) consortium|Davies|United Kingdom
>Spike|hCoV-19/Canada/ON-PHL-20-02334/2020|2020-09-00|EPI_ISL_671566|Original|hCoV-19^^Ontario|Human|Public Health Ontario Laboratory|Public Health Ontario Laboratory|Guthrie|Canada
>Spike|hCoV-19/Australia/VIC7293/2020|2020-07-18|EPI_ISL_564330|Original|hCoV-19^^Victoria|Human|Victorian Infectious Diseases Reference Laboratory (VIDRL)|VIDRL and MDU-PHL|Schultz|Australia
>Spike|hCoV-19/Japan/PG-7451/2020|2020-07-00|EPI_ISL_688553|Original|hCoV-19^^Japan|Human|Pathogen Genomics Center|Pathogen Genomics Center|Sekizuka|Japan
>Spike|hCoV-19/Spain/AR-IBV-98008076/2020|2020-09-29|EPI_ISL_654451|Original|hCoV-19^^Aragon|Human|Servicio de Microbiolog\u00eda|SeqCOVID-SPAIN consortium/IBV(CSIC)|Comas|Spain
>Spike|hCoV-19/USA/NY-NYCPHL-000513/2020|2020-03-23|EPI_ISL_631974|Original|hCoV-19^^New York|Human|NYC HH Coney Island Hospital|New York City Public Health Laboratory|Wang|USA
>Spike|hCoV-19/England/ALDP-B1BACD/2020|2020-11-01|EPI_ISL_645641|Original|hCoV-19^^England|Human|Lighthouse Lab in Alderley Park|Wellcome Sanger Institute for the COVID-19 Genomics UK (COG-UK) Consortium|Davies|United Kingdom
>Spike|hCoV-19/Wales/PHWC-484219/2020|2020-10-16|EPI_ISL_627978|Original|hCoV-19^^Wales|Human|Wales Specialist Virology Centre Sequencing lab|COVID-19 Genomics UK (COG-UK) Consortium|Moore|United Kingdom

>Spike|hCoV-19/Spain/CT-IBV-99009876/2020|2020-08-02|EPI_ISL_660273|Original|hCoV-19^^Catalunya|Human|Servicio de Microbiolog\u00eda|SeqCOVID-SPAIN consortium/IBV(CSIC)|Comas|Spain
>Spike|hCoV-19/Scotland/CVR2084/2020|2020-04-10|EPI_ISL_484558|Original|hCoV-19^^Scotland|Human|West of Scotland Specialist Virology Centre|COVID-19 Genomics UK (COG-UK) Consortium|Robertson|United Kingdom
>Spike|hCoV-19/Germany/SH-ChVir3052/2020|2020-03-20|EPI_ISL_729522|Original|hCoV-19^^Schleswig-Holstein|Human|A. Krumbholz|Charit\u00e9 Universit\u00e4tsmedizin Berlin|Muehlemann|Germany
>Spike|hCoV-19/USA/TX-HMH-MCoV-8943/2020|2020-07-02|EPI_ISL_780849|Original|hCoV-19^^Texas|Human|Houston Methodist Hospital|Houston Methodist Hospital|Long|USA
>Spike|hCoV-19/France/ARA93759/2020|2020-10-24|EPI_ISL_640009|Original|hCoV-19^^Auvergne-Rhone-Alpes|Human|CNR Virus des Infections Respiratoires - France SUD|CNR Virus des Infections Respiratoires - France SUD|Josset|France
>Spike|hCoV-19/England/CAMB-7BB96/2020|2020-04-04|EPI_ISL_442160|Original|hCoV-19^^England|Human|Department of Pathology|Wellcome Sanger Institute for the COVID-19 Genomics UK (COG-UK) consortium|Amato|United Kingdom
>Spike|hCoV-19/Wuhan/IVDC-HB-04/2020|2020-01-01|EPI_ISL_402120|Original|hCoV-19^^Hubei|Human|National Institute for Viral Disease Control and Prevention|National Institute for Viral Disease Control and Prevention|Tan|China
>Spike|hCoV-19/Australia/VIC6034/2020|2020-07-24|EPI_ISL_518075|Original|hCoV-19^^Victoria|Human|Microbiological Diagnostic Unit - Public Health Laboratory (MDU-PHL)|MDU-PHL|Schultz|Australia
>Spike|hCoV-19/Australia/VIC15370/2020|2020-08-06|EPI_ISL_593267|Original|hCoV-19^^Victoria|Human|Microbiological Diagnostic Unit - Public Health Laboratory (MDU-PHL)|MDU-PHL|Schultz|Australia
>Spike|hCoV-19/Wales/PHWC-2EED5/2020|2020-04-07|EPI_ISL_446171|Original|hCoV-19^^Wales|Human|Wales Specialist Virology Centre|Public Health Wales Microbiology Cardiff|Moore|United Kingdom
>Spike|hCoV-19/USA/HI-H200701/2020|2020-11-26|EPI_ISL_753222|Original|hCoV-19^^Hawaii|Human|State Laboratories Division|State Laboratories Division|Sultana|USA
>Spike|hCoV-19/Wales/PHWC-4A0A88/2020|2020-12-10|EPI_ISL_743536|Original|hCoV-19^^Wales|Human|Wales Specialist Virology Centre Sequencing lab|COVID-19 Genomics UK (COG-UK) Consortium|Moore|United Kingdom
>Spike|hCoV-19/Denmark/DCGC-11547/2020|2020-11-02|EPI_ISL_670988|Original|hCoV-19^^Nordjylland|Human|Department of Virus and Microbiological Special Diagnostics|Albertsen Lab|Michaelsen|Denmark
>Spike|hCoV-19/England/CAMC-B3485B/2020|2020-11-05|EPI_ISL_659463|Original|hCoV-19^^England|Human|Lighthouse Lab in Cambridge|Wellcome Sanger Institute for the COVID-19 Genomics UK (COG-UK) Consortium|Davies|United Kingdom
>Spike|hCoV-19/Scotland/EDB620/2020|2020-03-31|EPI_ISL_433217|Original|hCoV-19^^Scotland|Human|Virology Department|COVID-19 Genomics UK (COG-UK) Consortium|Williams|United Kingdom
>Spike|hCoV-19/USA/OR-OHSU-2824/2020|2020-09-02|EPI_ISL_667332|Original|hCoV-19^^Oregon|Human|OHSU Lab Services Molecular Microbiology Lab|Oregon SARS-CoV-2 Genome Sequencing Center|O'Roak|USA
>Spike|hCoV-19/Australia/VIC5577/2020|2020-07-16|EPI_ISL_518395|Original|hCoV-19^^Victoria|Human|Victorian Infectious Diseases Reference Laboratory (VIDRL)|VIDRL and MDU-PHL|Schultz|Australia
>Spike|hCoV-19/England/MILK-C53A04/2020|2020-12-12|EPI_ISL_769682|Original|hCoV-19^^England|Human|Lighthouse Lab in Milton Keynes|Wellcome Sanger Institute for the COVID-19 Genomics UK (COG-UK) Consortium|Davies|United Kingdom
>Spike|hCoV-19/USA/VA-DCLS-0254/2020|2020-04-20|EPI_ISL_452828|Original|hCoV-19^^Virginia|Human|Virginia DCLS|Virginia DCLS|St. Jacques|USA
>Spike|hCoV-19/France/PDL-SC601/2020|2020-03-12|EPI_ISL_645217|Original|hCoV-19^^Pays-de-la-Loire|Human|CHU Nantes|CNR Virus des Infections Respiratoires - France SUD|Bal|France
>Spike|hCoV-19/England/ALDP-9512F2/2020|2020-06-13|EPI_ISL_554155|Original|hCoV-19^^England|Human|Lighthouse Lab in Alderley Park|Wellcome Sanger Institute for the COVID-19 Genomics UK (COG-UK) consortium|Davies|United Kingdom

>Spike|hCoV-19/Gambia/GC19-3533/2020|2020-07-27|EPI_ISL_561193|Original|hCoV-19^^Kombo|Human|MRCG at LSHTM Genomics lab|MRCG at LSHTM Genomics lab|Sesay|Gambia
>Spike|hCoV-19/England/QEUH-CCCDD0/2020|2020-12-21|EPI_ISL_782419|Original|hCoV-19^^England|Human|Lighthouse Lab in Glasgow|Wellcome Sanger Institute for the COVID-19 Genomics UK (COG-UK) Consortium|Davies|United Kingdom
>Spike|hCoV-19/England/QEUH-AF51F2/2020|2020-10-30|EPI_ISL_663190|Original|hCoV-19^^England|Human|Lighthouse Lab in Glasgow|Wellcome Sanger Institute for the COVID-19 Genomics UK (COG-UK) Consortium|Davies|United Kingdom
>Spike|hCoV-19/Australia/VIC3009/2020|2020-07-08|EPI_ISL_521839|Original|hCoV-19^^Victoria|Human|Microbiological Diagnostic Unit - Public Health Laboratory (MDU-PHL)|MDU-PHL|Schultz|Australia
>Spike|hCoV-19/England/ALDP-953C98/2020|2020-06-13|EPI_ISL_554450|Original|hCoV-19^^England|Human|Lighthouse Lab in Alderley Park|Wellcome Sanger Institute for the COVID-19 Genomics UK (COG-UK) consortium|Davies|United Kingdom
>Spike|hCoV-19/England/BRIS-18531A6/2020|2020-04-01|EPI_ISL_492407|Original|hCoV-19^^England|Human|PHE South West Regional Laboratory|Wellcome Sanger Institute for the COVID-19 Genomics UK (COG-UK) consortium|Davies|United Kingdom
>Spike|hCoV-19/USA/CA-CZB-15105/2020|2020-11-20|EPI_ISL_739085|Original|hCoV-19^^California|Human|Santa Clara County Public Health Laboratory|Chan-Zuckerberg Biohub|Kamm|USA
>Spike|hCoV-19/Japan/PG-1531/2020|2020-04-00|EPI_ISL_685137|Original|hCoV-19^^Japan|Human|Pathogen Genomics Center|Pathogen Genomics Center|Sekizuka|Japan
>Spike|hCoV-19/USA/VA-DCLS-0557/2020|2020-03-18|EPI_ISL_485858|Original|hCoV-19^^Virginia|Human|Virginia DCLS|Virginia DCLS|St. Jacques|USA
>Spike|hCoV-19/Japan/PG-4026/2020|2020-07-00|EPI_ISL_692267|Original|hCoV-19^^Japan|Human|Pathogen Genomics Center|Pathogen Genomics Center|Sekizuka|Japan
>Spike|hCoV-19/England/QEUH-B68232/2020|2020-11-11|EPI_ISL_655006|Original|hCoV-19^^England|Human|Lighthouse Lab in Glasgow|Wellcome Sanger Institute for the COVID-19 Genomics UK (COG-UK) Consortium|Davies|United Kingdom
>Spike|hCoV-19/England/ALDP-B733AA/2020|2020-11-12|EPI_ISL_662429|Original|hCoV-19^^England|Human|Lighthouse Lab in Alderley Park|Wellcome Sanger Institute for the COVID-19 Genomics UK (COG-UK) Consortium|Davies|United Kingdom
>Spike|hCoV-19/England/MILK-A5604D/2020|2020-10-08|EPI_ISL_595129|Original|hCoV-19^^England|Human|Quadram Institute Bioscience|COVID-19 Genomics UK (COG-UK) Consortium|Alikhan|United Kingdom
>Spike|hCoV-19/Iceland/489/2020|2020-03-20|EPI_ISL_424511|Original|hCoV-19^^Reykjavik|Human|The National University Hospital of Iceland|deCODE genetics|J\u00f3nsson|Iceland
>Spike|hCoV-19/Australia/NSW2038/2020|2020-03-22|EPI_ISL_500610|Original|hCoV-19^^New South Wales|Human|Area of Virology|Area of Virology|Bull|Australia
>Spike|hCoV-19/Canada/QC-LSPQ-L00237109/2020|2020-03-25|EPI_ISL_536163|Original|hCoV-19^^Quebec|Human|H\u00f4pital du Suro\u00eet|Laboratoire de sant\u00e9 publique du Qu\u00e9bec|Moreira|Canada
>Spike|hCoV-19/USA/WA-OHSU-0506/2020|2020-06-05|EPI_ISL_526092|Original|hCoV-19^^Washington|Human|OHSU Lab Services Molecular Microbiology Lab|Ginkgo Bioworks Clinical Laboratory|O'Roak|USA
>Spike|hCoV-19/Belgium/ULG-10247/2020|2020-06-18|EPI_ISL_498629|Original|hCoV-19^^Li\u00e8ge|Human|Department of Clinical Microbiology|GIGA Medical Genomics|Durkin|Belgium
>Spike|hCoV-19/USA/CA-CZB-15067/2020|2020-11-17|EPI_ISL_739365|Original|hCoV-19^^California|Human|Santa Clara County Public Health Laboratory|Chan-Zuckerberg Biohub|Kamm|USA
>Spike|hCoV-19/Denmark/DCGC-13971/2020|2020-11-30|EPI_ISL_711485|Original|hCoV-19^^Hovedstaden|Human|Department of Virus and Microbiological Special Diagnostics|Albertsen Lab|Michaelsen|Denmark
>Spike|hCoV-19/England/MILK-B970B8/2020|2020-11-13|EPI_ISL_674877|Original|hCoV-19^^England|Human|Lighthouse Lab in Milton Keynes|Wellcome Sanger Institute for the COVID-19 Genomics UK (COG-UK) Consortium|Davies|United Kingdom
>Spike|hCoV-19/USA/WA-UW-5201/2020|2020-04-02|EPI_ISL_570063|Original|hCoV-19^^Washington|Human|UW Virology Lab|UW Virology Lab|Roychoudhury|USA

>Spike|hCoV-19/England/ALDP-B74761/2020|2020-11-12|EPI_ISL_662191|Original|hCoV-19^^England|Human|Lighthouse Lab in Alderley Park|Wellcome Sanger Institute for the COVID-19 Genomics UK (COG-UK) Consortium|Davies|United Kingdom
>Spike|hCoV-19/Switzerland/VD-ETHZ-280112/2020|2020-09-08|EPI_ISL_560497|Original|hCoV-19^^Vaud|Human|Viollier AG|Department of Biosystems Science and Engineering|Nadeau|Switzerland
>Spike|hCoV-19/Finland/23SS9F1/2020|2020-09-23|EPI_ISL_759808|Original|hCoV-19^^Uusimaa|Human|Department of Virology and Immunology|Department of Virology|Smura|Finland
>Spike|hCoV-19/England/MILK-9572DE/2020|2020-06-10|EPI_ISL_554208|Original|hCoV-19^^England|Human|Lighthouse Lab in Milton Keynes|Wellcome Sanger Institute for the COVID-19 Genomics UK (COG-UK) consortium|Davies|United Kingdom
>Spike|hCoV-19/Denmark/DCGC-10339/2020|2020-11-02|EPI_ISL_669909|Original|hCoV-19^^Sjaelland|Human|Department of Virus and Microbiological Special Diagnostics|Albertsen Lab|Michaelsen|Denmark
>Spike|hCoV-19/England/LOND-D5778/2020|2020-04-17|EPI_ISL_449719|Original|hCoV-19^^England|Human|University College London|COVID-19 Genomics UK (COG-UK) Consortium|Roy|United Kingdom
>Spike|hCoV-19/Norway/4640/2020|2020-10-18|EPI_ISL_635161|Original|hCoV-19^^Agder|Human|Hospital of Southern Norway - Kristiansand|Norwegian Institute of Public Health|Vollan|Norway
>Spike|hCoV-19/Scotland/CVR5944/2020|2020-11-22|EPI_ISL_705685|Original|hCoV-19^^Scotland|Human|West of Scotland Specialist Virology Centre|COVID-19 Genomics UK (COG-UK) Consortium|Robertson|United Kingdom
>Spike|hCoV-19/Panama/336377/2020|2020-04-04|EPI_ISL_496844|Original|hCoV-19^^Panama West|Human|Gorgas Memorial Laboratory of Health Studies|Gorgas Memorial Laboratory of Health Studies|Martinez|Panama
>Spike|hCoV-19/Thailand/CO63-1504-3/2020|2020-03-23|EPI_ISL_450979|Original|hCoV-19^^Thailand|Human|Center of Excellence in Clinical Virology|Center of Excellence in Clinical Virology|Poovorawan|Thailand
>Spike|hCoV-19/South Africa/KRISP-K001083/2020|2020-07-03|EPI_ISL_509336|Original|hCoV-19^^KwaZulu-Natal|Human|NHLS-IALCH|KRISP|de Oliveira|South Africa
>Spike|hCoV-19/USA/MN-MDH-1732/2020|2020-09-16|EPI_ISL_576241|Original|hCoV-19^^Minnesota|Human|Minnesota Department of Health|Minnesota Department of Health|Garfin|USA
>Spike|hCoV-19/Belgium/rega-09231187/2020|2020-09-23|EPI_ISL_735034|Original|hCoV-19^^Nossegem|Human|UZ Leuven|KU Leuven|Maes|Belgium
>Spike|hCoV-19/Brazil/SP-133/2020|2020-04-04|EPI_ISL_523957|Original|hCoV-19^^S\u00e3o Paulo|Human|Hospital Itamaraty|Instituto Adolfo Lutz|Sacchi|Brazil
>Spike|hCoV-19/USA/NY-NYUMC36/2020|2020-03-18|EPI_ISL_419697|Original|hCoV-19^^New York|Human|NYU Langone Health|Departments of Pathology and Medicine|Maurano|USA
>Spike|hCoV-19/England/QEUH-98FE8E/2020|2020-08-30|EPI_ISL_539968|Original|hCoV-19^^England|Human|Lighthouse Lab in Glasgow|Wellcome Sanger Institute for the COVID-19 Genomics UK (COG-UK) consortium|Davies|United Kingdom
>Spike|hCoV-19/England/SHEF-C65DA/2020|2020-04-14|EPI_ISL_453719|Original|hCoV-19^^England|Human|Virology Department|COVID-19 Genomics UK (COG-UK) Consortium|de Silva|United Kingdom
>Spike|hCoV-19/England/QEUH-B80656/2020|2020-11-14|EPI_ISL_668380|Original|hCoV-19^^England|Human|Lighthouse Lab in Glasgow|Wellcome Sanger Institute for the COVID-19 Genomics UK (COG-UK) Consortium|Davies|United Kingdom
>Spike|hCoV-19/Australia/VIC15912/2020|2020-07-31|EPI_ISL_641284|Original|hCoV-19^^Victoria|Human|Microbiological Diagnostic Unit - Public Health Laboratory (MDU-PHL)|MDU-PHL|Schultz|Australia
>Spike|hCoV-19/USA/WA-UW357/2020|2020-03-14|EPI_ISL_418924|Original|hCoV-19^^Washington|Human|UW Virology Lab|UW Virology Lab|Roychoudhury|USA
>Spike|hCoV-19/Japan/PG-1649/2020|2020-04-00|EPI_ISL_685255|Original|hCoV-19^^Japan|Human|Pathogen Genomics Center|Pathogen Genomics Center|Sekizuka|Japan

>Spike|hCoV-19/USA/MI-UM-10036386923/2020|2020-10-11|EPI_ISL_610069|Original|hCoV-19^^Michigan|Human|University of Michigan Clinical Microbiology Laboratory|Lauring Lab|Valesano|USA
>Spike|hCoV-19/England/LIVE-DB3FD5/2020|2020-10-21|EPI_ISL_651375|Original|hCoV-19^^England|Human|Liverpool Clinical Laboratories|COVID-19 Genomics UK (COG-UK) Consortium|Haldenby|United Kingdom
>Spike|hCoV-19/Kenya/C8454/2020|2020-05-20|EPI_ISL_568865|Original|hCoV-19^^Kwale|Human|KEMRI-Wellcome Trust Research Programme/KEMRI-CGMR-C Kilifi|KEMRI-Wellcome Trust Research Programme/KEMRI-CGMR-C Kilifi|Githinji|Kenya
>Spike|hCoV-19/England/QEUH-9DA59E/2020|2020-09-22|EPI_ISL_601421|Original|hCoV-19^^England|Human|Lighthouse Lab in Glasgow|Wellcome Sanger Institute for the COVID-19 Genomics UK (COG-UK) consortium|Davies|United Kingdom
>Spike|hCoV-19/Australia/VIC5966/2020|2020-00-00|EPI_ISL_518741|Original|hCoV-19^^Victoria|Human|Microbiological Diagnostic Unit - Public Health Laboratory (MDU-PHL)|MDU-PHL|Schultz|Australia
>Spike|hCoV-19/Denmark/DCGC-20920/2020|2020-12-21|EPI_ISL_758784|Original|hCoV-19^^Hovedstaden|Human|Department of Virus and Microbiological Special Diagnostics|Albertsen Lab|Michaelsen|Denmark
>Spike|hCoV-19/England/204691665/2020|2020-11-13|EPI_ISL_686600|Original|hCoV-19^^England|Human|Respiratory Virus Unit|COVID-19 Genomics UK (COG-UK) Consortium|Platt|United Kingdom
>Spike|hCoV-19/Russia/SVE-RII-MH4669S/2020|2020-09-08|EPI_ISL_733229|Original|hCoV-19^^Sverdlovsk|Human|UMMC-Health|WHO National Influenza Centre Russian Federation|Komissarov|Russia
>Spike|hCoV-19/Wales/PHWC-481A8D/2020|2020-10-19|EPI_ISL_613139|Original|hCoV-19^^Wales|Human|Wales Specialist Virology Centre Sequencing lab|COVID-19 Genomics UK (COG-UK) Consortium|Moore|United Kingdom
>Spike|hCoV-19/England/ALDP-B45DA3/2020|2020-11-07|EPI_ISL_656234|Original|hCoV-19^^England|Human|Lighthouse Lab in Alderley Park|Wellcome Sanger Institute for the COVID-19 Genomics UK (COG-UK) Consortium|Davies|United Kingdom
>Spike|hCoV-19/Denmark/DCGC-13837/2020|2020-11-30|EPI_ISL_713053|Original|hCoV-19^^Nordjylland|Human|Department of Virus and Microbiological Special Diagnostics|Albertsen Lab|Michaelsen|Denmark
>Spike|hCoV-19/Wales/PHWC-49AC9A/2020|2020-12-03|EPI_ISL_727671|Original|hCoV-19^^Wales|Human|Originating lab|COVID-19 Genomics UK (COG-UK) Consortium|Moore|United Kingdom
>Spike|hCoV-19/England/20164079304/2020|2020-04-15|EPI_ISL_466482|Original|hCoV-19^^England|Human|Respiratory Virus Unit|Respiratory Virus Unit|Platt|United Kingdom
>Spike|hCoV-19/Belgium/un-EMC-3/2020|2020-05-02|EPI_ISL_577745|Original|hCoV-19^^Belgium|Human|Dutch COVID-19 response team|Erasmus Medical Center|Oude Munnink|Belgium
>Spike|hCoV-19/Scotland/QEUH-9DE688/2020|2020-09-21|EPI_ISL_601199|Original|hCoV-19^^Scotland|Human|Lighthouse Lab in Glasgow|Wellcome Sanger Institute for the COVID-19 Genomics UK (COG-UK) consortium|Davies|United Kingdom
>Spike|hCoV-19/Germany/BY-MVP-0173/2020|2020-04-08|EPI_ISL_451947|Original|hCoV-19^^Bavaria|Human|Max von Pettenkofer Institute|Laboratory for Functional Genome Analysis|Graf|Germany
>Spike|hCoV-19/England/LOND-D6470/2020|2020-04-22|EPI_ISL_487564|Original|hCoV-19^^England|Human|University College London|Wellcome Sanger Institute for the COVID-19 Genomics UK (COG-UK) consortium|Davies|United Kingdom
>Spike|hCoV-19/Wales/PHWC-493DE9/2020|2020-11-20|EPI_ISL_726028|Original|hCoV-19^^Wales|Human|Originating lab|COVID-19 Genomics UK (COG-UK) Consortium|Moore|United Kingdom
>Spike|hCoV-19/England/OXON-AFFD9/2020|2020-04-18|EPI_ISL_478843|Original|hCoV-19^^England|Human|Oxford Viromics|COVID-19 Genomics UK (COG-UK) Consortium|Golubchik|United Kingdom
>Spike|hCoV-19/England/CAMC-B20EBA/2020|2020-11-02|EPI_ISL_647521|Original|hCoV-19^^England|Human|Lighthouse Lab in Cambridge|Wellcome Sanger Institute for the COVID-19 Genomics UK (COG-UK) Consortium|Davies|United Kingdom

>Spike|hCoV-19/England/LIVE-DB4095/2020|2020-10-19|EPI_ISL_650671|Original|hCoV-19^^England|Human|Liverpool Clinical Laboratories|COVID-19 Genomics UK (COG-UK) Consortium|Haldenby|United Kingdom
>Spike|hCoV-19/England/ALDP-6D08ED/2020|2020-07-02|EPI_ISL_556086|Original|hCoV-19^^England|Human|Lighthouse Lab in Alderley Park|Wellcome Sanger Institute for the COVID-19 Genomics UK (COG-UK) consortium|Davies|United Kingdom
>Spike|hCoV-19/USA/WA-UW-4592/2020|2020-03-31|EPI_ISL_515276|Original|hCoV-19^^Washington|Human|University of Washington Virology Lab|University of Washington Virology Lab|Roychoudhury|USA
>Spike|hCoV-19/England/MILK-A91A48/2020|2020-10-17|EPI_ISL_741353|Original|hCoV-19^^England|Human|Oxford Viromics|COVID-19 Genomics UK (COG-UK) Consortium|Golubchik|United Kingdom
>Spike|hCoV-19/England/MILK-ACEA60/2020|2020-10-21|EPI_ISL_629524|Original|hCoV-19^^England|Human|Lighthouse Lab in Milton Keynes|Wellcome Sanger Institute for the COVID-19 Genomics UK (COG-UK) consortium|Davies|United Kingdom
>Spike|hCoV-19/Denmark/DCGC-20657/2020|2020-12-21|EPI_ISL_759280|Original|hCoV-19^^Hovedstaden|Human|Department of Virus and Microbiological Special Diagnostics|Albertsen Lab|Michaelsen|Denmark
>Spike|hCoV-19/England/LOND-DAE7E/2020|2020-11-16|EPI_ISL_724721|Original|hCoV-19^^England|Human|University College London|COVID-19 Genomics UK (COG-UK) Consortium|Roy|United Kingdom
>Spike|hCoV-19/England/MILK-A7C779/2020|2020-10-16|EPI_ISL_607384|Original|hCoV-19^^England|Human|Lighthouse Lab in Milton Keynes|Wellcome Sanger Institute for the COVID-19 Genomics UK (COG-UK) consortium|Davies|United Kingdom
>Spike|hCoV-19/England/MILK-B8C98E/2020|2020-11-13|EPI_ISL_675849|Original|hCoV-19^^England|Human|Lighthouse Lab in Milton Keynes|Wellcome Sanger Institute for the COVID-19 Genomics UK (COG-UK) Consortium|Davies|United Kingdom
>Spike|hCoV-19/USA/MI-MDHHS-SC21705/2020|2020-06-17|EPI_ISL_529824|Original|hCoV-19^^Michigan|Human|Michigan Department of Health and Human Services|Michigan Department of Health and Human Services|Blankenship|USA
>Spike|hCoV-19/Denmark/DCGC-13058/2020|2020-11-30|EPI_ISL_712557|Original|hCoV-19^^Syddanmark|Human|Department of Virus and Microbiological Special Diagnostics|Albertsen Lab|Michaelsen|Denmark
>Spike|hCoV-19/Finland/23LS6F7/2020|2020-10-23|EPI_ISL_759785|Original|hCoV-19^^Uusimaa|Human|Department of Virology and Immunology|Department of Virology|Smura|Finland
>Spike|hCoV-19/Australia/WA108/2020|2020-03-18|EPI_ISL_512761|Original|hCoV-19^^Western Australia|Human|PathWest Laboratory Medicine WA|PathWest Laboratory Medicine WA Microbial Surveillance Unit|Sikazwe|Australia
>Spike|hCoV-19/Wales/PHWC-24BF6/2020|2020-03-20|EPI_ISL_419499|Original|hCoV-19^^Wales|Human|Wales Specialist Virology Centre|Public Health Wales Microbiology Cardiff|Moore|United Kingdom
>Spike|hCoV-19/England/CAMC-BBBBDB/2020|2020-11-21|EPI_ISL_724097|Original|hCoV-19^^England|Human|Northumbria University / South Tees Hospitals NHS Foundation Trust / North Cumbria Integrated Care NHS Foundation Trust / North Tees and Hartlepool NHS Foundation Trust / Newcastle Hospitals NHS Foundation Trust|COVID-19 Genomics UK (COG-UK) Consortium|Bashton|United Kingdom
>Spike|hCoV-19/Scotland/EDB721/2020|2020-04-02|EPI_ISL_433228|Original|hCoV-19^^Scotland|Human|Virology Department|COVID-19 Genomics UK (COG-UK) Consortium|Williams|United Kingdom
>Spike|hCoV-19/England/ALDP-9BDDB7/2020|2020-09-09|EPI_ISL_568061|Original|hCoV-19^^England|Human|Lighthouse Lab in Alderley Park|Wellcome Sanger Institute for the COVID-19 Genomics UK (COG-UK) consortium|Davies|United Kingdom
>Spike|hCoV-19/Australia/NT49/2020|2020-00-00|EPI_ISL_480696|Original|hCoV-19^^Northern Territory|Human|Royal Darwin Hospital Pathology|MDU-PHL|Schultz|Australia
>Spike|hCoV-19/USA/TX-HMH0372/2020|2020-03-30|EPI_ISL_434963|Original|hCoV-19^^Texas|Human|Houston Methodist Hospital|Houston Methodist Hospital|Long|USA

>Spike|hCoV-19/England/MILK-81F80E/2020|2020-07-17|EPI_ISL_557169|Original|hCoV-19^^England|Human|Lighthouse Lab in Milton Keynes|Wellcome Sanger Institute for the COVID-19 Genomics UK (COG-UK) consortium|Davies|United Kingdom
>Spike|hCoV-19/Germany/BE-ChVir-D1978-4478/2020|2020-10-10|EPI_ISL_753817|Original|hCoV-19^^Berlin|Human|Charit\u00e9 Universit\u00e4tsmedizin Berlin|Charit\u00e9 Universit\u00e4tsmedizin Berlin|Muehlemann|Germany
>Spike|hCoV-19/USA/CA-ALSR-1961/2020|2020-06-26|EPI_ISL_512264|Original|hCoV-19^^California|Human|San Diego County Public Health Laboratory|Andersen lab at Scripps Research|Gangavarapu|USA
>Spike|hCoV-19/USA/AK-PHL786/2020|2020-07-01|EPI_ISL_528524|Original|hCoV-19^^Alaska|Human|Alaska State Virology Laboratory|Alaska State Virology Laboratory|Drown|USA
>Spike|hCoV-19/Belgium/rega-12141398/2020|2020-12-14|EPI_ISL_738450|Original|hCoV-19^^Mol|Human|UZ Leuven|KU Leuven|Maes|Belgium
>Spike|hCoV-19/Australia/VIC17545/2020|2020-08-23|EPI_ISL_663916|Original|hCoV-19^^Victoria|Human|Microbiological Diagnostic Unit - Public Health Laboratory (MDU-PHL)|MDU-PHL|Schultz|Australia
>Spike|hCoV-19/USA/MA-MASPHL-00169/2020|2020-04-03|EPI_ISL_791634|Original|hCoV-19^^Massachusetts|Human|Massachusetts State Public Health Laboratory|Infectious Disease Program|Park|USA
>Spike|hCoV-19/USA/TX-HMH-MCoV-15080/2020|2020-10-17|EPI_ISL_786564|Original|hCoV-19^^Texas|Human|Houston Methodist Hospital|Houston Methodist Hospital|Long|USA
>Spike|hCoV-19/USA/WA-S2198/2020|2020-06-08|EPI_ISL_497626|Original|hCoV-19^^Washington|Human|Washington State Department of Health|Seattle Flu Study|Lee|USA
>Spike|hCoV-19/Germany/HH-hpi-p1321/2020|2020-03-22|EPI_ISL_775870|Original|hCoV-19^^Hamburg|Human|University Medical Center Hamburg Eppendorf|Heinrich Pette Institute|Robitaille|Germany
>Spike|hCoV-19/Wales/PHWC-162CE9/2020|2020-05-27|EPI_ISL_473188|Original|hCoV-19^^Wales|Human|Wales Specialist Virology Centre Sequencing lab|COVID-19 Genomics UK (COG-UK) Consortium|Moore|United Kingdom
>Spike|hCoV-19/England/MILK-9A55AB/2020|2020-09-03|EPI_ISL_550028|Original|hCoV-19^^England|Human|Lighthouse Lab in Milton Keynes|Wellcome Sanger Institute for the COVID-19 Genomics UK (COG-UK) consortium|Davies|United Kingdom
>Spike|hCoV-19/Belgium/Rega-0328190/2020|2020-03-28|EPI_ISL_458160|Original|hCoV-19^^Colfontaine|Human|KU Leuven|KU Leuven|Maes|Belgium
>Spike|hCoV-19/England/ALDP-6C66C2/2020|2020-07-02|EPI_ISL_556168|Original|hCoV-19^^England|Human|Lighthouse Lab in Alderley Park|Wellcome Sanger Institute for the COVID-19 Genomics UK (COG-UK) consortium|Davies|United Kingdom
>Spike|hCoV-19/USA/LA-GBCL-26791776/2020|2020-04-04|EPI_ISL_485383|Original|hCoV-19^^Louisiana|Human|River Road Testing Lab|Ginkgo Bioworks Clinical Laboratory|Smith|USA
>Spike|hCoV-19/USA/TX-HMH-MCoV-15452/2020|2020-10-24|EPI_ISL_786936|Original|hCoV-19^^Texas|Human|Houston Methodist Hospital|Houston Methodist Hospital|Long|USA
>Spike|hCoV-19/England/QEUH-B67585/2020|2020-11-11|EPI_ISL_662618|Original|hCoV-19^^England|Human|Lighthouse Lab in Glasgow|Wellcome Sanger Institute for the COVID-19 Genomics UK (COG-UK) Consortium|Davies|United Kingdom
>Spike|hCoV-19/USA/NY-Wadsworth-281268-01/2020|2020-11-29|EPI_ISL_754504|Original|hCoV-19^^New York|Human|Wadsworth Center|Wadsworth Center|Lasek-Nesselquist|USA
>Spike|hCoV-19/England/CAMB-1B7B42/2020|2020-10-20|EPI_ISL_638362|Original|hCoV-19^^England|Human|Department of Pathology|COVID-19 Genomics UK (COG-UK) Consortium|Goodfellow|United Kingdom
>Spike|hCoV-19/England/CAMB-71F2B/2020|2020-03-28|EPI_ISL_439951|Original|hCoV-19^^England|Human|Department of Pathology|Wellcome Sanger Institute for the COVID-19 Genomics UK (COG-UK) consortium|Amato|United Kingdom
>Spike|hCoV-19/England/CAMB-1BC59A/2020|2020-11-26|EPI_ISL_723881|Original|hCoV-19^^England|Human|Department of Pathology|COVID-19 Genomics UK (COG-UK) Consortium|Goodfellow|United Kingdom

>Spike|hCoV-19/Wales/PHWC-485F20/2020|2020-10-20|EPI_ISL_628293|Original|hCoV-19^^Wales|Human|Wales Specialist Virology Centre Sequencing lab|COVID-19 Genomics UK (COG-UK) Consortium|Moore|United Kingdom
>Spike|hCoV-19/England/QEUH-A212DC/2020|2020-10-01|EPI_ISL_587600|Original|hCoV-19^^England|Human|Lighthouse Lab in Glasgow|Wellcome Sanger Institute for the COVID-19 Genomics UK (COG-UK) consortium|Davies|United Kingdom
>Spike|hCoV-19/USA/TX-HMH-MCoV-12273/2020|2020-09-24|EPI_ISL_789855|Original|hCoV-19^^Texas|Human|Houston Methodist Hospital|Houston Methodist Hospital|Long|USA
>Spike|hCoV-19/Belgium/ULG-10271/2020|2020-07-18|EPI_ISL_515078|Original|hCoV-19^^Liege|Human|Department of Clinical Microbiology|GIGA Medical Genomics|Durkin|Belgium
>Spike|hCoV-19/England/MILK-A05362/2020|2020-09-30|EPI_ISL_599921|Original|hCoV-19^^England|Human|Lighthouse Lab in Milton Keynes|Wellcome Sanger Institute for the COVID-19 Genomics UK (COG-UK) consortium|Davies|United Kingdom
>Spike|hCoV-19/Scotland/GCVR-173B42/2020|2020-04-07|EPI_ISL_459161|Original|hCoV-19^^Scotland|Human|NHSGGC West of Scotland Specialist Virology Centre / MRC-University of Glasgow Centre for Virus Research|Wellcome Sanger Institute for the COVID-19 Genomics UK (COG-UK) consortium|Davies|United Kingdom
>Spike|hCoV-19/Wales/PHWC-163D30/2020|2020-05-09|EPI_ISL_474074|Original|hCoV-19^^Wales|Human|Originating lab|COVID-19 Genomics UK (COG-UK) Consortium|Moore|United Kingdom
>Spike|hCoV-19/Denmark/DCGC-19055/2020|2020-12-07|EPI_ISL_748726|Original|hCoV-19^^Sjaelland|Human|Department of Virus and Microbiological Special Diagnostics|Albertsen Lab|Michaelsen|Denmark
>Spike|hCoV-19/England/SHEF-D1606/2020|2020-05-15|EPI_ISL_462078|Original|hCoV-19^^England|Human|Virology Department|COVID-19 Genomics UK (COG-UK) Consortium|de Silva|United Kingdom
>Spike|hCoV-19/England/MILK-9C6FF1/2020|2020-09-08|EPI_ISL_572909|Original|hCoV-19^^England|Human|Northumbria University / South Tees Hospitals NHS Foundation Trust / North Cumbria Integrated Care NHS Foundation Trust / North Tees and Hartlepool NHS Foundation Trust / Newcastle Hospitals NHS Foundation Trust|COVID-19 Genomics UK (COG-UK) Consortium|Bashton|United Kingdom
>Spike|hCoV-19/England/LIVE-9F394/2020|2020-05-01|EPI_ISL_453451|Original|hCoV-19^^England|Human|Liverpool Clinical Laboratories|COVID-19 Genomics UK (COG-UK) Consortium|Haldenby|United Kingdom
>Spike|hCoV-19/Singapore/705/2020|2020-06-11|EPI_ISL_498603|Original|hCoV-19^^Singapore|Human|National Public Health Laboratory|National Public Health Laboratory|Mak|Singapore
>Spike|hCoV-19/USA/NY-NYUMC584/2020|2020-03-30|EPI_ISL_444701|Original|hCoV-19^^New York|Human|NYU Langone Health|Departments of Pathology and Medicine|Maurano|USA
>Spike|hCoV-19/Australia/VIC581/2020|2020-03-25|EPI_ISL_426838|Original|hCoV-19^^Victoria|Human|Victorian Infectious Diseases Reference Laboratory (VIDRL)|Microbiological Diagnostic Unit Public Health Laboratory and Victorian Infectious Diseases Reference Laboratory|Schultz|Australia
>Spike|hCoV-19/England/NORT-29B1BA/2020|2020-05-28|EPI_ISL_493491|Original|hCoV-19^^England|Human|Northumbria University / South Tees Hospitals NHS Foundation Trust / North Cumbria Integrated Care NHS Foundation Trust / North Tees and Hartlepool NHS Foundation Trust / Newcastle Hospitals NHS Foundation Trust|COVID-19 Genomics UK (COG-UK) Consortium|Bashton|United Kingdom
>Spike|hCoV-19/Denmark/DCGC-5615/2020|2020-09-28|EPI_ISL_620940|Original|hCoV-19^^Syddanmark|Human|Department of Virus and Microbiological Special Diagnostics|Albertsen lab|Michaelsen|Denmark
>Spike|hCoV-19/Switzerland/VD-ETHZ-310496/2020|2020-10-08|EPI_ISL_603565|Original|hCoV-19^^Vaud|Human|Viollier AG|Department of Biosystems Science and Engineering|Nadeau|Switzerland
>Spike|hCoV-19/Spain/AS-IBV-98006621/2020|2020-03-19|EPI_ISL_654675|Original|hCoV-19^^Asturias|Human|Servicio de Microbiolog\u00eda|SeqCOVID-SPAIN consortium/IBV(CSIC)|Comas|Spain

>Spike|hCoV-19/England/MILK-A23BB1/2020|2020-10-03|EPI_ISL_595103|Original|hCoV-19^^England|Human|Centre for Enzyme Innovation|COVID-19 Genomics UK (COG-UK) Consortium|Robson|United Kingdom
>Spike|hCoV-19/USA/WA-UW-5169/2020|2020-04-02|EPI_ISL_429654|Original|hCoV-19^^Washington|Human|UW Virology Lab|UW Virology Lab|Roychoudhury|USA
>Spike|hCoV-19/Italy/VEN-IZSVe-20RS2293-6_VI/2020|2020-12-21|EPI_ISL_766578|Original|hCoV-19^^Veneto|Human|ULSS 8 Berica|Istituto Zooprofilattico Sperimentale delle Venezie|Quaranta|Italy
>Spike|hCoV-19/Brazil/SP-L22-CD487/2020|2020-04-05|EPI_ISL_476383|Original|hCoV-19^^S\u00e3o Paulo|Human|Hospital da Cl\u00ednicas da Faculdade de Medicina da Universidade de S\u00e3o Paulo|Instituto de Medicina Tropical da Univesidade de S\u00e3o Paulo|Sabino|Brazil
>Spike|hCoV-19/USA/TX-HMH-MCoV-16008/2020|2020-07-10|EPI_ISL_785692|Original|hCoV-19^^Texas|Human|Houston Methodist Hospital|Houston Methodist Hospital|Long|USA
>Spike|hCoV-19/England/CAMC-AB1E46/2020|2020-10-23|EPI_ISL_623810|Original|hCoV-19^^England|Human|Lighthouse Lab in Cambridge|Wellcome Sanger Institute for the COVID-19 Genomics UK (COG-UK) consortium|Davies|United Kingdom
>Spike|hCoV-19/USA/WI-GMF-30820/2020|2020-11-02|EPI_ISL_661056|Original|hCoV-19^^Wisconsin|Human|Gundersen Molecular Diagnostics Laboratory|Kabara Cancer Research Institute|Kenny|USA
>Spike|hCoV-19/USA/FL-BPHL-0483/2020|2020-03-17|EPI_ISL_495389|Original|hCoV-19^^Florida|Human|Florida Bureau of Public Health Laboratories|Florida Bureau of Public Health Laboratories|Blanton|USA
>Spike|hCoV-19/USA/TX-HMH-MCoV-9076/2020|2020-07-02|EPI_ISL_780930|Original|hCoV-19^^Texas|Human|Houston Methodist Hospital|Houston Methodist Hospital|Long|USA
>Spike|hCoV-19/England/LIVE-1E1F16/2020|2020-09-28|EPI_ISL_594855|Original|hCoV-19^^England|Human|Liverpool Clinical Laboratories|COVID-19 Genomics UK (COG-UK) Consortium|Haldenby|United Kingdom
>Spike|hCoV-19/England/PORT-2D22C3/2020|2020-03-23|EPI_ISL_499307|Original|hCoV-19^^England|Human|Centre for Enzyme Innovation|COVID-19 Genomics UK (COG-UK) Consortium|Robson|United Kingdom
>Spike|hCoV-19/Australia/NSW680/2020|2020-07-16|EPI_ISL_513323|Original|hCoV-19^^New South Wales|Human|Sydney South West Pathology Service (SSWPS) - Liverpool Hospital - NSW Health Pathology|NSW Health Pathology - Institute of Clinical Pathology and Medical Research; Westmead Hospital; University of Sydney|Gall|Australia
>Spike|hCoV-19/USA/WA-UW-45088/2020|2020-12-08|EPI_ISL_747206|Original|hCoV-19^^Washington|Human|UW Virology Lab|UW Virology Lab|Roychoudhury|USA
>Spike|hCoV-19/England/TBSD-2488E84/2020|2020-04-26|EPI_ISL_482064|Original|hCoV-19^^England|Human|The Department of Microbiology|Wellcome Sanger Institute for the COVID-19 Genomics UK (COG-UK) consortium|Davies|United Kingdom
>Spike|hCoV-19/England/SHEF-10D35F5/2020|2020-12-21|EPI_ISL_765190|Original|hCoV-19^^England|Human|Virology Department|COVID-19 Genomics UK (COG-UK) Consortium|de Silva|United Kingdom
>Spike|hCoV-19/England/NORT-29370B/2020|2020-06-30|EPI_ISL_499602|Original|hCoV-19^^England|Human|Northumbria University / South Tees Hospitals NHS Foundation Trust / North Cumbria Integrated Care NHS Foundation Trust / North Tees and Hartlepool NHS Foundation Trust / Newcastle Hospitals NHS Foundation Trust|COVID-19 Genomics UK (COG-UK) Consortium|Bashton|United Kingdom
>Spike|hCoV-19/Ireland/MN-NVRL-20IRL51449/2020|2020-09-30|EPI_ISL_639892|Original|hCoV-19^^Monaghan|Human|National Virus Reference Laboratory|National Virus Reference Laboratory|Gonzalez|Ireland
>Spike|hCoV-19/Japan/PG-4176/2020|2020-07-00|EPI_ISL_692415|Original|hCoV-19^^Japan|Human|Pathogen Genomics Center|Pathogen Genomics Center|Sekizuka|Japan
>Spike|hCoV-19/Denmark/DCGC-5759/2020|2020-09-28|EPI_ISL_619908|Original|hCoV-19^^Hovedstaden|Human|Department of Virus and Microbiological Special Diagnostics|Albertsen lab|Michaelsen|Denmark
>Spike|hCoV-19/England/ALDP-9562B1/2020|2020-06-08|EPI_ISL_553969|Original|hCoV-19^^England|Human|Lighthouse Lab in Alderley Park|Wellcome Sanger Institute for the COVID-19 Genomics UK (COG-UK) consortium|Davies|United Kingdom

>Spike|hCoV-19/England/QEUH-BCCB04/2020|2020-11-25|EPI_ISL_704316|Original|hCoV-19^^England|Human|Lighthouse Lab in Glasgow|Wellcome Sanger Institute for the COVID-19 Genomics UK (COG-UK) Consortium|Davies|United Kingdom
>Spike|hCoV-19/England/QEUH-A3102A/2020|2020-10-05|EPI_ISL_595854|Original|hCoV-19^^England|Human|Quadram Institute Bioscience|COVID-19 Genomics UK (COG-UK) Consortium|Alikhan|United Kingdom
>Spike|hCoV-19/USA/WI-UW-915/2020|2020-07-10|EPI_ISL_509916|Original|hCoV-19^^Wisconsin|Human|University of Wisconsin-Madison AIDS Vaccine Research Laboratories|University of Wisconsin-Madison AIDS Vaccine Research Laboratories|Baczenas|USA
>Spike|hCoV-19/England/MILK-B059AB/2020|2020-10-24|EPI_ISL_651585|Original|hCoV-19^^England|Human|Queens Medical Centre|COVID-19 Genomics UK (COG-UK) Consortium|Loose|United Kingdom
>Spike|hCoV-19/USA/TX-HMH-MCoV-18345/2020|2020-11-18|EPI_ISL_784693|Original|hCoV-19^^Texas|Human|Houston Methodist Hospital|Houston Methodist Hospital|Long|USA
>Spike|hCoV-19/USA/NM-DOH-2020327025/2020|2020-09-03|EPI_ISL_575304|Original|hCoV-19^^New Mexico|Human|Molecular Biology|Molecular Biology|Johnson|USA
>Spike|hCoV-19/England/MILK-ACE21D/2020|2020-10-21|EPI_ISL_629369|Original|hCoV-19^^England|Human|Lighthouse Lab in Milton Keynes|Wellcome Sanger Institute for the COVID-19 Genomics UK (COG-UK) consortium|Davies|United Kingdom
>Spike|hCoV-19/Finland/5A10S4A10/2020|2020-04-05|EPI_ISL_481682|Original|hCoV-19^^Uusimaa|Human|Department of Virology and Immunology|Department of Virology|Smura|Finland
>Spike|hCoV-19/England/MILK-ACCB8D/2020|2020-10-20|EPI_ISL_634344|Original|hCoV-19^^England|Human|Lighthouse Lab in Milton Keynes|Wellcome Sanger Institute for the COVID-19 Genomics UK (COG-UK) consortium|Davies|United Kingdom
>Spike|hCoV-19/Luxembourg/LNS2718564/2020|2020-12-28|EPI_ISL_771123|Original|hCoV-19^^Luxembourg|Human|Laboratoire national de sant\u00e9|Laboratoire national de sant\u00e9|Wienecke-Baldacchino|Luxembourg
>Spike|hCoV-19/Scotland/QEUH-95EE97/2020|2020-08-08|EPI_ISL_532356|Original|hCoV-19^^Scotland|Human|NHSGGC West of Scotland Specialist Virology Centre / MRC-University of Glasgow Centre for Virus Research|Wellcome Sanger Institute for the COVID-19 Genomics UK (COG-UK) consortium|Davies|United Kingdom
>Spike|hCoV-19/England/ALDP-B6352F/2020|2020-11-09|EPI_ISL_656597|Original|hCoV-19^^England|Human|Lighthouse Lab in Alderley Park|Wellcome Sanger Institute for the COVID-19 Genomics UK (COG-UK) Consortium|Davies|United Kingdom
>Spike|hCoV-19/Netherlands/NA_601/2020|2020-03-25|EPI_ISL_460764|Original|hCoV-19^^Netherlands|Human|Dutch COVID-19 response team|Erasmus Medical Center|Oude Munnink|Netherlands
>Spike|hCoV-19/Australia/VIC3262/2020|2020-07-13|EPI_ISL_520941|Original|hCoV-19^^Victoria|Human|Microbiological Diagnostic Unit - Public Health Laboratory (MDU-PHL)|MDU-PHL|Schultz|Australia
>Spike|hCoV-19/England/BRIS-1239ED/2020|2020-04-07|EPI_ISL_442712|Original|hCoV-19^^England|Human|PHE South West Regional Laboratory|Wellcome Sanger Institute for the COVID-19 Genomics UK (COG-UK) consortium|Amato|United Kingdom
>Spike|hCoV-19/Scotland/CVR4561/2020|2020-09-22|EPI_ISL_585419|Original|hCoV-19^^Scotland|Human|Lighthouse Lab in Glasgow / MRC-University of Glasgow Centre for Virus Research|COVID-19 Genomics UK (COG-UK) Consortium|Robertson|United Kingdom
>Spike|hCoV-19/England/MILK-A05F5F/2020|2020-09-30|EPI_ISL_599934|Original|hCoV-19^^England|Human|Lighthouse Lab in Milton Keynes|Wellcome Sanger Institute for the COVID-19 Genomics UK (COG-UK) consortium|Davies|United Kingdom
>Spike|hCoV-19/Switzerland/ZH-ETHZ-101134/2020|2020-03-30|EPI_ISL_451801|Original|hCoV-19^^Zurich|Human|Viollier AG|Department of Biosystems Science and Engineering|Nadeau|Switzerland
>Spike|hCoV-19/Scotland/QEUH-A20950/2020|2020-10-01|EPI_ISL_599063|Original|hCoV-19^^Scotland|Human|Lighthouse Lab in Glasgow|Wellcome Sanger Institute for the COVID-19 Genomics UK (COG-UK) consortium|Davies|United Kingdom
>Spike|hCoV-19/Brazil/RJ-00458/2020|2020-07-01|EPI_ISL_717853|Original|hCoV-19^^Rio de Janeiro|Human|Laboratorio de Virologia Molecular / UFRJ|Bioinformatics Laboratory / LNCC|Almeida|Brazil

>Spike|hCoV-19/France/PAC-IHU-0132/2020|2020-03-13|EPI_ISL_569328|Original|hCoV-19^^Provence-Alpes-C\u00f4te-d\u2019Azur|Human|MEPHI|MEPHI|Levasseur|France
>Spike|hCoV-19/Australia/VIC1960/2020|2020-06-10|EPI_ISL_480683|Original|hCoV-19^^Victoria|Human|Victorian Infectious Diseases Reference Laboratory (VIDRL)|VIDRL and MDU-PHL|Schultz|Australia
>Spike|hCoV-19/England/QEUH-A1E955/2020|2020-10-01|EPI_ISL_587548|Original|hCoV-19^^England|Human|Lighthouse Lab in Glasgow|Wellcome Sanger Institute for the COVID-19 Genomics UK (COG-UK) consortium|Davies|United Kingdom
>Spike|hCoV-19/Wales/CVR4815/2020|2020-09-21|EPI_ISL_627358|Original|hCoV-19^^Wales|Human|West of Scotland Specialist Virology Centre|COVID-19 Genomics UK (COG-UK) Consortium|Robertson|United Kingdom
>Spike|hCoV-19/USA/MN-MDH-1289/2020|2020-03-21|EPI_ISL_484692|Original|hCoV-19^^Minnesota|Human|Minnesota Department of Health|Minnesota Department of Health|Garfin|USA
>Spike|hCoV-19/England/OXON-AE0B9/2020|2020-00-00|EPI_ISL_448661|Original|hCoV-19^^England|Human|Oxford Viromics|COVID-19 Genomics UK (COG-UK) Consortium|Golubchik|United Kingdom
>Spike|hCoV-19/Japan/PG-0397/2020|2020-03-29|EPI_ISL_480066|Original|hCoV-19^^Kansai|Human|Sakai City Institute of Public Health|Pathogen Genomics Center|Sekizuka|Japan
>Spike|hCoV-19/USA/CA-QDX-2691/2020|2020-03-16|EPI_ISL_604998|Original|hCoV-19^^California|Human|Quest Diagnostics|Quest Diagnostics|Kagan|USA
>Spike|hCoV-19/India/MH-NIV-64850/2020|2020-06-16|EPI_ISL_479556|Original|hCoV-19^^Maharashtra|Human|NIV Influenza|NIV Influenza|Potdar|India
>Spike|hCoV-19/Australia/NSW1137/2020|2020-07-27|EPI_ISL_593748|Original|hCoV-19^^New South Wales|Human|South Eastern Area Laboratory Services (SEALS)|NSW Health Pathology - Institute of Clinical Pathology and Medical Research; Westmead Hospital; University of Sydney|Gall|Australia
>Spike|hCoV-19/England/MILK-BDB70E/2020|2020-11-26|EPI_ISL_709772|Original|hCoV-19^^England|Human|Lighthouse Lab in Milton Keynes|Wellcome Sanger Institute for the COVID-19 Genomics UK (COG-UK) Consortium|Davies|United Kingdom
>Spike|hCoV-19/USA/WI-GMF-26669/2020|2020-10-19|EPI_ISL_660959|Original|hCoV-19^^Wisconsin|Human|Gundersen Molecular Diagnostics Laboratory|Kabara Cancer Research Institute|Kenny|USA
>Spike|hCoV-19/Northern Ireland/NIRE-FB396/2020|2020-03-19|EPI_ISL_441415|Original|hCoV-19^^Northern Ireland|Human|Regional Virus Laboratory|COVID-19 Genomics UK (COG-UK) Consortium|Fairley|United Kingdom
>Spike|hCoV-19/England/ALDP-C3C8CB/2020|2020-12-10|EPI_ISL_730982|Original|hCoV-19^^England|Human|Lighthouse Lab in Alderley Park|Wellcome Sanger Institute for the COVID-19 Genomics UK (COG-UK) Consortium|Davies|United Kingdom
>Spike|hCoV-19/Denmark/DCGC-18989/2020|2020-12-07|EPI_ISL_748684|Original|hCoV-19^^Sjaelland|Human|Department of Virus and Microbiological Special Diagnostics|Albertsen Lab|Michaelsen|Denmark
>Spike|hCoV-19/USA/TX-HMH-MCoV-10525/2020|2020-07-09|EPI_ISL_789518|Original|hCoV-19^^Texas|Human|Houston Methodist Hospital|Houston Methodist Hospital|Long|USA
>Spike|hCoV-19/Scotland/EDB1855/2020|2020-04-17|EPI_ISL_491524|Original|hCoV-19^^Scotland|Human|Virology Department|Wellcome Sanger Institute for the COVID-19 Genomics UK (COG-UK) consortium|Davies|United Kingdom
>Spike|hCoV-19/France/BRE-IPP9971/2020|2020-09-26|EPI_ISL_718230|Original|hCoV-19^^Bretagne|Human|Hospital|National Reference Center for Viruses of Respiratory Infections|Enouf|France
>Spike|hCoV-19/South Africa/KRISP-K002772/2020|2020-08-21|EPI_ISL_529767|Original|hCoV-19^^KwaZulu-Natal|Human|NHLS-IALCH|KRISP|de Oliveira|South Africa
>Spike|hCoV-19/England/MILK-97107A/2020|2020-08-16|EPI_ISL_553067|Original|hCoV-19^^England|Human|Lighthouse Lab in Milton Keynes|Wellcome Sanger Institute for the COVID-19 Genomics UK (COG-UK) consortium|Davies|United Kingdom
>Spike|hCoV-19/USA/MI-MDHHS-SC20870/2020|2020-04-21|EPI_ISL_471891|Original|hCoV-19^^Michigan|Human|Michigan Department of Health and Human Services|Michigan Department of Health and Human Services|Blankenship|USA

>Spike|hCoV-19/England/SHEF-10CFC7F/2020|2020-12-21|EPI_ISL_765174|Original|hCoV-19^^England|Human|Virology Department|COVID-19 Genomics UK (COG-UK) Consortium|de Silva|United Kingdom
>Spike|hCoV-19/England/QEUH-BCBB7E/2020|2020-11-27|EPI_ISL_704517|Original|hCoV-19^^England|Human|Lighthouse Lab in Glasgow|Wellcome Sanger Institute for the COVID-19 Genomics UK (COG-UK) Consortium|Davies|United Kingdom
>Spike|hCoV-19/Luxembourg/LNS4788360/2020|2020-12-18|EPI_ISL_740546|Original|hCoV-19^^Luxembourg|Human|Laboratoire national de sant\u00e9|Laboratoire national de sant\u00e9|Wienecke-Baldacchino|Luxembourg
>Spike|hCoV-19/England/ALDP-AA1C8B/2020|2020-10-22|EPI_ISL_624302|Original|hCoV-19^^England|Human|Lighthouse Lab in Alderley Park|Wellcome Sanger Institute for the COVID-19 Genomics UK (COG-UK) consortium|Davies|United Kingdom
>Spike|hCoV-19/England/ALDP-BB97F7/2020|2020-11-20|EPI_ISL_702805|Original|hCoV-19^^England|Human|Lighthouse Lab in Alderley Park|Wellcome Sanger Institute for the COVID-19 Genomics UK (COG-UK) Consortium|Davies|United Kingdom
>Spike|hCoV-19/Japan/PG-4897/2020|2020-08-00|EPI_ISL_690018|Original|hCoV-19^^Japan|Human|Sakai City Institute of Public Health|Pathogen Genomics Center|Sekizuka|Japan
>Spike|hCoV-19/England/QEUH-B0DC7D/2020|2020-11-02|EPI_ISL_642078|Original|hCoV-19^^England|Human|Lighthouse Lab in Glasgow|Wellcome Sanger Institute for the COVID-19 Genomics UK (COG-UK) Consortium|Davies|United Kingdom
>Spike|hCoV-19/Japan/PG-7842/2020|2020-08-00|EPI_ISL_686943|Original|hCoV-19^^Japan|Human|Pathogen Genomics Center|Pathogen Genomics Center|Sekizuka|Japan
>Spike|hCoV-19/England/MILK-B8D56B/2020|2020-11-13|EPI_ISL_673945|Original|hCoV-19^^England|Human|Lighthouse Lab in Milton Keynes|Wellcome Sanger Institute for the COVID-19 Genomics UK (COG-UK) Consortium|Davies|United Kingdom
>Spike|hCoV-19/England/CAMB-7FD05/2020|2020-04-19|EPI_ISL_444405|Original|hCoV-19^^England|Human|Department of Pathology|COVID-19 Genomics UK (COG-UK) Consortium|Meredith|United Kingdom
>Spike|hCoV-19/Denmark/DCGC-1738/2020|2020-03-09|EPI_ISL_622242|Original|hCoV-19^^Syddanmark|Human|Department of Virus and Microbiological Special Diagnostics|Albertsen lab|Michaelsen|Denmark
>Spike|hCoV-19/England/QEUH-9DDD84/2020|2020-09-21|EPI_ISL_599731|Original|hCoV-19^^England|Human|Lighthouse Lab in Glasgow|Wellcome Sanger Institute for the COVID-19 Genomics UK (COG-UK) consortium|Davies|United Kingdom
>Spike|hCoV-19/USA/NM-QDX-211/2020|2020-04-30|EPI_ISL_494534|Original|hCoV-19^^New Mexico|Human|Quest Diagnostics|Quest Diagnostics|Kagan|USA
>Spike|hCoV-19/USA/TX-HMH-MCoV-18756/2020|2020-11-23|EPI_ISL_785093|Original|hCoV-19^^Texas|Human|Houston Methodist Hospital|Houston Methodist Hospital|Long|USA
>Spike|hCoV-19/England/BRIS-12A6C1/2020|2020-04-30|EPI_ISL_458322|Original|hCoV-19^^England|Human|PHE South West Regional Laboratory|Wellcome Sanger Institute for the COVID-19 Genomics UK (COG-UK) consortium|Davies|United Kingdom
>Spike|hCoV-19/England/NORW-221C12/2020|2020-12-16|EPI_ISL_764470|Original|hCoV-19^^England|Human|Quadram Institute Bioscience|COVID-19 Genomics UK (COG-UK) Consortium|Alikhan|United Kingdom
>Spike|hCoV-19/USA/MA-MGH-01182/2020|2020-04-29|EPI_ISL_791899|Original|hCoV-19^^Massachusetts|Human|Massachusetts General Hospital|Infectious Disease Program|Park|USA
>Spike|hCoV-19/England/MILK-55D209/2020|2020-06-10|EPI_ISL_558248|Original|hCoV-19^^England|Human|Lighthouse Lab in Milton Keynes|Wellcome Sanger Institute for the COVID-19 Genomics UK (COG-UK) consortium|Davies|United Kingdom
>Spike|hCoV-19/England/MILK-B06C65/2020|2020-10-29|EPI_ISL_652269|Original|hCoV-19^^England|Human|Quadram Institute Bioscience|COVID-19 Genomics UK (COG-UK) Consortium|Alikhan|United Kingdom
>Spike|hCoV-19/England/MILK-A7B8B3/2020|2020-10-15|EPI_ISL_607590|Original|hCoV-19^^England|Human|Lighthouse Lab in Milton Keynes|Wellcome Sanger Institute for the COVID-19 Genomics UK (COG-UK) consortium|Davies|United Kingdom
>Spike|hCoV-19/USA/WA-UW-4406/2020|2020-03-31|EPI_ISL_426136|Original|hCoV-19^^Washington|Human|UW Virology Lab|UW Virology Lab|Roychoudhury|USA

>Spike|hCoV-19/Scotland/QEUH-A4B61C/2020|2020-10-08|EPI_ISL_637847|Original|hCoV-19^^Scotland|Human|Department of Pathology|COVID-19 Genomics UK (COG-UK) Consortium|Goodfellow|United Kingdom
>Spike|hCoV-19/USA/TX-HMH-MCoV-16041/2020|2020-10-27|EPI_ISL_785725|Original|hCoV-19^^Texas|Human|Houston Methodist Hospital|Houston Methodist Hospital|Long|USA
>Spike|hCoV-19/England/20169003804/2020|2020-04-15|EPI_ISL_465175|Original|hCoV-19^^England|Human|Respiratory Virus Unit|Respiratory Virus Unit|Platt|United Kingdom
>Spike|hCoV-19/USA/RI-Yale-445/2020|2020-04-15|EPI_ISL_738244|Original|hCoV-19^^Rhode Island|Human|Connecticut Veterans' Affairs Hospital|Grubaugh Lab - Yale School of Public Health|Grubaugh|USA
>Spike|hCoV-19/Denmark/DCGC-11020/2020|2020-11-02|EPI_ISL_669829|Original|hCoV-19^^Hovedstaden|Human|Department of Virus and Microbiological Special Diagnostics|Albertsen Lab|Michaelsen|Denmark
>Spike|hCoV-19/England/205020882/2020|2020-00-00|EPI_ISL_710519|Original|hCoV-19^^England|Human|Respiratory Virus Unit|COVID-19 Genomics UK (COG-UK) Consortium|Platt|United Kingdom
>Spike|hCoV-19/Japan/PG-0903/2020|2020-03-00|EPI_ISL_684513|Original|hCoV-19^^Japan|Human|Tokyo Metropolitan Institute of Public Health|Pathogen Genomics Center|Sekizuka|Japan
>Spike|hCoV-19/England/QEUH-AACF69/2020|2020-10-21|EPI_ISL_652930|Original|hCoV-19^^England|Human|Oxford Viromics|COVID-19 Genomics UK (COG-UK) Consortium|Golubchik|United Kingdom
>Spike|hCoV-19/England/QEUH-9CF945/2020|2020-09-16|EPI_ISL_590019|Original|hCoV-19^^England|Human|Lighthouse Lab in Glasgow|Wellcome Sanger Institute for the COVID-19 Genomics UK (COG-UK) consortium|Davies|United Kingdom
>Spike|hCoV-19/England/CAMB-80E2C/2020|2020-04-10|EPI_ISL_442844|Original|hCoV-19^^England|Human|Department of Pathology|Wellcome Sanger Institute for the COVID-19 Genomics UK (COG-UK) consortium|Amato|United Kingdom
>Spike|hCoV-19/Japan/PG-6918/2020|2020-09-00|EPI_ISL_688025|Original|hCoV-19^^Japan|Human|Pathogen Genomics Center|Pathogen Genomics Center|Sekizuka|Japan
>Spike|hCoV-19/USA/WI-UW-604/2020|2020-06-25|EPI_ISL_484979|Original|hCoV-19^^Wisconsin|Human|University of Wisconsin-Madison AIDS Vaccine Research Laboratories|University of Wisconsin-Madison AIDS Vaccine Research Laboratories|Baczenas|USA
>Spike|hCoV-19/England/CAMC-9E0058/2020|2020-09-19|EPI_ISL_576954|Original|hCoV-19^^England|Human|Oxford Viromics|COVID-19 Genomics UK (COG-UK) Consortium|Golubchik|United Kingdom
>Spike|hCoV-19/Spain/AN-IBV-98001643/2020|2020-04-07|EPI_ISL_538121|Original|hCoV-19^^Andalucia|Human|Servicio de Microbiolog\u00eda y Parasitolog\u00eda cl\u00ednica. UCEIMP. Hospital Universitario Virgen del Roc\u00edo/IBIS/CSIC/US|SeqCOVID-SPAIN consortium/IBV(CSIC)|Comas|Spain
>Spike|hCoV-19/England/QEUH-9DE2B1/2020|2020-09-21|EPI_ISL_599582|Original|hCoV-19^^England|Human|Lighthouse Lab in Glasgow|Wellcome Sanger Institute for the COVID-19 Genomics UK (COG-UK) consortium|Davies|United Kingdom
>Spike|hCoV-19/Netherlands/UT-RIVM-21003/2020|2020-12-19|EPI_ISL_791020|Original|hCoV-19^^Utrecht|Human|Dutch COVID-19 response team|National Institute for Public Health and the Environment (RIVM)|Meijer|Netherlands
>Spike|hCoV-19/Scotland/QEUH-A4B89E/2020|2020-10-08|EPI_ISL_628487|Original|hCoV-19^^Scotland|Human|Oxford Viromics|COVID-19 Genomics UK (COG-UK) Consortium|Golubchik|United Kingdom
>Spike|hCoV-19/England/QEUH-9B4EAE/2020|2020-09-04|EPI_ISL_590344|Original|hCoV-19^^England|Human|Lighthouse Lab in Glasgow|Wellcome Sanger Institute for the COVID-19 Genomics UK (COG-UK) consortium|Davies|United Kingdom
>Spike|hCoV-19/Iceland/407/2020|2020-03-19|EPI_ISL_424431|Original|hCoV-19^^Reykjavik|Human|The National University Hospital of Iceland|deCODE genetics|J\u00f3nsson|Iceland
>Spike|hCoV-19/Portugal/PT2087/2020|2020-11-08|EPI_ISL_738119|Original|hCoV-19^^Portugal|Human|Instituto Nacional de Saude (INSA)|Instituto Nacional de Saude (INSA)|Borges|Portugal

>Spike|hCoV-19/England/MILK-99D7EC/2020|2020-08-30|EPI_ISL_550681|Original|hCoV-19^^England|Human|Lighthouse Lab in Milton Keynes|Wellcome Sanger Institute for the COVID-19 Genomics UK (COG-UK) consortium|Davies|United Kingdom
>Spike|hCoV-19/England/MILK-A7A3B3/2020|2020-10-15|EPI_ISL_606722|Original|hCoV-19^^England|Human|Lighthouse Lab in Milton Keynes|Wellcome Sanger Institute for the COVID-19 Genomics UK (COG-UK) consortium|Davies|United Kingdom
>Spike|hCoV-19/England/QEUH-960A43/2020|2020-08-06|EPI_ISL_532052|Original|hCoV-19^^England|Human|Lighthouse Lab in Glasgow|Wellcome Sanger Institute for the COVID-19 Genomics UK (COG-UK) consortium|Davies|United Kingdom
>Spike|hCoV-19/England/MILK-9C43BB/2020|2020-09-09|EPI_ISL_566818|Original|hCoV-19^^England|Human|Lighthouse Lab in Milton Keynes|Wellcome Sanger Institute for the COVID-19 Genomics UK (COG-UK) consortium|Davies|United Kingdom
>Spike|hCoV-19/United Arab Emirates/0732/2020|2020-05-15|EPI_ISL_698883|Original|hCoV-19^^Abu Dhabi|Human|Group 42 (G42) Healthcare|G42 Healthcare|Ogrodzki|United Arab Emirates
>Spike|hCoV-19/Wales/PHWC-165FED/2020|2020-05-15|EPI_ISL_490824|Original|hCoV-19^^Wales|Human|Wales Specialist Virology Centre Sequencing lab|COVID-19 Genomics UK (COG-UK) Consortium|Moore|United Kingdom
>Spike|hCoV-19/USA/TX-HMH-4048/2020|2020-06-17|EPI_ISL_543325|Original|hCoV-19^^Texas|Human|Houston Methodist Hospital|Houston Methodist Hospital|Long|USA
>Spike|hCoV-19/Japan/IC-0374/2020|2020-12-00|EPI_ISL_721573|Original|hCoV-19^^Japan|Human|Pathogen Genomics Center|Pathogen Genomics Center|Sekizuka|Japan
>Spike|hCoV-19/England/LIVE-DC2D15/2020|2020-11-24|EPI_ISL_702956|Original|hCoV-19^^England|Human|Liverpool Clinical Laboratories|COVID-19 Genomics UK (COG-UK) Consortium|Haldenby|United Kingdom
>Spike|hCoV-19/England/CAMC-942FB1/2020|2020-07-27|EPI_ISL_556873|Original|hCoV-19^^England|Human|Lighthouse Lab in Cambridge|Wellcome Sanger Institute for the COVID-19 Genomics UK (COG-UK) consortium|Davies|United Kingdom
>Spike|hCoV-19/United Arab Emirates/1096/2020|2020-05-16|EPI_ISL_698169|Original|hCoV-19^^Abu Dhabi|Human|Group 42 (G42) Healthcare|G42 Healthcare|Ogrodzki|United Arab Emirates
>Spike|hCoV-19/England/LIVE-9AA58/2020|2020-04-01|EPI_ISL_449381|Original|hCoV-19^^England|Human|Liverpool Clinical Laboratories|COVID-19 Genomics UK (COG-UK) Consortium|Haldenby|United Kingdom
>Spike|hCoV-19/England/ALDP-9E87C4/2020|2020-09-24|EPI_ISL_637415|Original|hCoV-19^^England|Human|Department of Pathology|COVID-19 Genomics UK (COG-UK) Consortium|Goodfellow|United Kingdom
>Spike|hCoV-19/Denmark/DCGC-12272/2020|2020-11-16|EPI_ISL_682453|Original|hCoV-19^^Nordjylland|Human|Department of Virus and Microbiological Special Diagnostics|Albertsen Lab|Michaelsen|Denmark
>Spike|hCoV-19/England/MILK-99C8EA/2020|2020-08-30|EPI_ISL_550561|Original|hCoV-19^^England|Human|Lighthouse Lab in Milton Keynes|Wellcome Sanger Institute for the COVID-19 Genomics UK (COG-UK) consortium|Davies|United Kingdom
>Spike|hCoV-19/England/ALDP-A50368/2020|2020-10-07|EPI_ISL_597700|Original|hCoV-19^^England|Human|Lighthouse Lab in Alderley Park|Wellcome Sanger Institute for the COVID-19 Genomics UK (COG-UK) consortium|Davies|United Kingdom
>Spike|hCoV-19/Scotland/EDB058/2020|2020-03-18|EPI_ISL_425862|Original|hCoV-19^^Scotland|Human|Virology Department|COVID-19 Genomics UK (COG-UK) Consortium|Williams|United Kingdom
>Spike|hCoV-19/Wales/PHWC-48A4E0/2020|2020-11-04|EPI_ISL_652562|Original|hCoV-19^^Wales|Human|Wales Specialist Virology Centre Sequencing lab|COVID-19 Genomics UK (COG-UK) Consortium|Moore|United Kingdom
>Spike|hCoV-19/Switzerland/VD-ETHZ-100807/2020|2020-03-25|EPI_ISL_451757|Original|hCoV-19^^Vaud|Human|Viollier AG|Department of Biosystems Science and Engineering|Nadeau|Switzerland
>Spike|hCoV-19/England/20146062104/2020|2020-04-01|EPI_ISL_421988|Original|hCoV-19^^England|Human|Respiratory Virus Unit|Respiratory Virus Unit|Platt|United Kingdom
>Spike|hCoV-19/Australia/VIC3442/2020|2020-07-06|EPI_ISL_521108|Original|hCoV-19^^Victoria|Human|Victorian Infectious Diseases Reference Laboratory (VIDRL)|VIDRL and MDU-PHL|Schultz|Australia

>Spike|hCoV-19/England/CAMC-9BFE49/2020|2020-09-09|EPI_ISL_573155|Original|hCoV-19^^England|Human|Oxford Viromics|COVID-19 Genomics UK (COG-UK) Consortium|Golubchik|United Kingdom
>Spike|hCoV-19/USA/MA-MGH-00827/2020|2020-04-13|EPI_ISL_791783|Original|hCoV-19^^Massachusetts|Human|Massachusetts General Hospital|Infectious Disease Program|Park|USA
>Spike|hCoV-19/Wales/PHWC-486DCB/2020|2020-10-14|EPI_ISL_639134|Original|hCoV-19^^Wales|Human|Wales Specialist Virology Centre Sequencing lab|COVID-19 Genomics UK (COG-UK) Consortium|Moore|United Kingdom
>Spike|hCoV-19/USA/DE-DHSS-F970997/2020|2020-06-18|EPI_ISL_693705|Original|hCoV-19^^Delaware|Human|Delaware Public Health Laboratory|Delaware Public Health Laboratory|Bajwa|USA
>Spike|hCoV-19/Austria/CeMM0386/2020|2020-04-03|EPI_ISL_475828|Original|hCoV-19^^Mayrhofen|Human|Institut f\u00fcr Virologie am Department f\u00fcr Hygiene|Bergthaler laboratory|Bergthaler|Austria
>Spike|hCoV-19/Wales/PHWC-25348/2020|2020-03-23|EPI_ISL_432323|Original|hCoV-19^^Wales|Human|Wales Specialist Virology Centre|Public Health Wales Microbiology Cardiff|Moore|United Kingdom
>Spike|hCoV-19/England/ALDP-C3BF8B/2020|2020-12-10|EPI_ISL_731579|Original|hCoV-19^^England|Human|Lighthouse Lab in Alderley Park|Wellcome Sanger Institute for the COVID-19 Genomics UK (COG-UK) Consortium|Davies|United Kingdom
>Spike|hCoV-19/USA/CA-ALSR-4145/2020|2020-06-24|EPI_ISL_648849|Original|hCoV-19^^California|Human|San Diego County Public Health Laboratory|Andersen lab at Scripps Research|Gangavarapu|USA
>Spike|hCoV-19/England/MILK-B56A11/2020|2020-11-05|EPI_ISL_656867|Original|hCoV-19^^England|Human|Lighthouse Lab in Milton Keynes|Wellcome Sanger Institute for the COVID-19 Genomics UK (COG-UK) Consortium|Davies|United Kingdom
>Spike|hCoV-19/England/QEUH-B66FB5/2020|2020-11-11|EPI_ISL_661347|Original|hCoV-19^^England|Human|Lighthouse Lab in Glasgow|Wellcome Sanger Institute for the COVID-19 Genomics UK (COG-UK) Consortium|Davies|United Kingdom
>Spike|hCoV-19/England/QEUH-C14BB1/2020|2020-12-06|EPI_ISL_709530|Original|hCoV-19^^England|Human|Lighthouse Lab in Glasgow|Wellcome Sanger Institute for the COVID-19 Genomics UK (COG-UK) Consortium|Davies|United Kingdom
>Spike|hCoV-19/England/QEUH-96BBDC/2020|2020-08-14|EPI_ISL_531319|Original|hCoV-19^^England|Human|Lighthouse Lab in Glasgow|Wellcome Sanger Institute for the COVID-19 Genomics UK (COG-UK) consortium|Davies|United Kingdom
>Spike|hCoV-19/Scotland/EDB2659/2020|2020-04-22|EPI_ISL_439251|Original|hCoV-19^^Scotland|Human|Virology Department|COVID-19 Genomics UK (COG-UK) Consortium|Williams|United Kingdom
>Spike|hCoV-19/England/CAMB-7DD25/2020|2020-04-06|EPI_ISL_442308|Original|hCoV-19^^England|Human|Department of Pathology|Wellcome Sanger Institute for the COVID-19 Genomics UK (COG-UK) consortium|Amato|United Kingdom
>Spike|hCoV-19/Australia/VIC14150/2020|2020-07-30|EPI_ISL_592353|Original|hCoV-19^^Victoria|Human|Microbiological Diagnostic Unit - Public Health Laboratory (MDU-PHL)|MDU-PHL|Schultz|Australia
>Spike|hCoV-19/Australia/VIC14379/2020|2020-09-21|EPI_ISL_591809|Original|hCoV-19^^Victoria|Human|Microbiological Diagnostic Unit - Public Health Laboratory (MDU-PHL)|MDU-PHL|Schultz|Australia
>Spike|hCoV-19/England/MILK-995140/2020|2020-08-24|EPI_ISL_552393|Original|hCoV-19^^England|Human|Lighthouse Lab in Milton Keynes|Wellcome Sanger Institute for the COVID-19 Genomics UK (COG-UK) consortium|Davies|United Kingdom
>Spike|hCoV-19/Wales/PHWC-49AE49/2020|2020-12-04|EPI_ISL_727695|Original|hCoV-19^^Wales|Human|Originating lab|COVID-19 Genomics UK (COG-UK) Consortium|Moore|United Kingdom
>Spike|hCoV-19/USA/MN131-MDH131/2020|2020-03-16|EPI_ISL_421687|Original|hCoV-19^^Minnesota|Human|Minnesota Department of Health|Minnesota Department of Health|Wang|USA
>Spike|hCoV-19/England/BRIS-12AB77/2020|2020-05-01|EPI_ISL_458337|Original|hCoV-19^^England|Human|PHE South West Regional Laboratory|Wellcome Sanger Institute for the COVID-19 Genomics UK (COG-UK) consortium|Davies|United Kingdom

>Spike|hCoV-19/England/ALDP-956318/2020|2020-06-08|EPI_ISL_553925|Original|hCoV-19^^England|Human|Lighthouse Lab in Alderley Park|Wellcome Sanger Institute for the COVID-19 Genomics UK (COG-UK) consortium|Davies|United Kingdom
>Spike|hCoV-19/England/QEUH-B9E9B6/2020|2020-11-18|EPI_ISL_719893|Original|hCoV-19^^England|Human|Lighthouse Lab in Glasgow|Wellcome Sanger Institute for the COVID-19 Genomics UK (COG-UK) Consortium|Davies|United Kingdom
>Spike|hCoV-19/Japan/PG-6050/2020|2020-07-00|EPI_ISL_689162|Original|hCoV-19^^Japan|Human|Pathogen Genomics Center|Pathogen Genomics Center|Sekizuka|Japan
>Spike|hCoV-19/USA/MA-MGH-03317/2020|2020-11-01|EPI_ISL_765784|Original|hCoV-19^^Massachusetts|Human|Massachusetts General Hospital|Infectious Disease Program|Park|USA
>Spike|hCoV-19/England/ALDP-A66D43/2020|2020-10-12|EPI_ISL_630412|Original|hCoV-19^^England|Human|Lighthouse Lab in Alderley Park|Wellcome Sanger Institute for the COVID-19 Genomics UK (COG-UK) consortium|Davies|United Kingdom
>Spike|hCoV-19/England/MILK-A7CA52/2020|2020-10-15|EPI_ISL_606361|Original|hCoV-19^^England|Human|Lighthouse Lab in Milton Keynes|Wellcome Sanger Institute for the COVID-19 Genomics UK (COG-UK) consortium|Davies|United Kingdom
>Spike|hCoV-19/Wales/PHWC-487AA5/2020|2020-10-30|EPI_ISL_639269|Original|hCoV-19^^Wales|Human|Wales Specialist Virology Centre Sequencing lab|COVID-19 Genomics UK (COG-UK) Consortium|Moore|United Kingdom
>Spike|hCoV-19/USA/WA-UW-13085/2020|2020-06-30|EPI_ISL_570952|Original|hCoV-19^^Washington|Human|UW Virology Lab|UW Virology Lab|Roychoudhury|USA
>Spike|hCoV-19/USA/TX-HMH-4274/2020|2020-06-18|EPI_ISL_543486|Original|hCoV-19^^Texas|Human|Houston Methodist Hospital|Houston Methodist Hospital|Long|USA
>Spike|hCoV-19/England/CAMC-B0C634/2020|2020-10-30|EPI_ISL_643569|Original|hCoV-19^^England|Human|Lighthouse Lab in Cambridge|Wellcome Sanger Institute for the COVID-19 Genomics UK (COG-UK) Consortium|Davies|United Kingdom
>Spike|hCoV-19/England/ALDP-9EA003/2020|2020-09-24|EPI_ISL_580013|Original|hCoV-19^^England|Human|Lighthouse Lab in Alderley Park|Wellcome Sanger Institute for the COVID-19 Genomics UK (COG-UK) consortium|Davies|United Kingdom
>Spike|hCoV-19/Wales/PHWC-491F8B/2020|2020-11-16|EPI_ISL_706801|Original|hCoV-19^^Wales|Human|Originating lab|COVID-19 Genomics UK (COG-UK) Consortium|Moore|United Kingdom
>Spike|hCoV-19/England/QEUH-BC3790/2020|2020-11-25|EPI_ISL_704007|Original|hCoV-19^^England|Human|Lighthouse Lab in Glasgow|Wellcome Sanger Institute for the COVID-19 Genomics UK (COG-UK) Consortium|Davies|United Kingdom
>Spike|hCoV-19/England/MILK-9579BB/2020|2020-06-10|EPI_ISL_554133|Original|hCoV-19^^England|Human|Lighthouse Lab in Milton Keynes|Wellcome Sanger Institute for the COVID-19 Genomics UK (COG-UK) consortium|Davies|United Kingdom
>Spike|hCoV-19/England/ALDP-B47DED/2020|2020-11-07|EPI_ISL_649242|Original|hCoV-19^^England|Human|Lighthouse Lab in Alderley Park|Wellcome Sanger Institute for the COVID-19 Genomics UK (COG-UK) Consortium|Davies|United Kingdom
>Spike|hCoV-19/England/MILK-CA62CE/2020|2020-12-18|EPI_ISL_761475|Original|hCoV-19^^England|Human|Lighthouse Lab in Milton Keynes|Wellcome Sanger Institute for the COVID-19 Genomics UK (COG-UK) Consortium|Davies|United Kingdom
>Spike|hCoV-19/Wales/QEUH-A001F4/2020|2020-09-30|EPI_ISL_600479|Original|hCoV-19^^Wales|Human|Lighthouse Lab in Glasgow|Wellcome Sanger Institute for the COVID-19 Genomics UK (COG-UK) consortium|Davies|United Kingdom
>Spike|hCoV-19/England/QEUH-AD1C82/2020|2020-10-26|EPI_ISL_629992|Original|hCoV-19^^England|Human|Lighthouse Lab in Glasgow|Wellcome Sanger Institute for the COVID-19 Genomics UK (COG-UK) consortium|Davies|United Kingdom
>Spike|hCoV-19/Australia/VIC4423/2020|2020-07-17|EPI_ISL_519719|Original|hCoV-19^^Victoria|Human|Microbiological Diagnostic Unit - Public Health Laboratory (MDU-PHL)|MDU-PHL|Schultz|Australia
>Spike|hCoV-19/England/QEUH-997CB6/2020|2020-08-23|EPI_ISL_559759|Original|hCoV-19^^England|Human|Oxford Viromics|COVID-19 Genomics UK (COG-UK) Consortium|Golubchik|United Kingdom
>Spike|hCoV-19/USA/TX-HMH-MCoV-14447/2020|2020-10-07|EPI_ISL_787732|Original|hCoV-19^^Texas|Human|Houston Methodist Hospital|Houston Methodist Hospital|Long|USA

>Spike|hCoV-19/South Africa/NHLS-UCT-GP-5557/2020|2020-05-08|EPI_ISL_700513|Original|hCoV-19^^Western Cape|Human|Dr Abdurahman CDC wc DAC|NHLS/UCT|Iranzadeh|South Africa
>Spike|hCoV-19/England/20140005304/2020|2020-03-29|EPI_ISL_423380|Original|hCoV-19^^England|Human|Respiratory Virus Unit|Respiratory Virus Unit|Platt|United Kingdom
>Spike|hCoV-19/England/LIVE-AA606/2020|2020-03-24|EPI_ISL_517475|Original|hCoV-19^^England|Human|Liverpool Clinical Laboratories|COVID-19 Genomics UK (COG-UK) Consortium|Haldenby|United Kingdom
>Spike|hCoV-19/England/CAMC-94BC84/2020|2020-07-14|EPI_ISL_556769|Original|hCoV-19^^England|Human|Lighthouse Lab in Cambridge|Wellcome Sanger Institute for the COVID-19 Genomics UK (COG-UK) consortium|Davies|United Kingdom
>Spike|hCoV-19/England/ALDP-B8386C/2020|2020-11-14|EPI_ISL_675349|Original|hCoV-19^^England|Human|Lighthouse Lab in Alderley Park|Wellcome Sanger Institute for the COVID-19 Genomics UK (COG-UK) Consortium|Davies|United Kingdom
>Spike|hCoV-19/Denmark/DCGC-22245/2020|2020-12-14|EPI_ISL_758633|Original|hCoV-19^^Syddanmark|Human|Department of Virus and Microbiological Special Diagnostics|Albertsen Lab|Michaelsen|Denmark
>Spike|hCoV-19/England/CAMC-B09594/2020|2020-10-30|EPI_ISL_673154|Original|hCoV-19^^England|Human|Lighthouse Lab in Cambridge|Wellcome Sanger Institute for the COVID-19 Genomics UK (COG-UK) Consortium|Davies|United Kingdom
>Spike|hCoV-19/England/CAMC-AEA66A/2020|2020-10-25|EPI_ISL_633114|Original|hCoV-19^^England|Human|Lighthouse Lab in Cambridge|Wellcome Sanger Institute for the COVID-19 Genomics UK (COG-UK) consortium|Davies|United Kingdom
>Spike|hCoV-19/England/CAMB-1B8394/2020|2020-10-19|EPI_ISL_638386|Original|hCoV-19^^England|Human|Department of Pathology|COVID-19 Genomics UK (COG-UK) Consortium|Goodfellow|United Kingdom
>Spike|hCoV-19/USA/TX-HMH-MCoV-14376/2020|2020-10-06|EPI_ISL_787661|Original|hCoV-19^^Texas|Human|Houston Methodist Hospital|Houston Methodist Hospital|Long|USA
>Spike|hCoV-19/USA/CA-ALSR-3882/2020|2020-06-30|EPI_ISL_636259|Original|hCoV-19^^California|Human|San Diego County Public Health Laboratory|Andersen lab at Scripps Research|Gangavarapu|USA
>Spike|hCoV-19/England/ALDP-B627EE/2020|2020-11-08|EPI_ISL_656566|Original|hCoV-19^^England|Human|Lighthouse Lab in Alderley Park|Wellcome Sanger Institute for the COVID-19 Genomics UK (COG-UK) Consortium|Davies|United Kingdom
>Spike|hCoV-19/Wales/PHWC-4A63C3/2020|2020-12-16|EPI_ISL_765032|Original|hCoV-19^^Wales|Human|Wales Specialist Virology Centre Sequencing lab|COVID-19 Genomics UK (COG-UK) Consortium|Moore|United Kingdom
>Spike|hCoV-19/Ireland/WH-NVRL-MPP69791/2020|2020-04-11|EPI_ISL_767834|Original|hCoV-19^^Westmeath|Human|National Virus Reference Laboratory|Irish Coronavirus Sequencing Consortium - Teagasc Moorepark|Walsh|Ireland
>Spike|hCoV-19/Denmark/DCGC-12265/2020|2020-11-16|EPI_ISL_682446|Original|hCoV-19^^Hovedstaden|Human|Department of Virus and Microbiological Special Diagnostics|Albertsen Lab|Michaelsen|Denmark
>Spike|hCoV-19/Norway/5766/2020|2020-10-27|EPI_ISL_708167|Original|hCoV-19^^Vestfold og Telemark|Human|Vestfold Hospital|Norwegian Institute of Public Health|Kopperud Riis|Norway
>Spike|hCoV-19/England/QEUH-9A475B/2020|2020-09-01|EPI_ISL_540180|Original|hCoV-19^^England|Human|Lighthouse Lab in Glasgow|Wellcome Sanger Institute for the COVID-19 Genomics UK (COG-UK) consortium|Davies|United Kingdom
>Spike|hCoV-19/USA/LA-EVTL086/2020|2020-05-04|EPI_ISL_451247|Original|hCoV-19^^Louisiana|Human|LSUHS Emerging Viral Threat Laboratory|Microbial Genome Sequencing Center|Kamil|USA
>Spike|hCoV-19/England/ALDP-A51EC0/2020|2020-10-07|EPI_ISL_598093|Original|hCoV-19^^England|Human|Lighthouse Lab in Alderley Park|Wellcome Sanger Institute for the COVID-19 Genomics UK (COG-UK) consortium|Davies|United Kingdom
>Spike|hCoV-19/Denmark/ALAB-SSI-797/2020|2020-03-30|EPI_ISL_444911|Original|hCoV-19^^Denmark|Human|Department of Virus and Microbiological Special Diagnostics|Albertsen lab|Kirkegaard|Denmark
>Spike|hCoV-19/USA/TX-HMH-MCoV-11503/2020|2020-09-08|EPI_ISL_790034|Original|hCoV-19^^Texas|Human|Houston Methodist Hospital|Houston Methodist Hospital|Long|USA

>Spike|hCoV-19/USA/WA-S1600/2020|2020-05-26|EPI_ISL_496026|Original|hCoV-19^^Washington|Human|Washington State Department of Health|Seattle Flu Study|Lee|USA
>Spike|hCoV-19/England/ALDP-B48520/2020|2020-11-07|EPI_ISL_649311|Original|hCoV-19^^England|Human|Lighthouse Lab in Alderley Park|Wellcome Sanger Institute for the COVID-19 Genomics UK (COG-UK) Consortium|Davies|United Kingdom
>Spike|hCoV-19/Japan/PG-3079/2020|2020-04-00|EPI_ISL_691202|Original|hCoV-19^^Japan|Human|Pathogen Genomics Center|Pathogen Genomics Center|Sekizuka|Japan
>Spike|hCoV-19/England/ALDP-920BA6/2020|2020-06-23|EPI_ISL_552877|Original|hCoV-19^^England|Human|Lighthouse Lab in Alderley Park|Wellcome Sanger Institute for the COVID-19 Genomics UK (COG-UK) consortium|Davies|United Kingdom
>Spike|hCoV-19/England/MILK-A91A39/2020|2020-10-19|EPI_ISL_706005|Original|hCoV-19^^England|Human|Oxford Viromics|COVID-19 Genomics UK (COG-UK) Consortium|Golubchik|United Kingdom
>Spike|hCoV-19/USA/MN-QDX-1753/2020|2020-03-17|EPI_ISL_604083|Original|hCoV-19^^Minnesota|Human|Quest Diagnostics|Quest Diagnostics|Kagan|USA
>Spike|hCoV-19/England/20186006004/2020|2020-04-29|EPI_ISL_465465|Original|hCoV-19^^England|Human|Respiratory Virus Unit|Respiratory Virus Unit|Platt|United Kingdom
>Spike|hCoV-19/England/MILK-991A76/2020|2020-08-26|EPI_ISL_577017|Original|hCoV-19^^England|Human|Northumbria University / South Tees Hospitals NHS Foundation Trust / North Cumbria Integrated Care NHS Foundation Trust / North Tees and Hartlepool NHS Foundation Trust / Newcastle Hospitals NHS Foundation Trust|COVID-19 Genomics UK (COG-UK) Consortium|Bashton|United Kingdom
>Spike|hCoV-19/Netherlands/un-EMC-819/2020|2020-11-17|EPI_ISL_763172|Original|hCoV-19^^Netherlands|Human|Dutch COVID-19 response team|Erasmus Medical Center|Oude Munnink|Netherlands
>Spike|hCoV-19/Netherlands/ZH-EMC-725/2020|2020-09-29|EPI_ISL_632410|Original|hCoV-19^^Zuid Holland|Human|Dutch COVID-19 response team|Erasmus Medical Center|Oude Munnink|Netherlands
>Spike|hCoV-19/England/QEUH-A8C512/2020|2020-10-18|EPI_ISL_643182|Original|hCoV-19^^England|Human|Lighthouse Lab in Glasgow|Wellcome Sanger Institute for the COVID-19 Genomics UK (COG-UK) Consortium|Davies|United Kingdom
>Spike|hCoV-19/USA/AZ-TG280883/2020|2020-04-24|EPI_ISL_694212|Original|hCoV-19^^Arizona|Human|AZ SPHL|TGen North|French|USA
>Spike|hCoV-19/USA/TX-HMH-2711/2020|2020-06-06|EPI_ISL_545495|Original|hCoV-19^^Texas|Human|Houston Methodist Hospital|Houston Methodist Hospital|Long|USA
>Spike|hCoV-19/Scotland/QEUH-9DD276/2020|2020-09-22|EPI_ISL_599597|Original|hCoV-19^^Scotland|Human|Lighthouse Lab in Glasgow|Wellcome Sanger Institute for the COVID-19 Genomics UK (COG-UK) consortium|Davies|United Kingdom
>Spike|hCoV-19/Netherlands/Zeeland_9/2020|2020-03-22|EPI_ISL_461291|Original|hCoV-19^^Zeeland|Human|Dutch COVID-19 response team|Erasmus Medical Center|Oude Munnink|Netherlands
>Spike|hCoV-19/Wales/PHWC-497750/2020|2020-11-21|EPI_ISL_726875|Original|hCoV-19^^Wales|Human|Originating lab|COVID-19 Genomics UK (COG-UK) Consortium|Moore|United Kingdom
>Spike|hCoV-19/USA/WA-S1157/2020|2020-05-04|EPI_ISL_463647|Original|hCoV-19^^Washington|Human|Washington State Department of Health|Seattle Flu Study|Bedford|USA
>Spike|hCoV-19/USA/TX-HMH-MCoV-12474/2020|2020-09-28|EPI_ISL_790356|Original|hCoV-19^^Texas|Human|Houston Methodist Hospital|Houston Methodist Hospital|Long|USA
>Spike|hCoV-19/Peru/LOR-INS-082/2020|2020-04-22|EPI_ISL_514342|Original|hCoV-19^^Loreto|Human|Laboratorio de Referencia Nacional de Virus Respiratorio. Instituto Nacional de Salud Per\u00fa|Laboratorio de Referencia Nacional de Biotecnolog\u00eda y Biolog\u00eda Molecular. Instituto Nacional de Salud Per\u00fa|Padilla Rojas|Peru
>Spike|hCoV-19/India/MH-ACTREC-588/2020|2020-09-04|EPI_ISL_700227|Original|hCoV-19^^Maharashtra|Human|Hematopathology Laboratory|Hematopathology Laboratory|Patkar|India
>Spike|hCoV-19/USA/MN-QDX-1003/2020|2020-03-14|EPI_ISL_571730|Original|hCoV-19^^Minnesota|Human|Quest Diagnostics|Quest Diagnostics|Kagan|USA
>Spike|hCoV-19/Scotland/GCVR-173B24/2020|2020-04-07|EPI_ISL_459107|Original|hCoV-19^^Scotland|Human|NHSGGC West of Scotland Specialist Virology Centre / MRC-

University of Glasgow Centre for Virus Research|Wellcome Sanger Institute for the COVID-19 Genomics UK (COG-UK) consortium|Davies|United Kingdom
>Spike|hCoV-19/Scotland/EDB3521/2020|2020-04-13|EPI_ISL_487803|Original|hCoV-19^^Scotland|Human|Virology Department|Wellcome Sanger Institute for the COVID-19 Genomics UK (COG-UK) consortium|Davies|United Kingdom
>Spike|hCoV-19/Australia/VIC5892/2020|2020-07-21|EPI_ISL_518674|Original|hCoV-19^^Victoria|Human|Microbiological Diagnostic Unit - Public Health Laboratory (MDU-PHL)|MDU-PHL|Schultz|Australia
>Spike|hCoV-19/England/MILK-B55748/2020|2020-11-06|EPI_ISL_657558|Original|hCoV-19^^England|Human|Lighthouse Lab in Milton Keynes|Wellcome Sanger Institute for the COVID-19 Genomics UK (COG-UK) Consortium|Davies|United Kingdom
>Spike|hCoV-19/England/ALDP-B6206B/2020|2020-11-06|EPI_ISL_656604|Original|hCoV-19^^England|Human|Lighthouse Lab in Alderley Park|Wellcome Sanger Institute for the COVID-19 Genomics UK (COG-UK) Consortium|Davies|United Kingdom
>Spike|hCoV-19/USA/MI-MDHHS-SC21251/2020|2020-04-02|EPI_ISL_507506|Original|hCoV-19^^Michigan|Human|Michigan Department of Health and Human Services|Michigan Department of Health and Human Services|Blankenship|USA
>Spike|hCoV-19/England/QEUH-96BA48/2020|2020-08-14|EPI_ISL_531275|Original|hCoV-19^^England|Human|Lighthouse Lab in Glasgow|Wellcome Sanger Institute for the COVID-19 Genomics UK (COG-UK) consortium|Davies|United Kingdom
>Spike|hCoV-19/USA/MN-MDH-1428/2020|2020-03-25|EPI_ISL_514647|Original|hCoV-19^^Minnesota|Human|Allina Health Laboratory|Minnesota Department of Health|Garfin|USA
>Spike|hCoV-19/Wales/PHWC-499C22/2020|2020-12-02|EPI_ISL_727419|Original|hCoV-19^^Wales|Human|Originating lab|COVID-19 Genomics UK (COG-UK) Consortium|Moore|United Kingdom
>Spike|hCoV-19/England/QEUH-9CF0C8/2020|2020-09-15|EPI_ISL_590033|Original|hCoV-19^^England|Human|Lighthouse Lab in Glasgow|Wellcome Sanger Institute for the COVID-19 Genomics UK (COG-UK) consortium|Davies|United Kingdom
>Spike|hCoV-19/Wales/PHWC-49E2C1/2020|2020-12-07|EPI_ISL_742981|Original|hCoV-19^^Wales|Human|Wales Specialist Virology Centre Sequencing lab|COVID-19 Genomics UK (COG-UK) Consortium|Moore|United Kingdom
>Spike|hCoV-19/USA/CA-ALSR-3360/2020|2020-09-18|EPI_ISL_635981|Original|hCoV-19^^California|Human|San Diego County Public Health Laboratory|Andersen lab at Scripps Research|Gangavarapu|USA
>Spike|hCoV-19/Japan/PG-4517/2020|2020-07-00|EPI_ISL_689656|Original|hCoV-19^^Japan|Human|Pathogen Genomics Center|Pathogen Genomics Center|Sekizuka|Japan
>Spike|hCoV-19/Spain/AN-IBV-001169/2020|2020-03-26|EPI_ISL_452570|Original|hCoV-19^^Andalucia|Human|Servicio de Microbiolog\u00eda y Parasitolog\u00eda cl\u00ednica. UCEIMP. Hospital Universitario Virgen del Roc\u00edo/IBIS/CSIC/US.|SeqCOVID-SPAIN consortium/IBV(CSIC)|Comas|Spain
>Spike|hCoV-19/England/MILK-B56237/2020|2020-11-06|EPI_ISL_657605|Original|hCoV-19^^England|Human|Lighthouse Lab in Milton Keynes|Wellcome Sanger Institute for the COVID-19 Genomics UK (COG-UK) Consortium|Davies|United Kingdom
>Spike|hCoV-19/USA/MA-MASPHL-00197/2020|2020-04-07|EPI_ISL_791652|Original|hCoV-19^^Massachusetts|Human|Massachusetts State Public Health Laboratory|Infectious Disease Program|Park|USA
>Spike|hCoV-19/Northern Ireland/QEUH-964E70/2020|2020-08-13|EPI_ISL_531128|Original|hCoV-19^^Northern Ireland|Human|Lighthouse Lab in Glasgow|Wellcome Sanger Institute for the COVID-19 Genomics UK (COG-UK) consortium|Davies|United Kingdom
>Spike|hCoV-19/England/BRIS-12AB1D/2020|2020-04-30|EPI_ISL_458403|Original|hCoV-19^^England|Human|PHE South West Regional Laboratory|Wellcome Sanger Institute for the COVID-19 Genomics UK (COG-UK) consortium|Davies|United Kingdom
>Spike|hCoV-19/England/LEED-2A967E/2020|2020-04-08|EPI_ISL_538753|Original|hCoV-19^^England|Human|Leeds Teaching Hospitals NHS Trust and Public Health England|Wellcome Sanger Institute for the COVID-19 Genomics UK (COG-UK) consortium|Davies|United Kingdom

>Spike|hCoV-19/England/QEUH-9BA136/2020|2020-09-07|EPI_ISL_573581|Original|hCoV-19^^England|Human|Oxford Viromics|COVID-19 Genomics UK (COG-UK) Consortium|Golubchik|United Kingdom
>Spike|hCoV-19/England/QEUH-B665E0/2020|2020-11-11|EPI_ISL_661473|Original|hCoV-19^^England|Human|Lighthouse Lab in Glasgow|Wellcome Sanger Institute for the COVID-19 Genomics UK (COG-UK) Consortium|Davies|United Kingdom
>Spike|hCoV-19/USA/AZ-TG280824/2020|2020-04-21|EPI_ISL_694170|Original|hCoV-19^^Arizona|Human|AZ SPHL|TGen North|French|USA
>Spike|hCoV-19/Australia/VIC4267/2020|2020-07-15|EPI_ISL_519595|Original|hCoV-19^^Victoria|Human|Microbiological Diagnostic Unit - Public Health Laboratory (MDU-PHL)|MDU-PHL|Schultz|Australia
>Spike|hCoV-19/Sweden/20-53292/2020|2020-10-26|EPI_ISL_661296|Original|hCoV-19^^Sormland|Human|Unilabs|The Public Health Agency of Sweden|Svartstr\u00f6m|Sweden
>Spike|hCoV-19/Japan/IC-0395/2020|2020-12-00|EPI_ISL_736906|Original|hCoV-19^^Japan|Human|Pathogen Genomics Center|Pathogen Genomics Center|Sekizuka|Japan
>Spike|hCoV-19/USA/OR-UW-4409/2020|2020-03-30|EPI_ISL_443185|Original|hCoV-19^^Oregon|Human|UW Virology Lab|UW Virology Lab|Roychoudhury|USA
>Spike|hCoV-19/USA/CA-ALSR-1833/2020|2020-05-28|EPI_ISL_512202|Original|hCoV-19^^California|Human|San Diego County Public Health Laboratory|Andersen lab at Scripps Research|Gangavarapu|USA
>Spike|hCoV-19/USA/MI-MDHHS-SC20224/2020|2020-03-15|EPI_ISL_447194|Original|hCoV-19^^Michigan|Human|Michigan Department of Health and Human Services|Michigan Department of Health and Human Services|Blankenship|USA
>Spike|hCoV-19/England/QEUH-B140D1/2020|2020-11-03|EPI_ISL_641906|Original|hCoV-19^^England|Human|Lighthouse Lab in Glasgow|Wellcome Sanger Institute for the COVID-19 Genomics UK (COG-UK) Consortium|Davies|United Kingdom
>Spike|hCoV-19/Australia/VIC13631/2020|2020-09-08|EPI_ISL_563790|Original|hCoV-19^^Victoria|Human|Victorian Infectious Diseases Reference Laboratory (VIDRL)|VIDRL and MDU-PHL|Schultz|Australia
>Spike|hCoV-19/England/QEUH-B6A485/2020|2020-11-11|EPI_ISL_662523|Original|hCoV-19^^England|Human|Lighthouse Lab in Glasgow|Wellcome Sanger Institute for the COVID-19 Genomics UK (COG-UK) Consortium|Davies|United Kingdom
>Spike|hCoV-19/Wales/PHWC-2872B/2020|2020-03-31|EPI_ISL_432417|Original|hCoV-19^^Wales|Human|Wales Specialist Virology Centre|Public Health Wales Microbiology Cardiff|Moore|United Kingdom
>Spike|hCoV-19/Netherlands/ZH-EMC-784/2020|2020-09-27|EPI_ISL_632621|Original|hCoV-19^^Zuid Holland|Human|Dutch COVID-19 response team|Erasmus Medical Center|Oude Munnink|Netherlands
>Spike|hCoV-19/Australia/VIC4839/2020|2020-07-17|EPI_ISL_519223|Original|hCoV-19^^Victoria|Human|Microbiological Diagnostic Unit - Public Health Laboratory (MDU-PHL)|MDU-PHL|Schultz|Australia
>Spike|hCoV-19/France/ARA-60758/2020|2020-08-14|EPI_ISL_683373|Original|hCoV-19^^Auvergne-Rh\u00f4ne-Alpes|Human|CNR Virus des Infections Respiratoires - France SUD|CNR Virus des Infections Respiratoires - France SUD|Bal|France
>Spike|hCoV-19/USA/CA-CHLA-PLM67956098/2020|2020-11-09|EPI_ISL_753328|Original|hCoV-19^^California|Human|Clinical virology Laboratory|Center for Personalized Medicine|Dien Bard|USA
>Spike|hCoV-19/England/CAMC-B2337C/2020|2020-11-02|EPI_ISL_665034|Original|hCoV-19^^England|Human|Virology Department|COVID-19 Genomics UK (COG-UK) Consortium|de Silva|United Kingdom
>Spike|hCoV-19/USA/MI-MDHHS-SC21454/2020|2020-03-21|EPI_ISL_507708|Original|hCoV-19^^Michigan|Human|Michigan Department of Health and Human Services|Michigan Department of Health and Human Services|Blankenship|USA
>Spike|hCoV-19/Denmark/DCGC-9505/2020|2020-11-09|EPI_ISL_669007|Original|hCoV-19^^Nordjylland|Human|Department of Virus and Microbiological Special Diagnostics|Albertsen Lab|Michaelsen|Denmark
>Spike|hCoV-19/Denmark/DCGC-22094/2020|2020-12-14|EPI_ISL_757933|Original|hCoV-19^^Midtjylland|Human|Department of Virus and Microbiological Special Diagnostics|Albertsen Lab|Michaelsen|Denmark

>Spike|hCoV-19/Denmark/DCGC-20365/2020|2020-12-21|EPI_ISL_759106|Original|hCoV-19^^Sjaelland|Human|Department of Virus and Microbiological Special Diagnostics|Albertsen Lab|Michaelsen|Denmark
>Spike|hCoV-19/England/ALDP-B1C051/2020|2020-10-31|EPI_ISL_645844|Original|hCoV-19^^England|Human|Lighthouse Lab in Alderley Park|Wellcome Sanger Institute for the COVID-19 Genomics UK (COG-UK) Consortium|Davies|United Kingdom
>Spike|hCoV-19/Wales/PHWC-16AC5A/2020|2020-09-08|EPI_ISL_560176|Original|hCoV-19^^Wales|Human|Wales Specialist Virology Centre Sequencing lab|COVID-19 Genomics UK (COG-UK) Consortium|Moore|United Kingdom
>Spike|hCoV-19/Germany/BE-ChVir-D715-D799-17/2020|2020-03-06|EPI_ISL_516645|Original|hCoV-19^^Berlin|Human|Charite\u0301 Universita\u0308tsmedizin Berlin|Charite\u0301 Universita\u0308tsmedizin Berlin|Muehlemann|Germany
>Spike|hCoV-19/England/MILK-ABB6F9/2020|2020-10-21|EPI_ISL_623269|Original|hCoV-19^^England|Human|Lighthouse Lab in Milton Keynes|Wellcome Sanger Institute for the COVID-19 Genomics UK (COG-UK) consortium|Davies|United Kingdom
>Spike|hCoV-19/Japan/PG-5579/2020|2020-08-00|EPI_ISL_688704|Original|hCoV-19^^Japan|Human|Sakai City Institute of Public Health|Pathogen Genomics Center|Sekizuka|Japan
>Spike|hCoV-19/Scotland/QEUH-B0F121/2020|2020-11-02|EPI_ISL_649498|Original|hCoV-19^^Scotland|Human|Lighthouse Lab in Glasgow|Wellcome Sanger Institute for the COVID-19 Genomics UK (COG-UK) Consortium|Davies|United Kingdom
>Spike|hCoV-19/USA/UT-02910/2020|2020-04-30|EPI_ISL_509537|Original|hCoV-19^^Utah|Human|Utah Public Health Laboratory|Utah Public Health Laboratory|Butz|USA
>Spike|hCoV-19/England/CAMB-745BD/2020|2020-03-27|EPI_ISL_425311|Original|hCoV-19^^England|Human|Department of Pathology|COVID-19 Genomics UK (COG-UK) Consortium|Meredith|United Kingdom
>Spike|hCoV-19/England/20172046404/2020|2020-04-16|EPI_ISL_465351|Original|hCoV-19^^England|Human|Respiratory Virus Unit|Respiratory Virus Unit|Platt|United Kingdom
>Spike|hCoV-19/England/CAMC-AEB07A/2020|2020-10-25|EPI_ISL_633325|Original|hCoV-19^^England|Human|Lighthouse Lab in Cambridge|Wellcome Sanger Institute for the COVID-19 Genomics UK (COG-UK) consortium|Davies|United Kingdom
>Spike|hCoV-19/England/NOTT-10ED28/2020|2020-03-28|EPI_ISL_432916|Original|hCoV-19^^England|Human|Queens Medical Centre|COVID-19 Genomics UK (COG-UK) Consortium|Loose|United Kingdom
>Spike|hCoV-19/DRC/D07-1216/2020|2020-05-21|EPI_ISL_636982|Original|hCoV-19^^Kisantu|Human|Virology Lab|Project group Epidemology of Highly Pathogenic Microorganisms|Calvignac-Spencer|Democratic Republic of the Congo
>Spike|hCoV-19/USA/MN-MDH-1783/2020|2020-04-13|EPI_ISL_590785|Original|hCoV-19^^Minnesota|Human|Minnesota Department of Health|Minnesota Department of Health|Lorentz|USA
>Spike|hCoV-19/England/QEUH-A01BC8/2020|2020-10-01|EPI_ISL_595827|Original|hCoV-19^^England|Human|Virology Department|COVID-19 Genomics UK (COG-UK) Consortium|de Silva|United Kingdom
>Spike|hCoV-19/USA/MA-MGH-03374/2020|2020-11-09|EPI_ISL_765809|Original|hCoV-19^^Massachusetts|Human|Massachusetts General Hospital|Infectious Disease Program|Park|USA
>Spike|hCoV-19/USA/UT-02846/2020|2020-04-29|EPI_ISL_513655|Original|hCoV-19^^Utah|Human|Utah Public Health Laboratory|Utah Public Health Laboratory|Butz|USA
>Spike|hCoV-19/Switzerland/SH-ETHZ-380203/2020|2020-11-23|EPI_ISL_721858|Original|hCoV-19^^Schaffhausen|Human|Viollier AG|Department of Biosystems Science and Engineering|Nadeau|Switzerland
>Spike|hCoV-19/England/BIRM-67571/2020|2020-10-23|EPI_ISL_651616|Original|hCoV-19^^England|Human|University of Birmingham|COVID-19 Genomics UK (COG-UK) Consortium|Loman|United Kingdom
>Spike|hCoV-19/Wales/PHWC-160999/2020|2020-05-26|EPI_ISL_473000|Original|hCoV-19^^Wales|Human|Wales Specialist Virology Centre Sequencing lab|COVID-19 Genomics UK (COG-UK) Consortium|Moore|United Kingdom

>Spike|hCoV-19/USA/MI-MDHHS-SC20785/2020|2020-04-22|EPI_ISL_471806|Original|hCoV-19^^Michigan|Human|Michigan Department of Health and Human Services|Michigan Department of Health and Human Services|Blankenship|USA
>Spike|hCoV-19/USA/MA-MGH-00673/2020|2020-04-05|EPI_ISL_460341|Original|hCoV-19^^Massachusetts|Human|Massachusetts General Hospital|Infectious Disease Program|Lemieux|USA
>Spike|hCoV-19/England/QEUH-B100B7/2020|2020-11-02|EPI_ISL_642557|Original|hCoV-19^^England|Human|Lighthouse Lab in Glasgow|Wellcome Sanger Institute for the COVID-19 Genomics UK (COG-UK) Consortium|Davies|United Kingdom
>Spike|hCoV-19/USA/FL-BPHL-0160/2020|2020-04-01|EPI_ISL_480811|Original|hCoV-19^^Florida|Human|Florida Bureau of Public Health Laboratories|Florida Bureau of Public Health Laboratories|Blanton|USA
>Spike|hCoV-19/Northern Ireland/QEUH-8A8855/2020|2020-07-24|EPI_ISL_532773|Original|hCoV-19^^Northern Ireland|Human|Lighthouse Lab in Glasgow|Wellcome Sanger Institute for the COVID-19 Genomics UK (COG-UK) consortium|Davies|United Kingdom
>Spike|hCoV-19/England/MILK-B3DAF2/2020|2020-11-02|EPI_ISL_741388|Original|hCoV-19^^England|Human|Oxford Viromics|COVID-19 Genomics UK (COG-UK) Consortium|Golubchik|United Kingdom
>Spike|hCoV-19/England/MILK-B8DDEB/2020|2020-11-13|EPI_ISL_674721|Original|hCoV-19^^England|Human|Lighthouse Lab in Milton Keynes|Wellcome Sanger Institute for the COVID-19 Genomics UK (COG-UK) Consortium|Davies|United Kingdom
>Spike|hCoV-19/England/ALDP-B1C060/2020|2020-10-31|EPI_ISL_646043|Original|hCoV-19^^England|Human|Lighthouse Lab in Alderley Park|Wellcome Sanger Institute for the COVID-19 Genomics UK (COG-UK) Consortium|Davies|United Kingdom
>Spike|hCoV-19/England/QEUH-AAC206/2020|2020-10-21|EPI_ISL_652849|Original|hCoV-19^^England|Human|Oxford Viromics|COVID-19 Genomics UK (COG-UK) Consortium|Golubchik|United Kingdom
>Spike|hCoV-19/Wales/ALDP-B1E259/2020|2020-11-01|EPI_ISL_710974|Original|hCoV-19^^Wales|Human|Lighthouse Lab in Alderley Park|Wellcome Sanger Institute for the COVID-19 Genomics UK (COG-UK) Consortium|Davies|United Kingdom
>Spike|hCoV-19/England/MILK-50DDF8/2020|2020-06-10|EPI_ISL_558993|Original|hCoV-19^^England|Human|Lighthouse Lab in Milton Keynes|Wellcome Sanger Institute for the COVID-19 Genomics UK (COG-UK) consortium|Davies|United Kingdom
>Spike|hCoV-19/Scotland/EDB9456/2020|2020-10-31|EPI_ISL_652058|Original|hCoV-19^^Scotland|Human|Virology Department|COVID-19 Genomics UK (COG-UK) Consortium|McHugh|United Kingdom
>Spike|hCoV-19/USA/WA-UW-8831/2020|2020-05-08|EPI_ISL_570221|Original|hCoV-19^^Washington|Human|UW Virology Lab|UW Virology Lab|Roychoudhury|USA
>Spike|hCoV-19/England/BRIS-129C48/2020|2020-04-15|EPI_ISL_458416|Original|hCoV-19^^England|Human|PHE South West Regional Laboratory|Wellcome Sanger Institute for the COVID-19 Genomics UK (COG-UK) consortium|Davies|United Kingdom
>Spike|hCoV-19/Guangzhou/GZMU0095/2020|2020-02-17|EPI_ISL_457695|Original|hCoV-19^^Guangzhou|Human|The First Affiliated Hospital of Guangzhou Medical University|BGI-shenzhen & The First Affiliated Hospital of Guangzhou Medical University|Ji|China
>Spike|hCoV-19/Wales/PHWC-16E49A/2020|2020-09-30|EPI_ISL_585722|Original|hCoV-19^^Wales|Human|Wales Specialist Virology Centre Sequencing lab|COVID-19 Genomics UK (COG-UK) Consortium|Moore|United Kingdom
>Spike|hCoV-19/USA/WI-GMF-32685/2020|2020-11-09|EPI_ISL_661163|Original|hCoV-19^^Wisconsin|Human|Gundersen Molecular Diagnostics Laboratory|Kabara Cancer Research Institute|Kenny|USA
>Spike|hCoV-19/Wales/PHWC-2763E/2020|2020-03-30|EPI_ISL_432402|Original|hCoV-19^^Wales|Human|Wales Specialist Virology Centre|Public Health Wales Microbiology Cardiff|Moore|United Kingdom
>Spike|hCoV-19/England/ALDP-A87C4F/2020|2020-10-17|EPI_ISL_605942|Original|hCoV-19^^England|Human|Lighthouse Lab in Alderley Park|Wellcome Sanger Institute for the COVID-19 Genomics UK (COG-UK) consortium|Davies|United Kingdom
>Spike|hCoV-19/Japan/PG-8702/2020|2020-08-00|EPI_ISL_686298|Original|hCoV-19^^Japan|Human|Pathogen Genomics Center|Pathogen Genomics Center|Sekizuka|Japan

>Spike|hCoV-19/India/TG-CDFD-C1330/2020|2020-06-06|EPI_ISL_528557|Original|hCoV-19^^Telangana|Human|National Genomics Core-Center for DNA Fingerprinting and Diagnostics|National Genomics Core- Center for DNA Fingerprinting and Diagnostics (NGC-CDFD)- DBT's PAN-INDIA-1000 Genome consortium|Vashisht|India
>Spike|hCoV-19/Wales/ALDP-B24B91/2020|2020-11-04|EPI_ISL_711022|Original|hCoV-19^^Wales|Human|Lighthouse Lab in Alderley Park|Wellcome Sanger Institute for the COVID-19 Genomics UK (COG-UK) Consortium|Davies|United Kingdom
>Spike|hCoV-19/England/ALDP-B76170/2020|2020-11-12|EPI_ISL_723740|Original|hCoV-19^^England|Human|Oxford Viromics|COVID-19 Genomics UK (COG-UK) Consortium|Golubchik|United Kingdom
>Spike|hCoV-19/England/ALDP-CEB46D/2020|2020-12-21|EPI_ISL_781456|Original|hCoV-19^^England|Human|Lighthouse Lab in Alderley Park|Wellcome Sanger Institute for the COVID-19 Genomics UK (COG-UK) Consortium|Davies|United Kingdom
>Spike|hCoV-19/Wales/PHWC-28020/2020|2020-03-25|EPI_ISL_432107|Original|hCoV-19^^Wales|Human|Wales Specialist Virology Centre|Public Health Wales Microbiology Cardiff|Moore|United Kingdom
>Spike|hCoV-19/Australia/VIC10238/2020|2020-08-28|EPI_ISL_562836|Original|hCoV-19^^Victoria|Human|Microbiological Diagnostic Unit - Public Health Laboratory (MDU-PHL)|MDU-PHL|Schultz|Australia
>Spike|hCoV-19/England/QEUH-9F327A/2020|2020-09-22|EPI_ISL_588710|Original|hCoV-19^^England|Human|Lighthouse Lab in Glasgow|Wellcome Sanger Institute for the COVID-19 Genomics UK (COG-UK) consortium|Davies|United Kingdom
>Spike|hCoV-19/Denmark/DCGC-22482/2020|2020-12-14|EPI_ISL_757427|Original|hCoV-19^^Midtjylland|Human|Department of Virus and Microbiological Special Diagnostics|Albertsen Lab|Michaelsen|Denmark
>Spike|hCoV-19/England/CAMC-B780DB/2020|2020-11-12|EPI_ISL_668140|Original|hCoV-19^^England|Human|Lighthouse Lab in Cambridge|Wellcome Sanger Institute for the COVID-19 Genomics UK (COG-UK) Consortium|Davies|United Kingdom
>Spike|hCoV-19/Nigeria/OS085-CV14/2020|2020-03-29|EPI_ISL_455424|Original|hCoV-19^^Osun State|Human|Nigeria Centre for Disease Control (NCDC)|African Centre of Excellence for Genomics of Infectious Diseases (ACEGID)|Oluniyi|Nigeria
>Spike|hCoV-19/Denmark/DCGC-19094/2020|2020-12-07|EPI_ISL_748755|Original|hCoV-19^^Hovedstaden|Human|Department of Virus and Microbiological Special Diagnostics|Albertsen Lab|Michaelsen|Denmark
>Spike|hCoV-19/USA/TX-HMH-MCoV-14231/2020|2020-10-05|EPI_ISL_787516|Original|hCoV-19^^Texas|Human|Houston Methodist Hospital|Houston Methodist Hospital|Long|USA
>Spike|hCoV-19/USA/TX-DSHS-0566/2020|2020-05-14|EPI_ISL_574468|Original|hCoV-19^^Texas|Human|Texas Department of State Health Services|Texas Department of State Health Services|Rahman|USA
>Spike|hCoV-19/Wales/PHWC-4A26CE/2020|2020-12-16|EPI_ISL_743928|Original|hCoV-19^^Wales|Human|Wales Specialist Virology Centre Sequencing lab|COVID-19 Genomics UK (COG-UK) Consortium|Moore|United Kingdom
>Spike|hCoV-19/England/ALDP-9E9602/2020|2020-09-22|EPI_ISL_584226|Original|hCoV-19^^England|Human|Virology Department|COVID-19 Genomics UK (COG-UK) Consortium|de Silva|United Kingdom
>Spike|hCoV-19/England/ALDP-AA1F55/2020|2020-10-22|EPI_ISL_624436|Original|hCoV-19^^England|Human|Lighthouse Lab in Alderley Park|Wellcome Sanger Institute for the COVID-19 Genomics UK (COG-UK) consortium|Davies|United Kingdom
>Spike|hCoV-19/USA/MN-QDX-2030/2020|2020-03-15|EPI_ISL_604349|Original|hCoV-19^^Minnesota|Human|Quest Diagnostics|Quest Diagnostics|Kagan|USA
>Spike|hCoV-19/USA/CA-ALSR-4587/2020|2020-07-11|EPI_ISL_667023|Original|hCoV-19^^California|Human|San Diego County Public Health Laboratory|Andersen lab at Scripps Research|Gangavarapu|USA
>Spike|hCoV-19/Australia/VIC1675/2020|2020-05-09|EPI_ISL_456574|Original|hCoV-19^^Victoria|Human|Victorian Infectious Diseases Reference Laboratory (VIDRL)|Microbiological Diagnostic Unit Public Health Laboratory and Victorian Infectious Diseases Reference Laboratory|Schultz|Australia
>Spike|hCoV-19/USA/NY-NYCPHL-000524/2020|2020-04-07|EPI_ISL_631985|Original|hCoV-19^^New York|Human|St Barnabas Hospital|New York City Public Health Laboratory|Wang|USA

>Spike|hCoV-19/USA/VA-DCLS-0197/2020|2020-04-09|EPI_ISL_437412|Original|hCoV-19^^Virginia|Human|Virginia DCLS|Virginia DCLS|St. Jacques|USA
>Spike|hCoV-19/USA/AZ-TG502522/2020|2020-07-15|EPI_ISL_695284|Original|hCoV-19^^Arizona|Human|TGen North|TGen North|French|USA
>Spike|hCoV-19/Japan/PG-8200/2020|2020-08-00|EPI_ISL_687299|Original|hCoV-19^^Japan|Human|Pathogen Genomics Center|Pathogen Genomics Center|Sekizuka|Japan
>Spike|hCoV-19/England/CAMC-B21B76/2020|2020-11-01|EPI_ISL_647259|Original|hCoV-19^^England|Human|Lighthouse Lab in Cambridge|Wellcome Sanger Institute for the COVID-19 Genomics UK (COG-UK) Consortium|Davies|United Kingdom
>Spike|hCoV-19/England/CAMC-A56F7D/2020|2020-10-09|EPI_ISL_597287|Original|hCoV-19^^England|Human|Lighthouse Lab in Cambridge|Wellcome Sanger Institute for the COVID-19 Genomics UK (COG-UK) consortium|Davies|United Kingdom
>Spike|hCoV-19/Germany/HH-hpi-p2364/2020|2020-03-22|EPI_ISL_776452|Original|hCoV-19^^Hamburg|Human|University Medical Center Hamburg Eppendorf|Heinrich Pette Institute|Robitaille|Germany
>Spike|hCoV-19/USA/FL-BPHL-1594/2020|2020-05-21|EPI_ISL_594284|Original|hCoV-19^^Florida|Human|Florida Bureau of Public Health Laboratories|Florida Bureau of Public Health Laboratories|Blanton|USA
>Spike|hCoV-19/USA/WA-S1776/2020|2020-06-01|EPI_ISL_496202|Original|hCoV-19^^Washington|Human|Washington State Department of Health|Seattle Flu Study|Lee|USA
>Spike|hCoV-19/Australia/NSW533/2020|2020-05-07|EPI_ISL_455072|Original|hCoV-19^^New South Wales|Human|Sullivan Nicolaides Pathology|NSW Health Pathology - Institute of Clinical Pathology and Medical Research; Westmead Hospital; University of Sydney|Gall|Australia
>Spike|hCoV-19/England/ALDP-B5D8E3/2020|2020-11-08|EPI_ISL_705529|Original|hCoV-19^^England|Human|Department of Pathology|COVID-19 Genomics UK (COG-UK) Consortium|Goodfellow|United Kingdom
>Spike|hCoV-19/Japan/PG-3409/2020|2020-04-00|EPI_ISL_691523|Original|hCoV-19^^Japan|Human|Sakai City Institute of Public Health|Pathogen Genomics Center|Sekizuka|Japan
>Spike|hCoV-19/England/QEUH-AD48E5/2020|2020-10-26|EPI_ISL_634191|Original|hCoV-19^^England|Human|Lighthouse Lab in Glasgow|Wellcome Sanger Institute for the COVID-19 Genomics UK (COG-UK) consortium|Davies|United Kingdom
>Spike|hCoV-19/Australia/VIC3187/2020|2020-07-04|EPI_ISL_520874|Original|hCoV-19^^Victoria|Human|Victorian Infectious Diseases Reference Laboratory (VIDRL)|VIDRL and MDU-PHL|Schultz|Australia
>Spike|hCoV-19/England/QEUH-9F54BE/2020|2020-09-25|EPI_ISL_588643|Original|hCoV-19^^England|Human|Lighthouse Lab in Glasgow|Wellcome Sanger Institute for the COVID-19 Genomics UK (COG-UK) consortium|Davies|United Kingdom
>Spike|hCoV-19/USA/WA-UW-2192/2020|2020-03-25|EPI_ISL_426109|Original|hCoV-19^^Washington|Human|UW Virology Lab|UW Virology Lab|Roychoudhury|USA
>Spike|hCoV-19/France/OCC-170/2020|2020-10-24|EPI_ISL_751489|Original|hCoV-19^^Occitanie|Human|CHU Purpan - Laboratoire de Virologie - Institut F\u00e9d\u00e9ratif de Biologie|CHU Purpan - Laboratoire de Virologie - Institut F\u00e9d\u00e9ratif de Biologie|Latour|France
>Spike|hCoV-19/mink/Netherlands/NB-EMC-11-3/2020|2020-06-07|EPI_ISL_522998|Original|hCoV-19^^Noord Brabant|Neovison vison|Dutch COVID-19 response team|Erasmus Medical Center|Oude Munnink|Netherlands
>Spike|hCoV-19/England/ALDP-B5B78E/2020|2020-11-09|EPI_ISL_655317|Original|hCoV-19^^England|Human|Lighthouse Lab in Alderley Park|Wellcome Sanger Institute for the COVID-19 Genomics UK (COG-UK) Consortium|Davies|United Kingdom
>Spike|hCoV-19/England/C200028999/2020|2020-10-09|EPI_ISL_636698|Original|hCoV-19^^England|Human|Respiratory Virus Unit|Respiratory Virus Unit|Platt|United Kingdom
>Spike|hCoV-19/England/NORW-ECEC4/2020|2020-05-27|EPI_ISL_472349|Original|hCoV-19^^England|Human|Quadram Institute Bioscience|COVID-19 Genomics UK (COG-UK) Consortium|Alikhan|United Kingdom
>Spike|hCoV-19/Canada/QC-LSPQ-L00240479/2020|2020-03-27|EPI_ISL_536272|Original|hCoV-19^^Quebec|Human|CSSS Haut-Richelieu/Rouville (H\u00f4pital)|Laboratoire de sant\u00e9 publique du Qu\u00e9bec|Moreira|Canada

>Spike|hCoV-19/Belgium/ULG-10374/2020|2020-08-07|EPI_ISL_540541|Original|hCoV-19^^Li\u00e8ge|Human|Department of Clinical Microbiology|GIGA Medical Genomics|Durkin|Belgium
>Spike|hCoV-19/England/NORT-2A5FA4/2020|2020-10-11|EPI_ISL_637996|Original|hCoV-19^^England|Human|Northumbria University / South Tees Hospitals NHS Foundation Trust / North Cumbria Integrated Care NHS Foundation Trust / North Tees and Hartlepool NHS Foundation Trust / Newcastle Hospitals NHS Foundation Trust|COVID-19 Genomics UK (COG-UK) Consortium|Bashton|United Kingdom
>Spike|hCoV-19/South Korea/KDCA0422/2020|2020-11-11|EPI_ISL_760206|Original|hCoV-19^^South Korea|Human|Division of Emerging Infectious Diseases|Division of Emerging Infectious Diseases|Park|South Korea
>Spike|hCoV-19/Ireland/CO-NVRL-78IRL73482/2020|2020-11-22|EPI_ISL_752564|Original|hCoV-19^^Cork|Human|National Virus Reference Laboratory|National Virus Reference Laboratory|Gonzalez|Ireland
>Spike|hCoV-19/Denmark/DCGC-17864/2020|2020-12-14|EPI_ISL_749607|Original|hCoV-19^^Sjaelland|Human|Department of Virus and Microbiological Special Diagnostics|Albertsen Lab|Michaelsen|Denmark
>Spike|hCoV-19/USA/MI-MDHHS-SC21738/2020|2020-08-11|EPI_ISL_529857|Original|hCoV-19^^Michigan|Human|Michigan Department of Health and Human Services|Michigan Department of Health and Human Services|Blankenship|USA
>Spike|hCoV-19/Denmark/DCGC-7706/2020|2020-10-19|EPI_ISL_618580|Original|hCoV-19^^Syddanmark|Human|Department of Virus and Microbiological Special Diagnostics|Albertsen lab|Michaelsen|Denmark
>Spike|hCoV-19/England/MILK-9AB350/2020|2020-08-31|EPI_ISL_551254|Original|hCoV-19^^England|Human|Lighthouse Lab in Milton Keynes|Wellcome Sanger Institute for the COVID-19 Genomics UK (COG-UK) consortium|Davies|United Kingdom
>Spike|hCoV-19/Russia/Novy_Urengoy-RII24775S/2020|2020-05-00|EPI_ISL_507218|Original|hCoV-19^^Yamalo-Nenets|Human|WHO National Influenza Centre Russian Federation|WHO National Influenza Centre Russian Federation|Komissarov|Russia
>Spike|hCoV-19/England/201881261/2020|2020-04-30|EPI_ISL_500953|Original|hCoV-19^^England|Human|Respiratory Virus Unit|Respiratory Virus Unit|Platt|United Kingdom
>Spike|hCoV-19/Canada/BC_01594615/2020|2020-04-00|EPI_ISL_466711|Original|hCoV-19^^British Columbia|Human|BCCDC Public Health Laboratory|BCCDC Public Health Laboratory|Harrigan|Canada
>Spike|hCoV-19/USA/UT-UPHL-2012248379/2020|2020-08-13|EPI_ISL_746181|Original|hCoV-19^^Utah|Human|Ginkgo Bioworks Clinical Laboratory|Utah Public Health Laboratory|Young|USA
>Spike|hCoV-19/USA/UT-QDX-1829/2020|2020-03-16|EPI_ISL_604147|Original|hCoV-19^^Utah|Human|Quest Diagnostics|Quest Diagnostics|Kagan|USA
>Spike|hCoV-19/Scotland/EDB11011/2020|2020-12-13|EPI_ISL_742190|Original|hCoV-19^^Scotland|Human|Virology Department|COVID-19 Genomics UK (COG-UK) Consortium|McHugh|United Kingdom
>Spike|hCoV-19/South Africa/NHLS-UCT-GS-1856/2020|2020-09-10|EPI_ISL_640116|Original|hCoV-19^^Western Cape|Human|False Bay Hospital wc FBH|NHLS/UCT|Iranzadeh|South Africa
>Spike|hCoV-19/USA/TX-HMH-MCoV-18300/2020|2020-11-17|EPI_ISL_784648|Original|hCoV-19^^Texas|Human|Houston Methodist Hospital|Houston Methodist Hospital|Long|USA
>Spike|hCoV-19/Spain/CM-IBV-003760/2020|2020-04-02|EPI_ISL_474803|Original|hCoV-19^^Castilla La Mancha|Human|Complejo Hospitalario Universitario de Albacete|SeqCOVID-SPAIN consortium/IBV(CSIC)|Comas|Spain
>Spike|hCoV-19/Australia/VIC5360/2020|2020-07-18|EPI_ISL_520123|Original|hCoV-19^^Victoria|Human|Microbiological Diagnostic Unit - Public Health Laboratory (MDU-PHL)|MDU-PHL|Schultz|Australia
>Spike|hCoV-19/England/BIRM-5ECA5/2020|2020-04-28|EPI_ISL_447938|Original|hCoV-19^^England|Human|University of Birmingham|COVID-19 Genomics UK (COG-UK) Consortium|Loman|United Kingdom
>Spike|hCoV-19/England/QEUH-96FAEA/2020|2020-08-18|EPI_ISL_540319|Original|hCoV-19^^England|Human|Lighthouse Lab in Glasgow|Wellcome Sanger Institute for the COVID-19 Genomics UK (COG-UK) consortium|Davies|United Kingdom

>Spike|hCoV-19/Denmark/DCGC-21965/2020|2020-12-21|EPI_ISL_757926|Original|hCoV-19^^Nordjylland|Human|Department of Virus and Microbiological Special Diagnostics|Albertsen Lab|Michaelsen|Denmark
>Spike|hCoV-19/Denmark/DCGC-10078/2020|2020-11-09|EPI_ISL_668464|Original|hCoV-19^^Hovedstaden|Human|Department of Virus and Microbiological Special Diagnostics|Albertsen Lab|Michaelsen|Denmark
>Spike|hCoV-19/Costa Rica/INC-0080/2020|2020-07-05|EPI_ISL_527748|Original|hCoV-19^^Limon|Human|Area De Salud Corredores|Inciensa|Soto-Garita|Costa Rica
>Spike|hCoV-19/England/NORT-28374A/2020|2020-03-31|EPI_ISL_478510|Original|hCoV-19^^England|Human|Northumbria University / South Tees Hospitals NHS Foundation Trust / North Cumbria Integrated Care NHS Foundation Trust / North Tees and Hartlepool NHS Foundation Trust / Newcastle Hospitals NHS Foundation Trust|COVID-19 Genomics UK (COG-UK) Consortium|Bashton|United Kingdom
>Spike|hCoV-19/USA/MA-MGH-02947/2020|2020-07-03|EPI_ISL_765661|Original|hCoV-19^^Massachusetts|Human|Massachusetts General Hospital|Infectious Disease Program|Park|USA
>Spike|hCoV-19/England/BRIS-18538CF/2020|2020-04-15|EPI_ISL_492361|Original|hCoV-19^^England|Human|PHE South West Regional Laboratory|Wellcome Sanger Institute for the COVID-19 Genomics UK (COG-UK) consortium|Davies|United Kingdom
>Spike|hCoV-19/Kenya/C3427/2020|2020-04-29|EPI_ISL_457906|Original|hCoV-19^^Kenya|Human|KEMRI-CGMR-C|KEMRI-Wellcome Trust Research Programme/KEMRI-CGMR-C Kilifi|Githinji|Kenya
>Spike|hCoV-19/USA/MA-QDX-1456/2020|2020-03-16|EPI_ISL_603823|Original|hCoV-19^^Massachusetts|Human|Quest Diagnostics|Quest Diagnostics|Kagan|USA
>Spike|hCoV-19/USA/WA-S1183/2020|2020-05-05|EPI_ISL_463673|Original|hCoV-19^^Washington|Human|Washington State Department of Health|Seattle Flu Study|Bedford|USA
>Spike|hCoV-19/Canada/NS-NML-1349/2020|2020-04-13|EPI_ISL_579726|Original|hCoV-19^^Nova Scotia|Human|QEII Health Sciences Centre|National Microbiology Laboratory (NML)|Majer|Canada
>Spike|hCoV-19/Wales/QEUH-B12404/2020|2020-11-02|EPI_ISL_647872|Original|hCoV-19^^Wales|Human|Lighthouse Lab in Glasgow|Wellcome Sanger Institute for the COVID-19 Genomics UK (COG-UK) Consortium|Davies|United Kingdom
>Spike|hCoV-19/Finland/26LS3DF9/2020|2020-10-26|EPI_ISL_759936|Original|hCoV-19^^Uusimaa|Human|Department of Virology and Immunology|Department of Virology|Smura|Finland
>Spike|hCoV-19/Scotland/CVR3880/2020|2020-05-31|EPI_ISL_484668|Original|hCoV-19^^Scotland|Human|West of Scotland Specialist Virology Centre|COVID-19 Genomics UK (COG-UK) Consortium|Robertson|United Kingdom
>Spike|hCoV-19/England/QEUH-9F63ED/2020|2020-09-24|EPI_ISL_588731|Original|hCoV-19^^England|Human|Lighthouse Lab in Glasgow|Wellcome Sanger Institute for the COVID-19 Genomics UK (COG-UK) consortium|Davies|United Kingdom
>Spike|hCoV-19/USA/TX-HMH-4365/2020|2020-06-19|EPI_ISL_543563|Original|hCoV-19^^Texas|Human|Houston Methodist Hospital|Houston Methodist Hospital|Long|USA
>Spike|hCoV-19/Israel/CVL-n17925/2020|2020-06-03|EPI_ISL_516890|Original|hCoV-19^^Tel Aviv|Human|Israel Central Virology laboratory|Israel Central Virology laboratory|Zuckerman|Israel
>Spike|hCoV-19/Australia/VIC7788/2020|2020-08-02|EPI_ISL_564658|Original|hCoV-19^^Victoria|Human|Microbiological Diagnostic Unit - Public Health Laboratory (MDU-PHL)|MDU-PHL|Schultz|Australia
>Spike|hCoV-19/South Korea/KCDC2167/2020|2020-03-09|EPI_ISL_506961|Original|hCoV-19^^South Korea|Human|Division of Viral Diseases|Division of Viral Diseases|Kim|South Korea
>Spike|hCoV-19/Northern Ireland/QEUH-943D98/2020|2020-07-28|EPI_ISL_532614|Original|hCoV-19^^Northern Ireland|Human|Lighthouse Lab in Glasgow|Wellcome Sanger Institute for the COVID-19 Genomics UK (COG-UK) consortium|Davies|United Kingdom
>Spike|hCoV-19/USA/AZ-TG654694/2020|2020-04-01|EPI_ISL_696015|Original|hCoV-19^^Arizona|Human|Sonora Quest Laboratories|TGen North|French|USA

>Spike|hCoV-19/Spain/PV-IBV-003246/2020|2020-03-17|EPI_ISL_480976|Original|hCoV-19^^Basque Country|Human|Servicio de Microbiolog\u00eda. Hospital Universitario Donostia. OSI Donostialdea. \u00c1rea de Enfermedades Infecciosas|SeqCOVID-SPAIN consortium/IBV(CSIC)|Comas|Spain
>Spike|hCoV-19/Denmark/DCGC-22120/2020|2020-12-14|EPI_ISL_758565|Original|hCoV-19^^Hovedstaden|Human|Department of Virus and Microbiological Special Diagnostics|Albertsen Lab|Michaelsen|Denmark
>Spike|hCoV-19/England/205221139/2020|2020-12-18|EPI_ISL_766234|Original|hCoV-19^^England|Human|Respiratory Virus Unit|COVID-19 Genomics UK (COG-UK) Consortium|Platt|United Kingdom
>Spike|hCoV-19/Denmark/DCGC-6868/2020|2020-10-12|EPI_ISL_619713|Original|hCoV-19^^Midtjylland|Human|Department of Virus and Microbiological Special Diagnostics|Albertsen lab|Michaelsen|Denmark
>Spike|hCoV-19/England/QEUH-9C87D9/2020|2020-09-09|EPI_ISL_567150|Original|hCoV-19^^England|Human|Lighthouse Lab in Glasgow|Wellcome Sanger Institute for the COVID-19 Genomics UK (COG-UK) consortium|Davies|United Kingdom
>Spike|hCoV-19/England/MILK-BDB6A7/2020|2020-11-26|EPI_ISL_709735|Original|hCoV-19^^England|Human|Lighthouse Lab in Milton Keynes|Wellcome Sanger Institute for the COVID-19 Genomics UK (COG-UK) Consortium|Davies|United Kingdom
>Spike|hCoV-19/Singapore/259/2020|2020-04-07|EPI_ISL_462377|Original|hCoV-19^^Singapore|Human|National Public Health Laboratory|National Public Health Laboratory|Mak|Singapore
>Spike|hCoV-19/England/MILK-9C36E0/2020|2020-09-10|EPI_ISL_566709|Original|hCoV-19^^England|Human|Lighthouse Lab in Milton Keynes|Wellcome Sanger Institute for the COVID-19 Genomics UK (COG-UK) consortium|Davies|United Kingdom
>Spike|hCoV-19/USA/CO-CSU-D0376/2020|2020-04-08|EPI_ISL_527415|Original|hCoV-19^^Colorado|Human|Colorado State University - Ebel Lab|Colorado State University - Ebel Lab|Cline|USA
>Spike|hCoV-19/Poland/Masovia-12865/2020|2020-08-28|EPI_ISL_754904|Original|hCoV-19^^Masovia|Human|Laboratory Diagnostics and Clinical Immunology of Developmental Age|genXone SA|Kaszuba|Poland
>Spike|hCoV-19/England/MILK-B3D48B/2020|2020-11-01|EPI_ISL_645329|Original|hCoV-19^^England|Human|Lighthouse Lab in Milton Keynes|Wellcome Sanger Institute for the COVID-19 Genomics UK (COG-UK) Consortium|Davies|United Kingdom
>Spike|hCoV-19/England/QEUH-B43604/2020|2020-11-08|EPI_ISL_657334|Original|hCoV-19^^England|Human|Lighthouse Lab in Glasgow|Wellcome Sanger Institute for the COVID-19 Genomics UK (COG-UK) Consortium|Davies|United Kingdom
>Spike|hCoV-19/England/CAMC-B22D7F/2020|2020-11-01|EPI_ISL_647420|Original|hCoV-19^^England|Human|Lighthouse Lab in Cambridge|Wellcome Sanger Institute for the COVID-19 Genomics UK (COG-UK) Consortium|Davies|United Kingdom
>Spike|hCoV-19/Belgium/VDLH-030548-2/2020|2020-03-05|EPI_ISL_418797|Original|hCoV-19^^Kalmthout|Human|KU Leuven|KU Leuven|Maes|Belgium
>Spike|hCoV-19/England/BIRM-67711/2020|2020-10-31|EPI_ISL_650770|Original|hCoV-19^^England|Human|University of Birmingham|COVID-19 Genomics UK (COG-UK) Consortium|Loman|United Kingdom
>Spike|hCoV-19/India/KA-nimh-2873/2020|2020-04-21|EPI_ISL_436137|Original|hCoV-19^^Karnataka|Human|District Surveillance Unit|Department of Neurovirology|Pattabiraman|India
>Spike|hCoV-19/England/QEUH-5DD18C/2020|2020-06-17|EPI_ISL_533310|Original|hCoV-19^^England|Human|Lighthouse Lab in Glasgow|Wellcome Sanger Institute for the COVID-19 Genomics UK (COG-UK) consortium|Davies|United Kingdom
>Spike|hCoV-19/Australia/VIC10182/2020|2020-08-27|EPI_ISL_562817|Original|hCoV-19^^Victoria|Human|Microbiological Diagnostic Unit - Public Health Laboratory (MDU-PHL)|MDU-PHL|Schultz|Australia
>Spike|hCoV-19/South Africa/KRISP-0255/2020|2020-06-14|EPI_ISL_482728|Original|hCoV-19^^KwaZulu-Natal|Human|NHLS-IALCH|KRISP|de Oliveira|South Africa
>Spike|hCoV-19/USA/CT-Yale-283/2020|2020-04-02|EPI_ISL_469205|Original|hCoV-19^^Connecticut|Human|Yale Clinical Virology Laboratory|Grubaugh Lab - Yale School of Public Health|Grubaugh|USA

>Spike|hCoV-19/England/LIVE-DC4A1C/2020|2020-11-10|EPI_ISL_702647|Original|hCoV-19^^England|Human|Liverpool Clinical Laboratories|COVID-19 Genomics UK (COG-UK) Consortium|Haldenby|United Kingdom
>Spike|hCoV-19/Scotland/CVR45/2020|2020-03-13|EPI_ISL_425773|Original|hCoV-19^^Scotland|Human|West of Scotland Specialist Virology Centre|COVID-19 Genomics UK (COG-UK) Consortium|Robertson|United Kingdom
>Spike|hCoV-19/Australia/VIC131/2020|2020-03-21|EPI_ISL_419824|Original|hCoV-19^^Victoria|Human|Microbiological Diagnostic Unit Public Health Laboratory|Microbiological Diagnostic Unit Public Health Laboratory|Schultz|Australia
>Spike|hCoV-19/USA/TX-HMH-2109/2020|2020-05-23|EPI_ISL_545126|Original|hCoV-19^^Texas|Human|Houston Methodist Hospital|Houston Methodist Hospital|Long|USA
>Spike|hCoV-19/Northern Ireland/NIRE-10633D/2020|2020-05-02|EPI_ISL_500972|Original|hCoV-19^^Northern Ireland|Human|Regional Virus Laboratory|Wellcome Sanger Institute for the COVID-19 Genomics UK (COG-UK) consortium|Davies|United Kingdom
>Spike|hCoV-19/England/GSTT-265E19B/2020|2020-03-30|EPI_ISL_484163|Original|hCoV-19^^England|Human|Centre for Clinical Infection and Diagnostics Research and Genomics Innovation Unit|COVID-19 Genomics UK (COG-UK) Consortium|Awan|United Kingdom
>Spike|hCoV-19/England/QEUH-A4CA97/2020|2020-10-07|EPI_ISL_596014|Original|hCoV-19^^England|Human|Quadram Institute Bioscience|COVID-19 Genomics UK (COG-UK) Consortium|Alikhan|United Kingdom
>Spike|hCoV-19/England/QEUH-B670A2/2020|2020-11-11|EPI_ISL_661514|Original|hCoV-19^^England|Human|Lighthouse Lab in Glasgow|Wellcome Sanger Institute for the COVID-19 Genomics UK (COG-UK) Consortium|Davies|United Kingdom
>Spike|hCoV-19/South Korea/KCDC2489/2020|2020-06-12|EPI_ISL_514892|Original|hCoV-19^^South Korea|Human|Division of Viral Diseases|Division of Viral Diseases|Kim|South Korea
>Spike|hCoV-19/England/NOTT-11A11E/2020|2020-11-05|EPI_ISL_637558|Original|hCoV-19^^England|Human|Queens Medical Centre|COVID-19 Genomics UK (COG-UK) Consortium|Loose|United Kingdom
>Spike|hCoV-19/Australia/VIC4833/2020|2020-07-19|EPI_ISL_519217|Original|hCoV-19^^Victoria|Human|Microbiological Diagnostic Unit - Public Health Laboratory (MDU-PHL)|MDU-PHL|Schultz|Australia
>Spike|hCoV-19/Netherlands/ZH-EMC-778/2020|2020-09-26|EPI_ISL_632615|Original|hCoV-19^^Zuid Holland|Human|Dutch COVID-19 response team|Erasmus Medical Center|Oude Munnink|Netherlands
>Spike|hCoV-19/Denmark/DCGC-15147/2020|2020-11-23|EPI_ISL_713659|Original|hCoV-19^^Sjaelland|Human|Department of Virus and Microbiological Special Diagnostics|Albertsen Lab|Michaelsen|Denmark
>Spike|hCoV-19/Australia/VIC3669/2020|2020-07-08|EPI_ISL_520407|Original|hCoV-19^^Victoria|Human|Microbiological Diagnostic Unit - Public Health Laboratory (MDU-PHL)|MDU-PHL|Schultz|Australia
>Spike|hCoV-19/USA/WA-UW-4611/2020|2020-03-26|EPI_ISL_429613|Original|hCoV-19^^Washington|Human|UW Virology Lab|UW Virology Lab|Roychoudhury|USA
>Spike|hCoV-19/Switzerland/BS-UHB-42192008/2020|2020-03-15|EPI_ISL_528120|Original|hCoV-19^^Basel-Stadt|Human|University Hospital Basel|University Hospital Basel|Roloff Handschin|Switzerland
>Spike|hCoV-19/USA/WI-UW-2517/2020|2020-12-30|EPI_ISL_779348|Original|hCoV-19^^Wisconsin|Human|University of Wisconsin-Madison AIDS Vaccine Research Laboratories|University of Wisconsin-Madison AIDS Vaccine Research Laboratories|Moreno|USA
>Spike|hCoV-19/USA/WI-UW-1148/2020|2020-08-12|EPI_ISL_522904|Original|hCoV-19^^Wisconsin|Human|University of Wisconsin-Madison AIDS Vaccine Research Laboratories|University of Wisconsin-Madison AIDS Vaccine Research Laboratories|Baczenas|USA
>Spike|hCoV-19/Japan/PG-7550/2020|2020-08-00|EPI_ISL_686652|Original|hCoV-19^^Japan|Human|Pathogen Genomics Center|Pathogen Genomics Center|Sekizuka|Japan
>Spike|hCoV-19/England/CAMB-1B7F28/2020|2020-10-13|EPI_ISL_638183|Original|hCoV-19^^England|Human|Department of Pathology|COVID-19 Genomics UK (COG-UK) Consortium|Goodfellow|United Kingdom

>Spike|hCoV-19/England/BRIS-1853E09/2020|2020-04-27|EPI_ISL_492232|Original|hCoV-19^^England|Human|PHE South West Regional Laboratory|Wellcome Sanger Institute for the COVID-19 Genomics UK (COG-UK) consortium|Davies|United Kingdom
>Spike|hCoV-19/USA/HI-H200502/2020|2020-08-02|EPI_ISL_753041|Original|hCoV-19^^Hawaii|Human|State Laboratories Division|State Laboratories Division|Sultana|USA
>Spike|hCoV-19/Japan/PG-2881/2020|2020-04-00|EPI_ISL_691007|Original|hCoV-19^^Japan|Human|Pathogen Genomics Center|Pathogen Genomics Center|Sekizuka|Japan
>Spike|hCoV-19/USA/WA-S1553/2020|2020-05-26|EPI_ISL_495979|Original|hCoV-19^^Washington|Human|Washington State Department of Health|Seattle Flu Study|Lee|USA
>Spike|hCoV-19/USA/UN-PB-Wuhan-Hu-1-6/2020|2020-04-21|EPI_ISL_759865|Original|hCoV-19^^USA|Human|Molecular|Molecular|Papoutsis,A.J.|USA
>Spike|hCoV-19/England/ALDP-94AEDA/2020|2020-06-11|EPI_ISL_556389|Original|hCoV-19^^England|Human|Lighthouse Lab in Alderley Park|Wellcome Sanger Institute for the COVID-19 Genomics UK (COG-UK) consortium|Davies|United Kingdom
>Spike|hCoV-19/England/QEUH-B11274/2020|2020-11-02|EPI_ISL_642563|Original|hCoV-19^^England|Human|Lighthouse Lab in Glasgow|Wellcome Sanger Institute for the COVID-19 Genomics UK (COG-UK) Consortium|Davies|United Kingdom
>Spike|hCoV-19/England/MILK-B88463/2020|2020-11-13|EPI_ISL_674210|Original|hCoV-19^^England|Human|Lighthouse Lab in Milton Keynes|Wellcome Sanger Institute for the COVID-19 Genomics UK (COG-UK) Consortium|Davies|United Kingdom
>Spike|hCoV-19/England/QEUH-AD0913/2020|2020-10-26|EPI_ISL_629924|Original|hCoV-19^^England|Human|Lighthouse Lab in Glasgow|Wellcome Sanger Institute for the COVID-19 Genomics UK (COG-UK) consortium|Davies|United Kingdom
>Spike|hCoV-19/England/MILK-95799D/2020|2020-06-09|EPI_ISL_554185|Original|hCoV-19^^England|Human|Lighthouse Lab in Milton Keynes|Wellcome Sanger Institute for the COVID-19 Genomics UK (COG-UK) consortium|Davies|United Kingdom
>Spike|hCoV-19/Scotland/QEUH-9CFAD9/2020|2020-09-17|EPI_ISL_602026|Original|hCoV-19^^Scotland|Human|Lighthouse Lab in Glasgow|Wellcome Sanger Institute for the COVID-19 Genomics UK (COG-UK) consortium|Davies|United Kingdom
>Spike|hCoV-19/England/QEUH-9FB10C/2020|2020-09-29|EPI_ISL_588087|Original|hCoV-19^^England|Human|Lighthouse Lab in Glasgow|Wellcome Sanger Institute for the COVID-19 Genomics UK (COG-UK) consortium|Davies|United Kingdom
>Spike|hCoV-19/England/ALDP-91E69B/2020|2020-06-23|EPI_ISL_553324|Original|hCoV-19^^England|Human|Lighthouse Lab in Alderley Park|Wellcome Sanger Institute for the COVID-19 Genomics UK (COG-UK) consortium|Davies|United Kingdom
>Spike|hCoV-19/Singapore/152/2020|2020-04-08|EPI_ISL_443244|Original|hCoV-19^^Singapore|Human|National Public Health Laboratory|National Public Health Laboratory|Mak|Singapore
>Spike|hCoV-19/England/ALDP-496AD0/2020|2020-06-07|EPI_ISL_559311|Original|hCoV-19^^England|Human|Lighthouse Lab in Alderley Park|Wellcome Sanger Institute for the COVID-19 Genomics UK (COG-UK) consortium|Davies|United Kingdom
>Spike|hCoV-19/France/IDF-0386-islP1/2020|2020-01-28|EPI_ISL_411219|Vero E6 P1|hCoV-19^^\u00cele-de-France|Human|Department of Infectious and Tropical Diseases|Laboratoire Virpath|Fouret|France
>Spike|hCoV-19/USA/CA-CZB-1770/2020|2020-03-23|EPI_ISL_476787|Original|hCoV-19^^California|Human|Stanford clinical virology lab|Chan-Zuckerberg Biohub|Kamm|USA
>Spike|hCoV-19/Switzerland/ZH-ETHZ-410076/2020|2020-12-14|EPI_ISL_737574|Original|hCoV-19^^Zurich|Human|Viollier AG|Department of Biosystems Science and Engineering|Nadeau|Switzerland
>Spike|hCoV-19/Luxembourg/LNS0876336/2020|2020-11-03|EPI_ISL_739844|Original|hCoV-19^^Luxembourg|Human|Laboratoire national de sant\u00e9|Laboratoire national de sant\u00e9|Wienecke-Baldacchino|Luxembourg
>Spike|hCoV-19/USA/MI-MDHHS-SC21882/2020|2020-04-15|EPI_ISL_565943|Original|hCoV-19^^Michigan|Human|Michigan Department of Health and Human Services|Michigan Department of Health and Human Services|Blankenship|USA
>Spike|hCoV-19/Sweden/20-52418/2020|2020-07-20|EPI_ISL_534244|Original|hCoV-19^^Vasternorrland|Human|Sundsvalls sjukhus|The Public Health Agency of Sweden|Svartstr\u00f6m|Sweden

>Spike|hCoV-19/USA/CA-ALSR-1899/2020|2020-04-22|EPI_ISL_512223|Original|hCoV-19^^California|Human|San Diego County Public Health Laboratory|Andersen lab at Scripps Research|Gangavarapu|USA
>Spike|hCoV-19/England/ALDP-B1C491/2020|2020-11-01|EPI_ISL_645936|Original|hCoV-19^^England|Human|Lighthouse Lab in Alderley Park|Wellcome Sanger Institute for the COVID-19 Genomics UK (COG-UK) Consortium|Davies|United Kingdom
>Spike|hCoV-19/England/201360298/2020|2020-03-25|EPI_ISL_421777|Original|hCoV-19^^England|Human|Respiratory Virus Unit|Respiratory Virus Unit|Platt|United Kingdom
>Spike|hCoV-19/Japan/PG-7270/2020|2020-08-00|EPI_ISL_688373|Original|hCoV-19^^Japan|Human|Saitama Prefectural Institute of Public Health|Pathogen Genomics Center|Sekizuka|Japan
>Spike|hCoV-19/Netherlands/OV-RIVM-20093/2020|2020-11-11|EPI_ISL_723271|Orignal|hCoV-19^^Overijssel|Human|Dutch COVID-19 response team|National Institute for Public Health and the Environment (RIVM)|Meijer|Netherlands
>Spike|hCoV-19/Scotland/EDB7791/2020|2020-09-16|EPI_ISL_577354|Original|hCoV-19^^Scotland|Human|Virology Department|COVID-19 Genomics UK (COG-UK) Consortium|Williams|United Kingdom
>Spike|hCoV-19/USA/TX-HMH-2628/2020|2020-06-04|EPI_ISL_545436|Original|hCoV-19^^Texas|Human|Houston Methodist Hospital|Houston Methodist Hospital|Long|USA
>Spike|hCoV-19/England/MILK-B02319/2020|2020-10-23|EPI_ISL_642778|Original|hCoV-19^^England|Human|Lighthouse Lab in Milton Keynes|Wellcome Sanger Institute for the COVID-19 Genomics UK (COG-UK) Consortium|Davies|United Kingdom
>Spike|hCoV-19/Australia/QLDID931/2020|2020-03-25|EPI_ISL_420879|Original|hCoV-19^^Queensland|Human|Mater Pathology|Public Health Virology Laboratory|Warrilow|Australia
>Spike|hCoV-19/USA/WA-S873/2020|2020-04-16|EPI_ISL_463363|Original|hCoV-19^^Washington|Human|Washington State Department of Health|Seattle Flu Study|Bedford|USA
>Spike|hCoV-19/England/MILK-C60CB3/2020|2020-12-13|EPI_ISL_769342|Original|hCoV-19^^England|Human|Lighthouse Lab in Milton Keynes|Wellcome Sanger Institute for the COVID-19 Genomics UK (COG-UK) Consortium|Davies|United Kingdom
>Spike|hCoV-19/South Africa/Tygerberg_362_CC2/2020|2020-10-10|EPI_ISL_660156|Original|hCoV-19^^Western Cape|Human|PathCare|National Health Laboratory Service (NHLS)|de Oliveira|South Africa
>Spike|hCoV-19/Belgium/ULG-10733/2020|2020-09-18|EPI_ISL_641575|Original|hCoV-19^^Li\u00e8ge|Human|Department of Clinical Microbiology|GIGA Medical Genomics|Durkin|Belgium
>Spike|hCoV-19/USA/WI-UW-1650/2020|2020-09-10|EPI_ISL_605524|Original|hCoV-19^^Wisconsin|Human|University of Wisconsin-Madison AIDS Vaccine Research Laboratories|University of Wisconsin-Madison AIDS Vaccine Research Laboratories|Baczenas|USA
>Spike|hCoV-19/Wales/PHWC-484FD6/2020|2020-10-21|EPI_ISL_628134|Original|hCoV-19^^Wales|Human|Wales Specialist Virology Centre Sequencing lab|COVID-19 Genomics UK (COG-UK) Consortium|Moore|United Kingdom
>Spike|hCoV-19/England/QEUH-96BCF7/2020|2020-08-14|EPI_ISL_531254|Original|hCoV-19^^England|Human|Lighthouse Lab in Glasgow|Wellcome Sanger Institute for the COVID-19 Genomics UK (COG-UK) consortium|Davies|United Kingdom
>Spike|hCoV-19/USA/CA-CZB-2050/2020|2020-04-21|EPI_ISL_486294|Original|hCoV-19^^California|Human|San Joaquin County Public Health Lab|Chan-Zuckerberg Biohub|McGeever|USA
>Spike|hCoV-19/Denmark/DCGC-19618/2020|2020-12-07|EPI_ISL_748264|Original|hCoV-19^^Midtjylland|Human|Department of Virus and Microbiological Special Diagnostics|Albertsen Lab|Michaelsen|Denmark
>Spike|hCoV-19/Scotland/EDB3309/2020|2020-04-08|EPI_ISL_489017|Original|hCoV-19^^Scotland|Human|Virology Department|Wellcome Sanger Institute for the COVID-19 Genomics UK (COG-UK) consortium|Davies|United Kingdom
>Spike|hCoV-19/England/ALDP-953B9B/2020|2020-06-08|EPI_ISL_553868|Original|hCoV-19^^England|Human|Lighthouse Lab in Alderley Park|Wellcome Sanger Institute for the COVID-19 Genomics UK (COG-UK) consortium|Davies|United Kingdom

>Spike|hCoV-19/England/20099072104/2020|2020-03-01|EPI_ISL_464222|Original|hCoV-19^^England|Human|Respiratory Virus Unit|Respiratory Virus Unit|Platt|United Kingdom
>Spike|hCoV-19/England/CAMC-B210A4/2020|2020-11-02|EPI_ISL_647768|Original|hCoV-19^^England|Human|Lighthouse Lab in Cambridge|Wellcome Sanger Institute for the COVID-19 Genomics UK (COG-UK) Consortium|Davies|United Kingdom
>Spike|hCoV-19/Peru/LAM-UPCH-0006/2020|2020-06-08|EPI_ISL_529067|Original|hCoV-19^^Lambayeque|Human|Laboratorio de Referencia Nacional de Virus Respiratorios|Laboratorio de Gen\u00f3mica Microbiana|Tsukayama|Peru
>Spike|hCoV-19/England/MILK-81E3A4/2020|2020-07-17|EPI_ISL_557273|Original|hCoV-19^^England|Human|Lighthouse Lab in Milton Keynes|Wellcome Sanger Institute for the COVID-19 Genomics UK (COG-UK) consortium|Davies|United Kingdom
>Spike|hCoV-19/Gibraltar/205000693/2020|2020-12-00|EPI_ISL_710017|Original|hCoV-19^^Gibraltar|Human|Microbiology|Respiratory Virus Unit|Platt|Gibraltar
>Spike|hCoV-19/Netherlands/NA_572/2020|2020-04-13|EPI_ISL_460736|Original|hCoV-19^^Netherlands|Human|Dutch COVID-19 response team|Erasmus Medical Center|Oude Munnink|Netherlands
>Spike|hCoV-19/USA/LA-EVTL1179/2020|2020-12-11|EPI_ISL_768448|Original|hCoV-19^^Louisiana|Human|LSUHS Emerging Viral Threat Laboratory|Microbial Genome Sequencing Center|Kamil|USA
>Spike|hCoV-19/England/ALDP-AA1571/2020|2020-10-22|EPI_ISL_624551|Original|hCoV-19^^England|Human|Lighthouse Lab in Alderley Park|Wellcome Sanger Institute for the COVID-19 Genomics UK (COG-UK) consortium|Davies|United Kingdom
>Spike|hCoV-19/Scotland/QEUH-9CE278/2020|2020-09-16|EPI_ISL_601889|Original|hCoV-19^^Scotland|Human|Lighthouse Lab in Glasgow|Wellcome Sanger Institute for the COVID-19 Genomics UK (COG-UK) consortium|Davies|United Kingdom
>Spike|hCoV-19/USA/MA-MGH-03167/2020|2020-08-29|EPI_ISL_765727|Original|hCoV-19^^Massachusetts|Human|Massachusetts General Hospital|Infectious Disease Program|Park|USA
>Spike|hCoV-19/USA/LA-GBCL-26556539/2020|2020-04-15|EPI_ISL_485177|Original|hCoV-19^^Louisiana|Human|River Road Testing Lab|Ginkgo Bioworks Clinical Laboratory|Smith|USA
>Spike|hCoV-19/USA/TX-HMH-5327/2020|2020-06-29|EPI_ISL_544684|Original|hCoV-19^^Texas|Human|Houston Methodist Hospital|Houston Methodist Hospital|Long|USA
>Spike|hCoV-19/New Zealand/20VR2944/2020|2020-00-00|EPI_ISL_579365|Original|hCoV-19^^Auckland|Human|LabPLUS|Institute of Environmental Science and Research (ESR)|Wang|New Zealand
>Spike|hCoV-19/Japan/PG-7545/2020|2020-08-00|EPI_ISL_686647|Original|hCoV-19^^Japan|Human|Pathogen Genomics Center|Pathogen Genomics Center|Sekizuka|Japan
>Spike|hCoV-19/USA/TX-HMH-1537/2020|2020-04-16|EPI_ISL_542779|Original|hCoV-19^^Texas|Human|Houston Methodist Hospital|Houston Methodist Hospital|Long|USA
>Spike|hCoV-19/England/ALDP-BC6715/2020|2020-11-21|EPI_ISL_721273|Original|hCoV-19^^England|Human|Lighthouse Lab in Alderley Park|Wellcome Sanger Institute for the COVID-19 Genomics UK (COG-UK) Consortium|Davies|United Kingdom
>Spike|hCoV-19/England/QEUH-A78A0B/2020|2020-10-14|EPI_ISL_638773|Original|hCoV-19^^England|Human|Oxford Viromics|COVID-19 Genomics UK (COG-UK) Consortium|Golubchik|United Kingdom
>Spike|hCoV-19/USA/WA-UW-2747/2020|2020-04-15|EPI_ISL_486027|Original|hCoV-19^^Washington|Human|UW Virology Lab|UW Virology Lab|Roychoudhury|USA
>Spike|hCoV-19/England/MILK-938561/2020|2020-06-21|EPI_ISL_552987|Original|hCoV-19^^England|Human|Lighthouse Lab in Milton Keynes|Wellcome Sanger Institute for the COVID-19 Genomics UK (COG-UK) consortium|Davies|United Kingdom
>Spike|hCoV-19/Wales/PHWC-47FEFF/2020|2020-10-08|EPI_ISL_612828|Original|hCoV-19^^Wales|Human|Wales Specialist Virology Centre Sequencing lab|COVID-19 Genomics UK (COG-UK) Consortium|Moore|United Kingdom
>Spike|hCoV-19/England/ALDP-CB5D8F/2020|2020-12-17|EPI_ISL_761839|Original|hCoV-19^^England|Human|Lighthouse Lab in Alderley Park|Wellcome Sanger Institute for the COVID-19 Genomics UK (COG-UK) Consortium|Davies|United Kingdom
>Spike|hCoV-19/England/QEUH-AD3ED5/2020|2020-10-26|EPI_ISL_629931|Original|hCoV-19^^England|Human|Lighthouse Lab in Glasgow|Wellcome Sanger Institute for the COVID-19 Genomics UK (COG-UK) consortium|Davies|United Kingdom

>Spike|hCoV-19/Denmark/DCGC-17353/2020|2020-12-14|EPI_ISL_750401|Original|hCoV-19^^Midtjylland|Human|Department of Virus and Microbiological Special Diagnostics|Albertsen Lab|Michaelsen|Denmark
>Spike|hCoV-19/Ireland/KY-NVRL-20G35628/2020|2020-02-20|EPI_ISL_605797|Original|hCoV-19^^Kerry|Human|National Virus Reference Laboratory|Irish Coronavirus Sequencing Consortium - Helixworks|Chalapati|Ireland
>Spike|hCoV-19/USA/TX-HMH-7081/2020|2020-06-28|EPI_ISL_546488|Original|hCoV-19^^Texas|Human|Houston Methodist Hospital|Houston Methodist Hospital|Long|USA
>Spike|hCoV-19/England/SHEF-10D703C/2020|2020-12-12|EPI_ISL_727984|Original|hCoV-19^^England|Human|Virology Department|COVID-19 Genomics UK (COG-UK) Consortium|de Silva|United Kingdom
>Spike|hCoV-19/USA/WI-WSLH-200136/2020|2020-04-24|EPI_ISL_578364|Original|hCoV-19^^Wisconsin|Human|Wisconsin State Laboratory of Hygiene Communicable Disease Division|Wisconsin State Laboratory of Hygiene Communicable Disease Division|Florek|USA
>Spike|hCoV-19/Scotland/QEUH-AD0184/2020|2020-10-26|EPI_ISL_649759|Original|hCoV-19^^Scotland|Human|Lighthouse Lab in Glasgow|Wellcome Sanger Institute for the COVID-19 Genomics UK (COG-UK) Consortium|Davies|United Kingdom
>Spike|hCoV-19/England/CAMC-B1FBD5/2020|2020-11-01|EPI_ISL_647686|Original|hCoV-19^^England|Human|Lighthouse Lab in Cambridge|Wellcome Sanger Institute for the COVID-19 Genomics UK (COG-UK) Consortium|Davies|United Kingdom
>Spike|hCoV-19/USA/FL-BPHL-2110/2020|2020-03-10|EPI_ISL_653263|Original|hCoV-19^^Florida|Human|Florida Bureau of Public Health Laboratories|Florida Bureau of Public Health Laboratories|Blanton|USA
>Spike|hCoV-19/England/QEUH-9FF38A/2020|2020-10-02|EPI_ISL_600162|Original|hCoV-19^^England|Human|Lighthouse Lab in Glasgow|Wellcome Sanger Institute for the COVID-19 Genomics UK (COG-UK) consortium|Davies|United Kingdom
>Spike|hCoV-19/England/NORT-296FE2/2020|2020-04-23|EPI_ISL_488145|Original|hCoV-19^^England|Human|NU-OMICS DNA Sequencing research facility|Wellcome Sanger Institute for the COVID-19 Genomics UK (COG-UK) consortium|Davies|United Kingdom
>Spike|hCoV-19/USA/WA-UW249/2020|2020-03-16|EPI_ISL_418034|Original|hCoV-19^^Washington|Human|UW Virology Lab|UW Virology Lab|Roychoudhury|USA
>Spike|hCoV-19/Denmark/DCGC-19482/2020|2020-12-07|EPI_ISL_748980|Original|hCoV-19^^Syddanmark|Human|Department of Virus and Microbiological Special Diagnostics|Albertsen Lab|Michaelsen|Denmark
>Spike|hCoV-19/England/20139048804/2020|2020-03-28|EPI_ISL_420698|Original|hCoV-19^^England|Human|Respiratory Virus Unit|Respiratory Virus Unit|Platt|United Kingdom
>Spike|hCoV-19/Japan/PG-1106/2020|2020-03-00|EPI_ISL_684716|Original|hCoV-19^^Japan|Human|Pathogen Genomics Center|Pathogen Genomics Center|Sekizuka|Japan
>Spike|hCoV-19/England/QEUH-B418A6/2020|2020-11-08|EPI_ISL_658614|Original|hCoV-19^^England|Human|Lighthouse Lab in Glasgow|Wellcome Sanger Institute for the COVID-19 Genomics UK (COG-UK) Consortium|Davies|United Kingdom
>Spike|hCoV-19/USA/TX-HMH-MCoV-15306/2020|2020-10-19|EPI_ISL_786790|Original|hCoV-19^^Texas|Human|Houston Methodist Hospital|Houston Methodist Hospital|Long|USA
>Spike|hCoV-19/England/MILK-ABC1BB/2020|2020-10-21|EPI_ISL_631255|Original|hCoV-19^^England|Human|Lighthouse Lab in Milton Keynes|Wellcome Sanger Institute for the COVID-19 Genomics UK (COG-UK) consortium|Davies|United Kingdom
>Spike|hCoV-19/USA/WI-GMF-26493/2020|2020-10-18|EPI_ISL_660952|Original|hCoV-19^^Wisconsin|Human|Gundersen Molecular Diagnostics Laboratory|Kabara Cancer Research Institute|Kenny|USA
>Spike|hCoV-19/England/LIVE-A20B5/2020|2020-05-18|EPI_ISL_490376|Original|hCoV-19^^England|Human|Liverpool Clinical Laboratories|COVID-19 Genomics UK (COG-UK) Consortium|Haldenby|United Kingdom
>Spike|hCoV-19/England/MILK-B55098/2020|2020-11-06|EPI_ISL_657878|Original|hCoV-19^^England|Human|Lighthouse Lab in Milton Keynes|Wellcome Sanger Institute for the COVID-19 Genomics UK (COG-UK) Consortium|Davies|United Kingdom
>Spike|hCoV-19/England/LIVE-DCC09A/2020|2020-11-23|EPI_ISL_724486|Original|hCoV-19^^England|Human|Liverpool Clinical Laboratories|COVID-19 Genomics UK (COG-UK) Consortium|Haldenby|United Kingdom

>Spike|hCoV-19/England/ALDP-B5D6DA/2020|2020-11-09|EPI_ISL_703462|Original|hCoV-19^^England|Human|Department of Pathology|COVID-19 Genomics UK (COG-UK) Consortium|Goodfellow|United Kingdom
>Spike|hCoV-19/India/DL-NCDC7629-CSIR-IGIB/2020|2020-05-28|EPI_ISL_482651|Original|hCoV-19^^Delhi|Human|National Centre for Disease control (NCDC)|NCDC/CSIR-IGIB|Singh|India
>Spike|hCoV-19/England/MILK-B8C326/2020|2020-11-13|EPI_ISL_674167|Original|hCoV-19^^England|Human|Lighthouse Lab in Milton Keynes|Wellcome Sanger Institute for the COVID-19 Genomics UK (COG-UK) Consortium|Davies|United Kingdom
>Spike|hCoV-19/USA/MI-MDHHS-SC20463/2020|2020-03-30|EPI_ISL_460002|Original|hCoV-19^^Michigan|Human|Michigan Department of Health and Human Services|Michigan Department of Health and Human Services|Blankenship|USA
>Spike|hCoV-19/France/NAQ-SC587/2020|2020-05-11|EPI_ISL_663237|Original|hCoV-19^^Nouvelle-Aquitaine|Human|CHU Poitiers|CNR Virus des Infections Respiratoires - France SUD|Bal|France
>Spike|hCoV-19/Canada/BC_07312838/2020|2020-04-00|EPI_ISL_466815|Original|hCoV-19^^British Columbia|Human|BCCDC Public Health Laboratory|BCCDC Public Health Laboratory|Harrigan|Canada
>Spike|hCoV-19/England/CAMC-A81A3C/2020|2020-10-14|EPI_ISL_610649|Original|hCoV-19^^England|Human|Lighthouse Lab in Cambridge|Wellcome Sanger Institute for the COVID-19 Genomics UK (COG-UK) consortium|Davies|United Kingdom
>Spike|hCoV-19/Australia/VIC8195/2020|2020-08-13|EPI_ISL_564893|Original|hCoV-19^^Victoria|Human|Microbiological Diagnostic Unit - Public Health Laboratory (MDU-PHL)|MDU-PHL|Schultz|Australia
>Spike|hCoV-19/USA/NY-NYCPHL-001325/2020|2020-11-12|EPI_ISL_683967|Original|hCoV-19^^New York|Human|DOHMH Chelsea|New York City Public Health Laboratory|Wang|USA
>Spike|hCoV-19/England/ALDP-B5DF93/2020|2020-11-09|EPI_ISL_723618|Original|hCoV-19^^England|Human|Northumbria University / South Tees Hospitals NHS Foundation Trust / North Cumbria Integrated Care NHS Foundation Trust / North Tees and Hartlepool NHS Foundation Trust / Newcastle Hospitals NHS Foundation Trust|COVID-19 Genomics UK (COG-UK) Consortium|Bashton|United Kingdom
>Spike|hCoV-19/Thailand/SI201712-NT/2020|2020-02-03|EPI_ISL_437617|unknown|hCoV-19^^Thailand|Human|unknown|Faculty of Medicine|Submitter|Thailand
>Spike|hCoV-19/England/LEED-2A8888/2020|2020-04-01|EPI_ISL_539047|Original|hCoV-19^^England|Human|Leeds Teaching Hospitals NHS Trust and Public Health England|Wellcome Sanger Institute for the COVID-19 Genomics UK (COG-UK) consortium|Davies|United Kingdom
>Spike|hCoV-19/USA/FL-BPHL-0314/2020|2020-06-02|EPI_ISL_489723|Original|hCoV-19^^Florida|Human|Florida Bureau of Public Health Laboratories|Florida Bureau of Public Health Laboratories|Blanton|USA
>Spike|hCoV-19/USA/TX-HMH-MCoV-19224/2020|2020-11-27|EPI_ISL_783702|Original|hCoV-19^^Texas|Human|Houston Methodist Hospital|Houston Methodist Hospital|Long|USA
>Spike|hCoV-19/England/MILK-99A688/2020|2020-08-29|EPI_ISL_550406|Original|hCoV-19^^England|Human|Lighthouse Lab in Milton Keynes|Wellcome Sanger Institute for the COVID-19 Genomics UK (COG-UK) consortium|Davies|United Kingdom
>Spike|hCoV-19/Spain/IB-IBV-99010786/2020|2020-10-17|EPI_ISL_691675|Original|hCoV-19^^Balear Islands|Human|Servicio de Microbiolog\u00eda|SeqCOVID-SPAIN consortium/IBV(CSIC)|Comas|Spain
>Spike|hCoV-19/England/CAMB-1BCFBA/2020|2020-00-00|EPI_ISL_723917|Original|hCoV-19^^England|Human|Department of Pathology|COVID-19 Genomics UK (COG-UK) Consortium|Goodfellow|United Kingdom
>Spike|hCoV-19/England/QEUH-A5E300/2020|2020-10-12|EPI_ISL_597362|Original|hCoV-19^^England|Human|Lighthouse Lab in Glasgow|Wellcome Sanger Institute for the COVID-19 Genomics UK (COG-UK) consortium|Davies|United Kingdom
>Spike|hCoV-19/England/ALDP-A963DF/2020|2020-10-20|EPI_ISL_624633|Original|hCoV-19^^England|Human|Lighthouse Lab in Alderley Park|Wellcome Sanger Institute for the COVID-19 Genomics UK (COG-UK) consortium|Davies|United Kingdom
>Spike|hCoV-19/Wuhan/HB-WHCM-109/2020|2020-02-27|EPI_ISL_454992|Original|hCoV-19^^Hubei|Human|Wuhan Chain Medical Labs (CMLabs)|State Key Laboratory of Biotherapy of Sichuan University|Tang|China

>Spike|hCoV-19/England/ALDP-B47BB6/2020|2020-11-07|EPI_ISL_649263|Original|hCoV-19^^England|Human|Lighthouse Lab in Alderley Park|Wellcome Sanger Institute for the COVID-19 Genomics UK (COG-UK) Consortium|Davies|United Kingdom
>Spike|hCoV-19/India/MH-ACTREC-388/2020|2020-07-28|EPI_ISL_700037|Original|hCoV-19^^Maharashtra|Human|Hematopathology Laboratory|Hematopathology Laboratory|Patkar|India
>Spike|hCoV-19/England/ALDP-BC7D21/2020|2020-11-23|EPI_ISL_721414|Original|hCoV-19^^England|Human|Lighthouse Lab in Alderley Park|Wellcome Sanger Institute for the COVID-19 Genomics UK (COG-UK) Consortium|Davies|United Kingdom
>Spike|hCoV-19/Belgium/rega-0417524/2020|2020-04-17|EPI_ISL_734615|Original|hCoV-19^^Mouscron|Human|UZ Leuven|KU Leuven|Maes|Belgium
>Spike|hCoV-19/England/QEUH-548B8E/2020|2020-05-14|EPI_ISL_533431|Original|hCoV-19^^England|Human|Lighthouse Lab in Glasgow|Wellcome Sanger Institute for the COVID-19 Genomics UK (COG-UK) consortium|Davies|United Kingdom
>Spike|hCoV-19/Denmark/DCGC-14098/2020|2020-11-30|EPI_ISL_711227|Original|hCoV-19^^Hovedstaden|Human|Department of Virus and Microbiological Special Diagnostics|Albertsen Lab|Michaelsen|Denmark
>Spike|hCoV-19/England/MILK-B387D2/2020|2020-11-01|EPI_ISL_658999|Original|hCoV-19^^England|Human|Lighthouse Lab in Milton Keynes|Wellcome Sanger Institute for the COVID-19 Genomics UK (COG-UK) Consortium|Davies|United Kingdom
>Spike|hCoV-19/England/ALDP-CB61EC/2020|2020-12-18|EPI_ISL_761806|Original|hCoV-19^^England|Human|Lighthouse Lab in Alderley Park|Wellcome Sanger Institute for the COVID-19 Genomics UK (COG-UK) Consortium|Davies|United Kingdom
>Spike|hCoV-19/Germany/BE-ChVir-W1649-7416/2020|2020-07-16|EPI_ISL_753981|Original|hCoV-19^^Berlin|Human|Charit\u00e9 Universit\u00e4tsmedizin Berlin|Charit\u00e9 Universit\u00e4tsmedizin Berlin|Muehlemann|Germany
>Spike|hCoV-19/USA/ME-HETL-J0371/2020|2020-12-09|EPI_ISL_755493|Original|hCoV-19^^Maine|Human|Maine Health and Environmental Testing Laboratory|Tewhey Lab|Tewhey|USA
>Spike|hCoV-19/England/20149009004/2020|2020-03-30|EPI_ISL_466142|Original|hCoV-19^^England|Human|Respiratory Virus Unit|Respiratory Virus Unit|Platt|United Kingdom
>Spike|hCoV-19/England/NORW-EBC07/2020|2020-05-06|EPI_ISL_457490|Original|hCoV-19^^England|Human|Quadram Institute Bioscience|COVID-19 Genomics UK (COG-UK) Consortium|Alikhan|United Kingdom
>Spike|hCoV-19/Denmark/DCGC-9014/2020|2020-10-26|EPI_ISL_626187|Original|hCoV-19^^Midtjylland|Human|Department of Virus and Microbiological Special Diagnostics|Albertsen lab|Michaelsen|Denmark
>Spike|hCoV-19/USA/MI-UM-2006S000852/2020|2020-03-30|EPI_ISL_484711|Original|hCoV-19^^Michigan|Human|University of Michigan Clinical Microbiology Laboratory|Lauring Lab|Valesano|USA
>Spike|hCoV-19/Scotland/EDB3497/2020|2020-04-11|EPI_ISL_439270|Original|hCoV-19^^Scotland|Human|Virology Department|COVID-19 Genomics UK (COG-UK) Consortium|Williams|United Kingdom
>Spike|hCoV-19/England/ALDP-9B6695/2020|2020-08-28|EPI_ISL_573027|Original|hCoV-19^^England|Human|Oxford Viromics|COVID-19 Genomics UK (COG-UK) Consortium|Golubchik|United Kingdom
>Spike|hCoV-19/Wales/PHWC-2A088/2020|2020-04-04|EPI_ISL_432251|Original|hCoV-19^^Wales|Human|Wales Specialist Virology Centre|Public Health Wales Microbiology Cardiff|Moore|United Kingdom
>Spike|hCoV-19/Germany/NW-HHU-483/2020|2020-11-30|EPI_ISL_770717|Original|hCoV-19^^North Rhine-Westphalia|Human|ZOTZ KLIMAS MVZ D\u00fcsseldorf-Centrum GbR \u00dcBAG f\u00fcr Labormedizin|Center of Medical Microbiology|Wienemann|Germany
>Spike|hCoV-19/Japan/PG-7722/2020|2020-08-00|EPI_ISL_686824|Original|hCoV-19^^Japan|Human|Chiba Prefectural Institute of Public Health|Pathogen Genomics Center|Sekizuka|Japan
>Spike|hCoV-19/Luxembourg/LNS9162230/2020|2020-10-29|EPI_ISL_744862|Original|hCoV-19^^Luxembourg|Human|Laboratoire national de sant\u00e9|Laboratoire national de sant\u00e9|Wienecke-Baldacchino|Luxembourg

>Spike|hCoV-19/USA/VA-DCLS-1587/2020|2020-08-14|EPI_ISL_610142|Original|hCoV-19^^Virginia|Human|Virginia DCLS|Virginia DCLS|St. Jacques|USA
>Spike|hCoV-19/USA/VA-DCLS-0204/2020|2020-04-13|EPI_ISL_437419|Original|hCoV-19^^Virginia|Human|Virginia DCLS|Virginia DCLS|St. Jacques|USA
>Spike|hCoV-19/Denmark/DCGC-11518/2020|2020-11-02|EPI_ISL_670972|Original|hCoV-19^^Midtjylland|Human|Department of Virus and Microbiological Special Diagnostics|Albertsen Lab|Michaelsen|Denmark
>Spike|hCoV-19/England/ALDP-4AA990/2020|2020-06-13|EPI_ISL_559576|Original|hCoV-19^^England|Human|Lighthouse Lab in Alderley Park|Wellcome Sanger Institute for the COVID-19 Genomics UK (COG-UK) Consortium|Davies|United Kingdom
>Spike|hCoV-19/England/MILK-ACBF46/2020|2020-10-20|EPI_ISL_629257|Original|hCoV-19^^England|Human|Lighthouse Lab in Milton Keynes|Wellcome Sanger Institute for the COVID-19 Genomics UK (COG-UK) consortium|Davies|United Kingdom
>Spike|hCoV-19/Denmark/DCGC-17634/2020|2020-12-14|EPI_ISL_750388|Original|hCoV-19^^Hovedstaden|Human|Department of Virus and Microbiological Special Diagnostics|Albertsen Lab|Michaelsen|Denmark
>Spike|hCoV-19/Wales/PHWC-2C9C7/2020|2020-03-31|EPI_ISL_445931|Original|hCoV-19^^Wales|Human|Wales Specialist Virology Centre|Public Health Wales Microbiology Cardiff|Moore|United Kingdom
>Spike|hCoV-19/USA/CA-CZB-13448/2020|2020-09-08|EPI_ISL_672471|Original|hCoV-19^^California|Human|Alameda County Public Health Lab|Chan-Zuckerberg Biohub|Bhatt|USA
>Spike|hCoV-19/USA/NY-NYU-VC-009b/2020|2020-03-16|EPI_ISL_479666|Original|hCoV-19^^New York|Human|Center for Genomics and System Biology|Center for Genomics and System Biology|Ghedin|USA
>Spike|hCoV-19/Portugal/PT0266/2020|2020-04-04|EPI_ISL_453980|Original|hCoV-19^^Portugal|Human|unknown|Instituto Nacional de Saude (INSA)|Borges|Portugal
>Spike|hCoV-19/USA/FL-BPHL-1672/2020|2020-07-05|EPI_ISL_594361|Original|hCoV-19^^Florida|Human|Florida Bureau of Public Health Laboratories|Florida Bureau of Public Health Laboratories|Blanton|USA
>Spike|hCoV-19/England/CAMB-785F5/2020|2020-04-06|EPI_ISL_433755|Original|hCoV-19^^England|Human|Department of Pathology|COVID-19 Genomics UK (COG-UK) Consortium|Meredith|United Kingdom
>Spike|hCoV-19/USA/UT-UPHL-2008863/2020|2020-05-28|EPI_ISL_524980|Original|hCoV-19^^Utah|Human|Utah Public Health Laboratory|Utah Public Health Laboratory|Young|USA
>Spike|hCoV-19/England/ALDP-98CE63/2020|2020-07-23|EPI_ISL_529305|Original|hCoV-19^^England|Human|Liverpool Clinical Laboratories|COVID-19 Genomics UK (COG-UK) Consortium|Haldenby|United Kingdom
>Spike|hCoV-19/France/PAC-IHU-0168/2020|2020-03-25|EPI_ISL_569345|Original|hCoV-19^^Provence-Alpes-C\u00f4te-d\u2019Azur|Human|MEPHI|MEPHI|Levasseur|France
>Spike|hCoV-19/England/ALDP-A964FA/2020|2020-10-20|EPI_ISL_624760|Original|hCoV-19^^England|Human|Lighthouse Lab in Alderley Park|Wellcome Sanger Institute for the COVID-19 Genomics UK (COG-UK) consortium|Davies|United Kingdom
>Spike|hCoV-19/Wales/PHWC-165EA4/2020|2020-05-14|EPI_ISL_490810|Original|hCoV-19^^Wales|Human|Wales Specialist Virology Centre Sequencing lab|COVID-19 Genomics UK (COG-UK) Consortium|Moore|United Kingdom
>Spike|hCoV-19/Wales/PHWC-24279/2020|2020-03-16|EPI_ISL_418158|Original|hCoV-19^^Wales|Human|Wales Specialist Virology Centre|Public Health Wales Microbiology Cardiff|Moore|United Kingdom
>Spike|hCoV-19/India/MH-ACTREC-518/2020|2020-08-21|EPI_ISL_700164|Original|hCoV-19^^Maharashtra|Human|Hematopathology Laboratory|Hematopathology Laboratory|Patkar|India
>Spike|hCoV-19/England/CAMC-A41FA2/2020|2020-10-05|EPI_ISL_609370|Original|hCoV-19^^England|Human|Lighthouse Lab in Cambridge|Wellcome Sanger Institute for the COVID-19 Genomics UK (COG-UK) consortium|Davies|United Kingdom
>Spike|hCoV-19/Denmark/DCGC-6080/2020|2020-10-05|EPI_ISL_620274|Original|hCoV-19^^Hovedstaden|Human|Department of Virus and Microbiological Special Diagnostics|Albertsen lab|Michaelsen|Denmark

>Spike|hCoV-19/Switzerland/GR-ETHZ-400293/2020|2020-12-07|EPI_ISL_729060|Original|hCoV-19^^Graub\u00fcnden|Human|Viollier AG|Department of Biosystems Science and Engineering|Nadeau|Switzerland
>Spike|hCoV-19/Jordan/AM-ALSR-3912/2020|2020-09-30|EPI_ISL_636278|Original|hCoV-19^^Amman|Human|Biolab Diagnostic Laboratories|Andersen lab at Scripps Research|Gangavarapu|Jordan
>Spike|hCoV-19/England/NORW-ED26D/2020|2020-05-25|EPI_ISL_472357|Original|hCoV-19^^England|Human|Quadram Institute Bioscience|COVID-19 Genomics UK (COG-UK) Consortium|Alikhan|United Kingdom
>Spike|hCoV-19/Canada/ON-S860/2020|2020-04-00|EPI_ISL_586462|Original|hCoV-19^^Ontario|Human|Toronto Invasive Bacterial Diseases Network|McMaster University|McArthur|Canada
>Spike|hCoV-19/England/EXET-1389D6/2020|2020-10-21|EPI_ISL_626777|Original|hCoV-19^^England|Human|University of Exeter|COVID-19 Genomics UK (COG-UK) Consortium|Temperton|United Kingdom
>Spike|hCoV-19/England/ALDP-9CB79A/2020|2020-09-16|EPI_ISL_575599|Original|hCoV-19^^England|Human|Lighthouse Lab in Alderley Park|Wellcome Sanger Institute for the COVID-19 Genomics UK (COG-UK) consortium|Davies|United Kingdom
>Spike|hCoV-19/England/QEUH-AEFD24/2020|2020-10-29|EPI_ISL_662726|Original|hCoV-19^^England|Human|Lighthouse Lab in Glasgow|Wellcome Sanger Institute for the COVID-19 Genomics UK (COG-UK) Consortium|Davies|United Kingdom
>Spike|hCoV-19/Denmark/DCGC-8988/2020|2020-10-26|EPI_ISL_626192|Original|hCoV-19^^Midtjylland|Human|Department of Virus and Microbiological Special Diagnostics|Albertsen lab|Michaelsen|Denmark
>Spike|hCoV-19/Singapore/1287/2020|2020-05-09|EPI_ISL_648772|Original|hCoV-19^^Singapore|Human|Department of Laboratory Medicine|Department of Laboratory Medicine|Chen|Singapore
>Spike|hCoV-19/England/ALDP-497899/2020|2020-06-04|EPI_ISL_559430|Original|hCoV-19^^England|Human|Lighthouse Lab in Alderley Park|Wellcome Sanger Institute for the COVID-19 Genomics UK (COG-UK) consortium|Davies|United Kingdom
>Spike|hCoV-19/USA/ME-HETL-J0363/2020|2020-11-30|EPI_ISL_755486|Original|hCoV-19^^Maine|Human|Maine Health and Environmental Testing Laboratory|Tewhey Lab|Tewhey|USA
>Spike|hCoV-19/Australia/VIC5418/2020|2020-07-08|EPI_ISL_520180|Original|hCoV-19^^Victoria|Human|Microbiological Diagnostic Unit - Public Health Laboratory (MDU-PHL)|MDU-PHL|Schultz|Australia
>Spike|hCoV-19/England/ALDP-BB310E/2020|2020-11-19|EPI_ISL_702796|Original|hCoV-19^^England|Human|Lighthouse Lab in Alderley Park|Wellcome Sanger Institute for the COVID-19 Genomics UK (COG-UK) Consortium|Davies|United Kingdom
>Spike|hCoV-19/Germany/BY-MVP-V2010837/2020|2020-03-12|EPI_ISL_420900|Original|hCoV-19^^Bavaria|Human|Max von Pettenkofer Institute|Laboratory for Functional Genome Analysis|Graf|Germany
>Spike|hCoV-19/USA/GA-CDC-7676/2020|2020-10-27|EPI_ISL_681823|Original|hCoV-19^^Georgia|Human|GA Department of Public Health Laboratory|Pathogen Discovery|Queen|USA
>Spike|hCoV-19/Australia/TAS103/2020|2020-03-24|EPI_ISL_522569|Original|hCoV-19^^Tasmania|Human|Royal Hobart Hospital Microbiology Department|MDU-PHL|Schultz|Australia
>Spike|hCoV-19/Japan/PG-2912/2020|2020-04-00|EPI_ISL_691038|Original|hCoV-19^^Japan|Human|Pathogen Genomics Center|Pathogen Genomics Center|Sekizuka|Japan
>Spike|hCoV-19/England/QEUH-B2EDDD/2020|2020-11-03|EPI_ISL_659930|Original|hCoV-19^^England|Human|Lighthouse Lab in Glasgow|Wellcome Sanger Institute for the COVID-19 Genomics UK (COG-UK) Consortium|Davies|United Kingdom
>Spike|hCoV-19/Scotland/QEUH-9DAD87/2020|2020-09-22|EPI_ISL_601149|Original|hCoV-19^^Scotland|Human|Lighthouse Lab in Glasgow|Wellcome Sanger Institute for the COVID-19 Genomics UK (COG-UK) consortium|Davies|United Kingdom
>Spike|hCoV-19/Northern Ireland/QEUH-95EB45/2020|2020-08-09|EPI_ISL_532498|Original|hCoV-19^^Northern Ireland|Human|Lighthouse Lab in Glasgow|Wellcome Sanger Institute for the COVID-19 Genomics UK (COG-UK) consortium|Davies|United Kingdom

>Spike|hCoV-19/India/UN-2020777/2020|2020-00-00|EPI_ISL_420552|Vero CCL81 P1|hCoV-19^^India|Human|Indian Council of Medical Research-National Institute of Virology|Indian Council of Medical Research-National Institute of Virology|Yadav|India
>Spike|hCoV-19/England/CAMC-B51EFC/2020|2020-11-08|EPI_ISL_657852|Original|hCoV-19^^England|Human|Lighthouse Lab in Cambridge|Wellcome Sanger Institute for the COVID-19 Genomics UK (COG-UK) Consortium|Davies|United Kingdom
>Spike|hCoV-19/England/ALDP-B49413/2020|2020-11-07|EPI_ISL_649196|Original|hCoV-19^^England|Human|Lighthouse Lab in Alderley Park|Wellcome Sanger Institute for the COVID-19 Genomics UK (COG-UK) Consortium|Davies|United Kingdom
>Spike|hCoV-19/England/QEUH-9B4CC2/2020|2020-09-01|EPI_ISL_590236|Original|hCoV-19^^England|Human|Lighthouse Lab in Glasgow|Wellcome Sanger Institute for the COVID-19 Genomics UK (COG-UK) consortium|Davies|United Kingdom
>Spike|hCoV-19/Wales/MILK-9C2405/2020|2020-09-09|EPI_ISL_566585|Original|hCoV-19^^Wales|Human|Lighthouse Lab in Milton Keynes|Wellcome Sanger Institute for the COVID-19 Genomics UK (COG-UK) consortium|Davies|United Kingdom
>Spike|hCoV-19/Czech Republic/NRL-9964/2020|2020-09-22|EPI_ISL_660597|Original|hCoV-19^^South Moravian Region|Human|The National Institute of Public Health|State Veterinary Institute Prague|Nagy|Czech Republic
>Spike|hCoV-19/England/ALDP-BB9ED4/2020|2020-11-20|EPI_ISL_701974|Original|hCoV-19^^England|Human|Lighthouse Lab in Alderley Park|Wellcome Sanger Institute for the COVID-19 Genomics UK (COG-UK) Consortium|Davies|United Kingdom
>Spike|hCoV-19/England/ALDP-B26063/2020|2020-11-04|EPI_ISL_646610|Original|hCoV-19^^England|Human|Lighthouse Lab in Alderley Park|Wellcome Sanger Institute for the COVID-19 Genomics UK (COG-UK) Consortium|Davies|United Kingdom
>Spike|hCoV-19/Spain/CT-IBV-005623/2020|2020-04-27|EPI_ISL_508660|Original|hCoV-19^^Catalunya|Human|Departamento de Microbiolog\u00eda|SeqCOVID-SPAIN consortium/IBV(CSIC)|Comas|Spain
>Spike|hCoV-19/Northern Ireland/NIRE-FB4EE/2020|2020-03-16|EPI_ISL_441434|Original|hCoV-19^^Northern Ireland|Human|Regional Virus Laboratory|COVID-19 Genomics UK (COG-UK) Consortium|Fairley|United Kingdom
>Spike|hCoV-19/USA/WI-GMF-27490/2020|2020-10-24|EPI_ISL_660978|Original|hCoV-19^^Wisconsin|Human|Gundersen Molecular Diagnostics Laboratory|Kabara Cancer Research Institute|Kenny|USA
>Spike|hCoV-19/Japan/PG-1854/2020|2020-05-00|EPI_ISL_685460|Original|hCoV-19^^Japan|Human|Tokyo Metropolitan Institute of Public Health|Pathogen Genomics Center|Sekizuka|Japan
>Spike|hCoV-19/USA/NM-DOH-2020318805/2020|2020-08-27|EPI_ISL_732740|Original|hCoV-19^^New Mexico|Human|New Mexico Department of Health Scientific Laboratory|New Mexico Department of Health Scientific Laboratory|Johnson|USA
>Spike|hCoV-19/England/QEUH-96D19C/2020|2020-08-19|EPI_ISL_530629|Original|hCoV-19^^England|Human|Lighthouse Lab in Glasgow|Wellcome Sanger Institute for the COVID-19 Genomics UK (COG-UK) consortium|Davies|United Kingdom
>Spike|hCoV-19/Wales/PHWC-48E1E5/2020|2020-11-10|EPI_ISL_680822|Original|hCoV-19^^Wales|Human|Wales Specialist Virology Centre Sequencing lab|COVID-19 Genomics UK (COG-UK) Consortium|Moore|United Kingdom
>Spike|hCoV-19/England/ALDP-A2E1C3/2020|2020-10-06|EPI_ISL_586830|Original|hCoV-19^^England|Human|Lighthouse Lab in Alderley Park|Wellcome Sanger Institute for the COVID-19 Genomics UK (COG-UK) consortium|Davies|United Kingdom
>Spike|hCoV-19/Spain/GA-ISCIII-201863/2020|2020-03-10|EPI_ISL_455331|Original|hCoV-19^^Galicia|Human|Complejo Hospitalario Universitario La Coruna|Instituto de Salud Carlos III|Caballero|Spain
>Spike|hCoV-19/Wales/PHWC-367C0/2020|2020-04-28|EPI_ISL_474712|Original|hCoV-19^^Wales|Human|Originating lab|COVID-19 Genomics UK (COG-UK) Consortium|Moore|United Kingdom
>Spike|hCoV-19/USA/NY-NYCPHL-001072/2020|2020-10-15|EPI_ISL_633073|Original|hCoV-19^^New York|Human|DOHMH Fort Greene|New York City Public Health Laboratory|Wang|USA
>Spike|hCoV-19/England/CAMB-1AA650/2020|2020-04-17|EPI_ISL_470206|Original|hCoV-19^^England|Human|Department of Pathology|Wellcome Sanger Institute for the COVID-19 Genomics UK (COG-UK) consortium|Davies|United Kingdom

>Spike|hCoV-19/USA/WA-S208/2020|2020-03-23|EPI_ISL_430246|Original|hCoV-19^^Washington|Human|Washington State Department of Health|Seattle Flu Study|Bedford|USA
>Spike|hCoV-19/USA/OR-OHSU-2857/2020|2020-09-03|EPI_ISL_667360|Original|hCoV-19^^Oregon|Human|OHSU Lab Services Molecular Microbiology Lab|Oregon SARS-CoV-2 Genome Sequencing Center|O'Roak|USA
>Spike|hCoV-19/Bangladesh/GRMDRC-8/2020|2020-09-05|EPI_ISL_775217|Original|hCoV-19^^Dhaka|Human|Gonoshasthya-RNA Molecular Research Center|Gonoshasthya-RNA Molecular Research Center|Adnan|Bangladesh
>Spike|hCoV-19/England/ALDP-5A1F69/2020|2020-06-14|EPI_ISL_558682|Original|hCoV-19^^England|Human|Lighthouse Lab in Alderley Park|Wellcome Sanger Institute for the COVID-19 Genomics UK (COG-UK) consortium|Davies|United Kingdom
>Spike|hCoV-19/USA/MN-QDX-988/2020|2020-03-14|EPI_ISL_571786|Original|hCoV-19^^Minnesota|Human|Quest Diagnostics|Quest Diagnostics|Kagan|USA
>Spike|hCoV-19/Wales/PHWC-16EBC2/2020|2020-09-30|EPI_ISL_585833|Original|hCoV-19^^Wales|Human|Wales Specialist Virology Centre Sequencing lab|COVID-19 Genomics UK (COG-UK) Consortium|Moore|United Kingdom
>Spike|hCoV-19/Denmark/DCGC-21676/2020|2020-12-21|EPI_ISL_758302|Original|hCoV-19^^Hovedstaden|Human|Department of Virus and Microbiological Special Diagnostics|Albertsen Lab|Michaelsen|Denmark
>Spike|hCoV-19/England/ALDP-A86615/2020|2020-10-17|EPI_ISL_606206|Original|hCoV-19^^England|Human|Lighthouse Lab in Alderley Park|Wellcome Sanger Institute for the COVID-19 Genomics UK (COG-UK) consortium|Davies|United Kingdom
>Spike|hCoV-19/England/NORW-F1C2C/2020|2020-10-21|EPI_ISL_627625|Original|hCoV-19^^England|Human|Quadram Institute Bioscience|COVID-19 Genomics UK (COG-UK) Consortium|Alikhan|United Kingdom
>Spike|hCoV-19/Wales/PHWC-48CDB6/2020|2020-11-03|EPI_ISL_666539|Original|hCoV-19^^Wales|Human|Wales Specialist Virology Centre Sequencing lab|COVID-19 Genomics UK (COG-UK) Consortium|Moore|United Kingdom
>Spike|hCoV-19/Wales/PHWC-2D5B3/2020|2020-04-04|EPI_ISL_445999|Original|hCoV-19^^Wales|Human|Wales Specialist Virology Centre|Public Health Wales Microbiology Cardiff|Moore|United Kingdom
>Spike|hCoV-19/Ireland/CN-NVRL-20IRL82829/2020|2020-10-05|EPI_ISL_639904|Original|hCoV-19^^Cavan|Human|National Virus Reference Laboratory|National Virus Reference Laboratory|Gonzalez|Ireland
>Spike|hCoV-19/Luxembourg/LNS6516073/2020|2020-11-11|EPI_ISL_744425|Original|hCoV-19^^Luxembourg|Human|Laboratoire national de sant\u00e9|Laboratoire national de sant\u00e9|Wienecke-Baldacchino|Luxembourg
>Spike|hCoV-19/Scotland/QEUH-964960/2020|2020-08-13|EPI_ISL_531123|Original|hCoV-19^^Scotland|Human|NHSGGC West of Scotland Specialist Virology Centre / MRC-University of Glasgow Centre for Virus Research|Wellcome Sanger Institute for the COVID-19 Genomics UK (COG-UK) consortium|Davies|United Kingdom
>Spike|hCoV-19/England/ALDP-A66B2B/2020|2020-10-12|EPI_ISL_630390|Original|hCoV-19^^England|Human|Lighthouse Lab in Alderley Park|Wellcome Sanger Institute for the COVID-19 Genomics UK (COG-UK) consortium|Davies|United Kingdom
>Spike|hCoV-19/Australia/VIC13960/2020|2020-07-22|EPI_ISL_563894|Original|hCoV-19^^Victoria|Human|Microbiological Diagnostic Unit - Public Health Laboratory (MDU-PHL)|MDU-PHL|Schultz|Australia
>Spike|hCoV-19/England/ALDP-9CCCA9/2020|2020-09-15|EPI_ISL_575477|Original|hCoV-19^^England|Human|Lighthouse Lab in Alderley Park|Wellcome Sanger Institute for the COVID-19 Genomics UK (COG-UK) consortium|Davies|United Kingdom
>Spike|hCoV-19/USA/FL-USAFSAM-S035/2020|2020-03-27|EPI_ISL_512803|Original|hCoV-19^^Florida|Human|Public Health|Public Health|Fries|USA
>Spike|hCoV-19/USA/CA-ALSR-4369/2020|2020-07-31|EPI_ISL_648993|Original|hCoV-19^^California|Human|San Diego County Public Health Laboratory|Andersen lab at Scripps Research|Gangavarapu|USA
>Spike|hCoV-19/Denmark/DCGC-4828/2020|2020-09-21|EPI_ISL_621438|Original|hCoV-19^^Nordjylland|Human|Department of Virus and Microbiological Special Diagnostics|Albertsen lab|Michaelsen|Denmark

>Spike|hCoV-19/England/ALDP-64B48F/2020|2020-07-04|EPI_ISL_558067|Original|hCoV-19^^England|Human|Lighthouse Lab in Alderley Park|Wellcome Sanger Institute for the COVID-19 Genomics UK (COG-UK) consortium|Davies|United Kingdom
>Spike|hCoV-19/USA/UT-UPHL-2009681/2020|2020-07-25|EPI_ISL_605356|Original|hCoV-19^^Utah|Human|Utah Public Health Laboratory|Utah Public Health Laboratory|Young|USA
>Spike|hCoV-19/USA/NY-Wadsworth-34750-01/2020|2020-03-31|EPI_ISL_676795|Original|hCoV-19^^New York|Human|Wadsworth Center|Wadsworth Center|Lasek-Nesselquist|USA
>Spike|hCoV-19/Scotland/CVR4410/2020|2020-09-20|EPI_ISL_585284|Original|hCoV-19^^Scotland|Human|West of Scotland Specialist Virology Centre|COVID-19 Genomics UK (COG-UK) Consortium|Robertson|United Kingdom
>Spike|hCoV-19/Australia/VIC7365/2020|2020-07-26|EPI_ISL_564362|Original|hCoV-19^^Victoria|Human|Victorian Infectious Diseases Reference Laboratory (VIDRL)|VIDRL and MDU-PHL|Schultz|Australia
>Spike|hCoV-19/England/LIVE-9DB43/2020|2020-04-29|EPI_ISL_449618|Original|hCoV-19^^England|Human|Liverpool Clinical Laboratories|COVID-19 Genomics UK (COG-UK) Consortium|Haldenby|United Kingdom
>Spike|hCoV-19/USA/WA-S747/2020|2020-04-13|EPI_ISL_449917|Original|hCoV-19^^Washington|Human|Washington State Department of Health|Seattle Flu Study|Bedford|USA
>Spike|hCoV-19/USA/WI-WSLH-200225/2020|2020-04-13|EPI_ISL_578445|Original|hCoV-19^^Wisconsin|Human|Wisconsin State Laboratory of Hygiene Communicable Disease Division|Wisconsin State Laboratory of Hygiene Communicable Disease Division|Florek|USA
>Spike|hCoV-19/Oman/RESP-20-2181/2020|2020-03-17|EPI_ISL_457939|Original|hCoV-19^^Muscat|Human|Oman-NIC|Oman-NIC|Al Mahruqi|Oman
>Spike|hCoV-19/Northern Ireland/QEUH-96411D/2020|2020-08-12|EPI_ISL_536935|Original|hCoV-19^^Northern Ireland|Human|Lighthouse Lab in Glasgow|Wellcome Sanger Institute for the COVID-19 Genomics UK (COG-UK) consortium|Davies|United Kingdom
>Spike|hCoV-19/Spain/AR-IBV-2453/2020|2020-03-24|EPI_ISL_468831|Original|hCoV-19^^Aragon|Human|Servicio de Microbiolog\u00eda|SeqCOVID-SPAIN consortium/IBV(CSIC)|Comas|Spain
>Spike|hCoV-19/Wales/PHWC-49750B/2020|2020-11-30|EPI_ISL_726841|Original|hCoV-19^^Wales|Human|Originating lab|COVID-19 Genomics UK (COG-UK) Consortium|Moore|United Kingdom
>Spike|hCoV-19/Wales/PHWC-48512A/2020|2020-10-26|EPI_ISL_628149|Original|hCoV-19^^Wales|Human|Wales Specialist Virology Centre Sequencing lab|COVID-19 Genomics UK (COG-UK) Consortium|Moore|United Kingdom
>Spike|hCoV-19/Canada/ON-PHL-20-01177/2020|2020-06-00|EPI_ISL_548799|Original|hCoV-19^^Ontario|Human|Public Health Ontario Laboratory|Public Health Ontario Laboratory|Guthrie|Canada
>Spike|hCoV-19/England/ALDP-5A7868/2020|2020-06-13|EPI_ISL_558668|Original|hCoV-19^^England|Human|Lighthouse Lab in Alderley Park|Wellcome Sanger Institute for the COVID-19 Genomics UK (COG-UK) consortium|Davies|United Kingdom
>Spike|hCoV-19/USA/LA-EVTL824/2020|2020-07-28|EPI_ISL_653515|Original|hCoV-19^^Louisiana|Human|LSUHS Emerging Viral Threat Laboratory|Microbial Genome Sequencing Center|Kamil|USA
>Spike|hCoV-19/England/ALDP-9534EB/2020|2020-06-08|EPI_ISL_553815|Original|hCoV-19^^England|Human|Lighthouse Lab in Alderley Park|Wellcome Sanger Institute for the COVID-19 Genomics UK (COG-UK) consortium|Davies|United Kingdom
>Spike|hCoV-19/Czech Republic/NRL_9640/2020|2020-04-04|EPI_ISL_626593|Original|hCoV-19^^Moravian-Silesian Region|Human|The National Institute of Public Health|State Veterinary Institute Prague|Nagy|Czech Republic
>Spike|hCoV-19/Denmark/DCGC-11129/2020|2020-11-02|EPI_ISL_669750|Original|hCoV-19^^Midtjylland|Human|Department of Virus and Microbiological Special Diagnostics|Albertsen Lab|Michaelsen|Denmark
>Spike|hCoV-19/India/MH-NCCS-NR-5173/2020|2020-06-08|EPI_ISL_721658|Original|hCoV-19^^Maharashtra|Human|National Centre For Cell Science|National Centre For Cell Science|Paul|India

>Spike|hCoV-19/Scotland/CVR5491/2020|2020-10-17|EPI_ISL_651816|Original|hCoV-19^^Scotland|Human|West of Scotland Specialist Virology Centre|COVID-19 Genomics UK (COG-UK) Consortium|Robertson|United Kingdom
>Spike|hCoV-19/USA/VA-DCLS-1814/2020|2020-09-22|EPI_ISL_609985|Original|hCoV-19^^Virginia|Human|Virginia DCLS|Virginia DCLS|St. Jacques|USA
>Spike|hCoV-19/Denmark/DCGC-11683/2020|2020-10-26|EPI_ISL_671066|Original|hCoV-19^^Hovedstaden|Human|Department of Virus and Microbiological Special Diagnostics|Albertsen Lab|Michaelsen|Denmark
>Spike|hCoV-19/Australia/VIC9983/2020|2020-08-24|EPI_ISL_561992|Original|hCoV-19^^Victoria|Human|Microbiological Diagnostic Unit - Public Health Laboratory (MDU-PHL)|MDU-PHL|Schultz|Australia
>Spike|hCoV-19/England/LOND-1267424/2020|2020-12-11|EPI_ISL_741287|Original|hCoV-19^^England|Human|University College London|COVID-19 Genomics UK (COG-UK) Consortium|Roy|United Kingdom
>Spike|hCoV-19/England/ALDP-B73A0F/2020|2020-11-12|EPI_ISL_662622|Original|hCoV-19^^England|Human|Lighthouse Lab in Alderley Park|Wellcome Sanger Institute for the COVID-19 Genomics UK (COG-UK) Consortium|Davies|United Kingdom
>Spike|hCoV-19/England/QEUH-9CA7D7/2020|2020-09-10|EPI_ISL_567480|Original|hCoV-19^^England|Human|Lighthouse Lab in Glasgow|Wellcome Sanger Institute for the COVID-19 Genomics UK (COG-UK) consortium|Davies|United Kingdom
>Spike|hCoV-19/England/SHEF-C2495/2020|2020-10-08|EPI_ISL_596192|Original|hCoV-19^^England|Human|Virology Department|COVID-19 Genomics UK (COG-UK) Consortium|de Silva|United Kingdom
>Spike|hCoV-19/England/WSFT-25C1246/2020|2020-04-18|EPI_ISL_492903|Original|hCoV-19^^England|Human|Department of Medical Microbiology|Wellcome Sanger Institute for the COVID-19 Genomics UK (COG-UK) consortium|Davies|United Kingdom
>Spike|hCoV-19/England/LOND-126034C/2020|2020-03-15|EPI_ISL_524670|Original|hCoV-19^^England|Human|North West London Pathology|Wellcome Sanger Institute for the COVID-19 Genomics UK (COG-UK) consortium|Davies|United Kingdom
>Spike|hCoV-19/USA/TX-HMH-2264/2020|2020-05-24|EPI_ISL_545213|Original|hCoV-19^^Texas|Human|Houston Methodist Hospital|Houston Methodist Hospital|Long|USA
>Spike|hCoV-19/Scotland/QEUH-C01D84/2020|2020-12-05|EPI_ISL_757236|Original|hCoV-19^^Scotland|Human|Lighthouse Lab in Glasgow|Wellcome Sanger Institute for the COVID-19 Genomics UK (COG-UK) Consortium|Davies|United Kingdom
>Spike|hCoV-19/England/CAMC-A41F48/2020|2020-10-06|EPI_ISL_609332|Original|hCoV-19^^England|Human|Lighthouse Lab in Cambridge|Wellcome Sanger Institute for the COVID-19 Genomics UK (COG-UK) consortium|Davies|United Kingdom
>Spike|hCoV-19/USA/LA-SR0324/2020|2020-04-05|EPI_ISL_450178|Original|hCoV-19^^Louisiana|Human|Robert Garry lab|Andersen lab at Scripps Research|Gangavarapu|USA
>Spike|hCoV-19/USA/WA-UW-10110/2020|2020-05-29|EPI_ISL_570268|Original|hCoV-19^^Washington|Human|UW Virology Lab|UW Virology Lab|Roychoudhury|USA
>Spike|hCoV-19/Brazil/PE-IAM1254/2020|2020-05-09|EPI_ISL_572384|Original|hCoV-19^^Pernambuco|Human|LACEN/PE|WallauLab|Wallau|Brazil
>Spike|hCoV-19/Wales/PHWC-489FD1/2020|2020-11-03|EPI_ISL_652514|Original|hCoV-19^^Wales|Human|Wales Specialist Virology Centre Sequencing lab|COVID-19 Genomics UK (COG-UK) Consortium|Moore|United Kingdom
>Spike|hCoV-19/England/CAMB-79A9B/2020|2020-03-27|EPI_ISL_433818|Original|hCoV-19^^England|Human|Department of Pathology|COVID-19 Genomics UK (COG-UK) Consortium|Meredith|United Kingdom
>Spike|hCoV-19/England/BRIS-1247D3/2020|2020-03-25|EPI_ISL_443833|Original|hCoV-19^^England|Human|PHE South West Regional Laboratory|Wellcome Sanger Institute for the COVID-19 Genomics UK (COG-UK) consortium|Amato|United Kingdom
>Spike|hCoV-19/Jordan/IR-ALSR-5210/2020|2020-10-05|EPI_ISL_730448|Original|hCoV-19^^Irbid|Human|Biolab Diagnostic Laboratories|Andersen lab at Scripps Research|Gangavarapu|Jordan
>Spike|hCoV-19/England/MILK-A46A7F/2020|2020-10-06|EPI_ISL_598310|Original|hCoV-19^^England|Human|Lighthouse Lab in Milton Keynes|Wellcome Sanger Institute for the COVID-19 Genomics UK (COG-UK) consortium|Davies|United Kingdom

>Spike|hCoV-19/England/LIVE-DC30C0/2020|2020-11-21|EPI_ISL_705861|Original|hCoV-19^^England|Human|Liverpool Clinical Laboratories|COVID-19 Genomics UK (COG-UK) Consortium|Haldenby|United Kingdom
>Spike|hCoV-19/USA/NY-Wadsworth-281390-01/2020|2020-10-20|EPI_ISL_734459|Original|hCoV-19^^New York|Human|Masonic Medical Research Institute|Wadsworth Center|Lasek-Nesselquist|USA
>Spike|hCoV-19/Netherlands/NH-EMC-96/2020|2020-12-10|EPI_ISL_722455|Original|hCoV-19^^Noord Holland|Human|Dutch COVID-19 response team|Erasmus Medical Center|Oude Munnink|Netherlands
>Spike|hCoV-19/Canada/BC-BCCDC-8411/2020|2020-11-00|EPI_ISL_740783|Original|hCoV-19^^British Columbia|Human|BCCDC Public Health Laboratory|BCCDC Public Health Laboratory|Prystajecky|Canada
>Spike|hCoV-19/England/20129171904/2020|2020-03-21|EPI_ISL_424142|Original|hCoV-19^^England|Human|Respiratory Virus Unit|Respiratory Virus Unit|Platt|United Kingdom
>Spike|hCoV-19/Scotland/QEUH-B694C2/2020|2020-11-11|EPI_ISL_676329|Original|hCoV-19^^Scotland|Human|Lighthouse Lab in Glasgow|Wellcome Sanger Institute for the COVID-19 Genomics UK (COG-UK) Consortium|Davies|United Kingdom
>Spike|hCoV-19/Netherlands/ZH-EMC-661/2020|2020-09-27|EPI_ISL_632378|Original|hCoV-19^^Zuid Holland|Human|Dutch COVID-19 response team|Erasmus Medical Center|Oude Munnink|Netherlands
>Spike|hCoV-19/Spain/PV-IBV-001467/2020|2020-03-17|EPI_ISL_467101|Original|hCoV-19^^Basque Country|Human|Hospital Universitario Araba. Vitoria-Gasteiz|SeqCOVID-SPAIN consortium/IBV(CSIC)|Comas|Spain
>Spike|hCoV-19/Canada/BC_03959087/2020|2020-04-00|EPI_ISL_466758|Original|hCoV-19^^British Columbia|Human|BCCDC Public Health Laboratory|BCCDC Public Health Laboratory|Harrigan|Canada
>Spike|hCoV-19/England/LEED-2AA6AA/2020|2020-04-13|EPI_ISL_538953|Original|hCoV-19^^England|Human|Leeds Teaching Hospitals NHS Trust and Public Health England|Wellcome Sanger Institute for the COVID-19 Genomics UK (COG-UK) consortium|Davies|United Kingdom
>Spike|hCoV-19/USA/NY-NYCPHL-000911/2020|2020-03-19|EPI_ISL_632972|Original|hCoV-19^^New York|Human|Jamaica Hospital Medical Center|New York City Public Health Laboratory|Wang|USA
>Spike|hCoV-19/Spain/RI-IBV-99010995/2020|2020-10-14|EPI_ISL_732818|Original|hCoV-19^^La_Rioja|Human|Centro de Investigaci\u00f3n Biom\u00e9dica de La Rioja - Hospital San Pedro Logro\u00f1o|SeqCOVID-SPAIN consortium/IBV(CSIC)|Comas|Spain
>Spike|hCoV-19/England/QEUH-A9FA4A/2020|2020-10-20|EPI_ISL_665388|Original|hCoV-19^^England|Human|Northumbria University / South Tees Hospitals NHS Foundation Trust / North Cumbria Integrated Care NHS Foundation Trust / North Tees and Hartlepool NHS Foundation Trust / Newcastle Hospitals NHS Foundation Trust|COVID-19 Genomics UK (COG-UK) Consortium|Bashton|United Kingdom
>Spike|hCoV-19/Ukraine/203100329/2020|2020-05-27|EPI_ISL_512608|Original|hCoV-19^^Ukraine|Human|National Laboratory for Influenza/Virology reference laboratory|Respiratory Virus Unit|Platt|Ukraine
>Spike|hCoV-19/England/MILK-BAB93E/2020|2020-11-18|EPI_ISL_681426|Original|hCoV-19^^England|Human|Lighthouse Lab in Milton Keynes|Wellcome Sanger Institute for the COVID-19 Genomics UK (COG-UK) Consortium|Davies|United Kingdom
>Spike|hCoV-19/USA/TX-HMH-MCoV-11688/2020|2020-09-08|EPI_ISL_789981|Original|hCoV-19^^Texas|Human|Houston Methodist Hospital|Houston Methodist Hospital|Long|USA
>Spike|hCoV-19/USA/TX-HMH-MCoV-9304/2020|2020-07-03|EPI_ISL_781049|Original|hCoV-19^^Texas|Human|Houston Methodist Hospital|Houston Methodist Hospital|Long|USA
>Spike|hCoV-19/Scotland/CVR2863/2020|2020-04-21|EPI_ISL_473604|Original|hCoV-19^^Scotland|Human|West of Scotland Specialist Virology Centre|COVID-19 Genomics UK (COG-UK) Consortium|Robertson|United Kingdom
>Spike|hCoV-19/England/ALDP-94B38F/2020|2020-06-10|EPI_ISL_555806|Original|hCoV-19^^England|Human|Lighthouse Lab in Alderley Park|Wellcome Sanger Institute for the COVID-19 Genomics UK (COG-UK) consortium|Davies|United Kingdom
>Spike|hCoV-19/England/ALDP-8CF82B/2020|2020-06-24|EPI_ISL_557000|Original|hCoV-19^^England|Human|Lighthouse Lab in Alderley Park|Wellcome Sanger Institute for the COVID-19 Genomics UK (COG-UK) consortium|Davies|United Kingdom

>Spike|hCoV-19/England/ALDP-A6CDF2/2020|2020-10-15|EPI_ISL_608260|Original|hCoV-
19^^England|Human|Lighthouse Lab in Alderley Park|Wellcome Sanger Institute for the
COVID-19 Genomics UK (COG-UK) consortium|Davies|United Kingdom
>Spike|hCoV-19/Denmark/DCGC-10849/2020|2020-11-02|EPI_ISL_669603|Original|hCoV-
19^^Syddanmark|Human|Department of Virus and Microbiological Special
Diagnostics|Albertsen Lab|Michaelsen|Denmark
>Spike|hCoV-19/Northern Ireland/NIRE-23504C/2020|2020-10-
15|EPI_ISL_764406|Original|hCoV-19^^Northern Ireland|Human|Regional Virus
Laboratory|COVID-19 Genomics UK (COG-UK) Consortium|Fairley|United Kingdom
>Spike|hCoV-19/Belgium/UGent-252/2020|2020-10-27|EPI_ISL_717992|Original|hCoV-
19^^Ghent|Human|Lab voor klinische biologie|Onderzoeksgroep
Virologie|Lambrechts|Belgium
>Spike|hCoV-19/Russia/MOW-RII-MH1973S/2020|2020-05-20|EPI_ISL_596321|Original|hCoV-
19^^Moscow|Human|Pathogenic Microorganisms Variability Laboratory|WHO National
Influenza Centre Russian Federation|Komissarov|Russia
>Spike|hCoV-19/Singapore/296/2020|2020-04-09|EPI_ISL_462414|Original|hCoV-
19^^Singapore|Human|National Public Health Laboratory|National Public Health
Laboratory|Mak|Singapore
>Spike|hCoV-19/Netherlands/GE-RIVM-10322/2020|2020-03-
18|EPI_ISL_636531|Original|hCoV-19^^Gelderland|Human|Dutch COVID-19 response
team|National Institute for Public Health and the Environment
(RIVM)|Meijer|Netherlands
>Spike|hCoV-19/England/CAMB-80150/2020|2020-04-09|EPI_ISL_470409|Original|hCoV-
19^^England|Human|Department of Pathology|Wellcome Sanger Institute for the COVID-19
Genomics UK (COG-UK) consortium|Davies|United Kingdom
>Spike|hCoV-19/USA/WA-UW-5369/2020|2020-04-05|EPI_ISL_570066|Original|hCoV-
19^^Washington|Human|UW Virology Lab|UW Virology Lab|Roychoudhury|USA
>Spike|hCoV-19/Wales/PHWC-49D426/2020|2020-12-10|EPI_ISL_742781|Original|hCoV-
19^^Wales|Human|Wales Specialist Virology Centre Sequencing lab|COVID-19 Genomics UK
(COG-UK) Consortium|Moore|United Kingdom
>Spike|hCoV-19/Canada/ON-S926/2020|2020-04-13|EPI_ISL_591260|Original|hCoV-
19^^Ontario|Human|Toronto Invasive Bacterial Diseases Network|McMaster
University|McArthur|Canada
>Spike|hCoV-19/Scotland/CVR1145/2020|2020-03-29|EPI_ISL_433519|Original|hCoV-
19^^Scotland|Human|West of Scotland Specialist Virology Centre|COVID-19 Genomics UK
(COG-UK) Consortium|Robertson|United Kingdom
>Spike|hCoV-19/USA/MI-UM-10036399783/2020|2020-10-12|EPI_ISL_610053|Original|hCoV-
19^^Michigan|Human|University of Michigan Clinical Microbiology Laboratory|Lauring
Lab|Valesano|USA
>Spike|hCoV-19/England/LIVE-DC2B66/2020|2020-11-24|EPI_ISL_705385|Original|hCoV-
19^^England|Human|Liverpool Clinical Laboratories|COVID-19 Genomics UK (COG-UK)
Consortium|Haldenby|United Kingdom
>Spike|hCoV-19/Kenya/C5119/2020|2020-05-09|EPI_ISL_568750|Original|hCoV-
19^^Mombasa|Human|KEMRI-Wellcome Trust Research Programme/KEMRI-CGMR-C Kilifi|KEMRI-
Wellcome Trust Research Programme/KEMRI-CGMR-C Kilifi|Githinji|Kenya
>Spike|hCoV-19/Australia/QLD1261/2020|2020-03-24|EPI_ISL_693263|Original|hCoV-
19^^Australia|Human|unknown|Public Health Virology Laboratory|Nguyen|Australia
>Spike|hCoV-19/Spain/RI-IBV-99005926/2020|2020-03-12|EPI_ISL_537462|Original|hCoV-
19^^La Rioja|Human|Centro de Investigaci\u00f3n Biom\u00e9dica de La Rioja - Hospital
San Pedro Logro\u00f1o|SeqCOVID-SPAIN consortium/IBV(CSIC)|Comas|Spain
>Spike|hCoV-19/Brazil/RS-6190/2020|2020-05-11|EPI_ISL_729826|Original|hCoV-19^^Rio
Grande do Sul|Human|Laboratorio Central de Saude Publica do Estado do Rio Grande do
Sul (LACEN-RS)|Laboratory of Respiratory Viruses and Measles|Silva|Brazil
>Spike|hCoV-19/Sweden/20-14827/2020|2020-05-19|EPI_ISL_469063|Original|hCoV-
19^^Uppsala|Human|Ulltuna Vardcentral|The Public Health Agency of
Sweden|Svartstr\u00f6m|Sweden
>Spike|hCoV-19/USA/NY-Wadsworth-278037-01/2020|2020-11-
22|EPI_ISL_754596|Original|hCoV-19^^New York|Human|Wadsworth Center|Wadsworth
Center|Lasek-Nesselquist|USA

>Spike|hCoV-19/England/ALDP-AA2BAB/2020|2020-10-22|EPI_ISL_623979|Original|hCoV-19^^England|Human|Lighthouse Lab in Alderley Park|Wellcome Sanger Institute for the COVID-19 Genomics UK (COG-UK) consortium|Davies|United Kingdom
>Spike|hCoV-19/Switzerland/GR-ETHZ-100150/2020|2020-03-16|EPI_ISL_451734|Original|hCoV-19^^Graubunden|Human|Viollier AG|Department of Biosystems Science and Engineering|Nadeau|Switzerland
>Spike|hCoV-19/Spain/VC-IBV-98003554/2020|2020-03-30|EPI_ISL_537976|Original|hCoV-19^^Comunitat Valenciana|Human|Servicio de Microbiolog\u00eda. Hospital General Universitario de Castell\u00f3n|SeqCOVID-SPAIN consortium/IBV(CSIC)|Comas|Spain
>Spike|hCoV-19/Iran/K1r-139/2020|2020-06-00|EPI_ISL_767842|Original|hCoV-19^^Iran|Human|Virology|Virology|Ranjbar|Iran
>Spike|hCoV-19/Russia/OMS-ORINFI-9781S/2020|2020-05-21|EPI_ISL_639946|Original|hCoV-19^^Omsk|Human|Omsk Research Institute of Natural Focal Infections|WHO National Influenza Centre Russian Federation|Komissarov|Russia
>Spike|hCoV-19/Denmark/ALAB-SSI-905/2020|2020-03-30|EPI_ISL_444967|Original|hCoV-19^^Denmark|Human|Department of Virus and Microbiological Special Diagnostics|Albertsen lab|Kirkegaard|Denmark
>Spike|hCoV-19/USA/TX-HMH-MCoV-11521/2020|2020-09-01|EPI_ISL_790042|Original|hCoV-19^^Texas|Human|Houston Methodist Hospital|Houston Methodist Hospital|Long|USA
>Spike|hCoV-19/Netherlands/NA_418/2020|2020-03-28|EPI_ISL_455220|Original|hCoV-19^^Netherlands|Human|Dutch COVID-19 response team|Erasmus Medical Center|Oude Munnink|Netherlands
>Spike|hCoV-19/England/204491148/2020|2020-10-31|EPI_ISL_660766|Original|hCoV-19^^England|Human|Respiratory Virus Unit|COVID-19 Genomics UK (COG-UK) Consortium|Platt|United Kingdom
>Spike|hCoV-19/England/ALDP-925A2C/2020|2020-06-23|EPI_ISL_552801|Original|hCoV-19^^England|Human|Lighthouse Lab in Alderley Park|Wellcome Sanger Institute for the COVID-19 Genomics UK (COG-UK) consortium|Davies|United Kingdom
>Spike|hCoV-19/Denmark/DCGC-16959/2020|2020-12-14|EPI_ISL_750667|Original|hCoV-19^^Midtjylland|Human|Department of Virus and Microbiological Special Diagnostics|Albertsen Lab|Michaelsen|Denmark
>Spike|hCoV-19/Denmark/DCGC-21038/2020|2020-12-21|EPI_ISL_759519|Original|hCoV-19^^Syddanmark|Human|Department of Virus and Microbiological Special Diagnostics|Albertsen Lab|Michaelsen|Denmark
>Spike|hCoV-19/Spain/PV-IBV-000902/2020|2020-03-26|EPI_ISL_452664|Original|hCoV-19^^Basque Country|Human|Servicio de Microbiolog\u00eda. Hospital Universitario Donostia. OSI Donostialdea. \u00c1rea de Enfermedades Infecciosas|SeqCOVID-SPAIN consortium/IBV(CSIC)|Comas|Spain
>Spike|hCoV-19/England/ALDP-B5B760/2020|2020-11-09|EPI_ISL_655440|Original|hCoV-19^^England|Human|Lighthouse Lab in Alderley Park|Wellcome Sanger Institute for the COVID-19 Genomics UK (COG-UK) Consortium|Davies|United Kingdom
>Spike|hCoV-19/England/MILK-9C27C0/2020|2020-09-08|EPI_ISL_566494|Original|hCoV-19^^England|Human|Lighthouse Lab in Milton Keynes|Wellcome Sanger Institute for the COVID-19 Genomics UK (COG-UK) consortium|Davies|United Kingdom
>Spike|hCoV-19/Northern Ireland/NIRE-107220/2020|2020-03-30|EPI_ISL_585126|Original|hCoV-19^^Northern Ireland|Human|Regional Virus Laboratory|COVID-19 Genomics UK (COG-UK) Consortium|Fairley|United Kingdom
>Spike|hCoV-19/England/MILK-B55FE6/2020|2020-11-05|EPI_ISL_658297|Original|hCoV-19^^England|Human|Lighthouse Lab in Milton Keynes|Wellcome Sanger Institute for the COVID-19 Genomics UK (COG-UK) Consortium|Davies|United Kingdom
>Spike|hCoV-19/England/CAMB-1AEA6F/2020|2020-04-27|EPI_ISL_524585|Original|hCoV-19^^England|Human|Department of Pathology|Wellcome Sanger Institute for the COVID-19 Genomics UK (COG-UK) consortium|Davies|United Kingdom
>Spike|hCoV-19/Scotland/QEUH-96E984/2020|2020-08-19|EPI_ISL_534474|Original|hCoV-19^^Scotland|Human|NHSGGC West of Scotland Specialist Virology Centre / MRC-University of Glasgow Centre for Virus Research|Wellcome Sanger Institute for the COVID-19 Genomics UK (COG-UK) consortium|Davies|United Kingdom
>Spike|hCoV-19/Japan/PG-8683/2020|2020-09-00|EPI_ISL_686279|Original|hCoV-19^^Japan|Human|Pathogen Genomics Center|Pathogen Genomics Center|Sekizuka|Japan

>Spike|hCoV-19/Japan/PG-2638/2020|2020-04-00|EPI_ISL_690770|Original|hCoV-19^^Japan|Human|Pathogen Genomics Center|Pathogen Genomics Center|Sekizuka|Japan
>Spike|hCoV-19/USA/NY-NYCPHL-001556/2020|2020-12-02|EPI_ISL_745466|Original|hCoV-19^^New York|Human|DOHMH Corona|New York City Public Health Laboratory|Wang|USA
>Spike|hCoV-19/Ireland/WW-NVRL-20Z06466/2020|2020-04-17|EPI_ISL_671393|Original|hCoV-19^^Wicklow|Human|National Virus Reference Laboratory|Irish Coronavirus Sequencing Consortium - Teagasc Moorepark|Walsh|Ireland
>Spike|hCoV-19/Finland/15A50S5/2020|2020-04-15|EPI_ISL_481558|Original|hCoV-19^^Uusimaa|Human|Department of Virology and Immunology|Department of Virology|Smura|Finland
>Spike|hCoV-19/England/ALDP-A51C3F/2020|2020-10-07|EPI_ISL_598058|Original|hCoV-19^^England|Human|Lighthouse Lab in Alderley Park|Wellcome Sanger Institute for the COVID-19 Genomics UK (COG-UK) consortium|Davies|United Kingdom
>Spike|hCoV-19/England/QEUH-9F5023/2020|2020-09-27|EPI_ISL_638648|Original|hCoV-19^^England|Human|Department of Pathology|COVID-19 Genomics UK (COG-UK) Consortium|Goodfellow|United Kingdom
>Spike|hCoV-19/Canada/BC-BCCDC-8565/2020|2020-11-00|EPI_ISL_740824|Original|hCoV-19^^British Columbia|Human|BCCDC Public Health Laboratory|BCCDC Public Health Laboratory|Prystajecky|Canada
>Spike|hCoV-19/England/CAMB-7EEB8/2020|2020-04-07|EPI_ISL_442827|Original|hCoV-19^^England|Human|Department of Pathology|Wellcome Sanger Institute for the COVID-19 Genomics UK (COG-UK) consortium|Amato|United Kingdom
>Spike|hCoV-19/Switzerland/GE-SNRCI-3576/2020|2020-03-00|EPI_ISL_548169|Original|hCoV-19^^Geneve|Human|Laboratoire de Virologie|Swiss National Reference Centre for Influenza|Gon\u00e7alves Cabecinhas|Switzerland
>Spike|hCoV-19/England/ALDP-B848E3/2020|2020-11-14|EPI_ISL_675988|Original|hCoV-19^^England|Human|Lighthouse Lab in Alderley Park|Wellcome Sanger Institute for the COVID-19 Genomics UK (COG-UK) Consortium|Davies|United Kingdom
>Spike|hCoV-19/Australia/VIC14419/2020|2020-08-14|EPI_ISL_592493|Original|hCoV-19^^Victoria|Human|Victorian Infectious Diseases Reference Laboratory (VIDRL)|VIDRL and MDU-PHL|Schultz|Australia
>Spike|hCoV-19/Spain/NC-IBV-001362/2020|2020-03-10|EPI_ISL_452539|Original|hCoV-19^^Navarra|Human|Cl\u00ednica Universidad de Navarra. Servicio de Enfermedades Infecciosas y Microbiolog\u00eda cl\u00ednica|SeqCOVID-SPAIN consortium/IBV(CSIC)|Comas|Spain
>Spike|hCoV-19/Australia/QLD1027/2020|2020-08-01|EPI_ISL_530280|Original|hCoV-19^^Queensland|Human|Queensland Health Forensic and Scientific Services|Public Health Virology Laboratory|Nguyen|Australia
>Spike|hCoV-19/USA/WA-UW-3918/2020|2020-03-27|EPI_ISL_427209|Original|hCoV-19^^Washington|Human|UW Virology Lab|UW Virology Lab|Roychoudhury|USA
>Spike|hCoV-19/England/QEUH-9B8F4D/2020|2020-09-09|EPI_ISL_549583|Original|hCoV-19^^England|Human|Lighthouse Lab in Glasgow|Wellcome Sanger Institute for the COVID-19 Genomics UK (COG-UK) consortium|Davies|United Kingdom
>Spike|hCoV-19/England/QEUH-A89BE6/2020|2020-10-16|EPI_ISL_638814|Original|hCoV-19^^England|Human|Oxford Viromics|COVID-19 Genomics UK (COG-UK) Consortium|Golubchik|United Kingdom
>Spike|hCoV-19/India/TG-CCMB-R372/2020|2020-08-09|EPI_ISL_539648|Original|hCoV-19^^Telangana|Human|CSIR-Centre for Cellular and Molecular Biology|CSIR-Centre for Cellular and Molecular Biology|Mukherjee|India
>Spike|hCoV-19/USA/FL-04-UMTL-975H/2020|2020-04-07|EPI_ISL_479762|Original|hCoV-19^^Florida|Human|University of Miami Immunology and Histocompatibility Laboratory|University of Miami Immunology and Histocompatibility Laboratory|Margolles-Clark|USA
>Spike|hCoV-19/England/MILK-BB1D1B/2020|2020-11-17|EPI_ISL_720442|Original|hCoV-19^^England|Human|Lighthouse Lab in Milton Keynes|Wellcome Sanger Institute for the COVID-19 Genomics UK (COG-UK) Consortium|Davies|United Kingdom
>Spike|hCoV-19/England/QEUH-9603FD/2020|2020-08-05|EPI_ISL_531965|Original|hCoV-19^^England|Human|Lighthouse Lab in Glasgow|Wellcome Sanger Institute for the COVID-19 Genomics UK (COG-UK) consortium|Davies|United Kingdom

>Spike|hCoV-19/Canada/QC-LSPQ-L00232080/2020|2020-03-22|EPI_ISL_535974|Original|hCoV-19^^Quebec|Human|H\u00f4pital du Centre-de-la-Mauricie|Laboratoire de sant\u00e9 publique du Qu\u00e9bec|Moreira|Canada
>Spike|hCoV-19/Denmark/DCGC-10350/2020|2020-11-02|EPI_ISL_669919|Original|hCoV-19^^Syddanmark|Human|Department of Virus and Microbiological Special Diagnostics|Albertsen Lab|Michaelsen|Denmark
>Spike|hCoV-19/England/ALDP-AA1438/2020|2020-10-22|EPI_ISL_623897|Original|hCoV-19^^England|Human|Lighthouse Lab in Alderley Park|Wellcome Sanger Institute for the COVID-19 Genomics UK (COG-UK) consortium|Davies|United Kingdom
>Spike|hCoV-19/Sweden/20-50120/2020|2020-03-04|EPI_ISL_469076|Original|hCoV-19^^Uppsala|Human|Uppsala klinisk mikrobiologi|The Public Health Agency of Sweden|Svartstr\u00f6m|Sweden
>Spike|hCoV-19/England/MILK-B8F1A1/2020|2020-11-13|EPI_ISL_675131|Original|hCoV-19^^England|Human|Lighthouse Lab in Milton Keynes|Wellcome Sanger Institute for the COVID-19 Genomics UK (COG-UK) Consortium|Davies|United Kingdom
>Spike|hCoV-19/Norway/4545/2020|2020-10-07|EPI_ISL_635171|Original|hCoV-19^^Vestfold og Telemark|Human|Vestfold Hospital|Norwegian Institute of Public Health|Vollan|Norway
>Spike|hCoV-19/Australia/VIC1975/2020|2020-06-05|EPI_ISL_521894|Original|hCoV-19^^Victoria|Human|Victorian Infectious Diseases Reference Laboratory (VIDRL)|VIDRL and MDU-PHL|Schultz|Australia
>Spike|hCoV-19/Italy/TE.237004/2020|2020-11-04|EPI_ISL_730653|Original|hCoV-19^^Apulia|Human|University of Bari|Istituto Zooprofilattico Sperimentale dell'Abruzzo e del Molise \G. Caporale\.|De Sabato|Italy
>Spike|hCoV-19/England/NORW-F9820/2020|2020-12-02|EPI_ISL_704926|Original|hCoV-19^^England|Human|Quadram Institute Bioscience|COVID-19 Genomics UK (COG-UK) Consortium|Alikhan|United Kingdom
>Spike|hCoV-19/USA/MI-UM-10036613887/2020|2020-10-25|EPI_ISL_650006|Original|hCoV-19^^Michigan|Human|University of Michigan Clinical Microbiology Laboratory|Lauring Lab|Valesano|USA
>Spike|hCoV-19/Denmark/ALAB-SSI-1262/2020|2020-03-09|EPI_ISL_444834|Original|hCoV-19^^Denmark|Human|Department of Virus and Microbiological Special Diagnostics|Albertsen lab|Kirkegaard|Denmark
>Spike|hCoV-19/England/QEUH-A61E90/2020|2020-10-10|EPI_ISL_628657|Original|hCoV-19^^England|Human|Oxford Viromics|COVID-19 Genomics UK (COG-UK) Consortium|Golubchik|United Kingdom
>Spike|hCoV-19/Australia/TAS249/2020|2020-04-22|EPI_ISL_522676|Original|hCoV-19^^Tasmania|Human|Royal Hobart Hospital Microbiology Department|MDU-PHL|Schultz|Australia
>Spike|hCoV-19/Northern Ireland/NIRE-FC90C/2020|2020-04-22|EPI_ISL_459340|Original|hCoV-19^^Northern Ireland|Human|Regional Virus Laboratory|Wellcome Sanger Institute for the COVID-19 Genomics UK (COG-UK) consortium|Davies|United Kingdom
>Spike|hCoV-19/Chile/MA-ISPCH-4/2020|2020-03-19|EPI_ISL_445282|Original|hCoV-19^^Magallanes|Human|CLINICA MAGALLANES S.A.|Instituto de Salud Publica de Chile|Fernandez|Chile
>Spike|hCoV-19/USA/CA-CHLA-PLM54870960/2020|2020-12-09|EPI_ISL_753664|Original|hCoV-19^^California|Human|Clinical virology Laboratory|Center for Personalized Medicine|Dien Bard|USA
>Spike|hCoV-19/Netherlands/NH-EMC-69/2020|2020-09-11|EPI_ISL_577865|Original|hCoV-19^^Noord Holland|Human|Dutch COVID-19 response team|Erasmus Medical Center|Oude Munnink|Netherlands
>Spike|hCoV-19/England/QEUH-B7C08C/2020|2020-11-14|EPI_ISL_668312|Original|hCoV-19^^England|Human|Lighthouse Lab in Glasgow|Wellcome Sanger Institute for the COVID-19 Genomics UK (COG-UK) Consortium|Davies|United Kingdom
>Spike|hCoV-19/Japan/PG-4036/2020|2020-07-00|EPI_ISL_692277|Original|hCoV-19^^Japan|Human|Pathogen Genomics Center|Pathogen Genomics Center|Sekizuka|Japan
>Spike|hCoV-19/England/PORT-2EA44A/2020|2020-00-00|EPI_ISL_665698|Original|hCoV-19^^England|Human|Centre for Enzyme Innovation|COVID-19 Genomics UK (COG-UK) Consortium|Robson|United Kingdom

>Spike|hCoV-19/USA/NY-NYCPHL-001374/2020|2020-11-16|EPI_ISL_683930|Original|hCoV-19^^New York|Human|DOHMH Jamaica|New York City Public Health Laboratory|Wang|USA
>Spike|hCoV-19/Canada/ON-S10/2020|2020-03-14|EPI_ISL_586278|Original|hCoV-19^^Ontario|Human|Toronto Invasive Bacterial Diseases Network|McMaster University|McArthur|Canada
>Spike|hCoV-19/Canada/QC-LSPQ-L00237312/2020|2020-03-25|EPI_ISL_536173|Original|hCoV-19^^Quebec|Human|H\u00f4pital Pierre-Boucher|Laboratoire de sant\u00e9 publique du Qu\u00e9bec|Moreira|Canada
>Spike|hCoV-19/Colombia/DC-INS-093/2020|2020-06-16|EPI_ISL_526968|Original|hCoV-19^^Bogota|Human|Instituto Nacional de Salud|Instituto Nacional de Salud|Franco-Mu\u00f1oz|Colombia
>Spike|hCoV-19/England/204642475/2020|2020-11-11|EPI_ISL_700685|Original|hCoV-19^^England|Human|Respiratory Virus Unit|COVID-19 Genomics UK (COG-UK) Consortium|Platt|United Kingdom
>Spike|hCoV-19/USA/NY-Wadsworth-102659-01/2020|2020-05-15|EPI_ISL_676936|Original|hCoV-19^^New York|Human|Wadsworth Center|Wadsworth Center|Lasek-Nesselquist|USA
>Spike|hCoV-19/Denmark/DCGC-12502/2020|2020-11-16|EPI_ISL_682683|Original|hCoV-19^^Hovedstaden|Human|Department of Virus and Microbiological Special Diagnostics|Albertsen Lab|Michaelsen|Denmark
>Spike|hCoV-19/USA/OR-OHSU-1981/2020|2020-07-30|EPI_ISL_526082|Original|hCoV-19^^Oregon|Human|OHSU Lab Services Molecular Microbiology Lab|Oregon SARS-CoV-2 Genome Sequencing Center|O'Roak|USA
>Spike|hCoV-19/England/20166000104/2020|2020-04-15|EPI_ISL_466492|Original|hCoV-19^^England|Human|Respiratory Virus Unit|Respiratory Virus Unit|Platt|United Kingdom
>Spike|hCoV-19/USA/NY-Wadsworth-292056-01/2020|2020-12-20|EPI_ISL_767621|Original|hCoV-19^^New York|Human|WHITE PLAINS HOSPITAL CENTER LABORATORY|Wadsworth Center|Lasek-Nesselquist|USA
>Spike|hCoV-19/USA/MA-MGH-01110/2020|2020-05-01|EPI_ISL_791878|Original|hCoV-19^^Massachusetts|Human|Massachusetts General Hospital|Infectious Disease Program|Park|USA
>Spike|hCoV-19/England/NORT-28B153/2020|2020-04-17|EPI_ISL_469314|Original|hCoV-19^^England|Human|NU-OMICS DNA Sequencing research facility|Wellcome Sanger Institute for the COVID-19 Genomics UK (COG-UK) consortium|Davies|United Kingdom
>Spike|hCoV-19/England/LIVE-9FF81/2020|2020-06-08|EPI_ISL_472077|Original|hCoV-19^^England|Human|Liverpool Clinical Laboratories|COVID-19 Genomics UK (COG-UK) Consortium|Haldenby|United Kingdom
>Spike|hCoV-19/England/CAMB-1B2CDB/2020|2020-05-19|EPI_ISL_456719|Original|hCoV-19^^England|Human|Department of Pathology|COVID-19 Genomics UK (COG-UK) Consortium|Meredith|United Kingdom
>Spike|hCoV-19/England/ALDP-95C084/2020|2020-06-12|EPI_ISL_554890|Original|hCoV-19^^England|Human|Lighthouse Lab in Alderley Park|Wellcome Sanger Institute for the COVID-19 Genomics UK (COG-UK) consortium|Davies|United Kingdom
>Spike|hCoV-19/England/QEUH-B67B74/2020|2020-11-11|EPI_ISL_661556|Original|hCoV-19^^England|Human|Lighthouse Lab in Glasgow|Wellcome Sanger Institute for the COVID-19 Genomics UK (COG-UK) Consortium|Davies|United Kingdom
>Spike|hCoV-19/USA/CA-ALSR-0820-SAN/2020|2020-04-02|EPI_ISL_483380|Original|hCoV-19^^California|Human|UC San Diego Center for Advanced Laboratory Medicine|Andersen lab at Scripps Research|Gangavarapu|USA
>Spike|hCoV-19/Denmark/DCGC-17177/2020|2020-12-14|EPI_ISL_750804|Original|hCoV-19^^Midtjylland|Human|Department of Virus and Microbiological Special Diagnostics|Albertsen Lab|Michaelsen|Denmark
>Spike|hCoV-19/USA/MN-MDH-1457/2020|2020-06-24|EPI_ISL_515266|Original|hCoV-19^^Minnesota|Human|Mayo Clinic & Mayo Clinic Laboratories|Minnesota Department of Health|Garfin|USA
>Spike|hCoV-19/USA/NY-PV09006/2020|2020-03-20|EPI_ISL_421593|Original|hCoV-19^^New York|Human|MSHS Clinical Microbiology Laboratories|MSHS Pathogen Surveillance Program|van Bakel|USA

>Spike|hCoV-19/Switzerland/BS-UHB-42201217/2020|2020-03-
21|EPI_ISL_528282|Original|hCoV-19^^Basel-Stadt|Human|University Hospital
Basel|University Hospital Basel|Roloff Handschin|Switzerland
>Spike|hCoV-19/Egypt/EGY-045/2020|2020-08-22|EPI_ISL_527001|Original|hCoV-
19^^Egypt|Human|Biological prevention|Biological prevention|Seadawy|Egypt
>Spike|hCoV-19/Shanghai/SH0119/2020|2020-02-09|EPI_ISL_416404|Original|hCoV-
19^^Shanghai|Human|Shanghai Public Health Clinical Center|National Research Center
for Translational Medicine (Shanghai)|Zhang|China
>Spike|hCoV-19/USA/CA-CZB-15494/2020|2020-09-22|EPI_ISL_739017|Original|hCoV-
19^^California|Human|County of San Luis Obispo Public Health Laboratory|Chan-
Zuckerberg Biohub|Kamm|USA
>Spike|hCoV-19/England/QEUH-9B4907/2020|2020-09-02|EPI_ISL_590413|Original|hCoV-
19^^England|Human|Lighthouse Lab in Glasgow|Wellcome Sanger Institute for the COVID-
19 Genomics UK (COG-UK) consortium|Davies|United Kingdom
>Spike|hCoV-19/England/CAMC-B32E3D/2020|2020-11-05|EPI_ISL_659347|Original|hCoV-
19^^England|Human|Lighthouse Lab in Cambridge|Wellcome Sanger Institute for the
COVID-19 Genomics UK (COG-UK) Consortium|Davies|United Kingdom
>Spike|hCoV-19/Japan/PG-3864/2020|2020-07-00|EPI_ISL_692115|Original|hCoV-
19^^Japan|Human|Pathogen Genomics Center|Pathogen Genomics Center|Sekizuka|Japan
>Spike|hCoV-19/England/NORW-EE86A/2020|2020-00-00|EPI_ISL_549359|Original|hCoV-
19^^England|Human|Quadram Institute Bioscience|COVID-19 Genomics UK (COG-UK)
Consortium|Alikhan|United Kingdom
>Spike|hCoV-19/England/ALDP-AA3FEA/2020|2020-10-22|EPI_ISL_630809|Original|hCoV-
19^^England|Human|Lighthouse Lab in Alderley Park|Wellcome Sanger Institute for the
COVID-19 Genomics UK (COG-UK) consortium|Davies|United Kingdom
>Spike|hCoV-19/England/NORT-1B72496/2020|2020-11-30|EPI_ISL_724883|Original|hCoV-
19^^England|Human|Northumbria University / South Tees Hospitals NHS Foundation Trust
/ North Cumbria Integrated Care NHS Foundation Trust / North Tees and Hartlepool NHS
Foundation Trust / Newcastle Hospitals NHS Foundation Trust|COVID-19 Genomics UK
(COG-UK) Consortium|Bashton|United Kingdom
>Spike|hCoV-19/Spain/GA-IBV-002727/2020|2020-04-15|EPI_ISL_467276|Original|hCoV-
19^^Galicia|Human|Hospital Cl\u00ednico Universitario de Santiago de
Compostela|SeqCOVID-SPAIN consortium/IBV(CSIC)|Comas|Spain
>Spike|hCoV-19/USA/CO-CDPHE-2008170014/2020|2020-08-16|EPI_ISL_677302|Original|hCoV-
19^^Colorado|Human|Colorado Department of Public Health and Environment|Colorado
Department of Puplic Health and Environment|Bankers|USA
>Spike|hCoV-19/Switzerland/ZH-ETHZ-350458/2020|2020-11-
03|EPI_ISL_729206|Original|hCoV-19^^Zurich|Human|Viollier AG|Department of Biosystems
Science and Engineering|Nadeau|Switzerland
>Spike|hCoV-19/England/CAMC-B78879/2020|2020-11-12|EPI_ISL_783437|Original|hCoV-
19^^England|Human|Lighthouse Lab in Cambridge|Wellcome Sanger Institute for the
COVID-19 Genomics UK (COG-UK) Consortium|Davies|United Kingdom
>Spike|hCoV-19/England/QEUH-96B723/2020|2020-08-14|EPI_ISL_531350|Original|hCoV-
19^^England|Human|Lighthouse Lab in Glasgow|Wellcome Sanger Institute for the COVID-
19 Genomics UK (COG-UK) consortium|Davies|United Kingdom
>Spike|hCoV-19/Luxembourg/LNS8835096/2020|2020-11-29|EPI_ISL_770923|Original|hCoV-
19^^Luxembourg|Human|Laboratoire national de sant\u00e9|Laboratoire national de
sant\u00e9|Wienecke-Baldacchino|Luxembourg
>Spike|hCoV-19/Japan/PG-6384/2020|2020-08-00|EPI_ISL_689493|Original|hCoV-
19^^Japan|Human|Fukuoka Institute of Health and Environmental Sciences|Pathogen
Genomics Center|Sekizuka|Japan
>Spike|hCoV-19/Wales/PHWC-30CE5/2020|2020-04-12|EPI_ISL_446350|Original|hCoV-
19^^Wales|Human|Wales Specialist Virology Centre|Public Health Wales Microbiology
Cardiff|Moore|United Kingdom
>Spike|hCoV-19/England/GSTT-265D2D5/2020|2020-03-17|EPI_ISL_483937|Original|hCoV-
19^^England|Human|Centre for Clinical Infection and Diagnostics Research and Genomics
Innovation Unit|COVID-19 Genomics UK (COG-UK) Consortium|Awan|United Kingdom
>Spike|hCoV-19/Denmark/DCGC-19820/2020|2020-12-07|EPI_ISL_747809|Original|hCoV-
19^^Hovedstaden|Human|Department of Virus and Microbiological Special
Diagnostics|Albertsen Lab|Michaelsen|Denmark

>Spike|hCoV-19/Denmark/DCGC-21658/2020|2020-12-21|EPI_ISL_758291|Original|hCoV-19^^Hovedstaden|Human|Department of Virus and Microbiological Special Diagnostics|Albertsen Lab|Michaelsen|Denmark
>Spike|hCoV-19/England/ALDP-A693C6/2020|2020-10-12|EPI_ISL_608913|Original|hCoV-19^^England|Human|Lighthouse Lab in Alderley Park|Wellcome Sanger Institute for the COVID-19 Genomics UK (COG-UK) consortium|Davies|United Kingdom
>Spike|hCoV-19/England/CAMC-B548DD/2020|2020-11-09|EPI_ISL_658148|Original|hCoV-19^^England|Human|Lighthouse Lab in Cambridge|Wellcome Sanger Institute for the COVID-19 Genomics UK (COG-UK) Consortium|Davies|United Kingdom
>Spike|hCoV-19/Denmark/DCGC-7294/2020|2020-10-12|EPI_ISL_619254|Original|hCoV-19^^Midtjylland|Human|Department of Virus and Microbiological Special Diagnostics|Albertsen lab|Michaelsen|Denmark
>Spike|hCoV-19/Japan/PG-6974/2020|2020-07-00|EPI_ISL_688079|Original|hCoV-19^^Japan|Human|Pathogen Genomics Center|Pathogen Genomics Center|Sekizuka|Japan
>Spike|hCoV-19/Canada/ON-PHL-20-02304/2020|2020-09-00|EPI_ISL_671549|Original|hCoV-19^^Ontario|Human|Public Health Ontario Laboratory|Public Health Ontario Laboratory|Guthrie|Canada
>Spike|hCoV-19/USA/TX-DSHS-0647/2020|2020-07-16|EPI_ISL_576302|Original|hCoV-19^^Texas|Human|Texas Department of State Health Services|Texas Department of State Health Services|Rahman|USA
>Spike|hCoV-19/Bangladesh/CHRF-0024/2020|2020-06-14|EPI_ISL_477134|Original|hCoV-19^^Bangladesh|Human|Child Health Research Foundation|Child Health Research Foundation|Saha|Bangladesh
>Spike|hCoV-19/England/MILK-A46AD9/2020|2020-10-06|EPI_ISL_598387|Original|hCoV-19^^England|Human|Lighthouse Lab in Milton Keynes|Wellcome Sanger Institute for the COVID-19 Genomics UK (COG-UK) consortium|Davies|United Kingdom
>Spike|hCoV-19/England/ALDP-9E8533/2020|2020-09-24|EPI_ISL_637714|Original|hCoV-19^^England|Human|Centre for Enzyme Innovation|COVID-19 Genomics UK (COG-UK) Consortium|Robson|United Kingdom
>Spike|hCoV-19/Malaysia/2063/2020|2020-03-26|EPI_ISL_501188|Original|hCoV-19^^Kuala Lumpur|Human|Department of Medical Microbiology|Department of Medical Microbiology|Chan|Malaysia
>Spike|hCoV-19/USA/WA-S1906/2020|2020-05-29|EPI_ISL_496332|Original|hCoV-19^^Washington|Human|Washington State Department of Health|Seattle Flu Study|Lee|USA
>Spike|hCoV-19/Wales/PHWC-486411/2020|2020-10-31|EPI_ISL_638092|Original|hCoV-19^^Wales|Human|Wales Specialist Virology Centre Sequencing lab|COVID-19 Genomics UK (COG-UK) Consortium|Moore|United Kingdom
>Spike|hCoV-19/Brazil/RS-L10-CD175/2020|2020-03-19|EPI_ISL_476188|Original|hCoV-19^^Rio Grande do Sul|Human|DB Diagn\u00f3sticos do Brasil|Instituto de Medicina Tropical da Univesidade de S\u00e3o Paulo|Faria|Brazil
>Spike|hCoV-19/Netherlands/NH-RIVM-20029/2020|2020-11-20|EPI_ISL_723226|Orignal|hCoV-19^^Noord-Holland|Human|Dutch COVID-19 response team|National Institute for Public Health and the Environment (RIVM)|Meijer|Netherlands
>Spike|hCoV-19/England/LIVE-DC2EE5/2020|2020-11-25|EPI_ISL_702605|Original|hCoV-19^^England|Human|Liverpool Clinical Laboratories|COVID-19 Genomics UK (COG-UK) Consortium|Haldenby|United Kingdom
>Spike|hCoV-19/England/QEUH-969D23/2020|2020-08-12|EPI_ISL_530808|Original|hCoV-19^^England|Human|Lighthouse Lab in Glasgow|Wellcome Sanger Institute for the COVID-19 Genomics UK (COG-UK) consortium|Davies|United Kingdom
>Spike|hCoV-19/England/ALDP-A95D1E/2020|2020-10-20|EPI_ISL_624807|Original|hCoV-19^^England|Human|Lighthouse Lab in Alderley Park|Wellcome Sanger Institute for the COVID-19 Genomics UK (COG-UK) consortium|Davies|United Kingdom
>Spike|hCoV-19/Denmark/DCGC-18244/2020|2020-12-14|EPI_ISL_749838|Original|hCoV-19^^Hovedstaden|Human|Department of Virus and Microbiological Special Diagnostics|Albertsen Lab|Michaelsen|Denmark
>Spike|hCoV-19/England/MILK-ACD418/2020|2020-10-21|EPI_ISL_634519|Original|hCoV-19^^England|Human|Lighthouse Lab in Milton Keynes|Wellcome Sanger Institute for the COVID-19 Genomics UK (COG-UK) consortium|Davies|United Kingdom
>Spike|hCoV-19/Australia/VIC762/2020|2020-03-29|EPI_ISL_427038|Original|hCoV-19^^Victoria|Human|Victorian Infectious Diseases Reference Laboratory

(VIDRL)|Microbiological Diagnostic Unit Public Health Laboratory and Victorian Infectious Diseases Reference Laboratory|Schultz|Australia
>Spike|hCoV-19/Scotland/QEUH-9FB676/2020|2020-09-29|EPI_ISL_600563|Original|hCoV-19^^Scotland|Human|Lighthouse Lab in Glasgow|Wellcome Sanger Institute for the COVID-19 Genomics UK (COG-UK) consortium|Davies|United Kingdom
>Spike|hCoV-19/USA/WA-UW-4224/2020|2020-03-31|EPI_ISL_437823|Original|hCoV-19^^Washington|Human|UW Virology Lab|UW Virology Lab|Roychoudhury|USA
>Spike|hCoV-19/England/MILK-995C9A/2020|2020-08-24|EPI_ISL_551661|Original|hCoV-19^^England|Human|Lighthouse Lab in Milton Keynes|Wellcome Sanger Institute for the COVID-19 Genomics UK (COG-UK) consortium|Davies|United Kingdom
>Spike|hCoV-19/USA/TX-HMH-MCoV-18150/2020|2020-11-14|EPI_ISL_784498|Original|hCoV-19^^Texas|Human|Houston Methodist Hospital|Houston Methodist Hospital|Long|USA
>Spike|hCoV-19/England/MILK-9E2250/2020|2020-09-20|EPI_ISL_581255|Original|hCoV-19^^England|Human|Lighthouse Lab in Milton Keynes|Wellcome Sanger Institute for the COVID-19 Genomics UK (COG-UK) consortium|Davies|United Kingdom
>Spike|hCoV-19/England/ALDP-92248B/2020|2020-06-23|EPI_ISL_553288|Original|hCoV-19^^England|Human|Lighthouse Lab in Alderley Park|Wellcome Sanger Institute for the COVID-19 Genomics UK (COG-UK) consortium|Davies|United Kingdom
>Spike|hCoV-19/England/QEUH-96D017/2020|2020-08-21|EPI_ISL_530707|Original|hCoV-19^^England|Human|Lighthouse Lab in Glasgow|Wellcome Sanger Institute for the COVID-19 Genomics UK (COG-UK) consortium|Davies|United Kingdom
>Spike|hCoV-19/India/AP-CCMB-BB648/2020|2020-12-25|EPI_ISL_745201|Original|hCoV-19^^Andhra Pradesh|Human|Guntur General Hospital|CSIR-Centre for Cellular and Molecular Biology|Banu|India
>Spike|hCoV-19/England/MILK-9AA5C4/2020|2020-08-30|EPI_ISL_550829|Original|hCoV-19^^England|Human|Lighthouse Lab in Milton Keynes|Wellcome Sanger Institute for the COVID-19 Genomics UK (COG-UK) consortium|Davies|United Kingdom
>Spike|hCoV-19/England/LIVE-1DDE98/2020|2020-10-09|EPI_ISL_611635|Original|hCoV-19^^England|Human|Liverpool Clinical Laboratories|COVID-19 Genomics UK (COG-UK) Consortium|Haldenby|United Kingdom
>Spike|hCoV-19/Belarus/ChVir2072/2020|2020-03-00|EPI_ISL_419692|Original|hCoV-19^^Belarus|Human|The Republican Research and Practical Center for Epidemiology and Microbiology|Charite\u0301 Universita\u0308tsmedizin Berlin|Muehlemann|Belarus
>Spike|hCoV-19/England/LIVE-A58AB/2020|2020-03-20|EPI_ISL_499612|Original|hCoV-19^^England|Human|Liverpool Clinical Laboratories|COVID-19 Genomics UK (COG-UK) Consortium|Haldenby|United Kingdom
>Spike|hCoV-19/Switzerland/UR-ETHZ-321385/2020|2020-10-13|EPI_ISL_603628|Original|hCoV-19^^Uri|Human|Viollier AG|Department of Biosystems Science and Engineering|Nadeau|Switzerland
>Spike|hCoV-19/Japan/PG-2150/2020|2020-04-00|EPI_ISL_685755|Original|hCoV-19^^Japan|Human|Pathogen Genomics Center|Pathogen Genomics Center|Sekizuka|Japan
>Spike|hCoV-19/USA/TX-HMH-5103/2020|2020-06-24|EPI_ISL_544526|Original|hCoV-19^^Texas|Human|Houston Methodist Hospital|Houston Methodist Hospital|Long|USA
>Spike|hCoV-19/England/BIRM-6B3A0/2020|2020-11-14|EPI_ISL_679128|Original|hCoV-19^^England|Human|University of Birmingham|COVID-19 Genomics UK (COG-UK) Consortium|Loman|United Kingdom
>Spike|hCoV-19/England/NOTT-113E69/2020|2020-10-01|EPI_ISL_577118|Original|hCoV-19^^England|Human|Queens Medical Centre|COVID-19 Genomics UK (COG-UK) Consortium|Loose|United Kingdom
>Spike|hCoV-19/England/PORT-2D1BD8/2020|2020-04-02|EPI_ISL_507133|Original|hCoV-19^^England|Human|Centre for Enzyme Innovation|COVID-19 Genomics UK (COG-UK) Consortium|Robson|United Kingdom
>Spike|hCoV-19/Spain/CT-IBV-003167/2020|2020-03-16|EPI_ISL_510263|Original|hCoV-19^^Catalunya|Human|Hospital de la Santa Creu i Sant Pau. Servicio de Microbiolog\u00eda|SeqCOVID-SPAIN consortium/IBV(CSIC)|Comas|Spain
>Spike|hCoV-19/Guangdong/2020XN4243-P0035/2020|2020-01-30|EPI_ISL_413853|Original|hCoV-19^^Guangdong|Human|Guangdong Provincial Institution of Public Health|Guangdong Provincial Institution of Public Health|Lu|China

>Spike|hCoV-19/England/MILK-ACED67/2020|2020-10-21|EPI_ISL_629379|Original|hCoV-19^^England|Human|Lighthouse Lab in Milton Keynes|Wellcome Sanger Institute for the COVID-19 Genomics UK (COG-UK) consortium|Davies|United Kingdom
>Spike|hCoV-19/Australia/VIC8107/2020|2020-08-18|EPI_ISL_564853|Original|hCoV-19^^Victoria|Human|Microbiological Diagnostic Unit - Public Health Laboratory (MDU-PHL)|MDU-PHL|Schultz|Australia
>Spike|hCoV-19/Denmark/DCGC-18965/2020|2020-12-07|EPI_ISL_748670|Original|hCoV-19^^Hovedstaden|Human|Department of Virus and Microbiological Special Diagnostics|Albertsen Lab|Michaelsen|Denmark
>Spike|hCoV-19/England/ALDP-5A94AE/2020|2020-06-14|EPI_ISL_558704|Original|hCoV-19^^England|Human|Lighthouse Lab in Alderley Park|Wellcome Sanger Institute for the COVID-19 Genomics UK (COG-UK) consortium|Davies|United Kingdom
>Spike|hCoV-19/England/CAMC-B0853B/2020|2020-10-30|EPI_ISL_643535|Original|hCoV-19^^England|Human|Lighthouse Lab in Cambridge|Wellcome Sanger Institute for the COVID-19 Genomics UK (COG-UK) Consortium|Davies|United Kingdom
>Spike|hCoV-19/Australia/NSW58/2020|2020-03-16|EPI_ISL_417410|Original|hCoV-19^^New South Wales|Human|Centre for Infectious Diseases and Microbiology Public Health|NSW Health Pathology - Institute of Clinical Pathology and Medical Research; Westmead Hospital; University of Sydney|Eden|Australia
>Spike|hCoV-19/England/MILK-A7ADF1/2020|2020-10-16|EPI_ISL_606638|Original|hCoV-19^^England|Human|Lighthouse Lab in Milton Keynes|Wellcome Sanger Institute for the COVID-19 Genomics UK (COG-UK) consortium|Davies|United Kingdom
>Spike|hCoV-19/Gambia/NPHL-2444/2020|2020-07-21|EPI_ISL_561249|Original|hCoV-19^^Kombo|Human|MRCG at LSHTM Genomics lab|MRCG at LSHTM Genomics lab|Sesay|Gambia
>Spike|hCoV-19/Netherlands/GE-RIVM-20207/2020|2020-11-25|EPI_ISL_723360|Orignal|hCoV-19^^Gelderland|Human|Dutch COVID-19 response team|National Institute for Public Health and the Environment (RIVM)|Meijer|Netherlands
>Spike|hCoV-19/Faroe Islands/HFS-28/2020|2020-03-15|EPI_ISL_526942|Original|hCoV-19^^Faroe Islands|Human|Faroese National Reference Laboratory for Fish and Animal Diseases|Faroese National Reference Laboratory for Fish and Animal Diseases|Dahl|Faroe Islands
>Spike|hCoV-19/Portugal/PT0925/2020|2020-04-25|EPI_ISL_511182|Original|hCoV-19^^Portugal|Human|Instituto Nacional de Saude (INSA) and Instituto Gulbenkian de Ciencia (IGC)|Instituto Nacional de Saude (INSA) and Instituto Gulbenkian de Ciencia (IGC)|Borges|Portugal
>Spike|hCoV-19/USA/AK-PHL660/2020|2020-06-27|EPI_ISL_586252|Original|hCoV-19^^Alaska|Human|Alaska State Virology Laboratory|Alaska State Virology Laboratory|Chen|USA
>Spike|hCoV-19/USA/TX-DSHS-0064/2020|2020-05-09|EPI_ISL_517995|Original|hCoV-19^^Texas|Human|Texas Department of State Health Services|Texas Department of State Health Services|Rahman|USA
>Spike|hCoV-19/Scotland/QEUH-9CE3A2/2020|2020-09-16|EPI_ISL_601876|Original|hCoV-19^^Scotland|Human|Lighthouse Lab in Glasgow|Wellcome Sanger Institute for the COVID-19 Genomics UK (COG-UK) consortium|Davies|United Kingdom
>Spike|hCoV-19/USA/WA-UW-352/2020|2020-03-24|EPI_ISL_524441|Original|hCoV-19^^Washington|Human|University of Washington Virology Lab|Laboratory Medicine|Roychoudhury|USA
>Spike|hCoV-19/Denmark/DCGC-8092/2020|2020-10-26|EPI_ISL_617190|Original|hCoV-19^^Sjaelland|Human|Department of Virus and Microbiological Special Diagnostics|Albertsen lab|Michaelsen|Denmark
>Spike|hCoV-19/England/204161234/2020|2020-10-08|EPI_ISL_609854|Original|hCoV-19^^England|Human|Respiratory Virus Unit|Respiratory Virus Unit|Platt|United Kingdom
>Spike|hCoV-19/USA/TX-HMH-2865/2020|2020-06-08|EPI_ISL_543835|Original|hCoV-19^^Texas|Human|Houston Methodist Hospital|Houston Methodist Hospital|Long|USA
>Spike|hCoV-19/England/NOTT-121777/2020|2020-12-20|EPI_ISL_741653|Original|hCoV-19^^England|Human|Queens Medical Centre|COVID-19 Genomics UK (COG-UK) Consortium|Loose|United Kingdom
>Spike|hCoV-19/USA/CA-CZB-13023/2020|2020-05-11|EPI_ISL_672307|Original|hCoV-19^^California|Human|Alameda County Public Health Lab|Chan-Zuckerberg Biohub|Bhatt|USA

>Spike|hCoV-19/Switzerland/BE-ETHZ-190065/2020|2020-07-09|EPI_ISL_500925|Original|hCoV-19^^Bern|Human|Viollier AG|Department of Biosystems Science and Engineering|Nadeau|Switzerland
>Spike|hCoV-19/Scotland/QEUH-B7F2DE/2020|2020-11-14|EPI_ISL_710851|Original|hCoV-19^^Scotland|Human|Lighthouse Lab in Glasgow|Wellcome Sanger Institute for the COVID-19 Genomics UK (COG-UK) Consortium|Davies|United Kingdom
>Spike|hCoV-19/Scotland/EDB8306/2020|2020-10-12|EPI_ISL_594845|Original|hCoV-19^^Scotland|Human|Virology Department|COVID-19 Genomics UK (COG-UK) Consortium|McHugh|United Kingdom
>Spike|hCoV-19/Belgium/ULG-10203/2020|2020-05-23|EPI_ISL_471437|Original|hCoV-19^^Liege|Human|Department of Clinical Microbiology|GIGA Medical Genomics|Durkin|Belgium
>Spike|hCoV-19/USA/CA-CSMC8/2020|2020-03-27|EPI_ISL_475695|Original|hCoV-19^^California|Human|Cedars-Sinai Medical Center|Cedars-Sinai Medical Center|Zhang|USA
>Spike|hCoV-19/England/NORT-289AE1/2020|2020-04-03|EPI_ISL_453512|Original|hCoV-19^^England|Human|Northumbria University / South Tees Hospitals NHS Foundation Trust / North Cumbria Integrated Care NHS Foundation Trust / North Tees and Hartlepool NHS Foundation Trust / Newcastle Hospitals NHS Foundation Trust|COVID-19 Genomics UK (COG-UK) Consortium|Bashton|United Kingdom
>Spike|hCoV-19/England/MILK-A7B5AD/2020|2020-10-16|EPI_ISL_607437|Original|hCoV-19^^England|Human|Lighthouse Lab in Milton Keynes|Wellcome Sanger Institute for the COVID-19 Genomics UK (COG-UK) consortium|Davies|United Kingdom
>Spike|hCoV-19/England/QEUH-AD2911/2020|2020-10-26|EPI_ISL_634009|Original|hCoV-19^^England|Human|Lighthouse Lab in Glasgow|Wellcome Sanger Institute for the COVID-19 Genomics UK (COG-UK) consortium|Davies|United Kingdom
>Spike|hCoV-19/England/ALDP-95AFF2/2020|2020-06-15|EPI_ISL_555585|Original|hCoV-19^^England|Human|Lighthouse Lab in Alderley Park|Wellcome Sanger Institute for the COVID-19 Genomics UK (COG-UK) consortium|Davies|United Kingdom
>Spike|hCoV-19/England/CAMC-AAFC7E/2020|2020-10-22|EPI_ISL_623359|Original|hCoV-19^^England|Human|Lighthouse Lab in Cambridge|Wellcome Sanger Institute for the COVID-19 Genomics UK (COG-UK) consortium|Davies|United Kingdom
>Spike|hCoV-19/USA/MI-UM-10036483559/2020|2020-10-16|EPI_ISL_628821|Original|hCoV-19^^Michigan|Human|University of Michigan Clinical Microbiology Laboratory|Lauring Lab|Valesano|USA
>Spike|hCoV-19/Japan/PG-1300/2020|2020-03-00|EPI_ISL_684908|Original|hCoV-19^^Japan|Human|Saitama Prefectural Institute of Public Health|Pathogen Genomics Center|Sekizuka|Japan
>Spike|hCoV-19/England/CAMB-1AFFC9/2020|2020-00-00|EPI_ISL_448094|Original|hCoV-19^^England|Human|Department of Pathology|COVID-19 Genomics UK (COG-UK) Consortium|Meredith|United Kingdom
>Spike|hCoV-19/USA/TX-HMH-MCoV-8983/2020|2020-07-02|EPI_ISL_780873|Original|hCoV-19^^Texas|Human|Houston Methodist Hospital|Houston Methodist Hospital|Long|USA
>Spike|hCoV-19/Wales/PHWC-36186/2020|2020-04-29|EPI_ISL_474629|Original|hCoV-19^^Wales|Human|Originating lab|COVID-19 Genomics UK (COG-UK) Consortium|Moore|United Kingdom
>Spike|hCoV-19/England/C200029067/2020|2020-10-09|EPI_ISL_636700|Original|hCoV-19^^England|Human|Respiratory Virus Unit|Respiratory Virus Unit|Platt|United Kingdom
>Spike|hCoV-19/Australia/VIC2210/2020|2020-06-25|EPI_ISL_521950|Original|hCoV-19^^Victoria|Human|Victorian Infectious Diseases Reference Laboratory (VIDRL)|VIDRL and MDU-PHL|Schultz|Australia
>Spike|hCoV-19/England/QEUH-B7D8B0/2020|2020-11-14|EPI_ISL_727828|Original|hCoV-19^^England|Human|Oxford Viromics|COVID-19 Genomics UK (COG-UK) Consortium|Golubchik|United Kingdom
>Spike|hCoV-19/Japan/PG-5304/2020|2020-08-00|EPI_ISL_690407|Original|hCoV-19^^Japan|Human|Pathogen Genomics Center|Pathogen Genomics Center|Sekizuka|Japan
>Spike|hCoV-19/England/ALDP-AA0C9B/2020|2020-10-22|EPI_ISL_625144|Original|hCoV-19^^England|Human|Lighthouse Lab in Alderley Park|Wellcome Sanger Institute for the COVID-19 Genomics UK (COG-UK) consortium|Davies|United Kingdom

>Spike|hCoV-19/Portugal/PT1142/2020|2020-04-01|EPI_ISL_511689|Original|hCoV-19^^Portugal|Human|Instituto Nacional de Saude (INSA)|Instituto Nacional de Saude (INSA)|Borges|Portugal
>Spike|hCoV-19/USA/TX-HMH-5035/2020|2020-06-24|EPI_ISL_544475|Original|hCoV-19^^Texas|Human|Houston Methodist Hospital|Houston Methodist Hospital|Long|USA
>Spike|hCoV-19/Scotland/CVR1542/2020|2020-04-04|EPI_ISL_512439|Original|hCoV-19^^Scotland|Human|West of Scotland Specialist Virology Centre|COVID-19 Genomics UK (COG-UK) Consortium|Robertson|United Kingdom
>Spike|hCoV-19/USA/AZ-TG396393/2020|2020-06-12|EPI_ISL_694844|Original|hCoV-19^^Arizona|Human|AZ SPHL|TGen North|French|USA
>Spike|hCoV-19/USA/UT-UPHL-03073/2020|2020-05-04|EPI_ISL_560911|Original|hCoV-19^^Utah|Human|Utah Public Health Laboratory|Utah Public Health Laboratory|Young|USA
>Spike|hCoV-19/USA/NY-NYUMC728/2020|2020-03-26|EPI_ISL_451443|Original|hCoV-19^^New York|Human|NYU Langone Health|Departments of Pathology and Medicine|Maurano|USA
>Spike|hCoV-19/England/ALDP-950A73/2020|2020-06-14|EPI_ISL_554361|Original|hCoV-19^^England|Human|Lighthouse Lab in Alderley Park|Wellcome Sanger Institute for the COVID-19 Genomics UK (COG-UK) consortium|Davies|United Kingdom
>Spike|hCoV-19/England/201361013/2020|2020-03-26|EPI_ISL_421788|Original|hCoV-19^^England|Human|Respiratory Virus Unit|Respiratory Virus Unit|Platt|United Kingdom
>Spike|hCoV-19/England/QEUH-962DDF/2020|2020-08-11|EPI_ISL_531713|Original|hCoV-19^^England|Human|Lighthouse Lab in Glasgow|Wellcome Sanger Institute for the COVID-19 Genomics UK (COG-UK) consortium|Davies|United Kingdom
>Spike|hCoV-19/England/CAMC-C45EDC/2020|2020-12-10|EPI_ISL_718397|Original|hCoV-19^^England|Human|Lighthouse Lab in Cambridge|Wellcome Sanger Institute for the COVID-19 Genomics UK (COG-UK) Consortium|Davies|United Kingdom
>Spike|hCoV-19/USA/CA-SR0351/2020|2020-04-28|EPI_ISL_458238|Original|hCoV-19^^California|Human|Scripps Medical Laboratory|Andersen lab at Scripps Research|Gangavarapu|USA
>Spike|hCoV-19/Denmark/DCGC-14674/2020|2020-11-23|EPI_ISL_711903|Original|hCoV-19^^Hovedstaden|Human|Department of Virus and Microbiological Special Diagnostics|Albertsen Lab|Michaelsen|Denmark
>Spike|hCoV-19/Denmark/DCGC-3965/2020|2020-09-14|EPI_ISL_615878|Original|hCoV-19^^Sjaelland|Human|Department of Virus and Microbiological Special Diagnostics|Albertsen lab|Michaelsen|Denmark
>Spike|hCoV-19/England/ALDP-9EA838/2020|2020-09-24|EPI_ISL_580241|Original|hCoV-19^^England|Human|Lighthouse Lab in Alderley Park|Wellcome Sanger Institute for the COVID-19 Genomics UK (COG-UK) consortium|Davies|United Kingdom
>Spike|hCoV-19/Netherlands/NA_352/2020|2020-04-06|EPI_ISL_455157|Original|hCoV-19^^Netherlands|Human|Dutch COVID-19 response team|Erasmus Medical Center|Oude Munnink|Netherlands
>Spike|hCoV-19/USA/AZ-TG269863/2020|2020-03-16|EPI_ISL_426529|Original|hCoV-19^^Arizona|Human|AZ SPHL|TGen North|Lemmer|USA
>Spike|hCoV-19/England/ALDP-A51464/2020|2020-10-08|EPI_ISL_597934|Original|hCoV-19^^England|Human|Lighthouse Lab in Alderley Park|Wellcome Sanger Institute for the COVID-19 Genomics UK (COG-UK) consortium|Davies|United Kingdom
>Spike|hCoV-19/USA/CT-Yale-330/2020|2020-04-01|EPI_ISL_476557|Original|hCoV-19^^Connecticut|Human|Yale Clinical Virology Laboratory|Grubaugh Lab - Yale School of Public Health|Grubaugh|USA
>Spike|hCoV-19/England/LOND-D557E/2020|2020-04-16|EPI_ISL_444235|Original|hCoV-19^^England|Human|University College London|COVID-19 Genomics UK (COG-UK) Consortium|Roy|United Kingdom
>Spike|hCoV-19/England/20194106301/2020|2020-05-05|EPI_ISL_465616|Original|hCoV-19^^England|Human|Respiratory Virus Unit|Respiratory Virus Unit|Platt|United Kingdom
>Spike|hCoV-19/Australia/SAP050/2020|2020-03-26|EPI_ISL_451089|Original|hCoV-19^^South Australia|Human|SA Pathology|SA Pathology|Leong|Australia
>Spike|hCoV-19/USA/WA-QDX-526/2020|2020-03-18|EPI_ISL_571399|Original|hCoV-19^^Washington|Human|Quest Diagnostics|Quest Diagnostics|Kagan|USA
>Spike|hCoV-19/USA/WA-OHSU-0464/2020|2020-06-09|EPI_ISL_526089|Original|hCoV-19^^Washington|Human|OHSU Lab Services Molecular Microbiology Lab|Ginkgo Bioworks Clinical Laboratory|O'Roak|USA

>Spike|hCoV-19/USA/CA-CZB-4003/2020|2020-08-03|EPI_ISL_548669|Original|hCoV-19^^California|Human|County of Santa Clara Public Health Department|Chan-Zuckerberg Biohub|Bhatt|USA
>Spike|hCoV-19/England/QEUH-5F2716/2020|2020-06-16|EPI_ISL_533235|Original|hCoV-19^^England|Human|Lighthouse Lab in Glasgow|Wellcome Sanger Institute for the COVID-19 Genomics UK (COG-UK) consortium|Davies|United Kingdom
>Spike|hCoV-19/Australia/WA124/2020|2020-03-28|EPI_ISL_596687|Original|hCoV-19^^Western Australia|Human|PathWest Laboratory Medicine WA|PathWest Laboratory Medicine WA Microbial Surveillance Unit|Sikazwe|Australia
>Spike|hCoV-19/Bahrein/BAH-10/2020|2020-03-30|EPI_ISL_483551|Original|hCoV-19^^Bahrain|Human|Kingdom of Bahrein Ministry of Health|Erasmus Medical Center|Oude Munnink|Bahrain
>Spike|hCoV-19/South Korea/KCDC24/2020|2020-02-06|EPI_ISL_412873|Original|hCoV-19^^Chungcheongnam-do|Human|Division of Viral Diseases|Division of Viral Diseases|Kim|South Korea
>Spike|hCoV-19/Israel/2089723/2020|2020-04-01|EPI_ISL_447365|Original|hCoV-19^^Jerusalem District|Human|Clinical Virology Unit|Stern Lab|Miller|Israel
>Spike|hCoV-19/Australia/VIC3084/2020|2020-07-04|EPI_ISL_520791|Original|hCoV-19^^Victoria|Human|Victorian Infectious Diseases Reference Laboratory (VIDRL)|VIDRL and MDU-PHL|Schultz|Australia
>Spike|hCoV-19/England/ALDP-94E191/2020|2020-06-17|EPI_ISL_555717|Original|hCoV-19^^England|Human|Lighthouse Lab in Alderley Park|Wellcome Sanger Institute for the COVID-19 Genomics UK (COG-UK) consortium|Davies|United Kingdom
>Spike|hCoV-19/England/CAMC-A64D27/2020|2020-10-11|EPI_ISL_608426|Original|hCoV-19^^England|Human|Lighthouse Lab in Cambridge|Wellcome Sanger Institute for the COVID-19 Genomics UK (COG-UK) consortium|Davies|United Kingdom
>Spike|hCoV-19/USA/MI-MDHHS-SC22572/2020|2020-11-23|EPI_ISL_737141|Original|hCoV-19^^Michigan|Human|Michigan Department of Health and Human Services|Michigan Department of Health and Human Services|Blankenship|USA
>Spike|hCoV-19/England/MILK-9F0A75/2020|2020-09-24|EPI_ISL_589615|Original|hCoV-19^^England|Human|Lighthouse Lab in Milton Keynes|Wellcome Sanger Institute for the COVID-19 Genomics UK (COG-UK) consortium|Davies|United Kingdom
>Spike|hCoV-19/Canada/ON-S939/2020|2020-04-13|EPI_ISL_586496|Original|hCoV-19^^Ontario|Human|Toronto Invasive Bacterial Diseases Network|McMaster University|McArthur|Canada
>Spike|hCoV-19/Ireland/LH-NVRL-78IRL62518/2020|2020-11-22|EPI_ISL_752533|Original|hCoV-19^^Louth|Human|National Virus Reference Laboratory|National Virus Reference Laboratory|Gonzalez|Ireland
>Spike|hCoV-19/USA/OR-OHSU-0529/2020|2020-06-15|EPI_ISL_525833|Original|hCoV-19^^Oregon|Human|OHSU Lab Services Molecular Microbiology Lab|Oregon SARS-CoV-2 Genome Sequencing Center|O'Roak|USA
>Spike|hCoV-19/Denmark/DCGC-2518/2020|2020-08-10|EPI_ISL_617754|Original|hCoV-19^^Nordjylland|Human|Department of Virus and Microbiological Special Diagnostics|Albertsen lab|Michaelsen|Denmark
>Spike|hCoV-19/England/205090690/2020|2020-12-09|EPI_ISL_769878|Original|hCoV-19^^England|Human|Respiratory Virus Unit|COVID-19 Genomics UK (COG-UK) Consortium|Platt|United Kingdom
>Spike|hCoV-19/Vietnam/CM295/2020|2020-03-06|EPI_ISL_416430|Vero|hCoV-19^^Hanoi|Human|National Influenza Center|National Influenza Center|Ung|Vietnam
>Spike|hCoV-19/Austria/CeMM0427/2020|2020-03-13|EPI_ISL_475855|Original|hCoV-19^^Austria|Human|Austrian Agency for Health and Food Safety (AGES)|Bergthaler laboratory|Bergthaler|Austria
>Spike|hCoV-19/England/MILK-B8C195/2020|2020-11-13|EPI_ISL_674266|Original|hCoV-19^^England|Human|Lighthouse Lab in Milton Keynes|Wellcome Sanger Institute for the COVID-19 Genomics UK (COG-UK) Consortium|Davies|United Kingdom
>Spike|hCoV-19/USA/CA-CZB-3930/2020|2020-07-29|EPI_ISL_548534|Original|hCoV-19^^California|Human|Contra Costa Public Health Lab|Chan-Zuckerberg Biohub|Bhatt|USA
>Spike|hCoV-19/Canada/QC-LSPQ-L00238888/2020|2020-03-26|EPI_ISL_536225|Original|hCoV-19^^Quebec|Human|H\u00f4pital Charles-LeMoyne|Laboratoire de sant\u00e9 publique du Qu\u00e9bec|Moreira|Canada

>Spike|hCoV-19/England/QEUH-A0272D/2020|2020-09-29|EPI_ISL_584928|Original|hCoV-19^^England|Human|Quadram Institute Bioscience|COVID-19 Genomics UK (COG-UK) Consortium|Alikhan|United Kingdom
>Spike|hCoV-19/Japan/PG-6130/2020|2020-08-00|EPI_ISL_689242|Original|hCoV-19^^Japan|Human|Pathogen Genomics Center|Pathogen Genomics Center|Sekizuka|Japan
>Spike|hCoV-19/Wuhan/HB-WH5-229/2020|2020-02-22|EPI_ISL_454973|Original|hCoV-19^^Hubei|Human|Wuhan Chain Medical Labs (CMLabs)|State Key Laboratory of Biotherapy of Sichuan University|Tang|China
>Spike|hCoV-19/Wales/PHWC-16C5A8/2020|2020-09-25|EPI_ISL_574069|Original|hCoV-19^^Wales|Human|Wales Specialist Virology Centre Sequencing lab|COVID-19 Genomics UK (COG-UK) Consortium|Moore|United Kingdom
>Spike|hCoV-19/England/CAMB-1AA66F/2020|2020-04-17|EPI_ISL_470112|Original|hCoV-19^^England|Human|Department of Pathology|Wellcome Sanger Institute for the COVID-19 Genomics UK (COG-UK) consortium|Davies|United Kingdom
>Spike|hCoV-19/England/MILK-95711D/2020|2020-06-10|EPI_ISL_554213|Original|hCoV-19^^England|Human|Lighthouse Lab in Milton Keynes|Wellcome Sanger Institute for the COVID-19 Genomics UK (COG-UK) Consortium|Davies|United Kingdom
>Spike|hCoV-19/USA/FL-BPHL-1019/2020|2020-07-17|EPI_ISL_517909|Original|hCoV-19^^Florida|Human|Florida Bureau of Public Health Laboratories|Florida Bureau of Public Health Laboratories|Blanton|USA
>Spike|hCoV-19/Luxembourg/LNS6133647/2020|2020-06-29|EPI_ISL_744367|Original|hCoV-19^^Luxembourg|Human|Laboratoire national de sant\u00e9|Laboratoire national de sant\u00e9|Wienecke-Baldacchino|Luxembourg
>Spike|hCoV-19/Czech Republic/NRL_7918/2020|2020-07-27|EPI_ISL_545954|Original|hCoV-19^^Liberec Region|Human|The National Institute of Public Health|State Veterinary Institute Prague|Nagy|Czech Republic
>Spike|hCoV-19/USA/DC-HP00137/2020|2020-03-21|EPI_ISL_457823|Original|hCoV-19^^District of Columbia|Human|Johns Hopkins Hospital Department of Pathology|Johns Hopkins Hospital Department of Pathology|Ramakrishnan|USA
>Spike|hCoV-19/England/NORT-293F4F/2020|2020-07-21|EPI_ISL_572491|Original|hCoV-19^^England|Human|Northumbria University / South Tees Hospitals NHS Foundation Trust / North Cumbria Integrated Care NHS Foundation Trust / North Tees and Hartlepool NHS Foundation Trust / Newcastle Hospitals NHS Foundation Trust|COVID-19 Genomics UK (COG-UK) Consortium|Bashton|United Kingdom
>Spike|hCoV-19/Canada/NS-NML-1202/2020|2020-04-02|EPI_ISL_579603|Original|hCoV-19^^Nova Scotia|Human|QEII Health Sciences Centre|National Microbiology Laboratory (NML)|Majer|Canada
>Spike|hCoV-19/USA/CA-ALSR-4682/2020|2020-11-04|EPI_ISL_667074|Original|hCoV-19^^California|Human|San Diego County Public Health Laboratory|Andersen lab at Scripps Research|Gangavarapu|USA
>Spike|hCoV-19/Netherlands/ZH-EMC-552/2020|2020-09-08|EPI_ISL_577980|Original|hCoV-19^^Zuid Holland|Human|Dutch COVID-19 response team|Erasmus Medical Center|Oude Munnink|Netherlands
>Spike|hCoV-19/USA/NY-Wadsworth-264211-01/2020|2020-11-05|EPI_ISL_754520|Original|hCoV-19^^New York|Human|Wadsworth Center|Wadsworth Center|Lasek-Nesselquist|USA
>Spike|hCoV-19/India/GJ-GBRC259/2020|2020-06-11|EPI_ISL_483850|Original|hCoV-19^^Gujarat|Human|Department of Microbiology|Gujarat Biotechnology Research Centre|Joshi|India
>Spike|hCoV-19/USA/TX-HMH-MCoV-14834/2020|2020-10-13|EPI_ISL_786318|Original|hCoV-19^^Texas|Human|Houston Methodist Hospital|Houston Methodist Hospital|Long|USA
>Spike|hCoV-19/Poland/PL_MCB_3/2020|2020-04-22|EPI_ISL_645002|Original|hCoV-19^^Poland|Human|Human Genome Variation Research Group|Human Genome Variation Research Group||Poland
>Spike|hCoV-19/USA/CA-CSMC131/2020|2020-03-26|EPI_ISL_475609|Original|hCoV-19^^California|Human|Cedars-Sinai Medical Center|Cedars-Sinai Medical Center|Zhang|USA
>Spike|hCoV-19/USA/AZ-TG670188/2020|2020-05-01|EPI_ISL_696383|Original|hCoV-19^^Arizona|Human|Sonora Quest Laboratories|TGen North|French|USA

>Spike|hCoV-19/Wales/PHWC-49EB02/2020|2020-12-08|EPI_ISL_743095|Original|hCoV-19^^Wales|Human|Wales Specialist Virology Centre Sequencing lab|COVID-19 Genomics UK (COG-UK) Consortium|Moore|United Kingdom
>Spike|hCoV-19/USA/TX-HMH-7301/2020|2020-06-29|EPI_ISL_546658|Original|hCoV-19^^Texas|Human|Houston Methodist Hospital|Houston Methodist Hospital|Long|USA
>Spike|hCoV-19/England/QEUH-AF612E/2020|2020-10-30|EPI_ISL_662933|Original|hCoV-19^^England|Human|Lighthouse Lab in Glasgow|Wellcome Sanger Institute for the COVID-19 Genomics UK (COG-UK) Consortium|Davies|United Kingdom
>Spike|hCoV-19/France/PAC-IHU-0119/2020|2020-03-24|EPI_ISL_569319|Original|hCoV-19^^Provence-Alpes-C\u00f4te-d\u2019Azur|Human|MEPHI|MEPHI|Levasseur|France
>Spike|hCoV-19/England/QEUH-C33ABF/2020|2020-12-11|EPI_ISL_734142|Original|hCoV-19^^England|Human|Lighthouse Lab in Glasgow|Wellcome Sanger Institute for the COVID-19 Genomics UK (COG-UK) Consortium|Davies|United Kingdom
>Spike|hCoV-19/England/NORT-283D2A/2020|2020-04-04|EPI_ISL_488690|Original|hCoV-19^^England|Human|NU-OMICS DNA Sequencing research facility|Wellcome Sanger Institute for the COVID-19 Genomics UK (COG-UK) consortium|Davies|United Kingdom
>Spike|hCoV-19/Japan/PG-1016/2020|2020-03-00|EPI_ISL_684626|Original|hCoV-19^^Japan|Human|Tokyo Metropolitan Institute of Public Health|Pathogen Genomics Center|Sekizuka|Japan
>Spike|hCoV-19/USA/ME-HETL-J0006/2020|2020-03-14|EPI_ISL_513419|Original|hCoV-19^^Maine|Human|Maine HETL|Tewhey Lab|Tewhey|USA
>Spike|hCoV-19/USA/FL-BPHL-1839/2020|2020-04-09|EPI_ISL_613919|Original|hCoV-19^^Florida|Human|Florida Bureau of Public Health Laboratories|Florida Bureau of Public Health Laboratories|Blanton|USA
>Spike|hCoV-19/Wales/PHWC-48D034/2020|2020-11-08|EPI_ISL_666570|Original|hCoV-19^^Wales|Human|Wales Specialist Virology Centre Sequencing lab|COVID-19 Genomics UK (COG-UK) Consortium|Moore|United Kingdom
>Spike|hCoV-19/USA/AK-PHL1721/2020|2020-07-21|EPI_ISL_528500|Original|hCoV-19^^Alaska|Human|Alaska State Virology Laboratory|Alaska State Virology Laboratory|Drown|USA
>Spike|hCoV-19/Czech Republic/NRL_6849-11/2020|2020-05-16|EPI_ISL_577632|Original|hCoV-19^^Central Bohemia Region|Human|The National Institute of Public Health|State Veterinary Institute Prague|Nagy|Czech Republic
>Spike|hCoV-19/USA/CA-CDPH257/2020|2020-11-27|EPI_ISL_755024|Original|hCoV-19^^California|Human|California Department of Public Health|California Department of Public Health|Bell|USA
>Spike|hCoV-19/Australia/VIC10883/2020|2020-08-16|EPI_ISL_563137|Original|hCoV-19^^Victoria|Human|Victorian Infectious Diseases Reference Laboratory (VIDRL)|VIDRL and MDU-PHL|Schultz|Australia
>Spike|hCoV-19/Netherlands/GE-EMC-195/2020|2020-05-27|EPI_ISL_523177|Original|hCoV-19^^Gelderland|Human|Dutch COVID-19 response team|Erasmus Medical Center|Oude Munnink|Netherlands
>Spike|hCoV-19/Netherlands/NoordBrabant_11/2020|2020-03-02|EPI_ISL_414432|Original|hCoV-19^^Noord Brabant|Human|Dutch COVID-19 response team|Erasmus Medical Center|Oude Munnink|Netherlands
>Spike|hCoV-19/USA/TX-HMH-MCoV-13777/2020|2020-08-17|EPI_ISL_788870|Original|hCoV-19^^Texas|Human|Houston Methodist Hospital|Houston Methodist Hospital|Long|USA
>Spike|hCoV-19/Luxembourg/LNS2721373/2020|2020-11-10|EPI_ISL_740178|Original|hCoV-19^^Luxembourg|Human|Laboratoire national de sant\u00e9|Laboratoire national de sant\u00e9|Wienecke-Baldacchino|Luxembourg
>Spike|hCoV-19/Brazil/RJ-0251/2020|2020-03-27|EPI_ISL_470619|Original|hCoV-19^^Rio de Janeiro|Human|Laboratorio de Virologia Molecular / UFRJ|Bioinformatics Laboratory / LNCC|Almeida|Brazil
>Spike|hCoV-19/England/ALDP-6FB8FB/2020|2020-06-29|EPI_ISL_557356|Original|hCoV-19^^England|Human|Lighthouse Lab in Alderley Park|Wellcome Sanger Institute for the COVID-19 Genomics UK (COG-UK) consortium|Davies|United Kingdom
>Spike|hCoV-19/Sri Lanka/COV53/2020|2020-03-10|EPI_ISL_428671|Original|hCoV-19^^Sri Lanka|Human|Centre for Dengue Research|Centre for Dengue Research|Malavige|Sri Lanka

>Spike|hCoV-19/England/QEUH-C5BB81/2020|2020-12-14|EPI_ISL_769482|Original|hCoV-19^^England|Human|Lighthouse Lab in Glasgow|Wellcome Sanger Institute for the COVID-19 Genomics UK (COG-UK) Consortium|Davies|United Kingdom
>Spike|hCoV-19/New Zealand/20CV0146/2020|2020-08-22|EPI_ISL_548024|Original|hCoV-19^^Auckland|Human|LabPLUS|Institute of Environmental Science and Research (ESR)|Wang|New Zealand
>Spike|hCoV-19/USA/CA-IGI-0407/2020|2020-07-08|EPI_ISL_754889|Original|hCoV-19^^California|Human|Innovative Genomics Institute|Innovative Genomics Institute|Wyman|USA
>Spike|hCoV-19/England/MILK-A18A94/2020|2020-10-03|EPI_ISL_627477|Original|hCoV-19^^England|Human|University College London|COVID-19 Genomics UK (COG-UK) Consortium|Roy|United Kingdom
>Spike|hCoV-19/Japan/PG-4192/2020|2020-07-00|EPI_ISL_692431|Original|hCoV-19^^Japan|Human|Pathogen Genomics Center|Pathogen Genomics Center|Sekizuka|Japan
>Spike|hCoV-19/England/ALDP-AA07A9/2020|2020-10-22|EPI_ISL_623889|Original|hCoV-19^^England|Human|Lighthouse Lab in Alderley Park|Wellcome Sanger Institute for the COVID-19 Genomics UK (COG-UK) consortium|Davies|United Kingdom
>Spike|hCoV-19/USA/CA-CZB0746/2020|2020-04-03|EPI_ISL_429028|Original|hCoV-19^^California|Human|UCSF Clinical Microbiology Laboratory|Chan-Zuckerberg Biohub|Kamm|USA
>Spike|hCoV-19/Wales/QEUH-9B49BC/2020|2020-09-03|EPI_ISL_590432|Original|hCoV-19^^Wales|Human|Lighthouse Lab in Glasgow|Wellcome Sanger Institute for the COVID-19 Genomics UK (COG-UK) consortium|Davies|United Kingdom
>Spike|hCoV-19/France/PAC-IHU-1268/2020|2020-08-17|EPI_ISL_569102|Original|hCoV-19^^Provence-Alpes-C\u00f4te-d\u2019Azur|Human|MEPHI|MEPHI|Levasseur|France
>Spike|hCoV-19/England/MILK-B05A02/2020|2020-10-23|EPI_ISL_652214|Original|hCoV-19^^England|Human|Queens Medical Centre|COVID-19 Genomics UK (COG-UK) Consortium|Loose|United Kingdom
>Spike|hCoV-19/England/LIVE-A48AC/2020|2020-03-13|EPI_ISL_499929|Original|hCoV-19^^England|Human|Liverpool Clinical Laboratories|COVID-19 Genomics UK (COG-UK) Consortium|Haldenby|United Kingdom
>Spike|hCoV-19/Australia/VIC11817/2020|2020-08-08|EPI_ISL_591861|Original|hCoV-19^^Victoria|Human|Victorian Infectious Diseases Reference Laboratory (VIDRL)|VIDRL and MDU-PHL|Schultz|Australia
>Spike|hCoV-19/Canada/NS-NML-1219/2020|2020-04-03|EPI_ISL_579616|Original|hCoV-19^^Nova Scotia|Human|QEII Health Sciences Centre|National Microbiology Laboratory (NML)|Majer|Canada
>Spike|hCoV-19/Scotland/QEUH-A4BBD1/2020|2020-10-08|EPI_ISL_628514|Original|hCoV-19^^Scotland|Human|Oxford Viromics|COVID-19 Genomics UK (COG-UK) Consortium|Golubchik|United Kingdom
>Spike|hCoV-19/USA/TX-HMH-3744/2020|2020-06-15|EPI_ISL_543156|Original|hCoV-19^^Texas|Human|Houston Methodist Hospital|Houston Methodist Hospital|Long|USA
>Spike|hCoV-19/USA/UT-UPHL-2008965/2020|2020-05-11|EPI_ISL_525291|Original|hCoV-19^^Utah|Human|Utah Public Health Laboratory|Utah Public Health Laboratory|Young|USA
>Spike|hCoV-19/England/LOND-D89AC/2020|2020-04-14|EPI_ISL_492754|Original|hCoV-19^^England|Human|University College London|Wellcome Sanger Institute for the COVID-19 Genomics UK (COG-UK) consortium|Davies|United Kingdom
>Spike|hCoV-19/USA/TX-HMH-2734/2020|2020-06-06|EPI_ISL_545513|Original|hCoV-19^^Texas|Human|Houston Methodist Hospital|Houston Methodist Hospital|Long|USA
>Spike|hCoV-19/Jordan/AQ-ALSR-5272/2020|2020-10-06|EPI_ISL_730510|Original|hCoV-19^^Aqaba|Human|Biolab Diagnostic Laboratories|Andersen lab at Scripps Research|Gangavarapu|Jordan
>Spike|hCoV-19/USA/TX-HMH-2827/2020|2020-06-08|EPI_ISL_543801|Original|hCoV-19^^Texas|Human|Houston Methodist Hospital|Houston Methodist Hospital|Long|USA
>Spike|hCoV-19/England/ALDP-A856CB/2020|2020-10-17|EPI_ISL_610305|Original|hCoV-19^^England|Human|Lighthouse Lab in Alderley Park|Wellcome Sanger Institute for the COVID-19 Genomics UK (COG-UK) consortium|Davies|United Kingdom
>Spike|hCoV-19/Gibraltar/204462881/2020|2020-10-29|EPI_ISL_615048|Original|hCoV-19^^Gibraltar|Human|Microbiology|Respiratory Virus Unit|Platt|Gibraltar

>Spike|hCoV-19/Scotland/QEUH-98FAA8/2020|2020-08-30|EPI_ISL_530370|Original|hCoV-19^^Scotland|Human|NHSGGC West of Scotland Specialist Virology Centre / MRC-University of Glasgow Centre for Virus Research|Wellcome Sanger Institute for the COVID-19 Genomics UK (COG-UK) consortium|Davies|United Kingdom
>Spike|hCoV-19/USA/TX-HMH-MCoV-10228/2020|2020-07-07|EPI_ISL_789380|Original|hCoV-19^^Texas|Human|Houston Methodist Hospital|Houston Methodist Hospital|Long|USA
>Spike|hCoV-19/Netherlands/UT-EMC-57/2020|2020-07-11|EPI_ISL_523534|Original|hCoV-19^^Utrecht|Human|Dutch COVID-19 response team|Erasmus Medical Center|Oude Munnink|Netherlands
>Spike|hCoV-19/USA/WA-S2609/2020|2020-06-18|EPI_ISL_497241|Original|hCoV-19^^Washington|Human|Washington State Department of Health|Seattle Flu Study|Lee|USA
>Spike|hCoV-19/England/NORW-E988D/2020|2020-04-23|EPI_ISL_448340|Original|hCoV-19^^England|Human|Quadram Institute Bioscience|COVID-19 Genomics UK (COG-UK) Consortium|Alikhan|United Kingdom
>Spike|hCoV-19/Netherlands/ZH-EMC-449/2020|2020-08-12|EPI_ISL_523787|Original|hCoV-19^^Zuid Holland|Human|Dutch COVID-19 response team|Erasmus Medical Center|Oude Munnink|Netherlands
>Spike|hCoV-19/USA/IL-NM-1466/2020|2020-07-07|EPI_ISL_626525|Original|hCoV-19^^Illinois|Human|Northwestern Memorial Hospital|Ozer Lab|Ozer|USA
>Spike|hCoV-19/USA/WA-UW-12593/2020|2020-06-26|EPI_ISL_570869|Original|hCoV-19^^Washington|Human|UW Virology Lab|UW Virology Lab|Roychoudhury|USA
>Spike|hCoV-19/Spain/CL-IBV-97006216/2020|2020-04-13|EPI_ISL_537362|Original|hCoV-19^^Castilla y Leon|Human|Universidad de Le\u00f3n|SeqCOVID-SPAIN consortium/IBV(CSIC)|Comas|Spain
>Spike|hCoV-19/England/ALDP-9B606A/2020|2020-08-28|EPI_ISL_572985|Original|hCoV-19^^England|Human|Oxford Viromics|COVID-19 Genomics UK (COG-UK) Consortium|Golubchik|United Kingdom
>Spike|hCoV-19/England/ALDP-9BE81F/2020|2020-09-09|EPI_ISL_568050|Original|hCoV-19^^England|Human|Lighthouse Lab in Alderley Park|Wellcome Sanger Institute for the COVID-19 Genomics UK (COG-UK) consortium|Davies|United Kingdom
>Spike|hCoV-19/Netherlands/FL-EMC-32/2020|2020-10-27|EPI_ISL_632725|Original|hCoV-19^^Flevoland|Human|Dutch COVID-19 response team|Erasmus Medical Center|Oude Munnink|Netherlands
>Spike|hCoV-19/USA/LA-SR0318/2020|2020-04-04|EPI_ISL_450173|Original|hCoV-19^^Louisiana|Human|Robert Garry lab|Andersen lab at Scripps Research|Gangavarapu|USA
>Spike|hCoV-19/Australia/VIC15372/2020|2020-08-03|EPI_ISL_593269|Original|hCoV-19^^Victoria|Human|Microbiological Diagnostic Unit - Public Health Laboratory (MDU-PHL)|MDU-PHL|Schultz|Australia
>Spike|hCoV-19/Denmark/DCGC-18181/2020|2020-12-14|EPI_ISL_749213|Original|hCoV-19^^Hovedstaden|Human|Department of Virus and Microbiological Special Diagnostics|Albertsen Lab|Michaelsen|Denmark
>Spike|hCoV-19/USA/MA-MASPHL-00743/2020|2020-09-22|EPI_ISL_692930|Original|hCoV-19^^Massachusetts|Human|Massachusetts State Public Health Laboratory|Massachusetts State Public Health Laboratory|Lang|USA
>Spike|hCoV-19/USA/CA-CZB-10212/2020|2020-05-11|EPI_ISL_672260|Original|hCoV-19^^California|Human|The Ashley Laboratory|Chan-Zuckerberg Biohub|Bhatt|USA
>Spike|hCoV-19/Northern Ireland/QEUH-95FE3C/2020|2020-08-07|EPI_ISL_531895|Original|hCoV-19^^Northern Ireland|Human|Lighthouse Lab in Glasgow|Wellcome Sanger Institute for the COVID-19 Genomics UK (COG-UK) consortium|Davies|United Kingdom
>Spike|hCoV-19/England/ALDP-B735B3/2020|2020-11-12|EPI_ISL_662391|Original|hCoV-19^^England|Human|Lighthouse Lab in Alderley Park|Wellcome Sanger Institute for the COVID-19 Genomics UK (COG-UK) Consortium|Davies|United Kingdom
>Spike|hCoV-19/USA/TX-HMH-7108/2020|2020-06-29|EPI_ISL_546510|Original|hCoV-19^^Texas|Human|Houston Methodist Hospital|Houston Methodist Hospital|Long|USA
>Spike|hCoV-19/USA/IL-NM0112/2020|2020-03-20|EPI_ISL_444596|Original|hCoV-19^^Illinois|Human|Northwestern Memorial Hospital|Ozer Lab|Ozer|USA
>Spike|hCoV-19/England/CAMB-7DA3D/2020|2020-04-06|EPI_ISL_441666|Original|hCoV-19^^England|Human|Department of Pathology|Wellcome Sanger Institute for the COVID-19 Genomics UK (COG-UK) consortium|Amato|United Kingdom

>Spike|hCoV-19/Australia/VIC1631/2020|2020-05-14|EPI_ISL_456536|Original|hCoV-19^^Victoria|Human|Victorian Infectious Diseases Reference Laboratory (VIDRL)|Microbiological Diagnostic Unit Public Health Laboratory and Victorian Infectious Diseases Reference Laboratory|Schultz|Australia
>Spike|hCoV-19/England/CAMB-1BA08C/2020|2020-11-04|EPI_ISL_651712|Original|hCoV-19^^England|Human|Department of Pathology|COVID-19 Genomics UK (COG-UK) Consortium|Goodfellow|United Kingdom
>Spike|hCoV-19/Wales/PHWC-484176/2020|2020-10-23|EPI_ISL_627021|Original|hCoV-19^^Wales|Human|Wales Specialist Virology Centre Sequencing lab|COVID-19 Genomics UK (COG-UK) Consortium|Moore|United Kingdom
>Spike|hCoV-19/Spain/NC-IBV-001374/2020|2020-03-13|EPI_ISL_452527|Original|hCoV-19^^Navarra|Human|Cl\u00ednica Universidad de Navarra. Servicio de Enfermedades Infecciosas y Microbiolog\u00eda cl\u00ednica|SeqCOVID-SPAIN consortium/IBV(CSIC)|Comas|Spain
>Spike|hCoV-19/England/MILK-971818/2020|2020-08-18|EPI_ISL_553214|Original|hCoV-19^^England|Human|Lighthouse Lab in Milton Keynes|Wellcome Sanger Institute for the COVID-19 Genomics UK (COG-UK) consortium|Davies|United Kingdom
>Spike|hCoV-19/Australia/NSW581/2020|2020-03-30|EPI_ISL_478693|Original|hCoV-19^^New South Wales|Human|South Eastern Area Laboratory Services (SEALS)|NSW Health Pathology - Institute of Clinical Pathology and Medical Research; Westmead Hospital; University of Sydney|Gall|Australia
>Spike|hCoV-19/Denmark/DCGC-19209/2020|2020-12-07|EPI_ISL_748818|Original|hCoV-19^^Hovedstaden|Human|Department of Virus and Microbiological Special Diagnostics|Albertsen Lab|Michaelsen|Denmark
>Spike|hCoV-19/England/QEUH-A5E160/2020|2020-10-12|EPI_ISL_597420|Original|hCoV-19^^England|Human|Lighthouse Lab in Glasgow|Wellcome Sanger Institute for the COVID-19 Genomics UK (COG-UK) consortium|Davies|United Kingdom
>Spike|hCoV-19/Denmark/DCGC-16308/2020|2020-11-16|EPI_ISL_714695|Original|hCoV-19^^Hovedstaden|Human|Department of Virus and Microbiological Special Diagnostics|Albertsen Lab|Michaelsen|Denmark
>Spike|hCoV-19/India/GJ-GBRC360/2020|2020-06-15|EPI_ISL_514610|Original|hCoV-19^^Gujarat|Human|GMERS Medical College & Hospital|Gujarat Biotechnology Research Centre|Joshi|India
>Spike|hCoV-19/Panama/328933/2020|2020-03-09|EPI_ISL_496616|Original|hCoV-19^^Panama Center|Human|Gorgas Memorial Laboratory of Health Studies|Gorgas Memorial Laboratory of Health Studies|Martinez|Panama
>Spike|hCoV-19/Denmark/DCGC-12592/2020|2020-11-16|EPI_ISL_682773|Original|hCoV-19^^Nordjylland|Human|Department of Virus and Microbiological Special Diagnostics|Albertsen Lab|Michaelsen|Denmark
>Spike|hCoV-19/Australia/VIC15095/2020|2020-08-25|EPI_ISL_593134|Original|hCoV-19^^Victoria|Human|Microbiological Diagnostic Unit - Public Health Laboratory (MDU-PHL)|MDU-PHL|Schultz|Australia
>Spike|hCoV-19/Finland/9SS15B11/2020|2020-09-10|EPI_ISL_755966|Original|hCoV-19^^Uusimaa|Human|Department of Virology and Immunology|Department of Virology|Smura|Finland
>Spike|hCoV-19/England/ALDP-B85378/2020|2020-11-15|EPI_ISL_675699|Original|hCoV-19^^England|Human|Lighthouse Lab in Alderley Park|Wellcome Sanger Institute for the COVID-19 Genomics UK (COG-UK) Consortium|Davies|United Kingdom
>Spike|hCoV-19/Wales/MILK-A05B6A/2020|2020-09-30|EPI_ISL_599910|Original|hCoV-19^^Wales|Human|Lighthouse Lab in Milton Keynes|Wellcome Sanger Institute for the COVID-19 Genomics UK (COG-UK) consortium|Davies|United Kingdom
>Spike|hCoV-19/Wales/PHWC-26A15/2020|2020-03-29|EPI_ISL_422036|Original|hCoV-19^^Wales|Human|Wales Specialist Virology Centre|Public Health Wales Microbiology Cardiff|Moore|United Kingdom
>Spike|hCoV-19/England/QEUH-AF61C4/2020|2020-10-30|EPI_ISL_662919|Original|hCoV-19^^England|Human|Lighthouse Lab in Glasgow|Wellcome Sanger Institute for the COVID-19 Genomics UK (COG-UK) Consortium|Davies|United Kingdom
>Spike|hCoV-19/USA/WA-S2784/2020|2020-08-11|EPI_ISL_530141|Original|hCoV-19^^Washington|Human|Seattle Flu Study|Seattle Flu Study|Lee|USA

>Spike|hCoV-19/England/MILK-99481F/2020|2020-08-25|EPI_ISL_552297|Original|hCoV-19^^England|Human|Lighthouse Lab in Milton Keynes|Wellcome Sanger Institute for the COVID-19 Genomics UK (COG-UK) consortium|Davies|United Kingdom
>Spike|hCoV-19/Denmark/DCGC-18721/2020|2020-12-07|EPI_ISL_749171|Original|hCoV-19^^Hovedstaden|Human|Department of Virus and Microbiological Special Diagnostics|Albertsen Lab|Michaelsen|Denmark
>Spike|hCoV-19/Japan/PG-2297/2020|2020-04-00|EPI_ISL_685902|Original|hCoV-19^^Japan|Human|Pathogen Genomics Center|Pathogen Genomics Center|Sekizuka|Japan
>Spike|hCoV-19/USA/CA-CZB-2453/2020|2020-05-22|EPI_ISL_513861|Original|hCoV-19^^California|Human|San Francisco Public Health Laboratory|Chan-Zuckerberg Biohub|Kamm|USA
>Spike|hCoV-19/India/TG-CDFD-C3308/2020|2020-06-20|EPI_ISL_528597|Original|hCoV-19^^Telangana|Human|National Genomics Core-Center for DNA Fingerprinting and Diagnostics|National Genomics Core- Center for DNA Fingerprinting and Diagnostics (NGC-CDFD)- DBT's PAN-INDIA-1000 Genome consortium|Vashisht|India
>Spike|hCoV-19/Wales/PHWC-493A1F/2020|2020-11-30|EPI_ISL_725972|Original|hCoV-19^^Wales|Human|Originating lab|COVID-19 Genomics UK (COG-UK) Consortium|Moore|United Kingdom
>Spike|hCoV-19/Denmark/DCGC-7508/2020|2020-10-12|EPI_ISL_618413|Original|hCoV-19^^Sjaelland|Human|Department of Virus and Microbiological Special Diagnostics|Albertsen lab|Michaelsen|Denmark
>Spike|hCoV-19/England/ALDP-AC5D51/2020|2020-10-14|EPI_ISL_631012|Original|hCoV-19^^England|Human|Lighthouse Lab in Alderley Park|Wellcome Sanger Institute for the COVID-19 Genomics UK (COG-UK) consortium|Davies|United Kingdom
>Spike|hCoV-19/USA/TX-HMH-MCoV-16083/2020|2020-10-29|EPI_ISL_785767|Original|hCoV-19^^Texas|Human|Houston Methodist Hospital|Houston Methodist Hospital|Long|USA
>Spike|hCoV-19/Netherlands/GE-RIVM-20332/2020|2020-12-03|EPI_ISL_723460|Orignal|hCoV-19^^Gelderland|Human|Dutch COVID-19 response team|National Institute for Public Health and the Environment (RIVM)|Meijer|Netherlands
>Spike|hCoV-19/England/LOND-12F0790/2020|2020-11-16|EPI_ISL_679575|Original|hCoV-19^^England|Human|University College London|COVID-19 Genomics UK (COG-UK) Consortium|Roy|United Kingdom
>Spike|hCoV-19/Scotland/EDB7736/2020|2020-09-03|EPI_ISL_540880|Original|hCoV-19^^Scotland|Human|Virology Department|COVID-19 Genomics UK (COG-UK) Consortium|Williams|United Kingdom
>Spike|hCoV-19/England/NOTT-116367/2020|2020-10-19|EPI_ISL_595316|Original|hCoV-19^^England|Human|Queens Medical Centre|COVID-19 Genomics UK (COG-UK) Consortium|Loose|United Kingdom
>Spike|hCoV-19/Denmark/DCGC-1818/2020|2020-04-27|EPI_ISL_616923|Original|hCoV-19^^Hovedstaden|Human|Department of Virus and Microbiological Special Diagnostics|Albertsen lab|Michaelsen|Denmark
>Spike|hCoV-19/England/LOND-D6276/2020|2020-04-20|EPI_ISL_482191|Original|hCoV-19^^England|Human|University College London|Wellcome Sanger Institute for the COVID-19 Genomics UK (COG-UK) consortium|Davies|United Kingdom
>Spike|hCoV-19/England/ALDP-A9609C/2020|2020-10-19|EPI_ISL_624716|Original|hCoV-19^^England|Human|Lighthouse Lab in Alderley Park|Wellcome Sanger Institute for the COVID-19 Genomics UK (COG-UK) consortium|Davies|United Kingdom
>Spike|hCoV-19/England/CAMC-B22DBB/2020|2020-11-02|EPI_ISL_647299|Original|hCoV-19^^England|Human|Lighthouse Lab in Cambridge|Wellcome Sanger Institute for the COVID-19 Genomics UK (COG-UK) Consortium|Davies|United Kingdom
>Spike|hCoV-19/England/ALDP-B1BAFA/2020|2020-11-01|EPI_ISL_645670|Original|hCoV-19^^England|Human|Lighthouse Lab in Alderley Park|Wellcome Sanger Institute for the COVID-19 Genomics UK (COG-UK) Consortium|Davies|United Kingdom
>Spike|hCoV-19/England/CAMB-1B90E7/2020|2020-00-00|EPI_ISL_704406|Original|hCoV-19^^England|Human|Department of Pathology|COVID-19 Genomics UK (COG-UK) Consortium|Goodfellow|United Kingdom
>Spike|hCoV-19/USA/UT-UPHL-03763/2020|2020-04-05|EPI_ISL_575111|Original|hCoV-19^^Utah|Human|Utah Public Health Laboratory|Utah Public Health Laboratory|Young|USA

>Spike|hCoV-19/USA/FL-BPHL-1946/2020|2020-04-11|EPI_ISL_653099|Original|hCoV-19^^Florida|Human|Florida Bureau of Public Health Laboratories|Florida Bureau of Public Health Laboratories|Blanton|USA
>Spike|hCoV-19/USA/CA-CDPH029/2020|2020-05-26|EPI_ISL_515921|Original|hCoV-19^^California|Human|California Department of Public Health|California Department of Public Health|Bell|USA
>Spike|hCoV-19/England/QEUH-9F7E36/2020|2020-09-27|EPI_ISL_589189|Original|hCoV-19^^England|Human|Lighthouse Lab in Glasgow|Wellcome Sanger Institute for the COVID-19 Genomics UK (COG-UK) consortium|Davies|United Kingdom
>Spike|hCoV-19/France/BRE-IPP9985/2020|2020-10-10|EPI_ISL_734168|Original|hCoV-19^^Bretagne|Human|CHRU Pontchaillou - Laboratoire de Virologie|National Reference Center for Viruses of Respiratory Infections|Enouf|France
>Spike|hCoV-19/USA/WA-S327/2020|2020-04-05|EPI_ISL_434087|Original|hCoV-19^^Washington|Human|Washington State Department of Health|Seattle Flu Study|Bedford|USA
>Spike|hCoV-19/USA/MI-MDHHS-SC20185/2020|2020-03-27|EPI_ISL_436881|Original|hCoV-19^^Michigan|Human|Michigan Department of Health and Human Services|Michigan Department of Health and Human Services|Blankenship|USA
>Spike|hCoV-19/England/NORT-289F1F/2020|2020-04-07|EPI_ISL_453519|Original|hCoV-19^^England|Human|Northumbria University / South Tees Hospitals NHS Foundation Trust / North Cumbria Integrated Care NHS Foundation Trust / North Tees and Hartlepool NHS Foundation Trust / Newcastle Hospitals NHS Foundation Trust|COVID-19 Genomics UK (COG-UK) Consortium|Bashton|United Kingdom
>Spike|hCoV-19/England/MILK-A79FED/2020|2020-10-15|EPI_ISL_606760|Original|hCoV-19^^England|Human|Lighthouse Lab in Milton Keynes|Wellcome Sanger Institute for the COVID-19 Genomics UK (COG-UK) consortium|Davies|United Kingdom
>Spike|hCoV-19/USA/WA-UW-1472/2020|2020-03-17|EPI_ISL_424176|Original|hCoV-19^^Washington|Human|UW Virology Lab|UW Virology Lab|Roychoudhury|USA
>Spike|hCoV-19/England/20144072804/2020|2020-04-01|EPI_ISL_421962|Original|hCoV-19^^England|Human|Respiratory Virus Unit|Respiratory Virus Unit|Platt|United Kingdom
>Spike|hCoV-19/England/LEED-2A85BE/2020|2020-03-27|EPI_ISL_539031|Original|hCoV-19^^England|Human|Leeds Teaching Hospitals NHS Trust and Public Health England|Wellcome Sanger Institute for the COVID-19 Genomics UK (COG-UK) consortium|Davies|United Kingdom
>Spike|hCoV-19/England/CAMC-B7ADA5/2020|2020-11-13|EPI_ISL_723961|Original|hCoV-19^^England|Human|Oxford Viromics|COVID-19 Genomics UK (COG-UK) Consortium|Golubchik|United Kingdom
>Spike|hCoV-19/USA/CA-CZB-11129/2020|2020-05-30|EPI_ISL_625519|Original|hCoV-19^^California|Human|Alameda County Public Health Lab|Chan-Zuckerberg Biohub|Bhatt|USA
>Spike|hCoV-19/England/MILK-99369E/2020|2020-08-26|EPI_ISL_552099|Original|hCoV-19^^England|Human|Lighthouse Lab in Milton Keynes|Wellcome Sanger Institute for the COVID-19 Genomics UK (COG-UK) consortium|Davies|United Kingdom
>Spike|hCoV-19/Scotland/EDB4295/2020|2020-04-23|EPI_ISL_449290|Original|hCoV-19^^Scotland|Human|Virology Department|COVID-19 Genomics UK (COG-UK) Consortium|Williams|United Kingdom
>Spike|hCoV-19/Denmark/DCGC-15050/2020|2020-11-23|EPI_ISL_713300|Original|hCoV-19^^Syddanmark|Human|Department of Virus and Microbiological Special Diagnostics|Albertsen Lab|Michaelsen|Denmark
>Spike|hCoV-19/USA/FL-BPHL-1160/2020|2020-08-17|EPI_ISL_526646|Original|hCoV-19^^Florida|Human|Florida Bureau of Public Health Laboratories|Florida Bureau of Public Health Laboratories|Blanton|USA
>Spike|hCoV-19/England/QEUH-A63EF8/2020|2020-10-12|EPI_ISL_628676|Original|hCoV-19^^England|Human|Oxford Viromics|COVID-19 Genomics UK (COG-UK) Consortium|Golubchik|United Kingdom
>Spike|hCoV-19/USA/TX-HMH-MCoV-13476/2020|2020-08-21|EPI_ISL_788606|Original|hCoV-19^^Texas|Human|Houston Methodist Hospital|Houston Methodist Hospital|Long|USA
>Spike|hCoV-19/Canada/QC-LSPQ-L00236922/2020|2020-03-25|EPI_ISL_536152|Original|hCoV-19^^Quebec|Human|H\u00f4pital Pierre-Le Gardeur|Laboratoire de sant\u00e9 publique du Qu\u00e9bec|Moreira|Canada

>Spike|hCoV-19/Gambia/NPHL-2268/2020|2020-00-00|EPI_ISL_561242|Original|hCoV-19^^Kombo|Human|MRCG at LSHTM Genomics lab|MRCG at LSHTM Genomics lab|Sesay|Gambia
>Spike|hCoV-19/England/LEED-2AA033/2020|2020-04-08|EPI_ISL_538967|Original|hCoV-19^^England|Human|Leeds Teaching Hospitals NHS Trust and Public Health England|Wellcome Sanger Institute for the COVID-19 Genomics UK (COG-UK) consortium|Davies|United Kingdom
>Spike|hCoV-19/England/QEUH-AD28F6/2020|2020-10-25|EPI_ISL_634151|Original|hCoV-19^^England|Human|Lighthouse Lab in Glasgow|Wellcome Sanger Institute for the COVID-19 Genomics UK (COG-UK) consortium|Davies|United Kingdom
>Spike|hCoV-19/Russia/MOS-CRIE-13672647/2020|2020-11-09|EPI_ISL_754201|Original|hCoV-19^^Moscow|Human|Molecular diagnostic laboratory of Federal Budget Institution of Science \Central Research Institute of Epidemiology\ of The Federal Service on Customers' Rights Protection and Human Well-being Surveillance|Group of Genomics and Postgenomic Technologies of Central Research Institute of Epidemilolog
>y|Korneenko|Russia
>Spike|hCoV-19/England/NORW-E8666/2020|2020-04-17|EPI_ISL_449170|Original|hCoV-19^^England|Human|Quadram Institute Bioscience|COVID-19 Genomics UK (COG-UK) Consortium|Alikhan|United Kingdom
>Spike|hCoV-19/England/LIVE-A77F7/2020|2020-03-27|EPI_ISL_517074|Original|hCoV-19^^England|Human|Liverpool Clinical Laboratories|COVID-19 Genomics UK (COG-UK) Consortium|Haldenby|United Kingdom
>Spike|hCoV-19/USA/TX-HMH-MCoV-13888/2020|2020-08-17|EPI_ISL_787180|Original|hCoV-19^^Texas|Human|Houston Methodist Hospital|Houston Methodist Hospital|Long|USA
>Spike|hCoV-19/Australia/VIC17122/2020|2020-08-06|EPI_ISL_663744|Original|hCoV-19^^Victoria|Human|Microbiological Diagnostic Unit - Public Health Laboratory (MDU-PHL)|MDU-PHL|Schultz|Australia
>Spike|hCoV-19/England/MILK-AB8D42/2020|2020-10-22|EPI_ISL_623704|Original|hCoV-19^^England|Human|Lighthouse Lab in Milton Keynes|Wellcome Sanger Institute for the COVID-19 Genomics UK (COG-UK) consortium|Davies|United Kingdom
>Spike|hCoV-19/Canada/QC_AE2/2020|2020-04-05|EPI_ISL_515198|Original|hCoV-19^^Quebec|Human|Laboratoire de microbiologie|Smith Laboratory|Smith|Canada
>Spike|hCoV-19/Russia/AD-RII-MH1684S/2020|2020-09-09|EPI_ISL_596315|Original|hCoV-19^^Adygea|Human|HELIX LCC|WHO National Influenza Centre Russian Federation|Komissarov|Russia
>Spike|hCoV-19/Hong Kong/HKU-IHCE0511-009a/2020|2020-03-11|EPI_ISL_538535|Original|hCoV-19^^Hong Kong|Human|Department of Microbiology|Department of Microbiology|Chan|China
>Spike|hCoV-19/England/ALDP-A6D30E/2020|2020-10-14|EPI_ISL_608254|Original|hCoV-19^^England|Human|Lighthouse Lab in Alderley Park|Wellcome Sanger Institute for the COVID-19 Genomics UK (COG-UK) consortium|Davies|United Kingdom
>Spike|hCoV-19/Netherlands/ZH-EMC-642/2020|2020-09-21|EPI_ISL_578070|Original|hCoV-19^^Zuid Holland|Human|Dutch COVID-19 response team|Erasmus Medical Center|Oude Munnink|Netherlands
>Spike|hCoV-19/Spain/VC-IBV-98003094/2020|2020-03-14|EPI_ISL_538030|Original|hCoV-19^^Comunitat Valenciana|Human|Hospital Universitari i Polit\u00e8cnic La Fe de Val\u00e8ncia|SeqCOVID-SPAIN consortium/IBV(CSIC)|Comas|Spain
>Spike|hCoV-19/USA/TX-HMH-MCoV-18340/2020|2020-11-18|EPI_ISL_784688|Original|hCoV-19^^Texas|Human|Houston Methodist Hospital|Houston Methodist Hospital|Long|USA
>Spike|hCoV-19/England/20174004304/2020|2020-04-21|EPI_ISL_465384|Original|hCoV-19^^England|Human|Respiratory Virus Unit|Respiratory Virus Unit|Platt|United Kingdom
>Spike|hCoV-19/England/ALDP-9BEC40/2020|2020-09-09|EPI_ISL_568181|Original|hCoV-19^^England|Human|Lighthouse Lab in Alderley Park|Wellcome Sanger Institute for the COVID-19 Genomics UK (COG-UK) consortium|Davies|United Kingdom
>Spike|hCoV-19/Luxembourg/LNS9600094/2020|2020-11-02|EPI_ISL_744947|Original|hCoV-19^^Luxembourg|Human|Laboratoire national de sant\u00e9|Laboratoire national de sant\u00e9|Wienecke-Baldacchino|Luxembourg
>Spike|hCoV-19/Singapore/612/2020|2020-05-10|EPI_ISL_490062|Original|hCoV-19^^Singapore|Human|National Public Health Laboratory|National Public Health Laboratory|Mak|Singapore

>Spike|hCoV-19/England/QEUH-A00E48/2020|2020-09-30|EPI_ISL_584902|Original|hCoV-
19^^England|Human|University of Exeter|COVID-19 Genomics UK (COG-UK)
Consortium|Temperton|United Kingdom
>Spike|hCoV-19/England/MILK-9E1066/2020|2020-09-20|EPI_ISL_580913|Original|hCoV-
19^^England|Human|Lighthouse Lab in Milton Keynes|Wellcome Sanger Institute for the
COVID-19 Genomics UK (COG-UK) consortium|Davies|United Kingdom
>Spike|hCoV-19/England/QEUH-C93F63/2020|2020-12-17|EPI_ISL_777493|Original|hCoV-
19^^England|Human|Lighthouse Lab in Glasgow|Wellcome Sanger Institute for the COVID-
19 Genomics UK (COG-UK) Consortium|Davies|United Kingdom
>Spike|hCoV-19/Denmark/DCGC-3829/2020|2020-08-31|EPI_ISL_622705|Original|hCoV-
19^^Hovedstaden|Human|Department of Virus and Microbiological Special
Diagnostics|Albertsen lab|Michaelsen|Denmark
>Spike|hCoV-19/Belgium/ULG-10989/2020|2020-11-14|EPI_ISL_707758|Original|hCoV-
19^^Li\u00e8ge|Human|Department of Clinical Microbiology|GIGA Medical
Genomics|Durkin|Belgium
>Spike|hCoV-19/Ireland/CN-NVRL-20IRL82669/2020|2020-10-
05|EPI_ISL_639889|Original|hCoV-19^^Cavan|Human|National Virus Reference
Laboratory|National Virus Reference Laboratory|Gonzalez|Ireland
>Spike|hCoV-19/Wales/PHWC-48B546/2020|2020-11-05|EPI_ISL_666251|Original|hCoV-
19^^Wales|Human|Wales Specialist Virology Centre Sequencing lab|COVID-19 Genomics UK
(COG-UK) Consortium|Moore|United Kingdom
>Spike|hCoV-19/Luxembourg/LNS4280461/2020|2020-10-26|EPI_ISL_740442|Original|hCoV-
19^^Luxembourg|Human|Laboratoire national de sant\u00e9|Laboratoire national de
sant\u00e9|Wienecke-Baldacchino|Luxembourg
>Spike|hCoV-19/USA/DE-DHSS-F981902/2020|2020-08-11|EPI_ISL_708420|Original|hCoV-
19^^Delaware|Human|Delaware Public Health Lab|Delaware Public Health Lab|Bajwa|USA
>Spike|hCoV-19/England/ALDP-B84C32/2020|2020-11-15|EPI_ISL_675901|Original|hCoV-
19^^England|Human|Lighthouse Lab in Alderley Park|Wellcome Sanger Institute for the
COVID-19 Genomics UK (COG-UK) Consortium|Davies|United Kingdom
>Spike|hCoV-19/USA/CO-CDPHE-2008022512/2020|2020-07-31|EPI_ISL_771190|Original|hCoV-
19^^Colorado|Human|Colorado Department of Public Health and Environment|Colorado
Department of Puplic Health and Environment|Bankers|USA
>Spike|hCoV-19/Australia/VIC5756/2020|2020-07-21|EPI_ISL_518554|Original|hCoV-
19^^Victoria|Human|Microbiological Diagnostic Unit - Public Health Laboratory (MDU-
PHL)|MDU-PHL|Schultz|Australia
>Spike|hCoV-19/England/ALDP-5D9AB2/2020|2020-06-20|EPI_ISL_558514|Original|hCoV-
19^^England|Human|Lighthouse Lab in Alderley Park|Wellcome Sanger Institute for the
COVID-19 Genomics UK (COG-UK) consortium|Davies|United Kingdom
>Spike|hCoV-19/USA/LA-EVTL978/2020|2020-08-18|EPI_ISL_653669|Original|hCoV-
19^^Louisiana|Human|LSUHS Emerging Viral Threat Laboratory|Microbial Genome
Sequencing Center|Kamil|USA
>Spike|hCoV-19/Australia/VIC9987/2020|2020-08-24|EPI_ISL_562751|Original|hCoV-
19^^Victoria|Human|Microbiological Diagnostic Unit - Public Health Laboratory (MDU-
PHL)|MDU-PHL|Schultz|Australia
>Spike|hCoV-19/Israel/CVL-47-ngs/2020|2020-12-24|EPI_ISL_776634|Original|hCoV-
19^^Israel|Human|Israel Central Virology laboratory|Israel Central Virology
laboratory|Zuckerman|Israel
>Spike|hCoV-19/Portugal/PT0740/2020|2020-03-21|EPI_ISL_510999|Original|hCoV-
19^^Portugal|Human|Instituto Nacional de Saude (INSA)|Instituto Nacional de Saude
(INSA)|Borges|Portugal
>Spike|hCoV-19/USA/TX-HMH-MCoV-14037/2020|2020-08-14|EPI_ISL_787326|Original|hCoV-
19^^Texas|Human|Houston Methodist Hospital|Houston Methodist Hospital|Long|USA
>Spike|hCoV-19/Belgium/rega-0717846/2020|2020-06-17|EPI_ISL_734798|Original|hCoV-
19^^Diest|Human|UZ Leuven|KU Leuven|Maes|Belgium
>Spike|hCoV-19/USA/OR-OHSU-2799/2020|2020-08-31|EPI_ISL_667316|Original|hCoV-
19^^Oregon|Human|OHSU Lab Services Molecular Microbiology Lab|Oregon SARS-CoV-2
Genome Sequencing Center|O'Roak|USA
>Spike|hCoV-19/USA/TX-HMH-MCoV-8798/2020|2020-07-02|EPI_ISL_780762|Original|hCoV-
19^^Texas|Human|Houston Methodist Hospital|Houston Methodist Hospital|Long|USA

>Spike|hCoV-19/USA/TX-HMH-4408/2020|2020-06-17|EPI_ISL_543597|Original|hCoV-19^^Texas|Human|Houston Methodist Hospital|Houston Methodist Hospital|Long|USA
>Spike|hCoV-19/Sweden/20-53337/2020|2020-11-04|EPI_ISL_710613|Original|hCoV-19^^Halland|Human|Barnakuten|The Public Health Agency of Sweden|Svartstr\u00f6m|Sweden
>Spike|hCoV-19/England/QEUH-5424C5/2020|2020-05-14|EPI_ISL_533359|Original|hCoV-19^^England|Human|Lighthouse Lab in Glasgow|Wellcome Sanger Institute for the COVID-19 Genomics UK (COG-UK) consortium|Davies|United Kingdom
>Spike|hCoV-19/England/QEUH-B127A1/2020|2020-11-02|EPI_ISL_642221|Original|hCoV-19^^England|Human|Lighthouse Lab in Glasgow|Wellcome Sanger Institute for the COVID-19 Genomics UK (COG-UK) Consortium|Davies|United Kingdom
>Spike|hCoV-19/USA/TX-HMH-MCoV-8225/2020|2020-07-07|EPI_ISL_784276|Original|hCoV-19^^Texas|Human|Houston Methodist Hospital|Houston Methodist Hospital|Long|USA
>Spike|hCoV-19/England/QEUH-9A41A8/2020|2020-09-01|EPI_ISL_540222|Original|hCoV-19^^England|Human|Lighthouse Lab in Glasgow|Wellcome Sanger Institute for the COVID-19 Genomics UK (COG-UK) consortium|Davies|United Kingdom
>Spike|hCoV-19/England/205160929/2020|2020-12-12|EPI_ISL_766425|Original|hCoV-19^^England|Human|Respiratory Virus Unit|COVID-19 Genomics UK (COG-UK) Consortium|Platt|United Kingdom
>Spike|hCoV-19/Ireland/D-NVRL-MPH55349/2020|2020-04-13|EPI_ISL_767779|Original|hCoV-19^^Dublin|Human|National Virus Reference Laboratory|Irish Coronavirus Sequencing Consortium - Teagasc Moorepark|Walsh|Ireland
>Spike|hCoV-19/Japan/PG-6735/2020|2020-08-00|EPI_ISL_687846|Original|hCoV-19^^Japan|Human|Pathogen Genomics Center|Pathogen Genomics Center|Sekizuka|Japan
>Spike|hCoV-19/Scotland/QEUH-99822C/2020|2020-08-26|EPI_ISL_534440|Original|hCoV-19^^Scotland|Human|NHSGGC West of Scotland Specialist Virology Centre / MRC-University of Glasgow Centre for Virus Research|Wellcome Sanger Institute for the COVID-19 Genomics UK (COG-UK) consortium|Davies|United Kingdom
>Spike|hCoV-19/USA/LA-GBCL-26513025/2020|2020-04-22|EPI_ISL_485078|Original|hCoV-19^^Louisiana|Human|River Road Testing Lab|Ginkgo Bioworks Clinical Laboratory|Smith|USA
>Spike|hCoV-19/England/BRIS-1853B3F/2020|2020-04-26|EPI_ISL_492328|Original|hCoV-19^^England|Human|PHE South West Regional Laboratory|Wellcome Sanger Institute for the COVID-19 Genomics UK (COG-UK) consortium|Davies|United Kingdom
>Spike|hCoV-19/USA/MO-WUSTL043/2020|2020-06-19|EPI_ISL_493074|Original|hCoV-19^^Missouri|Human|Washington University in St. Louis|Washington University in St. Louis|Tahan|USA
>Spike|hCoV-19/Brazil/CE-L10-CD170/2020|2020-03-16|EPI_ISL_476183|Original|hCoV-19^^Cear\u00e1|Human|DB Diagn\u00f3sticos do Brasil|Instituto de Medicina Tropical da Univesidade de S\u00e3o Paulo|Faria|Brazil
>Spike|hCoV-19/England/ALDP-5B5E38/2020|2020-06-19|EPI_ISL_558649|Original|hCoV-19^^England|Human|Lighthouse Lab in Alderley Park|Wellcome Sanger Institute for the COVID-19 Genomics UK (COG-UK) consortium|Davies|United Kingdom
>Spike|hCoV-19/Australia/VIC5814/2020|2020-07-20|EPI_ISL_518609|Original|hCoV-19^^Victoria|Human|Microbiological Diagnostic Unit - Public Health Laboratory (MDU-PHL)|MDU-PHL|Schultz|Australia
>Spike|hCoV-19/USA/MI-UM-10036524165/2020|2020-10-19|EPI_ISL_628882|Original|hCoV-19^^Michigan|Human|University of Michigan Clinical Microbiology Laboratory|Lauring Lab|Valesano|USA
>Spike|hCoV-19/USA/MI-MDHHS-SC20137/2020|2020-03-20|EPI_ISL_436829|Original|hCoV-19^^Michigan|Human|Michigan Department of Health and Human Services|Michigan Department of Health and Human Services|Blankenship|USA
>Spike|hCoV-19/USA/VA-DCLS-1838/2020|2020-09-17|EPI_ISL_614130|Original|hCoV-19^^Virginia|Human|Virginia DCLS|Virginia DCLS|St. Jacques|USA
>Spike|hCoV-19/Wales/PHWC-496E6B/2020|2020-11-24|EPI_ISL_726738|Original|hCoV-19^^Wales|Human|Originating lab|COVID-19 Genomics UK (COG-UK) Consortium|Moore|United Kingdom
>Spike|hCoV-19/England/SHEF-10CF547/2020|2020-12-04|EPI_ISL_742016|Original|hCoV-19^^England|Human|Virology Department|COVID-19 Genomics UK (COG-UK) Consortium|de Silva|United Kingdom

>Spike|hCoV-19/USA/WA-UW-32820/2020|2020-10-15|EPI_ISL_737090|Original|hCoV-19^^Washington|Human|UW Virology Lab|UW Virology Lab|Roychoudhury|USA
>Spike|hCoV-19/Scotland/EDB9979/2020|2020-11-18|EPI_ISL_680555|Original|hCoV-19^^Scotland|Human|Virology Department|COVID-19 Genomics UK (COG-UK) Consortium|McHugh|United Kingdom
>Spike|hCoV-19/England/MILK-9D53E4/2020|2020-09-19|EPI_ISL_577055|Original|hCoV-19^^England|Human|Centre for Enzyme Innovation|COVID-19 Genomics UK (COG-UK) Consortium|Robson|United Kingdom
>Spike|hCoV-19/USA/IL-QDX-1098/2020|2020-03-19|EPI_ISL_571842|Original|hCoV-19^^Illinois|Human|Quest Diagnostics|Quest Diagnostics|Kagan|USA
>Spike|hCoV-19/England/CAMB-1B7CD6/2020|2020-10-19|EPI_ISL_705541|Original|hCoV-19^^England|Human|Department of Pathology|COVID-19 Genomics UK (COG-UK) Consortium|Goodfellow|United Kingdom
>Spike|hCoV-19/England/MILK-C2FD9F/2020|2020-12-07|EPI_ISL_782760|Original|hCoV-19^^England|Human|Lighthouse Lab in Milton Keynes|Wellcome Sanger Institute for the COVID-19 Genomics UK (COG-UK) Consortium|Davies|United Kingdom
>Spike|hCoV-19/England/NORT-1B725DF/2020|2020-11-30|EPI_ISL_724888|Original|hCoV-19^^England|Human|Northumbria University / South Tees Hospitals NHS Foundation Trust / North Cumbria Integrated Care NHS Foundation Trust / North Tees and Hartlepool NHS Foundation Trust / Newcastle Hospitals NHS Foundation Trust|COVID-19 Genomics UK (COG-UK) Consortium|Bashton|United Kingdom
>Spike|hCoV-19/USA/NY-NYCPHL-000544/2020|2020-03-22|EPI_ISL_632004|Original|hCoV-19^^New York|Human|Flushing Hospital Medical Center|New York City Public Health Laboratory|Wang|USA
>Spike|hCoV-19/USA/UT-UPHL-201226418/2020|2020-03-31|EPI_ISL_672541|Original|hCoV-19^^Utah|Human|Utah Public Health Laboratory|Utah Public Health Laboratory|Young|USA
>Spike|hCoV-19/USA/IA-GMF-18913/2020|2020-09-04|EPI_ISL_547788|Original|hCoV-19^^Iowa|Human|Gundersen Molecular Diagnostics Laboratory|Kabara Cancer Research Institute|Kenny|USA
>Spike|hCoV-19/USA/TX-HMH-2824/2020|2020-06-08|EPI_ISL_543799|Original|hCoV-19^^Texas|Human|Houston Methodist Hospital|Houston Methodist Hospital|Long|USA
>Spike|hCoV-19/Spain/CT-IBV-97007970/2020|2020-10-02|EPI_ISL_654526|Original|hCoV-19^^Catalunya|Human|Servicio de Microbiolog\u00eda|SeqCOVID-SPAIN consortium/IBV(CSIC)|Comas|Spain
>Spike|hCoV-19/England/ALDP-CB52F9/2020|2020-12-18|EPI_ISL_761865|Original|hCoV-19^^England|Human|Lighthouse Lab in Alderley Park|Wellcome Sanger Institute for the COVID-19 Genomics UK (COG-UK) Consortium|Davies|United Kingdom
>Spike|hCoV-19/England/NOTT-1155BD/2020|2020-10-14|EPI_ISL_595250|Original|hCoV-19^^England|Human|Queens Medical Centre|COVID-19 Genomics UK (COG-UK) Consortium|Loose|United Kingdom
>Spike|hCoV-19/England/20108007302/2020|2020-03-03|EPI_ISL_417256|Original|hCoV-19^^England|Human|Respiratory Virus Unit|Respiratory Virus Unit|Platt|United Kingdom
>Spike|hCoV-19/Denmark/DCGC-16089/2020|2020-11-16|EPI_ISL_714109|Original|hCoV-19^^Sjaelland|Human|Department of Virus and Microbiological Special Diagnostics|Albertsen Lab|Michaelsen|Denmark
>Spike|hCoV-19/USA/FL-BPHL-2129/2020|2020-04-08|EPI_ISL_653282|Original|hCoV-19^^Florida|Human|Florida Bureau of Public Health Laboratories|Florida Bureau of Public Health Laboratories|Blanton|USA
>Spike|hCoV-19/USA/WI-WSLH-200568/2020|2020-06-01|EPI_ISL_578680|Original|hCoV-19^^Wisconsin|Human|Wisconsin State Laboratory of Hygiene Communicable Disease Division|Wisconsin State Laboratory of Hygiene Communicable Disease Division|Florek|USA
>Spike|hCoV-19/USA/VA-DCLS-1963/2020|2020-10-07|EPI_ISL_644885|Original|hCoV-19^^Virginia|Human|Virginia DCLS|Virginia DCLS|St. Jacques|USA
>Spike|hCoV-19/England/QEUH-9628DE/2020|2020-08-10|EPI_ISL_531503|Original|hCoV-19^^England|Human|Lighthouse Lab in Glasgow|Wellcome Sanger Institute for the COVID-19 Genomics UK (COG-UK) consortium|Davies|United Kingdom
>Spike|hCoV-19/England/MILK-B045D5/2020|2020-10-24|EPI_ISL_652163|Original|hCoV-19^^England|Human|Department of Pathology|COVID-19 Genomics UK (COG-UK) Consortium|Goodfellow|United Kingdom

>Spike|hCoV-19/Wales/PHWC-489BDD/2020|2020-10-28|EPI_ISL_652466|Original|hCoV-19^^Wales|Human|Wales Specialist Virology Centre Sequencing lab|COVID-19 Genomics UK (COG-UK) Consortium|Moore|United Kingdom
>Spike|hCoV-19/USA/TX-HMH-519/2020|2020-04-03|EPI_ISL_544585|Original|hCoV-19^^Texas|Human|Houston Methodist Hospital|Houston Methodist Hospital|Long|USA
>Spike|hCoV-19/England/EXET-138F2F/2020|2020-10-29|EPI_ISL_665049|Original|hCoV-19^^England|Human|University of Exeter|COVID-19 Genomics UK (COG-UK) Consortium|Temperton|United Kingdom
>Spike|hCoV-19/USA/FL-BPHL-0331/2020|2020-04-13|EPI_ISL_489740|Original|hCoV-19^^Florida|Human|Florida Bureau of Public Health Laboratories|Florida Bureau of Public Health Laboratories|Blanton|USA
>Spike|hCoV-19/USA/CA-CZB-13938/2020|2020-06-22|EPI_ISL_739644|Original|hCoV-19^^California|Human|Alameda County Public Health Lab|Chan-Zuckerberg Biohub|Kamm|USA
>Spike|hCoV-19/USA/FL-BPHL-024/2020|2020-04-15|EPI_ISL_436528|Original|hCoV-19^^Florida|Human|Florida Bureau of Public Health Laboratories|Florida Bureau of Public Health Laboratories|Blanton|USA
>Spike|hCoV-19/USA/NY-NYCPHL-001290/2020|2020-11-09|EPI_ISL_683904|Original|hCoV-19^^New York|Human|DOHMH Crown Heights|New York City Public Health Laboratory|Wang|USA
>Spike|hCoV-19/USA/MI-UM-10036114947/2020|2020-09-24|EPI_ISL_583404|Original|hCoV-19^^Michigan|Human|University of Michigan Clinical Microbiology Laboratory|Lauring Lab|Valesano|USA
>Spike|hCoV-19/England/MILK-99CB0F/2020|2020-08-31|EPI_ISL_550785|Original|hCoV-19^^England|Human|Lighthouse Lab in Milton Keynes|Wellcome Sanger Institute for the COVID-19 Genomics UK (COG-UK) consortium|Davies|United Kingdom
>Spike|hCoV-19/England/ALDP-A2DC3C/2020|2020-10-05|EPI_ISL_586749|Original|hCoV-19^^England|Human|Lighthouse Lab in Alderley Park|Wellcome Sanger Institute for the COVID-19 Genomics UK (COG-UK) consortium|Davies|United Kingdom
>Spike|hCoV-19/USA/OR-OHSU-2543/2020|2020-08-19|EPI_ISL_667245|Original|hCoV-19^^Oregon|Human|OHSU Lab Services Molecular Microbiology Lab|Oregon SARS-CoV-2 Genome Sequencing Center|O'Roak|USA
>Spike|hCoV-19/USA/TX-HMH-MCoV-13484/2020|2020-08-22|EPI_ISL_788613|Original|hCoV-19^^Texas|Human|Houston Methodist Hospital|Houston Methodist Hospital|Long|USA
>Spike|hCoV-19/England/ALDP-95C96B/2020|2020-06-18|EPI_ISL_553535|Original|hCoV-19^^England|Human|Lighthouse Lab in Alderley Park|Wellcome Sanger Institute for the COVID-19 Genomics UK (COG-UK) consortium|Davies|United Kingdom
>Spike|hCoV-19/Luxembourg/LNS9941347/2020|2020-10-25|EPI_ISL_745017|Original|hCoV-19^^Luxembourg|Human|Laboratoire national de sant\u00e9|Laboratoire national de sant\u00e9|Wienecke-Baldacchino|Luxembourg
>Spike|hCoV-19/Denmark/DCGC-21653/2020|2020-12-21|EPI_ISL_758289|Original|hCoV-19^^Hovedstaden|Human|Department of Virus and Microbiological Special Diagnostics|Albertsen Lab|Michaelsen|Denmark
>Spike|hCoV-19/USA/MI-UM-10036796409/2020|2020-11-05|EPI_ISL_707851|Original|hCoV-19^^Michigan|Human|University of Michigan Clinical Microbiology Laboratory|Lauring Lab|Valesano|USA
>Spike|hCoV-19/USA/WI-UW-2620/2021|2021-01-03|EPI_ISL_791137|Original|hCoV-19^^Wisconsin|Human|University of Wisconsin-Madison AIDS Vaccine Research Laboratories|University of Wisconsin-Madison AIDS Vaccine Research Laboratories|Moreno|USA
>Spike|hCoV-19/Wales/PHWC-496557/2020|2020-11-27|EPI_ISL_726598|Original|hCoV-19^^Wales|Human|Originating lab|COVID-19 Genomics UK (COG-UK) Consortium|Moore|United Kingdom
>Spike|hCoV-19/England/MILK-A79ABF/2020|2020-10-15|EPI_ISL_606688|Original|hCoV-19^^England|Human|Lighthouse Lab in Milton Keynes|Wellcome Sanger Institute for the COVID-19 Genomics UK (COG-UK) consortium|Davies|United Kingdom
>Spike|hCoV-19/Spain/VC-FISABIO-581/2020|2020-03-28|EPI_ISL_447511|Original|hCoV-19^^Comunitat Valenciana|Human|Servicio de Microbiologia. Hospital Clinico Universitario de Valencia|Sequencing and Bioinformatics Service and Molecular Epidemiology Research Group. FISABIO-Public Health|Gonzalez-Candelas|Spain

>Spike|hCoV-19/England/CAMC-B0CD6C/2020|2020-10-30|EPI_ISL_643360|Original|hCoV-19^^England|Human|Lighthouse Lab in Cambridge|Wellcome Sanger Institute for the COVID-19 Genomics UK (COG-UK) Consortium|Davies|United Kingdom
>Spike|hCoV-19/USA/CA-ALSR-2386/2020|2020-07-06|EPI_ISL_635453|Original|hCoV-19^^California|Human|San Diego County Public Health Laboratory|Andersen lab at Scripps Research|Gangavarapu|USA
>Spike|hCoV-19/England/LOND-12614DF/2020|2020-04-02|EPI_ISL_524711|Original|hCoV-19^^England|Human|North West London Pathology|Wellcome Sanger Institute for the COVID-19 Genomics UK (COG-UK) consortium|Davies|United Kingdom
>Spike|hCoV-19/England/HECH-2599C76/2020|2020-05-05|EPI_ISL_482079|Original|hCoV-19^^England|Human|Microbiology Department|Wellcome Sanger Institute for the COVID-19 Genomics UK (COG-UK) consortium|Davies|United Kingdom
>Spike|hCoV-19/Wales/PHWC-166A72/2020|2020-06-27|EPI_ISL_494075|Original|hCoV-19^^Wales|Human|Originating lab|COVID-19 Genomics UK (COG-UK) Consortium|Moore|United Kingdom
>Spike|hCoV-19/England/MILK-B96B9A/2020|2020-11-13|EPI_ISL_675116|Original|hCoV-19^^England|Human|Lighthouse Lab in Milton Keynes|Wellcome Sanger Institute for the COVID-19 Genomics UK (COG-UK) Consortium|Davies|United Kingdom
>Spike|hCoV-19/England/CAMC-B07208/2020|2020-10-30|EPI_ISL_643665|Original|hCoV-19^^England|Human|Lighthouse Lab in Cambridge|Wellcome Sanger Institute for the COVID-19 Genomics UK (COG-UK) Consortium|Davies|United Kingdom
>Spike|hCoV-19/England/QEUH-BA078A/2020|2020-11-18|EPI_ISL_719770|Original|hCoV-19^^England|Human|Lighthouse Lab in Glasgow|Wellcome Sanger Institute for the COVID-19 Genomics UK (COG-UK) Consortium|Davies|United Kingdom
>Spike|hCoV-19/England/MILK-B8D325/2020|2020-11-13|EPI_ISL_673901|Original|hCoV-19^^England|Human|Lighthouse Lab in Milton Keynes|Wellcome Sanger Institute for the COVID-19 Genomics UK (COG-UK) Consortium|Davies|United Kingdom
>Spike|hCoV-19/USA/TX-HMH-MCoV-15948/2020|2020-07-10|EPI_ISL_785632|Original|hCoV-19^^Texas|Human|Houston Methodist Hospital|Houston Methodist Hospital|Long|USA
>Spike|hCoV-19/England/LOND-D667A/2020|2020-04-22|EPI_ISL_487631|Original|hCoV-19^^England|Human|University College London|Wellcome Sanger Institute for the COVID-19 Genomics UK (COG-UK) consortium|Davies|United Kingdom
>Spike|hCoV-19/USA/TX-HMH-4587/2020|2020-06-21|EPI_ISL_543737|Original|hCoV-19^^Texas|Human|Houston Methodist Hospital|Houston Methodist Hospital|Long|USA
>Spike|hCoV-19/Denmark/DCGC-15046/2020|2020-11-23|EPI_ISL_713603|Original|hCoV-19^^Hovedstaden|Human|Department of Virus and Microbiological Special Diagnostics|Albertsen Lab|Michaelsen|Denmark
>Spike|hCoV-19/USA/MI-MDHHS-SC20978/2020|2020-06-10|EPI_ISL_507766|Original|hCoV-19^^Michigan|Human|Michigan Department of Health and Human Services|Michigan Department of Health and Human Services|Blankenship|USA
>Spike|hCoV-19/Switzerland/BL-ETHZ-400101/2020|2020-12-07|EPI_ISL_729143|Original|hCoV-19^^Basel-Landschaft|Human|Viollier AG|Department of Biosystems Science and Engineering|Nadeau|Switzerland
>Spike|hCoV-19/USA/FL-BPHL-0344/2020|2020-04-11|EPI_ISL_489753|Original|hCoV-19^^Florida|Human|Florida Bureau of Public Health Laboratories|Florida Bureau of Public Health Laboratories|Blanton|USA
>Spike|hCoV-19/Wales/PHWC-49249A/2020|2020-11-17|EPI_ISL_706850|Original|hCoV-19^^Wales|Human|Originating lab|COVID-19 Genomics UK (COG-UK) Consortium|Moore|United Kingdom
>Spike|hCoV-19/Australia/NSW740/2020|2020-07-30|EPI_ISL_513383|Original|hCoV-19^^New South Wales|Human|Sydney South West Pathology Service (SSWPS) - Liverpool Hospital - NSW Health Pathology|NSW Health Pathology - Institute of Clinical Pathology and Medical Research; Westmead Hospital; University of Sydney|Gall|Australia
>Spike|hCoV-19/England/QEUH-A79053/2020|2020-10-10|EPI_ISL_638781|Original|hCoV-19^^England|Human|Oxford Viromics|COVID-19 Genomics UK (COG-UK) Consortium|Golubchik|United Kingdom
>Spike|hCoV-19/England/SHEF-C6EEE/2020|2020-03-25|EPI_ISL_442004|Original|hCoV-19^^England|Human|Virology Department|COVID-19 Genomics UK (COG-UK) Consortium|de Silva|United Kingdom

>Spike|hCoV-19/England/CAMC-B20C38/2020|2020-11-02|EPI_ISL_647529|Original|hCoV-19^^England|Human|Lighthouse Lab in Cambridge|Wellcome Sanger Institute for the COVID-19 Genomics UK (COG-UK) Consortium|Davies|United Kingdom
>Spike|hCoV-19/Scotland/GCVR-171063/2020|2020-03-27|EPI_ISL_459582|Original|hCoV-19^^Scotland|Human|NHSGGC West of Scotland Specialist Virology Centre / MRC-University of Glasgow Centre for Virus Research|Wellcome Sanger Institute for the COVID-19 Genomics UK (COG-UK) consortium|Davies|United Kingdom
>Spike|hCoV-19/Netherlands/NA_74/2020|2020-03-10|EPI_ISL_422834|Original|hCoV-19^^Netherlands|Human|Dutch COVID-19 response team|Erasmus Medical Center|Oude Munnink|Netherlands
>Spike|hCoV-19/Australia/VIC6875/2020|2020-07-28|EPI_ISL_564109|Original|hCoV-19^^Victoria|Human|Victorian Infectious Diseases Reference Laboratory (VIDRL)|VIDRL and MDU-PHL|Schultz|Australia
>Spike|hCoV-19/England/QEUH-9AE88B/2020|2020-09-02|EPI_ISL_577248|Original|hCoV-19^^England|Human|University of Exeter|COVID-19 Genomics UK (COG-UK) Consortium|Temperton|United Kingdom
>Spike|hCoV-19/USA/WA-S2566/2020|2020-06-18|EPI_ISL_497198|Original|hCoV-19^^Washington|Human|Washington State Department of Health|Seattle Flu Study|Lee|USA
>Spike|hCoV-19/Germany/HH-hpi-p2425/2020|2020-04-26|EPI_ISL_776494|Original|hCoV-19^^Hamburg|Human|University Medical Center Hamburg Eppendorf|Heinrich Pette Institute|Robitaille|Germany
>Spike|hCoV-19/USA/WA-S65/2020|2020-03-03|EPI_ISL_417118|Original|hCoV-19^^Washington|Human|Washington State Department of Health|Seattle Flu Study|Bedford|USA
>Spike|hCoV-19/Denmark/DCGC-20465/2020|2020-12-21|EPI_ISL_759168|Original|hCoV-19^^Midtjylland|Human|Department of Virus and Microbiological Special Diagnostics|Albertsen Lab|Michaelsen|Denmark
>Spike|hCoV-19/Denmark/DCGC-11122/2020|2020-11-02|EPI_ISL_670403|Original|hCoV-19^^Hovedstaden|Human|Department of Virus and Microbiological Special Diagnostics|Albertsen Lab|Michaelsen|Denmark
>Spike|hCoV-19/USA/DE-DHSS-F1014168/2020|2020-10-09|EPI_ISL_708385|Original|hCoV-19^^Delaware|Human|Delaware Public Health Lab|Delaware Public Health Lab|Bajwa|USA
>Spike|hCoV-19/USA/UT-UPHL-2012626021/2020|2020-08-11|EPI_ISL_745940|Original|hCoV-19^^Utah|Human|Ginkgo Bioworks Clinical Laboratory|Utah Public Health Laboratory|Young|USA
>Spike|hCoV-19/Australia/VIC9510/2020|2020-08-20|EPI_ISL_562742|Original|hCoV-19^^Victoria|Human|Microbiological Diagnostic Unit - Public Health Laboratory (MDU-PHL)|MDU-PHL|Schultz|Australia
>Spike|hCoV-19/Israel/CVL-28-ngs/2020|2020-12-24|EPI_ISL_776627|Original|hCoV-19^^Israel|Human|Israel Central Virology laboratory|Israel Central Virology laboratory|Zuckerman|Israel
>Spike|hCoV-19/Scotland/QEUH-B9E5D0/2020|2020-11-18|EPI_ISL_757204|Original|hCoV-19^^Scotland|Human|Lighthouse Lab in Glasgow|Wellcome Sanger Institute for the COVID-19 Genomics UK (COG-UK) Consortium|Davies|United Kingdom
>Spike|hCoV-19/Wales/PHWC-49E0C7/2020|2020-12-07|EPI_ISL_742952|Original|hCoV-19^^Wales|Human|Wales Specialist Virology Centre Sequencing lab|COVID-19 Genomics UK (COG-UK) Consortium|Moore|United Kingdom
>Spike|hCoV-19/India/GJ-GBRC163b/2020|2020-05-27|EPI_ISL_461498|Original|hCoV-19^^Gujarat|Human|B.J. Medical College and Civil hospital|Gujarat Biotechnology Research Centre|Joshi|India
>Spike|hCoV-19/England/ALDP-BB9BFB/2020|2020-11-21|EPI_ISL_702912|Original|hCoV-19^^England|Human|Lighthouse Lab in Alderley Park|Wellcome Sanger Institute for the COVID-19 Genomics UK (COG-UK) Consortium|Davies|United Kingdom
>Spike|hCoV-19/India/MH-ACTREC-118/2020|2020-05-25|EPI_ISL_699774|Original|hCoV-19^^Maharashtra|Human|Hematopathology Laboratory|Hematopathology Laboratory|Patkar|India
>Spike|hCoV-19/USA/NY-NYCPHL-000475/2020|2020-05-02|EPI_ISL_631943|Original|hCoV-19^^New York|Human|St Barnabas Hospital|New York City Public Health Laboratory|Wang|USA

>Spike|hCoV-19/England/MILK-63BB9C/2020|2020-07-04|EPI_ISL_557784|Original|hCoV-19^^England|Human|Lighthouse Lab in Milton Keynes|Wellcome Sanger Institute for the COVID-19 Genomics UK (COG-UK) consortium|Davies|United Kingdom
>Spike|hCoV-19/England/ALDP-A86ED1/2020|2020-10-16|EPI_ISL_605939|Original|hCoV-19^^England|Human|Lighthouse Lab in Alderley Park|Wellcome Sanger Institute for the COVID-19 Genomics UK (COG-UK) consortium|Davies|United Kingdom
>Spike|hCoV-19/England/ALDP-A2F98E/2020|2020-10-05|EPI_ISL_587050|Original|hCoV-19^^England|Human|Lighthouse Lab in Alderley Park|Wellcome Sanger Institute for the COVID-19 Genomics UK (COG-UK) consortium|Davies|United Kingdom
>Spike|hCoV-19/Brazil/SP-L6-CD54/2020|2020-03-18|EPI_ISL_476428|Original|hCoV-19^^S\u00e3o Paulo|Human|Hospital da Cl\u00ednicas da Faculdade de Medicina da Universidade de S\u00e3o Paulo|Instituto de Medicina Tropical da Univesidade de S\u00e3o Paulo|Sabino|Brazil
>Spike|hCoV-19/France/PAC-IHU-0146/2020|2020-03-21|EPI_ISL_569335|Original|hCoV-19^^Provence-Alpes-C\u00f4te-d\u2019Azur|Human|MEPHI|MEPHI|Levasseur|France
>Spike|hCoV-19/Wales/PHWC-47EAB0/2020|2020-10-12|EPI_ISL_595548|Original|hCoV-19^^Wales|Human|Originating lab|COVID-19 Genomics UK (COG-UK) Consortium|Moore|United Kingdom
>Spike|hCoV-19/England/QEUH-BA2E92/2020|2020-11-18|EPI_ISL_672853|Original|hCoV-19^^England|Human|Lighthouse Lab in Glasgow|Wellcome Sanger Institute for the COVID-19 Genomics UK (COG-UK) Consortium|Davies|United Kingdom
>Spike|hCoV-19/USA/TX-HMH-1351/2020|2020-04-14|EPI_ISL_542665|Original|hCoV-19^^Texas|Human|Houston Methodist Hospital|Houston Methodist Hospital|Long|USA
>Spike|hCoV-19/USA/CA-CZB-13296/2020|2020-10-31|EPI_ISL_672104|Original|hCoV-19^^California|Human|Santa Clara County Public Health Laboratory|Chan-Zuckerberg Biohub|Bhatt|USA
>Spike|hCoV-19/England/CAMC-B99FD7/2020|2020-11-15|EPI_ISL_673258|Original|hCoV-19^^England|Human|Lighthouse Lab in Cambridge|Wellcome Sanger Institute for the COVID-19 Genomics UK (COG-UK) Consortium|Davies|United Kingdom
>Spike|hCoV-19/England/201361008/2020|2020-03-25|EPI_ISL_421785|Original|hCoV-19^^England|Human|Respiratory Virus Unit|Respiratory Virus Unit|Platt|United Kingdom
>Spike|hCoV-19/USA/AZ-TG295777/2020|2020-05-07|EPI_ISL_694243|Original|hCoV-19^^Arizona|Human|TGen North|TGen North|French|USA
>Spike|hCoV-19/Wales/PHWC-4813A0/2020|2020-10-13|EPI_ISL_613062|Original|hCoV-19^^Wales|Human|Wales Specialist Virology Centre Sequencing lab|COVID-19 Genomics UK (COG-UK) Consortium|Moore|United Kingdom
>Spike|hCoV-19/England/BRIS-12E399/2020|2020-04-27|EPI_ISL_481990|Original|hCoV-19^^England|Human|PHE South West Regional Laboratory|Wellcome Sanger Institute for the COVID-19 Genomics UK (COG-UK) consortium|Davies|United Kingdom
>Spike|hCoV-19/England/SHEF-C591B/2020|2020-05-18|EPI_ISL_493682|Original|hCoV-19^^England|Human|Virology Department|COVID-19 Genomics UK (COG-UK) Consortium|de Silva|United Kingdom
>Spike|hCoV-19/Wales/ALDP-C58708/2020|2020-12-12|EPI_ISL_767132|Original|hCoV-19^^Wales|Human|Lighthouse Lab in Alderley Park|Wellcome Sanger Institute for the COVID-19 Genomics UK (COG-UK) Consortium|Davies|United Kingdom
>Spike|hCoV-19/England/QEUH-7A4C21/2020|2020-07-14|EPI_ISL_533017|Original|hCoV-19^^England|Human|Lighthouse Lab in Glasgow|Wellcome Sanger Institute for the COVID-19 Genomics UK (COG-UK) consortium|Davies|United Kingdom
>Spike|hCoV-19/England/ALDP-923F2F/2020|2020-06-23|EPI_ISL_553728|Original|hCoV-19^^England|Human|Lighthouse Lab in Alderley Park|Wellcome Sanger Institute for the COVID-19 Genomics UK (COG-UK) consortium|Davies|United Kingdom
>Spike|hCoV-19/Singapore/347/2020|2020-05-20|EPI_ISL_469102|Original|hCoV-19^^Singapore|Human|National Public Health Laboratory|National Public Health Laboratory|Mak|Singapore
>Spike|hCoV-19/Greece/218_35009/2020|2020-03-17|EPI_ISL_437886|Original|hCoV-19^^Athens|Human|Laboratory of Microbiology|Laboratory of Biology|Karakasiliotis|Greece
>Spike|hCoV-19/Portugal/PT0062/2020|2020-03-19|EPI_ISL_421465|Original|hCoV-19^^Portugal|Human|CHTMAD|Instituto Nacional de Saude (INSA)|Borges|Portugal

>Spike|hCoV-19/England/NOTT-1161F4/2020|2020-10-19|EPI_ISL_595310|Original|hCoV-19^^England|Human|Queens Medical Centre|COVID-19 Genomics UK (COG-UK) Consortium|Loose|United Kingdom
>Spike|hCoV-19/England/20144000204/2020|2020-03-31|EPI_ISL_423472|Original|hCoV-19^^England|Human|Respiratory Virus Unit|Respiratory Virus Unit|Platt|United Kingdom
>Spike|hCoV-19/England/QEUH-B437C5/2020|2020-11-08|EPI_ISL_657228|Original|hCoV-19^^England|Human|Lighthouse Lab in Glasgow|Wellcome Sanger Institute for the COVID-19 Genomics UK (COG-UK) Consortium|Davies|United Kingdom
>Spike|hCoV-19/England/MILK-BAC760/2020|2020-11-18|EPI_ISL_783262|Original|hCoV-19^^England|Human|Lighthouse Lab in Milton Keynes|Wellcome Sanger Institute for the COVID-19 Genomics UK (COG-UK) Consortium|Davies|United Kingdom
>Spike|hCoV-19/England/QEUH-98FD45/2020|2020-08-26|EPI_ISL_540097|Original|hCoV-19^^England|Human|Lighthouse Lab in Glasgow|Wellcome Sanger Institute for the COVID-19 Genomics UK (COG-UK) consortium|Davies|United Kingdom
>Spike|hCoV-19/SaudiArabia/KAUST-JEDDAH462/2020|2020-08-04|EPI_ISL_512920|Original|hCoV-19^^Jeddah|Human|Pathogen Genomics Lab King Abdullah University of Science and Technology(KAUST)|Pathogen Genomics Lab King Abdullah University of Science and Technology(KAUST)|Mohamed Ghazzali|Saudi Arabia
>Spike|hCoV-19/England/NORW-FA027/2020|2020-12-08|EPI_ISL_725460|Original|hCoV-19^^England|Human|Quadram Institute Bioscience|COVID-19 Genomics UK (COG-UK) Consortium|Alikhan|United Kingdom
>Spike|hCoV-19/Denmark/DCGC-17184/2020|2020-12-14|EPI_ISL_750809|Original|hCoV-19^^Hovedstaden|Human|Department of Virus and Microbiological Special Diagnostics|Albertsen Lab|Michaelsen|Denmark
>Spike|hCoV-19/England/ALDP-C3CA2F/2020|2020-12-10|EPI_ISL_731085|Original|hCoV-19^^England|Human|Lighthouse Lab in Alderley Park|Wellcome Sanger Institute for the COVID-19 Genomics UK (COG-UK) Consortium|Davies|United Kingdom
>Spike|hCoV-19/Australia/VIC5583/2020|2020-07-16|EPI_ISL_518401|Original|hCoV-19^^Victoria|Human|Victorian Infectious Diseases Reference Laboratory (VIDRL)|VIDRL and MDU-PHL|Schultz|Australia
>Spike|hCoV-19/USA/TX-HMH-MCoV-12622/2020|2020-08-31|EPI_ISL_790426|Original|hCoV-19^^Texas|Human|Houston Methodist Hospital|Houston Methodist Hospital|Long|USA
>Spike|hCoV-19/USA/CA-ALSR-3050/2020|2020-09-03|EPI_ISL_635814|Original|hCoV-19^^California|Human|San Diego County Public Health Laboratory|Andersen lab at Scripps Research|Gangavarapu|USA
>Spike|hCoV-19/England/QEUH-9F373F/2020|2020-09-24|EPI_ISL_588570|Original|hCoV-19^^England|Human|Lighthouse Lab in Glasgow|Wellcome Sanger Institute for the COVID-19 Genomics UK (COG-UK) consortium|Davies|United Kingdom
>Spike|hCoV-19/Australia/NSW131/2020|2020-03-23|EPI_ISL_427783|Original|hCoV-19^^New South Wales|Human|Centre for Infectious Diseases and Microbiology Public Health|NSW Health Pathology - Institute of Clinical Pathology and Medical Research; Westmead Hospital; University of Sydney|Gall|Australia
>Spike|hCoV-19/England/MILK-A7DDA3/2020|2020-10-13|EPI_ISL_638474|Original|hCoV-19^^England|Human|Oxford Viromics|COVID-19 Genomics UK (COG-UK) Consortium|Golubchik|United Kingdom
>Spike|hCoV-19/USA/CO-CDPHE-2011020202/2020|2020-11-01|EPI_ISL_710243|Original|hCoV-19^^Colorado|Human|Colorado Department of Public Health and Environment|Colorado Department of Puplic Health and Environment|Bankers|USA
>Spike|hCoV-19/England/QEUH-9CFD2B/2020|2020-09-15|EPI_ISL_589923|Original|hCoV-19^^England|Human|Lighthouse Lab in Glasgow|Wellcome Sanger Institute for the COVID-19 Genomics UK (COG-UK) consortium|Davies|United Kingdom
>Spike|hCoV-19/USA/TX-HMH-MCoV-9901/2020|2020-07-06|EPI_ISL_780332|Original|hCoV-19^^Texas|Human|Houston Methodist Hospital|Houston Methodist Hospital|Long|USA
>Spike|hCoV-19/England/QEUH-AAB29E/2020|2020-10-21|EPI_ISL_652780|Original|hCoV-19^^England|Human|Oxford Viromics|COVID-19 Genomics UK (COG-UK) Consortium|Golubchik|United Kingdom
>Spike|hCoV-19/Japan/PG-6312/2020|2020-08-00|EPI_ISL_689423|Original|hCoV-19^^Japan|Human|Pathogen Genomics Center|Pathogen Genomics Center|Sekizuka|Japan

>Spike|hCoV-19/England/CAMC-B09998/2020|2020-10-30|EPI_ISL_672915|Original|hCoV-19^^England|Human|Lighthouse Lab in Cambridge|Wellcome Sanger Institute for the COVID-19 Genomics UK (COG-UK) Consortium|Davies|United Kingdom
>Spike|hCoV-19/Denmark/DCGC-10648/2020|2020-11-02|EPI_ISL_670113|Original|hCoV-19^^Syddanmark|Human|Department of Virus and Microbiological Special Diagnostics|Albertsen Lab|Michaelsen|Denmark
>Spike|hCoV-19/Belgium/ULG-8541/2020|2020-03-19|EPI_ISL_418636|Original|hCoV-19^^Li\u00e8ge|Human|Department of Clinical Microbiology|GIGA Medical Genomics|Durkin|Belgium
>Spike|hCoV-19/England/QEUH-A605D8/2020|2020-10-12|EPI_ISL_665617|Original|hCoV-19^^England|Human|Northumbria University / South Tees Hospitals NHS Foundation Trust / North Cumbria Integrated Care NHS Foundation Trust / North Tees and Hartlepool NHS Foundation Trust / Newcastle Hospitals NHS Foundation Trust|COVID-19 Genomics UK (COG-UK) Consortium|Bashton|United Kingdom
>Spike|hCoV-19/USA/MI-UM-10036778541/2020|2020-11-04|EPI_ISL_707844|Original|hCoV-19^^Michigan|Human|University of Michigan Clinical Microbiology Laboratory|Lauring Lab|Valesano|USA
>Spike|hCoV-19/South Africa/LR00005/2020|2020-07-03|EPI_ISL_515136|Original|hCoV-19^^Gauteng|Human|National Institute for Communicable Diseases of the National Health Laboratory Service|National Institute for Communicable Diseases of the National Health Laboratory Service|Allam|South Africa
>Spike|hCoV-19/England/ALDP-A6D1D7/2020|2020-10-14|EPI_ISL_608247|Original|hCoV-19^^England|Human|Lighthouse Lab in Alderley Park|Wellcome Sanger Institute for the COVID-19 Genomics UK (COG-UK) consortium|Davies|United Kingdom
>Spike|hCoV-19/USA/WA-S1510/2020|2020-05-22|EPI_ISL_495936|Original|hCoV-19^^Washington|Human|Washington State Department of Health|Seattle Flu Study|Lee|USA
>Spike|hCoV-19/Scotland/EDB1835/2020|2020-04-16|EPI_ISL_491628|Original|hCoV-19^^Scotland|Human|Virology Department|Wellcome Sanger Institute for the COVID-19 Genomics UK (COG-UK) consortium|Davies|United Kingdom
>Spike|hCoV-19/Belgium/rega-09231006/2020|2020-09-23|EPI_ISL_735029|Original|hCoV-19^^Leest|Human|UZ Leuven|KU Leuven|Maes|Belgium
>Spike|hCoV-19/England/BRIS-1219B2/2020|2020-03-15|EPI_ISL_440140|Original|hCoV-19^^England|Human|PHE South West Regional Laboratory|Wellcome Sanger Institute for the COVID-19 Genomics UK (COG-UK) consortium|Amato|United Kingdom
>Spike|hCoV-19/England/ALDP-B83617/2020|2020-11-15|EPI_ISL_675406|Original|hCoV-19^^England|Human|Lighthouse Lab in Alderley Park|Wellcome Sanger Institute for the COVID-19 Genomics UK (COG-UK) Consortium|Davies|United Kingdom
>Spike|hCoV-19/Wales/MILK-9E0D8E/2020|2020-09-19|EPI_ISL_580900|Original|hCoV-19^^Wales|Human|Lighthouse Lab in Milton Keynes|Wellcome Sanger Institute for the COVID-19 Genomics UK (COG-UK) consortium|Davies|United Kingdom
>Spike|hCoV-19/USA/MI-UM-10036652539/2020|2020-10-27|EPI_ISL_650034|Original|hCoV-19^^Michigan|Human|University of Michigan Clinical Microbiology Laboratory|Lauring Lab|Valesano|USA
>Spike|hCoV-19/England/MILK-9A954D/2020|2020-09-01|EPI_ISL_551034|Original|hCoV-19^^England|Human|Lighthouse Lab in Milton Keynes|Wellcome Sanger Institute for the COVID-19 Genomics UK (COG-UK) consortium|Davies|United Kingdom
>Spike|hCoV-19/England/MILK-A918C6/2020|2020-10-19|EPI_ISL_705414|Original|hCoV-19^^England|Human|Oxford Viromics|COVID-19 Genomics UK (COG-UK) Consortium|Golubchik|United Kingdom
>Spike|hCoV-19/Australia/VIC3270/2020|2020-07-10|EPI_ISL_520949|Original|hCoV-19^^Victoria|Human|Microbiological Diagnostic Unit - Public Health Laboratory (MDU-PHL)|MDU-PHL|Schultz|Australia
>Spike|hCoV-19/Australia/VIC11112/2020|2020-08-31|EPI_ISL_563253|Original|hCoV-19^^Victoria|Human|Microbiological Diagnostic Unit - Public Health Laboratory (MDU-PHL)|MDU-PHL|Schultz|Australia
>Spike|hCoV-19/USA/WA-S2303/2020|2020-06-12|EPI_ISL_496935|Original|hCoV-19^^Washington|Human|Washington State Department of Health|Seattle Flu Study|Lee|USA
>Spike|hCoV-19/Canada/BC-BCCDC-8557/2020|2020-11-00|EPI_ISL_740816|Original|hCoV-19^^British Columbia|Human|BCCDC Public Health Laboratory|BCCDC Public Health Laboratory|Prystajecky|Canada

>Spike|hCoV-19/England/ALDP-A6EB41/2020|2020-10-15|EPI_ISL_607277|Original|hCoV-19^^England|Human|Lighthouse Lab in Alderley Park|Wellcome Sanger Institute for the COVID-19 Genomics UK (COG-UK) consortium|Davies|United Kingdom
>Spike|hCoV-19/USA/MI-UM-10035727656/2020|2020-08-31|EPI_ISL_583309|Original|hCoV-19^^Michigan|Human|University of Michigan Clinical Microbiology Laboratory|Lauring Lab|Valesano|USA
>Spike|hCoV-19/USA/WA-S1955/2020|2020-06-02|EPI_ISL_497383|Original|hCoV-19^^Washington|Human|Washington State Department of Health|Seattle Flu Study|Lee|USA
>Spike|hCoV-19/USA/NMDOH-2020324359/2020|2020-09-02|EPI_ISL_542095|Original|hCoV-19^^New Mexico|Human|New Mexico Department of Health Scientific Laboratory|New Mexico Department of Health Scientific Laboratory|Johnson|USA
>Spike|hCoV-19/England/LOND-D4C5C/2020|2020-04-14|EPI_ISL_449668|Original|hCoV-19^^England|Human|University College London|COVID-19 Genomics UK (COG-UK) Consortium|Roy|United Kingdom
>Spike|hCoV-19/USA/WY-WPHL-20044555/2020|2020-07-16|EPI_ISL_769912|Original|hCoV-19^^Wyoming|Human|Wyoming Public Health Laboratory|Wyoming Public Health Laboratory|Hull|USA
>Spike|hCoV-19/England/QEUH-A01612/2020|2020-09-30|EPI_ISL_679989|Original|hCoV-19^^England|Human|Virology Department|COVID-19 Genomics UK (COG-UK) Consortium|de Silva|United Kingdom
>Spike|hCoV-19/USA/UT-QDX-65/2020|2020-03-12|EPI_ISL_467922|Original|hCoV-19^^Utah|Human|Quest Diagnostics|Quest Diagnostics|Kagan|USA
>Spike|hCoV-19/Spain/VC-FISABIO-203/2020|2020-03-20|EPI_ISL_436298|Original|hCoV-19^^Comunitat Valenciana|Human|Servicio de Microbiologia. Hospital Clinico Universitario de Valencia|Sequencing and Bioinformatics Service and Molecular Epidemiology Research Group. FISABIO-Public Health|Gonzalez-Candelas|Spain
>Spike|hCoV-19/Norway/5607/2020|2020-11-09|EPI_ISL_708123|Original|hCoV-19^^Agder|Human|Hospital of Southern Norway - Kristiansand|Norwegian Institute of Public Health|Kopperud Riis|Norway
>Spike|hCoV-19/England/QEUH-A4D2F8/2020|2020-10-03|EPI_ISL_596108|Original|hCoV-19^^England|Human|Quadram Institute Bioscience|COVID-19 Genomics UK (COG-UK) Consortium|Alikhan|United Kingdom
>Spike|hCoV-19/USA/CA-CZB-6967/2020|2020-09-02|EPI_ISL_582902|Original|hCoV-19^^California|Human|County of Santa Clara Public Health Department|Chan-Zuckerberg Biohub|Bhatt|USA
>Spike|hCoV-19/USA/WA-S1636/2020|2020-05-27|EPI_ISL_496062|Original|hCoV-19^^Washington|Human|Washington State Department of Health|Seattle Flu Study|Lee|USA
>Spike|hCoV-19/Denmark/DCGC-7466/2020|2020-10-12|EPI_ISL_619401|Original|hCoV-19^^Sjaelland|Human|Department of Virus and Microbiological Special Diagnostics|Albertsen lab|Michaelsen|Denmark
>Spike|hCoV-19/Scotland/QEUH-9CA42B/2020|2020-09-13|EPI_ISL_576052|Original|hCoV-19^^Scotland|Human|Lighthouse Lab in Glasgow|Wellcome Sanger Institute for the COVID-19 Genomics UK (COG-UK) consortium|Davies|United Kingdom
>Spike|hCoV-19/Belgium/rega-11271174/2020|2020-11-27|EPI_ISL_735227|Original|hCoV-19^^Riemst|Human|UZ Leuven|KU Leuven|Maes|Belgium
>Spike|hCoV-19/USA/CA-CZB-15285/2020|2020-11-28|EPI_ISL_739214|Original|hCoV-19^^California|Human|Santa Clara County Public Health Laboratory|Chan-Zuckerberg Biohub|Kamm|USA
>Spike|hCoV-19/England/CAMC-C74706/2020|2020-12-15|EPI_ISL_768913|Original|hCoV-19^^England|Human|Lighthouse Lab in Cambridge|Wellcome Sanger Institute for the COVID-19 Genomics UK (COG-UK) Consortium|Davies|United Kingdom
>Spike|hCoV-19/Denmark/DCGC-18770/2020|2020-12-07|EPI_ISL_748283|Original|hCoV-19^^Midtjylland|Human|Department of Virus and Microbiological Special Diagnostics|Albertsen Lab|Michaelsen|Denmark
>Spike|hCoV-19/Wales/QEUH-AE20CE/2020|2020-10-26|EPI_ISL_647880|Original|hCoV-19^^Wales|Human|Lighthouse Lab in Glasgow|Wellcome Sanger Institute for the COVID-19 Genomics UK (COG-UK) Consortium|Davies|United Kingdom
>Spike|hCoV-19/Japan/PG-4075/2020|2020-07-00|EPI_ISL_692316|Original|hCoV-19^^Japan|Human|Gunma Prefectural Institute of Public Health and Environmental Sciences|Pathogen Genomics Center|Sekizuka|Japan

>Spike|hCoV-19/USA/TX-HMH-MCoV-9765/2020|2020-07-04|EPI_ISL_780261|Original|hCoV-19^^Texas|Human|Houston Methodist Hospital|Houston Methodist Hospital|Long|USA
>Spike|hCoV-19/USA/IL-NM-1229/2020|2020-06-23|EPI_ISL_626509|Original|hCoV-19^^Illinois|Human|Northwestern Memorial Hospital|Ozer Lab|Ozer|USA
>Spike|hCoV-19/Colombia/BOY-GVI-97795/2020|2020-04-04|EPI_ISL_447805|Original|hCoV-19^^Boyaca|Human|Instituto Nacional de Salud|Grupo de Investigaciones Microbiol\u00f3gicas-UR (GIMUR)|Ram\u00edrez|Colombia
>Spike|hCoV-19/England/MILK-A05BC4/2020|2020-09-30|EPI_ISL_599981|Original|hCoV-19^^England|Human|Lighthouse Lab in Milton Keynes|Wellcome Sanger Institute for the COVID-19 Genomics UK (COG-UK) consortium|Davies|United Kingdom
>Spike|hCoV-19/USA/TX-HMH-MCoV-13682/2020|2020-08-19|EPI_ISL_788779|Original|hCoV-19^^Texas|Human|Houston Methodist Hospital|Houston Methodist Hospital|Long|USA
>Spike|hCoV-19/England/ALDP-BB3195/2020|2020-11-19|EPI_ISL_702637|Original|hCoV-19^^England|Human|Lighthouse Lab in Alderley Park|Wellcome Sanger Institute for the COVID-19 Genomics UK (COG-UK) Consortium|Davies|United Kingdom
>Spike|hCoV-19/USA/WY-WPHL-20153670/2020|2020-11-30|EPI_ISL_770115|Original|hCoV-19^^Wyoming|Human|Wyoming Public Health Laboratory|Wyoming Public Health Laboratory|Hull|USA
>Spike|hCoV-19/USA/NY-Wadsworth-58877-01/2020|2020-04-21|EPI_ISL_676868|Original|hCoV-19^^New York|Human|Wadsworth Center|Wadsworth Center|Lasek-Nesselquist|USA
>Spike|hCoV-19/Netherlands/LI-EMC-86/2020|2020-03-19|EPI_ISL_523291|Original|hCoV-19^^Limburg|Human|Dutch COVID-19 response team|Erasmus Medical Center|Oude Munnink|Netherlands
>Spike|hCoV-19/Gibraltar/204841756/2020|2020-00-00|EPI_ISL_671323|Original|hCoV-19^^Gibraltar|Human|Microbiology|Respiratory Virus Unit|Platt|Gibraltar
>Spike|hCoV-19/England/NORT-28D625/2020|2020-11-18|EPI_ISL_705496|Original|hCoV-19^^England|Human|Northumbria University / South Tees Hospitals NHS Foundation Trust / North Cumbria Integrated Care NHS Foundation Trust / North Tees and Hartlepool NHS Foundation Trust / Newcastle Hospitals NHS Foundation Trust|COVID-19 Genomics UK (COG-UK) Consortium|Bashton|United Kingdom
>Spike|hCoV-19/England/ALDP-9FCF2F/2020|2020-10-01|EPI_ISL_587990|Original|hCoV-19^^England|Human|Lighthouse Lab in Alderley Park|Wellcome Sanger Institute for the COVID-19 Genomics UK (COG-UK) consortium|Davies|United Kingdom
>Spike|hCoV-19/England/QEUH-943A73/2020|2020-08-01|EPI_ISL_532539|Original|hCoV-19^^England|Human|Lighthouse Lab in Glasgow|Wellcome Sanger Institute for the COVID-19 Genomics UK (COG-UK) consortium|Davies|United Kingdom
>Spike|hCoV-19/Lithuania/MR-LUHS-Eilnr86/2020|2020-04-17|EPI_ISL_603104|Original|hCoV-19^^Marijampoles apskritis|Human|Lithuanian University of Health Sciences Hospital|Lithuanian University of Health Sciences|Zemaitis|Lithuania
>Spike|hCoV-19/England/QEUH-A8C293/2020|2020-10-17|EPI_ISL_643144|Original|hCoV-19^^England|Human|Lighthouse Lab in Glasgow|Wellcome Sanger Institute for the COVID-19 Genomics UK (COG-UK) Consortium|Davies|United Kingdom
>Spike|hCoV-19/India/MH-GA84/2020|2020-06-18|EPI_ISL_508279|Original|hCoV-19^^Maharashtra|Human|Government Medical College|National Institute of Biomedical Genomics|Maitra|India
>Spike|hCoV-19/Norway/2951/2020|2020-07-31|EPI_ISL_549165|Original|hCoV-19^^Viken|Human|Furst Medical Laboratory|Norwegian Institute of Public Health|Kopperud Riis|Norway
>Spike|hCoV-19/England/CAMC-A64AF3/2020|2020-10-09|EPI_ISL_608573|Original|hCoV-19^^England|Human|Lighthouse Lab in Cambridge|Wellcome Sanger Institute for the COVID-19 Genomics UK (COG-UK) consortium|Davies|United Kingdom
>Spike|hCoV-19/England/MILK-ACBE0D/2020|2020-10-21|EPI_ISL_629150|Original|hCoV-19^^England|Human|Lighthouse Lab in Milton Keynes|Wellcome Sanger Institute for the COVID-19 Genomics UK (COG-UK) consortium|Davies|United Kingdom
>Spike|hCoV-19/England/CAMB-1B5EB4/2020|2020-00-00|EPI_ISL_572699|Original|hCoV-19^^England|Human|Department of Pathology|COVID-19 Genomics UK (COG-UK) Consortium|Goodfellow|United Kingdom

>Spike|hCoV-19/England/ALDP-BB27EF/2020|2020-11-20|EPI_ISL_702938|Original|hCoV-
19^^England|Human|Lighthouse Lab in Alderley Park|Wellcome Sanger Institute for the
COVID-19 Genomics UK (COG-UK) Consortium|Davies|United Kingdom
>Spike|hCoV-19/Scotland/QEUH-A217EC/2020|2020-10-01|EPI_ISL_598902|Original|hCoV-
19^^Scotland|Human|Lighthouse Lab in Glasgow|Wellcome Sanger Institute for the COVID-
19 Genomics UK (COG-UK) consortium|Davies|United Kingdom
>Spike|hCoV-19/Scotland/GCVR-171AB0/2020|2020-03-25|EPI_ISL_489392|Original|hCoV-
19^^Scotland|Human|NHSGGC West of Scotland Specialist Virology Centre / MRC-
University of Glasgow Centre for Virus Research|Wellcome Sanger Institute for the
COVID-19 Genomics UK (COG-UK) consortium|Davies|United Kingdom
>Spike|hCoV-19/USA/TX-HMH-3373/2020|2020-06-13|EPI_ISL_544157|Original|hCoV-
19^^Texas|Human|Houston Methodist Hospital|Houston Methodist Hospital|Long|USA
>Spike|hCoV-19/Japan/PG-8598/2020|2020-08-00|EPI_ISL_686194|Original|hCoV-
19^^Japan|Human|Pathogen Genomics Center|Pathogen Genomics Center|Sekizuka|Japan
>Spike|hCoV-19/Australia/VIC17671/2020|2020-08-20|EPI_ISL_663989|Original|hCoV-
19^^Victoria|Human|Microbiological Diagnostic Unit - Public Health Laboratory (MDU-
PHL)|MDU-PHL|Schultz|Australia
>Spike|hCoV-19/Switzerland/SZ-ETHZ-390444/2020|2020-11-
04|EPI_ISL_729087|Original|hCoV-19^^Schwyz|Human|Viollier AG|Department of Biosystems
Science and Engineering|Nadeau|Switzerland
>Spike|hCoV-19/Denmark/DCGC-16059/2020|2020-11-16|EPI_ISL_714566|Original|hCoV-
19^^Hovedstaden|Human|Department of Virus and Microbiological Special
Diagnostics|Albertsen Lab|Michaelsen|Denmark
>Spike|hCoV-19/England/QEUH-AF57D2/2020|2020-10-30|EPI_ISL_663063|Original|hCoV-
19^^England|Human|Lighthouse Lab in Glasgow|Wellcome Sanger Institute for the COVID-
19 Genomics UK (COG-UK) Consortium|Davies|United Kingdom
>Spike|hCoV-19/India/DL-MN5/2020|2020-06-18|EPI_ISL_508421|Original|hCoV-
19^^Delhi|Human|Maulana Azad Medical College|National Institute of Biomedical
Genomics|Maitra|India
>Spike|hCoV-19/USA/MA-MGH-00657/2020|2020-04-03|EPI_ISL_460256|Original|hCoV-
19^^Massachusetts|Human|Massachusetts General Hospital|Infectious Disease
Program|Lemieux|USA
>Spike|hCoV-19/Hungary/US-33755w/2020|2020-09-03|EPI_ISL_677766|Original|hCoV-
19^^Csongr\u00e1d county|Human|University of Szeged|National Laboratory of
Virology|Kemenesi|Hungary
>Spike|hCoV-19/Scotland/QEUH-A48E53/2020|2020-10-08|EPI_ISL_595930|Original|hCoV-
19^^Scotland|Human|Quadram Institute Bioscience|COVID-19 Genomics UK (COG-UK)
Consortium|Alikhan|United Kingdom
>Spike|hCoV-19/USA/AZ-TG670192/2020|2020-04-30|EPI_ISL_696385|Original|hCoV-
19^^Arizona|Human|Sonora Quest Laboratories|TGen North|French|USA
>Spike|hCoV-19/Australia/VIC5218/2020|2020-07-18|EPI_ISL_519983|Original|hCoV-
19^^Victoria|Human|Microbiological Diagnostic Unit - Public Health Laboratory (MDU-
PHL)|MDU-PHL|Schultz|Australia
>Spike|hCoV-19/USA/TX-HMH-MCoV-14262/2020|2020-10-04|EPI_ISL_787547|Original|hCoV-
19^^Texas|Human|Houston Methodist Hospital|Houston Methodist Hospital|Long|USA
>Spike|hCoV-19/England/NORT-28273C/2020|2020-03-28|EPI_ISL_488498|Original|hCoV-
19^^England|Human|NU-OMICS DNA Sequencing research facility|Wellcome Sanger Institute
for the COVID-19 Genomics UK (COG-UK) consortium|Davies|United Kingdom
>Spike|hCoV-19/Australia/VIC14025/2020|2020-07-25|EPI_ISL_563935|Original|hCoV-
19^^Victoria|Human|Microbiological Diagnostic Unit - Public Health Laboratory (MDU-
PHL)|MDU-PHL|Schultz|Australia
>Spike|hCoV-19/Japan/PG-2167/2020|2020-04-00|EPI_ISL_685772|Original|hCoV-
19^^Japan|Human|Pathogen Genomics Center|Pathogen Genomics Center|Sekizuka|Japan
>Spike|hCoV-19/England/NOTT-11D3BB/2020|2020-11-22|EPI_ISL_665927|Original|hCoV-
19^^England|Human|Queens Medical Centre|COVID-19 Genomics UK (COG-UK)
Consortium|Loose|United Kingdom
>Spike|hCoV-19/England/QEUH-884804/2020|2020-07-23|EPI_ISL_532737|Original|hCoV-
19^^England|Human|Lighthouse Lab in Glasgow|Wellcome Sanger Institute for the COVID-
19 Genomics UK (COG-UK) consortium|Davies|United Kingdom

>Spike|hCoV-19/USA/MD-MDH-0408/2020|2020-11-05|EPI_ISL_666778|Original|hCoV-19^^Maryland|Human|Maryland Public Health Laboratory|Maryland Public Health Laboratory|Keller|USA
>Spike|hCoV-19/Hungary/UD-56412/2020|2020-09-01|EPI_ISL_671428|Original|hCoV-19^^Szabolcs-Szatm\u00e1r-Bereg county|Human|University of Debrecen|National Laboratory of Virology|Kemenesi|Hungary
>Spike|hCoV-19/Scotland/QEUH-9F614D/2020|2020-09-27|EPI_ISL_600850|Original|hCoV-19^^Scotland|Human|Lighthouse Lab in Glasgow|Wellcome Sanger Institute for the COVID-19 Genomics UK (COG-UK) consortium|Davies|United Kingdom
>Spike|hCoV-19/Kenya/C5019/2020|2020-05-08|EPI_ISL_568748|Original|hCoV-19^^Mombasa|Human|KEMRI-Wellcome Trust Research Programme/KEMRI-CGMR-C Kilifi|KEMRI-Wellcome Trust Research Programme/KEMRI-CGMR-C Kilifi|Githinji|Kenya
>Spike|hCoV-19/France/PAC-IHU-0256/2020|2020-03-30|EPI_ISL_569414|Original|hCoV-19^^Provence-Alpes-C\u00f4te-d\u2019Azur|Human|MEPHI|MEPHI|Levasseur|France
>Spike|hCoV-19/England/ALDP-B3EDE9/2020|2020-10-21|EPI_ISL_645452|Original|hCoV-19^^England|Human|Lighthouse Lab in Alderley Park|Wellcome Sanger Institute for the COVID-19 Genomics UK (COG-UK) Consortium|Davies|United Kingdom
>Spike|hCoV-19/Australia/VIC6523/2020|2020-07-23|EPI_ISL_521293|Original|hCoV-19^^Victoria|Human|Microbiological Diagnostic Unit - Public Health Laboratory (MDU-PHL)|MDU-PHL|Schultz|Australia
>Spike|hCoV-19/England/204600418/2020|2020-11-06|EPI_ISL_700635|Original|hCoV-19^^England|Human|Respiratory Virus Unit|COVID-19 Genomics UK (COG-UK) Consortium|Platt|United Kingdom
>Spike|hCoV-19/Israel/CVL-s2057/2020|2020-09-01|EPI_ISL_575333|Original|hCoV-19^^Central region|Human|Israel Central Virology laboratory|Israel Central Virology laboratory|Zuckerman|Israel
>Spike|hCoV-19/Australia/VIC3118/2020|2020-07-06|EPI_ISL_520825|Original|hCoV-19^^Victoria|Human|Victorian Infectious Diseases Reference Laboratory (VIDRL)|VIDRL and MDU-PHL|Schultz|Australia
>Spike|hCoV-19/England/CAMB-794CA/2020|2020-04-02|EPI_ISL_441829|Original|hCoV-19^^England|Human|Department of Pathology|Wellcome Sanger Institute for the COVID-19 Genomics UK (COG-UK) consortium|Amato|United Kingdom
>Spike|hCoV-19/England/ALDP-66F115/2020|2020-07-03|EPI_ISL_558226|Original|hCoV-19^^England|Human|Lighthouse Lab in Alderley Park|Wellcome Sanger Institute for the COVID-19 Genomics UK (COG-UK) consortium|Davies|United Kingdom
>Spike|hCoV-19/England/QEUH-AE2C33/2020|2020-10-26|EPI_ISL_633664|Original|hCoV-19^^England|Human|Lighthouse Lab in Glasgow|Wellcome Sanger Institute for the COVID-19 Genomics UK (COG-UK) consortium|Davies|United Kingdom
>Spike|hCoV-19/USA/CA-CDC-4699/2020|2020-06-04|EPI_ISL_535676|Original|hCoV-19^^California|Human|CDPH|Pathogen Discovery|Queen|USA
>Spike|hCoV-19/England/CAMC-BD17CC/2020|2020-11-27|EPI_ISL_702261|Original|hCoV-19^^England|Human|Lighthouse Lab in Cambridge|Wellcome Sanger Institute for the COVID-19 Genomics UK (COG-UK) Consortium|Davies|United Kingdom
>Spike|hCoV-19/England/CAMC-94B70B/2020|2020-07-26|EPI_ISL_556344|Original|hCoV-19^^England|Human|Lighthouse Lab in Cambridge|Wellcome Sanger Institute for the COVID-19 Genomics UK (COG-UK) consortium|Davies|United Kingdom
>Spike|hCoV-19/England/LIVE-A1283/2020|2020-05-19|EPI_ISL_499987|Original|hCoV-19^^England|Human|Liverpool Clinical Laboratories|COVID-19 Genomics UK (COG-UK) Consortium|Haldenby|United Kingdom
>Spike|hCoV-19/USA/WI-GMF-19271/2020|2020-09-08|EPI_ISL_547803|Original|hCoV-19^^Wisconsin|Human|Gundersen Molecular Diagnostics Laboratory|Kabara Cancer Research Institute|Kenny|USA
>Spike|hCoV-19/England/LIVE-99A95/2020|2020-04-12|EPI_ISL_439792|Original|hCoV-19^^England|Human|Liverpool Clinical Laboratories|COVID-19 Genomics UK (COG-UK) Consortium|Haldenby|United Kingdom
>Spike|hCoV-19/England/ALDP-A6896B/2020|2020-10-12|EPI_ISL_630469|Original|hCoV-19^^England|Human|Lighthouse Lab in Alderley Park|Wellcome Sanger Institute for the COVID-19 Genomics UK (COG-UK) consortium|Davies|United Kingdom

>Spike|hCoV-19/England/ALDP-A2EB7A/2020|2020-10-05|EPI_ISL_587129|Original|hCoV-19^^England|Human|Lighthouse Lab in Alderley Park|Wellcome Sanger Institute for the COVID-19 Genomics UK (COG-UK) consortium|Davies|United Kingdom
>Spike|hCoV-19/England/SHEF-CAF7E/2020|2020-03-29|EPI_ISL_432500|Original|hCoV-19^^England|Human|Virology Department|COVID-19 Genomics UK (COG-UK) Consortium|de Silva|United Kingdom
>Spike|hCoV-19/England/MILK-9C3264/2020|2020-09-10|EPI_ISL_566925|Original|hCoV-19^^England|Human|Lighthouse Lab in Milton Keynes|Wellcome Sanger Institute for the COVID-19 Genomics UK (COG-UK) consortium|Davies|United Kingdom
>Spike|hCoV-19/Wales/ALDP-C04B3C/2020|2020-12-03|EPI_ISL_767316|Original|hCoV-19^^Wales|Human|Lighthouse Lab in Alderley Park|Wellcome Sanger Institute for the COVID-19 Genomics UK (COG-UK) Consortium|Davies|United Kingdom
>Spike|hCoV-19/Australia/WA118/2020|2020-04-15|EPI_ISL_596681|Original|hCoV-19^^Western Australia|Human|PathWest Laboratory Medicine WA|PathWest Laboratory Medicine WA Microbial Surveillance Unit|Sikazwe|Australia
>Spike|hCoV-19/England/ALDP-B5D77D/2020|2020-11-09|EPI_ISL_703438|Original|hCoV-19^^England|Human|Department of Pathology|COVID-19 Genomics UK (COG-UK) Consortium|Goodfellow|United Kingdom
>Spike|hCoV-19/Denmark/DCGC-9338/2020|2020-11-16|EPI_ISL_668915|Original|hCoV-19^^Nordjylland|Human|Department of Virus and Microbiological Special Diagnostics|Albertsen Lab|Michaelsen|Denmark
>Spike|hCoV-19/England/ALDP-AA1122/2020|2020-10-21|EPI_ISL_624966|Original|hCoV-19^^England|Human|Lighthouse Lab in Alderley Park|Wellcome Sanger Institute for the COVID-19 Genomics UK (COG-UK) consortium|Davies|United Kingdom
>Spike|hCoV-19/Denmark/DCGC-8935/2020|2020-10-26|EPI_ISL_626160|Original|hCoV-19^^Hovedstaden|Human|Department of Virus and Microbiological Special Diagnostics|Albertsen lab|Michaelsen|Denmark
>Spike|hCoV-19/Canada/ON-UHTC_0018/2020|2020-03-30|EPI_ISL_464049|unknown|hCoV-19^^Ontario|Human|Unity Health Toronto|Ontario Institute for Cancer Research|de Borja|Canada
>Spike|hCoV-19/USA/UT-01823/2020|2020-04-10|EPI_ISL_509573|Original|hCoV-19^^Utah|Human|Utah Public Health Laboratory|Utah Public Health Laboratory|Butz|USA
>Spike|hCoV-19/England/QEUH-9AECF8/2020|2020-09-03|EPI_ISL_573496|Original|hCoV-19^^England|Human|University College London|COVID-19 Genomics UK (COG-UK) Consortium|Roy|United Kingdom
>Spike|hCoV-19/Russia/KDA-RII-MH9365S/2020|2020-10-14|EPI_ISL_733453|Original|hCoV-19^^Krasnodar|Human|HELIX LLC|WHO National Influenza Centre Russian Federation|Komissarov|Russia
>Spike|hCoV-19/Sweden/20-52728/2020|2020-08-06|EPI_ISL_648165|Original|hCoV-19^^Jonkoping|Human|The Public Health Agency of Sweden|The Public Health Agency of Sweden|Svartstr\u00f6m|Sweden
>Spike|hCoV-19/Wales/CAMC-AAF5CE/2020|2020-10-22|EPI_ISL_634798|Original|hCoV-19^^Wales|Human|Lighthouse Lab in Cambridge|Wellcome Sanger Institute for the COVID-19 Genomics UK (COG-UK) consortium|Davies|United Kingdom
>Spike|hCoV-19/Denmark/DCGC-16607/2020|2020-11-16|EPI_ISL_714867|Original|hCoV-19^^Midtjylland|Human|Department of Virus and Microbiological Special Diagnostics|Albertsen Lab|Michaelsen|Denmark
>Spike|hCoV-19/Scotland/MILK-9C263C/2020|2020-09-10|EPI_ISL_575984|Original|hCoV-19^^Scotland|Human|Lighthouse Lab in Milton Keynes|Wellcome Sanger Institute for the COVID-19 Genomics UK (COG-UK) consortium|Davies|United Kingdom
>Spike|hCoV-19/England/MILK-BB264C/2020|2020-11-18|EPI_ISL_720217|Original|hCoV-19^^England|Human|Lighthouse Lab in Milton Keynes|Wellcome Sanger Institute for the COVID-19 Genomics UK (COG-UK) Consortium|Davies|United Kingdom
>Spike|hCoV-19/England/ALDP-A67A2D/2020|2020-10-12|EPI_ISL_630377|Original|hCoV-19^^England|Human|Lighthouse Lab in Alderley Park|Wellcome Sanger Institute for the COVID-19 Genomics UK (COG-UK) consortium|Davies|United Kingdom
>Spike|hCoV-19/India/KA-nimh-11070/2020|2020-05-04|EPI_ISL_486401|Original|hCoV-19^^Karnataka|Human|DH|Department of Neurovirology|Pattabiraman|India

>Spike|hCoV-19/USA/TX-DSHS-0246/2020|2020-04-25|EPI_ISL_527822|Original|hCoV-19^^Texas|Human|Texas Department of State Health Services|Texas Department of State Health Services|Rahman|USA
>Spike|hCoV-19/SaudiArabia/KAUST-MAKKAH784/2020|2020-04-24|EPI_ISL_513252|Original|hCoV-19^^Makkah|Human|Pathogen Genomics Lab King Abdullah University of Science and Technology(KAUST)|Pathogen Genomics Lab King Abdullah University of Science and Technology(KAUST)|Mohamed Ghazzali|Saudi Arabia
>Spike|hCoV-19/England/CAMC-B99D91/2020|2020-11-15|EPI_ISL_673482|Original|hCoV-19^^England|Human|Lighthouse Lab in Cambridge|Wellcome Sanger Institute for the COVID-19 Genomics UK (COG-UK) Consortium|Davies|United Kingdom
>Spike|hCoV-19/Wales/PHWC-49FEDB/2020|2020-12-11|EPI_ISL_743366|Original|hCoV-19^^Wales|Human|Wales Specialist Virology Centre Sequencing lab|COVID-19 Genomics UK (COG-UK) Consortium|Moore|United Kingdom
>Spike|hCoV-19/USA/MI-MDHHS-SC22265/2020|2020-10-30|EPI_ISL_666931|Original|hCoV-19^^Michigan|Human|Michigan Department of Health and Human Services|Michigan Department of Health and Human Services|Jacob|USA
>Spike|hCoV-19/South Africa/Tygerberg_317_CC2/2020|2020-10-09|EPI_ISL_660126|Original|hCoV-19^^Western Cape|Human|Ampath|National Health Laboratory Service (NHLS)|de Oliveira|South Africa
>Spike|hCoV-19/England/ALDP-CBE603/2020|2020-12-18|EPI_ISL_760549|Original|hCoV-19^^England|Human|Lighthouse Lab in Alderley Park|Wellcome Sanger Institute for the COVID-19 Genomics UK (COG-UK) Consortium|Davies|United Kingdom
>Spike|hCoV-19/England/QEUH-9FA7CF/2020|2020-09-28|EPI_ISL_588251|Original|hCoV-19^^England|Human|Lighthouse Lab in Glasgow|Wellcome Sanger Institute for the COVID-19 Genomics UK (COG-UK) consortium|Davies|United Kingdom
>Spike|hCoV-19/Netherlands/NA_361/2020|2020-03-30|EPI_ISL_455165|Original|hCoV-19^^Netherlands|Human|Dutch COVID-19 response team|Erasmus Medical Center|Oude Munnink|Netherlands
>Spike|hCoV-19/Canada/NS-NML-1367/2020|2020-04-16|EPI_ISL_579743|Original|hCoV-19^^Nova Scotia|Human|QEII Health Sciences Centre|National Microbiology Laboratory (NML)|Majer|Canada
>Spike|hCoV-19/England/QEUH-B0FC02/2020|2020-11-02|EPI_ISL_641957|Original|hCoV-19^^England|Human|Lighthouse Lab in Glasgow|Wellcome Sanger Institute for the COVID-19 Genomics UK (COG-UK) Consortium|Davies|United Kingdom
>Spike|hCoV-19/USA/CA-ALSR-1948/2020|2020-06-26|EPI_ISL_512253|Original|hCoV-19^^California|Human|San Diego County Public Health Laboratory|Andersen lab at Scripps Research|Gangavarapu|USA
>Spike|hCoV-19/England/QEUH-A9F35E/2020|2020-10-20|EPI_ISL_611409|Original|hCoV-19^^England|Human|Lighthouse Lab in Glasgow|Wellcome Sanger Institute for the COVID-19 Genomics UK (COG-UK) consortium|Davies|United Kingdom
>Spike|hCoV-19/Denmark/DCGC-8732/2020|2020-10-26|EPI_ISL_626026|Original|hCoV-19^^Hovedstaden|Human|Department of Virus and Microbiological Special Diagnostics|Albertsen lab|Michaelsen|Denmark
>Spike|hCoV-19/Switzerland/LU-ETHZ-140064/2020|2020-04-30|EPI_ISL_476112|Original|hCoV-19^^Luzern|Human|Viollier AG|Department of Biosystems Science and Engineering|Nadeau|Switzerland
>Spike|hCoV-19/England/QEUH-B66EA9/2020|2020-11-11|EPI_ISL_662077|Original|hCoV-19^^England|Human|Lighthouse Lab in Glasgow|Wellcome Sanger Institute for the COVID-19 Genomics UK (COG-UK) Consortium|Davies|United Kingdom
>Spike|hCoV-19/England/QEUH-B4176D/2020|2020-11-08|EPI_ISL_658737|Original|hCoV-19^^England|Human|Lighthouse Lab in Glasgow|Wellcome Sanger Institute for the COVID-19 Genomics UK (COG-UK) Consortium|Davies|United Kingdom
>Spike|hCoV-19/England/NORT-2824BD/2020|2020-03-26|EPI_ISL_488683|Original|hCoV-19^^England|Human|NU-OMICS DNA Sequencing research facility|Wellcome Sanger Institute for the COVID-19 Genomics UK (COG-UK) consortium|Davies|United Kingdom
>Spike|hCoV-19/USA/TX-DSHS-0471/2020|2020-06-21|EPI_ISL_542002|Original|hCoV-19^^Texas|Human|Texas Department of State Health Services|Texas Department of State Health Services|Rahman|USA

>Spike|hCoV-19/USA/FL-BPHL-2187/2020|2020-08-22|EPI_ISL_653340|Original|hCoV-19^^Florida|Human|Florida Bureau of Public Health Laboratories|Florida Bureau of Public Health Laboratories|Blanton|USA
>Spike|hCoV-19/Sweden/20-04852/2020|2020-02-28|EPI_ISL_475547|Original|hCoV-19^^Stockholm|Human|Karolinska Universitetslaboratoriet|The Public Health Agency of Sweden|Svartstr\u00f6m|Sweden
>Spike|hCoV-19/England/QEUH-B66C27/2020|2020-11-11|EPI_ISL_662397|Original|hCoV-19^^England|Human|Lighthouse Lab in Glasgow|Wellcome Sanger Institute for the COVID-19 Genomics UK (COG-UK) Consortium|Davies|United Kingdom
>Spike|hCoV-19/Australia/VIC2551/2020|2020-06-27|EPI_ISL_522238|Original|hCoV-19^^Victoria|Human|Victorian Infectious Diseases Reference Laboratory (VIDRL)|VIDRL and MDU-PHL|Schultz|Australia
>Spike|hCoV-19/USA/MA-Yale-410/2020|2020-04-16|EPI_ISL_738282|Original|hCoV-19^^Massachusetts|Human|Connecticut Veterans' Affairs Hospital|Grubaugh Lab - Yale School of Public Health|Grubaugh|USA
>Spike|hCoV-19/England/QEUH-965440/2020|2020-08-11|EPI_ISL_531179|Original|hCoV-19^^England|Human|Lighthouse Lab in Glasgow|Wellcome Sanger Institute for the COVID-19 Genomics UK (COG-UK) consortium|Davies|United Kingdom
>Spike|hCoV-19/England/ALDP-AC120B/2020|2020-10-10|EPI_ISL_631096|Original|hCoV-19^^England|Human|Lighthouse Lab in Alderley Park|Wellcome Sanger Institute for the COVID-19 Genomics UK (COG-UK) consortium|Davies|United Kingdom
>Spike|hCoV-19/Japan/PG-2084/2020|2020-04-00|EPI_ISL_685689|Original|hCoV-19^^Japan|Human|Tokyo Metropolitan Institute of Public Health|Pathogen Genomics Center|Sekizuka|Japan
>Spike|hCoV-19/Chile/RM-112777/2020|2020-06-28|EPI_ISL_746593|Original|hCoV-19^^Region Metropolitana de Santiago|Human|Genetica Molecular and Subdepartamento de Virologia ISP Chile|Instituto de Salud Publica de Chile|Tognarelli|Chile
>Spike|hCoV-19/Sweden/20-08992/2020|2020-09-16|EPI_ISL_582774|Original|hCoV-19^^Ostergotland|Human|Klinsisk mikrobiologi Linkoping|The Public Health Agency of Sweden|Svartstr\u00f6m|Sweden
>Spike|hCoV-19/USA/OR-OHSU-2637/2020|2020-08-22|EPI_ISL_667269|Original|hCoV-19^^Oregon|Human|OHSU Lab Services Molecular Microbiology Lab|Oregon SARS-CoV-2 Genome Sequencing Center|O'Roak|USA
>Spike|hCoV-19/Uganda/UG124/2020|2020-09-01|EPI_ISL_738031|Original|hCoV-19^^Uganda|Human|Uganda Central Public Health Lab and Uganda Virus Research Institute|MRC/UVRI & LSHTM Uganda\u00a0Research Unit|Cotten|Uganda
>Spike|hCoV-19/England/QEUH-A1EE83/2020|2020-10-01|EPI_ISL_587439|Original|hCoV-19^^England|Human|Lighthouse Lab in Glasgow|Wellcome Sanger Institute for the COVID-19 Genomics UK (COG-UK) consortium|Davies|United Kingdom
>Spike|hCoV-19/USA/ME-HETL-J0333/2020|2020-11-24|EPI_ISL_755461|Original|hCoV-19^^Maine|Human|Maine Health and Environmental Testing Laboratory|Tewhey Lab|Tewhey|USA
>Spike|hCoV-19/Israel/CVL-144-ngs/2020|2020-12-27|EPI_ISL_776844|Original|hCoV-19^^Israel|Human|Israel Central Virology laboratory|Israel Central Virology laboratory|Zuckerman|Israel
>Spike|hCoV-19/Japan/PG-1156/2020|2020-04-00|EPI_ISL_684766|Original|hCoV-19^^Japan|Human|Pathogen Genomics Center|Pathogen Genomics Center|Sekizuka|Japan
>Spike|hCoV-19/England/CAMC-C439CE/2020|2020-12-10|EPI_ISL_719117|Original|hCoV-19^^England|Human|Lighthouse Lab in Cambridge|Wellcome Sanger Institute for the COVID-19 Genomics UK (COG-UK) Consortium|Davies|United Kingdom
>Spike|hCoV-19/USA/NY-NYCPHL-000185/2020|2020-04-22|EPI_ISL_631720|Original|hCoV-19^^New York|Human|Jamaica Hospital Medical Center|New York City Public Health Laboratory|Wang|USA
>Spike|hCoV-19/Faroe Islands/HFS-77/2020|2020-08-05|EPI_ISL_614312|Original|hCoV-19^^Faroe Islands|Human|Faroese National Reference Laboratory for Fish and Animal Diseases|Faroese National Reference Laboratory for Fish and Animal Diseases|Dahl|Faroe Islands
>Spike|hCoV-19/Japan/PG-0653/2020|2020-04-00|EPI_ISL_684263|Original|hCoV-19^^Japan|Human|Gunma Prefectural Institute of Public Health and Environmental Sciences|Pathogen Genomics Center|Sekizuka|Japan

>Spike|hCoV-19/England/MILK-995201/2020|2020-08-24|EPI_ISL_552340|Original|hCoV-19^^England|Human|Lighthouse Lab in Milton Keynes|Wellcome Sanger Institute for the COVID-19 Genomics UK (COG-UK) consortium|Davies|United Kingdom
>Spike|hCoV-19/Denmark/DCGC-15635/2020|2020-11-23|EPI_ISL_713933|Original|hCoV-19^^Sjaelland|Human|Department of Virus and Microbiological Special Diagnostics|Albertsen Lab|Michaelsen|Denmark
>Spike|hCoV-19/Japan/PG-5463/2020|2020-08-00|EPI_ISL_690566|Original|hCoV-19^^Japan|Human|Pathogen Genomics Center|Pathogen Genomics Center|Sekizuka|Japan
>Spike|hCoV-19/England/LEED-2A8CE6/2020|2020-03-25|EPI_ISL_539008|Original|hCoV-19^^England|Human|Leeds Teaching Hospitals NHS Trust and Public Health England|Wellcome Sanger Institute for the COVID-19 Genomics UK (COG-UK) consortium|Davies|United Kingdom
>Spike|hCoV-19/Finland/20MS4A4/2020|2020-03-20|EPI_ISL_757368|Original|hCoV-19^^Uusimaa|Human|Department of Virology and Immunology|Department of Virology|Smura|Finland
>Spike|hCoV-19/USA/NC_200468/2020|2020-04-00|EPI_ISL_458004|Original|hCoV-19^^North Carolina|Human|Dirk Dittmer|Dirk Dittmer|Moorad|USA
>Spike|hCoV-19/Canada/BC_03907778/2020|2020-04-00|EPI_ISL_466755|Original|hCoV-19^^British Columbia|Human|BCCDC Public Health Laboratory|BCCDC Public Health Laboratory|Harrigan|Canada
>Spike|hCoV-19/England/MILK-4AFFF3/2020|2020-06-05|EPI_ISL_559232|Original|hCoV-19^^England|Human|Lighthouse Lab in Milton Keynes|Wellcome Sanger Institute for the COVID-19 Genomics UK (COG-UK) consortium|Davies|United Kingdom
>Spike|hCoV-19/USA/CA-CZB-1018/2020|2020-04-10|EPI_ISL_436659|Original|hCoV-19^^California|Human|County of Santa Clara Public Health Department|Chan-Zuckerberg Biohub|Hao|USA
>Spike|hCoV-19/Netherlands/ZH-EMC-1010/2020|2020-11-16|EPI_ISL_722656|Original|hCoV-19^^Zuid Holland|Human|Dutch COVID-19 response team|Erasmus Medical Center|Oude Munnink|Netherlands
>Spike|hCoV-19/England/BIRM-6AAE8/2020|2020-11-11|EPI_ISL_651647|Original|hCoV-19^^England|Human|University of Birmingham|COVID-19 Genomics UK (COG-UK) Consortium|Loman|United Kingdom
>Spike|hCoV-19/Wales/PHWC-16E245/2020|2020-10-02|EPI_ISL_585688|Original|hCoV-19^^Wales|Human|Wales Specialist Virology Centre Sequencing lab|COVID-19 Genomics UK (COG-UK) Consortium|Moore|United Kingdom
>Spike|hCoV-19/India/MH-ACTREC-700/2020|2020-09-25|EPI_ISL_700325|Original|hCoV-19^^Maharashtra|Human|Hematopathology Laboratory|Hematopathology Laboratory|Patkar|India
>Spike|hCoV-19/USA/UT-UPHL-2012351195/2020|2020-10-23|EPI_ISL_746298|Original|hCoV-19^^Utah|Human|Ginkgo Bioworks Clinical Laboratory|Utah Public Health Laboratory|Young|USA
>Spike|hCoV-19/USA/WI-UW-934/2020|2020-05-07|EPI_ISL_509934|Original|hCoV-19^^Wisconsin|Human|University of Wisconsin-Madison AIDS Vaccine Research Laboratories|University of Wisconsin-Madison AIDS Vaccine Research Laboratories|Baczenas|USA
>Spike|hCoV-19/Cyprus/005/2020|2020-04-01|EPI_ISL_463745|Original|hCoV-19^^Cyprus|Human|Department of Molecular Virology|Department of Molecular Virology|Richter|Cyprus
>Spike|hCoV-19/England/204621520/2020|2020-11-10|EPI_ISL_700620|Original|hCoV-19^^England|Human|Respiratory Virus Unit|COVID-19 Genomics UK (COG-UK) Consortium|Platt|United Kingdom
>Spike|hCoV-19/England/MILK-A6ACF7/2020|2020-10-08|EPI_ISL_608768|Original|hCoV-19^^England|Human|Lighthouse Lab in Milton Keynes|Wellcome Sanger Institute for the COVID-19 Genomics UK (COG-UK) consortium|Davies|United Kingdom
>Spike|hCoV-19/Wales/PHWC-47E78C/2020|2020-10-11|EPI_ISL_595499|Original|hCoV-19^^Wales|Human|Originating lab|COVID-19 Genomics UK (COG-UK) Consortium|Moore|United Kingdom
>Spike|hCoV-19/England/CAMC-B097AD/2020|2020-10-31|EPI_ISL_672982|Original|hCoV-19^^England|Human|Lighthouse Lab in Cambridge|Wellcome Sanger Institute for the COVID-19 Genomics UK (COG-UK) Consortium|Davies|United Kingdom

>Spike|hCoV-19/England/ALDP-C3C1A2/2020|2020-12-10|EPI_ISL_730944|Original|hCoV-19^^England|Human|Lighthouse Lab in Alderley Park|Wellcome Sanger Institute for the COVID-19 Genomics UK (COG-UK) Consortium|Davies|United Kingdom
>Spike|hCoV-19/Australia/VIC8122/2020|2020-08-15|EPI_ISL_564861|Original|hCoV-19^^Victoria|Human|Microbiological Diagnostic Unit - Public Health Laboratory (MDU-PHL)|MDU-PHL|Schultz|Australia
>Spike|hCoV-19/England/201360047/2020|2020-03-26|EPI_ISL_423200|Original|hCoV-19^^England|Human|Respiratory Virus Unit|Respiratory Virus Unit|Platt|United Kingdom
>Spike|hCoV-19/USA/LA-EVTL1260/2020|2020-12-04|EPI_ISL_778926|Original|hCoV-19^^Louisiana|Human|LSUHS Emerging Viral Threat Laboratory|Microbial Genome Sequencing Center|Kamil|USA
>Spike|hCoV-19/England/MILK-AC7D5F/2020|2020-10-21|EPI_ISL_629284|Original|hCoV-19^^England|Human|Lighthouse Lab in Milton Keynes|Wellcome Sanger Institute for the COVID-19 Genomics UK (COG-UK) consortium|Davies|United Kingdom
>Spike|hCoV-19/Scotland/CVR5554/2020|2020-10-27|EPI_ISL_650734|Original|hCoV-19^^Scotland|Human|West of Scotland Specialist Virology Centre|COVID-19 Genomics UK (COG-UK) Consortium|Robertson|United Kingdom
>Spike|hCoV-19/England/20170030504/2020|2020-04-17|EPI_ISL_465272|Original|hCoV-19^^England|Human|Respiratory Virus Unit|Respiratory Virus Unit|Platt|United Kingdom
>Spike|hCoV-19/Wales/PHWC-48D2F2/2020|2020-11-15|EPI_ISL_680605|Original|hCoV-19^^Wales|Human|Wales Specialist Virology Centre Sequencing lab|COVID-19 Genomics UK (COG-UK) Consortium|Moore|United Kingdom
>Spike|hCoV-19/Wales/PHWC-16ECB0/2020|2020-10-05|EPI_ISL_585848|Original|hCoV-19^^Wales|Human|Wales Specialist Virology Centre Sequencing lab|COVID-19 Genomics UK (COG-UK) Consortium|Moore|United Kingdom
>Spike|hCoV-19/England/QEUH-A8BA14/2020|2020-10-16|EPI_ISL_643170|Original|hCoV-19^^England|Human|Lighthouse Lab in Glasgow|Wellcome Sanger Institute for the COVID-19 Genomics UK (COG-UK) Consortium|Davies|United Kingdom
>Spike|hCoV-19/Jordan/IR-ALSR-5226/2020|2020-10-12|EPI_ISL_730464|Original|hCoV-19^^Irbid|Human|Biolab Diagnostic Laboratories|Andersen lab at Scripps Research|Gangavarapu|Jordan
>Spike|hCoV-19/England/MILK-B5A2D9/2020|2020-11-06|EPI_ISL_658213|Original|hCoV-19^^England|Human|Lighthouse Lab in Milton Keynes|Wellcome Sanger Institute for the COVID-19 Genomics UK (COG-UK) Consortium|Davies|United Kingdom
>Spike|hCoV-19/England/MILK-CA4C86/2020|2020-12-16|EPI_ISL_762439|Original|hCoV-19^^England|Human|Lighthouse Lab in Milton Keynes|Wellcome Sanger Institute for the COVID-19 Genomics UK (COG-UK) Consortium|Davies|United Kingdom
>Spike|hCoV-19/USA/CA-IGI-0117/2020|2020-06-17|EPI_ISL_495139|Original|hCoV-19^^California|Human|Innovative Genomics Institute|Innovative Genomics Institute|Shivram|USA
>Spike|hCoV-19/Australia/SAP364/2020|2020-03-22|EPI_ISL_483135|Original|hCoV-19^^South Australia|Human|SA Pathology|SA Pathology|Leong|Australia
>Spike|hCoV-19/Scotland/QEUH-9980D7/2020|2020-08-27|EPI_ISL_568397|Original|hCoV-19^^Scotland|Human|Lighthouse Lab in Glasgow|Wellcome Sanger Institute for the COVID-19 Genomics UK (COG-UK) consortium|Davies|United Kingdom
>Spike|hCoV-19/England/NOTT-11767C/2020|2020-10-23|EPI_ISL_612578|Original|hCoV-19^^England|Human|Queens Medical Centre|COVID-19 Genomics UK (COG-UK) Consortium|Loose|United Kingdom
>Spike|hCoV-19/Wales/ALDP-94DFB6/2020|2020-06-16|EPI_ISL_555373|Original|hCoV-19^^Wales|Human|Lighthouse Lab in Alderley Park|Wellcome Sanger Institute for the COVID-19 Genomics UK (COG-UK) consortium|Davies|United Kingdom
>Spike|hCoV-19/Wales/PHWC-167FCD/2020|2020-03-31|EPI_ISL_494185|Original|hCoV-19^^Wales|Human|Originating lab|COVID-19 Genomics UK (COG-UK) Consortium|Moore|United Kingdom
>Spike|hCoV-19/USA/NY-Wadsworth-288918-01/2020|2020-12-18|EPI_ISL_767494|Original|hCoV-19^^New York|Human|Wadsworth Center|Wadsworth Center|Lasek-Nesselquist|USA
>Spike|hCoV-19/USA/AZ-TG402493/2020|2020-06-23|EPI_ISL_694857|Original|hCoV-19^^Arizona|Human|TGen North|TGen North|French|USA

>Spike|hCoV-19/England/LIVE-A68B9/2020|2020-03-16|EPI_ISL_499752|Original|hCoV-19^^England|Human|Liverpool Clinical Laboratories|COVID-19 Genomics UK (COG-UK) Consortium|Haldenby|United Kingdom
>Spike|hCoV-19/England/CAMB-1BCD92/2020|2020-12-06|EPI_ISL_723897|Original|hCoV-19^^England|Human|Department of Pathology|COVID-19 Genomics UK (COG-UK) Consortium|Goodfellow|United Kingdom
>Spike|hCoV-19/England/QEUH-B809D5/2020|2020-11-14|EPI_ISL_661851|Original|hCoV-19^^England|Human|Lighthouse Lab in Glasgow|Wellcome Sanger Institute for the COVID-19 Genomics UK (COG-UK) Consortium|Davies|United Kingdom
>Spike|hCoV-19/Australia/VIC809/2020|2020-03-28|EPI_ISL_427083|Original|hCoV-19^^Victoria|Human|Victorian Infectious Diseases Reference Laboratory (VIDRL)|Microbiological Diagnostic Unit Public Health Laboratory and Victorian Infectious Diseases Reference Laboratory|Schultz|Australia
>Spike|hCoV-19/England/CAMB-1AFB4D/2020|2020-05-02|EPI_ISL_459177|Original|hCoV-19^^England|Human|Department of Pathology|Wellcome Sanger Institute for the COVID-19 Genomics UK (COG-UK) consortium|Davies|United Kingdom
>Spike|hCoV-19/Wales/PHWC-4A3812/2020|2020-12-16|EPI_ISL_764579|Original|hCoV-19^^Wales|Human|Wales Specialist Virology Centre Sequencing lab|COVID-19 Genomics UK (COG-UK) Consortium|Moore|United Kingdom
>Spike|hCoV-19/England/CAMB-777F0/2020|2020-04-01|EPI_ISL_438514|Original|hCoV-19^^England|Human|Department of Pathology|Wellcome Sanger Institute for the COVID-19 Genomics UK (COG-UK) consortium|Amato|United Kingdom
>Spike|hCoV-19/England/ALDP-C48B87/2020|2020-12-11|EPI_ISL_736524|Original|hCoV-19^^England|Human|Lighthouse Lab in Alderley Park|Wellcome Sanger Institute for the COVID-19 Genomics UK (COG-UK) Consortium|Davies|United Kingdom
>Spike|hCoV-19/Germany/BY-ChVir-1017/2020|2020-01-30|EPI_ISL_450209|Original|hCoV-19^^Bavaria|Human|Department of Virology|Department of Virology|B\u00f6hmer|Germany
>Spike|hCoV-19/USA/TX-HMH-MCoV-12397/2020|2020-09-27|EPI_ISL_790326|Original|hCoV-19^^Texas|Human|Houston Methodist Hospital|Houston Methodist Hospital|Long|USA
>Spike|hCoV-19/Switzerland/VD-ETHZ-220002/2020|2020-07-24|EPI_ISL_511995|Original|hCoV-19^^Vaud|Human|Viollier AG|Department of Biosystems Science and Engineering|Nadeau|Switzerland
>Spike|hCoV-19/USA/WI-UW-839/2020|2020-05-05|EPI_ISL_509846|Original|hCoV-19^^Wisconsin|Human|University of Wisconsin-Madison AIDS Vaccine Research Laboratories|University of Wisconsin-Madison AIDS Vaccine Research Laboratories|Baczenas|USA
>Spike|hCoV-19/England/LIVE-A8583/2020|2020-03-26|EPI_ISL_517182|Original|hCoV-19^^England|Human|Liverpool Clinical Laboratories|COVID-19 Genomics UK (COG-UK) Consortium|Haldenby|United Kingdom
>Spike|hCoV-19/England/ALDP-B76A39/2020|2020-11-12|EPI_ISL_723798|Original|hCoV-19^^England|Human|Oxford Viromics|COVID-19 Genomics UK (COG-UK) Consortium|Golubchik|United Kingdom
>Spike|hCoV-19/Finland/15A84S16/2020|2020-04-15|EPI_ISL_481561|Original|hCoV-19^^Uusimaa|Human|Department of Virology and Immunology|Department of Virology|Smura|Finland
>Spike|hCoV-19/Germany/HH-hpi-p1589/2020|2020-03-16|EPI_ISL_776061|Original|hCoV-19^^Hamburg|Human|University Medical Center Hamburg Eppendorf|Heinrich Pette Institute|Robitaille|Germany
>Spike|hCoV-19/England/CAMB-1AE689/2020|2020-04-28|EPI_ISL_459472|Original|hCoV-19^^England|Human|Department of Pathology|Wellcome Sanger Institute for the COVID-19 Genomics UK (COG-UK) consortium|Davies|United Kingdom
>Spike|hCoV-19/USA/CA-CHLA-PLM57473032/2020|2020-11-30|EPI_ISL_753453|Original|hCoV-19^^California|Human|Clinical virology Laboratory|Center for Personalized Medicine|Dien Bard|USA
>Spike|hCoV-19/England/LIVE-9C810/2020|2020-03-23|EPI_ISL_449583|Original|hCoV-19^^England|Human|Liverpool Clinical Laboratories|COVID-19 Genomics UK (COG-UK) Consortium|Haldenby|United Kingdom
>Spike|hCoV-19/Netherlands/UT-RIVM-20782/2020|2020-12-21|EPI_ISL_790623|Original|hCoV-19^^Utrecht|Human|Dutch COVID-19 response

team|National Institute for Public Health and the Environment (RIVM)|Meijer|Netherlands
>Spike|hCoV-19/New Zealand/20VR2521/2020|2020-00-00|EPI_ISL_579267|Original|hCoV-19^^Auckland|Human|LabPLUS|Institute of Environmental Science and Research (ESR)|Wang|New Zealand
>Spike|hCoV-19/USA/TX-HMH-5451/2020|2020-06-26|EPI_ISL_544786|Original|hCoV-19^^Texas|Human|Houston Methodist Hospital|Houston Methodist Hospital|Long|USA
>Spike|hCoV-19/Denmark/DCGC-1372/2020|2020-03-30|EPI_ISL_622218|Original|hCoV-19^^Sjaelland|Human|Department of Virus and Microbiological Special Diagnostics|Albertsen lab|Michaelsen|Denmark
>Spike|hCoV-19/Austria/CeMM0901/2020|2020-03-30|EPI_ISL_583754|Original|hCoV-19^^Austria|Human|Dr. Gernot Walder GmbH|Bergthaler laboratory|Bergthaler|Austria
>Spike|hCoV-19/India/DL-NCDC2343-CSIR-IGIB/2020|2020-05-19|EPI_ISL_482562|Original|hCoV-19^^Delhi|Human|National Centre for Disease control (NCDC)|NCDC/CSIR-IGIB|Pandey|India
>Spike|hCoV-19/England/CAMC-948DC0/2020|2020-07-30|EPI_ISL_556528|Original|hCoV-19^^England|Human|Lighthouse Lab in Cambridge|Wellcome Sanger Institute for the COVID-19 Genomics UK (COG-UK) consortium|Davies|United Kingdom
>Spike|hCoV-19/England/ALDP-B1D0AB/2020|2020-11-01|EPI_ISL_645786|Original|hCoV-19^^England|Human|Lighthouse Lab in Alderley Park|Wellcome Sanger Institute for the COVID-19 Genomics UK (COG-UK) Consortium|Davies|United Kingdom
>Spike|hCoV-19/Wuhan/HB-WH3-179/2020|2020-03-13|EPI_ISL_454941|Original|hCoV-19^^Hubei|Human|Wuhan Chain Medical Labs (CMLabs)|State Key Laboratory of Biotherapy of Sichuan University|Tang|China
>Spike|hCoV-19/England/SHEF-D08D4/2020|2020-05-12|EPI_ISL_475488|Original|hCoV-19^^England|Human|Virology Department|COVID-19 Genomics UK (COG-UK) Consortium|de Silva|United Kingdom
>Spike|hCoV-19/USA/MI-MDHHS-SC21168/2020|2020-04-04|EPI_ISL_507425|Original|hCoV-19^^Michigan|Human|Michigan Department of Health and Human Services|Michigan Department of Health and Human Services|Blankenship|USA
>Spike|hCoV-19/USA/LA-EVTL578/2020|2020-06-30|EPI_ISL_578982|Original|hCoV-19^^Louisiana|Human|LSUHS Emerging Viral Threat Laboratory|Microbial Genome Sequencing Center|Kamil|USA
>Spike|hCoV-19/Bangladesh/DNAS_DRCIM_isl_KHL-1/2020|2020-06-30|EPI_ISL_774934|Original|hCoV-19^^Dhaka|Human|Designated Reference Institute for Chemical Measurements (DRICM)|DNA SOLUTION LTD.|Rabbi|Bangladesh
>Spike|hCoV-19/USA/LA-CDC-2103/2020|2020-05-27|EPI_ISL_535347|Original|hCoV-19^^Louisiana|Human|LA Office of Public Health Laboratories|Pathogen Discovery|Queen|USA
>Spike|hCoV-19/USA/NM-DOH-2020327107/2020|2020-09-03|EPI_ISL_575307|Original|hCoV-19^^New Mexico|Human|Molecular Biology|Molecular Biology|Johnson|USA
>Spike|hCoV-19/USA/CO-CDPHE-2008170075/2020|2020-08-16|EPI_ISL_677304|Original|hCoV-19^^Colorado|Human|Colorado Department of Public Health and Environment|Colorado Department of Puplic Health and Environment|Bankers|USA
>Spike|hCoV-19/Luxembourg/LNS3630121/2020|2020-10-22|EPI_ISL_740344|Original|hCoV-19^^Luxembourg|Human|Laboratoire national de sant\u00e9|Laboratoire national de sant\u00e9|Wienecke-Baldacchino|Luxembourg
>Spike|hCoV-19/USA/HI-H200462/2020|2020-10-08|EPI_ISL_753005|Original|hCoV-19^^Hawaii|Human|State Laboratories Division|State Laboratories Division|Sultana|USA
>Spike|hCoV-19/USA/TX-HMH-MCoV-12265/2020|2020-09-24|EPI_ISL_790275|Original|hCoV-19^^Texas|Human|Houston Methodist Hospital|Houston Methodist Hospital|Long|USA
>Spike|hCoV-19/England/ALDP-AA481B/2020|2020-10-22|EPI_ISL_624500|Original|hCoV-19^^England|Human|Lighthouse Lab in Alderley Park|Wellcome Sanger Institute for the COVID-19 Genomics UK (COG-UK) consortium|Davies|United Kingdom
>Spike|hCoV-19/England/QEUH-B6B0AE/2020|2020-11-11|EPI_ISL_662400|Original|hCoV-19^^England|Human|Lighthouse Lab in Glasgow|Wellcome Sanger Institute for the COVID-19 Genomics UK (COG-UK) Consortium|Davies|United Kingdom
>Spike|hCoV-19/England/CAMC-C2CF41/2020|2020-12-08|EPI_ISL_733787|Original|hCoV-19^^England|Human|Lighthouse Lab in Cambridge|Wellcome Sanger Institute for the COVID-19 Genomics UK (COG-UK) Consortium|Davies|United Kingdom

>Spike|hCoV-19/Wales/QEUH-A026D5/2020|2020-09-29|EPI_ISL_586241|Original|hCoV-19^^Wales|Human|Quadram Institute Bioscience|COVID-19 Genomics UK (COG-UK) Consortium|Alikhan|United Kingdom
>Spike|hCoV-19/England/ALDP-AA174E/2020|2020-10-22|EPI_ISL_624952|Original|hCoV-19^^England|Human|Lighthouse Lab in Alderley Park|Wellcome Sanger Institute for the COVID-19 Genomics UK (COG-UK) consortium|Davies|United Kingdom
>Spike|hCoV-19/England/LIVE-9FF09/2020|2020-06-01|EPI_ISL_472071|Original|hCoV-19^^England|Human|Liverpool Clinical Laboratories|COVID-19 Genomics UK (COG-UK) Consortium|Haldenby|United Kingdom
>Spike|hCoV-19/USA/MN-MDH-1929/2020|2020-10-09|EPI_ISL_613497|Original|hCoV-19^^Minnesota|Human|Public Health Laboratory - Infectious Disease Lab|Minnesota Department of Health|Lorentz|USA
>Spike|hCoV-19/England/QEUH-B3FBFD/2020|2020-11-08|EPI_ISL_658645|Original|hCoV-19^^England|Human|Lighthouse Lab in Glasgow|Wellcome Sanger Institute for the COVID-19 Genomics UK (COG-UK) Consortium|Davies|United Kingdom
>Spike|hCoV-19/England/BRIS-130FEB/2020|2020-03-25|EPI_ISL_501565|Original|hCoV-19^^England|Human|PHE South West Regional Laboratory|Wellcome Sanger Institute for the COVID-19 Genomics UK (COG-UK) consortium|Davies|United Kingdom
>Spike|hCoV-19/Switzerland/BE-ETHZ-190079/2020|2020-07-06|EPI_ISL_500936|Original|hCoV-19^^Bern|Human|Viollier AG|Department of Biosystems Science and Engineering|Nadeau|Switzerland
>Spike|hCoV-19/England/ALDP-A6E68C/2020|2020-10-15|EPI_ISL_606924|Original|hCoV-19^^England|Human|Lighthouse Lab in Alderley Park|Wellcome Sanger Institute for the COVID-19 Genomics UK (COG-UK) consortium|Davies|United Kingdom
>Spike|hCoV-19/Spain/PV-IBV-98008418/2020|2020-10-27|EPI_ISL_654596|Original|hCoV-19^^Basque Country|Human|Servicio de Microbiolog\u00eda. Hospital Universitario Donostia. OSI Donostialdea. \u00c1rea de Enfermedades Infecciosas|SeqCOVID-SPAIN consortium/IBV(CSIC)|Comas|Spain
>Spike|hCoV-19/England/ALDP-94DCDD/2020|2020-06-19|EPI_ISL_555725|Original|hCoV-19^^England|Human|Lighthouse Lab in Alderley Park|Wellcome Sanger Institute for the COVID-19 Genomics UK (COG-UK) consortium|Davies|United Kingdom
>Spike|hCoV-19/Scotland/EDB10775/2020|2020-12-07|EPI_ISL_724232|Original|hCoV-19^^Scotland|Human|Virology Department|COVID-19 Genomics UK (COG-UK) Consortium|McHugh|United Kingdom
>Spike|hCoV-19/USA/MI-MDHHS-SC21798/2020|2020-04-14|EPI_ISL_565841|Original|hCoV-19^^Michigan|Human|Michigan Department of Health and Human Services|Michigan Department of Health and Human Services|Blankenship|USA
>Spike|hCoV-19/Spain/CT-IBV-99009912/2020|2020-08-10|EPI_ISL_660309|Original|hCoV-19^^Catalunya|Human|Servicio de Microbiolog\u00eda|SeqCOVID-SPAIN consortium/IBV(CSIC)|Comas|Spain
>Spike|hCoV-19/England/CWAR-24FDF4B/2020|2020-06-10|EPI_ISL_584548|Original|hCoV-19^^England|Human|UHCW / University of Warwick|COVID-19 Genomics UK (COG-UK) Consortium|Stark|United Kingdom
>Spike|hCoV-19/Wales/PHWC-4A1331/2020|2020-12-09|EPI_ISL_743657|Original|hCoV-19^^Wales|Human|Wales Specialist Virology Centre Sequencing lab|COVID-19 Genomics UK (COG-UK) Consortium|Moore|United Kingdom
>Spike|hCoV-19/England/ALDP-91073C/2020|2020-06-24|EPI_ISL_553723|Original|hCoV-19^^England|Human|Lighthouse Lab in Alderley Park|Wellcome Sanger Institute for the COVID-19 Genomics UK (COG-UK) consortium|Davies|United Kingdom
>Spike|hCoV-19/England/QEUH-C32EF0/2020|2020-12-11|EPI_ISL_733973|Original|hCoV-19^^England|Human|Lighthouse Lab in Glasgow|Wellcome Sanger Institute for the COVID-19 Genomics UK (COG-UK) Consortium|Davies|United Kingdom
>Spike|hCoV-19/Finland/5May13S12/2020|2020-05-05|EPI_ISL_481694|Original|hCoV-19^^Uusimaa|Human|Department of Virology and Immunology|Department of Virology|Smura|Finland
>Spike|hCoV-19/Bangladesh/CHRF-0014/2020|2020-05-21|EPI_ISL_477127|Original|hCoV-19^^Bangladesh|Human|Child Health Research Foundation|Child Health Research Foundation|Saha|Bangladesh

>Spike|hCoV-19/Germany/HH-hpi-p2164/2020|2020-03-28|EPI_ISL_776394|Original|hCoV-19^^Hamburg|Human|University Medical Center Hamburg Eppendorf|Heinrich Pette Institute|Robitaille|Germany
>Spike|hCoV-19/Scotland/EDB2077/2020|2020-04-19|EPI_ISL_433176|Original|hCoV-19^^Scotland|Human|Virology Department|COVID-19 Genomics UK (COG-UK) Consortium|Williams|United Kingdom
>Spike|hCoV-19/England/MILK-970A04/2020|2020-08-18|EPI_ISL_553226|Original|hCoV-19^^England|Human|Lighthouse Lab in Milton Keynes|Wellcome Sanger Institute for the COVID-19 Genomics UK (COG-UK) consortium|Davies|United Kingdom
>Spike|hCoV-19/Netherlands/LI-EMC-118/2020|2020-11-06|EPI_ISL_722731|Original|hCoV-19^^Limburg|Human|Dutch COVID-19 response team|Erasmus Medical Center|Oude Munnink|Netherlands
>Spike|hCoV-19/USA/IL-QDX-2622/2020|2020-03-14|EPI_ISL_604933|Original|hCoV-19^^Illinois|Human|Quest Diagnostics|Quest Diagnostics|Kagan|USA
>Spike|hCoV-19/England/CAMB-7E340/2020|2020-04-17|EPI_ISL_433473|Original|hCoV-19^^England|Human|Department of Pathology|COVID-19 Genomics UK (COG-UK) Consortium|Meredith|United Kingdom
>Spike|hCoV-19/England/ALDP-B6271B/2020|2020-11-07|EPI_ISL_656661|Original|hCoV-19^^England|Human|Lighthouse Lab in Alderley Park|Wellcome Sanger Institute for the COVID-19 Genomics UK (COG-UK) Consortium|Davies|United Kingdom
>Spike|hCoV-19/Wales/QEUH-969488/2020|2020-08-12|EPI_ISL_531210|Original|hCoV-19^^Wales|Human|Lighthouse Lab in Glasgow|Wellcome Sanger Institute for the COVID-19 Genomics UK (COG-UK) consortium|Davies|United Kingdom
>Spike|hCoV-19/Germany/HH-hpi-p1622/2020|2020-03-17|EPI_ISL_776074|Original|hCoV-19^^Hamburg|Human|University Medical Center Hamburg Eppendorf|Heinrich Pette Institute|Robitaille|Germany
>Spike|hCoV-19/USA/TX-HMH-MCoV-12410/2020|2020-09-29|EPI_ISL_790333|Original|hCoV-19^^Texas|Human|Houston Methodist Hospital|Houston Methodist Hospital|Long|USA
>Spike|hCoV-19/USA/CA-ALSR-4413/2020|2020-06-22|EPI_ISL_649016|Original|hCoV-19^^California|Human|San Diego County Public Health Laboratory|Andersen lab at Scripps Research|Gangavarapu|USA
>Spike|hCoV-19/South Africa/R10178/2020|2020-05-20|EPI_ISL_490261|Original|hCoV-19^^Gauteng|Human|National Institute for Communicable Diseases of the National Health Laboratory Service|National Institute for Communicable Diseases of the National Health Laboratory Service|Allam|South Africa
>Spike|hCoV-19/Jordan/IR-ALSR-5233/2020|2020-10-19|EPI_ISL_730471|Original|hCoV-19^^Irbid|Human|Biolab Diagnostic Laboratories|Andersen lab at Scripps Research|Gangavarapu|Jordan
>Spike|hCoV-19/Wales/PHWC-15C09B/2020|2020-03-31|EPI_ISL_472432|Original|hCoV-19^^Wales|Human|Wales Specialist Virology Centre Sequencing lab|COVID-19 Genomics UK (COG-UK) Consortium|Moore|United Kingdom
>Spike|hCoV-19/England/NORW-F2AA9/2020|2020-11-12|EPI_ISL_650721|Original|hCoV-19^^England|Human|Quadram Institute Bioscience|COVID-19 Genomics UK (COG-UK) Consortium|Alikhan|United Kingdom
>Spike|hCoV-19/USA/TX-DSHS-1077/2020|2020-06-16|EPI_ISL_628728|Original|hCoV-19^^Texas|Human|Texas Department of State Health Services|Texas Department of State Health Services|Rahman|USA
>Spike|hCoV-19/England/ALDP-A7003E/2020|2020-10-15|EPI_ISL_608057|Original|hCoV-19^^England|Human|Lighthouse Lab in Alderley Park|Wellcome Sanger Institute for the COVID-19 Genomics UK (COG-UK) consortium|Davies|United Kingdom
>Spike|hCoV-19/England/NORW-E6DCD/2020|2020-04-21|EPI_ISL_448978|Original|hCoV-19^^England|Human|Quadram Institute Bioscience|COVID-19 Genomics UK (COG-UK) Consortium|Alikhan|United Kingdom
>Spike|hCoV-19/England/NORT-29EDC2/2020|2020-05-11|EPI_ISL_490484|Original|hCoV-19^^England|Human|Northumbria University / South Tees Hospitals NHS Foundation Trust / North Cumbria Integrated Care NHS Foundation Trust / North Tees and Hartlepool NHS Foundation Trust / Newcastle Hospitals NHS Foundation Trust|COVID-19 Genomics UK (COG-UK) Consortium|Bashton|United Kingdom

>Spike|hCoV-19/USA/LA-EVTL980/2020|2020-08-18|EPI_ISL_653671|Original|hCoV-19^^Louisiana|Human|LSUHS Emerging Viral Threat Laboratory|Microbial Genome Sequencing Center|Kamil|USA
>Spike|hCoV-19/USA/MN-MDH-1615/2020|2020-04-25|EPI_ISL_530211|Original|hCoV-19^^Minnesota|Human|Minnesota Department of Health|Minnesota Department of Health|Garfin|USA
>Spike|hCoV-19/England/LCST-24D6A0B/2020|2020-06-03|EPI_ISL_478319|Original|hCoV-19^^England|Human|University Hospitals Of Leicester NHS Trust and DeepSeq Nottingham|COVID-19 Genomics UK (COG-UK) Consortium|Loose|United Kingdom
>Spike|hCoV-19/England/OXON-AFF8E/2020|2020-04-10|EPI_ISL_478840|Original|hCoV-19^^England|Human|Oxford Viromics|COVID-19 Genomics UK (COG-UK) Consortium|Golubchik|United Kingdom
>Spike|hCoV-19/Australia/VIC6862/2020|2020-07-26|EPI_ISL_564102|Original|hCoV-19^^Victoria|Human|Victorian Infectious Diseases Reference Laboratory (VIDRL)|VIDRL and MDU-PHL|Schultz|Australia
>Spike|hCoV-19/USA/UT-08359/2020|2020-05-13|EPI_ISL_524142|Original|hCoV-19^^Utah|Human|Utah Public Health Laboratory|Utah Public Health Laboratory|Young|USA
>Spike|hCoV-19/France/GES-2720/2020|2020-03-12|EPI_ISL_420039|Original|hCoV-19^^Grand-Est|Human|L'Air du Temps|National Reference Center for Viruses of Respiratory Infections|Enouf|France
>Spike|hCoV-19/England/MILK-ABBF49/2020|2020-10-22|EPI_ISL_631211|Original|hCoV-19^^England|Human|Lighthouse Lab in Milton Keynes|Wellcome Sanger Institute for the COVID-19 Genomics UK (COG-UK) consortium|Davies|United Kingdom
>Spike|hCoV-19/Scotland/EDB1046/2020|2020-04-06|EPI_ISL_433378|Original|hCoV-19^^Scotland|Human|Virology Department|COVID-19 Genomics UK (COG-UK) Consortium|Williams|United Kingdom
>Spike|hCoV-19/USA/MI-MDHHS-SC21086/2020|2020-03-27|EPI_ISL_507862|Original|hCoV-19^^Michigan|Human|Michigan Department of Health and Human Services|Michigan Department of Health and Human Services|Blankenship|USA
>Spike|hCoV-19/United Arab Emirates/0209/2020|2020-05-18|EPI_ISL_698395|Original|hCoV-19^^Abu Dhabi|Human|Group 42 (G42) Healthcare|G42 Healthcare|Ogrodzki|United Arab Emirates
>Spike|hCoV-19/England/SHEF-D316D/2020|2020-09-22|EPI_ISL_573757|Original|hCoV-19^^England|Human|Virology Department|COVID-19 Genomics UK (COG-UK) Consortium|de Silva|United Kingdom
>Spike|hCoV-19/Belgium/un-EMC-4/2020|2020-04-27|EPI_ISL_577746|Original|hCoV-19^^Belgium|Human|Dutch COVID-19 response team|Erasmus Medical Center|Oude Munnink|Belgium
>Spike|hCoV-19/USA/NY-NYCPHL-001174/2020|2020-10-29|EPI_ISL_671754|Original|hCoV-19^^New York|Human|DOHMH Morrisania|New York City Public Health Laboratory|Wang|USA
>Spike|hCoV-19/Senegal/64969/2020|2020-06-13|EPI_ISL_476570|Original|hCoV-19^^Dakar|Human|Institut Pasteur Dakar|Institut Pasteur de Dakar|Dia|Senegal
>Spike|hCoV-19/England/MILK-B01D58/2020|2020-10-24|EPI_ISL_643036|Original|hCoV-19^^England|Human|Lighthouse Lab in Milton Keynes|Wellcome Sanger Institute for the COVID-19 Genomics UK (COG-UK) Consortium|Davies|United Kingdom
>Spike|hCoV-19/South Africa/KRISP-K002729/2020|2020-08-14|EPI_ISL_529728|Original|hCoV-19^^KwaZulu-Natal|Human|NHLS-IALCH|KRISP|de Oliveira|South Africa
>Spike|hCoV-19/USA/CA-QDX-2155/2020|2020-03-18|EPI_ISL_604432|Original|hCoV-19^^California|Human|Quest Diagnostics|Quest Diagnostics|Kagan|USA
>Spike|hCoV-19/South Africa/KRISP-K003746/2020|2020-09-30|EPI_ISL_602920|Original|hCoV-19^^KwaZulu-Natal|Human|NHLS-IALCH|KRISP|de Oliveira|South Africa
>Spike|hCoV-19/England/NORT-297B1A/2020|2020-04-24|EPI_ISL_488101|Original|hCoV-19^^England|Human|NU-OMICS DNA Sequencing research facility|Wellcome Sanger Institute for the COVID-19 Genomics UK (COG-UK) consortium|Davies|United Kingdom
>Spike|hCoV-19/England/20422147004/2020|2020-09-00|EPI_ISL_584058|Original|hCoV-19^^England|Human|Respiratory Virus Unit|Respiratory Virus Unit|Platt|United Kingdom

>Spike|hCoV-19/England/MILK-9A8CFE/2020|2020-09-01|EPI_ISL_550178|Original|hCoV-19^^England|Human|Lighthouse Lab in Milton Keynes|Wellcome Sanger Institute for the COVID-19 Genomics UK (COG-UK) consortium|Davies|United Kingdom
>Spike|hCoV-19/USA/WI-UW-1336/2020|2020-09-04|EPI_ISL_536707|Original|hCoV-19^^Wisconsin|Human|University of Wisconsin-Madison AIDS Vaccine Research Laboratories|University of Wisconsin-Madison AIDS Vaccine Research Laboratories|Baczenas|USA
>Spike|hCoV-19/England/CAMC-B33485/2020|2020-11-05|EPI_ISL_659354|Original|hCoV-19^^England|Human|Lighthouse Lab in Cambridge|Wellcome Sanger Institute for the COVID-19 Genomics UK (COG-UK) Consortium|Davies|United Kingdom
>Spike|hCoV-19/USA/WA-UW-2964/2020|2020-03-26|EPI_ISL_486063|Original|hCoV-19^^Washington|Human|UW Virology Lab|UW Virology Lab|Roychoudhury|USA
>Spike|hCoV-19/Spain/MD-IBV-99012430/2020|2020-11-28|EPI_ISL_780071|Original|hCoV-19^^Madrid|Human|Hospital General Universitario Gregorio Mara\u00f1\u00f3n|SeqCOVID-SPAIN consortium/IBV(CSIC)|Comas|Spain
>Spike|hCoV-19/Scotland/EDB3418/2020|2020-04-10|EPI_ISL_487935|Original|hCoV-19^^Scotland|Human|Virology Department|Wellcome Sanger Institute for the COVID-19 Genomics UK (COG-UK) consortium|Davies|United Kingdom
>Spike|hCoV-19/England/ALDP-94D9B8/2020|2020-06-20|EPI_ISL_555168|Original|hCoV-19^^England|Human|Lighthouse Lab in Alderley Park|Wellcome Sanger Institute for the COVID-19 Genomics UK (COG-UK) consortium|Davies|United Kingdom
>Spike|hCoV-19/India/OR-ILSCV27725/2020|2020-05-21|EPI_ISL_481122|Original|hCoV-19^^Odisha|Human|Immunogenomics lab|Immunogenomics lab|Raghav|India
>Spike|hCoV-19/Denmark/ALAB-SSI-1126/2020|2020-04-02|EPI_ISL_436962|Original|hCoV-19^^Denmark|Human|Department of Virus and Microbiological Special Diagnostics|Albertsen lab|Kirkegaard|Denmark
>Spike|hCoV-19/Denmark/DCGC-13139/2020|2020-11-30|EPI_ISL_712609|Original|hCoV-19^^Midtjylland|Human|Department of Virus and Microbiological Special Diagnostics|Albertsen Lab|Michaelsen|Denmark
>Spike|hCoV-19/Denmark/DCGC-14968/2020|2020-11-23|EPI_ISL_713555|Original|hCoV-19^^Hovedstaden|Human|Department of Virus and Microbiological Special Diagnostics|Albertsen Lab|Michaelsen|Denmark
>Spike|hCoV-19/United Arab Emirates/0127/2020|2020-05-18|EPI_ISL_698317|Original|hCoV-19^^Abu Dhabi|Human|Group 42 (G42) Healthcare|G42 Healthcare|Ogrodzki|United Arab Emirates
>Spike|hCoV-19/Australia/VIC4736/2020|2020-07-14|EPI_ISL_519124|Original|hCoV-19^^Victoria|Human|Microbiological Diagnostic Unit - Public Health Laboratory (MDU-PHL)|MDU-PHL|Schultz|Australia
>Spike|hCoV-19/Denmark/DCGC-14356/2020|2020-11-30|EPI_ISL_711183|Original|hCoV-19^^Hovedstaden|Human|Department of Virus and Microbiological Special Diagnostics|Albertsen Lab|Michaelsen|Denmark
>Spike|hCoV-19/South Korea/KCDC2759/2020|2020-07-20|EPI_ISL_522538|Original|hCoV-19^^South Korea|Human|Division of Viral Diseases|Division of Viral Diseases|Kim|South Korea
>Spike|hCoV-19/Australia/VIC10622/2020|2020-08-04|EPI_ISL_562997|Original|hCoV-19^^Victoria|Human|Microbiological Diagnostic Unit - Public Health Laboratory (MDU-PHL)|MDU-PHL|Schultz|Australia
>Spike|hCoV-19/England/NORT-28157F/2020|2020-03-17|EPI_ISL_488766|Original|hCoV-19^^England|Human|NU-OMICS DNA Sequencing research facility|Wellcome Sanger Institute for the COVID-19 Genomics UK (COG-UK) consortium|Davies|United Kingdom
>Spike|hCoV-19/Japan/NF-13/2020|2020-04-00|EPI_ISL_591483|Original|hCoV-19^^Japan|Human|Pathogen Genomics Center|Pathogen Genomics Center|Sekizuka|Japan
>Spike|hCoV-19/England/QEUH-9B8E7D/2020|2020-09-07|EPI_ISL_549590|Original|hCoV-19^^England|Human|Lighthouse Lab in Glasgow|Wellcome Sanger Institute for the COVID-19 Genomics UK (COG-UK) consortium|Davies|United Kingdom
>Spike|hCoV-19/England/204781669/2020|2020-11-20|EPI_ISL_683571|Original|hCoV-19^^England|Human|Respiratory Virus Unit|COVID-19 Genomics UK (COG-UK) Consortium|Platt|United Kingdom

>Spike|hCoV-19/USA/NY-Wadsworth-290720-01/2020|2020-12-21|EPI_ISL_767550|Original|hCoV-19^^New York|Human|URMC LABS|Wadsworth Center|Lasek-Nesselquist|USA
>Spike|hCoV-19/Australia/VIC9422/2020|2020-08-19|EPI_ISL_562698|Original|hCoV-19^^Victoria|Human|Microbiological Diagnostic Unit - Public Health Laboratory (MDU-PHL)|MDU-PHL|Schultz|Australia
>Spike|hCoV-19/Wales/PHWC-16E1FD/2020|2020-09-29|EPI_ISL_585683|Original|hCoV-19^^Wales|Human|Wales Specialist Virology Centre Sequencing lab|COVID-19 Genomics UK (COG-UK) Consortium|Moore|United Kingdom
>Spike|hCoV-19/England/CAMB-764EB/2020|2020-03-30|EPI_ISL_438288|Original|hCoV-19^^England|Human|Department of Pathology|Wellcome Sanger Institute for the COVID-19 Genomics UK (COG-UK) consortium|Amato|United Kingdom
>Spike|hCoV-19/USA/TX-HMH-MCoV-10928/2020|2020-07-09|EPI_ISL_789688|Original|hCoV-19^^Texas|Human|Houston Methodist Hospital|Houston Methodist Hospital|Long|USA
>Spike|hCoV-19/England/MILK-BDC318/2020|2020-11-26|EPI_ISL_709608|Original|hCoV-19^^England|Human|Lighthouse Lab in Milton Keynes|Wellcome Sanger Institute for the COVID-19 Genomics UK (COG-UK) Consortium|Davies|United Kingdom
>Spike|hCoV-19/USA/CA-CZB-4082/2020|2020-08-11|EPI_ISL_548591|Original|hCoV-19^^California|Human|Orange County Public Health Laboratory|Chan-Zuckerberg Biohub|Bhatt|USA
>Spike|hCoV-19/USA/MD-HP00031/2020|2020-03-16|EPI_ISL_457789|Original|hCoV-19^^Maryland|Human|Johns Hopkins Hospital Department of Pathology|Johns Hopkins Hospital Department of Pathology|Ramakrishnan|USA
>Spike|hCoV-19/France/HDF-2234/2020|2020-03-10|EPI_ISL_416495|Original|hCoV-19^^Hauts-de-France|Human|Centre Hospitalier Compi\u00e8gne Laboratoire de Biologie|National Reference Center for Viruses of Respiratory Infections|Enouf|France
>Spike|hCoV-19/England/ALDP-9CD859/2020|2020-09-16|EPI_ISL_566331|Original|hCoV-19^^England|Human|Lighthouse Lab in Alderley Park|Wellcome Sanger Institute for the COVID-19 Genomics UK (COG-UK) consortium|Davies|United Kingdom
>Spike|hCoV-19/Denmark/DCGC-13723/2020|2020-11-30|EPI_ISL_712984|Original|hCoV-19^^Midtjylland|Human|Department of Virus and Microbiological Special Diagnostics|Albertsen Lab|Michaelsen|Denmark
>Spike|hCoV-19/Brazil/RN-IEC-162277/2020|2020-03-14|EPI_ISL_524798|Original|hCoV-19^^Rio Grande do Norte|Human|\ufeffEvandro Chagas Institute|\ufeffEvandro Chagas Institute|Sousa J\u00fanior|Brazil
>Spike|hCoV-19/England/CAMC-A81AF0/2020|2020-10-14|EPI_ISL_610902|Original|hCoV-19^^England|Human|Lighthouse Lab in Cambridge|Wellcome Sanger Institute for the COVID-19 Genomics UK (COG-UK) consortium|Davies|United Kingdom
>Spike|hCoV-19/England/CAMC-BC006B/2020|2020-11-22|EPI_ISL_704988|Original|hCoV-19^^England|Human|Lighthouse Lab in Cambridge|Wellcome Sanger Institute for the COVID-19 Genomics UK (COG-UK) Consortium|Davies|United Kingdom
>Spike|hCoV-19/USA/NY-NYUMC909/2020|2020-04-30|EPI_ISL_467402|Original|hCoV-19^^New York|Human|NYU Langone Health|Departments of Pathology and Medicine|Maurano|USA
>Spike|hCoV-19/Denmark/DCGC-7304/2020|2020-10-12|EPI_ISL_618920|Original|hCoV-19^^Hovedstaden|Human|Department of Virus and Microbiological Special Diagnostics|Albertsen lab|Michaelsen|Denmark
>Spike|hCoV-19/England/MILK-9A68A1/2020|2020-09-01|EPI_ISL_550147|Original|hCoV-19^^England|Human|Lighthouse Lab in Milton Keynes|Wellcome Sanger Institute for the COVID-19 Genomics UK (COG-UK) consortium|Davies|United Kingdom
>Spike|hCoV-19/Northern Ireland/NIRE-FC359/2020|2020-04-12|EPI_ISL_459353|Original|hCoV-19^^Northern Ireland|Human|Regional Virus Laboratory|Wellcome Sanger Institute for the COVID-19 Genomics UK (COG-UK) consortium|Davies|United Kingdom
>Spike|hCoV-19/USA/NY-Wadsworth-250986-01/2020|2020-10-21|EPI_ISL_754839|Original|hCoV-19^^New York|Human|Wadsworth Center|Wadsworth Center|Lasek-Nesselquist|USA
>Spike|hCoV-19/England/CAMC-A583BB/2020|2020-10-08|EPI_ISL_596990|Original|hCoV-19^^England|Human|Lighthouse Lab in Cambridge|Wellcome Sanger Institute for the COVID-19 Genomics UK (COG-UK) consortium|Davies|United Kingdom

>Spike|hCoV-19/New Zealand/20VR3902/2020|2020-08-13|EPI_ISL_548094|Original|hCoV-19^^Auckland|Human|Middlemore Hospital|Institute of Environmental Science and Research (ESR)|Wang|New Zealand
>Spike|hCoV-19/England/LOND-D66A7/2020|2020-04-23|EPI_ISL_487558|Original|hCoV-19^^England|Human|University College London|Wellcome Sanger Institute for the COVID-19 Genomics UK (COG-UK) consortium|Davies|United Kingdom
>Spike|hCoV-19/England/204441529/2020|2020-10-18|EPI_ISL_622885|Original|hCoV-19^^England|Human|Respiratory Virus Unit|Respiratory Virus Unit|Platt|United Kingdom
>Spike|hCoV-19/England/NORT-28C84E/2020|2020-05-16|EPI_ISL_469338|Original|hCoV-19^^England|Human|NU-OMICS DNA Sequencing research facility|Wellcome Sanger Institute for the COVID-19 Genomics UK (COG-UK) consortium|Davies|United Kingdom
>Spike|hCoV-19/Bahrein/BAH-08/2020|2020-03-18|EPI_ISL_483549|Original|hCoV-19^^Bahrain|Human|Kingdom of Bahrein Ministry of Health|Erasmus Medical Center|Oude Munnink|Bahrain
>Spike|hCoV-19/USA/CA-CHLA-PLM26469289/2020|2020-12-03|EPI_ISL_753597|Original|hCoV-19^^California|Human|Clinical virology Laboratory|Center for Personalized Medicine|Dien Bard|USA
>Spike|hCoV-19/Scotland/QEUH-9DA84A/2020|2020-09-22|EPI_ISL_575390|Original|hCoV-19^^Scotland|Human|Lighthouse Lab in Glasgow|Wellcome Sanger Institute for the COVID-19 Genomics UK (COG-UK) consortium|Davies|United Kingdom
>Spike|hCoV-19/Scotland/QEUH-98F6A4/2020|2020-08-27|EPI_ISL_540356|Original|hCoV-19^^Scotland|Human|Lighthouse Lab in Glasgow|Wellcome Sanger Institute for the COVID-19 Genomics UK (COG-UK) consortium|Davies|United Kingdom
>Spike|hCoV-19/Wales/PHWC-48F6B8/2020|2020-11-10|EPI_ISL_681125|Original|hCoV-19^^Wales|Human|Wales Specialist Virology Centre Sequencing lab|COVID-19 Genomics UK (COG-UK) Consortium|Moore|United Kingdom
>Spike|hCoV-19/mink/Netherlands/NB-EMC-36-3/2020|2020-08-20|EPI_ISL_577779|Original|hCoV-19^^Noord Brabant|Neovison vison|Dutch COVID-19 response team|Erasmus Medical Center|Oude Munnink|Netherlands
>Spike|hCoV-19/Northern Ireland/NIRE-2332FD/2020|2020-09-24|EPI_ISL_680279|Original|hCoV-19^^Northern Ireland|Human|Regional Virus Laboratory|COVID-19 Genomics UK (COG-UK) Consortium|Fairley|United Kingdom
>Spike|hCoV-19/England/ALDP-C3AC1C/2020|2020-12-09|EPI_ISL_731641|Original|hCoV-19^^England|Human|Lighthouse Lab in Alderley Park|Wellcome Sanger Institute for the COVID-19 Genomics UK (COG-UK) Consortium|Davies|United Kingdom
>Spike|hCoV-19/Australia/TAS12/2020|2020-04-15|EPI_ISL_522585|Original|hCoV-19^^Tasmania|Human|Royal Hobart Hospital Microbiology Department|MDU-PHL|Schultz|Australia
>Spike|hCoV-19/Norway/3195/2020|2020-08-26|EPI_ISL_549028|Original|hCoV-19^^Viken|Human|Furst Medical Laboratory|Norwegian Institute of Public Health|Kopperud Riis|Norway
>Spike|hCoV-19/England/NORT-28D40D/2020|2020-11-16|EPI_ISL_703390|Original|hCoV-19^^England|Human|Northumbria University / South Tees Hospitals NHS Foundation Trust / North Cumbria Integrated Care NHS Foundation Trust / North Tees and Hartlepool NHS Foundation Trust / Newcastle Hospitals NHS Foundation Trust|COVID-19 Genomics UK (COG-UK) Consortium|Bashton|United Kingdom
>Spike|hCoV-19/USA/TX-HMH-MCoV-13031/2020|2020-08-28|EPI_ISL_788182|Original|hCoV-19^^Texas|Human|Houston Methodist Hospital|Houston Methodist Hospital|Long|USA
>Spike|hCoV-19/England/CAMB-7BDFA/2020|2020-04-04|EPI_ISL_442163|Original|hCoV-19^^England|Human|Department of Pathology|Wellcome Sanger Institute for the COVID-19 Genomics UK (COG-UK) consortium|Amato|United Kingdom
>Spike|hCoV-19/Australia/TAS64/2020|2020-03-14|EPI_ISL_522736|Original|hCoV-19^^Tasmania|Human|Royal Hobart Hospital Microbiology Department|MDU-PHL|Schultz|Australia
>Spike|hCoV-19/USA/ME-QDX-689/2020|2020-03-17|EPI_ISL_571437|Original|hCoV-19^^Maine|Human|Quest Diagnostics|Quest Diagnostics|Kagan|USA
>Spike|hCoV-19/USA/HI-H200700/2020|2020-11-29|EPI_ISL_753221|Original|hCoV-19^^Hawaii|Human|State Laboratories Division|State Laboratories Division|Sultana|USA

>Spike|hCoV-19/Wales/PHWC-31701/2020|2020-04-12|EPI_ISL_446477|Original|hCoV-19^^Wales|Human|Wales Specialist Virology Centre|Public Health Wales Microbiology Cardiff|Moore|United Kingdom
>Spike|hCoV-19/England/MILK-992A1B/2020|2020-08-27|EPI_ISL_551942|Original|hCoV-19^^England|Human|Lighthouse Lab in Milton Keynes|Wellcome Sanger Institute for the COVID-19 Genomics UK (COG-UK) consortium|Davies|United Kingdom
>Spike|hCoV-19/England/ALDP-A2F2A1/2020|2020-10-05|EPI_ISL_587064|Original|hCoV-19^^England|Human|Lighthouse Lab in Alderley Park|Wellcome Sanger Institute for the COVID-19 Genomics UK (COG-UK) consortium|Davies|United Kingdom
>Spike|hCoV-19/England/ALDP-A8777B/2020|2020-10-16|EPI_ISL_606257|Original|hCoV-19^^England|Human|Lighthouse Lab in Alderley Park|Wellcome Sanger Institute for the COVID-19 Genomics UK (COG-UK) consortium|Davies|United Kingdom
>Spike|hCoV-19/England/LEED-2A9A90/2020|2020-04-08|EPI_ISL_538806|Original|hCoV-19^^England|Human|Leeds Teaching Hospitals NHS Trust and Public Health England|Wellcome Sanger Institute for the COVID-19 Genomics UK (COG-UK) consortium|Davies|United Kingdom
>Spike|hCoV-19/USA/WI-UW-1126/2020|2020-08-13|EPI_ISL_522884|Original|hCoV-19^^Wisconsin|Human|University of Wisconsin-Madison AIDS Vaccine Research Laboratories|University of Wisconsin-Madison AIDS Vaccine Research Laboratories|Baczenas|USA
>Spike|hCoV-19/England/CAMB-7C1DF/2020|2020-04-15|EPI_ISL_434008|Original|hCoV-19^^England|Human|Department of Pathology|COVID-19 Genomics UK (COG-UK) Consortium|Meredith|United Kingdom
>Spike|hCoV-19/England/ALDP-A684D3/2020|2020-10-12|EPI_ISL_608630|Original|hCoV-19^^England|Human|Lighthouse Lab in Alderley Park|Wellcome Sanger Institute for the COVID-19 Genomics UK (COG-UK) consortium|Davies|United Kingdom
>Spike|hCoV-19/USA/WA-S2119/2020|2020-06-07|EPI_ISL_497547|Original|hCoV-19^^Washington|Human|Washington State Department of Health|Seattle Flu Study|Lee|USA
>Spike|hCoV-19/England/ALDP-9E9963/2020|2020-09-24|EPI_ISL_584269|Original|hCoV-19^^England|Human|Virology Department|COVID-19 Genomics UK (COG-UK) Consortium|de Silva|United Kingdom
>Spike|hCoV-19/Scotland/CVR5416/2020|2020-09-22|EPI_ISL_651810|Original|hCoV-19^^Scotland|Human|Lighthouse Lab in Glasgow / MRC-University of Glasgow Centre for Virus Research|COVID-19 Genomics UK (COG-UK) Consortium|Robertson|United Kingdom
>Spike|hCoV-19/England/QEUH-9FF88B/2020|2020-09-29|EPI_ISL_600317|Original|hCoV-19^^England|Human|Lighthouse Lab in Glasgow|Wellcome Sanger Institute for the COVID-19 Genomics UK (COG-UK) consortium|Davies|United Kingdom
>Spike|hCoV-19/Japan/PG-5159/2020|2020-08-00|EPI_ISL_690266|Original|hCoV-19^^Japan|Human|Pathogen Genomics Center|Pathogen Genomics Center|Sekizuka|Japan
>Spike|hCoV-19/England/EXET-135669/2020|2020-03-28|EPI_ISL_457082|Original|hCoV-19^^England|Human|University of Exeter|COVID-19 Genomics UK (COG-UK) Consortium|Temperton|United Kingdom
>Spike|hCoV-19/Switzerland/VD-ETHZ-180052/2020|2020-06-30|EPI_ISL_489986|Original|hCoV-19^^Vaud|Human|Viollier AG|Department of Biosystems Science and Engineering|Nadeau|Switzerland
>Spike|hCoV-19/Scotland/QEUH-AADAFE/2020|2020-10-22|EPI_ISL_639002|Original|hCoV-19^^Scotland|Human|Liverpool Clinical Laboratories|COVID-19 Genomics UK (COG-UK) Consortium|Haldenby|United Kingdom
>Spike|hCoV-19/Mexico/BCN-ALSR-4927/2020|2020-08-25|EPI_ISL_730216|Original|hCoV-19^^Baja California|Human|Genomica Lab Molecular|Andersen lab at Scripps Research|Gangavarapu|Mexico
>Spike|hCoV-19/Netherlands/LI-EMC-149/2020|2020-11-21|EPI_ISL_763117|Original|hCoV-19^^Limburg|Human|Dutch COVID-19 response team|Erasmus Medical Center|Oude Munnink|Netherlands
>Spike|hCoV-19/Singapore/387/2020|2020-04-17|EPI_ISL_469136|Original|hCoV-19^^Singapore|Human|National Public Health Laboratory|National Public Health Laboratory|Mak|Singapore
>Spike|hCoV-19/Belgium/rega-09221146/2020|2020-09-22|EPI_ISL_738234|Original|hCoV-19^^Zolder|Human|UZ Leuven|KU Leuven|Maes|Belgium

>Spike|hCoV-19/England/NORT-283DEE/2020|2020-04-04|EPI_ISL_472255|Original|hCoV-19^^England|Human|Northumbria University / South Tees Hospitals NHS Foundation Trust / North Cumbria Integrated Care NHS Foundation Trust / North Tees and Hartlepool NHS Foundation Trust / Newcastle Hospitals NHS Foundation Trust|COVID-19 Genomics UK (COG-UK) Consortium|Bashton|United Kingdom
>Spike|hCoV-19/England/ALDP-A703CC/2020|2020-10-15|EPI_ISL_607753|Original|hCoV-19^^England|Human|Lighthouse Lab in Alderley Park|Wellcome Sanger Institute for the COVID-19 Genomics UK (COG-UK) consortium|Davies|United Kingdom
>Spike|hCoV-19/England/QEUH-9F6C4C/2020|2020-09-27|EPI_ISL_595621|Original|hCoV-19^^England|Human|Oxford Viromics|COVID-19 Genomics UK (COG-UK) Consortium|Golubchik|United Kingdom
>Spike|hCoV-19/England/OXON-B2A3C/2020|2020-04-12|EPI_ISL_534787|Original|hCoV-19^^England|Human|Oxford Viromics|COVID-19 Genomics UK (COG-UK) Consortium|Golubchik|United Kingdom
>Spike|hCoV-19/USA/NY-NYCPHL-000029/2020|2020-03-09|EPI_ISL_631525|Original|hCoV-19^^New York|Human|Mount Sinai Hospital|New York City Public Health Laboratory|Wang|USA
>Spike|hCoV-19/DRC/4282/2020|2020-04-08|EPI_ISL_514103|Original|hCoV-19^^Kinshasa|Human|Viral Respiratory Lab|Pathogen Sequencing Lab|Lokilo|Democratic Republic of the Congo
>Spike|hCoV-19/Japan/PG-1267/2020|2020-00-00|EPI_ISL_684877|Original|hCoV-19^^Japan|Human|Pathogen Genomics Center|Pathogen Genomics Center|Sekizuka|Japan
>Spike|hCoV-19/England/NORT-2A70FA/2020|2020-10-28|EPI_ISL_637523|Original|hCoV-19^^England|Human|Northumbria University / South Tees Hospitals NHS Foundation Trust / North Cumbria Integrated Care NHS Foundation Trust / North Tees and Hartlepool NHS Foundation Trust / Newcastle Hospitals NHS Foundation Trust|COVID-19 Genomics UK (COG-UK) Consortium|Bashton|United Kingdom
>Spike|hCoV-19/Sweden/20-52767/2020|2020-08-05|EPI_ISL_654946|Original|hCoV-19^^Kronoberg|Human|Klinisk mikrobiologi|The Public Health Agency of Sweden|Svartstr\u00f6m|Sweden
>Spike|hCoV-19/Australia/VIC1339/2020|2020-04-13|EPI_ISL_430707|Original|hCoV-19^^Victoria|Human|Victorian Infectious Diseases Reference Laboratory (VIDRL)|Microbiological Diagnostic Unit Public Health Laboratory and Victorian Infectious Diseases Reference Laboratory|Schultz|Australia
>Spike|hCoV-19/Australia/NSW1155/2020|2020-10-27|EPI_ISL_629008|Original|hCoV-19^^New South Wales|Human|Laverty Pathology|NSW Health Pathology - Institute of Clinical Pathology and Medical Research; Westmead Hospital; University of Sydney|Gall|Australia
>Spike|hCoV-19/England/ALDP-B740B1/2020|2020-11-12|EPI_ISL_661980|Original|hCoV-19^^England|Human|Lighthouse Lab in Alderley Park|Wellcome Sanger Institute for the COVID-19 Genomics UK (COG-UK) Consortium|Davies|United Kingdom
>Spike|hCoV-19/England/SHEF-10B25DE/2020|2020-04-13|EPI_ISL_469927|Original|hCoV-19^^England|Human|Virology Department|Wellcome Sanger Institute for the COVID-19 Genomics UK (COG-UK) consortium|Davies|United Kingdom
>Spike|hCoV-19/England/CAMC-9C172B/2020|2020-09-10|EPI_ISL_573198|Original|hCoV-19^^England|Human|Oxford Viromics|COVID-19 Genomics UK (COG-UK) Consortium|Golubchik|United Kingdom
>Spike|hCoV-19/England/CAMB-1AB7D4/2020|2020-04-20|EPI_ISL_470242|Original|hCoV-19^^England|Human|Department of Pathology|Wellcome Sanger Institute for the COVID-19 Genomics UK (COG-UK) consortium|Davies|United Kingdom
>Spike|hCoV-19/England/EXET-138E31/2020|2020-10-30|EPI_ISL_664327|Original|hCoV-19^^England|Human|University of Exeter|COVID-19 Genomics UK (COG-UK) Consortium|Temperton|United Kingdom
>Spike|hCoV-19/USA/MI-MDHHS-SC22448/2020|2020-11-08|EPI_ISL_708541|Original|hCoV-19^^Michigan|Human|Michigan Department of Health and Human Services|Michigan Department of Health and Human Services|Jacob|USA
>Spike|hCoV-19/Australia/VIC2241/2020|2020-06-24|EPI_ISL_563983|Original|hCoV-19^^Victoria|Human|Microbiological Diagnostic Unit - Public Health Laboratory (MDU-PHL)|MDU-PHL|Schultz|Australia

>Spike|hCoV-19/USA/CA-CZB-1417/2020|2020-03-19|EPI_ISL_468391|Original|hCoV-19^^California|Human|County of San Luis Obispo Public Health Laboratory|Chan-Zuckerberg Biohub|Hao|USA
>Spike|hCoV-19/Slovakia/UKBA-407/2020|2020-11-30|EPI_ISL_718255|Original|hCoV-19^^Bratislava|Human|Institute of Virology|Faculty of Natural Sciences|Vinar|Slovakia
>Spike|hCoV-19/Colombia/ANT-INS-103573/2020|2020-10-21|EPI_ISL_739676|Original|hCoV-19^^Antioquia|Human|Instituto Nacional de Salud|Instituto Nacional de Salud|Franco-Mu\u00f1oz|Colombia
>Spike|hCoV-19/Wales/PHWC-495F0F/2020|2020-11-22|EPI_ISL_726504|Original|hCoV-19^^Wales|Human|Originating lab|COVID-19 Genomics UK (COG-UK) Consortium|Moore|United Kingdom
>Spike|hCoV-19/England/QEUH-AD046D/2020|2020-10-25|EPI_ISL_634176|Original|hCoV-19^^England|Human|Lighthouse Lab in Glasgow|Wellcome Sanger Institute for the COVID-19 Genomics UK (COG-UK) consortium|Davies|United Kingdom
>Spike|hCoV-19/USA/OR-OHSU-2667/2020|2020-08-24|EPI_ISL_667277|Original|hCoV-19^^Oregon|Human|OHSU Lab Services Molecular Microbiology Lab|Oregon SARS-CoV-2 Genome Sequencing Center|O'Roak|USA
>Spike|hCoV-19/Canada/ON-S1813/2020|2020-11-24|EPI_ISL_729282|Original|hCoV-19^^Ontario|Human|Toronto Invasive Bacterial Diseases Network|McMaster University|McArthur|Canada
>Spike|hCoV-19/Australia/VIC8903/2020|2020-07-22|EPI_ISL_565274|Original|hCoV-19^^Victoria|Human|Microbiological Diagnostic Unit - Public Health Laboratory (MDU-PHL)|MDU-PHL|Schultz|Australia
>Spike|hCoV-19/England/ALDP-5B8DDE/2020|2020-06-15|EPI_ISL_558728|Original|hCoV-19^^England|Human|Lighthouse Lab in Alderley Park|Wellcome Sanger Institute for the COVID-19 Genomics UK (COG-UK) consortium|Davies|United Kingdom
>Spike|hCoV-19/USA/MI-MDHHS-SC20415/2020|2020-03-16|EPI_ISL_452289|Original|hCoV-19^^Michigan|Human|Michigan Department of Health and Human Services|Michigan Department of Health and Human Services|Blankenship|USA
>Spike|hCoV-19/USA/LA-GBCL-26544538/2020|2020-04-18|EPI_ISL_485119|Original|hCoV-19^^Louisiana|Human|River Road Testing Lab|Ginkgo Bioworks Clinical Laboratory|Smith|USA
>Spike|hCoV-19/England/QEUH-B825DF/2020|2020-11-14|EPI_ISL_661632|Original|hCoV-19^^England|Human|Lighthouse Lab in Glasgow|Wellcome Sanger Institute for the COVID-19 Genomics UK (COG-UK) Consortium|Davies|United Kingdom
>Spike|hCoV-19/England/MILK-A7D392/2020|2020-10-13|EPI_ISL_606549|Original|hCoV-19^^England|Human|Lighthouse Lab in Milton Keynes|Wellcome Sanger Institute for the COVID-19 Genomics UK (COG-UK) consortium|Davies|United Kingdom
>Spike|hCoV-19/Spain/VC-FISABIO-247/2020|2020-03-25|EPI_ISL_436342|Original|hCoV-19^^Comunitat Valenciana|Human|Servicio de Microbiologia. Hospital Clinico Universitario de Valencia|Sequencing and Bioinformatics Service and Molecular Epidemiology Research Group. FISABIO-Public Health|Gonzalez-Candelas|Spain
>Spike|hCoV-19/England/ALDP-9E7798/2020|2020-09-24|EPI_ISL_638218|Original|hCoV-19^^England|Human|Department of Pathology|COVID-19 Genomics UK (COG-UK) Consortium|Goodfellow|United Kingdom
>Spike|hCoV-19/Scotland/CVR1402/2020|2020-04-01|EPI_ISL_438798|Original|hCoV-19^^Scotland|Human|West of Scotland Specialist Virology Centre|COVID-19 Genomics UK (COG-UK) Consortium|Robertson|United Kingdom
>Spike|hCoV-19/United Arab Emirates/L920632662/2020|2020-03-30|EPI_ISL_520721|Original|hCoV-19^^United Arab Emirates|Human|Mohammed Bin Rashid University of Medicine and Health Sciences|Al Jalila Genomics Center|Ramaswamy|United Arab Emirates
>Spike|hCoV-19/Brazil/PR-5619/2020|2020-03-16|EPI_ISL_541342|Original|hCoV-19^^Paran\u00e1|Human|LACEN/PR|Laboratory of Respiratory Viruses and Measles|Silva|Brazil
>Spike|hCoV-19/England/TBSD-2488FEB/2020|2020-04-27|EPI_ISL_482058|Original|hCoV-19^^England|Human|The Department of Microbiology|Wellcome Sanger Institute for the COVID-19 Genomics UK (COG-UK) consortium|Davies|United Kingdom
>Spike|hCoV-19/Belgium/rega-0423597/2020|2020-04-23|EPI_ISL_734644|Original|hCoV-19^^Anderlecht|Human|UZ Leuven|KU Leuven|Maes|Belgium

>Spike|hCoV-19/Brazil/SP-L24-CD533/2020|2020-04-14|EPI_ISL_672693|Original|hCoV-19^^S\u00e3o Paulo|Human|Hospital das Cl\u00ednicas da Faculdade de Medicina da Universidade de S\u00e3o Paulo (HC-FMUSP)|Laborat\u00f3rio de Parasitologia M\u00e9dica - Instituto de Medicina Tropical - Universidade de S\u00e3o Paulo|de Jesus|Brazil
>Spike|hCoV-19/England/MILK-9AA245/2020|2020-09-01|EPI_ISL_550805|Original|hCoV-19^^England|Human|Lighthouse Lab in Milton Keynes|Wellcome Sanger Institute for the COVID-19 Genomics UK (COG-UK) consortium|Davies|United Kingdom
>Spike|hCoV-19/Australia/VIC7959/2020|2020-08-07|EPI_ISL_564770|Original|hCoV-19^^Victoria|Human|Microbiological Diagnostic Unit - Public Health Laboratory (MDU-PHL)|MDU-PHL|Schultz|Australia
>Spike|hCoV-19/Saudi Arabia/KAUST-MADINAH1653/2020|2020-08-23|EPI_ISL_751236|Original|hCoV-19^^Madinah|Human|Pathogen Genomics Lab King Abdullah University of Science and Technology(KAUST)|Pathogen Genomics Lab King Abdullah University of Science and Technology(KAUST)|Mohamed Ghazzali|Saudi Arabia
>Spike|hCoV-19/Canada/MB-NML-1015/2020|2020-07-18|EPI_ISL_582381|Original|hCoV-19^^Manitoba|Human|Cadham Provincial Laboratory|National Microbiology Laboratory (NML)|Majer|Canada
>Spike|hCoV-19/Japan/PG-5170/2020|2020-08-00|EPI_ISL_690273|Original|hCoV-19^^Japan|Human|Pathogen Genomics Center|Pathogen Genomics Center|Sekizuka|Japan
>Spike|hCoV-19/Denmark/DCGC-14911/2020|2020-11-23|EPI_ISL_713129|Original|hCoV-19^^Hovedstaden|Human|Department of Virus and Microbiological Special Diagnostics|Albertsen Lab|Michaelsen|Denmark
>Spike|hCoV-19/Australia/NSW49/2020|2020-03-10|EPI_ISL_427659|Original|hCoV-19^^New South Wales|Human|Centre for Infectious Diseases and Microbiology Public Health|NSW Health Pathology - Institute of Clinical Pathology and Medical Research; Westmead Hospital; University of Sydney|Gall|Australia
>Spike|hCoV-19/England/MILK-99E350/2020|2020-08-31|EPI_ISL_550659|Original|hCoV-19^^England|Human|Lighthouse Lab in Milton Keynes|Wellcome Sanger Institute for the COVID-19 Genomics UK (COG-UK) consortium|Davies|United Kingdom
>Spike|hCoV-19/Portugal/PT0997/2020|2020-03-22|EPI_ISL_511545|Original|hCoV-19^^Portugal|Human|Instituto Nacional de Saude (INSA)|Instituto Nacional de Saude (INSA)|Borges|Portugal
>Spike|hCoV-19/England/MILK-ACE013/2020|2020-10-21|EPI_ISL_630089|Original|hCoV-19^^England|Human|Lighthouse Lab in Milton Keynes|Wellcome Sanger Institute for the COVID-19 Genomics UK (COG-UK) consortium|Davies|United Kingdom
>Spike|hCoV-19/Denmark/DCGC-8827/2020|2020-10-26|EPI_ISL_626085|Original|hCoV-19^^Hovedstaden|Human|Department of Virus and Microbiological Special Diagnostics|Albertsen lab|Michaelsen|Denmark
>Spike|hCoV-19/England/MILK-60B70A/2020|2020-07-07|EPI_ISL_581434|Original|hCoV-19^^England|Human|Lighthouse Lab in Milton Keynes|Wellcome Sanger Institute for the COVID-19 Genomics UK (COG-UK) consortium|Davies|United Kingdom
>Spike|hCoV-19/USA/CO-CDPHE-2006280582/2020|2020-06-26|EPI_ISL_677268|Original|hCoV-19^^Colorado|Human|Colorado Department of Public Health and Environment|Colorado Department of Puplic Health and Environment|Bankers|USA
>Spike|hCoV-19/Australia/VIC17772/2020|2020-08-25|EPI_ISL_779438|Original|hCoV-19^^Victoria|Human|Microbiological Diagnostic Unit - Public Health Laboratory (MDU-PHL)|MDU-PHL|Beck|Australia
>Spike|hCoV-19/USA/TX-HMH-MCoV-9726/2020|2020-07-04|EPI_ISL_780108|Original|hCoV-19^^Texas|Human|Houston Methodist Hospital|Houston Methodist Hospital|Long|USA
>Spike|hCoV-19/England/20139057204/2020|2020-03-27|EPI_ISL_420718|Original|hCoV-19^^England|Human|Respiratory Virus Unit|Respiratory Virus Unit|Platt|United Kingdom
>Spike|hCoV-19/Spain/PV-IBV-98007612/2020|2020-09-07|EPI_ISL_582061|Original|hCoV-19^^Basque Country|Human|Servicio de Microbiolog\u00eda. Hospital Universitario Donostia. OSI Donostialdea. \u00c1rea de Enfermedades Infecciosas|SeqCOVID-SPAIN consortium/IBV(CSIC)|Comas|Spain
>Spike|hCoV-19/England/MILK-B3B687/2020|2020-11-01|EPI_ISL_706075|Original|hCoV-19^^England|Human|Oxford Viromics|COVID-19 Genomics UK (COG-UK) Consortium|Golubchik|United Kingdom

>Spike|hCoV-19/England/WSFT-25C10A6/2020|2020-05-07|EPI_ISL_492910|Original|hCoV-19^^England|Human|Department of Medical Microbiology|Wellcome Sanger Institute for the COVID-19 Genomics UK (COG-UK) consortium|Davies|United Kingdom
>Spike|hCoV-19/England/QEUH-B7C5F6/2020|2020-11-14|EPI_ISL_741791|Original|hCoV-19^^England|Human|Oxford Viromics|COVID-19 Genomics UK (COG-UK) Consortium|Golubchik|United Kingdom
>Spike|hCoV-19/USA/VA-DCLS-2231/2020|2020-11-03|EPI_ISL_734212|Original|hCoV-19^^Virginia|Human|Virginia Division of Consolidated Laboratory Services (DCLS)|Virginia Division of Consolidated Laboratory Services (DCLS)|St. Jacques|USA
>Spike|hCoV-19/Colombia/VAC-GVI-93477/2020|2020-04-02|EPI_ISL_447783|Original|hCoV-19^^Valle del Cauca|Human|Instituto Nacional de Salud|Grupo de Investigaciones Microbiol\u00f3gicas-UR (GIMUR)|Ram\u00edrez|Colombia
>Spike|hCoV-19/Sweden/20-52801/2020|2020-06-29|EPI_ISL_623095|Original|hCoV-19^^Gavleborg|Human|Gavle klinisk mikrobiologi|The Public Health Agency of Sweden|Svartstr\u00f6m|Sweden
>Spike|hCoV-19/Norway/3618/2020|2020-09-24|EPI_ISL_635099|Original|hCoV-19^^Viken|Human|Medical Microbiology Unit|Norwegian Institute of Public Health|Vollan|Norway
>Spike|hCoV-19/India/HR-TF20/2020|2020-06-17|EPI_ISL_508497|Original|hCoV-19^^Haryana|Human|Translational Health Science and Technology Institute|National Institute of Biomedical Genomics|Maitra|India
>Spike|hCoV-19/England/201390489/2020|2020-03-27|EPI_ISL_465847|Original|hCoV-19^^England|Human|Respiratory Virus Unit|Respiratory Virus Unit|Platt|United Kingdom
>Spike|hCoV-19/Northern Ireland/NIRE-108EC1/2020|2020-09-18|EPI_ISL_612547|Original|hCoV-19^^Northern Ireland|Human|Regional Virus Laboratory|COVID-19 Genomics UK (COG-UK) Consortium|Fairley|United Kingdom
>Spike|hCoV-19/Denmark/DCGC-3301/2020|2020-08-31|EPI_ISL_616053|Original|hCoV-19^^Hovedstaden|Human|Department of Virus and Microbiological Special Diagnostics|Albertsen lab|Michaelsen|Denmark
>Spike|hCoV-19/England/MILK-C500F6/2020|2020-12-11|EPI_ISL_769574|Original|hCoV-19^^England|Human|Lighthouse Lab in Milton Keynes|Wellcome Sanger Institute for the COVID-19 Genomics UK (COG-UK) Consortium|Davies|United Kingdom
>Spike|hCoV-19/Australia/WA95/2020|2020-04-09|EPI_ISL_512748|Original|hCoV-19^^Western Australia|Human|PathWest Laboratory Medicine WA|PathWest Laboratory Medicine WA Microbial Surveillance Unit|Sikazwe|Australia
>Spike|hCoV-19/Sweden/20-52817/2020|2020-06-28|EPI_ISL_615103|Original|hCoV-19^^Gavleborg|Human|Gavle klinisk mikrobiologi|The Public Health Agency of Sweden|Svartstr\u00f6m|Sweden
>Spike|hCoV-19/Bangladesh/DNAS-isl-6300/2020|2020-05-18|EPI_ISL_605918|Original|hCoV-19^^Bangladesh|Human|NGS Lab|NGS Lab|Rabbi|Bangladesh
>Spike|hCoV-19/Japan/PG-3069/2020|2020-04-00|EPI_ISL_691192|Original|hCoV-19^^Japan|Human|Pathogen Genomics Center|Pathogen Genomics Center|Sekizuka|Japan
>Spike|hCoV-19/England/MILK-992D7C/2020|2020-08-26|EPI_ISL_551957|Original|hCoV-19^^England|Human|Lighthouse Lab in Milton Keynes|Wellcome Sanger Institute for the COVID-19 Genomics UK (COG-UK) consortium|Davies|United Kingdom
>Spike|hCoV-19/Wales/PHWC-3083C/2020|2020-04-08|EPI_ISL_446287|Original|hCoV-19^^Wales|Human|Wales Specialist Virology Centre|Public Health Wales Microbiology Cardiff|Moore|United Kingdom
>Spike|hCoV-19/South Africa/R12716-20/2020|2020-06-16|EPI_ISL_504241|Original|hCoV-19^^North West|Human|National Institute for Communicable Diseases of the National Health Laboratory Service|National Institute for Communicable Diseases of the National Health Laboratory Service|Allam|South Africa
>Spike|hCoV-19/England/ALDP-A6FD95/2020|2020-10-14|EPI_ISL_607149|Original|hCoV-19^^England|Human|Lighthouse Lab in Alderley Park|Wellcome Sanger Institute for the COVID-19 Genomics UK (COG-UK) consortium|Davies|United Kingdom
>Spike|hCoV-19/USA/MN-QDX-998/2020|2020-03-14|EPI_ISL_571795|Original|hCoV-19^^Minnesota|Human|Quest Diagnostics|Quest Diagnostics|Kagan|USA
>Spike|hCoV-19/India/MH-NIV-207312/2020|2020-09-11|EPI_ISL_577668|Original|hCoV-19^^Maharashtra|Human|NIV Influenza|NIV Influenza|Potdar|India

>Spike|hCoV-19/England/LIVE-DB89B4/2020|2020-10-26|EPI_ISL_651400|Original|hCoV-19^^England|Human|Liverpool Clinical Laboratories|COVID-19 Genomics UK (COG-UK) Consortium|Haldenby|United Kingdom
>Spike|hCoV-19/Ukraine/203100362/2020|2020-05-15|EPI_ISL_512810|Original|hCoV-19^^Ukraine|Human|National Laboratory for Influenza/Virology reference laboratory|Respiratory Virus Unit|Platt|Ukraine
>Spike|hCoV-19/England/SHEF-D068F/2020|2020-04-17|EPI_ISL_448905|Original|hCoV-19^^England|Human|Virology Department|COVID-19 Genomics UK (COG-UK) Consortium|de Silva|United Kingdom
>Spike|hCoV-19/USA/CA-CDPH119/2020|2020-09-20|EPI_ISL_754928|Original|hCoV-19^^California|Human|California Department of Public Health|California Department of Public Health|Bell|USA
>Spike|hCoV-19/England/MILK-BAB707/2020|2020-11-18|EPI_ISL_783352|Original|hCoV-19^^England|Human|Lighthouse Lab in Milton Keynes|Wellcome Sanger Institute for the COVID-19 Genomics UK (COG-UK) Consortium|Davies|United Kingdom
>Spike|hCoV-19/Japan/PG-5823/2020|2020-08-00|EPI_ISL_688940|Original|hCoV-19^^Japan|Human|Fukuoka Institute of Health and Environmental Sciences|Pathogen Genomics Center|Sekizuka|Japan
>Spike|hCoV-19/USA/TX-HMH-MCoV-8881/2020|2020-07-02|EPI_ISL_780808|Original|hCoV-19^^Texas|Human|Houston Methodist Hospital|Houston Methodist Hospital|Long|USA
>Spike|hCoV-19/USA/CA-ALSR-4340/2020|2020-10-12|EPI_ISL_648980|Original|hCoV-19^^California|Human|San Diego County Public Health Laboratory|Andersen lab at Scripps Research|Gangavarapu|USA
>Spike|hCoV-19/Denmark/DCGC-6393/2020|2020-09-28|EPI_ISL_622323|Original|hCoV-19^^Hovedstaden|Human|Department of Virus and Microbiological Special Diagnostics|Albertsen lab|Michaelsen|Denmark
>Spike|hCoV-19/USA/OR-OHSU-1603/2020|2020-07-21|EPI_ISL_525987|Original|hCoV-19^^Oregon|Human|OHSU Lab Services Molecular Microbiology Lab|Oregon SARS-CoV-2 Genome Sequencing Center|O'Roak|USA
>Spike|hCoV-19/Switzerland/BS-42204899/2020|2020-03-24|EPI_ISL_581783|Original|hCoV-19^^Basel-Stadt|Human|University Hospital Basel|University Hospital Basel|Roloff Handschin|Switzerland
>Spike|hCoV-19/Scotland/EDB6263/2020|2020-04-08|EPI_ISL_487714|Original|hCoV-19^^Scotland|Human|Virology Department|Wellcome Sanger Institute for the COVID-19 Genomics UK (COG-UK) consortium|Davies|United Kingdom
>Spike|hCoV-19/Wales/PHWC-494E12/2020|2020-11-21|EPI_ISL_726258|Original|hCoV-19^^Wales|Human|Originating lab|COVID-19 Genomics UK (COG-UK) Consortium|Moore|United Kingdom
>Spike|hCoV-19/England/CAMC-B228AB/2020|2020-11-01|EPI_ISL_647254|Original|hCoV-19^^England|Human|Lighthouse Lab in Cambridge|Wellcome Sanger Institute for the COVID-19 Genomics UK (COG-UK) Consortium|Davies|United Kingdom
>Spike|hCoV-19/England/ALDP-A8792A/2020|2020-10-17|EPI_ISL_606148|Original|hCoV-19^^England|Human|Lighthouse Lab in Alderley Park|Wellcome Sanger Institute for the COVID-19 Genomics UK (COG-UK) consortium|Davies|United Kingdom
>Spike|hCoV-19/England/MILK-B568CC/2020|2020-11-05|EPI_ISL_656913|Original|hCoV-19^^England|Human|Lighthouse Lab in Milton Keynes|Wellcome Sanger Institute for the COVID-19 Genomics UK (COG-UK) Consortium|Davies|United Kingdom
>Spike|hCoV-19/England/QEUH-9411CB/2020|2020-07-29|EPI_ISL_532638|Original|hCoV-19^^England|Human|Lighthouse Lab in Glasgow|Wellcome Sanger Institute for the COVID-19 Genomics UK (COG-UK) consortium|Davies|United Kingdom
>Spike|hCoV-19/England/NORT-29E3B1/2020|2020-05-05|EPI_ISL_493514|Original|hCoV-19^^England|Human|Northumbria University / South Tees Hospitals NHS Foundation Trust / North Cumbria Integrated Care NHS Foundation Trust / North Tees and Hartlepool NHS Foundation Trust / Newcastle Hospitals NHS Foundation Trust|COVID-19 Genomics UK (COG-UK) Consortium|Bashton|United Kingdom
>Spike|hCoV-19/United Arab Emirates/0542/2020|2020-05-17|EPI_ISL_698703|Original|hCoV-19^^Abu Dhabi|Human|Group 42 (G42) Healthcare|G42 Healthcare|Ogrodzki|United Arab Emirates

>Spike|hCoV-19/USA/FL-BPHL-1402/2020|2020-07-30|EPI_ISL_549184|Original|hCoV-19^^Florida|Human|Florida Bureau of Public Health Laboratories|Florida Bureau of Public Health Laboratories|Blanton|USA
>Spike|hCoV-19/USA/WA-UW-11823/2020|2020-06-25|EPI_ISL_570474|Original|hCoV-19^^Washington|Human|UW Virology Lab|UW Virology Lab|Roychoudhury|USA
>Spike|hCoV-19/Denmark/DCGC-6984/2020|2020-10-12|EPI_ISL_619830|Original|hCoV-19^^Syddanmark|Human|Department of Virus and Microbiological Special Diagnostics|Albertsen lab|Michaelsen|Denmark
>Spike|hCoV-19/England/QEUH-9FF724/2020|2020-10-01|EPI_ISL_600355|Original|hCoV-19^^England|Human|Lighthouse Lab in Glasgow|Wellcome Sanger Institute for the COVID-19 Genomics UK (COG-UK) consortium|Davies|United Kingdom
>Spike|hCoV-19/England/BRIS-128317/2020|2020-04-16|EPI_ISL_469791|Original|hCoV-19^^England|Human|PHE South West Regional Laboratory|Wellcome Sanger Institute for the COVID-19 Genomics UK (COG-UK) consortium|Davies|United Kingdom
>Spike|hCoV-19/Scotland/EDB8377/2020|2020-10-14|EPI_ISL_612348|Original|hCoV-19^^Scotland|Human|Virology Department|COVID-19 Genomics UK (COG-UK) Consortium|McHugh|United Kingdom
>Spike|hCoV-19/Ireland/D-NVRL-AIIDV0141v3/2020|2020-04-21|EPI_ISL_644304|Original|hCoV-19^^Dublin|Human|CEPHR / Vincent's Hospital|Irish Coronavirus Sequencing Consortium - National Virus Reference Laboratory|Gonzalez|Ireland
>Spike|hCoV-19/USA/SC-DHEC-0921/2020|2020-06-26|EPI_ISL_538416|Original|hCoV-19^^South Carolina|Human|Microbiology Division|Microbiology Division|Flores|USA
>Spike|hCoV-19/England/MILK-AB7790/2020|2020-10-21|EPI_ISL_623429|Original|hCoV-19^^England|Human|Lighthouse Lab in Milton Keynes|Wellcome Sanger Institute for the COVID-19 Genomics UK (COG-UK) consortium|Davies|United Kingdom
>Spike|hCoV-19/USA/AZ-TG276130/2020|2020-04-08|EPI_ISL_694086|Original|hCoV-19^^Arizona|Human|AZ SPHL|TGen North|French|USA
>Spike|hCoV-19/Kenya/C3381/2020|2020-04-30|EPI_ISL_568714|Original|hCoV-19^^Mombasa|Human|KEMRI-Wellcome Trust Research Programme/KEMRI-CGMR-C Kilifi|KEMRI-Wellcome Trust Research Programme/KEMRI-CGMR-C Kilifi|Githinji|Kenya
>Spike|hCoV-19/England/QEUH-AAC28E/2020|2020-10-21|EPI_ISL_652851|Original|hCoV-19^^England|Human|Oxford Viromics|COVID-19 Genomics UK (COG-UK) Consortium|Golubchik|United Kingdom
>Spike|hCoV-19/England/LIVE-A9D8A/2020|2020-03-23|EPI_ISL_517359|Original|hCoV-19^^England|Human|Liverpool Clinical Laboratories|COVID-19 Genomics UK (COG-UK) Consortium|Haldenby|United Kingdom
>Spike|hCoV-19/Wales/ALDP-B1DD68/2020|2020-11-01|EPI_ISL_723543|Original|hCoV-19^^Wales|Human|Centre for Enzyme Innovation|COVID-19 Genomics UK (COG-UK) Consortium|Robson|United Kingdom
>Spike|hCoV-19/Scotland/QEUH-A0B4C3/2020|2020-10-03|EPI_ISL_599206|Original|hCoV-19^^Scotland|Human|Lighthouse Lab in Glasgow|Wellcome Sanger Institute for the COVID-19 Genomics UK (COG-UK) consortium|Davies|United Kingdom
>Spike|hCoV-19/England/QEUH-AD4E5C/2020|2020-10-25|EPI_ISL_633937|Original|hCoV-19^^England|Human|Lighthouse Lab in Glasgow|Wellcome Sanger Institute for the COVID-19 Genomics UK (COG-UK) consortium|Davies|United Kingdom
>Spike|hCoV-19/Scotland/EDB2382/2020|2020-04-22|EPI_ISL_439196|Original|hCoV-19^^Scotland|Human|Virology Department|COVID-19 Genomics UK (COG-UK) Consortium|Williams|United Kingdom
>Spike|hCoV-19/Belgium/GJ-030458/2020|2020-03-04|EPI_ISL_420319|Original|hCoV-19^^Montigny-le-Tilleul|Human|KU Leuven|KU Leuven|Maes|Belgium
>Spike|hCoV-19/Spain/VC-FISABIO-220/2020|2020-03-27|EPI_ISL_436315|Original|hCoV-19^^Comunitat Valenciana|Human|Servicio de Microbiologia. Hospital Clinico Universitario de Valencia|Sequencing and Bioinformatics Service and Molecular Epidemiology Research Group. FISABIO-Public Health|Gonzalez-Candelas|Spain
>Spike|hCoV-19/USA/FL-BPHL-0389/2020|2020-04-29|EPI_ISL_489798|Original|hCoV-19^^Florida|Human|Florida Bureau of Public Health Laboratories|Florida Bureau of Public Health Laboratories|Blanton|USA

>Spike|hCoV-19/Australia/VIC3521/2020|2020-07-08|EPI_ISL_520273|Original|hCoV-19^^Victoria|Human|Victorian Infectious Diseases Reference Laboratory (VIDRL)|VIDRL and MDU-PHL|Schultz|Australia
>Spike|hCoV-19/England/GSTT-265D439/2020|2020-03-22|EPI_ISL_483959|Original|hCoV-19^^England|Human|Centre for Clinical Infection and Diagnostics Research and Genomics Innovation Unit|COVID-19 Genomics UK (COG-UK) Consortium|Awan|United Kingdom
>Spike|hCoV-19/England/CAMC-CB7664/2020|2020-12-19|EPI_ISL_777703|Original|hCoV-19^^England|Human|Lighthouse Lab in Cambridge|Wellcome Sanger Institute for the COVID-19 Genomics UK (COG-UK) Consortium|Davies|United Kingdom
>Spike|hCoV-19/England/ALDP-B5C1DA/2020|2020-11-10|EPI_ISL_655910|Original|hCoV-19^^England|Human|Lighthouse Lab in Alderley Park|Wellcome Sanger Institute for the COVID-19 Genomics UK (COG-UK) Consortium|Davies|United Kingdom
>Spike|hCoV-19/Gibraltar/204841722/2020|2020-00-00|EPI_ISL_671291|Original|hCoV-19^^Gibraltar|Human|Microbiology|Respiratory Virus Unit|Platt|Gibraltar
>Spike|hCoV-19/Scotland/EDB11095/2020|2020-12-11|EPI_ISL_764147|Original|hCoV-19^^Scotland|Human|Virology Department|COVID-19 Genomics UK (COG-UK) Consortium|McHugh|United Kingdom
>Spike|hCoV-19/USA/HI-H200049/2020|2020-04-20|EPI_ISL_752634|Original|hCoV-19^^Hawaii|Human|State Laboratories Division|State Laboratories Division|Sultana|USA
>Spike|hCoV-19/USA/TX-HMH-MCoV-10711/2020|2020-07-08|EPI_ISL_789597|Original|hCoV-19^^Texas|Human|Houston Methodist Hospital|Houston Methodist Hospital|Long|USA
>Spike|hCoV-19/Wales/PHWC-254FA/2020|2020-03-24|EPI_ISL_420913|Original|hCoV-19^^Wales|Human|Wales Specialist Virology Centre|Public Health Wales Microbiology Cardiff|Moore|United Kingdom
>Spike|hCoV-19/USA/MD-HP12134/2020|2020-12-27|EPI_ISL_791444|Original|hCoV-19^^Maryland|Human|Johns Hopkins Hospital Department of Pathology|Johns Hopkins Hospital Department of Pathology|Morris|USA
>Spike|hCoV-19/South Africa/KRISP-K002833/2020|2020-09-01|EPI_ISL_602652|Original|hCoV-19^^KwaZulu-Natal|Human|NHLS-IALCH|KRISP|de Oliveira|South Africa
>Spike|hCoV-19/USA/NY-Wadsworth-142754-01/2020|2020-04-17|EPI_ISL_676967|Original|hCoV-19^^New York|Human|Wadsworth Center|Wadsworth Center|Lasek-Nesselquist|USA
>Spike|hCoV-19/England/ALDP-B5AF87/2020|2020-11-08|EPI_ISL_655542|Original|hCoV-19^^England|Human|Lighthouse Lab in Alderley Park|Wellcome Sanger Institute for the COVID-19 Genomics UK (COG-UK) Consortium|Davies|United Kingdom
[truncated: 574,035 more chars]
