# Supplementary material for: Epitope Classification and RBD Binding Properties of Neutralizing Antibodies Against SARS-CoV-2 Variants of Concern
Source: Front Immunol. 2021 Jun 4;12:691715. doi: 10.3389/fimmu.2021.691715 (PMC8212047; doi:10.3389/fimmu.2021.691715)
Supplement: Supplementary file 4 [file DataSheet_4.pdf]

Supplementary Table 1. GISAID sequence headers (150,000-225,000)for each SARS-CoV-2 S amino acid sequence used in the frequency analysis.
>Spike|hCoV-19/Canada/ON-OICR-SLB2796/2020|2020-03-28|EPI_ISL_538328|Original|hCoV-19^^Ontario|Human|Kingston Health Sciences Centre / Queen's University|Ontario Institute for Cancer Research|de Borja|Canada
>Spike|hCoV-19/Germany/HH-hpi-p1336/2020|2020-03-24|EPI_ISL_775667|Original|hCoV-19^^Hamburg|Human|University Medical Center Hamburg Eppendorf|Heinrich Pette Institute|Robitaille|Germany
>Spike|hCoV-19/Scotland/CVR3092/2020|2020-04-24|EPI_ISL_461668|Original|hCoV-19^^Scotland|Human|West of Scotland Specialist Virology Centre|COVID-19 Genomics UK (COG-UK) Consortium|Robertson|United Kingdom
>Spike|hCoV-19/Australia/NSW2206/2020|2020-03-21|EPI_ISL_500664|Original|hCoV-19^^New South Wales|Human|Area of Virology|Area of Virology|Bull|Australia
>Spike|hCoV-19/Wales/PHWC-47FCAA/2020|2020-10-08|EPI_ISL_612796|Original|hCoV-19^^Wales|Human|Wales Specialist Virology Centre Sequencing lab|COVID-19 Genomics UK (COG-UK) Consortium|Moore|United Kingdom
>Spike|hCoV-19/Scotland/CVR1792/2020|2020-04-06|EPI_ISL_438848|Original|hCoV-19^^Scotland|Human|West of Scotland Specialist Virology Centre|COVID-19 Genomics UK (COG-UK) Consortium|Robertson|United Kingdom
>Spike|hCoV-19/USA/MI-MDHHS-SC20493/2020|2020-05-05|EPI_ISL_460031|Original|hCoV-19^^Michigan|Human|Michigan Department of Health and Human Services|Michigan Department of Health and Human Services|Blankenship|USA
>Spike|hCoV-19/England/ALDP-9BDC50/2020|2020-09-09|EPI_ISL_568179|Original|hCoV-19^^England|Human|Lighthouse Lab in Alderley Park|Wellcome Sanger Institute for the COVID-19 Genomics UK (COG-UK) consortium|Davies|United Kingdom
>Spike|hCoV-19/Belgium/ULG-10330/2020|2020-07-30|EPI_ISL_540497|Original|hCoV-19^^Li\u00e8ge|Human|Department of Clinical Microbiology|GIGA Medical Genomics|Durkin|Belgium
>Spike|hCoV-19/England/ALDP-5D210F/2020|2020-06-19|EPI_ISL_558544|Original|hCoV-19^^England|Human|Lighthouse Lab in Alderley Park|Wellcome Sanger Institute for the COVID-19 Genomics UK (COG-UK) consortium|Davies|United Kingdom
>Spike|hCoV-19/mink/Netherlands/NB-EMC-8-9/2020|2020-06-01|EPI_ISL_523118|Original|hCoV-19^^Limburg|Neovison vison|Dutch COVID-19 response team|Erasmus Medical Center|Oude Munnink|Netherlands
>Spike|hCoV-19/England/ALDP-A6FA16/2020|2020-10-14|EPI_ISL_607799|Original|hCoV-19^^England|Human|Lighthouse Lab in Alderley Park|Wellcome Sanger Institute for the COVID-19 Genomics UK (COG-UK) consortium|Davies|United Kingdom
>Spike|hCoV-19/England/QEUH-9C82F6/2020|2020-09-11|EPI_ISL_567149|Original|hCoV-19^^England|Human|Lighthouse Lab in Glasgow|Wellcome Sanger Institute for the COVID-19 Genomics UK (COG-UK) consortium|Davies|United Kingdom
>Spike|hCoV-19/USA/TX-HMH-3425/2020|2020-06-11|EPI_ISL_544183|Original|hCoV-19^^Texas|Human|Houston Methodist Hospital|Houston Methodist Hospital|Long|USA
>Spike|hCoV-19/Singapore/837/2020|2020-07-29|EPI_ISL_518001|Original|hCoV-19^^Singapore|Human|Singapore General Hospital|Department of Microbiology|Abdul Rahman|Singapore
>Spike|hCoV-19/England/MILK-9EF921/2020|2020-09-23|EPI_ISL_594958|Original|hCoV-19^^England|Human|Queens Medical Centre|COVID-19 Genomics UK (COG-UK) Consortium|Loose|United Kingdom
>Spike|hCoV-19/England/CAMB-1AF961/2020|2020-05-01|EPI_ISL_459277|Original|hCoV-19^^England|Human|Department of Pathology|Wellcome Sanger Institute for the COVID-19 Genomics UK (COG-UK) consortium|Davies|United Kingdom
>Spike|hCoV-19/England/ALDP-9554CB/2020|2020-06-12|EPI_ISL_553436|Original|hCoV-19^^England|Human|Lighthouse Lab in Alderley Park|Wellcome Sanger Institute for the COVID-19 Genomics UK (COG-UK) consortium|Davies|United Kingdom
>Spike|hCoV-19/England/QEUH-C93130/2020|2020-12-17|EPI_ISL_762161|Original|hCoV-19^^England|Human|Lighthouse Lab in Glasgow|Wellcome Sanger Institute for the COVID-19 Genomics UK (COG-UK) Consortium|Davies|United Kingdom
>Spike|hCoV-19/USA/MI-MDHHS-SC22050/2020|2020-09-21|EPI_ISL_583507|Original|hCoV-19^^Michigan|Human|Michigan Department of Health and Human Services|Michigan Department of Health and Human Services|Blankenship|USA

>Spike|hCoV-19/England/204742198/2020|2020-00-00|EPI_ISL_649909|Original|hCoV-19^^England|Human|Respiratory Virus Unit|COVID-19 Genomics UK (COG-UK) Consortium|Platt|United Kingdom
>Spike|hCoV-19/England/ALDP-8BA0A3/2020|2020-06-28|EPI_ISL_557122|Original|hCoV-19^^England|Human|Lighthouse Lab in Alderley Park|Wellcome Sanger Institute for the COVID-19 Genomics UK (COG-UK) consortium|Davies|United Kingdom
>Spike|hCoV-19/England/ALDP-A6E525/2020|2020-10-15|EPI_ISL_607896|Original|hCoV-19^^England|Human|Lighthouse Lab in Alderley Park|Wellcome Sanger Institute for the COVID-19 Genomics UK (COG-UK) consortium|Davies|United Kingdom
>Spike|hCoV-19/India/MH-GA14/2020|2020-06-17|EPI_ISL_508210|Original|hCoV-19^^Maharashtra|Human|Government Medical College|National Institute of Biomedical Genomics|Maitra|India
>Spike|hCoV-19/USA/MI-MDHHS-SC22525/2020|2020-11-17|EPI_ISL_708228|Original|hCoV-19^^Michigan|Human|Michigan Department of Health and Human Services|Michigan Department of Health and Human Services|Jacob|USA
>Spike|hCoV-19/Russia/StPetersburg-RII7501S/2020|2020-04-20|EPI_ISL_450254|Original|hCoV-19^^Saint Petersburg|Human|WHO National Influenza Centre Russian Federation|WHO National Influenza Centre Russian Federation|Komissarov|Russia
>Spike|hCoV-19/Ireland/G-NVRL-MPW79025/2020|2020-05-20|EPI_ISL_767794|Original|hCoV-19^^Galway|Human|National Virus Reference Laboratory|Irish Coronavirus Sequencing Consortium - Teagasc Moorepark|Walsh|Ireland
>Spike|hCoV-19/Wales/PHWC-2F4D2/2020|2020-04-17|EPI_ISL_446258|Original|hCoV-19^^Wales|Human|Wales Specialist Virology Centre|Public Health Wales Microbiology Cardiff|Moore|United Kingdom
>Spike|hCoV-19/Scotland/QEUH-95E459/2020|2020-08-10|EPI_ISL_534410|Original|hCoV-19^^Scotland|Human|NHSGGC West of Scotland Specialist Virology Centre / MRC-University of Glasgow Centre for Virus Research|Wellcome Sanger Institute for the COVID-19 Genomics UK (COG-UK) consortium|Davies|United Kingdom
>Spike|hCoV-19/Northern Ireland/NIRE-10541D/2020|2020-03-29|EPI_ISL_461786|Original|hCoV-19^^Northern Ireland|Human|Regional Virus Laboratory|COVID-19 Genomics UK (COG-UK) Consortium|Fairley|United Kingdom
>Spike|hCoV-19/England/SHEF-CA1F0/2020|2020-03-30|EPI_ISL_432487|Original|hCoV-19^^England|Human|Virology Department|COVID-19 Genomics UK (COG-UK) Consortium|de Silva|United Kingdom
>Spike|hCoV-19/England/NOTT-121952/2020|2020-12-23|EPI_ISL_764524|Original|hCoV-19^^England|Human|Queens Medical Centre|COVID-19 Genomics UK (COG-UK) Consortium|Loose|United Kingdom
>Spike|hCoV-19/Denmark/DCGC-19974/2020|2020-12-07|EPI_ISL_747901|Original|hCoV-19^^Sjaelland|Human|Department of Virus and Microbiological Special Diagnostics|Albertsen Lab|Michaelsen|Denmark
>Spike|hCoV-19/England/ALDP-AA4D1C/2020|2020-10-21|EPI_ISL_630901|Original|hCoV-19^^England|Human|Lighthouse Lab in Alderley Park|Wellcome Sanger Institute for the COVID-19 Genomics UK (COG-UK) consortium|Davies|United Kingdom
>Spike|hCoV-19/USA/WI-UW-1654/2020|2020-09-08|EPI_ISL_605528|Original|hCoV-19^^Wisconsin|Human|University of Wisconsin-Madison AIDS Vaccine Research Laboratories|University of Wisconsin-Madison AIDS Vaccine Research Laboratories|Baczenas|USA
>Spike|hCoV-19/England/ALDP-B1BB9D/2020|2020-11-01|EPI_ISL_645568|Original|hCoV-19^^England|Human|Lighthouse Lab in Alderley Park|Wellcome Sanger Institute for the COVID-19 Genomics UK (COG-UK) Consortium|Davies|United Kingdom
>Spike|hCoV-19/Scotland/EDB894/2020|2020-04-05|EPI_ISL_432868|Original|hCoV-19^^Scotland|Human|Virology Department|COVID-19 Genomics UK (COG-UK) Consortium|Williams|United Kingdom
>Spike|hCoV-19/England/MILK-B963A1/2020|2020-11-13|EPI_ISL_676156|Original|hCoV-19^^England|Human|Lighthouse Lab in Milton Keynes|Wellcome Sanger Institute for the COVID-19 Genomics UK (COG-UK) Consortium|Davies|United Kingdom
>Spike|hCoV-19/England/OXON-AE31A/2020|2020-00-00|EPI_ISL_448682|Original|hCoV-19^^England|Human|Oxford Viromics|COVID-19 Genomics UK (COG-UK) Consortium|Golubchik|United Kingdom

>Spike|hCoV-19/Australia/WA143/2020|2020-03-22|EPI_ISL_596706|Original|hCoV-
19^^Western Australia|Human|PathWest Laboratory Medicine WA|PathWest Laboratory
Medicine WA Microbial Surveillance Unit|Sikazwe|Australia
>Spike|hCoV-19/USA/WA-OHSU-1799/2020|2020-07-25|EPI_ISL_526108|Original|hCoV-
19^^Washington|Human|OHSU Lab Services Molecular Microbiology Lab|Oregon SARS-CoV-2
Genome Sequencing Center|O'Roak|USA
>Spike|hCoV-19/Denmark/DCGC-852/2020|2020-05-11|EPI_ISL_614785|Original|hCoV-
19^^Nordjylland|Human|Department of Virus and Microbiological Special
Diagnostics|Albertsen lab|Michaelsen|Denmark
>Spike|hCoV-19/Australia/VIC2018/2020|2020-05-15|EPI_ISL_480660|Original|hCoV-
19^^Victoria|Human|Victorian Infectious Diseases Reference Laboratory (VIDRL)|VIDRL
and MDU-PHL|Schultz|Australia
>Spike|hCoV-19/Wales/PHWC-480B6D/2020|2020-10-11|EPI_ISL_612977|Original|hCoV-
19^^Wales|Human|Wales Specialist Virology Centre Sequencing lab|COVID-19 Genomics UK
(COG-UK) Consortium|Moore|United Kingdom
>Spike|hCoV-19/Iran/SA-Isf-11/2020|2020-05-10|EPI_ISL_582003|Original|hCoV-
19^^Iran|Human|Infectious Diseases and Tropical Medicine Research Center|Infectious
Diseases and Tropical Medicine Research Center|Haghjoo javanmard,S.|Iran
>Spike|hCoV-19/Canada/BC-BCCDC-6398/2020|2020-10-00|EPI_ISL_740554|Original|hCoV-
19^^British Columbia|Human|BCCDC Public Health Laboratory|BCCDC Public Health
Laboratory|Prystajecky|Canada
>Spike|hCoV-19/England/QEUH-B7FDEC/2020|2020-11-14|EPI_ISL_668363|Original|hCoV-
19^^England|Human|Lighthouse Lab in Glasgow|Wellcome Sanger Institute for the COVID-
19 Genomics UK (COG-UK) Consortium|Davies|United Kingdom
>Spike|hCoV-19/USA/TX-HMH-MCoV-18237/2020|2020-09-08|EPI_ISL_784585|Original|hCoV-
19^^Texas|Human|Houston Methodist Hospital|Houston Methodist Hospital|Long|USA
>Spike|hCoV-19/USA/MA-MGH-00741/2020|2020-04-09|EPI_ISL_791739|Original|hCoV-
19^^Massachusetts|Human|Massachusetts General Hospital|Infectious Disease
Program|Park|USA
>Spike|hCoV-19/Spain/RI-IBV-99010936/2020|2020-06-19|EPI_ISL_732773|Original|hCoV-
19^^La_Rioja|Human|Centro de Investigaci\u00f3n Biom\u00e9dica de La Rioja - Hospital
San Pedro Logro\u00f1o|SeqCOVID-SPAIN consortium/IBV(CSIC)|Comas|Spain
>Spike|hCoV-19/England/ALDP-B46917/2020|2020-11-07|EPI_ISL_656137|Original|hCoV-
19^^England|Human|Lighthouse Lab in Alderley Park|Wellcome Sanger Institute for the
COVID-19 Genomics UK (COG-UK) Consortium|Davies|United Kingdom
>Spike|hCoV-19/England/CAMC-B08FC4/2020|2020-10-29|EPI_ISL_672956|Original|hCoV-
19^^England|Human|Lighthouse Lab in Cambridge|Wellcome Sanger Institute for the
COVID-19 Genomics UK (COG-UK) Consortium|Davies|United Kingdom
>Spike|hCoV-19/England/MILK-B8D2CE/2020|2020-11-13|EPI_ISL_674141|Original|hCoV-
19^^England|Human|Lighthouse Lab in Milton Keynes|Wellcome Sanger Institute for the
COVID-19 Genomics UK (COG-UK) Consortium|Davies|United Kingdom
>Spike|hCoV-19/USA/NY-SURV0472/2020|2020-03-09|EPI_ISL_436068|Original|hCoV-19^^New
York|Human|NYC Department of Health and Mental Hygiene|Pathogen Discovery|Queen|USA
>Spike|hCoV-19/England/LIVE-DC389B/2020|2020-11-22|EPI_ISL_702718|Original|hCoV-
19^^England|Human|Liverpool Clinical Laboratories|COVID-19 Genomics UK (COG-UK)
Consortium|Haldenby|United Kingdom
>Spike|hCoV-19/USA/ME-HETL-J0260/2020|2020-09-29|EPI_ISL_755408|Original|hCoV-
19^^Maine|Human|Maine Health and Environmental Testing Laboratory|Tewhey
Lab|Tewhey|USA
>Spike|hCoV-19/England/CAMB-795F4/2020|2020-04-04|EPI_ISL_441102|Original|hCoV-
19^^England|Human|Department of Pathology|Wellcome Sanger Institute for the COVID-19
Genomics UK (COG-UK) consortium|Amato|United Kingdom
>Spike|hCoV-19/England/ALDP-A92704/2020|2020-10-19|EPI_ISL_611036|Original|hCoV-
19^^England|Human|Lighthouse Lab in Alderley Park|Wellcome Sanger Institute for the
COVID-19 Genomics UK (COG-UK) consortium|Davies|United Kingdom
>Spike|hCoV-19/Australia/VIC18033/2020|2020-12-31|EPI_ISL_779641|Original|hCoV-
19^^Victoria|Human|Microbiological Diagnostic Unit - Public Health Laboratory (MDU-
PHL)|MDU-PHL|Beck|Australia

>Spike|hCoV-19/India/GJ-GBRC284b/2020|2020-06-30|EPI_ISL_495022|Original|hCoV-19^^Gujarat|Human|Government Medical College|Gujarat Biotechnology Research Centre|Joshi|India
>Spike|hCoV-19/Canada/ON-PHL-0654/2020|2020-03-00|EPI_ISL_418370|Original|hCoV-19^^Ontario|Human|Public Health Ontario Laboratories|Public Health Ontario Laboratories|Eshaghi|Canada
>Spike|hCoV-19/Bosnia and Herzegovina/ChVir7402/2020|2020-04-22|EPI_ISL_462470|Original|hCoV-19^^Sarajevo|Human|Clinical Center|Charite Universitatsmedizin Berlin|Muehlemann|Bosnia and Herzegovina
>Spike|hCoV-19/Singapore/37/2020|2020-03-13|EPI_ISL_422430|Original|hCoV-19^^Singapore|Human|National Public Health Laboratory|National Public Health Laboratory|Mak|Singapore
>Spike|hCoV-19/Russia/MOW-RII-MH2003S/2020|2020-06-19|EPI_ISL_596345|Original|hCoV-19^^Moscow|Human|Pathogenic Microorganisms Variability Laboratory|WHO National Influenza Centre Russian Federation|Komissarov|Russia
>Spike|hCoV-19/England/QEUH-9FF751/2020|2020-10-02|EPI_ISL_600226|Original|hCoV-19^^England|Human|Lighthouse Lab in Glasgow|Wellcome Sanger Institute for the COVID-19 Genomics UK (COG-UK) consortium|Davies|United Kingdom
>Spike|hCoV-19/United Arab Emirates/0189/2020|2020-05-18|EPI_ISL_698375|Original|hCoV-19^^Abu Dhabi|Human|Group 42 (G42) Healthcare|G42 Healthcare|Ogrodzki|United Arab Emirates
>Spike|hCoV-19/USA/TX-HMH-488/2020|2020-04-05|EPI_ISL_544398|Original|hCoV-19^^Texas|Human|Houston Methodist Hospital|Houston Methodist Hospital|Long|USA
>Spike|hCoV-19/USA/HI-H200027/2020|2020-03-18|EPI_ISL_752614|Original|hCoV-19^^Hawaii|Human|State Laboratories Division|State Laboratories Division|Sultana|USA
>Spike|hCoV-19/Denmark/DCGC-20979/2020|2020-12-21|EPI_ISL_759486|Original|hCoV-19^^Nordjylland|Human|Department of Virus and Microbiological Special Diagnostics|Albertsen Lab|Michaelsen|Denmark
>Spike|hCoV-19/United Arab Emirates/0503/2020|2020-05-18|EPI_ISL_698670|Original|hCoV-19^^Abu Dhabi|Human|Group 42 (G42) Healthcare|G42 Healthcare|Ogrodzki|United Arab Emirates
>Spike|hCoV-19/Spain/MD-IBV-003716/2020|2020-04-03|EPI_ISL_467238|Original|hCoV-19^^Madrid|Human|Hospital General Universitario Gregorio Mara\u00f1\u00f3n|SeqCOVID-SPAIN consortium/IBV(CSIC)|Comas|Spain
>Spike|hCoV-19/Ireland/D-SVUH-R9582/2020|2020-04-29|EPI_ISL_596640|Original|hCoV-19^^Dublin|Human|St.Vincent's University Hospital|St.Vincent's University Hospital|Gonzalez|Ireland
>Spike|hCoV-19/Belgium/AF-0327321/2020|2020-03-27|EPI_ISL_462175|Original|hCoV-19^^Marcinelle|Human|KU Leuven|KU Leuven|Maes|Belgium
>Spike|hCoV-19/England/ALDP-94D420/2020|2020-06-15|EPI_ISL_556065|Original|hCoV-19^^England|Human|Lighthouse Lab in Alderley Park|Wellcome Sanger Institute for the COVID-19 Genomics UK (COG-UK) consortium|Davies|United Kingdom
>Spike|hCoV-19/England/20119023104/2020|2020-03-13|EPI_ISL_465034|Original|hCoV-19^^England|Human|Respiratory Virus Unit|Respiratory Virus Unit|Platt|United Kingdom
>Spike|hCoV-19/Switzerland/ZH-ETHZ-240071/2020|2020-08-09|EPI_ISL_523909|Original|hCoV-19^^Zurich|Human|Viollier AG|Department of Biosystems Science and Engineering|Nadeau|Switzerland
>Spike|hCoV-19/England/ALDP-BBAAB1/2020|2020-11-20|EPI_ISL_702445|Original|hCoV-19^^England|Human|Lighthouse Lab in Alderley Park|Wellcome Sanger Institute for the COVID-19 Genomics UK (COG-UK) Consortium|Davies|United Kingdom
>Spike|hCoV-19/India/MH-ACTREC-615/2020|2020-09-09|EPI_ISL_723066|Original|hCoV-19^^Maharashtra|Human|Hematopathology Laboratory|Hematopathology Laboratory|Patkar|India
>Spike|hCoV-19/Wales/PHWC-486DBC/2020|2020-10-28|EPI_ISL_639133|Original|hCoV-19^^Wales|Human|Wales Specialist Virology Centre Sequencing lab|COVID-19 Genomics UK (COG-UK) Consortium|Moore|United Kingdom
>Spike|hCoV-19/USA/SC-CDC-2-3714370/2020|2020-12-01|EPI_ISL_751744|Original|hCoV-19^^South Carolina|Human|SC Dept of Health and Env. Control-Bureau of Laboratories|Genomics and Discovery|Queen|USA

>Spike|hCoV-19/USA/WA-UW-3871/2020|2020-03-26|EPI_ISL_427198|Original|hCoV-
19^^Washington|Human|UW Virology Lab|UW Virology Lab|Roychoudhury|USA
>Spike|hCoV-19/Denmark/DCGC-764/2020|2020-03-09|EPI_ISL_614790|Original|hCoV-
19^^Syddanmark|Human|Department of Virus and Microbiological Special
Diagnostics|Albertsen lab|Michaelsen|Denmark
>Spike|hCoV-19/South Africa/R13300-20/2020|2020-06-30|EPI_ISL_622909|Original|hCoV-
19^^KwaZulu-Natal|Human|National Institute for Communicable Diseases of the National
Health Laboratory Service|National Institute for Communicable Diseases of the
National Health Laboratory Service|Allam|South Africa
>Spike|hCoV-19/USA/LA-EVTL880/2020|2020-07-06|EPI_ISL_653571|Original|hCoV-
19^^Louisiana|Human|LSUHS Emerging Viral Threat Laboratory|Microbial Genome
Sequencing Center|Kamil|USA
>Spike|hCoV-19/Japan/PG-2347/2020|2020-05-00|EPI_ISL_685952|Original|hCoV-
19^^Japan|Human|Pathogen Genomics Center|Pathogen Genomics Center|Sekizuka|Japan
>Spike|hCoV-19/USA/NY-QDX-2043/2020|2020-03-17|EPI_ISL_604364|Original|hCoV-19^^New
York|Human|Quest Diagnostics|Quest Diagnostics|Kagan|USA
>Spike|hCoV-19/Australia/VIC12521/2020|2020-07-29|EPI_ISL_640335|Original|hCoV-
19^^Victoria|Human|Microbiological Diagnostic Unit - Public Health Laboratory (MDU-
PHL)|MDU-PHL|Schultz|Australia
>Spike|hCoV-19/Spain/MD-H12-63/2020|2020-04-12|EPI_ISL_529994|Original|hCoV-
19^^Madrid|Human|Hospital Universitario 12 de Octubre|Hospital Universitario 12 de
Octubre|Viedma|Spain
>Spike|hCoV-19/Japan/PG-6931/2020|2020-09-00|EPI_ISL_688037|Original|hCoV-
19^^Japan|Human|Pathogen Genomics Center|Pathogen Genomics Center|Sekizuka|Japan
>Spike|hCoV-19/USA/TX-HMH-MCoV-18936/2020|2020-11-25|EPI_ISL_785232|Original|hCoV-
19^^Texas|Human|Houston Methodist Hospital|Houston Methodist Hospital|Long|USA
>Spike|hCoV-19/South Korea/KCDC2567/2020|2020-06-25|EPI_ISL_514970|Original|hCoV-
19^^South Korea|Human|Division of Viral Diseases|Division of Viral Diseases|Kim|South
Korea
>Spike|hCoV-19/USA/TX-HMH-MCoV-16398/2020|2020-11-01|EPI_ISL_786082|Original|hCoV-
19^^Texas|Human|Houston Methodist Hospital|Houston Methodist Hospital|Long|USA
>Spike|hCoV-19/Wales/PHWC-499C40/2020|2020-12-02|EPI_ISL_727421|Original|hCoV-
19^^Wales|Human|Originating lab|COVID-19 Genomics UK (COG-UK) Consortium|Moore|United
Kingdom
>Spike|hCoV-19/Luxembourg/LNS2346680/2020|2020-11-18|EPI_ISL_740102|Original|hCoV-
19^^Luxembourg|Human|Laboratoire national de sant\u00e9|Laboratoire national de
sant\u00e9|Wienecke-Baldacchino|Luxembourg
>Spike|hCoV-19/Australia/TAS83/2020|2020-03-27|EPI_ISL_522754|Original|hCoV-
19^^Tasmania|Human|Royal Hobart Hospital Microbiology Department|MDU-
PHL|Schultz|Australia
>Spike|hCoV-19/Japan/Donner89/2020|2020-08-16|EPI_ISL_644993|Original|hCoV-
19^^Kanto|Human|Department of Infectious Diseases|Center for Medical
Genetics|Kosaki|Japan
>Spike|hCoV-19/USA/LA-EVTL079/2020|2020-05-01|EPI_ISL_451240|Original|hCoV-
19^^Louisiana|Human|LSUHS Emerging Viral Threat Laboratory|Microbial Genome
Sequencing Center|Kamil|USA
>Spike|hCoV-19/England/QEUH-B9F5EE/2020|2020-11-18|EPI_ISL_721064|Original|hCoV-
19^^England|Human|Lighthouse Lab in Glasgow|Wellcome Sanger Institute for the COVID-
19 Genomics UK (COG-UK) Consortium|Davies|United Kingdom
>Spike|hCoV-19/England/ALDP-950B07/2020|2020-06-14|EPI_ISL_554562|Original|hCoV-
19^^England|Human|Lighthouse Lab in Alderley Park|Wellcome Sanger Institute for the
COVID-19 Genomics UK (COG-UK) consortium|Davies|United Kingdom
>Spike|hCoV-19/Switzerland/VD-ETHZ-100795/2020|2020-03-
25|EPI_ISL_451745|Original|hCoV-19^^Vaud|Human|Viollier AG|Department of Biosystems
Science and Engineering|Nadeau|Switzerland
>Spike|hCoV-19/Wales/PHWC-329CE/2020|2020-04-14|EPI_ISL_474377|Original|hCoV-
19^^Wales|Human|Originating lab|COVID-19 Genomics UK (COG-UK) Consortium|Moore|United
Kingdom

>Spike|hCoV-19/England/QEUH-AAD37B/2020|2020-10-20|EPI_ISL_623908|Original|hCoV-
19^^England|Human|Lighthouse Lab in Glasgow|Wellcome Sanger Institute for the COVID-
19 Genomics UK (COG-UK) consortium|Davies|United Kingdom
>Spike|hCoV-19/England/ALDP-A505BD/2020|2020-10-07|EPI_ISL_597712|Original|hCoV-
19^^England|Human|Lighthouse Lab in Alderley Park|Wellcome Sanger Institute for the
COVID-19 Genomics UK (COG-UK) consortium|Davies|United Kingdom
>Spike|hCoV-19/England/ALDP-9E71D6/2020|2020-09-24|EPI_ISL_580442|Original|hCoV-
19^^England|Human|Lighthouse Lab in Alderley Park|Wellcome Sanger Institute for the
COVID-19 Genomics UK (COG-UK) consortium|Davies|United Kingdom
>Spike|hCoV-19/Russia/MOS-CRIE7160676/2020|2020-08-24|EPI_ISL_653923|Original|hCoV-
19^^Moscow|Human|Molecular diagnostic laboratory of Federal Budget Institution of
Science \Central Research Institute of Epidemiology\ of The Federal Service on
Customers' Rights Protection and Human Well-being Surveillance|Group of Genomics and
Postgenomic Technologies of Central Research Institute of Epidemilology|
>Samoilov|Russia
>Spike|hCoV-19/England/MILK-A8F069/2020|2020-10-16|EPI_ISL_650279|Original|hCoV-
19^^England|Human|Northumbria University / South Tees Hospitals NHS Foundation Trust
/ North Cumbria Integrated Care NHS Foundation Trust / North Tees and Hartlepool NHS
Foundation Trust / Newcastle Hospitals NHS Foundation Trust|COVID-19 Genomics UK
(COG-UK) Consortium|Bashton|United Kingdom
>Spike|hCoV-19/Wales/PHWC-495989/2020|2020-11-24|EPI_ISL_726420|Original|hCoV-
19^^Wales|Human|Originating lab|COVID-19 Genomics UK (COG-UK) Consortium|Moore|United
Kingdom
>Spike|hCoV-19/England/SHEF-CE402/2020|2020-11-26|EPI_ISL_707003|Original|hCoV-
19^^England|Human|Virology Department|COVID-19 Genomics UK (COG-UK) Consortium|de
Silva|United Kingdom
>Spike|hCoV-19/England/ALDP-B62809/2020|2020-11-09|EPI_ISL_656384|Original|hCoV-
19^^England|Human|Lighthouse Lab in Alderley Park|Wellcome Sanger Institute for the
COVID-19 Genomics UK (COG-UK) Consortium|Davies|United Kingdom
>Spike|hCoV-19/USA/MA-MGH-00464/2020|2020-04-04|EPI_ISL_460177|Original|hCoV-
19^^Massachusetts|Human|Massachusetts General Hospital|Infectious Disease
Program|Lemieux|USA
>Spike|hCoV-19/USA/LA-EVTL418/2020|2020-07-28|EPI_ISL_578824|Original|hCoV-
19^^Louisiana|Human|LSUHS Emerging Viral Threat Laboratory|Microbial Genome
Sequencing Center|Kamil|USA
>Spike|hCoV-19/Denmark/DCGC-16221/2020|2020-11-16|EPI_ISL_714647|Original|hCoV-
19^^Hovedstaden|Human|Department of Virus and Microbiological Special
Diagnostics|Albertsen Lab|Michaelsen|Denmark
>Spike|hCoV-19/England/NORT-1B72C8F/2020|2020-12-08|EPI_ISL_724923|Original|hCoV-
19^^England|Human|Northumbria University / South Tees Hospitals NHS Foundation Trust
/ North Cumbria Integrated Care NHS Foundation Trust / North Tees and Hartlepool NHS
Foundation Trust / Newcastle Hospitals NHS Foundation Trust|COVID-19 Genomics UK
(COG-UK) Consortium|Bashton|United Kingdom
>Spike|hCoV-19/USA/OR-OHSU-1233/2020|2020-07-13|EPI_ISL_509166|Original|hCoV-
19^^Oregon|Human|OHSU Lab Services Molecular Microbiology Lab|Oregon SARS-CoV-2
Genome Sequencing Center|O'Roak|USA
>Spike|hCoV-19/England/ALDP-A69636/2020|2020-10-12|EPI_ISL_609089|Original|hCoV-
19^^England|Human|Lighthouse Lab in Alderley Park|Wellcome Sanger Institute for the
COVID-19 Genomics UK (COG-UK) consortium|Davies|United Kingdom
>Spike|hCoV-19/England/QEUH-A8CA13/2020|2020-10-16|EPI_ISL_643107|Original|hCoV-
19^^England|Human|Lighthouse Lab in Glasgow|Wellcome Sanger Institute for the COVID-
19 Genomics UK (COG-UK) Consortium|Davies|United Kingdom
>Spike|hCoV-19/Brazil/SP-L7-CD74/2020|2020-03-25|EPI_ISL_476441|Original|hCoV-
19^^S\u00e3o Paulo|Human|Hospital da Cl\u00ednicas da Faculdade de Medicina da
Universidade de S\u00e3o Paulo|Instituto de Medicina Tropical da Univesidade de
S\u00e3o Paulo|Faria|Brazil
>Spike|hCoV-19/England/EXET-13546F/2020|2020-04-14|EPI_ISL_457062|Original|hCoV-
19^^England|Human|University of Exeter|COVID-19 Genomics UK (COG-UK)
Consortium|Temperton|United Kingdom

>Spike|hCoV-19/England/ALDP-A2ECFF/2020|2020-10-05|EPI_ISL_587039|Original|hCoV-
19^^England|Human|Lighthouse Lab in Alderley Park|Wellcome Sanger Institute for the
COVID-19 Genomics UK (COG-UK) consortium|Davies|United Kingdom
>Spike|hCoV-19/Scotland/CVR2734/2020|2020-04-16|EPI_ISL_453073|Original|hCoV-
19^^Scotland|Human|West of Scotland Specialist Virology Centre|COVID-19 Genomics UK
(COG-UK) Consortium|Robertson|United Kingdom
>Spike|hCoV-19/Switzerland/GL-ETHZ-110455/2020|2020-04-
07|EPI_ISL_451875|Original|hCoV-19^^Glarus|Human|Viollier AG|Department of Biosystems
Science and Engineering|Nadeau|Switzerland
>Spike|hCoV-19/England/EXET-13F1D7/2020|2020-12-15|EPI_ISL_763712|Original|hCoV-
19^^England|Human|University of Exeter|COVID-19 Genomics UK (COG-UK)
Consortium|Temperton|United Kingdom
>Spike|hCoV-19/England/ALDP-B762C8/2020|2020-11-12|EPI_ISL_723752|Original|hCoV-
19^^England|Human|Oxford Viromics|COVID-19 Genomics UK (COG-UK)
Consortium|Golubchik|United Kingdom
>Spike|hCoV-19/England/BIRM-67C21/2020|2020-11-06|EPI_ISL_650743|Original|hCoV-
19^^England|Human|University of Birmingham|COVID-19 Genomics UK (COG-UK)
Consortium|Loman|United Kingdom
>Spike|hCoV-19/England/20169019604/2020|2020-04-17|EPI_ISL_465205|Original|hCoV-
19^^England|Human|Respiratory Virus Unit|Respiratory Virus Unit|Platt|United Kingdom
>Spike|hCoV-19/Denmark/DCGC-14046/2020|2020-11-30|EPI_ISL_711535|Original|hCoV-
19^^Hovedstaden|Human|Department of Virus and Microbiological Special
Diagnostics|Albertsen Lab|Michaelsen|Denmark
>Spike|hCoV-19/England/LIVE-DC297B/2020|2020-11-24|EPI_ISL_702674|Original|hCoV-
19^^England|Human|Liverpool Clinical Laboratories|COVID-19 Genomics UK (COG-UK)
Consortium|Haldenby|United Kingdom
>Spike|hCoV-19/Wales/PHWC-165E86/2020|2020-06-19|EPI_ISL_490809|Original|hCoV-
19^^Wales|Human|Wales Specialist Virology Centre Sequencing lab|COVID-19 Genomics UK
(COG-UK) Consortium|Moore|United Kingdom
>Spike|hCoV-19/England/NORW-EAE20/2020|2020-04-29|EPI_ISL_457382|Original|hCoV-
19^^England|Human|Quadram Institute Bioscience|COVID-19 Genomics UK (COG-UK)
Consortium|Alikhan|United Kingdom
>Spike|hCoV-19/Israel/990059233/2020|2020-03-25|EPI_ISL_447273|Original|hCoV-
19^^South Coast District|Human|Microbiology laboratory|Stern Lab|Miller|Israel
>Spike|hCoV-19/Denmark/DCGC-20539/2020|2020-12-21|EPI_ISL_758904|Original|hCoV-
19^^Hovedstaden|Human|Department of Virus and Microbiological Special
Diagnostics|Albertsen Lab|Michaelsen|Denmark
>Spike|hCoV-19/United Arab Emirates/1070/2020|2020-06-
11|EPI_ISL_698144|Original|hCoV-19^^Abu Dhabi|Human|Group 42 (G42) Healthcare|G42
Healthcare|Ogrodzki|United Arab Emirates
>Spike|hCoV-19/England/QEUH-B2DCC2/2020|2020-11-03|EPI_ISL_646840|Original|hCoV-
19^^England|Human|Lighthouse Lab in Glasgow|Wellcome Sanger Institute for the COVID-
19 Genomics UK (COG-UK) Consortium|Davies|United Kingdom
>Spike|hCoV-19/England/LOND-1261590/2020|2020-03-29|EPI_ISL_524696|Original|hCoV-
19^^England|Human|North West London Pathology|Wellcome Sanger Institute for the
COVID-19 Genomics UK (COG-UK) consortium|Davies|United Kingdom
>Spike|hCoV-19/England/ALDP-94DF7A/2020|2020-06-16|EPI_ISL_555601|Original|hCoV-
19^^England|Human|Lighthouse Lab in Alderley Park|Wellcome Sanger Institute for the
COVID-19 Genomics UK (COG-UK) consortium|Davies|United Kingdom
>Spike|hCoV-19/England/QEUH-BCD981/2020|2020-11-26|EPI_ISL_704469|Original|hCoV-
19^^England|Human|Lighthouse Lab in Glasgow|Wellcome Sanger Institute for the COVID-
19 Genomics UK (COG-UK) Consortium|Davies|United Kingdom
>Spike|hCoV-19/Portugal/PT1103/2020|2020-03-28|EPI_ISL_511650|Original|hCoV-
19^^Portugal|Human|Instituto Nacional de Saude (INSA)|Instituto Nacional de Saude
(INSA)|Borges|Portugal
>Spike|hCoV-19/USA/AZ-QDX-2310/2020|2020-03-15|EPI_ISL_604503|Original|hCoV-
19^^Arizona|Human|Quest Diagnostics|Quest Diagnostics|Kagan|USA
>Spike|hCoV-19/USA/RI-CDC-2-3714565/2020|2020-12-03|EPI_ISL_751695|Original|hCoV-
19^^Rhode Island|Human|RI State Health Laboratories|Genomics and Discovery|Queen|USA

>Spike|hCoV-19/Luxembourg/LNS0958161/2020|2020-10-19|EPI_ISL_739862|Original|hCoV-19^^Luxembourg|Human|Laboratoire national de sant\u00e9|Laboratoire national de sant\u00e9|Wienecke-Baldacchino|Luxembourg
>Spike|hCoV-19/Luxembourg/LNS4516866/2020|2020-10-23|EPI_ISL_740485|Original|hCoV-19^^Luxembourg|Human|Laboratoire national de sant\u00e9|Laboratoire national de sant\u00e9|Wienecke-Baldacchino|Luxembourg
>Spike|hCoV-19/Scotland/EDB10633/2020|2020-12-02|EPI_ISL_705788|Original|hCoV-19^^Scotland|Human|Virology Department|COVID-19 Genomics UK (COG-UK) Consortium|McHugh|United Kingdom
>Spike|hCoV-19/USA/MA-Yale-425/2020|2020-04-08|EPI_ISL_738269|Original|hCoV-19^^Massachusetts|Human|Connecticut Veterans' Affairs Hospital|Grubaugh Lab - Yale School of Public Health|Grubaugh|USA
>Spike|hCoV-19/USA/CA-ALSR-4599/2020|2020-07-10|EPI_ISL_667031|Original|hCoV-19^^California|Human|San Diego County Public Health Laboratory|Andersen lab at Scripps Research|Gangavarapu|USA
>Spike|hCoV-19/England/MILK-CA9D24/2020|2020-12-18|EPI_ISL_762216|Original|hCoV-19^^England|Human|Lighthouse Lab in Milton Keynes|Wellcome Sanger Institute for the COVID-19 Genomics UK (COG-UK) Consortium|Davies|United Kingdom
>Spike|hCoV-19/Wales/PHWC-490B1F/2020|2020-11-16|EPI_ISL_706538|Original|hCoV-19^^Wales|Human|Originating lab|COVID-19 Genomics UK (COG-UK) Consortium|Moore|United Kingdom
>Spike|hCoV-19/Belgium/UGent-278/2020|2020-11-23|EPI_ISL_718013|Original|hCoV-19^^Ghent|Human|Lab voor klinische biologie|Onderzoeksgroep Virologie|Lambrechts|Belgium
>Spike|hCoV-19/Chile/RM-ISPCH-43/2020|2020-03-16|EPI_ISL_445322|Original|hCoV-19^^Region Metropolitana de Santiago|Human|UNIV.DE CHILE HOSP.CLINICO|Instituto de Salud Publica de Chile|Fernandez|Chile
>Spike|hCoV-19/England/201140037/2020|2020-03-10|EPI_ISL_464701|Original|hCoV-19^^England|Human|Respiratory Virus Unit|Respiratory Virus Unit|Platt|United Kingdom
>Spike|hCoV-19/England/ALDP-B83213/2020|2020-11-14|EPI_ISL_675529|Original|hCoV-19^^England|Human|Lighthouse Lab in Alderley Park|Wellcome Sanger Institute for the COVID-19 Genomics UK (COG-UK) Consortium|Davies|United Kingdom
>Spike|hCoV-19/USA/NY-Wadsworth-10715-01/2020|2020-03-05|EPI_ISL_525577|Original|hCoV-19^^New York|Human|Wadsworth Center|Wadsworth Center|Plitnick|USA
>Spike|hCoV-19/USA/AZ-TG396363/2020|2020-06-11|EPI_ISL_694820|Original|hCoV-19^^Arizona|Human|AZ SPHL|TGen North|French|USA
>Spike|hCoV-19/England/MILK-9E3301/2020|2020-09-21|EPI_ISL_581299|Original|hCoV-19^^England|Human|Lighthouse Lab in Milton Keynes|Wellcome Sanger Institute for the COVID-19 Genomics UK (COG-UK) consortium|Davies|United Kingdom
>Spike|hCoV-19/USA/WI-UW-1263/2020|2020-08-23|EPI_ISL_536636|Original|hCoV-19^^Wisconsin|Human|University of Wisconsin-Madison AIDS Vaccine Research Laboratories|University of Wisconsin-Madison AIDS Vaccine Research Laboratories|Baczenas|USA
>Spike|hCoV-19/USA/MN-MDH-2227/2020|2020-12-05|EPI_ISL_770801|Original|hCoV-19^^Minnesota|Human|Vault Health|Minnesota Department of Health|Lorentz|USA
>Spike|hCoV-19/England/20144075304/2020|2020-04-01|EPI_ISL_466008|Original|hCoV-19^^England|Human|Respiratory Virus Unit|Respiratory Virus Unit|Platt|United Kingdom
>Spike|hCoV-19/England/ALDP-B638AE/2020|2020-11-09|EPI_ISL_656603|Original|hCoV-19^^England|Human|Lighthouse Lab in Alderley Park|Wellcome Sanger Institute for the COVID-19 Genomics UK (COG-UK) Consortium|Davies|United Kingdom
>Spike|hCoV-19/England/ALDP-9EDC57/2020|2020-05-08|EPI_ISL_580720|Original|hCoV-19^^England|Human|Lighthouse Lab in Alderley Park|Wellcome Sanger Institute for the COVID-19 Genomics UK (COG-UK) consortium|Davies|United Kingdom
>Spike|hCoV-19/Australia/VIC10397/2020|2020-08-25|EPI_ISL_562899|Original|hCoV-19^^Victoria|Human|Microbiological Diagnostic Unit - Public Health Laboratory (MDU-PHL)|MDU-PHL|Schultz|Australia
>Spike|hCoV-19/Wales/PHWC-1638F3/2020|2020-05-07|EPI_ISL_474015|Original|hCoV-19^^Wales|Human|Originating lab|COVID-19 Genomics UK (COG-UK) Consortium|Moore|United Kingdom

>Spike|hCoV-19/USA/HI-H200595/2020|2020-11-02|EPI_ISL_753121|Original|hCoV-19^^Hawaii|Human|State Laboratories Division|State Laboratories Division|Sultana|USA
>Spike|hCoV-19/USA/CA-SR0259/2020|2020-03-25|EPI_ISL_437553|Original|hCoV-19^^California|Human|Scripps Medical Laboratory|Andersen lab at Scripps Research|Gangavarapu|USA
>Spike|hCoV-19/Denmark/DCGC-21935/2020|2020-12-21|EPI_ISL_758100|Original|hCoV-19^^Syddanmark|Human|Department of Virus and Microbiological Special Diagnostics|Albertsen Lab|Michaelsen|Denmark
>Spike|hCoV-19/USA/TX-HMH-1802/2020|2020-05-16|EPI_ISL_542850|Original|hCoV-19^^Texas|Human|Houston Methodist Hospital|Houston Methodist Hospital|Long|USA
>Spike|hCoV-19/Scotland/EDB2359/2020|2020-04-22|EPI_ISL_439189|Original|hCoV-19^^Scotland|Human|Virology Department|COVID-19 Genomics UK (COG-UK) Consortium|Williams|United Kingdom
>Spike|hCoV-19/Japan/PG-4565/2020|2020-07-00|EPI_ISL_689702|Original|hCoV-19^^Japan|Human|Pathogen Genomics Center|Pathogen Genomics Center|Sekizuka|Japan
>Spike|hCoV-19/England/ALDP-AA2455/2020|2020-10-22|EPI_ISL_624392|Original|hCoV-19^^England|Human|Lighthouse Lab in Alderley Park|Wellcome Sanger Institute for the COVID-19 Genomics UK (COG-UK) consortium|Davies|United Kingdom
>Spike|hCoV-19/Luxembourg/LNS6619597/2020|2020-12-01|EPI_ISL_771004|Original|hCoV-19^^Luxembourg|Human|Laboratoire national de sant\u00e9|Laboratoire national de sant\u00e9|Wienecke-Baldacchino|Luxembourg
>Spike|hCoV-19/Belgium/ULG-10958/2020|2020-09-07|EPI_ISL_707737|Original|hCoV-19^^Li\u00e8ge|Human|Department of Clinical Microbiology|GIGA Medical Genomics|Durkin|Belgium
>Spike|hCoV-19/Switzerland/BE-ETHZ-350183/2020|2020-11-04|EPI_ISL_693832|Original|hCoV-19^^Bern|Human|Viollier AG|Department of Biosystems Science and Engineering|Nadeau|Switzerland
>Spike|hCoV-19/USA/FL-BPHL-1606/2020|2020-07-05|EPI_ISL_594296|Original|hCoV-19^^Florida|Human|Florida Bureau of Public Health Laboratories|Florida Bureau of Public Health Laboratories|Blanton|USA
>Spike|hCoV-19/Spain/MD-IBV-99007126/2020|2020-07-31|EPI_ISL_541900|Original|hCoV-19^^Madrid|Human|Hospital General Universitario Gregorio Mara\u00f1\u00f3n|SeqCOVID-SPAIN consortium/IBV(CSIC)|Comas|Spain
>Spike|hCoV-19/USA/WA-S678/2020|2020-04-07|EPI_ISL_449848|Original|hCoV-19^^Washington|Human|Washington State Department of Health|Seattle Flu Study|Bedford|USA
>Spike|hCoV-19/England/BRIS-131339/2020|2020-05-21|EPI_ISL_467702|Original|hCoV-19^^England|Human|PHE South West Regional Laboratory|Wellcome Sanger Institute for the COVID-19 Genomics UK (COG-UK) consortium|Davies|United Kingdom
>Spike|hCoV-19/USA/TX-HMH-4139/2020|2020-06-18|EPI_ISL_543403|Original|hCoV-19^^Texas|Human|Houston Methodist Hospital|Houston Methodist Hospital|Long|USA
>Spike|hCoV-19/Australia/VIC2339/2020|2020-06-25|EPI_ISL_522060|Original|hCoV-19^^Victoria|Human|Victorian Infectious Diseases Reference Laboratory (VIDRL)|VIDRL and MDU-PHL|Schultz|Australia
>Spike|hCoV-19/USA/MI-MDHHS-SC21195/2020|2020-04-01|EPI_ISL_507452|Original|hCoV-19^^Michigan|Human|Michigan Department of Health and Human Services|Michigan Department of Health and Human Services|Blankenship|USA
>Spike|hCoV-19/Scotland/EDB3496/2020|2020-04-13|EPI_ISL_439269|Original|hCoV-19^^Scotland|Human|Virology Department|COVID-19 Genomics UK (COG-UK) Consortium|Williams|United Kingdom
>Spike|hCoV-19/England/QEUH-B2F659/2020|2020-11-04|EPI_ISL_660023|Original|hCoV-19^^England|Human|Lighthouse Lab in Glasgow|Wellcome Sanger Institute for the COVID-19 Genomics UK (COG-UK) Consortium|Davies|United Kingdom
>Spike|hCoV-19/England/CAMB-1ACD4A/2020|2020-04-22|EPI_ISL_489467|Original|hCoV-19^^England|Human|Department of Pathology|Wellcome Sanger Institute for the COVID-19 Genomics UK (COG-UK) consortium|Davies|United Kingdom
>Spike|hCoV-19/USA/NY-Wadsworth-142785-01/2020|2020-04-18|EPI_ISL_676978|Original|hCoV-19^^New York|Human|Wadsworth Center|Wadsworth Center|Lasek-Nesselquist|USA

>Spike|hCoV-19/Netherlands/NB-EMC-258/2020|2020-07-08|EPI_ISL_523381|Original|hCoV-19^^Noord Brabant|Human|Dutch COVID-19 response team|Erasmus Medical Center|Oude Munnink|Netherlands
>Spike|hCoV-19/England/LEED-2AA358/2020|2020-04-08|EPI_ISL_538908|Original|hCoV-19^^England|Human|Leeds Teaching Hospitals NHS Trust and Public Health England|Wellcome Sanger Institute for the COVID-19 Genomics UK (COG-UK) consortium|Davies|United Kingdom
>Spike|hCoV-19/England/ALDP-AA49FA/2020|2020-10-21|EPI_ISL_624925|Original|hCoV-19^^England|Human|Lighthouse Lab in Alderley Park|Wellcome Sanger Institute for the COVID-19 Genomics UK (COG-UK) consortium|Davies|United Kingdom
>Spike|hCoV-19/England/BRIS-12C067/2020|2020-05-10|EPI_ISL_469365|Original|hCoV-19^^England|Human|PHE South West Regional Laboratory|Wellcome Sanger Institute for the COVID-19 Genomics UK (COG-UK) consortium|Davies|United Kingdom
>Spike|hCoV-19/Israel/CVL-280-ngs/2020|2020-12-27|EPI_ISL_776812|Original|hCoV-19^^Israel|Human|Israel Central Virology laboratory|Israel Central Virology laboratory|Zuckerman|Israel
>Spike|hCoV-19/USA/CA-CZB-10011/2020|2020-06-03|EPI_ISL_672083|Original|hCoV-19^^California|Human|The Ashley Laboratory|Chan-Zuckerberg Biohub|Bhatt|USA
>Spike|hCoV-19/USA/DE-DHSS-F918424/2020|2020-03-31|EPI_ISL_593960|Original|hCoV-19^^Delaware|Human|Delaware Public Health Lab|Delaware Public Health Lab|Bajwa|USA
>Spike|hCoV-19/England/ALDP-A859E0/2020|2020-10-17|EPI_ISL_610355|Original|hCoV-19^^England|Human|Lighthouse Lab in Alderley Park|Wellcome Sanger Institute for the COVID-19 Genomics UK (COG-UK) consortium|Davies|United Kingdom
>Spike|hCoV-19/USA/WA-S118/2020|2020-03-01|EPI_ISL_417171|Original|hCoV-19^^Washington|Human|Washington State Department of Health|Seattle Flu Study|Bedford|USA
>Spike|hCoV-19/England/QEUH-B14208/2020|2020-11-02|EPI_ISL_641852|Original|hCoV-19^^England|Human|Lighthouse Lab in Glasgow|Wellcome Sanger Institute for the COVID-19 Genomics UK (COG-UK) Consortium|Davies|United Kingdom
>Spike|hCoV-19/England/20192017904/2020|2020-05-04|EPI_ISL_528416|Original|hCoV-19^^England|Human|Respiratory Virus Unit|Respiratory Virus Unit|Miah|United Kingdom
>Spike|hCoV-19/Denmark/DCGC-5149/2020|2020-09-21|EPI_ISL_621687|Original|hCoV-19^^Syddanmark|Human|Department of Virus and Microbiological Special Diagnostics|Albertsen lab|Michaelsen|Denmark
>Spike|hCoV-19/England/SHEF-10C5F16/2020|2020-12-21|EPI_ISL_741938|Original|hCoV-19^^England|Human|Virology Department|COVID-19 Genomics UK (COG-UK) Consortium|de Silva|United Kingdom
>Spike|hCoV-19/USA/MI-MDHHS-SC21620/2020|2020-03-29|EPI_ISL_516347|Original|hCoV-19^^Michigan|Human|Michigan Department of Health and Human Services|Michigan Department of Health and Human Services|Blankenship|USA
>Spike|hCoV-19/Scotland/EDB6111/2020|2020-05-12|EPI_ISL_478228|Original|hCoV-19^^Scotland|Human|Virology Department|COVID-19 Genomics UK (COG-UK) Consortium|Williams|United Kingdom
>Spike|hCoV-19/Canada/QC_AA3/2020|2020-04-05|EPI_ISL_444495|Original|hCoV-19^^Quebec|Human|Laboratoire de microbiologie|Smith Laboratory|Smith|Canada
>Spike|hCoV-19/Denmark/DCGC-8617/2020|2020-10-26|EPI_ISL_625999|Original|hCoV-19^^Nordjylland|Human|Department of Virus and Microbiological Special Diagnostics|Albertsen lab|Michaelsen|Denmark
>Spike|hCoV-19/Gibraltar/204421241/2020|2020-10-27|EPI_ISL_613700|Original|hCoV-19^^Gibraltar|Human|Microbiology|Respiratory Virus Unit|Platt|Gibraltar
>Spike|hCoV-19/England/MILK-BDB4E9/2020|2020-11-26|EPI_ISL_709655|Original|hCoV-19^^England|Human|Lighthouse Lab in Milton Keynes|Wellcome Sanger Institute for the COVID-19 Genomics UK (COG-UK) Consortium|Davies|United Kingdom
>Spike|hCoV-19/Denmark/DCGC-16537/2020|2020-11-16|EPI_ISL_714824|Original|hCoV-19^^Hovedstaden|Human|Department of Virus and Microbiological Special Diagnostics|Albertsen Lab|Michaelsen|Denmark
>Spike|hCoV-19/South Africa/KRISP-0553/2020|2020-07-14|EPI_ISL_498092|Original|hCoV-19^^KwaZulu-Natal|Human|NHLS-IALCH|KRISP|de Oliveira|South Africa

>Spike|hCoV-19/USA/UT-UPHL-2012729714/2020|2020-08-17|EPI_ISL_746193|Original|hCoV-
19^^Utah|Human|Ginkgo Bioworks Clinical Laboratory|Utah Public Health
Laboratory|Young|USA
>Spike|hCoV-19/USA/FL-BPHL-0299/2020|2020-05-14|EPI_ISL_480950|Original|hCoV-
19^^Florida|Human|Florida Bureau of Public Health Laboratories|Florida Bureau of
Public Health Laboratories|Blanton|USA
>Spike|hCoV-19/England/LIVE-A57AE/2020|2020-03-21|EPI_ISL_500150|Original|hCoV-
19^^England|Human|Liverpool Clinical Laboratories|COVID-19 Genomics UK (COG-UK)
Consortium|Haldenby|United Kingdom
>Spike|hCoV-19/England/LOND-D37F2/2020|2020-03-31|EPI_ISL_440990|Original|hCoV-
19^^England|Human|University College London|COVID-19 Genomics UK (COG-UK)
Consortium|Roy|United Kingdom
>Spike|hCoV-19/Australia/WA293/2020|2020-03-21|EPI_ISL_596836|Original|hCoV-
19^^Western Australia|Human|PathWest Laboratory Medicine WA|PathWest Laboratory
Medicine WA Microbial Surveillance Unit|Sikazwe|Australia
>Spike|hCoV-19/Scotland/QEUH-9E4CF6/2020|2020-09-23|EPI_ISL_600955|Original|hCoV-
19^^Scotland|Human|Lighthouse Lab in Glasgow|Wellcome Sanger Institute for the COVID-
19 Genomics UK (COG-UK) consortium|Davies|United Kingdom
>Spike|hCoV-19/England/CAMC-B5370E/2020|2020-11-08|EPI_ISL_656919|Original|hCoV-
19^^England|Human|Lighthouse Lab in Cambridge|Wellcome Sanger Institute for the
COVID-19 Genomics UK (COG-UK) Consortium|Davies|United Kingdom
>Spike|hCoV-19/England/201140079/2020|2020-03-09|EPI_ISL_464714|Original|hCoV-
19^^England|Human|Respiratory Virus Unit|Respiratory Virus Unit|Platt|United Kingdom
>Spike|hCoV-19/USA/CA-CZB-2164/2020|2020-05-22|EPI_ISL_513714|Original|hCoV-
19^^California|Human|Orange County Public Health Lab|Chan-Zuckerberg Biohub|Kamm|USA
>Spike|hCoV-19/England/QEUH-B3F638/2020|2020-11-08|EPI_ISL_658429|Original|hCoV-
19^^England|Human|Lighthouse Lab in Glasgow|Wellcome Sanger Institute for the COVID-
19 Genomics UK (COG-UK) Consortium|Davies|United Kingdom
>Spike|hCoV-19/Switzerland/BS-ETHZ-110000/2020|2020-04-
01|EPI_ISL_486442|Original|hCoV-19^^Basel-Stadt|Human|Viollier AG|Department of
Biosystems Science and Engineering|Nadeau|Switzerland
>Spike|hCoV-19/USA/OR-PROV-218/2020|2020-04-04|EPI_ISL_482453|Original|hCoV-
19^^Oregon|Human|Providence St. Joseph Health Molecular Genomics
Laboratory|Providence St. Joseph Health Molecular Genomics Laboratory|Dowdell|USA
>Spike|hCoV-19/Switzerland/BE-ETHZ-120030/2020|2020-04-
18|EPI_ISL_468222|Original|hCoV-19^^Bern|Human|Viollier AG|Department of Biosystems
Science and Engineering|Nadeau|Switzerland
>Spike|hCoV-19/England/LOND-124F361/2020|2020-04-24|EPI_ISL_664471|Original|hCoV-
19^^England|Human|University College London Hospital|COVID-19 Genomics UK (COG-UK)
Consortium|Byott|United Kingdom
>Spike|hCoV-19/USA/UT-UPHL-2012997217/2020|2020-06-17|EPI_ISL_746388|Original|hCoV-
19^^Utah|Human|Utah Public Health Laboratory|Utah Public Health
Laboratory|Gallagher|USA
>Spike|hCoV-19/Scotland/GCVR-173DE2/2020|2020-04-06|EPI_ISL_459115|Original|hCoV-
19^^Scotland|Human|NHSGGC West of Scotland Specialist Virology Centre / MRC-
University of Glasgow Centre for Virus Research|Wellcome Sanger Institute for the
COVID-19 Genomics UK (COG-UK) consortium|Davies|United Kingdom
>Spike|hCoV-19/Wales/MILK-A05818/2020|2020-09-30|EPI_ISL_599955|Original|hCoV-
19^^Wales|Human|Lighthouse Lab in Milton Keynes|Wellcome Sanger Institute for the
COVID-19 Genomics UK (COG-UK) consortium|Davies|United Kingdom
>Spike|hCoV-19/England/C200047041/2020|2020-11-13|EPI_ISL_668442|Original|hCoV-
19^^England|Human|Respiratory Virus Unit|COVID-19 Genomics UK (COG-UK)
Consortium|Platt|United Kingdom
>Spike|hCoV-19/India/KA-IB14/2020|2020-06-18|EPI_ISL_508292|Original|hCoV-
19^^Karnataka|Human|Indian Institute of Science|National Institute of Biomedical
Genomics|Maitra|India
>Spike|hCoV-19/Netherlands/FR-RIVM-20554/2020|2020-12-
15|EPI_ISL_790641|Original|hCoV-19^^Friesland|Human|Dutch COVID-19 response
team|National Institute for Public Health and the Environment
(RIVM)|Meijer|Netherlands

>Spike|hCoV-19/England/CAMC-B3476D/2020|2020-11-05|EPI_ISL_659495|Original|hCoV-19^^England|Human|Lighthouse Lab in Cambridge|Wellcome Sanger Institute for the COVID-19 Genomics UK (COG-UK) Consortium|Davies|United Kingdom
>Spike|hCoV-19/Wales/PHWC-15E4BB/2020|2020-05-15|EPI_ISL_472817|Original|hCoV-19^^Wales|Human|Wales Specialist Virology Centre Sequencing lab|COVID-19 Genomics UK (COG-UK) Consortium|Moore|United Kingdom
>Spike|hCoV-19/Denmark/DCGC-12555/2020|2020-11-16|EPI_ISL_682736|Original|hCoV-19^^Sjaelland|Human|Department of Virus and Microbiological Special Diagnostics|Albertsen Lab|Michaelsen|Denmark
>Spike|hCoV-19/England/MILK-9760B1/2020|2020-08-17|EPI_ISL_552755|Original|hCoV-19^^England|Human|Lighthouse Lab in Milton Keynes|Wellcome Sanger Institute for the COVID-19 Genomics UK (COG-UK) consortium|Davies|United Kingdom
>Spike|hCoV-19/USA/ME-HETL-J0037/2020|2020-03-16|EPI_ISL_513449|Original|hCoV-19^^Maine|Human|Maine HETL|Tewhey Lab|Tewhey|USA
>Spike|hCoV-19/Wales/CVR4749/2020|2020-09-20|EPI_ISL_627339|Original|hCoV-19^^Wales|Human|West of Scotland Specialist Virology Centre|COVID-19 Genomics UK (COG-UK) Consortium|Robertson|United Kingdom
>Spike|hCoV-19/Italy/CAM-IZSM-87503-NIKY/2020|2020-10-23|EPI_ISL_738121|Original|hCoV-19^^Campania|Human|IZSM-U.O.C. Virologia|Istituto Zooprofilattico Sperimentale del Mezzogiorno|Viscardi|Italy
>Spike|hCoV-19/England/QEUH-7532A7/2020|2020-07-19|EPI_ISL_532980|Original|hCoV-19^^England|Human|Lighthouse Lab in Glasgow|Wellcome Sanger Institute for the COVID-19 Genomics UK (COG-UK) consortium|Davies|United Kingdom
>Spike|hCoV-19/USA/NY-NYUMC516/2020|2020-03-30|EPI_ISL_444633|Original|hCoV-19^^New York|Human|NYU Langone Health|Departments of Pathology and Medicine|Maurano|USA
>Spike|hCoV-19/Denmark/DCGC-2021/2020|2020-07-27|EPI_ISL_618231|Original|hCoV-19^^Hovedstaden|Human|Department of Virus and Microbiological Special Diagnostics|Albertsen lab|Michaelsen|Denmark
>Spike|hCoV-19/England/MILK-B3999F/2020|2020-11-02|EPI_ISL_679652|Original|hCoV-19^^England|Human|Oxford Viromics|COVID-19 Genomics UK (COG-UK) Consortium|Golubchik|United Kingdom
>Spike|hCoV-19/Scotland/EDB9320/2020|2020-11-05|EPI_ISL_665790|Original|hCoV-19^^Scotland|Human|Virology Department|COVID-19 Genomics UK (COG-UK) Consortium|McHugh|United Kingdom
>Spike|hCoV-19/England/EXET-13EAFB/2020|2020-12-13|EPI_ISL_763707|Original|hCoV-19^^England|Human|University of Exeter|COVID-19 Genomics UK (COG-UK) Consortium|Temperton|United Kingdom
>Spike|hCoV-19/USA/AZ-TG305807/2020|2020-04-28|EPI_ISL_694282|Original|hCoV-19^^Arizona|Human|AZ SPHL|TGen North|French|USA
>Spike|hCoV-19/USA/LA-EVTL1100/2020|2020-12-02|EPI_ISL_768370|Original|hCoV-19^^Louisiana|Human|LSUHS Emerging Viral Threat Laboratory|Microbial Genome Sequencing Center|Kamil|USA
>Spike|hCoV-19/USA/AZ-TG408125/2020|2020-06-12|EPI_ISL_694888|Original|hCoV-19^^Arizona|Human|AZ SPHL|TGen North|French|USA
>Spike|hCoV-19/Denmark/DCGC-18693/2020|2020-12-07|EPI_ISL_749256|Original|hCoV-19^^Sjaelland|Human|Department of Virus and Microbiological Special Diagnostics|Albertsen Lab|Michaelsen|Denmark
>Spike|hCoV-19/England/ALDP-49BB50/2020|2020-06-11|EPI_ISL_559322|Original|hCoV-19^^England|Human|Lighthouse Lab in Alderley Park|Wellcome Sanger Institute for the COVID-19 Genomics UK (COG-UK) consortium|Davies|United Kingdom
>Spike|hCoV-19/Netherlands/OV-EMC-38/2020|2020-04-17|EPI_ISL_523422|Original|hCoV-19^^Overijssel|Human|Dutch COVID-19 response team|Erasmus Medical Center|Oude Munnink|Netherlands
>Spike|hCoV-19/USA/TX-DSHS-1835/2020|2020-06-23|EPI_ISL_745265|Original|hCoV-19^^Texas|Human|Texas Department of State Health Services|Texas Department of State Health Services|Rahman|USA
>Spike|hCoV-19/England/QEUH-9DCB9A/2020|2020-09-21|EPI_ISL_581082|Original|hCoV-19^^England|Human|Lighthouse Lab in Glasgow|Wellcome Sanger Institute for the COVID-19 Genomics UK (COG-UK) consortium|Davies|United Kingdom

>Spike|hCoV-19/South Africa/KRISP-K001323/2020|2020-07-18|EPI_ISL_515808|Original|hCoV-19^^KwaZulu-Natal|Human|Medical Disagnostics Services (MDS)|KRISP|de Oliveira|South Africa
>Spike|hCoV-19/Sweden/20-13139/2020|2020-12-23|EPI_ISL_789025|Original|hCoV-19^^Stockholm|Human|ABC|The Public Health Agency of Sweden|Svartstr\u00f6m|Sweden
>Spike|hCoV-19/England/ALDP-94C45E/2020|2020-06-15|EPI_ISL_555848|Original|hCoV-19^^England|Human|Lighthouse Lab in Alderley Park|Wellcome Sanger Institute for the COVID-19 Genomics UK (COG-UK) consortium|Davies|United Kingdom
>Spike|hCoV-19/England/ALDP-4FF97E/2020|2020-06-23|EPI_ISL_559191|Original|hCoV-19^^England|Human|Lighthouse Lab in Alderley Park|Wellcome Sanger Institute for the COVID-19 Genomics UK (COG-UK) consortium|Davies|United Kingdom
>Spike|hCoV-19/Wales/PHWC-30FCE/2020|2020-04-09|EPI_ISL_446389|Original|hCoV-19^^Wales|Human|Wales Specialist Virology Centre|Public Health Wales Microbiology Cardiff|Moore|United Kingdom
>Spike|hCoV-19/England/EXET-1359F7/2020|2020-03-17|EPI_ISL_457124|Original|hCoV-19^^England|Human|University of Exeter|COVID-19 Genomics UK (COG-UK) Consortium|Temperton|United Kingdom
>Spike|hCoV-19/USA/TX-HMH-MCoV-19372/2020|2020-11-27|EPI_ISL_783801|Original|hCoV-19^^Texas|Human|Houston Methodist Hospital|Houston Methodist Hospital|Long|USA
>Spike|hCoV-19/England/ALDP-A6FFAE/2020|2020-10-15|EPI_ISL_608293|Original|hCoV-19^^England|Human|Lighthouse Lab in Alderley Park|Wellcome Sanger Institute for the COVID-19 Genomics UK (COG-UK) consortium|Davies|United Kingdom
>Spike|hCoV-19/USA/CA-ALSR-2984/2020|2020-08-30|EPI_ISL_635772|Original|hCoV-19^^California|Human|San Diego County Public Health Laboratory|Andersen lab at Scripps Research|Gangavarapu|USA
>Spike|hCoV-19/Wales/PHWC-48C81F/2020|2020-11-07|EPI_ISL_666473|Original|hCoV-19^^Wales|Human|Wales Specialist Virology Centre Sequencing lab|COVID-19 Genomics UK (COG-UK) Consortium|Moore|United Kingdom
>Spike|hCoV-19/Wales/PHWC-4993E1/2020|2020-12-03|EPI_ISL_727300|Original|hCoV-19^^Wales|Human|Originating lab|COVID-19 Genomics UK (COG-UK) Consortium|Moore|United Kingdom
>Spike|hCoV-19/Denmark/DCGC-13953/2020|2020-11-30|EPI_ISL_711473|Original|hCoV-19^^Syddanmark|Human|Department of Virus and Microbiological Special Diagnostics|Albertsen Lab|Michaelsen|Denmark
>Spike|hCoV-19/Costa Rica/INC-0141/2020|2020-09-03|EPI_ISL_770010|Original|hCoV-19^^San Jose|Human|Area De Salud Escazu (Coopesana)|Inciensa|Soto-Garita|Costa Rica
>Spike|hCoV-19/Singapore/1447/2020|2020-12-07|EPI_ISL_728183|Original|hCoV-19^^Singapore|Human|National Public Health Laboratory|National Public Health Laboratory|Mak|Singapore
>Spike|hCoV-19/USA/IL-UW-1518/2020|2020-09-16|EPI_ISL_541547|Original|hCoV-19^^Illinois|Human|University of Wisconsin-Madison AIDS Vaccine Research Laboratories|University of Wisconsin-Madison AIDS Vaccine Research Laboratories|Baczenas|USA
>Spike|hCoV-19/England/BRIS-1228F0/2020|2020-04-03|EPI_ISL_440771|Original|hCoV-19^^England|Human|PHE South West Regional Laboratory|Wellcome Sanger Institute for the COVID-19 Genomics UK (COG-UK) consortium|Amato|United Kingdom
>Spike|hCoV-19/Denmark/DCGC-5596/2020|2020-09-21|EPI_ISL_620921|Original|hCoV-19^^Sjaelland|Human|Department of Virus and Microbiological Special Diagnostics|Albertsen lab|Michaelsen|Denmark
>Spike|hCoV-19/England/ALDP-A6F2F0/2020|2020-10-15|EPI_ISL_606946|Original|hCoV-19^^England|Human|Lighthouse Lab in Alderley Park|Wellcome Sanger Institute for the COVID-19 Genomics UK (COG-UK) consortium|Davies|United Kingdom
>Spike|hCoV-19/Wales/PHWC-2E1CD/2020|2020-04-07|EPI_ISL_446069|Original|hCoV-19^^Wales|Human|Wales Specialist Virology Centre|Public Health Wales Microbiology Cardiff|Moore|United Kingdom
>Spike|hCoV-19/England/SHEF-CD588/2020|2020-05-26|EPI_ISL_475457|Original|hCoV-19^^England|Human|Virology Department|COVID-19 Genomics UK (COG-UK) Consortium|de Silva|United Kingdom

>Spike|hCoV-19/England/NORT-29E9EC/2020|2020-05-11|EPI_ISL_488176|Original|hCoV-
19^^England|Human|NU-OMICS DNA Sequencing research facility|Wellcome Sanger Institute
for the COVID-19 Genomics UK (COG-UK) consortium|Davies|United Kingdom
>Spike|hCoV-19/USA/WA-S2572/2020|2020-06-17|EPI_ISL_497204|Original|hCoV-
19^^Washington|Human|Washington State Department of Health|Seattle Flu Study|Lee|USA
>Spike|hCoV-19/Denmark/DCGC-17852/2020|2020-12-14|EPI_ISL_749600|Original|hCoV-
19^^Hovedstaden|Human|Department of Virus and Microbiological Special
Diagnostics|Albertsen Lab|Michaelsen|Denmark
>Spike|hCoV-19/England/QEUH-9C844B/2020|2020-09-11|EPI_ISL_567025|Original|hCoV-
19^^England|Human|Lighthouse Lab in Glasgow|Wellcome Sanger Institute for the COVID-
19 Genomics UK (COG-UK) consortium|Davies|United Kingdom
>Spike|hCoV-19/England/NORW-F6243/2020|2020-11-26|EPI_ISL_703733|Original|hCoV-
19^^England|Human|Quadram Institute Bioscience|COVID-19 Genomics UK (COG-UK)
Consortium|Alikhan|United Kingdom
>Spike|hCoV-19/Australia/VIC8751/2020|2020-07-28|EPI_ISL_565174|Original|hCoV-
19^^Victoria|Human|Victorian Infectious Diseases Reference Laboratory (VIDRL)|VIDRL
and MDU-PHL|Schultz|Australia
>Spike|hCoV-19/England/ALDP-AA39EC/2020|2020-10-22|EPI_ISL_624387|Original|hCoV-
19^^England|Human|Lighthouse Lab in Alderley Park|Wellcome Sanger Institute for the
COVID-19 Genomics UK (COG-UK) consortium|Davies|United Kingdom
>Spike|hCoV-19/Japan/PG-0089/2020|2020-03-05|EPI_ISL_479836|Original|hCoV-
19^^Hokkaido|Human|Sapporo City Institute of Public Health|Pathogen Genomics
Center|Sekizuka|Japan
>Spike|hCoV-19/Australia/VIC10787/2020|2020-07-30|EPI_ISL_563089|Original|hCoV-
19^^Victoria|Human|Microbiological Diagnostic Unit - Public Health Laboratory (MDU-
PHL)|MDU-PHL|Schultz|Australia
>Spike|hCoV-19/England/QEUH-AF4AE9/2020|2020-10-30|EPI_ISL_663166|Original|hCoV-
19^^England|Human|Lighthouse Lab in Glasgow|Wellcome Sanger Institute for the COVID-
19 Genomics UK (COG-UK) Consortium|Davies|United Kingdom
>Spike|hCoV-19/USA/CA-CZB-1043/2020|2020-04-01|EPI_ISL_437043|Original|hCoV-
19^^California|Human|County of Santa Clara Public Health|Chan-Zuckerberg
Biohub|Hao|USA
>Spike|hCoV-19/Denmark/DCGC-1084/2020|2020-05-11|EPI_ISL_614509|Original|hCoV-
19^^Hovedstaden|Human|Department of Virus and Microbiological Special
Diagnostics|Albertsen lab|Michaelsen|Denmark
>Spike|hCoV-19/England/QEUH-9B7940/2020|2020-09-08|EPI_ISL_590477|Original|hCoV-
19^^England|Human|Lighthouse Lab in Glasgow|Wellcome Sanger Institute for the COVID-
19 Genomics UK (COG-UK) consortium|Davies|United Kingdom
>Spike|hCoV-19/England/ALDP-B1DF35/2020|2020-11-01|EPI_ISL_723549|Original|hCoV-
19^^England|Human|Centre for Enzyme Innovation|COVID-19 Genomics UK (COG-UK)
Consortium|Robson|United Kingdom
>Spike|hCoV-19/Belgium/rega-0417908/2020|2020-04-17|EPI_ISL_734619|Original|hCoV-
19^^Mont-Saint-Guibert|Human|UZ Leuven|KU Leuven|Maes|Belgium
>Spike|hCoV-19/USA/CA-CZB-14130/2020|2020-05-23|EPI_ISL_739576|Original|hCoV-
19^^California|Human|Alameda County Public Health Lab|Chan-Zuckerberg Biohub|Kamm|USA
>Spike|hCoV-19/USA/FL-BPHL-1195/2020|2020-08-21|EPI_ISL_526681|Original|hCoV-
19^^Florida|Human|Florida Bureau of Public Health Laboratories|Florida Bureau of
Public Health Laboratories|Blanton|USA
>Spike|hCoV-19/Denmark/DCGC-2160/2020|2020-08-10|EPI_ISL_617459|Original|hCoV-
19^^Midtjylland|Human|Department of Virus and Microbiological Special
Diagnostics|Albertsen lab|Michaelsen|Denmark
>Spike|hCoV-19/Wales/PHWC-290F2/2020|2020-04-02|EPI_ISL_445481|Original|hCoV-
19^^Wales|Human|Wales Specialist Virology Centre|Public Health Wales Microbiology
Cardiff|Moore|United Kingdom
>Spike|hCoV-19/England/MILK-99D6A3/2020|2020-08-31|EPI_ISL_549937|Original|hCoV-
19^^England|Human|Lighthouse Lab in Milton Keynes|Wellcome Sanger Institute for the
COVID-19 Genomics UK (COG-UK) consortium|Davies|United Kingdom
>Spike|hCoV-19/Wales/PHWC-3563D/2020|2020-04-24|EPI_ISL_474456|Original|hCoV-
19^^Wales|Human|Originating lab|COVID-19 Genomics UK (COG-UK) Consortium|Moore|United
Kingdom

>Spike|hCoV-19/Australia/VIC11796/2020|2020-08-01|EPI_ISL_591824|Original|hCoV-19^^Victoria|Human|Victorian Infectious Diseases Reference Laboratory (VIDRL)|VIDRL and MDU-PHL|Schultz|Australia
>Spike|hCoV-19/England/CAMB-7B62C/2020|2020-04-03|EPI_ISL_441195|Original|hCoV-19^^England|Human|Department of Pathology|Wellcome Sanger Institute for the COVID-19 Genomics UK (COG-UK) consortium|Amato|United Kingdom
>Spike|hCoV-19/England/LIVE-99989/2020|2020-04-09|EPI_ISL_439779|Original|hCoV-19^^England|Human|Liverpool Clinical Laboratories|COVID-19 Genomics UK (COG-UK) Consortium|Haldenby|United Kingdom
>Spike|hCoV-19/Scotland/EDB5108/2020|2020-03-31|EPI_ISL_453188|Original|hCoV-19^^Scotland|Human|Virology Department|COVID-19 Genomics UK (COG-UK) Consortium|Williams|United Kingdom
>Spike|hCoV-19/USA/CA-QDX-1598/2020|2020-03-18|EPI_ISL_603899|Original|hCoV-19^^California|Human|Quest Diagnostics|Quest Diagnostics|Kagan|USA
>Spike|hCoV-19/England/204742210/2020|2020-00-00|EPI_ISL_649917|Original|hCoV-19^^England|Human|Respiratory Virus Unit|COVID-19 Genomics UK (COG-UK) Consortium|Platt|United Kingdom
>Spike|hCoV-19/Denmark/DCGC-1771/2020|2020-07-13|EPI_ISL_618210|Original|hCoV-19^^Hovedstaden|Human|Department of Virus and Microbiological Special Diagnostics|Albertsen lab|Michaelsen|Denmark
>Spike|hCoV-19/USA/TX-HMH-2608/2020|2020-06-05|EPI_ISL_545424|Original|hCoV-19^^Texas|Human|Houston Methodist Hospital|Houston Methodist Hospital|Long|USA
>Spike|hCoV-19/England/NORW-EC6F9/2020|2020-05-11|EPI_ISL_457553|Original|hCoV-19^^England|Human|Quadram Institute Bioscience|COVID-19 Genomics UK (COG-UK) Consortium|Alikhan|United Kingdom
>Spike|hCoV-19/South Africa/NHLS-UCT-GS-0070/2020|2020-03-27|EPI_ISL_640063|Original|hCoV-19^^Western Cape|Human|CoVid WC Cape Town Metro|NHLS/UCT|Iranzadeh|South Africa
>Spike|hCoV-19/USA/DC-HP00043/2020|2020-03-22|EPI_ISL_434639|Original|hCoV-19^^District of Columbia|Human|Johns Hopkins Hospital Department of Pathology|Johns Hopkins Hospital Department of Pathology|Ramakrishnan|USA
>Spike|hCoV-19/England/MILK-9E2AFE/2020|2020-09-21|EPI_ISL_581210|Original|hCoV-19^^England|Human|Lighthouse Lab in Milton Keynes|Wellcome Sanger Institute for the COVID-19 Genomics UK (COG-UK) consortium|Davies|United Kingdom
>Spike|hCoV-19/Australia/VIC16653/2020|2020-08-08|EPI_ISL_640989|Original|hCoV-19^^Victoria|Human|Microbiological Diagnostic Unit - Public Health Laboratory (MDU-PHL)|MDU-PHL|Schultz|Australia
>Spike|hCoV-19/Denmark/DCGC-2338/2020|2020-08-10|EPI_ISL_617676|Original|hCoV-19^^Hovedstaden|Human|Department of Virus and Microbiological Special Diagnostics|Albertsen lab|Michaelsen|Denmark
>Spike|hCoV-19/England/QEUH-C340F8/2020|2020-12-11|EPI_ISL_733998|Original|hCoV-19^^England|Human|Lighthouse Lab in Glasgow|Wellcome Sanger Institute for the COVID-19 Genomics UK (COG-UK) Consortium|Davies|United Kingdom
>Spike|hCoV-19/England/CAMC-A6560D/2020|2020-10-11|EPI_ISL_608504|Original|hCoV-19^^England|Human|Lighthouse Lab in Cambridge|Wellcome Sanger Institute for the COVID-19 Genomics UK (COG-UK) consortium|Davies|United Kingdom
>Spike|hCoV-19/Japan/PG-7482/2020|2020-08-00|EPI_ISL_688584|Original|hCoV-19^^Japan|Human|Tokyo Metropolitan Institute of Public Health|Pathogen Genomics Center|Sekizuka|Japan
>Spike|hCoV-19/USA/CA-QDX-1550/2020|2020-03-18|EPI_ISL_603796|Original|hCoV-19^^California|Human|Quest Diagnostics|Quest Diagnostics|Kagan|USA
>Spike|hCoV-19/Germany/HH-hpi-p2360/2020|2020-03-19|EPI_ISL_775825|Original|hCoV-19^^Hamburg|Human|University Medical Center Hamburg Eppendorf|Heinrich Pette Institute|Robitaille|Germany
>Spike|hCoV-19/England/EXET-13E439/2020|2020-12-06|EPI_ISL_724322|Original|hCoV-19^^England|Human|University of Exeter|COVID-19 Genomics UK (COG-UK) Consortium|Temperton|United Kingdom
>Spike|hCoV-19/USA/CA-ALSR-4557/2020|2020-07-08|EPI_ISL_667005|Original|hCoV-19^^California|Human|San Diego County Public Health Laboratory|Andersen lab at Scripps Research|Gangavarapu|USA

>Spike|hCoV-19/Wales/PHWC-48C994/2020|2020-11-04|EPI_ISL_665803|Original|hCoV-19^^Wales|Human|Wales Specialist Virology Centre Sequencing lab|COVID-19 Genomics UK (COG-UK) Consortium|Moore|United Kingdom
>Spike|hCoV-19/USA/UT-UPHL-2012859839/2020|2020-08-08|EPI_ISL_746049|Original|hCoV-19^^Utah|Human|Ginkgo Bioworks Clinical Laboratory|Utah Public Health Laboratory|Young|USA
>Spike|hCoV-19/USA/WI-UW-585/2020|2020-06-22|EPI_ISL_484960|Original|hCoV-19^^Wisconsin|Human|University of Wisconsin-Madison AIDS Vaccine Research Laboratories|University of Wisconsin-Madison AIDS Vaccine Research Laboratories|Baczenas|USA
>Spike|hCoV-19/Netherlands/NA_153/2020|2020-03-20|EPI_ISL_422688|Original|hCoV-19^^Netherlands|Human|Dutch COVID-19 response team|Erasmus Medical Center|Oude Munnink|Netherlands
>Spike|hCoV-19/USA/CA-CSMC121/2020|2020-04-05|EPI_ISL_475598|Original|hCoV-19^^California|Human|Cedars-Sinai Medical Center|Cedars-Sinai Medical Center|Zhang|USA
>Spike|hCoV-19/USA/NM-UNM-00507/2020|2020-05-08|EPI_ISL_542937|Original|hCoV-19^^New Mexico|Human|TriCore Reference Laboratories|Center for Global Health|Domman|USA
>Spike|hCoV-19/USA/TX-HMH-MCoV-15479/2020|2020-10-23|EPI_ISL_786963|Original|hCoV-19^^Texas|Human|Houston Methodist Hospital|Houston Methodist Hospital|Long|USA
>Spike|hCoV-19/England/ALDP-51F5CE/2020|2020-06-02|EPI_ISL_558908|Original|hCoV-19^^England|Human|Lighthouse Lab in Alderley Park|Wellcome Sanger Institute for the COVID-19 Genomics UK (COG-UK) consortium|Davies|United Kingdom
>Spike|hCoV-19/England/QEUH-B6827E/2020|2020-11-11|EPI_ISL_655262|Original|hCoV-19^^England|Human|Lighthouse Lab in Glasgow|Wellcome Sanger Institute for the COVID-19 Genomics UK (COG-UK) Consortium|Davies|United Kingdom
>Spike|hCoV-19/England/QEUH-AD196D/2020|2020-10-26|EPI_ISL_634601|Original|hCoV-19^^England|Human|Lighthouse Lab in Glasgow|Wellcome Sanger Institute for the COVID-19 Genomics UK (COG-UK) consortium|Davies|United Kingdom
>Spike|hCoV-19/Scotland/QEUH-9E4652/2020|2020-09-23|EPI_ISL_600974|Original|hCoV-19^^Scotland|Human|Lighthouse Lab in Glasgow|Wellcome Sanger Institute for the COVID-19 Genomics UK (COG-UK) consortium|Davies|United Kingdom
>Spike|hCoV-19/United Arab Emirates/0569/2020|2020-05-15|EPI_ISL_698728|Original|hCoV-19^^Abu Dhabi|Human|Group 42 (G42) Healthcare|G42 Healthcare|Ogrodzki|United Arab Emirates
>Spike|hCoV-19/South Africa/Tygerberg_337_CC2/2020|2020-10-09|EPI_ISL_660144|Original|hCoV-19^^Western Cape|Human|PathCare|National Health Laboratory Service (NHLS)|de Oliveira|South Africa
>Spike|hCoV-19/England/CAMC-B77C50/2020|2020-11-12|EPI_ISL_783521|Original|hCoV-19^^England|Human|Lighthouse Lab in Cambridge|Wellcome Sanger Institute for the COVID-19 Genomics UK (COG-UK) Consortium|Davies|United Kingdom
>Spike|hCoV-19/USA/CA-CHLA-PLM57057181/2020|2020-11-22|EPI_ISL_753410|Original|hCoV-19^^California|Human|Clinical virology Laboratory|Center for Personalized Medicine|Dien Bard|USA
>Spike|hCoV-19/North Macedonia/5003/2020|2020-06-25|EPI_ISL_677711|Original|hCoV-19^^Ohrid|Human|General Hospital - Ohrid|Research Center for Genetic Engineering and Biotechnology \Georgi D. Efremov\u201d|Dimovski|North Macedonia
>Spike|hCoV-19/England/QEUH-9FA60E/2020|2020-09-26|EPI_ISL_588233|Original|hCoV-19^^England|Human|Lighthouse Lab in Glasgow|Wellcome Sanger Institute for the COVID-19 Genomics UK (COG-UK) consortium|Davies|United Kingdom
>Spike|hCoV-19/England/204481795/2020|2020-10-28|EPI_ISL_653995|Original|hCoV-19^^England|Human|Respiratory Virus Unit|COVID-19 Genomics UK (COG-UK) Consortium|Platt|United Kingdom
>Spike|hCoV-19/USA/LA-GBCL-26492458/2020|2020-04-28|EPI_ISL_485036|Original|hCoV-19^^Louisiana|Human|River Road Testing Lab|Ginkgo Bioworks Clinical Laboratory|Smith|USA
>Spike|hCoV-19/England/ALDP-A872F2/2020|2020-10-14|EPI_ISL_606283|Original|hCoV-19^^England|Human|Lighthouse Lab in Alderley Park|Wellcome Sanger Institute for the COVID-19 Genomics UK (COG-UK) consortium|Davies|United Kingdom

>Spike|hCoV-19/USA/AZ-TG664524/2020|2020-04-20|EPI_ISL_696141|Original|hCoV-19^^Arizona|Human|Sonora Quest Laboratories|TGen North|French|USA
>Spike|hCoV-19/USA/TX-HMH-1822/2020|2020-05-15|EPI_ISL_542864|Original|hCoV-19^^Texas|Human|Houston Methodist Hospital|Houston Methodist Hospital|Long|USA
>Spike|hCoV-19/England/MILK-B8FA5B/2020|2020-11-13|EPI_ISL_674712|Original|hCoV-19^^England|Human|Lighthouse Lab in Milton Keynes|Wellcome Sanger Institute for the COVID-19 Genomics UK (COG-UK) Consortium|Davies|United Kingdom
>Spike|hCoV-19/USA/VA-DCLS-1793/2020|2020-09-16|EPI_ISL_644876|Original|hCoV-19^^Virginia|Human|Virginia DCLS|Virginia DCLS|St. Jacques|USA
>Spike|hCoV-19/Canada/QC-LSPQ-L00239501/2020|2020-03-26|EPI_ISL_536254|Original|hCoV-19^^Quebec|Human|H\u00f4pital du Suro\u00eet|Laboratoire de sant\u00e9 publique du Qu\u00e9bec|Moreira|Canada
>Spike|hCoV-19/Denmark/DCGC-2528/2020|2020-08-10|EPI_ISL_617798|Original|hCoV-19^^Hovedstaden|Human|Department of Virus and Microbiological Special Diagnostics|Albertsen lab|Michaelsen|Denmark
>Spike|hCoV-19/USA/WA-CDC-8374/2020|2020-03-03|EPI_ISL_594447|original|hCoV-19^^Washington|Human|Washington State Public Health Laboratories|Pathogen Discovery|Queen|USA
>Spike|hCoV-19/England/CAMC-A57EE8/2020|2020-10-09|EPI_ISL_597012|Original|hCoV-19^^England|Human|Lighthouse Lab in Cambridge|Wellcome Sanger Institute for the COVID-19 Genomics UK (COG-UK) consortium|Davies|United Kingdom
>Spike|hCoV-19/England/LIVE-DCB7F0/2020|2020-11-24|EPI_ISL_724429|Original|hCoV-19^^England|Human|Liverpool Clinical Laboratories|COVID-19 Genomics UK (COG-UK) Consortium|Haldenby|United Kingdom
>Spike|hCoV-19/England/ALDP-943F74/2020|2020-06-07|EPI_ISL_554767|Original|hCoV-19^^England|Human|Lighthouse Lab in Alderley Park|Wellcome Sanger Institute for the COVID-19 Genomics UK (COG-UK) consortium|Davies|United Kingdom
>Spike|hCoV-19/England/MILK-ACC033/2020|2020-10-21|EPI_ISL_629329|Original|hCoV-19^^England|Human|Lighthouse Lab in Milton Keynes|Wellcome Sanger Institute for the COVID-19 Genomics UK (COG-UK) consortium|Davies|United Kingdom
>Spike|hCoV-19/Denmark/DCGC-22418/2020|2020-12-14|EPI_ISL_757552|Original|hCoV-19^^Midtjylland|Human|Department of Virus and Microbiological Special Diagnostics|Albertsen Lab|Michaelsen|Denmark
>Spike|hCoV-19/Netherlands/NB-EMC-185/2020|2020-05-07|EPI_ISL_523308|Original|hCoV-19^^Noord Brabant|Human|Dutch COVID-19 response team|Erasmus Medical Center|Oude Munnink|Netherlands
>Spike|hCoV-19/Austria/CeMM0274/2020|2020-03-24|EPI_ISL_438101|Original|hCoV-19^^Vienna|Human|Center for Virology|Bergthaler laboratory|Bergthaler|Austria
>Spike|hCoV-19/Wales/PHWC-16AA6F/2020|2020-09-13|EPI_ISL_560145|Original|hCoV-19^^Wales|Human|Wales Specialist Virology Centre Sequencing lab|COVID-19 Genomics UK (COG-UK) Consortium|Moore|United Kingdom
>Spike|hCoV-19/Scotland/QEUH-9611F2/2020|2020-08-04|EPI_ISL_532118|Original|hCoV-19^^Scotland|Human|NHSGGC West of Scotland Specialist Virology Centre / MRC-University of Glasgow Centre for Virus Research|Wellcome Sanger Institute for the COVID-19 Genomics UK (COG-UK) consortium|Davies|United Kingdom
>Spike|hCoV-19/England/MILK-A45F26/2020|2020-10-06|EPI_ISL_598729|Original|hCoV-19^^England|Human|Lighthouse Lab in Milton Keynes|Wellcome Sanger Institute for the COVID-19 Genomics UK (COG-UK) consortium|Davies|United Kingdom
>Spike|hCoV-19/India/MP-DRDE115/2020|2020-03-31|EPI_ISL_476023|Original|hCoV-19^^Madhya Pradesh|Human|Defence Research & Development Establishment (DRDE)|Defence Research & Development Establishment (DRDE)|Dash|India
>Spike|hCoV-19/Denmark/DCGC-2086/2020|2020-08-03|EPI_ISL_618343|Original|hCoV-19^^Syddanmark|Human|Department of Virus and Microbiological Special Diagnostics|Albertsen lab|Michaelsen|Denmark
>Spike|hCoV-19/USA/MI-MDHHS-SC21646/2020|2020-03-31|EPI_ISL_516373|Original|hCoV-19^^Michigan|Human|Michigan Department of Health and Human Services|Michigan Department of Health and Human Services|Blankenship|USA
>Spike|hCoV-19/Denmark/DCGC-21624/2020|2020-12-21|EPI_ISL_758269|Original|hCoV-19^^Denmark|Human|Department of Virus and Microbiological Special Diagnostics|Albertsen Lab|Michaelsen|Denmark

>Spike|hCoV-19/USA/TX-HMH-MCoV-14546/2020|2020-10-09|EPI_ISL_787831|Original|hCoV-
19^^Texas|Human|Houston Methodist Hospital|Houston Methodist Hospital|Long|USA
>Spike|hCoV-19/England/ALDP-CB53F6/2020|2020-12-19|EPI_ISL_761799|Original|hCoV-
19^^England|Human|Lighthouse Lab in Alderley Park|Wellcome Sanger Institute for the
COVID-19 Genomics UK (COG-UK) Consortium|Davies|United Kingdom
>Spike|hCoV-19/Denmark/DCGC-1654/2020|2020-03-09|EPI_ISL_622403|Original|hCoV-
19^^Hovedstaden|Human|Department of Virus and Microbiological Special
Diagnostics|Albertsen lab|Michaelsen|Denmark
>Spike|hCoV-19/USA/MI-MDHHS-SC21753/2020|2020-07-22|EPI_ISL_529871|Original|hCoV-
19^^Michigan|Human|Michigan Department of Health and Human Services|Michigan
Department of Health and Human Services|Blankenship|USA
>Spike|hCoV-19/England/ALDP-97CF90/2020|2020-07-18|EPI_ISL_526318|Original|hCoV-
19^^England|Human|University of Birmingham|COVID-19 Genomics UK (COG-UK)
Consortium|Loman|United Kingdom
>Spike|hCoV-19/Canada/BC_03385157/2020|2020-04-00|EPI_ISL_466737|Original|hCoV-
19^^British Columbia|Human|BCCDC Public Health Laboratory|BCCDC Public Health
Laboratory|Harrigan|Canada
>Spike|hCoV-19/USA/WI-WSLH-200536/2020|2020-05-29|EPI_ISL_578673|Original|hCoV-
19^^Wisconsin|Human|Wisconsin State Laboratory of Hygiene Communicable Disease
Division|Wisconsin State Laboratory of Hygiene Communicable Disease
Division|Florek|USA
>Spike|hCoV-19/USA/WA-S2593/2020|2020-06-18|EPI_ISL_497225|Original|hCoV-
19^^Washington|Human|Washington State Department of Health|Seattle Flu Study|Lee|USA
>Spike|hCoV-19/England/ALDP-9EE096/2020|2020-05-06|EPI_ISL_589361|Original|hCoV-
19^^England|Human|Lighthouse Lab in Alderley Park|Wellcome Sanger Institute for the
COVID-19 Genomics UK (COG-UK) consortium|Davies|United Kingdom
>Spike|hCoV-19/USA/LA-GBCL-26629255/2020|2020-04-21|EPI_ISL_485238|Original|hCoV-
19^^Louisiana|Human|River Road Testing Lab|Ginkgo Bioworks Clinical
Laboratory|Smith|USA
>Spike|hCoV-19/England/ALDP-92B112/2020|2020-06-22|EPI_ISL_551389|Original|hCoV-
19^^England|Human|Lighthouse Lab in Alderley Park|Wellcome Sanger Institute for the
COVID-19 Genomics UK (COG-UK) consortium|Davies|United Kingdom
>Spike|hCoV-19/England/201140293/2020|2020-03-08|EPI_ISL_464720|Original|hCoV-
19^^England|Human|Respiratory Virus Unit|Respiratory Virus Unit|Platt|United Kingdom
>Spike|hCoV-19/England/QEUH-A223C9/2020|2020-10-01|EPI_ISL_587345|Original|hCoV-
19^^England|Human|Lighthouse Lab in Glasgow|Wellcome Sanger Institute for the COVID-
19 Genomics UK (COG-UK) consortium|Davies|United Kingdom
>Spike|hCoV-19/Wales/PHWC-2AE42/2020|2020-04-07|EPI_ISL_432269|Original|hCoV-
19^^Wales|Human|Wales Specialist Virology Centre|Public Health Wales Microbiology
Cardiff|Moore|United Kingdom
>Spike|hCoV-19/Denmark/DCGC-22136/2020|2020-12-14|EPI_ISL_758577|Original|hCoV-
19^^Sjaelland|Human|Department of Virus and Microbiological Special
Diagnostics|Albertsen Lab|Michaelsen|Denmark
>Spike|hCoV-19/USA/NY-NYUMC358/2020|2020-04-08|EPI_ISL_430385|Original|hCoV-19^^New
York|Human|NYU Langone Health|Departments of Pathology and Medicine|Maurano|USA
>Spike|hCoV-19/Northern Ireland/QEUH-95EA93/2020|2020-08-
09|EPI_ISL_532303|Original|hCoV-19^^Northern Ireland|Human|Lighthouse Lab in
Glasgow|Wellcome Sanger Institute for the COVID-19 Genomics UK (COG-UK)
consortium|Davies|United Kingdom
>Spike|hCoV-19/Denmark/DCGC-10831/2020|2020-11-02|EPI_ISL_669825|Original|hCoV-
19^^Midtjylland|Human|Department of Virus and Microbiological Special
Diagnostics|Albertsen Lab|Michaelsen|Denmark
>Spike|hCoV-19/South Africa/LR00133/2020|2020-07-20|EPI_ISL_514364|Original|hCoV-
19^^Gauteng|Human|National Institute for Communicable Diseases of the National Health
Laboratory Service|National Institute for Communicable Diseases of the National
Health Laboratory Service|Allam|South Africa
>Spike|hCoV-19/England/QEUH-B69BAF/2020|2020-11-11|EPI_ISL_655197|Original|hCoV-
19^^England|Human|Lighthouse Lab in Glasgow|Wellcome Sanger Institute for the COVID-
19 Genomics UK (COG-UK) Consortium|Davies|United Kingdom

>Spike|hCoV-19/Spain/PV-IBV-003262/2020|2020-03-17|EPI_ISL_481032|Original|hCoV-19^^Basque Country|Human|Servicio de Microbiolog\u00eda. Hospital Universitario Donostia. OSI Donostialdea. \u00c1rea de Enfermedades Infecciosas|SeqCOVID-SPAIN consortium/IBV(CSIC)|Comas|Spain
>Spike|hCoV-19/Poland/1154942/2020|2020-06-07|EPI_ISL_512299|Original|hCoV-19^^Pomorskie|Human|Hematology Laboratory|Department of Virology|Smura|Poland
>Spike|hCoV-19/Wales/QEUH-A8B777/2020|2020-10-16|EPI_ISL_647860|Original|hCoV-19^^Wales|Human|Lighthouse Lab in Glasgow|Wellcome Sanger Institute for the COVID-19 Genomics UK (COG-UK) Consortium|Davies|United Kingdom
>Spike|hCoV-19/Germany/BE-ChVir8991/2020|2020-09-21|EPI_ISL_729600|Original|hCoV-19^^Berlin|Human|Charit\u00e9 Universit\u00e4tsmedizin Berlin|Charit\u00e9 Universit\u00e4tsmedizin Berlin|Muehlemann|Germany
>Spike|hCoV-19/USA/FL-BPHL-1225/2020|2020-07-28|EPI_ISL_541198|Original|hCoV-19^^Florida|Human|Florida Bureau of Public Health Laboratories|Florida Bureau of Public Health Laboratories|Blanton|USA
>Spike|hCoV-19/Netherlands/Flevoland_3/2020|2020-03-31|EPI_ISL_455112|Original|hCoV-19^^Flevoland|Human|Dutch COVID-19 response team|Erasmus Medical Center|Oude Munnink|Netherlands
>Spike|hCoV-19/Australia/VIC2395/2020|2020-06-29|EPI_ISL_522112|Original|hCoV-19^^Victoria|Human|Microbiological Diagnostic Unit - Public Health Laboratory (MDU-PHL)|MDU-PHL|Schultz|Australia
>Spike|hCoV-19/England/CAMB-1ACCC5/2020|2020-04-23|EPI_ISL_489515|Original|hCoV-19^^England|Human|Department of Pathology|Wellcome Sanger Institute for the COVID-19 Genomics UK (COG-UK) consortium|Davies|United Kingdom
>Spike|hCoV-19/England/PRIN-254BB49/2020|2020-04-26|EPI_ISL_509442|Original|hCoV-19^^England|Human|The Princess Alexandra Hospital|Wellcome Sanger Institute for the COVID-19 Genomics UK (COG-UK) consortium|Davies|United Kingdom
>Spike|hCoV-19/Wales/PHWC-47E79B/2020|2020-09-26|EPI_ISL_595500|Original|hCoV-19^^Wales|Human|Originating lab|COVID-19 Genomics UK (COG-UK) Consortium|Moore|United Kingdom
>Spike|hCoV-19/USA/TX-HMH-MCoV-15790/2020|2020-07-10|EPI_ISL_785474|Original|hCoV-19^^Texas|Human|Houston Methodist Hospital|Houston Methodist Hospital|Long|USA
>Spike|hCoV-19/Netherlands/ZH-EMC-1161/2020|2020-11-23|EPI_ISL_763236|Original|hCoV-19^^Zuid Holland|Human|Dutch COVID-19 response team|Erasmus Medical Center|Oude Munnink|Netherlands
>Spike|hCoV-19/Netherlands/Gelderland_122/2020|2020-05-22|EPI_ISL_460883|Original|hCoV-19^^Gelderland|Human|Dutch COVID-19 response team|Erasmus Medical Center|Oude Munnink|Netherlands
>Spike|hCoV-19/England/QEUH-9DDFAC/2020|2020-09-21|EPI_ISL_599533|Original|hCoV-19^^England|Human|Lighthouse Lab in Glasgow|Wellcome Sanger Institute for the COVID-19 Genomics UK (COG-UK) consortium|Davies|United Kingdom
>Spike|hCoV-19/Japan/PG-1032/2020|2020-04-00|EPI_ISL_684642|Original|hCoV-19^^Japan|Human|Fukuoka Institute of Health and Environmental Sciences|Pathogen Genomics Center|Sekizuka|Japan
>Spike|hCoV-19/England/ALDP-B1C042/2020|2020-11-01|EPI_ISL_645931|Original|hCoV-19^^England|Human|Lighthouse Lab in Alderley Park|Wellcome Sanger Institute for the COVID-19 Genomics UK (COG-UK) Consortium|Davies|United Kingdom
>Spike|hCoV-19/Scotland/CVR32/2020|2020-03-13|EPI_ISL_425761|Original|hCoV-19^^Scotland|Human|West of Scotland Specialist Virology Centre|COVID-19 Genomics UK (COG-UK) Consortium|Robertson|United Kingdom
>Spike|hCoV-19/India/TG-CDFD-C1486/2020|2020-06-10|EPI_ISL_528564|Original|hCoV-19^^Telangana|Human|National Genomics Core-Center for DNA Fingerprinting and Diagnostics|National Genomics Core- Center for DNA Fingerprinting and Diagnostics (NGC-CDFD)- DBT's PAN-INDIA-1000 Genome consortium|Vashisht|India
>Spike|hCoV-19/England/QEUH-9E52B7/2020|2020-09-23|EPI_ISL_579886|Original|hCoV-19^^England|Human|Lighthouse Lab in Glasgow|Wellcome Sanger Institute for the COVID-19 Genomics UK (COG-UK) consortium|Davies|United Kingdom
>Spike|hCoV-19/Finland/S20AE7/2020|2020-10-11|EPI_ISL_737076|Original|hCoV-19^^Uusimaa|Human|Department of Virology and Immunology|Department of Virology|Smura|Finland

>Spike|hCoV-19/Australia/VIC11678/2020|2020-07-22|EPI_ISL_563456|Original|hCoV-19^^Victoria|Human|Microbiological Diagnostic Unit - Public Health Laboratory (MDU-PHL)|MDU-PHL|Schultz|Australia
>Spike|hCoV-19/USA/TX-HMH-MCoV-13721/2020|2020-08-18|EPI_ISL_788814|Original|hCoV-19^^Texas|Human|Houston Methodist Hospital|Houston Methodist Hospital|Long|USA
>Spike|hCoV-19/Russia/MOS-CRIE-13679342/2020|2020-11-09|EPI_ISL_745251|Original|hCoV-19^^Moscow|Human|Molecular diagnostic laboratory of Federal Budget Institution of Science \Central Research Institute of Epidemiology\ of The Federal Service on Customers' Rights Protection and Human Well-being Surveillance|Group of Genomics and Postgenomic Technologies of Central Research Institute of Epidemilolog
>y|Korneenko|Russia
>Spike|hCoV-19/England/MILK-631F9A/2020|2020-07-05|EPI_ISL_556195|Original|hCoV-19^^England|Human|Lighthouse Lab in Milton Keynes|Wellcome Sanger Institute for the COVID-19 Genomics UK (COG-UK) consortium|Davies|United Kingdom
>Spike|hCoV-19/England/MILK-A79FFC/2020|2020-10-15|EPI_ISL_606821|Original|hCoV-19^^England|Human|Lighthouse Lab in Milton Keynes|Wellcome Sanger Institute for the COVID-19 Genomics UK (COG-UK) consortium|Davies|United Kingdom
>Spike|hCoV-19/England/CAMC-C44EFB/2020|2020-12-10|EPI_ISL_719160|Original|hCoV-19^^England|Human|Lighthouse Lab in Cambridge|Wellcome Sanger Institute for the COVID-19 Genomics UK (COG-UK) Consortium|Davies|United Kingdom
>Spike|hCoV-19/Spain/CT-IBV-98006563/2020|2020-06-30|EPI_ISL_541076|Original|hCoV-19^^Catalunya|Human|Hospital de la Santa Creu i Sant Pau. Servicio de Microbiolog\u00eda|SeqCOVID-SPAIN consortium/Institute of Biomedicine of Valencia|Comas|Spain
>Spike|hCoV-19/Denmark/DCGC-15658/2020|2020-11-23|EPI_ISL_713944|Original|hCoV-19^^Midtjylland|Human|Department of Virus and Microbiological Special Diagnostics|Albertsen Lab|Michaelsen|Denmark
>Spike|hCoV-19/England/MILK-B04EF8/2020|2020-10-24|EPI_ISL_650631|Original|hCoV-19^^England|Human|Department of Pathology|COVID-19 Genomics UK (COG-UK) Consortium|Goodfellow|United Kingdom
>Spike|hCoV-19/Pakistan/KP-RMI-01/2020|2020-04-01|EPI_ISL_632908|Original|hCoV-19^^Pakistan|Human|Genomic Sciences|Genomic Sciences|Haider|Pakistan
>Spike|hCoV-19/England/CAMB-1B3CE9/2020|2020-05-29|EPI_ISL_461583|Original|hCoV-19^^England|Human|Department of Pathology|COVID-19 Genomics UK (COG-UK) Consortium|Meredith|United Kingdom
>Spike|hCoV-19/Denmark/DCGC-3223/2020|2020-08-31|EPI_ISL_616883|Original|hCoV-19^^Syddanmark|Human|Department of Virus and Microbiological Special Diagnostics|Albertsen lab|Michaelsen|Denmark
>Spike|hCoV-19/England/CAMB-1AED57/2020|2020-05-07|EPI_ISL_452857|Original|hCoV-19^^England|Human|Department of Pathology|COVID-19 Genomics UK (COG-UK) Consortium|Meredith|United Kingdom
>Spike|hCoV-19/England/CAMC-B3666E/2020|2020-11-05|EPI_ISL_645268|Original|hCoV-19^^England|Human|Lighthouse Lab in Cambridge|Wellcome Sanger Institute for the COVID-19 Genomics UK (COG-UK) Consortium|Davies|United Kingdom
>Spike|hCoV-19/Hungary/MBL-465/2020|2020-03-27|EPI_ISL_435423|Original|hCoV-19^^Balassagyarmat|Human|Virological Research Group|Bioinformatics Research Group|Herczeg|Hungary
>Spike|hCoV-19/Denmark/DCGC-12997/2020|2020-12-07|EPI_ISL_712517|Original|hCoV-19^^Hovedstaden|Human|Department of Virus and Microbiological Special Diagnostics|Albertsen Lab|Michaelsen|Denmark
>Spike|hCoV-19/England/CAMC-B20B1D/2020|2020-11-02|EPI_ISL_647681|Original|hCoV-19^^England|Human|Lighthouse Lab in Cambridge|Wellcome Sanger Institute for the COVID-19 Genomics UK (COG-UK) Consortium|Davies|United Kingdom
>Spike|hCoV-19/Denmark/DCGC-2305/2020|2020-08-10|EPI_ISL_617730|Original|hCoV-19^^Hovedstaden|Human|Department of Virus and Microbiological Special Diagnostics|Albertsen lab|Michaelsen|Denmark
>Spike|hCoV-19/England/CAMB-74F0A/2020|2020-03-16|EPI_ISL_440418|Original|hCoV-19^^England|Human|Department of Pathology|Wellcome Sanger Institute for the COVID-19 Genomics UK (COG-UK) consortium|Amato|United Kingdom

>Spike|hCoV-19/Japan/PG-4586/2020|2020-08-00|EPI_ISL_689723|Original|hCoV-19^^Japan|Human|Pathogen Genomics Center|Pathogen Genomics Center|Sekizuka|Japan
>Spike|hCoV-19/Iceland/108/2020|2020-03-11|EPI_ISL_417708|Original|hCoV-19^^Reykjavik|Human|The National University Hospital of Iceland|deCODE genetics|J\u00f3nsson|Iceland
>Spike|hCoV-19/England/CAMB-7FD6F/2020|2020-00-00|EPI_ISL_444409|Original|hCoV-19^^England|Human|Department of Pathology|COVID-19 Genomics UK (COG-UK) Consortium|Meredith|United Kingdom
>Spike|hCoV-19/England/LIVE-A5C90/2020|2020-03-17|EPI_ISL_499657|Original|hCoV-19^^England|Human|Liverpool Clinical Laboratories|COVID-19 Genomics UK (COG-UK) Consortium|Haldenby|United Kingdom
>Spike|hCoV-19/Wales/PHWC-2C1B0/2020|2020-04-04|EPI_ISL_474287|Original|hCoV-19^^Wales|Human|Originating lab|COVID-19 Genomics UK (COG-UK) Consortium|Moore|United Kingdom
>Spike|hCoV-19/USA/TX-HMH-MCoV-15341/2020|2020-10-19|EPI_ISL_786825|Original|hCoV-19^^Texas|Human|Houston Methodist Hospital|Houston Methodist Hospital|Long|USA
>Spike|hCoV-19/Australia/VIC14661/2020|2020-08-06|EPI_ISL_592940|Original|hCoV-19^^Victoria|Human|Microbiological Diagnostic Unit - Public Health Laboratory (MDU-PHL)|MDU-PHL|Schultz|Australia
>Spike|hCoV-19/France/PAC-IHU-0369/2020|2020-03-25|EPI_ISL_569476|Original|hCoV-19^^Provence-Alpes-C\u00f4te-d\u2019Azur|Human|MEPHI|MEPHI|Levasseur|France
>Spike|hCoV-19/USA/TX-HMH-7569/2020|2020-06-25|EPI_ISL_546860|Original|hCoV-19^^Texas|Human|Houston Methodist Hospital|Houston Methodist Hospital|Long|USA
>Spike|hCoV-19/England/ALDP-C3A20B/2020|2020-12-09|EPI_ISL_731385|Original|hCoV-19^^England|Human|Lighthouse Lab in Alderley Park|Wellcome Sanger Institute for the COVID-19 Genomics UK (COG-UK) Consortium|Davies|United Kingdom
>Spike|hCoV-19/Wales/PHWC-4A2118/2020|2020-12-14|EPI_ISL_743848|Original|hCoV-19^^Wales|Human|Wales Specialist Virology Centre Sequencing lab|COVID-19 Genomics UK (COG-UK) Consortium|Moore|United Kingdom
>Spike|hCoV-19/Ireland/CE-NVRL-AIIDV1105v2/2020|2020-04-26|EPI_ISL_644269|Original|hCoV-19^^Clare|Human|CEPHR / Vincent's Hospital|Irish Coronavirus Sequencing Consortium - National Virus Reference Laboratory|Gonzalez|Ireland
>Spike|hCoV-19/Vietnam/40127/2020|2020-07-00|EPI_ISL_759877|Original|hCoV-19^^Vietnam|Human|National Influenza Center|National Influenza Center|Ung|Vietnam
>Spike|hCoV-19/England/SHEF-C9BA8/2020|2020-03-21|EPI_ISL_479237|Original|hCoV-19^^England|Human|Virology Department|COVID-19 Genomics UK (COG-UK) Consortium|de Silva|United Kingdom
>Spike|hCoV-19/Australia/VIC8067/2020|2020-08-18|EPI_ISL_564829|Original|hCoV-19^^Victoria|Human|Microbiological Diagnostic Unit - Public Health Laboratory (MDU-PHL)|MDU-PHL|Schultz|Australia
>Spike|hCoV-19/India/TG-CCMB-J131/2020|2020-04-01|EPI_ISL_447566|Original|hCoV-19^^Telangana|Human|CSIR-Centre for Cellular and Molecular Biology|CSIR-Centre for Cellular and Molecular Biology|Banu|India
>Spike|hCoV-19/England/QEUH-B2CD1B/2020|2020-11-03|EPI_ISL_659922|Original|hCoV-19^^England|Human|Lighthouse Lab in Glasgow|Wellcome Sanger Institute for the COVID-19 Genomics UK (COG-UK) Consortium|Davies|United Kingdom
>Spike|hCoV-19/England/LIVE-DCB417/2020|2020-11-22|EPI_ISL_724403|Original|hCoV-19^^England|Human|Liverpool Clinical Laboratories|COVID-19 Genomics UK (COG-UK) Consortium|Haldenby|United Kingdom
>Spike|hCoV-19/Denmark/DCGC-7222/2020|2020-10-12|EPI_ISL_619088|Original|hCoV-19^^Midtjylland|Human|Department of Virus and Microbiological Special Diagnostics|Albertsen lab|Michaelsen|Denmark
>Spike|hCoV-19/England/LIVE-A56A1/2020|2020-03-19|EPI_ISL_499967|Original|hCoV-19^^England|Human|Liverpool Clinical Laboratories|COVID-19 Genomics UK (COG-UK) Consortium|Haldenby|United Kingdom
>Spike|hCoV-19/Japan/PG-0686/2020|2020-00-00|EPI_ISL_684296|Original|hCoV-19^^Japan|Human|Pathogen Genomics Center|Pathogen Genomics Center|Sekizuka|Japan

>Spike|hCoV-19/USA/CA-CZB-13479/2020|2020-10-21|EPI_ISL_672525|Original|hCoV-19^^California|Human|Madera County Department of Public Health|Chan-Zuckerberg Biohub|Bhatt|USA
>Spike|hCoV-19/USA/LA-EVTL1201/2020|2020-12-12|EPI_ISL_768470|Original|hCoV-19^^Louisiana|Human|LSUHS Emerging Viral Threat Laboratory|Microbial Genome Sequencing Center|Kamil|USA
>Spike|hCoV-19/Wales/PHWC-4931B0/2020|2020-11-28|EPI_ISL_725850|Original|hCoV-19^^Wales|Human|Originating lab|COVID-19 Genomics UK (COG-UK) Consortium|Moore|United Kingdom
>Spike|hCoV-19/mink/Netherlands/NB-EMC-55-20/2020|2020-09-23|EPI_ISL_632380|Original|hCoV-19^^Noord Brabant|Neovison vison|Dutch COVID-19 response team|Erasmus Medical Center|Oude Munnink|Netherlands
>Spike|hCoV-19/USA/NY-Wadsworth-236998-01/2020|2020-10-05|EPI_ISL_734317|Original|hCoV-19^^New York|Human|Wadsworth Center|Wadsworth Center|Lasek-Nesselquist|USA
>Spike|hCoV-19/Switzerland/ZG-ETHZ-150018plus/2020|2020-06-08|EPI_ISL_483657|Original|hCoV-19^^Zug|Human|Viollier AG|Department of Biosystems Science and Engineering|Nadeau|Switzerland
>Spike|hCoV-19/England/NOTT-114C04/2020|2020-10-10|EPI_ISL_595234|Original|hCoV-19^^England|Human|Queens Medical Centre|COVID-19 Genomics UK (COG-UK) Consortium|Loose|United Kingdom
>Spike|hCoV-19/Kenya/C3457/2020|2020-04-30|EPI_ISL_568715|Original|hCoV-19^^Mombasa|Human|KEMRI-Wellcome Trust Research Programme/KEMRI-CGMR-C Kilifi|KEMRI-Wellcome Trust Research Programme/KEMRI-CGMR-C Kilifi|Githinji|Kenya
>Spike|hCoV-19/USA/UT-UPHL-2012535481/2020|2020-08-15|EPI_ISL_746130|Original|hCoV-19^^Utah|Human|Ginkgo Bioworks Clinical Laboratory|Utah Public Health Laboratory|Young|USA
>Spike|hCoV-19/USA/NM-UNM-00317/2020|2020-05-13|EPI_ISL_508097|Original|hCoV-19^^New Mexico|Human|New Mexico Department of Health Scientific Laboratory Division|Center for Global Health|Domman|USA
>Spike|hCoV-19/USA/TX-HMH-MCoV-12940/2020|2020-08-18|EPI_ISL_788092|Original|hCoV-19^^Texas|Human|Houston Methodist Hospital|Houston Methodist Hospital|Long|USA
>Spike|hCoV-19/USA/MI-MDHHS-SC21457/2020|2020-04-08|EPI_ISL_507711|Original|hCoV-19^^Michigan|Human|Michigan Department of Health and Human Services|Michigan Department of Health and Human Services|Blankenship|USA
>Spike|hCoV-19/Denmark/DCGC-8639/2020|2020-10-26|EPI_ISL_625987|Original|hCoV-19^^Syddanmark|Human|Department of Virus and Microbiological Special Diagnostics|Albertsen lab|Michaelsen|Denmark
>Spike|hCoV-19/Australia/NSW1062/2020|2020-07-28|EPI_ISL_593671|Original|hCoV-19^^New South Wales|Human|Pathology West - NSW Health Pathology|NSW Health Pathology - Institute of Clinical Pathology and Medical Research; Westmead Hospital; University of Sydney|Gall|Australia
>Spike|hCoV-19/USA/UT-UPHL-2009200/2020|2020-05-27|EPI_ISL_525182|Original|hCoV-19^^Utah|Human|Utah Public Health Laboratory|Utah Public Health Laboratory|Young|USA
>Spike|hCoV-19/Japan/PG-7113/2020|2020-08-00|EPI_ISL_688218|Original|hCoV-19^^Japan|Human|Pathogen Genomics Center|Pathogen Genomics Center|Sekizuka|Japan
>Spike|hCoV-19/Belgium/ULG-10915/2020|2020-11-24|EPI_ISL_678456|Original|hCoV-19^^Li\u00e8ge|Human|Department of Clinical Microbiology|GIGA Medical Genomics|Durkin|Belgium
>Spike|hCoV-19/South Africa/KRISP-K002486/2020|2020-08-07|EPI_ISL_535393|Original|hCoV-19^^KwaZulu-Natal|Human|NHLS-IALCH|KRISP|de Oliveira|South Africa
>Spike|hCoV-19/Australia/VIC8196/2020|2020-08-13|EPI_ISL_564894|Original|hCoV-19^^Victoria|Human|Microbiological Diagnostic Unit - Public Health Laboratory (MDU-PHL)|MDU-PHL|Schultz|Australia
>Spike|hCoV-19/England/CAMC-BBC800/2020|2020-11-20|EPI_ISL_705598|Original|hCoV-19^^England|Human|Quadram Institute Bioscience|COVID-19 Genomics UK (COG-UK) Consortium|Alikhan|United Kingdom
>Spike|hCoV-19/USA/WI-UW-2534/2020|2020-12-29|EPI_ISL_779365|Original|hCoV-19^^Wisconsin|Human|University of Wisconsin-Madison AIDS Vaccine Research

Laboratories|University of Wisconsin-Madison AIDS Vaccine Research Laboratories|Moreno|USA
>Spike|hCoV-19/Scotland/QEUH-96A95B/2020|2020-08-17|EPI_ISL_531382|Original|hCoV-19^^Scotland|Human|NHSGGC West of Scotland Specialist Virology Centre / MRC-University of Glasgow Centre for Virus Research|Wellcome Sanger Institute for the COVID-19 Genomics UK (COG-UK) consortium|Davies|United Kingdom
>Spike|hCoV-19/England/LIVE-9B037/2020|2020-03-28|EPI_ISL_449404|Original|hCoV-19^^England|Human|Liverpool Clinical Laboratories|COVID-19 Genomics UK (COG-UK) Consortium|Haldenby|United Kingdom
>Spike|hCoV-19/Australia/VIC8831/2020|2020-07-27|EPI_ISL_565222|Original|hCoV-19^^Victoria|Human|Victorian Infectious Diseases Reference Laboratory (VIDRL)|VIDRL and MDU-PHL|Schultz|Australia
>Spike|hCoV-19/Wales/PHWC-485AE3/2020|2020-10-23|EPI_ISL_628251|Original|hCoV-19^^Wales|Human|Wales Specialist Virology Centre Sequencing lab|COVID-19 Genomics UK (COG-UK) Consortium|Moore|United Kingdom
>Spike|hCoV-19/England/BRIS-12573C/2020|2020-03-28|EPI_ISL_443779|Original|hCoV-19^^England|Human|PHE South West Regional Laboratory|Wellcome Sanger Institute for the COVID-19 Genomics UK (COG-UK) consortium|Amato|United Kingdom
>Spike|hCoV-19/USA/VA-DCLS-2144/2020|2020-10-28|EPI_ISL_677163|Original|hCoV-19^^Virginia|Human|Virginia Division of Consolidated Laboratory Services|Virginia Division of Consolidated Laboratory Services|St. Jacques|USA
>Spike|hCoV-19/England/MILK-A194D1/2020|2020-10-03|EPI_ISL_612511|Original|hCoV-19^^England|Human|Wales Specialist Virology Centre Sequencing lab|COVID-19 Genomics UK (COG-UK) Consortium|Moore|United Kingdom
>Spike|hCoV-19/India/MH-ACTREC-516/2020|2020-08-19|EPI_ISL_700163|Original|hCoV-19^^Maharashtra|Human|Hematopathology Laboratory|Hematopathology Laboratory|Patkar|India
>Spike|hCoV-19/England/QEUH-9B7E50/2020|2020-09-07|EPI_ISL_590672|Original|hCoV-19^^England|Human|Lighthouse Lab in Glasgow|Wellcome Sanger Institute for the COVID-19 Genomics UK (COG-UK) consortium|Davies|United Kingdom
>Spike|hCoV-19/England/MILK-A79D4D/2020|2020-10-15|EPI_ISL_606711|Original|hCoV-19^^England|Human|Lighthouse Lab in Milton Keynes|Wellcome Sanger Institute for the COVID-19 Genomics UK (COG-UK) consortium|Davies|United Kingdom
>Spike|hCoV-19/Switzerland/FR-ETHZ-350302/2020|2020-10-30|EPI_ISL_728931|Original|hCoV-19^^Fribourg|Human|Viollier AG|Department of Biosystems Science and Engineering|Nadeau|Switzerland
>Spike|hCoV-19/Costa Rica/INC-0032/2020|2020-03-15|EPI_ISL_491444|Original|hCoV-19^^San Jose|Human|Area de Salud Escazu (Coopesana)|Inciensa|Brenes Porras|Costa Rica
>Spike|hCoV-19/USA/TX-HMH-MCoV-19588/2020|2020-11-21|EPI_ISL_783959|Original|hCoV-19^^Texas|Human|Houston Methodist Hospital|Houston Methodist Hospital|Long|USA
>Spike|hCoV-19/USA/CA-CZB-10952/2020|2020-09-16|EPI_ISL_583036|Original|hCoV-19^^California|Human|Orange County Public Health Lab|Chan-Zuckerberg Biohub|Bhatt|USA
>Spike|hCoV-19/USA/FL-BPHL-0911/2020|2020-06-30|EPI_ISL_517801|Original|hCoV-19^^Florida|Human|Florida Bureau of Public Health Laboratories|Florida Bureau of Public Health Laboratories|Blanton|USA
>Spike|hCoV-19/Belgium/rega-10131440/2020|2020-10-13|EPI_ISL_738492|Original|hCoV-19^^Nossegem|Human|UZ Leuven|KU Leuven|Maes|Belgium
>Spike|hCoV-19/England/CAMB-72467/2020|2020-03-24|EPI_ISL_439621|Original|hCoV-19^^England|Human|Department of Pathology|Wellcome Sanger Institute for the COVID-19 Genomics UK (COG-UK) consortium|Amato|United Kingdom
>Spike|hCoV-19/Denmark/DCGC-6985/2020|2020-10-12|EPI_ISL_619831|Original|hCoV-19^^Hovedstaden|Human|Department of Virus and Microbiological Special Diagnostics|Albertsen lab|Michaelsen|Denmark
>Spike|hCoV-19/Belgium/ULG-0190/2020|2020-03-23|EPI_ISL_447130|Original|hCoV-19^^Liege|Human|Department of Clinical Microbiology|GIGA Medical Genomics|Durkin|Belgium
>Spike|hCoV-19/USA/MA-QDX-2015/2020|2020-03-17|EPI_ISL_604345|Original|hCoV-19^^Massachusetts|Human|Quest Diagnostics|Quest Diagnostics|Kagan|USA

>Spike|hCoV-19/Denmark/DCGC-15562/2020|2020-11-23|EPI_ISL_713895|Original|hCoV-19^^Hovedstaden|Human|Department of Virus and Microbiological Special Diagnostics|Albertsen Lab|Michaelsen|Denmark
>Spike|hCoV-19/England/20124030003/2020|2020-03-17|EPI_ISL_418707|Original|hCoV-19^^England|Human|Respiratory Virus Unit|Respiratory Virus Unit|Platt|United Kingdom
>Spike|hCoV-19/England/CAMC-B7BD68/2020|2020-11-12|EPI_ISL_724055|Original|hCoV-19^^England|Human|Oxford Viromics|COVID-19 Genomics UK (COG-UK) Consortium|Golubchik|United Kingdom
>Spike|hCoV-19/England/ALDP-94DBA3/2020|2020-06-17|EPI_ISL_555542|Original|hCoV-19^^England|Human|Lighthouse Lab in Alderley Park|Wellcome Sanger Institute for the COVID-19 Genomics UK (COG-UK) consortium|Davies|United Kingdom
>Spike|hCoV-19/South Africa/R13287-20/2020|2020-07-02|EPI_ISL_622928|Original|hCoV-19^^KwaZulu-Natal|Human|National Institute for Communicable Diseases of the National Health Laboratory Service|National Institute for Communicable Diseases of the National Health Laboratory Service|Allam|South Africa
>Spike|hCoV-19/DRC/300/2020|2020-03-22|EPI_ISL_420841|Original|hCoV-19^^Sud Kivu|Human|Viral Respiratory Lab|Pathogen Sequencing Lab|Pratt|Democratic Republic of the Congo
>Spike|hCoV-19/Scotland/QEUH-AACBB0/2020|2020-10-21|EPI_ISL_652903|Original|hCoV-19^^Scotland|Human|Oxford Viromics|COVID-19 Genomics UK (COG-UK) Consortium|Golubchik|United Kingdom
>Spike|hCoV-19/Scotland/EDB3025/2020|2020-04-03|EPI_ISL_488979|Original|hCoV-19^^Scotland|Human|Virology Department|Wellcome Sanger Institute for the COVID-19 Genomics UK (COG-UK) consortium|Davies|United Kingdom
>Spike|hCoV-19/Denmark/DCGC-3753/2020|2020-04-20|EPI_ISL_622627|Original|hCoV-19^^Hovedstaden|Human|Department of Virus and Microbiological Special Diagnostics|Albertsen lab|Michaelsen|Denmark
>Spike|hCoV-19/England/QEUH-9AEFFF/2020|2020-09-03|EPI_ISL_573525|Original|hCoV-19^^England|Human|University College London|COVID-19 Genomics UK (COG-UK) Consortium|Roy|United Kingdom
>Spike|hCoV-19/England/ALDP-49A30E/2020|2020-06-08|EPI_ISL_559394|Original|hCoV-19^^England|Human|Lighthouse Lab in Alderley Park|Wellcome Sanger Institute for the COVID-19 Genomics UK (COG-UK) consortium|Davies|United Kingdom
>Spike|hCoV-19/Denmark/DCGC-6385/2020|2020-09-28|EPI_ISL_622322|Original|hCoV-19^^Sjaelland|Human|Department of Virus and Microbiological Special Diagnostics|Albertsen lab|Michaelsen|Denmark
>Spike|hCoV-19/Denmark/DCGC-8963/2020|2020-10-26|EPI_ISL_625869|Original|hCoV-19^^Nordjylland|Human|Department of Virus and Microbiological Special Diagnostics|Albertsen lab|Michaelsen|Denmark
>Spike|hCoV-19/England/ALDP-A6DF64/2020|2020-10-14|EPI_ISL_607710|Original|hCoV-19^^England|Human|Lighthouse Lab in Alderley Park|Wellcome Sanger Institute for the COVID-19 Genomics UK (COG-UK) consortium|Davies|United Kingdom
>Spike|hCoV-19/England/ALDP-A84A2A/2020|2020-10-18|EPI_ISL_610440|Original|hCoV-19^^England|Human|Lighthouse Lab in Alderley Park|Wellcome Sanger Institute for the COVID-19 Genomics UK (COG-UK) consortium|Davies|United Kingdom
>Spike|hCoV-19/Scotland/EDB740/2020|2020-04-02|EPI_ISL_705796|Original|hCoV-19^^Scotland|Human|Virology Department|COVID-19 Genomics UK (COG-UK) Consortium|McHugh|United Kingdom
>Spike|hCoV-19/Palestine/46/2020|2020-08-17|EPI_ISL_596529|Original|hCoV-19^^Jerusalem|Human|Palestinian Ministry of Health|Molecular Genetics Lab|van Dorp|Palestine
>Spike|hCoV-19/USA/VA-DCLS-1170/2020|2020-07-18|EPI_ISL_525197|Original|hCoV-19^^Virginia|Human|Virginia DCLS|Virginia DCLS|St. Jacques|USA
>Spike|hCoV-19/Scotland/GCVR-1724C0/2020|2020-03-30|EPI_ISL_469964|Original|hCoV-19^^Scotland|Human|NHSGGC West of Scotland Specialist Virology Centre / MRC-University of Glasgow Centre for Virus Research|Wellcome Sanger Institute for the COVID-19 Genomics UK (COG-UK) consortium|Davies|United Kingdom
>Spike|hCoV-19/England/MILK-ABBD8B/2020|2020-10-21|EPI_ISL_631198|Original|hCoV-19^^England|Human|Lighthouse Lab in Milton Keynes|Wellcome Sanger Institute for the COVID-19 Genomics UK (COG-UK) consortium|Davies|United Kingdom

>Spike|hCoV-19/South Africa/KRISP-0080/2020|2020-04-01|EPI_ISL_467454|Original|hCoV-19^^KwaZulu-Natal|Human|AMPATH-DBN|KRISP|de Oliveira|South Africa
>Spike|hCoV-19/Wales/PHWC-48A213/2020|2020-10-27|EPI_ISL_650914|Original|hCoV-19^^Wales|Human|Wales Specialist Virology Centre Sequencing lab|COVID-19 Genomics UK (COG-UK) Consortium|Moore|United Kingdom
>Spike|hCoV-19/Denmark/DCGC-14145/2020|2020-11-30|EPI_ISL_711288|Original|hCoV-19^^Syddanmark|Human|Department of Virus and Microbiological Special Diagnostics|Albertsen Lab|Michaelsen|Denmark
>Spike|hCoV-19/Switzerland/SG-ETHZ-331457/2020|2020-10-21|EPI_ISL_614945|Original|hCoV-19^^St Gallen|Human|Viollier AG|Department of Biosystems Science and Engineering|Nadeau|Switzerland
>Spike|hCoV-19/England/QEUH-A4D823/2020|2020-10-07|EPI_ISL_628612|Original|hCoV-19^^England|Human|Oxford Viromics|COVID-19 Genomics UK (COG-UK) Consortium|Golubchik|United Kingdom
>Spike|hCoV-19/USA/TX-HMH-5236/2020|2020-06-26|EPI_ISL_544614|Original|hCoV-19^^Texas|Human|Houston Methodist Hospital|Houston Methodist Hospital|Long|USA
>Spike|hCoV-19/USA/WA-UW65/2020|2020-03-10|EPI_ISL_415593|Original|hCoV-19^^Washington|Human|UW Virology Lab|UW Virology Lab|Roychoudhury|USA
>Spike|hCoV-19/India/DL-NCDC7557-CSIR-IGIB/2020|2020-05-27|EPI_ISL_482643|Original|hCoV-19^^Delhi|Human|National Centre for Disease control (NCDC)|NCDC/CSIR-IGIB|Singh|India
>Spike|hCoV-19/Japan/IC-0127/2020|2020-08-00|EPI_ISL_591468|Original|hCoV-19^^Japan|Human|Pathogen Genomics Center|Pathogen Genomics Center|Sekizuka|Japan
>Spike|hCoV-19/South Africa/KRISP-K002826/2020|2020-09-01|EPI_ISL_602646|Original|hCoV-19^^KwaZulu-Natal|Human|NHLS-IALCH|KRISP|de Oliveira|South Africa
>Spike|hCoV-19/England/NORW-E88E8/2020|2020-04-11|EPI_ISL_448248|Original|hCoV-19^^England|Human|Quadram Institute Bioscience|COVID-19 Genomics UK (COG-UK) Consortium|Alikhan|United Kingdom
>Spike|hCoV-19/Germany/HH-hpi-p1664/2020|2020-03-18|EPI_ISL_775646|Original|hCoV-19^^Hamburg|Human|University Medical Center Hamburg Eppendorf|Heinrich Pette Institute|Robitaille|Germany
>Spike|hCoV-19/Spain/AR-IBV-005036/2020|2020-04-20|EPI_ISL_500340|Original|hCoV-19^^Aragon|Human|Servicio de Microbiolog\u00eda|SeqCOVID-SPAIN consortium/IBV(CSIC)|Comas|Spain
>Spike|hCoV-19/Australia/VIC11899/2020|2020-08-03|EPI_ISL_591887|Original|hCoV-19^^Victoria|Human|Victorian Infectious Diseases Reference Laboratory (VIDRL)|VIDRL and MDU-PHL|Schultz|Australia
>Spike|hCoV-19/Italy/APU-IZSPB-172APT/2020|2020-08-20|EPI_ISL_649191|Vero|hCoV-19^^Apulia|Human|Istituto Zooprofilattico Sperimentale della Puglia e della Basilicata|Istituto Zooprofilattico Sperimentale della Puglia e della Basilicata|Parisi|Italy
>Spike|hCoV-19/England/20109002404/2020|2020-03-03|EPI_ISL_464504|Original|hCoV-19^^England|Human|Respiratory Virus Unit|Respiratory Virus Unit|Platt|United Kingdom
>Spike|hCoV-19/England/SHEF-CD737/2020|2020-03-29|EPI_ISL_432548|Original|hCoV-19^^England|Human|Virology Department|COVID-19 Genomics UK (COG-UK) Consortium|de Silva|United Kingdom
>Spike|hCoV-19/USA/WI-UW-971/2020|2020-07-03|EPI_ISL_509969|Original|hCoV-19^^Wisconsin|Human|University of Wisconsin-Madison AIDS Vaccine Research Laboratories|University of Wisconsin-Madison AIDS Vaccine Research Laboratories|Baczenas|USA
>Spike|hCoV-19/Japan/PG-0673/2020|2020-04-00|EPI_ISL_684283|Original|hCoV-19^^Japan|Human|Gunma Prefectural Institute of Public Health and Environmental Sciences|Pathogen Genomics Center|Sekizuka|Japan
>Spike|hCoV-19/USA/FL-BPHL-1057/2020|2020-06-04|EPI_ISL_517947|Original|hCoV-19^^Florida|Human|Florida Bureau of Public Health Laboratories|Florida Bureau of Public Health Laboratories|Blanton|USA
>Spike|hCoV-19/Denmark/DCGC-8950/2020|2020-10-26|EPI_ISL_626195|Original|hCoV-19^^Midtjylland|Human|Department of Virus and Microbiological Special Diagnostics|Albertsen lab|Michaelsen|Denmark

>Spike|hCoV-19/USA/UT-08716/2020|2020-05-19|EPI_ISL_524773|Original|hCoV-19^^Utah|Human|Utah Public Health Laboratory|Utah Public Health Laboratory|Young|USA
>Spike|hCoV-19/Brazil/SP-678/2020|2020-06-12|EPI_ISL_693233|Original|hCoV-19^^S\u00e3o Paulo|Human|Hospital Santa Cruz|Instituto Adolfo Lutz|Sacchi|Brazil
>Spike|hCoV-19/Hong Kong/VM20135296/2020|2020-10-20|EPI_ISL_730616|Original|hCoV-19^^Hong Kong|Human|United Christian Hospital|Hong Kong Department of Health|Mak|China
>Spike|hCoV-19/Japan/PG-0856/2020|2020-04-00|EPI_ISL_684466|Original|hCoV-19^^Japan|Human|Fukuoka Institute of Health and Environmental Sciences|Pathogen Genomics Center|Sekizuka|Japan
>Spike|hCoV-19/USA/TX-HMH-7751/2020|2020-06-26|EPI_ISL_545615|Original|hCoV-19^^Texas|Human|Houston Methodist Hospital|Houston Methodist Hospital|Long|USA
>Spike|hCoV-19/England/ALDP-A8525E/2020|2020-10-16|EPI_ISL_610506|Original|hCoV-19^^England|Human|Lighthouse Lab in Alderley Park|Wellcome Sanger Institute for the COVID-19 Genomics UK (COG-UK) consortium|Davies|United Kingdom
>Spike|hCoV-19/England/ALDP-9EA355/2020|2020-09-24|EPI_ISL_579942|Original|hCoV-19^^England|Human|Lighthouse Lab in Alderley Park|Wellcome Sanger Institute for the COVID-19 Genomics UK (COG-UK) consortium|Davies|United Kingdom
>Spike|hCoV-19/USA/CA-QDX-2361/2020|2020-03-17|EPI_ISL_604655|Original|hCoV-19^^California|Human|Quest Diagnostics|Quest Diagnostics|Kagan|USA
>Spike|hCoV-19/Australia/VIC4196/2020|2020-07-15|EPI_ISL_519524|Original|hCoV-19^^Victoria|Human|Microbiological Diagnostic Unit - Public Health Laboratory (MDU-PHL)|MDU-PHL|Schultz|Australia
>Spike|hCoV-19/England/20144057404/2020|2020-03-31|EPI_ISL_466003|Original|hCoV-19^^England|Human|Respiratory Virus Unit|Respiratory Virus Unit|Platt|United Kingdom
>Spike|hCoV-19/England/MILK-C7B5D8/2020|2020-12-15|EPI_ISL_756762|Original|hCoV-19^^England|Human|Lighthouse Lab in Milton Keynes|Wellcome Sanger Institute for the COVID-19 Genomics UK (COG-UK) Consortium|Davies|United Kingdom
>Spike|hCoV-19/Netherlands/NA_333/2020|2020-04-03|EPI_ISL_455138|Original|hCoV-19^^Netherlands|Human|Dutch COVID-19 response team|Erasmus Medical Center|Oude Munnink|Netherlands
>Spike|hCoV-19/Belgium/rega-12111435/2020|2020-12-11|EPI_ISL_738487|Original|hCoV-19^^Mol|Human|UZ Leuven|KU Leuven|Maes|Belgium
>Spike|hCoV-19/USA/WA-S740/2020|2020-04-13|EPI_ISL_449910|Original|hCoV-19^^Washington|Human|Washington State Department of Health|Seattle Flu Study|Bedford|USA
>Spike|hCoV-19/England/ALDP-AA0A91/2020|2020-10-22|EPI_ISL_625196|Original|hCoV-19^^England|Human|Lighthouse Lab in Alderley Park|Wellcome Sanger Institute for the COVID-19 Genomics UK (COG-UK) consortium|Davies|United Kingdom
>Spike|hCoV-19/Australia/NSW2796/2020|2020-09-20|EPI_ISL_678348|Original|hCoV-19^^New South Wales|Human|Area of Virology|Virology Research Laboratory; Area of Virology|Foster|Australia
>Spike|hCoV-19/England/QEUH-9E426D/2020|2020-09-22|EPI_ISL_579811|Original|hCoV-19^^England|Human|Lighthouse Lab in Glasgow|Wellcome Sanger Institute for the COVID-19 Genomics UK (COG-UK) consortium|Davies|United Kingdom
>Spike|hCoV-19/England/QEUH-B6ADD5/2020|2020-11-11|EPI_ISL_662377|Original|hCoV-19^^England|Human|Lighthouse Lab in Glasgow|Wellcome Sanger Institute for the COVID-19 Genomics UK (COG-UK) Consortium|Davies|United Kingdom
>Spike|hCoV-19/USA/NY-NYCPHL-001424/2020|2020-11-20|EPI_ISL_683794|Original|hCoV-19^^New York|Human|DOHMH Corona|New York City Public Health Laboratory|Wang|USA
>Spike|hCoV-19/England/20126095704/2020|2020-03-18|EPI_ISL_423904|Original|hCoV-19^^England|Human|Respiratory Virus Unit|Respiratory Virus Unit|Platt|United Kingdom
>Spike|hCoV-19/Canada/QC_AT9/2020|2020-04-05|EPI_ISL_463944|Original|hCoV-19^^Quebec|Human|Laboratoire de microbiologie|Smith Laboratory|Smith|Canada
>Spike|hCoV-19/England/CAMC-BBFF17/2020|2020-11-21|EPI_ISL_704830|Original|hCoV-19^^England|Human|Lighthouse Lab in Cambridge|Wellcome Sanger Institute for the COVID-19 Genomics UK (COG-UK) Consortium|Davies|United Kingdom
>Spike|hCoV-19/France/ARA-SC043/2020|2020-03-31|EPI_ISL_666663|Original|hCoV-19^^Auvergne-Rh\u00f4ne-Alpes|Human|Laboratoire du Centre Hospitalier Annecy Genevois|CNR Virus des Infections Respiratoires - France SUD|Bal|France

>Spike|hCoV-19/Australia/VIC4571/2020|2020-07-13|EPI_ISL_518977|Original|hCoV-19^^Victoria|Human|Microbiological Diagnostic Unit - Public Health Laboratory (MDU-PHL)|MDU-PHL|Schultz|Australia
>Spike|hCoV-19/Denmark/DCGC-17447/2020|2020-12-14|EPI_ISL_750990|Original|hCoV-19^^Nordjylland|Human|Department of Virus and Microbiological Special Diagnostics|Albertsen Lab|Michaelsen|Denmark
>Spike|hCoV-19/Portugal/PT0415/2020|2020-03-23|EPI_ISL_454139|Original|hCoV-19^^Portugal|Human|unknown|Instituto Nacional de Saude (INSA)|Borges|Portugal
>Spike|hCoV-19/Mexico/AGS-InDRE-79/2020|2020-11-02|EPI_ISL_658865|Original|hCoV-19^^Aguascalientes|Human|Instituto de Diagnostico y Referencia Epidemiologicos (INDRE)|Instituto de Diagnostico y Referencia Epidemiologicos (INDRE)|Rodriguez|Mexico
>Spike|hCoV-19/England/ALDP-9CC16E/2020|2020-09-16|EPI_ISL_573088|Original|hCoV-19^^England|Human|Quadram Institute Bioscience|COVID-19 Genomics UK (COG-UK) Consortium|Alikhan|United Kingdom
>Spike|hCoV-19/USA/TX-HMH-MCoV-12745/2020|2020-08-29|EPI_ISL_790502|Original|hCoV-19^^Texas|Human|Houston Methodist Hospital|Houston Methodist Hospital|Long|USA
>Spike|hCoV-19/England/QEUH-9B7E32/2020|2020-09-07|EPI_ISL_590587|Original|hCoV-19^^England|Human|Lighthouse Lab in Glasgow|Wellcome Sanger Institute for the COVID-19 Genomics UK (COG-UK) consortium|Davies|United Kingdom
>Spike|hCoV-19/Denmark/DCGC-15267/2020|2020-11-23|EPI_ISL_713732|Original|hCoV-19^^Syddanmark|Human|Department of Virus and Microbiological Special Diagnostics|Albertsen Lab|Michaelsen|Denmark
>Spike|hCoV-19/USA/FL-BPHL-0730/2020|2020-07-14|EPI_ISL_509788|Original|hCoV-19^^Florida|Human|Florida Bureau of Public Health Laboratories|Florida Bureau of Public Health Laboratories|Blanton|USA
>Spike|hCoV-19/USA/CA-CZB-1911/2020|2020-04-22|EPI_ISL_486204|Original|hCoV-19^^California|Human|Orange County Public Health Laboratory|Chan-Zuckerberg Biohub|McGeever|USA
>Spike|hCoV-19/England/NORT-284DCF/2020|2020-03-27|EPI_ISL_478595|Original|hCoV-19^^England|Human|Northumbria University / South Tees Hospitals NHS Foundation Trust / North Cumbria Integrated Care NHS Foundation Trust / North Tees and Hartlepool NHS Foundation Trust / Newcastle Hospitals NHS Foundation Trust|COVID-19 Genomics UK (COG-UK) Consortium|Bashton|United Kingdom
>Spike|hCoV-19/India/HR-NIBG-TF77/2020|2020-05-25|EPI_ISL_653917|Original|hCoV-19^^Haryana|Human|Translational Health Science and Technology Institute|National Institute of Biomedical Genomics|Maitra|India
>Spike|hCoV-19/USA/FL-BPHL-1684/2020|2020-08-12|EPI_ISL_594373|Original|hCoV-19^^Florida|Human|Florida Bureau of Public Health Laboratories|Florida Bureau of Public Health Laboratories|Blanton|USA
>Spike|hCoV-19/Canada/QC-LSPQ-L00234639/2020|2020-03-23|EPI_ISL_536065|Original|hCoV-19^^Quebec|Human|H\u00f4pital Pierre-Le Gardeur|Laboratoire de sant\u00e9 publique du Qu\u00e9bec|Moreira|Canada
>Spike|hCoV-19/Japan/PG-4441/2020|2020-07-00|EPI_ISL_692664|Original|hCoV-19^^Japan|Human|Sakai City Institute of Public Health|Pathogen Genomics Center|Sekizuka|Japan
>Spike|hCoV-19/Denmark/DCGC-12614/2020|2020-11-16|EPI_ISL_682795|Original|hCoV-19^^Syddanmark|Human|Department of Virus and Microbiological Special Diagnostics|Albertsen Lab|Michaelsen|Denmark
>Spike|hCoV-19/Australia/VIC3304/2020|2020-07-11|EPI_ISL_520983|Original|hCoV-19^^Victoria|Human|Microbiological Diagnostic Unit - Public Health Laboratory (MDU-PHL)|MDU-PHL|Schultz|Australia
>Spike|hCoV-19/England/QEUH-AA03F0/2020|2020-10-19|EPI_ISL_611454|Original|hCoV-19^^England|Human|Lighthouse Lab in Glasgow|Wellcome Sanger Institute for the COVID-19 Genomics UK (COG-UK) consortium|Davies|United Kingdom
>Spike|hCoV-19/Australia/NSW852/2020|2020-08-12|EPI_ISL_526194|Original|hCoV-19^^New South Wales|Human|St Vincent's Pathology (SydPath)|NSW Health Pathology - Institute of Clinical Pathology and Medical Research; Westmead Hospital; University of Sydney|Gall|Australia

>Spike|hCoV-19/USA/WA-UW-5750/2020|2020-04-06|EPI_ISL_430918|Original|hCoV-
19^^Washington|Human|UW Virology Lab|UW Virology Lab|Roychoudhury|USA
>Spike|hCoV-19/USA/TX-HMH-MCoV-12587/2020|2020-08-31|EPI_ISL_790408|Original|hCoV-
19^^Texas|Human|Houston Methodist Hospital|Houston Methodist Hospital|Long|USA
>Spike|hCoV-19/Australia/VIC6697/2020|2020-07-20|EPI_ISL_563999|Original|hCoV-
19^^Victoria|Human|Victorian Infectious Diseases Reference Laboratory (VIDRL)|VIDRL
and MDU-PHL|Schultz|Australia
>Spike|hCoV-19/England/QEUH-9C8241/2020|2020-09-11|EPI_ISL_567062|Original|hCoV-
19^^England|Human|Lighthouse Lab in Glasgow|Wellcome Sanger Institute for the COVID-
19 Genomics UK (COG-UK) consortium|Davies|United Kingdom
>Spike|hCoV-19/Denmark/DCGC-15113/2020|2020-11-23|EPI_ISL_713638|Original|hCoV-
19^^Hovedstaden|Human|Department of Virus and Microbiological Special
Diagnostics|Albertsen Lab|Michaelsen|Denmark
>Spike|hCoV-19/England/CAMC-BC1EBB/2020|2020-11-22|EPI_ISL_705634|Original|hCoV-
19^^England|Human|Department of Pathology|COVID-19 Genomics UK (COG-UK)
Consortium|Goodfellow|United Kingdom
>Spike|hCoV-19/Australia/VIC10756/2020|2020-00-00|EPI_ISL_563073|Original|hCoV-
19^^Victoria|Human|Microbiological Diagnostic Unit - Public Health Laboratory (MDU-
PHL)|MDU-PHL|Schultz|Australia
>Spike|hCoV-19/Wales/PHWC-16DB5A/2020|2020-09-22|EPI_ISL_577496|Original|hCoV-
19^^Wales|Human|Wales Specialist Virology Centre Sequencing lab|COVID-19 Genomics UK
(COG-UK) Consortium|Moore|United Kingdom
>Spike|hCoV-19/England/CAMC-BD1B57/2020|2020-11-27|EPI_ISL_702190|Original|hCoV-
19^^England|Human|Lighthouse Lab in Cambridge|Wellcome Sanger Institute for the
COVID-19 Genomics UK (COG-UK) Consortium|Davies|United Kingdom
>Spike|hCoV-19/England/MILK-A0613A/2020|2020-10-01|EPI_ISL_600116|Original|hCoV-
19^^England|Human|Lighthouse Lab in Milton Keynes|Wellcome Sanger Institute for the
COVID-19 Genomics UK (COG-UK) consortium|Davies|United Kingdom
>Spike|hCoV-19/England/ALDP-B235FE/2020|2020-11-04|EPI_ISL_646941|Original|hCoV-
19^^England|Human|Lighthouse Lab in Alderley Park|Wellcome Sanger Institute for the
COVID-19 Genomics UK (COG-UK) Consortium|Davies|United Kingdom
>Spike|hCoV-19/England/ALDP-B73F5B/2020|2020-11-12|EPI_ISL_664638|Original|hCoV-
19^^England|Human|Quadram Institute Bioscience|COVID-19 Genomics UK (COG-UK)
Consortium|Alikhan|United Kingdom
>Spike|hCoV-19/Canada/QC_BC6/2020|2020-04-11|EPI_ISL_515245|Original|hCoV-
19^^Quebec|Human|Laboratoire de microbiologie|Smith Laboratory|Smith|Canada
>Spike|hCoV-19/England/MILK-B39908/2020|2020-11-01|EPI_ISL_741360|Original|hCoV-
19^^England|Human|University of Exeter|COVID-19 Genomics UK (COG-UK)
Consortium|Temperton|United Kingdom
>Spike|hCoV-19/England/LOND-12F0E31/2020|2020-12-03|EPI_ISL_705331|Original|hCoV-
19^^England|Human|University College London|COVID-19 Genomics UK (COG-UK)
Consortium|Roy|United Kingdom
>Spike|hCoV-19/England/GSTT-265DD89/2020|2020-03-28|EPI_ISL_484102|Original|hCoV-
19^^England|Human|Centre for Clinical Infection and Diagnostics Research and Genomics
Innovation Unit|COVID-19 Genomics UK (COG-UK) Consortium|Awan|United Kingdom
>Spike|hCoV-19/USA/ME-HETL-J0337/2020|2020-11-24|EPI_ISL_755465|Original|hCoV-
19^^Maine|Human|Maine Health and Environmental Testing Laboratory|Tewhey
Lab|Tewhey|USA
>Spike|hCoV-19/England/CAMB-1AD383/2020|2020-04-25|EPI_ISL_489488|Original|hCoV-
19^^England|Human|Department of Pathology|Wellcome Sanger Institute for the COVID-19
Genomics UK (COG-UK) consortium|Davies|United Kingdom
>Spike|hCoV-19/France/OCC-154/2020|2020-11-18|EPI_ISL_751474|Original|hCoV-
19^^Occitanie|Human|CHU Purpan - Laboratoire de Virologie - Institut
F\u00e9d\u00e9ratif de Biologie|CHU Purpan - Laboratoire de Virologie - Institut
F\u00e9d\u00e9ratif de Biologie|Latour|France
>Spike|hCoV-19/DRC/3440/2020|2020-04-22|EPI_ISL_447597|Original|hCoV-
19^^Kinshasa|Human|Viral Respiratory Lab|Pathogen Sequencing Lab|Lusamaki|Democratic
Republic of the Congo

>Spike|hCoV-19/England/OXON-B0281/2020|2020-04-10|EPI_ISL_478870|Original|hCoV-
19^^England|Human|Oxford Viromics|COVID-19 Genomics UK (COG-UK)
Consortium|Golubchik|United Kingdom
>Spike|hCoV-19/England/BIRM-5F0A8/2020|2020-05-05|EPI_ISL_473308|Original|hCoV-
19^^England|Human|University of Birmingham|COVID-19 Genomics UK (COG-UK)
Consortium|Loman|United Kingdom
>Spike|hCoV-19/USA/TX-HMH-MCoV-18260/2020|2020-11-17|EPI_ISL_784608|Original|hCoV-
19^^Texas|Human|Houston Methodist Hospital|Houston Methodist Hospital|Long|USA
>Spike|hCoV-19/England/ALDP-A67A4B/2020|2020-10-12|EPI_ISL_630478|Original|hCoV-
19^^England|Human|Lighthouse Lab in Alderley Park|Wellcome Sanger Institute for the
COVID-19 Genomics UK (COG-UK) consortium|Davies|United Kingdom
>Spike|hCoV-19/Norway/2952/2020|2020-07-31|EPI_ISL_549027|Original|hCoV-
19^^Oslo|Human|Furst Medical Laboratory|Norwegian Institute of Public Health|Kopperud
Riis|Norway
>Spike|hCoV-19/USA/AZ-TG347264/2020|2020-06-02|EPI_ISL_694396|Original|hCoV-
19^^Arizona|Human|TGen North|TGen North|French|USA
>Spike|hCoV-19/England/CAMC-B37FAE/2020|2020-11-05|EPI_ISL_659084|Original|hCoV-
19^^England|Human|Lighthouse Lab in Cambridge|Wellcome Sanger Institute for the
COVID-19 Genomics UK (COG-UK) Consortium|Davies|United Kingdom
>Spike|hCoV-19/England/ALDP-AA19FD/2020|2020-10-22|EPI_ISL_624525|Original|hCoV-
19^^England|Human|Lighthouse Lab in Alderley Park|Wellcome Sanger Institute for the
COVID-19 Genomics UK (COG-UK) consortium|Davies|United Kingdom
>Spike|hCoV-19/England/QEUH-9B41E1/2020|2020-09-03|EPI_ISL_559881|Original|hCoV-
19^^England|Human|Oxford Viromics|COVID-19 Genomics UK (COG-UK)
Consortium|Golubchik|United Kingdom
>Spike|hCoV-19/Wales/PHWC-48C985/2020|2020-11-08|EPI_ISL_666489|Original|hCoV-
19^^Wales|Human|Wales Specialist Virology Centre Sequencing lab|COVID-19 Genomics UK
(COG-UK) Consortium|Moore|United Kingdom
>Spike|hCoV-19/England/CAMB-768FE/2020|2020-03-29|EPI_ISL_438462|Original|hCoV-
19^^England|Human|Department of Pathology|Wellcome Sanger Institute for the COVID-19
Genomics UK (COG-UK) consortium|Amato|United Kingdom
>Spike|hCoV-19/South Korea/KCDC2758/2020|2020-07-20|EPI_ISL_522537|Original|hCoV-
19^^South Korea|Human|Division of Viral Diseases|Division of Viral Diseases|Kim|South
Korea
>Spike|hCoV-19/Singapore/1443/2020|2020-12-05|EPI_ISL_728179|Original|hCoV-
19^^Singapore|Human|National Public Health Laboratory|National Public Health
Laboratory|Mak|Singapore
>Spike|hCoV-19/USA/MD-HP12084/2020|2020-11-18|EPI_ISL_791409|Original|hCoV-
19^^Maryland|Human|Johns Hopkins Hospital Department of Pathology|Johns Hopkins
Hospital Department of Pathology|Morris|USA
>Spike|hCoV-19/Australia/NSW261/2020|2020-03-27|EPI_ISL_451526|Original|hCoV-19^^New
South Wales|Human|Pathology West - NSW Health Pathology|NSW Health Pathology -
Institute of Clinical Pathology and Medical Research; Westmead Hospital; University
of Sydney|Gall|Australia
>Spike|hCoV-19/England/204961138/2020|2020-12-03|EPI_ISL_700624|Original|hCoV-
19^^England|Human|Respiratory Virus Unit|COVID-19 Genomics UK (COG-UK)
Consortium|Platt|United Kingdom
>Spike|hCoV-19/England/QEUH-B7E503/2020|2020-11-14|EPI_ISL_668053|Original|hCoV-
19^^England|Human|Lighthouse Lab in Glasgow|Wellcome Sanger Institute for the COVID-
19 Genomics UK (COG-UK) Consortium|Davies|United Kingdom
>Spike|hCoV-19/England/QEUH-A79509/2020|2020-10-12|EPI_ISL_638808|Original|hCoV-
19^^England|Human|Oxford Viromics|COVID-19 Genomics UK (COG-UK)
Consortium|Golubchik|United Kingdom
>Spike|hCoV-19/Australia/VIC8127/2020|2020-08-06|EPI_ISL_564865|Original|hCoV-
19^^Victoria|Human|Microbiological Diagnostic Unit - Public Health Laboratory (MDU-
PHL)|MDU-PHL|Schultz|Australia
>Spike|hCoV-19/Denmark/DCGC-15999/2020|2020-11-23|EPI_ISL_714121|Original|hCoV-
19^^Syddanmark|Human|Department of Virus and Microbiological Special
Diagnostics|Albertsen Lab|Michaelsen|Denmark

>Spike|hCoV-19/England/QEUH-9984CC/2020|2020-08-24|EPI_ISL_540004|Original|hCoV-19^^England|Human|Lighthouse Lab in Glasgow|Wellcome Sanger Institute for the COVID-19 Genomics UK (COG-UK) consortium|Davies|United Kingdom
>Spike|hCoV-19/Scotland/CVR2730/2020|2020-04-18|EPI_ISL_473550|Original|hCoV-19^^Scotland|Human|West of Scotland Specialist Virology Centre|COVID-19 Genomics UK (COG-UK) Consortium|Robertson|United Kingdom
>Spike|hCoV-19/USA/UT-QDX-2480/2020|2020-03-13|EPI_ISL_604850|Original|hCoV-19^^Utah|Human|Quest Diagnostics|Quest Diagnostics|Kagan|USA
>Spike|hCoV-19/England/NORT-295B94/2020|2020-04-03|EPI_ISL_488489|Original|hCoV-19^^England|Human|NU-OMICS DNA Sequencing research facility|Wellcome Sanger Institute for the COVID-19 Genomics UK (COG-UK) consortium|Davies|United Kingdom
>Spike|hCoV-19/England/OXON-AC428/2020|2020-04-07|EPI_ISL_448449|Original|hCoV-19^^England|Human|Oxford Viromics|COVID-19 Genomics UK (COG-UK) Consortium|Golubchik|United Kingdom
>Spike|hCoV-19/England/SHEF-CDE14/2020|2020-05-27|EPI_ISL_535082|Original|hCoV-19^^England|Human|Virology Department|COVID-19 Genomics UK (COG-UK) Consortium|de Silva|United Kingdom
>Spike|hCoV-19/Denmark/DCGC-11474/2020|2020-11-02|EPI_ISL_670939|Original|hCoV-19^^Hovedstaden|Human|Department of Virus and Microbiological Special Diagnostics|Albertsen Lab|Michaelsen|Denmark
>Spike|hCoV-19/Denmark/DCGC-5897/2020|2020-10-05|EPI_ISL_620162|Original|hCoV-19^^Midtjylland|Human|Department of Virus and Microbiological Special Diagnostics|Albertsen lab|Michaelsen|Denmark
>Spike|hCoV-19/England/CAMB-82CE4/2020|2020-04-13|EPI_ISL_443468|Original|hCoV-19^^England|Human|Department of Pathology|Wellcome Sanger Institute for the COVID-19 Genomics UK (COG-UK) consortium|Amato|United Kingdom
>Spike|hCoV-19/England/ALDP-9CD358/2020|2020-09-15|EPI_ISL_566130|Original|hCoV-19^^England|Human|Lighthouse Lab in Alderley Park|Wellcome Sanger Institute for the COVID-19 Genomics UK (COG-UK) consortium|Davies|United Kingdom
>Spike|hCoV-19/Japan/PG-7658/2020|2020-08-00|EPI_ISL_686760|Original|hCoV-19^^Japan|Human|Chiba Prefectural Institute of Public Health|Pathogen Genomics Center|Sekizuka|Japan
>Spike|hCoV-19/Scotland/CVR4926/2020|2020-10-01|EPI_ISL_594692|Original|hCoV-19^^Scotland|Human|West of Scotland Specialist Virology Centre|COVID-19 Genomics UK (COG-UK) Consortium|Robertson|United Kingdom
>Spike|hCoV-19/Northern Ireland/NIRE-2344E7/2020|2020-10-19|EPI_ISL_680398|Original|hCoV-19^^Northern Ireland|Human|Regional Virus Laboratory|COVID-19 Genomics UK (COG-UK) Consortium|Fairley|United Kingdom
>Spike|hCoV-19/Scotland/EDB3157/2020|2020-04-06|EPI_ISL_489063|Original|hCoV-19^^Scotland|Human|Virology Department|Wellcome Sanger Institute for the COVID-19 Genomics UK (COG-UK) consortium|Davies|United Kingdom
>Spike|hCoV-19/Germany/BY-ChVir20731/2020|2020-11-18|EPI_ISL_729673|Original|hCoV-19^^Bavaria|Human|A. Krumbholz|Charit\u00e9 Universit\u00e4tsmedizin Berlin|Muehlemann|Germany
>Spike|hCoV-19/Canada/NS-NML-1371/2020|2020-04-15|EPI_ISL_579747|Original|hCoV-19^^Nova Scotia|Human|QEII Health Sciences Centre|National Microbiology Laboratory (NML)|Majer|Canada
>Spike|hCoV-19/England/QEUH-A49A12/2020|2020-10-08|EPI_ISL_628445|Original|hCoV-19^^England|Human|Oxford Viromics|COVID-19 Genomics UK (COG-UK) Consortium|Golubchik|United Kingdom
>Spike|hCoV-19/Scotland/QEUH-96E8B4/2020|2020-08-17|EPI_ISL_534468|Original|hCoV-19^^Scotland|Human|NHSGGC West of Scotland Specialist Virology Centre / MRC-University of Glasgow Centre for Virus Research|Wellcome Sanger Institute for the COVID-19 Genomics UK (COG-UK) consortium|Davies|United Kingdom
>Spike|hCoV-19/Spain/MD-IBV-004968/2020|2020-05-07|EPI_ISL_481041|Original|hCoV-19^^Madrid|Human|Hospital General Universitario Gregorio Mara\u00f1\u00f3n|SeqCOVID-SPAIN consortium/IBV(CSIC)|Comas|Spain
>Spike|hCoV-19/Japan/PG-8669/2020|2020-09-00|EPI_ISL_686265|Original|hCoV-19^^Japan|Human|Pathogen Genomics Center|Pathogen Genomics Center|Sekizuka|Japan

>Spike|hCoV-19/Denmark/DCGC-16852/2020|2020-12-14|EPI_ISL_750526|Original|hCoV-19^^Nordjylland|Human|Department of Virus and Microbiological Special Diagnostics|Albertsen Lab|Michaelsen|Denmark
>Spike|hCoV-19/South Africa/NHLS-UCT-GS-1026/2020|2020-10-27|EPI_ISL_700497|Original|hCoV-19^^Western Cape|Human|George Hospital wc GRH|NHLS/UCT|Iranzadeh|South Africa
>Spike|hCoV-19/England/QEUH-B9EC08/2020|2020-11-18|EPI_ISL_719618|Original|hCoV-19^^England|Human|Lighthouse Lab in Glasgow|Wellcome Sanger Institute for the COVID-19 Genomics UK (COG-UK) Consortium|Davies|United Kingdom
>Spike|hCoV-19/England/CAMC-BBB79B/2020|2020-11-21|EPI_ISL_704486|Original|hCoV-19^^England|Human|Department of Pathology|COVID-19 Genomics UK (COG-UK) Consortium|Goodfellow|United Kingdom
>Spike|hCoV-19/England/LOND-1261554/2020|2020-03-28|EPI_ISL_524679|Original|hCoV-19^^England|Human|North West London Pathology|Wellcome Sanger Institute for the COVID-19 Genomics UK (COG-UK) consortium|Davies|United Kingdom
>Spike|hCoV-19/England/MILK-802B23/2020|2020-07-16|EPI_ISL_557207|Original|hCoV-19^^England|Human|Lighthouse Lab in Milton Keynes|Wellcome Sanger Institute for the COVID-19 Genomics UK (COG-UK) consortium|Davies|United Kingdom
>Spike|hCoV-19/England/ALDP-A6EC20/2020|2020-10-15|EPI_ISL_607259|Original|hCoV-19^^England|Human|Lighthouse Lab in Alderley Park|Wellcome Sanger Institute for the COVID-19 Genomics UK (COG-UK) consortium|Davies|United Kingdom
>Spike|hCoV-19/USA/HI-H200342/2020|2020-08-27|EPI_ISL_752898|Original|hCoV-19^^Hawaii|Human|State Laboratories Division|State Laboratories Division|Sultana|USA
>Spike|hCoV-19/England/ALDP-B23910/2020|2020-11-04|EPI_ISL_646812|Original|hCoV-19^^England|Human|Lighthouse Lab in Alderley Park|Wellcome Sanger Institute for the COVID-19 Genomics UK (COG-UK) Consortium|Davies|United Kingdom
>Spike|hCoV-19/England/SHEF-10D5FF5/2020|2020-12-19|EPI_ISL_742083|Original|hCoV-19^^England|Human|Virology Department|COVID-19 Genomics UK (COG-UK) Consortium|de Silva|United Kingdom
>Spike|hCoV-19/England/QEUH-A61E18/2020|2020-10-12|EPI_ISL_706938|Original|hCoV-19^^England|Human|Oxford Viromics|COVID-19 Genomics UK (COG-UK) Consortium|Golubchik|United Kingdom
>Spike|hCoV-19/Australia/VIC17566/2020|2020-08-21|EPI_ISL_663925|Original|hCoV-19^^Victoria|Human|Microbiological Diagnostic Unit - Public Health Laboratory (MDU-PHL)|MDU-PHL|Schultz|Australia
>Spike|hCoV-19/Spain/VC-FISABIO-627/2020|2020-03-31|EPI_ISL_455739|Original|hCoV-19^^Comunitat Valenciana|Human|Servicio de Microbiologia. Hospital Clinico Universitario de Valencia|Sequencing and Bioinformatics Service and Molecular Epidemiology Research Group. FISABIO-Public Health|Gonzalez-Candelas|Spain
>Spike|hCoV-19/Australia/VIC12250/2020|2020-07-26|EPI_ISL_640500|Original|hCoV-19^^Victoria|Human|Microbiological Diagnostic Unit - Public Health Laboratory (MDU-PHL)|MDU-PHL|Schultz|Australia
>Spike|hCoV-19/USA/WA-S2187/2020|2020-06-08|EPI_ISL_497615|Original|hCoV-19^^Washington|Human|Washington State Department of Health|Seattle Flu Study|Lee|USA
>Spike|hCoV-19/Denmark/DCGC-8503/2020|2020-10-26|EPI_ISL_625898|Original|hCoV-19^^Syddanmark|Human|Department of Virus and Microbiological Special Diagnostics|Albertsen lab|Michaelsen|Denmark
>Spike|hCoV-19/Sweden/20-53236/2020|2020-10-26|EPI_ISL_676496|Original|hCoV-19^^Orebro|Human|Klinisk mikrobiologi|The Public Health Agency of Sweden|Svartstr\u00f6m|Sweden
>Spike|hCoV-19/Denmark/DCGC-15517/2020|2020-11-23|EPI_ISL_713864|Original|hCoV-19^^Hovedstaden|Human|Department of Virus and Microbiological Special Diagnostics|Albertsen Lab|Michaelsen|Denmark
>Spike|hCoV-19/England/QEUH-B11C1C/2020|2020-11-02|EPI_ISL_642209|Original|hCoV-19^^England|Human|Lighthouse Lab in Glasgow|Wellcome Sanger Institute for the COVID-19 Genomics UK (COG-UK) Consortium|Davies|United Kingdom
>Spike|hCoV-19/Finland/18ES5AH5/2020|2020-08-18|EPI_ISL_756283|Original|hCoV-19^^Uusimaa|Human|Department of Virology and Immunology|Department of Virology|Smura|Finland

>Spike|hCoV-19/England/ALDP-A8630F/2020|2020-10-17|EPI_ISL_606258|Original|hCoV-
19^^England|Human|Lighthouse Lab in Alderley Park|Wellcome Sanger Institute for the
COVID-19 Genomics UK (COG-UK) consortium|Davies|United Kingdom
>Spike|hCoV-19/Japan/PG-8083/2020|2020-08-00|EPI_ISL_687184|Original|hCoV-
19^^Japan|Human|Pathogen Genomics Center|Pathogen Genomics Center|Sekizuka|Japan
>Spike|hCoV-19/England/NORT-293E60/2020|2020-07-20|EPI_ISL_573331|Original|hCoV-
19^^England|Human|Northumbria University / South Tees Hospitals NHS Foundation Trust
/ North Cumbria Integrated Care NHS Foundation Trust / North Tees and Hartlepool NHS
Foundation Trust / Newcastle Hospitals NHS Foundation Trust|COVID-19 Genomics UK
(COG-UK) Consortium|Bashton|United Kingdom
>Spike|hCoV-19/England/MILK-9C3941/2020|2020-09-10|EPI_ISL_566867|Original|hCoV-
19^^England|Human|Lighthouse Lab in Milton Keynes|Wellcome Sanger Institute for the
COVID-19 Genomics UK (COG-UK) consortium|Davies|United Kingdom
>Spike|hCoV-19/England/CAMC-C458A1/2020|2020-12-10|EPI_ISL_719545|Original|hCoV-
19^^England|Human|Lighthouse Lab in Cambridge|Wellcome Sanger Institute for the
COVID-19 Genomics UK (COG-UK) Consortium|Davies|United Kingdom
>Spike|hCoV-19/England/MILK-C7B59C/2020|2020-12-15|EPI_ISL_756786|Original|hCoV-
19^^England|Human|Lighthouse Lab in Milton Keynes|Wellcome Sanger Institute for the
COVID-19 Genomics UK (COG-UK) Consortium|Davies|United Kingdom
>Spike|hCoV-19/England/NORW-EC5A1/2020|2020-05-10|EPI_ISL_457535|Original|hCoV-
19^^England|Human|Quadram Institute Bioscience|COVID-19 Genomics UK (COG-UK)
Consortium|Alikhan|United Kingdom
>Spike|hCoV-19/England/MILK-A6B25E/2020|2020-10-09|EPI_ISL_608779|Original|hCoV-
19^^England|Human|Lighthouse Lab in Milton Keynes|Wellcome Sanger Institute for the
COVID-19 Genomics UK (COG-UK) consortium|Davies|United Kingdom
>Spike|hCoV-19/England/MILK-9F03F2/2020|2020-09-24|EPI_ISL_580634|Original|hCoV-
19^^England|Human|Lighthouse Lab in Milton Keynes|Wellcome Sanger Institute for the
COVID-19 Genomics UK (COG-UK) consortium|Davies|United Kingdom
>Spike|hCoV-19/England/BHRT-24B37EF/2020|2020-05-16|EPI_ISL_528803|Original|hCoV-
19^^England|Human|Microbiology Department|Wellcome Sanger Institute for the COVID-19
Genomics UK (COG-UK) consortium|Davies|United Kingdom
>Spike|hCoV-19/England/CAMC-B78220/2020|2020-11-12|EPI_ISL_783401|Original|hCoV-
19^^England|Human|Lighthouse Lab in Cambridge|Wellcome Sanger Institute for the
COVID-19 Genomics UK (COG-UK) Consortium|Davies|United Kingdom
>Spike|hCoV-19/Bangladesh/BCSIR-NILMRC-383/2020|2020-07-
21|EPI_ISL_514251|Original|hCoV-19^^Chittagong|Human|National Institute of Laboratory
Medicine and Referral Center|Genomic Research Lab|Khan|Bangladesh
>Spike|hCoV-19/Denmark/DCGC-13874/2020|2020-11-30|EPI_ISL_713075|Original|hCoV-
19^^Midtjylland|Human|Department of Virus and Microbiological Special
Diagnostics|Albertsen Lab|Michaelsen|Denmark
>Spike|hCoV-19/USA/MA-MGH-03205/2020|2020-10-01|EPI_ISL_765746|Original|hCoV-
19^^Massachusetts|Human|Massachusetts General Hospital|Infectious Disease
Program|Park|USA
>Spike|hCoV-19/USA/MI-QDX-902/2020|2020-03-19|EPI_ISL_571623|Original|hCoV-
19^^Michigan|Human|Quest Diagnostics|Quest Diagnostics|Kagan|USA
>Spike|hCoV-19/USA/WI-UW-2602/2021|2021-01-03|EPI_ISL_791119|Original|hCoV-
19^^Wisconsin|Human|University of Wisconsin-Madison AIDS Vaccine Research
Laboratories|University of Wisconsin-Madison AIDS Vaccine Research
Laboratories|Moreno|USA
>Spike|hCoV-19/USA/AZ-TG618045/2020|2020-07-20|EPI_ISL_695687|Original|hCoV-
19^^Arizona|Human|AZ SPHL|TGen North|French|USA
>Spike|hCoV-19/Switzerland/GR-ETHZ-390381/2020|2020-12-
01|EPI_ISL_721818|Original|hCoV-19^^Graub\u00fcnden|Human|Viollier AG|Department of
Biosystems Science and Engineering|Nadeau|Switzerland
>Spike|hCoV-19/Wales/PHWC-4800B9/2020|2020-10-21|EPI_ISL_612849|Original|hCoV-
19^^Wales|Human|Wales Specialist Virology Centre Sequencing lab|COVID-19 Genomics UK
(COG-UK) Consortium|Moore|United Kingdom
>Spike|hCoV-19/England/CAMC-A5801E/2020|2020-10-09|EPI_ISL_596937|Original|hCoV-
19^^England|Human|Lighthouse Lab in Cambridge|Wellcome Sanger Institute for the
COVID-19 Genomics UK (COG-UK) consortium|Davies|United Kingdom

>Spike|hCoV-19/England/MILK-ABB608/2020|2020-10-21|EPI_ISL_623471|Original|hCoV-19^^England|Human|Lighthouse Lab in Milton Keynes|Wellcome Sanger Institute for the COVID-19 Genomics UK (COG-UK) consortium|Davies|United Kingdom
>Spike|hCoV-19/United Kingdom/USAFSAM-S026/2020|2020-03-18|EPI_ISL_513310|Original|hCoV-19^^United Kingdom|Human|Public Health|Public Health|Fries|United Kingdom
>Spike|hCoV-19/Indonesia/JB-WJHL-ITB-A02050/2020|2020-10-21|EPI_ISL_747235|Original|hCoV-19^^West Java|Human|Sukabumi Health Laboratory|West Java Health Laboratory; School of Life Sciences and Technology|Fibriani|Indonesia
>Spike|hCoV-19/France/ARA-10580/2020|2020-03-18|EPI_ISL_508999|Original|hCoV-19^^Auvergne-Rh\u00f4ne-Alpes|Human|CNR Virus des Infections Respiratoires - France SUD|CNR Virus des Infections Respiratoires - France SUD|Josset|France
>Spike|hCoV-19/Northern Ireland/NIRE-FC74E/2020|2020-04-21|EPI_ISL_459349|Original|hCoV-19^^Northern Ireland|Human|Regional Virus Laboratory|Wellcome Sanger Institute for the COVID-19 Genomics UK (COG-UK) consortium|Davies|United Kingdom
>Spike|hCoV-19/Scotland/QEUH-96BF49/2020|2020-08-17|EPI_ISL_534673|Original|hCoV-19^^Scotland|Human|NHSGGC West of Scotland Specialist Virology Centre / MRC-University of Glasgow Centre for Virus Research|Wellcome Sanger Institute for the COVID-19 Genomics UK (COG-UK) consortium|Davies|United Kingdom
>Spike|hCoV-19/USA/TX-HMH-MCoV-11831/2020|2020-09-17|EPI_ISL_790118|Original|hCoV-19^^Texas|Human|Houston Methodist Hospital|Houston Methodist Hospital|Long|USA
>Spike|hCoV-19/England/CAMB-83C3E/2020|2020-04-16|EPI_ISL_443376|Original|hCoV-19^^England|Human|Department of Pathology|Wellcome Sanger Institute for the COVID-19 Genomics UK (COG-UK) consortium|Amato|United Kingdom
>Spike|hCoV-19/England/QEUH-AF61D3/2020|2020-10-30|EPI_ISL_663189|Original|hCoV-19^^England|Human|Lighthouse Lab in Glasgow|Wellcome Sanger Institute for the COVID-19 Genomics UK (COG-UK) Consortium|Davies|United Kingdom
>Spike|hCoV-19/Germany/HH-hpi-p1528/2020|2020-03-15|EPI_ISL_775905|Original|hCoV-19^^Hamburg|Human|University Medical Center Hamburg Eppendorf|Heinrich Pette Institute|Robitaille|Germany
>Spike|hCoV-19/Peru/LIM-UPCH-0191/2020|2020-10-15|EPI_ISL_729887|Original|hCoV-19^^Lima|Human|Laboratorio de Referencia Nacional de Virus Respiratorios|Laboratorio de Gen\u00f3mica Microbiana|Tsukayama|Peru
>Spike|hCoV-19/England/MILK-B8F1DE/2020|2020-11-13|EPI_ISL_675185|Original|hCoV-19^^England|Human|Lighthouse Lab in Milton Keynes|Wellcome Sanger Institute for the COVID-19 Genomics UK (COG-UK) Consortium|Davies|United Kingdom
>Spike|hCoV-19/Scotland/QEUH-96A27E/2020|2020-08-15|EPI_ISL_534376|Original|hCoV-19^^Scotland|Human|NHSGGC West of Scotland Specialist Virology Centre / MRC-University of Glasgow Centre for Virus Research|Wellcome Sanger Institute for the COVID-19 Genomics UK (COG-UK) consortium|Davies|United Kingdom
>Spike|hCoV-19/South Africa/KRISP-K002390/2020|2020-07-25|EPI_ISL_515755|Original|hCoV-19^^KwaZulu-Natal|Human|NHLS-IALCH|KRISP|de Oliveira|South Africa
>Spike|hCoV-19/England/QEUH-A004EC/2020|2020-09-30|EPI_ISL_600406|Original|hCoV-19^^England|Human|Lighthouse Lab in Glasgow|Wellcome Sanger Institute for the COVID-19 Genomics UK (COG-UK) consortium|Davies|United Kingdom
>Spike|hCoV-19/Palestine/95/2020|2020-07-11|EPI_ISL_596565|Original|hCoV-19^^Nablus|Human|Palestinian Ministry of Health|Molecular Genetics Lab|van Dorp|Palestine
>Spike|hCoV-19/Wales/PHWC-26110/2020|2020-03-24|EPI_ISL_422210|Original|hCoV-19^^Wales|Human|Wales Specialist Virology Centre|Public Health Wales Microbiology Cardiff|Moore|United Kingdom
>Spike|hCoV-19/USA/CA-QDX-2116/2020|2020-03-13|EPI_ISL_604417|Original|hCoV-19^^California|Human|Quest Diagnostics|Quest Diagnostics|Kagan|USA
>Spike|hCoV-19/Australia/VIC4470/2020|2020-07-16|EPI_ISL_519766|Original|hCoV-19^^Victoria|Human|Microbiological Diagnostic Unit - Public Health Laboratory (MDU-PHL)|MDU-PHL|Schultz|Australia
>Spike|hCoV-19/England/20136006402/2020|2020-03-25|EPI_ISL_420519|Original|hCoV-19^^England|Human|Respiratory Virus Unit|Respiratory Virus Unit|Platt|United Kingdom

>Spike|hCoV-19/Colombia/CAQ-INS-007007/2020|2020-10-17|EPI_ISL_739679|Original|hCoV-19^^Caqueta|Human|Instituto Nacional de Salud|Instituto Nacional de Salud|Franco-Mu\u00f1oz|Colombia
>Spike|hCoV-19/Australia/WA367/2020|2020-10-23|EPI_ISL_605844|Original|hCoV-19^^Western Australia|Human|PathWest Laboratory Medicine WA|PathWest Laboratory Medicine WA Microbial Surveillance Unit|Sikazwe|Australia
>Spike|hCoV-19/USA/MN-MDH-1775/2020|2020-04-13|EPI_ISL_590778|Original|hCoV-19^^Minnesota|Human|Minnesota Department of Health|Minnesota Department of Health|Lorentz|USA
>Spike|hCoV-19/Japan/PG-5619/2020|2020-08-00|EPI_ISL_688744|Original|hCoV-19^^Japan|Human|Pathogen Genomics Center|Pathogen Genomics Center|Sekizuka|Japan
>Spike|hCoV-19/Latvia/206/2020|2020-09-16|EPI_ISL_770042|Original|hCoV-19^^Latvia|Human|Centr\u0101l\u0101 Laboratorija|Latvian Biomedical Research and Study Centre|Zrelovs|Latvia
>Spike|hCoV-19/England/ALDP-AA2B50/2020|2020-10-22|EPI_ISL_624088|Original|hCoV-19^^England|Human|Lighthouse Lab in Alderley Park|Wellcome Sanger Institute for the COVID-19 Genomics UK (COG-UK) consortium|Davies|United Kingdom
>Spike|hCoV-19/USA/IL-NM091/2020|2020-03-19|EPI_ISL_444581|Original|hCoV-19^^Illinois|Human|Northwestern Memorial Hospital|Ozer Lab|Ozer|USA
>Spike|hCoV-19/Australia/VIC2236/2020|2020-06-22|EPI_ISL_521973|Original|hCoV-19^^Victoria|Human|Microbiological Diagnostic Unit - Public Health Laboratory (MDU-PHL)|MDU-PHL|Schultz|Australia
>Spike|hCoV-19/England/20156041804/2020|2020-04-08|EPI_ISL_466312|Original|hCoV-19^^England|Human|Respiratory Virus Unit|Respiratory Virus Unit|Platt|United Kingdom
>Spike|hCoV-19/USA/VA-DCLS-0694/2020|2020-04-17|EPI_ISL_500855|Original|hCoV-19^^Virginia|Human|Virginia DCLS|Virginia DCLS|St. Jacques|USA
>Spike|hCoV-19/Russia/MOS-CRIE-13373431/2020|2020-11-07|EPI_ISL_745237|Original|hCoV-19^^Moscow|Human|Molecular diagnostic laboratory of Federal Budget Institution of Science \Central Research Institute of Epidemiology\ of The Federal Service on Customers' Rights Protection and Human Well-being Surveillance|Group of Genomics and Postgenomic Technologies of Central Research Institute of Epidemilolog
>y|Korneenko|Russia
>Spike|hCoV-19/Denmark/DCGC-1780/2020|2020-07-13|EPI_ISL_618183|Original|hCoV-19^^Nordjylland|Human|Department of Virus and Microbiological Special Diagnostics|Albertsen lab|Michaelsen|Denmark
>Spike|hCoV-19/England/QEUH-B418D3/2020|2020-11-08|EPI_ISL_658726|Original|hCoV-19^^England|Human|Lighthouse Lab in Glasgow|Wellcome Sanger Institute for the COVID-19 Genomics UK (COG-UK) Consortium|Davies|United Kingdom
>Spike|hCoV-19/USA/WI-GMF-07491/2020|2020-06-23|EPI_ISL_489921|Original|hCoV-19^^Wisconsin|Human|Gundersen Molecular Diagnostics Laboratory|Kabara Cancer Research Institute|Kenny|USA
>Spike|hCoV-19/England/202822188/2020|2020-06-14|EPI_ISL_490244|Original|hCoV-19^^England|Human|Respiratory Virus Unit|Respiratory Virus Unit|Platt|United Kingdom
>Spike|hCoV-19/Denmark/DCGC-22156/2020|2020-12-14|EPI_ISL_757820|Original|hCoV-19^^Hovedstaden|Human|Department of Virus and Microbiological Special Diagnostics|Albertsen Lab|Michaelsen|Denmark
>Spike|hCoV-19/England/CAMC-BD147A/2020|2020-11-29|EPI_ISL_702336|Original|hCoV-19^^England|Human|Lighthouse Lab in Cambridge|Wellcome Sanger Institute for the COVID-19 Genomics UK (COG-UK) Consortium|Davies|United Kingdom
>Spike|hCoV-19/England/CAMB-7A748/2020|2020-04-02|EPI_ISL_441094|Original|hCoV-19^^England|Human|Department of Pathology|Wellcome Sanger Institute for the COVID-19 Genomics UK (COG-UK) consortium|Amato|United Kingdom
>Spike|hCoV-19/USA/AZ-TG491507/2020|2020-06-18|EPI_ISL_695131|Original|hCoV-19^^Arizona|Human|AZ SPHL|TGen North|French|USA
>Spike|hCoV-19/Finland/8LS12D10/2020|2020-10-08|EPI_ISL_755971|Original|hCoV-19^^Uusimaa|Human|Department of Virology and Immunology|Department of Virology|Smura|Finland
>Spike|hCoV-19/Australia/NSW102/2020|2020-03-11|EPI_ISL_427656|Original|hCoV-19^^New South Wales|Human|Centre for Infectious Diseases and Microbiology Public Health|NSW

Health Pathology - Institute of Clinical Pathology and Medical Research; Westmead Hospital; University of Sydney|Gall|Australia
>Spike|hCoV-19/Portugal/PT0249/2020|2020-04-02|EPI_ISL_453963|Original|hCoV-19^^Portugal|Human|unknown|Instituto Nacional de Saude (INSA)|Borges|Portugal
>Spike|hCoV-19/Belgium/UGent-52/2020|2020-03-31|EPI_ISL_451165|Original|hCoV-19^^Ghent|Human|Lab voor klinische biologie|Onderzoeksgroep Virologie|Lambrechts|Belgium
>Spike|hCoV-19/Canada/NS-NML-1377/2020|2020-04-15|EPI_ISL_579752|Original|hCoV-19^^Nova Scotia|Human|QEII Health Sciences Centre|National Microbiology Laboratory (NML)|Majer|Canada
>Spike|hCoV-19/Australia/VIC11143/2020|2020-07-25|EPI_ISL_563268|Original|hCoV-19^^Victoria|Human|Microbiological Diagnostic Unit - Public Health Laboratory (MDU-PHL)|MDU-PHL|Schultz|Australia
>Spike|hCoV-19/USA/AZ-TG395932/2020|2020-06-08|EPI_ISL_694768|Original|hCoV-19^^Arizona|Human|AZ SPHL|TGen North|French|USA
>Spike|hCoV-19/Wales/PHWC-4851A2/2020|2020-10-25|EPI_ISL_628155|Original|hCoV-19^^Wales|Human|Wales Specialist Virology Centre Sequencing lab|COVID-19 Genomics UK (COG-UK) Consortium|Moore|United Kingdom
>Spike|hCoV-19/USA/CA-CZB-14762/2020|2020-11-05|EPI_ISL_739292|Original|hCoV-19^^California|Human|Humboldt County Public Health Laboratory|Chan-Zuckerberg Biohub|Kamm|USA
>Spike|hCoV-19/Japan/PG-7673/2020|2020-08-00|EPI_ISL_686775|Original|hCoV-19^^Japan|Human|Chiba Prefectural Institute of Public Health|Pathogen Genomics Center|Sekizuka|Japan
>Spike|hCoV-19/Denmark/DCGC-19017/2020|2020-12-07|EPI_ISL_748700|Original|hCoV-19^^Hovedstaden|Human|Department of Virus and Microbiological Special Diagnostics|Albertsen Lab|Michaelsen|Denmark
>Spike|hCoV-19/England/BRIS-1233FE/2020|2020-04-05|EPI_ISL_442721|Original|hCoV-19^^England|Human|PHE South West Regional Laboratory|Wellcome Sanger Institute for the COVID-19 Genomics UK (COG-UK) consortium|Amato|United Kingdom
>Spike|hCoV-19/England/OXON-B389B/2020|2020-04-07|EPI_ISL_534892|Original|hCoV-19^^England|Human|Oxford Viromics|COVID-19 Genomics UK (COG-UK) Consortium|Golubchik|United Kingdom
>Spike|hCoV-19/Australia/VIC15727/2020|2020-00-00|EPI_ISL_593426|Original|hCoV-19^^Victoria|Human|Microbiological Diagnostic Unit - Public Health Laboratory (MDU-PHL)|MDU-PHL|Schultz|Australia
>Spike|hCoV-19/USA/CA-CZB-13728/2020|2020-11-09|EPI_ISL_739477|Original|hCoV-19^^California|Human|County of San Luis Obispo Public Health Laboratory|Chan-Zuckerberg Biohub|Kamm|USA
>Spike|hCoV-19/Germany/SH-ChVir8333/2020|2020-08-05|EPI_ISL_729586|Original|hCoV-19^^Schleswig-Holstein|Human|A. Krumbholz|Charit\u00e9 Universit\u00e4tsmedizin Berlin|Muehlemann|Germany
>Spike|hCoV-19/England/ALDP-A95A35/2020|2020-10-19|EPI_ISL_624783|Original|hCoV-19^^England|Human|Lighthouse Lab in Alderley Park|Wellcome Sanger Institute for the COVID-19 Genomics UK (COG-UK) consortium|Davies|United Kingdom
>Spike|hCoV-19/Canada/BC_07924910/2020|2020-04-00|EPI_ISL_477102|Original|hCoV-19^^British Columbia|Human|BCCDC Public Health Laboratory|BCCDC Public Health Laboratory|Harrigan|Canada
>Spike|hCoV-19/England/QEUH-B822AB/2020|2020-11-14|EPI_ISL_661861|Original|hCoV-19^^England|Human|Lighthouse Lab in Glasgow|Wellcome Sanger Institute for the COVID-19 Genomics UK (COG-UK) Consortium|Davies|United Kingdom
>Spike|hCoV-19/England/ALDP-9BCE4C/2020|2020-09-09|EPI_ISL_573079|Original|hCoV-19^^England|Human|Oxford Viromics|COVID-19 Genomics UK (COG-UK) Consortium|Golubchik|United Kingdom
>Spike|hCoV-19/England/C200028755/2020|2020-10-09|EPI_ISL_733586|Original|hCoV-19^^England|Human|Respiratory Virus Unit|COVID-19 Genomics UK (COG-UK) Consortium|Lackenby|United Kingdom
>Spike|hCoV-19/USA/WA-S1959/2020|2020-06-04|EPI_ISL_497387|Original|hCoV-19^^Washington|Human|Washington State Department of Health|Seattle Flu Study|Lee|USA

>Spike|hCoV-19/USA/NV-NSPHL-A0108/2020|2020-04-17|EPI_ISL_515381|Original|hCoV-19^^Nevada|Human|Nevada State Public Health Laboratory|Nevada State Public Health Laboratory|Sevinsky|USA
>Spike|hCoV-19/England/CAMB-7D04A/2020|2020-04-03|EPI_ISL_442811|Original|hCoV-19^^England|Human|Department of Pathology|Wellcome Sanger Institute for the COVID-19 Genomics UK (COG-UK) consortium|Amato|United Kingdom
>Spike|hCoV-19/England/LOND-126041C/2020|2020-03-16|EPI_ISL_524653|Original|hCoV-19^^England|Human|North West London Pathology|Wellcome Sanger Institute for the COVID-19 Genomics UK (COG-UK) consortium|Davies|United Kingdom
>Spike|hCoV-19/England/CAMB-848B1/2020|2020-03-04|EPI_ISL_440512|Original|hCoV-19^^England|Human|Department of Pathology|Wellcome Sanger Institute for the COVID-19 Genomics UK (COG-UK) consortium|Amato|United Kingdom
>Spike|hCoV-19/USA/DE-DHSS-F917709/2020|2020-03-23|EPI_ISL_560307|Original|hCoV-19^^Delaware|Human|Delaware Public Health Lab|Delaware Public Health Lab|Bajwa|USA
>Spike|hCoV-19/USA/TX-DSHS-0357/2020|2020-06-13|EPI_ISL_538294|Original|hCoV-19^^Texas|Human|Texas Department of State Health Services|Texas Department of State Health Services|Rahman|USA
>Spike|hCoV-19/Wales/PHWC-498A56/2020|2020-12-05|EPI_ISL_727163|Original|hCoV-19^^Wales|Human|Originating lab|COVID-19 Genomics UK (COG-UK) Consortium|Moore|United Kingdom
>Spike|hCoV-19/USA/LA-EVTL268/2020|2020-08-22|EPI_ISL_574402|Original|hCoV-19^^Louisiana|Human|LSUHS Emerging Viral Threat Laboratory|Microbial Genome Sequencing Center|Kamil|USA
>Spike|hCoV-19/England/ALDP-A6E543/2020|2020-10-15|EPI_ISL_607193|Original|hCoV-19^^England|Human|Lighthouse Lab in Alderley Park|Wellcome Sanger Institute for the COVID-19 Genomics UK (COG-UK) consortium|Davies|United Kingdom
>Spike|hCoV-19/USA/TX-HMH-6930/2020|2020-06-27|EPI_ISL_546360|Original|hCoV-19^^Texas|Human|Houston Methodist Hospital|Houston Methodist Hospital|Long|USA
>Spike|hCoV-19/England/NORW-E7D62/2020|2020-05-06|EPI_ISL_449089|Original|hCoV-19^^England|Human|Quadram Institute Bioscience|COVID-19 Genomics UK (COG-UK) Consortium|Alikhan|United Kingdom
>Spike|hCoV-19/Wales/PHWC-2CB1C/2020|2020-04-01|EPI_ISL_445950|Original|hCoV-19^^Wales|Human|Wales Specialist Virology Centre|Public Health Wales Microbiology Cardiff|Moore|United Kingdom
>Spike|hCoV-19/Singapore/564nan/2020|2020-04-02|EPI_ISL_538471|Original|hCoV-19^^Singapore|Human|Department of Laboratory Medicine|Department of Laboratory Medicine|Chen|Singapore
>Spike|hCoV-19/USA/TX-HMH-MCoV-14841/2020|2020-10-13|EPI_ISL_786325|Original|hCoV-19^^Texas|Human|Houston Methodist Hospital|Houston Methodist Hospital|Long|USA
>Spike|hCoV-19/England/NOTT-11CC1B/2020|2020-11-19|EPI_ISL_665740|Original|hCoV-19^^England|Human|Queens Medical Centre|COVID-19 Genomics UK (COG-UK) Consortium|Loose|United Kingdom
>Spike|hCoV-19/England/CAMC-C98D28/2020|2020-12-18|EPI_ISL_777332|Original|hCoV-19^^England|Human|Lighthouse Lab in Cambridge|Wellcome Sanger Institute for the COVID-19 Genomics UK (COG-UK) Consortium|Davies|United Kingdom
>Spike|hCoV-19/Australia/VIC4229/2020|2020-07-14|EPI_ISL_519557|Original|hCoV-19^^Victoria|Human|Microbiological Diagnostic Unit - Public Health Laboratory (MDU-PHL)|MDU-PHL|Schultz|Australia
>Spike|hCoV-19/Wales/PHWC-29292/2020|2020-04-03|EPI_ISL_445502|Original|hCoV-19^^Wales|Human|Wales Specialist Virology Centre|Public Health Wales Microbiology Cardiff|Moore|United Kingdom
>Spike|hCoV-19/England/QEUH-AD2FD3/2020|2020-10-26|EPI_ISL_630004|Original|hCoV-19^^England|Human|Lighthouse Lab in Glasgow|Wellcome Sanger Institute for the COVID-19 Genomics UK (COG-UK) consortium|Davies|United Kingdom
>Spike|hCoV-19/England/20129177404/2020|2020-03-22|EPI_ISL_424161|Original|hCoV-19^^England|Human|Respiratory Virus Unit|Respiratory Virus Unit|Platt|United Kingdom
>Spike|hCoV-19/England/ALDP-94482D/2020|2020-06-07|EPI_ISL_555599|Original|hCoV-19^^England|Human|Lighthouse Lab in Alderley Park|Wellcome Sanger Institute for the COVID-19 Genomics UK (COG-UK) consortium|Davies|United Kingdom

>Spike|hCoV-19/England/BIRM-65A56/2020|2020-10-01|EPI_ISL_594605|Original|hCoV-
19^^England|Human|University of Birmingham|COVID-19 Genomics UK (COG-UK)
Consortium|Loman|United Kingdom
>Spike|hCoV-19/England/MILK-ACD4AF/2020|2020-10-22|EPI_ISL_634510|Original|hCoV-
19^^England|Human|Lighthouse Lab in Milton Keynes|Wellcome Sanger Institute for the
COVID-19 Genomics UK (COG-UK) consortium|Davies|United Kingdom
>Spike|hCoV-19/USA/TX-DSHS-1094/2020|2020-05-22|EPI_ISL_615133|Original|hCoV-
19^^Texas|Human|Texas Department of State Health Services|Texas Department of State
Health Services|Rahman|USA
>Spike|hCoV-19/Australia/NSW1308/2020|2020-12-18|EPI_ISL_767874|Original|hCoV-19^^New
South Wales|Human|Histopath|NSW Health Pathology - Institute of Clinical Pathology
and Medical Research; Westmead Hospital; University of Sydney|Gall|Australia
>Spike|hCoV-19/England/MILK-A7D71E/2020|2020-10-13|EPI_ISL_606497|Original|hCoV-
19^^England|Human|Lighthouse Lab in Milton Keynes|Wellcome Sanger Institute for the
COVID-19 Genomics UK (COG-UK) consortium|Davies|United Kingdom
>Spike|hCoV-19/USA/WI-GMF-13999/2020|2020-08-05|EPI_ISL_547664|Original|hCoV-
19^^Wisconsin|Human|Gundersen Molecular Diagnostics Laboratory|Kabara Cancer Research
Institute|Kenny|USA
>Spike|hCoV-19/Scotland/EDB031/2020|2020-03-13|EPI_ISL_425837|Original|hCoV-
19^^Scotland|Human|Virology Department|COVID-19 Genomics UK (COG-UK)
Consortium|Williams|United Kingdom
>Spike|hCoV-19/England/QEUH-BC4BC0/2020|2020-11-25|EPI_ISL_704114|Original|hCoV-
19^^England|Human|Lighthouse Lab in Glasgow|Wellcome Sanger Institute for the COVID-
19 Genomics UK (COG-UK) Consortium|Davies|United Kingdom
>Spike|hCoV-19/USA/TX-HMH-4100/2020|2020-06-18|EPI_ISL_543370|Original|hCoV-
19^^Texas|Human|Houston Methodist Hospital|Houston Methodist Hospital|Long|USA
>Spike|hCoV-19/England/MILK-BEE39B/2020|2020-11-30|EPI_ISL_721526|Original|hCoV-
19^^England|Human|Lighthouse Lab in Milton Keynes|Wellcome Sanger Institute for the
COVID-19 Genomics UK (COG-UK) Consortium|Davies|United Kingdom
>Spike|hCoV-19/England/ALDP-A2E15A/2020|2020-10-06|EPI_ISL_586917|Original|hCoV-
19^^England|Human|Lighthouse Lab in Alderley Park|Wellcome Sanger Institute for the
COVID-19 Genomics UK (COG-UK) consortium|Davies|United Kingdom
>Spike|hCoV-19/England/CAMC-A57B87/2020|2020-10-10|EPI_ISL_597250|Original|hCoV-
19^^England|Human|Lighthouse Lab in Cambridge|Wellcome Sanger Institute for the
COVID-19 Genomics UK (COG-UK) consortium|Davies|United Kingdom
>Spike|hCoV-19/England/ALDP-B45AE8/2020|2020-11-07|EPI_ISL_656023|Original|hCoV-
19^^England|Human|Lighthouse Lab in Alderley Park|Wellcome Sanger Institute for the
COVID-19 Genomics UK (COG-UK) Consortium|Davies|United Kingdom
>Spike|hCoV-19/Wales/MILK-B048DC/2020|2020-10-24|EPI_ISL_651031|Original|hCoV-
19^^Wales|Human|Department of Pathology|COVID-19 Genomics UK (COG-UK)
Consortium|Goodfellow|United Kingdom
>Spike|hCoV-19/England/201090513/2020|2020-03-07|EPI_ISL_464577|Original|hCoV-
19^^England|Human|Respiratory Virus Unit|Respiratory Virus Unit|Platt|United Kingdom
>Spike|hCoV-19/England/QEUH-9B4952/2020|2020-09-03|EPI_ISL_590242|Original|hCoV-
19^^England|Human|Lighthouse Lab in Glasgow|Wellcome Sanger Institute for the COVID-
19 Genomics UK (COG-UK) consortium|Davies|United Kingdom
>Spike|hCoV-19/Denmark/DCGC-2647/2020|2020-08-17|EPI_ISL_617866|Original|hCoV-
19^^Syddanmark|Human|Department of Virus and Microbiological Special
Diagnostics|Albertsen lab|Michaelsen|Denmark
>Spike|hCoV-19/USA/CA-CZB-3629/2020|2020-07-23|EPI_ISL_548351|Original|hCoV-
19^^California|Human|County of San Luis Obispo Public Health Laboratory|Chan-
Zuckerberg Biohub|Bhatt|USA
>Spike|hCoV-19/England/CAMB-7BE60/2020|2020-04-05|EPI_ISL_470497|Original|hCoV-
19^^England|Human|Department of Pathology|Wellcome Sanger Institute for the COVID-19
Genomics UK (COG-UK) consortium|Davies|United Kingdom
>Spike|hCoV-19/Japan/PG-6316/2020|2020-08-00|EPI_ISL_689427|Original|hCoV-
19^^Japan|Human|Pathogen Genomics Center|Pathogen Genomics Center|Sekizuka|Japan
>Spike|hCoV-19/Denmark/DCGC-10108/2020|2020-11-09|EPI_ISL_669398|Original|hCoV-
19^^Hovedstaden|Human|Department of Virus and Microbiological Special
Diagnostics|Albertsen Lab|Michaelsen|Denmark

>Spike|hCoV-19/USA/CA-CZB-4067/2020|2020-08-05|EPI_ISL_548654|Original|hCoV-19^^California|Human|Orange County Public Health Laboratory|Chan-Zuckerberg Biohub|Bhatt|USA
>Spike|hCoV-19/Denmark/DCGC-20391/2020|2020-12-21|EPI_ISL_758720|Original|hCoV-19^^Hovedstaden|Human|Department of Virus and Microbiological Special Diagnostics|Albertsen Lab|Michaelsen|Denmark
>Spike|hCoV-19/England/MILK-BFF485/2020|2020-12-02|EPI_ISL_720826|Original|hCoV-19^^England|Human|Lighthouse Lab in Milton Keynes|Wellcome Sanger Institute for the COVID-19 Genomics UK (COG-UK) Consortium|Davies|United Kingdom
>Spike|hCoV-19/Denmark/DCGC-9634/2020|2020-11-09|EPI_ISL_669078|Original|hCoV-19^^Sjaelland|Human|Department of Virus and Microbiological Special Diagnostics|Albertsen Lab|Michaelsen|Denmark
>Spike|hCoV-19/England/MILK-4674DA/2020|2020-05-21|EPI_ISL_559719|Original|hCoV-19^^England|Human|Lighthouse Lab in Milton Keynes|Wellcome Sanger Institute for the COVID-19 Genomics UK (COG-UK) consortium|Davies|United Kingdom
>Spike|hCoV-19/USA/CT-UW-4238/2020|2020-03-30|EPI_ISL_427162|Original|hCoV-19^^Connecticut|Human|UW Virology Lab|UW Virology Lab|Roychoudhury|USA
>Spike|hCoV-19/England/QEUH-B2BA06/2020|2020-11-02|EPI_ISL_646539|Original|hCoV-19^^England|Human|Lighthouse Lab in Glasgow|Wellcome Sanger Institute for the COVID-19 Genomics UK (COG-UK) Consortium|Davies|United Kingdom
>Spike|hCoV-19/England/QEUH-B68618/2020|2020-11-11|EPI_ISL_662257|Original|hCoV-19^^England|Human|Lighthouse Lab in Glasgow|Wellcome Sanger Institute for the COVID-19 Genomics UK (COG-UK) Consortium|Davies|United Kingdom
>Spike|hCoV-19/England/CAMC-B08CCD/2020|2020-10-30|EPI_ISL_643674|Original|hCoV-19^^England|Human|Lighthouse Lab in Cambridge|Wellcome Sanger Institute for the COVID-19 Genomics UK (COG-UK) Consortium|Davies|United Kingdom
>Spike|hCoV-19/England/QEUH-9DE284/2020|2020-09-21|EPI_ISL_599764|Original|hCoV-19^^England|Human|Lighthouse Lab in Glasgow|Wellcome Sanger Institute for the COVID-19 Genomics UK (COG-UK) consortium|Davies|United Kingdom
>Spike|hCoV-19/Denmark/DCGC-2743/2020|2020-08-17|EPI_ISL_616488|Original|hCoV-19^^Midtjylland|Human|Department of Virus and Microbiological Special Diagnostics|Albertsen lab|Michaelsen|Denmark
>Spike|hCoV-19/USA/AZ-TG670225/2020|2020-04-30|EPI_ISL_696408|Original|hCoV-19^^Arizona|Human|Sonora Quest Laboratories|TGen North|French|USA
>Spike|hCoV-19/USA/CA-CZB-1036/2020|2020-04-02|EPI_ISL_436677|Original|hCoV-19^^California|Human|County of Santa Clara Public Health Department|Chan-Zuckerberg Biohub|Hao|USA
>Spike|hCoV-19/Switzerland/ZH-ETHZ-300299/2020|2020-09-25|EPI_ISL_603331|Original|hCoV-19^^Zurich|Human|Viollier AG|Department of Biosystems Science and Engineering|Nadeau|Switzerland
>Spike|hCoV-19/Wales/PHWC-3465C/2020|2020-04-20|EPI_ISL_446953|Original|hCoV-19^^Wales|Human|Wales Specialist Virology Centre|Public Health Wales Microbiology Cardiff|Moore|United Kingdom
>Spike|hCoV-19/USA/FL-BPHL-0432/2020|2020-03-22|EPI_ISL_495338|Original|hCoV-19^^Florida|Human|Florida Bureau of Public Health Laboratories|Florida Bureau of Public Health Laboratories|Blanton|USA
>Spike|hCoV-19/USA/CA-CZB-1283/2020|2020-04-02|EPI_ISL_454641|Original|hCoV-19^^California|Human|Humboldt County Public Health Laboratory|Chan-Zuckerberg Biohub|Hao|USA
>Spike|hCoV-19/England/MILK-BDB72C/2020|2020-11-26|EPI_ISL_709570|Original|hCoV-19^^England|Human|Lighthouse Lab in Milton Keynes|Wellcome Sanger Institute for the COVID-19 Genomics UK (COG-UK) Consortium|Davies|United Kingdom
>Spike|hCoV-19/United Arab Emirates/0467/2020|2020-05-18|EPI_ISL_698637|Original|hCoV-19^^Abu Dhabi|Human|Group 42 (G42) Healthcare|G42 Healthcare|Ogrodzki|United Arab Emirates
>Spike|hCoV-19/Denmark/DCGC-11324/2020|2020-11-02|EPI_ISL_670840|Original|hCoV-19^^Syddanmark|Human|Department of Virus and Microbiological Special Diagnostics|Albertsen Lab|Michaelsen|Denmark

>Spike|hCoV-19/England/ALDP-5B4938/2020|2020-06-18|EPI_ISL_558718|Original|hCoV-19^^England|Human|Lighthouse Lab in Alderley Park|Wellcome Sanger Institute for the COVID-19 Genomics UK (COG-UK) consortium|Davies|United Kingdom
>Spike|hCoV-19/England/QEUH-9DC6F3/2020|2020-09-21|EPI_ISL_581052|Original|hCoV-19^^England|Human|Lighthouse Lab in Glasgow|Wellcome Sanger Institute for the COVID-19 Genomics UK (COG-UK) consortium|Davies|United Kingdom
>Spike|hCoV-19/England/MILK-BB1258/2020|2020-11-17|EPI_ISL_719995|Original|hCoV-19^^England|Human|Lighthouse Lab in Milton Keynes|Wellcome Sanger Institute for the COVID-19 Genomics UK (COG-UK) Consortium|Davies|United Kingdom
>Spike|hCoV-19/England/CAMC-B083AA/2020|2020-10-30|EPI_ISL_643880|Original|hCoV-19^^England|Human|Lighthouse Lab in Cambridge|Wellcome Sanger Institute for the COVID-19 Genomics UK (COG-UK) Consortium|Davies|United Kingdom
>Spike|hCoV-19/USA/CA-IGI-0101/2020|2020-06-03|EPI_ISL_495123|Original|hCoV-19^^California|Human|Innovative Genomics Institute|Innovative Genomics Institute|Shivram|USA
>Spike|hCoV-19/Wales/PHWC-49E407/2020|2020-12-07|EPI_ISL_743000|Original|hCoV-19^^Wales|Human|Wales Specialist Virology Centre Sequencing lab|COVID-19 Genomics UK (COG-UK) Consortium|Moore|United Kingdom
>Spike|hCoV-19/Australia/VIC5107/2020|2020-07-19|EPI_ISL_519910|Original|hCoV-19^^Victoria|Human|Microbiological Diagnostic Unit - Public Health Laboratory (MDU-PHL)|MDU-PHL|Schultz|Australia
>Spike|hCoV-19/Spain/AR-IBV-97010749/2020|2020-10-02|EPI_ISL_779740|Original|hCoV-19^^Aragon|Human|Servicio de Microbiolog\u00eda|SeqCOVID-SPAIN consortium/IBV(CSIC)|Comas|Spain
>Spike|hCoV-19/England/QEUH-B6ACAB/2020|2020-11-11|EPI_ISL_655166|Original|hCoV-19^^England|Human|Lighthouse Lab in Glasgow|Wellcome Sanger Institute for the COVID-19 Genomics UK (COG-UK) Consortium|Davies|United Kingdom
>Spike|hCoV-19/USA/TX-HMH-MCoV-13655/2020|2020-08-20|EPI_ISL_788757|Original|hCoV-19^^Texas|Human|Houston Methodist Hospital|Houston Methodist Hospital|Long|USA
>Spike|hCoV-19/England/ALDP-95CB74/2020|2020-06-19|EPI_ISL_553471|Original|hCoV-19^^England|Human|Lighthouse Lab in Alderley Park|Wellcome Sanger Institute for the COVID-19 Genomics UK (COG-UK) consortium|Davies|United Kingdom
>Spike|hCoV-19/France/PAC-IHU-0136/2020|2020-03-15|EPI_ISL_569331|Original|hCoV-19^^Provence-Alpes-C\u00f4te-d\u2019Azur|Human|MEPHI|MEPHI|Levasseur|France
>Spike|hCoV-19/USA/NY-Wadsworth-46358-01/2020|2020-04-14|EPI_ISL_676846|Original|hCoV-19^^New York|Human|Wadsworth Center|Wadsworth Center|Lasek-Nesselquist|USA
>Spike|hCoV-19/USA/CA-ALSR-1793/2020|2020-05-18|EPI_ISL_512188|Original|hCoV-19^^California|Human|San Diego County Public Health Laboratory|Andersen lab at Scripps Research|Gangavarapu|USA
>Spike|hCoV-19/Australia/VIC9635/2020|2020-08-21|EPI_ISL_562746|Original|hCoV-19^^Victoria|Human|Microbiological Diagnostic Unit - Public Health Laboratory (MDU-PHL)|MDU-PHL|Schultz|Australia
>Spike|hCoV-19/Russia/MOS-CRIE-13385721/2020|2020-11-06|EPI_ISL_732672|Original|hCoV-19^^Moscow|Human|Molecular diagnostic laboratory of Federal Budget Institution of Science \Central Research Institute of Epidemiology\ of The Federal Service on Customers' Rights Protection and Human Well-being Surveillance|Group of Genomics and Postgenomic Technologies of Central Research Institute of Epidemilolog
>y|Korneenko|Russia
>Spike|hCoV-19/England/QEUH-AD2F5B/2020|2020-10-26|EPI_ISL_629894|Original|hCoV-19^^England|Human|Lighthouse Lab in Glasgow|Wellcome Sanger Institute for the COVID-19 Genomics UK (COG-UK) consortium|Davies|United Kingdom
>Spike|hCoV-19/England/BRIS-12A685/2020|2020-04-30|EPI_ISL_458469|Original|hCoV-19^^England|Human|PHE South West Regional Laboratory|Wellcome Sanger Institute for the COVID-19 Genomics UK (COG-UK) consortium|Davies|United Kingdom
>Spike|hCoV-19/Russia/ROS-RII-MH2820S/2020|2020-09-24|EPI_ISL_639965|Original|hCoV-19^^Rostov|Human|HELIX LLC|WHO National Influenza Centre Russian Federation|Komissarov|Russia

>Spike|hCoV-19/England/QEUH-B9F548/2020|2020-11-18|EPI_ISL_721036|Original|hCoV-
19^^England|Human|Lighthouse Lab in Glasgow|Wellcome Sanger Institute for the COVID-
19 Genomics UK (COG-UK) Consortium|Davies|United Kingdom
>Spike|hCoV-19/USA/TX-HMH-4487/2020|2020-06-19|EPI_ISL_543660|Original|hCoV-
19^^Texas|Human|Houston Methodist Hospital|Houston Methodist Hospital|Long|USA
>Spike|hCoV-19/England/QEUH-B3FC09/2020|2020-11-08|EPI_ISL_658451|Original|hCoV-
19^^England|Human|Lighthouse Lab in Glasgow|Wellcome Sanger Institute for the COVID-
19 Genomics UK (COG-UK) Consortium|Davies|United Kingdom
>Spike|hCoV-19/USA/UT-08596/2020|2020-05-26|EPI_ISL_524343|Original|hCoV-
19^^Utah|Human|Utah Public Health Laboratory|Utah Public Health Laboratory|Young|USA
>Spike|hCoV-19/England/NOTT-1112F6/2020|2020-05-11|EPI_ISL_453646|Original|hCoV-
19^^England|Human|Queens Medical Centre|COVID-19 Genomics UK (COG-UK)
Consortium|Loose|United Kingdom
>Spike|hCoV-19/India/GJ-GBRC-419b/2020|2020-07-14|EPI_ISL_586550|Original|hCoV-
19^^Gujarat|Human|CCC,Veraval|Gujarat Biotechnology Research Centre|Joshi|India
>Spike|hCoV-19/USA/MN-MDH-1338/2020|2020-03-25|EPI_ISL_496924|Original|hCoV-
19^^Minnesota|Human|Minnesota Department of Health|Minnesota Department of
Health|Garfin|USA
>Spike|hCoV-19/Denmark/DCGC-7279/2020|2020-10-12|EPI_ISL_619248|Original|hCoV-
19^^NA|Human|Department of Virus and Microbiological Special Diagnostics|Albertsen
lab|Michaelsen|Denmark
>Spike|hCoV-19/Spain/PV-IBV-98007653/2020|2020-09-10|EPI_ISL_582044|Original|hCoV-
19^^Basque Country|Human|Servicio de Microbiolog\u00eda. Hospital Universitario
Donostia. OSI Donostialdea. \u00c1rea de Enfermedades Infecciosas|SeqCOVID-SPAIN
consortium/IBV(CSIC)|Comas|Spain
>Spike|hCoV-19/England/BRIS-12332B/2020|2020-04-05|EPI_ISL_442804|Original|hCoV-
19^^England|Human|PHE South West Regional Laboratory|Wellcome Sanger Institute for
the COVID-19 Genomics UK (COG-UK) consortium|Amato|United Kingdom
>Spike|hCoV-19/England/QEUH-A1DBE7/2020|2020-09-29|EPI_ISL_587309|Original|hCoV-
19^^England|Human|Lighthouse Lab in Glasgow|Wellcome Sanger Institute for the COVID-
19 Genomics UK (COG-UK) consortium|Davies|United Kingdom
>Spike|hCoV-19/Australia/VIC4500/2020|2020-07-14|EPI_ISL_518911|Original|hCoV-
19^^Victoria|Human|Microbiological Diagnostic Unit - Public Health Laboratory (MDU-
PHL)|MDU-PHL|Schultz|Australia
>Spike|hCoV-19/England/ALDP-95B094/2020|2020-06-15|EPI_ISL_555531|Original|hCoV-
19^^England|Human|Lighthouse Lab in Alderley Park|Wellcome Sanger Institute for the
COVID-19 Genomics UK (COG-UK) consortium|Davies|United Kingdom
>Spike|hCoV-19/England/QEUH-9C88C7/2020|2020-09-10|EPI_ISL_567253|Original|hCoV-
19^^England|Human|Lighthouse Lab in Glasgow|Wellcome Sanger Institute for the COVID-
19 Genomics UK (COG-UK) consortium|Davies|United Kingdom
>Spike|hCoV-19/England/QEUH-AAF4B2/2020|2020-10-17|EPI_ISL_653026|Original|hCoV-
19^^England|Human|Oxford Viromics|COVID-19 Genomics UK (COG-UK)
Consortium|Golubchik|United Kingdom
>Spike|hCoV-19/Wales/PHWC-4813FB/2020|2020-10-19|EPI_ISL_613066|Original|hCoV-
19^^Wales|Human|Wales Specialist Virology Centre Sequencing lab|COVID-19 Genomics UK
(COG-UK) Consortium|Moore|United Kingdom
>Spike|hCoV-19/England/MILK-9E21F9/2020|2020-09-20|EPI_ISL_581114|Original|hCoV-
19^^England|Human|Lighthouse Lab in Milton Keynes|Wellcome Sanger Institute for the
COVID-19 Genomics UK (COG-UK) consortium|Davies|United Kingdom
>Spike|hCoV-19/USA/TX-HMH-5807/2020|2020-06-28|EPI_ISL_547029|Original|hCoV-
19^^Texas|Human|Houston Methodist Hospital|Houston Methodist Hospital|Long|USA
>Spike|hCoV-19/England/ALDP-A86E59/2020|2020-10-16|EPI_ISL_605980|Original|hCoV-
19^^England|Human|Lighthouse Lab in Alderley Park|Wellcome Sanger Institute for the
COVID-19 Genomics UK (COG-UK) consortium|Davies|United Kingdom
>Spike|hCoV-19/Scotland/QEUH-96E33B/2020|2020-08-20|EPI_ISL_534457|Original|hCoV-
19^^Scotland|Human|NHSGGC West of Scotland Specialist Virology Centre / MRC-
University of Glasgow Centre for Virus Research|Wellcome Sanger Institute for the
COVID-19 Genomics UK (COG-UK) consortium|Davies|United Kingdom

>Spike|hCoV-19/Netherlands/ZH-EMC-367/2020|2020-08-02|EPI_ISL_523707|Original|hCoV-19^^Zuid Holland|Human|Dutch COVID-19 response team|Erasmus Medical Center|Oude Munnink|Netherlands
>Spike|hCoV-19/England/QEUH-C15F96/2020|2020-12-06|EPI_ISL_709380|Original|hCoV-19^^England|Human|Lighthouse Lab in Glasgow|Wellcome Sanger Institute for the COVID-19 Genomics UK (COG-UK) Consortium|Davies|United Kingdom
>Spike|hCoV-19/USA/TX-HMH-MCoV-10040/2020|2020-07-06|EPI_ISL_789300|Original|hCoV-19^^Texas|Human|Houston Methodist Hospital|Houston Methodist Hospital|Long|USA
>Spike|hCoV-19/England/QEUH-A00D0F/2020|2020-10-02|EPI_ISL_584900|Original|hCoV-19^^England|Human|University of Exeter|COVID-19 Genomics UK (COG-UK) Consortium|Temperton|United Kingdom
>Spike|hCoV-19/England/CAMC-B08058/2020|2020-10-29|EPI_ISL_643364|Original|hCoV-19^^England|Human|Lighthouse Lab in Cambridge|Wellcome Sanger Institute for the COVID-19 Genomics UK (COG-UK) Consortium|Davies|United Kingdom
>Spike|hCoV-19/England/LIVE-DB106B/2020|2020-10-23|EPI_ISL_637954|Original|hCoV-19^^England|Human|Liverpool Clinical Laboratories|COVID-19 Genomics UK (COG-UK) Consortium|Haldenby|United Kingdom
>Spike|hCoV-19/England/QEUH-B66322/2020|2020-11-10|EPI_ISL_661617|Original|hCoV-19^^England|Human|Lighthouse Lab in Glasgow|Wellcome Sanger Institute for the COVID-19 Genomics UK (COG-UK) Consortium|Davies|United Kingdom
>Spike|hCoV-19/USA/WI-GMF-00744/2020|2020-04-03|EPI_ISL_422462|Original|hCoV-19^^Iowa|Human|Gundersen Molecular Diagnostics Laboratory|Kabara Cancer Research Institute|Kenny|USA
>Spike|hCoV-19/Australia/VIC12975/2020|2020-07-28|EPI_ISL_563614|Original|hCoV-19^^Victoria|Human|Microbiological Diagnostic Unit - Public Health Laboratory (MDU-PHL)|MDU-PHL|Schultz|Australia
>Spike|hCoV-19/USA/TX-HMH-6763/2020|2020-06-25|EPI_ISL_546226|Original|hCoV-19^^Texas|Human|Houston Methodist Hospital|Houston Methodist Hospital|Long|USA
>Spike|hCoV-19/Scotland/QEUH-A00B7E/2020|2020-10-02|EPI_ISL_585553|Original|hCoV-19^^Scotland|Human|University of Exeter|COVID-19 Genomics UK (COG-UK) Consortium|Temperton|United Kingdom
>Spike|hCoV-19/India/TG-CCMB-W866/2020|2020-07-14|EPI_ISL_528829|Original|hCoV-19^^Telangana|Human|CSIR-Centre for Cellular and Molecular Biology|CSIR-Centre for Cellular and Molecular Biology|Banu|India
>Spike|hCoV-19/England/QEUH-C149C6/2020|2020-12-06|EPI_ISL_709565|Original|hCoV-19^^England|Human|Lighthouse Lab in Glasgow|Wellcome Sanger Institute for the COVID-19 Genomics UK (COG-UK) Consortium|Davies|United Kingdom
>Spike|hCoV-19/England/SHEF-CAF41/2020|2020-05-12|EPI_ISL_479244|Original|hCoV-19^^England|Human|Virology Department|COVID-19 Genomics UK (COG-UK) Consortium|de Silva|United Kingdom
>Spike|hCoV-19/England/NORT-28945C/2020|2020-03-29|EPI_ISL_458667|Original|hCoV-19^^England|Human|NU-OMICS DNA Sequencing research facility|Wellcome Sanger Institute for the COVID-19 Genomics UK (COG-UK) consortium|Davies|United Kingdom
>Spike|hCoV-19/Denmark/ALAB-SSI119/2020|2020-03-08|EPI_ISL_429348|Original|hCoV-19^^Denmark|Human|Department of Virus and Microbiological Special Diagnostics|Albertsen lab|Kirkegaard|Denmark
>Spike|hCoV-19/England/ALDP-AA383A/2020|2020-10-22|EPI_ISL_624948|Original|hCoV-19^^England|Human|Lighthouse Lab in Alderley Park|Wellcome Sanger Institute for the COVID-19 Genomics UK (COG-UK) consortium|Davies|United Kingdom
>Spike|hCoV-19/France/OCC-SC566/2020|2020-05-20|EPI_ISL_663243|Original|hCoV-19^^Occitanie|Human|CHU N\u00eemes|CNR Virus des Infections Respiratoires - France SUD|Bal|France
>Spike|hCoV-19/USA/AZ-TG666165/2020|2020-05-01|EPI_ISL_696245|Original|hCoV-19^^Arizona|Human|Sonora Quest Laboratories|TGen North|French|USA
>Spike|hCoV-19/Wales/PHWC-481F51/2020|2020-10-16|EPI_ISL_613188|Original|hCoV-19^^Wales|Human|Wales Specialist Virology Centre Sequencing lab|COVID-19 Genomics UK (COG-UK) Consortium|Moore|United Kingdom
>Spike|hCoV-19/England/CAMC-AB215B/2020|2020-10-23|EPI_ISL_623809|Original|hCoV-19^^England|Human|Lighthouse Lab in Cambridge|Wellcome Sanger Institute for the COVID-19 Genomics UK (COG-UK) consortium|Davies|United Kingdom

>Spike|hCoV-19/USA/WA-UW50/2020|2020-03-08|EPI_ISL_415615|Original|hCoV-19^^Washington|Human|UW Virology Lab|UW Virology Lab|Roychoudhury|USA
>Spike|hCoV-19/Australia/QLD1083/2020|2020-03-26|EPI_ISL_593602|Original|hCoV-19^^Australia|Human|unknown|Public Health Virology Laboratory|Nguyen|Australia
>Spike|hCoV-19/England/LIVE-1D2E93/2020|2020-04-01|EPI_ISL_612431|Original|hCoV-19^^England|Human|Liverpool Clinical Laboratories|COVID-19 Genomics UK (COG-UK) Consortium|Haldenby|United Kingdom
>Spike|hCoV-19/USA/MI-MDHHS-SC22596/2020|2020-11-18|EPI_ISL_737169|Original|hCoV-19^^Michigan|Human|Michigan Department of Health and Human Services|Michigan Department of Health and Human Services|Blankenship|USA
>Spike|hCoV-19/Australia/NSW1366/2020|2020-12-24|EPI_ISL_767932|Original|hCoV-19^^New South Wales|Human|Southern. IML Pathology|NSW Health Pathology - Institute of Clinical Pathology and Medical Research; Westmead Hospital; University of Sydney|Gall|Australia
>Spike|hCoV-19/USA/TX-DSHS-2032/2020|2020-07-30|EPI_ISL_766681|Original|hCoV-19^^Texas|Human|Texas Department of State Health Services|Texas Department of State Health Services|Rahman|USA
>Spike|hCoV-19/England/MILK-B3B62D/2020|2020-11-01|EPI_ISL_706071|Original|hCoV-19^^England|Human|Oxford Viromics|COVID-19 Genomics UK (COG-UK) Consortium|Golubchik|United Kingdom
>Spike|hCoV-19/England/MILK-992CBB/2020|2020-08-26|EPI_ISL_552154|Original|hCoV-19^^England|Human|Lighthouse Lab in Milton Keynes|Wellcome Sanger Institute for the COVID-19 Genomics UK (COG-UK) consortium|Davies|United Kingdom
>Spike|hCoV-19/England/ALDP-9BCD30/2020|2020-09-09|EPI_ISL_573072|Original|hCoV-19^^England|Human|Oxford Viromics|COVID-19 Genomics UK (COG-UK) Consortium|Golubchik|United Kingdom
>Spike|hCoV-19/Wales/PHWC-486183/2020|2020-10-15|EPI_ISL_639013|Original|hCoV-19^^Wales|Human|Wales Specialist Virology Centre Sequencing lab|COVID-19 Genomics UK (COG-UK) Consortium|Moore|United Kingdom
>Spike|hCoV-19/USA/TX-HMH-7912/2020|2020-06-30|EPI_ISL_545701|Original|hCoV-19^^Texas|Human|Houston Methodist Hospital|Houston Methodist Hospital|Long|USA
>Spike|hCoV-19/England/QEUH-B7C884/2020|2020-11-14|EPI_ISL_741795|Original|hCoV-19^^England|Human|Oxford Viromics|COVID-19 Genomics UK (COG-UK) Consortium|Golubchik|United Kingdom
>Spike|hCoV-19/Scotland/QEUH-A4B2CD/2020|2020-10-08|EPI_ISL_637826|Original|hCoV-19^^Scotland|Human|Department of Pathology|COVID-19 Genomics UK (COG-UK) Consortium|Goodfellow|United Kingdom
>Spike|hCoV-19/USA/CA-CZB-14306/2020|2020-08-24|EPI_ISL_739179|Original|hCoV-19^^California|Human|Alameda County Public Health Lab|Chan-Zuckerberg Biohub|Kamm|USA
>Spike|hCoV-19/USA/UT-UPHL-2012118130/2020|2020-08-08|EPI_ISL_745784|Original|hCoV-19^^Utah|Human|Ginkgo Bioworks Clinical Laboratory|Utah Public Health Laboratory|Young|USA
>Spike|hCoV-19/Japan/PG-5389/2020|2020-08-00|EPI_ISL_690492|Original|hCoV-19^^Japan|Human|Fukuoka Institute of Health and Environmental Sciences|Pathogen Genomics Center|Sekizuka|Japan
>Spike|hCoV-19/USA/WI-WSLH-200180/2020|2020-04-06|EPI_ISL_578405|Original|hCoV-19^^Wisconsin|Human|Wisconsin State Laboratory of Hygiene Communicable Disease Division|Wisconsin State Laboratory of Hygiene Communicable Disease Division|Florek|USA
>Spike|hCoV-19/England/CAMB-1AE908/2020|2020-04-29|EPI_ISL_524587|Original|hCoV-19^^England|Human|Department of Pathology|Wellcome Sanger Institute for the COVID-19 Genomics UK (COG-UK) consortium|Davies|United Kingdom
>Spike|hCoV-19/Sweden/20-50257/2020|2020-03-08|EPI_ISL_455895|Original|hCoV-19^^Stockholm|Human|Karolinska Universitetslaboratoriet|The Public Health Agency of Sweden|Svartstr\u00f6m|Sweden
>Spike|hCoV-19/England/ALDP-A680EE/2020|2020-10-10|EPI_ISL_609078|Original|hCoV-19^^England|Human|Lighthouse Lab in Alderley Park|Wellcome Sanger Institute for the COVID-19 Genomics UK (COG-UK) consortium|Davies|United Kingdom

>Spike|hCoV-19/England/ALDP-A6D034/2020|2020-10-14|EPI_ISL_608324|Original|hCoV-19^^England|Human|Lighthouse Lab in Alderley Park|Wellcome Sanger Institute for the COVID-19 Genomics UK (COG-UK) consortium|Davies|United Kingdom
>Spike|hCoV-19/USA/OR-OHSU-1425/2020|2020-07-16|EPI_ISL_509197|Original|hCoV-19^^Oregon|Human|OHSU Lab Services Molecular Microbiology Lab|Oregon SARS-CoV-2 Genome Sequencing Center|O'Roak|USA
>Spike|hCoV-19/Scotland/EDB413/2020|2020-03-29|EPI_ISL_426004|Original|hCoV-19^^Scotland|Human|Virology Department|COVID-19 Genomics UK (COG-UK) Consortium|Williams|United Kingdom
>Spike|hCoV-19/England/CAMC-CB736D/2020|2020-12-18|EPI_ISL_778185|Original|hCoV-19^^England|Human|Lighthouse Lab in Cambridge|Wellcome Sanger Institute for the COVID-19 Genomics UK (COG-UK) Consortium|Davies|United Kingdom
>Spike|hCoV-19/Wales/PHWC-24419/2020|2020-03-17|EPI_ISL_419409|Original|hCoV-19^^Wales|Human|Wales Specialist Virology Centre|Public Health Wales Microbiology Cardiff|Moore|United Kingdom
>Spike|hCoV-19/England/MILK-ACE981/2020|2020-10-21|EPI_ISL_629202|Original|hCoV-19^^England|Human|Lighthouse Lab in Milton Keynes|Wellcome Sanger Institute for the COVID-19 Genomics UK (COG-UK) consortium|Davies|United Kingdom
>Spike|hCoV-19/England/NORT-1B7A021/2020|2020-11-23|EPI_ISL_724950|Original|hCoV-19^^England|Human|Northumbria University / South Tees Hospitals NHS Foundation Trust / North Cumbria Integrated Care NHS Foundation Trust / North Tees and Hartlepool NHS Foundation Trust / Newcastle Hospitals NHS Foundation Trust|COVID-19 Genomics UK (COG-UK) Consortium|Bashton|United Kingdom
>Spike|hCoV-19/Denmark/DCGC-19909/2020|2020-12-07|EPI_ISL_747535|Original|hCoV-19^^Hovedstaden|Human|Department of Virus and Microbiological Special Diagnostics|Albertsen Lab|Michaelsen|Denmark
>Spike|hCoV-19/England/NOTT-113212/2020|2020-09-13|EPI_ISL_540711|Original|hCoV-19^^England|Human|Queens Medical Centre|COVID-19 Genomics UK (COG-UK) Consortium|Loose|United Kingdom
>Spike|hCoV-19/USA/OR-OHSU-0184/2020|2020-05-09|EPI_ISL_468126|Original|hCoV-19^^Oregon|Human|OHSU Lab Services Molecular Microbiology Lab|Oregon SARS-CoV-2 Genome Sequencing Center|O'Roak|USA
>Spike|hCoV-19/England/ALDP-BB3669/2020|2020-11-19|EPI_ISL_702638|Original|hCoV-19^^England|Human|Lighthouse Lab in Alderley Park|Wellcome Sanger Institute for the COVID-19 Genomics UK (COG-UK) Consortium|Davies|United Kingdom
>Spike|hCoV-19/Scotland/QEUH-9C92C8/2020|2020-09-14|EPI_ISL_576062|Original|hCoV-19^^Scotland|Human|Lighthouse Lab in Glasgow|Wellcome Sanger Institute for the COVID-19 Genomics UK (COG-UK) consortium|Davies|United Kingdom
>Spike|hCoV-19/USA/WA-UW99/2020|2020-03-12|EPI_ISL_416637|Original|hCoV-19^^Washington|Human|UW Virology Lab|UW Virology Lab|Roychoudhury|USA
>Spike|hCoV-19/England/PORT-2D3A06/2020|2020-04-17|EPI_ISL_529350|Original|hCoV-19^^England|Human|Centre for Enzyme Innovation|COVID-19 Genomics UK (COG-UK) Consortium|Robson|United Kingdom
>Spike|hCoV-19/Guangdong/20SF4051/2020|2020-02-17|EPI_ISL_428463|Original|hCoV-19^^Guangdong|Human|Guangdong Provincial Center for Diseases Control and Prevention;Guangdong Provincial Institute of Public Health|School of Public Health|Gu|China
>Spike|hCoV-19/England/205260868/2020|2020-12-20|EPI_ISL_754371|Original|hCoV-19^^England|Human|Respiratory Virus Unit|COVID-19 Genomics UK (COG-UK) Consortium|Platt|United Kingdom
>Spike|hCoV-19/USA/UT-UPHL-2012527816/2020|2020-12-03|EPI_ISL_747030|Original|hCoV-19^^Utah|Human|Utah Public Health Laboratory|Utah Public Health Laboratory|Gallagher|USA
>Spike|hCoV-19/Australia/VIC9614/2020|2020-07-29|EPI_ISL_565626|Original|hCoV-19^^Victoria|Human|Microbiological Diagnostic Unit - Public Health Laboratory (MDU-PHL)|MDU-PHL|Schultz|Australia
>Spike|hCoV-19/England/BRIS-133C5A/2020|2020-08-20|EPI_ISL_529655|Original|hCoV-19^^England|Human|University of Birmingham|COVID-19 Genomics UK (COG-UK) Consortium|Loman|United Kingdom

>Spike|hCoV-19/England/QEUH-963372/2020|2020-08-10|EPI_ISL_531786|Original|hCoV-19^^England|Human|Lighthouse Lab in Glasgow|Wellcome Sanger Institute for the COVID-19 Genomics UK (COG-UK) consortium|Davies|United Kingdom
>Spike|hCoV-19/Greece/54_37163/2020|2020-03-31|EPI_ISL_447835|unknown|hCoV-19^^Athens|Human|unknown|Department of Medicine|Submitter|Greece
>Spike|hCoV-19/England/QEUH-C321BB/2020|2020-12-11|EPI_ISL_782558|Original|hCoV-19^^England|Human|Lighthouse Lab in Glasgow|Wellcome Sanger Institute for the COVID-19 Genomics UK (COG-UK) Consortium|Davies|United Kingdom
>Spike|hCoV-19/South Africa/NHLS-UCT-GS-1374/2020|2020-11-13|EPI_ISL_700434|Original|hCoV-19^^Western Cape|Human|Plettenberg Bay Clinic wc PLC|NHLS/UCT|Iranzadeh|South Africa
>Spike|hCoV-19/England/ALDP-B76DA9/2020|2020-11-12|EPI_ISL_723811|Original|hCoV-19^^England|Human|Oxford Viromics|COVID-19 Genomics UK (COG-UK) Consortium|Golubchik|United Kingdom
>Spike|hCoV-19/Denmark/DCGC-8903/2020|2020-10-26|EPI_ISL_625886|Original|hCoV-19^^Hovedstaden|Human|Department of Virus and Microbiological Special Diagnostics|Albertsen lab|Michaelsen|Denmark
>Spike|hCoV-19/England/ALDP-C48307/2020|2020-12-11|EPI_ISL_736642|Original|hCoV-19^^England|Human|Lighthouse Lab in Alderley Park|Wellcome Sanger Institute for the COVID-19 Genomics UK (COG-UK) Consortium|Davies|United Kingdom
>Spike|hCoV-19/Switzerland/BE-ETHZ-280140/2020|2020-09-08|EPI_ISL_560438|Original|hCoV-19^^Bern|Human|Viollier AG|Department of Biosystems Science and Engineering|Nadeau|Switzerland
>Spike|hCoV-19/Northern Ireland/NIRE-23322A/2020|2020-09-24|EPI_ISL_680268|Original|hCoV-19^^Northern Ireland|Human|Regional Virus Laboratory|COVID-19 Genomics UK (COG-UK) Consortium|Fairley|United Kingdom
>Spike|hCoV-19/Sweden/20-06165/2020|2020-03-23|EPI_ISL_452230|Original|hCoV-19^^Vastra Gotaland|Human|Narhalsan Backa vardcentral|The Public Health Agency of Sweden|Svartstr\u00f6m|Sweden
>Spike|hCoV-19/USA/MA-MGH-01018/2020|2020-04-30|EPI_ISL_791845|Original|hCoV-19^^Massachusetts|Human|Massachusetts General Hospital|Infectious Disease Program|Park|USA
>Spike|hCoV-19/England/ALDP-A9384C/2020|2020-10-20|EPI_ISL_611045|Original|hCoV-19^^England|Human|Lighthouse Lab in Alderley Park|Wellcome Sanger Institute for the COVID-19 Genomics UK (COG-UK) consortium|Davies|United Kingdom
>Spike|hCoV-19/Denmark/DCGC-11158/2020|2020-11-02|EPI_ISL_670426|Original|hCoV-19^^Syddanmark|Human|Department of Virus and Microbiological Special Diagnostics|Albertsen Lab|Michaelsen|Denmark
>Spike|hCoV-19/Denmark/DCGC-2615/2020|2020-08-17|EPI_ISL_617852|Original|hCoV-19^^Midtjylland|Human|Department of Virus and Microbiological Special Diagnostics|Albertsen lab|Michaelsen|Denmark
>Spike|hCoV-19/USA/UT-UPHL-201121906/2020|2020-04-23|EPI_ISL_634898|Original|hCoV-19^^Utah|Human|Utah Public Health Laboratory|Utah Public Health Laboratory|Young|USA
>Spike|hCoV-19/England/MILK-B8780E/2020|2020-11-13|EPI_ISL_724803|Original|hCoV-19^^England|Human|Oxford Viromics|COVID-19 Genomics UK (COG-UK) Consortium|Golubchik|United Kingdom
>Spike|hCoV-19/England/ALDP-B62C49/2020|2020-11-08|EPI_ISL_655511|Original|hCoV-19^^England|Human|Lighthouse Lab in Alderley Park|Wellcome Sanger Institute for the COVID-19 Genomics UK (COG-UK) Consortium|Davies|United Kingdom
>Spike|hCoV-19/USA/MN-MDH-162/2020|2020-03-23|EPI_ISL_460057|Original|hCoV-19^^Minnesota|Human|Minnesota Department of Health|Minnesota Department of Health|Garfin|USA
>Spike|hCoV-19/Hong Kong/VM20072469/2020|2020-07-23|EPI_ISL_602601|Original|hCoV-19^^Hong Kong|Human|Tseung Kwan O Hospital|Hong Kong Department of Health|Mak|China
>Spike|hCoV-19/Spain/PV-IBV-000754/2020|2020-03-10|EPI_ISL_452735|Original|hCoV-19^^Basque Country|Human|Hospital Universitario Araba. Vitoria-Gasteiz,|SeqCOVID-SPAIN consortium/IBV(CSIC)|Comas|Spain
>Spike|hCoV-19/USA/MI-UM-10036535365/2020|2020-10-20|EPI_ISL_640148|Original|hCoV-19^^Michigan|Human|University of Michigan Clinical Microbiology Laboratory|Lauring Lab|Valesano|USA

>Spike|hCoV-19/USA/UT-01888/2020|2020-04-13|EPI_ISL_470743|Original|hCoV-19^^Utah|Human|Utah Public Health Laboratory|Utah Public Health Laboratory|Butz|USA
>Spike|hCoV-19/USA/AZ-TG274748/2020|2020-04-07|EPI_ISL_694068|Original|hCoV-19^^Arizona|Human|AZ SPHL|TGen North|French|USA
>Spike|hCoV-19/England/CAMB-83FEA/2020|2020-04-27|EPI_ISL_438721|Original|hCoV-19^^England|Human|Department of Pathology|COVID-19 Genomics UK (COG-UK) Consortium|Meredith|United Kingdom
>Spike|hCoV-19/England/PORT-2E9C43/2020|2020-00-00|EPI_ISL_706875|Original|hCoV-19^^England|Human|Centre for Enzyme Innovation|COVID-19 Genomics UK (COG-UK) Consortium|Robson|United Kingdom
>Spike|hCoV-19/Italy/LAZ-INMI-128/2020|2020-12-22|EPI_ISL_746835|Original|hCoV-19^^Lazio|Human|National Institute for Infectious Diseases|National Institute for Infectious Diseases|Gruber|Italy
>Spike|hCoV-19/USA/LA-EVTL648/2020|2020-08-06|EPI_ISL_579052|Original|hCoV-19^^Louisiana|Human|LSUHS Emerging Viral Threat Laboratory|Microbial Genome Sequencing Center|Kamil|USA
>Spike|hCoV-19/South Africa/LR00105/2020|2020-07-15|EPI_ISL_514402|Original|hCoV-19^^Gauteng|Human|National Institute for Communicable Diseases of the National Health Laboratory Service|National Institute for Communicable Diseases of the National Health Laboratory Service|Allam|South Africa
>Spike|hCoV-19/England/MILK-C52F42/2020|2020-12-12|EPI_ISL_736214|Original|hCoV-19^^England|Human|Lighthouse Lab in Milton Keynes|Wellcome Sanger Institute for the COVID-19 Genomics UK (COG-UK) Consortium|Davies|United Kingdom
>Spike|hCoV-19/Wales/PHWC-483A12/2020|2020-10-20|EPI_ISL_627884|Original|hCoV-19^^Wales|Human|Wales Specialist Virology Centre Sequencing lab|COVID-19 Genomics UK (COG-UK) Consortium|Moore|United Kingdom
>Spike|hCoV-19/USA/HI-QDX-1288/2020|2020-03-16|EPI_ISL_571927|Original|hCoV-19^^Hawaii|Human|Quest Diagnostics|Quest Diagnostics|Kagan|USA
>Spike|hCoV-19/Scotland/EDB6740/2020|2020-04-13|EPI_ISL_491618|Original|hCoV-19^^Scotland|Human|Virology Department|Wellcome Sanger Institute for the COVID-19 Genomics UK (COG-UK) consortium|Davies|United Kingdom
>Spike|hCoV-19/Switzerland/BS-42222420/2020|2020-04-06|EPI_ISL_581683|Original|hCoV-19^^Basel-Stadt|Human|University Hospital Basel|University Hospital Basel|Roloff Handschin|Switzerland
>Spike|hCoV-19/Switzerland/BS-UHB-42197025/2020|2020-03-18|EPI_ISL_528202|Original|hCoV-19^^Basel-Stadt|Human|University Hospital Basel|University Hospital Basel|Roloff Handschin|Switzerland
>Spike|hCoV-19/USA/WI-UW-1358/2020|2020-09-08|EPI_ISL_536729|Original|hCoV-19^^Wisconsin|Human|University of Wisconsin-Madison AIDS Vaccine Research Laboratories|University of Wisconsin-Madison AIDS Vaccine Research Laboratories|Baczenas|USA
>Spike|hCoV-19/USA/MD-MDH-0407/2020|2020-11-05|EPI_ISL_666777|Original|hCoV-19^^Maryland|Human|Maryland Public Health Laboratory|Maryland Public Health Laboratory|Keller|USA
>Spike|hCoV-19/Northern Ireland/QEUH-5E535E/2020|2020-06-17|EPI_ISL_533281|Original|hCoV-19^^Northern Ireland|Human|Lighthouse Lab in Glasgow|Wellcome Sanger Institute for the COVID-19 Genomics UK (COG-UK) consortium|Davies|United Kingdom
>Spike|hCoV-19/USA/WA-S626/2020|2020-04-18|EPI_ISL_438179|Original|hCoV-19^^Washington|Human|Washington State Department of Health|Seattle Flu Study|Bedford|USA
>Spike|hCoV-19/USA/WI-UW-2595/2021|2021-01-01|EPI_ISL_791112|Original|hCoV-19^^Wisconsin|Human|University of Wisconsin-Madison AIDS Vaccine Research Laboratories|University of Wisconsin-Madison AIDS Vaccine Research Laboratories|Moreno|USA
>Spike|hCoV-19/Ireland/D-NVRL-20G35482/2020|2020-03-20|EPI_ISL_605118|Original|hCoV-19^^Dublin|Human|National Virus Reference Laboratory|Irish Coronavirus Sequencing Consortium - Helixworks|Chalapati|Ireland

>Spike|hCoV-19/USA/TX-DSHS-0237/2020|2020-04-25|EPI_ISL_525788|Original|hCoV-19^^Texas|Human|Texas Department of State Health Services|Texas Department of State Health Services|Rahman|USA
>Spike|hCoV-19/England/LIVE-99400/2020|2020-04-08|EPI_ISL_439720|Original|hCoV-19^^England|Human|Liverpool Clinical Laboratories|COVID-19 Genomics UK (COG-UK) Consortium|Haldenby|United Kingdom
>Spike|hCoV-19/Portugal/PT0858/2020|2020-03-27|EPI_ISL_511117|Original|hCoV-19^^Portugal|Human|Instituto Nacional de Saude (INSA)|Instituto Nacional de Saude (INSA)|Borges|Portugal
>Spike|hCoV-19/England/ALDP-A6D377/2020|2020-10-15|EPI_ISL_608277|Original|hCoV-19^^England|Human|Lighthouse Lab in Alderley Park|Wellcome Sanger Institute for the COVID-19 Genomics UK (COG-UK) consortium|Davies|United Kingdom
>Spike|hCoV-19/USA/AZ-TG480916/2020|2020-07-08|EPI_ISL_695069|Original|hCoV-19^^Arizona|Human|TGen North|TGen North|French|USA
>Spike|hCoV-19/England/QEUH-A9F9C5/2020|2020-10-20|EPI_ISL_651143|Original|hCoV-19^^England|Human|University of Exeter|COVID-19 Genomics UK (COG-UK) Consortium|Temperton|United Kingdom
>Spike|hCoV-19/New Zealand/20VR2081/2020|2020-04-11|EPI_ISL_456367|Original|hCoV-19^^Canterbury|Human|Canterbury Health Laboratories|Institute of Environmental Science and Research (ESR)|de Ligt|New Zealand
>Spike|hCoV-19/Scotland/EDB4438/2020|2020-05-04|EPI_ISL_493900|Original|hCoV-19^^Scotland|Human|Virology Department|COVID-19 Genomics UK (COG-UK) Consortium|Williams|United Kingdom
>Spike|hCoV-19/England/MILK-9D52D8/2020|2020-09-18|EPI_ISL_637781|Original|hCoV-19^^England|Human|Centre for Enzyme Innovation|COVID-19 Genomics UK (COG-UK) Consortium|Robson|United Kingdom
>Spike|hCoV-19/Wales/PHWC-49B6AA/2020|2020-12-03|EPI_ISL_742368|Original|hCoV-19^^Wales|Human|Wales Specialist Virology Centre Sequencing lab|COVID-19 Genomics UK (COG-UK) Consortium|Moore|United Kingdom
>Spike|hCoV-19/England/NORW-E7272/2020|2020-04-19|EPI_ISL_449015|Original|hCoV-19^^England|Human|Quadram Institute Bioscience|COVID-19 Genomics UK (COG-UK) Consortium|Alikhan|United Kingdom
>Spike|hCoV-19/Australia/VIC2831/2020|2020-07-01|EPI_ISL_521662|Original|hCoV-19^^Victoria|Human|Microbiological Diagnostic Unit - Public Health Laboratory (MDU-PHL)|MDU-PHL|Schultz|Australia
>Spike|hCoV-19/USA/CA-CZB-1485/2020|2020-05-06|EPI_ISL_468436|Original|hCoV-19^^California|Human|County of San Luis Obispo Public Health Laboratory|Chan-Zuckerberg Biohub|Hao|USA
>Spike|hCoV-19/USA/NM-NMDOH-2020135000/2020|2020-06-18|EPI_ISL_535287|Original|hCoV-19^^New Mexico|Human|New Mexico Department of Health Scientific Laboratory|New Mexico Department of Health Scientific Laboratory|Johnson|USA
>Spike|hCoV-19/India/DL-SK-IGIB-CSIR-IGIB/2020|2020-08-10|EPI_ISL_636832|Original|hCoV-19^^Delhi|Human|National Centre for Disease control (NCDC)|NCDC/CSIR-IGIB|Faruq|India
>Spike|hCoV-19/USA/NM-NMDOH-2020320996/2020|2020-09-01|EPI_ISL_632823|Original|hCoV-19^^New Mexico|Human|New Mexico Department of Health Scientific Laboratory|New Mexico Department of Health Scientific Laboratory|Johnson|USA
>Spike|hCoV-19/England/QEUH-969F69/2020|2020-08-12|EPI_ISL_530820|Original|hCoV-19^^England|Human|Lighthouse Lab in Glasgow|Wellcome Sanger Institute for the COVID-19 Genomics UK (COG-UK) consortium|Davies|United Kingdom
>Spike|hCoV-19/England/CAMC-946CC5/2020|2020-07-14|EPI_ISL_556807|Original|hCoV-19^^England|Human|Lighthouse Lab in Cambridge|Wellcome Sanger Institute for the COVID-19 Genomics UK (COG-UK) consortium|Davies|United Kingdom
>Spike|hCoV-19/England/CAMB-1B1155/2020|2020-05-07|EPI_ISL_459204|Original|hCoV-19^^England|Human|Department of Pathology|Wellcome Sanger Institute for the COVID-19 Genomics UK (COG-UK) consortium|Davies|United Kingdom
>Spike|hCoV-19/Spain/AN-IBV-004091/2020|2020-04-17|EPI_ISL_510050|Original|hCoV-19^^Andalucia|Human|Servicio de Microbiolog\u00eda. HRU de M\u00e1laga. Servicio Andaluz de Salud|SeqCOVID-SPAIN consortium/IBV(CSIC)|Comas|Spain

>Spike|hCoV-19/Russia/MOS-CRIE-13677408/2020|2020-11-09|EPI_ISL_745229|Original|hCoV-19^^Moscow|Human|Molecular diagnostic laboratory of Federal Budget Institution of Science \Central Research Institute of Epidemiology\ of The Federal Service on Customers' Rights Protection and Human Well-being Surveillance|Group of Genomics and Postgenomic Technologies of Central Research Institute of Epidemilolog
>y|Korneenko|Russia
>Spike|hCoV-19/England/ALDP-9315C2/2020|2020-06-21|EPI_ISL_552816|Original|hCoV-19^^England|Human|Lighthouse Lab in Alderley Park|Wellcome Sanger Institute for the COVID-19 Genomics UK (COG-UK) consortium|Davies|United Kingdom
>Spike|hCoV-19/Netherlands/OV-RIVM-20464/2020|2020-11-17|EPI_ISL_728679|Original|hCoV-19^^Overijssel|Human|Dutch COVID-19 response team|National Institute for Public Health and the Environment (RIVM)|Meijer|Netherlands
>Spike|hCoV-19/Scotland/EDB9503/2020|2020-10-25|EPI_ISL_650815|Original|hCoV-19^^Scotland|Human|Virology Department|COVID-19 Genomics UK (COG-UK) Consortium|McHugh|United Kingdom
>Spike|hCoV-19/Scotland/GCVR-171036/2020|2020-03-27|EPI_ISL_459701|Original|hCoV-19^^Scotland|Human|NHSGGC West of Scotland Specialist Virology Centre / MRC-University of Glasgow Centre for Virus Research|Wellcome Sanger Institute for the COVID-19 Genomics UK (COG-UK) consortium|Davies|United Kingdom
>Spike|hCoV-19/England/20119073604/2020|2020-03-13|EPI_ISL_465101|Original|hCoV-19^^England|Human|Respiratory Virus Unit|Respiratory Virus Unit|Platt|United Kingdom
>Spike|hCoV-19/South Africa/N00089/2020|2020-08-06|EPI_ISL_623039|Original|hCoV-19^^Eastern Cape|Human|National Health Laboratory Service|National Institute for Communicable Diseases of the National Health Laboratory Service|Allam|South Africa
>Spike|hCoV-19/Spain/VC-FISABIO-105/2020|2020-03-18|EPI_ISL_436200|Original|hCoV-19^^Comunitat Valenciana|Human|Servicio de Microbiolog\u00eda. Consorcio Hospital General Universitario de Valencia|Sequencing and Bioinformatics Service and Molecular Epidemiology Research Group. FISABIO-Public Health|Gonzalez-Candelas|Spain
>Spike|hCoV-19/Scotland/QEUH-9F6CE2/2020|2020-09-27|EPI_ISL_595625|Original|hCoV-19^^Scotland|Human|Oxford Viromics|COVID-19 Genomics UK (COG-UK) Consortium|Golubchik|United Kingdom
>Spike|hCoV-19/Denmark/DCGC-14217/2020|2020-11-30|EPI_ISL_711631|Original|hCoV-19^^Nordjylland|Human|Department of Virus and Microbiological Special Diagnostics|Albertsen Lab|Michaelsen|Denmark
>Spike|hCoV-19/England/BRIS-1256E4/2020|2020-03-28|EPI_ISL_443783|Original|hCoV-19^^England|Human|PHE South West Regional Laboratory|Wellcome Sanger Institute for the COVID-19 Genomics UK (COG-UK) consortium|Amato|United Kingdom
>Spike|hCoV-19/England/QEUH-95DE6B/2020|2020-08-07|EPI_ISL_537036|Original|hCoV-19^^England|Human|Lighthouse Lab in Glasgow|Wellcome Sanger Institute for the COVID-19 Genomics UK (COG-UK) consortium|Davies|United Kingdom
>Spike|hCoV-19/England/CAMC-9EC58A/2020|2020-09-24|EPI_ISL_580499|Original|hCoV-19^^England|Human|Lighthouse Lab in Cambridge|Wellcome Sanger Institute for the COVID-19 Genomics UK (COG-UK) consortium|Davies|United Kingdom
>Spike|hCoV-19/India/MH-NIV-155669/2020|2020-08-08|EPI_ISL_541705|Original|hCoV-19^^Maharashtra|Human|National Institute of Virology|National Institute of Virology|Potdar|India
>Spike|hCoV-19/USA/FL-BPHL-1503/2020|2020-07-23|EPI_ISL_568601|Original|hCoV-19^^Florida|Human|Florida Bureau of Public Health Laboratories|Florida Bureau of Public Health Laboratories|Blanton|USA
>Spike|hCoV-19/England/MILK-B56AF3/2020|2020-11-05|EPI_ISL_656955|Original|hCoV-19^^England|Human|Lighthouse Lab in Milton Keynes|Wellcome Sanger Institute for the COVID-19 Genomics UK (COG-UK) Consortium|Davies|United Kingdom
>Spike|hCoV-19/Scotland/EDB1437/2020|2020-04-12|EPI_ISL_433448|Original|hCoV-19^^Scotland|Human|Virology Department|COVID-19 Genomics UK (COG-UK) Consortium|Williams|United Kingdom
>Spike|hCoV-19/USA/TX-DSHS-1858/2020|2020-06-24|EPI_ISL_745294|Original|hCoV-19^^Texas|Human|Texas Department of State Health Services|Texas Department of State Health Services|Rahman|USA

>Spike|hCoV-19/Northern Ireland/QEUH-96D5AF/2020|2020-08-
20|EPI_ISL_530792|Original|hCoV-19^^Northern Ireland|Human|Lighthouse Lab in
Glasgow|Wellcome Sanger Institute for the COVID-19 Genomics UK (COG-UK)
consortium|Davies|United Kingdom
>Spike|hCoV-19/England/ALDP-BB36B4/2020|2020-11-19|EPI_ISL_693332|Original|hCoV-
19^^England|Human|Lighthouse Lab in Alderley Park|Wellcome Sanger Institute for the
COVID-19 Genomics UK (COG-UK) Consortium|Davies|United Kingdom
>Spike|hCoV-19/Norway/3715/2020|2020-09-03|EPI_ISL_590987|Original|hCoV-
19^^Viken|Human|Ostfold Hospital Trust - Kalnes|Norwegian Institute of Public
Health|Vollan|Norway
>Spike|hCoV-19/Denmark/DCGC-18538/2020|2020-12-07|EPI_ISL_750028|Original|hCoV-
19^^Midtjylland|Human|Department of Virus and Microbiological Special
Diagnostics|Albertsen Lab|Michaelsen|Denmark
>Spike|hCoV-19/England/NOTT-11284E/2020|2020-04-14|EPI_ISL_493552|Original|hCoV-
19^^England|Human|Lincolnshire Hospitals and DeepSeq Nottingham|COVID-19 Genomics UK
(COG-UK) Consortium|Loose|United Kingdom
>Spike|hCoV-19/Wales/PHWC-16491D/2020|2020-06-09|EPI_ISL_479323|Original|hCoV-
19^^Wales|Human|Wales Specialist Virology Centre Sequencing lab|COVID-19 Genomics UK
(COG-UK) Consortium|Moore|United Kingdom
>Spike|hCoV-19/South Korea/KCDC2270/2020|2020-05-14|EPI_ISL_510608|Original|hCoV-
19^^South Korea|Human|Division of Viral Diseases|Division of Viral Diseases|Kim|South
Korea
>Spike|hCoV-19/USA/MI-MDHHS-SC21303/2020|2020-04-07|EPI_ISL_507558|Original|hCoV-
19^^Michigan|Human|Michigan Department of Health and Human Services|Michigan
Department of Health and Human Services|Blankenship|USA
>Spike|hCoV-19/England/SHEF-C965C/2020|2020-04-18|EPI_ISL_462040|Original|hCoV-
19^^England|Human|Virology Department|COVID-19 Genomics UK (COG-UK) Consortium|de
Silva|United Kingdom
>Spike|hCoV-19/England/ALDP-AA5179/2020|2020-10-22|EPI_ISL_624178|Original|hCoV-
19^^England|Human|Lighthouse Lab in Alderley Park|Wellcome Sanger Institute for the
COVID-19 Genomics UK (COG-UK) consortium|Davies|United Kingdom
>Spike|hCoV-19/USA/VA-DCLS-0777/2020|2020-06-11|EPI_ISL_526891|Original|hCoV-
19^^Virginia|Human|Virginia DCLS|Virginia DCLS|St. Jacques|USA
>Spike|hCoV-19/Denmark/DCGC-13322/2020|2020-11-30|EPI_ISL_712264|Original|hCoV-
19^^Sjaelland|Human|Department of Virus and Microbiological Special
Diagnostics|Albertsen Lab|Michaelsen|Denmark
>Spike|hCoV-19/England/MILK-A068DB/2020|2020-10-01|EPI_ISL_599919|Original|hCoV-
19^^England|Human|Lighthouse Lab in Milton Keynes|Wellcome Sanger Institute for the
COVID-19 Genomics UK (COG-UK) consortium|Davies|United Kingdom
>Spike|hCoV-19/England/LIVE-DCC5AA/2020|2020-11-28|EPI_ISL_724517|Original|hCoV-
19^^England|Human|Liverpool Clinical Laboratories|COVID-19 Genomics UK (COG-UK)
Consortium|Haldenby|United Kingdom
>Spike|hCoV-19/Northern Ireland/QEUH-9F9915/2020|2020-09-
25|EPI_ISL_589102|Original|hCoV-19^^Northern Ireland|Human|Lighthouse Lab in
Glasgow|Wellcome Sanger Institute for the COVID-19 Genomics UK (COG-UK)
consortium|Davies|United Kingdom
>Spike|hCoV-19/India/MH-ACTREC-649/2020|2020-09-14|EPI_ISL_700279|Original|hCoV-
19^^Maharashtra|Human|Hematopathology Laboratory|Hematopathology
Laboratory|Patkar|India
>Spike|hCoV-19/USA/TX-HMH-MCoV-15574/2020|2020-10-26|EPI_ISL_787058|Original|hCoV-
19^^Texas|Human|Houston Methodist Hospital|Houston Methodist Hospital|Long|USA
>Spike|hCoV-19/USA/CA-QDX-2005/2020|2020-03-17|EPI_ISL_604302|Original|hCoV-
19^^California|Human|Quest Diagnostics|Quest Diagnostics|Kagan|USA
>Spike|hCoV-19/England/LIVE-DC6D3F/2020|2020-11-27|EPI_ISL_705387|Original|hCoV-
19^^England|Human|Liverpool Clinical Laboratories|COVID-19 Genomics UK (COG-UK)
Consortium|Haldenby|United Kingdom
>Spike|hCoV-19/England/NOTT-11368F/2020|2020-09-13|EPI_ISL_549371|Original|hCoV-
19^^England|Human|Lincolnshire Hospitals and DeepSeq Nottingham|COVID-19 Genomics UK
(COG-UK) Consortium|Loose|United Kingdom

>Spike|hCoV-19/USA/IL-UW-627/2020|2020-06-26|EPI_ISL_491308|Original|hCoV-19^^Iowa|Human|University of Wisconsin-Madison AIDS Vaccine Research Laboratories|University of Wisconsin-Madison AIDS Vaccine Research Laboratories|Baczenas|USA
>Spike|hCoV-19/Brazil/RS-00661/2020|2020-11-26|EPI_ISL_770604|Original|hCoV-19^^Rio Grande do Sul|Human|Laborat\u00f3rio de Microbiologia Molecular - Universidade FEEVALE|Bioinformatics Laboratory / LNCC|Almeida|Brazil
>Spike|hCoV-19/England/CAMC-A647CF/2020|2020-10-10|EPI_ISL_608868|Original|hCoV-19^^England|Human|Lighthouse Lab in Cambridge|Wellcome Sanger Institute for the COVID-19 Genomics UK (COG-UK) consortium|Davies|United Kingdom
>Spike|hCoV-19/USA/VA-DCLS-2258/2020|2020-11-07|EPI_ISL_723505|Original|hCoV-19^^Virginia|Human|Virginia Division of Consolidated Laboratory Services (DCLS)|Virginia Division of Consolidated Laboratory Services (DCLS)|St. Jacques|USA
>Spike|hCoV-19/England/BRIS-1283EA/2020|2020-04-16|EPI_ISL_469564|Original|hCoV-19^^England|Human|PHE South West Regional Laboratory|Wellcome Sanger Institute for the COVID-19 Genomics UK (COG-UK) consortium|Davies|United Kingdom
>Spike|hCoV-19/Brazil/RJ-2100/2020|2020-04-16|EPI_ISL_541353|Original|hCoV-19^^Rio de Janeiro|Human|Laboratory of Respiratory Viruses and Measles|Laboratory of Respiratory Viruses and Measles|Silva|Brazil
>Spike|hCoV-19/USA/MN-MDH-1683/2020|2020-04-08|EPI_ISL_560770|Original|hCoV-19^^Minnesota|Human|Minnesota Department of Health|Minnesota Department of Health|Garfin|USA
>Spike|hCoV-19/England/MILK-A7D286/2020|2020-10-13|EPI_ISL_606558|Original|hCoV-19^^England|Human|Lighthouse Lab in Milton Keynes|Wellcome Sanger Institute for the COVID-19 Genomics UK (COG-UK) consortium|Davies|United Kingdom
>Spike|hCoV-19/Singapore/587/2020|2020-03-27|EPI_ISL_483610|Original|hCoV-19^^Singapore|Human|National Public Health Laboratory|National Public Health Laboratory|Mak|Singapore
>Spike|hCoV-19/England/NORT-282787/2020|2020-03-27|EPI_ISL_488482|Original|hCoV-19^^England|Human|NU-OMICS DNA Sequencing research facility|Wellcome Sanger Institute for the COVID-19 Genomics UK (COG-UK) consortium|Davies|United Kingdom
>Spike|hCoV-19/England/ALDP-A70827/2020|2020-10-15|EPI_ISL_608262|Original|hCoV-19^^England|Human|Lighthouse Lab in Alderley Park|Wellcome Sanger Institute for the COVID-19 Genomics UK (COG-UK) consortium|Davies|United Kingdom
>Spike|hCoV-19/USA/SC-DHEC-1116/2020|2020-07-09|EPI_ISL_574570|Original|hCoV-19^^South Carolina|Human|Microbiology Division|Microbiology Division|Flores|USA
>Spike|hCoV-19/England/ALDP-4FBAAC/2020|2020-06-23|EPI_ISL_559160|Original|hCoV-19^^England|Human|Lighthouse Lab in Alderley Park|Wellcome Sanger Institute for the COVID-19 Genomics UK (COG-UK) consortium|Davies|United Kingdom
>Spike|hCoV-19/Australia/VIC11312/2020|2020-08-14|EPI_ISL_563325|Original|hCoV-19^^Victoria|Human|Microbiological Diagnostic Unit - Public Health Laboratory (MDU-PHL)|MDU-PHL|Schultz|Australia
>Spike|hCoV-19/Spain/PV-IBV-2157/2020|2020-04-12|EPI_ISL_468937|Original|hCoV-19^^Basque Country|Human|Servicio de Microbiolog\u00eda. Hospital Universitario Donostia. OSI Donostialdea. \u00c1rea de Enfermedades Infecciosas|SeqCOVID-SPAIN consortium/IBV(CSIC)|Comas|Spain
>Spike|hCoV-19/USA/LA-BIE-092/2020|2020-04-05|EPI_ISL_436959|Original|hCoV-19^^Louisiana|Human|Ochsner Health|Bioinfoexperts|Cross|USA
>Spike|hCoV-19/England/ALDP-5B4859/2020|2020-06-18|EPI_ISL_558784|Original|hCoV-19^^England|Human|Lighthouse Lab in Alderley Park|Wellcome Sanger Institute for the COVID-19 Genomics UK (COG-UK) consortium|Davies|United Kingdom
>Spike|hCoV-19/USA/WI-UW-1989/2020|2020-09-26|EPI_ISL_677397|Original|hCoV-19^^Wisconsin|Human|University of Wisconsin-Madison AIDS Vaccine Research Laboratories|University of Wisconsin-Madison AIDS Vaccine Research Laboratories|Baczenas|USA
>Spike|hCoV-19/Canada/QC-LSPQ-L00237113/2020|2020-03-25|EPI_ISL_536164|Original|hCoV-19^^Quebec|Human|CSSS Haut-Richelieu/Rouville (H\u00f4pital)|Laboratoire de sant\u00e9 publique du Qu\u00e9bec|Moreira|Canada

>Spike|hCoV-19/India/DL-NCDC5723-CSIR-IGIB/2020|2020-05-
23|EPI_ISL_482611|Original|hCoV-19^^Delhi|Human|National Centre for Disease control
(NCDC)|NCDC/CSIR-IGIB|Singh|India
>Spike|hCoV-19/England/ALDP-BB562B/2020|2020-11-20|EPI_ISL_701921|Original|hCoV-
19^^England|Human|Lighthouse Lab in Alderley Park|Wellcome Sanger Institute for the
COVID-19 Genomics UK (COG-UK) Consortium|Davies|United Kingdom
>Spike|hCoV-19/USA/WI-UW-04/2020|2020-03-15|EPI_ISL_416491|Original|hCoV-
19^^Wisconsin|Human|University of Wisconsin-Madison AIDS Vaccine Research
Laboratories|University of Wisconsin-Madison AIDS Vaccine Research
Laboratories|Braun|USA
>Spike|hCoV-19/England/LIVE-A61DC/2020|2020-03-21|EPI_ISL_499692|Original|hCoV-
19^^England|Human|Liverpool Clinical Laboratories|COVID-19 Genomics UK (COG-UK)
Consortium|Haldenby|United Kingdom
>Spike|hCoV-19/Denmark/DCGC-15106/2020|2020-11-23|EPI_ISL_713633|Original|hCoV-
19^^Sjaelland|Human|Department of Virus and Microbiological Special
Diagnostics|Albertsen Lab|Michaelsen|Denmark
>Spike|hCoV-19/Australia/NSW2508/2020|2020-07-04|EPI_ISL_527029|Original|hCoV-19^^New
South Wales|Human|Area of Virology|Area of Virology|Yeang|Australia
>Spike|hCoV-19/England/MILK-4691FF/2020|2020-05-22|EPI_ISL_559738|Original|hCoV-
19^^England|Human|Lighthouse Lab in Milton Keynes|Wellcome Sanger Institute for the
COVID-19 Genomics UK (COG-UK) consortium|Davies|United Kingdom
>Spike|hCoV-19/England/MILK-957AD6/2020|2020-06-11|EPI_ISL_554152|Original|hCoV-
19^^England|Human|Lighthouse Lab in Milton Keynes|Wellcome Sanger Institute for the
COVID-19 Genomics UK (COG-UK) consortium|Davies|United Kingdom
>Spike|hCoV-19/Japan/PG-3929/2020|2020-07-00|EPI_ISL_692180|Original|hCoV-
19^^Japan|Human|Pathogen Genomics Center|Pathogen Genomics Center|Sekizuka|Japan
>Spike|hCoV-19/England/QEUH-A5E3E2/2020|2020-10-12|EPI_ISL_597322|Original|hCoV-
19^^England|Human|Lighthouse Lab in Glasgow|Wellcome Sanger Institute for the COVID-
19 Genomics UK (COG-UK) consortium|Davies|United Kingdom
>Spike|hCoV-19/Japan/PG-5735/2020|2020-08-00|EPI_ISL_688855|Original|hCoV-
19^^Japan|Human|Pathogen Genomics Center|Pathogen Genomics Center|Sekizuka|Japan
>Spike|hCoV-19/USA/TX-HMH-MCoV-15996/2020|2020-07-10|EPI_ISL_785680|Original|hCoV-
19^^Texas|Human|Houston Methodist Hospital|Houston Methodist Hospital|Long|USA
>Spike|hCoV-19/USA/TX-HMH-7065/2020|2020-06-28|EPI_ISL_546475|Original|hCoV-
19^^Texas|Human|Houston Methodist Hospital|Houston Methodist Hospital|Long|USA
>Spike|hCoV-19/mink/Netherlands/NB-EMC-12-23/2020|2020-06-
07|EPI_ISL_523007|Original|hCoV-19^^Limburg|Neovison vison|Dutch COVID-19 response
team|Erasmus Medical Center|Oude Munnink|Netherlands
>Spike|hCoV-19/United Arab Emirates/0399/2020|2020-05-
19|EPI_ISL_698574|Original|hCoV-19^^Abu Dhabi|Human|Group 42 (G42) Healthcare|G42
Healthcare|Ogrodzki|United Arab Emirates
>Spike|hCoV-19/Wales/PHWC-4833DB/2020|2020-10-23|EPI_ISL_627813|Original|hCoV-
19^^Wales|Human|Wales Specialist Virology Centre Sequencing lab|COVID-19 Genomics UK
(COG-UK) Consortium|Moore|United Kingdom
>Spike|hCoV-19/England/ALDP-AA5319/2020|2020-10-21|EPI_ISL_625015|Original|hCoV-
19^^England|Human|Lighthouse Lab in Alderley Park|Wellcome Sanger Institute for the
COVID-19 Genomics UK (COG-UK) consortium|Davies|United Kingdom
>Spike|hCoV-19/Scotland/QEUH-96B3A7/2020|2020-08-17|EPI_ISL_534652|Original|hCoV-
19^^Scotland|Human|NHSGGC West of Scotland Specialist Virology Centre / MRC-
University of Glasgow Centre for Virus Research|Wellcome Sanger Institute for the
COVID-19 Genomics UK (COG-UK) consortium|Davies|United Kingdom
>Spike|hCoV-19/USA/MA-MASPHL-00877/2020|2020-10-13|EPI_ISL_692855|Original|hCoV-
19^^Massachusetts|Human|Massachusetts State Public Health Laboratory|Massachusetts
State Public Health Laboratory|Lang|USA
>Spike|hCoV-19/England/CAMB-77778/2020|2020-03-30|EPI_ISL_439457|Original|hCoV-
19^^England|Human|Department of Pathology|Wellcome Sanger Institute for the COVID-19
Genomics UK (COG-UK) consortium|Amato|United Kingdom
>Spike|hCoV-19/England/QEUH-C14EE5/2020|2020-12-06|EPI_ISL_709336|Original|hCoV-
19^^England|Human|Lighthouse Lab in Glasgow|Wellcome Sanger Institute for the COVID-
19 Genomics UK (COG-UK) Consortium|Davies|United Kingdom

>Spike|hCoV-19/USA/LA-SR0161/2020|2020-03-28|EPI_ISL_435464|Original|hCoV-19^^Louisiana|Human|Robert Garry lab|Andersen lab at Scripps Research|Gangavarapu|USA
>Spike|hCoV-19/England/QEUH-A1DACC/2020|2020-09-29|EPI_ISL_587409|Original|hCoV-19^^England|Human|Lighthouse Lab in Glasgow|Wellcome Sanger Institute for the COVID-19 Genomics UK (COG-UK) consortium|Davies|United Kingdom
>Spike|hCoV-19/Chile/RM-170122/2020|2020-09-04|EPI_ISL_746710|Original|hCoV-19^^Region Metropolitana de Santiago|Human|Genetica Molecular and Subdepartamento de Virologia ISP Chile|Instituto de Salud Publica de Chile|Tognarelli|Chile
>Spike|hCoV-19/USA/TX-HMH-MCoV-9353/2020|2020-07-03|EPI_ISL_781074|Original|hCoV-19^^Texas|Human|Houston Methodist Hospital|Houston Methodist Hospital|Long|USA
>Spike|hCoV-19/England/MILK-BA9DDA/2020|2020-11-17|EPI_ISL_783198|Original|hCoV-19^^England|Human|Lighthouse Lab in Milton Keynes|Wellcome Sanger Institute for the COVID-19 Genomics UK (COG-UK) Consortium|Davies|United Kingdom
>Spike|hCoV-19/Sweden/20-53904/2020|2020-12-16|EPI_ISL_789056|Original|hCoV-19^^Stockholm|Human|Klinisk mikrobiologi|The Public Health Agency of Sweden|Svartstr\u00f6m|Sweden
>Spike|hCoV-19/England/BRIS-185373B/2020|2020-04-13|EPI_ISL_492267|Original|hCoV-19^^England|Human|PHE South West Regional Laboratory|Wellcome Sanger Institute for the COVID-19 Genomics UK (COG-UK) consortium|Davies|United Kingdom
>Spike|hCoV-19/USA/LA-GBCL-26544563/2020|2020-04-18|EPI_ISL_485137|Original|hCoV-19^^Louisiana|Human|River Road Testing Lab|Ginkgo Bioworks Clinical Laboratory|Smith|USA
>Spike|hCoV-19/England/QEUH-A4BCCF/2020|2020-10-05|EPI_ISL_628522|Original|hCoV-19^^England|Human|Oxford Viromics|COVID-19 Genomics UK (COG-UK) Consortium|Golubchik|United Kingdom
>Spike|hCoV-19/England/QEUH-9F5ABC/2020|2020-09-27|EPI_ISL_588332|Original|hCoV-19^^England|Human|Lighthouse Lab in Glasgow|Wellcome Sanger Institute for the COVID-19 Genomics UK (COG-UK) consortium|Davies|United Kingdom
>Spike|hCoV-19/Scotland/EDB8603/2020|2020-10-22|EPI_ISL_651978|Original|hCoV-19^^Scotland|Human|Virology Department|COVID-19 Genomics UK (COG-UK) Consortium|McHugh|United Kingdom
>Spike|hCoV-19/USA/TX-HMH-4253/2020|2020-06-18|EPI_ISL_543465|Original|hCoV-19^^Texas|Human|Houston Methodist Hospital|Houston Methodist Hospital|Long|USA
>Spike|hCoV-19/Luxembourg/LNS5453199/2020|2020-12-01|EPI_ISL_770934|Original|hCoV-19^^Luxembourg|Human|Laboratoire national de sant\u00e9|Laboratoire national de sant\u00e9|Wienecke-Baldacchino|Luxembourg
>Spike|hCoV-19/Wales/PHWC-160489/2020|2020-05-24|EPI_ISL_472937|Original|hCoV-19^^Wales|Human|Wales Specialist Virology Centre Sequencing lab|COVID-19 Genomics UK (COG-UK) Consortium|Moore|United Kingdom
>Spike|hCoV-19/USA/UT-UPHL-201021581/2020|2020-04-18|EPI_ISL_594110|Original|hCoV-19^^Utah|Human|Utah Public Health Laboratory|Utah Public Health Laboratory|Young|USA
>Spike|hCoV-19/England/20189164301/2020|2020-05-01|EPI_ISL_465568|Original|hCoV-19^^England|Human|Respiratory Virus Unit|Respiratory Virus Unit|Platt|United Kingdom
>Spike|hCoV-19/USA/UT-QDX-2549/2020|2020-03-12|EPI_ISL_604864|Original|hCoV-19^^Utah|Human|Quest Diagnostics|Quest Diagnostics|Kagan|USA
>Spike|hCoV-19/Scotland/CVR2784/2020|2020-04-20|EPI_ISL_473564|Original|hCoV-19^^Scotland|Human|West of Scotland Specialist Virology Centre|COVID-19 Genomics UK (COG-UK) Consortium|Robertson|United Kingdom
>Spike|hCoV-19/England/ALDP-9D95BD/2020|2020-09-18|EPI_ISL_576743|Original|hCoV-19^^England|Human|Oxford Viromics|COVID-19 Genomics UK (COG-UK) Consortium|Golubchik|United Kingdom
>Spike|hCoV-19/England/CAMC-AAF616/2020|2020-10-23|EPI_ISL_631148|Original|hCoV-19^^England|Human|Lighthouse Lab in Cambridge|Wellcome Sanger Institute for the COVID-19 Genomics UK (COG-UK) consortium|Davies|United Kingdom
>Spike|hCoV-19/Luxembourg/LNS8287433/2020|2020-11-02|EPI_ISL_744712|Original|hCoV-19^^Luxembourg|Human|Laboratoire national de sant\u00e9|Laboratoire national de sant\u00e9|Wienecke-Baldacchino|Luxembourg
>Spike|hCoV-19/England/QEUH-AA01BA/2020|2020-10-19|EPI_ISL_611484|Original|hCoV-19^^England|Human|Lighthouse Lab in Glasgow|Wellcome Sanger Institute for the COVID-19 Genomics UK (COG-UK) consortium|Davies|United Kingdom

>Spike|hCoV-19/Australia/VIC3274/2020|2020-07-07|EPI_ISL_520953|Original|hCoV-19^^Victoria|Human|Microbiological Diagnostic Unit - Public Health Laboratory (MDU-PHL)|MDU-PHL|Schultz|Australia
>Spike|hCoV-19/England/CAMC-B36947/2020|2020-11-05|EPI_ISL_645524|Original|hCoV-19^^England|Human|Lighthouse Lab in Cambridge|Wellcome Sanger Institute for the COVID-19 Genomics UK (COG-UK) Consortium|Davies|United Kingdom
>Spike|hCoV-19/India/GJ-GBRC233b/2020|2020-06-12|EPI_ISL_476875|Original|hCoV-19^^Gujarat|Human|Department of MicroBiology|Gujarat Biotechnology Research Centre|Joshi|India
>Spike|hCoV-19/England/CAMB-7C37F/2020|2020-04-14|EPI_ISL_434033|Original|hCoV-19^^England|Human|Department of Pathology|COVID-19 Genomics UK (COG-UK) Consortium|Meredith|United Kingdom
>Spike|hCoV-19/England/ALDP-59A974/2020|2020-06-15|EPI_ISL_558350|Original|hCoV-19^^England|Human|Lighthouse Lab in Alderley Park|Wellcome Sanger Institute for the COVID-19 Genomics UK (COG-UK) consortium|Davies|United Kingdom
>Spike|hCoV-19/USA/TX-HMH-MCoV-15583/2020|2020-10-25|EPI_ISL_787067|Original|hCoV-19^^Texas|Human|Houston Methodist Hospital|Houston Methodist Hospital|Long|USA
>Spike|hCoV-19/USA/MA-MGH-00580/2020|2020-03-28|EPI_ISL_460273|Original|hCoV-19^^Massachusetts|Human|Massachusetts General Hospital|Infectious Disease Program|Lemieux|USA
>Spike|hCoV-19/England/MILK-ACD463/2020|2020-10-22|EPI_ISL_634356|Original|hCoV-19^^England|Human|Lighthouse Lab in Milton Keynes|Wellcome Sanger Institute for the COVID-19 Genomics UK (COG-UK) consortium|Davies|United Kingdom
>Spike|hCoV-19/England/QEUH-B6C7D5/2020|2020-11-10|EPI_ISL_655735|Original|hCoV-19^^England|Human|Lighthouse Lab in Glasgow|Wellcome Sanger Institute for the COVID-19 Genomics UK (COG-UK) Consortium|Davies|United Kingdom
>Spike|hCoV-19/England/MILK-9D5144/2020|2020-09-15|EPI_ISL_577049|Original|hCoV-19^^England|Human|Centre for Enzyme Innovation|COVID-19 Genomics UK (COG-UK) Consortium|Robson|United Kingdom
>Spike|hCoV-19/England/MILK-A6A5A1/2020|2020-10-09|EPI_ISL_627512|Original|hCoV-19^^England|Human|Virology Department|COVID-19 Genomics UK (COG-UK) Consortium|de Silva|United Kingdom
>Spike|hCoV-19/Denmark/DCGC-18518/2020|2020-12-07|EPI_ISL_750017|Original|hCoV-19^^Nordjylland|Human|Department of Virus and Microbiological Special Diagnostics|Albertsen Lab|Michaelsen|Denmark
>Spike|hCoV-19/Mexico/GUA-InDRE-20/2020|2020-04-02|EPI_ISL_493335|Original|hCoV-19^^Guanajuato|Human|Instituto de Diagnostico y Referencia Epidemiologicos (INDRE)|Instituto de Diagnostico y Referencia Epidemiologicos (INDRE)|Rodriguez|Mexico
>Spike|hCoV-19/Italy/LAZ-INMI-131/2020|2020-12-21|EPI_ISL_765569|Original|hCoV-19^^Lazio|Human|National Institute for Infectious Diseases|National Institute for Infectious Diseases|Gruber|Italy
>Spike|hCoV-19/USA/CA-CDPH124/2020|2020-09-21|EPI_ISL_754951|Original|hCoV-19^^California|Human|California Department of Public Health|California Department of Public Health|Bell|USA
>Spike|hCoV-19/South Africa/NHLS-UCT-GS-0030/2020|2020-08-19|EPI_ISL_640031|Original|hCoV-19^^Western Cape|Human|Beaufort West Hospital wc BWH|NHLS/UCT|Iranzadeh|South Africa
>Spike|hCoV-19/Denmark/DCGC-9734/2020|2020-11-09|EPI_ISL_669135|Original|hCoV-19^^Sjaelland|Human|Department of Virus and Microbiological Special Diagnostics|Albertsen Lab|Michaelsen|Denmark
>Spike|hCoV-19/Scotland/CVR4067/2020|2020-08-31|EPI_ISL_535158|Original|hCoV-19^^Scotland|Human|West of Scotland Specialist Virology Centre|COVID-19 Genomics UK (COG-UK) Consortium|Robertson|United Kingdom
>Spike|hCoV-19/Hungary/MH-6074/2020|2020-04-30|EPI_ISL_526219|Original|hCoV-19^^Pest county|Human|Hungarian Defence Forces Military Medical Centre|National Laboratory of Virology|Kemenesi|Hungary
>Spike|hCoV-19/USA/MI-MDHHS-SC21860/2020|2020-05-10|EPI_ISL_565903|Original|hCoV-19^^Michigan|Human|Michigan Department of Health and Human Services|Michigan Department of Health and Human Services|Blankenship|USA

>Spike|hCoV-19/Australia/SAP170/2020|2020-03-13|EPI_ISL_467985|Original|hCoV-19^^South Australia|Human|SA Pathology|SA Pathology|Leong|Australia
>Spike|hCoV-19/Australia/VIC17532/2020|2020-08-19|EPI_ISL_663911|Original|hCoV-19^^Victoria|Human|Microbiological Diagnostic Unit - Public Health Laboratory (MDU-PHL)|MDU-PHL|Schultz|Australia
>Spike|hCoV-19/USA/NY-NYCPHL-000839/2020|2020-03-29|EPI_ISL_632216|Original|hCoV-19^^New York|Human|Richmond University Medical Center|New York City Public Health Laboratory|Wang|USA
>Spike|hCoV-19/England/MILK-BAFEC2/2020|2020-11-17|EPI_ISL_720584|Original|hCoV-19^^England|Human|Lighthouse Lab in Milton Keynes|Wellcome Sanger Institute for the COVID-19 Genomics UK (COG-UK) Consortium|Davies|United Kingdom
>Spike|hCoV-19/Denmark/DCGC-21797/2020|2020-12-21|EPI_ISL_758368|Original|hCoV-19^^Midtjylland|Human|Department of Virus and Microbiological Special Diagnostics|Albertsen Lab|Michaelsen|Denmark
>Spike|hCoV-19/Canada/ON-PHL-20-02482/2020|2020-09-00|EPI_ISL_671603|Original|hCoV-19^^Ontario|Human|Public Health Ontario Laboratory|Public Health Ontario Laboratory|Guthrie|Canada
>Spike|hCoV-19/England/CAMC-B211B0/2020|2020-11-03|EPI_ISL_647475|Original|hCoV-19^^England|Human|Lighthouse Lab in Cambridge|Wellcome Sanger Institute for the COVID-19 Genomics UK (COG-UK) Consortium|Davies|United Kingdom
>Spike|hCoV-19/England/BRIS-129167/2020|2020-04-19|EPI_ISL_458782|Original|hCoV-19^^England|Human|PHE South West Regional Laboratory|Wellcome Sanger Institute for the COVID-19 Genomics UK (COG-UK) consortium|Davies|United Kingdom
>Spike|hCoV-19/USA/TX-HMH0375/2020|2020-03-31|EPI_ISL_434964|Original|hCoV-19^^Texas|Human|Houston Methodist Hospital|Houston Methodist Hospital|Long|USA
>Spike|hCoV-19/Spain/CL-IBV-97006208/2020|2020-04-13|EPI_ISL_537354|Original|hCoV-19^^Castilla y Leon|Human|Universidad de Le\u00f3n|SeqCOVID-SPAIN consortium/IBV(CSIC)|Comas|Spain
>Spike|hCoV-19/Australia/NSW241/2020|2020-03-25|EPI_ISL_451507|Original|hCoV-19^^New South Wales|Human|Pathology West - NSW Health Pathology|NSW Health Pathology - Institute of Clinical Pathology and Medical Research; Westmead Hospital; University of Sydney|Gall|Australia
>Spike|hCoV-19/England/MILK-BFF4B2/2020|2020-12-02|EPI_ISL_720890|Original|hCoV-19^^England|Human|Lighthouse Lab in Milton Keynes|Wellcome Sanger Institute for the COVID-19 Genomics UK (COG-UK) Consortium|Davies|United Kingdom
>Spike|hCoV-19/England/ALDP-C3D068/2020|2020-12-10|EPI_ISL_730961|Original|hCoV-19^^England|Human|Lighthouse Lab in Alderley Park|Wellcome Sanger Institute for the COVID-19 Genomics UK (COG-UK) Consortium|Davies|United Kingdom
>Spike|hCoV-19/USA/HI-H200091/2020|2020-06-21|EPI_ISL_752658|Original|hCoV-19^^Hawaii|Human|State Laboratories Division|State Laboratories Division|Sultana|USA
>Spike|hCoV-19/Japan/PG-6381/2020|2020-08-00|EPI_ISL_689491|Original|hCoV-19^^Japan|Human|Fukuoka Institute of Health and Environmental Sciences|Pathogen Genomics Center|Sekizuka|Japan
>Spike|hCoV-19/England/ALDP-BC7565/2020|2020-11-22|EPI_ISL_721226|Original|hCoV-19^^England|Human|Lighthouse Lab in Alderley Park|Wellcome Sanger Institute for the COVID-19 Genomics UK (COG-UK) Consortium|Davies|United Kingdom
>Spike|hCoV-19/England/QEUH-9644F6/2020|2020-08-11|EPI_ISL_536819|Original|hCoV-19^^England|Human|Lighthouse Lab in Glasgow|Wellcome Sanger Institute for the COVID-19 Genomics UK (COG-UK) consortium|Davies|United Kingdom
>Spike|hCoV-19/Scotland/QEUH-A60F9E/2020|2020-10-13|EPI_ISL_665618|Original|hCoV-19^^Scotland|Human|Northumbria University / South Tees Hospitals NHS Foundation Trust / North Cumbria Integrated Care NHS Foundation Trust / North Tees and Hartlepool NHS Foundation Trust / Newcastle Hospitals NHS Foundation Trust|COVID-19 Genomics UK (COG-UK) Consortium|Bashton|United Kingdom
>Spike|hCoV-19/Iran/K1r-64/2020|2020-03-25|EPI_ISL_568498|Original|hCoV-19^^Iran|Human|Virology|Virology|Ranjbar|Iran
>Spike|hCoV-19/England/QEUH-AD1DCB/2020|2020-10-24|EPI_ISL_629964|Original|hCoV-19^^England|Human|Lighthouse Lab in Glasgow|Wellcome Sanger Institute for the COVID-19 Genomics UK (COG-UK) consortium|Davies|United Kingdom

>Spike|hCoV-19/England/LEED-2A8C5F/2020|2020-04-01|EPI_ISL_539090|Original|hCoV-19^^England|Human|Leeds Teaching Hospitals NHS Trust and Public Health England|Wellcome Sanger Institute for the COVID-19 Genomics UK (COG-UK) consortium|Davies|United Kingdom
>Spike|hCoV-19/USA/UT-UPHL-2012683062/2020|2020-06-19|EPI_ISL_746458|Original|hCoV-19^^Utah|Human|Utah Public Health Laboratory|Utah Public Health Laboratory|Gallagher|USA
>Spike|hCoV-19/USA/NY-SBU-A58597/2020|2020-04-25|EPI_ISL_602563|Original|hCoV-19^^New York|Human|Department of Microbiology and Immunology|Department of Microbiology and Immunology|Nascimento Conde|USA
>Spike|hCoV-19/Denmark/DCGC-4126/2020|2020-09-21|EPI_ISL_621946|Original|hCoV-19^^Hovedstaden|Human|Department of Virus and Microbiological Special Diagnostics|Albertsen lab|Michaelsen|Denmark
>Spike|hCoV-19/England/MILK-A7C436/2020|2020-10-15|EPI_ISL_606572|Original|hCoV-19^^England|Human|Lighthouse Lab in Milton Keynes|Wellcome Sanger Institute for the COVID-19 Genomics UK (COG-UK) consortium|Davies|United Kingdom
>Spike|hCoV-19/Spain/RI-IBV-99010951/2020|2020-08-25|EPI_ISL_732782|Original|hCoV-19^^La_Rioja|Human|Centro de Investigaci\u00f3n Biom\u00e9dica de La Rioja - Hospital San Pedro Logro\u00f1o|SeqCOVID-SPAIN consortium/IBV(CSIC)|Comas|Spain
>Spike|hCoV-19/Malaysia/MGI-G873/2020|2020-04-07|EPI_ISL_528738|Original|hCoV-19^^Selangor|Human|Malaysia Genome Institute|Malaysia Genome Institute|Mat Isa|Malaysia
>Spike|hCoV-19/Wuhan/HB-WH3-185/2020|2020-03-15|EPI_ISL_454945|Original|hCoV-19^^Hubei|Human|Wuhan Chain Medical Labs (CMLabs)|State Key Laboratory of Biotherapy of Sichuan University|Tang|China
>Spike|hCoV-19/USA/TX-HMH-4013/2020|2020-06-16|EPI_ISL_543295|Original|hCoV-19^^Texas|Human|Houston Methodist Hospital|Houston Methodist Hospital|Long|USA
>Spike|hCoV-19/USA/MN-MDH-1268/2020|2020-03-10|EPI_ISL_482989|Original|hCoV-19^^Minnesota|Human|Minnesota Department of Health|Minnesota Department of Health|Garfin|USA
>Spike|hCoV-19/England/MILK-991ED4/2020|2020-08-27|EPI_ISL_552085|Original|hCoV-19^^England|Human|Lighthouse Lab in Milton Keynes|Wellcome Sanger Institute for the COVID-19 Genomics UK (COG-UK) consortium|Davies|United Kingdom
>Spike|hCoV-19/Netherlands/GE-EMC-323/2020|2020-10-07|EPI_ISL_632655|Original|hCoV-19^^Gelderland|Human|Dutch COVID-19 response team|Erasmus Medical Center|Oude Munnink|Netherlands
>Spike|hCoV-19/Scotland/QEUH-A1EDD1/2020|2020-10-01|EPI_ISL_598861|Original|hCoV-19^^Scotland|Human|Lighthouse Lab in Glasgow|Wellcome Sanger Institute for the COVID-19 Genomics UK (COG-UK) consortium|Davies|United Kingdom
>Spike|hCoV-19/Jordan/AM-ALSR-3978/2020|2020-10-02|EPI_ISL_636341|Original|hCoV-19^^Amman|Human|Biolab Diagnostic Laboratories|Andersen lab at Scripps Research|Gangavarapu|Jordan
>Spike|hCoV-19/Switzerland/BS-UHB-42200965/2020|2020-03-20|EPI_ISL_528280|Original|hCoV-19^^Basel-Stadt|Human|University Hospital Basel|University Hospital Basel|Roloff Handschin|Switzerland
>Spike|hCoV-19/USA/CA-CZB-12795/2020|2020-10-14|EPI_ISL_648450|Original|hCoV-19^^California|Human|Santa Clara County Public Health Laboratory|Chan-Zuckerberg Biohub|Bhatt|USA
>Spike|hCoV-19/Switzerland/BS-ETHZ-321468/2020|2020-10-14|EPI_ISL_603682|Original|hCoV-19^^Basel-Stadt|Human|Viollier AG|Department of Biosystems Science and Engineering|Nadeau|Switzerland
>Spike|hCoV-19/Denmark/DCGC-15073/2020|2020-11-23|EPI_ISL_713619|Original|hCoV-19^^Midtjylland|Human|Department of Virus and Microbiological Special Diagnostics|Albertsen Lab|Michaelsen|Denmark
>Spike|hCoV-19/Morocco/6899/2020|2020-04-21|EPI_ISL_459977|Original|hCoV-19^^Morocco|Human|Institut Pasteur du Maroc|Institut Pasteur du Maroc|Enouf|Morocco
>Spike|hCoV-19/USA/FL-BPHL-017/2020|2020-04-08|EPI_ISL_436521|Original|hCoV-19^^Florida|Human|Florida Bureau of Public Health Laboratories|Florida Bureau of Public Health Laboratories|Blanton|USA

>Spike|hCoV-19/USA/MN-MDH-1933/2020|2020-10-06|EPI_ISL_613493|Original|hCoV-19^^Minnesota|Human|Public Health Laboratory - Infectious Disease Lab|Minnesota Department of Health|Lorentz|USA
>Spike|hCoV-19/United Arab Emirates/0518/2020|2020-05-18|EPI_ISL_698681|Original|hCoV-19^^Abu Dhabi|Human|Group 42 (G42) Healthcare|G42 Healthcare|Ogrodzki|United Arab Emirates
>Spike|hCoV-19/Denmark/DCGC-17822/2020|2020-12-14|EPI_ISL_749586|Original|hCoV-19^^Hovedstaden|Human|Department of Virus and Microbiological Special Diagnostics|Albertsen Lab|Michaelsen|Denmark
>Spike|hCoV-19/USA/CA-CZB-16177/2020|2020-11-30|EPI_ISL_739348|Original|hCoV-19^^California|Human|Renegade|Chan-Zuckerberg Biohub|Kamm|USA
>Spike|hCoV-19/England/EXET-13F386/2020|2020-12-16|EPI_ISL_764196|Original|hCoV-19^^England|Human|University of Exeter|COVID-19 Genomics UK (COG-UK) Consortium|Temperton|United Kingdom
>Spike|hCoV-19/England/ALDP-B74F3C/2020|2020-11-12|EPI_ISL_664916|Original|hCoV-19^^England|Human|Quadram Institute Bioscience|COVID-19 Genomics UK (COG-UK) Consortium|Alikhan|United Kingdom
>Spike|hCoV-19/England/BIRM-5F794/2020|2020-05-11|EPI_ISL_473329|Original|hCoV-19^^England|Human|University of Birmingham|COVID-19 Genomics UK (COG-UK) Consortium|Loman|United Kingdom
>Spike|hCoV-19/USA/WA-S2871/2020|2020-09-22|EPI_ISL_596402|Original|hCoV-19^^Washington|Human|Seattle Flu Study|Seattle Flu Study|Lee|USA
>Spike|hCoV-19/USA/WI-GMF-31613/2020|2020-11-05|EPI_ISL_661102|Original|hCoV-19^^Wisconsin|Human|Gundersen Molecular Diagnostics Laboratory|Kabara Cancer Research Institute|Kenny|USA
>Spike|hCoV-19/USA/WA-UW-4210/2020|2020-03-31|EPI_ISL_437821|Original|hCoV-19^^Washington|Human|UW Virology Lab|UW Virology Lab|Roychoudhury|USA
>Spike|hCoV-19/England/CAMB-1BD64B/2020|2020-11-30|EPI_ISL_764325|Original|hCoV-19^^England|Human|Department of Pathology|COVID-19 Genomics UK (COG-UK) Consortium|Goodfellow|United Kingdom
>Spike|hCoV-19/Australia/VIC2205/2020|2020-06-25|EPI_ISL_521947|Original|hCoV-19^^Victoria|Human|Victorian Infectious Diseases Reference Laboratory (VIDRL)|VIDRL and MDU-PHL|Schultz|Australia
>Spike|hCoV-19/USA/MI-MDHHS-SC20230/2020|2020-03-18|EPI_ISL_447200|Original|hCoV-19^^Michigan|Human|Michigan Department of Health and Human Services|Michigan Department of Health and Human Services|Blankenship|USA
>Spike|hCoV-19/Latvia/061/2020|2020-07-07|EPI_ISL_492996|Original|hCoV-19^^Latvia|Human|E. Gulbja Laboratorija|Latvian Biomedical Research and Study Centre|Zrelovs|Latvia
>Spike|hCoV-19/Australia/VIC355/2020|2020-03-21|EPI_ISL_426663|Original|hCoV-19^^Victoria|Human|Victorian Infectious Diseases Reference Laboratory (VIDRL)|Microbiological Diagnostic Unit Public Health Laboratory and Victorian Infectious Diseases Reference Laboratory|Schultz|Australia
>Spike|hCoV-19/Australia/VIC4930/2020|2020-07-14|EPI_ISL_519304|Original|hCoV-19^^Victoria|Human|Victorian Infectious Diseases Reference Laboratory (VIDRL)|VIDRL and MDU-PHL|Schultz|Australia
>Spike|hCoV-19/Wales/PHWC-4895FD/2020|2020-10-28|EPI_ISL_639594|Original|hCoV-19^^Wales|Human|Wales Specialist Virology Centre Sequencing lab|COVID-19 Genomics UK (COG-UK) Consortium|Moore|United Kingdom
>Spike|hCoV-19/Saudi Arabia/85790/2020|2020-03-15|EPI_ISL_469245|Original|hCoV-19^^Jeddah|Human|Special Infectious Agents Unit|Special Infectious Agents Unit|Submitter|Saudi Arabia
>Spike|hCoV-19/Wales/PHWC-491149/2020|2020-11-10|EPI_ISL_706616|Original|hCoV-19^^Wales|Human|Originating lab|COVID-19 Genomics UK (COG-UK) Consortium|Moore|United Kingdom
>Spike|hCoV-19/Iceland/225/2020|2020-03-16|EPI_ISL_417554|Original|hCoV-19^^Reykjavik|Human|The National University Hospital of Iceland|deCODE genetics|J\u00f3nsson|Iceland

>Spike|hCoV-19/England/ALDP-9ED282/2020|2020-05-05|EPI_ISL_589490|Original|hCoV-19^^England|Human|Lighthouse Lab in Alderley Park|Wellcome Sanger Institute for the COVID-19 Genomics UK (COG-UK) consortium|Davies|United Kingdom
>Spike|hCoV-19/Switzerland/BS-UHB-42188149/2020|2020-03-12|EPI_ISL_528033|Original|hCoV-19^^Basel-Stadt|Human|University Hospital Basel|University Hospital Basel|Roloff Handschin|Switzerland
>Spike|hCoV-19/England/ALDP-9BEDB6/2020|2020-09-10|EPI_ISL_568073|Original|hCoV-19^^England|Human|Lighthouse Lab in Alderley Park|Wellcome Sanger Institute for the COVID-19 Genomics UK (COG-UK) consortium|Davies|United Kingdom
>Spike|hCoV-19/England/ALDP-B75FD1/2020|2020-11-12|EPI_ISL_723721|Original|hCoV-19^^England|Human|Oxford Viromics|COVID-19 Genomics UK (COG-UK) Consortium|Golubchik|United Kingdom
>Spike|hCoV-19/England/QEUH-BF0B8F/2020|2020-12-05|EPI_ISL_709190|Original|hCoV-19^^England|Human|Lighthouse Lab in Glasgow|Wellcome Sanger Institute for the COVID-19 Genomics UK (COG-UK) Consortium|Davies|United Kingdom
>Spike|hCoV-19/England/QEUH-AD2771/2020|2020-10-24|EPI_ISL_629785|Original|hCoV-19^^England|Human|Lighthouse Lab in Glasgow|Wellcome Sanger Institute for the COVID-19 Genomics UK (COG-UK) consortium|Davies|United Kingdom
>Spike|hCoV-19/England/20130087004/2020|2020-03-20|EPI_ISL_423673|Original|hCoV-19^^England|Human|Respiratory Virus Unit|Respiratory Virus Unit|Platt|United Kingdom
>Spike|hCoV-19/USA/TX-HMH-MCoV-12845/2020|2020-08-19|EPI_ISL_787998|Original|hCoV-19^^Texas|Human|Houston Methodist Hospital|Houston Methodist Hospital|Long|USA
>Spike|hCoV-19/England/QEUH-96E052/2020|2020-08-18|EPI_ISL_530458|Original|hCoV-19^^England|Human|Lighthouse Lab in Glasgow|Wellcome Sanger Institute for the COVID-19 Genomics UK (COG-UK) consortium|Davies|United Kingdom
>Spike|hCoV-19/USA/AZ-TG305735/2020|2020-04-27|EPI_ISL_694259|Original|hCoV-19^^Arizona|Human|AZ SPHL|TGen North|French|USA
>Spike|hCoV-19/USA/AZ-TG491470/2020|2020-06-17|EPI_ISL_695105|Original|hCoV-19^^Arizona|Human|AZ SPHL|TGen North|French|USA
>Spike|hCoV-19/USA/FL-BPHL-0285/2020|2020-05-13|EPI_ISL_480936|Original|hCoV-19^^Florida|Human|Florida Bureau of Public Health Laboratories|Florida Bureau of Public Health Laboratories|Blanton|USA
>Spike|hCoV-19/USA/TX-HMH-5711/2020|2020-06-29|EPI_ISL_546961|Original|hCoV-19^^Texas|Human|Houston Methodist Hospital|Houston Methodist Hospital|Long|USA
>Spike|hCoV-19/England/MILK-9705B8/2020|2020-08-18|EPI_ISL_553237|Original|hCoV-19^^England|Human|Lighthouse Lab in Milton Keynes|Wellcome Sanger Institute for the COVID-19 Genomics UK (COG-UK) consortium|Davies|United Kingdom
>Spike|hCoV-19/England/MILK-C307D9/2020|2020-12-07|EPI_ISL_782935|Original|hCoV-19^^England|Human|Lighthouse Lab in Milton Keynes|Wellcome Sanger Institute for the COVID-19 Genomics UK (COG-UK) Consortium|Davies|United Kingdom
>Spike|hCoV-19/England/ALDP-937C7C/2020|2020-06-21|EPI_ISL_552881|Original|hCoV-19^^England|Human|Lighthouse Lab in Alderley Park|Wellcome Sanger Institute for the COVID-19 Genomics UK (COG-UK) consortium|Davies|United Kingdom
>Spike|hCoV-19/USA/TX-HMH-MCoV-14412/2020|2020-10-07|EPI_ISL_787697|Original|hCoV-19^^Texas|Human|Houston Methodist Hospital|Houston Methodist Hospital|Long|USA
>Spike|hCoV-19/England/CAMC-B32C9D/2020|2020-11-05|EPI_ISL_659713|Original|hCoV-19^^England|Human|Lighthouse Lab in Cambridge|Wellcome Sanger Institute for the COVID-19 Genomics UK (COG-UK) Consortium|Davies|United Kingdom
>Spike|hCoV-19/USA/TX-HMH-MCoV-18079/2020|2020-11-15|EPI_ISL_784427|Original|hCoV-19^^Texas|Human|Houston Methodist Hospital|Houston Methodist Hospital|Long|USA
>Spike|hCoV-19/England/NOTT-1113D5/2020|2020-05-15|EPI_ISL_453658|Original|hCoV-19^^England|Human|Queens Medical Centre|COVID-19 Genomics UK (COG-UK) Consortium|Loose|United Kingdom
>Spike|hCoV-19/USA/CA-CZB-3830/2020|2020-07-21|EPI_ISL_548451|Original|hCoV-19^^California|Human|County of Santa Clara Public Health Department|Chan-Zuckerberg Biohub|Bhatt|USA
>Spike|hCoV-19/France/PAC-IHU-1169/2020|2020-08-14|EPI_ISL_569072|Original|hCoV-19^^Provence-Alpes-C\u00f4te-d\u2019Azur|Human|MEPHI|MEPHI|Levasseur|France
>Spike|hCoV-19/Australia/VIC1603/2020|2020-05-11|EPI_ISL_456515|Original|hCoV-19^^Victoria|Human|Victorian Infectious Diseases Reference Laboratory

(VIDRL)|Microbiological Diagnostic Unit Public Health Laboratory and Victorian Infectious Diseases Reference Laboratory|Schultz|Australia
>Spike|hCoV-19/Ireland/KE-NVRL-73IRL13514/2020|2020-09-16|EPI_ISL_578288|Original|hCoV-19^^Kildare|Human|National Virus Reference Laboratory|National Virus Reference Laboratory|Gonzalez|Ireland
>Spike|hCoV-19/Wales/PHWC-493790/2020|2020-11-29|EPI_ISL_725939|Original|hCoV-19^^Wales|Human|Originating lab|COVID-19 Genomics UK (COG-UK) Consortium|Moore|United Kingdom
>Spike|hCoV-19/England/205141686/2020|2020-12-15|EPI_ISL_766223|Original|hCoV-19^^England|Human|Respiratory Virus Unit|COVID-19 Genomics UK (COG-UK) Consortium|Platt|United Kingdom
>Spike|hCoV-19/Wales/PHWC-48BD20/2020|2020-11-03|EPI_ISL_666349|Original|hCoV-19^^Wales|Human|Wales Specialist Virology Centre Sequencing lab|COVID-19 Genomics UK (COG-UK) Consortium|Moore|United Kingdom
>Spike|hCoV-19/Scotland/EDB1730/2020|2020-04-15|EPI_ISL_433130|Original|hCoV-19^^Scotland|Human|Virology Department|COVID-19 Genomics UK (COG-UK) Consortium|Williams|United Kingdom
>Spike|hCoV-19/England/CAMC-C45F06/2020|2020-12-10|EPI_ISL_718532|Original|hCoV-19^^England|Human|Lighthouse Lab in Cambridge|Wellcome Sanger Institute for the COVID-19 Genomics UK (COG-UK) Consortium|Davies|United Kingdom
>Spike|hCoV-19/South Korea/KCDC2257/2020|2020-05-12|EPI_ISL_510595|Original|hCoV-19^^South Korea|Human|Division of Viral Diseases|Division of Viral Diseases|Kim|South Korea
>Spike|hCoV-19/England/SHEF-10D2C97/2020|2020-12-18|EPI_ISL_742048|Original|hCoV-19^^England|Human|Virology Department|COVID-19 Genomics UK (COG-UK) Consortium|de Silva|United Kingdom
>Spike|hCoV-19/England/LOND-D622B/2020|2020-04-20|EPI_ISL_482167|Original|hCoV-19^^England|Human|University College London|Wellcome Sanger Institute for the COVID-19 Genomics UK (COG-UK) consortium|Davies|United Kingdom
>Spike|hCoV-19/Denmark/DCGC-18995/2020|2020-12-07|EPI_ISL_748186|Original|hCoV-19^^Hovedstaden|Human|Department of Virus and Microbiological Special Diagnostics|Albertsen Lab|Michaelsen|Denmark
>Spike|hCoV-19/USA/HI-H200294/2020|2020-08-11|EPI_ISL_752853|Original|hCoV-19^^Hawaii|Human|State Laboratories Division|State Laboratories Division|Sultana|USA
>Spike|hCoV-19/England/MILK-CA9FD3/2020|2020-12-17|EPI_ISL_761587|Original|hCoV-19^^England|Human|Lighthouse Lab in Milton Keynes|Wellcome Sanger Institute for the COVID-19 Genomics UK (COG-UK) Consortium|Davies|United Kingdom
>Spike|hCoV-19/Australia/VIC11975/2020|2020-08-11|EPI_ISL_640924|Original|hCoV-19^^Victoria|Human|Microbiological Diagnostic Unit - Public Health Laboratory (MDU-PHL)|MDU-PHL|Schultz|Australia
>Spike|hCoV-19/England/ALDP-A93A0A/2020|2020-10-19|EPI_ISL_611223|Original|hCoV-19^^England|Human|Lighthouse Lab in Alderley Park|Wellcome Sanger Institute for the COVID-19 Genomics UK (COG-UK) consortium|Davies|United Kingdom
>Spike|hCoV-19/Northern Ireland/NIRE-108CB8/2020|2020-10-05|EPI_ISL_627550|Original|hCoV-19^^Northern Ireland|Human|Regional Virus Laboratory|COVID-19 Genomics UK (COG-UK) Consortium|Fairley|United Kingdom
>Spike|hCoV-19/England/ALDP-9CCD97/2020|2020-09-16|EPI_ISL_566146|Original|hCoV-19^^England|Human|Lighthouse Lab in Alderley Park|Wellcome Sanger Institute for the COVID-19 Genomics UK (COG-UK) consortium|Davies|United Kingdom
>Spike|hCoV-19/USA/TX-HMH-7270/2020|2020-06-29|EPI_ISL_546639|Original|hCoV-19^^Texas|Human|Houston Methodist Hospital|Houston Methodist Hospital|Long|USA
>Spike|hCoV-19/Australia/VIC12277/2020|2020-08-08|EPI_ISL_591946|Original|hCoV-19^^Victoria|Human|Victorian Infectious Diseases Reference Laboratory (VIDRL)|VIDRL and MDU-PHL|Schultz|Australia
>Spike|hCoV-19/USA/CT-QDX-2695/2020|2020-03-16|EPI_ISL_605023|Original|hCoV-19^^Connecticut|Human|Quest Diagnostics|Quest Diagnostics|Kagan|USA
>Spike|hCoV-19/England/ALDP-B45B21/2020|2020-11-07|EPI_ISL_656322|Original|hCoV-19^^England|Human|Lighthouse Lab in Alderley Park|Wellcome Sanger Institute for the COVID-19 Genomics UK (COG-UK) Consortium|Davies|United Kingdom

>Spike|hCoV-19/USA/LA-GBCL-26492443/2020|2020-04-28|EPI_ISL_485026|Original|hCoV-19^^Louisiana|Human|River Road Testing Lab|Ginkgo Bioworks Clinical Laboratory|Smith|USA
>Spike|hCoV-19/Scotland/EDB5077/2020|2020-04-23|EPI_ISL_487820|Original|hCoV-19^^Scotland|Human|Virology Department|Wellcome Sanger Institute for the COVID-19 Genomics UK (COG-UK) consortium|Davies|United Kingdom
>Spike|hCoV-19/England/CAMC-B51C01/2020|2020-11-09|EPI_ISL_657994|Original|hCoV-19^^England|Human|Lighthouse Lab in Cambridge|Wellcome Sanger Institute for the COVID-19 Genomics UK (COG-UK) Consortium|Davies|United Kingdom
>Spike|hCoV-19/Denmark/DCGC-15413/2020|2020-11-23|EPI_ISL_713812|Original|hCoV-19^^Nordjylland|Human|Department of Virus and Microbiological Special Diagnostics|Albertsen Lab|Michaelsen|Denmark
>Spike|hCoV-19/England/ALDP-B83B45/2020|2020-11-14|EPI_ISL_675650|Original|hCoV-19^^England|Human|Lighthouse Lab in Alderley Park|Wellcome Sanger Institute for the COVID-19 Genomics UK (COG-UK) Consortium|Davies|United Kingdom
>Spike|hCoV-19/USA/MA-MGH-00028/2020|2020-03-25|EPI_ISL_460469|Original|hCoV-19^^Massachusetts|Human|Massachusetts General Hospital|Infectious Disease Program|Lemieux|USA
>Spike|hCoV-19/Australia/VIC5281/2020|2020-07-17|EPI_ISL_520046|Original|hCoV-19^^Victoria|Human|Microbiological Diagnostic Unit - Public Health Laboratory (MDU-PHL)|MDU-PHL|Schultz|Australia
>Spike|hCoV-19/Denmark/DCGC-5518/2020|2020-09-21|EPI_ISL_620843|Original|hCoV-19^^Hovedstaden|Human|Department of Virus and Microbiological Special Diagnostics|Albertsen lab|Michaelsen|Denmark
>Spike|hCoV-19/Denmark/DCGC-10499/2020|2020-11-02|EPI_ISL_670018|Original|hCoV-19^^Hovedstaden|Human|Department of Virus and Microbiological Special Diagnostics|Albertsen Lab|Michaelsen|Denmark
>Spike|hCoV-19/England/CAMC-A81D60/2020|2020-10-13|EPI_ISL_610727|Original|hCoV-19^^England|Human|Lighthouse Lab in Cambridge|Wellcome Sanger Institute for the COVID-19 Genomics UK (COG-UK) consortium|Davies|United Kingdom
>Spike|hCoV-19/USA/TX-HMH-MCoV-14092/2020|2020-08-14|EPI_ISL_787378|Original|hCoV-19^^Texas|Human|Houston Methodist Hospital|Houston Methodist Hospital|Long|USA
>Spike|hCoV-19/USA/FL-BPHL-2179/2020|2020-08-19|EPI_ISL_653332|Original|hCoV-19^^Florida|Human|Florida Bureau of Public Health Laboratories|Florida Bureau of Public Health Laboratories|Blanton|USA
>Spike|hCoV-19/USA/TX-HMH-MCoV-11476/2020|2020-09-03|EPI_ISL_790028|Original|hCoV-19^^Texas|Human|Houston Methodist Hospital|Houston Methodist Hospital|Long|USA
>Spike|hCoV-19/USA/NV-NSPHL-A0083/2020|2020-04-13|EPI_ISL_515360|Original|hCoV-19^^Nevada|Human|Nevada State Public Health Laboratory|Nevada State Public Health Laboratory|Sevinsky|USA
>Spike|hCoV-19/USA/WA-UW-22757/2020|2020-08-06|EPI_ISL_570712|Original|hCoV-19^^Washington|Human|UW Virology Lab|UW Virology Lab|Roychoudhury|USA
>Spike|hCoV-19/Gibraltar/204421216/2020|2020-10-27|EPI_ISL_613677|Original|hCoV-19^^Gibraltar|Human|Microbiology|Respiratory Virus Unit|Platt|Gibraltar
>Spike|hCoV-19/England/QEUH-C31BCD/2020|2020-12-11|EPI_ISL_782530|Original|hCoV-19^^England|Human|Lighthouse Lab in Glasgow|Wellcome Sanger Institute for the COVID-19 Genomics UK (COG-UK) Consortium|Davies|United Kingdom
>Spike|hCoV-19/USA/UT-UPHL-2012523098/2020|2020-08-03|EPI_ISL_745751|Original|hCoV-19^^Utah|Human|Ginkgo Bioworks Clinical Laboratory|Utah Public Health Laboratory|Young|USA
>Spike|hCoV-19/Scotland/QEUH-B135A6/2020|2020-11-03|EPI_ISL_649608|Original|hCoV-19^^Scotland|Human|Lighthouse Lab in Glasgow|Wellcome Sanger Institute for the COVID-19 Genomics UK (COG-UK) Consortium|Davies|United Kingdom
>Spike|hCoV-19/Wales/PHWC-499606/2020|2020-12-04|EPI_ISL_727331|Original|hCoV-19^^Wales|Human|Originating lab|COVID-19 Genomics UK (COG-UK) Consortium|Moore|United Kingdom
>Spike|hCoV-19/England/NORT-28937D/2020|2020-03-29|EPI_ISL_458693|Original|hCoV-19^^England|Human|NU-OMICS DNA Sequencing research facility|Wellcome Sanger Institute for the COVID-19 Genomics UK (COG-UK) consortium|Davies|United Kingdom

>Spike|hCoV-19/Denmark/DCGC-20870/2020|2020-12-21|EPI_ISL_759420|Original|hCoV-19^^Nordjylland|Human|Department of Virus and Microbiological Special Diagnostics|Albertsen Lab|Michaelsen|Denmark
>Spike|hCoV-19/Japan/PG-5602/2020|2020-08-00|EPI_ISL_688727|Original|hCoV-19^^Japan|Human|Pathogen Genomics Center|Pathogen Genomics Center|Sekizuka|Japan
>Spike|hCoV-19/England/ALDP-B8493B/2020|2020-11-14|EPI_ISL_675206|Original|hCoV-19^^England|Human|Lighthouse Lab in Alderley Park|Wellcome Sanger Institute for the COVID-19 Genomics UK (COG-UK) Consortium|Davies|United Kingdom
>Spike|hCoV-19/England/NORW-E8A4C/2020|2020-04-11|EPI_ISL_448267|Original|hCoV-19^^England|Human|Quadram Institute Bioscience|COVID-19 Genomics UK (COG-UK) Consortium|Alikhan|United Kingdom
>Spike|hCoV-19/Germany/BE-ChVir-D2016-5933/2020|2020-10-18|EPI_ISL_753901|Original|hCoV-19^^Berlin|Human|Charit\u00e9 Universit\u00e4tsmedizin Berlin|Charit\u00e9 Universit\u00e4tsmedizin Berlin|Muehlemann|Germany
>Spike|hCoV-19/Wales/PHWC-16F323/2020|2020-10-05|EPI_ISL_585948|Original|hCoV-19^^Wales|Human|Wales Specialist Virology Centre Sequencing lab|COVID-19 Genomics UK (COG-UK) Consortium|Moore|United Kingdom
>Spike|hCoV-19/England/BRIS-12A2DC/2020|2020-04-16|EPI_ISL_458396|Original|hCoV-19^^England|Human|PHE South West Regional Laboratory|Wellcome Sanger Institute for the COVID-19 Genomics UK (COG-UK) consortium|Davies|United Kingdom
>Spike|hCoV-19/England/LIVE-DB40A4/2020|2020-10-19|EPI_ISL_650331|Original|hCoV-19^^England|Human|Liverpool Clinical Laboratories|COVID-19 Genomics UK (COG-UK) Consortium|Haldenby|United Kingdom
>Spike|hCoV-19/USA/NY-Wadsworth-251944-01/2020|2020-10-22|EPI_ISL_734390|Original|hCoV-19^^New York|Human|Wadsworth Center|Wadsworth Center|Lasek-Nesselquist|USA
>Spike|hCoV-19/England/OXON-AC789/2020|2020-04-07|EPI_ISL_448482|Original|hCoV-19^^England|Human|Oxford Viromics|COVID-19 Genomics UK (COG-UK) Consortium|Golubchik|United Kingdom
>Spike|hCoV-19/England/CAMC-A64FE5/2020|2020-10-11|EPI_ISL_608447|Original|hCoV-19^^England|Human|Lighthouse Lab in Cambridge|Wellcome Sanger Institute for the COVID-19 Genomics UK (COG-UK) consortium|Davies|United Kingdom
>Spike|hCoV-19/Scotland/CVR5072/2020|2020-10-11|EPI_ISL_594805|Original|hCoV-19^^Scotland|Human|West of Scotland Specialist Virology Centre|COVID-19 Genomics UK (COG-UK) Consortium|Robertson|United Kingdom
>Spike|hCoV-19/Spain/AN-ISCIII-201616/2020|2020-03-05|EPI_ISL_455319|Original|hCoV-19^^Andalucia|Human|Hospital Virgen de las Nieves|Instituto de Salud Carlos III|Caballero|Spain
>Spike|hCoV-19/England/LIVE-DC9CB7/2020|2020-12-03|EPI_ISL_705347|Original|hCoV-19^^England|Human|Liverpool Clinical Laboratories|COVID-19 Genomics UK (COG-UK) Consortium|Haldenby|United Kingdom
>Spike|hCoV-19/Greece/9057_HPI/2020|2020-04-04|EPI_ISL_487379|Original|hCoV-19^^Athens|Human|Hellenic Pasteur Institute|Hellenic Pasteur Institute|Kossyvakis|Greece
>Spike|hCoV-19/Wales/PHWC-49A5CC/2020|2020-12-02|EPI_ISL_727569|Original|hCoV-19^^Wales|Human|Originating lab|COVID-19 Genomics UK (COG-UK) Consortium|Moore|United Kingdom
>Spike|hCoV-19/USA/NY-NYUMC191/2020|2020-04-01|EPI_ISL_427486|Original|hCoV-19^^New York|Human|NYU Langone Health|Departments of Pathology and Medicine|Maurano|USA
>Spike|hCoV-19/Russia/OMS-ORINFI-MH10285M/2020|2020-11-12|EPI_ISL_733489|Original|hCoV-19^^Omsk|Human|Omsk Research Institute of Natural Focal Infections|WHO National Influenza Centre Russian Federation|Komissarov|Russia
>Spike|hCoV-19/England/CAMC-A5948A/2020|2020-10-08|EPI_ISL_596954|Original|hCoV-19^^England|Human|Lighthouse Lab in Cambridge|Wellcome Sanger Institute for the COVID-19 Genomics UK (COG-UK) consortium|Davies|United Kingdom
>Spike|hCoV-19/Scotland/CVR6021/2020|2020-11-29|EPI_ISL_680458|Original|hCoV-19^^Scotland|Human|West of Scotland Specialist Virology Centre|COVID-19 Genomics UK (COG-UK) Consortium|Robertson|United Kingdom

>Spike|hCoV-19/England/CAMB-1B616F/2020|2020-09-17|EPI_ISL_573149|Original|hCoV-19^^England|Human|Department of Pathology|COVID-19 Genomics UK (COG-UK) Consortium|Goodfellow|United Kingdom
>Spike|hCoV-19/England/LIVE-1DDFD1/2020|2020-10-09|EPI_ISL_611675|Original|hCoV-19^^England|Human|Liverpool Clinical Laboratories|COVID-19 Genomics UK (COG-UK) Consortium|Haldenby|United Kingdom
>Spike|hCoV-19/England/MILK-597072/2020|2020-06-13|EPI_ISL_558241|Original|hCoV-19^^England|Human|Lighthouse Lab in Milton Keynes|Wellcome Sanger Institute for the COVID-19 Genomics UK (COG-UK) consortium|Davies|United Kingdom
>Spike|hCoV-19/Australia/VIC14141/2020|2020-09-20|EPI_ISL_591815|Original|hCoV-19^^Victoria|Human|Microbiological Diagnostic Unit - Public Health Laboratory (MDU-PHL)|MDU-PHL|Schultz|Australia
>Spike|hCoV-19/England/QEUH-B9E8B9/2020|2020-11-18|EPI_ISL_719832|Original|hCoV-19^^England|Human|Lighthouse Lab in Glasgow|Wellcome Sanger Institute for the COVID-19 Genomics UK (COG-UK) Consortium|Davies|United Kingdom
>Spike|hCoV-19/USA/WA-UW-4326/2020|2020-03-31|EPI_ISL_427263|Original|hCoV-19^^Washington|Human|UW Virology Lab|UW Virology Lab|Roychoudhury|USA
>Spike|hCoV-19/Wales/PHWC-47F7C7/2020|2020-10-09|EPI_ISL_612725|Original|hCoV-19^^Wales|Human|Wales Specialist Virology Centre Sequencing lab|COVID-19 Genomics UK (COG-UK) Consortium|Moore|United Kingdom
>Spike|hCoV-19/Denmark/DCGC-2972/2020|2020-08-24|EPI_ISL_616734|Original|hCoV-19^^Midtjylland|Human|Department of Virus and Microbiological Special Diagnostics|Albertsen lab|Michaelsen|Denmark
>Spike|hCoV-19/Austria/CeMM0285/2020|2020-03-26|EPI_ISL_438111|Original|hCoV-19^^Burgenland|Human|Center for Virology|Bergthaler laboratory|Bergthaler|Austria
>Spike|hCoV-19/Spain/MD-HLP-56/2020|2020-04-20|EPI_ISL_530059|Original|hCoV-19^^Madrid|Human|Hospital Universitario La Paz|Hospital Universitario La Paz|Viedma|Spain
>Spike|hCoV-19/Taiwan/CGMH-CGU-29/2020|2020-04-02|EPI_ISL_464093|Vero E6|hCoV-19^^Taoyuan|Human|Laboratory Medicine|Department of Laboratory Medicine|Yi Chun|Taiwan
>Spike|hCoV-19/Japan/PG-1042/2020|2020-04-00|EPI_ISL_684652|Original|hCoV-19^^Japan|Human|Fukuoka Institute of Health and Environmental Sciences|Pathogen Genomics Center|Sekizuka|Japan
>Spike|hCoV-19/England/204261293/2020|2020-10-15|EPI_ISL_613582|Original|hCoV-19^^England|Human|Respiratory Virus Unit|Respiratory Virus Unit|Platt|United Kingdom
>Spike|hCoV-19/USA/CA-ALSR-1036-SAN/2020|2020-06-21|EPI_ISL_483476|Original|hCoV-19^^California|Human|UC San Diego Center for Advanced Laboratory Medicine|Andersen lab at Scripps Research|Gangavarapu|USA
>Spike|hCoV-19/Scotland/EDB4546/2020|2020-05-05|EPI_ISL_461720|Original|hCoV-19^^Scotland|Human|Virology Department|COVID-19 Genomics UK (COG-UK) Consortium|Williams|United Kingdom
>Spike|hCoV-19/England/CAMB-74016/2020|2020-03-18|EPI_ISL_440303|Original|hCoV-19^^England|Human|Department of Pathology|Wellcome Sanger Institute for the COVID-19 Genomics UK (COG-UK) consortium|Amato|United Kingdom
>Spike|hCoV-19/Canada/MB-NML-864/2020|2020-03-30|EPI_ISL_582345|Original|hCoV-19^^Manitoba|Human|Cadham Provincial Laboratory|National Microbiology Laboratory (NML)|Majer|Canada
>Spike|hCoV-19/England/BIRM-62901/2020|2020-08-21|EPI_ISL_529628|Original|hCoV-19^^England|Human|University of Birmingham|COVID-19 Genomics UK (COG-UK) Consortium|Loman|United Kingdom
>Spike|hCoV-19/USA/TX-HMH-MCoV-18842/2020|2020-11-25|EPI_ISL_785161|Original|hCoV-19^^Texas|Human|Houston Methodist Hospital|Houston Methodist Hospital|Long|USA
>Spike|hCoV-19/United Arab Emirates/0105/2020|2020-05-15|EPI_ISL_698295|Original|hCoV-19^^Abu Dhabi|Human|Group 42 (G42) Healthcare|G42 Healthcare|Ogrodzki|United Arab Emirates
>Spike|hCoV-19/Denmark/DCGC-21334/2020|2020-12-21|EPI_ISL_759686|Original|hCoV-19^^Nordjylland|Human|Department of Virus and Microbiological Special Diagnostics|Albertsen Lab|Michaelsen|Denmark

>Spike|hCoV-19/England/EXET-13E536/2020|2020-00-00|EPI_ISL_724327|Original|hCoV-19^^England|Human|University of Exeter|COVID-19 Genomics UK (COG-UK) Consortium|Temperton|United Kingdom
>Spike|hCoV-19/Denmark/DCGC-2335/2020|2020-08-10|EPI_ISL_617673|Original|hCoV-19^^Nordjylland|Human|Department of Virus and Microbiological Special Diagnostics|Albertsen lab|Michaelsen|Denmark
>Spike|hCoV-19/Scotland/ALDP-9FDD42/2020|2020-10-01|EPI_ISL_600523|Original|hCoV-19^^Scotland|Human|Lighthouse Lab in Alderley Park|Wellcome Sanger Institute for the COVID-19 Genomics UK (COG-UK) consortium|Davies|United Kingdom
>Spike|hCoV-19/Australia/VIC4347/2020|2020-07-13|EPI_ISL_519643|Original|hCoV-19^^Victoria|Human|Microbiological Diagnostic Unit - Public Health Laboratory (MDU-PHL)|MDU-PHL|Schultz|Australia
>Spike|hCoV-19/USA/WA-S1945/2020|2020-06-02|EPI_ISL_497373|Original|hCoV-19^^Washington|Human|Washington State Department of Health|Seattle Flu Study|Lee|USA
>Spike|hCoV-19/USA/LA-EVTL318/2020|2020-05-15|EPI_ISL_578725|Original|hCoV-19^^Louisiana|Human|LSUHS Emerging Viral Threat Laboratory|Microbial Genome Sequencing Center|Kamil|USA
>Spike|hCoV-19/Australia/VIC15232/2020|2020-08-04|EPI_ISL_593208|Original|hCoV-19^^Victoria|Human|Microbiological Diagnostic Unit - Public Health Laboratory (MDU-PHL)|MDU-PHL|Schultz|Australia
>Spike|hCoV-19/United Arab Emirates/0225/2020|2020-05-17|EPI_ISL_698175|Original|hCoV-19^^Abu Dhabi|Human|Group 42 (G42) Healthcare|G42 Healthcare|Ogrodzki|United Arab Emirates
>Spike|hCoV-19/USA/LA-EVTL022/2020|2020-04-24|EPI_ISL_435560|Original|hCoV-19^^Louisiana|Human|LSUHS Emerging Viral Threat Laboratory|Microbial Genome Sequencing Center|Kamil|USA
>Spike|hCoV-19/England/QEUH-B4230E/2020|2020-11-08|EPI_ISL_657336|Original|hCoV-19^^England|Human|Lighthouse Lab in Glasgow|Wellcome Sanger Institute for the COVID-19 Genomics UK (COG-UK) Consortium|Davies|United Kingdom
>Spike|hCoV-19/USA/TX-HMH-6331/2020|2020-06-29|EPI_ISL_547395|Original|hCoV-19^^Texas|Human|Houston Methodist Hospital|Houston Methodist Hospital|Long|USA
>Spike|hCoV-19/Netherlands/NA_553/2020|2020-03-19|EPI_ISL_460721|Original|hCoV-19^^Netherlands|Human|Dutch COVID-19 response team|Erasmus Medical Center|Oude Munnink|Netherlands
>Spike|hCoV-19/USA/MA-MGH-00521/2020|2020-03-16|EPI_ISL_460224|Original|hCoV-19^^Massachusetts|Human|Massachusetts General Hospital|Infectious Disease Program|Lemieux|USA
>Spike|hCoV-19/India/GJ-GBRC348a/2020|2020-06-18|EPI_ISL_514591|Original|hCoV-19^^Gujarat|Human|B.J. Medical College and Civil hospital|Gujarat Biotechnology Research Centre|Joshi|India
>Spike|hCoV-19/Australia/NSW1314/2020|2020-12-21|EPI_ISL_767880|Original|hCoV-19^^New South Wales|Human|Laverty Pathology|NSW Health Pathology - Institute of Clinical Pathology and Medical Research; Westmead Hospital; University of Sydney|Gall|Australia
>Spike|hCoV-19/Denmark/DCGC-2758/2020|2020-08-24|EPI_ISL_616592|Original|hCoV-19^^Hovedstaden|Human|Department of Virus and Microbiological Special Diagnostics|Albertsen lab|Michaelsen|Denmark
>Spike|hCoV-19/Switzerland/LU-ETHZ-110029/2020|2020-04-01|EPI_ISL_486466|Original|hCoV-19^^Luzern|Human|Viollier AG|Department of Biosystems Science and Engineering|Nadeau|Switzerland
>Spike|hCoV-19/India/MH-AFCO-15275/2020|2020-07-25|EPI_ISL_721632|Original|hCoV-19^^Maharashtra|Human|Armed Forces Medical College|National Centre For Cell Science|Paul|India
>Spike|hCoV-19/England/MILK-B54B5C/2020|2020-11-05|EPI_ISL_657981|Original|hCoV-19^^England|Human|Lighthouse Lab in Milton Keynes|Wellcome Sanger Institute for the COVID-19 Genomics UK (COG-UK) Consortium|Davies|United Kingdom
>Spike|hCoV-19/England/CAMC-A65403/2020|2020-10-11|EPI_ISL_608359|Original|hCoV-19^^England|Human|Lighthouse Lab in Cambridge|Wellcome Sanger Institute for the COVID-19 Genomics UK (COG-UK) consortium|Davies|United Kingdom

>Spike|hCoV-19/England/ALDP-C48440/2020|2020-12-12|EPI_ISL_736562|Original|hCoV-19^^England|Human|Lighthouse Lab in Alderley Park|Wellcome Sanger Institute for the COVID-19 Genomics UK (COG-UK) Consortium|Davies|United Kingdom
>Spike|hCoV-19/USA/HI-H200584/2020|2020-11-04|EPI_ISL_753110|Original|hCoV-19^^Hawaii|Human|State Laboratories Division|State Laboratories Division|Sultana|USA
>Spike|hCoV-19/DRC/81/2020|2020-03-18|EPI_ISL_417944|Original|hCoV-19^^Kinshasa|Human|Viral Respiratory Lab|Pathogen Sequencing Lab|Pratt|Democratic Republic of the Congo
>Spike|hCoV-19/England/CAMC-B9971B/2020|2020-11-16|EPI_ISL_673705|Original|hCoV-19^^England|Human|Lighthouse Lab in Cambridge|Wellcome Sanger Institute for the COVID-19 Genomics UK (COG-UK) Consortium|Davies|United Kingdom
>Spike|hCoV-19/Wales/PHWC-163389/2020|2020-04-24|EPI_ISL_473285|Original|hCoV-19^^Wales|Human|Wales Specialist Virology Centre Sequencing lab|COVID-19 Genomics UK (COG-UK) Consortium|Moore|United Kingdom
>Spike|hCoV-19/England/QEUH-9F88EC/2020|2020-09-27|EPI_ISL_588733|Original|hCoV-19^^England|Human|Lighthouse Lab in Glasgow|Wellcome Sanger Institute for the COVID-19 Genomics UK (COG-UK) consortium|Davies|United Kingdom
>Spike|hCoV-19/Scotland/EDB901/2020|2020-04-05|EPI_ISL_432873|Original|hCoV-19^^Scotland|Human|Virology Department|COVID-19 Genomics UK (COG-UK) Consortium|Williams|United Kingdom
>Spike|hCoV-19/Denmark/DCGC-9291/2020|2020-11-16|EPI_ISL_668889|Original|hCoV-19^^Nordjylland|Human|Department of Virus and Microbiological Special Diagnostics|Albertsen Lab|Michaelsen|Denmark
>Spike|hCoV-19/England/SHEF-CD1DF/2020|2020-04-23|EPI_ISL_453749|Original|hCoV-19^^England|Human|Virology Department|COVID-19 Genomics UK (COG-UK) Consortium|de Silva|United Kingdom
>Spike|hCoV-19/England/MILK-A47425/2020|2020-10-06|EPI_ISL_598471|Original|hCoV-19^^England|Human|Lighthouse Lab in Milton Keynes|Wellcome Sanger Institute for the COVID-19 Genomics UK (COG-UK) consortium|Davies|United Kingdom
>Spike|hCoV-19/USA/CA-IGI-0205/2020|2020-07-09|EPI_ISL_569889|Original|hCoV-19^^California|Human|Innovative Genomics Institute|Innovative Genomics Institute|Wyman|USA
>Spike|hCoV-19/India/DL-NCDC2004-CSIR-IGIB/2020|2020-05-18|EPI_ISL_482524|Original|hCoV-19^^Delhi|Human|National Centre for Disease control (NCDC)|NCDC/CSIR-IGIB|Pandey|India
>Spike|hCoV-19/USA/VA-DCLS-1621/2020|2020-08-19|EPI_ISL_614028|Original|hCoV-19^^Virginia|Human|Virginia DCLS|Virginia DCLS|St. Jacques|USA
>Spike|hCoV-19/Luxembourg/LNS9459476/2020|2020-10-27|EPI_ISL_744919|Original|hCoV-19^^Luxembourg|Human|Laboratoire national de sant\u00e9|Laboratoire national de sant\u00e9|Wienecke-Baldacchino|Luxembourg
>Spike|hCoV-19/Denmark/DCGC-20982/2020|2020-12-21|EPI_ISL_759488|Original|hCoV-19^^Midtjylland|Human|Department of Virus and Microbiological Special Diagnostics|Albertsen Lab|Michaelsen|Denmark
>Spike|hCoV-19/USA/OR-OHSU-1827/2020|2020-07-27|EPI_ISL_526040|Original|hCoV-19^^Oregon|Human|OHSU Lab Services Molecular Microbiology Lab|Oregon SARS-CoV-2 Genome Sequencing Center|O'Roak|USA
>Spike|hCoV-19/USA/UT-02234/2020|2020-04-14|EPI_ISL_436931|Original|hCoV-19^^Utah|Human|Utah Public Health Laboratory|Utah Public Health Laboratory|Young|USA
>Spike|hCoV-19/England/204241459/2020|2020-10-14|EPI_ISL_622854|Original|hCoV-19^^England|Human|Respiratory Virus Unit|Respiratory Virus Unit|Platt|United Kingdom
>Spike|hCoV-19/Denmark/DCGC-11278/2020|2020-11-02|EPI_ISL_670809|Original|hCoV-19^^Midtjylland|Human|Department of Virus and Microbiological Special Diagnostics|Albertsen Lab|Michaelsen|Denmark
>Spike|hCoV-19/USA/TX-HMH-921/2020|2020-04-17|EPI_ISL_545920|Original|hCoV-19^^Texas|Human|Houston Methodist Hospital|Houston Methodist Hospital|Long|USA
>Spike|hCoV-19/England/QEUH-9F480E/2020|2020-09-27|EPI_ISL_588365|Original|hCoV-19^^England|Human|Lighthouse Lab in Glasgow|Wellcome Sanger Institute for the COVID-19 Genomics UK (COG-UK) consortium|Davies|United Kingdom
>Spike|hCoV-19/USA/NY-NYUMC396/2020|2020-04-11|EPI_ISL_430423|Original|hCoV-19^^New York|Human|NYU Langone Health|Departments of Pathology and Medicine|Maurano|USA

>Spike|hCoV-19/Romania/ROSV_4995/2020|2020-04-27|EPI_ISL_491080|Original|hCoV-19^^Suceava|Human|Suceava County Emergency Hospital|\Stefan cel Mare\ University Metagenomics Lab|Lobiuc|Romania
>Spike|hCoV-19/England/MILK-C62D18/2020|2020-12-13|EPI_ISL_735681|Original|hCoV-19^^England|Human|Lighthouse Lab in Milton Keynes|Wellcome Sanger Institute for the COVID-19 Genomics UK (COG-UK) Consortium|Davies|United Kingdom
>Spike|hCoV-19/Denmark/DCGC-3460/2020|2020-08-31|EPI_ISL_616224|Original|hCoV-19^^Hovedstaden|Human|Department of Virus and Microbiological Special Diagnostics|Albertsen lab|Michaelsen|Denmark
>Spike|hCoV-19/England/MILK-99D755/2020|2020-08-31|EPI_ISL_549913|Original|hCoV-19^^England|Human|Lighthouse Lab in Milton Keynes|Wellcome Sanger Institute for the COVID-19 Genomics UK (COG-UK) consortium|Davies|United Kingdom
>Spike|hCoV-19/USA/DE-DHSS-F918378/2020|2020-03-30|EPI_ISL_593951|Original|hCoV-19^^Delaware|Human|Delaware Public Health Lab|Delaware Public Health Lab|Bajwa|USA
>Spike|hCoV-19/Norway/3143/2020|2020-08-09|EPI_ISL_549044|Original|hCoV-19^^Viken|Human|Ostfold Hospital Trust - Kalnes|Norwegian Institute of Public Health|Kopperud Riis|Norway
>Spike|hCoV-19/Turkey/Ankara_GUMV_22790/2020|2020-08-26|EPI_ISL_730571|Original|hCoV-19^^Ankara|Human|Gazi University Faculty of Medicine|Gazi University Faculty of Medicine|Sahin|Turkey
>Spike|hCoV-19/England/ALDP-C57F4D/2020|2020-12-14|EPI_ISL_736189|Original|hCoV-19^^England|Human|Lighthouse Lab in Alderley Park|Wellcome Sanger Institute for the COVID-19 Genomics UK (COG-UK) Consortium|Davies|United Kingdom
>Spike|hCoV-19/England/NORW-EB00B/2020|2020-04-30|EPI_ISL_457405|Original|hCoV-19^^England|Human|Quadram Institute Bioscience|COVID-19 Genomics UK (COG-UK) Consortium|Alikhan|United Kingdom
>Spike|hCoV-19/England/ALDP-B83671/2020|2020-11-15|EPI_ISL_675213|Original|hCoV-19^^England|Human|Lighthouse Lab in Alderley Park|Wellcome Sanger Institute for the COVID-19 Genomics UK (COG-UK) Consortium|Davies|United Kingdom
>Spike|hCoV-19/USA/TX-HMH-MCoV-10504/2020|2020-07-08|EPI_ISL_789504|Original|hCoV-19^^Texas|Human|Houston Methodist Hospital|Houston Methodist Hospital|Long|USA
>Spike|hCoV-19/England/ALDP-9ED158/2020|2020-05-05|EPI_ISL_589364|Original|hCoV-19^^England|Human|Lighthouse Lab in Alderley Park|Wellcome Sanger Institute for the COVID-19 Genomics UK (COG-UK) consortium|Davies|United Kingdom
>Spike|hCoV-19/USA/MN-MDH-1255/2020|2020-06-09|EPI_ISL_482976|Original|hCoV-19^^Minnesota|Human|Mayo Clinic & Mayo Clinic Laboratories|Minnesota Department of Health|Garfin|USA
>Spike|hCoV-19/England/MILK-9A9237/2020|2020-08-31|EPI_ISL_551277|Original|hCoV-19^^England|Human|Lighthouse Lab in Milton Keynes|Wellcome Sanger Institute for the COVID-19 Genomics UK (COG-UK) consortium|Davies|United Kingdom
>Spike|hCoV-19/Japan/PG-3242/2020|2020-04-00|EPI_ISL_691358|Original|hCoV-19^^Japan|Human|Pathogen Genomics Center|Pathogen Genomics Center|Sekizuka|Japan
>Spike|hCoV-19/England/MILK-ACFC3C/2020|2020-10-22|EPI_ISL_652128|Original|hCoV-19^^England|Human|Oxford Viromics|COVID-19 Genomics UK (COG-UK) Consortium|Golubchik|United Kingdom
>Spike|hCoV-19/England/NORW-F9FC1/2020|2020-12-14|EPI_ISL_741606|Original|hCoV-19^^England|Human|Quadram Institute Bioscience|COVID-19 Genomics UK (COG-UK) Consortium|Alikhan|United Kingdom
>Spike|hCoV-19/South Africa/KRISP-K002351/2020|2020-07-21|EPI_ISL_515723|Original|hCoV-19^^KwaZulu-Natal|Human|NHLS-IALCH|KRISP|de Oliveira|South Africa
>Spike|hCoV-19/India/MH-NIV-10435/2020|2020-04-16|EPI_ISL_541683|Original|hCoV-19^^Maharashtra|Human|National Institute of Virology|National Institute of Virology|Potdar|India
>Spike|hCoV-19/Malaysia/IMR-WC272624/2020|2020-11-12|EPI_ISL_728247|Original|hCoV-19^^Malaysia|Human|Institute for Medical Research|Institute for Medical Research|Suppiah|Malaysia
>Spike|hCoV-19/USA/MN-GMF-28226/2020|2020-10-27|EPI_ISL_661013|Original|hCoV-19^^Minnesota|Human|Gundersen Molecular Diagnostics Laboratory|Kabara Cancer Research Institute|Kenny|USA

>Spike|hCoV-19/United Arab Emirates/L5621/2020|2020-03-11|EPI_ISL_435136|Original|hCoV-19^^United Arab Emirates|Human|Mohammed Bin Rashid University of Medicine and Health Sciences|Al Jalila Genomics Center|Ramaswamy|United Arab Emirates
>Spike|hCoV-19/India/UP-NBRI-N14-TCR324/2020|2020-04-02|EPI_ISL_516943|Original|hCoV-19^^Uttar Pradesh|Human|King Georges Medical University|CSIR-National Botanical Research Institute|Bag|India
>Spike|hCoV-19/England/QEUH-9F322F/2020|2020-09-23|EPI_ISL_588502|Original|hCoV-19^^England|Human|Lighthouse Lab in Glasgow|Wellcome Sanger Institute for the COVID-19 Genomics UK (COG-UK) consortium|Davies|United Kingdom
>Spike|hCoV-19/USA/IL-NM-0488/2020|2020-04-23|EPI_ISL_626428|Original|hCoV-19^^Illinois|Human|Northwestern Memorial Hospital|Ozer Lab|Ozer|USA
>Spike|hCoV-19/USA/TX-HMH-MCoV-8360/2020|2020-07-07|EPI_ISL_784351|Original|hCoV-19^^Texas|Human|Houston Methodist Hospital|Houston Methodist Hospital|Long|USA
>Spike|hCoV-19/Wales/QEUH-95E978/2020|2020-08-06|EPI_ISL_532427|Original|hCoV-19^^Wales|Human|Lighthouse Lab in Glasgow|Wellcome Sanger Institute for the COVID-19 Genomics UK (COG-UK) consortium|Davies|United Kingdom
>Spike|hCoV-19/England/CAMB-74641/2020|2020-03-20|EPI_ISL_425318|Original|hCoV-19^^England|Human|Department of Pathology|COVID-19 Genomics UK (COG-UK) Consortium|Meredith|United Kingdom
>Spike|hCoV-19/Japan/PG-6340/2020|2020-08-00|EPI_ISL_689451|Original|hCoV-19^^Japan|Human|Pathogen Genomics Center|Pathogen Genomics Center|Sekizuka|Japan
>Spike|hCoV-19/Scotland/EDB2278/2020|2020-04-21|EPI_ISL_491507|Original|hCoV-19^^Scotland|Human|Virology Department|Wellcome Sanger Institute for the COVID-19 Genomics UK (COG-UK) consortium|Davies|United Kingdom
>Spike|hCoV-19/Wales/PHWC-491AC6/2020|2020-11-10|EPI_ISL_706738|Original|hCoV-19^^Wales|Human|Originating lab|COVID-19 Genomics UK (COG-UK) Consortium|Moore|United Kingdom
>Spike|hCoV-19/Norway/5885/2020|2020-11-13|EPI_ISL_775346|Original|hCoV-19^^Vestland|Human|Department of medical microbiology|Norwegian Institute of Public Health|Vollan|Norway
>Spike|hCoV-19/Denmark/DCGC-8973/2020|2020-10-26|EPI_ISL_626190|Original|hCoV-19^^Syddanmark|Human|Department of Virus and Microbiological Special Diagnostics|Albertsen lab|Michaelsen|Denmark
>Spike|hCoV-19/Wales/PHWC-491E33/2020|2020-11-16|EPI_ISL_706785|Original|hCoV-19^^Wales|Human|Originating lab|COVID-19 Genomics UK (COG-UK) Consortium|Moore|United Kingdom
>Spike|hCoV-19/England/ALDP-4996AC/2020|2020-05-27|EPI_ISL_559432|Original|hCoV-19^^England|Human|Lighthouse Lab in Alderley Park|Wellcome Sanger Institute for the COVID-19 Genomics UK (COG-UK) consortium|Davies|United Kingdom
>Spike|hCoV-19/England/MILK-AC8A1B/2020|2020-10-22|EPI_ISL_629616|Original|hCoV-19^^England|Human|Lighthouse Lab in Milton Keynes|Wellcome Sanger Institute for the COVID-19 Genomics UK (COG-UK) consortium|Davies|United Kingdom
>Spike|hCoV-19/Spain/MD-IBV-005331/2020|2020-03-17|EPI_ISL_510209|Original|hCoV-19^^Madrid|Human|Hospital General Universitario Gregorio Mara\u00f1\u00f3n|SeqCOVID-SPAIN consortium/IBV(CSIC)|Comas|Spain
>Spike|hCoV-19/Israel/CVL-46381/2020|2020-12-15|EPI_ISL_745070|Original|hCoV-19^^Tel Aviv|Human|Israel Central Virology laboratory|Israel Central Virology laboratory|Zuckerman|Israel
>Spike|hCoV-19/England/QEUH-9DC769/2020|2020-09-19|EPI_ISL_581024|Original|hCoV-19^^England|Human|Lighthouse Lab in Glasgow|Wellcome Sanger Institute for the COVID-19 Genomics UK (COG-UK) consortium|Davies|United Kingdom
>Spike|hCoV-19/England/ALDP-4FC786/2020|2020-06-23|EPI_ISL_559137|Original|hCoV-19^^England|Human|Lighthouse Lab in Alderley Park|Wellcome Sanger Institute for the COVID-19 Genomics UK (COG-UK) consortium|Davies|United Kingdom
>Spike|hCoV-19/Denmark/DCGC-7248/2020|2020-10-12|EPI_ISL_619098|Original|hCoV-19^^Sjaelland|Human|Department of Virus and Microbiological Special Diagnostics|Albertsen lab|Michaelsen|Denmark

>Spike|hCoV-19/Canada/MB-NML-1141/2020|2020-08-03|EPI_ISL_582471|Original|hCoV-19^^Manitoba|Human|Cadham Provincial Laboratory|National Microbiology Laboratory (NML)|Majer|Canada
>Spike|hCoV-19/Denmark/DCGC-4592/2020|2020-09-21|EPI_ISL_622261|Original|hCoV-19^^Sjaelland|Human|Department of Virus and Microbiological Special Diagnostics|Albertsen lab|Michaelsen|Denmark
>Spike|hCoV-19/Wales/PHWC-16B6D3/2020|2020-09-17|EPI_ISL_573889|Original|hCoV-19^^Wales|Human|Wales Specialist Virology Centre Sequencing lab|COVID-19 Genomics UK (COG-UK) Consortium|Moore|United Kingdom
>Spike|hCoV-19/England/ALDP-9EA522/2020|2020-09-24|EPI_ISL_579947|Original|hCoV-19^^England|Human|Lighthouse Lab in Alderley Park|Wellcome Sanger Institute for the COVID-19 Genomics UK (COG-UK) consortium|Davies|United Kingdom
>Spike|hCoV-19/Wales/PHWC-47FD01/2020|2020-10-13|EPI_ISL_612800|Original|hCoV-19^^Wales|Human|Wales Specialist Virology Centre Sequencing lab|COVID-19 Genomics UK (COG-UK) Consortium|Moore|United Kingdom
>Spike|hCoV-19/USA/UT-08719/2020|2020-05-21|EPI_ISL_524776|Original|hCoV-19^^Utah|Human|Utah Public Health Laboratory|Utah Public Health Laboratory|Young|USA
>Spike|hCoV-19/Saudi Arabia/562/2020|2020-02-16|EPI_ISL_490003|Original|hCoV-19^^Saudi Arabia|Human|King Fahad Medical City|King Fahad Medical City|Naeem|Saudi Arabia
>Spike|hCoV-19/USA/NY-Wadsworth-289756-01/2020|2020-12-21|EPI_ISL_767503|Original|hCoV-19^^New York|Human|Wadsworth Center|Wadsworth Center|Lasek-Nesselquist|USA
>Spike|hCoV-19/England/NOTT-113BCC/2020|2020-09-28|EPI_ISL_573466|Original|hCoV-19^^England|Human|Queens Medical Centre|COVID-19 Genomics UK (COG-UK) Consortium|Loose|United Kingdom
>Spike|hCoV-19/Belgium/rega-0417510/2020|2020-04-17|EPI_ISL_734602|Original|hCoV-19^^Gent|Human|UZ Leuven|KU Leuven|Maes|Belgium
>Spike|hCoV-19/USA/CA-CZB-2353/2020|2020-05-13|EPI_ISL_513800|Original|hCoV-19^^California|Human|Orange County Public Health Laboratory|Chan-Zuckerberg Biohub|Kamm|USA
>Spike|hCoV-19/Latvia/065/2020|2020-07-09|EPI_ISL_493000|Original|hCoV-19^^Latvia|Human|E. Gulbja Laboratorija|Latvian Biomedical Research and Study Centre|Zrelovs|Latvia
>Spike|hCoV-19/USA/TX-HMH-6881/2020|2020-06-25|EPI_ISL_546316|Original|hCoV-19^^Texas|Human|Houston Methodist Hospital|Houston Methodist Hospital|Long|USA
>Spike|hCoV-19/USA/WA-S745/2020|2020-04-13|EPI_ISL_449915|Original|hCoV-19^^Washington|Human|Washington State Department of Health|Seattle Flu Study|Bedford|USA
>Spike|hCoV-19/England/ALDP-520032/2020|2020-06-02|EPI_ISL_559064|Original|hCoV-19^^England|Human|Lighthouse Lab in Alderley Park|Wellcome Sanger Institute for the COVID-19 Genomics UK (COG-UK) consortium|Davies|United Kingdom
>Spike|hCoV-19/USA/TX-HMH-MCoV-14869/2020|2020-10-13|EPI_ISL_786353|Original|hCoV-19^^Texas|Human|Houston Methodist Hospital|Houston Methodist Hospital|Long|USA
>Spike|hCoV-19/Denmark/DCGC-1996/2020|2020-07-20|EPI_ISL_618297|Original|hCoV-19^^Nordjylland|Human|Department of Virus and Microbiological Special Diagnostics|Albertsen lab|Michaelsen|Denmark
>Spike|hCoV-19/Poland/PL_MCB_50/2020|2020-05-10|EPI_ISL_645049|Original|hCoV-19^^Poland|Human|Human Genome Variation Research Group|Human Genome Variation Research Group||Poland
>Spike|hCoV-19/USA/MN-QDX-2054/2020|2020-03-15|EPI_ISL_604355|Original|hCoV-19^^Minnesota|Human|Quest Diagnostics|Quest Diagnostics|Kagan|USA
>Spike|hCoV-19/Denmark/DCGC-3555/2020|2020-08-31|EPI_ISL_616348|Original|hCoV-19^^Sjaelland|Human|Department of Virus and Microbiological Special Diagnostics|Albertsen lab|Michaelsen|Denmark
>Spike|hCoV-19/USA/HI-H200217/2020|2020-08-10|EPI_ISL_752776|Original|hCoV-19^^Hawaii|Human|State Laboratories Division|State Laboratories Division|Sultana|USA
>Spike|hCoV-19/Scotland/QEUH-9AE43C/2020|2020-09-05|EPI_ISL_638907|Original|hCoV-19^^Scotland|Human|Department of Pathology|COVID-19 Genomics UK (COG-UK) Consortium|Goodfellow|United Kingdom

>Spike|hCoV-19/Japan/PG-8361/2020|2020-07-00|EPI_ISL_687460|Original|hCoV-19^^Japan|Human|Pathogen Genomics Center|Pathogen Genomics Center|Sekizuka|Japan
>Spike|hCoV-19/England/SHEF-C50BC/2020|2020-04-18|EPI_ISL_462017|Original|hCoV-19^^England|Human|Virology Department|COVID-19 Genomics UK (COG-UK) Consortium|de Silva|United Kingdom
>Spike|hCoV-19/England/QEUH-AF27B7/2020|2020-10-30|EPI_ISL_662677|Original|hCoV-19^^England|Human|Lighthouse Lab in Glasgow|Wellcome Sanger Institute for the COVID-19 Genomics UK (COG-UK) Consortium|Davies|United Kingdom
>Spike|hCoV-19/England/ALDP-9D8E77/2020|2020-09-18|EPI_ISL_576678|Original|hCoV-19^^England|Human|Oxford Viromics|COVID-19 Genomics UK (COG-UK) Consortium|Golubchik|United Kingdom
>Spike|hCoV-19/England/QEUH-BA316B/2020|2020-11-18|EPI_ISL_672786|Original|hCoV-19^^England|Human|Lighthouse Lab in Glasgow|Wellcome Sanger Institute for the COVID-19 Genomics UK (COG-UK) Consortium|Davies|United Kingdom
>Spike|hCoV-19/England/ALDP-A52560/2020|2020-10-07|EPI_ISL_598018|Original|hCoV-19^^England|Human|Lighthouse Lab in Alderley Park|Wellcome Sanger Institute for the COVID-19 Genomics UK (COG-UK) consortium|Davies|United Kingdom
>Spike|hCoV-19/Australia/VIC7363/2020|2020-07-21|EPI_ISL_564360|Original|hCoV-19^^Victoria|Human|Victorian Infectious Diseases Reference Laboratory (VIDRL)|VIDRL and MDU-PHL|Schultz|Australia
>Spike|hCoV-19/England/QEUH-B41123/2020|2020-11-08|EPI_ISL_658432|Original|hCoV-19^^England|Human|Lighthouse Lab in Glasgow|Wellcome Sanger Institute for the COVID-19 Genomics UK (COG-UK) Consortium|Davies|United Kingdom
>Spike|hCoV-19/USA/MI-UM-10036819118/2020|2020-11-06|EPI_ISL_728396|Original|hCoV-19^^Michigan|Human|University of Michigan Clinical Microbiology Laboratory|Lauring Lab|Valesano|USA
>Spike|hCoV-19/Belgium/rega-10181068/2020|2020-10-18|EPI_ISL_735108|Original|hCoV-19^^Sint-Stevens-Woluwe|Human|UZ Leuven|KU Leuven|Maes|Belgium
>Spike|hCoV-19/USA/CA-ALSR-3259/2020|2020-09-05|EPI_ISL_635935|Original|hCoV-19^^California|Human|San Diego County Public Health Laboratory|Andersen lab at Scripps Research|Gangavarapu|USA
>Spike|hCoV-19/Switzerland/BE-ETHZ-120221/2020|2020-04-30|EPI_ISL_468284|Original|hCoV-19^^Bern|Human|Viollier AG|Department of Biosystems Science and Engineering|Nadeau|Switzerland
>Spike|hCoV-19/Sweden/20-08044/2020|2020-03-09|EPI_ISL_454439|Original|hCoV-19^^Halland|Human|Halmstad klinisk mikrobiologi|The Public Health Agency of Sweden|Svartstr\u00f6m|Sweden
>Spike|hCoV-19/England/QEUH-9C98E4/2020|2020-09-10|EPI_ISL_567468|Original|hCoV-19^^England|Human|Lighthouse Lab in Glasgow|Wellcome Sanger Institute for the COVID-19 Genomics UK (COG-UK) consortium|Davies|United Kingdom
>Spike|hCoV-19/England/CAMB-1B0350/2020|2020-05-01|EPI_ISL_459253|Original|hCoV-19^^England|Human|Department of Pathology|Wellcome Sanger Institute for the COVID-19 Genomics UK (COG-UK) consortium|Davies|United Kingdom
>Spike|hCoV-19/Wales/PHWC-28A5F/2020|2020-04-02|EPI_ISL_445391|Original|hCoV-19^^Wales|Human|Wales Specialist Virology Centre|Public Health Wales Microbiology Cardiff|Moore|United Kingdom
>Spike|hCoV-19/USA/NY-Wadsworth-213738-01/2020|2020-09-09|EPI_ISL_765501|Original|hCoV-19^^New York|Human|Wadsworth Center|Wadsworth Center|Lasek-Nesselquist|USA
>Spike|hCoV-19/England/CAMC-A592CC/2020|2020-10-07|EPI_ISL_597226|Original|hCoV-19^^England|Human|Lighthouse Lab in Cambridge|Wellcome Sanger Institute for the COVID-19 Genomics UK (COG-UK) consortium|Davies|United Kingdom
>Spike|hCoV-19/Australia/VIC9520/2020|2020-08-19|EPI_ISL_565584|Original|hCoV-19^^Victoria|Human|Microbiological Diagnostic Unit - Public Health Laboratory (MDU-PHL)|MDU-PHL|Schultz|Australia
>Spike|hCoV-19/USA/NY-WCMP5H02P/2020|2020-03-13|EPI_ISL_427542|Original|hCoV-19^^New York|Human|NewYork-Presbyterian & Mason Lab|Mason Lab|Mason|USA
>Spike|hCoV-19/USA/IA-GMF-21220/2020|2020-09-19|EPI_ISL_660829|Original|hCoV-19^^Iowa|Human|Gundersen Molecular Diagnostics Laboratory|Kabara Cancer Research Institute|Kenny|USA

>Spike|hCoV-19/England/QEUH-A9B668/2020|2020-10-20|EPI_ISL_611451|Original|hCoV-19^^England|Human|Lighthouse Lab in Glasgow|Wellcome Sanger Institute for the COVID-19 Genomics UK (COG-UK) consortium|Davies|United Kingdom
>Spike|hCoV-19/England/MILK-ACDB9B/2020|2020-10-21|EPI_ISL_630072|Original|hCoV-19^^England|Human|Lighthouse Lab in Milton Keynes|Wellcome Sanger Institute for the COVID-19 Genomics UK (COG-UK) consortium|Davies|United Kingdom
>Spike|hCoV-19/England/CAMB-784DA/2020|2020-04-06|EPI_ISL_433742|Original|hCoV-19^^England|Human|Department of Pathology|COVID-19 Genomics UK (COG-UK) Consortium|Meredith|United Kingdom
>Spike|hCoV-19/England/MILK-A47BD5/2020|2020-10-05|EPI_ISL_598345|Original|hCoV-19^^England|Human|Lighthouse Lab in Milton Keynes|Wellcome Sanger Institute for the COVID-19 Genomics UK (COG-UK) consortium|Davies|United Kingdom
>Spike|hCoV-19/Oman/24774/2020|2020-04-17|EPI_ISL_491151|Original|hCoV-19^^South Batinah|Human|Oman-National Influenza Center|Biotechnology & OMICs Laboratory|Al Mahruqi|Oman
>Spike|hCoV-19/Hong Kong/VM20001776/2020|2020-01-29|EPI_ISL_409002|Original|hCoV-19^^Hong Kong|Human|Queen Mary Hospital|Hong Kong Department of Health|Mak|China
>Spike|hCoV-19/USA/AZ-TG670178/2020|2020-04-29|EPI_ISL_696375|Original|hCoV-19^^Arizona|Human|Sonora Quest Laboratories|TGen North|French|USA
>Spike|hCoV-19/Uruguay/BUN-M151/2020|2020-07-20|EPI_ISL_751185|Original|hCoV-19^^Bella Union|Human|CENUR Litoral Norte \u2013 UdelaR|Institut Pasteur de Montevideo|Spangenberg|Uruguay
>Spike|hCoV-19/USA/MI-MDHHS-SC20268/2020|2020-03-27|EPI_ISL_447066|Original|hCoV-19^^Michigan|Human|Michigan Department of Health and Human Services|Michigan Department of Health and Human Services|Blankenship|USA
>Spike|hCoV-19/USA/MI-UM-10036818548/2020|2020-11-06|EPI_ISL_728435|Original|hCoV-19^^Michigan|Human|University of Michigan Clinical Microbiology Laboratory|Lauring Lab|Valesano|USA
>Spike|hCoV-19/Austria/CeMM0993/2020|2020-04-11|EPI_ISL_583837|Original|hCoV-19^^Austria|Human|Dr. Gernot Walder GmbH|Bergthaler laboratory|Bergthaler|Austria
>Spike|hCoV-19/South Africa/LR00022/2020|2020-07-02|EPI_ISL_515151|Original|hCoV-19^^Gauteng|Human|National Institute for Communicable Diseases of the National Health Laboratory Service|National Institute for Communicable Diseases of the National Health Laboratory Service|Allam|South Africa
>Spike|hCoV-19/Singapore/1047/2020|2020-03-22|EPI_ISL_574532|Original|hCoV-19^^Singapore|Human|National Public Health Laboratory|National Public Health Laboratory|Mak|Singapore
>Spike|hCoV-19/England/20118087504/2020|2020-03-12|EPI_ISL_464946|Original|hCoV-19^^England|Human|Respiratory Virus Unit|Respiratory Virus Unit|Platt|United Kingdom
>Spike|hCoV-19/USA/MI-MDHHS-SC21192/2020|2020-03-30|EPI_ISL_507449|Original|hCoV-19^^Michigan|Human|Michigan Department of Health and Human Services|Michigan Department of Health and Human Services|Blankenship|USA
>Spike|hCoV-19/England/CAMB-1B0086/2020|2020-00-00|EPI_ISL_448105|Original|hCoV-19^^England|Human|Department of Pathology|COVID-19 Genomics UK (COG-UK) Consortium|Meredith|United Kingdom
>Spike|hCoV-19/USA/WY-WYPHL007/2020|2020-03-15|EPI_ISL_421544|Original|hCoV-19^^Wyoming|Human|Wyoming Public Health Laboratory|Center for Global Health|Domman|USA
>Spike|hCoV-19/USA/CA-ALSR-5032/2020|2020-12-02|EPI_ISL_730303|Original|hCoV-19^^California|Human|San Diego County Public Health Laboratory|Andersen lab at Scripps Research|Gangavarapu|USA
>Spike|hCoV-19/USA/TX-HMH-MCoV-10892/2020|2020-07-09|EPI_ISL_789673|Original|hCoV-19^^Texas|Human|Houston Methodist Hospital|Houston Methodist Hospital|Long|USA
>Spike|hCoV-19/Austria/CeMM0875/2020|2020-04-30|EPI_ISL_583738|Original|hCoV-19^^Austria|Human|Dr. Gernot Walder GmbH|Bergthaler laboratory|Bergthaler|Austria
>Spike|hCoV-19/USA/VA-DCLS-0092/2020|2020-04-01|EPI_ISL_429989|Original|hCoV-19^^Virginia|Human|Virginia DCLS|Virginia DCLS|St. Jacques|USA
>Spike|hCoV-19/Switzerland/BS-42216240/2020|2020-04-01|EPI_ISL_581669|Original|hCoV-19^^Basel-Stadt|Human|University Hospital Basel|University Hospital Basel|Roloff Handschin|Switzerland

>Spike|hCoV-19/England/ALDP-B859EF/2020|2020-11-14|EPI_ISL_675833|Original|hCoV-19^^England|Human|Lighthouse Lab in Alderley Park|Wellcome Sanger Institute for the COVID-19 Genomics UK (COG-UK) Consortium|Davies|United Kingdom
>Spike|hCoV-19/England/CAMB-76445/2020|2020-03-30|EPI_ISL_438273|Original|hCoV-19^^England|Human|Department of Pathology|Wellcome Sanger Institute for the COVID-19 Genomics UK (COG-UK) consortium|Amato|United Kingdom
>Spike|hCoV-19/Denmark/DCGC-22532/2020|2020-12-14|EPI_ISL_757620|Original|hCoV-19^^Nordjylland|Human|Department of Virus and Microbiological Special Diagnostics|Albertsen Lab|Michaelsen|Denmark
>Spike|hCoV-19/USA/UT-UPHL-201117690/2020|2020-04-09|EPI_ISL_623195|Original|hCoV-19^^Utah|Human|Utah Public Health Laboratory|Utah Public Health Laboratory|Young|USA
>Spike|hCoV-19/Denmark/DCGC-3394/2020|2020-08-31|EPI_ISL_616153|Original|hCoV-19^^Hovedstaden|Human|Department of Virus and Microbiological Special Diagnostics|Albertsen lab|Michaelsen|Denmark
>Spike|hCoV-19/England/205190041/2020|2020-12-18|EPI_ISL_791218|Original|hCoV-19^^England|Human|Respiratory Virus Unit|COVID-19 Genomics UK (COG-UK) Consortium|Platt|United Kingdom
>Spike|hCoV-19/England/CAMC-B9A2A0/2020|2020-11-15|EPI_ISL_673521|Original|hCoV-19^^England|Human|Lighthouse Lab in Cambridge|Wellcome Sanger Institute for the COVID-19 Genomics UK (COG-UK) Consortium|Davies|United Kingdom
>Spike|hCoV-19/Australia/VIC6154/2020|2020-07-22|EPI_ISL_518163|Original|hCoV-19^^Victoria|Human|Victorian Infectious Diseases Reference Laboratory (VIDRL)|VIDRL and MDU-PHL|Schultz|Australia
>Spike|hCoV-19/England/QEUH-A4D4E3/2020|2020-10-05|EPI_ISL_628602|Original|hCoV-19^^England|Human|Oxford Viromics|COVID-19 Genomics UK (COG-UK) Consortium|Golubchik|United Kingdom
>Spike|hCoV-19/England/OXON-AF416/2020|2020-04-13|EPI_ISL_478736|Original|hCoV-19^^England|Human|Oxford Viromics|COVID-19 Genomics UK (COG-UK) Consortium|Golubchik|United Kingdom
>Spike|hCoV-19/Spain/MD-IBV-006427/2020|2020-04-01|EPI_ISL_510141|Original|hCoV-19^^Madrid|Human|Hospital General Universitario Gregorio Mara\u00f1\u00f3n|SeqCOVID-SPAIN consortium/IBV(CSIC)|Comas|Spain
>Spike|hCoV-19/England/ALDP-8C95B1/2020|2020-06-27|EPI_ISL_556619|Original|hCoV-19^^England|Human|Lighthouse Lab in Alderley Park|Wellcome Sanger Institute for the COVID-19 Genomics UK (COG-UK) consortium|Davies|United Kingdom
>Spike|hCoV-19/Australia/VIC6011/2020|2020-07-23|EPI_ISL_518786|Original|hCoV-19^^Victoria|Human|Microbiological Diagnostic Unit - Public Health Laboratory (MDU-PHL)|MDU-PHL|Schultz|Australia
>Spike|hCoV-19/USA/AZ-TG569502/2020|2020-07-10|EPI_ISL_695586|Original|hCoV-19^^Arizona|Human|AZ SPHL|TGen North|French|USA
>Spike|hCoV-19/USA/TX-HMH-MCoV-18293/2020|2020-11-17|EPI_ISL_784641|Original|hCoV-19^^Texas|Human|Houston Methodist Hospital|Houston Methodist Hospital|Long|USA
>Spike|hCoV-19/England/CAMB-1B9DB3/2020|2020-10-31|EPI_ISL_651557|Original|hCoV-19^^England|Human|Department of Pathology|COVID-19 Genomics UK (COG-UK) Consortium|Goodfellow|United Kingdom
>Spike|hCoV-19/USA/UT-UPHL-2012405767/2020|2020-08-11|EPI_ISL_745932|Original|hCoV-19^^Utah|Human|Ginkgo Bioworks Clinical Laboratory|Utah Public Health Laboratory|Young|USA
>Spike|hCoV-19/USA/AZ-TG654314/2020|2020-04-12|EPI_ISL_695969|Original|hCoV-19^^Arizona|Human|Sonora Quest Laboratories|TGen North|French|USA
>Spike|hCoV-19/England/QEUH-A3141F/2020|2020-10-05|EPI_ISL_595886|Original|hCoV-19^^England|Human|Quadram Institute Bioscience|COVID-19 Genomics UK (COG-UK) Consortium|Alikhan|United Kingdom
>Spike|hCoV-19/Denmark/DCGC-14652/2020|2020-11-23|EPI_ISL_711892|Original|hCoV-19^^Nordjylland|Human|Department of Virus and Microbiological Special Diagnostics|Albertsen Lab|Michaelsen|Denmark
>Spike|hCoV-19/Australia/VIC5031/2020|2020-07-17|EPI_ISL_519835|Original|hCoV-19^^Victoria|Human|Microbiological Diagnostic Unit - Public Health Laboratory (MDU-PHL)|MDU-PHL|Schultz|Australia

>Spike|hCoV-19/England/ALDP-B1D542/2020|2020-11-01|EPI_ISL_645858|Original|hCoV-19^^England|Human|Lighthouse Lab in Alderley Park|Wellcome Sanger Institute for the COVID-19 Genomics UK (COG-UK) Consortium|Davies|United Kingdom
>Spike|hCoV-19/New Zealand/20VR1833/2020|2020-03-30|EPI_ISL_456276|Original|hCoV-19^^Southern|Human|Southern Community Labs Dunedin|Institute of Environmental Science and Research (ESR)|de Ligt|New Zealand
>Spike|hCoV-19/England/CAMC-A57176/2020|2020-10-08|EPI_ISL_597233|Original|hCoV-19^^England|Human|Lighthouse Lab in Cambridge|Wellcome Sanger Institute for the COVID-19 Genomics UK (COG-UK) consortium|Davies|United Kingdom
>Spike|hCoV-19/USA/TX-HMH-MCoV-10036/2020|2020-07-08|EPI_ISL_789298|Original|hCoV-19^^Texas|Human|Houston Methodist Hospital|Houston Methodist Hospital|Long|USA
>Spike|hCoV-19/Northern Ireland/NIRE-10338B/2020|2020-04-17|EPI_ISL_489377|Original|hCoV-19^^Northern Ireland|Human|Regional Virus Laboratory|Wellcome Sanger Institute for the COVID-19 Genomics UK (COG-UK) consortium|Davies|United Kingdom
>Spike|hCoV-19/England/CAMB-72BEA/2020|2020-03-22|EPI_ISL_439996|Original|hCoV-19^^England|Human|Department of Pathology|Wellcome Sanger Institute for the COVID-19 Genomics UK (COG-UK) consortium|Amato|United Kingdom
>Spike|hCoV-19/Denmark/DCGC-6940/2020|2020-10-12|EPI_ISL_619786|Original|hCoV-19^^Sjaelland|Human|Department of Virus and Microbiological Special Diagnostics|Albertsen lab|Michaelsen|Denmark
>Spike|hCoV-19/Wales/PHWC-16BDA1/2020|2020-09-17|EPI_ISL_573972|Original|hCoV-19^^Wales|Human|Wales Specialist Virology Centre Sequencing lab|COVID-19 Genomics UK (COG-UK) Consortium|Moore|United Kingdom
>Spike|hCoV-19/Denmark/DCGC-15254/2020|2020-11-23|EPI_ISL_713722|Original|hCoV-19^^Sjaelland|Human|Department of Virus and Microbiological Special Diagnostics|Albertsen Lab|Michaelsen|Denmark
>Spike|hCoV-19/Luxembourg/LNS1055944/2020|2020-03-23|EPI_ISL_429768|Original|hCoV-19^^Luxembourg|Human|Laboratoire National de Sante|Laboratoire National de Sante|Wienecke-Baldacchino|Luxembourg
>Spike|hCoV-19/USA/CA-QRTP-Donor14/2020|2020-04-01|EPI_ISL_490218|Original|hCoV-19^^California|Human|Quest Diagnostics|Q Squared Solutions - QRTP facility|Weigman|USA
>Spike|hCoV-19/Northern Ireland/QEUH-943493/2020|2020-08-01|EPI_ISL_532561|Original|hCoV-19^^Northern Ireland|Human|Lighthouse Lab in Glasgow|Wellcome Sanger Institute for the COVID-19 Genomics UK (COG-UK) consortium|Davies|United Kingdom
>Spike|hCoV-19/Beijing/DT-travelIT05/2020|2020-03-19|EPI_ISL_452334|Original|hCoV-19^^Beijing|Human|Laboratory of Infectious Diseases Center of Beijing Ditan Hospital|Laboratory of Infectious Diseases Center of Beijing Ditan Hospital|Yang|China
>Spike|hCoV-19/England/QEUH-B9E1EB/2020|2020-11-17|EPI_ISL_720916|Original|hCoV-19^^England|Human|Lighthouse Lab in Glasgow|Wellcome Sanger Institute for the COVID-19 Genomics UK (COG-UK) Consortium|Davies|United Kingdom
>Spike|hCoV-19/Switzerland/BS-ETHZ-100020/2020|2020-03-09|EPI_ISL_466944|Original|hCoV-19^^Basel-Stadt|Human|Viollier AG|Department of Biosystems Science and Engineering|Nadeau|Switzerland
>Spike|hCoV-19/USA/CA-ALSR-3650/2020|2020-09-28|EPI_ISL_636127|Original|hCoV-19^^California|Human|San Diego County Public Health Laboratory|Andersen lab at Scripps Research|Gangavarapu|USA
>Spike|hCoV-19/Netherlands/Overijssel_15/2020|2020-04-13|EPI_ISL_460804|Original|hCoV-19^^Overijssel|Human|Dutch COVID-19 response team|Erasmus Medical Center|Oude Munnink|Netherlands
>Spike|hCoV-19/Scotland/QEUH-AA0660/2020|2020-10-21|EPI_ISL_666096|Original|hCoV-19^^Scotland|Human|Northumbria University / South Tees Hospitals NHS Foundation Trust / North Cumbria Integrated Care NHS Foundation Trust / North Tees and Hartlepool NHS Foundation Trust / Newcastle Hospitals NHS Foundation Trust|COVID-19 Genomics UK (COG-UK) Consortium|Bashton|United Kingdom
>Spike|hCoV-19/USA/NY-NYCPHL-001516/2020|2020-11-30|EPI_ISL_745433|Original|hCoV-19^^New York|Human|DOHMH Morrisania|New York City Public Health Laboratory|Wang|USA

>Spike|hCoV-19/England/20124020802/2020|2020-03-16|EPI_ISL_418702|Original|hCoV-
19^^England|Human|Respiratory Virus Unit|Respiratory Virus Unit|Platt|United Kingdom
>Spike|hCoV-19/Singapore/1220/2020|2020-04-23|EPI_ISL_648636|Original|hCoV-
19^^Singapore|Human|Department of Laboratory Medicine|Department of Laboratory
Medicine|Chen|Singapore
>Spike|hCoV-19/England/ALDP-A68B65/2020|2020-10-12|EPI_ISL_608676|Original|hCoV-
19^^England|Human|Lighthouse Lab in Alderley Park|Wellcome Sanger Institute for the
COVID-19 Genomics UK (COG-UK) consortium|Davies|United Kingdom
>Spike|hCoV-19/England/MILK-BABFC3/2020|2020-11-18|EPI_ISL_720544|Original|hCoV-
19^^England|Human|Lighthouse Lab in Milton Keynes|Wellcome Sanger Institute for the
COVID-19 Genomics UK (COG-UK) Consortium|Davies|United Kingdom
>Spike|hCoV-19/USA/WA-S730/2020|2020-04-11|EPI_ISL_449900|Original|hCoV-
19^^Washington|Human|Washington State Department of Health|Seattle Flu
Study|Bedford|USA
>Spike|hCoV-19/England/CAMB-1AC25A/2020|2020-04-27|EPI_ISL_444385|Original|hCoV-
19^^England|Human|Department of Pathology|COVID-19 Genomics UK (COG-UK)
Consortium|Meredith|United Kingdom
>Spike|hCoV-19/England/ALDP-9C596D/2020|2020-09-10|EPI_ISL_567720|Original|hCoV-
19^^England|Human|Lighthouse Lab in Alderley Park|Wellcome Sanger Institute for the
COVID-19 Genomics UK (COG-UK) consortium|Davies|United Kingdom
>Spike|hCoV-19/Germany/BW-UKT-a020/2020|2020-04-04|EPI_ISL_723111|Original|hCoV-
19^^Baden-Wurttemberg|Human|Institute of Medical Genetics and Applied
Genomics|Institute of Medical Genetics and Applied Genomics|Gross|Germany
>Spike|hCoV-19/Czech Republic/NRL-10733/2020|2020-11-03|EPI_ISL_693675|Original|hCoV-
19^^Czech Republic|Human|The National Institute of Public Health|State Veterinary
Institute Prague|Nagy|Czech Republic
>Spike|hCoV-19/England/EXET-1370F0/2020|2020-04-21|EPI_ISL_471949|Original|hCoV-
19^^England|Human|University of Exeter|COVID-19 Genomics UK (COG-UK)
Consortium|Temperton|United Kingdom
>Spike|hCoV-19/Scotland/EDB10674/2020|2020-12-03|EPI_ISL_705793|Original|hCoV-
19^^Scotland|Human|Virology Department|COVID-19 Genomics UK (COG-UK)
Consortium|McHugh|United Kingdom
>Spike|hCoV-19/Wales/PHWC-167D87/2020|2020-04-17|EPI_ISL_494154|Original|hCoV-
19^^Wales|Human|Originating lab|COVID-19 Genomics UK (COG-UK) Consortium|Moore|United
Kingdom
>Spike|hCoV-19/USA/NY-Wadsworth-32317-01/2020|2020-03-
29|EPI_ISL_676782|Original|hCoV-19^^New York|Human|Wadsworth Center|Wadsworth
Center|Lasek-Nesselquist|USA
>Spike|hCoV-19/Australia/VIC3909/2020|2020-07-11|EPI_ISL_520621|Original|hCoV-
19^^Victoria|Human|Microbiological Diagnostic Unit - Public Health Laboratory (MDU-
PHL)|MDU-PHL|Schultz|Australia
>Spike|hCoV-19/Mexico/BCN-ALSR-2571/2020|2020-07-08|EPI_ISL_635572|Original|hCoV-
19^^Baja California|Human|Centro de Diagn\u221a\u2265stico COVID-19 UABC
Tijuana|Andersen lab at Scripps Research|Gangavarapu|Mexico
>Spike|hCoV-19/England/CAMC-B372A5/2020|2020-11-05|EPI_ISL_645273|Original|hCoV-
19^^England|Human|Lighthouse Lab in Cambridge|Wellcome Sanger Institute for the
COVID-19 Genomics UK (COG-UK) Consortium|Davies|United Kingdom
>Spike|hCoV-19/Japan/PG-3237/2020|2020-04-00|EPI_ISL_691353|Original|hCoV-
19^^Japan|Human|Pathogen Genomics Center|Pathogen Genomics Center|Sekizuka|Japan
>Spike|hCoV-19/Denmark/DCGC-14821/2020|2020-11-23|EPI_ISL_712009|Original|hCoV-
19^^Sjaelland|Human|Department of Virus and Microbiological Special
Diagnostics|Albertsen Lab|Michaelsen|Denmark
>Spike|hCoV-19/Belgium/ULG-10483/2020|2020-09-23|EPI_ISL_581648|Original|hCoV-
19^^Li\u00e8ge|Human|Department of Clinical Microbiology|GIGA Medical
Genomics|Durkin|Belgium
>Spike|hCoV-19/USA/IA-GMF-28410/2020|2020-10-28|EPI_ISL_661018|Original|hCoV-
19^^Iowa|Human|Gundersen Molecular Diagnostics Laboratory|Kabara Cancer Research
Institute|Kenny|USA
>Spike|hCoV-19/USA/WA-UW-21786/2020|2020-08-05|EPI_ISL_571006|Original|hCoV-
19^^Washington|Human|UW Virology Lab|UW Virology Lab|Roychoudhury|USA

>Spike|hCoV-19/Denmark/DCGC-3503/2020|2020-08-31|EPI_ISL_616292|Original|hCoV-19^^Syddanmark|Human|Department of Virus and Microbiological Special Diagnostics|Albertsen lab|Michaelsen|Denmark
>Spike|hCoV-19/Australia/VIC9767/2020|2020-07-17|EPI_ISL_565703|Original|hCoV-19^^Victoria|Human|Microbiological Diagnostic Unit - Public Health Laboratory (MDU-PHL)|MDU-PHL|Schultz|Australia
>Spike|hCoV-19/England/MILK-B3CF6D/2020|2020-11-02|EPI_ISL_706209|Original|hCoV-19^^England|Human|Oxford Viromics|COVID-19 Genomics UK (COG-UK) Consortium|Golubchik|United Kingdom
>Spike|hCoV-19/England/ALDP-B240B0/2020|2020-11-04|EPI_ISL_646323|Original|hCoV-19^^England|Human|Lighthouse Lab in Alderley Park|Wellcome Sanger Institute for the COVID-19 Genomics UK (COG-UK) Consortium|Davies|United Kingdom
>Spike|hCoV-19/Wales/PHWC-4A0147/2020|2020-12-02|EPI_ISL_743401|Original|hCoV-19^^Wales|Human|Wales Specialist Virology Centre Sequencing lab|COVID-19 Genomics UK (COG-UK) Consortium|Moore|United Kingdom
>Spike|hCoV-19/Israel/CVL-265-ngs/2020|2020-12-27|EPI_ISL_776828|Original|hCoV-19^^Israel|Human|Israel Central Virology laboratory|Israel Central Virology laboratory|Zuckerman|Israel
>Spike|hCoV-19/England/LEED-2AA1F4/2020|2020-03-30|EPI_ISL_538932|Original|hCoV-19^^England|Human|Leeds Teaching Hospitals NHS Trust and Public Health England|Wellcome Sanger Institute for the COVID-19 Genomics UK (COG-UK) consortium|Davies|United Kingdom
>Spike|hCoV-19/USA/CA-CZB-4050/2020|2020-08-03|EPI_ISL_548616|Original|hCoV-19^^California|Human|County of Santa Clara Public Health Department|Chan-Zuckerberg Biohub|Bhatt|USA
>Spike|hCoV-19/Netherlands/NoordBrabant_71/2020|2020-03-11|EPI_ISL_422867|Original|hCoV-19^^Noord Brabant|Human|Dutch COVID-19 response team|Erasmus Medical Center|Oude Munnink|Netherlands
>Spike|hCoV-19/Denmark/DCGC-11265/2020|2020-11-02|EPI_ISL_670802|Original|hCoV-19^^Syddanmark|Human|Department of Virus and Microbiological Special Diagnostics|Albertsen Lab|Michaelsen|Denmark
>Spike|hCoV-19/USA/UT-UPHL-2012566391/2020|2020-08-11|EPI_ISL_745541|Original|hCoV-19^^Utah|Human|Ginkgo Bioworks Clinical Laboratory|Utah Public Health Laboratory|Young|USA
>Spike|hCoV-19/Thailand/Bangkok_2864/2020|2020-03-00|EPI_ISL_515475|Vero P2|hCoV-19^^Bangkok|Human|National Institute of Health|National Institute of Health|Phuygun|Thailand
>Spike|hCoV-19/England/20132031404/2020|2020-03-22|EPI_ISL_423769|Original|hCoV-19^^England|Human|Respiratory Virus Unit|Respiratory Virus Unit|Platt|United Kingdom
>Spike|hCoV-19/USA/FL-BPHL-1969/2020|2020-04-15|EPI_ISL_653122|Original|hCoV-19^^Florida|Human|Florida Bureau of Public Health Laboratories|Florida Bureau of Public Health Laboratories|Blanton|USA
>Spike|hCoV-19/USA/WI-WSLH-200346/2020|2020-04-20|EPI_ISL_578520|Original|hCoV-19^^Wisconsin|Human|Wisconsin State Laboratory of Hygiene Communicable Disease Division|Wisconsin State Laboratory of Hygiene Communicable Disease Division|Florek|USA
>Spike|hCoV-19/USA/TX-HMH-MCoV-14749/2020|2020-10-12|EPI_ISL_786234|Original|hCoV-19^^Texas|Human|Houston Methodist Hospital|Houston Methodist Hospital|Long|USA
>Spike|hCoV-19/Lithuania/MR-LUHS-Eilnr39/2020|2020-04-06|EPI_ISL_603093|Original|hCoV-19^^Marijampoles apskritis|Human|Lithuanian University of Health Sciences Hospital|Lithuanian University of Health Sciences|Zemaitis|Lithuania
>Spike|hCoV-19/USA/AZ-TG325106/2020|2020-05-22|EPI_ISL_694343|Original|hCoV-19^^Arizona|Human|TGen North|TGen North|French|USA
>Spike|hCoV-19/Scotland/CVR151/2020|2020-03-17|EPI_ISL_425694|Original|hCoV-19^^Scotland|Human|West of Scotland Specialist Virology Centre|COVID-19 Genomics UK (COG-UK) Consortium|Robertson|United Kingdom
>Spike|hCoV-19/England/CAMB-73DC5/2020|2020-04-01|EPI_ISL_433698|Original|hCoV-19^^England|Human|Department of Pathology|COVID-19 Genomics UK (COG-UK) Consortium|Meredith|United Kingdom

>Spike|hCoV-19/USA/TX-HMH-MCoV-10837/2020|2020-07-05|EPI_ISL_789652|Original|hCoV-19^^Texas|Human|Houston Methodist Hospital|Houston Methodist Hospital|Long|USA
>Spike|hCoV-19/Australia/VIC7046/2020|2020-07-19|EPI_ISL_564207|Original|hCoV-19^^Victoria|Human|Victorian Infectious Diseases Reference Laboratory (VIDRL)|VIDRL and MDU-PHL|Schultz|Australia
>Spike|hCoV-19/Japan/PG-0847/2020|2020-04-00|EPI_ISL_684457|Original|hCoV-19^^Japan|Human|Fukuoka Institute of Health and Environmental Sciences|Pathogen Genomics Center|Sekizuka|Japan
>Spike|hCoV-19/Wales/PHWC-27B02/2020|2020-03-30|EPI_ISL_432321|Original|hCoV-19^^Wales|Human|Wales Specialist Virology Centre|Public Health Wales Microbiology Cardiff|Moore|United Kingdom
>Spike|hCoV-19/Denmark/DCGC-11821/2020|2020-10-26|EPI_ISL_671154|Original|hCoV-19^^Syddanmark|Human|Department of Virus and Microbiological Special Diagnostics|Albertsen Lab|Michaelsen|Denmark
>Spike|hCoV-19/Denmark/DCGC-1336/2020|2020-06-01|EPI_ISL_618010|Original|hCoV-19^^Hovedstaden|Human|Department of Virus and Microbiological Special Diagnostics|Albertsen lab|Michaelsen|Denmark
>Spike|hCoV-19/Switzerland/ZH-UZH-IMV110/2020|2020-12-20|EPI_ISL_730627|Original|hCoV-19^^Zurich|Human|M\u00e4nnedorf Hospital|Institute of Medical Virology|Schmutz|Switzerland
>Spike|hCoV-19/England/QEUH-9F7C78/2020|2020-09-27|EPI_ISL_588964|Original|hCoV-19^^England|Human|Lighthouse Lab in Glasgow|Wellcome Sanger Institute for the COVID-19 Genomics UK (COG-UK) consortium|Davies|United Kingdom
>Spike|hCoV-19/England/GSTT-5781/2020|2020-03-23|EPI_ISL_560713|Original|hCoV-19^^England|Human|Centre for Clinical Infection and Diagnostics Research and Genomics Innovation Unit|Centre for Clinical Infection and Diagnostics Research and Genomics Innovation Unit|Awan|United Kingdom
>Spike|hCoV-19/USA/CA-ALSR-2266/2020|2020-06-29|EPI_ISL_635400|Original|hCoV-19^^California|Human|San Diego County Public Health Laboratory|Andersen lab at Scripps Research|Gangavarapu|USA
>Spike|hCoV-19/England/BIRM-5F6A6/2020|2020-00-00|EPI_ISL_461525|Original|hCoV-19^^England|Human|University of Birmingham|COVID-19 Genomics UK (COG-UK) Consortium|Loman|United Kingdom
>Spike|hCoV-19/Kenya/C4616/2020|2020-05-08|EPI_ISL_568731|Original|hCoV-19^^Mombasa|Human|KEMRI-Wellcome Trust Research Programme/KEMRI-CGMR-C Kilifi|KEMRI-Wellcome Trust Research Programme/KEMRI-CGMR-C Kilifi|Githinji|Kenya
>Spike|hCoV-19/Northern Ireland/NIRE-107466/2020|2020-03-31|EPI_ISL_585152|Original|hCoV-19^^Northern Ireland|Human|Regional Virus Laboratory|COVID-19 Genomics UK (COG-UK) Consortium|Fairley|United Kingdom
>Spike|hCoV-19/England/MILK-99376E/2020|2020-08-26|EPI_ISL_551998|Original|hCoV-19^^England|Human|Lighthouse Lab in Milton Keynes|Wellcome Sanger Institute for the COVID-19 Genomics UK (COG-UK) consortium|Davies|United Kingdom
>Spike|hCoV-19/England/CAMB-7B6FF/2020|2020-04-03|EPI_ISL_442259|Original|hCoV-19^^England|Human|Department of Pathology|Wellcome Sanger Institute for the COVID-19 Genomics UK (COG-UK) consortium|Amato|United Kingdom
>Spike|hCoV-19/Denmark/DCGC-5751/2020|2020-09-28|EPI_ISL_621256|Original|hCoV-19^^Hovedstaden|Human|Department of Virus and Microbiological Special Diagnostics|Albertsen lab|Michaelsen|Denmark
>Spike|hCoV-19/England/SHEF-C3B80/2020|2020-05-04|EPI_ISL_475373|Original|hCoV-19^^England|Human|Virology Department|COVID-19 Genomics UK (COG-UK) Consortium|de Silva|United Kingdom
>Spike|hCoV-19/USA/NY-NYCPHL-000735/2020|2020-03-22|EPI_ISL_632117|Original|hCoV-19^^New York|Human|Richmond University Medical Center|New York City Public Health Laboratory|Wang|USA
>Spike|hCoV-19/England/ALDP-94DF98/2020|2020-06-16|EPI_ISL_555566|Original|hCoV-19^^England|Human|Lighthouse Lab in Alderley Park|Wellcome Sanger Institute for the COVID-19 Genomics UK (COG-UK) consortium|Davies|United Kingdom
>Spike|hCoV-19/Wales/PHWC-16CBD3/2020|2020-09-24|EPI_ISL_574145|Original|hCoV-19^^Wales|Human|Wales Specialist Virology Centre Sequencing lab|COVID-19 Genomics UK (COG-UK) Consortium|Moore|United Kingdom

>Spike|hCoV-19/Spain/MD-HLP-R81/2020|2020-05-07|EPI_ISL_530114|Original|hCoV-19^^Madrid|Human|Hospital Universitario Ram\u00f3n y Cajal|Hospital Universitario La Paz|Viedma|Spain
>Spike|hCoV-19/Australia/NSW776/2020|2020-07-29|EPI_ISL_526118|Original|hCoV-19^^New South Wales|Human|Pathology West - NSW Health Pathology|NSW Health Pathology - Institute of Clinical Pathology and Medical Research; Westmead Hospital; University of Sydney|Gall|Australia
>Spike|hCoV-19/Netherlands/ZH-EMC-1088/2020|2020-12-13|EPI_ISL_722769|Original|hCoV-19^^Zuid Holland|Human|Dutch COVID-19 response team|Erasmus Medical Center|Oude Munnink|Netherlands
>Spike|hCoV-19/England/CAMB-72EE1/2020|2020-03-23|EPI_ISL_440055|Original|hCoV-19^^England|Human|Department of Pathology|Wellcome Sanger Institute for the COVID-19 Genomics UK (COG-UK) consortium|Amato|United Kingdom
>Spike|hCoV-19/England/GSTT-265DC40/2020|2020-03-21|EPI_ISL_484084|Original|hCoV-19^^England|Human|Centre for Clinical Infection and Diagnostics Research and Genomics Innovation Unit|COVID-19 Genomics UK (COG-UK) Consortium|Awan|United Kingdom
>Spike|hCoV-19/England/NOTT-117C4D/2020|2020-10-25|EPI_ISL_612588|Original|hCoV-19^^England|Human|Queens Medical Centre|COVID-19 Genomics UK (COG-UK) Consortium|Loose|United Kingdom
>Spike|hCoV-19/USA/WA-QDX-1060/2020|2020-03-17|EPI_ISL_571802|Original|hCoV-19^^Washington|Human|Quest Diagnostics|Quest Diagnostics|Kagan|USA
>Spike|hCoV-19/England/MILK-B95278/2020|2020-11-13|EPI_ISL_673801|Original|hCoV-19^^England|Human|Lighthouse Lab in Milton Keynes|Wellcome Sanger Institute for the COVID-19 Genomics UK (COG-UK) Consortium|Davies|United Kingdom
>Spike|hCoV-19/Wales/PHWC-2B1EE/2020|2020-04-07|EPI_ISL_432272|Original|hCoV-19^^Wales|Human|Wales Specialist Virology Centre|Public Health Wales Microbiology Cardiff|Moore|United Kingdom
>Spike|hCoV-19/England/ALDP-4A78C3/2020|2020-06-08|EPI_ISL_559608|Original|hCoV-19^^England|Human|Lighthouse Lab in Alderley Park|Wellcome Sanger Institute for the COVID-19 Genomics UK (COG-UK) consortium|Davies|United Kingdom
>Spike|hCoV-19/Luxembourg/LNS0927824/2020|2020-11-22|EPI_ISL_739858|Original|hCoV-19^^Luxembourg|Human|Laboratoire national de sant\u00e9|Laboratoire national de sant\u00e9|Wienecke-Baldacchino|Luxembourg
>Spike|hCoV-19/USA/CA-ALSR-3175/2020|2020-06-03|EPI_ISL_635901|Original|hCoV-19^^California|Human|San Diego County Public Health Laboratory|Andersen lab at Scripps Research|Gangavarapu|USA
>Spike|hCoV-19/England/20134004304/2020|2020-03-23|EPI_ISL_423062|Original|hCoV-19^^England|Human|Respiratory Virus Unit|Respiratory Virus Unit|Platt|United Kingdom
>Spike|hCoV-19/England/LCST-24D6D8A/2020|2020-06-10|EPI_ISL_478341|Original|hCoV-19^^England|Human|University Hospitals Of Leicester NHS Trust and DeepSeq Nottingham|COVID-19 Genomics UK (COG-UK) Consortium|Loose|United Kingdom
>Spike|hCoV-19/England/BRIS-1305F8/2020|2020-04-12|EPI_ISL_492530|Original|hCoV-19^^England|Human|PHE South West Regional Laboratory|Wellcome Sanger Institute for the COVID-19 Genomics UK (COG-UK) consortium|Davies|United Kingdom
>Spike|hCoV-19/Luxembourg/LNS6434733/2020|2020-10-26|EPI_ISL_744413|Original|hCoV-19^^Luxembourg|Human|Laboratoire national de sant\u00e9|Laboratoire national de sant\u00e9|Wienecke-Baldacchino|Luxembourg
>Spike|hCoV-19/Wales/PHWC-485D35/2020|2020-10-21|EPI_ISL_628275|Original|hCoV-19^^Wales|Human|Wales Specialist Virology Centre Sequencing lab|COVID-19 Genomics UK (COG-UK) Consortium|Moore|United Kingdom
>Spike|hCoV-19/USA/WA-S1037/2020|2020-05-03|EPI_ISL_463527|Original|hCoV-19^^Washington|Human|Washington State Department of Health|Seattle Flu Study|Bedford|USA
>Spike|hCoV-19/USA/NM-UNM-00570/2020|2020-08-08|EPI_ISL_542984|Original|hCoV-19^^New Mexico|Human|TriCore Reference Laboratories|Center for Global Health|Domman|USA
>Spike|hCoV-19/DRC/3841/2020|2020-04-25|EPI_ISL_447249|Original|hCoV-19^^Kinshasa|Human|Viral Respiratory Lab|Pathogen Sequencing Lab|Amuri|Democratic Republic of the Congo
>Spike|hCoV-19/USA/WA-UW324/2020|2020-03-24|EPI_ISL_418891|Original|hCoV-19^^Washington|Human|UW Virology Lab|UW Virology Lab|Roychoudhury|USA

>Spike|hCoV-19/England/ALDP-A2FB1F/2020|2020-10-05|EPI_ISL_586955|Original|hCoV-
19^^England|Human|Lighthouse Lab in Alderley Park|Wellcome Sanger Institute for the
COVID-19 Genomics UK (COG-UK) consortium|Davies|United Kingdom
>Spike|hCoV-19/Japan/PG-1348/2020|2020-04-00|EPI_ISL_684956|Original|hCoV-
19^^Japan|Human|Saitama Prefectural Institute of Public Health|Pathogen Genomics
Center|Sekizuka|Japan
>Spike|hCoV-19/Russia/SPE-RII-7388V/2020|2020-04-20|EPI_ISL_524049|Vero P1|hCoV-
19^^Saint Petersburg|Human|WHO National Influenza Centre Russian Federation|WHO
National Influenza Centre Russian Federation|Komissarov|Russia
>Spike|hCoV-19/Australia/VIC9018/2020|2020-07-24|EPI_ISL_565355|Original|hCoV-
19^^Victoria|Human|Microbiological Diagnostic Unit - Public Health Laboratory (MDU-
PHL)|MDU-PHL|Schultz|Australia
>Spike|hCoV-19/France/PAC-IHU-0199/2020|2020-03-27|EPI_ISL_569366|Original|hCoV-
19^^Provence-Alpes-C\u00f4te-d\u2019Azur|Human|MEPHI|MEPHI|Levasseur|France
>Spike|hCoV-19/England/205320747/2020|2020-12-28|EPI_ISL_770469|Original|hCoV-
19^^England|Human|Respiratory Virus Unit|COVID-19 Genomics UK (COG-UK)
Consortium|Platt|United Kingdom
>Spike|hCoV-19/England/QEUH-9B553F/2020|2020-09-03|EPI_ISL_590353|Original|hCoV-
19^^England|Human|Lighthouse Lab in Glasgow|Wellcome Sanger Institute for the COVID-
19 Genomics UK (COG-UK) consortium|Davies|United Kingdom
>Spike|hCoV-19/England/204221433/2020|2020-00-00|EPI_ISL_593809|Original|hCoV-
19^^England|Human|Respiratory Virus Unit|Respiratory Virus Unit|Platt|United Kingdom
>Spike|hCoV-19/USA/TX-HMH-MCoV-8155/2020|2020-06-30|EPI_ISL_784236|Original|hCoV-
19^^Texas|Human|Houston Methodist Hospital|Houston Methodist Hospital|Long|USA
>Spike|hCoV-19/England/NORW-F8092/2020|2020-11-13|EPI_ISL_650387|Original|hCoV-
19^^England|Human|Quadram Institute Bioscience|COVID-19 Genomics UK (COG-UK)
Consortium|Alikhan|United Kingdom
>Spike|hCoV-19/Wales/PHWC-15CBB8/2020|2020-03-31|EPI_ISL_472591|Original|hCoV-
19^^Wales|Human|Wales Specialist Virology Centre Sequencing lab|COVID-19 Genomics UK
(COG-UK) Consortium|Moore|United Kingdom
>Spike|hCoV-19/USA/TX-DSHS-0030/2020|2020-05-07|EPI_ISL_517963|Original|hCoV-
19^^Texas|Human|Texas Department of State Health Services|Texas Department of State
Health Services|Rahman|USA
>Spike|hCoV-19/Denmark/DCGC-18475/2020|2020-12-07|EPI_ISL_749988|Original|hCoV-
19^^Syddanmark|Human|Department of Virus and Microbiological Special
Diagnostics|Albertsen Lab|Michaelsen|Denmark
>Spike|hCoV-19/USA/LA-GBCL-26629248/2020|2020-04-21|EPI_ISL_485894|Original|hCoV-
19^^Louisiana|Human|River Road Testing Lab|Ginkgo Bioworks Clinical
Laboratory|Smith|USA
>Spike|hCoV-19/Wales/PHWC-4862BD/2020|2020-10-16|EPI_ISL_637619|Original|hCoV-
19^^Wales|Human|Wales Specialist Virology Centre Sequencing lab|COVID-19 Genomics UK
(COG-UK) Consortium|Moore|United Kingdom
>Spike|hCoV-19/Denmark/DCGC-3141/2020|2020-08-31|EPI_ISL_615960|Original|hCoV-
19^^NA|Human|Department of Virus and Microbiological Special Diagnostics|Albertsen
lab|Michaelsen|Denmark
>Spike|hCoV-19/England/ALDP-B84537/2020|2020-11-14|EPI_ISL_675455|Original|hCoV-
19^^England|Human|Lighthouse Lab in Alderley Park|Wellcome Sanger Institute for the
COVID-19 Genomics UK (COG-UK) Consortium|Davies|United Kingdom
>Spike|hCoV-19/USA/CA-CZB-15278/2020|2020-11-28|EPI_ISL_738610|Original|hCoV-
19^^California|Human|Santa Clara County Public Health Laboratory|Chan-Zuckerberg
Biohub|Kamm|USA
>Spike|hCoV-19/England/ALDP-AA28E0/2020|2020-10-21|EPI_ISL_630758|Original|hCoV-
19^^England|Human|Lighthouse Lab in Alderley Park|Wellcome Sanger Institute for the
COVID-19 Genomics UK (COG-UK) consortium|Davies|United Kingdom
>Spike|hCoV-19/Denmark/DCGC-20645/2020|2020-12-21|EPI_ISL_759273|Original|hCoV-
19^^Hovedstaden|Human|Department of Virus and Microbiological Special
Diagnostics|Albertsen Lab|Michaelsen|Denmark
>Spike|hCoV-19/England/SHEF-10D7E05/2020|2020-12-09|EPI_ISL_742110|Original|hCoV-
19^^England|Human|Virology Department|COVID-19 Genomics UK (COG-UK) Consortium|de
Silva|United Kingdom

>Spike|hCoV-19/England/SHEF-C8ACA/2020|2020-11-05|EPI_ISL_680156|Original|hCoV-19^^England|Human|Virology Department|COVID-19 Genomics UK (COG-UK) Consortium|de Silva|United Kingdom
>Spike|hCoV-19/Canada/BC_05845391/2020|2020-04-00|EPI_ISL_466791|Original|hCoV-19^^British Columbia|Human|BCCDC Public Health Laboratory|BCCDC Public Health Laboratory|Harrigan|Canada
>Spike|hCoV-19/Denmark/DCGC-7409/2020|2020-10-12|EPI_ISL_619359|Original|hCoV-19^^Midtjylland|Human|Department of Virus and Microbiological Special Diagnostics|Albertsen lab|Michaelsen|Denmark
>Spike|hCoV-19/England/NORW-ED564/2020|2020-00-00|EPI_ISL_472374|Original|hCoV-19^^England|Human|Quadram Institute Bioscience|COVID-19 Genomics UK (COG-UK) Consortium|Alikhan|United Kingdom
>Spike|hCoV-19/Finland/23LS5C4/2020|2020-10-23|EPI_ISL_759776|Original|hCoV-19^^Uusimaa|Human|Department of Virology and Immunology|Department of Virology|Smura|Finland
>Spike|hCoV-19/Spain/PV-IBV-005154/2020|2020-06-22|EPI_ISL_509642|Original|hCoV-19^^Basque Country|Human|Servicio de Microbiolog\u00eda. Hospital Universitario Donostia. OSI Donostialdea. \u00c1rea de Enfermedades Infecciosas|SeqCOVID-SPAIN consortium/IBV(CSIC)|Comas|Spain
>Spike|hCoV-19/Switzerland/ZH-ETHZ-150041plus/2020|2020-06-02|EPI_ISL_483666|Original|hCoV-19^^Zurich|Human|Viollier AG|Department of Biosystems Science and Engineering|Nadeau|Switzerland
>Spike|hCoV-19/England/QEUH-AD2243/2020|2020-10-26|EPI_ISL_629938|Original|hCoV-19^^England|Human|Lighthouse Lab in Glasgow|Wellcome Sanger Institute for the COVID-19 Genomics UK (COG-UK) consortium|Davies|United Kingdom
>Spike|hCoV-19/England/QEUH-BA331A/2020|2020-11-18|EPI_ISL_721127|Original|hCoV-19^^England|Human|Lighthouse Lab in Glasgow|Wellcome Sanger Institute for the COVID-19 Genomics UK (COG-UK) Consortium|Davies|United Kingdom
>Spike|hCoV-19/England/ALDP-AA2060/2020|2020-10-22|EPI_ISL_624300|Original|hCoV-19^^England|Human|Lighthouse Lab in Alderley Park|Wellcome Sanger Institute for the COVID-19 Genomics UK (COG-UK) consortium|Davies|United Kingdom
>Spike|hCoV-19/USA/TX-HMH-MCoV-19275/2020|2020-11-23|EPI_ISL_783734|Original|hCoV-19^^Texas|Human|Houston Methodist Hospital|Houston Methodist Hospital|Long|USA
>Spike|hCoV-19/USA/AZ-TG519153/2020|2020-06-30|EPI_ISL_695357|Original|hCoV-19^^Arizona|Human|AZ SPHL|TGen North|French|USA
>Spike|hCoV-19/Belgium/rega-09181047/2020|2020-09-18|EPI_ISL_734991|Original|hCoV-19^^Wijgmaal|Human|UZ Leuven|KU Leuven|Maes|Belgium
>Spike|hCoV-19/USA/NY-SURV0983/2020|2020-03-19|EPI_ISL_436078|Original|hCoV-19^^New York|Human|NYC Department of Health and Mental Hygiene|Pathogen Discovery|Queen|USA
>Spike|hCoV-19/USA/TX-HMH-MCoV-8634/2020|2020-07-01|EPI_ISL_780664|Original|hCoV-19^^Texas|Human|Houston Methodist Hospital|Houston Methodist Hospital|Long|USA
>Spike|hCoV-19/Spain/GA-IBV-002723/2020|2020-04-07|EPI_ISL_467295|Original|hCoV-19^^Galicia|Human|Hospital Cl\u00ednico Universitario de Santiago de Compostela|SeqCOVID-SPAIN consortium/IBV(CSIC)|Comas|Spain
>Spike|hCoV-19/England/MILK-A7AE76/2020|2020-10-15|EPI_ISL_606376|Original|hCoV-19^^England|Human|Lighthouse Lab in Milton Keynes|Wellcome Sanger Institute for the COVID-19 Genomics UK (COG-UK) consortium|Davies|United Kingdom
>Spike|hCoV-19/England/20108165204/2020|2020-03-05|EPI_ISL_452253|Original|hCoV-19^^England|Human|Respiratory Virus Unit|Respiratory Virus Unit|Platt|United Kingdom
>Spike|hCoV-19/Wales/PHWC-47D9B4/2020|2020-10-08|EPI_ISL_586219|Original|hCoV-19^^Wales|Human|Wales Specialist Virology Centre Sequencing lab|COVID-19 Genomics UK (COG-UK) Consortium|Moore|United Kingdom
>Spike|hCoV-19/Russia/SVE-RII-MH1924S/2020|2020-09-16|EPI_ISL_602367|Original|hCoV-19^^Sverdlovsk|Human|HELIX LLC|WHO National Influenza Centre Russian Federation|Komissarov|Russia
>Spike|hCoV-19/England/CAMC-9DF845/2020|2020-09-21|EPI_ISL_638427|Original|hCoV-19^^England|Human|Department of Pathology|COVID-19 Genomics UK (COG-UK) Consortium|Goodfellow|United Kingdom
>Spike|hCoV-19/USA/TX-HMH-4792/2020|2020-06-22|EPI_ISL_544335|Original|hCoV-19^^Texas|Human|Houston Methodist Hospital|Houston Methodist Hospital|Long|USA

>Spike|hCoV-19/USA/TX-HMH-MCoV-12302/2020|2020-09-25|EPI_ISL_790290|Original|hCoV-19^^Texas|Human|Houston Methodist Hospital|Houston Methodist Hospital|Long|USA
>Spike|hCoV-19/Wales/PHWC-48F8FE/2020|2020-11-10|EPI_ISL_681157|Original|hCoV-19^^Wales|Human|Wales Specialist Virology Centre Sequencing lab|COVID-19 Genomics UK (COG-UK) Consortium|Moore|United Kingdom
>Spike|hCoV-19/England/CAMB-7FB74/2020|2020-04-17|EPI_ISL_433483|Original|hCoV-19^^England|Human|Department of Pathology|COVID-19 Genomics UK (COG-UK) Consortium|Meredith|United Kingdom
>Spike|hCoV-19/Vietnam/PIHCM-372/2020|2020-03-20|EPI_ISL_654872|Vero E6|hCoV-19^^Ho Chi Minh city|Human|Pasteur Institute in Ho Chi Minh city|Department of Microbiology and Immunology - Pasteur Institute in Ho Chi Minh city|Vu|Viet Nam
>Spike|hCoV-19/England/MILK-BAB743/2020|2020-11-18|EPI_ISL_783362|Original|hCoV-19^^England|Human|Lighthouse Lab in Milton Keynes|Wellcome Sanger Institute for the COVID-19 Genomics UK (COG-UK) Consortium|Davies|United Kingdom
>Spike|hCoV-19/Russia/MOW-RII-MH2917S/2020|2020-09-23|EPI_ISL_733069|Original|hCoV-19^^Moscow|Human|HELIX LLC|WHO National Influenza Centre Russian Federation|Komissarov|Russia
>Spike|hCoV-19/England/ALDP-B5D93B/2020|2020-11-10|EPI_ISL_704446|Original|hCoV-19^^England|Human|Department of Pathology|COVID-19 Genomics UK (COG-UK) Consortium|Goodfellow|United Kingdom
>Spike|hCoV-19/Wales/PHWC-4A630F/2020|2020-12-16|EPI_ISL_765025|Original|hCoV-19^^Wales|Human|Wales Specialist Virology Centre Sequencing lab|COVID-19 Genomics UK (COG-UK) Consortium|Moore|United Kingdom
>Spike|hCoV-19/Israel/CVL-11-ngs/2020|2020-12-24|EPI_ISL_776581|Original|hCoV-19^^Israel|Human|Israel Central Virology laboratory|Israel Central Virology laboratory|Zuckerman|Israel
>Spike|hCoV-19/Wales/PHWC-49E759/2020|2020-12-08|EPI_ISL_743042|Original|hCoV-19^^Wales|Human|Wales Specialist Virology Centre Sequencing lab|COVID-19 Genomics UK (COG-UK) Consortium|Moore|United Kingdom
>Spike|hCoV-19/South Korea/KCDC2066/2020|2020-02-20|EPI_ISL_497967|Vero E6 P1|hCoV-19^^South Korea|Human|Division of Viral Diseases|Division of Viral Diseases|Kim|South Korea
>Spike|hCoV-19/Denmark/DCGC-22339/2020|2020-12-14|EPI_ISL_757848|Original|hCoV-19^^Sjaelland|Human|Department of Virus and Microbiological Special Diagnostics|Albertsen Lab|Michaelsen|Denmark
>Spike|hCoV-19/USA/TX-HMH-MCoV-8758/2020|2020-07-01|EPI_ISL_780737|Original|hCoV-19^^Texas|Human|Houston Methodist Hospital|Houston Methodist Hospital|Long|USA
>Spike|hCoV-19/South Korea/KDCA0294/2020|2020-10-26|EPI_ISL_760088|Original|hCoV-19^^South Korea|Human|Division of Emerging Infectious Diseases|Division of Emerging Infectious Diseases|Park|South Korea
>Spike|hCoV-19/England/ALDP-C3AD91/2020|2020-12-09|EPI_ISL_731265|Original|hCoV-19^^England|Human|Lighthouse Lab in Alderley Park|Wellcome Sanger Institute for the COVID-19 Genomics UK (COG-UK) Consortium|Davies|United Kingdom
>Spike|hCoV-19/Scotland/QEUH-BEB04C/2020|2020-12-03|EPI_ISL_710646|Original|hCoV-19^^Scotland|Human|Lighthouse Lab in Glasgow|Wellcome Sanger Institute for the COVID-19 Genomics UK (COG-UK) Consortium|Davies|United Kingdom
>Spike|hCoV-19/Netherlands/FR-RIVM-20101/2020|2020-12-02|EPI_ISL_723278|Orignal|hCoV-19^^Friesland|Human|Dutch COVID-19 response team|National Institute for Public Health and the Environment (RIVM)|Meijer|Netherlands
>Spike|hCoV-19/Wales/PHWC-164BAE/2020|2020-06-01|EPI_ISL_479355|Original|hCoV-19^^Wales|Human|Wales Specialist Virology Centre Sequencing lab|COVID-19 Genomics UK (COG-UK) Consortium|Moore|United Kingdom
>Spike|hCoV-19/Austria/CeMM0213/2020|2020-03-16|EPI_ISL_438049|Original|hCoV-19^^Vienna|Human|Center for Virology|Bergthaler laboratory|Bergthaler|Austria
>Spike|hCoV-19/Hong Kong/VM20123559/2020|2020-09-18|EPI_ISL_760035|Original|hCoV-19^^Hong Kong|Human|Hong Kong Department of Health|School of Public Health|Chu|China
>Spike|hCoV-19/USA/CA-QDX-2397/2020|2020-03-17|EPI_ISL_604671|Original|hCoV-19^^California|Human|Quest Diagnostics|Quest Diagnostics|Kagan|USA

>Spike|hCoV-19/Netherlands/ZH-EMC-405/2020|2020-07-23|EPI_ISL_523745|Original|hCoV-19^^Zuid Holland|Human|Dutch COVID-19 response team|Erasmus Medical Center|Oude Munnink|Netherlands
>Spike|hCoV-19/Wales/PHWC-16874C/2020|2020-04-19|EPI_ISL_494280|Original|hCoV-19^^Wales|Human|Originating lab|COVID-19 Genomics UK (COG-UK) Consortium|Moore|United Kingdom
>Spike|hCoV-19/Netherlands/Friesland_9/2020|2020-03-30|EPI_ISL_422573|Original|hCoV-19^^Friesland|Human|Dutch COVID-19 response team|Erasmus Medical Center|Oude Munnink|Netherlands
>Spike|hCoV-19/South Africa/R10639/2020|2020-06-11|EPI_ISL_490291|Original|hCoV-19^^Free State|Human|National Institute for Communicable Diseases of the National Health Laboratory Service|National Institute for Communicable Diseases of the National Health Laboratory Service|Allam|South Africa
>Spike|hCoV-19/England/SHEF-C6D4B/2020|2020-04-23|EPI_ISL_457615|Original|hCoV-19^^England|Human|Virology Department|COVID-19 Genomics UK (COG-UK) Consortium|de Silva|United Kingdom
>Spike|hCoV-19/USA/IL-NM058/2020|2020-03-18|EPI_ISL_444554|Original|hCoV-19^^Illinois|Human|Northwestern Memorial Hospital|Ozer Lab|Ozer|USA
>Spike|hCoV-19/England/QEUH-AD4FFF/2020|2020-10-26|EPI_ISL_633854|Original|hCoV-19^^England|Human|Lighthouse Lab in Glasgow|Wellcome Sanger Institute for the COVID-19 Genomics UK (COG-UK) consortium|Davies|United Kingdom
>Spike|hCoV-19/England/MILK-9943D2/2020|2020-08-26|EPI_ISL_551682|Original|hCoV-19^^England|Human|Lighthouse Lab in Milton Keynes|Wellcome Sanger Institute for the COVID-19 Genomics UK (COG-UK) consortium|Davies|United Kingdom
>Spike|hCoV-19/Canada/NS_40/2020|2020-03-05|EPI_ISL_482469|Original|hCoV-19^^Nova Scotia|Human|Queen Elizabeth II Health Science Centre|National Microbiology Laboratory|Majer|Canada
>Spike|hCoV-19/Wales/PHWC-498889/2020|2020-11-27|EPI_ISL_727134|Original|hCoV-19^^Wales|Human|Originating lab|COVID-19 Genomics UK (COG-UK) Consortium|Moore|United Kingdom
>Spike|hCoV-19/USA/LA-EVTL380/2020|2020-06-03|EPI_ISL_578786|Original|hCoV-19^^Louisiana|Human|LSUHS Emerging Viral Threat Laboratory|Microbial Genome Sequencing Center|Kamil|USA
>Spike|hCoV-19/USA/TX-HMH-MCoV-18096/2020|2020-11-13|EPI_ISL_784444|Original|hCoV-19^^Texas|Human|Houston Methodist Hospital|Houston Methodist Hospital|Long|USA
>Spike|hCoV-19/USA/AZ-TG640829/2020|2020-04-27|EPI_ISL_695783|Original|hCoV-19^^Arizona|Human|Sonora Quest Laboratories|TGen North|French|USA
>Spike|hCoV-19/England/QEUH-9601C6/2020|2020-08-06|EPI_ISL_531868|Original|hCoV-19^^England|Human|Lighthouse Lab in Glasgow|Wellcome Sanger Institute for the COVID-19 Genomics UK (COG-UK) consortium|Davies|United Kingdom
>Spike|hCoV-19/England/MILK-6395CD/2020|2020-07-04|EPI_ISL_557740|Original|hCoV-19^^England|Human|Lighthouse Lab in Milton Keynes|Wellcome Sanger Institute for the COVID-19 Genomics UK (COG-UK) consortium|Davies|United Kingdom
>Spike|hCoV-19/Australia/VIC13784/2020|2020-09-16|EPI_ISL_563835|Original|hCoV-19^^Victoria|Human|Microbiological Diagnostic Unit - Public Health Laboratory (MDU-PHL)|MDU-PHL|Schultz|Australia
>Spike|hCoV-19/Scotland/QEUH-9E4898/2020|2020-09-23|EPI_ISL_600950|Original|hCoV-19^^Scotland|Human|Lighthouse Lab in Glasgow|Wellcome Sanger Institute for the COVID-19 Genomics UK (COG-UK) consortium|Davies|United Kingdom
>Spike|hCoV-19/Japan/PG-3177/2020|2020-03-00|EPI_ISL_691297|Original|hCoV-19^^Japan|Human|Pathogen Genomics Center|Pathogen Genomics Center|Sekizuka|Japan
>Spike|hCoV-19/England/ALDP-A68A2C/2020|2020-10-12|EPI_ISL_608972|Original|hCoV-19^^England|Human|Lighthouse Lab in Alderley Park|Wellcome Sanger Institute for the COVID-19 Genomics UK (COG-UK) consortium|Davies|United Kingdom
>Spike|hCoV-19/Denmark/DCGC-1702/2020|2020-06-29|EPI_ISL_618169|Original|hCoV-19^^Nordjylland|Human|Department of Virus and Microbiological Special Diagnostics|Albertsen lab|Michaelsen|Denmark
>Spike|hCoV-19/England/MILK-99DBC2/2020|2020-08-31|EPI_ISL_550601|Original|hCoV-19^^England|Human|Lighthouse Lab in Milton Keynes|Wellcome Sanger Institute for the COVID-19 Genomics UK (COG-UK) consortium|Davies|United Kingdom

>Spike|hCoV-19/England/QEUH-BA31C5/2020|2020-11-18|EPI_ISL_672897|Original|hCoV-19^^England|Human|Lighthouse Lab in Glasgow|Wellcome Sanger Institute for the COVID-19 Genomics UK (COG-UK) Consortium|Davies|United Kingdom
>Spike|hCoV-19/Scotland/CVR2777/2020|2020-04-20|EPI_ISL_478036|Original|hCoV-19^^Scotland|Human|West of Scotland Specialist Virology Centre|COVID-19 Genomics UK (COG-UK) Consortium|Robertson|United Kingdom
>Spike|hCoV-19/Bangladesh/BCSIR-NILMRC-318/2020|2020-05-31|EPI_ISL_498789|Original|hCoV-19^^Mymensingh|Human|National Institute of Laboratory Medicine and Referral Center|Genomic Research Lab|Khan|Bangladesh
>Spike|hCoV-19/France/ARA-SC346/2020|2020-03-17|EPI_ISL_641543|Original|hCoV-19^^Auvergne-Rhone-Alpes|Human|CHU de Saint-\u00c9tienne H\u00f4pital Nord|CNR Virus des Infections Respiratoires - France SUD|Bal|France
>Spike|hCoV-19/England/20146006804/2020|2020-04-01|EPI_ISL_466018|Original|hCoV-19^^England|Human|Respiratory Virus Unit|Respiratory Virus Unit|Platt|United Kingdom
>Spike|hCoV-19/Wales/PHWC-47F1C9/2020|2020-10-08|EPI_ISL_612642|Original|hCoV-19^^Wales|Human|Wales Specialist Virology Centre Sequencing lab|COVID-19 Genomics UK (COG-UK) Consortium|Moore|United Kingdom
>Spike|hCoV-19/USA/HI-H200605/2020|2020-11-23|EPI_ISL_753131|Original|hCoV-19^^Hawaii|Human|State Laboratories Division|State Laboratories Division|Sultana|USA
>Spike|hCoV-19/Scotland/QEUH-996929/2020|2020-08-29|EPI_ISL_559990|Original|hCoV-19^^Scotland|Human|Oxford Viromics|COVID-19 Genomics UK (COG-UK) Consortium|Golubchik|United Kingdom
>Spike|hCoV-19/Wales/MILK-B02470/2020|2020-10-24|EPI_ISL_647851|Original|hCoV-19^^Wales|Human|Lighthouse Lab in Milton Keynes|Wellcome Sanger Institute for the COVID-19 Genomics UK (COG-UK) Consortium|Davies|United Kingdom
>Spike|hCoV-19/Denmark/DCGC-20107/2020|2020-12-07|EPI_ISL_747986|Original|hCoV-19^^Nordjylland|Human|Department of Virus and Microbiological Special Diagnostics|Albertsen Lab|Michaelsen|Denmark
>Spike|hCoV-19/Denmark/DCGC-11526/2020|2020-11-02|EPI_ISL_670529|Original|hCoV-19^^Hovedstaden|Human|Department of Virus and Microbiological Special Diagnostics|Albertsen Lab|Michaelsen|Denmark
>Spike|hCoV-19/India/MH-ACTREC-146/2020|2020-05-29|EPI_ISL_699802|Original|hCoV-19^^Maharashtra|Human|Hematopathology Laboratory|Hematopathology Laboratory|Patkar|India
>Spike|hCoV-19/Japan/PG-5193/2020|2020-08-00|EPI_ISL_690296|Original|hCoV-19^^Japan|Human|Pathogen Genomics Center|Pathogen Genomics Center|Sekizuka|Japan
>Spike|hCoV-19/Wales/PHWC-48CC8C/2020|2020-10-31|EPI_ISL_666525|Original|hCoV-19^^Wales|Human|Wales Specialist Virology Centre Sequencing lab|COVID-19 Genomics UK (COG-UK) Consortium|Moore|United Kingdom
>Spike|hCoV-19/Italy/LAZ-INMI-50/2020|2020-06-02|EPI_ISL_603157|Original|hCoV-19^^Lazio|Human|INMI Lazzaro Spallanzani IRCCS|INMI Lazzaro Spallanzani IRCCS|Gruber|Italy
>Spike|hCoV-19/Spain/PV-IBV-2103/2020|2020-04-11|EPI_ISL_468890|Original|hCoV-19^^Basque Country|Human|Servicio de Microbiolog\u00eda. Hospital Universitario Donostia. OSI Donostialdea. \u00c1rea de Enfermedades Infecciosas|SeqCOVID-SPAIN consortium/IBV(CSIC)|Comas|Spain
>Spike|hCoV-19/USA/CA-ALSR-2055/2020|2020-05-07|EPI_ISL_635321|Original|hCoV-19^^California|Human|San Diego County Public Health Laboratory|Andersen lab at Scripps Research|Gangavarapu|USA
>Spike|hCoV-19/USA/TX-HMH-MCoV-18371/2020|2020-11-18|EPI_ISL_784719|Original|hCoV-19^^Texas|Human|Houston Methodist Hospital|Houston Methodist Hospital|Long|USA
>Spike|hCoV-19/USA/WI-UW-2389/2020|2020-10-04|EPI_ISL_754628|Original|hCoV-19^^Wisconsin|Human|University of Wisconsin-Madison AIDS Vaccine Research Laboratories|University of Wisconsin-Madison AIDS Vaccine Research Laboratories|Moreno|USA
>Spike|hCoV-19/Switzerland/BE-UNIBE-200-5795/2020|2020-03-26|EPI_ISL_574838|Original|hCoV-19^^Bern|Human|Institute for Infectious Diseases|Institute for Infectious Diseases|Koch|Switzerland

>Spike|hCoV-19/Scotland/QEUH-96C6BC/2020|2020-08-07|EPI_ISL_530869|Original|hCoV-19^^Scotland|Human|Lighthouse Lab in Glasgow|Wellcome Sanger Institute for the COVID-19 Genomics UK (COG-UK) consortium|Davies|United Kingdom
>Spike|hCoV-19/India/GJ-GBRC149/2020|2020-05-17|EPI_ISL_458111|Original|hCoV-19^^Gujarat|Human|Gujarat Biotechnology Research Centre|Gujarat Biotechnology Research Centre|Joshi|India
>Spike|hCoV-19/Japan/PG-5370/2020|2020-08-00|EPI_ISL_690473|Original|hCoV-19^^Japan|Human|Fukuoka Institute of Health and Environmental Sciences|Pathogen Genomics Center|Sekizuka|Japan
>Spike|hCoV-19/England/LOND-124F765/2020|2020-03-25|EPI_ISL_665170|Original|hCoV-19^^England|Human|University College London Hospital|COVID-19 Genomics UK (COG-UK) Consortium|Byott|United Kingdom
>Spike|hCoV-19/USA/MA-MASPHL-00186/2020|2020-04-06|EPI_ISL_791645|Original|hCoV-19^^Massachusetts|Human|Massachusetts State Public Health Laboratory|Infectious Disease Program|Park|USA
>Spike|hCoV-19/England/LIVE-9F1C7/2020|2020-05-02|EPI_ISL_453435|Original|hCoV-19^^England|Human|Liverpool Clinical Laboratories|COVID-19 Genomics UK (COG-UK) Consortium|Haldenby|United Kingdom
>Spike|hCoV-19/England/204841912/2020|2020-11-25|EPI_ISL_683581|Original|hCoV-19^^England|Human|Respiratory Virus Unit|COVID-19 Genomics UK (COG-UK) Consortium|Platt|United Kingdom
>Spike|hCoV-19/Ecuador/USFQ-171/2020|2020-07-29|EPI_ISL_516652|Original|hCoV-19^^Orellana|Human|Institute of Microbiology|Institute of Microbiology|Cardenas|Ecuador
>Spike|hCoV-19/Wales/PHWC-48DB51/2020|2020-11-01|EPI_ISL_680731|Original|hCoV-19^^Wales|Human|Wales Specialist Virology Centre Sequencing lab|COVID-19 Genomics UK (COG-UK) Consortium|Moore|United Kingdom
>Spike|hCoV-19/USA/CA-CZB-3616/2020|2020-07-28|EPI_ISL_548339|Original|hCoV-19^^California|Human|County of Santa Clara Public Health Department|Chan-Zuckerberg Biohub|Bhatt|USA
>Spike|hCoV-19/England/ALDP-B23F2D/2020|2020-11-04|EPI_ISL_646309|Original|hCoV-19^^England|Human|Lighthouse Lab in Alderley Park|Wellcome Sanger Institute for the COVID-19 Genomics UK (COG-UK) Consortium|Davies|United Kingdom
>Spike|hCoV-19/England/ALDP-B1AEE0/2020|2020-11-01|EPI_ISL_645853|Original|hCoV-19^^England|Human|Lighthouse Lab in Alderley Park|Wellcome Sanger Institute for the COVID-19 Genomics UK (COG-UK) Consortium|Davies|United Kingdom
>Spike|hCoV-19/England/BRIS-12FA0C/2020|2020-04-03|EPI_ISL_488293|Original|hCoV-19^^England|Human|PHE South West Regional Laboratory|Wellcome Sanger Institute for the COVID-19 Genomics UK (COG-UK) consortium|Davies|United Kingdom
>Spike|hCoV-19/Denmark/DCGC-2105/2020|2020-07-27|EPI_ISL_618367|Original|hCoV-19^^Midtjylland|Human|Department of Virus and Microbiological Special Diagnostics|Albertsen lab|Michaelsen|Denmark
>Spike|hCoV-19/England/LIVE-1D5012/2020|2020-09-17|EPI_ISL_572479|Original|hCoV-19^^England|Human|Liverpool Clinical Laboratories|COVID-19 Genomics UK (COG-UK) Consortium|Haldenby|United Kingdom
>Spike|hCoV-19/England/MILK-A91723/2020|2020-10-19|EPI_ISL_705979|Original|hCoV-19^^England|Human|Oxford Viromics|COVID-19 Genomics UK (COG-UK) Consortium|Golubchik|United Kingdom
>Spike|hCoV-19/Denmark/DCGC-4178/2020|2020-09-14|EPI_ISL_621998|Original|hCoV-19^^Hovedstaden|Human|Department of Virus and Microbiological Special Diagnostics|Albertsen lab|Michaelsen|Denmark
>Spike|hCoV-19/Scotland/EDB6256/2020|2020-06-02|EPI_ISL_478238|Original|hCoV-19^^Scotland|Human|Virology Department|COVID-19 Genomics UK (COG-UK) Consortium|Williams|United Kingdom
>Spike|hCoV-19/England/SHEF-10C5963/2020|2020-12-14|EPI_ISL_765149|Original|hCoV-19^^England|Human|Virology Department|COVID-19 Genomics UK (COG-UK) Consortium|de Silva|United Kingdom
>Spike|hCoV-19/England/EXET-13998A/2020|2020-00-00|EPI_ISL_724255|Original|hCoV-19^^England|Human|University of Exeter|COVID-19 Genomics UK (COG-UK) Consortium|Temperton|United Kingdom

>Spike|hCoV-19/Spain/MD-IBV-99012386/2020|2020-10-29|EPI_ISL_779961|Original|hCoV-19^^Madrid|Human|Hospital General Universitario Gregorio Mara\u00f1\u00f3n|SeqCOVID-SPAIN consortium/IBV(CSIC)|Comas|Spain
>Spike|hCoV-19/Japan/PG-4904/2020|2020-08-00|EPI_ISL_690025|Original|hCoV-19^^Japan|Human|Sakai City Institute of Public Health|Pathogen Genomics Center|Sekizuka|Japan
>Spike|hCoV-19/England/QEUH-A4D726/2020|2020-10-05|EPI_ISL_628609|Original|hCoV-19^^England|Human|Oxford Viromics|COVID-19 Genomics UK (COG-UK) Consortium|Golubchik|United Kingdom
>Spike|hCoV-19/USA/TX-HMH-MCoV-17995/2020|2020-11-16|EPI_ISL_786143|Original|hCoV-19^^Texas|Human|Houston Methodist Hospital|Houston Methodist Hospital|Long|USA
>Spike|hCoV-19/England/QEUH-B6C5AE/2020|2020-11-10|EPI_ISL_661537|Original|hCoV-19^^England|Human|Lighthouse Lab in Glasgow|Wellcome Sanger Institute for the COVID-19 Genomics UK (COG-UK) Consortium|Davies|United Kingdom
>Spike|hCoV-19/England/NORT-29DB23/2020|2020-05-07|EPI_ISL_488173|Original|hCoV-19^^England|Human|NU-OMICS DNA Sequencing research facility|Wellcome Sanger Institute for the COVID-19 Genomics UK (COG-UK) consortium|Davies|United Kingdom
>Spike|hCoV-19/England/ALDP-A2FA30/2020|2020-10-05|EPI_ISL_587227|Original|hCoV-19^^England|Human|Lighthouse Lab in Alderley Park|Wellcome Sanger Institute for the COVID-19 Genomics UK (COG-UK) consortium|Davies|United Kingdom
>Spike|hCoV-19/England/MILK-B38550/2020|2020-11-01|EPI_ISL_658990|Original|hCoV-19^^England|Human|Lighthouse Lab in Milton Keynes|Wellcome Sanger Institute for the COVID-19 Genomics UK (COG-UK) Consortium|Davies|United Kingdom
>Spike|hCoV-19/Wales/PHWC-48E5E9/2020|2020-11-12|EPI_ISL_680883|Original|hCoV-19^^Wales|Human|Wales Specialist Virology Centre Sequencing lab|COVID-19 Genomics UK (COG-UK) Consortium|Moore|United Kingdom
>Spike|hCoV-19/Wales/PHWC-49F80A/2020|2020-12-08|EPI_ISL_743273|Original|hCoV-19^^Wales|Human|Wales Specialist Virology Centre Sequencing lab|COVID-19 Genomics UK (COG-UK) Consortium|Moore|United Kingdom
>Spike|hCoV-19/Scotland/CVR6106/2020|2020-12-03|EPI_ISL_703233|Original|hCoV-19^^Scotland|Human|West of Scotland Specialist Virology Centre|COVID-19 Genomics UK (COG-UK) Consortium|Robertson|United Kingdom
>Spike|hCoV-19/USA/FL-BPHL-0155/2020|2020-03-31|EPI_ISL_480806|Original|hCoV-19^^Florida|Human|Florida Bureau of Public Health Laboratories|Florida Bureau of Public Health Laboratories|Blanton|USA
>Spike|hCoV-19/Japan/PG-4575/2020|2020-07-00|EPI_ISL_689712|Original|hCoV-19^^Japan|Human|Pathogen Genomics Center|Pathogen Genomics Center|Sekizuka|Japan
>Spike|hCoV-19/USA/NY-Wadsworth-281872-01/2020|2020-12-01|EPI_ISL_754426|Original|hCoV-19^^New York|Human|Wadsworth Center|Wadsworth Center|Lasek-Nesselquist|USA
>Spike|hCoV-19/Switzerland/ZH-ETHZ-240091/2020|2020-08-13|EPI_ISL_523926|Original|hCoV-19^^Zurich|Human|Viollier AG|Department of Biosystems Science and Engineering|Nadeau|Switzerland
>Spike|hCoV-19/Japan/PG-0082/2020|2020-03-02|EPI_ISL_479833|Original|hCoV-19^^Hokkaido|Human|Sapporo City Institute of Public Health|Pathogen Genomics Center|Sekizuka|Japan
>Spike|hCoV-19/Canada/NS-NML-1268/2020|2020-04-16|EPI_ISL_579656|Original|hCoV-19^^Nova Scotia|Human|QEII Health Sciences Centre|National Microbiology Laboratory (NML)|Majer|Canada
>Spike|hCoV-19/Germany/BE-ChVir-W1693-4382/2020|2020-08-04|EPI_ISL_753988|Original|hCoV-19^^Berlin|Human|Charit\u00e9 Universit\u00e4tsmedizin Berlin|Charit\u00e9 Universit\u00e4tsmedizin Berlin|Muehlemann|Germany
>Spike|hCoV-19/England/CAMB-8067F/2020|2020-04-10|EPI_ISL_470442|Original|hCoV-19^^England|Human|Department of Pathology|Wellcome Sanger Institute for the COVID-19 Genomics UK (COG-UK) consortium|Davies|United Kingdom
>Spike|hCoV-19/France/PDL-SC646/2020|2020-04-12|EPI_ISL_663283|Original|hCoV-19^^Pays-de-la-Loire|Human|CHU Nantes|CNR Virus des Infections Respiratoires - France SUD|Bal|France

>Spike|hCoV-19/England/QEUH-964C67/2020|2020-08-13|EPI_ISL_530987|Original|hCoV-19^^England|Human|Lighthouse Lab in Glasgow|Wellcome Sanger Institute for the COVID-19 Genomics UK (COG-UK) consortium|Davies|United Kingdom
>Spike|hCoV-19/England/204291438/2020|2020-10-16|EPI_ISL_622875|Original|hCoV-19^^England|Human|Respiratory Virus Unit|Respiratory Virus Unit|Platt|United Kingdom
>Spike|hCoV-19/USA/TX-HMH-MCoV-10712/2020|2020-07-09|EPI_ISL_789598|Original|hCoV-19^^Texas|Human|Houston Methodist Hospital|Houston Methodist Hospital|Long|USA
>Spike|hCoV-19/USA/NY-NYCPHL-000818/2020|2020-04-02|EPI_ISL_632195|Original|hCoV-19^^New York|Human|Jamaica Hospital Medical Center|New York City Public Health Laboratory|Wang|USA
>Spike|hCoV-19/France/ARA-34744/2020|2020-05-06|EPI_ISL_636481|Original|hCoV-19^^Auvergne-Rhone-Alpes|Human|Centre Hospitalier de Bourg en Bresse|CNR Virus des Infections Respiratoires - France SUD|Josset|France
>Spike|hCoV-19/Germany/BY-MGZ-02/2020|2020-04-21|EPI_ISL_490206|Original|hCoV-19^^Bavaria|Human|M\u00fcnchen Klinik Schwabing|MGZ Medical Genetics Center|Wolf|Germany
>Spike|hCoV-19/Australia/VIC13639/2020|2020-09-13|EPI_ISL_563792|Original|hCoV-19^^Victoria|Human|Victorian Infectious Diseases Reference Laboratory (VIDRL)|VIDRL and MDU-PHL|Schultz|Australia
>Spike|hCoV-19/Denmark/DCGC-6025/2020|2020-09-21|EPI_ISL_621134|Original|hCoV-19^^Midtjylland|Human|Department of Virus and Microbiological Special Diagnostics|Albertsen lab|Michaelsen|Denmark
>Spike|hCoV-19/USA/VA-DCLS-2036/2020|2020-10-13|EPI_ISL_677178|Original|hCoV-19^^Virginia|Human|Virginia Division of Consolidated Laboratory Services|Virginia Division of Consolidated Laboratory Services|St. Jacques|USA
>Spike|hCoV-19/Wales/PHWC-25A34/2020|2020-03-22|EPI_ISL_422301|Original|hCoV-19^^Wales|Human|Wales Specialist Virology Centre|Public Health Wales Microbiology Cardiff|Moore|United Kingdom
>Spike|hCoV-19/England/CAMC-AEB0C5/2020|2020-10-25|EPI_ISL_633129|Original|hCoV-19^^England|Human|Lighthouse Lab in Cambridge|Wellcome Sanger Institute for the COVID-19 Genomics UK (COG-UK) consortium|Davies|United Kingdom
>Spike|hCoV-19/Scotland/EDB7079/2020|2020-08-02|EPI_ISL_514518|Original|hCoV-19^^Scotland|Human|Virology Department|COVID-19 Genomics UK (COG-UK) Consortium|Williams|United Kingdom
>Spike|hCoV-19/USA/FL-BPHL-2160/2020|2020-05-21|EPI_ISL_653313|Original|hCoV-19^^Florida|Human|Florida Bureau of Public Health Laboratories|Florida Bureau of Public Health Laboratories|Blanton|USA
>Spike|hCoV-19/Canada/ON-PHL-20-01260/2020|2020-06-00|EPI_ISL_548875|Original|hCoV-19^^Ontario|Human|Public Health Ontario Laboratory|Public Health Ontario Laboratory|Guthrie|Canada
>Spike|hCoV-19/Japan/PG-8134/2020|2020-08-00|EPI_ISL_687233|Original|hCoV-19^^Japan|Human|Pathogen Genomics Center|Pathogen Genomics Center|Sekizuka|Japan
>Spike|hCoV-19/Japan/PG-1361/2020|2020-04-00|EPI_ISL_684969|Original|hCoV-19^^Japan|Human|Saitama Prefectural Institute of Public Health|Pathogen Genomics Center|Sekizuka|Japan
>Spike|hCoV-19/Northern Ireland/NIRE-100DAE/2020|2020-05-02|EPI_ISL_482146|Original|hCoV-19^^Northern Ireland|Human|Regional Virus Laboratory|Wellcome Sanger Institute for the COVID-19 Genomics UK (COG-UK) consortium|Davies|United Kingdom
>Spike|hCoV-19/England/MILK-C7B3CF/2020|2020-12-15|EPI_ISL_756863|Original|hCoV-19^^England|Human|Lighthouse Lab in Milton Keynes|Wellcome Sanger Institute for the COVID-19 Genomics UK (COG-UK) Consortium|Davies|United Kingdom
>Spike|hCoV-19/England/204591848/2020|2020-00-00|EPI_ISL_637185|Original|hCoV-19^^England|Human|Respiratory Virus Unit|COVID-19 Genomics UK (COG-UK) Consortium|Platt|United Kingdom
>Spike|hCoV-19/Wales/PHWC-2A808/2020|2020-04-07|EPI_ISL_445622|Original|hCoV-19^^Wales|Human|Wales Specialist Virology Centre|Public Health Wales Microbiology Cardiff|Moore|United Kingdom

>Spike|hCoV-19/Belgium/ULG-10560/2020|2020-10-23|EPI_ISL_626328|Original|hCoV-19^^Li\u00e8ge|Human|Department of Clinical Microbiology|GIGA Medical Genomics|Durkin|Belgium
>Spike|hCoV-19/USA/CA-SCCPHD-UC130/2020|2020-03-06|EPI_ISL_435609|Original|hCoV-19^^California|Human|Santa Clara County Public Health Department|Chiu Laboratory|Chiu|USA
>Spike|hCoV-19/USA/NY-NYCPHL-001256/2020|2020-11-06|EPI_ISL_683895|Original|hCoV-19^^New York|Human|DOHMH Riverside|New York City Public Health Laboratory|Wang|USA
>Spike|hCoV-19/England/ALDP-B472DF/2020|2020-11-07|EPI_ISL_656303|Original|hCoV-19^^England|Human|Lighthouse Lab in Alderley Park|Wellcome Sanger Institute for the COVID-19 Genomics UK (COG-UK) Consortium|Davies|United Kingdom
>Spike|hCoV-19/England/BRIS-18535AA/2020|2020-04-08|EPI_ISL_492357|Original|hCoV-19^^England|Human|PHE South West Regional Laboratory|Wellcome Sanger Institute for the COVID-19 Genomics UK (COG-UK) consortium|Davies|United Kingdom
>Spike|hCoV-19/England/BRIS-12358F/2020|2020-04-06|EPI_ISL_442763|Original|hCoV-19^^England|Human|PHE South West Regional Laboratory|Wellcome Sanger Institute for the COVID-19 Genomics UK (COG-UK) consortium|Amato|United Kingdom
>Spike|hCoV-19/England/CAMB-7E1EC/2020|2020-04-15|EPI_ISL_434050|Original|hCoV-19^^England|Human|Department of Pathology|COVID-19 Genomics UK (COG-UK) Consortium|Meredith|United Kingdom
>Spike|hCoV-19/Belgium/ULG-10953/2020|2020-09-06|EPI_ISL_707732|Original|hCoV-19^^Li\u00e8ge|Human|Department of Clinical Microbiology|GIGA Medical Genomics|Durkin|Belgium
>Spike|hCoV-19/USA/FL-BPHL-0373/2020|2020-04-21|EPI_ISL_489782|Original|hCoV-19^^Florida|Human|Florida Bureau of Public Health Laboratories|Florida Bureau of Public Health Laboratories|Blanton|USA
>Spike|hCoV-19/England/ALDP-B1A572/2020|2020-11-01|EPI_ISL_647618|Original|hCoV-19^^England|Human|Lighthouse Lab in Alderley Park|Wellcome Sanger Institute for the COVID-19 Genomics UK (COG-UK) Consortium|Davies|United Kingdom
>Spike|hCoV-19/USA/VA-DCLS-0414/2020|2020-04-26|EPI_ISL_467808|Original|hCoV-19^^Virginia|Human|Virginia DCLS|Virginia DCLS|St. Jacques|USA
>Spike|hCoV-19/Netherlands/UT-EMC-77/2020|2020-08-27|EPI_ISL_577893|Original|hCoV-19^^Utrecht|Human|Dutch COVID-19 response team|Erasmus Medical Center|Oude Munnink|Netherlands
>Spike|hCoV-19/England/CAMC-B98D1A/2020|2020-11-15|EPI_ISL_673582|Original|hCoV-19^^England|Human|Lighthouse Lab in Cambridge|Wellcome Sanger Institute for the COVID-19 Genomics UK (COG-UK) Consortium|Davies|United Kingdom
>Spike|hCoV-19/England/QEUH-B0F947/2020|2020-11-02|EPI_ISL_642050|Original|hCoV-19^^England|Human|Lighthouse Lab in Glasgow|Wellcome Sanger Institute for the COVID-19 Genomics UK (COG-UK) Consortium|Davies|United Kingdom
>Spike|hCoV-19/USA/WI-UW-1577/2020|2020-09-17|EPI_ISL_605454|Original|hCoV-19^^Wisconsin|Human|University of Wisconsin-Madison AIDS Vaccine Research Laboratories|University of Wisconsin-Madison AIDS Vaccine Research Laboratories|Baczenas|USA
>Spike|hCoV-19/Denmark/DCGC-22439/2020|2020-12-14|EPI_ISL_757563|Original|hCoV-19^^Hovedstaden|Human|Department of Virus and Microbiological Special Diagnostics|Albertsen Lab|Michaelsen|Denmark
>Spike|hCoV-19/England/CAMC-9D731F/2020|2020-09-18|EPI_ISL_584362|Original|hCoV-19^^England|Human|Virology Department|COVID-19 Genomics UK (COG-UK) Consortium|de Silva|United Kingdom
>Spike|hCoV-19/Spain/MD-IBV-005323/2020|2020-03-17|EPI_ISL_510202|Original|hCoV-19^^Madrid|Human|Hospital General Universitario Gregorio Mara\u00f1\u00f3n|SeqCOVID-SPAIN consortium/IBV(CSIC)|Comas|Spain
>Spike|hCoV-19/England/CAMB-71E2E/2020|2020-03-29|EPI_ISL_438435|Original|hCoV-19^^England|Human|Department of Pathology|Wellcome Sanger Institute for the COVID-19 Genomics UK (COG-UK) consortium|Amato|United Kingdom
>Spike|hCoV-19/England/PORT-2D2506/2020|2020-03-26|EPI_ISL_507152|Original|hCoV-19^^England|Human|Centre for Enzyme Innovation|COVID-19 Genomics UK (COG-UK) Consortium|Robson|United Kingdom

>Spike|hCoV-19/England/CAMB-77EA0/2020|2020-04-01|EPI_ISL_441185|Original|hCoV-19^^England|Human|Department of Pathology|Wellcome Sanger Institute for the COVID-19 Genomics UK (COG-UK) consortium|Amato|United Kingdom
>Spike|hCoV-19/Wales/CAMC-A58AF2/2020|2020-10-09|EPI_ISL_609162|Original|hCoV-19^^Wales|Human|Lighthouse Lab in Cambridge|Wellcome Sanger Institute for the COVID-19 Genomics UK (COG-UK) consortium|Davies|United Kingdom
>Spike|hCoV-19/Australia/QLD1148/2020|2020-03-29|EPI_ISL_639751|Original|hCoV-19^^Australia|Human|unknown|Public Health Virology Laboratory|Nguyen|Australia
>Spike|hCoV-19/USA/WI-GMF-19746/2020|2020-09-11|EPI_ISL_547845|Original|hCoV-19^^Wisconsin|Human|Gundersen Molecular Diagnostics Laboratory|Kabara Cancer Research Institute|Kenny|USA
>Spike|hCoV-19/USA/WA-UW-3186/2020|2020-03-28|EPI_ISL_491009|Original|hCoV-19^^Washington|Human|UW Virology Lab|UW Virology Lab|Roychoudhury|USA
>Spike|hCoV-19/England/QEUH-A8BE45/2020|2020-10-16|EPI_ISL_643051|Original|hCoV-19^^England|Human|Lighthouse Lab in Glasgow|Wellcome Sanger Institute for the COVID-19 Genomics UK (COG-UK) Consortium|Davies|United Kingdom
>Spike|hCoV-19/England/CAMB-78C5A/2020|2020-04-03|EPI_ISL_441138|Original|hCoV-19^^England|Human|Department of Pathology|Wellcome Sanger Institute for the COVID-19 Genomics UK (COG-UK) consortium|Amato|United Kingdom
>Spike|hCoV-19/USA/TX-HMH-MCoV-10549/2020|2020-07-09|EPI_ISL_789530|Original|hCoV-19^^Texas|Human|Houston Methodist Hospital|Houston Methodist Hospital|Long|USA
>Spike|hCoV-19/England/QEUH-5DB986/2020|2020-06-17|EPI_ISL_533275|Original|hCoV-19^^England|Human|Lighthouse Lab in Glasgow|Wellcome Sanger Institute for the COVID-19 Genomics UK (COG-UK) consortium|Davies|United Kingdom
>Spike|hCoV-19/Denmark/DCGC-7742/2020|2020-10-19|EPI_ISL_618677|Original|hCoV-19^^Syddanmark|Human|Department of Virus and Microbiological Special Diagnostics|Albertsen lab|Michaelsen|Denmark
>Spike|hCoV-19/Wales/PHWC-487920/2020|2020-10-30|EPI_ISL_639254|Original|hCoV-19^^Wales|Human|Wales Specialist Virology Centre Sequencing lab|COVID-19 Genomics UK (COG-UK) Consortium|Moore|United Kingdom
>Spike|hCoV-19/USA/VA-DCLS-1039/2020|2020-06-03|EPI_ISL_526923|Original|hCoV-19^^Virginia|Human|Virginia DCLS|Virginia DCLS|St. Jacques|USA
>Spike|hCoV-19/England/204740425/2020|2020-11-17|EPI_ISL_693465|Original|hCoV-19^^England|Human|Respiratory Virus Unit|COVID-19 Genomics UK (COG-UK) Consortium|Platt|United Kingdom
>Spike|hCoV-19/England/QEUH-B12088/2020|2020-11-02|EPI_ISL_642608|Original|hCoV-19^^England|Human|Lighthouse Lab in Glasgow|Wellcome Sanger Institute for the COVID-19 Genomics UK (COG-UK) Consortium|Davies|United Kingdom
>Spike|hCoV-19/USA/NY-NYUMC653/2020|2020-04-10|EPI_ISL_444770|Original|hCoV-19^^New York|Human|NYU Langone Health|Departments of Pathology and Medicine|Maurano|USA
>Spike|hCoV-19/USA/MN-MDH-1704/2020|2020-04-10|EPI_ISL_560791|Original|hCoV-19^^Minnesota|Human|Minnesota Department of Health|Minnesota Department of Health|Garfin|USA
>Spike|hCoV-19/Canada/ON-PHL-20-01323/2020|2020-06-00|EPI_ISL_548926|Original|hCoV-19^^Ontario|Human|Public Health Ontario Laboratory|Public Health Ontario Laboratory|Guthrie|Canada
>Spike|hCoV-19/USA/WA-S2582/2020|2020-06-18|EPI_ISL_497214|Original|hCoV-19^^Washington|Human|Washington State Department of Health|Seattle Flu Study|Lee|USA
>Spike|hCoV-19/Colombia/DC-INS-2553/2020|2020-08-06|EPI_ISL_653745|Original|hCoV-19^^Bogota|Human|Instituto Nacional de Salud|Instituto Nacional de Salud|Franco-Mu\u00f1oz|Colombia
>Spike|hCoV-19/Russia/OMS-ORINFI-4822S/2020|2020-07-10|EPI_ISL_569800|Original|hCoV-19^^Omsk|Human|Omsk Research Institute of Natural Focal Infections|WHO National Influenza Centre Russian Federation|Komissarov|Russia
>Spike|hCoV-19/Switzerland/SZ-ETHZ-420546/2020|2020-12-22|EPI_ISL_768233|Original|hCoV-19^^Schwyz|Human|Viollier AG|Department of Biosystems Science and Engineering|Nadeau|Switzerland
>Spike|hCoV-19/Wales/PHWC-16B4BB/2020|2020-09-14|EPI_ISL_560294|Original|hCoV-19^^Wales|Human|Wales Specialist Virology Centre Sequencing lab|COVID-19 Genomics UK (COG-UK) Consortium|Moore|United Kingdom

>Spike|hCoV-19/England/CAMB-1B1DCA/2020|2020-05-09|EPI_ISL_458552|Original|hCoV-19^^England|Human|Department of Pathology|Wellcome Sanger Institute for the COVID-19 Genomics UK (COG-UK) consortium|Davies|United Kingdom
>Spike|hCoV-19/Scotland/CVR896/2020|2020-03-27|EPI_ISL_439131|Original|hCoV-19^^Scotland|Human|West of Scotland Specialist Virology Centre|COVID-19 Genomics UK (COG-UK) Consortium|Robertson|United Kingdom
>Spike|hCoV-19/England/CAMB-84927/2020|2020-03-06|EPI_ISL_440481|Original|hCoV-19^^England|Human|Department of Pathology|Wellcome Sanger Institute for the COVID-19 Genomics UK (COG-UK) consortium|Amato|United Kingdom
>Spike|hCoV-19/Australia/NT53/2020|2020-06-30|EPI_ISL_521862|Original|hCoV-19^^Northern Territory|Human|Royal Darwin Hospital Pathology|MDU-PHL|Schultz|Australia
>Spike|hCoV-19/USA/WI-UW-660/2020|2020-06-26|EPI_ISL_491347|Original|hCoV-19^^Wisconsin|Human|University of Wisconsin-Madison AIDS Vaccine Research Laboratories|University of Wisconsin-Madison AIDS Vaccine Research Laboratories|Baczenas|USA
>Spike|hCoV-19/England/LIVE-A263B/2020|2020-05-20|EPI_ISL_500000|Original|hCoV-19^^England|Human|Liverpool Clinical Laboratories|COVID-19 Genomics UK (COG-UK) Consortium|Haldenby|United Kingdom
>Spike|hCoV-19/England/MILK-843D20/2020|2020-07-15|EPI_ISL_557310|Original|hCoV-19^^England|Human|Lighthouse Lab in Milton Keynes|Wellcome Sanger Institute for the COVID-19 Genomics UK (COG-UK) consortium|Davies|United Kingdom
>Spike|hCoV-19/USA/WI-UW-236/2020|2020-03-27|EPI_ISL_428332|Original|hCoV-19^^Wisconsin|Human|University of Wisconsin-Madison AIDS Vaccine Research Laboratories|University of Wisconsin-Madison AIDS Vaccine Research Laboratories|Moreno|USA
>Spike|hCoV-19/Denmark/DCGC-7647/2020|2020-10-19|EPI_ISL_618539|Original|hCoV-19^^Nordjylland|Human|Department of Virus and Microbiological Special Diagnostics|Albertsen lab|Michaelsen|Denmark
>Spike|hCoV-19/England/ALDP-9BE3B4/2020|2020-09-10|EPI_ISL_567971|Original|hCoV-19^^England|Human|Lighthouse Lab in Alderley Park|Wellcome Sanger Institute for the COVID-19 Genomics UK (COG-UK) consortium|Davies|United Kingdom
>Spike|hCoV-19/England/ALDP-B73FC4/2020|2020-11-12|EPI_ISL_662239|Original|hCoV-19^^England|Human|Lighthouse Lab in Alderley Park|Wellcome Sanger Institute for the COVID-19 Genomics UK (COG-UK) Consortium|Davies|United Kingdom
>Spike|hCoV-19/England/MILK-A7D13E/2020|2020-10-13|EPI_ISL_606517|Original|hCoV-19^^England|Human|Lighthouse Lab in Milton Keynes|Wellcome Sanger Institute for the COVID-19 Genomics UK (COG-UK) consortium|Davies|United Kingdom
>Spike|hCoV-19/England/QEUH-C4B577/2020|2020-12-13|EPI_ISL_769724|Original|hCoV-19^^England|Human|Lighthouse Lab in Glasgow|Wellcome Sanger Institute for the COVID-19 Genomics UK (COG-UK) Consortium|Davies|United Kingdom
>Spike|hCoV-19/England/QEUH-A01CB6/2020|2020-10-02|EPI_ISL_595836|Original|hCoV-19^^England|Human|Virology Department|COVID-19 Genomics UK (COG-UK) Consortium|de Silva|United Kingdom
>Spike|hCoV-19/Denmark/DCGC-16294/2020|2020-11-16|EPI_ISL_714285|Original|hCoV-19^^Midtjylland|Human|Department of Virus and Microbiological Special Diagnostics|Albertsen Lab|Michaelsen|Denmark
>Spike|hCoV-19/England/20132022304/2020|2020-03-23|EPI_ISL_423745|Original|hCoV-19^^England|Human|Respiratory Virus Unit|Respiratory Virus Unit|Platt|United Kingdom
>Spike|hCoV-19/USA/NY-QDX-865/2020|2020-03-17|EPI_ISL_571672|Original|hCoV-19^^New York|Human|Quest Diagnostics|Quest Diagnostics|Kagan|USA
>Spike|hCoV-19/USA/CA-CZB-5154/2020|2020-08-22|EPI_ISL_672028|Original|hCoV-19^^California|Human|County of San Luis Obispo Public Health Laboratory|Chan-Zuckerberg Biohub|Bhatt|USA
>Spike|hCoV-19/England/LOND-D9A6C/2020|2020-09-09|EPI_ISL_584674|Original|hCoV-19^^England|Human|University College London|COVID-19 Genomics UK (COG-UK) Consortium|Roy|United Kingdom
>Spike|hCoV-19/Kenya/C6876/2020|2020-05-15|EPI_ISL_568812|Original|hCoV-19^^Taita Taveta|Human|KEMRI-Wellcome Trust Research Programme/KEMRI-CGMR-C Kilifi|KEMRI-Wellcome Trust Research Programme/KEMRI-CGMR-C Kilifi|Githinji|Kenya

>Spike|hCoV-19/England/ALDP-CB4D9F/2020|2020-12-17|EPI_ISL_761203|Original|hCoV-19^^England|Human|Lighthouse Lab in Alderley Park|Wellcome Sanger Institute for the COVID-19 Genomics UK (COG-UK) Consortium|Davies|United Kingdom
>Spike|hCoV-19/USA/HI-H200117/2020|2020-06-10|EPI_ISL_752680|Original|hCoV-19^^Hawaii|Human|State Laboratories Division|State Laboratories Division|Sultana|USA
>Spike|hCoV-19/Wales/PHWC-48AF3A/2020|2020-11-04|EPI_ISL_666175|Original|hCoV-19^^Wales|Human|Wales Specialist Virology Centre Sequencing lab|COVID-19 Genomics UK (COG-UK) Consortium|Moore|United Kingdom
>Spike|hCoV-19/Japan/PG-8497/2020|2020-06-00|EPI_ISL_687596|Original|hCoV-19^^Japan|Human|Pathogen Genomics Center|Pathogen Genomics Center|Sekizuka|Japan
>Spike|hCoV-19/Australia/VIC259/2020|2020-03-21|EPI_ISL_419952|Original|hCoV-19^^Victoria|Human|Victorian Infectious Diseases Reference Laboratory (VIDRL)|Victorian Infectious Diseases Reference Laboratory and Microbiological Diagnostic Unit Public Health Laboratory|Schultz|Australia
>Spike|hCoV-19/Iran/SA-Isf-38/2020|2020-06-10|EPI_ISL_672590|Original|hCoV-19^^Iran|Human|Infectious Diseases and Tropical Medicine Research Center|Infectious Diseases and Tropical Medicine Research Center|Ahangarzadeh,S.|Iran
>Spike|hCoV-19/Denmark/DCGC-2102/2020|2020-07-20|EPI_ISL_618361|Original|hCoV-19^^Hovedstaden|Human|Department of Virus and Microbiological Special Diagnostics|Albertsen lab|Michaelsen|Denmark
>Spike|hCoV-19/England/MILK-B3AA13/2020|2020-11-02|EPI_ISL_679702|Original|hCoV-19^^England|Human|Oxford Viromics|COVID-19 Genomics UK (COG-UK) Consortium|Golubchik|United Kingdom
>Spike|hCoV-19/England/20166019504/2020|2020-04-16|EPI_ISL_528400|Original|hCoV-19^^England|Human|Respiratory Virus Unit|Respiratory Virus Unit|Miah|United Kingdom
>Spike|hCoV-19/USA/FL-BPHL-040/2020|2020-04-16|EPI_ISL_436544|Original|hCoV-19^^Florida|Human|Florida Bureau of Public Health Laboratories|Florida Bureau of Public Health Laboratories|Blanton|USA
>Spike|hCoV-19/USA/TX-HMH-MCoV-14528/2020|2020-10-08|EPI_ISL_787813|Original|hCoV-19^^Texas|Human|Houston Methodist Hospital|Houston Methodist Hospital|Long|USA
>Spike|hCoV-19/mink/Netherlands/NB03_index/2020|2020-05-06|EPI_ISL_447633|Original|hCoV-19^^Netherlands|Mustela lutreola|Virology|Virology|Oreshkova|Netherlands
>Spike|hCoV-19/England/QEUH-B68E7A/2020|2020-11-11|EPI_ISL_662284|Original|hCoV-19^^England|Human|Lighthouse Lab in Glasgow|Wellcome Sanger Institute for the COVID-19 Genomics UK (COG-UK) Consortium|Davies|United Kingdom
>Spike|hCoV-19/Australia/NSW2738/2020|2020-09-02|EPI_ISL_530336|Original|hCoV-19^^New South Wales|Human|Area of Virology|Area of Virology|Yeang|Australia
>Spike|hCoV-19/England/SHEF-10CCEE5/2020|2020-12-23|EPI_ISL_764213|Original|hCoV-19^^England|Human|Virology Department|COVID-19 Genomics UK (COG-UK) Consortium|de Silva|United Kingdom
>Spike|hCoV-19/Belgium/rega-12111338/2020|2020-12-11|EPI_ISL_738391|Original|hCoV-19^^Mol|Human|UZ Leuven|KU Leuven|Maes|Belgium
>Spike|hCoV-19/USA/WA-UW-11115/2020|2020-06-18|EPI_ISL_570376|Original|hCoV-19^^Washington|Human|UW Virology Lab|UW Virology Lab|Roychoudhury|USA
>Spike|hCoV-19/England/ALDP-68397D/2020|2020-07-02|EPI_ISL_551755|Original|hCoV-19^^England|Human|Lighthouse Lab in Alderley Park|Wellcome Sanger Institute for the COVID-19 Genomics UK (COG-UK) consortium|Davies|United Kingdom
>Spike|hCoV-19/England/ALDP-A9268F/2020|2020-10-19|EPI_ISL_610949|Original|hCoV-19^^England|Human|Lighthouse Lab in Alderley Park|Wellcome Sanger Institute for the COVID-19 Genomics UK (COG-UK) consortium|Davies|United Kingdom
>Spike|hCoV-19/England/ALDP-50631B/2020|2020-06-25|EPI_ISL_559272|Original|hCoV-19^^England|Human|Lighthouse Lab in Alderley Park|Wellcome Sanger Institute for the COVID-19 Genomics UK (COG-UK) consortium|Davies|United Kingdom
>Spike|hCoV-19/Switzerland/ZH-UZH-IMV4/2020|2020-03-11|EPI_ISL_590826|Vero P2|hCoV-19^^Zurich|Human|Institute of Medical Virology|Institute of Medical Virology|Kufner|Switzerland
>Spike|hCoV-19/Portugal/PT0361/2020|2020-03-19|EPI_ISL_454077|Original|hCoV-19^^Portugal|Human|unknown|Instituto Nacional de Saude (INSA)|Borges|Portugal

>Spike|hCoV-19/USA/MA-MASPHL-00928/2020|2020-05-28|EPI_ISL_692825|Original|hCoV-19^^Massachusetts|Human|Massachusetts State Public Health Laboratory|Massachusetts State Public Health Laboratory|Lang|USA
>Spike|hCoV-19/Russia/SPE-RII-MH1500S/2020|2020-09-11|EPI_ISL_596282|Original|hCoV-19^^Saint Petersburg|Human|HELIX LCC|WHO National Influenza Centre Russian Federation|Komissarov|Russia
>Spike|hCoV-19/Japan/PG-5187/2020|2020-08-00|EPI_ISL_690290|Original|hCoV-19^^Japan|Human|Pathogen Genomics Center|Pathogen Genomics Center|Sekizuka|Japan
>Spike|hCoV-19/Beijing/DT-travelUSA04/2020|2020-03-22|EPI_ISL_452339|Original|hCoV-19^^Beijing|Human|Laboratory of Infectious Diseases Center of Beijing Ditan Hospital|Laboratory of Infectious Diseases Center of Beijing Ditan Hospital|Yang|China
>Spike|hCoV-19/England/EXET-139F5B/2020|2020-00-00|EPI_ISL_679354|Original|hCoV-19^^England|Human|University of Exeter|COVID-19 Genomics UK (COG-UK) Consortium|Temperton|United Kingdom
>Spike|hCoV-19/USA/VA-DCLS-0743/2020|2020-06-12|EPI_ISL_526863|Original|hCoV-19^^Virginia|Human|Virginia DCLS|Virginia DCLS|St. Jacques|USA
>Spike|hCoV-19/England/QEUH-B68EB6/2020|2020-11-11|EPI_ISL_662037|Original|hCoV-19^^England|Human|Lighthouse Lab in Glasgow|Wellcome Sanger Institute for the COVID-19 Genomics UK (COG-UK) Consortium|Davies|United Kingdom
>Spike|hCoV-19/USA/NC-QDX-253/2020|2020-04-30|EPI_ISL_498732|Original|hCoV-19^^North Carolina|Human|Quest Diagnostics|Quest Diagnostics|Kagan|USA
>Spike|hCoV-19/Netherlands/NA_308/2020|2020-04-02|EPI_ISL_422617|Original|hCoV-19^^Netherlands|Human|Dutch COVID-19 response team|Erasmus Medical Center|Oude Munnink|Netherlands
>Spike|hCoV-19/Denmark/DCGC-12706/2020|2020-11-16|EPI_ISL_682887|Original|hCoV-19^^Sjaelland|Human|Department of Virus and Microbiological Special Diagnostics|Albertsen Lab|Michaelsen|Denmark
>Spike|hCoV-19/England/CAMC-BD2172/2020|2020-11-28|EPI_ISL_702087|Original|hCoV-19^^England|Human|Lighthouse Lab in Cambridge|Wellcome Sanger Institute for the COVID-19 Genomics UK (COG-UK) Consortium|Davies|United Kingdom
>Spike|hCoV-19/Wales/PHWC-2ECDB/2020|2020-04-06|EPI_ISL_446139|Original|hCoV-19^^Wales|Human|Wales Specialist Virology Centre|Public Health Wales Microbiology Cardiff|Moore|United Kingdom
>Spike|hCoV-19/Scotland/QEUH-A769A6/2020|2020-10-15|EPI_ISL_613340|Original|hCoV-19^^Scotland|Human|Queens Medical Centre|COVID-19 Genomics UK (COG-UK) Consortium|Loose|United Kingdom
>Spike|hCoV-19/England/EXET-13DF0C/2020|2020-12-03|EPI_ISL_724300|Original|hCoV-19^^England|Human|University of Exeter|COVID-19 Genomics UK (COG-UK) Consortium|Temperton|United Kingdom
>Spike|hCoV-19/Switzerland/BL-ETHZ-240022/2020|2020-08-10|EPI_ISL_523873|Original|hCoV-19^^Basel-Landschaft|Human|Viollier AG|Department of Biosystems Science and Engineering|Nadeau|Switzerland
>Spike|hCoV-19/England/MILK-ACCFDC/2020|2020-10-20|EPI_ISL_630337|Original|hCoV-19^^England|Human|Lighthouse Lab in Milton Keynes|Wellcome Sanger Institute for the COVID-19 Genomics UK (COG-UK) consortium|Davies|United Kingdom
>Spike|hCoV-19/Denmark/DCGC-22078/2020|2020-12-14|EPI_ISL_758540|Original|hCoV-19^^Hovedstaden|Human|Department of Virus and Microbiological Special Diagnostics|Albertsen Lab|Michaelsen|Denmark
>Spike|hCoV-19/Russia/VOR-RII-MH2793S/2020|2020-09-28|EPI_ISL_602455|Original|hCoV-19^^Voronezh|Human|HELIX LLC|WHO National Influenza Centre Russian Federation|Komissarov|Russia
>Spike|hCoV-19/USA/FL-BPHL-0670/2020|2020-03-13|EPI_ISL_509728|Original|hCoV-19^^Florida|Human|Florida Bureau of Public Health Laboratories|Florida Bureau of Public Health Laboratories|Blanton|USA
>Spike|hCoV-19/England/MILK-9AAA6B/2020|2020-08-31|EPI_ISL_550908|Original|hCoV-19^^England|Human|Lighthouse Lab in Milton Keynes|Wellcome Sanger Institute for the COVID-19 Genomics UK (COG-UK) consortium|Davies|United Kingdom

>Spike|hCoV-19/England/MILK-B8EA5C/2020|2020-11-13|EPI_ISL_674663|Original|hCoV-19^^England|Human|Lighthouse Lab in Milton Keynes|Wellcome Sanger Institute for the COVID-19 Genomics UK (COG-UK) Consortium|Davies|United Kingdom
>Spike|hCoV-19/Luxembourg/LNS5121437/2020|2020-08-31|EPI_ISL_744187|Original|hCoV-19^^Luxembourg|Human|Laboratoire national de sant\u00e9|Laboratoire national de sant\u00e9|Wienecke-Baldacchino|Luxembourg
>Spike|hCoV-19/USA/CA-CZB-14279/2020|2020-08-27|EPI_ISL_739426|Original|hCoV-19^^California|Human|Alameda County Public Health Lab|Chan-Zuckerberg Biohub|Kamm|USA
>Spike|hCoV-19/Scotland/EDB112/2020|2020-03-22|EPI_ISL_433407|Original|hCoV-19^^Scotland|Human|Virology Department|COVID-19 Genomics UK (COG-UK) Consortium|Williams|United Kingdom
>Spike|hCoV-19/England/CAMC-B77F1B/2020|2020-11-12|EPI_ISL_783503|Original|hCoV-19^^England|Human|Lighthouse Lab in Cambridge|Wellcome Sanger Institute for the COVID-19 Genomics UK (COG-UK) Consortium|Davies|United Kingdom
>Spike|hCoV-19/USA/NY-Wadsworth-251061-01/2020|2020-10-21|EPI_ISL_754693|Original|hCoV-19^^New York|Human|Wadsworth Center|Wadsworth Center|Lasek-Nesselquist|USA
>Spike|hCoV-19/England/CAMB-1ACF9F/2020|2020-04-23|EPI_ISL_489522|Original|hCoV-19^^England|Human|Department of Pathology|Wellcome Sanger Institute for the COVID-19 Genomics UK (COG-UK) consortium|Davies|United Kingdom
>Spike|hCoV-19/England/CAMC-AEACE0/2020|2020-10-25|EPI_ISL_633267|Original|hCoV-19^^England|Human|Lighthouse Lab in Cambridge|Wellcome Sanger Institute for the COVID-19 Genomics UK (COG-UK) consortium|Davies|United Kingdom
>Spike|hCoV-19/USA/MN-MDH-1437/2020|2020-03-31|EPI_ISL_514656|Original|hCoV-19^^Minnesota|Human|Mayo Clinic & Mayo Clinic Laboratories|Minnesota Department of Health|Garfin|USA
>Spike|hCoV-19/USA/UT-UPHL-2009331/2020|2020-05-16|EPI_ISL_524932|Original|hCoV-19^^Utah|Human|Utah Public Health Laboratory|Utah Public Health Laboratory|Young|USA
>Spike|hCoV-19/Belgium/ULG-10146/2020|2020-04-17|EPI_ISL_447138|Original|hCoV-19^^Liege|Human|Department of Clinical Microbiology|GIGA Medical Genomics|Durkin|Belgium
>Spike|hCoV-19/USA/NY-WCMP12E06P/2020|2020-03-16|EPI_ISL_427607|Original|hCoV-19^^New York|Human|NewYork-Presbyterian & Mason Lab|Mason Lab|Mason|USA
>Spike|hCoV-19/Australia/VIC15176/2020|2020-08-11|EPI_ISL_593172|Original|hCoV-19^^Victoria|Human|Microbiological Diagnostic Unit - Public Health Laboratory (MDU-PHL)|MDU-PHL|Schultz|Australia
>Spike|hCoV-19/Denmark/DCGC-19682/2020|2020-12-07|EPI_ISL_749108|Original|hCoV-19^^Sjaelland|Human|Department of Virus and Microbiological Special Diagnostics|Albertsen Lab|Michaelsen|Denmark
>Spike|hCoV-19/Luxembourg/LNS5638426/2020|2020-11-24|EPI_ISL_744267|Original|hCoV-19^^Luxembourg|Human|Laboratoire national de sant\u00e9|Laboratoire national de sant\u00e9|Wienecke-Baldacchino|Luxembourg
>Spike|hCoV-19/England/NOTT-112161/2020|2020-06-20|EPI_ISL_484348|Original|hCoV-19^^England|Human|Queens Medical Centre|COVID-19 Genomics UK (COG-UK) Consortium|Loose|United Kingdom
>Spike|hCoV-19/Wales/MILK-9C1E53/2020|2020-09-10|EPI_ISL_566471|Original|hCoV-19^^Wales|Human|Lighthouse Lab in Milton Keynes|Wellcome Sanger Institute for the COVID-19 Genomics UK (COG-UK) consortium|Davies|United Kingdom
>Spike|hCoV-19/Scotland/QEUH-99B4D8/2020|2020-08-30|EPI_ISL_559996|Original|hCoV-19^^Scotland|Human|Oxford Viromics|COVID-19 Genomics UK (COG-UK) Consortium|Golubchik|United Kingdom
>Spike|hCoV-19/Scotland/QEUH-9D0431/2020|2020-09-16|EPI_ISL_601811|Original|hCoV-19^^Scotland|Human|Lighthouse Lab in Glasgow|Wellcome Sanger Institute for the COVID-19 Genomics UK (COG-UK) consortium|Davies|United Kingdom
>Spike|hCoV-19/Iran/SA-Isf-33/2020|2020-04-12|EPI_ISL_672585|Original|hCoV-19^^Iran|Human|Infectious Diseases and Tropical Medicine Research Center|Infectious Diseases and Tropical Medicine Research Center|Haghjooy Javanmard,S.|Iran
>Spike|hCoV-19/England/LIVE-A1AF4/2020|2020-06-15|EPI_ISL_490353|Original|hCoV-19^^England|Human|Liverpool Clinical Laboratories|COVID-19 Genomics UK (COG-UK) Consortium|Haldenby|United Kingdom

>Spike|hCoV-19/Germany/BE-ChVir-W1048-4346/2020|2020-03-27|EPI_ISL_753926|Original|hCoV-19^^Berlin|Human|Charit\u00e9 Universit\u00e4tsmedizin Berlin|Charit\u00e9 Universit\u00e4tsmedizin Berlin|Muehlemann|Germany
>Spike|hCoV-19/Jordan/AM-ALSR-5307/2020|2020-11-02|EPI_ISL_730543|Original|hCoV-19^^Amman|Human|Biolab Diagnostic Laboratories|Andersen lab at Scripps Research|Gangavarapu|Jordan
>Spike|hCoV-19/England/204390706/2020|2020-10-22|EPI_ISL_649873|Original|hCoV-19^^England|Human|Respiratory Virus Unit|COVID-19 Genomics UK (COG-UK) Consortium|Platt|United Kingdom
>Spike|hCoV-19/Spain/PV-IBV-98008428/2020|2020-10-21|EPI_ISL_654602|Original|hCoV-19^^Basque Country|Human|Servicio de Microbiolog\u00eda. Hospital Universitario Donostia. OSI Donostialdea. \u00c1rea de Enfermedades Infecciosas|SeqCOVID-SPAIN consortium/IBV(CSIC)|Comas|Spain
>Spike|hCoV-19/Luxembourg/LNS8187762/2020|2020-10-22|EPI_ISL_744699|Original|hCoV-19^^Luxembourg|Human|Laboratoire national de sant\u00e9|Laboratoire national de sant\u00e9|Wienecke-Baldacchino|Luxembourg
>Spike|hCoV-19/Japan/PG-8484/2020|2020-08-00|EPI_ISL_687583|Original|hCoV-19^^Japan|Human|Pathogen Genomics Center|Pathogen Genomics Center|Sekizuka|Japan
>Spike|hCoV-19/Wales/PHWC-48DABE/2020|2020-11-01|EPI_ISL_680722|Original|hCoV-19^^Wales|Human|Wales Specialist Virology Centre Sequencing lab|COVID-19 Genomics UK (COG-UK) Consortium|Moore|United Kingdom
>Spike|hCoV-19/Wales/ALDP-BB9D22/2020|2020-11-20|EPI_ISL_710739|Original|hCoV-19^^Wales|Human|Lighthouse Lab in Alderley Park|Wellcome Sanger Institute for the COVID-19 Genomics UK (COG-UK) Consortium|Davies|United Kingdom
>Spike|hCoV-19/Scotland/CVR1952/2020|2020-04-08|EPI_ISL_438861|Original|hCoV-19^^Scotland|Human|West of Scotland Specialist Virology Centre|COVID-19 Genomics UK (COG-UK) Consortium|Robertson|United Kingdom
>Spike|hCoV-19/England/OXON-AFCE1/2020|2020-00-00|EPI_ISL_478821|Original|hCoV-19^^England|Human|Oxford Viromics|COVID-19 Genomics UK (COG-UK) Consortium|Golubchik|United Kingdom
>Spike|hCoV-19/Latvia/095/2020|2020-07-21|EPI_ISL_512650|Original|hCoV-19^^Latvia|Human|Latvijas Infektolo\u0123ijas centrs|Latvian Biomedical Research and Study Centre|Zrelovs|Latvia
>Spike|hCoV-19/England/LOND-12E85CE/2020|2020-12-15|EPI_ISL_741331|Original|hCoV-19^^England|Human|University College London|COVID-19 Genomics UK (COG-UK) Consortium|Roy|United Kingdom
>Spike|hCoV-19/Denmark/DCGC-14855/2020|2020-11-23|EPI_ISL_712030|Original|hCoV-19^^Hovedstaden|Human|Department of Virus and Microbiological Special Diagnostics|Albertsen Lab|Michaelsen|Denmark
>Spike|hCoV-19/France/ARA-17747/2020|2020-03-31|EPI_ISL_508960|Original|hCoV-19^^Auvergne-Rh\u00f4ne-Alpes|Human|Centre Hospitalier Saint Joseph Saint Luc|CNR Virus des Infections Respiratoires - France SUD|Josset|France
>Spike|hCoV-19/England/ALDP-9EA9EA/2020|2020-09-24|EPI_ISL_580028|Original|hCoV-19^^England|Human|Lighthouse Lab in Alderley Park|Wellcome Sanger Institute for the COVID-19 Genomics UK (COG-UK) consortium|Davies|United Kingdom
>Spike|hCoV-19/England/20149051704/2020|2020-04-02|EPI_ISL_466205|Original|hCoV-19^^England|Human|Respiratory Virus Unit|Respiratory Virus Unit|Platt|United Kingdom
>Spike|hCoV-19/USA/MA-MGH-00231/2020|2020-04-06|EPI_ISL_460345|Original|hCoV-19^^Massachusetts|Human|Massachusetts General Hospital|Infectious Disease Program|Lemieux|USA
>Spike|hCoV-19/South Africa/NHLS-UCT-GP-5597/2020|2020-05-12|EPI_ISL_700595|Original|hCoV-19^^Western Cape|Human|Heideveld Emergency Centre|NHLS/UCT|Iranzadeh|South Africa
>Spike|hCoV-19/England/QEUH-B68F86/2020|2020-11-11|EPI_ISL_661976|Original|hCoV-19^^England|Human|Lighthouse Lab in Glasgow|Wellcome Sanger Institute for the COVID-19 Genomics UK (COG-UK) Consortium|Davies|United Kingdom
>Spike|hCoV-19/England/MILK-B8C757/2020|2020-11-13|EPI_ISL_675896|Original|hCoV-19^^England|Human|Lighthouse Lab in Milton Keynes|Wellcome Sanger Institute for the COVID-19 Genomics UK (COG-UK) Consortium|Davies|United Kingdom

>Spike|hCoV-19/Switzerland/NE-ETHZ-350329/2020|2020-11-03|EPI_ISL_729194|Original|hCoV-19^^Neuch\u00e2tel|Human|Viollier AG|Department of Biosystems Science and Engineering|Nadeau|Switzerland
>Spike|hCoV-19/England/MILK-AB84A7/2020|2020-10-21|EPI_ISL_623435|Original|hCoV-19^^England|Human|Lighthouse Lab in Milton Keynes|Wellcome Sanger Institute for the COVID-19 Genomics UK (COG-UK) consortium|Davies|United Kingdom
>Spike|hCoV-19/Finland/21SS13C8/2020|2020-09-21|EPI_ISL_757380|Original|hCoV-19^^Uusimaa|Human|Department of Virology and Immunology|Department of Virology|Smura|Finland
>Spike|hCoV-19/Australia/VIC14708/2020|2020-08-06|EPI_ISL_592962|Original|hCoV-19^^Victoria|Human|Microbiological Diagnostic Unit - Public Health Laboratory (MDU-PHL)|MDU-PHL|Schultz|Australia
>Spike|hCoV-19/USA/AZ-TG410819/2020|2020-06-24|EPI_ISL_694970|Original|hCoV-19^^Arizona|Human|TGen North|TGen North|French|USA
>Spike|hCoV-19/USA/AZ-TG547955/2020|2020-07-27|EPI_ISL_695523|Original|hCoV-19^^Arizona|Human|TGen North|TGen North|French|USA
>Spike|hCoV-19/USA/TX-HMH-4136/2020|2020-06-18|EPI_ISL_543400|Original|hCoV-19^^Texas|Human|Houston Methodist Hospital|Houston Methodist Hospital|Long|USA
>Spike|hCoV-19/USA/MN-MDH-1779/2020|2020-04-13|EPI_ISL_590781|Original|hCoV-19^^Minnesota|Human|Minnesota Department of Health|Minnesota Department of Health|Lorentz|USA
>Spike|hCoV-19/USA/CA-CHLA-PLM14086335/2020|2020-12-01|EPI_ISL_753574|Original|hCoV-19^^California|Human|Clinical virology Laboratory|Center for Personalized Medicine|Dien Bard|USA
>Spike|hCoV-19/Japan/PG-2911/2020|2020-04-00|EPI_ISL_691037|Original|hCoV-19^^Japan|Human|Pathogen Genomics Center|Pathogen Genomics Center|Sekizuka|Japan
>Spike|hCoV-19/England/ALDP-9B5915/2020|2020-08-28|EPI_ISL_590284|Original|hCoV-19^^England|Human|Lighthouse Lab in Alderley Park|Wellcome Sanger Institute for the COVID-19 Genomics UK (COG-UK) consortium|Davies|United Kingdom
>Spike|hCoV-19/Northern Ireland/QEUH-96220D/2020|2020-08-10|EPI_ISL_531627|Original|hCoV-19^^Northern Ireland|Human|Lighthouse Lab in Glasgow|Wellcome Sanger Institute for the COVID-19 Genomics UK (COG-UK) consortium|Davies|United Kingdom
>Spike|hCoV-19/India/OR-ILSCV30220/2020|2020-05-23|EPI_ISL_481143|Original|hCoV-19^^Odisha|Human|Immunogenomics lab|Immunogenomics lab|Raghav|India
>Spike|hCoV-19/Switzerland/BS-ETHZ-250129/2020|2020-08-15|EPI_ISL_535636|Original|hCoV-19^^Basel-Stadt|Human|Viollier AG|Department of Biosystems Science and Engineering|Nadeau|Switzerland
>Spike|hCoV-19/USA/TX-HMH-MCoV-16273/2020|2020-10-27|EPI_ISL_785957|Original|hCoV-19^^Texas|Human|Houston Methodist Hospital|Houston Methodist Hospital|Long|USA
>Spike|hCoV-19/USA/AZ-TG668390/2020|2020-04-20|EPI_ISL_696296|Original|hCoV-19^^Arizona|Human|Sonora Quest Laboratories|TGen North|French|USA
>Spike|hCoV-19/England/SHEF-10CBFD4/2020|2020-12-20|EPI_ISL_741986|Original|hCoV-19^^England|Human|Virology Department|COVID-19 Genomics UK (COG-UK) Consortium|de Silva|United Kingdom
>Spike|hCoV-19/USA/TX-HMH-MCoV-15470/2020|2020-10-23|EPI_ISL_786954|Original|hCoV-19^^Texas|Human|Houston Methodist Hospital|Houston Methodist Hospital|Long|USA
>Spike|hCoV-19/England/ALDP-B485B7/2020|2020-11-07|EPI_ISL_649397|Original|hCoV-19^^England|Human|Lighthouse Lab in Alderley Park|Wellcome Sanger Institute for the COVID-19 Genomics UK (COG-UK) Consortium|Davies|United Kingdom
>Spike|hCoV-19/India/UT-AR17/2020|2020-05-27|EPI_ISL_508164|Original|hCoV-19^^Uttarakhand|Human|All india institute of Medical Sciences Rishikesh|National Institute of Biomedical Genomics|Maitra|India
>Spike|hCoV-19/Australia/VIC10118/2020|2020-08-11|EPI_ISL_562795|Original|hCoV-19^^Victoria|Human|Microbiological Diagnostic Unit - Public Health Laboratory (MDU-PHL)|MDU-PHL|Schultz|Australia
>Spike|hCoV-19/USA/CA-ALSR-4261/2020|2020-08-21|EPI_ISL_648921|Original|hCoV-19^^California|Human|San Diego County Public Health Laboratory|Andersen lab at Scripps Research|Gangavarapu|USA

>Spike|hCoV-19/Singapore/409/2020|2020-04-22|EPI_ISL_475940|Original|hCoV-19^^Singapore|Human|National Public Health Laboratory|National Public Health Laboratory|Mak|Singapore
>Spike|hCoV-19/Iceland/79/2020|2020-03-09|EPI_ISL_417679|Original|hCoV-19^^Reykjavik|Human|The National University Hospital of Iceland|deCODE genetics|J\u00f3nsson|Iceland
>Spike|hCoV-19/Spain/CT-IBV-99009896/2020|2020-08-07|EPI_ISL_660293|Original|hCoV-19^^Catalunya|Human|Servicio de Microbiolog\u00eda|SeqCOVID-SPAIN consortium/IBV(CSIC)|Comas|Spain
>Spike|hCoV-19/Australia/VIC13755/2020|2020-08-30|EPI_ISL_563826|Original|hCoV-19^^Victoria|Human|Microbiological Diagnostic Unit - Public Health Laboratory (MDU-PHL)|MDU-PHL|Schultz|Australia
>Spike|hCoV-19/USA/WA-S1265/2020|2020-05-13|EPI_ISL_495715|Original|hCoV-19^^Washington|Human|Washington State Department of Health|Seattle Flu Study|Lee|USA
>Spike|hCoV-19/England/204521588/2020|2020-11-02|EPI_ISL_637183|Original|hCoV-19^^England|Human|Respiratory Virus Unit|COVID-19 Genomics UK (COG-UK) Consortium|Platt|United Kingdom
>Spike|hCoV-19/Australia/VIC8461/2020|2020-07-27|EPI_ISL_565031|Original|hCoV-19^^Victoria|Human|Microbiological Diagnostic Unit - Public Health Laboratory (MDU-PHL)|MDU-PHL|Schultz|Australia
>Spike|hCoV-19/Wales/PHWC-3182C/2020|2020-04-10|EPI_ISL_446494|Original|hCoV-19^^Wales|Human|Wales Specialist Virology Centre|Public Health Wales Microbiology Cardiff|Moore|United Kingdom
>Spike|hCoV-19/Wales/PHWC-494D15/2020|2020-11-22|EPI_ISL_726244|Original|hCoV-19^^Wales|Human|Originating lab|COVID-19 Genomics UK (COG-UK) Consortium|Moore|United Kingdom
>Spike|hCoV-19/Germany/HH-hpi-p2447/2020|2020-04-21|EPI_ISL_776510|Original|hCoV-19^^Hamburg|Human|University Medical Center Hamburg Eppendorf|Heinrich Pette Institute|Robitaille|Germany
>Spike|hCoV-19/Spain/CL-IBV-97006342/2020|2020-05-26|EPI_ISL_537689|Original|hCoV-19^^Castilla y Leon|Human|Universidad de Le\u00f3n|SeqCOVID-SPAIN consortium/IBV(CSIC)|Comas|Spain
>Spike|hCoV-19/Puerto Rico/CDC-S19/2020|2020-04-01|EPI_ISL_434553|Original|hCoV-19^^Puerto Rico|Human|Puerto Rico Department of Health|Centers for Disease Control and Prevention|Santiago|USA
>Spike|hCoV-19/USA/NY-WCMP12F07P/2020|2020-03-16|EPI_ISL_427612|Original|hCoV-19^^New York|Human|NewYork-Presbyterian & Mason Lab|Mason Lab|Mason|USA
>Spike|hCoV-19/Scotland/EDB8405/2020|2020-10-15|EPI_ISL_612354|Original|hCoV-19^^Scotland|Human|Virology Department|COVID-19 Genomics UK (COG-UK) Consortium|McHugh|United Kingdom
>Spike|hCoV-19/England/ALDP-5B30AD/2020|2020-06-18|EPI_ISL_558630|Original|hCoV-19^^England|Human|Lighthouse Lab in Alderley Park|Wellcome Sanger Institute for the COVID-19 Genomics UK (COG-UK) consortium|Davies|United Kingdom
>Spike|hCoV-19/USA/TX-DSHS-1135/2020|2020-06-17|EPI_ISL_634939|Original|hCoV-19^^Texas|Human|Texas Department of State Health Services|Texas Department of State Health Services|Rahman|USA
>Spike|hCoV-19/England/QEUH-9C9D6D/2020|2020-09-10|EPI_ISL_567280|Original|hCoV-19^^England|Human|Lighthouse Lab in Glasgow|Wellcome Sanger Institute for the COVID-19 Genomics UK (COG-UK) consortium|Davies|United Kingdom
>Spike|hCoV-19/Denmark/DCGC-8787/2020|2020-10-26|EPI_ISL_626073|Original|hCoV-19^^Hovedstaden|Human|Department of Virus and Microbiological Special Diagnostics|Albertsen lab|Michaelsen|Denmark
>Spike|hCoV-19/Denmark/DCGC-795/2020|2020-04-13|EPI_ISL_614711|Original|hCoV-19^^Hovedstaden|Human|Department of Virus and Microbiological Special Diagnostics|Albertsen lab|Michaelsen|Denmark
>Spike|hCoV-19/England/NORW-EC7F6/2020|2020-05-13|EPI_ISL_457564|Original|hCoV-19^^England|Human|Quadram Institute Bioscience|COVID-19 Genomics UK (COG-UK) Consortium|Alikhan|United Kingdom

>Spike|hCoV-19/Scotland/CVR3941/2020|2020-07-03|EPI_ISL_490705|Original|hCoV-19^^Scotland|Human|West of Scotland Specialist Virology Centre|COVID-19 Genomics UK (COG-UK) Consortium|Robertson|United Kingdom
>Spike|hCoV-19/Japan/PG-1050/2020|2020-04-00|EPI_ISL_684660|Original|hCoV-19^^Japan|Human|Fukuoka Institute of Health and Environmental Sciences|Pathogen Genomics Center|Sekizuka|Japan
>Spike|hCoV-19/Mali/M002673/2020|2020-04-10|EPI_ISL_487455|Original|hCoV-19^^Bamako|Human|CICM-Mali|Bundeswehr Institut of Microbiology|Antwerpen|Mali
>Spike|hCoV-19/Italy/LAZ-INMI-37/2020|2020-06-12|EPI_ISL_603144|Original|hCoV-19^^Lazio|Human|INMI Lazzaro Spallanzani IRCCS|INMI Lazzaro Spallanzani IRCCS|Gruber|Italy
>Spike|hCoV-19/Japan/PG-7253/2020|2020-08-00|EPI_ISL_688356|Original|hCoV-19^^Japan|Human|Saitama Prefectural Institute of Public Health|Pathogen Genomics Center|Sekizuka|Japan
>Spike|hCoV-19/South Korea/KCDC2709/2020|2020-07-08|EPI_ISL_522488|Original|hCoV-19^^South Korea|Human|Division of Viral Diseases|Division of Viral Diseases|Kim|South Korea
>Spike|hCoV-19/England/204742214/2020|2020-00-00|EPI_ISL_649920|Original|hCoV-19^^England|Human|Respiratory Virus Unit|COVID-19 Genomics UK (COG-UK) Consortium|Platt|United Kingdom
>Spike|hCoV-19/England/ALDP-BB3C58/2020|2020-11-20|EPI_ISL_701828|Original|hCoV-19^^England|Human|Lighthouse Lab in Alderley Park|Wellcome Sanger Institute for the COVID-19 Genomics UK (COG-UK) Consortium|Davies|United Kingdom
>Spike|hCoV-19/Nigeria/AK-CV319/2020|2020-07-17|EPI_ISL_729990|Original|hCoV-19^^Akwa-Ibom State|Human|Nigeria Centre for Disease Control (NCDC)|African Centre of Excellence for Genomics of Infectious Diseases (ACEGID)|Oluniyi|Nigeria
>Spike|hCoV-19/England/MILK-C5263E/2020|2020-12-11|EPI_ISL_736308|Original|hCoV-19^^England|Human|Lighthouse Lab in Milton Keynes|Wellcome Sanger Institute for the COVID-19 Genomics UK (COG-UK) Consortium|Davies|United Kingdom
>Spike|hCoV-19/England/CAMB-75BBA/2020|2020-03-29|EPI_ISL_438338|Original|hCoV-19^^England|Human|Department of Pathology|Wellcome Sanger Institute for the COVID-19 Genomics UK (COG-UK) consortium|Amato|United Kingdom
>Spike|hCoV-19/England/QEUH-9C7AFF/2020|2020-09-11|EPI_ISL_567160|Original|hCoV-19^^England|Human|Lighthouse Lab in Glasgow|Wellcome Sanger Institute for the COVID-19 Genomics UK (COG-UK) consortium|Davies|United Kingdom
>Spike|hCoV-19/England/MILK-9E18E6/2020|2020-09-19|EPI_ISL_577091|Original|hCoV-19^^England|Human|Oxford Viromics|COVID-19 Genomics UK (COG-UK) Consortium|Golubchik|United Kingdom
>Spike|hCoV-19/Australia/VIC3641/2020|2020-07-08|EPI_ISL_520381|Original|hCoV-19^^Victoria|Human|Victorian Infectious Diseases Reference Laboratory (VIDRL)|VIDRL and MDU-PHL|Schultz|Australia
>Spike|hCoV-19/Japan/PG-0970/2020|2020-04-00|EPI_ISL_684580|Original|hCoV-19^^Japan|Human|Pathogen Genomics Center|Pathogen Genomics Center|Sekizuka|Japan
>Spike|hCoV-19/USA/NY-Wadsworth-16833-01/2020|2020-03-15|EPI_ISL_426316|Original|hCoV-19^^New York|Human|Wadsworth Center|Wadsworth Center|Lasek-Nesselquist|USA
>Spike|hCoV-19/England/SHEF-CFB39/2020|2020-10-15|EPI_ISL_611783|Original|hCoV-19^^England|Human|Virology Department|COVID-19 Genomics UK (COG-UK) Consortium|de Silva|United Kingdom
>Spike|hCoV-19/Wales/PHWC-4A642A/2020|2020-12-16|EPI_ISL_765036|Original|hCoV-19^^Wales|Human|Wales Specialist Virology Centre Sequencing lab|COVID-19 Genomics UK (COG-UK) Consortium|Moore|United Kingdom
>Spike|hCoV-19/USA/WI-GMF-10913/2020|2020-07-15|EPI_ISL_547620|Original|hCoV-19^^Wisconsin|Human|Gundersen Molecular Diagnostics Laboratory|Kabara Cancer Research Institute|Kenny|USA
>Spike|hCoV-19/England/ALDP-B25204/2020|2020-11-04|EPI_ISL_646493|Original|hCoV-19^^England|Human|Lighthouse Lab in Alderley Park|Wellcome Sanger Institute for the COVID-19 Genomics UK (COG-UK) Consortium|Davies|United Kingdom
>Spike|hCoV-19/France/OCC-110/2020|2020-10-23|EPI_ISL_671956|Original|hCoV-19^^Occitanie|Human|CHU Purpan - Laboratoire de Virologie - Institut

F\u00e9d\u00e9ratif de Biologie|CHU Purpan - Laboratoire de Virologie - Institut F\u00e9d\u00e9ratif de Biologie|Latour|France
>Spike|hCoV-19/England/LEED-2A8633/2020|2020-03-20|EPI_ISL_539131|Original|hCoV-19^^England|Human|Leeds Teaching Hospitals NHS Trust and Public Health England|Wellcome Sanger Institute for the COVID-19 Genomics UK (COG-UK) consortium|Davies|United Kingdom
>Spike|hCoV-19/Denmark/DCGC-10300/2020|2020-11-02|EPI_ISL_669885|Original|hCoV-19^^Syddanmark|Human|Department of Virus and Microbiological Special Diagnostics|Albertsen Lab|Michaelsen|Denmark
>Spike|hCoV-19/Uganda/UG065/2020|2020-08-26|EPI_ISL_737981|Original|hCoV-19^^Uganda|Human|Uganda Central Public Health Lab and Uganda Virus Research Institute|MRC/UVRI & LSHTM Uganda\u00a0Research Unit|Cotten|Uganda
>Spike|hCoV-19/England/ALDP-94E0FE/2020|2020-06-16|EPI_ISL_555338|Original|hCoV-19^^England|Human|Lighthouse Lab in Alderley Park|Wellcome Sanger Institute for the COVID-19 Genomics UK (COG-UK) consortium|Davies|United Kingdom
>Spike|hCoV-19/Germany/SH-ChVir3196/2020|2020-03-22|EPI_ISL_729540|Original|hCoV-19^^Schleswig-Holstein|Human|A. Krumbholz|Charit\u00e9 Universit\u00e4tsmedizin Berlin|Muehlemann|Germany
>Spike|hCoV-19/India/TG-CCMB-OM5/2020|2020-05-14|EPI_ISL_495292|Original|hCoV-19^^Telangana|Human|Osmania Medical College|CSIR-Centre for Cellular and Molecular Biology|Banu|India
>Spike|hCoV-19/England/CAMC-A82224/2020|2020-10-12|EPI_ISL_610830|Original|hCoV-19^^England|Human|Lighthouse Lab in Cambridge|Wellcome Sanger Institute for the COVID-19 Genomics UK (COG-UK) consortium|Davies|United Kingdom
>Spike|hCoV-19/Netherlands/Utrecht_30/2020|2020-04-20|EPI_ISL_461228|Original|hCoV-19^^Utrecht|Human|Dutch COVID-19 response team|Erasmus Medical Center|Oude Munnink|Netherlands
>Spike|hCoV-19/USA/NY-Wadsworth-41097-01/2020|2020-04-08|EPI_ISL_676817|Original|hCoV-19^^New York|Human|Wadsworth Center|Wadsworth Center|Lasek-Nesselquist|USA
>Spike|hCoV-19/England/ALDP-BBA116/2020|2020-11-20|EPI_ISL_701817|Original|hCoV-19^^England|Human|Lighthouse Lab in Alderley Park|Wellcome Sanger Institute for the COVID-19 Genomics UK (COG-UK) Consortium|Davies|United Kingdom
>Spike|hCoV-19/USA/TX-HMH-MCoV-13052/2020|2020-08-27|EPI_ISL_788203|Original|hCoV-19^^Texas|Human|Houston Methodist Hospital|Houston Methodist Hospital|Long|USA
>Spike|hCoV-19/Japan/PG-2346/2020|2020-05-00|EPI_ISL_685951|Original|hCoV-19^^Japan|Human|Pathogen Genomics Center|Pathogen Genomics Center|Sekizuka|Japan
>Spike|hCoV-19/Northern Ireland/OXON-F40533/2020|2020-12-08|EPI_ISL_764532|Original|hCoV-19^^Northern Ireland|Human|Oxford Viromics|COVID-19 Genomics UK (COG-UK) Consortium|Golubchik|United Kingdom
>Spike|hCoV-19/Singapore/36nan/2020|2020-02-16|EPI_ISL_538435|Original|hCoV-19^^Singapore|Human|Department of Laboratory Medicine|Department of Laboratory Medicine|Chen|Singapore
>Spike|hCoV-19/USA/FL-BPHL-1212/2020|2020-07-25|EPI_ISL_541185|Original|hCoV-19^^Florida|Human|Florida Bureau of Public Health Laboratories|Florida Bureau of Public Health Laboratories|Blanton|USA
>Spike|hCoV-19/England/20170036004/2020|2020-04-14|EPI_ISL_465284|Original|hCoV-19^^England|Human|Respiratory Virus Unit|Respiratory Virus Unit|Platt|United Kingdom
>Spike|hCoV-19/England/QEUH-C31B45/2020|2020-12-11|EPI_ISL_782675|Original|hCoV-19^^England|Human|Lighthouse Lab in Glasgow|Wellcome Sanger Institute for the COVID-19 Genomics UK (COG-UK) Consortium|Davies|United Kingdom
>Spike|hCoV-19/USA/TX-HMH-MCoV-15180/2020|2020-10-19|EPI_ISL_786664|Original|hCoV-19^^Texas|Human|Houston Methodist Hospital|Houston Methodist Hospital|Long|USA
>Spike|hCoV-19/USA/MI-MDHHS-SC22125/2020|2020-10-06|EPI_ISL_614176|Original|hCoV-19^^Michigan|Human|Michigan Department of Health and Human Services|Michigan Department of Health and Human Services|Jacob|USA
>Spike|hCoV-19/Denmark/DCGC-1840/2020|2020-07-13|EPI_ISL_622605|Original|hCoV-19^^Hovedstaden|Human|Department of Virus and Microbiological Special Diagnostics|Albertsen lab|Michaelsen|Denmark

>Spike|hCoV-19/Wales/PHWC-33F43/2020|2020-04-17|EPI_ISL_446844|Original|hCoV-19^^Wales|Human|Wales Specialist Virology Centre|Public Health Wales Microbiology Cardiff|Moore|United Kingdom
>Spike|hCoV-19/England/LIVE-A88E4/2020|2020-03-26|EPI_ISL_517229|Original|hCoV-19^^England|Human|Liverpool Clinical Laboratories|COVID-19 Genomics UK (COG-UK) Consortium|Haldenby|United Kingdom
>Spike|hCoV-19/England/ALDP-95CAB3/2020|2020-06-21|EPI_ISL_554933|Original|hCoV-19^^England|Human|Lighthouse Lab in Alderley Park|Wellcome Sanger Institute for the COVID-19 Genomics UK (COG-UK) consortium|Davies|United Kingdom
>Spike|hCoV-19/England/SHEF-10D2D1C/2020|2020-12-14|EPI_ISL_742050|Original|hCoV-19^^England|Human|Virology Department|COVID-19 Genomics UK (COG-UK) Consortium|de Silva|United Kingdom
>Spike|hCoV-19/Canada/BC-BCCDC-8621/2020|2020-11-00|EPI_ISL_740856|Original|hCoV-19^^British Columbia|Human|BCCDC Public Health Laboratory|BCCDC Public Health Laboratory|Prystajecky|Canada
>Spike|hCoV-19/USA/CA-SEARCH-5573/2020|2020-11-25|EPI_ISL_755300|Original|hCoV-19^^California|Human|San Diego County Public Health Laboratory|Andersen lab at Scripps Research|Gangavarapu|USA
>Spike|hCoV-19/USA/NY-Wadsworth-287495-01/2020|2020-12-15|EPI_ISL_767462|Original|hCoV-19^^New York|Human|Wadsworth Center|Wadsworth Center|Lasek-Nesselquist|USA
>Spike|hCoV-19/USA/UT-UPHL-201225377/2020|2020-12-01|EPI_ISL_684007|Original|hCoV-19^^Utah|Human|Utah Public Health Laboratory|Utah Public Health Laboratory|Young|USA
>Spike|hCoV-19/England/LIVE-DCB30B/2020|2020-11-23|EPI_ISL_724397|Original|hCoV-19^^England|Human|Liverpool Clinical Laboratories|COVID-19 Genomics UK (COG-UK) Consortium|Haldenby|United Kingdom
>Spike|hCoV-19/England/QEUH-9F6E91/2020|2020-09-27|EPI_ISL_595640|Original|hCoV-19^^England|Human|Oxford Viromics|COVID-19 Genomics UK (COG-UK) Consortium|Golubchik|United Kingdom
>Spike|hCoV-19/England/ALDP-95AD43/2020|2020-06-16|EPI_ISL_555680|Original|hCoV-19^^England|Human|Lighthouse Lab in Alderley Park|Wellcome Sanger Institute for the COVID-19 Genomics UK (COG-UK) consortium|Davies|United Kingdom
>Spike|hCoV-19/England/QEUH-9F51B7/2020|2020-09-25|EPI_ISL_637856|Original|hCoV-19^^England|Human|Department of Pathology|COVID-19 Genomics UK (COG-UK) Consortium|Goodfellow|United Kingdom
>Spike|hCoV-19/England/NORT-294B86/2020|2020-03-28|EPI_ISL_490467|Original|hCoV-19^^England|Human|Northumbria University / South Tees Hospitals NHS Foundation Trust / North Cumbria Integrated Care NHS Foundation Trust / North Tees and Hartlepool NHS Foundation Trust / Newcastle Hospitals NHS Foundation Trust|COVID-19 Genomics UK (COG-UK) Consortium|Bashton|United Kingdom
>Spike|hCoV-19/Scotland/CVR3911/2020|2020-06-15|EPI_ISL_512461|Original|hCoV-19^^Scotland|Human|West of Scotland Specialist Virology Centre|COVID-19 Genomics UK (COG-UK) Consortium|Robertson|United Kingdom
>Spike|hCoV-19/England/QEUH-A8A9AF/2020|2020-10-16|EPI_ISL_638866|Original|hCoV-19^^England|Human|Oxford Viromics|COVID-19 Genomics UK (COG-UK) Consortium|Golubchik|United Kingdom
>Spike|hCoV-19/England/QEUH-C30FFF/2020|2020-12-08|EPI_ISL_731398|Original|hCoV-19^^England|Human|Lighthouse Lab in Glasgow|Wellcome Sanger Institute for the COVID-19 Genomics UK (COG-UK) Consortium|Davies|United Kingdom
>Spike|hCoV-19/England/CAMB-7EA0F/2020|2020-04-08|EPI_ISL_442609|Original|hCoV-19^^England|Human|Department of Pathology|Wellcome Sanger Institute for the COVID-19 Genomics UK (COG-UK) consortium|Amato|United Kingdom
>Spike|hCoV-19/Lithuania/KL-LUHS-Eilnr59/2020|2020-04-10|EPI_ISL_603095|Original|hCoV-19^^Klaipedos apskritis|Human|Lithuanian University of Health Sciences Hospital|Lithuanian University of Health Sciences|Zemaitis|Lithuania
>Spike|hCoV-19/England/ALDP-A6C4A2/2020|2020-10-14|EPI_ISL_608207|Original|hCoV-19^^England|Human|Lighthouse Lab in Alderley Park|Wellcome Sanger Institute for the COVID-19 Genomics UK (COG-UK) consortium|Davies|United Kingdom

>Spike|hCoV-19/Sweden/20-53305/2020|2020-10-27|EPI_ISL_676516|Original|hCoV-19^^Gavleborg|Human|Laboratoriemedicin|The Public Health Agency of Sweden|Svartstr\u00f6m|Sweden
>Spike|hCoV-19/England/BRIS-12F5ED/2020|2020-03-03|EPI_ISL_488202|Original|hCoV-19^^England|Human|PHE South West Regional Laboratory|Wellcome Sanger Institute for the COVID-19 Genomics UK (COG-UK) consortium|Davies|United Kingdom
>Spike|hCoV-19/India/GJ-GBRC65b/2020|2020-05-03|EPI_ISL_447041|Original|hCoV-19^^Gujarat|Human|B.J. Medical College and Civil hospital|Gujarat Biotechnology Research Centre|Joshi|India
>Spike|hCoV-19/Scotland/CVR5704/2020|2020-11-06|EPI_ISL_651922|Original|hCoV-19^^Scotland|Human|West of Scotland Specialist Virology Centre|COVID-19 Genomics UK (COG-UK) Consortium|Robertson|United Kingdom
>Spike|hCoV-19/Spain/PV-IBV-005172/2020|2020-06-26|EPI_ISL_509651|Original|hCoV-19^^Basque Country|Human|Servicio de Microbiolog\u00eda. Hospital Universitario Donostia. OSI Donostialdea. \u00c1rea de Enfermedades Infecciosas|SeqCOVID-SPAIN consortium/IBV(CSIC)|Comas|Spain
>Spike|hCoV-19/Australia/VIC3494/2020|2020-07-04|EPI_ISL_520250|Original|hCoV-19^^Victoria|Human|Victorian Infectious Diseases Reference Laboratory (VIDRL)|VIDRL and MDU-PHL|Schultz|Australia
>Spike|hCoV-19/USA/CA-CZB-14690/2020|2020-11-17|EPI_ISL_738603|Original|hCoV-19^^California|Human|Orange County Public Health Lab|Chan-Zuckerberg Biohub|Kamm|USA
>Spike|hCoV-19/England/EXET-136B69/2020|2020-04-24|EPI_ISL_457250|Original|hCoV-19^^England|Human|University of Exeter|COVID-19 Genomics UK (COG-UK) Consortium|Temperton|United Kingdom
>Spike|hCoV-19/Kenya/C4717/2020|2020-05-08|EPI_ISL_568737|Original|hCoV-19^^Mombasa|Human|KEMRI-Wellcome Trust Research Programme/KEMRI-CGMR-C Kilifi|KEMRI-Wellcome Trust Research Programme/KEMRI-CGMR-C Kilifi|Githinji|Kenya
>Spike|hCoV-19/England/LOND-126377A/2020|2020-11-01|EPI_ISL_679475|Original|hCoV-19^^England|Human|University College London|COVID-19 Genomics UK (COG-UK) Consortium|Roy|United Kingdom
>Spike|hCoV-19/Wales/PHWC-4A3038/2020|2020-12-14|EPI_ISL_744043|Original|hCoV-19^^Wales|Human|Wales Specialist Virology Centre Sequencing lab|COVID-19 Genomics UK (COG-UK) Consortium|Moore|United Kingdom
>Spike|hCoV-19/Wales/QEUH-A5DE6A/2020|2020-10-12|EPI_ISL_609206|Original|hCoV-19^^Wales|Human|Lighthouse Lab in Glasgow|Wellcome Sanger Institute for the COVID-19 Genomics UK (COG-UK) consortium|Davies|United Kingdom
>Spike|hCoV-19/England/CAMB-7D1DE/2020|2020-04-06|EPI_ISL_442051|Original|hCoV-19^^England|Human|Department of Pathology|Wellcome Sanger Institute for the COVID-19 Genomics UK (COG-UK) consortium|Amato|United Kingdom
>Spike|hCoV-19/USA/MI-MDHHS-SC22566/2020|2020-11-10|EPI_ISL_737138|Original|hCoV-19^^Michigan|Human|Michigan Department of Health and Human Services|Michigan Department of Health and Human Services|Blankenship|USA
>Spike|hCoV-19/England/QEUH-B806ED/2020|2020-11-14|EPI_ISL_668360|Original|hCoV-19^^England|Human|Lighthouse Lab in Glasgow|Wellcome Sanger Institute for the COVID-19 Genomics UK (COG-UK) Consortium|Davies|United Kingdom
>Spike|hCoV-19/England/ALDP-B5C2D7/2020|2020-11-10|EPI_ISL_655681|Original|hCoV-19^^England|Human|Lighthouse Lab in Alderley Park|Wellcome Sanger Institute for the COVID-19 Genomics UK (COG-UK) Consortium|Davies|United Kingdom
>Spike|hCoV-19/England/QEUH-98F677/2020|2020-08-27|EPI_ISL_539988|Original|hCoV-19^^England|Human|Lighthouse Lab in Glasgow|Wellcome Sanger Institute for the COVID-19 Genomics UK (COG-UK) consortium|Davies|United Kingdom
>Spike|hCoV-19/Portugal/PT2061/2020|2020-11-10|EPI_ISL_732275|Original|hCoV-19^^Portugal|Human|Instituto Nacional de Saude (INSA) and Instituto Gulbenkian de Ciencia (IGC)|Instituto Nacional de Saude (INSA) and Instituto Gulbenkian de Ciencia (IGC)|Borges|Portugal
>Spike|hCoV-19/England/NORW-F1961/2020|2020-11-15|EPI_ISL_725125|Original|hCoV-19^^England|Human|Quadram Institute Bioscience|COVID-19 Genomics UK (COG-UK) Consortium|Alikhan|United Kingdom
>Spike|hCoV-19/England/20144012904/2020|2020-03-31|EPI_ISL_421931|Original|hCoV-19^^England|Human|Respiratory Virus Unit|Respiratory Virus Unit|Platt|United Kingdom

>Spike|hCoV-19/England/QEUH-AD1D34/2020|2020-10-26|EPI_ISL_629996|Original|hCoV-
19^^England|Human|Lighthouse Lab in Glasgow|Wellcome Sanger Institute for the COVID-
19 Genomics UK (COG-UK) consortium|Davies|United Kingdom
>Spike|hCoV-19/Wales/PHWC-4898B8/2020|2020-10-27|EPI_ISL_652420|Original|hCoV-
19^^Wales|Human|Wales Specialist Virology Centre Sequencing lab|COVID-19 Genomics UK
(COG-UK) Consortium|Moore|United Kingdom
>Spike|hCoV-19/England/BRIS-125383/2020|2020-03-26|EPI_ISL_443907|Original|hCoV-
19^^England|Human|PHE South West Regional Laboratory|Wellcome Sanger Institute for
the COVID-19 Genomics UK (COG-UK) consortium|Amato|United Kingdom
>Spike|hCoV-19/England/MILK-B54F7E/2020|2020-11-06|EPI_ISL_657862|Original|hCoV-
19^^England|Human|Lighthouse Lab in Milton Keynes|Wellcome Sanger Institute for the
COVID-19 Genomics UK (COG-UK) Consortium|Davies|United Kingdom
>Spike|hCoV-19/USA/TX-HMH-MCoV-9992/2020|2020-07-06|EPI_ISL_780372|Original|hCoV-
19^^Texas|Human|Houston Methodist Hospital|Houston Methodist Hospital|Long|USA
>Spike|hCoV-19/USA/UT-UPHL-2012889221/2020|2020-08-13|EPI_ISL_745915|Original|hCoV-
19^^Utah|Human|Ginkgo Bioworks Clinical Laboratory|Utah Public Health
Laboratory|Young|USA
>Spike|hCoV-19/England/QEUH-A1DBF6/2020|2020-09-29|EPI_ISL_587453|Original|hCoV-
19^^England|Human|Lighthouse Lab in Glasgow|Wellcome Sanger Institute for the COVID-
19 Genomics UK (COG-UK) consortium|Davies|United Kingdom
>Spike|hCoV-19/USA/TX-HMH-MCoV-8650/2020|2020-07-01|EPI_ISL_780677|Original|hCoV-
19^^Texas|Human|Houston Methodist Hospital|Houston Methodist Hospital|Long|USA
>Spike|hCoV-19/England/CAMB-1B3232/2020|2020-05-21|EPI_ISL_456754|Original|hCoV-
19^^England|Human|Department of Pathology|COVID-19 Genomics UK (COG-UK)
Consortium|Meredith|United Kingdom
>Spike|hCoV-19/Spain/CT-IBV-005604/2020|2020-03-26|EPI_ISL_508650|Original|hCoV-
19^^Catalunya|Human|Departamento de Microbiolog\u00eda|SeqCOVID-SPAIN
consortium/IBV(CSIC)|Comas|Spain
>Spike|hCoV-19/Switzerland/ZH-ETHZ-260055/2020|2020-08-
24|EPI_ISL_539382|Original|hCoV-19^^Zurich|Human|Viollier AG|Department of Biosystems
Science and Engineering|Nadeau|Switzerland
>Spike|hCoV-19/Denmark/DCGC-17080/2020|2020-12-14|EPI_ISL_750746|Original|hCoV-
19^^Midtjylland|Human|Department of Virus and Microbiological Special
Diagnostics|Albertsen Lab|Michaelsen|Denmark
>Spike|hCoV-19/England/QEUH-969F4B/2020|2020-08-13|EPI_ISL_530916|Original|hCoV-
19^^England|Human|Lighthouse Lab in Glasgow|Wellcome Sanger Institute for the COVID-
19 Genomics UK (COG-UK) consortium|Davies|United Kingdom
>Spike|hCoV-19/USA/WA-S427/2020|2020-03-28|EPI_ISL_434187|Original|hCoV-
19^^Washington|Human|Washington State Department of Health|Seattle Flu
Study|Bedford|USA
>Spike|hCoV-19/Denmark/DCGC-5740/2020|2020-09-28|EPI_ISL_621245|Original|hCoV-
19^^Sjaelland|Human|Department of Virus and Microbiological Special
Diagnostics|Albertsen lab|Michaelsen|Denmark
>Spike|hCoV-19/Netherlands/ZH-RIVM-10133/2020|2020-00-
00|EPI_ISL_547568|Original|hCoV-19^^Zuid Holland|Human|Dutch COVID-19 response
team|National Institute for Public Health and the Environment
(RIVM)|Meijer|Netherlands
>Spike|hCoV-19/England/QEUH-9C900A/2020|2020-09-11|EPI_ISL_567217|Original|hCoV-
19^^England|Human|Lighthouse Lab in Glasgow|Wellcome Sanger Institute for the COVID-
19 Genomics UK (COG-UK) consortium|Davies|United Kingdom
>Spike|hCoV-19/Portugal/PT0975/2020|2020-03-27|EPI_ISL_511524|Original|hCoV-
19^^Portugal|Human|Instituto Nacional de Saude (INSA)|Instituto Nacional de Saude
(INSA)|Borges|Portugal
>Spike|hCoV-19/USA/TX-HMH-1278/2020|2020-04-15|EPI_ISL_542625|Original|hCoV-
19^^Texas|Human|Houston Methodist Hospital|Houston Methodist Hospital|Long|USA
>Spike|hCoV-19/England/MILK-ACBF82/2020|2020-10-20|EPI_ISL_629267|Original|hCoV-
19^^England|Human|Lighthouse Lab in Milton Keynes|Wellcome Sanger Institute for the
COVID-19 Genomics UK (COG-UK) consortium|Davies|United Kingdom

>Spike|hCoV-19/England/C200049042/2020|2020-00-00|EPI_ISL_683456|Original|hCoV-19^^England|Human|Respiratory Virus Unit|COVID-19 Genomics UK (COG-UK) Consortium|Platt|United Kingdom
>Spike|hCoV-19/England/MILK-512376/2020|2020-06-10|EPI_ISL_559043|Original|hCoV-19^^England|Human|Lighthouse Lab in Milton Keynes|Wellcome Sanger Institute for the COVID-19 Genomics UK (COG-UK) consortium|Davies|United Kingdom
>Spike|hCoV-19/England/ALDP-9535F7/2020|2020-06-08|EPI_ISL_553867|Original|hCoV-19^^England|Human|Lighthouse Lab in Alderley Park|Wellcome Sanger Institute for the COVID-19 Genomics UK (COG-UK) consortium|Davies|United Kingdom
>Spike|hCoV-19/England/20124097004/2020|2020-03-13|EPI_ISL_465794|Original|hCoV-19^^England|Human|Respiratory Virus Unit|Respiratory Virus Unit|Platt|United Kingdom
>Spike|hCoV-19/England/QEUH-B302BB/2020|2020-11-04|EPI_ISL_659846|Original|hCoV-19^^England|Human|Lighthouse Lab in Glasgow|Wellcome Sanger Institute for the COVID-19 Genomics UK (COG-UK) Consortium|Davies|United Kingdom
>Spike|hCoV-19/England/MILK-B88700/2020|2020-11-14|EPI_ISL_674359|Original|hCoV-19^^England|Human|Lighthouse Lab in Milton Keynes|Wellcome Sanger Institute for the COVID-19 Genomics UK (COG-UK) Consortium|Davies|United Kingdom
>Spike|hCoV-19/Belgium/ULG-10552/2020|2020-10-23|EPI_ISL_626304|Original|hCoV-19^^Li\u00e8ge|Human|Department of Clinical Microbiology|GIGA Medical Genomics|Durkin|Belgium
>Spike|hCoV-19/England/QEUH-9F57A6/2020|2020-09-25|EPI_ISL_588824|Original|hCoV-19^^England|Human|Lighthouse Lab in Glasgow|Wellcome Sanger Institute for the COVID-19 Genomics UK (COG-UK) consortium|Davies|United Kingdom
>Spike|hCoV-19/Denmark/ALAB-SSI215/2020|2020-03-11|EPI_ISL_429407|Original|hCoV-19^^Denmark|Human|Department of Virus and Microbiological Special Diagnostics|Albertsen lab|Kirkegaard|Denmark
>Spike|hCoV-19/England/201140359/2020|2020-03-11|EPI_ISL_464728|Original|hCoV-19^^England|Human|Respiratory Virus Unit|Respiratory Virus Unit|Platt|United Kingdom
>Spike|hCoV-19/Japan/TK-YE611821/2020|2020-04-00|EPI_ISL_486891|Original|hCoV-19^^Tokyo|Human|Tokyo Metoropolitan Institute of Public Health|Tokyo Metoropolitan Institute of Public Health|Nagashima|Japan
>Spike|hCoV-19/USA/FL-BPHL-1573/2020|2020-09-15|EPI_ISL_568671|Original|hCoV-19^^Florida|Human|Florida Bureau of Public Health Laboratories|Florida Bureau of Public Health Laboratories|Blanton|USA
>Spike|hCoV-19/Canada/ON-PHL-20-01237/2020|2020-06-00|EPI_ISL_548859|Original|hCoV-19^^Ontario|Human|Public Health Ontario Laboratory|Public Health Ontario Laboratory|Guthrie|Canada
>Spike|hCoV-19/Scotland/CVR4018/2020|2020-08-29|EPI_ISL_535117|Original|hCoV-19^^Scotland|Human|West of Scotland Specialist Virology Centre|COVID-19 Genomics UK (COG-UK) Consortium|Robertson|United Kingdom
>Spike|hCoV-19/USA/MI-MDHHS-SC21421/2020|2020-03-25|EPI_ISL_507675|Original|hCoV-19^^Michigan|Human|Michigan Department of Health and Human Services|Michigan Department of Health and Human Services|Blankenship|USA
>Spike|hCoV-19/Denmark/DCGC-15230/2020|2020-11-23|EPI_ISL_713710|Original|hCoV-19^^Hovedstaden|Human|Department of Virus and Microbiological Special Diagnostics|Albertsen Lab|Michaelsen|Denmark
>Spike|hCoV-19/USA/AZ-TG670148/2020|2020-04-29|EPI_ISL_696352|Original|hCoV-19^^Arizona|Human|Sonora Quest Laboratories|TGen North|French|USA
>Spike|hCoV-19/England/QEUH-B136A3/2020|2020-11-02|EPI_ISL_642272|Original|hCoV-19^^England|Human|Lighthouse Lab in Glasgow|Wellcome Sanger Institute for the COVID-19 Genomics UK (COG-UK) Consortium|Davies|United Kingdom
>Spike|hCoV-19/Switzerland/BS-42210896/2020|2020-03-28|EPI_ISL_581807|Original|hCoV-19^^Basel-Stadt|Human|University Hospital Basel|University Hospital Basel|Roloff Handschin|Switzerland
>Spike|hCoV-19/Scotland/EDB5781/2020|2020-04-27|EPI_ISL_487890|Original|hCoV-19^^Scotland|Human|Virology Department|Wellcome Sanger Institute for the COVID-19 Genomics UK (COG-UK) consortium|Davies|United Kingdom
>Spike|hCoV-19/England/CAMB-1AFB7A/2020|2020-05-02|EPI_ISL_459194|Original|hCoV-19^^England|Human|Department of Pathology|Wellcome Sanger Institute for the COVID-19 Genomics UK (COG-UK) consortium|Davies|United Kingdom

>Spike|hCoV-19/Switzerland/BE-ETHZ-100054/2020|2020-03-
12|EPI_ISL_466976|Original|hCoV-19^^Bern|Human|Viollier AG|Department of Biosystems
Science and Engineering|Nadeau|Switzerland
>Spike|hCoV-19/USA/NM-DOH-2020631397/2020|2020-12-19|EPI_ISL_766899|Original|hCoV-
19^^New Mexico|Human|New Mexico Department of Health Scientific Laboratory|New Mexico
Department of Health Scientific Laboratory|Malone|USA
>Spike|hCoV-19/England/QEUH-B2B3A1/2020|2020-11-03|EPI_ISL_646766|Original|hCoV-
19^^England|Human|Lighthouse Lab in Glasgow|Wellcome Sanger Institute for the COVID-
19 Genomics UK (COG-UK) Consortium|Davies|United Kingdom
>Spike|hCoV-19/USA/FL-BPHL-0513/2020|2020-06-12|EPI_ISL_508716|Original|hCoV-
19^^Florida|Human|Florida Bureau of Public Health Laboratories|Florida Bureau of
Public Health Laboratories|Blanton|USA
>Spike|hCoV-19/Germany/BY-MVP-0076/2020|2020-03-30|EPI_ISL_437271|Original|hCoV-
19^^Bavaria|Human|Max von Pettenkofer Institute|Laboratory for Functional Genome
Analysis|Graf|Germany
>Spike|hCoV-19/England/CAMB-81BF7/2020|2020-04-11|EPI_ISL_442541|Original|hCoV-
19^^England|Human|Department of Pathology|Wellcome Sanger Institute for the COVID-19
Genomics UK (COG-UK) consortium|Amato|United Kingdom
>Spike|hCoV-19/Spain/IB-IBV-99010835/2020|2020-08-24|EPI_ISL_691645|Original|hCoV-
19^^Balear Islands|Human|Servicio de Microbiolog\u00eda|SeqCOVID-SPAIN
consortium/IBV(CSIC)|Comas|Spain
>Spike|hCoV-19/USA/MI-MDHHS-SC20874/2020|2020-05-15|EPI_ISL_471895|Original|hCoV-
19^^Michigan|Human|Michigan Department of Health and Human Services|Michigan
Department of Health and Human Services|Blankenship|USA
>Spike|hCoV-19/South Africa/KRISP-K004636/2020|2020-11-
01|EPI_ISL_660652|Original|hCoV-19^^KwaZulu-Natal|Human|NHLS-IALCH|KRISP|de
Oliveira|South Africa
>Spike|hCoV-19/USA/TX-HMH-MCoV-12033/2020|2020-09-19|EPI_ISL_790176|Original|hCoV-
19^^Texas|Human|Houston Methodist Hospital|Houston Methodist Hospital|Long|USA
>Spike|hCoV-19/USA/DE-DHSS-F917887/2020|2020-03-25|EPI_ISL_584013|Original|hCoV-
19^^Delaware|Human|Delaware Public Health Lab|Delaware Public Health Lab|Bajwa|USA
>Spike|hCoV-19/Denmark/DCGC-16321/2020|2020-11-16|EPI_ISL_714702|Original|hCoV-
19^^Hovedstaden|Human|Department of Virus and Microbiological Special
Diagnostics|Albertsen Lab|Michaelsen|Denmark
>Spike|hCoV-19/France/PAC-IHU-0617/2020|2020-04-12|EPI_ISL_568890|Original|hCoV-
19^^Provence-Alpes-C\u00f4te-d\u2019Azur|Human|MEPHI|MEPHI|Levasseur|France
>Spike|hCoV-19/Denmark/DCGC-17094/2020|2020-12-14|EPI_ISL_750753|Original|hCoV-
19^^Midtjylland|Human|Department of Virus and Microbiological Special
Diagnostics|Albertsen Lab|Michaelsen|Denmark
>Spike|hCoV-19/South Africa/KRISP-K002848/2020|2020-09-
01|EPI_ISL_602664|Original|hCoV-19^^KwaZulu-Natal|Human|NHLS-IALCH|KRISP|de
Oliveira|South Africa
>Spike|hCoV-19/England/NORT-29349B/2020|2020-06-22|EPI_ISL_499502|Original|hCoV-
19^^England|Human|Northumbria University / South Tees Hospitals NHS Foundation Trust
/ North Cumbria Integrated Care NHS Foundation Trust / North Tees and Hartlepool NHS
Foundation Trust / Newcastle Hospitals NHS Foundation Trust|COVID-19 Genomics UK
(COG-UK) Consortium|Bashton|United Kingdom
>Spike|hCoV-19/England/LEED-2AA07F/2020|2020-04-08|EPI_ISL_538921|Original|hCoV-
19^^England|Human|Leeds Teaching Hospitals NHS Trust and Public Health
England|Wellcome Sanger Institute for the COVID-19 Genomics UK (COG-UK)
consortium|Davies|United Kingdom
>Spike|hCoV-19/England/ALDP-94C5C4/2020|2020-06-15|EPI_ISL_555874|Original|hCoV-
19^^England|Human|Lighthouse Lab in Alderley Park|Wellcome Sanger Institute for the
COVID-19 Genomics UK (COG-UK) consortium|Davies|United Kingdom
>Spike|hCoV-19/England/CAMB-79460/2020|2020-04-03|EPI_ISL_441114|Original|hCoV-
19^^England|Human|Department of Pathology|Wellcome Sanger Institute for the COVID-19
Genomics UK (COG-UK) consortium|Amato|United Kingdom
>Spike|hCoV-19/Portugal/PT0915/2020|2020-03-28|EPI_ISL_511172|Original|hCoV-
19^^Portugal|Human|Instituto Nacional de Saude (INSA)|Instituto Nacional de Saude
(INSA)|Borges|Portugal

>Spike|hCoV-19/Scotland/CVR143/2020|2020-03-17|EPI_ISL_425687|Original|hCoV-19^^Scotland|Human|West of Scotland Specialist Virology Centre|COVID-19 Genomics UK (COG-UK) Consortium|Robertson|United Kingdom
>Spike|hCoV-19/England/MILK-B3D676/2020|2020-11-02|EPI_ISL_645438|Original|hCoV-19^^England|Human|Lighthouse Lab in Milton Keynes|Wellcome Sanger Institute for the COVID-19 Genomics UK (COG-UK) Consortium|Davies|United Kingdom
>Spike|hCoV-19/England/204590464/2020|2020-11-06|EPI_ISL_683505|Original|hCoV-19^^England|Human|Respiratory Virus Unit|COVID-19 Genomics UK (COG-UK) Consortium|Platt|United Kingdom
>Spike|hCoV-19/Northern Ireland/QEUH-963EF9/2020|2020-08-12|EPI_ISL_536843|Original|hCoV-19^^Northern Ireland|Human|Lighthouse Lab in Glasgow|Wellcome Sanger Institute for the COVID-19 Genomics UK (COG-UK) consortium|Davies|United Kingdom
>Spike|hCoV-19/Japan/PG-7846/2020|2020-08-00|EPI_ISL_686947|Original|hCoV-19^^Japan|Human|Pathogen Genomics Center|Pathogen Genomics Center|Sekizuka|Japan
>Spike|hCoV-19/Luxembourg/LNS9388829/2020|2020-10-26|EPI_ISL_744901|Original|hCoV-19^^Luxembourg|Human|Laboratoire national de sant\u00e9|Laboratoire national de sant\u00e9|Wienecke-Baldacchino|Luxembourg
>Spike|hCoV-19/England/PORT-2D46D4/2020|2020-10-08|EPI_ISL_613291|Original|hCoV-19^^England|Human|Centre for Enzyme Innovation|COVID-19 Genomics UK (COG-UK) Consortium|Robson|United Kingdom
>Spike|hCoV-19/England/EXET-136A7B/2020|2020-04-27|EPI_ISL_457245|Original|hCoV-19^^England|Human|University of Exeter|COVID-19 Genomics UK (COG-UK) Consortium|Temperton|United Kingdom
>Spike|hCoV-19/Wales/PHWC-15D197/2020|2020-03-30|EPI_ISL_472677|Original|hCoV-19^^Wales|Human|Wales Specialist Virology Centre Sequencing lab|COVID-19 Genomics UK (COG-UK) Consortium|Moore|United Kingdom
>Spike|hCoV-19/USA/MA-MGH-03417/2020|2020-11-11|EPI_ISL_765827|Original|hCoV-19^^Massachusetts|Human|Massachusetts General Hospital|Infectious Disease Program|Park|USA
>Spike|hCoV-19/USA/WI-UW-1499/2020|2020-09-11|EPI_ISL_541626|Original|hCoV-19^^Wisconsin|Human|University of Wisconsin-Madison AIDS Vaccine Research Laboratories|University of Wisconsin-Madison AIDS Vaccine Research Laboratories|Baczenas|USA
>Spike|hCoV-19/England/QEUH-BA0429/2020|2020-11-18|EPI_ISL_719730|Original|hCoV-19^^England|Human|Lighthouse Lab in Glasgow|Wellcome Sanger Institute for the COVID-19 Genomics UK (COG-UK) Consortium|Davies|United Kingdom
>Spike|hCoV-19/England/QEUH-B3FBC0/2020|2020-11-08|EPI_ISL_658489|Original|hCoV-19^^England|Human|Lighthouse Lab in Glasgow|Wellcome Sanger Institute for the COVID-19 Genomics UK (COG-UK) Consortium|Davies|United Kingdom
>Spike|hCoV-19/France/OCC-117/2020|2020-10-22|EPI_ISL_671962|Original|hCoV-19^^Occitanie|Human|CHU Purpan - Laboratoire de Virologie - Institut F\u00e9d\u00e9ratif de Biologie|CHU Purpan - Laboratoire de Virologie - Institut F\u00e9d\u00e9ratif de Biologie|Latour|France
>Spike|hCoV-19/Wales/QEUH-9648DC/2020|2020-08-11|EPI_ISL_531127|Original|hCoV-19^^Wales|Human|Lighthouse Lab in Glasgow|Wellcome Sanger Institute for the COVID-19 Genomics UK (COG-UK) consortium|Davies|United Kingdom
>Spike|hCoV-19/England/MILK-99DFB7/2020|2020-08-31|EPI_ISL_550544|Original|hCoV-19^^England|Human|Lighthouse Lab in Milton Keynes|Wellcome Sanger Institute for the COVID-19 Genomics UK (COG-UK) consortium|Davies|United Kingdom
>Spike|hCoV-19/England/CAMB-1BC1C3/2020|2020-11-21|EPI_ISL_723855|Original|hCoV-19^^England|Human|Department of Pathology|COVID-19 Genomics UK (COG-UK) Consortium|Goodfellow|United Kingdom
>Spike|hCoV-19/USA/WA-S1025/2020|2020-05-01|EPI_ISL_463515|Original|hCoV-19^^Washington|Human|Washington State Department of Health|Seattle Flu Study|Bedford|USA
>Spike|hCoV-19/Germany/SH-ChVir8315/2020|2020-07-26|EPI_ISL_729574|Original|hCoV-19^^Schleswig-Holstein|Human|A. Krumbholz|Charit\u00e9 Universit\u00e4tsmedizin Berlin|Muehlemann|Germany

>Spike|hCoV-19/England/CAMC-941899/2020|2020-07-23|EPI_ISL_556856|Original|hCoV-19^^England|Human|Lighthouse Lab in Cambridge|Wellcome Sanger Institute for the COVID-19 Genomics UK (COG-UK) consortium|Davies|United Kingdom
>Spike|hCoV-19/England/NOTT-11F60B/2020|2020-11-25|EPI_ISL_725547|Original|hCoV-19^^England|Human|Lincolnshire Hospitals and DeepSeq Nottingham|COVID-19 Genomics UK (COG-UK) Consortium|Loose|United Kingdom
>Spike|hCoV-19/USA/CA-ALSR-3764/2020|2020-05-27|EPI_ISL_636189|Original|hCoV-19^^California|Human|San Diego County Public Health Laboratory|Andersen lab at Scripps Research|Gangavarapu|USA
>Spike|hCoV-19/Japan/PG-4444/2020|2020-07-00|EPI_ISL_692667|Original|hCoV-19^^Japan|Human|Sakai City Institute of Public Health|Pathogen Genomics Center|Sekizuka|Japan
>Spike|hCoV-19/England/OXON-B0F3F/2020|2020-04-09|EPI_ISL_478979|Original|hCoV-19^^England|Human|Oxford Viromics|COVID-19 Genomics UK (COG-UK) Consortium|Golubchik|United Kingdom
>Spike|hCoV-19/Scotland/CVR4909/2020|2020-10-05|EPI_ISL_594675|Original|hCoV-19^^Scotland|Human|West of Scotland Specialist Virology Centre|COVID-19 Genomics UK (COG-UK) Consortium|Robertson|United Kingdom
>Spike|hCoV-19/Netherlands/ZuidHolland_34/2020|2020-03-16|EPI_ISL_422906|Original|hCoV-19^^Zuid Holland|Human|Dutch COVID-19 response team|Erasmus Medical Center|Oude Munnink|Netherlands
>Spike|hCoV-19/England/ALDP-A2DBB7/2020|2020-10-05|EPI_ISL_586668|Original|hCoV-19^^England|Human|Lighthouse Lab in Alderley Park|Wellcome Sanger Institute for the COVID-19 Genomics UK (COG-UK) consortium|Davies|United Kingdom
>Spike|hCoV-19/Japan/IC-0537/2020|2020-09-00|EPI_ISL_779220|Original|hCoV-19^^Japan|Human|Pathogen Genomics Center|Pathogen Genomics Center|Sekizuka|Japan
>Spike|hCoV-19/India/MH-GA38/2020|2020-06-20|EPI_ISL_508232|Original|hCoV-19^^Maharashtra|Human|Government Medical College|National Institute of Biomedical Genomics|Maitra|India
>Spike|hCoV-19/Scotland/EDB4536/2020|2020-05-06|EPI_ISL_461719|Original|hCoV-19^^Scotland|Human|Virology Department|COVID-19 Genomics UK (COG-UK) Consortium|Williams|United Kingdom
>Spike|hCoV-19/USA/CA-CZB-10293/2020|2020-09-08|EPI_ISL_583020|Original|hCoV-19^^California|Human|Orange County Public Health Lab|Chan-Zuckerberg Biohub|Bhatt|USA
>Spike|hCoV-19/mink/Netherlands/NB-EMC-55-17/2020|2020-09-23|EPI_ISL_632420|Original|hCoV-19^^Noord Brabant|Neovison vison|Dutch COVID-19 response team|Erasmus Medical Center|Oude Munnink|Netherlands
>Spike|hCoV-19/England/QEUH-9FF618/2020|2020-09-30|EPI_ISL_600342|Original|hCoV-19^^England|Human|Lighthouse Lab in Glasgow|Wellcome Sanger Institute for the COVID-19 Genomics UK (COG-UK) consortium|Davies|United Kingdom
>Spike|hCoV-19/Jordan/AM-ALSR-4071/2020|2020-10-03|EPI_ISL_636431|Original|hCoV-19^^Amman|Human|Biolab Diagnostic Laboratories|Andersen lab at Scripps Research|Gangavarapu|Jordan
>Spike|hCoV-19/Uruguay/UY-AT/2020|2020-05-20|EPI_ISL_480347|Original|hCoV-19^^Montevideo|Human|Microbial Genomics Laboratory|Microbial Genomics Laboratory|Iraola|Uruguay
>Spike|hCoV-19/Spain/VC-IBV-98003451/2020|2020-04-03|EPI_ISL_539258|Original|hCoV-19^^Comunitat Valenciana|Human|Hospital Universitario de La Ribera (Alzira|SeqCOVID-SPAIN consortium/IBV(CSIC)|Comas|Spain
>Spike|hCoV-19/Wales/ALDP-A851F7/2020|2020-10-16|EPI_ISL_625275|Original|hCoV-19^^Wales|Human|Lighthouse Lab in Alderley Park|Wellcome Sanger Institute for the COVID-19 Genomics UK (COG-UK) consortium|Davies|United Kingdom
>Spike|hCoV-19/England/MILK-BB1E72/2020|2020-11-18|EPI_ISL_720052|Original|hCoV-19^^England|Human|Lighthouse Lab in Milton Keynes|Wellcome Sanger Institute for the COVID-19 Genomics UK (COG-UK) Consortium|Davies|United Kingdom
>Spike|hCoV-19/England/NORT-29DF81/2020|2020-05-06|EPI_ISL_493503|Original|hCoV-19^^England|Human|Northumbria University / South Tees Hospitals NHS Foundation Trust / North Cumbria Integrated Care NHS Foundation Trust / North Tees and Hartlepool NHS Foundation Trust / Newcastle Hospitals NHS Foundation Trust|COVID-19 Genomics UK (COG-UK) Consortium|Bashton|United Kingdom

>Spike|hCoV-19/USA/TX-HMH-MCoV-18897/2020|2020-11-25|EPI_ISL_785205|Original|hCoV-19^^Texas|Human|Houston Methodist Hospital|Houston Methodist Hospital|Long|USA
>Spike|hCoV-19/DRC/11632/2020|2020-05-25|EPI_ISL_527493|Original|hCoV-19^^Kinshasa|Human|Viral Respiratory Lab|Pathogen Sequencing Lab|Lokilo|Democratic Republic of the Congo
>Spike|hCoV-19/England/ALDP-A85FC0/2020|2020-10-17|EPI_ISL_610456|Original|hCoV-19^^England|Human|Lighthouse Lab in Alderley Park|Wellcome Sanger Institute for the COVID-19 Genomics UK (COG-UK) consortium|Davies|United Kingdom
>Spike|hCoV-19/Japan/PG-4520/2020|2020-07-00|EPI_ISL_689659|Original|hCoV-19^^Japan|Human|Pathogen Genomics Center|Pathogen Genomics Center|Sekizuka|Japan
>Spike|hCoV-19/Singapore/1291/2020|2020-05-11|EPI_ISL_648776|Original|hCoV-19^^Singapore|Human|Department of Laboratory Medicine|Department of Laboratory Medicine|Chen|Singapore
>Spike|hCoV-19/England/MILK-B8C8BE/2020|2020-11-12|EPI_ISL_675792|Original|hCoV-19^^England|Human|Lighthouse Lab in Milton Keynes|Wellcome Sanger Institute for the COVID-19 Genomics UK (COG-UK) Consortium|Davies|United Kingdom
>Spike|hCoV-19/Wales/PHWC-492B0E/2020|2020-11-26|EPI_ISL_725749|Original|hCoV-19^^Wales|Human|Originating lab|COVID-19 Genomics UK (COG-UK) Consortium|Moore|United Kingdom
>Spike|hCoV-19/USA/NY-Wadsworth-251095-01/2020|2020-10-21|EPI_ISL_734301|Original|hCoV-19^^New York|Human|Wadsworth Center|Wadsworth Center|Lasek-Nesselquist|USA
>Spike|hCoV-19/USA/MI-QDX-1308/2020|2020-03-17|EPI_ISL_571963|Original|hCoV-19^^Michigan|Human|Quest Diagnostics|Quest Diagnostics|Kagan|USA
>Spike|hCoV-19/England/ALDP-B8418E/2020|2020-11-14|EPI_ISL_675177|Original|hCoV-19^^England|Human|Lighthouse Lab in Alderley Park|Wellcome Sanger Institute for the COVID-19 Genomics UK (COG-UK) Consortium|Davies|United Kingdom
>Spike|hCoV-19/Wales/PHWC-29FC8/2020|2020-04-03|EPI_ISL_432313|Original|hCoV-19^^Wales|Human|Wales Specialist Virology Centre|Public Health Wales Microbiology Cardiff|Moore|United Kingdom
>Spike|hCoV-19/USA/MI-QDX-345/2020|2020-03-23|EPI_ISL_571089|Original|hCoV-19^^Michigan|Human|Quest Diagnostics|Quest Diagnostics|Kagan|USA
>Spike|hCoV-19/Wales/PHWC-36809/2020|2020-04-29|EPI_ISL_474716|Original|hCoV-19^^Wales|Human|Originating lab|COVID-19 Genomics UK (COG-UK) Consortium|Moore|United Kingdom
>Spike|hCoV-19/India/UP-NBRI-N16-TCR338/2020|2020-04-01|EPI_ISL_516945|Original|hCoV-19^^Uttar Pradesh|Human|King Georges Medical University|CSIR-National Botanical Research Institute|Bag|India
>Spike|hCoV-19/USA/CT-Yale-205/2020|2020-03-30|EPI_ISL_463143|Original|hCoV-19^^Connecticut|Human|Yale Clinical Virology Laboratory|Grubaugh Lab - Yale School of Public Health|Grubaugh|USA
>Spike|hCoV-19/USA/GA-CDC-7556/2020|2020-10-23|EPI_ISL_681795|Original|hCoV-19^^Georgia|Human|GA Department of Public Health Laboratory|Pathogen Discovery|Queen|USA
>Spike|hCoV-19/France/OCC-SC423/2020|2020-04-12|EPI_ISL_660376|Original|hCoV-19^^Occitanie|Human|CHU Toulouse|CNR Virus des Infections Respiratoires - France SUD|Bal|France
>Spike|hCoV-19/South Africa/KRISP-005/2020|2020-04-29|EPI_ISL_455630|Original|hCoV-19^^KwaZulu-Natal|Human|KRISP|KRISP|de Oliveira|South Africa
>Spike|hCoV-19/Wales/PHWC-31E0C/2020|2020-04-11|EPI_ISL_446571|Original|hCoV-19^^Wales|Human|Wales Specialist Virology Centre|Public Health Wales Microbiology Cardiff|Moore|United Kingdom
>Spike|hCoV-19/England/20142115101/2020|2020-03-30|EPI_ISL_465954|Original|hCoV-19^^England|Human|Respiratory Virus Unit|Respiratory Virus Unit|Platt|United Kingdom
>Spike|hCoV-19/Australia/VIC5668/2020|2020-07-19|EPI_ISL_518475|Original|hCoV-19^^Victoria|Human|Microbiological Diagnostic Unit - Public Health Laboratory (MDU-PHL)|MDU-PHL|Schultz|Australia
>Spike|hCoV-19/Scotland/CVR3517/2020|2020-05-12|EPI_ISL_473657|Original|hCoV-19^^Scotland|Human|West of Scotland Specialist Virology Centre|COVID-19 Genomics UK (COG-UK) Consortium|Robertson|United Kingdom

>Spike|hCoV-19/England/MILK-A7C2E1/2020|2020-10-15|EPI_ISL_606351|Original|hCoV-19^^England|Human|Lighthouse Lab in Milton Keynes|Wellcome Sanger Institute for the COVID-19 Genomics UK (COG-UK) consortium|Davies|United Kingdom
>Spike|hCoV-19/England/SHEF-10B2D30/2020|2020-05-15|EPI_ISL_537269|Original|hCoV-19^^England|Human|Virology Department|Wellcome Sanger Institute for the COVID-19 Genomics UK (COG-UK) consortium|Davies|United Kingdom
>Spike|hCoV-19/Australia/VIC6487/2020|2020-00-00|EPI_ISL_521265|Original|hCoV-19^^Victoria|Human|Microbiological Diagnostic Unit - Public Health Laboratory (MDU-PHL)|MDU-PHL|Schultz|Australia
>Spike|hCoV-19/England/CAMC-B09E6C/2020|2020-10-29|EPI_ISL_672961|Original|hCoV-19^^England|Human|Lighthouse Lab in Cambridge|Wellcome Sanger Institute for the COVID-19 Genomics UK (COG-UK) Consortium|Davies|United Kingdom
>Spike|hCoV-19/Canada/BC_21462515/2020|2020-03-00|EPI_ISL_462815|Original|hCoV-19^^British Columbia|Human|BCCDC Public Health Laboratory|BCCDC Public Health Laboratory|Harrigan|Canada
>Spike|hCoV-19/Denmark/DCGC-4602/2020|2020-09-21|EPI_ISL_621271|Original|hCoV-19^^Nordjylland|Human|Department of Virus and Microbiological Special Diagnostics|Albertsen lab|Michaelsen|Denmark
>Spike|hCoV-19/Australia/VIC7711/2020|2020-08-04|EPI_ISL_564596|Original|hCoV-19^^Victoria|Human|Microbiological Diagnostic Unit - Public Health Laboratory (MDU-PHL)|MDU-PHL|Schultz|Australia
>Spike|hCoV-19/United Arab Emirates/1026/2020|2020-06-11|EPI_ISL_699158|Original|hCoV-19^^Abu Dhabi|Human|Group 42 (G42) Healthcare|G42 Healthcare|Ogrodzki|United Arab Emirates
>Spike|hCoV-19/England/SHEF-C06BF/2020|2020-03-25|EPI_ISL_420248|Original|hCoV-19^^England|Human|Virology Department|Department of Infection|de Silva|United Kingdom
>Spike|hCoV-19/England/QEUH-9F70F4/2020|2020-09-27|EPI_ISL_595661|Original|hCoV-19^^England|Human|Oxford Viromics|COVID-19 Genomics UK (COG-UK) Consortium|Golubchik|United Kingdom
>Spike|hCoV-19/Netherlands/ZH-EMC-475/2020|2020-08-25|EPI_ISL_574787|Original|hCoV-19^^Zuid Holland|Human|Dutch COVID-19 response team|Erasmus Medical Center|Oude Munnink|Netherlands
>Spike|hCoV-19/England/ALDP-95CCEA/2020|2020-06-17|EPI_ISL_554912|Original|hCoV-19^^England|Human|Lighthouse Lab in Alderley Park|Wellcome Sanger Institute for the COVID-19 Genomics UK (COG-UK) consortium|Davies|United Kingdom
>Spike|hCoV-19/Netherlands/NB-RIVM-20269/2020|2020-11-20|EPI_ISL_723412|Orignal|hCoV-19^^Noord-Brabant|Human|Dutch COVID-19 response team|National Institute for Public Health and the Environment (RIVM)|Meijer|Netherlands
>Spike|hCoV-19/England/CAMB-8214F/2020|2020-04-21|EPI_ISL_438623|Original|hCoV-19^^England|Human|Department of Pathology|COVID-19 Genomics UK (COG-UK) Consortium|Meredith|United Kingdom
>Spike|hCoV-19/Israel/CVL-85-ngs/2020|2020-12-27|EPI_ISL_776653|Original|hCoV-19^^Israel|Human|Israel Central Virology laboratory|Israel Central Virology laboratory|Zuckerman|Israel
>Spike|hCoV-19/England/ALDP-649463/2020|2020-06-18|EPI_ISL_558076|Original|hCoV-19^^England|Human|Lighthouse Lab in Alderley Park|Wellcome Sanger Institute for the COVID-19 Genomics UK (COG-UK) consortium|Davies|United Kingdom
>Spike|hCoV-19/England/ALDP-9EAA32/2020|2020-09-24|EPI_ISL_580165|Original|hCoV-19^^England|Human|Lighthouse Lab in Alderley Park|Wellcome Sanger Institute for the COVID-19 Genomics UK (COG-UK) consortium|Davies|United Kingdom
>Spike|hCoV-19/England/CAMB-7D262/2020|2020-04-06|EPI_ISL_442123|Original|hCoV-19^^England|Human|Department of Pathology|Wellcome Sanger Institute for the COVID-19 Genomics UK (COG-UK) consortium|Amato|United Kingdom
>Spike|hCoV-19/Denmark/DCGC-884/2020|2020-05-11|EPI_ISL_614800|Original|hCoV-19^^Hovedstaden|Human|Department of Virus and Microbiological Special Diagnostics|Albertsen lab|Michaelsen|Denmark
>Spike|hCoV-19/France/PAC-SC444/2020|2020-04-22|EPI_ISL_660735|Original|hCoV-19^^Provence-Alpes-C\u00f4te-d\u2019Azur|Human|Unit\u00e9 des Virus \u00c9mergents|CNR Virus des Infections Respiratoires - France SUD|Bal|France

>Spike|hCoV-19/England/MILK-9D4B1A/2020|2020-09-19|EPI_ISL_601653|Original|hCoV-19^^England|Human|Lighthouse Lab in Milton Keynes|Wellcome Sanger Institute for the COVID-19 Genomics UK (COG-UK) consortium|Davies|United Kingdom
>Spike|hCoV-19/Denmark/DCGC-1731/2020|2020-05-11|EPI_ISL_617950|Original|hCoV-19^^Hovedstaden|Human|Department of Virus and Microbiological Special Diagnostics|Albertsen lab|Michaelsen|Denmark
>Spike|hCoV-19/USA/CA-CZB-3269/2020|2020-07-15|EPI_ISL_548267|Original|hCoV-19^^California|Human|County of Santa Clara Public Health Department|Chan-Zuckerberg Biohub|Bhatt|USA
>Spike|hCoV-19/Japan/PG-5899/2020|2020-08-00|EPI_ISL_689014|Original|hCoV-19^^Japan|Human|Pathogen Genomics Center|Pathogen Genomics Center|Sekizuka|Japan
>Spike|hCoV-19/USA/TX-HMH-MCoV-8120/2020|2020-06-30|EPI_ISL_784211|Original|hCoV-19^^Texas|Human|Houston Methodist Hospital|Houston Methodist Hospital|Long|USA
>Spike|hCoV-19/India/KA-nimh-12171/2020|2020-05-06|EPI_ISL_515953|Original|hCoV-19^^Karnataka|Human|VICTORIA HOSPITAL|Department of Neurovirology|Pattabiraman|India
>Spike|hCoV-19/Denmark/DCGC-17131/2020|2020-12-14|EPI_ISL_750331|Original|hCoV-19^^Midtjylland|Human|Department of Virus and Microbiological Special Diagnostics|Albertsen Lab|Michaelsen|Denmark
>Spike|hCoV-19/USA/CA-CHLA-PLM03763789/2020|2020-11-30|EPI_ISL_753484|Original|hCoV-19^^California|Human|Clinical virology Laboratory|Center for Personalized Medicine|Dien Bard|USA
>Spike|hCoV-19/Scotland/EDB842/2020|2020-04-04|EPI_ISL_433254|Original|hCoV-19^^Scotland|Human|Virology Department|COVID-19 Genomics UK (COG-UK) Consortium|Williams|United Kingdom
>Spike|hCoV-19/Wales/PHWC-49C9EC/2020|2020-11-19|EPI_ISL_742629|Original|hCoV-19^^Wales|Human|Wales Specialist Virology Centre Sequencing lab|COVID-19 Genomics UK (COG-UK) Consortium|Moore|United Kingdom
>Spike|hCoV-19/Switzerland/BE-UNIBE-197-5085/2020|2020-03-24|EPI_ISL_574835|Original|hCoV-19^^Bern|Human|Institute for Infectious Diseases|Institute for Infectious Diseases|Koch|Switzerland
>Spike|hCoV-19/England/MILK-99B4F6/2020|2020-08-30|EPI_ISL_550402|Original|hCoV-19^^England|Human|Lighthouse Lab in Milton Keynes|Wellcome Sanger Institute for the COVID-19 Genomics UK (COG-UK) consortium|Davies|United Kingdom
>Spike|hCoV-19/Ireland/SO-NVRL-73IRL69650/2020|2020-09-13|EPI_ISL_639846|Original|hCoV-19^^Sligo|Human|National Virus Reference Laboratory|National Virus Reference Laboratory|Gonzalez|Ireland
>Spike|hCoV-19/England/QEUH-9F37E4/2020|2020-09-24|EPI_ISL_588511|Original|hCoV-19^^England|Human|Lighthouse Lab in Glasgow|Wellcome Sanger Institute for the COVID-19 Genomics UK (COG-UK) consortium|Davies|United Kingdom
>Spike|hCoV-19/USA/MI-MDHHS-SC21739/2020|2020-03-25|EPI_ISL_529858|Original|hCoV-19^^Michigan|Human|Michigan Department of Health and Human Services|Michigan Department of Health and Human Services|Blankenship|USA
>Spike|hCoV-19/England/CAMC-9D1EE7/2020|2020-09-18|EPI_ISL_601700|Original|hCoV-19^^England|Human|Lighthouse Lab in Cambridge|Wellcome Sanger Institute for the COVID-19 Genomics UK (COG-UK) consortium|Davies|United Kingdom
>Spike|hCoV-19/Netherlands/NoordBrabant_30/2020|2020-03-08|EPI_ISL_414539|Original|hCoV-19^^Noord Brabant|Human|Dutch COVID-19 response team|Erasmus Medical Center|Oude Munnink|Netherlands
>Spike|hCoV-19/Russia/SPE-RII-10167V/2020|2020-04-25|EPI_ISL_523996|Vero P1|hCoV-19^^Saint Petersburg|Human|WHO National Influenza Centre Russian Federation|WHO National Influenza Centre Russian Federation|Komissarov|Russia
>Spike|hCoV-19/England/OXON-AF364/2020|2020-00-00|EPI_ISL_478730|Original|hCoV-19^^England|Human|Oxford Viromics|COVID-19 Genomics UK (COG-UK) Consortium|Golubchik|United Kingdom
>Spike|hCoV-19/USA/TX-HMH-MCoV-9133/2020|2020-07-02|EPI_ISL_780959|Original|hCoV-19^^Texas|Human|Houston Methodist Hospital|Houston Methodist Hospital|Long|USA
>Spike|hCoV-19/Australia/VIC2649/2020|2020-07-01|EPI_ISL_521495|Original|hCoV-19^^Victoria|Human|Victorian Infectious Diseases Reference Laboratory (VIDRL)|VIDRL and MDU-PHL|Schultz|Australia

>Spike|hCoV-19/England/ALDP-4F7D89/2020|2020-06-24|EPI_ISL_559279|Original|hCoV-
19^^England|Human|Lighthouse Lab in Alderley Park|Wellcome Sanger Institute for the
COVID-19 Genomics UK (COG-UK) consortium|Davies|United Kingdom
>Spike|hCoV-19/England/CAMC-AAF959/2020|2020-10-23|EPI_ISL_623273|Original|hCoV-
19^^England|Human|Lighthouse Lab in Cambridge|Wellcome Sanger Institute for the
COVID-19 Genomics UK (COG-UK) consortium|Davies|United Kingdom
>Spike|hCoV-19/England/NORW-EB40F/2020|2020-05-01|EPI_ISL_457429|Original|hCoV-
19^^England|Human|Quadram Institute Bioscience|COVID-19 Genomics UK (COG-UK)
Consortium|Alikhan|United Kingdom
>Spike|hCoV-19/Luxembourg/LNS6212793/2020|2020-10-22|EPI_ISL_744382|Original|hCoV-
19^^Luxembourg|Human|Laboratoire national de sant\u00e9|Laboratoire national de
sant\u00e9|Wienecke-Baldacchino|Luxembourg
>Spike|hCoV-19/Wales/PHWC-30F37/2020|2020-04-09|EPI_ISL_446381|Original|hCoV-
19^^Wales|Human|Wales Specialist Virology Centre|Public Health Wales Microbiology
Cardiff|Moore|United Kingdom
>Spike|hCoV-19/USA/WA-S1531/2020|2020-05-25|EPI_ISL_495957|Original|hCoV-
19^^Washington|Human|Washington State Department of Health|Seattle Flu Study|Lee|USA
>Spike|hCoV-19/Cote dIvoire/BKE0349/2020|2020-06-29|EPI_ISL_681832|Original|hCoV-
19^^Boauk\u00e9|Human|Molecular diagnostic unit for viral haemorrhagic fevers and
emerging viruses|Project group Epidemology of Highly Pathogenic
Microorganisms|Schubert|C\u00f4te d'Ivoire
>Spike|hCoV-19/USA/AZ-TG334281/2020|2020-05-26|EPI_ISL_694364|Original|hCoV-
19^^Arizona|Human|TGen North|TGen North|French|USA
>Spike|hCoV-19/Netherlands/ZH-EMC-282/2020|2020-06-19|EPI_ISL_523624|Original|hCoV-
19^^Zuid Holland|Human|Dutch COVID-19 response team|Erasmus Medical Center|Oude
Munnink|Netherlands
>Spike|hCoV-19/Scotland/EDB7819/2020|2020-09-21|EPI_ISL_573795|Original|hCoV-
19^^Scotland|Human|Virology Department|COVID-19 Genomics UK (COG-UK)
Consortium|Williams|United Kingdom
>Spike|hCoV-19/England/BIRM-65CC9/2020|2020-10-09|EPI_ISL_612219|Original|hCoV-
19^^England|Human|University of Birmingham|COVID-19 Genomics UK (COG-UK)
Consortium|Loman|United Kingdom
>Spike|hCoV-19/Denmark/DCGC-5089/2020|2020-09-21|EPI_ISL_621627|Original|hCoV-
19^^Sjaelland|Human|Department of Virus and Microbiological Special
Diagnostics|Albertsen lab|Michaelsen|Denmark
>Spike|hCoV-19/USA/AZ-TG381372/2020|2020-06-15|EPI_ISL_694661|Original|hCoV-
19^^Arizona|Human|TGen North|TGen North|French|USA
>Spike|hCoV-19/USA/CA-LACPHL-AF00009/2020|2020-12-01|EPI_ISL_737244|Original|hCoV-
19^^California|Human|Los Angeles County PHL|Los Angeles County PHL|Hemarajata|USA
>Spike|hCoV-19/Wales/PHWC-16C283/2020|2020-09-17|EPI_ISL_574031|Original|hCoV-
19^^Wales|Human|Wales Specialist Virology Centre Sequencing lab|COVID-19 Genomics UK
(COG-UK) Consortium|Moore|United Kingdom
>Spike|hCoV-19/Japan/PG-2535/2020|2020-05-00|EPI_ISL_690670|Original|hCoV-
19^^Japan|Human|Saitama Prefectural Institute of Public Health|Pathogen Genomics
Center|Sekizuka|Japan
>Spike|hCoV-19/Japan/PG-1180/2020|2020-04-00|EPI_ISL_684790|Original|hCoV-
19^^Japan|Human|Pathogen Genomics Center|Pathogen Genomics Center|Sekizuka|Japan
>Spike|hCoV-19/England/ALDP-AA0976/2020|2020-10-22|EPI_ISL_630865|Original|hCoV-
19^^England|Human|Lighthouse Lab in Alderley Park|Wellcome Sanger Institute for the
COVID-19 Genomics UK (COG-UK) consortium|Davies|United Kingdom
>Spike|hCoV-19/USA/PR-B3SB/2020|2020-08-06|EPI_ISL_534694|Original|hCoV-19^^Puerto
Rico|Human|University of Miami Immunology and Histocompatibility
Laboratory|University of Miami Immunology and Histocompatibility
Laboratory|Margolles-Clark|USA
>Spike|hCoV-19/Singapore/765/2020|2020-06-11|EPI_ISL_509375|Original|hCoV-
19^^Singapore|Human|Singapore General Hospital|Department of Microbiology|Abdul
Rahman|Singapore
>Spike|hCoV-19/New Zealand/20CV0435/2020|2020-10-16|EPI_ISL_622777|Original|hCoV-
19^^Auckland|Human|LabPLUS|Institute of Environmental Science and Research
(ESR)|Wang|New Zealand

>Spike|hCoV-19/England/NOTT-115043/2020|2020-10-13|EPI_ISL_595237|Original|hCoV-19^^England|Human|Queens Medical Centre|COVID-19 Genomics UK (COG-UK) Consortium|Loose|United Kingdom
>Spike|hCoV-19/USA/MI-UM-10036629769/2020|2020-10-26|EPI_ISL_650055|Original|hCoV-19^^Michigan|Human|University of Michigan Clinical Microbiology Laboratory|Lauring Lab|Valesano|USA
>Spike|hCoV-19/Wales/PHWC-49A483/2020|2020-12-02|EPI_ISL_727550|Original|hCoV-19^^Wales|Human|Originating lab|COVID-19 Genomics UK (COG-UK) Consortium|Moore|United Kingdom
>Spike|hCoV-19/Belgium/rega-10031031/2020|2020-10-03|EPI_ISL_735048|Original|hCoV-19^^Rotselaar|Human|UZ Leuven|KU Leuven|Maes|Belgium
>Spike|hCoV-19/Bangladesh/DNAS_DRICM_isl_MH-25/2020|2020-06-28|EPI_ISL_775008|Original|hCoV-19^^Dhaka|Human|Designated Reference Institute for Chemical Measurements (DRICM)|DNA SOLUTION LTD.|Rabbi|Bangladesh
>Spike|hCoV-19/Brazil/RJ-00308/2020|2020-05-21|EPI_ISL_623111|Original|hCoV-19^^Rio de Janeiro|Human|Laboratorio de Virologia Molecular / UFRJ|Bioinformatics Laboratory / LNCC|Almeida|Brazil
>Spike|hCoV-19/USA/TX-HMH-MCoV-8862/2020|2020-07-02|EPI_ISL_780534|Original|hCoV-19^^Texas|Human|Houston Methodist Hospital|Houston Methodist Hospital|Long|USA
>Spike|hCoV-19/New Zealand/20CV0291/2020|2020-03-27|EPI_ISL_579081|Original|hCoV-19^^Canterbury|Human|Canterbury Health Laboratories|Institute of Environmental Science and Research (ESR)|Wang|New Zealand
>Spike|hCoV-19/Saudi Arabia/KAUST-Makkah124/2020|2020-03-24|EPI_ISL_437691|Original|hCoV-19^^Makkah|Human|Pathogen Genomics Lab King Abdullah University of Science and Technology(KAUST)|Pathogen Genomics Lab King Abdullah University of Science and Technology(KAUST)|Mohamed Ghazzali|Saudi Arabia
>Spike|hCoV-19/USA/HI-H200514/2020|2020-08-16|EPI_ISL_753050|Original|hCoV-19^^Hawaii|Human|State Laboratories Division|State Laboratories Division|Sultana|USA
>Spike|hCoV-19/England/CAMB-73C5F/2020|2020-04-02|EPI_ISL_433680|Original|hCoV-19^^England|Human|Department of Pathology|COVID-19 Genomics UK (COG-UK) Consortium|Meredith|United Kingdom
>Spike|hCoV-19/England/NORT-292E16/2020|2020-05-20|EPI_ISL_489140|Original|hCoV-19^^England|Human|NU-OMICS DNA Sequencing research facility|Wellcome Sanger Institute for the COVID-19 Genomics UK (COG-UK) consortium|Davies|United Kingdom
>Spike|hCoV-19/Scotland/EDB1077/2020|2020-04-07|EPI_ISL_433385|Original|hCoV-19^^Scotland|Human|Virology Department|COVID-19 Genomics UK (COG-UK) Consortium|Williams|United Kingdom
>Spike|hCoV-19/USA/LA-CDC-1271/2020|2020-06-10|EPI_ISL_535312|Vero P1|hCoV-19^^Louisiana|Human|LA Office of Public Health Laboratories|Pathogen Discovery|Queen|USA
>Spike|hCoV-19/Denmark/DCGC-18912/2020|2020-12-07|EPI_ISL_748636|Original|hCoV-19^^Hovedstaden|Human|Department of Virus and Microbiological Special Diagnostics|Albertsen Lab|Michaelsen|Denmark
>Spike|hCoV-19/Luxembourg/LNS4845603/2020|2020-03-15|EPI_ISL_419589|Original|hCoV-19^^Luxembourg|Human|Laboratoire National de Sant\u00e9|Laboratoire National de Sant\u00e9|Wienecke-Baldacchino|Luxembourg
>Spike|hCoV-19/USA/MI-MDHHS-SC20004/2020|2020-03-10|EPI_ISL_424982|unknown|hCoV-19^^Michigan|Human|Bureau of Laboratories|Michigan Department of Health and Human Services|Blankenship|USA
>Spike|hCoV-19/Italy/VEN-UniVR-98/2020|2020-03-25|EPI_ISL_751404|Original|hCoV-19^^Veneto|Human|IRCCS Sacro Cuore Don Calabria Hospital|University of Verona|Mori|Italy
>Spike|hCoV-19/USA/TX-HMH-3795/2020|2020-06-11|EPI_ISL_543199|Original|hCoV-19^^Texas|Human|Houston Methodist Hospital|Houston Methodist Hospital|Long|USA
>Spike|hCoV-19/USA/TX-DSHS-0618/2020|2020-06-06|EPI_ISL_569728|Original|hCoV-19^^Texas|Human|Texas Department of State Health Services|Texas Department of State Health Services|Rahman|USA
>Spike|hCoV-19/Japan/PG-4756/2020|2020-08-00|EPI_ISL_689892|Original|hCoV-19^^Japan|Human|Pathogen Genomics Center|Pathogen Genomics Center|Sekizuka|Japan

>Spike|hCoV-19/Wales/PHWC-168D86/2020|2020-04-20|EPI_ISL_494363|Original|hCoV-19^^Wales|Human|Originating lab|COVID-19 Genomics UK (COG-UK) Consortium|Moore|United Kingdom
>Spike|hCoV-19/Jordan/AM-ALSR-5326/2020|2020-11-03|EPI_ISL_730562|Original|hCoV-19^^Amman|Human|Biolab Diagnostic Laboratories|Andersen lab at Scripps Research|Gangavarapu|Jordan
>Spike|hCoV-19/USA/WI-GMF-20295/2020|2020-09-15|EPI_ISL_547862|Original|hCoV-19^^Wisconsin|Human|Gundersen Molecular Diagnostics Laboratory|Kabara Cancer Research Institute|Kenny|USA
>Spike|hCoV-19/Denmark/DCGC-15662/2020|2020-11-23|EPI_ISL_713947|Original|hCoV-19^^Sjaelland|Human|Department of Virus and Microbiological Special Diagnostics|Albertsen Lab|Michaelsen|Denmark
>Spike|hCoV-19/Luxembourg/LNS7385509/2020|2020-11-20|EPI_ISL_744558|Original|hCoV-19^^Luxembourg|Human|Laboratoire national de sant\u00e9|Laboratoire national de sant\u00e9|Wienecke-Baldacchino|Luxembourg
>Spike|hCoV-19/England/20134025204/2020|2020-03-23|EPI_ISL_423129|Original|hCoV-19^^England|Human|Respiratory Virus Unit|Respiratory Virus Unit|Platt|United Kingdom
>Spike|hCoV-19/India/MH-NIV-122530/2020|2020-07-18|EPI_ISL_541690|Original|hCoV-19^^Maharashtra|Human|National Institute of Virology|National Institute of Virology|Potdar|India
>Spike|hCoV-19/England/ALDP-A708EB/2020|2020-10-14|EPI_ISL_608144|Original|hCoV-19^^England|Human|Lighthouse Lab in Alderley Park|Wellcome Sanger Institute for the COVID-19 Genomics UK (COG-UK) consortium|Davies|United Kingdom
>Spike|hCoV-19/Denmark/ALAB-SSI-1398/2020|2020-04-03|EPI_ISL_444858|Original|hCoV-19^^Denmark|Human|Department of Virus and Microbiological Special Diagnostics|Albertsen lab|Kirkegaard|Denmark
>Spike|hCoV-19/England/ALDP-6FCA6D/2020|2020-06-29|EPI_ISL_557399|Original|hCoV-19^^England|Human|Lighthouse Lab in Alderley Park|Wellcome Sanger Institute for the COVID-19 Genomics UK (COG-UK) consortium|Davies|United Kingdom
>Spike|hCoV-19/Switzerland/BL-ETHZ-110427/2020|2020-04-07|EPI_ISL_451857|Original|hCoV-19^^Basel-Landschaft|Human|Viollier AG|Department of Biosystems Science and Engineering|Nadeau|Switzerland
>Spike|hCoV-19/USA/CA-QRTP-Donor18/2020|2020-04-01|EPI_ISL_490221|Original|hCoV-19^^California|Human|Quest Diagnostics|Q Squared Solutions - QRTP facility|Weigman|USA
>Spike|hCoV-19/France/ARA-32195/2020|2020-04-29|EPI_ISL_678539|Original|hCoV-19^^Auvergne-Rh\u00f4ne-Alpes|Human|CNR Virus des Infections Respiratoires - France SUD|CNR Virus des Infections Respiratoires - France SUD|Bal|France
>Spike|hCoV-19/England/ALDP-BB3DBF/2020|2020-11-20|EPI_ISL_701809|Original|hCoV-19^^England|Human|Lighthouse Lab in Alderley Park|Wellcome Sanger Institute for the COVID-19 Genomics UK (COG-UK) Consortium|Davies|United Kingdom
>Spike|hCoV-19/England/ALDP-9BC723/2020|2020-09-08|EPI_ISL_568022|Original|hCoV-19^^England|Human|Lighthouse Lab in Alderley Park|Wellcome Sanger Institute for the COVID-19 Genomics UK (COG-UK) consortium|Davies|United Kingdom
>Spike|hCoV-19/England/QEUH-CC2DBC/2020|2020-12-21|EPI_ISL_778156|Original|hCoV-19^^England|Human|Lighthouse Lab in Glasgow|Wellcome Sanger Institute for the COVID-19 Genomics UK (COG-UK) Consortium|Davies|United Kingdom
>Spike|hCoV-19/Australia/VIC10142/2020|2020-08-14|EPI_ISL_562806|Original|hCoV-19^^Victoria|Human|Victorian Infectious Diseases Reference Laboratory (VIDRL)|VIDRL and MDU-PHL|Schultz|Australia
>Spike|hCoV-19/Portugal/PT0391a/2020|2020-04-14|EPI_ISL_454110|Original|hCoV-19^^Portugal|Human|unknown|Instituto Nacional de Saude (INSA)|Borges|Portugal
>Spike|hCoV-19/South Africa/KRISP-K003511/2020|2020-09-26|EPI_ISL_602833|Original|hCoV-19^^KwaZulu-Natal|Human|NHLS-IALCH|KRISP|de Oliveira|South Africa
>Spike|hCoV-19/Netherlands/UT-RIVM-20382/2020|2020-12-07|EPI_ISL_728602|Original|hCoV-19^^Utrecht|Human|Dutch COVID-19 response team|National Institute for Public Health and the Environment (RIVM)|Meijer|Netherlands

>Spike|hCoV-19/Brazil/SP-L25-CD665/2020|2020-04-14|EPI_ISL_672717|Original|hCoV-19^^S\u00e3o Paulo|Human|Institute of Tropical Medicine at the University of S\u00e3o Paulo (IMT-USP)|Laborat\u00f3rio de Parasitologia M\u00e9dica - Instituto de Medicina Tropical - Universidade de S\u00e3o Paulo|de Jesus|Brazil
>Spike|hCoV-19/Australia/VIC3962/2020|2020-07-15|EPI_ISL_519355|Original|hCoV-19^^Victoria|Human|Microbiological Diagnostic Unit - Public Health Laboratory (MDU-PHL)|MDU-PHL|Schultz|Australia
>Spike|hCoV-19/Scotland/CVR1928/2020|2020-04-08|EPI_ISL_484524|Original|hCoV-19^^Scotland|Human|West of Scotland Specialist Virology Centre|COVID-19 Genomics UK (COG-UK) Consortium|Robertson|United Kingdom
>Spike|hCoV-19/England/MILK-B96101/2020|2020-11-14|EPI_ISL_676203|Original|hCoV-19^^England|Human|Lighthouse Lab in Milton Keynes|Wellcome Sanger Institute for the COVID-19 Genomics UK (COG-UK) Consortium|Davies|United Kingdom
>Spike|hCoV-19/Scotland/EDB1812/2020|2020-04-16|EPI_ISL_433136|Original|hCoV-19^^Scotland|Human|Virology Department|COVID-19 Genomics UK (COG-UK) Consortium|Williams|United Kingdom
>Spike|hCoV-19/India/MH-NCCS-NR2523/2020|2020-05-24|EPI_ISL_496584|Original|hCoV-19^^Maharashtra|Human|National Centre For Cell Science|National Centre For Cell Science|Paul|India
>Spike|hCoV-19/Iceland/329/2020|2020-03-14|EPI_ISL_417658|Original|hCoV-19^^Reykjavik|Human|deCODE genetics|deCODE genetics|J\u00f3nsson|Iceland
>Spike|hCoV-19/England/MILK-BA9B49/2020|2020-11-17|EPI_ISL_681651|Original|hCoV-19^^England|Human|Lighthouse Lab in Milton Keynes|Wellcome Sanger Institute for the COVID-19 Genomics UK (COG-UK) Consortium|Davies|United Kingdom
>Spike|hCoV-19/USA/CA-ALSR-1268/2020|2020-05-04|EPI_ISL_494468|Original|hCoV-19^^California|Human|San Diego County Public Health Laboratory|Andersen lab at Scripps Research|Gangavarapu|USA
>Spike|hCoV-19/Hong Kong/VM20040263/2020|2020-07-14|EPI_ISL_523847|Original|hCoV-19^^Hong Kong|Human|Queen Elizabeth Hospital|Hong Kong Department of Health|Mak|China
>Spike|hCoV-19/Hong Kong/HKPU-1362/2020|2020-07-09|EPI_ISL_610179|Original|hCoV-19^^Hong Kong|Human|Department of Health Technology and Informatics|Department of Health Technology and Informatics|Leung|China
>Spike|hCoV-19/Australia/VIC17787/2020|2020-07-28|EPI_ISL_779452|Original|hCoV-19^^Victoria|Human|Microbiological Diagnostic Unit - Public Health Laboratory (MDU-PHL)|MDU-PHL|Beck|Australia
>Spike|hCoV-19/Denmark/DCGC-15755/2020|2020-11-23|EPI_ISL_714002|Original|hCoV-19^^Hovedstaden|Human|Department of Virus and Microbiological Special Diagnostics|Albertsen Lab|Michaelsen|Denmark
>Spike|hCoV-19/England/LOND-D61F1/2020|2020-04-20|EPI_ISL_482161|Original|hCoV-19^^England|Human|University College London|Wellcome Sanger Institute for the COVID-19 Genomics UK (COG-UK) consortium|Davies|United Kingdom
>Spike|hCoV-19/England/QEUH-A01B5F/2020|2020-10-01|EPI_ISL_680008|Original|hCoV-19^^England|Human|Virology Department|COVID-19 Genomics UK (COG-UK) Consortium|de Silva|United Kingdom
>Spike|hCoV-19/Australia/VIC1342/2020|2020-04-14|EPI_ISL_430683|Original|hCoV-19^^Victoria|Human|Microbiological Diagnostic Unit Public Health Laboratory|Microbiological Diagnostic Unit Public Health Laboratory|Schultz|Australia
>Spike|hCoV-19/Wales/PHWC-33839/2020|2020-04-18|EPI_ISL_446755|Original|hCoV-19^^Wales|Human|Wales Specialist Virology Centre|Public Health Wales Microbiology Cardiff|Moore|United Kingdom
>Spike|hCoV-19/Singapore/11/2020|2020-02-02|EPI_ISL_410719|Original|hCoV-19^^Singapore|Human|National Public Health Laboratory|National Public Health Laboratory|Mak|Singapore
>Spike|hCoV-19/England/CAMC-9B1BD7/2020|2020-09-03|EPI_ISL_566445|Original|hCoV-19^^England|Human|Lighthouse Lab in Cambridge|Wellcome Sanger Institute for the COVID-19 Genomics UK (COG-UK) consortium|Davies|United Kingdom
>Spike|hCoV-19/Scotland/EDB4043/2020|2020-04-15|EPI_ISL_473793|Original|hCoV-19^^Scotland|Human|Virology Department|COVID-19 Genomics UK (COG-UK) Consortium|Williams|United Kingdom

>Spike|hCoV-19/England/MILK-A05BE2/2020|2020-09-30|EPI_ISL_599962|Original|hCoV-19^^England|Human|Lighthouse Lab in Milton Keynes|Wellcome Sanger Institute for the COVID-19 Genomics UK (COG-UK) consortium|Davies|United Kingdom
>Spike|hCoV-19/Canada/NS-NML-1361/2020|2020-04-14|EPI_ISL_579737|Original|hCoV-19^^Nova Scotia|Human|QEII Health Sciences Centre|National Microbiology Laboratory (NML)|Majer|Canada
>Spike|hCoV-19/Portugal/PT0190/2020|2020-03-29|EPI_ISL_453906|Original|hCoV-19^^Portugal|Human|unknown|Instituto Nacional de Saude (INSA)|Borges|Portugal
>Spike|hCoV-19/England/ALDP-A6F6E5/2020|2020-10-15|EPI_ISL_607823|Original|hCoV-19^^England|Human|Lighthouse Lab in Alderley Park|Wellcome Sanger Institute for the COVID-19 Genomics UK (COG-UK) consortium|Davies|United Kingdom
>Spike|hCoV-19/Burkina Faso/2-068-2228/2020|2020-09-06|EPI_ISL_660457|Original|hCoV-19^^Bobo Dioulasso|Human|Laboratoire de Microbiologie CHU Souro Sanou|Centre Muraz|Ouedraogo|Burkina Faso
>Spike|hCoV-19/USA/IA-CDC-4052/2020|2020-03-09|EPI_ISL_647998|Original|hCoV-19^^Iowa|Human|IA State Hygienic Laboratory|Pathogen Discovery|Queen|USA
>Spike|hCoV-19/England/NORW-E8AB5/2020|2020-04-13|EPI_ISL_448274|Original|hCoV-19^^England|Human|Quadram Institute Bioscience|COVID-19 Genomics UK (COG-UK) Consortium|Alikhan|United Kingdom
>Spike|hCoV-19/Scotland/QEUH-B7DEDC/2020|2020-11-13|EPI_ISL_742255|Original|hCoV-19^^Scotland|Human|Oxford Viromics|COVID-19 Genomics UK (COG-UK) Consortium|Golubchik|United Kingdom
>Spike|hCoV-19/England/ALDP-B2741B/2020|2020-11-04|EPI_ISL_647072|Original|hCoV-19^^England|Human|Lighthouse Lab in Alderley Park|Wellcome Sanger Institute for the COVID-19 Genomics UK (COG-UK) Consortium|Davies|United Kingdom
>Spike|hCoV-19/Brazil/SP-376/2020|2020-06-07|EPI_ISL_603023|Original|hCoV-19^^S\u00e3o Paulo|Human|Vigil\u00e2ncia em Sa\u00fade Visa Sul|Instituto Adolfo Lutz|Sacchi|Brazil
>Spike|hCoV-19/Scotland/CVR2096/2020|2020-04-10|EPI_ISL_490662|Original|hCoV-19^^Scotland|Human|West of Scotland Specialist Virology Centre|COVID-19 Genomics UK (COG-UK) Consortium|Robertson|United Kingdom
>Spike|hCoV-19/USA/TX-HMH-2812/2020|2020-06-08|EPI_ISL_545574|Original|hCoV-19^^Texas|Human|Houston Methodist Hospital|Houston Methodist Hospital|Long|USA
>Spike|hCoV-19/Denmark/DCGC-8844/2020|2020-10-26|EPI_ISL_626114|Original|hCoV-19^^Sjaelland|Human|Department of Virus and Microbiological Special Diagnostics|Albertsen lab|Michaelsen|Denmark
>Spike|hCoV-19/USA/OR-OHSU-2441/2020|2020-08-14|EPI_ISL_667208|Original|hCoV-19^^Oregon|Human|OHSU Lab Services Molecular Microbiology Lab|Oregon SARS-CoV-2 Genome Sequencing Center|O'Roak|USA
>Spike|hCoV-19/England/ALDP-A6E30D/2020|2020-10-15|EPI_ISL_606964|Original|hCoV-19^^England|Human|Lighthouse Lab in Alderley Park|Wellcome Sanger Institute for the COVID-19 Genomics UK (COG-UK) consortium|Davies|United Kingdom
>Spike|hCoV-19/USA/TX-HMH-MCoV-13946/2020|2020-08-15|EPI_ISL_787238|Original|hCoV-19^^Texas|Human|Houston Methodist Hospital|Houston Methodist Hospital|Long|USA
>Spike|hCoV-19/England/WSFT-25C0F9B/2020|2020-05-02|EPI_ISL_492881|Original|hCoV-19^^England|Human|Department of Medical Microbiology|Wellcome Sanger Institute for the COVID-19 Genomics UK (COG-UK) consortium|Davies|United Kingdom
>Spike|hCoV-19/Wales/PHWC-4A483F/2020|2020-12-17|EPI_ISL_764737|Original|hCoV-19^^Wales|Human|Wales Specialist Virology Centre Sequencing lab|COVID-19 Genomics UK (COG-UK) Consortium|Moore|United Kingdom
>Spike|hCoV-19/England/QEUH-BC4AE1/2020|2020-11-25|EPI_ISL_704008|Original|hCoV-19^^England|Human|Lighthouse Lab in Glasgow|Wellcome Sanger Institute for the COVID-19 Genomics UK (COG-UK) Consortium|Davies|United Kingdom
>Spike|hCoV-19/England/20150041004/2020|2020-04-06|EPI_ISL_466231|Original|hCoV-19^^England|Human|Respiratory Virus Unit|Respiratory Virus Unit|Platt|United Kingdom
>Spike|hCoV-19/England/CAMB-1B1568/2020|2020-05-08|EPI_ISL_458560|Original|hCoV-19^^England|Human|Department of Pathology|Wellcome Sanger Institute for the COVID-19 Genomics UK (COG-UK) consortium|Davies|United Kingdom

>Spike|hCoV-19/Wales/PHWC-2A0D3/2020|2020-04-04|EPI_ISL_432391|Original|hCoV-19^^Wales|Human|Wales Specialist Virology Centre|Public Health Wales Microbiology Cardiff|Moore|United Kingdom
>Spike|hCoV-19/England/ALDP-9509A3/2020|2020-06-14|EPI_ISL_554439|Original|hCoV-19^^England|Human|Lighthouse Lab in Alderley Park|Wellcome Sanger Institute for the COVID-19 Genomics UK (COG-UK) consortium|Davies|United Kingdom
>Spike|hCoV-19/Australia/VIC10651/2020|2020-07-31|EPI_ISL_563016|Original|hCoV-19^^Victoria|Human|Microbiological Diagnostic Unit - Public Health Laboratory (MDU-PHL)|MDU-PHL|Schultz|Australia
>Spike|hCoV-19/Germany/HH-hpi-p1462/2020|2020-03-12|EPI_ISL_775975|Original|hCoV-19^^Hamburg|Human|University Medical Center Hamburg Eppendorf|Heinrich Pette Institute|Robitaille|Germany
>Spike|hCoV-19/England/ALDP-982B75/2020|2020-08-10|EPI_ISL_552579|Original|hCoV-19^^England|Human|Lighthouse Lab in Alderley Park|Wellcome Sanger Institute for the COVID-19 Genomics UK (COG-UK) consortium|Davies|United Kingdom
>Spike|hCoV-19/England/ALDP-B634C8/2020|2020-11-09|EPI_ISL_656487|Original|hCoV-19^^England|Human|Lighthouse Lab in Alderley Park|Wellcome Sanger Institute for the COVID-19 Genomics UK (COG-UK) Consortium|Davies|United Kingdom
>Spike|hCoV-19/Switzerland/SO-ETHZ-340102/2020|2020-10-26|EPI_ISL_721868|Original|hCoV-19^^Solothurn|Human|Viollier AG|Department of Biosystems Science and Engineering|Nadeau|Switzerland
>Spike|hCoV-19/Scotland/QEUH-9B9D8E/2020|2020-09-07|EPI_ISL_568239|Original|hCoV-19^^Scotland|Human|Lighthouse Lab in Glasgow|Wellcome Sanger Institute for the COVID-19 Genomics UK (COG-UK) consortium|Davies|United Kingdom
>Spike|hCoV-19/England/MILK-A6B3F1/2020|2020-10-08|EPI_ISL_608806|Original|hCoV-19^^England|Human|Lighthouse Lab in Milton Keynes|Wellcome Sanger Institute for the COVID-19 Genomics UK (COG-UK) consortium|Davies|United Kingdom
>Spike|hCoV-19/USA/TX-HMH-1826/2020|2020-05-16|EPI_ISL_542867|Original|hCoV-19^^Texas|Human|Houston Methodist Hospital|Houston Methodist Hospital|Long|USA
>Spike|hCoV-19/South Korea/KDCA0004/2020|2020-09-08|EPI_ISL_747245|Original|hCoV-19^^South Korea|Human|Division of Emerging Infectious Diseases|Division of Emerging Infectious Diseases|Park|South Korea
>Spike|hCoV-19/Australia/VIC9225/2020|2020-08-20|EPI_ISL_565453|Original|hCoV-19^^Victoria|Human|Microbiological Diagnostic Unit - Public Health Laboratory (MDU-PHL)|MDU-PHL|Schultz|Australia
>Spike|hCoV-19/England/ALDP-CEC0C2/2020|2020-12-21|EPI_ISL_781378|Original|hCoV-19^^England|Human|Lighthouse Lab in Alderley Park|Wellcome Sanger Institute for the COVID-19 Genomics UK (COG-UK) Consortium|Davies|United Kingdom
>Spike|hCoV-19/England/QEUH-B662CB/2020|2020-11-09|EPI_ISL_661348|Original|hCoV-19^^England|Human|Lighthouse Lab in Glasgow|Wellcome Sanger Institute for the COVID-19 Genomics UK (COG-UK) Consortium|Davies|United Kingdom
>Spike|hCoV-19/Scotland/QEUH-9E35DE/2020|2020-09-24|EPI_ISL_585509|Original|hCoV-19^^Scotland|Human|Northumbria University / South Tees Hospitals NHS Foundation Trust / North Cumbria Integrated Care NHS Foundation Trust / North Tees and Hartlepool NHS Foundation Trust / Newcastle Hospitals NHS Foundation Trust|COVID-19 Genomics UK (COG-UK) Consortium|Bashton|United Kingdom
>Spike|hCoV-19/USA/LA-CDC-1266/2020|2020-06-10|EPI_ISL_535307|Vero P1|hCoV-19^^Louisiana|Human|LA Office of Public Health Laboratories|Pathogen Discovery|Queen|USA
>Spike|hCoV-19/England/ALDP-B4856C/2020|2020-11-07|EPI_ISL_649415|Original|hCoV-19^^England|Human|Lighthouse Lab in Alderley Park|Wellcome Sanger Institute for the COVID-19 Genomics UK (COG-UK) Consortium|Davies|United Kingdom
>Spike|hCoV-19/Japan/PG-3392/2020|2020-05-00|EPI_ISL_691506|Original|hCoV-19^^Japan|Human|Pathogen Genomics Center|Pathogen Genomics Center|Sekizuka|Japan
>Spike|hCoV-19/USA/TX-HMH-MCoV-16279/2020|2020-10-30|EPI_ISL_785963|Original|hCoV-19^^Texas|Human|Houston Methodist Hospital|Houston Methodist Hospital|Long|USA
>Spike|hCoV-19/England/MILK-A7E012/2020|2020-10-12|EPI_ISL_638482|Original|hCoV-19^^England|Human|Oxford Viromics|COVID-19 Genomics UK (COG-UK) Consortium|Golubchik|United Kingdom

>Spike|hCoV-19/England/CAMC-9EC5C6/2020|2020-09-24|EPI_ISL_580341|Original|hCoV-
19^^England|Human|Lighthouse Lab in Cambridge|Wellcome Sanger Institute for the
COVID-19 Genomics UK (COG-UK) consortium|Davies|United Kingdom
>Spike|hCoV-19/England/GSTT-265D509/2020|2020-03-23|EPI_ISL_483971|Original|hCoV-
19^^England|Human|Centre for Clinical Infection and Diagnostics Research and Genomics
Innovation Unit|COVID-19 Genomics UK (COG-UK) Consortium|Awan|United Kingdom
>Spike|hCoV-19/South Korea/KCDC2171/2020|2020-03-23|EPI_ISL_506965|Original|hCoV-
19^^South Korea|Human|Division of Viral Diseases|Division of Viral Diseases|Kim|South
Korea
>Spike|hCoV-19/USA/TX-HMH-1981/2020|2020-05-18|EPI_ISL_542934|Original|hCoV-
19^^Texas|Human|Houston Methodist Hospital|Houston Methodist Hospital|Long|USA
>Spike|hCoV-19/USA/CA-CZB-14334/2020|2020-07-23|EPI_ISL_739407|Original|hCoV-
19^^California|Human|Alameda County Public Health Lab|Chan-Zuckerberg Biohub|Kamm|USA
>Spike|hCoV-19/USA/TX-HMH-4115/2020|2020-06-18|EPI_ISL_543382|Original|hCoV-
19^^Texas|Human|Houston Methodist Hospital|Houston Methodist Hospital|Long|USA
>Spike|hCoV-19/USA/TX-HMH-MCoV-13302/2020|2020-08-25|EPI_ISL_788450|Original|hCoV-
19^^Texas|Human|Houston Methodist Hospital|Houston Methodist Hospital|Long|USA
>Spike|hCoV-19/USA/TX-HMH-4952/2020|2020-06-20|EPI_ISL_544426|Original|hCoV-
19^^Texas|Human|Houston Methodist Hospital|Houston Methodist Hospital|Long|USA
>Spike|hCoV-19/England/QEUH-C342F2/2020|2020-12-11|EPI_ISL_733926|Original|hCoV-
19^^England|Human|Lighthouse Lab in Glasgow|Wellcome Sanger Institute for the COVID-
19 Genomics UK (COG-UK) Consortium|Davies|United Kingdom
>Spike|hCoV-19/England/QEUH-B3219E/2020|2020-11-04|EPI_ISL_646425|Original|hCoV-
19^^England|Human|Lighthouse Lab in Glasgow|Wellcome Sanger Institute for the COVID-
19 Genomics UK (COG-UK) Consortium|Davies|United Kingdom
>Spike|hCoV-19/Germany/SH-ChVir9275/2020|2020-09-28|EPI_ISL_729616|Original|hCoV-
19^^Schleswig-Holstein|Human|A. Krumbholz|Charit\u00e9 Universit\u00e4tsmedizin
Berlin|Muehlemann|Germany
>Spike|hCoV-19/Netherlands/NB-EMC-410/2020|2020-00-00|EPI_ISL_722484|Original|hCoV-
19^^Noord Brabant|Human|Dutch COVID-19 response team|Erasmus Medical Center|Oude
Munnink|Netherlands
>Spike|hCoV-19/England/ALDP-9E9F16/2020|2020-09-24|EPI_ISL_580212|Original|hCoV-
19^^England|Human|Lighthouse Lab in Alderley Park|Wellcome Sanger Institute for the
COVID-19 Genomics UK (COG-UK) consortium|Davies|United Kingdom
>Spike|hCoV-19/Jordan/AQ-ALSR-5261/2020|2020-10-06|EPI_ISL_730499|Original|hCoV-
19^^Aqaba|Human|Biolab Diagnostic Laboratories|Andersen lab at Scripps
Research|Gangavarapu|Jordan
>Spike|hCoV-19/Germany/NW-UBI-10/2020|2020-04-03|EPI_ISL_602291|original|hCoV-
19^^North Rhine-Westphalia|Human|Evangelisches Klinikum Bethel|Bielefeld
University|Brandt|Germany
>Spike|hCoV-19/Northern Ireland/OXON-F40885/2020|2020-12-
09|EPI_ISL_764539|Original|hCoV-19^^Northern Ireland|Human|Oxford Viromics|COVID-19
Genomics UK (COG-UK) Consortium|Golubchik|United Kingdom
>Spike|hCoV-19/Panama/330754/2020|2020-03-12|EPI_ISL_496671|Original|hCoV-19^^Panama
West|Human|Gorgas Memorial Laboratory of Health Studies|Gorgas Memorial Laboratory of
Health Studies|Martinez|Panama
>Spike|hCoV-19/England/ALDP-C57E5F/2020|2020-12-13|EPI_ISL_736061|Original|hCoV-
19^^England|Human|Lighthouse Lab in Alderley Park|Wellcome Sanger Institute for the
COVID-19 Genomics UK (COG-UK) Consortium|Davies|United Kingdom
>Spike|hCoV-19/Wales/PHWC-49C7C4/2020|2020-11-19|EPI_ISL_742603|Original|hCoV-
19^^Wales|Human|Wales Specialist Virology Centre Sequencing lab|COVID-19 Genomics UK
(COG-UK) Consortium|Moore|United Kingdom
>Spike|hCoV-19/England/ALDP-A2DEDC/2020|2020-10-06|EPI_ISL_586657|Original|hCoV-
19^^England|Human|Lighthouse Lab in Alderley Park|Wellcome Sanger Institute for the
COVID-19 Genomics UK (COG-UK) consortium|Davies|United Kingdom
>Spike|hCoV-19/England/QEUH-9F898F/2020|2020-09-27|EPI_ISL_588788|Original|hCoV-
19^^England|Human|Lighthouse Lab in Glasgow|Wellcome Sanger Institute for the COVID-
19 Genomics UK (COG-UK) consortium|Davies|United Kingdom

>Spike|hCoV-19/Brazil/SP-L10-CD143/2020|2020-03-13|EPI_ISL_476173|Original|hCoV-19^^S\u00e3o Paulo|Human|DB Diagn\u00f3sticos do Brasil|Instituto de Medicina Tropical da Univesidade de S\u00e3o Paulo|Sabino|Brazil
>Spike|hCoV-19/Wales/PHWC-4855F1/2020|2020-10-26|EPI_ISL_628198|Original|hCoV-19^^Wales|Human|Wales Specialist Virology Centre Sequencing lab|COVID-19 Genomics UK (COG-UK) Consortium|Moore|United Kingdom
>Spike|hCoV-19/USA/AZ-TG640919/2020|2020-04-27|EPI_ISL_695816|Original|hCoV-19^^Arizona|Human|Sonora Quest Laboratories|TGen North|French|USA
>Spike|hCoV-19/Wales/PHWC-4805AB/2020|2020-10-18|EPI_ISL_611887|Original|hCoV-19^^Wales|Human|Wales Specialist Virology Centre Sequencing lab|COVID-19 Genomics UK (COG-UK) Consortium|Moore|United Kingdom
>Spike|hCoV-19/England/MILK-993661/2020|2020-08-26|EPI_ISL_551928|Original|hCoV-19^^England|Human|Lighthouse Lab in Milton Keynes|Wellcome Sanger Institute for the COVID-19 Genomics UK (COG-UK) consortium|Davies|United Kingdom
>Spike|hCoV-19/England/EXET-1353EA/2020|2020-04-13|EPI_ISL_457055|Original|hCoV-19^^England|Human|University of Exeter|COVID-19 Genomics UK (COG-UK) Consortium|Temperton|United Kingdom
>Spike|hCoV-19/USA/TX-HMH-MCoV-19600/2020|2020-11-24|EPI_ISL_783970|Original|hCoV-19^^Texas|Human|Houston Methodist Hospital|Houston Methodist Hospital|Long|USA
>Spike|hCoV-19/Israel/CVL-n-7422/2020|2020-04-11|EPI_ISL_475023|Original|hCoV-19^^Israel|Human|Israel Central Virology laboratory|Israel Central Virology laboratory|Zuckerman|Israel
>Spike|hCoV-19/England/ALDP-5C78EA/2020|2020-06-19|EPI_ISL_558559|Original|hCoV-19^^England|Human|Lighthouse Lab in Alderley Park|Wellcome Sanger Institute for the COVID-19 Genomics UK (COG-UK) consortium|Davies|United Kingdom
>Spike|hCoV-19/USA/UT-02593/2020|2020-04-26|EPI_ISL_522259|Original|hCoV-19^^Utah|Human|Utah Public Health Laboratory|Utah Public Health Laboratory|Young|USA
>Spike|hCoV-19/England/20164010104/2020|2020-04-14|EPI_ISL_528397|Original|hCoV-19^^England|Human|Respiratory Virus Unit|Respiratory Virus Unit|Miah|United Kingdom
>Spike|hCoV-19/USA/NM-UNM-00575/2020|2020-08-07|EPI_ISL_542989|Original|hCoV-19^^New Mexico|Human|TriCore Reference Laboratories|Center for Global Health|Domman|USA
>Spike|hCoV-19/England/NOTT-10E0E3/2020|2020-03-19|EPI_ISL_425497|Original|hCoV-19^^England|Human|Queens Medical Centre|COVID-19 Genomics UK (COG-UK) Consortium|Loose|United Kingdom
>Spike|hCoV-19/England/ALDP-951F28/2020|2020-06-09|EPI_ISL_551322|Original|hCoV-19^^England|Human|Lighthouse Lab in Alderley Park|Wellcome Sanger Institute for the COVID-19 Genomics UK (COG-UK) consortium|Davies|United Kingdom
>Spike|hCoV-19/England/ALDP-BE963D/2020|2020-12-03|EPI_ISL_708882|Original|hCoV-19^^England|Human|Lighthouse Lab in Alderley Park|Wellcome Sanger Institute for the COVID-19 Genomics UK (COG-UK) Consortium|Davies|United Kingdom
>Spike|hCoV-19/USA/WI-UW-614/2020|2020-06-23|EPI_ISL_484989|Original|hCoV-19^^Wisconsin|Human|University of Wisconsin-Madison AIDS Vaccine Research Laboratories|University of Wisconsin-Madison AIDS Vaccine Research Laboratories|Baczenas|USA
>Spike|hCoV-19/USA/NY-Wadsworth-288623-01/2020|2020-12-16|EPI_ISL_767483|Original|hCoV-19^^New York|Human|Wadsworth Center|Wadsworth Center|Lasek-Nesselquist|USA
>Spike|hCoV-19/England/MILK-B55A9A/2020|2020-11-05|EPI_ISL_657367|Original|hCoV-19^^England|Human|Lighthouse Lab in Milton Keynes|Wellcome Sanger Institute for the COVID-19 Genomics UK (COG-UK) Consortium|Davies|United Kingdom
>Spike|hCoV-19/USA/WA-S2690/2020|2020-06-22|EPI_ISL_497322|Original|hCoV-19^^Washington|Human|Washington State Department of Health|Seattle Flu Study|Lee|USA
>Spike|hCoV-19/USA/TX-HMH-MCoV-14354/2020|2020-10-06|EPI_ISL_787639|Original|hCoV-19^^Texas|Human|Houston Methodist Hospital|Houston Methodist Hospital|Long|USA
>Spike|hCoV-19/England/CAMB-79AF5/2020|2020-03-27|EPI_ISL_433823|Original|hCoV-19^^England|Human|Department of Pathology|COVID-19 Genomics UK (COG-UK) Consortium|Meredith|United Kingdom
>Spike|hCoV-19/Scotland/QEUH-9A3FCD/2020|2020-09-01|EPI_ISL_568380|Original|hCoV-19^^Scotland|Human|Lighthouse Lab in Glasgow|Wellcome Sanger Institute for the COVID-19 Genomics UK (COG-UK) consortium|Davies|United Kingdom

>Spike|hCoV-19/Canada/ON_MU-S679/2020|2020-04-09|EPI_ISL_463992|Original|hCoV-19^^Ontario|Human|Toronto Invasive Bacterial Diseases Network|McMaster University|McArthur|Canada
>Spike|hCoV-19/England/ALDP-C3C6A3/2020|2020-12-10|EPI_ISL_731820|Original|hCoV-19^^England|Human|Lighthouse Lab in Alderley Park|Wellcome Sanger Institute for the COVID-19 Genomics UK (COG-UK) Consortium|Davies|United Kingdom
>Spike|hCoV-19/USA/TX-HMH-2197/2020|2020-05-21|EPI_ISL_545175|Original|hCoV-19^^Texas|Human|Houston Methodist Hospital|Houston Methodist Hospital|Long|USA
>Spike|hCoV-19/England/MILK-A1927D/2020|2020-09-30|EPI_ISL_679627|Original|hCoV-19^^England|Human|University College London|COVID-19 Genomics UK (COG-UK) Consortium|Roy|United Kingdom
>Spike|hCoV-19/Denmark/DCGC-18231/2020|2020-12-14|EPI_ISL_749829|Original|hCoV-19^^Hovedstaden|Human|Department of Virus and Microbiological Special Diagnostics|Albertsen Lab|Michaelsen|Denmark
>Spike|hCoV-19/Spain/MD-IBV-99008682/2020|2020-10-06|EPI_ISL_654237|Original|hCoV-19^^Madrid|Human|Hospital General Universitario Gregorio Mara\u00f1\u00f3n|SeqCOVID-SPAIN consortium/IBV(CSIC)|Comas|Spain
>Spike|hCoV-19/Japan/IC-0180/2020|2020-09-00|EPI_ISL_667609|Original|hCoV-19^^Japan|Human|Pathogen Genomics Center|Pathogen Genomics Center|Sekizuka|Japan
>Spike|hCoV-19/Wales/PHWC-498ED2/2020|2020-11-30|EPI_ISL_727226|Original|hCoV-19^^Wales|Human|Originating lab|COVID-19 Genomics UK (COG-UK) Consortium|Moore|United Kingdom
>Spike|hCoV-19/Japan/PG-0993/2020|2020-04-00|EPI_ISL_684603|Original|hCoV-19^^Japan|Human|Pathogen Genomics Center|Pathogen Genomics Center|Sekizuka|Japan
>Spike|hCoV-19/USA/CA-ALSR-4848/2020|2020-11-04|EPI_ISL_730176|Original|hCoV-19^^California|Human|San Diego County Public Health Laboratory|Andersen lab at Scripps Research|Gangavarapu|USA
>Spike|hCoV-19/England/OXON-B2D06/2020|2020-04-14|EPI_ISL_534808|Original|hCoV-19^^England|Human|Oxford Viromics|COVID-19 Genomics UK (COG-UK) Consortium|Golubchik|United Kingdom
>Spike|hCoV-19/England/LIVE-9C300/2020|2020-03-30|EPI_ISL_449529|Original|hCoV-19^^England|Human|Liverpool Clinical Laboratories|COVID-19 Genomics UK (COG-UK) Consortium|Haldenby|United Kingdom
>Spike|hCoV-19/Netherlands/Zeeland_35/2020|2020-04-27|EPI_ISL_461274|Original|hCoV-19^^Zeeland|Human|Dutch COVID-19 response team|Erasmus Medical Center|Oude Munnink|Netherlands
>Spike|hCoV-19/USA/WI-UW-792/2020|2020-07-05|EPI_ISL_495496|Original|hCoV-19^^Wisconsin|Human|University of Wisconsin-Madison AIDS Vaccine Research Laboratories|University of Wisconsin-Madison AIDS Vaccine Research Laboratories|Baczenas|USA
>Spike|hCoV-19/Japan/PG-7650/2020|2020-08-00|EPI_ISL_686752|Original|hCoV-19^^Japan|Human|Chiba Prefectural Institute of Public Health|Pathogen Genomics Center|Sekizuka|Japan
>Spike|hCoV-19/England/CAMB-1B6518/2020|2020-09-22|EPI_ISL_576919|Original|hCoV-19^^England|Human|Department of Pathology|COVID-19 Genomics UK (COG-UK) Consortium|Goodfellow|United Kingdom
>Spike|hCoV-19/Scotland/EDB8349/2020|2020-10-05|EPI_ISL_612337|Original|hCoV-19^^Scotland|Human|Virology Department|COVID-19 Genomics UK (COG-UK) Consortium|McHugh|United Kingdom
>Spike|hCoV-19/Indonesia/JT-NIHRD-PME3225/2020|2020-07-17|EPI_ISL_791987|Original|hCoV-19^^Center Java|Human|RSUD dr. Loekmono Hadi Kudus|National Institute of Health Research and Development|Setiawati|Indonesia
>Spike|hCoV-19/England/ALDP-9CD112/2020|2020-09-16|EPI_ISL_566407|Original|hCoV-19^^England|Human|Lighthouse Lab in Alderley Park|Wellcome Sanger Institute for the COVID-19 Genomics UK (COG-UK) consortium|Davies|United Kingdom
>Spike|hCoV-19/USA/TX-HMH-MCoV-19215/2020|2020-11-24|EPI_ISL_783695|Original|hCoV-19^^Texas|Human|Houston Methodist Hospital|Houston Methodist Hospital|Long|USA
>Spike|hCoV-19/England/QEUH-AF48A3/2020|2020-10-30|EPI_ISL_663040|Original|hCoV-19^^England|Human|Lighthouse Lab in Glasgow|Wellcome Sanger Institute for the COVID-19 Genomics UK (COG-UK) Consortium|Davies|United Kingdom

>Spike|hCoV-19/England/PORT-2F5707/2020|2020-00-00|EPI_ISL_611762|Original|hCoV-19^^England|Human|Centre for Enzyme Innovation|COVID-19 Genomics UK (COG-UK) Consortium|Robson|United Kingdom
>Spike|hCoV-19/USA/AZ-TG664242/2020|2020-04-28|EPI_ISL_696046|Original|hCoV-19^^Arizona|Human|Sonora Quest Laboratories|TGen North|French|USA
>Spike|hCoV-19/Wales/PHWC-3660F/2020|2020-05-01|EPI_ISL_474690|Original|hCoV-19^^Wales|Human|Originating lab|COVID-19 Genomics UK (COG-UK) Consortium|Moore|United Kingdom
>Spike|hCoV-19/Portugal/PT1429/2020|2020-03-28|EPI_ISL_511388|Original|hCoV-19^^Portugal|Human|Instituto Nacional de Saude (INSA)|Instituto Nacional de Saude (INSA)|Borges|Portugal
>Spike|hCoV-19/England/ALDP-A67841/2020|2020-10-09|EPI_ISL_630644|Original|hCoV-19^^England|Human|Lighthouse Lab in Alderley Park|Wellcome Sanger Institute for the COVID-19 Genomics UK (COG-UK) consortium|Davies|United Kingdom
>Spike|hCoV-19/Norway/CS-28/2020|2020-04-21|EPI_ISL_666608|Original|hCoV-19^^Viken|Human|Dept. of Microbiology and Infection Control|Dept. of Microbiology and Infection Control|Hesselberg L\u00f8vestad|Norway
>Spike|hCoV-19/England/MILK-A6A6BD/2020|2020-10-09|EPI_ISL_627514|Original|hCoV-19^^England|Human|Virology Department|COVID-19 Genomics UK (COG-UK) Consortium|de Silva|United Kingdom
>Spike|hCoV-19/England/CAMB-7E322/2020|2020-04-17|EPI_ISL_433471|Original|hCoV-19^^England|Human|Department of Pathology|COVID-19 Genomics UK (COG-UK) Consortium|Meredith|United Kingdom
>Spike|hCoV-19/England/CAMB-1B5CE7/2020|2020-08-20|EPI_ISL_529524|Original|hCoV-19^^England|Human|Department of Pathology|COVID-19 Genomics UK (COG-UK) Consortium|Meredith|United Kingdom
>Spike|hCoV-19/Australia/VIC4731/2020|2020-07-14|EPI_ISL_519119|Original|hCoV-19^^Victoria|Human|Microbiological Diagnostic Unit - Public Health Laboratory (MDU-PHL)|MDU-PHL|Schultz|Australia
>Spike|hCoV-19/England/ALDP-66A9D6/2020|2020-07-02|EPI_ISL_558205|Original|hCoV-19^^England|Human|Lighthouse Lab in Alderley Park|Wellcome Sanger Institute for the COVID-19 Genomics UK (COG-UK) consortium|Davies|United Kingdom
>Spike|hCoV-19/France/NAQ-SC251/2020|2020-03-16|EPI_ISL_645174|Original|hCoV-19^^Nouvelle-Aquitaine|Human|CHU de Limoges|CNR Virus des Infections Respiratoires - France SUD|Bal|France
>Spike|hCoV-19/USA/FL-BPHL-2025/2020|2020-03-13|EPI_ISL_653178|Original|hCoV-19^^Florida|Human|Florida Bureau of Public Health Laboratories|Florida Bureau of Public Health Laboratories|Blanton|USA
>Spike|hCoV-19/England/CAMC-A58667/2020|2020-10-09|EPI_ISL_597046|Original|hCoV-19^^England|Human|Lighthouse Lab in Cambridge|Wellcome Sanger Institute for the COVID-19 Genomics UK (COG-UK) consortium|Davies|United Kingdom
>Spike|hCoV-19/England/CAMC-B079D6/2020|2020-10-30|EPI_ISL_643963|Original|hCoV-19^^England|Human|Lighthouse Lab in Cambridge|Wellcome Sanger Institute for the COVID-19 Genomics UK (COG-UK) Consortium|Davies|United Kingdom
>Spike|hCoV-19/England/ALDP-B24AB2/2020|2020-11-04|EPI_ISL_647098|Original|hCoV-19^^England|Human|Lighthouse Lab in Alderley Park|Wellcome Sanger Institute for the COVID-19 Genomics UK (COG-UK) Consortium|Davies|United Kingdom
>Spike|hCoV-19/USA/AR-CDC-4299/2020|2020-07-17|EPI_ISL_527648|Original|hCoV-19^^Arkansas|Human|AR Dept. of Health-Public Health Lab|Pathogen Discovery|Queen|USA
>Spike|hCoV-19/England/CAMC-BC197E/2020|2020-11-22|EPI_ISL_704302|Original|hCoV-19^^England|Human|Department of Pathology|COVID-19 Genomics UK (COG-UK) Consortium|Goodfellow|United Kingdom
>Spike|hCoV-19/Japan/PG-7957/2020|2020-05-00|EPI_ISL_687058|Original|hCoV-19^^Japan|Human|Kanagawa Prefectural Institute of Public Health|Pathogen Genomics Center|Sekizuka|Japan
>Spike|hCoV-19/England/QEUH-C32F75/2020|2020-12-11|EPI_ISL_734089|Original|hCoV-19^^England|Human|Lighthouse Lab in Glasgow|Wellcome Sanger Institute for the COVID-19 Genomics UK (COG-UK) Consortium|Davies|United Kingdom
>Spike|hCoV-19/Netherlands/NH-RIVM-20700/2020|2020-12-19|EPI_ISL_790767|Original|hCoV-19^^Noord-Holland|Human|Dutch COVID-19 response

team|National Institute for Public Health and the Environment (RIVM)|Meijer|Netherlands
>Spike|hCoV-19/England/QEUH-9F3535/2020|2020-09-24|EPI_ISL_588631|Original|hCoV-19^^England|Human|Lighthouse Lab in Glasgow|Wellcome Sanger Institute for the COVID-19 Genomics UK (COG-UK) consortium|Davies|United Kingdom
>Spike|hCoV-19/Australia/VIC2278/2020|2020-06-27|EPI_ISL_522000|Original|hCoV-19^^Victoria|Human|Microbiological Diagnostic Unit - Public Health Laboratory (MDU-PHL)|MDU-PHL|Schultz|Australia
>Spike|hCoV-19/Australia/VIC3099/2020|2020-07-04|EPI_ISL_520806|Original|hCoV-19^^Victoria|Human|Victorian Infectious Diseases Reference Laboratory (VIDRL)|VIDRL and MDU-PHL|Schultz|Australia
>Spike|hCoV-19/England/SHEF-CB9AC/2020|2020-04-24|EPI_ISL_479249|Original|hCoV-19^^England|Human|Virology Department|COVID-19 Genomics UK (COG-UK) Consortium|de Silva|United Kingdom
>Spike|hCoV-19/Portugal/PT1947/2020|2020-11-11|EPI_ISL_732174|Original|hCoV-19^^Portugal|Human|Instituto Nacional de Saude (INSA) and Instituto Gulbenkian de Ciencia (IGC)|Instituto Nacional de Saude (INSA) and Instituto Gulbenkian de Ciencia (IGC)|Borges|Portugal
>Spike|hCoV-19/England/NORW-EED89/2020|2020-11-29|EPI_ISL_704908|Original|hCoV-19^^England|Human|Quadram Institute Bioscience|COVID-19 Genomics UK (COG-UK) Consortium|Alikhan|United Kingdom
>Spike|hCoV-19/USA/TX-HMH-6185/2020|2020-07-04|EPI_ISL_547297|Original|hCoV-19^^Texas|Human|Houston Methodist Hospital|Houston Methodist Hospital|Long|USA
>Spike|hCoV-19/Australia/VIC8586/2020|2020-08-15|EPI_ISL_565101|Original|hCoV-19^^Victoria|Human|Microbiological Diagnostic Unit - Public Health Laboratory (MDU-PHL)|MDU-PHL|Schultz|Australia
>Spike|hCoV-19/Wales/PHWC-4A1665/2020|2020-12-10|EPI_ISL_743706|Original|hCoV-19^^Wales|Human|Wales Specialist Virology Centre Sequencing lab|COVID-19 Genomics UK (COG-UK) Consortium|Moore|United Kingdom
>Spike|hCoV-19/Switzerland/VS-ETHZ-320408/2020|2020-10-12|EPI_ISL_737833|Original|hCoV-19^^Valais|Human|Viollier AG|Department of Biosystems Science and Engineering|Nadeau|Switzerland
>Spike|hCoV-19/USA/MA-MGH-00097/2020|2020-03-24|EPI_ISL_460326|Original|hCoV-19^^Massachusetts|Human|Massachusetts General Hospital|Infectious Disease Program|Lemieux|USA
>Spike|hCoV-19/England/CAMB-7B510/2020|2020-04-13|EPI_ISL_433970|Original|hCoV-19^^England|Human|Department of Pathology|COVID-19 Genomics UK (COG-UK) Consortium|Meredith|United Kingdom
>Spike|hCoV-19/USA/MI-MDHHS-SC21813/2020|2020-06-29|EPI_ISL_565856|Original|hCoV-19^^Michigan|Human|Michigan Department of Health and Human Services|Michigan Department of Health and Human Services|Blankenship|USA
>Spike|hCoV-19/Chile/RM-CMM-0007M/2020|2020-05-01|EPI_ISL_629020|Original|hCoV-19^^Region Metropolitana de Santiago|Human|Centro de Biotecnolog\u00eda Vegetal|Center for Mathematical Modeling and Center for Genome Regulation. Santiago|Palma V\u00e9jares|Chile
>Spike|hCoV-19/Denmark/DCGC-3371/2020|2020-08-31|EPI_ISL_616128|Original|hCoV-19^^Midtjylland|Human|Department of Virus and Microbiological Special Diagnostics|Albertsen lab|Michaelsen|Denmark
>Spike|hCoV-19/England/QEUH-B69B36/2020|2020-11-11|EPI_ISL_661547|Original|hCoV-19^^England|Human|Lighthouse Lab in Glasgow|Wellcome Sanger Institute for the COVID-19 Genomics UK (COG-UK) Consortium|Davies|United Kingdom
>Spike|hCoV-19/Denmark/DCGC-4969/2020|2020-09-21|EPI_ISL_621507|Original|hCoV-19^^Hovedstaden|Human|Department of Virus and Microbiological Special Diagnostics|Albertsen lab|Michaelsen|Denmark
>Spike|hCoV-19/England/NORT-28B205/2020|2020-04-18|EPI_ISL_469305|Original|hCoV-19^^England|Human|NU-OMICS DNA Sequencing research facility|Wellcome Sanger Institute for the COVID-19 Genomics UK (COG-UK) consortium|Davies|United Kingdom
>Spike|hCoV-19/USA/MI-UM-2006S001095/2020|2020-04-18|EPI_ISL_495578|Original|hCoV-19^^Michigan|Human|University of Michigan Clinical Microbiology Laboratory|Lauring Lab|Valesano|USA

>Spike|hCoV-19/Italy/UMB-IZSUM-48682-6/2020|2020-10-29|EPI_ISL_763324|Original|hCoV-19^^Umbria|Human|Istituto Zooprofilattico Sperimentale dell' Umbria e delle Marche - Togo Rosati|Istituto Superiore di Sanit\u00e0|De Sabato|Italy
>Spike|hCoV-19/England/LEED-2A9D1F/2020|2020-03-29|EPI_ISL_538968|Original|hCoV-19^^England|Human|Leeds Teaching Hospitals NHS Trust and Public Health England|Wellcome Sanger Institute for the COVID-19 Genomics UK (COG-UK) consortium|Davies|United Kingdom
>Spike|hCoV-19/England/PORT-2D4B20/2020|2020-10-15|EPI_ISL_611983|Original|hCoV-19^^England|Human|Centre for Enzyme Innovation|COVID-19 Genomics UK (COG-UK) Consortium|Robson|United Kingdom
>Spike|hCoV-19/Japan/PG-1502/2020|2020-04-00|EPI_ISL_685109|Original|hCoV-19^^Japan|Human|Saitama Prefectural Institute of Public Health|Pathogen Genomics Center|Sekizuka|Japan
>Spike|hCoV-19/England/204291476/2020|2020-10-16|EPI_ISL_649810|Original|hCoV-19^^England|Human|Respiratory Virus Unit|COVID-19 Genomics UK (COG-UK) Consortium|Platt|United Kingdom
>Spike|hCoV-19/England/ALDP-A6E419/2020|2020-10-15|EPI_ISL_607874|Original|hCoV-19^^England|Human|Lighthouse Lab in Alderley Park|Wellcome Sanger Institute for the COVID-19 Genomics UK (COG-UK) consortium|Davies|United Kingdom
>Spike|hCoV-19/USA/WI-UW-837/2020|2020-05-03|EPI_ISL_509844|Original|hCoV-19^^Wisconsin|Human|University of Wisconsin-Madison AIDS Vaccine Research Laboratories|University of Wisconsin-Madison AIDS Vaccine Research Laboratories|Baczenas|USA
>Spike|hCoV-19/Japan/PG-5573/2020|2020-08-00|EPI_ISL_688698|Original|hCoV-19^^Japan|Human|Pathogen Genomics Center|Pathogen Genomics Center|Sekizuka|Japan
>Spike|hCoV-19/England/ALDP-B251BC/2020|2020-11-04|EPI_ISL_646787|Original|hCoV-19^^England|Human|Lighthouse Lab in Alderley Park|Wellcome Sanger Institute for the COVID-19 Genomics UK (COG-UK) Consortium|Davies|United Kingdom
>Spike|hCoV-19/India/GJ-GBRC228b/2020|2020-06-11|EPI_ISL_476866|Original|hCoV-19^^Gujarat|Human|GMERS Medical College and Hospital|Gujarat Biotechnology Research Centre|Joshi|India
>Spike|hCoV-19/England/CAMC-C994B9/2020|2020-12-17|EPI_ISL_762183|Original|hCoV-19^^England|Human|Lighthouse Lab in Cambridge|Wellcome Sanger Institute for the COVID-19 Genomics UK (COG-UK) Consortium|Davies|United Kingdom
>Spike|hCoV-19/England/QEUH-CAD8E3/2020|2020-12-20|EPI_ISL_760985|Original|hCoV-19^^England|Human|Lighthouse Lab in Glasgow|Wellcome Sanger Institute for the COVID-19 Genomics UK (COG-UK) Consortium|Davies|United Kingdom
>Spike|hCoV-19/France/PAC-IHU-0525/2020|2020-04-07|EPI_ISL_569571|Original|hCoV-19^^Provence-Alpes-C\u00f4te-d\u2019Azur|Human|MEPHI|MEPHI|Levasseur|France
>Spike|hCoV-19/India/TG-CCMB-M950/2020|2020-06-13|EPI_ISL_495266|Original|hCoV-19^^Telangana|Human|CSIR-Centre for Cellular and Molecular Biology|CSIR-Centre for Cellular and Molecular Biology|Banu|India
>Spike|hCoV-19/Wales/PHWC-16C98E/2020|2020-06-01|EPI_ISL_572612|Original|hCoV-19^^Wales|Human|Wales Specialist Virology Centre Sequencing lab|COVID-19 Genomics UK (COG-UK) Consortium|Moore|United Kingdom
>Spike|hCoV-19/England/CAMC-B322E6/2020|2020-11-05|EPI_ISL_659210|Original|hCoV-19^^England|Human|Lighthouse Lab in Cambridge|Wellcome Sanger Institute for the COVID-19 Genomics UK (COG-UK) Consortium|Davies|United Kingdom
>Spike|hCoV-19/USA/UT-UPHL-2008893/2020|2020-05-26|EPI_ISL_525007|Original|hCoV-19^^Utah|Human|Utah Public Health Laboratory|Utah Public Health Laboratory|Young|USA
>Spike|hCoV-19/USA/SC-DHEC-0550/2020|2020-06-19|EPI_ISL_529089|Original|hCoV-19^^South Carolina|Human|Microbiology Division|Microbiology Division|Flores,H.|USA
>Spike|hCoV-19/England/CAMC-9EBD83/2020|2020-09-24|EPI_ISL_584419|Original|hCoV-19^^England|Human|Queens Medical Centre|COVID-19 Genomics UK (COG-UK) Consortium|Loose|United Kingdom
>Spike|hCoV-19/USA/CA-CZB-10176/2020|2020-05-12|EPI_ISL_672245|Original|hCoV-19^^California|Human|The Ashley Laboratory|Chan-Zuckerberg Biohub|Bhatt|USA
>Spike|hCoV-19/England/205141190/2020|2020-12-16|EPI_ISL_756144|Original|hCoV-19^^England|Human|Respiratory Virus Unit|COVID-19 Genomics UK (COG-UK) Consortium|Miah|United Kingdom

>Spike|hCoV-19/England/SHEF-C3F84/2020|2020-04-03|EPI_ISL_432785|Original|hCoV-19^^England|Human|Virology Department|COVID-19 Genomics UK (COG-UK) Consortium|de Silva|United Kingdom
>Spike|hCoV-19/Wales/PHWC-4A3D6E/2020|2020-12-12|EPI_ISL_764626|Original|hCoV-19^^Wales|Human|Wales Specialist Virology Centre Sequencing lab|COVID-19 Genomics UK (COG-UK) Consortium|Moore|United Kingdom
>Spike|hCoV-19/Scotland/QEUH-AD2B0C/2020|2020-10-27|EPI_ISL_644033|Original|hCoV-19^^Scotland|Human|Lighthouse Lab in Glasgow|Wellcome Sanger Institute for the COVID-19 Genomics UK (COG-UK) Consortium|Davies|United Kingdom
>Spike|hCoV-19/England/ALDP-B1CA71/2020|2020-11-02|EPI_ISL_664843|Original|hCoV-19^^England|Human|Centre for Enzyme Innovation|COVID-19 Genomics UK (COG-UK) Consortium|Robson|United Kingdom
>Spike|hCoV-19/France/OCC-37/2020|2020-06-23|EPI_ISL_591545|Original|hCoV-19^^Occitanie|Human|CHU Purpan - Laboratoire de Virologie - Institut F\u00e9d\u00e9ratif de Biologie|CHU Purpan - Laboratoire de Virologie - Institut F\u00e9d\u00e9ratif de Biologie|Latour|France
>Spike|hCoV-19/England/SHEF-C517D/2020|2020-04-27|EPI_ISL_475381|Original|hCoV-19^^England|Human|Virology Department|COVID-19 Genomics UK (COG-UK) Consortium|de Silva|United Kingdom
>Spike|hCoV-19/England/OXON-B070A/2020|2020-04-10|EPI_ISL_478904|Original|hCoV-19^^England|Human|Oxford Viromics|COVID-19 Genomics UK (COG-UK) Consortium|Golubchik|United Kingdom
>Spike|hCoV-19/England/CAMC-B21332/2020|2020-11-03|EPI_ISL_647163|Original|hCoV-19^^England|Human|Lighthouse Lab in Cambridge|Wellcome Sanger Institute for the COVID-19 Genomics UK (COG-UK) Consortium|Davies|United Kingdom
>Spike|hCoV-19/England/ALDP-A4F988/2020|2020-10-07|EPI_ISL_597857|Original|hCoV-19^^England|Human|Lighthouse Lab in Alderley Park|Wellcome Sanger Institute for the COVID-19 Genomics UK (COG-UK) consortium|Davies|United Kingdom
>Spike|hCoV-19/England/QEUH-B0F8E0/2020|2020-11-02|EPI_ISL_641974|Original|hCoV-19^^England|Human|Lighthouse Lab in Glasgow|Wellcome Sanger Institute for the COVID-19 Genomics UK (COG-UK) Consortium|Davies|United Kingdom
>Spike|hCoV-19/Japan/PG-0703/2020|2020-04-00|EPI_ISL_684313|Original|hCoV-19^^Japan|Human|Pathogen Genomics Center|Pathogen Genomics Center|Sekizuka|Japan
>Spike|hCoV-19/Northern Ireland/QEUH-96DCAA/2020|2020-08-20|EPI_ISL_530650|Original|hCoV-19^^Northern Ireland|Human|Lighthouse Lab in Glasgow|Wellcome Sanger Institute for the COVID-19 Genomics UK (COG-UK) consortium|Davies|United Kingdom
>Spike|hCoV-19/England/ALDP-94F910/2020|2020-06-16|EPI_ISL_555222|Original|hCoV-19^^England|Human|Lighthouse Lab in Alderley Park|Wellcome Sanger Institute for the COVID-19 Genomics UK (COG-UK) consortium|Davies|United Kingdom
>Spike|hCoV-19/England/NOTT-11C914/2020|2020-11-17|EPI_ISL_665543|Original|hCoV-19^^England|Human|Queens Medical Centre|COVID-19 Genomics UK (COG-UK) Consortium|Loose|United Kingdom
>Spike|hCoV-19/Australia/VIC12647/2020|2020-07-25|EPI_ISL_592043|Original|hCoV-19^^Victoria|Human|Microbiological Diagnostic Unit - Public Health Laboratory (MDU-PHL)|MDU-PHL|Schultz|Australia
>Spike|hCoV-19/England/QEUH-A4CD70/2020|2020-10-05|EPI_ISL_596052|Original|hCoV-19^^England|Human|Quadram Institute Bioscience|COVID-19 Genomics UK (COG-UK) Consortium|Alikhan|United Kingdom
>Spike|hCoV-19/Netherlands/ZH-RIVM-20491/2020|2020-10-26|EPI_ISL_728706|Original|hCoV-19^^Zuid-holland|Human|Dutch COVID-19 response team|National Institute for Public Health and the Environment (RIVM)|Meijer|Netherlands
>Spike|hCoV-19/England/CAMC-BBCE59/2020|2020-11-21|EPI_ISL_705613|Original|hCoV-19^^England|Human|Queens Medical Centre|COVID-19 Genomics UK (COG-UK) Consortium|Loose|United Kingdom
>Spike|hCoV-19/England/ALDP-8E5397/2020|2020-06-25|EPI_ISL_556597|Original|hCoV-19^^England|Human|Lighthouse Lab in Alderley Park|Wellcome Sanger Institute for the COVID-19 Genomics UK (COG-UK) consortium|Davies|United Kingdom

>Spike|hCoV-19/England/20154083704/2020|2020-04-07|EPI_ISL_466273|Original|hCoV-
19^^England|Human|Respiratory Virus Unit|Respiratory Virus Unit|Platt|United Kingdom
>Spike|hCoV-19/Romania/ROSV_4730/2020|2020-04-26|EPI_ISL_491079|Original|hCoV-
19^^Suceava|Human|Suceava County Emergency Hospital|\Stefan cel Mare\ University
Metagenomics Lab|Lobiuc|Romania
>Spike|hCoV-19/Denmark/DCGC-10104/2020|2020-11-09|EPI_ISL_669395|Original|hCoV-
19^^Hovedstaden|Human|Department of Virus and Microbiological Special
Diagnostics|Albertsen Lab|Michaelsen|Denmark
>Spike|hCoV-19/England/20118172104/2020|2020-03-13|EPI_ISL_464995|Original|hCoV-
19^^England|Human|Respiratory Virus Unit|Respiratory Virus Unit|Platt|United Kingdom
>Spike|hCoV-19/USA/NY-Yale-025/2020|2020-03-19|EPI_ISL_420093|Original|hCoV-19^^New
York|Human|Yale Clinical Virology Laboratory|Grubaugh Lab - Yale School of Public
Health|Grubaugh|USA
>Spike|hCoV-19/USA/WA-S413/2020|2020-03-26|EPI_ISL_434173|Original|hCoV-
19^^Washington|Human|Washington State Department of Health|Seattle Flu
Study|Bedford|USA
>Spike|hCoV-19/England/ALDP-CB52DB/2020|2020-12-18|EPI_ISL_761765|Original|hCoV-
19^^England|Human|Lighthouse Lab in Alderley Park|Wellcome Sanger Institute for the
COVID-19 Genomics UK (COG-UK) Consortium|Davies|United Kingdom
>Spike|hCoV-19/England/MILK-957AC7/2020|2020-06-09|EPI_ISL_554223|Original|hCoV-
19^^England|Human|Lighthouse Lab in Milton Keynes|Wellcome Sanger Institute for the
COVID-19 Genomics UK (COG-UK) consortium|Davies|United Kingdom
>Spike|hCoV-19/USA/FL-04-UMTL-95QV/2020|2020-04-03|EPI_ISL_479759|Original|hCoV-
19^^Florida|Human|University of Miami Immunology and Histocompatibility
Laboratory|University of Miami Immunology and Histocompatibility
Laboratory|Margolles-Clark|USA
>Spike|hCoV-19/England/BRIS-18542EE/2020|2020-04-28|EPI_ISL_492274|Original|hCoV-
19^^England|Human|PHE South West Regional Laboratory|Wellcome Sanger Institute for
the COVID-19 Genomics UK (COG-UK) consortium|Davies|United Kingdom
>Spike|hCoV-19/Greece/135/2020|2020-03-10|EPI_ISL_434476|Original|hCoV-
19^^Athens|Human|Laboratory of Microbiology|Laboratory of
Biology|Karakasiliotis|Greece
>Spike|hCoV-19/Wales/PHWC-351A5/2020|2020-04-25|EPI_ISL_474436|Original|hCoV-
19^^Wales|Human|Originating lab|COVID-19 Genomics UK (COG-UK) Consortium|Moore|United
Kingdom
>Spike|hCoV-19/USA/WI-UW-916/2020|2020-07-12|EPI_ISL_509917|Original|hCoV-
19^^Wisconsin|Human|University of Wisconsin-Madison AIDS Vaccine Research
Laboratories|University of Wisconsin-Madison AIDS Vaccine Research
Laboratories|Baczenas|USA
>Spike|hCoV-19/Netherlands/ZH-EMC-972/2020|2020-10-05|EPI_ISL_722595|Original|hCoV-
19^^Zuid Holland|Human|Dutch COVID-19 response team|Erasmus Medical Center|Oude
Munnink|Netherlands
>Spike|hCoV-19/Spain/PV-IBV-001542/2020|2020-03-15|EPI_ISL_509618|Original|hCoV-
19^^Basque Country|Human|Hospital Universitario Araba. Vitoria-Gasteiz|SeqCOVID-SPAIN
consortium/IBV(CSIC)|Comas|Spain
>Spike|hCoV-19/England/20132096004/2020|2020-03-23|EPI_ISL_423829|Original|hCoV-
19^^England|Human|Respiratory Virus Unit|Respiratory Virus Unit|Platt|United Kingdom
>Spike|hCoV-19/Ireland/LH-NVRL-20IRL51314/2020|2020-09-
30|EPI_ISL_639893|Original|hCoV-19^^Louth|Human|National Virus Reference
Laboratory|National Virus Reference Laboratory|Gonzalez|Ireland
>Spike|hCoV-19/England/ALDP-A2ED1A/2020|2020-10-05|EPI_ISL_587242|Original|hCoV-
19^^England|Human|Lighthouse Lab in Alderley Park|Wellcome Sanger Institute for the
COVID-19 Genomics UK (COG-UK) consortium|Davies|United Kingdom
>Spike|hCoV-19/England/MILK-B8F7EB/2020|2020-11-13|EPI_ISL_674753|Original|hCoV-
19^^England|Human|Lighthouse Lab in Milton Keynes|Wellcome Sanger Institute for the
COVID-19 Genomics UK (COG-UK) Consortium|Davies|United Kingdom
>Spike|hCoV-19/USA/NM-NMDOH-2020214860/2020|2020-07-16|EPI_ISL_510878|Original|hCoV-
19^^New Mexico|Human|New Mexico Department of Health Scientific Laboratory|New Mexico
Department of Health Scientific Laboratory|Johnson|USA

>Spike|hCoV-19/USA/OR-OHSU-2151/2020|2020-08-05|EPI_ISL_667128|Original|hCoV-19^^Oregon|Human|OHSU Lab Services Molecular Microbiology Lab|Oregon SARS-CoV-2 Genome Sequencing Center|O'Roak|USA
>Spike|hCoV-19/Brazil/SP-L17-CD328/2020|2020-03-27|EPI_ISL_476300|Original|hCoV-19^^S\u00e3o Paulo|Human|DB Diagn\u00f3sticos do Brasil|Instituto de Medicina Tropical da Univesidade de S\u00e3o Paulo|Sabino|Brazil
>Spike|hCoV-19/England/QEUH-9F9DA0/2020|2020-09-26|EPI_ISL_595765|Original|hCoV-19^^England|Human|Oxford Viromics|COVID-19 Genomics UK (COG-UK) Consortium|Golubchik|United Kingdom
>Spike|hCoV-19/USA/MA-MGH-03452/2020|2020-11-16|EPI_ISL_765844|Original|hCoV-19^^Massachusetts|Human|Massachusetts General Hospital|Infectious Disease Program|Park|USA
>Spike|hCoV-19/England/QEUH-9605CA/2020|2020-08-03|EPI_ISL_531961|Original|hCoV-19^^England|Human|Lighthouse Lab in Glasgow|Wellcome Sanger Institute for the COVID-19 Genomics UK (COG-UK) consortium|Davies|United Kingdom
>Spike|hCoV-19/England/ALDP-C240BF/2020|2020-12-06|EPI_ISL_733808|Original|hCoV-19^^England|Human|Lighthouse Lab in Alderley Park|Wellcome Sanger Institute for the COVID-19 Genomics UK (COG-UK) Consortium|Davies|United Kingdom
>Spike|hCoV-19/England/EXET-13A242/2020|2020-11-03|EPI_ISL_664398|Original|hCoV-19^^England|Human|University of Exeter|COVID-19 Genomics UK (COG-UK) Consortium|Temperton|United Kingdom
>Spike|hCoV-19/USA/AR-CDC-2-3714076/2020|2020-09-02|EPI_ISL_751731|Original|hCoV-19^^Arkansas|Human|AR Dept. of Health-Public Health Lab|Genomics and Discovery|Queen|USA
>Spike|hCoV-19/Switzerland/GR-ETHZ-340106/2020|2020-10-26|EPI_ISL_721815|Original|hCoV-19^^Graub\u00fcnden|Human|Viollier AG|Department of Biosystems Science and Engineering|Nadeau|Switzerland
>Spike|hCoV-19/Belgium/ITM-C5185/2020|2020-09-16|EPI_ISL_582130|Original|hCoV-19^^Edegem|Human|Antwerp University Hospital|Institute of Tropical Medicine|Selhorst|Belgium
>Spike|hCoV-19/England/CAMB-7325d/2020|2020-03-22|EPI_ISL_440176|Original|hCoV-19^^England|Human|Department of Pathology|Wellcome Sanger Institute for the COVID-19 Genomics UK (COG-UK) consortium|Amato|United Kingdom
>Spike|hCoV-19/Scotland/CVR1085/2020|2020-03-29|EPI_ISL_433508|Original|hCoV-19^^Scotland|Human|West of Scotland Specialist Virology Centre|COVID-19 Genomics UK (COG-UK) Consortium|Robertson|United Kingdom
>Spike|hCoV-19/USA/VA-DCLS-2277/2020|2020-10-24|EPI_ISL_723516|Original|hCoV-19^^Virginia|Human|Virginia Division of Consolidated Laboratory Services (DCLS)|Virginia Division of Consolidated Laboratory Services (DCLS)|St. Jacques|USA
>Spike|hCoV-19/England/NORT-28928F/2020|2020-03-28|EPI_ISL_458640|Original|hCoV-19^^England|Human|NU-OMICS DNA Sequencing research facility|Wellcome Sanger Institute for the COVID-19 Genomics UK (COG-UK) consortium|Davies|United Kingdom
>Spike|hCoV-19/Spain/MD-HLP-108/2020|2020-04-22|EPI_ISL_530037|Original|hCoV-19^^Madrid|Human|Hospital Universitario La Paz|Hospital Universitario La Paz|Viedma|Spain
>Spike|hCoV-19/USA/WI-UW-1930/2020|2020-09-24|EPI_ISL_677339|Original|hCoV-19^^Wisconsin|Human|University of Wisconsin-Madison AIDS Vaccine Research Laboratories|University of Wisconsin-Madison AIDS Vaccine Research Laboratories|Baczenas|USA
>Spike|hCoV-19/Japan/IC-0603/2021|2021-01-00|EPI_ISL_779689|Original|hCoV-19^^Japan|Human|Pathogen Genomics Center|Pathogen Genomics Center|Sekizuka|Japan
>Spike|hCoV-19/England/BRIS-12A61C/2020|2020-04-29|EPI_ISL_458345|Original|hCoV-19^^England|Human|PHE South West Regional Laboratory|Wellcome Sanger Institute for the COVID-19 Genomics UK (COG-UK) consortium|Davies|United Kingdom
>Spike|hCoV-19/Denmark/DCGC-19857/2020|2020-12-07|EPI_ISL_747832|Original|hCoV-19^^Sjaelland|Human|Department of Virus and Microbiological Special Diagnostics|Albertsen Lab|Michaelsen|Denmark
>Spike|hCoV-19/England/MILK-AC89D2/2020|2020-10-22|EPI_ISL_629652|Original|hCoV-19^^England|Human|Lighthouse Lab in Milton Keynes|Wellcome Sanger Institute for the COVID-19 Genomics UK (COG-UK) consortium|Davies|United Kingdom

>Spike|hCoV-19/Norway/3053/2020|2020-08-07|EPI_ISL_549082|Original|hCoV-19^^Oslo|Human|Furst Medical Laboratory|Norwegian Institute of Public Health|Kopperud Riis|Norway
>Spike|hCoV-19/USA/MI-MDHHS-SC20720/2020|2020-04-20|EPI_ISL_471744|Original|hCoV-19^^Michigan|Human|Michigan Department of Health and Human Services|Michigan Department of Health and Human Services|Blankenship|USA
>Spike|hCoV-19/Denmark/DCGC-5132/2020|2020-09-21|EPI_ISL_621670|Original|hCoV-19^^Syddanmark|Human|Department of Virus and Microbiological Special Diagnostics|Albertsen lab|Michaelsen|Denmark
>Spike|hCoV-19/England/ALDP-B5DAFC/2020|2020-11-10|EPI_ISL_704395|Original|hCoV-19^^England|Human|Department of Pathology|COVID-19 Genomics UK (COG-UK) Consortium|Goodfellow|United Kingdom
>Spike|hCoV-19/Scotland/CVR1695/2020|2020-04-05|EPI_ISL_433655|Original|hCoV-19^^Scotland|Human|West of Scotland Specialist Virology Centre|COVID-19 Genomics UK (COG-UK) Consortium|Robertson|United Kingdom
>Spike|hCoV-19/England/QEUH-B69C33/2020|2020-11-11|EPI_ISL_655012|Original|hCoV-19^^England|Human|Lighthouse Lab in Glasgow|Wellcome Sanger Institute for the COVID-19 Genomics UK (COG-UK) Consortium|Davies|United Kingdom
>Spike|hCoV-19/USA/CT-Yale-508/2020|2020-11-05|EPI_ISL_729785|Original|hCoV-19^^Connecticut|Human|Grubaugh Lab - Yale School of Public Health|Grubaugh Lab - Yale School of Public Health|Grubaugh|USA
>Spike|hCoV-19/Wales/PHWC-47E31F/2020|2020-10-11|EPI_ISL_595438|Original|hCoV-19^^Wales|Human|Originating lab|COVID-19 Genomics UK (COG-UK) Consortium|Moore|United Kingdom
>Spike|hCoV-19/Switzerland/ZH-ETHZ-350473/2020|2020-11-03|EPI_ISL_728836|Original|hCoV-19^^Zurich|Human|Viollier AG|Department of Biosystems Science and Engineering|Nadeau|Switzerland
>Spike|hCoV-19/Saudi Arabia/KAUST-jeddah839/2020|2020-05-24|EPI_ISL_636972|Original|hCoV-19^^Jeddah|Human|Pathogen Genomics Lab King Abdullah University of Science and Technology (KAUST)|Pathogen Genomics Lab King Abdullah University of Science and Technology (KAUST)|Mohamed Ghazzali|Saudi Arabia
>Spike|hCoV-19/Wales/PHWC-48ABAF/2020|2020-11-05|EPI_ISL_666129|Original|hCoV-19^^Wales|Human|Wales Specialist Virology Centre Sequencing lab|COVID-19 Genomics UK (COG-UK) Consortium|Moore|United Kingdom
>Spike|hCoV-19/Japan/PG-7594/2020|2020-07-00|EPI_ISL_686696|Original|hCoV-19^^Japan|Human|Chiba Prefectural Institute of Public Health|Pathogen Genomics Center|Sekizuka|Japan
>Spike|hCoV-19/Japan/PG-1820/2020|2020-04-00|EPI_ISL_685426|Original|hCoV-19^^Japan|Human|Tokyo Metropolitan Institute of Public Health|Pathogen Genomics Center|Sekizuka|Japan
>Spike|hCoV-19/USA/AZ-TG412678/2020|2020-06-25|EPI_ISL_694984|Original|hCoV-19^^Arizona|Human|TGen North|TGen North|French|USA
>Spike|hCoV-19/England/LIVE-A6093/2020|2020-03-21|EPI_ISL_499681|Original|hCoV-19^^England|Human|Liverpool Clinical Laboratories|COVID-19 Genomics UK (COG-UK) Consortium|Haldenby|United Kingdom
>Spike|hCoV-19/England/QEUH-AF5B7C/2020|2020-10-30|EPI_ISL_663184|Original|hCoV-19^^England|Human|Lighthouse Lab in Glasgow|Wellcome Sanger Institute for the COVID-19 Genomics UK (COG-UK) Consortium|Davies|United Kingdom
>Spike|hCoV-19/Denmark/DCGC-2576/2020|2020-08-17|EPI_ISL_616407|Original|hCoV-19^^Syddanmark|Human|Department of Virus and Microbiological Special Diagnostics|Albertsen lab|Michaelsen|Denmark
>Spike|hCoV-19/Luxembourg/LNS8672733/2020|2020-11-01|EPI_ISL_744783|Original|hCoV-19^^Luxembourg|Human|Laboratoire national de sant\u00e9|Laboratoire national de sant\u00e9|Wienecke-Baldacchino|Luxembourg
>Spike|hCoV-19/England/NOTT-1104B5/2020|2020-04-19|EPI_ISL_441521|Original|hCoV-19^^England|Human|Queens Medical Centre|COVID-19 Genomics UK (COG-UK) Consortium|Loose|United Kingdom
>Spike|hCoV-19/Sweden/20-52279/2020|2020-07-05|EPI_ISL_560982|Original|hCoV-19^^Stockholm|Human|Karolinska universitetslaboratoriet SOLNA|The Public Health Agency of Sweden|Svartstr\u00f6m|Sweden

>Spike|hCoV-19/Spain/AN-IBV-002983/2020|2020-03-31|EPI_ISL_474908|Original|hCoV-
19^^Andalucia|Human|Hospital Universitario Virgen de las Nieves de Granada-
SAS|SeqCOVID-SPAIN consortium/IBV(CSIC)|Comas|Spain
>Spike|hCoV-19/Australia/VIC8974/2020|2020-07-28|EPI_ISL_565325|Original|hCoV-
19^^Victoria|Human|Microbiological Diagnostic Unit - Public Health Laboratory (MDU-
PHL)|MDU-PHL|Schultz|Australia
>Spike|hCoV-19/Spain/CL-IBV-97006232/2020|2020-04-14|EPI_ISL_537378|Original|hCoV-
19^^Castilla y Leon|Human|Universidad de Le\u00f3n|SeqCOVID-SPAIN
consortium/IBV(CSIC)|Comas|Spain
>Spike|hCoV-19/Guangdong/20SF616/2020|2020-01-24|EPI_ISL_428465|Original|hCoV-
19^^Guangdong|Human|Guangdong Provincial Center for Diseases Control and
Prevention;Guangdong Provincial Institute of Public Health|School of Public
Health|Gu|China
>Spike|hCoV-19/England/BRIS-127381/2020|2020-04-22|EPI_ISL_469779|Original|hCoV-
19^^England|Human|PHE South West Regional Laboratory|Wellcome Sanger Institute for
the COVID-19 Genomics UK (COG-UK) consortium|Davies|United Kingdom
>Spike|hCoV-19/South Africa/R12723-20/2020|2020-06-16|EPI_ISL_504243|Original|hCoV-
19^^North West|Human|National Institute for Communicable Diseases of the National
Health Laboratory Service|National Institute for Communicable Diseases of the
National Health Laboratory Service|Allam|South Africa
>Spike|hCoV-19/Australia/VIC15214/2020|2020-08-03|EPI_ISL_593193|Original|hCoV-
19^^Victoria|Human|Microbiological Diagnostic Unit - Public Health Laboratory (MDU-
PHL)|MDU-PHL|Schultz|Australia
>Spike|hCoV-19/England/202822193/2020|2020-05-28|EPI_ISL_490249|Original|hCoV-
19^^England|Human|Respiratory Virus Unit|Respiratory Virus Unit|Platt|United Kingdom
>Spike|hCoV-19/Wales/PHWC-4A6484/2020|2020-12-16|EPI_ISL_765040|Original|hCoV-
19^^Wales|Human|Wales Specialist Virology Centre Sequencing lab|COVID-19 Genomics UK
(COG-UK) Consortium|Moore|United Kingdom
>Spike|hCoV-19/England/MILK-97788B/2020|2020-07-25|EPI_ISL_550506|Original|hCoV-
19^^England|Human|Lighthouse Lab in Milton Keynes|Wellcome Sanger Institute for the
COVID-19 Genomics UK (COG-UK) consortium|Davies|United Kingdom
>Spike|hCoV-19/India/OR-ILSCV16514/2020|2020-05-12|EPI_ISL_463052|Original|hCoV-
19^^Odisha|Human|Institute of Life Sciences|Immunogenomics lab|Raghav|India
>Spike|hCoV-19/England/CAMC-9D23C9/2020|2020-09-18|EPI_ISL_601604|Original|hCoV-
19^^England|Human|Lighthouse Lab in Cambridge|Wellcome Sanger Institute for the
COVID-19 Genomics UK (COG-UK) consortium|Davies|United Kingdom
>Spike|hCoV-19/Northern Ireland/NIRE-FCD4C/2020|2020-04-
27|EPI_ISL_489326|Original|hCoV-19^^Northern Ireland|Human|Regional Virus
Laboratory|Wellcome Sanger Institute for the COVID-19 Genomics UK (COG-UK)
consortium|Davies|United Kingdom
>Spike|hCoV-19/USA/MI-UM-10036438245/2020|2020-10-14|EPI_ISL_610111|Original|hCoV-
19^^Michigan|Human|University of Michigan Clinical Microbiology Laboratory|Lauring
Lab|Valesano|USA
>Spike|hCoV-19/USA/CA-CZB-1785/2020|2020-06-05|EPI_ISL_486118|Original|hCoV-
19^^California|Human|County of Santa Clara Public Health Department|Chan-Zuckerberg
Biohub|McGeever|USA
>Spike|hCoV-19/England/ALDP-6D1A04/2020|2020-07-02|EPI_ISL_556143|Original|hCoV-
19^^England|Human|Lighthouse Lab in Alderley Park|Wellcome Sanger Institute for the
COVID-19 Genomics UK (COG-UK) consortium|Davies|United Kingdom
>Spike|hCoV-19/Netherlands/FR-RIVM-20306/2020|2020-11-21|EPI_ISL_723445|Orignal|hCoV-
19^^Friesland|Human|Dutch COVID-19 response team|National Institute for Public Health
and the Environment (RIVM)|Meijer|Netherlands
>Spike|hCoV-19/Spain/VC-FISABIO-118/2020|2020-03-13|EPI_ISL_436213|Original|hCoV-
19^^Comunitat Valenciana|Human|Servicio de Microbiolog\u00eda. Consorcio Hospital
General Universitario de Valencia|Sequencing and Bioinformatics Service and Molecular
Epidemiology Research Group. FISABIO-Public Health|Gonzalez-Candelas|Spain
>Spike|hCoV-19/England/QEUH-B7C839/2020|2020-11-14|EPI_ISL_741793|Original|hCoV-
19^^England|Human|Oxford Viromics|COVID-19 Genomics UK (COG-UK)
Consortium|Golubchik|United Kingdom

>Spike|hCoV-19/England/QEUH-CDE306/2020|2020-12-26|EPI_ISL_782306|Original|hCoV-19^^England|Human|Lighthouse Lab in Glasgow|Wellcome Sanger Institute for the COVID-19 Genomics UK (COG-UK) Consortium|Davies|United Kingdom
>Spike|hCoV-19/England/ALDP-AA317B/2020|2020-10-22|EPI_ISL_630961|Original|hCoV-19^^England|Human|Lighthouse Lab in Alderley Park|Wellcome Sanger Institute for the COVID-19 Genomics UK (COG-UK) consortium|Davies|United Kingdom
>Spike|hCoV-19/England/NORT-28406C/2020|2020-05-04|EPI_ISL_488622|Original|hCoV-19^^England|Human|NU-OMICS DNA Sequencing research facility|Wellcome Sanger Institute for the COVID-19 Genomics UK (COG-UK) consortium|Davies|United Kingdom
>Spike|hCoV-19/Denmark/DCGC-16450/2020|2020-11-16|EPI_ISL_714774|Original|hCoV-19^^Sjaelland|Human|Department of Virus and Microbiological Special Diagnostics|Albertsen Lab|Michaelsen|Denmark
>Spike|hCoV-19/Canada/ON-UHTC_0022/2020|2020-03-31|EPI_ISL_464055|unknown|hCoV-19^^Ontario|Human|Unity Health Toronto|Ontario Institute for Cancer Research|de Borja|Canada
>Spike|hCoV-19/USA/MI-MDHHS-SC20912/2020|2020-04-29|EPI_ISL_476048|Original|hCoV-19^^Michigan|Human|Michigan Department of Health and Human Services|Michigan Department of Health and Human Services|Blankenship|USA
>Spike|hCoV-19/England/SHEF-10C9F12/2020|2020-12-19|EPI_ISL_741967|Original|hCoV-19^^England|Human|Virology Department|COVID-19 Genomics UK (COG-UK) Consortium|de Silva|United Kingdom
>Spike|hCoV-19/Northern Ireland/NIRE-106AA1/2020|2020-05-20|EPI_ISL_501048|Original|hCoV-19^^Northern Ireland|Human|Regional Virus Laboratory|Wellcome Sanger Institute for the COVID-19 Genomics UK (COG-UK) consortium|Davies|United Kingdom
>Spike|hCoV-19/England/ALDP-A935FA/2020|2020-10-20|EPI_ISL_611056|Original|hCoV-19^^England|Human|Lighthouse Lab in Alderley Park|Wellcome Sanger Institute for the COVID-19 Genomics UK (COG-UK) consortium|Davies|United Kingdom
>Spike|hCoV-19/USA/TX-HMH-MCoV-8269/2020|2020-07-07|EPI_ISL_784300|Original|hCoV-19^^Texas|Human|Houston Methodist Hospital|Houston Methodist Hospital|Long|USA
>Spike|hCoV-19/Denmark/DCGC-6461/2020|2020-10-12|EPI_ISL_620361|Original|hCoV-19^^Hovedstaden|Human|Department of Virus and Microbiological Special Diagnostics|Albertsen lab|Michaelsen|Denmark
>Spike|hCoV-19/DRC/1779/2020|2020-04-10|EPI_ISL_435158|Original|hCoV-19^^Kinshasa|Human|Viral Respiratory Lab|Pathogen Sequencing Lab|Pratt|Democratic Republic of the Congo
>Spike|hCoV-19/USA/FL-BPHL-0356/2020|2020-04-13|EPI_ISL_489765|Original|hCoV-19^^Florida|Human|Florida Bureau of Public Health Laboratories|Florida Bureau of Public Health Laboratories|Blanton|USA
>Spike|hCoV-19/England/ALDP-A9477B/2020|2020-10-19|EPI_ISL_624675|Original|hCoV-19^^England|Human|Lighthouse Lab in Alderley Park|Wellcome Sanger Institute for the COVID-19 Genomics UK (COG-UK) consortium|Davies|United Kingdom
>Spike|hCoV-19/USA/FL-BPHL-1190/2020|2020-08-20|EPI_ISL_526676|Original|hCoV-19^^Florida|Human|Florida Bureau of Public Health Laboratories|Florida Bureau of Public Health Laboratories|Blanton|USA
>Spike|hCoV-19/England/QEUH-9B4A8C/2020|2020-09-03|EPI_ISL_590185|Original|hCoV-19^^England|Human|Lighthouse Lab in Glasgow|Wellcome Sanger Institute for the COVID-19 Genomics UK (COG-UK) consortium|Davies|United Kingdom
>Spike|hCoV-19/USA/WA-UW-47430/2020|2020-12-11|EPI_ISL_776742|Original|hCoV-19^^Washington|Human|UW Virology Lab|UW Virology Lab|Roychoudhury|USA
>Spike|hCoV-19/Australia/VIC15633/2020|2020-07-30|EPI_ISL_593388|Original|hCoV-19^^Victoria|Human|Microbiological Diagnostic Unit - Public Health Laboratory (MDU-PHL)|MDU-PHL|Schultz|Australia
>Spike|hCoV-19/USA/OR-OHSU-2971/2020|2020-09-07|EPI_ISL_667438|Original|hCoV-19^^Oregon|Human|OHSU Lab Services Molecular Microbiology Lab|Oregon SARS-CoV-2 Genome Sequencing Center|O'Roak|USA
>Spike|hCoV-19/Denmark/DCGC-3060/2020|2020-08-24|EPI_ISL_616840|Original|hCoV-19^^Hovedstaden|Human|Department of Virus and Microbiological Special Diagnostics|Albertsen lab|Michaelsen|Denmark

>Spike|hCoV-19/Canada/QC-LSPQ-L00231860/2020|2020-03-20|EPI_ISL_535965|Original|hCoV-19^^Quebec|Human|H\u00f4pital de Chicoutimi|Laboratoire de sant\u00e9 publique du Qu\u00e9bec|Moreira|Canada
>Spike|hCoV-19/Australia/VIC14014/2020|2020-09-16|EPI_ISL_563929|Original|hCoV-19^^Victoria|Human|Microbiological Diagnostic Unit - Public Health Laboratory (MDU-PHL)|MDU-PHL|Schultz|Australia
>Spike|hCoV-19/USA/UT-UPHL-2012922250/2020|2020-06-18|EPI_ISL_746474|Original|hCoV-19^^Utah|Human|Utah Public Health Laboratory|Utah Public Health Laboratory|Gallagher|USA
>Spike|hCoV-19/England/QEUH-77ADF4/2020|2020-07-15|EPI_ISL_533172|Original|hCoV-19^^England|Human|Lighthouse Lab in Glasgow|Wellcome Sanger Institute for the COVID-19 Genomics UK (COG-UK) consortium|Davies|United Kingdom
>Spike|hCoV-19/England/QEUH-B41AA0/2020|2020-11-08|EPI_ISL_658530|Original|hCoV-19^^England|Human|Lighthouse Lab in Glasgow|Wellcome Sanger Institute for the COVID-19 Genomics UK (COG-UK) Consortium|Davies|United Kingdom
>Spike|hCoV-19/England/CAMC-AAF91D/2020|2020-10-23|EPI_ISL_631171|Original|hCoV-19^^England|Human|Lighthouse Lab in Cambridge|Wellcome Sanger Institute for the COVID-19 Genomics UK (COG-UK) consortium|Davies|United Kingdom
>Spike|hCoV-19/Belgium/rega-12141311/2020|2020-12-14|EPI_ISL_738365|Original|hCoV-19^^Mol|Human|UZ Leuven|KU Leuven|Maes|Belgium
>Spike|hCoV-19/Singapore/95/2020|2020-03-18|EPI_ISL_435700|Original|hCoV-19^^Singapore|Human|National Public Health Laboratory|National Public Health Laboratory|Mak|Singapore
>Spike|hCoV-19/Serbia/Sremska Mitrovica-011821207/2020|2020-07-11|EPI_ISL_676575|Original|hCoV-19^^Sremska Mitrovica|Human|Scientific Veterinary Institute Novi Sad|Veterinary Specialized Institute \Kraljevo\|Vidanovic|Serbia
>Spike|hCoV-19/Finland/24ES15B12/2020|2020-08-24|EPI_ISL_759809|Original|hCoV-19^^Uusimaa|Human|Department of Virology and Immunology|Department of Virology|Smura|Finland
>Spike|hCoV-19/France/PDL-SC627/2020|2020-04-01|EPI_ISL_649178|Original|hCoV-19^^Pays-de-la-Loire|Human|CHU Nantes|CNR Virus des Infections Respiratoires - France SUD|Bal|France
>Spike|hCoV-19/England/MILK-62D81E/2020|2020-07-05|EPI_ISL_557953|Original|hCoV-19^^England|Human|Lighthouse Lab in Milton Keynes|Wellcome Sanger Institute for the COVID-19 Genomics UK (COG-UK) consortium|Davies|United Kingdom
>Spike|hCoV-19/USA/TX-HMH-MCoV-15270/2020|2020-10-19|EPI_ISL_786754|Original|hCoV-19^^Texas|Human|Houston Methodist Hospital|Houston Methodist Hospital|Long|USA
>Spike|hCoV-19/England/ALDP-4A723E/2020|2020-06-13|EPI_ISL_559463|Original|hCoV-19^^England|Human|Lighthouse Lab in Alderley Park|Wellcome Sanger Institute for the COVID-19 Genomics UK (COG-UK) consortium|Davies|United Kingdom
>Spike|hCoV-19/Singapore/647/2020|2020-05-06|EPI_ISL_493407|Original|hCoV-19^^Singapore|Human|National Public Health Laboratory|National Public Health Laboratory|Mak|Singapore
>Spike|hCoV-19/Portugal/PT1442/2020|2020-03-25|EPI_ISL_511401|Original|hCoV-19^^Portugal|Human|Instituto Nacional de Saude (INSA)|Instituto Nacional de Saude (INSA)|Borges|Portugal
>Spike|hCoV-19/England/BRIS-122F46/2020|2020-04-01|EPI_ISL_440786|Original|hCoV-19^^England|Human|PHE South West Regional Laboratory|Wellcome Sanger Institute for the COVID-19 Genomics UK (COG-UK) consortium|Amato|United Kingdom
>Spike|hCoV-19/Australia/VIC4394/2020|2020-07-13|EPI_ISL_519690|Original|hCoV-19^^Victoria|Human|Microbiological Diagnostic Unit - Public Health Laboratory (MDU-PHL)|MDU-PHL|Schultz|Australia
>Spike|hCoV-19/USA/WA-S1299/2020|2020-05-14|EPI_ISL_495749|Original|hCoV-19^^Washington|Human|Washington State Department of Health|Seattle Flu Study|Lee|USA
>Spike|hCoV-19/Spain/NC-IBV-001373/2020|2020-03-13|EPI_ISL_452528|Original|hCoV-19^^Navarra|Human|Cl\u00ednica Universidad de Navarra. Servicio de Enfermedades Infecciosas y Microbiolog\u00eda cl\u00ednica|SeqCOVID-SPAIN consortium/IBV(CSIC)|Comas|Spain
>Spike|hCoV-19/Belgium/rega-09221148/2020|2020-09-22|EPI_ISL_738236|Original|hCoV-19^^Tervuren|Human|UZ Leuven|KU Leuven|Maes|Belgium

>Spike|hCoV-19/USA/IL-NM012/2020|2020-03-17|EPI_ISL_444524|Original|hCoV-19^^Illinois|Human|Northwestern Memorial Hospital|Ozer Lab|Ozer|USA
>Spike|hCoV-19/USA/UT-UPHL-2012105352/2020|2020-07-27|EPI_ISL_745797|Original|hCoV-19^^Utah|Human|Ginkgo Bioworks Clinical Laboratory|Utah Public Health Laboratory|Young|USA
>Spike|hCoV-19/Japan/PG-7450/2020|2020-07-00|EPI_ISL_688552|Original|hCoV-19^^Japan|Human|Pathogen Genomics Center|Pathogen Genomics Center|Sekizuka|Japan
>Spike|hCoV-19/USA/NY-Wadsworth-292753-01/2020|2020-12-19|EPI_ISL_769931|Original|hCoV-19^^New York|Human|Albany Medical Center Hospital Clinical Laboratories|Wadsworth Center|Lasek-Nesselquist|USA
>Spike|hCoV-19/Portugal/PT0640/2020|2020-03-29|EPI_ISL_510901|Original|hCoV-19^^Portugal|Human|Instituto Nacional de Saude (INSA)|Instituto Nacional de Saude (INSA)|Borges|Portugal
>Spike|hCoV-19/Switzerland/GE-SNRCI-6699/2020|2020-03-00|EPI_ISL_548178|Original|hCoV-19^^Geneve|Human|Laboratoire de Virologie|Swiss National Reference Centre for Influenza|Gon\u00e7alves Cabecinhas|Switzerland
>Spike|hCoV-19/Australia/VIC4512/2020|2020-07-19|EPI_ISL_518923|Original|hCoV-19^^Victoria|Human|Microbiological Diagnostic Unit - Public Health Laboratory (MDU-PHL)|MDU-PHL|Schultz|Australia
>Spike|hCoV-19/England/QEUH-C14665/2020|2020-12-06|EPI_ISL_709404|Original|hCoV-19^^England|Human|Lighthouse Lab in Glasgow|Wellcome Sanger Institute for the COVID-19 Genomics UK (COG-UK) Consortium|Davies|United Kingdom
>Spike|hCoV-19/Netherlands/Flevoland_17/2020|2020-05-22|EPI_ISL_460840|Original|hCoV-19^^Flevoland|Human|Dutch COVID-19 response team|Erasmus Medical Center|Oude Munnink|Netherlands
>Spike|hCoV-19/Australia/VIC4242/2020|2020-00-00|EPI_ISL_519570|Original|hCoV-19^^Victoria|Human|Microbiological Diagnostic Unit - Public Health Laboratory (MDU-PHL)|MDU-PHL|Schultz|Australia
>Spike|hCoV-19/Austria/CeMM0198/2020|2020-03-14|EPI_ISL_438039|Original|hCoV-19^^Vienna|Human|Center for Virology|Bergthaler laboratory|Bergthaler|Austria
>Spike|hCoV-19/USA/CA-CZB-13331/2020|2020-11-02|EPI_ISL_672437|Original|hCoV-19^^California|Human|Santa Clara County Public Health Laboratory|Chan-Zuckerberg Biohub|Bhatt|USA
>Spike|hCoV-19/Denmark/DCGC-17593/2020|2020-12-14|EPI_ISL_751092|Original|hCoV-19^^Sjaelland|Human|Department of Virus and Microbiological Special Diagnostics|Albertsen Lab|Michaelsen|Denmark
>Spike|hCoV-19/England/MILK-9C212C/2020|2020-09-11|EPI_ISL_566438|Original|hCoV-19^^England|Human|Lighthouse Lab in Milton Keynes|Wellcome Sanger Institute for the COVID-19 Genomics UK (COG-UK) consortium|Davies|United Kingdom
>Spike|hCoV-19/England/CAMC-B3235C/2020|2020-11-03|EPI_ISL_659167|Original|hCoV-19^^England|Human|Lighthouse Lab in Cambridge|Wellcome Sanger Institute for the COVID-19 Genomics UK (COG-UK) Consortium|Davies|United Kingdom
>Spike|hCoV-19/Denmark/DCGC-22267/2020|2020-12-14|EPI_ISL_757838|Original|hCoV-19^^Hovedstaden|Human|Department of Virus and Microbiological Special Diagnostics|Albertsen Lab|Michaelsen|Denmark
>Spike|hCoV-19/Wales/PHWC-49E698/2020|2020-12-07|EPI_ISL_743032|Original|hCoV-19^^Wales|Human|Wales Specialist Virology Centre Sequencing lab|COVID-19 Genomics UK (COG-UK) Consortium|Moore|United Kingdom
>Spike|hCoV-19/England/QEUH-B0F349/2020|2020-11-02|EPI_ISL_642204|Original|hCoV-19^^England|Human|Lighthouse Lab in Glasgow|Wellcome Sanger Institute for the COVID-19 Genomics UK (COG-UK) Consortium|Davies|United Kingdom
>Spike|hCoV-19/USA/AZ-TG491612/2020|2020-06-24|EPI_ISL_695194|Original|hCoV-19^^Arizona|Human|AZ SPHL|TGen North|French|USA
>Spike|hCoV-19/USA/FL-BPHL-1397/2020|2020-04-13|EPI_ISL_549179|Original|hCoV-19^^Florida|Human|Florida Bureau of Public Health Laboratories|Florida Bureau of Public Health Laboratories|Blanton|USA
>Spike|hCoV-19/England/BRIS-12377A/2020|2020-04-02|EPI_ISL_442784|Original|hCoV-19^^England|Human|PHE South West Regional Laboratory|Wellcome Sanger Institute for the COVID-19 Genomics UK (COG-UK) consortium|Amato|United Kingdom

>Spike|hCoV-19/Netherlands/ZH-EMC-1173/2020|2020-12-20|EPI_ISL_763248|Original|hCoV-19^^Zuid Holland|Human|Dutch COVID-19 response team|Erasmus Medical Center|Oude Munnink|Netherlands
>Spike|hCoV-19/USA/OH-UW-2397/2020|2020-12-05|EPI_ISL_754667|Original|hCoV-19^^Ohio|Human|University of Wisconsin-Madison AIDS Vaccine Research Laboratories|University of Wisconsin-Madison AIDS Vaccine Research Laboratories|Moreno|USA
>Spike|hCoV-19/Denmark/DCGC-6522/2020|2020-10-05|EPI_ISL_619422|Original|hCoV-19^^Midtjylland|Human|Department of Virus and Microbiological Special Diagnostics|Albertsen lab|Michaelsen|Denmark
>Spike|hCoV-19/England/MILK-B8682D/2020|2020-11-13|EPI_ISL_674072|Original|hCoV-19^^England|Human|Lighthouse Lab in Milton Keynes|Wellcome Sanger Institute for the COVID-19 Genomics UK (COG-UK) Consortium|Davies|United Kingdom
>Spike|hCoV-19/USA/TX-HMH-MCoV-8942/2020|2020-07-02|EPI_ISL_780848|Original|hCoV-19^^Texas|Human|Houston Methodist Hospital|Houston Methodist Hospital|Long|USA
>Spike|hCoV-19/India/DL-IGIB-1130078/2020|2020-06-00|EPI_ISL_641354|Original|hCoV-19^^Delhi|Human|Devki Devi Foundation|CSIR Institute of Genomics and Integrative Biology (CSIR-IGIB) / Max|Pandey|India
>Spike|hCoV-19/Australia/SAP559/2020|2020-11-16|EPI_ISL_654802|Original|hCoV-19^^South Australia|Human|SA Pathology|SA Pathology|Leong|Australia
>Spike|hCoV-19/USA/TX-HMH-MCoV-13029/2020|2020-08-28|EPI_ISL_788180|Original|hCoV-19^^Texas|Human|Houston Methodist Hospital|Houston Methodist Hospital|Long|USA
>Spike|hCoV-19/USA/HI-H200337/2020|2020-08-16|EPI_ISL_752895|Original|hCoV-19^^Hawaii|Human|State Laboratories Division|State Laboratories Division|Sultana|USA
>Spike|hCoV-19/Japan/PG-1779/2020|2020-04-00|EPI_ISL_685385|Original|hCoV-19^^Japan|Human|Pathogen Genomics Center|Pathogen Genomics Center|Sekizuka|Japan
>Spike|hCoV-19/Wales/PHWC-16625C/2020|2020-06-22|EPI_ISL_490856|Original|hCoV-19^^Wales|Human|Wales Specialist Virology Centre Sequencing lab|COVID-19 Genomics UK (COG-UK) Consortium|Moore|United Kingdom
>Spike|hCoV-19/Australia/VIC5773/2020|2020-07-21|EPI_ISL_518571|Original|hCoV-19^^Victoria|Human|Microbiological Diagnostic Unit - Public Health Laboratory (MDU-PHL)|MDU-PHL|Schultz|Australia
>Spike|hCoV-19/Denmark/DCGC-2907/2020|2020-08-17|EPI_ISL_616541|Original|hCoV-19^^Syddanmark|Human|Department of Virus and Microbiological Special Diagnostics|Albertsen lab|Michaelsen|Denmark
>Spike|hCoV-19/USA/IL-NM0116/2020|2020-03-19|EPI_ISL_444599|Original|hCoV-19^^Illinois|Human|Northwestern Memorial Hospital|Ozer Lab|Ozer|USA
>Spike|hCoV-19/Hungary/MH-6633/2020|2020-05-11|EPI_ISL_526220|Original|hCoV-19^^Pest county|Human|Hungarian Defence Forces Military Medical Centre|National Laboratory of Virology|Kemenesi|Hungary
>Spike|hCoV-19/England/QEUH-9F5FEA/2020|2020-09-25|EPI_ISL_588400|Original|hCoV-19^^England|Human|Lighthouse Lab in Glasgow|Wellcome Sanger Institute for the COVID-19 Genomics UK (COG-UK) consortium|Davies|United Kingdom
>Spike|hCoV-19/USA/TX-HMH-MCoV-15011/2020|2020-10-16|EPI_ISL_786495|Original|hCoV-19^^Texas|Human|Houston Methodist Hospital|Houston Methodist Hospital|Long|USA
>Spike|hCoV-19/Denmark/DCGC-14401/2020|2020-11-30|EPI_ISL_711745|Original|hCoV-19^^Syddanmark|Human|Department of Virus and Microbiological Special Diagnostics|Albertsen Lab|Michaelsen|Denmark
>Spike|hCoV-19/England/MILK-BDC4AC/2020|2020-11-26|EPI_ISL_709696|Original|hCoV-19^^England|Human|Lighthouse Lab in Milton Keynes|Wellcome Sanger Institute for the COVID-19 Genomics UK (COG-UK) Consortium|Davies|United Kingdom
>Spike|hCoV-19/Wales/PHWC-48B494/2020|2020-11-04|EPI_ISL_666240|Original|hCoV-19^^Wales|Human|Wales Specialist Virology Centre Sequencing lab|COVID-19 Genomics UK (COG-UK) Consortium|Moore|United Kingdom
>Spike|hCoV-19/England/SHEF-CF96C/2020|2020-05-23|EPI_ISL_475481|Original|hCoV-19^^England|Human|Virology Department|COVID-19 Genomics UK (COG-UK) Consortium|de Silva|United Kingdom
>Spike|hCoV-19/England/LOND-126169D/2020|2020-03-29|EPI_ISL_524706|Original|hCoV-19^^England|Human|North West London Pathology|Wellcome Sanger Institute for the COVID-19 Genomics UK (COG-UK) consortium|Davies|United Kingdom

>Spike|hCoV-19/England/ALDP-951BBB/2020|2020-06-10|EPI_ISL_551285|Original|hCoV-
19^^England|Human|Lighthouse Lab in Alderley Park|Wellcome Sanger Institute for the
COVID-19 Genomics UK (COG-UK) consortium|Davies|United Kingdom
>Spike|hCoV-19/England/MILK-9F0CE8/2020|2020-09-24|EPI_ISL_589573|Original|hCoV-
19^^England|Human|Lighthouse Lab in Milton Keynes|Wellcome Sanger Institute for the
COVID-19 Genomics UK (COG-UK) consortium|Davies|United Kingdom
>Spike|hCoV-19/Australia/VIC4719/2020|2020-07-13|EPI_ISL_519107|Original|hCoV-
19^^Victoria|Human|Microbiological Diagnostic Unit - Public Health Laboratory (MDU-
PHL)|MDU-PHL|Schultz|Australia
>Spike|hCoV-19/USA/MD-HP00129/2020|2020-03-30|EPI_ISL_457818|Original|hCoV-
19^^Maryland|Human|Johns Hopkins Hospital Department of Pathology|Johns Hopkins
Hospital Department of Pathology|Ramakrishnan|USA
>Spike|hCoV-19/Cote dIvoire/BKE0880/2020|2020-08-17|EPI_ISL_614387|Original|hCoV-
19^^Boauk\u00e9|Human|Molecular diagnostic unit for viral haemorrhagic fevers and
emerging viruses|Project group Epidemology of Highly Pathogenic
Microorganisms|Calvignac-Spencer|C\u00f4te d'Ivoire
>Spike|hCoV-19/SaudiArabia/KAUST-MADINAH1038/2020|2020-06-
13|EPI_ISL_513074|Original|hCoV-19^^Madinah|Human|Pathogen Genomics Lab King Abdullah
University of Science and Technology(KAUST)|Pathogen Genomics Lab King Abdullah
University of Science and Technology(KAUST)|Mohamed Ghazzali|Saudi Arabia
>Spike|hCoV-19/USA/AK-PHL640/2020|2020-06-26|EPI_ISL_586248|Original|hCoV-
19^^Alaska|Human|Alaska State Virology Laboratory|Alaska State Virology
Laboratory|Chen|USA
>Spike|hCoV-19/England/MILK-ACD032/2020|2020-10-21|EPI_ISL_634445|Original|hCoV-
19^^England|Human|Lighthouse Lab in Milton Keynes|Wellcome Sanger Institute for the
COVID-19 Genomics UK (COG-UK) consortium|Davies|United Kingdom
>Spike|hCoV-19/England/LIVE-9B592/2020|2020-04-02|EPI_ISL_449464|Original|hCoV-
19^^England|Human|Liverpool Clinical Laboratories|COVID-19 Genomics UK (COG-UK)
Consortium|Haldenby|United Kingdom
>Spike|hCoV-19/England/CAMB-1AB992/2020|2020-04-20|EPI_ISL_470194|Original|hCoV-
19^^England|Human|Department of Pathology|Wellcome Sanger Institute for the COVID-19
Genomics UK (COG-UK) consortium|Davies|United Kingdom
>Spike|hCoV-19/USA/MN-MDH-78/2020|2020-03-17|EPI_ISL_460065|Original|hCoV-
19^^Minnesota|Human|Minnesota Department of Health|Minnesota Department of
Health|Garfin|USA
>Spike|hCoV-19/England/CAMB-77C79/2020|2020-04-01|EPI_ISL_440815|Original|hCoV-
19^^England|Human|Department of Pathology|Wellcome Sanger Institute for the COVID-19
Genomics UK (COG-UK) consortium|Amato|United Kingdom
>Spike|hCoV-19/Japan/NGS-CD-365/2020|2020-04-24|EPI_ISL_481263|Vero|hCoV-
19^^Nagasaki|Human|Department of Emerging Infectious Diseases|Department of Emerging
Infectious Diseases|Abe|Japan
>Spike|hCoV-19/England/QEUH-9FB649/2020|2020-09-29|EPI_ISL_588077|Original|hCoV-
19^^England|Human|Lighthouse Lab in Glasgow|Wellcome Sanger Institute for the COVID-
19 Genomics UK (COG-UK) consortium|Davies|United Kingdom
>Spike|hCoV-19/England/CAMC-9EC371/2020|2020-09-21|EPI_ISL_580550|Original|hCoV-
19^^England|Human|Lighthouse Lab in Cambridge|Wellcome Sanger Institute for the
COVID-19 Genomics UK (COG-UK) consortium|Davies|United Kingdom
>Spike|hCoV-19/England/QEUH-BCCADA/2020|2020-11-25|EPI_ISL_704544|Original|hCoV-
19^^England|Human|Lighthouse Lab in Glasgow|Wellcome Sanger Institute for the COVID-
19 Genomics UK (COG-UK) Consortium|Davies|United Kingdom
>Spike|hCoV-19/Luxembourg/LNS4648424/2020|2020-11-09|EPI_ISL_740515|Original|hCoV-
19^^Luxembourg|Human|Laboratoire national de sant\u00e9|Laboratoire national de
sant\u00e9|Wienecke-Baldacchino|Luxembourg
>Spike|hCoV-19/England/QEUH-96A8B8/2020|2020-08-12|EPI_ISL_530807|Original|hCoV-
19^^England|Human|Lighthouse Lab in Glasgow|Wellcome Sanger Institute for the COVID-
19 Genomics UK (COG-UK) consortium|Davies|United Kingdom
>Spike|hCoV-19/Switzerland/GE-HUG-2164/2020|2020-03-17|EPI_ISL_429208|Original|hCoV-
19^^Geneve|Human|University Hospitals of Geneva Laboratory of Virology|University
Hospitals of Geneva Laboratory of Virology|Cordey|Switzerland

>Spike|hCoV-19/England/NORW-EBEC5/2020|2020-05-07|EPI_ISL_457519|Original|hCoV-19^^England|Human|Quadram Institute Bioscience|COVID-19 Genomics UK (COG-UK) Consortium|Alikhan|United Kingdom
>Spike|hCoV-19/Japan/PG-0708/2020|2020-04-00|EPI_ISL_684318|Original|hCoV-19^^Japan|Human|Pathogen Genomics Center|Pathogen Genomics Center|Sekizuka|Japan
>Spike|hCoV-19/Switzerland/GE-SNRCI-7836/2020|2020-03-00|EPI_ISL_548204|Original|hCoV-19^^Geneve|Human|Laboratoire de Virologie|Swiss National Reference Centre for Influenza|Gon\u00e7alves Cabecinhas|Switzerland
>Spike|hCoV-19/USA/CA-CZB-10796/2020|2020-06-25|EPI_ISL_583056|Original|hCoV-19^^California|Human|Humboldt County Public Health Laboratory|Chan-Zuckerberg Biohub|Bhatt|USA
>Spike|hCoV-19/USA/MD-MDH-0242/2020|2020-10-02|EPI_ISL_602265|Original|hCoV-19^^Maryland|Human|MD PHL|MD PHL|Keller|USA
>Spike|hCoV-19/England/BIRM-61340/2020|2020-04-28|EPI_ISL_477763|Original|hCoV-19^^England|Human|University of Birmingham|COVID-19 Genomics UK (COG-UK) Consortium|Loman|United Kingdom
>Spike|hCoV-19/Australia/VIC5752/2020|2020-07-21|EPI_ISL_518550|Original|hCoV-19^^Victoria|Human|Microbiological Diagnostic Unit - Public Health Laboratory (MDU-PHL)|MDU-PHL|Schultz|Australia
>Spike|hCoV-19/USA/NY-NYUMC340/2020|2020-04-07|EPI_ISL_430367|Original|hCoV-19^^New York|Human|NYU Langone Health|Departments of Pathology and Medicine|Maurano|USA
>Spike|hCoV-19/Scotland/EDB025/2020|2020-03-15|EPI_ISL_425831|Original|hCoV-19^^Scotland|Human|Virology Department|COVID-19 Genomics UK (COG-UK) Consortium|Williams|United Kingdom
>Spike|hCoV-19/England/ALDP-95581A/2020|2020-06-12|EPI_ISL_554523|Original|hCoV-19^^England|Human|Lighthouse Lab in Alderley Park|Wellcome Sanger Institute for the COVID-19 Genomics UK (COG-UK) consortium|Davies|United Kingdom
>Spike|hCoV-19/England/ALDP-B76EB5/2020|2020-11-12|EPI_ISL_723823|Original|hCoV-19^^England|Human|Oxford Viromics|COVID-19 Genomics UK (COG-UK) Consortium|Golubchik|United Kingdom
>Spike|hCoV-19/USA/WA-S1634/2020|2020-05-27|EPI_ISL_496060|Original|hCoV-19^^Washington|Human|Washington State Department of Health|Seattle Flu Study|Lee|USA
>Spike|hCoV-19/Scotland/QEUH-BA14DD/2020|2020-11-18|EPI_ISL_757213|Original|hCoV-19^^Scotland|Human|Lighthouse Lab in Glasgow|Wellcome Sanger Institute for the COVID-19 Genomics UK (COG-UK) Consortium|Davies|United Kingdom
>Spike|hCoV-19/England/ALDP-A52797/2020|2020-10-07|EPI_ISL_598068|Original|hCoV-19^^England|Human|Lighthouse Lab in Alderley Park|Wellcome Sanger Institute for the COVID-19 Genomics UK (COG-UK) consortium|Davies|United Kingdom
>Spike|hCoV-19/England/CAMC-991584/2020|2020-08-28|EPI_ISL_552166|Original|hCoV-19^^England|Human|Lighthouse Lab in Cambridge|Wellcome Sanger Institute for the COVID-19 Genomics UK (COG-UK) consortium|Davies|United Kingdom
>Spike|hCoV-19/USA/CA-SCCPHD-UC113/2020|2020-03-03|EPI_ISL_435592|Original|hCoV-19^^California|Human|Santa Clara County Public Health Department|Chiu Laboratory|Chiu|USA
>Spike|hCoV-19/England/MILK-C78A18/2020|2020-12-15|EPI_ISL_756818|Original|hCoV-19^^England|Human|Lighthouse Lab in Milton Keynes|Wellcome Sanger Institute for the COVID-19 Genomics UK (COG-UK) Consortium|Davies|United Kingdom
>Spike|hCoV-19/Singapore/630/2020|2020-02-06|EPI_ISL_493390|Original|hCoV-19^^Singapore|Human|National Public Health Laboratory|National Public Health Laboratory|Mak|Singapore
>Spike|hCoV-19/Italy/ABR-IZSGC-TE5547/2020|2020-03-20|EPI_ISL_436722|Original|hCoV-19^^Abruzzo|Human|Ospedale Civile S. Liberatore di Atri|Istituto Zooprofilattico Sperimentale dell'Abruzzo e Molise \G.Caporale\|Lorusso|Italy
>Spike|hCoV-19/USA/WA-UW-4779/2020|2020-03-29|EPI_ISL_461432|Original|hCoV-19^^Washington|Human|UW Virology Lab|UW Virology Lab|Roychoudhury|USA
>Spike|hCoV-19/Wales/PHWC-16B54F/2020|2020-09-15|EPI_ISL_560301|Original|hCoV-19^^Wales|Human|Wales Specialist Virology Centre Sequencing lab|COVID-19 Genomics UK (COG-UK) Consortium|Moore|United Kingdom
>Spike|hCoV-19/USA/TX-HMH-7950/2020|2020-06-26|EPI_ISL_545733|Original|hCoV-19^^Texas|Human|Houston Methodist Hospital|Houston Methodist Hospital|Long|USA

>Spike|hCoV-19/England/SHEF-CA55E/2020|2020-05-05|EPI_ISL_493695|Original|hCoV-19^^England|Human|Virology Department|COVID-19 Genomics UK (COG-UK) Consortium|de Silva|United Kingdom
>Spike|hCoV-19/Northern Ireland/NIRE-234D91/2020|2020-10-19|EPI_ISL_764395|Original|hCoV-19^^Northern Ireland|Human|Regional Virus Laboratory|COVID-19 Genomics UK (COG-UK) Consortium|Fairley|United Kingdom
>Spike|hCoV-19/Portugal/PT1146/2020|2020-04-08|EPI_ISL_511693|Original|hCoV-19^^Portugal|Human|Instituto Nacional de Saude (INSA)|Instituto Nacional de Saude (INSA)|Borges|Portugal
>Spike|hCoV-19/England/BRIS-12ECCB/2020|2020-04-21|EPI_ISL_488313|Original|hCoV-19^^England|Human|PHE South West Regional Laboratory|Wellcome Sanger Institute for the COVID-19 Genomics UK (COG-UK) consortium|Davies|United Kingdom
>Spike|hCoV-19/Ireland/OY-NVRL-20G31858/2020|2020-03-17|EPI_ISL_605079|Original|hCoV-19^^Offaly|Human|National Virus Reference Laboratory|Irish Coronavirus Sequencing Consortium - Helixworks|Chalapati|Ireland
>Spike|hCoV-19/USA/WA-S1417/2020|2020-05-19|EPI_ISL_495867|Original|hCoV-19^^Washington|Human|Washington State Department of Health|Seattle Flu Study|Lee|USA
>Spike|hCoV-19/Denmark/DCGC-14705/2020|2020-11-23|EPI_ISL_711919|Original|hCoV-19^^Hovedstaden|Human|Department of Virus and Microbiological Special Diagnostics|Albertsen Lab|Michaelsen|Denmark
>Spike|hCoV-19/USA/UT-QDX-2313/2020|2020-03-13|EPI_ISL_604620|Original|hCoV-19^^Utah|Human|Quest Diagnostics|Quest Diagnostics|Kagan|USA
>Spike|hCoV-19/England/20126045602/2020|2020-03-18|EPI_ISL_418767|Original|hCoV-19^^England|Human|Respiratory Virus Unit|Respiratory Virus Unit|Platt|United Kingdom
>Spike|hCoV-19/Brazil/SP-619/2020|2020-04-13|EPI_ISL_515562|Original|hCoV-19^^S\u00e3o Paulo|Human|Hospital Municipal Doutor Alexandre Zaio|Instituto Adolfo Lutz|Sacchi|Brazil
>Spike|hCoV-19/Denmark/DCGC-9916/2020|2020-11-09|EPI_ISL_669270|Original|hCoV-19^^Hovedstaden|Human|Department of Virus and Microbiological Special Diagnostics|Albertsen Lab|Michaelsen|Denmark
>Spike|hCoV-19/England/ALDP-C58902/2020|2020-12-14|EPI_ISL_736028|Original|hCoV-19^^England|Human|Lighthouse Lab in Alderley Park|Wellcome Sanger Institute for the COVID-19 Genomics UK (COG-UK) Consortium|Davies|United Kingdom
>Spike|hCoV-19/USA/LA-EVTL519/2020|2020-08-03|EPI_ISL_578923|Original|hCoV-19^^Louisiana|Human|LSUHS Emerging Viral Threat Laboratory|Microbial Genome Sequencing Center|Kamil|USA
>Spike|hCoV-19/England/NOTT-10FEAC/2020|2020-04-09|EPI_ISL_441453|Original|hCoV-19^^England|Human|Queens Medical Centre|COVID-19 Genomics UK (COG-UK) Consortium|Loose|United Kingdom
>Spike|hCoV-19/Switzerland/BS-UHB-42195540/2020|2020-03-17|EPI_ISL_528178|Original|hCoV-19^^Basel-Stadt|Human|University Hospital Basel|University Hospital Basel|Roloff Handschin|Switzerland
>Spike|hCoV-19/England/QEUH-B68D7D/2020|2020-11-11|EPI_ISL_662119|Original|hCoV-19^^England|Human|Lighthouse Lab in Glasgow|Wellcome Sanger Institute for the COVID-19 Genomics UK (COG-UK) Consortium|Davies|United Kingdom
>Spike|hCoV-19/USA/UT-UPHL-2009217/2020|2020-05-15|EPI_ISL_525217|Original|hCoV-19^^Utah|Human|Utah Public Health Laboratory|Utah Public Health Laboratory|Young|USA
>Spike|hCoV-19/USA/CO-CDPHE-2100023473/2020|2020-11-19|EPI_ISL_771332|Original|hCoV-19^^Colorado|Human|Colorado Department of Public Health and Environment|Colorado Department of Puplic Health and Environment|Bankers|USA
>Spike|hCoV-19/Brazil/SP-L32-CD839/2020|2020-04-16|EPI_ISL_722033|Original|hCoV-19^^S\u00e3o Paulo|Human|Hospital das Cl\u00ednicas Universidade de S\u00e3o Paulo Medical School|Laborat\u00f3rio de Parasitologia M\u00e9dica - Instituto de Medicina Tropical - Universidade de S\u00e3o Paulo|de Jesus|Brazil
>Spike|hCoV-19/England/ALDP-951007/2020|2020-06-13|EPI_ISL_554280|Original|hCoV-19^^England|Human|Lighthouse Lab in Alderley Park|Wellcome Sanger Institute for the COVID-19 Genomics UK (COG-UK) consortium|Davies|United Kingdom
>Spike|hCoV-19/India/MH-NIV-QC-800/2020|2020-04-26|EPI_ISL_452217|Original|hCoV-19^^Maharashtra|Human|NIV Influenza|NIV Influenza|Potdar|India

>Spike|hCoV-19/England/NORW-E8727/2020|2020-00-00|EPI_ISL_448228|Original|hCoV-19^^England|Human|Quadram Institute Bioscience|COVID-19 Genomics UK (COG-UK) Consortium|Alikhan|United Kingdom
>Spike|hCoV-19/USA/DE-DHSS-F1013965/2020|2020-10-12|EPI_ISL_708387|Original|hCoV-19^^Delaware|Human|Delaware Public Health Lab|Delaware Public Health Lab|Bajwa|USA
>Spike|hCoV-19/England/20134068904/2020|2020-03-24|EPI_ISL_423160|Original|hCoV-19^^England|Human|Respiratory Virus Unit|Respiratory Virus Unit|Platt|United Kingdom
>Spike|hCoV-19/England/MILK-9A8AC7/2020|2020-08-31|EPI_ISL_550171|Original|hCoV-19^^England|Human|Lighthouse Lab in Milton Keynes|Wellcome Sanger Institute for the COVID-19 Genomics UK (COG-UK) consortium|Davies|United Kingdom
>Spike|hCoV-19/England/ALDP-A2D0A9/2020|2020-10-06|EPI_ISL_586857|Original|hCoV-19^^England|Human|Lighthouse Lab in Alderley Park|Wellcome Sanger Institute for the COVID-19 Genomics UK (COG-UK) consortium|Davies|United Kingdom
>Spike|hCoV-19/USA/GA-EHC-086H/2020|2020-03-10|EPI_ISL_455356|Original|hCoV-19^^Georgia|Human|Emory Molecular Diagnostics Laboratory|Piantadosi Lab|Piantadosi|USA
>Spike|hCoV-19/South Africa/KRISP-K003758/2020|2020-09-28|EPI_ISL_602929|Original|hCoV-19^^KwaZulu-Natal|Human|NHLS-IALCH|KRISP|de Oliveira|South Africa
>Spike|hCoV-19/Italy/SIC-IZSBM193-67/2020|2020-06-00|EPI_ISL_582846|Original|hCoV-19^^Sicily|Human|Laboratorio di Riferimento Regionale della Sicilia Occidentale per l'Emergenza COVID-19|Istituto Zooprofilattico Sperimentale della Sicilia|Reale|Italy
>Spike|hCoV-19/USA/CA-ALSR-2382/2020|2020-07-06|EPI_ISL_635451|Original|hCoV-19^^California|Human|San Diego County Public Health Laboratory|Andersen lab at Scripps Research|Gangavarapu|USA
>Spike|hCoV-19/Scotland/QEUH-9F856D/2020|2020-09-27|EPI_ISL_600878|Original|hCoV-19^^Scotland|Human|Lighthouse Lab in Glasgow|Wellcome Sanger Institute for the COVID-19 Genomics UK (COG-UK) consortium|Davies|United Kingdom
>Spike|hCoV-19/Japan/PG-0541/2020|2020-04-03|EPI_ISL_480197|Original|hCoV-19^^HokurikuShinsyu|Human|Toyama Institute of Health|Pathogen Genomics Center|Sekizuka|Japan
>Spike|hCoV-19/Luxembourg/LNS9580764/2020|2020-11-09|EPI_ISL_744943|Original|hCoV-19^^Luxembourg|Human|Laboratoire national de sant\u00e9|Laboratoire national de sant\u00e9|Wienecke-Baldacchino|Luxembourg
>Spike|hCoV-19/Scotland/CVR2284/2020|2020-04-13|EPI_ISL_448175|Original|hCoV-19^^Scotland|Human|West of Scotland Specialist Virology Centre|COVID-19 Genomics UK (COG-UK) Consortium|Robertson|United Kingdom
>Spike|hCoV-19/England/QEUH-9DDBC6/2020|2020-09-21|EPI_ISL_599777|Original|hCoV-19^^England|Human|Lighthouse Lab in Glasgow|Wellcome Sanger Institute for the COVID-19 Genomics UK (COG-UK) consortium|Davies|United Kingdom
>Spike|hCoV-19/Netherlands/GE-RIVM-10385/2020|2020-03-23|EPI_ISL_636501|Original|hCoV-19^^Gelderland|Human|Dutch COVID-19 response team|National Institute for Public Health and the Environment (RIVM)|Meijer|Netherlands
>Spike|hCoV-19/Australia/VIC4724/2020|2020-07-14|EPI_ISL_519112|Original|hCoV-19^^Victoria|Human|Microbiological Diagnostic Unit - Public Health Laboratory (MDU-PHL)|MDU-PHL|Schultz|Australia
>Spike|hCoV-19/Singapore/1364/2020|2020-06-25|EPI_ISL_648715|Original|hCoV-19^^Singapore|Human|Department of Laboratory Medicine|Department of Laboratory Medicine|Chen|Singapore
>Spike|hCoV-19/England/MILK-A6BA74/2020|2020-10-09|EPI_ISL_608755|Original|hCoV-19^^England|Human|Lighthouse Lab in Milton Keynes|Wellcome Sanger Institute for the COVID-19 Genomics UK (COG-UK) consortium|Davies|United Kingdom
>Spike|hCoV-19/Denmark/DCGC-9455/2020|2020-11-09|EPI_ISL_668980|Original|hCoV-19^^Hovedstaden|Human|Department of Virus and Microbiological Special Diagnostics|Albertsen Lab|Michaelsen|Denmark
>Spike|hCoV-19/Portugal/PT1602/2020|2020-07-05|EPI_ISL_693605|Original|hCoV-19^^Portugal|Human|Instituto Nacional de Saude (INSA)|Instituto Nacional de Saude (INSA)|Borges|Portugal

>Spike|hCoV-19/Wales/MILK-973175/2020|2020-08-20|EPI_ISL_575842|Original|hCoV-19^^Wales|Human|Lighthouse Lab in Milton Keynes|Wellcome Sanger Institute for the COVID-19 Genomics UK (COG-UK) consortium|Davies|United Kingdom
>Spike|hCoV-19/England/LIVE-9E948/2020|2020-04-11|EPI_ISL_453345|Original|hCoV-19^^England|Human|Liverpool Clinical Laboratories|COVID-19 Genomics UK (COG-UK) Consortium|Haldenby|United Kingdom
>Spike|hCoV-19/South Africa/KRISP-0579/2020|2020-07-08|EPI_ISL_498117|Original|hCoV-19^^KwaZulu-Natal|Human|NHLS-IALCH|KRISP|de Oliveira|South Africa
>Spike|hCoV-19/England/20180032304/2020|2020-04-24|EPI_ISL_465436|Original|hCoV-19^^England|Human|Respiratory Virus Unit|Respiratory Virus Unit|Platt|United Kingdom
>Spike|hCoV-19/Japan/PG-5874/2020|2020-08-00|EPI_ISL_688989|Original|hCoV-19^^Japan|Human|Pathogen Genomics Center|Pathogen Genomics Center|Sekizuka|Japan
>Spike|hCoV-19/Wales/PHWC-166E94/2020|2020-06-11|EPI_ISL_494124|Original|hCoV-19^^Wales|Human|Originating lab|COVID-19 Genomics UK (COG-UK) Consortium|Moore|United Kingdom
>Spike|hCoV-19/Wales/PHWC-499FA1/2020|2020-11-25|EPI_ISL_727475|Original|hCoV-19^^Wales|Human|Originating lab|COVID-19 Genomics UK (COG-UK) Consortium|Moore|United Kingdom
>Spike|hCoV-19/South Africa/KRISP-K002175/2020|2020-07-09|EPI_ISL_515577|Original|hCoV-19^^KwaZulu-Natal|Human|NHLS-IALCH|KRISP|de Oliveira|South Africa
>Spike|hCoV-19/USA/AZ-TG395900/2020|2020-06-05|EPI_ISL_694744|Original|hCoV-19^^Arizona|Human|AZ SPHL|TGen North|French|USA
>Spike|hCoV-19/USA/CA-CZB-2459/2020|2020-05-21|EPI_ISL_513865|Original|hCoV-19^^California|Human|San Francisco Public Health Laboratory|Chan-Zuckerberg Biohub|Kamm|USA
>Spike|hCoV-19/USA/WA-S2442/2020|2020-06-16|EPI_ISL_497074|Original|hCoV-19^^Washington|Human|Washington State Department of Health|Seattle Flu Study|Lee|USA
>Spike|hCoV-19/Netherlands/NA_354/2020|2020-04-07|EPI_ISL_455158|Original|hCoV-19^^Netherlands|Human|Dutch COVID-19 response team|Erasmus Medical Center|Oude Munnink|Netherlands
>Spike|hCoV-19/Denmark/DCGC-8020/2020|2020-10-26|EPI_ISL_617071|Original|hCoV-19^^Nordjylland|Human|Department of Virus and Microbiological Special Diagnostics|Albertsen lab|Michaelsen|Denmark
>Spike|hCoV-19/India/MH-ACTREC-225/2020|2020-07-01|EPI_ISL_699879|Original|hCoV-19^^Maharashtra|Human|Hematopathology Laboratory|Hematopathology Laboratory|Patkar|India
>Spike|hCoV-19/USA/TX-HMH-MCoV-12554/2020|2020-09-30|EPI_ISL_790387|Original|hCoV-19^^Texas|Human|Houston Methodist Hospital|Houston Methodist Hospital|Long|USA
>Spike|hCoV-19/Denmark/DCGC-19095/2020|2020-12-07|EPI_ISL_748294|Original|hCoV-19^^Midtjylland|Human|Department of Virus and Microbiological Special Diagnostics|Albertsen Lab|Michaelsen|Denmark
>Spike|hCoV-19/Canada/ON-PHL-20-00033/2020|2020-00-00|EPI_ISL_548714|Original|hCoV-19^^Ontario|Human|Public Health Ontario Laboratory|Public Health Ontario Laboratory|Guthrie|Canada
>Spike|hCoV-19/South Africa/Tygerberg_21/2020|2020-04-09|EPI_ISL_464132|Original|hCoV-19^^Western Cape|Human|National Health Laboratory Service (NHLS)|Division of Medical Virology|Engelbrecht|South Africa
>Spike|hCoV-19/Portugal/PT1544/2020|2020-06-21|EPI_ISL_693558|Original|hCoV-19^^Portugal|Human|Instituto Nacional de Saude (INSA)|Instituto Nacional de Saude (INSA)|Borges|Portugal
>Spike|hCoV-19/England/MILK-C30654/2020|2020-12-07|EPI_ISL_782990|Original|hCoV-19^^England|Human|Lighthouse Lab in Milton Keynes|Wellcome Sanger Institute for the COVID-19 Genomics UK (COG-UK) Consortium|Davies|United Kingdom
>Spike|hCoV-19/England/CAMB-1B493C/2020|2020-06-04|EPI_ISL_473487|Original|hCoV-19^^England|Human|Department of Pathology|COVID-19 Genomics UK (COG-UK) Consortium|Meredith|United Kingdom
>Spike|hCoV-19/England/204291949/2020|2020-10-18|EPI_ISL_660743|Original|hCoV-19^^England|Human|Respiratory Virus Unit|COVID-19 Genomics UK (COG-UK) Consortium|Platt|United Kingdom

>Spike|hCoV-19/Japan/PG-8822/2020|2020-08-00|EPI_ISL_686404|Original|hCoV-19^^Japan|Human|Pathogen Genomics Center|Pathogen Genomics Center|Sekizuka|Japan
>Spike|hCoV-19/Northern Ireland/NIRE-102708/2020|2020-04-18|EPI_ISL_469857|Original|hCoV-19^^Northern Ireland|Human|Regional Virus Laboratory|Wellcome Sanger Institute for the COVID-19 Genomics UK (COG-UK) consortium|Davies|United Kingdom
>Spike|hCoV-19/Netherlands/ZH-EMC-483/2020|2020-08-09|EPI_ISL_577913|Original|hCoV-19^^Zuid Holland|Human|Dutch COVID-19 response team|Erasmus Medical Center|Oude Munnink|Netherlands
>Spike|hCoV-19/Latvia/120/2020|2020-07-31|EPI_ISL_534209|Original|hCoV-19^^Latvia|Human|Latvijas Infektolo\u0123ijas centrs|Latvian Biomedical Research and Study Centre|Zrelovs|Latvia
>Spike|hCoV-19/France/PAC-MEPHI-2221/2020|2020-10-02|EPI_ISL_644528|Original|hCoV-19^^Provence-Alpes-Cote-d\u2019Azur|Human|MEPHI|MEPHI|Levasseur|France
>Spike|hCoV-19/USA/TX-HMH-MCoV-18504/2020|2020-11-20|EPI_ISL_784852|Original|hCoV-19^^Texas|Human|Houston Methodist Hospital|Houston Methodist Hospital|Long|USA
>Spike|hCoV-19/England/20144014504/2020|2020-03-31|EPI_ISL_423509|Original|hCoV-19^^England|Human|Respiratory Virus Unit|Respiratory Virus Unit|Platt|United Kingdom
>Spike|hCoV-19/England/QEUH-A6357B/2020|2020-10-12|EPI_ISL_596120|Original|hCoV-19^^England|Human|Quadram Institute Bioscience|COVID-19 Genomics UK (COG-UK) Consortium|Alikhan|United Kingdom
>Spike|hCoV-19/USA/WA-UW-9600/2020|2020-05-22|EPI_ISL_570267|Original|hCoV-19^^Washington|Human|UW Virology Lab|UW Virology Lab|Roychoudhury|USA
>Spike|hCoV-19/USA/LA-EVTL1061/2020|2020-11-27|EPI_ISL_768331|Original|hCoV-19^^Louisiana|Human|LSUHS Emerging Viral Threat Laboratory|Microbial Genome Sequencing Center|Kamil|USA
>Spike|hCoV-19/Luxembourg/LNS0829711/2020|2020-11-05|EPI_ISL_739836|Original|hCoV-19^^Luxembourg|Human|Laboratoire national de sant\u00e9|Laboratoire national de sant\u00e9|Wienecke-Baldacchino|Luxembourg
>Spike|hCoV-19/India/MH-ACTREC-093/2020|2020-05-22|EPI_ISL_699749|Original|hCoV-19^^Maharashtra|Human|Hematopathology Laboratory|Hematopathology Laboratory|Patkar|India
>Spike|hCoV-19/USA/TX-HMH-MCoV-19537/2020|2020-11-17|EPI_ISL_783913|Original|hCoV-19^^Texas|Human|Houston Methodist Hospital|Houston Methodist Hospital|Long|USA
>Spike|hCoV-19/England/CAMB-1AC15D/2020|2020-04-28|EPI_ISL_444377|Original|hCoV-19^^England|Human|Department of Pathology|COVID-19 Genomics UK (COG-UK) Consortium|Meredith|United Kingdom
>Spike|hCoV-19/USA/TX-DSHS-1210/2020|2020-03-18|EPI_ISL_631671|Original|hCoV-19^^Texas|Human|Texas Department of State Health Services|Texas Department of State Health Services|Rahman|USA
>Spike|hCoV-19/USA/TX-HMH-MCoV-15717/2020|2020-07-09|EPI_ISL_785401|Original|hCoV-19^^Texas|Human|Houston Methodist Hospital|Houston Methodist Hospital|Long|USA
>Spike|hCoV-19/England/ALDP-9B5FE6/2020|2020-08-28|EPI_ISL_572981|Original|hCoV-19^^England|Human|Oxford Viromics|COVID-19 Genomics UK (COG-UK) Consortium|Golubchik|United Kingdom
>Spike|hCoV-19/Gibraltar/204841769/2020|2020-00-00|EPI_ISL_671334|Original|hCoV-19^^Gibraltar|Human|Microbiology|Respiratory Virus Unit|Platt|Gibraltar
>Spike|hCoV-19/Denmark/DCGC-4079/2020|2020-09-14|EPI_ISL_622035|Original|hCoV-19^^Hovedstaden|Human|Department of Virus and Microbiological Special Diagnostics|Albertsen lab|Michaelsen|Denmark
>Spike|hCoV-19/Denmark/DCGC-14091/2020|2020-11-30|EPI_ISL_711561|Original|hCoV-19^^Nordjylland|Human|Department of Virus and Microbiological Special Diagnostics|Albertsen Lab|Michaelsen|Denmark
>Spike|hCoV-19/Scotland/CAMC-A82ED5/2020|2020-10-13|EPI_ISL_625294|Original|hCoV-19^^Scotland|Human|Lighthouse Lab in Cambridge|Wellcome Sanger Institute for the COVID-19 Genomics UK (COG-UK) consortium|Davies|United Kingdom
>Spike|hCoV-19/USA/OR-OHSU-2884/2020|2020-09-04|EPI_ISL_667385|Original|hCoV-19^^Oregon|Human|OHSU Lab Services Molecular Microbiology Lab|Oregon SARS-CoV-2 Genome Sequencing Center|O'Roak|USA

>Spike|hCoV-19/England/NOTT-114C22/2020|2020-10-10|EPI_ISL_584765|Original|hCoV-19^^England|Human|Queens Medical Centre|COVID-19 Genomics UK (COG-UK) Consortium|Loose|United Kingdom
>Spike|hCoV-19/USA/MI-MDHHS-SC22267/2020|2020-11-02|EPI_ISL_666933|Original|hCoV-19^^Michigan|Human|Michigan Department of Health and Human Services|Michigan Department of Health and Human Services|Jacob|USA
>Spike|hCoV-19/Beijing/ICDC-DT005/2020|2020-02-05|EPI_ISL_412386|Original|hCoV-19^^Beijing|Human|Beijing Ditan Hospital|National Institute for Communicable Disease Control and Prevention|Lu|China
>Spike|hCoV-19/Australia/VIC6340/2020|2020-07-20|EPI_ISL_518322|Original|hCoV-19^^Victoria|Human|Microbiological Diagnostic Unit - Public Health Laboratory (MDU-PHL)|MDU-PHL|Schultz|Australia
>Spike|hCoV-19/Australia/VIC15084/2020|2020-08-11|EPI_ISL_593130|Original|hCoV-19^^Victoria|Human|Microbiological Diagnostic Unit - Public Health Laboratory (MDU-PHL)|MDU-PHL|Schultz|Australia
>Spike|hCoV-19/USA/WA-UW-1718/2020|2020-03-20|EPI_ISL_424210|Original|hCoV-19^^Washington|Human|UW Virology Lab|UW Virology Lab|Roychoudhury|USA
>Spike|hCoV-19/USA/VA-DCLS-2165/2020|2020-11-06|EPI_ISL_677194|Original|hCoV-19^^Virginia|Human|Virginia Division of Consolidated Laboratory Services|Virginia Division of Consolidated Laboratory Services|St. Jacques|USA
>Spike|hCoV-19/India/GJ-GBRC24b/2020|2020-04-26|EPI_ISL_437454|Original|hCoV-19^^Gujarat|Human|B.J. Medical College and Civil hospital|Gujarat Biotechnology Research Centre|Joshi|India
>Spike|hCoV-19/USA/TX-HMH-5689/2020|2020-06-26|EPI_ISL_546946|Original|hCoV-19^^Texas|Human|Houston Methodist Hospital|Houston Methodist Hospital|Long|USA
>Spike|hCoV-19/England/CAMC-B33A47/2020|2020-11-05|EPI_ISL_659231|Original|hCoV-19^^England|Human|Lighthouse Lab in Cambridge|Wellcome Sanger Institute for the COVID-19 Genomics UK (COG-UK) Consortium|Davies|United Kingdom
>Spike|hCoV-19/USA/FL-BPHL-0260/2020|2020-05-04|EPI_ISL_480911|Original|hCoV-19^^Florida|Human|Florida Bureau of Public Health Laboratories|Florida Bureau of Public Health Laboratories|Blanton|USA
>Spike|hCoV-19/USA/CA-ALSR-4331/2020|2020-10-06|EPI_ISL_648976|Original|hCoV-19^^California|Human|San Diego County Public Health Laboratory|Andersen lab at Scripps Research|Gangavarapu|USA
>Spike|hCoV-19/Japan/IC-0611/2020|2020-12-00|EPI_ISL_779697|Original|hCoV-19^^Japan|Human|Pathogen Genomics Center|Pathogen Genomics Center|Sekizuka|Japan
>Spike|hCoV-19/England/QEUH-B2D406/2020|2020-11-04|EPI_ISL_659968|Original|hCoV-19^^England|Human|Lighthouse Lab in Glasgow|Wellcome Sanger Institute for the COVID-19 Genomics UK (COG-UK) Consortium|Davies|United Kingdom
>Spike|hCoV-19/USA/MI-MDHHS-SC20421/2020|2020-03-17|EPI_ISL_452295|Original|hCoV-19^^Michigan|Human|Michigan Department of Health and Human Services|Michigan Department of Health and Human Services|Blankenship|USA
>Spike|hCoV-19/England/ALDP-9CB85B/2020|2020-09-16|EPI_ISL_575621|Original|hCoV-19^^England|Human|Lighthouse Lab in Alderley Park|Wellcome Sanger Institute for the COVID-19 Genomics UK (COG-UK) consortium|Davies|United Kingdom
>Spike|hCoV-19/England/QEUH-A4BB2C/2020|2020-10-05|EPI_ISL_628510|Original|hCoV-19^^England|Human|Oxford Viromics|COVID-19 Genomics UK (COG-UK) Consortium|Golubchik|United Kingdom
>Spike|hCoV-19/Canada/ON-S246/2020|2020-03-27|EPI_ISL_755831|Original|hCoV-19^^Ontario|Human|Toronto Invasive Bacterial Diseases Network|McMaster University|McArthur|Canada
>Spike|hCoV-19/England/MILK-B952B4/2020|2020-11-13|EPI_ISL_673633|Original|hCoV-19^^England|Human|Lighthouse Lab in Milton Keynes|Wellcome Sanger Institute for the COVID-19 Genomics UK (COG-UK) Consortium|Davies|United Kingdom
>Spike|hCoV-19/England/ALDP-AA4839/2020|2020-10-21|EPI_ISL_624523|Original|hCoV-19^^England|Human|Lighthouse Lab in Alderley Park|Wellcome Sanger Institute for the COVID-19 Genomics UK (COG-UK) consortium|Davies|United Kingdom
>Spike|hCoV-19/USA/WI-UW-1527/2020|2020-09-03|EPI_ISL_605405|Original|hCoV-19^^Wisconsin|Human|University of Wisconsin-Madison AIDS Vaccine Research

Laboratories|University of Wisconsin-Madison AIDS Vaccine Research Laboratories|Baczenas|USA
>Spike|hCoV-19/England/LOND-124DA7D/2020|2020-00-00|EPI_ISL_584636|Original|hCoV-19^^England|Human|University College London Hospital|COVID-19 Genomics UK (COG-UK) Consortium|Byott|United Kingdom
>Spike|hCoV-19/England/QEUH-A029AF/2020|2020-09-29|EPI_ISL_584939|Original|hCoV-19^^England|Human|Quadram Institute Bioscience|COVID-19 Genomics UK (COG-UK) Consortium|Alikhan|United Kingdom
>Spike|hCoV-19/Brazil/RS-00667/2020|2020-11-26|EPI_ISL_770609|Original|hCoV-19^^Rio Grande do Sul|Human|Laborat\u00f3rio de Microbiologia Molecular - Universidade FEEVALE|Bioinformatics Laboratory / LNCC|Almeida|Brazil
>Spike|hCoV-19/England/CAMC-B325ED/2020|2020-11-05|EPI_ISL_659362|Original|hCoV-19^^England|Human|Lighthouse Lab in Cambridge|Wellcome Sanger Institute for the COVID-19 Genomics UK (COG-UK) Consortium|Davies|United Kingdom
>Spike|hCoV-19/England/ALDP-B270CC/2020|2020-11-04|EPI_ISL_664386|Original|hCoV-19^^England|Human|Northumbria University / South Tees Hospitals NHS Foundation Trust / North Cumbria Integrated Care NHS Foundation Trust / North Tees and Hartlepool NHS Foundation Trust / Newcastle Hospitals NHS Foundation Trust|COVID-19 Genomics UK (COG-UK) Consortium|Bashton|United Kingdom
>Spike|hCoV-19/USA/CT-Yale-032/2020|2020-03-18|EPI_ISL_428370|Original|hCoV-19^^Connecticut|Human|Yale Clinical Virology Laboratory|Grubaugh Lab - Yale School of Public Health|Grubaugh|USA
>Spike|hCoV-19/USA/LA-EVTL1023/2020|2020-08-25|EPI_ISL_653714|Original|hCoV-19^^Louisiana|Human|LSUHS Emerging Viral Threat Laboratory|Microbial Genome Sequencing Center|Kamil|USA
>Spike|hCoV-19/Canada/ON-OICR-SLB2830/2020|2020-04-03|EPI_ISL_538356|Original|hCoV-19^^Ontario|Human|Kingston Health Sciences Centre / Queen's University|Ontario Institute for Cancer Research|de Borja|Canada
>Spike|hCoV-19/Netherlands/UT-EMC-53/2020|2020-05-22|EPI_ISL_523530|Original|hCoV-19^^Utrecht|Human|Dutch COVID-19 response team|Erasmus Medical Center|Oude Munnink|Netherlands
>Spike|hCoV-19/USA/CA-SEARCH-5545/2020|2020-12-01|EPI_ISL_755286|Original|hCoV-19^^California|Human|San Diego County Public Health Laboratory|Andersen lab at Scripps Research|Gangavarapu|USA
>Spike|hCoV-19/DRC/4084/2020|2020-04-27|EPI_ISL_471407|Original|hCoV-19^^Kinshasa|Human|Viral Respiratory Lab|Pathogen Sequencing Lab|Lusamaki|Democratic Republic of the Congo
>Spike|hCoV-19/Denmark/DCGC-4727/2020|2020-09-14|EPI_ISL_622166|Original|hCoV-19^^Syddanmark|Human|Department of Virus and Microbiological Special Diagnostics|Albertsen lab|Michaelsen|Denmark
>Spike|hCoV-19/England/ALDP-A84C06/2020|2020-10-16|EPI_ISL_610604|Original|hCoV-19^^England|Human|Lighthouse Lab in Alderley Park|Wellcome Sanger Institute for the COVID-19 Genomics UK (COG-UK) consortium|Davies|United Kingdom
>Spike|hCoV-19/England/ALDP-B75A85/2020|2020-11-12|EPI_ISL_664931|Original|hCoV-19^^England|Human|Quadram Institute Bioscience|COVID-19 Genomics UK (COG-UK) Consortium|Alikhan|United Kingdom
>Spike|hCoV-19/Australia/VIC6461/2020|2020-07-22|EPI_ISL_521239|Original|hCoV-19^^Victoria|Human|Microbiological Diagnostic Unit - Public Health Laboratory (MDU-PHL)|MDU-PHL|Schultz|Australia
>Spike|hCoV-19/England/QEUH-A60BF4/2020|2020-10-13|EPI_ISL_665387|Original|hCoV-19^^England|Human|Northumbria University / South Tees Hospitals NHS Foundation Trust / North Cumbria Integrated Care NHS Foundation Trust / North Tees and Hartlepool NHS Foundation Trust / Newcastle Hospitals NHS Foundation Trust|COVID-19 Genomics UK (COG-UK) Consortium|Bashton|United Kingdom
>Spike|hCoV-19/England/20194008304/2020|2020-05-04|EPI_ISL_465607|Original|hCoV-19^^England|Human|Respiratory Virus Unit|Respiratory Virus Unit|Platt|United Kingdom
>Spike|hCoV-19/Canada/MB-NML-583/2020|2020-03-31|EPI_ISL_582273|Original|hCoV-19^^Manitoba|Human|Cadham Provincial Laboratory|National Microbiology Laboratory (NML)|Majer|Canada

>Spike|hCoV-19/USA/MI-MDHHS-SC21078/2020|2020-03-29|EPI_ISL_507854|Original|hCoV-
19^^Michigan|Human|Michigan Department of Health and Human Services|Michigan
Department of Health and Human Services|Blankenship|USA
>Spike|hCoV-19/England/CAMC-C8391D/2020|2020-12-17|EPI_ISL_762549|Original|hCoV-
19^^England|Human|Lighthouse Lab in Cambridge|Wellcome Sanger Institute for the
COVID-19 Genomics UK (COG-UK) Consortium|Davies|United Kingdom
>Spike|hCoV-19/Singapore/113nan/2020|2020-03-17|EPI_ISL_538448|Original|hCoV-
19^^Singapore|Human|Department of Laboratory Medicine|Department of Laboratory
Medicine|Chen|Singapore
>Spike|hCoV-19/Japan/PG-4157/2020|2020-07-00|EPI_ISL_692396|Original|hCoV-
19^^Japan|Human|Pathogen Genomics Center|Pathogen Genomics Center|Sekizuka|Japan
>Spike|hCoV-19/England/ALDP-B61FD8/2020|2020-11-08|EPI_ISL_656347|Original|hCoV-
19^^England|Human|Lighthouse Lab in Alderley Park|Wellcome Sanger Institute for the
COVID-19 Genomics UK (COG-UK) Consortium|Davies|United Kingdom
>Spike|hCoV-19/Spain/EX-IBV-005006/2020|2020-03-13|EPI_ISL_510315|Original|hCoV-
19^^Extremadura|Human|Hospital San Pedro de Alc\u00e1ntara (C\u00e1ceres)|SeqCOVID-
SPAIN consortium/IBV(CSIC)|Comas|Spain
>Spike|hCoV-19/Wales/PHWC-16A267/2020|2020-09-06|EPI_ISL_535222|Original|hCoV-
19^^Wales|Human|Wales Specialist Virology Centre Sequencing lab|COVID-19 Genomics UK
(COG-UK) Consortium|Moore|United Kingdom
>Spike|hCoV-19/England/QEUH-768A5C/2020|2020-07-15|EPI_ISL_532969|Original|hCoV-
19^^England|Human|Lighthouse Lab in Glasgow|Wellcome Sanger Institute for the COVID-
19 Genomics UK (COG-UK) consortium|Davies|United Kingdom
>Spike|hCoV-19/Australia/VIC2686/2020|2020-07-01|EPI_ISL_521527|Original|hCoV-
19^^Victoria|Human|Victorian Infectious Diseases Reference Laboratory (VIDRL)|VIDRL
and MDU-PHL|Schultz|Australia
>Spike|hCoV-19/USA/IL-UW-2079/2020|2020-10-19|EPI_ISL_677484|Original|hCoV-
19^^Illinois|Human|University of Wisconsin-Madison AIDS Vaccine Research
Laboratories|University of Wisconsin-Madison AIDS Vaccine Research
Laboratories|Baczenas|USA
>Spike|hCoV-19/USA/TX-HMH-3371/2020|2020-06-12|EPI_ISL_544155|Original|hCoV-
19^^Texas|Human|Houston Methodist Hospital|Houston Methodist Hospital|Long|USA
>Spike|hCoV-19/Denmark/DCGC-18540/2020|2020-12-07|EPI_ISL_750030|Original|hCoV-
19^^Hovedstaden|Human|Department of Virus and Microbiological Special
Diagnostics|Albertsen Lab|Michaelsen|Denmark
>Spike|hCoV-19/Netherlands/NA_587/2020|2020-00-00|EPI_ISL_460751|Original|hCoV-
19^^Netherlands|Human|Dutch COVID-19 response team|Erasmus Medical Center|Oude
Munnink|Netherlands
>Spike|hCoV-19/Wales/PHWC-33EEC/2020|2020-04-16|EPI_ISL_446839|Original|hCoV-
19^^Wales|Human|Wales Specialist Virology Centre|Public Health Wales Microbiology
Cardiff|Moore|United Kingdom
>Spike|hCoV-19/England/ALDP-9C539C/2020|2020-09-10|EPI_ISL_567875|Original|hCoV-
19^^England|Human|Lighthouse Lab in Alderley Park|Wellcome Sanger Institute for the
COVID-19 Genomics UK (COG-UK) consortium|Davies|United Kingdom
>Spike|hCoV-19/England/ALDP-AA48FD/2020|2020-10-21|EPI_ISL_624280|Original|hCoV-
19^^England|Human|Lighthouse Lab in Alderley Park|Wellcome Sanger Institute for the
COVID-19 Genomics UK (COG-UK) consortium|Davies|United Kingdom
>Spike|hCoV-19/Wales/PHWC-49C23C/2020|2020-12-07|EPI_ISL_742528|Original|hCoV-
19^^Wales|Human|Wales Specialist Virology Centre Sequencing lab|COVID-19 Genomics UK
(COG-UK) Consortium|Moore|United Kingdom
>Spike|hCoV-19/USA/LA-EVTL1130/2020|2020-12-07|EPI_ISL_768399|Original|hCoV-
19^^Louisiana|Human|LSUHS Emerging Viral Threat Laboratory|Microbial Genome
Sequencing Center|Kamil|USA
>Spike|hCoV-19/England/OXON-ACB05/2020|2020-04-06|EPI_ISL_448509|Original|hCoV-
19^^England|Human|Oxford Viromics|COVID-19 Genomics UK (COG-UK)
Consortium|Golubchik|United Kingdom
>Spike|hCoV-19/England/LIVE-1DDAC1/2020|2020-09-27|EPI_ISL_611743|Original|hCoV-
19^^England|Human|Liverpool Clinical Laboratories|COVID-19 Genomics UK (COG-UK)
Consortium|Haldenby|United Kingdom

>Spike|hCoV-19/USA/TX-HMH-MCoV-18067/2020|2020-11-16|EPI_ISL_784415|Original|hCoV-19^^Texas|Human|Houston Methodist Hospital|Houston Methodist Hospital|Long|USA
>Spike|hCoV-19/England/QEUH-B7C8C0/2020|2020-11-14|EPI_ISL_741798|Original|hCoV-19^^England|Human|Oxford Viromics|COVID-19 Genomics UK (COG-UK) Consortium|Golubchik|United Kingdom
>Spike|hCoV-19/Denmark/DCGC-4315/2020|2020-09-07|EPI_ISL_615752|Original|hCoV-19^^Nordjylland|Human|Department of Virus and Microbiological Special Diagnostics|Albertsen lab|Michaelsen|Denmark
>Spike|hCoV-19/USA/WA-UW-1574/2020|2020-03-19|EPI_ISL_423019|Original|hCoV-19^^Washington|Human|UW Virology Lab|UW Virology Lab|Roychoudhury|USA
>Spike|hCoV-19/Weifang/WF0012/2020|2020-02-00|EPI_ISL_413697|Original|hCoV-19^^Weifang|Human|Weifang Center for Disease Control and Prevention|Weifang Center for Disease Control and Prevention & BGI-Shenzhen|Li|China
>Spike|hCoV-19/England/QEUH-A76320/2020|2020-10-13|EPI_ISL_613317|Original|hCoV-19^^England|Human|Queens Medical Centre|COVID-19 Genomics UK (COG-UK) Consortium|Loose|United Kingdom
>Spike|hCoV-19/USA/TX-HMH-4078/2020|2020-06-18|EPI_ISL_543349|Original|hCoV-19^^Texas|Human|Houston Methodist Hospital|Houston Methodist Hospital|Long|USA
>Spike|hCoV-19/England/LIVE-9C0DC/2020|2020-04-01|EPI_ISL_449508|Original|hCoV-19^^England|Human|Liverpool Clinical Laboratories|COVID-19 Genomics UK (COG-UK) Consortium|Haldenby|United Kingdom
>Spike|hCoV-19/Denmark/DCGC-4064/2020|2020-09-14|EPI_ISL_622020|Original|hCoV-19^^Hovedstaden|Human|Department of Virus and Microbiological Special Diagnostics|Albertsen lab|Michaelsen|Denmark
>Spike|hCoV-19/England/ALDP-A68770/2020|2020-10-12|EPI_ISL_608691|Original|hCoV-19^^England|Human|Lighthouse Lab in Alderley Park|Wellcome Sanger Institute for the COVID-19 Genomics UK (COG-UK) consortium|Davies|United Kingdom
>Spike|hCoV-19/England/CAMC-B22911/2020|2020-11-01|EPI_ISL_647452|Original|hCoV-19^^England|Human|Lighthouse Lab in Cambridge|Wellcome Sanger Institute for the COVID-19 Genomics UK (COG-UK) Consortium|Davies|United Kingdom
>Spike|hCoV-19/Australia/VIC8417/2020|2020-07-24|EPI_ISL_564998|Original|hCoV-19^^Victoria|Human|Microbiological Diagnostic Unit - Public Health Laboratory (MDU-PHL)|MDU-PHL|Schultz|Australia
>Spike|hCoV-19/England/MILK-A7C9A0/2020|2020-10-16|EPI_ISL_607412|Original|hCoV-19^^England|Human|Lighthouse Lab in Milton Keynes|Wellcome Sanger Institute for the COVID-19 Genomics UK (COG-UK) consortium|Davies|United Kingdom
>Spike|hCoV-19/Australia/VIC15219/2020|2020-08-03|EPI_ISL_593198|Original|hCoV-19^^Victoria|Human|Microbiological Diagnostic Unit - Public Health Laboratory (MDU-PHL)|MDU-PHL|Schultz|Australia
>Spike|hCoV-19/England/ALDP-6CE92B/2020|2020-07-02|EPI_ISL_556215|Original|hCoV-19^^England|Human|Lighthouse Lab in Alderley Park|Wellcome Sanger Institute for the COVID-19 Genomics UK (COG-UK) consortium|Davies|United Kingdom
>Spike|hCoV-19/USA/FL-BPHL-1650/2020|2020-07-03|EPI_ISL_594339|Original|hCoV-19^^Florida|Human|Florida Bureau of Public Health Laboratories|Florida Bureau of Public Health Laboratories|Blanton|USA
>Spike|hCoV-19/Japan/PG-5933/2020|2020-00-00|EPI_ISL_689048|Original|hCoV-19^^Japan|Human|Gunma Prefectural Institute of Public Health and Environmental Sciences|Pathogen Genomics Center|Sekizuka|Japan
>Spike|hCoV-19/USA/TX-HMH-MCoV-19530/2020|2020-11-30|EPI_ISL_783906|Original|hCoV-19^^Texas|Human|Houston Methodist Hospital|Houston Methodist Hospital|Long|USA
>Spike|hCoV-19/England/ALDP-4F43A8/2020|2020-06-24|EPI_ISL_559225|Original|hCoV-19^^England|Human|Lighthouse Lab in Alderley Park|Wellcome Sanger Institute for the COVID-19 Genomics UK (COG-UK) consortium|Davies|United Kingdom
>Spike|hCoV-19/USA/TX-HMH-MCoV-18566/2020|2020-11-20|EPI_ISL_784914|Original|hCoV-19^^Texas|Human|Houston Methodist Hospital|Houston Methodist Hospital|Long|USA
>Spike|hCoV-19/USA/MD-MDH-0038/2020|2020-04-09|EPI_ISL_486917|Original|hCoV-19^^Maryland|Human|Maryland Department of Health|Maryland Department of Health|Keller|USA

>Spike|hCoV-19/Wales/PHWC-4A488A/2020|2020-12-14|EPI_ISL_764741|Original|hCoV-
19^^Wales|Human|Wales Specialist Virology Centre Sequencing lab|COVID-19 Genomics UK
(COG-UK) Consortium|Moore|United Kingdom
>Spike|hCoV-19/USA/WA-UW-22094/2020|2020-08-03|EPI_ISL_570624|Original|hCoV-
19^^Washington|Human|UW Virology Lab|UW Virology Lab|Roychoudhury|USA
>Spike|hCoV-19/South Africa/KRISP-K002661/2020|2020-08-
03|EPI_ISL_535529|Original|hCoV-19^^KwaZulu-Natal|Human|NHLS-IALCH|KRISP|de
Oliveira|South Africa
>Spike|hCoV-19/USA/CA-CZB-1427/2020|2020-03-21|EPI_ISL_468398|Original|hCoV-
19^^California|Human|County of San Luis Obispo Public Health Laboratory|Chan-
Zuckerberg Biohub|Hao|USA
>Spike|hCoV-19/England/CAMC-9C05AA/2020|2020-09-08|EPI_ISL_573191|Original|hCoV-
19^^England|Human|Oxford Viromics|COVID-19 Genomics UK (COG-UK)
Consortium|Golubchik|United Kingdom
>Spike|hCoV-19/England/MILK-BB0189/2020|2020-11-18|EPI_ISL_720215|Original|hCoV-
19^^England|Human|Lighthouse Lab in Milton Keynes|Wellcome Sanger Institute for the
COVID-19 Genomics UK (COG-UK) Consortium|Davies|United Kingdom
>Spike|hCoV-19/England/ALDP-C57FB6/2020|2020-12-14|EPI_ISL_735943|Original|hCoV-
19^^England|Human|Lighthouse Lab in Alderley Park|Wellcome Sanger Institute for the
COVID-19 Genomics UK (COG-UK) Consortium|Davies|United Kingdom
>Spike|hCoV-19/England/PORT-2D54E8/2020|2020-00-00|EPI_ISL_765118|Original|hCoV-
19^^England|Human|Centre for Enzyme Innovation|COVID-19 Genomics UK (COG-UK)
Consortium|Robson|United Kingdom
>Spike|hCoV-19/USA/OR-OHSU-0540/2020|2020-06-15|EPI_ISL_525837|Original|hCoV-
19^^Oregon|Human|OHSU Lab Services Molecular Microbiology Lab|Ginkgo Bioworks
Clinical Laboratory|O'Roak|USA
>Spike|hCoV-19/England/QEUH-B82557/2020|2020-11-14|EPI_ISL_661686|Original|hCoV-
19^^England|Human|Lighthouse Lab in Glasgow|Wellcome Sanger Institute for the COVID-
19 Genomics UK (COG-UK) Consortium|Davies|United Kingdom
>Spike|hCoV-19/Spain/CN-IBV-97006520/2020|2020-04-05|EPI_ISL_537725|Original|hCoV-
19^^Canary Islands|Human|Hospital Universitario de Gran Canaria Dr.
Negr\u00edn|SeqCOVID-SPAIN consortium/IBV(CSIC)|Comas|Spain
>Spike|hCoV-19/Lithuania/MR-LUHS-Eilnrj22/2020|2020-11-
00|EPI_ISL_770541|Original|hCoV-19^^Jonavos apskritis|Human|Private lab|Lithuanian
University of Health Sciences|Zemaitis|Lithuania
>Spike|hCoV-19/Scotland/QEUH-A1FBF4/2020|2020-10-01|EPI_ISL_599105|Original|hCoV-
19^^Scotland|Human|Lighthouse Lab in Glasgow|Wellcome Sanger Institute for the COVID-
19 Genomics UK (COG-UK) consortium|Davies|United Kingdom
>Spike|hCoV-19/USA/FL-BPHL-1102/2020|2020-08-04|EPI_ISL_526588|Original|hCoV-
19^^Florida|Human|Florida Bureau of Public Health Laboratories|Florida Bureau of
Public Health Laboratories|Blanton|USA
>Spike|hCoV-19/Bangladesh/CHRF-0010/2020|2020-05-03|EPI_ISL_468078|Original|hCoV-
19^^Dhaka|Human|Child Health Research Foundation|Child Health Research
Foundation|Saha|Bangladesh
>Spike|hCoV-19/England/ALDP-5ADA02/2020|2020-06-16|EPI_ISL_558721|Original|hCoV-
19^^England|Human|Lighthouse Lab in Alderley Park|Wellcome Sanger Institute for the
COVID-19 Genomics UK (COG-UK) consortium|Davies|United Kingdom
>Spike|hCoV-19/England/QEUH-AA0017/2020|2020-10-20|EPI_ISL_652700|Original|hCoV-
19^^England|Human|University of Exeter|COVID-19 Genomics UK (COG-UK)
Consortium|Temperton|United Kingdom
>Spike|hCoV-19/England/ALDP-AA4F25/2020|2020-10-21|EPI_ISL_630839|Original|hCoV-
19^^England|Human|Lighthouse Lab in Alderley Park|Wellcome Sanger Institute for the
COVID-19 Genomics UK (COG-UK) consortium|Davies|United Kingdom
>Spike|hCoV-19/England/CAMC-B9A914/2020|2020-11-15|EPI_ISL_673323|Original|hCoV-
19^^England|Human|Lighthouse Lab in Cambridge|Wellcome Sanger Institute for the
COVID-19 Genomics UK (COG-UK) Consortium|Davies|United Kingdom
>Spike|hCoV-19/Kenya/C7603/2020|2020-05-16|EPI_ISL_568845|Original|hCoV-
19^^Kwale|Human|KEMRI-Wellcome Trust Research Programme/KEMRI-CGMR-C Kilifi|KEMRI-
Wellcome Trust Research Programme/KEMRI-CGMR-C Kilifi|Githinji|Kenya

>Spike|hCoV-19/England/MILK-99D807/2020|2020-08-30|EPI_ISL_550703|Original|hCoV-19^^England|Human|Lighthouse Lab in Milton Keynes|Wellcome Sanger Institute for the COVID-19 Genomics UK (COG-UK) consortium|Davies|United Kingdom
>Spike|hCoV-19/Australia/VIC2300/2020|2020-06-24|EPI_ISL_522021|Original|hCoV-19^^Victoria|Human|Microbiological Diagnostic Unit - Public Health Laboratory (MDU-PHL)|MDU-PHL|Schultz|Australia
>Spike|hCoV-19/Australia/VIC2069/2020|2020-06-14|EPI_ISL_480758|Original|hCoV-19^^Victoria|Human|Microbiological Diagnostic Unit - Public Health Laboratory (MDU-PHL)|MDU-PHL|Schultz|Australia
>Spike|hCoV-19/England/QEUH-C4B771/2020|2020-12-13|EPI_ISL_769773|Original|hCoV-19^^England|Human|Lighthouse Lab in Glasgow|Wellcome Sanger Institute for the COVID-19 Genomics UK (COG-UK) Consortium|Davies|United Kingdom
>Spike|hCoV-19/Finland/24MS5H7/2020|2020-03-24|EPI_ISL_759817|Original|hCoV-19^^Uusimaa|Human|Department of Virology and Immunology|Department of Virology|Smura|Finland
>Spike|hCoV-19/England/CAMC-B98CE0/2020|2020-11-15|EPI_ISL_673626|Original|hCoV-19^^England|Human|Lighthouse Lab in Cambridge|Wellcome Sanger Institute for the COVID-19 Genomics UK (COG-UK) Consortium|Davies|United Kingdom
>Spike|hCoV-19/England/ALDP-501ED3/2020|2020-06-25|EPI_ISL_559178|Original|hCoV-19^^England|Human|Lighthouse Lab in Alderley Park|Wellcome Sanger Institute for the COVID-19 Genomics UK (COG-UK) consortium|Davies|United Kingdom
>Spike|hCoV-19/England/ALDP-A6EA53/2020|2020-10-15|EPI_ISL_607913|Original|hCoV-19^^England|Human|Lighthouse Lab in Alderley Park|Wellcome Sanger Institute for the COVID-19 Genomics UK (COG-UK) consortium|Davies|United Kingdom
>Spike|hCoV-19/Wales/PHWC-31F18/2020|2020-04-07|EPI_ISL_446585|Original|hCoV-19^^Wales|Human|Wales Specialist Virology Centre|Public Health Wales Microbiology Cardiff|Moore|United Kingdom
>Spike|hCoV-19/USA/MI-MDHHS-SC20819/2020|2020-05-13|EPI_ISL_471840|Original|hCoV-19^^Michigan|Human|Michigan Department of Health and Human Services|Michigan Department of Health and Human Services|Blankenship|USA
>Spike|hCoV-19/Beijing/Wuhan_IME-BJ02/2020|2020-01-25|EPI_ISL_424356|unknown|hCoV-19^^Beijing|Human|Beijing Institute of Microbiology and Epidemiology|Beijing Institute of Microbiology and Epidemiology|Submitter|China
>Spike|hCoV-19/Denmark/DCGC-2046/2020|2020-04-13|EPI_ISL_619337|Original|hCoV-19^^Midtjylland|Human|Department of Virus and Microbiological Special Diagnostics|Albertsen lab|Michaelsen|Denmark
>Spike|hCoV-19/USA/AZ-TG664512/2020|2020-04-20|EPI_ISL_696134|Original|hCoV-19^^Arizona|Human|Sonora Quest Laboratories|TGen North|French|USA
>Spike|hCoV-19/Sweden/20-07044/2020|2020-04-05|EPI_ISL_445230|Original|hCoV-19^^Uppsala|Human|Uppsala Narakut Aleris|The Public Health Agency of Sweden|Svartstr\u00f6m|Sweden
>Spike|hCoV-19/Spain/MD-IBV-99012439/2020|2020-11-27|EPI_ISL_780052|Original|hCoV-19^^Madrid|Human|Hospital General Universitario Gregorio Mara\u00f1\u00f3n|SeqCOVID-SPAIN consortium/IBV(CSIC)|Comas|Spain
>Spike|hCoV-19/England/QEUH-AD4C8F/2020|2020-10-26|EPI_ISL_634652|Original|hCoV-19^^England|Human|Lighthouse Lab in Glasgow|Wellcome Sanger Institute for the COVID-19 Genomics UK (COG-UK) consortium|Davies|United Kingdom
>Spike|hCoV-19/Portugal/PT0680/2020|2020-03-25|EPI_ISL_510939|Original|hCoV-19^^Portugal|Human|Instituto Nacional de Saude (INSA)|Instituto Nacional de Saude (INSA)|Borges|Portugal
>Spike|hCoV-19/USA/CA-ALSR-4649/2020|2020-09-04|EPI_ISL_667056|Original|hCoV-19^^California|Human|San Diego County Public Health Laboratory|Andersen lab at Scripps Research|Gangavarapu|USA
>Spike|hCoV-19/England/CAMC-B20C0B/2020|2020-11-02|EPI_ISL_647729|Original|hCoV-19^^England|Human|Lighthouse Lab in Cambridge|Wellcome Sanger Institute for the COVID-19 Genomics UK (COG-UK) Consortium|Davies|United Kingdom
>Spike|hCoV-19/England/MILK-BAAFA6/2020|2020-11-17|EPI_ISL_681614|Original|hCoV-19^^England|Human|Lighthouse Lab in Milton Keynes|Wellcome Sanger Institute for the COVID-19 Genomics UK (COG-UK) Consortium|Davies|United Kingdom

>Spike|hCoV-19/Australia/VIC16199/2020|2020-10-03|EPI_ISL_640588|Original|hCoV-
19^^Victoria|Human|Victorian Infectious Diseases Reference Laboratory (VIDRL)|VIDRL
and MDU-PHL|Schultz|Australia
>Spike|hCoV-19/England/QEUH-795FB1/2020|2020-07-15|EPI_ISL_533019|Original|hCoV-
19^^England|Human|Lighthouse Lab in Glasgow|Wellcome Sanger Institute for the COVID-
19 Genomics UK (COG-UK) consortium|Davies|United Kingdom
>Spike|hCoV-19/England/CAMB-7AF6E/2020|2020-04-10|EPI_ISL_433899|Original|hCoV-
19^^England|Human|Department of Pathology|COVID-19 Genomics UK (COG-UK)
Consortium|Meredith|United Kingdom
>Spike|hCoV-19/USA/CA-CDC-4730/2020|2020-06-16|EPI_ISL_535701|Original|hCoV-
19^^California|Human|CDPH|Pathogen Discovery|Queen|USA
>Spike|hCoV-19/England/LOND-124D1B4/2020|2020-03-23|EPI_ISL_507063|Original|hCoV-
19^^England|Human|University College London Hospital|COVID-19 Genomics UK (COG-UK)
Consortium|Byott|United Kingdom
>Spike|hCoV-19/Germany/ChVir20311/2020|2020-11-16|EPI_ISL_729646|Original|hCoV-
19^^Germany|Human|A. Krumbholz|Charit\u00e9 Universit\u00e4tsmedizin
Berlin|Muehlemann|Germany
>Spike|hCoV-19/Switzerland/TG-ETHZ-210010/2020|2020-07-
20|EPI_ISL_511985|Original|hCoV-19^^Thurgau|Human|Viollier AG|Department of
Biosystems Science and Engineering|Nadeau|Switzerland
>Spike|hCoV-19/Canada/BC_12246693/2020|2020-04-00|EPI_ISL_467333|Original|hCoV-
19^^British Columbia|Human|BCCDC Public Health Laboratory|BCCDC Public Health
Laboratory|Harrigan|Canada
>Spike|hCoV-19/Hong Kong/VM20010467/2020|2020-04-08|EPI_ISL_463322|Original|hCoV-
19^^Hong Kong|Human|North Lantau Hospital|Hong Kong Department of Health|Mak|China
>Spike|hCoV-19/Denmark/DCGC-19048/2020|2020-12-07|EPI_ISL_748720|Original|hCoV-
19^^Hovedstaden|Human|Department of Virus and Microbiological Special
Diagnostics|Albertsen Lab|Michaelsen|Denmark
>Spike|hCoV-19/England/LIVE-A0758/2020|2020-06-03|EPI_ISL_472132|Original|hCoV-
19^^England|Human|Liverpool Clinical Laboratories|COVID-19 Genomics UK (COG-UK)
Consortium|Haldenby|United Kingdom
>Spike|hCoV-19/England/20170000704/2020|2020-04-20|EPI_ISL_465262|Original|hCoV-
19^^England|Human|Respiratory Virus Unit|Respiratory Virus Unit|Platt|United Kingdom
>Spike|hCoV-19/Canada/ON-S1227/2020|2020-04-02|EPI_ISL_586302|Original|hCoV-
19^^Ontario|Human|Toronto Invasive Bacterial Diseases Network|McMaster
University|McArthur|Canada
>Spike|hCoV-19/USA/MA-MASPHL-00527/2020|2020-05-05|EPI_ISL_693051|Original|hCoV-
19^^Massachusetts|Human|Massachusetts State Public Health Laboratory|Massachusetts
State Public Health Laboratory|Lang|USA
>Spike|hCoV-19/USA/MI-MDHHS-SC20663/2020|2020-04-29|EPI_ISL_471687|Original|hCoV-
19^^Michigan|Human|Michigan Department of Health and Human Services|Michigan
Department of Health and Human Services|Blankenship|USA
>Spike|hCoV-19/England/ALDP-C3BD36/2020|2020-12-11|EPI_ISL_731728|Original|hCoV-
19^^England|Human|Lighthouse Lab in Alderley Park|Wellcome Sanger Institute for the
COVID-19 Genomics UK (COG-UK) Consortium|Davies|United Kingdom
>Spike|hCoV-19/England/EXET-137843/2020|2020-05-03|EPI_ISL_471988|Original|hCoV-
19^^England|Human|University of Exeter|COVID-19 Genomics UK (COG-UK)
Consortium|Temperton|United Kingdom
>Spike|hCoV-19/USA/WI-UW-1070/2020|2020-08-05|EPI_ISL_516496|Original|hCoV-
19^^Wisconsin|Human|University of Wisconsin-Madison AIDS Vaccine Research
Laboratories|University of Wisconsin-Madison AIDS Vaccine Research
Laboratories|Baczenas|USA
>Spike|hCoV-19/Australia/VIC4287/2020|2020-07-14|EPI_ISL_519615|Original|hCoV-
19^^Victoria|Human|Microbiological Diagnostic Unit - Public Health Laboratory (MDU-
PHL)|MDU-PHL|Schultz|Australia
>Spike|hCoV-19/England/EXET-13B038/2020|2020-11-10|EPI_ISL_665107|Original|hCoV-
19^^England|Human|University of Exeter|COVID-19 Genomics UK (COG-UK)
Consortium|Temperton|United Kingdom

>Spike|hCoV-19/United Arab Emirates/0244/2020|2020-05-17|EPI_ISL_698426|Original|hCoV-19^^Abu Dhabi|Human|Group 42 (G42) Healthcare|G42 Healthcare|Ogrodzki|United Arab Emirates
>Spike|hCoV-19/Luxembourg/LNS0613111/2020|2020-11-17|EPI_ISL_739805|Original|hCoV-19^^Luxembourg|Human|Laboratoire national de sant\u00e9|Laboratoire national de sant\u00e9|Wienecke-Baldacchino|Luxembourg
>Spike|hCoV-19/England/ALDP-A2E05D/2020|2020-10-06|EPI_ISL_586908|Original|hCoV-19^^England|Human|Lighthouse Lab in Alderley Park|Wellcome Sanger Institute for the COVID-19 Genomics UK (COG-UK) consortium|Davies|United Kingdom
>Spike|hCoV-19/England/C200029520/2020|2020-10-09|EPI_ISL_636703|Original|hCoV-19^^England|Human|Respiratory Virus Unit|Respiratory Virus Unit|Platt|United Kingdom
>Spike|hCoV-19/Denmark/DCGC-18902/2020|2020-12-07|EPI_ISL_748178|Original|hCoV-19^^Hovedstaden|Human|Department of Virus and Microbiological Special Diagnostics|Albertsen Lab|Michaelsen|Denmark
>Spike|hCoV-19/USA/AK-PHL648/2020|2020-06-26|EPI_ISL_586251|Original|hCoV-19^^Alaska|Human|Alaska State Virology Laboratory|Alaska State Virology Laboratory|Chen|USA
>Spike|hCoV-19/England/NORW-22233A/2020|2020-12-21|EPI_ISL_764487|Original|hCoV-19^^England|Human|Quadram Institute Bioscience|COVID-19 Genomics UK (COG-UK) Consortium|Alikhan|United Kingdom
>Spike|hCoV-19/Oman/205036690/2020|2020-05-17|EPI_ISL_491981|Original|hCoV-19^^Muscat|Human|Oman-NIC|Department of\u00a0Microbiology\u00a0and Immunology-SQUH|AL Zadjali|Oman
>Spike|hCoV-19/Latvia/103/2020|2020-08-05|EPI_ISL_515190|Original|hCoV-19^^Latvia|Human|E. Gulbja Laboratorija|Latvian Biomedical Research and Study Centre|Zrelovs|Latvia
>Spike|hCoV-19/Denmark/DCGC-14185/2020|2020-11-30|EPI_ISL_711104|Original|hCoV-19^^Sjaelland|Human|Department of Virus and Microbiological Special Diagnostics|Albertsen Lab|Michaelsen|Denmark
>Spike|hCoV-19/Denmark/DCGC-17062/2020|2020-12-14|EPI_ISL_750218|Original|hCoV-19^^Sjaelland|Human|Department of Virus and Microbiological Special Diagnostics|Albertsen Lab|Michaelsen|Denmark
>Spike|hCoV-19/England/QEUH-AD49C4/2020|2020-10-26|EPI_ISL_634159|Original|hCoV-19^^England|Human|Lighthouse Lab in Glasgow|Wellcome Sanger Institute for the COVID-19 Genomics UK (COG-UK) consortium|Davies|United Kingdom
>Spike|hCoV-19/Turkey/6224-Ankara1034/2020|2020-03-17|EPI_ISL_417413|Original|hCoV-19^^Turkey|Human|Ministry of Health Turkey|Ministry of Health Turkey|Bayrakdar|Turkey
>Spike|hCoV-19/Denmark/DCGC-2195/2020|2020-08-10|EPI_ISL_617496|Original|hCoV-19^^Nordjylland|Human|Department of Virus and Microbiological Special Diagnostics|Albertsen lab|Michaelsen|Denmark
>Spike|hCoV-19/USA/CA-ALSR-1498/2020|2020-04-01|EPI_ISL_494623|Original|hCoV-19^^California|Human|San Diego County Public Health Laboratory|Andersen lab at Scripps Research|Gangavarapu|USA
>Spike|hCoV-19/England/MILK-975920/2020|2020-08-20|EPI_ISL_552674|Original|hCoV-19^^England|Human|Lighthouse Lab in Milton Keynes|Wellcome Sanger Institute for the COVID-19 Genomics UK (COG-UK) consortium|Davies|United Kingdom
>Spike|hCoV-19/England/CAMC-BBCD10/2020|2020-11-21|EPI_ISL_679276|Original|hCoV-19^^England|Human|Queens Medical Centre|COVID-19 Genomics UK (COG-UK) Consortium|Loose|United Kingdom
>Spike|hCoV-19/Denmark/DCGC-16570/2020|2020-11-16|EPI_ISL_714843|Original|hCoV-19^^Sjaelland|Human|Department of Virus and Microbiological Special Diagnostics|Albertsen Lab|Michaelsen|Denmark
>Spike|hCoV-19/Indonesia/JK-EIJK42/2020|2020-06-03|EPI_ISL_574613|Original|hCoV-19^^Jakarta|Human|RS Harapan Bunda|Eijkman Institute for Molecular Biology|Soebandrio|Indonesia
>Spike|hCoV-19/Wales/PHWC-48E394/2020|2020-11-12|EPI_ISL_680849|Original|hCoV-19^^Wales|Human|Wales Specialist Virology Centre Sequencing lab|COVID-19 Genomics UK (COG-UK) Consortium|Moore|United Kingdom

>Spike|hCoV-19/England/LIVE-DC982C/2020|2020-12-04|EPI_ISL_705304|Original|hCoV-19^^England|Human|Liverpool Clinical Laboratories|COVID-19 Genomics UK (COG-UK) Consortium|Haldenby|United Kingdom
>Spike|hCoV-19/USA/NY-NYUMC182/2020|2020-04-01|EPI_ISL_427478|Original|hCoV-19^^New York|Human|NYU Langone Health|Departments of Pathology and Medicine|Maurano|USA
>Spike|hCoV-19/USA/LA-EVTL181/2020|2020-05-15|EPI_ISL_574316|Original|hCoV-19^^Louisiana|Human|LSUHS Emerging Viral Threat Laboratory|Microbial Genome Sequencing Center|Kamil|USA
>Spike|hCoV-19/France/PAC-IHU-1524/2020|2020-08-12|EPI_ISL_569188|Original|hCoV-19^^Provence-Alpes-C\u00f4te-d\u2019Azur|Human|MEPHI|MEPHI|Levasseur|France
>Spike|hCoV-19/England/CAMC-B7AF18/2020|2020-11-12|EPI_ISL_723969|Original|hCoV-19^^England|Human|Oxford Viromics|COVID-19 Genomics UK (COG-UK) Consortium|Golubchik|United Kingdom
>Spike|hCoV-19/Netherlands/ZH-EMC-838/2020|2020-10-05|EPI_ISL_632344|Original|hCoV-19^^Zuid Holland|Human|Dutch COVID-19 response team|Erasmus Medical Center|Oude Munnink|Netherlands
>Spike|hCoV-19/England/CAMB-1BBB3F/2020|2020-11-17|EPI_ISL_679196|Original|hCoV-19^^England|Human|Department of Pathology|COVID-19 Genomics UK (COG-UK) Consortium|Goodfellow|United Kingdom
>Spike|hCoV-19/Wales/PHWC-4833BD/2020|2020-10-21|EPI_ISL_627812|Original|hCoV-19^^Wales|Human|Wales Specialist Virology Centre Sequencing lab|COVID-19 Genomics UK (COG-UK) Consortium|Moore|United Kingdom
>Spike|hCoV-19/Kazakhstan/21927/2020|2020-04-22|EPI_ISL_454503|Original|hCoV-19^^Nur-Sultan|Human|RSE \National Center for Biotechnology\|RSE \National Center for Biotechnology\|Kalendar|Kazakhstan
>Spike|hCoV-19/Denmark/DCGC-11745/2020|2020-10-26|EPI_ISL_671109|Original|hCoV-19^^Syddanmark|Human|Department of Virus and Microbiological Special Diagnostics|Albertsen Lab|Michaelsen|Denmark
>Spike|hCoV-19/England/20104014004/2020|2020-03-02|EPI_ISL_464330|Original|hCoV-19^^England|Human|Respiratory Virus Unit|Respiratory Virus Unit|Platt|United Kingdom
>Spike|hCoV-19/England/CAMC-B34A55/2020|2020-11-05|EPI_ISL_659654|Original|hCoV-19^^England|Human|Lighthouse Lab in Cambridge|Wellcome Sanger Institute for the COVID-19 Genomics UK (COG-UK) Consortium|Davies|United Kingdom
>Spike|hCoV-19/Spain/PV-IBV-001574/2020|2020-03-14|EPI_ISL_467180|Original|hCoV-19^^Basque Country|Human|Hospital Universitario Araba. Vitoria-Gasteiz|SeqCOVID-SPAIN consortium/IBV(CSIC)|Comas|Spain
>Spike|hCoV-19/USA/WA-UW-4664/2020|2020-04-06|EPI_ISL_513635|Original|hCoV-19^^Washington|Human|University of Washington Virology Lab|University of Washington Virology Lab|Roychoudhury|USA
>Spike|hCoV-19/Belgium/ULG-10367/2020|2020-08-07|EPI_ISL_540534|Original|hCoV-19^^Li\u00e8ge|Human|Department of Clinical Microbiology|GIGA Medical Genomics|Durkin|Belgium
>Spike|hCoV-19/Denmark/DCGC-21441/2020|2020-12-21|EPI_ISL_758167|Original|hCoV-19^^Hovedstaden|Human|Department of Virus and Microbiological Special Diagnostics|Albertsen Lab|Michaelsen|Denmark
>Spike|hCoV-19/Japan/PG-1647/2020|2020-04-00|EPI_ISL_685253|Original|hCoV-19^^Japan|Human|Pathogen Genomics Center|Pathogen Genomics Center|Sekizuka|Japan
>Spike|hCoV-19/USA/WI-UW-645/2020|2020-06-26|EPI_ISL_491336|Original|hCoV-19^^Wisconsin|Human|University of Wisconsin-Madison AIDS Vaccine Research Laboratories|University of Wisconsin-Madison AIDS Vaccine Research Laboratories|Baczenas|USA
>Spike|hCoV-19/Japan/PG-2778/2020|2020-06-00|EPI_ISL_690905|Original|hCoV-19^^Japan|Human|Tokyo Metropolitan Institute of Public Health|Pathogen Genomics Center|Sekizuka|Japan
>Spike|hCoV-19/Australia/VIC9133/2020|2020-08-09|EPI_ISL_565403|Original|hCoV-19^^Victoria|Human|Victorian Infectious Diseases Reference Laboratory (VIDRL)|VIDRL and MDU-PHL|Schultz|Australia
>Spike|hCoV-19/USA/TX-HMH-3138/2020|2020-06-11|EPI_ISL_544020|Original|hCoV-19^^Texas|Human|Houston Methodist Hospital|Houston Methodist Hospital|Long|USA

>Spike|hCoV-19/USA/MI-MDHHS-SC21178/2020|2020-07-06|EPI_ISL_507435|Original|hCoV-19^^Michigan|Human|Michigan Department of Health and Human Services|Michigan Department of Health and Human Services|Blankenship|USA
>Spike|hCoV-19/USA/WI-UW-1558/2020|2020-09-15|EPI_ISL_605436|Original|hCoV-19^^Wisconsin|Human|University of Wisconsin-Madison AIDS Vaccine Research Laboratories|University of Wisconsin-Madison AIDS Vaccine Research Laboratories|Baczenas|USA
>Spike|hCoV-19/England/MILK-957A7C/2020|2020-06-11|EPI_ISL_554231|Original|hCoV-19^^England|Human|Lighthouse Lab in Milton Keynes|Wellcome Sanger Institute for the COVID-19 Genomics UK (COG-UK) consortium|Davies|United Kingdom
>Spike|hCoV-19/England/ALDP-B5C43B/2020|2020-11-10|EPI_ISL_655968|Original|hCoV-19^^England|Human|Lighthouse Lab in Alderley Park|Wellcome Sanger Institute for the COVID-19 Genomics UK (COG-UK) Consortium|Davies|United Kingdom
>Spike|hCoV-19/Wales/PHWC-48F1F3/2020|2020-11-07|EPI_ISL_681059|Original|hCoV-19^^Wales|Human|Wales Specialist Virology Centre Sequencing lab|COVID-19 Genomics UK (COG-UK) Consortium|Moore|United Kingdom
>Spike|hCoV-19/England/NOTT-111EE3/2020|2020-05-02|EPI_ISL_472422|Original|hCoV-19^^England|Human|Queens Medical Centre|COVID-19 Genomics UK (COG-UK) Consortium|Loose|United Kingdom
>Spike|hCoV-19/USA/TX-HMH-MCoV-19563/2020|2020-11-30|EPI_ISL_783935|Original|hCoV-19^^Texas|Human|Houston Methodist Hospital|Houston Methodist Hospital|Long|USA
>Spike|hCoV-19/England/MILK-9AB657/2020|2020-08-31|EPI_ISL_549451|Original|hCoV-19^^England|Human|Oxford Viromics|COVID-19 Genomics UK (COG-UK) Consortium|Golubchik|United Kingdom
>Spike|hCoV-19/Scotland/EDB4507/2020|2020-05-05|EPI_ISL_456946|Original|hCoV-19^^Scotland|Human|Virology Department|COVID-19 Genomics UK (COG-UK) Consortium|Williams|United Kingdom
>Spike|hCoV-19/Scotland/QEUH-A0BD9B/2020|2020-10-03|EPI_ISL_599385|Original|hCoV-19^^Scotland|Human|Lighthouse Lab in Glasgow|Wellcome Sanger Institute for the COVID-19 Genomics UK (COG-UK) consortium|Davies|United Kingdom
>Spike|hCoV-19/Scotland/EDB9048/2020|2020-10-31|EPI_ISL_652038|Original|hCoV-19^^Scotland|Human|Virology Department|COVID-19 Genomics UK (COG-UK) Consortium|McHugh|United Kingdom
>Spike|hCoV-19/USA/MI-MDHHS-SC22134/2020|2020-10-19|EPI_ISL_614185|Original|hCoV-19^^Michigan|Human|Michigan Department of Health and Human Services|Michigan Department of Health and Human Services|Jacob|USA
>Spike|hCoV-19/USA/AZ-TG276476/2020|2020-04-13|EPI_ISL_694105|Original|hCoV-19^^Arizona|Human|AZ SPHL|TGen North|French|USA
>Spike|hCoV-19/England/LIVE-DB7D9B/2020|2020-10-26|EPI_ISL_651288|Original|hCoV-19^^England|Human|Liverpool Clinical Laboratories|COVID-19 Genomics UK (COG-UK) Consortium|Haldenby|United Kingdom
>Spike|hCoV-19/Scotland/CVR2298/2020|2020-04-12|EPI_ISL_484574|Original|hCoV-19^^Scotland|Human|West of Scotland Specialist Virology Centre|COVID-19 Genomics UK (COG-UK) Consortium|Robertson|United Kingdom
>Spike|hCoV-19/Japan/PG-5308/2020|2020-08-00|EPI_ISL_690411|Original|hCoV-19^^Japan|Human|Pathogen Genomics Center|Pathogen Genomics Center|Sekizuka|Japan
>Spike|hCoV-19/England/LIVE-DCA472/2020|2020-12-06|EPI_ISL_724364|Original|hCoV-19^^England|Human|Liverpool Clinical Laboratories|COVID-19 Genomics UK (COG-UK) Consortium|Haldenby|United Kingdom
>Spike|hCoV-19/Belgium/ULG-10305/2020|2020-07-23|EPI_ISL_540472|Original|hCoV-19^^Li\u00e8ge|Human|Department of Clinical Microbiology|GIGA Medical Genomics|Durkin|Belgium
>Spike|hCoV-19/England/CAMC-B234E2/2020|2020-11-02|EPI_ISL_651730|Original|hCoV-19^^England|Human|Virology Department|COVID-19 Genomics UK (COG-UK) Consortium|de Silva|United Kingdom
>Spike|hCoV-19/England/LIVE-A695C/2020|2020-03-16|EPI_ISL_499757|Original|hCoV-19^^England|Human|Liverpool Clinical Laboratories|COVID-19 Genomics UK (COG-UK) Consortium|Haldenby|United Kingdom

>Spike|hCoV-19/England/MILK-A7D374/2020|2020-10-13|EPI_ISL_606420|Original|hCoV-19^^England|Human|Lighthouse Lab in Milton Keynes|Wellcome Sanger Institute for the COVID-19 Genomics UK (COG-UK) consortium|Davies|United Kingdom
>Spike|hCoV-19/England/CAMB-84848/2020|2020-03-04|EPI_ISL_440518|Original|hCoV-19^^England|Human|Department of Pathology|Wellcome Sanger Institute for the COVID-19 Genomics UK (COG-UK) consortium|Amato|United Kingdom
>Spike|hCoV-19/England/20148044604/2020|2020-04-02|EPI_ISL_423592|Original|hCoV-19^^England|Human|Respiratory Virus Unit|Respiratory Virus Unit|Platt|United Kingdom
>Spike|hCoV-19/New Zealand/20VR3202/2020|2020-03-31|EPI_ISL_579487|Original|hCoV-19^^Canterbury|Human|Canterbury Health Laboratories|Institute of Environmental Science and Research (ESR)|Wang|New Zealand
>Spike|hCoV-19/Switzerland/ZH-ETHZ-300235/2020|2020-09-30|EPI_ISL_603268|Original|hCoV-19^^Zurich|Human|Viollier AG|Department of Biosystems Science and Engineering|Nadeau|Switzerland
>Spike|hCoV-19/Australia/VIC16732/2020|2020-08-08|EPI_ISL_663557|Original|hCoV-19^^Victoria|Human|Microbiological Diagnostic Unit - Public Health Laboratory (MDU-PHL)|MDU-PHL|Schultz|Australia
>Spike|hCoV-19/England/QEUH-B68B46/2020|2020-11-11|EPI_ISL_655224|Original|hCoV-19^^England|Human|Lighthouse Lab in Glasgow|Wellcome Sanger Institute for the COVID-19 Genomics UK (COG-UK) Consortium|Davies|United Kingdom
>Spike|hCoV-19/England/20139057704/2020|2020-03-28|EPI_ISL_420722|Original|hCoV-19^^England|Human|Respiratory Virus Unit|Respiratory Virus Unit|Platt|United Kingdom
>Spike|hCoV-19/Scotland/QEUH-A9D174/2020|2020-10-20|EPI_ISL_652660|Original|hCoV-19^^Scotland|Human|Queens Medical Centre|COVID-19 Genomics UK (COG-UK) Consortium|Loose|United Kingdom
>Spike|hCoV-19/USA/UT-098/2020|2020-03-14|EPI_ISL_430036|Original|hCoV-19^^Utah|Human|Utah Public Health Laboratory|Utah Public Health Laboratory|Young|USA
>Spike|hCoV-19/England/NOTT-112A84/2020|2020-04-23|EPI_ISL_493579|Original|hCoV-19^^England|Human|Lincolnshire Hospitals and DeepSeq Nottingham|COVID-19 Genomics UK (COG-UK) Consortium|Loose|United Kingdom
>Spike|hCoV-19/England/BRIS-13009D/2020|2020-04-11|EPI_ISL_492625|Original|hCoV-19^^England|Human|PHE South West Regional Laboratory|Wellcome Sanger Institute for the COVID-19 Genomics UK (COG-UK) consortium|Davies|United Kingdom
>Spike|hCoV-19/Australia/VIC12056/2020|2020-07-23|EPI_ISL_591910|Original|hCoV-19^^Victoria|Human|Microbiological Diagnostic Unit - Public Health Laboratory (MDU-PHL)|MDU-PHL|Schultz|Australia
>Spike|hCoV-19/England/QEUH-AD0861/2020|2020-10-24|EPI_ISL_633706|Original|hCoV-19^^England|Human|Lighthouse Lab in Glasgow|Wellcome Sanger Institute for the COVID-19 Genomics UK (COG-UK) consortium|Davies|United Kingdom
>Spike|hCoV-19/England/CAMB-7C755/2020|2020-04-05|EPI_ISL_442211|Original|hCoV-19^^England|Human|Department of Pathology|Wellcome Sanger Institute for the COVID-19 Genomics UK (COG-UK) consortium|Amato|United Kingdom
>Spike|hCoV-19/Scotland/CVR1397/2020|2020-04-01|EPI_ISL_526455|Original|hCoV-19^^Scotland|Human|West of Scotland Specialist Virology Centre|COVID-19 Genomics UK (COG-UK) Consortium|Robertson|United Kingdom
>Spike|hCoV-19/Denmark/DCGC-15714/2020|2020-11-23|EPI_ISL_713113|Original|hCoV-19^^Hovedstaden|Human|Department of Virus and Microbiological Special Diagnostics|Albertsen Lab|Michaelsen|Denmark
>Spike|hCoV-19/Scotland/QEUH-A4B315/2020|2020-10-08|EPI_ISL_628465|Original|hCoV-19^^Scotland|Human|Oxford Viromics|COVID-19 Genomics UK (COG-UK) Consortium|Golubchik|United Kingdom
>Spike|hCoV-19/USA/HI-H200412/2020|2020-08-06|EPI_ISL_752956|Original|hCoV-19^^Hawaii|Human|State Laboratories Division|State Laboratories Division|Sultana|USA
>Spike|hCoV-19/Denmark/DCGC-1269/2020|2020-06-08|EPI_ISL_618058|Original|hCoV-19^^Nordjylland|Human|Department of Virus and Microbiological Special Diagnostics|Albertsen lab|Michaelsen|Denmark
>Spike|hCoV-19/Scotland/EDB8759/2020|2020-10-25|EPI_ISL_651366|Original|hCoV-19^^Scotland|Human|Virology Department|COVID-19 Genomics UK (COG-UK) Consortium|McHugh|United Kingdom

>Spike|hCoV-19/England/ALDP-94FC62/2020|2020-06-15|EPI_ISL_555298|Original|hCoV-19^^England|Human|Lighthouse Lab in Alderley Park|Wellcome Sanger Institute for the COVID-19 Genomics UK (COG-UK) consortium|Davies|United Kingdom
>Spike|hCoV-19/England/CAMC-AAFB62/2020|2020-10-23|EPI_ISL_623444|Original|hCoV-19^^England|Human|Lighthouse Lab in Cambridge|Wellcome Sanger Institute for the COVID-19 Genomics UK (COG-UK) consortium|Davies|United Kingdom
>Spike|hCoV-19/USA/ID-UW-2173/2020|2020-03-23|EPI_ISL_529160|Original|hCoV-19^^Idaho|Human|University of Washington|University of Washington|Roychoudhury|USA
>Spike|hCoV-19/England/QEUH-AD669E/2020|2020-10-26|EPI_ISL_634211|Original|hCoV-19^^England|Human|Lighthouse Lab in Glasgow|Wellcome Sanger Institute for the COVID-19 Genomics UK (COG-UK) consortium|Davies|United Kingdom
>Spike|hCoV-19/Spain/AN-IBV-001892/2020|2020-03-12|EPI_ISL_452456|Original|hCoV-19^^Andalucia|Human|Hospital Universitario Puerta del Mar de C\u00e1diz - INIBICA|SeqCOVID-SPAIN consortium/IBV(CSIC)|Comas|Spain
>Spike|hCoV-19/United Arab Emirates/0151/2020|2020-05-18|EPI_ISL_698338|Original|hCoV-19^^Abu Dhabi|Human|Group 42 (G42) Healthcare|G42 Healthcare|Ogrodzki|United Arab Emirates
>Spike|hCoV-19/USA/CA-CZB-11821/2020|2020-10-02|EPI_ISL_625659|Original|hCoV-19^^California|Human|Orange County Public Health Lab|Chan-Zuckerberg Biohub|Bhatt|USA
>Spike|hCoV-19/England/GSTT-265D28A/2020|2020-03-16|EPI_ISL_483934|Original|hCoV-19^^England|Human|Centre for Clinical Infection and Diagnostics Research and Genomics Innovation Unit|COVID-19 Genomics UK (COG-UK) Consortium|Awan|United Kingdom
>Spike|hCoV-19/Gibraltar/205000669/2020|2020-12-00|EPI_ISL_709995|Original|hCoV-19^^Gibraltar|Human|Microbiology|Respiratory Virus Unit|Platt|Gibraltar
>Spike|hCoV-19/Japan/PG-8720/2020|2020-08-00|EPI_ISL_686316|Original|hCoV-19^^Japan|Human|Pathogen Genomics Center|Pathogen Genomics Center|Sekizuka|Japan
>Spike|hCoV-19/USA/TX-HMH-MCoV-8425/2020|2020-07-01|EPI_ISL_784390|Original|hCoV-19^^Texas|Human|Houston Methodist Hospital|Houston Methodist Hospital|Long|USA
>Spike|hCoV-19/USA/TX-HMH-MCoV-15295/2020|2020-10-19|EPI_ISL_786779|Original|hCoV-19^^Texas|Human|Houston Methodist Hospital|Houston Methodist Hospital|Long|USA
>Spike|hCoV-19/England/CAMC-B52286/2020|2020-11-08|EPI_ISL_656722|Original|hCoV-19^^England|Human|Lighthouse Lab in Cambridge|Wellcome Sanger Institute for the COVID-19 Genomics UK (COG-UK) Consortium|Davies|United Kingdom
>Spike|hCoV-19/Japan/PG-1132/2020|2020-04-00|EPI_ISL_684742|Original|hCoV-19^^Japan|Human|Pathogen Genomics Center|Pathogen Genomics Center|Sekizuka|Japan
>Spike|hCoV-19/Denmark/DCGC-17012/2020|2020-12-14|EPI_ISL_750708|Original|hCoV-19^^Midtjylland|Human|Department of Virus and Microbiological Special Diagnostics|Albertsen Lab|Michaelsen|Denmark
>Spike|hCoV-19/Scotland/QEUH-A1FE19/2020|2020-10-01|EPI_ISL_599136|Original|hCoV-19^^Scotland|Human|Lighthouse Lab in Glasgow|Wellcome Sanger Institute for the COVID-19 Genomics UK (COG-UK) consortium|Davies|United Kingdom
>Spike|hCoV-19/Denmark/DCGC-1437/2020|2020-03-23|EPI_ISL_622445|Original|hCoV-19^^Hovedstaden|Human|Department of Virus and Microbiological Special Diagnostics|Albertsen lab|Michaelsen|Denmark
>Spike|hCoV-19/Canada/MB-NML-421/2020|2020-03-29|EPI_ISL_582255|Original|hCoV-19^^Manitoba|Human|Cadham Provincial Laboratory|National Microbiology Laboratory (NML)|Majer|Canada
>Spike|hCoV-19/Scotland/CVR2416/2020|2020-04-14|EPI_ISL_477933|Original|hCoV-19^^Scotland|Human|West of Scotland Specialist Virology Centre|COVID-19 Genomics UK (COG-UK) Consortium|Robertson|United Kingdom
>Spike|hCoV-19/Netherlands/NH-EMC-63/2020|2020-08-18|EPI_ISL_577859|Original|hCoV-19^^Noord Holland|Human|Dutch COVID-19 response team|Erasmus Medical Center|Oude Munnink|Netherlands
>Spike|hCoV-19/USA/UT-UPHL-2012089916/2020|2020-10-27|EPI_ISL_745654|Original|hCoV-19^^Utah|Human|Ginkgo Bioworks Clinical Laboratory|Utah Public Health Laboratory|Young|USA
>Spike|hCoV-19/Scotland/EDB1080/2020|2020-04-07|EPI_ISL_439146|Original|hCoV-19^^Scotland|Human|Virology Department|COVID-19 Genomics UK (COG-UK) Consortium|Williams|United Kingdom

>Spike|hCoV-19/Scotland/GCVR-17040E/2020|2020-03-24|EPI_ISL_459679|Original|hCoV-19^^Scotland|Human|NHSGGC West of Scotland Specialist Virology Centre / MRC-University of Glasgow Centre for Virus Research|Wellcome Sanger Institute for the COVID-19 Genomics UK (COG-UK) consortium|Davies|United Kingdom
>Spike|hCoV-19/Denmark/DCGC-18062/2020|2020-12-14|EPI_ISL_749362|Original|hCoV-19^^Hovedstaden|Human|Department of Virus and Microbiological Special Diagnostics|Albertsen Lab|Michaelsen|Denmark
>Spike|hCoV-19/Netherlands/NA_185/2020|2020-03-23|EPI_ISL_422720|Original|hCoV-19^^Netherlands|Human|Dutch COVID-19 response team|Erasmus Medical Center|Oude Munnink|Netherlands
>Spike|hCoV-19/Japan/PG-4927/2020|2020-07-00|EPI_ISL_690047|Original|hCoV-19^^Japan|Human|Pathogen Genomics Center|Pathogen Genomics Center|Sekizuka|Japan
>Spike|hCoV-19/England/LOND-12E8670/2020|2020-12-15|EPI_ISL_741340|Original|hCoV-19^^England|Human|University College London|COVID-19 Genomics UK (COG-UK) Consortium|Roy|United Kingdom
>Spike|hCoV-19/USA/WA-S169/2020|2020-03-18|EPI_ISL_430207|Original|hCoV-19^^Washington|Human|Washington State Department of Health|Seattle Flu Study|Bedford|USA
>Spike|hCoV-19/Wales/PHWC-48361E/2020|2020-10-23|EPI_ISL_627838|Original|hCoV-19^^Wales|Human|Wales Specialist Virology Centre Sequencing lab|COVID-19 Genomics UK (COG-UK) Consortium|Moore|United Kingdom
>Spike|hCoV-19/England/ALDP-B62818/2020|2020-11-09|EPI_ISL_656440|Original|hCoV-19^^England|Human|Lighthouse Lab in Alderley Park|Wellcome Sanger Institute for the COVID-19 Genomics UK (COG-UK) Consortium|Davies|United Kingdom
>Spike|hCoV-19/Denmark/DCGC-17942/2020|2020-12-14|EPI_ISL_749658|Original|hCoV-19^^Sjaelland|Human|Department of Virus and Microbiological Special Diagnostics|Albertsen Lab|Michaelsen|Denmark
>Spike|hCoV-19/Scotland/EDB2287/2020|2020-04-21|EPI_ISL_439179|Original|hCoV-19^^Scotland|Human|Virology Department|COVID-19 Genomics UK (COG-UK) Consortium|Williams|United Kingdom
>Spike|hCoV-19/USA/AZ-TG666095/2020|2020-05-02|EPI_ISL_696185|Original|hCoV-19^^Arizona|Human|Sonora Quest Laboratories|TGen North|French|USA
>Spike|hCoV-19/USA/UT-QDX-1786/2020|2020-03-16|EPI_ISL_604118|Original|hCoV-19^^Utah|Human|Quest Diagnostics|Quest Diagnostics|Kagan|USA
>Spike|hCoV-19/Australia/VIC6117/2020|2020-07-22|EPI_ISL_518135|Original|hCoV-19^^Victoria|Human|Victorian Infectious Diseases Reference Laboratory (VIDRL)|VIDRL and MDU-PHL|Schultz|Australia
>Spike|hCoV-19/England/MILK-99200A/2020|2020-08-27|EPI_ISL_540643|Original|hCoV-19^^England|Human|Queens Medical Centre|COVID-19 Genomics UK (COG-UK) Consortium|Loose|United Kingdom
>Spike|hCoV-19/England/ALDP-AA1298/2020|2020-10-22|EPI_ISL_624082|Original|hCoV-19^^England|Human|Lighthouse Lab in Alderley Park|Wellcome Sanger Institute for the COVID-19 Genomics UK (COG-UK) consortium|Davies|United Kingdom
>Spike|hCoV-19/Australia/VIC8899/2020|2020-07-23|EPI_ISL_565271|Original|hCoV-19^^Victoria|Human|Microbiological Diagnostic Unit - Public Health Laboratory (MDU-PHL)|MDU-PHL|Schultz|Australia
>Spike|hCoV-19/Scotland/CVR5029/2020|2020-10-08|EPI_ISL_594763|Original|hCoV-19^^Scotland|Human|West of Scotland Specialist Virology Centre|COVID-19 Genomics UK (COG-UK) Consortium|Robertson|United Kingdom
>Spike|hCoV-19/England/MILK-AB7402/2020|2020-10-20|EPI_ISL_623223|Original|hCoV-19^^England|Human|Lighthouse Lab in Milton Keynes|Wellcome Sanger Institute for the COVID-19 Genomics UK (COG-UK) consortium|Davies|United Kingdom
>Spike|hCoV-19/England/MILK-9718CD/2020|2020-08-18|EPI_ISL_553007|Original|hCoV-19^^England|Human|Lighthouse Lab in Milton Keynes|Wellcome Sanger Institute for the COVID-19 Genomics UK (COG-UK) consortium|Davies|United Kingdom
>Spike|hCoV-19/USA/UT-UPHL-2012105116/2020|2020-08-14|EPI_ISL_745964|Original|hCoV-19^^Utah|Human|Ginkgo Bioworks Clinical Laboratory|Utah Public Health Laboratory|Young|USA
>Spike|hCoV-19/USA/TX-HMH-MCoV-8289/2020|2020-07-07|EPI_ISL_784312|Original|hCoV-19^^Texas|Human|Houston Methodist Hospital|Houston Methodist Hospital|Long|USA

>Spike|hCoV-19/England/QEUH-9AD27F/2020|2020-09-05|EPI_ISL_559823|Original|hCoV-19^^England|Human|Oxford Viromics|COVID-19 Genomics UK (COG-UK) Consortium|Golubchik|United Kingdom
>Spike|hCoV-19/Italy/APU-IZSPB_308PT/2020|2020-10-24|EPI_ISL_653802|Original|hCoV-19^^Apulia|Human|I.R.C.C.S. \S. De Bellis\ - Ente Ospedaliero|Istituto Zooprofilattico Sperimentale della Puglia e della Basilicata|Parisi|Italy
>Spike|hCoV-19/USA/VA_NIDDL_2768/2020|2020-03-29|EPI_ISL_491916|Original|hCoV-19^^Virginia|Human|Naval Infectious Diseases Diagnostic Laboratory|Naval Medical Research Center Biological Defense Research Directorate|Voegtly|USA
>Spike|hCoV-19/USA/CA-CZB-14299/2020|2020-08-24|EPI_ISL_738592|Original|hCoV-19^^California|Human|Alameda County Public Health Lab|Chan-Zuckerberg Biohub|Kamm|USA
>Spike|hCoV-19/USA/TX-HMH-6739/2020|2020-06-25|EPI_ISL_546206|Original|hCoV-19^^Texas|Human|Houston Methodist Hospital|Houston Methodist Hospital|Long|USA
>Spike|hCoV-19/USA/SC-DHEC-1143/2020|2020-07-10|EPI_ISL_574564|Original|hCoV-19^^South Carolina|Human|Microbiology Division|Microbiology Division|Flores|USA
>Spike|hCoV-19/Ghana/84260_S28/2020|2020-05-25|EPI_ISL_515107|Original|hCoV-19^^Ghana|Human|Department of Biochemistry|WACCBIP|Amuzu|Ghana
>Spike|hCoV-19/South Africa/UFS-VIRO-NGS-31/2020|2020-09-23|EPI_ISL_682338|Original|hCoV-19^^Free State|Human|NHLS Universitas Academic|UFS Virology|Bester|South Africa
>Spike|hCoV-19/England/QEUH-9F6AAC/2020|2020-09-27|EPI_ISL_588319|Original|hCoV-19^^England|Human|Lighthouse Lab in Glasgow|Wellcome Sanger Institute for the COVID-19 Genomics UK (COG-UK) consortium|Davies|United Kingdom
>Spike|hCoV-19/Australia/VIC13186/2020|2020-08-14|EPI_ISL_563634|Original|hCoV-19^^Victoria|Human|Microbiological Diagnostic Unit - Public Health Laboratory (MDU-PHL)|MDU-PHL|Schultz|Australia
>Spike|hCoV-19/England/ALDP-49CD86/2020|2020-06-14|EPI_ISL_559471|Original|hCoV-19^^England|Human|Lighthouse Lab in Alderley Park|Wellcome Sanger Institute for the COVID-19 Genomics UK (COG-UK) consortium|Davies|United Kingdom
>Spike|hCoV-19/France/ARA-70122/2020|2020-09-09|EPI_ISL_693490|Original|hCoV-19^^Auvergne-Rh\u00f4ne-Alpes|Human|CNR Virus des Infections Respiratoires - France SUD|CNR Virus des Infections Respiratoires - France SUD|Bal|France
>Spike|hCoV-19/South Korea/KDCA0318/2020|2020-10-16|EPI_ISL_760104|Original|hCoV-19^^South Korea|Human|Division of Emerging Infectious Diseases|Division of Emerging Infectious Diseases|Park|South Korea
>Spike|hCoV-19/USA/WA-S625/2020|2020-04-19|EPI_ISL_438178|Original|hCoV-19^^Washington|Human|Washington State Department of Health|Seattle Flu Study|Bedford|USA
>Spike|hCoV-19/Denmark/DCGC-3072/2020|2020-08-24|EPI_ISL_616852|Original|hCoV-19^^Hovedstaden|Human|Department of Virus and Microbiological Special Diagnostics|Albertsen lab|Michaelsen|Denmark
>Spike|hCoV-19/Switzerland/ZH-ETHZ-300272/2020|2020-10-01|EPI_ISL_603304|Original|hCoV-19^^Zurich|Human|Viollier AG|Department of Biosystems Science and Engineering|Nadeau|Switzerland
>Spike|hCoV-19/Hungary/UD-56307/2020|2020-08-31|EPI_ISL_671426|Original|hCoV-19^^Hajd\u00fa-Bihar county|Human|University of Debrecen|National Laboratory of Virology|Kemenesi|Hungary
>Spike|hCoV-19/England/LOND-D64BC/2020|2020-04-22|EPI_ISL_487615|Original|hCoV-19^^England|Human|University College London|Wellcome Sanger Institute for the COVID-19 Genomics UK (COG-UK) consortium|Davies|United Kingdom
>Spike|hCoV-19/USA/CA-CZB042/2020|2020-03-25|EPI_ISL_429035|Original|hCoV-19^^California|Human|UCSF Clinical Microbiology Laboratory|Chan-Zuckerberg Biohub|Kamm|USA
>Spike|hCoV-19/England/ALDP-AA2DA5/2020|2020-10-22|EPI_ISL_623894|Original|hCoV-19^^England|Human|Lighthouse Lab in Alderley Park|Wellcome Sanger Institute for the COVID-19 Genomics UK (COG-UK) consortium|Davies|United Kingdom
>Spike|hCoV-19/Japan/PG-7310/2020|2020-08-00|EPI_ISL_688413|Original|hCoV-19^^Japan|Human|Pathogen Genomics Center|Pathogen Genomics Center|Sekizuka|Japan

>Spike|hCoV-19/Canada/MB-NML-1080/2020|2020-08-04|EPI_ISL_582432|Original|hCoV-19^^Manitoba|Human|Cadham Provincial Laboratory|National Microbiology Laboratory (NML)|Majer|Canada
>Spike|hCoV-19/USA/UT-02009/2020|2020-04-16|EPI_ISL_454727|Original|hCoV-19^^Utah|Human|Utah Public Health Laboratory|Utah Public Health Laboratory|Young|USA
>Spike|hCoV-19/USA/CA-UCSF-UC41/2020|2020-03-14|EPI_ISL_450233|Original|hCoV-19^^California|Human|UCSF Clinical Microbiology Laboratory|Chiu Laboratory|Chiu|USA
>Spike|hCoV-19/Israel/CVL-n14268/2020|2020-03-17|EPI_ISL_514308|Original|hCoV-19^^Central District|Human|Israel Central Virology laboratory|Israel Central Virology laboratory|Zuckerman|Israel
>Spike|hCoV-19/England/ALDP-9C603A/2020|2020-09-10|EPI_ISL_567776|Original|hCoV-19^^England|Human|Lighthouse Lab in Alderley Park|Wellcome Sanger Institute for the COVID-19 Genomics UK (COG-UK) consortium|Davies|United Kingdom
>Spike|hCoV-19/USA/CA-ALSR-1165/2020|2020-04-05|EPI_ISL_494407|Original|hCoV-19^^California|Human|San Diego County Public Health Laboratory|Andersen lab at Scripps Research|Gangavarapu|USA
>Spike|hCoV-19/USA/IL-NM026/2020|2020-03-16|EPI_ISL_444536|Original|hCoV-19^^Illinois|Human|Northwestern Memorial Hospital|Ozer Lab|Ozer|USA
>Spike|hCoV-19/India/GJ-GBRC-433/2020|2020-09-01|EPI_ISL_586570|Original|hCoV-19^^Gujarat|Human|GCRI,Ahmedabad|Gujarat Biotechnology Research Centre|Joshi|India
>Spike|hCoV-19/Singapore/1438/2020|2020-11-23|EPI_ISL_693326|Original|hCoV-19^^Singapore|Human|National Public Health Laboratory|National Public Health Laboratory|Mak|Singapore
>Spike|hCoV-19/USA/MA-MASPHL-00639/2020|2020-05-13|EPI_ISL_692990|Original|hCoV-19^^Massachusetts|Human|Massachusetts State Public Health Laboratory|Massachusetts State Public Health Laboratory|Lang|USA
>Spike|hCoV-19/England/ALDP-BE9011/2020|2020-12-03|EPI_ISL_708845|Original|hCoV-19^^England|Human|Lighthouse Lab in Alderley Park|Wellcome Sanger Institute for the COVID-19 Genomics UK (COG-UK) Consortium|Davies|United Kingdom
>Spike|hCoV-19/England/QEUH-A8B786/2020|2020-10-16|EPI_ISL_643217|Original|hCoV-19^^England|Human|Lighthouse Lab in Glasgow|Wellcome Sanger Institute for the COVID-19 Genomics UK (COG-UK) Consortium|Davies|United Kingdom
>Spike|hCoV-19/USA/FL-BPHL-1517/2020|2020-07-27|EPI_ISL_568615|Original|hCoV-19^^Florida|Human|Florida Bureau of Public Health Laboratories|Florida Bureau of Public Health Laboratories|Blanton|USA
>Spike|hCoV-19/Mexico/BCN-ALSR-2520/2020|2020-06-16|EPI_ISL_635524|Original|hCoV-19^^Baja California|Human|Centro de Diagn\u221a\u2265stico COVID-19 UABC Tijuana|Andersen lab at Scripps Research|Gangavarapu|Mexico
>Spike|hCoV-19/Switzerland/AG-ETHZ-260149/2020|2020-08-26|EPI_ISL_539445|Original|hCoV-19^^Aargau|Human|Viollier AG|Department of Biosystems Science and Engineering|Nadeau|Switzerland
>Spike|hCoV-19/England/LOND-D36D7/2020|2020-03-31|EPI_ISL_440975|Original|hCoV-19^^England|Human|University College London|COVID-19 Genomics UK (COG-UK) Consortium|Roy|United Kingdom
>Spike|hCoV-19/USA/CA-CZB-7149/2020|2020-09-09|EPI_ISL_582933|Original|hCoV-19^^California|Human|County of Santa Clara Public Health Department|Chan-Zuckerberg Biohub|Bhatt|USA
>Spike|hCoV-19/Brazil/RJ-UFRJ-54093/2020|2020-04-16|EPI_ISL_492042|Original|hCoV-19^^Rio de Janeiro|Human|Instituto de Biologia do Ex\u00e9rcito|Laborat\u00f3rio Metabolismo Macromolecular FirminoTorres de Castro|Moura-Neto|Brazil
>Spike|hCoV-19/England/MILK-9C43D9/2020|2020-09-09|EPI_ISL_566777|Original|hCoV-19^^England|Human|Lighthouse Lab in Milton Keynes|Wellcome Sanger Institute for the COVID-19 Genomics UK (COG-UK) consortium|Davies|United Kingdom
>Spike|hCoV-19/USA/MI-UM-10036721426/2020|2020-10-31|EPI_ISL_682136|Original|hCoV-19^^Michigan|Human|University of Michigan Clinical Microbiology Laboratory|Lauring Lab|Valesano|USA
>Spike|hCoV-19/England/ALDP-BBAC7F/2020|2020-11-21|EPI_ISL_701769|Original|hCoV-19^^England|Human|Lighthouse Lab in Alderley Park|Wellcome Sanger Institute for the COVID-19 Genomics UK (COG-UK) Consortium|Davies|United Kingdom

>Spike|hCoV-19/Russia/OMS-ORINFI-916S/2020|2020-06-26|EPI_ISL_639945|Original|hCoV-19^^Omsk|Human|Omsk Research Institute of Natural Focal Infections|WHO National Influenza Centre Russian Federation|Komissarov|Russia
>Spike|hCoV-19/USA/CO-CDPHE-2008063525/2020|2020-08-05|EPI_ISL_710321|Original|hCoV-19^^Colorado|Human|Colorado Department of Public Health and Environment|Colorado Department of Puplic Health and Environment|Bankers|USA
>Spike|hCoV-19/England/ALDP-C3CB0E/2020|2020-12-10|EPI_ISL_731702|Original|hCoV-19^^England|Human|Lighthouse Lab in Alderley Park|Wellcome Sanger Institute for the COVID-19 Genomics UK (COG-UK) Consortium|Davies|United Kingdom
>Spike|hCoV-19/USA/CA-ALSR-2787/2020|2020-07-22|EPI_ISL_635679|Original|hCoV-19^^California|Human|San Diego County Public Health Laboratory|Andersen lab at Scripps Research|Gangavarapu|USA
>Spike|hCoV-19/Peru/LIM-UPCH-0155/2020|2020-08-27|EPI_ISL_568548|Original|hCoV-19^^Lima|Human|Laboratorio de Referencia Nacional de Virus Respiratorios|Laboratorio de Gen\u00f3mica Microbiana|Tsukayama|Peru
>Spike|hCoV-19/USA/CA-ALSR-4539/2020|2020-09-16|EPI_ISL_666997|Original|hCoV-19^^California|Human|San Diego County Public Health Laboratory|Andersen lab at Scripps Research|Gangavarapu|USA
>Spike|hCoV-19/USA/CA-CZB-14886/2020|2020-11-24|EPI_ISL_738893|Original|hCoV-19^^California|Human|Orange County Public Health Lab|Chan-Zuckerberg Biohub|Kamm|USA
>Spike|hCoV-19/USA/TX-HMH-MCoV-15734/2020|2020-07-09|EPI_ISL_785418|Original|hCoV-19^^Texas|Human|Houston Methodist Hospital|Houston Methodist Hospital|Long|USA
>Spike|hCoV-19/Japan/PG-3377/2020|2020-04-00|EPI_ISL_691492|Original|hCoV-19^^Japan|Human|Pathogen Genomics Center|Pathogen Genomics Center|Sekizuka|Japan
>Spike|hCoV-19/England/ALDP-9CE050/2020|2020-09-16|EPI_ISL_566192|Original|hCoV-19^^England|Human|Lighthouse Lab in Alderley Park|Wellcome Sanger Institute for the COVID-19 Genomics UK (COG-UK) consortium|Davies|United Kingdom
>Spike|hCoV-19/USA/AZ-TG377726/2020|2020-06-13|EPI_ISL_694624|Original|hCoV-19^^Arizona|Human|TGen North|TGen North|French|USA
>Spike|hCoV-19/Scotland/QEUH-A00C8A/2020|2020-10-02|EPI_ISL_585566|Original|hCoV-19^^Scotland|Human|University of Exeter|COVID-19 Genomics UK (COG-UK) Consortium|Temperton|United Kingdom
>Spike|hCoV-19/Switzerland/AG-ETHZ-340139/2020|2020-10-27|EPI_ISL_721708|Original|hCoV-19^^Aargau|Human|Viollier AG|Department of Biosystems Science and Engineering|Nadeau|Switzerland
>Spike|hCoV-19/USA/MI-UM-10036787921/2020|2020-11-04|EPI_ISL_707860|Original|hCoV-19^^Michigan|Human|University of Michigan Clinical Microbiology Laboratory|Lauring Lab|Valesano|USA
>Spike|hCoV-19/England/CAMB-79390/2020|2020-04-03|EPI_ISL_441832|Original|hCoV-19^^England|Human|Department of Pathology|Wellcome Sanger Institute for the COVID-19 Genomics UK (COG-UK) consortium|Amato|United Kingdom
>Spike|hCoV-19/Denmark/DCGC-19538/2020|2020-12-07|EPI_ISL_749015|Original|hCoV-19^^Hovedstaden|Human|Department of Virus and Microbiological Special Diagnostics|Albertsen Lab|Michaelsen|Denmark
>Spike|hCoV-19/England/ALDP-508F33/2020|2020-06-25|EPI_ISL_559161|Original|hCoV-19^^England|Human|Lighthouse Lab in Alderley Park|Wellcome Sanger Institute for the COVID-19 Genomics UK (COG-UK) consortium|Davies|United Kingdom
>Spike|hCoV-19/England/MILK-A7C849/2020|2020-10-15|EPI_ISL_607605|Original|hCoV-19^^England|Human|Lighthouse Lab in Milton Keynes|Wellcome Sanger Institute for the COVID-19 Genomics UK (COG-UK) consortium|Davies|United Kingdom
>Spike|hCoV-19/Japan/PG-6573/2020|2020-09-00|EPI_ISL_687685|Original|hCoV-19^^Japan|Human|Pathogen Genomics Center|Pathogen Genomics Center|Sekizuka|Japan
>Spike|hCoV-19/Croatia/1146_Split/2020|2020-03-18|EPI_ISL_454581|Original|hCoV-19^^Split|Human|University Hospital for Infectious Diseases \Dr. Fran Mihaljevi\u0107\|University of Zagreb|Ivancic Jelecki|Croatia
>Spike|hCoV-19/USA/ID-QDX-1294/2020|2020-03-16|EPI_ISL_571933|Original|hCoV-19^^Idaho|Human|Quest Diagnostics|Quest Diagnostics|Kagan|USA
>Spike|hCoV-19/Sweden/20-06817/2020|2020-04-01|EPI_ISL_445229|Original|hCoV-19^^Vastra Gotaland|Human|Narhalsan Backa vardcentral|The Public Health Agency of Sweden|Svartstr\u00f6m|Sweden

>Spike|hCoV-19/England/CAMB-1B3074/2020|2020-05-25|EPI_ISL_461547|Original|hCoV-19^^England|Human|Department of Pathology|COVID-19 Genomics UK (COG-UK) Consortium|Meredith|United Kingdom
>Spike|hCoV-19/Japan/PG-6998/2020|2020-09-00|EPI_ISL_688103|Original|hCoV-19^^Japan|Human|Pathogen Genomics Center|Pathogen Genomics Center|Sekizuka|Japan
>Spike|hCoV-19/England/CAMC-A5802D/2020|2020-10-08|EPI_ISL_596928|Original|hCoV-19^^England|Human|Lighthouse Lab in Cambridge|Wellcome Sanger Institute for the COVID-19 Genomics UK (COG-UK) consortium|Davies|United Kingdom
>Spike|hCoV-19/England/LOND-12F1717/2020|2020-12-15|EPI_ISL_763544|Original|hCoV-19^^England|Human|University College London|COVID-19 Genomics UK (COG-UK) Consortium|Roy|United Kingdom
>Spike|hCoV-19/USA/NM-DOH-2020315751/2020|2020-08-27|EPI_ISL_732714|Original|hCoV-19^^New Mexico|Human|New Mexico Department of Health Scientific Laboratory|New Mexico Department of Health Scientific Laboratory|Johnson|USA
>Spike|hCoV-19/Chile/MA-ISPCH-13/2020|2020-03-21|EPI_ISL_445294|Original|hCoV-19^^Magallanes|Human|HOSPITAL REG.LAUTARO NAVARRO AVARIA|Instituto de Salud Publica de Chile|Fernandez|Chile
>Spike|hCoV-19/England/LIVE-DBEA15/2020|2020-10-29|EPI_ISL_664632|Original|hCoV-19^^England|Human|Liverpool Clinical Laboratories|COVID-19 Genomics UK (COG-UK) Consortium|Haldenby|United Kingdom
>Spike|hCoV-19/USA/CA-ALSR-4220/2020|2020-10-02|EPI_ISL_648894|Original|hCoV-19^^California|Human|San Diego County Public Health Laboratory|Andersen lab at Scripps Research|Gangavarapu|USA
>Spike|hCoV-19/Wales/QEUH-9B7D17/2020|2020-09-04|EPI_ISL_590487|Original|hCoV-19^^Wales|Human|Lighthouse Lab in Glasgow|Wellcome Sanger Institute for the COVID-19 Genomics UK (COG-UK) consortium|Davies|United Kingdom
>Spike|hCoV-19/England/20144064402/2020|2020-03-30|EPI_ISL_421955|Original|hCoV-19^^England|Human|Respiratory Virus Unit|Respiratory Virus Unit|Platt|United Kingdom
>Spike|hCoV-19/Denmark/DCGC-2579/2020|2020-08-17|EPI_ISL_616415|Original|hCoV-19^^Hovedstaden|Human|Department of Virus and Microbiological Special Diagnostics|Albertsen lab|Michaelsen|Denmark
>Spike|hCoV-19/England/LIVE-1D2A80/2020|2020-03-29|EPI_ISL_612404|Original|hCoV-19^^England|Human|Liverpool Clinical Laboratories|COVID-19 Genomics UK (COG-UK) Consortium|Haldenby|United Kingdom
>Spike|hCoV-19/England/CAMB-79372/2020|2020-04-03|EPI_ISL_441165|Original|hCoV-19^^England|Human|Department of Pathology|Wellcome Sanger Institute for the COVID-19 Genomics UK (COG-UK) consortium|Amato|United Kingdom
>Spike|hCoV-19/Scotland/CVR5675/2020|2020-11-09|EPI_ISL_651011|Original|hCoV-19^^Scotland|Human|West of Scotland Specialist Virology Centre|COVID-19 Genomics UK (COG-UK) Consortium|Robertson|United Kingdom
>Spike|hCoV-19/Belgium/rega-0319860/2020|2020-03-19|EPI_ISL_734509|Original|hCoV-19^^Auderghem|Human|UZ Leuven|KU Leuven|Maes|Belgium
>Spike|hCoV-19/South Africa/KRISP-K001360/2020|2020-07-16|EPI_ISL_515845|Original|hCoV-19^^KwaZulu-Natal|Human|Medical Disagnostics Services (MDS)|KRISP|de Oliveira|South Africa
>Spike|hCoV-19/England/ALDP-B5E484/2020|2020-11-09|EPI_ISL_723659|Original|hCoV-19^^England|Human|Oxford Viromics|COVID-19 Genomics UK (COG-UK) Consortium|Golubchik|United Kingdom
>Spike|hCoV-19/Belgium/ULG-10866/2020|2020-11-13|EPI_ISL_678410|Original|hCoV-19^^Li\u00e8ge|Human|Department of Clinical Microbiology|GIGA Medical Genomics|Durkin|Belgium
>Spike|hCoV-19/England/MILK-559F06/2020|2020-06-10|EPI_ISL_558280|Original|hCoV-19^^England|Human|Lighthouse Lab in Milton Keynes|Wellcome Sanger Institute for the COVID-19 Genomics UK (COG-UK) consortium|Davies|United Kingdom
>Spike|hCoV-19/Wuhan/IME-WH05/2020|2019-12-30|EPI_ISL_529217|Original|hCoV-19^^Hubei|Human|Beijing Institute of Microbiology and Epidemiology|Beijing Institute of Microbiology and Epidemiology|Fan|China
>Spike|hCoV-19/Denmark/DCGC-1135/2020|2020-03-16|EPI_ISL_614619|Original|hCoV-19^^Syddanmark|Human|Department of Virus and Microbiological Special Diagnostics|Albertsen lab|Michaelsen|Denmark

>Spike|hCoV-19/England/QEUH-B12A02/2020|2020-10-31|EPI_ISL_642655|Original|hCoV-19^^England|Human|Lighthouse Lab in Glasgow|Wellcome Sanger Institute for the COVID-19 Genomics UK (COG-UK) Consortium|Davies|United Kingdom
>Spike|hCoV-19/Scotland/EDB6737/2020|2020-04-13|EPI_ISL_491551|Original|hCoV-19^^Scotland|Human|Virology Department|Wellcome Sanger Institute for the COVID-19 Genomics UK (COG-UK) consortium|Davies|United Kingdom
>Spike|hCoV-19/Luxembourg/LNS4377160/2020|2020-10-24|EPI_ISL_740460|Original|hCoV-19^^Luxembourg|Human|Laboratoire national de sant\u00e9|Laboratoire national de sant\u00e9|Wienecke-Baldacchino|Luxembourg
>Spike|hCoV-19/England/ALDP-B6354D/2020|2020-11-09|EPI_ISL_656506|Original|hCoV-19^^England|Human|Lighthouse Lab in Alderley Park|Wellcome Sanger Institute for the COVID-19 Genomics UK (COG-UK) Consortium|Davies|United Kingdom
>Spike|hCoV-19/England/CAMB-82BAB/2020|2020-04-25|EPI_ISL_438666|Original|hCoV-19^^England|Human|Department of Pathology|COVID-19 Genomics UK (COG-UK) Consortium|Meredith|United Kingdom
>Spike|hCoV-19/Northern Ireland/NIRE-FCEFE/2020|2020-04-24|EPI_ISL_489334|Original|hCoV-19^^Northern Ireland|Human|Regional Virus Laboratory|Wellcome Sanger Institute for the COVID-19 Genomics UK (COG-UK) consortium|Davies|United Kingdom
>Spike|hCoV-19/Australia/SAP562/2020|2020-11-16|EPI_ISL_654805|Original|hCoV-19^^South Australia|Human|SA Pathology|SA Pathology|Leong|Australia
>Spike|hCoV-19/England/ALDP-9D9034/2020|2020-09-18|EPI_ISL_576693|Original|hCoV-19^^England|Human|Oxford Viromics|COVID-19 Genomics UK (COG-UK) Consortium|Golubchik|United Kingdom
>Spike|hCoV-19/England/BIRM-62AC2/2020|2020-09-02|EPI_ISL_529641|Original|hCoV-19^^England|Human|University of Birmingham|COVID-19 Genomics UK (COG-UK) Consortium|Loman|United Kingdom
>Spike|hCoV-19/USA/FL-BPHL-1630/2020|2020-06-29|EPI_ISL_594320|Original|hCoV-19^^Florida|Human|Florida Bureau of Public Health Laboratories|Florida Bureau of Public Health Laboratories|Blanton|USA
>Spike|hCoV-19/Finland/18SS12E4/2020|2020-09-18|EPI_ISL_756289|Original|hCoV-19^^Uusimaa|Human|Department of Virology and Immunology|Department of Virology|Smura|Finland
>Spike|hCoV-19/Denmark/DCGC-7030/2020|2020-10-12|EPI_ISL_619877|Original|hCoV-19^^Hovedstaden|Human|Department of Virus and Microbiological Special Diagnostics|Albertsen lab|Michaelsen|Denmark
>Spike|hCoV-19/Spain/AN-ISCIII-201373/2020|2020-03-02|EPI_ISL_418244|Original|hCoV-19^^Andalucia|Human|HOSPITAL UNIVERSITARIO VIRGEN DE LAS NIEVES|Instituto de Salud Carlos III|Caballero|Spain
>Spike|hCoV-19/Canada/ON-S215/2020|2020-03-26|EPI_ISL_586380|Original|hCoV-19^^Ontario|Human|Toronto Invasive Bacterial Diseases Network|McMaster University|McArthur|Canada
>Spike|hCoV-19/England/MILK-BB0DD0/2020|2020-11-18|EPI_ISL_720507|Original|hCoV-19^^England|Human|Lighthouse Lab in Milton Keynes|Wellcome Sanger Institute for the COVID-19 Genomics UK (COG-UK) Consortium|Davies|United Kingdom
>Spike|hCoV-19/Australia/VIC13485/2020|2020-08-03|EPI_ISL_592260|Original|hCoV-19^^Victoria|Human|Microbiological Diagnostic Unit - Public Health Laboratory (MDU-PHL)|MDU-PHL|Schultz|Australia
>Spike|hCoV-19/USA/WA-S2114/2020|2020-06-08|EPI_ISL_497542|Original|hCoV-19^^Washington|Human|Washington State Department of Health|Seattle Flu Study|Lee|USA
>Spike|hCoV-19/England/SHEF-C98DE/2020|2020-04-17|EPI_ISL_462041|Original|hCoV-19^^England|Human|Virology Department|COVID-19 Genomics UK (COG-UK) Consortium|de Silva|United Kingdom
>Spike|hCoV-19/Wales/MILK-9D55C0/2020|2020-09-19|EPI_ISL_577397|Original|hCoV-19^^Wales|Human|Centre for Enzyme Innovation|COVID-19 Genomics UK (COG-UK) Consortium|Robson|United Kingdom
>Spike|hCoV-19/Denmark/DCGC-6801/2020|2020-10-12|EPI_ISL_619498|Original|hCoV-19^^Hovedstaden|Human|Department of Virus and Microbiological Special Diagnostics|Albertsen lab|Michaelsen|Denmark

>Spike|hCoV-19/England/LOND-12673FA/2020|2020-12-08|EPI_ISL_741284|Original|hCoV-19^^England|Human|University College London|COVID-19 Genomics UK (COG-UK) Consortium|Roy|United Kingdom
>Spike|hCoV-19/Canada/ON-PHL-20-01291/2020|2020-06-00|EPI_ISL_548895|Original|hCoV-19^^Ontario|Human|Public Health Ontario Laboratory|Public Health Ontario Laboratory|Guthrie|Canada
>Spike|hCoV-19/Denmark/DCGC-21282/2020|2020-12-21|EPI_ISL_759652|Original|hCoV-19^^Nordjylland|Human|Department of Virus and Microbiological Special Diagnostics|Albertsen Lab|Michaelsen|Denmark
>Spike|hCoV-19/England/QEUH-9C8C8F/2020|2020-09-10|EPI_ISL_567186|Original|hCoV-19^^England|Human|Lighthouse Lab in Glasgow|Wellcome Sanger Institute for the COVID-19 Genomics UK (COG-UK) consortium|Davies|United Kingdom
>Spike|hCoV-19/England/NORT-283C0F/2020|2020-04-03|EPI_ISL_472250|Original|hCoV-19^^England|Human|Northumbria University / South Tees Hospitals NHS Foundation Trust / North Cumbria Integrated Care NHS Foundation Trust / North Tees and Hartlepool NHS Foundation Trust / Newcastle Hospitals NHS Foundation Trust|COVID-19 Genomics UK (COG-UK) Consortium|Bashton|United Kingdom
>Spike|hCoV-19/Netherlands/LI-EMC-144/2020|2020-11-20|EPI_ISL_763112|Original|hCoV-19^^Limburg|Human|Dutch COVID-19 response team|Erasmus Medical Center|Oude Munnink|Netherlands
>Spike|hCoV-19/Spain/AN-IBV-006086/2020|2020-04-27|EPI_ISL_510426|Original|hCoV-19^^Andalucia|Human|Hospital Universitario Virgen de las Nieves de Granada-SAS|SeqCOVID-SPAIN consortium/IBV(CSIC)|Comas|Spain
>Spike|hCoV-19/England/LIVE-1D2DF0/2020|2020-03-31|EPI_ISL_612425|Original|hCoV-19^^England|Human|Liverpool Clinical Laboratories|COVID-19 Genomics UK (COG-UK) Consortium|Haldenby|United Kingdom
>Spike|hCoV-19/England/20134078004/2020|2020-03-24|EPI_ISL_423172|Original|hCoV-19^^England|Human|Respiratory Virus Unit|Respiratory Virus Unit|Platt|United Kingdom
>Spike|hCoV-19/England/QEUH-A9F38B/2020|2020-10-20|EPI_ISL_611390|Original|hCoV-19^^England|Human|Lighthouse Lab in Glasgow|Wellcome Sanger Institute for the COVID-19 Genomics UK (COG-UK) consortium|Davies|United Kingdom
>Spike|hCoV-19/Chile/RM-83613/2020|2020-05-30|EPI_ISL_746800|Original|hCoV-19^^Region Metropolitana de Santiago|Human|Genetica Molecular and Subdepartamento de Virologia ISP Chile|Instituto de Salud Publica de Chile|Tognarelli|Chile
>Spike|hCoV-19/England/ALDP-A9287A/2020|2020-10-20|EPI_ISL_610926|Original|hCoV-19^^England|Human|Lighthouse Lab in Alderley Park|Wellcome Sanger Institute for the COVID-19 Genomics UK (COG-UK) consortium|Davies|United Kingdom
>Spike|hCoV-19/England/CAMC-B36455/2020|2020-11-05|EPI_ISL_645230|Original|hCoV-19^^England|Human|Lighthouse Lab in Cambridge|Wellcome Sanger Institute for the COVID-19 Genomics UK (COG-UK) Consortium|Davies|United Kingdom
>Spike|hCoV-19/England/QEUH-9F33B3/2020|2020-09-24|EPI_ISL_588695|Original|hCoV-19^^England|Human|Lighthouse Lab in Glasgow|Wellcome Sanger Institute for the COVID-19 Genomics UK (COG-UK) consortium|Davies|United Kingdom
>Spike|hCoV-19/Canada/ON-PHL-20-01791/2020|2020-07-00|EPI_ISL_636941|Original|hCoV-19^^Ontario|Human|Public Health Ontario Laboratory|Public Health Ontario Laboratory|Guthrie|Canada
>Spike|hCoV-19/South Africa/R09103/2020|2020-04-28|EPI_ISL_470879|Original|hCoV-19^^Eastern Cape|Human|National Institute for Communicable Diseases of the National Health Laboratory Service|National Institute for Communicable Diseases of the National Health Laboratory Service|Allam|South Africa
>Spike|hCoV-19/Denmark/DCGC-2187/2020|2020-08-10|EPI_ISL_617488|Original|hCoV-19^^Hovedstaden|Human|Department of Virus and Microbiological Special Diagnostics|Albertsen lab|Michaelsen|Denmark
>Spike|hCoV-19/England/QEUH-9F4C7B/2020|2020-09-25|EPI_ISL_638174|Original|hCoV-19^^England|Human|Department of Pathology|COVID-19 Genomics UK (COG-UK) Consortium|Goodfellow|United Kingdom
>Spike|hCoV-19/Japan/PG-1809/2020|2020-04-00|EPI_ISL_685415|Original|hCoV-19^^Japan|Human|Tokyo Metropolitan Institute of Public Health|Pathogen Genomics Center|Sekizuka|Japan

>Spike|hCoV-19/Scotland/EDB4832/2020|2020-04-03|EPI_ISL_453159|Original|hCoV-19^^Scotland|Human|Virology Department|COVID-19 Genomics UK (COG-UK) Consortium|Williams|United Kingdom
>Spike|hCoV-19/HongKong/CM20000489/2020|2020-12-20|EPI_ISL_759892|Original|hCoV-19^^Hong Kong|Human|Temporary Specimen Collection Centre|Hong Kong Department of Health|Tsang|China
>Spike|hCoV-19/USA/TX-HMH-6395/2020|2020-07-05|EPI_ISL_545968|Original|hCoV-19^^Texas|Human|Houston Methodist Hospital|Houston Methodist Hospital|Long|USA
>Spike|hCoV-19/England/201620591/2020|2020-04-14|EPI_ISL_466427|Original|hCoV-19^^England|Human|Respiratory Virus Unit|Respiratory Virus Unit|Platt|United Kingdom
>Spike|hCoV-19/Japan/PG-8445/2020|2020-09-00|EPI_ISL_687544|Original|hCoV-19^^Japan|Human|Fukuoka Institute of Health and Environmental Sciences|Pathogen Genomics Center|Sekizuka|Japan
>Spike|hCoV-19/Netherlands/Gelderland_35/2020|2020-04-29|EPI_ISL_460935|Original|hCoV-19^^Gelderland|Human|Dutch COVID-19 response team|Erasmus Medical Center|Oude Munnink|Netherlands
>Spike|hCoV-19/Wales/PHWC-2517B/2020|2020-03-22|EPI_ISL_420975|Original|hCoV-19^^Wales|Human|Wales Specialist Virology Centre|Public Health Wales Microbiology Cardiff|Moore|United Kingdom
>Spike|hCoV-19/USA/TX-HMH-MCoV-15288/2020|2020-10-21|EPI_ISL_786772|Original|hCoV-19^^Texas|Human|Houston Methodist Hospital|Houston Methodist Hospital|Long|USA
>Spike|hCoV-19/Denmark/DCGC-19462/2020|2020-12-07|EPI_ISL_748962|Original|hCoV-19^^Syddanmark|Human|Department of Virus and Microbiological Special Diagnostics|Albertsen Lab|Michaelsen|Denmark
>Spike|hCoV-19/Colombia/SAN-INS-80123/2020|2020-03-15|EPI_ISL_456149|Original|hCoV-19^^Santander|Human|Instituto Nacional de Salud - Unidad de Secuenciaci\u00f3n y An\u00e1lisis Gen\u00f3mico|Instituto Nacional de Salud|D\u00edaz|Colombia
>Spike|hCoV-19/Denmark/DCGC-3086/2020|2020-08-31|EPI_ISL_616866|Original|hCoV-19^^Hovedstaden|Human|Department of Virus and Microbiological Special Diagnostics|Albertsen lab|Michaelsen|Denmark
>Spike|hCoV-19/England/ALDP-5A219F/2020|2020-06-12|EPI_ISL_558816|Original|hCoV-19^^England|Human|Lighthouse Lab in Alderley Park|Wellcome Sanger Institute for the COVID-19 Genomics UK (COG-UK) consortium|Davies|United Kingdom
>Spike|hCoV-19/USA/TX-HMH-MCoV-12896/2020|2020-08-18|EPI_ISL_788048|Original|hCoV-19^^Texas|Human|Houston Methodist Hospital|Houston Methodist Hospital|Long|USA
>Spike|hCoV-19/South Korea/KCDC2462/2020|2020-06-08|EPI_ISL_514865|Original|hCoV-19^^South Korea|Human|Division of Viral Diseases|Division of Viral Diseases|Kim|South Korea
>Spike|hCoV-19/USA/MI-MDHHS-SC20857/2020|2020-04-29|EPI_ISL_471878|Original|hCoV-19^^Michigan|Human|Michigan Department of Health and Human Services|Michigan Department of Health and Human Services|Blankenship|USA
>Spike|hCoV-19/Iceland/202/2020|2020-03-16|EPI_ISL_417750|Original|hCoV-19^^Reykjavik|Human|The National University Hospital of Iceland|deCODE genetics|J\u00f3nsson|Iceland
>Spike|hCoV-19/USA/MA-MGH-03537/2020|2020-11-27|EPI_ISL_765877|Original|hCoV-19^^Massachusetts|Human|Massachusetts General Hospital|Infectious Disease Program|Park|USA
>Spike|hCoV-19/Wales/PHWC-4930E0/2020|2020-11-28|EPI_ISL_725837|Original|hCoV-19^^Wales|Human|Originating lab|COVID-19 Genomics UK (COG-UK) Consortium|Moore|United Kingdom
>Spike|hCoV-19/Japan/PG-0804/2020|2020-04-00|EPI_ISL_684414|Original|hCoV-19^^Japan|Human|Fukuoka Institute of Health and Environmental Sciences|Pathogen Genomics Center|Sekizuka|Japan
>Spike|hCoV-19/USA/WI-WSLH-200159/2020|2020-03-31|EPI_ISL_578386|Original|hCoV-19^^Wisconsin|Human|Wisconsin State Laboratory of Hygiene Communicable Disease Division|Wisconsin State Laboratory of Hygiene Communicable Disease Division|Florek|USA
>Spike|hCoV-19/USA/WI-WSLH-200027/2020|2020-04-17|EPI_ISL_471192|Original|hCoV-19^^Wisconsin|Human|Wisconsin State Laboratory of Hygiene Communicable Disease

Division|Wisconsin State Laboratory of Hygiene Communicable Disease Division|Florek|USA
>Spike|hCoV-19/England/204891537/2020|2020-11-27|EPI_ISL_723020|Original|hCoV-19^^England|Human|Respiratory Virus Unit|COVID-19 Genomics UK (COG-UK) Consortium|Platt|United Kingdom
>Spike|hCoV-19/Netherlands/UT-RIVM-10138/2020|2020-04-22|EPI_ISL_547538|Original|hCoV-19^^Utrecht|Human|Dutch COVID-19 response team|National Institute for Public Health and the Environment (RIVM)|Meijer|Netherlands
>Spike|hCoV-19/England/NOTT-1129E1/2020|2020-04-22|EPI_ISL_493572|Original|hCoV-19^^England|Human|Lincolnshire Hospitals and DeepSeq Nottingham|COVID-19 Genomics UK (COG-UK) Consortium|Loose|United Kingdom
>Spike|hCoV-19/England/LIVE-DC0EBA/2020|2020-11-03|EPI_ISL_705411|Original|hCoV-19^^England|Human|Liverpool Clinical Laboratories|COVID-19 Genomics UK (COG-UK) Consortium|Haldenby|United Kingdom
>Spike|hCoV-19/England/20148049704/2020|2020-04-02|EPI_ISL_466071|Original|hCoV-19^^England|Human|Respiratory Virus Unit|Respiratory Virus Unit|Platt|United Kingdom
>Spike|hCoV-19/USA/NY-NYCPHL-000774/2020|2020-03-16|EPI_ISL_632154|Original|hCoV-19^^New York|Human|Richmond University Medical Center|New York City Public Health Laboratory|Wang|USA
>Spike|hCoV-19/England/MILK-AC8064/2020|2020-10-22|EPI_ISL_629732|Original|hCoV-19^^England|Human|Lighthouse Lab in Milton Keynes|Wellcome Sanger Institute for the COVID-19 Genomics UK (COG-UK) consortium|Davies|United Kingdom
>Spike|hCoV-19/Singapore/937/2020|2020-04-02|EPI_ISL_538470|Original|hCoV-19^^Singapore|Human|Department of Laboratory Medicine|Department of Laboratory Medicine|Chen|Singapore
>Spike|hCoV-19/New Zealand/20CV0516/2020|2020-10-30|EPI_ISL_626354|Original|hCoV-19^^Auckland|Human|LabPLUS|Institute of Environmental Science and Research (ESR)|Wang|New Zealand
>Spike|hCoV-19/Australia/VIC3601/2020|2020-07-08|EPI_ISL_520346|Original|hCoV-19^^Victoria|Human|Victorian Infectious Diseases Reference Laboratory (VIDRL)|VIDRL and MDU-PHL|Schultz|Australia
>Spike|hCoV-19/England/QEUH-9DE3DC/2020|2020-09-21|EPI_ISL_599636|Original|hCoV-19^^England|Human|Lighthouse Lab in Glasgow|Wellcome Sanger Institute for the COVID-19 Genomics UK (COG-UK) consortium|Davies|United Kingdom
>Spike|hCoV-19/England/20110015106/2020|2020-03-08|EPI_ISL_417306|Original|hCoV-19^^England|Human|Respiratory Virus Unit|Respiratory Virus Unit|Platt|United Kingdom
>Spike|hCoV-19/Japan/PG-7742/2020|2020-09-00|EPI_ISL_686844|Original|hCoV-19^^Japan|Human|Chiba Prefectural Institute of Public Health|Pathogen Genomics Center|Sekizuka|Japan
>Spike|hCoV-19/England/ALDP-B24AEF/2020|2020-11-04|EPI_ISL_646884|Original|hCoV-19^^England|Human|Lighthouse Lab in Alderley Park|Wellcome Sanger Institute for the COVID-19 Genomics UK (COG-UK) Consortium|Davies|United Kingdom
>Spike|hCoV-19/England/MILK-B5A3C7/2020|2020-11-06|EPI_ISL_658140|Original|hCoV-19^^England|Human|Lighthouse Lab in Milton Keynes|Wellcome Sanger Institute for the COVID-19 Genomics UK (COG-UK) Consortium|Davies|United Kingdom
>Spike|hCoV-19/England/MILK-95D7D9/2020|2020-06-21|EPI_ISL_556170|Original|hCoV-19^^England|Human|Lighthouse Lab in Milton Keynes|Wellcome Sanger Institute for the COVID-19 Genomics UK (COG-UK) consortium|Davies|United Kingdom
>Spike|hCoV-19/England/CAMB-1B08D9/2020|2020-05-04|EPI_ISL_459224|Original|hCoV-19^^England|Human|Department of Pathology|Wellcome Sanger Institute for the COVID-19 Genomics UK (COG-UK) consortium|Davies|United Kingdom
>Spike|hCoV-19/Wales/PHWC-24015/2020|2020-03-15|EPI_ISL_418138|Original|hCoV-19^^Wales|Human|Wales Specialist Virology Centre|Public Health Wales Microbiology Cardiff|Moore|United Kingdom
>Spike|hCoV-19/England/QEUH-A366BA/2020|2020-10-05|EPI_ISL_706913|Original|hCoV-19^^England|Human|Oxford Viromics|COVID-19 Genomics UK (COG-UK) Consortium|Golubchik|United Kingdom
>Spike|hCoV-19/England/20188020404/2020|2020-04-03|EPI_ISL_465499|Original|hCoV-19^^England|Human|Respiratory Virus Unit|Respiratory Virus Unit|Platt|United Kingdom

>Spike|hCoV-19/England/ALDP-9CB080/2020|2020-09-16|EPI_ISL_575402|Original|hCoV-19^^England|Human|Lighthouse Lab in Alderley Park|Wellcome Sanger Institute for the COVID-19 Genomics UK (COG-UK) consortium|Davies|United Kingdom
>Spike|hCoV-19/USA/WA-S1641/2020|2020-05-26|EPI_ISL_496067|Original|hCoV-19^^Washington|Human|Washington State Department of Health|Seattle Flu Study|Lee|USA
>Spike|hCoV-19/England/BRIS-128E34/2020|2020-04-18|EPI_ISL_458892|Original|hCoV-19^^England|Human|PHE South West Regional Laboratory|Wellcome Sanger Institute for the COVID-19 Genomics UK (COG-UK) consortium|Davies|United Kingdom
>Spike|hCoV-19/Norway/5840/2020|2020-11-05|EPI_ISL_775487|Original|hCoV-19^^Nordland|Human|Nordland Hospital - Bodo|Norwegian Institute of Public Health|Vollan|Norway
>Spike|hCoV-19/England/ALDP-9EA1E2/2020|2020-09-24|EPI_ISL_580101|Original|hCoV-19^^England|Human|Lighthouse Lab in Alderley Park|Wellcome Sanger Institute for the COVID-19 Genomics UK (COG-UK) consortium|Davies|United Kingdom
>Spike|hCoV-19/Denmark/DCGC-1221/2020|2020-05-18|EPI_ISL_614864|Original|hCoV-19^^Hovedstaden|Human|Department of Virus and Microbiological Special Diagnostics|Albertsen lab|Michaelsen|Denmark
>Spike|hCoV-19/Singapore/456/2020|2020-03-19|EPI_ISL_475987|Original|hCoV-19^^Singapore|Human|National Public Health Laboratory|National Public Health Laboratory|Mak|Singapore
>Spike|hCoV-19/USA/TX-HMH-MCoV-8671/2020|2020-07-01|EPI_ISL_780481|Original|hCoV-19^^Texas|Human|Houston Methodist Hospital|Houston Methodist Hospital|Long|USA
>Spike|hCoV-19/USA/MI-QDX-373/2020|2020-03-23|EPI_ISL_571116|Original|hCoV-19^^Michigan|Human|Quest Diagnostics|Quest Diagnostics|Kagan|USA
>Spike|hCoV-19/USA/NY-NYCPHL-000490/2020|2020-04-01|EPI_ISL_631953|Original|hCoV-19^^New York|Human|NYC HH Lincoln Medical And Mental Health Center|New York City Public Health Laboratory|Wang|USA
>Spike|hCoV-19/England/SHEF-D2007/2020|2020-09-14|EPI_ISL_559944|Original|hCoV-19^^England|Human|Virology Department|COVID-19 Genomics UK (COG-UK) Consortium|de Silva|United Kingdom
>Spike|hCoV-19/USA/VA-DCLS-0100/2020|2020-04-10|EPI_ISL_434594|Original|hCoV-19^^Virginia|Human|Virginia DCLS|Virginia DCLS|St. Jacques|USA
>Spike|hCoV-19/USA/CA-CZB-1455/2020|2020-04-10|EPI_ISL_468416|Original|hCoV-19^^California|Human|County of San Luis Obispo Public Health Laboratory|Chan-Zuckerberg Biohub|Hao|USA
>Spike|hCoV-19/Denmark/DCGC-17466/2020|2020-12-14|EPI_ISL_750355|Original|hCoV-19^^Nordjylland|Human|Department of Virus and Microbiological Special Diagnostics|Albertsen Lab|Michaelsen|Denmark
>Spike|hCoV-19/Australia/VIC9584/2020|2020-08-21|EPI_ISL_565613|Original|hCoV-19^^Victoria|Human|Microbiological Diagnostic Unit - Public Health Laboratory (MDU-PHL)|MDU-PHL|Schultz|Australia
>Spike|hCoV-19/Scotland/EDB7860/2020|2020-09-22|EPI_ISL_572543|Original|hCoV-19^^Scotland|Human|Virology Department|COVID-19 Genomics UK (COG-UK) Consortium|Williams|United Kingdom
>Spike|hCoV-19/Oman/C-6324/2020|2020-04-25|EPI_ISL_491126|Original|hCoV-19^^Muscat|Human|Oman-National Influenza Center|Biotechnology & OMICs Laboratory|Al Mahruqi|Oman
>Spike|hCoV-19/Japan/PG-6470/2020|2020-08-00|EPI_ISL_689575|Original|hCoV-19^^Japan|Human|Pathogen Genomics Center|Pathogen Genomics Center|Sekizuka|Japan
>Spike|hCoV-19/Netherlands/NB-RIVM-20607/2020|2020-12-10|EPI_ISL_790690|Original|hCoV-19^^Noord-Brabant|Human|Dutch COVID-19 response team|National Institute for Public Health and the Environment (RIVM)|Meijer|Netherlands
>Spike|hCoV-19/USA/CO-CDPHE-2011210342/2020|2020-11-19|EPI_ISL_771273|Original|hCoV-19^^Colorado|Human|Colorado Department of Public Health and Environment|Colorado Department of Puplic Health and Environment|Bankers|USA
>Spike|hCoV-19/England/QEUH-CAD1E8/2020|2020-12-20|EPI_ISL_760934|Original|hCoV-19^^England|Human|Lighthouse Lab in Glasgow|Wellcome Sanger Institute for the COVID-19 Genomics UK (COG-UK) Consortium|Davies|United Kingdom

>Spike|hCoV-19/USA/NY-NYCPHL-000552/2020|2020-03-24|EPI_ISL_632012|Original|hCoV-19^^New York|Human|Jamaica Hospital Medical Center|New York City Public Health Laboratory|Wang|USA
>Spike|hCoV-19/USA/CA-CZB-1235/2020|2020-04-11|EPI_ISL_454655|Original|hCoV-19^^California|Human|County of Santa Clara Public Health Department|Chan-Zuckerberg Biohub|Hao|USA
>Spike|hCoV-19/England/ALDP-9DA0F7/2020|2020-09-18|EPI_ISL_576846|Original|hCoV-19^^England|Human|Oxford Viromics|COVID-19 Genomics UK (COG-UK) Consortium|Golubchik|United Kingdom
>Spike|hCoV-19/England/BRIS-12ED13/2020|2020-04-22|EPI_ISL_488252|Original|hCoV-19^^England|Human|PHE South West Regional Laboratory|Wellcome Sanger Institute for the COVID-19 Genomics UK (COG-UK) consortium|Davies|United Kingdom
>Spike|hCoV-19/England/ALDP-691C19/2020|2020-07-02|EPI_ISL_558209|Original|hCoV-19^^England|Human|Lighthouse Lab in Alderley Park|Wellcome Sanger Institute for the COVID-19 Genomics UK (COG-UK) consortium|Davies|United Kingdom
>Spike|hCoV-19/USA/TX-HMH-2063/2020|2020-05-11|EPI_ISL_545097|Original|hCoV-19^^Texas|Human|Houston Methodist Hospital|Houston Methodist Hospital|Long|USA
>Spike|hCoV-19/United Arab Emirates/0139/2020|2020-05-18|EPI_ISL_698326|Original|hCoV-19^^Abu Dhabi|Human|Group 42 (G42) Healthcare|G42 Healthcare|Ogrodzki|United Arab Emirates
>Spike|hCoV-19/Canada/ON-PHL-20-03770/2020|2020-11-00|EPI_ISL_775079|Original|hCoV-19^^Ontario|Human|Public Health Ontario Laboratory|Public Health Ontario Laboratory|Guthrie|Canada
>Spike|hCoV-19/England/QEUH-B417B8/2020|2020-11-08|EPI_ISL_658503|Original|hCoV-19^^England|Human|Lighthouse Lab in Glasgow|Wellcome Sanger Institute for the COVID-19 Genomics UK (COG-UK) Consortium|Davies|United Kingdom
>Spike|hCoV-19/USA/UT-08435/2020|2020-05-15|EPI_ISL_524211|Original|hCoV-19^^Utah|Human|Utah Public Health Laboratory|Utah Public Health Laboratory|Young|USA
>Spike|hCoV-19/DRC/5957/2020|2020-05-07|EPI_ISL_496499|Original|hCoV-19^^Kinshasa|Human|Viral Respiratory Lab|Pathogen Sequencing Lab|Lokilo|Democratic Republic of the Congo
>Spike|hCoV-19/Scotland/QEUH-9ADF69/2020|2020-09-05|EPI_ISL_637955|Original|hCoV-19^^Scotland|Human|Department of Pathology|COVID-19 Genomics UK (COG-UK) Consortium|Goodfellow|United Kingdom
>Spike|hCoV-19/Wales/PHWC-2B4C7/2020|2020-04-11|EPI_ISL_445718|Original|hCoV-19^^Wales|Human|Wales Specialist Virology Centre|Public Health Wales Microbiology Cardiff|Moore|United Kingdom
>Spike|hCoV-19/Japan/PG-1652/2020|2020-04-00|EPI_ISL_685258|Original|hCoV-19^^Japan|Human|Pathogen Genomics Center|Pathogen Genomics Center|Sekizuka|Japan
>Spike|hCoV-19/USA/AZ-TG664493/2020|2020-04-20|EPI_ISL_696119|Original|hCoV-19^^Arizona|Human|Sonora Quest Laboratories|TGen North|French|USA
>Spike|hCoV-19/Australia/VIC12154/2020|2020-08-09|EPI_ISL_641289|Original|hCoV-19^^Victoria|Human|Microbiological Diagnostic Unit - Public Health Laboratory (MDU-PHL)|MDU-PHL|Schultz|Australia
>Spike|hCoV-19/USA/CA-SEARCH-5392/2020|2020-12-01|EPI_ISL_755203|Original|hCoV-19^^California|Human|UCSD EXCITE lab|Andersen lab at Scripps Research|Gangavarapu|USA
>Spike|hCoV-19/USA/MI-UM-10036626781/2020|2020-10-26|EPI_ISL_650024|Original|hCoV-19^^Michigan|Human|University of Michigan Clinical Microbiology Laboratory|Lauring Lab|Valesano|USA
>Spike|hCoV-19/England/SHEF-CF93F/2020|2020-04-13|EPI_ISL_448898|Original|hCoV-19^^England|Human|Virology Department|COVID-19 Genomics UK (COG-UK) Consortium|de Silva|United Kingdom
>Spike|hCoV-19/Spain/MD-IBV-99008482/2020|2020-08-11|EPI_ISL_654035|Original|hCoV-19^^Madrid|Human|Hospital General Universitario Gregorio Mara\u00f1\u00f3n|SeqCOVID-SPAIN consortium/IBV(CSIC)|Comas|Spain
>Spike|hCoV-19/France/ARA-CDP81800/2020|2020-09-16|EPI_ISL_582116|Original|hCoV-19^^Auvergne-Rh\u00f4ne-Alpes|Human|CNR Virus des Infections Respiratoires - France SUD|CNR Virus des Infections Respiratoires - France SUD|Josset|France

>Spike|hCoV-19/South Africa/KRISP-K001041/2020|2020-07-16|EPI_ISL_509304|Original|hCoV-19^^KwaZulu-Natal|Human|NHLS-IALCH|KRISP|de Oliveira|South Africa
>Spike|hCoV-19/England/QEUH-B685B1/2020|2020-11-11|EPI_ISL_654962|Original|hCoV-19^^England|Human|Lighthouse Lab in Glasgow|Wellcome Sanger Institute for the COVID-19 Genomics UK (COG-UK) Consortium|Davies|United Kingdom
>Spike|hCoV-19/England/CAMC-9488ED/2020|2020-07-21|EPI_ISL_556961|Original|hCoV-19^^England|Human|Lighthouse Lab in Cambridge|Wellcome Sanger Institute for the COVID-19 Genomics UK (COG-UK) consortium|Davies|United Kingdom
>Spike|hCoV-19/England/CAMC-9D1DAE/2020|2020-09-18|EPI_ISL_601771|Original|hCoV-19^^England|Human|Lighthouse Lab in Cambridge|Wellcome Sanger Institute for the COVID-19 Genomics UK (COG-UK) consortium|Davies|United Kingdom
>Spike|hCoV-19/England/205261879/2020|2020-12-18|EPI_ISL_766560|Original|hCoV-19^^England|Human|Respiratory Virus Unit|COVID-19 Genomics UK (COG-UK) Consortium|Platt|United Kingdom
>Spike|hCoV-19/Romania/ROSV_2794/2020|2020-04-16|EPI_ISL_491060|Original|hCoV-19^^Suceava|Human|Suceava County Emergency Hospital|\Stefan cel Mare\ University Metagenomics Lab|Lobiuc|Romania
>Spike|hCoV-19/England/MILK-B391D3/2020|2020-11-01|EPI_ISL_652295|Original|hCoV-19^^England|Human|University of Exeter|COVID-19 Genomics UK (COG-UK) Consortium|Temperton|United Kingdom
>Spike|hCoV-19/England/ALDP-A6C32D/2020|2020-10-15|EPI_ISL_607983|Original|hCoV-19^^England|Human|Lighthouse Lab in Alderley Park|Wellcome Sanger Institute for the COVID-19 Genomics UK (COG-UK) consortium|Davies|United Kingdom
>Spike|hCoV-19/England/QEUH-9F4D5A/2020|2020-09-25|EPI_ISL_588385|Original|hCoV-19^^England|Human|Lighthouse Lab in Glasgow|Wellcome Sanger Institute for the COVID-19 Genomics UK (COG-UK) consortium|Davies|United Kingdom
>Spike|hCoV-19/Egypt/CUNCI-HGC010/2020|2020-05-02|EPI_ISL_468055|Original|hCoV-19^^Egypt|Human|Egyptian National Cancer Institute (ENCI)|Egyptian National Cancer Institute (ENCI)|Zekri|Egypt
>Spike|hCoV-19/Canada/QC-LSPQ-L00236905/2020|2020-03-25|EPI_ISL_536151|Original|hCoV-19^^Quebec|Human|Centre Hospitalier R\u00e9gional de Lanaudi\u00e8re|Laboratoire de sant\u00e9 publique du Qu\u00e9bec|Moreira|Canada
>Spike|hCoV-19/USA/TX-HMH-MCoV-13475/2020|2020-08-21|EPI_ISL_788605|Original|hCoV-19^^Texas|Human|Houston Methodist Hospital|Houston Methodist Hospital|Long|USA
>Spike|hCoV-19/Hong Kong/VM20097391/2020|2020-08-03|EPI_ISL_602621|Original|hCoV-19^^Hong Kong|Human|GLENEAGLES HONG KONG (CLINICAL LAB)|Hong Kong Department of Health|Mak|China
>Spike|hCoV-19/England/201390708/2020|2020-03-29|EPI_ISL_465863|Original|hCoV-19^^England|Human|Respiratory Virus Unit|Respiratory Virus Unit|Platt|United Kingdom
>Spike|hCoV-19/Netherlands/NB-EMC-347/2020|2020-10-25|EPI_ISL_722287|Original|hCoV-19^^Noord Brabant|Human|Dutch COVID-19 response team|Erasmus Medical Center|Oude Munnink|Netherlands
>Spike|hCoV-19/Scotland/QEUH-AE1D7D/2020|2020-10-24|EPI_ISL_649640|Original|hCoV-19^^Scotland|Human|Lighthouse Lab in Glasgow|Wellcome Sanger Institute for the COVID-19 Genomics UK (COG-UK) Consortium|Davies|United Kingdom
>Spike|hCoV-19/England/QEUH-BA357E/2020|2020-11-18|EPI_ISL_721037|Original|hCoV-19^^England|Human|Lighthouse Lab in Glasgow|Wellcome Sanger Institute for the COVID-19 Genomics UK (COG-UK) Consortium|Davies|United Kingdom
>Spike|hCoV-19/England/ALDP-B47EBD/2020|2020-11-07|EPI_ISL_649345|Original|hCoV-19^^England|Human|Lighthouse Lab in Alderley Park|Wellcome Sanger Institute for the COVID-19 Genomics UK (COG-UK) Consortium|Davies|United Kingdom
>Spike|hCoV-19/England/NOTT-11E2EA/2020|2020-11-27|EPI_ISL_725523|Original|hCoV-19^^England|Human|Queens Medical Centre|COVID-19 Genomics UK (COG-UK) Consortium|Loose|United Kingdom
>Spike|hCoV-19/Australia/VIC10279/2020|2020-07-28|EPI_ISL_562848|Original|hCoV-19^^Victoria|Human|Microbiological Diagnostic Unit - Public Health Laboratory (MDU-PHL)|MDU-PHL|Schultz|Australia

>Spike|hCoV-19/England/ALDP-C3BC57/2020|2020-12-11|EPI_ISL_731723|Original|hCoV-19^^England|Human|Lighthouse Lab in Alderley Park|Wellcome Sanger Institute for the COVID-19 Genomics UK (COG-UK) Consortium|Davies|United Kingdom
>Spike|hCoV-19/USA/CA-IGI-0363/2020|2020-06-29|EPI_ISL_576547|Original|hCoV-19^^California|Human|Innovative Genomics Institute|Innovative Genomics Institute|Wyman|USA
>Spike|hCoV-19/Japan/PG-7235/2020|2020-08-00|EPI_ISL_688339|Original|hCoV-19^^Japan|Human|Saitama Prefectural Institute of Public Health|Pathogen Genomics Center|Sekizuka|Japan
>Spike|hCoV-19/USA/NM-NMDOH-2020322589/2020|2020-09-03|EPI_ISL_569635|Original|hCoV-19^^New Mexico|Human|New Mexico Department of Health Scientific Laboratory|New Mexico Department of Health Scientific Laboratory|Johnson|USA
>Spike|hCoV-19/Denmark/DCGC-9817/2020|2020-11-09|EPI_ISL_668486|Original|hCoV-19^^Hovedstaden|Human|Department of Virus and Microbiological Special Diagnostics|Albertsen Lab|Michaelsen|Denmark
>Spike|hCoV-19/England/MILK-B3A9AD/2020|2020-11-02|EPI_ISL_679696|Original|hCoV-19^^England|Human|Oxford Viromics|COVID-19 Genomics UK (COG-UK) Consortium|Golubchik|United Kingdom
>Spike|hCoV-19/USA/WI-WSLH-200202/2020|2020-03-18|EPI_ISL_578425|Original|hCoV-19^^Wisconsin|Human|Wisconsin State Laboratory of Hygiene Communicable Disease Division|Wisconsin State Laboratory of Hygiene Communicable Disease Division|Florek|USA
>Spike|hCoV-19/England/MILK-95DAEF/2020|2020-06-19|EPI_ISL_556285|Original|hCoV-19^^England|Human|Lighthouse Lab in Milton Keynes|Wellcome Sanger Institute for the COVID-19 Genomics UK (COG-UK) consortium|Davies|United Kingdom
>Spike|hCoV-19/England/QEUH-B7FE43/2020|2020-11-14|EPI_ISL_661745|Original|hCoV-19^^England|Human|Lighthouse Lab in Glasgow|Wellcome Sanger Institute for the COVID-19 Genomics UK (COG-UK) Consortium|Davies|United Kingdom
>Spike|hCoV-19/England/ALDP-C06086/2020|2020-12-04|EPI_ISL_709437|Original|hCoV-19^^England|Human|Lighthouse Lab in Alderley Park|Wellcome Sanger Institute for the COVID-19 Genomics UK (COG-UK) Consortium|Davies|United Kingdom
>Spike|hCoV-19/Netherlands/GE-EMC-221/2020|2020-05-12|EPI_ISL_523203|Original|hCoV-19^^Gelderland|Human|Dutch COVID-19 response team|Erasmus Medical Center|Oude Munnink|Netherlands
>Spike|hCoV-19/USA/CA-CHLA-PLM02905782/2020|2020-11-30|EPI_ISL_753448|Original|hCoV-19^^California|Human|Clinical virology Laboratory|Center for Personalized Medicine|Dien Bard|USA
>Spike|hCoV-19/USA/CA-UCLA-18/2020|2020-05-01|EPI_ISL_537506|Original|hCoV-19^^California|Human|UCLA Pathology Clinical Microbiology Lab|Kruglyak Lab|Boocock|USA
>Spike|hCoV-19/USA/FL-BPHL-1303/2020|2020-08-31|EPI_ISL_541235|Original|hCoV-19^^Florida|Human|Florida Bureau of Public Health Laboratories|Florida Bureau of Public Health Laboratories|Blanton|USA
>Spike|hCoV-19/Australia/VIC3945/2020|2020-07-14|EPI_ISL_520656|Original|hCoV-19^^Victoria|Human|Microbiological Diagnostic Unit - Public Health Laboratory (MDU-PHL)|MDU-PHL|Schultz|Australia
>Spike|hCoV-19/New Zealand/20VR3481/2020|2020-04-03|EPI_ISL_579499|Original|hCoV-19^^Canterbury|Human|Canterbury Health Laboratories|Institute of Environmental Science and Research (ESR)|Wang|New Zealand
>Spike|hCoV-19/Australia/VIC7724/2020|2020-08-04|EPI_ISL_564607|Original|hCoV-19^^Victoria|Human|Microbiological Diagnostic Unit - Public Health Laboratory (MDU-PHL)|MDU-PHL|Schultz|Australia
>Spike|hCoV-19/England/QEUH-B66C18/2020|2020-11-11|EPI_ISL_662615|Original|hCoV-19^^England|Human|Lighthouse Lab in Glasgow|Wellcome Sanger Institute for the COVID-19 Genomics UK (COG-UK) Consortium|Davies|United Kingdom
>Spike|hCoV-19/USA/TX-DSHS-1962/2020|2020-06-23|EPI_ISL_765931|Original|hCoV-19^^Texas|Human|TXDSHS|TXDSHS|Rahman|USA
>Spike|hCoV-19/England/20134016504/2020|2020-03-23|EPI_ISL_423089|Original|hCoV-19^^England|Human|Respiratory Virus Unit|Respiratory Virus Unit|Platt|United Kingdom

>Spike|hCoV-19/Canada/QC_AO9/2020|2020-04-09|EPI_ISL_463923|Original|hCoV-19^^Quebec|Human|Laboratoire de microbiologie|Smith Laboratory|Smith|Canada
>Spike|hCoV-19/Australia/VIC2695/2020|2020-07-02|EPI_ISL_521535|Original|hCoV-19^^Victoria|Human|Victorian Infectious Diseases Reference Laboratory (VIDRL)|VIDRL and MDU-PHL|Schultz|Australia
>Spike|hCoV-19/Northern Ireland/NIRE-101F7A/2020|2020-03-23|EPI_ISL_441360|Original|hCoV-19^^Northern Ireland|Human|Regional Virus Laboratory|COVID-19 Genomics UK (COG-UK) Consortium|Fairley|United Kingdom
>Spike|hCoV-19/England/QEUH-AD1736/2020|2020-10-26|EPI_ISL_634666|Original|hCoV-19^^England|Human|Lighthouse Lab in Glasgow|Wellcome Sanger Institute for the COVID-19 Genomics UK (COG-UK) consortium|Davies|United Kingdom
>Spike|hCoV-19/Thailand/Bangkok-0042/2020|2020-03-28|EPI_ISL_429178|Original|hCoV-19^^Bangkok|Human|Ramathibodi Hospital|COVID-19 Network Investigations (CONI) Alliance|Batty|Thailand
>Spike|hCoV-19/USA/UT-UPHL-2008915/2020|2020-05-18|EPI_ISL_525021|Original|hCoV-19^^Utah|Human|Utah Public Health Laboratory|Utah Public Health Laboratory|Young|USA
>Spike|hCoV-19/England/CAMB-72C23/2020|2020-03-22|EPI_ISL_439963|Original|hCoV-19^^England|Human|Department of Pathology|Wellcome Sanger Institute for the COVID-19 Genomics UK (COG-UK) consortium|Amato|United Kingdom
>Spike|hCoV-19/England/MILK-B95A61/2020|2020-11-13|EPI_ISL_673767|Original|hCoV-19^^England|Human|Lighthouse Lab in Milton Keynes|Wellcome Sanger Institute for the COVID-19 Genomics UK (COG-UK) Consortium|Davies|United Kingdom
>Spike|hCoV-19/England/MILK-56DF0F/2020|2020-06-11|EPI_ISL_558365|Original|hCoV-19^^England|Human|Lighthouse Lab in Milton Keynes|Wellcome Sanger Institute for the COVID-19 Genomics UK (COG-UK) consortium|Davies|United Kingdom
>Spike|hCoV-19/Australia/VIC4691/2020|2020-07-17|EPI_ISL_519079|Original|hCoV-19^^Victoria|Human|Microbiological Diagnostic Unit - Public Health Laboratory (MDU-PHL)|MDU-PHL|Schultz|Australia
>Spike|hCoV-19/Japan/PG-4145/2020|2020-07-00|EPI_ISL_692384|Original|hCoV-19^^Japan|Human|Pathogen Genomics Center|Pathogen Genomics Center|Sekizuka|Japan
>Spike|hCoV-19/Japan/PG-5162/2020|2020-08-00|EPI_ISL_690268|Original|hCoV-19^^Japan|Human|Pathogen Genomics Center|Pathogen Genomics Center|Sekizuka|Japan
>Spike|hCoV-19/Wales/PHWC-163B54/2020|2020-04-29|EPI_ISL_474046|Original|hCoV-19^^Wales|Human|Originating lab|COVID-19 Genomics UK (COG-UK) Consortium|Moore|United Kingdom
>Spike|hCoV-19/Denmark/DCGC-16310/2020|2020-11-16|EPI_ISL_714696|Original|hCoV-19^^Hovedstaden|Human|Department of Virus and Microbiological Special Diagnostics|Albertsen Lab|Michaelsen|Denmark
>Spike|hCoV-19/Denmark/DCGC-2775/2020|2020-08-24|EPI_ISL_616610|Original|hCoV-19^^Midtjylland|Human|Department of Virus and Microbiological Special Diagnostics|Albertsen lab|Michaelsen|Denmark
>Spike|hCoV-19/England/CAMC-BC1BB4/2020|2020-11-22|EPI_ISL_704767|Original|hCoV-19^^England|Human|Lighthouse Lab in Cambridge|Wellcome Sanger Institute for the COVID-19 Genomics UK (COG-UK) Consortium|Davies|United Kingdom
>Spike|hCoV-19/England/SHEF-C1F86/2020|2020-04-26|EPI_ISL_457589|Original|hCoV-19^^England|Human|Virology Department|COVID-19 Genomics UK (COG-UK) Consortium|de Silva|United Kingdom
>Spike|hCoV-19/Denmark/DCGC-12589/2020|2020-11-16|EPI_ISL_682770|Original|hCoV-19^^Midtjylland|Human|Department of Virus and Microbiological Special Diagnostics|Albertsen Lab|Michaelsen|Denmark
>Spike|hCoV-19/England/NORT-293E24/2020|2020-07-20|EPI_ISL_573330|Original|hCoV-19^^England|Human|Northumbria University / South Tees Hospitals NHS Foundation Trust / North Cumbria Integrated Care NHS Foundation Trust / North Tees and Hartlepool NHS Foundation Trust / Newcastle Hospitals NHS Foundation Trust|COVID-19 Genomics UK (COG-UK) Consortium|Bashton|United Kingdom
>Spike|hCoV-19/England/205120838/2020|2020-12-12|EPI_ISL_766109|Original|hCoV-19^^England|Human|Respiratory Virus Unit|COVID-19 Genomics UK (COG-UK) Consortium|Platt|United Kingdom

>Spike|hCoV-19/USA/CA-ALSR-1687/2020|2020-03-25|EPI_ISL_494744|Original|hCoV-19^^California|Human|San Diego County Public Health Laboratory|Andersen lab at Scripps Research|Gangavarapu|USA
>Spike|hCoV-19/Netherlands/NH-RIVM-20005/2020|2020-11-20|EPI_ISL_723204|Orignal|hCoV-19^^Noord-Holland|Human|Dutch COVID-19 response team|National Institute for Public Health and the Environment (RIVM)|Meijer|Netherlands
>Spike|hCoV-19/Belgium/rega-10191305/2020|2020-10-19|EPI_ISL_735208|Original|hCoV-19^^Heverlee|Human|UZ Leuven|KU Leuven|Maes|Belgium
>Spike|hCoV-19/USA/ID-UW-4255/2020|2020-03-30|EPI_ISL_430940|Original|hCoV-19^^Idaho|Human|UW Virology Lab|UW Virology Lab|Roychoudhury|USA
>Spike|hCoV-19/Japan/PG-7202/2020|2020-07-00|EPI_ISL_688306|Original|hCoV-19^^Japan|Human|Saitama Prefectural Institute of Public Health|Pathogen Genomics Center|Sekizuka|Japan
>Spike|hCoV-19/Scotland/CVR3094/2020|2020-04-25|EPI_ISL_439034|Original|hCoV-19^^Scotland|Human|West of Scotland Specialist Virology Centre|COVID-19 Genomics UK (COG-UK) Consortium|Robertson|United Kingdom
>Spike|hCoV-19/Scotland/EDB10459/2020|2020-11-09|EPI_ISL_705766|Original|hCoV-19^^Scotland|Human|Virology Department|COVID-19 Genomics UK (COG-UK) Consortium|McHugh|United Kingdom
>Spike|hCoV-19/Luxembourg/LNS9375963/2020|2020-10-31|EPI_ISL_744895|Original|hCoV-19^^Luxembourg|Human|Laboratoire national de sant\u00e9|Laboratoire national de sant\u00e9|Wienecke-Baldacchino|Luxembourg
>Spike|hCoV-19/England/QEUH-9DA73E/2020|2020-09-22|EPI_ISL_601318|Original|hCoV-19^^England|Human|Lighthouse Lab in Glasgow|Wellcome Sanger Institute for the COVID-19 Genomics UK (COG-UK) consortium|Davies|United Kingdom
>Spike|hCoV-19/Wales/PHWC-23D88/2020|2020-03-13|EPI_ISL_418112|Original|hCoV-19^^Wales|Human|Wales Specialist Virology Centre|Public Health Wales Microbiology Cardiff|Moore|United Kingdom
>Spike|hCoV-19/Australia/NSW943/2020|2020-04-15|EPI_ISL_545015|Original|hCoV-19^^New South Wales|Human|South Eastern Area Laboratory Services (SEALS)|NSW Health Pathology - Institute of Clinical Pathology and Medical Research; Westmead Hospital; University of Sydney|Gall|Australia
>Spike|hCoV-19/USA/MA-MASPHL-00414/2020|2020-04-26|EPI_ISL_693136|Original|hCoV-19^^Massachusetts|Human|Massachusetts State Public Health Laboratory|Massachusetts State Public Health Laboratory|Lang|USA
>Spike|hCoV-19/England/ALDP-A855BF/2020|2020-10-17|EPI_ISL_610306|Original|hCoV-19^^England|Human|Lighthouse Lab in Alderley Park|Wellcome Sanger Institute for the COVID-19 Genomics UK (COG-UK) consortium|Davies|United Kingdom
>Spike|hCoV-19/USA/TX-HMH-MCoV-14571/2020|2020-10-08|EPI_ISL_787856|Original|hCoV-19^^Texas|Human|Houston Methodist Hospital|Houston Methodist Hospital|Long|USA
>Spike|hCoV-19/England/ALDP-A6FA8F/2020|2020-10-15|EPI_ISL_607056|Original|hCoV-19^^England|Human|Lighthouse Lab in Alderley Park|Wellcome Sanger Institute for the COVID-19 Genomics UK (COG-UK) consortium|Davies|United Kingdom
>Spike|hCoV-19/Denmark/DCGC-18409/2020|2020-12-07|EPI_ISL_749949|Original|hCoV-19^^Hovedstaden|Human|Department of Virus and Microbiological Special Diagnostics|Albertsen Lab|Michaelsen|Denmark
>Spike|hCoV-19/Canada/ON-S1835/2020|2020-11-24|EPI_ISL_729299|Original|hCoV-19^^Ontario|Human|Toronto Invasive Bacterial Diseases Network|McMaster University|McArthur|Canada
>Spike|hCoV-19/USA/CA-ALSR-1461/2020|2020-03-23|EPI_ISL_494617|Original|hCoV-19^^California|Human|Scripps Medical Laboratory|Andersen lab at Scripps Research|Gangavarapu|USA
>Spike|hCoV-19/Spain/RI-IBV-99010987/2020|2020-08-28|EPI_ISL_732810|Original|hCoV-19^^La_Rioja|Human|Centro de Investigaci\u00f3n Biom\u00e9dica de La Rioja - Hospital San Pedro Logro\u00f1o|SeqCOVID-SPAIN consortium/IBV(CSIC)|Comas|Spain
>Spike|hCoV-19/Wales/PHWC-491CA2/2020|2020-11-12|EPI_ISL_706765|Original|hCoV-19^^Wales|Human|Originating lab|COVID-19 Genomics UK (COG-UK) Consortium|Moore|United Kingdom

>Spike|hCoV-19/England/MILK-9C4A3E/2020|2020-09-10|EPI_ISL_573263|Original|hCoV-19^^England|Human|Centre for Enzyme Innovation|COVID-19 Genomics UK (COG-UK) Consortium|Robson|United Kingdom
>Spike|hCoV-19/Japan/PG-5625/2020|2020-08-00|EPI_ISL_688750|Original|hCoV-19^^Japan|Human|Pathogen Genomics Center|Pathogen Genomics Center|Sekizuka|Japan
>Spike|hCoV-19/England/ALDP-9BE8A6/2020|2020-09-09|EPI_ISL_568100|Original|hCoV-19^^England|Human|Lighthouse Lab in Alderley Park|Wellcome Sanger Institute for the COVID-19 Genomics UK (COG-UK) consortium|Davies|United Kingdom
>Spike|hCoV-19/Denmark/DCGC-1103/2020|2020-03-16|EPI_ISL_614612|Original|hCoV-19^^Hovedstaden|Human|Department of Virus and Microbiological Special Diagnostics|Albertsen lab|Michaelsen|Denmark
>Spike|hCoV-19/Denmark/DCGC-16623/2020|2020-11-16|EPI_ISL_714877|Original|hCoV-19^^Hovedstaden|Human|Department of Virus and Microbiological Special Diagnostics|Albertsen Lab|Michaelsen|Denmark
>Spike|hCoV-19/Japan/PG-0449/2020|2020-03-21|EPI_ISL_480112|Original|hCoV-19^^Kyusyu|Human|Oita Prefectural Institute of Public Health and Environmental Science|Pathogen Genomics Center|Sekizuka|Japan
>Spike|hCoV-19/Canada/ON-S907/2020|2020-04-13|EPI_ISL_586480|Original|hCoV-19^^Ontario|Human|Toronto Invasive Bacterial Diseases Network|McMaster University|McArthur|Canada
>Spike|hCoV-19/Denmark/DCGC-11856/2020|2020-10-26|EPI_ISL_671180|Original|hCoV-19^^Hovedstaden|Human|Department of Virus and Microbiological Special Diagnostics|Albertsen Lab|Michaelsen|Denmark
>Spike|hCoV-19/Scotland/EDB2137/2020|2020-04-20|EPI_ISL_439167|Original|hCoV-19^^Scotland|Human|Virology Department|COVID-19 Genomics UK (COG-UK) Consortium|Williams|United Kingdom
>Spike|hCoV-19/Belgium/UGent-243/2020|2020-11-13|EPI_ISL_717628|Original|hCoV-19^^Ghent|Human|Lab voor klinische biologie|Onderzoeksgroep Virologie|Lambrechts|Belgium
>Spike|hCoV-19/Bangladesh/BCSIR-NILMRC-141/2020|2020-06-18|EPI_ISL_480417|Original|hCoV-19^^Dhaka|Human|National Institute of Laboratory Medicine and Referral Center|Bangladesh Council of Scientific and Industrial Research|Khan|Bangladesh
>Spike|hCoV-19/Wales/PHWC-2B579/2020|2020-04-10|EPI_ISL_445727|Original|hCoV-19^^Wales|Human|Wales Specialist Virology Centre|Public Health Wales Microbiology Cardiff|Moore|United Kingdom
>Spike|hCoV-19/England/ALDP-9FDA78/2020|2020-10-01|EPI_ISL_587761|Original|hCoV-19^^England|Human|Lighthouse Lab in Alderley Park|Wellcome Sanger Institute for the COVID-19 Genomics UK (COG-UK) consortium|Davies|United Kingdom
>Spike|hCoV-19/England/ALDP-B2642B/2020|2020-11-04|EPI_ISL_646512|Original|hCoV-19^^England|Human|Lighthouse Lab in Alderley Park|Wellcome Sanger Institute for the COVID-19 Genomics UK (COG-UK) Consortium|Davies|United Kingdom
>Spike|hCoV-19/England/QEUH-AD3B83/2020|2020-10-26|EPI_ISL_634230|Original|hCoV-19^^England|Human|Lighthouse Lab in Glasgow|Wellcome Sanger Institute for the COVID-19 Genomics UK (COG-UK) consortium|Davies|United Kingdom
>Spike|hCoV-19/Denmark/DCGC-4146/2020|2020-09-21|EPI_ISL_621966|Original|hCoV-19^^Hovedstaden|Human|Department of Virus and Microbiological Special Diagnostics|Albertsen lab|Michaelsen|Denmark
>Spike|hCoV-19/Germany/BY-UHR-7ZK/2020|2020-03-22|EPI_ISL_513304|Viral culture|hCoV-19^^Bavaria|Human|Department of Infection Prevention and Infectious Diseases|University Hospital Regensburg|Fritsch|Germany
>Spike|hCoV-19/England/QEUH-AF0537/2020|2020-10-29|EPI_ISL_662779|Original|hCoV-19^^England|Human|Lighthouse Lab in Glasgow|Wellcome Sanger Institute for the COVID-19 Genomics UK (COG-UK) Consortium|Davies|United Kingdom
>Spike|hCoV-19/England/ALDP-B48362/2020|2020-11-07|EPI_ISL_649294|Original|hCoV-19^^England|Human|Lighthouse Lab in Alderley Park|Wellcome Sanger Institute for the COVID-19 Genomics UK (COG-UK) Consortium|Davies|United Kingdom
>Spike|hCoV-19/USA/CA-ALSR-2417/2020|2020-05-27|EPI_ISL_635469|Original|hCoV-19^^California|Human|San Diego County Public Health Laboratory|Andersen lab at Scripps Research|Gangavarapu|USA

>Spike|hCoV-19/Denmark/DCGC-2981/2020|2020-08-24|EPI_ISL_616743|Original|hCoV-19^^Hovedstaden|Human|Department of Virus and Microbiological Special Diagnostics|Albertsen lab|Michaelsen|Denmark
>Spike|hCoV-19/England/CAMC-B32CBB/2020|2020-11-06|EPI_ISL_659580|Original|hCoV-19^^England|Human|Lighthouse Lab in Cambridge|Wellcome Sanger Institute for the COVID-19 Genomics UK (COG-UK) Consortium|Davies|United Kingdom
>Spike|hCoV-19/USA/TX-HMH-MCoV-15274/2020|2020-10-21|EPI_ISL_786758|Original|hCoV-19^^Texas|Human|Houston Methodist Hospital|Houston Methodist Hospital|Long|USA
>Spike|hCoV-19/Indonesia/BT-MRINUPH-200624-349/2020|2020-06-22|EPI_ISL_779395|Original|hCoV-19^^Banten|Human|Siloam Hospital Lippo Village|Mochtar Riady Institute for Nanotechnology-Universitas Pelita Harapan|Suriapranata|Indonesia
>Spike|hCoV-19/USA/CA-QDX-223/2020|2020-04-11|EPI_ISL_498699|Original|hCoV-19^^California|Human|Quest Diagnostics|Quest Diagnostics|Kagan|USA
>Spike|hCoV-19/Denmark/DCGC-14987/2020|2020-11-23|EPI_ISL_713564|Original|hCoV-19^^Midtjylland|Human|Department of Virus and Microbiological Special Diagnostics|Albertsen Lab|Michaelsen|Denmark
>Spike|hCoV-19/England/SHEF-CA7B2/2020|2020-03-31|EPI_ISL_442363|Original|hCoV-19^^England|Human|Virology Department|COVID-19 Genomics UK (COG-UK) Consortium|de Silva|United Kingdom
>Spike|hCoV-19/Wales/PHWC-48FB9B/2020|2020-11-11|EPI_ISL_681196|Original|hCoV-19^^Wales|Human|Wales Specialist Virology Centre Sequencing lab|COVID-19 Genomics UK (COG-UK) Consortium|Moore|United Kingdom
>Spike|hCoV-19/USA/MI-QDX-933/2020|2020-03-20|EPI_ISL_571637|Original|hCoV-19^^Michigan|Human|Quest Diagnostics|Quest Diagnostics|Kagan|USA
>Spike|hCoV-19/England/ALDP-A693D5/2020|2020-10-12|EPI_ISL_609134|Original|hCoV-19^^England|Human|Lighthouse Lab in Alderley Park|Wellcome Sanger Institute for the COVID-19 Genomics UK (COG-UK) consortium|Davies|United Kingdom
>Spike|hCoV-19/Portugal/PT0571/2020|2020-03-26|EPI_ISL_454294|Original|hCoV-19^^Portugal|Human|unknown|Instituto Nacional de Saude (INSA)|Borges|Portugal
>Spike|hCoV-19/Denmark/DCGC-783/2020|2020-04-27|EPI_ISL_614754|Original|hCoV-19^^Syddanmark|Human|Department of Virus and Microbiological Special Diagnostics|Albertsen lab|Michaelsen|Denmark
>Spike|hCoV-19/England/LIVE-1D8C0C/2020|2020-09-25|EPI_ISL_576982|Original|hCoV-19^^England|Human|Liverpool Clinical Laboratories|COVID-19 Genomics UK (COG-UK) Consortium|Haldenby|United Kingdom
>Spike|hCoV-19/USA/TX-DSHS-1799/2020|2020-06-13|EPI_ISL_738172|Original|hCoV-19^^Texas|Human|Texas Department of State Health Services|Texas Department of State Health Services|Rahman|USA
>Spike|hCoV-19/USA/HI-H200224/2020|2020-08-11|EPI_ISL_752783|Original|hCoV-19^^Hawaii|Human|State Laboratories Division|State Laboratories Division|Sultana|USA
>Spike|hCoV-19/England/MILK-A45DD1/2020|2020-10-06|EPI_ISL_598608|Original|hCoV-19^^England|Human|Lighthouse Lab in Milton Keynes|Wellcome Sanger Institute for the COVID-19 Genomics UK (COG-UK) consortium|Davies|United Kingdom
>Spike|hCoV-19/England/203290153/2020|2020-08-06|EPI_ISL_534721|Original|hCoV-19^^England|Human|Respiratory Virus Unit|Respiratory Virus Unit|Platt|United Kingdom
>Spike|hCoV-19/USA/LA-GBCL-26684517/2020|2020-04-10|EPI_ISL_485319|Original|hCoV-19^^Louisiana|Human|River Road Testing Lab|Ginkgo Bioworks Clinical Laboratory|Smith|USA
>Spike|hCoV-19/England/SHEF-CBF04/2020|2020-11-14|EPI_ISL_705276|Original|hCoV-19^^England|Human|Virology Department|COVID-19 Genomics UK (COG-UK) Consortium|de Silva|United Kingdom
>Spike|hCoV-19/Japan/PG-5800/2020|2020-08-00|EPI_ISL_688919|Original|hCoV-19^^Japan|Human|Fukuoka Institute of Health and Environmental Sciences|Pathogen Genomics Center|Sekizuka|Japan
>Spike|hCoV-19/Netherlands/LI-RIVM-20447/2020|2020-12-09|EPI_ISL_728666|Original|hCoV-19^^Limburg|Human|Dutch COVID-19 response team|National Institute for Public Health and the Environment (RIVM)|Meijer|Netherlands

>Spike|hCoV-19/England/QEUH-A636A5/2020|2020-10-12|EPI_ISL_596132|Original|hCoV-19^^England|Human|Quadram Institute Bioscience|COVID-19 Genomics UK (COG-UK) Consortium|Alikhan|United Kingdom
>Spike|hCoV-19/Denmark/DCGC-9696/2020|2020-11-09|EPI_ISL_669115|Original|hCoV-19^^Syddanmark|Human|Department of Virus and Microbiological Special Diagnostics|Albertsen Lab|Michaelsen|Denmark
>Spike|hCoV-19/Australia/VIC14265/2020|2020-07-29|EPI_ISL_592426|Original|hCoV-19^^Victoria|Human|Microbiological Diagnostic Unit - Public Health Laboratory (MDU-PHL)|MDU-PHL|Schultz|Australia
>Spike|hCoV-19/France/PAC-MEPHI-2132/2020|2020-09-25|EPI_ISL_644505|Original|hCoV-19^^Provence-Alpes-Cote-d\u2019Azur|Human|MEPHI|MEPHI|Levasseur|France
>Spike|hCoV-19/England/ALDP-AA1975/2020|2020-10-21|EPI_ISL_623971|Original|hCoV-19^^England|Human|Lighthouse Lab in Alderley Park|Wellcome Sanger Institute for the COVID-19 Genomics UK (COG-UK) consortium|Davies|United Kingdom
>Spike|hCoV-19/Netherlands/NoordBrabant_10/2020|2020-03-02|EPI_ISL_414431|Original|hCoV-19^^Noord Brabant|Human|Dutch COVID-19 response team|Erasmus Medical Center|Oude Munnink|Netherlands
>Spike|hCoV-19/England/MILK-A7AA90/2020|2020-10-15|EPI_ISL_606414|Original|hCoV-19^^England|Human|Lighthouse Lab in Milton Keynes|Wellcome Sanger Institute for the COVID-19 Genomics UK (COG-UK) consortium|Davies|United Kingdom
>Spike|hCoV-19/England/QEUH-AF505F/2020|2020-10-30|EPI_ISL_663066|Original|hCoV-19^^England|Human|Lighthouse Lab in Glasgow|Wellcome Sanger Institute for the COVID-19 Genomics UK (COG-UK) Consortium|Davies|United Kingdom
>Spike|hCoV-19/Japan/PG-5123/2020|2020-08-00|EPI_ISL_689617|Original|hCoV-19^^Japan|Human|Gunma Prefectural Institute of Public Health and Environmental Sciences|Pathogen Genomics Center|Sekizuka|Japan
>Spike|hCoV-19/England/ALDP-AA4259/2020|2020-10-22|EPI_ISL_624287|Original|hCoV-19^^England|Human|Lighthouse Lab in Alderley Park|Wellcome Sanger Institute for the COVID-19 Genomics UK (COG-UK) consortium|Davies|United Kingdom
>Spike|hCoV-19/England/CAMC-B34202/2020|2020-11-05|EPI_ISL_659726|Original|hCoV-19^^England|Human|Lighthouse Lab in Cambridge|Wellcome Sanger Institute for the COVID-19 Genomics UK (COG-UK) Consortium|Davies|United Kingdom
>Spike|hCoV-19/England/QEUH-9DE3FA/2020|2020-09-21|EPI_ISL_599609|Original|hCoV-19^^England|Human|Lighthouse Lab in Glasgow|Wellcome Sanger Institute for the COVID-19 Genomics UK (COG-UK) consortium|Davies|United Kingdom
>Spike|hCoV-19/Denmark/DCGC-6101/2020|2020-10-05|EPI_ISL_620296|Original|hCoV-19^^Hovedstaden|Human|Department of Virus and Microbiological Special Diagnostics|Albertsen lab|Michaelsen|Denmark
>Spike|hCoV-19/England/MILK-BACD9B/2020|2020-11-17|EPI_ISL_681635|Original|hCoV-19^^England|Human|Lighthouse Lab in Milton Keynes|Wellcome Sanger Institute for the COVID-19 Genomics UK (COG-UK) Consortium|Davies|United Kingdom
>Spike|hCoV-19/England/QEUH-AD43B7/2020|2020-10-26|EPI_ISL_634673|Original|hCoV-19^^England|Human|Lighthouse Lab in Glasgow|Wellcome Sanger Institute for the COVID-19 Genomics UK (COG-UK) consortium|Davies|United Kingdom
>Spike|hCoV-19/England/CAMC-A41959/2020|2020-10-07|EPI_ISL_609392|Original|hCoV-19^^England|Human|Lighthouse Lab in Cambridge|Wellcome Sanger Institute for the COVID-19 Genomics UK (COG-UK) consortium|Davies|United Kingdom
>Spike|hCoV-19/England/SHEF-C1083/2020|2020-11-15|EPI_ISL_706991|Original|hCoV-19^^England|Human|Virology Department|COVID-19 Genomics UK (COG-UK) Consortium|de Silva|United Kingdom
>Spike|hCoV-19/England/ALDP-A52700/2020|2020-10-07|EPI_ISL_598050|Original|hCoV-19^^England|Human|Lighthouse Lab in Alderley Park|Wellcome Sanger Institute for the COVID-19 Genomics UK (COG-UK) consortium|Davies|United Kingdom
>Spike|hCoV-19/Norway/3743/2020|2020-09-01|EPI_ISL_590998|Original|hCoV-19^^Viken|Human|Ostfold Hospital Trust - Kalnes|Norwegian Institute of Public Health|Vollan|Norway
>Spike|hCoV-19/Luxembourg/LNS8387253/2020|2020-11-02|EPI_ISL_744732|Original|hCoV-19^^Luxembourg|Human|Laboratoire national de sant\u00e9|Laboratoire national de sant\u00e9|Wienecke-Baldacchino|Luxembourg

>Spike|hCoV-19/England/CAMC-9D2235/2020|2020-09-19|EPI_ISL_601555|Original|hCoV-19^^England|Human|Lighthouse Lab in Cambridge|Wellcome Sanger Institute for the COVID-19 Genomics UK (COG-UK) consortium|Davies|United Kingdom

>Spike|hCoV-19/Australia/VIC15729/2020|2020-10-04|EPI_ISL_593427|Original|hCoV-19^^Victoria|Human|Microbiological Diagnostic Unit - Public Health Laboratory (MDU-PHL)|MDU-PHL|Schultz|Australia

>Spike|hCoV-19/England/NORT-29DE0C/2020|2020-05-06|EPI_ISL_484318|Original|hCoV-19^^England|Human|Northumbria University / South Tees Hospitals NHS Foundation Trust / North Cumbria Integrated Care NHS Foundation Trust / North Tees and Hartlepool NHS Foundation Trust / Newcastle Hospitals NHS Foundation Trust|COVID-19 Genomics UK (COG-UK) Consortium|Bashton|United Kingdom

>Spike|hCoV-19/England/ALDP-9E99AF/2020|2020-09-24|EPI_ISL_584272|Original|hCoV-19^^England|Human|Virology Department|COVID-19 Genomics UK (COG-UK) Consortium|de Silva|United Kingdom

>Spike|hCoV-19/Australia/VIC15047/2020|2020-07-29|EPI_ISL_593122|Original|hCoV-19^^Victoria|Human|Microbiological Diagnostic Unit - Public Health Laboratory (MDU-PHL)|MDU-PHL|Schultz|Australia

>Spike|hCoV-19/Portugal/PT0505/2020|2020-03-18|EPI_ISL_454229|Original|hCoV-19^^Portugal|Human|unknown|Instituto Nacional de Saude (INSA)|Borges|Portugal

>Spike|hCoV-19/Japan/PG-6129/2020|2020-08-00|EPI_ISL_689241|Original|hCoV-19^^Japan|Human|Pathogen Genomics Center|Pathogen Genomics Center|Sekizuka|Japan

>Spike|hCoV-19/USA/FL-BPHL-0199/2020|2020-04-07|EPI_ISL_480850|Original|hCoV-19^^Florida|Human|Florida Bureau of Public Health Laboratories|Florida Bureau of Public Health Laboratories|Blanton|USA

>Spike|hCoV-19/England/QEUH-B2B65D/2020|2020-11-01|EPI_ISL_646507|Original|hCoV-19^^England|Human|Lighthouse Lab in Glasgow|Wellcome Sanger Institute for the COVID-19 Genomics UK (COG-UK) Consortium|Davies|United Kingdom

>Spike|hCoV-19/Canada/QC-LSPQ-L00238906/2020|2020-03-26|EPI_ISL_536226|Original|hCoV-19^^Quebec|Human|H\u00f4pital du Suro\u00eet|Laboratoire de sant\u00e9 publique du Qu\u00e9bec|Moreira|Canada

>Spike|hCoV-19/Denmark/DCGC-16718/2020|2020-10-19|EPI_ISL_714430|Original|hCoV-19^^Hovedstaden|Human|Department of Virus and Microbiological Special Diagnostics|Albertsen Lab|Michaelsen|Denmark

>Spike|hCoV-19/England/BRIS-126795/2020|2020-04-08|EPI_ISL_459015|Original|hCoV-19^^England|Human|PHE South West Regional Laboratory|Wellcome Sanger Institute for the COVID-19 Genomics UK (COG-UK) consortium|Davies|United Kingdom

>Spike|hCoV-19/England/SHEF-BFF21/2020|2020-03-25|EPI_ISL_418309|Original|hCoV-19^^England|Human|Virology Department|Department of Infection|de Silva|United Kingdom

>Spike|hCoV-19/India/TG-CCMB-AB223/2020|2020-08-14|EPI_ISL_539640|Original|hCoV-19^^Telangana|Human|CSIR-Centre for Cellular and Molecular Biology|CSIR-Centre for Cellular and Molecular Biology|Mukherjee|India

>Spike|hCoV-19/Japan/PG-1586/2020|2020-04-00|EPI_ISL_685192|Original|hCoV-19^^Japan|Human|Pathogen Genomics Center|Pathogen Genomics Center|Sekizuka|Japan

>Spike|hCoV-19/USA/CA-CHLA-PLM07768214/2020|2020-11-28|EPI_ISL_753531|Original|hCoV-19^^California|Human|Clinical virology Laboratory|Center for Personalized Medicine|Dien Bard|USA

>Spike|hCoV-19/England/CAMB-1AB24C/2020|2020-04-19|EPI_ISL_470111|Original|hCoV-19^^England|Human|Department of Pathology|Wellcome Sanger Institute for the COVID-19 Genomics UK (COG-UK) consortium|Davies|United Kingdom

>Spike|hCoV-19/Canada/BC_26650347/2020|2020-03-00|EPI_ISL_463250|Original|hCoV-19^^British Columbia|Human|BCCDC Public Health Laboratory|BCCDC Public Health Laboratory|Harrigan|Canada

>Spike|hCoV-19/USA/UT-01857/2020|2020-04-13|EPI_ISL_498631|Original|hCoV-19^^Utah|Human|Utah Public Health Laboratory|Utah Public Health Laboratory|Butz|USA

>Spike|hCoV-19/England/LIVE-DB3D62/2020|2020-10-20|EPI_ISL_650656|Original|hCoV-19^^England|Human|Liverpool Clinical Laboratories|COVID-19 Genomics UK (COG-UK) Consortium|Haldenby|United Kingdom

>Spike|hCoV-19/England/204682412/2020|2020-11-13|EPI_ISL_693426|Original|hCoV-19^^England|Human|Respiratory Virus Unit|COVID-19 Genomics UK (COG-UK) Consortium|Platt|United Kingdom

>Spike|hCoV-19/Denmark/DCGC-14287/2020|2020-11-30|EPI_ISL_711420|Original|hCoV-19^^Hovedstaden|Human|Department of Virus and Microbiological Special Diagnostics|Albertsen Lab|Michaelsen|Denmark
>Spike|hCoV-19/England/EXET-135EBC/2020|2020-03-22|EPI_ISL_457177|Original|hCoV-19^^England|Human|University of Exeter|COVID-19 Genomics UK (COG-UK) Consortium|Temperton|United Kingdom
>Spike|hCoV-19/Japan/PG-7135/2020|2020-08-00|EPI_ISL_688240|Original|hCoV-19^^Japan|Human|Pathogen Genomics Center|Pathogen Genomics Center|Sekizuka|Japan
>Spike|hCoV-19/Australia/WA368/2020|2020-10-18|EPI_ISL_605845|Original|hCoV-19^^Western Australia|Human|PathWest Laboratory Medicine WA|PathWest Laboratory Medicine WA Microbial Surveillance Unit|Sikazwe|Australia
>Spike|hCoV-19/Switzerland/BE-ETHZ-280109/2020|2020-09-07|EPI_ISL_560432|Original|hCoV-19^^Bern|Human|Viollier AG|Department of Biosystems Science and Engineering|Nadeau|Switzerland
>Spike|hCoV-19/Australia/NSW919/2020|2020-09-02|EPI_ISL_545000|Original|hCoV-19^^New South Wales|Human|St Vincent's Pathology (SydPath)|NSW Health Pathology - Institute of Clinical Pathology and Medical Research; Westmead Hospital; University of Sydney|Gall|Australia
>Spike|hCoV-19/Guatemala/CDC-1228/2020|2020-03-15|EPI_ISL_509696|Original|hCoV-19^^Guatemala|Human|Guatemala Ministry of Public Health|Pathogen Discovery|Queen|Guatemala
>Spike|hCoV-19/Japan/PG-5473/2020|2020-08-00|EPI_ISL_690576|Original|hCoV-19^^Japan|Human|Pathogen Genomics Center|Pathogen Genomics Center|Sekizuka|Japan
>Spike|hCoV-19/Spain/PV-IBV-004304/2020|2020-04-09|EPI_ISL_510419|Original|hCoV-19^^Basque Country|Human|Servicio de Microbiolog\u00eda. Hospital Universitario Donostia. OSI Donostialdea. \u00c1rea de Enfermedades Infecciosas|SeqCOVID-SPAIN consortium/IBV(CSIC)|Comas|Spain
>Spike|hCoV-19/Wales/PHWC-48E4EC/2020|2020-11-12|EPI_ISL_680868|Original|hCoV-19^^Wales|Human|Wales Specialist Virology Centre Sequencing lab|COVID-19 Genomics UK (COG-UK) Consortium|Moore|United Kingdom
>Spike|hCoV-19/USA/AZ-TG274711/2020|2020-04-05|EPI_ISL_694062|Original|hCoV-19^^Arizona|Human|AZ SPHL|TGen North|French|USA
>Spike|hCoV-19/Denmark/DCGC-16600/2020|2020-11-16|EPI_ISL_714862|Original|hCoV-19^^Midtjylland|Human|Department of Virus and Microbiological Special Diagnostics|Albertsen Lab|Michaelsen|Denmark
>Spike|hCoV-19/USA/NY-Wadsworth-251067-01/2020|2020-10-21|EPI_ISL_754822|Original|hCoV-19^^New York|Human|Wadsworth Center|Wadsworth Center|Lasek-Nesselquist|USA
>Spike|hCoV-19/France/ARA-23092/2020|2020-04-10|EPI_ISL_508925|Original|hCoV-19^^Auvergne-Rh\u00f4ne-Alpes|Human|CNR Virus des Infections Respiratoires - France SUD|CNR Virus des Infections Respiratoires - France SUD|Josset|France
>Spike|hCoV-19/USA/WA-S918/2020|2020-04-24|EPI_ISL_463408|Original|hCoV-19^^Washington|Human|Washington State Department of Health|Seattle Flu Study|Bedford|USA
>Spike|hCoV-19/England/LOND-D4B5F/2020|2020-04-11|EPI_ISL_444181|Original|hCoV-19^^England|Human|University College London|COVID-19 Genomics UK (COG-UK) Consortium|Roy|United Kingdom
>Spike|hCoV-19/USA/CA-IGI-0355/2020|2020-07-20|EPI_ISL_754875|Original|hCoV-19^^California|Human|Innovative Genomics Institute|Innovative Genomics Institute|Wyman|USA
>Spike|hCoV-19/England/MILK-A240EE/2020|2020-10-04|EPI_ISL_652108|Original|hCoV-19^^England|Human|Centre for Enzyme Innovation|COVID-19 Genomics UK (COG-UK) Consortium|Robson|United Kingdom
>Spike|hCoV-19/Scotland/QEUH-A4CC46/2020|2020-10-08|EPI_ISL_596036|Original|hCoV-19^^Scotland|Human|Quadram Institute Bioscience|COVID-19 Genomics UK (COG-UK) Consortium|Alikhan|United Kingdom
>Spike|hCoV-19/South Korea/KDCA0421/2020|2020-10-04|EPI_ISL_760205|Original|hCoV-19^^South Korea|Human|Division of Emerging Infectious Diseases|Division of Emerging Infectious Diseases|Park|South Korea

>Spike|hCoV-19/England/MILK-B39AF6/2020|2020-11-02|EPI_ISL_651058|Original|hCoV-19^^England|Human|University of Exeter|COVID-19 Genomics UK (COG-UK) Consortium|Temperton|United Kingdom
>Spike|hCoV-19/Jordan/SEARCH-5485/2020|2020-09-13|EPI_ISL_755123|Original|hCoV-19^^Amman|Human|Biolab Diagnostic Laboratories|Andersen lab at Scripps Research|Gangavarapu|Jordan
>Spike|hCoV-19/Wales/PHWC-49AF28/2020|2020-12-05|EPI_ISL_742273|Original|hCoV-19^^Wales|Human|Wales Specialist Virology Centre Sequencing lab|COVID-19 Genomics UK (COG-UK) Consortium|Moore|United Kingdom
>Spike|hCoV-19/England/CAMC-B0A5A2/2020|2020-10-29|EPI_ISL_672978|Original|hCoV-19^^England|Human|Lighthouse Lab in Cambridge|Wellcome Sanger Institute for the COVID-19 Genomics UK (COG-UK) Consortium|Davies|United Kingdom
>Spike|hCoV-19/England/LEED-2A99FD/2020|2020-04-10|EPI_ISL_538919|Original|hCoV-19^^England|Human|Leeds Teaching Hospitals NHS Trust and Public Health England|Wellcome Sanger Institute for the COVID-19 Genomics UK (COG-UK) consortium|Davies|United Kingdom
>Spike|hCoV-19/England/MILK-A7BE66/2020|2020-10-15|EPI_ISL_607511|Original|hCoV-19^^England|Human|Lighthouse Lab in Milton Keynes|Wellcome Sanger Institute for the COVID-19 Genomics UK (COG-UK) consortium|Davies|United Kingdom
>Spike|hCoV-19/USA/TX-HMH-MCoV-15833/2020|2020-07-10|EPI_ISL_785517|Original|hCoV-19^^Texas|Human|Houston Methodist Hospital|Houston Methodist Hospital|Long|USA
>Spike|hCoV-19/England/BRIS-122CC7/2020|2020-04-03|EPI_ISL_440775|Original|hCoV-19^^England|Human|PHE South West Regional Laboratory|Wellcome Sanger Institute for the COVID-19 Genomics UK (COG-UK) consortium|Amato|United Kingdom
>Spike|hCoV-19/USA/VI-CDC-1993/2020|2020-04-01|EPI_ISL_436046|Original|hCoV-19^^Virgin Islands|Human|US VI Department of Health|Pathogen Discovery|Queen|USA
>Spike|hCoV-19/Denmark/DCGC-7721/2020|2020-10-19|EPI_ISL_618663|Original|hCoV-19^^Sjaelland|Human|Department of Virus and Microbiological Special Diagnostics|Albertsen lab|Michaelsen|Denmark
>Spike|hCoV-19/England/NORW-EBCAD/2020|2020-05-06|EPI_ISL_457498|Original|hCoV-19^^England|Human|Quadram Institute Bioscience|COVID-19 Genomics UK (COG-UK) Consortium|Alikhan|United Kingdom
>Spike|hCoV-19/Denmark/DCGC-18864/2020|2020-12-07|EPI_ISL_748605|Original|hCoV-19^^Hovedstaden|Human|Department of Virus and Microbiological Special Diagnostics|Albertsen Lab|Michaelsen|Denmark
>Spike|hCoV-19/Australia/VIC13317/2020|2020-07-28|EPI_ISL_563668|Original|hCoV-19^^Victoria|Human|Microbiological Diagnostic Unit - Public Health Laboratory (MDU-PHL)|MDU-PHL|Schultz|Australia
>Spike|hCoV-19/England/ALDP-CF159E/2020|2020-12-18|EPI_ISL_781954|Original|hCoV-19^^England|Human|Lighthouse Lab in Alderley Park|Wellcome Sanger Institute for the COVID-19 Genomics UK (COG-UK) Consortium|Davies|United Kingdom
>Spike|hCoV-19/England/CAMC-9D74C1/2020|2020-09-18|EPI_ISL_584376|Original|hCoV-19^^England|Human|Virology Department|COVID-19 Genomics UK (COG-UK) Consortium|de Silva|United Kingdom
>Spike|hCoV-19/United Arab Emirates/0868/2020|2020-05-19|EPI_ISL_699007|Original|hCoV-19^^Abu Dhabi|Human|Group 42 (G42) Healthcare|G42 Healthcare|Ogrodzki|United Arab Emirates
>Spike|hCoV-19/England/ALDP-A6F3FD/2020|2020-10-15|EPI_ISL_607943|Original|hCoV-19^^England|Human|Lighthouse Lab in Alderley Park|Wellcome Sanger Institute for the COVID-19 Genomics UK (COG-UK) consortium|Davies|United Kingdom
>Spike|hCoV-19/Iceland/89/2020|2020-03-10|EPI_ISL_417738|Original|hCoV-19^^Reykjavik|Human|The National University Hospital of Iceland|deCODE genetics|J\u00f3nsson|Iceland
>Spike|hCoV-19/Wales/PHWC-2B807/2020|2020-04-09|EPI_ISL_445765|Original|hCoV-19^^Wales|Human|Wales Specialist Virology Centre|Public Health Wales Microbiology Cardiff|Moore|United Kingdom
>Spike|hCoV-19/Germany/TH-RKI-NP-0012/2020|2020-12-03|EPI_ISL_763008|Original|hCoV-19^^Thuringia|Human|Unit 17|Project group Epidemiology of Highly Pathogenic Microorganisms|Schubert|Germany

>Spike|hCoV-19/USA/CA-CZB-13922/2020|2020-08-06|EPI_ISL_739572|Original|hCoV-19^^California|Human|Alameda County Public Health Lab|Chan-Zuckerberg Biohub|Kamm|USA
>Spike|hCoV-19/USA/VA-DCLS-1753/2020|2020-09-10|EPI_ISL_644853|Original|hCoV-19^^Virginia|Human|Virginia DCLS|Virginia DCLS|St. Jacques|USA
>Spike|hCoV-19/Japan/PG-0549/2020|2020-04-00|EPI_ISL_684179|Original|hCoV-19^^Japan|Human|Pathogen Genomics Center|Pathogen Genomics Center|Sekizuka|Japan
>Spike|hCoV-19/USA/WI-UW-2183/2020|2020-10-21|EPI_ISL_677587|Original|hCoV-19^^Wisconsin|Human|University of Wisconsin-Madison AIDS Vaccine Research Laboratories|University of Wisconsin-Madison AIDS Vaccine Research Laboratories|Baczenas|USA
>Spike|hCoV-19/England/MILK-A6B203/2020|2020-10-08|EPI_ISL_608820|Original|hCoV-19^^England|Human|Lighthouse Lab in Milton Keynes|Wellcome Sanger Institute for the COVID-19 Genomics UK (COG-UK) consortium|Davies|United Kingdom
>Spike|hCoV-19/England/ALDP-94E841/2020|2020-06-16|EPI_ISL_555048|Original|hCoV-19^^England|Human|Lighthouse Lab in Alderley Park|Wellcome Sanger Institute for the COVID-19 Genomics UK (COG-UK) consortium|Davies|United Kingdom
>Spike|hCoV-19/mink/Netherlands/NB-EMC-64-5/2020|2020-10-14|EPI_ISL_632439|Original|hCoV-19^^Noord Brabant|Neovison vison|Dutch COVID-19 response team|Erasmus Medical Center|Oude Munnink|Netherlands
>Spike|hCoV-19/England/CAMC-A5936F/2020|2020-10-09|EPI_ISL_597164|Original|hCoV-19^^England|Human|Lighthouse Lab in Cambridge|Wellcome Sanger Institute for the COVID-19 Genomics UK (COG-UK) consortium|Davies|United Kingdom
>Spike|hCoV-19/England/ALDP-BB9F68/2020|2020-11-20|EPI_ISL_701887|Original|hCoV-19^^England|Human|Lighthouse Lab in Alderley Park|Wellcome Sanger Institute for the COVID-19 Genomics UK (COG-UK) Consortium|Davies|United Kingdom
>Spike|hCoV-19/USA/TX-DSHS-1608/2020|2020-09-14|EPI_ISL_700734|Original|hCoV-19^^Texas|Human|Texas Department of State Health Services|Texas Department of State Health Services|Rahman|USA
>Spike|hCoV-19/USA/AZ-TG547283/2020|2020-07-02|EPI_ISL_695462|Original|hCoV-19^^Arizona|Human|AZ SPHL|TGen North|French|USA
>Spike|hCoV-19/Wales/PHWC-248C2/2020|2020-03-19|EPI_ISL_419454|Original|hCoV-19^^Wales|Human|Wales Specialist Virology Centre|Public Health Wales Microbiology Cardiff|Moore|United Kingdom
>Spike|hCoV-19/Australia/VIC8385/2020|2020-08-12|EPI_ISL_564975|Original|hCoV-19^^Victoria|Human|Microbiological Diagnostic Unit - Public Health Laboratory (MDU-PHL)|MDU-PHL|Schultz|Australia
>Spike|hCoV-19/USA/WA-S864/2020|2020-04-23|EPI_ISL_463354|Original|hCoV-19^^Washington|Human|Washington State Department of Health|Seattle Flu Study|Bedford|USA
>Spike|hCoV-19/USA/CA-QDX-1442/2020|2020-03-18|EPI_ISL_572093|Original|hCoV-19^^California|Human|Quest Diagnostics|Quest Diagnostics|Kagan|USA
>Spike|hCoV-19/Scotland/QEUH-9E5572/2020|2020-09-24|EPI_ISL_600951|Original|hCoV-19^^Scotland|Human|Lighthouse Lab in Glasgow|Wellcome Sanger Institute for the COVID-19 Genomics UK (COG-UK) consortium|Davies|United Kingdom
>Spike|hCoV-19/England/QEUH-9986D5/2020|2020-08-24|EPI_ISL_540114|Original|hCoV-19^^England|Human|Lighthouse Lab in Glasgow|Wellcome Sanger Institute for the COVID-19 Genomics UK (COG-UK) consortium|Davies|United Kingdom
>Spike|hCoV-19/USA/FL-BPHL-1310/2020|2020-07-06|EPI_ISL_541248|Original|hCoV-19^^Florida|Human|Florida Bureau of Public Health Laboratories|Florida Bureau of Public Health Laboratories|Blanton|USA
>Spike|hCoV-19/England/MILK-9D52E7/2020|2020-09-18|EPI_ISL_637494|Original|hCoV-19^^England|Human|Centre for Enzyme Innovation|COVID-19 Genomics UK (COG-UK) Consortium|Robson|United Kingdom
>Spike|hCoV-19/England/BRIS-128EBC/2020|2020-04-19|EPI_ISL_458847|Original|hCoV-19^^England|Human|PHE South West Regional Laboratory|Wellcome Sanger Institute for the COVID-19 Genomics UK (COG-UK) consortium|Davies|United Kingdom
>Spike|hCoV-19/Wales/PHWC-162979/2020|2020-05-13|EPI_ISL_473145|Original|hCoV-19^^Wales|Human|Wales Specialist Virology Centre Sequencing lab|COVID-19 Genomics UK (COG-UK) Consortium|Moore|United Kingdom

>Spike|hCoV-19/Spain/VC-IBV-98003581/2020|2020-03-31|EPI_ISL_537994|Original|hCoV-19^^Comunitat Valenciana|Human|Servicio de Microbiolog\u00eda. Hospital General Universitario de Castell\u00f3n|SeqCOVID-SPAIN consortium/IBV(CSIC)|Comas|Spain
>Spike|hCoV-19/Russia/STA-RII-MH2787S/2020|2020-09-19|EPI_ISL_602452|Original|hCoV-19^^Stavropol|Human|HELIX LLC|WHO National Influenza Centre Russian Federation|Komissarov|Russia
>Spike|hCoV-19/Brazil/SP-218/2020|2020-04-22|EPI_ISL_527865|Original|hCoV-19^^S\u00e3o Paulo|Human|Hospital e Maternidade S\u00e3o Crist\u00f3v\u00e3o|Instituto Adolfo Lutz|Sacchi|Brazil
>Spike|hCoV-19/Russia/MOW-RII-MH2890S/2020|2020-09-22|EPI_ISL_733057|Original|hCoV-19^^Moscow|Human|HELIX LLC|WHO National Influenza Centre Russian Federation|Komissarov|Russia
>Spike|hCoV-19/USA/CA-IGI-0765/2020|2020-07-31|EPI_ISL_677886|Original|hCoV-19^^California|Human|Innovative Genomics Institute|Innovative Genomics Institute|Wyman|USA
>Spike|hCoV-19/Sweden/20-53340/2020|2020-11-01|EPI_ISL_710598|Original|hCoV-19^^Halland|Human|Klinisk mikrobiologi|The Public Health Agency of Sweden|Svartstr\u00f6m|Sweden
>Spike|hCoV-19/Colombia/ANT-GUV-92252/2020|2020-04-01|EPI_ISL_447767|Original|hCoV-19^^Antioquia|Human|Instituto Nacional de Salud|Grupo de Investigaciones Microbiol\u00f3gicas-UR (GIMUR)|Ram\u00edrez|Colombia
>Spike|hCoV-19/South Africa/Tygerberg_37/2020|2020-04-08|EPI_ISL_464148|Original|hCoV-19^^Western Cape|Human|National Health Laboratory Service (NHLS)|Division of Medical Virology|Engelbrecht|South Africa
>Spike|hCoV-19/England/CAMC-A80D52/2020|2020-10-13|EPI_ISL_610712|Original|hCoV-19^^England|Human|Lighthouse Lab in Cambridge|Wellcome Sanger Institute for the COVID-19 Genomics UK (COG-UK) consortium|Davies|United Kingdom
>Spike|hCoV-19/Japan/IC-0249/2020|2020-10-00|EPI_ISL_667678|Original|hCoV-19^^Japan|Human|Pathogen Genomics Center|Pathogen Genomics Center|Sekizuka|Japan
>Spike|hCoV-19/USA/OR-OHSU-1277/2020|2020-07-14|EPI_ISL_509177|Original|hCoV-19^^Oregon|Human|OHSU Lab Services Molecular Microbiology Lab|Oregon SARS-CoV-2 Genome Sequencing Center|O'Roak|USA
>Spike|hCoV-19/Peru/LIM-UPCH-0117/2020|2020-06-09|EPI_ISL_568514|Original|hCoV-19^^Lima|Human|Laboratorio de Referencia Nacional de Virus Respiratorios|Laboratorio de Gen\u00f3mica Microbiana|Tsukayama|Peru
>Spike|hCoV-19/Jordan/AM-ALSR-5163/2020|2020-10-16|EPI_ISL_730402|Original|hCoV-19^^Amman|Human|Biolab Diagnostic Laboratories|Andersen lab at Scripps Research|Gangavarapu|Jordan
>Spike|hCoV-19/Portugal/PT0274/2020|2020-04-05|EPI_ISL_453988|Original|hCoV-19^^Portugal|Human|unknown|Instituto Nacional de Saude (INSA)|Borges|Portugal
>Spike|hCoV-19/England/ALDP-9E6D88/2020|2020-09-24|EPI_ISL_580289|Original|hCoV-19^^England|Human|Lighthouse Lab in Alderley Park|Wellcome Sanger Institute for the COVID-19 Genomics UK (COG-UK) consortium|Davies|United Kingdom
>Spike|hCoV-19/Northern Ireland/NIRE-FD024/2020|2020-04-24|EPI_ISL_489166|Original|hCoV-19^^Northern Ireland|Human|Regional Virus Laboratory|Wellcome Sanger Institute for the COVID-19 Genomics UK (COG-UK) consortium|Davies|United Kingdom
>Spike|hCoV-19/England/ALDP-9E7112/2020|2020-09-24|EPI_ISL_580372|Original|hCoV-19^^England|Human|Lighthouse Lab in Alderley Park|Wellcome Sanger Institute for the COVID-19 Genomics UK (COG-UK) consortium|Davies|United Kingdom
>Spike|hCoV-19/Luxembourg/LNS5936010/2020|2020-11-14|EPI_ISL_744326|Original|hCoV-19^^Luxembourg|Human|Laboratoire national de sant\u00e9|Laboratoire national de sant\u00e9|Wienecke-Baldacchino|Luxembourg
>Spike|hCoV-19/England/BRIS-1264AD/2020|2020-04-10|EPI_ISL_459057|Original|hCoV-19^^England|Human|PHE South West Regional Laboratory|Wellcome Sanger Institute for the COVID-19 Genomics UK (COG-UK) consortium|Davies|United Kingdom
>Spike|hCoV-19/Germany/BY-MVP-V2012612/2020|2020-03-19|EPI_ISL_420908|Original|hCoV-19^^Bavaria|Human|Max von Pettenkofer Institute|Laboratory for Functional Genome Analysis|Graf|Germany

>Spike|hCoV-19/Belgium/rega-0804726/2020|2020-08-04|EPI_ISL_734907|Original|hCoV-19^^Deurne|Human|UZ Leuven|KU Leuven|Maes|Belgium
>Spike|hCoV-19/England/204590738/2020|2020-11-06|EPI_ISL_700672|Original|hCoV-19^^England|Human|Respiratory Virus Unit|COVID-19 Genomics UK (COG-UK) Consortium|Platt|United Kingdom
>Spike|hCoV-19/Japan/PG-3423/2020|2020-04-00|EPI_ISL_691535|Original|hCoV-19^^Japan|Human|Sakai City Institute of Public Health|Pathogen Genomics Center|Sekizuka|Japan
>Spike|hCoV-19/Northern Ireland/NIRE-1017EB/2020|2020-05-09|EPI_ISL_489286|Original|hCoV-19^^Northern Ireland|Human|Regional Virus Laboratory|Wellcome Sanger Institute for the COVID-19 Genomics UK (COG-UK) consortium|Davies|United Kingdom
>Spike|hCoV-19/England/MILK-9584E6/2020|2020-06-11|EPI_ISL_554858|Original|hCoV-19^^England|Human|Lighthouse Lab in Milton Keynes|Wellcome Sanger Institute for the COVID-19 Genomics UK (COG-UK) consortium|Davies|United Kingdom
>Spike|hCoV-19/Denmark/DCGC-16390/2020|2020-11-16|EPI_ISL_714177|Original|hCoV-19^^Syddanmark|Human|Department of Virus and Microbiological Special Diagnostics|Albertsen Lab|Michaelsen|Denmark
>Spike|hCoV-19/England/QEUH-AA0545/2020|2020-10-19|EPI_ISL_611350|Original|hCoV-19^^England|Human|Lighthouse Lab in Glasgow|Wellcome Sanger Institute for the COVID-19 Genomics UK (COG-UK) consortium|Davies|United Kingdom
>Spike|hCoV-19/England/LOND-D44CD/2020|2020-04-08|EPI_ISL_444129|Original|hCoV-19^^England|Human|University College London|COVID-19 Genomics UK (COG-UK) Consortium|Roy|United Kingdom
>Spike|hCoV-19/England/ALDP-9BE6D9/2020|2020-09-09|EPI_ISL_567963|Original|hCoV-19^^England|Human|Lighthouse Lab in Alderley Park|Wellcome Sanger Institute for the COVID-19 Genomics UK (COG-UK) consortium|Davies|United Kingdom
>Spike|hCoV-19/Switzerland/TI-ETHZ-100098/2020|2020-03-13|EPI_ISL_451690|Original|hCoV-19^^Ticino|Human|Viollier AG|Department of Biosystems Science and Engineering|Nadeau|Switzerland
>Spike|hCoV-19/Spain/MD-IBV-99012315/2020|2020-11-17|EPI_ISL_779975|Original|hCoV-19^^Madrid|Human|Hospital General Universitario Gregorio Mara\u00f1\u00f3n|SeqCOVID-SPAIN consortium/IBV(CSIC)|Comas|Spain
>Spike|hCoV-19/Sweden/20-50486/2020|2020-03-26|EPI_ISL_475143|Original|hCoV-19^^Vasterbotten|Human|Umea klinisk mikrobiologi|The Public Health Agency of Sweden|Svartstr\u00f6m|Sweden
>Spike|hCoV-19/USA/LA-EVTL241/2020|2020-08-22|EPI_ISL_574375|Original|hCoV-19^^Louisiana|Human|LSUHS Emerging Viral Threat Laboratory|Microbial Genome Sequencing Center|Kamil|USA
>Spike|hCoV-19/Hungary/SRC-00175/2020|2020-03-22|EPI_ISL_435406|Original|hCoV-19^^Baranya|Human|Virological Research Group|Bioinformatics Research Group|Herczeg|Hungary
>Spike|hCoV-19/England/CAMB-1AFC95/2020|2020-05-13|EPI_ISL_452872|Original|hCoV-19^^England|Human|Department of Pathology|COVID-19 Genomics UK (COG-UK) Consortium|Meredith|United Kingdom
>Spike|hCoV-19/England/MILK-9C3A8A/2020|2020-09-09|EPI_ISL_566962|Original|hCoV-19^^England|Human|Lighthouse Lab in Milton Keynes|Wellcome Sanger Institute for the COVID-19 Genomics UK (COG-UK) consortium|Davies|United Kingdom
>Spike|hCoV-19/England/MILK-A6AD5E/2020|2020-10-08|EPI_ISL_608787|Original|hCoV-19^^England|Human|Lighthouse Lab in Milton Keynes|Wellcome Sanger Institute for the COVID-19 Genomics UK (COG-UK) consortium|Davies|United Kingdom
>Spike|hCoV-19/Netherlands/NB-EMC-262/2020|2020-06-15|EPI_ISL_523385|Original|hCoV-19^^Noord Brabant|Human|Dutch COVID-19 response team|Erasmus Medical Center|Oude Munnink|Netherlands
>Spike|hCoV-19/USA/PR-B6BV/2020|2020-08-11|EPI_ISL_527394|Original|hCoV-19^^Puerto Rico|Human|University of Miami Immunology and Histocompatibility Laboratory|University of Miami Immunology and Histocompatibility Laboratory|Margolles-Clark|USA

>Spike|hCoV-19/Lithuania/MR-LUHS-Eilnr235905/2020|2020-12-18|EPI_ISL_770504|Original|hCoV-19^^Kauno apskritis|Human|Lithuanian University of Health Sciences Hospital|Lithuanian University of Health Sciences|Zemaitis|Lithuania
>Spike|hCoV-19/Wales/PHWC-49AEA3/2020|2020-12-07|EPI_ISL_742266|Original|hCoV-19^^Wales|Human|Wales Specialist Virology Centre Sequencing lab|COVID-19 Genomics UK (COG-UK) Consortium|Moore|United Kingdom
>Spike|hCoV-19/USA/TX-HMH-MCoV-18659/2020|2020-11-17|EPI_ISL_785007|Original|hCoV-19^^Texas|Human|Houston Methodist Hospital|Houston Methodist Hospital|Long|USA
>Spike|hCoV-19/England/ALDP-B24690/2020|2020-11-04|EPI_ISL_646844|Original|hCoV-19^^England|Human|Lighthouse Lab in Alderley Park|Wellcome Sanger Institute for the COVID-19 Genomics UK (COG-UK) Consortium|Davies|United Kingdom
>Spike|hCoV-19/USA/TX-HMH-MCoV-16320/2020|2020-11-02|EPI_ISL_786004|Original|hCoV-19^^Texas|Human|Houston Methodist Hospital|Houston Methodist Hospital|Long|USA
>Spike|hCoV-19/USA/MI-MDHHS-SC22043/2020|2020-09-16|EPI_ISL_577593|Original|hCoV-19^^Michigan|Human|Michigan Department of Health and Human Services|Michigan Department of Health and Human Services|Jacob|USA
>Spike|hCoV-19/Scotland/EDB4168/2020|2020-04-20|EPI_ISL_456930|Original|hCoV-19^^Scotland|Human|Virology Department|COVID-19 Genomics UK (COG-UK) Consortium|Williams|United Kingdom
>Spike|hCoV-19/USA/WA-UW-22900/2020|2020-08-06|EPI_ISL_570732|Original|hCoV-19^^Washington|Human|UW Virology Lab|UW Virology Lab|Roychoudhury|USA
>Spike|hCoV-19/England/CAMB-1B0244/2020|2020-04-30|EPI_ISL_459259|Original|hCoV-19^^England|Human|Department of Pathology|Wellcome Sanger Institute for the COVID-19 Genomics UK (COG-UK) consortium|Davies|United Kingdom
>Spike|hCoV-19/England/205141159/2020|2020-12-16|EPI_ISL_756135|Original|hCoV-19^^England|Human|Respiratory Virus Unit|COVID-19 Genomics UK (COG-UK) Consortium|Miah|United Kingdom
>Spike|hCoV-19/Australia/VIC4380/2020|2020-07-14|EPI_ISL_519676|Original|hCoV-19^^Victoria|Human|Microbiological Diagnostic Unit - Public Health Laboratory (MDU-PHL)|MDU-PHL|Schultz|Australia
>Spike|hCoV-19/Luxembourg/LNS0972408/2020|2020-10-28|EPI_ISL_739866|Original|hCoV-19^^Luxembourg|Human|Laboratoire national de sant\u00e9|Laboratoire national de sant\u00e9|Wienecke-Baldacchino|Luxembourg
>Spike|hCoV-19/England/NOTT-121804/2020|2020-12-21|EPI_ISL_763819|Original|hCoV-19^^England|Human|Queens Medical Centre|COVID-19 Genomics UK (COG-UK) Consortium|Loose|United Kingdom
>Spike|hCoV-19/Denmark/DCGC-9764/2020|2020-11-09|EPI_ISL_669157|Original|hCoV-19^^Sjaelland|Human|Department of Virus and Microbiological Special Diagnostics|Albertsen Lab|Michaelsen|Denmark
>Spike|hCoV-19/England/MILK-ACBD00/2020|2020-10-20|EPI_ISL_629117|Original|hCoV-19^^England|Human|Lighthouse Lab in Milton Keynes|Wellcome Sanger Institute for the COVID-19 Genomics UK (COG-UK) consortium|Davies|United Kingdom
>Spike|hCoV-19/Japan/PG-6727/2020|2020-08-00|EPI_ISL_687838|Original|hCoV-19^^Japan|Human|Pathogen Genomics Center|Pathogen Genomics Center|Sekizuka|Japan
>Spike|hCoV-19/Denmark/DCGC-7047/2020|2020-10-12|EPI_ISL_619896|Original|hCoV-19^^Syddanmark|Human|Department of Virus and Microbiological Special Diagnostics|Albertsen lab|Michaelsen|Denmark
>Spike|hCoV-19/Wales/ALDP-9C5772/2020|2020-09-10|EPI_ISL_567782|Original|hCoV-19^^Wales|Human|Lighthouse Lab in Alderley Park|Wellcome Sanger Institute for the COVID-19 Genomics UK (COG-UK) consortium|Davies|United Kingdom
>Spike|hCoV-19/USA/TX-HMH-2591/2020|2020-06-02|EPI_ISL_545414|Original|hCoV-19^^Texas|Human|Houston Methodist Hospital|Houston Methodist Hospital|Long|USA
>Spike|hCoV-19/Wales/ALDP-C3AEBC/2020|2020-12-08|EPI_ISL_767202|Original|hCoV-19^^Wales|Human|Lighthouse Lab in Alderley Park|Wellcome Sanger Institute for the COVID-19 Genomics UK (COG-UK) Consortium|Davies|United Kingdom
>Spike|hCoV-19/Japan/PG-2655/2020|2020-04-00|EPI_ISL_690787|Original|hCoV-19^^Japan|Human|Pathogen Genomics Center|Pathogen Genomics Center|Sekizuka|Japan
>Spike|hCoV-19/England/QEUH-C5D8F1/2020|2020-12-14|EPI_ISL_769360|Original|hCoV-19^^England|Human|Lighthouse Lab in Glasgow|Wellcome Sanger Institute for the COVID-19 Genomics UK (COG-UK) Consortium|Davies|United Kingdom

>Spike|hCoV-19/USA/MN-MDH-2231/2020|2020-12-02|EPI_ISL_770805|Original|hCoV-19^^Minnesota|Human|Vault Health|Minnesota Department of Health|Lorentz|USA
>Spike|hCoV-19/England/CAMB-1B2488/2020|2020-05-15|EPI_ISL_456682|Original|hCoV-19^^England|Human|Department of Pathology|COVID-19 Genomics UK (COG-UK) Consortium|Meredith|United Kingdom
>Spike|hCoV-19/Russia/Sverdlovsk-71905/2020|2020-03-19|EPI_ISL_428879|Vero|hCoV-19^^Sverdlovsk|Human|State Research Center of Virology and Biotechnology VECTOR|State Research Center of Virology and Biotechnology VECTOR|Shvalov|Russia
>Spike|hCoV-19/USA/TX-HMH-4353/2020|2020-06-17|EPI_ISL_543554|Original|hCoV-19^^Texas|Human|Houston Methodist Hospital|Houston Methodist Hospital|Long|USA
>Spike|hCoV-19/Scotland/QEUH-A4C569/2020|2020-10-07|EPI_ISL_595977|Original|hCoV-19^^Scotland|Human|Quadram Institute Bioscience|COVID-19 Genomics UK (COG-UK) Consortium|Alikhan|United Kingdom
>Spike|hCoV-19/Australia/VIC15150/2020|2020-08-27|EPI_ISL_593160|Original|hCoV-19^^Victoria|Human|Microbiological Diagnostic Unit - Public Health Laboratory (MDU-PHL)|MDU-PHL|Schultz|Australia
>Spike|hCoV-19/Canada/ON-S1591/2020|2020-09-04|EPI_ISL_755932|Original|hCoV-19^^Ontario|Human|Toronto Invasive Bacterial Diseases Network|McMaster University|McArthur|Canada
>Spike|hCoV-19/Switzerland/BS-42341001/2020|2020-07-06|EPI_ISL_581897|Original|hCoV-19^^Basel-Stadt|Human|University Hospital Basel|University Hospital Basel|Roloff Handschin|Switzerland
>Spike|hCoV-19/Denmark/DCGC-13546/2020|2020-11-30|EPI_ISL_712239|Original|hCoV-19^^Midtjylland|Human|Department of Virus and Microbiological Special Diagnostics|Albertsen Lab|Michaelsen|Denmark
>Spike|hCoV-19/Switzerland/BL-42209561/2020|2020-03-27|EPI_ISL_581798|Original|hCoV-19^^Basel-Land|Human|University Hospital Basel|University Hospital Basel|Roloff Handschin|Switzerland
>Spike|hCoV-19/USA/CA-CZB-11914/2020|2020-10-04|EPI_ISL_672289|Original|hCoV-19^^California|Human|Santa Clara County Public Health Laboratory|Chan-Zuckerberg Biohub|Bhatt|USA
>Spike|hCoV-19/USA/TX-HMH-7967/2020|2020-06-29|EPI_ISL_545749|Original|hCoV-19^^Texas|Human|Houston Methodist Hospital|Houston Methodist Hospital|Long|USA
>Spike|hCoV-19/England/ALDP-9CE032/2020|2020-09-16|EPI_ISL_566286|Original|hCoV-19^^England|Human|Lighthouse Lab in Alderley Park|Wellcome Sanger Institute for the COVID-19 Genomics UK (COG-UK) consortium|Davies|United Kingdom
>Spike|hCoV-19/Australia/VIC9382/2020|2020-08-17|EPI_ISL_565533|Original|hCoV-19^^Victoria|Human|Microbiological Diagnostic Unit - Public Health Laboratory (MDU-PHL)|MDU-PHL|Schultz|Australia
>Spike|hCoV-19/Canada/MB_8/2020|2020-03-12|EPI_ISL_418812|Original|hCoV-19^^Manitoba|Human|Cadham Provincial Laboratory|National Microbiology Laboratory|Majer|Canada
>Spike|hCoV-19/England/CAMC-C80AA4/2020|2020-12-17|EPI_ISL_762717|Original|hCoV-19^^England|Human|Lighthouse Lab in Cambridge|Wellcome Sanger Institute for the COVID-19 Genomics UK (COG-UK) Consortium|Davies|United Kingdom
>Spike|hCoV-19/England/ALDP-A667CD/2020|2020-10-12|EPI_ISL_630620|Original|hCoV-19^^England|Human|Lighthouse Lab in Alderley Park|Wellcome Sanger Institute for the COVID-19 Genomics UK (COG-UK) consortium|Davies|United Kingdom
>Spike|hCoV-19/England/QEUH-9B8EC8/2020|2020-09-06|EPI_ISL_602142|Original|hCoV-19^^England|Human|Lighthouse Lab in Glasgow|Wellcome Sanger Institute for the COVID-19 Genomics UK (COG-UK) consortium|Davies|United Kingdom
>Spike|hCoV-19/Belgium/ULG-10570/2020|2020-10-23|EPI_ISL_626316|Original|hCoV-19^^Li\u00e8ge|Human|Department of Clinical Microbiology|GIGA Medical Genomics|Durkin|Belgium
>Spike|hCoV-19/England/ALDP-B23C35/2020|2020-11-04|EPI_ISL_646429|Original|hCoV-19^^England|Human|Lighthouse Lab in Alderley Park|Wellcome Sanger Institute for the COVID-19 Genomics UK (COG-UK) Consortium|Davies|United Kingdom
>Spike|hCoV-19/Iceland/312/2020|2020-03-18|EPI_ISL_417641|Original|hCoV-19^^Reykjavik|Human|The National University Hospital of Iceland|deCODE genetics|J\u00f3nsson|Iceland

>Spike|hCoV-19/Singapore/447/2020|2020-04-15|EPI_ISL_475978|Original|hCoV-
19^^Singapore|Human|National Public Health Laboratory|National Public Health
Laboratory|Mak|Singapore
>Spike|hCoV-19/Norway/4601/2020|2020-10-14|EPI_ISL_775478|Original|hCoV-19^^Vestfold
og Telemark|Human|Unilabs Laboratory Medicine|Norwegian Institute of Public
Health|Vollan|Norway
>Spike|hCoV-19/USA/CT-Yale-098/2020|2020-04-02|EPI_ISL_428749|Original|hCoV-
19^^Connecticut|Human|Yale COVID-19 Biorepository|Grubaugh Lab - Yale School of
Public Health|Grubaugh|USA
>Spike|hCoV-19/England/QEUH-C31204/2020|2020-12-10|EPI_ISL_731429|Original|hCoV-
19^^England|Human|Lighthouse Lab in Glasgow|Wellcome Sanger Institute for the COVID-
19 Genomics UK (COG-UK) Consortium|Davies|United Kingdom
>Spike|hCoV-19/Japan/IC-0007/2020|2020-06-00|EPI_ISL_591353|Original|hCoV-
19^^Japan|Human|Pathogen Genomics Center|Pathogen Genomics Center|Sekizuka|Japan
>Spike|hCoV-19/SaudiArabia/KAUST-JEDDAH1018/2020|2020-06-
17|EPI_ISL_512889|Original|hCoV-19^^Jeddah|Human|Pathogen Genomics Lab King Abdullah
University of Science and Technology(KAUST)|Pathogen Genomics Lab King Abdullah
University of Science and Technology(KAUST)|Mohamed Ghazzali|Saudi Arabia
>Spike|hCoV-19/England/20186006704/2020|2020-04-29|EPI_ISL_465466|Original|hCoV-
19^^England|Human|Respiratory Virus Unit|Respiratory Virus Unit|Platt|United Kingdom
>Spike|hCoV-19/USA/UT-UPHL-201113557/2020|2020-04-23|EPI_ISL_634901|Original|hCoV-
19^^Utah|Human|Utah Public Health Laboratory|Utah Public Health Laboratory|Young|USA
>Spike|hCoV-19/England/ALDP-B5B42D/2020|2020-11-08|EPI_ISL_655488|Original|hCoV-
19^^England|Human|Lighthouse Lab in Alderley Park|Wellcome Sanger Institute for the
COVID-19 Genomics UK (COG-UK) Consortium|Davies|United Kingdom
>Spike|hCoV-19/Scotland/EDB3631/2020|2020-04-27|EPI_ISL_439301|Original|hCoV-
19^^Scotland|Human|Virology Department|COVID-19 Genomics UK (COG-UK)
Consortium|Williams|United Kingdom
>Spike|hCoV-19/USA/TX-HMH-7250/2020|2020-06-29|EPI_ISL_546626|Original|hCoV-
19^^Texas|Human|Houston Methodist Hospital|Houston Methodist Hospital|Long|USA
>Spike|hCoV-19/Scotland/QEUH-A369DF/2020|2020-10-04|EPI_ISL_638932|Original|hCoV-
19^^Scotland|Human|Wales Specialist Virology Centre Sequencing lab|COVID-19 Genomics
UK (COG-UK) Consortium|Moore|United Kingdom
>Spike|hCoV-19/England/NOTT-110C35/2020|2020-05-01|EPI_ISL_448396|Original|hCoV-
19^^England|Human|Queens Medical Centre|COVID-19 Genomics UK (COG-UK)
Consortium|Loose|United Kingdom
>Spike|hCoV-19/USA/CA-CZB-2413/2020|2020-05-22|EPI_ISL_513835|Original|hCoV-
19^^California|Human|Orange County Public Health Laboratory|Chan-Zuckerberg
Biohub|Kamm|USA
>Spike|hCoV-19/USA/AZ-TG280882/2020|2020-04-23|EPI_ISL_694211|Original|hCoV-
19^^Arizona|Human|AZ SPHL|TGen North|French|USA
>Spike|hCoV-19/Japan/PG-6696/2020|2020-07-00|EPI_ISL_687807|Original|hCoV-
19^^Japan|Human|Pathogen Genomics Center|Pathogen Genomics Center|Sekizuka|Japan
>Spike|hCoV-19/India/MH-ACTREC-174/2020|2020-06-13|EPI_ISL_699829|Original|hCoV-
19^^Maharashtra|Human|Hematopathology Laboratory|Hematopathology
Laboratory|Patkar|India
>Spike|hCoV-19/USA/MA-MGH-01504/2020|2020-05-07|EPI_ISL_791961|Original|hCoV-
19^^Massachusetts|Human|Massachusetts General Hospital|Infectious Disease
Program|Park|USA
>Spike|hCoV-19/Spain/CL-IBV-97006162/2020|2020-04-05|EPI_ISL_537309|Original|hCoV-
19^^Castilla y Leon|Human|Universidad de Le\u00f3n|SeqCOVID-SPAIN
consortium/IBV(CSIC)|Comas|Spain
>Spike|hCoV-19/England/ALDP-955373/2020|2020-06-12|EPI_ISL_553427|Original|hCoV-
19^^England|Human|Lighthouse Lab in Alderley Park|Wellcome Sanger Institute for the
COVID-19 Genomics UK (COG-UK) consortium|Davies|United Kingdom
>Spike|hCoV-19/USA/CA-ALSR-0682-SAN/2020|2020-03-28|EPI_ISL_483299|Original|hCoV-
19^^California|Human|UC San Diego Center for Advanced Laboratory Medicine|Andersen
lab at Scripps Research|Gangavarapu|USA

>Spike|hCoV-19/England/ALDP-66DEF2/2020|2020-07-02|EPI_ISL_558050|Original|hCoV-19^^England|Human|Lighthouse Lab in Alderley Park|Wellcome Sanger Institute for the COVID-19 Genomics UK (COG-UK) consortium|Davies|United Kingdom
>Spike|hCoV-19/Australia/VIC15225/2020|2020-08-03|EPI_ISL_593202|Original|hCoV-19^^Victoria|Human|Microbiological Diagnostic Unit - Public Health Laboratory (MDU-PHL)|MDU-PHL|Schultz|Australia
>Spike|hCoV-19/Singapore/846/2020|2020-07-12|EPI_ISL_518010|Original|hCoV-19^^Singapore|Human|Singapore General Hospital|Department of Microbiology|Abdul Rahman|Singapore
>Spike|hCoV-19/England/ALDP-AC0F8D/2020|2020-10-08|EPI_ISL_649312|Original|hCoV-19^^England|Human|Lighthouse Lab in Alderley Park|Wellcome Sanger Institute for the COVID-19 Genomics UK (COG-UK) Consortium|Davies|United Kingdom
>Spike|hCoV-19/Netherlands/NoordHolland_7/2020|2020-04-01|EPI_ISL_460796|Original|hCoV-19^^Noord Holland|Human|Dutch COVID-19 response team|Erasmus Medical Center|Oude Munnink|Netherlands
>Spike|hCoV-19/Japan/Donner93/2020|2020-08-20|EPI_ISL_644997|Original|hCoV-19^^Kanto|Human|Department of Infectious Diseases|Center for Medical Genetics|Kosaki|Japan
>Spike|hCoV-19/England/MILK-B88A9E/2020|2020-11-14|EPI_ISL_675940|Original|hCoV-19^^England|Human|Lighthouse Lab in Milton Keynes|Wellcome Sanger Institute for the COVID-19 Genomics UK (COG-UK) Consortium|Davies|United Kingdom
>Spike|hCoV-19/England/CAMB-71D9A/2020|2020-03-30|EPI_ISL_438300|Original|hCoV-19^^England|Human|Department of Pathology|Wellcome Sanger Institute for the COVID-19 Genomics UK (COG-UK) consortium|Amato|United Kingdom
>Spike|hCoV-19/England/ALDP-B5D986/2020|2020-11-09|EPI_ISL_704452|Original|hCoV-19^^England|Human|Department of Pathology|COVID-19 Genomics UK (COG-UK) Consortium|Goodfellow|United Kingdom
>Spike|hCoV-19/England/ALDP-950D2F/2020|2020-06-14|EPI_ISL_554566|Original|hCoV-19^^England|Human|Lighthouse Lab in Alderley Park|Wellcome Sanger Institute for the COVID-19 Genomics UK (COG-UK) consortium|Davies|United Kingdom
>Spike|hCoV-19/USA/MA-MGH-00705/2020|2020-04-06|EPI_ISL_460293|Original|hCoV-19^^Massachusetts|Human|Massachusetts General Hospital|Infectious Disease Program|Lemieux|USA
>Spike|hCoV-19/England/ALDP-955BB7/2020|2020-06-12|EPI_ISL_555489|Original|hCoV-19^^England|Human|Lighthouse Lab in Alderley Park|Wellcome Sanger Institute for the COVID-19 Genomics UK (COG-UK) consortium|Davies|United Kingdom
>Spike|hCoV-19/Spain/RI-IBV-005934/2020|2020-03-20|EPI_ISL_500370|Original|hCoV-19^^La Rioja|Human|Centro de Investigaci\u00f3n Biom\u00e9dica de La Rioja - Hospital San Pedro Logro\u00f1o|SeqCOVID-SPAIN consortium/IBV(CSIC)|Comas|Spain
>Spike|hCoV-19/Australia/VIC17056/2020|2020-08-04|EPI_ISL_663710|Original|hCoV-19^^Victoria|Human|Microbiological Diagnostic Unit - Public Health Laboratory (MDU-PHL)|MDU-PHL|Schultz|Australia
>Spike|hCoV-19/England/ALDP-B626E1/2020|2020-11-08|EPI_ISL_656436|Original|hCoV-19^^England|Human|Lighthouse Lab in Alderley Park|Wellcome Sanger Institute for the COVID-19 Genomics UK (COG-UK) Consortium|Davies|United Kingdom
>Spike|hCoV-19/England/LIVE-DC11FC/2020|2020-11-01|EPI_ISL_664354|Original|hCoV-19^^England|Human|Liverpool Clinical Laboratories|COVID-19 Genomics UK (COG-UK) Consortium|Haldenby|United Kingdom
>Spike|hCoV-19/USA/TX-HMH-4628/2020|2020-06-22|EPI_ISL_543765|Original|hCoV-19^^Texas|Human|Houston Methodist Hospital|Houston Methodist Hospital|Long|USA
>Spike|hCoV-19/Finland/8May30S7/2020|2020-05-08|EPI_ISL_481723|Original|hCoV-19^^Uusimaa|Human|Department of Virology and Immunology|Department of Virology|Smura|Finland
>Spike|hCoV-19/Wales/PHWC-368BE/2020|2020-04-28|EPI_ISL_474725|Original|hCoV-19^^Wales|Human|Originating lab|COVID-19 Genomics UK (COG-UK) Consortium|Moore|United Kingdom
>Spike|hCoV-19/Denmark/DCGC-20254/2020|2020-11-30|EPI_ISL_748077|Original|hCoV-19^^Sjaelland|Human|Department of Virus and Microbiological Special Diagnostics|Albertsen Lab|Michaelsen|Denmark

>Spike|hCoV-19/Australia/VIC11436/2020|2020-08-22|EPI_ISL_562643|Original|hCoV-19^^Victoria|Human|Microbiological Diagnostic Unit - Public Health Laboratory (MDU-PHL)|MDU-PHL|Schultz|Australia
>Spike|hCoV-19/USA/NY-Wadsworth-290635-01/2020|2020-12-24|EPI_ISL_767536|Original|hCoV-19^^New York|Human|Wadsworth Center|Wadsworth Center|Lasek-Nesselquist|USA
>Spike|hCoV-19/England/SHEF-C5B8E/2020|2020-11-24|EPI_ISL_680142|Original|hCoV-19^^England|Human|Virology Department|COVID-19 Genomics UK (COG-UK) Consortium|de Silva|United Kingdom
>Spike|hCoV-19/England/ALDP-94C755/2020|2020-06-15|EPI_ISL_556061|Original|hCoV-19^^England|Human|Lighthouse Lab in Alderley Park|Wellcome Sanger Institute for the COVID-19 Genomics UK (COG-UK) consortium|Davies|United Kingdom
>Spike|hCoV-19/Wales/PHWC-16667E/2020|2020-06-23|EPI_ISL_494027|Original|hCoV-19^^Wales|Human|Originating lab|COVID-19 Genomics UK (COG-UK) Consortium|Moore|United Kingdom
>Spike|hCoV-19/England/20189054101/2020|2020-04-30|EPI_ISL_465535|Original|hCoV-19^^England|Human|Respiratory Virus Unit|Respiratory Virus Unit|Platt|United Kingdom
>Spike|hCoV-19/England/QEUH-9DCA7F/2020|2020-09-21|EPI_ISL_580947|Original|hCoV-19^^England|Human|Lighthouse Lab in Glasgow|Wellcome Sanger Institute for the COVID-19 Genomics UK (COG-UK) consortium|Davies|United Kingdom
>Spike|hCoV-19/Wales/PHWC-490168/2020|2020-11-05|EPI_ISL_706450|Original|hCoV-19^^Wales|Human|Originating lab|COVID-19 Genomics UK (COG-UK) Consortium|Moore|United Kingdom
>Spike|hCoV-19/United Arab Emirates/0419/2020|2020-05-15|EPI_ISL_698594|Original|hCoV-19^^Abu Dhabi|Human|Group 42 (G42) Healthcare|G42 Healthcare|Ogrodzki|United Arab Emirates
>Spike|hCoV-19/England/QEUH-9DE4BB/2020|2020-09-21|EPI_ISL_599727|Original|hCoV-19^^England|Human|Lighthouse Lab in Glasgow|Wellcome Sanger Institute for the COVID-19 Genomics UK (COG-UK) consortium|Davies|United Kingdom
>Spike|hCoV-19/Japan/PG-0055/2020|2020-03-07|EPI_ISL_479818|Original|hCoV-19^^Hokkaido|Human|Hokkaido Institute of Public Health|Pathogen Genomics Center|Sekizuka|Japan
>Spike|hCoV-19/Spain/VC-FISABIO-236/2020|2020-03-25|EPI_ISL_436331|Original|hCoV-19^^Comunitat Valenciana|Human|Servicio de Microbiologia. Hospital Clinico Universitario de Valencia|Sequencing and Bioinformatics Service and Molecular Epidemiology Research Group. FISABIO-Public Health|Gonzalez-Candelas|Spain
>Spike|hCoV-19/Denmark/DCGC-9647/2020|2020-11-09|EPI_ISL_669086|Original|hCoV-19^^Sjaelland|Human|Department of Virus and Microbiological Special Diagnostics|Albertsen Lab|Michaelsen|Denmark
>Spike|hCoV-19/Hong Kong/HKU-200723-025/2020|2020-02-25|EPI_ISL_497792|Original|hCoV-19^^Hong Kong|Human|Department of Microbiology|Department of Microbiology|Chan|China
>Spike|hCoV-19/Australia/SAP317/2020|2020-03-26|EPI_ISL_483097|Original|hCoV-19^^South Australia|Human|SA Pathology|SA Pathology|Leong|Australia
>Spike|hCoV-19/USA/MI-MDHHS-SC20550/2020|2020-03-24|EPI_ISL_460598|Original|hCoV-19^^Michigan|Human|Michigan Department of Health and Human Services|Michigan Department of Health and Human Services|Blankenship|USA
>Spike|hCoV-19/England/QEUH-B2EEF8/2020|2020-11-03|EPI_ISL_660018|Original|hCoV-19^^England|Human|Lighthouse Lab in Glasgow|Wellcome Sanger Institute for the COVID-19 Genomics UK (COG-UK) Consortium|Davies|United Kingdom
>Spike|hCoV-19/Netherlands/Gelderland_78/2020|2020-05-19|EPI_ISL_460977|Original|hCoV-19^^Gelderland|Human|Dutch COVID-19 response team|Erasmus Medical Center|Oude Munnink|Netherlands
>Spike|hCoV-19/England/QEUH-9647B1/2020|2020-08-11|EPI_ISL_536851|Original|hCoV-19^^England|Human|Lighthouse Lab in Glasgow|Wellcome Sanger Institute for the COVID-19 Genomics UK (COG-UK) consortium|Davies|United Kingdom
>Spike|hCoV-19/England/MILK-B02DB1/2020|2020-10-24|EPI_ISL_642770|Original|hCoV-19^^England|Human|Lighthouse Lab in Milton Keynes|Wellcome Sanger Institute for the COVID-19 Genomics UK (COG-UK) Consortium|Davies|United Kingdom

>Spike|hCoV-19/Wales/PHWC-165800/2020|2020-06-13|EPI_ISL_490728|Original|hCoV-19^^Wales|Human|Wales Specialist Virology Centre Sequencing lab|COVID-19 Genomics UK (COG-UK) Consortium|Moore|United Kingdom
>Spike|hCoV-19/England/CAMC-9D20E0/2020|2020-09-18|EPI_ISL_601580|Original|hCoV-19^^England|Human|Lighthouse Lab in Cambridge|Wellcome Sanger Institute for the COVID-19 Genomics UK (COG-UK) consortium|Davies|United Kingdom
>Spike|hCoV-19/England/OXON-B328E/2020|2020-03-27|EPI_ISL_534846|Original|hCoV-19^^England|Human|Oxford Viromics|COVID-19 Genomics UK (COG-UK) Consortium|Golubchik|United Kingdom
>Spike|hCoV-19/Israel/CVL-76-ngs/2020|2020-12-24|EPI_ISL_776580|Original|hCoV-19^^Israel|Human|Israel Central Virology laboratory|Israel Central Virology laboratory|Zuckerman|Israel
>Spike|hCoV-19/England/MILK-99A460/2020|2020-08-29|EPI_ISL_550387|Original|hCoV-19^^England|Human|Lighthouse Lab in Milton Keynes|Wellcome Sanger Institute for the COVID-19 Genomics UK (COG-UK) consortium|Davies|United Kingdom
>Spike|hCoV-19/England/LOND-124D424/2020|2020-04-27|EPI_ISL_507097|Original|hCoV-19^^England|Human|University College London Hospital|COVID-19 Genomics UK (COG-UK) Consortium|Byott|United Kingdom
>Spike|hCoV-19/England/MILK-9A802F/2020|2020-09-02|EPI_ISL_572514|Original|hCoV-19^^England|Human|Virology Department|COVID-19 Genomics UK (COG-UK) Consortium|Williams|United Kingdom
>Spike|hCoV-19/USA/UT-UPHL-2012584910/2020|2020-08-10|EPI_ISL_745854|Original|hCoV-19^^Utah|Human|Ginkgo Bioworks Clinical Laboratory|Utah Public Health Laboratory|Young|USA
>Spike|hCoV-19/USA/WI-GMF-11750/2020|2020-07-21|EPI_ISL_547632|Original|hCoV-19^^Wisconsin|Human|Gundersen Molecular Diagnostics Laboratory|Kabara Cancer Research Institute|Kenny|USA
>Spike|hCoV-19/Ireland/MN-NVRL-MPL10032/2020|2020-04-17|EPI_ISL_767822|Original|hCoV-19^^Monaghan|Human|National Virus Reference Laboratory|Irish Coronavirus Sequencing Consortium - Teagasc Moorepark|Walsh|Ireland
>Spike|hCoV-19/Belgium/rega-10191289/2020|2020-10-19|EPI_ISL_735195|Original|hCoV-19^^Nossegem|Human|UZ Leuven|KU Leuven|Maes|Belgium
>Spike|hCoV-19/USA/TX-HMH-MCoV-12794/2020|2020-08-19|EPI_ISL_790527|Original|hCoV-19^^Texas|Human|Houston Methodist Hospital|Houston Methodist Hospital|Long|USA
>Spike|hCoV-19/USA/CA-CZB-2342/2020|2020-05-12|EPI_ISL_513792|Original|hCoV-19^^California|Human|Orange County Public Health Laboratory|Chan-Zuckerberg Biohub|Kamm|USA
>Spike|hCoV-19/England/CAMC-B09F2D/2020|2020-10-30|EPI_ISL_673142|Original|hCoV-19^^England|Human|Lighthouse Lab in Cambridge|Wellcome Sanger Institute for the COVID-19 Genomics UK (COG-UK) Consortium|Davies|United Kingdom
>Spike|hCoV-19/USA/NY-Wadsworth-290320-01/2020|2020-00-00|EPI_ISL_765521|Original|hCoV-19^^New York|Human|SARATOGA HOSPITAL LABORATORY|Wadsworth Center|Lasek-Nesselquist|USA
>Spike|hCoV-19/Japan/Donner78/2020|2020-08-01|EPI_ISL_644982|Original|hCoV-19^^Kanto|Human|Department of Infectious Diseases|Center for Medical Genetics|Kosaki|Japan
>Spike|hCoV-19/Iran/SA-Isf-55/2020|2020-08-06|EPI_ISL_591346|Original|hCoV-19^^Iran|Human|Infectious Diseases and Tropical Medicine Research Center|Infectious Diseases and Tropical Medicine Research Center|Haghjooy Javanmard,S.|Iran
>Spike|hCoV-19/Wales/PHWC-16F772/2020|2020-10-04|EPI_ISL_586016|Original|hCoV-19^^Wales|Human|Wales Specialist Virology Centre Sequencing lab|COVID-19 Genomics UK (COG-UK) Consortium|Moore|United Kingdom
>Spike|hCoV-19/Iran/SA-Isf-61/2020|2020-05-13|EPI_ISL_582007|Original|hCoV-19^^Iran|Human|Infectious Diseases and Tropical Medicine Research Center|Infectious Diseases and Tropical Medicine Research Center|Ahangarzadeh,S.|Iran
>Spike|hCoV-19/USA/UT-02574/2020|2020-04-25|EPI_ISL_487220|Original|hCoV-19^^Utah|Human|Utah Public Health Laboratory|Utah Public Health Laboratory|Butz|USA
>Spike|hCoV-19/England/MILK-B39FAC/2020|2020-10-31|EPI_ISL_706013|Original|hCoV-19^^England|Human|Oxford Viromics|COVID-19 Genomics UK (COG-UK) Consortium|Golubchik|United Kingdom

>Spike|hCoV-19/Luxembourg/LNS9117076/2020|2020-10-26|EPI_ISL_744855|Original|hCoV-19^^Luxembourg|Human|Laboratoire national de sant\u00e9|Laboratoire national de sant\u00e9|Wienecke-Baldacchino|Luxembourg
>Spike|hCoV-19/England/CAMC-CFC28D/2020|2020-12-21|EPI_ISL_781737|Original|hCoV-19^^England|Human|Lighthouse Lab in Cambridge|Wellcome Sanger Institute for the COVID-19 Genomics UK (COG-UK) Consortium|Davies|United Kingdom
>Spike|hCoV-19/Spain/MD-H12-53/2020|2020-04-12|EPI_ISL_529985|Vero|hCoV-19^^Madrid|Human|Hospital Universitario 12 de Octubre|Hospital Universitario 12 de Octubre|Viedma|Spain
>Spike|hCoV-19/USA/MA-CDC-STM-P056/2020|2020-12-19|EPI_ISL_755617|Original|hCoV-19^^Massachusetts|Human|Helix/Illumina|Genomics and Discovery|Cook|USA
>Spike|hCoV-19/USA/AZ-TG491542/2020|2020-06-21|EPI_ISL_695158|Original|hCoV-19^^Arizona|Human|AZ SPHL|TGen North|French|USA
>Spike|hCoV-19/Canada/ON-S1561/2020|2020-09-02|EPI_ISL_755918|Original|hCoV-19^^Ontario|Human|Toronto Invasive Bacterial Diseases Network|McMaster University|McArthur|Canada
>Spike|hCoV-19/USA/WA-UW-11602/2020|2020-06-17|EPI_ISL_570445|Original|hCoV-19^^Washington|Human|UW Virology Lab|UW Virology Lab|Roychoudhury|USA
>Spike|hCoV-19/USA/LA-EVTL1244/2020|2020-12-18|EPI_ISL_768513|Original|hCoV-19^^Louisiana|Human|LSUHS Emerging Viral Threat Laboratory|Microbial Genome Sequencing Center|Kamil|USA
>Spike|hCoV-19/Ireland/D-NVRL-AIIDV0053v2/2020|2020-04-09|EPI_ISL_644335|Original|hCoV-19^^Dublin|Human|CEPHR / Vincent's Hospital|Irish Coronavirus Sequencing Consortium - National Virus Reference Laboratory|Gonzalez|Ireland
>Spike|hCoV-19/Wales/PHWC-480FE9/2020|2020-10-20|EPI_ISL_613025|Original|hCoV-19^^Wales|Human|Wales Specialist Virology Centre Sequencing lab|COVID-19 Genomics UK (COG-UK) Consortium|Moore|United Kingdom
>Spike|hCoV-19/England/ALDP-BC7B72/2020|2020-11-23|EPI_ISL_721328|Original|hCoV-19^^England|Human|Lighthouse Lab in Alderley Park|Wellcome Sanger Institute for the COVID-19 Genomics UK (COG-UK) Consortium|Davies|United Kingdom
>Spike|hCoV-19/USA/NY-NYUMC694/2020|2020-03-26|EPI_ISL_451409|Original|hCoV-19^^New York|Human|NYU Langone Health|Departments of Pathology and Medicine|Maurano|USA
>Spike|hCoV-19/England/ALDP-A849F0/2020|2020-10-18|EPI_ISL_610523|Original|hCoV-19^^England|Human|Lighthouse Lab in Alderley Park|Wellcome Sanger Institute for the COVID-19 Genomics UK (COG-UK) consortium|Davies|United Kingdom
>Spike|hCoV-19/Spain/RI-IBV-99005875/2020|2020-03-07|EPI_ISL_537417|Original|hCoV-19^^La Rioja|Human|Centro de Investigaci\u00f3n Biom\u00e9dica de La Rioja - Hospital San Pedro Logro\u00f1o|SeqCOVID-SPAIN consortium/IBV(CSIC)|Comas|Spain
>Spike|hCoV-19/Australia/VIC2136/2020|2020-06-20|EPI_ISL_480737|Original|hCoV-19^^Victoria|Human|Victorian Infectious Diseases Reference Laboratory (VIDRL)|VIDRL and MDU-PHL|Schultz|Australia
>Spike|hCoV-19/England/LIVE-1D16AB/2020|2020-05-14|EPI_ISL_572815|Original|hCoV-19^^England|Human|Liverpool Clinical Laboratories|COVID-19 Genomics UK (COG-UK) Consortium|Haldenby|United Kingdom
>Spike|hCoV-19/India/TG-CCMB-J021/2020|2020-03-30|EPI_ISL_447556|Original|hCoV-19^^Telangana|Human|CSIR-Centre for Cellular and Molecular Biology|CSIR-Centre for Cellular and Molecular Biology|Banu|India
>Spike|hCoV-19/England/MILK-9A9C0C/2020|2020-09-01|EPI_ISL_551135|Original|hCoV-19^^England|Human|Lighthouse Lab in Milton Keynes|Wellcome Sanger Institute for the COVID-19 Genomics UK (COG-UK) consortium|Davies|United Kingdom
>Spike|hCoV-19/Bangladesh/DNAS-CPH-isl-479/2020|2020-04-29|EPI_ISL_605890|Original|hCoV-19^^Bangladesh|Human|NGS Lab|NGS Lab|Rabbi|Bangladesh
>Spike|hCoV-19/England/ALDP-95CD32/2020|2020-06-16|EPI_ISL_555124|Original|hCoV-19^^England|Human|Lighthouse Lab in Alderley Park|Wellcome Sanger Institute for the COVID-19 Genomics UK (COG-UK) consortium|Davies|United Kingdom
>Spike|hCoV-19/USA/TX-DSHS-1307/2020|2020-03-31|EPI_ISL_648255|Original|hCoV-19^^Texas|Human|Texas Department of State Health Services|Texas Department of State Health Services|Rahman|USA

>Spike|hCoV-19/England/LIVE-DC302A/2020|2020-11-25|EPI_ISL_703807|Original|hCoV-19^^England|Human|Liverpool Clinical Laboratories|COVID-19 Genomics UK (COG-UK) Consortium|Haldenby|United Kingdom
>Spike|hCoV-19/USA/TX-HMH-MCoV-9368/2020|2020-07-03|EPI_ISL_781081|Original|hCoV-19^^Texas|Human|Houston Methodist Hospital|Houston Methodist Hospital|Long|USA
>Spike|hCoV-19/Wales/PHWC-49AD88/2020|2020-12-03|EPI_ISL_727683|Original|hCoV-19^^Wales|Human|Originating lab|COVID-19 Genomics UK (COG-UK) Consortium|Moore|United Kingdom
>Spike|hCoV-19/Australia/SAP254/2020|2020-03-26|EPI_ISL_483082|Original|hCoV-19^^South Australia|Human|SA Pathology|SA Pathology|Leong|Australia
>Spike|hCoV-19/USA/IL-NM-0437/2020|2020-04-10|EPI_ISL_626408|Original|hCoV-19^^Illinois|Human|Northwestern Memorial Hospital|Ozer Lab|Ozer|USA
>Spike|hCoV-19/USA/CO-CDPHE-2005110165/2020|2020-05-11|EPI_ISL_710211|Original|hCoV-19^^Colorado|Human|Colorado Department of Public Health and Environment|Colorado Department of Puplic Health and Environment|Bankers|USA
>Spike|hCoV-19/England/BRIS-12CFB5/2020|2020-04-25|EPI_ISL_481839|Original|hCoV-19^^England|Human|PHE South West Regional Laboratory|Wellcome Sanger Institute for the COVID-19 Genomics UK (COG-UK) consortium|Davies|United Kingdom
>Spike|hCoV-19/South Korea/KCDC2377/2020|2020-05-31|EPI_ISL_514780|Original|hCoV-19^^South Korea|Human|Division of Viral Diseases|Division of Viral Diseases|Kim|South Korea
>Spike|hCoV-19/Turkey/HSGM-1495/2020|2020-03-19|EPI_ISL_437325|Vero P1|hCoV-19^^Istanbul|Human|Ministry of Health Turkey|Ministry of Health Turkey|Bayrakdar|Turkey
>Spike|hCoV-19/Canada/BC_07833956/2020|2020-04-00|EPI_ISL_466822|Original|hCoV-19^^British Columbia|Human|BCCDC Public Health Laboratory|BCCDC Public Health Laboratory|Harrigan|Canada
>Spike|hCoV-19/Denmark/DCGC-3007/2020|2020-08-24|EPI_ISL_616783|Original|hCoV-19^^Syddanmark|Human|Department of Virus and Microbiological Special Diagnostics|Albertsen lab|Michaelsen|Denmark
>Spike|hCoV-19/South Africa/KRISP-0411/2020|2020-07-02|EPI_ISL_495536|Original|hCoV-19^^KwaZulu-Natal|Human|Medical Disagnostics Services (MDS)|KRISP|de Oliveira|South Africa
>Spike|hCoV-19/England/ALDP-936ED1/2020|2020-06-21|EPI_ISL_552932|Original|hCoV-19^^England|Human|Lighthouse Lab in Alderley Park|Wellcome Sanger Institute for the COVID-19 Genomics UK (COG-UK) consortium|Davies|United Kingdom
>Spike|hCoV-19/Wales/PHWC-36906/2020|2020-04-28|EPI_ISL_474730|Original|hCoV-19^^Wales|Human|Originating lab|COVID-19 Genomics UK (COG-UK) Consortium|Moore|United Kingdom
>Spike|hCoV-19/Netherlands/GE-EMC-421/2020|2020-11-27|EPI_ISL_722693|Original|hCoV-19^^Gelderland|Human|Dutch COVID-19 response team|Erasmus Medical Center|Oude Munnink|Netherlands
>Spike|hCoV-19/England/20140074104/2020|2020-03-29|EPI_ISL_423420|Original|hCoV-19^^England|Human|Respiratory Virus Unit|Respiratory Virus Unit|Platt|United Kingdom
>Spike|hCoV-19/Portugal/PT2053/2020|2020-11-09|EPI_ISL_732267|Original|hCoV-19^^Portugal|Human|Instituto Nacional de Saude (INSA) and Instituto Gulbenkian de Ciencia (IGC)|Instituto Nacional de Saude (INSA) and Instituto Gulbenkian de Ciencia (IGC)|Borges|Portugal
>Spike|hCoV-19/England/MILK-B3D306/2020|2020-11-01|EPI_ISL_679835|Original|hCoV-19^^England|Human|Oxford Viromics|COVID-19 Genomics UK (COG-UK) Consortium|Golubchik|United Kingdom
>Spike|hCoV-19/England/ALDP-950A28/2020|2020-06-14|EPI_ISL_554420|Original|hCoV-19^^England|Human|Lighthouse Lab in Alderley Park|Wellcome Sanger Institute for the COVID-19 Genomics UK (COG-UK) consortium|Davies|United Kingdom
>Spike|hCoV-19/India/MH-ACTREC-003/2020|2020-05-01|EPI_ISL_699660|Original|hCoV-19^^Maharashtra|Human|Hematopathology Laboratory|Hematopathology Laboratory|Patkar|India
>Spike|hCoV-19/Denmark/DCGC-6410/2020|2020-10-12|EPI_ISL_620310|Original|hCoV-19^^Sjaelland|Human|Department of Virus and Microbiological Special Diagnostics|Albertsen lab|Michaelsen|Denmark

>Spike|hCoV-19/Netherlands/GE-EMC-198/2020|2020-05-04|EPI_ISL_523180|Original|hCoV-19^^Gelderland|Human|Dutch COVID-19 response team|Erasmus Medical Center|Oude Munnink|Netherlands
>Spike|hCoV-19/Australia/VIC7364/2020|2020-07-21|EPI_ISL_564361|Original|hCoV-19^^Victoria|Human|Victorian Infectious Diseases Reference Laboratory (VIDRL)|VIDRL and MDU-PHL|Schultz|Australia
>Spike|hCoV-19/Belgium/rega-10181069/2020|2020-10-18|EPI_ISL_735109|Original|hCoV-19^^Bierbeek|Human|UZ Leuven|KU Leuven|Maes|Belgium
>Spike|hCoV-19/England/MILK-976637/2020|2020-08-20|EPI_ISL_575826|Original|hCoV-19^^England|Human|Lighthouse Lab in Milton Keynes|Wellcome Sanger Institute for the COVID-19 Genomics UK (COG-UK) consortium|Davies|United Kingdom
>Spike|hCoV-19/USA/CA-CZB-15145/2020|2020-11-23|EPI_ISL_738763|Original|hCoV-19^^California|Human|Santa Clara County Public Health Laboratory|Chan-Zuckerberg Biohub|Kamm|USA
>Spike|hCoV-19/England/ALDP-9D8E86/2020|2020-09-18|EPI_ISL_576679|Original|hCoV-19^^England|Human|Oxford Viromics|COVID-19 Genomics UK (COG-UK) Consortium|Golubchik|United Kingdom
>Spike|hCoV-19/Portugal/PT0456/2020|2020-04-18|EPI_ISL_454180|Original|hCoV-19^^Portugal|Human|unknown|Instituto Nacional de Saude (INSA)|Borges|Portugal
>Spike|hCoV-19/USA/NY-Wadsworth-286666-01/2020|2020-12-14|EPI_ISL_767451|Original|hCoV-19^^New York|Human|Wadsworth Center|Wadsworth Center|Lasek-Nesselquist|USA
>Spike|hCoV-19/England/ALDP-C3B1A3/2020|2020-12-09|EPI_ISL_730741|Original|hCoV-19^^England|Human|Lighthouse Lab in Alderley Park|Wellcome Sanger Institute for the COVID-19 Genomics UK (COG-UK) Consortium|Davies|United Kingdom
>Spike|hCoV-19/Australia/VIC9408/2020|2020-08-19|EPI_ISL_565538|Original|hCoV-19^^Victoria|Human|Microbiological Diagnostic Unit - Public Health Laboratory (MDU-PHL)|MDU-PHL|Schultz|Australia
>Spike|hCoV-19/England/SHEF-C4F56/2020|2020-04-19|EPI_ISL_462016|Original|hCoV-19^^England|Human|Virology Department|COVID-19 Genomics UK (COG-UK) Consortium|de Silva|United Kingdom
>Spike|hCoV-19/England/MILK-B95928/2020|2020-11-13|EPI_ISL_674975|Original|hCoV-19^^England|Human|Lighthouse Lab in Milton Keynes|Wellcome Sanger Institute for the COVID-19 Genomics UK (COG-UK) Consortium|Davies|United Kingdom
>Spike|hCoV-19/USA/CA-SR0370/2020|2020-04-29|EPI_ISL_458253|Original|hCoV-19^^California|Human|Scripps Medical Laboratory|Andersen lab at Scripps Research|Gangavarapu|USA
>Spike|hCoV-19/Japan/PG-8642/2020|2020-09-00|EPI_ISL_686238|Original|hCoV-19^^Japan|Human|Pathogen Genomics Center|Pathogen Genomics Center|Sekizuka|Japan
>Spike|hCoV-19/Australia/VIC4634/2020|2020-07-10|EPI_ISL_519025|Original|hCoV-19^^Victoria|Human|Victorian Infectious Diseases Reference Laboratory (VIDRL)|VIDRL and MDU-PHL|Schultz|Australia
>Spike|hCoV-19/Iceland/389/2020|2020-03-19|EPI_ISL_424413|Original|hCoV-19^^Reykjavik|Human|The National University Hospital of Iceland|deCODE genetics|J\u00f3nsson|Iceland
>Spike|hCoV-19/Italy/LAZ-INMI-41/2020|2020-06-12|EPI_ISL_603148|Original|hCoV-19^^Lazio|Human|INMI Lazzaro Spallanzani IRCCS|INMI Lazzaro Spallanzani IRCCS|Gruber|Italy
>Spike|hCoV-19/USA/NY-NYCPHL-001461/2020|2020-11-25|EPI_ISL_683822|Original|hCoV-19^^New York|Human|DOHMH Jamaica|New York City Public Health Laboratory|Wang|USA
>Spike|hCoV-19/Spain/CL-IBV-97006266/2020|2020-04-21|EPI_ISL_537638|Original|hCoV-19^^Castilla y Leon|Human|Universidad de Le\u00f3n|SeqCOVID-SPAIN consortium/IBV(CSIC)|Comas|Spain
>Spike|hCoV-19/USA/FL-BPHL-0527/2020|2020-06-19|EPI_ISL_508730|Original|hCoV-19^^Florida|Human|Florida Bureau of Public Health Laboratories|Florida Bureau of Public Health Laboratories|Blanton|USA
>Spike|hCoV-19/Luxembourg/LNS3694003/2020|2020-03-18|EPI_ISL_417528|Original|hCoV-19^^Luxembourg|Human|Laboratoire Nationale de Sant\u00e9|Laboratoire Nationale de Sant\u00e9|Wienecke-Baldacchino|Luxembourg

>Spike|hCoV-19/England/CAMB-77611/2020|2020-03-31|EPI_ISL_439388|Original|hCoV-19^^England|Human|Department of Pathology|Wellcome Sanger Institute for the COVID-19 Genomics UK (COG-UK) consortium|Amato|United Kingdom
>Spike|hCoV-19/Wales/PHWC-4A5AFC/2020|2020-12-11|EPI_ISL_764953|Original|hCoV-19^^Wales|Human|Wales Specialist Virology Centre Sequencing lab|COVID-19 Genomics UK (COG-UK) Consortium|Moore|United Kingdom
>Spike|hCoV-19/England/NORT-1B72B19/2020|2020-12-06|EPI_ISL_724913|Original|hCoV-19^^England|Human|Northumbria University / South Tees Hospitals NHS Foundation Trust / North Cumbria Integrated Care NHS Foundation Trust / North Tees and Hartlepool NHS Foundation Trust / Newcastle Hospitals NHS Foundation Trust|COVID-19 Genomics UK (COG-UK) Consortium|Bashton|United Kingdom
>Spike|hCoV-19/Australia/VIC11740/2020|2020-07-22|EPI_ISL_563478|Original|hCoV-19^^Victoria|Human|Microbiological Diagnostic Unit - Public Health Laboratory (MDU-PHL)|MDU-PHL|Schultz|Australia
>Spike|hCoV-19/England/LIVE-DC6BEA/2020|2020-11-29|EPI_ISL_702974|Original|hCoV-19^^England|Human|Liverpool Clinical Laboratories|COVID-19 Genomics UK (COG-UK) Consortium|Haldenby|United Kingdom
>Spike|hCoV-19/Japan/PG-5058/2020|2020-08-00|EPI_ISL_690169|Original|hCoV-19^^Japan|Human|Pathogen Genomics Center|Pathogen Genomics Center|Sekizuka|Japan
>Spike|hCoV-19/USA/MI-QDX-732/2020|2020-03-21|EPI_ISL_571465|Original|hCoV-19^^Michigan|Human|Quest Diagnostics|Quest Diagnostics|Kagan|USA
>Spike|hCoV-19/USA/MN-QDX-1695/2020|2020-03-16|EPI_ISL_604009|Original|hCoV-19^^Minnesota|Human|Quest Diagnostics|Quest Diagnostics|Kagan|USA
>Spike|hCoV-19/England/QEUH-BA3550/2020|2020-11-18|EPI_ISL_721054|Original|hCoV-19^^England|Human|Lighthouse Lab in Glasgow|Wellcome Sanger Institute for the COVID-19 Genomics UK (COG-UK) Consortium|Davies|United Kingdom
>Spike|hCoV-19/USA/AZ-TG637292/2020|2020-07-27|EPI_ISL_695721|Original|hCoV-19^^Arizona|Human|AZ SPHL|TGen North|French|USA
>Spike|hCoV-19/USA/TX-HMH-4415/2020|2020-06-17|EPI_ISL_543602|Original|hCoV-19^^Texas|Human|Houston Methodist Hospital|Houston Methodist Hospital|Long|USA
>Spike|hCoV-19/USA/UT-02595/2020|2020-04-26|EPI_ISL_522261|Original|hCoV-19^^Utah|Human|Utah Public Health Laboratory|Utah Public Health Laboratory|Young|USA
>Spike|hCoV-19/Wales/PHWC-48A231/2020|2020-11-04|EPI_ISL_651543|Original|hCoV-19^^Wales|Human|Wales Specialist Virology Centre Sequencing lab|COVID-19 Genomics UK (COG-UK) Consortium|Moore|United Kingdom
>Spike|hCoV-19/South Korea/KDCA0189/2020|2020-11-22|EPI_ISL_747425|Original|hCoV-19^^South Korea|Human|Division of Emerging Infectious Diseases|Division of Emerging Infectious Diseases|Park|South Korea
>Spike|hCoV-19/Canada/QC-LSPQ-L00236526/2020|2020-03-24|EPI_ISL_536132|Original|hCoV-19^^Quebec|Human|H\u00f4pital de Hull|Laboratoire de sant\u00e9 publique du Qu\u00e9bec|Moreira|Canada
>Spike|hCoV-19/England/NORT-28171F/2020|2020-03-19|EPI_ISL_488789|Original|hCoV-19^^England|Human|NU-OMICS DNA Sequencing research facility|Wellcome Sanger Institute for the COVID-19 Genomics UK (COG-UK) consortium|Davies|United Kingdom
>Spike|hCoV-19/India/UN-777/2020|2020-03-03|EPI_ISL_420551|Original|hCoV-19^^India|Human|National Influenza Center|Indian Council of Medical Research-National Institute of Virology|Yadav|India
>Spike|hCoV-19/England/BIRM-5E971/2020|2020-04-26|EPI_ISL_447925|Original|hCoV-19^^England|Human|University of Birmingham|COVID-19 Genomics UK (COG-UK) Consortium|Loman|United Kingdom
>Spike|hCoV-19/Canada/ON-S846/2020|2020-04-11|EPI_ISL_591169|Original|hCoV-19^^Ontario|Human|Toronto Invasive Bacterial Diseases Network|McMaster University|McArthur|Canada
>Spike|hCoV-19/England/QEUH-9B0D0F/2020|2020-09-03|EPI_ISL_559861|Original|hCoV-19^^England|Human|Oxford Viromics|COVID-19 Genomics UK (COG-UK) Consortium|Golubchik|United Kingdom
>Spike|hCoV-19/Denmark/DCGC-8708/2020|2020-10-26|EPI_ISL_625699|Original|hCoV-19^^Hovedstaden|Human|Department of Virus and Microbiological Special Diagnostics|Albertsen lab|Michaelsen|Denmark

>Spike|hCoV-19/USA/UT-UPHL-201117349/2020|2020-04-01|EPI_ISL_623181|Original|hCoV-19^^Utah|Human|Utah Public Health Laboratory|Utah Public Health Laboratory|Young|USA
>Spike|hCoV-19/Denmark/DCGC-8634/2020|2020-10-26|EPI_ISL_625996|Original|hCoV-19^^Sjaelland|Human|Department of Virus and Microbiological Special Diagnostics|Albertsen lab|Michaelsen|Denmark
>Spike|hCoV-19/USA/WA-S494/2020|2020-04-01|EPI_ISL_434254|Original|hCoV-19^^Washington|Human|Washington State Department of Health|Seattle Flu Study|Bedford|USA
>Spike|hCoV-19/Denmark/DCGC-2709/2020|2020-08-17|EPI_ISL_616442|Original|hCoV-19^^Midtjylland|Human|Department of Virus and Microbiological Special Diagnostics|Albertsen lab|Michaelsen|Denmark
>Spike|hCoV-19/USA/FL-BPHL-0326/2020|2020-04-13|EPI_ISL_489735|Original|hCoV-19^^Florida|Human|Florida Bureau of Public Health Laboratories|Florida Bureau of Public Health Laboratories|Blanton|USA
>Spike|hCoV-19/Australia/NSW1015/2020|2020-03-22|EPI_ISL_593660|Original|hCoV-19^^New South Wales|Human|Pathology West - NSW Health Pathology|NSW Health Pathology - Institute of Clinical Pathology and Medical Research; Westmead Hospital; University of Sydney|Gall|Australia
>Spike|hCoV-19/England/MILK-9A95A7/2020|2020-08-31|EPI_ISL_551249|Original|hCoV-19^^England|Human|Lighthouse Lab in Milton Keynes|Wellcome Sanger Institute for the COVID-19 Genomics UK (COG-UK) consortium|Davies|United Kingdom
>Spike|hCoV-19/England/ALDP-A6D016/2020|2020-10-14|EPI_ISL_608169|Original|hCoV-19^^England|Human|Lighthouse Lab in Alderley Park|Wellcome Sanger Institute for the COVID-19 Genomics UK (COG-UK) consortium|Davies|United Kingdom
>Spike|hCoV-19/USA/MN-MDH-1227/2020|2020-05-18|EPI_ISL_482948|Original|hCoV-19^^Minnesota|Human|Minnesota Department of Health|Minnesota Department of Health|Garfin|USA
>Spike|hCoV-19/USA/IL-UW-2119/2020|2020-10-02|EPI_ISL_677524|Original|hCoV-19^^Illinois|Human|University of Wisconsin-Madison AIDS Vaccine Research Laboratories|University of Wisconsin-Madison AIDS Vaccine Research Laboratories|Baczenas|USA
>Spike|hCoV-19/USA/WA-S951/2020|2020-04-27|EPI_ISL_463441|Original|hCoV-19^^Washington|Human|Washington State Department of Health|Seattle Flu Study|Bedford|USA
>Spike|hCoV-19/England/QEUH-C5BEC4/2020|2020-12-14|EPI_ISL_769460|Original|hCoV-19^^England|Human|Lighthouse Lab in Glasgow|Wellcome Sanger Institute for the COVID-19 Genomics UK (COG-UK) Consortium|Davies|United Kingdom
>Spike|hCoV-19/USA/MA-MASPHL-00820/2020|2020-05-27|EPI_ISL_692881|Original|hCoV-19^^Massachusetts|Human|Massachusetts State Public Health Laboratory|Massachusetts State Public Health Laboratory|Lang|USA
>Spike|hCoV-19/England/QEUH-96D26C/2020|2020-08-18|EPI_ISL_530697|Original|hCoV-19^^England|Human|Lighthouse Lab in Glasgow|Wellcome Sanger Institute for the COVID-19 Genomics UK (COG-UK) consortium|Davies|United Kingdom
>Spike|hCoV-19/USA/VA-DCLS-1606/2020|2020-08-20|EPI_ISL_614016|Original|hCoV-19^^Virginia|Human|Virginia DCLS|Virginia DCLS|St. Jacques|USA
>Spike|hCoV-19/Wales/PHWC-48C93A/2020|2020-11-02|EPI_ISL_665584|Original|hCoV-19^^Wales|Human|Wales Specialist Virology Centre Sequencing lab|COVID-19 Genomics UK (COG-UK) Consortium|Moore|United Kingdom
>Spike|hCoV-19/Spain/PV-IBV-98008265/2020|2020-10-01|EPI_ISL_654553|Original|hCoV-19^^Basque Country|Human|Servicio de Microbiolog\u00eda. Hospital Universitario Donostia. OSI Donostialdea. \u00c1rea de Enfermedades Infecciosas|SeqCOVID-SPAIN consortium/IBV(CSIC)|Comas|Spain
>Spike|hCoV-19/Switzerland/VS-ETHZ-321402/2020|2020-10-14|EPI_ISL_603643|Original|hCoV-19^^Valais|Human|Viollier AG|Department of Biosystems Science and Engineering|Nadeau|Switzerland
>Spike|hCoV-19/England/LIVE-DB61AF/2020|2020-11-06|EPI_ISL_650542|Original|hCoV-19^^England|Human|Liverpool Clinical Laboratories|COVID-19 Genomics UK (COG-UK) Consortium|Haldenby|United Kingdom

>Spike|hCoV-19/England/ALDP-A85A29/2020|2020-10-17|EPI_ISL_610502|Original|hCoV-19^^England|Human|Lighthouse Lab in Alderley Park|Wellcome Sanger Institute for the COVID-19 Genomics UK (COG-UK) Consortium|Davies|United Kingdom
>Spike|hCoV-19/England/PORT-2EA95A/2020|2020-00-00|EPI_ISL_704588|Original|hCoV-19^^England|Human|Centre for Enzyme Innovation|COVID-19 Genomics UK (COG-UK) Consortium|Robson|United Kingdom
>Spike|hCoV-19/USA/TX-HMH-MCoV-10869/2020|2020-07-09|EPI_ISL_789667|Original|hCoV-19^^Texas|Human|Houston Methodist Hospital|Houston Methodist Hospital|Long|USA
>Spike|hCoV-19/USA/GA-CDC-7407/2020|2020-10-22|EPI_ISL_681820|Original|hCoV-19^^Georgia|Human|GA Department of Public Health Laboratory|Pathogen Discovery|Queen|USA
>Spike|hCoV-19/Japan/TKYE622387/2020|2020-07-00|EPI_ISL_649128|Original|hCoV-19^^Japan|Human|Tokyo Metropolitan Institute of Public Health|Tokyo Metropolitan Institute of Public Health|Ryota|Japan
>Spike|hCoV-19/England/PORT-2D3F9E/2020|2020-00-00|EPI_ISL_507158|Original|hCoV-19^^England|Human|Centre for Enzyme Innovation|COVID-19 Genomics UK (COG-UK) Consortium|Robson|United Kingdom
>Spike|hCoV-19/USA/IL-NM-1071/2020|2020-06-06|EPI_ISL_626494|Original|hCoV-19^^Illinois|Human|Northwestern Memorial Hospital|Ozer Lab|Ozer|USA
>Spike|hCoV-19/Israel/CVL-46394/2020|2020-12-15|EPI_ISL_745071|Original|hCoV-19^^Tel Aviv|Human|Israel Central Virology laboratory|Israel Central Virology laboratory|Zuckerman|Israel
>Spike|hCoV-19/Iceland/210/2020|2020-03-16|EPI_ISL_417829|Original|hCoV-19^^Reykjavik|Human|The National University Hospital of Iceland|deCODE genetics|J\u00f3nsson|Iceland
>Spike|hCoV-19/Canada/NS-NML-1261/2020|2020-04-06|EPI_ISL_579649|Original|hCoV-19^^Nova Scotia|Human|QEII Health Sciences Centre|National Microbiology Laboratory (NML)|Majer|Canada
>Spike|hCoV-19/England/MILK-A22DE9/2020|2020-10-03|EPI_ISL_611951|Original|hCoV-19^^England|Human|Northumbria University / South Tees Hospitals NHS Foundation Trust / North Cumbria Integrated Care NHS Foundation Trust / North Tees and Hartlepool NHS Foundation Trust / Newcastle Hospitals NHS Foundation Trust|COVID-19 Genomics UK (COG-UK) Consortium|Bashton|United Kingdom
>Spike|hCoV-19/USA/TX-HMH-7402/2020|2020-06-29|EPI_ISL_546741|Original|hCoV-19^^Texas|Human|Houston Methodist Hospital|Houston Methodist Hospital|Long|USA
>Spike|hCoV-19/Japan/PG-2527/2020|2020-05-00|EPI_ISL_690662|Original|hCoV-19^^Japan|Human|Saitama Prefectural Institute of Public Health|Pathogen Genomics Center|Sekizuka|Japan
>Spike|hCoV-19/England/20118109104/2020|2020-03-12|EPI_ISL_464952|Original|hCoV-19^^England|Human|Respiratory Virus Unit|Respiratory Virus Unit|Platt|United Kingdom
>Spike|hCoV-19/Wales/PHWC-48AE5B/2020|2020-11-06|EPI_ISL_665361|Original|hCoV-19^^Wales|Human|Wales Specialist Virology Centre Sequencing lab|COVID-19 Genomics UK (COG-UK) Consortium|Moore|United Kingdom
>Spike|hCoV-19/Denmark/DCGC-16490/2020|2020-11-16|EPI_ISL_714799|Original|hCoV-19^^Midtjylland|Human|Department of Virus and Microbiological Special Diagnostics|Albertsen Lab|Michaelsen|Denmark
>Spike|hCoV-19/Canada/ON-S1806/2020|2020-11-24|EPI_ISL_729278|Original|hCoV-19^^Ontario|Human|Toronto Invasive Bacterial Diseases Network|McMaster University|McArthur|Canada
>Spike|hCoV-19/Wales/PHWC-487EA9/2020|2020-10-29|EPI_ISL_639318|Original|hCoV-19^^Wales|Human|Wales Specialist Virology Centre Sequencing lab|COVID-19 Genomics UK (COG-UK) Consortium|Moore|United Kingdom
>Spike|hCoV-19/England/CAMC-BC0F22/2020|2020-11-22|EPI_ISL_704793|Original|hCoV-19^^England|Human|Lighthouse Lab in Cambridge|Wellcome Sanger Institute for the COVID-19 Genomics UK (COG-UK) Consortium|Davies|United Kingdom
>Spike|hCoV-19/USA/TX-HMH-MCoV-18365/2020|2020-11-18|EPI_ISL_784713|Original|hCoV-19^^Texas|Human|Houston Methodist Hospital|Houston Methodist Hospital|Long|USA
>Spike|hCoV-19/France/PAC-IHU-0170/2020|2020-03-25|EPI_ISL_569346|Original|hCoV-19^^Provence-Alpes-C\u00f4te-d\u2019Azur|Human|MEPHI|MEPHI|Levasseur|France

>Spike|hCoV-19/England/MILK-AB7E6E/2020|2020-10-21|EPI_ISL_623327|Original|hCoV-19^^England|Human|Lighthouse Lab in Milton Keynes|Wellcome Sanger Institute for the COVID-19 Genomics UK (COG-UK) consortium|Davies|United Kingdom
>Spike|hCoV-19/England/LOND-D62C1/2020|2020-04-21|EPI_ISL_482204|Original|hCoV-19^^England|Human|University College London|Wellcome Sanger Institute for the COVID-19 Genomics UK (COG-UK) consortium|Davies|United Kingdom
>Spike|hCoV-19/Denmark/DCGC-16204/2020|2020-11-16|EPI_ISL_714367|Original|hCoV-19^^Midtjylland|Human|Department of Virus and Microbiological Special Diagnostics|Albertsen Lab|Michaelsen|Denmark
>Spike|hCoV-19/USA/CA-IGI-0133/2020|2020-06-10|EPI_ISL_495154|Original|hCoV-19^^California|Human|Innovative Genomics Institute|Innovative Genomics Institute|Shivram|USA
>Spike|hCoV-19/USA/AR-CDC-2-3693681/2020|2020-10-29|EPI_ISL_747066|Original|hCoV-19^^Arkansas|Human|Respiratory Viruses Branch|Respiratory Viruses Branch|Queen|USA
>Spike|hCoV-19/England/ALDP-A506BA/2020|2020-10-07|EPI_ISL_597838|Original|hCoV-19^^England|Human|Lighthouse Lab in Alderley Park|Wellcome Sanger Institute for the COVID-19 Genomics UK (COG-UK) consortium|Davies|United Kingdom
>Spike|hCoV-19/Luxembourg/LNS1667297/2020|2020-04-17|EPI_ISL_445065|Original|hCoV-19^^Luxembourg|Human|Laboratoire National de Sante|Laboratoire National de Sante|Wienecke-Baldacchino|Luxembourg
>Spike|hCoV-19/Wales/PHWC-48D1D7/2020|2020-11-07|EPI_ISL_680588|Original|hCoV-19^^Wales|Human|Wales Specialist Virology Centre Sequencing lab|COVID-19 Genomics UK (COG-UK) Consortium|Moore|United Kingdom
>Spike|hCoV-19/Ireland/MH-NVRL-MPK48258/2020|2020-05-20|EPI_ISL_767816|Original|hCoV-19^^Meath|Human|National Virus Reference Laboratory|Irish Coronavirus Sequencing Consortium - Teagasc Moorepark|Walsh|Ireland
>Spike|hCoV-19/USA/UT-UPHL-2009665/2020|2020-07-27|EPI_ISL_605340|Original|hCoV-19^^Utah|Human|Utah Public Health Laboratory|Utah Public Health Laboratory|Young|USA
>Spike|hCoV-19/Denmark/ALAB-SSI-840/2020|2020-03-29|EPI_ISL_444933|Original|hCoV-19^^Denmark|Human|Department of Virus and Microbiological Special Diagnostics|Albertsen lab|Kirkegaard|Denmark
>Spike|hCoV-19/Wales/PHWC-36371/2020|2020-04-29|EPI_ISL_474657|Original|hCoV-19^^Wales|Human|Originating lab|COVID-19 Genomics UK (COG-UK) Consortium|Moore|United Kingdom
>Spike|hCoV-19/England/ALDP-B46856/2020|2020-11-06|EPI_ISL_656041|Original|hCoV-19^^England|Human|Lighthouse Lab in Alderley Park|Wellcome Sanger Institute for the COVID-19 Genomics UK (COG-UK) Consortium|Davies|United Kingdom
>Spike|hCoV-19/England/PORT-2D1E66/2020|2020-04-05|EPI_ISL_499287|Original|hCoV-19^^England|Human|Centre for Enzyme Innovation|COVID-19 Genomics UK (COG-UK) Consortium|Robson|United Kingdom
>Spike|hCoV-19/England/ALDP-A66AF1/2020|2020-10-12|EPI_ISL_630683|Original|hCoV-19^^England|Human|Lighthouse Lab in Alderley Park|Wellcome Sanger Institute for the COVID-19 Genomics UK (COG-UK) consortium|Davies|United Kingdom
>Spike|hCoV-19/USA/WA-S1578/2020|2020-05-26|EPI_ISL_496004|Original|hCoV-19^^Washington|Human|Washington State Department of Health|Seattle Flu Study|Lee|USA
>Spike|hCoV-19/England/MILK-BB1B20/2020|2020-11-18|EPI_ISL_720618|Original|hCoV-19^^England|Human|Lighthouse Lab in Milton Keynes|Wellcome Sanger Institute for the COVID-19 Genomics UK (COG-UK) Consortium|Davies|United Kingdom
>Spike|hCoV-19/England/NORT-28AD8D/2020|2020-04-15|EPI_ISL_492942|Original|hCoV-19^^England|Human|NU-OMICS DNA Sequencing research facility|Wellcome Sanger Institute for the COVID-19 Genomics UK (COG-UK) consortium|Davies|United Kingdom
>Spike|hCoV-19/Wales/PHWC-499B25/2020|2020-12-05|EPI_ISL_727404|Original|hCoV-19^^Wales|Human|Originating lab|COVID-19 Genomics UK (COG-UK) Consortium|Moore|United Kingdom
>Spike|hCoV-19/England/NORT-29E71F/2020|2020-04-30|EPI_ISL_488097|Original|hCoV-19^^England|Human|NU-OMICS DNA Sequencing research facility|Wellcome Sanger Institute for the COVID-19 Genomics UK (COG-UK) consortium|Davies|United Kingdom
>Spike|hCoV-19/Canada/ON-PHL-20-02292/2020|2020-09-00|EPI_ISL_671522|Original|hCoV-19^^Ontario|Human|Public Health Ontario Laboratory|Public Health Ontario Laboratory|Guthrie|Canada

>Spike|hCoV-19/India/TG-CDFD-C673/2020|2020-05-12|EPI_ISL_528602|Original|hCoV-
19^^Telangana|Human|National Genomics Core-Center for DNA Fingerprinting and
Diagnostics|National Genomics Core- Center for DNA Fingerprinting and Diagnostics
(NGC-CDFD)- DBT's PAN-INDIA-1000 Genome consortium|Vashisht|India
>Spike|hCoV-19/USA/WA-UW216/2020|2020-03-14|EPI_ISL_417365|Original|hCoV-
19^^Washington|Human|UW Virology Lab|UW Virology Lab|Roychoudhury|USA
>Spike|hCoV-19/Denmark/DCGC-5691/2020|2020-09-21|EPI_ISL_621196|Original|hCoV-
19^^Hovedstaden|Human|Department of Virus and Microbiological Special
Diagnostics|Albertsen lab|Michaelsen|Denmark
>Spike|hCoV-19/USA/CA-CZB-3735/2020|2020-07-22|EPI_ISL_548438|Original|hCoV-
19^^California|Human|Orange County Public Health Laboratory|Chan-Zuckerberg
Biohub|Bhatt|USA
>Spike|hCoV-19/Australia/VIC6847/2020|2020-07-25|EPI_ISL_564094|Original|hCoV-
19^^Victoria|Human|Victorian Infectious Diseases Reference Laboratory (VIDRL)|VIDRL
and MDU-PHL|Schultz|Australia
>Spike|hCoV-19/USA/CA-CZB-9789/2020|2020-05-17|EPI_ISL_672177|Original|hCoV-
19^^California|Human|The Ashley Laboratory|Chan-Zuckerberg Biohub|Bhatt|USA
>Spike|hCoV-19/USA/FL_9655/2020|2020-03-05|EPI_ISL_424854|Original|hCoV-
19^^Florida|Human|FL Bureau of Public Health Laboratories-Miami|Pathogen
Discovery|Queen|USA
>Spike|hCoV-19/England/QEUH-C32FEE/2020|2020-12-11|EPI_ISL_782653|Original|hCoV-
19^^England|Human|Lighthouse Lab in Glasgow|Wellcome Sanger Institute for the COVID-
19 Genomics UK (COG-UK) Consortium|Davies|United Kingdom
>Spike|hCoV-19/Australia/NSW796/2020|2020-08-04|EPI_ISL_526138|Original|hCoV-19^^New
South Wales|Human|Pathology West - NSW Health Pathology|NSW Health Pathology -
Institute of Clinical Pathology and Medical Research; Westmead Hospital; University
of Sydney|Gall|Australia
>Spike|hCoV-19/Iceland/252/2020|2020-03-17|EPI_ISL_417580|Original|hCoV-
19^^Reykjavik|Human|The National University Hospital of Iceland|deCODE
genetics|J\u00f3nsson|Iceland
>Spike|hCoV-19/England/BRIS-1250F5/2020|2020-03-23|EPI_ISL_443693|Original|hCoV-
19^^England|Human|PHE South West Regional Laboratory|Wellcome Sanger Institute for
the COVID-19 Genomics UK (COG-UK) consortium|Amato|United Kingdom
>Spike|hCoV-19/Scotland/EDB10083/2020|2020-11-20|EPI_ISL_680466|Original|hCoV-
19^^Scotland|Human|Virology Department|COVID-19 Genomics UK (COG-UK)
Consortium|McHugh|United Kingdom
>Spike|hCoV-19/Canada/ON-S901/2020|2020-04-12|EPI_ISL_586474|Original|hCoV-
19^^Ontario|Human|Toronto Invasive Bacterial Diseases Network|McMaster
University|McArthur|Canada
>Spike|hCoV-19/Denmark/ALAB-SSI490/2020|2020-03-21|EPI_ISL_429567|Original|hCoV-
19^^Denmark|Human|Department of Virus and Microbiological Special
Diagnostics|Albertsen lab|Kirkegaard|Denmark
>Spike|hCoV-19/USA/TX-HMH-MCoV-10224/2020|2020-07-07|EPI_ISL_789377|Original|hCoV-
19^^Texas|Human|Houston Methodist Hospital|Houston Methodist Hospital|Long|USA
>Spike|hCoV-19/Scotland/CVR2580/2020|2020-04-16|EPI_ISL_473518|Original|hCoV-
19^^Scotland|Human|West of Scotland Specialist Virology Centre|COVID-19 Genomics UK
(COG-UK) Consortium|Robertson|United Kingdom
>Spike|hCoV-19/Netherlands/ZH-EMC-577/2020|2020-09-19|EPI_ISL_578005|Original|hCoV-
19^^Zuid Holland|Human|Dutch COVID-19 response team|Erasmus Medical Center|Oude
Munnink|Netherlands
>Spike|hCoV-19/England/CAMC-A42035/2020|2020-10-05|EPI_ISL_609478|Original|hCoV-
19^^England|Human|Lighthouse Lab in Cambridge|Wellcome Sanger Institute for the
COVID-19 Genomics UK (COG-UK) consortium|Davies|United Kingdom
>Spike|hCoV-19/Scotland/EDB3500/2020|2020-04-13|EPI_ISL_439273|Original|hCoV-
19^^Scotland|Human|Virology Department|COVID-19 Genomics UK (COG-UK)
Consortium|Williams|United Kingdom
>Spike|hCoV-19/England/QEUH-A8A277/2020|2020-10-16|EPI_ISL_638840|Original|hCoV-
19^^England|Human|Oxford Viromics|COVID-19 Genomics UK (COG-UK)
Consortium|Golubchik|United Kingdom

>Spike|hCoV-19/Japan/PG-1567/2020|2020-04-00|EPI_ISL_685173|Original|hCoV-
19^^Japan|Human|Pathogen Genomics Center|Pathogen Genomics Center|Sekizuka|Japan
>Spike|hCoV-19/USA/CT-CDC-2-3693464/2020|2020-11-05|EPI_ISL_747135|Original|hCoV-
19^^Connecticut|Human|Respiratory Viruses Branch|Respiratory Viruses Branch|Queen|USA
>Spike|hCoV-19/Japan/PG-2294/2020|2020-04-00|EPI_ISL_685899|Original|hCoV-
19^^Japan|Human|Pathogen Genomics Center|Pathogen Genomics Center|Sekizuka|Japan
>Spike|hCoV-19/England/MILK-A7AA36/2020|2020-10-15|EPI_ISL_606343|Original|hCoV-
19^^England|Human|Lighthouse Lab in Milton Keynes|Wellcome Sanger Institute for the
COVID-19 Genomics UK (COG-UK) consortium|Davies|United Kingdom
>Spike|hCoV-19/USA/NY-PV09071/2020|2020-03-20|EPI_ISL_421618|Original|hCoV-19^^New
York|Human|MSHS Clinical Microbiology Laboratories|MSHS Pathogen Surveillance
Program|van Bakel|USA
>Spike|hCoV-19/Denmark/DCGC-4788/2020|2020-09-21|EPI_ISL_621398|Original|hCoV-
19^^Sjaelland|Human|Department of Virus and Microbiological Special
Diagnostics|Albertsen lab|Michaelsen|Denmark
>Spike|hCoV-19/England/QEUH-C93CA8/2020|2020-12-17|EPI_ISL_762002|Original|hCoV-
19^^England|Human|Lighthouse Lab in Glasgow|Wellcome Sanger Institute for the COVID-
19 Genomics UK (COG-UK) Consortium|Davies|United Kingdom
>Spike|hCoV-19/Latvia/094/2020|2020-07-21|EPI_ISL_512649|Original|hCoV-
19^^Latvia|Human|Latvijas Infektolo\u0123ijas centrs|Latvian Biomedical Research and
Study Centre|Zrelovs|Latvia
>Spike|hCoV-19/Switzerland/BE-ETHZ-360193/2020|2020-11-
10|EPI_ISL_728866|Original|hCoV-19^^Bern|Human|Viollier AG|Department of Biosystems
Science and Engineering|Nadeau|Switzerland
>Spike|hCoV-19/USA/WA-S639/2020|2020-04-16|EPI_ISL_438192|Original|hCoV-
19^^Washington|Human|Washington State Department of Health|Seattle Flu
Study|Bedford|USA
>Spike|hCoV-19/England/LEED-2A8ADD/2020|2020-04-04|EPI_ISL_539044|Original|hCoV-
19^^England|Human|Leeds Teaching Hospitals NHS Trust and Public Health
England|Wellcome Sanger Institute for the COVID-19 Genomics UK (COG-UK)
consortium|Davies|United Kingdom
>Spike|hCoV-19/England/LIVE-AB9C0/2020|2020-05-31|EPI_ISL_612446|Original|hCoV-
19^^England|Human|Liverpool Clinical Laboratories|COVID-19 Genomics UK (COG-UK)
Consortium|Haldenby|United Kingdom
>Spike|hCoV-19/France/ARA-SC402/2020|2020-04-15|EPI_ISL_660364|Original|hCoV-
19^^Auvergne-Rh\u00f4ne-Alpes|Human|CHU Clermont-Ferrand|CNR Virus des Infections
Respiratoires - France SUD|Bal|France
>Spike|hCoV-19/USA/NY-Wadsworth-194570-01/2020|2020-08-
07|EPI_ISL_677034|Original|hCoV-19^^New York|Human|Wadsworth Center|Wadsworth
Center|Lasek-Nesselquist|USA
>Spike|hCoV-19/England/ALDP-B63C75/2020|2020-11-08|EPI_ISL_656417|Original|hCoV-
19^^England|Human|Lighthouse Lab in Alderley Park|Wellcome Sanger Institute for the
COVID-19 Genomics UK (COG-UK) Consortium|Davies|United Kingdom
>Spike|hCoV-19/Denmark/DCGC-8675/2020|2020-10-26|EPI_ISL_625983|Original|hCoV-
19^^Hovedstaden|Human|Department of Virus and Microbiological Special
Diagnostics|Albertsen lab|Michaelsen|Denmark
>Spike|hCoV-19/South Korea/KCDC2481/2020|2020-06-11|EPI_ISL_514884|Original|hCoV-
19^^South Korea|Human|Division of Viral Diseases|Division of Viral Diseases|Kim|South
Korea
>Spike|hCoV-19/Australia/NSW596/2020|2020-03-27|EPI_ISL_478708|Original|hCoV-19^^New
South Wales|Human|South Eastern Area Laboratory Services (SEALS)|NSW Health Pathology
- Institute of Clinical Pathology and Medical Research; Westmead Hospital; University
of Sydney|Gall|Australia
>Spike|hCoV-19/USA/WA-S2602/2020|2020-06-18|EPI_ISL_497234|Original|hCoV-
19^^Washington|Human|Washington State Department of Health|Seattle Flu Study|Lee|USA
>Spike|hCoV-19/Denmark/DCGC-5860/2020|2020-09-28|EPI_ISL_621047|Original|hCoV-
19^^Midtjylland|Human|Department of Virus and Microbiological Special
Diagnostics|Albertsen lab|Michaelsen|Denmark

>Spike|hCoV-19/USA/MA-MASPHL-00317/2020|2020-04-18|EPI_ISL_791723|Original|hCoV-19^^Massachusetts|Human|Massachusetts State Public Health Laboratory|Infectious Disease Program|Park|USA
>Spike|hCoV-19/England/QEUH-9F380F/2020|2020-09-24|EPI_ISL_588529|Original|hCoV-19^^England|Human|Lighthouse Lab in Glasgow|Wellcome Sanger Institute for the COVID-19 Genomics UK (COG-UK) consortium|Davies|United Kingdom
>Spike|hCoV-19/England/ALDP-94FAC2/2020|2020-06-16|EPI_ISL_555777|Original|hCoV-19^^England|Human|Lighthouse Lab in Alderley Park|Wellcome Sanger Institute for the COVID-19 Genomics UK (COG-UK) consortium|Davies|United Kingdom
>Spike|hCoV-19/Brazil/SP-317/2020|2020-05-04|EPI_ISL_534314|Original|hCoV-19^^S\u00e3o Paulo|Human|Hospital Universitario da USP de SP|Instituto Adolfo Lutz|Sacchi|Brazil
>Spike|hCoV-19/England/QEUH-BA0D00/2020|2020-11-18|EPI_ISL_719781|Original|hCoV-19^^England|Human|Lighthouse Lab in Glasgow|Wellcome Sanger Institute for the COVID-19 Genomics UK (COG-UK) Consortium|Davies|United Kingdom
>Spike|hCoV-19/USA/NY-PV09122/2020|2020-03-19|EPI_ISL_422497|Original|hCoV-19^^New York|Human|MSHS Clinical Microbiology Laboratories|MSHS Pathogen Surveillance Program|van Bakel|USA
>Spike|hCoV-19/England/ALDP-94F5C1/2020|2020-06-18|EPI_ISL_556322|Original|hCoV-19^^England|Human|Lighthouse Lab in Alderley Park|Wellcome Sanger Institute for the COVID-19 Genomics UK (COG-UK) consortium|Davies|United Kingdom
>Spike|hCoV-19/England/ALDP-B45EDD/2020|2020-11-07|EPI_ISL_655997|Original|hCoV-19^^England|Human|Lighthouse Lab in Alderley Park|Wellcome Sanger Institute for the COVID-19 Genomics UK (COG-UK) Consortium|Davies|United Kingdom
>Spike|hCoV-19/USA/MI-MDHHS-SC21049/2020|2020-03-25|EPI_ISL_507825|Original|hCoV-19^^Michigan|Human|Michigan Department of Health and Human Services|Michigan Department of Health and Human Services|Blankenship|USA
>Spike|hCoV-19/USA/WA-S101/2020|2020-02-28|EPI_ISL_417154|Original|hCoV-19^^Washington|Human|Washington State Department of Health|Seattle Flu Study|Bedford|USA
>Spike|hCoV-19/USA/CT-Yale-309/2020|2020-03-30|EPI_ISL_476537|Original|hCoV-19^^Connecticut|Human|Yale Clinical Virology Laboratory|Grubaugh Lab - Yale School of Public Health|Grubaugh|USA
>Spike|hCoV-19/England/CAMC-B2228F/2020|2020-11-02|EPI_ISL_645866|Original|hCoV-19^^England|Human|Lighthouse Lab in Cambridge|Wellcome Sanger Institute for the COVID-19 Genomics UK (COG-UK) Consortium|Davies|United Kingdom
>Spike|hCoV-19/Denmark/DCGC-3011/2020|2020-08-24|EPI_ISL_616788|Original|hCoV-19^^Hovedstaden|Human|Department of Virus and Microbiological Special Diagnostics|Albertsen lab|Michaelsen|Denmark
>Spike|hCoV-19/England/QEUH-B68724/2020|2020-11-11|EPI_ISL_662157|Original|hCoV-19^^England|Human|Lighthouse Lab in Glasgow|Wellcome Sanger Institute for the COVID-19 Genomics UK (COG-UK) Consortium|Davies|United Kingdom
>Spike|hCoV-19/Belgium/rega-0411455/2020|2020-04-11|EPI_ISL_476965|Original|hCoV-19^^Mouscron|Human|KU Leuven|KU Leuven|Maes|Belgium
>Spike|hCoV-19/Germany/BY-MVP-0221/2020|2020-04-22|EPI_ISL_466894|Original|hCoV-19^^Bavaria|Human|Max von Pettenkofer Institute|Laboratory for Functional Genome Analysis|Graf|Germany
>Spike|hCoV-19/Luxembourg/LNS7630398/2020|2020-10-24|EPI_ISL_744602|Original|hCoV-19^^Luxembourg|Human|Laboratoire national de sant\u00e9|Laboratoire national de sant\u00e9|Wienecke-Baldacchino|Luxembourg
>Spike|hCoV-19/Japan/PG-7542/2020|2020-08-00|EPI_ISL_686644|Original|hCoV-19^^Japan|Human|Pathogen Genomics Center|Pathogen Genomics Center|Sekizuka|Japan
>Spike|hCoV-19/Italy/LOM-INMI-BG-9225/2020|2020-02-26|EPI_ISL_460083|Original|hCoV-19^^Lombardy|Human|Molecular Virology Unit|Laboratory of Virology|Gruber|Italy
>Spike|hCoV-19/USA/VA-DCLS-0177/2020|2020-04-06|EPI_ISL_437393|Original|hCoV-19^^Virginia|Human|Virginia DCLS|Virginia DCLS|St. Jacques|USA
>Spike|hCoV-19/Scotland/QEUH-A4C305/2020|2020-10-05|EPI_ISL_628561|Original|hCoV-19^^Scotland|Human|Oxford Viromics|COVID-19 Genomics UK (COG-UK) Consortium|Golubchik|United Kingdom

>Spike|hCoV-19/Scotland/CVR6022/2020|2020-11-30|EPI_ISL_680459|Original|hCoV-19^^Scotland|Human|West of Scotland Specialist Virology Centre|COVID-19 Genomics UK (COG-UK) Consortium|Robertson|United Kingdom
>Spike|hCoV-19/England/QEUH-7A0C25/2020|2020-07-12|EPI_ISL_533108|Original|hCoV-19^^England|Human|Lighthouse Lab in Glasgow|Wellcome Sanger Institute for the COVID-19 Genomics UK (COG-UK) consortium|Davies|United Kingdom
>Spike|hCoV-19/Austria/CeMM0796/2020|2020-05-08|EPI_ISL_583706|Original|hCoV-19^^Austria|Human|Center for Virology|Bergthaler laboratory|Bergthaler|Austria
>Spike|hCoV-19/Denmark/DCGC-7431/2020|2020-10-12|EPI_ISL_619353|Original|hCoV-19^^Syddanmark|Human|Department of Virus and Microbiological Special Diagnostics|Albertsen lab|Michaelsen|Denmark
>Spike|hCoV-19/USA/TX-HMH-MCoV-14006/2020|2020-08-15|EPI_ISL_787297|Original|hCoV-19^^Texas|Human|Houston Methodist Hospital|Houston Methodist Hospital|Long|USA
>Spike|hCoV-19/England/CAMC-B32E88/2020|2020-11-05|EPI_ISL_659313|Original|hCoV-19^^England|Human|Lighthouse Lab in Cambridge|Wellcome Sanger Institute for the COVID-19 Genomics UK (COG-UK) Consortium|Davies|United Kingdom
>Spike|hCoV-19/Denmark/DCGC-13655/2020|2020-11-30|EPI_ISL_712941|Original|hCoV-19^^Hovedstaden|Human|Department of Virus and Microbiological Special Diagnostics|Albertsen Lab|Michaelsen|Denmark
>Spike|hCoV-19/England/BRIS-12C7CC/2020|2020-05-13|EPI_ISL_469518|Original|hCoV-19^^England|Human|PHE South West Regional Laboratory|Wellcome Sanger Institute for the COVID-19 Genomics UK (COG-UK) consortium|Davies|United Kingdom
>Spike|hCoV-19/USA/FL-BPHL-0459/2020|2020-03-24|EPI_ISL_495365|Original|hCoV-19^^Florida|Human|Florida Bureau of Public Health Laboratories|Florida Bureau of Public Health Laboratories|Blanton|USA
>Spike|hCoV-19/South Korea/KCDC2436/2020|2020-06-04|EPI_ISL_514839|Original|hCoV-19^^South Korea|Human|Division of Viral Diseases|Division of Viral Diseases|Kim|South Korea
>Spike|hCoV-19/England/QEUH-9CE8B2/2020|2020-09-17|EPI_ISL_590142|Original|hCoV-19^^England|Human|Lighthouse Lab in Glasgow|Wellcome Sanger Institute for the COVID-19 Genomics UK (COG-UK) consortium|Davies|United Kingdom
>Spike|hCoV-19/England/LIVE-9DFDE/2020|2020-04-09|EPI_ISL_453258|Original|hCoV-19^^England|Human|Liverpool Clinical Laboratories|COVID-19 Genomics UK (COG-UK) Consortium|Haldenby|United Kingdom
>Spike|hCoV-19/England/QEUH-B0DE2C/2020|2020-11-02|EPI_ISL_642070|Original|hCoV-19^^England|Human|Lighthouse Lab in Glasgow|Wellcome Sanger Institute for the COVID-19 Genomics UK (COG-UK) Consortium|Davies|United Kingdom
>Spike|hCoV-19/Scotland/QEUH-9F6875/2020|2020-09-27|EPI_ISL_600836|Original|hCoV-19^^Scotland|Human|Lighthouse Lab in Glasgow|Wellcome Sanger Institute for the COVID-19 Genomics UK (COG-UK) consortium|Davies|United Kingdom
>Spike|hCoV-19/England/QEUH-A63FC8/2020|2020-10-12|EPI_ISL_628681|Original|hCoV-19^^England|Human|Oxford Viromics|COVID-19 Genomics UK (COG-UK) Consortium|Golubchik|United Kingdom
>Spike|hCoV-19/England/ALDP-8D8379/2020|2020-06-24|EPI_ISL_556995|Original|hCoV-19^^England|Human|Lighthouse Lab in Alderley Park|Wellcome Sanger Institute for the COVID-19 Genomics UK (COG-UK) consortium|Davies|United Kingdom
>Spike|hCoV-19/Scotland/EDB8490/2020|2020-10-18|EPI_ISL_612372|Original|hCoV-19^^Scotland|Human|Virology Department|COVID-19 Genomics UK (COG-UK) Consortium|McHugh|United Kingdom
>Spike|hCoV-19/USA/LA-EVTL1249/2020|2020-12-18|EPI_ISL_768518|Original|hCoV-19^^Louisiana|Human|LSUHS Emerging Viral Threat Laboratory|Microbial Genome Sequencing Center|Kamil|USA
>Spike|hCoV-19/England/ALDP-A87AAF/2020|2020-10-17|EPI_ISL_606292|Original|hCoV-19^^England|Human|Lighthouse Lab in Alderley Park|Wellcome Sanger Institute for the COVID-19 Genomics UK (COG-UK) consortium|Davies|United Kingdom
>Spike|hCoV-19/Canada/QC-LSPQ-L00215981/2020|2020-03-12|EPI_ISL_535743|Original|hCoV-19^^Quebec|Human|Centre de SSS La Pommeraie|Laboratoire de sant\u00e9 publique du Qu\u00e9bec|Moreira|Canada

>Spike|hCoV-19/Denmark/DCGC-5744/2020|2020-09-28|EPI_ISL_621249|Original|hCoV-19^^Hovedstaden|Human|Department of Virus and Microbiological Special Diagnostics|Albertsen lab|Michaelsen|Denmark
>Spike|hCoV-19/Wales/PHWC-163A39/2020|2020-05-10|EPI_ISL_474030|Original|hCoV-19^^Wales|Human|Originating lab|COVID-19 Genomics UK (COG-UK) Consortium|Moore|United Kingdom
>Spike|hCoV-19/Spain/VC-IBV-98003477/2020|2020-04-07|EPI_ISL_539275|Original|hCoV-19^^Comunitat Valenciana|Human|Hospital Universitario de La Ribera (Alzira|SeqCOVID-SPAIN consortium/IBV(CSIC)|Comas|Spain
>Spike|hCoV-19/Netherlands/LI-RIVM-20588/2020|2020-12-13|EPI_ISL_790671|Original|hCoV-19^^Limburg|Human|Dutch COVID-19 response team|National Institute for Public Health and the Environment (RIVM)|Meijer|Netherlands
>Spike|hCoV-19/England/ALDP-C586DE/2020|2020-12-13|EPI_ISL_736073|Original|hCoV-19^^England|Human|Lighthouse Lab in Alderley Park|Wellcome Sanger Institute for the COVID-19 Genomics UK (COG-UK) Consortium|Davies|United Kingdom
>Spike|hCoV-19/USA/MN-MDH-1190/2020|2020-05-05|EPI_ISL_477280|Original|hCoV-19^^Minnesota|Human|M Health Fairview|Minnesota Department of Health|Garfin|USA
>Spike|hCoV-19/Germany/HH-hpi-p1541/2020|2020-03-15|EPI_ISL_776033|Original|hCoV-19^^Hamburg|Human|University Medical Center Hamburg Eppendorf|Heinrich Pette Institute|Robitaille|Germany
>Spike|hCoV-19/Canada/ON-E71b/2020|2020-04-03|EPI_ISL_770824|Original|hCoV-19^^Ontario|Human|Eastern Ontario Regional Laboratory Association|McMaster University|McArthur|Canada
>Spike|hCoV-19/USA/LA-EVTL479/2020|2020-06-23|EPI_ISL_578883|Original|hCoV-19^^Louisiana|Human|LSUHS Emerging Viral Threat Laboratory|Microbial Genome Sequencing Center|Kamil|USA
>Spike|hCoV-19/USA/AZ-TG396346/2020|2020-06-09|EPI_ISL_694805|Original|hCoV-19^^Arizona|Human|AZ SPHL|TGen North|French|USA
>Spike|hCoV-19/USA/WA-S1660/2020|2020-05-29|EPI_ISL_496086|Original|hCoV-19^^Washington|Human|Washington State Department of Health|Seattle Flu Study|Lee|USA
>Spike|hCoV-19/Colombia/CAU-INS-89002/2020|2020-03-31|EPI_ISL_498159|Original|hCoV-19^^Cauca|Human|Instituto Nacional de Salud|Instituto Nacional de Salud|Franco-Mu\u00f1oz|Colombia
>Spike|hCoV-19/Switzerland/BL-ETHZ-110036/2020|2020-04-01|EPI_ISL_486472|Original|hCoV-19^^Basel-Landschaft|Human|Viollier AG|Department of Biosystems Science and Engineering|Nadeau|Switzerland
>Spike|hCoV-19/USA/CO-CDPHE-2100038277/2020|2020-11-17|EPI_ISL_771172|Original|hCoV-19^^Colorado|Human|Colorado Department of Public Health and Environment|Colorado Department of Puplic Health and Environment|Bankers|USA
>Spike|hCoV-19/Switzerland/ZH-ETHZ-120013/2020|2020-04-17|EPI_ISL_468212|Original|hCoV-19^^Zurich|Human|Viollier AG|Department of Biosystems Science and Engineering|Nadeau|Switzerland
>Spike|hCoV-19/England/CAMC-B08FF1/2020|2020-10-29|EPI_ISL_681656|Original|hCoV-19^^England|Human|Lighthouse Lab in Cambridge|Wellcome Sanger Institute for the COVID-19 Genomics UK (COG-UK) Consortium|Davies|United Kingdom
>Spike|hCoV-19/England/BRIS-130BAB/2020|2020-03-14|EPI_ISL_492592|Original|hCoV-19^^England|Human|PHE South West Regional Laboratory|Wellcome Sanger Institute for the COVID-19 Genomics UK (COG-UK) consortium|Davies|United Kingdom
>Spike|hCoV-19/France/PAC-IHU-0977/2020|2020-07-24|EPI_ISL_569024|Original|hCoV-19^^Provence-Alpes-C\u00f4te-d\u2019Azur|Human|MEPHI|MEPHI|Levasseur|France
>Spike|hCoV-19/England/CAMB-7C852/2020|2020-04-05|EPI_ISL_441613|Original|hCoV-19^^England|Human|Department of Pathology|Wellcome Sanger Institute for the COVID-19 Genomics UK (COG-UK) consortium|Amato|United Kingdom
>Spike|hCoV-19/Bahrain/340861247_S22/2020|2020-10-14|EPI_ISL_632255|Original|hCoV-19^^Bahrain|Human|Communicable Disease Laboratory|Communicable Disease Laboratory|Altaif|Bahrain
>Spike|hCoV-19/USA/CA-ALSR-3851/2020|2020-06-29|EPI_ISL_636244|Original|hCoV-19^^California|Human|San Diego County Public Health Laboratory|Andersen lab at Scripps Research|Gangavarapu|USA

>Spike|hCoV-19/England/ALDP-AA3542/2020|2020-10-22|EPI_ISL_623912|Original|hCoV-19^^England|Human|Lighthouse Lab in Alderley Park|Wellcome Sanger Institute for the COVID-19 Genomics UK (COG-UK) consortium|Davies|United Kingdom
>Spike|hCoV-19/USA/LA-EVTL036/2020|2020-04-24|EPI_ISL_435574|Original|hCoV-19^^Louisiana|Human|LSUHS Emerging Viral Threat Laboratory|Microbial Genome Sequencing Center|Kamil|USA
>Spike|hCoV-19/England/MILK-A05784/2020|2020-09-30|EPI_ISL_600032|Original|hCoV-19^^England|Human|Lighthouse Lab in Milton Keynes|Wellcome Sanger Institute for the COVID-19 Genomics UK (COG-UK) consortium|Davies|United Kingdom
>Spike|hCoV-19/USA/AZ-TG280795/2020|2020-04-18|EPI_ISL_694152|Original|hCoV-19^^Arizona|Human|AZ SPHL|TGen North|French|USA
>Spike|hCoV-19/Denmark/DCGC-13174/2020|2020-11-30|EPI_ISL_712145|Original|hCoV-19^^Hovedstaden|Human|Department of Virus and Microbiological Special Diagnostics|Albertsen Lab|Michaelsen|Denmark
>Spike|hCoV-19/Portugal/PT1411/2020|2020-03-28|EPI_ISL_511370|Original|hCoV-19^^Portugal|Human|Instituto Nacional de Saude (INSA)|Instituto Nacional de Saude (INSA)|Borges|Portugal
>Spike|hCoV-19/USA/CA-CZB-3659/2020|2020-07-18|EPI_ISL_516734|Original|hCoV-19^^California|Human|UCSF Clinical Microbiology Laboratory|Chan-Zuckerberg Biohub|Kamm|USA
>Spike|hCoV-19/USA/UT-UPHL-2012135808/2020|2020-08-15|EPI_ISL_746305|Original|hCoV-19^^Utah|Human|Ginkgo Bioworks Clinical Laboratory|Utah Public Health Laboratory|Young|USA
>Spike|hCoV-19/England/ALDP-A6E33A/2020|2020-10-15|EPI_ISL_607173|Original|hCoV-19^^England|Human|Lighthouse Lab in Alderley Park|Wellcome Sanger Institute for the COVID-19 Genomics UK (COG-UK) consortium|Davies|United Kingdom
>Spike|hCoV-19/India/MH-CMP-34/2020|2020-09-09|EPI_ISL_779708|Original|hCoV-19^^Maharashtra|Human|The Foundation for Medical Research|The Foundation for Medical Research|Mandal|India
>Spike|hCoV-19/England/ALDP-CC061D/2020|2020-12-18|EPI_ISL_760587|Original|hCoV-19^^England|Human|Lighthouse Lab in Alderley Park|Wellcome Sanger Institute for the COVID-19 Genomics UK (COG-UK) Consortium|Davies|United Kingdom
>Spike|hCoV-19/England/ALDP-A6CC40/2020|2020-10-14|EPI_ISL_607088|Original|hCoV-19^^England|Human|Lighthouse Lab in Alderley Park|Wellcome Sanger Institute for the COVID-19 Genomics UK (COG-UK) consortium|Davies|United Kingdom
>Spike|hCoV-19/Japan/PG-8758/2020|2020-09-00|EPI_ISL_686354|Original|hCoV-19^^Japan|Human|Pathogen Genomics Center|Pathogen Genomics Center|Sekizuka|Japan
>Spike|hCoV-19/USA/TX-HMH-3720/2020|2020-06-16|EPI_ISL_543136|Original|hCoV-19^^Texas|Human|Houston Methodist Hospital|Houston Methodist Hospital|Long|USA
>Spike|hCoV-19/England/MILK-59460A/2020|2020-06-11|EPI_ISL_558255|Original|hCoV-19^^England|Human|Lighthouse Lab in Milton Keynes|Wellcome Sanger Institute for the COVID-19 Genomics UK (COG-UK) consortium|Davies|United Kingdom
>Spike|hCoV-19/Australia/VIC6217/2020|2020-07-23|EPI_ISL_518215|Original|hCoV-19^^Victoria|Human|Victorian Infectious Diseases Reference Laboratory (VIDRL)|VIDRL and MDU-PHL|Schultz|Australia
>Spike|hCoV-19/SaudiArabia/KAUST-MADINAH1066/2020|2020-06-13|EPI_ISL_513087|Original|hCoV-19^^Madinah|Human|Pathogen Genomics Lab King Abdullah University of Science and Technology(KAUST)|Pathogen Genomics Lab King Abdullah University of Science and Technology(KAUST)|Mohamed Ghazzali|Saudi Arabia
>Spike|hCoV-19/England/MILK-BAA0A3/2020|2020-11-18|EPI_ISL_783173|Original|hCoV-19^^England|Human|Lighthouse Lab in Milton Keynes|Wellcome Sanger Institute for the COVID-19 Genomics UK (COG-UK) Consortium|Davies|United Kingdom
>Spike|hCoV-19/England/SHEF-C658F/2020|2020-04-25|EPI_ISL_479219|Original|hCoV-19^^England|Human|Virology Department|COVID-19 Genomics UK (COG-UK) Consortium|de Silva|United Kingdom
>Spike|hCoV-19/England/ALDP-B26160/2020|2020-11-04|EPI_ISL_646691|Original|hCoV-19^^England|Human|Lighthouse Lab in Alderley Park|Wellcome Sanger Institute for the COVID-19 Genomics UK (COG-UK) Consortium|Davies|United Kingdom

>Spike|hCoV-19/USA/MI-MDHHS-SC21205/2020|2020-04-01|EPI_ISL_507462|Original|hCoV-19^^Michigan|Human|Michigan Department of Health and Human Services|Michigan Department of Health and Human Services|Blankenship|USA
>Spike|hCoV-19/England/CAMC-B52338/2020|2020-11-08|EPI_ISL_656760|Original|hCoV-19^^England|Human|Lighthouse Lab in Cambridge|Wellcome Sanger Institute for the COVID-19 Genomics UK (COG-UK) Consortium|Davies|United Kingdom
>Spike|hCoV-19/England/204241470/2020|2020-10-13|EPI_ISL_622855|Original|hCoV-19^^England|Human|Respiratory Virus Unit|Respiratory Virus Unit|Platt|United Kingdom
>Spike|hCoV-19/England/CAMC-C70B68/2020|2020-12-16|EPI_ISL_769207|Original|hCoV-19^^England|Human|Lighthouse Lab in Cambridge|Wellcome Sanger Institute for the COVID-19 Genomics UK (COG-UK) Consortium|Davies|United Kingdom
>Spike|hCoV-19/England/MILK-C61362/2020|2020-12-13|EPI_ISL_735560|Original|hCoV-19^^England|Human|Lighthouse Lab in Milton Keynes|Wellcome Sanger Institute for the COVID-19 Genomics UK (COG-UK) Consortium|Davies|United Kingdom
>Spike|hCoV-19/England/NOTT-113B08/2020|2020-09-26|EPI_ISL_573458|Original|hCoV-19^^England|Human|Queens Medical Centre|COVID-19 Genomics UK (COG-UK) Consortium|Loose|United Kingdom
>Spike|hCoV-19/USA/CA-CZB-13943/2020|2020-06-23|EPI_ISL_739056|Original|hCoV-19^^California|Human|Alameda County Public Health Lab|Chan-Zuckerberg Biohub|Kamm|USA
>Spike|hCoV-19/Scotland/EDB1541/2020|2020-04-12|EPI_ISL_433077|Original|hCoV-19^^Scotland|Human|Virology Department|COVID-19 Genomics UK (COG-UK) Consortium|Williams|United Kingdom
>Spike|hCoV-19/England/ALDP-BC76BD/2020|2020-11-22|EPI_ISL_721185|Original|hCoV-19^^England|Human|Lighthouse Lab in Alderley Park|Wellcome Sanger Institute for the COVID-19 Genomics UK (COG-UK) Consortium|Davies|United Kingdom
>Spike|hCoV-19/England/MILK-B8C07A/2020|2020-11-13|EPI_ISL_674213|Original|hCoV-19^^England|Human|Lighthouse Lab in Milton Keynes|Wellcome Sanger Institute for the COVID-19 Genomics UK (COG-UK) Consortium|Davies|United Kingdom
>Spike|hCoV-19/USA/WA-UW89/2020|2020-03-10|EPI_ISL_416445|Original|hCoV-19^^Washington|Human|UW Virology Lab|UW Virology Lab|Roychoudhury|USA
>Spike|hCoV-19/Austria/CeMM0376/2020|2020-03-28|EPI_ISL_475820|Original|hCoV-19^^Mayrhofen|Human|Institut f\u00fcr Virologie am Department f\u00fcr Hygiene|Bergthaler laboratory|Bergthaler|Austria
>Spike|hCoV-19/England/MILK-C2FB2C/2020|2020-12-07|EPI_ISL_733872|Original|hCoV-19^^England|Human|Lighthouse Lab in Milton Keynes|Wellcome Sanger Institute for the COVID-19 Genomics UK (COG-UK) Consortium|Davies|United Kingdom
>Spike|hCoV-19/Canada/ON-PHL-20-03814/2020|2020-11-00|EPI_ISL_775025|Original|hCoV-19^^Ontario|Human|Public Health Ontario Laboratory|Public Health Ontario Laboratory|Guthrie|Canada
>Spike|hCoV-19/Portugal/PT2065/2020|2020-11-11|EPI_ISL_732279|Original|hCoV-19^^Portugal|Human|Instituto Nacional de Saude (INSA) and Instituto Gulbenkian de Ciencia (IGC)|Instituto Nacional de Saude (INSA) and Instituto Gulbenkian de Ciencia (IGC)|Borges|Portugal
>Spike|hCoV-19/Australia/NSW2538/2020|2020-04-04|EPI_ISL_527040|Original|hCoV-19^^New South Wales|Human|Area of Virology|Area of Virology|Yeang|Australia
>Spike|hCoV-19/England/EXET-13DA92/2020|2020-00-00|EPI_ISL_724275|Original|hCoV-19^^England|Human|University of Exeter|COVID-19 Genomics UK (COG-UK) Consortium|Temperton|United Kingdom
>Spike|hCoV-19/Spain/CT-IBV-97010114/2020|2020-11-14|EPI_ISL_683641|Original|hCoV-19^^Catalunya|Human|Servicio de Microbiolog\u00eda|SeqCOVID-SPAIN consortium/IBV(CSIC)|Comas|Spain
>Spike|hCoV-19/Australia/VIC17272/2020|2020-07-31|EPI_ISL_663814|Original|hCoV-19^^Victoria|Human|Microbiological Diagnostic Unit - Public Health Laboratory (MDU-PHL)|MDU-PHL|Schultz|Australia
>Spike|hCoV-19/Denmark/DCGC-2297/2020|2020-08-10|EPI_ISL_617721|Original|hCoV-19^^Midtjylland|Human|Department of Virus and Microbiological Special Diagnostics|Albertsen lab|Michaelsen|Denmark
>Spike|hCoV-19/England/CAMB-1AA553/2020|2020-04-17|EPI_ISL_443604|Original|hCoV-19^^England|Human|Department of Pathology|Wellcome Sanger Institute for the COVID-19 Genomics UK (COG-UK) consortium|Amato|United Kingdom

>Spike|hCoV-19/Belgium/IMJ-0401373/2020|2020-04-01|EPI_ISL_462227|Original|hCoV-19^^Bornem|Human|KU Leuven|KU Leuven|Maes|Belgium
>Spike|hCoV-19/Portugal/PT0037/2020|2020-03-15|EPI_ISL_418022|Original|hCoV-19^^Portugal|Human|H Braga|Instituto Nacional de Saude (INSA)|Borges|Portugal
>Spike|hCoV-19/Netherlands/NA_227/2020|2020-03-27|EPI_ISL_422762|Original|hCoV-19^^Netherlands|Human|Dutch COVID-19 response team|Erasmus Medical Center|Oude Munnink|Netherlands
>Spike|hCoV-19/Denmark/DCGC-8169/2020|2020-10-26|EPI_ISL_617224|Original|hCoV-19^^Hovedstaden|Human|Department of Virus and Microbiological Special Diagnostics|Albertsen lab|Michaelsen|Denmark
>Spike|hCoV-19/Belgium/ULG-10628/2020|2020-10-25|EPI_ISL_636607|Original|hCoV-19^^Li\u00e8ge|Human|Department of Clinical Microbiology|GIGA Medical Genomics|Durkin|Belgium
>Spike|hCoV-19/England/QEUH-B6895B/2020|2020-11-11|EPI_ISL_661517|Original|hCoV-19^^England|Human|Lighthouse Lab in Glasgow|Wellcome Sanger Institute for the COVID-19 Genomics UK (COG-UK) Consortium|Davies|United Kingdom
>Spike|hCoV-19/Scotland/QEUH-A0B2D8/2020|2020-10-03|EPI_ISL_599294|Original|hCoV-19^^Scotland|Human|Lighthouse Lab in Glasgow|Wellcome Sanger Institute for the COVID-19 Genomics UK (COG-UK) consortium|Davies|United Kingdom
>Spike|hCoV-19/Australia/VIC4830/2020|2020-07-18|EPI_ISL_519214|Original|hCoV-19^^Victoria|Human|Microbiological Diagnostic Unit - Public Health Laboratory (MDU-PHL)|MDU-PHL|Schultz|Australia
>Spike|hCoV-19/USA/FL-BPHL-0143/2020|2020-03-06|EPI_ISL_480794|Original|hCoV-19^^Florida|Human|Florida Bureau of Public Health Laboratories|Florida Bureau of Public Health Laboratories|Blanton|USA
>Spike|hCoV-19/England/PORT-2D0E85/2020|2020-03-31|EPI_ISL_475297|Original|hCoV-19^^England|Human|Centre for Enzyme Innovation|COVID-19 Genomics UK (COG-UK) Consortium|Robson|United Kingdom
>Spike|hCoV-19/England/MILK-9C463A/2020|2020-09-09|EPI_ISL_566956|Original|hCoV-19^^England|Human|Lighthouse Lab in Milton Keynes|Wellcome Sanger Institute for the COVID-19 Genomics UK (COG-UK) consortium|Davies|United Kingdom
>Spike|hCoV-19/Wales/PHWC-47D8D5/2020|2020-10-09|EPI_ISL_586206|Original|hCoV-19^^Wales|Human|Wales Specialist Virology Centre Sequencing lab|COVID-19 Genomics UK (COG-UK) Consortium|Moore|United Kingdom
>Spike|hCoV-19/Japan/PG-1553/2020|2020-04-00|EPI_ISL_685159|Original|hCoV-19^^Japan|Human|Pathogen Genomics Center|Pathogen Genomics Center|Sekizuka|Japan
>Spike|hCoV-19/England/GSTT-265D8F1/2020|2020-03-25|EPI_ISL_484033|Original|hCoV-19^^England|Human|Centre for Clinical Infection and Diagnostics Research and Genomics Innovation Unit|COVID-19 Genomics UK (COG-UK) Consortium|Awan|United Kingdom
>Spike|hCoV-19/Denmark/DCGC-13205/2020|2020-11-30|EPI_ISL_712150|Original|hCoV-19^^Hovedstaden|Human|Department of Virus and Microbiological Special Diagnostics|Albertsen Lab|Michaelsen|Denmark
>Spike|hCoV-19/Japan/PG-5045/2020|2020-07-00|EPI_ISL_690156|Original|hCoV-19^^Japan|Human|Pathogen Genomics Center|Pathogen Genomics Center|Sekizuka|Japan
>Spike|hCoV-19/Scotland/QEUH-A1FE64/2020|2020-10-01|EPI_ISL_598971|Original|hCoV-19^^Scotland|Human|Lighthouse Lab in Glasgow|Wellcome Sanger Institute for the COVID-19 Genomics UK (COG-UK) consortium|Davies|United Kingdom
>Spike|hCoV-19/England/NOTT-113027/2020|2020-09-07|EPI_ISL_534757|Original|hCoV-19^^England|Human|Queens Medical Centre|COVID-19 Genomics UK (COG-UK) Consortium|Loose|United Kingdom
>Spike|hCoV-19/England/MILK-AC8617/2020|2020-10-21|EPI_ISL_629426|Original|hCoV-19^^England|Human|Lighthouse Lab in Milton Keynes|Wellcome Sanger Institute for the COVID-19 Genomics UK (COG-UK) consortium|Davies|United Kingdom
>Spike|hCoV-19/England/MILK-A0639E/2020|2020-10-01|EPI_ISL_600027|Original|hCoV-19^^England|Human|Lighthouse Lab in Milton Keynes|Wellcome Sanger Institute for the COVID-19 Genomics UK (COG-UK) consortium|Davies|United Kingdom
>Spike|hCoV-19/Netherlands/ZuidHolland_95/2020|2020-03-31|EPI_ISL_422631|Original|hCoV-19^^Zuid Holland|Human|Dutch COVID-19 response team|Erasmus Medical Center|Oude Munnink|Netherlands

>Spike|hCoV-19/England/QEUH-CC77D7/2020|2020-12-20|EPI_ISL_760377|Original|hCoV-
19^^England|Human|Lighthouse Lab in Glasgow|Wellcome Sanger Institute for the COVID-
19 Genomics UK (COG-UK) Consortium|Davies|United Kingdom
>Spike|hCoV-19/Australia/VIC126/2020|2020-03-20|EPI_ISL_419728|Original|hCoV-
19^^Victoria|Human|Microbiological Diagnostic Unit Public Health
Laboratory|Microbiological Diagnostic Unit Public Health Laboratory|Schultz|Australia
>Spike|hCoV-19/Australia/VIC16938/2020|2020-08-05|EPI_ISL_663648|Original|hCoV-
19^^Victoria|Human|Microbiological Diagnostic Unit - Public Health Laboratory (MDU-
PHL)|MDU-PHL|Schultz|Australia
>Spike|hCoV-19/Japan/IC-0535/2021|2021-01-00|EPI_ISL_779218|Original|hCoV-
19^^Japan|Human|Pathogen Genomics Center|Pathogen Genomics Center|Sekizuka|Japan
>Spike|hCoV-19/England/ALDP-A958B3/2020|2020-10-19|EPI_ISL_624846|Original|hCoV-
19^^England|Human|Lighthouse Lab in Alderley Park|Wellcome Sanger Institute for the
COVID-19 Genomics UK (COG-UK) consortium|Davies|United Kingdom
>Spike|hCoV-19/USA/TX-HMH-MCoV-15781/2020|2020-07-10|EPI_ISL_785465|Original|hCoV-
19^^Texas|Human|Houston Methodist Hospital|Houston Methodist Hospital|Long|USA
>Spike|hCoV-19/USA/WA-UW-12903/2020|2020-06-29|EPI_ISL_570809|Original|hCoV-
19^^Washington|Human|UW Virology Lab|UW Virology Lab|Roychoudhury|USA
>Spike|hCoV-19/England/MILK-B384BD/2020|2020-11-01|EPI_ISL_658751|Original|hCoV-
19^^England|Human|Lighthouse Lab in Milton Keynes|Wellcome Sanger Institute for the
COVID-19 Genomics UK (COG-UK) Consortium|Davies|United Kingdom
>Spike|hCoV-19/USA/UT-UPHL-2009308/2020|2020-05-14|EPI_ISL_524914|Original|hCoV-
19^^Utah|Human|Utah Public Health Laboratory|Utah Public Health Laboratory|Young|USA
>Spike|hCoV-19/USA/MI-QDX-467/2020|2020-03-23|EPI_ISL_571202|Original|hCoV-
19^^Michigan|Human|Quest Diagnostics|Quest Diagnostics|Kagan|USA
>Spike|hCoV-19/Australia/VIC705/2020|2020-03-30|EPI_ISL_426992|Original|hCoV-
19^^Victoria|Human|Victorian Infectious Diseases Reference Laboratory
(VIDRL)|Microbiological Diagnostic Unit Public Health Laboratory and Victorian
Infectious Diseases Reference Laboratory|Schultz|Australia
>Spike|hCoV-19/USA/UN-UW-1407/2020|2020-03-17|EPI_ISL_422983|Original|hCoV-
19^^USA|Human|UW Virology Lab|UW Virology Lab|Roychoudhury|USA
>Spike|hCoV-19/Denmark/DCGC-2846/2020|2020-08-24|EPI_ISL_616673|Original|hCoV-
19^^Hovedstaden|Human|Department of Virus and Microbiological Special
Diagnostics|Albertsen lab|Michaelsen|Denmark
>Spike|hCoV-19/USA/FL-BPHL-1490/2020|2020-08-10|EPI_ISL_568588|Original|hCoV-
19^^Florida|Human|Florida Bureau of Public Health Laboratories|Florida Bureau of
Public Health Laboratories|Blanton|USA
>Spike|hCoV-19/England/QEUH-96216A/2020|2020-08-10|EPI_ISL_531747|Original|hCoV-
19^^England|Human|Lighthouse Lab in Glasgow|Wellcome Sanger Institute for the COVID-
19 Genomics UK (COG-UK) consortium|Davies|United Kingdom
>Spike|hCoV-19/England/20100122107/2020|2020-03-02|EPI_ISL_415143|Original|hCoV-
19^^England|Human|Respiratory Virus Unit|Respiratory Virus Unit|Platt|United Kingdom
>Spike|hCoV-19/USA/CA-CZB-2036/2020|2020-05-13|EPI_ISL_486282|Original|hCoV-
19^^California|Human|Humboldt County Public Health Laboratory|Chan-Zuckerberg
Biohub|McGeever|USA
>Spike|hCoV-19/England/QEUH-B10D56/2020|2020-11-02|EPI_ISL_642433|Original|hCoV-
19^^England|Human|Lighthouse Lab in Glasgow|Wellcome Sanger Institute for the COVID-
19 Genomics UK (COG-UK) Consortium|Davies|United Kingdom
>Spike|hCoV-19/France/ARA75981/2020|2020-09-21|EPI_ISL_639985|Original|hCoV-
19^^Auvergne-Rhone-Alpes|Human|CNR Virus des Infections Respiratoires - France
SUD|CNR Virus des Infections Respiratoires - France SUD|Josset|France
>Spike|hCoV-19/Denmark/DCGC-15975/2020|2020-11-23|EPI_ISL_714515|Original|hCoV-
19^^Syddanmark|Human|Department of Virus and Microbiological Special
Diagnostics|Albertsen Lab|Michaelsen|Denmark
>Spike|hCoV-19/England/QEUH-95F537/2020|2020-08-06|EPI_ISL_532312|Original|hCoV-
19^^England|Human|Lighthouse Lab in Glasgow|Wellcome Sanger Institute for the COVID-
19 Genomics UK (COG-UK) consortium|Davies|United Kingdom
>Spike|hCoV-19/England/ALDP-953612/2020|2020-06-08|EPI_ISL_553771|Original|hCoV-
19^^England|Human|Lighthouse Lab in Alderley Park|Wellcome Sanger Institute for the
COVID-19 Genomics UK (COG-UK) consortium|Davies|United Kingdom

>Spike|hCoV-19/England/PORT-2D7510/2020|2020-10-27|EPI_ISL_651277|Original|hCoV-19^^England|Human|Centre for Enzyme Innovation|COVID-19 Genomics UK (COG-UK) Consortium|Robson|United Kingdom
>Spike|hCoV-19/England/QEUH-B66A1E/2020|2020-11-11|EPI_ISL_655266|Original|hCoV-19^^England|Human|Lighthouse Lab in Glasgow|Wellcome Sanger Institute for the COVID-19 Genomics UK (COG-UK) Consortium|Davies|United Kingdom
>Spike|hCoV-19/England/QEUH-9C80FC/2020|2020-09-11|EPI_ISL_567140|Original|hCoV-19^^England|Human|Lighthouse Lab in Glasgow|Wellcome Sanger Institute for the COVID-19 Genomics UK (COG-UK) consortium|Davies|United Kingdom
>Spike|hCoV-19/England/LCST-24D7071/2020|2020-06-06|EPI_ISL_478358|Original|hCoV-19^^England|Human|University Hospitals Of Leicester NHS Trust and DeepSeq Nottingham|COVID-19 Genomics UK (COG-UK) Consortium|Loose|United Kingdom
>Spike|hCoV-19/England/BRIS-12ADBD/2020|2020-05-02|EPI_ISL_458427|Original|hCoV-19^^England|Human|PHE South West Regional Laboratory|Wellcome Sanger Institute for the COVID-19 Genomics UK (COG-UK) consortium|Davies|United Kingdom
>Spike|hCoV-19/Australia/VIC2148/2020|2020-06-20|EPI_ISL_521917|Original|hCoV-19^^Victoria|Human|Victorian Infectious Diseases Reference Laboratory (VIDRL)|VIDRL and MDU-PHL|Schultz|Australia
>Spike|hCoV-19/India/MH-AFMC-5309/2020|2020-05-21|EPI_ISL_496538|Original|hCoV-19^^Maharashtra|Human|Armed Forces Medical College|National Centre For Cell Science|Paul|India
>Spike|hCoV-19/England/205091067/2020|2020-12-11|EPI_ISL_766299|Original|hCoV-19^^England|Human|Respiratory Virus Unit|COVID-19 Genomics UK (COG-UK) Consortium|Platt|United Kingdom
>Spike|hCoV-19/USA/MN-MDH-2141/2020|2020-11-09|EPI_ISL_708479|Original|hCoV-19^^Minnesota|Human|Minnesota Department of Health|Minnesota Department of Health|Lorentz|USA
>Spike|hCoV-19/Belgium/rega-10191293/2020|2020-10-19|EPI_ISL_735198|Original|hCoV-19^^Nossegem|Human|UZ Leuven|KU Leuven|Maes|Belgium
>Spike|hCoV-19/Denmark/DCGC-6003/2020|2020-09-28|EPI_ISL_621112|Original|hCoV-19^^Midtjylland|Human|Department of Virus and Microbiological Special Diagnostics|Albertsen lab|Michaelsen|Denmark
>Spike|hCoV-19/England/MILK-B894CC/2020|2020-11-13|EPI_ISL_675958|Original|hCoV-19^^England|Human|Lighthouse Lab in Milton Keynes|Wellcome Sanger Institute for the COVID-19 Genomics UK (COG-UK) Consortium|Davies|United Kingdom
>Spike|hCoV-19/Spain/MD-IBV-99008615/2020|2020-09-28|EPI_ISL_654194|Original|hCoV-19^^Madrid|Human|Hospital General Universitario Gregorio Mara\u00f1\u00f3n|SeqCOVID-SPAIN consortium/IBV(CSIC)|Comas|Spain
>Spike|hCoV-19/Netherlands/NA_313/2020|2020-04-02|EPI_ISL_422622|Original|hCoV-19^^Netherlands|Human|Dutch COVID-19 response team|Erasmus Medical Center|Oude Munnink|Netherlands
>Spike|hCoV-19/England/SHEF-C8990/2020|2020-10-12|EPI_ISL_613375|Original|hCoV-19^^England|Human|Virology Department|COVID-19 Genomics UK (COG-UK) Consortium|de Silva|United Kingdom
>Spike|hCoV-19/England/CAMC-B366B9/2020|2020-11-05|EPI_ISL_645339|Original|hCoV-19^^England|Human|Lighthouse Lab in Cambridge|Wellcome Sanger Institute for the COVID-19 Genomics UK (COG-UK) Consortium|Davies|United Kingdom
>Spike|hCoV-19/England/LEED-2A992A/2020|2020-04-12|EPI_ISL_538878|Original|hCoV-19^^England|Human|Leeds Teaching Hospitals NHS Trust and Public Health England|Wellcome Sanger Institute for the COVID-19 Genomics UK (COG-UK) consortium|Davies|United Kingdom
>Spike|hCoV-19/England/EXET-13BC25/2020|2020-00-00|EPI_ISL_679361|Original|hCoV-19^^England|Human|University of Exeter|COVID-19 Genomics UK (COG-UK) Consortium|Temperton|United Kingdom
>Spike|hCoV-19/India/UN-15348/2020|2020-00-00|EPI_ISL_491479|Original|hCoV-19^^India|Human|CSIR-CDRI/SGPGI|CSIR-CDRI/SGPGI|Singh|India
>Spike|hCoV-19/USA/TX-HMH-MCoV-18857/2020|2020-11-24|EPI_ISL_785173|Original|hCoV-19^^Texas|Human|Houston Methodist Hospital|Houston Methodist Hospital|Long|USA

>Spike|hCoV-19/USA/CA-ALSR-2666/2020|2020-07-09|EPI_ISL_635617|Original|hCoV-
19^^California|Human|San Diego County Public Health Laboratory|Andersen lab at
Scripps Research|Gangavarapu|USA
>Spike|hCoV-19/England/QEUH-B68C70/2020|2020-11-11|EPI_ISL_655128|Original|hCoV-
19^^England|Human|Lighthouse Lab in Glasgow|Wellcome Sanger Institute for the COVID-
19 Genomics UK (COG-UK) Consortium|Davies|United Kingdom
>Spike|hCoV-19/Australia/VIC138/2020|2020-03-23|EPI_ISL_419834|Original|hCoV-
19^^Victoria|Human|Victorian Infectious Diseases Reference Laboratory
(VIDRL)|Victorian Infectious Diseases Reference Laboratory and Microbiological
Diagnostic Unit Public Health Laboratory|Schultz|Australia
>Spike|hCoV-19/Australia/VIC3831/2020|2020-07-13|EPI_ISL_520544|Original|hCoV-
19^^Victoria|Human|Microbiological Diagnostic Unit - Public Health Laboratory (MDU-
PHL)|MDU-PHL|Schultz|Australia
>Spike|hCoV-19/England/ALDP-9C4B4A/2020|2020-09-11|EPI_ISL_567718|Original|hCoV-
19^^England|Human|Lighthouse Lab in Alderley Park|Wellcome Sanger Institute for the
COVID-19 Genomics UK (COG-UK) consortium|Davies|United Kingdom
>Spike|hCoV-19/England/MILK-B95DC2/2020|2020-11-14|EPI_ISL_709897|Original|hCoV-
19^^England|Human|Lighthouse Lab in Milton Keynes|Wellcome Sanger Institute for the
COVID-19 Genomics UK (COG-UK) Consortium|Davies|United Kingdom
>Spike|hCoV-19/USA/TX-DSHS-0556/2020|2020-05-14|EPI_ISL_574459|Original|hCoV-
19^^Texas|Human|Texas Department of State Health Services|Texas Department of State
Health Services|Rahman|USA
>Spike|hCoV-19/England/ALDP-9CAC05/2020|2020-09-16|EPI_ISL_575705|Original|hCoV-
19^^England|Human|Lighthouse Lab in Alderley Park|Wellcome Sanger Institute for the
COVID-19 Genomics UK (COG-UK) consortium|Davies|United Kingdom
>Spike|hCoV-19/Poland/PL_MCB_65/2020|2020-06-06|EPI_ISL_645064|Original|hCoV-
19^^Poland|Human|Human Genome Variation Research Group|Human Genome Variation
Research Group||Poland
>Spike|hCoV-19/England/QEUH-9CFBB8/2020|2020-09-16|EPI_ISL_589892|Original|hCoV-
19^^England|Human|Lighthouse Lab in Glasgow|Wellcome Sanger Institute for the COVID-
19 Genomics UK (COG-UK) consortium|Davies|United Kingdom
>Spike|hCoV-19/Scotland/QEUH-9E5396/2020|2020-09-24|EPI_ISL_601035|Original|hCoV-
19^^Scotland|Human|Lighthouse Lab in Glasgow|Wellcome Sanger Institute for the COVID-
19 Genomics UK (COG-UK) consortium|Davies|United Kingdom
>Spike|hCoV-19/Wales/PHWC-35B6B/2020|2020-04-27|EPI_ISL_474533|Original|hCoV-
19^^Wales|Human|Originating lab|COVID-19 Genomics UK (COG-UK) Consortium|Moore|United
Kingdom
>Spike|hCoV-19/Denmark/DCGC-22447/2020|2020-12-14|EPI_ISL_757416|Original|hCoV-
19^^Hovedstaden|Human|Department of Virus and Microbiological Special
Diagnostics|Albertsen Lab|Michaelsen|Denmark
>Spike|hCoV-19/Wales/PHWC-249DE/2020|2020-03-19|EPI_ISL_419466|Original|hCoV-
19^^Wales|Human|Wales Specialist Virology Centre|Public Health Wales Microbiology
Cardiff|Moore|United Kingdom
>Spike|hCoV-19/England/ALDP-B62906/2020|2020-11-09|EPI_ISL_656620|Original|hCoV-
19^^England|Human|Lighthouse Lab in Alderley Park|Wellcome Sanger Institute for the
COVID-19 Genomics UK (COG-UK) Consortium|Davies|United Kingdom
>Spike|hCoV-19/USA/MI-UM-10035977253/2020|2020-09-16|EPI_ISL_583356|Original|hCoV-
19^^Michigan|Human|University of Michigan Clinical Microbiology Laboratory|Lauring
Lab|Valesano|USA
>Spike|hCoV-19/Netherlands/ZH-EMC-1089/2020|2020-12-13|EPI_ISL_722770|Original|hCoV-
19^^Zuid Holland|Human|Dutch COVID-19 response team|Erasmus Medical Center|Oude
Munnink|Netherlands
>Spike|hCoV-19/Australia/VIC9349/2020|2020-07-30|EPI_ISL_565514|Original|hCoV-
19^^Victoria|Human|Microbiological Diagnostic Unit - Public Health Laboratory (MDU-
PHL)|MDU-PHL|Schultz|Australia
>Spike|hCoV-19/USA/CA-QDX-1784/2020|2020-03-17|EPI_ISL_604098|Original|hCoV-
19^^California|Human|Quest Diagnostics|Quest Diagnostics|Kagan|USA
>Spike|hCoV-19/England/MILK-A7AA09/2020|2020-10-15|EPI_ISL_606471|Original|hCoV-
19^^England|Human|Lighthouse Lab in Milton Keynes|Wellcome Sanger Institute for the
COVID-19 Genomics UK (COG-UK) consortium|Davies|United Kingdom

>Spike|hCoV-19/Denmark/DCGC-2885/2020|2020-08-24|EPI_ISL_616580|Original|hCoV-19^^Hovedstaden|Human|Department of Virus and Microbiological Special Diagnostics|Albertsen lab|Michaelsen|Denmark
>Spike|hCoV-19/England/LOND-D3BC9/2020|2020-04-04|EPI_ISL_449637|Original|hCoV-19^^England|Human|University College London|COVID-19 Genomics UK (COG-UK) Consortium|Roy|United Kingdom
>Spike|hCoV-19/USA/TX-HMH-4185/2020|2020-06-18|EPI_ISL_543420|Original|hCoV-19^^Texas|Human|Houston Methodist Hospital|Houston Methodist Hospital|Long|USA
>Spike|hCoV-19/England/ALDP-C8B47E/2020|2020-12-16|EPI_ISL_756619|Original|hCoV-19^^England|Human|Lighthouse Lab in Alderley Park|Wellcome Sanger Institute for the COVID-19 Genomics UK (COG-UK) Consortium|Davies|United Kingdom
>Spike|hCoV-19/England/MILK-9581B2/2020|2020-06-09|EPI_ISL_554770|Original|hCoV-19^^England|Human|Lighthouse Lab in Milton Keynes|Wellcome Sanger Institute for the COVID-19 Genomics UK (COG-UK) consortium|Davies|United Kingdom
>Spike|hCoV-19/Panama/333271/2020|2020-02-23|EPI_ISL_496736|Original|hCoV-19^^Panama Center|Human|Gorgas Memorial Laboratory of Health Studies|Gorgas Memorial Laboratory of Health Studies|Martinez|Panama
>Spike|hCoV-19/England/ALDP-9EDBD2/2020|2020-05-09|EPI_ISL_580650|Original|hCoV-19^^England|Human|Lighthouse Lab in Alderley Park|Wellcome Sanger Institute for the COVID-19 Genomics UK (COG-UK) consortium|Davies|United Kingdom
>Spike|hCoV-19/USA/UT-UPHL-201108499/2020|2020-05-12|EPI_ISL_628988|Original|hCoV-19^^Utah|Human|Utah Public Health Laboratory|Utah Public Health Laboratory|Young|USA
>Spike|hCoV-19/India/MH-CSIR-IGIB-patE-F/2020|2020-09-04|EPI_ISL_676509|Original|hCoV-19^^Maharashtra|Human|TNMC & Nair ch. Hospital|CSIR-Institute of Genomics and Integrative Biology|Pandey|India
>Spike|hCoV-19/USA/WA-UW-10420/2020|2020-06-05|EPI_ISL_501089|Original|hCoV-19^^Washington|Human|University of Washington Virology Lab|University of Washington Virology Lab|Roychoudhury|USA
>Spike|hCoV-19/England/QEUH-98EF6E/2020|2020-08-28|EPI_ISL_540395|Original|hCoV-19^^England|Human|Lighthouse Lab in Glasgow|Wellcome Sanger Institute for the COVID-19 Genomics UK (COG-UK) consortium|Davies|United Kingdom
>Spike|hCoV-19/USA/WA-UW39/2020|2020-03-06|EPI_ISL_416456|Original|hCoV-19^^Washington|Human|UW Virology Lab|UW Virology Lab|Roychoudhury|USA
>Spike|hCoV-19/England/NOTT-110F3C/2020|2020-04-20|EPI_ISL_448438|Original|hCoV-19^^England|Human|Queens Medical Centre|COVID-19 Genomics UK (COG-UK) Consortium|Loose|United Kingdom
>Spike|hCoV-19/Switzerland/UR-ETHZ-240077/2020|2020-08-10|EPI_ISL_523915|Original|hCoV-19^^Uri|Human|Viollier AG|Department of Biosystems Science and Engineering|Nadeau|Switzerland
>Spike|hCoV-19/Singapore/171/2020|2020-04-15|EPI_ISL_462289|Original|hCoV-19^^Singapore|Human|National Public Health Laboratory|National Public Health Laboratory|Mak|Singapore
>Spike|hCoV-19/England/ALDP-9E8A34/2020|2020-09-24|EPI_ISL_638273|Original|hCoV-19^^England|Human|Centre for Enzyme Innovation|COVID-19 Genomics UK (COG-UK) Consortium|Robson|United Kingdom
>Spike|hCoV-19/Russia/NVS-RII-34320S/2020|2020-00-00|EPI_ISL_605792|Original|hCoV-19^^Novosibirsk|Human|Department of Experimental Modeling and Pathogenesis of Infectious Diseases|WHO National Influenza Centre Russian Federation|Komissarov|Russia
>Spike|hCoV-19/Belgium/ULG-10072/2020|2020-04-03|EPI_ISL_424631|Original|hCoV-19^^Li\u00e8ge|Human|Department of Clinical Microbiology|GIGA Medical Genomics|Durkin|Belgium
>Spike|hCoV-19/England/CAMC-B21E7D/2020|2020-11-02|EPI_ISL_646067|Original|hCoV-19^^England|Human|Lighthouse Lab in Cambridge|Wellcome Sanger Institute for the COVID-19 Genomics UK (COG-UK) Consortium|Davies|United Kingdom
>Spike|hCoV-19/Australia/VIC9216/2020|2020-08-21|EPI_ISL_565447|Original|hCoV-19^^Victoria|Human|Microbiological Diagnostic Unit - Public Health Laboratory (MDU-PHL)|MDU-PHL|Schultz|Australia
>Spike|hCoV-19/England/20144005404/2020|2020-03-31|EPI_ISL_423490|Original|hCoV-19^^England|Human|Respiratory Virus Unit|Respiratory Virus Unit|Platt|United Kingdom

>Spike|hCoV-19/England/QEUH-9C9DE5/2020|2020-09-10|EPI_ISL_567516|Original|hCoV-19^^England|Human|Lighthouse Lab in Glasgow|Wellcome Sanger Institute for the COVID-19 Genomics UK (COG-UK) consortium|Davies|United Kingdom
>Spike|hCoV-19/England/LIVE-DC0C65/2020|2020-11-03|EPI_ISL_705340|Original|hCoV-19^^England|Human|Liverpool Clinical Laboratories|COVID-19 Genomics UK (COG-UK) Consortium|Haldenby|United Kingdom
>Spike|hCoV-19/Netherlands/ZH-EMC-769/2020|2020-10-10|EPI_ISL_632605|Original|hCoV-19^^Zuid Holland|Human|Dutch COVID-19 response team|Erasmus Medical Center|Oude Munnink|Netherlands
>Spike|hCoV-19/Belgium/SC-0325380/2020|2020-03-25|EPI_ISL_462234|Original|hCoV-19^^Aisemont|Human|KU Leuven|KU Leuven|Maes|Belgium
>Spike|hCoV-19/Switzerland/ZH-ETHZ-400052/2020|2020-12-05|EPI_ISL_729109|Original|hCoV-19^^Zurich|Human|Viollier AG|Department of Biosystems Science and Engineering|Nadeau|Switzerland
>Spike|hCoV-19/England/CAMB-7B237/2020|2020-04-11|EPI_ISL_433933|Original|hCoV-19^^England|Human|Department of Pathology|COVID-19 Genomics UK (COG-UK) Consortium|Meredith|United Kingdom
>Spike|hCoV-19/Jordan/AM-ALSR-5147/2020|2020-10-16|EPI_ISL_730386|Original|hCoV-19^^Amman|Human|Biolab Diagnostic Laboratories|Andersen lab at Scripps Research|Gangavarapu|Jordan
>Spike|hCoV-19/England/QEUH-9F7BD5/2020|2020-09-27|EPI_ISL_589096|Original|hCoV-19^^England|Human|Lighthouse Lab in Glasgow|Wellcome Sanger Institute for the COVID-19 Genomics UK (COG-UK) consortium|Davies|United Kingdom
>Spike|hCoV-19/USA/NY-PV09075/2020|2020-03-20|EPI_ISL_421621|Original|hCoV-19^^New York|Human|MSHS Clinical Microbiology Laboratories|MSHS Pathogen Surveillance Program|van Bakel|USA
>Spike|hCoV-19/England/LIVE-9F929/2020|2020-06-07|EPI_ISL_472025|Original|hCoV-19^^England|Human|Liverpool Clinical Laboratories|COVID-19 Genomics UK (COG-UK) Consortium|Haldenby|United Kingdom
>Spike|hCoV-19/USA/TX-HMH-MCoV-8639/2020|2020-07-01|EPI_ISL_780668|Original|hCoV-19^^Texas|Human|Houston Methodist Hospital|Houston Methodist Hospital|Long|USA
>Spike|hCoV-19/Senegal/1749/2020|2020-03-31|EPI_ISL_480787|Original|hCoV-19^^Dakar|Human|Institut Pasteur Dakar|Institut Pasteur de Dakar|Dia|Senegal
>Spike|hCoV-19/USA/MI-MDHHS-SC20897/2020|2020-04-24|EPI_ISL_476034|Original|hCoV-19^^Michigan|Human|Michigan Department of Health and Human Services|Michigan Department of Health and Human Services|Blankenship|USA
>Spike|hCoV-19/USA/CT-Yale-604/2020|2020-05-05|EPI_ISL_730066|Original|hCoV-19^^Connecticut|Human|Yale Clinical Virology Laboratory|Grubaugh Lab - Yale School of Public Health|Grubaugh|USA
>Spike|hCoV-19/Poland/76089/2020|2020-11-00|EPI_ISL_732860|Original|hCoV-19^^Poland|Human|genXone SA|genXone SA|Kaszuba|Poland
>Spike|hCoV-19/USA/WI-UW-1438/2020|2020-09-11|EPI_ISL_541571|Original|hCoV-19^^Wisconsin|Human|University of Wisconsin-Madison AIDS Vaccine Research Laboratories|University of Wisconsin-Madison AIDS Vaccine Research Laboratories|Baczenas|USA
>Spike|hCoV-19/Denmark/DCGC-9865/2020|2020-11-09|EPI_ISL_669238|Original|hCoV-19^^Midtjylland|Human|Department of Virus and Microbiological Special Diagnostics|Albertsen Lab|Michaelsen|Denmark
>Spike|hCoV-19/USA/AZ-TG547246/2020|2020-07-03|EPI_ISL_695445|Original|hCoV-19^^Arizona|Human|AZ SPHL|TGen North|French|USA
>Spike|hCoV-19/England/MILK-56133C/2020|2020-06-10|EPI_ISL_558300|Original|hCoV-19^^England|Human|Lighthouse Lab in Milton Keynes|Wellcome Sanger Institute for the COVID-19 Genomics UK (COG-UK) consortium|Davies|United Kingdom
>Spike|hCoV-19/England/MILK-99A66A/2020|2020-08-29|EPI_ISL_550322|Original|hCoV-19^^England|Human|Lighthouse Lab in Milton Keynes|Wellcome Sanger Institute for the COVID-19 Genomics UK (COG-UK) consortium|Davies|United Kingdom
>Spike|hCoV-19/England/QEUH-AD39F2/2020|2020-10-26|EPI_ISL_629872|Original|hCoV-19^^England|Human|Lighthouse Lab in Glasgow|Wellcome Sanger Institute for the COVID-19 Genomics UK (COG-UK) consortium|Davies|United Kingdom

>Spike|hCoV-19/England/202881397/2020|2020-07-03|EPI_ISL_491731|Original|hCoV-19^^England|Human|Respiratory Virus Unit|Respiratory Virus Unit|Platt|United Kingdom
>Spike|hCoV-19/USA/NY-NYUMC365/2020|2020-04-08|EPI_ISL_430392|Original|hCoV-19^^New York|Human|NYU Langone Health|Departments of Pathology and Medicine|Maurano|USA
>Spike|hCoV-19/England/MILK-ACE040/2020|2020-10-21|EPI_ISL_630317|Original|hCoV-19^^England|Human|Lighthouse Lab in Milton Keynes|Wellcome Sanger Institute for the COVID-19 Genomics UK (COG-UK) consortium|Davies|United Kingdom
>Spike|hCoV-19/Australia/NSW1321/2020|2020-12-16|EPI_ISL_767887|Original|hCoV-19^^New South Wales|Human|South Eastern Area Laboratory Services (SEALS)|NSW Health Pathology - Institute of Clinical Pathology and Medical Research; Westmead Hospital; University of Sydney|Gall|Australia
>Spike|hCoV-19/USA/WI-UW-587/2020|2020-06-24|EPI_ISL_484962|Original|hCoV-19^^Wisconsin|Human|University of Wisconsin-Madison AIDS Vaccine Research Laboratories|University of Wisconsin-Madison AIDS Vaccine Research Laboratories|Baczenas|USA
>Spike|hCoV-19/England/NORW-EF966/2020|2020-09-26|EPI_ISL_572651|Original|hCoV-19^^England|Human|Quadram Institute Bioscience|COVID-19 Genomics UK (COG-UK) Consortium|Alikhan|United Kingdom
>Spike|hCoV-19/England/QEUH-BF1975/2020|2020-12-05|EPI_ISL_709288|Original|hCoV-19^^England|Human|Lighthouse Lab in Glasgow|Wellcome Sanger Institute for the COVID-19 Genomics UK (COG-UK) Consortium|Davies|United Kingdom
>Spike|hCoV-19/England/QEUH-B6885E/2020|2020-11-11|EPI_ISL_655260|Original|hCoV-19^^England|Human|Lighthouse Lab in Glasgow|Wellcome Sanger Institute for the COVID-19 Genomics UK (COG-UK) Consortium|Davies|United Kingdom
>Spike|hCoV-19/England/CAMB-79B6B/2020|2020-04-02|EPI_ISL_441305|Original|hCoV-19^^England|Human|Department of Pathology|Wellcome Sanger Institute for the COVID-19 Genomics UK (COG-UK) consortium|Amato|United Kingdom
>Spike|hCoV-19/Finland/S11F9/2020|2020-09-20|EPI_ISL_732629|Original|hCoV-19^^Uusimaa|Human|Department of Virology and Immunology|Department of Virology|Smura|Finland
>Spike|hCoV-19/England/MILK-B391B5/2020|2020-11-01|EPI_ISL_658858|Original|hCoV-19^^England|Human|Lighthouse Lab in Milton Keynes|Wellcome Sanger Institute for the COVID-19 Genomics UK (COG-UK) Consortium|Davies|United Kingdom
>Spike|hCoV-19/USA/TX-HMH-6547/2020|2020-06-23|EPI_ISL_546073|Original|hCoV-19^^Texas|Human|Houston Methodist Hospital|Houston Methodist Hospital|Long|USA
>Spike|hCoV-19/England/20139009504/2020|2020-03-27|EPI_ISL_421868|Original|hCoV-19^^England|Human|Respiratory Virus Unit|Respiratory Virus Unit|Platt|United Kingdom
>Spike|hCoV-19/England/CAMC-B5315B/2020|2020-11-09|EPI_ISL_657634|Original|hCoV-19^^England|Human|Lighthouse Lab in Cambridge|Wellcome Sanger Institute for the COVID-19 Genomics UK (COG-UK) Consortium|Davies|United Kingdom
>Spike|hCoV-19/USA/OR-OHSU-0859/2020|2020-06-28|EPI_ISL_525913|Original|hCoV-19^^Oregon|Human|OHSU Lab Services Molecular Microbiology Lab|Oregon SARS-CoV-2 Genome Sequencing Center|O'Roak|USA
>Spike|hCoV-19/Japan/PG-6593/2020|2020-08-00|EPI_ISL_687705|Original|hCoV-19^^Japan|Human|Niigata Prefectural Institute of Public Health and Environmental Sciences|Pathogen Genomics Center|Sekizuka|Japan
>Spike|hCoV-19/Denmark/DCGC-21328/2020|2020-12-21|EPI_ISL_759681|Original|hCoV-19^^Sjaelland|Human|Department of Virus and Microbiological Special Diagnostics|Albertsen Lab|Michaelsen|Denmark
>Spike|hCoV-19/USA/MI-MDHHS-SC21495/2020|2020-07-30|EPI_ISL_516222|Original|hCoV-19^^Michigan|Human|Michigan Department of Health and Human Services|Michigan Department of Health and Human Services|Blankenship|USA
>Spike|hCoV-19/Wales/QEUH-B2CA05/2020|2020-11-02|EPI_ISL_711006|Original|hCoV-19^^Wales|Human|Lighthouse Lab in Glasgow|Wellcome Sanger Institute for the COVID-19 Genomics UK (COG-UK) Consortium|Davies|United Kingdom
>Spike|hCoV-19/Canada/MB-NML-859/2020|2020-03-30|EPI_ISL_582342|Original|hCoV-19^^Manitoba|Human|Cadham Provincial Laboratory|National Microbiology Laboratory (NML)|Majer|Canada

>Spike|hCoV-19/USA/OR-OHSU-1153/2020|2020-07-10|EPI_ISL_509150|Original|hCoV-19^^Oregon|Human|OHSU Lab Services Molecular Microbiology Lab|Oregon SARS-CoV-2 Genome Sequencing Center|O'Roak|USA
>Spike|hCoV-19/Japan/PG-1045/2020|2020-04-00|EPI_ISL_684655|Original|hCoV-19^^Japan|Human|Fukuoka Institute of Health and Environmental Sciences|Pathogen Genomics Center|Sekizuka|Japan
>Spike|hCoV-19/Germany/NW-HHU-313/2020|2020-10-06|EPI_ISL_631316|Orginal|hCoV-19^^North Rhine-Westphalia|Human|ZOTZ KLIMAS MVZ D\u00fcsseldorf-Centrum GbR \u00dcBAG f\u00fcr Labormedizin|Center of Medical Microbiology|Wienemann|Germany
>Spike|hCoV-19/Denmark/DCGC-5526/2020|2020-09-28|EPI_ISL_620851|Original|hCoV-19^^Hovedstaden|Human|Department of Virus and Microbiological Special Diagnostics|Albertsen lab|Michaelsen|Denmark
>Spike|hCoV-19/USA/NY-NYCPHL-001036/2020|2020-09-29|EPI_ISL_633049|Original|hCoV-19^^New York|Human|DOHMH Morrisania|New York City Public Health Laboratory|Wang|USA
>Spike|hCoV-19/Wales/PHWC-4873AA/2020|2020-10-14|EPI_ISL_639187|Original|hCoV-19^^Wales|Human|Wales Specialist Virology Centre Sequencing lab|COVID-19 Genomics UK (COG-UK) Consortium|Moore|United Kingdom
>Spike|hCoV-19/USA/SC-DHEC-0238/2020|2020-06-03|EPI_ISL_529199|Original|hCoV-19^^South Carolina|Human|South Carolina Department of Health and Environmental Control|South Carolina Department of Health and Environmental Control|Flores|USA
>Spike|hCoV-19/USA/TX-HMH-MCoV-9153/2020|2020-07-02|EPI_ISL_780972|Original|hCoV-19^^Texas|Human|Houston Methodist Hospital|Houston Methodist Hospital|Long|USA
>Spike|hCoV-19/USA/MN-MDH-1839/2020|2020-09-21|EPI_ISL_602974|Original|hCoV-19^^Minnesota|Human|Minnesota Department of Health|Minnesota Department of Health|Garfin|USA
>Spike|hCoV-19/Australia/VIC12037/2020|2020-07-13|EPI_ISL_563512|Original|hCoV-19^^Victoria|Human|Microbiological Diagnostic Unit - Public Health Laboratory (MDU-PHL)|MDU-PHL|Schultz|Australia
>Spike|hCoV-19/England/MILK-C006E4/2020|2020-12-02|EPI_ISL_720868|Original|hCoV-19^^England|Human|Lighthouse Lab in Milton Keynes|Wellcome Sanger Institute for the COVID-19 Genomics UK (COG-UK) Consortium|Davies|United Kingdom
>Spike|hCoV-19/England/ALDP-6EA6C8/2020|2020-06-30|EPI_ISL_557568|Original|hCoV-19^^England|Human|Lighthouse Lab in Alderley Park|Wellcome Sanger Institute for the COVID-19 Genomics UK (COG-UK) consortium|Davies|United Kingdom
>Spike|hCoV-19/England/ALDP-C25FC0/2020|2020-12-06|EPI_ISL_782791|Original|hCoV-19^^England|Human|Lighthouse Lab in Alderley Park|Wellcome Sanger Institute for the COVID-19 Genomics UK (COG-UK) Consortium|Davies|United Kingdom
>Spike|hCoV-19/Wales/PHWC-4A5212/2020|2020-12-15|EPI_ISL_764839|Original|hCoV-19^^Wales|Human|Wales Specialist Virology Centre Sequencing lab|COVID-19 Genomics UK (COG-UK) Consortium|Moore|United Kingdom
>Spike|hCoV-19/Costa Rica/INC-0059/2020|2020-06-18|EPI_ISL_512665|Original|hCoV-19^^San Jose|Human|Hospital De Las Mujeres Dr. Adolfo Carit|Inciensa|Brenes Porras|Costa Rica
>Spike|hCoV-19/England/QEUH-A2236F/2020|2020-10-01|EPI_ISL_587459|Original|hCoV-19^^England|Human|Lighthouse Lab in Glasgow|Wellcome Sanger Institute for the COVID-19 Genomics UK (COG-UK) consortium|Davies|United Kingdom
>Spike|hCoV-19/England/QEUH-9B0C7B/2020|2020-09-03|EPI_ISL_559858|Original|hCoV-19^^England|Human|Oxford Viromics|COVID-19 Genomics UK (COG-UK) Consortium|Golubchik|United Kingdom
>Spike|hCoV-19/Norway/3142/2020|2020-08-09|EPI_ISL_549043|Original|hCoV-19^^Viken|Human|Ostfold Hospital Trust - Kalnes|Norwegian Institute of Public Health|Kopperud Riis|Norway
>Spike|hCoV-19/England/MILK-BABF87/2020|2020-11-18|EPI_ISL_720487|Original|hCoV-19^^England|Human|Lighthouse Lab in Milton Keynes|Wellcome Sanger Institute for the COVID-19 Genomics UK (COG-UK) Consortium|Davies|United Kingdom
>Spike|hCoV-19/Kenya/C1162/2020|2020-04-19|EPI_ISL_457850|Original|hCoV-19^^Kenya|Human|KEMRI-CGMR-C|KEMRI-Wellcome Trust Research Programme/KEMRI-CGMR-C Kilifi|Githinji|Kenya

>Spike|hCoV-19/Russia/OMS-ORINFI-3868S/2020|2020-07-08|EPI_ISL_569781|Original|hCoV-19^^Omsk|Human|Omsk Research Institute of Natural Focal Infections|WHO National Influenza Centre Russian Federation|Komissarov|Russia
>Spike|hCoV-19/USA/UT-02245/2020|2020-04-15|EPI_ISL_436936|Original|hCoV-19^^Utah|Human|Utah Public Health Laboratory|Utah Public Health Laboratory|Young|USA
>Spike|hCoV-19/England/CAMC-946560/2020|2020-07-14|EPI_ISL_556689|Original|hCoV-19^^England|Human|Lighthouse Lab in Cambridge|Wellcome Sanger Institute for the COVID-19 Genomics UK (COG-UK) consortium|Davies|United Kingdom
>Spike|hCoV-19/England/ALDP-5BB61C/2020|2020-06-15|EPI_ISL_558756|Original|hCoV-19^^England|Human|Lighthouse Lab in Alderley Park|Wellcome Sanger Institute for the COVID-19 Genomics UK (COG-UK) consortium|Davies|United Kingdom
>Spike|hCoV-19/USA/MN-MDH-564/2020|2020-04-14|EPI_ISL_437380|Original|hCoV-19^^Minnesota|Human|Minnesota Department of Health|Minnesota Department of Health|Garfin|USA
>Spike|hCoV-19/USA/WI-UW-456/2020|2020-06-16|EPI_ISL_484831|Original|hCoV-19^^Wisconsin|Human|University of Wisconsin-Madison AIDS Vaccine Research Laboratories|University of Wisconsin-Madison AIDS Vaccine Research Laboratories|Baczenas|USA
>Spike|hCoV-19/England/CAMB-7A678/2020|2020-04-01|EPI_ISL_441256|Original|hCoV-19^^England|Human|Department of Pathology|Wellcome Sanger Institute for the COVID-19 Genomics UK (COG-UK) consortium|Amato|United Kingdom
>Spike|hCoV-19/England/MILK-ABB592/2020|2020-10-22|EPI_ISL_623400|Original|hCoV-19^^England|Human|Lighthouse Lab in Milton Keynes|Wellcome Sanger Institute for the COVID-19 Genomics UK (COG-UK) consortium|Davies|United Kingdom
>Spike|hCoV-19/Northern Ireland/NIRE-FC4DE/2020|2020-04-12|EPI_ISL_459385|Original|hCoV-19^^Northern Ireland|Human|Regional Virus Laboratory|Wellcome Sanger Institute for the COVID-19 Genomics UK (COG-UK) consortium|Davies|United Kingdom
>Spike|hCoV-19/USA/NJ-CDC-7300/2020|2020-03-10|EPI_ISL_452117|Original|hCoV-19^^New Jersey|Human|NJ Public Health and Environmental Laboratories|Pathogen Discovery|Queen|USA
>Spike|hCoV-19/England/ALDP-C3CA3E/2020|2020-12-10|EPI_ISL_731072|Original|hCoV-19^^England|Human|Lighthouse Lab in Alderley Park|Wellcome Sanger Institute for the COVID-19 Genomics UK (COG-UK) Consortium|Davies|United Kingdom
>Spike|hCoV-19/USA/WA-UW-3843/2020|2020-03-27|EPI_ISL_427191|Original|hCoV-19^^Washington|Human|UW Virology Lab|UW Virology Lab|Roychoudhury|USA
>Spike|hCoV-19/Denmark/DCGC-16728/2020|2020-10-19|EPI_ISL_714440|Original|hCoV-19^^Hovedstaden|Human|Department of Virus and Microbiological Special Diagnostics|Albertsen Lab|Michaelsen|Denmark
>Spike|hCoV-19/England/QEUH-AD1CCE/2020|2020-10-26|EPI_ISL_629921|Original|hCoV-19^^England|Human|Lighthouse Lab in Glasgow|Wellcome Sanger Institute for the COVID-19 Genomics UK (COG-UK) consortium|Davies|United Kingdom
>Spike|hCoV-19/USA/TX-HMH-MCoV-13355/2020|2020-08-24|EPI_ISL_788502|Original|hCoV-19^^Texas|Human|Houston Methodist Hospital|Houston Methodist Hospital|Long|USA
>Spike|hCoV-19/England/MILK-A69C9E/2020|2020-10-09|EPI_ISL_608838|Original|hCoV-19^^England|Human|Lighthouse Lab in Milton Keynes|Wellcome Sanger Institute for the COVID-19 Genomics UK (COG-UK) consortium|Davies|United Kingdom
>Spike|hCoV-19/England/QEUH-9F8D47/2020|2020-09-27|EPI_ISL_588957|Original|hCoV-19^^England|Human|Lighthouse Lab in Glasgow|Wellcome Sanger Institute for the COVID-19 Genomics UK (COG-UK) consortium|Davies|United Kingdom
>Spike|hCoV-19/Denmark/DCGC-19373/2020|2020-12-07|EPI_ISL_748912|Original|hCoV-19^^Syddanmark|Human|Department of Virus and Microbiological Special Diagnostics|Albertsen Lab|Michaelsen|Denmark
>Spike|hCoV-19/Wales/PHWC-486AA6/2020|2020-10-30|EPI_ISL_639105|Original|hCoV-19^^Wales|Human|Wales Specialist Virology Centre Sequencing lab|COVID-19 Genomics UK (COG-UK) Consortium|Moore|United Kingdom
>Spike|hCoV-19/Denmark/DCGC-14381/2020|2020-11-30|EPI_ISL_711732|Original|hCoV-19^^Hovedstaden|Human|Department of Virus and Microbiological Special Diagnostics|Albertsen Lab|Michaelsen|Denmark

>Spike|hCoV-19/USA/DE-DHSS-F986750/2020|2020-11-17|EPI_ISL_767001|Original|hCoV-19^^Delaware|Human|Delaware Public Health Laboratory|Delaware Public Health Laboratory|Bajwa|USA
>Spike|hCoV-19/Japan/PG-7355/2020|2020-08-00|EPI_ISL_688457|Original|hCoV-19^^Japan|Human|Pathogen Genomics Center|Pathogen Genomics Center|Sekizuka|Japan
>Spike|hCoV-19/England/ALDP-B62EDA/2020|2020-11-08|EPI_ISL_655609|Original|hCoV-19^^England|Human|Lighthouse Lab in Alderley Park|Wellcome Sanger Institute for the COVID-19 Genomics UK (COG-UK) Consortium|Davies|United Kingdom
>Spike|hCoV-19/USA/VA-DCLS-0410/2020|2020-04-24|EPI_ISL_467804|Original|hCoV-19^^Virginia|Human|Virginia DCLS|Virginia DCLS|St. Jacques|USA
>Spike|hCoV-19/Netherlands/NH-RIVM-10315/2020|2020-00-00|EPI_ISL_636563|Original|hCoV-19^^NoordHolland|Human|Dutch COVID-19 response team|National Institute for Public Health and the Environment (RIVM)|Meijer|Netherlands
>Spike|hCoV-19/USA/GA_2070/2020|2020-03-07|EPI_ISL_426417|Original|hCoV-19^^Georgia|Human|GA Department of Public Health Laboratory|Pathogen Discovery|Queen|USA
>Spike|hCoV-19/Australia/SAP033/2020|2020-03-19|EPI_ISL_451078|Original|hCoV-19^^South Australia|Human|SA Pathology|SA Pathology|Leong|Australia
>Spike|hCoV-19/India/MH-CCMB-NIV4/2020|2020-03-26|EPI_ISL_450324|Original|hCoV-19^^Maharashtra|Human|NIV Pune|CSIR-Centre for Cellular and Molecular Biology|Banu|India
>Spike|hCoV-19/Wales/ALDP-94D7AF/2020|2020-06-16|EPI_ISL_555103|Original|hCoV-19^^Wales|Human|Lighthouse Lab in Alderley Park|Wellcome Sanger Institute for the COVID-19 Genomics UK (COG-UK) consortium|Davies|United Kingdom
>Spike|hCoV-19/England/LIVE-99840/2020|2020-04-09|EPI_ISL_439765|Original|hCoV-19^^England|Human|Liverpool Clinical Laboratories|COVID-19 Genomics UK (COG-UK) Consortium|Haldenby|United Kingdom
>Spike|hCoV-19/England/ALDP-B3EC91/2020|2020-10-21|EPI_ISL_645418|Original|hCoV-19^^England|Human|Lighthouse Lab in Alderley Park|Wellcome Sanger Institute for the COVID-19 Genomics UK (COG-UK) Consortium|Davies|United Kingdom
>Spike|hCoV-19/USA/WI-WSLH-200037/2020|2020-03-25|EPI_ISL_471202|Original|hCoV-19^^Wisconsin|Human|Wisconsin State Laboratory of Hygiene Communicable Disease Division|Wisconsin State Laboratory of Hygiene Communicable Disease Division|Florek|USA
>Spike|hCoV-19/England/20149012004/2020|2020-04-03|EPI_ISL_466152|Original|hCoV-19^^England|Human|Respiratory Virus Unit|Respiratory Virus Unit|Platt|United Kingdom
>Spike|hCoV-19/Wales/PHWC-47F67F/2020|2020-10-06|EPI_ISL_612708|Original|hCoV-19^^Wales|Human|Wales Specialist Virology Centre Sequencing lab|COVID-19 Genomics UK (COG-UK) Consortium|Moore|United Kingdom
>Spike|hCoV-19/Wales/PHWC-48E024/2020|2020-11-09|EPI_ISL_680795|Original|hCoV-19^^Wales|Human|Wales Specialist Virology Centre Sequencing lab|COVID-19 Genomics UK (COG-UK) Consortium|Moore|United Kingdom
>Spike|hCoV-19/England/20129042204/2020|2020-03-12|EPI_ISL_424043|Original|hCoV-19^^England|Human|Respiratory Virus Unit|Respiratory Virus Unit|Platt|United Kingdom
>Spike|hCoV-19/USA/AZ-TG666138/2020|2020-03-25|EPI_ISL_696221|Original|hCoV-19^^Arizona|Human|Sonora Quest Laboratories|TGen North|French|USA
>Spike|hCoV-19/England/QEUH-A314A6/2020|2020-10-05|EPI_ISL_595894|Original|hCoV-19^^England|Human|Quadram Institute Bioscience|COVID-19 Genomics UK (COG-UK) Consortium|Alikhan|United Kingdom
>Spike|hCoV-19/England/ALDP-8E1762/2020|2020-06-25|EPI_ISL_557052|Original|hCoV-19^^England|Human|Lighthouse Lab in Alderley Park|Wellcome Sanger Institute for the COVID-19 Genomics UK (COG-UK) consortium|Davies|United Kingdom
>Spike|hCoV-19/England/CAMC-990A95/2020|2020-08-26|EPI_ISL_551845|Original|hCoV-19^^England|Human|Lighthouse Lab in Cambridge|Wellcome Sanger Institute for the COVID-19 Genomics UK (COG-UK) consortium|Davies|United Kingdom
>Spike|hCoV-19/Russia/SPE-RII-MH1624S/2020|2020-09-09|EPI_ISL_602341|Original|hCoV-19^^Saint Petersburg|Human|HELIX LLC|WHO National Influenza Centre Russian Federation|Komissarov|Russia

>Spike|hCoV-19/New Zealand/20VR1817/2020|2020-03-27|EPI_ISL_456264|Original|hCoV-
19^^Southern|Human|Southern Community Labs Dunedin|Institute of Environmental Science
and Research (ESR)|de Ligt|New Zealand
>Spike|hCoV-19/Australia/VIC5023/2020|2020-07-15|EPI_ISL_519827|Original|hCoV-
19^^Victoria|Human|Microbiological Diagnostic Unit - Public Health Laboratory (MDU-
PHL)|MDU-PHL|Schultz|Australia
>Spike|hCoV-19/USA/CA-ALSR-3128/2020|2020-08-26|EPI_ISL_635861|Original|hCoV-
19^^California|Human|San Diego County Public Health Laboratory|Andersen lab at
Scripps Research|Gangavarapu|USA
>Spike|hCoV-19/USA/MN-MDH-2162/2020|2020-12-03|EPI_ISL_708500|Original|hCoV-
19^^Minnesota|Human|Minnesota Department of Health|Minnesota Department of
Health|Lorentz|USA
>Spike|hCoV-19/England/QEUH-78EC5A/2020|2020-07-15|EPI_ISL_533199|Original|hCoV-
19^^England|Human|Lighthouse Lab in Glasgow|Wellcome Sanger Institute for the COVID-
19 Genomics UK (COG-UK) consortium|Davies|United Kingdom
>Spike|hCoV-19/Switzerland/AG-ETHZ-420517/2020|2020-12-
22|EPI_ISL_767956|Original|hCoV-19^^Aargau|Human|Viollier AG|Department of Biosystems
Science and Engineering|Nadeau|Switzerland
>Spike|hCoV-19/England/ALDP-9FCE7D/2020|2020-10-01|EPI_ISL_587786|Original|hCoV-
19^^England|Human|Lighthouse Lab in Alderley Park|Wellcome Sanger Institute for the
COVID-19 Genomics UK (COG-UK) consortium|Davies|United Kingdom
>Spike|hCoV-19/Spain/GA-ISCIII-202282/2020|2020-03-11|EPI_ISL_455334|Original|hCoV-
19^^Galicia|Human|Complejo Hospitalario de Orense|Instituto de Salud Carlos
III|Caballero|Spain
>Spike|hCoV-19/Germany/SH-ChVir9939/2020|2020-10-30|EPI_ISL_729426|Original|hCoV-
19^^Schleswig-Holstein|Human|A. Krumbholz|Charit\u00e9 Universit\u00e4tsmedizin
Berlin|Muehlemann|Germany
>Spike|hCoV-19/England/CAMB-1AF891/2020|2020-05-01|EPI_ISL_459307|Original|hCoV-
19^^England|Human|Department of Pathology|Wellcome Sanger Institute for the COVID-19
Genomics UK (COG-UK) consortium|Davies|United Kingdom
>Spike|hCoV-19/USA/UT-UPHL-201211368/2020|2020-04-07|EPI_ISL_678512|Original|hCoV-
19^^Utah|Human|Utah Public Health Laboratory|Utah Public Health Laboratory|Young|USA
>Spike|hCoV-19/USA/CA-IGI-0125/2020|2020-06-08|EPI_ISL_495147|Original|hCoV-
19^^California|Human|Innovative Genomics Institute|Innovative Genomics
Institute|Shivram|USA
>Spike|hCoV-19/England/QEUH-B7D452/2020|2020-11-14|EPI_ISL_741862|Original|hCoV-
19^^England|Human|Oxford Viromics|COVID-19 Genomics UK (COG-UK)
Consortium|Golubchik|United Kingdom
>Spike|hCoV-19/England/QEUH-9CFABB/2020|2020-09-16|EPI_ISL_589805|Original|hCoV-
19^^England|Human|Lighthouse Lab in Glasgow|Wellcome Sanger Institute for the COVID-
19 Genomics UK (COG-UK) consortium|Davies|United Kingdom
>Spike|hCoV-19/USA/AZ-TG670174/2020|2020-04-29|EPI_ISL_696372|Original|hCoV-
19^^Arizona|Human|Sonora Quest Laboratories|TGen North|French|USA
>Spike|hCoV-19/Scotland/CVR23/2020|2020-03-12|EPI_ISL_425749|Original|hCoV-
19^^Scotland|Human|West of Scotland Specialist Virology Centre|COVID-19 Genomics UK
(COG-UK) Consortium|Robertson|United Kingdom
>Spike|hCoV-19/England/QEUH-B2E186/2020|2020-11-03|EPI_ISL_646243|Original|hCoV-
19^^England|Human|Lighthouse Lab in Glasgow|Wellcome Sanger Institute for the COVID-
19 Genomics UK (COG-UK) Consortium|Davies|United Kingdom
>Spike|hCoV-19/India/MH-ACTREC-668/2020|2020-09-16|EPI_ISL_700294|Original|hCoV-
19^^Maharashtra|Human|Hematopathology Laboratory|Hematopathology
Laboratory|Patkar|India
>Spike|hCoV-19/Scotland/QEUH-C33958/2020|2020-12-11|EPI_ISL_757184|Original|hCoV-
19^^Scotland|Human|Lighthouse Lab in Glasgow|Wellcome Sanger Institute for the COVID-
19 Genomics UK (COG-UK) Consortium|Davies|United Kingdom
>Spike|hCoV-19/Japan/IC-0398/2020|2020-12-00|EPI_ISL_736908|Original|hCoV-
19^^Japan|Human|Pathogen Genomics Center|Pathogen Genomics Center|Sekizuka|Japan
>Spike|hCoV-19/France/PAC-IHU-0187/2020|2020-03-27|EPI_ISL_569355|Original|hCoV-
19^^Provence-Alpes-C\u00f4te-d\u2019Azur|Human|MEPHI|MEPHI|Levasseur|France

>Spike|hCoV-19/South Africa/Tygerberg_41/2020|2020-04-08|EPI_ISL_464150|Original|hCoV-19^^Western Cape|Human|National Health Laboratory Service (NHLS)|Division of Medical Virology|Engelbrecht|South Africa
>Spike|hCoV-19/England/MILK-AC73D5/2020|2020-10-22|EPI_ISL_630075|Original|hCoV-19^^England|Human|Lighthouse Lab in Milton Keynes|Wellcome Sanger Institute for the COVID-19 Genomics UK (COG-UK) consortium|Davies|United Kingdom
>Spike|hCoV-19/Denmark/DCGC-10528/2020|2020-11-02|EPI_ISL_670035|Original|hCoV-19^^Hovedstaden|Human|Department of Virus and Microbiological Special Diagnostics|Albertsen Lab|Michaelsen|Denmark
>Spike|hCoV-19/USA/UT-UPHL-201015242/2020|2020-04-08|EPI_ISL_594091|Original|hCoV-19^^Utah|Human|Utah Public Health Laboratory|Utah Public Health Laboratory|Young|USA
>Spike|hCoV-19/Jordan/IR-ALSR-3902/2020|2020-09-30|EPI_ISL_636268|Original|hCoV-19^^Irbid|Human|Biolab Diagnostic Laboratories|Andersen lab at Scripps Research|Gangavarapu|Jordan
>Spike|hCoV-19/Spain/IB-IBV-99011559/2020|2020-12-14|EPI_ISL_779896|Original|hCoV-19^^Balear Islands|Human|Servicio de Microbiolog\u00eda|SeqCOVID-SPAIN consortium/IBV(CSIC)|Comas|Spain
>Spike|hCoV-19/USA/CA-CZB-2158/2020|2020-05-22|EPI_ISL_513708|Original|hCoV-19^^California|Human|Orange County Public Health Lab|Chan-Zuckerberg Biohub|Kamm|USA
>Spike|hCoV-19/England/MILK-9730C3/2020|2020-08-20|EPI_ISL_575820|Original|hCoV-19^^England|Human|Lighthouse Lab in Milton Keynes|Wellcome Sanger Institute for the COVID-19 Genomics UK (COG-UK) consortium|Davies|United Kingdom
>Spike|hCoV-19/England/MILK-991B64/2020|2020-08-27|EPI_ISL_577026|Original|hCoV-19^^England|Human|Northumbria University / South Tees Hospitals NHS Foundation Trust / North Cumbria Integrated Care NHS Foundation Trust / North Tees and Hartlepool NHS Foundation Trust / Newcastle Hospitals NHS Foundation Trust|COVID-19 Genomics UK (COG-UK) Consortium|Bashton|United Kingdom
>Spike|hCoV-19/USA/VA-DCLS-2537/2020|2020-11-14|EPI_ISL_734253|Original|hCoV-19^^Virginia|Human|Virginia Division of Consolidated Laboratory Services (DCLS)|Virginia Division of Consolidated Laboratory Services (DCLS)|St. Jacques|USA
>Spike|hCoV-19/USA/WA-UW-10272/2020|2020-06-03|EPI_ISL_570287|Original|hCoV-19^^Washington|Human|UW Virology Lab|UW Virology Lab|Roychoudhury|USA
>Spike|hCoV-19/England/ALDP-A681FA/2020|2020-10-12|EPI_ISL_608584|Original|hCoV-19^^England|Human|Lighthouse Lab in Alderley Park|Wellcome Sanger Institute for the COVID-19 Genomics UK (COG-UK) consortium|Davies|United Kingdom
>Spike|hCoV-19/England/ALDP-52C0FA/2020|2020-06-01|EPI_ISL_558935|Original|hCoV-19^^England|Human|Lighthouse Lab in Alderley Park|Wellcome Sanger Institute for the COVID-19 Genomics UK (COG-UK) consortium|Davies|United Kingdom
>Spike|hCoV-19/USA/WI-WSLH-200220/2020|2020-04-10|EPI_ISL_578440|Original|hCoV-19^^Wisconsin|Human|Wisconsin State Laboratory of Hygiene Communicable Disease Division|Wisconsin State Laboratory of Hygiene Communicable Disease Division|Florek|USA
>Spike|hCoV-19/Scotland/CVR5569/2020|2020-10-28|EPI_ISL_651844|Original|hCoV-19^^Scotland|Human|West of Scotland Specialist Virology Centre|COVID-19 Genomics UK (COG-UK) Consortium|Robertson|United Kingdom
>Spike|hCoV-19/USA/AZ-TG271882/2020|2020-03-20|EPI_ISL_426544|Original|hCoV-19^^Arizona|Human|AZ SPHL|TGen North|Lemmer|USA
>Spike|hCoV-19/Scotland/EDB3032/2020|2020-04-03|EPI_ISL_489038|Original|hCoV-19^^Scotland|Human|Virology Department|Wellcome Sanger Institute for the COVID-19 Genomics UK (COG-UK) consortium|Davies|United Kingdom
>Spike|hCoV-19/USA/CA-CZB-2366/2020|2020-05-15|EPI_ISL_513807|Original|hCoV-19^^California|Human|Orange County Public Health Laboratory|Chan-Zuckerberg Biohub|Kamm|USA
>Spike|hCoV-19/Japan/PG-0619/2020|2020-04-00|EPI_ISL_684229|Original|hCoV-19^^Japan|Human|Gunma Prefectural Institute of Public Health and Environmental Sciences|Pathogen Genomics Center|Sekizuka|Japan
>Spike|hCoV-19/Australia/VIC2944/2020|2020-07-11|EPI_ISL_521774|Original|hCoV-19^^Victoria|Human|Microbiological Diagnostic Unit - Public Health Laboratory (MDU-PHL)|MDU-PHL|Schultz|Australia

>Spike|hCoV-19/Slovakia/UKBA-207/2020|2020-04-29|EPI_ISL_577738|Original|hCoV-19^^Pezinok|Human|Institute of Virology|Faculty of Natural Sciences|Vinar|Slovakia
>Spike|hCoV-19/USA/CA-CZB-4005/2020|2020-08-01|EPI_ISL_548630|Original|hCoV-19^^California|Human|County of Santa Clara Public Health Department|Chan-Zuckerberg Biohub|Bhatt|USA
>Spike|hCoV-19/Japan/PG-6332/2020|2020-07-00|EPI_ISL_689443|Original|hCoV-19^^Japan|Human|Pathogen Genomics Center|Pathogen Genomics Center|Sekizuka|Japan
>Spike|hCoV-19/USA/WI-UW-800/2020|2020-07-05|EPI_ISL_495504|Original|hCoV-19^^Wisconsin|Human|University of Wisconsin-Madison AIDS Vaccine Research Laboratories|University of Wisconsin-Madison AIDS Vaccine Research Laboratories|Baczenas|USA
>Spike|hCoV-19/England/OXON-B4083/2020|2020-04-21|EPI_ISL_534927|Original|hCoV-19^^England|Human|Oxford Viromics|COVID-19 Genomics UK (COG-UK) Consortium|Golubchik|United Kingdom
>Spike|hCoV-19/Canada/BC_41851/2020|2020-03-02|EPI_ISL_415584|Original|hCoV-19^^British Columbia|Human|BCCDC Public Health Laboratory|BCCDC Public Health Laboratory|Harrigan|Canada
>Spike|hCoV-19/Turkey/IST-GLAB-248/2020|2020-05-18|EPI_ISL_735314|Original|hCoV-19^^Istanbul|Human|Genomic Laboratory (GLAB) (Conjoint lab of Health Directorate of Istanbul and Istanbul Technical University)|Genomic Laboratory (GLAB)|Karacan|Turkey
>Spike|hCoV-19/Australia/VIC6006/2020|2020-07-25|EPI_ISL_518781|Original|hCoV-19^^Victoria|Human|Microbiological Diagnostic Unit - Public Health Laboratory (MDU-PHL)|MDU-PHL|Schultz|Australia
>Spike|hCoV-19/USA/MI-MDHHS-SC22229/2020|2020-10-25|EPI_ISL_666895|Original|hCoV-19^^Michigan|Human|Michigan Department of Health and Human Services|Michigan Department of Health and Human Services|Jacob|USA
>Spike|hCoV-19/USA/TX-HMH-5219/2020|2020-06-24|EPI_ISL_544603|Original|hCoV-19^^Texas|Human|Houston Methodist Hospital|Houston Methodist Hospital|Long|USA
>Spike|hCoV-19/Wuhan/HB-WHCM-117/2020|2020-03-02|EPI_ISL_454997|Original|hCoV-19^^Hubei|Human|Wuhan Chain Medical Labs (CMLabs)|State Key Laboratory of Biotherapy of Sichuan University|Tang|China
>Spike|hCoV-19/Scotland/QEUH-96D67F/2020|2020-08-20|EPI_ISL_534628|Original|hCoV-19^^Scotland|Human|NHSGGC West of Scotland Specialist Virology Centre / MRC-University of Glasgow Centre for Virus Research|Wellcome Sanger Institute for the COVID-19 Genomics UK (COG-UK) consortium|Davies|United Kingdom
>Spike|hCoV-19/England/QEUH-9FF94C/2020|2020-09-30|EPI_ISL_600158|Original|hCoV-19^^England|Human|Lighthouse Lab in Glasgow|Wellcome Sanger Institute for the COVID-19 Genomics UK (COG-UK) consortium|Davies|United Kingdom
>Spike|hCoV-19/USA/HI-H200442/2020|2020-10-07|EPI_ISL_752985|Original|hCoV-19^^Hawaii|Human|State Laboratories Division|State Laboratories Division|Sultana|USA
>Spike|hCoV-19/Luxembourg/LNS9804369/2020|2020-11-14|EPI_ISL_744989|Original|hCoV-19^^Luxembourg|Human|Laboratoire national de sant\u00e9|Laboratoire national de sant\u00e9|Wienecke-Baldacchino|Luxembourg
>Spike|hCoV-19/England/QEUH-9C8539/2020|2020-09-11|EPI_ISL_567091|Original|hCoV-19^^England|Human|Lighthouse Lab in Glasgow|Wellcome Sanger Institute for the COVID-19 Genomics UK (COG-UK) consortium|Davies|United Kingdom
>Spike|hCoV-19/England/CAMB-1B9ADA/2020|2020-11-08|EPI_ISL_679150|Original|hCoV-19^^England|Human|Department of Pathology|COVID-19 Genomics UK (COG-UK) Consortium|Goodfellow|United Kingdom
>Spike|hCoV-19/USA/NJ-QDX-1221/2020|2020-03-16|EPI_ISL_571977|Original|hCoV-19^^New Jersey|Human|Quest Diagnostics|Quest Diagnostics|Kagan|USA
>Spike|hCoV-19/England/QEUH-795F48/2020|2020-07-15|EPI_ISL_533131|Original|hCoV-19^^England|Human|Lighthouse Lab in Glasgow|Wellcome Sanger Institute for the COVID-19 Genomics UK (COG-UK) consortium|Davies|United Kingdom
>Spike|hCoV-19/Australia/VIC17433/2020|2020-08-17|EPI_ISL_663885|Original|hCoV-19^^Victoria|Human|Microbiological Diagnostic Unit - Public Health Laboratory (MDU-PHL)|MDU-PHL|Schultz|Australia
>Spike|hCoV-19/England/ALDP-9CB3C3/2020|2020-09-17|EPI_ISL_575589|Original|hCoV-19^^England|Human|Lighthouse Lab in Alderley Park|Wellcome Sanger Institute for the COVID-19 Genomics UK (COG-UK) consortium|Davies|United Kingdom

>Spike|hCoV-19/Scotland/QEUH-A35EB3/2020|2020-10-05|EPI_ISL_706898|Original|hCoV-19^^Scotland|Human|Oxford Viromics|COVID-19 Genomics UK (COG-UK) Consortium|Golubchik|United Kingdom
>Spike|hCoV-19/France/ARA-118811/2020|2020-12-11|EPI_ISL_745382|Original|hCoV-19^^Auvergne-Rh\u00f4ne-Alpes|Human|CNR Virus des Infections Respiratoires - France SUD|CNR Virus des Infections Respiratoires - France SUD|Bal|France
>Spike|hCoV-19/England/20188069004/2020|2020-04-30|EPI_ISL_465512|Original|hCoV-19^^England|Human|Respiratory Virus Unit|Respiratory Virus Unit|Platt|United Kingdom
>Spike|hCoV-19/South Africa/N00358/2020|2020-09-28|EPI_ISL_712092|Original|hCoV-19^^Eastern Cape|Human|East London NHLS\u00a0Laboratory|National Institute for Communicable Diseases of the National Health Laboratory Service|Bhiman|South Africa
>Spike|hCoV-19/USA/NY-MH001832/2020|2020-05-06|EPI_ISL_526688|Original|hCoV-19^^New York|Human|Faith Laboratory|van Bakel Laboratory|van Bakel|USA
>Spike|hCoV-19/England/QEUH-5480AD/2020|2020-05-14|EPI_ISL_533434|Original|hCoV-19^^England|Human|Lighthouse Lab in Glasgow|Wellcome Sanger Institute for the COVID-19 Genomics UK (COG-UK) consortium|Davies|United Kingdom
>Spike|hCoV-19/USA/NY-QDX-2370/2020|2020-03-17|EPI_ISL_604711|Original|hCoV-19^^New York|Human|Quest Diagnostics|Quest Diagnostics|Kagan|USA
>Spike|hCoV-19/USA/WA-S684/2020|2020-04-11|EPI_ISL_449854|Original|hCoV-19^^Washington|Human|Washington State Department of Health|Seattle Flu Study|Bedford|USA
>Spike|hCoV-19/England/BIRM-65CBA/2020|2020-10-10|EPI_ISL_612218|Original|hCoV-19^^England|Human|University of Birmingham|COVID-19 Genomics UK (COG-UK) Consortium|Loman|United Kingdom
>Spike|hCoV-19/Wales/PHWC-16F806/2020|2020-10-01|EPI_ISL_586024|Original|hCoV-19^^Wales|Human|Wales Specialist Virology Centre Sequencing lab|COVID-19 Genomics UK (COG-UK) Consortium|Moore|United Kingdom
>Spike|hCoV-19/Japan/PG-8198/2020|2020-08-00|EPI_ISL_687297|Original|hCoV-19^^Japan|Human|Pathogen Genomics Center|Pathogen Genomics Center|Sekizuka|Japan
>Spike|hCoV-19/Japan/PG-6906/2020|2020-09-00|EPI_ISL_688014|Original|hCoV-19^^Japan|Human|Pathogen Genomics Center|Pathogen Genomics Center|Sekizuka|Japan
>Spike|hCoV-19/USA/NY-PV08464/2020|2020-03-18|EPI_ISL_421410|Original|hCoV-19^^New York|Human|MSHS Clinical Microbiology Laboratories|MSHS Pathogen Surveillance Program|van Bakel|USA
>Spike|hCoV-19/USA/FL-BPHL-1546/2020|2020-09-03|EPI_ISL_568644|Original|hCoV-19^^Florida|Human|Florida Bureau of Public Health Laboratories|Florida Bureau of Public Health Laboratories|Blanton|USA
>Spike|hCoV-19/USA/WA-UW-10027/2020|2020-05-29|EPI_ISL_461450|Original|hCoV-19^^Washington|Human|UW Virology Lab|UW Virology Lab|Roychoudhury|USA
>Spike|hCoV-19/England/CAMB-735CD/2020|2020-03-20|EPI_ISL_440073|Original|hCoV-19^^England|Human|Department of Pathology|Wellcome Sanger Institute for the COVID-19 Genomics UK (COG-UK) consortium|Amato|United Kingdom
>Spike|hCoV-19/Denmark/DCGC-7266/2020|2020-10-12|EPI_ISL_619239|Original|hCoV-19^^Midtjylland|Human|Department of Virus and Microbiological Special Diagnostics|Albertsen lab|Michaelsen|Denmark
>Spike|hCoV-19/England/MILK-97061F/2020|2020-08-18|EPI_ISL_553258|Original|hCoV-19^^England|Human|Lighthouse Lab in Milton Keynes|Wellcome Sanger Institute for the COVID-19 Genomics UK (COG-UK) consortium|Davies|United Kingdom
>Spike|hCoV-19/USA/NY-Wadsworth-32670-01/2020|2020-03-30|EPI_ISL_676785|Original|hCoV-19^^New York|Human|Wadsworth Center|Wadsworth Center|Lasek-Nesselquist|USA
>Spike|hCoV-19/Norway/4698/2020|2020-10-15|EPI_ISL_635070|Original|hCoV-19^^Troms og Finnmark|Human|University Hospital of Northern Norway|Norwegian Institute of Public Health|Vollan|Norway
>Spike|hCoV-19/USA/FL-BPHL-0664/2020|2020-03-14|EPI_ISL_509722|Original|hCoV-19^^Florida|Human|Florida Bureau of Public Health Laboratories|Florida Bureau of Public Health Laboratories|Blanton|USA
>Spike|hCoV-19/Nigeria/FC-CV247/2020|2020-07-08|EPI_ISL_729932|Original|hCoV-19^^Abuja|Human|Nigeria Centre for Disease Control (NCDC)|African Centre of Excellence for Genomics of Infectious Diseases (ACEGID)|Oluniyi|Nigeria

>Spike|hCoV-19/India/GJ-GBRC-402b/2020|2020-06-25|EPI_ISL_586521|Original|hCoV-19^^Gujarat|Human|B.J. Medical College and Civil hospital|Gujarat Biotechnology Research Centre|Joshi|India
>Spike|hCoV-19/USA/TX-HMH-MCoV-16030/2020|2020-10-27|EPI_ISL_785714|Original|hCoV-19^^Texas|Human|Houston Methodist Hospital|Houston Methodist Hospital|Long|USA
>Spike|hCoV-19/Denmark/DCGC-14290/2020|2020-11-30|EPI_ISL_711674|Original|hCoV-19^^Sjaelland|Human|Department of Virus and Microbiological Special Diagnostics|Albertsen Lab|Michaelsen|Denmark
>Spike|hCoV-19/USA/UT-08578/2020|2020-05-20|EPI_ISL_524332|Original|hCoV-19^^Utah|Human|Utah Public Health Laboratory|Utah Public Health Laboratory|Young|USA
>Spike|hCoV-19/England/QEUH-CC5B64/2020|2020-12-22|EPI_ISL_760503|Original|hCoV-19^^England|Human|Lighthouse Lab in Glasgow|Wellcome Sanger Institute for the COVID-19 Genomics UK (COG-UK) Consortium|Davies|United Kingdom
>Spike|hCoV-19/England/QEUH-B7D6D4/2020|2020-11-14|EPI_ISL_674589|Original|hCoV-19^^England|Human|Lighthouse Lab in Glasgow|Wellcome Sanger Institute for the COVID-19 Genomics UK (COG-UK) Consortium|Davies|United Kingdom
>Spike|hCoV-19/South Africa/LR00127/2020|2020-07-20|EPI_ISL_514359|Original|hCoV-19^^Gauteng|Human|National Institute for Communicable Diseases of the National Health Laboratory Service|National Institute for Communicable Diseases of the National Health Laboratory Service|Allam|South Africa
>Spike|hCoV-19/Japan/PG-5118/2020|2020-08-00|EPI_ISL_690227|Original|hCoV-19^^Japan|Human|Gunma Prefectural Institute of Public Health and Environmental Sciences|Pathogen Genomics Center|Sekizuka|Japan
>Spike|hCoV-19/Jordan/AM-ALSR-4065/2020|2020-10-03|EPI_ISL_636425|Original|hCoV-19^^Amman|Human|Biolab Diagnostic Laboratories|Andersen lab at Scripps Research|Gangavarapu|Jordan
>Spike|hCoV-19/USA/CA-CZB-13454/2020|2020-09-09|EPI_ISL_672474|Original|hCoV-19^^California|Human|Alameda County Public Health Lab|Chan-Zuckerberg Biohub|Bhatt|USA
>Spike|hCoV-19/Australia/VIC8389/2020|2020-08-10|EPI_ISL_564978|Original|hCoV-19^^Victoria|Human|Microbiological Diagnostic Unit - Public Health Laboratory (MDU-PHL)|MDU-PHL|Schultz|Australia
>Spike|hCoV-19/England/NORT-1B63225/2020|2020-12-01|EPI_ISL_741425|Original|hCoV-19^^England|Human|Northumbria University / South Tees Hospitals NHS Foundation Trust / North Cumbria Integrated Care NHS Foundation Trust / North Tees and Hartlepool NHS Foundation Trust / Newcastle Hospitals NHS Foundation Trust|COVID-19 Genomics UK (COG-UK) Consortium|Bashton|United Kingdom
>Spike|hCoV-19/USA/CT-UW-4347/2020|2020-03-30|EPI_ISL_426054|Original|hCoV-19^^Connecticut|Human|UW Virology Lab|UW Virology Lab|Roychoudhury|USA
>Spike|hCoV-19/Italy/CAM-IZSM-119235/2020|2020-08-26|EPI_ISL_738128|Original|hCoV-19^^Campania|Human|IZSM-U.O.C. Virologia|Istituto Zooprofilattico Sperimentale del Mezzogiorno|Viscardi|Italy
>Spike|hCoV-19/India/TG-CCMB-K499/2020|2020-04-14|EPI_ISL_447865|Original|hCoV-19^^Telangana|Human|CSIR-Centre for Cellular and Molecular Biology|CSIR-Centre for Cellular and Molecular Biology|Banu|India
>Spike|hCoV-19/Wales/PHWC-48CCF5/2020|2020-11-07|EPI_ISL_666529|Original|hCoV-19^^Wales|Human|Wales Specialist Virology Centre Sequencing lab|COVID-19 Genomics UK (COG-UK) Consortium|Moore|United Kingdom
>Spike|hCoV-19/England/MILK-CA3E72/2020|2020-12-16|EPI_ISL_762452|Original|hCoV-19^^England|Human|Lighthouse Lab in Milton Keynes|Wellcome Sanger Institute for the COVID-19 Genomics UK (COG-UK) Consortium|Davies|United Kingdom
>Spike|hCoV-19/USA/UN-UW-2-2069/2020|2020-04-16|EPI_ISL_485949|Original|hCoV-19^^USA|Human|UW Virology Lab|UW Virology Lab|Roychoudhury|USA
>Spike|hCoV-19/USA/UT-02713/2020|2020-04-26|EPI_ISL_522272|Original|hCoV-19^^Utah|Human|Utah Public Health Laboratory|Utah Public Health Laboratory|Young|USA
>Spike|hCoV-19/Russia/OMS-ORINFI-1612S/2020|2020-07-06|EPI_ISL_639914|Original|hCoV-19^^Omsk|Human|Omsk Research Institute of Natural Focal Infections|WHO National Influenza Centre Russian Federation|Komissarov|Russia

>Spike|hCoV-19/USA/OR-OHSU-0969/2020|2020-06-30|EPI_ISL_525939|Original|hCoV-19^^Oregon|Human|OHSU Lab Services Molecular Microbiology Lab|Oregon SARS-CoV-2 Genome Sequencing Center|O'Roak|USA
>Spike|hCoV-19/Ireland/LK-NVRL-MPT39150/2020|2020-05-20|EPI_ISL_767810|Original|hCoV-19^^Limerick|Human|National Virus Reference Laboratory|Irish Coronavirus Sequencing Consortium - Teagasc Moorepark|Walsh|Ireland
>Spike|hCoV-19/England/NORT-2837FF/2020|2020-03-31|EPI_ISL_488529|Original|hCoV-19^^England|Human|NU-OMICS DNA Sequencing research facility|Wellcome Sanger Institute for the COVID-19 Genomics UK (COG-UK) consortium|Davies|United Kingdom
>Spike|hCoV-19/Switzerland/BL-ETHZ-100816/2020|2020-03-26|EPI_ISL_451766|Original|hCoV-19^^Basel-Landschaft|Human|Viollier AG|Department of Biosystems Science and Engineering|Nadeau|Switzerland
>Spike|hCoV-19/England/QEUH-95F467/2020|2020-08-06|EPI_ISL_532383|Original|hCoV-19^^England|Human|Lighthouse Lab in Glasgow|Wellcome Sanger Institute for the COVID-19 Genomics UK (COG-UK) consortium|Davies|United Kingdom
>Spike|hCoV-19/England/ALDP-920B1F/2020|2020-06-23|EPI_ISL_552853|Original|hCoV-19^^England|Human|Lighthouse Lab in Alderley Park|Wellcome Sanger Institute for the COVID-19 Genomics UK (COG-UK) consortium|Davies|United Kingdom
>Spike|hCoV-19/England/QEUH-96AD31/2020|2020-08-14|EPI_ISL_531436|Original|hCoV-19^^England|Human|Lighthouse Lab in Glasgow|Wellcome Sanger Institute for the COVID-19 Genomics UK (COG-UK) consortium|Davies|United Kingdom
>Spike|hCoV-19/England/ALDP-A6D2D4/2020|2020-10-14|EPI_ISL_608029|Original|hCoV-19^^England|Human|Lighthouse Lab in Alderley Park|Wellcome Sanger Institute for the COVID-19 Genomics UK (COG-UK) consortium|Davies|United Kingdom
>Spike|hCoV-19/Australia/VIC15268/2020|2020-08-04|EPI_ISL_593225|Original|hCoV-19^^Victoria|Human|Microbiological Diagnostic Unit - Public Health Laboratory (MDU-PHL)|MDU-PHL|Schultz|Australia
>Spike|hCoV-19/England/CAMC-A660A4/2020|2020-10-10|EPI_ISL_637424|Original|hCoV-19^^England|Human|Department of Pathology|COVID-19 Genomics UK (COG-UK) Consortium|Goodfellow|United Kingdom
>Spike|hCoV-19/Peru/LIM-UPCH-0103/2020|2020-06-17|EPI_ISL_540981|Original|hCoV-19^^Lima|Human|Laboratorio de Referencia Nacional de Virus Respiratorios|Laboratorio de Gen\u00f3mica Microbiana|Tsukayama|Peru
>Spike|hCoV-19/USA/FL-UW-1387/2020|2020-09-05|EPI_ISL_536758|Original|hCoV-19^^Florida|Human|University of Wisconsin-Madison AIDS Vaccine Research Laboratories|University of Wisconsin-Madison AIDS Vaccine Research Laboratories|Baczenas|USA
>Spike|hCoV-19/England/ALDP-B462D0/2020|2020-11-07|EPI_ISL_656188|Original|hCoV-19^^England|Human|Lighthouse Lab in Alderley Park|Wellcome Sanger Institute for the COVID-19 Genomics UK (COG-UK) Consortium|Davies|United Kingdom
>Spike|hCoV-19/USA/AZ-TG448671/2020|2020-06-26|EPI_ISL_695018|Original|hCoV-19^^Arizona|Human|TGen North|TGen North|French|USA
>Spike|hCoV-19/Japan/PG-2198/2020|2020-04-00|EPI_ISL_685803|Original|hCoV-19^^Japan|Human|Pathogen Genomics Center|Pathogen Genomics Center|Sekizuka|Japan
>Spike|hCoV-19/England/BRIS-12D1AF/2020|2020-05-04|EPI_ISL_481897|Original|hCoV-19^^England|Human|PHE South West Regional Laboratory|Wellcome Sanger Institute for the COVID-19 Genomics UK (COG-UK) consortium|Davies|United Kingdom
>Spike|hCoV-19/England/BRIS-1303FE/2020|2020-04-11|EPI_ISL_492739|Original|hCoV-19^^England|Human|PHE South West Regional Laboratory|Wellcome Sanger Institute for the COVID-19 Genomics UK (COG-UK) consortium|Davies|United Kingdom
>Spike|hCoV-19/Wales/PHWC-497A57/2020|2020-11-23|EPI_ISL_726923|Original|hCoV-19^^Wales|Human|Originating lab|COVID-19 Genomics UK (COG-UK) Consortium|Moore|United Kingdom
>Spike|hCoV-19/USA/TX-HMH-MCoV-15902/2020|2020-07-10|EPI_ISL_785586|Original|hCoV-19^^Texas|Human|Houston Methodist Hospital|Houston Methodist Hospital|Long|USA
>Spike|hCoV-19/USA/AZ-TG379993/2020|2020-06-16|EPI_ISL_694641|Original|hCoV-19^^Arizona|Human|TGen North|TGen North|French|USA
>Spike|hCoV-19/Switzerland/BE-ETHZ-310523/2020|2020-10-08|EPI_ISL_603591|Original|hCoV-19^^Bern|Human|Viollier AG|Department of Biosystems Science and Engineering|Nadeau|Switzerland

>Spike|hCoV-19/Scotland/CVR5237/2020|2020-10-20|EPI_ISL_626849|Original|hCoV-
19^^Scotland|Human|West of Scotland Specialist Virology Centre|COVID-19 Genomics UK
(COG-UK) Consortium|Robertson|United Kingdom
>Spike|hCoV-19/Canada/ON-UHTC_0041/2020|2020-04-03|EPI_ISL_464037|unknown|hCoV-
19^^Ontario|Human|Unity Health Toronto|Ontario Institute for Cancer Research|de
Borja|Canada
>Spike|hCoV-19/England/CAMB-1B9FAE/2020|2020-11-04|EPI_ISL_651571|Original|hCoV-
19^^England|Human|Department of Pathology|COVID-19 Genomics UK (COG-UK)
Consortium|Goodfellow|United Kingdom
>Spike|hCoV-19/USA/MN-QDX-1362/2020|2020-03-15|EPI_ISL_572151|Original|hCoV-
19^^Minnesota|Human|Quest Diagnostics|Quest Diagnostics|Kagan|USA
>Spike|hCoV-19/France/PAC-IHU-0604/2020|2020-04-10|EPI_ISL_568884|Original|hCoV-
19^^Provence-Alpes-C\u00f4te-d\u2019Azur|Human|MEPHI|MEPHI|Levasseur|France
>Spike|hCoV-19/Italy/ABR-IZSGC-TE5166/2020|2020-03-18|EPI_ISL_420563|Original|hCoV-
19^^Abruzzo|Human|Ospedale Civile Giuseppe Mazzini|Istituto Zooprofilattico
Sperimentale dell'Abruzzo e Molise \G. Caporale\|Lorusso|Italy
>Spike|hCoV-19/USA/WA-S1864/2020|2020-05-27|EPI_ISL_496290|Original|hCoV-
19^^Washington|Human|Washington State Department of Health|Seattle Flu Study|Lee|USA
>Spike|hCoV-19/Russia/PER-RII-MH1950S/2020|2020-09-16|EPI_ISL_602375|Original|hCoV-
19^^Perm|Human|HELIX LLC|WHO National Influenza Centre Russian
Federation|Komissarov|Russia
>Spike|hCoV-19/England/20109008404/2020|2020-03-05|EPI_ISL_464532|Original|hCoV-
19^^England|Human|Respiratory Virus Unit|Respiratory Virus Unit|Platt|United Kingdom
>Spike|hCoV-19/England/ALDP-8DE30A/2020|2020-06-25|EPI_ISL_557066|Original|hCoV-
19^^England|Human|Lighthouse Lab in Alderley Park|Wellcome Sanger Institute for the
COVID-19 Genomics UK (COG-UK) consortium|Davies|United Kingdom
>Spike|hCoV-19/Scotland/CVR4630/2020|2020-09-07|EPI_ISL_612284|Original|hCoV-
19^^Scotland|Human|West of Scotland Specialist Virology Centre|COVID-19 Genomics UK
(COG-UK) Consortium|Robertson|United Kingdom
>Spike|hCoV-19/USA/UT-QDX-1954/2020|2020-03-12|EPI_ISL_604288|Original|hCoV-
19^^Utah|Human|Quest Diagnostics|Quest Diagnostics|Kagan|USA
>Spike|hCoV-19/England/CAMC-9DF8FA/2020|2020-09-20|EPI_ISL_638435|Original|hCoV-
19^^England|Human|Department of Pathology|COVID-19 Genomics UK (COG-UK)
Consortium|Goodfellow|United Kingdom
>Spike|hCoV-19/Canada/ON-PHL-20-03656/2020|2020-11-00|EPI_ISL_775095|Original|hCoV-
19^^Ontario|Human|Public Health Ontario Laboratory|Public Health Ontario
Laboratory|Guthrie|Canada
>Spike|hCoV-19/Australia/VIC17283/2020|2020-08-16|EPI_ISL_663819|Original|hCoV-
19^^Victoria|Human|Microbiological Diagnostic Unit - Public Health Laboratory (MDU-
PHL)|MDU-PHL|Schultz|Australia
>Spike|hCoV-19/Netherlands/UT-EMC-220/2020|2020-12-04|EPI_ISL_722385|Original|hCoV-
19^^Utrecht|Human|Dutch COVID-19 response team|Erasmus Medical Center|Oude
Munnink|Netherlands
>Spike|hCoV-19/England/QEUH-96E09E/2020|2020-08-18|EPI_ISL_530618|Original|hCoV-
19^^England|Human|Lighthouse Lab in Glasgow|Wellcome Sanger Institute for the COVID-
19 Genomics UK (COG-UK) consortium|Davies|United Kingdom
>Spike|hCoV-19/USA/CA-QDX-811/2020|2020-03-23|EPI_ISL_571504|Original|hCoV-
19^^California|Human|Quest Diagnostics|Quest Diagnostics|Kagan|USA
>Spike|hCoV-19/USA/TX-HMH-6156/2020|2020-07-04|EPI_ISL_547277|Original|hCoV-
19^^Texas|Human|Houston Methodist Hospital|Houston Methodist Hospital|Long|USA
>Spike|hCoV-19/Luxembourg/LNS2068754/2020|2020-10-31|EPI_ISL_740053|Original|hCoV-
19^^Luxembourg|Human|Laboratoire national de sant\u00e9|Laboratoire national de
sant\u00e9|Wienecke-Baldacchino|Luxembourg
>Spike|hCoV-19/England/20139044504/2020|2020-03-28|EPI_ISL_420683|Original|hCoV-
19^^England|Human|Respiratory Virus Unit|Respiratory Virus Unit|Platt|United Kingdom
>Spike|hCoV-19/England/NORT-28D6DA/2020|2020-11-18|EPI_ISL_702815|Original|hCoV-
19^^England|Human|Northumbria University / South Tees Hospitals NHS Foundation Trust
/ North Cumbria Integrated Care NHS Foundation Trust / North Tees and Hartlepool NHS
Foundation Trust / Newcastle Hospitals NHS Foundation Trust|COVID-19 Genomics UK
(COG-UK) Consortium|Bashton|United Kingdom

>Spike|hCoV-19/Wales/PHWC-48031A/2020|2020-10-11|EPI_ISL_612164|Original|hCoV-19^^Wales|Human|Wales Specialist Virology Centre Sequencing lab|COVID-19 Genomics UK (COG-UK) Consortium|Moore|United Kingdom
>Spike|hCoV-19/USA/TX-HMH-MCoV-13940/2020|2020-08-15|EPI_ISL_787232|Original|hCoV-19^^Texas|Human|Houston Methodist Hospital|Houston Methodist Hospital|Long|USA
>Spike|hCoV-19/USA/NY-NYCPHL-000127/2020|2020-04-02|EPI_ISL_631618|Original|hCoV-19^^New York|Human|OCME Office Of Chief Medical Examiner|New York City Public Health Laboratory|Wang|USA
>Spike|hCoV-19/Australia/VIC16932/2020|2020-08-06|EPI_ISL_663645|Original|hCoV-19^^Victoria|Human|Microbiological Diagnostic Unit - Public Health Laboratory (MDU-PHL)|MDU-PHL|Schultz|Australia
>Spike|hCoV-19/USA/CA-CZB-7140/2020|2020-09-02|EPI_ISL_583015|Original|hCoV-19^^California|Human|Orange County Public Health Lab|Chan-Zuckerberg Biohub|Bhatt|USA
>Spike|hCoV-19/England/CAMC-B32BFA/2020|2020-11-05|EPI_ISL_659105|Original|hCoV-19^^England|Human|Lighthouse Lab in Cambridge|Wellcome Sanger Institute for the COVID-19 Genomics UK (COG-UK) Consortium|Davies|United Kingdom
>Spike|hCoV-19/USA/MA-MGH-00055/2020|2020-03-25|EPI_ISL_460175|Original|hCoV-19^^Massachusetts|Human|Massachusetts General Hospital|Infectious Disease Program|Lemieux|USA
>Spike|hCoV-19/USA/WA-UW108/2020|2020-03-11|EPI_ISL_416646|Original|hCoV-19^^Washington|Human|UW Virology Lab|UW Virology Lab|Roychoudhury|USA
>Spike|hCoV-19/USA/TX-HMH-MCoV-13597/2020|2020-08-20|EPI_ISL_788707|Original|hCoV-19^^Texas|Human|Houston Methodist Hospital|Houston Methodist Hospital|Long|USA
>Spike|hCoV-19/Northern Ireland/NIRE-FBCB9/2020|2020-04-10|EPI_ISL_470084|Original|hCoV-19^^Northern Ireland|Human|Regional Virus Laboratory|Wellcome Sanger Institute for the COVID-19 Genomics UK (COG-UK) consortium|Davies|United Kingdom
>Spike|hCoV-19/USA/TX-HMH-MCoV-14299/2020|2020-10-05|EPI_ISL_787584|Original|hCoV-19^^Texas|Human|Houston Methodist Hospital|Houston Methodist Hospital|Long|USA
>Spike|hCoV-19/USA/VA-DCLS-0725/2020|2020-07-09|EPI_ISL_526848|Original|hCoV-19^^Virginia|Human|Virginia DCLS|Virginia DCLS|St. Jacques|USA
>Spike|hCoV-19/France/PAC-IHU-0545/2020|2020-04-06|EPI_ISL_569584|Original|hCoV-19^^Provence-Alpes-C\u00f4te-d\u2019Azur|Human|MEPHI|MEPHI|Levasseur|France
>Spike|hCoV-19/England/ALDP-B49185/2020|2020-11-07|EPI_ISL_649314|Original|hCoV-19^^England|Human|Lighthouse Lab in Alderley Park|Wellcome Sanger Institute for the COVID-19 Genomics UK (COG-UK) Consortium|Davies|United Kingdom
>Spike|hCoV-19/England/QEUH-B31DB9/2020|2020-11-04|EPI_ISL_646197|Original|hCoV-19^^England|Human|Lighthouse Lab in Glasgow|Wellcome Sanger Institute for the COVID-19 Genomics UK (COG-UK) Consortium|Davies|United Kingdom
>Spike|hCoV-19/England/CAMB-1AF336/2020|2020-04-30|EPI_ISL_459260|Original|hCoV-19^^England|Human|Department of Pathology|Wellcome Sanger Institute for the COVID-19 Genomics UK (COG-UK) consortium|Davies|United Kingdom
>Spike|hCoV-19/England/201160049/2020|2020-03-11|EPI_ISL_464781|Original|hCoV-19^^England|Human|Respiratory Virus Unit|Respiratory Virus Unit|Platt|United Kingdom
>Spike|hCoV-19/England/NORW-F57EB/2020|2020-10-31|EPI_ISL_665458|Original|hCoV-19^^England|Human|Quadram Institute Bioscience|COVID-19 Genomics UK (COG-UK) Consortium|Alikhan|United Kingdom
>Spike|hCoV-19/England/MILK-99C6B3/2020|2020-08-30|EPI_ISL_550571|Original|hCoV-19^^England|Human|Lighthouse Lab in Milton Keynes|Wellcome Sanger Institute for the COVID-19 Genomics UK (COG-UK) consortium|Davies|United Kingdom
>Spike|hCoV-19/Spain/MD-ISCIII-2014599/2020|2020-08-19|EPI_ISL_691702|Original|hCoV-19^^Madrid|Human|Hospital Cl\u00ednico San Carlos|Instituto de Salud Carlos III|Caballero|Spain
>Spike|hCoV-19/England/PORT-2E9F1D/2020|2020-00-00|EPI_ISL_679975|Original|hCoV-19^^England|Human|Centre for Enzyme Innovation|COVID-19 Genomics UK (COG-UK) Consortium|Robson|United Kingdom
>Spike|hCoV-19/Scotland/EDB2096/2020|2020-04-19|EPI_ISL_433194|Original|hCoV-19^^Scotland|Human|Virology Department|COVID-19 Genomics UK (COG-UK) Consortium|Williams|United Kingdom

>Spike|hCoV-19/Russia/OMS-ORINFI-6680S/2020|2020-05-12|EPI_ISL_639935|Original|hCoV-19^^Omsk|Human|Omsk Research Institute of Natural Focal Infections|WHO National Influenza Centre Russian Federation|Komissarov|Russia
>Spike|hCoV-19/England/QEUH-9F5867/2020|2020-09-25|EPI_ISL_588462|Original|hCoV-19^^England|Human|Lighthouse Lab in Glasgow|Wellcome Sanger Institute for the COVID-19 Genomics UK (COG-UK) consortium|Davies|United Kingdom
>Spike|hCoV-19/USA/NY-Wadsworth-11330-01/2020|2020-03-07|EPI_ISL_426328|Original|hCoV-19^^New York|Human|Wadsworth Center|Wadsworth Center|Lasek-Nesselquist|USA
>Spike|hCoV-19/England/ALDP-B1C9CF/2020|2020-11-01|EPI_ISL_645622|Original|hCoV-19^^England|Human|Lighthouse Lab in Alderley Park|Wellcome Sanger Institute for the COVID-19 Genomics UK (COG-UK) Consortium|Davies|United Kingdom
>Spike|hCoV-19/England/QEUH-B1370A/2020|2020-11-02|EPI_ISL_641633|Original|hCoV-19^^England|Human|Lighthouse Lab in Glasgow|Wellcome Sanger Institute for the COVID-19 Genomics UK (COG-UK) Consortium|Davies|United Kingdom
>Spike|hCoV-19/Japan/PG-7428/2020|2020-07-00|EPI_ISL_688530|Original|hCoV-19^^Japan|Human|Pathogen Genomics Center|Pathogen Genomics Center|Sekizuka|Japan
>Spike|hCoV-19/USA/TX-HMH-MCoV-8988/2020|2020-07-02|EPI_ISL_780478|Original|hCoV-19^^Texas|Human|Houston Methodist Hospital|Houston Methodist Hospital|Long|USA
>Spike|hCoV-19/Denmark/DCGC-909/2020|2020-05-18|EPI_ISL_614472|Original|hCoV-19^^Hovedstaden|Human|Department of Virus and Microbiological Special Diagnostics|Albertsen lab|Michaelsen|Denmark
>Spike|hCoV-19/Ukraine/Kharkiv-879/2020|2020-08-07|EPI_ISL_582511|Original|hCoV-19^^Kharkiv|Human|Department of Respiratory and other Viral Infections of L.V.Gromashevsky Institute of Epidemiology & Infectious Diseases NAMS of Ukrain\u0435|Department of Respiratory and other Viral Infections of L.V.Gromashevsky Institute of Epidemiology & Infectious Diseases NAMS of Ukrain\u0435|Mironenko|Ukraine
>Spike|hCoV-19/England/ALDP-9C5323/2020|2020-09-10|EPI_ISL_567809|Original|hCoV-19^^England|Human|Lighthouse Lab in Alderley Park|Wellcome Sanger Institute for the COVID-19 Genomics UK (COG-UK) consortium|Davies|United Kingdom
>Spike|hCoV-19/Wales/PHWC-4987F5/2020|2020-11-26|EPI_ISL_727126|Original|hCoV-19^^Wales|Human|Originating lab|COVID-19 Genomics UK (COG-UK) Consortium|Moore|United Kingdom
>Spike|hCoV-19/England/ALDP-BB4D54/2020|2020-11-20|EPI_ISL_702514|Original|hCoV-19^^England|Human|Lighthouse Lab in Alderley Park|Wellcome Sanger Institute for the COVID-19 Genomics UK (COG-UK) Consortium|Davies|United Kingdom
>Spike|hCoV-19/England/MILK-B55933/2020|2020-11-05|EPI_ISL_657682|Original|hCoV-19^^England|Human|Lighthouse Lab in Milton Keynes|Wellcome Sanger Institute for the COVID-19 Genomics UK (COG-UK) Consortium|Davies|United Kingdom
>Spike|hCoV-19/Wales/PHWC-47DC51/2020|2020-10-13|EPI_ISL_595344|Original|hCoV-19^^Wales|Human|Originating lab|COVID-19 Genomics UK (COG-UK) Consortium|Moore|United Kingdom
>Spike|hCoV-19/Denmark/DCGC-19310/2020|2020-12-07|EPI_ISL_748875|Original|hCoV-19^^Hovedstaden|Human|Department of Virus and Microbiological Special Diagnostics|Albertsen Lab|Michaelsen|Denmark
>Spike|hCoV-19/England/QEUH-BF0B25/2020|2020-12-05|EPI_ISL_709283|Original|hCoV-19^^England|Human|Lighthouse Lab in Glasgow|Wellcome Sanger Institute for the COVID-19 Genomics UK (COG-UK) Consortium|Davies|United Kingdom
>Spike|hCoV-19/Netherlands/GE-EMC-273/2020|2020-06-06|EPI_ISL_523252|Original|hCoV-19^^Gelderland|Human|Dutch COVID-19 response team|Erasmus Medical Center|Oude Munnink|Netherlands
>Spike|hCoV-19/USA/TX-DSHS-1527/2020|2020-06-23|EPI_ISL_683442|Original|hCoV-19^^Texas|Human|Texas Department of State Health Services|Texas Department of State Health Services|Rahman|USA
>Spike|hCoV-19/England/MILK-B964BD/2020|2020-11-14|EPI_ISL_674947|Original|hCoV-19^^England|Human|Lighthouse Lab in Milton Keynes|Wellcome Sanger Institute for the COVID-19 Genomics UK (COG-UK) Consortium|Davies|United Kingdom

>Spike|hCoV-19/England/CAMB-8283B/2020|2020-04-13|EPI_ISL_443407|Original|hCoV-19^^England|Human|Department of Pathology|Wellcome Sanger Institute for the COVID-19 Genomics UK (COG-UK) consortium|Amato|United Kingdom
>Spike|hCoV-19/Northern Ireland/NIRE-FBB9E/2020|2020-04-13|EPI_ISL_470039|Original|hCoV-19^^Northern Ireland|Human|Regional Virus Laboratory|Wellcome Sanger Institute for the COVID-19 Genomics UK (COG-UK) consortium|Davies|United Kingdom
>Spike|hCoV-19/Canada/NB-226/2020|2020-03-23|EPI_ISL_479642|Original|hCoV-19^^New Brunswick|Human|Dr. Georges-L.-Dumont University Hospital Centre|National Microbiology Laboratory|Majer|Canada
>Spike|hCoV-19/South Africa/LR00066/2020|2020-07-13|EPI_ISL_514378|Original|hCoV-19^^Gauteng|Human|National Institute for Communicable Diseases of the National Health Laboratory Service|National Institute for Communicable Diseases of the National Health Laboratory Service|Allam|South Africa
>Spike|hCoV-19/USA/CA-ALSR-4472/2020|2020-10-20|EPI_ISL_649049|Original|hCoV-19^^California|Human|San Diego County Public Health Laboratory|Andersen lab at Scripps Research|Gangavarapu|USA
>Spike|hCoV-19/Netherlands/ZH-EMC-417/2020|2020-08-10|EPI_ISL_523757|Original|hCoV-19^^Zuid Holland|Human|Dutch COVID-19 response team|Erasmus Medical Center|Oude Munnink|Netherlands
>Spike|hCoV-19/Ireland/D-NVRL-AIIDV0030v1/2020|2020-04-03|EPI_ISL_644326|Original|hCoV-19^^Dublin|Human|CEPHR / Vincent's Hospital|Irish Coronavirus Sequencing Consortium - National Virus Reference Laboratory|Gonzalez|Ireland
>Spike|hCoV-19/Wales/PHWC-16881C/2020|2020-04-21|EPI_ISL_494292|Original|hCoV-19^^Wales|Human|Originating lab|COVID-19 Genomics UK (COG-UK) Consortium|Moore|United Kingdom
>Spike|hCoV-19/Wales/PHWC-49AA72/2020|2020-12-03|EPI_ISL_727638|Original|hCoV-19^^Wales|Human|Originating lab|COVID-19 Genomics UK (COG-UK) Consortium|Moore|United Kingdom
>Spike|hCoV-19/England/CAMC-9DF748/2020|2020-09-21|EPI_ISL_638420|Original|hCoV-19^^England|Human|Department of Pathology|COVID-19 Genomics UK (COG-UK) Consortium|Goodfellow|United Kingdom
>Spike|hCoV-19/England/20109007304/2020|2020-03-07|EPI_ISL_464527|Original|hCoV-19^^England|Human|Respiratory Virus Unit|Respiratory Virus Unit|Platt|United Kingdom
>Spike|hCoV-19/England/BRIS-1854002/2020|2020-04-27|EPI_ISL_492294|Original|hCoV-19^^England|Human|PHE South West Regional Laboratory|Wellcome Sanger Institute for the COVID-19 Genomics UK (COG-UK) consortium|Davies|United Kingdom
>Spike|hCoV-19/England/QEUH-B68557/2020|2020-11-11|EPI_ISL_655070|Original|hCoV-19^^England|Human|Lighthouse Lab in Glasgow|Wellcome Sanger Institute for the COVID-19 Genomics UK (COG-UK) Consortium|Davies|United Kingdom
>Spike|hCoV-19/USA/TX-HMH-3788/2020|2020-06-12|EPI_ISL_543193|Original|hCoV-19^^Texas|Human|Houston Methodist Hospital|Houston Methodist Hospital|Long|USA
>Spike|hCoV-19/USA/TX-HMH-4285/2020|2020-06-18|EPI_ISL_543496|Original|hCoV-19^^Texas|Human|Houston Methodist Hospital|Houston Methodist Hospital|Long|USA
>Spike|hCoV-19/USA/MI-UM-10036117605/2020|2020-09-24|EPI_ISL_590717|Original|hCoV-19^^Michigan|Human|University of Michigan Clinical Microbiology Laboratory|Lauring Lab|Valesano|USA
>Spike|hCoV-19/Northern Ireland/NIRE-103B47/2020|2020-04-16|EPI_ISL_489217|Original|hCoV-19^^Northern Ireland|Human|Regional Virus Laboratory|Wellcome Sanger Institute for the COVID-19 Genomics UK (COG-UK) consortium|Davies|United Kingdom
>Spike|hCoV-19/USA/TX-DSHS-1345/2020|2020-04-07|EPI_ISL_654926|Original|hCoV-19^^Texas|Human|Texas Department of State Health Services|Texas Department of State Health Services|Rahman|USA
>Spike|hCoV-19/England/CAMC-B3632B/2020|2020-11-05|EPI_ISL_645261|Original|hCoV-19^^England|Human|Lighthouse Lab in Cambridge|Wellcome Sanger Institute for the COVID-19 Genomics UK (COG-UK) Consortium|Davies|United Kingdom

>Spike|hCoV-19/England/ALDP-AA1526/2020|2020-10-22|EPI_ISL_624307|Original|hCoV-19^^England|Human|Lighthouse Lab in Alderley Park|Wellcome Sanger Institute for the COVID-19 Genomics UK (COG-UK) consortium|Davies|United Kingdom
>Spike|hCoV-19/Switzerland/SO-ETHZ-380286/2020|2020-11-25|EPI_ISL_721877|Original|hCoV-19^^Solothurn|Human|Viollier AG|Department of Biosystems Science and Engineering|Nadeau|Switzerland
>Spike|hCoV-19/USA/CT-Yale-395/2020|2020-05-05|EPI_ISL_738312|Original|hCoV-19^^Connecticut|Human|Connecticut Veterans' Affairs Hospital|Grubaugh Lab - Yale School of Public Health|Grubaugh|USA
>Spike|hCoV-19/USA/TX-HMH-7078/2020|2020-06-28|EPI_ISL_546486|Original|hCoV-19^^Texas|Human|Houston Methodist Hospital|Houston Methodist Hospital|Long|USA
>Spike|hCoV-19/England/SHEF-C82B3/2020|2020-11-12|EPI_ISL_680151|Original|hCoV-19^^England|Human|Virology Department|COVID-19 Genomics UK (COG-UK) Consortium|de Silva|United Kingdom
>Spike|hCoV-19/Wales/ALDP-94CA10/2020|2020-06-15|EPI_ISL_556072|Original|hCoV-19^^Wales|Human|Lighthouse Lab in Alderley Park|Wellcome Sanger Institute for the COVID-19 Genomics UK (COG-UK) consortium|Davies|United Kingdom
>Spike|hCoV-19/England/ALDP-5A30DD/2020|2020-06-14|EPI_ISL_558748|Original|hCoV-19^^England|Human|Lighthouse Lab in Alderley Park|Wellcome Sanger Institute for the COVID-19 Genomics UK (COG-UK) consortium|Davies|United Kingdom
>Spike|hCoV-19/England/CAMB-1B823D/2020|2020-10-19|EPI_ISL_638198|Original|hCoV-19^^England|Human|Department of Pathology|COVID-19 Genomics UK (COG-UK) Consortium|Goodfellow|United Kingdom
>Spike|hCoV-19/England/EXET-13E985/2020|2020-12-12|EPI_ISL_764170|Original|hCoV-19^^England|Human|University of Exeter|COVID-19 Genomics UK (COG-UK) Consortium|Temperton|United Kingdom
>Spike|hCoV-19/England/ALDP-A6FC89/2020|2020-10-15|EPI_ISL_607090|Original|hCoV-19^^England|Human|Lighthouse Lab in Alderley Park|Wellcome Sanger Institute for the COVID-19 Genomics UK (COG-UK) consortium|Davies|United Kingdom
>Spike|hCoV-19/Japan/PG-7189/2020|2020-07-00|EPI_ISL_688293|Original|hCoV-19^^Japan|Human|Saitama Prefectural Institute of Public Health|Pathogen Genomics Center|Sekizuka|Japan
>Spike|hCoV-19/Ireland/KK-NVRL-72IRL21404/2020|2020-08-13|EPI_ISL_528483|Original|hCoV-19^^Kilkenny|Human|National Virus Reference Laboratory|National Virus Reference Laboratory|Gonzalez|Ireland
>Spike|hCoV-19/England/ALDP-94E902/2020|2020-06-20|EPI_ISL_555122|Original|hCoV-19^^England|Human|Lighthouse Lab in Alderley Park|Wellcome Sanger Institute for the COVID-19 Genomics UK (COG-UK) consortium|Davies|United Kingdom
>Spike|hCoV-19/England/QEUH-BC3F98/2020|2020-11-24|EPI_ISL_703801|Original|hCoV-19^^England|Human|Lighthouse Lab in Glasgow|Wellcome Sanger Institute for the COVID-19 Genomics UK (COG-UK) Consortium|Davies|United Kingdom
>Spike|hCoV-19/Denmark/DCGC-15766/2020|2020-11-23|EPI_ISL_714008|Original|hCoV-19^^Hovedstaden|Human|Department of Virus and Microbiological Special Diagnostics|Albertsen Lab|Michaelsen|Denmark
>Spike|hCoV-19/England/QEUH-9DE99E/2020|2020-09-21|EPI_ISL_584816|Original|hCoV-19^^England|Human|University College London|COVID-19 Genomics UK (COG-UK) Consortium|Roy|United Kingdom
>Spike|hCoV-19/England/CAMB-1B4671/2020|2020-05-27|EPI_ISL_473479|Original|hCoV-19^^England|Human|Department of Pathology|COVID-19 Genomics UK (COG-UK) Consortium|Meredith|United Kingdom
>Spike|hCoV-19/England/OXON-AD0BA/2020|2020-00-00|EPI_ISL_448560|Original|hCoV-19^^England|Human|Oxford Viromics|COVID-19 Genomics UK (COG-UK) Consortium|Golubchik|United Kingdom
>Spike|hCoV-19/USA/TX-HMH-4988/2020|2020-06-22|EPI_ISL_544439|Original|hCoV-19^^Texas|Human|Houston Methodist Hospital|Houston Methodist Hospital|Long|USA
>Spike|hCoV-19/USA/IN-CDC-2-3713960/2020|2020-12-04|EPI_ISL_751600|Original|hCoV-19^^Indiana|Human|IN State Department of Health Laboratory Services|Genomics and Discovery|Queen|USA

>Spike|hCoV-19/Wales/PHWC-49887A/2020|2020-11-27|EPI_ISL_727133|Original|hCoV-19^^Wales|Human|Originating lab|COVID-19 Genomics UK (COG-UK) Consortium|Moore|United Kingdom
>Spike|hCoV-19/England/BRIS-12A9B9/2020|2020-04-30|EPI_ISL_458491|Original|hCoV-19^^England|Human|PHE South West Regional Laboratory|Wellcome Sanger Institute for the COVID-19 Genomics UK (COG-UK) Consortium|Davies|United Kingdom
>Spike|hCoV-19/Scotland/QEUH-A1FE73/2020|2020-10-01|EPI_ISL_599082|Original|hCoV-19^^Scotland|Human|Lighthouse Lab in Glasgow|Wellcome Sanger Institute for the COVID-19 Genomics UK (COG-UK) consortium|Davies|United Kingdom
>Spike|hCoV-19/USA/CA-CZB-13342/2020|2020-05-02|EPI_ISL_672440|Original|hCoV-19^^California|Human|Alameda County Public Health Lab|Chan-Zuckerberg Biohub|Bhatt|USA
>Spike|hCoV-19/Ireland/D-NVRL-AIIDV0091v1/2020|2020-04-14|EPI_ISL_644333|Original|hCoV-19^^Dublin|Human|CEPHR / Vincent's Hospital|Irish Coronavirus Sequencing Consortium - National Virus Reference Laboratory|Gonzalez|Ireland
>Spike|hCoV-19/England/CAMC-B226B0/2020|2020-11-02|EPI_ISL_647183|Original|hCoV-19^^England|Human|Lighthouse Lab in Cambridge|Wellcome Sanger Institute for the COVID-19 Genomics UK (COG-UK) Consortium|Davies|United Kingdom
>Spike|hCoV-19/Singapore/7/2020|2020-01-27|EPI_ISL_410713|Original|hCoV-19^^Singapore|Human|National Public Health Laboratory|National Public Health Laboratory|Mak|Singapore
>Spike|hCoV-19/England/QEUH-AE5016/2020|2020-10-27|EPI_ISL_633259|Original|hCoV-19^^England|Human|Lighthouse Lab in Glasgow|Wellcome Sanger Institute for the COVID-19 Genomics UK (COG-UK) consortium|Davies|United Kingdom
>Spike|hCoV-19/England/20170037804/2020|2020-04-09|EPI_ISL_465290|Original|hCoV-19^^England|Human|Respiratory Virus Unit|Respiratory Virus Unit|Platt|United Kingdom
>Spike|hCoV-19/England/ALDP-A2D8A1/2020|2020-10-06|EPI_ISL_586648|Original|hCoV-19^^England|Human|Lighthouse Lab in Alderley Park|Wellcome Sanger Institute for the COVID-19 Genomics UK (COG-UK) consortium|Davies|United Kingdom
>Spike|hCoV-19/England/NOTT-11974A/2020|2020-11-02|EPI_ISL_627304|Original|hCoV-19^^England|Human|Queens Medical Centre|COVID-19 Genomics UK (COG-UK) Consortium|Loose|United Kingdom
>Spike|hCoV-19/England/LIVE-DC199A/2020|2020-11-19|EPI_ISL_705812|Original|hCoV-19^^England|Human|Liverpool Clinical Laboratories|COVID-19 Genomics UK (COG-UK) Consortium|Haldenby|United Kingdom
>Spike|hCoV-19/Japan/PG-6703/2020|2020-07-00|EPI_ISL_687814|Original|hCoV-19^^Japan|Human|Pathogen Genomics Center|Pathogen Genomics Center|Sekizuka|Japan
>Spike|hCoV-19/England/QEUH-B6AC7E/2020|2020-11-11|EPI_ISL_655123|Original|hCoV-19^^England|Human|Lighthouse Lab in Glasgow|Wellcome Sanger Institute for the COVID-19 Genomics UK (COG-UK) Consortium|Davies|United Kingdom
>Spike|hCoV-19/India/MH-CMP-30/2020|2020-09-09|EPI_ISL_779705|Original|hCoV-19^^Maharashtra|Human|The Foundation for Medical Research|The Foundation for Medical Research|Mandal|India
>Spike|hCoV-19/Singapore/1069/2020|2020-10-05|EPI_ISL_596459|Original|hCoV-19^^Singapore|Human|National Public Health Laboratory|National Public Health Laboratory|Mak|Singapore
>Spike|hCoV-19/England/205081168/2020|2020-12-09|EPI_ISL_766292|Original|hCoV-19^^England|Human|Respiratory Virus Unit|COVID-19 Genomics UK (COG-UK) Consortium|Platt|United Kingdom
>Spike|hCoV-19/Netherlands/ZH-EMC-522/2020|2020-09-01|EPI_ISL_577950|Original|hCoV-19^^Zuid Holland|Human|Dutch COVID-19 response team|Erasmus Medical Center|Oude Munnink|Netherlands
>Spike|hCoV-19/Canada/ON-UHTC_0101/2020|2020-05-10|EPI_ISL_526257|Original|hCoV-19^^Ontario|Human|Unity Health Toronto|Ontario Institute for Cancer Research|de Borja|Canada
>Spike|hCoV-19/England/QEUH-9F8D38/2020|2020-09-27|EPI_ISL_588898|Original|hCoV-19^^England|Human|Lighthouse Lab in Glasgow|Wellcome Sanger Institute for the COVID-19 Genomics UK (COG-UK) consortium|Davies|United Kingdom

>Spike|hCoV-19/England/CAMC-942EB4/2020|2020-07-22|EPI_ISL_556876|Original|hCoV-19^^England|Human|Lighthouse Lab in Cambridge|Wellcome Sanger Institute for the COVID-19 Genomics UK (COG-UK) consortium|Davies|United Kingdom
>Spike|hCoV-19/Denmark/DCGC-2318/2020|2020-08-10|EPI_ISL_617655|Original|hCoV-19^^Midtjylland|Human|Department of Virus and Microbiological Special Diagnostics|Albertsen lab|Michaelsen|Denmark
>Spike|hCoV-19/England/ALDP-AA0ED1/2020|2020-10-22|EPI_ISL_623920|Original|hCoV-19^^England|Human|Lighthouse Lab in Alderley Park|Wellcome Sanger Institute for the COVID-19 Genomics UK (COG-UK) consortium|Davies|United Kingdom
>Spike|hCoV-19/South Africa/KRISP-K002276/2020|2020-07-19|EPI_ISL_515656|Original|hCoV-19^^KwaZulu-Natal|Human|NHLS-IALCH|KRISP|de Oliveira|South Africa
>Spike|hCoV-19/USA/CA-ALSR-4358/2020|2020-08-04|EPI_ISL_648988|Original|hCoV-19^^California|Human|San Diego County Public Health Laboratory|Andersen lab at Scripps Research|Gangavarapu|USA
>Spike|hCoV-19/Japan/PG-7795/2020|2020-07-00|EPI_ISL_686897|Original|hCoV-19^^Japan|Human|Pathogen Genomics Center|Pathogen Genomics Center|Sekizuka|Japan
>Spike|hCoV-19/United Arab Emirates/1079/2020|2020-06-12|EPI_ISL_698153|Original|hCoV-19^^Abu Dhabi|Human|Group 42 (G42) Healthcare|G42 Healthcare|Ogrodzki|United Arab Emirates
>Spike|hCoV-19/USA/WA-UW-8601/2020|2020-05-06|EPI_ISL_776659|Original|hCoV-19^^Washington|Human|UW Virology Lab|UW Virology Lab|Roychoudhury|USA
>Spike|hCoV-19/England/QEUH-9CA731/2020|2020-09-10|EPI_ISL_567471|Original|hCoV-19^^England|Human|Lighthouse Lab in Glasgow|Wellcome Sanger Institute for the COVID-19 Genomics UK (COG-UK) consortium|Davies|United Kingdom
>Spike|hCoV-19/Switzerland/BE-ETHZ-270038/2020|2020-09-02|EPI_ISL_541416|Original|hCoV-19^^Bern|Human|Viollier AG|Department of Biosystems Science and Engineering|Nadeau|Switzerland
>Spike|hCoV-19/USA/MD-MDH-0508/2020|2020-11-13|EPI_ISL_778889|Original|hCoV-19^^Maryland|Human|Maryland Public Health Laboratory|Maryland Public Health Laboratory|Keller|USA
>Spike|hCoV-19/USA/WI-GMF-21221/2020|2020-09-19|EPI_ISL_660830|Original|hCoV-19^^Wisconsin|Human|Gundersen Molecular Diagnostics Laboratory|Kabara Cancer Research Institute|Kenny|USA
>Spike|hCoV-19/England/MILK-A79C13/2020|2020-10-15|EPI_ISL_606718|Original|hCoV-19^^England|Human|Lighthouse Lab in Milton Keynes|Wellcome Sanger Institute for the COVID-19 Genomics UK (COG-UK) consortium|Davies|United Kingdom
>Spike|hCoV-19/India/KA-IB57/2020|2020-05-13|EPI_ISL_511948|Original|hCoV-19^^Karnataka|Human|Indian Institute of Science|National Institute of Biomedical Genomics -\u00a0DBT's PAN-INDIA 1000 SARS--CoV-2 RNA Genome Sequencing Consortium|Maitra|India
>Spike|hCoV-19/Wales/PHWC-2D3D7/2020|2020-04-03|EPI_ISL_445982|Original|hCoV-19^^Wales|Human|Wales Specialist Virology Centre|Public Health Wales Microbiology Cardiff|Moore|United Kingdom
>Spike|hCoV-19/England/EXET-13E9D0/2020|2020-12-12|EPI_ISL_763705|Original|hCoV-19^^England|Human|University of Exeter|COVID-19 Genomics UK (COG-UK) Consortium|Temperton|United Kingdom
>Spike|hCoV-19/England/204221459/2020|2020-09-19|EPI_ISL_593824|Original|hCoV-19^^England|Human|Respiratory Virus Unit|Respiratory Virus Unit|Platt|United Kingdom
>Spike|hCoV-19/England/CAMB-1AB80E/2020|2020-04-26|EPI_ISL_444334|Original|hCoV-19^^England|Human|Department of Pathology|COVID-19 Genomics UK (COG-UK) Consortium|Meredith|United Kingdom
>Spike|hCoV-19/Scotland/QEUH-96EDE2/2020|2020-08-19|EPI_ISL_534490|Original|hCoV-19^^Scotland|Human|NHSGGC West of Scotland Specialist Virology Centre / MRC-University of Glasgow Centre for Virus Research|Wellcome Sanger Institute for the COVID-19 Genomics UK (COG-UK) consortium|Davies|United Kingdom
>Spike|hCoV-19/England/ALDP-9FD95D/2020|2020-10-02|EPI_ISL_587979|Original|hCoV-19^^England|Human|Lighthouse Lab in Alderley Park|Wellcome Sanger Institute for the COVID-19 Genomics UK (COG-UK) consortium|Davies|United Kingdom

>Spike|hCoV-19/USA/MI-MDHHS-SC22512/2020|2020-11-17|EPI_ISL_708215|Original|hCoV-19^^Michigan|Human|Michigan Department of Health and Human Services|Michigan Department of Health and Human Services|Jacob|USA
>Spike|hCoV-19/France/ARA-120740/2020|2020-12-16|EPI_ISL_779787|Original|hCoV-19^^Auvergne-Rh\u00f4ne-Alpes|Human|CNR Virus des Infections Respiratoires - France SUD|CNR Virus des Infections Respiratoires - France SUD|Bal|France
>Spike|hCoV-19/USA/IN-QDX-964/2020|2020-03-16|EPI_ISL_571720|Original|hCoV-19^^Indiana|Human|Quest Diagnostics|Quest Diagnostics|Kagan|USA
>Spike|hCoV-19/England/ALDP-A5172F/2020|2020-10-07|EPI_ISL_597888|Original|hCoV-19^^England|Human|Lighthouse Lab in Alderley Park|Wellcome Sanger Institute for the COVID-19 Genomics UK (COG-UK) consortium|Davies|United Kingdom
>Spike|hCoV-19/Wales/PHWC-163820/2020|2020-04-29|EPI_ISL_474002|Original|hCoV-19^^Wales|Human|Originating lab|COVID-19 Genomics UK (COG-UK) Consortium|Moore|United Kingdom
>Spike|hCoV-19/Spain/RI-IBV-99005855/2020|2020-03-24|EPI_ISL_537398|Original|hCoV-19^^La Rioja|Human|Centro de Investigaci\u00f3n Biom\u00e9dica de La Rioja - Hospital San Pedro Logro\u00f1o|SeqCOVID-SPAIN consortium/IBV(CSIC)|Comas|Spain
>Spike|hCoV-19/Denmark/DCGC-2828/2020|2020-08-24|EPI_ISL_616654|Original|hCoV-19^^Hovedstaden|Human|Department of Virus and Microbiological Special Diagnostics|Albertsen lab|Michaelsen|Denmark
>Spike|hCoV-19/USA/MN-MDH-1907/2020|2020-10-18|EPI_ISL_613519|Original|hCoV-19^^Minnesota|Human|Public Health Laboratory - Infectious Disease Lab|Minnesota Department of Health|Lorentz|USA
>Spike|hCoV-19/Panama/329862/2020|2020-03-14|EPI_ISL_496651|Original|hCoV-19^^Panama Center|Human|Gorgas Memorial Laboratory of Health Studies|Gorgas Memorial Laboratory of Health Studies|Martinez|Panama
>Spike|hCoV-19/England/ALDP-95A61B/2020|2020-06-10|EPI_ISL_554918|Original|hCoV-19^^England|Human|Lighthouse Lab in Alderley Park|Wellcome Sanger Institute for the COVID-19 Genomics UK (COG-UK) consortium|Davies|United Kingdom
>Spike|hCoV-19/Wales/PHWC-2EFE1/2020|2020-04-04|EPI_ISL_446185|Original|hCoV-19^^Wales|Human|Wales Specialist Virology Centre|Public Health Wales Microbiology Cardiff|Moore|United Kingdom
>Spike|hCoV-19/Denmark/DCGC-7256/2020|2020-10-12|EPI_ISL_619232|Original|hCoV-19^^Midtjylland|Human|Department of Virus and Microbiological Special Diagnostics|Albertsen lab|Michaelsen|Denmark
>Spike|hCoV-19/England/CAMC-B32FD0/2020|2020-11-05|EPI_ISL_659272|Original|hCoV-19^^England|Human|Lighthouse Lab in Cambridge|Wellcome Sanger Institute for the COVID-19 Genomics UK (COG-UK) Consortium|Davies|United Kingdom
>Spike|hCoV-19/England/204540536/2020|2020-11-03|EPI_ISL_678651|Original|hCoV-19^^England|Human|Respiratory Virus Unit|COVID-19 Genomics UK (COG-UK) Consortium|Platt|United Kingdom
>Spike|hCoV-19/England/ALDP-983215/2020|2020-07-21|EPI_ISL_529553|Original|hCoV-19^^England|Human|Queens Medical Centre|COVID-19 Genomics UK (COG-UK) Consortium|Loose|United Kingdom
>Spike|hCoV-19/England/QEUH-B0FC11/2020|2020-11-02|EPI_ISL_642111|Original|hCoV-19^^England|Human|Lighthouse Lab in Glasgow|Wellcome Sanger Institute for the COVID-19 Genomics UK (COG-UK) Consortium|Davies|United Kingdom
>Spike|hCoV-19/Northern Ireland/NIRE-108DE2/2020|2020-10-12|EPI_ISL_627563|Original|hCoV-19^^Northern Ireland|Human|Regional Virus Laboratory|COVID-19 Genomics UK (COG-UK) Consortium|Fairley|United Kingdom
>Spike|hCoV-19/England/QEUH-9CF5AB/2020|2020-09-15|EPI_ISL_590023|Original|hCoV-19^^England|Human|Lighthouse Lab in Glasgow|Wellcome Sanger Institute for the COVID-19 Genomics UK (COG-UK) consortium|Davies|United Kingdom
>Spike|hCoV-19/England/CAMB-71C15/2020|2020-03-31|EPI_ISL_439927|Original|hCoV-19^^England|Human|Department of Pathology|Wellcome Sanger Institute for the COVID-19 Genomics UK (COG-UK) consortium|Amato|United Kingdom
>Spike|hCoV-19/USA/WI-UW-164/2020|2020-03-16|EPI_ISL_428260|Original|hCoV-19^^Wisconsin|Human|University of Wisconsin-Madison AIDS Vaccine Research Laboratories|University of Wisconsin-Madison AIDS Vaccine Research Laboratories|Moreno|USA

>Spike|hCoV-19/USA/NY-Wadsworth-292840-01/2020|2020-12-
28|EPI_ISL_769979|Original|hCoV-19^^New York|Human|Albany Medical Center Hospital
Clinical Laboratories|Wadsworth Center|Lasek-Nesselquist|USA
>Spike|hCoV-19/USA/NM-DOH-2020315317/2020|2020-08-27|EPI_ISL_574288|Original|hCoV-
19^^New Mexico|Human|New Mexico Department of Health Scientific Laboratory|New Mexico
Department of Health Scientific Laboratory|Johnson|USA
>Spike|hCoV-19/Scotland/QEUH-AD14A8/2020|2020-10-27|EPI_ISL_649686|Original|hCoV-
19^^Scotland|Human|Lighthouse Lab in Glasgow|Wellcome Sanger Institute for the COVID-
19 Genomics UK (COG-UK) Consortium|Davies|United Kingdom
>Spike|hCoV-19/Wales/PHWC-47F712/2020|2020-10-06|EPI_ISL_612716|Original|hCoV-
19^^Wales|Human|Wales Specialist Virology Centre Sequencing lab|COVID-19 Genomics UK
(COG-UK) Consortium|Moore|United Kingdom
>Spike|hCoV-19/Canada/QC-LSPQ-L00237883/2020|2020-03-25|EPI_ISL_536197|Original|hCoV-
19^^Quebec|Human|CSSS Haut-Richelieu/Rouville (H\u00f4pital)|Laboratoire de
sant\u00e9 publique du Qu\u00e9bec|Moreira|Canada
>Spike|hCoV-19/Denmark/DCGC-8960/2020|2020-10-26|EPI_ISL_626189|Original|hCoV-
19^^Hovedstaden|Human|Department of Virus and Microbiological Special
Diagnostics|Albertsen lab|Michaelsen|Denmark
>Spike|hCoV-19/USA/TX-HMH-6346/2020|2020-06-28|EPI_ISL_547405|Original|hCoV-
19^^Texas|Human|Houston Methodist Hospital|Houston Methodist Hospital|Long|USA
>Spike|hCoV-19/Canada/BC-BCCDC-6481/2020|2020-10-00|EPI_ISL_740621|Original|hCoV-
19^^British Columbia|Human|BCCDC Public Health Laboratory|BCCDC Public Health
Laboratory|Prystajecky|Canada
>Spike|hCoV-19/USA/CT-Yale-480/2020|2020-09-18|EPI_ISL_729765|Original|hCoV-
19^^Connecticut|Human|Connecticut Department of Health|Grubaugh Lab - Yale School of
Public Health|Grubaugh|USA
>Spike|hCoV-19/England/QEUH-BCD24A/2020|2020-11-25|EPI_ISL_704375|Original|hCoV-
19^^England|Human|Lighthouse Lab in Glasgow|Wellcome Sanger Institute for the COVID-
19 Genomics UK (COG-UK) Consortium|Davies|United Kingdom
>Spike|hCoV-19/Netherlands/NB-EMC-417/2020|2020-11-09|EPI_ISL_722312|Original|hCoV-
19^^Noord Brabant|Human|Dutch COVID-19 response team|Erasmus Medical Center|Oude
Munnink|Netherlands
>Spike|hCoV-19/USA/WA-S1477/2020|2020-05-11|EPI_ISL_495652|Original|hCoV-
19^^Washington|Human|Washington State Department of Health|Seattle Flu Study|Lee|USA
>Spike|hCoV-19/Denmark/DCGC-6494/2020|2020-10-05|EPI_ISL_620394|Original|hCoV-
19^^Hovedstaden|Human|Department of Virus and Microbiological Special
Diagnostics|Albertsen lab|Michaelsen|Denmark
>Spike|hCoV-19/Belgium/ULG-11006/2020|2020-11-14|EPI_ISL_707720|Original|hCoV-
19^^Li\u00e8ge|Human|Department of Clinical Microbiology|GIGA Medical
Genomics|Durkin|Belgium
>Spike|hCoV-19/England/ALDP-9E83FD/2020|2020-09-24|EPI_ISL_638247|Original|hCoV-
19^^England|Human|Department of Pathology|COVID-19 Genomics UK (COG-UK)
Consortium|Goodfellow|United Kingdom
>Spike|hCoV-19/USA/WA-S7/2020|2020-02-24|EPI_ISL_416462|Original|hCoV-
19^^Washington|Human|Seattle Flu Study|Seattle Flu Study|Bedford|USA
>Spike|hCoV-19/England/BIRM-67043/2020|2020-10-28|EPI_ISL_651606|Original|hCoV-
19^^England|Human|University of Birmingham|COVID-19 Genomics UK (COG-UK)
Consortium|Loman|United Kingdom
>Spike|hCoV-19/USA/OR-OHSU-2301/2020|2020-08-10|EPI_ISL_667175|Original|hCoV-
19^^Oregon|Human|OHSU Lab Services Molecular Microbiology Lab|Oregon SARS-CoV-2
Genome Sequencing Center|O'Roak|USA
>Spike|hCoV-19/England/MILK-A6A9E1/2020|2020-10-08|EPI_ISL_627135|Original|hCoV-
19^^England|Human|Liverpool Clinical Laboratories|COVID-19 Genomics UK (COG-UK)
Consortium|Haldenby|United Kingdom
>Spike|hCoV-19/Scotland/CVR3248/2020|2020-04-27|EPI_ISL_449255|Original|hCoV-
19^^Scotland|Human|West of Scotland Specialist Virology Centre|COVID-19 Genomics UK
(COG-UK) Consortium|Robertson|United Kingdom
>Spike|hCoV-19/USA/WI-UW-2177/2020|2020-10-28|EPI_ISL_677581|Original|hCoV-
19^^Wisconsin|Human|University of Wisconsin-Madison AIDS Vaccine Research

Laboratories|University of Wisconsin-Madison AIDS Vaccine Research Laboratories|Baczenas|USA
>Spike|hCoV-19/Australia/VIC14735/2020|2020-08-22|EPI_ISL_592976|Original|hCoV-19^^Victoria|Human|Victorian Infectious Diseases Reference Laboratory (VIDRL)|VIDRL and MDU-PHL|Schultz|Australia
>Spike|hCoV-19/USA/OR-OHSU-3071/2020|2020-09-30|EPI_ISL_667470|Original|hCoV-19^^Oregon|Human|OHSU Lab Services Molecular Microbiology Lab|Oregon SARS-CoV-2 Genome Sequencing Center|O'Roak|USA
>Spike|hCoV-19/England/ALDP-5BA01F/2020|2020-06-15|EPI_ISL_558878|Original|hCoV-19^^England|Human|Lighthouse Lab in Alderley Park|Wellcome Sanger Institute for the COVID-19 Genomics UK (COG-UK) consortium|Davies|United Kingdom
>Spike|hCoV-19/England/MILK-B8DE7F/2020|2020-11-13|EPI_ISL_674731|Original|hCoV-19^^England|Human|Lighthouse Lab in Milton Keynes|Wellcome Sanger Institute for the COVID-19 Genomics UK (COG-UK) Consortium|Davies|United Kingdom
>Spike|hCoV-19/Denmark/DCGC-5420/2020|2020-09-14|EPI_ISL_615352|Original|hCoV-19^^Syddanmark|Human|Department of Virus and Microbiological Special Diagnostics|Albertsen lab|Michaelsen|Denmark
>Spike|hCoV-19/Australia/VIC461/2020|2020-03-27|EPI_ISL_426754|Original|hCoV-19^^Victoria|Human|Victorian Infectious Diseases Reference Laboratory (VIDRL)|Microbiological Diagnostic Unit Public Health Laboratory and Victorian Infectious Diseases Reference Laboratory|Schultz|Australia
>Spike|hCoV-19/France/ARA-18783/2020|2020-04-03|EPI_ISL_508974|Original|hCoV-19^^Auvergne-Rh\u00f4ne-Alpes|Human|CNR Virus des Infections Respiratoires - France SUD|CNR Virus des Infections Respiratoires - France SUD|Josset|France
>Spike|hCoV-19/USA/NY-NYUMC2/2020|2020-03-16|EPI_ISL_416830|Original|hCoV-19^^New York|Human|NYU Langone Health|Department of Pathology and Medicine|Maurano|USA
>Spike|hCoV-19/USA/CA-CHLA-PLM28814725/2020|2020-11-15|EPI_ISL_753338|Original|hCoV-19^^California|Human|Clinical virology Laboratory|Center for Personalized Medicine|Dien Bard|USA
>Spike|hCoV-19/England/BRIS-121994/2020|2020-03-15|EPI_ISL_440196|Original|hCoV-19^^England|Human|PHE South West Regional Laboratory|Wellcome Sanger Institute for the COVID-19 Genomics UK (COG-UK) consortium|Amato|United Kingdom
>Spike|hCoV-19/Wales/PHWC-48E2F1/2020|2020-11-12|EPI_ISL_680839|Original|hCoV-19^^Wales|Human|Wales Specialist Virology Centre Sequencing lab|COVID-19 Genomics UK (COG-UK) Consortium|Moore|United Kingdom
>Spike|hCoV-19/England/MILK-AC9203/2020|2020-10-22|EPI_ISL_629552|Original|hCoV-19^^England|Human|Lighthouse Lab in Milton Keynes|Wellcome Sanger Institute for the COVID-19 Genomics UK (COG-UK) consortium|Davies|United Kingdom
>Spike|hCoV-19/England/ALDP-95069D/2020|2020-06-18|EPI_ISL_555240|Original|hCoV-19^^England|Human|Lighthouse Lab in Alderley Park|Wellcome Sanger Institute for the COVID-19 Genomics UK (COG-UK) consortium|Davies|United Kingdom
>Spike|hCoV-19/India/TG-GMC-RK100/2020|2020-03-01|EPI_ISL_431101|Original|hCoV-19^^Telangana|Human|Department of Microbiology,Gandhi Medical College and Hospital|Virus Research Laboratory|Muttineni|India
>Spike|hCoV-19/mink/Netherlands/NB-EMC-63-2/2020|2020-10-06|EPI_ISL_632334|Original|hCoV-19^^Noord Brabant|Neovison vison|Dutch COVID-19 response team|Erasmus Medical Center|Oude Munnink|Netherlands
>Spike|hCoV-19/USA/MA-MASPHL-00299/2020|2020-04-17|EPI_ISL_791711|Original|hCoV-19^^Massachusetts|Human|Massachusetts State Public Health Laboratory|Infectious Disease Program|Park|USA
>Spike|hCoV-19/USA/TX-HMH-4603/2020|2020-06-21|EPI_ISL_543749|Original|hCoV-19^^Texas|Human|Houston Methodist Hospital|Houston Methodist Hospital|Long|USA
>Spike|hCoV-19/England/SHEF-C3081/2020|2020-06-04|EPI_ISL_484442|Original|hCoV-19^^England|Human|Virology Department|COVID-19 Genomics UK (COG-UK) Consortium|de Silva|United Kingdom
>Spike|hCoV-19/USA/IA-GMF-20217/2020|2020-09-14|EPI_ISL_547857|Original|hCoV-19^^Iowa|Human|Gundersen Molecular Diagnostics Laboratory|Kabara Cancer Research Institute|Kenny|USA
>Spike|hCoV-19/Cote dIvoire/BKE0104/2020|2020-06-08|EPI_ISL_614353|Original|hCoV-19^^Boauk\u00e9|Human|Molecular diagnostic unit for viral haemorrhagic fevers and

emerging viruses|Project group Epidemology of Highly Pathogenic
Microorganisms|Calvignac-Spencer|C\u00f4te d'Ivoire
>Spike|hCoV-19/Denmark/DCGC-1389/2020|2020-05-11|EPI_ISL_622420|Original|hCoV-
19^^Hovedstaden|Human|Department of Virus and Microbiological Special
Diagnostics|Albertsen lab|Michaelsen|Denmark
>Spike|hCoV-19/England/NOTT-10E881/2020|2020-03-25|EPI_ISL_425604|Original|hCoV-
19^^England|Human|Queens Medical Centre|COVID-19 Genomics UK (COG-UK)
Consortium|Loose|United Kingdom
>Spike|hCoV-19/England/20109005704/2020|2020-03-06|EPI_ISL_464518|Original|hCoV-
19^^England|Human|Respiratory Virus Unit|Respiratory Virus Unit|Platt|United Kingdom
>Spike|hCoV-19/USA/UT-08352/2020|2020-05-15|EPI_ISL_524137|Original|hCoV-
19^^Utah|Human|Utah Public Health Laboratory|Utah Public Health Laboratory|Young|USA
>Spike|hCoV-19/Wales/PHWC-49940C/2020|2020-12-03|EPI_ISL_727302|Original|hCoV-
19^^Wales|Human|Originating lab|COVID-19 Genomics UK (COG-UK) Consortium|Moore|United
Kingdom
>Spike|hCoV-19/USA/TX-HMH-MCoV-10531/2020|2020-07-08|EPI_ISL_789522|Original|hCoV-
19^^Texas|Human|Houston Methodist Hospital|Houston Methodist Hospital|Long|USA
>Spike|hCoV-19/Wales/PHWC-49D374/2020|2020-12-10|EPI_ISL_742770|Original|hCoV-
19^^Wales|Human|Wales Specialist Virology Centre Sequencing lab|COVID-19 Genomics UK
(COG-UK) Consortium|Moore|United Kingdom
>Spike|hCoV-19/Netherlands/ZuidHolland_178/2020|2020-04-
20|EPI_ISL_461350|Original|hCoV-19^^Zuid Holland|Human|Dutch COVID-19 response
team|Erasmus Medical Center|Oude Munnink|Netherlands
>Spike|hCoV-19/USA/MI-QDX-496/2020|2020-03-23|EPI_ISL_571230|Original|hCoV-
19^^Michigan|Human|Quest Diagnostics|Quest Diagnostics|Kagan|USA
>Spike|hCoV-19/Denmark/DCGC-16992/2020|2020-12-14|EPI_ISL_750696|Original|hCoV-
19^^Midtjylland|Human|Department of Virus and Microbiological Special
Diagnostics|Albertsen Lab|Michaelsen|Denmark
>Spike|hCoV-19/Wales/PHWC-47F25D/2020|2020-10-05|EPI_ISL_612650|Original|hCoV-
19^^Wales|Human|Wales Specialist Virology Centre Sequencing lab|COVID-19 Genomics UK
(COG-UK) Consortium|Moore|United Kingdom
>Spike|hCoV-19/USA/MI-MDHHS-SC20133/2020|2020-03-24|EPI_ISL_436851|Original|hCoV-
19^^Michigan|Human|Michigan Department of Health and Human Services|Michigan
Department of Health and Human Services|Blankenship|USA
>Spike|hCoV-19/England/MILK-ACEA42/2020|2020-10-22|EPI_ISL_629749|Original|hCoV-
19^^England|Human|Lighthouse Lab in Milton Keynes|Wellcome Sanger Institute for the
COVID-19 Genomics UK (COG-UK) consortium|Davies|United Kingdom
>Spike|hCoV-19/England/MILK-B8DF9A/2020|2020-11-13|EPI_ISL_674597|Original|hCoV-
19^^England|Human|Lighthouse Lab in Milton Keynes|Wellcome Sanger Institute for the
COVID-19 Genomics UK (COG-UK) Consortium|Davies|United Kingdom
>Spike|hCoV-19/England/ALDP-A2F5C6/2020|2020-10-05|EPI_ISL_587086|Original|hCoV-
19^^England|Human|Lighthouse Lab in Alderley Park|Wellcome Sanger Institute for the
COVID-19 Genomics UK (COG-UK) consortium|Davies|United Kingdom
>Spike|hCoV-19/Denmark/DCGC-14768/2020|2020-11-23|EPI_ISL_711965|Original|hCoV-
19^^Hovedstaden|Human|Department of Virus and Microbiological Special
Diagnostics|Albertsen Lab|Michaelsen|Denmark
>Spike|hCoV-19/England/205260459/2020|2020-12-20|EPI_ISL_754382|Original|hCoV-
19^^England|Human|Respiratory Virus Unit|COVID-19 Genomics UK (COG-UK)
Consortium|Platt|United Kingdom
>Spike|hCoV-19/Australia/VIC5545/2020|2020-07-20|EPI_ISL_518374|Original|hCoV-
19^^Victoria|Human|Microbiological Diagnostic Unit - Public Health Laboratory (MDU-
PHL)|MDU-PHL|Schultz|Australia
>Spike|hCoV-19/Spain/AN-IBV-001161/2020|2020-03-25|EPI_ISL_452575|Original|hCoV-
19^^Andalucia|Human|Servicio de Microbiolog\u00eda y Parasitolog\u00eda cl\u00ednica.
UCEIMP. Hospital Universitario Virgen del Roc\u00edo/IBIS/CSIC/US.|SeqCOVID-SPAIN
consortium/IBV(CSIC)|Comas|Spain
>Spike|hCoV-19/England/ALDP-9CD75C/2020|2020-09-15|EPI_ISL_566280|Original|hCoV-
19^^England|Human|Lighthouse Lab in Alderley Park|Wellcome Sanger Institute for the
COVID-19 Genomics UK (COG-UK) consortium|Davies|United Kingdom

>Spike|hCoV-19/Canada/QC-LSPQ-L00242662/2020|2020-03-29|EPI_ISL_536331|Original|hCoV-
19^^Quebec|Human|H\u00f4pital Charles-LeMoyne|Laboratoire de sant\u00e9 publique du
Qu\u00e9bec|Moreira|Canada
>Spike|hCoV-19/England/NORT-29498C/2020|2020-03-27|EPI_ISL_490459|Original|hCoV-
19^^England|Human|Northumbria University / South Tees Hospitals NHS Foundation Trust
/ North Cumbria Integrated Care NHS Foundation Trust / North Tees and Hartlepool NHS
Foundation Trust / Newcastle Hospitals NHS Foundation Trust|COVID-19 Genomics UK
(COG-UK) Consortium|Bashton|United Kingdom
>Spike|hCoV-19/England/QEUH-A223D8/2020|2020-10-01|EPI_ISL_587366|Original|hCoV-
19^^England|Human|Lighthouse Lab in Glasgow|Wellcome Sanger Institute for the COVID-
19 Genomics UK (COG-UK) consortium|Davies|United Kingdom
>Spike|hCoV-19/DRC/10375/2020|2020-05-22|EPI_ISL_527499|Original|hCoV-
19^^Kinshasa|Human|Viral Respiratory Lab|Pathogen Sequencing Lab|Lokilo|Democratic
Republic of the Congo
>Spike|hCoV-19/Netherlands/NA_539/2020|2020-00-00|EPI_ISL_460712|Original|hCoV-
19^^Netherlands|Human|Dutch COVID-19 response team|Erasmus Medical Center|Oude
Munnink|Netherlands
>Spike|hCoV-19/USA/NY-Wadsworth-10131-01/2020|2020-02-
29|EPI_ISL_525575|Original|hCoV-19^^New York|Human|Wadsworth Center|Wadsworth
Center|Plitnick|USA
>Spike|hCoV-19/England/CAMB-1BAC00/2020|2020-11-12|EPI_ISL_664786|Original|hCoV-
19^^England|Human|Department of Pathology|COVID-19 Genomics UK (COG-UK)
Consortium|Goodfellow|United Kingdom
>Spike|hCoV-19/USA/MA-MASPHL-00684/2020|2020-04-15|EPI_ISL_692962|Original|hCoV-
19^^Massachusetts|Human|Massachusetts State Public Health Laboratory|Massachusetts
State Public Health Laboratory|Lang|USA
>Spike|hCoV-19/England/GSTT-265D6F7/2020|2020-03-24|EPI_ISL_484001|Original|hCoV-
19^^England|Human|Centre for Clinical Infection and Diagnostics Research and Genomics
Innovation Unit|COVID-19 Genomics UK (COG-UK) Consortium|Awan|United Kingdom
>Spike|hCoV-19/Wales/PHWC-47D028/2020|2020-10-09|EPI_ISL_586087|Original|hCoV-
19^^Wales|Human|Wales Specialist Virology Centre Sequencing lab|COVID-19 Genomics UK
(COG-UK) Consortium|Moore|United Kingdom
>Spike|hCoV-19/South Africa/KRISP-K002573/2020|2020-07-
29|EPI_ISL_535439|Original|hCoV-19^^KwaZulu-Natal|Human|NHLS-IALCH|KRISP|de
Oliveira|South Africa
>Spike|hCoV-19/England/CAMC-B5182B/2020|2020-11-08|EPI_ISL_658186|Original|hCoV-
19^^England|Human|Lighthouse Lab in Cambridge|Wellcome Sanger Institute for the
COVID-19 Genomics UK (COG-UK) Consortium|Davies|United Kingdom
>Spike|hCoV-19/Italy/VEN-UniVR-83/2020|2020-03-23|EPI_ISL_751392|Original|hCoV-
19^^Veneto|Human|IRCCS Sacro Cuore Don Calabria Hospital|University of
Verona|Mori|Italy
>Spike|hCoV-19/USA/AZ-TG366706/2020|2020-06-01|EPI_ISL_694470|Original|hCoV-
19^^Arizona|Human|AZ SPHL|TGen North|French|USA
>Spike|hCoV-19/Wales/PHWC-483D37/2020|2020-10-21|EPI_ISL_627924|Original|hCoV-
19^^Wales|Human|Wales Specialist Virology Centre Sequencing lab|COVID-19 Genomics UK
(COG-UK) Consortium|Moore|United Kingdom
>Spike|hCoV-19/Scotland/EDB4457/2020|2020-05-05|EPI_ISL_461713|Original|hCoV-
19^^Scotland|Human|Virology Department|COVID-19 Genomics UK (COG-UK)
Consortium|Williams|United Kingdom
>Spike|hCoV-19/Iran/K1r-135/2020|2020-05-27|EPI_ISL_568507|Original|hCoV-
19^^Iran|Human|Virology|Virology|Ranjbar|Iran
>Spike|hCoV-19/Scotland/CVR213/2020|2020-03-19|EPI_ISL_425742|Original|hCoV-
19^^Scotland|Human|West of Scotland Specialist Virology Centre|COVID-19 Genomics UK
(COG-UK) Consortium|Robertson|United Kingdom
>Spike|hCoV-19/Spain/CL-IBV-97006263/2020|2020-04-20|EPI_ISL_537635|Original|hCoV-
19^^Castilla y Leon|Human|Universidad de Le\u00f3n|SeqCOVID-SPAIN
consortium/IBV(CSIC)|Comas|Spain
>Spike|hCoV-19/England/CAMB-1AE8A1/2020|2020-04-30|EPI_ISL_524574|Original|hCoV-
19^^England|Human|Department of Pathology|Wellcome Sanger Institute for the COVID-19
Genomics UK (COG-UK) consortium|Davies|United Kingdom

>Spike|hCoV-19/USA/MN-MDH-1595/2020|2020-05-13|EPI_ISL_530191|Original|hCoV-19^^Minnesota|Human|Minnesota Department of Health|Minnesota Department of Health|Garfin|USA
>Spike|hCoV-19/Denmark/DCGC-5540/2020|2020-09-28|EPI_ISL_620865|Original|hCoV-19^^Midtjylland|Human|Department of Virus and Microbiological Special Diagnostics|Albertsen lab|Michaelsen|Denmark
>Spike|hCoV-19/USA/WA-UW141/2020|2020-03-11|EPI_ISL_416679|Original|hCoV-19^^Washington|Human|UW Virology Lab|UW Virology Lab|Roychoudhury|USA
>Spike|hCoV-19/Scotland/CVR3940/2020|2020-07-03|EPI_ISL_490704|Original|hCoV-19^^Scotland|Human|West of Scotland Specialist Virology Centre|COVID-19 Genomics UK (COG-UK) Consortium|Robertson|United Kingdom
>Spike|hCoV-19/England/QEUH-9B9E12/2020|2020-09-07|EPI_ISL_549692|Original|hCoV-19^^England|Human|Lighthouse Lab in Glasgow|Wellcome Sanger Institute for the COVID-19 Genomics UK (COG-UK) consortium|Davies|United Kingdom
>Spike|hCoV-19/England/ALDP-AA0B52/2020|2020-10-22|EPI_ISL_625071|Original|hCoV-19^^England|Human|Lighthouse Lab in Alderley Park|Wellcome Sanger Institute for the COVID-19 Genomics UK (COG-UK) consortium|Davies|United Kingdom
>Spike|hCoV-19/Luxembourg/LNS1419064/2020|2020-11-03|EPI_ISL_739933|Original|hCoV-19^^Luxembourg|Human|Laboratoire national de sant\u00e9|Laboratoire national de sant\u00e9|Wienecke-Baldacchino|Luxembourg
>Spike|hCoV-19/Wales/PHWC-16E078/2020|2020-09-30|EPI_ISL_585661|Original|hCoV-19^^Wales|Human|Wales Specialist Virology Centre Sequencing lab|COVID-19 Genomics UK (COG-UK) Consortium|Moore|United Kingdom
>Spike|hCoV-19/Chile/RM-157312/2020|2020-08-21|EPI_ISL_746494|Original|hCoV-19^^Region Metropolitana de Santiago|Human|Genetica Molecular and Subdepartamento de Virologia ISP Chile|Instituto de Salud Publica de Chile|Tognarelli|Chile
>Spike|hCoV-19/Italy/APU-PODV-15711018/2020|2020-04-19|EPI_ISL_788964|Original|hCoV-19^^Apulia|Human|Ospedale \Di Venere\|Beaconlab (Bioinformatics|Chiara|Italy
>Spike|hCoV-19/England/ALDP-94DF10/2020|2020-06-17|EPI_ISL_555255|Original|hCoV-19^^England|Human|Lighthouse Lab in Alderley Park|Wellcome Sanger Institute for the COVID-19 Genomics UK (COG-UK) consortium|Davies|United Kingdom
>Spike|hCoV-19/Wales/PHWC-16A540/2020|2020-09-06|EPI_ISL_540903|Original|hCoV-19^^Wales|Human|Wales Specialist Virology Centre Sequencing lab|COVID-19 Genomics UK (COG-UK) Consortium|Moore|United Kingdom
>Spike|hCoV-19/USA/TX-HMH-6596/2020|2020-06-24|EPI_ISL_546114|Original|hCoV-19^^Texas|Human|Houston Methodist Hospital|Houston Methodist Hospital|Long|USA
>Spike|hCoV-19/Japan/PG-4917/2020|2020-08-00|EPI_ISL_690037|Original|hCoV-19^^Japan|Human|Pathogen Genomics Center|Pathogen Genomics Center|Sekizuka|Japan
>Spike|hCoV-19/Luxembourg/LNS9838378/2020|2020-11-04|EPI_ISL_744997|Original|hCoV-19^^Luxembourg|Human|Laboratoire national de sant\u00e9|Laboratoire national de sant\u00e9|Wienecke-Baldacchino|Luxembourg
>Spike|hCoV-19/USA/HI-H200131/2020|2020-07-06|EPI_ISL_752694|Original|hCoV-19^^Hawaii|Human|State Laboratories Division|State Laboratories Division|Sultana|USA
>Spike|hCoV-19/England/20172048804/2020|2020-04-17|EPI_ISL_465354|Original|hCoV-19^^England|Human|Respiratory Virus Unit|Respiratory Virus Unit|Platt|United Kingdom
>Spike|hCoV-19/Denmark/DCGC-11958/2020|2020-09-21|EPI_ISL_671231|Original|hCoV-19^^Midtjylland|Human|Department of Virus and Microbiological Special Diagnostics|Albertsen Lab|Michaelsen|Denmark
>Spike|hCoV-19/Switzerland/VD-ETHZ-100168/2020|2020-03-16|EPI_ISL_574979|Original|hCoV-19^^Vaud|Human|Viollier AG|Department of Biosystems Science and Engineering|Nadeau|Switzerland
>Spike|hCoV-19/USA/MN-CDC-6302/2020|2020-05-08|EPI_ISL_527697|Original|hCoV-19^^Minnesota|Human|MN PHL Division|Pathogen Discovery|Queen|USA
>Spike|hCoV-19/England/MILK-AC787C/2020|2020-10-21|EPI_ISL_634439|Original|hCoV-19^^England|Human|Lighthouse Lab in Milton Keynes|Wellcome Sanger Institute for the COVID-19 Genomics UK (COG-UK) consortium|Davies|United Kingdom
>Spike|hCoV-19/England/BRIS-124CF2/2020|2020-03-26|EPI_ISL_443939|Original|hCoV-19^^England|Human|PHE South West Regional Laboratory|Wellcome Sanger Institute for the COVID-19 Genomics UK (COG-UK) consortium|Amato|United Kingdom

>Spike|hCoV-19/USA/CA-ALSR-2975/2020|2020-09-02|EPI_ISL_635765|Original|hCoV-19^^California|Human|San Diego County Public Health Laboratory|Andersen lab at Scripps Research|Gangavarapu|USA
>Spike|hCoV-19/USA/HI-H200539/2020|2020-11-05|EPI_ISL_753070|Original|hCoV-19^^Hawaii|Human|State Laboratories Division|State Laboratories Division|Sultana|USA
>Spike|hCoV-19/USA/TX-HMH-3660/2020|2020-06-15|EPI_ISL_543104|Original|hCoV-19^^Texas|Human|Houston Methodist Hospital|Houston Methodist Hospital|Long|USA
>Spike|hCoV-19/England/LOND-DA069/2020|2020-11-07|EPI_ISL_665669|Original|hCoV-19^^England|Human|University College London|COVID-19 Genomics UK (COG-UK) Consortium|Roy|United Kingdom
>Spike|hCoV-19/Wales/PHWC-30B24/2020|2020-04-10|EPI_ISL_446323|Original|hCoV-19^^Wales|Human|Wales Specialist Virology Centre|Public Health Wales Microbiology Cardiff|Moore|United Kingdom
>Spike|hCoV-19/England/ALDP-9E6F73/2020|2020-09-24|EPI_ISL_580374|Original|hCoV-19^^England|Human|Lighthouse Lab in Alderley Park|Wellcome Sanger Institute for the COVID-19 Genomics UK (COG-UK) consortium|Davies|United Kingdom
>Spike|hCoV-19/England/QEUH-BCB75C/2020|2020-11-27|EPI_ISL_704665|Original|hCoV-19^^England|Human|Lighthouse Lab in Glasgow|Wellcome Sanger Institute for the COVID-19 Genomics UK (COG-UK) Consortium|Davies|United Kingdom
>Spike|hCoV-19/England/CAMC-BBE21F/2020|2020-11-22|EPI_ISL_702798|Original|hCoV-19^^England|Human|Virology Department|COVID-19 Genomics UK (COG-UK) Consortium|de Silva|United Kingdom
>Spike|hCoV-19/Wales/PHWC-4A06FD/2020|2020-12-08|EPI_ISL_743484|Original|hCoV-19^^Wales|Human|Wales Specialist Virology Centre Sequencing lab|COVID-19 Genomics UK (COG-UK) Consortium|Moore|United Kingdom
>Spike|hCoV-19/USA/MA-MASPHL-01028/2020|2020-11-15|EPI_ISL_692783|Original|hCoV-19^^Massachusetts|Human|Massachusetts State Public Health Laboratory|Massachusetts State Public Health Laboratory|Lang|USA
>Spike|hCoV-19/England/EXET-1386EE/2020|2020-10-15|EPI_ISL_627425|Original|hCoV-19^^England|Human|University of Exeter|COVID-19 Genomics UK (COG-UK) Consortium|Temperton|United Kingdom
>Spike|hCoV-19/USA/CA-ALSR-4187/2020|2020-08-21|EPI_ISL_648873|Original|hCoV-19^^California|Human|San Diego County Public Health Laboratory|Andersen lab at Scripps Research|Gangavarapu|USA
>Spike|hCoV-19/England/ALDP-A9559E/2020|2020-10-20|EPI_ISL_624695|Original|hCoV-19^^England|Human|Lighthouse Lab in Alderley Park|Wellcome Sanger Institute for the COVID-19 Genomics UK (COG-UK) consortium|Davies|United Kingdom
>Spike|hCoV-19/Australia/NSW712/2020|2020-07-26|EPI_ISL_513355|Original|hCoV-19^^New South Wales|Human|St Vincent's Pathology (SydPath)|NSW Health Pathology - Institute of Clinical Pathology and Medical Research; Westmead Hospital; University of Sydney|Gall|Australia
>Spike|hCoV-19/Wales/PHWC-496645/2020|2020-11-19|EPI_ISL_726613|Original|hCoV-19^^Wales|Human|Originating lab|COVID-19 Genomics UK (COG-UK) Consortium|Moore|United Kingdom
>Spike|hCoV-19/England/CAMB-7DB1C/2020|2020-04-07|EPI_ISL_441767|Original|hCoV-19^^England|Human|Department of Pathology|Wellcome Sanger Institute for the COVID-19 Genomics UK (COG-UK) consortium|Amato|United Kingdom
>Spike|hCoV-19/England/CAMC-A3EFD5/2020|2020-10-06|EPI_ISL_609780|Original|hCoV-19^^England|Human|Lighthouse Lab in Cambridge|Wellcome Sanger Institute for the COVID-19 Genomics UK (COG-UK) consortium|Davies|United Kingdom
>Spike|hCoV-19/Gibraltar/205121231/2020|2020-00-00|EPI_ISL_766356|Original|hCoV-19^^Gibraltar|Human|Microbiology|Respiratory Virus Unit|Platt|Gibraltar
>Spike|hCoV-19/USA/MA-MGH-00425/2020|2020-03-07|EPI_ISL_460313|Original|hCoV-19^^Massachusetts|Human|Massachusetts General Hospital|Infectious Disease Program|Lemieux|USA
>Spike|hCoV-19/England/QEUH-54C6C5/2020|2020-05-12|EPI_ISL_532224|Original|hCoV-19^^England|Human|Lighthouse Lab in Glasgow|Wellcome Sanger Institute for the COVID-19 Genomics UK (COG-UK) consortium|Davies|United Kingdom

>Spike|hCoV-19/Wales/PHWC-31F72/2020|2020-04-14|EPI_ISL_446591|Original|hCoV-19^^Wales|Human|Wales Specialist Virology Centre|Public Health Wales Microbiology Cardiff|Moore|United Kingdom
>Spike|hCoV-19/England/ALDP-952358/2020|2020-06-09|EPI_ISL_553647|Original|hCoV-19^^England|Human|Lighthouse Lab in Alderley Park|Wellcome Sanger Institute for the COVID-19 Genomics UK (COG-UK) consortium|Davies|United Kingdom
>Spike|hCoV-19/Denmark/DCGC-16711/2020|2020-10-19|EPI_ISL_714423|Original|hCoV-19^^Hovedstaden|Human|Department of Virus and Microbiological Special Diagnostics|Albertsen Lab|Michaelsen|Denmark
>Spike|hCoV-19/Northern Ireland/NIRE-23472A/2020|2020-10-14|EPI_ISL_680429|Original|hCoV-19^^Northern Ireland|Human|Regional Virus Laboratory|COVID-19 Genomics UK (COG-UK) Consortium|Fairley|United Kingdom
>Spike|hCoV-19/Australia/VIC9188/2020|2020-08-18|EPI_ISL_565431|Original|hCoV-19^^Victoria|Human|Microbiological Diagnostic Unit - Public Health Laboratory (MDU-PHL)|MDU-PHL|Schultz|Australia
>Spike|hCoV-19/USA/UT-UPHL-05395/2020|2020-07-10|EPI_ISL_560873|Original|hCoV-19^^Utah|Human|Utah Public Health Laboratory|Utah Public Health Laboratory|Young|USA
>Spike|hCoV-19/England/ALDP-CB3589/2020|2020-12-20|EPI_ISL_761240|Original|hCoV-19^^England|Human|Lighthouse Lab in Alderley Park|Wellcome Sanger Institute for the COVID-19 Genomics UK (COG-UK) Consortium|Davies|United Kingdom
>Spike|hCoV-19/England/MILK-B8C4D8/2020|2020-11-13|EPI_ISL_674196|Original|hCoV-19^^England|Human|Lighthouse Lab in Milton Keynes|Wellcome Sanger Institute for the COVID-19 Genomics UK (COG-UK) Consortium|Davies|United Kingdom
>Spike|hCoV-19/Indonesia/BT-MRINUPH-200620-312/2020|2020-06-17|EPI_ISL_779393|Original|hCoV-19^^Banten|Human|Siloam Hospital Lippo Village|Mochtar Riady Institute for Nanotechnology-Universitas Pelita Harapan|Suriapranata|Indonesia
>Spike|hCoV-19/England/CAMB-1AC5E8/2020|2020-04-23|EPI_ISL_489432|Original|hCoV-19^^England|Human|Department of Pathology|Wellcome Sanger Institute for the COVID-19 Genomics UK (COG-UK) consortium|Davies|United Kingdom
>Spike|hCoV-19/England/LIVE-995D0/2020|2020-04-08|EPI_ISL_439739|Original|hCoV-19^^England|Human|Liverpool Clinical Laboratories|COVID-19 Genomics UK (COG-UK) Consortium|Haldenby|United Kingdom
>Spike|hCoV-19/England/LIVE-DCBA51/2020|2020-11-25|EPI_ISL_724448|Original|hCoV-19^^England|Human|Liverpool Clinical Laboratories|COVID-19 Genomics UK (COG-UK) Consortium|Haldenby|United Kingdom
>Spike|hCoV-19/England/ALDP-95134A/2020|2020-06-13|EPI_ISL_554203|Original|hCoV-19^^England|Human|Lighthouse Lab in Alderley Park|Wellcome Sanger Institute for the COVID-19 Genomics UK (COG-UK) consortium|Davies|United Kingdom
>Spike|hCoV-19/New Zealand/20VR1857/2020|2020-03-30|EPI_ISL_456285|Original|hCoV-19^^Southern|Human|Southern Community Labs Dunedin|Institute of Environmental Science and Research (ESR)|de Ligt|New Zealand
>Spike|hCoV-19/Japan/PG-5277/2020|2020-08-00|EPI_ISL_690380|Original|hCoV-19^^Japan|Human|Pathogen Genomics Center|Pathogen Genomics Center|Sekizuka|Japan
>Spike|hCoV-19/Australia/NSW1281/2020|2020-12-17|EPI_ISL_745096|Original|hCoV-19^^New South Wales|Human|Pathology North - Hunter - NSW Health Pathology|CIDM-PH|Gall|Australia
>Spike|hCoV-19/USA/TX-HMH-MCoV-19441/2020|2020-11-30|EPI_ISL_783841|Original|hCoV-19^^Texas|Human|Houston Methodist Hospital|Houston Methodist Hospital|Long|USA
>Spike|hCoV-19/Jordan/AQ-ALSR-5277/2020|2020-10-05|EPI_ISL_730515|Original|hCoV-19^^Aqaba|Human|Biolab Diagnostic Laboratories|Andersen lab at Scripps Research|Gangavarapu|Jordan
>Spike|hCoV-19/England/BRIS-12CB1B/2020|2020-04-18|EPI_ISL_481842|Original|hCoV-19^^England|Human|PHE South West Regional Laboratory|Wellcome Sanger Institute for the COVID-19 Genomics UK (COG-UK) consortium|Davies|United Kingdom
>Spike|hCoV-19/India/GJ-GBRC302a/2020|2020-06-15|EPI_ISL_495048|Original|hCoV-19^^Gujarat|Human|GMERS Medical College & Hospital|Gujarat Biotechnology Research Centre|Joshi|India

>Spike|hCoV-19/England/NOTT-1109C5/2020|2020-04-30|EPI_ISL_441882|Original|hCoV-19^^England|Human|Queens Medical Centre|COVID-19 Genomics UK (COG-UK) Consortium|Loose|United Kingdom
>Spike|hCoV-19/Netherlands/FR-RIVM-20197/2020|2020-11-29|EPI_ISL_723351|Orignal|hCoV-19^^Friesland|Human|Dutch COVID-19 response team|National Institute for Public Health and the Environment (RIVM)|Meijer|Netherlands
>Spike|hCoV-19/Spain/RI-IBV-99010993/2020|2020-11-06|EPI_ISL_732816|Original|hCoV-19^^La_Rioja|Human|Centro de Investigaci\u00f3n Biom\u00e9dica de La Rioja - Hospital San Pedro Logro\u00f1o|SeqCOVID-SPAIN consortium/IBV(CSIC)|Comas|Spain
>Spike|hCoV-19/England/CAMB-1BBE63/2020|2020-11-14|EPI_ISL_679244|Original|hCoV-19^^England|Human|Department of Pathology|COVID-19 Genomics UK (COG-UK) Consortium|Goodfellow|United Kingdom
>Spike|hCoV-19/England/QEUH-C013EC/2020|2020-12-04|EPI_ISL_720704|Original|hCoV-19^^England|Human|Lighthouse Lab in Glasgow|Wellcome Sanger Institute for the COVID-19 Genomics UK (COG-UK) Consortium|Davies|United Kingdom
>Spike|hCoV-19/USA/WA-UW262/2020|2020-03-16|EPI_ISL_418047|Original|hCoV-19^^Washington|Human|UW Virology Lab|UW Virology Lab|Roychoudhury|USA
>Spike|hCoV-19/England/ALDP-C06DE9/2020|2020-12-06|EPI_ISL_709305|Original|hCoV-19^^England|Human|Lighthouse Lab in Alderley Park|Wellcome Sanger Institute for the COVID-19 Genomics UK (COG-UK) Consortium|Davies|United Kingdom
>Spike|hCoV-19/Australia/WA421/2020|2020-03-20|EPI_ISL_672633|Original|hCoV-19^^Western Australia|Human|PathWest Laboratory Medicine WA|PathWest Laboratory Medicine WA Microbial Surveillance Unit|Sikazwe|Australia
>Spike|hCoV-19/USA/FL-BPHL-1777/2020|2020-07-29|EPI_ISL_613755|Original|hCoV-19^^Florida|Human|Florida Bureau of Public Health Laboratories|Florida Bureau of Public Health Laboratories|Blanton|USA
>Spike|hCoV-19/USA/AK-CDC-2-3714567/2020|2020-11-16|EPI_ISL_751793|Original|hCoV-19^^Alaska|Human|AK State Public Health Lab|Genomics and Discovery|Queen|USA
>Spike|hCoV-19/Switzerland/BL-ETHZ-140063/2020|2020-04-30|EPI_ISL_476111|Original|hCoV-19^^Basel-Landschaft|Human|Viollier AG|Department of Biosystems Science and Engineering|Nadeau|Switzerland
>Spike|hCoV-19/Switzerland/BL-UHB-42186686/2020|2020-03-11|EPI_ISL_528015|Original|hCoV-19^^Basel-Land|Human|University Hospital Basel|University Hospital Basel|Roloff Handschin|Switzerland
>Spike|hCoV-19/England/PRIN-254BC91/2020|2020-04-29|EPI_ISL_509436|Original|hCoV-19^^England|Human|The Princess Alexandra Hospital|Wellcome Sanger Institute for the COVID-19 Genomics UK (COG-UK) consortium|Davies|United Kingdom
>Spike|hCoV-19/England/20114052703/2020|2020-03-11|EPI_ISL_420458|Original|hCoV-19^^England|Human|Respiratory Virus Unit|Respiratory Virus Unit|Platt|United Kingdom
>Spike|hCoV-19/Singapore/472cul/2020|2020-06-03|EPI_ISL_574492|VeroE6 P1|hCoV-19^^Singapore|Human|Programme in Emerging Infectious Diseases|National Public Health Laboratory|Mak|Singapore
>Spike|hCoV-19/England/LIVE-DC4D40/2020|2020-11-12|EPI_ISL_703992|Original|hCoV-19^^England|Human|Liverpool Clinical Laboratories|COVID-19 Genomics UK (COG-UK) Consortium|Haldenby|United Kingdom
>Spike|hCoV-19/USA/TX-HMH-MCoV-8200/2020|2020-07-07|EPI_ISL_784259|Original|hCoV-19^^Texas|Human|Houston Methodist Hospital|Houston Methodist Hospital|Long|USA
>Spike|hCoV-19/England/MILK-99CEAC/2020|2020-08-31|EPI_ISL_549899|Original|hCoV-19^^England|Human|Lighthouse Lab in Milton Keynes|Wellcome Sanger Institute for the COVID-19 Genomics UK (COG-UK) consortium|Davies|United Kingdom
>Spike|hCoV-19/England/BRIS-12F134/2020|2020-04-13|EPI_ISL_488385|Original|hCoV-19^^England|Human|PHE South West Regional Laboratory|Wellcome Sanger Institute for the COVID-19 Genomics UK (COG-UK) consortium|Davies|United Kingdom
>Spike|hCoV-19/Norway/4968/2020|2020-10-26|EPI_ISL_635084|Original|hCoV-19^^Vestlandet|Human|Haukeland University Hospital|Norwegian Institute of Public Health|Vollan|Norway
>Spike|hCoV-19/United Arab Emirates/0695/2020|2020-05-15|EPI_ISL_698846|Original|hCoV-19^^Abu Dhabi|Human|Group 42 (G42) Healthcare|G42 Healthcare|Ogrodzki|United Arab Emirates

>Spike|hCoV-19/Germany/SH-ChVir8607/2020|2020-08-24|EPI_ISL_729594|Original|hCoV-
19^^Schleswig-Holstein|Human|A. Krumbholz|Charit\u00e9 Universit\u00e4tsmedizin
Berlin|Muehlemann|Germany
>Spike|hCoV-19/England/NORW-F205C/2020|2020-10-19|EPI_ISL_627024|Original|hCoV-
19^^England|Human|Quadram Institute Bioscience|COVID-19 Genomics UK (COG-UK)
Consortium|Alikhan|United Kingdom
>Spike|hCoV-19/USA/MI-MDHHS-SC20886/2020|2020-05-18|EPI_ISL_471907|Original|hCoV-
19^^Michigan|Human|Michigan Department of Health and Human Services|Michigan
Department of Health and Human Services|Blankenship|USA
>Spike|hCoV-19/Uganda/UG035/2020|2020-03-27|EPI_ISL_737955|Original|hCoV-
19^^Uganda|Human|Uganda Central Public Health Lab and Uganda Virus Research
Institute|MRC/UVRI & LSHTM Uganda\u00a0Research Unit|Cotten|Uganda
>Spike|hCoV-19/Switzerland/VD-ETHZ-100845/2020|2020-03-
25|EPI_ISL_451792|Original|hCoV-19^^Vaud|Human|Viollier AG|Department of Biosystems
Science and Engineering|Nadeau|Switzerland
>Spike|hCoV-19/Germany/HH-hpi-p2496/2020|2020-06-26|EPI_ISL_776546|Original|hCoV-
19^^Hamburg|Human|University Medical Center Hamburg Eppendorf|Heinrich Pette
Institute|Robitaille|Germany
>Spike|hCoV-19/Scotland/QEUH-AD53B6/2020|2020-10-26|EPI_ISL_634580|Original|hCoV-
19^^Scotland|Human|Lighthouse Lab in Glasgow|Wellcome Sanger Institute for the COVID-
19 Genomics UK (COG-UK) consortium|Davies|United Kingdom
>Spike|hCoV-19/England/20130065604/2020|2020-03-21|EPI_ISL_423655|Original|hCoV-
19^^England|Human|Respiratory Virus Unit|Respiratory Virus Unit|Platt|United Kingdom
>Spike|hCoV-19/Denmark/DCGC-3522/2020|2020-08-31|EPI_ISL_616313|Original|hCoV-
19^^Midtjylland|Human|Department of Virus and Microbiological Special
Diagnostics|Albertsen lab|Michaelsen|Denmark
>Spike|hCoV-19/USA/UT-UPHL-2012337841/2020|2020-08-10|EPI_ISL_745892|Original|hCoV-
19^^Utah|Human|Ginkgo Bioworks Clinical Laboratory|Utah Public Health
Laboratory|Young|USA
>Spike|hCoV-19/Australia/NSW217/2020|2020-03-02|EPI_ISL_427644|Original|hCoV-19^^New
South Wales|Human|Centre for Infectious Diseases and Microbiology Public Health|NSW
Health Pathology - Institute of Clinical Pathology and Medical Research; Westmead
Hospital; University of Sydney|Gall|Australia
>Spike|hCoV-19/Singapore/49/2020|2020-03-08|EPI_ISL_428828|Original|hCoV-
19^^Singapore|Human|National Public Health Laboratory|National Public Health
Laboratory|Mak|Singapore
>Spike|hCoV-19/USA/TX-HMH-1341/2020|2020-04-14|EPI_ISL_542659|Original|hCoV-
19^^Texas|Human|Houston Methodist Hospital|Houston Methodist Hospital|Long|USA
>Spike|hCoV-19/England/OXON-B01A2/2020|2020-00-00|EPI_ISL_478862|Original|hCoV-
19^^England|Human|Oxford Viromics|COVID-19 Genomics UK (COG-UK)
Consortium|Golubchik|United Kingdom
>Spike|hCoV-19/USA/TX-HMH-2720/2020|2020-06-06|EPI_ISL_545503|Original|hCoV-
19^^Texas|Human|Houston Methodist Hospital|Houston Methodist Hospital|Long|USA
>Spike|hCoV-19/Denmark/DCGC-1588/2020|2020-04-13|EPI_ISL_618632|Original|hCoV-
19^^Hovedstaden|Human|Department of Virus and Microbiological Special
Diagnostics|Albertsen lab|Michaelsen|Denmark
>Spike|hCoV-19/South Africa/KRISP-0475/2020|2020-07-06|EPI_ISL_495558|Original|hCoV-
19^^KwaZulu-Natal|Human|NHLS-IALCH|KRISP|de Oliveira|South Africa
>Spike|hCoV-19/USA/TX-HMH-MCoV-10649/2020|2020-07-08|EPI_ISL_789575|Original|hCoV-
19^^Texas|Human|Houston Methodist Hospital|Houston Methodist Hospital|Long|USA
>Spike|hCoV-19/Wales/PHWC-16A971/2020|2020-09-09|EPI_ISL_560131|Original|hCoV-
19^^Wales|Human|Wales Specialist Virology Centre Sequencing lab|COVID-19 Genomics UK
(COG-UK) Consortium|Moore|United Kingdom
>Spike|hCoV-19/Scotland/EDB3588/2020|2020-04-27|EPI_ISL_439294|Original|hCoV-
19^^Scotland|Human|Virology Department|COVID-19 Genomics UK (COG-UK)
Consortium|Williams|United Kingdom
>Spike|hCoV-19/Japan/IC-0081/2020|2020-07-00|EPI_ISL_591423|Original|hCoV-
19^^Japan|Human|Pathogen Genomics Center|Pathogen Genomics Center|Sekizuka|Japan

>Spike|hCoV-19/Spain/AS-IBV-98006597/2020|2020-03-12|EPI_ISL_654641|Original|hCoV-19^^Asturias|Human|Servicio de Microbiolog\u00eda|SeqCOVID-SPAIN consortium/IBV(CSIC)|Comas|Spain
>Spike|hCoV-19/England/CAMC-B0CC50/2020|2020-10-30|EPI_ISL_643511|Original|hCoV-19^^England|Human|Lighthouse Lab in Cambridge|Wellcome Sanger Institute for the COVID-19 Genomics UK (COG-UK) Consortium|Davies|United Kingdom
>Spike|hCoV-19/Belgium/rega-0320861/2020|2020-03-20|EPI_ISL_734511|Original|hCoV-19^^Genval|Human|UZ Leuven|KU Leuven|Maes|Belgium
>Spike|hCoV-19/South Africa/R10852/2020|2020-06-17|EPI_ISL_490310|Original|hCoV-19^^Free State|Human|National Institute for Communicable Diseases of the National Health Laboratory Service|National Institute for Communicable Diseases of the National Health Laboratory Service|Allam|South Africa
>Spike|hCoV-19/India/KA-IB51/2020|2020-06-19|EPI_ISL_508329|Original|hCoV-19^^Karnataka|Human|Indian Institute of Science|National Institute of Biomedical Genomics|Maitra|India
>Spike|hCoV-19/England/20194103201/2020|2020-05-05|EPI_ISL_465613|Original|hCoV-19^^England|Human|Respiratory Virus Unit|Respiratory Virus Unit|Platt|United Kingdom
>Spike|hCoV-19/England/SHEF-10C6610/2020|2020-12-06|EPI_ISL_702487|Original|hCoV-19^^England|Human|Virology Department|COVID-19 Genomics UK (COG-UK) Consortium|de Silva|United Kingdom
>Spike|hCoV-19/South Africa/KRISP-K002386/2020|2020-07-21|EPI_ISL_515752|Original|hCoV-19^^KwaZulu-Natal|Human|NHLS-IALCH|KRISP|de Oliveira|South Africa
>Spike|hCoV-19/Poland/PL_P1/2020|2020-03-03|EPI_ISL_416488|Vero E6|hCoV-19^^Zielonogorskie|Human|ViroGenetics - BSL3 Laboratory of Virology; Human Genome Variation Research Group & Genomics Centre MCB; Bioinformatics Research Group Department of Virology|ViroGenetics - BSL3 Laboratory of Virology; Human Genome Variation Research Group & Genomics Centre MCB; Bioinformatics Research Group Departmen
>t of Virology||Poland
>Spike|hCoV-19/England/QEUH-9C8584/2020|2020-09-11|EPI_ISL_567070|Original|hCoV-19^^England|Human|Lighthouse Lab in Glasgow|Wellcome Sanger Institute for the COVID-19 Genomics UK (COG-UK) consortium|Davies|United Kingdom
>Spike|hCoV-19/Wales/PHWC-48B670/2020|2020-10-31|EPI_ISL_665576|Original|hCoV-19^^Wales|Human|Wales Specialist Virology Centre Sequencing lab|COVID-19 Genomics UK (COG-UK) Consortium|Moore|United Kingdom
>Spike|hCoV-19/England/LOND-12624ED/2020|2020-09-27|EPI_ISL_594861|Original|hCoV-19^^England|Human|University College London|COVID-19 Genomics UK (COG-UK) Consortium|Roy|United Kingdom
>Spike|hCoV-19/Scotland/QEUH-A219AA/2020|2020-10-01|EPI_ISL_598852|Original|hCoV-19^^Scotland|Human|Lighthouse Lab in Glasgow|Wellcome Sanger Institute for the COVID-19 Genomics UK (COG-UK) consortium|Davies|United Kingdom
>Spike|hCoV-19/England/ALDP-9FD683/2020|2020-10-01|EPI_ISL_588049|Original|hCoV-19^^England|Human|Lighthouse Lab in Alderley Park|Wellcome Sanger Institute for the COVID-19 Genomics UK (COG-UK) consortium|Davies|United Kingdom
>Spike|hCoV-19/Sweden/20-53293/2020|2020-10-27|EPI_ISL_648166|Original|hCoV-19^^Sweden|Human|Eskilstuna|The Public Health Agency of Sweden|Svartstr\u00f6m|Sweden
>Spike|hCoV-19/France/ARA-100617/2020|2020-11-03|EPI_ISL_683334|Original|hCoV-19^^Auvergne-Rh\u00f4ne-Alpes|Human|CNR Virus des Infections Respiratoires - France SUD|CNR Virus des Infections Respiratoires - France SUD|Bal|France
>Spike|hCoV-19/Scotland/CVR3174/2020|2020-04-26|EPI_ISL_449202|Original|hCoV-19^^Scotland|Human|West of Scotland Specialist Virology Centre|COVID-19 Genomics UK (COG-UK) Consortium|Robertson|United Kingdom
>Spike|hCoV-19/England/QEUH-AA02D5/2020|2020-10-20|EPI_ISL_611314|Original|hCoV-19^^England|Human|Lighthouse Lab in Glasgow|Wellcome Sanger Institute for the COVID-19 Genomics UK (COG-UK) consortium|Davies|United Kingdom
>Spike|hCoV-19/England/MILK-B8EA2F/2020|2020-11-13|EPI_ISL_674766|Original|hCoV-19^^England|Human|Lighthouse Lab in Milton Keynes|Wellcome Sanger Institute for the COVID-19 Genomics UK (COG-UK) Consortium|Davies|United Kingdom

>Spike|hCoV-19/Switzerland/ZH-ETHZ-250070/2020|2020-08-
18|EPI_ISL_535595|Original|hCoV-19^^Zurich|Human|Viollier AG|Department of Biosystems
Science and Engineering|Nadeau|Switzerland
>Spike|hCoV-19/England/ALDP-AA1447/2020|2020-10-22|EPI_ISL_623899|Original|hCoV-
19^^England|Human|Lighthouse Lab in Alderley Park|Wellcome Sanger Institute for the
COVID-19 Genomics UK (COG-UK) consortium|Davies|United Kingdom
>Spike|hCoV-19/USA/NY-NYCPHL-001081/2020|2020-10-18|EPI_ISL_633082|Original|hCoV-
19^^New York|Human|DOHMH Morrisania|New York City Public Health Laboratory|Wang|USA
>Spike|hCoV-19/Canada/BC_04806819/2020|2020-05-00|EPI_ISL_477056|Original|hCoV-
19^^British Columbia|Human|BCCDC Public Health Laboratory|BCCDC Public Health
Laboratory|Harrigan|Canada
>Spike|hCoV-19/England/ALDP-B5C705/2020|2020-11-09|EPI_ISL_655640|Original|hCoV-
19^^England|Human|Lighthouse Lab in Alderley Park|Wellcome Sanger Institute for the
COVID-19 Genomics UK (COG-UK) Consortium|Davies|United Kingdom
>Spike|hCoV-19/USA/WA-UW-24556/2020|2020-08-17|EPI_ISL_576485|Original|hCoV-
19^^Washington|Human|UW Virology Lab|UW Virology Lab|Roychoudhury|USA
>Spike|hCoV-19/USA/TX-HMH-MCoV-16230/2020|2020-10-30|EPI_ISL_785914|Original|hCoV-
19^^Texas|Human|Houston Methodist Hospital|Houston Methodist Hospital|Long|USA
>Spike|hCoV-19/England/CAMB-1B97A6/2020|2020-11-04|EPI_ISL_664577|Original|hCoV-
19^^England|Human|Department of Pathology|COVID-19 Genomics UK (COG-UK)
Consortium|Goodfellow|United Kingdom
>Spike|hCoV-19/India/WB-IK7/2020|2020-04-03|EPI_ISL_508394|Original|hCoV-19^^West
Bengal|Human|Institute of Post Graduate Medical Education & Research|National
Institute of Biomedical Genomics|Maitra|India
>Spike|hCoV-19/England/MILK-ACFA32/2020|2020-10-22|EPI_ISL_629658|Original|hCoV-
19^^England|Human|Lighthouse Lab in Milton Keynes|Wellcome Sanger Institute for the
COVID-19 Genomics UK (COG-UK) consortium|Davies|United Kingdom
>Spike|hCoV-19/USA/AZ-TG323186/2020|2020-05-21|EPI_ISL_694339|Original|hCoV-
19^^Arizona|Human|TGen North|TGen North|French|USA
>Spike|hCoV-19/USA/TX-HMH-994/2020|2020-04-07|EPI_ISL_545951|Original|hCoV-
19^^Texas|Human|Houston Methodist Hospital|Houston Methodist Hospital|Long|USA
>Spike|hCoV-19/Netherlands/OV-RIVM-20678/2020|2020-12-
07|EPI_ISL_790747|Original|hCoV-19^^Overijssel|Human|Dutch COVID-19 response
team|National Institute for Public Health and the Environment
(RIVM)|Meijer|Netherlands
>Spike|hCoV-19/Denmark/DCGC-18645/2020|2020-12-07|EPI_ISL_749508|Original|hCoV-
19^^Hovedstaden|Human|Department of Virus and Microbiological Special
Diagnostics|Albertsen Lab|Michaelsen|Denmark
>Spike|hCoV-19/England/QEUH-A1ED95/2020|2020-09-29|EPI_ISL_587492|Original|hCoV-
19^^England|Human|Lighthouse Lab in Glasgow|Wellcome Sanger Institute for the COVID-
19 Genomics UK (COG-UK) consortium|Davies|United Kingdom
>Spike|hCoV-19/Canada/BC-BCCDC-7309/2020|2020-00-00|EPI_ISL_740676|Original|hCoV-
19^^British Columbia|Human|BCCDC Public Health Laboratory|BCCDC Public Health
Laboratory|Prystajecky|Canada
>Spike|hCoV-19/England/CAMC-B789FE/2020|2020-11-12|EPI_ISL_668041|Original|hCoV-
19^^England|Human|Lighthouse Lab in Cambridge|Wellcome Sanger Institute for the
COVID-19 Genomics UK (COG-UK) Consortium|Davies|United Kingdom
>Spike|hCoV-19/England/QEUH-C3317E/2020|2020-12-11|EPI_ISL_733951|Original|hCoV-
19^^England|Human|Lighthouse Lab in Glasgow|Wellcome Sanger Institute for the COVID-
19 Genomics UK (COG-UK) Consortium|Davies|United Kingdom
>Spike|hCoV-19/England/ALDP-A69223/2020|2020-10-10|EPI_ISL_608922|Original|hCoV-
19^^England|Human|Lighthouse Lab in Alderley Park|Wellcome Sanger Institute for the
COVID-19 Genomics UK (COG-UK) consortium|Davies|United Kingdom
>Spike|hCoV-19/South Africa/KRISP-K005313/2020|2020-10-
29|EPI_ISL_678606|Original|hCoV-19^^KwaZulu-Natal|Human|NHLS-IALCH|KRISP|de
Oliveira|South Africa
>Spike|hCoV-19/Singapore/1161/2020|2020-04-03|EPI_ISL_648088|Original|hCoV-
19^^Singapore|Human|Department of Laboratory Medicine|Department of Laboratory
Medicine|Chen|Singapore

>Spike|hCoV-19/USA/MI-MDHHS-SC20150/2020|2020-03-13|EPI_ISL_436813|Original|hCoV-19^^Michigan|Human|Michigan Department of Health and Human Services|Michigan Department of Health and Human Services|Blankenship|USA
>Spike|hCoV-19/Australia/VIC5839/2020|2020-07-21|EPI_ISL_518633|Original|hCoV-19^^Victoria|Human|Microbiological Diagnostic Unit - Public Health Laboratory (MDU-PHL)|MDU-PHL|Schultz|Australia
>Spike|hCoV-19/Japan/PG-7670/2020|2020-08-00|EPI_ISL_686772|Original|hCoV-19^^Japan|Human|Chiba Prefectural Institute of Public Health|Pathogen Genomics Center|Sekizuka|Japan
>Spike|hCoV-19/USA/OR-OHSU-2890/2020|2020-09-04|EPI_ISL_667391|Original|hCoV-19^^Oregon|Human|OHSU Lab Services Molecular Microbiology Lab|Oregon SARS-CoV-2 Genome Sequencing Center|O'Roak|USA
>Spike|hCoV-19/Denmark/DCGC-1633/2020|2020-04-06|EPI_ISL_619185|Original|hCoV-19^^Hovedstaden|Human|Department of Virus and Microbiological Special Diagnostics|Albertsen lab|Michaelsen|Denmark
>Spike|hCoV-19/USA/WI-WSLH-200184/2020|2020-04-06|EPI_ISL_578409|Original|hCoV-19^^Wisconsin|Human|Wisconsin State Laboratory of Hygiene Communicable Disease Division|Wisconsin State Laboratory of Hygiene Communicable Disease Division|Florek|USA
>Spike|hCoV-19/England/CAMC-A584F4/2020|2020-10-06|EPI_ISL_597135|Original|hCoV-19^^England|Human|Lighthouse Lab in Cambridge|Wellcome Sanger Institute for the COVID-19 Genomics UK (COG-UK) consortium|Davies|United Kingdom
>Spike|hCoV-19/Japan/PG-3003/2020|2020-04-00|EPI_ISL_691127|Original|hCoV-19^^Japan|Human|Pathogen Genomics Center|Pathogen Genomics Center|Sekizuka|Japan
>Spike|hCoV-19/Denmark/DCGC-1873/2020|2020-07-13|EPI_ISL_622599|Original|hCoV-19^^Hovedstaden|Human|Department of Virus and Microbiological Special Diagnostics|Albertsen lab|Michaelsen|Denmark
>Spike|hCoV-19/Australia/VIC160/2020|2020-03-18|EPI_ISL_419857|Original|hCoV-19^^Victoria|Human|Victorian Infectious Diseases Reference Laboratory (VIDRL)|Victorian Infectious Diseases Reference Laboratory and Microbiological Diagnostic Unit Public Health Laboratory|Schultz|Australia
>Spike|hCoV-19/Russia/StPetersburg-RII8417S/2020|2020-04-22|EPI_ISL_450283|Original|hCoV-19^^Saint Petersburg|Human|WHO National Influenza Centre Russian Federation|WHO National Influenza Centre Russian Federation|Komissarov|Russia
>Spike|hCoV-19/England/ALDP-A501F5/2020|2020-10-07|EPI_ISL_597862|Original|hCoV-19^^England|Human|Lighthouse Lab in Alderley Park|Wellcome Sanger Institute for the COVID-19 Genomics UK (COG-UK) consortium|Davies|United Kingdom
>Spike|hCoV-19/Finland/18SS6E7/2020|2020-09-18|EPI_ISL_756291|Original|hCoV-19^^Uusimaa|Human|Department of Virology and Immunology|Department of Virology|Smura|Finland
>Spike|hCoV-19/USA/SC-20-0946-T14/2020|2020-06-24|EPI_ISL_516751|Original|hCoV-19^^South Carolina|Human|van Bakel Laboratory|van Bakel Laboratory|van Bakel|USA
>Spike|hCoV-19/Netherlands/NH-RIVM-20018/2020|2020-11-21|EPI_ISL_723217|Orignal|hCoV-19^^Noord-Holland|Human|Dutch COVID-19 response team|National Institute for Public Health and the Environment (RIVM)|Meijer|Netherlands
>Spike|hCoV-19/Denmark/DCGC-20244/2020|2020-11-30|EPI_ISL_747539|Original|hCoV-19^^Hovedstaden|Human|Department of Virus and Microbiological Special Diagnostics|Albertsen Lab|Michaelsen|Denmark
>Spike|hCoV-19/Russia/Khabarovsk-71403/2020|2020-03-19|EPI_ISL_428880|Vero|hCoV-19^^Khabarovsk|Human|State Research Center of Virology and Biotechnology VECTOR|State Research Center of Virology and Biotechnology VECTOR|Shvalov|Russia
>Spike|hCoV-19/Denmark/DCGC-761/2020|2020-03-09|EPI_ISL_614752|Original|hCoV-19^^Syddanmark|Human|Department of Virus and Microbiological Special Diagnostics|Albertsen lab|Michaelsen|Denmark
>Spike|hCoV-19/England/MILK-B8F262/2020|2020-11-13|EPI_ISL_675331|Original|hCoV-19^^England|Human|Lighthouse Lab in Milton Keynes|Wellcome Sanger Institute for the COVID-19 Genomics UK (COG-UK) Consortium|Davies|United Kingdom
>Spike|hCoV-19/Australia/NSW645/2020|2020-07-12|EPI_ISL_498764|Original|hCoV-19^^New South Wales|Human|Sydney South West Pathology Service (SSWPS) - Liverpool Hospital -

NSW Health Pathology|NSW Health Pathology - Institute of Clinical Pathology and Medical Research; Westmead Hospital; University of Sydney|Gall|Australia
>Spike|hCoV-19/USA/WA-UW-7776/2020|2020-04-19|EPI_ISL_570193|Original|hCoV-19^^Washington|Human|UW Virology Lab|UW Virology Lab|Roychoudhury|USA
>Spike|hCoV-19/England/CAMC-CB8B82/2020|2020-12-19|EPI_ISL_778134|Original|hCoV-19^^England|Human|Lighthouse Lab in Cambridge|Wellcome Sanger Institute for the COVID-19 Genomics UK (COG-UK) Consortium|Davies|United Kingdom
>Spike|hCoV-19/England/BRIS-12C498/2020|2020-05-12|EPI_ISL_469493|Original|hCoV-19^^England|Human|PHE South West Regional Laboratory|Wellcome Sanger Institute for the COVID-19 Genomics UK (COG-UK) consortium|Davies|United Kingdom
>Spike|hCoV-19/Wales/PHWC-1661E6/2020|2020-06-13|EPI_ISL_490849|Original|hCoV-19^^Wales|Human|Wales Specialist Virology Centre Sequencing lab|COVID-19 Genomics UK (COG-UK) Consortium|Moore|United Kingdom
>Spike|hCoV-19/Chile/RM-112467/2020|2020-06-28|EPI_ISL_746585|Original|hCoV-19^^Region Metropolitana de Santiago|Human|Genetica Molecular and Subdepartamento de Virologia ISP Chile|Instituto de Salud Publica de Chile|Tognarelli|Chile
>Spike|hCoV-19/Spain/IB-IBV-99011588/2020|2020-12-08|EPI_ISL_779862|Original|hCoV-19^^Balear Islands|Human|Servicio de Microbiolog\u00eda|SeqCOVID-SPAIN consortium/IBV(CSIC)|Comas|Spain
>Spike|hCoV-19/Sweden/20-08106/2020|2020-03-10|EPI_ISL_454477|Original|hCoV-19^^Stockholm|Human|Karolinska Universitetslaboratoriet|The Public Health Agency of Sweden|Svartstr\u00f6m|Sweden
>Spike|hCoV-19/Wales/PHWC-16BA04/2020|2020-09-14|EPI_ISL_573929|Original|hCoV-19^^Wales|Human|Wales Specialist Virology Centre Sequencing lab|COVID-19 Genomics UK (COG-UK) Consortium|Moore|United Kingdom
>Spike|hCoV-19/Scotland/EDB10517/2020|2020-11-29|EPI_ISL_705775|Original|hCoV-19^^Scotland|Human|Virology Department|COVID-19 Genomics UK (COG-UK) Consortium|McHugh|United Kingdom
>Spike|hCoV-19/England/MILK-BB0365/2020|2020-11-17|EPI_ISL_720347|Original|hCoV-19^^England|Human|Lighthouse Lab in Milton Keynes|Wellcome Sanger Institute for the COVID-19 Genomics UK (COG-UK) Consortium|Davies|United Kingdom
>Spike|hCoV-19/USA/NM-DOH-2020313085/2020|2020-08-26|EPI_ISL_574262|Original|hCoV-19^^New Mexico|Human|New Mexico Department of Health Scientific Laboratory|New Mexico Department of Health Scientific Laboratory|Johnson|USA
>Spike|hCoV-19/England/204561386/2020|2020-11-04|EPI_ISL_678684|Original|hCoV-19^^England|Human|Respiratory Virus Unit|COVID-19 Genomics UK (COG-UK) Consortium|Platt|United Kingdom
>Spike|hCoV-19/Poland/PL_P45/2020|2020-04-13|EPI_ISL_492070|Original|hCoV-19^^Mazowieckie|Human|1. ViroGenetics - BSL3 Laboratory of Virology|1. ViroGenetics - BSL3 Laboratory of Virology|Pyrc|Poland
>Spike|hCoV-19/USA/HI-H200680/2020|2020-12-01|EPI_ISL_753203|Original|hCoV-19^^Hawaii|Human|State Laboratories Division|State Laboratories Division|Sultana|USA
>Spike|hCoV-19/South Africa/KRISP-K002579/2020|2020-07-29|EPI_ISL_535444|Original|hCoV-19^^KwaZulu-Natal|Human|NHLS-IALCH|KRISP|de Oliveira|South Africa
>Spike|hCoV-19/South Africa/KRISP-K004555/2020|2020-10-19|EPI_ISL_660252|Original|hCoV-19^^KwaZulu-Natal|Human|NHLS-IALCH|KRISP|de Oliveira|South Africa
>Spike|hCoV-19/USA/CA-ALSR-1309/2020|2020-05-11|EPI_ISL_494488|Original|hCoV-19^^California|Human|San Diego County Public Health Laboratory|Andersen lab at Scripps Research|Gangavarapu|USA
>Spike|hCoV-19/England/ALDP-B63AD5/2020|2020-11-09|EPI_ISL_656521|Original|hCoV-19^^England|Human|Lighthouse Lab in Alderley Park|Wellcome Sanger Institute for the COVID-19 Genomics UK (COG-UK) Consortium|Davies|United Kingdom
>Spike|hCoV-19/Australia/NSW1130/2020|2020-09-05|EPI_ISL_593743|Original|hCoV-19^^New South Wales|Human|South Eastern Area Laboratory Services (SEALS)|NSW Health Pathology - Institute of Clinical Pathology and Medical Research; Westmead Hospital; University of Sydney|Gall|Australia
>Spike|hCoV-19/England/20140003204/2020|2020-03-28|EPI_ISL_465870|Original|hCoV-19^^England|Human|Respiratory Virus Unit|Respiratory Virus Unit|Platt|United Kingdom

>Spike|hCoV-19/Australia/VIC760/2020|2020-03-29|EPI_ISL_427036|Original|hCoV-19^^Victoria|Human|Victorian Infectious Diseases Reference Laboratory (VIDRL)|Microbiological Diagnostic Unit Public Health Laboratory and Victorian Infectious Diseases Reference Laboratory|Schultz|Australia
>Spike|hCoV-19/England/CAMB-75E2A/2020|2020-03-29|EPI_ISL_438344|Original|hCoV-19^^England|Human|Department of Pathology|Wellcome Sanger Institute for the COVID-19 Genomics UK (COG-UK) consortium|Amato|United Kingdom
>Spike|hCoV-19/Wales/PHWC-48F1A8/2020|2020-11-09|EPI_ISL_681054|Original|hCoV-19^^Wales|Human|Wales Specialist Virology Centre Sequencing lab|COVID-19 Genomics UK (COG-UK) Consortium|Moore|United Kingdom
>Spike|hCoV-19/England/QEUH-9D089F/2020|2020-09-16|EPI_ISL_589873|Original|hCoV-19^^England|Human|Lighthouse Lab in Glasgow|Wellcome Sanger Institute for the COVID-19 Genomics UK (COG-UK) consortium|Davies|United Kingdom
>Spike|hCoV-19/Denmark/DCGC-22100/2020|2020-12-14|EPI_ISL_757818|Original|hCoV-19^^Hovedstaden|Human|Department of Virus and Microbiological Special Diagnostics|Albertsen Lab|Michaelsen|Denmark
>Spike|hCoV-19/England/LIVE-DC2CFA/2020|2020-11-25|EPI_ISL_705845|Original|hCoV-19^^England|Human|Liverpool Clinical Laboratories|COVID-19 Genomics UK (COG-UK) Consortium|Haldenby|United Kingdom
>Spike|hCoV-19/England/LOND-D3947/2020|2020-04-03|EPI_ISL_441011|Original|hCoV-19^^England|Human|University College London|COVID-19 Genomics UK (COG-UK) Consortium|Roy|United Kingdom
>Spike|hCoV-19/USA/UT-UPHL-08764/2020|2020-08-13|EPI_ISL_525350|Original|hCoV-19^^Utah|Human|Utah Public Health Laboratory|Utah Public Health Laboratory|Young|USA
>Spike|hCoV-19/England/ALDP-C063D8/2020|2020-12-06|EPI_ISL_709499|Original|hCoV-19^^England|Human|Lighthouse Lab in Alderley Park|Wellcome Sanger Institute for the COVID-19 Genomics UK (COG-UK) Consortium|Davies|United Kingdom
>Spike|hCoV-19/England/OXON-B2E12/2020|2020-03-31|EPI_ISL_534817|Original|hCoV-19^^England|Human|Oxford Viromics|COVID-19 Genomics UK (COG-UK) Consortium|Golubchik|United Kingdom
>Spike|hCoV-19/Wales/PHWC-24B32/2020|2020-03-20|EPI_ISL_419487|Original|hCoV-19^^Wales|Human|Wales Specialist Virology Centre|Public Health Wales Microbiology Cardiff|Moore|United Kingdom
>Spike|hCoV-19/USA/UT-01694/2020|2020-04-13|EPI_ISL_454715|Original|hCoV-19^^Utah|Human|Utah Public Health Laboratory|Utah Public Health Laboratory|Young|USA
>Spike|hCoV-19/Australia/VIC11645/2020|2020-08-14|EPI_ISL_641029|Original|hCoV-19^^Victoria|Human|Microbiological Diagnostic Unit - Public Health Laboratory (MDU-PHL)|MDU-PHL|Schultz|Australia
>Spike|hCoV-19/USA/TX-HMH-MCoV-18879/2020|2020-11-23|EPI_ISL_785192|Original|hCoV-19^^Texas|Human|Houston Methodist Hospital|Houston Methodist Hospital|Long|USA
>Spike|hCoV-19/England/QEUH-961A15/2020|2020-08-03|EPI_ISL_537010|Original|hCoV-19^^England|Human|Lighthouse Lab in Glasgow|Wellcome Sanger Institute for the COVID-19 Genomics UK (COG-UK) consortium|Davies|United Kingdom
>Spike|hCoV-19/India/TN-NCDC-02332/2020|2020-03-29|EPI_ISL_435096|Original|hCoV-19^^Tamil Nadu|Human|National Centre for Disease control (NCDC)|NCDC/CSIR-IGIB|Faruq|India
>Spike|hCoV-19/Sweden/20-50181/2020|2020-03-08|EPI_ISL_429154|Original|hCoV-19^^Varmland|Human|Klinisk mikrobiologi Orebro|The Public Health Agency of Sweden|Svartstr\u00f6m|Sweden
>Spike|hCoV-19/USA/CA-IGI-0639/2020|2020-08-03|EPI_ISL_677855|Original|hCoV-19^^California|Human|Innovative Genomics Institute|Innovative Genomics Institute|Wyman|USA
>Spike|hCoV-19/Hong Kong/VM20118387/2020|2020-09-05|EPI_ISL_730582|Original|hCoV-19^^Hong Kong|Human|Hong Kong Adventist Hospital \u2013 Tsuen Wan|Hong Kong Department of Health|Mak|China
>Spike|hCoV-19/England/CAMB-79679/2020|2020-04-03|EPI_ISL_441815|Original|hCoV-19^^England|Human|Department of Pathology|Wellcome Sanger Institute for the COVID-19 Genomics UK (COG-UK) consortium|Amato|United Kingdom

>Spike|hCoV-19/USA/MI-UM-10036847408/2020|2020-11-09|EPI_ISL_728451|Original|hCoV-
19^^Michigan|Human|University of Michigan Clinical Microbiology Laboratory|Lauring
Lab|Valesano|USA
>Spike|hCoV-19/Denmark/DCGC-12719/2020|2020-11-16|EPI_ISL_682900|Original|hCoV-
19^^Hovedstaden|Human|Department of Virus and Microbiological Special
Diagnostics|Albertsen Lab|Michaelsen|Denmark
>Spike|hCoV-19/England/QEUH-98F9F6/2020|2020-08-26|EPI_ISL_540029|Original|hCoV-
19^^England|Human|Lighthouse Lab in Glasgow|Wellcome Sanger Institute for the COVID-
19 Genomics UK (COG-UK) consortium|Davies|United Kingdom
>Spike|hCoV-19/USA/MN-MDH-1845/2020|2020-09-29|EPI_ISL_602980|Original|hCoV-
19^^Minnesota|Human|Minnesota Department of Health|Minnesota Department of
Health|Garfin|USA
>Spike|hCoV-19/Australia/VIC511/2020|2020-03-23|EPI_ISL_426781|Original|hCoV-
19^^Victoria|Human|Victorian Infectious Diseases Reference Laboratory
(VIDRL)|Microbiological Diagnostic Unit Public Health Laboratory and Victorian
Infectious Diseases Reference Laboratory|Schultz|Australia
>Spike|hCoV-19/England/ALDP-BE8F42/2020|2020-12-03|EPI_ISL_708924|Original|hCoV-
19^^England|Human|Lighthouse Lab in Alderley Park|Wellcome Sanger Institute for the
COVID-19 Genomics UK (COG-UK) Consortium|Davies|United Kingdom
>Spike|hCoV-19/Northern Ireland/QEUH-961BF4/2020|2020-08-
02|EPI_ISL_537143|Original|hCoV-19^^Northern Ireland|Human|Lighthouse Lab in
Glasgow|Wellcome Sanger Institute for the COVID-19 Genomics UK (COG-UK)
consortium|Davies|United Kingdom
>Spike|hCoV-19/England/NORT-28943E/2020|2020-03-29|EPI_ISL_458595|Original|hCoV-
19^^England|Human|NU-OMICS DNA Sequencing research facility|Wellcome Sanger Institute
for the COVID-19 Genomics UK (COG-UK) consortium|Davies|United Kingdom
>Spike|hCoV-19/USA/NY-NYCPHL-000969/2020|2020-08-25|EPI_ISL_633000|Original|hCoV-
19^^New York|Human|DOHMH Jamaica|New York City Public Health Laboratory|Wang|USA
>Spike|hCoV-19/Poland/Sla2/2020|2020-05-05|EPI_ISL_451656|Original|hCoV-
19^^Slask|Human|State Sanitary Inspectorate|Laboratory of Recombinant
Vaccines|Rabalski|Poland
>Spike|hCoV-19/England/LEED-2A8AA0/2020|2020-04-02|EPI_ISL_539021|Original|hCoV-
19^^England|Human|Leeds Teaching Hospitals NHS Trust and Public Health
England|Wellcome Sanger Institute for the COVID-19 Genomics UK (COG-UK)
consortium|Davies|United Kingdom
>Spike|hCoV-19/Sweden/20-50371/2020|2020-03-16|EPI_ISL_475138|Original|hCoV-
19^^Kalmar|Human|Kalmar klinisk mikrobiologi|The Public Health Agency of
Sweden|Svartstr\u00f6m|Sweden
>Spike|hCoV-19/USA/MI-MDHHS-SC20983/2020|2020-05-27|EPI_ISL_507770|Original|hCoV-
19^^Michigan|Human|Michigan Department of Health and Human Services|Michigan
Department of Health and Human Services|Blankenship|USA
>Spike|hCoV-19/Scotland/EDB1580/2020|2020-04-13|EPI_ISL_433085|Original|hCoV-
19^^Scotland|Human|Virology Department|COVID-19 Genomics UK (COG-UK)
Consortium|Williams|United Kingdom
>Spike|hCoV-19/Latvia/015/2020|2020-03-23|EPI_ISL_437094|Original|hCoV-
19^^Latvia|Human|Latvijas Infektolo\u0123ijas centrs|Latvian Biomedical Research and
Study Centre|Zrelovs|Latvia
>Spike|hCoV-19/England/CAMC-CFC33F/2020|2020-12-21|EPI_ISL_781688|Original|hCoV-
19^^England|Human|Lighthouse Lab in Cambridge|Wellcome Sanger Institute for the
COVID-19 Genomics UK (COG-UK) Consortium|Davies|United Kingdom
>Spike|hCoV-19/England/MILK-B046E1/2020|2020-10-24|EPI_ISL_652166|Original|hCoV-
19^^England|Human|Department of Pathology|COVID-19 Genomics UK (COG-UK)
Consortium|Goodfellow|United Kingdom
>Spike|hCoV-19/Belgium/CGP-0325334/2020|2020-03-25|EPI_ISL_462188|Original|hCoV-
19^^Bottelare|Human|KU Leuven|KU Leuven|Maes|Belgium
>Spike|hCoV-19/USA/TX-HMH-MCoV-13487/2020|2020-08-21|EPI_ISL_788616|Original|hCoV-
19^^Texas|Human|Houston Methodist Hospital|Houston Methodist Hospital|Long|USA
>Spike|hCoV-19/England/LIVE-9B5A1/2020|2020-04-02|EPI_ISL_449465|Original|hCoV-
19^^England|Human|Liverpool Clinical Laboratories|COVID-19 Genomics UK (COG-UK)
Consortium|Haldenby|United Kingdom

>Spike|hCoV-19/Germany/HH-hpi-p1834/2020|2020-03-22|EPI_ISL_776187|Original|hCoV-19^^Hamburg|Human|University Medical Center Hamburg Eppendorf|Heinrich Pette Institute|Robitaille|Germany
>Spike|hCoV-19/England/QEUH-CC301C/2020|2020-12-21|EPI_ISL_778058|Original|hCoV-19^^England|Human|Lighthouse Lab in Glasgow|Wellcome Sanger Institute for the COVID-19 Genomics UK (COG-UK) Consortium|Davies|United Kingdom
>Spike|hCoV-19/Scotland/CVR5544/2020|2020-10-26|EPI_ISL_651533|Original|hCoV-19^^Scotland|Human|West of Scotland Specialist Virology Centre|COVID-19 Genomics UK (COG-UK) Consortium|Robertson|United Kingdom
>Spike|hCoV-19/Denmark/DCGC-2064/2020|2020-07-13|EPI_ISL_618308|Original|hCoV-19^^Hovedstaden|Human|Department of Virus and Microbiological Special Diagnostics|Albertsen lab|Michaelsen|Denmark
>Spike|hCoV-19/Netherlands/GE-EMC-356/2020|2020-10-21|EPI_ISL_722522|Original|hCoV-19^^Gelderland|Human|Dutch COVID-19 response team|Erasmus Medical Center|Oude Munnink|Netherlands
>Spike|hCoV-19/England/ALDP-C88426/2020|2020-12-17|EPI_ISL_762562|Original|hCoV-19^^England|Human|Lighthouse Lab in Alderley Park|Wellcome Sanger Institute for the COVID-19 Genomics UK (COG-UK) Consortium|Davies|United Kingdom
>Spike|hCoV-19/England/ALDP-A2EE53/2020|2020-10-05|EPI_ISL_587115|Original|hCoV-19^^England|Human|Lighthouse Lab in Alderley Park|Wellcome Sanger Institute for the COVID-19 Genomics UK (COG-UK) consortium|Davies|United Kingdom
>Spike|hCoV-19/England/MILK-B04C85/2020|2020-10-23|EPI_ISL_651036|Original|hCoV-19^^England|Human|Department of Pathology|COVID-19 Genomics UK (COG-UK) Consortium|Goodfellow|United Kingdom
>Spike|hCoV-19/New Zealand/20VR1290/2020|2020-03-21|EPI_ISL_456212|Original|hCoV-19^^Auckland|Human|LabPLUS|Institute of Environmental Science and Research (ESR)|de Ligt|New Zealand
>Spike|hCoV-19/Japan/PG-6012/2020|2020-07-00|EPI_ISL_689125|Original|hCoV-19^^Japan|Human|Pathogen Genomics Center|Pathogen Genomics Center|Sekizuka|Japan
>Spike|hCoV-19/England/MILK-9570B6/2020|2020-06-12|EPI_ISL_554294|Original|hCoV-19^^England|Human|Lighthouse Lab in Milton Keynes|Wellcome Sanger Institute for the COVID-19 Genomics UK (COG-UK) consortium|Davies|United Kingdom
>Spike|hCoV-19/England/QEUH-A4C9F4/2020|2020-10-05|EPI_ISL_628586|Original|hCoV-19^^England|Human|Oxford Viromics|COVID-19 Genomics UK (COG-UK) Consortium|Golubchik|United Kingdom
>Spike|hCoV-19/England/CAMC-BC0BB5/2020|2020-11-22|EPI_ISL_705107|Original|hCoV-19^^England|Human|Lighthouse Lab in Cambridge|Wellcome Sanger Institute for the COVID-19 Genomics UK (COG-UK) Consortium|Davies|United Kingdom
>Spike|hCoV-19/USA/WA-UW-2305/2020|2020-03-30|EPI_ISL_485982|Original|hCoV-19^^Washington|Human|UW Virology Lab|UW Virology Lab|Roychoudhury|USA
>Spike|hCoV-19/Wales/PHWC-2CA88/2020|2020-04-01|EPI_ISL_445942|Original|hCoV-19^^Wales|Human|Wales Specialist Virology Centre|Public Health Wales Microbiology Cardiff|Moore|United Kingdom
>Spike|hCoV-19/Scotland/EDB7746/2020|2020-09-09|EPI_ISL_540885|Original|hCoV-19^^Scotland|Human|Virology Department|COVID-19 Genomics UK (COG-UK) Consortium|Williams|United Kingdom
>Spike|hCoV-19/USA/UT-UPHL-04311_2/2020|2020-05-15|EPI_ISL_591302|Original|hCoV-19^^Utah|Human|Utah Public Health Laboratory|Utah Public Health Laboratory|Young|USA
>Spike|hCoV-19/England/CAMC-A64853/2020|2020-10-10|EPI_ISL_608908|Original|hCoV-19^^England|Human|Lighthouse Lab in Cambridge|Wellcome Sanger Institute for the COVID-19 Genomics UK (COG-UK) consortium|Davies|United Kingdom
>Spike|hCoV-19/England/QEUH-B2BF52/2020|2020-11-04|EPI_ISL_680040|Original|hCoV-19^^England|Human|Department of Pathology|COVID-19 Genomics UK (COG-UK) Consortium|Goodfellow|United Kingdom
>Spike|hCoV-19/Wales/PHWC-489065/2020|2020-11-02|EPI_ISL_639530|Original|hCoV-19^^Wales|Human|Wales Specialist Virology Centre Sequencing lab|COVID-19 Genomics UK (COG-UK) Consortium|Moore|United Kingdom
>Spike|hCoV-19/England/LOND-12D4CFD/2020|2020-10-09|EPI_ISL_679570|Original|hCoV-19^^England|Human|University College London|COVID-19 Genomics UK (COG-UK) Consortium|Roy|United Kingdom

>Spike|hCoV-19/mink/Denmark/AL25-Farm1/2020|2020-06-14|EPI_ISL_626347|Original|hCoV-19^^Denmark|Neovison vison|Statens Serum Institute|Statens Serum Institute|Rasmussen|Denmark
>Spike|hCoV-19/USA/WA-S2262/2020|2020-06-10|EPI_ISL_497690|Original|hCoV-19^^Washington|Human|Washington State Department of Health|Seattle Flu Study|Lee|USA
>Spike|hCoV-19/England/MILK-9D4E5D/2020|2020-09-19|EPI_ISL_601699|Original|hCoV-19^^England|Human|Lighthouse Lab in Milton Keynes|Wellcome Sanger Institute for the COVID-19 Genomics UK (COG-UK) consortium|Davies|United Kingdom
>Spike|hCoV-19/USA/WI-GMF-M00026/2020|2020-08-02|EPI_ISL_547676|Original|hCoV-19^^Wisconsin|Human|Gundersen Clinical Microbiology Laboratory|Kabara Cancer Research Institute|Kenny|USA
>Spike|hCoV-19/England/MILK-A7D013/2020|2020-10-13|EPI_ISL_606485|Original|hCoV-19^^England|Human|Lighthouse Lab in Milton Keynes|Wellcome Sanger Institute for the COVID-19 Genomics UK (COG-UK) consortium|Davies|United Kingdom
>Spike|hCoV-19/Wales/PHWC-16B7C1/2020|2020-09-14|EPI_ISL_573903|Original|hCoV-19^^Wales|Human|Wales Specialist Virology Centre Sequencing lab|COVID-19 Genomics UK (COG-UK) Consortium|Moore|United Kingdom
>Spike|hCoV-19/Australia/NSW1027/2020|2020-03-15|EPI_ISL_593756|Original|hCoV-19^^New South Wales|Human|Sydney South West Pathology Service (SSWPS) - Liverpool Hospital - NSW Health Pathology|NSW Health Pathology - Institute of Clinical Pathology and Medical Research; Westmead Hospital; University of Sydney|Gall|Australia
>Spike|hCoV-19/Scotland/GCVR-16FBEF/2020|2020-03-21|EPI_ISL_489603|Original|hCoV-19^^Scotland|Human|NHSGGC West of Scotland Specialist Virology Centre / MRC-University of Glasgow Centre for Virus Research|Wellcome Sanger Institute for the COVID-19 Genomics UK (COG-UK) consortium|Davies|United Kingdom
>Spike|hCoV-19/USA/UT-UPHL-2012297777/2020|2020-11-27|EPI_ISL_746940|Original|hCoV-19^^Utah|Human|Utah Public Health Laboratory|Utah Public Health Laboratory|Gallagher|USA
>Spike|hCoV-19/England/204561543/2020|2020-11-05|EPI_ISL_678691|Original|hCoV-19^^England|Human|Respiratory Virus Unit|COVID-19 Genomics UK (COG-UK) Consortium|Platt|United Kingdom
>Spike|hCoV-19/England/MILK-B8D158/2020|2020-11-13|EPI_ISL_674047|Original|hCoV-19^^England|Human|Lighthouse Lab in Milton Keynes|Wellcome Sanger Institute for the COVID-19 Genomics UK (COG-UK) Consortium|Davies|United Kingdom
>Spike|hCoV-19/England/BRIS-1854239/2020|2020-04-28|EPI_ISL_492385|Original|hCoV-19^^England|Human|PHE South West Regional Laboratory|Wellcome Sanger Institute for the COVID-19 Genomics UK (COG-UK) consortium|Davies|United Kingdom
>Spike|hCoV-19/England/QEUH-B31AA3/2020|2020-11-04|EPI_ISL_646220|Original|hCoV-19^^England|Human|Lighthouse Lab in Glasgow|Wellcome Sanger Institute for the COVID-19 Genomics UK (COG-UK) Consortium|Davies|United Kingdom
>Spike|hCoV-19/Argentina/Heritas-HG023/2020|2020-05-26|EPI_ISL_615121|Original|hCoV-19^^Ciudad Autonoma de Buenos Aires|Human|Hospital de Pediatr\u00eda \Prof. Dr. Juan P Garrahan\|H\u00e9ritas|Pereyra|Argentina
>Spike|hCoV-19/Japan/PG-4059/2020|2020-07-00|EPI_ISL_692300|Original|hCoV-19^^Japan|Human|Sakai City Institute of Public Health|Pathogen Genomics Center|Sekizuka|Japan
>Spike|hCoV-19/USA/MA-MASPHL-00866/2020|2020-05-18|EPI_ISL_692859|Original|hCoV-19^^Massachusetts|Human|Massachusetts State Public Health Laboratory|Massachusetts State Public Health Laboratory|Lang|USA
>Spike|hCoV-19/USA/NM-DOH-2020317191/2020|2020-08-26|EPI_ISL_732726|Original|hCoV-19^^New Mexico|Human|New Mexico Department of Health Scientific Laboratory|New Mexico Department of Health Scientific Laboratory|Johnson|USA
>Spike|hCoV-19/England/OXON-AF513/2020|2020-04-13|EPI_ISL_478747|Original|hCoV-19^^England|Human|Oxford Viromics|COVID-19 Genomics UK (COG-UK) Consortium|Golubchik|United Kingdom
>Spike|hCoV-19/England/QEUH-98F7B0/2020|2020-08-27|EPI_ISL_540081|Original|hCoV-19^^England|Human|Lighthouse Lab in Glasgow|Wellcome Sanger Institute for the COVID-19 Genomics UK (COG-UK) consortium|Davies|United Kingdom

>Spike|hCoV-19/England/QEUH-B68A76/2020|2020-11-11|EPI_ISL_654964|Original|hCoV-19^^England|Human|Lighthouse Lab in Glasgow|Wellcome Sanger Institute for the COVID-19 Genomics UK (COG-UK) Consortium|Davies|United Kingdom
>Spike|hCoV-19/England/ALDP-CB68C9/2020|2020-12-17|EPI_ISL_761122|Original|hCoV-19^^England|Human|Lighthouse Lab in Alderley Park|Wellcome Sanger Institute for the COVID-19 Genomics UK (COG-UK) Consortium|Davies|United Kingdom
>Spike|hCoV-19/Netherlands/NB-EMC-292/2020|2020-08-24|EPI_ISL_577841|Original|hCoV-19^^Noord Brabant|Human|Dutch COVID-19 response team|Erasmus Medical Center|Oude Munnink|Netherlands
>Spike|hCoV-19/France/PAC-IHU-1519/2020|2020-08-12|EPI_ISL_569185|Original|hCoV-19^^Provence-Alpes-C\u00f4te-d\u2019Azur|Human|MEPHI|MEPHI|Levasseur|France
>Spike|hCoV-19/England/204590468/2020|2020-11-06|EPI_ISL_686556|Original|hCoV-19^^England|Human|Respiratory Virus Unit|COVID-19 Genomics UK (COG-UK) Consortium|Platt|United Kingdom
>Spike|hCoV-19/Scotland/EDB6261/2020|2020-06-02|EPI_ISL_478242|Original|hCoV-19^^Scotland|Human|Virology Department|COVID-19 Genomics UK (COG-UK) Consortium|Williams|United Kingdom
>Spike|hCoV-19/Ireland/MN-NVRL-73IRL72912/2020|2020-09-14|EPI_ISL_639831|Original|hCoV-19^^Monaghan|Human|National Virus Reference Laboratory|National Virus Reference Laboratory|Gonzalez|Ireland
>Spike|hCoV-19/Scotland/EDB1610/2020|2020-04-14|EPI_ISL_433090|Original|hCoV-19^^Scotland|Human|Virology Department|COVID-19 Genomics UK (COG-UK) Consortium|Williams|United Kingdom
>Spike|hCoV-19/USA/CA-ALSR-3033/2020|2020-08-28|EPI_ISL_635802|Original|hCoV-19^^California|Human|San Diego County Public Health Laboratory|Andersen lab at Scripps Research|Gangavarapu|USA
>Spike|hCoV-19/Switzerland/BL-ETHZ-400095/2020|2020-12-07|EPI_ISL_729140|Original|hCoV-19^^Basel-Landschaft|Human|Viollier AG|Department of Biosystems Science and Engineering|Nadeau|Switzerland
>Spike|hCoV-19/England/ALDP-B1BC12/2020|2020-11-01|EPI_ISL_645726|Original|hCoV-19^^England|Human|Lighthouse Lab in Alderley Park|Wellcome Sanger Institute for the COVID-19 Genomics UK (COG-UK) Consortium|Davies|United Kingdom
>Spike|hCoV-19/USA/MI-MDHHS-SC20526/2020|2020-05-15|EPI_ISL_460574|Original|hCoV-19^^Michigan|Human|Michigan Department of Health and Human Services|Michigan Department of Health and Human Services|Blankenship|USA
>Spike|hCoV-19/Denmark/DCGC-21562/2020|2020-12-21|EPI_ISL_758237|Original|hCoV-19^^Hovedstaden|Human|Department of Virus and Microbiological Special Diagnostics|Albertsen Lab|Michaelsen|Denmark
>Spike|hCoV-19/USA/CA-CZB-4095/2020|2020-08-14|EPI_ISL_548646|Original|hCoV-19^^California|Human|Orange County Public Health Laboratory|Chan-Zuckerberg Biohub|Bhatt|USA
>Spike|hCoV-19/Denmark/DCGC-1718/2020|2020-03-30|EPI_ISL_615577|Original|hCoV-19^^Sjaelland|Human|Department of Virus and Microbiological Special Diagnostics|Albertsen lab|Michaelsen|Denmark
>Spike|hCoV-19/England/NOTT-10FE8E/2020|2020-04-09|EPI_ISL_441452|Original|hCoV-19^^England|Human|Queens Medical Centre|COVID-19 Genomics UK (COG-UK) Consortium|Loose|United Kingdom
>Spike|hCoV-19/Germany/HH-hpi-p1839/2020|2020-03-22|EPI_ISL_776192|Original|hCoV-19^^Hamburg|Human|University Medical Center Hamburg Eppendorf|Heinrich Pette Institute|Robitaille|Germany
>Spike|hCoV-19/Wales/ALDP-BCA7C6/2020|2020-11-23|EPI_ISL_710750|Original|hCoV-19^^Wales|Human|Lighthouse Lab in Alderley Park|Wellcome Sanger Institute for the COVID-19 Genomics UK (COG-UK) Consortium|Davies|United Kingdom
>Spike|hCoV-19/Scotland/QEUH-89C0D4/2020|2020-07-28|EPI_ISL_532811|Original|hCoV-19^^Scotland|Human|NHSGGC West of Scotland Specialist Virology Centre / MRC-University of Glasgow Centre for Virus Research|Wellcome Sanger Institute for the COVID-19 Genomics UK (COG-UK) consortium|Davies|United Kingdom
>Spike|hCoV-19/Australia/VIC4031/2020|2020-07-11|EPI_ISL_519416|Original|hCoV-19^^Victoria|Human|Microbiological Diagnostic Unit - Public Health Laboratory (MDU-PHL)|MDU-PHL|Schultz|Australia

>Spike|hCoV-19/Scotland/QEUH-A00B8D/2020|2020-10-02|EPI_ISL_585554|Original|hCoV-19^^Scotland|Human|University of Exeter|COVID-19 Genomics UK (COG-UK) Consortium|Temperton|United Kingdom
>Spike|hCoV-19/England/QEUH-B2D66A/2020|2020-11-04|EPI_ISL_660049|Original|hCoV-19^^England|Human|Lighthouse Lab in Glasgow|Wellcome Sanger Institute for the COVID-19 Genomics UK (COG-UK) Consortium|Davies|United Kingdom
>Spike|hCoV-19/Netherlands/Gelderland_26/2020|2020-04-26|EPI_ISL_460926|Original|hCoV-19^^Gelderland|Human|Dutch COVID-19 response team|Erasmus Medical Center|Oude Munnink|Netherlands
>Spike|hCoV-19/Australia/VIC12966/2020|2020-07-29|EPI_ISL_563613|Original|hCoV-19^^Victoria|Human|Microbiological Diagnostic Unit - Public Health Laboratory (MDU-PHL)|MDU-PHL|Schultz|Australia
>Spike|hCoV-19/England/QEUH-B2DFAB/2020|2020-11-04|EPI_ISL_659956|Original|hCoV-19^^England|Human|Lighthouse Lab in Glasgow|Wellcome Sanger Institute for the COVID-19 Genomics UK (COG-UK) Consortium|Davies|United Kingdom
>Spike|hCoV-19/Japan/PG-4631/2020|2020-07-00|EPI_ISL_689768|Original|hCoV-19^^Japan|Human|Fukuoka Institute of Health and Environmental Sciences|Pathogen Genomics Center|Sekizuka|Japan
>Spike|hCoV-19/Scotland/QEUH-9B9DCA/2020|2020-09-07|EPI_ISL_568264|Original|hCoV-19^^Scotland|Human|Lighthouse Lab in Glasgow|Wellcome Sanger Institute for the COVID-19 Genomics UK (COG-UK) consortium|Davies|United Kingdom
>Spike|hCoV-19/USA/SC-DHEC-1081/2020|2020-07-06|EPI_ISL_541665|Original|hCoV-19^^South Carolina|Human|Microbiology Division|Microbiology Division|Flores|USA
>Spike|hCoV-19/England/LEED-2A8642/2020|2020-03-26|EPI_ISL_539089|Original|hCoV-19^^England|Human|Leeds Teaching Hospitals NHS Trust and Public Health England|Wellcome Sanger Institute for the COVID-19 Genomics UK (COG-UK) consortium|Davies|United Kingdom
>Spike|hCoV-19/England/ALDP-951DE2/2020|2020-06-10|EPI_ISL_551291|Original|hCoV-19^^England|Human|Lighthouse Lab in Alderley Park|Wellcome Sanger Institute for the COVID-19 Genomics UK (COG-UK) consortium|Davies|United Kingdom
>Spike|hCoV-19/England/QEUH-AABCFA/2020|2020-10-21|EPI_ISL_652824|Original|hCoV-19^^England|Human|Oxford Viromics|COVID-19 Genomics UK (COG-UK) Consortium|Golubchik|United Kingdom
>Spike|hCoV-19/USA/VA-DCLS-1655/2020|2020-08-29|EPI_ISL_644828|Original|hCoV-19^^Virginia|Human|Virginia DCLS|Virginia DCLS|St. Jacques|USA
>Spike|hCoV-19/England/20106145903/2020|2020-03-03|EPI_ISL_417246|Original|hCoV-19^^England|Human|Respiratory Virus Unit|Respiratory Virus Unit|Platt|United Kingdom
>Spike|hCoV-19/Australia/QLD1248/2020|2020-03-22|EPI_ISL_693252|Original|hCoV-19^^Australia|Human|unknown|Public Health Virology Laboratory|Nguyen|Australia
>Spike|hCoV-19/USA/AZ-TG408168/2020|2020-06-16|EPI_ISL_694919|Original|hCoV-19^^Arizona|Human|AZ SPHL|TGen North|French|USA
>Spike|hCoV-19/Denmark/DCGC-11586/2020|2020-11-02|EPI_ISL_670692|Original|hCoV-19^^Midtjylland|Human|Department of Virus and Microbiological Special Diagnostics|Albertsen Lab|Michaelsen|Denmark
>Spike|hCoV-19/USA/MI-UM-10036690355/2020|2020-10-29|EPI_ISL_682130|Original|hCoV-19^^Michigan|Human|University of Michigan Clinical Microbiology Laboratory|Lauring Lab|Valesano|USA
>Spike|hCoV-19/England/ALDP-A68075/2020|2020-10-10|EPI_ISL_608994|Original|hCoV-19^^England|Human|Lighthouse Lab in Alderley Park|Wellcome Sanger Institute for the COVID-19 Genomics UK (COG-UK) consortium|Davies|United Kingdom
>Spike|hCoV-19/England/ALDP-BCA104/2020|2020-11-23|EPI_ISL_703386|Original|hCoV-19^^England|Human|Lighthouse Lab in Alderley Park|Wellcome Sanger Institute for the COVID-19 Genomics UK (COG-UK) Consortium|Davies|United Kingdom
>Spike|hCoV-19/Finland/20A49S7/2020|2020-04-20|EPI_ISL_481600|Original|hCoV-19^^Uusimaa|Human|Department of Virology and Immunology|Department of Virology|Smura|Finland
>Spike|hCoV-19/England/201380063/2020|2020-03-25|EPI_ISL_421815|Original|hCoV-19^^England|Human|Respiratory Virus Unit|Respiratory Virus Unit|Platt|United Kingdom
>Spike|hCoV-19/Brazil/PE-IAM1447/2020|2020-05-13|EPI_ISL_572394|Original|hCoV-19^^Pernambuco|Human|LACEN/PE|WallauLab|Wallau|Brazil

>Spike|hCoV-19/Scotland/EDB5731/2020|2020-04-09|EPI_ISL_473848|Original|hCoV-19^^Scotland|Human|Virology Department|COVID-19 Genomics UK (COG-UK) Consortium|Williams|United Kingdom
>Spike|hCoV-19/USA/CA-CZB-10810/2020|2020-07-06|EPI_ISL_583063|Original|hCoV-19^^California|Human|Humboldt County Public Health Laboratory|Chan-Zuckerberg Biohub|Bhatt|USA
>Spike|hCoV-19/Scotland/EDB6890/2020|2020-04-30|EPI_ISL_559976|Original|hCoV-19^^Scotland|Human|Virology Department|COVID-19 Genomics UK (COG-UK) Consortium|Williams|United Kingdom
>Spike|hCoV-19/Japan/PG-3068/2020|2020-04-00|EPI_ISL_691191|Original|hCoV-19^^Japan|Human|Pathogen Genomics Center|Pathogen Genomics Center|Sekizuka|Japan
>Spike|hCoV-19/England/CAMB-1AC0AB/2020|2020-04-28|EPI_ISL_444368|Original|hCoV-19^^England|Human|Department of Pathology|COVID-19 Genomics UK (COG-UK) Consortium|Meredith|United Kingdom
>Spike|hCoV-19/Malaysia/IMR-CV140338/2020|2020-09-01|EPI_ISL_718305|Original|hCoV-19^^Malaysia|Human|Institute for Medical Research|Institute for Medical Research|Suppiah|Malaysia
>Spike|hCoV-19/Netherlands/NA_23/2020|2020-03-09|EPI_ISL_415480|Original|hCoV-19^^Netherlands|Human|Dutch COVID-19 response team|Erasmus Medical Center|Oude Munnink|Netherlands
>Spike|hCoV-19/England/QEUH-A5E17F/2020|2020-10-12|EPI_ISL_597486|Original|hCoV-19^^England|Human|Lighthouse Lab in Glasgow|Wellcome Sanger Institute for the COVID-19 Genomics UK (COG-UK) consortium|Davies|United Kingdom
>Spike|hCoV-19/England/NORW-E9085/2020|2020-04-25|EPI_ISL_453580|Original|hCoV-19^^England|Human|Quadram Institute Bioscience|COVID-19 Genomics UK (COG-UK) Consortium|Alikhan|United Kingdom
>Spike|hCoV-19/Northern Ireland/QEUH-96AAFE/2020|2020-08-17|EPI_ISL_531239|Original|hCoV-19^^Northern Ireland|Human|Lighthouse Lab in Glasgow|Wellcome Sanger Institute for the COVID-19 Genomics UK (COG-UK) consortium|Davies|United Kingdom
>Spike|hCoV-19/USA/NY-NYCPHL-001548/2020|2020-12-02|EPI_ISL_745463|Original|hCoV-19^^New York|Human|DOHMH Fort Greene|New York City Public Health Laboratory|Wang|USA
>Spike|hCoV-19/Denmark/DCGC-5566/2020|2020-09-28|EPI_ISL_620891|Original|hCoV-19^^Hovedstaden|Human|Department of Virus and Microbiological Special Diagnostics|Albertsen lab|Michaelsen|Denmark
>Spike|hCoV-19/Spain/PV-IBV-2085/2020|2020-04-10|EPI_ISL_468876|Original|hCoV-19^^Basque Country|Human|Servicio de Microbiolog\u00eda. Hospital Universitario Donostia. OSI Donostialdea. \u00c1rea de Enfermedades Infecciosas|SeqCOVID-SPAIN consortium/IBV(CSIC)|Comas|Spain
>Spike|hCoV-19/England/QEUH-9612D1/2020|2020-08-03|EPI_ISL_532089|Original|hCoV-19^^England|Human|Lighthouse Lab in Glasgow|Wellcome Sanger Institute for the COVID-19 Genomics UK (COG-UK) consortium|Davies|United Kingdom
>Spike|hCoV-19/Wales/PHWC-47F035/2020|2020-10-14|EPI_ISL_612619|Original|hCoV-19^^Wales|Human|Wales Specialist Virology Centre Sequencing lab|COVID-19 Genomics UK (COG-UK) Consortium|Moore|United Kingdom
>Spike|hCoV-19/Australia/WA204/2020|2020-03-25|EPI_ISL_596767|Original|hCoV-19^^Western Australia|Human|PathWest Laboratory Medicine WA|PathWest Laboratory Medicine WA Microbial Surveillance Unit|Sikazwe|Australia
>Spike|hCoV-19/USA/MA-MGH-02757/2020|2020-09-23|EPI_ISL_765619|Original|hCoV-19^^Massachusetts|Human|Massachusetts General Hospital|Infectious Disease Program|Park|USA
>Spike|hCoV-19/England/ALDP-9C5F6B/2020|2020-09-10|EPI_ISL_567770|Original|hCoV-19^^England|Human|Lighthouse Lab in Alderley Park|Wellcome Sanger Institute for the COVID-19 Genomics UK (COG-UK) consortium|Davies|United Kingdom
>Spike|hCoV-19/Uruguay/TYT-M64/2020|2020-06-21|EPI_ISL_749155|Original|hCoV-19^^Treinta y Tres|Human|Sanatorio Americano|Institut Pasteur de Montevideo|Spangenberg|Uruguay
>Spike|hCoV-19/Hong Kong/VM20060398/2020|2020-07-19|EPI_ISL_539811|Original|hCoV-19^^Hong Kong|Human|Asiaworld Expo Command Post|Hong Kong Department of Health|Tsang|China

>Spike|hCoV-19/USA/MI-QDX-1143/2020|2020-03-23|EPI_ISL_571873|Original|hCoV-
19^^Michigan|Human|Quest Diagnostics|Quest Diagnostics|Kagan|USA
>Spike|hCoV-19/England/CAMB-7706E/2020|2020-03-31|EPI_ISL_425456|Original|hCoV-
19^^England|Human|Department of Pathology|COVID-19 Genomics UK (COG-UK)
Consortium|Meredith|United Kingdom
>Spike|hCoV-19/Belgium/ULG-10231/2020|2020-05-16|EPI_ISL_498137|Original|hCoV-
19^^Li\u00e8ge|Human|Department of Clinical Microbiology|GIGA Medical
Genomics|Durkin|Belgium
>Spike|hCoV-19/Japan/PG-6937/2020|2020-09-00|EPI_ISL_688043|Original|hCoV-
19^^Japan|Human|Pathogen Genomics Center|Pathogen Genomics Center|Sekizuka|Japan
>Spike|hCoV-19/USA/CA-ALSR-4320/2020|2020-10-02|EPI_ISL_648969|Original|hCoV-
19^^California|Human|San Diego County Public Health Laboratory|Andersen lab at
Scripps Research|Gangavarapu|USA
>Spike|hCoV-19/Scotland/QEUH-96E580/2020|2020-08-19|EPI_ISL_530533|Original|hCoV-
19^^Scotland|Human|NHSGGC West of Scotland Specialist Virology Centre / MRC-
University of Glasgow Centre for Virus Research|Wellcome Sanger Institute for the
COVID-19 Genomics UK (COG-UK) consortium|Davies|United Kingdom
>Spike|hCoV-19/England/ALDP-A6CFA1/2020|2020-10-14|EPI_ISL_608270|Original|hCoV-
19^^England|Human|Lighthouse Lab in Alderley Park|Wellcome Sanger Institute for the
COVID-19 Genomics UK (COG-UK) consortium|Davies|United Kingdom
>Spike|hCoV-19/England/SHEF-CEE40/2020|2020-09-28|EPI_ISL_585070|Original|hCoV-
19^^England|Human|Virology Department|COVID-19 Genomics UK (COG-UK) Consortium|de
Silva|United Kingdom
>Spike|hCoV-19/USA/FL-BPHL-2000/2020|2020-03-21|EPI_ISL_653153|Original|hCoV-
19^^Florida|Human|Florida Bureau of Public Health Laboratories|Florida Bureau of
Public Health Laboratories|Blanton|USA
>Spike|hCoV-19/Denmark/DCGC-5133/2020|2020-09-21|EPI_ISL_621671|Original|hCoV-
19^^Syddanmark|Human|Department of Virus and Microbiological Special
Diagnostics|Albertsen lab|Michaelsen|Denmark
>Spike|hCoV-19/Switzerland/SZ-ETHZ-280242/2020|2020-09-
10|EPI_ISL_560482|Original|hCoV-19^^Schwyz|Human|Viollier AG|Department of Biosystems
Science and Engineering|Nadeau|Switzerland
>Spike|hCoV-19/USA/VA-DCLS-1490/2020|2020-07-28|EPI_ISL_581564|Original|hCoV-
19^^Virginia|Human|Virginia DCLS|Virginia DCLS|St. Jacques|USA
>Spike|hCoV-19/England/CAMB-8307E/2020|2020-04-14|EPI_ISL_443522|Original|hCoV-
19^^England|Human|Department of Pathology|Wellcome Sanger Institute for the COVID-19
Genomics UK (COG-UK) consortium|Amato|United Kingdom
>Spike|hCoV-19/Spain/CL-IBV-97006150/2020|2020-04-05|EPI_ISL_537297|Original|hCoV-
19^^Castilla y Leon|Human|Universidad de Le\u00f3n|SeqCOVID-SPAIN
consortium/IBV(CSIC)|Comas|Spain
>Spike|hCoV-19/Canada/BC_13871523/2020|2020-03-00|EPI_ISL_460608|Original|hCoV-
19^^British Columbia|Human|BCCDC Public Health Laboratory|BCCDC Public Health
Laboratory|Harrigan|Canada
>Spike|hCoV-19/Scotland/EDB1156/2020|2020-04-07|EPI_ISL_456891|Original|hCoV-
19^^Scotland|Human|Virology Department|COVID-19 Genomics UK (COG-UK)
Consortium|Williams|United Kingdom
>Spike|hCoV-19/USA/AZ-TG408200/2020|2020-06-17|EPI_ISL_694938|Original|hCoV-
19^^Arizona|Human|AZ SPHL|TGen North|French|USA
>Spike|hCoV-19/Austria/CeMM0916/2020|2020-03-16|EPI_ISL_583768|Original|hCoV-
19^^Austria|Human|Dr. Gernot Walder GmbH|Bergthaler laboratory|Bergthaler|Austria
>Spike|hCoV-19/Spain/CT-IBV-97007954/2020|2020-10-07|EPI_ISL_654517|Original|hCoV-
19^^Catalunya|Human|Servicio de Microbiolog\u00eda|SeqCOVID-SPAIN
consortium/IBV(CSIC)|Comas|Spain
>Spike|hCoV-19/Estonia/ChVir4266/2020|2020-03-00|EPI_ISL_457722|Original|hCoV-
19^^Estonia|Human|SYNLAB Eesti OU\u0308|Charite Universitatsmedizin
Berlin|Muehlemann|Estonia
>Spike|hCoV-19/USA/CA-QDX-1247/2020|2020-03-16|EPI_ISL_571920|Original|hCoV-
19^^California|Human|Quest Diagnostics|Quest Diagnostics|Kagan|USA

>Spike|hCoV-19/Switzerland/BL-ETHZ-350001/2020|2020-11-
03|EPI_ISL_693776|Original|hCoV-19^^Basel-Landschaft|Human|Viollier AG|Department of
Biosystems Science and Engineering|Nadeau|Switzerland
>Spike|hCoV-19/Singapore/738/2020|2020-07-06|EPI_ISL_500558|Original|hCoV-
19^^Singapore|Human|Singapore General Hospital|Department of Microbiology|Abdul
Rahman|Singapore
>Spike|hCoV-19/England/ALDP-B743A9/2020|2020-11-12|EPI_ISL_661983|Original|hCoV-
19^^England|Human|Lighthouse Lab in Alderley Park|Wellcome Sanger Institute for the
COVID-19 Genomics UK (COG-UK) Consortium|Davies|United Kingdom
>Spike|hCoV-19/USA/LA-EVTL651/2020|2020-08-06|EPI_ISL_579055|Original|hCoV-
19^^Louisiana|Human|LSUHS Emerging Viral Threat Laboratory|Microbial Genome
Sequencing Center|Kamil|USA
>Spike|hCoV-19/USA/WI-UW-1875/2020|2020-09-11|EPI_ISL_605747|Original|hCoV-
19^^Wisconsin|Human|University of Wisconsin-Madison AIDS Vaccine Research
Laboratories|University of Wisconsin-Madison AIDS Vaccine Research
Laboratories|Baczenas|USA
>Spike|hCoV-19/England/ALDP-BC7C24/2020|2020-11-23|EPI_ISL_721446|Original|hCoV-
19^^England|Human|Lighthouse Lab in Alderley Park|Wellcome Sanger Institute for the
COVID-19 Genomics UK (COG-UK) Consortium|Davies|United Kingdom
>Spike|hCoV-19/Spain/MD-IBV-006380/2020|2020-04-02|EPI_ISL_510111|Original|hCoV-
19^^Madrid|Human|Hospital General Universitario Gregorio Mara\u00f1\u00f3n|SeqCOVID-
SPAIN consortium/IBV(CSIC)|Comas|Spain
>Spike|hCoV-19/Scotland/QEUH-A2199B/2020|2020-10-01|EPI_ISL_598859|Original|hCoV-
19^^Scotland|Human|Lighthouse Lab in Glasgow|Wellcome Sanger Institute for the COVID-
19 Genomics UK (COG-UK) consortium|Davies|United Kingdom
>Spike|hCoV-19/England/QEUH-BC3AA6/2020|2020-11-25|EPI_ISL_704074|Original|hCoV-
19^^England|Human|Lighthouse Lab in Glasgow|Wellcome Sanger Institute for the COVID-
19 Genomics UK (COG-UK) Consortium|Davies|United Kingdom
>Spike|hCoV-19/USA/CA-ALSR-4208/2020|2020-08-11|EPI_ISL_648887|Original|hCoV-
19^^California|Human|San Diego County Public Health Laboratory|Andersen lab at
Scripps Research|Gangavarapu|USA
>Spike|hCoV-19/England/ALDP-9E9C1F/2020|2020-09-23|EPI_ISL_580060|Original|hCoV-
19^^England|Human|Lighthouse Lab in Alderley Park|Wellcome Sanger Institute for the
COVID-19 Genomics UK (COG-UK) consortium|Davies|United Kingdom
>Spike|hCoV-19/England/QEUH-9DA69B/2020|2020-09-21|EPI_ISL_601253|Original|hCoV-
19^^England|Human|Lighthouse Lab in Glasgow|Wellcome Sanger Institute for the COVID-
19 Genomics UK (COG-UK) consortium|Davies|United Kingdom
>Spike|hCoV-19/Netherlands/GE-RIVM-20523/2020|2020-12-
03|EPI_ISL_728738|Original|hCoV-19^^Gelderland|Human|Dutch COVID-19 response
team|National Institute for Public Health and the Environment
(RIVM)|Meijer|Netherlands
>Spike|hCoV-19/England/MILK-B3CD90/2020|2020-11-01|EPI_ISL_706198|Original|hCoV-
19^^England|Human|Oxford Viromics|COVID-19 Genomics UK (COG-UK)
Consortium|Golubchik|United Kingdom
>Spike|hCoV-19/Japan/PG-2868/2020|2020-04-00|EPI_ISL_690994|Original|hCoV-
19^^Japan|Human|Pathogen Genomics Center|Pathogen Genomics Center|Sekizuka|Japan
>Spike|hCoV-19/England/ALDP-644F1C/2020|2020-07-04|EPI_ISL_558173|Original|hCoV-
19^^England|Human|Lighthouse Lab in Alderley Park|Wellcome Sanger Institute for the
COVID-19 Genomics UK (COG-UK) consortium|Davies|United Kingdom
>Spike|hCoV-19/England/QEUH-B143AB/2020|2020-11-03|EPI_ISL_642646|Original|hCoV-
19^^England|Human|Lighthouse Lab in Glasgow|Wellcome Sanger Institute for the COVID-
19 Genomics UK (COG-UK) Consortium|Davies|United Kingdom
>Spike|hCoV-19/England/QEUH-B2C908/2020|2020-11-03|EPI_ISL_646657|Original|hCoV-
19^^England|Human|Lighthouse Lab in Glasgow|Wellcome Sanger Institute for the COVID-
19 Genomics UK (COG-UK) Consortium|Davies|United Kingdom
>Spike|hCoV-19/England/204441570/2020|2020-10-00|EPI_ISL_622893|Original|hCoV-
19^^England|Human|Respiratory Virus Unit|Respiratory Virus Unit|Platt|United Kingdom
>Spike|hCoV-19/USA/TX-HMH-2725/2020|2020-06-05|EPI_ISL_545508|Original|hCoV-
19^^Texas|Human|Houston Methodist Hospital|Houston Methodist Hospital|Long|USA

>Spike|hCoV-19/USA/NY-NYCPHL-000670/2020|2020-03-18|EPI_ISL_632088|Original|hCoV-19^^New York|Human|Jamaica Hospital Medical Center|New York City Public Health Laboratory|Wang|USA
>Spike|hCoV-19/Sweden/20-07856/2020|2020-04-23|EPI_ISL_452237|Original|hCoV-19^^Stockholm|Human|Huddinge VC|The Public Health Agency of Sweden|Svartstr\u00f6m|Sweden
>Spike|hCoV-19/Wales/PHWC-16D185/2020|2020-09-26|EPI_ISL_627731|Original|hCoV-19^^Wales|Human|Wales Specialist Virology Centre Sequencing lab|COVID-19 Genomics UK (COG-UK) Consortium|Moore|United Kingdom
>Spike|hCoV-19/Japan/PG-3675/2020|2020-04-00|EPI_ISL_691928|Original|hCoV-19^^Japan|Human|Pathogen Genomics Center|Pathogen Genomics Center|Sekizuka|Japan
>Spike|hCoV-19/USA/OR-PROV-020/2020|2020-03-26|EPI_ISL_482306|Original|hCoV-19^^Oregon|Human|Providence St. Joseph Health Molecular Genomics Laboratory|Providence St. Joseph Health Molecular Genomics Laboratory|Dowdell|USA
>Spike|hCoV-19/Denmark/DCGC-3931/2020|2020-09-14|EPI_ISL_615842|Original|hCoV-19^^Hovedstaden|Human|Department of Virus and Microbiological Special Diagnostics|Albertsen lab|Michaelsen|Denmark
>Spike|hCoV-19/Scotland/QEUH-B3185E/2020|2020-11-04|EPI_ISL_676427|Original|hCoV-19^^Scotland|Human|Lighthouse Lab in Glasgow|Wellcome Sanger Institute for the COVID-19 Genomics UK (COG-UK) Consortium|Davies|United Kingdom
>Spike|hCoV-19/England/ALDP-BB4E60/2020|2020-11-20|EPI_ISL_702392|Original|hCoV-19^^England|Human|Lighthouse Lab in Alderley Park|Wellcome Sanger Institute for the COVID-19 Genomics UK (COG-UK) Consortium|Davies|United Kingdom
>Spike|hCoV-19/England/MILK-9C26D2/2020|2020-09-10|EPI_ISL_566439|Original|hCoV-19^^England|Human|Lighthouse Lab in Milton Keynes|Wellcome Sanger Institute for the COVID-19 Genomics UK (COG-UK) consortium|Davies|United Kingdom
>Spike|hCoV-19/Scotland/CVR6097/2020|2020-11-26|EPI_ISL_703924|Original|hCoV-19^^Scotland|Human|West of Scotland Specialist Virology Centre|COVID-19 Genomics UK (COG-UK) Consortium|Robertson|United Kingdom
>Spike|hCoV-19/Canada/MB-NML-1035/2020|2020-07-17|EPI_ISL_582397|Original|hCoV-19^^Manitoba|Human|Cadham Provincial Laboratory|National Microbiology Laboratory (NML)|Majer|Canada
>Spike|hCoV-19/USA/WA-S775/2020|2020-04-14|EPI_ISL_449945|Original|hCoV-19^^Washington|Human|Washington State Department of Health|Seattle Flu Study|Bedford|USA
>Spike|hCoV-19/England/QEUH-9FAD36/2020|2020-09-28|EPI_ISL_588259|Original|hCoV-19^^England|Human|Lighthouse Lab in Glasgow|Wellcome Sanger Institute for the COVID-19 Genomics UK (COG-UK) consortium|Davies|United Kingdom
>Spike|hCoV-19/England/ALDP-C5860B/2020|2020-12-14|EPI_ISL_736008|Original|hCoV-19^^England|Human|Lighthouse Lab in Alderley Park|Wellcome Sanger Institute for the COVID-19 Genomics UK (COG-UK) Consortium|Davies|United Kingdom
>Spike|hCoV-19/England/ALDP-9D7D7B/2020|2020-09-18|EPI_ISL_576628|Original|hCoV-19^^England|Human|Oxford Viromics|COVID-19 Genomics UK (COG-UK) Consortium|Golubchik|United Kingdom
>Spike|hCoV-19/Netherlands/NH-RIVM-20850/2020|2020-12-21|EPI_ISL_790905|Original|hCoV-19^^Noord-Holland|Human|Dutch COVID-19 response team|National Institute for Public Health and the Environment (RIVM)|Meijer|Netherlands
>Spike|hCoV-19/USA/CA-QDX-795/2020|2020-03-21|EPI_ISL_571489|Original|hCoV-19^^California|Human|Quest Diagnostics|Quest Diagnostics|Kagan|USA
>Spike|hCoV-19/USA/MA-MASPHL-00649/2020|2020-05-14|EPI_ISL_692983|Original|hCoV-19^^Massachusetts|Human|Massachusetts State Public Health Laboratory|Massachusetts State Public Health Laboratory|Lang|USA
>Spike|hCoV-19/South Africa/KRISP-0584/2020|2020-07-07|EPI_ISL_498122|Original|hCoV-19^^KwaZulu-Natal|Human|NHLS-IALCH|KRISP|de Oliveira|South Africa
>Spike|hCoV-19/Colombia/VAC-GVI-97212/2020|2020-04-04|EPI_ISL_447795|Original|hCoV-19^^Valle del Cauca|Human|Instituto Nacional de Salud|Grupo de Investigaciones Microbiol\u00f3gicas-UR (GIMUR)|Ram\u00edrez|Colombia

>Spike|hCoV-19/England/QEUH-961C5B/2020|2020-08-01|EPI_ISL_537031|Original|hCoV-19^^England|Human|Lighthouse Lab in Glasgow|Wellcome Sanger Institute for the COVID-19 Genomics UK (COG-UK) consortium|Davies|United Kingdom
>Spike|hCoV-19/USA/MI-MDHHS-SC21685/2020|2020-07-08|EPI_ISL_516412|Original|hCoV-19^^Michigan|Human|Michigan Department of Health and Human Services|Michigan Department of Health and Human Services|Blankenship|USA
>Spike|hCoV-19/Switzerland/BS-UHB-42203098/2020|2020-03-23|EPI_ISL_528343|Original|hCoV-19^^Basel-Stadt|Human|University Hospital Basel|University Hospital Basel|Roloff Handschin|Switzerland
>Spike|hCoV-19/England/QEUH-AD3910/2020|2020-10-25|EPI_ISL_633898|Original|hCoV-19^^England|Human|Lighthouse Lab in Glasgow|Wellcome Sanger Institute for the COVID-19 Genomics UK (COG-UK) consortium|Davies|United Kingdom
>Spike|hCoV-19/Mexico/NLE-UANL-035/2020|2020-11-16|EPI_ISL_779196|Original|hCoV-19^^Nuevo Leon|Human|Laboratorio Estatal de Salud P\u00fablica de Nuevo Le\u00f3n|Laboratorio de Infectolog\u00eda Molecular|Gal\u00e1n|Mexico
>Spike|hCoV-19/South Korea/KCDC2214/2020|2020-05-08|EPI_ISL_510552|Original|hCoV-19^^South Korea|Human|Division of Viral Diseases|Division of Viral Diseases|Kim|South Korea
>Spike|hCoV-19/England/QEUH-A5DFD0/2020|2020-10-12|EPI_ISL_597276|Original|hCoV-19^^England|Human|Lighthouse Lab in Glasgow|Wellcome Sanger Institute for the COVID-19 Genomics UK (COG-UK) consortium|Davies|United Kingdom
>Spike|hCoV-19/Australia/VIC7835/2020|2020-07-28|EPI_ISL_564688|Original|hCoV-19^^Victoria|Human|Microbiological Diagnostic Unit - Public Health Laboratory (MDU-PHL)|MDU-PHL|Schultz|Australia
>Spike|hCoV-19/England/CAMC-B32592/2020|2020-11-05|EPI_ISL_659286|Original|hCoV-19^^England|Human|Lighthouse Lab in Cambridge|Wellcome Sanger Institute for the COVID-19 Genomics UK (COG-UK) Consortium|Davies|United Kingdom
>Spike|hCoV-19/USA/NY-NYUMC217/2020|2020-04-03|EPI_ISL_427512|Original|hCoV-19^^New York|Human|NYU Langone Health|Departments of Pathology and Medicine|Maurano|USA
>Spike|hCoV-19/Scotland/QEUH-9D1C47/2020|2020-09-16|EPI_ISL_572684|Original|hCoV-19^^Scotland|Human|University College London|COVID-19 Genomics UK (COG-UK) Consortium|Roy|United Kingdom
>Spike|hCoV-19/USA/CA-CDPH071/2020|2020-07-01|EPI_ISL_754969|Original|hCoV-19^^California|Human|California Department of Public Health|California Department of Public Health|Bell|USA
>Spike|hCoV-19/Denmark/DCGC-10575/2020|2020-11-02|EPI_ISL_670062|Original|hCoV-19^^Hovedstaden|Human|Department of Virus and Microbiological Special Diagnostics|Albertsen Lab|Michaelsen|Denmark
>Spike|hCoV-19/Germany/BE-ChVir-W2202-4448/2020|2020-11-21|EPI_ISL_753828|Original|hCoV-19^^Berlin|Human|Charit\u00e9 Universit\u00e4tsmedizin Berlin|Charit\u00e9 Universit\u00e4tsmedizin Berlin|Muehlemann|Germany
>Spike|hCoV-19/USA/CA-CZB-1984/2020|2020-05-07|EPI_ISL_486251|Original|hCoV-19^^California|Human|Orange County Public Health Laboratory|Chan-Zuckerberg Biohub|McGeever|USA
>Spike|hCoV-19/Brazil/RJ-00485/2020|2020-10-26|EPI_ISL_717879|Original|hCoV-19^^Rio de Janeiro|Human|Laboratorio de Virologia Molecular / UFRJ|Bioinformatics Laboratory / LNCC|Almeida|Brazil
>Spike|hCoV-19/England/ALDP-8E1DCA/2020|2020-06-25|EPI_ISL_557156|Original|hCoV-19^^England|Human|Lighthouse Lab in Alderley Park|Wellcome Sanger Institute for the COVID-19 Genomics UK (COG-UK) consortium|Davies|United Kingdom
>Spike|hCoV-19/USA/CA-CDPH017/2020|2020-02-27|EPI_ISL_515910|Original|hCoV-19^^California|Human|California Department of Public Health|California Department of Public Health|Bell|USA
>Spike|hCoV-19/USA/NM-UNM-00792/2020|2020-05-05|EPI_ISL_610258|Original|hCoV-19^^New Mexico|Human|TriCore Reference Laboratories|Center for Global Health|Domman|USA
>Spike|hCoV-19/England/CAMC-B51FDB/2020|2020-11-08|EPI_ISL_657953|Original|hCoV-19^^England|Human|Lighthouse Lab in Cambridge|Wellcome Sanger Institute for the COVID-19 Genomics UK (COG-UK) Consortium|Davies|United Kingdom

>Spike|hCoV-19/England/QEUH-B69BDC/2020|2020-11-11|EPI_ISL_655155|Original|hCoV-19^^England|Human|Lighthouse Lab in Glasgow|Wellcome Sanger Institute for the COVID-19 Genomics UK (COG-UK) Consortium|Davies|United Kingdom
>Spike|hCoV-19/Canada/QC-LSPQ-L00218559/2020|2020-03-14|EPI_ISL_535785|Original|hCoV-19^^Quebec|Human|CHUL-LABO MULTI / MICRO|Laboratoire de sant\u00e9 publique du Qu\u00e9bec|Moreira|Canada
>Spike|hCoV-19/South Korea/KDCA0050/2020|2020-09-15|EPI_ISL_747291|Original|hCoV-19^^South Korea|Human|Division of Emerging Infectious Diseases|Division of Emerging Infectious Diseases|Park|South Korea
>Spike|hCoV-19/Australia/VIC14746/2020|2020-08-19|EPI_ISL_592982|Original|hCoV-19^^Victoria|Human|Victorian Infectious Diseases Reference Laboratory (VIDRL)|VIDRL and MDU-PHL|Schultz|Australia
>Spike|hCoV-19/Denmark/DCGC-6460/2020|2020-10-12|EPI_ISL_620360|Original|hCoV-19^^Hovedstaden|Human|Department of Virus and Microbiological Special Diagnostics|Albertsen lab|Michaelsen|Denmark
>Spike|hCoV-19/England/CAMC-A3F3E7/2020|2020-10-06|EPI_ISL_609761|Original|hCoV-19^^England|Human|Lighthouse Lab in Cambridge|Wellcome Sanger Institute for the COVID-19 Genomics UK (COG-UK) consortium|Davies|United Kingdom
>Spike|hCoV-19/USA/MD-HP00079/2020|2020-03-17|EPI_ISL_438236|Original|hCoV-19^^Maryland|Human|Johns Hopkins Hospital Department of Pathology|Johns Hopkins Hospital Department of Pathology|Mehoke|USA
>Spike|hCoV-19/Netherlands/ZH-EMC-761/2020|2020-10-01|EPI_ISL_632598|Original|hCoV-19^^Zuid Holland|Human|Dutch COVID-19 response team|Erasmus Medical Center|Oude Munnink|Netherlands
>Spike|hCoV-19/USA/CA-SCCPHD-UC200/2020|2020-03-10|EPI_ISL_671768|Original|hCoV-19^^California|Human|Santa Clara County Public Health Department|Chiu Laboratory|Chiu|USA
>Spike|hCoV-19/Canada/ON-S38/2020|2020-03-17|EPI_ISL_591138|Original|hCoV-19^^Ontario|Human|Toronto Invasive Bacterial Diseases Network|McMaster University|McArthur|Canada
>Spike|hCoV-19/Scotland/CVR1644/2020|2020-04-06|EPI_ISL_433641|Original|hCoV-19^^Scotland|Human|West of Scotland Specialist Virology Centre|COVID-19 Genomics UK (COG-UK) Consortium|Robertson|United Kingdom
>Spike|hCoV-19/England/CAMC-B35492/2020|2020-11-05|EPI_ISL_659530|Original|hCoV-19^^England|Human|Lighthouse Lab in Cambridge|Wellcome Sanger Institute for the COVID-19 Genomics UK (COG-UK) Consortium|Davies|United Kingdom
>Spike|hCoV-19/England/MILK-B878C2/2020|2020-11-13|EPI_ISL_724811|Original|hCoV-19^^England|Human|Oxford Viromics|COVID-19 Genomics UK (COG-UK) Consortium|Golubchik|United Kingdom
>Spike|hCoV-19/India/DL-NCDC6082-CSIR-IGIB/2020|2020-05-25|EPI_ISL_482616|Original|hCoV-19^^Delhi|Human|National Centre for Disease control (NCDC)|NCDC/CSIR-IGIB|Singh|India
>Spike|hCoV-19/Spain/PV-IBV-000778/2020|2020-03-09|EPI_ISL_452727|Original|hCoV-19^^Basque Country|Human|Hospital Universitario Araba. Vitoria-Gasteiz,|SeqCOVID-SPAIN consortium/IBV(CSIC)|Comas|Spain
>Spike|hCoV-19/DRC/2942/2020|2020-04-17|EPI_ISL_437355|Original|hCoV-19^^Kinshasa|Human|Viral Respiratory Lab|Pathogen Sequencing Lab|Amuri|Democratic Republic of the Congo
>Spike|hCoV-19/Japan/PG-6290/2020|2020-07-00|EPI_ISL_689401|Original|hCoV-19^^Japan|Human|Pathogen Genomics Center|Pathogen Genomics Center|Sekizuka|Japan
>Spike|hCoV-19/England/QEUH-AD1FD4/2020|2020-10-26|EPI_ISL_634160|Original|hCoV-19^^England|Human|Lighthouse Lab in Glasgow|Wellcome Sanger Institute for the COVID-19 Genomics UK (COG-UK) consortium|Davies|United Kingdom
>Spike|hCoV-19/Australia/VIC4039/2020|2020-07-11|EPI_ISL_519422|Original|hCoV-19^^Victoria|Human|Microbiological Diagnostic Unit - Public Health Laboratory (MDU-PHL)|MDU-PHL|Schultz|Australia
>Spike|hCoV-19/England/CAMC-B7750A/2020|2020-11-12|EPI_ISL_783418|Original|hCoV-19^^England|Human|Lighthouse Lab in Cambridge|Wellcome Sanger Institute for the COVID-19 Genomics UK (COG-UK) Consortium|Davies|United Kingdom

>Spike|hCoV-19/Japan/NF-10/2020|2020-04-00|EPI_ISL_591480|Original|hCoV-
19^^Japan|Human|Pathogen Genomics Center|Pathogen Genomics Center|Sekizuka|Japan
>Spike|hCoV-19/USA/CA-CHLA-PLM50317441/2020|2020-11-28|EPI_ISL_753529|Original|hCoV-
19^^California|Human|Clinical virology Laboratory|Center for Personalized
Medicine|Dien Bard|USA
>Spike|hCoV-19/England/ALDP-9DA455/2020|2020-09-18|EPI_ISL_576874|Original|hCoV-
19^^England|Human|Oxford Viromics|COVID-19 Genomics UK (COG-UK)
Consortium|Golubchik|United Kingdom
>Spike|hCoV-19/USA/WA-S1989/2020|2020-06-04|EPI_ISL_497417|Original|hCoV-
19^^Washington|Human|Washington State Department of Health|Seattle Flu Study|Lee|USA
>Spike|hCoV-19/England/NORW-F0883/2020|2020-10-17|EPI_ISL_595194|Original|hCoV-
19^^England|Human|Quadram Institute Bioscience|COVID-19 Genomics UK (COG-UK)
Consortium|Alikhan|United Kingdom
>Spike|hCoV-19/Denmark/DCGC-5635/2020|2020-09-28|EPI_ISL_619922|Original|hCoV-
19^^Hovedstaden|Human|Department of Virus and Microbiological Special
Diagnostics|Albertsen lab|Michaelsen|Denmark
>Spike|hCoV-19/USA/NY-NYCPHL-000269/2020|2020-03-17|EPI_ISL_631795|Original|hCoV-
19^^New York|Human|Jamaica Hospital Medical Center|New York City Public Health
Laboratory|Wang|USA
>Spike|hCoV-19/England/QEUH-AD1D70/2020|2020-10-26|EPI_ISL_629949|Original|hCoV-
19^^England|Human|Lighthouse Lab in Glasgow|Wellcome Sanger Institute for the COVID-
19 Genomics UK (COG-UK) consortium|Davies|United Kingdom
>Spike|hCoV-19/Denmark/DCGC-14435/2020|2020-11-30|EPI_ISL_711765|Original|hCoV-
19^^Hovedstaden|Human|Department of Virus and Microbiological Special
Diagnostics|Albertsen Lab|Michaelsen|Denmark
>Spike|hCoV-19/USA/CA-CZB-15493/2020|2020-09-18|EPI_ISL_739038|Original|hCoV-
19^^California|Human|County of San Luis Obispo Public Health Laboratory|Chan-
Zuckerberg Biohub|Kamm|USA
>Spike|hCoV-19/England/MILK-A7C506/2020|2020-10-15|EPI_ISL_607603|Original|hCoV-
19^^England|Human|Lighthouse Lab in Milton Keynes|Wellcome Sanger Institute for the
COVID-19 Genomics UK (COG-UK) consortium|Davies|United Kingdom
>Spike|hCoV-19/England/WSFT-25C0D73/2020|2020-05-01|EPI_ISL_492855|Original|hCoV-
19^^England|Human|Department of Medical Microbiology|Wellcome Sanger Institute for
the COVID-19 Genomics UK (COG-UK) consortium|Davies|United Kingdom
>Spike|hCoV-19/Northern Ireland/NIRE-2358EA/2020|2020-11-
10|EPI_ISL_742161|Original|hCoV-19^^Northern Ireland|Human|Regional Virus
Laboratory|COVID-19 Genomics UK (COG-UK) Consortium|Fairley|United Kingdom
>Spike|hCoV-19/England/CAMC-BD1F79/2020|2020-11-28|EPI_ISL_702121|Original|hCoV-
19^^England|Human|Lighthouse Lab in Cambridge|Wellcome Sanger Institute for the
COVID-19 Genomics UK (COG-UK) Consortium|Davies|United Kingdom
>Spike|hCoV-19/England/MILK-A47EAF/2020|2020-10-06|EPI_ISL_598421|Original|hCoV-
19^^England|Human|Lighthouse Lab in Milton Keynes|Wellcome Sanger Institute for the
COVID-19 Genomics UK (COG-UK) consortium|Davies|United Kingdom
>Spike|hCoV-19/Switzerland/BL-UHB-42179323/2020|2020-03-
06|EPI_ISL_527946|Original|hCoV-19^^Basel-Land|Human|University Hospital
Basel|University Hospital Basel|Roloff Handschin|Switzerland
>Spike|hCoV-19/Netherlands/ZH-EMC-974/2020|2020-11-17|EPI_ISL_722364|Original|hCoV-
19^^Zuid Holland|Human|Dutch COVID-19 response team|Erasmus Medical Center|Oude
Munnink|Netherlands
>Spike|hCoV-19/USA/AZ-TG670229/2020|2020-05-01|EPI_ISL_696411|Original|hCoV-
19^^Arizona|Human|Sonora Quest Laboratories|TGen North|French|USA
>Spike|hCoV-19/Brazil/SP-138/2020|2020-04-08|EPI_ISL_468305|Original|hCoV-
19^^S\u00e3o Paulo|Human|Centro de Vigilancia a Saude de Diadema|Instituto Adolfo
Lutz|Sacchi|Brazil
>Spike|hCoV-19/India/DL-NCDC3820-CSIR-IGIB/2020|2020-05-
20|EPI_ISL_482574|Original|hCoV-19^^Delhi|Human|National Centre for Disease control
(NCDC)|NCDC/CSIR-IGIB|Pandey|India
>Spike|hCoV-19/Iceland/109/2020|2020-03-11|EPI_ISL_417748|Original|hCoV-
19^^Reykjavik|Human|The National University Hospital of Iceland|deCODE
genetics|J\u00f3nsson|Iceland

>Spike|hCoV-19/Australia/VIC1458/2020|2020-04-20|EPI_ISL_456454|Original|hCoV-19^^Victoria|Human|Victorian Infectious Diseases Reference Laboratory (VIDRL)|Microbiological Diagnostic Unit Public Health Laboratory and Victorian Infectious Diseases Reference Laboratory|Schultz|Australia
>Spike|hCoV-19/Gibraltar/204462817/2020|2020-10-29|EPI_ISL_614992|Original|hCoV-19^^Gibraltar|Human|Microbiology|Respiratory Virus Unit|Platt|Gibraltar
>Spike|hCoV-19/France/PAC-IHU-0291/2020|2020-03-31|EPI_ISL_569436|Original|hCoV-19^^Provence-Alpes-C\u00f4te-d\u2019Azur|Human|MEPHI|MEPHI|Levasseur|France
>Spike|hCoV-19/Wales/PHWC-32409/2020|2020-04-10|EPI_ISL_446645|Original|hCoV-19^^Wales|Human|Wales Specialist Virology Centre|Public Health Wales Microbiology Cardiff|Moore|United Kingdom
>Spike|hCoV-19/England/QEUH-AE523E/2020|2020-10-27|EPI_ISL_633343|Original|hCoV-19^^England|Human|Lighthouse Lab in Glasgow|Wellcome Sanger Institute for the COVID-19 Genomics UK (COG-UK) consortium|Davies|United Kingdom
>Spike|hCoV-19/England/LIVE-1D7511/2020|2020-09-26|EPI_ISL_584603|Original|hCoV-19^^England|Human|Liverpool Clinical Laboratories|COVID-19 Genomics UK (COG-UK) Consortium|Haldenby|United Kingdom
>Spike|hCoV-19/Belgium/rega-0916972/2020|2020-09-16|EPI_ISL_734962|Original|hCoV-19^^Wezembeek-Oppem|Human|UZ Leuven|KU Leuven|Maes|Belgium
>Spike|hCoV-19/Japan/PG-3440/2020|2020-07-00|EPI_ISL_691550|Original|hCoV-19^^Japan|Human|Fukuoka Institute of Health and Environmental Sciences|Pathogen Genomics Center|Sekizuka|Japan
>Spike|hCoV-19/Denmark/DCGC-14971/2020|2020-11-23|EPI_ISL_713556|Original|hCoV-19^^Hovedstaden|Human|Department of Virus and Microbiological Special Diagnostics|Albertsen Lab|Michaelsen|Denmark
>Spike|hCoV-19/France/ARA-9315/2020|2020-03-15|EPI_ISL_678494|Original|hCoV-19^^Auvergne-Rh\u00f4ne-Alpes|Human|CNR Virus des Infections Respiratoires - France SUD|CNR Virus des Infections Respiratoires - France SUD|Bal|France
>Spike|hCoV-19/England/QEUH-9D0D36/2020|2020-09-13|EPI_ISL_589983|Original|hCoV-19^^England|Human|Lighthouse Lab in Glasgow|Wellcome Sanger Institute for the COVID-19 Genomics UK (COG-UK) consortium|Davies|United Kingdom
>Spike|hCoV-19/Bahrain/340846472/2020|2020-10-11|EPI_ISL_682314|Original|hCoV-19^^Bahrain|Human|Communicable Disease Laboratory|Communicable Disease Laboratory|Altaif|Bahrain
>Spike|hCoV-19/Denmark/DCGC-17683/2020|2020-12-14|EPI_ISL_751142|Original|hCoV-19^^Sjaelland|Human|Department of Virus and Microbiological Special Diagnostics|Albertsen Lab|Michaelsen|Denmark
>Spike|hCoV-19/Wales/PHWC-168A52/2020|2020-04-20|EPI_ISL_494320|Original|hCoV-19^^Wales|Human|Originating lab|COVID-19 Genomics UK (COG-UK) Consortium|Moore|United Kingdom
>Spike|hCoV-19/Wales/PHWC-48C211/2020|2020-11-03|EPI_ISL_666407|Original|hCoV-19^^Wales|Human|Wales Specialist Virology Centre Sequencing lab|COVID-19 Genomics UK (COG-UK) Consortium|Moore|United Kingdom
>Spike|hCoV-19/Denmark/ALAB-SSI113/2020|2020-03-08|EPI_ISL_429344|Original|hCoV-19^^Denmark|Human|Department of Virus and Microbiological Special Diagnostics|Albertsen lab|Kirkegaard|Denmark
>Spike|hCoV-19/USA/ME-HETL-J0328/2020|2020-11-24|EPI_ISL_755456|Original|hCoV-19^^Maine|Human|Maine Health and Environmental Testing Laboratory|Tewhey Lab|Tewhey|USA
>Spike|hCoV-19/Belgium/AM-0328342/2020|2020-03-28|EPI_ISL_462196|Original|hCoV-19^^Genk|Human|KU Leuven|KU Leuven|Maes|Belgium
>Spike|hCoV-19/Italy/VEN-UniVR-153/2020|2020-04-16|EPI_ISL_751447|Original|hCoV-19^^Veneto|Human|IRCCS Sacro Cuore Don Calabria Hospital|University of Verona|Mori|Italy
>Spike|hCoV-19/England/CAMC-BBCC40/2020|2020-11-22|EPI_ISL_679270|Original|hCoV-19^^England|Human|Queens Medical Centre|COVID-19 Genomics UK (COG-UK) Consortium|Loose|United Kingdom
>Spike|hCoV-19/Chile/RM-170370/2020|2020-09-05|EPI_ISL_746717|Original|hCoV-19^^Region Metropolitana de Santiago|Human|Genetica Molecular and Subdepartamento de Virologia ISP Chile|Instituto de Salud Publica de Chile|Tognarelli|Chile

>Spike|hCoV-19/England/20149035404/2020|2020-04-03|EPI_ISL_466187|Original|hCoV-19^^England|Human|Respiratory Virus Unit|Respiratory Virus Unit|Platt|United Kingdom
>Spike|hCoV-19/France/ARA-532/2020|2020-03-04|EPI_ISL_416749|Original|hCoV-19^^Auvergne-Rh\u00f4ne-Alpes|Human|Centre Hospitalier de Valence|CNR Virus des Infections Respiratoires - France SUD|Josset|France
>Spike|hCoV-19/England/MILK-B8EE32/2020|2020-11-13|EPI_ISL_675243|Original|hCoV-19^^England|Human|Lighthouse Lab in Milton Keynes|Wellcome Sanger Institute for the COVID-19 Genomics UK (COG-UK) Consortium|Davies|United Kingdom
>Spike|hCoV-19/England/MILK-A7BB8D/2020|2020-10-15|EPI_ISL_607561|Original|hCoV-19^^England|Human|Lighthouse Lab in Milton Keynes|Wellcome Sanger Institute for the COVID-19 Genomics UK (COG-UK) consortium|Davies|United Kingdom
>Spike|hCoV-19/Scotland/CVR3377/2020|2020-05-07|EPI_ISL_456806|Original|hCoV-19^^Scotland|Human|West of Scotland Specialist Virology Centre|COVID-19 Genomics UK (COG-UK) Consortium|Robertson|United Kingdom
>Spike|hCoV-19/Canada/BC_04033837/2020|2020-04-00|EPI_ISL_466762|Original|hCoV-19^^British Columbia|Human|BCCDC Public Health Laboratory|BCCDC Public Health Laboratory|Harrigan|Canada
>Spike|hCoV-19/England/EXET-1390A3/2020|2020-10-28|EPI_ISL_665052|Original|hCoV-19^^England|Human|University of Exeter|COVID-19 Genomics UK (COG-UK) Consortium|Temperton|United Kingdom
>Spike|hCoV-19/Scotland/EDB6251/2020|2020-05-22|EPI_ISL_473931|Original|hCoV-19^^Scotland|Human|Virology Department|COVID-19 Genomics UK (COG-UK) Consortium|Williams|United Kingdom
>Spike|hCoV-19/Netherlands/GE-EMC-343/2020|2020-10-20|EPI_ISL_632342|Original|hCoV-19^^Gelderland|Human|Dutch COVID-19 response team|Erasmus Medical Center|Oude Munnink|Netherlands
>Spike|hCoV-19/USA/MI-MDHHS-SC22466/2020|2020-11-03|EPI_ISL_708568|Original|hCoV-19^^Michigan|Human|Michigan Department of Health and Human Services|Michigan Department of Health and Human Services|Jacob|USA
>Spike|hCoV-19/USA/TX-DSHS-0403/2020|2020-06-01|EPI_ISL_542463|Original|hCoV-19^^Texas|Human|Texas Department of State Health Services|Texas Department of State Health Services|Rahman|USA
>Spike|hCoV-19/England/ALDP-C57CDD/2020|2020-12-14|EPI_ISL_735963|Original|hCoV-19^^England|Human|Lighthouse Lab in Alderley Park|Wellcome Sanger Institute for the COVID-19 Genomics UK (COG-UK) Consortium|Davies|United Kingdom
>Spike|hCoV-19/USA/AZ-TG371281/2020|2020-05-16|EPI_ISL_694540|Original|hCoV-19^^Arizona|Human|AZ SPHL|TGen North|French|USA
>Spike|hCoV-19/Wales/PHWC-49106A/2020|2020-11-10|EPI_ISL_706604|Original|hCoV-19^^Wales|Human|Originating lab|COVID-19 Genomics UK (COG-UK) Consortium|Moore|United Kingdom
>Spike|hCoV-19/USA/CA-QDX-2419/2020|2020-03-17|EPI_ISL_604682|Original|hCoV-19^^California|Human|Quest Diagnostics|Quest Diagnostics|Kagan|USA
>Spike|hCoV-19/USA/CA-ALSR-0941-SAN/2020|2020-03-29|EPI_ISL_483426|Original|hCoV-19^^California|Human|UC San Diego Center for Advanced Laboratory Medicine|Andersen lab at Scripps Research|Gangavarapu|USA
>Spike|hCoV-19/Denmark/DCGC-21071/2020|2020-12-21|EPI_ISL_759536|Original|hCoV-19^^Nordjylland|Human|Department of Virus and Microbiological Special Diagnostics|Albertsen Lab|Michaelsen|Denmark
>Spike|hCoV-19/England/BRIS-1227D5/2020|2020-04-05|EPI_ISL_440656|Original|hCoV-19^^England|Human|PHE South West Regional Laboratory|Wellcome Sanger Institute for the COVID-19 Genomics UK (COG-UK) consortium|Amato|United Kingdom
>Spike|hCoV-19/Brazil/RJ-00451/2020|2020-06-01|EPI_ISL_717846|Original|hCoV-19^^Rio de Janeiro|Human|Laboratorio de Virologia Molecular / UFRJ|Bioinformatics Laboratory / LNCC|Almeida|Brazil
>Spike|hCoV-19/Japan/PG-5953/2020|2020-00-00|EPI_ISL_689068|Original|hCoV-19^^Japan|Human|Gunma Prefectural Institute of Public Health and Environmental Sciences|Pathogen Genomics Center|Sekizuka|Japan
>Spike|hCoV-19/USA/TX-HMH-MCoV-9432/2020|2020-07-03|EPI_ISL_781120|Original|hCoV-19^^Texas|Human|Houston Methodist Hospital|Houston Methodist Hospital|Long|USA

>Spike|hCoV-19/USA/CA-SR0410/2020|2020-04-21|EPI_ISL_458280|Original|hCoV-19^^California|Human|Scripps Medical Laboratory|Andersen lab at Scripps Research|Gangavarapu|USA
>Spike|hCoV-19/India/MH-ACTREC-219/2020|2020-06-29|EPI_ISL_699873|Original|hCoV-19^^Maharashtra|Human|Hematopathology Laboratory|Hematopathology Laboratory|Patkar|India
>Spike|hCoV-19/USA/CA-ALSR-1332/2020|2020-04-29|EPI_ISL_494496|Original|hCoV-19^^California|Human|San Diego County Public Health Laboratory|Andersen lab at Scripps Research|Gangavarapu|USA
>Spike|hCoV-19/India/OR-ILSCV20596/2020|2020-05-15|EPI_ISL_463079|Original|hCoV-19^^Odisha|Human|Institute of Life Sciences|Immunogenomics lab|Raghav|India
>Spike|hCoV-19/USA/OR-OHSU-0993/2020|2020-07-07|EPI_ISL_525944|Original|hCoV-19^^Oregon|Human|OHSU Lab Services Molecular Microbiology Lab|Oregon SARS-CoV-2 Genome Sequencing Center|O'Roak|USA
>Spike|hCoV-19/USA/TX-HMH-MCoV-18519/2020|2020-11-19|EPI_ISL_784867|Original|hCoV-19^^Texas|Human|Houston Methodist Hospital|Houston Methodist Hospital|Long|USA
>Spike|hCoV-19/Scotland/QEUH-AD1E40/2020|2020-10-27|EPI_ISL_644027|Original|hCoV-19^^Scotland|Human|Lighthouse Lab in Glasgow|Wellcome Sanger Institute for the COVID-19 Genomics UK (COG-UK) Consortium|Davies|United Kingdom
>Spike|hCoV-19/England/ALDP-A94FBF/2020|2020-10-19|EPI_ISL_624718|Original|hCoV-19^^England|Human|Lighthouse Lab in Alderley Park|Wellcome Sanger Institute for the COVID-19 Genomics UK (COG-UK) consortium|Davies|United Kingdom
>Spike|hCoV-19/England/ALDP-921E33/2020|2020-06-23|EPI_ISL_553298|Original|hCoV-19^^England|Human|Lighthouse Lab in Alderley Park|Wellcome Sanger Institute for the COVID-19 Genomics UK (COG-UK) consortium|Davies|United Kingdom
>Spike|hCoV-19/Netherlands/GR-RIVM-20421/2020|2020-11-30|EPI_ISL_728641|Original|hCoV-19^^Groningen|Human|Dutch COVID-19 response team|National Institute for Public Health and the Environment (RIVM)|Meijer|Netherlands
>Spike|hCoV-19/England/ALDP-9EA67A/2020|2020-09-24|EPI_ISL_580119|Original|hCoV-19^^England|Human|Lighthouse Lab in Alderley Park|Wellcome Sanger Institute for the COVID-19 Genomics UK (COG-UK) Consortium|Davies|United Kingdom
>Spike|hCoV-19/Wales/PHWC-168050/2020|2020-04-19|EPI_ISL_494193|Original|hCoV-19^^Wales|Human|Originating lab|COVID-19 Genomics UK (COG-UK) Consortium|Moore|United Kingdom
>Spike|hCoV-19/England/ALDP-A6D08F/2020|2020-10-14|EPI_ISL_608054|Original|hCoV-19^^England|Human|Lighthouse Lab in Alderley Park|Wellcome Sanger Institute for the COVID-19 Genomics UK (COG-UK) consortium|Davies|United Kingdom
>Spike|hCoV-19/USA/WA-S2691/2020|2020-06-22|EPI_ISL_497323|Original|hCoV-19^^Washington|Human|Washington State Department of Health|Seattle Flu Study|Lee|USA
>Spike|hCoV-19/England/QEUH-9F5375/2020|2020-09-25|EPI_ISL_638667|Original|hCoV-19^^England|Human|Department of Pathology|COVID-19 Genomics UK (COG-UK) Consortium|Goodfellow|United Kingdom
>Spike|hCoV-19/USA/WA-UW-31218/2020|2020-10-13|EPI_ISL_735462|Original|hCoV-19^^Washington|Human|UW Virology Lab|UW Virology Lab|Roychoudhury|USA
>Spike|hCoV-19/USA/CA-CZB-14512/2020|2020-11-11|EPI_ISL_739451|Original|hCoV-19^^California|Human|Santa Clara County Public Health Laboratory|Chan-Zuckerberg Biohub|Kamm|USA
>Spike|hCoV-19/USA/NY-NYCPHL-000023/2020|2020-03-10|EPI_ISL_631519|Original|hCoV-19^^New York|Human|New York Presbyterian-Brooklyn Methodist Hospital|New York City Public Health Laboratory|Wang|USA
>Spike|hCoV-19/England/MILK-9A5602/2020|2020-09-03|EPI_ISL_550095|Original|hCoV-19^^England|Human|Lighthouse Lab in Milton Keynes|Wellcome Sanger Institute for the COVID-19 Genomics UK (COG-UK) consortium|Davies|United Kingdom
>Spike|hCoV-19/USA/VA-DCLS-1087/2020|2020-06-18|EPI_ISL_528679|Original|hCoV-19^^Virginia|Human|Virginia Division of Consolidated Laboratory Services (DCLS)|Virginia Division of Consolidated Laboratory Services (DCLS)|St. Jacques|USA
>Spike|hCoV-19/USA/UT-UPHL-201026758/2020|2020-07-06|EPI_ISL_594044|Original|hCoV-19^^Utah|Human|Utah Public Health Laboratory|Utah Public Health Laboratory|Young|USA

>Spike|hCoV-19/Wales/PHWC-4A1304/2020|2020-12-09|EPI_ISL_743654|Original|hCoV-19^^Wales|Human|Wales Specialist Virology Centre Sequencing lab|COVID-19 Genomics UK (COG-UK) Consortium|Moore|United Kingdom
>Spike|hCoV-19/England/ALDP-9C5068/2020|2020-09-10|EPI_ISL_567855|Original|hCoV-19^^England|Human|Lighthouse Lab in Alderley Park|Wellcome Sanger Institute for the COVID-19 Genomics UK (COG-UK) consortium|Davies|United Kingdom
>Spike|hCoV-19/England/QEUH-B80A78/2020|2020-11-14|EPI_ISL_661847|Original|hCoV-19^^England|Human|Lighthouse Lab in Glasgow|Wellcome Sanger Institute for the COVID-19 Genomics UK (COG-UK) Consortium|Davies|United Kingdom
>Spike|hCoV-19/Belgium/rega-0409682/2020|2020-04-09|EPI_ISL_738208|Original|hCoV-19^^Sint-Niklaas|Human|UZ Leuven|KU Leuven|Maes|Belgium
>Spike|hCoV-19/USA/TX-HMH-MCoV-15576/2020|2020-10-26|EPI_ISL_787060|Original|hCoV-19^^Texas|Human|Houston Methodist Hospital|Houston Methodist Hospital|Long|USA
>Spike|hCoV-19/Japan/PG-7286/2020|2020-08-00|EPI_ISL_688389|Original|hCoV-19^^Japan|Human|Saitama Prefectural Institute of Public Health|Pathogen Genomics Center|Sekizuka|Japan
>Spike|hCoV-19/Ireland/OY-NVRL-76IRL27010/2020|2020-10-18|EPI_ISL_732523|Original|hCoV-19^^Offaly|Human|National Virus Reference Laboratory|National Virus Reference Laboratory|Gonzalez|Ireland
>Spike|hCoV-19/USA/CA-CZB-14430/2020|2020-11-04|EPI_ISL_738802|Original|hCoV-19^^California|Human|Alameda County Public Health Lab|Chan-Zuckerberg Biohub|Kamm|USA
>Spike|hCoV-19/Iran/SA-Isf-2/2020|2020-04-18|EPI_ISL_660111|Original|hCoV-19^^Iran|Human|Infectious Diseases and Tropical Medicine Research Center|Infectious Diseases and Tropical Medicine Research Center|Haghjooy Javanmard,S.|Iran
>Spike|hCoV-19/England/QEUH-96A205/2020|2020-08-15|EPI_ISL_536958|Original|hCoV-19^^England|Human|Lighthouse Lab in Glasgow|Wellcome Sanger Institute for the COVID-19 Genomics UK (COG-UK) consortium|Davies|United Kingdom
>Spike|hCoV-19/England/20172002704/2020|2020-04-18|EPI_ISL_465322|Original|hCoV-19^^England|Human|Respiratory Virus Unit|Respiratory Virus Unit|Platt|United Kingdom
>Spike|hCoV-19/Netherlands/NA_69/2020|2020-03-09|EPI_ISL_422829|Original|hCoV-19^^Netherlands|Human|Dutch COVID-19 response team|Erasmus Medical Center|Oude Munnink|Netherlands
>Spike|hCoV-19/USA/TX-HMH-2763/2020|2020-06-03|EPI_ISL_545537|Original|hCoV-19^^Texas|Human|Houston Methodist Hospital|Houston Methodist Hospital|Long|USA
>Spike|hCoV-19/England/QEUH-76CAB2/2020|2020-07-14|EPI_ISL_533204|Original|hCoV-19^^England|Human|Lighthouse Lab in Glasgow|Wellcome Sanger Institute for the COVID-19 Genomics UK (COG-UK) consortium|Davies|United Kingdom
>Spike|hCoV-19/England/QEUH-B139F5/2020|2020-11-02|EPI_ISL_641826|Original|hCoV-19^^England|Human|Lighthouse Lab in Glasgow|Wellcome Sanger Institute for the COVID-19 Genomics UK (COG-UK) Consortium|Davies|United Kingdom
>Spike|hCoV-19/England/ALDP-981A1F/2020|2020-08-09|EPI_ISL_526358|Original|hCoV-19^^England|Human|Queens Medical Centre|COVID-19 Genomics UK (COG-UK) Consortium|Loose|United Kingdom
>Spike|hCoV-19/USA/UT-UPHL-2012193060/2020|2020-08-13|EPI_ISL_745652|Original|hCoV-19^^Utah|Human|Ginkgo Bioworks Clinical Laboratory|Utah Public Health Laboratory|Young|USA
>Spike|hCoV-19/England/QEUH-99078F/2020|2020-08-24|EPI_ISL_530354|Original|hCoV-19^^England|Human|Lighthouse Lab in Glasgow|Wellcome Sanger Institute for the COVID-19 Genomics UK (COG-UK) consortium|Davies|United Kingdom
>Spike|hCoV-19/Australia/VIC17340/2020|2020-00-00|EPI_ISL_663850|Original|hCoV-19^^Victoria|Human|Microbiological Diagnostic Unit - Public Health Laboratory (MDU-PHL)|MDU-PHL|Schultz|Australia
>Spike|hCoV-19/England/CAMC-B78CAA/2020|2020-11-12|EPI_ISL_667841|Original|hCoV-19^^England|Human|Lighthouse Lab in Cambridge|Wellcome Sanger Institute for the COVID-19 Genomics UK (COG-UK) Consortium|Davies|United Kingdom
>Spike|hCoV-19/Scotland/QEUH-A0CAC0/2020|2020-10-03|EPI_ISL_585617|Original|hCoV-19^^Scotland|Human|Quadram Institute Bioscience|COVID-19 Genomics UK (COG-UK) Consortium|Alikhan|United Kingdom
>Spike|hCoV-19/Australia/SAP191/2020|2020-03-21|EPI_ISL_492128|Original|hCoV-19^^South Australia|Human|SA Pathology|SA Pathology|Leong|Australia

>Spike|hCoV-19/USA/WI-UW-723/2020|2020-06-30|EPI_ISL_491398|Original|hCoV-19^^Wisconsin|Human|University of Wisconsin-Madison AIDS Vaccine Research Laboratories|University of Wisconsin-Madison AIDS Vaccine Research Laboratories|Baczenas|USA
>Spike|hCoV-19/England/QEUH-A0266C/2020|2020-09-28|EPI_ISL_584919|Original|hCoV-19^^England|Human|Quadram Institute Bioscience|COVID-19 Genomics UK (COG-UK) Consortium|Alikhan|United Kingdom
>Spike|hCoV-19/Wuhan/HB-WH3-181/2020|2020-03-02|EPI_ISL_454942|Original|hCoV-19^^Hubei|Human|Wuhan Chain Medical Labs (CMLabs)|State Key Laboratory of Biotherapy of Sichuan University|Tang|China
>Spike|hCoV-19/Colombia/AMA-INS-103039/2020|2020-04-23|EPI_ISL_456144|Original|hCoV-19^^Amazonas|Human|Instituto Nacional de Salud - Unidad de Secuenciaci\u00f3n y An\u00e1lisis Gen\u00f3mico|Instituto Nacional de Salud|D\u00edaz|Colombia
>Spike|hCoV-19/Japan/PG-0809/2020|2020-04-00|EPI_ISL_684419|Original|hCoV-19^^Japan|Human|Fukuoka Institute of Health and Environmental Sciences|Pathogen Genomics Center|Sekizuka|Japan
>Spike|hCoV-19/USA/WA-QDX-2176/2020|2020-03-21|EPI_ISL_604499|Original|hCoV-19^^Washington|Human|Quest Diagnostics|Quest Diagnostics|Kagan|USA
>Spike|hCoV-19/Spain/GA-IBV-004032/2020|2020-03-14|EPI_ISL_510273|Original|hCoV-19^^Galicia|Human|Hospital Cl\u00ednico Universitario de Santiago de Compostela|SeqCOVID-SPAIN consortium/IBV(CSIC)|Comas|Spain
>Spike|hCoV-19/Wales/PHWC-492A2F/2020|2020-11-23|EPI_ISL_725735|Original|hCoV-19^^Wales|Human|Originating lab|COVID-19 Genomics UK (COG-UK) Consortium|Moore|United Kingdom
>Spike|hCoV-19/England/MILK-BAAE9A/2020|2020-11-18|EPI_ISL_783137|Original|hCoV-19^^England|Human|Lighthouse Lab in Milton Keynes|Wellcome Sanger Institute for the COVID-19 Genomics UK (COG-UK) Consortium|Davies|United Kingdom
>Spike|hCoV-19/Germany/SH-ChVir20798/2020|2020-12-02|EPI_ISL_729706|Original|hCoV-19^^Schleswig-Holstein|Human|A. Krumbholz|Charit\u00e9 Universit\u00e4tsmedizin Berlin|Muehlemann|Germany
>Spike|hCoV-19/Ireland/D-NVRL-20G35042/2020|2020-03-19|EPI_ISL_605120|Original|hCoV-19^^Dublin|Human|National Virus Reference Laboratory|Irish Coronavirus Sequencing Consortium - Helixworks|Chalapati|Ireland
>Spike|hCoV-19/England/MILK-97098F/2020|2020-08-18|EPI_ISL_553083|Original|hCoV-19^^England|Human|Lighthouse Lab in Milton Keynes|Wellcome Sanger Institute for the COVID-19 Genomics UK (COG-UK) consortium|Davies|United Kingdom
>Spike|hCoV-19/England/MILK-A79B8F/2020|2020-10-15|EPI_ISL_606695|Original|hCoV-19^^England|Human|Lighthouse Lab in Milton Keynes|Wellcome Sanger Institute for the COVID-19 Genomics UK (COG-UK) consortium|Davies|United Kingdom
>Spike|hCoV-19/England/BIRM-5FC68/2020|2020-05-21|EPI_ISL_473393|Original|hCoV-19^^England|Human|University of Birmingham|COVID-19 Genomics UK (COG-UK) Consortium|Loman|United Kingdom
>Spike|hCoV-19/England/MILK-9F0D4F/2020|2020-09-24|EPI_ISL_589579|Original|hCoV-19^^England|Human|Lighthouse Lab in Milton Keynes|Wellcome Sanger Institute for the COVID-19 Genomics UK (COG-UK) consortium|Davies|United Kingdom
>Spike|hCoV-19/USA/TX-HMH-MCoV-8657/2020|2020-07-01|EPI_ISL_780682|Original|hCoV-19^^Texas|Human|Houston Methodist Hospital|Houston Methodist Hospital|Long|USA
>Spike|hCoV-19/USA/TX-HMH-MCoV-18072/2020|2020-11-13|EPI_ISL_784420|Original|hCoV-19^^Texas|Human|Houston Methodist Hospital|Houston Methodist Hospital|Long|USA
>Spike|hCoV-19/USA/WA-UW-1709/2020|2020-03-21|EPI_ISL_424230|Original|hCoV-19^^Washington|Human|UW Virology Lab|UW Virology Lab|Roychoudhury|USA
>Spike|hCoV-19/Denmark/DCGC-6757/2020|2020-04-20|EPI_ISL_622348|Original|hCoV-19^^Hovedstaden|Human|Department of Virus and Microbiological Special Diagnostics|Albertsen lab|Michaelsen|Denmark
>Spike|hCoV-19/Wales/PHWC-4939E5/2020|2020-11-30|EPI_ISL_725969|Original|hCoV-19^^Wales|Human|Originating lab|COVID-19 Genomics UK (COG-UK) Consortium|Moore|United Kingdom
>Spike|hCoV-19/England/20139058304/2020|2020-03-27|EPI_ISL_420724|Original|hCoV-19^^England|Human|Respiratory Virus Unit|Respiratory Virus Unit|Platt|United Kingdom

>Spike|hCoV-19/Switzerland/GE-SNRCI-2480/2020|2020-03-
00|EPI_ISL_548153|Original|hCoV-19^^Geneve|Human|Laboratoire de Virologie|Swiss
National Reference Centre for Influenza|Gon\u00e7alves Cabecinhas|Switzerland
>Spike|hCoV-19/USA/NC_200272/2020|2020-04-00|EPI_ISL_454811|Original|hCoV-19^^North
Carolina|Human|Dirk Dittmer|Dirk Dittmer|Moorad|USA
>Spike|hCoV-19/Chile/RM-ISPCH-60/2020|2020-03-21|EPI_ISL_445356|Original|hCoV-
19^^Region Metropolitana de Santiago|Human|PONTIFICIA U. CATOLICA SERV.
LABORATORIO|Instituto de Salud Publica de Chile|Fernandez|Chile
>Spike|hCoV-19/Northern Ireland/NIRE-104083/2020|2020-04-
08|EPI_ISL_482137|Original|hCoV-19^^Northern Ireland|Human|Regional Virus
Laboratory|Wellcome Sanger Institute for the COVID-19 Genomics UK (COG-UK)
consortium|Davies|United Kingdom
>Spike|hCoV-19/USA/MD-HP12067/2020|2020-11-25|EPI_ISL_791399|Original|hCoV-
19^^Maryland|Human|Johns Hopkins Hospital Department of Pathology|Johns Hopkins
Hospital Department of Pathology|Morris|USA
>Spike|hCoV-19/Japan/PG-1707/2020|2020-04-00|EPI_ISL_685313|Original|hCoV-
19^^Japan|Human|Pathogen Genomics Center|Pathogen Genomics Center|Sekizuka|Japan
>Spike|hCoV-19/USA/UT-UPHL-2012543435/2020|2020-08-13|EPI_ISL_746009|Original|hCoV-
19^^Utah|Human|Ginkgo Bioworks Clinical Laboratory|Utah Public Health
Laboratory|Young|USA
>Spike|hCoV-19/Australia/VIC2441/2020|2020-06-26|EPI_ISL_522147|Original|hCoV-
19^^Victoria|Human|Victorian Infectious Diseases Reference Laboratory (VIDRL)|VIDRL
and MDU-PHL|Schultz|Australia
>Spike|hCoV-19/England/CAMC-A3E861/2020|2020-10-04|EPI_ISL_609545|Original|hCoV-
19^^England|Human|Lighthouse Lab in Cambridge|Wellcome Sanger Institute for the
COVID-19 Genomics UK (COG-UK) consortium|Davies|United Kingdom
>Spike|hCoV-19/South Africa/KRISP-K002162/2020|2020-07-
08|EPI_ISL_515568|Original|hCoV-19^^KwaZulu-Natal|Human|NHLS-IALCH|KRISP|de
Oliveira|South Africa
>Spike|hCoV-19/USA/MI-MDHHS-SC21320/2020|2020-04-01|EPI_ISL_507575|Original|hCoV-
19^^Michigan|Human|Michigan Department of Health and Human Services|Michigan
Department of Health and Human Services|Blankenship|USA
>Spike|hCoV-19/USA/CA-CHLA-PLM61959420/2020|2020-11-30|EPI_ISL_753450|Original|hCoV-
19^^California|Human|Clinical virology Laboratory|Center for Personalized
Medicine|Dien Bard|USA
>Spike|hCoV-19/England/CAMC-B9C5E1/2020|2020-11-15|EPI_ISL_673245|Original|hCoV-
19^^England|Human|Lighthouse Lab in Cambridge|Wellcome Sanger Institute for the
COVID-19 Genomics UK (COG-UK) Consortium|Davies|United Kingdom
>Spike|hCoV-19/USA/ME-HETL-J0085/2020|2020-03-21|EPI_ISL_513495|Original|hCoV-
19^^Maine|Human|Maine HETL|Tewhey Lab|Tewhey|USA
>Spike|hCoV-19/USA/LA-GBCL-26513022/2020|2020-04-23|EPI_ISL_485076|Original|hCoV-
19^^Louisiana|Human|River Road Testing Lab|Ginkgo Bioworks Clinical
Laboratory|Smith|USA
>Spike|hCoV-19/England/ALDP-B24074/2020|2020-11-04|EPI_ISL_646283|Original|hCoV-
19^^England|Human|Lighthouse Lab in Alderley Park|Wellcome Sanger Institute for the
COVID-19 Genomics UK (COG-UK) Consortium|Davies|United Kingdom
>Spike|hCoV-19/Wales/PHWC-15C505/2020|2020-03-31|EPI_ISL_472499|Original|hCoV-
19^^Wales|Human|Wales Specialist Virology Centre Sequencing lab|COVID-19 Genomics UK
(COG-UK) Consortium|Moore|United Kingdom
>Spike|hCoV-19/England/QEUH-B11D64/2020|2020-11-02|EPI_ISL_642292|Original|hCoV-
19^^England|Human|Lighthouse Lab in Glasgow|Wellcome Sanger Institute for the COVID-
19 Genomics UK (COG-UK) Consortium|Davies|United Kingdom
>Spike|hCoV-19/Wales/MILK-B02CF0/2020|2020-10-24|EPI_ISL_647835|Original|hCoV-
19^^Wales|Human|Lighthouse Lab in Milton Keynes|Wellcome Sanger Institute for the
COVID-19 Genomics UK (COG-UK) Consortium|Davies|United Kingdom
>Spike|hCoV-19/South Korea/KCDC2080/2020|2020-02-21|EPI_ISL_497981|Original|hCoV-
19^^South Korea|Human|Division of Viral Diseases|Division of Viral Diseases|Kim|South
Korea

>Spike|hCoV-19/England/CAMC-A59280/2020|2020-10-08|EPI_ISL_597101|Original|hCoV-19^^England|Human|Lighthouse Lab in Cambridge|Wellcome Sanger Institute for the COVID-19 Genomics UK (COG-UK) consortium|Davies|United Kingdom
>Spike|hCoV-19/USA/OR-OHSU-0347/2020|2020-05-17|EPI_ISL_509112|Original|hCoV-19^^Oregon|Human|OHSU Lab Services Molecular Microbiology Lab|Oregon SARS-CoV-2 Genome Sequencing Center|O'Roak|USA
>Spike|hCoV-19/England/CAMB-73F74/2020|2020-03-20|EPI_ISL_440346|Original|hCoV-19^^England|Human|Department of Pathology|Wellcome Sanger Institute for the COVID-19 Genomics UK (COG-UK) consortium|Amato|United Kingdom
>Spike|hCoV-19/USA/CT-UW-2465/2020|2020-03-29|EPI_ISL_485921|Original|hCoV-19^^Connecticut|Human|UW Virology Lab|UW Virology Lab|Roychoudhury|USA
>Spike|hCoV-19/Scotland/CVR5641/2020|2020-10-20|EPI_ISL_651884|Original|hCoV-19^^Scotland|Human|West of Scotland Specialist Virology Centre|COVID-19 Genomics UK (COG-UK) Consortium|Robertson|United Kingdom
>Spike|hCoV-19/England/BRIS-12D682/2020|2020-05-08|EPI_ISL_481930|Original|hCoV-19^^England|Human|PHE South West Regional Laboratory|Wellcome Sanger Institute for the COVID-19 Genomics UK (COG-UK) consortium|Davies|United Kingdom
>Spike|hCoV-19/USA/AZ-TG648608/2020|2020-04-27|EPI_ISL_695855|Original|hCoV-19^^Arizona|Human|Sonora Quest Laboratories|TGen North|French|USA
>Spike|hCoV-19/England/BIRM-63821/2020|2020-08-19|EPI_ISL_529368|Original|hCoV-19^^England|Human|University of Birmingham|COVID-19 Genomics UK (COG-UK) Consortium|Loman|United Kingdom
>Spike|hCoV-19/England/MILK-B8F2F9/2020|2020-11-13|EPI_ISL_675305|Original|hCoV-19^^England|Human|Lighthouse Lab in Milton Keynes|Wellcome Sanger Institute for the COVID-19 Genomics UK (COG-UK) Consortium|Davies|United Kingdom
>Spike|hCoV-19/England/ALDP-A95859/2020|2020-10-20|EPI_ISL_624802|Original|hCoV-19^^England|Human|Lighthouse Lab in Alderley Park|Wellcome Sanger Institute for the COVID-19 Genomics UK (COG-UK) consortium|Davies|United Kingdom
>Spike|hCoV-19/Italy/CAM-INTPas-9/2020|2020-00-00|EPI_ISL_494763|Original|hCoV-19^^Campania|Human|INT Fondazione Pascale|INT Fondazione Pascale|Rachiglio|Italy
>Spike|hCoV-19/USA/LA-EVTL896/2020|2020-07-08|EPI_ISL_653587|Original|hCoV-19^^Louisiana|Human|LSUHS Emerging Viral Threat Laboratory|Microbial Genome Sequencing Center|Kamil|USA
>Spike|hCoV-19/USA/WA-S2704/2020|2020-06-22|EPI_ISL_497336|Original|hCoV-19^^Washington|Human|Washington State Department of Health|Seattle Flu Study|Lee|USA
>Spike|hCoV-19/India/MH-ACTREC-461/2020|2020-08-10|EPI_ISL_700109|Original|hCoV-19^^Maharashtra|Human|Hematopathology Laboratory|Hematopathology Laboratory|Patkar|India
>Spike|hCoV-19/USA/TX-HMH-6487/2020|2020-06-27|EPI_ISL_546031|Original|hCoV-19^^Texas|Human|Houston Methodist Hospital|Houston Methodist Hospital|Long|USA
>Spike|hCoV-19/England/CAMB-1BD60F/2020|2020-12-02|EPI_ISL_764322|Original|hCoV-19^^England|Human|Department of Pathology|COVID-19 Genomics UK (COG-UK) Consortium|Goodfellow|United Kingdom
>Spike|hCoV-19/England/NORT-29F5F6/2020|2020-04-30|EPI_ISL_490492|Original|hCoV-19^^England|Human|Northumbria University / South Tees Hospitals NHS Foundation Trust / North Cumbria Integrated Care NHS Foundation Trust / North Tees and Hartlepool NHS Foundation Trust / Newcastle Hospitals NHS Foundation Trust|COVID-19 Genomics UK (COG-UK) Consortium|Bashton|United Kingdom
>Spike|hCoV-19/England/ALDP-954462/2020|2020-06-13|EPI_ISL_553788|Original|hCoV-19^^England|Human|Lighthouse Lab in Alderley Park|Wellcome Sanger Institute for the COVID-19 Genomics UK (COG-UK) consortium|Davies|United Kingdom
>Spike|hCoV-19/USA/CA-CZB-1203/2020|2020-03-25|EPI_ISL_450452|Original|hCoV-19^^California|Human|Stanford clinical virology lab|Chan-Zuckerberg Biohub|Hao|USA
>Spike|hCoV-19/England/ALDP-AA3560/2020|2020-10-22|EPI_ISL_623943|Original|hCoV-19^^England|Human|Lighthouse Lab in Alderley Park|Wellcome Sanger Institute for the COVID-19 Genomics UK (COG-UK) consortium|Davies|United Kingdom
>Spike|hCoV-19/Switzerland/ZH-ETHZ-420606/2020|2020-12-22|EPI_ISL_768311|Original|hCoV-19^^Zurich|Human|Viollier AG|Department of Biosystems Science and Engineering|Nadeau|Switzerland

>Spike|hCoV-19/Spain/PV-IBV-000714/2020|2020-03-05|EPI_ISL_452754|Original|hCoV-19^^Basque Country|Human|Hospital Universitario Araba. Vitoria-Gasteiz,|SeqCOVID-SPAIN consortium/IBV(CSIC)|Comas|Spain
>Spike|hCoV-19/England/LIVE-AAEA4/2020|2020-07-28|EPI_ISL_540623|Original|hCoV-19^^England|Human|Liverpool Clinical Laboratories|COVID-19 Genomics UK (COG-UK) Consortium|Haldenby|United Kingdom
>Spike|hCoV-19/USA/NY-NYUMC58/2020|2020-03-18|EPI_ISL_420573|Original|hCoV-19^^New York|Human|NYU Langone Health|Departments of Pathology and Medicine|Maurano|USA
>Spike|hCoV-19/USA/TX-HMH-MCoV-19437/2020|2020-11-30|EPI_ISL_783839|Original|hCoV-19^^Texas|Human|Houston Methodist Hospital|Houston Methodist Hospital|Long|USA
>Spike|hCoV-19/Gibraltar/205000642/2020|2020-12-00|EPI_ISL_709977|Original|hCoV-19^^Gibraltar|Human|Microbiology|Respiratory Virus Unit|Platt|Gibraltar
>Spike|hCoV-19/USA/TX-DSHS-0457/2020|2020-06-18|EPI_ISL_541990|Original|hCoV-19^^Texas|Human|Texas Department of State Health Services|Texas Department of State Health Services|Rahman|USA
>Spike|hCoV-19/USA/TX-HMH-MCoV-12307/2020|2020-09-25|EPI_ISL_790292|Original|hCoV-19^^Texas|Human|Houston Methodist Hospital|Houston Methodist Hospital|Long|USA
>Spike|hCoV-19/USA/TX-HMH-4798/2020|2020-06-22|EPI_ISL_544337|Original|hCoV-19^^Texas|Human|Houston Methodist Hospital|Houston Methodist Hospital|Long|USA
>Spike|hCoV-19/USA/MI-MDHHS-SC20932/2020|2020-03-23|EPI_ISL_507722|Original|hCoV-19^^Michigan|Human|Michigan Department of Health and Human Services|Michigan Department of Health and Human Services|Blankenship|USA
>Spike|hCoV-19/Iceland/363/2020|2020-03-19|EPI_ISL_424387|Original|hCoV-19^^Reykjavik|Human|The National University Hospital of Iceland|deCODE genetics|J\u00f3nsson|Iceland
>Spike|hCoV-19/Austria/CeMM0475/2020|2020-03-16|EPI_ISL_475880|Original|hCoV-19^^Austria|Human|Austrian Agency for Health and Food Safety (AGES)|Bergthaler laboratory|Bergthaler|Austria
>Spike|hCoV-19/England/ALDP-C8EEC8/2020|2020-12-09|EPI_ISL_777169|Original|hCoV-19^^England|Human|Lighthouse Lab in Alderley Park|Wellcome Sanger Institute for the COVID-19 Genomics UK (COG-UK) Consortium|Davies|United Kingdom
>Spike|hCoV-19/Belgium/UGent-138/2020|2020-04-27|EPI_ISL_481230|Original|hCoV-19^^Ghent|Human|Lab voor klinische biologie|Onderzoeksgroep Virologie|Lambrechts|Belgium
>Spike|hCoV-19/Denmark/DCGC-16515/2020|2020-11-16|EPI_ISL_714809|Original|hCoV-19^^Hovedstaden|Human|Department of Virus and Microbiological Special Diagnostics|Albertsen Lab|Michaelsen|Denmark
>Spike|hCoV-19/USA/WA-S973/2020|2020-05-01|EPI_ISL_463463|Original|hCoV-19^^Washington|Human|Washington State Department of Health|Seattle Flu Study|Bedford|USA
>Spike|hCoV-19/Denmark/DCGC-19424/2020|2020-12-07|EPI_ISL_748936|Original|hCoV-19^^Sjaelland|Human|Department of Virus and Microbiological Special Diagnostics|Albertsen Lab|Michaelsen|Denmark
>Spike|hCoV-19/England/CAMC-B22C90/2020|2020-11-02|EPI_ISL_647335|Original|hCoV-19^^England|Human|Lighthouse Lab in Cambridge|Wellcome Sanger Institute for the COVID-19 Genomics UK (COG-UK) Consortium|Davies|United Kingdom
>Spike|hCoV-19/Canada/BC_20847524/2020|2020-03-00|EPI_ISL_462782|Original|hCoV-19^^British Columbia|Human|BCCDC Public Health Laboratory|BCCDC Public Health Laboratory|Harrigan|Canada
>Spike|hCoV-19/Wales/PHWC-166702/2020|2020-06-13|EPI_ISL_494031|Original|hCoV-19^^Wales|Human|Originating lab|COVID-19 Genomics UK (COG-UK) Consortium|Moore|United Kingdom
>Spike|hCoV-19/Japan/PG-1613/2020|2020-04-00|EPI_ISL_685219|Original|hCoV-19^^Japan|Human|Pathogen Genomics Center|Pathogen Genomics Center|Sekizuka|Japan
>Spike|hCoV-19/England/LIVE-DB6251/2020|2020-11-06|EPI_ISL_650560|Original|hCoV-19^^England|Human|Liverpool Clinical Laboratories|COVID-19 Genomics UK (COG-UK) Consortium|Haldenby|United Kingdom
>Spike|hCoV-19/USA/MI-MDHHS-SC22621/2020|2020-11-23|EPI_ISL_737194|Original|hCoV-19^^Michigan|Human|Michigan Department of Health and Human Services|Michigan Department of Health and Human Services|Blankenship|USA

>Spike|hCoV-19/Denmark/DCGC-2566/2020|2020-08-10|EPI_ISL_617781|Original|hCoV-19^^Midtjylland|Human|Department of Virus and Microbiological Special Diagnostics|Albertsen lab|Michaelsen|Denmark
>Spike|hCoV-19/USA/WI-UW-213/2020|2020-03-25|EPI_ISL_428309|Original|hCoV-19^^Wisconsin|Human|University of Wisconsin-Madison AIDS Vaccine Research Laboratories|University of Wisconsin-Madison AIDS Vaccine Research Laboratories|Moreno|USA
>Spike|hCoV-19/England/CAMC-9B1D1D/2020|2020-09-04|EPI_ISL_566519|Original|hCoV-19^^England|Human|Lighthouse Lab in Cambridge|Wellcome Sanger Institute for the COVID-19 Genomics UK (COG-UK) consortium|Davies|United Kingdom
>Spike|hCoV-19/Egypt/CUNCI-HGC4I029/2020|2020-06-02|EPI_ISL_479701|Original|hCoV-19^^Egypt|Human|Egyptian National Cancer Institute (ENCI)|Egyptian National Cancer Institute (ENCI)|Zekri|Egypt
>Spike|hCoV-19/Japan/PG-2208/2020|2020-04-00|EPI_ISL_685813|Original|hCoV-19^^Japan|Human|Pathogen Genomics Center|Pathogen Genomics Center|Sekizuka|Japan
>Spike|hCoV-19/Singapore/1019/2020|2020-03-23|EPI_ISL_574504|Original|hCoV-19^^Singapore|Human|National Public Health Laboratory|National Public Health Laboratory|Mak|Singapore
>Spike|hCoV-19/England/ALDP-9FD850/2020|2020-10-02|EPI_ISL_587810|Original|hCoV-19^^England|Human|Lighthouse Lab in Alderley Park|Wellcome Sanger Institute for the COVID-19 Genomics UK (COG-UK) consortium|Davies|United Kingdom
>Spike|hCoV-19/Netherlands/NB-EMC-188/2020|2020-00-00|EPI_ISL_523311|Original|hCoV-19^^Noord Brabant|Human|Dutch COVID-19 response team|Erasmus Medical Center|Oude Munnink|Netherlands
>Spike|hCoV-19/India/TG-CCMB-X94/2020|2020-07-14|EPI_ISL_528869|Original|hCoV-19^^Telangana|Human|CSIR-Centre for Cellular and Molecular Biology|CSIR-Centre for Cellular and Molecular Biology|Banu|India
>Spike|hCoV-19/Belgium/GC-0328313/2020|2020-03-28|EPI_ISL_462167|Original|hCoV-19^^Binche|Human|KU Leuven|KU Leuven|Maes|Belgium
>Spike|hCoV-19/USA/NY-NYCPHL-000595/2020|2020-03-10|EPI_ISL_632053|Original|hCoV-19^^New York|Human|New York Presbyterian Queens|New York City Public Health Laboratory|Wang|USA
>Spike|hCoV-19/Peru/AMZ-UPCH-0257/2020|2020-09-28|EPI_ISL_729903|Original|hCoV-19^^Amazonas|Human|Instituto de Medicina Tropical|Laboratorio de Gen\u00f3mica Microbiana|Tsukayama|Peru
>Spike|hCoV-19/Hungary/US-60432w/2020|2020-10-02|EPI_ISL_677807|Original|hCoV-19^^B\u00e9k\u00e9s county|Human|University of Szeged|National Laboratory of Virology|Kemenesi|Hungary
>Spike|hCoV-19/USA/WA-UW-1828/2020|2020-03-22|EPI_ISL_424269|Original|hCoV-19^^Washington|Human|UW Virology Lab|UW Virology Lab|Roychoudhury|USA
>Spike|hCoV-19/England/MILK-995993/2020|2020-08-23|EPI_ISL_551680|Original|hCoV-19^^England|Human|Lighthouse Lab in Milton Keynes|Wellcome Sanger Institute for the COVID-19 Genomics UK (COG-UK) consortium|Davies|United Kingdom
>Spike|hCoV-19/Scotland/EDB6157/2020|2020-05-14|EPI_ISL_473893|Original|hCoV-19^^Scotland|Human|Virology Department|COVID-19 Genomics UK (COG-UK) Consortium|Williams|United Kingdom
>Spike|hCoV-19/England/MILK-AC81E9/2020|2020-10-21|EPI_ISL_629486|Original|hCoV-19^^England|Human|Lighthouse Lab in Milton Keynes|Wellcome Sanger Institute for the COVID-19 Genomics UK (COG-UK) consortium|Davies|United Kingdom
>Spike|hCoV-19/England/ALDP-BB35C6/2020|2020-11-19|EPI_ISL_702001|Original|hCoV-19^^England|Human|Lighthouse Lab in Alderley Park|Wellcome Sanger Institute for the COVID-19 Genomics UK (COG-UK) Consortium|Davies|United Kingdom
>Spike|hCoV-19/Netherlands/NB-RIVM-20282/2020|2020-11-28|EPI_ISL_723422|Orignal|hCoV-19^^Noord-Brabant|Human|Dutch COVID-19 response team|National Institute for Public Health and the Environment (RIVM)|Meijer|Netherlands
>Spike|hCoV-19/England/20124097102/2020|2020-03-13|EPI_ISL_418734|Original|hCoV-19^^England|Human|Respiratory Virus Unit|Respiratory Virus Unit|Platt|United Kingdom
>Spike|hCoV-19/England/ALDP-9E5A19/2020|2020-09-24|EPI_ISL_589473|Original|hCoV-19^^England|Human|Lighthouse Lab in Alderley Park|Wellcome Sanger Institute for the COVID-19 Genomics UK (COG-UK) consortium|Davies|United Kingdom

>Spike|hCoV-19/Denmark/DCGC-22455/2020|2020-12-14|EPI_ISL_757418|Original|hCoV-19^^Hovedstaden|Human|Department of Virus and Microbiological Special Diagnostics|Albertsen Lab|Michaelsen|Denmark
>Spike|hCoV-19/England/ALDP-4A8570/2020|2020-06-11|EPI_ISL_559542|Original|hCoV-19^^England|Human|Lighthouse Lab in Alderley Park|Wellcome Sanger Institute for the COVID-19 Genomics UK (COG-UK) consortium|Davies|United Kingdom
>Spike|hCoV-19/USA/FL-BPHL-0942/2020|2020-07-17|EPI_ISL_517832|Original|hCoV-19^^Florida|Human|Florida Bureau of Public Health Laboratories|Florida Bureau of Public Health Laboratories|Blanton|USA
>Spike|hCoV-19/England/LOND-1264D59/2020|2020-11-23|EPI_ISL_724563|Original|hCoV-19^^England|Human|University College London|COVID-19 Genomics UK (COG-UK) Consortium|Roy|United Kingdom
>Spike|hCoV-19/Denmark/DCGC-6956/2020|2020-10-12|EPI_ISL_619802|Original|hCoV-19^^Syddanmark|Human|Department of Virus and Microbiological Special Diagnostics|Albertsen lab|Michaelsen|Denmark
>Spike|hCoV-19/Scotland/QEUH-AEFEF4/2020|2020-10-30|EPI_ISL_676454|Original|hCoV-19^^Scotland|Human|Lighthouse Lab in Glasgow|Wellcome Sanger Institute for the COVID-19 Genomics UK (COG-UK) Consortium|Davies|United Kingdom
>Spike|hCoV-19/England/QEUH-B6A4D0/2020|2020-11-11|EPI_ISL_662509|Original|hCoV-19^^England|Human|Lighthouse Lab in Glasgow|Wellcome Sanger Institute for the COVID-19 Genomics UK (COG-UK) Consortium|Davies|United Kingdom
>Spike|hCoV-19/India/KA-nimh-17585/2020|2020-05-12|EPI_ISL_515963|Original|hCoV-19^^Karnataka|Human|VICTORIA HOSPITAL|Department of Neurovirology|Pattabiraman|India
>Spike|hCoV-19/England/CAMC-B33476/2020|2020-11-05|EPI_ISL_659115|Original|hCoV-19^^England|Human|Lighthouse Lab in Cambridge|Wellcome Sanger Institute for the COVID-19 Genomics UK (COG-UK) Consortium|Davies|United Kingdom
>Spike|hCoV-19/England/MILK-9589BA/2020|2020-06-15|EPI_ISL_554690|Original|hCoV-19^^England|Human|Lighthouse Lab in Milton Keynes|Wellcome Sanger Institute for the COVID-19 Genomics UK (COG-UK) consortium|Davies|United Kingdom
>Spike|hCoV-19/Wales/PHWC-485412/2020|2020-10-25|EPI_ISL_628182|Original|hCoV-19^^Wales|Human|Wales Specialist Virology Centre Sequencing lab|COVID-19 Genomics UK (COG-UK) Consortium|Moore|United Kingdom
>Spike|hCoV-19/Australia/VIC1711/2020|2020-05-19|EPI_ISL_456616|Original|hCoV-19^^Victoria|Human|Victorian Infectious Diseases Reference Laboratory (VIDRL)|Microbiological Diagnostic Unit Public Health Laboratory and Victorian Infectious Diseases Reference Laboratory|Schultz|Australia
>Spike|hCoV-19/Japan/PG-6415/2020|2020-07-00|EPI_ISL_689521|Original|hCoV-19^^Japan|Human|Pathogen Genomics Center|Pathogen Genomics Center|Sekizuka|Japan
>Spike|hCoV-19/Australia/TAS75/2020|2020-03-20|EPI_ISL_522746|Original|hCoV-19^^Tasmania|Human|Royal Hobart Hospital Microbiology Department|MDU-PHL|Schultz|Australia
>Spike|hCoV-19/England/ALDP-955BE4/2020|2020-06-12|EPI_ISL_555329|Original|hCoV-19^^England|Human|Lighthouse Lab in Alderley Park|Wellcome Sanger Institute for the COVID-19 Genomics UK (COG-UK) consortium|Davies|United Kingdom
>Spike|hCoV-19/England/LOND-1260713/2020|2020-03-17|EPI_ISL_516833|Original|hCoV-19^^England|Human|North West London Pathology|Wellcome Sanger Institute for the COVID-19 Genomics UK (COG-UK) consortium|Davies|United Kingdom
>Spike|hCoV-19/Australia/VIC15241/2020|2020-08-02|EPI_ISL_593214|Original|hCoV-19^^Victoria|Human|Microbiological Diagnostic Unit - Public Health Laboratory (MDU-PHL)|MDU-PHL|Schultz|Australia
>Spike|hCoV-19/Northern Ireland/NIRE-10892A/2020|2020-09-10|EPI_ISL_585218|Original|hCoV-19^^Northern Ireland|Human|Regional Virus Laboratory|COVID-19 Genomics UK (COG-UK) Consortium|Fairley|United Kingdom
>Spike|hCoV-19/England/CAMC-A80D25/2020|2020-10-13|EPI_ISL_610821|Original|hCoV-19^^England|Human|Lighthouse Lab in Cambridge|Wellcome Sanger Institute for the COVID-19 Genomics UK (COG-UK) consortium|Davies|United Kingdom
>Spike|hCoV-19/Latvia/073/2020|2020-07-10|EPI_ISL_501289|Original|hCoV-19^^Latvia|Human|Centr\u0101l\u0101 laboratorija|Latvian Biomedical Research and Study Centre|Zrelovs|Latvia

>Spike|hCoV-19/England/QEUH-9DC06E/2020|2020-09-21|EPI_ISL_601366|Original|hCoV-19^^England|Human|Lighthouse Lab in Glasgow|Wellcome Sanger Institute for the COVID-19 Genomics UK (COG-UK) Consortium|Davies|United Kingdom
>Spike|hCoV-19/Scotland/EDB7104/2020|2020-08-03|EPI_ISL_514530|Original|hCoV-19^^Scotland|Human|Virology Department|COVID-19 Genomics UK (COG-UK) Consortium|Williams|United Kingdom
>Spike|hCoV-19/Scotland/CVR6052/2020|2020-11-26|EPI_ISL_705718|Original|hCoV-19^^Scotland|Human|West of Scotland Specialist Virology Centre|COVID-19 Genomics UK (COG-UK) Consortium|Robertson|United Kingdom
>Spike|hCoV-19/USA/MI-MDHHS-SC21995/2020|2020-09-26|EPI_ISL_576218|Original|hCoV-19^^Michigan|Human|Michigan Department of Health and Human Services|Michigan Department of Health and Human Services|Blankenship|USA
>Spike|hCoV-19/England/NORW-EE34B/2020|2020-00-00|EPI_ISL_490530|Original|hCoV-19^^England|Human|Quadram Institute Bioscience|COVID-19 Genomics UK (COG-UK) Consortium|Alikhan|United Kingdom
>Spike|hCoV-19/USA/UT-08475/2020|2020-05-20|EPI_ISL_524245|Original|hCoV-19^^Utah|Human|Utah Public Health Laboratory|Utah Public Health Laboratory|Young|USA
>Spike|hCoV-19/Panama/335862/2020|2020-04-03|EPI_ISL_496822|Original|hCoV-19^^Panama West|Human|Gorgas Memorial Laboratory of Health Studies|Gorgas Memorial Laboratory of Health Studies|Martinez|Panama
>Spike|hCoV-19/Scotland/QEUH-A1EA70/2020|2020-10-01|EPI_ISL_598897|Original|hCoV-19^^Scotland|Human|Lighthouse Lab in Glasgow|Wellcome Sanger Institute for the COVID-19 Genomics UK (COG-UK) consortium|Davies|United Kingdom
>Spike|hCoV-19/England/ALDP-931735/2020|2020-06-21|EPI_ISL_552999|Original|hCoV-19^^England|Human|Lighthouse Lab in Alderley Park|Wellcome Sanger Institute for the COVID-19 Genomics UK (COG-UK) consortium|Davies|United Kingdom
>Spike|hCoV-19/Thailand/NIH-3083/2020|2020-03-00|EPI_ISL_515292|Vero P2|hCoV-19^^Bangkok|Human|Ramkhamhaeng Hospital|National Institute of Health. Department of medical Sciences|Phuygun|Thailand
>Spike|hCoV-19/USA/CA-ALSR-3505/2020|2020-09-17|EPI_ISL_636057|Original|hCoV-19^^California|Human|San Diego County Public Health Laboratory|Andersen lab at Scripps Research|Gangavarapu|USA
>Spike|hCoV-19/Netherlands/Gelderland_75/2020|2020-05-18|EPI_ISL_460974|Original|hCoV-19^^Gelderland|Human|Dutch COVID-19 response team|Erasmus Medical Center|Oude Munnink|Netherlands
>Spike|hCoV-19/England/MILK-ACED0D/2020|2020-10-21|EPI_ISL_629325|Original|hCoV-19^^England|Human|Lighthouse Lab in Milton Keynes|Wellcome Sanger Institute for the COVID-19 Genomics UK (COG-UK) consortium|Davies|United Kingdom
>Spike|hCoV-19/USA/TX-HMH-1517/2020|2020-05-01|EPI_ISL_542767|Original|hCoV-19^^Texas|Human|Houston Methodist Hospital|Houston Methodist Hospital|Long|USA
>Spike|hCoV-19/England/ALDP-502652/2020|2020-06-25|EPI_ISL_559101|Original|hCoV-19^^England|Human|Lighthouse Lab in Alderley Park|Wellcome Sanger Institute for the COVID-19 Genomics UK (COG-UK) consortium|Davies|United Kingdom
>Spike|hCoV-19/Morocco/6903/2020|2020-04-19|EPI_ISL_459981|Original|hCoV-19^^Morocco|Human|Institut Pasteur du Maroc|Institut Pasteur du Maroc|Enouf|Morocco
>Spike|hCoV-19/USA/TX-HMH-MCoV-13203/2020|2020-08-26|EPI_ISL_788354|Original|hCoV-19^^Texas|Human|Houston Methodist Hospital|Houston Methodist Hospital|Long|USA
>Spike|hCoV-19/USA/CA-CZB-1572/2020|2020-04-12|EPI_ISL_468504|Original|hCoV-19^^California|Human|Ventura County Public Health Lab|Chan-Zuckerberg Biohub|Hao|USA
>Spike|hCoV-19/Germany/HH-hpi-p1870/2020|2020-03-23|EPI_ISL_775675|Original|hCoV-19^^Hamburg|Human|University Medical Center Hamburg Eppendorf|Heinrich Pette Institute|Robitaille|Germany
>Spike|hCoV-19/England/CAMC-991171/2020|2020-08-27|EPI_ISL_551874|Original|hCoV-19^^England|Human|Lighthouse Lab in Cambridge|Wellcome Sanger Institute for the COVID-19 Genomics UK (COG-UK) consortium|Davies|United Kingdom
>Spike|hCoV-19/USA/CA-CZB-14424/2020|2020-11-05|EPI_ISL_739606|Original|hCoV-19^^California|Human|Alameda County Public Health Lab|Chan-Zuckerberg Biohub|Kamm|USA
>Spike|hCoV-19/Spain/GA-IBV-002750/2020|2020-04-13|EPI_ISL_467282|Original|hCoV-19^^Galicia|Human|Hospital Cl\u00ednico Universitario de Santiago de Compostela|SeqCOVID-SPAIN consortium/IBV(CSIC)|Comas|Spain

>Spike|hCoV-19/England/OXON-B3B47/2020|2020-03-23|EPI_ISL_534903|Original|hCoV-19^^England|Human|Oxford Viromics|COVID-19 Genomics UK (COG-UK) Consortium|Golubchik|United Kingdom
>Spike|hCoV-19/USA/CA-CZB-13814/2020|2020-08-17|EPI_ISL_739303|Original|hCoV-19^^California|Human|Alameda County Public Health Lab|Chan-Zuckerberg Biohub|Kamm|USA
>Spike|hCoV-19/England/LIVE-DBEAD9/2020|2020-10-29|EPI_ISL_664652|Original|hCoV-19^^England|Human|Liverpool Clinical Laboratories|COVID-19 Genomics UK (COG-UK) Consortium|Haldenby|United Kingdom
>Spike|hCoV-19/England/LIVE-DB4A5B/2020|2020-10-24|EPI_ISL_651391|Original|hCoV-19^^England|Human|Liverpool Clinical Laboratories|COVID-19 Genomics UK (COG-UK) Consortium|Haldenby|United Kingdom
>Spike|hCoV-19/England/MILK-BFD5EE/2020|2020-12-03|EPI_ISL_720769|Original|hCoV-19^^England|Human|Lighthouse Lab in Milton Keynes|Wellcome Sanger Institute for the COVID-19 Genomics UK (COG-UK) Consortium|Davies|United Kingdom
>Spike|hCoV-19/Wales/PHWC-31BD8/2020|2020-04-12|EPI_ISL_446541|Original|hCoV-19^^Wales|Human|Wales Specialist Virology Centre|Public Health Wales Microbiology Cardiff|Moore|United Kingdom
>Spike|hCoV-19/England/QEUH-9C8399/2020|2020-09-11|EPI_ISL_567008|Original|hCoV-19^^England|Human|Lighthouse Lab in Glasgow|Wellcome Sanger Institute for the COVID-19 Genomics UK (COG-UK) consortium|Davies|United Kingdom
>Spike|hCoV-19/England/CAMC-BBCF47/2020|2020-11-18|EPI_ISL_704502|Original|hCoV-19^^England|Human|Department of Pathology|COVID-19 Genomics UK (COG-UK) Consortium|Goodfellow|United Kingdom
>Spike|hCoV-19/England/CAMB-1B8F72/2020|2020-11-02|EPI_ISL_651694|Original|hCoV-19^^England|Human|Department of Pathology|COVID-19 Genomics UK (COG-UK) Consortium|Goodfellow|United Kingdom
>Spike|hCoV-19/England/MILK-74A838/2020|2020-06-30|EPI_ISL_557435|Original|hCoV-19^^England|Human|Lighthouse Lab in Milton Keynes|Wellcome Sanger Institute for the COVID-19 Genomics UK (COG-UK) consortium|Davies|United Kingdom
>Spike|hCoV-19/USA/DE-DHSS-F917736/2020|2020-03-24|EPI_ISL_561346|Original|hCoV-19^^Delaware|Human|Delaware Public Health Lab|Delaware Public Health Lab|Bajwa|USA
>Spike|hCoV-19/England/QEUH-A9EDBB/2020|2020-10-20|EPI_ISL_611467|Original|hCoV-19^^England|Human|Lighthouse Lab in Glasgow|Wellcome Sanger Institute for the COVID-19 Genomics UK (COG-UK) consortium|Davies|United Kingdom
>Spike|hCoV-19/England/QEUH-BA2CA7/2020|2020-11-18|EPI_ISL_672802|Original|hCoV-19^^England|Human|Lighthouse Lab in Glasgow|Wellcome Sanger Institute for the COVID-19 Genomics UK (COG-UK) Consortium|Davies|United Kingdom
>Spike|hCoV-19/USA/TX-HMH-7353/2020|2020-06-29|EPI_ISL_546698|Original|hCoV-19^^Texas|Human|Houston Methodist Hospital|Houston Methodist Hospital|Long|USA
>Spike|hCoV-19/Spain/AN-IBV-001064/2020|2020-04-01|EPI_ISL_452612|Original|hCoV-19^^Andalucia|Human|Servicio de Microbiolog\u00eda y Parasitolog\u00eda cl\u00ednica. UCEIMP. Hospital Universitario Virgen del Roc\u00edo/IBIS/CSIC/US.|SeqCOVID-SPAIN consortium/IBV(CSIC)|Comas|Spain
>Spike|hCoV-19/Colombia/SAP-INS-SPSAN02/2020|2020-12-28|EPI_ISL_791088|Original|hCoV-19^^San Andres|Human|Instituto Nacional de Salud - Unidad de Secuenciaci\u00f3n y An\u00e1lisis Gen\u00f3mico|Instituto Nacional de Salud - Direcci\u00f3n de Investigaci\u00f3n en Salud P\u00fablica|D\u00edaz|Colombia
>Spike|hCoV-19/Netherlands/OV-EMC-97/2020|2020-09-09|EPI_ISL_722436|Original|hCoV-19^^Overijssel|Human|Dutch COVID-19 response team|Erasmus Medical Center|Oude Munnink|Netherlands
>Spike|hCoV-19/USA/TX-HMH-MCoV-9686/2020|2020-07-06|EPI_ISL_780225|Original|hCoV-19^^Texas|Human|Houston Methodist Hospital|Houston Methodist Hospital|Long|USA
>Spike|hCoV-19/England/QEUH-B67DAB/2020|2020-11-11|EPI_ISL_655133|Original|hCoV-19^^England|Human|Lighthouse Lab in Glasgow|Wellcome Sanger Institute for the COVID-19 Genomics UK (COG-UK) Consortium|Davies|United Kingdom
>Spike|hCoV-19/Belgium/CJE-0325178/2020|2020-03-25|EPI_ISL_420435|Original|hCoV-19^^Colfontaine|Human|KU Leuven|KU Leuven|Maes|Belgium
>Spike|hCoV-19/Wales/PHWC-493C0A/2020|2020-11-17|EPI_ISL_725998|Original|hCoV-19^^Wales|Human|Originating lab|COVID-19 Genomics UK (COG-UK) Consortium|Moore|United Kingdom

>Spike|hCoV-19/England/ALDP-94F330/2020|2020-06-22|EPI_ISL_556489|Original|hCoV-19^^England|Human|Lighthouse Lab in Alderley Park|Wellcome Sanger Institute for the COVID-19 Genomics UK (COG-UK) consortium|Davies|United Kingdom
>Spike|hCoV-19/Portugal/PT1121/2020|2020-03-31|EPI_ISL_511668|Original|hCoV-19^^Portugal|Human|Instituto Nacional de Saude (INSA)|Instituto Nacional de Saude (INSA)|Borges|Portugal
>Spike|hCoV-19/England/QEUH-BA2D59/2020|2020-11-18|EPI_ISL_721063|Original|hCoV-19^^England|Human|Lighthouse Lab in Glasgow|Wellcome Sanger Institute for the COVID-19 Genomics UK (COG-UK) Consortium|Davies|United Kingdom
>Spike|hCoV-19/England/CAMC-A8234F/2020|2020-10-12|EPI_ISL_610834|Original|hCoV-19^^England|Human|Lighthouse Lab in Cambridge|Wellcome Sanger Institute for the COVID-19 Genomics UK (COG-UK) consortium|Davies|United Kingdom
>Spike|hCoV-19/Denmark/DCGC-13269/2020|2020-11-30|EPI_ISL_712158|Original|hCoV-19^^Hovedstaden|Human|Department of Virus and Microbiological Special Diagnostics|Albertsen Lab|Michaelsen|Denmark
>Spike|hCoV-19/Finland/S11BD1/2020|2020-09-22|EPI_ISL_732627|Original|hCoV-19^^Uusimaa|Human|Department of Virology and Immunology|Department of Virology|Smura|Finland
>Spike|hCoV-19/England/CAMB-7FC35/2020|2020-04-16|EPI_ISL_433494|Original|hCoV-19^^England|Human|Department of Pathology|COVID-19 Genomics UK (COG-UK) Consortium|Meredith|United Kingdom
>Spike|hCoV-19/France/IDF-8707/2020|2020-06-29|EPI_ISL_560642|Original|hCoV-19^^\u00cele-de-France|Human|Labo Analyses Med|National Reference Center for Viruses of Respiratory Infections|Enouf|France
>Spike|hCoV-19/Canada/ON-PHL-20-02222/2020|2020-09-00|EPI_ISL_671500|Original|hCoV-19^^Ontario|Human|Public Health Ontario Laboratory|Public Health Ontario Laboratory|Guthrie|Canada
>Spike|hCoV-19/England/CAMC-B21912/2020|2020-11-01|EPI_ISL_647273|Original|hCoV-19^^England|Human|Lighthouse Lab in Cambridge|Wellcome Sanger Institute for the COVID-19 Genomics UK (COG-UK) Consortium|Davies|United Kingdom
>Spike|hCoV-19/Scotland/EDB2983/2020|2020-04-02|EPI_ISL_487733|Original|hCoV-19^^Scotland|Human|Virology Department|Wellcome Sanger Institute for the COVID-19 Genomics UK (COG-UK) consortium|Davies|United Kingdom
>Spike|hCoV-19/Wales/MILK-973148/2020|2020-08-20|EPI_ISL_575811|Original|hCoV-19^^Wales|Human|Lighthouse Lab in Milton Keynes|Wellcome Sanger Institute for the COVID-19 Genomics UK (COG-UK) consortium|Davies|United Kingdom
>Spike|hCoV-19/Spain/MD-H12-62/2020|2020-04-12|EPI_ISL_529993|Original|hCoV-19^^Madrid|Human|Hospital Universitario 12 de Octubre|Hospital Universitario 12 de Octubre|Viedma|Spain
>Spike|hCoV-19/Denmark/DCGC-5749/2020|2020-09-28|EPI_ISL_621254|Original|hCoV-19^^Nordjylland|Human|Department of Virus and Microbiological Special Diagnostics|Albertsen lab|Michaelsen|Denmark
>Spike|hCoV-19/Netherlands/ZH-EMC-1223/2020|2020-12-17|EPI_ISL_763292|Original|hCoV-19^^Zuid Holland|Human|Dutch COVID-19 response team|Erasmus Medical Center|Oude Munnink|Netherlands
>Spike|hCoV-19/Denmark/DCGC-9270/2020|2020-11-16|EPI_ISL_668880|Original|hCoV-19^^Nordjylland|Human|Department of Virus and Microbiological Special Diagnostics|Albertsen Lab|Michaelsen|Denmark
>Spike|hCoV-19/Australia/VIC1948/2020|2020-06-09|EPI_ISL_480672|Original|hCoV-19^^Victoria|Human|Victorian Infectious Diseases Reference Laboratory (VIDRL)|VIDRL and MDU-PHL|Schultz|Australia
>Spike|hCoV-19/Wales/PHWC-25393/2020|2020-03-23|EPI_ISL_432190|Original|hCoV-19^^Wales|Human|Wales Specialist Virology Centre|Public Health Wales Microbiology Cardiff|Moore|United Kingdom
>Spike|hCoV-19/Scotland/QEUH-9E4EB4/2020|2020-09-24|EPI_ISL_601011|Original|hCoV-19^^Scotland|Human|Lighthouse Lab in Glasgow|Wellcome Sanger Institute for the COVID-19 Genomics UK (COG-UK) consortium|Davies|United Kingdom
>Spike|hCoV-19/Northern Ireland/NIRE-109051/2020|2020-10-13|EPI_ISL_652310|Original|hCoV-19^^Northern Ireland|Human|Regional Virus Laboratory|COVID-19 Genomics UK (COG-UK) Consortium|Fairley|United Kingdom

>Spike|hCoV-19/USA/MI-UM-10036762197/2020|2020-11-03|EPI_ISL_682221|Original|hCoV-
19^^Michigan|Human|University of Michigan Clinical Microbiology Laboratory|Lauring
Lab|Valesano|USA
>Spike|hCoV-19/England/CAMB-7277D/2020|2020-03-24|EPI_ISL_439638|Original|hCoV-
19^^England|Human|Department of Pathology|Wellcome Sanger Institute for the COVID-19
Genomics UK (COG-UK) consortium|Amato|United Kingdom
>Spike|hCoV-19/Wales/PHWC-1638C6/2020|2020-05-02|EPI_ISL_474012|Original|hCoV-
19^^Wales|Human|Originating lab|COVID-19 Genomics UK (COG-UK) Consortium|Moore|United
Kingdom
>Spike|hCoV-19/England/MILK-9A67FF/2020|2020-08-30|EPI_ISL_576094|Original|hCoV-
19^^England|Human|Lighthouse Lab in Milton Keynes|Wellcome Sanger Institute for the
COVID-19 Genomics UK (COG-UK) consortium|Davies|United Kingdom
>Spike|hCoV-19/USA/MI-QDX-501/2020|2020-03-24|EPI_ISL_571235|Original|hCoV-
19^^Michigan|Human|Quest Diagnostics|Quest Diagnostics|Kagan|USA
>Spike|hCoV-19/Denmark/DCGC-22547/2020|2020-12-14|EPI_ISL_757432|Original|hCoV-
19^^Syddanmark|Human|Department of Virus and Microbiological Special
Diagnostics|Albertsen Lab|Michaelsen|Denmark
>Spike|hCoV-19/Scotland/QEUH-943703/2020|2020-08-02|EPI_ISL_532620|Original|hCoV-
19^^Scotland|Human|NHSGGC West of Scotland Specialist Virology Centre / MRC-
University of Glasgow Centre for Virus Research|Wellcome Sanger Institute for the
COVID-19 Genomics UK (COG-UK) consortium|Davies|United Kingdom
>Spike|hCoV-19/Portugal/PT0700/2020|2020-03-17|EPI_ISL_510959|Original|hCoV-
19^^Portugal|Human|Instituto Nacional de Saude (INSA)|Instituto Nacional de Saude
(INSA)|Borges|Portugal
>Spike|hCoV-19/Denmark/DCGC-18608/2020|2020-12-07|EPI_ISL_750076|Original|hCoV-
19^^Midtjylland|Human|Department of Virus and Microbiological Special
Diagnostics|Albertsen Lab|Michaelsen|Denmark
>Spike|hCoV-19/Chile/RM-ISPCH-46/2020|2020-03-21|EPI_ISL_445325|Original|hCoV-
19^^Region Metropolitana de Santiago|Human|HOSPITAL DR.SOTERO DEL RIO|Instituto de
Salud Publica de Chile|Fernandez|Chile
>Spike|hCoV-19/England/MILK-4882BB/2020|2020-05-07|EPI_ISL_559688|Original|hCoV-
19^^England|Human|Lighthouse Lab in Milton Keynes|Wellcome Sanger Institute for the
COVID-19 Genomics UK (COG-UK) consortium|Davies|United Kingdom
>Spike|hCoV-19/Switzerland/VS-ETHZ-110010/2020|2020-04-
01|EPI_ISL_486450|Original|hCoV-19^^Valais|Human|Viollier AG|Department of Biosystems
Science and Engineering|Nadeau|Switzerland
>Spike|hCoV-19/Luxembourg/LNS9562893/2020|2020-12-29|EPI_ISL_771150|Original|hCoV-
19^^Luxembourg|Human|Laboratoire national de sant\u00e9|Laboratoire national de
sant\u00e9|Wienecke-Baldacchino|Luxembourg
>Spike|hCoV-19/England/BIRM-6A08C/2020|2020-11-09|EPI_ISL_679018|Original|hCoV-
19^^England|Human|University of Birmingham|COVID-19 Genomics UK (COG-UK)
Consortium|Loman|United Kingdom
>Spike|hCoV-19/England/BIRM-64EB5/2020|2020-09-24|EPI_ISL_594527|Original|hCoV-
19^^England|Human|University of Birmingham|COVID-19 Genomics UK (COG-UK)
Consortium|Loman|United Kingdom
>Spike|hCoV-19/Denmark/DCGC-8964/2020|2020-10-26|EPI_ISL_625870|Original|hCoV-
19^^Nordjylland|Human|Department of Virus and Microbiological Special
Diagnostics|Albertsen lab|Michaelsen|Denmark
>Spike|hCoV-19/USA/UT-UPHL-04858/2020|2020-05-23|EPI_ISL_575179|Original|hCoV-
19^^Utah|Human|Utah Public Health Laboratory|Utah Public Health Laboratory|Young|USA
>Spike|hCoV-19/Denmark/DCGC-9761/2020|2020-11-09|EPI_ISL_669154|Original|hCoV-
19^^Midtjylland|Human|Department of Virus and Microbiological Special
Diagnostics|Albertsen Lab|Michaelsen|Denmark
>Spike|hCoV-19/USA/TX-HMH-1155/2020|2020-04-27|EPI_ISL_542556|Original|hCoV-
19^^Texas|Human|Houston Methodist Hospital|Houston Methodist Hospital|Long|USA
>Spike|hCoV-19/Australia/VIC2187/2020|2020-06-23|EPI_ISL_521933|Original|hCoV-
19^^Victoria|Human|Microbiological Diagnostic Unit - Public Health Laboratory (MDU-
PHL)|MDU-PHL|Schultz|Australia

>Spike|hCoV-19/Denmark/DCGC-6704/2020|2020-04-20|EPI_ISL_622386|Original|hCoV-19^^Hovedstaden|Human|Department of Virus and Microbiological Special Diagnostics|Albertsen lab|Michaelsen|Denmark
>Spike|hCoV-19/Spain/AR-IBV-2432/2020|2020-03-24|EPI_ISL_468788|Original|hCoV-19^^Aragon|Human|Servicio de Microbiolog\u00eda|SeqCOVID-SPAIN consortium/IBV(CSIC)|Comas|Spain
>Spike|hCoV-19/Denmark/DCGC-20333/2020|2020-11-16|EPI_ISL_748118|Original|hCoV-19^^Sjaelland|Human|Department of Virus and Microbiological Special Diagnostics|Albertsen Lab|Michaelsen|Denmark
>Spike|hCoV-19/England/MILK-ACCA80/2020|2020-10-21|EPI_ISL_629411|Original|hCoV-19^^England|Human|Lighthouse Lab in Milton Keynes|Wellcome Sanger Institute for the COVID-19 Genomics UK (COG-UK) consortium|Davies|United Kingdom
>Spike|hCoV-19/England/MILK-A79BE9/2020|2020-10-15|EPI_ISL_606708|Original|hCoV-19^^England|Human|Lighthouse Lab in Milton Keynes|Wellcome Sanger Institute for the COVID-19 Genomics UK (COG-UK) consortium|Davies|United Kingdom
>Spike|hCoV-19/England/NORW-E8FB6/2020|2020-04-24|EPI_ISL_453571|Original|hCoV-19^^England|Human|Quadram Institute Bioscience|COVID-19 Genomics UK (COG-UK) Consortium|Alikhan|United Kingdom
>Spike|hCoV-19/Sweden/20-08533/2020|2020-03-27|EPI_ISL_476143|Original|hCoV-19^^Vastra Gotaland|Human|Skovde/Unilabs|The Public Health Agency of Sweden|Svartstr\u00f6m|Sweden
>Spike|hCoV-19/Switzerland/VD-ETHZ-280181/2020|2020-09-09|EPI_ISL_560520|Original|hCoV-19^^Vaud|Human|Viollier AG|Department of Biosystems Science and Engineering|Nadeau|Switzerland
>Spike|hCoV-19/Sweden/20-50194/2020|2020-03-05|EPI_ISL_455863|Original|hCoV-19^^Stockholm|Human|Karolinska Universitetslaboratoriet|The Public Health Agency of Sweden|Svartstr\u00f6m|Sweden
>Spike|hCoV-19/Japan/PG-7249/2020|2020-08-00|EPI_ISL_688352|Original|hCoV-19^^Japan|Human|Saitama Prefectural Institute of Public Health|Pathogen Genomics Center|Sekizuka|Japan
>Spike|hCoV-19/USA/MI-MDHHS-SC22159/2020|2020-10-12|EPI_ISL_614210|Original|hCoV-19^^Michigan|Human|Michigan Department of Health and Human Services|Michigan Department of Health and Human Services|Jacob|USA
>Spike|hCoV-19/England/ALDP-B23673/2020|2020-11-04|EPI_ISL_647111|Original|hCoV-19^^England|Human|Lighthouse Lab in Alderley Park|Wellcome Sanger Institute for the COVID-19 Genomics UK (COG-UK) Consortium|Davies|United Kingdom
>Spike|hCoV-19/Denmark/DCGC-20113/2020|2020-12-07|EPI_ISL_747991|Original|hCoV-19^^Syddanmark|Human|Department of Virus and Microbiological Special Diagnostics|Albertsen Lab|Michaelsen|Denmark
>Spike|hCoV-19/England/ALDP-B2397A/2020|2020-11-04|EPI_ISL_646914|Original|hCoV-19^^England|Human|Lighthouse Lab in Alderley Park|Wellcome Sanger Institute for the COVID-19 Genomics UK (COG-UK) Consortium|Davies|United Kingdom
>Spike|hCoV-19/Denmark/DCGC-1307/2020|2020-06-08|EPI_ISL_618090|Original|hCoV-19^^Nordjylland|Human|Department of Virus and Microbiological Special Diagnostics|Albertsen lab|Michaelsen|Denmark
>Spike|hCoV-19/Wales/PHWC-492245/2020|2020-11-17|EPI_ISL_704038|Original|hCoV-19^^Wales|Human|Originating lab|COVID-19 Genomics UK (COG-UK) Consortium|Moore|United Kingdom
>Spike|hCoV-19/USA/MA-MASPHL-00914/2020|2020-10-22|EPI_ISL_692834|Original|hCoV-19^^Massachusetts|Human|Massachusetts State Public Health Laboratory|Massachusetts State Public Health Laboratory|Lang|USA
>Spike|hCoV-19/Australia/VIC7214/2020|2020-07-11|EPI_ISL_564287|Original|hCoV-19^^Victoria|Human|Microbiological Diagnostic Unit - Public Health Laboratory (MDU-PHL)|MDU-PHL|Schultz|Australia
>Spike|hCoV-19/Denmark/DCGC-13016/2020|2020-11-30|EPI_ISL_712531|Original|hCoV-19^^Sjaelland|Human|Department of Virus and Microbiological Special Diagnostics|Albertsen Lab|Michaelsen|Denmark
>Spike|hCoV-19/England/MILK-699FBE/2020|2020-07-01|EPI_ISL_551744|Original|hCoV-19^^England|Human|Lighthouse Lab in Milton Keynes|Wellcome Sanger Institute for the COVID-19 Genomics UK (COG-UK) consortium|Davies|United Kingdom

>Spike|hCoV-19/USA/MI-UM-10036853496/2020|2020-11-09|EPI_ISL_728494|Original|hCoV-19^^Michigan|Human|University of Michigan Clinical Microbiology Laboratory|Lauring Lab|Valesano|USA
>Spike|hCoV-19/USA/FL-BPHL-051/2020|2020-04-17|EPI_ISL_436555|Original|hCoV-19^^Florida|Human|Florida Bureau of Public Health Laboratories|Florida Bureau of Public Health Laboratories|Blanton|USA
>Spike|hCoV-19/USA/NY-SURV0144/2020|2020-03-02|EPI_ISL_436050|Original|hCoV-19^^New York|Human|NYC Department of Health and Mental Hygiene|Pathogen Discovery|Queen|USA
>Spike|hCoV-19/Wales/PHWC-48AE4C/2020|2020-11-03|EPI_ISL_666164|Original|hCoV-19^^Wales|Human|Wales Specialist Virology Centre Sequencing lab|COVID-19 Genomics UK (COG-UK) Consortium|Moore|United Kingdom
>Spike|hCoV-19/England/CAMC-B20A7A/2020|2020-11-01|EPI_ISL_647547|Original|hCoV-19^^England|Human|Lighthouse Lab in Cambridge|Wellcome Sanger Institute for the COVID-19 Genomics UK (COG-UK) Consortium|Davies|United Kingdom
>Spike|hCoV-19/England/204290933/2020|2020-10-17|EPI_ISL_622862|Original|hCoV-19^^England|Human|Respiratory Virus Unit|Respiratory Virus Unit|Platt|United Kingdom
>Spike|hCoV-19/Scotland/EDB4636/2020|2020-05-08|EPI_ISL_478172|Original|hCoV-19^^Scotland|Human|Virology Department|COVID-19 Genomics UK (COG-UK) Consortium|Williams|United Kingdom
>Spike|hCoV-19/USA/MI-QDX-641/2020|2020-03-17|EPI_ISL_571366|Original|hCoV-19^^Michigan|Human|Quest Diagnostics|Quest Diagnostics|Kagan|USA
>Spike|hCoV-19/Wales/PHWC-24DB4/2020|2020-03-20|EPI_ISL_419511|Original|hCoV-19^^Wales|Human|Wales Specialist Virology Centre|Public Health Wales Microbiology Cardiff|Moore|United Kingdom
>Spike|hCoV-19/Sichuan/SC-WCH2-006/2020|2020-02-08|EPI_ISL_451076|Original|hCoV-19^^Sichuan|Human|West China Hospital of Sichuan University|State Key Laboratory of Biotherapy of Sichuan University|Tang|China
>Spike|hCoV-19/Germany/NW-HHU-85/2020|2020-08-17|EPI_ISL_539601|Original|hCoV-19^^North Rhine-Westphalia|Human|ZOTZ KLIMAS MVZ D\u00fcsseldorf-Centrum GbR \u00dcBAG f\u00fcr Labormedizin|Center of Medical Microbiology|Wienemann|Germany
>Spike|hCoV-19/England/MILK-BAC171/2020|2020-11-18|EPI_ISL_783144|Original|hCoV-19^^England|Human|Lighthouse Lab in Milton Keynes|Wellcome Sanger Institute for the COVID-19 Genomics UK (COG-UK) Consortium|Davies|United Kingdom
>Spike|hCoV-19/England/CAMC-A5951E/2020|2020-10-09|EPI_ISL_597021|Original|hCoV-19^^England|Human|Lighthouse Lab in Cambridge|Wellcome Sanger Institute for the COVID-19 Genomics UK (COG-UK) consortium|Davies|United Kingdom
>Spike|hCoV-19/USA/TX-HMH-5943/2020|2020-06-25|EPI_ISL_547110|Original|hCoV-19^^Texas|Human|Houston Methodist Hospital|Houston Methodist Hospital|Long|USA
>Spike|hCoV-19/Canada/ON-PHL-3680/2020|2020-03-00|EPI_ISL_418348|Original|hCoV-19^^Ontario|Human|Public Health Ontario Laboratories|Public Health Ontario Laboratories|Eshaghi|Canada
>Spike|hCoV-19/USA/FL-BPHL-0865/2020|2020-07-02|EPI_ISL_514211|Original|hCoV-19^^Florida|Human|Florida Bureau of Public Health Laboratories|Florida Bureau of Public Health Laboratories|Blanton|USA
>Spike|hCoV-19/England/ALDP-955258/2020|2020-06-12|EPI_ISL_553615|Original|hCoV-19^^England|Human|Lighthouse Lab in Alderley Park|Wellcome Sanger Institute for the COVID-19 Genomics UK (COG-UK) consortium|Davies|United Kingdom
>Spike|hCoV-19/Switzerland/BS-UHB-42173111/2020|2020-03-02|EPI_ISL_527920|Original|hCoV-19^^Basel-Stadt|Human|University Hospital Basel|University Hospital Basel|Roloff Handschin|Switzerland
>Spike|hCoV-19/England/ALDP-C3B774/2020|2020-12-11|EPI_ISL_731043|Original|hCoV-19^^England|Human|Lighthouse Lab in Alderley Park|Wellcome Sanger Institute for the COVID-19 Genomics UK (COG-UK) Consortium|Davies|United Kingdom
>Spike|hCoV-19/Suriname/SR-33/2020|2020-06-25|EPI_ISL_517644|Original|hCoV-19^^Suriname|Human|Academic Hospital Paramaribo|Erasmus Medical Center|M\u00fcnger|Suriname
>Spike|hCoV-19/India/DL-NCDC1895-CSIR-IGIB/2020|2020-05-18|EPI_ISL_482512|Original|hCoV-19^^Delhi|Human|National Centre for Disease control (NCDC)|NCDC/CSIR-IGIB|Pandey|India

>Spike|hCoV-19/USA/CA-CZB-3870/2020|2020-07-17|EPI_ISL_548576|Original|hCoV-
19^^California|Human|County of Santa Clara Public Health Department|Chan-Zuckerberg
Biohub|Bhatt|USA
>Spike|hCoV-19/Austria/CeMM0829/2020|2020-04-15|EPI_ISL_583724|Original|hCoV-
19^^Austria|Human|Center for Virology|Bergthaler laboratory|Bergthaler|Austria
>Spike|hCoV-19/Denmark/DCGC-12416/2020|2020-11-16|EPI_ISL_682597|Original|hCoV-
19^^Hovedstaden|Human|Department of Virus and Microbiological Special
Diagnostics|Albertsen Lab|Michaelsen|Denmark
>Spike|hCoV-19/England/MILK-A6B731/2020|2020-10-09|EPI_ISL_608836|Original|hCoV-
19^^England|Human|Lighthouse Lab in Milton Keynes|Wellcome Sanger Institute for the
COVID-19 Genomics UK (COG-UK) consortium|Davies|United Kingdom
>Spike|hCoV-19/Japan/PG-3912/2020|2020-06-00|EPI_ISL_692163|Original|hCoV-
19^^Japan|Human|Tokyo Metropolitan Institute of Public Health|Pathogen Genomics
Center|Sekizuka|Japan
>Spike|hCoV-19/England/ALDP-CECD43/2020|2020-12-21|EPI_ISL_781348|Original|hCoV-
19^^England|Human|Lighthouse Lab in Alderley Park|Wellcome Sanger Institute for the
COVID-19 Genomics UK (COG-UK) Consortium|Davies|United Kingdom
>Spike|hCoV-19/Colombia/VAC-INS-79930/2020|2020-03-14|EPI_ISL_456137|Original|hCoV-
19^^Valle del Cauca|Human|Instituto Nacional de Salud - Unidad de Secuenciaci\u00f3n
y An\u00e1lisis Gen\u00f3mico|Instituto Nacional de Salud|D\u00edaz|Colombia
>Spike|hCoV-19/England/QEUH-BC3718/2020|2020-11-25|EPI_ISL_704085|Original|hCoV-
19^^England|Human|Lighthouse Lab in Glasgow|Wellcome Sanger Institute for the COVID-
19 Genomics UK (COG-UK) Consortium|Davies|United Kingdom
>Spike|hCoV-19/Australia/VIC17876/2020|2020-08-21|EPI_ISL_779524|Original|hCoV-
19^^Victoria|Human|Microbiological Diagnostic Unit - Public Health Laboratory (MDU-
PHL)|MDU-PHL|Beck|Australia
>Spike|hCoV-19/Denmark/DCGC-22138/2020|2020-12-14|EPI_ISL_758107|Original|hCoV-
19^^Hovedstaden|Human|Department of Virus and Microbiological Special
Diagnostics|Albertsen Lab|Michaelsen|Denmark
>Spike|hCoV-19/Singapore/1153/2020|2020-03-19|EPI_ISL_648067|Original|hCoV-
19^^Singapore|Human|Department of Laboratory Medicine|Department of Laboratory
Medicine|Chen|Singapore
>Spike|hCoV-19/Scotland/CVR5216/2020|2020-10-15|EPI_ISL_626687|Original|hCoV-
19^^Scotland|Human|West of Scotland Specialist Virology Centre|COVID-19 Genomics UK
(COG-UK) Consortium|Robertson|United Kingdom
>Spike|hCoV-19/England/QEUH-A005DA/2020|2020-10-02|EPI_ISL_584891|Original|hCoV-
19^^England|Human|University of Exeter|COVID-19 Genomics UK (COG-UK)
Consortium|Temperton|United Kingdom
>Spike|hCoV-19/USA/MI-MDHHS-SC22029/2020|2020-09-14|EPI_ISL_577585|Original|hCoV-
19^^Michigan|Human|Michigan Department of Health and Human Services|Michigan
Department of Health and Human Services|Jacob|USA
>Spike|hCoV-19/England/QEUH-89C2DE/2020|2020-07-27|EPI_ISL_532688|Original|hCoV-
19^^England|Human|Lighthouse Lab in Glasgow|Wellcome Sanger Institute for the COVID-
19 Genomics UK (COG-UK) consortium|Davies|United Kingdom
>Spike|hCoV-19/USA/UT-UPHL-2012575394/2020|2020-08-03|EPI_ISL_745767|Original|hCoV-
19^^Utah|Human|Ginkgo Bioworks Clinical Laboratory|Utah Public Health
Laboratory|Young|USA
>Spike|hCoV-19/England/CAMB-A98434/2020|2020-12-26|EPI_ISL_764344|Original|hCoV-
19^^England|Human|Department of Pathology|COVID-19 Genomics UK (COG-UK)
Consortium|Goodfellow|United Kingdom
>Spike|hCoV-19/England/SHEF-D125D/2020|2020-04-23|EPI_ISL_484514|Original|hCoV-
19^^England|Human|Virology Department|COVID-19 Genomics UK (COG-UK) Consortium|de
Silva|United Kingdom
>Spike|hCoV-19/Wales/PHWC-261D4/2020|2020-03-26|EPI_ISL_422316|Original|hCoV-
19^^Wales|Human|Wales Specialist Virology Centre|Public Health Wales Microbiology
Cardiff|Moore|United Kingdom
>Spike|hCoV-19/England/ALDP-9C5736/2020|2020-09-10|EPI_ISL_567794|Original|hCoV-
19^^England|Human|Lighthouse Lab in Alderley Park|Wellcome Sanger Institute for the
COVID-19 Genomics UK (COG-UK) consortium|Davies|United Kingdom

>Spike|hCoV-19/New Zealand/20CV0611/2020|2020-11-28|EPI_ISL_682289|Original|hCoV-19^^Counties Manukau|Human|Middlemore Hospital|Institute of Environmental Science and Research (ESR)|Wang|New Zealand
>Spike|hCoV-19/Wales/PHWC-48978E/2020|2020-11-03|EPI_ISL_639615|Original|hCoV-19^^Wales|Human|Wales Specialist Virology Centre Sequencing lab|COVID-19 Genomics UK (COG-UK) Consortium|Moore|United Kingdom
>Spike|hCoV-19/USA/UT-UPHL-201211132/2020|2020-04-06|EPI_ISL_678509|Original|hCoV-19^^Utah|Human|Utah Public Health Laboratory|Utah Public Health Laboratory|Young|USA
>Spike|hCoV-19/England/QEUH-9D19E6/2020|2020-09-14|EPI_ISL_573674|Original|hCoV-19^^England|Human|University College London|COVID-19 Genomics UK (COG-UK) Consortium|Roy|United Kingdom
>Spike|hCoV-19/Brazil/RJ-00440/2020|2020-06-03|EPI_ISL_717835|Original|hCoV-19^^Rio de Janeiro|Human|LACEN Dr. Francisco Rimolo Neto|Bioinformatics Laboratory / LNCC|Almeida|Brazil
>Spike|hCoV-19/USA/TX-DSHS-1376/2020|2020-05-11|EPI_ISL_654740|Original|hCoV-19^^Texas|Human|Texas Department of State Health Services|Texas Department of State Health Services|Rahman|USA
>Spike|hCoV-19/Wales/PHWC-2A440/2020|2020-04-01|EPI_ISL_445568|Original|hCoV-19^^Wales|Human|Wales Specialist Virology Centre|Public Health Wales Microbiology Cardiff|Moore|United Kingdom
>Spike|hCoV-19/Scotland/CVR5596/2020|2020-10-31|EPI_ISL_650751|Original|hCoV-19^^Scotland|Human|West of Scotland Specialist Virology Centre|COVID-19 Genomics UK (COG-UK) Consortium|Robertson|United Kingdom
>Spike|hCoV-19/USA/LA-EVTL403/2020|2020-06-01|EPI_ISL_578809|Original|hCoV-19^^Louisiana|Human|LSUHS Emerging Viral Threat Laboratory|Microbial Genome Sequencing Center|Kamil|USA
>Spike|hCoV-19/USA/TX-DSHS-0180/2020|2020-03-16|EPI_ISL_524061|Original|hCoV-19^^Texas|Human|Texas Department of State Health Services|Texas Department of State Health Services|Rahman|USA
>Spike|hCoV-19/USA/VA-DCLS-0750/2020|2020-06-15|EPI_ISL_526867|Original|hCoV-19^^Virginia|Human|Virginia DCLS|Virginia DCLS|St. Jacques|USA
>Spike|hCoV-19/Denmark/DCGC-15728/2020|2020-11-23|EPI_ISL_713476|Original|hCoV-19^^Nordjylland|Human|Department of Virus and Microbiological Special Diagnostics|Albertsen Lab|Michaelsen|Denmark
>Spike|hCoV-19/England/NORW-F6E4F/2020|2020-12-05|EPI_ISL_725305|Original|hCoV-19^^England|Human|Quadram Institute Bioscience|COVID-19 Genomics UK (COG-UK) Consortium|Alikhan|United Kingdom
>Spike|hCoV-19/England/CAMB-7E1BF/2020|2020-04-16|EPI_ISL_434047|Original|hCoV-19^^England|Human|Department of Pathology|COVID-19 Genomics UK (COG-UK) Consortium|Meredith|United Kingdom
>Spike|hCoV-19/England/20138027404/2020|2020-03-26|EPI_ISL_423317|Original|hCoV-19^^England|Human|Respiratory Virus Unit|Respiratory Virus Unit|Platt|United Kingdom
>Spike|hCoV-19/USA/CA-QDX-1438/2020|2020-03-18|EPI_ISL_572090|Original|hCoV-19^^California|Human|Quest Diagnostics|Quest Diagnostics|Kagan|USA
>Spike|hCoV-19/Ireland/un-NVRL-AIIDB0134v1/2020|2020-12-11|EPI_ISL_778831|Original|hCoV-19^^Ireland|Human|AIID|Irish Coronavirus Sequencing Consortium-Teagasc Grange|McCabe|Ireland
>Spike|hCoV-19/England/204360017/2020|2020-10-21|EPI_ISL_668445|Original|hCoV-19^^England|Human|Respiratory Virus Unit|COVID-19 Genomics UK (COG-UK) Consortium|Platt|United Kingdom
>Spike|hCoV-19/Scotland/QEUH-C30FD1/2020|2020-12-11|EPI_ISL_757122|Original|hCoV-19^^Scotland|Human|Lighthouse Lab in Glasgow|Wellcome Sanger Institute for the COVID-19 Genomics UK (COG-UK) Consortium|Davies|United Kingdom
>Spike|hCoV-19/New Zealand/20VR2922/2020|2020-04-25|EPI_ISL_456391|Original|hCoV-19^^Auckland|Human|LabPLUS|Institute of Environmental Science and Research (ESR)|de Ligt|New Zealand
>Spike|hCoV-19/Japan/PG-1488/2020|2020-04-00|EPI_ISL_685095|Original|hCoV-19^^Japan|Human|Saitama Prefectural Institute of Public Health|Pathogen Genomics Center|Sekizuka|Japan

>Spike|hCoV-19/Wales/MILK-B025AA/2020|2020-10-24|EPI_ISL_647853|Original|hCoV-19^^Wales|Human|Lighthouse Lab in Milton Keynes|Wellcome Sanger Institute for the COVID-19 Genomics UK (COG-UK) Consortium|Davies|United Kingdom
>Spike|hCoV-19/Spain/CN-IBV-97006483/2020|2020-03-25|EPI_ISL_537698|Original|hCoV-19^^Canary Islands|Human|Hospital Universitario de Gran Canaria Dr. Negr\u00edn|SeqCOVID-SPAIN consortium/IBV(CSIC)|Comas|Spain
>Spike|hCoV-19/Spain/MD-HLP-H11-40/2020|2020-03-12|EPI_ISL_417967|Original|hCoV-19^^Madrid|Human|Hospital Universitario 12 de Octubre|Hospital Universitario La Paz|Viedma|Spain
>Spike|hCoV-19/Burkina Faso/3103/2020|2020-09-28|EPI_ISL_660467|Original|hCoV-19^^Bobo Dioulasso|Human|Laboratoire de Microbiologie CHU Souro Sanou|Centre Muraz|Ouedraogo|Burkina Faso
>Spike|hCoV-19/Denmark/DCGC-21221/2020|2020-12-21|EPI_ISL_759618|Original|hCoV-19^^Sjaelland|Human|Department of Virus and Microbiological Special Diagnostics|Albertsen Lab|Michaelsen|Denmark
>Spike|hCoV-19/England/204221398/2020|2020-00-00|EPI_ISL_593793|Original|hCoV-19^^England|Human|Respiratory Virus Unit|Respiratory Virus Unit|Platt|United Kingdom
>Spike|hCoV-19/USA/TX-HMH-MCoV-13327/2020|2020-08-25|EPI_ISL_788475|Original|hCoV-19^^Texas|Human|Houston Methodist Hospital|Houston Methodist Hospital|Long|USA
>Spike|hCoV-19/England/MILK-B50534/2020|2020-10-19|EPI_ISL_649386|Original|hCoV-19^^England|Human|Lighthouse Lab in Milton Keynes|Wellcome Sanger Institute for the COVID-19 Genomics UK (COG-UK) Consortium|Davies|United Kingdom
>Spike|hCoV-19/Norway/4548/2020|2020-10-16|EPI_ISL_775451|Original|hCoV-19^^Vestfold og Telemark|Human|Vestfold Hospital|Norwegian Institute of Public Health|Vollan|Norway
>Spike|hCoV-19/Canada/ON-S1222/2020|2020-03-18|EPI_ISL_586299|Original|hCoV-19^^Ontario|Human|Toronto Invasive Bacterial Diseases Network|McMaster University|McArthur|Canada
>Spike|hCoV-19/Scotland/QEUH-9DC471/2020|2020-09-22|EPI_ISL_601157|Original|hCoV-19^^Scotland|Human|Lighthouse Lab in Glasgow|Wellcome Sanger Institute for the COVID-19 Genomics UK (COG-UK) consortium|Davies|United Kingdom
>Spike|hCoV-19/Denmark/DCGC-5090/2020|2020-09-21|EPI_ISL_621628|Original|hCoV-19^^Sjaelland|Human|Department of Virus and Microbiological Special Diagnostics|Albertsen lab|Michaelsen|Denmark
>Spike|hCoV-19/England/MILK-99D5C4/2020|2020-08-31|EPI_ISL_549930|Original|hCoV-19^^England|Human|Lighthouse Lab in Milton Keynes|Wellcome Sanger Institute for the COVID-19 Genomics UK (COG-UK) consortium|Davies|United Kingdom
>Spike|hCoV-19/Wales/PHWC-49F2B1/2020|2020-12-06|EPI_ISL_743191|Original|hCoV-19^^Wales|Human|Wales Specialist Virology Centre Sequencing lab|COVID-19 Genomics UK (COG-UK) Consortium|Moore|United Kingdom
>Spike|hCoV-19/Wales/MILK-9E11FA/2020|2020-09-20|EPI_ISL_581038|Original|hCoV-19^^Wales|Human|Lighthouse Lab in Milton Keynes|Wellcome Sanger Institute for the COVID-19 Genomics UK (COG-UK) consortium|Davies|United Kingdom
>Spike|hCoV-19/United Arab Emirates/0171/2020|2020-05-18|EPI_ISL_698358|Original|hCoV-19^^Abu Dhabi|Human|Group 42 (G42) Healthcare|G42 Healthcare|Ogrodzki|United Arab Emirates
>Spike|hCoV-19/England/QEUH-95E969/2020|2020-08-07|EPI_ISL_532486|Original|hCoV-19^^England|Human|Lighthouse Lab in Glasgow|Wellcome Sanger Institute for the COVID-19 Genomics UK (COG-UK) consortium|Davies|United Kingdom
>Spike|hCoV-19/England/CAMC-9D74B2/2020|2020-09-20|EPI_ISL_584375|Original|hCoV-19^^England|Human|Virology Department|COVID-19 Genomics UK (COG-UK) Consortium|de Silva|United Kingdom
>Spike|hCoV-19/England/QEUH-9644AB/2020|2020-08-12|EPI_ISL_536878|Original|hCoV-19^^England|Human|Lighthouse Lab in Glasgow|Wellcome Sanger Institute for the COVID-19 Genomics UK (COG-UK) consortium|Davies|United Kingdom
>Spike|hCoV-19/USA/NE-11331/2020|2020-05-04|EPI_ISL_475208|Original|hCoV-19^^Nebraska|Human|Nebraska Public Health Laboratory|UNMC COVID-19 Response Team|Pratt|USA
>Spike|hCoV-19/USA/TX-HMH-MCoV-15332/2020|2020-10-19|EPI_ISL_786816|Original|hCoV-19^^Texas|Human|Houston Methodist Hospital|Houston Methodist Hospital|Long|USA

>Spike|hCoV-19/USA/WA-UW-2670/2020|2020-03-30|EPI_ISL_486013|Original|hCoV-19^^Washington|Human|UW Virology Lab|UW Virology Lab|Roychoudhury|USA
>Spike|hCoV-19/Netherlands/un-EMC-788/2020|2020-00-00|EPI_ISL_577884|Original|hCoV-19^^Netherlands|Human|Dutch COVID-19 response team|Erasmus Medical Center|Oude Munnink|Netherlands
>Spike|hCoV-19/England/QEUH-AF6944/2020|2020-10-30|EPI_ISL_663161|Original|hCoV-19^^England|Human|Lighthouse Lab in Glasgow|Wellcome Sanger Institute for the COVID-19 Genomics UK (COG-UK) Consortium|Davies|United Kingdom
>Spike|hCoV-19/USA/FL-CDC-3582/2020|2020-04-07|EPI_ISL_594461|original|hCoV-19^^Florida|Human|FL Bureau of Public Health Laboratories|Pathogen Discovery|Queen|USA
>Spike|hCoV-19/USA/OR-OHSU-2289/2020|2020-08-10|EPI_ISL_667170|Original|hCoV-19^^Oregon|Human|OHSU Lab Services Molecular Microbiology Lab|Oregon SARS-CoV-2 Genome Sequencing Center|O'Roak|USA
>Spike|hCoV-19/Gibraltar/205121220/2020|2020-00-00|EPI_ISL_766346|Original|hCoV-19^^Gibraltar|Human|Microbiology|Respiratory Virus Unit|Platt|Gibraltar
>Spike|hCoV-19/Wales/PHWC-490432/2020|2020-10-30|EPI_ISL_706479|Original|hCoV-19^^Wales|Human|Originating lab|COVID-19 Genomics UK (COG-UK) Consortium|Moore|United Kingdom
>Spike|hCoV-19/Australia/VIC10690/2020|2020-09-03|EPI_ISL_563033|Original|hCoV-19^^Victoria|Human|Microbiological Diagnostic Unit - Public Health Laboratory (MDU-PHL)|MDU-PHL|Schultz|Australia
>Spike|hCoV-19/France/PAC-MEPHI-1208/2020|2020-08-17|EPI_ISL_644402|Original|hCoV-19^^Provence-Alpes-Cote-d\u2019Azur|Human|MEPHI|MEPHI|Levasseur|France
>Spike|hCoV-19/England/QEUH-787301/2020|2020-07-15|EPI_ISL_533182|Original|hCoV-19^^England|Human|Lighthouse Lab in Glasgow|Wellcome Sanger Institute for the COVID-19 Genomics UK (COG-UK) consortium|Davies|United Kingdom
>Spike|hCoV-19/Wales/PHWC-4A45DE/2020|2020-12-16|EPI_ISL_764712|Original|hCoV-19^^Wales|Human|Wales Specialist Virology Centre Sequencing lab|COVID-19 Genomics UK (COG-UK) Consortium|Moore|United Kingdom
>Spike|hCoV-19/Italy/APU-PODV-15934631/2020|2020-08-23|EPI_ISL_788974|Original|hCoV-19^^Apulia|Human|Ospedale \Di Venere\|Beaconlab (Bioinformatics|Chiara|Italy
>Spike|hCoV-19/USA/AZ-TG340910/2020|2020-06-01|EPI_ISL_694036|Original|hCoV-19^^Arizona|Human|TGen North|TGen North|French|USA
>Spike|hCoV-19/USA/NY-PV09379/2020|2020-03-19|EPI_ISL_450062|Original|hCoV-19^^New York|Human|MSHS Clinical Microbiology Laboratories|MSHS Pathogen Surveillance Program|van Bakel|USA
>Spike|hCoV-19/Scotland/EDB8205/2020|2020-10-08|EPI_ISL_594833|Original|hCoV-19^^Scotland|Human|Virology Department|COVID-19 Genomics UK (COG-UK) Consortium|McHugh|United Kingdom
>Spike|hCoV-19/Denmark/DCGC-11929/2020|2020-10-19|EPI_ISL_671221|Original|hCoV-19^^Midtjylland|Human|Department of Virus and Microbiological Special Diagnostics|Albertsen Lab|Michaelsen|Denmark
>Spike|hCoV-19/Chile/RM-204591/2020|2020-10-12|EPI_ISL_746764|Original|hCoV-19^^Region Metropolitana de Santiago|Human|Genetica Molecular and Subdepartamento de Virologia ISP Chile|Instituto de Salud Publica de Chile|Tognarelli|Chile
>Spike|hCoV-19/Switzerland/VS-ETHZ-100173/2020|2020-03-16|EPI_ISL_574969|Original|hCoV-19^^Valais|Human|Viollier AG|Department of Biosystems Science and Engineering|Nadeau|Switzerland
>Spike|hCoV-19/USA/MN-MDH-1871/2020|2020-10-13|EPI_ISL_603006|Original|hCoV-19^^Minnesota|Human|Minnesota Department of Health|Minnesota Department of Health|Garfin|USA
>Spike|hCoV-19/USA/NY-NYCPHL-000235/2020|2020-03-15|EPI_ISL_631764|Original|hCoV-19^^New York|Human|Mount Sinai Hospital|New York City Public Health Laboratory|Wang|USA
>Spike|hCoV-19/Canada/ON-UHTC_0355/2020|2020-10-03|EPI_ISL_609822|Original|hCoV-19^^Ontario|Human|Unity Health Toronto|Ontario Institute for Cancer Research|de Borja|Canada
>Spike|hCoV-19/USA/AZ-TG280923/2020|2020-04-27|EPI_ISL_694219|Original|hCoV-19^^Arizona|Human|TGen North|TGen North|French|USA

>Spike|hCoV-19/USA/AK-PHL550/2020|2020-06-20|EPI_ISL_522405|Original|hCoV-19^^Alaska|Human|Alaska State Virology Laboratory|Alaska State Virology Laboratory|Chen|USA
>Spike|hCoV-19/England/CAMC-B22AC3/2020|2020-11-02|EPI_ISL_647198|Original|hCoV-19^^England|Human|Lighthouse Lab in Cambridge|Wellcome Sanger Institute for the COVID-19 Genomics UK (COG-UK) Consortium|Davies|United Kingdom
>Spike|hCoV-19/England/QEUH-9D07ED/2020|2020-09-16|EPI_ISL_589882|Original|hCoV-19^^England|Human|Lighthouse Lab in Glasgow|Wellcome Sanger Institute for the COVID-19 Genomics UK (COG-UK) consortium|Davies|United Kingdom
>Spike|hCoV-19/England/ALDP-A6FA34/2020|2020-10-14|EPI_ISL_607832|Original|hCoV-19^^England|Human|Lighthouse Lab in Alderley Park|Wellcome Sanger Institute for the COVID-19 Genomics UK (COG-UK) consortium|Davies|United Kingdom
>Spike|hCoV-19/England/CAMB-1AE294/2020|2020-04-27|EPI_ISL_459436|Original|hCoV-19^^England|Human|Department of Pathology|Wellcome Sanger Institute for the COVID-19 Genomics UK (COG-UK) consortium|Davies|United Kingdom
>Spike|hCoV-19/Japan/PG-3336/2020|2020-04-00|EPI_ISL_691451|Original|hCoV-19^^Japan|Human|Pathogen Genomics Center|Pathogen Genomics Center|Sekizuka|Japan
>Spike|hCoV-19/South Korea/KDCA0264/2020|2020-08-14|EPI_ISL_760029|Original|hCoV-19^^South Korea|Human|Division of Emerging Infectious Diseases|Division of Emerging Infectious Diseases|Park|South Korea
>Spike|hCoV-19/Wales/PHWC-33769/2020|2020-04-17|EPI_ISL_446744|Original|hCoV-19^^Wales|Human|Wales Specialist Virology Centre|Public Health Wales Microbiology Cardiff|Moore|United Kingdom
>Spike|hCoV-19/Italy/ABR-IZSGC-TE5173/2020|2020-03-18|EPI_ISL_528924|Original|hCoV-19^^Abruzzo|Human|Ospedale \Giuseppe Mazzini\-Teramo|Istitituto Zooprofilattico Sperimentale dell'Abruzzo e Molise \G.Caporale\|Lorusso|Italy
>Spike|hCoV-19/England/MILK-9D47DA/2020|2020-09-17|EPI_ISL_601520|Original|hCoV-19^^England|Human|Lighthouse Lab in Milton Keynes|Wellcome Sanger Institute for the COVID-19 Genomics UK (COG-UK) consortium|Davies|United Kingdom
>Spike|hCoV-19/India/PB-IMT-L176/2020|2020-04-30|EPI_ISL_539486|Original|hCoV-19^^Punjab|Human|Civil Hospital|CSIR-Institute of Microbial Technology|Patil|India
>Spike|hCoV-19/USA/HI-H200285/2020|2020-07-07|EPI_ISL_752844|Original|hCoV-19^^Hawaii|Human|State Laboratories Division|State Laboratories Division|Sultana|USA
>Spike|hCoV-19/England/QEUH-C30A85/2020|2020-12-09|EPI_ISL_731421|Original|hCoV-19^^England|Human|Lighthouse Lab in Glasgow|Wellcome Sanger Institute for the COVID-19 Genomics UK (COG-UK) Consortium|Davies|United Kingdom
>Spike|hCoV-19/Luxembourg/LNS9095238/2020|2020-10-31|EPI_ISL_744848|Original|hCoV-19^^Luxembourg|Human|Laboratoire national de sant\u00e9|Laboratoire national de sant\u00e9|Wienecke-Baldacchino|Luxembourg
>Spike|hCoV-19/Switzerland/AG-ETHZ-331066/2020|2020-10-20|EPI_ISL_614893|Original|hCoV-19^^Aargau|Human|Viollier AG|Department of Biosystems Science and Engineering|Nadeau|Switzerland
>Spike|hCoV-19/England/ALDP-9D8A37/2020|2020-09-18|EPI_ISL_576645|Original|hCoV-19^^England|Human|Oxford Viromics|COVID-19 Genomics UK (COG-UK) Consortium|Golubchik|United Kingdom
>Spike|hCoV-19/France/PAC-IHU-0986/2020|2020-08-09|EPI_ISL_569032|Original|hCoV-19^^Provence-Alpes-C\u00f4te-d\u2019Azur|Human|MEPHI|MEPHI|Levasseur|France
>Spike|hCoV-19/Japan/PG-4523/2020|2020-08-00|EPI_ISL_689662|Original|hCoV-19^^Japan|Human|Pathogen Genomics Center|Pathogen Genomics Center|Sekizuka|Japan
>Spike|hCoV-19/USA/CA-CZB-14846/2020|2020-11-20|EPI_ISL_739106|Original|hCoV-19^^California|Human|Humboldt County Public Health Laboratory|Chan-Zuckerberg Biohub|Kamm|USA
>Spike|hCoV-19/Wales/PHWC-4A691C/2020|2020-12-17|EPI_ISL_765084|Original|hCoV-19^^Wales|Human|Wales Specialist Virology Centre Sequencing lab|COVID-19 Genomics UK (COG-UK) Consortium|Moore|United Kingdom
>Spike|hCoV-19/USA/CA-ALSR-4549/2020|2020-07-09|EPI_ISL_667002|Original|hCoV-19^^California|Human|San Diego County Public Health Laboratory|Andersen lab at Scripps Research|Gangavarapu|USA

>Spike|hCoV-19/England/QEUH-AD2D7F/2020|2020-10-26|EPI_ISL_629877|Original|hCoV-
19^^England|Human|Lighthouse Lab in Glasgow|Wellcome Sanger Institute for the COVID-
19 Genomics UK (COG-UK) consortium|Davies|United Kingdom
>Spike|hCoV-19/Australia/VIC10890/2020|2020-08-17|EPI_ISL_563141|Original|hCoV-
19^^Victoria|Human|Victorian Infectious Diseases Reference Laboratory (VIDRL)|VIDRL
and MDU-PHL|Schultz|Australia
>Spike|hCoV-19/USA/TX-HMH-1164/2020|2020-04-26|EPI_ISL_542562|Original|hCoV-
19^^Texas|Human|Houston Methodist Hospital|Houston Methodist Hospital|Long|USA
>Spike|hCoV-19/Scotland/CVR4871/2020|2020-10-03|EPI_ISL_650490|Original|hCoV-
19^^Scotland|Human|West of Scotland Specialist Virology Centre|COVID-19 Genomics UK
(COG-UK) Consortium|Robertson|United Kingdom
>Spike|hCoV-19/England/BRIS-13090B/2020|2020-04-13|EPI_ISL_492539|Original|hCoV-
19^^England|Human|PHE South West Regional Laboratory|Wellcome Sanger Institute for
the COVID-19 Genomics UK (COG-UK) consortium|Davies|United Kingdom
>Spike|hCoV-19/Denmark/DCGC-20814/2020|2020-12-21|EPI_ISL_759381|Original|hCoV-
19^^Syddanmark|Human|Department of Virus and Microbiological Special
Diagnostics|Albertsen Lab|Michaelsen|Denmark
>Spike|hCoV-19/Denmark/DCGC-17333/2020|2020-12-14|EPI_ISL_750915|Original|hCoV-
19^^Syddanmark|Human|Department of Virus and Microbiological Special
Diagnostics|Albertsen Lab|Michaelsen|Denmark
>Spike|hCoV-19/Scotland/MILK-A6B063/2020|2020-10-08|EPI_ISL_625416|Original|hCoV-
19^^Scotland|Human|Lighthouse Lab in Milton Keynes|Wellcome Sanger Institute for the
COVID-19 Genomics UK (COG-UK) consortium|Davies|United Kingdom
>Spike|hCoV-19/England/MILK-95DBFB/2020|2020-06-18|EPI_ISL_556187|Original|hCoV-
19^^England|Human|Lighthouse Lab in Milton Keynes|Wellcome Sanger Institute for the
COVID-19 Genomics UK (COG-UK) consortium|Davies|United Kingdom
>Spike|hCoV-19/USA/WA-UW-5756/2020|2020-04-07|EPI_ISL_430920|Original|hCoV-
19^^Washington|Human|UW Virology Lab|UW Virology Lab|Roychoudhury|USA
>Spike|hCoV-19/Mali/M002826/2020|2020-04-14|EPI_ISL_487465|Original|hCoV-
19^^Bamako|Human|CICM-Mali|Bundeswehr Institut of Microbiology|Antwerpen|Mali
>Spike|hCoV-19/Pakistan/NIH-44905/2020|2020-06-02|EPI_ISL_468159|unknown|hCoV-
19^^Islamabad|Human|unknown|Department of Virology|Umair|Pakistan
>Spike|hCoV-19/Luxembourg/LNS9510711/2020|2020-03-17|EPI_ISL_421762|Original|hCoV-
19^^Luxembourg|Human|Laboratoire National de Sante|Laboratoire National de
Sante|Wienecke-Baldacchino|Luxembourg
>Spike|hCoV-19/England/ALDP-A92731/2020|2020-10-19|EPI_ISL_611169|Original|hCoV-
19^^England|Human|Lighthouse Lab in Alderley Park|Wellcome Sanger Institute for the
COVID-19 Genomics UK (COG-UK) consortium|Davies|United Kingdom
>Spike|hCoV-19/Wales/PHWC-15C2A4/2020|2020-04-20|EPI_ISL_472463|Original|hCoV-
19^^Wales|Human|Wales Specialist Virology Centre Sequencing lab|COVID-19 Genomics UK
(COG-UK) Consortium|Moore|United Kingdom
>Spike|hCoV-19/Brazil/RJ-00412/2020|2020-09-03|EPI_ISL_717814|Original|hCoV-19^^Rio
de Janeiro|Human|Laboratorio de Virologia Molecular / UFRJ|Bioinformatics Laboratory
/ LNCC|Almeida|Brazil
>Spike|hCoV-19/England/NORT-2867CF/2020|2020-10-08|EPI_ISL_638568|Original|hCoV-
19^^England|Human|Northumbria University / South Tees Hospitals NHS Foundation Trust
/ North Cumbria Integrated Care NHS Foundation Trust / North Tees and Hartlepool NHS
Foundation Trust / Newcastle Hospitals NHS Foundation Trust|COVID-19 Genomics UK
(COG-UK) Consortium|Bashton|United Kingdom
>Spike|hCoV-19/England/ALDP-C0643F/2020|2020-12-06|EPI_ISL_709363|Original|hCoV-
19^^England|Human|Lighthouse Lab in Alderley Park|Wellcome Sanger Institute for the
COVID-19 Genomics UK (COG-UK) Consortium|Davies|United Kingdom
>Spike|hCoV-19/England/QEUH-A5E704/2020|2020-10-12|EPI_ISL_597348|Original|hCoV-
19^^England|Human|Lighthouse Lab in Glasgow|Wellcome Sanger Institute for the COVID-
19 Genomics UK (COG-UK) consortium|Davies|United Kingdom
>Spike|hCoV-19/England/QEUH-AE294B/2020|2020-10-27|EPI_ISL_633549|Original|hCoV-
19^^England|Human|Lighthouse Lab in Glasgow|Wellcome Sanger Institute for the COVID-
19 Genomics UK (COG-UK) consortium|Davies|United Kingdom
>Spike|hCoV-19/Australia/NSW2542/2020|2020-04-04|EPI_ISL_527043|Original|hCoV-19^^New
South Wales|Human|Area of Virology|Area of Virology|Yeang|Australia

>Spike|hCoV-19/England/MILK-9F0EB5/2020|2020-09-23|EPI_ISL_589663|Original|hCoV-
19^^England|Human|Lighthouse Lab in Milton Keynes|Wellcome Sanger Institute for the
COVID-19 Genomics UK (COG-UK) consortium|Davies|United Kingdom
>Spike|hCoV-19/USA/MA-MGH-03544/2020|2020-11-28|EPI_ISL_765880|Original|hCoV-
19^^Massachusetts|Human|Massachusetts General Hospital|Infectious Disease
Program|Park|USA
>Spike|hCoV-19/England/MILK-BDB9DB/2020|2020-11-27|EPI_ISL_709666|Original|hCoV-
19^^England|Human|Lighthouse Lab in Milton Keynes|Wellcome Sanger Institute for the
COVID-19 Genomics UK (COG-UK) Consortium|Davies|United Kingdom
>Spike|hCoV-19/Wales/PHWC-15C72D/2020|2020-03-31|EPI_ISL_472530|Original|hCoV-
19^^Wales|Human|Wales Specialist Virology Centre Sequencing lab|COVID-19 Genomics UK
(COG-UK) Consortium|Moore|United Kingdom
>Spike|hCoV-19/India/MH-ACTREC-323/2020|2020-07-17|EPI_ISL_699972|Original|hCoV-
19^^Maharashtra|Human|Hematopathology Laboratory|Hematopathology
Laboratory|Patkar|India
>Spike|hCoV-19/USA/TX-HMH-3783/2020|2020-06-12|EPI_ISL_543188|Original|hCoV-
19^^Texas|Human|Houston Methodist Hospital|Houston Methodist Hospital|Long|USA
>Spike|hCoV-19/Brazil/SP-387/2020|2020-06-20|EPI_ISL_603027|Original|hCoV-
19^^S\u00e3o Paulo|Human|Santa Casa de Misericordia de Ara\u00e7atuba|Instituto
Adolfo Lutz|Sacchi|Brazil
>Spike|hCoV-19/Australia/VIC7508/2020|2020-00-00|EPI_ISL_564456|Original|hCoV-
19^^Victoria|Human|Microbiological Diagnostic Unit - Public Health Laboratory (MDU-
PHL)|MDU-PHL|Schultz|Australia
>Spike|hCoV-19/USA/UT-08698/2020|2020-05-08|EPI_ISL_524416|Original|hCoV-
19^^Utah|Human|Utah Public Health Laboratory|Utah Public Health Laboratory|Young|USA
>Spike|hCoV-19/Japan/PG-0596/2020|2020-04-00|EPI_ISL_684206|Original|hCoV-
19^^Japan|Human|Pathogen Genomics Center|Pathogen Genomics Center|Sekizuka|Japan
>Spike|hCoV-19/USA/TX-HMH-2894/2020|2020-06-08|EPI_ISL_543862|Original|hCoV-
19^^Texas|Human|Houston Methodist Hospital|Houston Methodist Hospital|Long|USA
>Spike|hCoV-19/Canada/ON-UHTC_0344/2020|2020-09-11|EPI_ISL_609803|Original|hCoV-
19^^Ontario|Human|Unity Health Toronto|Ontario Institute for Cancer Research|de
Borja|Canada
>Spike|hCoV-19/England/MILK-AC83C5/2020|2020-10-21|EPI_ISL_629393|Original|hCoV-
19^^England|Human|Lighthouse Lab in Milton Keynes|Wellcome Sanger Institute for the
COVID-19 Genomics UK (COG-UK) consortium|Davies|United Kingdom
>Spike|hCoV-19/Denmark/ALAB-HH75/2020|2020-03-18|EPI_ISL_429316|Original|hCoV-
19^^Denmark|Human|Department of Clinical Microbiology|Albertsen
lab|Kirkegaard|Denmark
>Spike|hCoV-19/Belgium/rega-0912943/2020|2020-09-12|EPI_ISL_734930|Original|hCoV-
19^^Maasmechelen|Human|UZ Leuven|KU Leuven|Maes|Belgium
>Spike|hCoV-19/USA/WA-S375/2020|2020-03-26|EPI_ISL_434135|Original|hCoV-
19^^Washington|Human|Washington State Department of Health|Seattle Flu
Study|Bedford|USA
>Spike|hCoV-19/USA/LA-EVTL1277/2020|2020-12-04|EPI_ISL_778943|Original|hCoV-
19^^Louisiana|Human|LSUHS Emerging Viral Threat Laboratory|Microbial Genome
Sequencing Center|Kamil|USA
>Spike|hCoV-19/Wales/PHWC-492C92/2020|2020-11-27|EPI_ISL_725772|Original|hCoV-
19^^Wales|Human|Originating lab|COVID-19 Genomics UK (COG-UK) Consortium|Moore|United
Kingdom
>Spike|hCoV-19/Luxembourg/LNS5868219/2020|2020-08-10|EPI_ISL_744311|Original|hCoV-
19^^Luxembourg|Human|Laboratoire national de sant\u00e9|Laboratoire national de
sant\u00e9|Wienecke-Baldacchino|Luxembourg
>Spike|hCoV-19/Sweden/20-52118/2020|2020-05-07|EPI_ISL_615096|Original|hCoV-
19^^Vasterbotten|Human|Umea klinisk mikrobiologi|The Public Health Agency of
Sweden|Svartstr\u00f6m|Sweden
>Spike|hCoV-19/Denmark/DCGC-18483/2020|2020-12-07|EPI_ISL_749994|Original|hCoV-
19^^Midtjylland|Human|Department of Virus and Microbiological Special
Diagnostics|Albertsen Lab|Michaelsen|Denmark
>Spike|hCoV-19/USA/TX-HMH-MCoV-13868/2020|2020-08-17|EPI_ISL_787161|Original|hCoV-
19^^Texas|Human|Houston Methodist Hospital|Houston Methodist Hospital|Long|USA

>Spike|hCoV-19/England/ALDP-C3ADDD/2020|2020-12-09|EPI_ISL_731692|Original|hCoV-19^^England|Human|Lighthouse Lab in Alderley Park|Wellcome Sanger Institute for the COVID-19 Genomics UK (COG-UK) Consortium|Davies|United Kingdom
>Spike|hCoV-19/Wales/QEUH-AD58E4/2020|2020-10-25|EPI_ISL_647921|Original|hCoV-19^^Wales|Human|Lighthouse Lab in Glasgow|Wellcome Sanger Institute for the COVID-19 Genomics UK (COG-UK) Consortium|Davies|United Kingdom
>Spike|hCoV-19/England/NOTT-1107CB/2020|2020-04-20|EPI_ISL_441859|Original|hCoV-19^^England|Human|Queens Medical Centre|COVID-19 Genomics UK (COG-UK) Consortium|Loose|United Kingdom
>Spike|hCoV-19/Denmark/DCGC-19509/2020|2020-12-07|EPI_ISL_748322|Original|hCoV-19^^Syddanmark|Human|Department of Virus and Microbiological Special Diagnostics|Albertsen Lab|Michaelsen|Denmark
>Spike|hCoV-19/England/QEUH-9B5511/2020|2020-09-03|EPI_ISL_590451|Original|hCoV-19^^England|Human|Lighthouse Lab in Glasgow|Wellcome Sanger Institute for the COVID-19 Genomics UK (COG-UK) consortium|Davies|United Kingdom
>Spike|hCoV-19/USA/NY-Wadsworth-278519-01/2020|2020-11-23|EPI_ISL_754405|Original|hCoV-19^^New York|Human|Wadsworth Center|Wadsworth Center|Lasek-Nesselquist|USA
>Spike|hCoV-19/USA/NV-NSPHL-A0010/2020|2020-03-14|EPI_ISL_515299|Original|hCoV-19^^Nevada|Human|Nevada State Public Health Laboratory|Nevada State Public Health Laboratory|Sevinsky|USA
>Spike|hCoV-19/Netherlands/NA_497/2020|2020-04-06|EPI_ISL_460670|Original|hCoV-19^^Netherlands|Human|Dutch COVID-19 response team|Erasmus Medical Center|Oude Munnink|Netherlands
>Spike|hCoV-19/England/CAMB-7A48D/2020|2020-04-02|EPI_ISL_441253|Original|hCoV-19^^England|Human|Department of Pathology|Wellcome Sanger Institute for the COVID-19 Genomics UK (COG-UK) consortium|Amato|United Kingdom
>Spike|hCoV-19/England/MILK-AB7E31/2020|2020-10-21|EPI_ISL_623405|Original|hCoV-19^^England|Human|Lighthouse Lab in Milton Keynes|Wellcome Sanger Institute for the COVID-19 Genomics UK (COG-UK) consortium|Davies|United Kingdom
>Spike|hCoV-19/United Arab Emirates/0834/2020|2020-05-19|EPI_ISL_698973|Original|hCoV-19^^Abu Dhabi|Human|Group 42 (G42) Healthcare|G42 Healthcare|Ogrodzki|United Arab Emirates
>Spike|hCoV-19/Ireland/DL-NVRL-20G45123/2020|2020-04-02|EPI_ISL_666708|Original|hCoV-19^^Donegal|Human|National Virus Reference Lab (NVRL)|Irish Coronavirus Sequencing Consortium-Teagasc Grange|McCabe|Ireland
>Spike|hCoV-19/USA/TX-HMH-MCoV-15353/2020|2020-10-22|EPI_ISL_786837|Original|hCoV-19^^Texas|Human|Houston Methodist Hospital|Houston Methodist Hospital|Long|USA
>Spike|hCoV-19/England/ALDP-BB8F69/2020|2020-11-20|EPI_ISL_703210|Original|hCoV-19^^England|Human|Lighthouse Lab in Alderley Park|Wellcome Sanger Institute for the COVID-19 Genomics UK (COG-UK) Consortium|Davies|United Kingdom
>Spike|hCoV-19/England/LIVE-A9F39/2020|2020-03-23|EPI_ISL_517375|Original|hCoV-19^^England|Human|Liverpool Clinical Laboratories|COVID-19 Genomics UK (COG-UK) Consortium|Haldenby|United Kingdom
>Spike|hCoV-19/Norway/5415/2020|2020-10-28|EPI_ISL_708082|Original|hCoV-19^^Troms og Finnmark|Human|University Hospital of Northern Norway|Norwegian Institute of Public Health|Kopperud Riis|Norway
>Spike|hCoV-19/England/QEUH-99870F/2020|2020-08-24|EPI_ISL_540065|Original|hCoV-19^^England|Human|Lighthouse Lab in Glasgow|Wellcome Sanger Institute for the COVID-19 Genomics UK (COG-UK) consortium|Davies|United Kingdom
>Spike|hCoV-19/USA/TX-HMH-2471/2020|2020-05-29|EPI_ISL_545328|Original|hCoV-19^^Texas|Human|Houston Methodist Hospital|Houston Methodist Hospital|Long|USA
>Spike|hCoV-19/Denmark/DCGC-18264/2020|2020-12-14|EPI_ISL_749265|Original|hCoV-19^^Sjaelland|Human|Department of Virus and Microbiological Special Diagnostics|Albertsen Lab|Michaelsen|Denmark
>Spike|hCoV-19/USA/LA-GBCL-26791769/2020|2020-04-04|EPI_ISL_485379|Original|hCoV-19^^Louisiana|Human|River Road Testing Lab|Ginkgo Bioworks Clinical Laboratory|Smith|USA
>Spike|hCoV-19/India/DL-NCDC-02155/2020|2020-03-26|EPI_ISL_435072|Original|hCoV-19^^Delhi|Human|National Centre for Disease control (NCDC)|NCDC/CSIR-IGIB|Faruq|India

>Spike|hCoV-19/Germany/TH-IIMK-CaSe-9/2020|2020-05-24|EPI_ISL_728303|Original|hCoV-
19^^Thuringia|Human|Jena University Hospital|Institute of infectious medicine &
hospital hygiene|Brandt|Germany
>Spike|hCoV-19/Canada/ON-UHTC-0176/2020|2020-05-23|EPI_ISL_569970|Original|hCoV-
19^^Ontario|Human|Unity Health Toronto|Ontario Institute for Cancer Research|de
Borja|Canada
>Spike|hCoV-19/England/MILK-9F09E1/2020|2020-09-24|EPI_ISL_589725|Original|hCoV-
19^^England|Human|Lighthouse Lab in Milton Keynes|Wellcome Sanger Institute for the
COVID-19 Genomics UK (COG-UK) consortium|Davies|United Kingdom
>Spike|hCoV-19/Belgium/ULG-3000/2020|2020-03-05|EPI_ISL_417004|Original|hCoV-
19^^Li\u00e8ge|Human|Department of Clinical Microbiology|GIGA Medical
Genomics|Durkin|Belgium
>Spike|hCoV-19/Scotland/QEUH-9C8469/2020|2020-09-15|EPI_ISL_576008|Original|hCoV-
19^^Scotland|Human|Lighthouse Lab in Glasgow|Wellcome Sanger Institute for the COVID-
19 Genomics UK (COG-UK) consortium|Davies|United Kingdom
>Spike|hCoV-19/USA/UT-UPHL-2008998/2020|2020-05-11|EPI_ISL_525309|Original|hCoV-
19^^Utah|Human|Utah Public Health Laboratory|Utah Public Health Laboratory|Young|USA
>Spike|hCoV-19/England/204280144/2020|2020-10-15|EPI_ISL_613595|Original|hCoV-
19^^England|Human|Respiratory Virus Unit|Respiratory Virus Unit|Platt|United Kingdom
>Spike|hCoV-19/Northern Ireland/QEUH-5E542E/2020|2020-06-
17|EPI_ISL_533305|Original|hCoV-19^^Northern Ireland|Human|Lighthouse Lab in
Glasgow|Wellcome Sanger Institute for the COVID-19 Genomics UK (COG-UK)
consortium|Davies|United Kingdom
>Spike|hCoV-19/England/NORT-281A52/2020|2020-03-22|EPI_ISL_472173|Original|hCoV-
19^^England|Human|Northumbria University / South Tees Hospitals NHS Foundation Trust
/ North Cumbria Integrated Care NHS Foundation Trust / North Tees and Hartlepool NHS
Foundation Trust / Newcastle Hospitals NHS Foundation Trust|COVID-19 Genomics UK
(COG-UK) Consortium|Bashton|United Kingdom
>Spike|hCoV-19/USA/MI-MDHHS-SC20146/2020|2020-03-27|EPI_ISL_436875|Original|hCoV-
19^^Michigan|Human|Michigan Department of Health and Human Services|Michigan
Department of Health and Human Services|Blankenship|USA
>Spike|hCoV-19/Australia/VIC15763/2020|2020-08-01|EPI_ISL_593447|Original|hCoV-
19^^Victoria|Human|Microbiological Diagnostic Unit - Public Health Laboratory (MDU-
PHL)|MDU-PHL|Schultz|Australia
>Spike|hCoV-19/USA/TX-DSHS-1624/2020|2020-10-20|EPI_ISL_700703|Original|hCoV-
19^^Texas|Human|Texas Department of State Health Services|Texas Department of State
Health Services|Rahman|USA
>Spike|hCoV-19/USA/WA-UW-1334/2020|2020-03-17|EPI_ISL_422995|Original|hCoV-
19^^Washington|Human|UW Virology Lab|UW Virology Lab|Roychoudhury|USA
>Spike|hCoV-19/Wales/PHWC-16A3EC/2020|2020-09-06|EPI_ISL_535245|Original|hCoV-
19^^Wales|Human|Wales Specialist Virology Centre Sequencing lab|COVID-19 Genomics UK
(COG-UK) Consortium|Moore|United Kingdom
>Spike|hCoV-19/USA/TX-HMH-4379/2020|2020-06-17|EPI_ISL_543576|Original|hCoV-
19^^Texas|Human|Houston Methodist Hospital|Houston Methodist Hospital|Long|USA
>Spike|hCoV-19/England/QEUH-9F38E1/2020|2020-09-24|EPI_ISL_588724|Original|hCoV-
19^^England|Human|Lighthouse Lab in Glasgow|Wellcome Sanger Institute for the COVID-
19 Genomics UK (COG-UK) consortium|Davies|United Kingdom
>Spike|hCoV-19/Wales/PHWC-1633F2/2020|2020-05-10|EPI_ISL_473292|Original|hCoV-
19^^Wales|Human|Wales Specialist Virology Centre Sequencing lab|COVID-19 Genomics UK
(COG-UK) Consortium|Moore|United Kingdom
>Spike|hCoV-19/Canada/NS-NML-1356/2020|2020-04-14|EPI_ISL_579733|Original|hCoV-
19^^Nova Scotia|Human|QEII Health Sciences Centre|National Microbiology Laboratory
(NML)|Majer|Canada
>Spike|hCoV-19/USA/NY-NYUMC238/2020|2020-04-06|EPI_ISL_427637|Original|hCoV-19^^New
York|Human|NYU Langone Health|Departments of Pathology and Medicine|Maurano|USA
>Spike|hCoV-19/Belgium/VAG-03013/2020|2020-03-01|EPI_ISL_415155|Original|hCoV-
19^^Huldenberg|Human|KU Leuven|KU Leuven|Maes|Belgium
>Spike|hCoV-19/England/ALDP-9EA21C/2020|2020-09-24|EPI_ISL_580203|Original|hCoV-
19^^England|Human|Lighthouse Lab in Alderley Park|Wellcome Sanger Institute for the
COVID-19 Genomics UK (COG-UK) consortium|Davies|United Kingdom

>Spike|hCoV-19/USA/TX-HMH-842/2020|2020-04-09|EPI_ISL_545864|Original|hCoV-19^^Texas|Human|Houston Methodist Hospital|Houston Methodist Hospital|Long|USA
>Spike|hCoV-19/England/QEUH-B2EECB/2020|2020-11-03|EPI_ISL_659849|Original|hCoV-19^^England|Human|Lighthouse Lab in Glasgow|Wellcome Sanger Institute for the COVID-19 Genomics UK (COG-UK) Consortium|Davies|United Kingdom
>Spike|hCoV-19/Wales/PHWC-494E4F/2020|2020-11-21|EPI_ISL_726261|Original|hCoV-19^^Wales|Human|Originating lab|COVID-19 Genomics UK (COG-UK) Consortium|Moore|United Kingdom
>Spike|hCoV-19/England/NOTT-117FAE/2020|2020-10-26|EPI_ISL_612594|Original|hCoV-19^^England|Human|Queens Medical Centre|COVID-19 Genomics UK (COG-UK) Consortium|Loose|United Kingdom
>Spike|hCoV-19/England/QEUH-961514/2020|2020-08-03|EPI_ISL_537049|Original|hCoV-19^^England|Human|Lighthouse Lab in Glasgow|Wellcome Sanger Institute for the COVID-19 Genomics UK (COG-UK) consortium|Davies|United Kingdom
>Spike|hCoV-19/England/LOND-1250D32/2020|2020-11-08|EPI_ISL_665811|Original|hCoV-19^^England|Human|University College London Hospital|COVID-19 Genomics UK (COG-UK) Consortium|Byott|United Kingdom
>Spike|hCoV-19/Denmark/DCGC-4298/2020|2020-09-07|EPI_ISL_615735|Original|hCoV-19^^Hovedstaden|Human|Department of Virus and Microbiological Special Diagnostics|Albertsen lab|Michaelsen|Denmark
>Spike|hCoV-19/India/MH-NCCS-NR2347/2020|2020-05-22|EPI_ISL_496580|Original|hCoV-19^^Maharashtra|Human|National Centre For Cell Science|National Centre For Cell Science|Paul|India
>Spike|hCoV-19/England/MILK-B8C335/2020|2020-11-13|EPI_ISL_674356|Original|hCoV-19^^England|Human|Lighthouse Lab in Milton Keynes|Wellcome Sanger Institute for the COVID-19 Genomics UK (COG-UK) Consortium|Davies|United Kingdom
>Spike|hCoV-19/England/CAMC-B77D6C/2020|2020-11-12|EPI_ISL_668294|Original|hCoV-19^^England|Human|Lighthouse Lab in Cambridge|Wellcome Sanger Institute for the COVID-19 Genomics UK (COG-UK) Consortium|Davies|United Kingdom
>Spike|hCoV-19/England/CAMC-B0761B/2020|2020-10-30|EPI_ISL_643779|Original|hCoV-19^^England|Human|Lighthouse Lab in Cambridge|Wellcome Sanger Institute for the COVID-19 Genomics UK (COG-UK) Consortium|Davies|United Kingdom
>Spike|hCoV-19/Belgium/rega-0623853/2020|2020-06-23|EPI_ISL_734779|Original|hCoV-19^^Brugge|Human|UZ Leuven|KU Leuven|Maes|Belgium
>Spike|hCoV-19/Japan/PG-1735/2020|2020-04-00|EPI_ISL_685341|Original|hCoV-19^^Japan|Human|Pathogen Genomics Center|Pathogen Genomics Center|Sekizuka|Japan
>Spike|hCoV-19/Wales/ALDP-95AAF1/2020|2020-06-11|EPI_ISL_602160|Original|hCoV-19^^Wales|Human|Lighthouse Lab in Alderley Park|Wellcome Sanger Institute for the COVID-19 Genomics UK (COG-UK) consortium|Davies|United Kingdom
>Spike|hCoV-19/England/CAMB-7A335/2020|2020-04-02|EPI_ISL_441315|Original|hCoV-19^^England|Human|Department of Pathology|Wellcome Sanger Institute for the COVID-19 Genomics UK (COG-UK) consortium|Amato|United Kingdom
>Spike|hCoV-19/USA/UT-QDX-2643/2020|2020-03-12|EPI_ISL_604977|Original|hCoV-19^^Utah|Human|Quest Diagnostics|Quest Diagnostics|Kagan|USA
>Spike|hCoV-19/England/CAMC-A583AC/2020|2020-10-08|EPI_ISL_597000|Original|hCoV-19^^England|Human|Lighthouse Lab in Cambridge|Wellcome Sanger Institute for the COVID-19 Genomics UK (COG-UK) consortium|Davies|United Kingdom
>Spike|hCoV-19/England/CAMC-B07147/2020|2020-10-29|EPI_ISL_643925|Original|hCoV-19^^England|Human|Lighthouse Lab in Cambridge|Wellcome Sanger Institute for the COVID-19 Genomics UK (COG-UK) Consortium|Davies|United Kingdom
>Spike|hCoV-19/India/TG-CCMB-R249/2020|2020-08-09|EPI_ISL_539620|Original|hCoV-19^^Telangana|Human|CSIR-Centre for Cellular and Molecular Biology|CSIR-Centre for Cellular and Molecular Biology|Mukherjee|India
>Spike|hCoV-19/South Korea/KCDC2764/2020|2020-07-21|EPI_ISL_522543|Original|hCoV-19^^South Korea|Human|Division of Viral Diseases|Division of Viral Diseases|Kim|South Korea
>Spike|hCoV-19/England/CAMB-75D0F/2020|2020-03-29|EPI_ISL_438416|Original|hCoV-19^^England|Human|Department of Pathology|Wellcome Sanger Institute for the COVID-19 Genomics UK (COG-UK) consortium|Amato|United Kingdom

>Spike|hCoV-19/Israel/CVL-45936/2020|2020-12-10|EPI_ISL_745058|Original|hCoV-19^^Jerusalem|Human|Israel Central Virology laboratory|Israel Central Virology laboratory|Zuckerman|Israel
>Spike|hCoV-19/Luxembourg/LNS5800178/2020|2020-11-04|EPI_ISL_744304|Original|hCoV-19^^Luxembourg|Human|Laboratoire national de sant\u00e9|Laboratoire national de sant\u00e9|Wienecke-Baldacchino|Luxembourg
>Spike|hCoV-19/Canada/ON-E36/2020|2020-05-26|EPI_ISL_593514|Original|hCoV-19^^Ontario|Human|Eastern Ontario Regional Laboratory Association|McMaster University|McArthur|Canada
>Spike|hCoV-19/Ireland/D-SVUH-R6565/2020|2020-04-05|EPI_ISL_596659|Original|hCoV-19^^Dublin|Human|St.Vincent's University Hospital|St.Vincent's University Hospital|Gonzalez|Ireland
>Spike|hCoV-19/England/MILK-95839E/2020|2020-06-11|EPI_ISL_554775|Original|hCoV-19^^England|Human|Lighthouse Lab in Milton Keynes|Wellcome Sanger Institute for the COVID-19 Genomics UK (COG-UK) consortium|Davies|United Kingdom
>Spike|hCoV-19/England/204581874/2020|2020-11-05|EPI_ISL_700650|Original|hCoV-19^^England|Human|Respiratory Virus Unit|COVID-19 Genomics UK (COG-UK) Consortium|Platt|United Kingdom
>Spike|hCoV-19/USA/TX-HMH-MCoV-14003/2020|2020-08-15|EPI_ISL_787294|Original|hCoV-19^^Texas|Human|Houston Methodist Hospital|Houston Methodist Hospital|Long|USA
>Spike|hCoV-19/England/CAMB-79EAE/2020|2020-04-01|EPI_ISL_441246|Original|hCoV-19^^England|Human|Department of Pathology|Wellcome Sanger Institute for the COVID-19 Genomics UK (COG-UK) consortium|Amato|United Kingdom
>Spike|hCoV-19/Wales/PHWC-489D5F/2020|2020-10-28|EPI_ISL_652487|Original|hCoV-19^^Wales|Human|Wales Specialist Virology Centre Sequencing lab|COVID-19 Genomics UK (COG-UK) Consortium|Moore|United Kingdom
>Spike|hCoV-19/England/ALDP-952DD2/2020|2020-06-11|EPI_ISL_553634|Original|hCoV-19^^England|Human|Lighthouse Lab in Alderley Park|Wellcome Sanger Institute for the COVID-19 Genomics UK (COG-UK) consortium|Davies|United Kingdom
>Spike|hCoV-19/USA/TX-HMH-1025/2020|2020-04-08|EPI_ISL_542499|Original|hCoV-19^^Texas|Human|Houston Methodist Hospital|Houston Methodist Hospital|Long|USA
>Spike|hCoV-19/Scotland/CVR2554/2020|2020-04-16|EPI_ISL_477967|Original|hCoV-19^^Scotland|Human|West of Scotland Specialist Virology Centre|COVID-19 Genomics UK (COG-UK) Consortium|Robertson|United Kingdom
>Spike|hCoV-19/England/ALDP-A6D562/2020|2020-10-14|EPI_ISL_606975|Original|hCoV-19^^England|Human|Lighthouse Lab in Alderley Park|Wellcome Sanger Institute for the COVID-19 Genomics UK (COG-UK) consortium|Davies|United Kingdom
>Spike|hCoV-19/Spain/VC-IBV-98003604/2020|2020-03-14|EPI_ISL_538621|Original|hCoV-19^^Comunitat Valenciana|Human|Servicio de Microbiolog\u00eda. Hospital General Universitario de Castell\u00f3n|SeqCOVID-SPAIN consortium/IBV(CSIC)|Comas|Spain
>Spike|hCoV-19/USA/WI-WSLH-200521/2020|2020-05-27|EPI_ISL_578661|Original|hCoV-19^^Wisconsin|Human|Wisconsin State Laboratory of Hygiene Communicable Disease Division|Wisconsin State Laboratory of Hygiene Communicable Disease Division|Florek|USA
>Spike|hCoV-19/Canada/BC_02585169/2020|2020-04-00|EPI_ISL_466725|Original|hCoV-19^^British Columbia|Human|BCCDC Public Health Laboratory|BCCDC Public Health Laboratory|Harrigan|Canada
>Spike|hCoV-19/Belgium/rega-0502639/2020|2020-05-02|EPI_ISL_734697|Original|hCoV-19^^Kessel-Lo|Human|UZ Leuven|KU Leuven|Maes|Belgium
>Spike|hCoV-19/Scotland/QEUH-A21F6C/2020|2020-10-01|EPI_ISL_598834|Original|hCoV-19^^Scotland|Human|Lighthouse Lab in Glasgow|Wellcome Sanger Institute for the COVID-19 Genomics UK (COG-UK) consortium|Davies|United Kingdom
>Spike|hCoV-19/Switzerland/BE-ETHZ-370520/2020|2020-11-19|EPI_ISL_694002|Original|hCoV-19^^Bern|Human|Viollier AG|Department of Biosystems Science and Engineering|Nadeau|Switzerland
>Spike|hCoV-19/Wales/ALDP-9503D2/2020|2020-06-16|EPI_ISL_555191|Original|hCoV-19^^Wales|Human|Lighthouse Lab in Alderley Park|Wellcome Sanger Institute for the COVID-19 Genomics UK (COG-UK) consortium|Davies|United Kingdom

>Spike|hCoV-19/Australia/VIC8835/2020|2020-07-23|EPI_ISL_565225|Original|hCoV-19^^Victoria|Human|Microbiological Diagnostic Unit - Public Health Laboratory (MDU-PHL)|MDU-PHL|Schultz|Australia
>Spike|hCoV-19/England/LEED-2A98A5/2020|2020-04-10|EPI_ISL_538911|Original|hCoV-19^^England|Human|Leeds Teaching Hospitals NHS Trust and Public Health England|Wellcome Sanger Institute for the COVID-19 Genomics UK (COG-UK) consortium|Davies|United Kingdom
>Spike|hCoV-19/England/CAMC-B20CB0/2020|2020-11-02|EPI_ISL_647559|Original|hCoV-19^^England|Human|Lighthouse Lab in Cambridge|Wellcome Sanger Institute for the COVID-19 Genomics UK (COG-UK) Consortium|Davies|United Kingdom
>Spike|hCoV-19/Wales/PHWC-168D3B/2020|2020-04-01|EPI_ISL_494358|Original|hCoV-19^^Wales|Human|Originating lab|COVID-19 Genomics UK (COG-UK) Consortium|Moore|United Kingdom
>Spike|hCoV-19/Scotland/CVR3258/2020|2020-04-28|EPI_ISL_473646|Original|hCoV-19^^Scotland|Human|West of Scotland Specialist Virology Centre|COVID-19 Genomics UK (COG-UK) Consortium|Robertson|United Kingdom
>Spike|hCoV-19/Luxembourg/LNS6230945/2020|2020-11-08|EPI_ISL_744386|Original|hCoV-19^^Luxembourg|Human|Laboratoire national de sant\u00e9|Laboratoire national de sant\u00e9|Wienecke-Baldacchino|Luxembourg
>Spike|hCoV-19/England/CWAR-24FDDE7/2020|2020-06-07|EPI_ISL_584526|Original|hCoV-19^^England|Human|UHCW / University of Warwick|COVID-19 Genomics UK (COG-UK) Consortium|Stark|United Kingdom
>Spike|hCoV-19/USA/WA-S482/2020|2020-04-06|EPI_ISL_434242|Original|hCoV-19^^Washington|Human|Washington State Department of Health|Seattle Flu Study|Bedford|USA
>Spike|hCoV-19/Switzerland/AG-ETHZ-420598/2020|2020-12-22|EPI_ISL_767946|Original|hCoV-19^^Aargau|Human|Viollier AG|Department of Biosystems Science and Engineering|Nadeau|Switzerland
>Spike|hCoV-19/Portugal/PT2034/2020|2020-11-09|EPI_ISL_732250|Original|hCoV-19^^Portugal|Human|Instituto Nacional de Saude (INSA) and Instituto Gulbenkian de Ciencia (IGC)|Instituto Nacional de Saude (INSA) and Instituto Gulbenkian de Ciencia (IGC)|Borges|Portugal
>Spike|hCoV-19/England/QEUH-9B87EB/2020|2020-09-05|EPI_ISL_549689|Original|hCoV-19^^England|Human|Lighthouse Lab in Glasgow|Wellcome Sanger Institute for the COVID-19 Genomics UK (COG-UK) consortium|Davies|United Kingdom
>Spike|hCoV-19/Scotland/QEUH-AD0C56/2020|2020-10-26|EPI_ISL_649766|Original|hCoV-19^^Scotland|Human|Lighthouse Lab in Glasgow|Wellcome Sanger Institute for the COVID-19 Genomics UK (COG-UK) Consortium|Davies|United Kingdom
>Spike|hCoV-19/Germany/HH-hpi-p1851/2020|2020-03-23|EPI_ISL_776201|Original|hCoV-19^^Hamburg|Human|University Medical Center Hamburg Eppendorf|Heinrich Pette Institute|Robitaille|Germany
>Spike|hCoV-19/Denmark/DCGC-3392/2020|2020-08-31|EPI_ISL_616151|Original|hCoV-19^^Hovedstaden|Human|Department of Virus and Microbiological Special Diagnostics|Albertsen lab|Michaelsen|Denmark
>Spike|hCoV-19/England/ALDP-B46838/2020|2020-11-06|EPI_ISL_656111|Original|hCoV-19^^England|Human|Lighthouse Lab in Alderley Park|Wellcome Sanger Institute for the COVID-19 Genomics UK (COG-UK) Consortium|Davies|United Kingdom
>Spike|hCoV-19/England/MILK-C508D0/2020|2020-12-11|EPI_ISL_736744|Original|hCoV-19^^England|Human|Lighthouse Lab in Milton Keynes|Wellcome Sanger Institute for the COVID-19 Genomics UK (COG-UK) Consortium|Davies|United Kingdom
>Spike|hCoV-19/England/GSTT-265E0BC/2020|2020-03-30|EPI_ISL_484149|Original|hCoV-19^^England|Human|Centre for Clinical Infection and Diagnostics Research and Genomics Innovation Unit|COVID-19 Genomics UK (COG-UK) Consortium|Awan|United Kingdom
>Spike|hCoV-19/USA/TX-HMH-MCoV-12876/2020|2020-08-18|EPI_ISL_788028|Original|hCoV-19^^Texas|Human|Houston Methodist Hospital|Houston Methodist Hospital|Long|USA
>Spike|hCoV-19/England/BRIS-12E250/2020|2020-04-13|EPI_ISL_482009|Original|hCoV-19^^England|Human|PHE South West Regional Laboratory|Wellcome Sanger Institute for the COVID-19 Genomics UK (COG-UK) consortium|Davies|United Kingdom

>Spike|hCoV-19/England/QEUH-9F5991/2020|2020-09-25|EPI_ISL_588947|Original|hCoV-19^^England|Human|Lighthouse Lab in Glasgow|Wellcome Sanger Institute for the COVID-19 Genomics UK (COG-UK) consortium|Davies|United Kingdom
>Spike|hCoV-19/Denmark/DCGC-21598/2020|2020-12-21|EPI_ISL_758119|Original|hCoV-19^^Hovedstaden|Human|Department of Virus and Microbiological Special Diagnostics|Albertsen Lab|Michaelsen|Denmark
>Spike|hCoV-19/Wales/PHWC-48545E/2020|2020-10-25|EPI_ISL_626762|Original|hCoV-19^^Wales|Human|Wales Specialist Virology Centre Sequencing lab|COVID-19 Genomics UK (COG-UK) Consortium|Moore|United Kingdom
>Spike|hCoV-19/Chile/RM-ISPCH-2/2020|2020-03-05|EPI_ISL_414580|Original|hCoV-19^^Region Metropolitana de Santiago|Human|Clinica Santa Maria|Instituto de Salud Publica de Chile|Fernandez|Chile
>Spike|hCoV-19/England/NOTT-114475/2020|2020-10-08|EPI_ISL_577165|Original|hCoV-19^^England|Human|Queens Medical Centre|COVID-19 Genomics UK (COG-UK) Consortium|Loose|United Kingdom
>Spike|hCoV-19/USA/WI-UW-2465/2020|2020-12-18|EPI_ISL_759740|Original|hCoV-19^^Wisconsin|Human|University of Wisconsin-Madison AIDS Vaccine Research Laboratories|University of Wisconsin-Madison AIDS Vaccine Research Laboratories|Moreno|USA
>Spike|hCoV-19/Hong Kong/HKU-IHCE0511-004b/2020|2020-02-05|EPI_ISL_538543|Original|hCoV-19^^Hong Kong|Human|Department of Microbiology|Department of Microbiology|Chan|China
>Spike|hCoV-19/USA/CA-CZB-1654/2020|2020-04-18|EPI_ISL_468598|Original|hCoV-19^^California|Human|Orange County Public Health Lab|Chan-Zuckerberg Biohub|McGeever|USA
>Spike|hCoV-19/USA/CA-CSMC9/2020|2020-03-27|EPI_ISL_475706|Original|hCoV-19^^California|Human|Cedars-Sinai Medical Center|Cedars-Sinai Medical Center|Zhang|USA
>Spike|hCoV-19/Japan/PG-7920/2020|2020-07-00|EPI_ISL_687021|Original|hCoV-19^^Japan|Human|Pathogen Genomics Center|Pathogen Genomics Center|Sekizuka|Japan
>Spike|hCoV-19/England/ALDP-9C5C82/2020|2020-09-10|EPI_ISL_567671|Original|hCoV-19^^England|Human|Lighthouse Lab in Alderley Park|Wellcome Sanger Institute for the COVID-19 Genomics UK (COG-UK) consortium|Davies|United Kingdom
>Spike|hCoV-19/England/EXET-1360B5/2020|2020-04-17|EPI_ISL_457195|Original|hCoV-19^^England|Human|University of Exeter|COVID-19 Genomics UK (COG-UK) Consortium|Temperton|United Kingdom
>Spike|hCoV-19/Switzerland/SO-ETHZ-420716/2020|2020-12-24|EPI_ISL_768172|Original|hCoV-19^^Solothurn|Human|Viollier AG|Department of Biosystems Science and Engineering|Nadeau|Switzerland
>Spike|hCoV-19/USA/UT-02219/2020|2020-04-15|EPI_ISL_436919|Original|hCoV-19^^Utah|Human|Utah Public Health Laboratory|Utah Public Health Laboratory|Young|USA
>Spike|hCoV-19/Wales/PHWC-484914/2020|2020-10-23|EPI_ISL_628058|Original|hCoV-19^^Wales|Human|Wales Specialist Virology Centre Sequencing lab|COVID-19 Genomics UK (COG-UK) Consortium|Moore|United Kingdom
>Spike|hCoV-19/England/CAMB-A985AA/2020|2020-12-27|EPI_ISL_764141|Original|hCoV-19^^England|Human|Department of Pathology|COVID-19 Genomics UK (COG-UK) Consortium|Goodfellow|United Kingdom
>Spike|hCoV-19/Gibraltar/204641721/2020|2020-00-00|EPI_ISL_637199|Original|hCoV-19^^Gibraltar|Human|Microbiology|Respiratory Virus Unit|Platt|Gibraltar
>Spike|hCoV-19/England/MILK-994493/2020|2020-08-25|EPI_ISL_552404|Original|hCoV-19^^England|Human|Lighthouse Lab in Milton Keynes|Wellcome Sanger Institute for the COVID-19 Genomics UK (COG-UK) consortium|Davies|United Kingdom
>Spike|hCoV-19/England/204221405/2020|2020-00-00|EPI_ISL_593798|Original|hCoV-19^^England|Human|Respiratory Virus Unit|Respiratory Virus Unit|Platt|United Kingdom
>Spike|hCoV-19/England/MILK-ACC507/2020|2020-10-21|EPI_ISL_634329|Original|hCoV-19^^England|Human|Lighthouse Lab in Milton Keynes|Wellcome Sanger Institute for the COVID-19 Genomics UK (COG-UK) consortium|Davies|United Kingdom
>Spike|hCoV-19/Denmark/DCGC-6343/2020|2020-09-28|EPI_ISL_622325|Original|hCoV-19^^Hovedstaden|Human|Department of Virus and Microbiological Special Diagnostics|Albertsen lab|Michaelsen|Denmark

>Spike|hCoV-19/England/QEUH-AD54D1/2020|2020-10-26|EPI_ISL_633862|Original|hCoV-19^^England|Human|Lighthouse Lab in Glasgow|Wellcome Sanger Institute for the COVID-19 Genomics UK (COG-UK) consortium|Davies|United Kingdom
>Spike|hCoV-19/England/CAMB-1B1911/2020|2020-05-13|EPI_ISL_452981|Original|hCoV-19^^England|Human|Department of Pathology|COVID-19 Genomics UK (COG-UK) Consortium|Meredith|United Kingdom
>Spike|hCoV-19/USA/FL-BPHL-1230/2020|2020-04-10|EPI_ISL_541240|Original|hCoV-19^^Florida|Human|Florida Bureau of Public Health Laboratories|Florida Bureau of Public Health Laboratories|Blanton|USA
>Spike|hCoV-19/England/ALDP-95C066/2020|2020-06-14|EPI_ISL_556110|Original|hCoV-19^^England|Human|Lighthouse Lab in Alderley Park|Wellcome Sanger Institute for the COVID-19 Genomics UK (COG-UK) consortium|Davies|United Kingdom
>Spike|hCoV-19/England/BIRM-6128F/2020|2020-06-08|EPI_ISL_473448|Original|hCoV-19^^England|Human|University of Birmingham|COVID-19 Genomics UK (COG-UK) Consortium|Loman|United Kingdom
>Spike|hCoV-19/Wales/PHWC-47DD7C/2020|2020-10-10|EPI_ISL_595360|Original|hCoV-19^^Wales|Human|Originating lab|COVID-19 Genomics UK (COG-UK) Consortium|Moore|United Kingdom
>Spike|hCoV-19/USA/FL-BPHL-1543/2020|2020-08-27|EPI_ISL_568641|Original|hCoV-19^^Florida|Human|Florida Bureau of Public Health Laboratories|Florida Bureau of Public Health Laboratories|Blanton|USA
>Spike|hCoV-19/Scotland/EDB3783/2020|2020-04-29|EPI_ISL_439323|Original|hCoV-19^^Scotland|Human|Virology Department|COVID-19 Genomics UK (COG-UK) Consortium|Williams|United Kingdom
>Spike|hCoV-19/Denmark/DCGC-14045/2020|2020-11-30|EPI_ISL_711094|Original|hCoV-19^^Hovedstaden|Human|Department of Virus and Microbiological Special Diagnostics|Albertsen Lab|Michaelsen|Denmark
>Spike|hCoV-19/England/MILK-A69A49/2020|2020-10-09|EPI_ISL_665721|Original|hCoV-19^^England|Human|University of Exeter|COVID-19 Genomics UK (COG-UK) Consortium|Temperton|United Kingdom
>Spike|hCoV-19/USA/SC-DHEC-0886/2020|2020-06-26|EPI_ISL_538410|Original|hCoV-19^^South Carolina|Human|Microbiology Division|Microbiology Division|Flores|USA
>Spike|hCoV-19/Scotland/CVR5589/2020|2020-10-30|EPI_ISL_651854|Original|hCoV-19^^Scotland|Human|West of Scotland Specialist Virology Centre|COVID-19 Genomics UK (COG-UK) Consortium|Robertson|United Kingdom
>Spike|hCoV-19/Wales/QEUH-A60C88/2020|2020-10-12|EPI_ISL_666591|Original|hCoV-19^^Wales|Human|Northumbria University / South Tees Hospitals NHS Foundation Trust / North Cumbria Integrated Care NHS Foundation Trust / North Tees and Hartlepool NHS Foundation Trust / Newcastle Hospitals NHS Foundation Trust|COVID-19 Genomics UK (COG-UK) Consortium|Bashton|United Kingdom
>Spike|hCoV-19/Denmark/DCGC-13832/2020|2020-11-30|EPI_ISL_712224|Original|hCoV-19^^Syddanmark|Human|Department of Virus and Microbiological Special Diagnostics|Albertsen Lab|Michaelsen|Denmark
>Spike|hCoV-19/Japan/PG-2369/2020|2020-05-00|EPI_ISL_685974|Original|hCoV-19^^Japan|Human|Tokyo Metropolitan Institute of Public Health|Pathogen Genomics Center|Sekizuka|Japan
>Spike|hCoV-19/Netherlands/NA_395/2020|2020-04-05|EPI_ISL_455197|Original|hCoV-19^^Netherlands|Human|Dutch COVID-19 response team|Erasmus Medical Center|Oude Munnink|Netherlands
>Spike|hCoV-19/Oman/C-6069/2020|2020-04-26|EPI_ISL_491146|Original|hCoV-19^^Muscat|Human|Oman-National Influenza Center|Biotechnology & OMICs Laboratory|Al Mahruqi|Oman
>Spike|hCoV-19/USA/TX-DSHS-1115/2020|2020-06-21|EPI_ISL_615152|Original|hCoV-19^^Texas|Human|Texas Department of State Health Services|Texas Department of State Health Services|Rahman|USA
>Spike|hCoV-19/England/QEUH-B6C0CB/2020|2020-11-11|EPI_ISL_655112|Original|hCoV-19^^England|Human|Lighthouse Lab in Glasgow|Wellcome Sanger Institute for the COVID-19 Genomics UK (COG-UK) Consortium|Davies|United Kingdom
>Spike|hCoV-19/USA/TX-HMH-MCoV-9651/2020|2020-07-06|EPI_ISL_780204|Original|hCoV-19^^Texas|Human|Houston Methodist Hospital|Houston Methodist Hospital|Long|USA

>Spike|hCoV-19/Iceland/381/2020|2020-03-19|EPI_ISL_424405|Original|hCoV-19^^Reykjavik|Human|The National University Hospital of Iceland|deCODE genetics|J\u00f3nsson|Iceland
>Spike|hCoV-19/England/ALDP-A8733B/2020|2020-10-17|EPI_ISL_606213|Original|hCoV-19^^England|Human|Lighthouse Lab in Alderley Park|Wellcome Sanger Institute for the COVID-19 Genomics UK (COG-UK) consortium|Davies|United Kingdom
>Spike|hCoV-19/Scotland/QEUH-9C7CBD/2020|2020-09-15|EPI_ISL_576037|Original|hCoV-19^^Scotland|Human|Lighthouse Lab in Glasgow|Wellcome Sanger Institute for the COVID-19 Genomics UK (COG-UK) consortium|Davies|United Kingdom
>Spike|hCoV-19/USA/UT-UPHL-04008/2020|2020-05-07|EPI_ISL_575287|Original|hCoV-19^^Utah|Human|Utah Public Health Laboratory|Utah Public Health Laboratory|Young|USA
>Spike|hCoV-19/England/SHEF-D3297/2020|2020-04-29|EPI_ISL_475508|Original|hCoV-19^^England|Human|Virology Department|COVID-19 Genomics UK (COG-UK) Consortium|de Silva|United Kingdom
>Spike|hCoV-19/USA/AZ-TG664281/2020|2020-04-29|EPI_ISL_696082|Original|hCoV-19^^Arizona|Human|Sonora Quest Laboratories|TGen North|French|USA
>Spike|hCoV-19/Japan/PG-8577/2020|2020-08-00|EPI_ISL_686173|Original|hCoV-19^^Japan|Human|Pathogen Genomics Center|Pathogen Genomics Center|Sekizuka|Japan
>Spike|hCoV-19/England/SHEF-D04D0/2020|2020-04-17|EPI_ISL_448903|Original|hCoV-19^^England|Human|Virology Department|COVID-19 Genomics UK (COG-UK) Consortium|de Silva|United Kingdom
>Spike|hCoV-19/England/QEUH-9FA7FC/2020|2020-09-28|EPI_ISL_588143|Original|hCoV-19^^England|Human|Lighthouse Lab in Glasgow|Wellcome Sanger Institute for the COVID-19 Genomics UK (COG-UK) consortium|Davies|United Kingdom
>Spike|hCoV-19/Switzerland/BS-42205161/2020|2020-03-24|EPI_ISL_581738|Original|hCoV-19^^Basel-Stadt|Human|University Hospital Basel|University Hospital Basel|Roloff Handschin|Switzerland
>Spike|hCoV-19/England/PORT-2D3D85/2020|2020-04-22|EPI_ISL_514504|Original|hCoV-19^^England|Human|Centre for Enzyme Innovation|COVID-19 Genomics UK (COG-UK) Consortium|Robson|United Kingdom
>Spike|hCoV-19/Scotland/CVR4028/2020|2020-08-29|EPI_ISL_535127|Original|hCoV-19^^Scotland|Human|West of Scotland Specialist Virology Centre|COVID-19 Genomics UK (COG-UK) Consortium|Robertson|United Kingdom
>Spike|hCoV-19/Wales/PHWC-16BD74/2020|2020-09-15|EPI_ISL_573970|Original|hCoV-19^^Wales|Human|Wales Specialist Virology Centre Sequencing lab|COVID-19 Genomics UK (COG-UK) Consortium|Moore|United Kingdom
>Spike|hCoV-19/England/QEUH-9B8CDD/2020|2020-09-08|EPI_ISL_549621|Original|hCoV-19^^England|Human|Lighthouse Lab in Glasgow|Wellcome Sanger Institute for the COVID-19 Genomics UK (COG-UK) consortium|Davies|United Kingdom
>Spike|hCoV-19/USA/WI-UW-2481/2020|2020-12-25|EPI_ISL_759755|Original|hCoV-19^^Wisconsin|Human|University of Wisconsin-Madison AIDS Vaccine Research Laboratories|University of Wisconsin-Madison AIDS Vaccine Research Laboratories|Moreno|USA
>Spike|hCoV-19/England/NOTT-110DBA/2020|2020-05-05|EPI_ISL_448417|Original|hCoV-19^^England|Human|Queens Medical Centre|COVID-19 Genomics UK (COG-UK) Consortium|Loose|United Kingdom
>Spike|hCoV-19/USA/CA-CZB-1354/2020|2020-04-12|EPI_ISL_468368|Original|hCoV-19^^California|Human|Alameda County Public Health Lab|Chan-Zuckerberg Biohub|Hao|USA
>Spike|hCoV-19/India/MH-ACTREC-517/2020|2020-08-20|EPI_ISL_723055|Original|hCoV-19^^Maharashtra|Human|Hematopathology Laboratory|Hematopathology Laboratory|Patkar|India
>Spike|hCoV-19/Luxembourg/LNS1695013/2020|2020-10-23|EPI_ISL_739980|Original|hCoV-19^^Luxembourg|Human|Laboratoire national de sant\u00e9|Laboratoire national de sant\u00e9|Wienecke-Baldacchino|Luxembourg
>Spike|hCoV-19/England/PORT-2F534F/2020|2020-00-00|EPI_ISL_706888|Original|hCoV-19^^England|Human|Centre for Enzyme Innovation|COVID-19 Genomics UK (COG-UK) Consortium|Robson|United Kingdom
>Spike|hCoV-19/Japan/PG-2483/2020|2020-04-00|EPI_ISL_686088|Original|hCoV-19^^Japan|Human|Saitama Prefectural Institute of Public Health|Pathogen Genomics Center|Sekizuka|Japan

>Spike|hCoV-19/Norway/3361/2020|2020-09-08|EPI_ISL_590881|Original|hCoV-19^^Oslo|Human|Furst Medical Laboratory|Norwegian Institute of Public Health|Vollan|Norway
>Spike|hCoV-19/Australia/VIC9253/2020|2020-08-23|EPI_ISL_565468|Original|hCoV-19^^Victoria|Human|Microbiological Diagnostic Unit - Public Health Laboratory (MDU-PHL)|MDU-PHL|Schultz|Australia
>Spike|hCoV-19/England/ALDP-9444B0/2020|2020-06-07|EPI_ISL_554850|Original|hCoV-19^^England|Human|Lighthouse Lab in Alderley Park|Wellcome Sanger Institute for the COVID-19 Genomics UK (COG-UK) consortium|Davies|United Kingdom
>Spike|hCoV-19/Ireland/WW-NVRL-AIIDV1257v1/2020|2020-10-21|EPI_ISL_768782|Original|hCoV-19^^Wicklow|Human|AIID|Irish Coronavirus Sequencing Consortium - National Virus Reference Laboratory|Gonzalez|Ireland
>Spike|hCoV-19/England/QEUH-9CA081/2020|2020-09-10|EPI_ISL_567361|Original|hCoV-19^^England|Human|Lighthouse Lab in Glasgow|Wellcome Sanger Institute for the COVID-19 Genomics UK (COG-UK) consortium|Davies|United Kingdom
>Spike|hCoV-19/Australia/VIC11434/2020|2020-08-30|EPI_ISL_563370|Original|hCoV-19^^Victoria|Human|Microbiological Diagnostic Unit - Public Health Laboratory (MDU-PHL)|MDU-PHL|Schultz|Australia
>Spike|hCoV-19/Netherlands/ZH-EMC-585/2020|2020-09-18|EPI_ISL_578013|Original|hCoV-19^^Zuid Holland|Human|Dutch COVID-19 response team|Erasmus Medical Center|Oude Munnink|Netherlands
>Spike|hCoV-19/Australia/NSW315/2020|2020-03-27|EPI_ISL_451578|Original|hCoV-19^^New South Wales|Human|Pathology Sydney South West - NSW Health Pathology|NSW Health Pathology - Institute of Clinical Pathology and Medical Research; Westmead Hospital; University of Sydney|Gall|Australia
>Spike|hCoV-19/England/20149013604/2020|2020-04-03|EPI_ISL_466157|Original|hCoV-19^^England|Human|Respiratory Virus Unit|Respiratory Virus Unit|Platt|United Kingdom
>Spike|hCoV-19/Wuhan/0125-A145/2020|2020-01-25|EPI_ISL_493151|Original|hCoV-19^^Hubei|Human|National Virus Resource Center|Computational Virology Group|Liu|China
>Spike|hCoV-19/Scotland/EDB5112/2020|2020-04-21|EPI_ISL_453191|Original|hCoV-19^^Scotland|Human|Virology Department|COVID-19 Genomics UK (COG-UK) Consortium|Williams|United Kingdom
>Spike|hCoV-19/England/ALDP-9C4B2C/2020|2020-09-10|EPI_ISL_567664|Original|hCoV-19^^England|Human|Lighthouse Lab in Alderley Park|Wellcome Sanger Institute for the COVID-19 Genomics UK (COG-UK) consortium|Davies|United Kingdom
>Spike|hCoV-19/England/BIRM-65B53/2020|2020-10-11|EPI_ISL_612208|Original|hCoV-19^^England|Human|University of Birmingham|COVID-19 Genomics UK (COG-UK) Consortium|Loman|United Kingdom
>Spike|hCoV-19/England/QEUH-B10CB3/2020|2020-11-02|EPI_ISL_642304|Original|hCoV-19^^England|Human|Lighthouse Lab in Glasgow|Wellcome Sanger Institute for the COVID-19 Genomics UK (COG-UK) Consortium|Davies|United Kingdom
>Spike|hCoV-19/Cote dIvoire/BKE0011/2020|2020-05-30|EPI_ISL_614348|Original|hCoV-19^^Boauk\u00e9|Human|Molecular diagnostic unit for viral haemorrhagic fevers and emerging viruses|Project group Epidemology of Highly Pathogenic Microorganisms|Calvignac-Spencer|C\u00f4te d'Ivoire
>Spike|hCoV-19/Luxembourg/LNS3841557/2020|2020-11-01|EPI_ISL_740382|Original|hCoV-19^^Luxembourg|Human|Laboratoire national de sant\u00e9|Laboratoire national de sant\u00e9|Wienecke-Baldacchino|Luxembourg
>Spike|hCoV-19/Spain/MD-IBV-99008744/2020|2020-10-16|EPI_ISL_654308|Original|hCoV-19^^Madrid|Human|Hospital General Universitario Gregorio Mara\u00f1\u00f3n|SeqCOVID-SPAIN consortium/IBV(CSIC)|Comas|Spain
>Spike|hCoV-19/Australia/VIC7523/2020|2020-00-00|EPI_ISL_564469|Original|hCoV-19^^Victoria|Human|Microbiological Diagnostic Unit - Public Health Laboratory (MDU-PHL)|MDU-PHL|Schultz|Australia
>Spike|hCoV-19/USA/AZ-CDC-2-3693477/2020|2020-11-02|EPI_ISL_747123|Original|hCoV-19^^Arizona|Human|Respiratory Viruses Branch|Respiratory Viruses Branch|Queen|USA
>Spike|hCoV-19/USA/CA-CZB-4089/2020|2020-08-12|EPI_ISL_548620|Original|hCoV-19^^California|Human|Orange County Public Health Laboratory|Chan-Zuckerberg Biohub|Bhatt|USA

>Spike|hCoV-19/England/NORW-EE730/2020|2020-00-00|EPI_ISL_529243|Original|hCoV-19^^England|Human|Quadram Institute Bioscience|COVID-19 Genomics UK (COG-UK) Consortium|Alikhan|United Kingdom
>Spike|hCoV-19/Saudi Arabia/KAUST-MAKKAH26/2020|2020-03-26|EPI_ISL_678141|Original|hCoV-19^^Makkah|Human|Pathogen Genomics Lab King Abdullah University of Science and Technology(KAUST)|Pathogen Genomics Lab King Abdullah University of Science and Technology(KAUST)|Mohamed Ghazzali|Saudi Arabia
>Spike|hCoV-19/Australia/NSW2171/2020|2020-03-20|EPI_ISL_500642|Original|hCoV-19^^New South Wales|Human|Area of Virology|Area of Virology|Bull|Australia
>Spike|hCoV-19/Australia/VIC4844/2020|2020-07-15|EPI_ISL_519228|Original|hCoV-19^^Victoria|Human|Microbiological Diagnostic Unit - Public Health Laboratory (MDU-PHL)|MDU-PHL|Schultz|Australia
>Spike|hCoV-19/USA/WY-WYPHL-20092968/2020|2020-09-30|EPI_ISL_754182|Original|hCoV-19^^Wyoming|Human|Wyoming Public Health Laboratory|Wyoming Public Health Laboratory|Hull|USA
>Spike|hCoV-19/England/SHEF-CD069/2020|2020-06-21|EPI_ISL_493709|Original|hCoV-19^^England|Human|Virology Department|COVID-19 Genomics UK (COG-UK) Consortium|de Silva|United Kingdom
>Spike|hCoV-19/England/SHEF-10C762E/2020|2020-12-21|EPI_ISL_765155|Original|hCoV-19^^England|Human|Virology Department|COVID-19 Genomics UK (COG-UK) Consortium|de Silva|United Kingdom
>Spike|hCoV-19/Denmark/DCGC-22195/2020|2020-12-14|EPI_ISL_758607|Original|hCoV-19^^Midtjylland|Human|Department of Virus and Microbiological Special Diagnostics|Albertsen Lab|Michaelsen|Denmark
>Spike|hCoV-19/Spain/CN-IBV-97006526/2020|2020-06-18|EPI_ISL_561372|Original|hCoV-19^^Canary Islands|Human|Hospital Universitario de Gran Canaria Dr. Negr\u00edn|SeqCOVID-SPAIN consortium/IBV(CSIC)|Comas|Spain
>Spike|hCoV-19/USA/WI-UW-2415/2020|2020-12-12|EPI_ISL_754634|Original|hCoV-19^^Wisconsin|Human|University of Wisconsin-Madison AIDS Vaccine Research Laboratories|University of Wisconsin-Madison AIDS Vaccine Research Laboratories|Moreno|USA
>Spike|hCoV-19/USA/OR-QDX-1274/2020|2020-03-16|EPI_ISL_572033|Original|hCoV-19^^Oregon|Human|Quest Diagnostics|Quest Diagnostics|Kagan|USA
>Spike|hCoV-19/Costa Rica/INC-0045/2020|2020-03-23|EPI_ISL_491456|Original|hCoV-19^^San Jose|Human|Hospital San Juan de Dios|Inciensa|Brenes Porras|Costa Rica
>Spike|hCoV-19/Denmark/DCGC-11739/2020|2020-10-26|EPI_ISL_670535|Original|hCoV-19^^Midtjylland|Human|Department of Virus and Microbiological Special Diagnostics|Albertsen Lab|Michaelsen|Denmark
>Spike|hCoV-19/USA/MI-MDHHS-SC20449/2020|2020-03-16|EPI_ISL_452323|Original|hCoV-19^^Michigan|Human|Michigan Department of Health and Human Services|Michigan Department of Health and Human Services|Blankenship|USA
>Spike|hCoV-19/USA/LA-CDC-2-3693763/2020|2020-11-04|EPI_ISL_747070|Original|hCoV-19^^Louisiana|Human|Respiratory Viruses Branch|Respiratory Viruses Branch|Queen|USA
>Spike|hCoV-19/England/QEUH-B7D9EA/2020|2020-11-14|EPI_ISL_727840|Original|hCoV-19^^England|Human|Oxford Viromics|COVID-19 Genomics UK (COG-UK) Consortium|Golubchik|United Kingdom
>Spike|hCoV-19/USA/TX-HMH-4413/2020|2020-06-19|EPI_ISL_543601|Original|hCoV-19^^Texas|Human|Houston Methodist Hospital|Houston Methodist Hospital|Long|USA
>Spike|hCoV-19/England/QEUH-B7C07D/2020|2020-11-14|EPI_ISL_668203|Original|hCoV-19^^England|Human|Lighthouse Lab in Glasgow|Wellcome Sanger Institute for the COVID-19 Genomics UK (COG-UK) Consortium|Davies|United Kingdom
>Spike|hCoV-19/England/CAMC-A3E500/2020|2020-10-05|EPI_ISL_609660|Original|hCoV-19^^England|Human|Lighthouse Lab in Cambridge|Wellcome Sanger Institute for the COVID-19 Genomics UK (COG-UK) consortium|Davies|United Kingdom
>Spike|hCoV-19/USA/UT-UPHL-2012433715/2020|2020-11-30|EPI_ISL_746926|Original|hCoV-19^^Utah|Human|Utah Public Health Laboratory|Utah Public Health Laboratory|Gallagher|USA
>Spike|hCoV-19/England/PORT-2D1EB1/2020|2020-04-06|EPI_ISL_499291|Original|hCoV-19^^England|Human|Centre for Enzyme Innovation|COVID-19 Genomics UK (COG-UK) Consortium|Robson|United Kingdom

>Spike|hCoV-19/England/MILK-9E1DAB/2020|2020-09-20|EPI_ISL_581228|Original|hCoV-19^^England|Human|Lighthouse Lab in Milton Keynes|Wellcome Sanger Institute for the COVID-19 Genomics UK (COG-UK) consortium|Davies|United Kingdom
>Spike|hCoV-19/England/QEUH-9DA6AA/2020|2020-09-21|EPI_ISL_601347|Original|hCoV-19^^England|Human|Lighthouse Lab in Glasgow|Wellcome Sanger Institute for the COVID-19 Genomics UK (COG-UK) consortium|Davies|United Kingdom
>Spike|hCoV-19/England/CAMC-B9A12B/2020|2020-11-15|EPI_ISL_673465|Original|hCoV-19^^England|Human|Lighthouse Lab in Cambridge|Wellcome Sanger Institute for the COVID-19 Genomics UK (COG-UK) Consortium|Davies|United Kingdom
>Spike|hCoV-19/England/ALDP-9CC18C/2020|2020-09-16|EPI_ISL_584165|Original|hCoV-19^^England|Human|Quadram Institute Bioscience|COVID-19 Genomics UK (COG-UK) Consortium|Alikhan|United Kingdom
>Spike|hCoV-19/South Africa/KRISP-K000581/2020|2020-07-13|EPI_ISL_509237|Original|hCoV-19^^KwaZulu-Natal|Human|NHLS-IALCH|KRISP|de Oliveira|South Africa
>Spike|hCoV-19/England/QEUH-AD5B54/2020|2020-10-26|EPI_ISL_634639|Original|hCoV-19^^England|Human|Lighthouse Lab in Glasgow|Wellcome Sanger Institute for the COVID-19 Genomics UK (COG-UK) consortium|Davies|United Kingdom
>Spike|hCoV-19/England/ALDP-A6EC8A/2020|2020-10-15|EPI_ISL_607207|Original|hCoV-19^^England|Human|Lighthouse Lab in Alderley Park|Wellcome Sanger Institute for the COVID-19 Genomics UK (COG-UK) consortium|Davies|United Kingdom
>Spike|hCoV-19/USA/CA-ALSR-0543-SAN/2020|2020-05-04|EPI_ISL_483213|Original|hCoV-19^^California|Human|UC San Diego Center for Advanced Laboratory Medicine|Andersen lab at Scripps Research|Gangavarapu|USA
>Spike|hCoV-19/England/NORT-29DC20/2020|2020-05-07|EPI_ISL_488081|Original|hCoV-19^^England|Human|NU-OMICS DNA Sequencing research facility|Wellcome Sanger Institute for the COVID-19 Genomics UK (COG-UK) consortium|Davies|United Kingdom
>Spike|hCoV-19/England/LIVE-A883F/2020|2020-03-26|EPI_ISL_517219|Original|hCoV-19^^England|Human|Liverpool Clinical Laboratories|COVID-19 Genomics UK (COG-UK) Consortium|Haldenby|United Kingdom
>Spike|hCoV-19/England/CAMB-71E79/2020|2020-03-29|EPI_ISL_439633|Original|hCoV-19^^England|Human|Department of Pathology|Wellcome Sanger Institute for the COVID-19 Genomics UK (COG-UK) consortium|Amato|United Kingdom
>Spike|hCoV-19/USA/WA-S2381/2020|2020-06-12|EPI_ISL_497013|Original|hCoV-19^^Washington|Human|Washington State Department of Health|Seattle Flu Study|Lee|USA
>Spike|hCoV-19/USA/CA-CSMC3/2020|2020-03-27|EPI_ISL_475640|Original|hCoV-19^^California|Human|Cedars-Sinai Medical Center|Cedars-Sinai Medical Center|Zhang|USA
>Spike|hCoV-19/England/QEUH-B6793E/2020|2020-11-10|EPI_ISL_655850|Original|hCoV-19^^England|Human|Lighthouse Lab in Glasgow|Wellcome Sanger Institute for the COVID-19 Genomics UK (COG-UK) Consortium|Davies|United Kingdom
>Spike|hCoV-19/Canada/BC-BCCDC-8392/2020|2020-11-00|EPI_ISL_740769|Original|hCoV-19^^British Columbia|Human|BCCDC Public Health Laboratory|BCCDC Public Health Laboratory|Prystajecky|Canada
>Spike|hCoV-19/England/BRIS-1287FD/2020|2020-04-15|EPI_ISL_458755|Original|hCoV-19^^England|Human|PHE South West Regional Laboratory|Wellcome Sanger Institute for the COVID-19 Genomics UK (COG-UK) consortium|Davies|United Kingdom
>Spike|hCoV-19/USA/CA-ALSR-3655/2020|2020-09-25|EPI_ISL_636130|Original|hCoV-19^^California|Human|San Diego County Public Health Laboratory|Andersen lab at Scripps Research|Gangavarapu|USA
>Spike|hCoV-19/England/20144063304/2020|2020-03-31|EPI_ISL_466004|Original|hCoV-19^^England|Human|Respiratory Virus Unit|Respiratory Virus Unit|Platt|United Kingdom
>Spike|hCoV-19/USA/TX-HMH-1337/2020|2020-04-22|EPI_ISL_542657|Original|hCoV-19^^Texas|Human|Houston Methodist Hospital|Houston Methodist Hospital|Long|USA
>Spike|hCoV-19/England/ALDP-C8A10F/2020|2020-12-16|EPI_ISL_762847|Original|hCoV-19^^England|Human|Lighthouse Lab in Alderley Park|Wellcome Sanger Institute for the COVID-19 Genomics UK (COG-UK) Consortium|Davies|United Kingdom
>Spike|hCoV-19/England/QEUH-9C7BED/2020|2020-09-12|EPI_ISL_567265|Original|hCoV-19^^England|Human|Lighthouse Lab in Glasgow|Wellcome Sanger Institute for the COVID-19 Genomics UK (COG-UK) consortium|Davies|United Kingdom

>Spike|hCoV-19/England/PORT-2D0F82/2020|2020-04-23|EPI_ISL_475312|Original|hCoV-19^^England|Human|Centre for Enzyme Innovation|COVID-19 Genomics UK (COG-UK) Consortium|Robson|United Kingdom
>Spike|hCoV-19/USA/MI-MDHHS-SC20179/2020|2020-03-12|EPI_ISL_436808|Original|hCoV-19^^Michigan|Human|Michigan Department of Health and Human Services|Michigan Department of Health and Human Services|Blankenship|USA
>Spike|hCoV-19/England/NORT-2834DA/2020|2020-03-29|EPI_ISL_488633|Original|hCoV-19^^England|Human|NU-OMICS DNA Sequencing research facility|Wellcome Sanger Institute for the COVID-19 Genomics UK (COG-UK) consortium|Davies|United Kingdom
>Spike|hCoV-19/Wales/PHWC-49170B/2020|2020-11-05|EPI_ISL_706683|Original|hCoV-19^^Wales|Human|Originating lab|COVID-19 Genomics UK (COG-UK) Consortium|Moore|United Kingdom
>Spike|hCoV-19/England/NOTT-1207F5/2020|2020-12-10|EPI_ISL_725649|Original|hCoV-19^^England|Human|Queens Medical Centre|COVID-19 Genomics UK (COG-UK) Consortium|Loose|United Kingdom
>Spike|hCoV-19/USA/FL-BPHL-0954/2020|2020-07-07|EPI_ISL_517844|Original|hCoV-19^^Florida|Human|Florida Bureau of Public Health Laboratories|Florida Bureau of Public Health Laboratories|Blanton|USA
>Spike|hCoV-19/England/20166058904/2020|2020-04-15|EPI_ISL_466556|Original|hCoV-19^^England|Human|Respiratory Virus Unit|Respiratory Virus Unit|Platt|United Kingdom
>Spike|hCoV-19/England/BRIS-12EDD7/2020|2020-04-21|EPI_ISL_488336|Original|hCoV-19^^England|Human|PHE South West Regional Laboratory|Wellcome Sanger Institute for the COVID-19 Genomics UK (COG-UK) consortium|Davies|United Kingdom
>Spike|hCoV-19/USA/CA-CZB-3677/2020|2020-07-13|EPI_ISL_548479|Original|hCoV-19^^California|Human|University of California|Chan-Zuckerberg Biohub|Bhatt|USA
>Spike|hCoV-19/Wales/CAMC-9DF72A/2020|2020-09-20|EPI_ISL_639004|Original|hCoV-19^^Wales|Human|Department of Pathology|COVID-19 Genomics UK (COG-UK) Consortium|Goodfellow|United Kingdom
>Spike|hCoV-19/England/LIVE-A3FC6/2020|2020-06-25|EPI_ISL_490400|Original|hCoV-19^^England|Human|Liverpool Clinical Laboratories|COVID-19 Genomics UK (COG-UK) Consortium|Haldenby|United Kingdom
>Spike|hCoV-19/India/MH-NIV-157478/2020|2020-08-10|EPI_ISL_541707|Original|hCoV-19^^Maharashtra|Human|National Institute of Virology|National Institute of Virology|Potdar|India
>Spike|hCoV-19/England/MILK-BAA191/2020|2020-11-18|EPI_ISL_681628|Original|hCoV-19^^England|Human|Lighthouse Lab in Milton Keynes|Wellcome Sanger Institute for the COVID-19 Genomics UK (COG-UK) Consortium|Davies|United Kingdom
>Spike|hCoV-19/Wales/PHWC-33DD0/2020|2020-04-16|EPI_ISL_446823|Original|hCoV-19^^Wales|Human|Wales Specialist Virology Centre|Public Health Wales Microbiology Cardiff|Moore|United Kingdom
>Spike|hCoV-19/Wales/PHWC-4805C9/2020|2020-10-18|EPI_ISL_612906|Original|hCoV-19^^Wales|Human|Wales Specialist Virology Centre Sequencing lab|COVID-19 Genomics UK (COG-UK) Consortium|Moore|United Kingdom
>Spike|hCoV-19/Germany/NW-HHU-380/2020|2020-11-26|EPI_ISL_718048|Orginal|hCoV-19^^North Rhine-Westphalia|Human|ZOTZ KLIMAS MVZ D\u00fcsseldorf-Centrum GbR \u00dcBAG f\u00fcr Labormedizin|Center of Medical Microbiology|Wienemann|Germany
>Spike|hCoV-19/Italy/VEN-UniVR-67/2020|2020-03-14|EPI_ISL_751376|Original|hCoV-19^^Veneto|Human|IRCCS Sacro Cuore Don Calabria Hospital|University of Verona|Mori|Italy
>Spike|hCoV-19/England/PORT-2EA1CB/2020|2020-00-00|EPI_ISL_665483|Original|hCoV-19^^England|Human|Centre for Enzyme Innovation|COVID-19 Genomics UK (COG-UK) Consortium|Robson|United Kingdom
>Spike|hCoV-19/Norway/3708/2020|2020-09-21|EPI_ISL_590985|original|hCoV-19^^Vestland|Human|Foerde Hospital|Norwegian Institute of Public Health|Vollan|Norway
>Spike|hCoV-19/USA/VA-DCLS-0687/2020|2020-04-18|EPI_ISL_500849|Original|hCoV-19^^Virginia|Human|Virginia DCLS|Virginia DCLS|St. Jacques|USA
>Spike|hCoV-19/Spain/AN-IBV-004097/2020|2020-04-17|EPI_ISL_510052|Original|hCoV-19^^Andalucia|Human|Servicio de Microbiolog\u00eda. HRU de M\u00e1laga. Servicio Andaluz de Salud|SeqCOVID-SPAIN consortium/IBV(CSIC)|Comas|Spain

>Spike|hCoV-19/England/LOND-DA63A/2020|2020-11-11|EPI_ISL_724648|Original|hCoV-
19^^England|Human|University College London|COVID-19 Genomics UK (COG-UK)
Consortium|Roy|United Kingdom
>Spike|hCoV-19/England/LIVE-DC3163/2020|2020-11-20|EPI_ISL_704137|Original|hCoV-
19^^England|Human|Liverpool Clinical Laboratories|COVID-19 Genomics UK (COG-UK)
Consortium|Haldenby|United Kingdom
>Spike|hCoV-19/Japan/PG-1775/2020|2020-04-00|EPI_ISL_685381|Original|hCoV-
19^^Japan|Human|Pathogen Genomics Center|Pathogen Genomics Center|Sekizuka|Japan
>Spike|hCoV-19/Austria/CeMM0779/2020|2020-04-24|EPI_ISL_583696|Original|hCoV-
19^^Austria|Human|Center for Virology|Bergthaler laboratory|Bergthaler|Austria
>Spike|hCoV-19/England/ALDP-B84F2A/2020|2020-11-15|EPI_ISL_674396|Original|hCoV-
19^^England|Human|Lighthouse Lab in Alderley Park|Wellcome Sanger Institute for the
COVID-19 Genomics UK (COG-UK) Consortium|Davies|United Kingdom
>Spike|hCoV-19/India/MH-AFMC-5670/2020|2020-05-25|EPI_ISL_496540|Original|hCoV-
19^^Maharashtra|Human|Armed Forces Medical College|National Centre For Cell
Science|Paul|India
>Spike|hCoV-19/India/GJ-GBRC182b/2020|2020-06-03|EPI_ISL_467052|Original|hCoV-
19^^Gujarat|Human|B.J. Medical College and Civil hospital|Gujarat Biotechnology
Research Centre|Joshi|India
>Spike|hCoV-19/England/MILK-48213C/2020|2020-04-29|EPI_ISL_559669|Original|hCoV-
19^^England|Human|Lighthouse Lab in Milton Keynes|Wellcome Sanger Institute for the
COVID-19 Genomics UK (COG-UK) consortium|Davies|United Kingdom
>Spike|hCoV-19/England/MILK-9932E5/2020|2020-08-27|EPI_ISL_551944|Original|hCoV-
19^^England|Human|Lighthouse Lab in Milton Keynes|Wellcome Sanger Institute for the
COVID-19 Genomics UK (COG-UK) consortium|Davies|United Kingdom
>Spike|hCoV-19/Burkina Faso/2-042-1650/2020|2020-08-13|EPI_ISL_660493|Original|hCoV-
19^^Hound\u00e9|Human|Laboratoire de Microbiologie CHU Souro Sanou|Centre
Muraz|Ouedraogo|Burkina Faso
>Spike|hCoV-19/Norway/2090/2020|2020-03-16|EPI_ISL_420153|Original|hCoV-
19^^Norway|Human|University Hospital of Northern Norway|Norwegian Institute of Public
Health|Hungnes|Norway
>Spike|hCoV-19/England/OXON-B2FE2/2020|2020-05-24|EPI_ISL_534833|Original|hCoV-
19^^England|Human|Oxford Viromics|COVID-19 Genomics UK (COG-UK)
Consortium|Golubchik|United Kingdom
>Spike|hCoV-19/England/QEUH-9DDA14/2020|2020-09-21|EPI_ISL_599549|Original|hCoV-
19^^England|Human|Lighthouse Lab in Glasgow|Wellcome Sanger Institute for the COVID-
19 Genomics UK (COG-UK) consortium|Davies|United Kingdom
>Spike|hCoV-19/Japan/PG-4408/2020|2020-07-00|EPI_ISL_692634|Original|hCoV-
19^^Japan|Human|Pathogen Genomics Center|Pathogen Genomics Center|Sekizuka|Japan
>Spike|hCoV-19/Germany/HH-hpi-p1862/2020|2020-03-23|EPI_ISL_775919|Original|hCoV-
19^^Hamburg|Human|University Medical Center Hamburg Eppendorf|Heinrich Pette
Institute|Robitaille|Germany
>Spike|hCoV-19/Denmark/DCGC-22595/2020|2020-12-14|EPI_ISL_757660|Original|hCoV-
19^^Midtjylland|Human|Department of Virus and Microbiological Special
Diagnostics|Albertsen Lab|Michaelsen|Denmark
>Spike|hCoV-19/USA/FL-BPHL-0352/2020|2020-04-13|EPI_ISL_489761|Original|hCoV-
19^^Florida|Human|Florida Bureau of Public Health Laboratories|Florida Bureau of
Public Health Laboratories|Blanton|USA
>Spike|hCoV-19/Ireland/RN-NVRL-73IRL30636/2020|2020-09-
16|EPI_ISL_578319|Original|hCoV-19^^Roscommon|Human|National Virus Reference
Laboratory|National Virus Reference Laboratory|Gonzalez|Ireland
>Spike|hCoV-19/USA/MD-MDH-0462/2020|2020-11-11|EPI_ISL_682073|Original|hCoV-
19^^Maryland|Human|Maryland Public Health Laboratory|Maryland Public Health
Laboratory|Keller|USA
>Spike|hCoV-19/India/MH-ACTREC-426/2020|2020-07-31|EPI_ISL_700075|Original|hCoV-
19^^Maharashtra|Human|Hematopathology Laboratory|Hematopathology
Laboratory|Patkar|India
>Spike|hCoV-19/France/PAC-SC456/2020|2020-05-08|EPI_ISL_645212|Original|hCoV-
19^^Provence-Alpes-Cote-d\u2019Azur|Human|Unit\u00e9 des Virus \u00c9mergents|CNR
Virus des Infections Respiratoires - France SUD|Bal|France

>Spike|hCoV-19/England/CAMB-77B9A/2020|2020-04-03|EPI_ISL_440830|Original|hCoV-19^^England|Human|Department of Pathology|Wellcome Sanger Institute for the COVID-19 Genomics UK (COG-UK) consortium|Amato|United Kingdom
>Spike|hCoV-19/England/QEUH-BCD7F0/2020|2020-11-26|EPI_ISL_704681|Original|hCoV-19^^England|Human|Lighthouse Lab in Glasgow|Wellcome Sanger Institute for the COVID-19 Genomics UK (COG-UK) Consortium|Davies|United Kingdom
>Spike|hCoV-19/England/ALDP-954CE2/2020|2020-06-12|EPI_ISL_553514|Original|hCoV-19^^England|Human|Lighthouse Lab in Alderley Park|Wellcome Sanger Institute for the COVID-19 Genomics UK (COG-UK) consortium|Davies|United Kingdom
>Spike|hCoV-19/England/20132053902/2020|2020-03-22|EPI_ISL_420505|Original|hCoV-19^^England|Human|Respiratory Virus Unit|Respiratory Virus Unit|Platt|United Kingdom
>Spike|hCoV-19/Russia/OMS-ORINFI-543S/2020|2020-05-23|EPI_ISL_569751|Original|hCoV-19^^Omsk|Human|Omsk Research Institute of Natural Focal Infections|WHO National Influenza Centre Russian Federation|Komissarov|Russia
>Spike|hCoV-19/Scotland/QEUH-9D09AB/2020|2020-09-16|EPI_ISL_601849|Original|hCoV-19^^Scotland|Human|Lighthouse Lab in Glasgow|Wellcome Sanger Institute for the COVID-19 Genomics UK (COG-UK) consortium|Davies|United Kingdom
>Spike|hCoV-19/USA/CA-CZB-1129/2020|2020-03-31|EPI_ISL_445177|Original|hCoV-19^^California|Human|UCSF Clinical Microbiology Laboratory|Chan-Zuckerberg Biohub|Kamm|USA
>Spike|hCoV-19/Denmark/DCGC-21515/2020|2020-12-21|EPI_ISL_758206|Original|hCoV-19^^Hovedstaden|Human|Department of Virus and Microbiological Special Diagnostics|Albertsen Lab|Michaelsen|Denmark
>Spike|hCoV-19/England/20194020701/2020|2020-05-05|EPI_ISL_465608|Original|hCoV-19^^England|Human|Respiratory Virus Unit|Respiratory Virus Unit|Platt|United Kingdom
>Spike|hCoV-19/England/ALDP-B23BED/2020|2020-11-04|EPI_ISL_646342|Original|hCoV-19^^England|Human|Lighthouse Lab in Alderley Park|Wellcome Sanger Institute for the COVID-19 Genomics UK (COG-UK) Consortium|Davies|United Kingdom
>Spike|hCoV-19/England/NOTT-119A8D/2020|2020-11-03|EPI_ISL_627721|Original|hCoV-19^^England|Human|Queens Medical Centre|COVID-19 Genomics UK (COG-UK) Consortium|Loose|United Kingdom
>Spike|hCoV-19/Norway/2966/2020|2020-07-22|EPI_ISL_549093|Original|hCoV-19^^Oslo|Human|Ostfold Hospital Trust - Kalnes|Norwegian Institute of Public Health|Kopperud Riis|Norway
>Spike|hCoV-19/Cyprus/008/2020|2020-04-27|EPI_ISL_463748|Original|hCoV-19^^Cyprus|Human|Department of Molecular Virology|Department of Molecular Virology|Richter|Cyprus
>Spike|hCoV-19/USA/WI-GMF-27798/2020|2020-10-26|EPI_ISL_660992|Original|hCoV-19^^Wisconsin|Human|Gundersen Molecular Diagnostics Laboratory|Kabara Cancer Research Institute|Kenny|USA
>Spike|hCoV-19/USA/CA-CZB-13012/2020|2020-10-26|EPI_ISL_672065|Original|hCoV-19^^California|Human|Santa Clara County Public Health Laboratory|Chan-Zuckerberg Biohub|Bhatt|USA
>Spike|hCoV-19/USA/UT-QDX-2523/2020|2020-03-13|EPI_ISL_604859|Original|hCoV-19^^Utah|Human|Quest Diagnostics|Quest Diagnostics|Kagan|USA
>Spike|hCoV-19/Australia/VIC17944/2020|2020-08-16|EPI_ISL_779578|Original|hCoV-19^^Victoria|Human|Microbiological Diagnostic Unit - Public Health Laboratory (MDU-PHL)|MDU-PHL|Beck|Australia
>Spike|hCoV-19/Portugal/PT0371/2020|2020-03-19|EPI_ISL_454087|Original|hCoV-19^^Portugal|Human|unknown|Instituto Nacional de Saude (INSA)|Borges|Portugal
>Spike|hCoV-19/Scotland/QEUH-9B8253/2020|2020-09-10|EPI_ISL_568255|Original|hCoV-19^^Scotland|Human|Lighthouse Lab in Glasgow|Wellcome Sanger Institute for the COVID-19 Genomics UK (COG-UK) consortium|Davies|United Kingdom
>Spike|hCoV-19/England/ALDP-BBB151/2020|2020-11-20|EPI_ISL_701691|Original|hCoV-19^^England|Human|Lighthouse Lab in Alderley Park|Wellcome Sanger Institute for the COVID-19 Genomics UK (COG-UK) Consortium|Davies|United Kingdom
>Spike|hCoV-19/France/PAC-IHU-1292/2020|2020-08-18|EPI_ISL_569109|Original|hCoV-19^^Provence-Alpes-C\u00f4te-d\u2019Azur|Human|MEPHI|MEPHI|Levasseur|France

>Spike|hCoV-19/England/CAMC-A64E60/2020|2020-10-11|EPI_ISL_608485|Original|hCoV-19^^England|Human|Lighthouse Lab in Cambridge|Wellcome Sanger Institute for the COVID-19 Genomics UK (COG-UK) consortium|Davies|United Kingdom
>Spike|hCoV-19/USA/UT-UPHL-201002768/2020|2020-04-24|EPI_ISL_594428|Original|hCoV-19^^Utah|Human|Utah Public Health Laboratory|Utah Public Health Laboratory|Young|USA
>Spike|hCoV-19/USA/OR-OHSU-0698/2020|2020-06-18|EPI_ISL_525890|Original|hCoV-19^^Oregon|Human|OHSU Lab Services Molecular Microbiology Lab|Ginkgo Bioworks Clinical Laboratory|O'Roak|USA
>Spike|hCoV-19/DRC/4647/2020|2020-04-30|EPI_ISL_513609|Original|hCoV-19^^Kinshasa|Human|Viral Respiratory Lab|Pathogen Sequencing Lab|Lokilo|Democratic Republic of the Congo
>Spike|hCoV-19/USA/TX-HMH-781/2020|2020-04-09|EPI_ISL_545645|Original|hCoV-19^^Texas|Human|Houston Methodist Hospital|Houston Methodist Hospital|Long|USA
>Spike|hCoV-19/USA/WA-UW-1578/2020|2020-03-19|EPI_ISL_423016|Original|hCoV-19^^Washington|Human|UW Virology Lab|UW Virology Lab|Roychoudhury|USA
>Spike|hCoV-19/Luxembourg/LNS4470088/2020|2020-11-19|EPI_ISL_740476|Original|hCoV-19^^Luxembourg|Human|Laboratoire national de sant\u00e9|Laboratoire national de sant\u00e9|Wienecke-Baldacchino|Luxembourg
>Spike|hCoV-19/England/QEUH-C938E0/2020|2020-12-17|EPI_ISL_777059|Original|hCoV-19^^England|Human|Lighthouse Lab in Glasgow|Wellcome Sanger Institute for the COVID-19 Genomics UK (COG-UK) Consortium|Davies|United Kingdom
>Spike|hCoV-19/Wales/PHWC-2EE3F/2020|2020-04-06|EPI_ISL_446161|Original|hCoV-19^^Wales|Human|Wales Specialist Virology Centre|Public Health Wales Microbiology Cardiff|Moore|United Kingdom
>Spike|hCoV-19/England/QEUH-AE26EA/2020|2020-10-26|EPI_ISL_633467|Original|hCoV-19^^England|Human|Lighthouse Lab in Glasgow|Wellcome Sanger Institute for the COVID-19 Genomics UK (COG-UK) consortium|Davies|United Kingdom
>Spike|hCoV-19/USA/TX-HMH-MCoV-8959/2020|2020-07-02|EPI_ISL_780859|Original|hCoV-19^^Texas|Human|Houston Methodist Hospital|Houston Methodist Hospital|Long|USA
>Spike|hCoV-19/England/ALDP-B751EA/2020|2020-11-12|EPI_ISL_740899|Original|hCoV-19^^England|Human|Oxford Viromics|COVID-19 Genomics UK (COG-UK) Consortium|Golubchik|United Kingdom
>Spike|hCoV-19/Finland/S5CD7/2020|2020-10-30|EPI_ISL_732651|Original|hCoV-19^^Uusimaa|Human|Department of Virology and Immunology|Department of Virology|Smura|Finland
>Spike|hCoV-19/USA/LA-GBCL-26684506/2020|2020-04-10|EPI_ISL_485311|Original|hCoV-19^^Louisiana|Human|River Road Testing Lab|Ginkgo Bioworks Clinical Laboratory|Smith|USA
>Spike|hCoV-19/USA/TX-HMH-MCoV-10339/2020|2020-07-08|EPI_ISL_789428|Original|hCoV-19^^Texas|Human|Houston Methodist Hospital|Houston Methodist Hospital|Long|USA
>Spike|hCoV-19/USA/CA-ALSR-0907-IPL/2020|2020-04-27|EPI_ISL_483409|Original|hCoV-19^^California|Human|UC San Diego Center for Advanced Laboratory Medicine|Andersen lab at Scripps Research|Gangavarapu|USA
>Spike|hCoV-19/Denmark/DCGC-10589/2020|2020-11-02|EPI_ISL_670072|Original|hCoV-19^^Hovedstaden|Human|Department of Virus and Microbiological Special Diagnostics|Albertsen Lab|Michaelsen|Denmark
>Spike|hCoV-19/Iceland/241/2020|2020-03-17|EPI_ISL_417569|Original|hCoV-19^^Reykjavik|Human|The National University Hospital of Iceland|deCODE genetics|J\u00f3nsson|Iceland
>Spike|hCoV-19/England/ALDP-500469/2020|2020-06-25|EPI_ISL_559099|Original|hCoV-19^^England|Human|Lighthouse Lab in Alderley Park|Wellcome Sanger Institute for the COVID-19 Genomics UK (COG-UK) consortium|Davies|United Kingdom
>Spike|hCoV-19/Netherlands/ZH-EMC-462/2020|2020-08-12|EPI_ISL_523799|Original|hCoV-19^^Zuid Holland|Human|Dutch COVID-19 response team|Erasmus Medical Center|Oude Munnink|Netherlands
>Spike|hCoV-19/England/QEUH-AAED9A/2020|2020-10-22|EPI_ISL_653005|Original|hCoV-19^^England|Human|Oxford Viromics|COVID-19 Genomics UK (COG-UK) Consortium|Golubchik|United Kingdom

>Spike|hCoV-19/England/CAMC-BC2096/2020|2020-11-22|EPI_ISL_705123|Original|hCoV-19^^England|Human|Lighthouse Lab in Cambridge|Wellcome Sanger Institute for the COVID-19 Genomics UK (COG-UK) Consortium|Davies|United Kingdom
>Spike|hCoV-19/England/ALDP-9C5D25/2020|2020-09-10|EPI_ISL_567775|Original|hCoV-19^^England|Human|Lighthouse Lab in Alderley Park|Wellcome Sanger Institute for the COVID-19 Genomics UK (COG-UK) consortium|Davies|United Kingdom
>Spike|hCoV-19/England/MILK-A69803/2020|2020-10-08|EPI_ISL_665273|Original|hCoV-19^^England|Human|University of Exeter|COVID-19 Genomics UK (COG-UK) Consortium|Temperton|United Kingdom
>Spike|hCoV-19/USA/UT-0291/2020|2020-03-27|EPI_ISL_420811|Original|hCoV-19^^Utah|Human|Utah Public Health Laboratory|Utah Public Health Laboratory|Young|USA
>Spike|hCoV-19/Scotland/QEUH-9D31EC/2020|2020-09-20|EPI_ISL_577381|Original|hCoV-19^^Scotland|Human|Centre for Enzyme Innovation|COVID-19 Genomics UK (COG-UK) Consortium|Robson|United Kingdom
>Spike|hCoV-19/USA/ID-UW-6468/2020|2020-04-02|EPI_ISL_570120|Original|hCoV-19^^Idaho|Human|UW Virology Lab|UW Virology Lab|Roychoudhury|USA
>Spike|hCoV-19/England/SHEF-C27F6/2020|2020-05-01|EPI_ISL_457594|Original|hCoV-19^^England|Human|Virology Department|COVID-19 Genomics UK (COG-UK) Consortium|de Silva|United Kingdom
>Spike|hCoV-19/Belgium/ULG-11095/2020|2020-12-15|EPI_ISL_737344|Original|hCoV-19^^Li\u00e8ge|Human|Department of Clinical Microbiology|GIGA Medical Genomics|Durkin|Belgium
>Spike|hCoV-19/England/CAMC-B2187F/2020|2020-11-01|EPI_ISL_647205|Original|hCoV-19^^England|Human|Lighthouse Lab in Cambridge|Wellcome Sanger Institute for the COVID-19 Genomics UK (COG-UK) Consortium|Davies|United Kingdom
>Spike|hCoV-19/England/LIVE-A4332/2020|2020-06-16|EPI_ISL_490415|Original|hCoV-19^^England|Human|Liverpool Clinical Laboratories|COVID-19 Genomics UK (COG-UK) Consortium|Haldenby|United Kingdom
>Spike|hCoV-19/England/CAMC-D025B7/2020|2020-12-21|EPI_ISL_781757|Original|hCoV-19^^England|Human|Lighthouse Lab in Cambridge|Wellcome Sanger Institute for the COVID-19 Genomics UK (COG-UK) Consortium|Davies|United Kingdom
>Spike|hCoV-19/Wales/PHWC-497C24/2020|2020-11-25|EPI_ISL_726950|Original|hCoV-19^^Wales|Human|Originating lab|COVID-19 Genomics UK (COG-UK) Consortium|Moore|United Kingdom
>Spike|hCoV-19/Denmark/DCGC-19517/2020|2020-12-07|EPI_ISL_749003|Original|hCoV-19^^Sjaelland|Human|Department of Virus and Microbiological Special Diagnostics|Albertsen Lab|Michaelsen|Denmark
>Spike|hCoV-19/England/QEUH-9DE187/2020|2020-09-21|EPI_ISL_599752|Original|hCoV-19^^England|Human|Lighthouse Lab in Glasgow|Wellcome Sanger Institute for the COVID-19 Genomics UK (COG-UK) consortium|Davies|United Kingdom
>Spike|hCoV-19/Scotland/QEUH-B68BA0/2020|2020-11-11|EPI_ISL_676381|Original|hCoV-19^^Scotland|Human|Lighthouse Lab in Glasgow|Wellcome Sanger Institute for the COVID-19 Genomics UK (COG-UK) Consortium|Davies|United Kingdom
>Spike|hCoV-19/Switzerland/BS-42222049/2020|2020-04-06|EPI_ISL_581681|Original|hCoV-19^^Basel-Stadt|Human|University Hospital Basel|University Hospital Basel|Roloff Handschin|Switzerland
>Spike|hCoV-19/Denmark/DCGC-16726/2020|2020-10-19|EPI_ISL_714438|Original|hCoV-19^^Hovedstaden|Human|Department of Virus and Microbiological Special Diagnostics|Albertsen Lab|Michaelsen|Denmark
>Spike|hCoV-19/USA/CA-QDX-79/2020|2020-03-15|EPI_ISL_467843|Original|hCoV-19^^California|Human|Quest Diagnostics|Quest Diagnostics|Kagan|USA
>Spike|hCoV-19/England/QEUH-9E45EC/2020|2020-09-22|EPI_ISL_579907|Original|hCoV-19^^England|Human|Lighthouse Lab in Glasgow|Wellcome Sanger Institute for the COVID-19 Genomics UK (COG-UK) consortium|Davies|United Kingdom
>Spike|hCoV-19/United Arab Emirates/L920634181/2020|2020-03-31|EPI_ISL_520727|Original|hCoV-19^^United Arab Emirates|Human|Mohammed Bin Rashid University of Medicine and Health Sciences|Al Jalila Genomics Center|Ramaswamy|United Arab Emirates

>Spike|hCoV-19/England/QEUH-9F57D3/2020|2020-09-25|EPI_ISL_588910|Original|hCoV-19^^England|Human|Lighthouse Lab in Glasgow|Wellcome Sanger Institute for the COVID-19 Genomics UK (COG-UK) consortium|Davies|United Kingdom
>Spike|hCoV-19/Wales/PHWC-48895C/2020|2020-11-01|EPI_ISL_639447|Original|hCoV-19^^Wales|Human|Wales Specialist Virology Centre Sequencing lab|COVID-19 Genomics UK (COG-UK) Consortium|Moore|United Kingdom
>Spike|hCoV-19/England/CAMC-B0C86B/2020|2020-10-31|EPI_ISL_643347|Original|hCoV-19^^England|Human|Lighthouse Lab in Cambridge|Wellcome Sanger Institute for the COVID-19 Genomics UK (COG-UK) Consortium|Davies|United Kingdom
>Spike|hCoV-19/England/MILK-B04B5B/2020|2020-10-24|EPI_ISL_650951|Original|hCoV-19^^England|Human|Department of Pathology|COVID-19 Genomics UK (COG-UK) Consortium|Goodfellow|United Kingdom
>Spike|hCoV-19/Luxembourg/LNS1908786/2020|2020-10-26|EPI_ISL_740020|Original|hCoV-19^^Luxembourg|Human|Laboratoire national de sant\u00e9|Laboratoire national de sant\u00e9|Wienecke-Baldacchino|Luxembourg
>Spike|hCoV-19/England/ALDP-B854B1/2020|2020-11-12|EPI_ISL_675489|Original|hCoV-19^^England|Human|Lighthouse Lab in Alderley Park|Wellcome Sanger Institute for the COVID-19 Genomics UK (COG-UK) Consortium|Davies|United Kingdom
>Spike|hCoV-19/Wales/CAMC-AAF6DA/2020|2020-10-21|EPI_ISL_634795|Original|hCoV-19^^Wales|Human|Lighthouse Lab in Cambridge|Wellcome Sanger Institute for the COVID-19 Genomics UK (COG-UK) consortium|Davies|United Kingdom
>Spike|hCoV-19/England/MILK-9AAE23/2020|2020-08-26|EPI_ISL_551208|Original|hCoV-19^^England|Human|Lighthouse Lab in Milton Keynes|Wellcome Sanger Institute for the COVID-19 Genomics UK (COG-UK) consortium|Davies|United Kingdom
>Spike|hCoV-19/USA/TX-HMH-MCoV-12605/2020|2020-08-31|EPI_ISL_790414|Original|hCoV-19^^Texas|Human|Houston Methodist Hospital|Houston Methodist Hospital|Long|USA
>Spike|hCoV-19/Sweden/20-08561/2020|2020-03-29|EPI_ISL_475109|Original|hCoV-19^^Sormland|Human|Skovde/Unilabs|The Public Health Agency of Sweden|Svartstr\u00f6m|Sweden
>Spike|hCoV-19/France/ARA-SC326/2020|2020-03-24|EPI_ISL_660330|Original|hCoV-19^^Auvergne-Rh\u00f4ne-Alpes|Human|CHU de Saint-\u00c9tienne H\u00f4pital Nord|CNR Virus des Infections Respiratoires - France SUD|Bal|France
>Spike|hCoV-19/USA/MI-MDHHS-SC22535/2020|2020-11-02|EPI_ISL_708547|Original|hCoV-19^^Michigan|Human|Michigan Department of Health and Human Services|Michigan Department of Health and Human Services|Jacob|USA
>Spike|hCoV-19/England/ALDP-98243E/2020|2020-08-09|EPI_ISL_526362|Original|hCoV-19^^England|Human|Queens Medical Centre|COVID-19 Genomics UK (COG-UK) Consortium|Loose|United Kingdom
>Spike|hCoV-19/England/NORT-1B71BCF/2020|2020-11-19|EPI_ISL_704574|Original|hCoV-19^^England|Human|Northumbria University / South Tees Hospitals NHS Foundation Trust / North Cumbria Integrated Care NHS Foundation Trust / North Tees and Hartlepool NHS Foundation Trust / Newcastle Hospitals NHS Foundation Trust|COVID-19 Genomics UK (COG-UK) Consortium|Bashton|United Kingdom
>Spike|hCoV-19/USA/UT-UPHL-2012864126/2020|2020-08-11|EPI_ISL_745967|Original|hCoV-19^^Utah|Human|Ginkgo Bioworks Clinical Laboratory|Utah Public Health Laboratory|Young|USA
>Spike|hCoV-19/USA/TX-HMH-MCoV-13171/2020|2020-08-26|EPI_ISL_788322|Original|hCoV-19^^Texas|Human|Houston Methodist Hospital|Houston Methodist Hospital|Long|USA
>Spike|hCoV-19/England/ALDP-B8436A/2020|2020-11-14|EPI_ISL_674435|Original|hCoV-19^^England|Human|Lighthouse Lab in Alderley Park|Wellcome Sanger Institute for the COVID-19 Genomics UK (COG-UK) Consortium|Davies|United Kingdom
>Spike|hCoV-19/Canada/MB-NML-574/2020|2020-03-28|EPI_ISL_582269|Original|hCoV-19^^Manitoba|Human|Cadham Provincial Laboratory|National Microbiology Laboratory (NML)|Majer|Canada
>Spike|hCoV-19/Denmark/DCGC-1400/2020|2020-03-30|EPI_ISL_622479|Original|hCoV-19^^Hovedstaden|Human|Department of Virus and Microbiological Special Diagnostics|Albertsen lab|Michaelsen|Denmark
>Spike|hCoV-19/Canada/ON-S1820/2020|2020-11-24|EPI_ISL_729284|Original|hCoV-19^^Ontario|Human|Toronto Invasive Bacterial Diseases Network|McMaster University|McArthur|Canada

>Spike|hCoV-19/Switzerland/SO-ETHZ-420485/2020|2020-12-
21|EPI_ISL_768198|Original|hCoV-19^^Solothurn|Human|Viollier AG|Department of
Biosystems Science and Engineering|Nadeau|Switzerland
>Spike|hCoV-19/Wales/PHWC-27777/2020|2020-03-31|EPI_ISL_432008|Original|hCoV-
19^^Wales|Human|Wales Specialist Virology Centre|Public Health Wales Microbiology
Cardiff|Moore|United Kingdom
>Spike|hCoV-19/England/20172002104/2020|2020-04-20|EPI_ISL_465318|Original|hCoV-
19^^England|Human|Respiratory Virus Unit|Respiratory Virus Unit|Platt|United Kingdom
>Spike|hCoV-19/England/C200042043/2020|2020-11-02|EPI_ISL_649801|Original|hCoV-
19^^England|Human|Respiratory Virus Unit|COVID-19 Genomics UK (COG-UK)
Consortium|Platt|United Kingdom
>Spike|hCoV-19/Japan/PG-1511/2020|2020-04-00|EPI_ISL_685118|Original|hCoV-
19^^Japan|Human|Saitama Prefectural Institute of Public Health|Pathogen Genomics
Center|Sekizuka|Japan
>Spike|hCoV-19/England/MILK-A7DE46/2020|2020-10-15|EPI_ISL_638479|Original|hCoV-
19^^England|Human|Oxford Viromics|COVID-19 Genomics UK (COG-UK)
Consortium|Golubchik|United Kingdom
>Spike|hCoV-19/England/SHEF-CCC75/2020|2020-04-02|EPI_ISL_432532|Original|hCoV-
19^^England|Human|Virology Department|COVID-19 Genomics UK (COG-UK) Consortium|de
Silva|United Kingdom
>Spike|hCoV-19/USA/NY-NYCPHL-000886/2020|2020-03-27|EPI_ISL_632958|Original|hCoV-
19^^New York|Human|Jamaica Hospital Medical Center|New York City Public Health
Laboratory|Wang|USA
>Spike|hCoV-19/England/ALDP-9BC680/2020|2020-09-09|EPI_ISL_568122|Original|hCoV-
19^^England|Human|Lighthouse Lab in Alderley Park|Wellcome Sanger Institute for the
COVID-19 Genomics UK (COG-UK) consortium|Davies|United Kingdom
>Spike|hCoV-19/USA/CA-CZB-11114/2020|2020-05-28|EPI_ISL_625507|Original|hCoV-
19^^California|Human|Alameda County Public Health Lab|Chan-Zuckerberg
Biohub|Bhatt|USA
>Spike|hCoV-19/England/LIVE-DC38D7/2020|2020-11-11|EPI_ISL_705312|Original|hCoV-
19^^England|Human|Liverpool Clinical Laboratories|COVID-19 Genomics UK (COG-UK)
Consortium|Haldenby|United Kingdom
>Spike|hCoV-19/Denmark/DCGC-20673/2020|2020-12-21|EPI_ISL_759290|Original|hCoV-
19^^Nordjylland|Human|Department of Virus and Microbiological Special
Diagnostics|Albertsen Lab|Michaelsen|Denmark
>Spike|hCoV-19/England/ALDP-B1A378/2020|2020-11-01|EPI_ISL_646088|Original|hCoV-
19^^England|Human|Lighthouse Lab in Alderley Park|Wellcome Sanger Institute for the
COVID-19 Genomics UK (COG-UK) Consortium|Davies|United Kingdom
>Spike|hCoV-19/England/CAMC-A65315/2020|2020-10-11|EPI_ISL_608412|Original|hCoV-
19^^England|Human|Lighthouse Lab in Cambridge|Wellcome Sanger Institute for the
COVID-19 Genomics UK (COG-UK) consortium|Davies|United Kingdom
>Spike|hCoV-19/Spain/CT-IBV-97009570/2020|2020-07-29|EPI_ISL_683600|Original|hCoV-
19^^Catalunya|Human|Servicio de Microbiolog\u00eda|SeqCOVID-SPAIN
consortium/IBV(CSIC)|Comas|Spain
>Spike|hCoV-19/DRC/6163/2020|2020-05-08|EPI_ISL_513623|Original|hCoV-
19^^Kinshasa|Human|Viral Respiratory Lab|Pathogen Sequencing Lab|Lokilo|Democratic
Republic of the Congo
>Spike|hCoV-19/England/MILK-BB259A/2020|2020-11-18|EPI_ISL_720298|Original|hCoV-
19^^England|Human|Lighthouse Lab in Milton Keynes|Wellcome Sanger Institute for the
COVID-19 Genomics UK (COG-UK) Consortium|Davies|United Kingdom
>Spike|hCoV-19/USA/TX-HMH-MCoV-18228/2020|2020-11-12|EPI_ISL_784576|Original|hCoV-
19^^Texas|Human|Houston Methodist Hospital|Houston Methodist Hospital|Long|USA
>Spike|hCoV-19/USA/WI-UW-501/2020|2020-06-01|EPI_ISL_484876|Original|hCoV-
19^^Wisconsin|Human|University of Wisconsin-Madison AIDS Vaccine Research
Laboratories|University of Wisconsin-Madison AIDS Vaccine Research
Laboratories|Baczenas|USA
>Spike|hCoV-19/England/MILK-A6B494/2020|2020-10-09|EPI_ISL_608840|Original|hCoV-
19^^England|Human|Lighthouse Lab in Milton Keynes|Wellcome Sanger Institute for the
COVID-19 Genomics UK (COG-UK) consortium|Davies|United Kingdom

>Spike|hCoV-19/Wales/PHWC-49F406/2020|2020-12-07|EPI_ISL_743212|Original|hCoV-19^^Wales|Human|Wales Specialist Virology Centre Sequencing lab|COVID-19 Genomics UK (COG-UK) Consortium|Moore|United Kingdom
>Spike|hCoV-19/Colombia/VAC-INS-81271/2020|2020-03-18|EPI_ISL_456141|Original|hCoV-19^^Valle del Cauca|Human|Instituto Nacional de Salud - Unidad de Secuenciaci\u00f3n y An\u00e1lisis Gen\u00f3mico|Instituto Nacional de Salud|D\u00edaz|Colombia
>Spike|hCoV-19/Netherlands/ZH-RIVM-20710/2020|2020-12-22|EPI_ISL_790776|Original|hCoV-19^^Zuid-holland|Human|Dutch COVID-19 response team|National Institute for Public Health and the Environment (RIVM)|Meijer|Netherlands
>Spike|hCoV-19/England/CAMB-1AA887/2020|2020-04-17|EPI_ISL_470126|Original|hCoV-19^^England|Human|Department of Pathology|Wellcome Sanger Institute for the COVID-19 Genomics UK (COG-UK) consortium|Davies|United Kingdom
>Spike|hCoV-19/England/EXET-138AA6/2020|2020-10-18|EPI_ISL_626805|Original|hCoV-19^^England|Human|University of Exeter|COVID-19 Genomics UK (COG-UK) Consortium|Temperton|United Kingdom
>Spike|hCoV-19/England/ALDP-B5B6FA/2020|2020-11-09|EPI_ISL_655591|Original|hCoV-19^^England|Human|Lighthouse Lab in Alderley Park|Wellcome Sanger Institute for the COVID-19 Genomics UK (COG-UK) Consortium|Davies|United Kingdom
>Spike|hCoV-19/USA/WA-S2010/2020|2020-06-04|EPI_ISL_497438|Original|hCoV-19^^Washington|Human|Washington State Department of Health|Seattle Flu Study|Lee|USA
>Spike|hCoV-19/Australia/VIC2157/2020|2020-06-15|EPI_ISL_480768|Original|hCoV-19^^Victoria|Human|Microbiological Diagnostic Unit - Public Health Laboratory (MDU-PHL)|MDU-PHL|Schultz|Australia
>Spike|hCoV-19/USA/MD-HP00128/2020|2020-03-24|EPI_ISL_457817|Original|hCoV-19^^Maryland|Human|Johns Hopkins Hospital Department of Pathology|Johns Hopkins Hospital Department of Pathology|Ramakrishnan|USA
>Spike|hCoV-19/Spain/RI-IBV-005953/2020|2020-03-10|EPI_ISL_500387|Original|hCoV-19^^La Rioja|Human|Centro de Investigaci\u00f3n Biom\u00e9dica de La Rioja - Hospital San Pedro Logro\u00f1o|SeqCOVID-SPAIN consortium/IBV(CSIC)|Comas|Spain
>Spike|hCoV-19/England/ALDP-4F8061/2020|2020-06-24|EPI_ISL_559148|Original|hCoV-19^^England|Human|Lighthouse Lab in Alderley Park|Wellcome Sanger Institute for the COVID-19 Genomics UK (COG-UK) consortium|Davies|United Kingdom
>Spike|hCoV-19/England/MILK-B9507E/2020|2020-11-13|EPI_ISL_673616|Original|hCoV-19^^England|Human|Lighthouse Lab in Milton Keynes|Wellcome Sanger Institute for the COVID-19 Genomics UK (COG-UK) Consortium|Davies|United Kingdom
>Spike|hCoV-19/Japan/PG-6084/2020|2020-07-00|EPI_ISL_689196|Original|hCoV-19^^Japan|Human|Pathogen Genomics Center|Pathogen Genomics Center|Sekizuka|Japan
>Spike|hCoV-19/England/MILK-8009A3/2020|2020-07-16|EPI_ISL_557224|Original|hCoV-19^^England|Human|Lighthouse Lab in Milton Keynes|Wellcome Sanger Institute for the COVID-19 Genomics UK (COG-UK) consortium|Davies|United Kingdom
>Spike|hCoV-19/USA/AZ-TG398891/2020|2020-06-22|EPI_ISL_694851|Original|hCoV-19^^Arizona|Human|TGen North|TGen North|French|USA
>Spike|hCoV-19/Wales/PHWC-494C54/2020|2020-11-24|EPI_ISL_726236|Original|hCoV-19^^Wales|Human|Originating lab|COVID-19 Genomics UK (COG-UK) Consortium|Moore|United Kingdom
>Spike|hCoV-19/USA/TX-HMH-MCoV-8484/2020|2020-07-01|EPI_ISL_780434|Original|hCoV-19^^Texas|Human|Houston Methodist Hospital|Houston Methodist Hospital|Long|USA
>Spike|hCoV-19/England/QEUH-9B8499/2020|2020-09-07|EPI_ISL_549800|Original|hCoV-19^^England|Human|Lighthouse Lab in Glasgow|Wellcome Sanger Institute for the COVID-19 Genomics UK (COG-UK) consortium|Davies|United Kingdom
>Spike|hCoV-19/England/ALDP-B6249C/2020|2020-11-08|EPI_ISL_656377|Original|hCoV-19^^England|Human|Lighthouse Lab in Alderley Park|Wellcome Sanger Institute for the COVID-19 Genomics UK (COG-UK) Consortium|Davies|United Kingdom
>Spike|hCoV-19/Denmark/DCGC-19545/2020|2020-12-07|EPI_ISL_748233|Original|hCoV-19^^Hovedstaden|Human|Department of Virus and Microbiological Special Diagnostics|Albertsen Lab|Michaelsen|Denmark
>Spike|hCoV-19/England/ALDP-AA3393/2020|2020-10-22|EPI_ISL_624150|Original|hCoV-19^^England|Human|Lighthouse Lab in Alderley Park|Wellcome Sanger Institute for the COVID-19 Genomics UK (COG-UK) consortium|Davies|United Kingdom

>Spike|hCoV-19/England/MILK-A45AF8/2020|2020-10-06|EPI_ISL_598682|Original|hCoV-19^^England|Human|Lighthouse Lab in Milton Keynes|Wellcome Sanger Institute for the COVID-19 Genomics UK (COG-UK) consortium|Davies|United Kingdom
>Spike|hCoV-19/Germany/HH-hpi-p1611/2020|2020-03-16|EPI_ISL_776067|Original|hCoV-19^^Hamburg|Human|University Medical Center Hamburg Eppendorf|Heinrich Pette Institute|Robitaille|Germany
>Spike|hCoV-19/USA/AZ-TG532062/2020|2020-07-01|EPI_ISL_695402|Original|hCoV-19^^Arizona|Human|AZ SPHL|TGen North|French|USA
>Spike|hCoV-19/England/ALDP-B74628/2020|2020-11-12|EPI_ISL_678938|Original|hCoV-19^^England|Human|Queens Medical Centre|COVID-19 Genomics UK (COG-UK) Consortium|Loose|United Kingdom
>Spike|hCoV-19/Wales/PHWC-2465F/2020|2020-03-17|EPI_ISL_419434|Original|hCoV-19^^Wales|Human|Wales Specialist Virology Centre|Public Health Wales Microbiology Cardiff|Moore|United Kingdom
>Spike|hCoV-19/England/LIVE-9C458/2020|2020-03-30|EPI_ISL_449538|Original|hCoV-19^^England|Human|Liverpool Clinical Laboratories|COVID-19 Genomics UK (COG-UK) Consortium|Haldenby|United Kingdom
>Spike|hCoV-19/England/SHEF-C58E1/2020|2020-04-27|EPI_ISL_475385|Original|hCoV-19^^England|Human|Virology Department|COVID-19 Genomics UK (COG-UK) Consortium|de Silva|United Kingdom
>Spike|hCoV-19/England/ALDP-B838A8/2020|2020-11-14|EPI_ISL_675300|Original|hCoV-19^^England|Human|Lighthouse Lab in Alderley Park|Wellcome Sanger Institute for the COVID-19 Genomics UK (COG-UK) Consortium|Davies|United Kingdom
>Spike|hCoV-19/Switzerland/SG-ETHZ-101137/2020|2020-03-30|EPI_ISL_451804|Original|hCoV-19^^Sankt Gallen|Human|Viollier AG|Department of Biosystems Science and Engineering|Nadeau|Switzerland
>Spike|hCoV-19/England/QEUH-B6AE4B/2020|2020-11-11|EPI_ISL_662555|Original|hCoV-19^^England|Human|Lighthouse Lab in Glasgow|Wellcome Sanger Institute for the COVID-19 Genomics UK (COG-UK) Consortium|Davies|United Kingdom
>Spike|hCoV-19/England/CAMB-73F0B/2020|2020-03-19|EPI_ISL_440343|Original|hCoV-19^^England|Human|Department of Pathology|Wellcome Sanger Institute for the COVID-19 Genomics UK (COG-UK) consortium|Amato|United Kingdom
>Spike|hCoV-19/England/QEUH-AD1D43/2020|2020-10-26|EPI_ISL_629787|Original|hCoV-19^^England|Human|Lighthouse Lab in Glasgow|Wellcome Sanger Institute for the COVID-19 Genomics UK (COG-UK) consortium|Davies|United Kingdom
>Spike|hCoV-19/Spain/MD-IBV-99008665/2020|2020-09-30|EPI_ISL_654226|Original|hCoV-19^^Madrid|Human|Hospital General Universitario Gregorio Mara\u00f1\u00f3n|SeqCOVID-SPAIN consortium/IBV(CSIC)|Comas|Spain
>Spike|hCoV-19/USA/WI-UW-1054/2020|2020-07-30|EPI_ISL_516480|Original|hCoV-19^^Wisconsin|Human|University of Wisconsin-Madison AIDS Vaccine Research Laboratories|University of Wisconsin-Madison AIDS Vaccine Research Laboratories|Baczenas|USA
>Spike|hCoV-19/USA/WA-S2869/2020|2020-09-21|EPI_ISL_596400|Original|hCoV-19^^Washington|Human|Seattle Flu Study|Seattle Flu Study|Lee|USA
>Spike|hCoV-19/England/CAMC-B211DE/2020|2020-11-03|EPI_ISL_647782|Original|hCoV-19^^England|Human|Lighthouse Lab in Cambridge|Wellcome Sanger Institute for the COVID-19 Genomics UK (COG-UK) Consortium|Davies|United Kingdom
>Spike|hCoV-19/England/QEUH-CC5D8C/2020|2020-12-22|EPI_ISL_760336|Original|hCoV-19^^England|Human|Lighthouse Lab in Glasgow|Wellcome Sanger Institute for the COVID-19 Genomics UK (COG-UK) Consortium|Davies|United Kingdom
>Spike|hCoV-19/Singapore/492/2020|2020-04-26|EPI_ISL_477189|Original|hCoV-19^^Singapore|Human|Department of Laboratory Medicine Tan Tock Seng Hospital|Department of Laboratory Medicine Tan Tock Seng Hospital|Chen|Singapore
>Spike|hCoV-19/Wales/PHWC-486B85/2020|2020-10-16|EPI_ISL_637449|Original|hCoV-19^^Wales|Human|Wales Specialist Virology Centre Sequencing lab|COVID-19 Genomics UK (COG-UK) Consortium|Moore|United Kingdom
>Spike|hCoV-19/Scotland/QEUH-A0BBFB/2020|2020-10-01|EPI_ISL_599386|Original|hCoV-19^^Scotland|Human|Lighthouse Lab in Glasgow|Wellcome Sanger Institute for the COVID-19 Genomics UK (COG-UK) consortium|Davies|United Kingdom

>Spike|hCoV-19/USA/MS-CDC-6516/2020|2020-08-22|EPI_ISL_648004|Original|hCoV-19^^Mississippi|Human|MS Public Health Laboratory|Pathogen Discovery|Queen|USA
>Spike|hCoV-19/Denmark/DCGC-10160/2020|2020-11-09|EPI_ISL_669427|Original|hCoV-19^^Sjaelland|Human|Department of Virus and Microbiological Special Diagnostics|Albertsen Lab|Michaelsen|Denmark
>Spike|hCoV-19/USA/TX-HMH-5509/2020|2020-06-26|EPI_ISL_544832|Original|hCoV-19^^Texas|Human|Houston Methodist Hospital|Houston Methodist Hospital|Long|USA
>Spike|hCoV-19/USA/UT-QDX-2556/2020|2020-03-14|EPI_ISL_604866|Original|hCoV-19^^Utah|Human|Quest Diagnostics|Quest Diagnostics|Kagan|USA
>Spike|hCoV-19/USA/OR-OHSU-2067/2020|2020-08-03|EPI_ISL_667106|Original|hCoV-19^^Oregon|Human|OHSU Lab Services Molecular Microbiology Lab|Oregon SARS-CoV-2 Genome Sequencing Center|O'Roak|USA
>Spike|hCoV-19/England/MILK-B8C3BD/2020|2020-11-13|EPI_ISL_674247|Original|hCoV-19^^England|Human|Lighthouse Lab in Milton Keynes|Wellcome Sanger Institute for the COVID-19 Genomics UK (COG-UK) Consortium|Davies|United Kingdom
>Spike|hCoV-19/Belgium/ULG-10979/2020|2020-09-08|EPI_ISL_707753|Original|hCoV-19^^Li\u00e8ge|Human|Department of Clinical Microbiology|GIGA Medical Genomics|Durkin|Belgium
>Spike|hCoV-19/USA/WA-S255/2020|2020-03-08|EPI_ISL_430293|Original|hCoV-19^^Washington|Human|Washington State Department of Health|Seattle Flu Study|Bedford|USA
>Spike|hCoV-19/France/BRE-IPP9945/2020|2020-09-09|EPI_ISL_693759|Original|hCoV-19^^Bretagne|Human|Hospital|National Reference Center for Viruses of Respiratory Infections|Enouf|France
>Spike|hCoV-19/England/NORT-2963F5/2020|2020-05-13|EPI_ISL_484269|Original|hCoV-19^^England|Human|Northumbria University / South Tees Hospitals NHS Foundation Trust / North Cumbria Integrated Care NHS Foundation Trust / North Tees and Hartlepool NHS Foundation Trust / Newcastle Hospitals NHS Foundation Trust|COVID-19 Genomics UK (COG-UK) Consortium|Bashton|United Kingdom
>Spike|hCoV-19/England/QEUH-9F5CE3/2020|2020-09-24|EPI_ISL_588330|Original|hCoV-19^^England|Human|Lighthouse Lab in Glasgow|Wellcome Sanger Institute for the COVID-19 Genomics UK (COG-UK) consortium|Davies|United Kingdom
>Spike|hCoV-19/England/CAMC-A58F20/2020|2020-10-08|EPI_ISL_597042|Original|hCoV-19^^England|Human|Lighthouse Lab in Cambridge|Wellcome Sanger Institute for the COVID-19 Genomics UK (COG-UK) consortium|Davies|United Kingdom
>Spike|hCoV-19/Wales/ALDP-94F585/2020|2020-06-18|EPI_ISL_555884|Original|hCoV-19^^Wales|Human|Lighthouse Lab in Alderley Park|Wellcome Sanger Institute for the COVID-19 Genomics UK (COG-UK) consortium|Davies|United Kingdom
>Spike|hCoV-19/England/ALDP-CB3E51/2020|2020-12-19|EPI_ISL_761271|Original|hCoV-19^^England|Human|Lighthouse Lab in Alderley Park|Wellcome Sanger Institute for the COVID-19 Genomics UK (COG-UK) Consortium|Davies|United Kingdom
>Spike|hCoV-19/England/ALDP-B5B9B5/2020|2020-11-08|EPI_ISL_655584|Original|hCoV-19^^England|Human|Lighthouse Lab in Alderley Park|Wellcome Sanger Institute for the COVID-19 Genomics UK (COG-UK) Consortium|Davies|United Kingdom
>Spike|hCoV-19/Luxembourg/LNS7851926/2020|2020-11-08|EPI_ISL_744643|Original|hCoV-19^^Luxembourg|Human|Laboratoire national de sant\u00e9|Laboratoire national de sant\u00e9|Wienecke-Baldacchino|Luxembourg
>Spike|hCoV-19/Switzerland/ZH-ETHZ-210021/2020|2020-07-21|EPI_ISL_510766|Original|hCoV-19^^Zurich|Human|Viollier AG|Department of Biosystems Science and Engineering|Nadeau|Switzerland
>Spike|hCoV-19/England/CAMB-1AC9A0/2020|2020-04-23|EPI_ISL_489403|Original|hCoV-19^^England|Human|Department of Pathology|Wellcome Sanger Institute for the COVID-19 Genomics UK (COG-UK) consortium|Davies|United Kingdom
>Spike|hCoV-19/England/ALDP-9E6CC7/2020|2020-09-24|EPI_ISL_580617|Original|hCoV-19^^England|Human|Lighthouse Lab in Alderley Park|Wellcome Sanger Institute for the COVID-19 Genomics UK (COG-UK) consortium|Davies|United Kingdom
>Spike|hCoV-19/USA/TX-HMH-1546/2020|2020-04-13|EPI_ISL_542786|Original|hCoV-19^^Texas|Human|Houston Methodist Hospital|Houston Methodist Hospital|Long|USA

>Spike|hCoV-19/England/SHEF-C4E3B/2020|2020-10-28|EPI_ISL_638883|Original|hCoV-19^^England|Human|Virology Department|COVID-19 Genomics UK (COG-UK) Consortium|de Silva|United Kingdom
>Spike|hCoV-19/England/CAMC-BBCCF5/2020|2020-11-21|EPI_ISL_679275|Original|hCoV-19^^England|Human|Queens Medical Centre|COVID-19 Genomics UK (COG-UK) Consortium|Loose|United Kingdom
>Spike|hCoV-19/United Arab Emirates/0534/2020|2020-05-17|EPI_ISL_698696|Original|hCoV-19^^Abu Dhabi|Human|Group 42 (G42) Healthcare|G42 Healthcare|Ogrodzki|United Arab Emirates
>Spike|hCoV-19/Finland/13M69/2020|2020-03-13|EPI_ISL_418396|Original|hCoV-19^^Finland|Human|Department of Virology and Immunology|Department of Virology|Smura|Finland
>Spike|hCoV-19/Germany/BY-MVP-0149/2020|2020-03-30|EPI_ISL_451942|Original|hCoV-19^^Bavaria|Human|Max von Pettenkofer Institute|Laboratory for Functional Genome Analysis|Graf|Germany
>Spike|hCoV-19/Spain/MD-IBV-99009611/2020|2020-10-22|EPI_ISL_654360|Original|hCoV-19^^Madrid|Human|Hospital General Universitario Gregorio Mara\u00f1\u00f3n|SeqCOVID-SPAIN consortium/IBV(CSIC)|Comas|Spain
>Spike|hCoV-19/Switzerland/BS-42243953/2020|2020-04-24|EPI_ISL_581919|Original|hCoV-19^^Basel-Stadt|Human|University Hospital Basel|University Hospital Basel|Roloff Handschin|Switzerland
>Spike|hCoV-19/England/MILK-C61DA0/2020|2020-12-13|EPI_ISL_735669|Original|hCoV-19^^England|Human|Lighthouse Lab in Milton Keynes|Wellcome Sanger Institute for the COVID-19 Genomics UK (COG-UK) Consortium|Davies|United Kingdom
>Spike|hCoV-19/Belgium/rega-0404885/2020|2020-04-04|EPI_ISL_734535|Original|hCoV-19^^Moriensart|Human|UZ Leuven|KU Leuven|Maes|Belgium
>Spike|hCoV-19/England/MILK-BDB6C5/2020|2020-11-26|EPI_ISL_709631|Original|hCoV-19^^England|Human|Lighthouse Lab in Milton Keynes|Wellcome Sanger Institute for the COVID-19 Genomics UK (COG-UK) Consortium|Davies|United Kingdom
>Spike|hCoV-19/Denmark/DCGC-2697/2020|2020-08-17|EPI_ISL_616429|Original|hCoV-19^^Sjaelland|Human|Department of Virus and Microbiological Special Diagnostics|Albertsen lab|Michaelsen|Denmark
>Spike|hCoV-19/Australia/SAP373/2020|2020-03-20|EPI_ISL_492138|Original|hCoV-19^^South Australia|Human|SA Pathology|SA Pathology|Leong|Australia
>Spike|hCoV-19/Belgium/ULG-10681/2020|2020-10-26|EPI_ISL_636653|Original|hCoV-19^^Li\u00e8ge|Human|Department of Clinical Microbiology|GIGA Medical Genomics|Durkin|Belgium
>Spike|hCoV-19/Spain/VC-IBV-98006459/2020|2020-06-25|EPI_ISL_538663|Original|hCoV-19^^Comunitat Valenciana|Human|Servicio de Microbiolog\u00eda. Hospital General Universitario de Castell\u00f3n|SeqCOVID-SPAIN consortium/IBV(CSIC)|Comas|Spain
>Spike|hCoV-19/England/ALDP-A6D605/2020|2020-10-14|EPI_ISL_607132|Original|hCoV-19^^England|Human|Lighthouse Lab in Alderley Park|Wellcome Sanger Institute for the COVID-19 Genomics UK (COG-UK) consortium|Davies|United Kingdom
>Spike|hCoV-19/England/CAMB-783DD/2020|2020-04-05|EPI_ISL_433728|Original|hCoV-19^^England|Human|Department of Pathology|COVID-19 Genomics UK (COG-UK) Consortium|Meredith|United Kingdom
>Spike|hCoV-19/Spain/CL-IBV-97006283/2020|2020-04-21|EPI_ISL_537653|Original|hCoV-19^^Castilla y Leon|Human|Universidad de Le\u00f3n|SeqCOVID-SPAIN consortium/IBV(CSIC)|Comas|Spain
>Spike|hCoV-19/USA/ME-HETL-J0385/2020|2020-12-15|EPI_ISL_755500|Original|hCoV-19^^Maine|Human|Maine Health and Environmental Testing Laboratory|Tewhey Lab|Tewhey|USA
>Spike|hCoV-19/Wales/ALDP-B49237/2020|2020-11-07|EPI_ISL_710902|Original|hCoV-19^^Wales|Human|Lighthouse Lab in Alderley Park|Wellcome Sanger Institute for the COVID-19 Genomics UK (COG-UK) Consortium|Davies|United Kingdom
>Spike|hCoV-19/Japan/PG-8153/2020|2020-09-00|EPI_ISL_687252|Original|hCoV-19^^Japan|Human|Pathogen Genomics Center|Pathogen Genomics Center|Sekizuka|Japan
>Spike|hCoV-19/England/ALDP-C8E55A/2020|2020-12-15|EPI_ISL_777576|Original|hCoV-19^^England|Human|Lighthouse Lab in Alderley Park|Wellcome Sanger Institute for the COVID-19 Genomics UK (COG-UK) Consortium|Davies|United Kingdom

>Spike|hCoV-19/USA/MS-CDC-6558/2020|2020-09-03|EPI_ISL_648011|Original|hCoV-19^^Mississippi|Human|MS Public Health Laboratory|Pathogen Discovery|Queen|USA
>Spike|hCoV-19/USA/TX-HMH-MCoV-13758/2020|2020-08-18|EPI_ISL_788851|Original|hCoV-19^^Texas|Human|Houston Methodist Hospital|Houston Methodist Hospital|Long|USA
>Spike|hCoV-19/England/MILK-99E2BD/2020|2020-08-31|EPI_ISL_550528|Original|hCoV-19^^England|Human|Lighthouse Lab in Milton Keynes|Wellcome Sanger Institute for the COVID-19 Genomics UK (COG-UK) consortium|Davies|United Kingdom
>Spike|hCoV-19/England/ALDP-A85BAE/2020|2020-10-17|EPI_ISL_610487|Original|hCoV-19^^England|Human|Lighthouse Lab in Alderley Park|Wellcome Sanger Institute for the COVID-19 Genomics UK (COG-UK) consortium|Davies|United Kingdom
>Spike|hCoV-19/USA/WI-GMF-31832/2020|2020-11-06|EPI_ISL_661115|Original|hCoV-19^^Wisconsin|Human|Gundersen Molecular Diagnostics Laboratory|Kabara Cancer Research Institute|Kenny|USA
>Spike|hCoV-19/England/QEUH-9DDD75/2020|2020-09-21|EPI_ISL_599696|Original|hCoV-19^^England|Human|Lighthouse Lab in Glasgow|Wellcome Sanger Institute for the COVID-19 Genomics UK (COG-UK) consortium|Davies|United Kingdom
>Spike|hCoV-19/USA/CA-ALSR-5020/2020|2020-11-12|EPI_ISL_730292|Original|hCoV-19^^California|Human|San Diego County Public Health Laboratory|Andersen lab at Scripps Research|Gangavarapu|USA
>Spike|hCoV-19/Wales/PHWC-485F7B/2020|2020-10-25|EPI_ISL_628296|Original|hCoV-19^^Wales|Human|Wales Specialist Virology Centre Sequencing lab|COVID-19 Genomics UK (COG-UK) Consortium|Moore|United Kingdom
>Spike|hCoV-19/England/QEUH-A224B7/2020|2020-10-01|EPI_ISL_587605|Original|hCoV-19^^England|Human|Lighthouse Lab in Glasgow|Wellcome Sanger Institute for the COVID-19 Genomics UK (COG-UK) consortium|Davies|United Kingdom
>Spike|hCoV-19/USA/CA-CZB-14200/2020|2020-11-09|EPI_ISL_739151|Original|hCoV-19^^California|Human|Orange County Public Health Lab|Chan-Zuckerberg Biohub|Kamm|USA
>Spike|hCoV-19/England/SHEF-10CA46D/2020|2020-11-29|EPI_ISL_727895|Original|hCoV-19^^England|Human|Virology Department|COVID-19 Genomics UK (COG-UK) Consortium|de Silva|United Kingdom
>Spike|hCoV-19/Wales/PHWC-32AAD/2020|2020-04-15|EPI_ISL_474391|Original|hCoV-19^^Wales|Human|Originating lab|COVID-19 Genomics UK (COG-UK) Consortium|Moore|United Kingdom
>Spike|hCoV-19/Japan/PG-4328/2020|2020-07-00|EPI_ISL_692556|Original|hCoV-19^^Japan|Human|Pathogen Genomics Center|Pathogen Genomics Center|Sekizuka|Japan
>Spike|hCoV-19/England/CAMC-B348D3/2020|2020-11-05|EPI_ISL_659704|Original|hCoV-19^^England|Human|Lighthouse Lab in Cambridge|Wellcome Sanger Institute for the COVID-19 Genomics UK (COG-UK) Consortium|Davies|United Kingdom
>Spike|hCoV-19/England/MILK-9A670E/2020|2020-09-01|EPI_ISL_550090|Original|hCoV-19^^England|Human|Lighthouse Lab in Milton Keynes|Wellcome Sanger Institute for the COVID-19 Genomics UK (COG-UK) consortium|Davies|United Kingdom
>Spike|hCoV-19/England/201141550/2020|2020-03-09|EPI_ISL_464765|Original|hCoV-19^^England|Human|Respiratory Virus Unit|Respiratory Virus Unit|Platt|United Kingdom
>Spike|hCoV-19/USA/TX-HMH-MCoV-18990/2020|2020-11-25|EPI_ISL_785269|Original|hCoV-19^^Texas|Human|Houston Methodist Hospital|Houston Methodist Hospital|Long|USA
>Spike|hCoV-19/England/MILK-B8F8D9/2020|2020-11-13|EPI_ISL_674611|Original|hCoV-19^^England|Human|Lighthouse Lab in Milton Keynes|Wellcome Sanger Institute for the COVID-19 Genomics UK (COG-UK) Consortium|Davies|United Kingdom
>Spike|hCoV-19/SaudiArabia/KAUST-MAKKAH810/2020|2020-04-24|EPI_ISL_513260|Original|hCoV-19^^Makkah|Human|Pathogen Genomics Lab King Abdullah University of Science and Technology(KAUST)|Pathogen Genomics Lab King Abdullah University of Science and Technology(KAUST)|Mohamed Ghazzali|Saudi Arabia
>Spike|hCoV-19/USA/UT-01843/2020|2020-04-13|EPI_ISL_470685|Original|hCoV-19^^Utah|Human|Utah Public Health Laboratory|Utah Public Health Laboratory|Young|USA
>Spike|hCoV-19/England/CAMB-1AD972/2020|2020-05-02|EPI_ISL_448029|Original|hCoV-19^^England|Human|Department of Pathology|COVID-19 Genomics UK (COG-UK) Consortium|Meredith|United Kingdom
>Spike|hCoV-19/England/BRIS-12FBDC/2020|2020-04-04|EPI_ISL_492633|Original|hCoV-19^^England|Human|PHE South West Regional Laboratory|Wellcome Sanger Institute for the COVID-19 Genomics UK (COG-UK) consortium|Davies|United Kingdom

>Spike|hCoV-19/USA/TX-QDX-414/2020|2020-03-18|EPI_ISL_571256|Original|hCoV-19^^Texas|Human|Quest Diagnostics|Quest Diagnostics|Kagan|USA
>Spike|hCoV-19/India/GJ-GBRC235b/2020|2020-06-12|EPI_ISL_476877|Original|hCoV-19^^Gujarat|Human|Department of MicroBiology|Gujarat Biotechnology Research Centre|Joshi|India
>Spike|hCoV-19/England/QEUH-AF6926/2020|2020-10-30|EPI_ISL_662912|Original|hCoV-19^^England|Human|Lighthouse Lab in Glasgow|Wellcome Sanger Institute for the COVID-19 Genomics UK (COG-UK) Consortium|Davies|United Kingdom
>Spike|hCoV-19/USA/CO-CDPHE-2009051670/2020|2020-09-03|EPI_ISL_677244|Original|hCoV-19^^Colorado|Human|Colorado Department of Public Health and Environment|Colorado Department of Puplic Health and Environment|Bankers|USA
>Spike|hCoV-19/England/CAMB-1AC90A/2020|2020-04-24|EPI_ISL_489550|Original|hCoV-19^^England|Human|Department of Pathology|Wellcome Sanger Institute for the COVID-19 Genomics UK (COG-UK) consortium|Davies|United Kingdom
>Spike|hCoV-19/Gibraltar/204641730/2020|2020-00-00|EPI_ISL_637204|Original|hCoV-19^^Gibraltar|Human|Microbiology|Respiratory Virus Unit|Platt|Gibraltar
>Spike|hCoV-19/USA/HI-H200619/2020|2020-11-23|EPI_ISL_753145|Original|hCoV-19^^Hawaii|Human|State Laboratories Division|State Laboratories Division|Sultana|USA
>Spike|hCoV-19/England/ALDP-9E97D2/2020|2020-09-24|EPI_ISL_584250|Original|hCoV-19^^England|Human|Virology Department|COVID-19 Genomics UK (COG-UK) Consortium|de Silva|United Kingdom
>Spike|hCoV-19/Australia/VIC6325/2020|2020-07-23|EPI_ISL_518312|Original|hCoV-19^^Victoria|Human|Microbiological Diagnostic Unit - Public Health Laboratory (MDU-PHL)|MDU-PHL|Schultz|Australia
>Spike|hCoV-19/Wales/PHWC-15C839/2020|2020-04-09|EPI_ISL_472546|Original|hCoV-19^^Wales|Human|Wales Specialist Virology Centre Sequencing lab|COVID-19 Genomics UK (COG-UK) Consortium|Moore|United Kingdom
>Spike|hCoV-19/Wales/PHWC-48C3B4/2020|2020-11-05|EPI_ISL_666423|Original|hCoV-19^^Wales|Human|Wales Specialist Virology Centre Sequencing lab|COVID-19 Genomics UK (COG-UK) Consortium|Moore|United Kingdom
>Spike|hCoV-19/USA/OR-PROV-024/2020|2020-03-28|EPI_ISL_482308|Original|hCoV-19^^Oregon|Human|Providence St. Joseph Health Molecular Genomics Laboratory|Providence St. Joseph Health Molecular Genomics Laboratory|Dowdell|USA
>Spike|hCoV-19/Belgium/rega-0322864/2020|2020-03-22|EPI_ISL_734514|Original|hCoV-19^^Chaumont-gistoux|Human|UZ Leuven|KU Leuven|Maes|Belgium
>Spike|hCoV-19/England/205260437/2020|2020-00-00|EPI_ISL_766549|Original|hCoV-19^^England|Human|Respiratory Virus Unit|COVID-19 Genomics UK (COG-UK) Consortium|Platt|United Kingdom
>Spike|hCoV-19/England/ALDP-A84A93/2020|2020-10-18|EPI_ISL_610615|Original|hCoV-19^^England|Human|Lighthouse Lab in Alderley Park|Wellcome Sanger Institute for the COVID-19 Genomics UK (COG-UK) consortium|Davies|United Kingdom
>Spike|hCoV-19/USA/TX-HMH-MCoV-15859/2020|2020-07-10|EPI_ISL_785543|Original|hCoV-19^^Texas|Human|Houston Methodist Hospital|Houston Methodist Hospital|Long|USA
>Spike|hCoV-19/Italy/SIC-IZSBM169-66/2020|2020-06-00|EPI_ISL_582767|Original|hCoV-19^^Sicily|Human|Laboratorio di Riferimento Regionale della Sicilia Occidentale per l'Emergenza COVID-19|Istituto Zooprofilattico Sperimentale della Sicilia|Reale|Italy
>Spike|hCoV-19/USA/MN-MDH-1013/2020|2020-04-30|EPI_ISL_462858|Original|hCoV-19^^Minnesota|Human|Minnesota Department of Health|Minnesota Department of Health|Garfin|USA
>Spike|hCoV-19/Spain/AS-IBV-98006600/2020|2020-03-13|EPI_ISL_654644|Original|hCoV-19^^Asturias|Human|Servicio de Microbiolog\u00eda|SeqCOVID-SPAIN consortium/IBV(CSIC)|Comas|Spain
>Spike|hCoV-19/Scotland/QEUH-A0BC34/2020|2020-10-03|EPI_ISL_599404|Original|hCoV-19^^Scotland|Human|Lighthouse Lab in Glasgow|Wellcome Sanger Institute for the COVID-19 Genomics UK (COG-UK) consortium|Davies|United Kingdom
>Spike|hCoV-19/USA/NY-NYUMC878/2020|2020-03-18|EPI_ISL_458017|Original|hCoV-19^^New York|Human|NYU Langone Health|Departments of Pathology and Medicine|Maurano|USA
>Spike|hCoV-19/Denmark/DCGC-16599/2020|2020-11-16|EPI_ISL_714861|Original|hCoV-19^^Hovedstaden|Human|Department of Virus and Microbiological Special Diagnostics|Albertsen Lab|Michaelsen|Denmark

>Spike|hCoV-19/England/MILK-B3D9E6/2020|2020-11-01|EPI_ISL_741377|Original|hCoV-19^^England|Human|Oxford Viromics|COVID-19 Genomics UK (COG-UK) Consortium|Golubchik|United Kingdom
>Spike|hCoV-19/Netherlands/GE-RIVM-20521/2020|2020-11-24|EPI_ISL_728736|Original|hCoV-19^^Gelderland|Human|Dutch COVID-19 response team|National Institute for Public Health and the Environment (RIVM)|Meijer|Netherlands
>Spike|hCoV-19/Denmark/DCGC-17930/2020|2020-12-14|EPI_ISL_749650|Original|hCoV-19^^Midtjylland|Human|Department of Virus and Microbiological Special Diagnostics|Albertsen Lab|Michaelsen|Denmark
>Spike|hCoV-19/England/MILK-46DEF4/2020|2020-06-04|EPI_ISL_559724|Original|hCoV-19^^England|Human|Lighthouse Lab in Milton Keynes|Wellcome Sanger Institute for the COVID-19 Genomics UK (COG-UK) Consortium|Davies|United Kingdom
>Spike|hCoV-19/Scotland/GCVR-172105/2020|2020-03-31|EPI_ISL_469997|Original|hCoV-19^^Scotland|Human|NHSGGC West of Scotland Specialist Virology Centre / MRC-University of Glasgow Centre for Virus Research|Wellcome Sanger Institute for the COVID-19 Genomics UK (COG-UK) consortium|Davies|United Kingdom
>Spike|hCoV-19/Wales/PHWC-16296A/2020|2020-05-20|EPI_ISL_473042|Original|hCoV-19^^Wales|Human|Wales Specialist Virology Centre Sequencing lab|COVID-19 Genomics UK (COG-UK) Consortium|Moore|United Kingdom
>Spike|hCoV-19/USA/TX-HMH-MCoV-18194/2020|2020-11-15|EPI_ISL_784542|Original|hCoV-19^^Texas|Human|Houston Methodist Hospital|Houston Methodist Hospital|Long|USA
>Spike|hCoV-19/USA/AZ-TG670259/2020|2020-03-25|EPI_ISL_696435|Original|hCoV-19^^Arizona|Human|Sonora Quest Laboratories|TGen North|French|USA
>Spike|hCoV-19/Canada/ON-SLB2861/2020|2020-04-07|EPI_ISL_718105|Original|hCoV-19^^Ontario|Human|Kingston Health Sciences Centre and Queen's University|Ontario Institute for Cancer Research|de Borja|Canada
>Spike|hCoV-19/USA/UT-01967/2020|2020-04-15|EPI_ISL_452164|Original|hCoV-19^^Utah|Human|Utah Public Health Laboratory|Utah Public Health Laboratory|Young|USA
>Spike|hCoV-19/Luxembourg/LNS2321488/2020|2020-11-24|EPI_ISL_740096|Original|hCoV-19^^Luxembourg|Human|Laboratoire national de sant\u00e9|Laboratoire national de sant\u00e9|Wienecke-Baldacchino|Luxembourg
>Spike|hCoV-19/USA/MI-UM-10035702532/2020|2020-08-28|EPI_ISL_583263|Original|hCoV-19^^Michigan|Human|University of Michigan Clinical Microbiology Laboratory|Lauring Lab|Valesano|USA
>Spike|hCoV-19/USA/CT-Yale-393/2020|2020-06-13|EPI_ISL_594133|Original|hCoV-19^^Connecticut|Human|Yale COVID-19 Biorepository|Grubaugh Lab - Yale School of Public Health|Grubaugh|USA
>Spike|hCoV-19/Scotland/QEUH-9B4925/2020|2020-09-03|EPI_ISL_602050|Original|hCoV-19^^Scotland|Human|Lighthouse Lab in Glasgow|Wellcome Sanger Institute for the COVID-19 Genomics UK (COG-UK) consortium|Davies|United Kingdom
>Spike|hCoV-19/Wales/PHWC-4968E5/2020|2020-11-21|EPI_ISL_726654|Original|hCoV-19^^Wales|Human|Originating lab|COVID-19 Genomics UK (COG-UK) Consortium|Moore|United Kingdom
>Spike|hCoV-19/England/QEUH-98F668/2020|2020-08-27|EPI_ISL_540013|Original|hCoV-19^^England|Human|Lighthouse Lab in Glasgow|Wellcome Sanger Institute for the COVID-19 Genomics UK (COG-UK) consortium|Davies|United Kingdom
>Spike|hCoV-19/England/MILK-C610C5/2020|2020-12-12|EPI_ISL_735515|Original|hCoV-19^^England|Human|Lighthouse Lab in Milton Keynes|Wellcome Sanger Institute for the COVID-19 Genomics UK (COG-UK) Consortium|Davies|United Kingdom
>Spike|hCoV-19/Australia/VIC8145/2020|2020-08-05|EPI_ISL_564872|Original|hCoV-19^^Victoria|Human|Microbiological Diagnostic Unit - Public Health Laboratory (MDU-PHL)|MDU-PHL|Schultz|Australia
>Spike|hCoV-19/England/NOTT-10EFF5/2020|2020-03-30|EPI_ISL_432954|Original|hCoV-19^^England|Human|Queens Medical Centre|COVID-19 Genomics UK (COG-UK) Consortium|Loose|United Kingdom
>Spike|hCoV-19/Australia/VIC4057/2020|2020-07-10|EPI_ISL_519439|Original|hCoV-19^^Victoria|Human|Microbiological Diagnostic Unit - Public Health Laboratory (MDU-PHL)|MDU-PHL|Schultz|Australia

>Spike|hCoV-19/USA/TX-DSHS-1721/2020|2020-06-19|EPI_ISL_708611|Original|hCoV-19^^Texas|Human|Texas Department of State Health Services|Texas Department of State Health Services|Rahman|USA
>Spike|hCoV-19/England/ALDP-A50A72/2020|2020-10-07|EPI_ISL_597566|Original|hCoV-19^^England|Human|Lighthouse Lab in Alderley Park|Wellcome Sanger Institute for the COVID-19 Genomics UK (COG-UK) consortium|Davies|United Kingdom
>Spike|hCoV-19/Denmark/DCGC-9693/2020|2020-11-09|EPI_ISL_668541|Original|hCoV-19^^Syddanmark|Human|Department of Virus and Microbiological Special Diagnostics|Albertsen Lab|Michaelsen|Denmark
>Spike|hCoV-19/England/MILK-BAC5EE/2020|2020-11-18|EPI_ISL_720449|Original|hCoV-19^^England|Human|Lighthouse Lab in Milton Keynes|Wellcome Sanger Institute for the COVID-19 Genomics UK (COG-UK) Consortium|Davies|United Kingdom
>Spike|hCoV-19/England/204393980/2020|2020-10-25|EPI_ISL_649888|Original|hCoV-19^^England|Human|Respiratory Virus Unit|COVID-19 Genomics UK (COG-UK) Consortium|Platt|United Kingdom
>Spike|hCoV-19/USA/TX-HMH-MCoV-9799/2020|2020-07-04|EPI_ISL_780105|Original|hCoV-19^^Texas|Human|Houston Methodist Hospital|Houston Methodist Hospital|Long|USA
>Spike|hCoV-19/USA/MI-UM-10035862473/2020|2020-09-09|EPI_ISL_583330|Original|hCoV-19^^Michigan|Human|University of Michigan Clinical Microbiology Laboratory|Lauring Lab|Valesano|USA
>Spike|hCoV-19/England/QEUH-BC3E8C/2020|2020-11-25|EPI_ISL_704194|Original|hCoV-19^^England|Human|Lighthouse Lab in Glasgow|Wellcome Sanger Institute for the COVID-19 Genomics UK (COG-UK) Consortium|Davies|United Kingdom
>Spike|hCoV-19/England/20178032304/2020|2020-04-23|EPI_ISL_465418|Original|hCoV-19^^England|Human|Respiratory Virus Unit|Respiratory Virus Unit|Platt|United Kingdom
>Spike|hCoV-19/England/MILK-BAC43C/2020|2020-11-17|EPI_ISL_681333|Original|hCoV-19^^England|Human|Lighthouse Lab in Milton Keynes|Wellcome Sanger Institute for the COVID-19 Genomics UK (COG-UK) Consortium|Davies|United Kingdom
>Spike|hCoV-19/Canada/ON-PHL-20-01283/2020|2020-06-00|EPI_ISL_548888|Original|hCoV-19^^Ontario|Human|Public Health Ontario Laboratory|Public Health Ontario Laboratory|Guthrie|Canada
>Spike|hCoV-19/India/TG-CCMB-M844/2020|2020-06-12|EPI_ISL_495256|Original|hCoV-19^^Telangana|Human|CSIR-Centre for Cellular and Molecular Biology|CSIR-Centre for Cellular and Molecular Biology|Banu|India
>Spike|hCoV-19/USA/CA-CZB-1783/2020|2020-06-03|EPI_ISL_486117|Original|hCoV-19^^California|Human|County of Santa Clara Public Health Department|Chan-Zuckerberg Biohub|McGeever|USA
>Spike|hCoV-19/Wales/PHWC-48BB44/2020|2020-11-03|EPI_ISL_666328|Original|hCoV-19^^Wales|Human|Wales Specialist Virology Centre Sequencing lab|COVID-19 Genomics UK (COG-UK) Consortium|Moore|United Kingdom
>Spike|hCoV-19/Japan/PG-5165/2020|2020-08-00|EPI_ISL_689614|Original|hCoV-19^^Japan|Human|Pathogen Genomics Center|Pathogen Genomics Center|Sekizuka|Japan
>Spike|hCoV-19/USA/MI-MDHHS-SC20053/2020|2020-03-20|EPI_ISL_437140|Original|hCoV-19^^Michigan|Human|Michigan Department of Health and Human Services|Michigan Department of Health and Human Services|Blankenship|USA
>Spike|hCoV-19/England/MILK-A45B7D/2020|2020-10-06|EPI_ISL_598556|Original|hCoV-19^^England|Human|Lighthouse Lab in Milton Keynes|Wellcome Sanger Institute for the COVID-19 Genomics UK (COG-UK) consortium|Davies|United Kingdom
>Spike|hCoV-19/USA/TX-DSHS-1944/2020|2020-06-21|EPI_ISL_765918|Original|hCoV-19^^Texas|Human|TXDSHS|TXDSHS|Rahman|USA
>Spike|hCoV-19/USA/MA-JLL-17S/2020|2020-00-00|EPI_ISL_632288|Original|hCoV-19^^Massachusetts|Human|Brigham and Women's Hospital|Jonathan Li laboratory|Choudhary|USA
>Spike|hCoV-19/Denmark/DCGC-6100/2020|2020-10-05|EPI_ISL_620295|Original|hCoV-19^^Hovedstaden|Human|Department of Virus and Microbiological Special Diagnostics|Albertsen lab|Michaelsen|Denmark
>Spike|hCoV-19/England/QEUH-998E91/2020|2020-08-24|EPI_ISL_541816|Original|hCoV-19^^England|Human|Lighthouse Lab in Glasgow|Wellcome Sanger Institute for the COVID-19 Genomics UK (COG-UK) consortium|Davies|United Kingdom

>Spike|hCoV-19/England/ALDP-AA1A36/2020|2020-10-22|EPI_ISL_624461|Original|hCoV-19^^England|Human|Lighthouse Lab in Alderley Park|Wellcome Sanger Institute for the COVID-19 Genomics UK (COG-UK) consortium|Davies|United Kingdom
>Spike|hCoV-19/Australia/VIC15046/2020|2020-07-29|EPI_ISL_593121|Original|hCoV-19^^Victoria|Human|Microbiological Diagnostic Unit - Public Health Laboratory (MDU-PHL)|MDU-PHL|Schultz|Australia
>Spike|hCoV-19/England/NORW-F693F/2020|2020-11-06|EPI_ISL_652352|Original|hCoV-19^^England|Human|Quadram Institute Bioscience|COVID-19 Genomics UK (COG-UK) Consortium|Alikhan|United Kingdom
>Spike|hCoV-19/USA/WI-UW-1220/2020|2020-08-17|EPI_ISL_536593|Original|hCoV-19^^Wisconsin|Human|University of Wisconsin-Madison AIDS Vaccine Research Laboratories|University of Wisconsin-Madison AIDS Vaccine Research Laboratories|Baczenas|USA
>Spike|hCoV-19/Wales/PHWC-4851FD/2020|2020-10-25|EPI_ISL_628157|Original|hCoV-19^^Wales|Human|Wales Specialist Virology Centre Sequencing lab|COVID-19 Genomics UK (COG-UK) Consortium|Moore|United Kingdom
>Spike|hCoV-19/England/CAMC-A3E8BC/2020|2020-10-05|EPI_ISL_609574|Original|hCoV-19^^England|Human|Lighthouse Lab in Cambridge|Wellcome Sanger Institute for the COVID-19 Genomics UK (COG-UK) consortium|Davies|United Kingdom
>Spike|hCoV-19/USA/TX-HMH-MCoV-19192/2020|2020-11-23|EPI_ISL_783550|Original|hCoV-19^^Texas|Human|Houston Methodist Hospital|Houston Methodist Hospital|Long|USA
>Spike|hCoV-19/Scotland/QEUH-C14C63/2020|2020-12-06|EPI_ISL_710691|Original|hCoV-19^^Scotland|Human|Lighthouse Lab in Glasgow|Wellcome Sanger Institute for the COVID-19 Genomics UK (COG-UK) Consortium|Davies|United Kingdom
>Spike|hCoV-19/Wales/PHWC-16CF31/2020|2020-09-25|EPI_ISL_574187|Original|hCoV-19^^Wales|Human|Wales Specialist Virology Centre Sequencing lab|COVID-19 Genomics UK (COG-UK) Consortium|Moore|United Kingdom
>Spike|hCoV-19/Netherlands/ZE-RIVM-20013/2020|2020-10-10|EPI_ISL_723212|Orignal|hCoV-19^^Zeeland|Human|Dutch COVID-19 response team|National Institute for Public Health and the Environment (RIVM)|Meijer|Netherlands
>Spike|hCoV-19/USA/UT-UPHL-2012191595/2020|2020-06-17|EPI_ISL_746365|Original|hCoV-19^^Utah|Human|Utah Public Health Laboratory|Utah Public Health Laboratory|Gallagher|USA
>Spike|hCoV-19/USA/WA-UW-11538/2020|2020-06-21|EPI_ISL_501101|Original|hCoV-19^^Washington|Human|University of Washington Virology Lab|University of Washington Virology Lab|Roychoudhury|USA
>Spike|hCoV-19/Denmark/DCGC-5011/2020|2020-09-14|EPI_ISL_621791|Original|hCoV-19^^Hovedstaden|Human|Department of Virus and Microbiological Special Diagnostics|Albertsen lab|Michaelsen|Denmark
>Spike|hCoV-19/England/MILK-994448/2020|2020-08-26|EPI_ISL_551655|Original|hCoV-19^^England|Human|Lighthouse Lab in Milton Keynes|Wellcome Sanger Institute for the COVID-19 Genomics UK (COG-UK) consortium|Davies|United Kingdom
>Spike|hCoV-19/USA/UT-UPHL-2012645552/2020|2020-07-31|EPI_ISL_745730|Original|hCoV-19^^Utah|Human|Ginkgo Bioworks Clinical Laboratory|Utah Public Health Laboratory|Young|USA
>Spike|hCoV-19/Wales/PHWC-4840B5/2020|2020-10-25|EPI_ISL_627963|Original|hCoV-19^^Wales|Human|Wales Specialist Virology Centre Sequencing lab|COVID-19 Genomics UK (COG-UK) Consortium|Moore|United Kingdom
>Spike|hCoV-19/England/CAMC-B0A593/2020|2020-10-31|EPI_ISL_673129|Original|hCoV-19^^England|Human|Lighthouse Lab in Cambridge|Wellcome Sanger Institute for the COVID-19 Genomics UK (COG-UK) Consortium|Davies|United Kingdom
>Spike|hCoV-19/England/BRIS-12566C/2020|2020-03-28|EPI_ISL_443722|Original|hCoV-19^^England|Human|PHE South West Regional Laboratory|Wellcome Sanger Institute for the COVID-19 Genomics UK (COG-UK) consortium|Amato|United Kingdom
>Spike|hCoV-19/England/LIVE-DC0CCF/2020|2020-11-03|EPI_ISL_704129|Original|hCoV-19^^England|Human|Liverpool Clinical Laboratories|COVID-19 Genomics UK (COG-UK) Consortium|Haldenby|United Kingdom
>Spike|hCoV-19/Wales/ALDP-B1B6C9/2020|2020-11-01|EPI_ISL_710923|Original|hCoV-19^^Wales|Human|Lighthouse Lab in Alderley Park|Wellcome Sanger Institute for the COVID-19 Genomics UK (COG-UK) Consortium|Davies|United Kingdom

>Spike|hCoV-19/Ukraine/Chernivtsi_860/2020|2020-08-06|EPI_ISL_654818|Original|hCoV-
19^^Chernivtsi|Human|Department of Respiratory and other Viral Infections of
L.V.Gromashevsky Institute of Epidemiology & Infectious Diseases NAMS of
Ukrain\u0435|Department of Respiratory and other Viral Infections of L.V.Gromashevsky
Institute of Epidemiology & Infectious Diseases NAMS of Ukrain\u0435|Mironenko|Ukr
>aine
>Spike|hCoV-19/France/ARA-0873/2020|2020-03-14|EPI_ISL_508875|Original|hCoV-
19^^Auvergne-Rh\u00f4ne-Alpes|Human|Centre Hospitalier de Macon|CNR Virus des
Infections Respiratoires - France SUD|Josset|France
>Spike|hCoV-19/USA/WA-S1068/2020|2020-04-29|EPI_ISL_463558|Original|hCoV-
19^^Washington|Human|Washington State Department of Health|Seattle Flu
Study|Bedford|USA
>Spike|hCoV-19/Portugal/PT0453/2020|2020-04-04|EPI_ISL_454177|Original|hCoV-
19^^Portugal|Human|unknown|Instituto Nacional de Saude (INSA)|Borges|Portugal
>Spike|hCoV-19/USA/TX-HMH-MCoV-16361/2020|2020-11-01|EPI_ISL_786045|Original|hCoV-
19^^Texas|Human|Houston Methodist Hospital|Houston Methodist Hospital|Long|USA
>Spike|hCoV-19/USA/AZ-TG408181/2020|2020-06-16|EPI_ISL_694923|Original|hCoV-
19^^Arizona|Human|AZ SPHL|TGen North|French|USA
>Spike|hCoV-19/Australia/QLD1266/2020|2020-03-25|EPI_ISL_693268|Original|hCoV-
19^^Australia|Human|unknown|Public Health Virology Laboratory|Nguyen|Australia
>Spike|hCoV-19/England/QEUH-A36CB8/2020|2020-10-05|EPI_ISL_638705|Original|hCoV-
19^^England|Human|Wales Specialist Virology Centre Sequencing lab|COVID-19 Genomics
UK (COG-UK) Consortium|Moore|United Kingdom
>Spike|hCoV-19/Japan/PG-3883/2020|2020-07-00|EPI_ISL_692134|Original|hCoV-
19^^Japan|Human|Pathogen Genomics Center|Pathogen Genomics Center|Sekizuka|Japan
>Spike|hCoV-19/England/ALDP-B62AD6/2020|2020-11-08|EPI_ISL_655355|Original|hCoV-
19^^England|Human|Lighthouse Lab in Alderley Park|Wellcome Sanger Institute for the
COVID-19 Genomics UK (COG-UK) Consortium|Davies|United Kingdom
>Spike|hCoV-19/USA/TX-HMH-MCoV-15370/2020|2020-10-21|EPI_ISL_786854|Original|hCoV-
19^^Texas|Human|Houston Methodist Hospital|Houston Methodist Hospital|Long|USA
>Spike|hCoV-19/England/CAMC-C2C8EC/2020|2020-12-08|EPI_ISL_733812|Original|hCoV-
19^^England|Human|Lighthouse Lab in Cambridge|Wellcome Sanger Institute for the
COVID-19 Genomics UK (COG-UK) Consortium|Davies|United Kingdom
>Spike|hCoV-19/Wales/PHWC-49541F/2020|2020-11-21|EPI_ISL_726344|Original|hCoV-
19^^Wales|Human|Originating lab|COVID-19 Genomics UK (COG-UK) Consortium|Moore|United
Kingdom
>Spike|hCoV-19/Ireland/TA-UHL288/2020|2020-04-26|EPI_ISL_681770|Original|hCoV-
19^^Tipperary|Human|University Hospital Limerick|Irish Coronavirus Sequencing
Consortium - Teagasc Moorepark|Walsh|Ireland
>Spike|hCoV-19/England/MILK-A7D268/2020|2020-10-13|EPI_ISL_606551|Original|hCoV-
19^^England|Human|Lighthouse Lab in Milton Keynes|Wellcome Sanger Institute for the
COVID-19 Genomics UK (COG-UK) consortium|Davies|United Kingdom
>Spike|hCoV-19/Denmark/DCGC-4145/2020|2020-09-21|EPI_ISL_621965|Original|hCoV-
19^^Midtjylland|Human|Department of Virus and Microbiological Special
Diagnostics|Albertsen lab|Michaelsen|Denmark
>Spike|hCoV-19/Malaysia/IMR_WC1098/2020|2020-02-29|EPI_ISL_430442|Vero E6|hCoV-
19^^Malaysia|Human|Institute for Medical Research|Institute for Medical
Research|Suppiah|Malaysia
>Spike|hCoV-19/Wales/PHWC-48FB8C/2020|2020-11-11|EPI_ISL_681195|Original|hCoV-
19^^Wales|Human|Wales Specialist Virology Centre Sequencing lab|COVID-19 Genomics UK
(COG-UK) Consortium|Moore|United Kingdom
>Spike|hCoV-19/Scotland/QEUH-9DC356/2020|2020-09-22|EPI_ISL_601115|Original|hCoV-
19^^Scotland|Human|Lighthouse Lab in Glasgow|Wellcome Sanger Institute for the COVID-
19 Genomics UK (COG-UK) consortium|Davies|United Kingdom
>Spike|hCoV-19/Denmark/DCGC-19591/2020|2020-12-07|EPI_ISL_748341|Original|hCoV-
19^^Hovedstaden|Human|Department of Virus and Microbiological Special
Diagnostics|Albertsen Lab|Michaelsen|Denmark
>Spike|hCoV-19/England/CAMC-B072F9/2020|2020-10-30|EPI_ISL_643953|Original|hCoV-
19^^England|Human|Lighthouse Lab in Cambridge|Wellcome Sanger Institute for the
COVID-19 Genomics UK (COG-UK) Consortium|Davies|United Kingdom

>Spike|hCoV-19/England/MILK-AB90B1/2020|2020-10-21|EPI_ISL_623786|Original|hCoV-19^^England|Human|Lighthouse Lab in Milton Keynes|Wellcome Sanger Institute for the COVID-19 Genomics UK (COG-UK) consortium|Davies|United Kingdom
>Spike|hCoV-19/England/CAMC-B346CA/2020|2020-11-05|EPI_ISL_659583|Original|hCoV-19^^England|Human|Lighthouse Lab in Cambridge|Wellcome Sanger Institute for the COVID-19 Genomics UK (COG-UK) Consortium|Davies|United Kingdom
>Spike|hCoV-19/England/CAMC-AEAC1D/2020|2020-10-26|EPI_ISL_633372|Original|hCoV-19^^England|Human|Lighthouse Lab in Cambridge|Wellcome Sanger Institute for the COVID-19 Genomics UK (COG-UK) consortium|Davies|United Kingdom
>Spike|hCoV-19/Scotland/CVR3353/2020|2020-05-01|EPI_ISL_456785|Original|hCoV-19^^Scotland|Human|West of Scotland Specialist Virology Centre|COVID-19 Genomics UK (COG-UK) Consortium|Robertson|United Kingdom
>Spike|hCoV-19/Australia/VIC2309/2020|2020-06-26|EPI_ISL_522030|Original|hCoV-19^^Victoria|Human|Microbiological Diagnostic Unit - Public Health Laboratory (MDU-PHL)|MDU-PHL|Schultz|Australia
>Spike|hCoV-19/England/QEUH-9AEA85/2020|2020-09-05|EPI_ISL_577271|Original|hCoV-19^^England|Human|University of Exeter|COVID-19 Genomics UK (COG-UK) Consortium|Temperton|United Kingdom
>Spike|hCoV-19/England/NOTT-11369E/2020|2020-09-11|EPI_ISL_549520|Original|hCoV-19^^England|Human|Lincolnshire Hospitals and DeepSeq Nottingham|COVID-19 Genomics UK (COG-UK) Consortium|Loose|United Kingdom
>Spike|hCoV-19/Denmark/DCGC-4723/2020|2020-09-21|EPI_ISL_622162|Original|hCoV-19^^Syddanmark|Human|Department of Virus and Microbiological Special Diagnostics|Albertsen lab|Michaelsen|Denmark
>Spike|hCoV-19/USA/LA-EVTL514/2020|2020-06-25|EPI_ISL_578918|Original|hCoV-19^^Louisiana|Human|LSUHS Emerging Viral Threat Laboratory|Microbial Genome Sequencing Center|Kamil|USA
>Spike|hCoV-19/Switzerland/BE-ETHZ-190010/2020|2020-07-03|EPI_ISL_498627|Original|hCoV-19^^Bern|Human|Viollier AG|Department of Biosystems Science and Engineering|Nadeau|Switzerland
>Spike|hCoV-19/Japan/PG-2545/2020|2020-05-00|EPI_ISL_690680|Original|hCoV-19^^Japan|Human|Saitama Prefectural Institute of Public Health|Pathogen Genomics Center|Sekizuka|Japan
>Spike|hCoV-19/Argentina/PAIS-A0019/2020|2020-04-07|EPI_ISL_430810|Original|hCoV-19^^Ciudad Autonoma de Buenos Aires|Human|Laboratorio de Virolog\u00eda del Hospital de Ni\u00f1os Dr. Ricardo Gutierrez|\u00c1rea de Secuenciaci\u00f3n del Laboratorio de Virolog\u00eda del Hospital de Ni\u00f1os Dr. Ricardo Gutierrez|Viegas|Argentina
>Spike|hCoV-19/England/CAMC-B78387/2020|2020-11-12|EPI_ISL_661697|Original|hCoV-19^^England|Human|Lighthouse Lab in Cambridge|Wellcome Sanger Institute for the COVID-19 Genomics UK (COG-UK) Consortium|Davies|United Kingdom
>Spike|hCoV-19/England/CAMC-BD2AA4/2020|2020-11-28|EPI_ISL_702272|Original|hCoV-19^^England|Human|Lighthouse Lab in Cambridge|Wellcome Sanger Institute for the COVID-19 Genomics UK (COG-UK) Consortium|Davies|United Kingdom
>Spike|hCoV-19/France/IDF-9150/2020|2020-09-01|EPI_ISL_593930|Original|hCoV-19^^\u00cele-de-France|Human|Labo Analyses Med|National Reference Center for Viruses of Respiratory Infections|Enouf|France
>Spike|hCoV-19/England/ALDP-A92E2D/2020|2020-10-20|EPI_ISL_611059|Original|hCoV-19^^England|Human|Lighthouse Lab in Alderley Park|Wellcome Sanger Institute for the COVID-19 Genomics UK (COG-UK) consortium|Davies|United Kingdom
>Spike|hCoV-19/Japan/PG-8802/2020|2020-08-00|EPI_ISL_686389|Original|hCoV-19^^Japan|Human|Pathogen Genomics Center|Pathogen Genomics Center|Sekizuka|Japan
>Spike|hCoV-19/USA/PA-MGSC55-05/2020|2020-03-23|EPI_ISL_436502|Original|hCoV-19^^Pennsylvania|Human|UPMC Clinical Laboratory|Microbial Genome Sequencing Center|Cooper|USA
>Spike|hCoV-19/Wales/PHWC-4A23E5/2020|2020-12-13|EPI_ISL_743884|Original|hCoV-19^^Wales|Human|Wales Specialist Virology Centre Sequencing lab|COVID-19 Genomics UK (COG-UK) Consortium|Moore|United Kingdom
>Spike|hCoV-19/Scotland/QEUH-96FB7E/2020|2020-08-18|EPI_ISL_568406|Original|hCoV-19^^Scotland|Human|Lighthouse Lab in Glasgow|Wellcome Sanger Institute for the COVID-19 Genomics UK (COG-UK) consortium|Davies|United Kingdom

>Spike|hCoV-19/Russia/OMS-ORINFI-60S/2020|2020-05-13|EPI_ISL_569855|Original|hCoV-19^^Omsk|Human|Omsk Research Institute of Natural Focal Infections|WHO National Influenza Centre Russian Federation|Komissarov|Russia
>Spike|hCoV-19/DRC/1767/2020|2020-04-10|EPI_ISL_435157|Original|hCoV-19^^Kinshasa|Human|Viral Respiratory Lab|Pathogen Sequencing Lab|Pratt|Democratic Republic of the Congo
>Spike|hCoV-19/Portugal/PT1210/2020|2020-03-26|EPI_ISL_511753|Original|hCoV-19^^Portugal|Human|Instituto Nacional de Saude (INSA)|Instituto Nacional de Saude (INSA) and Instituto Gulbenkian de Ciencia (IGC)|Borges|Portugal
>Spike|hCoV-19/USA/LA-EVTL187/2020|2020-05-21|EPI_ISL_574321|Original|hCoV-19^^Louisiana|Human|LSUHS Emerging Viral Threat Laboratory|Microbial Genome Sequencing Center|Kamil|USA
>Spike|hCoV-19/England/ALDP-B74DC9/2020|2020-11-12|EPI_ISL_661998|Original|hCoV-19^^England|Human|Lighthouse Lab in Alderley Park|Wellcome Sanger Institute for the COVID-19 Genomics UK (COG-UK) Consortium|Davies|United Kingdom
>Spike|hCoV-19/England/ALDP-658829/2020|2020-07-03|EPI_ISL_558168|Original|hCoV-19^^England|Human|Lighthouse Lab in Alderley Park|Wellcome Sanger Institute for the COVID-19 Genomics UK (COG-UK) consortium|Davies|United Kingdom
>Spike|hCoV-19/France/ARA-117399/2020|2020-12-08|EPI_ISL_745373|Original|hCoV-19^^Auvergne-Rh\u00f4ne-Alpes|Human|CNR Virus des Infections Respiratoires - France SUD|CNR Virus des Infections Respiratoires - France SUD|Bal|France
>Spike|hCoV-19/Northern Ireland/NIRE-233952/2020|2020-10-04|EPI_ISL_680327|Original|hCoV-19^^Northern Ireland|Human|Regional Virus Laboratory|COVID-19 Genomics UK (COG-UK) Consortium|Fairley|United Kingdom
>Spike|hCoV-19/England/CAMC-B51AAD/2020|2020-11-08|EPI_ISL_658234|Original|hCoV-19^^England|Human|Lighthouse Lab in Cambridge|Wellcome Sanger Institute for the COVID-19 Genomics UK (COG-UK) Consortium|Davies|United Kingdom
>Spike|hCoV-19/USA/SD-SDPHL-0001/2020|2020-04-03|EPI_ISL_569609|Original|hCoV-19^^South Dakota|Human|Bennet County Hospital|South Dakota Public Health Laboratory|Carlson|USA
>Spike|hCoV-19/England/EXET-1350F2/2020|2020-04-04|EPI_ISL_457032|Original|hCoV-19^^England|Human|University of Exeter|COVID-19 Genomics UK (COG-UK) Consortium|Temperton|United Kingdom
>Spike|hCoV-19/England/CAMB-1AC102/2020|2020-04-29|EPI_ISL_444373|Original|hCoV-19^^England|Human|Department of Pathology|COVID-19 Genomics UK (COG-UK) Consortium|Meredith|United Kingdom
>Spike|hCoV-19/USA/WA-UW-12085/2020|2020-06-22|EPI_ISL_570486|Original|hCoV-19^^Washington|Human|UW Virology Lab|UW Virology Lab|Roychoudhury|USA
>Spike|hCoV-19/USA/CA-CZB-3666/2020|2020-07-08|EPI_ISL_516741|Original|hCoV-19^^California|Human|UCSF Clinical Microbiology Laboratory|Chan-Zuckerberg Biohub|Kamm|USA
>Spike|hCoV-19/England/LIVE-A0372/2020|2020-05-25|EPI_ISL_472105|Original|hCoV-19^^England|Human|Liverpool Clinical Laboratories|COVID-19 Genomics UK (COG-UK) Consortium|Haldenby|United Kingdom
>Spike|hCoV-19/England/CAMC-B9907A/2020|2020-11-15|EPI_ISL_673439|Original|hCoV-19^^England|Human|Lighthouse Lab in Cambridge|Wellcome Sanger Institute for the COVID-19 Genomics UK (COG-UK) Consortium|Davies|United Kingdom
>Spike|hCoV-19/South Africa/KRISP-K003522/2020|2020-09-26|EPI_ISL_602841|Original|hCoV-19^^KwaZulu-Natal|Human|NHLS-IALCH|KRISP|de Oliveira|South Africa
>Spike|hCoV-19/England/MILK-BAFC22/2020|2020-11-17|EPI_ISL_720652|Original|hCoV-19^^England|Human|Lighthouse Lab in Milton Keynes|Wellcome Sanger Institute for the COVID-19 Genomics UK (COG-UK) Consortium|Davies|United Kingdom
>Spike|hCoV-19/USA/TX-HMH-4088/2020|2020-06-17|EPI_ISL_543358|Original|hCoV-19^^Texas|Human|Houston Methodist Hospital|Houston Methodist Hospital|Long|USA
>Spike|hCoV-19/England/NORT-282B4F/2020|2020-03-28|EPI_ISL_488565|Original|hCoV-19^^England|Human|NU-OMICS DNA Sequencing research facility|Wellcome Sanger Institute for the COVID-19 Genomics UK (COG-UK) consortium|Davies|United Kingdom

>Spike|hCoV-19/Luxembourg/LNS0795813/2020|2020-10-28|EPI_ISL_739832|Original|hCoV-19^^Luxembourg|Human|Laboratoire national de sant\u00e9|Laboratoire national de sant\u00e9|Wienecke-Baldacchino|Luxembourg
>Spike|hCoV-19/Chile/RM-126534/2020|2020-07-16|EPI_ISL_746595|Original|hCoV-19^^Region Metropolitana de Santiago|Human|Genetica Molecular and Subdepartamento de Virologia ISP Chile|Instituto de Salud Publica de Chile|Tognarelli|Chile
>Spike|hCoV-19/France/ARA-09434/2020|2020-03-15|EPI_ISL_418414|Original|hCoV-19^^Auvergne-Rh\u00f4ne-Alpes|Human|Centre Hospitalier de Valence|CNR Virus des Infections Respiratoires - France SUD|Josset|France
>Spike|hCoV-19/England/MILK-ABBF67/2020|2020-10-22|EPI_ISL_631090|Original|hCoV-19^^England|Human|Lighthouse Lab in Milton Keynes|Wellcome Sanger Institute for the COVID-19 Genomics UK (COG-UK) Consortium|Davies|United Kingdom
>Spike|hCoV-19/USA/MN-MDH-1627/2020|2020-03-31|EPI_ISL_530223|Original|hCoV-19^^Minnesota|Human|Minnesota Department of Health|Minnesota Department of Health|Garfin|USA
>Spike|hCoV-19/USA/WI-UW-91/2020|2020-04-01|EPI_ISL_425148|Original|hCoV-19^^Wisconsin|Human|University of Wisconsin-Madison AIDS Vaccine Research Laboratories|University of Wisconsin-Madison AIDS Vaccine Research Laboratories|Baczenas|USA
>Spike|hCoV-19/England/ALDP-671A24/2020|2020-07-03|EPI_ISL_558086|Original|hCoV-19^^England|Human|Lighthouse Lab in Alderley Park|Wellcome Sanger Institute for the COVID-19 Genomics UK (COG-UK) consortium|Davies|United Kingdom
>Spike|hCoV-19/England/ALDP-9E9040/2020|2020-09-24|EPI_ISL_584184|Original|hCoV-19^^England|Human|Centre for Enzyme Innovation|COVID-19 Genomics UK (COG-UK) Consortium|Robson|United Kingdom
>Spike|hCoV-19/Japan/PG-1731/2020|2020-04-00|EPI_ISL_685337|Original|hCoV-19^^Japan|Human|Pathogen Genomics Center|Pathogen Genomics Center|Sekizuka|Japan
>Spike|hCoV-19/USA/FL-BPHL-1277/2020|2020-08-07|EPI_ISL_541209|Original|hCoV-19^^Florida|Human|Florida Bureau of Public Health Laboratories|Florida Bureau of Public Health Laboratories|Blanton|USA
>Spike|hCoV-19/Germany/NW-HHU-351/2020|2020-09-10|EPI_ISL_666638|Orginal|hCoV-19^^North Rhine-Westphalia|Human|ZOTZ KLIMAS MVZ D\u00fcsseldorf-Centrum GbR \u00dcBAG f\u00fcr Labormedizin|Center of Medical Microbiology|Wienemann|Germany
>Spike|hCoV-19/Japan/PG-6953/2020|2020-07-00|EPI_ISL_688058|Original|hCoV-19^^Japan|Human|Pathogen Genomics Center|Pathogen Genomics Center|Sekizuka|Japan
>Spike|hCoV-19/Australia/SAP446/2020|2020-03-20|EPI_ISL_508137|Original|hCoV-19^^South Australia|Human|SA Pathology|SA Pathology|Leong|Australia
>Spike|hCoV-19/Spain/PV-IBV-98007684/2020|2020-09-14|EPI_ISL_582079|Original|hCoV-19^^Basque Country|Human|Servicio de Microbiolog\u00eda. Hospital Universitario Donostia. OSI Donostialdea. \u00c1rea de Enfermedades Infecciosas|SeqCOVID-SPAIN consortium/IBV(CSIC)|Comas|Spain
>Spike|hCoV-19/USA/WI-UW-1132/2020|2020-08-13|EPI_ISL_522889|Original|hCoV-19^^Wisconsin|Human|University of Wisconsin-Madison AIDS Vaccine Research Laboratories|University of Wisconsin-Madison AIDS Vaccine Research Laboratories|Baczenas|USA
>Spike|hCoV-19/USA/WI-UW-1882/2020|2020-09-14|EPI_ISL_632897|Original|hCoV-19^^Wisconsin|Human|University of Wisconsin-Madison AIDS Vaccine Research Laboratories|University of Wisconsin-Madison AIDS Vaccine Research Laboratories|Baczenas|USA
>Spike|hCoV-19/Luxembourg/LNS2818424/2020|2020-04-15|EPI_ISL_434500|Original|hCoV-19^^Luxembourg|Human|Laboratoire National de Sante|Laboratoire National de Sante|Wienecke-Baldacchino|Luxembourg
>Spike|hCoV-19/Norway/4793/2020|2020-10-22|EPI_ISL_668407|Original|hCoV-19^^Rogaland|Human|Dept. of Medical Microbiology|Norwegian Institute of Public Health|Kopperud Riis|Norway
>Spike|hCoV-19/Switzerland/BE-ETHZ-110486/2020|2020-04-07|EPI_ISL_451898|Original|hCoV-19^^Bern|Human|Viollier AG|Department of Biosystems Science and Engineering|Nadeau|Switzerland

>Spike|hCoV-19/England/CAMC-A64BC3/2020|2020-10-11|EPI_ISL_608602|Original|hCoV-19^^England|Human|Lighthouse Lab in Cambridge|Wellcome Sanger Institute for the COVID-19 Genomics UK (COG-UK) consortium|Davies|United Kingdom
>Spike|hCoV-19/USA/NY-NYCPHL-001528/2020|2020-12-01|EPI_ISL_745447|Original|hCoV-19^^New York|Human|DOHMH Corona|New York City Public Health Laboratory|Wang|USA
>Spike|hCoV-19/Australia/VIC4656/2020|2020-07-13|EPI_ISL_519045|Original|hCoV-19^^Victoria|Human|Victorian Infectious Diseases Reference Laboratory (VIDRL)|VIDRL and MDU-PHL|Schultz|Australia
>Spike|hCoV-19/Denmark/DCGC-5168/2020|2020-09-21|EPI_ISL_621706|Original|hCoV-19^^Syddanmark|Human|Department of Virus and Microbiological Special Diagnostics|Albertsen lab|Michaelsen|Denmark
>Spike|hCoV-19/England/SHEF-D069E/2020|2020-07-03|EPI_ISL_526448|Original|hCoV-19^^England|Human|Virology Department|COVID-19 Genomics UK (COG-UK) Consortium|de Silva|United Kingdom
>Spike|hCoV-19/England/QEUH-9FAE60/2020|2020-09-27|EPI_ISL_588268|Original|hCoV-19^^England|Human|Lighthouse Lab in Glasgow|Wellcome Sanger Institute for the COVID-19 Genomics UK (COG-UK) consortium|Davies|United Kingdom
>Spike|hCoV-19/North Macedonia/3310/2020|2020-06-13|EPI_ISL_516423|Original|hCoV-19^^Skopje|Human|Center for public health - Skopje|Research Center for Genetic Engineering and Biotechnology \Georgi D. Efremov\u201d|Dimovski|North Macedonia
>Spike|hCoV-19/Greece/210_33942/2020|2020-03-14|EPI_ISL_437880|Original|hCoV-19^^Athens|Human|Laboratory of Microbiology|Laboratory of Biology|Karakasiliotis|Greece
>Spike|hCoV-19/India/MH-NIV-69925/2020|2020-06-19|EPI_ISL_541733|Original|hCoV-19^^Maharashtra|Human|National Institute of Virology|National Institute of Virology|Potdar|India
>Spike|hCoV-19/Wales/PHWC-27AAB/2020|2020-03-30|EPI_ISL_432227|Original|hCoV-19^^Wales|Human|Wales Specialist Virology Centre|Public Health Wales Microbiology Cardiff|Moore|United Kingdom
>Spike|hCoV-19/Russia/LEN-RII-MH3119S/2020|2020-10-01|EPI_ISL_733094|Original|hCoV-19^^Leningrad|Human|HELIX LLC|WHO National Influenza Centre Russian Federation|Komissarov|Russia
>Spike|hCoV-19/England/QEUH-9F4FDC/2020|2020-09-25|EPI_ISL_637805|Original|hCoV-19^^England|Human|Department of Pathology|COVID-19 Genomics UK (COG-UK) Consortium|Goodfellow|United Kingdom
>Spike|hCoV-19/Australia/VIC3944/2020|2020-07-14|EPI_ISL_520655|Original|hCoV-19^^Victoria|Human|Microbiological Diagnostic Unit - Public Health Laboratory (MDU-PHL)|MDU-PHL|Schultz|Australia
>Spike|hCoV-19/England/CAMB-1AD8EE/2020|2020-05-02|EPI_ISL_448022|Original|hCoV-19^^England|Human|Department of Pathology|COVID-19 Genomics UK (COG-UK) Consortium|Meredith|United Kingdom
>Spike|hCoV-19/Australia/VIC2667/2020|2020-07-02|EPI_ISL_521509|Original|hCoV-19^^Victoria|Human|Victorian Infectious Diseases Reference Laboratory (VIDRL)|VIDRL and MDU-PHL|Schultz|Australia
>Spike|hCoV-19/Wales/MILK-9EEB2C/2020|2020-09-23|EPI_ISL_580840|Original|hCoV-19^^Wales|Human|Lighthouse Lab in Milton Keynes|Wellcome Sanger Institute for the COVID-19 Genomics UK (COG-UK) Consortium|Davies|United Kingdom
>Spike|hCoV-19/England/LIVE-9E8D2/2020|2020-04-11|EPI_ISL_453341|Original|hCoV-19^^England|Human|Liverpool Clinical Laboratories|COVID-19 Genomics UK (COG-UK) Consortium|Haldenby|United Kingdom
>Spike|hCoV-19/England/CAMB-7CE05/2020|2020-04-05|EPI_ISL_442151|Original|hCoV-19^^England|Human|Department of Pathology|Wellcome Sanger Institute for the COVID-19 Genomics UK (COG-UK) consortium|Amato|United Kingdom
>Spike|hCoV-19/Ireland/G-NVRL-76IRL13817/2020|2020-10-16|EPI_ISL_671905|Original|hCoV-19^^Galway|Human|National Virus Reference Laboratory|National Virus Reference Laboratory|Gonzalez|Ireland
>Spike|hCoV-19/Belgium/ITM_C340/2020|2020-04-06|EPI_ISL_434367|Original|hCoV-19^^Antwerp|Human|Hospital AZ Rivierenland|Institute of Tropical Medicine|Selhorst|Belgium

>Spike|hCoV-19/Scotland/GCVR-171467/2020|2020-03-28|EPI_ISL_489606|Original|hCoV-19^^Scotland|Human|NHSGGC West of Scotland Specialist Virology Centre / MRC-University of Glasgow Centre for Virus Research|Wellcome Sanger Institute for the COVID-19 Genomics UK (COG-UK) consortium|Davies|United Kingdom
>Spike|hCoV-19/England/BRIS-1255D8/2020|2020-03-28|EPI_ISL_443708|Original|hCoV-19^^England|Human|PHE South West Regional Laboratory|Wellcome Sanger Institute for the COVID-19 Genomics UK (COG-UK) consortium|Amato|United Kingdom
>Spike|hCoV-19/Japan/PG-4409/2020|2020-07-00|EPI_ISL_691750|Original|hCoV-19^^Japan|Human|Pathogen Genomics Center|Pathogen Genomics Center|Sekizuka|Japan
>Spike|hCoV-19/England/LCST-24D69A4/2020|2020-06-02|EPI_ISL_478314|Original|hCoV-19^^England|Human|University Hospitals Of Leicester NHS Trust and DeepSeq Nottingham|COVID-19 Genomics UK (COG-UK) Consortium|Loose|United Kingdom
>Spike|hCoV-19/Denmark/DCGC-7063/2020|2020-10-12|EPI_ISL_619117|Original|hCoV-19^^Midtjylland|Human|Department of Virus and Microbiological Special Diagnostics|Albertsen lab|Michaelsen|Denmark
>Spike|hCoV-19/USA/TX-HMH-MCoV-8204/2020|2020-07-07|EPI_ISL_784263|Original|hCoV-19^^Texas|Human|Houston Methodist Hospital|Houston Methodist Hospital|Long|USA
>Spike|hCoV-19/England/CAMC-B32B81/2020|2020-11-05|EPI_ISL_659452|Original|hCoV-19^^England|Human|Lighthouse Lab in Cambridge|Wellcome Sanger Institute for the COVID-19 Genomics UK (COG-UK) Consortium|Davies|United Kingdom
>Spike|hCoV-19/Wales/PHWC-494841/2020|2020-11-20|EPI_ISL_726175|Original|hCoV-19^^Wales|Human|Originating lab|COVID-19 Genomics UK (COG-UK) Consortium|Moore|United Kingdom
>Spike|hCoV-19/England/MILK-A45DC2/2020|2020-10-06|EPI_ISL_598621|Original|hCoV-19^^England|Human|Lighthouse Lab in Milton Keynes|Wellcome Sanger Institute for the COVID-19 Genomics UK (COG-UK) consortium|Davies|United Kingdom
>Spike|hCoV-19/England/QEUH-B10BF2/2020|2020-11-02|EPI_ISL_642731|Original|hCoV-19^^England|Human|Lighthouse Lab in Glasgow|Wellcome Sanger Institute for the COVID-19 Genomics UK (COG-UK) Consortium|Davies|United Kingdom
>Spike|hCoV-19/USA/NY-Wadsworth-46242-01/2020|2020-04-12|EPI_ISL_676844|Original|hCoV-19^^New York|Human|Wadsworth Center|Wadsworth Center|Lasek-Nesselquist|USA
>Spike|hCoV-19/England/QEUH-9B70B4/2020|2020-09-04|EPI_ISL_590603|Original|hCoV-19^^England|Human|Lighthouse Lab in Glasgow|Wellcome Sanger Institute for the COVID-19 Genomics UK (COG-UK) consortium|Davies|United Kingdom
>Spike|hCoV-19/Gibraltar/204462917/2020|2020-10-29|EPI_ISL_632815|Original|hCoV-19^^Gibraltar|Human|Microbiology|Respiratory Virus Unit|Platt|Gibraltar
>Spike|hCoV-19/Japan/NGY-NNH-035/2020|2020-07-28|EPI_ISL_637001|Original|hCoV-19^^Aichi|Human|Department of Infectious Diseases and Immunology|Clinical Research Center|Iwatani|Japan
>Spike|hCoV-19/Denmark/DCGC-19306/2020|2020-12-07|EPI_ISL_748526|Original|hCoV-19^^Hovedstaden|Human|Department of Virus and Microbiological Special Diagnostics|Albertsen Lab|Michaelsen|Denmark
>Spike|hCoV-19/England/CAMC-BBCB07/2020|2020-11-21|EPI_ISL_705609|Original|hCoV-19^^England|Human|Quadram Institute Bioscience|COVID-19 Genomics UK (COG-UK) Consortium|Alikhan|United Kingdom
>Spike|hCoV-19/Wales/ALDP-6CE967/2020|2020-07-02|EPI_ISL_556088|Original|hCoV-19^^Wales|Human|Lighthouse Lab in Alderley Park|Wellcome Sanger Institute for the COVID-19 Genomics UK (COG-UK) consortium|Davies|United Kingdom
>Spike|hCoV-19/Japan/PG-1240/2020|2020-04-00|EPI_ISL_684850|Original|hCoV-19^^Japan|Human|Yamagata Prefectural Institute of Public Health|Pathogen Genomics Center|Sekizuka|Japan
>Spike|hCoV-19/England/CAMC-BFBA87/2020|2020-12-04|EPI_ISL_708989|Original|hCoV-19^^England|Human|Lighthouse Lab in Cambridge|Wellcome Sanger Institute for the COVID-19 Genomics UK (COG-UK) Consortium|Davies|United Kingdom
>Spike|hCoV-19/New Zealand/20VR3025/2020|2020-00-00|EPI_ISL_579391|Original|hCoV-19^^Combined Wellington|Human|Wellington SCL (WN)|Institute of Environmental Science and Research (ESR)|Wang|New Zealand

>Spike|hCoV-19/England/MILK-992231/2020|2020-08-26|EPI_ISL_540661|Original|hCoV-19^^England|Human|Queens Medical Centre|COVID-19 Genomics UK (COG-UK) Consortium|Loose|United Kingdom
>Spike|hCoV-19/Wales/PHWC-164564/2020|2020-05-09|EPI_ISL_474194|Original|hCoV-19^^Wales|Human|Originating lab|COVID-19 Genomics UK (COG-UK) Consortium|Moore|United Kingdom
>Spike|hCoV-19/USA/UT-UPHL-2012849927/2020|2020-12-01|EPI_ISL_779314|Original|hCoV-19^^Utah|Human|Utah Public Health Laboratory|Utah Public Health Laboratory|Gallagher|USA
>Spike|hCoV-19/USA/MI-MDHHS-SC21224/2020|2020-04-07|EPI_ISL_507480|Original|hCoV-19^^Michigan|Human|Michigan Department of Health and Human Services|Michigan Department of Health and Human Services|Blankenship|USA
>Spike|hCoV-19/England/ALDP-B260FA/2020|2020-11-04|EPI_ISL_646673|Original|hCoV-19^^England|Human|Lighthouse Lab in Alderley Park|Wellcome Sanger Institute for the COVID-19 Genomics UK (COG-UK) Consortium|Davies|United Kingdom
>Spike|hCoV-19/Hungary/US-636w/2020|2020-03-30|EPI_ISL_476078|Original|hCoV-19^^Csongr\u00e1d County|Human|University of Szeged|National Laboratory of Virology|Kemenesi|Hungary
>Spike|hCoV-19/England/ALDP-8D7592/2020|2020-06-24|EPI_ISL_557034|Original|hCoV-19^^England|Human|Lighthouse Lab in Alderley Park|Wellcome Sanger Institute for the COVID-19 Genomics UK (COG-UK) consortium|Davies|United Kingdom
>Spike|hCoV-19/England/ALDP-BB28FB/2020|2020-11-19|EPI_ISL_703111|Original|hCoV-19^^England|Human|Lighthouse Lab in Alderley Park|Wellcome Sanger Institute for the COVID-19 Genomics UK (COG-UK) Consortium|Davies|United Kingdom
>Spike|hCoV-19/England/MILK-956A13/2020|2020-06-05|EPI_ISL_554284|Original|hCoV-19^^England|Human|Lighthouse Lab in Milton Keynes|Wellcome Sanger Institute for the COVID-19 Genomics UK (COG-UK) consortium|Davies|United Kingdom
>Spike|hCoV-19/England/QEUH-9FF37B/2020|2020-10-02|EPI_ISL_600276|Original|hCoV-19^^England|Human|Lighthouse Lab in Glasgow|Wellcome Sanger Institute for the COVID-19 Genomics UK (COG-UK) consortium|Davies|United Kingdom
>Spike|hCoV-19/USA/MI-MDHHS-SC20740/2020|2020-04-29|EPI_ISL_471761|Original|hCoV-19^^Michigan|Human|Michigan Department of Health and Human Services|Michigan Department of Health and Human Services|Blankenship|USA
>Spike|hCoV-19/England/PORT-2D2269/2020|2020-03-23|EPI_ISL_507138|Original|hCoV-19^^England|Human|Centre for Enzyme Innovation|COVID-19 Genomics UK (COG-UK) Consortium|Robson|United Kingdom
>Spike|hCoV-19/England/PORT-2D74B9/2020|2020-00-00|EPI_ISL_665301|Original|hCoV-19^^England|Human|Centre for Enzyme Innovation|COVID-19 Genomics UK (COG-UK) Consortium|Robson|United Kingdom
>Spike|hCoV-19/Wales/QEUH-A9FB92/2020|2020-10-20|EPI_ISL_625341|Original|hCoV-19^^Wales|Human|Lighthouse Lab in Glasgow|Wellcome Sanger Institute for the COVID-19 Genomics UK (COG-UK) consortium|Davies|United Kingdom
>Spike|hCoV-19/USA/LA-BIE-031/2020|2020-04-04|EPI_ISL_463279|Original|hCoV-19^^Louisiana|Human|Ochsner Health|Bioinfoexperts|Cross|USA
>Spike|hCoV-19/Japan/PG-5500/2020|2020-08-00|EPI_ISL_690602|Original|hCoV-19^^Japan|Human|Pathogen Genomics Center|Pathogen Genomics Center|Sekizuka|Japan
>Spike|hCoV-19/USA/WA-UW-4630/2020|2020-03-28|EPI_ISL_570052|Original|hCoV-19^^Washington|Human|UW Virology Lab|UW Virology Lab|Roychoudhury|USA
>Spike|hCoV-19/Australia/QLD1254/2020|2020-03-23|EPI_ISL_693257|Original|hCoV-19^^Australia|Human|unknown|Public Health Virology Laboratory|Nguyen|Australia
>Spike|hCoV-19/England/LOND-12516EB/2020|2020-11-21|EPI_ISL_679426|Original|hCoV-19^^England|Human|University College London Hospital|COVID-19 Genomics UK (COG-UK) Consortium|Byott|United Kingdom
>Spike|hCoV-19/USA/FL-UW-MAYO68/2020|2020-03-18|EPI_ISL_490988|Original|hCoV-19^^Florida|Human|Mayo Clinic Laboratories|UW Virology Lab|Roychoudhury|USA
>Spike|hCoV-19/Netherlands/FR-RIVM-20810/2020|2020-12-13|EPI_ISL_790867|Original|hCoV-19^^Friesland|Human|Dutch COVID-19 response team|National Institute for Public Health and the Environment (RIVM)|Meijer|Netherlands

>Spike|hCoV-19/England/20189159304/2020|2020-05-02|EPI_ISL_465561|Original|hCoV-19^^England|Human|Respiratory Virus Unit|Respiratory Virus Unit|Platt|United Kingdom
>Spike|hCoV-19/England/LOND-D4612/2020|2020-04-08|EPI_ISL_444142|Original|hCoV-19^^England|Human|University College London|COVID-19 Genomics UK (COG-UK) Consortium|Roy|United Kingdom
>Spike|hCoV-19/Wales/PHWC-4801D4/2020|2020-10-21|EPI_ISL_612861|Original|hCoV-19^^Wales|Human|Wales Specialist Virology Centre Sequencing lab|COVID-19 Genomics UK (COG-UK) Consortium|Moore|United Kingdom
>Spike|hCoV-19/England/NOTT-115508/2020|2020-10-14|EPI_ISL_595245|Original|hCoV-19^^England|Human|Queens Medical Centre|COVID-19 Genomics UK (COG-UK) Consortium|Loose|United Kingdom
>Spike|hCoV-19/Northern Ireland/NIRE-1064FE/2020|2020-05-04|EPI_ISL_500993|Original|hCoV-19^^Northern Ireland|Human|Regional Virus Laboratory|Wellcome Sanger Institute for the COVID-19 Genomics UK (COG-UK) consortium|Davies|United Kingdom
>Spike|hCoV-19/USA/NY-Wadsworth-284794-01/2020|2020-12-08|EPI_ISL_754459|Original|hCoV-19^^New York|Human|Wadsworth Center|Wadsworth Center|Lasek-Nesselquist|USA
>Spike|hCoV-19/England/MILK-974DE9/2020|2020-08-18|EPI_ISL_552521|Original|hCoV-19^^England|Human|Lighthouse Lab in Milton Keynes|Wellcome Sanger Institute for the COVID-19 Genomics UK (COG-UK) consortium|Davies|United Kingdom
>Spike|hCoV-19/Japan/PG-7328/2020|2020-08-00|EPI_ISL_688431|Original|hCoV-19^^Japan|Human|Pathogen Genomics Center|Pathogen Genomics Center|Sekizuka|Japan
>Spike|hCoV-19/Wales/PHWC-485922/2020|2020-10-21|EPI_ISL_628235|Original|hCoV-19^^Wales|Human|Wales Specialist Virology Centre Sequencing lab|COVID-19 Genomics UK (COG-UK) Consortium|Moore|United Kingdom
>Spike|hCoV-19/Denmark/DCGC-15285/2020|2020-11-23|EPI_ISL_713743|Original|hCoV-19^^Hovedstaden|Human|Department of Virus and Microbiological Special Diagnostics|Albertsen Lab|Michaelsen|Denmark
>Spike|hCoV-19/England/QEUH-B0F798/2020|2020-11-02|EPI_ISL_641674|Original|hCoV-19^^England|Human|Lighthouse Lab in Glasgow|Wellcome Sanger Institute for the COVID-19 Genomics UK (COG-UK) Consortium|Davies|United Kingdom
>Spike|hCoV-19/USA/TX-HMH-MCoV-19112/2020|2020-11-23|EPI_ISL_783632|Original|hCoV-19^^Texas|Human|Houston Methodist Hospital|Houston Methodist Hospital|Long|USA
>Spike|hCoV-19/England/ALDP-B63413/2020|2020-11-09|EPI_ISL_656524|Original|hCoV-19^^England|Human|Lighthouse Lab in Alderley Park|Wellcome Sanger Institute for the COVID-19 Genomics UK (COG-UK) Consortium|Davies|United Kingdom
>Spike|hCoV-19/Netherlands/NB-EMC-416/2020|2020-11-09|EPI_ISL_722291|Original|hCoV-19^^Noord Brabant|Human|Dutch COVID-19 response team|Erasmus Medical Center|Oude Munnink|Netherlands
>Spike|hCoV-19/England/LEED-2A91B9/2020|2020-04-07|EPI_ISL_539177|Original|hCoV-19^^England|Human|Leeds Teaching Hospitals NHS Trust and Public Health England|Wellcome Sanger Institute for the COVID-19 Genomics UK (COG-UK) consortium|Davies|United Kingdom
>Spike|hCoV-19/Wales/PHWC-497152/2020|2020-11-26|EPI_ISL_726785|Original|hCoV-19^^Wales|Human|Originating lab|COVID-19 Genomics UK (COG-UK) Consortium|Moore|United Kingdom
>Spike|hCoV-19/England/LIVE-9CCE7/2020|2020-04-23|EPI_ISL_440942|Original|hCoV-19^^England|Human|Liverpool Clinical Laboratories|COVID-19 Genomics UK (COG-UK) Consortium|Haldenby|United Kingdom
>Spike|hCoV-19/Northern Ireland/NIRE-103118/2020|2020-04-20|EPI_ISL_489316|Original|hCoV-19^^Northern Ireland|Human|Regional Virus Laboratory|Wellcome Sanger Institute for the COVID-19 Genomics UK (COG-UK) consortium|Davies|United Kingdom
>Spike|hCoV-19/South Korea/KCDC2585/2020|2020-06-27|EPI_ISL_514988|Original|hCoV-19^^South Korea|Human|Division of Viral Diseases|Division of Viral Diseases|Kim|South Korea
>Spike|hCoV-19/Denmark/DCGC-19117/2020|2020-12-07|EPI_ISL_748165|Original|hCoV-19^^Sjaelland|Human|Department of Virus and Microbiological Special Diagnostics|Albertsen Lab|Michaelsen|Denmark

>Spike|hCoV-19/Japan/PG-6471/2020|2020-08-00|EPI_ISL_689576|Original|hCoV-
19^^Japan|Human|Pathogen Genomics Center|Pathogen Genomics Center|Sekizuka|Japan
>Spike|hCoV-19/Denmark/DCGC-19147/2020|2020-12-07|EPI_ISL_748780|Original|hCoV-
19^^Hovedstaden|Human|Department of Virus and Microbiological Special
Diagnostics|Albertsen Lab|Michaelsen|Denmark
>Spike|hCoV-19/England/ALDP-BCA2F2/2020|2020-11-23|EPI_ISL_703552|Original|hCoV-
19^^England|Human|Lighthouse Lab in Alderley Park|Wellcome Sanger Institute for the
COVID-19 Genomics UK (COG-UK) Consortium|Davies|United Kingdom
>Spike|hCoV-19/England/ALDP-A5019B/2020|2020-10-07|EPI_ISL_597858|Original|hCoV-
19^^England|Human|Lighthouse Lab in Alderley Park|Wellcome Sanger Institute for the
COVID-19 Genomics UK (COG-UK) consortium|Davies|United Kingdom
>Spike|hCoV-19/England/MILK-AB9075/2020|2020-10-21|EPI_ISL_623642|Original|hCoV-
19^^England|Human|Lighthouse Lab in Milton Keynes|Wellcome Sanger Institute for the
COVID-19 Genomics UK (COG-UK) consortium|Davies|United Kingdom
>Spike|hCoV-19/England/QEUH-95F0BE/2020|2020-08-07|EPI_ISL_532500|Original|hCoV-
19^^England|Human|Lighthouse Lab in Glasgow|Wellcome Sanger Institute for the COVID-
19 Genomics UK (COG-UK) consortium|Davies|United Kingdom
>Spike|hCoV-19/India/MH-MW23/2020|2020-05-16|EPI_ISL_511925|Original|hCoV-
19^^Maharashtra|Human|Mahatma Gandhi Institute of Medical Sciences|National Institute
of Biomedical Genomics -\u00a0DBT's PAN-INDIA 1000 SARS--CoV-2 RNA Genome Sequencing
Consortium|Maitra|India
>Spike|hCoV-19/England/MILK-9F28D3/2020|2020-09-23|EPI_ISL_589121|Original|hCoV-
19^^England|Human|Lighthouse Lab in Milton Keynes|Wellcome Sanger Institute for the
COVID-19 Genomics UK (COG-UK) consortium|Davies|United Kingdom
>Spike|hCoV-19/Denmark/DCGC-18175/2020|2020-12-14|EPI_ISL_749164|Original|hCoV-
19^^Sjaelland|Human|Department of Virus and Microbiological Special
Diagnostics|Albertsen Lab|Michaelsen|Denmark
>Spike|hCoV-19/Portugal/PT0491/2020|2020-04-29|EPI_ISL_454215|Original|hCoV-
19^^Portugal|Human|unknown|Instituto Nacional de Saude (INSA)|Borges|Portugal
>Spike|hCoV-19/England/CAMC-B537A4/2020|2020-11-08|EPI_ISL_657020|Original|hCoV-
19^^England|Human|Lighthouse Lab in Cambridge|Wellcome Sanger Institute for the
COVID-19 Genomics UK (COG-UK) Consortium|Davies|United Kingdom
>Spike|hCoV-19/USA/WI-WSLH-200212/2020|2020-03-28|EPI_ISL_578433|Original|hCoV-
19^^Wisconsin|Human|Wisconsin State Laboratory of Hygiene Communicable Disease
Division|Wisconsin State Laboratory of Hygiene Communicable Disease
Division|Florek|USA
>Spike|hCoV-19/USA/MI-MDHHS-SC22209/2020|2020-10-26|EPI_ISL_644361|Original|hCoV-
19^^Michigan|Human|Michigan Department of Health and Human Services|Michigan
Department of Health and Human Services|Jacob|USA
>Spike|hCoV-19/England/ALDP-9FD823/2020|2020-10-02|EPI_ISL_587914|Original|hCoV-
19^^England|Human|Lighthouse Lab in Alderley Park|Wellcome Sanger Institute for the
COVID-19 Genomics UK (COG-UK) consortium|Davies|United Kingdom
>Spike|hCoV-19/England/MILK-99486A/2020|2020-08-25|EPI_ISL_552282|Original|hCoV-
19^^England|Human|Lighthouse Lab in Milton Keynes|Wellcome Sanger Institute for the
COVID-19 Genomics UK (COG-UK) consortium|Davies|United Kingdom
>Spike|hCoV-19/England/ALDP-9060F2/2020|2020-06-24|EPI_ISL_551537|Original|hCoV-
19^^England|Human|Lighthouse Lab in Alderley Park|Wellcome Sanger Institute for the
COVID-19 Genomics UK (COG-UK) consortium|Davies|United Kingdom
>Spike|hCoV-19/USA/NY-NYCPHL-001534/2020|2020-12-01|EPI_ISL_745452|Original|hCoV-
19^^New York|Human|DOHMH Crown Heights|New York City Public Health
Laboratory|Wang|USA
>Spike|hCoV-19/Scotland/CVR2625/2020|2020-04-17|EPI_ISL_484621|Original|hCoV-
19^^Scotland|Human|West of Scotland Specialist Virology Centre|COVID-19 Genomics UK
(COG-UK) Consortium|Robertson|United Kingdom
>Spike|hCoV-19/England/NORT-2820B9/2020|2020-03-25|EPI_ISL_472192|Original|hCoV-
19^^England|Human|Northumbria University / South Tees Hospitals NHS Foundation Trust
/ North Cumbria Integrated Care NHS Foundation Trust / North Tees and Hartlepool NHS
Foundation Trust / Newcastle Hospitals NHS Foundation Trust|COVID-19 Genomics UK
(COG-UK) Consortium|Bashton|United Kingdom

>Spike|hCoV-19/USA/CA-CZB-9522/2020|2020-04-07|EPI_ISL_672117|Original|hCoV-19^^California|Human|The Ashley Laboratory|Chan-Zuckerberg Biohub|Bhatt|USA
>Spike|hCoV-19/England/NOTT-116112/2020|2020-10-18|EPI_ISL_595303|Original|hCoV-19^^England|Human|Queens Medical Centre|COVID-19 Genomics UK (COG-UK) Consortium|Loose|United Kingdom
>Spike|hCoV-19/USA/ME-HETL-J0163/2020|2020-09-14|EPI_ISL_755346|Original|hCoV-19^^Maine|Human|Maine Health and Environmental Testing Laboratory|Tewhey Lab|Tewhey|USA
>Spike|hCoV-19/USA/NM-UNM-00563/2020|2020-04-10|EPI_ISL_542979|Original|hCoV-19^^New Mexico|Human|TriCore Reference Laboratories|Center for Global Health|Domman|USA
>Spike|hCoV-19/Scotland/CVR4887/2020|2020-10-06|EPI_ISL_651738|Original|hCoV-19^^Scotland|Human|West of Scotland Specialist Virology Centre|COVID-19 Genomics UK (COG-UK) Consortium|Robertson|United Kingdom
>Spike|hCoV-19/Wales/PHWC-48BA74/2020|2020-11-05|EPI_ISL_666317|Original|hCoV-19^^Wales|Human|Wales Specialist Virology Centre Sequencing lab|COVID-19 Genomics UK (COG-UK) Consortium|Moore|United Kingdom
>Spike|hCoV-19/USA/NM-NMDOH-2020330308/2020|2020-09-03|EPI_ISL_644584|Original|hCoV-19^^New Mexico|Human|New Mexico Department of Health Scientific Laboratory|New Mexico Department of Health Scientific Laboratory|Johnson|USA
>Spike|hCoV-19/USA/AR-CDC-2-3713986/2020|2020-09-02|EPI_ISL_751652|Original|hCoV-19^^Arkansas|Human|AR Dept. of Health-Public Health Lab|Genomics and Discovery|Queen|USA
>Spike|hCoV-19/England/ALDP-94E46B/2020|2020-06-17|EPI_ISL_555475|Original|hCoV-19^^England|Human|Lighthouse Lab in Alderley Park|Wellcome Sanger Institute for the COVID-19 Genomics UK (COG-UK) consortium|Davies|United Kingdom
>Spike|hCoV-19/England/ALDP-AA0DE3/2020|2020-10-22|EPI_ISL_624008|Original|hCoV-19^^England|Human|Lighthouse Lab in Alderley Park|Wellcome Sanger Institute for the COVID-19 Genomics UK (COG-UK) consortium|Davies|United Kingdom
>Spike|hCoV-19/Scotland/QEUH-A61355/2020|2020-10-13|EPI_ISL_665314|Original|hCoV-19^^Scotland|Human|Northumbria University / South Tees Hospitals NHS Foundation Trust / North Cumbria Integrated Care NHS Foundation Trust / North Tees and Hartlepool NHS Foundation Trust / Newcastle Hospitals NHS Foundation Trust|COVID-19 Genomics UK (COG-UK) Consortium|Bashton|United Kingdom
>Spike|hCoV-19/Japan/PG-2239/2020|2020-04-00|EPI_ISL_685844|Original|hCoV-19^^Japan|Human|Pathogen Genomics Center|Pathogen Genomics Center|Sekizuka|Japan
>Spike|hCoV-19/Scotland/QEUH-96D4A2/2020|2020-08-20|EPI_ISL_534513|Original|hCoV-19^^Scotland|Human|NHSGGC West of Scotland Specialist Virology Centre / MRC-University of Glasgow Centre for Virus Research|Wellcome Sanger Institute for the COVID-19 Genomics UK (COG-UK) consortium|Davies|United Kingdom
>Spike|hCoV-19/Australia/VIC13735/2020|2020-09-01|EPI_ISL_592311|Original|hCoV-19^^Victoria|Human|Microbiological Diagnostic Unit - Public Health Laboratory (MDU-PHL)|MDU-PHL|Schultz|Australia
>Spike|hCoV-19/India/WB-S2/2020|2020-03-21|EPI_ISL_430468|Original|hCoV-19^^West Bengal|Human|ICMR-National Institute of Cholera and Enteric Diseases|National Institute of Biomedical Genomics|Maitra|India
>Spike|hCoV-19/England/SHEF-C2AFD/2020|2020-04-22|EPI_ISL_453702|Original|hCoV-19^^England|Human|Virology Department|COVID-19 Genomics UK (COG-UK) Consortium|de Silva|United Kingdom
>Spike|hCoV-19/Brazil/MA-L19-CD407/2020|2020-04-02|EPI_ISL_476330|Original|hCoV-19^^Maranh\u00e3o|Human|DB Diagn\u00f3sticos do Brasil|Instituto de Medicina Tropical da Univesidade de S\u00e3o Paulo|Faria|Brazil
>Spike|hCoV-19/Switzerland/TI-ETHZ-321395/2020|2020-10-13|EPI_ISL_603636|Original|hCoV-19^^Ticino|Human|Viollier AG|Department of Biosystems Science and Engineering|Nadeau|Switzerland
>Spike|hCoV-19/USA/CO-CDC-2-3713995/2020|2020-12-03|EPI_ISL_751701|Original|hCoV-19^^Colorado|Human|CO Dept. of Public Health and Environment|Genomics and Discovery|Queen|USA
>Spike|hCoV-19/Scotland/GCVR-1707E7/2020|2020-03-26|EPI_ISL_501635|Original|hCoV-19^^Scotland|Human|NHSGGC West of Scotland Specialist Virology Centre / MRC-

University of Glasgow Centre for Virus Research|Wellcome Sanger Institute for the COVID-19 Genomics UK (COG-UK) consortium|Davies|United Kingdom
>Spike|hCoV-19/Bangladesh/BCSIR-NILMRC-188/2020|2020-06-16|EPI_ISL_483633|Original|hCoV-19^^Chittagong|Human|National Institute of Laboratory Medicine and Referral Center|Genomic Research Lab|Khan|Bangladesh
>Spike|hCoV-19/India/GJ-GBRC137b/2020|2020-05-24|EPI_ISL_458095|Original|hCoV-19^^Gujarat|Human|B.J. Medical College and Civil hospital|Gujarat Biotechnology Research Centre|Joshi|India
>Spike|hCoV-19/South Africa/KRISP-0436/2020|2020-07-01|EPI_ISL_498055|Original|hCoV-19^^KwaZulu-Natal|Human|NHLS-IALCH|KRISP|de Oliveira|South Africa
>Spike|hCoV-19/Gibraltar/204462814/2020|2020-10-29|EPI_ISL_614989|Original|hCoV-19^^Gibraltar|Human|Microbiology|Respiratory Virus Unit|Platt|Gibraltar
>Spike|hCoV-19/USA/MN-MDH-1666/2020|2020-04-04|EPI_ISL_560753|Original|hCoV-19^^Minnesota|Human|Minnesota Department of Health|Minnesota Department of Health|Garfin|USA
>Spike|hCoV-19/Belgium/rega-0429623/2020|2020-04-29|EPI_ISL_734676|Original|hCoV-19^^Couvin|Human|UZ Leuven|KU Leuven|Maes|Belgium
>Spike|hCoV-19/USA/UT-UPHL-2012787431/2020|2020-08-15|EPI_ISL_746154|Original|hCoV-19^^Utah|Human|Ginkgo Bioworks Clinical Laboratory|Utah Public Health Laboratory|Young|USA
>Spike|hCoV-19/India/MH-ACTREC-405/2020|2020-07-29|EPI_ISL_700054|Original|hCoV-19^^Maharashtra|Human|Hematopathology Laboratory|Hematopathology Laboratory|Patkar|India
>Spike|hCoV-19/England/ALDP-950EFF/2020|2020-06-13|EPI_ISL_554171|Original|hCoV-19^^England|Human|Lighthouse Lab in Alderley Park|Wellcome Sanger Institute for the COVID-19 Genomics UK (COG-UK) consortium|Davies|United Kingdom
>Spike|hCoV-19/Russia/StPetersburg-RII15673S/2020|2020-05-15|EPI_ISL_507270|Original|hCoV-19^^Saint Petersburg|Human|WHO National Influenza Centre Russian Federation|WHO National Influenza Centre Russian Federation|Komissarov|Russia
>Spike|hCoV-19/Spain/ML-ISCIII-202449/2020|2020-03-14|EPI_ISL_455347|Original|hCoV-19^^Melilla|Human|Hospital Comarcal de Melilla|Instituto de Salud Carlos III|Caballero|Spain
>Spike|hCoV-19/England/ALDP-A93A55/2020|2020-10-19|EPI_ISL_610925|Original|hCoV-19^^England|Human|Lighthouse Lab in Alderley Park|Wellcome Sanger Institute for the COVID-19 Genomics UK (COG-UK) consortium|Davies|United Kingdom
>Spike|hCoV-19/Scotland/EDB6535/2020|2020-06-17|EPI_ISL_478252|Original|hCoV-19^^Scotland|Human|Virology Department|COVID-19 Genomics UK (COG-UK) Consortium|Williams|United Kingdom
>Spike|hCoV-19/Japan/PG-7397/2020|2020-07-00|EPI_ISL_688499|Original|hCoV-19^^Japan|Human|Pathogen Genomics Center|Pathogen Genomics Center|Sekizuka|Japan
>Spike|hCoV-19/England/QEUH-B12431/2020|2020-11-02|EPI_ISL_642597|Original|hCoV-19^^England|Human|Lighthouse Lab in Glasgow|Wellcome Sanger Institute for the COVID-19 Genomics UK (COG-UK) Consortium|Davies|United Kingdom
>Spike|hCoV-19/Canada/ON-OICR-SLB2859/2020|2020-04-07|EPI_ISL_538378|Original|hCoV-19^^Ontario|Human|Kingston Health Sciences Centre / Queen's University|Ontario Institute for Cancer Research|de Borja|Canada
>Spike|hCoV-19/Wales/MILK-B04960/2020|2020-10-24|EPI_ISL_651033|Original|hCoV-19^^Wales|Human|Department of Pathology|COVID-19 Genomics UK (COG-UK) Consortium|Goodfellow|United Kingdom
>Spike|hCoV-19/England/QEUH-B14059/2020|2020-11-03|EPI_ISL_641828|Original|hCoV-19^^England|Human|Lighthouse Lab in Glasgow|Wellcome Sanger Institute for the COVID-19 Genomics UK (COG-UK) Consortium|Davies|United Kingdom
>Spike|hCoV-19/New Zealand/20VR1450/2020|2020-03-24|EPI_ISL_456217|Original|hCoV-19^^Southern|Human|Southern Community Labs Dunedin|Institute of Environmental Science and Research (ESR)|de Ligt|New Zealand
>Spike|hCoV-19/England/ALDP-A6C581/2020|2020-10-15|EPI_ISL_607995|Original|hCoV-19^^England|Human|Lighthouse Lab in Alderley Park|Wellcome Sanger Institute for the COVID-19 Genomics UK (COG-UK) consortium|Davies|United Kingdom

>Spike|hCoV-19/England/MILK-B3DC1A/2020|2020-11-01|EPI_ISL_706249|Original|hCoV-19^^England|Human|Oxford Viromics|COVID-19 Genomics UK (COG-UK) Consortium|Golubchik|United Kingdom
>Spike|hCoV-19/Australia/VIC578/2020|2020-03-26|EPI_ISL_426836|Original|hCoV-19^^Victoria|Human|Victorian Infectious Diseases Reference Laboratory (VIDRL)|Microbiological Diagnostic Unit Public Health Laboratory and Victorian Infectious Diseases Reference Laboratory|Schultz|Australia
>Spike|hCoV-19/England/QEUH-B41105/2020|2020-11-08|EPI_ISL_658648|Original|hCoV-19^^England|Human|Lighthouse Lab in Glasgow|Wellcome Sanger Institute for the COVID-19 Genomics UK (COG-UK) Consortium|Davies|United Kingdom
>Spike|hCoV-19/Australia/VIC12949/2020|2020-07-28|EPI_ISL_563610|Original|hCoV-19^^Victoria|Human|Microbiological Diagnostic Unit - Public Health Laboratory (MDU-PHL)|MDU-PHL|Schultz|Australia
>Spike|hCoV-19/Hong Kong/HKU-IHCE0511-007b/2020|2020-02-15|EPI_ISL_538533|Original|hCoV-19^^Hong Kong|Human|Department of Microbiology|Department of Microbiology|Chan|China
>Spike|hCoV-19/England/NORT-289322/2020|2020-03-29|EPI_ISL_458663|Original|hCoV-19^^England|Human|NU-OMICS DNA Sequencing research facility|Wellcome Sanger Institute for the COVID-19 Genomics UK (COG-UK) consortium|Davies|United Kingdom
>Spike|hCoV-19/Denmark/DCGC-935/2020|2020-03-09|EPI_ISL_614695|Original|hCoV-19^^Sjaelland|Human|Department of Virus and Microbiological Special Diagnostics|Albertsen lab|Michaelsen|Denmark
>Spike|hCoV-19/United Arab Emirates/0456/2020|2020-05-18|EPI_ISL_698626|Original|hCoV-19^^Abu Dhabi|Human|Group 42 (G42) Healthcare|G42 Healthcare|Ogrodzki|United Arab Emirates
>Spike|hCoV-19/USA/CA-ALSR-4703/2020|2020-09-09|EPI_ISL_730091|Original|hCoV-19^^California|Human|San Diego County Public Health Laboratory|Andersen lab at Scripps Research|Gangavarapu|USA
>Spike|hCoV-19/England/NORT-2818EF/2020|2020-03-20|EPI_ISL_488752|Original|hCoV-19^^England|Human|NU-OMICS DNA Sequencing research facility|Wellcome Sanger Institute for the COVID-19 Genomics UK (COG-UK) consortium|Davies|United Kingdom
>Spike|hCoV-19/England/QEUH-9DBE0B/2020|2020-09-21|EPI_ISL_601429|Original|hCoV-19^^England|Human|Lighthouse Lab in Glasgow|Wellcome Sanger Institute for the COVID-19 Genomics UK (COG-UK) consortium|Davies|United Kingdom
>Spike|hCoV-19/Wales/PHWC-480268/2020|2020-10-05|EPI_ISL_612867|Original|hCoV-19^^Wales|Human|Wales Specialist Virology Centre Sequencing lab|COVID-19 Genomics UK (COG-UK) Consortium|Moore|United Kingdom
>Spike|hCoV-19/Denmark/DCGC-3244/2020|2020-08-24|EPI_ISL_616876|Original|hCoV-19^^Nordjylland|Human|Department of Virus and Microbiological Special Diagnostics|Albertsen lab|Michaelsen|Denmark
>Spike|hCoV-19/England/GSTT-6885/2020|2020-03-30|EPI_ISL_560740|Original|hCoV-19^^England|Human|Centre for Clinical Infection and Diagnostics Research and Genomics Innovation Unit|Centre for Clinical Infection and Diagnostics Research and Genomics Innovation Unit|Awan|United Kingdom
>Spike|hCoV-19/France/ARA-SC036/2020|2020-03-28|EPI_ISL_629094|Original|hCoV-19^^Auvergne-Rh\u00f4ne-Alpes|Human|Laboratoire du Centre Hospitalier Annecy Genevois|CNR Virus des Infections Respiratoires - France SUD|Bal|France
>Spike|hCoV-19/Austria/CeMM0474/2020|2020-03-10|EPI_ISL_475879|Original|hCoV-19^^Austria|Human|Austrian Agency for Health and Food Safety (AGES)|Bergthaler laboratory|Bergthaler|Austria
>Spike|hCoV-19/Denmark/DCGC-11741/2020|2020-10-26|EPI_ISL_671107|Original|hCoV-19^^Syddanmark|Human|Department of Virus and Microbiological Special Diagnostics|Albertsen Lab|Michaelsen|Denmark
>Spike|hCoV-19/England/MILK-A7C71F/2020|2020-10-15|EPI_ISL_606568|Original|hCoV-19^^England|Human|Lighthouse Lab in Milton Keynes|Wellcome Sanger Institute for the COVID-19 Genomics UK (COG-UK) consortium|Davies|United Kingdom
>Spike|hCoV-19/Australia/VIC10711/2020|2020-09-06|EPI_ISL_563048|Original|hCoV-19^^Victoria|Human|Microbiological Diagnostic Unit - Public Health Laboratory (MDU-PHL)|MDU-PHL|Schultz|Australia

>Spike|hCoV-19/USA/CA-CZB-1082/2020|2020-04-23|EPI_ISL_437082|Original|hCoV-19^^California|Human|County of Santa Clara Public Health|Chan-Zuckerberg Biohub|Hao|USA
>Spike|hCoV-19/South Africa/NHLS-UCT-GS-1378/2020|2020-11-13|EPI_ISL_700542|Original|hCoV-19^^Western Cape|Human|Knysna CDC wc WLC|NHLS/UCT|Iranzadeh|South Africa
>Spike|hCoV-19/England/MILK-9D45EF/2020|2020-09-17|EPI_ISL_601774|Original|hCoV-19^^England|Human|Lighthouse Lab in Milton Keynes|Wellcome Sanger Institute for the COVID-19 Genomics UK (COG-UK) consortium|Davies|United Kingdom
>Spike|hCoV-19/Bangladesh/109879/2020|2020-04-09|EPI_ISL_600428|Original|hCoV-19^^Barishal|Human|Institute of Epidemiology Disease Control And Research|Institute for Developing Science and Health Initiatives|Cowley|Bangladesh
>Spike|hCoV-19/USA/WA-S472/2020|2020-04-05|EPI_ISL_434232|Original|hCoV-19^^Washington|Human|Washington State Department of Health|Seattle Flu Study|Bedford|USA
>Spike|hCoV-19/England/ALDP-B620B6/2020|2020-11-08|EPI_ISL_656464|Original|hCoV-19^^England|Human|Lighthouse Lab in Alderley Park|Wellcome Sanger Institute for the COVID-19 Genomics UK (COG-UK) Consortium|Davies|United Kingdom
>Spike|hCoV-19/Scotland/EDB6329/2020|2020-06-04|EPI_ISL_473939|Original|hCoV-19^^Scotland|Human|Virology Department|COVID-19 Genomics UK (COG-UK) Consortium|Williams|United Kingdom
>Spike|hCoV-19/England/MILK-93CCA4/2020|2020-06-21|EPI_ISL_552975|Original|hCoV-19^^England|Human|Lighthouse Lab in Milton Keynes|Wellcome Sanger Institute for the COVID-19 Genomics UK (COG-UK) consortium|Davies|United Kingdom
>Spike|hCoV-19/Japan/PG-3180/2020|2020-04-00|EPI_ISL_691300|Original|hCoV-19^^Japan|Human|Pathogen Genomics Center|Pathogen Genomics Center|Sekizuka|Japan
>Spike|hCoV-19/India/MH-ACTREC-398/2020|2020-07-29|EPI_ISL_700047|Original|hCoV-19^^Maharashtra|Human|Hematopathology Laboratory|Hematopathology Laboratory|Patkar|India
>Spike|hCoV-19/England/CAMC-9466B8/2020|2020-07-16|EPI_ISL_556964|Original|hCoV-19^^England|Human|Lighthouse Lab in Cambridge|Wellcome Sanger Institute for the COVID-19 Genomics UK (COG-UK) consortium|Davies|United Kingdom
>Spike|hCoV-19/USA/CA-ALSR-0850-NBG/2020|2020-05-14|EPI_ISL_483398|Original|hCoV-19^^Louisiana|Human|UC San Diego Center for Advanced Laboratory Medicine|Andersen lab at Scripps Research|Gangavarapu|USA
>Spike|hCoV-19/USA/LA-EVTL517/2020|2020-06-25|EPI_ISL_578921|Original|hCoV-19^^Louisiana|Human|LSUHS Emerging Viral Threat Laboratory|Microbial Genome Sequencing Center|Kamil|USA
>Spike|hCoV-19/England/MILK-A45E74/2020|2020-10-06|EPI_ISL_598774|Original|hCoV-19^^England|Human|Lighthouse Lab in Milton Keynes|Wellcome Sanger Institute for the COVID-19 Genomics UK (COG-UK) consortium|Davies|United Kingdom
>Spike|hCoV-19/England/MILK-9922B9/2020|2020-08-27|EPI_ISL_540667|Original|hCoV-19^^England|Human|Queens Medical Centre|COVID-19 Genomics UK (COG-UK) Consortium|Loose|United Kingdom
>Spike|hCoV-19/England/QEUH-A4D1EC/2020|2020-10-07|EPI_ISL_628590|Original|hCoV-19^^England|Human|Oxford Viromics|COVID-19 Genomics UK (COG-UK) Consortium|Golubchik|United Kingdom
>Spike|hCoV-19/England/ALDP-CBDEDF/2020|2020-12-18|EPI_ISL_760498|Original|hCoV-19^^England|Human|Lighthouse Lab in Alderley Park|Wellcome Sanger Institute for the COVID-19 Genomics UK (COG-UK) Consortium|Davies|United Kingdom
>Spike|hCoV-19/USA/LA-SR0290/2020|2020-04-01|EPI_ISL_445145|Original|hCoV-19^^Louisiana|Human|Robert Garry lab|Andersen lab at Scripps Research|Gangavarapu|USA
>Spike|hCoV-19/Wales/MILK-970A5F/2020|2020-08-18|EPI_ISL_553023|Original|hCoV-19^^Wales|Human|Lighthouse Lab in Milton Keynes|Wellcome Sanger Institute for the COVID-19 Genomics UK (COG-UK) consortium|Davies|United Kingdom
>Spike|hCoV-19/USA/TX-DSHS-1041/2020|2020-06-15|EPI_ISL_628723|Original|hCoV-19^^Texas|Human|Texas Department of State Health Services|Texas Department of State Health Services|Rahman|USA

>Spike|hCoV-19/England/MILK-C5023C/2020|2020-12-11|EPI_ISL_769555|Original|hCoV-19^^England|Human|Lighthouse Lab in Milton Keynes|Wellcome Sanger Institute for the COVID-19 Genomics UK (COG-UK) Consortium|Davies|United Kingdom
>Spike|hCoV-19/England/EXET-137825/2020|2020-05-01|EPI_ISL_471986|Original|hCoV-19^^England|Human|University of Exeter|COVID-19 Genomics UK (COG-UK) Consortium|Temperton|United Kingdom
>Spike|hCoV-19/Australia/VIC14239/2020|2020-08-03|EPI_ISL_592409|Original|hCoV-19^^Victoria|Human|Microbiological Diagnostic Unit - Public Health Laboratory (MDU-PHL)|MDU-PHL|Schultz|Australia
>Spike|hCoV-19/USA/WI-UW-1763/2020|2020-09-14|EPI_ISL_605636|Original|hCoV-19^^Wisconsin|Human|University of Wisconsin-Madison AIDS Vaccine Research Laboratories|University of Wisconsin-Madison AIDS Vaccine Research Laboratories|Baczenas|USA
>Spike|hCoV-19/England/ALDP-AA557D/2020|2020-10-21|EPI_ISL_625149|Original|hCoV-19^^England|Human|Lighthouse Lab in Alderley Park|Wellcome Sanger Institute for the COVID-19 Genomics UK (COG-UK) consortium|Davies|United Kingdom
>Spike|hCoV-19/USA/CA-ALSR-3290/2020|2020-09-16|EPI_ISL_635952|Original|hCoV-19^^California|Human|San Diego County Public Health Laboratory|Andersen lab at Scripps Research|Gangavarapu|USA
>Spike|hCoV-19/England/QEUH-99930A/2020|2020-08-23|EPI_ISL_541788|Original|hCoV-19^^England|Human|Lighthouse Lab in Glasgow|Wellcome Sanger Institute for the COVID-19 Genomics UK (COG-UK) consortium|Davies|United Kingdom
>Spike|hCoV-19/Iceland/451/2020|2020-03-20|EPI_ISL_424475|Original|hCoV-19^^Reykjavik|Human|The National University Hospital of Iceland|deCODE genetics|J\u00f3nsson|Iceland
>Spike|hCoV-19/Luxembourg/LNS0061167/2020|2020-10-26|EPI_ISL_739704|Original|hCoV-19^^Luxembourg|Human|Laboratoire national de sant\u00e9|Laboratoire national de sant\u00e9|Wienecke-Baldacchino|Luxembourg
>Spike|hCoV-19/England/ALDP-C489E7/2020|2020-12-12|EPI_ISL_736587|Original|hCoV-19^^England|Human|Lighthouse Lab in Alderley Park|Wellcome Sanger Institute for the COVID-19 Genomics UK (COG-UK) Consortium|Davies|United Kingdom
>Spike|hCoV-19/Guangdong/20SF1159/2020|2020-01-31|EPI_ISL_428446|Original|hCoV-19^^Guangdong|Human|Guangdong Provincial Center for Diseases Control and Prevention;Guangdong Provincial Institute of Public Health|School of Public Health|Gu|China
>Spike|hCoV-19/Wales/PHWC-49E461/2020|2020-12-08|EPI_ISL_743004|Original|hCoV-19^^Wales|Human|Wales Specialist Virology Centre Sequencing lab|COVID-19 Genomics UK (COG-UK) Consortium|Moore|United Kingdom
>Spike|hCoV-19/USA/WA-S1204/2020|2020-05-06|EPI_ISL_463694|Original|hCoV-19^^Washington|Human|Washington State Department of Health|Seattle Flu Study|Bedford|USA
>Spike|hCoV-19/England/ALDP-94E35F/2020|2020-06-17|EPI_ISL_555588|Original|hCoV-19^^England|Human|Lighthouse Lab in Alderley Park|Wellcome Sanger Institute for the COVID-19 Genomics UK (COG-UK) consortium|Davies|United Kingdom
>Spike|hCoV-19/Japan/PG-5514/2020|2020-08-00|EPI_ISL_688639|Original|hCoV-19^^Japan|Human|Pathogen Genomics Center|Pathogen Genomics Center|Sekizuka|Japan
>Spike|hCoV-19/Wales/PHWC-4A58C5/2020|2020-12-16|EPI_ISL_764935|Original|hCoV-19^^Wales|Human|Wales Specialist Virology Centre Sequencing lab|COVID-19 Genomics UK (COG-UK) Consortium|Moore|United Kingdom
>Spike|hCoV-19/USA/NY-NYUMC887/2020|2020-04-30|EPI_ISL_467380|Original|hCoV-19^^New York|Human|NYU Langone Health|Departments of Pathology and Medicine|Maurano|USA
>Spike|hCoV-19/Scotland/CVR3992/2020|2020-09-02|EPI_ISL_540789|Original|hCoV-19^^Scotland|Human|West of Scotland Specialist Virology Centre|COVID-19 Genomics UK (COG-UK) Consortium|Robertson|United Kingdom
>Spike|hCoV-19/Denmark/DCGC-2394/2020|2020-08-10|EPI_ISL_617576|Original|hCoV-19^^Midtjylland|Human|Department of Virus and Microbiological Special Diagnostics|Albertsen lab|Michaelsen|Denmark
>Spike|hCoV-19/USA/TX-HMH-MCoV-15147/2020|2020-10-18|EPI_ISL_786631|Original|hCoV-19^^Texas|Human|Houston Methodist Hospital|Houston Methodist Hospital|Long|USA

>Spike|hCoV-19/England/LIVE-DC1ED7/2020|2020-11-22|EPI_ISL_679396|Original|hCoV-19^^England|Human|Liverpool Clinical Laboratories|COVID-19 Genomics UK (COG-UK) Consortium|Haldenby|United Kingdom
>Spike|hCoV-19/England/CAMC-AEAD1A/2020|2020-10-26|EPI_ISL_633218|Original|hCoV-19^^England|Human|Lighthouse Lab in Cambridge|Wellcome Sanger Institute for the COVID-19 Genomics UK (COG-UK) consortium|Davies|United Kingdom
>Spike|hCoV-19/England/QEUH-96F103/2020|2020-08-17|EPI_ISL_530442|Original|hCoV-19^^England|Human|Lighthouse Lab in Glasgow|Wellcome Sanger Institute for the COVID-19 Genomics UK (COG-UK) consortium|Davies|United Kingdom
>Spike|hCoV-19/Australia/NSW96/2020|2020-03-23|EPI_ISL_427678|Original|hCoV-19^^New South Wales|Human|Centre for Infectious Diseases and Microbiology Public Health|NSW Health Pathology - Institute of Clinical Pathology and Medical Research; Westmead Hospital; University of Sydney|Gall|Australia
>Spike|hCoV-19/Wales/MILK-A22824/2020|2020-10-02|EPI_ISL_611864|Original|hCoV-19^^Wales|Human|Northumbria University / South Tees Hospitals NHS Foundation Trust / North Cumbria Integrated Care NHS Foundation Trust / North Tees and Hartlepool NHS Foundation Trust / Newcastle Hospitals NHS Foundation Trust|COVID-19 Genomics UK (COG-UK) Consortium|Bashton|United Kingdom
>Spike|hCoV-19/England/ALDP-B5C3A7/2020|2020-11-10|EPI_ISL_655835|Original|hCoV-19^^England|Human|Lighthouse Lab in Alderley Park|Wellcome Sanger Institute for the COVID-19 Genomics UK (COG-UK) Consortium|Davies|United Kingdom
>Spike|hCoV-19/USA/WA-S1139/2020|2020-05-04|EPI_ISL_463629|Original|hCoV-19^^Washington|Human|Washington State Department of Health|Seattle Flu Study|Bedford|USA
>Spike|hCoV-19/USA/CA-CSMC16/2020|2020-04-13|EPI_ISL_475625|Original|hCoV-19^^California|Human|Cedars-Sinai Medical Center|Cedars-Sinai Medical Center|Zhang|USA
>Spike|hCoV-19/England/ALDP-954286/2020|2020-06-12|EPI_ISL_554589|Original|hCoV-19^^England|Human|Lighthouse Lab in Alderley Park|Wellcome Sanger Institute for the COVID-19 Genomics UK (COG-UK) consortium|Davies|United Kingdom
>Spike|hCoV-19/USA/TX-DSHS-0347/2020|2020-06-13|EPI_ISL_538285|Original|hCoV-19^^Texas|Human|Texas Department of State Health Services|Texas Department of State Health Services|Rahman|USA
>Spike|hCoV-19/Scotland/EDB5405/2020|2020-04-05|EPI_ISL_487762|Original|hCoV-19^^Scotland|Human|Virology Department|Wellcome Sanger Institute for the COVID-19 Genomics UK (COG-UK) consortium|Davies|United Kingdom
>Spike|hCoV-19/USA/NY-NYUMC487/2020|2020-03-18|EPI_ISL_436735|Original|hCoV-19^^New York|Human|NYU Langone Health|Departments of Pathology and Medicine|Maurano|USA
>Spike|hCoV-19/Japan/PG-6956/2020|2020-07-00|EPI_ISL_688061|Original|hCoV-19^^Japan|Human|Pathogen Genomics Center|Pathogen Genomics Center|Sekizuka|Japan
>Spike|hCoV-19/England/QEUH-A8BCF0/2020|2020-10-17|EPI_ISL_643233|Original|hCoV-19^^England|Human|Lighthouse Lab in Glasgow|Wellcome Sanger Institute for the COVID-19 Genomics UK (COG-UK) Consortium|Davies|United Kingdom
>Spike|hCoV-19/England/BRIS-1254DB/2020|2020-03-27|EPI_ISL_444017|Original|hCoV-19^^England|Human|PHE South West Regional Laboratory|Wellcome Sanger Institute for the COVID-19 Genomics UK (COG-UK) consortium|Amato|United Kingdom
>Spike|hCoV-19/Denmark/DCGC-17764/2020|2020-12-14|EPI_ISL_749297|Original|hCoV-19^^Syddanmark|Human|Department of Virus and Microbiological Special Diagnostics|Albertsen Lab|Michaelsen|Denmark
>Spike|hCoV-19/England/LEED-2A9EFE/2020|2020-04-10|EPI_ISL_538780|Original|hCoV-19^^England|Human|Leeds Teaching Hospitals NHS Trust and Public Health England|Wellcome Sanger Institute for the COVID-19 Genomics UK (COG-UK) consortium|Davies|United Kingdom
>Spike|hCoV-19/England/MILK-B04687/2020|2020-10-24|EPI_ISL_652165|Original|hCoV-19^^England|Human|Department of Pathology|COVID-19 Genomics UK (COG-UK) Consortium|Goodfellow|United Kingdom
>Spike|hCoV-19/USA/TX-DSHS-1015/2020|2020-04-09|EPI_ISL_644169|Original|hCoV-19^^Texas|Human|Texas Department of State Health Services|Texas Department of State Health Services|Rahman|USA

>Spike|hCoV-19/USA/TX-HMH-5186/2020|2020-06-23|EPI_ISL_544583|Original|hCoV-
19^^Texas|Human|Houston Methodist Hospital|Houston Methodist Hospital|Long|USA
>Spike|hCoV-19/Kazakhstan/26829/2020|2020-04-26|EPI_ISL_454518|Original|hCoV-19^^Nur-
Sultan|Human|RSE \National Center for Biotechnology\|RSE \National Center for
Biotechnology\|Kalendar|Kazakhstan
>Spike|hCoV-19/USA/WA-UW76/2020|2020-03-10|EPI_ISL_415604|Original|hCoV-
19^^Washington|Human|UW Virology Lab|UW Virology Lab|Roychoudhury|USA
>Spike|hCoV-19/Finland/23SS12G8/2020|2020-09-23|EPI_ISL_759798|Original|hCoV-
19^^Uusimaa|Human|Department of Virology and Immunology|Department of
Virology|Smura|Finland
>Spike|hCoV-19/USA/UT-04480/2020|2020-05-21|EPI_ISL_522341|Original|hCoV-
19^^Utah|Human|Utah Public Health Laboratory|Utah Public Health Laboratory|Young|USA
>Spike|hCoV-19/England/CAMB-1B2A4A/2020|2020-05-15|EPI_ISL_469913|Original|hCoV-
19^^England|Human|Department of Pathology|Wellcome Sanger Institute for the COVID-19
Genomics UK (COG-UK) consortium|Davies|United Kingdom
>Spike|hCoV-19/England/OXON-B4D5F/2020|2020-05-02|EPI_ISL_535011|Original|hCoV-
19^^England|Human|Oxford Viromics|COVID-19 Genomics UK (COG-UK)
Consortium|Golubchik|United Kingdom
>Spike|hCoV-19/Singapore/903/2020|2020-09-03|EPI_ISL_536444|Original|hCoV-
19^^Singapore|Human|National Public Health Laboratory|National Public Health
Laboratory|Mak|Singapore
>Spike|hCoV-19/Wales/PHWC-48F506/2020|2020-11-10|EPI_ISL_681104|Original|hCoV-
19^^Wales|Human|Wales Specialist Virology Centre Sequencing lab|COVID-19 Genomics UK
(COG-UK) Consortium|Moore|United Kingdom
>Spike|hCoV-19/Australia/VIC17984/2020|2020-08-21|EPI_ISL_779602|Original|hCoV-
19^^Victoria|Human|Microbiological Diagnostic Unit - Public Health Laboratory (MDU-
PHL)|MDU-PHL|Beck|Australia
>Spike|hCoV-19/Netherlands/NH-RIVM-20226/2020|2020-12-05|EPI_ISL_723378|Orignal|hCoV-
19^^Noord-Holland|Human|Dutch COVID-19 response team|National Institute for Public
Health and the Environment (RIVM)|Meijer|Netherlands
>Spike|hCoV-19/Wales/PHWC-1608C9/2020|2020-04-29|EPI_ISL_472990|Original|hCoV-
19^^Wales|Human|Wales Specialist Virology Centre Sequencing lab|COVID-19 Genomics UK
(COG-UK) Consortium|Moore|United Kingdom
>Spike|hCoV-19/USA/WI-GMF-24736/2020|2020-10-08|EPI_ISL_660908|Original|hCoV-
19^^Wisconsin|Human|Gundersen Molecular Diagnostics Laboratory|Kabara Cancer Research
Institute|Kenny|USA
>Spike|hCoV-19/England/ALDP-B5AF4B/2020|2020-11-09|EPI_ISL_655521|Original|hCoV-
19^^England|Human|Lighthouse Lab in Alderley Park|Wellcome Sanger Institute for the
COVID-19 Genomics UK (COG-UK) Consortium|Davies|United Kingdom
>Spike|hCoV-19/Portugal/PT1463/2020|2020-03-30|EPI_ISL_511422|Original|hCoV-
19^^Portugal|Human|Instituto Nacional de Saude (INSA)|Instituto Nacional de Saude
(INSA)|Borges|Portugal
>Spike|hCoV-19/Scotland/CVR3776/2020|2020-05-31|EPI_ISL_478109|Original|hCoV-
19^^Scotland|Human|West of Scotland Specialist Virology Centre|COVID-19 Genomics UK
(COG-UK) Consortium|Robertson|United Kingdom
>Spike|hCoV-19/Aruba/AW-RIVM-10390/2020|2020-08-06|EPI_ISL_636516|Original|hCoV-
19^^Aruba|Human|Dutch COVID-19 response team|National Institute for Public Health and
the Environment (RIVM)|Meijer|Aruba
>Spike|hCoV-19/Russia/LEN-RII-MH2050S/2020|2020-09-18|EPI_ISL_602389|Original|hCoV-
19^^Leningrad|Human|HELIX LLC|WHO National Influenza Centre Russian
Federation|Komissarov|Russia
>Spike|hCoV-19/USA/TX-HMH-MCoV-15013/2020|2020-10-17|EPI_ISL_786497|Original|hCoV-
19^^Texas|Human|Houston Methodist Hospital|Houston Methodist Hospital|Long|USA
>Spike|hCoV-19/USA/WI-UW-1348/2020|2020-09-08|EPI_ISL_536719|Original|hCoV-
19^^Wisconsin|Human|University of Wisconsin-Madison AIDS Vaccine Research
Laboratories|University of Wisconsin-Madison AIDS Vaccine Research
Laboratories|Baczenas|USA
>Spike|hCoV-19/USA/TX-HMH-MCoV-18646/2020|2020-11-21|EPI_ISL_784994|Original|hCoV-
19^^Texas|Human|Houston Methodist Hospital|Houston Methodist Hospital|Long|USA

>Spike|hCoV-19/Spain/CB-IBV-98007076/2020|2020-09-03|EPI_ISL_582106|Original|hCoV-19^^Cantabria|Human|Hospital Universitario Marqu\u00e9s de Valdecilla - IDIVAL (Santander|SeqCOVID-SPAIN consortium/IBV(CSIC)|Comas|Spain
>Spike|hCoV-19/England/CAMC-B22A3C/2020|2020-11-02|EPI_ISL_647364|Original|hCoV-19^^England|Human|Lighthouse Lab in Cambridge|Wellcome Sanger Institute for the COVID-19 Genomics UK (COG-UK) Consortium|Davies|United Kingdom
>Spike|hCoV-19/USA/MI-MDHHS-SC22199/2020|2020-10-26|EPI_ISL_644367|Original|hCoV-19^^Michigan|Human|Michigan Department of Health and Human Services|Michigan Department of Health and Human Services|Jacob|USA
>Spike|hCoV-19/Wales/PHWC-4A2822/2020|2020-12-08|EPI_ISL_743946|Original|hCoV-19^^Wales|Human|Wales Specialist Virology Centre Sequencing lab|COVID-19 Genomics UK (COG-UK) Consortium|Moore|United Kingdom
>Spike|hCoV-19/USA/TX-DSHS-0587/2020|2020-06-03|EPI_ISL_569698|Original|hCoV-19^^Texas|Human|Texas Department of State Health Services|Texas Department of State Health Services|Rahman|USA
>Spike|hCoV-19/Japan/PG-5140/2020|2020-08-00|EPI_ISL_690247|Original|hCoV-19^^Japan|Human|Gunma Prefectural Institute of Public Health and Environmental Sciences|Pathogen Genomics Center|Sekizuka|Japan
>Spike|hCoV-19/Northern Ireland/NIRE-FCBE8/2020|2020-04-22|EPI_ISL_470078|Original|hCoV-19^^Northern Ireland|Human|Regional Virus Laboratory|Wellcome Sanger Institute for the COVID-19 Genomics UK (COG-UK) consortium|Davies|United Kingdom
>Spike|hCoV-19/USA/UT-01696/2020|2020-04-13|EPI_ISL_454716|Original|hCoV-19^^Utah|Human|Utah Public Health Laboratory|Utah Public Health Laboratory|Young|USA
>Spike|hCoV-19/USA/CA-CHLA-PLM58846794/2020|2020-11-30|EPI_ISL_753458|Original|hCoV-19^^California|Human|Clinical virology Laboratory|Center for Personalized Medicine|Dien Bard|USA
>Spike|hCoV-19/Japan/PG-8313/2020|2020-08-00|EPI_ISL_687412|Original|hCoV-19^^Japan|Human|Pathogen Genomics Center|Pathogen Genomics Center|Sekizuka|Japan
>Spike|hCoV-19/Wales/PHWC-246AA/2020|2020-03-17|EPI_ISL_419439|Original|hCoV-19^^Wales|Human|Wales Specialist Virology Centre|Public Health Wales Microbiology Cardiff|Moore|United Kingdom
>Spike|hCoV-19/England/MILK-B5A628/2020|2020-11-06|EPI_ISL_658145|Original|hCoV-19^^England|Human|Lighthouse Lab in Milton Keynes|Wellcome Sanger Institute for the COVID-19 Genomics UK (COG-UK) Consortium|Davies|United Kingdom
>Spike|hCoV-19/England/BRIS-125013/2020|2020-03-26|EPI_ISL_444002|Original|hCoV-19^^England|Human|PHE South West Regional Laboratory|Wellcome Sanger Institute for the COVID-19 Genomics UK (COG-UK) consortium|Amato|United Kingdom
>Spike|hCoV-19/England/QEUH-9CF3ED/2020|2020-09-17|EPI_ISL_590048|Original|hCoV-19^^England|Human|Lighthouse Lab in Glasgow|Wellcome Sanger Institute for the COVID-19 Genomics UK (COG-UK) consortium|Davies|United Kingdom
>Spike|hCoV-19/England/CAMC-B52329/2020|2020-11-08|EPI_ISL_656970|Original|hCoV-19^^England|Human|Lighthouse Lab in Cambridge|Wellcome Sanger Institute for the COVID-19 Genomics UK (COG-UK) Consortium|Davies|United Kingdom
>Spike|hCoV-19/Australia/VIC7249/2020|2020-07-27|EPI_ISL_564312|Original|hCoV-19^^Victoria|Human|Microbiological Diagnostic Unit - Public Health Laboratory (MDU-PHL)|MDU-PHL|Schultz|Australia
>Spike|hCoV-19/England/MILK-974AB5/2020|2020-08-21|EPI_ISL_552461|Original|hCoV-19^^England|Human|Lighthouse Lab in Milton Keynes|Wellcome Sanger Institute for the COVID-19 Genomics UK (COG-UK) consortium|Davies|United Kingdom
>Spike|hCoV-19/USA/WA-UW-32904/2020|2020-10-17|EPI_ISL_737096|Original|hCoV-19^^Washington|Human|UW Virology Lab|UW Virology Lab|Roychoudhury|USA
>Spike|hCoV-19/USA/MN-MDH-1632/2020|2020-04-28|EPI_ISL_539826|Original|hCoV-19^^Minnesota|Human|Minnesota Department of Health|Minnesota Department of Health|Garfin|USA
>Spike|hCoV-19/England/QEUH-AD3224/2020|2020-10-25|EPI_ISL_633807|Original|hCoV-19^^England|Human|Lighthouse Lab in Glasgow|Wellcome Sanger Institute for the COVID-19 Genomics UK (COG-UK) consortium|Davies|United Kingdom

>Spike|hCoV-19/England/QEUH-BA0535/2020|2020-11-18|EPI_ISL_719636|Original|hCoV-19^^England|Human|Lighthouse Lab in Glasgow|Wellcome Sanger Institute for the COVID-19 Genomics UK (COG-UK) Consortium|Davies|United Kingdom
>Spike|hCoV-19/England/201380217/2020|2020-03-25|EPI_ISL_421822|Original|hCoV-19^^England|Human|Respiratory Virus Unit|Respiratory Virus Unit|Platt|United Kingdom
>Spike|hCoV-19/Vietnam/VNHN_1072/2020|2020-03-17|EPI_ISL_455717|Original|hCoV-19^^Hanoi|Human|National Hospital of Tropical Diseases|Oxford University Clinical Research Unit|Nguyen|Vietnam
>Spike|hCoV-19/England/QEUH-9F58D0/2020|2020-09-25|EPI_ISL_588423|Original|hCoV-19^^England|Human|Lighthouse Lab in Glasgow|Wellcome Sanger Institute for the COVID-19 Genomics UK (COG-UK) consortium|Davies|United Kingdom
>Spike|hCoV-19/USA/CA-ALSR-1829/2020|2020-05-19|EPI_ISL_512199|Original|hCoV-19^^California|Human|San Diego County Public Health Laboratory|Andersen lab at Scripps Research|Gangavarapu|USA
>Spike|hCoV-19/England/CAMC-B99265/2020|2020-11-15|EPI_ISL_673415|Original|hCoV-19^^England|Human|Lighthouse Lab in Cambridge|Wellcome Sanger Institute for the COVID-19 Genomics UK (COG-UK) Consortium|Davies|United Kingdom
>Spike|hCoV-19/Denmark/ALAB-SSI-885/2020|2020-03-30|EPI_ISL_444955|Original|hCoV-19^^Denmark|Human|Department of Virus and Microbiological Special Diagnostics|Albertsen lab|Kirkegaard|Denmark
>Spike|hCoV-19/England/MILK-BAB752/2020|2020-11-18|EPI_ISL_783331|Original|hCoV-19^^England|Human|Lighthouse Lab in Milton Keynes|Wellcome Sanger Institute for the COVID-19 Genomics UK (COG-UK) Consortium|Davies|United Kingdom
>Spike|hCoV-19/England/MILK-B39522/2020|2020-11-02|EPI_ISL_658812|Original|hCoV-19^^England|Human|Lighthouse Lab in Milton Keynes|Wellcome Sanger Institute for the COVID-19 Genomics UK (COG-UK) Consortium|Davies|United Kingdom
>Spike|hCoV-19/England/MILK-A7A429/2020|2020-10-15|EPI_ISL_606773|Original|hCoV-19^^England|Human|Lighthouse Lab in Milton Keynes|Wellcome Sanger Institute for the COVID-19 Genomics UK (COG-UK) consortium|Davies|United Kingdom
>Spike|hCoV-19/England/205160492/2020|2020-12-16|EPI_ISL_766423|Original|hCoV-19^^England|Human|Respiratory Virus Unit|COVID-19 Genomics UK (COG-UK) Consortium|Platt|United Kingdom
>Spike|hCoV-19/England/SHEF-D1A64/2020|2020-09-13|EPI_ISL_559941|Original|hCoV-19^^England|Human|Virology Department|COVID-19 Genomics UK (COG-UK) Consortium|de Silva|United Kingdom
>Spike|hCoV-19/England/ALDP-A670C1/2020|2020-10-12|EPI_ISL_630505|Original|hCoV-19^^England|Human|Lighthouse Lab in Alderley Park|Wellcome Sanger Institute for the COVID-19 Genomics UK (COG-UK) consortium|Davies|United Kingdom
>Spike|hCoV-19/USA/FL-BPHL-1840/2020|2020-04-09|EPI_ISL_613920|Original|hCoV-19^^Florida|Human|Florida Bureau of Public Health Laboratories|Florida Bureau of Public Health Laboratories|Blanton|USA
>Spike|hCoV-19/Wales/PHWC-165967/2020|2020-05-15|EPI_ISL_490745|Original|hCoV-19^^Wales|Human|Wales Specialist Virology Centre Sequencing lab|COVID-19 Genomics UK (COG-UK) Consortium|Moore|United Kingdom
>Spike|hCoV-19/Denmark/DCGC-11560/2020|2020-11-02|EPI_ISL_670995|Original|hCoV-19^^Nordjylland|Human|Department of Virus and Microbiological Special Diagnostics|Albertsen Lab|Michaelsen|Denmark
>Spike|hCoV-19/USA/CA-ALSR-4811/2020|2020-05-01|EPI_ISL_730155|Original|hCoV-19^^California|Human|Scripps Medical Laboratory|Andersen lab at Scripps Research|Gangavarapu|USA
>Spike|hCoV-19/Scotland/GCVR-1725BE/2020|2020-04-04|EPI_ISL_524535|Original|hCoV-19^^Scotland|Human|NHSGGC West of Scotland Specialist Virology Centre / MRC-University of Glasgow Centre for Virus Research|Wellcome Sanger Institute for the COVID-19 Genomics UK (COG-UK) consortium|Davies|United Kingdom
>Spike|hCoV-19/England/MILK-9944EE/2020|2020-08-25|EPI_ISL_552206|Original|hCoV-19^^England|Human|Lighthouse Lab in Milton Keynes|Wellcome Sanger Institute for the COVID-19 Genomics UK (COG-UK) consortium|Davies|United Kingdom
>Spike|hCoV-19/USA/CT-Yale-371/2020|2020-08-17|EPI_ISL_527785|Original|hCoV-19^^Connecticut|Human|Connecticut State Department of Public Health|Grubaugh Lab - Yale School of Public Health|Fauver|USA

>Spike|hCoV-19/England/QEUH-BCC06F/2020|2020-11-27|EPI_ISL_703984|Original|hCoV-19^^England|Human|Lighthouse Lab in Glasgow|Wellcome Sanger Institute for the COVID-19 Genomics UK (COG-UK) Consortium|Davies|United Kingdom
>Spike|hCoV-19/Singapore/141cul/2020|2020-03-23|EPI_ISL_574484|VeroE6 P1|hCoV-19^^Singapore|Human|Programme in Emerging Infectious Diseases|National Public Health Laboratory|Mak|Singapore
>Spike|hCoV-19/Australia/VIC4335/2020|2020-07-13|EPI_ISL_519631|Original|hCoV-19^^Victoria|Human|Microbiological Diagnostic Unit - Public Health Laboratory (MDU-PHL)|MDU-PHL|Schultz|Australia
>Spike|hCoV-19/Denmark/DCGC-2419/2020|2020-08-10|EPI_ISL_617601|Original|hCoV-19^^Midtjylland|Human|Department of Virus and Microbiological Special Diagnostics|Albertsen lab|Michaelsen|Denmark
>Spike|hCoV-19/England/ALDP-AA11C8/2020|2020-10-22|EPI_ISL_623974|Original|hCoV-19^^England|Human|Lighthouse Lab in Alderley Park|Wellcome Sanger Institute for the COVID-19 Genomics UK (COG-UK) Consortium|Davies|United Kingdom
>Spike|hCoV-19/USA/WA-UW-10159/2020|2020-06-02|EPI_ISL_570270|Original|hCoV-19^^Washington|Human|UW Virology Lab|UW Virology Lab|Roychoudhury|USA
>Spike|hCoV-19/England/LIVE-DCA393/2020|2020-12-06|EPI_ISL_724355|Original|hCoV-19^^England|Human|Liverpool Clinical Laboratories|COVID-19 Genomics UK (COG-UK) Consortium|Haldenby|United Kingdom
>Spike|hCoV-19/India/MH-NCCS-NR1290/2020|2020-05-15|EPI_ISL_496558|Original|hCoV-19^^Maharashtra|Human|National Centre For Cell Science|National Centre For Cell Science|Paul|India
>Spike|hCoV-19/England/QEUH-CABDE6/2020|2020-12-20|EPI_ISL_761058|Original|hCoV-19^^England|Human|Lighthouse Lab in Glasgow|Wellcome Sanger Institute for the COVID-19 Genomics UK (COG-UK) Consortium|Davies|United Kingdom
>Spike|hCoV-19/Timor-Leste/TL06/2020|2020-04-11|EPI_ISL_456598|Original|hCoV-19^^Timor-Leste|Human|National Health Laboratory|Microbiological Diagnostic Unit Public Health Laboratory|Schultz|Timor-Leste
>Spike|hCoV-19/Australia/VIC2763/2020|2020-07-07|EPI_ISL_521598|Original|hCoV-19^^Victoria|Human|Microbiological Diagnostic Unit - Public Health Laboratory (MDU-PHL)|MDU-PHL|Schultz|Australia
>Spike|hCoV-19/USA/HI-H200331/2020|2020-08-24|EPI_ISL_752889|Original|hCoV-19^^Hawaii|Human|State Laboratories Division|State Laboratories Division|Sultana|USA
>Spike|hCoV-19/Australia/VIC7844/2020|2020-07-29|EPI_ISL_564695|Original|hCoV-19^^Victoria|Human|Microbiological Diagnostic Unit - Public Health Laboratory (MDU-PHL)|MDU-PHL|Schultz|Australia
>Spike|hCoV-19/England/QEUH-9C9C60/2020|2020-09-10|EPI_ISL_567425|Original|hCoV-19^^England|Human|Lighthouse Lab in Glasgow|Wellcome Sanger Institute for the COVID-19 Genomics UK (COG-UK) consortium|Davies|United Kingdom
>Spike|hCoV-19/England/QEUH-A37103/2020|2020-10-04|EPI_ISL_706929|Original|hCoV-19^^England|Human|Oxford Viromics|COVID-19 Genomics UK (COG-UK) Consortium|Golubchik|United Kingdom
>Spike|hCoV-19/England/QEUH-9A40E7/2020|2020-09-01|EPI_ISL_540146|Original|hCoV-19^^England|Human|Lighthouse Lab in Glasgow|Wellcome Sanger Institute for the COVID-19 Genomics UK (COG-UK) consortium|Davies|United Kingdom
>Spike|hCoV-19/England/QEUH-A0B733/2020|2020-10-01|EPI_ISL_599309|Original|hCoV-19^^England|Human|Lighthouse Lab in Glasgow|Wellcome Sanger Institute for the COVID-19 Genomics UK (COG-UK) consortium|Davies|United Kingdom
>Spike|hCoV-19/England/ALDP-5D2390/2020|2020-06-19|EPI_ISL_558590|Original|hCoV-19^^England|Human|Lighthouse Lab in Alderley Park|Wellcome Sanger Institute for the COVID-19 Genomics UK (COG-UK) consortium|Davies|United Kingdom
>Spike|hCoV-19/England/CAMC-B32E00/2020|2020-11-05|EPI_ISL_659723|Original|hCoV-19^^England|Human|Lighthouse Lab in Cambridge|Wellcome Sanger Institute for the COVID-19 Genomics UK (COG-UK) Consortium|Davies|United Kingdom
>Spike|hCoV-19/England/LOND-D4BAA/2020|2020-04-11|EPI_ISL_449657|Original|hCoV-19^^England|Human|University College London|COVID-19 Genomics UK (COG-UK) Consortium|Roy|United Kingdom

>Spike|hCoV-19/Denmark/DCGC-10979/2020|2020-11-02|EPI_ISL_670322|Original|hCoV-19^^Sjaelland|Human|Department of Virus and Microbiological Special Diagnostics|Albertsen Lab|Michaelsen|Denmark
>Spike|hCoV-19/Japan/PG-8945/2020|2020-08-00|EPI_ISL_686491|Original|hCoV-19^^Japan|Human|Pathogen Genomics Center|Pathogen Genomics Center|Sekizuka|Japan
>Spike|hCoV-19/England/CAMC-A3F596/2020|2020-10-06|EPI_ISL_609692|Original|hCoV-19^^England|Human|Lighthouse Lab in Cambridge|Wellcome Sanger Institute for the COVID-19 Genomics UK (COG-UK) consortium|Davies|United Kingdom
>Spike|hCoV-19/Wales/PHWC-481267/2020|2020-10-19|EPI_ISL_613048|Original|hCoV-19^^Wales|Human|Wales Specialist Virology Centre Sequencing lab|COVID-19 Genomics UK (COG-UK) Consortium|Moore|United Kingdom
>Spike|hCoV-19/England/QEUH-AAEDE5/2020|2020-10-22|EPI_ISL_653008|Original|hCoV-19^^England|Human|Oxford Viromics|COVID-19 Genomics UK (COG-UK) Consortium|Golubchik|United Kingdom
>Spike|hCoV-19/Japan/PG-0788/2020|2020-04-00|EPI_ISL_684398|Original|hCoV-19^^Japan|Human|Pathogen Genomics Center|Pathogen Genomics Center|Sekizuka|Japan
>Spike|hCoV-19/Wales/PHWC-49EAD8/2020|2020-12-08|EPI_ISL_743093|Original|hCoV-19^^Wales|Human|Wales Specialist Virology Centre Sequencing lab|COVID-19 Genomics UK (COG-UK) Consortium|Moore|United Kingdom
>Spike|hCoV-19/England/ALDP-950958/2020|2020-06-14|EPI_ISL_554346|Original|hCoV-19^^England|Human|Lighthouse Lab in Alderley Park|Wellcome Sanger Institute for the COVID-19 Genomics UK (COG-UK) consortium|Davies|United Kingdom
>Spike|hCoV-19/Finland/15A32S5/2020|2020-04-15|EPI_ISL_481555|Original|hCoV-19^^Uusimaa|Human|Department of Virology and Immunology|Department of Virology|Smura|Finland
>Spike|hCoV-19/Canada/ON-PHL-20-01158/2020|2020-04-00|EPI_ISL_548781|Original|hCoV-19^^Ontario|Human|Public Health Ontario Laboratory|Public Health Ontario Laboratory|Guthrie|Canada
>Spike|hCoV-19/Scotland/CVR2621/2020|2020-04-17|EPI_ISL_484618|Original|hCoV-19^^Scotland|Human|West of Scotland Specialist Virology Centre|COVID-19 Genomics UK (COG-UK) Consortium|Robertson|United Kingdom
>Spike|hCoV-19/USA/NY-Wadsworth-285291-01/2020|2020-12-08|EPI_ISL_754475|Original|hCoV-19^^New York|Human|Wadsworth Center|Wadsworth Center|Lasek-Nesselquist|USA
>Spike|hCoV-19/Denmark/DCGC-1276/2020|2020-06-08|EPI_ISL_618069|Original|hCoV-19^^Nordjylland|Human|Department of Virus and Microbiological Special Diagnostics|Albertsen lab|Michaelsen|Denmark
>Spike|hCoV-19/England/LIVE-9BA39/2020|2020-04-19|EPI_ISL_440895|Original|hCoV-19^^England|Human|Liverpool Clinical Laboratories|COVID-19 Genomics UK (COG-UK) Consortium|Haldenby|United Kingdom
>Spike|hCoV-19/England/ALDP-BCA77B/2020|2020-11-23|EPI_ISL_703260|Original|hCoV-19^^England|Human|Lighthouse Lab in Alderley Park|Wellcome Sanger Institute for the COVID-19 Genomics UK (COG-UK) Consortium|Davies|United Kingdom
>Spike|hCoV-19/USA/MI-MDHHS-SC20552/2020|2020-04-29|EPI_ISL_460600|Original|hCoV-19^^Michigan|Human|Michigan Department of Health and Human Services|Michigan Department of Health and Human Services|Blankenship|USA
>Spike|hCoV-19/Japan/PG-1886/2020|2020-05-00|EPI_ISL_685492|Original|hCoV-19^^Japan|Human|Tokyo Metropolitan Institute of Public Health|Pathogen Genomics Center|Sekizuka|Japan
>Spike|hCoV-19/Denmark/DCGC-13987/2020|2020-11-30|EPI_ISL_711498|Original|hCoV-19^^Nordjylland|Human|Department of Virus and Microbiological Special Diagnostics|Albertsen Lab|Michaelsen|Denmark
>Spike|hCoV-19/England/CAMB-1AA6AB/2020|2020-04-17|EPI_ISL_470114|Original|hCoV-19^^England|Human|Department of Pathology|Wellcome Sanger Institute for the COVID-19 Genomics UK (COG-UK) consortium|Davies|United Kingdom
>Spike|hCoV-19/Switzerland/VD-ETHZ-100177/2020|2020-03-17|EPI_ISL_574985|Original|hCoV-19^^Vaud|Human|Viollier AG|Department of Biosystems Science and Engineering|Nadeau|Switzerland

>Spike|hCoV-19/Denmark/DCGC-6260/2020|2020-10-05|EPI_ISL_620020|Original|hCoV-19^^Syddanmark|Human|Department of Virus and Microbiological Special Diagnostics|Albertsen lab|Michaelsen|Denmark
>Spike|hCoV-19/England/QEUH-96BF76/2020|2020-08-14|EPI_ISL_531362|Original|hCoV-19^^England|Human|Lighthouse Lab in Glasgow|Wellcome Sanger Institute for the COVID-19 Genomics UK (COG-UK) consortium|Davies|United Kingdom
>Spike|hCoV-19/Switzerland/BL-ETHZ-200059/2020|2020-07-14|EPI_ISL_510741|Original|hCoV-19^^Basel-Landschaft|Human|Viollier AG|Department of Biosystems Science and Engineering|Nadeau|Switzerland
>Spike|hCoV-19/England/QEUH-A1DC11/2020|2020-09-29|EPI_ISL_587491|Original|hCoV-19^^England|Human|Lighthouse Lab in Glasgow|Wellcome Sanger Institute for the COVID-19 Genomics UK (COG-UK) consortium|Davies|United Kingdom
>Spike|hCoV-19/USA/UT-UPHL-2008808/2020|2020-05-23|EPI_ISL_524834|Original|hCoV-19^^Utah|Human|Utah Public Health Laboratory|Utah Public Health Laboratory|Young|USA
>Spike|hCoV-19/England/QEUH-A49B00/2020|2020-10-05|EPI_ISL_627281|Original|hCoV-19^^England|Human|Oxford Viromics|COVID-19 Genomics UK (COG-UK) Consortium|Golubchik|United Kingdom
>Spike|hCoV-19/Wales/PHWC-484404/2020|2020-10-23|EPI_ISL_628002|Original|hCoV-19^^Wales|Human|Wales Specialist Virology Centre Sequencing lab|COVID-19 Genomics UK (COG-UK) Consortium|Moore|United Kingdom
>Spike|hCoV-19/Denmark/DCGC-22377/2020|2020-12-14|EPI_ISL_757525|Original|hCoV-19^^Syddanmark|Human|Department of Virus and Microbiological Special Diagnostics|Albertsen Lab|Michaelsen|Denmark
>Spike|hCoV-19/USA/WA-UW-24557/2020|2020-08-17|EPI_ISL_576486|Original|hCoV-19^^Washington|Human|UW Virology Lab|UW Virology Lab|Roychoudhury|USA
>Spike|hCoV-19/USA/TX-DSHS-0235/2020|2020-04-25|EPI_ISL_525787|Original|hCoV-19^^Texas|Human|Texas Department of State Health Services|Texas Department of State Health Services|Rahman|USA
>Spike|hCoV-19/England/BIRM-66202/2020|2020-10-07|EPI_ISL_612247|Original|hCoV-19^^England|Human|University of Birmingham|COVID-19 Genomics UK (COG-UK) Consortium|Loman|United Kingdom
>Spike|hCoV-19/South Korea/KCDC2747/2020|2020-07-19|EPI_ISL_522526|Original|hCoV-19^^South Korea|Human|Division of Viral Diseases|Division of Viral Diseases|Kim|South Korea
>Spike|hCoV-19/England/BRIS-126DDF/2020|2020-04-09|EPI_ISL_459010|Original|hCoV-19^^England|Human|PHE South West Regional Laboratory|Wellcome Sanger Institute for the COVID-19 Genomics UK (COG-UK) consortium|Davies|United Kingdom
>Spike|hCoV-19/USA/VAN4606/2020|2020-05-11|EPI_ISL_677688|Original|hCoV-19^^New York|Human|Vanda Pharmaceuticals Clinical Site|Vanda Pharmaceuticals|Przychodzen|USA
>Spike|hCoV-19/Northern Ireland/NIRE-10675F/2020|2020-05-14|EPI_ISL_501054|Original|hCoV-19^^Northern Ireland|Human|Regional Virus Laboratory|Wellcome Sanger Institute for the COVID-19 Genomics UK (COG-UK) consortium|Davies|United Kingdom
>Spike|hCoV-19/England/MILK-AB902A/2020|2020-10-21|EPI_ISL_623728|Original|hCoV-19^^England|Human|Lighthouse Lab in Milton Keynes|Wellcome Sanger Institute for the COVID-19 Genomics UK (COG-UK) consortium|Davies|United Kingdom
>Spike|hCoV-19/USA/TX-HMH-MCoV-14561/2020|2020-10-08|EPI_ISL_787846|Original|hCoV-19^^Texas|Human|Houston Methodist Hospital|Houston Methodist Hospital|Long|USA
>Spike|hCoV-19/England/MILK-9C7039/2020|2020-09-08|EPI_ISL_573273|Original|hCoV-19^^England|Human|Northumbria University / South Tees Hospitals NHS Foundation Trust / North Cumbria Integrated Care NHS Foundation Trust / North Tees and Hartlepool NHS Foundation Trust / Newcastle Hospitals NHS Foundation Trust|COVID-19 Genomics UK (COG-UK) Consortium|Bashton|United Kingdom
>Spike|hCoV-19/Denmark/DCGC-16439/2020|2020-11-16|EPI_ISL_714768|Original|hCoV-19^^Sjaelland|Human|Department of Virus and Microbiological Special Diagnostics|Albertsen Lab|Michaelsen|Denmark
>Spike|hCoV-19/USA/MD-MDH-0162/2020|2020-07-14|EPI_ISL_524889|Original|hCoV-19^^Maryland|Human|MD PHL|MD PHL|Keller|USA

>Spike|hCoV-19/England/MILK-A79633/2020|2020-10-13|EPI_ISL_606910|Original|hCoV-19^^England|Human|Lighthouse Lab in Milton Keynes|Wellcome Sanger Institute for the COVID-19 Genomics UK (COG-UK) consortium|Davies|United Kingdom
>Spike|hCoV-19/England/SHEF-C7ADA/2020|2020-05-26|EPI_ISL_475401|Original|hCoV-19^^England|Human|Virology Department|COVID-19 Genomics UK (COG-UK) Consortium|de Silva|United Kingdom
>Spike|hCoV-19/Denmark/ALAB-SSI515/2020|2020-03-23|EPI_ISL_429583|Original|hCoV-19^^Denmark|Human|Department of Virus and Microbiological Special Diagnostics|Albertsen lab|Kirkegaard|Denmark
>Spike|hCoV-19/Japan/PG-4957/2020|2020-08-00|EPI_ISL_690076|Original|hCoV-19^^Japan|Human|Pathogen Genomics Center|Pathogen Genomics Center|Sekizuka|Japan
>Spike|hCoV-19/Morocco/RMPS-21/2020|2020-04-01|EPI_ISL_728235|Original|hCoV-19^^Morocco|Human|LNR National Reference Laboratory|Medical Biotechnology Laboratory|Kartti|Morocco
>Spike|hCoV-19/Wales/PHWC-4A1C81/2020|2020-12-11|EPI_ISL_743798|Original|hCoV-19^^Wales|Human|Wales Specialist Virology Centre Sequencing lab|COVID-19 Genomics UK (COG-UK) Consortium|Moore|United Kingdom
>Spike|hCoV-19/Denmark/DCGC-2484/2020|2020-08-10|EPI_ISL_617752|Original|hCoV-19^^Midtjylland|Human|Department of Virus and Microbiological Special Diagnostics|Albertsen lab|Michaelsen|Denmark
>Spike|hCoV-19/Denmark/DCGC-19789/2020|2020-12-07|EPI_ISL_747789|Original|hCoV-19^^Midtjylland|Human|Department of Virus and Microbiological Special Diagnostics|Albertsen Lab|Michaelsen|Denmark
>Spike|hCoV-19/England/ALDP-C588AB/2020|2020-12-14|EPI_ISL_735941|Original|hCoV-19^^England|Human|Lighthouse Lab in Alderley Park|Wellcome Sanger Institute for the COVID-19 Genomics UK (COG-UK) Consortium|Davies|United Kingdom
>Spike|hCoV-19/USA/UT-UPHL-2012159735/2020|2020-07-27|EPI_ISL_745665|Original|hCoV-19^^Utah|Human|Ginkgo Bioworks Clinical Laboratory|Utah Public Health Laboratory|Young|USA
>Spike|hCoV-19/Japan/PG-6505/2020|2020-07-00|EPI_ISL_689607|Original|hCoV-19^^Japan|Human|Pathogen Genomics Center|Pathogen Genomics Center|Sekizuka|Japan
>Spike|hCoV-19/Australia/VIC13901/2020|2020-09-14|EPI_ISL_563873|Original|hCoV-19^^Victoria|Human|Microbiological Diagnostic Unit - Public Health Laboratory (MDU-PHL)|MDU-PHL|Schultz|Australia
>Spike|hCoV-19/USA/VA-DCLS-2167/2020|2020-11-06|EPI_ISL_677196|Original|hCoV-19^^Virginia|Human|Virginia Division of Consolidated Laboratory Services|Virginia Division of Consolidated Laboratory Services|St. Jacques|USA
>Spike|hCoV-19/USA/UT-08643/2020|2020-05-26|EPI_ISL_524378|Original|hCoV-19^^Utah|Human|Utah Public Health Laboratory|Utah Public Health Laboratory|Young|USA
>Spike|hCoV-19/Denmark/DCGC-20118/2020|2020-12-07|EPI_ISL_747672|Original|hCoV-19^^Syddanmark|Human|Department of Virus and Microbiological Special Diagnostics|Albertsen Lab|Michaelsen|Denmark
>Spike|hCoV-19/Chile/RM-ISPCH-77/2020|2020-04-04|EPI_ISL_445374|Original|hCoV-19^^Region Metropolitana de Santiago|Human|HOSPITAL SAN JUAN DE DIOS|Instituto de Salud Publica de Chile|Fernandez|Chile
>Spike|hCoV-19/Australia/NSW583/2020|2020-03-14|EPI_ISL_478695|Original|hCoV-19^^New South Wales|Human|South Eastern Area Laboratory Services (SEALS)|NSW Health Pathology - Institute of Clinical Pathology and Medical Research; Westmead Hospital; University of Sydney|Gall|Australia
>Spike|hCoV-19/Denmark/DCGC-20782/2020|2020-12-21|EPI_ISL_759359|Original|hCoV-19^^Nordjylland|Human|Department of Virus and Microbiological Special Diagnostics|Albertsen Lab|Michaelsen|Denmark
>Spike|hCoV-19/Scotland/EDB10461/2020|2020-11-09|EPI_ISL_704187|Original|hCoV-19^^Scotland|Human|Virology Department|COVID-19 Genomics UK (COG-UK) Consortium|McHugh|United Kingdom
>Spike|hCoV-19/England/QEUH-AD3F1E/2020|2020-10-26|EPI_ISL_634663|Original|hCoV-19^^England|Human|Lighthouse Lab in Glasgow|Wellcome Sanger Institute for the COVID-19 Genomics UK (COG-UK) consortium|Davies|United Kingdom

>Spike|hCoV-19/Wales/PHWC-2A5D4/2020|2020-04-06|EPI_ISL_445591|Original|hCoV-
19^^Wales|Human|Wales Specialist Virology Centre|Public Health Wales Microbiology
Cardiff|Moore|United Kingdom
>Spike|hCoV-19/Jordan/SR-054/2020|2020-03-30|EPI_ISL_430012|Original|hCoV-
19^^Irbid|Human|Biolab Diagnostic Laboratories|Andersen lab at Scripps
Research|Gangavarapu|Jordan
>Spike|hCoV-19/England/QEUH-99922B/2020|2020-08-24|EPI_ISL_541805|Original|hCoV-
19^^England|Human|Lighthouse Lab in Glasgow|Wellcome Sanger Institute for the COVID-
19 Genomics UK (COG-UK) consortium|Davies|United Kingdom
>Spike|hCoV-19/South Korea/KCDC2678/2020|2020-06-22|EPI_ISL_522460|Original|hCoV-
19^^South Korea|Human|Center for Laboratory Control of Infectious Diseases|Center for
Laboratory Control of Infectious Diseases|Kim|South Korea
>Spike|hCoV-19/England/ALDP-953E29/2020|2020-06-13|EPI_ISL_553818|Original|hCoV-
19^^England|Human|Lighthouse Lab in Alderley Park|Wellcome Sanger Institute for the
COVID-19 Genomics UK (COG-UK) consortium|Davies|United Kingdom
>Spike|hCoV-19/England/QEUH-B2EA12/2020|2020-11-03|EPI_ISL_659839|Original|hCoV-
19^^England|Human|Lighthouse Lab in Glasgow|Wellcome Sanger Institute for the COVID-
19 Genomics UK (COG-UK) Consortium|Davies|United Kingdom
>Spike|hCoV-19/Scotland/EDB3426/2020|2020-04-10|EPI_ISL_487852|Original|hCoV-
19^^Scotland|Human|Virology Department|Wellcome Sanger Institute for the COVID-19
Genomics UK (COG-UK) consortium|Davies|United Kingdom
>Spike|hCoV-19/Luxembourg/LNS6690193/2020|2020-04-15|EPI_ISL_434506|Original|hCoV-
19^^Luxembourg|Human|Laboratoire National de Sante|Laboratoire National de
Sante|Wienecke-Baldacchino|Luxembourg
>Spike|hCoV-19/Switzerland/AG-ETHZ-101157/2020|2020-03-
31|EPI_ISL_451823|Original|hCoV-19^^Aargau|Human|Viollier AG|Department of Biosystems
Science and Engineering|Nadeau|Switzerland
>Spike|hCoV-19/England/ALDP-A6FBE6/2020|2020-10-15|EPI_ISL_607100|Original|hCoV-
19^^England|Human|Lighthouse Lab in Alderley Park|Wellcome Sanger Institute for the
COVID-19 Genomics UK (COG-UK) consortium|Davies|United Kingdom
>Spike|hCoV-19/Egypt/CUNCI-HGC3I013/2020|2020-06-02|EPI_ISL_479694|Original|hCoV-
19^^Egypt|Human|Egyptian National Cancer Institute (ENCI)|Egyptian National Cancer
Institute (ENCI)|Zekri|Egypt
>Spike|hCoV-19/England/CAMB-81E94/2020|2020-04-22|EPI_ISL_438588|Original|hCoV-
19^^England|Human|Department of Pathology|COVID-19 Genomics UK (COG-UK)
Consortium|Meredith|United Kingdom
>Spike|hCoV-19/USA/MI-MDHHS-SC21515/2020|2020-04-02|EPI_ISL_516242|Original|hCoV-
19^^Michigan|Human|Michigan Department of Health and Human Services|Michigan
Department of Health and Human Services|Blankenship|USA
>Spike|hCoV-19/USA/LA-GBCL-26684476/2020|2020-04-10|EPI_ISL_485903|Original|hCoV-
19^^Louisiana|Human|River Road Testing Lab|Ginkgo Bioworks Clinical
Laboratory|Smith|USA
>Spike|hCoV-19/England/ALDP-C3B4B9/2020|2020-12-09|EPI_ISL_731719|Original|hCoV-
19^^England|Human|Lighthouse Lab in Alderley Park|Wellcome Sanger Institute for the
COVID-19 Genomics UK (COG-UK) Consortium|Davies|United Kingdom
>Spike|hCoV-19/Wales/PHWC-495B92/2020|2020-11-26|EPI_ISL_726449|Original|hCoV-
19^^Wales|Human|Originating lab|COVID-19 Genomics UK (COG-UK) Consortium|Moore|United
Kingdom
>Spike|hCoV-19/England/ALDP-CEB4D6/2020|2020-12-21|EPI_ISL_781412|Original|hCoV-
19^^England|Human|Lighthouse Lab in Alderley Park|Wellcome Sanger Institute for the
COVID-19 Genomics UK (COG-UK) Consortium|Davies|United Kingdom
>Spike|hCoV-19/Japan/PG-1963/2020|2020-04-00|EPI_ISL_685569|Original|hCoV-
19^^Japan|Human|Pathogen Genomics Center|Pathogen Genomics Center|Sekizuka|Japan
>Spike|hCoV-19/England/QEUH-960955/2020|2020-08-07|EPI_ISL_532029|Original|hCoV-
19^^England|Human|Lighthouse Lab in Glasgow|Wellcome Sanger Institute for the COVID-
19 Genomics UK (COG-UK) consortium|Davies|United Kingdom
>Spike|hCoV-19/Scotland/QEUH-A1F59C/2020|2020-10-01|EPI_ISL_598917|Original|hCoV-
19^^Scotland|Human|Lighthouse Lab in Glasgow|Wellcome Sanger Institute for the COVID-
19 Genomics UK (COG-UK) consortium|Davies|United Kingdom

>Spike|hCoV-19/Japan/PG-0374/2020|2020-02-22|EPI_ISL_480044|Original|hCoV-19^^Chubu|Human|Nagoya City Public Health Research Institute|Pathogen Genomics Center|Sekizuka|Japan
>Spike|hCoV-19/USA/CA-ALSR-2603/2020|2020-07-11|EPI_ISL_635582|Original|hCoV-19^^California|Human|San Diego County Public Health Laboratory|Andersen lab at Scripps Research|Gangavarapu|USA
>Spike|hCoV-19/USA/NY-WC-13741-01/2020|2020-03-12|EPI_ISL_534731|Original|hCoV-19^^New York|Human|Wadsworth Center|Wadsworth Center|Plitnick|USA
>Spike|hCoV-19/Netherlands/UT-EMC-129/2020|2020-10-22|EPI_ISL_632723|Original|hCoV-19^^Utrecht|Human|Dutch COVID-19 response team|Erasmus Medical Center|Oude Munnink|Netherlands
>Spike|hCoV-19/Ireland/D-NVRL-AIIDV1472/2020|2020-12-14|EPI_ISL_791306|Original|hCoV-19^^Dublin|Human|National Virus Reference Laboratory|Irish Coronavirus Sequencing Consortium - Teagasc Moorepark|Walsh|Ireland
>Spike|hCoV-19/USA/NY-NYUMC440/2020|2020-04-15|EPI_ISL_435507|Original|hCoV-19^^New York|Human|NYU Langone Health|Departments of Pathology and Medicine|Maurano|USA
>Spike|hCoV-19/England/ALDP-A67D8E/2020|2020-10-10|EPI_ISL_608989|Original|hCoV-19^^England|Human|Lighthouse Lab in Alderley Park|Wellcome Sanger Institute for the COVID-19 Genomics UK (COG-UK) consortium|Davies|United Kingdom
>Spike|hCoV-19/USA/TX-HMH-1046/2020|2020-04-05|EPI_ISL_542507|Original|hCoV-19^^Texas|Human|Houston Methodist Hospital|Houston Methodist Hospital|Long|USA
>Spike|hCoV-19/Russia/PRI-RII-MH762S/2020|2020-08-11|EPI_ISL_733010|Original|hCoV-19^^Primorsky|Human|WHO National Influenza Centre Russian Federation|WHO National Influenza Centre Russian Federation|Komissarov|Russia
>Spike|hCoV-19/England/MILK-B94E39/2020|2020-11-14|EPI_ISL_676277|Original|hCoV-19^^England|Human|Lighthouse Lab in Milton Keynes|Wellcome Sanger Institute for the COVID-19 Genomics UK (COG-UK) Consortium|Davies|United Kingdom
>Spike|hCoV-19/Scotland/CVR2026/2020|2020-04-10|EPI_ISL_477844|Original|hCoV-19^^Scotland|Human|West of Scotland Specialist Virology Centre|COVID-19 Genomics UK (COG-UK) Consortium|Robertson|United Kingdom
>Spike|hCoV-19/USA/LA-EVTL1063/2020|2020-11-27|EPI_ISL_768333|Original|hCoV-19^^Louisiana|Human|LSUHS Emerging Viral Threat Laboratory|Microbial Genome Sequencing Center|Kamil|USA
>Spike|hCoV-19/Ireland/CE-NVRL-73IRL22902/2020|2020-09-16|EPI_ISL_578247|Original|hCoV-19^^Clare|Human|National Virus Reference Laboratory|National Virus Reference Laboratory|Gonzalez|Ireland
>Spike|hCoV-19/USA/AZ-TG388028/2020|2020-06-18|EPI_ISL_694697|Original|hCoV-19^^Arizona|Human|TGen North|TGen North|French|USA
>Spike|hCoV-19/Wales/PHWC-47E212/2020|2020-09-27|EPI_ISL_595424|Original|hCoV-19^^Wales|Human|Originating lab|COVID-19 Genomics UK (COG-UK) Consortium|Moore|United Kingdom
>Spike|hCoV-19/USA/UT-UPHL-2012698275/2020|2020-10-19|EPI_ISL_745590|Original|hCoV-19^^Utah|Human|Ginkgo Bioworks Clinical Laboratory|Utah Public Health Laboratory|Young|USA
>Spike|hCoV-19/Australia/VIC6915/2020|2020-07-24|EPI_ISL_564136|Original|hCoV-19^^Victoria|Human|Microbiological Diagnostic Unit - Public Health Laboratory (MDU-PHL)|MDU-PHL|Schultz|Australia
>Spike|hCoV-19/Italy/APU-IZSPB_326PT/2020|2020-11-04|EPI_ISL_653819|Original|hCoV-19^^Apulia|Human|I.R.C.C.S. \S. De Bellis\ - Ente Ospedaliero|Istituto Zooprofilattico Sperimentale della Puglia e della Basilicata|Parisi|Italy
>Spike|hCoV-19/USA/IL-NM-0693/2020|2020-05-10|EPI_ISL_626470|Original|hCoV-19^^Illinois|Human|Northwestern Memorial Hospital|Ozer Lab|Ozer|USA
>Spike|hCoV-19/USA/HI-H200572/2020|2020-10-24|EPI_ISL_753099|Original|hCoV-19^^Hawaii|Human|State Laboratories Division|State Laboratories Division|Sultana|USA
>Spike|hCoV-19/Scotland/QEUH-96CDF3/2020|2020-08-21|EPI_ISL_534582|Original|hCoV-19^^Scotland|Human|NHSGGC West of Scotland Specialist Virology Centre / MRC-University of Glasgow Centre for Virus Research|Wellcome Sanger Institute for the COVID-19 Genomics UK (COG-UK) consortium|Davies|United Kingdom

>Spike|hCoV-19/England/BRIS-1276B5/2020|2020-04-02|EPI_ISL_469636|Original|hCoV-19^^England|Human|PHE South West Regional Laboratory|Wellcome Sanger Institute for the COVID-19 Genomics UK (COG-UK) consortium|Davies|United Kingdom
>Spike|hCoV-19/Portugal/PT1635/2020|2020-05-28|EPI_ISL_731945|Original|hCoV-19^^Portugal|Human|Instituto Nacional de Saude (INSA)|Instituto Nacional de Saude (INSA)|Borges|Portugal
>Spike|hCoV-19/Denmark/DCGC-2479/2020|2020-08-10|EPI_ISL_617747|Original|hCoV-19^^Midtjylland|Human|Department of Virus and Microbiological Special Diagnostics|Albertsen lab|Michaelsen|Denmark
>Spike|hCoV-19/England/ALDP-95031E/2020|2020-06-18|EPI_ISL_555201|Original|hCoV-19^^England|Human|Lighthouse Lab in Alderley Park|Wellcome Sanger Institute for the COVID-19 Genomics UK (COG-UK) consortium|Davies|United Kingdom
>Spike|hCoV-19/Portugal/PT1289/2020|2020-03-24|EPI_ISL_511250|Original|hCoV-19^^Portugal|Human|Instituto Nacional de Saude (INSA) and Instituto Gulbenkian de Ciencia (IGC)|Instituto Nacional de Saude (INSA) and Instituto Gulbenkian de Ciencia (IGC)|Borges|Portugal
>Spike|hCoV-19/USA/CA-CZB-15488/2020|2020-09-15|EPI_ISL_739588|Original|hCoV-19^^California|Human|County of San Luis Obispo Public Health Laboratory|Chan-Zuckerberg Biohub|Kamm|USA
>Spike|hCoV-19/England/MILK-9E201A/2020|2020-09-20|EPI_ISL_581321|Original|hCoV-19^^England|Human|Lighthouse Lab in Milton Keynes|Wellcome Sanger Institute for the COVID-19 Genomics UK (COG-UK) consortium|Davies|United Kingdom
>Spike|hCoV-19/England/MILK-A24084/2020|2020-10-04|EPI_ISL_650949|Original|hCoV-19^^England|Human|Centre for Enzyme Innovation|COVID-19 Genomics UK (COG-UK) Consortium|Robson|United Kingdom
>Spike|hCoV-19/Australia/WA418/2020|2020-07-11|EPI_ISL_672630|Original|hCoV-19^^Western Australia|Human|PathWest Laboratory Medicine WA|PathWest Laboratory Medicine WA Microbial Surveillance Unit|Sikazwe|Australia
>Spike|hCoV-19/Japan/PG-7884/2020|2020-07-00|EPI_ISL_686985|Original|hCoV-19^^Japan|Human|Pathogen Genomics Center|Pathogen Genomics Center|Sekizuka|Japan
>Spike|hCoV-19/England/CAMB-7A168/2020|2020-04-03|EPI_ISL_441236|Original|hCoV-19^^England|Human|Department of Pathology|Wellcome Sanger Institute for the COVID-19 Genomics UK (COG-UK) consortium|Amato|United Kingdom
>Spike|hCoV-19/USA/TX-HMH-MCoV-15624/2020|2020-10-26|EPI_ISL_785308|Original|hCoV-19^^Texas|Human|Houston Methodist Hospital|Houston Methodist Hospital|Long|USA
>Spike|hCoV-19/Jordan/SEARCH-5432/2020|2020-09-07|EPI_ISL_755239|Original|hCoV-19^^Amman|Human|Biolab Diagnostic Laboratories|Andersen lab at Scripps Research|Gangavarapu|Jordan
>Spike|hCoV-19/USA/WA-UW-2940/2020|2020-03-28|EPI_ISL_486057|Original|hCoV-19^^Washington|Human|UW Virology Lab|UW Virology Lab|Roychoudhury|USA
>Spike|hCoV-19/Denmark/DCGC-13645/2020|2020-11-30|EPI_ISL_712932|Original|hCoV-19^^Hovedstaden|Human|Department of Virus and Microbiological Special Diagnostics|Albertsen Lab|Michaelsen|Denmark
>Spike|hCoV-19/England/QEUH-AE2F76/2020|2020-10-28|EPI_ISL_633591|Original|hCoV-19^^England|Human|Lighthouse Lab in Glasgow|Wellcome Sanger Institute for the COVID-19 Genomics UK (COG-UK) consortium|Davies|United Kingdom
>Spike|hCoV-19/England/ALDP-B5CF85/2020|2020-11-09|EPI_ISL_655656|Original|hCoV-19^^England|Human|Lighthouse Lab in Alderley Park|Wellcome Sanger Institute for the COVID-19 Genomics UK (COG-UK) Consortium|Davies|United Kingdom
>Spike|hCoV-19/Wales/PHWC-4953F4/2020|2020-11-21|EPI_ISL_726342|Original|hCoV-19^^Wales|Human|Originating lab|COVID-19 Genomics UK (COG-UK) Consortium|Moore|United Kingdom
>Spike|hCoV-19/Mexico/MIC-InDRE-73/2020|2020-08-20|EPI_ISL_576276|Original|hCoV-19^^Michoacan|Human|Instituto de Diagnostico y Referencia Epidemiologicos (INDRE)|Instituto de Diagnostico y Referencia Epidemiologicos (INDRE)|Rodriguez|Mexico
>Spike|hCoV-19/USA/GA-CDC-7327/2020|2020-10-22|EPI_ISL_681776|Original|hCoV-19^^Georgia|Human|GA Department of Public Health Laboratory|Pathogen Discovery|Queen|USA

>Spike|hCoV-19/USA/TX-HMH-3738/2020|2020-06-16|EPI_ISL_543150|Original|hCoV-19^^Texas|Human|Houston Methodist Hospital|Houston Methodist Hospital|Long|USA
>Spike|hCoV-19/Switzerland/BS-42418442/2020|2020-08-31|EPI_ISL_581996|Original|hCoV-19^^Basel-Stadt|Human|University Hospital Basel|University Hospital Basel|Roloff Handschin|Switzerland
>Spike|hCoV-19/England/ALDP-B271F6/2020|2020-11-04|EPI_ISL_664561|Original|hCoV-19^^England|Human|Northumbria University / South Tees Hospitals NHS Foundation Trust / North Cumbria Integrated Care NHS Foundation Trust / North Tees and Hartlepool NHS Foundation Trust / Newcastle Hospitals NHS Foundation Trust|COVID-19 Genomics UK (COG-UK) Consortium|Bashton|United Kingdom
>Spike|hCoV-19/United Arab Emirates/0902/2020|2020-06-25|EPI_ISL_699040|Original|hCoV-19^^Abu Dhabi|Human|Group 42 (G42) Healthcare|G42 Healthcare|Ogrodzki|United Arab Emirates
>Spike|hCoV-19/Australia/VIC2953/2020|2020-07-07|EPI_ISL_521783|Original|hCoV-19^^Victoria|Human|Microbiological Diagnostic Unit - Public Health Laboratory (MDU-PHL)|MDU-PHL|Schultz|Australia
>Spike|hCoV-19/Switzerland/SO-ETHZ-360303/2020|2020-11-06|EPI_ISL_729221|Original|hCoV-19^^Solothurn|Human|Viollier AG|Department of Biosystems Science and Engineering|Nadeau|Switzerland
>Spike|hCoV-19/Netherlands/ZH-RIVM-20683/2020|2020-12-23|EPI_ISL_790751|Original|hCoV-19^^Zuid-holland|Human|Dutch COVID-19 response team|National Institute for Public Health and the Environment (RIVM)|Meijer|Netherlands
>Spike|hCoV-19/New Zealand/20VR2647/2020|2020-00-00|EPI_ISL_579315|Original|hCoV-19^^Auckland|Human|LabPLUS|Institute of Environmental Science and Research (ESR)|Wang|New Zealand
>Spike|hCoV-19/Scotland/CVR2748/2020|2020-04-18|EPI_ISL_438946|Original|hCoV-19^^Scotland|Human|West of Scotland Specialist Virology Centre|COVID-19 Genomics UK (COG-UK) Consortium|Robertson|United Kingdom
>Spike|hCoV-19/Japan/PG-1004/2020|2020-04-00|EPI_ISL_684614|Original|hCoV-19^^Japan|Human|Pathogen Genomics Center|Pathogen Genomics Center|Sekizuka|Japan
>Spike|hCoV-19/England/LOND-12650C8/2020|2020-11-26|EPI_ISL_724584|Original|hCoV-19^^England|Human|University College London|COVID-19 Genomics UK (COG-UK) Consortium|Roy|United Kingdom
>Spike|hCoV-19/England/BRIS-1853847/2020|2020-04-14|EPI_ISL_492398|Original|hCoV-19^^England|Human|PHE South West Regional Laboratory|Wellcome Sanger Institute for the COVID-19 Genomics UK (COG-UK) consortium|Davies|United Kingdom
>Spike|hCoV-19/England/ALDP-B1A2B7/2020|2020-11-01|EPI_ISL_645967|Original|hCoV-19^^England|Human|Lighthouse Lab in Alderley Park|Wellcome Sanger Institute for the COVID-19 Genomics UK (COG-UK) Consortium|Davies|United Kingdom
>Spike|hCoV-19/Denmark/DCGC-2032/2020|2020-07-27|EPI_ISL_618253|Original|hCoV-19^^Midtjylland|Human|Department of Virus and Microbiological Special Diagnostics|Albertsen lab|Michaelsen|Denmark
>Spike|hCoV-19/USA/MI-MDHHS-SC21148/2020|2020-03-26|EPI_ISL_507924|Original|hCoV-19^^Michigan|Human|Michigan Department of Health and Human Services|Michigan Department of Health and Human Services|Blankenship|USA
>Spike|hCoV-19/Northern Ireland/NIRE-23358B/2020|2020-09-09|EPI_ISL_680300|Original|hCoV-19^^Northern Ireland|Human|Regional Virus Laboratory|COVID-19 Genomics UK (COG-UK) Consortium|Fairley|United Kingdom
>Spike|hCoV-19/Australia/VIC1849/2020|2020-03-24|EPI_ISL_480560|Original|hCoV-19^^Victoria|Human|Microbiological Diagnostic Unit - Public Health Laboratory (MDU-PHL)|MDU-PHL|Schultz|Australia
>Spike|hCoV-19/USA/AZ-TG351918/2020|2020-05-24|EPI_ISL_694405|Original|hCoV-19^^Arizona|Human|AZ SPHL|TGen North|French|USA
>Spike|hCoV-19/Canada/ON_MU-S401/2020|2020-03-30|EPI_ISL_463985|Original|hCoV-19^^Ontario|Human|Toronto Invasive Bacterial Diseases Network|McMaster University|McArthur|Canada
>Spike|hCoV-19/England/ALDP-4A7D88/2020|2020-06-11|EPI_ISL_559645|Original|hCoV-19^^England|Human|Lighthouse Lab in Alderley Park|Wellcome Sanger Institute for the COVID-19 Genomics UK (COG-UK) consortium|Davies|United Kingdom

>Spike|hCoV-19/England/MILK-ACF7C2/2020|2020-10-22|EPI_ISL_629461|Original|hCoV-19^^England|Human|Lighthouse Lab in Milton Keynes|Wellcome Sanger Institute for the COVID-19 Genomics UK (COG-UK) consortium|Davies|United Kingdom
>Spike|hCoV-19/England/ALDP-932734/2020|2020-06-20|EPI_ISL_551362|Original|hCoV-19^^England|Human|Lighthouse Lab in Alderley Park|Wellcome Sanger Institute for the COVID-19 Genomics UK (COG-UK) consortium|Davies|United Kingdom
>Spike|hCoV-19/SaudiArabia/KAUST-JEDDAH1007/2020|2020-06-17|EPI_ISL_512878|Original|hCoV-19^^Jeddah|Human|Pathogen Genomics Lab King Abdullah University of Science and Technology(KAUST)|Pathogen Genomics Lab King Abdullah University of Science and Technology(KAUST)|Mohamed Ghazzali|Saudi Arabia
>Spike|hCoV-19/Senegal/6430/2020|2020-04-11|EPI_ISL_486872|Original|hCoV-19^^Dakar|Human|Institut Pasteur Dakar|Institut Pasteur de Dakar|Dia|Senegal
>Spike|hCoV-19/USA/FL-BPHL-1356/2020|2020-07-17|EPI_ISL_541293|Original|hCoV-19^^Florida|Human|Florida Bureau of Public Health Laboratories|Florida Bureau of Public Health Laboratories|Blanton|USA
>Spike|hCoV-19/England/201160010/2020|2020-03-11|EPI_ISL_464776|Original|hCoV-19^^England|Human|Respiratory Virus Unit|Respiratory Virus Unit|Platt|United Kingdom
>Spike|hCoV-19/USA/AZ-TG664248/2020|2020-04-28|EPI_ISL_696051|Original|hCoV-19^^Arizona|Human|Sonora Quest Laboratories|TGen North|French|USA
>Spike|hCoV-19/USA/WI-UW-1269/2020|2020-08-25|EPI_ISL_536641|Original|hCoV-19^^Wisconsin|Human|University of Wisconsin-Madison AIDS Vaccine Research Laboratories|University of Wisconsin-Madison AIDS Vaccine Research Laboratories|Baczenas|USA
>Spike|hCoV-19/USA/WI-UW-548/2020|2020-06-22|EPI_ISL_484923|Original|hCoV-19^^Wisconsin|Human|University of Wisconsin-Madison AIDS Vaccine Research Laboratories|University of Wisconsin-Madison AIDS Vaccine Research Laboratories|Baczenas|USA
>Spike|hCoV-19/USA/TX-DSHS-1677/2020|2020-06-24|EPI_ISL_710046|Original|hCoV-19^^Texas|Human|Texas Department of State Health Services|Texas Department of State Health Services|Rahman|USA
>Spike|hCoV-19/Denmark/DCGC-10970/2020|2020-11-02|EPI_ISL_670316|Original|hCoV-19^^Syddanmark|Human|Department of Virus and Microbiological Special Diagnostics|Albertsen Lab|Michaelsen|Denmark
>Spike|hCoV-19/USA/LA-EVTL1436/2020|2020-12-15|EPI_ISL_779102|Original|hCoV-19^^Louisiana|Human|LSUHS Emerging Viral Threat Laboratory|Microbial Genome Sequencing Center|Kamil|USA
>Spike|hCoV-19/USA/CA-CZB-3766/2020|2020-06-28|EPI_ISL_548455|Original|hCoV-19^^California|Human|Ventura County Public Health Lab|Chan-Zuckerberg Biohub|Bhatt|USA
>Spike|hCoV-19/Australia/VIC7260/2020|2020-07-23|EPI_ISL_564319|Original|hCoV-19^^Victoria|Human|Victorian Infectious Diseases Reference Laboratory (VIDRL)|VIDRL and MDU-PHL|Schultz|Australia
>Spike|hCoV-19/England/QEUH-9614EA/2020|2020-08-03|EPI_ISL_537018|Original|hCoV-19^^England|Human|Lighthouse Lab in Glasgow|Wellcome Sanger Institute for the COVID-19 Genomics UK (COG-UK) consortium|Davies|United Kingdom
>Spike|hCoV-19/England/MILK-629C52/2020|2020-07-05|EPI_ISL_557828|Original|hCoV-19^^England|Human|Lighthouse Lab in Milton Keynes|Wellcome Sanger Institute for the COVID-19 Genomics UK (COG-UK) consortium|Davies|United Kingdom
>Spike|hCoV-19/SaudiArabia/KAUST-MADINAH857/2020|2020-05-26|EPI_ISL_513193|Original|hCoV-19^^Madinah|Human|Pathogen Genomics Lab King Abdullah University of Science and Technology(KAUST)|Pathogen Genomics Lab King Abdullah University of Science and Technology(KAUST)|Mohamed Ghazzali|Saudi Arabia
>Spike|hCoV-19/England/ALDP-9E974B/2020|2020-09-24|EPI_ISL_584243|Original|hCoV-19^^England|Human|Virology Department|COVID-19 Genomics UK (COG-UK) Consortium|de Silva|United Kingdom
>Spike|hCoV-19/England/QEUH-9B929E/2020|2020-09-05|EPI_ISL_549806|Original|hCoV-19^^England|Human|Lighthouse Lab in Glasgow|Wellcome Sanger Institute for the COVID-19 Genomics UK (COG-UK) consortium|Davies|United Kingdom

>Spike|hCoV-19/England/ALDP-9E57D6/2020|2020-09-24|EPI_ISL_589429|Original|hCoV-19^^England|Human|Lighthouse Lab in Alderley Park|Wellcome Sanger Institute for the COVID-19 Genomics UK (COG-UK) consortium|Davies|United Kingdom
>Spike|hCoV-19/USA/TX-HMH-4318/2020|2020-06-18|EPI_ISL_543527|Original|hCoV-19^^Texas|Human|Houston Methodist Hospital|Houston Methodist Hospital|Long|USA
>Spike|hCoV-19/Scotland/CVR2964/2020|2020-04-22|EPI_ISL_478051|Original|hCoV-19^^Scotland|Human|West of Scotland Specialist Virology Centre|COVID-19 Genomics UK (COG-UK) Consortium|Robertson|United Kingdom
>Spike|hCoV-19/Japan/PG-3300/2020|2020-04-00|EPI_ISL_691415|Original|hCoV-19^^Japan|Human|Pathogen Genomics Center|Pathogen Genomics Center|Sekizuka|Japan
>Spike|hCoV-19/USA/TX-HMH-MCoV-19800/2020|2020-11-26|EPI_ISL_783562|Original|hCoV-19^^Texas|Human|Houston Methodist Hospital|Houston Methodist Hospital|Long|USA
>Spike|hCoV-19/Wales/PHWC-4A60AE/2020|2020-12-16|EPI_ISL_764996|Original|hCoV-19^^Wales|Human|Wales Specialist Virology Centre Sequencing lab|COVID-19 Genomics UK (COG-UK) Consortium|Moore|United Kingdom
>Spike|hCoV-19/USA/TX-HMH-MCoV-9454/2020|2020-07-03|EPI_ISL_780561|Original|hCoV-19^^Texas|Human|Houston Methodist Hospital|Houston Methodist Hospital|Long|USA
>Spike|hCoV-19/England/QEUH-AD2902/2020|2020-10-25|EPI_ISL_634214|Original|hCoV-19^^England|Human|Lighthouse Lab in Glasgow|Wellcome Sanger Institute for the COVID-19 Genomics UK (COG-UK) consortium|Davies|United Kingdom
>Spike|hCoV-19/England/NOTT-11CFF4/2020|2020-11-20|EPI_ISL_665913|Original|hCoV-19^^England|Human|Queens Medical Centre|COVID-19 Genomics UK (COG-UK) Consortium|Loose|United Kingdom
>Spike|hCoV-19/England/NORT-29ADC6/2020|2020-05-25|EPI_ISL_492493|Original|hCoV-19^^England|Human|NU-OMICS DNA Sequencing research facility|Wellcome Sanger Institute for the COVID-19 Genomics UK (COG-UK) consortium|Davies|United Kingdom
>Spike|hCoV-19/USA/TX-HMH-7860/2020|2020-06-30|EPI_ISL_545669|Original|hCoV-19^^Texas|Human|Houston Methodist Hospital|Houston Methodist Hospital|Long|USA
>Spike|hCoV-19/USA/FL-BPHL-0775/2020|2020-06-29|EPI_ISL_512579|Original|hCoV-19^^Florida|Human|Florida Bureau of Public Health Laboratories|Florida Bureau of Public Health Laboratories|Blanton|USA
>Spike|hCoV-19/Scotland/QEUH-9F6471/2020|2020-09-25|EPI_ISL_600864|Original|hCoV-19^^Scotland|Human|Lighthouse Lab in Glasgow|Wellcome Sanger Institute for the COVID-19 Genomics UK (COG-UK) consortium|Davies|United Kingdom
>Spike|hCoV-19/England/202521344/2020|2020-06-13|EPI_ISL_566046|Original|hCoV-19^^England|Human|Respiratory Virus Unit|Respiratory Virus Unit|Platt|United Kingdom
>Spike|hCoV-19/USA/MI-QDX-560/2020|2020-03-17|EPI_ISL_571316|Original|hCoV-19^^Michigan|Human|Quest Diagnostics|Quest Diagnostics|Kagan|USA
>Spike|hCoV-19/USA/WA-UW-862/2020|2020-03-14|EPI_ISL_476937|Original|hCoV-19^^Washington|Human|UW Virology Lab|UW Virology Lab|Roychoudhury|USA
>Spike|hCoV-19/England/ALDP-9CB105/2020|2020-09-16|EPI_ISL_575602|Original|hCoV-19^^England|Human|Lighthouse Lab in Alderley Park|Wellcome Sanger Institute for the COVID-19 Genomics UK (COG-UK) consortium|Davies|United Kingdom
>Spike|hCoV-19/Denmark/DCGC-21667/2020|2020-12-21|EPI_ISL_758297|Original|hCoV-19^^Hovedstaden|Human|Department of Virus and Microbiological Special Diagnostics|Albertsen Lab|Michaelsen|Denmark
>Spike|hCoV-19/England/CAMC-B07280/2020|2020-10-29|EPI_ISL_643974|Original|hCoV-19^^England|Human|Lighthouse Lab in Cambridge|Wellcome Sanger Institute for the COVID-19 Genomics UK (COG-UK) Consortium|Davies|United Kingdom
>Spike|hCoV-19/England/CAMC-990F78/2020|2020-08-28|EPI_ISL_551886|Original|hCoV-19^^England|Human|Lighthouse Lab in Cambridge|Wellcome Sanger Institute for the COVID-19 Genomics UK (COG-UK) consortium|Davies|United Kingdom
>Spike|hCoV-19/USA/WA-S1554/2020|2020-05-26|EPI_ISL_495980|Original|hCoV-19^^Washington|Human|Washington State Department of Health|Seattle Flu Study|Lee|USA
>Spike|hCoV-19/Iceland/516/2020|2020-03-24|EPI_ISL_424538|Original|hCoV-19^^Reykjavik|Human|deCODE genetics|deCODE genetics|J\u00f3nsson|Iceland
>Spike|hCoV-19/Morocco/6895/2020|2020-03-20|EPI_ISL_459973|Original|hCoV-19^^Morocco|Human|Institut Pasteur du Maroc|Institut Pasteur du Maroc|Enouf|Morocco

>Spike|hCoV-19/USA/MI-MDHHS-SC22679/2020|2020-11-26|EPI_ISL_737293|Original|hCoV-19^^Michigan|Human|Michigan Department of Health and Human Services|Michigan Department of Health and Human Services|Blankenship|USA
>Spike|hCoV-19/Australia/VIC6890/2020|2020-07-11|EPI_ISL_564117|Original|hCoV-19^^Victoria|Human|Microbiological Diagnostic Unit - Public Health Laboratory (MDU-PHL)|MDU-PHL|Schultz|Australia
>Spike|hCoV-19/USA/TX-HMH-2760/2020|2020-06-04|EPI_ISL_545534|Original|hCoV-19^^Texas|Human|Houston Methodist Hospital|Houston Methodist Hospital|Long|USA
>Spike|hCoV-19/England/QEUH-C940E7/2020|2020-12-18|EPI_ISL_777436|Original|hCoV-19^^England|Human|Lighthouse Lab in Glasgow|Wellcome Sanger Institute for the COVID-19 Genomics UK (COG-UK) Consortium|Davies|United Kingdom
>Spike|hCoV-19/England/MILK-A8EEF7/2020|2020-10-16|EPI_ISL_650968|Original|hCoV-19^^England|Human|Northumbria University / South Tees Hospitals NHS Foundation Trust / North Cumbria Integrated Care NHS Foundation Trust / North Tees and Hartlepool NHS Foundation Trust / Newcastle Hospitals NHS Foundation Trust|COVID-19 Genomics UK (COG-UK) Consortium|Bashton|United Kingdom
>Spike|hCoV-19/USA/TX-HMH019/2020|2020-03-17|EPI_ISL_434729|Original|hCoV-19^^Texas|Human|Houston Methodist Hospital|Houston Methodist Hospital|Long|USA
>Spike|hCoV-19/England/SHEF-10B234D/2020|2020-04-13|EPI_ISL_469918|Original|hCoV-19^^England|Human|Virology Department|Wellcome Sanger Institute for the COVID-19 Genomics UK (COG-UK) consortium|Davies|United Kingdom
>Spike|hCoV-19/England/CAMB-1BBA9C/2020|2020-11-13|EPI_ISL_679186|Original|hCoV-19^^England|Human|Department of Pathology|COVID-19 Genomics UK (COG-UK) Consortium|Goodfellow|United Kingdom
>Spike|hCoV-19/England/20139021304/2020|2020-03-26|EPI_ISL_421881|Original|hCoV-19^^England|Human|Respiratory Virus Unit|Respiratory Virus Unit|Platt|United Kingdom
>Spike|hCoV-19/England/MILK-A44F90/2020|2020-10-06|EPI_ISL_598571|Original|hCoV-19^^England|Human|Lighthouse Lab in Milton Keynes|Wellcome Sanger Institute for the COVID-19 Genomics UK (COG-UK) consortium|Davies|United Kingdom
>Spike|hCoV-19/Scotland/EDB2767/2020|2020-03-29|EPI_ISL_488905|Original|hCoV-19^^Scotland|Human|Virology Department|Wellcome Sanger Institute for the COVID-19 Genomics UK (COG-UK) consortium|Davies|United Kingdom
>Spike|hCoV-19/Australia/VIC5817/2020|2020-07-20|EPI_ISL_518611|Original|hCoV-19^^Victoria|Human|Microbiological Diagnostic Unit - Public Health Laboratory (MDU-PHL)|MDU-PHL|Schultz|Australia
>Spike|hCoV-19/England/QEUH-C14A0F/2020|2020-12-06|EPI_ISL_709439|Original|hCoV-19^^England|Human|Lighthouse Lab in Glasgow|Wellcome Sanger Institute for the COVID-19 Genomics UK (COG-UK) Consortium|Davies|United Kingdom
>Spike|hCoV-19/England/LIVE-A8680/2020|2020-03-26|EPI_ISL_517197|Original|hCoV-19^^England|Human|Liverpool Clinical Laboratories|COVID-19 Genomics UK (COG-UK) Consortium|Haldenby|United Kingdom
>Spike|hCoV-19/England/BRIS-132F61/2020|2020-06-20|EPI_ISL_511980|Original|hCoV-19^^England|Human|PHE South West Regional Laboratory|Wellcome Sanger Institute for the COVID-19 Genomics UK (COG-UK) consortium|Davies|United Kingdom
>Spike|hCoV-19/Israel/CVL-57-ngs/2020|2020-12-24|EPI_ISL_776571|Original|hCoV-19^^Israel|Human|Israel Central Virology laboratory|Israel Central Virology laboratory|Zuckerman|Israel
>Spike|hCoV-19/India/MH-NCCS-NR-7614/2020|2020-06-26|EPI_ISL_721661|Original|hCoV-19^^Maharashtra|Human|National Centre For Cell Science|National Centre For Cell Science|Paul|India
>Spike|hCoV-19/England/ALDP-9CABF9/2020|2020-09-16|EPI_ISL_575744|Original|hCoV-19^^England|Human|Lighthouse Lab in Alderley Park|Wellcome Sanger Institute for the COVID-19 Genomics UK (COG-UK) consortium|Davies|United Kingdom
>Spike|hCoV-19/USA/TX-HMH-5804/2020|2020-06-27|EPI_ISL_547026|Original|hCoV-19^^Texas|Human|Houston Methodist Hospital|Houston Methodist Hospital|Long|USA
>Spike|hCoV-19/USA/WI-WSLH-200315/2020|2020-04-20|EPI_ISL_578496|Original|hCoV-19^^Wisconsin|Human|Wisconsin State Laboratory of Hygiene Communicable Disease Division|Wisconsin State Laboratory of Hygiene Communicable Disease Division|Florek|USA

>Spike|hCoV-19/Denmark/DCGC-1941/2020|2020-03-30|EPI_ISL_622506|Original|hCoV-19^^Hovedstaden|Human|Department of Virus and Microbiological Special Diagnostics|Albertsen lab|Michaelsen|Denmark
>Spike|hCoV-19/Russia/SPE-RII-16011V/2020|2020-05-19|EPI_ISL_524011|Vero P1|hCoV-19^^Saint Petersburg|Human|WHO National Influenza Centre Russian Federation|WHO National Influenza Centre Russian Federation|Komissarov|Russia
>Spike|hCoV-19/USA/TX-HMH-2682/2020|2020-06-05|EPI_ISL_545472|Original|hCoV-19^^Texas|Human|Houston Methodist Hospital|Houston Methodist Hospital|Long|USA
>Spike|hCoV-19/Australia/VIC5235/2020|2020-07-17|EPI_ISL_520000|Original|hCoV-19^^Victoria|Human|Microbiological Diagnostic Unit - Public Health Laboratory (MDU-PHL)|MDU-PHL|Schultz|Australia
>Spike|hCoV-19/England/SHEF-C6F27/2020|2020-04-29|EPI_ISL_475396|Original|hCoV-19^^England|Human|Virology Department|COVID-19 Genomics UK (COG-UK) Consortium|de Silva|United Kingdom
>Spike|hCoV-19/United Arab Emirates/skmc-004/2020|2020-02-21|EPI_ISL_582613|Original|hCoV-19^^United Arab Emirates|Human|Sheikh Khalifa Medical City|Molecular/Surveillance lab\nSheikh Khalifa Medical City|Amirtharaj|United Arab Emirates
>Spike|hCoV-19/USA/AR-QDX-679/2020|2020-03-18|EPI_ISL_571408|Original|hCoV-19^^Arkansas|Human|Quest Diagnostics|Quest Diagnostics|Kagan|USA
>Spike|hCoV-19/USA/CO-CSU-D1657-3/2020|2020-04-15|EPI_ISL_527444|Original|hCoV-19^^Colorado|Human|Colorado State University - Ebel Lab|Colorado State University - Ebel Lab|Cline|USA
>Spike|hCoV-19/Denmark/DCGC-18049/2020|2020-12-14|EPI_ISL_749724|Original|hCoV-19^^Midtjylland|Human|Department of Virus and Microbiological Special Diagnostics|Albertsen Lab|Michaelsen|Denmark
>Spike|hCoV-19/Sweden/20-08081/2020|2020-03-05|EPI_ISL_454455|Original|hCoV-19^^Stockholm|Human|Karolinska Universitetslaboratoriet|The Public Health Agency of Sweden|Svartstr\u00f6m|Sweden
>Spike|hCoV-19/England/QEUH-B7E4F7/2020|2020-11-14|EPI_ISL_667904|Original|hCoV-19^^England|Human|Lighthouse Lab in Glasgow|Wellcome Sanger Institute for the COVID-19 Genomics UK (COG-UK) Consortium|Davies|United Kingdom
>Spike|hCoV-19/USA/MA-MASPHL-00176/2020|2020-04-03|EPI_ISL_791637|Original|hCoV-19^^Massachusetts|Human|Massachusetts State Public Health Laboratory|Infectious Disease Program|Park|USA
>Spike|hCoV-19/Switzerland/VD-ETHZ-310505/2020|2020-10-08|EPI_ISL_603573|Original|hCoV-19^^Vaud|Human|Viollier AG|Department of Biosystems Science and Engineering|Nadeau|Switzerland
>Spike|hCoV-19/England/CAMC-C448C0/2020|2020-12-10|EPI_ISL_719426|Original|hCoV-19^^England|Human|Lighthouse Lab in Cambridge|Wellcome Sanger Institute for the COVID-19 Genomics UK (COG-UK) Consortium|Davies|United Kingdom
>Spike|hCoV-19/England/BIRM-5F837/2020|2020-05-13|EPI_ISL_473338|Original|hCoV-19^^England|Human|University of Birmingham|COVID-19 Genomics UK (COG-UK) Consortium|Loman|United Kingdom
>Spike|hCoV-19/Wales/PHWC-481F33/2020|2020-10-16|EPI_ISL_613187|Original|hCoV-19^^Wales|Human|Wales Specialist Virology Centre Sequencing lab|COVID-19 Genomics UK (COG-UK) Consortium|Moore|United Kingdom
>Spike|hCoV-19/USA/WI-UW-2668/2020|2020-12-31|EPI_ISL_791181|Original|hCoV-19^^Wisconsin|Human|University of Wisconsin-Madison AIDS Vaccine Research Laboratories|University of Wisconsin-Madison AIDS Vaccine Research Laboratories|Moreno|USA
>Spike|hCoV-19/Scotland/CVR3315/2020|2020-05-05|EPI_ISL_493868|Original|hCoV-19^^Scotland|Human|West of Scotland Specialist Virology Centre|COVID-19 Genomics UK (COG-UK) Consortium|Robertson|United Kingdom
>Spike|hCoV-19/Australia/SAP217/2020|2020-03-03|EPI_ISL_483073|Original|hCoV-19^^South Australia|Human|SA Pathology|SA Pathology|Leong|Australia
>Spike|hCoV-19/England/ALDP-B242F6/2020|2020-11-04|EPI_ISL_647027|Original|hCoV-19^^England|Human|Lighthouse Lab in Alderley Park|Wellcome Sanger Institute for the COVID-19 Genomics UK (COG-UK) Consortium|Davies|United Kingdom

>Spike|hCoV-19/Japan/PG-7159/2020|2020-09-00|EPI_ISL_688264|Original|hCoV-19^^Japan|Human|Pathogen Genomics Center|Pathogen Genomics Center|Sekizuka|Japan
>Spike|hCoV-19/USA/WA-S190/2020|2020-03-23|EPI_ISL_430228|Original|hCoV-19^^Washington|Human|Washington State Department of Health|Seattle Flu Study|Bedford|USA
>Spike|hCoV-19/England/MILK-B969AF/2020|2020-11-13|EPI_ISL_676310|Original|hCoV-19^^England|Human|Lighthouse Lab in Milton Keynes|Wellcome Sanger Institute for the COVID-19 Genomics UK (COG-UK) Consortium|Davies|United Kingdom
>Spike|hCoV-19/Japan/PG-7379/2020|2020-07-00|EPI_ISL_688481|Original|hCoV-19^^Japan|Human|Pathogen Genomics Center|Pathogen Genomics Center|Sekizuka|Japan
>Spike|hCoV-19/Belgium/UGent-220/2020|2020-10-19|EPI_ISL_717619|Original|hCoV-19^^Ghent|Human|Lab voor klinische biologie|Onderzoeksgroep Virologie|Lambrechts|Belgium
>Spike|hCoV-19/England/SHEF-C9470/2020|2020-05-27|EPI_ISL_475420|Original|hCoV-19^^England|Human|Virology Department|COVID-19 Genomics UK (COG-UK) Consortium|de Silva|United Kingdom
>Spike|hCoV-19/USA/CA-ALSR-0839-IPL/2020|2020-04-21|EPI_ISL_483393|Original|hCoV-19^^California|Human|UC San Diego Center for Advanced Laboratory Medicine|Andersen lab at Scripps Research|Gangavarapu|USA
>Spike|hCoV-19/England/210100383/2021|2021-01-04|EPI_ISL_791261|Original|hCoV-19^^England|Human|Respiratory Virus Unit|COVID-19 Genomics UK (COG-UK) Consortium|Platt|United Kingdom
>Spike|hCoV-19/England/MILK-BEE36E/2020|2020-11-30|EPI_ISL_708984|Original|hCoV-19^^England|Human|Lighthouse Lab in Milton Keynes|Wellcome Sanger Institute for the COVID-19 Genomics UK (COG-UK) Consortium|Davies|United Kingdom
>Spike|hCoV-19/Japan/PG-7079/2020|2020-08-00|EPI_ISL_688184|Original|hCoV-19^^Japan|Human|Pathogen Genomics Center|Pathogen Genomics Center|Sekizuka|Japan
>Spike|hCoV-19/USA/CA-LACPHL-AB00001/2020|2020-09-29|EPI_ISL_694046|Original|hCoV-19^^California|Human|Los Angeles County Public Health Laboratory|Los Angeles County Public Health Laboratory|Hemarajata|USA
>Spike|hCoV-19/England/ALDP-A6D41A/2020|2020-10-15|EPI_ISL_608104|Original|hCoV-19^^England|Human|Lighthouse Lab in Alderley Park|Wellcome Sanger Institute for the COVID-19 Genomics UK (COG-UK) consortium|Davies|United Kingdom
>Spike|hCoV-19/France/PAC-IHU-0397/2020|2020-03-29|EPI_ISL_569488|Original|hCoV-19^^Provence-Alpes-C\u00f4te-d\u2019Azur|Human|MEPHI|MEPHI|Levasseur|France
>Spike|hCoV-19/USA/TX-HMH-6687/2020|2020-06-24|EPI_ISL_546174|Original|hCoV-19^^Texas|Human|Houston Methodist Hospital|Houston Methodist Hospital|Long|USA
>Spike|hCoV-19/Spain/MD-IBV-006423/2020|2020-04-11|EPI_ISL_510138|Original|hCoV-19^^Madrid|Human|Hospital General Universitario Gregorio Mara\u00f1\u00f3n|SeqCOVID-SPAIN consortium/IBV(CSIC)|Comas|Spain
>Spike|hCoV-19/Denmark/DCGC-1314/2020|2020-06-08|EPI_ISL_618048|Original|hCoV-19^^Syddanmark|Human|Department of Virus and Microbiological Special Diagnostics|Albertsen lab|Michaelsen|Denmark
>Spike|hCoV-19/USA/LA-EVTL321/2020|2020-05-18|EPI_ISL_578698|Original|hCoV-19^^Louisiana|Human|LSUHS Emerging Viral Threat Laboratory|Microbial Genome Sequencing Center|Kamil|USA
>Spike|hCoV-19/USA/TX-HMH-MCoV-19708/2020|2020-11-18|EPI_ISL_784060|Original|hCoV-19^^Texas|Human|Houston Methodist Hospital|Houston Methodist Hospital|Long|USA
>Spike|hCoV-19/England/NORW-ECBEB/2020|2020-06-03|EPI_ISL_472339|Original|hCoV-19^^England|Human|Quadram Institute Bioscience|COVID-19 Genomics UK (COG-UK) Consortium|Alikhan|United Kingdom
>Spike|hCoV-19/Spain/PV-IBV-004194/2020|2020-04-02|EPI_ISL_510480|Original|hCoV-19^^Basque Country|Human|Servicio de Microbiolog\u00eda. Hospital Universitario Donostia. OSI Donostialdea. \u00c1rea de Enfermedades Infecciosas|SeqCOVID-SPAIN consortium/IBV(CSIC)|Comas|Spain
>Spike|hCoV-19/Denmark/DCGC-3489/2020|2020-08-31|EPI_ISL_616277|Original|hCoV-19^^Hovedstaden|Human|Department of Virus and Microbiological Special Diagnostics|Albertsen lab|Michaelsen|Denmark

>Spike|hCoV-19/Iceland/36/2020|2020-03-04|EPI_ISL_417844|Original|hCoV-19^^Reykjavik|Human|The National University Hospital of Iceland|deCODE genetics|J\u00f3nsson|Iceland
>Spike|hCoV-19/England/QEUH-B66638/2020|2020-11-11|EPI_ISL_661618|Original|hCoV-19^^England|Human|Lighthouse Lab in Glasgow|Wellcome Sanger Institute for the COVID-19 Genomics UK (COG-UK) Consortium|Davies|United Kingdom
>Spike|hCoV-19/England/CAMB-761D5/2020|2020-03-30|EPI_ISL_438452|Original|hCoV-19^^England|Human|Department of Pathology|Wellcome Sanger Institute for the COVID-19 Genomics UK (COG-UK) consortium|Amato|United Kingdom
>Spike|hCoV-19/Portugal/PT0880/2020|2020-04-24|EPI_ISL_511139|Original|hCoV-19^^Portugal|Human|Instituto Nacional de Saude (INSA)|Instituto Nacional de Saude (INSA)|Borges|Portugal
>Spike|hCoV-19/USA/HI-H200467/2020|2020-10-09|EPI_ISL_753010|Original|hCoV-19^^Hawaii|Human|State Laboratories Division|State Laboratories Division|Sultana|USA
>Spike|hCoV-19/Switzerland/AG-ETHZ-380337/2020|2020-11-26|EPI_ISL_721732|Original|hCoV-19^^Aargau|Human|Viollier AG|Department of Biosystems Science and Engineering|Nadeau|Switzerland
>Spike|hCoV-19/England/OXON-AEF8F/2020|2020-04-08|EPI_ISL_448799|Original|hCoV-19^^England|Human|Oxford Viromics|COVID-19 Genomics UK (COG-UK) Consortium|Golubchik|United Kingdom
>Spike|hCoV-19/Australia/VIC5967/2020|2020-00-00|EPI_ISL_518742|Original|hCoV-19^^Victoria|Human|Microbiological Diagnostic Unit - Public Health Laboratory (MDU-PHL)|MDU-PHL|Schultz|Australia
>Spike|hCoV-19/Taiwan/170/2020|2020-03-21|EPI_ISL_420084|Original|hCoV-19^^Taiwan|Human|Centers for Disease Control|Centers for Disease Control|Yang|Taiwan
>Spike|hCoV-19/USA/NM-DOH-2020327831/2020|2020-09-03|EPI_ISL_575312|Original|hCoV-19^^New Mexico|Human|Molecular Biology|Molecular Biology|Johnson|USA
>Spike|hCoV-19/Luxembourg/LNS3537918/2020|2020-11-30|EPI_ISL_770930|Original|hCoV-19^^Luxembourg|Human|Laboratoire national de sant\u00e9|Laboratoire national de sant\u00e9|Wienecke-Baldacchino|Luxembourg
>Spike|hCoV-19/Thailand/Bangkok-0079/2020|2020-04-01|EPI_ISL_447020|Original|hCoV-19^^Bangkok|Human|Ramathibodi Hospital|COVID-19 Network Investigations (CONI) Alliance|Batty|Thailand
>Spike|hCoV-19/USA/UT-01560/2020|2020-04-07|EPI_ISL_449815|Original|hCoV-19^^Utah|Human|Utah Public Health Laboratory|Utah Public Health Laboratory|Young|USA
>Spike|hCoV-19/USA/UT-02512/2020|2020-04-23|EPI_ISL_493100|Original|hCoV-19^^Utah|Human|Utah Public Health Laboratory|Utah Public Health Laboratory|Butz|USA
>Spike|hCoV-19/USA/HI-H200113/2020|2020-06-24|EPI_ISL_752676|Original|hCoV-19^^Hawaii|Human|State Laboratories Division|State Laboratories Division|Sultana|USA
>Spike|hCoV-19/Japan/PG-7001/2020|2020-09-00|EPI_ISL_688106|Original|hCoV-19^^Japan|Human|Pathogen Genomics Center|Pathogen Genomics Center|Sekizuka|Japan
>Spike|hCoV-19/England/QEUH-AAE565/2020|2020-10-22|EPI_ISL_623884|Original|hCoV-19^^England|Human|Lighthouse Lab in Glasgow|Wellcome Sanger Institute for the COVID-19 Genomics UK (COG-UK) consortium|Davies|United Kingdom
>Spike|hCoV-19/England/NORT-282B21/2020|2020-03-28|EPI_ISL_488486|Original|hCoV-19^^England|Human|NU-OMICS DNA Sequencing research facility|Wellcome Sanger Institute for the COVID-19 Genomics UK (COG-UK) consortium|Davies|United Kingdom
>Spike|hCoV-19/Estonia/ChVir4298/2020|2020-03-00|EPI_ISL_457734|Original|hCoV-19^^Estonia|Human|SYNLAB Eesti OU\u0308|Charite Universitatsmedizin Berlin|Muehlemann|Estonia
>Spike|hCoV-19/England/CAMC-B782C6/2020|2020-11-12|EPI_ISL_661928|Original|hCoV-19^^England|Human|Lighthouse Lab in Cambridge|Wellcome Sanger Institute for the COVID-19 Genomics UK (COG-UK) Consortium|Davies|United Kingdom
>Spike|hCoV-19/Brazil/RS-6177/2020|2020-05-06|EPI_ISL_729820|Original|hCoV-19^^Rio Grande do Sul|Human|Laboratorio Central de Saude Publica do Estado do Rio Grande do Sul (LACEN-RS)|Laboratory of Respiratory Viruses and Measles|Silva|Brazil
>Spike|hCoV-19/England/CAMB-84F9E/2020|2020-03-13|EPI_ISL_440464|Original|hCoV-19^^England|Human|Department of Pathology|Wellcome Sanger Institute for the COVID-19 Genomics UK (COG-UK) consortium|Amato|United Kingdom

>Spike|hCoV-19/Wales/PHWC-48B634/2020|2020-11-04|EPI_ISL_666263|Original|hCoV-19^^Wales|Human|Wales Specialist Virology Centre Sequencing lab|COVID-19 Genomics UK (COG-UK) Consortium|Moore|United Kingdom
>Spike|hCoV-19/England/CAMC-C45373/2020|2020-12-10|EPI_ISL_718418|Original|hCoV-19^^England|Human|Lighthouse Lab in Cambridge|Wellcome Sanger Institute for the COVID-19 Genomics UK (COG-UK) Consortium|Davies|United Kingdom
>Spike|hCoV-19/USA/WA-UW-11193/2020|2020-06-17|EPI_ISL_570380|Original|hCoV-19^^Washington|Human|UW Virology Lab|UW Virology Lab|Roychoudhury|USA
>Spike|hCoV-19/Denmark/DCGC-2894/2020|2020-08-17|EPI_ISL_616410|Original|hCoV-19^^Syddanmark|Human|Department of Virus and Microbiological Special Diagnostics|Albertsen lab|Michaelsen|Denmark
>Spike|hCoV-19/Wales/PHWC-29CFE/2020|2020-04-02|EPI_ISL_474246|Original|hCoV-19^^Wales|Human|Originating lab|COVID-19 Genomics UK (COG-UK) Consortium|Moore|United Kingdom
>Spike|hCoV-19/India/WB-S44/2020|2020-05-01|EPI_ISL_455665|Original|hCoV-19^^West Bengal|Human|ICMR-National Institute of Cholera and Enteric Diseases|National Institute of Biomedical Genomics|Maitra|India
>Spike|hCoV-19/Denmark/DCGC-19801/2020|2020-12-07|EPI_ISL_747797|Original|hCoV-19^^Hovedstaden|Human|Department of Virus and Microbiological Special Diagnostics|Albertsen Lab|Michaelsen|Denmark
>Spike|hCoV-19/England/ALDP-5CCD22/2020|2020-06-19|EPI_ISL_558460|Original|hCoV-19^^England|Human|Lighthouse Lab in Alderley Park|Wellcome Sanger Institute for the COVID-19 Genomics UK (COG-UK) consortium|Davies|United Kingdom
>Spike|hCoV-19/USA/LA-EVTL639/2020|2020-08-06|EPI_ISL_579043|Original|hCoV-19^^Louisiana|Human|LSUHS Emerging Viral Threat Laboratory|Microbial Genome Sequencing Center|Kamil|USA
>Spike|hCoV-19/Australia/NSW332/2020|2020-04-11|EPI_ISL_451594|Original|hCoV-19^^New South Wales|Human|Childrens Hospital Westmead|NSW Health Pathology - Institute of Clinical Pathology and Medical Research; Westmead Hospital; University of Sydney|Gall|Australia
>Spike|hCoV-19/USA/CO-CDPHE-2100014440/2020|2020-11-20|EPI_ISL_771304|Original|hCoV-19^^Colorado|Human|Colorado Department of Public Health and Environment|Colorado Department of Puplic Health and Environment|Bankers|USA
>Spike|hCoV-19/USA/NY-Wadsworth-249773-01/2020|2020-10-20|EPI_ISL_754736|Original|hCoV-19^^New York|Human|Wadsworth Center|Wadsworth Center|Lasek-Nesselquist|USA
>Spike|hCoV-19/USA/NY-NYUMC611/2020|2020-04-01|EPI_ISL_444728|Original|hCoV-19^^New York|Human|NYU Langone Health|Departments of Pathology and Medicine|Maurano|USA
>Spike|hCoV-19/USA/LA-EVTL511/2020|2020-06-25|EPI_ISL_578915|Original|hCoV-19^^Louisiana|Human|LSUHS Emerging Viral Threat Laboratory|Microbial Genome Sequencing Center|Kamil|USA
>Spike|hCoV-19/Denmark/DCGC-17077/2020|2020-12-14|EPI_ISL_750222|Original|hCoV-19^^Hovedstaden|Human|Department of Virus and Microbiological Special Diagnostics|Albertsen Lab|Michaelsen|Denmark
>Spike|hCoV-19/Denmark/DCGC-18407/2020|2020-12-07|EPI_ISL_749947|Original|hCoV-19^^Hovedstaden|Human|Department of Virus and Microbiological Special Diagnostics|Albertsen Lab|Michaelsen|Denmark
>Spike|hCoV-19/Australia/VIC13374/2020|2020-08-11|EPI_ISL_563699|Original|hCoV-19^^Victoria|Human|Microbiological Diagnostic Unit - Public Health Laboratory (MDU-PHL)|MDU-PHL|Schultz|Australia
>Spike|hCoV-19/Denmark/ALAB-SSI-1421/2020|2020-03-26|EPI_ISL_437653|Original|hCoV-19^^Denmark|Human|Department of Virus and Microbiological Special Diagnostics|Albertsen lab|Kirkegaard|Denmark
>Spike|hCoV-19/USA/HI-H200564/2020|2020-10-24|EPI_ISL_753092|Original|hCoV-19^^Hawaii|Human|State Laboratories Division|State Laboratories Division|Sultana|USA
>Spike|hCoV-19/Japan/PG-8796/2020|2020-08-00|EPI_ISL_686384|Original|hCoV-19^^Japan|Human|Pathogen Genomics Center|Pathogen Genomics Center|Sekizuka|Japan
>Spike|hCoV-19/Spain/CT-HUVH-VH7458/2020|2020-04-06|EPI_ISL_444974|Original|hCoV-19^^Catalunya|Human|Hospital Universitari Vall d'Hebron - Vall d'Hebron Institut de Recerca|Hospital Universitari Vall d'Hebron|Anton|Spain

>Spike|hCoV-19/Australia/VIC6035/2020|2020-07-24|EPI_ISL_518076|Original|hCoV-
19^^Victoria|Human|Microbiological Diagnostic Unit - Public Health Laboratory (MDU-
PHL)|MDU-PHL|Schultz|Australia
>Spike|hCoV-19/England/ALDP-A96427/2020|2020-10-20|EPI_ISL_624724|Original|hCoV-
19^^England|Human|Lighthouse Lab in Alderley Park|Wellcome Sanger Institute for the
COVID-19 Genomics UK (COG-UK) consortium|Davies|United Kingdom
>Spike|hCoV-19/USA/NV-NSPHL-A0033/2020|2020-03-21|EPI_ISL_515319|Original|hCoV-
19^^Nevada|Human|Nevada State Public Health Laboratory|Nevada State Public Health
Laboratory|Sevinsky|USA
>Spike|hCoV-19/England/QEUH-AEFAF0/2020|2020-10-29|EPI_ISL_662699|Original|hCoV-
19^^England|Human|Lighthouse Lab in Glasgow|Wellcome Sanger Institute for the COVID-
19 Genomics UK (COG-UK) Consortium|Davies|United Kingdom
>Spike|hCoV-19/Scotland/QEUH-96310F/2020|2020-08-11|EPI_ISL_531477|Original|hCoV-
19^^Scotland|Human|NHSGGC West of Scotland Specialist Virology Centre / MRC-
University of Glasgow Centre for Virus Research|Wellcome Sanger Institute for the
COVID-19 Genomics UK (COG-UK) consortium|Davies|United Kingdom
>Spike|hCoV-19/England/CAMB-82B50/2020|2020-04-22|EPI_ISL_438663|Original|hCoV-
19^^England|Human|Department of Pathology|COVID-19 Genomics UK (COG-UK)
Consortium|Meredith|United Kingdom
>Spike|hCoV-19/England/QEUH-AE4B61/2020|2020-10-26|EPI_ISL_633171|Original|hCoV-
19^^England|Human|Lighthouse Lab in Glasgow|Wellcome Sanger Institute for the COVID-
19 Genomics UK (COG-UK) consortium|Davies|United Kingdom
>Spike|hCoV-19/USA/TX-HMH-MCoV-12830/2020|2020-08-19|EPI_ISL_790554|Original|hCoV-
19^^Texas|Human|Houston Methodist Hospital|Houston Methodist Hospital|Long|USA
>Spike|hCoV-19/England/CAMC-B0A5DF/2020|2020-10-29|EPI_ISL_673131|Original|hCoV-
19^^England|Human|Lighthouse Lab in Cambridge|Wellcome Sanger Institute for the
COVID-19 Genomics UK (COG-UK) Consortium|Davies|United Kingdom
>Spike|hCoV-19/USA/WA-UW-1603/2020|2020-03-19|EPI_ISL_423005|Original|hCoV-
19^^Washington|Human|UW Virology Lab|UW Virology Lab|Roychoudhury|USA
>Spike|hCoV-19/USA/MI-MDHHS-SC21877/2020|2020-08-21|EPI_ISL_565938|Original|hCoV-
19^^Michigan|Human|Michigan Department of Health and Human Services|Michigan
Department of Health and Human Services|Blankenship|USA
>Spike|hCoV-19/Northern Ireland/NIRE-1083D1/2020|2020-09-
09|EPI_ISL_585188|Original|hCoV-19^^Northern Ireland|Human|Regional Virus
Laboratory|COVID-19 Genomics UK (COG-UK) Consortium|Fairley|United Kingdom
>Spike|hCoV-19/England/ALDP-C3CBD1/2020|2020-12-10|EPI_ISL_731712|Original|hCoV-
19^^England|Human|Lighthouse Lab in Alderley Park|Wellcome Sanger Institute for the
COVID-19 Genomics UK (COG-UK) Consortium|Davies|United Kingdom
>Spike|hCoV-19/USA/NY-NYCPHL-001271/2020|2020-11-09|EPI_ISL_683870|Original|hCoV-
19^^New York|Human|DOHMH Corona|New York City Public Health Laboratory|Wang|USA
>Spike|hCoV-19/England/NOTT-11831A/2020|2020-10-26|EPI_ISL_627674|Original|hCoV-
19^^England|Human|Queens Medical Centre|COVID-19 Genomics UK (COG-UK)
Consortium|Loose|United Kingdom
>Spike|hCoV-19/England/ALDP-B85A55/2020|2020-11-14|EPI_ISL_675629|Original|hCoV-
19^^England|Human|Lighthouse Lab in Alderley Park|Wellcome Sanger Institute for the
COVID-19 Genomics UK (COG-UK) Consortium|Davies|United Kingdom
>Spike|hCoV-19/Switzerland/BL-65240238/2020|2020-04-06|EPI_ISL_581873|Original|hCoV-
19^^Basel-Land|Human|University Hospital Basel|University Hospital Basel|Roloff
Handschin|Switzerland
>Spike|hCoV-19/USA/CA-CZB-10825/2020|2020-07-13|EPI_ISL_583075|Original|hCoV-
19^^California|Human|Humboldt County Public Health Laboratory|Chan-Zuckerberg
Biohub|Bhatt|USA
>Spike|hCoV-19/USA/FL-BPHL-1636/2020|2020-09-11|EPI_ISL_594325|Original|hCoV-
19^^Florida|Human|Florida Bureau of Public Health Laboratories|Florida Bureau of
Public Health Laboratories|Blanton|USA
>Spike|hCoV-19/England/LIVE-9C133/2020|2020-03-31|EPI_ISL_449514|Original|hCoV-
19^^England|Human|Liverpool Clinical Laboratories|COVID-19 Genomics UK (COG-UK)
Consortium|Haldenby|United Kingdom

>Spike|hCoV-19/England/CAMC-BBE4CE/2020|2020-11-21|EPI_ISL_703575|Original|hCoV-19^^England|Human|Virology Department|COVID-19 Genomics UK (COG-UK) Consortium|de Silva|United Kingdom
>Spike|hCoV-19/England/CAMC-B32B45/2020|2020-11-05|EPI_ISL_659460|Original|hCoV-19^^England|Human|Lighthouse Lab in Cambridge|Wellcome Sanger Institute for the COVID-19 Genomics UK (COG-UK) Consortium|Davies|United Kingdom
>Spike|hCoV-19/Gambia/3673/2020|2020-07-30|EPI_ISL_560995|Original|hCoV-19^^Kombo|Human|MRCG at LSHTM Genomics lab|MRCG at LSHTM Genomics lab|Sesay|Gambia
>Spike|hCoV-19/Netherlands/ZuidHolland_85/2020|2020-03-27|EPI_ISL_422953|Original|hCoV-19^^Zuid Holland|Human|Dutch COVID-19 response team|Erasmus Medical Center|Oude Munnink|Netherlands
>Spike|hCoV-19/Wales/PHWC-163161/2020|2020-05-27|EPI_ISL_473254|Original|hCoV-19^^Wales|Human|Wales Specialist Virology Centre Sequencing lab|COVID-19 Genomics UK (COG-UK) Consortium|Moore|United Kingdom
>Spike|hCoV-19/Japan/PG-3367/2020|2020-04-00|EPI_ISL_691482|Original|hCoV-19^^Japan|Human|Pathogen Genomics Center|Pathogen Genomics Center|Sekizuka|Japan
>Spike|hCoV-19/England/QEUH-BA2FAE/2020|2020-11-18|EPI_ISL_672867|Original|hCoV-19^^England|Human|Lighthouse Lab in Glasgow|Wellcome Sanger Institute for the COVID-19 Genomics UK (COG-UK) Consortium|Davies|United Kingdom
>Spike|hCoV-19/Pakistan/JRCGR-KHI15/2020|2020-08-10|EPI_ISL_779275|Original|hCoV-19^^Karachi|Human|Jamil-ur-Rahman Center for Genome Research|Jamil-ur-Rahman Center for Genome Research|Shakeel|Pakistan
>Spike|hCoV-19/England/QEUH-C32704/2020|2020-12-11|EPI_ISL_782614|Original|hCoV-19^^England|Human|Lighthouse Lab in Glasgow|Wellcome Sanger Institute for the COVID-19 Genomics UK (COG-UK) Consortium|Davies|United Kingdom
>Spike|hCoV-19/USA/UT-0366/2020|2020-03-28|EPI_ISL_435354|Original|hCoV-19^^Utah|Human|Utah Public Health Laboratory|Utah Public Health Laboratory|Young|USA
>Spike|hCoV-19/USA/CA-CZB-13735/2020|2020-09-09|EPI_ISL_739288|Original|hCoV-19^^California|Human|Alameda County Public Health Lab|Chan-Zuckerberg Biohub|Kamm|USA
>Spike|hCoV-19/Brazil/SP-0104/2020|2020-03-13|EPI_ISL_417928|Original|hCoV-19^^S\u00e3o Paulo|Human|Laborat\u00f3rio Hermes Pardini|Bioinformatics Laboratory - LNCC|Almeida|Brazil
>Spike|hCoV-19/Japan/PG-2325/2020|2020-03-00|EPI_ISL_685930|Original|hCoV-19^^Japan|Human|Pathogen Genomics Center|Pathogen Genomics Center|Sekizuka|Japan
>Spike|hCoV-19/England/PORT-2D1534/2020|2020-04-01|EPI_ISL_512400|Original|hCoV-19^^England|Human|Centre for Enzyme Innovation|COVID-19 Genomics UK (COG-UK) Consortium|Robson|United Kingdom
>Spike|hCoV-19/England/QEUH-78634E/2020|2020-07-15|EPI_ISL_533023|Original|hCoV-19^^England|Human|Lighthouse Lab in Glasgow|Wellcome Sanger Institute for the COVID-19 Genomics UK (COG-UK) consortium|Davies|United Kingdom
>Spike|hCoV-19/USA/TX-DSHS-0256/2020|2020-04-25|EPI_ISL_527829|Original|hCoV-19^^Texas|Human|Texas Department of State Health Services|Texas Department of State Health Services|Rahman|USA
>Spike|hCoV-19/Netherlands/ZH-EMC-570/2020|2020-09-20|EPI_ISL_577998|Original|hCoV-19^^Zuid Holland|Human|Dutch COVID-19 response team|Erasmus Medical Center|Oude Munnink|Netherlands
>Spike|hCoV-19/England/20128036004/2020|2020-03-19|EPI_ISL_423952|Original|hCoV-19^^England|Human|Respiratory Virus Unit|Respiratory Virus Unit|Platt|United Kingdom
>Spike|hCoV-19/England/MILK-A916DB/2020|2020-10-19|EPI_ISL_704866|Original|hCoV-19^^England|Human|Oxford Viromics|COVID-19 Genomics UK (COG-UK) Consortium|Golubchik|United Kingdom
>Spike|hCoV-19/England/LOND-124C3A0/2020|2020-04-16|EPI_ISL_492905|Original|hCoV-19^^England|Human|Royal Free Hospital / Health Services Laboratories|Wellcome Sanger Institute for the COVID-19 Genomics UK (COG-UK) consortium|Davies|United Kingdom
>Spike|hCoV-19/England/CAMC-B99669/2020|2020-11-15|EPI_ISL_673489|Original|hCoV-19^^England|Human|Lighthouse Lab in Cambridge|Wellcome Sanger Institute for the COVID-19 Genomics UK (COG-UK) Consortium|Davies|United Kingdom
>Spike|hCoV-19/Scotland/CVR5670/2020|2020-11-02|EPI_ISL_650146|Original|hCoV-19^^Scotland|Human|West of Scotland Specialist Virology Centre|COVID-19 Genomics UK (COG-UK) Consortium|Robertson|United Kingdom

>Spike|hCoV-19/Luxembourg/LNS1932776/2020|2020-10-31|EPI_ISL_740026|Original|hCoV-19^^Luxembourg|Human|Laboratoire national de sant\u00e9|Laboratoire national de sant\u00e9|Wienecke-Baldacchino|Luxembourg
>Spike|hCoV-19/Wales/PHWC-36AB8/2020|2020-05-01|EPI_ISL_474756|Original|hCoV-19^^Wales|Human|Originating lab|COVID-19 Genomics UK (COG-UK) Consortium|Moore|United Kingdom
>Spike|hCoV-19/Denmark/DCGC-3346/2020|2020-08-31|EPI_ISL_616100|Original|hCoV-19^^Hovedstaden|Human|Department of Virus and Microbiological Special Diagnostics|Albertsen lab|Michaelsen|Denmark
>Spike|hCoV-19/USA/NY-NYCPHL-001019/2020|2020-09-16|EPI_ISL_633037|Original|hCoV-19^^New York|Human|DOHMH Central Harlem|New York City Public Health Laboratory|Wang|USA
>Spike|hCoV-19/USA/VA-DCLS-0300/2020|2020-05-18|EPI_ISL_463103|Original|hCoV-19^^Virginia|Human|Virginia DCLS|Virginia DCLS|St. Jacques|USA
>Spike|hCoV-19/Denmark/DCGC-20440/2020|2020-12-21|EPI_ISL_759149|Original|hCoV-19^^Syddanmark|Human|Department of Virus and Microbiological Special Diagnostics|Albertsen Lab|Michaelsen|Denmark
>Spike|hCoV-19/USA/UT-UPHL-201025399/2020|2020-06-22|EPI_ISL_594263|Original|hCoV-19^^Utah|Human|Utah Public Health Laboratory|Utah Public Health Laboratory|Young|USA
>Spike|hCoV-19/Australia/VIC17147/2020|2020-08-15|EPI_ISL_663763|Original|hCoV-19^^Victoria|Human|Microbiological Diagnostic Unit - Public Health Laboratory (MDU-PHL)|MDU-PHL|Schultz|Australia
>Spike|hCoV-19/Guangdong/20SF013/2020|2020-01-15|EPI_ISL_403933|Original|hCoV-19^^Guangdong|Human|Guangdong Provincial Center for Diseases Control and Prevention; Guangdong Provincial Public Health|Department of Microbiology|Lu|China
>Spike|hCoV-19/England/CAMB-78F9D/2020|2020-04-03|EPI_ISL_441175|Original|hCoV-19^^England|Human|Department of Pathology|Wellcome Sanger Institute for the COVID-19 Genomics UK (COG-UK) consortium|Amato|United Kingdom
>Spike|hCoV-19/Australia/NSW556/2020|2020-04-14|EPI_ISL_455095|Original|hCoV-19^^New South Wales|Human|Pathology West - NSW Health Pathology|NSW Health Pathology - Institute of Clinical Pathology and Medical Research; Westmead Hospital; University of Sydney|Gall|Australia
>Spike|hCoV-19/Hong Kong/HKU-200723-086/2020|2020-07-12|EPI_ISL_497853|Original|hCoV-19^^Hong Kong|Human|Department of Microbiology|Department of Microbiology|Chan|China
>Spike|hCoV-19/USA/MN-MDH-148/2020|2020-03-21|EPI_ISL_460053|Original|hCoV-19^^Minnesota|Human|Minnesota Department of Health|Minnesota Department of Health|Garfin|USA
>Spike|hCoV-19/England/QEUH-9968A4/2020|2020-08-29|EPI_ISL_549538|Original|hCoV-19^^England|Human|Oxford Viromics|COVID-19 Genomics UK (COG-UK) Consortium|Golubchik|United Kingdom
>Spike|hCoV-19/England/CAMB-1B2CF9/2020|2020-05-20|EPI_ISL_456720|Original|hCoV-19^^England|Human|Department of Pathology|COVID-19 Genomics UK (COG-UK) Consortium|Meredith|United Kingdom
>Spike|hCoV-19/England/ALDP-C049E7/2020|2020-12-04|EPI_ISL_720816|Original|hCoV-19^^England|Human|Lighthouse Lab in Alderley Park|Wellcome Sanger Institute for the COVID-19 Genomics UK (COG-UK) Consortium|Davies|United Kingdom
>Spike|hCoV-19/England/QEUH-A78151/2020|2020-10-12|EPI_ISL_638145|Original|hCoV-19^^England|Human|Oxford Viromics|COVID-19 Genomics UK (COG-UK) Consortium|Golubchik|United Kingdom
>Spike|hCoV-19/Singapore/1382/2020|2020-07-18|EPI_ISL_648736|Original|hCoV-19^^Singapore|Human|Department of Laboratory Medicine|Department of Laboratory Medicine|Chen|Singapore
>Spike|hCoV-19/Canada/ON-S1427/2020|2020-08-24|EPI_ISL_591193|Original|hCoV-19^^Ontario|Human|Toronto Invasive Bacterial Diseases Network|McMaster University|McArthur|Canada
>Spike|hCoV-19/Scotland/QEUH-96D949/2020|2020-08-20|EPI_ISL_534521|Original|hCoV-19^^Scotland|Human|NHSGGC West of Scotland Specialist Virology Centre / MRC-University of Glasgow Centre for Virus Research|Wellcome Sanger Institute for the COVID-19 Genomics UK (COG-UK) consortium|Davies|United Kingdom

>Spike|hCoV-19/Australia/VIC11872/2020|2020-08-04|EPI_ISL_591879|Original|hCoV-19^^Victoria|Human|Victorian Infectious Diseases Reference Laboratory (VIDRL)|VIDRL and MDU-PHL|Schultz|Australia
>Spike|hCoV-19/Russia/MOW-RII-MH1988S/2020|2020-05-25|EPI_ISL_596332|Original|hCoV-19^^Moscow|Human|Pathogenic Microorganisms Variability Laboratory|WHO National Influenza Centre Russian Federation|Komissarov|Russia
>Spike|hCoV-19/USA/PA-MGEL92/2020|2020-04-01|EPI_ISL_454374|Original|hCoV-19^^Pennsylvania|Human|UPMC Clinical Microbiology Laboratory|Microbial Genome Sequencing Center|Mustapha|USA
>Spike|hCoV-19/Wales/PHWC-48B740/2020|2020-10-29|EPI_ISL_666276|Original|hCoV-19^^Wales|Human|Wales Specialist Virology Centre Sequencing lab|COVID-19 Genomics UK (COG-UK) Consortium|Moore|United Kingdom
>Spike|hCoV-19/England/QEUH-98FD90/2020|2020-08-25|EPI_ISL_539941|Original|hCoV-19^^England|Human|Lighthouse Lab in Glasgow|Wellcome Sanger Institute for the COVID-19 Genomics UK (COG-UK) consortium|Davies|United Kingdom
>Spike|hCoV-19/Australia/VIC7195/2020|2020-07-11|EPI_ISL_564275|Original|hCoV-19^^Victoria|Human|Microbiological Diagnostic Unit - Public Health Laboratory (MDU-PHL)|MDU-PHL|Schultz|Australia
>Spike|hCoV-19/USA/MI-MDHHS-SC20369/2020|2020-03-15|EPI_ISL_450580|Original|hCoV-19^^Michigan|Human|Michigan Department of Health and Human Services|Michigan Department of Health and Human Services|Blankenship|USA
>Spike|hCoV-19/England/MILK-9C4AD4/2020|2020-09-10|EPI_ISL_549360|Original|hCoV-19^^England|Human|Centre for Enzyme Innovation|COVID-19 Genomics UK (COG-UK) Consortium|Robson|United Kingdom
>Spike|hCoV-19/USA/WI-WSLH-200706/2020|2020-06-26|EPI_ISL_631463|Original|hCoV-19^^Wisconsin|Human|Wisconsin State Laboratory of Hygiene Communicable Disease Division|Wisconsin State Laboratory of Hygiene Communicable Disease Division|Florek|USA
>Spike|hCoV-19/England/CAMC-C3FAC2/2020|2020-12-10|EPI_ISL_719254|Original|hCoV-19^^England|Human|Lighthouse Lab in Cambridge|Wellcome Sanger Institute for the COVID-19 Genomics UK (COG-UK) Consortium|Davies|United Kingdom
>Spike|hCoV-19/USA/VA-DCLS-0636/2020|2020-04-11|EPI_ISL_495308|Original|hCoV-19^^Virginia|Human|Virginia DCLS|Virginia DCLS|St. Jacques|USA
>Spike|hCoV-19/Scotland/EDB10890/2020|2020-12-10|EPI_ISL_742177|Original|hCoV-19^^Scotland|Human|Virology Department|COVID-19 Genomics UK (COG-UK) Consortium|McHugh|United Kingdom
>Spike|hCoV-19/Portugal/PT0378/2020|2020-03-20|EPI_ISL_454094|Original|hCoV-19^^Portugal|Human|unknown|Instituto Nacional de Saude (INSA)|Borges|Portugal
>Spike|hCoV-19/Denmark/DCGC-20068/2020|2020-12-07|EPI_ISL_747963|Original|hCoV-19^^Midtjylland|Human|Department of Virus and Microbiological Special Diagnostics|Albertsen Lab|Michaelsen|Denmark
>Spike|hCoV-19/Canada/ON-S3/2020|2020-03-13|EPI_ISL_586390|Original|hCoV-19^^Ontario|Human|Toronto Invasive Bacterial Diseases Network|McMaster University|McArthur|Canada
>Spike|hCoV-19/USA/LA-ALSR-0059-NBG/2020|2020-03-23|EPI_ISL_434518|Original|hCoV-19^^Louisiana|Human|Robert Garry lab|Andersen lab at Scripps Research|Gangavarapu|USA
>Spike|hCoV-19/Wales/PHWC-49EB4E/2020|2020-12-08|EPI_ISL_743098|Original|hCoV-19^^Wales|Human|Wales Specialist Virology Centre Sequencing lab|COVID-19 Genomics UK (COG-UK) Consortium|Moore|United Kingdom
>Spike|hCoV-19/USA/WA-S1118/2020|2020-05-04|EPI_ISL_463608|Original|hCoV-19^^Washington|Human|Washington State Department of Health|Seattle Flu Study|Bedford|USA
>Spike|hCoV-19/Denmark/DCGC-3943/2020|2020-09-14|EPI_ISL_615854|Original|hCoV-19^^Hovedstaden|Human|Department of Virus and Microbiological Special Diagnostics|Albertsen lab|Michaelsen|Denmark
>Spike|hCoV-19/England/NORW-E9216/2020|2020-04-26|EPI_ISL_453583|Original|hCoV-19^^England|Human|Quadram Institute Bioscience|COVID-19 Genomics UK (COG-UK) Consortium|Alikhan|United Kingdom
>Spike|hCoV-19/USA/HI-H200169/2020|2020-07-11|EPI_ISL_752730|Original|hCoV-19^^Hawaii|Human|State Laboratories Division|State Laboratories Division|Sultana|USA

>Spike|hCoV-19/England/NORT-2A4B1A/2020|2020-09-16|EPI_ISL_573396|Original|hCoV-19^^England|Human|Northumbria University / South Tees Hospitals NHS Foundation Trust / North Cumbria Integrated Care NHS Foundation Trust / North Tees and Hartlepool NHS Foundation Trust / Newcastle Hospitals NHS Foundation Trust|COVID-19 Genomics UK (COG-UK) Consortium|Bashton|United Kingdom
>Spike|hCoV-19/USA/TX-HMH-MCoV-15126/2020|2020-10-16|EPI_ISL_786610|Original|hCoV-19^^Texas|Human|Houston Methodist Hospital|Houston Methodist Hospital|Long|USA
>Spike|hCoV-19/USA/TX-HMH-MCoV-10479/2020|2020-07-08|EPI_ISL_789493|Original|hCoV-19^^Texas|Human|Houston Methodist Hospital|Houston Methodist Hospital|Long|USA
>Spike|hCoV-19/Netherlands/Friesland_42/2020|2020-04-29|EPI_ISL_460857|Original|hCoV-19^^Friesland|Human|Dutch COVID-19 response team|Erasmus Medical Center|Oude Munnink|Netherlands
>Spike|hCoV-19/New Zealand/20CV0594/2020|2020-11-20|EPI_ISL_661264|Original|hCoV-19^^Auckland|Human|LabPLUS|Institute of Environmental Science and Research (ESR)|Wang|New Zealand
>Spike|hCoV-19/Wales/PHWC-49FFAB/2020|2020-12-12|EPI_ISL_743378|Original|hCoV-19^^Wales|Human|Wales Specialist Virology Centre Sequencing lab|COVID-19 Genomics UK (COG-UK) Consortium|Moore|United Kingdom
>Spike|hCoV-19/England/SHEF-D0388/2020|2020-05-02|EPI_ISL_457672|Original|hCoV-19^^England|Human|Virology Department|COVID-19 Genomics UK (COG-UK) Consortium|de Silva|United Kingdom
>Spike|hCoV-19/USA/UT-QDX-18/2020|2020-03-13|EPI_ISL_467916|Original|hCoV-19^^Utah|Human|Quest Diagnostics|Quest Diagnostics|Kagan|USA
>Spike|hCoV-19/Wales/PHWC-276C5/2020|2020-03-31|EPI_ISL_432200|Original|hCoV-19^^Wales|Human|Wales Specialist Virology Centre|Public Health Wales Microbiology Cardiff|Moore|United Kingdom
>Spike|hCoV-19/England/ALDP-CB4F8A/2020|2020-12-17|EPI_ISL_761904|Original|hCoV-19^^England|Human|Lighthouse Lab in Alderley Park|Wellcome Sanger Institute for the COVID-19 Genomics UK (COG-UK) Consortium|Davies|United Kingdom
>Spike|hCoV-19/Jordan/AM-ALSR-3929/2020|2020-09-30|EPI_ISL_636294|Original|hCoV-19^^Amman|Human|Biolab Diagnostic Laboratories|Andersen lab at Scripps Research|Gangavarapu|Jordan
>Spike|hCoV-19/England/CAMC-BBB898/2020|2020-11-21|EPI_ISL_702561|Original|hCoV-19^^England|Human|Department of Pathology|COVID-19 Genomics UK (COG-UK) Consortium|Goodfellow|United Kingdom
>Spike|hCoV-19/England/BRIS-12218C/2020|2020-03-23|EPI_ISL_439978|Original|hCoV-19^^England|Human|PHE South West Regional Laboratory|Wellcome Sanger Institute for the COVID-19 Genomics UK (COG-UK) consortium|Amato|United Kingdom
>Spike|hCoV-19/England/QEUH-C579F4/2020|2020-12-12|EPI_ISL_736343|Original|hCoV-19^^England|Human|Lighthouse Lab in Glasgow|Wellcome Sanger Institute for the COVID-19 Genomics UK (COG-UK) Consortium|Davies|United Kingdom
>Spike|hCoV-19/England/QEUH-B302D9/2020|2020-11-04|EPI_ISL_659795|Original|hCoV-19^^England|Human|Lighthouse Lab in Glasgow|Wellcome Sanger Institute for the COVID-19 Genomics UK (COG-UK) Consortium|Davies|United Kingdom
>Spike|hCoV-19/Ireland/D-NVRL-73IRL14028/2020|2020-09-16|EPI_ISL_578259|Original|hCoV-19^^Dublin|Human|National Virus Reference Laboratory|National Virus Reference Laboratory|Gonzalez|Ireland
>Spike|hCoV-19/Scotland/QEUH-A21861/2020|2020-10-01|EPI_ISL_598909|Original|hCoV-19^^Scotland|Human|Lighthouse Lab in Glasgow|Wellcome Sanger Institute for the COVID-19 Genomics UK (COG-UK) consortium|Davies|United Kingdom
>Spike|hCoV-19/Northern Ireland/NIRE-FB23F/2020|2020-03-24|EPI_ISL_448952|Original|hCoV-19^^Northern Ireland|Human|Regional Virus Laboratory|COVID-19 Genomics UK (COG-UK) Consortium|Fairley|United Kingdom
>Spike|hCoV-19/England/QEUH-A5E69E/2020|2020-10-12|EPI_ISL_597260|Original|hCoV-19^^England|Human|Lighthouse Lab in Glasgow|Wellcome Sanger Institute for the COVID-19 Genomics UK (COG-UK) consortium|Davies|United Kingdom
>Spike|hCoV-19/USA/TX-HMH-MCoV-18393/2020|2020-11-18|EPI_ISL_784741|Original|hCoV-19^^Texas|Human|Houston Methodist Hospital|Houston Methodist Hospital|Long|USA

>Spike|hCoV-19/England/ALDP-9CD437/2020|2020-09-16|EPI_ISL_566197|Original|hCoV-19^^England|Human|Lighthouse Lab in Alderley Park|Wellcome Sanger Institute for the COVID-19 Genomics UK (COG-UK) consortium|Davies|United Kingdom
>Spike|hCoV-19/Austria/CeMM0264/2020|2020-03-23|EPI_ISL_438093|Original|hCoV-19^^Vienna|Human|Center for Virology|Bergthaler laboratory|Bergthaler|Austria
>Spike|hCoV-19/Australia/VIC8977/2020|2020-07-28|EPI_ISL_565327|Original|hCoV-19^^Victoria|Human|Microbiological Diagnostic Unit - Public Health Laboratory (MDU-PHL)|MDU-PHL|Schultz|Australia
>Spike|hCoV-19/England/ALDP-954AAC/2020|2020-06-11|EPI_ISL_553454|Original|hCoV-19^^England|Human|Lighthouse Lab in Alderley Park|Wellcome Sanger Institute for the COVID-19 Genomics UK (COG-UK) consortium|Davies|United Kingdom
>Spike|hCoV-19/USA/TX-HMH-MCoV-13675/2020|2020-08-20|EPI_ISL_788772|Original|hCoV-19^^Texas|Human|Houston Methodist Hospital|Houston Methodist Hospital|Long|USA
>Spike|hCoV-19/USA/MA-MGH-00240/2020|2020-04-04|EPI_ISL_460405|Original|hCoV-19^^Massachusetts|Human|Massachusetts General Hospital|Infectious Disease Program|Lemieux|USA
>Spike|hCoV-19/Luxembourg/LNS3352091/2020|2020-11-02|EPI_ISL_740295|Original|hCoV-19^^Luxembourg|Human|Laboratoire national de sant\u00e9|Laboratoire national de sant\u00e9|Wienecke-Baldacchino|Luxembourg
>Spike|hCoV-19/Switzerland/GE-SNRCI-8820/2020|2020-03-00|EPI_ISL_548232|Original|hCoV-19^^Geneve|Human|Laboratoire de Virologie|Swiss National Reference Centre for Influenza|Gon\u00e7alves Cabecinhas|Switzerland
>Spike|hCoV-19/England/LIVE-DC0B2C/2020|2020-11-04|EPI_ISL_705198|Original|hCoV-19^^England|Human|Liverpool Clinical Laboratories|COVID-19 Genomics UK (COG-UK) Consortium|Haldenby|United Kingdom
>Spike|hCoV-19/Scotland/EDB4432/2020|2020-05-04|EPI_ISL_559975|Original|hCoV-19^^Scotland|Human|Virology Department|COVID-19 Genomics UK (COG-UK) Consortium|Williams|United Kingdom
>Spike|hCoV-19/USA/CA-CDPH117/2020|2020-09-20|EPI_ISL_755029|Original|hCoV-19^^California|Human|California Department of Public Health|California Department of Public Health|Bell|USA
>Spike|hCoV-19/USA/TX-DSHS-1117/2020|2020-06-21|EPI_ISL_615154|Original|hCoV-19^^Texas|Human|Texas Department of State Health Services|Texas Department of State Health Services|Rahman|USA
>Spike|hCoV-19/Beijing/DT-travelFR01/2020|2020-03-21|EPI_ISL_452330|Original|hCoV-19^^Beijing|Human|Laboratory of Infectious Diseases Center of Beijing Ditan Hospital|Laboratory of Infectious Diseases Center of Beijing Ditan Hospital|Yang|China
>Spike|hCoV-19/England/ALDP-AA215E/2020|2020-10-22|EPI_ISL_624486|Original|hCoV-19^^England|Human|Lighthouse Lab in Alderley Park|Wellcome Sanger Institute for the COVID-19 Genomics UK (COG-UK) consortium|Davies|United Kingdom
>Spike|hCoV-19/USA/CO-CDPHE-2011020331/2020|2020-11-01|EPI_ISL_710373|Original|hCoV-19^^Colorado|Human|Colorado Department of Public Health and Environment|Colorado Department of Puplic Health and Environment|Bankers|USA
>Spike|hCoV-19/Turkey/KU-044/2020|2020-05-17|EPI_ISL_495454|Original|hCoV-19^^Kars Province|Human|Kafkas University|Kafkas University|Karamese|Turkey
>Spike|hCoV-19/USA/CO-CSU-D1490-1/2020|2020-04-08|EPI_ISL_527439|Original|hCoV-19^^Colorado|Human|Colorado State University - Ebel Lab|Colorado State University - Ebel Lab|Cline|USA
>Spike|hCoV-19/USA/WA-UW-22885/2020|2020-08-06|EPI_ISL_570729|Original|hCoV-19^^Washington|Human|UW Virology Lab|UW Virology Lab|Roychoudhury|USA
>Spike|hCoV-19/USA/MI-MDHHS-SC22036/2020|2020-09-01|EPI_ISL_577588|Original|hCoV-19^^Michigan|Human|Michigan Department of Health and Human Services|Michigan Department of Health and Human Services|Jacob|USA
>Spike|hCoV-19/England/MILK-B85EB3/2020|2020-11-13|EPI_ISL_673899|Original|hCoV-19^^England|Human|Lighthouse Lab in Milton Keynes|Wellcome Sanger Institute for the COVID-19 Genomics UK (COG-UK) Consortium|Davies|United Kingdom
>Spike|hCoV-19/England/QEUH-B13B2C/2020|2020-11-02|EPI_ISL_641611|Original|hCoV-19^^England|Human|Lighthouse Lab in Glasgow|Wellcome Sanger Institute for the COVID-19 Genomics UK (COG-UK) Consortium|Davies|United Kingdom

>Spike|hCoV-19/Iceland/336/2020|2020-03-14|EPI_ISL_417665|Original|hCoV-19^^Reykjavik|Human|deCODE genetics|deCODE genetics|J\u00f3nsson|Iceland
>Spike|hCoV-19/Denmark/DCGC-6336/2020|2020-08-17|EPI_ISL_621193|Original|hCoV-19^^Midtjylland|Human|Department of Virus and Microbiological Special Diagnostics|Albertsen lab|Michaelsen|Denmark
>Spike|hCoV-19/USA/TX-HMH-MCoV-13330/2020|2020-08-24|EPI_ISL_788478|Original|hCoV-19^^Texas|Human|Houston Methodist Hospital|Houston Methodist Hospital|Long|USA
>Spike|hCoV-19/England/MILK-B95FCC/2020|2020-11-13|EPI_ISL_676283|Original|hCoV-19^^England|Human|Lighthouse Lab in Milton Keynes|Wellcome Sanger Institute for the COVID-19 Genomics UK (COG-UK) Consortium|Davies|United Kingdom
>Spike|hCoV-19/England/MILK-51856B/2020|2020-06-10|EPI_ISL_558923|Original|hCoV-19^^England|Human|Lighthouse Lab in Milton Keynes|Wellcome Sanger Institute for the COVID-19 Genomics UK (COG-UK) consortium|Davies|United Kingdom
>Spike|hCoV-19/England/ALDP-A707DF/2020|2020-10-15|EPI_ISL_608097|Original|hCoV-19^^England|Human|Lighthouse Lab in Alderley Park|Wellcome Sanger Institute for the COVID-19 Genomics UK (COG-UK) consortium|Davies|United Kingdom
>Spike|hCoV-19/South Africa/KRISP-K000567/2020|2020-07-13|EPI_ISL_509224|Original|hCoV-19^^KwaZulu-Natal|Human|NHLS-IALCH|KRISP|de Oliveira|South Africa
>Spike|hCoV-19/Japan/PG-1772/2020|2020-04-00|EPI_ISL_685378|Original|hCoV-19^^Japan|Human|Pathogen Genomics Center|Pathogen Genomics Center|Sekizuka|Japan
>Spike|hCoV-19/Italy/APU-UniMI-5436/2020|2020-04-11|EPI_ISL_525568|Original|hCoV-19^^Apulia|Human|Istituto Zooprofilattico Sperimentale Puglia e Basilicata; Dipartimento di Bioscienze|Beaconlab (Bioinformatics|Chiara|Italy
>Spike|hCoV-19/USA/HI-H200309/2020|2020-08-12|EPI_ISL_752868|Original|hCoV-19^^Hawaii|Human|State Laboratories Division|State Laboratories Division|Sultana|USA
>Spike|hCoV-19/England/MILK-BAB28E/2020|2020-11-18|EPI_ISL_681369|Original|hCoV-19^^England|Human|Lighthouse Lab in Milton Keynes|Wellcome Sanger Institute for the COVID-19 Genomics UK (COG-UK) Consortium|Davies|United Kingdom
>Spike|hCoV-19/England/ALDP-95A7AF/2020|2020-06-13|EPI_ISL_554910|Original|hCoV-19^^England|Human|Lighthouse Lab in Alderley Park|Wellcome Sanger Institute for the COVID-19 Genomics UK (COG-UK) consortium|Davies|United Kingdom
>Spike|hCoV-19/England/QEUH-C801BE/2020|2020-12-16|EPI_ISL_756879|Original|hCoV-19^^England|Human|Lighthouse Lab in Glasgow|Wellcome Sanger Institute for the COVID-19 Genomics UK (COG-UK) Consortium|Davies|United Kingdom
>Spike|hCoV-19/USA/MI-UM-10033903914/2020|2020-04-10|EPI_ISL_578153|Original|hCoV-19^^Michigan|Human|University of Michigan Clinical Microbiology Laboratory|Lauring Lab|Valesano|USA
>Spike|hCoV-19/Australia/WA170/2020|2020-04-07|EPI_ISL_596733|Original|hCoV-19^^Western Australia|Human|PathWest Laboratory Medicine WA|PathWest Laboratory Medicine WA Microbial Surveillance Unit|Sikazwe|Australia
>Spike|hCoV-19/England/BRIS-123C3F/2020|2020-04-07|EPI_ISL_442779|Original|hCoV-19^^England|Human|PHE South West Regional Laboratory|Wellcome Sanger Institute for the COVID-19 Genomics UK (COG-UK) consortium|Amato|United Kingdom
>Spike|hCoV-19/Germany/BW-UKT-046/2020|2020-03-30|EPI_ISL_581463|Original|hCoV-19^^Baden-W\u00fcrttemberg|Human|Medizinische Klinik Innere Medizin I|NGS Competence Center T\u00fcbingen|Angelov|Germany
>Spike|hCoV-19/England/CAMB-1AC1C6/2020|2020-04-28|EPI_ISL_444381|Original|hCoV-19^^England|Human|Department of Pathology|COVID-19 Genomics UK (COG-UK) Consortium|Meredith|United Kingdom
>Spike|hCoV-19/England/CAMB-71B27/2020|2020-04-01|EPI_ISL_439620|Original|hCoV-19^^England|Human|Department of Pathology|Wellcome Sanger Institute for the COVID-19 Genomics UK (COG-UK) consortium|Amato|United Kingdom
>Spike|hCoV-19/Denmark/DCGC-3564/2020|2020-08-31|EPI_ISL_616359|Original|hCoV-19^^Sjaelland|Human|Department of Virus and Microbiological Special Diagnostics|Albertsen lab|Michaelsen|Denmark
>Spike|hCoV-19/USA/NY-PV09186/2020|2020-03-20|EPI_ISL_422543|Original|hCoV-19^^New York|Human|MSHS Clinical Microbiology Laboratories|MSHS Pathogen Surveillance Program|van Bakel|USA

>Spike|hCoV-19/USA/UT-UPHL-2012790266/2020|2020-06-18|EPI_ISL_746382|Original|hCoV-19^^Utah|Human|Utah Public Health Laboratory|Utah Public Health Laboratory|Gallagher|USA
>Spike|hCoV-19/Denmark/DCGC-19360/2020|2020-12-07|EPI_ISL_748904|Original|hCoV-19^^Syddanmark|Human|Department of Virus and Microbiological Special Diagnostics|Albertsen Lab|Michaelsen|Denmark
>Spike|hCoV-19/India/GJ-GBRC165/2020|2020-05-27|EPI_ISL_461500|Original|hCoV-19^^Gujarat|Human|B.J. Medical College and Civil hospital|Gujarat Biotechnology Research Centre|Joshi|India
>Spike|hCoV-19/England/QEUH-9B4E26/2020|2020-09-04|EPI_ISL_590178|Original|hCoV-19^^England|Human|Lighthouse Lab in Glasgow|Wellcome Sanger Institute for the COVID-19 Genomics UK (COG-UK) consortium|Davies|United Kingdom
>Spike|hCoV-19/USA/TX-HMH-MCoV-10213/2020|2020-07-07|EPI_ISL_789372|Original|hCoV-19^^Texas|Human|Houston Methodist Hospital|Houston Methodist Hospital|Long|USA
>Spike|hCoV-19/England/QEUH-9C7C17/2020|2020-09-12|EPI_ISL_567092|Original|hCoV-19^^England|Human|Lighthouse Lab in Glasgow|Wellcome Sanger Institute for the COVID-19 Genomics UK (COG-UK) consortium|Davies|United Kingdom
>Spike|hCoV-19/England/NOTT-10F8F9/2020|2020-04-04|EPI_ISL_433058|Original|hCoV-19^^England|Human|Queens Medical Centre|COVID-19 Genomics UK (COG-UK) Consortium|Loose|United Kingdom
>Spike|hCoV-19/Switzerland/BS-UHB-42199797/2020|2020-03-20|EPI_ISL_528251|Original|hCoV-19^^Basel-Stadt|Human|University Hospital Basel|University Hospital Basel|Roloff Handschin|Switzerland
>Spike|hCoV-19/USA/MA-MGH-03384/2020|2020-11-10|EPI_ISL_765811|Original|hCoV-19^^Massachusetts|Human|Massachusetts General Hospital|Infectious Disease Program|Park|USA
>Spike|hCoV-19/Germany/NW-HHU-58/2020|2020-03-27|EPI_ISL_523929|Original|hCoV-19^^North Rhine-Westphalia|Human|Center of Medical Microbiology|Center of Medical Microbiology|Wienemann|Germany
>Spike|hCoV-19/Poland/PL_P23/2020|2020-03-29|EPI_ISL_451984|Original|hCoV-19^^Malopolska|Human|1. ViroGenetics - BSL3 Laboratory of Virology|1. ViroGenetics - BSL3 Laboratory of Virology|Pyrc|Poland
>Spike|hCoV-19/England/ALDP-9C58F7/2020|2020-09-11|EPI_ISL_567677|Original|hCoV-19^^England|Human|Lighthouse Lab in Alderley Park|Wellcome Sanger Institute for the COVID-19 Genomics UK (COG-UK) consortium|Davies|United Kingdom
>Spike|hCoV-19/Japan/PG-5274/2020|2020-08-00|EPI_ISL_690377|Original|hCoV-19^^Japan|Human|Pathogen Genomics Center|Pathogen Genomics Center|Sekizuka|Japan
>Spike|hCoV-19/Denmark/DCGC-1905/2020|2020-05-25|EPI_ISL_622495|Original|hCoV-19^^Hovedstaden|Human|Department of Virus and Microbiological Special Diagnostics|Albertsen lab|Michaelsen|Denmark
>Spike|hCoV-19/Scotland/QEUH-A5E643/2020|2020-10-11|EPI_ISL_609178|Original|hCoV-19^^Scotland|Human|Lighthouse Lab in Glasgow|Wellcome Sanger Institute for the COVID-19 Genomics UK (COG-UK) consortium|Davies|United Kingdom
>Spike|hCoV-19/USA/WI-UW-2624/2021|2021-01-02|EPI_ISL_791141|Original|hCoV-19^^Wisconsin|Human|University of Wisconsin-Madison AIDS Vaccine Research Laboratories|University of Wisconsin-Madison AIDS Vaccine Research Laboratories|Moreno|USA
>Spike|hCoV-19/Wales/PHWC-2BEC9/2020|2020-04-10|EPI_ISL_445864|Original|hCoV-19^^Wales|Human|Wales Specialist Virology Centre|Public Health Wales Microbiology Cardiff|Moore|United Kingdom
>Spike|hCoV-19/USA/NY-Wadsworth-192895-01/2020|2020-07-06|EPI_ISL_677669|Original|hCoV-19^^New York|Human|Masonic Medical Research Institute|Wadsworth Center|Lasek-Nesselquist|USA
>Spike|hCoV-19/England/ALDP-A2E59A/2020|2020-10-05|EPI_ISL_586791|Original|hCoV-19^^England|Human|Lighthouse Lab in Alderley Park|Wellcome Sanger Institute for the COVID-19 Genomics UK (COG-UK) consortium|Davies|United Kingdom
>Spike|hCoV-19/Spain/MD-HLP-59/2020|2020-04-20|EPI_ISL_530062|Original|hCoV-19^^Madrid|Human|Hospital Universitario La Paz|Hospital Universitario La Paz|Viedma|Spain

>Spike|hCoV-19/Denmark/DCGC-17769/2020|2020-12-14|EPI_ISL_749557|Original|hCoV-19^^Midtjylland|Human|Department of Virus and Microbiological Special Diagnostics|Albertsen Lab|Michaelsen|Denmark
>Spike|hCoV-19/USA/LA-EVTL710/2020|2020-07-15|EPI_ISL_653402|Original|hCoV-19^^Louisiana|Human|LSUHS Emerging Viral Threat Laboratory|Microbial Genome Sequencing Center|Kamil|USA
>Spike|hCoV-19/Guadeloupe/IPG-7344/2020|2020-03-26|EPI_ISL_613442|Original|hCoV-19^^Saint-Fran\u00e7ois|Human|Institut Pasteur de la Guadeloupe|Institut Pasteur de la Guadeloupe|Enouf|Guadeloupe
>Spike|hCoV-19/Spain/PV-IBV-000698/2020|2020-03-05|EPI_ISL_452766|Original|hCoV-19^^Basque Country|Human|Hospital Universitario Araba. Vitoria-Gasteiz,|SeqCOVID-SPAIN consortium/IBV(CSIC)|Comas|Spain
>Spike|hCoV-19/England/ALDP-C49852/2020|2020-12-12|EPI_ISL_718707|Original|hCoV-19^^England|Human|Lighthouse Lab in Alderley Park|Wellcome Sanger Institute for the COVID-19 Genomics UK (COG-UK) Consortium|Davies|United Kingdom
>Spike|hCoV-19/England/CAMB-1B9D1D/2020|2020-11-06|EPI_ISL_651553|Original|hCoV-19^^England|Human|Department of Pathology|COVID-19 Genomics UK (COG-UK) Consortium|Goodfellow|United Kingdom
>Spike|hCoV-19/USA/UT-UPHL-2009282/2020|2020-05-14|EPI_ISL_525263|Original|hCoV-19^^Utah|Human|Utah Public Health Laboratory|Utah Public Health Laboratory|Young|USA
>Spike|hCoV-19/Wales/PHWC-4A5009/2020|2020-12-13|EPI_ISL_764810|Original|hCoV-19^^Wales|Human|Wales Specialist Virology Centre Sequencing lab|COVID-19 Genomics UK (COG-UK) Consortium|Moore|United Kingdom
>Spike|hCoV-19/England/BIRM-5EC3C/2020|2020-04-28|EPI_ISL_447935|Original|hCoV-19^^England|Human|University of Birmingham|COVID-19 Genomics UK (COG-UK) Consortium|Loman|United Kingdom
>Spike|hCoV-19/England/MILK-B398ED/2020|2020-11-02|EPI_ISL_651056|Original|hCoV-19^^England|Human|University of Exeter|COVID-19 Genomics UK (COG-UK) Consortium|Temperton|United Kingdom
>Spike|hCoV-19/Switzerland/GE-ETHZ-360077/2020|2020-11-09|EPI_ISL_693890|Original|hCoV-19^^Geneva|Human|Viollier AG|Department of Biosystems Science and Engineering|Nadeau|Switzerland
>Spike|hCoV-19/South Africa/KRISP-K002759/2020|2020-08-15|EPI_ISL_529755|Original|hCoV-19^^KwaZulu-Natal|Human|NHLS-IALCH|KRISP|de Oliveira|South Africa
>Spike|hCoV-19/England/MILK-A6B86B/2020|2020-10-10|EPI_ISL_709945|Original|hCoV-19^^England|Human|Lighthouse Lab in Milton Keynes|Wellcome Sanger Institute for the COVID-19 Genomics UK (COG-UK) Consortium|Davies|United Kingdom
>Spike|hCoV-19/USA/CA-IGI-0058/2020|2020-05-20|EPI_ISL_511813|Original|hCoV-19^^California|Human|Innovative Genomics Institute|Innovative Genomics Institute|Wyman|USA
>Spike|hCoV-19/Hong Kong/HKU-200723-037/2020|2020-03-25|EPI_ISL_497804|Original|hCoV-19^^Hong Kong|Human|Department of Microbiology|Department of Microbiology|Chan|China
>Spike|hCoV-19/USA/LA-EVTL1418/2020|2020-12-14|EPI_ISL_779084|Original|hCoV-19^^Louisiana|Human|LSUHS Emerging Viral Threat Laboratory|Microbial Genome Sequencing Center|Kamil|USA
>Spike|hCoV-19/Canada/BC-BCCDC-6420/2020|2020-10-00|EPI_ISL_740574|Original|hCoV-19^^British Columbia|Human|BCCDC Public Health Laboratory|BCCDC Public Health Laboratory|Prystajecky|Canada
>Spike|hCoV-19/South Africa/NHLS-UCT-GS-2215/2020|2020-08-05|EPI_ISL_700534|Original|hCoV-19^^Western Cape|Human|Groote Schuur Hospital wc GSH|NHLS/UCT|Iranzadeh|South Africa
>Spike|hCoV-19/USA/AZ-TG519187/2020|2020-07-01|EPI_ISL_695367|Original|hCoV-19^^Arizona|Human|AZ SPHL|TGen North|French|USA
>Spike|hCoV-19/Japan/PG-5689/2020|2020-05-00|EPI_ISL_688813|Original|hCoV-19^^Japan|Human|Pathogen Genomics Center|Pathogen Genomics Center|Sekizuka|Japan
>Spike|hCoV-19/Spain/AN-ISCIII-201623/2020|2020-03-05|EPI_ISL_455322|Original|hCoV-19^^Andalucia|Human|Hospital Virgen de las Nieves|Instituto de Salud Carlos III|Caballero|Spain

>Spike|hCoV-19/Spain/CT-ISCIII-2013592/2020|2020-06-11|EPI_ISL_539544|Original|hCoV-19^^Catalunya|Human|Hospital Cl\u00ednic|Instituto de Salud Carlos III|Caballero|Spain
>Spike|hCoV-19/England/QEUH-BA2A61/2020|2020-11-18|EPI_ISL_672782|Original|hCoV-19^^England|Human|Lighthouse Lab in Glasgow|Wellcome Sanger Institute for the COVID-19 Genomics UK (COG-UK) Consortium|Davies|United Kingdom
>Spike|hCoV-19/USA/AK-PHL587/2020|2020-06-22|EPI_ISL_512152|Original|hCoV-19^^Alaska|Human|Alaska State Virology Laboratory|Alaska State Virology Laboratory|Drown|USA
>Spike|hCoV-19/USA/UT-UPHL-2012988511/2020|2020-08-04|EPI_ISL_746077|Original|hCoV-19^^Utah|Human|Ginkgo Bioworks Clinical Laboratory|Utah Public Health Laboratory|Young|USA
>Spike|hCoV-19/USA/VA-DCLS-1821/2020|2020-09-22|EPI_ISL_614140|Original|hCoV-19^^Virginia|Human|Virginia DCLS|Virginia DCLS|St. Jacques|USA
>Spike|hCoV-19/England/ALDP-9ED71A/2020|2020-05-09|EPI_ISL_580825|Original|hCoV-19^^England|Human|Lighthouse Lab in Alderley Park|Wellcome Sanger Institute for the COVID-19 Genomics UK (COG-UK) consortium|Davies|United Kingdom
>Spike|hCoV-19/United Arab Emirates/0665/2020|2020-05-15|EPI_ISL_698819|Original|hCoV-19^^Abu Dhabi|Human|Group 42 (G42) Healthcare|G42 Healthcare|Ogrodzki|United Arab Emirates
>Spike|hCoV-19/England/CAMB-1B71B9/2020|2020-10-13|EPI_ISL_638309|Original|hCoV-19^^England|Human|Department of Pathology|COVID-19 Genomics UK (COG-UK) Consortium|Goodfellow|United Kingdom
>Spike|hCoV-19/Singapore/418/2020|2020-03-18|EPI_ISL_475949|Original|hCoV-19^^Singapore|Human|National Public Health Laboratory|National Public Health Laboratory|Mak|Singapore
>Spike|hCoV-19/England/ALDP-A68BFC/2020|2020-10-12|EPI_ISL_608587|Original|hCoV-19^^England|Human|Lighthouse Lab in Alderley Park|Wellcome Sanger Institute for the COVID-19 Genomics UK (COG-UK) consortium|Davies|United Kingdom
>Spike|hCoV-19/England/ALDP-52165D/2020|2020-06-02|EPI_ISL_559060|Original|hCoV-19^^England|Human|Lighthouse Lab in Alderley Park|Wellcome Sanger Institute for the COVID-19 Genomics UK (COG-UK) consortium|Davies|United Kingdom
>Spike|hCoV-19/Australia/VIC4627/2020|2020-07-12|EPI_ISL_519020|Original|hCoV-19^^Victoria|Human|Victorian Infectious Diseases Reference Laboratory (VIDRL)|VIDRL and MDU-PHL|Schultz|Australia
>Spike|hCoV-19/USA/UT-UPHL-201118084/2020|2020-06-21|EPI_ISL_640300|Original|hCoV-19^^Utah|Human|Utah Public Health Laboratory|Utah Public Health Laboratory|Young|USA
>Spike|hCoV-19/England/CAMB-81113/2020|2020-04-19|EPI_ISL_438569|Original|hCoV-19^^England|Human|Department of Pathology|COVID-19 Genomics UK (COG-UK) Consortium|Meredith|United Kingdom
>Spike|hCoV-19/Wales/PHWC-25ABC/2020|2020-03-24|EPI_ISL_422053|Original|hCoV-19^^Wales|Human|Wales Specialist Virology Centre|Public Health Wales Microbiology Cardiff|Moore|United Kingdom
>Spike|hCoV-19/Belgium/ULG-10768/2020|2020-11-02|EPI_ISL_644614|Original|hCoV-19^^Li\u00e8ge|Human|Department of Clinical Microbiology|GIGA Medical Genomics|Durkin|Belgium
>Spike|hCoV-19/USA/WI-UW-1634/2020|2020-09-09|EPI_ISL_605508|Original|hCoV-19^^Wisconsin|Human|University of Wisconsin-Madison AIDS Vaccine Research Laboratories|University of Wisconsin-Madison AIDS Vaccine Research Laboratories|Baczenas|USA
>Spike|hCoV-19/Portugal/PT1576/2020|2020-06-29|EPI_ISL_693584|Original|hCoV-19^^Portugal|Human|Instituto Nacional de Saude (INSA)|Instituto Nacional de Saude (INSA)|Borges|Portugal
>Spike|hCoV-19/USA/NM-NMDOH-2020126501/2020|2020-06-15|EPI_ISL_535269|Original|hCoV-19^^New Mexico|Human|New Mexico Department of Health Scientific Laboratory|New Mexico Department of Health Scientific Laboratory|Johnson|USA
>Spike|hCoV-19/USA/WA-S2082/2020|2020-06-06|EPI_ISL_497510|Original|hCoV-19^^Washington|Human|Washington State Department of Health|Seattle Flu Study|Lee|USA

>Spike|hCoV-19/Wales/PHWC-48D7B7/2020|2020-11-07|EPI_ISL_680678|Original|hCoV-
19^^Wales|Human|Wales Specialist Virology Centre Sequencing lab|COVID-19 Genomics UK
(COG-UK) Consortium|Moore|United Kingdom
>Spike|hCoV-19/Denmark/DCGC-5922/2020|2020-10-05|EPI_ISL_620187|Original|hCoV-
19^^Hovedstaden|Human|Department of Virus and Microbiological Special
Diagnostics|Albertsen lab|Michaelsen|Denmark
>Spike|hCoV-19/England/QEUH-9DCE64/2020|2020-09-21|EPI_ISL_599518|Original|hCoV-
19^^England|Human|Lighthouse Lab in Glasgow|Wellcome Sanger Institute for the COVID-
19 Genomics UK (COG-UK) consortium|Davies|United Kingdom
>Spike|hCoV-19/Australia/VIC3303/2020|2020-07-07|EPI_ISL_520982|Original|hCoV-
19^^Victoria|Human|Microbiological Diagnostic Unit - Public Health Laboratory (MDU-
PHL)|MDU-PHL|Schultz|Australia
>Spike|hCoV-19/Japan/PG-7683/2020|2020-08-00|EPI_ISL_686785|Original|hCoV-
19^^Japan|Human|Chiba Prefectural Institute of Public Health|Pathogen Genomics
Center|Sekizuka|Japan
>Spike|hCoV-19/England/CAMC-990EE4/2020|2020-08-27|EPI_ISL_551915|Original|hCoV-
19^^England|Human|Lighthouse Lab in Cambridge|Wellcome Sanger Institute for the
COVID-19 Genomics UK (COG-UK) consortium|Davies|United Kingdom
>Spike|hCoV-19/Oman/11374/2020|2020-04-07|EPI_ISL_491155|Original|hCoV-19^^South
Batinah|Human|Oman-National Influenza Center|Biotechnology & OMICs Laboratory|Al
Mahruqi|Oman
>Spike|hCoV-19/USA/HI-H200673/2020|2020-11-25|EPI_ISL_753196|Original|hCoV-
19^^Hawaii|Human|State Laboratories Division|State Laboratories Division|Sultana|USA
>Spike|hCoV-19/Australia/VIC16986/2020|2020-08-17|EPI_ISL_663672|Original|hCoV-
19^^Victoria|Human|Microbiological Diagnostic Unit - Public Health Laboratory (MDU-
PHL)|MDU-PHL|Schultz|Australia
>Spike|hCoV-19/India/MH-GA36/2020|2020-06-20|EPI_ISL_508230|Original|hCoV-
19^^Maharashtra|Human|Government Medical College|National Institute of Biomedical
Genomics|Maitra|India
>Spike|hCoV-19/Luxembourg/LNS0988203/2020|2020-10-20|EPI_ISL_739868|Original|hCoV-
19^^Luxembourg|Human|Laboratoire national de sant\u00e9|Laboratoire national de
sant\u00e9|Wienecke-Baldacchino|Luxembourg
>Spike|hCoV-19/England/MILK-9D4901/2020|2020-09-19|EPI_ISL_601644|Original|hCoV-
19^^England|Human|Lighthouse Lab in Milton Keynes|Wellcome Sanger Institute for the
COVID-19 Genomics UK (COG-UK) consortium|Davies|United Kingdom
>Spike|hCoV-19/USA/TX-HMH-MCoV-10022/2020|2020-07-06|EPI_ISL_789289|Original|hCoV-
19^^Texas|Human|Houston Methodist Hospital|Houston Methodist Hospital|Long|USA
>Spike|hCoV-19/Wales/PHWC-4A004A/2020|2020-11-30|EPI_ISL_743387|Original|hCoV-
19^^Wales|Human|Wales Specialist Virology Centre Sequencing lab|COVID-19 Genomics UK
(COG-UK) Consortium|Moore|United Kingdom
>Spike|hCoV-19/Norway/4018/2020|2020-09-21|EPI_ISL_590969|original|hCoV-
19^^Rogaland|Human|Dept. of Medical Microbiology|Norwegian Institute of Public
Health|Vollan|Norway
>Spike|hCoV-19/Israel/CVL-70-ngs/2020|2020-12-24|EPI_ISL_776644|Original|hCoV-
19^^Israel|Human|Israel Central Virology laboratory|Israel Central Virology
laboratory|Zuckerman|Israel
>Spike|hCoV-19/England/ALDP-B5D5BF/2020|2020-11-10|EPI_ISL_702971|Original|hCoV-
19^^England|Human|Department of Pathology|COVID-19 Genomics UK (COG-UK)
Consortium|Goodfellow|United Kingdom
>Spike|hCoV-19/India/MH-ACTREC-531/2020|2020-08-25|EPI_ISL_700177|Original|hCoV-
19^^Maharashtra|Human|Hematopathology Laboratory|Hematopathology
Laboratory|Patkar|India
>Spike|hCoV-19/USA/CA-CHLA-PLM87208720/2020|2020-11-03|EPI_ISL_753276|Original|hCoV-
19^^California|Human|Clinical virology Laboratory|Center for Personalized
Medicine|Dien Bard|USA
>Spike|hCoV-19/England/LOND-DA324/2020|2020-11-16|EPI_ISL_724619|Original|hCoV-
19^^England|Human|University College London|COVID-19 Genomics UK (COG-UK)
Consortium|Roy|United Kingdom

>Spike|hCoV-19/Spain/AR-IBV-005046/2020|2020-04-21|EPI_ISL_500341|Original|hCoV-19^^Aragon|Human|Servicio de Microbiolog\u00eda|SeqCOVID-SPAIN consortium/IBV(CSIC)|Comas|Spain
>Spike|hCoV-19/Belgium/ULG-10416/2020|2020-08-14|EPI_ISL_581598|Original|hCoV-19^^Li\u00e8ge|Human|Department of Clinical Microbiology|GIGA Medical Genomics|Durkin|Belgium
>Spike|hCoV-19/USA/MI-UM-10036188357/2020|2020-09-29|EPI_ISL_590735|Original|hCoV-19^^Michigan|Human|University of Michigan Clinical Microbiology Laboratory|Lauring Lab|Valesano|USA
>Spike|hCoV-19/England/BIRM-5F916/2020|2020-05-22|EPI_ISL_473350|Original|hCoV-19^^England|Human|University of Birmingham|COVID-19 Genomics UK (COG-UK) Consortium|Loman|United Kingdom
>Spike|hCoV-19/Canada/BC-BCCDC-7318/2020|2020-10-00|EPI_ISL_740681|Original|hCoV-19^^British Columbia|Human|BCCDC Public Health Laboratory|BCCDC Public Health Laboratory|Prystajecky|Canada
>Spike|hCoV-19/Australia/VIC2636/2020|2020-06-28|EPI_ISL_521482|Original|hCoV-19^^Victoria|Human|Victorian Infectious Diseases Reference Laboratory (VIDRL)|VIDRL and MDU-PHL|Schultz|Australia
>Spike|hCoV-19/Wales/PHWC-492272/2020|2020-11-17|EPI_ISL_706833|Original|hCoV-19^^Wales|Human|Originating lab|COVID-19 Genomics UK (COG-UK) Consortium|Moore|United Kingdom
>Spike|hCoV-19/England/ALDP-AA2DB4/2020|2020-10-22|EPI_ISL_624325|Original|hCoV-19^^England|Human|Lighthouse Lab in Alderley Park|Wellcome Sanger Institute for the COVID-19 Genomics UK (COG-UK) consortium|Davies|United Kingdom
>Spike|hCoV-19/Finland/6A962SIIC/2020|2020-04-06|EPI_ISL_481712|Original|hCoV-19^^Uusimaa|Human|Department of Virology and Immunology|Department of Virology|Smura|Finland
>Spike|hCoV-19/England/PORT-2D320E/2020|2020-04-09|EPI_ISL_628316|Original|hCoV-19^^England|Human|Centre for Enzyme Innovation|COVID-19 Genomics UK (COG-UK) Consortium|Robson|United Kingdom
>Spike|hCoV-19/India/GJ-GBRC189/2020|2020-06-02|EPI_ISL_469032|Original|hCoV-19^^Gujarat|Human|Government Medical College|Gujarat Biotechnology Research Centre|Joshi|India
>Spike|hCoV-19/USA/CA-CZB0719/2020|2020-03-31|EPI_ISL_429072|Original|hCoV-19^^California|Human|UCSF Clinical Microbiology Laboratory|Chan-Zuckerberg Biohub|Kamm|USA
>Spike|hCoV-19/Peru/LIM-UPCH-0170/2020|2020-10-14|EPI_ISL_729877|Original|hCoV-19^^Lima|Human|Laboratorio de Referencia Nacional de Virus Respiratorios|Laboratorio de Gen\u00f3mica Microbiana|Tsukayama|Peru
>Spike|hCoV-19/Belgium/ULG-10573/2020|2020-10-23|EPI_ISL_626274|Original|hCoV-19^^Li\u00e8ge|Human|Department of Clinical Microbiology|GIGA Medical Genomics|Durkin|Belgium
>Spike|hCoV-19/USA/CA-ALSR-0767-IPL/2020|2020-04-12|EPI_ISL_483346|Original|hCoV-19^^California|Human|UC San Diego Center for Advanced Laboratory Medicine|Andersen lab at Scripps Research|Gangavarapu|USA
>Spike|hCoV-19/England/BRIS-128441/2020|2020-04-16|EPI_ISL_469537|Original|hCoV-19^^England|Human|PHE South West Regional Laboratory|Wellcome Sanger Institute for the COVID-19 Genomics UK (COG-UK) consortium|Davies|United Kingdom
>Spike|hCoV-19/England/LOND-D44AF/2020|2020-04-08|EPI_ISL_444127|Original|hCoV-19^^England|Human|University College London|COVID-19 Genomics UK (COG-UK) Consortium|Roy|United Kingdom
>Spike|hCoV-19/England/CAMB-7EFB5/2020|2020-04-08|EPI_ISL_442930|Original|hCoV-19^^England|Human|Department of Pathology|Wellcome Sanger Institute for the COVID-19 Genomics UK (COG-UK) consortium|Amato|United Kingdom
>Spike|hCoV-19/USA/WA-UW-2203/2020|2020-03-25|EPI_ISL_426111|Original|hCoV-19^^Washington|Human|UW Virology Lab|UW Virology Lab|Roychoudhury|USA
>Spike|hCoV-19/USA/WI-WSLH-200036/2020|2020-03-27|EPI_ISL_471201|Original|hCoV-19^^Wisconsin|Human|Wisconsin State Laboratory of Hygiene Communicable Disease Division|Wisconsin State Laboratory of Hygiene Communicable Disease Division|Florek|USA

>Spike|hCoV-19/England/C200044569/2020|2020-11-06|EPI_ISL_664038|Original|hCoV-19^^England|Human|Respiratory Virus Unit|COVID-19 Genomics UK (COG-UK) Consortium|Platt|United Kingdom
>Spike|hCoV-19/Canada/ON-E65/2020|2020-06-07|EPI_ISL_593538|Original|hCoV-19^^Ontario|Human|Eastern Ontario Regional Laboratory Association|McMaster University|McArthur|Canada
>Spike|hCoV-19/Mexico/CMX-INMEGEN-15/2020|2020-08-06|EPI_ISL_522980|Original|hCoV-19^^Mexico City|Human|Instituto Nacional de Medicina Gen\u00f3mica|Instituto Nacional de Medicina Gen\u00f3mica|Hidalgo|Mexico
>Spike|hCoV-19/USA/ME-HETL-J0357/2020|2020-11-24|EPI_ISL_755480|Original|hCoV-19^^Maine|Human|Maine Health and Environmental Testing Laboratory|Tewhey Lab|Tewhey|USA
>Spike|hCoV-19/Italy/BAS-IZSPB-372PT/2020|2020-10-28|EPI_ISL_722905|Original|hCoV-19^^Basilicata|Human|Istituto Zooprofilattico Sperimentale della Puglia e della Basilicata|Istituto Zooprofilattico Sperimentale della Puglia e della Basilicata|Parisi|Italy
>Spike|hCoV-19/Denmark/DCGC-16629/2020|2020-11-16|EPI_ISL_714299|Original|hCoV-19^^Midtjylland|Human|Department of Virus and Microbiological Special Diagnostics|Albertsen Lab|Michaelsen|Denmark
>Spike|hCoV-19/USA/FL-BPHL-1959/2020|2020-04-13|EPI_ISL_653112|Original|hCoV-19^^Florida|Human|Florida Bureau of Public Health Laboratories|Florida Bureau of Public Health Laboratories|Blanton|USA
>Spike|hCoV-19/USA/LA-EVTL137/2020|2020-03-28|EPI_ISL_455980|Original|hCoV-19^^Louisiana|Human|LSUHS Emerging Viral Threat Laboratory|Microbial Genome Sequencing Center|Kamil|USA
>Spike|hCoV-19/Singapore/1038/2020|2020-03-22|EPI_ISL_574523|Original|hCoV-19^^Singapore|Human|National Public Health Laboratory|National Public Health Laboratory|Mak|Singapore
>Spike|hCoV-19/England/QEUH-BCE4F8/2020|2020-11-26|EPI_ISL_704368|Original|hCoV-19^^England|Human|Lighthouse Lab in Glasgow|Wellcome Sanger Institute for the COVID-19 Genomics UK (COG-UK) Consortium|Davies|United Kingdom
>Spike|hCoV-19/Wales/PHWC-169B6D/2020|2020-08-03|EPI_ISL_517596|Original|hCoV-19^^Wales|Human|Wales Specialist Virology Centre Sequencing lab|COVID-19 Genomics UK (COG-UK) Consortium|Moore|United Kingdom
>Spike|hCoV-19/England/ALDP-AA1993/2020|2020-10-21|EPI_ISL_624047|Original|hCoV-19^^England|Human|Lighthouse Lab in Alderley Park|Wellcome Sanger Institute for the COVID-19 Genomics UK (COG-UK) consortium|Davies|United Kingdom
>Spike|hCoV-19/New Zealand/20VR2957/2020|2020-00-00|EPI_ISL_579376|Original|hCoV-19^^Auckland|Human|LabPLUS|Institute of Environmental Science and Research (ESR)|Wang|New Zealand
>Spike|hCoV-19/England/QEUH-B14587/2020|2020-11-03|EPI_ISL_642685|Original|hCoV-19^^England|Human|Lighthouse Lab in Glasgow|Wellcome Sanger Institute for the COVID-19 Genomics UK (COG-UK) Consortium|Davies|United Kingdom
>Spike|hCoV-19/England/MILK-56A4E5/2020|2020-06-10|EPI_ISL_558250|Original|hCoV-19^^England|Human|Lighthouse Lab in Milton Keynes|Wellcome Sanger Institute for the COVID-19 Genomics UK (COG-UK) consortium|Davies|United Kingdom
>Spike|hCoV-19/Australia/NSW99/2020|2020-03-25|EPI_ISL_427777|Original|hCoV-19^^New South Wales|Human|Centre for Infectious Diseases and Microbiology Public Health|NSW Health Pathology - Institute of Clinical Pathology and Medical Research; Westmead Hospital; University of Sydney|Gall|Australia
>Spike|hCoV-19/USA/LA-GBCL-26556517/2020|2020-04-16|EPI_ISL_485164|Original|hCoV-19^^Louisiana|Human|River Road Testing Lab|Ginkgo Bioworks Clinical Laboratory|Smith|USA
>Spike|hCoV-19/England/LIVE-DCB7D2/2020|2020-11-24|EPI_ISL_724428|Original|hCoV-19^^England|Human|Liverpool Clinical Laboratories|COVID-19 Genomics UK (COG-UK) Consortium|Haldenby|United Kingdom
>Spike|hCoV-19/Wales/PHWC-486E6E/2020|2020-10-28|EPI_ISL_639139|Original|hCoV-19^^Wales|Human|Wales Specialist Virology Centre Sequencing lab|COVID-19 Genomics UK (COG-UK) Consortium|Moore|United Kingdom

>Spike|hCoV-19/Denmark/DCGC-16731/2020|2020-10-19|EPI_ISL_714443|Original|hCoV-19^^Hovedstaden|Human|Department of Virus and Microbiological Special Diagnostics|Albertsen Lab|Michaelsen|Denmark
>Spike|hCoV-19/England/MILK-AC7690/2020|2020-10-21|EPI_ISL_630247|Original|hCoV-19^^England|Human|Lighthouse Lab in Milton Keynes|Wellcome Sanger Institute for the COVID-19 Genomics UK (COG-UK) consortium|Davies|United Kingdom
>Spike|hCoV-19/USA/MI-MDHHS-SC21001/2020|2020-06-11|EPI_ISL_507787|Original|hCoV-19^^Michigan|Human|Michigan Department of Health and Human Services|Michigan Department of Health and Human Services|Blankenship|USA
>Spike|hCoV-19/Brazil/RJ-00303/2020|2020-04-24|EPI_ISL_623106|Original|hCoV-19^^Rio de Janeiro|Human|Laboratorio de Virologia Molecular / UFRJ|Bioinformatics Laboratory / LNCC|Almeida|Brazil
>Spike|hCoV-19/Wales/PHWC-36D37/2020|2020-05-03|EPI_ISL_474796|Original|hCoV-19^^Wales|Human|Originating lab|COVID-19 Genomics UK (COG-UK) Consortium|Moore|United Kingdom
>Spike|hCoV-19/England/MILK-808AF3/2020|2020-07-16|EPI_ISL_557333|Original|hCoV-19^^England|Human|Lighthouse Lab in Milton Keynes|Wellcome Sanger Institute for the COVID-19 Genomics UK (COG-UK) consortium|Davies|United Kingdom
>Spike|hCoV-19/USA/WI-UW-683/2020|2020-06-26|EPI_ISL_491364|Original|hCoV-19^^Wisconsin|Human|University of Wisconsin-Madison AIDS Vaccine Research Laboratories|University of Wisconsin-Madison AIDS Vaccine Research Laboratories|Baczenas|USA
>Spike|hCoV-19/USA/NM-NMDOH-2020242037/2020|2020-07-22|EPI_ISL_510885|Original|hCoV-19^^New Mexico|Human|New Mexico Department of Health Scientific Laboratory|New Mexico Department of Health Scientific Laboratory|Johnson|USA
>Spike|hCoV-19/Israel/51146355/2020|2020-04-04|EPI_ISL_447315|Original|hCoV-19^^South District|Human|Clinical Virology Laboratory|Stern Lab|Miller|Israel
>Spike|hCoV-19/England/ALDP-505DF0/2020|2020-06-25|EPI_ISL_559251|Original|hCoV-19^^England|Human|Lighthouse Lab in Alderley Park|Wellcome Sanger Institute for the COVID-19 Genomics UK (COG-UK) consortium|Davies|United Kingdom
>Spike|hCoV-19/Denmark/DCGC-3237/2020|2020-08-31|EPI_ISL_616897|Original|hCoV-19^^Hovedstaden|Human|Department of Virus and Microbiological Special Diagnostics|Albertsen lab|Michaelsen|Denmark
>Spike|hCoV-19/England/ALDP-94F94D/2020|2020-06-15|EPI_ISL_555262|Original|hCoV-19^^England|Human|Lighthouse Lab in Alderley Park|Wellcome Sanger Institute for the COVID-19 Genomics UK (COG-UK) consortium|Davies|United Kingdom
>Spike|hCoV-19/Peru/LIM-UPCH-0034/2020|2020-06-17|EPI_ISL_540934|Original|hCoV-19^^Lima|Human|Laboratorio de Referencia Nacional de Virus Respiratorios|Laboratorio de Gen\u00f3mica Microbiana|Tsukayama|Peru
>Spike|hCoV-19/England/ALDP-9ECF6E/2020|2020-05-05|EPI_ISL_589360|Original|hCoV-19^^England|Human|Lighthouse Lab in Alderley Park|Wellcome Sanger Institute for the COVID-19 Genomics UK (COG-UK) consortium|Davies|United Kingdom
>Spike|hCoV-19/United Arab Emirates/0352/2020|2020-05-15|EPI_ISL_698527|Original|hCoV-19^^Abu Dhabi|Human|Group 42 (G42) Healthcare|G42 Healthcare|Ogrodzki|United Arab Emirates
>Spike|hCoV-19/England/ALDP-A85EA5/2020|2020-10-17|EPI_ISL_610585|Original|hCoV-19^^England|Human|Lighthouse Lab in Alderley Park|Wellcome Sanger Institute for the COVID-19 Genomics UK (COG-UK) consortium|Davies|United Kingdom
>Spike|hCoV-19/mink/Netherlands/NB-EMC-20-21/2020|2020-07-09|EPI_ISL_523054|Original|hCoV-19^^Limburg|Neovison vison|Dutch COVID-19 response team|Erasmus Medical Center|Oude Munnink|Netherlands
>Spike|hCoV-19/England/CAMC-A6598C/2020|2020-10-11|EPI_ISL_608456|Original|hCoV-19^^England|Human|Lighthouse Lab in Cambridge|Wellcome Sanger Institute for the COVID-19 Genomics UK (COG-UK) consortium|Davies|United Kingdom
>Spike|hCoV-19/England/MILK-99386B/2020|2020-08-26|EPI_ISL_552323|Original|hCoV-19^^England|Human|Lighthouse Lab in Milton Keynes|Wellcome Sanger Institute for the COVID-19 Genomics UK (COG-UK) consortium|Davies|United Kingdom
>Spike|hCoV-19/Australia/VIC7497/2020|2020-07-23|EPI_ISL_564450|Original|hCoV-19^^Victoria|Human|Microbiological Diagnostic Unit - Public Health Laboratory (MDU-PHL)|MDU-PHL|Schultz|Australia

>Spike|hCoV-19/Northern Ireland/NIRE-10966E/2020|2020-10-
22|EPI_ISL_680211|Original|hCoV-19^^Northern Ireland|Human|Regional Virus
Laboratory|COVID-19 Genomics UK (COG-UK) Consortium|Fairley|United Kingdom
>Spike|hCoV-19/England/ALDP-94EFD3/2020|2020-06-19|EPI_ISL_556332|Original|hCoV-
19^^England|Human|Lighthouse Lab in Alderley Park|Wellcome Sanger Institute for the
COVID-19 Genomics UK (COG-UK) consortium|Davies|United Kingdom
>Spike|hCoV-19/Wales/PHWC-16F3D8/2020|2020-10-05|EPI_ISL_585959|Original|hCoV-
19^^Wales|Human|Wales Specialist Virology Centre Sequencing lab|COVID-19 Genomics UK
(COG-UK) Consortium|Moore|United Kingdom
>Spike|hCoV-19/England/ALDP-A6E5DA/2020|2020-10-15|EPI_ISL_607030|Original|hCoV-
19^^England|Human|Lighthouse Lab in Alderley Park|Wellcome Sanger Institute for the
COVID-19 Genomics UK (COG-UK) consortium|Davies|United Kingdom
>Spike|hCoV-19/Ukraine/Kyiv-785/2020|2020-08-03|EPI_ISL_576148|Original|hCoV-
19^^Kyiv|Human|Department of Respiratory & Other Viral Infections of L.V.
Gromashevsky Institute of Epidemiology & Infectious Diseases NAMS of
Ukraine|Department of Respiratory & Other Viral Infections of L.V. Gromashevsky
Institute of Epidemiology & Infectious Diseases NAMS of Ukraine|Mironenko|Ukraine
>Spike|hCoV-19/USA/VA-DCLS-1023/2020|2020-06-03|EPI_ISL_526913|Original|hCoV-
19^^Virginia|Human|Virginia DCLS|Virginia DCLS|St. Jacques|USA
>Spike|hCoV-19/USA/NY-SURV0331/2020|2020-03-09|EPI_ISL_436062|Original|hCoV-19^^New
York|Human|NYC Department of Health and Mental Hygiene|Pathogen Discovery|Queen|USA
>Spike|hCoV-19/England/ALDP-94E6A1/2020|2020-06-22|EPI_ISL_555487|Original|hCoV-
19^^England|Human|Lighthouse Lab in Alderley Park|Wellcome Sanger Institute for the
COVID-19 Genomics UK (COG-UK) consortium|Davies|United Kingdom
>Spike|hCoV-19/USA/TX-HMH-MCoV-15849/2020|2020-07-10|EPI_ISL_785533|Original|hCoV-
19^^Texas|Human|Houston Methodist Hospital|Houston Methodist Hospital|Long|USA
>Spike|hCoV-19/England/MILK-A7AF82/2020|2020-10-14|EPI_ISL_607535|Original|hCoV-
19^^England|Human|Lighthouse Lab in Milton Keynes|Wellcome Sanger Institute for the
COVID-19 Genomics UK (COG-UK) consortium|Davies|United Kingdom
>Spike|hCoV-19/Switzerland/BL-42237708/2020|2020-04-19|EPI_ISL_581714|Original|hCoV-
19^^Basel-Land|Human|University Hospital Basel|University Hospital Basel|Roloff
Handschin|Switzerland
>Spike|hCoV-19/England/CAMC-B5281B/2020|2020-11-08|EPI_ISL_657637|Original|hCoV-
19^^England|Human|Lighthouse Lab in Cambridge|Wellcome Sanger Institute for the
COVID-19 Genomics UK (COG-UK) Consortium|Davies|United Kingdom
>Spike|hCoV-19/Wales/PHWC-493BD0/2020|2020-11-16|EPI_ISL_725995|Original|hCoV-
19^^Wales|Human|Originating lab|COVID-19 Genomics UK (COG-UK) Consortium|Moore|United
Kingdom
>Spike|hCoV-19/England/ALDP-65DB2B/2020|2020-07-03|EPI_ISL_555634|Original|hCoV-
19^^England|Human|Lighthouse Lab in Alderley Park|Wellcome Sanger Institute for the
COVID-19 Genomics UK (COG-UK) consortium|Davies|United Kingdom
>Spike|hCoV-19/USA/MI-MDHHS-SC21415/2020|2020-03-21|EPI_ISL_507669|Original|hCoV-
19^^Michigan|Human|Michigan Department of Health and Human Services|Michigan
Department of Health and Human Services|Blankenship|USA
>Spike|hCoV-19/England/NORT-292BD3/2020|2020-05-27|EPI_ISL_472280|Original|hCoV-
19^^England|Human|Northumbria University / South Tees Hospitals NHS Foundation Trust
/ North Cumbria Integrated Care NHS Foundation Trust / North Tees and Hartlepool NHS
Foundation Trust / Newcastle Hospitals NHS Foundation Trust|COVID-19 Genomics UK
(COG-UK) Consortium|Bashton|United Kingdom
>Spike|hCoV-19/USA/NY-Wadsworth-38057-01/2020|2020-04-
07|EPI_ISL_676711|Original|hCoV-19^^New York|Human|Wadsworth Center|Wadsworth
Center|Lasek-Nesselquist|USA
>Spike|hCoV-19/Portugal/PT1401/2020|2020-03-29|EPI_ISL_511360|Original|hCoV-
19^^Portugal|Human|Instituto Nacional de Saude (INSA)|Instituto Nacional de Saude
(INSA)|Borges|Portugal
>Spike|hCoV-19/Portugal/PT0081/2020|2020-03-19|EPI_ISL_421484|Original|hCoV-
19^^Portugal|Human|CH VN Gaia - Espinho|Instituto Nacional de Saude
(INSA)|Borges|Portugal

>Spike|hCoV-19/USA/TX-DSHS-1889/2020|2020-07-08|EPI_ISL_751540|Original|hCoV-
19^^Texas|Human|Texas Department of State Health Services|Texas Department of State
Health Services|Rahman|USA
>Spike|hCoV-19/Denmark/DCGC-6548/2020|2020-10-05|EPI_ISL_619448|Original|hCoV-
19^^Hovedstaden|Human|Department of Virus and Microbiological Special
Diagnostics|Albertsen lab|Michaelsen|Denmark
>Spike|hCoV-19/France/OCC-SC516/2020|2020-04-29|EPI_ISL_644696|Original|hCoV-
19^^Occitanie|Human|CHU Montpellier|CNR Virus des Infections Respiratoires - France
SUD|Bal|France
>Spike|hCoV-19/Wales/MILK-9C44E5/2020|2020-09-09|EPI_ISL_566800|Original|hCoV-
19^^Wales|Human|Lighthouse Lab in Milton Keynes|Wellcome Sanger Institute for the
COVID-19 Genomics UK (COG-UK) consortium|Davies|United Kingdom
>Spike|hCoV-19/Denmark/DCGC-8781/2020|2020-10-26|EPI_ISL_626045|Original|hCoV-
19^^Hovedstaden|Human|Department of Virus and Microbiological Special
Diagnostics|Albertsen lab|Michaelsen|Denmark
>Spike|hCoV-19/Russia/MOW-RII-MH1679V/2020|2020-09-06|EPI_ISL_733393|Vero|hCoV-
19^^Moscow|Human|HELIX LLC|WHO National Influenza Centre Russian
Federation|Komissarov|Russia
>Spike|hCoV-19/England/QEUH-AF0DA8/2020|2020-10-29|EPI_ISL_666005|Original|hCoV-
19^^England|Human|Northumbria University / South Tees Hospitals NHS Foundation Trust
/ North Cumbria Integrated Care NHS Foundation Trust / North Tees and Hartlepool NHS
Foundation Trust / Newcastle Hospitals NHS Foundation Trust|COVID-19 Genomics UK
(COG-UK) Consortium|Bashton|United Kingdom
>Spike|hCoV-19/Japan/PG-5540/2020|2020-08-00|EPI_ISL_688665|Original|hCoV-
19^^Japan|Human|Pathogen Genomics Center|Pathogen Genomics Center|Sekizuka|Japan
>Spike|hCoV-19/England/CAMC-CF987F/2020|2020-12-18|EPI_ISL_781471|Original|hCoV-
19^^England|Human|Lighthouse Lab in Cambridge|Wellcome Sanger Institute for the
COVID-19 Genomics UK (COG-UK) Consortium|Davies|United Kingdom
>Spike|hCoV-19/USA/TX-GCID-192000254/2020|2020-04-07|EPI_ISL_501171|Original|hCoV-
19^^Texas|Human|Baylor College of Medicine|Baylor College of Medicine|Skinner|USA
>Spike|hCoV-19/United Arab Emirates/0101/2020|2020-05-
19|EPI_ISL_698291|Original|hCoV-19^^Abu Dhabi|Human|Group 42 (G42) Healthcare|G42
Healthcare|Ogrodzki|United Arab Emirates
>Spike|hCoV-19/Scotland/QEUH-A20E60/2020|2020-10-01|EPI_ISL_599022|Original|hCoV-
19^^Scotland|Human|Lighthouse Lab in Glasgow|Wellcome Sanger Institute for the COVID-
19 Genomics UK (COG-UK) consortium|Davies|United Kingdom
>Spike|hCoV-19/Italy/LAZ-INMI-65/2020|2020-06-19|EPI_ISL_603172|Original|hCoV-
19^^Lazio|Human|INMI Lazzaro Spallanzani IRCCS|INMI Lazzaro Spallanzani
IRCCS|Gruber|Italy
>Spike|hCoV-19/England/ALDP-A864A2/2020|2020-10-17|EPI_ISL_605965|Original|hCoV-
19^^England|Human|Lighthouse Lab in Alderley Park|Wellcome Sanger Institute for the
COVID-19 Genomics UK (COG-UK) consortium|Davies|United Kingdom
>Spike|hCoV-19/England/CAMB-7CF4E/2020|2020-04-05|EPI_ISL_442256|Original|hCoV-
19^^England|Human|Department of Pathology|Wellcome Sanger Institute for the COVID-19
Genomics UK (COG-UK) consortium|Amato|United Kingdom
>Spike|hCoV-19/Australia/VIC12152/2020|2020-07-25|EPI_ISL_641063|Original|hCoV-
19^^Victoria|Human|Microbiological Diagnostic Unit - Public Health Laboratory (MDU-
PHL)|MDU-PHL|Schultz|Australia
>Spike|hCoV-19/Portugal/PT0195/2020|2020-03-29|EPI_ISL_453911|Original|hCoV-
19^^Portugal|Human|unknown|Instituto Nacional de Saude (INSA)|Borges|Portugal
>Spike|hCoV-19/Mexico/BCN-ALSR-1715/2020|2020-04-29|EPI_ISL_496376|Original|hCoV-
19^^Baja California|Human|Infectolab|Andersen lab at Scripps
Research|Gangavarapu|Mexico
>Spike|hCoV-19/Lithuania/MR-LUHS-Eilnr55/2020|2020-04-
09|EPI_ISL_541866|Original|hCoV-19^^Marijampoles apskritis|Human|Lithuanian
University of Health Sciences Hospital|Lithuanian University of Health
Sciences|Zemaitis|Lithuania
>Spike|hCoV-19/South Africa/R13561-20/2020|2020-07-13|EPI_ISL_622903|Original|hCoV-
19^^North West|Human|National Institute for Communicable Diseases of the National

Health Laboratory Service|National Institute for Communicable Diseases of the National Health Laboratory Service|Allam|South Africa
>Spike|hCoV-19/Belgium/ULG-10724/2020|2020-09-15|EPI_ISL_641566|Original|hCoV-19^^Li\u00e8ge|Human|Department of Clinical Microbiology|GIGA Medical Genomics|Durkin|Belgium
>Spike|hCoV-19/England/ALDP-744449/2020|2020-06-29|EPI_ISL_557485|Original|hCoV-19^^England|Human|Lighthouse Lab in Alderley Park|Wellcome Sanger Institute for the COVID-19 Genomics UK (COG-UK) consortium|Davies|United Kingdom
>Spike|hCoV-19/Singapore/69/2020|2020-04-15|EPI_ISL_428848|Original|hCoV-19^^Singapore|Human|National Public Health Laboratory|National Public Health Laboratory|Mak|Singapore
>Spike|hCoV-19/USA/TX-DSHS-1619/2020|2020-10-14|EPI_ISL_700710|Original|hCoV-19^^Texas|Human|Texas Department of State Health Services|Texas Department of State Health Services|Rahman|USA
>Spike|hCoV-19/Brazil/SP-626/2020|2020-03-24|EPI_ISL_515529|Original|hCoV-19^^S\u00e3o Paulo|Human|Pronto Socorro Municipal Julio Tupy|Instituto Adolfo Lutz|Sacchi|Brazil
>Spike|hCoV-19/England/SHEF-C02CA/2020|2020-03-27|EPI_ISL_420180|Original|hCoV-19^^England|Human|Virology Department|Department of Infection|de Silva|United Kingdom
>Spike|hCoV-19/Japan/PG-1746/2020|2020-05-00|EPI_ISL_685352|Original|hCoV-19^^Japan|Human|Pathogen Genomics Center|Pathogen Genomics Center|Sekizuka|Japan
>Spike|hCoV-19/France/ARA-11228/2020|2020-03-18|EPI_ISL_525542|Original|hCoV-19^^Auvergne-Rh\u00f4ne-Alpes|Human|CNR Virus des Infections Respiratoires - France SUD|CNR Virus des Infections Respiratoires - France SUD|Josset|France
>Spike|hCoV-19/USA/CA-CZB-1861/2020|2020-04-28|EPI_ISL_486167|Original|hCoV-19^^California|Human|Orange County Public Health Laboratory|Chan-Zuckerberg Biohub|McGeever|USA
>Spike|hCoV-19/USA/WI-UW-586/2020|2020-06-19|EPI_ISL_484961|Original|hCoV-19^^Wisconsin|Human|University of Wisconsin-Madison AIDS Vaccine Research Laboratories|University of Wisconsin-Madison AIDS Vaccine Research Laboratories|Baczenas|USA
>Spike|hCoV-19/Scotland/EDB4708/2020|2020-04-27|EPI_ISL_449324|Original|hCoV-19^^Scotland|Human|Virology Department|COVID-19 Genomics UK (COG-UK) Consortium|Williams|United Kingdom
>Spike|hCoV-19/USA/CA-CZB-14826/2020|2020-11-19|EPI_ISL_739405|Original|hCoV-19^^California|Human|Humboldt County Public Health Laboratory|Chan-Zuckerberg Biohub|Kamm|USA
>Spike|hCoV-19/England/CAMB-76DB3/2020|2020-03-31|EPI_ISL_425428|Original|hCoV-19^^England|Human|Department of Pathology|COVID-19 Genomics UK (COG-UK) Consortium|Meredith|United Kingdom
>Spike|hCoV-19/England/SHEF-C3959/2020|2020-04-02|EPI_ISL_432778|Original|hCoV-19^^England|Human|Virology Department|COVID-19 Genomics UK (COG-UK) Consortium|de Silva|United Kingdom
>Spike|hCoV-19/USA/VA-DCLS-1933/2020|2020-09-28|EPI_ISL_644924|Original|hCoV-19^^Virginia|Human|Virginia DCLS|Virginia DCLS|St. Jacques|USA
>Spike|hCoV-19/USA/MI-UM-10035869177/2020|2020-09-09|EPI_ISL_583340|Original|hCoV-19^^Michigan|Human|University of Michigan Clinical Microbiology Laboratory|Lauring Lab|Valesano|USA
>Spike|hCoV-19/England/CAMC-B513DF/2020|2020-11-08|EPI_ISL_657771|Original|hCoV-19^^England|Human|Lighthouse Lab in Cambridge|Wellcome Sanger Institute for the COVID-19 Genomics UK (COG-UK) Consortium|Davies|United Kingdom
>Spike|hCoV-19/Luxembourg/LNS5351629/2020|2020-12-29|EPI_ISL_771099|Original|hCoV-19^^Luxembourg|Human|Laboratoire national de sant\u00e9|Laboratoire national de sant\u00e9|Wienecke-Baldacchino|Luxembourg
>Spike|hCoV-19/USA/TX-HMH-MCoV-18627/2020|2020-11-19|EPI_ISL_784975|Original|hCoV-19^^Texas|Human|Houston Methodist Hospital|Houston Methodist Hospital|Long|USA
>Spike|hCoV-19/Scotland/CVR3426/2020|2020-05-07|EPI_ISL_456847|Original|hCoV-19^^Scotland|Human|West of Scotland Specialist Virology Centre|COVID-19 Genomics UK (COG-UK) Consortium|Robertson|United Kingdom

>Spike|hCoV-19/England/CAMB-1B97E2/2020|2020-11-04|EPI_ISL_664945|Original|hCoV-19^^England|Human|Department of Pathology|COVID-19 Genomics UK (COG-UK) Consortium|Goodfellow|United Kingdom
>Spike|hCoV-19/England/MILK-B04EE9/2020|2020-10-24|EPI_ISL_650630|Original|hCoV-19^^England|Human|Department of Pathology|COVID-19 Genomics UK (COG-UK) Consortium|Goodfellow|United Kingdom
>Spike|hCoV-19/Switzerland/BL-UHB-42192884/2020|2020-03-16|EPI_ISL_528133|Original|hCoV-19^^Basel-Land|Human|University Hospital Basel|University Hospital Basel|Roloff Handschin|Switzerland
>Spike|hCoV-19/Australia/VIC6920/2020|2020-07-21|EPI_ISL_564140|Original|hCoV-19^^Victoria|Human|Microbiological Diagnostic Unit - Public Health Laboratory (MDU-PHL)|MDU-PHL|Schultz|Australia
>Spike|hCoV-19/England/NOTT-10EA8B/2020|2020-03-29|EPI_ISL_425633|Original|hCoV-19^^England|Human|Queens Medical Centre|COVID-19 Genomics UK (COG-UK) Consortium|Loose|United Kingdom
>Spike|hCoV-19/USA/AK-PHL549/2020|2020-06-20|EPI_ISL_525759|Original|hCoV-19^^Alaska|Human|Alaska State Virology Laboratory|Alaska State Virology Laboratory|Chen|USA
>Spike|hCoV-19/Japan/PG-3690/2020|2020-04-00|EPI_ISL_691943|Original|hCoV-19^^Japan|Human|Pathogen Genomics Center|Pathogen Genomics Center|Sekizuka|Japan
>Spike|hCoV-19/India/OR-ILSCV32652/2020|2020-06-03|EPI_ISL_481160|Original|hCoV-19^^Odisha|Human|Immunogenomics lab|Immunogenomics lab|Raghav|India
>Spike|hCoV-19/USA/WA-S1804/2020|2020-06-02|EPI_ISL_496230|Original|hCoV-19^^Washington|Human|Washington State Department of Health|Seattle Flu Study|Lee|USA
>Spike|hCoV-19/USA/MN-MDH-1543/2020|2020-08-03|EPI_ISL_527608|Original|hCoV-19^^Minnesota|Human|Minnesota Department of Health|Minnesota Department of Health|Garfin|USA
>Spike|hCoV-19/England/CAMC-C836DA/2020|2020-12-17|EPI_ISL_762580|Original|hCoV-19^^England|Human|Lighthouse Lab in Cambridge|Wellcome Sanger Institute for the COVID-19 Genomics UK (COG-UK) Consortium|Davies|United Kingdom
>Spike|hCoV-19/USA/UT-UPHL-2009490/2020|2020-07-19|EPI_ISL_605174|Original|hCoV-19^^Utah|Human|Utah Public Health Laboratory|Utah Public Health Laboratory|Young|USA
>Spike|hCoV-19/USA/MI-QDX-612/2020|2020-03-18|EPI_ISL_571341|Original|hCoV-19^^Michigan|Human|Quest Diagnostics|Quest Diagnostics|Kagan|USA
>Spike|hCoV-19/England/EXET-13E90D/2020|2020-12-13|EPI_ISL_764178|Original|hCoV-19^^England|Human|University of Exeter|COVID-19 Genomics UK (COG-UK) Consortium|Temperton|United Kingdom
>Spike|hCoV-19/Switzerland/ZH-ETHZ-200033/2020|2020-07-15|EPI_ISL_510722|Original|hCoV-19^^Zurich|Human|Viollier AG|Department of Biosystems Science and Engineering|Nadeau|Switzerland
>Spike|hCoV-19/Luxembourg/LNS7643605/2020|2020-10-26|EPI_ISL_744607|Original|hCoV-19^^Luxembourg|Human|Laboratoire national de sant\u00e9|Laboratoire national de sant\u00e9|Wienecke-Baldacchino|Luxembourg
>Spike|hCoV-19/England/LEED-2A83E1/2020|2020-03-30|EPI_ISL_538995|Original|hCoV-19^^England|Human|Leeds Teaching Hospitals NHS Trust and Public Health England|Wellcome Sanger Institute for the COVID-19 Genomics UK (COG-UK) consortium|Davies|United Kingdom
>Spike|hCoV-19/England/ALDP-CB3835/2020|2020-12-20|EPI_ISL_761235|Original|hCoV-19^^England|Human|Lighthouse Lab in Alderley Park|Wellcome Sanger Institute for the COVID-19 Genomics UK (COG-UK) Consortium|Davies|United Kingdom
>Spike|hCoV-19/Switzerland/LU-ETHZ-350450/2020|2020-11-04|EPI_ISL_729257|Original|hCoV-19^^Lucerne|Human|Viollier AG|Department of Biosystems Science and Engineering|Nadeau|Switzerland
>Spike|hCoV-19/Denmark/DCGC-18649/2020|2020-12-07|EPI_ISL_750102|Original|hCoV-19^^Midtjylland|Human|Department of Virus and Microbiological Special Diagnostics|Albertsen Lab|Michaelsen|Denmark
>Spike|hCoV-19/Wales/PHWC-496760/2020|2020-11-20|EPI_ISL_726631|Original|hCoV-19^^Wales|Human|Originating lab|COVID-19 Genomics UK (COG-UK) Consortium|Moore|United Kingdom

>Spike|hCoV-19/Italy/ABR-IZSGC-484/2020|2020-02-27|EPI_ISL_457749|Vero E6|hCoV-19^^Abruzzo|Human|Department of Infectious Diseases|Army Medical and Veterinary Research Center|Lista|Italy
>Spike|hCoV-19/Wales/PHWC-2B737/2020|2020-04-13|EPI_ISL_445754|Original|hCoV-19^^Wales|Human|Wales Specialist Virology Centre|Public Health Wales Microbiology Cardiff|Moore|United Kingdom
>Spike|hCoV-19/USA/WA-UW-1977/2020|2020-03-24|EPI_ISL_424337|Original|hCoV-19^^Washington|Human|UW Virology Lab|UW Virology Lab|Roychoudhury|USA
>Spike|hCoV-19/England/ALDP-9E8991/2020|2020-09-24|EPI_ISL_638270|Original|hCoV-19^^England|Human|Centre for Enzyme Innovation|COVID-19 Genomics UK (COG-UK) Consortium|Robson|United Kingdom
>Spike|hCoV-19/USA/DC-BBI2/2020|2020-10-14|EPI_ISL_605791|Original|hCoV-19^^District of Columbia|Human|Brotman Baty Institute for Precision Medicine|Brotman Baty Institute for Precision Medicine|Bedford|USA
>Spike|hCoV-19/England/SHEF-C039A/2020|2020-03-28|EPI_ISL_420195|Original|hCoV-19^^England|Human|Virology Department|Department of Infection|de Silva|United Kingdom
>Spike|hCoV-19/Denmark/DCGC-13299/2020|2020-11-30|EPI_ISL_712718|Original|hCoV-19^^Hovedstaden|Human|Department of Virus and Microbiological Special Diagnostics|Albertsen Lab|Michaelsen|Denmark
>Spike|hCoV-19/Denmark/DCGC-6207/2020|2020-10-05|EPI_ISL_620110|Original|hCoV-19^^Midtjylland|Human|Department of Virus and Microbiological Special Diagnostics|Albertsen lab|Michaelsen|Denmark
>Spike|hCoV-19/England/20138080104/2020|2020-03-26|EPI_ISL_423325|Original|hCoV-19^^England|Human|Respiratory Virus Unit|Respiratory Virus Unit|Platt|United Kingdom
>Spike|hCoV-19/USA/VA-DCLS-2171/2020|2020-11-06|EPI_ISL_677200|Original|hCoV-19^^Virginia|Human|Virginia Division of Consolidated Laboratory Services|Virginia Division of Consolidated Laboratory Services|St. Jacques|USA
>Spike|hCoV-19/England/BRIS-123394/2020|2020-04-06|EPI_ISL_442746|Original|hCoV-19^^England|Human|PHE South West Regional Laboratory|Wellcome Sanger Institute for the COVID-19 Genomics UK (COG-UK) consortium|Amato|United Kingdom
>Spike|hCoV-19/Northern Ireland/NIRE-FAF2A/2020|2020-04-14|EPI_ISL_470024|Original|hCoV-19^^Northern Ireland|Human|Regional Virus Laboratory|Wellcome Sanger Institute for the COVID-19 Genomics UK (COG-UK) consortium|Davies|United Kingdom
>Spike|hCoV-19/Australia/VIC5860/2020|2020-07-18|EPI_ISL_518653|Original|hCoV-19^^Victoria|Human|Microbiological Diagnostic Unit - Public Health Laboratory (MDU-PHL)|MDU-PHL|Schultz|Australia
>Spike|hCoV-19/Australia/SAP063/2020|2020-03-28|EPI_ISL_451102|Original|hCoV-19^^South Australia|Human|SA Pathology|SA Pathology|Leong|Australia
>Spike|hCoV-19/DRC/191/2020|2020-03-21|EPI_ISL_417955|Original|hCoV-19^^Kinshasa|Human|Viral Respiratory Lab|Pathogen Sequencing Lab|Pratt|Democratic Republic of the Congo
>Spike|hCoV-19/France/ARA78036/2020|2020-09-25|EPI_ISL_639995|Original|hCoV-19^^Auvergne-Rhone-Alpes|Human|CNR Virus des Infections Respiratoires - France SUD|CNR Virus des Infections Respiratoires - France SUD|Josset|France
>Spike|hCoV-19/USA/WA-UW-1805/2020|2020-03-22|EPI_ISL_424264|Original|hCoV-19^^Washington|Human|UW Virology Lab|UW Virology Lab|Roychoudhury|USA
>Spike|hCoV-19/England/201140309/2020|2020-03-11|EPI_ISL_464721|Original|hCoV-19^^England|Human|Respiratory Virus Unit|Respiratory Virus Unit|Platt|United Kingdom
>Spike|hCoV-19/Northern Ireland/NIRE-108D00/2020|2020-10-09|EPI_ISL_627554|Original|hCoV-19^^Northern Ireland|Human|Regional Virus Laboratory|COVID-19 Genomics UK (COG-UK) Consortium|Fairley|United Kingdom
>Spike|hCoV-19/Wales/PHWC-49FA5F/2020|2020-12-09|EPI_ISL_743310|Original|hCoV-19^^Wales|Human|Wales Specialist Virology Centre Sequencing lab|COVID-19 Genomics UK (COG-UK) Consortium|Moore|United Kingdom
>Spike|hCoV-19/England/LIVE-1D28FF/2020|2020-08-28|EPI_ISL_534747|Original|hCoV-19^^England|Human|Liverpool Clinical Laboratories|COVID-19 Genomics UK (COG-UK) Consortium|Haldenby|United Kingdom

>Spike|hCoV-19/England/QEUH-9CA564/2020|2020-09-09|EPI_ISL_567446|Original|hCoV-19^^England|Human|Lighthouse Lab in Glasgow|Wellcome Sanger Institute for the COVID-19 Genomics UK (COG-UK) consortium|Davies|United Kingdom
>Spike|hCoV-19/Denmark/DCGC-21354/2020|2020-12-21|EPI_ISL_759697|Original|hCoV-19^^Hovedstaden|Human|Department of Virus and Microbiological Special Diagnostics|Albertsen Lab|Michaelsen|Denmark
>Spike|hCoV-19/USA/TX-HMH-1183/2020|2020-04-01|EPI_ISL_542571|Original|hCoV-19^^Texas|Human|Houston Methodist Hospital|Houston Methodist Hospital|Long|USA
>Spike|hCoV-19/USA/TX-HMH-MCoV-15041/2020|2020-10-15|EPI_ISL_786525|Original|hCoV-19^^Texas|Human|Houston Methodist Hospital|Houston Methodist Hospital|Long|USA
>Spike|hCoV-19/Denmark/DCGC-11541/2020|2020-11-02|EPI_ISL_670519|Original|hCoV-19^^Hovedstaden|Human|Department of Virus and Microbiological Special Diagnostics|Albertsen Lab|Michaelsen|Denmark
>Spike|hCoV-19/USA/MI-MDHHS-SC22186/2020|2020-10-20|EPI_ISL_614237|Original|hCoV-19^^Michigan|Human|Michigan Department of Health and Human Services|Michigan Department of Health and Human Services|Jacob|USA
>Spike|hCoV-19/USA/WA-S2159/2020|2020-06-08|EPI_ISL_497587|Original|hCoV-19^^Washington|Human|Washington State Department of Health|Seattle Flu Study|Lee|USA
>Spike|hCoV-19/South Korea/KDCA0293/2020|2020-10-26|EPI_ISL_760087|Original|hCoV-19^^South Korea|Human|Division of Emerging Infectious Diseases|Division of Emerging Infectious Diseases|Park|South Korea
>Spike|hCoV-19/England/ALDP-9BEDD4/2020|2020-09-10|EPI_ISL_568020|Original|hCoV-19^^England|Human|Lighthouse Lab in Alderley Park|Wellcome Sanger Institute for the COVID-19 Genomics UK (COG-UK) consortium|Davies|United Kingdom
>Spike|hCoV-19/Japan/PG-5545/2020|2020-08-00|EPI_ISL_688670|Original|hCoV-19^^Japan|Human|Pathogen Genomics Center|Pathogen Genomics Center|Sekizuka|Japan
>Spike|hCoV-19/England/OXON-ADD95/2020|2020-00-00|EPI_ISL_448635|Original|hCoV-19^^England|Human|Oxford Viromics|COVID-19 Genomics UK (COG-UK) Consortium|Golubchik|United Kingdom
>Spike|hCoV-19/USA/CA-ALSR-1021-IPL/2020|2020-04-23|EPI_ISL_483467|Original|hCoV-19^^California|Human|UC San Diego Center for Advanced Laboratory Medicine|Andersen lab at Scripps Research|Gangavarapu|USA
>Spike|hCoV-19/Wales/PHWC-49919C/2020|2020-12-02|EPI_ISL_727266|Original|hCoV-19^^Wales|Human|Originating lab|COVID-19 Genomics UK (COG-UK) Consortium|Moore|United Kingdom
>Spike|hCoV-19/Wales/PHWC-487EE5/2020|2020-10-31|EPI_ISL_639322|Original|hCoV-19^^Wales|Human|Wales Specialist Virology Centre Sequencing lab|COVID-19 Genomics UK (COG-UK) Consortium|Moore|United Kingdom
>Spike|hCoV-19/Germany/HH-hpi-p1770/2020|2020-03-19|EPI_ISL_776144|Original|hCoV-19^^Hamburg|Human|University Medical Center Hamburg Eppendorf|Heinrich Pette Institute|Robitaille|Germany
>Spike|hCoV-19/USA/WA-S2020/2020|2020-06-05|EPI_ISL_497448|Original|hCoV-19^^Washington|Human|Washington State Department of Health|Seattle Flu Study|Lee|USA
>Spike|hCoV-19/USA/WA-S347/2020|2020-04-01|EPI_ISL_434107|Original|hCoV-19^^Washington|Human|Washington State Department of Health|Seattle Flu Study|Bedford|USA
>Spike|hCoV-19/England/C200044549/2020|2020-11-06|EPI_ISL_664033|Original|hCoV-19^^England|Human|Respiratory Virus Unit|COVID-19 Genomics UK (COG-UK) Consortium|Platt|United Kingdom
>Spike|hCoV-19/Wales/PHWC-483A30/2020|2020-10-06|EPI_ISL_626875|Original|hCoV-19^^Wales|Human|Wales Specialist Virology Centre Sequencing lab|COVID-19 Genomics UK (COG-UK) Consortium|Moore|United Kingdom
>Spike|hCoV-19/Japan/TKYT22342/2020|2020-07-00|EPI_ISL_649126|Original|hCoV-19^^Japan|Human|Tokyo Metropolitan Institute of Public Health|Tokyo Metropolitan Institute of Public Health|Ryota|Japan
>Spike|hCoV-19/Netherlands/ZH-EMC-296/2020|2020-07-03|EPI_ISL_523638|Original|hCoV-19^^Zuid Holland|Human|Dutch COVID-19 response team|Erasmus Medical Center|Oude Munnink|Netherlands

>Spike|hCoV-19/USA/WA-S679/2020|2020-04-07|EPI_ISL_449849|Original|hCoV-19^^Washington|Human|Washington State Department of Health|Seattle Flu Study|Bedford|USA
>Spike|hCoV-19/England/ALDP-A6E73E/2020|2020-10-15|EPI_ISL_607953|Original|hCoV-19^^England|Human|Lighthouse Lab in Alderley Park|Wellcome Sanger Institute for the COVID-19 Genomics UK (COG-UK) consortium|Davies|United Kingdom
>Spike|hCoV-19/England/ALDP-4FF659/2020|2020-06-23|EPI_ISL_559138|Original|hCoV-19^^England|Human|Lighthouse Lab in Alderley Park|Wellcome Sanger Institute for the COVID-19 Genomics UK (COG-UK) consortium|Davies|United Kingdom
>Spike|hCoV-19/Denmark/DCGC-3450/2020|2020-08-31|EPI_ISL_616214|Original|hCoV-19^^Syddanmark|Human|Department of Virus and Microbiological Special Diagnostics|Albertsen lab|Michaelsen|Denmark
>Spike|hCoV-19/Scotland/QEUH-9DAB41/2020|2020-09-22|EPI_ISL_601144|Original|hCoV-19^^Scotland|Human|Lighthouse Lab in Glasgow|Wellcome Sanger Institute for the COVID-19 Genomics UK (COG-UK) consortium|Davies|United Kingdom
>Spike|hCoV-19/Australia/VIC15984/2020|2020-10-09|EPI_ISL_641104|Original|hCoV-19^^Victoria|Human|Microbiological Diagnostic Unit - Public Health Laboratory (MDU-PHL)|MDU-PHL|Schultz|Australia
>Spike|hCoV-19/England/CAMC-B08A87/2020|2020-10-29|EPI_ISL_643902|Original|hCoV-19^^England|Human|Lighthouse Lab in Cambridge|Wellcome Sanger Institute for the COVID-19 Genomics UK (COG-UK) Consortium|Davies|United Kingdom
>Spike|hCoV-19/Switzerland/ZH-ETHZ-160008/2020|2020-06-19|EPI_ISL_486505|Original|hCoV-19^^Zurich|Human|Viollier AG|Department of Biosystems Science and Engineering|Nadeau|Switzerland
>Spike|hCoV-19/Scotland/EDB4727/2020|2020-04-28|EPI_ISL_487659|Original|hCoV-19^^Scotland|Human|Virology Department|Wellcome Sanger Institute for the COVID-19 Genomics UK (COG-UK) consortium|Davies|United Kingdom
>Spike|hCoV-19/Japan/PG-2125/2020|2020-05-00|EPI_ISL_685730|Original|hCoV-19^^Japan|Human|Pathogen Genomics Center|Pathogen Genomics Center|Sekizuka|Japan
>Spike|hCoV-19/Spain/VC-FISABIO-3/2020|2020-03-05|EPI_ISL_414598|Original|hCoV-19^^Comunitat Valenciana|Human|Servicio Microbiologia|Sequencing and Bioinformatics Service and Molecular Epidemiology Research Group. FISABIO-Public Health.|Gonzalez-Candelas|Spain
>Spike|hCoV-19/Germany/SN-RKI-NP-0028/2020|2020-12-08|EPI_ISL_763018|Original|hCoV-19^^Saxony|Human|Unit 17|Project group Epidemiology of Highly Pathogenic Microorganisms|Schubert|Germany
>Spike|hCoV-19/England/LIVE-DC2D06/2020|2020-11-24|EPI_ISL_702894|Original|hCoV-19^^England|Human|Liverpool Clinical Laboratories|COVID-19 Genomics UK (COG-UK) Consortium|Haldenby|United Kingdom
>Spike|hCoV-19/Scotland/CVR3233/2020|2020-04-26|EPI_ISL_449243|Original|hCoV-19^^Scotland|Human|West of Scotland Specialist Virology Centre|COVID-19 Genomics UK (COG-UK) Consortium|Robertson|United Kingdom
>Spike|hCoV-19/Denmark/DCGC-18685/2020|2020-12-07|EPI_ISL_750123|Original|hCoV-19^^Hovedstaden|Human|Department of Virus and Microbiological Special Diagnostics|Albertsen Lab|Michaelsen|Denmark
>Spike|hCoV-19/Denmark/DCGC-16540/2020|2020-11-16|EPI_ISL_714825|Original|hCoV-19^^Sjaelland|Human|Department of Virus and Microbiological Special Diagnostics|Albertsen Lab|Michaelsen|Denmark
>Spike|hCoV-19/Scotland/QEUH-96D79A/2020|2020-08-20|EPI_ISL_534635|Original|hCoV-19^^Scotland|Human|NHSGGC West of Scotland Specialist Virology Centre / MRC-University of Glasgow Centre for Virus Research|Wellcome Sanger Institute for the COVID-19 Genomics UK (COG-UK) consortium|Davies|United Kingdom
>Spike|hCoV-19/England/CAMB-812D4/2020|2020-04-11|EPI_ISL_442986|Original|hCoV-19^^England|Human|Department of Pathology|Wellcome Sanger Institute for the COVID-19 Genomics UK (COG-UK) consortium|Amato|United Kingdom
>Spike|hCoV-19/Denmark/DCGC-14858/2020|2020-11-23|EPI_ISL_712032|Original|hCoV-19^^Hovedstaden|Human|Department of Virus and Microbiological Special Diagnostics|Albertsen Lab|Michaelsen|Denmark

>Spike|hCoV-19/France/ARA-1811/2020|2020-04-01|EPI_ISL_508962|Original|hCoV-19^^Auvergne-Rh\u00f4ne-Alpes|Human|CNR Virus des Infections Respiratoires - France SUD|CNR Virus des Infections Respiratoires - France SUD|Josset|France
>Spike|hCoV-19/Denmark/DCGC-8147/2020|2020-10-26|EPI_ISL_617215|Original|hCoV-19^^Hovedstaden|Human|Department of Virus and Microbiological Special Diagnostics|Albertsen lab|Michaelsen|Denmark
>Spike|hCoV-19/England/ALDP-A6EADB/2020|2020-10-15|EPI_ISL_606952|Original|hCoV-19^^England|Human|Lighthouse Lab in Alderley Park|Wellcome Sanger Institute for the COVID-19 Genomics UK (COG-UK) consortium|Davies|United Kingdom
>Spike|hCoV-19/England/CAMB-1B1498/2020|2020-05-08|EPI_ISL_458556|Original|hCoV-19^^England|Human|Department of Pathology|Wellcome Sanger Institute for the COVID-19 Genomics UK (COG-UK) consortium|Davies|United Kingdom
>Spike|hCoV-19/Spain/NC-IBV-001403/2020|2020-03-19|EPI_ISL_452498|Original|hCoV-19^^Navarra|Human|Cl\u00ednica Universidad de Navarra. Servicio de Enfermedades Infecciosas y Microbiolog\u00eda cl\u00ednica|SeqCOVID-SPAIN consortium/IBV(CSIC)|Comas|Spain
>Spike|hCoV-19/England/CAMB-84BB8/2020|2020-03-11|EPI_ISL_440485|Original|hCoV-19^^England|Human|Department of Pathology|Wellcome Sanger Institute for the COVID-19 Genomics UK (COG-UK) consortium|Amato|United Kingdom
>Spike|hCoV-19/Spain/MD-IBV-99012307/2020|2020-11-14|EPI_ISL_779970|Original|hCoV-19^^Madrid|Human|Hospital General Universitario Gregorio Mara\u00f1\u00f3n|SeqCOVID-SPAIN consortium/IBV(CSIC)|Comas|Spain
>Spike|hCoV-19/England/QEUH-997CC5/2020|2020-08-25|EPI_ISL_559760|Original|hCoV-19^^England|Human|Oxford Viromics|COVID-19 Genomics UK (COG-UK) Consortium|Golubchik|United Kingdom
>Spike|hCoV-19/England/QEUH-CDF7FA/2020|2020-12-26|EPI_ISL_782446|Original|hCoV-19^^England|Human|Lighthouse Lab in Glasgow|Wellcome Sanger Institute for the COVID-19 Genomics UK (COG-UK) Consortium|Davies|United Kingdom
>Spike|hCoV-19/USA/CT-Yale-095/2020|2020-04-08|EPI_ISL_428398|Original|hCoV-19^^Connecticut|Human|Yale COVID-19 Biorepository|Grubaugh Lab - Yale School of Public Health|Grubaugh|USA
>Spike|hCoV-19/USA/IL-NM-0487/2020|2020-04-23|EPI_ISL_626427|Original|hCoV-19^^Illinois|Human|Northwestern Memorial Hospital|Ozer Lab|Ozer|USA
>Spike|hCoV-19/Australia/VIC7518/2020|2020-00-00|EPI_ISL_564464|Original|hCoV-19^^Victoria|Human|Microbiological Diagnostic Unit - Public Health Laboratory (MDU-PHL)|MDU-PHL|Schultz|Australia
>Spike|hCoV-19/Luxembourg/LNS7342327/2020|2020-05-12|EPI_ISL_459905|Original|hCoV-19^^Luxembourg|Human|Laboratoire National de Sante|Laboratoire National de Sante|Wienecke-Baldacchino|Luxembourg
>Spike|hCoV-19/England/MILK-A6B573/2020|2020-10-09|EPI_ISL_608782|Original|hCoV-19^^England|Human|Lighthouse Lab in Milton Keynes|Wellcome Sanger Institute for the COVID-19 Genomics UK (COG-UK) consortium|Davies|United Kingdom
>Spike|hCoV-19/USA/WI-UW-48/2020|2020-03-25|EPI_ISL_421306|Original|hCoV-19^^Wisconsin|Human|University of Wisconsin-Madison AIDS Vaccine Research Laboratories|University of Wisconsin-Madison AIDS Vaccine Research Laboratories|Baczenas|USA
>Spike|hCoV-19/Scotland/QEUH-5F6624/2020|2020-06-25|EPI_ISL_533271|Original|hCoV-19^^Scotland|Human|NHSGGC West of Scotland Specialist Virology Centre / MRC-University of Glasgow Centre for Virus Research|Wellcome Sanger Institute for the COVID-19 Genomics UK (COG-UK) consortium|Davies|United Kingdom
>Spike|hCoV-19/Wales/PHWC-4878D8/2020|2020-10-28|EPI_ISL_639250|Original|hCoV-19^^Wales|Human|Wales Specialist Virology Centre Sequencing lab|COVID-19 Genomics UK (COG-UK) Consortium|Moore|United Kingdom
>Spike|hCoV-19/Finland/S8AC11/2020|2020-10-10|EPI_ISL_737056|Original|hCoV-19^^Uusimaa|Human|Department of Virology and Immunology|Department of Virology|Smura|Finland
>Spike|hCoV-19/Luxembourg/LNS4260242/2020|2020-11-21|EPI_ISL_740439|Original|hCoV-19^^Luxembourg|Human|Laboratoire national de sant\u00e9|Laboratoire national de sant\u00e9|Wienecke-Baldacchino|Luxembourg

>Spike|hCoV-19/Denmark/DCGC-12921/2020|2020-12-07|EPI_ISL_712464|Original|hCoV-
19^^Midtjylland|Human|Department of Virus and Microbiological Special
Diagnostics|Albertsen Lab|Michaelsen|Denmark
>Spike|hCoV-19/Australia/QLD1012/2020|2020-03-26|EPI_ISL_530272|Original|hCoV-
19^^Queensland|Human|Queensland Health Forensic and Scientific Services|Public Health
Virology Laboratory|Nguyen|Australia
>Spike|hCoV-19/Denmark/DCGC-16128/2020|2020-11-16|EPI_ISL_714599|Original|hCoV-
19^^Midtjylland|Human|Department of Virus and Microbiological Special
Diagnostics|Albertsen Lab|Michaelsen|Denmark
>Spike|hCoV-19/Wales/PHWC-483D73/2020|2020-10-21|EPI_ISL_627927|Original|hCoV-
19^^Wales|Human|Wales Specialist Virology Centre Sequencing lab|COVID-19 Genomics UK
(COG-UK) Consortium|Moore|United Kingdom
>Spike|hCoV-19/Denmark/DCGC-10210/2020|2020-11-09|EPI_ISL_669457|Original|hCoV-
19^^Hovedstaden|Human|Department of Virus and Microbiological Special
Diagnostics|Albertsen Lab|Michaelsen|Denmark
>Spike|hCoV-19/England/ALDP-94DF01/2020|2020-06-17|EPI_ISL_555305|Original|hCoV-
19^^England|Human|Lighthouse Lab in Alderley Park|Wellcome Sanger Institute for the
COVID-19 Genomics UK (COG-UK) consortium|Davies|United Kingdom
>Spike|hCoV-19/Australia/VIC11735/2020|2020-07-23|EPI_ISL_563476|Original|hCoV-
19^^Victoria|Human|Microbiological Diagnostic Unit - Public Health Laboratory (MDU-
PHL)|MDU-PHL|Schultz|Australia
>Spike|hCoV-19/Australia/VIC16840/2020|2020-08-06|EPI_ISL_663599|Original|hCoV-
19^^Victoria|Human|Microbiological Diagnostic Unit - Public Health Laboratory (MDU-
PHL)|MDU-PHL|Schultz|Australia
>Spike|hCoV-19/England/QEUH-9BA2F7/2020|2020-09-07|EPI_ISL_573587|Original|hCoV-
19^^England|Human|Oxford Viromics|COVID-19 Genomics UK (COG-UK)
Consortium|Golubchik|United Kingdom
>Spike|hCoV-19/Denmark/ALAB-SSI-832/2020|2020-03-30|EPI_ISL_444928|Original|hCoV-
19^^Denmark|Human|Department of Virus and Microbiological Special
Diagnostics|Albertsen lab|Kirkegaard|Denmark
>Spike|hCoV-19/England/MILK-A7CE0B/2020|2020-10-13|EPI_ISL_606801|Original|hCoV-
19^^England|Human|Lighthouse Lab in Milton Keynes|Wellcome Sanger Institute for the
COVID-19 Genomics UK (COG-UK) consortium|Davies|United Kingdom
>Spike|hCoV-19/Wales/PHWC-48C897/2020|2020-11-06|EPI_ISL_665464|Original|hCoV-
19^^Wales|Human|Wales Specialist Virology Centre Sequencing lab|COVID-19 Genomics UK
(COG-UK) Consortium|Moore|United Kingdom
>Spike|hCoV-19/Wales/MILK-9E227E/2020|2020-09-20|EPI_ISL_581225|Original|hCoV-
19^^Wales|Human|Lighthouse Lab in Milton Keynes|Wellcome Sanger Institute for the
COVID-19 Genomics UK (COG-UK) consortium|Davies|United Kingdom
>Spike|hCoV-19/South Africa/KRISP-K003478/2020|2020-09-
12|EPI_ISL_602810|Original|hCoV-19^^KwaZulu-Natal|Human|NHLS-IALCH|KRISP|de
Oliveira|South Africa
>Spike|hCoV-19/USA/WA-UW-5629/2020|2020-04-08|EPI_ISL_430912|Original|hCoV-
19^^Washington|Human|UW Virology Lab|UW Virology Lab|Roychoudhury|USA
>Spike|hCoV-19/Chile/RM-CMM-IN233883987/2020|2020-08-24|EPI_ISL_591531|Original|hCoV-
19^^Region Metropolitana de Santiago|Human|Laboratorio de Infectologia y virologia
molecular|Center for Mathematical Modeling and Center for Genome Regulation.
Santiago|Palma V\u00e9jares|Chile
>Spike|hCoV-19/Wales/ALDP-C39D56/2020|2020-12-09|EPI_ISL_767207|Original|hCoV-
19^^Wales|Human|Lighthouse Lab in Alderley Park|Wellcome Sanger Institute for the
COVID-19 Genomics UK (COG-UK) Consortium|Davies|United Kingdom
>Spike|hCoV-19/England/CAMC-949C68/2020|2020-07-19|EPI_ISL_556750|Original|hCoV-
19^^England|Human|Lighthouse Lab in Cambridge|Wellcome Sanger Institute for the
COVID-19 Genomics UK (COG-UK) consortium|Davies|United Kingdom
>Spike|hCoV-19/Spain/CL-IBV-97006187/2020|2020-04-13|EPI_ISL_537333|Original|hCoV-
19^^Castilla y Leon|Human|Universidad de Le\u00f3n|SeqCOVID-SPAIN
consortium/IBV(CSIC)|Comas|Spain
>Spike|hCoV-19/USA/AZ-TG305912/2020|2020-04-29|EPI_ISL_694296|Original|hCoV-
19^^Arizona|Human|AZ SPHL|TGen North|French|USA

>Spike|hCoV-19/England/MILK-A8EF9A/2020|2020-10-16|EPI_ISL_650247|Original|hCoV-
19^^England|Human|Northumbria University / South Tees Hospitals NHS Foundation Trust
/ North Cumbria Integrated Care NHS Foundation Trust / North Tees and Hartlepool NHS
Foundation Trust / Newcastle Hospitals NHS Foundation Trust|COVID-19 Genomics UK
(COG-UK) Consortium|Bashton|United Kingdom
>Spike|hCoV-19/India/MH-ACTREC-157/2020|2020-06-04|EPI_ISL_699813|Original|hCoV-
19^^Maharashtra|Human|Hematopathology Laboratory|Hematopathology
Laboratory|Patkar|India
>Spike|hCoV-19/Netherlands/ZH-EMC-652/2020|2020-09-28|EPI_ISL_632494|Original|hCoV-
19^^Zuid Holland|Human|Dutch COVID-19 response team|Erasmus Medical Center|Oude
Munnink|Netherlands
>Spike|hCoV-19/England/CAMC-B98794/2020|2020-11-15|EPI_ISL_673788|Original|hCoV-
19^^England|Human|Lighthouse Lab in Cambridge|Wellcome Sanger Institute for the
COVID-19 Genomics UK (COG-UK) Consortium|Davies|United Kingdom
>Spike|hCoV-19/USA/CA-ALSR-1841/2020|2020-05-18|EPI_ISL_512204|Original|hCoV-
19^^California|Human|San Diego County Public Health Laboratory|Andersen lab at
Scripps Research|Gangavarapu|USA
>Spike|hCoV-19/Wales/PHWC-4896DC/2020|2020-11-02|EPI_ISL_639606|Original|hCoV-
19^^Wales|Human|Wales Specialist Virology Centre Sequencing lab|COVID-19 Genomics UK
(COG-UK) Consortium|Moore|United Kingdom
>Spike|hCoV-19/Lithuania/MR-LUHS-Eilnr25/2020|2020-04-
06|EPI_ISL_541852|Original|hCoV-19^^Alytaus apskritis|Human|Lithuanian University of
Health Sciences Hospital|Lithuanian University of Health Sciences|Zemaitis|Lithuania
>Spike|hCoV-19/Denmark/DCGC-14769/2020|2020-11-23|EPI_ISL_711966|Original|hCoV-
19^^Hovedstaden|Human|Department of Virus and Microbiological Special
Diagnostics|Albertsen Lab|Michaelsen|Denmark
>Spike|hCoV-19/England/205260457/2020|2020-12-18|EPI_ISL_754381|Original|hCoV-
19^^England|Human|Respiratory Virus Unit|COVID-19 Genomics UK (COG-UK)
Consortium|Platt|United Kingdom
>Spike|hCoV-19/USA/NY-NYCPHL-001228/2020|2020-11-05|EPI_ISL_683891|Original|hCoV-
19^^New York|Human|DOHMH Crown Heights|New York City Public Health
Laboratory|Wang|USA
>Spike|hCoV-19/USA/CA-CZB-13425/2020|2020-09-03|EPI_ISL_672469|Original|hCoV-
19^^California|Human|Alameda County Public Health Lab|Chan-Zuckerberg
Biohub|Bhatt|USA
>Spike|hCoV-19/USA/MI-MDHHS-SC21245/2020|2020-04-04|EPI_ISL_507500|Original|hCoV-
19^^Michigan|Human|Michigan Department of Health and Human Services|Michigan
Department of Health and Human Services|Blankenship|USA
>Spike|hCoV-19/England/CAMC-A40E4C/2020|2020-10-06|EPI_ISL_609530|Original|hCoV-
19^^England|Human|Lighthouse Lab in Cambridge|Wellcome Sanger Institute for the
COVID-19 Genomics UK (COG-UK) consortium|Davies|United Kingdom
>Spike|hCoV-19/England/QEUH-9648BE/2020|2020-08-11|EPI_ISL_536938|Original|hCoV-
19^^England|Human|Lighthouse Lab in Glasgow|Wellcome Sanger Institute for the COVID-
19 Genomics UK (COG-UK) consortium|Davies|United Kingdom
>Spike|hCoV-19/Sweden/20-51952/2020|2020-06-05|EPI_ISL_560974|Original|hCoV-
19^^Stockholm|Human|Karolinska Universitetslaboratoriet|The Public Health Agency of
Sweden|Svartstr\u00f6m|Sweden
>Spike|hCoV-19/England/QEUH-C5B750/2020|2020-12-14|EPI_ISL_736057|Original|hCoV-
19^^England|Human|Lighthouse Lab in Glasgow|Wellcome Sanger Institute for the COVID-
19 Genomics UK (COG-UK) Consortium|Davies|United Kingdom
>Spike|hCoV-19/England/ALDP-B62942/2020|2020-11-09|EPI_ISL_656602|Original|hCoV-
19^^England|Human|Lighthouse Lab in Alderley Park|Wellcome Sanger Institute for the
COVID-19 Genomics UK (COG-UK) Consortium|Davies|United Kingdom
>Spike|hCoV-19/Australia/VIC6059/2020|2020-07-26|EPI_ISL_518097|Original|hCoV-
19^^Victoria|Human|Microbiological Diagnostic Unit - Public Health Laboratory (MDU-
PHL)|MDU-PHL|Schultz|Australia
>Spike|hCoV-19/Denmark/DCGC-7245/2020|2020-10-12|EPI_ISL_619224|Original|hCoV-
19^^Hovedstaden|Human|Department of Virus and Microbiological Special
Diagnostics|Albertsen lab|Michaelsen|Denmark

>Spike|hCoV-19/SaudiArabia/KAUST-MADINAH955/2020|2020-05-12|EPI_ISL_513205|Original|hCoV-19^^Madinah|Human|Pathogen Genomics Lab King Abdullah University of Science and Technology(KAUST)|Pathogen Genomics Lab King Abdullah University of Science and Technology(KAUST)|Mohamed Ghazzali|Saudi Arabia
>Spike|hCoV-19/Canada/BC_0554880/2020|2020-03-13|EPI_ISL_418817|Original|hCoV-19^^British Columbia|Human|BCCDC Public Health Laboratory|BCCDC Public Health Laboratory|Harrigan|Canada
>Spike|hCoV-19/England/ALDP-BB3D64/2020|2020-11-20|EPI_ISL_701798|Original|hCoV-19^^England|Human|Lighthouse Lab in Alderley Park|Wellcome Sanger Institute for the COVID-19 Genomics UK (COG-UK) Consortium|Davies|United Kingdom
>Spike|hCoV-19/Luxembourg/LNS7025344/2020|2020-10-15|EPI_ISL_744495|Original|hCoV-19^^Luxembourg|Human|Laboratoire national de sant\u00e9|Laboratoire national de sant\u00e9|Wienecke-Baldacchino|Luxembourg
>Spike|hCoV-19/Wales/PHWC-162A67/2020|2020-05-13|EPI_ISL_473155|Original|hCoV-19^^Wales|Human|Wales Specialist Virology Centre Sequencing lab|COVID-19 Genomics UK (COG-UK) Consortium|Moore|United Kingdom
>Spike|hCoV-19/USA/UT-03002/2020|2020-05-01|EPI_ISL_513700|Original|hCoV-19^^Utah|Human|Utah Public Health Laboratory|Utah Public Health Laboratory|Butz|USA
>Spike|hCoV-19/USA/TX-HMH-493/2020|2020-04-07|EPI_ISL_544410|Original|hCoV-19^^Texas|Human|Houston Methodist Hospital|Houston Methodist Hospital|Long|USA
>Spike|hCoV-19/Denmark/DCGC-16807/2020|2020-12-14|EPI_ISL_750575|Original|hCoV-19^^Hovedstaden|Human|Department of Virus and Microbiological Special Diagnostics|Albertsen Lab|Michaelsen|Denmark
>Spike|hCoV-19/Palestine/52/2020|2020-08-19|EPI_ISL_596536|Original|hCoV-19^^Ramallah|Human|Palestinian Ministry of Health|Molecular Genetics Lab|van Dorp|Palestine
>Spike|hCoV-19/USA/TX-DSHS-0380/2020|2020-06-14|EPI_ISL_538316|Original|hCoV-19^^Texas|Human|Texas Department of State Health Services|Texas Department of State Health Services|Rahman|USA
>Spike|hCoV-19/England/ALDP-94E2E9/2020|2020-06-19|EPI_ISL_555746|Original|hCoV-19^^England|Human|Lighthouse Lab in Alderley Park|Wellcome Sanger Institute for the COVID-19 Genomics UK (COG-UK) consortium|Davies|United Kingdom
>Spike|hCoV-19/USA/AZ-TG371276/2020|2020-05-15|EPI_ISL_694535|Original|hCoV-19^^Arizona|Human|AZ SPHL|TGen North|French|USA
>Spike|hCoV-19/Scotland/EDB11046/2020|2020-12-14|EPI_ISL_742196|Original|hCoV-19^^Scotland|Human|Virology Department|COVID-19 Genomics UK (COG-UK) Consortium|McHugh|United Kingdom
>Spike|hCoV-19/USA/MD-MDH-0424/2020|2020-11-03|EPI_ISL_666792|Original|hCoV-19^^Maryland|Human|Maryland Public Health Laboratory|Maryland Public Health Laboratory|Keller|USA
>Spike|hCoV-19/England/CAMB-77B21/2020|2020-04-01|EPI_ISL_439425|Original|hCoV-19^^England|Human|Department of Pathology|Wellcome Sanger Institute for the COVID-19 Genomics UK (COG-UK) consortium|Amato|United Kingdom
>Spike|hCoV-19/Netherlands/ZH-EMC-228/2020|2020-05-17|EPI_ISL_523570|Original|hCoV-19^^Zuid Holland|Human|Dutch COVID-19 response team|Erasmus Medical Center|Oude Munnink|Netherlands
>Spike|hCoV-19/USA/MN-MDH-1232/2020|2020-05-18|EPI_ISL_482953|Original|hCoV-19^^Minnesota|Human|Minnesota Department of Health|Minnesota Department of Health|Garfin|USA
>Spike|hCoV-19/Wales/PHWC-16A601/2020|2020-09-08|EPI_ISL_540915|Original|hCoV-19^^Wales|Human|Wales Specialist Virology Centre Sequencing lab|COVID-19 Genomics UK (COG-UK) Consortium|Moore|United Kingdom
>Spike|hCoV-19/Italy/VEN-UniVR-135/2020|2020-04-02|EPI_ISL_751432|Original|hCoV-19^^Veneto|Human|IRCCS Sacro Cuore Don Calabria Hospital|University of Verona|Mori|Italy
>Spike|hCoV-19/England/BRIS-12BBFE/2020|2020-05-16|EPI_ISL_469371|Original|hCoV-19^^England|Human|PHE South West Regional Laboratory|Wellcome Sanger Institute for the COVID-19 Genomics UK (COG-UK) consortium|Davies|United Kingdom
>Spike|hCoV-19/USA/UT-03434/2020|2020-04-20|EPI_ISL_498663|Original|hCoV-19^^Utah|Human|Utah Public Health Laboratory|Utah Public Health Laboratory|Butz|USA

>Spike|hCoV-19/England/LIVE-9F77A/2020|2020-04-21|EPI_ISL_472012|Original|hCoV-19^^England|Human|Liverpool Clinical Laboratories|COVID-19 Genomics UK (COG-UK) Consortium|Haldenby|United Kingdom
>Spike|hCoV-19/England/ALDP-4F5BAF/2020|2020-06-24|EPI_ISL_559190|Original|hCoV-19^^England|Human|Lighthouse Lab in Alderley Park|Wellcome Sanger Institute for the COVID-19 Genomics UK (COG-UK) consortium|Davies|United Kingdom
>Spike|hCoV-19/England/ALDP-9FD304/2020|2020-10-02|EPI_ISL_587894|Original|hCoV-19^^England|Human|Lighthouse Lab in Alderley Park|Wellcome Sanger Institute for the COVID-19 Genomics UK (COG-UK) consortium|Davies|United Kingdom
>Spike|hCoV-19/USA/TX-HMH-MCoV-14526/2020|2020-10-08|EPI_ISL_787811|Original|hCoV-19^^Texas|Human|Houston Methodist Hospital|Houston Methodist Hospital|Long|USA
>Spike|hCoV-19/USA/WA-UW-22415/2020|2020-08-07|EPI_ISL_570591|Original|hCoV-19^^Washington|Human|UW Virology Lab|UW Virology Lab|Roychoudhury|USA
>Spike|hCoV-19/Denmark/DCGC-21584/2020|2020-12-21|EPI_ISL_758249|Original|hCoV-19^^Hovedstaden|Human|Department of Virus and Microbiological Special Diagnostics|Albertsen Lab|Michaelsen|Denmark
>Spike|hCoV-19/Denmark/DCGC-19611/2020|2020-12-07|EPI_ISL_749059|Original|hCoV-19^^Hovedstaden|Human|Department of Virus and Microbiological Special Diagnostics|Albertsen Lab|Michaelsen|Denmark
>Spike|hCoV-19/Bangladesh/BARJ-CVASU-CTG-501/2020|2020-05-10|EPI_ISL_450339|Original|hCoV-19^^Chittagong|Human|Bangladesh Institute of Tropical & Infectious Diseases|Basic and Applied Research on Jute Project|Ahmed|Bangladesh
>Spike|hCoV-19/Japan/PG-4049/2020|2020-07-00|EPI_ISL_692290|Original|hCoV-19^^Japan|Human|Sakai City Institute of Public Health|Pathogen Genomics Center|Sekizuka|Japan
>Spike|hCoV-19/England/ALDP-A95A08/2020|2020-10-19|EPI_ISL_624563|Original|hCoV-19^^England|Human|Lighthouse Lab in Alderley Park|Wellcome Sanger Institute for the COVID-19 Genomics UK (COG-UK) consortium|Davies|United Kingdom
>Spike|hCoV-19/England/GSTT-265D3C3/2020|2020-03-18|EPI_ISL_483952|Original|hCoV-19^^England|Human|Centre for Clinical Infection and Diagnostics Research and Genomics Innovation Unit|COVID-19 Genomics UK (COG-UK) Consortium|Awan|United Kingdom
>Spike|hCoV-19/England/LIVE-9996B/2020|2020-04-09|EPI_ISL_439778|Original|hCoV-19^^England|Human|Liverpool Clinical Laboratories|COVID-19 Genomics UK (COG-UK) Consortium|Haldenby|United Kingdom
>Spike|hCoV-19/England/CAMB-76991/2020|2020-03-30|EPI_ISL_438424|Original|hCoV-19^^England|Human|Department of Pathology|Wellcome Sanger Institute for the COVID-19 Genomics UK (COG-UK) consortium|Amato|United Kingdom
>Spike|hCoV-19/USA/FL-BPHL-1074/2020|2020-04-24|EPI_ISL_526560|Original|hCoV-19^^Florida|Human|Florida Bureau of Public Health Laboratories|Florida Bureau of Public Health Laboratories|Blanton|USA
>Spike|hCoV-19/USA/TX-HMH-MCoV-14158/2020|2020-10-02|EPI_ISL_787443|Original|hCoV-19^^Texas|Human|Houston Methodist Hospital|Houston Methodist Hospital|Long|USA
>Spike|hCoV-19/USA/LA-EVTL717/2020|2020-07-14|EPI_ISL_653409|Original|hCoV-19^^Louisiana|Human|LSUHS Emerging Viral Threat Laboratory|Microbial Genome Sequencing Center|Kamil|USA
>Spike|hCoV-19/Japan/PG-2544/2020|2020-05-00|EPI_ISL_690679|Original|hCoV-19^^Japan|Human|Saitama Prefectural Institute of Public Health|Pathogen Genomics Center|Sekizuka|Japan
>Spike|hCoV-19/England/CAMC-A4191D/2020|2020-10-04|EPI_ISL_609476|Original|hCoV-19^^England|Human|Lighthouse Lab in Cambridge|Wellcome Sanger Institute for the COVID-19 Genomics UK (COG-UK) consortium|Davies|United Kingdom
>Spike|hCoV-19/Denmark/DCGC-18710/2020|2020-12-07|EPI_ISL_750136|Original|hCoV-19^^Midtjylland|Human|Department of Virus and Microbiological Special Diagnostics|Albertsen Lab|Michaelsen|Denmark
>Spike|hCoV-19/Scotland/CVR2572/2020|2020-04-16|EPI_ISL_473516|Original|hCoV-19^^Scotland|Human|West of Scotland Specialist Virology Centre|COVID-19 Genomics UK (COG-UK) Consortium|Robertson|United Kingdom
>Spike|hCoV-19/Wales/CAMC-9B1AAD/2020|2020-09-04|EPI_ISL_590173|Original|hCoV-19^^Wales|Human|Lighthouse Lab in Cambridge|Wellcome Sanger Institute for the COVID-19 Genomics UK (COG-UK) consortium|Davies|United Kingdom

>Spike|hCoV-19/Israel/ISR_NH_GD3_0726/2020|2020-07-26|EPI_ISL_594162|Original|hCoV-19^^Israel|Human|Israel Institute for Biological Research|Israel Institute for Biological Research|Zaide|Israel
>Spike|hCoV-19/Scotland/QEUH-964308/2020|2020-08-12|EPI_ISL_534398|Original|hCoV-19^^Scotland|Human|NHSGGC West of Scotland Specialist Virology Centre / MRC-University of Glasgow Centre for Virus Research|Wellcome Sanger Institute for the COVID-19 Genomics UK (COG-UK) consortium|Davies|United Kingdom
>Spike|hCoV-19/England/204691279/2020|2020-11-13|EPI_ISL_686595|Original|hCoV-19^^England|Human|Respiratory Virus Unit|COVID-19 Genomics UK (COG-UK) Consortium|Platt|United Kingdom
>Spike|hCoV-19/England/BIRM-67034/2020|2020-10-28|EPI_ISL_651605|Original|hCoV-19^^England|Human|University of Birmingham|COVID-19 Genomics UK (COG-UK) Consortium|Loman|United Kingdom
>Spike|hCoV-19/Denmark/DCGC-16975/2020|2020-12-14|EPI_ISL_750680|Original|hCoV-19^^Nordjylland|Human|Department of Virus and Microbiological Special Diagnostics|Albertsen Lab|Michaelsen|Denmark
>Spike|hCoV-19/Spain/VC-IBV-98000396/2020|2020-03-23|EPI_ISL_539244|Original|hCoV-19^^Comunitat Valenciana|Human|Servicio de Microbiologia. Hospital Clinico Universitario de Valencia|SeqCOVID-SPAIN consortium/IBV(CSIC)|Comas|Spain
>Spike|hCoV-19/USA/CA-ALSR-4505/2020|2020-11-02|EPI_ISL_666973|Original|hCoV-19^^California|Human|San Diego County Public Health Laboratory|Andersen lab at Scripps Research|Gangavarapu|USA
>Spike|hCoV-19/England/CAMC-A40F3A/2020|2020-10-06|EPI_ISL_609525|Original|hCoV-19^^England|Human|Lighthouse Lab in Cambridge|Wellcome Sanger Institute for the COVID-19 Genomics UK (COG-UK) consortium|Davies|United Kingdom
>Spike|hCoV-19/England/CAMC-A658DA/2020|2020-10-11|EPI_ISL_608679|Original|hCoV-19^^England|Human|Lighthouse Lab in Cambridge|Wellcome Sanger Institute for the COVID-19 Genomics UK (COG-UK) consortium|Davies|United Kingdom
>Spike|hCoV-19/Australia/VIC12435/2020|2020-08-01|EPI_ISL_641042|Original|hCoV-19^^Victoria|Human|Microbiological Diagnostic Unit - Public Health Laboratory (MDU-PHL)|MDU-PHL|Schultz|Australia
>Spike|hCoV-19/Denmark/DCGC-19143/2020|2020-12-07|EPI_ISL_748256|Original|hCoV-19^^Hovedstaden|Human|Department of Virus and Microbiological Special Diagnostics|Albertsen Lab|Michaelsen|Denmark
>Spike|hCoV-19/England/ALDP-B5BD7D/2020|2020-11-09|EPI_ISL_655848|Original|hCoV-19^^England|Human|Lighthouse Lab in Alderley Park|Wellcome Sanger Institute for the COVID-19 Genomics UK (COG-UK) Consortium|Davies|United Kingdom
>Spike|hCoV-19/Northern Ireland/QEUH-9634CA/2020|2020-08-11|EPI_ISL_531763|Original|hCoV-19^^Northern Ireland|Human|Lighthouse Lab in Glasgow|Wellcome Sanger Institute for the COVID-19 Genomics UK (COG-UK) consortium|Davies|United Kingdom
>Spike|hCoV-19/USA/TX-HMH-3584/2020|2020-06-15|EPI_ISL_543041|Original|hCoV-19^^Texas|Human|Houston Methodist Hospital|Houston Methodist Hospital|Long|USA
>Spike|hCoV-19/Mexico/BCN-ALSR-1696/2020|2020-04-24|EPI_ISL_496357|Original|hCoV-19^^Baja California|Human|Infectolab|Andersen lab at Scripps Research|Gangavarapu|Mexico
>Spike|hCoV-19/Wales/PHWC-3177A/2020|2020-04-13|EPI_ISL_446483|Original|hCoV-19^^Wales|Human|Wales Specialist Virology Centre|Public Health Wales Microbiology Cardiff|Moore|United Kingdom
>Spike|hCoV-19/England/QEUH-AE49FE/2020|2020-10-27|EPI_ISL_633185|Original|hCoV-19^^England|Human|Lighthouse Lab in Glasgow|Wellcome Sanger Institute for the COVID-19 Genomics UK (COG-UK) consortium|Davies|United Kingdom
>Spike|hCoV-19/USA/UT-UPHL-03104/2020|2020-05-05|EPI_ISL_576367|Original|hCoV-19^^Utah|Human|Utah Public Health Laboratory|Utah Public Health Laboratory|Young|USA
>Spike|hCoV-19/Canada/QC-LSPQ-L00242573/2020|2020-03-29|EPI_ISL_536327|Original|hCoV-19^^Quebec|Human|H\u00f4pital Charles-LeMoyne|Laboratoire de sant\u00e9 publique du Qu\u00e9bec|Moreira|Canada
>Spike|hCoV-19/USA/WI-UW-167/2020|2020-03-18|EPI_ISL_428263|Original|hCoV-19^^Wisconsin|Human|University of Wisconsin-Madison AIDS Vaccine Research

Laboratories|University of Wisconsin-Madison AIDS Vaccine Research Laboratories|Moreno|USA
>Spike|hCoV-19/Brazil/RS-00644/2020|2020-11-25|EPI_ISL_779161|Original|hCoV-19^^Rio Grande do Sul|Human|Laborat\u00f3rio de Microbiologia Molecular - Universidade FEEVALE|Bioinformatics Laboratory / LNCC|Almeida|Brazil
>Spike|hCoV-19/Luxembourg/LNS6889202/2020|2020-06-26|EPI_ISL_744480|Original|hCoV-19^^Luxembourg|Human|Laboratoire national de sant\u00e9|Laboratoire national de sant\u00e9|Wienecke-Baldacchino|Luxembourg
>Spike|hCoV-19/Scotland/QEUH-AD1499/2020|2020-10-27|EPI_ISL_649685|Original|hCoV-19^^Scotland|Human|Lighthouse Lab in Glasgow|Wellcome Sanger Institute for the COVID-19 Genomics UK (COG-UK) Consortium|Davies|United Kingdom
>Spike|hCoV-19/Denmark/DCGC-13480/2020|2020-11-30|EPI_ISL_712823|Original|hCoV-19^^Midtjylland|Human|Department of Virus and Microbiological Special Diagnostics|Albertsen Lab|Michaelsen|Denmark
>Spike|hCoV-19/Australia/VIC9366/2020|2020-08-17|EPI_ISL_565523|Original|hCoV-19^^Victoria|Human|Microbiological Diagnostic Unit - Public Health Laboratory (MDU-PHL)|MDU-PHL|Schultz|Australia
>Spike|hCoV-19/Denmark/DCGC-18590/2020|2020-12-07|EPI_ISL_750065|Original|hCoV-19^^Hovedstaden|Human|Department of Virus and Microbiological Special Diagnostics|Albertsen Lab|Michaelsen|Denmark
>Spike|hCoV-19/Brazil/RJ-1119/2020|2020-03-25|EPI_ISL_456077|Original|hCoV-19^^Rio de Janeiro|Human|LACEN RJ - Laborat\u00f3rio Central de Sa\u00fade P\u00fablica Noel Nutels|Laboratory of Respiratory Viruses and Measles|Silva|Brazil
>Spike|hCoV-19/England/201140250/2020|2020-03-09|EPI_ISL_464715|Original|hCoV-19^^England|Human|Respiratory Virus Unit|Respiratory Virus Unit|Platt|United Kingdom
>Spike|hCoV-19/England/LOND-DC3B9/2020|2020-10-26|EPI_ISL_651253|Original|hCoV-19^^England|Human|University College London|COVID-19 Genomics UK (COG-UK) Consortium|Roy|United Kingdom
>Spike|hCoV-19/South Africa/KRISP-K000643/2020|2020-07-10|EPI_ISL_509292|Original|hCoV-19^^KwaZulu-Natal|Human|NHLS-IALCH|KRISP|de Oliveira|South Africa
>Spike|hCoV-19/Denmark/DCGC-10184/2020|2020-11-09|EPI_ISL_669442|Original|hCoV-19^^Hovedstaden|Human|Department of Virus and Microbiological Special Diagnostics|Albertsen Lab|Michaelsen|Denmark
>Spike|hCoV-19/Switzerland/AG-ETHZ-410275/2020|2020-12-15|EPI_ISL_737546|Original|hCoV-19^^Aargau|Human|Viollier AG|Department of Biosystems Science and Engineering|Nadeau|Switzerland
>Spike|hCoV-19/Northern Ireland/QEUH-96DD5C/2020|2020-08-20|EPI_ISL_530762|Original|hCoV-19^^Northern Ireland|Human|Lighthouse Lab in Glasgow|Wellcome Sanger Institute for the COVID-19 Genomics UK (COG-UK) consortium|Davies|United Kingdom
>Spike|hCoV-19/Jordan/AM-ALSR-3909/2020|2020-09-30|EPI_ISL_636275|Original|hCoV-19^^Amman|Human|Biolab Diagnostic Laboratories|Andersen lab at Scripps Research|Gangavarapu|Jordan
>Spike|hCoV-19/Denmark/DCGC-2018/2020|2020-07-06|EPI_ISL_618230|Original|hCoV-19^^Syddanmark|Human|Department of Virus and Microbiological Special Diagnostics|Albertsen lab|Michaelsen|Denmark
>Spike|hCoV-19/Germany/BE-ChVir-D1914-8251/2020|2020-10-05|EPI_ISL_753862|Original|hCoV-19^^Berlin|Human|Charit\u00e9 Universit\u00e4tsmedizin Berlin|Charit\u00e9 Universit\u00e4tsmedizin Berlin|Muehlemann|Germany
>Spike|hCoV-19/France/ARA33273/2020|2020-05-02|EPI_ISL_636490|Original|hCoV-19^^Auvergne-Rhone-Alpes|Human|CNR Virus des Infections Respiratoires - France SUD|CNR Virus des Infections Respiratoires - France SUD|Josset|France
>Spike|hCoV-19/Spain/MD-IBV-97010077/2020|2020-03-17|EPI_ISL_779739|Original|hCoV-19^^Madrid|Human|IdISSC/Hospital Cl\u00ednico San Carlos de Madrid\u200b|SeqCOVID-SPAIN consortium/IBV(CSIC)|Comas|Spain
>Spike|hCoV-19/England/203020503/2020|2020-07-10|EPI_ISL_500709|Original|hCoV-19^^England|Human|Respiratory Virus Unit|Respiratory Virus Unit|Platt|United Kingdom

>Spike|hCoV-19/Spain/CL-IBV-97006179/2020|2020-04-10|EPI_ISL_537326|Original|hCoV-19^^Castilla y Leon|Human|Universidad de Le\u00f3n|SeqCOVID-SPAIN consortium/IBV(CSIC)|Comas|Spain
>Spike|hCoV-19/Chile/RM-133147/2020|2020-07-25|EPI_ISL_746631|Original|hCoV-19^^Region Metropolitana de Santiago|Human|Genetica Molecular and Subdepartamento de Virologia ISP Chile|Instituto de Salud Publica de Chile|Tognarelli|Chile
>Spike|hCoV-19/England/MILK-976910/2020|2020-08-18|EPI_ISL_552754|Original|hCoV-19^^England|Human|Lighthouse Lab in Milton Keynes|Wellcome Sanger Institute for the COVID-19 Genomics UK (COG-UK) consortium|Davies|United Kingdom
>Spike|hCoV-19/England/QEUH-9DE11E/2020|2020-09-21|EPI_ISL_599506|Original|hCoV-19^^England|Human|Lighthouse Lab in Glasgow|Wellcome Sanger Institute for the COVID-19 Genomics UK (COG-UK) consortium|Davies|United Kingdom
>Spike|hCoV-19/USA/TX-DSHS-1381/2020|2020-05-13|EPI_ISL_654746|Original|hCoV-19^^Texas|Human|Texas Department of State Health Services|Texas Department of State Health Services|Rahman|USA
>Spike|hCoV-19/Denmark/DCGC-798/2020|2020-04-20|EPI_ISL_614706|Original|hCoV-19^^Hovedstaden|Human|Department of Virus and Microbiological Special Diagnostics|Albertsen lab|Michaelsen|Denmark
>Spike|hCoV-19/Scotland/CVR5784/2020|2020-11-10|EPI_ISL_666024|Original|hCoV-19^^Scotland|Human|West of Scotland Specialist Virology Centre|COVID-19 Genomics UK (COG-UK) Consortium|Robertson|United Kingdom
>Spike|hCoV-19/England/QEUH-75E998/2020|2020-07-16|EPI_ISL_532981|Original|hCoV-19^^England|Human|Lighthouse Lab in Glasgow|Wellcome Sanger Institute for the COVID-19 Genomics UK (COG-UK) consortium|Davies|United Kingdom
>Spike|hCoV-19/Denmark/DCGC-10514/2020|2020-11-02|EPI_ISL_670028|Original|hCoV-19^^Sjaelland|Human|Department of Virus and Microbiological Special Diagnostics|Albertsen Lab|Michaelsen|Denmark
>Spike|hCoV-19/Wales/PHWC-168409/2020|2020-04-01|EPI_ISL_494239|Original|hCoV-19^^Wales|Human|Originating lab|COVID-19 Genomics UK (COG-UK) Consortium|Moore|United Kingdom
>Spike|hCoV-19/England/ALDP-94C8BC/2020|2020-06-16|EPI_ISL_555925|Original|hCoV-19^^England|Human|Lighthouse Lab in Alderley Park|Wellcome Sanger Institute for the COVID-19 Genomics UK (COG-UK) consortium|Davies|United Kingdom
>Spike|hCoV-19/England/BRIS-132CA6/2020|2020-06-15|EPI_ISL_511974|Original|hCoV-19^^England|Human|PHE South West Regional Laboratory|Wellcome Sanger Institute for the COVID-19 Genomics UK (COG-UK) consortium|Davies|United Kingdom
>Spike|hCoV-19/England/202822171/2020|2020-05-30|EPI_ISL_490228|Original|hCoV-19^^England|Human|Respiratory Virus Unit|Respiratory Virus Unit|Platt|United Kingdom
>Spike|hCoV-19/South Africa/R12681-20/2020|2020-06-16|EPI_ISL_504222|Original|hCoV-19^^North West|Human|National Institute for Communicable Diseases of the National Health Laboratory Service|National Institute for Communicable Diseases of the National Health Laboratory Service|Allam|South Africa
>Spike|hCoV-19/Denmark/DCGC-1545/2020|2020-06-01|EPI_ISL_617966|Original|hCoV-19^^Hovedstaden|Human|Department of Virus and Microbiological Special Diagnostics|Albertsen lab|Michaelsen|Denmark
>Spike|hCoV-19/Japan/PG-4419/2020|2020-08-00|EPI_ISL_692644|Original|hCoV-19^^Japan|Human|Pathogen Genomics Center|Pathogen Genomics Center|Sekizuka|Japan
>Spike|hCoV-19/USA/TX-DSHS-0177/2020|2020-03-13|EPI_ISL_524058|Original|hCoV-19^^Texas|Human|Texas Department of State Health Services|Texas Department of State Health Services|Rahman|USA
>Spike|hCoV-19/Australia/VIC8994/2020|2020-07-27|EPI_ISL_565344|Original|hCoV-19^^Victoria|Human|Microbiological Diagnostic Unit - Public Health Laboratory (MDU-PHL)|MDU-PHL|Schultz|Australia
>Spike|hCoV-19/Japan/PG-1038/2020|2020-04-00|EPI_ISL_684648|Original|hCoV-19^^Japan|Human|Fukuoka Institute of Health and Environmental Sciences|Pathogen Genomics Center|Sekizuka|Japan
>Spike|hCoV-19/England/MILK-99E235/2020|2020-08-31|EPI_ISL_550687|Original|hCoV-19^^England|Human|Lighthouse Lab in Milton Keynes|Wellcome Sanger Institute for the COVID-19 Genomics UK (COG-UK) consortium|Davies|United Kingdom

>Spike|hCoV-19/England/MILK-B01EEC/2020|2020-10-24|EPI_ISL_642775|Original|hCoV-19^^England|Human|Lighthouse Lab in Milton Keynes|Wellcome Sanger Institute for the COVID-19 Genomics UK (COG-UK) Consortium|Davies|United Kingdom
>Spike|hCoV-19/USA/WA-UW313/2020|2020-03-14|EPI_ISL_418880|Original|hCoV-19^^Washington|Human|UW Virology Lab|UW Virology Lab|Roychoudhury|USA
>Spike|hCoV-19/England/QEUH-A36A63/2020|2020-10-05|EPI_ISL_638690|Original|hCoV-19^^England|Human|Wales Specialist Virology Centre Sequencing lab|COVID-19 Genomics UK (COG-UK) Consortium|Moore|United Kingdom
>Spike|hCoV-19/Switzerland/BL-ETHZ-260114/2020|2020-08-26|EPI_ISL_539423|Original|hCoV-19^^Basel-Landschaft|Human|Viollier AG|Department of Biosystems Science and Engineering|Nadeau|Switzerland
>Spike|hCoV-19/Australia/VIC787/2020|2020-03-30|EPI_ISL_427062|Original|hCoV-19^^Victoria|Human|Microbiological Diagnostic Unit Public Health Laboratory|Microbiological Diagnostic Unit Public Health Laboratory|Schultz|Australia
>Spike|hCoV-19/USA/MA-MGH-00170/2020|2020-03-27|EPI_ISL_460283|Original|hCoV-19^^Massachusetts|Human|Massachusetts General Hospital|Infectious Disease Program|Lemieux|USA
>Spike|hCoV-19/England/NOTT-11F331/2020|2020-11-24|EPI_ISL_679963|Original|hCoV-19^^England|Human|Lincolnshire Hospitals and DeepSeq Nottingham|COVID-19 Genomics UK (COG-UK) Consortium|Loose|United Kingdom
>Spike|hCoV-19/Luxembourg/LNS6358751/2020|2020-10-30|EPI_ISL_744402|Original|hCoV-19^^Luxembourg|Human|Laboratoire national de sant\u00e9|Laboratoire national de sant\u00e9|Wienecke-Baldacchino|Luxembourg
>Spike|hCoV-19/Wales/PHWC-4907EE/2020|2020-11-13|EPI_ISL_706513|Original|hCoV-19^^Wales|Human|Originating lab|COVID-19 Genomics UK (COG-UK) Consortium|Moore|United Kingdom
>Spike|hCoV-19/USA/CA-QDX-1117/2020|2020-03-20|EPI_ISL_571813|Original|hCoV-19^^California|Human|Quest Diagnostics|Quest Diagnostics|Kagan|USA
>Spike|hCoV-19/USA/TX-HMH-MCoV-15109/2020|2020-10-21|EPI_ISL_786593|Original|hCoV-19^^Texas|Human|Houston Methodist Hospital|Houston Methodist Hospital|Long|USA
>Spike|hCoV-19/Scotland/QEUH-A4B588/2020|2020-10-08|EPI_ISL_637841|Original|hCoV-19^^Scotland|Human|Department of Pathology|COVID-19 Genomics UK (COG-UK) Consortium|Goodfellow|United Kingdom
>Spike|hCoV-19/Germany/HH-hpi-p1299/2020|2020-03-19|EPI_ISL_775887|Original|hCoV-19^^Hamburg|Human|University Medical Center Hamburg Eppendorf|Heinrich Pette Institute|Robitaille|Germany
>Spike|hCoV-19/USA/MN-QDX-1778/2020|2020-03-13|EPI_ISL_604090|Original|hCoV-19^^Minnesota|Human|Quest Diagnostics|Quest Diagnostics|Kagan|USA
>Spike|hCoV-19/USA/NY-Wadsworth-288188-01/2020|2020-12-15|EPI_ISL_767475|Original|hCoV-19^^New York|Human|Wadsworth Center|Wadsworth Center|Lasek-Nesselquist|USA
>Spike|hCoV-19/USA/CA-ALSR-0578-SAN/2020|2020-05-13|EPI_ISL_483234|Original|hCoV-19^^California|Human|UC San Diego Center for Advanced Laboratory Medicine|Andersen lab at Scripps Research|Gangavarapu|USA
>Spike|hCoV-19/England/MILK-B55AB8/2020|2020-11-05|EPI_ISL_657391|Original|hCoV-19^^England|Human|Lighthouse Lab in Milton Keynes|Wellcome Sanger Institute for the COVID-19 Genomics UK (COG-UK) Consortium|Davies|United Kingdom
>Spike|hCoV-19/England/ALDP-A6EEB1/2020|2020-10-15|EPI_ISL_607220|Original|hCoV-19^^England|Human|Lighthouse Lab in Alderley Park|Wellcome Sanger Institute for the COVID-19 Genomics UK (COG-UK) consortium|Davies|United Kingdom
>Spike|hCoV-19/USA/MN-MDH-1152/2020|2020-05-01|EPI_ISL_476737|Original|hCoV-19^^Minnesota|Human|Minnesota Department of Health|Minnesota Department of Health|Garfin|USA
>Spike|hCoV-19/Wales/ALDP-94E568/2020|2020-06-16|EPI_ISL_555392|Original|hCoV-19^^Wales|Human|Lighthouse Lab in Alderley Park|Wellcome Sanger Institute for the COVID-19 Genomics UK (COG-UK) consortium|Davies|United Kingdom
>Spike|hCoV-19/England/MILK-B939EE/2020|2020-11-13|EPI_ISL_676157|Original|hCoV-19^^England|Human|Lighthouse Lab in Milton Keynes|Wellcome Sanger Institute for the COVID-19 Genomics UK (COG-UK) Consortium|Davies|United Kingdom

>Spike|hCoV-19/England/20170041504/2020|2020-04-17|EPI_ISL_465293|Original|hCoV-19^^England|Human|Respiratory Virus Unit|Respiratory Virus Unit|Platt|United Kingdom
>Spike|hCoV-19/Australia/VIC697/2020|2020-03-28|EPI_ISL_426989|Original|hCoV-19^^Victoria|Human|Victorian Infectious Diseases Reference Laboratory (VIDRL)|Microbiological Diagnostic Unit Public Health Laboratory and Victorian Infectious Diseases Reference Laboratory|Schultz|Australia
>Spike|hCoV-19/Japan/PG-3334/2020|2020-04-00|EPI_ISL_691449|Original|hCoV-19^^Japan|Human|Pathogen Genomics Center|Pathogen Genomics Center|Sekizuka|Japan
>Spike|hCoV-19/Denmark/DCGC-13293/2020|2020-11-30|EPI_ISL_712713|Original|hCoV-19^^Sjaelland|Human|Department of Virus and Microbiological Special Diagnostics|Albertsen Lab|Michaelsen|Denmark
>Spike|hCoV-19/England/CAMC-9E001C/2020|2020-09-21|EPI_ISL_576952|Original|hCoV-19^^England|Human|Oxford Viromics|COVID-19 Genomics UK (COG-UK) Consortium|Golubchik|United Kingdom
>Spike|hCoV-19/England/QEUH-AD6379/2020|2020-10-24|EPI_ISL_629760|Original|hCoV-19^^England|Human|Lighthouse Lab in Glasgow|Wellcome Sanger Institute for the COVID-19 Genomics UK (COG-UK) consortium|Davies|United Kingdom
>Spike|hCoV-19/Australia/NSW1282/2020|2020-12-17|EPI_ISL_745106|Original|hCoV-19^^New South Wales|Human|Pathology North - Royal North Shore Hospital - NSW Health Pathology|CIDM-PH|Gall|Australia
>Spike|hCoV-19/Denmark/DCGC-3864/2020|2020-09-07|EPI_ISL_622741|Original|hCoV-19^^Hovedstaden|Human|Department of Virus and Microbiological Special Diagnostics|Albertsen lab|Michaelsen|Denmark
>Spike|hCoV-19/USA/WI-WSLH-200607/2020|2020-08-18|EPI_ISL_631400|Original|hCoV-19^^Wisconsin|Human|Wisconsin State Laboratory of Hygiene Communicable Disease Division|Wisconsin State Laboratory of Hygiene Communicable Disease Division|Florek|USA
>Spike|hCoV-19/USA/LA-QDX-1194/2020|2020-03-16|EPI_ISL_571940|Original|hCoV-19^^Louisiana|Human|Quest Diagnostics|Quest Diagnostics|Kagan|USA
>Spike|hCoV-19/Switzerland/SO-ETHZ-380245/2020|2020-11-21|EPI_ISL_721874|Original|hCoV-19^^Solothurn|Human|Viollier AG|Department of Biosystems Science and Engineering|Nadeau|Switzerland
>Spike|hCoV-19/England/CAMC-947259/2020|2020-07-21|EPI_ISL_556574|Original|hCoV-19^^England|Human|Lighthouse Lab in Cambridge|Wellcome Sanger Institute for the COVID-19 Genomics UK (COG-UK) consortium|Davies|United Kingdom
>Spike|hCoV-19/USA/TX-HMH-MCoV-13887/2020|2020-08-17|EPI_ISL_787179|Original|hCoV-19^^Texas|Human|Houston Methodist Hospital|Houston Methodist Hospital|Long|USA
>Spike|hCoV-19/England/ALDP-94B8EA/2020|2020-06-11|EPI_ISL_556391|Original|hCoV-19^^England|Human|Lighthouse Lab in Alderley Park|Wellcome Sanger Institute for the COVID-19 Genomics UK (COG-UK) consortium|Davies|United Kingdom
>Spike|hCoV-19/Portugal/PT0594/2020|2020-04-03|EPI_ISL_454317|Original|hCoV-19^^Portugal|Human|unknown|Instituto Nacional de Saude (INSA)|Borges|Portugal
>Spike|hCoV-19/England/CAMC-B783B4/2020|2020-11-11|EPI_ISL_661651|Original|hCoV-19^^England|Human|Lighthouse Lab in Cambridge|Wellcome Sanger Institute for the COVID-19 Genomics UK (COG-UK) Consortium|Davies|United Kingdom
>Spike|hCoV-19/Russia/MOW-RII-MH2010S/2020|2020-06-05|EPI_ISL_596351|Original|hCoV-19^^Moscow|Human|Pathogenic Microorganisms Variability Laboratory|WHO National Influenza Centre Russian Federation|Komissarov|Russia
>Spike|hCoV-19/Japan/PG-5240/2020|2020-08-00|EPI_ISL_690343|Original|hCoV-19^^Japan|Human|Pathogen Genomics Center|Pathogen Genomics Center|Sekizuka|Japan
>Spike|hCoV-19/England/MILK-BB1FF7/2020|2020-11-18|EPI_ISL_720427|Original|hCoV-19^^England|Human|Lighthouse Lab in Milton Keynes|Wellcome Sanger Institute for the COVID-19 Genomics UK (COG-UK) Consortium|Davies|United Kingdom
>Spike|hCoV-19/Wales/PHWC-24F18/2020|2020-03-20|EPI_ISL_420927|Original|hCoV-19^^Wales|Human|Wales Specialist Virology Centre|Public Health Wales Microbiology Cardiff|Moore|United Kingdom
>Spike|hCoV-19/USA/MN-MDH-2016/2020|2020-09-28|EPI_ISL_654697|Original|hCoV-19^^Minnesota|Human|Essentia Health-St. Mary's Medical Center|Minnesota Department of Health|Lorentz|USA

>Spike|hCoV-19/South Korea/KDCA0378/2020|2020-11-04|EPI_ISL_760162|Original|hCoV-19^^South Korea|Human|Division of Emerging Infectious Diseases|Division of Emerging Infectious Diseases|Park|South Korea
>Spike|hCoV-19/England/ALDP-B1CB7E/2020|2020-11-02|EPI_ISL_678831|Original|hCoV-19^^England|Human|Centre for Enzyme Innovation|COVID-19 Genomics UK (COG-UK) Consortium|Robson|United Kingdom
>Spike|hCoV-19/South Korea/KCDC2382/2020|2020-05-31|EPI_ISL_514785|Original|hCoV-19^^South Korea|Human|Division of Viral Diseases|Division of Viral Diseases|Kim|South Korea
>Spike|hCoV-19/England/QEUH-96F90B/2020|2020-08-17|EPI_ISL_540320|Original|hCoV-19^^England|Human|Lighthouse Lab in Glasgow|Wellcome Sanger Institute for the COVID-19 Genomics UK (COG-UK) consortium|Davies|United Kingdom
>Spike|hCoV-19/Scotland/CVR3537/2020|2020-05-14|EPI_ISL_473674|Original|hCoV-19^^Scotland|Human|West of Scotland Specialist Virology Centre|COVID-19 Genomics UK (COG-UK) Consortium|Robertson|United Kingdom
>Spike|hCoV-19/England/NOTT-10EAD6/2020|2020-03-30|EPI_ISL_425638|Original|hCoV-19^^England|Human|Queens Medical Centre|COVID-19 Genomics UK (COG-UK) Consortium|Loose|United Kingdom
>Spike|hCoV-19/Poland/Wro-02/2020|2020-03-18|EPI_ISL_437625|Original|hCoV-19^^Dolnoslakie|Human|Laboratory of Genomics & Bioinformatics|Laboratory of Genomics & Bioinformatics|Laczmanski|Poland
>Spike|hCoV-19/USA/TX-HMH-MCoV-9982/2020|2020-07-06|EPI_ISL_780365|Original|hCoV-19^^Texas|Human|Houston Methodist Hospital|Houston Methodist Hospital|Long|USA
>Spike|hCoV-19/England/ALDP-B638CC/2020|2020-11-09|EPI_ISL_656426|Original|hCoV-19^^England|Human|Lighthouse Lab in Alderley Park|Wellcome Sanger Institute for the COVID-19 Genomics UK (COG-UK) Consortium|Davies|United Kingdom
>Spike|hCoV-19/Denmark/DCGC-5862/2020|2020-09-28|EPI_ISL_621049|Original|hCoV-19^^Midtjylland|Human|Department of Virus and Microbiological Special Diagnostics|Albertsen lab|Michaelsen|Denmark
>Spike|hCoV-19/Denmark/DCGC-21985/2020|2020-12-14|EPI_ISL_758493|Original|hCoV-19^^Sjaelland|Human|Department of Virus and Microbiological Special Diagnostics|Albertsen Lab|Michaelsen|Denmark
>Spike|hCoV-19/England/CAMB-7BC66/2020|2020-04-04|EPI_ISL_442046|Original|hCoV-19^^England|Human|Department of Pathology|Wellcome Sanger Institute for the COVID-19 Genomics UK (COG-UK) consortium|Amato|United Kingdom
>Spike|hCoV-19/Canada/NS-NML-160/2020|2020-04-18|EPI_ISL_579530|Original|hCoV-19^^Nova Scotia|Human|QEII Health Sciences Centre|National Microbiology Laboratory (NML)|Majer|Canada
>Spike|hCoV-19/England/210100343/2021|2021-01-04|EPI_ISL_791228|Original|hCoV-19^^England|Human|Respiratory Virus Unit|COVID-19 Genomics UK (COG-UK) Consortium|Platt|United Kingdom
>Spike|hCoV-19/Japan/PG-0423/2020|2020-04-02|EPI_ISL_480091|Original|hCoV-19^^Kansai|Human|Department of Infectious Diseases|Pathogen Genomics Center|Sekizuka|Japan
>Spike|hCoV-19/England/ALDP-9ED817/2020|2020-05-05|EPI_ISL_589285|Original|hCoV-19^^England|Human|Lighthouse Lab in Alderley Park|Wellcome Sanger Institute for the COVID-19 Genomics UK (COG-UK) consortium|Davies|United Kingdom
>Spike|hCoV-19/Scotland/EDB3181/2020|2020-04-06|EPI_ISL_488959|Original|hCoV-19^^Scotland|Human|Virology Department|Wellcome Sanger Institute for the COVID-19 Genomics UK (COG-UK) consortium|Davies|United Kingdom
>Spike|hCoV-19/USA/OR-OHSU-1453/2020|2020-07-17|EPI_ISL_509205|Original|hCoV-19^^Oregon|Human|OHSU Lab Services Molecular Microbiology Lab|Oregon SARS-CoV-2 Genome Sequencing Center|O'Roak|USA
>Spike|hCoV-19/Hong Kong/VM20010007/2020|2020-04-07|EPI_ISL_444040|Original|hCoV-19^^Hong Kong|Human|Queen Elizabeth Hospital|Hong Kong Department of Health|Mak|China
>Spike|hCoV-19/Wales/PHWC-16F6C0/2020|2020-10-05|EPI_ISL_586006|Original|hCoV-19^^Wales|Human|Wales Specialist Virology Centre Sequencing lab|COVID-19 Genomics UK (COG-UK) Consortium|Moore|United Kingdom
>Spike|hCoV-19/Belgium/rega-0616834/2020|2020-06-16|EPI_ISL_734761|Original|hCoV-19^^Hulste|Human|UZ Leuven|KU Leuven|Maes|Belgium

>Spike|hCoV-19/USA/TX-HMH-2383/2020|2020-05-25|EPI_ISL_545276|Original|hCoV-19^^Texas|Human|Houston Methodist Hospital|Houston Methodist Hospital|Long|USA
>Spike|hCoV-19/England/NORT-28AD50/2020|2020-04-15|EPI_ISL_492966|Original|hCoV-19^^England|Human|NU-OMICS DNA Sequencing research facility|Wellcome Sanger Institute for the COVID-19 Genomics UK (COG-UK) consortium|Davies|United Kingdom
>Spike|hCoV-19/USA/MA-Yale-439/2020|2020-04-10|EPI_ISL_738257|Original|hCoV-19^^Massachusetts|Human|Connecticut Veterans' Affairs Hospital|Grubaugh Lab - Yale School of Public Health|Grubaugh|USA
>Spike|hCoV-19/Brazil/CE-L13-CD207/2020|2020-03-16|EPI_ISL_476227|Original|hCoV-19^^Cear\u00e1|Human|DB Diagn\u00f3sticos do Brasil|Instituto de Medicina Tropical da Univesidade de S\u00e3o Paulo|Sabino|Brazil
>Spike|hCoV-19/Australia/VIC3552/2020|2020-07-08|EPI_ISL_520301|Original|hCoV-19^^Victoria|Human|Victorian Infectious Diseases Reference Laboratory (VIDRL)|VIDRL and MDU-PHL|Schultz|Australia
>Spike|hCoV-19/England/MILK-9AB156/2020|2020-08-31|EPI_ISL_551076|Original|hCoV-19^^England|Human|Lighthouse Lab in Milton Keynes|Wellcome Sanger Institute for the COVID-19 Genomics UK (COG-UK) consortium|Davies|United Kingdom
>Spike|hCoV-19/England/QEUH-9F7953/2020|2020-09-27|EPI_ISL_589165|Original|hCoV-19^^England|Human|Lighthouse Lab in Glasgow|Wellcome Sanger Institute for the COVID-19 Genomics UK (COG-UK) consortium|Davies|United Kingdom
>Spike|hCoV-19/England/MILK-BB0EEC/2020|2020-11-17|EPI_ISL_720561|Original|hCoV-19^^England|Human|Lighthouse Lab in Milton Keynes|Wellcome Sanger Institute for the COVID-19 Genomics UK (COG-UK) Consortium|Davies|United Kingdom
>Spike|hCoV-19/France/ARA-111014/2020|2020-11-23|EPI_ISL_728543|Original|hCoV-19^^Auvergne-Rh\u00f4ne-Alpes|Human|CNR Virus des Infections Respiratoires - France SUD|CNR Virus des Infections Respiratoires - France SUD|Bal|France
>Spike|hCoV-19/Denmark/DCGC-3611/2020|2020-09-07|EPI_ISL_616378|Original|hCoV-19^^Hovedstaden|Human|Department of Virus and Microbiological Special Diagnostics|Albertsen lab|Michaelsen|Denmark
>Spike|hCoV-19/England/20124095002/2020|2020-03-13|EPI_ISL_418727|Original|hCoV-19^^England|Human|Respiratory Virus Unit|Respiratory Virus Unit|Platt|United Kingdom
>Spike|hCoV-19/Wales/PHWC-497732/2020|2020-11-21|EPI_ISL_726873|Original|hCoV-19^^Wales|Human|Originating lab|COVID-19 Genomics UK (COG-UK) Consortium|Moore|United Kingdom
>Spike|hCoV-19/Scotland/QEUH-A4B263/2020|2020-10-08|EPI_ISL_638937|Original|hCoV-19^^Scotland|Human|Department of Pathology|COVID-19 Genomics UK (COG-UK) Consortium|Goodfellow|United Kingdom
>Spike|hCoV-19/Denmark/DCGC-9282/2020|2020-11-16|EPI_ISL_668886|Original|hCoV-19^^Nordjylland|Human|Department of Virus and Microbiological Special Diagnostics|Albertsen Lab|Michaelsen|Denmark
>Spike|hCoV-19/England/ALDP-4AC1E1/2020|2020-06-14|EPI_ISL_559551|Original|hCoV-19^^England|Human|Lighthouse Lab in Alderley Park|Wellcome Sanger Institute for the COVID-19 Genomics UK (COG-UK) consortium|Davies|United Kingdom
>Spike|hCoV-19/England/MILK-AB83F5/2020|2020-10-21|EPI_ISL_623303|Original|hCoV-19^^England|Human|Lighthouse Lab in Milton Keynes|Wellcome Sanger Institute for the COVID-19 Genomics UK (COG-UK) consortium|Davies|United Kingdom
>Spike|hCoV-19/South Africa/KRISP-K003327/2020|2020-09-07|EPI_ISL_602720|Original|hCoV-19^^KwaZulu-Natal|Human|NHLS-IALCH|KRISP|de Oliveira|South Africa
>Spike|hCoV-19/France/PAC-IHU-0195/2020|2020-03-27|EPI_ISL_569362|Original|hCoV-19^^Provence-Alpes-C\u00f4te-d\u2019Azur|Human|MEPHI|MEPHI|Levasseur|France
>Spike|hCoV-19/South Korea/KDCA0313/2020|2020-10-29|EPI_ISL_760099|Original|hCoV-19^^South Korea|Human|Division of Emerging Infectious Diseases|Division of Emerging Infectious Diseases|Park|South Korea
>Spike|hCoV-19/USA/MA-MGH-00466/2020|2020-04-04|EPI_ISL_460176|Original|hCoV-19^^Massachusetts|Human|Massachusetts General Hospital|Infectious Disease Program|Lemieux|USA
>Spike|hCoV-19/Denmark/DCGC-22247/2020|2020-12-14|EPI_ISL_758635|Original|hCoV-19^^Sjaelland|Human|Department of Virus and Microbiological Special Diagnostics|Albertsen Lab|Michaelsen|Denmark

>Spike|hCoV-19/Romania/Bucuresti-14658/2020|2020-04-17|EPI_ISL_468154|Original|hCoV-19^^Bucuresti|Human|[Romania|[Romania|Paraschiv|Romania

>Spike|hCoV-19/USA/NY-QDX-1225/2020|2020-03-17|EPI_ISL_572001|Original|hCoV-19^^New York|Human|Quest Diagnostics|Quest Diagnostics|Kagan|USA

>Spike|hCoV-19/New Zealand/20VR1899/2020|2020-03-31|EPI_ISL_456300|Original|hCoV-19^^Waikato|Human|LabPLUS|Institute of Environmental Science and Research (ESR)|de Ligt|New Zealand

>Spike|hCoV-19/SaudiArabia/KAUST-MADINAH521/2020|2020-04-06|EPI_ISL_513167|Original|hCoV-19^^Madinah|Human|Pathogen Genomics Lab King Abdullah University of Science and Technology(KAUST)|Pathogen Genomics Lab King Abdullah University of Science and Technology(KAUST)|Mohamed Ghazzali|Saudi Arabia

>Spike|hCoV-19/Ireland/D-NVRL-20G44542/2020|2020-03-31|EPI_ISL_605084|Original|hCoV-19^^Dublin|Human|National Virus Reference Laboratory|Irish Coronavirus Sequencing Consortium - Helixworks|Chalapati|Ireland

>Spike|hCoV-19/Crimea/RII-MH2937S/2020|2020-09-21|EPI_ISL_733078|Original|hCoV-19^^Alushta|Human|HELIX LLC|WHO National Influenza Centre Russian Federation|Komissarov|Crimea

>Spike|hCoV-19/England/BRIS-127B2F/2020|2020-04-17|EPI_ISL_469802|Original|hCoV-19^^England|Human|PHE South West Regional Laboratory|Wellcome Sanger Institute for the COVID-19 Genomics UK (COG-UK) consortium|Davies|United Kingdom

>Spike|hCoV-19/France/PAC-IHU-1195/2020|2020-08-17|EPI_ISL_569082|Original|hCoV-19^^Provence-Alpes-C\u00f4te-d\u2019Azur|Human|MEPHI|MEPHI|Levasseur|France

>Spike|hCoV-19/England/QEUH-B814C4/2020|2020-11-14|EPI_ISL_668231|Original|hCoV-19^^England|Human|Lighthouse Lab in Glasgow|Wellcome Sanger Institute for the COVID-19 Genomics UK (COG-UK) Consortium|Davies|United Kingdom

>Spike|hCoV-19/India/MH-ACTREC-067/2020|2020-05-06|EPI_ISL_699723|Original|hCoV-19^^Maharashtra|Human|Hematopathology Laboratory|Hematopathology Laboratory|Patkar|India

>Spike|hCoV-19/Japan/PG-8558/2020|2020-08-00|EPI_ISL_686154|Original|hCoV-19^^Japan|Human|Pathogen Genomics Center|Pathogen Genomics Center|Sekizuka|Japan

>Spike|hCoV-19/England/MILK-9C3835/2020|2020-09-10|EPI_ISL_566704|Original|hCoV-19^^England|Human|Lighthouse Lab in Milton Keynes|Wellcome Sanger Institute for the COVID-19 Genomics UK (COG-UK) consortium|Davies|United Kingdom

>Spike|hCoV-19/France/OCC-SC539/2020|2020-03-28|EPI_ISL_660716|Original|hCoV-19^^Occitanie|Human|CHU N\u00eemes|CNR Virus des Infections Respiratoires - France SUD|Bal|France

>Spike|hCoV-19/England/MILK-9E088D/2020|2020-09-20|EPI_ISL_601375|Original|hCoV-19^^England|Human|Lighthouse Lab in Milton Keynes|Wellcome Sanger Institute for the COVID-19 Genomics UK (COG-UK) consortium|Davies|United Kingdom

>Spike|hCoV-19/Switzerland/AG-ETHZ-260131/2020|2020-08-25|EPI_ISL_539436|Original|hCoV-19^^Aargau|Human|Viollier AG|Department of Biosystems Science and Engineering|Nadeau|Switzerland

>Spike|hCoV-19/USA/CA-CZB-1566/2020|2020-04-05|EPI_ISL_468502|Original|hCoV-19^^California|Human|Ventura County Public Health Lab|Chan-Zuckerberg Biohub|Hao|USA

>Spike|hCoV-19/Luxembourg/LNS7753431/2020|2020-03-12|EPI_ISL_419595|Original|hCoV-19^^Luxembourg|Human|Laboratoire National de Sant\u00e9|Laboratoire National de Sant\u00e9|Wienecke-Baldacchino|Luxembourg

>Spike|hCoV-19/Canada/MB-NML-620/2020|2020-04-03|EPI_ISL_632917|Original|hCoV-19^^Manitoba|Human|Cadham Provincial laboratory|Cadham Provincial laboratory|Majer|Canada

>Spike|hCoV-19/Lithuania/MR-LUHS-Eilnr479/2020|2020-09-12|EPI_ISL_636906|Original|hCoV-19^^Kauno apskritis|Human|Lithuanian University of Health Sciences Hospital|Lithuanian University of Health Sciences|Zemaitis|Lithuania

>Spike|hCoV-19/England/ALDP-989D69/2020|2020-08-01|EPI_ISL_529271|Original|hCoV-19^^England|Human|Liverpool Clinical Laboratories|COVID-19 Genomics UK (COG-UK) Consortium|Haldenby|United Kingdom

>Spike|hCoV-19/England/ALDP-B5B8D6/2020|2020-11-08|EPI_ISL_655563|Original|hCoV-19^^England|Human|Lighthouse Lab in Alderley Park|Wellcome Sanger Institute for the COVID-19 Genomics UK (COG-UK) Consortium|Davies|United Kingdom

>Spike|hCoV-19/England/QEUH-B145C3/2020|2020-11-03|EPI_ISL_642633|Original|hCoV-19^^England|Human|Lighthouse Lab in Glasgow|Wellcome Sanger Institute for the COVID-19 Genomics UK (COG-UK) Consortium|Davies|United Kingdom
>Spike|hCoV-19/Wales/PHWC-494182/2020|2020-11-16|EPI_ISL_726077|Original|hCoV-19^^Wales|Human|Originating lab|COVID-19 Genomics UK (COG-UK) Consortium|Moore|United Kingdom
>Spike|hCoV-19/South Korea/KDCA0181/2020|2020-07-03|EPI_ISL_747414|Original|hCoV-19^^South Korea|Human|Division of Emerging Infectious Diseases|Division of Emerging Infectious Diseases|Park|South Korea
>Spike|hCoV-19/Canada/QC-LSPQ-L00235638/2020|2020-03-24|EPI_ISL_536103|Original|hCoV-19^^Quebec|Human|H\u00f4pital Pierre-Boucher|Laboratoire de sant\u00e9 publique du Qu\u00e9bec|Moreira|Canada
>Spike|hCoV-19/Gambia/NPHL-1208/2020|2020-06-30|EPI_ISL_561213|Original|hCoV-19^^Kombo|Human|MRCG at LSHTM Genomics lab|MRCG at LSHTM Genomics lab|Sesay|Gambia
>Spike|hCoV-19/England/MILK-B39337/2020|2020-11-01|EPI_ISL_658903|Original|hCoV-19^^England|Human|Lighthouse Lab in Milton Keynes|Wellcome Sanger Institute for the COVID-19 Genomics UK (COG-UK) Consortium|Davies|United Kingdom
>Spike|hCoV-19/England/ALDP-94E717/2020|2020-06-18|EPI_ISL_555269|Original|hCoV-19^^England|Human|Lighthouse Lab in Alderley Park|Wellcome Sanger Institute for the COVID-19 Genomics UK (COG-UK) consortium|Davies|United Kingdom
>Spike|hCoV-19/England/BRIS-12607C/2020|2020-04-09|EPI_ISL_458986|Original|hCoV-19^^England|Human|PHE South West Regional Laboratory|Wellcome Sanger Institute for the COVID-19 Genomics UK (COG-UK) consortium|Davies|United Kingdom
>Spike|hCoV-19/India/DL-NCDC4592-CSIR-IGIB/2020|2020-05-23|EPI_ISL_482596|Original|hCoV-19^^Delhi|Human|National Centre for Disease control (NCDC)|NCDC/CSIR-IGIB|Singh|India
>Spike|hCoV-19/USA/WI-UW-1860/2020|2020-09-14|EPI_ISL_605732|Original|hCoV-19^^Wisconsin|Human|University of Wisconsin-Madison AIDS Vaccine Research Laboratories|University of Wisconsin-Madison AIDS Vaccine Research Laboratories|Baczenas|USA
>Spike|hCoV-19/Brazil/RS-6242/2020|2020-05-31|EPI_ISL_729843|Original|hCoV-19^^Rio Grande do Sul|Human|Laboratorio Central de Saude Publica do Estado do Rio Grande do Sul (LACEN-RS)|Laboratory of Respiratory Viruses and Measles|Silva|Brazil
>Spike|hCoV-19/USA/WI-UW-1505/2020|2020-09-14|EPI_ISL_541631|Original|hCoV-19^^Wisconsin|Human|University of Wisconsin-Madison AIDS Vaccine Research Laboratories|University of Wisconsin-Madison AIDS Vaccine Research Laboratories|Baczenas|USA
>Spike|hCoV-19/Australia/VIC7036/2020|2020-07-14|EPI_ISL_564203|Original|hCoV-19^^Victoria|Human|Victorian Infectious Diseases Reference Laboratory (VIDRL)|VIDRL and MDU-PHL|Schultz|Australia
>Spike|hCoV-19/USA/MA-MGH-00829/2020|2020-04-13|EPI_ISL_791785|Original|hCoV-19^^Massachusetts|Human|Massachusetts General Hospital|Infectious Disease Program|Park|USA
>Spike|hCoV-19/Wales/ALDP-5C23DF/2020|2020-06-15|EPI_ISL_558851|Original|hCoV-19^^Wales|Human|Lighthouse Lab in Alderley Park|Wellcome Sanger Institute for the COVID-19 Genomics UK (COG-UK) consortium|Davies|United Kingdom
>Spike|hCoV-19/USA/TX-HMH-2465/2020|2020-05-29|EPI_ISL_545325|Original|hCoV-19^^Texas|Human|Houston Methodist Hospital|Houston Methodist Hospital|Long|USA
>Spike|hCoV-19/England/QEUH-9647DF/2020|2020-08-11|EPI_ISL_536854|Original|hCoV-19^^England|Human|Lighthouse Lab in Glasgow|Wellcome Sanger Institute for the COVID-19 Genomics UK (COG-UK) consortium|Davies|United Kingdom
>Spike|hCoV-19/USA/LA-EVTL732/2020|2020-07-16|EPI_ISL_653423|Original|hCoV-19^^Louisiana|Human|LSUHS Emerging Viral Threat Laboratory|Microbial Genome Sequencing Center|Kamil|USA
>Spike|hCoV-19/USA/WA-S706/2020|2020-04-08|EPI_ISL_449876|Original|hCoV-19^^Washington|Human|Washington State Department of Health|Seattle Flu Study|Bedford|USA
>Spike|hCoV-19/Israel/CVL-45201/2020|2020-12-03|EPI_ISL_745040|Original|hCoV-19^^Haifa|Human|Israel Central Virology laboratory|Israel Central Virology laboratory|Zuckerman|Israel

>Spike|hCoV-19/England/BRIS-12ABE0/2020|2020-05-01|EPI_ISL_458331|Original|hCoV-
19^^England|Human|PHE South West Regional Laboratory|Wellcome Sanger Institute for
the COVID-19 Genomics UK (COG-UK) consortium|Davies|United Kingdom
>Spike|hCoV-19/Australia/SAP309/2020|2020-03-27|EPI_ISL_483092|Original|hCoV-
19^^South Australia|Human|SA Pathology|SA Pathology|Leong|Australia
>Spike|hCoV-19/Kenya/C1357/2020|2020-04-20|EPI_ISL_457858|Original|hCoV-
19^^Kenya|Human|KEMRI-CGMR-C|KEMRI-Wellcome Trust Research Programme/KEMRI-CGMR-C
Kilifi|Githinji|Kenya
>Spike|hCoV-19/Japan/PG-4696/2020|2020-08-00|EPI_ISL_689833|Original|hCoV-
19^^Japan|Human|Pathogen Genomics Center|Pathogen Genomics Center|Sekizuka|Japan
>Spike|hCoV-19/England/PORT-2D4917/2020|2020-10-13|EPI_ISL_611960|Original|hCoV-
19^^England|Human|Centre for Enzyme Innovation|COVID-19 Genomics UK (COG-UK)
Consortium|Robson|United Kingdom
>Spike|hCoV-19/SaudiArabia/KAUST-MADINAH1072/2020|2020-06-
11|EPI_ISL_513089|Original|hCoV-19^^Madinah|Human|Pathogen Genomics Lab King Abdullah
University of Science and Technology(KAUST)|Pathogen Genomics Lab King Abdullah
University of Science and Technology(KAUST)|Mohamed Ghazzali|Saudi Arabia
>Spike|hCoV-19/England/MILK-B3BC3A/2020|2020-10-31|EPI_ISL_679797|Original|hCoV-
19^^England|Human|Oxford Viromics|COVID-19 Genomics UK (COG-UK)
Consortium|Golubchik|United Kingdom
>Spike|hCoV-19/Ireland/WW-NVRL-AIIDV0063v1/2020|2020-04-
08|EPI_ISL_644303|Original|hCoV-19^^Wicklow|Human|CEPHR / Vincent's Hospital|Irish
Coronavirus Sequencing Consortium - National Virus Reference
Laboratory|Gonzalez|Ireland
>Spike|hCoV-19/USA/AZ-TG271922/2020|2020-03-26|EPI_ISL_426555|Original|hCoV-
19^^Arizona|Human|AZ SPHL|TGen North|Lemmer|USA
>Spike|hCoV-19/USA/WA-S2375/2020|2020-06-11|EPI_ISL_497007|Original|hCoV-
19^^Washington|Human|Washington State Department of Health|Seattle Flu Study|Lee|USA
>Spike|hCoV-19/England/NOTT-112143/2020|2020-06-18|EPI_ISL_484346|Original|hCoV-
19^^England|Human|Queens Medical Centre|COVID-19 Genomics UK (COG-UK)
Consortium|Loose|United Kingdom
>Spike|hCoV-19/Denmark/DCGC-15600/2020|2020-11-23|EPI_ISL_713179|Original|hCoV-
19^^Hovedstaden|Human|Department of Virus and Microbiological Special
Diagnostics|Albertsen Lab|Michaelsen|Denmark
>Spike|hCoV-19/England/QEUH-C149E4/2020|2020-12-06|EPI_ISL_709443|Original|hCoV-
19^^England|Human|Lighthouse Lab in Glasgow|Wellcome Sanger Institute for the COVID-
19 Genomics UK (COG-UK) Consortium|Davies|United Kingdom
>Spike|hCoV-19/USA/AK-PHL462/2020|2020-06-10|EPI_ISL_512140|Original|hCoV-
19^^Alaska|Human|Alaska State Virology Laboratory|Alaska State Virology
Laboratory|Drown|USA
>Spike|hCoV-19/Denmark/DCGC-17439/2020|2020-12-14|EPI_ISL_750983|Original|hCoV-
19^^Midtjylland|Human|Department of Virus and Microbiological Special
Diagnostics|Albertsen Lab|Michaelsen|Denmark
>Spike|hCoV-19/Wales/PHWC-4842A0/2020|2020-10-18|EPI_ISL_627983|Original|hCoV-
19^^Wales|Human|Wales Specialist Virology Centre Sequencing lab|COVID-19 Genomics UK
(COG-UK) Consortium|Moore|United Kingdom
>Spike|hCoV-19/England/QEUH-9B9304/2020|2020-09-07|EPI_ISL_549766|Original|hCoV-
19^^England|Human|Lighthouse Lab in Glasgow|Wellcome Sanger Institute for the COVID-
19 Genomics UK (COG-UK) consortium|Davies|United Kingdom
>Spike|hCoV-19/Bahrain/340864151_S32/2020|2020-10-14|EPI_ISL_632268|Original|hCoV-
19^^Bahrain|Human|Communicable Disease Laboratory|Communicable Disease
Laboratory|Altaif|Bahrain
>Spike|hCoV-19/Australia/VIC776/2020|2020-03-30|EPI_ISL_427051|Original|hCoV-
19^^Victoria|Human|Victorian Infectious Diseases Reference Laboratory
(VIDRL)|Microbiological Diagnostic Unit Public Health Laboratory and Victorian
Infectious Diseases Reference Laboratory|Schultz|Australia
>Spike|hCoV-19/Wales/PHWC-3131C/2020|2020-04-10|EPI_ISL_446432|Original|hCoV-
19^^Wales|Human|Wales Specialist Virology Centre|Public Health Wales Microbiology
Cardiff|Moore|United Kingdom

>Spike|hCoV-19/USA/NY-NYCPHL-000461/2020|2020-05-05|EPI_ISL_631932|Original|hCoV-19^^New York|Human|OCME Office Of Chief Medical Examiner|New York City Public Health Laboratory|Wang|USA
>Spike|hCoV-19/Finland/2A72SVIIIA/2020|2020-04-02|EPI_ISL_481653|Original|hCoV-19^^Uusimaa|Human|Department of Virology and Immunology|Department of Virology|Smura|Finland
>Spike|hCoV-19/Scotland/CVR5292/2020|2020-10-21|EPI_ISL_650854|Original|hCoV-19^^Scotland|Human|West of Scotland Specialist Virology Centre|COVID-19 Genomics UK (COG-UK) Consortium|Robertson|United Kingdom
>Spike|hCoV-19/England/PORT-2D1E48/2020|2020-04-02|EPI_ISL_499285|Original|hCoV-19^^England|Human|Centre for Enzyme Innovation|COVID-19 Genomics UK (COG-UK) Consortium|Robson|United Kingdom
>Spike|hCoV-19/Australia/VIC14566/2020|2020-08-27|EPI_ISL_592897|Original|hCoV-19^^Victoria|Human|Microbiological Diagnostic Unit - Public Health Laboratory (MDU-PHL)|MDU-PHL|Schultz|Australia
>Spike|hCoV-19/England/MILK-BB1CF0/2020|2020-11-17|EPI_ISL_731252|Original|hCoV-19^^England|Human|Lighthouse Lab in Milton Keynes|Wellcome Sanger Institute for the COVID-19 Genomics UK (COG-UK) Consortium|Davies|United Kingdom
>Spike|hCoV-19/England/QEUH-960BB9/2020|2020-08-04|EPI_ISL_532067|Original|hCoV-19^^England|Human|Lighthouse Lab in Glasgow|Wellcome Sanger Institute for the COVID-19 Genomics UK (COG-UK) consortium|Davies|United Kingdom
>Spike|hCoV-19/England/LIVE-DC4117/2020|2020-11-08|EPI_ISL_703530|Original|hCoV-19^^England|Human|Liverpool Clinical Laboratories|COVID-19 Genomics UK (COG-UK) Consortium|Haldenby|United Kingdom
>Spike|hCoV-19/England/QEUH-B69152/2020|2020-11-11|EPI_ISL_662039|Original|hCoV-19^^England|Human|Lighthouse Lab in Glasgow|Wellcome Sanger Institute for the COVID-19 Genomics UK (COG-UK) Consortium|Davies|United Kingdom
>Spike|hCoV-19/Spain/PV-IBV-2132/2020|2020-04-12|EPI_ISL_468898|Original|hCoV-19^^Basque Country|Human|Servicio de Microbiolog\u00eda. Hospital Universitario Donostia. OSI Donostialdea. \u00c1rea de Enfermedades Infecciosas|SeqCOVID-SPAIN consortium/IBV(CSIC)|Comas|Spain
>Spike|hCoV-19/South Korea/KCDC2714/2020|2020-07-12|EPI_ISL_522493|Original|hCoV-19^^South Korea|Human|Center for Laboratory Control of Infectious Diseases|Center for Laboratory Control of Infectious Diseases|Kim|South Korea
>Spike|hCoV-19/England/ALDP-A668AC/2020|2020-10-12|EPI_ISL_630561|Original|hCoV-19^^England|Human|Lighthouse Lab in Alderley Park|Wellcome Sanger Institute for the COVID-19 Genomics UK (COG-UK) consortium|Davies|United Kingdom
>Spike|hCoV-19/England/ALDP-B23E7B/2020|2020-11-04|EPI_ISL_646252|Original|hCoV-19^^England|Human|Lighthouse Lab in Alderley Park|Wellcome Sanger Institute for the COVID-19 Genomics UK (COG-UK) Consortium|Davies|United Kingdom
>Spike|hCoV-19/Israel/2115964/2020|2020-04-16|EPI_ISL_447338|Original|hCoV-19^^Tel Aviv District|Human|Clinical Virology Unit|Stern Lab|Miller|Israel
>Spike|hCoV-19/England/CAMC-947F16/2020|2020-07-22|EPI_ISL_556915|Original|hCoV-19^^England|Human|Lighthouse Lab in Cambridge|Wellcome Sanger Institute for the COVID-19 Genomics UK (COG-UK) consortium|Davies|United Kingdom
>Spike|hCoV-19/Wales/PHWC-49DAAC/2020|2020-12-08|EPI_ISL_742870|Original|hCoV-19^^Wales|Human|Wales Specialist Virology Centre Sequencing lab|COVID-19 Genomics UK (COG-UK) Consortium|Moore|United Kingdom
>Spike|hCoV-19/Denmark/DCGC-11247/2020|2020-11-02|EPI_ISL_670792|Original|hCoV-19^^Denmark|Human|Department of Virus and Microbiological Special Diagnostics|Albertsen Lab|Michaelsen|Denmark
>Spike|hCoV-19/USA/TX-HMH-MCoV-12649/2020|2020-08-31|EPI_ISL_790442|Original|hCoV-19^^Texas|Human|Houston Methodist Hospital|Houston Methodist Hospital|Long|USA
>Spike|hCoV-19/Australia/NSW370/2020|2020-03-26|EPI_ISL_451631|Original|hCoV-19^^New South Wales|Human|Laverty Pathology|NSW Health Pathology - Institute of Clinical Pathology and Medical Research; Westmead Hospital; University of Sydney|Gall|Australia
>Spike|hCoV-19/Wales/PHWC-486059/2020|2020-10-26|EPI_ISL_628304|Original|hCoV-19^^Wales|Human|Wales Specialist Virology Centre Sequencing lab|COVID-19 Genomics UK (COG-UK) Consortium|Moore|United Kingdom

>Spike|hCoV-19/Spain/RI-IBV-98002384/2020|2020-03-11|EPI_ISL_537873|Original|hCoV-19^^La Rioja|Human|Centro de Investigaci\u00f3n Biom\u00e9dica de La Rioja - Hospital San Pedro Logro\u00f1o|SeqCOVID-SPAIN consortium/IBV(CSIC)|Comas|Spain
>Spike|hCoV-19/England/CAMC-B382E0/2020|2020-11-05|EPI_ISL_659073|Original|hCoV-19^^England|Human|Lighthouse Lab in Cambridge|Wellcome Sanger Institute for the COVID-19 Genomics UK (COG-UK) Consortium|Davies|United Kingdom
>Spike|hCoV-19/Australia/VIC11914/2020|2020-08-02|EPI_ISL_591894|Original|hCoV-19^^Victoria|Human|Victorian Infectious Diseases Reference Laboratory (VIDRL)|VIDRL and MDU-PHL|Schultz|Australia
>Spike|hCoV-19/England/CAMB-1B625D/2020|2020-09-16|EPI_ISL_572471|Original|hCoV-19^^England|Human|Department of Pathology|COVID-19 Genomics UK (COG-UK) Consortium|Goodfellow|United Kingdom
>Spike|hCoV-19/England/NORW-E80E0/2020|2020-04-08|EPI_ISL_449107|Original|hCoV-19^^England|Human|Quadram Institute Bioscience|COVID-19 Genomics UK (COG-UK) Consortium|Alikhan|United Kingdom
>Spike|hCoV-19/England/ALDP-BB2907/2020|2020-11-19|EPI_ISL_703166|Original|hCoV-19^^England|Human|Lighthouse Lab in Alderley Park|Wellcome Sanger Institute for the COVID-19 Genomics UK (COG-UK) Consortium|Davies|United Kingdom
>Spike|hCoV-19/England/LIVE-AAD3E/2020|2020-07-29|EPI_ISL_540616|Original|hCoV-19^^England|Human|Liverpool Clinical Laboratories|COVID-19 Genomics UK (COG-UK) Consortium|Haldenby|United Kingdom
>Spike|hCoV-19/Australia/VIC6766/2020|2020-07-26|EPI_ISL_562085|Original|hCoV-19^^Victoria|Human|Victorian Infectious Diseases Reference Laboratory (VIDRL)|VIDRL and MDU-PHL|Schultz|Australia
>Spike|hCoV-19/South Africa/KRISP-K002839/2020|2020-09-01|EPI_ISL_602655|Original|hCoV-19^^KwaZulu-Natal|Human|NHLS-IALCH|KRISP|de Oliveira|South Africa
>Spike|hCoV-19/England/CAMC-B089B7/2020|2020-10-30|EPI_ISL_643549|Original|hCoV-19^^England|Human|Lighthouse Lab in Cambridge|Wellcome Sanger Institute for the COVID-19 Genomics UK (COG-UK) Consortium|Davies|United Kingdom
>Spike|hCoV-19/England/QEUH-B2B453/2020|2020-11-03|EPI_ISL_646604|Original|hCoV-19^^England|Human|Lighthouse Lab in Glasgow|Wellcome Sanger Institute for the COVID-19 Genomics UK (COG-UK) Consortium|Davies|United Kingdom
>Spike|hCoV-19/England/NORT-295DCB/2020|2020-05-17|EPI_ISL_488723|Original|hCoV-19^^England|Human|NU-OMICS DNA Sequencing research facility|Wellcome Sanger Institute for the COVID-19 Genomics UK (COG-UK) consortium|Davies|United Kingdom
>Spike|hCoV-19/USA/UT-08407/2020|2020-05-20|EPI_ISL_524185|Original|hCoV-19^^Utah|Human|Utah Public Health Laboratory|Utah Public Health Laboratory|Young|USA
>Spike|hCoV-19/Guadeloupe/IPG-7353/2020|2020-03-31|EPI_ISL_613445|Original|hCoV-19^^M\u00e9tropole|Human|Institut Pasteur de la Guadeloupe|Institut Pasteur de la Guadeloupe|Enouf|Guadeloupe
>Spike|hCoV-19/USA/NY-NYCPHL-000609/2020|2020-03-11|EPI_ISL_632066|Original|hCoV-19^^New York|Human|Jamaica Hospital Medical Center|New York City Public Health Laboratory|Wang|USA
>Spike|hCoV-19/USA/MI-QDX-656/2020|2020-03-19|EPI_ISL_571379|Original|hCoV-19^^Michigan|Human|Quest Diagnostics|Quest Diagnostics|Kagan|USA
>Spike|hCoV-19/Hong Kong/HKU-IHCE0511-003a/2020|2020-01-27|EPI_ISL_538542|Original|hCoV-19^^Hong Kong|Human|Department of Microbiology|Department of Microbiology|Chan|China
>Spike|hCoV-19/Northern Ireland/NIRE-233336/2020|2020-09-24|EPI_ISL_680281|Original|hCoV-19^^Northern Ireland|Human|Regional Virus Laboratory|COVID-19 Genomics UK (COG-UK) Consortium|Fairley|United Kingdom
>Spike|hCoV-19/Japan/PG-2221/2020|2020-04-00|EPI_ISL_685826|Original|hCoV-19^^Japan|Human|Pathogen Genomics Center|Pathogen Genomics Center|Sekizuka|Japan
>Spike|hCoV-19/England/MILK-AC7EF2/2020|2020-10-22|EPI_ISL_629600|Original|hCoV-19^^England|Human|Lighthouse Lab in Milton Keynes|Wellcome Sanger Institute for the COVID-19 Genomics UK (COG-UK) consortium|Davies|United Kingdom
>Spike|hCoV-19/USA/MI-UM-10036310581/2020|2020-10-06|EPI_ISL_596587|Original|hCoV-19^^Michigan|Human|University of Michigan Clinical Microbiology Laboratory|Lauring Lab|Valesano|USA

>Spike|hCoV-19/Denmark/ALAB-SSI488/2020|2020-03-24|EPI_ISL_429565|Original|hCoV-19^^Denmark|Human|Department of Virus and Microbiological Special Diagnostics|Albertsen lab|Kirkegaard|Denmark
>Spike|hCoV-19/Sichuan/SC-WCH4-287/2020|2020-01-30|EPI_ISL_451389|Original|hCoV-19^^Sichuan|Human|West China Hospital of Sichuan University|State Key Laboratory of Biotherapy of Sichuan University|Tang|China
>Spike|hCoV-19/Denmark/DCGC-3029/2020|2020-08-24|EPI_ISL_616807|Original|hCoV-19^^NA|Human|Department of Virus and Microbiological Special Diagnostics|Albertsen lab|Michaelsen|Denmark
>Spike|hCoV-19/Japan/PG-7038/2020|2020-08-00|EPI_ISL_688143|Original|hCoV-19^^Japan|Human|Pathogen Genomics Center|Pathogen Genomics Center|Sekizuka|Japan
>Spike|hCoV-19/Hungary/UD-90512/2020|2020-10-04|EPI_ISL_671473|Original|hCoV-19^^Pest county|Human|University of Debrecen|National Laboratory of Virology|Kemenesi|Hungary
>Spike|hCoV-19/USA/WI-WSLH-200675/2020|2020-06-23|EPI_ISL_631433|Original|hCoV-19^^Wisconsin|Human|Wisconsin State Laboratory of Hygiene Communicable Disease Division|Wisconsin State Laboratory of Hygiene Communicable Disease Division|Florek|USA
>Spike|hCoV-19/Wales/PHWC-47FE2C/2020|2020-10-04|EPI_ISL_612816|Original|hCoV-19^^Wales|Human|Wales Specialist Virology Centre Sequencing lab|COVID-19 Genomics UK (COG-UK) Consortium|Moore|United Kingdom
>Spike|hCoV-19/England/ALDP-94DA00/2020|2020-06-17|EPI_ISL_555309|Original|hCoV-19^^England|Human|Lighthouse Lab in Alderley Park|Wellcome Sanger Institute for the COVID-19 Genomics UK (COG-UK) consortium|Davies|United Kingdom
>Spike|hCoV-19/Ireland/D-NVRL-73IRL62905/2020|2020-09-12|EPI_ISL_596893|Original|hCoV-19^^Dublin|Human|National Virus Reference Laboratory|National Virus Reference Laboratory|Gonzalez|Ireland
>Spike|hCoV-19/USA/CA-CZB-5681/2020|2020-07-23|EPI_ISL_582973|Original|hCoV-19^^California|Human|San Luis Obispo Public Health Department|Chan-Zuckerberg Biohub|Bhatt|USA
>Spike|hCoV-19/Norway/5733/2020|2020-11-12|EPI_ISL_708152|Original|hCoV-19^^Innlandet|Human|Innlandet Hospital Trust|Norwegian Institute of Public Health|Kopperud Riis|Norway
>Spike|hCoV-19/England/MILK-C5FFCD/2020|2020-12-13|EPI_ISL_769531|Original|hCoV-19^^England|Human|Lighthouse Lab in Milton Keynes|Wellcome Sanger Institute for the COVID-19 Genomics UK (COG-UK) Consortium|Davies|United Kingdom
>Spike|hCoV-19/Switzerland/BE-ETHZ-260084/2020|2020-08-25|EPI_ISL_539405|Original|hCoV-19^^Bern|Human|Viollier AG|Department of Biosystems Science and Engineering|Nadeau|Switzerland
>Spike|hCoV-19/England/NORW-F562A/2020|2020-10-30|EPI_ISL_665887|Original|hCoV-19^^England|Human|Quadram Institute Bioscience|COVID-19 Genomics UK (COG-UK) Consortium|Alikhan|United Kingdom
>Spike|hCoV-19/England/NOTT-11EC64/2020|2020-11-30|EPI_ISL_679945|Original|hCoV-19^^England|Human|Queens Medical Centre|COVID-19 Genomics UK (COG-UK) Consortium|Loose|United Kingdom
>Spike|hCoV-19/England/NORW-21F761/2020|2020-12-08|EPI_ISL_725047|Original|hCoV-19^^England|Human|Quadram Institute Bioscience|COVID-19 Genomics UK (COG-UK) Consortium|Alikhan|United Kingdom
>Spike|hCoV-19/Canada/QC-LSPQ-L00243875/2020|2020-03-31|EPI_ISL_536383|Original|hCoV-19^^Quebec|Human|CSSS Haut-Richelieu/Rouville (H\u00f4pital)|Laboratoire de sant\u00e9 publique du Qu\u00e9bec|Moreira|Canada
>Spike|hCoV-19/England/ALDP-A2F951/2020|2020-10-05|EPI_ISL_587034|Original|hCoV-19^^England|Human|Lighthouse Lab in Alderley Park|Wellcome Sanger Institute for the COVID-19 Genomics UK (COG-UK) consortium|Davies|United Kingdom
>Spike|hCoV-19/USA/MI-MDHHS-SC20448/2020|2020-03-16|EPI_ISL_452322|Original|hCoV-19^^Michigan|Human|Michigan Department of Health and Human Services|Michigan Department of Health and Human Services|Blankenship|USA
>Spike|hCoV-19/Denmark/DCGC-1856/2020|2020-07-06|EPI_ISL_622569|Original|hCoV-19^^Hovedstaden|Human|Department of Virus and Microbiological Special Diagnostics|Albertsen lab|Michaelsen|Denmark

>Spike|hCoV-19/England/ALDP-9E6614/2020|2020-09-24|EPI_ISL_580295|Original|hCoV-
19^^England|Human|Lighthouse Lab in Alderley Park|Wellcome Sanger Institute for the
COVID-19 Genomics UK (COG-UK) consortium|Davies|United Kingdom
>Spike|hCoV-19/England/QEUH-B7D4AD/2020|2020-11-14|EPI_ISL_741866|Original|hCoV-
19^^England|Human|Oxford Viromics|COVID-19 Genomics UK (COG-UK)
Consortium|Golubchik|United Kingdom
>Spike|hCoV-19/Hong Kong/VM20001794/2020|2020-01-29|EPI_ISL_409001|Original|hCoV-
19^^Hong Kong|Human|Queen Mary Hospital|Hong Kong Department of Health|Mak|China
>Spike|hCoV-19/Australia/VIC3306/2020|2020-07-07|EPI_ISL_520985|Original|hCoV-
19^^Victoria|Human|Microbiological Diagnostic Unit - Public Health Laboratory (MDU-
PHL)|MDU-PHL|Schultz|Australia
>Spike|hCoV-19/USA/WA-UW138/2020|2020-03-11|EPI_ISL_416676|Original|hCoV-
19^^Washington|Human|UW Virology Lab|UW Virology Lab|Roychoudhury|USA
>Spike|hCoV-19/USA/NM-DOH-2020321223/2020|2020-09-03|EPI_ISL_575293|Original|hCoV-
19^^New Mexico|Human|Molecular Biology|Molecular Biology|Johnson|USA
>Spike|hCoV-19/Wales/PHWC-4915A7/2020|2020-10-28|EPI_ISL_706668|Original|hCoV-
19^^Wales|Human|Originating lab|COVID-19 Genomics UK (COG-UK) Consortium|Moore|United
Kingdom
>Spike|hCoV-19/USA/NY-Wadsworth-11344-01/2020|2020-03-
06|EPI_ISL_426045|Original|hCoV-19^^New York|Human|Wadsworth Center|Wadsworth
Center|Lasek-Nesselquist|USA
>Spike|hCoV-19/USA/WA-UW-11761/2020|2020-06-24|EPI_ISL_570466|Original|hCoV-
19^^Washington|Human|UW Virology Lab|UW Virology Lab|Roychoudhury|USA
>Spike|hCoV-19/England/NOTT-121830/2020|2020-12-21|EPI_ISL_764503|Original|hCoV-
19^^England|Human|Queens Medical Centre|COVID-19 Genomics UK (COG-UK)
Consortium|Loose|United Kingdom
>Spike|hCoV-19/England/MILK-9569F8/2020|2020-06-07|EPI_ISL_554308|Original|hCoV-
19^^England|Human|Lighthouse Lab in Milton Keynes|Wellcome Sanger Institute for the
COVID-19 Genomics UK (COG-UK) consortium|Davies|United Kingdom
>Spike|hCoV-19/England/MILK-BB1B5D/2020|2020-11-18|EPI_ISL_720552|Original|hCoV-
19^^England|Human|Lighthouse Lab in Milton Keynes|Wellcome Sanger Institute for the
COVID-19 Genomics UK (COG-UK) Consortium|Davies|United Kingdom
>Spike|hCoV-19/Japan/PG-3627/2020|2020-04-00|EPI_ISL_691880|Original|hCoV-
19^^Japan|Human|Pathogen Genomics Center|Pathogen Genomics Center|Sekizuka|Japan
>Spike|hCoV-19/USA/WI-UW-1015/2020|2020-07-22|EPI_ISL_516441|Original|hCoV-
19^^Wisconsin|Human|University of Wisconsin-Madison AIDS Vaccine Research
Laboratories|University of Wisconsin-Madison AIDS Vaccine Research
Laboratories|Baczenas|USA
>Spike|hCoV-19/USA/CA-CDPH155/2020|2020-09-15|EPI_ISL_754987|Original|hCoV-
19^^California|Human|California Department of Public Health|California Department of
Public Health|Bell|USA
>Spike|hCoV-19/USA/NY-PV08450/2020|2020-03-17|EPI_ISL_421401|Original|hCoV-19^^New
York|Human|MSHS Clinical Microbiology Laboratories|MSHS Pathogen Surveillance
Program|van Bakel|USA
>Spike|hCoV-19/Iran/K1r-13/2020|2020-03-00|EPI_ISL_582024|Original|hCoV-
19^^Iran|Human|Virology|Virology|Ranjbar|Iran
>Spike|hCoV-19/Singapore/514/2020|2020-05-24|EPI_ISL_479592|Original|hCoV-
19^^Singapore|Human|National Public Health Laboratory|National Public Health
Laboratory|Mak|Singapore
>Spike|hCoV-19/USA/TX-HMH-3307/2020|2020-06-11|EPI_ISL_544119|Original|hCoV-
19^^Texas|Human|Houston Methodist Hospital|Houston Methodist Hospital|Long|USA
>Spike|hCoV-19/England/MILK-B3A72B/2020|2020-11-01|EPI_ISL_706030|Original|hCoV-
19^^England|Human|Oxford Viromics|COVID-19 Genomics UK (COG-UK)
Consortium|Golubchik|United Kingdom
>Spike|hCoV-19/USA/CA-IGI-0509/2020|2020-07-27|EPI_ISL_756332|Original|hCoV-
19^^California|Human|Innovative Genomics Institute|Innovative Genomics
Institute|Wyman|USA
>Spike|hCoV-19/Canada/ON-S341/2020|2020-03-29|EPI_ISL_755868|Original|hCoV-
19^^Ontario|Human|Toronto Invasive Bacterial Diseases Network|McMaster
University|McArthur|Canada

>Spike|hCoV-19/Japan/PG-2228/2020|2020-04-00|EPI_ISL_685833|Original|hCoV-19^^Japan|Human|Pathogen Genomics Center|Pathogen Genomics Center|Sekizuka|Japan
>Spike|hCoV-19/England/MILK-9C70DF/2020|2020-09-08|EPI_ISL_573280|Original|hCoV-19^^England|Human|Northumbria University / South Tees Hospitals NHS Foundation Trust / North Cumbria Integrated Care NHS Foundation Trust / North Tees and Hartlepool NHS Foundation Trust / Newcastle Hospitals NHS Foundation Trust|COVID-19 Genomics UK (COG-UK) Consortium|Bashton|United Kingdom
>Spike|hCoV-19/England/CAMB-80D98/2020|2020-04-10|EPI_ISL_442937|Original|hCoV-19^^England|Human|Department of Pathology|Wellcome Sanger Institute for the COVID-19 Genomics UK (COG-UK) consortium|Amato|United Kingdom
>Spike|hCoV-19/England/HECH-259A158/2020|2020-05-18|EPI_ISL_488839|Original|hCoV-19^^England|Human|Microbiology Department|Wellcome Sanger Institute for the COVID-19 Genomics UK (COG-UK) consortium|Davies|United Kingdom
>Spike|hCoV-19/England/QEUH-B6636E/2020|2020-11-10|EPI_ISL_661597|Original|hCoV-19^^England|Human|Lighthouse Lab in Glasgow|Wellcome Sanger Institute for the COVID-19 Genomics UK (COG-UK) Consortium|Davies|United Kingdom
>Spike|hCoV-19/Peru/AMZ-UPCH-0269/2020|2020-10-01|EPI_ISL_729870|Original|hCoV-19^^Amazonas|Human|Instituto de Medicina Tropical|Laboratorio de Gen\u00f3mica Microbiana|Tsukayama|Peru
>Spike|hCoV-19/England/PORT-2D522A/2020|2020-00-00|EPI_ISL_741687|Original|hCoV-19^^England|Human|Centre for Enzyme Innovation|COVID-19 Genomics UK (COG-UK) Consortium|Robson|United Kingdom
>Spike|hCoV-19/England/CAMC-AAF670/2020|2020-10-23|EPI_ISL_631123|Original|hCoV-19^^England|Human|Lighthouse Lab in Cambridge|Wellcome Sanger Institute for the COVID-19 Genomics UK (COG-UK) consortium|Davies|United Kingdom
>Spike|hCoV-19/USA/WI-UW-1086/2020|2020-07-25|EPI_ISL_516512|Original|hCoV-19^^Wisconsin|Human|University of Wisconsin-Madison AIDS Vaccine Research Laboratories|University of Wisconsin-Madison AIDS Vaccine Research Laboratories|Baczenas|USA
>Spike|hCoV-19/USA/CT-Yale-208/2020|2020-03-30|EPI_ISL_463146|Original|hCoV-19^^Connecticut|Human|Yale Clinical Virology Laboratory|Grubaugh Lab - Yale School of Public Health|Grubaugh|USA
>Spike|hCoV-19/England/201421117/2020|2020-03-30|EPI_ISL_465951|Original|hCoV-19^^England|Human|Respiratory Virus Unit|Respiratory Virus Unit|Platt|United Kingdom
>Spike|hCoV-19/England/CAMC-B0A40F/2020|2020-10-29|EPI_ISL_673032|Original|hCoV-19^^England|Human|Lighthouse Lab in Cambridge|Wellcome Sanger Institute for the COVID-19 Genomics UK (COG-UK) Consortium|Davies|United Kingdom
>Spike|hCoV-19/Australia/NSW363/2020|2020-04-19|EPI_ISL_451624|Original|hCoV-19^^New South Wales|Human|Pathology West - NSW Health Pathology|NSW Health Pathology - Institute of Clinical Pathology and Medical Research; Westmead Hospital; University of Sydney|Gall|Australia
>Spike|hCoV-19/USA/FL-BPHL-1138/2020|2020-08-17|EPI_ISL_526624|Original|hCoV-19^^Florida|Human|Florida Bureau of Public Health Laboratories|Florida Bureau of Public Health Laboratories|Blanton|USA
>Spike|hCoV-19/USA/WA-S1714/2020|2020-06-01|EPI_ISL_496140|Original|hCoV-19^^Washington|Human|Washington State Department of Health|Seattle Flu Study|Lee|USA
>Spike|hCoV-19/Australia/VIC7982/2020|2020-08-13|EPI_ISL_564788|Original|hCoV-19^^Victoria|Human|Microbiological Diagnostic Unit - Public Health Laboratory (MDU-PHL)|MDU-PHL|Schultz|Australia
>Spike|hCoV-19/Belgium/ULG-10849/2020|2020-10-29|EPI_ISL_666884|Original|hCoV-19^^Li\u00e8ge|Human|Department of Clinical Microbiology|GIGA Medical Genomics|Durkin|Belgium
>Spike|hCoV-19/England/CAMC-BBB6F8/2020|2020-11-22|EPI_ISL_703476|Original|hCoV-19^^England|Human|Department of Pathology|COVID-19 Genomics UK (COG-UK) Consortium|Goodfellow|United Kingdom
>Spike|hCoV-19/Japan/PG-6045/2020|2020-07-00|EPI_ISL_689157|Original|hCoV-19^^Japan|Human|Pathogen Genomics Center|Pathogen Genomics Center|Sekizuka|Japan
>Spike|hCoV-19/England/BRIS-12549F/2020|2020-03-26|EPI_ISL_443936|Original|hCoV-19^^England|Human|PHE South West Regional Laboratory|Wellcome Sanger Institute for the COVID-19 Genomics UK (COG-UK) consortium|Amato|United Kingdom

>Spike|hCoV-19/Denmark/DCGC-14636/2020|2020-11-23|EPI_ISL_711884|Original|hCoV-19^^Sjaelland|Human|Department of Virus and Microbiological Special Diagnostics|Albertsen Lab|Michaelsen|Denmark
>Spike|hCoV-19/England/ALDP-A66C19/2020|2020-10-12|EPI_ISL_630427|Original|hCoV-19^^England|Human|Lighthouse Lab in Alderley Park|Wellcome Sanger Institute for the COVID-19 Genomics UK (COG-UK) consortium|Davies|United Kingdom
>Spike|hCoV-19/USA/CA-CZB-1602/2020|2020-04-01|EPI_ISL_468531|Original|hCoV-19^^California|Human|San Joaquin County Public Health Lab|Chan-Zuckerberg Biohub|Hao|USA
>Spike|hCoV-19/England/QEUH-B7E07B/2020|2020-11-13|EPI_ISL_741935|Original|hCoV-19^^England|Human|Oxford Viromics|COVID-19 Genomics UK (COG-UK) Consortium|Golubchik|United Kingdom
>Spike|hCoV-19/England/SHEF-CB1E0/2020|2020-04-08|EPI_ISL_442380|Original|hCoV-19^^England|Human|Virology Department|COVID-19 Genomics UK (COG-UK) Consortium|de Silva|United Kingdom
>Spike|hCoV-19/Japan/PG-0628/2020|2020-04-00|EPI_ISL_684238|Original|hCoV-19^^Japan|Human|Gunma Prefectural Institute of Public Health and Environmental Sciences|Pathogen Genomics Center|Sekizuka|Japan
>Spike|hCoV-19/England/NORW-EB01A/2020|2020-04-30|EPI_ISL_457406|Original|hCoV-19^^England|Human|Quadram Institute Bioscience|COVID-19 Genomics UK (COG-UK) Consortium|Alikhan|United Kingdom
>Spike|hCoV-19/England/OXON-AEC79/2020|2020-04-08|EPI_ISL_448774|Original|hCoV-19^^England|Human|Oxford Viromics|COVID-19 Genomics UK (COG-UK) Consortium|Golubchik|United Kingdom
>Spike|hCoV-19/England/204691946/2020|2020-11-15|EPI_ISL_693452|Original|hCoV-19^^England|Human|Respiratory Virus Unit|COVID-19 Genomics UK (COG-UK) Consortium|Platt|United Kingdom
>Spike|hCoV-19/England/NORT-296FA6/2020|2020-04-27|EPI_ISL_484282|Original|hCoV-19^^England|Human|Northumbria University / South Tees Hospitals NHS Foundation Trust / North Cumbria Integrated Care NHS Foundation Trust / North Tees and Hartlepool NHS Foundation Trust / Newcastle Hospitals NHS Foundation Trust|COVID-19 Genomics UK (COG-UK) Consortium|Bashton|United Kingdom
>Spike|hCoV-19/England/LIVE-DCC1B5/2020|2020-11-23|EPI_ISL_724492|Original|hCoV-19^^England|Human|Liverpool Clinical Laboratories|COVID-19 Genomics UK (COG-UK) Consortium|Haldenby|United Kingdom
>Spike|hCoV-19/USA/MI-MDHHS-SC20760/2020|2020-04-20|EPI_ISL_471781|Original|hCoV-19^^Michigan|Human|Michigan Department of Health and Human Services|Michigan Department of Health and Human Services|Blankenship|USA
>Spike|hCoV-19/USA/TX-HMH-MCoV-14796/2020|2020-10-13|EPI_ISL_786281|Original|hCoV-19^^Texas|Human|Houston Methodist Hospital|Houston Methodist Hospital|Long|USA
>Spike|hCoV-19/South Africa/NHLS-UCT-GS-0958/2020|2020-10-15|EPI_ISL_700519|Original|hCoV-19^^Western Cape|Human|Clinic-in-Asla|NHLS/UCT|Iranzadeh|South Africa
>Spike|hCoV-19/USA/NM-DOH-2020643097/2020|2020-12-28|EPI_ISL_767087|Original|hCoV-19^^New Mexico|Human|New Mexico Department of Health Scientific Laboratory|New Mexico Department of Health Scientific Laboratory|Malone|USA
>Spike|hCoV-19/England/CAMC-B513FD/2020|2020-11-08|EPI_ISL_657763|Original|hCoV-19^^England|Human|Lighthouse Lab in Cambridge|Wellcome Sanger Institute for the COVID-19 Genomics UK (COG-UK) Consortium|Davies|United Kingdom
>Spike|hCoV-19/USA/CA-ALSR-1428/2020|2020-03-28|EPI_ISL_494596|Original|hCoV-19^^California|Human|Scripps Medical Laboratory|Andersen lab at Scripps Research|Gangavarapu|USA
>Spike|hCoV-19/Japan/PG-0515/2020|2020-04-03|EPI_ISL_480173|Original|hCoV-19^^Kanto|Human|Gunma Prefectural Institute of Public Health and Environmental Sciences|Pathogen Genomics Center|Sekizuka|Japan
>Spike|hCoV-19/England/MILK-956D38/2020|2020-06-10|EPI_ISL_554184|Original|hCoV-19^^England|Human|Lighthouse Lab in Milton Keynes|Wellcome Sanger Institute for the COVID-19 Genomics UK (COG-UK) consortium|Davies|United Kingdom

>Spike|hCoV-19/USA/OR-OHSU-3045/2020|2020-09-30|EPI_ISL_667455|Original|hCoV-19^^Oregon|Human|OHSU Lab Services Molecular Microbiology Lab|Oregon SARS-CoV-2 Genome Sequencing Center|O'Roak|USA
>Spike|hCoV-19/England/ALDP-A5117C/2020|2020-10-07|EPI_ISL_598177|Original|hCoV-19^^England|Human|Lighthouse Lab in Alderley Park|Wellcome Sanger Institute for the COVID-19 Genomics UK (COG-UK) consortium|Davies|United Kingdom
>Spike|hCoV-19/Denmark/DCGC-1255/2020|2020-06-01|EPI_ISL_618018|Original|hCoV-19^^Hovedstaden|Human|Department of Virus and Microbiological Special Diagnostics|Albertsen lab|Michaelsen|Denmark
>Spike|hCoV-19/Spain/AN-IBV-001965/2020|2020-03-13|EPI_ISL_452416|Original|hCoV-19^^Andalucia|Human|Servicio de Microbiolog\u00eda. HRU de M\u00e1laga. Servicio Andaluz de Salud|SeqCOVID-SPAIN consortium/IBV(CSIC)|Comas|Spain
>Spike|hCoV-19/England/MILK-B599C6/2020|2020-11-06|EPI_ISL_679894|Original|hCoV-19^^England|Human|Virology Department|COVID-19 Genomics UK (COG-UK) Consortium|de Silva|United Kingdom
>Spike|hCoV-19/England/QEUH-9B4A40/2020|2020-09-03|EPI_ISL_590450|Original|hCoV-19^^England|Human|Lighthouse Lab in Glasgow|Wellcome Sanger Institute for the COVID-19 Genomics UK (COG-UK) consortium|Davies|United Kingdom
>Spike|hCoV-19/England/QEUH-B2DE08/2020|2020-11-03|EPI_ISL_646396|Original|hCoV-19^^England|Human|Lighthouse Lab in Glasgow|Wellcome Sanger Institute for the COVID-19 Genomics UK (COG-UK) Consortium|Davies|United Kingdom
>Spike|hCoV-19/Spain/MD-IBV-99007162/2020|2020-08-19|EPI_ISL_541932|Original|hCoV-19^^Madrid|Human|Hospital General Universitario Gregorio Mara\u00f1\u00f3n|SeqCOVID-SPAIN consortium/IBV(CSIC)|Comas|Spain
>Spike|hCoV-19/USA/TX-HMH-3717/2020|2020-06-16|EPI_ISL_543134|Original|hCoV-19^^Texas|Human|Houston Methodist Hospital|Houston Methodist Hospital|Long|USA
>Spike|hCoV-19/USA/UN-PB-Wuhan-Hu-1-5/2020|2020-04-05|EPI_ISL_759864|Original|hCoV-19^^USA|Human|Molecular|Molecular|Papoutsis,A.J.|USA
>Spike|hCoV-19/England/MILK-B06D9F/2020|2020-10-29|EPI_ISL_652276|Original|hCoV-19^^England|Human|Quadram Institute Bioscience|COVID-19 Genomics UK (COG-UK) Consortium|Alikhan|United Kingdom
>Spike|hCoV-19/Bangladesh/DNAS_DRICM_isl_MH-32/2020|2020-07-11|EPI_ISL_774971|Original|hCoV-19^^Dhaka|Human|Designated Reference Institute for Chemical Measurements (DRICM)|DNA SOLUTION LTD.|Rabbi|Bangladesh
>Spike|hCoV-19/New Zealand/20VR2951/2020|2020-00-00|EPI_ISL_579371|Original|hCoV-19^^Auckland|Human|LabPLUS|Institute of Environmental Science and Research (ESR)|Wang|New Zealand
>Spike|hCoV-19/England/ALDP-AA125C/2020|2020-10-22|EPI_ISL_624040|Original|hCoV-19^^England|Human|Lighthouse Lab in Alderley Park|Wellcome Sanger Institute for the COVID-19 Genomics UK (COG-UK) consortium|Davies|United Kingdom
>Spike|hCoV-19/Scotland/EDB3607/2020|2020-04-27|EPI_ISL_491586|Original|hCoV-19^^Scotland|Human|Virology Department|Wellcome Sanger Institute for the COVID-19 Genomics UK (COG-UK) consortium|Davies|United Kingdom
>Spike|hCoV-19/England/205160993/2020|2020-12-17|EPI_ISL_766086|Original|hCoV-19^^England|Human|Respiratory Virus Unit|COVID-19 Genomics UK (COG-UK) Consortium|Platt|United Kingdom
>Spike|hCoV-19/England/MILK-ACD73D/2020|2020-10-21|EPI_ISL_634302|Original|hCoV-19^^England|Human|Lighthouse Lab in Milton Keynes|Wellcome Sanger Institute for the COVID-19 Genomics UK (COG-UK) consortium|Davies|United Kingdom
>Spike|hCoV-19/Denmark/DCGC-11029/2020|2020-11-02|EPI_ISL_670353|Original|hCoV-19^^Sjaelland|Human|Department of Virus and Microbiological Special Diagnostics|Albertsen Lab|Michaelsen|Denmark
>Spike|hCoV-19/Switzerland/ZH-ETHZ-120004/2020|2020-04-17|EPI_ISL_468205|Original|hCoV-19^^Zurich|Human|Viollier AG|Department of Biosystems Science and Engineering|Nadeau|Switzerland
>Spike|hCoV-19/Japan/PG-0258/2020|2020-03-24|EPI_ISL_479942|Original|hCoV-19^^Kanto|Human|Ibaraki Prefectural Institute of Public Health|Pathogen Genomics Center|Sekizuka|Japan
>Spike|hCoV-19/SaudiArabia/KAUST-JEDDAH1009/2020|2020-06-17|EPI_ISL_512880|Original|hCoV-19^^Jeddah|Human|Pathogen Genomics Lab King Abdullah

University of Science and Technology(KAUST)|Pathogen Genomics Lab King Abdullah University of Science and Technology(KAUST)|Mohamed Ghazzali|Saudi Arabia
>Spike|hCoV-19/Netherlands/NA_556/2020|2020-00-00|EPI_ISL_460722|Original|hCoV-19^^Netherlands|Human|Dutch COVID-19 response team|Erasmus Medical Center|Oude Munnink|Netherlands
>Spike|hCoV-19/England/CAMC-B33E96/2020|2020-11-05|EPI_ISL_659257|Original|hCoV-19^^England|Human|Lighthouse Lab in Cambridge|Wellcome Sanger Institute for the COVID-19 Genomics UK (COG-UK) Consortium|Davies|United Kingdom
>Spike|hCoV-19/Scotland/EDB3657/2020|2020-04-28|EPI_ISL_439308|Original|hCoV-19^^Scotland|Human|Virology Department|COVID-19 Genomics UK (COG-UK) Consortium|Williams|United Kingdom
>Spike|hCoV-19/USA/UT-UPHL-2009310/2020|2020-05-14|EPI_ISL_524916|Original|hCoV-19^^Utah|Human|Utah Public Health Laboratory|Utah Public Health Laboratory|Young|USA
>Spike|hCoV-19/USA/TX-DSHS-1596/2020|2020-06-22|EPI_ISL_700717|Original|hCoV-19^^Texas|Human|Texas Department of State Health Services|Texas Department of State Health Services|Rahman|USA
>Spike|hCoV-19/Russia/StPetersburg-RII4694S/2020|2020-04-07|EPI_ISL_427326|Original|hCoV-19^^Saint Petersburg|Human|WHO National Influenza Centre Russian Federation|WHO National Influenza Centre Russian Federation|Komissarov|Russia
>Spike|hCoV-19/England/EXET-13A7E9/2020|2020-11-09|EPI_ISL_664153|Original|hCoV-19^^England|Human|University of Exeter|COVID-19 Genomics UK (COG-UK) Consortium|Temperton|United Kingdom
>Spike|hCoV-19/Wales/MILK-9F0635/2020|2020-09-24|EPI_ISL_580712|Original|hCoV-19^^Wales|Human|Lighthouse Lab in Milton Keynes|Wellcome Sanger Institute for the COVID-19 Genomics UK (COG-UK) consortium|Davies|United Kingdom
>Spike|hCoV-19/England/QEUH-BCBCB7/2020|2020-11-26|EPI_ISL_703948|Original|hCoV-19^^England|Human|Lighthouse Lab in Glasgow|Wellcome Sanger Institute for the COVID-19 Genomics UK (COG-UK) Consortium|Davies|United Kingdom
>Spike|hCoV-19/Netherlands/NB-EMC-404/2020|2020-00-00|EPI_ISL_722481|Original|hCoV-19^^Noord Brabant|Human|Dutch COVID-19 response team|Erasmus Medical Center|Oude Munnink|Netherlands
>Spike|hCoV-19/England/QEUH-9F6AE8/2020|2020-09-26|EPI_ISL_588288|Original|hCoV-19^^England|Human|Lighthouse Lab in Glasgow|Wellcome Sanger Institute for the COVID-19 Genomics UK (COG-UK) consortium|Davies|United Kingdom
>Spike|hCoV-19/USA/CA-CZB-2487/2020|2020-04-15|EPI_ISL_513881|Original|hCoV-19^^California|Human|San Francisco Public Health Laboratory|Chan-Zuckerberg Biohub|Kamm|USA
>Spike|hCoV-19/USA/CA-ALSR-0482-SAN/2020|2020-04-01|EPI_ISL_483170|Original|hCoV-19^^California|Human|UC San Diego Center for Advanced Laboratory Medicine|Andersen lab at Scripps Research|Gangavarapu|USA
>Spike|hCoV-19/England/CAMC-B22B75/2020|2020-11-02|EPI_ISL_647421|Original|hCoV-19^^England|Human|Lighthouse Lab in Cambridge|Wellcome Sanger Institute for the COVID-19 Genomics UK (COG-UK) Consortium|Davies|United Kingdom
>Spike|hCoV-19/Denmark/DCGC-22256/2020|2020-12-14|EPI_ISL_758639|Original|hCoV-19^^Syddanmark|Human|Department of Virus and Microbiological Special Diagnostics|Albertsen Lab|Michaelsen|Denmark
>Spike|hCoV-19/England/ALDP-B8427C/2020|2020-11-14|EPI_ISL_675343|Original|hCoV-19^^England|Human|Lighthouse Lab in Alderley Park|Wellcome Sanger Institute for the COVID-19 Genomics UK (COG-UK) Consortium|Davies|United Kingdom
>Spike|hCoV-19/Australia/WA79/2020|2020-07-13|EPI_ISL_512732|Original|hCoV-19^^Western Australia|Human|PathWest Laboratory Medicine WA|PathWest Laboratory Medicine WA Microbial Surveillance Unit|Sikazwe|Australia
>Spike|hCoV-19/England/MILK-975D33/2020|2020-08-20|EPI_ISL_552772|Original|hCoV-19^^England|Human|Lighthouse Lab in Milton Keynes|Wellcome Sanger Institute for the COVID-19 Genomics UK (COG-UK) consortium|Davies|United Kingdom
>Spike|hCoV-19/Switzerland/BE-ETHZ-270126/2020|2020-09-03|EPI_ISL_541433|Original|hCoV-19^^Bern|Human|Viollier AG|Department of Biosystems Science and Engineering|Nadeau|Switzerland

>Spike|hCoV-19/South Africa/KRISP-0259/2020|2020-06-15|EPI_ISL_482729|Original|hCoV-19^^KwaZulu-Natal|Human|NHLS-IALCH|KRISP|de Oliveira|South Africa
>Spike|hCoV-19/England/QEUH-9D35A4/2020|2020-09-18|EPI_ISL_577340|Original|hCoV-19^^England|Human|Centre for Enzyme Innovation|COVID-19 Genomics UK (COG-UK) Consortium|Robson|United Kingdom
>Spike|hCoV-19/Wales/PHWC-49C7F1/2020|2020-11-18|EPI_ISL_742606|Original|hCoV-19^^Wales|Human|Wales Specialist Virology Centre Sequencing lab|COVID-19 Genomics UK (COG-UK) Consortium|Moore|United Kingdom
>Spike|hCoV-19/Japan/PG-8105/2020|2020-08-00|EPI_ISL_687204|Original|hCoV-19^^Japan|Human|Pathogen Genomics Center|Pathogen Genomics Center|Sekizuka|Japan
>Spike|hCoV-19/England/QEUH-9CA160/2020|2020-09-10|EPI_ISL_567454|Original|hCoV-19^^England|Human|Lighthouse Lab in Glasgow|Wellcome Sanger Institute for the COVID-19 Genomics UK (COG-UK) consortium|Davies|United Kingdom
>Spike|hCoV-19/USA/NY-NYUMC167/2020|2020-04-01|EPI_ISL_426622|Original|hCoV-19^^New York|Human|NYU Langone Health|Departments of Pathology and Medicine|Maurano|USA
>Spike|hCoV-19/Wales/MILK-A6AC60/2020|2020-10-09|EPI_ISL_625417|Original|hCoV-19^^Wales|Human|Lighthouse Lab in Milton Keynes|Wellcome Sanger Institute for the COVID-19 Genomics UK (COG-UK) consortium|Davies|United Kingdom
>Spike|hCoV-19/England/QEUH-BF0D3E/2020|2020-12-04|EPI_ISL_709231|Original|hCoV-19^^England|Human|Lighthouse Lab in Glasgow|Wellcome Sanger Institute for the COVID-19 Genomics UK (COG-UK) Consortium|Davies|United Kingdom
>Spike|hCoV-19/USA/CA-QDX-1542/2020|2020-03-18|EPI_ISL_603790|Original|hCoV-19^^California|Human|Quest Diagnostics|Quest Diagnostics|Kagan|USA
>Spike|hCoV-19/England/QEUH-CC5F86/2020|2020-12-21|EPI_ISL_760375|Original|hCoV-19^^England|Human|Lighthouse Lab in Glasgow|Wellcome Sanger Institute for the COVID-19 Genomics UK (COG-UK) Consortium|Davies|United Kingdom
>Spike|hCoV-19/England/20129010104/2020|2020-03-20|EPI_ISL_423980|Original|hCoV-19^^England|Human|Respiratory Virus Unit|Respiratory Virus Unit|Platt|United Kingdom
>Spike|hCoV-19/USA/OR-OHSU-0871/2020|2020-06-29|EPI_ISL_525917|Original|hCoV-19^^Oregon|Human|OHSU Lab Services Molecular Microbiology Lab|Oregon SARS-CoV-2 Genome Sequencing Center|O'Roak|USA
>Spike|hCoV-19/Scotland/CVR336/2020|2020-03-21|EPI_ISL_439055|Original|hCoV-19^^Scotland|Human|West of Scotland Specialist Virology Centre|COVID-19 Genomics UK (COG-UK) Consortium|Robertson|United Kingdom
>Spike|hCoV-19/USA/GA-QDX-2259/2020|2020-03-13|EPI_ISL_604531|Original|hCoV-19^^Georgia|Human|Quest Diagnostics|Quest Diagnostics|Kagan|USA
>Spike|hCoV-19/USA/CA-UW-MAYO55/2020|2020-03-27|EPI_ISL_500534|Original|hCoV-19^^California|Human|Mayo Clinic Laboratories|University of Washington Virology Lab|Roychoudhury|USA
>Spike|hCoV-19/Thailand/Bangkok-CONI-0323/2020|2020-12-05|EPI_ISL_707710|Original|hCoV-19^^Bangkok|Human|Hospital for Tropical Diseases|COVID-19 Network Investigations (CONI) Alliance|Batty|Thailand
>Spike|hCoV-19/France/PAC-IHU-1475/2020|2020-08-11|EPI_ISL_569175|Original|hCoV-19^^Provence-Alpes-C\u00f4te-d\u2019Azur|Human|MEPHI|MEPHI|Levasseur|France
>Spike|hCoV-19/England/MILK-B3C4B9/2020|2020-11-02|EPI_ISL_706149|Original|hCoV-19^^England|Human|Oxford Viromics|COVID-19 Genomics UK (COG-UK) Consortium|Golubchik|United Kingdom
>Spike|hCoV-19/Canada/NS-NML-951/2020|2020-03-26|EPI_ISL_579581|Original|hCoV-19^^Nova Scotia|Human|QEII Health Sciences Centre|National Microbiology Laboratory (NML)|Majer|Canada
>Spike|hCoV-19/USA/AZ-TG664535/2020|2020-04-20|EPI_ISL_696152|Original|hCoV-19^^Arizona|Human|Sonora Quest Laboratories|TGen North|French|USA
>Spike|hCoV-19/Norway/5452/2020|2020-11-03|EPI_ISL_708096|Original|hCoV-19^^Norway|Human|Oslo University Hospital|Norwegian Institute of Public Health|Kopperud Riis|Norway
>Spike|hCoV-19/England/201120454/2020|2020-03-09|EPI_ISL_464675|Original|hCoV-19^^England|Human|Respiratory Virus Unit|Respiratory Virus Unit|Platt|United Kingdom
>Spike|hCoV-19/England/QEUH-AAC707/2020|2020-10-21|EPI_ISL_652869|Original|hCoV-19^^England|Human|Oxford Viromics|COVID-19 Genomics UK (COG-UK) Consortium|Golubchik|United Kingdom

>Spike|hCoV-19/USA/LA-CDC-0499/2020|2020-03-10|EPI_ISL_452106|Original|hCoV-
19^^Louisiana|Human|LA Office of Public Health Laboratories|Pathogen
Discovery|Queen|USA
>Spike|hCoV-19/England/ALDP-B1C473/2020|2020-11-01|EPI_ISL_646063|Original|hCoV-
19^^England|Human|Lighthouse Lab in Alderley Park|Wellcome Sanger Institute for the
COVID-19 Genomics UK (COG-UK) Consortium|Davies|United Kingdom
>Spike|hCoV-19/England/MILK-956A31/2020|2020-06-05|EPI_ISL_554191|Original|hCoV-
19^^England|Human|Lighthouse Lab in Milton Keynes|Wellcome Sanger Institute for the
COVID-19 Genomics UK (COG-UK) consortium|Davies|United Kingdom
>Spike|hCoV-19/Scotland/QEUH-9E386C/2020|2020-09-23|EPI_ISL_585519|Original|hCoV-
19^^Scotland|Human|Northumbria University / South Tees Hospitals NHS Foundation Trust
/ North Cumbria Integrated Care NHS Foundation Trust / North Tees and Hartlepool NHS
Foundation Trust / Newcastle Hospitals NHS Foundation Trust|COVID-19 Genomics UK
(COG-UK) Consortium|Bashton|United Kingdom
>Spike|hCoV-19/Scotland/CVR3826/2020|2020-05-28|EPI_ISL_478152|Original|hCoV-
19^^Scotland|Human|West of Scotland Specialist Virology Centre|COVID-19 Genomics UK
(COG-UK) Consortium|Robertson|United Kingdom
>Spike|hCoV-19/Australia/VIC2326/2020|2020-06-25|EPI_ISL_522047|Original|hCoV-
19^^Victoria|Human|Microbiological Diagnostic Unit - Public Health Laboratory (MDU-
PHL)|MDU-PHL|Schultz|Australia
>Spike|hCoV-19/Australia/VIC3134/2020|2020-07-04|EPI_ISL_520841|Original|hCoV-
19^^Victoria|Human|Victorian Infectious Diseases Reference Laboratory (VIDRL)|VIDRL
and MDU-PHL|Schultz|Australia
>Spike|hCoV-19/USA/MN-MDH-1714/2020|2020-08-25|EPI_ISL_560801|Original|hCoV-
19^^Minnesota|Human|M Health Fairview|Minnesota Department of Health|Garfin|USA
>Spike|hCoV-19/Scotland/QEUH-9DAA44/2020|2020-09-22|EPI_ISL_575357|Original|hCoV-
19^^Scotland|Human|Lighthouse Lab in Glasgow|Wellcome Sanger Institute for the COVID-
19 Genomics UK (COG-UK) consortium|Davies|United Kingdom
>Spike|hCoV-19/England/CAMC-B08A69/2020|2020-10-30|EPI_ISL_643924|Original|hCoV-
19^^England|Human|Lighthouse Lab in Cambridge|Wellcome Sanger Institute for the
COVID-19 Genomics UK (COG-UK) Consortium|Davies|United Kingdom
>Spike|hCoV-19/Vietnam/40227/2020|2020-08-00|EPI_ISL_759871|Original|hCoV-
19^^Vietnam|Human|National Influenza Center|National Influenza Center|Ung|Vietnam
>Spike|hCoV-19/USA/TX-HMH-MCoV-16289/2020|2020-10-30|EPI_ISL_785973|Original|hCoV-
19^^Texas|Human|Houston Methodist Hospital|Houston Methodist Hospital|Long|USA
>Spike|hCoV-19/Denmark/DCGC-2664/2020|2020-08-17|EPI_ISL_617884|Original|hCoV-
19^^Hovedstaden|Human|Department of Virus and Microbiological Special
Diagnostics|Albertsen lab|Michaelsen|Denmark
>Spike|hCoV-19/USA/GA-CDC-7731/2020|2020-10-27|EPI_ISL_681808|Original|hCoV-
19^^Georgia|Human|GA Department of Public Health Laboratory|Pathogen
Discovery|Queen|USA
>Spike|hCoV-19/Canada/ON-S267/2020|2020-03-27|EPI_ISL_755842|Original|hCoV-
19^^Ontario|Human|Toronto Invasive Bacterial Diseases Network|McMaster
University|McArthur|Canada
>Spike|hCoV-19/England/QEUH-BCE2D0/2020|2020-11-26|EPI_ISL_704345|Original|hCoV-
19^^England|Human|Lighthouse Lab in Glasgow|Wellcome Sanger Institute for the COVID-
19 Genomics UK (COG-UK) Consortium|Davies|United Kingdom
>Spike|hCoV-19/England/MILK-9C2E16/2020|2020-09-10|EPI_ISL_566513|Original|hCoV-
19^^England|Human|Lighthouse Lab in Milton Keynes|Wellcome Sanger Institute for the
COVID-19 Genomics UK (COG-UK) consortium|Davies|United Kingdom
>Spike|hCoV-19/Egypt/MASRI-018/2020|2020-05-10|EPI_ISL_483036|Original|hCoV-
19^^Cairo|Human|Medical Ain Shams Research Institute (MASRI)|Medical Ain Shams
Research Institute (MASRI)|Moustafa|Egypt
>Spike|hCoV-19/England/QEUH-A00631/2020|2020-10-01|EPI_ISL_600283|Original|hCoV-
19^^England|Human|Lighthouse Lab in Glasgow|Wellcome Sanger Institute for the COVID-
19 Genomics UK (COG-UK) consortium|Davies|United Kingdom
>Spike|hCoV-19/USA/TX-HMH-MCoV-12318/2020|2020-09-25|EPI_ISL_790298|Original|hCoV-
19^^Texas|Human|Houston Methodist Hospital|Houston Methodist Hospital|Long|USA

>Spike|hCoV-19/Denmark/ALAB-SSI-1033/2020|2020-03-31|EPI_ISL_437629|Original|hCoV-19^^Denmark|Human|Department of Virus and Microbiological Special Diagnostics|Albertsen lab|Kirkegaard|Denmark
>Spike|hCoV-19/England/NORT-29D516/2020|2020-05-08|EPI_ISL_484297|Original|hCoV-19^^England|Human|Northumbria University / South Tees Hospitals NHS Foundation Trust / North Cumbria Integrated Care NHS Foundation Trust / North Tees and Hartlepool NHS Foundation Trust / Newcastle Hospitals NHS Foundation Trust|COVID-19 Genomics UK (COG-UK) Consortium|Bashton|United Kingdom
>Spike|hCoV-19/England/BRIS-12A236/2020|2020-04-16|EPI_ISL_458395|Original|hCoV-19^^England|Human|PHE South West Regional Laboratory|Wellcome Sanger Institute for the COVID-19 Genomics UK (COG-UK) consortium|Davies|United Kingdom
>Spike|hCoV-19/Denmark/DCGC-1550/2020|2020-04-13|EPI_ISL_618655|Original|hCoV-19^^Syddanmark|Human|Department of Virus and Microbiological Special Diagnostics|Albertsen lab|Michaelsen|Denmark
>Spike|hCoV-19/England/CAMC-A82CBD/2020|2020-10-13|EPI_ISL_610677|Original|hCoV-19^^England|Human|Lighthouse Lab in Cambridge|Wellcome Sanger Institute for the COVID-19 Genomics UK (COG-UK) consortium|Davies|United Kingdom
>Spike|hCoV-19/France/PAC-IHU-1615/2020|2020-09-04|EPI_ISL_569228|Original|hCoV-19^^Provence-Alpes-C\u00f4te-d\u2019Azur|Human|MEPHI|MEPHI|Levasseur|France
>Spike|hCoV-19/USA/TX-HMH-4289/2020|2020-06-18|EPI_ISL_543499|Original|hCoV-19^^Texas|Human|Houston Methodist Hospital|Houston Methodist Hospital|Long|USA
>Spike|hCoV-19/Denmark/DCGC-5588/2020|2020-09-28|EPI_ISL_620913|Original|hCoV-19^^Sjaelland|Human|Department of Virus and Microbiological Special Diagnostics|Albertsen lab|Michaelsen|Denmark
>Spike|hCoV-19/England/ALDP-506041/2020|2020-06-25|EPI_ISL_559243|Original|hCoV-19^^England|Human|Lighthouse Lab in Alderley Park|Wellcome Sanger Institute for the COVID-19 Genomics UK (COG-UK) consortium|Davies|United Kingdom
>Spike|hCoV-19/USA/TX-HMH-MCoV-18624/2020|2020-11-21|EPI_ISL_784972|Original|hCoV-19^^Texas|Human|Houston Methodist Hospital|Houston Methodist Hospital|Long|USA
>Spike|hCoV-19/Northern Ireland/NIRE-102DAC/2020|2020-04-20|EPI_ISL_489218|Original|hCoV-19^^Northern Ireland|Human|Regional Virus Laboratory|Wellcome Sanger Institute for the COVID-19 Genomics UK (COG-UK) consortium|Davies|United Kingdom
>Spike|hCoV-19/Switzerland/ZH-ETHZ-331074/2020|2020-10-20|EPI_ISL_614969|Original|hCoV-19^^Zurich|Human|Viollier AG|Department of Biosystems Science and Engineering|Nadeau|Switzerland
>Spike|hCoV-19/USA/WA-UW-22228/2020|2020-08-02|EPI_ISL_570642|Original|hCoV-19^^Washington|Human|UW Virology Lab|UW Virology Lab|Roychoudhury|USA
>Spike|hCoV-19/Peru/LIM-UPCH-0025/2020|2020-06-17|EPI_ISL_540926|Original|hCoV-19^^Lima|Human|Laboratorio de Referencia Nacional de Virus Respiratorios|Laboratorio de Gen\u00f3mica Microbiana|Tsukayama|Peru
>Spike|hCoV-19/Palestine/16/2020|2020-03-16|EPI_ISL_596505|Original|hCoV-19^^Bethlehem|Human|Palestinian Ministry of Health|Molecular Genetics Lab|van Dorp|Palestine
>Spike|hCoV-19/USA/WI-GMF-28108/2020|2020-10-27|EPI_ISL_661005|Original|hCoV-19^^Wisconsin|Human|Gundersen Molecular Diagnostics Laboratory|Kabara Cancer Research Institute|Kenny|USA
>Spike|hCoV-19/Austria/CeMM0037/2020|2020-03-09|EPI_ISL_437926|Original|hCoV-19^^Ischgl|Human|Institut f\u00fcr Virologie am Department f\u00fcr Hygiene|Bergthaler laboratory|Bergthaler|Austria
>Spike|hCoV-19/DRC/3483/2020|2020-04-22|EPI_ISL_447602|Original|hCoV-19^^Kinshasa|Human|Viral Respiratory Lab|Pathogen Sequencing Lab|Lusamaki|Democratic Republic of the Congo
>Spike|hCoV-19/Gambia/1277/2020|2020-05-07|EPI_ISL_471171|Original|hCoV-19^^Kombo|Human|MRCG at LSHTM Genomics lab|MRCG at LSHTM Genomics lab|Sesay|Gambia
>Spike|hCoV-19/Australia/VIC3011/2020|2020-07-05|EPI_ISL_521840|Original|hCoV-19^^Victoria|Human|Victorian Infectious Diseases Reference Laboratory (VIDRL)|VIDRL and MDU-PHL|Schultz|Australia

>Spike|hCoV-19/USA/MI-MDHHS-SC22641/2020|2020-12-01|EPI_ISL_737123|Original|hCoV-19^^Michigan|Human|Michigan Department of Health and Human Services|Michigan Department of Health and Human Services|Blankenship|USA
>Spike|hCoV-19/USA/NY-QDX-19/2020|2020-03-16|EPI_ISL_467897|Original|hCoV-19^^New York|Human|Quest Diagnostics|Quest Diagnostics|Kagan|USA
>Spike|hCoV-19/Scotland/CVR5879/2020|2020-11-16|EPI_ISL_705641|Original|hCoV-19^^Scotland|Human|West of Scotland Specialist Virology Centre|COVID-19 Genomics UK (COG-UK) Consortium|Robertson|United Kingdom
>Spike|hCoV-19/USA/TX-HMH-MCoV-10373/2020|2020-07-08|EPI_ISL_789441|Original|hCoV-19^^Texas|Human|Houston Methodist Hospital|Houston Methodist Hospital|Long|USA
>Spike|hCoV-19/Switzerland/SZ-ETHZ-310391/2020|2020-10-08|EPI_ISL_603465|Original|hCoV-19^^Schwyz|Human|Viollier AG|Department of Biosystems Science and Engineering|Nadeau|Switzerland
>Spike|hCoV-19/USA/WA-S2152/2020|2020-06-08|EPI_ISL_497580|Original|hCoV-19^^Washington|Human|Washington State Department of Health|Seattle Flu Study|Lee|USA
>Spike|hCoV-19/England/LOND-D3F90/2020|2020-04-04|EPI_ISL_441049|Original|hCoV-19^^England|Human|University College London|COVID-19 Genomics UK (COG-UK) Consortium|Roy|United Kingdom
>Spike|hCoV-19/Scotland/EDB5444/2020|2020-04-13|EPI_ISL_473824|Original|hCoV-19^^Scotland|Human|Virology Department|COVID-19 Genomics UK (COG-UK) Consortium|Williams|United Kingdom
>Spike|hCoV-19/United Arab Emirates/0286/2020|2020-05-17|EPI_ISL_698466|Original|hCoV-19^^Abu Dhabi|Human|Group 42 (G42) Healthcare|G42 Healthcare|Ogrodzki|United Arab Emirates
>Spike|hCoV-19/England/201380026/2020|2020-03-26|EPI_ISL_465828|Original|hCoV-19^^England|Human|Respiratory Virus Unit|Respiratory Virus Unit|Platt|United Kingdom
>Spike|hCoV-19/India/GJ-GBRC207a/2020|2020-06-11|EPI_ISL_475033|Original|hCoV-19^^Gujarat|Human|Department of MicroBiology|Gujarat Biotechnology Research Centre|Joshi|India
>Spike|hCoV-19/Netherlands/NA_447/2020|2020-04-01|EPI_ISL_455249|Original|hCoV-19^^Netherlands|Human|Dutch COVID-19 response team|Erasmus Medical Center|Oude Munnink|Netherlands
>Spike|hCoV-19/South Africa/NHLS-UCT-GS-0784/2020|2020-07-23|EPI_ISL_640070|Original|hCoV-19^^Western Cape|Human|Guguletu CHC wc GDH|NHLS/UCT|Iranzadeh|South Africa
>Spike|hCoV-19/England/MILK-A7CF44/2020|2020-10-12|EPI_ISL_606827|Original|hCoV-19^^England|Human|Lighthouse Lab in Milton Keynes|Wellcome Sanger Institute for the COVID-19 Genomics UK (COG-UK) consortium|Davies|United Kingdom
>Spike|hCoV-19/Norway/3598/2020|2020-09-23|EPI_ISL_590944|Original|hCoV-19^^Norway|Human|University Hospital of Northern Norway|Norwegian Institute of Public Health|Vollan|Norway
>Spike|hCoV-19/England/LOND-125FF1F/2020|2020-03-08|EPI_ISL_516839|Original|hCoV-19^^England|Human|North West London Pathology|Wellcome Sanger Institute for the COVID-19 Genomics UK (COG-UK) consortium|Davies|United Kingdom
>Spike|hCoV-19/England/ALDP-BE8F51/2020|2020-12-03|EPI_ISL_708892|Original|hCoV-19^^England|Human|Lighthouse Lab in Alderley Park|Wellcome Sanger Institute for the COVID-19 Genomics UK (COG-UK) Consortium|Davies|United Kingdom
>Spike|hCoV-19/Russia/MOS-CRIE-13644986/2020|2020-11-09|EPI_ISL_745257|Original|hCoV-19^^Moscow|Human|Molecular diagnostic laboratory of Federal Budget Institution of Science \Central Research Institute of Epidemiology\ of The Federal Service on Customers' Rights Protection and Human Well-being Surveillance|Group of Genomics and Postgenomic Technologies of Central Research Institute of Epidemilolog
>y|Korneenko|Russia
>Spike|hCoV-19/Japan/PG-2684/2020|2020-06-00|EPI_ISL_690816|Original|hCoV-19^^Japan|Human|Tokyo Metropolitan Institute of Public Health|Pathogen Genomics Center|Sekizuka|Japan
>Spike|hCoV-19/Sweden/20-53909/2020|2020-12-14|EPI_ISL_766725|Original|hCoV-19^^Uppsala|Human|Klinisk mikrobiologi|The Public Health Agency of Sweden|Svartstr\u00f6m|Sweden

>Spike|hCoV-19/England/MILK-ABBA84/2020|2020-10-22|EPI_ISL_623542|Original|hCoV-19^^England|Human|Lighthouse Lab in Milton Keynes|Wellcome Sanger Institute for the COVID-19 Genomics UK (COG-UK) consortium|Davies|United Kingdom
>Spike|hCoV-19/USA/TX-HMH-6312/2020|2020-06-30|EPI_ISL_547383|Original|hCoV-19^^Texas|Human|Houston Methodist Hospital|Houston Methodist Hospital|Long|USA
>Spike|hCoV-19/USA/CA-CZB-14831/2020|2020-11-18|EPI_ISL_739417|Original|hCoV-19^^California|Human|Humboldt County Public Health Laboratory|Chan-Zuckerberg Biohub|Kamm|USA
>Spike|hCoV-19/Spain/PV-IBV-001514/2020|2020-03-19|EPI_ISL_467093|Original|hCoV-19^^Basque Country|Human|Hospital Universitario Araba. Vitoria-Gasteiz|SeqCOVID-SPAIN consortium/IBV(CSIC)|Comas|Spain
>Spike|hCoV-19/Scotland/CVR2267/2020|2020-04-10|EPI_ISL_477888|Original|hCoV-19^^Scotland|Human|West of Scotland Specialist Virology Centre|COVID-19 Genomics UK (COG-UK) Consortium|Robertson|United Kingdom
>Spike|hCoV-19/Australia/NSW1330/2020|2020-12-24|EPI_ISL_767896|Original|hCoV-19^^New South Wales|Human|Australian Clinical Labs|NSW Health Pathology - Institute of Clinical Pathology and Medical Research; Westmead Hospital; University of Sydney|Gall|Australia
>Spike|hCoV-19/England/QEUH-B319D3/2020|2020-11-04|EPI_ISL_646435|Original|hCoV-19^^England|Human|Lighthouse Lab in Glasgow|Wellcome Sanger Institute for the COVID-19 Genomics UK (COG-UK) Consortium|Davies|United Kingdom
>Spike|hCoV-19/Spain/AR-IBV-98008124/2020|2020-10-13|EPI_ISL_654428|Original|hCoV-19^^Aragon|Human|Servicio de Microbiolog\u00eda|SeqCOVID-SPAIN consortium/IBV(CSIC)|Comas|Spain
>Spike|hCoV-19/England/CAMC-B98C59/2020|2020-11-15|EPI_ISL_673713|Original|hCoV-19^^England|Human|Lighthouse Lab in Cambridge|Wellcome Sanger Institute for the COVID-19 Genomics UK (COG-UK) Consortium|Davies|United Kingdom
>Spike|hCoV-19/Italy/TUS-DBM-21/2020|2020-04-27|EPI_ISL_583958|Original|hCoV-19^^Tuscany|Human|UOC Microbiologia e Virologia|Dipartimento di Biotecnologie Mediche|Pinzauti|Italy
>Spike|hCoV-19/Scotland/EDB2834/2020|2020-03-31|EPI_ISL_489016|Original|hCoV-19^^Scotland|Human|Virology Department|Wellcome Sanger Institute for the COVID-19 Genomics UK (COG-UK) consortium|Davies|United Kingdom
>Spike|hCoV-19/USA/CA-QDX-1045/2020|2020-03-17|EPI_ISL_571710|Original|hCoV-19^^California|Human|Quest Diagnostics|Quest Diagnostics|Kagan|USA
>Spike|hCoV-19/Japan/PG-8614/2020|2020-08-00|EPI_ISL_686210|Original|hCoV-19^^Japan|Human|Pathogen Genomics Center|Pathogen Genomics Center|Sekizuka|Japan
>Spike|hCoV-19/Scotland/GCVR-173C4F/2020|2020-04-07|EPI_ISL_459127|Original|hCoV-19^^Scotland|Human|NHSGGC West of Scotland Specialist Virology Centre / MRC-University of Glasgow Centre for Virus Research|Wellcome Sanger Institute for the COVID-19 Genomics UK (COG-UK) consortium|Davies|United Kingdom
>Spike|hCoV-19/Scotland/QEUH-C4BF2E/2020|2020-12-14|EPI_ISL_757117|Original|hCoV-19^^Scotland|Human|Lighthouse Lab in Glasgow|Wellcome Sanger Institute for the COVID-19 Genomics UK (COG-UK) Consortium|Davies|United Kingdom
>Spike|hCoV-19/India/GJ-GBRC249b/2020|2020-06-12|EPI_ISL_483832|Original|hCoV-19^^Gujarat|Human|Pandit Deendayal Upadhyay Government Medical College|Gujarat Biotechnology Research Centre|Joshi|India
>Spike|hCoV-19/USA/MN-MDH-1897/2020|2020-10-23|EPI_ISL_613529|Original|hCoV-19^^Minnesota|Human|Public Health Laboratory - Infectious Disease Lab|Minnesota Department of Health|Lorentz|USA
>Spike|hCoV-19/England/NORW-EEEFF/2020|2020-09-29|EPI_ISL_584716|Original|hCoV-19^^England|Human|Quadram Institute Bioscience|COVID-19 Genomics UK (COG-UK) Consortium|Alikhan|United Kingdom
>Spike|hCoV-19/Scotland/QEUH-9DD531/2020|2020-09-21|EPI_ISL_599588|Original|hCoV-19^^Scotland|Human|Lighthouse Lab in Glasgow|Wellcome Sanger Institute for the COVID-19 Genomics UK (COG-UK) consortium|Davies|United Kingdom
>Spike|hCoV-19/Morocco/OUA677-19/2020|2020-04-23|EPI_ISL_451400|Original|hCoV-19^^Ouarzazate|Human|Laboratoire de Recherche et d'Analyse M\u00e9dicale de la Gendarmerie Royale|Laboratoire de Recherche et d'Analyse M\u00e9dicale de la Gendarmerie Royale|Lemriss|Morocco

>Spike|hCoV-19/Wales/PHWC-4884B5/2020|2020-10-30|EPI_ISL_639398|Original|hCoV-19^^Wales|Human|Wales Specialist Virology Centre Sequencing lab|COVID-19 Genomics UK (COG-UK) Consortium|Moore|United Kingdom
>Spike|hCoV-19/Netherlands/ZH-RIVM-10126/2020|2020-00-00|EPI_ISL_547566|Original|hCoV-19^^Zuid Holland|Human|Dutch COVID-19 response team|National Institute for Public Health and the Environment (RIVM)|Meijer|Netherlands
>Spike|hCoV-19/England/ALDP-95A4B7/2020|2020-06-12|EPI_ISL_553549|Original|hCoV-19^^England|Human|Lighthouse Lab in Alderley Park|Wellcome Sanger Institute for the COVID-19 Genomics UK (COG-UK) consortium|Davies|United Kingdom
>Spike|hCoV-19/England/QEUH-5DE288/2020|2020-06-17|EPI_ISL_533276|Original|hCoV-19^^England|Human|Lighthouse Lab in Glasgow|Wellcome Sanger Institute for the COVID-19 Genomics UK (COG-UK) consortium|Davies|United Kingdom
>Spike|hCoV-19/England/CAMC-B0B301/2020|2020-10-29|EPI_ISL_643557|Original|hCoV-19^^England|Human|Lighthouse Lab in Cambridge|Wellcome Sanger Institute for the COVID-19 Genomics UK (COG-UK) Consortium|Davies|United Kingdom
>Spike|hCoV-19/England/LIVE-9F060/2020|2020-04-15|EPI_ISL_453423|Original|hCoV-19^^England|Human|Liverpool Clinical Laboratories|COVID-19 Genomics UK (COG-UK) Consortium|Haldenby|United Kingdom
>Spike|hCoV-19/USA/NY-NYCPHL-000243/2020|2020-04-12|EPI_ISL_631770|Original|hCoV-19^^New York|Human|Flushing Hospital Medical Center|New York City Public Health Laboratory|Wang|USA
>Spike|hCoV-19/Wales/PHWC-15C56F/2020|2020-05-04|EPI_ISL_472505|Original|hCoV-19^^Wales|Human|Wales Specialist Virology Centre Sequencing lab|COVID-19 Genomics UK (COG-UK) Consortium|Moore|United Kingdom
>Spike|hCoV-19/Thailand/Bangkok-CONI-0137/2020|2020-03-13|EPI_ISL_455931|Original|hCoV-19^^Bangkok|Human|Ramathibodi Hospital|COVID-19 Network Investigations (CONI) Alliance|Batty|Thailand
>Spike|hCoV-19/Scotland/QEUH-960560/2020|2020-08-05|EPI_ISL_531990|Original|hCoV-19^^Scotland|Human|NHSGGC West of Scotland Specialist Virology Centre / MRC-University of Glasgow Centre for Virus Research|Wellcome Sanger Institute for the COVID-19 Genomics UK (COG-UK) consortium|Davies|United Kingdom
>Spike|hCoV-19/Australia/VIC17292/2020|2020-07-29|EPI_ISL_663822|Original|hCoV-19^^Victoria|Human|Microbiological Diagnostic Unit - Public Health Laboratory (MDU-PHL)|MDU-PHL|Schultz|Australia
>Spike|hCoV-19/USA/TX-HMH-6992/2020|2020-06-28|EPI_ISL_546415|Original|hCoV-19^^Texas|Human|Houston Methodist Hospital|Houston Methodist Hospital|Long|USA
>Spike|hCoV-19/Belgium/rega-12141328/2020|2020-12-14|EPI_ISL_738381|Original|hCoV-19^^Mol|Human|UZ Leuven|KU Leuven|Maes|Belgium
>Spike|hCoV-19/USA/CA-CZB-12353/2020|2020-10-12|EPI_ISL_648425|Original|hCoV-19^^California|Human|Santa Clara County Public Health Laboratory|Chan-Zuckerberg Biohub|Bhatt|USA
>Spike|hCoV-19/Canada/BC_15448387/2020|2020-04-00|EPI_ISL_468667|Original|hCoV-19^^British Columbia|Human|BCCDC Public Health Laboratory|BCCDC Public Health Laboratory|Harrigan|Canada
>Spike|hCoV-19/Germany/BY-ChVir1980/2020|2020-03-06|EPI_ISL_729494|Original|hCoV-19^^Bavaria|Human|Charit\u00e9 Universit\u00e4tsmedizin Berlin|Charit\u00e9 Universit\u00e4tsmedizin Berlin|Muehlemann|Germany
>Spike|hCoV-19/Gambia/GC19-3418/2020|2020-07-27|EPI_ISL_561176|Original|hCoV-19^^Kombo|Human|MRCG at LSHTM Genomics lab|MRCG at LSHTM Genomics lab|Sesay|Gambia
>Spike|hCoV-19/England/QEUH-9F80E4/2020|2020-09-27|EPI_ISL_595729|Original|hCoV-19^^England|Human|Oxford Viromics|COVID-19 Genomics UK (COG-UK) Consortium|Golubchik|United Kingdom
>Spike|hCoV-19/Scotland/CVR5698/2020|2020-11-07|EPI_ISL_651918|Original|hCoV-19^^Scotland|Human|West of Scotland Specialist Virology Centre|COVID-19 Genomics UK (COG-UK) Consortium|Robertson|United Kingdom
>Spike|hCoV-19/England/CAMC-B081BF/2020|2020-10-29|EPI_ISL_643905|Original|hCoV-19^^England|Human|Lighthouse Lab in Cambridge|Wellcome Sanger Institute for the COVID-19 Genomics UK (COG-UK) Consortium|Davies|United Kingdom

>Spike|hCoV-19/England/ALDP-CF1552/2020|2020-12-18|EPI_ISL_781943|Original|hCoV-
19^^England|Human|Lighthouse Lab in Alderley Park|Wellcome Sanger Institute for the
COVID-19 Genomics UK (COG-UK) Consortium|Davies|United Kingdom
>Spike|hCoV-19/Australia/VIC5092/2020|2020-07-19|EPI_ISL_519895|Original|hCoV-
19^^Victoria|Human|Microbiological Diagnostic Unit - Public Health Laboratory (MDU-
PHL)|Microbiological Diagnostic Unit - Public Health Laboratory (MDU-
PHL)|Schultz|Australia
>Spike|hCoV-19/England/QEUH-9F47B6/2020|2020-09-24|EPI_ISL_588352|Original|hCoV-
19^^England|Human|Lighthouse Lab in Glasgow|Wellcome Sanger Institute for the COVID-
19 Genomics UK (COG-UK) consortium|Davies|United Kingdom
>Spike|hCoV-19/England/ALDP-A509FD/2020|2020-10-07|EPI_ISL_597525|Original|hCoV-
19^^England|Human|Lighthouse Lab in Alderley Park|Wellcome Sanger Institute for the
COVID-19 Genomics UK (COG-UK) consortium|Davies|United Kingdom
>Spike|hCoV-19/Denmark/DCGC-18877/2020|2020-12-07|EPI_ISL_748612|Original|hCoV-
19^^Midtjylland|Human|Department of Virus and Microbiological Special
Diagnostics|Albertsen Lab|Michaelsen|Denmark
>Spike|hCoV-19/Wales/PHWC-3225A/2020|2020-04-09|EPI_ISL_446622|Original|hCoV-
19^^Wales|Human|Wales Specialist Virology Centre|Public Health Wales Microbiology
Cardiff|Moore|United Kingdom
>Spike|hCoV-19/New Zealand/20VR2730/2020|2020-00-00|EPI_ISL_579345|Original|hCoV-
19^^Auckland|Human|LabPLUS|Institute of Environmental Science and Research
(ESR)|Wang|New Zealand
>Spike|hCoV-19/Germany/HH-hpi-p1769/2020|2020-03-19|EPI_ISL_776143|Original|hCoV-
19^^Hamburg|Human|University Medical Center Hamburg Eppendorf|Heinrich Pette
Institute|Robitaille|Germany
>Spike|hCoV-19/Spain/AR-IBV-98010596/2020|2020-10-03|EPI_ISL_722250|Original|hCoV-
19^^Aragon|Human|Servicio de Microbiolog\u00eda|SeqCOVID-SPAIN
consortium/IBV(CSIC)|Comas|Spain
>Spike|hCoV-19/Wales/PHWC-48661B/2020|2020-10-21|EPI_ISL_639059|Original|hCoV-
19^^Wales|Human|Wales Specialist Virology Centre Sequencing lab|COVID-19 Genomics UK
(COG-UK) Consortium|Moore|United Kingdom
>Spike|hCoV-19/England/BIRM-64E97/2020|2020-09-24|EPI_ISL_594526|Original|hCoV-
19^^England|Human|University of Birmingham|COVID-19 Genomics UK (COG-UK)
Consortium|Loman|United Kingdom
>Spike|hCoV-19/Denmark/DCGC-4439/2020|2020-09-21|EPI_ISL_621860|Original|hCoV-
19^^Nordjylland|Human|Department of Virus and Microbiological Special
Diagnostics|Albertsen lab|Michaelsen|Denmark
>Spike|hCoV-19/Spain/VC-IBV-98003567/2020|2020-03-14|EPI_ISL_537983|Original|hCoV-
19^^Comunitat Valenciana|Human|Servicio de Microbiolog\u00eda. Hospital General
Universitario de Castell\u00f3n|SeqCOVID-SPAIN consortium/IBV(CSIC)|Comas|Spain
>Spike|hCoV-19/France/PAC-IHU-0955/2020|2020-07-13|EPI_ISL_569007|Original|hCoV-
19^^Provence-Alpes-C\u00f4te-d\u2019Azur|Human|MEPHI|MEPHI|Levasseur|France
>Spike|hCoV-19/Scotland/CVR5300/2020|2020-10-22|EPI_ISL_651744|Original|hCoV-
19^^Scotland|Human|West of Scotland Specialist Virology Centre|COVID-19 Genomics UK
(COG-UK) Consortium|Robertson|United Kingdom
>Spike|hCoV-19/Germany/BY-ChVir-929-2/2020|2020-01-28|EPI_ISL_450198|Original|hCoV-
19^^Bavaria|Human|Department of Virology|Department of Virology|B\u00f6hmer|Germany
>Spike|hCoV-19/Netherlands/NB-RIVM-20263/2020|2020-11-17|EPI_ISL_723409|Orignal|hCoV-
19^^Noord-Brabant|Human|Dutch COVID-19 response team|National Institute for Public
Health and the Environment (RIVM)|Meijer|Netherlands
>Spike|hCoV-19/USA/TX-HMH-MCoV-15467/2020|2020-10-23|EPI_ISL_786951|Original|hCoV-
19^^Texas|Human|Houston Methodist Hospital|Houston Methodist Hospital|Long|USA
>Spike|hCoV-19/Luxembourg/LNS2761686/2020|2020-07-16|EPI_ISL_740186|Original|hCoV-
19^^Luxembourg|Human|Laboratoire national de sant\u00e9|Laboratoire national de
sant\u00e9|Wienecke-Baldacchino|Luxembourg
>Spike|hCoV-19/Spain/VC-IBV-98003447/2020|2020-04-03|EPI_ISL_539256|Original|hCoV-
19^^Comunitat Valenciana|Human|Hospital Universitario de La Ribera (Alzira|SeqCOVID-
SPAIN consortium/IBV(CSIC)|Comas|Spain

>Spike|hCoV-19/USA/MI-MDHHS-SC20142/2020|2020-03-24|EPI_ISL_436855|Original|hCoV-19^^Michigan|Human|Michigan Department of Health and Human Services|Michigan Department of Health and Human Services|Blankenship|USA
>Spike|hCoV-19/England/20138024004/2020|2020-03-25|EPI_ISL_423303|Original|hCoV-19^^England|Human|Respiratory Virus Unit|Respiratory Virus Unit|Platt|United Kingdom
>Spike|hCoV-19/USA/WA-S2771/2020|2020-08-01|EPI_ISL_530128|Original|hCoV-19^^Washington|Human|Seattle Flu Study|Seattle Flu Study|Lee|USA
>Spike|hCoV-19/USA/NY-NYCPHL-001265/2020|2020-11-09|EPI_ISL_683896|Original|hCoV-19^^New York|Human|DOHMH Corona|New York City Public Health Laboratory|Wang|USA
>Spike|hCoV-19/England/CAMB-7FC71/2020|2020-04-19|EPI_ISL_444398|Original|hCoV-19^^England|Human|Department of Pathology|COVID-19 Genomics UK (COG-UK) Consortium|Meredith|United Kingdom
>Spike|hCoV-19/France/ARA-36389/2020|2020-05-11|EPI_ISL_636482|Original|hCoV-19^^Auvergne-Rhone-Alpes|Human|Centre Hospitalier Pierre Oudot|CNR Virus des Infections Respiratoires - France SUD|Josset|France
>Spike|hCoV-19/England/ALDP-6C43DC/2020|2020-07-01|EPI_ISL_556252|Original|hCoV-19^^England|Human|Lighthouse Lab in Alderley Park|Wellcome Sanger Institute for the COVID-19 Genomics UK (COG-UK) consortium|Davies|United Kingdom
>Spike|hCoV-19/Australia/VIC7614/2020|2020-07-21|EPI_ISL_564530|Original|hCoV-19^^Victoria|Human|Microbiological Diagnostic Unit - Public Health Laboratory (MDU-PHL)|MDU-PHL|Schultz|Australia
>Spike|hCoV-19/Brazil/SP-L7-CD82/2020|2020-03-24|EPI_ISL_476447|Original|hCoV-19^^S\u00e3o Paulo|Human|Hospital da Cl\u00ednicas da Faculdade de Medicina da Universidade de S\u00e3o Paulo|Instituto de Medicina Tropical da Univesidade de S\u00e3o Paulo|Faria|Brazil
>Spike|hCoV-19/England/ALDP-A6E6B9/2020|2020-10-15|EPI_ISL_607150|Original|hCoV-19^^England|Human|Lighthouse Lab in Alderley Park|Wellcome Sanger Institute for the COVID-19 Genomics UK (COG-UK) consortium|Davies|United Kingdom
>Spike|hCoV-19/USA/TX_GCID_192000052/2020|2020-03-23|EPI_ISL_445079|Original|hCoV-19^^Texas|Human|Baylor College of Medicine|Baylor College of Medicine|Skinner|USA
>Spike|hCoV-19/England/QEUH-5DF339/2020|2020-06-17|EPI_ISL_533325|Original|hCoV-19^^England|Human|Lighthouse Lab in Glasgow|Wellcome Sanger Institute for the COVID-19 Genomics UK (COG-UK) consortium|Davies|United Kingdom
>Spike|hCoV-19/USA/CA-ALSR-4727/2020|2020-09-21|EPI_ISL_730110|Original|hCoV-19^^California|Human|San Diego County Public Health Laboratory|Andersen lab at Scripps Research|Gangavarapu|USA
>Spike|hCoV-19/England/EXET-139DE8/2020|2020-00-00|EPI_ISL_679344|Original|hCoV-19^^England|Human|University of Exeter|COVID-19 Genomics UK (COG-UK) Consortium|Temperton|United Kingdom
>Spike|hCoV-19/Poland/19876/2020|2020-11-00|EPI_ISL_732916|Original|hCoV-19^^Poland|Human|genXone SA|genXone SA|Kaszuba|Poland
>Spike|hCoV-19/USA/MI-UM-10036455460/2020|2020-10-15|EPI_ISL_628843|Original|hCoV-19^^Michigan|Human|University of Michigan Clinical Microbiology Laboratory|Lauring Lab|Valesano|USA
>Spike|hCoV-19/Germany/NW-HHU-282/2020|2020-09-29|EPI_ISL_631326|Orginal|hCoV-19^^North Rhine-Westphalia|Human|ZOTZ KLIMAS MVZ D\u00fcsseldorf-Centrum GbR \u00dcBAG f\u00fcr Labormedizin|Center of Medical Microbiology|Wienemann|Germany
>Spike|hCoV-19/Denmark/DCGC-8812/2020|2020-10-26|EPI_ISL_626076|Original|hCoV-19^^Hovedstaden|Human|Department of Virus and Microbiological Special Diagnostics|Albertsen lab|Michaelsen|Denmark
>Spike|hCoV-19/England/MILK-A7AE3A/2020|2020-10-16|EPI_ISL_606806|Original|hCoV-19^^England|Human|Lighthouse Lab in Milton Keynes|Wellcome Sanger Institute for the COVID-19 Genomics UK (COG-UK) consortium|Davies|United Kingdom
>Spike|hCoV-19/Denmark/DCGC-20524/2020|2020-12-21|EPI_ISL_758902|Original|hCoV-19^^Syddanmark|Human|Department of Virus and Microbiological Special Diagnostics|Albertsen Lab|Michaelsen|Denmark
>Spike|hCoV-19/United Arab Emirates/L3290161092/2020|2020-05-13|EPI_ISL_520681|Original|hCoV-19^^United Arab Emirates|Human|Mohammed Bin Rashid University of Medicine and Health Sciences|Al Jalila Genomics Center|Ramaswamy|United Arab Emirates

>Spike|hCoV-19/South Africa/NHLS-UCT-GS-0045/2020|2020-03-
22|EPI_ISL_640051|Original|hCoV-19^^Western Cape|Human|Groote Schuur Hospital wc
GSH|NHLS/UCT|Iranzadeh|South Africa
>Spike|hCoV-19/Netherlands/NA_466/2020|2020-04-10|EPI_ISL_455268|Original|hCoV-
19^^Netherlands|Human|Dutch COVID-19 response team|Erasmus Medical Center|Oude
Munnink|Netherlands
>Spike|hCoV-19/Denmark/DCGC-13378/2020|2020-11-30|EPI_ISL_712166|Original|hCoV-
19^^Syddanmark|Human|Department of Virus and Microbiological Special
Diagnostics|Albertsen Lab|Michaelsen|Denmark
>Spike|hCoV-19/Canada/QC-LSPQ-L00244068/2020|2020-03-31|EPI_ISL_536388|Original|hCoV-
19^^Quebec|Human|H\u00f4pital Pierre-Boucher|Laboratoire de sant\u00e9 publique du
Qu\u00e9bec|Moreira|Canada
>Spike|hCoV-19/Australia/VIC3644/2020|2020-07-08|EPI_ISL_520384|Original|hCoV-
19^^Victoria|Human|Victorian Infectious Diseases Reference Laboratory (VIDRL)|VIDRL
and MDU-PHL|Schultz|Australia
>Spike|hCoV-19/USA/OR-CDC-2-3714482/2020|2020-11-30|EPI_ISL_751693|Original|hCoV-
19^^Oregon|Human|OR State PHL-Virology/Immunology Section|Genomics and
Discovery|Queen|USA
>Spike|hCoV-19/England/PORT-2D1B14/2020|2020-04-02|EPI_ISL_493650|Original|hCoV-
19^^England|Human|Centre for Enzyme Innovation|COVID-19 Genomics UK (COG-UK)
Consortium|Robson|United Kingdom
>Spike|hCoV-19/England/CAMC-A3F8F7/2020|2020-10-07|EPI_ISL_609791|Original|hCoV-
19^^England|Human|Lighthouse Lab in Cambridge|Wellcome Sanger Institute for the
COVID-19 Genomics UK (COG-UK) consortium|Davies|United Kingdom
>Spike|hCoV-19/England/ALDP-9FCFA7/2020|2020-10-01|EPI_ISL_587721|Original|hCoV-
19^^England|Human|Lighthouse Lab in Alderley Park|Wellcome Sanger Institute for the
COVID-19 Genomics UK (COG-UK) consortium|Davies|United Kingdom
>Spike|hCoV-19/England/MILK-9767BC/2020|2020-08-17|EPI_ISL_575969|Original|hCoV-
19^^England|Human|Lighthouse Lab in Milton Keynes|Wellcome Sanger Institute for the
COVID-19 Genomics UK (COG-UK) consortium|Davies|United Kingdom
>Spike|hCoV-19/Australia/VIC8749/2020|2020-07-28|EPI_ISL_565172|Original|hCoV-
19^^Victoria|Human|Victorian Infectious Diseases Reference Laboratory (VIDRL)|VIDRL
and MDU-PHL|Schultz|Australia
>Spike|hCoV-19/Wuhan/HB-WH2-157/2020|2020-02-28|EPI_ISL_454925|Original|hCoV-
19^^Hubei|Human|Wuhan Chain Medical Labs (CMLabs)|State Key Laboratory of Biotherapy
of Sichuan University|Tang|China
>Spike|hCoV-19/USA/MI-UM-10036684811/2020|2020-10-29|EPI_ISL_682086|Original|hCoV-
19^^Michigan|Human|University of Michigan Clinical Microbiology Laboratory|Lauring
Lab|Valesano|USA
>Spike|hCoV-19/India/MH-ACTREC-431/2020|2020-08-04|EPI_ISL_700080|Original|hCoV-
19^^Maharashtra|Human|Hematopathology Laboratory|Hematopathology
Laboratory|Patkar|India
>Spike|hCoV-19/Denmark/DCGC-1262/2020|2020-06-08|EPI_ISL_618039|Original|hCoV-
19^^Nordjylland|Human|Department of Virus and Microbiological Special
Diagnostics|Albertsen lab|Michaelsen|Denmark
>Spike|hCoV-19/Wales/PHWC-24D5A/2020|2020-03-20|EPI_ISL_419505|Original|hCoV-
19^^Wales|Human|Wales Specialist Virology Centre|Public Health Wales Microbiology
Cardiff|Moore|United Kingdom
>Spike|hCoV-19/England/QEUH-9F3B15/2020|2020-09-23|EPI_ISL_588642|Original|hCoV-
19^^England|Human|Lighthouse Lab in Glasgow|Wellcome Sanger Institute for the COVID-
19 Genomics UK (COG-UK) consortium|Davies|United Kingdom
>Spike|hCoV-19/Luxembourg/LNS3497064/2020|2020-11-13|EPI_ISL_740320|Original|hCoV-
19^^Luxembourg|Human|Laboratoire national de sant\u00e9|Laboratoire national de
sant\u00e9|Wienecke-Baldacchino|Luxembourg
>Spike|hCoV-19/Canada/NS-NML-1267/2020|2020-04-16|EPI_ISL_579655|Original|hCoV-
19^^Nova Scotia|Human|QEII Health Sciences Centre|National Microbiology Laboratory
(NML)|Majer|Canada
>Spike|hCoV-19/USA/CA-ALSR-2987/2020|2020-08-30|EPI_ISL_635774|Original|hCoV-
19^^California|Human|San Diego County Public Health Laboratory|Andersen lab at
Scripps Research|Gangavarapu|USA

>Spike|hCoV-19/India/MH-ACTREC-301/2020|2020-07-14|EPI_ISL_699952|Original|hCoV-19^^Maharashtra|Human|Hematopathology Laboratory|Hematopathology Laboratory|Patkar|India
>Spike|hCoV-19/Northern Ireland/NIRE-232F24/2020|2020-09-25|EPI_ISL_680237|Original|hCoV-19^^Northern Ireland|Human|Regional Virus Laboratory|COVID-19 Genomics UK (COG-UK) Consortium|Fairley|United Kingdom
>Spike|hCoV-19/England/CAMC-A57D45/2020|2020-10-09|EPI_ISL_597088|Original|hCoV-19^^England|Human|Lighthouse Lab in Cambridge|Wellcome Sanger Institute for the COVID-19 Genomics UK (COG-UK) consortium|Davies|United Kingdom
>Spike|hCoV-19/USA/UT-UPHL-2012280511/2020|2020-10-20|EPI_ISL_746260|Original|hCoV-19^^Utah|Human|Ginkgo Bioworks Clinical Laboratory|Utah Public Health Laboratory|Young|USA
>Spike|hCoV-19/Australia/VIC7532/2020|2020-07-23|EPI_ISL_564476|Original|hCoV-19^^Victoria|Human|Microbiological Diagnostic Unit - Public Health Laboratory (MDU-PHL)|MDU-PHL|Schultz|Australia
>Spike|hCoV-19/USA/TX-HMH-4758/2020|2020-06-21|EPI_ISL_544309|Original|hCoV-19^^Texas|Human|Houston Methodist Hospital|Houston Methodist Hospital|Long|USA
>Spike|hCoV-19/Wales/PHWC-2B0F0/2020|2020-04-07|EPI_ISL_432055|Original|hCoV-19^^Wales|Human|Wales Specialist Virology Centre|Public Health Wales Microbiology Cardiff|Moore|United Kingdom
>Spike|hCoV-19/England/CAMB-74553/2020|2020-03-28|EPI_ISL_425305|Original|hCoV-19^^England|Human|Department of Pathology|COVID-19 Genomics UK (COG-UK) Consortium|Meredith|United Kingdom
>Spike|hCoV-19/England/BIRM-63DAA/2020|2020-09-04|EPI_ISL_576884|Original|hCoV-19^^England|Human|University of Birmingham|COVID-19 Genomics UK (COG-UK) Consortium|Loman|United Kingdom
>Spike|hCoV-19/USA/CA-ALSR-1080-SAN/2020|2020-06-23|EPI_ISL_483507|Original|hCoV-19^^California|Human|UC San Diego Center for Advanced Laboratory Medicine|Andersen lab at Scripps Research|Gangavarapu|USA
>Spike|hCoV-19/USA/WI-UW-1168/2020|2020-08-08|EPI_ISL_522924|Original|hCoV-19^^Wisconsin|Human|University of Wisconsin-Madison AIDS Vaccine Research Laboratories|University of Wisconsin-Madison AIDS Vaccine Research Laboratories|Baczenas|USA
>Spike|hCoV-19/Denmark/DCGC-4781/2020|2020-09-21|EPI_ISL_621391|Original|hCoV-19^^Hovedstaden|Human|Department of Virus and Microbiological Special Diagnostics|Albertsen lab|Michaelsen|Denmark
>Spike|hCoV-19/Netherlands/FR-RIVM-20147/2020|2020-11-23|EPI_ISL_723317|Orignal|hCoV-19^^Friesland|Human|Dutch COVID-19 response team|National Institute for Public Health and the Environment (RIVM)|Meijer|Netherlands
>Spike|hCoV-19/Spain/MD-IBV-006439/2020|2020-03-17|EPI_ISL_510149|Original|hCoV-19^^Madrid|Human|Hospital General Universitario Gregorio Mara\u00f1\u00f3n|SeqCOVID-SPAIN consortium/IBV(CSIC)|Comas|Spain
>Spike|hCoV-19/England/GSTT-265E052/2020|2020-03-30|EPI_ISL_484143|Original|hCoV-19^^England|Human|Centre for Clinical Infection and Diagnostics Research and Genomics Innovation Unit|COVID-19 Genomics UK (COG-UK) Consortium|Awan|United Kingdom
>Spike|hCoV-19/Denmark/DCGC-14841/2020|2020-11-23|EPI_ISL_712020|Original|hCoV-19^^Midtjylland|Human|Department of Virus and Microbiological Special Diagnostics|Albertsen Lab|Michaelsen|Denmark
>Spike|hCoV-19/England/CAMB-7EBA2/2020|2020-04-06|EPI_ISL_442994|Original|hCoV-19^^England|Human|Department of Pathology|Wellcome Sanger Institute for the COVID-19 Genomics UK (COG-UK) consortium|Amato|United Kingdom
>Spike|hCoV-19/USA/CA-IGI-0371/2020|2020-07-22|EPI_ISL_754877|Original|hCoV-19^^California|Human|Innovative Genomics Institute|Innovative Genomics Institute|Wyman|USA
>Spike|hCoV-19/Wales/PHWC-4823AF/2020|2020-10-15|EPI_ISL_613216|Original|hCoV-19^^Wales|Human|Wales Specialist Virology Centre Sequencing lab|COVID-19 Genomics UK (COG-UK) Consortium|Moore|United Kingdom
>Spike|hCoV-19/South Korea/KCDC2024/2020|2020-02-19|EPI_ISL_471440|Original|hCoV-19^^South Korea|Human|Division of Viral Diseases|Division of Viral Diseases|Kim|South Korea

>Spike|hCoV-19/Denmark/DCGC-17820/2020|2020-12-14|EPI_ISL_749303|Original|hCoV-19^^Sjaelland|Human|Department of Virus and Microbiological Special Diagnostics|Albertsen Lab|Michaelsen|Denmark
>Spike|hCoV-19/Ireland/G-NVRL-78IRL74215/2020|2020-11-23|EPI_ISL_752565|Original|hCoV-19^^Galway|Human|National Virus Reference Laboratory|National Virus Reference Laboratory|Gonzalez|Ireland
>Spike|hCoV-19/USA/CA-CHLA-PLM71317172/2020|2020-12-04|EPI_ISL_753639|Original|hCoV-19^^California|Human|Clinical virology Laboratory|Center for Personalized Medicine|Dien Bard|USA
>Spike|hCoV-19/Scotland/CVR878/2020|2020-03-27|EPI_ISL_439127|Original|hCoV-19^^Scotland|Human|West of Scotland Specialist Virology Centre|COVID-19 Genomics UK (COG-UK) Consortium|Robertson|United Kingdom
>Spike|hCoV-19/Scotland/QEUH-9BA8E6/2020|2020-09-09|EPI_ISL_573829|Original|hCoV-19^^Scotland|Human|Oxford Viromics|COVID-19 Genomics UK (COG-UK) Consortium|Golubchik|United Kingdom
>Spike|hCoV-19/USA/UT-03889/2020|2020-04-13|EPI_ISL_450566|Original|hCoV-19^^Utah|Human|Utah Public Health Laboratory|Utah Public Health Laboratory|Young|USA
>Spike|hCoV-19/Denmark/DCGC-7711/2020|2020-10-19|EPI_ISL_618581|Original|hCoV-19^^Midtjylland|Human|Department of Virus and Microbiological Special Diagnostics|Albertsen lab|Michaelsen|Denmark
>Spike|hCoV-19/USA/WA-UW-5205/2020|2020-04-02|EPI_ISL_430900|Original|hCoV-19^^Washington|Human|UW Virology Lab|UW Virology Lab|Roychoudhury|USA
>Spike|hCoV-19/Spain/CL-IBV-97006174/2020|2020-04-06|EPI_ISL_537321|Original|hCoV-19^^Castilla y Leon|Human|Universidad de Le\u00f3n|SeqCOVID-SPAIN consortium/IBV(CSIC)|Comas|Spain
>Spike|hCoV-19/England/QEUH-9DDB20/2020|2020-09-21|EPI_ISL_599501|Original|hCoV-19^^England|Human|Lighthouse Lab in Glasgow|Wellcome Sanger Institute for the COVID-19 Genomics UK (COG-UK) consortium|Davies|United Kingdom
>Spike|hCoV-19/Denmark/DCGC-14028/2020|2020-11-30|EPI_ISL_711526|Original|hCoV-19^^Hovedstaden|Human|Department of Virus and Microbiological Special Diagnostics|Albertsen Lab|Michaelsen|Denmark
>Spike|hCoV-19/Australia/NSW223/2020|2020-03-26|EPI_ISL_451489|Original|hCoV-19^^New South Wales|Human|Laverty Pathology|NSW Health Pathology - Institute of Clinical Pathology and Medical Research; Westmead Hospital; University of Sydney|Gall|Australia
>Spike|hCoV-19/Switzerland/ZH-ETHZ-110442/2020|2020-04-07|EPI_ISL_451866|Original|hCoV-19^^Zurich|Human|Viollier AG|Department of Biosystems Science and Engineering|Nadeau|Switzerland
>Spike|hCoV-19/India/MH-ACTREC-145/2020|2020-05-29|EPI_ISL_699801|Original|hCoV-19^^Maharashtra|Human|Hematopathology Laboratory|Hematopathology Laboratory|Patkar|India
>Spike|hCoV-19/England/MILK-974790/2020|2020-08-21|EPI_ISL_575788|Original|hCoV-19^^England|Human|Lighthouse Lab in Milton Keynes|Wellcome Sanger Institute for the COVID-19 Genomics UK (COG-UK) consortium|Davies|United Kingdom
>Spike|hCoV-19/New Zealand/20CV0610/2020|2020-11-27|EPI_ISL_682288|Original|hCoV-19^^Counties Manukau|Human|Middlemore Hospital|Institute of Environmental Science and Research (ESR)|Wang|New Zealand
>Spike|hCoV-19/Denmark/DCGC-17552/2020|2020-12-14|EPI_ISL_751063|Original|hCoV-19^^Midtjylland|Human|Department of Virus and Microbiological Special Diagnostics|Albertsen Lab|Michaelsen|Denmark
>Spike|hCoV-19/USA/NY-Wadsworth-33281-01/2020|2020-03-30|EPI_ISL_525639|Original|hCoV-19^^New York|Human|Wadsworth Center|Wadsworth Center|Plitnick|USA
>Spike|hCoV-19/England/MILK-99485B/2020|2020-08-25|EPI_ISL_552256|Original|hCoV-19^^England|Human|Lighthouse Lab in Milton Keynes|Wellcome Sanger Institute for the COVID-19 Genomics UK (COG-UK) consortium|Davies|United Kingdom
>Spike|hCoV-19/Switzerland/VS-ETHZ-280128/2020|2020-09-07|EPI_ISL_560534|Original|hCoV-19^^Valais|Human|Viollier AG|Department of Biosystems Science and Engineering|Nadeau|Switzerland

>Spike|hCoV-19/England/QEUH-B7C462/2020|2020-11-14|EPI_ISL_741777|Original|hCoV-19^^England|Human|Oxford Viromics|COVID-19 Genomics UK (COG-UK) Consortium|Golubchik|United Kingdom
>Spike|hCoV-19/England/ALDP-4A5889/2020|2020-06-09|EPI_ISL_559621|Original|hCoV-19^^England|Human|Lighthouse Lab in Alderley Park|Wellcome Sanger Institute for the COVID-19 Genomics UK (COG-UK) consortium|Davies|United Kingdom
>Spike|hCoV-19/USA/MI-MDHHS-SC21787/2020|2020-08-14|EPI_ISL_529905|Original|hCoV-19^^Michigan|Human|Michigan Department of Health and Human Services|Michigan Department of Health and Human Services|Blankenship|USA
>Spike|hCoV-19/Japan/PG-0326/2020|2020-03-26|EPI_ISL_480001|Original|hCoV-19^^HokurikuShinsyu|Human|Nagano Environmental Conservation Research Institute|Pathogen Genomics Center|Sekizuka|Japan
>Spike|hCoV-19/USA/NMDOH-2020305747/2020|2020-08-21|EPI_ISL_542047|Original|hCoV-19^^New Mexico|Human|New Mexico Department of Health Scientific Laboratory|New Mexico Department of Health Scientific Laboratory|Johnson|USA
>Spike|hCoV-19/England/ALDP-9CABEA/2020|2020-09-16|EPI_ISL_575468|Original|hCoV-19^^England|Human|Lighthouse Lab in Alderley Park|Wellcome Sanger Institute for the COVID-19 Genomics UK (COG-UK) consortium|Davies|United Kingdom
>Spike|hCoV-19/Netherlands/ZH-RIVM-20413/2020|2020-12-11|EPI_ISL_728633|Original|hCoV-19^^Zuid-holland|Human|Dutch COVID-19 response team|National Institute for Public Health and the Environment (RIVM)|Meijer|Netherlands
>Spike|hCoV-19/USA/MI-MDHHS-SC20570/2020|2020-04-30|EPI_ISL_462652|Original|hCoV-19^^Michigan|Human|Michigan Department of Health and Human Services|Michigan Department of Health and Human Services|Blankenship|USA
>Spike|hCoV-19/England/LIVE-1DDCCB/2020|2020-10-10|EPI_ISL_612019|Original|hCoV-19^^England|Human|Liverpool Clinical Laboratories|COVID-19 Genomics UK (COG-UK) Consortium|Haldenby|United Kingdom
>Spike|hCoV-19/Luxembourg/LNS2783388/2020|2020-10-22|EPI_ISL_740193|Original|hCoV-19^^Luxembourg|Human|Laboratoire national de sant\u00e9|Laboratoire national de sant\u00e9|Wienecke-Baldacchino|Luxembourg
>Spike|hCoV-19/England/QEUH-9CE75B/2020|2020-09-17|EPI_ISL_590027|Original|hCoV-19^^England|Human|Lighthouse Lab in Glasgow|Wellcome Sanger Institute for the COVID-19 Genomics UK (COG-UK) consortium|Davies|United Kingdom
>Spike|hCoV-19/England/LIVE-A8750/2020|2020-03-27|EPI_ISL_517206|Original|hCoV-19^^England|Human|Liverpool Clinical Laboratories|COVID-19 Genomics UK (COG-UK) Consortium|Haldenby|United Kingdom
>Spike|hCoV-19/India/TG-CCMB-M359/2020|2020-06-09|EPI_ISL_495200|Original|hCoV-19^^Telangana|Human|CSIR-Centre for Cellular and Molecular Biology|CSIR-Centre for Cellular and Molecular Biology|Banu|India
>Spike|hCoV-19/USA/FL-UF-15/2020|2020-04-17|EPI_ISL_468721|Original|hCoV-19^^Florida|Human|University of Florida|University of Florida|Lednicky|USA
>Spike|hCoV-19/Wales/PHWC-16C247/2020|2020-09-22|EPI_ISL_572674|Original|hCoV-19^^Wales|Human|Wales Specialist Virology Centre Sequencing lab|COVID-19 Genomics UK (COG-UK) Consortium|Moore|United Kingdom
>Spike|hCoV-19/England/QEUH-9B3AD8/2020|2020-09-04|EPI_ISL_573528|Original|hCoV-19^^England|Human|Virology Department|COVID-19 Genomics UK (COG-UK) Consortium|de Silva|United Kingdom
>Spike|hCoV-19/England/204540597/2020|2020-11-03|EPI_ISL_678655|Original|hCoV-19^^England|Human|Respiratory Virus Unit|COVID-19 Genomics UK (COG-UK) Consortium|Platt|United Kingdom
>Spike|hCoV-19/USA/MI-MDHHS-SC20029/2020|2020-03-15|EPI_ISL_437116|Original|hCoV-19^^Michigan|Human|Michigan Department of Health and Human Services|Michigan Department of Health and Human Services|Blankenship|USA
>Spike|hCoV-19/England/QEUH-AF69DB/2020|2020-10-30|EPI_ISL_663033|Original|hCoV-19^^England|Human|Lighthouse Lab in Glasgow|Wellcome Sanger Institute for the COVID-19 Genomics UK (COG-UK) Consortium|Davies|United Kingdom
>Spike|hCoV-19/Japan/PG-4292/2020|2020-07-00|EPI_ISL_692521|Original|hCoV-19^^Japan|Human|Pathogen Genomics Center|Pathogen Genomics Center|Sekizuka|Japan

>Spike|hCoV-19/England/QEUH-95EE6A/2020|2020-08-06|EPI_ISL_532331|Original|hCoV-19^^England|Human|Lighthouse Lab in Glasgow|Wellcome Sanger Institute for the COVID-19 Genomics UK (COG-UK) consortium|Davies|United Kingdom
>Spike|hCoV-19/USA/TX-HMH-MCoV-11188/2020|2020-09-02|EPI_ISL_789728|Original|hCoV-19^^Texas|Human|Houston Methodist Hospital|Houston Methodist Hospital|Long|USA
>Spike|hCoV-19/Australia/VIC13535/2020|2020-08-04|EPI_ISL_563739|Original|hCoV-19^^Victoria|Human|Microbiological Diagnostic Unit - Public Health Laboratory (MDU-PHL)|MDU-PHL|Schultz|Australia
>Spike|hCoV-19/England/CAMC-AAFD8A/2020|2020-10-21|EPI_ISL_623393|Original|hCoV-19^^England|Human|Lighthouse Lab in Cambridge|Wellcome Sanger Institute for the COVID-19 Genomics UK (COG-UK) consortium|Davies|United Kingdom
>Spike|hCoV-19/Germany/HH-hpi-p1499/2020|2020-03-14|EPI_ISL_776005|Original|hCoV-19^^Hamburg|Human|University Medical Center Hamburg Eppendorf|Heinrich Pette Institute|Robitaille|Germany
>Spike|hCoV-19/England/CAMB-1B2594/2020|2020-05-15|EPI_ISL_456695|Original|hCoV-19^^England|Human|Department of Pathology|COVID-19 Genomics UK (COG-UK) Consortium|Meredith|United Kingdom
>Spike|hCoV-19/USA/UT-UPHL-05584/2020|2020-06-01|EPI_ISL_575230|Original|hCoV-19^^Utah|Human|Utah Public Health Laboratory|Utah Public Health Laboratory|Young|USA
>Spike|hCoV-19/Luxembourg/LNS8585990/2020|2020-11-10|EPI_ISL_744767|Original|hCoV-19^^Luxembourg|Human|Laboratoire national de sant\u00e9|Laboratoire national de sant\u00e9|Wienecke-Baldacchino|Luxembourg
>Spike|hCoV-19/Switzerland/BL-ETHZ-100236/2020|2020-03-18|EPI_ISL_574886|Original|hCoV-19^^Basel-Landschaft|Human|Viollier AG|Department of Biosystems Science and Engineering|Nadeau|Switzerland
>Spike|hCoV-19/USA/MN-MDH-301/2020|2020-03-30|EPI_ISL_450768|Original|hCoV-19^^Minnesota|Human|Minnesota Department of Health|Minnesota Department of Health|Garfin|USA
>Spike|hCoV-19/England/ALDP-95C628/2020|2020-06-20|EPI_ISL_555075|Original|hCoV-19^^England|Human|Lighthouse Lab in Alderley Park|Wellcome Sanger Institute for the COVID-19 Genomics UK (COG-UK) consortium|Davies|United Kingdom
>Spike|hCoV-19/England/CAMC-B9A219/2020|2020-11-15|EPI_ISL_673718|Original|hCoV-19^^England|Human|Lighthouse Lab in Cambridge|Wellcome Sanger Institute for the COVID-19 Genomics UK (COG-UK) Consortium|Davies|United Kingdom
>Spike|hCoV-19/England/ALDP-9FD39B/2020|2020-10-02|EPI_ISL_587942|Original|hCoV-19^^England|Human|Lighthouse Lab in Alderley Park|Wellcome Sanger Institute for the COVID-19 Genomics UK (COG-UK) consortium|Davies|United Kingdom
>Spike|hCoV-19/England/CAMC-BBB7B9/2020|2020-11-21|EPI_ISL_724067|Original|hCoV-19^^England|Human|Northumbria University / South Tees Hospitals NHS Foundation Trust / North Cumbria Integrated Care NHS Foundation Trust / North Tees and Hartlepool NHS Foundation Trust / Newcastle Hospitals NHS Foundation Trust|COVID-19 Genomics UK (COG-UK) Consortium|Bashton|United Kingdom
>Spike|hCoV-19/Wales/PHWC-16CC76/2020|2020-09-23|EPI_ISL_574153|Original|hCoV-19^^Wales|Human|Wales Specialist Virology Centre Sequencing lab|COVID-19 Genomics UK (COG-UK) Consortium|Moore|United Kingdom
>Spike|hCoV-19/USA/MN-MDH-288/2020|2020-03-31|EPI_ISL_450757|Original|hCoV-19^^Minnesota|Human|Minnesota Department of Health|Minnesota Department of Health|Garfin|USA
>Spike|hCoV-19/USA/MN-MDH-1904/2020|2020-10-18|EPI_ISL_613522|Original|hCoV-19^^Minnesota|Human|Public Health Laboratory - Infectious Disease Lab|Minnesota Department of Health|Lorentz|USA
>Spike|hCoV-19/England/MILK-B95BE6/2020|2020-11-14|EPI_ISL_676150|Original|hCoV-19^^England|Human|Lighthouse Lab in Milton Keynes|Wellcome Sanger Institute for the COVID-19 Genomics UK (COG-UK) Consortium|Davies|United Kingdom
>Spike|hCoV-19/Japan/TK-YV12689/2020|2020-04-00|EPI_ISL_486894|Original|hCoV-19^^Tokyo|Human|Tokyo Metoropolitan Institute of Public Health|Tokyo Metoropolitan Institute of Public Health|Nagashima|Japan
>Spike|hCoV-19/USA/CA-CSMC105/2020|2020-04-02|EPI_ISL_475580|Original|hCoV-19^^California|Human|Cedars-Sinai Medical Center|Cedars-Sinai Medical Center|Zhang|USA

>Spike|hCoV-19/Belgium/ULG-10526/2020|2020-10-23|EPI_ISL_626301|Original|hCoV-19^^Li\u00e8ge|Human|Department of Clinical Microbiology|GIGA Medical Genomics|Durkin|Belgium
>Spike|hCoV-19/England/205090680/2020|2020-12-10|EPI_ISL_766296|Original|hCoV-19^^England|Human|Respiratory Virus Unit|COVID-19 Genomics UK (COG-UK) Consortium|Platt|United Kingdom
>Spike|hCoV-19/India/MH-ACTREC-075/2020|2020-05-18|EPI_ISL_699731|Original|hCoV-19^^Maharashtra|Human|Hematopathology Laboratory|Hematopathology Laboratory|Patkar|India
>Spike|hCoV-19/USA/TX-HMH-6543/2020|2020-06-22|EPI_ISL_546069|Original|hCoV-19^^Texas|Human|Houston Methodist Hospital|Houston Methodist Hospital|Long|USA
>Spike|hCoV-19/South Africa/KRISP-0170/2020|2020-05-31|EPI_ISL_467520|Original|hCoV-19^^KwaZulu-Natal|Human|NHLS-IALCH|KRISP|de Oliveira|South Africa
>Spike|hCoV-19/Denmark/DCGC-3285/2020|2020-08-31|EPI_ISL_616037|Original|hCoV-19^^Hovedstaden|Human|Department of Virus and Microbiological Special Diagnostics|Albertsen lab|Michaelsen|Denmark
>Spike|hCoV-19/USA/NY-Wadsworth-119711-01/2020|2020-05-26|EPI_ISL_676708|Original|hCoV-19^^New York|Human|Wadsworth Center|Wadsworth Center|Lasek-Nesselquist|USA
>Spike|hCoV-19/England/CAMB-73A37/2020|2020-03-31|EPI_ISL_425269|Original|hCoV-19^^England|Human|Department of Pathology|COVID-19 Genomics UK (COG-UK) Consortium|Meredith|United Kingdom
>Spike|hCoV-19/Spain/AN-IBV-001078/2020|2020-03-26|EPI_ISL_452606|Original|hCoV-19^^Andalucia|Human|Servicio de Microbiolog\u00eda y Parasitolog\u00eda cl\u00ednica. UCEIMP. Hospital Universitario Virgen del Roc\u00edo/IBIS/CSIC/US.|SeqCOVID-SPAIN consortium/IBV(CSIC)|Comas|Spain
>Spike|hCoV-19/France/PAC-IHU-0204/2020|2020-03-27|EPI_ISL_569370|Original|hCoV-19^^Provence-Alpes-C\u00f4te-d\u2019Azur|Human|MEPHI|MEPHI|Levasseur|France
>Spike|hCoV-19/England/QEUH-AD58C6/2020|2020-10-26|EPI_ISL_634544|Original|hCoV-19^^England|Human|Lighthouse Lab in Glasgow|Wellcome Sanger Institute for the COVID-19 Genomics UK (COG-UK) consortium|Davies|United Kingdom
>Spike|hCoV-19/England/QEUH-B2DE62/2020|2020-11-03|EPI_ISL_646266|Original|hCoV-19^^England|Human|Lighthouse Lab in Glasgow|Wellcome Sanger Institute for the COVID-19 Genomics UK (COG-UK) Consortium|Davies|United Kingdom
>Spike|hCoV-19/Scotland/QEUH-9E527B/2020|2020-09-23|EPI_ISL_601087|Original|hCoV-19^^Scotland|Human|Lighthouse Lab in Glasgow|Wellcome Sanger Institute for the COVID-19 Genomics UK (COG-UK) consortium|Davies|United Kingdom
>Spike|hCoV-19/England/20131103104/2020|2020-03-23|EPI_ISL_423691|Original|hCoV-19^^England|Human|Respiratory Virus Unit|Respiratory Virus Unit|Platt|United Kingdom
>Spike|hCoV-19/Indonesia/JI-NIHRD-PME0999/2020|2020-00-00|EPI_ISL_538498|Original|hCoV-19^^East Java|Human|RSUD Soediono Madiun East Java|National Institute of Health Research and Development|Setiawati|Indonesia
>Spike|hCoV-19/Australia/NSW90/2020|2020-03-22|EPI_ISL_427680|Original|hCoV-19^^New South Wales|Human|Centre for Infectious Diseases and Microbiology Public Health|NSW Health Pathology - Institute of Clinical Pathology and Medical Research; Westmead Hospital; University of Sydney|Gall|Australia
>Spike|hCoV-19/England/QEUH-A61838/2020|2020-10-10|EPI_ISL_628635|Original|hCoV-19^^England|Human|Oxford Viromics|COVID-19 Genomics UK (COG-UK) Consortium|Golubchik|United Kingdom
>Spike|hCoV-19/South Africa/KRISP-K002680/2020|2020-08-03|EPI_ISL_535544|Original|hCoV-19^^KwaZulu-Natal|Human|NHLS-IALCH|KRISP|de Oliveira|South Africa
>Spike|hCoV-19/Netherlands/ZH-RIVM-20844/2020|2020-12-17|EPI_ISL_790899|Original|hCoV-19^^Zuid-holland|Human|Dutch COVID-19 response team|National Institute for Public Health and the Environment (RIVM)|Meijer|Netherlands
>Spike|hCoV-19/Japan/PG-4898/2020|2020-08-00|EPI_ISL_690019|Original|hCoV-19^^Japan|Human|Sakai City Institute of Public Health|Pathogen Genomics Center|Sekizuka|Japan

>Spike|hCoV-19/England/QEUH-9AE4A5/2020|2020-09-02|EPI_ISL_637547|Original|hCoV-19^^England|Human|Department of Pathology|COVID-19 Genomics UK (COG-UK) Consortium|Goodfellow|United Kingdom
>Spike|hCoV-19/USA/UT-UPHL-2009545/2020|2020-07-18|EPI_ISL_605225|Original|hCoV-19^^Utah|Human|Utah Public Health Laboratory|Utah Public Health Laboratory|Young|USA
>Spike|hCoV-19/Wales/PHWC-488D6F/2020|2020-11-01|EPI_ISL_639498|Original|hCoV-19^^Wales|Human|Wales Specialist Virology Centre Sequencing lab|COVID-19 Genomics UK (COG-UK) Consortium|Moore|United Kingdom
>Spike|hCoV-19/Portugal/PT2051/2020|2020-11-13|EPI_ISL_732265|Original|hCoV-19^^Portugal|Human|Instituto Nacional de Saude (INSA) and Instituto Gulbenkian de Ciencia (IGC)|Instituto Nacional de Saude (INSA) and Instituto Gulbenkian de Ciencia (IGC)|Borges|Portugal
>Spike|hCoV-19/England/ALDP-9CDF81/2020|2020-09-16|EPI_ISL_566340|Original|hCoV-19^^England|Human|Lighthouse Lab in Alderley Park|Wellcome Sanger Institute for the COVID-19 Genomics UK (COG-UK) consortium|Davies|United Kingdom
>Spike|hCoV-19/Spain/IB-IBV-99010756/2020|2020-05-11|EPI_ISL_691640|Original|hCoV-19^^Balear Islands|Human|Servicio de Microbiolog\u00eda|SeqCOVID-SPAIN consortium/IBV(CSIC)|Comas|Spain
>Spike|hCoV-19/England/CAMC-BC1668/2020|2020-11-20|EPI_ISL_704516|Original|hCoV-19^^England|Human|Department of Pathology|COVID-19 Genomics UK (COG-UK) Consortium|Goodfellow|United Kingdom
>Spike|hCoV-19/USA/TX-HMH-4635/2020|2020-06-23|EPI_ISL_543772|Original|hCoV-19^^Texas|Human|Houston Methodist Hospital|Houston Methodist Hospital|Long|USA
>Spike|hCoV-19/Switzerland/VD-ETHZ-100302/2020|2020-03-19|EPI_ISL_575001|Original|hCoV-19^^Vaud|Human|Viollier AG|Department of Biosystems Science and Engineering|Nadeau|Switzerland
>Spike|hCoV-19/Australia/QLD1142/2020|2020-08-29|EPI_ISL_593643|Original|hCoV-19^^Australia|Human|unknown|Public Health Virology Laboratory|Nguyen|Australia
>Spike|hCoV-19/England/MILK-B8C1A4/2020|2020-11-13|EPI_ISL_674457|Original|hCoV-19^^England|Human|Lighthouse Lab in Milton Keynes|Wellcome Sanger Institute for the COVID-19 Genomics UK (COG-UK) Consortium|Davies|United Kingdom
>Spike|hCoV-19/England/BRIS-1257F0/2020|2020-03-29|EPI_ISL_443917|Original|hCoV-19^^England|Human|PHE South West Regional Laboratory|Wellcome Sanger Institute for the COVID-19 Genomics UK (COG-UK) consortium|Amato|United Kingdom
>Spike|hCoV-19/Turkey/HSGM-1458/2020|2020-03-19|EPI_ISL_437327|Vero P1|hCoV-19^^Agri|Human|Ministry of Health Turkey|Ministry of Health Turkey|Bayrakdar|Turkey
>Spike|hCoV-19/Switzerland/VS-ETHZ-350360/2020|2020-10-31|EPI_ISL_728850|Original|hCoV-19^^Valais|Human|Viollier AG|Department of Biosystems Science and Engineering|Nadeau|Switzerland
>Spike|hCoV-19/Canada/ON-UHTC_0039/2020|2020-04-10|EPI_ISL_464026|unknown|hCoV-19^^Ontario|Human|Unity Health Toronto|Ontario Institute for Cancer Research|de Borja|Canada
>Spike|hCoV-19/Norway/6246/2020|2020-11-16|EPI_ISL_775364|Original|hCoV-19^^Vestland|Human|Foerde Hospital|Norwegian Institute of Public Health|Vollan|Norway
>Spike|hCoV-19/England/ALDP-720B8A/2020|2020-07-01|EPI_ISL_557572|Original|hCoV-19^^England|Human|Lighthouse Lab in Alderley Park|Wellcome Sanger Institute for the COVID-19 Genomics UK (COG-UK) consortium|Davies|United Kingdom
>Spike|hCoV-19/England/ALDP-CB3D81/2020|2020-12-19|EPI_ISL_760832|Original|hCoV-19^^England|Human|Lighthouse Lab in Alderley Park|Wellcome Sanger Institute for the COVID-19 Genomics UK (COG-UK) Consortium|Davies|United Kingdom
>Spike|hCoV-19/USA/TX-HMH-5260/2020|2020-06-26|EPI_ISL_544633|Original|hCoV-19^^Texas|Human|Houston Methodist Hospital|Houston Methodist Hospital|Long|USA
>Spike|hCoV-19/Spain/PV-IBV-2154/2020|2020-04-12|EPI_ISL_468942|Original|hCoV-19^^Basque Country|Human|Servicio de Microbiolog\u00eda. Hospital Universitario Donostia. OSI Donostialdea. \u00c1rea de Enfermedades Infecciosas|SeqCOVID-SPAIN consortium/IBV(CSIC)|Comas|Spain
>Spike|hCoV-19/Portugal/PT1927/2020|2020-11-15|EPI_ISL_732138|Original|hCoV-19^^Portugal|Human|Instituto Nacional de Saude (INSA) and Instituto Gulbenkian de Ciencia (IGC)|Instituto Nacional de Saude (INSA) and Instituto Gulbenkian de Ciencia (IGC)|Borges|Portugal

>Spike|hCoV-19/Denmark/DCGC-17781/2020|2020-12-14|EPI_ISL_749564|Original|hCoV-19^^Sjaelland|Human|Department of Virus and Microbiological Special Diagnostics|Albertsen Lab|Michaelsen|Denmark
>Spike|hCoV-19/Wales/PHWC-4999B2/2020|2020-12-04|EPI_ISL_727384|Original|hCoV-19^^Wales|Human|Originating lab|COVID-19 Genomics UK (COG-UK) Consortium|Moore|United Kingdom
>Spike|hCoV-19/England/ALDP-AA266E/2020|2020-10-22|EPI_ISL_630854|Original|hCoV-19^^England|Human|Lighthouse Lab in Alderley Park|Wellcome Sanger Institute for the COVID-19 Genomics UK (COG-UK) consortium|Davies|United Kingdom
>Spike|hCoV-19/England/ALDP-9C5AE2/2020|2020-09-10|EPI_ISL_567644|Original|hCoV-19^^England|Human|Lighthouse Lab in Alderley Park|Wellcome Sanger Institute for the COVID-19 Genomics UK (COG-UK) consortium|Davies|United Kingdom
>Spike|hCoV-19/USA/CA-IGI-0390/2020|2020-07-17|EPI_ISL_756313|Original|hCoV-19^^California|Human|Innovative Genomics Institute|Innovative Genomics Institute|Wyman|USA
>Spike|hCoV-19/England/MILK-ACEEEC/2020|2020-10-22|EPI_ISL_629634|Original|hCoV-19^^England|Human|Lighthouse Lab in Milton Keynes|Wellcome Sanger Institute for the COVID-19 Genomics UK (COG-UK) consortium|Davies|United Kingdom
>Spike|hCoV-19/USA/MA-MGH-00855/2020|2020-04-13|EPI_ISL_791797|Original|hCoV-19^^Massachusetts|Human|Massachusetts General Hospital|Infectious Disease Program|Park|USA
>Spike|hCoV-19/USA/CA-ALSR-5073/2020|2020-06-25|EPI_ISL_730334|Original|hCoV-19^^California|Human|San Diego County Public Health Laboratory|Andersen lab at Scripps Research|Gangavarapu|USA
>Spike|hCoV-19/USA/WI-UW-1496/2020|2020-09-14|EPI_ISL_541623|Original|hCoV-19^^Wisconsin|Human|University of Wisconsin-Madison AIDS Vaccine Research Laboratories|University of Wisconsin-Madison AIDS Vaccine Research Laboratories|Baczenas|USA
>Spike|hCoV-19/USA/CA-CZB0141/2020|2020-04-08|EPI_ISL_429054|Original|hCoV-19^^California|Human|UCSF Clinical Microbiology Laboratory|Chan-Zuckerberg Biohub|Kamm|USA
>Spike|hCoV-19/England/EXET-1368F9/2020|2020-04-29|EPI_ISL_457240|Original|hCoV-19^^England|Human|University of Exeter|COVID-19 Genomics UK (COG-UK) Consortium|Temperton|United Kingdom
>Spike|hCoV-19/England/SHEF-D15DC/2020|2020-03-30|EPI_ISL_432632|Original|hCoV-19^^England|Human|Virology Department|COVID-19 Genomics UK (COG-UK) Consortium|de Silva|United Kingdom
>Spike|hCoV-19/Scotland/CVR1825/2020|2020-04-07|EPI_ISL_448132|Original|hCoV-19^^Scotland|Human|West of Scotland Specialist Virology Centre|COVID-19 Genomics UK (COG-UK) Consortium|Robertson|United Kingdom
>Spike|hCoV-19/England/BRIS-12C902/2020|2020-05-13|EPI_ISL_469511|Original|hCoV-19^^England|Human|PHE South West Regional Laboratory|Wellcome Sanger Institute for the COVID-19 Genomics UK (COG-UK) consortium|Davies|United Kingdom
>Spike|hCoV-19/Australia/VIC3031/2020|2020-07-01|EPI_ISL_520745|Original|hCoV-19^^Victoria|Human|Victorian Infectious Diseases Reference Laboratory (VIDRL)|VIDRL and MDU-PHL|Schultz|Australia
>Spike|hCoV-19/England/PORT-2D47C2/2020|2020-10-11|EPI_ISL_613294|Original|hCoV-19^^England|Human|Centre for Enzyme Innovation|COVID-19 Genomics UK (COG-UK) Consortium|Robson|United Kingdom
>Spike|hCoV-19/Canada/ON-UHTC_0393/2020|2020-11-24|EPI_ISL_698086|Original|hCoV-19^^Ontario|Human|Unity Health Toronto|Ontario Institute for Cancer Research|de Borja|Canada
>Spike|hCoV-19/Portugal/PT0085/2020|2020-03-22|EPI_ISL_421488|Original|hCoV-19^^Portugal|Human|H Santarem|Instituto Nacional de Saude (INSA)|Borges|Portugal
>Spike|hCoV-19/USA/TX-HMH-MCoV-19023/2020|2020-11-25|EPI_ISL_785289|Original|hCoV-19^^Texas|Human|Houston Methodist Hospital|Houston Methodist Hospital|Long|USA
>Spike|hCoV-19/Wales/PHWC-4A35C0/2020|2020-12-15|EPI_ISL_744115|Original|hCoV-19^^Wales|Human|Wales Specialist Virology Centre Sequencing lab|COVID-19 Genomics UK (COG-UK) Consortium|Moore|United Kingdom

>Spike|hCoV-19/England/MILK-9A6B7B/2020|2020-09-02|EPI_ISL_550070|Original|hCoV-19^^England|Human|Lighthouse Lab in Milton Keynes|Wellcome Sanger Institute for the COVID-19 Genomics UK (COG-UK) consortium|Davies|United Kingdom
>Spike|hCoV-19/Wales/PHWC-3118B/2020|2020-04-10|EPI_ISL_446413|Original|hCoV-19^^Wales|Human|Wales Specialist Virology Centre|Public Health Wales Microbiology Cardiff|Moore|United Kingdom
>Spike|hCoV-19/Scotland/EDB084/2020|2020-03-21|EPI_ISL_425876|Original|hCoV-19^^Scotland|Human|Virology Department|COVID-19 Genomics UK (COG-UK) Consortium|Williams|United Kingdom
>Spike|hCoV-19/Wales/MILK-99C9C9/2020|2020-08-31|EPI_ISL_550575|Original|hCoV-19^^Wales|Human|Lighthouse Lab in Milton Keynes|Wellcome Sanger Institute for the COVID-19 Genomics UK (COG-UK) consortium|Davies|United Kingdom
>Spike|hCoV-19/India/TG-CCMB-L1967/2020|2020-06-04|EPI_ISL_495177|Original|hCoV-19^^Telangana|Human|CSIR-Centre for Cellular and Molecular Biology|CSIR-Centre for Cellular and Molecular Biology|Banu|India
>Spike|hCoV-19/Scotland/EDB3546/2020|2020-04-13|EPI_ISL_487880|Original|hCoV-19^^Scotland|Human|Virology Department|Wellcome Sanger Institute for the COVID-19 Genomics UK (COG-UK) consortium|Davies|United Kingdom
>Spike|hCoV-19/USA/LA-GBCL-26513042/2020|2020-04-21|EPI_ISL_485086|Original|hCoV-19^^Louisiana|Human|River Road Testing Lab|Ginkgo Bioworks Clinical Laboratory|Smith|USA
>Spike|hCoV-19/Scotland/CVR51/2020|2020-03-13|EPI_ISL_425779|Original|hCoV-19^^Scotland|Human|West of Scotland Specialist Virology Centre|COVID-19 Genomics UK (COG-UK) Consortium|Robertson|United Kingdom
>Spike|hCoV-19/Canada/QC-LSPQ-L00234537/2020|2020-03-23|EPI_ISL_536057|Original|hCoV-19^^Quebec|Human|H\u00f4pital Sainte-Croix|Laboratoire de sant\u00e9 publique du Qu\u00e9bec|Moreira|Canada
>Spike|hCoV-19/England/LIVE-AA5FA/2020|2020-03-24|EPI_ISL_517474|Original|hCoV-19^^England|Human|Liverpool Clinical Laboratories|COVID-19 Genomics UK (COG-UK) Consortium|Haldenby|United Kingdom
>Spike|hCoV-19/Japan/PG-1900/2020|2020-04-00|EPI_ISL_685506|Original|hCoV-19^^Japan|Human|Pathogen Genomics Center|Pathogen Genomics Center|Sekizuka|Japan
>Spike|hCoV-19/USA/WA-UW104/2020|2020-03-11|EPI_ISL_416642|Original|hCoV-19^^Washington|Human|UW Virology Lab|UW Virology Lab|Roychoudhury|USA
>Spike|hCoV-19/England/MILK-A79CD7/2020|2020-10-15|EPI_ISL_606736|Original|hCoV-19^^England|Human|Lighthouse Lab in Milton Keynes|Wellcome Sanger Institute for the COVID-19 Genomics UK (COG-UK) consortium|Davies|United Kingdom
>Spike|hCoV-19/Belgium/ULG-10826/2020|2020-11-13|EPI_ISL_661215|Original|hCoV-19^^Li\u00e8ge|Human|Department of Clinical Microbiology|GIGA Medical Genomics|Durkin|Belgium
>Spike|hCoV-19/USA/VA-DCLS-2048/2020|2020-10-26|EPI_ISL_677219|Original|hCoV-19^^Virginia|Human|Virginia Division of Consolidated Laboratory Services|Virginia Division of Consolidated Laboratory Services|St. Jacques|USA
>Spike|hCoV-19/USA/MA-MASPHL-00025/2020|2020-04-02|EPI_ISL_791531|Original|hCoV-19^^Massachusetts|Human|Massachusetts State Public Health Laboratory|Infectious Disease Program|Park|USA
>Spike|hCoV-19/England/NORT-2A5C8F/2020|2020-10-04|EPI_ISL_584708|Original|hCoV-19^^England|Human|Northumbria University / South Tees Hospitals NHS Foundation Trust / North Cumbria Integrated Care NHS Foundation Trust / North Tees and Hartlepool NHS Foundation Trust / Newcastle Hospitals NHS Foundation Trust|COVID-19 Genomics UK (COG-UK) Consortium|Bashton|United Kingdom
>Spike|hCoV-19/England/NORT-287D71/2020|2020-11-11|EPI_ISL_724998|Original|hCoV-19^^England|Human|Northumbria University / South Tees Hospitals NHS Foundation Trust / North Cumbria Integrated Care NHS Foundation Trust / North Tees and Hartlepool NHS Foundation Trust / Newcastle Hospitals NHS Foundation Trust|COVID-19 Genomics UK (COG-UK) Consortium|Bashton|United Kingdom
>Spike|hCoV-19/England/CAMC-B0CB44/2020|2020-10-30|EPI_ISL_643486|Original|hCoV-19^^England|Human|Lighthouse Lab in Cambridge|Wellcome Sanger Institute for the COVID-19 Genomics UK (COG-UK) Consortium|Davies|United Kingdom

>Spike|hCoV-19/United Arab Emirates/0010/2020|2020-05-
07|EPI_ISL_698220|Original|hCoV-19^^Abu Dhabi|Human|Group 42 (G42) Healthcare|G42
Healthcare|Ogrodzki|United Arab Emirates
>Spike|hCoV-19/USA/CA-CSMC61/2020|2020-04-01|EPI_ISL_475675|Original|hCoV-
19^^California|Human|Cedars-Sinai Medical Center|Cedars-Sinai Medical
Center|Zhang|USA
>Spike|hCoV-19/Switzerland/SH-ETHZ-370458/2020|2020-11-
16|EPI_ISL_693958|Original|hCoV-19^^Schaffhausen|Human|Viollier AG|Department of
Biosystems Science and Engineering|Nadeau|Switzerland
>Spike|hCoV-19/Equatorial Guinea/12462/2020|2020-05-29|EPI_ISL_648311|Original|hCoV-
19^^Bioko Norte|Human|Laboratorio de Investigaciones de Baney|University Hospital
Basel|Hosch|Equatorial Guinea
>Spike|hCoV-19/England/NORT-28ADBA/2020|2020-04-16|EPI_ISL_461795|Original|hCoV-
19^^England|Human|Northumbria University / South Tees Hospitals NHS Foundation Trust
/ North Cumbria Integrated Care NHS Foundation Trust / North Tees and Hartlepool NHS
Foundation Trust / Newcastle Hospitals NHS Foundation Trust|COVID-19 Genomics UK
(COG-UK) Consortium|Bashton|United Kingdom
>Spike|hCoV-19/USA/TX-HMH-4954/2020|2020-06-20|EPI_ISL_544428|Original|hCoV-
19^^Texas|Human|Houston Methodist Hospital|Houston Methodist Hospital|Long|USA
>Spike|hCoV-19/England/OXON-AD23C/2020|2020-00-00|EPI_ISL_448571|Original|hCoV-
19^^England|Human|Oxford Viromics|COVID-19 Genomics UK (COG-UK)
Consortium|Golubchik|United Kingdom
>Spike|hCoV-19/England/QEUH-9F6F07/2020|2020-09-27|EPI_ISL_595645|Original|hCoV-
19^^England|Human|Oxford Viromics|COVID-19 Genomics UK (COG-UK)
Consortium|Golubchik|United Kingdom
>Spike|hCoV-19/England/SHEF-10D32EF/2020|2020-12-10|EPI_ISL_742055|Original|hCoV-
19^^England|Human|Virology Department|COVID-19 Genomics UK (COG-UK) Consortium|de
Silva|United Kingdom
>Spike|hCoV-19/USA/LA-EVTL700/2020|2020-07-14|EPI_ISL_653392|Original|hCoV-
19^^Louisiana|Human|LSUHS Emerging Viral Threat Laboratory|Microbial Genome
Sequencing Center|Kamil|USA
>Spike|hCoV-19/Thailand/Bangkok-CONI-0274/2020|2020-03-
14|EPI_ISL_693352|Original|hCoV-19^^Bangkok|Human|Ramathibodi Hospital|COVID-19
Network Investigations (CONI) Alliance|Batty|Thailand
>Spike|hCoV-19/Japan/PG-6924/2020|2020-09-00|EPI_ISL_688031|Original|hCoV-
19^^Japan|Human|Pathogen Genomics Center|Pathogen Genomics Center|Sekizuka|Japan
>Spike|hCoV-19/Belgium/ULG-10239/2020|2020-06-26|EPI_ISL_498145|Original|hCoV-
19^^Li\u00e8ge|Human|Department of Clinical Microbiology|GIGA Medical
Genomics|Durkin|Belgium
>Spike|hCoV-19/Brazil/RS-00636/2020|2020-11-24|EPI_ISL_770584|Original|hCoV-19^^Rio
Grande do Sul|Human|Laborat\u00f3rio de Microbiologia Molecular - Universidade
FEEVALE|Bioinformatics Laboratory / LNCC|Almeida|Brazil
>Spike|hCoV-19/England/QEUH-9F965A/2020|2020-09-25|EPI_ISL_589031|Original|hCoV-
19^^England|Human|Lighthouse Lab in Glasgow|Wellcome Sanger Institute for the COVID-
19 Genomics UK (COG-UK) consortium|Davies|United Kingdom
>Spike|hCoV-19/USA/MN-MDH-1546/2020|2020-08-03|EPI_ISL_527611|Original|hCoV-
19^^Minnesota|Human|Minnesota Department of Health|Minnesota Department of
Health|Garfin|USA
>Spike|hCoV-19/England/MILK-9756B0/2020|2020-08-20|EPI_ISL_552697|Original|hCoV-
19^^England|Human|Lighthouse Lab in Milton Keynes|Wellcome Sanger Institute for the
COVID-19 Genomics UK (COG-UK) consortium|Davies|United Kingdom
>Spike|hCoV-19/England/BRIS-130E48/2020|2020-03-23|EPI_ISL_501596|Original|hCoV-
19^^England|Human|PHE South West Regional Laboratory|Wellcome Sanger Institute for
the COVID-19 Genomics UK (COG-UK) consortium|Davies|United Kingdom
>Spike|hCoV-19/England/LIVE-DB3E23/2020|2020-10-21|EPI_ISL_650694|Original|hCoV-
19^^England|Human|Liverpool Clinical Laboratories|COVID-19 Genomics UK (COG-UK)
Consortium|Haldenby|United Kingdom
>Spike|hCoV-19/England/QEUH-9C9D9A/2020|2020-09-10|EPI_ISL_567354|Original|hCoV-
19^^England|Human|Lighthouse Lab in Glasgow|Wellcome Sanger Institute for the COVID-
19 Genomics UK (COG-UK) consortium|Davies|United Kingdom

>Spike|hCoV-19/USA/WA-S2042/2020|2020-06-05|EPI_ISL_497470|Original|hCoV-19^^Washington|Human|Washington State Department of Health|Seattle Flu Study|Lee|USA
>Spike|hCoV-19/Luxembourg/LNS9425421/2020|2020-10-29|EPI_ISL_744911|Original|hCoV-19^^Luxembourg|Human|Laboratoire national de sant\u00e9|Laboratoire national de sant\u00e9|Wienecke-Baldacchino|Luxembourg
>Spike|hCoV-19/England/QEUH-9F81A5/2020|2020-09-27|EPI_ISL_588967|Original|hCoV-19^^England|Human|Lighthouse Lab in Glasgow|Wellcome Sanger Institute for the COVID-19 Genomics UK (COG-UK) consortium|Davies|United Kingdom
>Spike|hCoV-19/England/20189054401/2020|2020-04-30|EPI_ISL_465537|Original|hCoV-19^^England|Human|Respiratory Virus Unit|Respiratory Virus Unit|Platt|United Kingdom
>Spike|hCoV-19/Canada/QC-LSPQ-L00229794/2020|2020-03-20|EPI_ISL_535903|Original|hCoV-19^^Quebec|Human|H\u00f4tel-Dieu de Sorel|Laboratoire de sant\u00e9 publique du Qu\u00e9bec|Moreira|Canada
>Spike|hCoV-19/USA/WI-UW-2486/2020|2020-12-03|EPI_ISL_759760|Original|hCoV-19^^Wisconsin|Human|University of Wisconsin-Madison AIDS Vaccine Research Laboratories|University of Wisconsin-Madison AIDS Vaccine Research Laboratories|Moreno|USA
>Spike|hCoV-19/Canada/ON-S842/2020|2020-04-00|EPI_ISL_586457|Original|hCoV-19^^Ontario|Human|Toronto Invasive Bacterial Diseases Network|McMaster University|McArthur|Canada
>Spike|hCoV-19/England/QEUH-AD3A2C/2020|2020-10-26|EPI_ISL_629993|Original|hCoV-19^^England|Human|Lighthouse Lab in Glasgow|Wellcome Sanger Institute for the COVID-19 Genomics UK (COG-UK) consortium|Davies|United Kingdom
>Spike|hCoV-19/England/ALDP-B1B5DB/2020|2020-11-01|EPI_ISL_645775|Original|hCoV-19^^England|Human|Lighthouse Lab in Alderley Park|Wellcome Sanger Institute for the COVID-19 Genomics UK (COG-UK) Consortium|Davies|United Kingdom
>Spike|hCoV-19/England/ALDP-97CCD5/2020|2020-07-17|EPI_ISL_526315|Original|hCoV-19^^England|Human|University of Birmingham|COVID-19 Genomics UK (COG-UK) Consortium|Loman|United Kingdom
>Spike|hCoV-19/Spain/IB-IBV-99010845/2020|2020-09-05|EPI_ISL_691655|Original|hCoV-19^^Balear Islands|Human|Servicio de Microbiolog\u00eda|SeqCOVID-SPAIN consortium/IBV(CSIC)|Comas|Spain
>Spike|hCoV-19/England/MILK-B87525/2020|2020-11-13|EPI_ISL_724779|Original|hCoV-19^^England|Human|Oxford Viromics|COVID-19 Genomics UK (COG-UK) Consortium|Golubchik|United Kingdom
>Spike|hCoV-19/Wales/ALDP-BB4F30/2020|2020-11-20|EPI_ISL_710703|Original|hCoV-19^^Wales|Human|Lighthouse Lab in Alderley Park|Wellcome Sanger Institute for the COVID-19 Genomics UK (COG-UK) Consortium|Davies|United Kingdom
>Spike|hCoV-19/USA/NY-NYCPHL-001251/2020|2020-11-06|EPI_ISL_683947|Original|hCoV-19^^New York|Human|DOHMH Morrisania|New York City Public Health Laboratory|Wang|USA
>Spike|hCoV-19/India/GJ-GBRC286/2020|2020-07-03|EPI_ISL_495024|Original|hCoV-19^^Gujarat|Human|Government Medical College|Gujarat Biotechnology Research Centre|Joshi|India
>Spike|hCoV-19/South Africa/KRISP-K001339/2020|2020-07-10|EPI_ISL_515824|Original|hCoV-19^^KwaZulu-Natal|Human|Medical Disagnostics Services (MDS)|KRISP|de Oliveira|South Africa
>Spike|hCoV-19/Denmark/DCGC-18369/2020|2020-12-07|EPI_ISL_749923|Original|hCoV-19^^Hovedstaden|Human|Department of Virus and Microbiological Special Diagnostics|Albertsen Lab|Michaelsen|Denmark
>Spike|hCoV-19/England/LOND-D5E19/2020|2020-04-19|EPI_ISL_444249|Original|hCoV-19^^England|Human|University College London|COVID-19 Genomics UK (COG-UK) Consortium|Roy|United Kingdom
>Spike|hCoV-19/England/CAMB-7DED7/2020|2020-04-06|EPI_ISL_470481|Original|hCoV-19^^England|Human|Department of Pathology|Wellcome Sanger Institute for the COVID-19 Genomics UK (COG-UK) consortium|Davies|United Kingdom
>Spike|hCoV-19/USA/WA-S2727/2020|2020-07-08|EPI_ISL_525717|Original|hCoV-19^^Washington|Human|Seattle Flu Study|Seattle Flu Study|Lee|USA
>Spike|hCoV-19/Wales/PHWC-496056/2020|2020-11-19|EPI_ISL_726522|Original|hCoV-19^^Wales|Human|Originating lab|COVID-19 Genomics UK (COG-UK) Consortium|Moore|United Kingdom

>Spike|hCoV-19/USA/CA-ALSR-2843/2020|2020-08-06|EPI_ISL_635709|Original|hCoV-19^^California|Human|San Diego County Public Health Laboratory|Andersen lab at Scripps Research|Gangavarapu|USA
>Spike|hCoV-19/Belarus/ChVir2070/2020|2020-03-00|EPI_ISL_451948|Original|hCoV-19^^Belarus|Human|The Republican Research and Practical Center for Epidemiology and Microbiology|Charite Universitatsmedizin Berlin|Muehlemann|Belarus
>Spike|hCoV-19/England/20109041904/2020|2020-03-04|EPI_ISL_464561|Original|hCoV-19^^England|Human|Respiratory Virus Unit|Respiratory Virus Unit|Platt|United Kingdom
>Spike|hCoV-19/Belgium/rega-0428425/2020|2020-04-28|EPI_ISL_476993|Original|hCoV-19^^Leuven|Human|KU Leuven|KU Leuven|Maes|Belgium
>Spike|hCoV-19/USA/CA-CZB-16178/2020|2020-12-01|EPI_ISL_739355|Original|hCoV-19^^California|Human|Renegade|Chan-Zuckerberg Biohub|Kamm|USA
>Spike|hCoV-19/Scotland/CVR3380/2020|2020-05-08|EPI_ISL_456809|Original|hCoV-19^^Scotland|Human|West of Scotland Specialist Virology Centre|COVID-19 Genomics UK (COG-UK) Consortium|Robertson|United Kingdom
>Spike|hCoV-19/USA/TX-QDX-745/2020|2020-03-19|EPI_ISL_571557|Original|hCoV-19^^Texas|Human|Quest Diagnostics|Quest Diagnostics|Kagan|USA
>Spike|hCoV-19/Sweden/20-52881/2020|2020-09-09|EPI_ISL_648148|Original|hCoV-19^^Halland|Human|Halmstad klinisk mikrobiologi|The Public Health Agency of Sweden|Svartstr\u00f6m|Sweden
>Spike|hCoV-19/England/NOTT-11F4B6/2020|2020-11-25|EPI_ISL_702846|Original|hCoV-19^^England|Human|Queens Medical Centre|COVID-19 Genomics UK (COG-UK) Consortium|Loose|United Kingdom
>Spike|hCoV-19/USA/WA-S1246/2020|2020-05-11|EPI_ISL_495696|Original|hCoV-19^^Washington|Human|Washington State Department of Health|Seattle Flu Study|Lee|USA
>Spike|hCoV-19/USA/WI-UW-1893/2020|2020-09-23|EPI_ISL_605762|Original|hCoV-19^^Wisconsin|Human|University of Wisconsin-Madison AIDS Vaccine Research Laboratories|University of Wisconsin-Madison AIDS Vaccine Research Laboratories|Baczenas|USA
>Spike|hCoV-19/England/OXON-B4C8F/2020|2020-04-30|EPI_ISL_535003|Original|hCoV-19^^England|Human|Oxford Viromics|COVID-19 Genomics UK (COG-UK) Consortium|Golubchik|United Kingdom
>Spike|hCoV-19/Denmark/DCGC-12362/2020|2020-11-16|EPI_ISL_682543|Original|hCoV-19^^Hovedstaden|Human|Department of Virus and Microbiological Special Diagnostics|Albertsen Lab|Michaelsen|Denmark
>Spike|hCoV-19/USA/UT-UPHL-2012365234/2020|2020-10-21|EPI_ISL_745648|Original|hCoV-19^^Utah|Human|Ginkgo Bioworks Clinical Laboratory|Utah Public Health Laboratory|Young|USA
>Spike|hCoV-19/Australia/NSW1365/2020|2020-12-25|EPI_ISL_767931|Original|hCoV-19^^New South Wales|Human|4Cyte Pathology|NSW Health Pathology - Institute of Clinical Pathology and Medical Research; Westmead Hospital; University of Sydney|Gall|Australia
>Spike|hCoV-19/England/201060055/2020|2020-03-04|EPI_ISL_464384|Original|hCoV-19^^England|Human|Respiratory Virus Unit|Respiratory Virus Unit|Platt|United Kingdom
>Spike|hCoV-19/Portugal/PT2006/2020|2020-11-13|EPI_ISL_732227|Original|hCoV-19^^Portugal|Human|Instituto Nacional de Saude (INSA) and Instituto Gulbenkian de Ciencia (IGC)|Instituto Nacional de Saude (INSA) and Instituto Gulbenkian de Ciencia (IGC)|Borges|Portugal
>Spike|hCoV-19/Brazil/SP-162/2020|2020-04-15|EPI_ISL_523958|Original|hCoV-19^^S\u00e3o Paulo|Human|Pronto Socorro Municipal de Perus|Instituto Adolfo Lutz|Sacchi|Brazil
>Spike|hCoV-19/USA/FL-BPHL-0754/2020|2020-06-23|EPI_ISL_512560|Original|hCoV-19^^Florida|Human|Florida Bureau of Public Health Laboratories|Florida Bureau of Public Health Laboratories|Blanton|USA
>Spike|hCoV-19/USA/WA-QDX-1080/2020|2020-03-19|EPI_ISL_571905|Original|hCoV-19^^Washington|Human|Quest Diagnostics|Quest Diagnostics|Kagan|USA
>Spike|hCoV-19/England/QEUH-B68DC8/2020|2020-11-11|EPI_ISL_662241|Original|hCoV-19^^England|Human|Lighthouse Lab in Glasgow|Wellcome Sanger Institute for the COVID-19 Genomics UK (COG-UK) Consortium|Davies|United Kingdom

>Spike|hCoV-19/England/MILK-9937B9/2020|2020-08-27|EPI_ISL_552025|Original|hCoV-19^^England|Human|Lighthouse Lab in Milton Keynes|Wellcome Sanger Institute for the COVID-19 Genomics UK (COG-UK) consortium|Davies|United Kingdom
>Spike|hCoV-19/England/MILK-6EF195/2020|2020-06-30|EPI_ISL_557368|Original|hCoV-19^^England|Human|Lighthouse Lab in Milton Keynes|Wellcome Sanger Institute for the COVID-19 Genomics UK (COG-UK) consortium|Davies|United Kingdom
>Spike|hCoV-19/USA/TX-HMH-MCoV-9201/2020|2020-07-02|EPI_ISL_780997|Original|hCoV-19^^Texas|Human|Houston Methodist Hospital|Houston Methodist Hospital|Long|USA
>Spike|hCoV-19/United Arab Emirates/L3290324509/2020|2020-05-27|EPI_ISL_520707|Original|hCoV-19^^United Arab Emirates|Human|Mohammed Bin Rashid University of Medicine and Health Sciences|Al Jalila Genomics Center|Ramaswamy|United Arab Emirates
>Spike|hCoV-19/USA/MN-MDH-1856/2020|2020-10-06|EPI_ISL_602991|Original|hCoV-19^^Minnesota|Human|Minnesota Department of Health|Minnesota Department of Health|Garfin|USA
>Spike|hCoV-19/England/QEUH-A0114E/2020|2020-10-02|EPI_ISL_584903|Original|hCoV-19^^England|Human|University of Exeter|COVID-19 Genomics UK (COG-UK) Consortium|Temperton|United Kingdom
>Spike|hCoV-19/England/NOTT-10F9C9/2020|2020-04-05|EPI_ISL_432670|Original|hCoV-19^^England|Human|Queens Medical Centre|COVID-19 Genomics UK (COG-UK) Consortium|Loose|United Kingdom
>Spike|hCoV-19/USA/TX-HMH-6188/2020|2020-07-04|EPI_ISL_547299|Original|hCoV-19^^Texas|Human|Houston Methodist Hospital|Houston Methodist Hospital|Long|USA
>Spike|hCoV-19/Wales/PHWC-1640EB/2020|2020-05-11|EPI_ISL_474128|Original|hCoV-19^^Wales|Human|Originating lab|COVID-19 Genomics UK (COG-UK) Consortium|Moore|United Kingdom
>Spike|hCoV-19/USA/WA-UW30/2020|2020-03-08|EPI_ISL_414617|Original|hCoV-19^^Washington|Human|UW Virology Lab|UW Virology Lab|Roychoudhury|USA
>Spike|hCoV-19/Denmark/DCGC-14980/2020|2020-11-23|EPI_ISL_713559|Original|hCoV-19^^Hovedstaden|Human|Department of Virus and Microbiological Special Diagnostics|Albertsen Lab|Michaelsen|Denmark
>Spike|hCoV-19/England/BIRM-60086/2020|2020-00-00|EPI_ISL_493436|Original|hCoV-19^^England|Human|University of Birmingham|COVID-19 Genomics UK (COG-UK) Consortium|Loman|United Kingdom
>Spike|hCoV-19/USA/CA-LACPHL-AF00002/2020|2020-12-18|EPI_ISL_737237|Original|hCoV-19^^California|Human|Los Angeles County PHL|Los Angeles County PHL|Hemarajata|USA
>Spike|hCoV-19/England/CAMB-7EE5E/2020|2020-04-08|EPI_ISL_443002|Original|hCoV-19^^England|Human|Department of Pathology|Wellcome Sanger Institute for the COVID-19 Genomics UK (COG-UK) consortium|Amato|United Kingdom
>Spike|hCoV-19/USA/WI-GMF-00707/2020|2020-04-06|EPI_ISL_422461|Original|hCoV-19^^Iowa|Human|Gundersen Molecular Diagnostics Laboratory|Kabara Cancer Research Institute|Kenny|USA
>Spike|hCoV-19/England/LIVE-A34A9/2020|2020-05-20|EPI_ISL_490383|Original|hCoV-19^^England|Human|Liverpool Clinical Laboratories|COVID-19 Genomics UK (COG-UK) Consortium|Haldenby|United Kingdom
>Spike|hCoV-19/USA/NY-QDX-1946/2020|2020-03-15|EPI_ISL_604266|Original|hCoV-19^^New York|Human|Quest Diagnostics|Quest Diagnostics|Kagan|USA
>Spike|hCoV-19/England/LIVE-A2C93/2020|2020-05-19|EPI_ISL_499917|Original|hCoV-19^^England|Human|Liverpool Clinical Laboratories|COVID-19 Genomics UK (COG-UK) Consortium|Haldenby|United Kingdom
>Spike|hCoV-19/Italy/SAR-ASSL-06/2020|2020-04-28|EPI_ISL_614397|Original|hCoV-19^^Sardinia|Human|Laboratorio Biologia Molecolare Sars Cov2 - UOC Laboratorio Analisi - Servizio Medicina di Laboratorio|Laboratorio specialistico UOC Ematologia - Ospedale \San Francesco\ - ATS-ASSL Nuoro|Piras|Italy
>Spike|hCoV-19/USA/WI-UW-287/2020|2020-04-06|EPI_ISL_436581|Original|hCoV-19^^Wisconsin|Human|University of Wisconsin-Madison AIDS Vaccine Research Laboratories|University of Wisconsin-Madison AIDS Vaccine Research Laboratories|Baczenas|USA
>Spike|hCoV-19/USA/TX-HMH-3757/2020|2020-06-16|EPI_ISL_543168|Original|hCoV-19^^Texas|Human|Houston Methodist Hospital|Houston Methodist Hospital|Long|USA

>Spike|hCoV-19/Mexico/BCN-ALSR-2499/2020|2020-06-01|EPI_ISL_635507|Original|hCoV-19^^Baja California|Human|Centro de Diagn\u221a\u2265stico COVID-19 UABC Tijuana|Andersen lab at Scripps Research|Gangavarapu|Mexico
>Spike|hCoV-19/Scotland/EDB8971/2020|2020-10-30|EPI_ISL_651229|Original|hCoV-19^^Scotland|Human|Virology Department|COVID-19 Genomics UK (COG-UK) Consortium|McHugh|United Kingdom
>Spike|hCoV-19/England/204690983/2020|2020-11-13|EPI_ISL_766269|Original|hCoV-19^^England|Human|Respiratory Virus Unit|COVID-19 Genomics UK (COG-UK) Consortium|Platt|United Kingdom
>Spike|hCoV-19/England/ALDP-94B8DB/2020|2020-06-11|EPI_ISL_556462|Original|hCoV-19^^England|Human|Lighthouse Lab in Alderley Park|Wellcome Sanger Institute for the COVID-19 Genomics UK (COG-UK) consortium|Davies|United Kingdom
>Spike|hCoV-19/Wales/PHWC-48B959/2020|2020-11-05|EPI_ISL_666303|Original|hCoV-19^^Wales|Human|Wales Specialist Virology Centre Sequencing lab|COVID-19 Genomics UK (COG-UK) Consortium|Moore|United Kingdom
>Spike|hCoV-19/India/TG-CCMB-L1781/2020|2020-06-02|EPI_ISL_471633|Original|hCoV-19^^Telangana|Human|CSIR-Centre for Cellular and Molecular Biology|CSIR-Centre for Cellular and Molecular Biology|Banu|India
>Spike|hCoV-19/Scotland/QEUH-B317E8/2020|2020-11-04|EPI_ISL_676417|Original|hCoV-19^^Scotland|Human|Lighthouse Lab in Glasgow|Wellcome Sanger Institute for the COVID-19 Genomics UK (COG-UK) Consortium|Davies|United Kingdom
>Spike|hCoV-19/England/ALDP-9C5AB5/2020|2020-09-11|EPI_ISL_567755|Original|hCoV-19^^England|Human|Lighthouse Lab in Alderley Park|Wellcome Sanger Institute for the COVID-19 Genomics UK (COG-UK) consortium|Davies|United Kingdom
>Spike|hCoV-19/England/QEUH-9FA3E9/2020|2020-09-28|EPI_ISL_588269|Original|hCoV-19^^England|Human|Lighthouse Lab in Glasgow|Wellcome Sanger Institute for the COVID-19 Genomics UK (COG-UK) consortium|Davies|United Kingdom
>Spike|hCoV-19/USA/WA-S1427/2020|2020-05-20|EPI_ISL_495877|Original|hCoV-19^^Washington|Human|Washington State Department of Health|Seattle Flu Study|Lee|USA
>Spike|hCoV-19/England/CAMB-8422C/2020|2020-04-15|EPI_ISL_443454|Original|hCoV-19^^England|Human|Department of Pathology|Wellcome Sanger Institute for the COVID-19 Genomics UK (COG-UK) consortium|Amato|United Kingdom
>Spike|hCoV-19/Italy/SAR-ASSL-01/2020|2020-04-04|EPI_ISL_613560|Original|hCoV-19^^Sardinia|Human|Laboratorio Biologia Molecolare Sars Cov2 - UOC Laboratorio Analisi - Servizio Medicina di Laboratorio|Laboratorio specialistico UOC Ematologia - Ospedale \San Francesco\ - ATS-ASSL Nuoro Nuoro|Piras|Italy
>Spike|hCoV-19/Wales/PHWC-499387/2020|2020-12-03|EPI_ISL_727295|Original|hCoV-19^^Wales|Human|Originating lab|COVID-19 Genomics UK (COG-UK) Consortium|Moore|United Kingdom
>Spike|hCoV-19/USA/WI-UW-29/2020|2020-03-24|EPI_ISL_421287|Original|hCoV-19^^Wisconsin|Human|University of Wisconsin-Madison AIDS Vaccine Research Laboratories|University of Wisconsin-Madison AIDS Vaccine Research Laboratories|Baczenas|USA
>Spike|hCoV-19/USA/TX-DSHS-1496/2020|2020-06-22|EPI_ISL_683411|Original|hCoV-19^^Texas|Human|Texas Department of State Health Services|Texas Department of State Health Services|Rahman|USA
>Spike|hCoV-19/Netherlands/GE-EMC-219/2020|2020-05-27|EPI_ISL_523201|Original|hCoV-19^^Gelderland|Human|Dutch COVID-19 response team|Erasmus Medical Center|Oude Munnink|Netherlands
>Spike|hCoV-19/England/MILK-863DFD/2020|2020-07-17|EPI_ISL_557463|Original|hCoV-19^^England|Human|Lighthouse Lab in Milton Keynes|Wellcome Sanger Institute for the COVID-19 Genomics UK (COG-UK) consortium|Davies|United Kingdom
>Spike|hCoV-19/USA/LA-EVTL471/2020|2020-06-23|EPI_ISL_578875|Original|hCoV-19^^Louisiana|Human|LSUHS Emerging Viral Threat Laboratory|Microbial Genome Sequencing Center|Kamil|USA
>Spike|hCoV-19/England/MILK-9715A8/2020|2020-08-18|EPI_ISL_553177|Original|hCoV-19^^England|Human|Lighthouse Lab in Milton Keynes|Wellcome Sanger Institute for the COVID-19 Genomics UK (COG-UK) consortium|Davies|United Kingdom
>Spike|hCoV-19/Germany/BE-ChVir-D1979-4481/2020|2020-10-09|EPI_ISL_753818|Original|hCoV-19^^Berlin|Human|Charit\u00e9

Universit\u00e4tsmedizin Berlin|Charit\u00e9 Universit\u00e4tsmedizin Berlin|Muehlemann|Germany
>Spike|hCoV-19/England/LIVE-A81BC/2020|2020-03-26|EPI_ISL_517126|Original|hCoV-19^^England|Human|Liverpool Clinical Laboratories|COVID-19 Genomics UK (COG-UK) Consortium|Haldenby|United Kingdom
>Spike|hCoV-19/Denmark/DCGC-18956/2020|2020-12-07|EPI_ISL_748291|Original|hCoV-19^^Syddanmark|Human|Department of Virus and Microbiological Special Diagnostics|Albertsen Lab|Michaelsen|Denmark
>Spike|hCoV-19/England/204441477/2020|2020-10-28|EPI_ISL_653972|Original|hCoV-19^^England|Human|Respiratory Virus Unit|COVID-19 Genomics UK (COG-UK) Consortium|Platt|United Kingdom
>Spike|hCoV-19/Denmark/DCGC-21784/2020|2020-12-21|EPI_ISL_758360|Original|hCoV-19^^Hovedstaden|Human|Department of Virus and Microbiological Special Diagnostics|Albertsen Lab|Michaelsen|Denmark
>Spike|hCoV-19/Japan/IC-0178/2020|2020-09-00|EPI_ISL_667607|Original|hCoV-19^^Japan|Human|Pathogen Genomics Center|Pathogen Genomics Center|Sekizuka|Japan
>Spike|hCoV-19/England/SHEF-C1609/2020|2020-05-19|EPI_ISL_529677|Original|hCoV-19^^England|Human|Virology Department|COVID-19 Genomics UK (COG-UK) Consortium|de Silva|United Kingdom
>Spike|hCoV-19/Spain/MD-IBV-99007095/2020|2020-04-14|EPI_ISL_541880|Original|hCoV-19^^Madrid|Human|Hospital General Universitario Gregorio Mara\u00f1\u00f3n|SeqCOVID-SPAIN consortium/IBV(CSIC)|Comas|Spain
>Spike|hCoV-19/England/QEUH-C4BDAC/2020|2020-12-14|EPI_ISL_736580|Original|hCoV-19^^England|Human|Lighthouse Lab in Glasgow|Wellcome Sanger Institute for the COVID-19 Genomics UK (COG-UK) Consortium|Davies|United Kingdom
>Spike|hCoV-19/USA/NY-NYCPHL-000477/2020|2020-05-05|EPI_ISL_631944|Original|hCoV-19^^New York|Human|St Barnabas Hospital|New York City Public Health Laboratory|Wang|USA
>Spike|hCoV-19/Denmark/DCGC-5938/2020|2020-10-05|EPI_ISL_620203|Original|hCoV-19^^Midtjylland|Human|Department of Virus and Microbiological Special Diagnostics|Albertsen lab|Michaelsen|Denmark
>Spike|hCoV-19/Russia/SVE-RII-MH2841S/2020|2020-09-26|EPI_ISL_733028|Original|hCoV-19^^Sverdlovsk|Human|HELIX LLC|WHO National Influenza Centre Russian Federation|Komissarov|Russia
>Spike|hCoV-19/England/CAMB-75D1E/2020|2020-03-29|EPI_ISL_438355|Original|hCoV-19^^England|Human|Department of Pathology|Wellcome Sanger Institute for the COVID-19 Genomics UK (COG-UK) consortium|Amato|United Kingdom
>Spike|hCoV-19/Luxembourg/LNS4369621/2020|2020-06-24|EPI_ISL_740456|Original|hCoV-19^^Luxembourg|Human|Laboratoire national de sant\u00e9|Laboratoire national de sant\u00e9|Wienecke-Baldacchino|Luxembourg
>Spike|hCoV-19/Denmark/DCGC-3321/2020|2020-08-31|EPI_ISL_616075|Original|hCoV-19^^Hovedstaden|Human|Department of Virus and Microbiological Special Diagnostics|Albertsen lab|Michaelsen|Denmark
>Spike|hCoV-19/Denmark/DCGC-18806/2020|2020-12-07|EPI_ISL_748171|Original|hCoV-19^^Syddanmark|Human|Department of Virus and Microbiological Special Diagnostics|Albertsen Lab|Michaelsen|Denmark
>Spike|hCoV-19/England/ALDP-9CADA8/2020|2020-09-16|EPI_ISL_575415|Original|hCoV-19^^England|Human|Lighthouse Lab in Alderley Park|Wellcome Sanger Institute for the COVID-19 Genomics UK (COG-UK) consortium|Davies|United Kingdom
>Spike|hCoV-19/USA/TX-HMH-4096/2020|2020-06-18|EPI_ISL_543366|Original|hCoV-19^^Texas|Human|Houston Methodist Hospital|Houston Methodist Hospital|Long|USA
>Spike|hCoV-19/England/QEUH-9FA8CC/2020|2020-09-28|EPI_ISL_588134|Original|hCoV-19^^England|Human|Lighthouse Lab in Glasgow|Wellcome Sanger Institute for the COVID-19 Genomics UK (COG-UK) consortium|Davies|United Kingdom
>Spike|hCoV-19/England/CAMB-1B477E/2020|2020-06-03|EPI_ISL_473482|Original|hCoV-19^^England|Human|Department of Pathology|COVID-19 Genomics UK (COG-UK) Consortium|Meredith|United Kingdom
>Spike|hCoV-19/England/NOTT-10E39F/2020|2020-03-21|EPI_ISL_425535|Original|hCoV-19^^England|Human|Queens Medical Centre|COVID-19 Genomics UK (COG-UK) Consortium|Loose|United Kingdom

>Spike|hCoV-19/England/PORT-2D22D2/2020|2020-03-23|EPI_ISL_499308|Original|hCoV-19^^England|Human|Centre for Enzyme Innovation|COVID-19 Genomics UK (COG-UK) Consortium|Robson|United Kingdom
>Spike|hCoV-19/USA/TX-HMH-MCoV-12097/2020|2020-09-22|EPI_ISL_790199|Original|hCoV-19^^Texas|Human|Houston Methodist Hospital|Houston Methodist Hospital|Long|USA
>Spike|hCoV-19/USA/FL-BPHL-2130/2020|2020-04-09|EPI_ISL_653283|Original|hCoV-19^^Florida|Human|Florida Bureau of Public Health Laboratories|Florida Bureau of Public Health Laboratories|Blanton|USA
>Spike|hCoV-19/England/NORT-288DBC/2020|2020-03-22|EPI_ISL_458591|Original|hCoV-19^^England|Human|NU-OMICS DNA Sequencing research facility|Wellcome Sanger Institute for the COVID-19 Genomics UK (COG-UK) consortium|Davies|United Kingdom
>Spike|hCoV-19/Denmark/DCGC-14394/2020|2020-11-30|EPI_ISL_711740|Original|hCoV-19^^Sjaelland|Human|Department of Virus and Microbiological Special Diagnostics|Albertsen Lab|Michaelsen|Denmark
>Spike|hCoV-19/England/MILK-B54C95/2020|2020-11-05|EPI_ISL_657797|Original|hCoV-19^^England|Human|Lighthouse Lab in Milton Keynes|Wellcome Sanger Institute for the COVID-19 Genomics UK (COG-UK) Consortium|Davies|United Kingdom
>Spike|hCoV-19/Wales/PHWC-25BF5/2020|2020-03-24|EPI_ISL_422291|Original|hCoV-19^^Wales|Human|Wales Specialist Virology Centre|Public Health Wales Microbiology Cardiff|Moore|United Kingdom
>Spike|hCoV-19/Denmark/DCGC-13258/2020|2020-11-30|EPI_ISL_712315|Original|hCoV-19^^Hovedstaden|Human|Department of Virus and Microbiological Special Diagnostics|Albertsen Lab|Michaelsen|Denmark
>Spike|hCoV-19/USA/CA-IGI-0809/2020|2020-06-30|EPI_ISL_756344|Original|hCoV-19^^California|Human|Innovative Genomics Institute|Innovative Genomics Institute|Wyman|USA
>Spike|hCoV-19/England/201620535/2020|2020-03-26|EPI_ISL_466425|Original|hCoV-19^^England|Human|Respiratory Virus Unit|Respiratory Virus Unit|Platt|United Kingdom
>Spike|hCoV-19/England/MILK-9A557E/2020|2020-09-03|EPI_ISL_549959|Original|hCoV-19^^England|Human|Lighthouse Lab in Milton Keynes|Wellcome Sanger Institute for the COVID-19 Genomics UK (COG-UK) consortium|Davies|United Kingdom
>Spike|hCoV-19/USA/WI-UW-918/2020|2020-07-10|EPI_ISL_509919|Original|hCoV-19^^Wisconsin|Human|University of Wisconsin-Madison AIDS Vaccine Research Laboratories|University of Wisconsin-Madison AIDS Vaccine Research Laboratories|Baczenas|USA
>Spike|hCoV-19/England/NORT-291C95/2020|2020-05-11|EPI_ISL_489119|Original|hCoV-19^^England|Human|NU-OMICS DNA Sequencing research facility|Wellcome Sanger Institute for the COVID-19 Genomics UK (COG-UK) consortium|Davies|United Kingdom
>Spike|hCoV-19/Germany/BW-MVZ-LS-04/2020|2020-04-14|EPI_ISL_640222|Original|hCoV-19^^Baden-W\u00fcrttemberg|Human|MVZ Labor\u00e4rzte Singen|MVZ Labor\u00e4rzte Singen|Schmidt|Germany
>Spike|hCoV-19/Japan/PG-6393/2020|2020-08-00|EPI_ISL_689499|Original|hCoV-19^^Japan|Human|Pathogen Genomics Center|Pathogen Genomics Center|Sekizuka|Japan
>Spike|hCoV-19/Luxembourg/LNS4090457/2020|2020-11-29|EPI_ISL_770929|Original|hCoV-19^^Luxembourg|Human|Laboratoire national de sant\u00e9|Laboratoire national de sant\u00e9|Wienecke-Baldacchino|Luxembourg
>Spike|hCoV-19/England/ALDP-9081DF/2020|2020-06-22|EPI_ISL_551465|Original|hCoV-19^^England|Human|Lighthouse Lab in Alderley Park|Wellcome Sanger Institute for the COVID-19 Genomics UK (COG-UK) consortium|Davies|United Kingdom
>Spike|hCoV-19/Australia/NSW2249/2020|2020-03-23|EPI_ISL_500688|Original|hCoV-19^^New South Wales|Human|Area of Virology|Area of Virology|Bull|Australia
>Spike|hCoV-19/USA/CA-CZB-13422/2020|2020-09-03|EPI_ISL_672045|Original|hCoV-19^^California|Human|Alameda County Public Health Lab|Chan-Zuckerberg Biohub|Bhatt|USA
>Spike|hCoV-19/England/MILK-A7A623/2020|2020-10-15|EPI_ISL_606627|Original|hCoV-19^^England|Human|Lighthouse Lab in Milton Keynes|Wellcome Sanger Institute for the COVID-19 Genomics UK (COG-UK) consortium|Davies|United Kingdom
>Spike|hCoV-19/England/BRIS-131023/2020|2020-03-21|EPI_ISL_501569|Original|hCoV-19^^England|Human|PHE South West Regional Laboratory|Wellcome Sanger Institute for the COVID-19 Genomics UK (COG-UK) consortium|Davies|United Kingdom

>Spike|hCoV-19/USA/UT-UPHL-2009207/2020|2020-05-11|EPI_ISL_525207|Original|hCoV-19^^Utah|Human|Utah Public Health Laboratory|Utah Public Health Laboratory|Young|USA
>Spike|hCoV-19/Denmark/ALAB-SSI-824/2020|2020-03-28|EPI_ISL_437675|Original|hCoV-19^^Denmark|Human|Department of Virus and Microbiological Special Diagnostics|Albertsen lab|Kirkegaard|Denmark
>Spike|hCoV-19/Rwanda/U66912-RD/2020|2020-07-00|EPI_ISL_615063|Original|hCoV-19^^Rwanda|Human|Rwanda National Reference Laboratory|Rwanda National Reference Laboratory|Mukantwari|Rwanda
>Spike|hCoV-19/England/NORW-E85C3/2020|2020-04-18|EPI_ISL_449164|Original|hCoV-19^^England|Human|Quadram Institute Bioscience|COVID-19 Genomics UK (COG-UK) Consortium|Alikhan|United Kingdom
>Spike|hCoV-19/USA/UN-UW-12776/2020|2020-06-29|EPI_ISL_576403|Original|hCoV-19^^USA|Human|UW Virology Lab|UW Virology Lab|Roychoudhury|USA
>Spike|hCoV-19/India/GJ-GBRC311b/2020|2020-06-20|EPI_ISL_495066|Original|hCoV-19^^Gujarat|Human|GAIMS & G K General Hospital|Gujarat Biotechnology Research Centre|Joshi|India
>Spike|hCoV-19/England/204580253/2020|2020-11-05|EPI_ISL_686547|Original|hCoV-19^^England|Human|Respiratory Virus Unit|COVID-19 Genomics UK (COG-UK) Consortium|Platt|United Kingdom
>Spike|hCoV-19/England/QEUH-9F334A/2020|2020-09-24|EPI_ISL_588577|Original|hCoV-19^^England|Human|Lighthouse Lab in Glasgow|Wellcome Sanger Institute for the COVID-19 Genomics UK (COG-UK) consortium|Davies|United Kingdom
>Spike|hCoV-19/England/ALDP-C57DDA/2020|2020-12-13|EPI_ISL_735979|Original|hCoV-19^^England|Human|Lighthouse Lab in Alderley Park|Wellcome Sanger Institute for the COVID-19 Genomics UK (COG-UK) Consortium|Davies|United Kingdom
>Spike|hCoV-19/Scotland/EDB300/2020|2020-03-29|EPI_ISL_425967|Original|hCoV-19^^Scotland|Human|Virology Department|COVID-19 Genomics UK (COG-UK) Consortium|Williams|United Kingdom
>Spike|hCoV-19/Wales/PHWC-489BEC/2020|2020-10-24|EPI_ISL_652467|Original|hCoV-19^^Wales|Human|Wales Specialist Virology Centre Sequencing lab|COVID-19 Genomics UK (COG-UK) Consortium|Moore|United Kingdom
>Spike|hCoV-19/Canada/ON-OICR-SLB2860/2020|2020-04-07|EPI_ISL_538379|Original|hCoV-19^^Ontario|Human|Kingston Health Sciences Centre / Queen's University|Ontario Institute for Cancer Research|de Borja|Canada
>Spike|hCoV-19/USA/CA-ALSR-3048/2020|2020-07-31|EPI_ISL_635813|Original|hCoV-19^^California|Human|San Diego County Public Health Laboratory|Andersen lab at Scripps Research|Gangavarapu|USA
>Spike|hCoV-19/England/QEUH-9F87EF/2020|2020-09-27|EPI_ISL_588998|Original|hCoV-19^^England|Human|Lighthouse Lab in Glasgow|Wellcome Sanger Institute for the COVID-19 Genomics UK (COG-UK) consortium|Davies|United Kingdom
>Spike|hCoV-19/USA/FL-BPHL-1847/2020|2020-04-09|EPI_ISL_613927|Original|hCoV-19^^Florida|Human|Florida Bureau of Public Health Laboratories|Florida Bureau of Public Health Laboratories|Blanton|USA
>Spike|hCoV-19/England/SHEF-D2B33/2020|2020-09-11|EPI_ISL_559946|Original|hCoV-19^^England|Human|Virology Department|COVID-19 Genomics UK (COG-UK) Consortium|de Silva|United Kingdom
>Spike|hCoV-19/Scotland/EDB733/2020|2020-04-02|EPI_ISL_433232|Original|hCoV-19^^Scotland|Human|Virology Department|COVID-19 Genomics UK (COG-UK) Consortium|Williams|United Kingdom
>Spike|hCoV-19/USA/LA-EVTL456/2020|2020-06-22|EPI_ISL_578860|Original|hCoV-19^^Louisiana|Human|LSUHS Emerging Viral Threat Laboratory|Microbial Genome Sequencing Center|Kamil|USA
>Spike|hCoV-19/Portugal/PT1862/2020|2020-11-09|EPI_ISL_732081|Original|hCoV-19^^Portugal|Human|Instituto Nacional de Saude (INSA)|Instituto Nacional de Saude (INSA)|Borges|Portugal
>Spike|hCoV-19/Scotland/QEUH-960F44/2020|2020-08-05|EPI_ISL_532004|Original|hCoV-19^^Scotland|Human|NHSGGC West of Scotland Specialist Virology Centre / MRC-University of Glasgow Centre for Virus Research|Wellcome Sanger Institute for the COVID-19 Genomics UK (COG-UK) consortium|Davies|United Kingdom

>Spike|hCoV-19/Russia/MOS-CRIE-13378928/2020|2020-11-06|EPI_ISL_732664|Original|hCoV-19^^Moscow|Human|Molecular diagnostic laboratory of Federal Budget Institution of Science \Central Research Institute of Epidemiology\ of The Federal Service on Customers' Rights Protection and Human Well-being Surveillance|Group of Genomics and Postgenomic Technologies of Central Research Institute of Epidemilolog
>y|Korneenko|Russia
>Spike|hCoV-19/England/CAMC-B37384/2020|2020-11-05|EPI_ISL_645361|Original|hCoV-19^^England|Human|Lighthouse Lab in Cambridge|Wellcome Sanger Institute for the COVID-19 Genomics UK (COG-UK) Consortium|Davies|United Kingdom
>Spike|hCoV-19/England/20144023404/2020|2020-03-27|EPI_ISL_423526|Original|hCoV-19^^England|Human|Respiratory Virus Unit|Respiratory Virus Unit|Platt|United Kingdom
>Spike|hCoV-19/Sweden/20-07616/2020|2020-04-20|EPI_ISL_434676|Original|hCoV-19^^Stockholm|Human|Surbrunns VC|The Public Health Agency of Sweden|Svartstr\u00f6m|Sweden
>Spike|hCoV-19/Netherlands/GE-EMC-166/2020|2020-05-22|EPI_ISL_523149|Original|hCoV-19^^Gelderland|Human|Dutch COVID-19 response team|Erasmus Medical Center|Oude Munnink|Netherlands
>Spike|hCoV-19/France/ARA-SC139/2020|2020-04-27|EPI_ISL_700403|Original|hCoV-19^^Auvergne-Rh\u00f4ne-Alpes|Human|Centre hospitalier M\u00e9tropole Savoie|CNR Virus des Infections Respiratoires - France SUD|Bal|France
>Spike|hCoV-19/Luxembourg/LNS4281216/2020|2020-10-26|EPI_ISL_740443|Original|hCoV-19^^Luxembourg|Human|Laboratoire national de sant\u00e9|Laboratoire national de sant\u00e9|Wienecke-Baldacchino|Luxembourg
>Spike|hCoV-19/Denmark/DCGC-2281/2020|2020-08-10|EPI_ISL_617639|Original|hCoV-19^^NA|Human|Department of Virus and Microbiological Special Diagnostics|Albertsen lab|Michaelsen|Denmark
>Spike|hCoV-19/England/ALDP-9E72E2/2020|2020-09-24|EPI_ISL_580406|Original|hCoV-19^^England|Human|Lighthouse Lab in Alderley Park|Wellcome Sanger Institute for the COVID-19 Genomics UK (COG-UK) consortium|Davies|United Kingdom
>Spike|hCoV-19/England/NORT-292924/2020|2020-05-31|EPI_ISL_472273|Original|hCoV-19^^England|Human|Northumbria University / South Tees Hospitals NHS Foundation Trust / North Cumbria Integrated Care NHS Foundation Trust / North Tees and Hartlepool NHS Foundation Trust / Newcastle Hospitals NHS Foundation Trust|COVID-19 Genomics UK (COG-UK) Consortium|Bashton|United Kingdom
>Spike|hCoV-19/Canada/ON-UHTC-0289/2020|2020-07-28|EPI_ISL_569990|Original|hCoV-19^^Ontario|Human|Unity Health Toronto|Ontario Institute for Cancer Research|de Borja|Canada
>Spike|hCoV-19/Greece/234_31670/2020|2020-03-05|EPI_ISL_437894|Original|hCoV-19^^Athens|Human|Laboratory of Microbiology|Laboratory of Biology|Karakasiliotis|Greece
>Spike|hCoV-19/Denmark/DCGC-18147/2020|2020-12-14|EPI_ISL_749780|Original|hCoV-19^^Sjaelland|Human|Department of Virus and Microbiological Special Diagnostics|Albertsen Lab|Michaelsen|Denmark
>Spike|hCoV-19/England/LIVE-A43AB/2020|2020-06-17|EPI_ISL_490420|Original|hCoV-19^^England|Human|Liverpool Clinical Laboratories|COVID-19 Genomics UK (COG-UK) Consortium|Haldenby|United Kingdom
>Spike|hCoV-19/USA/TX-DSHS-0360/2020|2020-06-13|EPI_ISL_538297|Original|hCoV-19^^Texas|Human|Texas Department of State Health Services|Texas Department of State Health Services|Rahman|USA
>Spike|hCoV-19/USA/VA-DCLS-1556/2020|2020-08-08|EPI_ISL_610120|Original|hCoV-19^^Virginia|Human|Virginia DCLS|Virginia DCLS|St. Jacques|USA
>Spike|hCoV-19/USA/WA-UW-24459/2020|2020-08-14|EPI_ISL_776688|Original|hCoV-19^^Washington|Human|UW Virology Lab|UW Virology Lab|Roychoudhury|USA
>Spike|hCoV-19/USA/FL-BPHL-1418/2020|2020-08-13|EPI_ISL_549200|Original|hCoV-19^^Florida|Human|Florida Bureau of Public Health Laboratories|Florida Bureau of Public Health Laboratories|Blanton|USA
>Spike|hCoV-19/Wales/PHWC-499F0B/2020|2020-11-24|EPI_ISL_727465|Original|hCoV-19^^Wales|Human|Originating lab|COVID-19 Genomics UK (COG-UK) Consortium|Moore|United Kingdom

>Spike|hCoV-19/USA/WA-S2300/2020|2020-06-12|EPI_ISL_496932|Original|hCoV-19^^Washington|Human|Washington State Department of Health|Seattle Flu Study|Lee|USA
>Spike|hCoV-19/England/ALDP-A68FF0/2020|2020-10-12|EPI_ISL_608919|Original|hCoV-19^^England|Human|Lighthouse Lab in Alderley Park|Wellcome Sanger Institute for the COVID-19 Genomics UK (COG-UK) consortium|Davies|United Kingdom
>Spike|hCoV-19/England/QEUH-B2C08B/2020|2020-11-04|EPI_ISL_680051|Original|hCoV-19^^England|Human|Department of Pathology|COVID-19 Genomics UK (COG-UK) Consortium|Goodfellow|United Kingdom
>Spike|hCoV-19/USA/UT-UPHL-03109/2020|2020-05-04|EPI_ISL_575258|Original|hCoV-19^^Utah|Human|Utah Public Health Laboratory|Utah Public Health Laboratory|Young|USA
>Spike|hCoV-19/England/ALDP-AA22A6/2020|2020-10-22|EPI_ISL_625206|Original|hCoV-19^^England|Human|Lighthouse Lab in Alderley Park|Wellcome Sanger Institute for the COVID-19 Genomics UK (COG-UK) consortium|Davies|United Kingdom
>Spike|hCoV-19/Japan/PG-5133/2020|2020-08-00|EPI_ISL_690240|Original|hCoV-19^^Japan|Human|Gunma Prefectural Institute of Public Health and Environmental Sciences|Pathogen Genomics Center|Sekizuka|Japan
>Spike|hCoV-19/Scotland/EDB8778/2020|2020-10-26|EPI_ISL_650280|Original|hCoV-19^^Scotland|Human|Virology Department|COVID-19 Genomics UK (COG-UK) Consortium|McHugh|United Kingdom
>Spike|hCoV-19/USA/MI-UM-10036861068/2020|2020-11-09|EPI_ISL_728506|Original|hCoV-19^^Michigan|Human|University of Michigan Clinical Microbiology Laboratory|Lauring Lab|Valesano|USA
>Spike|hCoV-19/England/ALDP-6CE985/2020|2020-07-02|EPI_ISL_556292|Original|hCoV-19^^England|Human|Lighthouse Lab in Alderley Park|Wellcome Sanger Institute for the COVID-19 Genomics UK (COG-UK) consortium|Davies|United Kingdom
>Spike|hCoV-19/Denmark/DCGC-8603/2020|2020-10-26|EPI_ISL_625703|Original|hCoV-19^^Syddanmark|Human|Department of Virus and Microbiological Special Diagnostics|Albertsen lab|Michaelsen|Denmark
>Spike|hCoV-19/England/ALDP-B84133/2020|2020-11-14|EPI_ISL_675388|Original|hCoV-19^^England|Human|Lighthouse Lab in Alderley Park|Wellcome Sanger Institute for the COVID-19 Genomics UK (COG-UK) Consortium|Davies|United Kingdom
>Spike|hCoV-19/Canada/BC-BCCDC-7559/2020|2020-11-00|EPI_ISL_740721|Original|hCoV-19^^British Columbia|Human|BCCDC Public Health Laboratory|BCCDC Public Health Laboratory|Prystajecky|Canada
>Spike|hCoV-19/Australia/VIC2474/2020|2020-06-27|EPI_ISL_522177|Original|hCoV-19^^Victoria|Human|Victorian Infectious Diseases Reference Laboratory (VIDRL)|VIDRL and MDU-PHL|Schultz|Australia
>Spike|hCoV-19/USA/OR-OHSU-2895/2020|2020-09-04|EPI_ISL_667395|Original|hCoV-19^^Oregon|Human|OHSU Lab Services Molecular Microbiology Lab|Oregon SARS-CoV-2 Genome Sequencing Center|O'Roak|USA
>Spike|hCoV-19/Italy/ABR-IZSGC-TE4953/2020|2020-03-14|EPI_ISL_418258|Original|hCoV-19^^Abruzzo|Human|Presidio ospedaliero \Santo Spirito\|Istituto Zooprofilattico Sperimentale dell'Abruzzo e Molise \G. Caporale\|Lorusso|Italy
>Spike|hCoV-19/Belgium/Rega-0330248/2020|2020-03-30|EPI_ISL_458218|Original|hCoV-19^^Bury|Human|KU Leuven|KU Leuven|Maes|Belgium
>Spike|hCoV-19/Luxembourg/LNS0511956/2020|2020-10-30|EPI_ISL_739794|Original|hCoV-19^^Luxembourg|Human|Laboratoire national de sant\u00e9|Laboratoire national de sant\u00e9|Wienecke-Baldacchino|Luxembourg
>Spike|hCoV-19/England/ALDP-C48686/2020|2020-12-13|EPI_ISL_736517|Original|hCoV-19^^England|Human|Lighthouse Lab in Alderley Park|Wellcome Sanger Institute for the COVID-19 Genomics UK (COG-UK) Consortium|Davies|United Kingdom
>Spike|hCoV-19/Shanghai/SH0088/2020|2020-02-01|EPI_ISL_416387|Original|hCoV-19^^Shanghai|Human|Shanghai Public Health Clinical Center|National Research Center for Translational Medicine (Shanghai)|Zhang|China
>Spike|hCoV-19/USA/NY-QDX-2701/2020|2020-03-15|EPI_ISL_605043|Original|hCoV-19^^New York|Human|Quest Diagnostics|Quest Diagnostics|Kagan|USA
>Spike|hCoV-19/Germany/SH-ChVir9991/2020|2020-11-04|EPI_ISL_729665|Original|hCoV-19^^Schleswig-Holstein|Human|A. Krumbholz|Charit\u00e9 Universit\u00e4tsmedizin Berlin|Muehlemann|Germany

>Spike|hCoV-19/England/ALDP-BB4F21/2020|2020-11-20|EPI_ISL_702388|Original|hCoV-19^^England|Human|Lighthouse Lab in Alderley Park|Wellcome Sanger Institute for the COVID-19 Genomics UK (COG-UK) Consortium|Davies|United Kingdom
>Spike|hCoV-19/USA/CA-CZB-14110/2020|2020-05-27|EPI_ISL_739171|Original|hCoV-19^^California|Human|Alameda County Public Health Lab|Chan-Zuckerberg Biohub|Kamm|USA
>Spike|hCoV-19/England/NORT-1B6C013/2020|2020-09-30|EPI_ISL_611746|Original|hCoV-19^^England|Human|Northumbria University / South Tees Hospitals NHS Foundation Trust / North Cumbria Integrated Care NHS Foundation Trust / North Tees and Hartlepool NHS Foundation Trust / Newcastle Hospitals NHS Foundation Trust|COVID-19 Genomics UK (COG-UK) Consortium|Bashton|United Kingdom
>Spike|hCoV-19/USA/TX-HMH-MCoV-18062/2020|2020-11-17|EPI_ISL_784410|Original|hCoV-19^^Texas|Human|Houston Methodist Hospital|Houston Methodist Hospital|Long|USA
>Spike|hCoV-19/India/MH-MW18/2020|2020-05-08|EPI_ISL_508426|Original|hCoV-19^^Maharashtra|Human|Mahatma Gandhi Institute of Medical Sciences|National Institute of Biomedical Genomics|Maitra|India
>Spike|hCoV-19/Wales/PHWC-494A0F/2020|2020-11-22|EPI_ISL_726203|Original|hCoV-19^^Wales|Human|Originating lab|COVID-19 Genomics UK (COG-UK) Consortium|Moore|United Kingdom
>Spike|hCoV-19/Switzerland/VD-ETHZ-100835/2020|2020-03-25|EPI_ISL_451783|Original|hCoV-19^^Vaud|Human|Viollier AG|Department of Biosystems Science and Engineering|Nadeau|Switzerland
>Spike|hCoV-19/USA/MI-MDHHS-SC21681/2020|2020-06-29|EPI_ISL_516408|Original|hCoV-19^^Michigan|Human|Michigan Department of Health and Human Services|Michigan Department of Health and Human Services|Blankenship|USA
>Spike|hCoV-19/England/LOND-D3D87/2020|2020-04-04|EPI_ISL_449650|Original|hCoV-19^^England|Human|University College London|COVID-19 Genomics UK (COG-UK) Consortium|Roy|United Kingdom
>Spike|hCoV-19/Australia/VIC3348/2020|2020-07-08|EPI_ISL_521027|Original|hCoV-19^^Victoria|Human|Microbiological Diagnostic Unit - Public Health Laboratory (MDU-PHL)|MDU-PHL|Schultz|Australia
>Spike|hCoV-19/USA/MA-MASPHL-00626/2020|2020-05-12|EPI_ISL_692999|Original|hCoV-19^^Massachusetts|Human|Massachusetts State Public Health Laboratory|Massachusetts State Public Health Laboratory|Lang|USA
>Spike|hCoV-19/England/ALDP-A2EE26/2020|2020-10-05|EPI_ISL_586996|Original|hCoV-19^^England|Human|Lighthouse Lab in Alderley Park|Wellcome Sanger Institute for the COVID-19 Genomics UK (COG-UK) consortium|Davies|United Kingdom
>Spike|hCoV-19/Japan/PG-1320/2020|2020-04-00|EPI_ISL_684928|Original|hCoV-19^^Japan|Human|Saitama Prefectural Institute of Public Health|Pathogen Genomics Center|Sekizuka|Japan
>Spike|hCoV-19/Wales/PHWC-3603E/2020|2020-04-28|EPI_ISL_474609|Original|hCoV-19^^Wales|Human|Originating lab|COVID-19 Genomics UK (COG-UK) Consortium|Moore|United Kingdom
>Spike|hCoV-19/England/CAMB-1BD511/2020|2020-12-01|EPI_ISL_764312|Original|hCoV-19^^England|Human|Department of Pathology|COVID-19 Genomics UK (COG-UK) Consortium|Goodfellow|United Kingdom
>Spike|hCoV-19/England/CAMB-78A14/2020|2020-00-00|EPI_ISL_433807|Original|hCoV-19^^England|Human|Department of Pathology|COVID-19 Genomics UK (COG-UK) Consortium|Meredith|United Kingdom
>Spike|hCoV-19/USA/CA-CZB-1213/2020|2020-03-23|EPI_ISL_450462|Original|hCoV-19^^California|Human|Stanford clinical virology lab|Chan-Zuckerberg Biohub|Hao|USA
>Spike|hCoV-19/Spain/CT-IBV-99009872/2020|2020-07-31|EPI_ISL_660269|Original|hCoV-19^^Catalunya|Human|Servicio de Microbiolog\u00eda|SeqCOVID-SPAIN consortium/IBV(CSIC)|Comas|Spain
>Spike|hCoV-19/India/GJ-GBRC216a/2020|2020-06-08|EPI_ISL_475050|Original|hCoV-19^^Gujarat|Human|GMERS Medical College & Hospital|Gujarat Biotechnology Research Centre|Joshi|India
>Spike|hCoV-19/England/20122018304/2020|2020-03-16|EPI_ISL_465744|Original|hCoV-19^^England|Human|Respiratory Virus Unit|Respiratory Virus Unit|Platt|United Kingdom
>Spike|hCoV-19/USA/TX-HMH-MCoV-8179/2020|2020-06-30|EPI_ISL_784248|Original|hCoV-19^^Texas|Human|Houston Methodist Hospital|Houston Methodist Hospital|Long|USA

>Spike|hCoV-19/Denmark/DCGC-8437/2020|2020-10-26|EPI_ISL_617909|Original|hCoV-19^^Syddanmark|Human|Department of Virus and Microbiological Special Diagnostics|Albertsen lab|Michaelsen|Denmark
>Spike|hCoV-19/USA/NY-NYUMC545/2020|2020-03-31|EPI_ISL_444662|Original|hCoV-19^^New York|Human|NYU Langone Health|Departments of Pathology and Medicine|Maurano|USA
>Spike|hCoV-19/Wales/PHWC-164DE4/2020|2020-05-22|EPI_ISL_479383|Original|hCoV-19^^Wales|Human|Wales Specialist Virology Centre Sequencing lab|COVID-19 Genomics UK (COG-UK) Consortium|Moore|United Kingdom
>Spike|hCoV-19/England/CAMC-A57158/2020|2020-10-09|EPI_ISL_597489|Original|hCoV-19^^England|Human|Lighthouse Lab in Cambridge|Wellcome Sanger Institute for the COVID-19 Genomics UK (COG-UK) consortium|Davies|United Kingdom
>Spike|hCoV-19/England/NORT-293D54/2020|2020-07-18|EPI_ISL_573327|Original|hCoV-19^^England|Human|Northumbria University / South Tees Hospitals NHS Foundation Trust / North Cumbria Integrated Care NHS Foundation Trust / North Tees and Hartlepool NHS Foundation Trust / Newcastle Hospitals NHS Foundation Trust|COVID-19 Genomics UK (COG-UK) Consortium|Bashton|United Kingdom
>Spike|hCoV-19/Ireland/TA-NVRL-78IRL62510/2020|2020-11-22|EPI_ISL_752532|Original|hCoV-19^^Tipperary|Human|National Virus Reference Laboratory|National Virus Reference Laboratory|Gonzalez|Ireland
>Spike|hCoV-19/England/ALDP-9CD65F/2020|2020-09-15|EPI_ISL_566328|Original|hCoV-19^^England|Human|Lighthouse Lab in Alderley Park|Wellcome Sanger Institute for the COVID-19 Genomics UK (COG-UK) consortium|Davies|United Kingdom
>Spike|hCoV-19/South Africa/KRISP-K002807/2020|2020-08-25|EPI_ISL_529801|Original|hCoV-19^^KwaZulu-Natal|Human|NHLS-IALCH|KRISP|de Oliveira|South Africa
>Spike|hCoV-19/England/NOTT-1116DC/2020|2020-05-22|EPI_ISL_461909|Original|hCoV-19^^England|Human|Queens Medical Centre|COVID-19 Genomics UK (COG-UK) Consortium|Loose|United Kingdom
>Spike|hCoV-19/USA/UT-08521/2020|2020-05-17|EPI_ISL_524284|Original|hCoV-19^^Utah|Human|Utah Public Health Laboratory|Utah Public Health Laboratory|Young|USA
>Spike|hCoV-19/DRC/11676/2020|2020-05-26|EPI_ISL_527531|Original|hCoV-19^^Kinshasa|Human|Viral Respiratory Lab|Pathogen Sequencing Lab|Lokilo|Democratic Republic of the Congo
>Spike|hCoV-19/USA/TX-DSHS-0813/2020|2020-06-03|EPI_ISL_590874|Original|hCoV-19^^Texas|Human|Texas Department of State Health Services|Texas Department of State Health Services|Rahman|USA
>Spike|hCoV-19/Scotland/QEUH-A4CEC8/2020|2020-10-05|EPI_ISL_596063|Original|hCoV-19^^Scotland|Human|Quadram Institute Bioscience|COVID-19 Genomics UK (COG-UK) Consortium|Alikhan|United Kingdom
>Spike|hCoV-19/Denmark/DCGC-18747/2020|2020-12-07|EPI_ISL_749354|Original|hCoV-19^^Midtjylland|Human|Department of Virus and Microbiological Special Diagnostics|Albertsen Lab|Michaelsen|Denmark
>Spike|hCoV-19/Netherlands/NA_181/2020|2020-03-22|EPI_ISL_422716|Original|hCoV-19^^Netherlands|Human|Dutch COVID-19 response team|Erasmus Medical Center|Oude Munnink|Netherlands
>Spike|hCoV-19/USA/CA-CZB-4068/2020|2020-08-05|EPI_ISL_548668|Original|hCoV-19^^California|Human|Orange County Public Health Laboratory|Chan-Zuckerberg Biohub|Bhatt|USA
>Spike|hCoV-19/Ukraine/203100338/2020|2020-07-10|EPI_ISL_512617|Original|hCoV-19^^Ukraine|Human|National Laboratory for Influenza/Virology reference laboratory|Respiratory Virus Unit|Platt|Ukraine
>Spike|hCoV-19/England/QEUH-A9FEA8/2020|2020-10-20|EPI_ISL_665397|Original|hCoV-19^^England|Human|Northumbria University / South Tees Hospitals NHS Foundation Trust / North Cumbria Integrated Care NHS Foundation Trust / North Tees and Hartlepool NHS Foundation Trust / Newcastle Hospitals NHS Foundation Trust|COVID-19 Genomics UK (COG-UK) Consortium|Bashton|United Kingdom
>Spike|hCoV-19/USA/MA-MASPHL-01035/2020|2020-11-17|EPI_ISL_692778|Original|hCoV-19^^Massachusetts|Human|Massachusetts State Public Health Laboratory|Massachusetts State Public Health Laboratory|Lang|USA

>Spike|hCoV-19/England/ALDP-5AF9B8/2020|2020-06-19|EPI_ISL_558799|Original|hCoV-19^^England|Human|Lighthouse Lab in Alderley Park|Wellcome Sanger Institute for the COVID-19 Genomics UK (COG-UK) consortium|Davies|United Kingdom
>Spike|hCoV-19/Gambia/NPHL-2529/2020|2020-07-21|EPI_ISL_561269|Original|hCoV-19^^Kombo|Human|MRCG at LSHTM Genomics lab|MRCG at LSHTM Genomics lab|Sesay|Gambia
>Spike|hCoV-19/England/BRIS-12175E/2020|2020-03-02|EPI_ISL_440225|Original|hCoV-19^^England|Human|PHE South West Regional Laboratory|Wellcome Sanger Institute for the COVID-19 Genomics UK (COG-UK) consortium|Amato|United Kingdom
>Spike|hCoV-19/Wales/PHWC-48C378/2020|2020-11-07|EPI_ISL_666422|Original|hCoV-19^^Wales|Human|Wales Specialist Virology Centre Sequencing lab|COVID-19 Genomics UK (COG-UK) Consortium|Moore|United Kingdom
>Spike|hCoV-19/England/QEUH-B822D8/2020|2020-11-14|EPI_ISL_661683|Original|hCoV-19^^England|Human|Lighthouse Lab in Glasgow|Wellcome Sanger Institute for the COVID-19 Genomics UK (COG-UK) Consortium|Davies|United Kingdom
>Spike|hCoV-19/mink/Netherlands/NB-EMC-31-6/2020|2020-08-15|EPI_ISL_577760|Original|hCoV-19^^Noord Brabant|Neovison vison|Dutch COVID-19 response team|Erasmus Medical Center|Oude Munnink|Netherlands
>Spike|hCoV-19/England/205260436/2020|2020-00-00|EPI_ISL_766548|Original|hCoV-19^^England|Human|Respiratory Virus Unit|COVID-19 Genomics UK (COG-UK) Consortium|Platt|United Kingdom
>Spike|hCoV-19/Australia/VIC205/2020|2020-03-19|EPI_ISL_419900|Original|hCoV-19^^Victoria|Human|Victorian Infectious Diseases Reference Laboratory (VIDRL)|Victorian Infectious Diseases Reference Laboratory and Microbiological Diagnostic Unit Public Health Laboratory|Schultz|Australia
>Spike|hCoV-19/England/MILK-9A862D/2020|2020-09-02|EPI_ISL_550203|Original|hCoV-19^^England|Human|Lighthouse Lab in Milton Keynes|Wellcome Sanger Institute for the COVID-19 Genomics UK (COG-UK) consortium|Davies|United Kingdom
>Spike|hCoV-19/USA/UT-02548/2020|2020-04-23|EPI_ISL_493119|Original|hCoV-19^^Utah|Human|Utah Public Health Laboratory|Utah Public Health Laboratory|Butz|USA
>Spike|hCoV-19/USA/TX-HMH-5996/2020|2020-06-27|EPI_ISL_547153|Original|hCoV-19^^Texas|Human|Houston Methodist Hospital|Houston Methodist Hospital|Long|USA
>Spike|hCoV-19/England/MILK-9C22DE/2020|2020-09-11|EPI_ISL_566570|Original|hCoV-19^^England|Human|Lighthouse Lab in Milton Keynes|Wellcome Sanger Institute for the COVID-19 Genomics UK (COG-UK) consortium|Davies|United Kingdom
>Spike|hCoV-19/Denmark/DCGC-19528/2020|2020-12-07|EPI_ISL_749009|Original|hCoV-19^^Midtjylland|Human|Department of Virus and Microbiological Special Diagnostics|Albertsen Lab|Michaelsen|Denmark
>Spike|hCoV-19/USA/CT-Yale-465/2020|2020-09-12|EPI_ISL_729736|Original|hCoV-19^^Connecticut|Human|Connecticut Department of Health|Grubaugh Lab - Yale School of Public Health|Grubaugh|USA
>Spike|hCoV-19/Portugal/PT0117/2020|2020-03-25|EPI_ISL_453833|Original|hCoV-19^^Portugal|Human|unknown|Instituto Nacional de Saude (INSA)|Borges|Portugal
>Spike|hCoV-19/Denmark/DCGC-9995/2020|2020-11-09|EPI_ISL_669319|Original|hCoV-19^^Midtjylland|Human|Department of Virus and Microbiological Special Diagnostics|Albertsen Lab|Michaelsen|Denmark
>Spike|hCoV-19/South Korea/KCDC2354/2020|2020-05-28|EPI_ISL_514757|Original|hCoV-19^^South Korea|Human|Division of Viral Diseases|Division of Viral Diseases|Kim|South Korea
>Spike|hCoV-19/Australia/NSW265/2020|2020-03-28|EPI_ISL_451530|Original|hCoV-19^^New South Wales|Human|Pathology Sydney South West - NSW Health Pathology|NSW Health Pathology - Institute of Clinical Pathology and Medical Research; Westmead Hospital; University of Sydney|Gall|Australia
>Spike|hCoV-19/England/QEUH-963521/2020|2020-08-10|EPI_ISL_531580|Original|hCoV-19^^England|Human|Lighthouse Lab in Glasgow|Wellcome Sanger Institute for the COVID-19 Genomics UK (COG-UK) consortium|Davies|United Kingdom
>Spike|hCoV-19/England/ALDP-B1B8C3/2020|2020-11-01|EPI_ISL_646000|Original|hCoV-19^^England|Human|Lighthouse Lab in Alderley Park|Wellcome Sanger Institute for the COVID-19 Genomics UK (COG-UK) Consortium|Davies|United Kingdom

>Spike|hCoV-19/England/QEUH-AE2A93/2020|2020-10-27|EPI_ISL_633583|Original|hCoV-19^^England|Human|Lighthouse Lab in Glasgow|Wellcome Sanger Institute for the COVID-19 Genomics UK (COG-UK) consortium|Davies|United Kingdom
>Spike|hCoV-19/USA/CA-ALSR-4126/2020|2020-07-06|EPI_ISL_648835|Original|hCoV-19^^California|Human|San Diego County Public Health Laboratory|Andersen lab at Scripps Research|Gangavarapu|USA
>Spike|hCoV-19/USA/VA-DCLS-0631/2020|2020-04-09|EPI_ISL_495304|Original|hCoV-19^^Virginia|Human|Virginia DCLS|Virginia DCLS|St. Jacques|USA
>Spike|hCoV-19/USA/VA-DCLS-2285/2020|2020-10-30|EPI_ISL_723518|Original|hCoV-19^^Virginia|Human|Virginia Division of Consolidated Laboratory Services (DCLS)|Virginia Division of Consolidated Laboratory Services (DCLS)|St. Jacques|USA
>Spike|hCoV-19/England/SHEF-D186A/2020|2020-05-27|EPI_ISL_535094|Original|hCoV-19^^England|Human|Virology Department|COVID-19 Genomics UK (COG-UK) Consortium|de Silva|United Kingdom
>Spike|hCoV-19/Ireland/TA-UHL11/2020|2020-03-31|EPI_ISL_681764|Original|hCoV-19^^Tipperary|Human|University Hospital Limerick|Irish Coronavirus Sequencing Consortium - Teagasc Moorepark|Walsh|Ireland
>Spike|hCoV-19/England/205120686/2020|2020-12-15|EPI_ISL_766310|Original|hCoV-19^^England|Human|Respiratory Virus Unit|COVID-19 Genomics UK (COG-UK) Consortium|Platt|United Kingdom
>Spike|hCoV-19/Ireland/WD-NVRL-20IRL03398/2020|2020-08-17|EPI_ISL_578240|Original|hCoV-19^^Waterford|Human|National Virus Reference Laboratory|National Virus Reference Laboratory|Gonzalez|Ireland
>Spike|hCoV-19/Spain/IB-IBV-002495/2020|2020-03-19|EPI_ISL_467058|Original|hCoV-19^^Illes Balears|Human|Servicio de Microbiolog\u00eda|SeqCOVID-SPAIN consortium/IBV(CSIC)|Comas|Spain
>Spike|hCoV-19/England/ALDP-B73498/2020|2020-11-12|EPI_ISL_662390|Original|hCoV-19^^England|Human|Lighthouse Lab in Alderley Park|Wellcome Sanger Institute for the COVID-19 Genomics UK (COG-UK) Consortium|Davies|United Kingdom
>Spike|hCoV-19/England/ALDP-9447A8/2020|2020-06-07|EPI_ISL_555650|Original|hCoV-19^^England|Human|Lighthouse Lab in Alderley Park|Wellcome Sanger Institute for the COVID-19 Genomics UK (COG-UK) Consortium|Davies|United Kingdom
>Spike|hCoV-19/Russia/NVS-RII-MH2810S/2020|2020-09-25|EPI_ISL_639959|Original|hCoV-19^^Novosibirsk|Human|HELIX LLC|WHO National Influenza Centre Russian Federation|Komissarov|Russia
>Spike|hCoV-19/USA/CA-CZB-15001/2020|2020-11-18|EPI_ISL_738780|Original|hCoV-19^^California|Human|Santa Clara County Public Health Laboratory|Chan-Zuckerberg Biohub|Kamm|USA
>Spike|hCoV-19/USA/TX-HMH-549/2020|2020-04-06|EPI_ISL_544812|Original|hCoV-19^^Texas|Human|Houston Methodist Hospital|Houston Methodist Hospital|Long|USA
>Spike|hCoV-19/England/ALDP-94E5E0/2020|2020-06-17|EPI_ISL_555355|Original|hCoV-19^^England|Human|Lighthouse Lab in Alderley Park|Wellcome Sanger Institute for the COVID-19 Genomics UK (COG-UK) consortium|Davies|United Kingdom
>Spike|hCoV-19/England/205141230/2020|2020-12-16|EPI_ISL_756160|Original|hCoV-19^^England|Human|Respiratory Virus Unit|COVID-19 Genomics UK (COG-UK) Consortium|Miah|United Kingdom
>Spike|hCoV-19/England/CAMC-94BD45/2020|2020-07-17|EPI_ISL_556700|Original|hCoV-19^^England|Human|Lighthouse Lab in Cambridge|Wellcome Sanger Institute for the COVID-19 Genomics UK (COG-UK) consortium|Davies|United Kingdom
>Spike|hCoV-19/USA/ID-UW-4307/2020|2020-03-31|EPI_ISL_430942|Original|hCoV-19^^Idaho|Human|UW Virology Lab|UW Virology Lab|Roychoudhury|USA
>Spike|hCoV-19/USA/TX-HMH-2148/2020|2020-05-20|EPI_ISL_545144|Original|hCoV-19^^Texas|Human|Houston Methodist Hospital|Houston Methodist Hospital|Long|USA
>Spike|hCoV-19/England/ALDP-A50B9D/2020|2020-10-07|EPI_ISL_597873|Original|hCoV-19^^England|Human|Lighthouse Lab in Alderley Park|Wellcome Sanger Institute for the COVID-19 Genomics UK (COG-UK) consortium|Davies|United Kingdom
>Spike|hCoV-19/Scotland/QEUH-9CF30B/2020|2020-09-17|EPI_ISL_601910|Original|hCoV-19^^Scotland|Human|Lighthouse Lab in Glasgow|Wellcome Sanger Institute for the COVID-19 Genomics UK (COG-UK) consortium|Davies|United Kingdom

>Spike|hCoV-19/England/EXET-13511D/2020|2020-04-06|EPI_ISL_457033|Original|hCoV-19^^England|Human|University of Exeter|COVID-19 Genomics UK (COG-UK) Consortium|Temperton|United Kingdom
>Spike|hCoV-19/Netherlands/ZH-EMC-448/2020|2020-08-12|EPI_ISL_523786|Original|hCoV-19^^Zuid Holland|Human|Dutch COVID-19 response team|Erasmus Medical Center|Oude Munnink|Netherlands
>Spike|hCoV-19/USA/NM-UNM-00814/2020|2020-05-13|EPI_ISL_610257|Original|hCoV-19^^New Mexico|Human|TriCore Reference Laboratories|Center for Global Health|Domman|USA
>Spike|hCoV-19/Scotland/CVR2134/2020|2020-04-10|EPI_ISL_453022|Original|hCoV-19^^Scotland|Human|West of Scotland Specialist Virology Centre|COVID-19 Genomics UK (COG-UK) Consortium|Robertson|United Kingdom
>Spike|hCoV-19/Australia/VIC5828/2020|2020-07-22|EPI_ISL_518622|Original|hCoV-19^^Victoria|Human|Microbiological Diagnostic Unit - Public Health Laboratory (MDU-PHL)|MDU-PHL|Schultz|Australia
>Spike|hCoV-19/England/SHEF-CB0E3/2020|2020-04-19|EPI_ISL_462048|Original|hCoV-19^^England|Human|Virology Department|COVID-19 Genomics UK (COG-UK) Consortium|de Silva|United Kingdom
>Spike|hCoV-19/Hong Kong/VM20003891/2020|2020-03-19|EPI_ISL_463314|Original|hCoV-19^^Hong Kong|Human|Queen Elizabeth Hospital|Hong Kong Department of Health|Mak|China
>Spike|hCoV-19/Denmark/DCGC-2777/2020|2020-08-24|EPI_ISL_616612|Original|hCoV-19^^Midtjylland|Human|Department of Virus and Microbiological Special Diagnostics|Albertsen lab|Michaelsen|Denmark
>Spike|hCoV-19/Wales/PHWC-4A0E5F/2020|2020-12-10|EPI_ISL_743584|Original|hCoV-19^^Wales|Human|Wales Specialist Virology Centre Sequencing lab|COVID-19 Genomics UK (COG-UK) Consortium|Moore|United Kingdom
>Spike|hCoV-19/England/CAMB-7C193/2020|2020-04-14|EPI_ISL_434004|Original|hCoV-19^^England|Human|Department of Pathology|COVID-19 Genomics UK (COG-UK) Consortium|Meredith|United Kingdom
>Spike|hCoV-19/USA/MI-MDHHS-SC20129/2020|2020-03-10|EPI_ISL_436802|Original|hCoV-19^^Michigan|Human|Michigan Department of Health and Human Services|Michigan Department of Health and Human Services|Blankenship|USA
>Spike|hCoV-19/USA/UT-UPHL-2012203505/2020|2020-08-11|EPI_ISL_745479|Original|hCoV-19^^Utah|Human|Ginkgo Bioworks Clinical Laboratory|Utah Public Health Laboratory|Young|USA
>Spike|hCoV-19/USA/TX-HMH-4291/2020|2020-06-19|EPI_ISL_543501|Original|hCoV-19^^Texas|Human|Houston Methodist Hospital|Houston Methodist Hospital|Long|USA
>Spike|hCoV-19/England/QEUH-9F513F/2020|2020-09-27|EPI_ISL_638657|Original|hCoV-19^^England|Human|Department of Pathology|COVID-19 Genomics UK (COG-UK) Consortium|Goodfellow|United Kingdom
>Spike|hCoV-19/Denmark/ALAB-HH-144/2020|2020-03-11|EPI_ISL_452023|Original|hCoV-19^^Denmark|Human|Department of Clinical Microbiology|Albertsen lab|Kirkegaard|Denmark
>Spike|hCoV-19/Canada/QC-LSPQ-L00243460/2020|2020-03-30|EPI_ISL_536362|Original|hCoV-19^^Quebec|Human|H\u00f4pital du Suro\u00eet|Laboratoire de sant\u00e9 publique du Qu\u00e9bec|Moreira|Canada
>Spike|hCoV-19/England/QEUH-B82681/2020|2020-11-14|EPI_ISL_661877|Original|hCoV-19^^England|Human|Lighthouse Lab in Glasgow|Wellcome Sanger Institute for the COVID-19 Genomics UK (COG-UK) Consortium|Davies|United Kingdom
>Spike|hCoV-19/Austria/CeMM0562/2020|2020-03-12|EPI_ISL_475912|Original|hCoV-19^^Ischgl|Human|Klinikum Wels-Grieskirchen|Bergthaler laboratory|Bergthaler|Austria
>Spike|hCoV-19/USA/CA-CZB-14088/2020|2020-06-22|EPI_ISL_739164|Original|hCoV-19^^California|Human|Alameda County Public Health Lab|Chan-Zuckerberg Biohub|Kamm|USA
>Spike|hCoV-19/USA/MI-MDHHS-SC20882/2020|2020-05-16|EPI_ISL_471903|Original|hCoV-19^^Michigan|Human|Michigan Department of Health and Human Services|Michigan Department of Health and Human Services|Blankenship|USA
>Spike|hCoV-19/England/MILK-A7D3ED/2020|2020-10-13|EPI_ISL_606403|Original|hCoV-19^^England|Human|Lighthouse Lab in Milton Keynes|Wellcome Sanger Institute for the COVID-19 Genomics UK (COG-UK) consortium|Davies|United Kingdom

>Spike|hCoV-19/England/CAMB-1AA3A4/2020|2020-04-16|EPI_ISL_443538|Original|hCoV-19^^England|Human|Department of Pathology|Wellcome Sanger Institute for the COVID-19 Genomics UK (COG-UK) consortium|Amato|United Kingdom
>Spike|hCoV-19/England/CAMC-B3618B/2020|2020-11-05|EPI_ISL_645440|Original|hCoV-19^^England|Human|Lighthouse Lab in Cambridge|Wellcome Sanger Institute for the COVID-19 Genomics UK (COG-UK) Consortium|Davies|United Kingdom
>Spike|hCoV-19/England/MILK-B05A3F/2020|2020-10-25|EPI_ISL_652215|Original|hCoV-19^^England|Human|Queens Medical Centre|COVID-19 Genomics UK (COG-UK) Consortium|Loose|United Kingdom
>Spike|hCoV-19/England/20132000804/2020|2020-03-23|EPI_ISL_423702|Original|hCoV-19^^England|Human|Respiratory Virus Unit|Respiratory Virus Unit|Platt|United Kingdom
>Spike|hCoV-19/Japan/PG-8739/2020|2020-08-00|EPI_ISL_686335|Original|hCoV-19^^Japan|Human|Pathogen Genomics Center|Pathogen Genomics Center|Sekizuka|Japan
>Spike|hCoV-19/USA/MI-MDHHS-SC20416/2020|2020-03-16|EPI_ISL_452290|Original|hCoV-19^^Michigan|Human|Michigan Department of Health and Human Services|Michigan Department of Health and Human Services|Blankenship|USA
>Spike|hCoV-19/Switzerland/BS-42414282/2020|2020-08-27|EPI_ISL_581991|Original|hCoV-19^^Basel-Stadt|Human|University Hospital Basel|University Hospital Basel|Roloff Handschin|Switzerland
>Spike|hCoV-19/Scotland/QEUH-96D91C/2020|2020-08-20|EPI_ISL_534538|Original|hCoV-19^^Scotland|Human|NHSGGC West of Scotland Specialist Virology Centre / MRC-University of Glasgow Centre for Virus Research|Wellcome Sanger Institute for the COVID-19 Genomics UK (COG-UK) consortium|Davies|United Kingdom
>Spike|hCoV-19/Spain/IB-IBV-99007192/2020|2020-03-08|EPI_ISL_541951|Original|hCoV-19^^Balear Islands|Human|Servicio de Microbiolog\u00eda|SeqCOVID-SPAIN consortium/IBV(CSIC)|Comas|Spain
>Spike|hCoV-19/Japan/PG-4191/2020|2020-07-00|EPI_ISL_692430|Original|hCoV-19^^Japan|Human|Pathogen Genomics Center|Pathogen Genomics Center|Sekizuka|Japan
>Spike|hCoV-19/Denmark/DCGC-10334/2020|2020-11-02|EPI_ISL_669906|Original|hCoV-19^^Nordjylland|Human|Department of Virus and Microbiological Special Diagnostics|Albertsen Lab|Michaelsen|Denmark
>Spike|hCoV-19/USA/MN-MDH-1315/2020|2020-03-24|EPI_ISL_495614|Original|hCoV-19^^Minnesota|Human|Minnesota Department of Health|Minnesota Department of Health|Garfin|USA
>Spike|hCoV-19/England/LIVE-1DD7D9/2020|2020-10-02|EPI_ISL_611600|Original|hCoV-19^^England|Human|Liverpool Clinical Laboratories|COVID-19 Genomics UK (COG-UK) Consortium|Haldenby|United Kingdom
>Spike|hCoV-19/Scotland/GCVR-1722F3/2020|2020-03-30|EPI_ISL_469983|Original|hCoV-19^^Scotland|Human|NHSGGC West of Scotland Specialist Virology Centre / MRC-University of Glasgow Centre for Virus Research|Wellcome Sanger Institute for the COVID-19 Genomics UK (COG-UK) consortium|Davies|United Kingdom
>Spike|hCoV-19/England/EXET-139252/2020|2020-10-26|EPI_ISL_664686|Original|hCoV-19^^England|Human|University of Exeter|COVID-19 Genomics UK (COG-UK) Consortium|Temperton|United Kingdom
>Spike|hCoV-19/Australia/VIC6192/2020|2020-07-22|EPI_ISL_518194|Original|hCoV-19^^Victoria|Human|Victorian Infectious Diseases Reference Laboratory (VIDRL)|VIDRL and MDU-PHL|Schultz|Australia
>Spike|hCoV-19/England/NORW-EFCC7/2020|2020-00-00|EPI_ISL_549379|Original|hCoV-19^^England|Human|Quadram Institute Bioscience|COVID-19 Genomics UK (COG-UK) Consortium|Alikhan|United Kingdom
>Spike|hCoV-19/USA/OR-OHSU-2969/2020|2020-09-07|EPI_ISL_667436|Original|hCoV-19^^Oregon|Human|OHSU Lab Services Molecular Microbiology Lab|Oregon SARS-CoV-2 Genome Sequencing Center|O'Roak|USA
>Spike|hCoV-19/USA/UT-QDX-2491/2020|2020-03-12|EPI_ISL_604853|Original|hCoV-19^^Utah|Human|Quest Diagnostics|Quest Diagnostics|Kagan|USA
>Spike|hCoV-19/England/SHEF-D2C5E/2020|2020-11-10|EPI_ISL_680195|Original|hCoV-19^^England|Human|Virology Department|COVID-19 Genomics UK (COG-UK) Consortium|de Silva|United Kingdom
>Spike|hCoV-19/USA/CA-QDX-1855/2020|2020-03-17|EPI_ISL_604104|Original|hCoV-19^^California|Human|Quest Diagnostics|Quest Diagnostics|Kagan|USA

>Spike|hCoV-19/England/OXON-AF197/2020|2020-03-17|EPI_ISL_448818|Original|hCoV-19^^England|Human|Oxford Viromics|COVID-19 Genomics UK (COG-UK) Consortium|Golubchik|United Kingdom
>Spike|hCoV-19/England/NORW-F9073/2020|2020-11-24|EPI_ISL_705332|Original|hCoV-19^^England|Human|Quadram Institute Bioscience|COVID-19 Genomics UK (COG-UK) Consortium|Alikhan|United Kingdom
>Spike|hCoV-19/USA/CA-ALSR-3087/2020|2020-09-06|EPI_ISL_635839|Original|hCoV-19^^California|Human|San Diego County Public Health Laboratory|Andersen lab at Scripps Research|Gangavarapu|USA
>Spike|hCoV-19/USA/MI-MDHHS-SC22460/2020|2020-11-04|EPI_ISL_708558|Original|hCoV-19^^Michigan|Human|Michigan Department of Health and Human Services|Michigan Department of Health and Human Services|Jacob|USA
>Spike|hCoV-19/Russia/KLU-RII-MH3423S/2020|2020-10-06|EPI_ISL_733144|Original|hCoV-19^^Kaluga|Human|HELIX LLC|WHO National Influenza Centre Russian Federation|Komissarov|Russia
>Spike|hCoV-19/England/CAMC-A6545E/2020|2020-10-11|EPI_ISL_608432|Original|hCoV-19^^England|Human|Lighthouse Lab in Cambridge|Wellcome Sanger Institute for the COVID-19 Genomics UK (COG-UK) consortium|Davies|United Kingdom
>Spike|hCoV-19/Germany/HH-hpi-p1918/2020|2020-03-25|EPI_ISL_776249|Original|hCoV-19^^Hamburg|Human|University Medical Center Hamburg Eppendorf|Heinrich Pette Institute|Robitaille|Germany
>Spike|hCoV-19/England/ALDP-B7529C/2020|2020-11-12|EPI_ISL_661999|Original|hCoV-19^^England|Human|Lighthouse Lab in Alderley Park|Wellcome Sanger Institute for the COVID-19 Genomics UK (COG-UK) Consortium|Davies|United Kingdom
>Spike|hCoV-19/England/MILK-9954FC/2020|2020-08-25|EPI_ISL_552347|Original|hCoV-19^^England|Human|Lighthouse Lab in Milton Keynes|Wellcome Sanger Institute for the COVID-19 Genomics UK (COG-UK) consortium|Davies|United Kingdom
>Spike|hCoV-19/England/MILK-B3E147/2020|2020-11-01|EPI_ISL_706262|Original|hCoV-19^^England|Human|Oxford Viromics|COVID-19 Genomics UK (COG-UK) Consortium|Golubchik|United Kingdom
>Spike|hCoV-19/USA/MI-MDHHS-SC21589/2020|2020-03-26|EPI_ISL_516316|Original|hCoV-19^^Michigan|Human|Michigan Department of Health and Human Services|Michigan Department of Health and Human Services|Blankenship|USA
>Spike|hCoV-19/Wales/PHWC-15C8C0/2020|2020-03-31|EPI_ISL_472552|Original|hCoV-19^^Wales|Human|Wales Specialist Virology Centre Sequencing lab|COVID-19 Genomics UK (COG-UK) Consortium|Moore|United Kingdom
>Spike|hCoV-19/USA/MD-MDH-0361/2020|2020-10-22|EPI_ISL_648297|Original|hCoV-19^^Maryland|Human|MD PHL|MD PHL|Keller|USA
>Spike|hCoV-19/Sweden/RV-FOI-5/2020|2020-03-19|EPI_ISL_549024|Original|hCoV-19^^Vasterbotten|Human|Klinisk mikrobiologi|Unit for Biological Agents|Sjodin|Sweden
>Spike|hCoV-19/USA/CA-ALSR-2929/2020|2020-07-17|EPI_ISL_635736|Original|hCoV-19^^California|Human|San Diego County Public Health Laboratory|Andersen lab at Scripps Research|Gangavarapu|USA
>Spike|hCoV-19/Netherlands/ZH-EMC-1246/2020|2020-00-00|EPI_ISL_763315|Original|hCoV-19^^Zuid Holland|Human|Dutch COVID-19 response team|Erasmus Medical Center|Oude Munnink|Netherlands
>Spike|hCoV-19/USA/NY-Wadsworth-97873-01/2020|2020-05-13|EPI_ISL_676929|Original|hCoV-19^^New York|Human|Wadsworth Center|Wadsworth Center|Lasek-Nesselquist|USA
>Spike|hCoV-19/Northern Ireland/QEUH-963FE7/2020|2020-08-12|EPI_ISL_536886|Original|hCoV-19^^Northern Ireland|Human|Lighthouse Lab in Glasgow|Wellcome Sanger Institute for the COVID-19 Genomics UK (COG-UK) consortium|Davies|United Kingdom
>Spike|hCoV-19/Switzerland/UR-ETHZ-310469/2020|2020-10-07|EPI_ISL_603540|Original|hCoV-19^^Uri|Human|Viollier AG|Department of Biosystems Science and Engineering|Nadeau|Switzerland
>Spike|hCoV-19/Denmark/DCGC-20934/2020|2020-12-21|EPI_ISL_759455|Original|hCoV-19^^Syddanmark|Human|Department of Virus and Microbiological Special Diagnostics|Albertsen Lab|Michaelsen|Denmark

>Spike|hCoV-19/England/NORT-1B79B03/2020|2020-11-25|EPI_ISL_741449|Original|hCoV-19^^England|Human|Northumbria University / South Tees Hospitals NHS Foundation Trust / North Cumbria Integrated Care NHS Foundation Trust / North Tees and Hartlepool NHS Foundation Trust / Newcastle Hospitals NHS Foundation Trust|COVID-19 Genomics UK (COG-UK) Consortium|Bashton|United Kingdom
>Spike|hCoV-19/Japan/PG-3297/2020|2020-04-00|EPI_ISL_691412|Original|hCoV-19^^Japan|Human|Pathogen Genomics Center|Pathogen Genomics Center|Sekizuka|Japan
>Spike|hCoV-19/England/QEUH-C321F7/2020|2020-12-11|EPI_ISL_730831|Original|hCoV-19^^England|Human|Lighthouse Lab in Glasgow|Wellcome Sanger Institute for the COVID-19 Genomics UK (COG-UK) Consortium|Davies|United Kingdom
>Spike|hCoV-19/England/CAMC-B0752D/2020|2020-10-31|EPI_ISL_643820|Original|hCoV-19^^England|Human|Lighthouse Lab in Cambridge|Wellcome Sanger Institute for the COVID-19 Genomics UK (COG-UK) Consortium|Davies|United Kingdom
>Spike|hCoV-19/Denmark/DCGC-15834/2020|2020-11-23|EPI_ISL_714050|Original|hCoV-19^^Hovedstaden|Human|Department of Virus and Microbiological Special Diagnostics|Albertsen Lab|Michaelsen|Denmark
>Spike|hCoV-19/USA/FL-ADNH/2020|2020-06-25|EPI_ISL_513274|Original|hCoV-19^^Florida|Human|University of Miami Immunology and Histocompatibility Laboratory|University of Miami Immunology and Histocompatibility Laboratory|Margolles-Clark|USA
>Spike|hCoV-19/USA/TX-HMH-5712/2020|2020-06-20|EPI_ISL_546962|Original|hCoV-19^^Texas|Human|Houston Methodist Hospital|Houston Methodist Hospital|Long|USA
>Spike|hCoV-19/England/CAMB-7C940/2020|2020-04-05|EPI_ISL_442819|Original|hCoV-19^^England|Human|Department of Pathology|Wellcome Sanger Institute for the COVID-19 Genomics UK (COG-UK) consortium|Amato|United Kingdom
>Spike|hCoV-19/USA/NY-NYUMC455/2020|2020-04-20|EPI_ISL_435521|Original|hCoV-19^^New York|Human|NYU Langone Health|Departments of Pathology and Medicine|Maurano|USA
>Spike|hCoV-19/England/CAMB-1AF55E/2020|2020-05-08|EPI_ISL_448080|Original|hCoV-19^^England|Human|Department of Pathology|COVID-19 Genomics UK (COG-UK) Consortium|Meredith|United Kingdom
>Spike|hCoV-19/Canada/ON-PHL-20-01178/2020|2020-06-00|EPI_ISL_548800|Original|hCoV-19^^Ontario|Human|Public Health Ontario Laboratory|Public Health Ontario Laboratory|Guthrie|Canada
>Spike|hCoV-19/England/CAMC-B22683/2020|2020-11-02|EPI_ISL_647286|Original|hCoV-19^^England|Human|Lighthouse Lab in Cambridge|Wellcome Sanger Institute for the COVID-19 Genomics UK (COG-UK) Consortium|Davies|United Kingdom
>Spike|hCoV-19/France/PAC-MEPHI-2288/2020|2020-10-06|EPI_ISL_644533|Original|hCoV-19^^Provence-Alpes-Cote-d\u2019Azur|Human|MEPHI|MEPHI|Levasseur|France
>Spike|hCoV-19/Australia/VIC14241/2020|2020-08-04|EPI_ISL_592410|Original|hCoV-19^^Victoria|Human|Microbiological Diagnostic Unit - Public Health Laboratory (MDU-PHL)|MDU-PHL|Schultz|Australia
>Spike|hCoV-19/England/NORT-2952AE/2020|2020-03-31|EPI_ISL_488545|Original|hCoV-19^^England|Human|NU-OMICS DNA Sequencing research facility|Wellcome Sanger Institute for the COVID-19 Genomics UK (COG-UK) consortium|Davies|United Kingdom
>Spike|hCoV-19/USA/WA-UW-1860/2020|2020-03-23|EPI_ISL_424289|Original|hCoV-19^^Washington|Human|UW Virology Lab|UW Virology Lab|Roychoudhury|USA
>Spike|hCoV-19/USA/UT-UPHL-2012038111/2020|2020-07-27|EPI_ISL_745705|Original|hCoV-19^^Utah|Human|Ginkgo Bioworks Clinical Laboratory|Utah Public Health Laboratory|Young|USA
>Spike|hCoV-19/France/ARA74567/2020|2020-09-17|EPI_ISL_639978|Original|hCoV-19^^Auvergne-Rhone-Alpes|Human|Centre Hospitalier Saint Joseph Saint Luc|CNR Virus des Infections Respiratoires - France SUD|Josset|France
>Spike|hCoV-19/USA/WI-WSLH-200300/2020|2020-04-17|EPI_ISL_578484|Original|hCoV-19^^Wisconsin|Human|Wisconsin State Laboratory of Hygiene Communicable Disease Division|Wisconsin State Laboratory of Hygiene Communicable Disease Division|Florek|USA
>Spike|hCoV-19/England/QEUH-9A4551/2020|2020-09-01|EPI_ISL_540228|Original|hCoV-19^^England|Human|Lighthouse Lab in Glasgow|Wellcome Sanger Institute for the COVID-19 Genomics UK (COG-UK) consortium|Davies|United Kingdom

>Spike|hCoV-19/Brazil/RJ-00462/2020|2020-07-02|EPI_ISL_717857|Original|hCoV-19^^Rio
de Janeiro|Human|Laboratorio de Virologia Molecular / UFRJ|Bioinformatics Laboratory
/ LNCC|Almeida|Brazil
>Spike|hCoV-19/Scotland/CVR20/2020|2020-03-13|EPI_ISL_425730|Original|hCoV-
19^^Scotland|Human|West of Scotland Specialist Virology Centre|COVID-19 Genomics UK
(COG-UK) Consortium|Robertson|United Kingdom
>Spike|hCoV-19/Scotland/EDB5376/2020|2020-03-31|EPI_ISL_457012|Original|hCoV-
19^^Scotland|Human|Virology Department|COVID-19 Genomics UK (COG-UK)
Consortium|Williams|United Kingdom
>Spike|hCoV-19/USA/TX-HMH-MCoV-19565/2020|2020-11-30|EPI_ISL_783937|Original|hCoV-
19^^Texas|Human|Houston Methodist Hospital|Houston Methodist Hospital|Long|USA
>Spike|hCoV-19/South Africa/LR00077/2020|2020-07-13|EPI_ISL_514388|Original|hCoV-
19^^Gauteng|Human|National Institute for Communicable Diseases of the National Health
Laboratory Service|National Institute for Communicable Diseases of the National
Health Laboratory Service|Allam|South Africa
>Spike|hCoV-19/England/QEUH-AD2F88/2020|2020-10-26|EPI_ISL_629786|Original|hCoV-
19^^England|Human|Lighthouse Lab in Glasgow|Wellcome Sanger Institute for the COVID-
19 Genomics UK (COG-UK) consortium|Davies|United Kingdom
>Spike|hCoV-19/Northern Ireland/QEUH-969DBA/2020|2020-08-
14|EPI_ISL_530809|Original|hCoV-19^^Northern Ireland|Human|Lighthouse Lab in
Glasgow|Wellcome Sanger Institute for the COVID-19 Genomics UK (COG-UK)
consortium|Davies|United Kingdom
>Spike|hCoV-19/USA/TX-HMH0384/2020|2020-03-31|EPI_ISL_434972|Original|hCoV-
19^^Texas|Human|Houston Methodist Hospital|Houston Methodist Hospital|Long|USA
>Spike|hCoV-19/USA/NY-NYUMC539/2020|2020-03-30|EPI_ISL_444656|Original|hCoV-19^^New
York|Human|NYU Langone Health|Departments of Pathology and Medicine|Maurano|USA
>Spike|hCoV-19/England/20154089204/2020|2020-04-05|EPI_ISL_466276|Original|hCoV-
19^^England|Human|Respiratory Virus Unit|Respiratory Virus Unit|Platt|United Kingdom
>Spike|hCoV-19/Czech Republic/NRL_5553/2020|2020-03-18|EPI_ISL_530350|Original|hCoV-
19^^Brno|Human|The National Institute of Public Health|State Veterinary Institute
Prague|Nagy|Czech Republic
>Spike|hCoV-19/England/ALDP-9E5D01/2020|2020-09-24|EPI_ISL_589325|Original|hCoV-
19^^England|Human|Lighthouse Lab in Alderley Park|Wellcome Sanger Institute for the
COVID-19 Genomics UK (COG-UK) consortium|Davies|United Kingdom
>Spike|hCoV-19/England/QEUH-B41378/2020|2020-11-08|EPI_ISL_658642|Original|hCoV-
19^^England|Human|Lighthouse Lab in Glasgow|Wellcome Sanger Institute for the COVID-
19 Genomics UK (COG-UK) Consortium|Davies|United Kingdom
>Spike|hCoV-19/England/QEUH-C347E4/2020|2020-12-11|EPI_ISL_734024|Original|hCoV-
19^^England|Human|Lighthouse Lab in Glasgow|Wellcome Sanger Institute for the COVID-
19 Genomics UK (COG-UK) Consortium|Davies|United Kingdom
>Spike|hCoV-19/England/SHEF-10B30DC/2020|2020-06-04|EPI_ISL_537211|Original|hCoV-
19^^England|Human|Virology Department|Wellcome Sanger Institute for the COVID-19
Genomics UK (COG-UK) consortium|Davies|United Kingdom
>Spike|hCoV-19/England/20124002002/2020|2020-03-17|EPI_ISL_418687|Original|hCoV-
19^^England|Human|Respiratory Virus Unit|Respiratory Virus Unit|Platt|United Kingdom
>Spike|hCoV-19/USA/CA-CZB-3633/2020|2020-07-23|EPI_ISL_548359|Original|hCoV-
19^^California|Human|County of San Luis Obispo Public Health Laboratory|Chan-
Zuckerberg Biohub|Bhatt|USA
>Spike|hCoV-19/USA/WA-S1561/2020|2020-05-26|EPI_ISL_495987|Original|hCoV-
19^^Washington|Human|Washington State Department of Health|Seattle Flu Study|Lee|USA
>Spike|hCoV-19/England/LOND-D50B9/2020|2020-04-15|EPI_ISL_444200|Original|hCoV-
19^^England|Human|University College London|COVID-19 Genomics UK (COG-UK)
Consortium|Roy|United Kingdom
>Spike|hCoV-19/England/ALDP-8DAA09/2020|2020-06-25|EPI_ISL_557096|Original|hCoV-
19^^England|Human|Lighthouse Lab in Alderley Park|Wellcome Sanger Institute for the
COVID-19 Genomics UK (COG-UK) consortium|Davies|United Kingdom
>Spike|hCoV-19/Denmark/DCGC-3269/2020|2020-08-31|EPI_ISL_616265|Original|hCoV-
19^^Hovedstaden|Human|Department of Virus and Microbiological Special
Diagnostics|Albertsen lab|Michaelsen|Denmark

>Spike|hCoV-19/India/OR-ILSCV29666/2020|2020-05-23|EPI_ISL_481130|Original|hCoV-19^^Odisha|Human|Immunogenomics lab|Immunogenomics lab|Raghav|India
>Spike|hCoV-19/Netherlands/ZH-EMC-307/2020|2020-07-04|EPI_ISL_523649|Original|hCoV-19^^Zuid Holland|Human|Dutch COVID-19 response team|Erasmus Medical Center|Oude Munnink|Netherlands
>Spike|hCoV-19/Denmark/DCGC-21656/2020|2020-12-21|EPI_ISL_758290|Original|hCoV-19^^Syddanmark|Human|Department of Virus and Microbiological Special Diagnostics|Albertsen Lab|Michaelsen|Denmark
>Spike|hCoV-19/England/MILK-9A6B8A/2020|2020-09-02|EPI_ISL_550237|Original|hCoV-19^^England|Human|Lighthouse Lab in Milton Keynes|Wellcome Sanger Institute for the COVID-19 Genomics UK (COG-UK) consortium|Davies|United Kingdom
>Spike|hCoV-19/USA/WA-S130/2020|2020-03-13|EPI_ISL_430167|Original|hCoV-19^^Washington|Human|Washington State Department of Health|Seattle Flu Study|Bedford|USA
>Spike|hCoV-19/England/MILK-BDC7A3/2020|2020-11-27|EPI_ISL_709578|Original|hCoV-19^^England|Human|Lighthouse Lab in Milton Keynes|Wellcome Sanger Institute for the COVID-19 Genomics UK (COG-UK) Consortium|Davies|United Kingdom
>Spike|hCoV-19/England/BRIS-124463/2020|2020-04-08|EPI_ISL_492774|Original|hCoV-19^^England|Human|PHE South West Regional Laboratory|Wellcome Sanger Institute for the COVID-19 Genomics UK (COG-UK) consortium|Davies|United Kingdom
>Spike|hCoV-19/Germany/NW-HHU-80/2020|2020-08-16|EPI_ISL_539596|Original|hCoV-19^^North Rhine-Westphalia|Human|ZOTZ KLIMAS MVZ D\u00fcsseldorf-Centrum GbR \u00dcBAG f\u00fcr Labormedizin|Center of Medical Microbiology|Wienemann|Germany
>Spike|hCoV-19/Japan/PG-1638/2020|2020-04-00|EPI_ISL_685244|Original|hCoV-19^^Japan|Human|Pathogen Genomics Center|Pathogen Genomics Center|Sekizuka|Japan
>Spike|hCoV-19/USA/WA-QDX-2548/2020|2020-03-16|EPI_ISL_604872|Original|hCoV-19^^Washington|Human|Quest Diagnostics|Quest Diagnostics|Kagan|USA
>Spike|hCoV-19/England/PORT-2CF751/2020|2020-05-19|EPI_ISL_461980|Original|hCoV-19^^England|Human|Centre for Enzyme Innovation|COVID-19 Genomics UK (COG-UK) Consortium|Robson|United Kingdom
>Spike|hCoV-19/Denmark/DCGC-6555/2020|2020-10-12|EPI_ISL_619603|Original|hCoV-19^^Syddanmark|Human|Department of Virus and Microbiological Special Diagnostics|Albertsen lab|Michaelsen|Denmark
>Spike|hCoV-19/England/ALDP-B1BC4F/2020|2020-11-01|EPI_ISL_645781|Original|hCoV-19^^England|Human|Lighthouse Lab in Alderley Park|Wellcome Sanger Institute for the COVID-19 Genomics UK (COG-UK) Consortium|Davies|United Kingdom
>Spike|hCoV-19/Denmark/DCGC-8573/2020|2020-10-26|EPI_ISL_625954|Original|hCoV-19^^Hovedstaden|Human|Department of Virus and Microbiological Special Diagnostics|Albertsen lab|Michaelsen|Denmark
>Spike|hCoV-19/Scotland/CVR2982/2020|2020-04-23|EPI_ISL_439002|Original|hCoV-19^^Scotland|Human|West of Scotland Specialist Virology Centre|COVID-19 Genomics UK (COG-UK) Consortium|Robertson|United Kingdom
>Spike|hCoV-19/England/CAMC-B1FDC0/2020|2020-11-01|EPI_ISL_647796|Original|hCoV-19^^England|Human|Lighthouse Lab in Cambridge|Wellcome Sanger Institute for the COVID-19 Genomics UK (COG-UK) Consortium|Davies|United Kingdom
>Spike|hCoV-19/USA/NY-QDX-2198/2020|2020-03-15|EPI_ISL_604566|Original|hCoV-19^^New York|Human|Quest Diagnostics|Quest Diagnostics|Kagan|USA
>Spike|hCoV-19/Netherlands/OV-EMC-68/2020|2020-06-13|EPI_ISL_523452|Original|hCoV-19^^Overijssel|Human|Dutch COVID-19 response team|Erasmus Medical Center|Oude Munnink|Netherlands
>Spike|hCoV-19/Singapore/115/2020|2020-03-18|EPI_ISL_443207|Original|hCoV-19^^Singapore|Human|National Public Health Laboratory|National Public Health Laboratory|Mak|Singapore
>Spike|hCoV-19/Canada/ON-PHL-20-01193/2020|2020-06-00|EPI_ISL_548815|Original|hCoV-19^^Ontario|Human|Public Health Ontario Laboratory|Public Health Ontario Laboratory|Guthrie|Canada
>Spike|hCoV-19/USA/WA-S1088/2020|2020-04-29|EPI_ISL_463578|Original|hCoV-19^^Washington|Human|Washington State Department of Health|Seattle Flu Study|Bedford|USA

>Spike|hCoV-19/Wales/PHWC-2815A/2020|2020-03-25|EPI_ISL_432170|Original|hCoV-19^^Wales|Human|Wales Specialist Virology Centre|Public Health Wales Microbiology Cardiff|Moore|United Kingdom
>Spike|hCoV-19/Scotland/EDB6608/2020|2020-06-22|EPI_ISL_484683|Original|hCoV-19^^Scotland|Human|Virology Department|COVID-19 Genomics UK (COG-UK) Consortium|Williams|United Kingdom
>Spike|hCoV-19/England/ALDP-94DC19/2020|2020-06-18|EPI_ISL_555584|Original|hCoV-19^^England|Human|Lighthouse Lab in Alderley Park|Wellcome Sanger Institute for the COVID-19 Genomics UK (COG-UK) consortium|Davies|United Kingdom
>Spike|hCoV-19/Japan/Donner9/2020|2020-04-06|EPI_ISL_438954|Original|hCoV-19^^Kanto|Human|Keio University School of Medicine|Keio University School of Medicine|Kosaki|Japan
>Spike|hCoV-19/Russia/SPE-RII-17942V/2020|2020-05-27|EPI_ISL_524016|Vero P1|hCoV-19^^Saint Petersburg|Human|WHO National Influenza Centre Russian Federation|WHO National Influenza Centre Russian Federation|Komissarov|Russia
>Spike|hCoV-19/USA/ME-HETL-J0387/2020|2020-12-15|EPI_ISL_755501|Original|hCoV-19^^Maine|Human|Maine Health and Environmental Testing Laboratory|Tewhey Lab|Tewhey|USA
>Spike|hCoV-19/Japan/NGY-NNH-005/2020|2020-04-07|EPI_ISL_568560|Original|hCoV-19^^Aichi|Human|Department of Infectious Diseases and Immunology|Clinical Research Center|Iwatani|Japan
>Spike|hCoV-19/Luxembourg/LNS0084824/2020|2020-11-16|EPI_ISL_739708|Original|hCoV-19^^Luxembourg|Human|Laboratoire national de sant\u00e9|Laboratoire national de sant\u00e9|Wienecke-Baldacchino|Luxembourg
>Spike|hCoV-19/England/ALDP-B5C9B4/2020|2020-11-08|EPI_ISL_655884|Original|hCoV-19^^England|Human|Lighthouse Lab in Alderley Park|Wellcome Sanger Institute for the COVID-19 Genomics UK (COG-UK) Consortium|Davies|United Kingdom
>Spike|hCoV-19/Netherlands/NA_273/2020|2020-03-30|EPI_ISL_422584|Original|hCoV-19^^Netherlands|Human|Dutch COVID-19 response team|Erasmus Medical Center|Oude Munnink|Netherlands
>Spike|hCoV-19/England/EXET-13A770/2020|2020-11-09|EPI_ISL_664401|Original|hCoV-19^^England|Human|University of Exeter|COVID-19 Genomics UK (COG-UK) Consortium|Temperton|United Kingdom
>Spike|hCoV-19/England/ALDP-AA2C6C/2020|2020-10-22|EPI_ISL_625218|Original|hCoV-19^^England|Human|Lighthouse Lab in Alderley Park|Wellcome Sanger Institute for the COVID-19 Genomics UK (COG-UK) consortium|Davies|United Kingdom
>Spike|hCoV-19/Wales/PHWC-32F44/2020|2020-04-14|EPI_ISL_446712|Original|hCoV-19^^Wales|Human|Wales Specialist Virology Centre|Public Health Wales Microbiology Cardiff|Moore|United Kingdom
>Spike|hCoV-19/England/20139069404/2020|2020-03-29|EPI_ISL_423360|Original|hCoV-19^^England|Human|Respiratory Virus Unit|Respiratory Virus Unit|Platt|United Kingdom
>Spike|hCoV-19/Wales/MILK-9F0617/2020|2020-09-24|EPI_ISL_580745|Original|hCoV-19^^Wales|Human|Lighthouse Lab in Milton Keynes|Wellcome Sanger Institute for the COVID-19 Genomics UK (COG-UK) consortium|Davies|United Kingdom
>Spike|hCoV-19/USA/MI-UM-10035812504/2020|2020-09-04|EPI_ISL_583310|Original|hCoV-19^^Michigan|Human|University of Michigan Clinical Microbiology Laboratory|Lauring Lab|Valesano|USA
>Spike|hCoV-19/USA/UT-UPHL-201005514/2020|2020-06-22|EPI_ISL_594040|Original|hCoV-19^^Utah|Human|Utah Public Health Laboratory|Utah Public Health Laboratory|Young|USA
>Spike|hCoV-19/Saudi Arabia/KAUST-Makkah12/2020|2020-03-23|EPI_ISL_437465|Original|hCoV-19^^Makkah|Human|Pathogen Genomics Lab King Abdullah University of Science and Technology(KAUST)|Pathogen Genomics Lab King Abdullah University of Science and Technology(KAUST)|Mohamed Ghazzali|Saudi Arabia
>Spike|hCoV-19/Japan/PG-0215/2020|2020-03-01|EPI_ISL_479915|Original|hCoV-19^^Kanto|Human|Niigata City Public Health Research Institute|Pathogen Genomics Center|Sekizuka|Japan
>Spike|hCoV-19/Wales/PHWC-16C3BD/2020|2020-09-23|EPI_ISL_574049|Original|hCoV-19^^Wales|Human|Wales Specialist Virology Centre Sequencing lab|COVID-19 Genomics UK (COG-UK) Consortium|Moore|United Kingdom

>Spike|hCoV-19/England/ALDP-9EDEAC/2020|2020-05-03|EPI_ISL_589568|Original|hCoV-19^^England|Human|Lighthouse Lab in Alderley Park|Wellcome Sanger Institute for the COVID-19 Genomics UK (COG-UK) consortium|Davies|United Kingdom
>Spike|hCoV-19/Australia/VIC11291/2020|2020-07-18|EPI_ISL_563319|Original|hCoV-19^^Victoria|Human|Microbiological Diagnostic Unit - Public Health Laboratory (MDU-PHL)|MDU-PHL|Schultz|Australia
>Spike|hCoV-19/Denmark/DCGC-14965/2020|2020-11-23|EPI_ISL_713552|Original|hCoV-19^^Hovedstaden|Human|Department of Virus and Microbiological Special Diagnostics|Albertsen Lab|Michaelsen|Denmark
>Spike|hCoV-19/England/202600045/2020|2020-06-05|EPI_ISL_566054|Original|hCoV-19^^England|Human|Respiratory Virus Unit|Respiratory Virus Unit|Platt|United Kingdom
>Spike|hCoV-19/England/BRIS-123710/2020|2020-04-06|EPI_ISL_442650|Original|hCoV-19^^England|Human|PHE South West Regional Laboratory|Wellcome Sanger Institute for the COVID-19 Genomics UK (COG-UK) consortium|Amato|United Kingdom
>Spike|hCoV-19/Australia/VIC4034/2020|2020-07-08|EPI_ISL_519419|Original|hCoV-19^^Victoria|Human|Microbiological Diagnostic Unit - Public Health Laboratory (MDU-PHL)|MDU-PHL|Schultz|Australia
>Spike|hCoV-19/USA/TX-HMH-602/2020|2020-04-09|EPI_ISL_547172|Original|hCoV-19^^Texas|Human|Houston Methodist Hospital|Houston Methodist Hospital|Long|USA
>Spike|hCoV-19/Scotland/EDB5519/2020|2020-04-22|EPI_ISL_487932|Original|hCoV-19^^Scotland|Human|Virology Department|Wellcome Sanger Institute for the COVID-19 Genomics UK (COG-UK) consortium|Davies|United Kingdom
>Spike|hCoV-19/England/LOND-D6C3C/2020|2020-03-30|EPI_ISL_478415|Original|hCoV-19^^England|Human|University College London|COVID-19 Genomics UK (COG-UK) Consortium|Roy|United Kingdom
>Spike|hCoV-19/England/MILK-A0689F/2020|2020-10-01|EPI_ISL_599876|Original|hCoV-19^^England|Human|Lighthouse Lab in Milton Keynes|Wellcome Sanger Institute for the COVID-19 Genomics UK (COG-UK) consortium|Davies|United Kingdom
>Spike|hCoV-19/England/ALDP-CB55A5/2020|2020-12-20|EPI_ISL_761775|Original|hCoV-19^^England|Human|Lighthouse Lab in Alderley Park|Wellcome Sanger Institute for the COVID-19 Genomics UK (COG-UK) Consortium|Davies|United Kingdom
>Spike|hCoV-19/USA/AR-CDC-0837/2020|2020-07-07|EPI_ISL_535650|Original|hCoV-19^^Arkansas|Human|AR Dept. of Health-Public Health Lab|Pathogen Discovery|Queen|USA
>Spike|hCoV-19/Zimbabwe/ZW-25/2020|2020-03-01|EPI_ISL_647977|Original|hCoV-19^^Zimbabwe|Human|National Microbiology Reference Laboratory|Quadram Institute Bioscience|Alikhan|Zimbabwe
>Spike|hCoV-19/England/MILK-BAC1EA/2020|2020-11-18|EPI_ISL_783126|Original|hCoV-19^^England|Human|Lighthouse Lab in Milton Keynes|Wellcome Sanger Institute for the COVID-19 Genomics UK (COG-UK) Consortium|Davies|United Kingdom
>Spike|hCoV-19/England/NORT-291DFC/2020|2020-05-05|EPI_ISL_489107|Original|hCoV-19^^England|Human|NU-OMICS DNA Sequencing research facility|Wellcome Sanger Institute for the COVID-19 Genomics UK (COG-UK) consortium|Davies|United Kingdom
>Spike|hCoV-19/England/CAMB-1B8A08/2020|2020-10-29|EPI_ISL_651674|Original|hCoV-19^^England|Human|Department of Pathology|COVID-19 Genomics UK (COG-UK) Consortium|Goodfellow|United Kingdom
>Spike|hCoV-19/Sweden/20-52048/2020|2020-06-09|EPI_ISL_510868|Original|hCoV-19^^Vastra Gotaland|Human|Klinisk mikrobiologi NAL Trollhattan|The Public Health Agency of Sweden|Svartstr\u00f6m|Sweden
>Spike|hCoV-19/Wales/PHWC-49A31D/2020|2020-12-01|EPI_ISL_727528|Original|hCoV-19^^Wales|Human|Originating lab|COVID-19 Genomics UK (COG-UK) Consortium|Moore|United Kingdom
>Spike|hCoV-19/Australia/SAP374/2020|2020-03-20|EPI_ISL_492139|Original|hCoV-19^^South Australia|Human|SA Pathology|SA Pathology|Leong|Australia
>Spike|hCoV-19/England/CAMB-77550/2020|2020-04-01|EPI_ISL_439454|Original|hCoV-19^^England|Human|Department of Pathology|Wellcome Sanger Institute for the COVID-19 Genomics UK (COG-UK) consortium|Amato|United Kingdom
>Spike|hCoV-19/Canada/BC_04346268/2020|2020-04-00|EPI_ISL_466766|Original|hCoV-19^^British Columbia|Human|BCCDC Public Health Laboratory|BCCDC Public Health Laboratory|Harrigan|Canada

>Spike|hCoV-19/Wales/PHWC-498546/2020|2020-12-01|EPI_ISL_727090|Original|hCoV-19^^Wales|Human|Originating lab|COVID-19 Genomics UK (COG-UK) Consortium|Moore|United Kingdom
>Spike|hCoV-19/USA/LA-EVTL1035/2020|2020-08-25|EPI_ISL_653725|Original|hCoV-19^^Louisiana|Human|LSUHS Emerging Viral Threat Laboratory|Microbial Genome Sequencing Center|Kamil|USA
>Spike|hCoV-19/USA/MI-MDHHS-SC21701/2020|2020-03-25|EPI_ISL_529820|Original|hCoV-19^^Michigan|Human|Michigan Department of Health and Human Services|Michigan Department of Health and Human Services|Blankenship|USA
>Spike|hCoV-19/Germany/HH-hpi-p2429/2020|2020-04-20|EPI_ISL_776498|Original|hCoV-19^^Hamburg|Human|University Medical Center Hamburg Eppendorf|Heinrich Pette Institute|Robitaille|Germany
>Spike|hCoV-19/Wales/PHWC-4960CF/2020|2020-11-25|EPI_ISL_726529|Original|hCoV-19^^Wales|Human|Originating lab|COVID-19 Genomics UK (COG-UK) Consortium|Moore|United Kingdom
>Spike|hCoV-19/Germany/BE-ChVir-W1139-8123/2020|2020-04-06|EPI_ISL_753940|Original|hCoV-19^^Berlin|Human|Charit\u00e9 Universit\u00e4tsmedizin Berlin|Charit\u00e9 Universit\u00e4tsmedizin Berlin|Muehlemann|Germany
>Spike|hCoV-19/England/PORT-2E9180/2020|2020-12-18|EPI_ISL_763624|Original|hCoV-19^^England|Human|Centre for Enzyme Innovation|COVID-19 Genomics UK (COG-UK) Consortium|Robson|United Kingdom
>Spike|hCoV-19/Slovenia/4499/2020|2020-03-05|EPI_ISL_635207|Original|hCoV-19^^Slovenia|Human|Institute of Microbiology and Immunology|Institute of Microbiology and Immunology|Zakotnik|Slovenia
>Spike|hCoV-19/England/QEUH-C33B16/2020|2020-12-11|EPI_ISL_734031|Original|hCoV-19^^England|Human|Lighthouse Lab in Glasgow|Wellcome Sanger Institute for the COVID-19 Genomics UK (COG-UK) Consortium|Davies|United Kingdom
>Spike|hCoV-19/USA/TX-DSHS-0125/2020|2020-06-11|EPI_ISL_522952|Original|hCoV-19^^Texas|Human|Texas Department of State Health Services|Texas Department of State Health Services|Rahman|USA
>Spike|hCoV-19/USA/HI-H200567/2020|2020-10-24|EPI_ISL_753095|Original|hCoV-19^^Hawaii|Human|State Laboratories Division|State Laboratories Division|Sultana|USA
>Spike|hCoV-19/England/ALDP-B23E20/2020|2020-11-04|EPI_ISL_646343|Original|hCoV-19^^England|Human|Lighthouse Lab in Alderley Park|Wellcome Sanger Institute for the COVID-19 Genomics UK (COG-UK) Consortium|Davies|United Kingdom
>Spike|hCoV-19/Netherlands/ZH-EMC-931/2020|2020-10-15|EPI_ISL_722507|Original|hCoV-19^^Zuid Holland|Human|Dutch COVID-19 response team|Erasmus Medical Center|Oude Munnink|Netherlands
>Spike|hCoV-19/USA/OR-PROV-035/2020|2020-03-26|EPI_ISL_482317|Original|hCoV-19^^Oregon|Human|Providence St. Joseph Health Molecular Genomics Laboratory|Providence St. Joseph Health Molecular Genomics Laboratory|Dowdell|USA
>Spike|hCoV-19/USA/NC-CDC-2-4693552/2020|2020-11-09|EPI_ISL_747175|Original|hCoV-19^^North Carolina|Human|Respiratory Viruses Branch|Respiratory Viruses Branch|Queen|USA
>Spike|hCoV-19/Australia/SAP057/2020|2020-03-27|EPI_ISL_451096|Original|hCoV-19^^South Australia|Human|SA Pathology|SA Pathology|Leong|Australia
>Spike|hCoV-19/Switzerland/LU-ETHZ-350472/2020|2020-11-03|EPI_ISL_728826|Original|hCoV-19^^Lucerne|Human|Viollier AG|Department of Biosystems Science and Engineering|Nadeau|Switzerland
>Spike|hCoV-19/England/204961136/2020|2020-12-03|EPI_ISL_700691|Original|hCoV-19^^England|Human|Respiratory Virus Unit|COVID-19 Genomics UK (COG-UK) Consortium|Platt|United Kingdom
>Spike|hCoV-19/USA/TX-HMH-3797/2020|2020-06-13|EPI_ISL_543201|Original|hCoV-19^^Texas|Human|Houston Methodist Hospital|Houston Methodist Hospital|Long|USA
>Spike|hCoV-19/England/SHEF-C9267/2020|2020-05-23|EPI_ISL_475418|Original|hCoV-19^^England|Human|Virology Department|COVID-19 Genomics UK (COG-UK) Consortium|de Silva|United Kingdom

>Spike|hCoV-19/South Africa/KRISP-K004591/2020|2020-11-07|EPI_ISL_660621|Original|hCoV-19^^KwaZulu-Natal|Human|NHLS-IALCH|KRISP|de Oliveira|South Africa
>Spike|hCoV-19/USA/AZ-TG666102/2020|2020-03-27|EPI_ISL_696192|Original|hCoV-19^^Arizona|Human|Sonora Quest Laboratories|TGen North|French|USA
>Spike|hCoV-19/Denmark/DCGC-15933/2020|2020-11-23|EPI_ISL_714491|Original|hCoV-19^^Hovedstaden|Human|Department of Virus and Microbiological Special Diagnostics|Albertsen Lab|Michaelsen|Denmark
>Spike|hCoV-19/England/ALDP-AA08F1/2020|2020-10-21|EPI_ISL_625029|Original|hCoV-19^^England|Human|Lighthouse Lab in Alderley Park|Wellcome Sanger Institute for the COVID-19 Genomics UK (COG-UK) consortium|Davies|United Kingdom
>Spike|hCoV-19/Wales/PHWC-16DEF7/2020|2020-09-28|EPI_ISL_585639|Original|hCoV-19^^Wales|Human|Wales Specialist Virology Centre Sequencing lab|COVID-19 Genomics UK (COG-UK) Consortium|Moore|United Kingdom
>Spike|hCoV-19/Northern Ireland/QEUH-7A4D4C/2020|2020-07-14|EPI_ISL_533177|Original|hCoV-19^^Northern Ireland|Human|Lighthouse Lab in Glasgow|Wellcome Sanger Institute for the COVID-19 Genomics UK (COG-UK) consortium|Davies|United Kingdom
>Spike|hCoV-19/England/C200044478/2020|2020-11-06|EPI_ISL_666761|Original|hCoV-19^^England|Human|Respiratory Virus Unit|COVID-19 Genomics UK (COG-UK) Consortium|Platt|United Kingdom
>Spike|hCoV-19/England/201090820/2020|2020-03-04|EPI_ISL_464608|Original|hCoV-19^^England|Human|Respiratory Virus Unit|Respiratory Virus Unit|Platt|United Kingdom
>Spike|hCoV-19/Singapore/607/2020|2020-07-01|EPI_ISL_490057|Original|hCoV-19^^Singapore|Human|National Public Health Laboratory|National Public Health Laboratory|Mak|Singapore
>Spike|hCoV-19/Luxembourg/LNS9726231/2020|2020-11-24|EPI_ISL_744972|Original|hCoV-19^^Luxembourg|Human|Laboratoire national de sant\u00e9|Laboratoire national de sant\u00e9|Wienecke-Baldacchino|Luxembourg
>Spike|hCoV-19/England/CAMC-BBB75F/2020|2020-11-21|EPI_ISL_705561|Original|hCoV-19^^England|Human|Department of Pathology|COVID-19 Genomics UK (COG-UK) Consortium|Goodfellow|United Kingdom
>Spike|hCoV-19/England/ALDP-9EE0D2/2020|2020-05-06|EPI_ISL_589557|Original|hCoV-19^^England|Human|Lighthouse Lab in Alderley Park|Wellcome Sanger Institute for the COVID-19 Genomics UK (COG-UK) consortium|Davies|United Kingdom
>Spike|hCoV-19/Bangladesh/DNAS_DRCIM_isl_SM-01/2020|2020-07-04|EPI_ISL_774919|Original|hCoV-19^^Dhaka|Human|Designated Reference Institute for Chemical Measurements (DRICM)|DNA SOLUTION LTD.|Rabbi|Bangladesh
>Spike|hCoV-19/Denmark/DCGC-13965/2020|2020-11-30|EPI_ISL_711481|Original|hCoV-19^^Midtjylland|Human|Department of Virus and Microbiological Special Diagnostics|Albertsen Lab|Michaelsen|Denmark
>Spike|hCoV-19/Wales/PHWC-24F27/2020|2020-03-22|EPI_ISL_420956|Original|hCoV-19^^Wales|Human|Wales Specialist Virology Centre|Public Health Wales Microbiology Cardiff|Moore|United Kingdom
>Spike|hCoV-19/USA/AZ-TG312514/2020|2020-05-16|EPI_ISL_694323|Original|hCoV-19^^Arizona|Human|TGen North|TGen North|French|USA
>Spike|hCoV-19/England/ALDP-94FF96/2020|2020-06-15|EPI_ISL_555150|Original|hCoV-19^^England|Human|Lighthouse Lab in Alderley Park|Wellcome Sanger Institute for the COVID-19 Genomics UK (COG-UK) consortium|Davies|United Kingdom
>Spike|hCoV-19/USA/CA-QDX-1668/2020|2020-03-18|EPI_ISL_603919|Original|hCoV-19^^California|Human|Quest Diagnostics|Quest Diagnostics|Kagan|USA
>Spike|hCoV-19/France/ARA-27286/2020|2020-04-19|EPI_ISL_676570|Original|hCoV-19^^Auvergne-Rh\u00f4ne-Alpes|Human|CNR Virus des Infections Respiratoires - France SUD|CNR Virus des Infections Respiratoires - France SUD|Bal|France
>Spike|hCoV-19/England/CAMB-80967/2020|2020-04-10|EPI_ISL_443036|Original|hCoV-19^^England|Human|Department of Pathology|Wellcome Sanger Institute for the COVID-19 Genomics UK (COG-UK) consortium|Amato|United Kingdom
>Spike|hCoV-19/England/OXON-B28E7/2020|2020-04-10|EPI_ISL_534780|Original|hCoV-19^^England|Human|Oxford Viromics|COVID-19 Genomics UK (COG-UK) Consortium|Golubchik|United Kingdom

>Spike|hCoV-19/Germany/NW-HHU-368/2020|2020-11-26|EPI_ISL_718049|Orginal|hCoV-19^^North Rhine-Westphalia|Human|ZOTZ KLIMAS MVZ D\u00fcsseldorf-Centrum GbR \u00dcBAG f\u00fcr Labormedizin|Center of Medical Microbiology|Wienemann|Germany
>Spike|hCoV-19/England/QEUH-9E36AE/2020|2020-09-24|EPI_ISL_584864|Original|hCoV-19^^England|Human|Northumbria University / South Tees Hospitals NHS Foundation Trust / North Cumbria Integrated Care NHS Foundation Trust / North Tees and Hartlepool NHS Foundation Trust / Newcastle Hospitals NHS Foundation Trust|COVID-19 Genomics UK (COG-UK) Consortium|Bashton|United Kingdom
>Spike|hCoV-19/USA/CA-CZB-14939/2020|2020-11-29|EPI_ISL_738736|Original|hCoV-19^^California|Human|Orange County Public Health Lab|Chan-Zuckerberg Biohub|Kamm|USA
>Spike|hCoV-19/Wales/PHWC-15DA9C/2020|2020-05-14|EPI_ISL_472757|Original|hCoV-19^^Wales|Human|Wales Specialist Virology Centre Sequencing lab|COVID-19 Genomics UK (COG-UK) Consortium|Moore|United Kingdom
>Spike|hCoV-19/South Korea/KCDC2512/2020|2020-06-17|EPI_ISL_514915|Original|hCoV-19^^South Korea|Human|Division of Viral Diseases|Division of Viral Diseases|Kim|South Korea
>Spike|hCoV-19/England/QEUH-B67B83/2020|2020-11-11|EPI_ISL_661493|Original|hCoV-19^^England|Human|Lighthouse Lab in Glasgow|Wellcome Sanger Institute for the COVID-19 Genomics UK (COG-UK) Consortium|Davies|United Kingdom
>Spike|hCoV-19/USA/PA-QDX-30/2020|2020-03-13|EPI_ISL_467904|Original|hCoV-19^^Pennsylvania|Human|Quest Diagnostics|Quest Diagnostics|Kagan|USA
>Spike|hCoV-19/Belgium/rega-09221140/2020|2020-09-22|EPI_ISL_738233|Original|hCoV-19^^Kumtich|Human|UZ Leuven|KU Leuven|Maes|Belgium
>Spike|hCoV-19/Scotland/CVR453/2020|2020-03-23|EPI_ISL_439080|Original|hCoV-19^^Scotland|Human|West of Scotland Specialist Virology Centre|COVID-19 Genomics UK (COG-UK) Consortium|Robertson|United Kingdom
>Spike|hCoV-19/England/CAMB-1B7B06/2020|2020-10-20|EPI_ISL_638359|Original|hCoV-19^^England|Human|Department of Pathology|COVID-19 Genomics UK (COG-UK) Consortium|Goodfellow|United Kingdom
>Spike|hCoV-19/USA/NM-UNM-00610/2020|2020-05-11|EPI_ISL_560325|Original|hCoV-19^^New Mexico|Human|TriCore Reference Laboratories|Center for Global Health|Domman|USA
>Spike|hCoV-19/South Korea/KCDC2641/2020|2020-07-09|EPI_ISL_515043|Original|hCoV-19^^South Korea|Human|Division of Viral Diseases|Division of Viral Diseases|Kim|South Korea
>Spike|hCoV-19/USA/WA-S771/2020|2020-04-13|EPI_ISL_449941|Original|hCoV-19^^Washington|Human|Washington State Department of Health|Seattle Flu Study|Bedford|USA
>Spike|hCoV-19/Northern Ireland/NIRE-107299/2020|2020-03-31|EPI_ISL_585132|Original|hCoV-19^^Northern Ireland|Human|Regional Virus Laboratory|COVID-19 Genomics UK (COG-UK) Consortium|Fairley|United Kingdom
>Spike|hCoV-19/Australia/VIC12258/2020|2020-07-23|EPI_ISL_591938|Original|hCoV-19^^Victoria|Human|Microbiological Diagnostic Unit - Public Health Laboratory (MDU-PHL)|MDU-PHL|Schultz|Australia
>Spike|hCoV-19/Denmark/DCGC-17600/2020|2020-12-14|EPI_ISL_751097|Original|hCoV-19^^Hovedstaden|Human|Department of Virus and Microbiological Special Diagnostics|Albertsen Lab|Michaelsen|Denmark
>Spike|hCoV-19/USA/MI-MDHHS-SC22425/2020|2020-11-09|EPI_ISL_708258|Original|hCoV-19^^Michigan|Human|Michigan Department of Health and Human Services|Michigan Department of Health and Human Services|Jacob|USA
>Spike|hCoV-19/England/CAMC-B7B0C9/2020|2020-11-12|EPI_ISL_668108|Original|hCoV-19^^England|Human|Lighthouse Lab in Cambridge|Wellcome Sanger Institute for the COVID-19 Genomics UK (COG-UK) Consortium|Davies|United Kingdom
>Spike|hCoV-19/Denmark/DCGC-13379/2020|2020-11-30|EPI_ISL_712762|Original|hCoV-19^^Sjaelland|Human|Department of Virus and Microbiological Special Diagnostics|Albertsen Lab|Michaelsen|Denmark
>Spike|hCoV-19/Wales/PHWC-16D149/2020|2020-09-24|EPI_ISL_572932|Original|hCoV-19^^Wales|Human|Wales Specialist Virology Centre Sequencing lab|COVID-19 Genomics UK (COG-UK) Consortium|Moore|United Kingdom

>Spike|hCoV-19/Netherlands/Friesland_30/2020|2020-04-18|EPI_ISL_460845|Original|hCoV-19^^Friesland|Human|Dutch COVID-19 response team|Erasmus Medical Center|Oude Munnink|Netherlands
>Spike|hCoV-19/England/MILK-B967A5/2020|2020-11-14|EPI_ISL_676126|Original|hCoV-19^^England|Human|Lighthouse Lab in Milton Keynes|Wellcome Sanger Institute for the COVID-19 Genomics UK (COG-UK) Consortium|Davies|United Kingdom
>Spike|hCoV-19/England/ALDP-4A8376/2020|2020-06-11|EPI_ISL_559535|Original|hCoV-19^^England|Human|Lighthouse Lab in Alderley Park|Wellcome Sanger Institute for the COVID-19 Genomics UK (COG-UK) consortium|Davies|United Kingdom
>Spike|hCoV-19/Wales/PHWC-47EF75/2020|2020-10-06|EPI_ISL_612608|Original|hCoV-19^^Wales|Human|Wales Specialist Virology Centre Sequencing lab|COVID-19 Genomics UK (COG-UK) Consortium|Moore|United Kingdom
>Spike|hCoV-19/USA/WA-UW-7670/2020|2020-04-17|EPI_ISL_570189|Original|hCoV-19^^Washington|Human|UW Virology Lab|UW Virology Lab|Roychoudhury|USA
>Spike|hCoV-19/England/QEUH-96BED3/2020|2020-08-13|EPI_ISL_531295|Original|hCoV-19^^England|Human|Lighthouse Lab in Glasgow|Wellcome Sanger Institute for the COVID-19 Genomics UK (COG-UK) consortium|Davies|United Kingdom
>Spike|hCoV-19/England/ALDP-B26027/2020|2020-11-04|EPI_ISL_646715|Original|hCoV-19^^England|Human|Lighthouse Lab in Alderley Park|Wellcome Sanger Institute for the COVID-19 Genomics UK (COG-UK) Consortium|Davies|United Kingdom
>Spike|hCoV-19/Spain/AN-IBV-002054/2020|2020-03-15|EPI_ISL_452375|Original|hCoV-19^^Andalucia|Human|Servicio de Microbiolog\u00eda. HRU de M\u00e1laga. Servicio Andaluz de Salud|SeqCOVID-SPAIN consortium/IBV(CSIC)|Comas|Spain
>Spike|hCoV-19/USA/TX-HMH-MCoV-8191/2020|2020-07-07|EPI_ISL_784256|Original|hCoV-19^^Texas|Human|Houston Methodist Hospital|Houston Methodist Hospital|Long|USA
>Spike|hCoV-19/England/CAMC-B51E56/2020|2020-11-06|EPI_ISL_657957|Original|hCoV-19^^England|Human|Lighthouse Lab in Cambridge|Wellcome Sanger Institute for the COVID-19 Genomics UK (COG-UK) Consortium|Davies|United Kingdom
>Spike|hCoV-19/England/BRIS-124F44/2020|2020-03-26|EPI_ISL_443832|Original|hCoV-19^^England|Human|PHE South West Regional Laboratory|Wellcome Sanger Institute for the COVID-19 Genomics UK (COG-UK) consortium|Amato|United Kingdom
>Spike|hCoV-19/USA/AZ-TG670099/2020|2020-05-02|EPI_ISL_696321|Original|hCoV-19^^Arizona|Human|Sonora Quest Laboratories|TGen North|French|USA
>Spike|hCoV-19/USA/WA-S1618/2020|2020-05-26|EPI_ISL_496044|Original|hCoV-19^^Washington|Human|Washington State Department of Health|Seattle Flu Study|Lee|USA
>Spike|hCoV-19/England/CAMB-82B6F/2020|2020-04-22|EPI_ISL_438664|Original|hCoV-19^^England|Human|Department of Pathology|COVID-19 Genomics UK (COG-UK) Consortium|Meredith|United Kingdom
>Spike|hCoV-19/Australia/VIC17846/2020|2020-07-27|EPI_ISL_779497|Original|hCoV-19^^Victoria|Human|Microbiological Diagnostic Unit - Public Health Laboratory (MDU-PHL)|MDU-PHL|Beck|Australia
>Spike|hCoV-19/USA/WA-S1057/2020|2020-04-30|EPI_ISL_463547|Original|hCoV-19^^Washington|Human|Washington State Department of Health|Seattle Flu Study|Bedford|USA
>Spike|hCoV-19/Switzerland/AG-ETHZ-260158/2020|2020-08-26|EPI_ISL_539452|Original|hCoV-19^^Aargau|Human|Viollier AG|Department of Biosystems Science and Engineering|Nadeau|Switzerland
>Spike|hCoV-19/England/QEUH-A5DDF4/2020|2020-10-12|EPI_ISL_597389|Original|hCoV-19^^England|Human|Lighthouse Lab in Glasgow|Wellcome Sanger Institute for the COVID-19 Genomics UK (COG-UK) consortium|Davies|United Kingdom
>Spike|hCoV-19/France/ARA-21154/2020|2020-04-07|EPI_ISL_508991|Original|hCoV-19^^Auvergne-Rh\u00f4ne-Alpes|Human|CNR Virus des Infections Respiratoires - France SUD|CNR Virus des Infections Respiratoires - France SUD|Josset|France
>Spike|hCoV-19/env/Qingdao/IVDC-05-10/2020|2020-09-24|EPI_ISL_591274|Original|hCoV-19^^Shandong|Environment|National Institute for Viral Disease Control and Prevention|National Institute for Viral Disease Control and Prevention|Chen|China
>Spike|hCoV-19/England/ALDP-9BE484/2020|2020-09-09|EPI_ISL_567928|Original|hCoV-19^^England|Human|Lighthouse Lab in Alderley Park|Wellcome Sanger Institute for the COVID-19 Genomics UK (COG-UK) consortium|Davies|United Kingdom

>Spike|hCoV-19/USA/NY-Wadsworth-243231-01/2020|2020-10-11|EPI_ISL_734346|Original|hCoV-19^^New York|Human|Wadsworth Center|Wadsworth Center|Lasek-Nesselquist|USA
>Spike|hCoV-19/Australia/VIC5474/2020|2020-07-20|EPI_ISL_520223|Original|hCoV-19^^Victoria|Human|Microbiological Diagnostic Unit - Public Health Laboratory (MDU-PHL)|MDU-PHL|Schultz|Australia
>Spike|hCoV-19/Canada/ON-A27/2020|2020-11-24|EPI_ISL_755725|Original|hCoV-19^^Ontario|Human|Toronto Invasive Bacterial Diseases Network|McMaster University|McArthur|Canada
>Spike|hCoV-19/England/QEUH-B9EC35/2020|2020-11-18|EPI_ISL_719756|Original|hCoV-19^^England|Human|Lighthouse Lab in Glasgow|Wellcome Sanger Institute for the COVID-19 Genomics UK (COG-UK) Consortium|Davies|United Kingdom
>Spike|hCoV-19/England/NORW-F7E02/2020|2020-11-13|EPI_ISL_650502|Original|hCoV-19^^England|Human|Quadram Institute Bioscience|COVID-19 Genomics UK (COG-UK) Consortium|Alikhan|United Kingdom
>Spike|hCoV-19/England/LIVE-DB2CDF/2020|2020-11-01|EPI_ISL_637976|Original|hCoV-19^^England|Human|Liverpool Clinical Laboratories|COVID-19 Genomics UK (COG-UK) Consortium|Haldenby|United Kingdom
>Spike|hCoV-19/India/TG-CCMB-AA586/2020|2020-08-12|EPI_ISL_539649|Original|hCoV-19^^Telangana|Human|CSIR-Centre for Cellular and Molecular Biology|CSIR-Centre for Cellular and Molecular Biology|Mukherjee|India
>Spike|hCoV-19/England/BRIS-12EF68/2020|2020-04-25|EPI_ISL_488271|Original|hCoV-19^^England|Human|PHE South West Regional Laboratory|Wellcome Sanger Institute for the COVID-19 Genomics UK (COG-UK) consortium|Davies|United Kingdom
>Spike|hCoV-19/Switzerland/BE-ETHZ-360261/2020|2020-11-09|EPI_ISL_729238|Original|hCoV-19^^Bern|Human|Viollier AG|Department of Biosystems Science and Engineering|Nadeau|Switzerland
>Spike|hCoV-19/England/QEUH-9B89E5/2020|2020-09-07|EPI_ISL_549582|Original|hCoV-19^^England|Human|Lighthouse Lab in Glasgow|Wellcome Sanger Institute for the COVID-19 Genomics UK (COG-UK) consortium|Davies|United Kingdom
>Spike|hCoV-19/USA/NY-NYUMC670/2020|2020-04-14|EPI_ISL_444787|Original|hCoV-19^^New York|Human|NYU Langone Health|Departments of Pathology and Medicine|Maurano|USA
>Spike|hCoV-19/Hong Kong/VM20002588/2020|2020-02-13|EPI_ISL_413497|Original|hCoV-19^^Hong Kong|Human|Ruttonjee Hospital|Hong Kong Department of Health|Mak|China
>Spike|hCoV-19/India/DL-NCDC5643-CSIR-IGIB/2020|2020-05-25|EPI_ISL_482607|Original|hCoV-19^^Delhi|Human|National Centre for Disease control (NCDC)|NCDC/CSIR-IGIB|Singh|India
>Spike|hCoV-19/USA/UT-UPHL-2009244/2020|2020-05-12|EPI_ISL_525233|Original|hCoV-19^^Utah|Human|Utah Public Health Laboratory|Utah Public Health Laboratory|Young|USA
>Spike|hCoV-19/USA/LA-EVTL1316/2020|2020-12-11|EPI_ISL_778982|Original|hCoV-19^^Louisiana|Human|LSUHS Emerging Viral Threat Laboratory|Microbial Genome Sequencing Center|Kamil|USA
>Spike|hCoV-19/USA/WA-UW398/2020|2020-03-29|EPI_ISL_529200|Original|hCoV-19^^Washington|Human|University of Washington|University of Washington|Roychoudhury|USA
>Spike|hCoV-19/Denmark/DCGC-13276/2020|2020-11-30|EPI_ISL_712699|Original|hCoV-19^^Sjaelland|Human|Department of Virus and Microbiological Special Diagnostics|Albertsen Lab|Michaelsen|Denmark
>Spike|hCoV-19/England/LIVE-DC6E3C/2020|2020-11-24|EPI_ISL_705926|Original|hCoV-19^^England|Human|Liverpool Clinical Laboratories|COVID-19 Genomics UK (COG-UK) Consortium|Haldenby|United Kingdom
>Spike|hCoV-19/Portugal/PT0169/2020|2020-03-28|EPI_ISL_453885|Original|hCoV-19^^Portugal|Human|unknown|Instituto Nacional de Saude (INSA)|Borges|Portugal
>Spike|hCoV-19/England/QEUH-BCC1B7/2020|2020-11-27|EPI_ISL_704390|Original|hCoV-19^^England|Human|Lighthouse Lab in Glasgow|Wellcome Sanger Institute for the COVID-19 Genomics UK (COG-UK) Consortium|Davies|United Kingdom
>Spike|hCoV-19/USA/MD-MDH-0350/2020|2020-10-20|EPI_ISL_648289|Original|hCoV-19^^Maryland|Human|MD PHL|MD PHL|Keller|USA

>Spike|hCoV-19/England/MILK-ACBEB2/2020|2020-10-20|EPI_ISL_709915|Original|hCoV-19^^England|Human|Lighthouse Lab in Milton Keynes|Wellcome Sanger Institute for the COVID-19 Genomics UK (COG-UK) Consortium|Davies|United Kingdom
>Spike|hCoV-19/USA/WA-S167/2020|2020-03-18|EPI_ISL_430205|Original|hCoV-19^^Washington|Human|Washington State Department of Health|Seattle Flu Study|Bedford|USA
>Spike|hCoV-19/England/BRIS-1853B20/2020|2020-04-25|EPI_ISL_492416|Original|hCoV-19^^England|Human|PHE South West Regional Laboratory|Wellcome Sanger Institute for the COVID-19 Genomics UK (COG-UK) consortium|Davies|United Kingdom
>Spike|hCoV-19/DRC/1486/2020|2020-04-08|EPI_ISL_434711|Original|hCoV-19^^Kinshasa|Human|Viral Respiratory Lab|Pathogen Sequencing Lab|Amuri|Democratic Republic of the Congo
>Spike|hCoV-19/England/PORT-2E9CBC/2020|2020-00-00|EPI_ISL_706876|Original|hCoV-19^^England|Human|Centre for Enzyme Innovation|COVID-19 Genomics UK (COG-UK) Consortium|Robson|United Kingdom
>Spike|hCoV-19/England/NORW-F77BC/2020|2020-12-02|EPI_ISL_704070|Original|hCoV-19^^England|Human|Quadram Institute Bioscience|COVID-19 Genomics UK (COG-UK) Consortium|Alikhan|United Kingdom
>Spike|hCoV-19/Japan/P4-1/2020|2020-03-11|EPI_ISL_419301|Original|hCoV-19^^Saitama|Human|Saitama Prefectural Institute of Public Health|Pathogen Genomics Center|Sekizuka|Japan
>Spike|hCoV-19/England/MILK-9A564E/2020|2020-09-03|EPI_ISL_550155|Original|hCoV-19^^England|Human|Lighthouse Lab in Milton Keynes|Wellcome Sanger Institute for the COVID-19 Genomics UK (COG-UK) consortium|Davies|United Kingdom
>Spike|hCoV-19/England/QEUH-9B51E0/2020|2020-09-03|EPI_ISL_590195|Original|hCoV-19^^England|Human|Lighthouse Lab in Glasgow|Wellcome Sanger Institute for the COVID-19 Genomics UK (COG-UK) consortium|Davies|United Kingdom
>Spike|hCoV-19/USA/MA-MASPHL-00488/2020|2020-05-02|EPI_ISL_693079|Original|hCoV-19^^Massachusetts|Human|Massachusetts State Public Health Laboratory|Massachusetts State Public Health Laboratory|Lang|USA
>Spike|hCoV-19/England/MILK-A47461/2020|2020-10-05|EPI_ISL_598304|Original|hCoV-19^^England|Human|Lighthouse Lab in Milton Keynes|Wellcome Sanger Institute for the COVID-19 Genomics UK (COG-UK) consortium|Davies|United Kingdom
>Spike|hCoV-19/USA/CA-CZB-10815/2020|2020-07-06|EPI_ISL_583067|Original|hCoV-19^^California|Human|Humboldt County Public Health Laboratory|Chan-Zuckerberg Biohub|Bhatt|USA
>Spike|hCoV-19/Luxembourg/LNS3343013/2020|2020-10-26|EPI_ISL_740292|Original|hCoV-19^^Luxembourg|Human|Laboratoire national de sant\u00e9|Laboratoire national de sant\u00e9|Wienecke-Baldacchino|Luxembourg
>Spike|hCoV-19/United Arab Emirates/L5857/2020|2020-03-31|EPI_ISL_469279|Original|hCoV-19^^United Arab Emirates|Human|Mohammed Bin Rashid University of Medicine and Health Sciences|Al Jalila Genomics Center|Ramaswamy|United Arab Emirates
>Spike|hCoV-19/Netherlands/NA_27/2020|2020-03-13|EPI_ISL_415484|Original|hCoV-19^^Netherlands|Human|Dutch COVID-19 response team|Erasmus Medical Center|Oude Munnink|Netherlands
>Spike|hCoV-19/Wales/PHWC-164E4B/2020|2020-05-23|EPI_ISL_479388|Original|hCoV-19^^Wales|Human|Wales Specialist Virology Centre Sequencing lab|COVID-19 Genomics UK (COG-UK) Consortium|Moore|United Kingdom
>Spike|hCoV-19/Denmark/DCGC-21036/2020|2020-12-21|EPI_ISL_759518|Original|hCoV-19^^Sjaelland|Human|Department of Virus and Microbiological Special Diagnostics|Albertsen Lab|Michaelsen|Denmark
>Spike|hCoV-19/Australia/VIC966/2020|2020-03-27|EPI_ISL_430653|Original|hCoV-19^^Victoria|Human|Microbiological Diagnostic Unit Public Health Laboratory|Microbiological Diagnostic Unit Public Health Laboratory|Schultz|Australia
>Spike|hCoV-19/Japan/PG-1789/2020|2020-05-00|EPI_ISL_685395|Original|hCoV-19^^Japan|Human|Pathogen Genomics Center|Pathogen Genomics Center|Sekizuka|Japan
>Spike|hCoV-19/USA/AZ-TG496036/2020|2020-07-10|EPI_ISL_695264|Original|hCoV-19^^Arizona|Human|TGen North|TGen North|French|USA

>Spike|hCoV-19/India/MH-ACTREC-484/2020|2020-08-13|EPI_ISL_700132|Original|hCoV-19^^Maharashtra|Human|Hematopathology Laboratory|Hematopathology Laboratory|Patkar|India
>Spike|hCoV-19/USA/NY-Wadsworth-278038-01/2020|2020-11-22|EPI_ISL_754597|Original|hCoV-19^^New York|Human|Wadsworth Center|Wadsworth Center|Lasek-Nesselquist|USA
>Spike|hCoV-19/England/ALDP-5D1F15/2020|2020-06-19|EPI_ISL_558521|Original|hCoV-19^^England|Human|Lighthouse Lab in Alderley Park|Wellcome Sanger Institute for the COVID-19 Genomics UK (COG-UK) consortium|Davies|United Kingdom
>Spike|hCoV-19/Denmark/DCGC-15348/2020|2020-11-23|EPI_ISL_713776|Original|hCoV-19^^Hovedstaden|Human|Department of Virus and Microbiological Special Diagnostics|Albertsen Lab|Michaelsen|Denmark
>Spike|hCoV-19/USA/VA-DCLS-2235/2020|2020-11-03|EPI_ISL_734215|Original|hCoV-19^^Virginia|Human|Virginia Division of Consolidated Laboratory Services (DCLS)|Virginia Division of Consolidated Laboratory Services (DCLS)|St. Jacques|USA
>Spike|hCoV-19/USA/WA-S1309/2020|2020-05-14|EPI_ISL_495759|Original|hCoV-19^^Washington|Human|Washington State Department of Health|Seattle Flu Study|Lee|USA
>Spike|hCoV-19/England/CAMB-7DC55/2020|2020-04-05|EPI_ISL_441679|Original|hCoV-19^^England|Human|Department of Pathology|Wellcome Sanger Institute for the COVID-19 Genomics UK (COG-UK) consortium|Amato|United Kingdom
>Spike|hCoV-19/Scotland/QEUH-A4B2AF/2020|2020-10-08|EPI_ISL_637825|Original|hCoV-19^^Scotland|Human|Department of Pathology|COVID-19 Genomics UK (COG-UK) Consortium|Goodfellow|United Kingdom
>Spike|hCoV-19/England/MILK-ABC009/2020|2020-10-22|EPI_ISL_631032|Original|hCoV-19^^England|Human|Lighthouse Lab in Milton Keynes|Wellcome Sanger Institute for the COVID-19 Genomics UK (COG-UK) consortium|Davies|United Kingdom
>Spike|hCoV-19/England/MILK-B3B64B/2020|2020-11-02|EPI_ISL_706072|Original|hCoV-19^^England|Human|Oxford Viromics|COVID-19 Genomics UK (COG-UK) Consortium|Golubchik|United Kingdom
>Spike|hCoV-19/Japan/PG-3827/2020|2020-07-00|EPI_ISL_692078|Original|hCoV-19^^Japan|Human|Pathogen Genomics Center|Pathogen Genomics Center|Sekizuka|Japan
>Spike|hCoV-19/Denmark/DCGC-12682/2020|2020-11-16|EPI_ISL_682863|Original|hCoV-19^^Syddanmark|Human|Department of Virus and Microbiological Special Diagnostics|Albertsen Lab|Michaelsen|Denmark
>Spike|hCoV-19/Canada/ON-PHL-20-01274/2020|2020-06-00|EPI_ISL_548882|Original|hCoV-19^^Ontario|Human|Public Health Ontario Laboratory|Public Health Ontario Laboratory|Guthrie|Canada
>Spike|hCoV-19/South Korea/KCDC2497/2020|2020-06-15|EPI_ISL_514900|Original|hCoV-19^^South Korea|Human|Division of Viral Diseases|Division of Viral Diseases|Kim|South Korea
>Spike|hCoV-19/England/20176041404/2020|2020-04-21|EPI_ISL_465412|Original|hCoV-19^^England|Human|Respiratory Virus Unit|Respiratory Virus Unit|Platt|United Kingdom
>Spike|hCoV-19/USA/CA-CZB-15987/2020|2020-11-23|EPI_ISL_738723|Original|hCoV-19^^California|Human|Renegade|Chan-Zuckerberg Biohub|Kamm|USA
>Spike|hCoV-19/USA/TX-HMH-MCoV-18948/2020|2020-11-25|EPI_ISL_785242|Original|hCoV-19^^Texas|Human|Houston Methodist Hospital|Houston Methodist Hospital|Long|USA
>Spike|hCoV-19/United Arab Emirates/skmc-3891274/2020|2020-09-23|EPI_ISL_582683|Original|hCoV-19^^United Arab Emirates|Human|Sheikh Khalifa Medical City|Molecular/Surveillance lab\nSheikh Khalifa Medical City|Amirtharaj|United Arab Emirates
>Spike|hCoV-19/USA/TX-DSHS-1515/2020|2020-06-22|EPI_ISL_683430|Original|hCoV-19^^Texas|Human|Texas Department of State Health Services|Texas Department of State Health Services|Rahman|USA
>Spike|hCoV-19/USA/NY-PV09142/2020|2020-03-20|EPI_ISL_422513|Original|hCoV-19^^New York|Human|MSHS Clinical Microbiology Laboratories|MSHS Pathogen Surveillance Program|van Bakel|USA
>Spike|hCoV-19/USA/CA-SCCPHD-UC164/2020|2020-03-12|EPI_ISL_435643|Original|hCoV-19^^California|Human|Santa Clara County Public Health Department|Chiu Laboratory|Chiu|USA

>Spike|hCoV-19/England/ALDP-59977B/2020|2020-06-08|EPI_ISL_558379|Original|hCoV-
19^^England|Human|Lighthouse Lab in Alderley Park|Wellcome Sanger Institute for the
COVID-19 Genomics UK (COG-UK) consortium|Davies|United Kingdom
>Spike|hCoV-19/England/MILK-974596/2020|2020-08-17|EPI_ISL_575769|Original|hCoV-
19^^England|Human|Lighthouse Lab in Milton Keynes|Wellcome Sanger Institute for the
COVID-19 Genomics UK (COG-UK) consortium|Davies|United Kingdom
>Spike|hCoV-19/Scotland/QEUH-96F3D0/2020|2020-08-17|EPI_ISL_534475|Original|hCoV-
19^^Scotland|Human|NHSGGC West of Scotland Specialist Virology Centre / MRC-
University of Glasgow Centre for Virus Research|Wellcome Sanger Institute for the
COVID-19 Genomics UK (COG-UK) consortium|Davies|United Kingdom
>Spike|hCoV-19/Wales/PHWC-4A0A6A/2020|2020-12-10|EPI_ISL_743534|Original|hCoV-
19^^Wales|Human|Wales Specialist Virology Centre Sequencing lab|COVID-19 Genomics UK
(COG-UK) Consortium|Moore|United Kingdom
>Spike|hCoV-19/Australia/VIC5913/2020|2020-07-21|EPI_ISL_518692|Original|hCoV-
19^^Victoria|Human|Microbiological Diagnostic Unit - Public Health Laboratory (MDU-
PHL)|MDU-PHL|Schultz|Australia
>Spike|hCoV-19/England/MILK-A44F81/2020|2020-10-06|EPI_ISL_598612|Original|hCoV-
19^^England|Human|Lighthouse Lab in Milton Keynes|Wellcome Sanger Institute for the
COVID-19 Genomics UK (COG-UK) consortium|Davies|United Kingdom
>Spike|hCoV-19/USA/AZ-TG547344/2020|2020-07-06|EPI_ISL_695492|Original|hCoV-
19^^Arizona|Human|AZ SPHL|TGen North|French|USA
>Spike|hCoV-19/USA/FL-BPHL-1234/2020|2020-05-28|EPI_ISL_541158|Original|hCoV-
19^^Florida|Human|Florida Bureau of Public Health Laboratories|Florida Bureau of
Public Health Laboratories|Blanton|USA
>Spike|hCoV-19/Portugal/PT0009/2020|2020-03-08|EPI_ISL_417994|Original|hCoV-
19^^Portugal|Human|CHULC - H Curry Cabral|Instituto Nacional de Saude
(INSA)|Borges|Portugal
>Spike|hCoV-19/USA/TX-DSHS-1427/2020|2020-06-23|EPI_ISL_671639|Original|hCoV-
19^^Texas|Human|Texas Department of State Health Services|Texas Department of State
Health Services|Rahman|USA
>Spike|hCoV-19/USA/WI-UW-1730/2020|2020-09-09|EPI_ISL_605603|Original|hCoV-
19^^Wisconsin|Human|University of Wisconsin-Madison AIDS Vaccine Research
Laboratories|University of Wisconsin-Madison AIDS Vaccine Research
Laboratories|Baczenas|USA
>Spike|hCoV-19/New Zealand/20CV0369/2020|2020-09-24|EPI_ISL_579111|Original|hCoV-
19^^Auckland|Human|LabPLUS|Institute of Environmental Science and Research
(ESR)|Wang|New Zealand
>Spike|hCoV-19/USA/TX-HMH-MCoV-19080/2020|2020-11-24|EPI_ISL_783614|Original|hCoV-
19^^Texas|Human|Houston Methodist Hospital|Houston Methodist Hospital|Long|USA
>Spike|hCoV-19/Denmark/DCGC-8536/2020|2020-10-26|EPI_ISL_625896|Original|hCoV-
19^^Midtjylland|Human|Department of Virus and Microbiological Special
Diagnostics|Albertsen lab|Michaelsen|Denmark
>Spike|hCoV-19/England/MILK-978662/2020|2020-07-31|EPI_ISL_550510|Original|hCoV-
19^^England|Human|Lighthouse Lab in Milton Keynes|Wellcome Sanger Institute for the
COVID-19 Genomics UK (COG-UK) consortium|Davies|United Kingdom
>Spike|hCoV-19/USA/AZ-TG640815/2020|2020-04-27|EPI_ISL_695772|Original|hCoV-
19^^Arizona|Human|Sonora Quest Laboratories|TGen North|French|USA
>Spike|hCoV-19/England/ALDP-BBAFD0/2020|2020-11-20|EPI_ISL_701778|Original|hCoV-
19^^England|Human|Lighthouse Lab in Alderley Park|Wellcome Sanger Institute for the
COVID-19 Genomics UK (COG-UK) Consortium|Davies|United Kingdom
>Spike|hCoV-19/England/ALDP-9EA980/2020|2020-09-24|EPI_ISL_580124|Original|hCoV-
19^^England|Human|Lighthouse Lab in Alderley Park|Wellcome Sanger Institute for the
COVID-19 Genomics UK (COG-UK) consortium|Davies|United Kingdom
>Spike|hCoV-19/USA/TX-HMH-MCoV-11543/2020|2020-09-11|EPI_ISL_790050|Original|hCoV-
19^^Texas|Human|Houston Methodist Hospital|Houston Methodist Hospital|Long|USA
>Spike|hCoV-19/United Arab Emirates/1043/2020|2020-06-
11|EPI_ISL_698118|Original|hCoV-19^^Abu Dhabi|Human|Group 42 (G42) Healthcare|G42
Healthcare|Ogrodzki|United Arab Emirates

>Spike|hCoV-19/Scotland/CVR5827/2020|2020-11-14|EPI_ISL_665439|Original|hCoV-19^^Scotland|Human|West of Scotland Specialist Virology Centre|COVID-19 Genomics UK (COG-UK) Consortium|Robertson|United Kingdom
>Spike|hCoV-19/New Zealand/20VR3084/2020|2020-00-00|EPI_ISL_579414|Original|hCoV-19^^Canterbury|Human|Canterbury Health Laboratories|Institute of Environmental Science and Research (ESR)|Wang|New Zealand
>Spike|hCoV-19/England/CAMC-A41F0C/2020|2020-10-06|EPI_ISL_609335|Original|hCoV-19^^England|Human|Lighthouse Lab in Cambridge|Wellcome Sanger Institute for the COVID-19 Genomics UK (COG-UK) consortium|Davies|United Kingdom
>Spike|hCoV-19/Canada/ON-PHL-20-01286/2020|2020-06-00|EPI_ISL_548891|Original|hCoV-19^^Ontario|Human|Public Health Ontario Laboratory|Public Health Ontario Laboratory|Guthrie|Canada
>Spike|hCoV-19/Wales/PHWC-4A4978/2020|2020-12-14|EPI_ISL_763413|Original|hCoV-19^^Wales|Human|Wales Specialist Virology Centre Sequencing lab|COVID-19 Genomics UK (COG-UK) Consortium|Moore|United Kingdom
>Spike|hCoV-19/England/SHEF-10D0FEB/2020|2020-12-18|EPI_ISL_742030|Original|hCoV-19^^England|Human|Virology Department|COVID-19 Genomics UK (COG-UK) Consortium|de Silva|United Kingdom
>Spike|hCoV-19/Japan/IC-0139/2020|2020-09-00|EPI_ISL_667568|Original|hCoV-19^^Japan|Human|Pathogen Genomics Center|Pathogen Genomics Center|Sekizuka|Japan
>Spike|hCoV-19/Australia/VIC4509/2020|2020-07-17|EPI_ISL_518920|Original|hCoV-19^^Victoria|Human|Microbiological Diagnostic Unit - Public Health Laboratory (MDU-PHL)|MDU-PHL|Schultz|Australia
>Spike|hCoV-19/England/QEUH-AAD117/2020|2020-10-21|EPI_ISL_652941|Original|hCoV-19^^England|Human|Oxford Viromics|COVID-19 Genomics UK (COG-UK) Consortium|Golubchik|United Kingdom
>Spike|hCoV-19/Croatia/Zagreb-10/2020|2020-09-30|EPI_ISL_710568|Original|hCoV-19^^Zagreb|Human|University Hospital Dubrava|Ru\u0111er Bo\u0161kovic Institute; Forensic Science Centre Ivan Vu\u010deti\u0107; University of Zagreb Faculty of Science|Karlic|Croatia
>Spike|hCoV-19/USA/CA-IGI-0703/2020|2020-07-28|EPI_ISL_677876|Original|hCoV-19^^California|Human|Innovative Genomics Institute|Innovative Genomics Institute|Wyman|USA
>Spike|hCoV-19/USA/AZ-TG268770/2020|2020-03-19|EPI_ISL_426511|Original|hCoV-19^^Arizona|Human|TGen North|TGen North|Lemmer|USA
>Spike|hCoV-19/England/SHEF-C0AFF/2020|2020-09-27|EPI_ISL_573687|Original|hCoV-19^^England|Human|Virology Department|COVID-19 Genomics UK (COG-UK) Consortium|de Silva|United Kingdom
>Spike|hCoV-19/England/CAMC-A59754/2020|2020-10-08|EPI_ISL_597129|Original|hCoV-19^^England|Human|Lighthouse Lab in Cambridge|Wellcome Sanger Institute for the COVID-19 Genomics UK (COG-UK) consortium|Davies|United Kingdom
>Spike|hCoV-19/USA/CA-CDPH230/2020|2020-11-15|EPI_ISL_755080|Original|hCoV-19^^California|Human|California Department of Public Health|California Department of Public Health|Bell|USA
>Spike|hCoV-19/Peru/LIM-INS-009/2020|2020-03-08|EPI_ISL_489987|Original|hCoV-19^^Lima|Human|Laboratorio de Referencia Nacional de Virus Respiratorio. Instituto Nacional de Salud Per\u00fa|Laboratorio de Referencia Nacional de Biotecnolog\u00eda y Biolog\u00eda Molecular. Instituto Nacional de Salud Per\u00fa|Padilla Rojas|Peru
>Spike|hCoV-19/Japan/PG-1072/2020|2020-04-00|EPI_ISL_684682|Original|hCoV-19^^Japan|Human|Fukuoka Institute of Health and Environmental Sciences|Pathogen Genomics Center|Sekizuka|Japan
>Spike|hCoV-19/Belgium/ULG-10097/2020|2020-04-06|EPI_ISL_427348|Original|hCoV-19^^Liege|Human|Department of Clinical Microbiology|GIGA Medical Genomics|Durkin|Belgium
>Spike|hCoV-19/USA/MD-MDH-0078/2020|2020-05-25|EPI_ISL_509482|Original|hCoV-19^^Maryland|Human|Maryland Department of Health|Maryland Department of Health|Keller|USA
>Spike|hCoV-19/Japan/PG-2630/2020|2020-04-00|EPI_ISL_690762|Original|hCoV-19^^Japan|Human|Pathogen Genomics Center|Pathogen Genomics Center|Sekizuka|Japan

>Spike|hCoV-19/Morocco/FMP-13/2020|2020-11-00|EPI_ISL_728297|Original|hCoV-19^^Rabat|Human|Laboratoire Biolife|Laboratoire de Biotechnologie|Ouadghiri|Morocco
>Spike|hCoV-19/Iceland/163/2020|2020-03-14|EPI_ISL_417775|Original|hCoV-19^^Reykjavik|Human|The National University Hospital of Iceland|deCODE genetics|J\u00f3nsson|Iceland
>Spike|hCoV-19/Wales/PHWC-2B588/2020|2020-04-10|EPI_ISL_445728|Original|hCoV-19^^Wales|Human|Wales Specialist Virology Centre|Public Health Wales Microbiology Cardiff|Moore|United Kingdom
>Spike|hCoV-19/USA/NY-Wadsworth-290723-01/2020|2020-12-20|EPI_ISL_767551|Original|hCoV-19^^New York|Human|URMC LABS|Wadsworth Center|Lasek-Nesselquist|USA
>Spike|hCoV-19/England/BIRM-5FF9C/2020|2020-00-00|EPI_ISL_461543|Original|hCoV-19^^England|Human|University of Birmingham|COVID-19 Genomics UK (COG-UK) Consortium|Loman|United Kingdom
>Spike|hCoV-19/England/BRIS-123938/2020|2020-04-07|EPI_ISL_442669|Original|hCoV-19^^England|Human|PHE South West Regional Laboratory|Wellcome Sanger Institute for the COVID-19 Genomics UK (COG-UK) consortium|Amato|United Kingdom
>Spike|hCoV-19/Portugal/PT1443/2020|2020-03-24|EPI_ISL_511402|Original|hCoV-19^^Portugal|Human|Instituto Nacional de Saude (INSA)|Instituto Nacional de Saude (INSA)|Borges|Portugal
>Spike|hCoV-19/Netherlands/GE-RIVM-10382/2020|2020-10-09|EPI_ISL_636536|Original|hCoV-19^^Gelderland|Human|Dutch COVID-19 response team|National Institute for Public Health and the Environment (RIVM)|Meijer|Netherlands
>Spike|hCoV-19/England/LIVE-A2B69/2020|2020-05-19|EPI_ISL_499925|Original|hCoV-19^^England|Human|Liverpool Clinical Laboratories|COVID-19 Genomics UK (COG-UK) Consortium|Haldenby|United Kingdom
>Spike|hCoV-19/USA/CA-ALSR-3012/2020|2020-07-12|EPI_ISL_635786|Original|hCoV-19^^California|Human|San Diego County Public Health Laboratory|Andersen lab at Scripps Research|Gangavarapu|USA
>Spike|hCoV-19/Japan/PG-2257/2020|2020-04-00|EPI_ISL_685862|Original|hCoV-19^^Japan|Human|Pathogen Genomics Center|Pathogen Genomics Center|Sekizuka|Japan
>Spike|hCoV-19/England/ALDP-9C4EE7/2020|2020-09-10|EPI_ISL_567634|Original|hCoV-19^^England|Human|Lighthouse Lab in Alderley Park|Wellcome Sanger Institute for the COVID-19 Genomics UK (COG-UK) consortium|Davies|United Kingdom
>Spike|hCoV-19/Denmark/DCGC-15935/2020|2020-11-23|EPI_ISL_714272|Original|hCoV-19^^Hovedstaden|Human|Department of Virus and Microbiological Special Diagnostics|Albertsen Lab|Michaelsen|Denmark
>Spike|hCoV-19/Japan/PG-1529/2020|2020-03-00|EPI_ISL_685135|Original|hCoV-19^^Japan|Human|Pathogen Genomics Center|Pathogen Genomics Center|Sekizuka|Japan
>Spike|hCoV-19/England/MILK-B02337/2020|2020-10-23|EPI_ISL_643008|Original|hCoV-19^^England|Human|Lighthouse Lab in Milton Keynes|Wellcome Sanger Institute for the COVID-19 Genomics UK (COG-UK) Consortium|Davies|United Kingdom
>Spike|hCoV-19/England/MILK-ACC7C5/2020|2020-10-21|EPI_ISL_634467|Original|hCoV-19^^England|Human|Lighthouse Lab in Milton Keynes|Wellcome Sanger Institute for the COVID-19 Genomics UK (COG-UK) consortium|Davies|United Kingdom
>Spike|hCoV-19/Scotland/CVR2862/2020|2020-04-21|EPI_ISL_473603|Original|hCoV-19^^Scotland|Human|West of Scotland Specialist Virology Centre|COVID-19 Genomics UK (COG-UK) Consortium|Robertson|United Kingdom
>Spike|hCoV-19/DRC/3453/2020|2020-04-22|EPI_ISL_447599|Original|hCoV-19^^Kinshasa|Human|Viral Respiratory Lab|Pathogen Sequencing Lab|Lusamaki|Democratic Republic of the Congo
>Spike|hCoV-19/USA/TX-HMH-MCoV-10635/2020|2020-07-08|EPI_ISL_789569|Original|hCoV-19^^Texas|Human|Houston Methodist Hospital|Houston Methodist Hospital|Long|USA
>Spike|hCoV-19/England/MILK-992170/2020|2020-08-27|EPI_ISL_540652|Original|hCoV-19^^England|Human|Queens Medical Centre|COVID-19 Genomics UK (COG-UK) Consortium|Loose|United Kingdom
>Spike|hCoV-19/USA/WA-OHSU-0504/2020|2020-06-05|EPI_ISL_526090|Original|hCoV-19^^Washington|Human|OHSU Lab Services Molecular Microbiology Lab|Ginkgo Bioworks Clinical Laboratory|O'Roak|USA

>Spike|hCoV-19/England/CAMC-A81B93/2020|2020-10-14|EPI_ISL_610903|Original|hCoV-19^^England|Human|Lighthouse Lab in Cambridge|Wellcome Sanger Institute for the COVID-19 Genomics UK (COG-UK) consortium|Davies|United Kingdom
>Spike|hCoV-19/Wales/ALDP-950F56/2020|2020-06-14|EPI_ISL_554157|Original|hCoV-19^^Wales|Human|Lighthouse Lab in Alderley Park|Wellcome Sanger Institute for the COVID-19 Genomics UK (COG-UK) consortium|Davies|United Kingdom
>Spike|hCoV-19/Wales/PHWC-48E525/2020|2020-11-12|EPI_ISL_680872|Original|hCoV-19^^Wales|Human|Wales Specialist Virology Centre Sequencing lab|COVID-19 Genomics UK (COG-UK) Consortium|Moore|United Kingdom
>Spike|hCoV-19/England/ALDP-9CB615/2020|2020-09-16|EPI_ISL_575574|Original|hCoV-19^^England|Human|Lighthouse Lab in Alderley Park|Wellcome Sanger Institute for the COVID-19 Genomics UK (COG-UK) consortium|Davies|United Kingdom
>Spike|hCoV-19/England/MILK-ACE5D8/2020|2020-10-21|EPI_ISL_629519|Original|hCoV-19^^England|Human|Lighthouse Lab in Milton Keynes|Wellcome Sanger Institute for the COVID-19 Genomics UK (COG-UK) consortium|Davies|United Kingdom
>Spike|hCoV-19/Switzerland/ZH-ETHZ-400346/2020|2020-12-08|EPI_ISL_729176|Original|hCoV-19^^Zurich|Human|Viollier AG|Department of Biosystems Science and Engineering|Nadeau|Switzerland
>Spike|hCoV-19/England/EXET-138569/2020|2020-10-18|EPI_ISL_612400|Original|hCoV-19^^England|Human|University of Exeter|COVID-19 Genomics UK (COG-UK) Consortium|Temperton|United Kingdom
>Spike|hCoV-19/England/QEUH-95F1BB/2020|2020-08-07|EPI_ISL_532290|Original|hCoV-19^^England|Human|Lighthouse Lab in Glasgow|Wellcome Sanger Institute for the COVID-19 Genomics UK (COG-UK) consortium|Davies|United Kingdom
>Spike|hCoV-19/Japan/PG-4250/2020|2020-07-00|EPI_ISL_692480|Original|hCoV-19^^Japan|Human|Fukuoka Institute of Health and Environmental Sciences|Pathogen Genomics Center|Sekizuka|Japan
>Spike|hCoV-19/Canada/QC_AE6/2020|2020-04-06|EPI_ISL_463906|Original|hCoV-19^^Quebec|Human|Laboratoire de microbiologie|Smith Laboratory|Smith|Canada
>Spike|hCoV-19/Brazil/SP-L14-CD250/2020|2020-03-27|EPI_ISL_476250|Original|hCoV-19^^S\u00e3o Paulo|Human|Hospital da Cl\u00ednicas da Faculdade de Medicina da Universidade de S\u00e3o Paulo|Instituto de Medicina Tropical da Univesidade de S\u00e3o Paulo|Sabino|Brazil
>Spike|hCoV-19/South Africa/N00017/2020|2020-08-06|EPI_ISL_623029|Original|hCoV-19^^Eastern Cape|Human|National Health Laboratory Service|National Institute for Communicable Diseases of the National Health Laboratory Service|Allam|South Africa
>Spike|hCoV-19/USA/IL-NM022/2020|2020-03-15|EPI_ISL_444532|Original|hCoV-19^^Illinois|Human|Northwestern Memorial Hospital|Ozer Lab|Ozer|USA
>Spike|hCoV-19/Japan/PG-5394/2020|2020-08-00|EPI_ISL_690497|Original|hCoV-19^^Japan|Human|Fukuoka Institute of Health and Environmental Sciences|Pathogen Genomics Center|Sekizuka|Japan
>Spike|hCoV-19/Wales/PHWC-48BB08/2020|2020-11-06|EPI_ISL_666324|Original|hCoV-19^^Wales|Human|Wales Specialist Virology Centre Sequencing lab|COVID-19 Genomics UK (COG-UK) Consortium|Moore|United Kingdom
>Spike|hCoV-19/Luxembourg/LNS1146047/2020|2020-10-26|EPI_ISL_739896|Original|hCoV-19^^Luxembourg|Human|Laboratoire national de sant\u00e9|Laboratoire national de sant\u00e9|Wienecke-Baldacchino|Luxembourg
>Spike|hCoV-19/Denmark/DCGC-13688/2020|2020-11-30|EPI_ISL_712194|Original|hCoV-19^^Midtjylland|Human|Department of Virus and Microbiological Special Diagnostics|Albertsen Lab|Michaelsen|Denmark
>Spike|hCoV-19/Japan/PG-5082/2020|2020-08-00|EPI_ISL_690192|Original|hCoV-19^^Japan|Human|Pathogen Genomics Center|Pathogen Genomics Center|Sekizuka|Japan
>Spike|hCoV-19/England/NOTT-112DA9/2020|2020-07-17|EPI_ISL_499269|Original|hCoV-19^^England|Human|Queens Medical Centre|COVID-19 Genomics UK (COG-UK) Consortium|Loose|United Kingdom
>Spike|hCoV-19/USA/FL-BPHL-1450/2020|2020-04-07|EPI_ISL_549232|Original|hCoV-19^^Florida|Human|Florida Bureau of Public Health Laboratories|Florida Bureau of Public Health Laboratories|Blanton|USA

>Spike|hCoV-19/Italy/VEN-UniVR-54/2020|2020-03-12|EPI_ISL_751364|Original|hCoV-19^^Veneto|Human|IRCCS Sacro Cuore Don Calabria Hospital|University of Verona|Mori|Italy
>Spike|hCoV-19/England/QEUH-B69AA2/2020|2020-11-11|EPI_ISL_661480|Original|hCoV-19^^England|Human|Lighthouse Lab in Glasgow|Wellcome Sanger Institute for the COVID-19 Genomics UK (COG-UK) Consortium|Davies|United Kingdom
>Spike|hCoV-19/England/ALDP-6CBA2B/2020|2020-07-02|EPI_ISL_556140|Original|hCoV-19^^England|Human|Lighthouse Lab in Alderley Park|Wellcome Sanger Institute for the COVID-19 Genomics UK (COG-UK) consortium|Davies|United Kingdom
>Spike|hCoV-19/England/ALDP-BCA91B/2020|2020-11-23|EPI_ISL_703339|Original|hCoV-19^^England|Human|Lighthouse Lab in Alderley Park|Wellcome Sanger Institute for the COVID-19 Genomics UK (COG-UK) Consortium|Davies|United Kingdom
>Spike|hCoV-19/England/MILK-AC82D7/2020|2020-10-21|EPI_ISL_629207|Original|hCoV-19^^England|Human|Lighthouse Lab in Milton Keynes|Wellcome Sanger Institute for the COVID-19 Genomics UK (COG-UK) consortium|Davies|United Kingdom
>Spike|hCoV-19/England/CAMC-A64BB4/2020|2020-10-11|EPI_ISL_608624|Original|hCoV-19^^England|Human|Lighthouse Lab in Cambridge|Wellcome Sanger Institute for the COVID-19 Genomics UK (COG-UK) consortium|Davies|United Kingdom
>Spike|hCoV-19/USA/CO-CDPHE-2003120922/2020|2020-03-11|EPI_ISL_677269|Original|hCoV-19^^Colorado|Human|Colorado Department of Public Health and Environment|Colorado Department of Puplic Health and Environment|Bankers|USA
>Spike|hCoV-19/USA/WA-UW-47087/2020|2020-12-15|EPI_ISL_756225|Original|hCoV-19^^Washington|Human|UW Virology Lab|UW Virology Lab|Roychoudhury|USA
>Spike|hCoV-19/Equatorial Guinea/16819/2020|2020-06-10|EPI_ISL_648361|Original|hCoV-19^^Bioko Norte|Human|Laboratorio de Investigaciones de Baney|University Hospital Basel|Hosch|Equatorial Guinea
>Spike|hCoV-19/USA/MN-CDC-6568/2020|2020-04-19|EPI_ISL_527732|Original|hCoV-19^^Minnesota|Human|MN PHL Division|Pathogen Discovery|Queen|USA
>Spike|hCoV-19/Switzerland/BL-ETHZ-360156/2020|2020-11-06|EPI_ISL_693928|Original|hCoV-19^^Basel-Landschaft|Human|Viollier AG|Department of Biosystems Science and Engineering|Nadeau|Switzerland
>Spike|hCoV-19/Japan/PG-5169/2020|2020-08-00|EPI_ISL_690272|Original|hCoV-19^^Japan|Human|Pathogen Genomics Center|Pathogen Genomics Center|Sekizuka|Japan
>Spike|hCoV-19/Japan/PG-8021/2020|2020-08-00|EPI_ISL_687122|Original|hCoV-19^^Japan|Human|Pathogen Genomics Center|Pathogen Genomics Center|Sekizuka|Japan
>Spike|hCoV-19/USA/CA-CSMC128/2020|2020-04-09|EPI_ISL_475605|Original|hCoV-19^^California|Human|Cedars-Sinai Medical Center|Cedars-Sinai Medical Center|Zhang|USA
>Spike|hCoV-19/Germany/BE-ChVir-D1952-5748/2020|2020-10-09|EPI_ISL_753882|Original|hCoV-19^^Berlin|Human|Charit\u00e9 Universit\u00e4tsmedizin Berlin|Charit\u00e9 Universit\u00e4tsmedizin Berlin|Muehlemann|Germany
>Spike|hCoV-19/Netherlands/UT-EMC-105/2020|2020-09-28|EPI_ISL_632461|Original|hCoV-19^^Utrecht|Human|Dutch COVID-19 response team|Erasmus Medical Center|Oude Munnink|Netherlands
>Spike|hCoV-19/Switzerland/BS-42221749/2020|2020-04-06|EPI_ISL_581867|Original|hCoV-19^^Basel-Stadt|Human|University Hospital Basel|University Hospital Basel|Roloff Handschin|Switzerland
>Spike|hCoV-19/USA/TX-DSHS-0955/2020|2020-07-16|EPI_ISL_602252|Original|hCoV-19^^Texas|Human|Texas Department of State Health Services|Texas Department of State Health Services|Rahman|USA
>Spike|hCoV-19/New Zealand/20VR2108/2020|2020-04-15|EPI_ISL_456377|Original|hCoV-19^^MidCentral|Human|MedLab Central Ltd|Institute of Environmental Science and Research (ESR)|de Ligt|New Zealand
>Spike|hCoV-19/Wales/PHWC-49B74D/2020|2020-12-04|EPI_ISL_742378|Original|hCoV-19^^Wales|Human|Wales Specialist Virology Centre Sequencing lab|COVID-19 Genomics UK (COG-UK) Consortium|Moore|United Kingdom
>Spike|hCoV-19/England/MILK-CA5159/2020|2020-12-17|EPI_ISL_762407|Original|hCoV-19^^England|Human|Lighthouse Lab in Milton Keynes|Wellcome Sanger Institute for the COVID-19 Genomics UK (COG-UK) Consortium|Davies|United Kingdom

>Spike|hCoV-19/Scotland/EDB4655/2020|2020-05-08|EPI_ISL_493910|Original|hCoV-
19^^Scotland|Human|Virology Department|COVID-19 Genomics UK (COG-UK)
Consortium|Williams|United Kingdom
>Spike|hCoV-19/England/QEUH-AD3637/2020|2020-10-26|EPI_ISL_633780|Original|hCoV-
19^^England|Human|Lighthouse Lab in Glasgow|Wellcome Sanger Institute for the COVID-
19 Genomics UK (COG-UK) consortium|Davies|United Kingdom
>Spike|hCoV-19/England/NORT-1B71FA5/2020|2020-11-22|EPI_ISL_706329|Original|hCoV-
19^^England|Human|Northumbria University / South Tees Hospitals NHS Foundation Trust
/ North Cumbria Integrated Care NHS Foundation Trust / North Tees and Hartlepool NHS
Foundation Trust / Newcastle Hospitals NHS Foundation Trust|COVID-19 Genomics UK
(COG-UK) Consortium|Bashton|United Kingdom
>Spike|hCoV-19/USA/CO-CDPHE-2008210131/2020|2020-08-20|EPI_ISL_710325|Original|hCoV-
19^^Colorado|Human|Colorado Department of Public Health and Environment|Colorado
Department of Puplic Health and Environment|Bankers|USA
>Spike|hCoV-19/Netherlands/ZuidHolland_79/2020|2020-03-
26|EPI_ISL_422947|Original|hCoV-19^^Zuid Holland|Human|Dutch COVID-19 response
team|Erasmus Medical Center|Oude Munnink|Netherlands
>Spike|hCoV-19/Scotland/QEUH-B127CF/2020|2020-11-02|EPI_ISL_649585|Original|hCoV-
19^^Scotland|Human|Lighthouse Lab in Glasgow|Wellcome Sanger Institute for the COVID-
19 Genomics UK (COG-UK) Consortium|Davies|United Kingdom
>Spike|hCoV-19/USA/NM-NMDOH-2020229805/2020|2020-07-20|EPI_ISL_535297|Original|hCoV-
19^^New Mexico|Human|New Mexico Department of Health Scientific Laboratory|New Mexico
Department of Health Scientific Laboratory|Johnson|USA
>Spike|hCoV-19/Scotland/CVR2770/2020|2020-04-20|EPI_ISL_438978|Original|hCoV-
19^^Scotland|Human|West of Scotland Specialist Virology Centre|COVID-19 Genomics UK
(COG-UK) Consortium|Robertson|United Kingdom
>Spike|hCoV-19/England/MILK-74AF7F/2020|2020-06-30|EPI_ISL_557444|Original|hCoV-
19^^England|Human|Lighthouse Lab in Milton Keynes|Wellcome Sanger Institute for the
COVID-19 Genomics UK (COG-UK) consortium|Davies|United Kingdom
>Spike|hCoV-19/Canada/ON-PHL-20-01217/2020|2020-06-00|EPI_ISL_548839|Original|hCoV-
19^^Ontario|Human|Public Health Ontario Laboratory|Public Health Ontario
Laboratory|Guthrie|Canada
>Spike|hCoV-19/England/CAMC-A64A5D/2020|2020-10-10|EPI_ISL_608996|Original|hCoV-
19^^England|Human|Lighthouse Lab in Cambridge|Wellcome Sanger Institute for the
COVID-19 Genomics UK (COG-UK) consortium|Davies|United Kingdom
>Spike|hCoV-19/Sweden/20-05469/2020|2020-03-10|EPI_ISL_434644|Original|hCoV-
19^^Stockholm|Human|Kungsholmsdoktorn|The Public Health Agency of
Sweden|Svartstr\u00f6m|Sweden
>Spike|hCoV-19/England/MILK-9E046B/2020|2020-09-20|EPI_ISL_601302|Original|hCoV-
19^^England|Human|Lighthouse Lab in Milton Keynes|Wellcome Sanger Institute for the
COVID-19 Genomics UK (COG-UK) consortium|Davies|United Kingdom
>Spike|hCoV-19/Norway/4613/2020|2020-09-30|EPI_ISL_635098|Original|hCoV-
19^^Vestland|Human|Foerde Hospital|Norwegian Institute of Public Health|Vollan|Norway
>Spike|hCoV-19/Wales/PHWC-48C150/2020|2020-11-03|EPI_ISL_666399|Original|hCoV-
19^^Wales|Human|Wales Specialist Virology Centre Sequencing lab|COVID-19 Genomics UK
(COG-UK) Consortium|Moore|United Kingdom
>Spike|hCoV-19/England/ALDP-9CE041/2020|2020-09-16|EPI_ISL_566276|Original|hCoV-
19^^England|Human|Lighthouse Lab in Alderley Park|Wellcome Sanger Institute for the
COVID-19 Genomics UK (COG-UK) consortium|Davies|United Kingdom
>Spike|hCoV-19/USA/WA-S581/2020|2020-03-30|EPI_ISL_434341|Original|hCoV-
19^^Washington|Human|Washington State Department of Health|Seattle Flu
Study|Bedford|USA
>Spike|hCoV-19/Australia/VIC14276/2020|2020-08-04|EPI_ISL_592429|Original|hCoV-
19^^Victoria|Human|Microbiological Diagnostic Unit - Public Health Laboratory (MDU-
PHL)|MDU-PHL|Schultz|Australia
>Spike|hCoV-19/USA/NY-NYUMC323/2020|2020-04-02|EPI_ISL_430350|Original|hCoV-19^^New
York|Human|NYU Langone Health|Departments of Pathology and Medicine|Maurano|USA
>Spike|hCoV-19/Scotland/EDB4674/2020|2020-05-09|EPI_ISL_478181|Original|hCoV-
19^^Scotland|Human|Virology Department|COVID-19 Genomics UK (COG-UK)
Consortium|Williams|United Kingdom

>Spike|hCoV-19/USA/WA-UW-12886/2020|2020-06-29|EPI_ISL_570923|Original|hCoV-19^^Washington|Human|UW Virology Lab|UW Virology Lab|Roychoudhury|USA
>Spike|hCoV-19/Australia/NSW317/2020|2020-03-31|EPI_ISL_451580|Original|hCoV-19^^New South Wales|Human|Pathology West - NSW Health Pathology|NSW Health Pathology - Institute of Clinical Pathology and Medical Research; Westmead Hospital; University of Sydney|Gall|Australia
>Spike|hCoV-19/Scotland/EDB4881/2020|2020-05-13|EPI_ISL_453169|Original|hCoV-19^^Scotland|Human|Virology Department|COVID-19 Genomics UK (COG-UK) Consortium|Williams|United Kingdom
>Spike|hCoV-19/England/MILK-AC7BDD/2020|2020-10-22|EPI_ISL_634472|Original|hCoV-19^^England|Human|Lighthouse Lab in Milton Keynes|Wellcome Sanger Institute for the COVID-19 Genomics UK (COG-UK) consortium|Davies|United Kingdom
>Spike|hCoV-19/USA/MI-MDHHS-SC20509/2020|2020-05-08|EPI_ISL_460557|Original|hCoV-19^^Michigan|Human|Michigan Department of Health and Human Services|Michigan Department of Health and Human Services|Blankenship|USA
>Spike|hCoV-19/Switzerland/BS-42378086/2020|2020-08-03|EPI_ISL_581934|Original|hCoV-19^^Basel-Stadt|Human|University Hospital Basel|University Hospital Basel|Roloff Handschin|Switzerland
>Spike|hCoV-19/USA/WA-S2740/2020|2020-07-16|EPI_ISL_525730|Original|hCoV-19^^Washington|Human|Seattle Flu Study|Seattle Flu Study|Lee|USA
>Spike|hCoV-19/Australia/VIC9899/2020|2020-08-25|EPI_ISL_565770|Original|hCoV-19^^Victoria|Human|Microbiological Diagnostic Unit - Public Health Laboratory (MDU-PHL)|MDU-PHL|Schultz|Australia
>Spike|hCoV-19/USA/WI-UW-811/2020|2020-06-22|EPI_ISL_509996|Original|hCoV-19^^Wisconsin|Human|University of Wisconsin-Madison AIDS Vaccine Research Laboratories|University of Wisconsin-Madison AIDS Vaccine Research Laboratories|Baczenas|USA
>Spike|hCoV-19/England/MILK-ACDF26/2020|2020-10-20|EPI_ISL_630286|Original|hCoV-19^^England|Human|Lighthouse Lab in Milton Keynes|Wellcome Sanger Institute for the COVID-19 Genomics UK (COG-UK) consortium|Davies|United Kingdom
>Spike|hCoV-19/Scotland/EDB6579/2020|2020-05-10|EPI_ISL_478261|Original|hCoV-19^^Scotland|Human|Virology Department|COVID-19 Genomics UK (COG-UK) Consortium|Williams|United Kingdom
>Spike|hCoV-19/England/204661388/2020|2020-11-11|EPI_ISL_686575|Original|hCoV-19^^England|Human|Respiratory Virus Unit|COVID-19 Genomics UK (COG-UK) Consortium|Platt|United Kingdom
>Spike|hCoV-19/England/CAMC-A3FB0D/2020|2020-10-05|EPI_ISL_609776|Original|hCoV-19^^England|Human|Lighthouse Lab in Cambridge|Wellcome Sanger Institute for the COVID-19 Genomics UK (COG-UK) consortium|Davies|United Kingdom
>Spike|hCoV-19/England/CAMB-1AA948/2020|2020-04-18|EPI_ISL_470243|Original|hCoV-19^^England|Human|Department of Pathology|Wellcome Sanger Institute for the COVID-19 Genomics UK (COG-UK) consortium|Davies|United Kingdom
>Spike|hCoV-19/England/CAMC-A598D9/2020|2020-10-09|EPI_ISL_596984|Original|hCoV-19^^England|Human|Lighthouse Lab in Cambridge|Wellcome Sanger Institute for the COVID-19 Genomics UK (COG-UK) consortium|Davies|United Kingdom
>Spike|hCoV-19/USA/TX-HMH-3808/2020|2020-06-12|EPI_ISL_543212|Original|hCoV-19^^Texas|Human|Houston Methodist Hospital|Houston Methodist Hospital|Long|USA
>Spike|hCoV-19/England/CAMB-77CA6/2020|2020-04-01|EPI_ISL_440822|Original|hCoV-19^^England|Human|Department of Pathology|Wellcome Sanger Institute for the COVID-19 Genomics UK (COG-UK) consortium|Amato|United Kingdom
>Spike|hCoV-19/Australia/VIC15476/2020|2020-08-02|EPI_ISL_593323|Original|hCoV-19^^Victoria|Human|Microbiological Diagnostic Unit - Public Health Laboratory (MDU-PHL)|MDU-PHL|Schultz|Australia
>Spike|hCoV-19/Kenya/C4895/2020|2020-05-08|EPI_ISL_568742|Original|hCoV-19^^Mombasa|Human|KEMRI-Wellcome Trust Research Programme/KEMRI-CGMR-C Kilifi|KEMRI-Wellcome Trust Research Programme/KEMRI-CGMR-C Kilifi|Githinji|Kenya
>Spike|hCoV-19/France/ARA-64293/2020|2020-08-26|EPI_ISL_683380|Original|hCoV-19^^Auvergne-Rh\u00f4ne-Alpes|Human|CNR Virus des Infections Respiratoires - France SUD|CNR Virus des Infections Respiratoires - France SUD|Bal|France

>Spike|hCoV-19/England/MILK-BEE42F/2020|2020-11-30|EPI_ISL_708912|Original|hCoV-19^^England|Human|Lighthouse Lab in Milton Keynes|Wellcome Sanger Institute for the COVID-19 Genomics UK (COG-UK) Consortium|Davies|United Kingdom
>Spike|hCoV-19/USA/MN-MDH-1472/2020|2020-03-27|EPI_ISL_518866|Original|hCoV-19^^Minnesota|Human|Mayo Clinic & Mayo Clinic Laboratories|Minnesota Department of Health|Garfin|USA
>Spike|hCoV-19/Australia/VIC14638/2020|2020-08-02|EPI_ISL_592928|Original|hCoV-19^^Victoria|Human|Microbiological Diagnostic Unit - Public Health Laboratory (MDU-PHL)|MDU-PHL|Schultz|Australia
>Spike|hCoV-19/USA/CA-UCLA-11/2020|2020-04-17|EPI_ISL_537470|Original|hCoV-19^^California|Human|UCLA Pathology Clinical Microbiology Lab|Kruglyak Lab|Boocock|USA
>Spike|hCoV-19/England/LIVE-DC2119/2020|2020-11-21|EPI_ISL_679400|Original|hCoV-19^^England|Human|Liverpool Clinical Laboratories|COVID-19 Genomics UK (COG-UK) Consortium|Haldenby|United Kingdom
>Spike|hCoV-19/USA/TX-HMH-MCoV-15928/2020|2020-07-10|EPI_ISL_785612|Original|hCoV-19^^Texas|Human|Houston Methodist Hospital|Houston Methodist Hospital|Long|USA
>Spike|hCoV-19/USA/OR-OHSU-0674/2020|2020-06-17|EPI_ISL_525882|Original|hCoV-19^^Oregon|Human|OHSU Lab Services Molecular Microbiology Lab|Ginkgo Bioworks Clinical Laboratory|O'Roak|USA
>Spike|hCoV-19/England/ALDP-9CCA09/2020|2020-09-16|EPI_ISL_573124|Original|hCoV-19^^England|Human|Quadram Institute Bioscience|COVID-19 Genomics UK (COG-UK) Consortium|Alikhan|United Kingdom
>Spike|hCoV-19/England/ALDP-9829F3/2020|2020-08-10|EPI_ISL_552507|Original|hCoV-19^^England|Human|Lighthouse Lab in Alderley Park|Wellcome Sanger Institute for the COVID-19 Genomics UK (COG-UK) consortium|Davies|United Kingdom
>Spike|hCoV-19/Canada/QC-LSPQ-L00232073/2020|2020-03-22|EPI_ISL_535973|Original|hCoV-19^^Quebec|Human|H\u00f4pital Charles-LeMoyne|Laboratoire de sant\u00e9 publique du Qu\u00e9bec|Moreira|Canada
>Spike|hCoV-19/England/MILK-C2FEC9/2020|2020-12-07|EPI_ISL_782753|Original|hCoV-19^^England|Human|Lighthouse Lab in Milton Keynes|Wellcome Sanger Institute for the COVID-19 Genomics UK (COG-UK) Consortium|Davies|United Kingdom
>Spike|hCoV-19/USA/VA-DCLS-1455/2020|2020-08-19|EPI_ISL_572198|Original|hCoV-19^^Virginia|Human|Virginia DCLS|Virginia DCLS|St. Jacques|USA
>Spike|hCoV-19/England/ALDP-9CBCC8/2020|2020-09-16|EPI_ISL_575603|Original|hCoV-19^^England|Human|Lighthouse Lab in Alderley Park|Wellcome Sanger Institute for the COVID-19 Genomics UK (COG-UK) consortium|Davies|United Kingdom
>Spike|hCoV-19/USA/TX-HMH-3759/2020|2020-06-16|EPI_ISL_543170|Original|hCoV-19^^Texas|Human|Houston Methodist Hospital|Houston Methodist Hospital|Long|USA
>Spike|hCoV-19/Northern Ireland/QEUH-96209A/2020|2020-08-10|EPI_ISL_531652|Original|hCoV-19^^Northern Ireland|Human|Lighthouse Lab in Glasgow|Wellcome Sanger Institute for the COVID-19 Genomics UK (COG-UK) consortium|Davies|United Kingdom
>Spike|hCoV-19/England/QEUH-9D1184/2020|2020-09-16|EPI_ISL_589994|Original|hCoV-19^^England|Human|Lighthouse Lab in Glasgow|Wellcome Sanger Institute for the COVID-19 Genomics UK (COG-UK) consortium|Davies|United Kingdom
>Spike|hCoV-19/England/MILK-99E5C3/2020|2020-08-31|EPI_ISL_550734|Original|hCoV-19^^England|Human|Lighthouse Lab in Milton Keynes|Wellcome Sanger Institute for the COVID-19 Genomics UK (COG-UK) consortium|Davies|United Kingdom
>Spike|hCoV-19/England/ALDP-C5A047/2020|2020-12-13|EPI_ISL_736173|Original|hCoV-19^^England|Human|Lighthouse Lab in Alderley Park|Wellcome Sanger Institute for the COVID-19 Genomics UK (COG-UK) Consortium|Davies|United Kingdom
>Spike|hCoV-19/New Zealand/20VR2716/2020|2020-00-00|EPI_ISL_579335|Original|hCoV-19^^Auckland|Human|LabPLUS|Institute of Environmental Science and Research (ESR)|Wang|New Zealand
>Spike|hCoV-19/England/ALDP-AA2DE1/2020|2020-10-22|EPI_ISL_624004|Original|hCoV-19^^England|Human|Lighthouse Lab in Alderley Park|Wellcome Sanger Institute for the COVID-19 Genomics UK (COG-UK) consortium|Davies|United Kingdom

>Spike|hCoV-19/England/QEUH-B2EF8C/2020|2020-11-03|EPI_ISL_660055|Original|hCoV-19^^England|Human|Lighthouse Lab in Glasgow|Wellcome Sanger Institute for the COVID-19 Genomics UK (COG-UK) Consortium|Davies|United Kingdom
>Spike|hCoV-19/England/QEUH-9AE487/2020|2020-09-02|EPI_ISL_637345|Original|hCoV-19^^England|Human|Department of Pathology|COVID-19 Genomics UK (COG-UK) Consortium|Goodfellow|United Kingdom
>Spike|hCoV-19/Denmark/DCGC-7302/2020|2020-10-12|EPI_ISL_619300|Original|hCoV-19^^Hovedstaden|Human|Department of Virus and Microbiological Special Diagnostics|Albertsen lab|Michaelsen|Denmark
>Spike|hCoV-19/USA/OR-OHSU-2093/2020|2020-08-04|EPI_ISL_667114|Original|hCoV-19^^Oregon|Human|OHSU Lab Services Molecular Microbiology Lab|Oregon SARS-CoV-2 Genome Sequencing Center|O'Roak|USA
>Spike|hCoV-19/England/MILK-AC9A47/2020|2020-10-21|EPI_ISL_630204|Original|hCoV-19^^England|Human|Lighthouse Lab in Milton Keynes|Wellcome Sanger Institute for the COVID-19 Genomics UK (COG-UK) consortium|Davies|United Kingdom
>Spike|hCoV-19/England/CAMC-947D67/2020|2020-07-20|EPI_ISL_556529|Original|hCoV-19^^England|Human|Lighthouse Lab in Cambridge|Wellcome Sanger Institute for the COVID-19 Genomics UK (COG-UK) consortium|Davies|United Kingdom
>Spike|hCoV-19/Wales/PHWC-4930FF/2020|2020-11-28|EPI_ISL_725838|Original|hCoV-19^^Wales|Human|Originating lab|COVID-19 Genomics UK (COG-UK) Consortium|Moore|United Kingdom
>Spike|hCoV-19/Switzerland/BS-UHB-42201258/2020|2020-03-21|EPI_ISL_528285|Original|hCoV-19^^Basel-Stadt|Human|University Hospital Basel|University Hospital Basel|Roloff Handschin|Switzerland
>Spike|hCoV-19/Finland/23LS8F3/2020|2020-10-23|EPI_ISL_759792|Original|hCoV-19^^Uusimaa|Human|Department of Virology and Immunology|Department of Virology|Smura|Finland
>Spike|hCoV-19/Russia/MOW-RII-MH4105S/2020|2020-05-12|EPI_ISL_733163|Original|hCoV-19^^Moscow|Human|Pathogenic Microorganisms Variability Laboratory|WHO National Influenza Centre Russian Federation|Komissarov|Russia
>Spike|hCoV-19/USA/MA-MGH-00798/2020|2020-04-13|EPI_ISL_791770|Original|hCoV-19^^Massachusetts|Human|Massachusetts General Hospital|Infectious Disease Program|Park|USA
>Spike|hCoV-19/England/ALDP-AA459C/2020|2020-10-21|EPI_ISL_624359|Original|hCoV-19^^England|Human|Lighthouse Lab in Alderley Park|Wellcome Sanger Institute for the COVID-19 Genomics UK (COG-UK) consortium|Davies|United Kingdom
>Spike|hCoV-19/England/MILK-C2FFB7/2020|2020-12-07|EPI_ISL_733889|Original|hCoV-19^^England|Human|Lighthouse Lab in Milton Keynes|Wellcome Sanger Institute for the COVID-19 Genomics UK (COG-UK) Consortium|Davies|United Kingdom
>Spike|hCoV-19/Scotland/QEUH-AACA0E/2020|2020-10-21|EPI_ISL_652885|Original|hCoV-19^^Scotland|Human|Oxford Viromics|COVID-19 Genomics UK (COG-UK) Consortium|Golubchik|United Kingdom
>Spike|hCoV-19/England/CAMB-76F26/2020|2020-04-01|EPI_ISL_439447|Original|hCoV-19^^England|Human|Department of Pathology|Wellcome Sanger Institute for the COVID-19 Genomics UK (COG-UK) consortium|Amato|United Kingdom
>Spike|hCoV-19/Switzerland/AG-ETHZ-190081/2020|2020-07-06|EPI_ISL_500937|Original|hCoV-19^^Aargau|Human|Viollier AG|Department of Biosystems Science and Engineering|Nadeau|Switzerland
>Spike|hCoV-19/England/MILK-BFF50A/2020|2020-12-02|EPI_ISL_720727|Original|hCoV-19^^England|Human|Lighthouse Lab in Milton Keynes|Wellcome Sanger Institute for the COVID-19 Genomics UK (COG-UK) Consortium|Davies|United Kingdom
>Spike|hCoV-19/Netherlands/NA_515/2020|2020-04-01|EPI_ISL_460688|Original|hCoV-19^^Netherlands|Human|Dutch COVID-19 response team|Erasmus Medical Center|Oude Munnink|Netherlands
>Spike|hCoV-19/USA/WA-S2635/2020|2020-06-20|EPI_ISL_497267|Original|hCoV-19^^Washington|Human|Washington State Department of Health|Seattle Flu Study|Lee|USA
>Spike|hCoV-19/Belgium/ULG-10665/2020|2020-10-26|EPI_ISL_636641|Original|hCoV-19^^Li\u00e8ge|Human|Department of Clinical Microbiology|GIGA Medical Genomics|Durkin|Belgium

>Spike|hCoV-19/USA/WA-S2555/2020|2020-06-17|EPI_ISL_497187|Original|hCoV-19^^Washington|Human|Washington State Department of Health|Seattle Flu Study|Lee|USA
>Spike|hCoV-19/Brazil/RJ-00511/2020|2020-07-27|EPI_ISL_717902|Original|hCoV-19^^Rio de Janeiro|Human|LACEN RJ - Noel Nutels|Bioinformatics Laboratory / LNCC|Almeida|Brazil
>Spike|hCoV-19/England/ALDP-9E5888/2020|2020-09-24|EPI_ISL_589270|Original|hCoV-19^^England|Human|Lighthouse Lab in Alderley Park|Wellcome Sanger Institute for the COVID-19 Genomics UK (COG-UK) consortium|Davies|United Kingdom
>Spike|hCoV-19/USA/TX-HMH-6489/2020|2020-06-27|EPI_ISL_546033|Original|hCoV-19^^Texas|Human|Houston Methodist Hospital|Houston Methodist Hospital|Long|USA
>Spike|hCoV-19/Japan/PG-4997/2020|2020-08-00|EPI_ISL_690110|Original|hCoV-19^^Japan|Human|Pathogen Genomics Center|Pathogen Genomics Center|Sekizuka|Japan
>Spike|hCoV-19/Portugal/PT0420/2020|2020-03-25|EPI_ISL_454144|Original|hCoV-19^^Portugal|Human|unknown|Instituto Nacional de Saude (INSA)|Borges|Portugal
>Spike|hCoV-19/Malaysia/5760/2020|2020-03-26|EPI_ISL_506999|Original|hCoV-19^^Kuala Lumpur|Human|Department of Medical Microbiology|Department of Medical Microbiology|Chan|Malaysia
>Spike|hCoV-19/England/ALDP-4AD362/2020|2020-06-14|EPI_ISL_559341|Original|hCoV-19^^England|Human|Lighthouse Lab in Alderley Park|Wellcome Sanger Institute for the COVID-19 Genomics UK (COG-UK) consortium|Davies|United Kingdom
>Spike|hCoV-19/Australia/SAP220/2020|2020-03-15|EPI_ISL_467995|Original|hCoV-19^^South Australia|Human|SA Pathology|SA Pathology|Leong|Australia
>Spike|hCoV-19/USA/VA-DCLS-1274/2020|2020-08-12|EPI_ISL_529960|Original|hCoV-19^^Virginia|Human|Virginia DCLS|Virginia DCLS|St. Jacques|USA
>Spike|hCoV-19/Denmark/DCGC-1919/2020|2020-04-20|EPI_ISL_622512|Original|hCoV-19^^Hovedstaden|Human|Department of Virus and Microbiological Special Diagnostics|Albertsen lab|Michaelsen|Denmark
>Spike|hCoV-19/USA/CA-ALSR-1975/2020|2020-04-22|EPI_ISL_512273|Original|hCoV-19^^California|Human|San Diego County Public Health Laboratory|Andersen lab at Scripps Research|Gangavarapu|USA
>Spike|hCoV-19/England/SHEF-CBD91/2020|2020-11-10|EPI_ISL_653080|Original|hCoV-19^^England|Human|Virology Department|COVID-19 Genomics UK (COG-UK) Consortium|de Silva|United Kingdom
>Spike|hCoV-19/Wales/PHWC-498F2A/2020|2020-11-30|EPI_ISL_727230|Original|hCoV-19^^Wales|Human|Originating lab|COVID-19 Genomics UK (COG-UK) Consortium|Moore|United Kingdom
>Spike|hCoV-19/England/20098017104/2020|2020-02-27|EPI_ISL_464192|Original|hCoV-19^^England|Human|Respiratory Virus Unit|Respiratory Virus Unit|Platt|United Kingdom
>Spike|hCoV-19/England/ALDP-B2618E/2020|2020-11-04|EPI_ISL_646784|Original|hCoV-19^^England|Human|Lighthouse Lab in Alderley Park|Wellcome Sanger Institute for the COVID-19 Genomics UK (COG-UK) Consortium|Davies|United Kingdom
>Spike|hCoV-19/England/BRIS-1234BF/2020|2020-04-06|EPI_ISL_442710|Original|hCoV-19^^England|Human|PHE South West Regional Laboratory|Wellcome Sanger Institute for the COVID-19 Genomics UK (COG-UK) consortium|Amato|United Kingdom
>Spike|hCoV-19/South Africa/R13452-20/2020|2020-07-07|EPI_ISL_622940|Original|hCoV-19^^KwaZulu-Natal|Human|National Institute for Communicable Diseases of the National Health Laboratory Service|National Institute for Communicable Diseases of the National Health Laboratory Service|Allam|South Africa
>Spike|hCoV-19/Spain/VC-IBV-98000395/2020|2020-03-13|EPI_ISL_539226|Original|hCoV-19^^Comunitat Valenciana|Human|Servicio de Microbiologia. Hospital Clinico Universitario de Valencia|SeqCOVID-SPAIN consortium/IBV(CSIC)|Comas|Spain
>Spike|hCoV-19/USA/MI-MDHHS-SC22298/2020|2020-11-10|EPI_ISL_708274|Original|hCoV-19^^Michigan|Human|Michigan Department of Health and Human Services|Michigan Department of Health and Human Services|Jacob|USA
>Spike|hCoV-19/Denmark/DCGC-3537/2020|2020-08-31|EPI_ISL_616330|Original|hCoV-19^^Hovedstaden|Human|Department of Virus and Microbiological Special Diagnostics|Albertsen lab|Michaelsen|Denmark
>Spike|hCoV-19/USA/WA-S1146/2020|2020-05-05|EPI_ISL_463636|Original|hCoV-19^^Washington|Human|Washington State Department of Health|Seattle Flu Study|Bedford|USA

>Spike|hCoV-19/Denmark/DCGC-9810/2020|2020-11-09|EPI_ISL_669198|Original|hCoV-19^^Sjaelland|Human|Department of Virus and Microbiological Special Diagnostics|Albertsen Lab|Michaelsen|Denmark
>Spike|hCoV-19/Australia/VIC8479/2020|2020-07-27|EPI_ISL_565044|Original|hCoV-19^^Victoria|Human|Microbiological Diagnostic Unit - Public Health Laboratory (MDU-PHL)|MDU-PHL|Schultz|Australia
>Spike|hCoV-19/USA/TX-HMH-MCoV-14407/2020|2020-10-07|EPI_ISL_787692|Original|hCoV-19^^Texas|Human|Houston Methodist Hospital|Houston Methodist Hospital|Long|USA
>Spike|hCoV-19/Greece/42_36236/2020|2020-03-23|EPI_ISL_437899|Original|hCoV-19^^Athens|Human|Laboratory of Microbiology|Laboratory of Biology|Karakasiliotis|Greece
>Spike|hCoV-19/Switzerland/BE-ETHZ-410244/2020|2020-11-27|EPI_ISL_737599|Original|hCoV-19^^Bern|Human|Viollier AG|Department of Biosystems Science and Engineering|Nadeau|Switzerland
>Spike|hCoV-19/England/BIRM-60FD4/2020|2020-05-15|EPI_ISL_473427|Original|hCoV-19^^England|Human|University of Birmingham|COVID-19 Genomics UK (COG-UK) Consortium|Loman|United Kingdom
>Spike|hCoV-19/England/CAMB-1AF05D/2020|2020-04-30|EPI_ISL_459296|Original|hCoV-19^^England|Human|Department of Pathology|Wellcome Sanger Institute for the COVID-19 Genomics UK (COG-UK) consortium|Davies|United Kingdom
>Spike|hCoV-19/England/QEUH-9A4481/2020|2020-09-01|EPI_ISL_540173|Original|hCoV-19^^England|Human|Lighthouse Lab in Glasgow|Wellcome Sanger Institute for the COVID-19 Genomics UK (COG-UK) consortium|Davies|United Kingdom
>Spike|hCoV-19/USA/NM-UNM-00555/2020|2020-05-09|EPI_ISL_542975|Original|hCoV-19^^New Mexico|Human|TriCore Reference Laboratories|Center for Global Health|Domman|USA
>Spike|hCoV-19/USA/WI-UW-2144/2020|2020-10-15|EPI_ISL_677548|Original|hCoV-19^^Wisconsin|Human|University of Wisconsin-Madison AIDS Vaccine Research Laboratories|University of Wisconsin-Madison AIDS Vaccine Research Laboratories|Baczenas|USA
>Spike|hCoV-19/Belgium/ULG-10195/2020|2020-05-18|EPI_ISL_471430|Original|hCoV-19^^Liege|Human|Department of Clinical Microbiology|GIGA Medical Genomics|Durkin|Belgium
>Spike|hCoV-19/England/CAMC-B98F23/2020|2020-11-15|EPI_ISL_673559|Original|hCoV-19^^England|Human|Lighthouse Lab in Cambridge|Wellcome Sanger Institute for the COVID-19 Genomics UK (COG-UK) Consortium|Davies|United Kingdom
>Spike|hCoV-19/England/ALDP-9ED7FC/2020|2020-05-05|EPI_ISL_589336|Original|hCoV-19^^England|Human|Lighthouse Lab in Alderley Park|Wellcome Sanger Institute for the COVID-19 Genomics UK (COG-UK) consortium|Davies|United Kingdom
>Spike|hCoV-19/Wales/PHWC-15D1C4/2020|2020-03-26|EPI_ISL_472680|Original|hCoV-19^^Wales|Human|Wales Specialist Virology Centre Sequencing lab|COVID-19 Genomics UK (COG-UK) Consortium|Moore|United Kingdom
>Spike|hCoV-19/USA/TX-HMH-6090/2020|2020-06-29|EPI_ISL_547229|Original|hCoV-19^^Texas|Human|Houston Methodist Hospital|Houston Methodist Hospital|Long|USA
>Spike|hCoV-19/Denmark/DCGC-20659/2020|2020-12-21|EPI_ISL_759282|Original|hCoV-19^^Hovedstaden|Human|Department of Virus and Microbiological Special Diagnostics|Albertsen Lab|Michaelsen|Denmark
>Spike|hCoV-19/England/BRIS-12EE7A/2020|2020-04-22|EPI_ISL_488405|Original|hCoV-19^^England|Human|PHE South West Regional Laboratory|Wellcome Sanger Institute for the COVID-19 Genomics UK (COG-UK) consortium|Davies|United Kingdom
>Spike|hCoV-19/Beijing/IVDC-BJ-005/2020|2020-01-18|EPI_ISL_408485|Original|hCoV-19^^Beijing|Human|National Institute for Viral Disease Control and Prevention|National Institute for Viral Disease Control & Prevention|Tan|China
>Spike|hCoV-19/Portugal/PT1217/2020|2020-03-28|EPI_ISL_511760|Original|hCoV-19^^Portugal|Human|Instituto Nacional de Saude (INSA)|Instituto Nacional de Saude (INSA) and Instituto Gulbenkian de Ciencia (IGC)|Borges|Portugal
>Spike|hCoV-19/Japan/PG-8504/2020|2020-09-00|EPI_ISL_687603|Original|hCoV-19^^Japan|Human|Pathogen Genomics Center|Pathogen Genomics Center|Sekizuka|Japan
>Spike|hCoV-19/Denmark/DCGC-15058/2020|2020-11-23|EPI_ISL_713609|Original|hCoV-19^^Hovedstaden|Human|Department of Virus and Microbiological Special Diagnostics|Albertsen Lab|Michaelsen|Denmark

>Spike|hCoV-19/Netherlands/NH-EMC-52/2020|2020-08-31|EPI_ISL_577848|Original|hCoV-19^^Noord Holland|Human|Dutch COVID-19 response team|Erasmus Medical Center|Oude Munnink|Netherlands
>Spike|hCoV-19/England/QEUH-C93929/2020|2020-12-17|EPI_ISL_777127|Original|hCoV-19^^England|Human|Lighthouse Lab in Glasgow|Wellcome Sanger Institute for the COVID-19 Genomics UK (COG-UK) Consortium|Davies|United Kingdom
>Spike|hCoV-19/Japan/PG-3647/2020|2020-04-00|EPI_ISL_691900|Original|hCoV-19^^Japan|Human|Pathogen Genomics Center|Pathogen Genomics Center|Sekizuka|Japan
>Spike|hCoV-19/Vietnam/40230/2020|2020-08-00|EPI_ISL_759888|Original|hCoV-19^^Vietnam|Human|National Influenza Center|National Influenza Center|Ung|Vietnam
>Spike|hCoV-19/Scotland/EDB6697/2020|2020-05-07|EPI_ISL_507189|Original|hCoV-19^^Scotland|Human|Virology Department|COVID-19 Genomics UK (COG-UK) Consortium|Williams|United Kingdom
>Spike|hCoV-19/USA/NY-NYCPHL-000180/2020|2020-04-15|EPI_ISL_631715|Original|hCoV-19^^New York|Human|Flushing Hospital Medical Center|New York City Public Health Laboratory|Wang|USA
>Spike|hCoV-19/USA/TX-HMH-MCoV-19493/2020|2020-11-30|EPI_ISL_783877|Original|hCoV-19^^Texas|Human|Houston Methodist Hospital|Houston Methodist Hospital|Long|USA
>Spike|hCoV-19/USA/UT-UPHL-201103306/2020|2020-05-13|EPI_ISL_628975|Original|hCoV-19^^Utah|Human|Utah Public Health Laboratory|Utah Public Health Laboratory|Young|USA
>Spike|hCoV-19/USA/CA-ALSR-1504/2020|2020-04-02|EPI_ISL_494627|Original|hCoV-19^^California|Human|San Diego County Public Health Laboratory|Andersen lab at Scripps Research|Gangavarapu|USA
>Spike|hCoV-19/Wales/PHWC-49CEDE/2020|2020-12-09|EPI_ISL_742704|Original|hCoV-19^^Wales|Human|Wales Specialist Virology Centre Sequencing lab|COVID-19 Genomics UK (COG-UK) Consortium|Moore|United Kingdom
>Spike|hCoV-19/Scotland/CVR1416/2020|2020-04-01|EPI_ISL_438801|Original|hCoV-19^^Scotland|Human|West of Scotland Specialist Virology Centre|COVID-19 Genomics UK (COG-UK) Consortium|Robertson|United Kingdom
>Spike|hCoV-19/Australia/VIC6865/2020|2020-07-27|EPI_ISL_564103|Original|hCoV-19^^Victoria|Human|Victorian Infectious Diseases Reference Laboratory (VIDRL)|VIDRL and MDU-PHL|Schultz|Australia
>Spike|hCoV-19/USA/IA-GMF-25157/2020|2020-10-11|EPI_ISL_660917|Original|hCoV-19^^Iowa|Human|Gundersen Molecular Diagnostics Laboratory|Kabara Cancer Research Institute|Kenny|USA
>Spike|hCoV-19/USA/MI-MDHHS-SC22673/2020|2020-11-18|EPI_ISL_737287|Original|hCoV-19^^Michigan|Human|Michigan Department of Health and Human Services|Michigan Department of Health and Human Services|Blankenship|USA
>Spike|hCoV-19/Scotland/GCVR-170AB1/2020|2020-03-25|EPI_ISL_459588|Original|hCoV-19^^Scotland|Human|NHSGGC West of Scotland Specialist Virology Centre / MRC-University of Glasgow Centre for Virus Research|Wellcome Sanger Institute for the COVID-19 Genomics UK (COG-UK) consortium|Davies|United Kingdom
>Spike|hCoV-19/England/ALDP-CBF444/2020|2020-12-20|EPI_ISL_778044|Original|hCoV-19^^England|Human|Lighthouse Lab in Alderley Park|Wellcome Sanger Institute for the COVID-19 Genomics UK (COG-UK) Consortium|Davies|United Kingdom
>Spike|hCoV-19/England/20172027504/2020|2020-04-20|EPI_ISL_465335|Original|hCoV-19^^England|Human|Respiratory Virus Unit|Respiratory Virus Unit|Platt|United Kingdom
>Spike|hCoV-19/Wales/PHWC-1640AF/2020|2020-05-10|EPI_ISL_474125|Original|hCoV-19^^Wales|Human|Originating lab|COVID-19 Genomics UK (COG-UK) Consortium|Moore|United Kingdom
>Spike|hCoV-19/England/QEUH-C5BBAF/2020|2020-12-14|EPI_ISL_769254|Original|hCoV-19^^England|Human|Lighthouse Lab in Glasgow|Wellcome Sanger Institute for the COVID-19 Genomics UK (COG-UK) Consortium|Davies|United Kingdom
>Spike|hCoV-19/Switzerland/GR-ETHZ-420534/2020|2020-12-22|EPI_ISL_768108|Original|hCoV-19^^Graub\u00fcnden|Human|Viollier AG|Department of Biosystems Science and Engineering|Nadeau|Switzerland
>Spike|hCoV-19/USA/WA-S137/2020|2020-03-13|EPI_ISL_430174|Original|hCoV-19^^Washington|Human|Washington State Department of Health|Seattle Flu Study|Bedford|USA

>Spike|hCoV-19/South Africa/KRISP-K002881/2020|2020-09-04|EPI_ISL_602693|Original|hCoV-19^^KwaZulu-Natal|Human|NHLS-IALCH|KRISP|de Oliveira|South Africa
>Spike|hCoV-19/USA/TX-HMH-4248/2020|2020-06-18|EPI_ISL_543460|Original|hCoV-19^^Texas|Human|Houston Methodist Hospital|Houston Methodist Hospital|Long|USA
>Spike|hCoV-19/Denmark/DCGC-20931/2020|2020-12-21|EPI_ISL_758786|Original|hCoV-19^^Hovedstaden|Human|Department of Virus and Microbiological Special Diagnostics|Albertsen Lab|Michaelsen|Denmark
>Spike|hCoV-19/USA/MI-MDHHS-SC22352/2020|2020-11-17|EPI_ISL_708332|Original|hCoV-19^^Michigan|Human|Michigan Department of Health and Human Services|Michigan Department of Health and Human Services|Jacob|USA
>Spike|hCoV-19/Luxembourg/LNS3670718/2020|2020-11-17|EPI_ISL_740350|Original|hCoV-19^^Luxembourg|Human|Laboratoire national de sant\u00e9|Laboratoire national de sant\u00e9|Wienecke-Baldacchino|Luxembourg
>Spike|hCoV-19/Belgium/ULG-10980/2020|2020-09-09|EPI_ISL_707754|Original|hCoV-19^^Li\u00e8ge|Human|Department of Clinical Microbiology|GIGA Medical Genomics|Durkin|Belgium
>Spike|hCoV-19/Australia/VIC1489/2020|2020-04-27|EPI_ISL_456456|Original|hCoV-19^^Victoria|Human|Victorian Infectious Diseases Reference Laboratory (VIDRL)|Microbiological Diagnostic Unit Public Health Laboratory and Victorian Infectious Diseases Reference Laboratory|Schultz|Australia
>Spike|hCoV-19/France/PAC-IHU-1347/2020|2020-08-19|EPI_ISL_569131|Original|hCoV-19^^Provence-Alpes-C\u00f4te-d\u2019Azur|Human|MEPHI|MEPHI|Levasseur|France
>Spike|hCoV-19/Denmark/DCGC-20286/2020|2020-11-30|EPI_ISL_748092|Original|hCoV-19^^Syddanmark|Human|Department of Virus and Microbiological Special Diagnostics|Albertsen Lab|Michaelsen|Denmark
>Spike|hCoV-19/England/20139010904/2020|2020-03-27|EPI_ISL_420660|Original|hCoV-19^^England|Human|Respiratory Virus Unit|Respiratory Virus Unit|Platt|United Kingdom
>Spike|hCoV-19/England/MILK-AB7AE2/2020|2020-10-21|EPI_ISL_623455|Original|hCoV-19^^England|Human|Lighthouse Lab in Milton Keynes|Wellcome Sanger Institute for the COVID-19 Genomics UK (COG-UK) consortium|Davies|United Kingdom
>Spike|hCoV-19/Costa Rica/01/2020|2020-03-16|EPI_ISL_434533|Original|hCoV-19^^Costa Rica|Human|Area de Salud Alajuela Sur|Inciensa|Brenes Porras|Costa Rica
>Spike|hCoV-19/Netherlands/NA_345/2020|2020-04-05|EPI_ISL_455150|Original|hCoV-19^^Netherlands|Human|Dutch COVID-19 response team|Erasmus Medical Center|Oude Munnink|Netherlands
>Spike|hCoV-19/Australia/SAP137/2020|2020-03-26|EPI_ISL_451141|Original|hCoV-19^^South Australia|Human|SA Pathology|SA Pathology|Leong|Australia
>Spike|hCoV-19/SaudiArabia/KAUST-MADINAH715/2020|2020-04-01|EPI_ISL_513187|Original|hCoV-19^^Madinah|Human|Pathogen Genomics Lab King Abdullah University of Science and Technology(KAUST)|Pathogen Genomics Lab King Abdullah University of Science and Technology(KAUST)|Mohamed Ghazzali|Saudi Arabia
>Spike|hCoV-19/Scotland/QEUH-96EA54/2020|2020-08-19|EPI_ISL_534459|Original|hCoV-19^^Scotland|Human|NHSGGC West of Scotland Specialist Virology Centre / MRC-University of Glasgow Centre for Virus Research|Wellcome Sanger Institute for the COVID-19 Genomics UK (COG-UK) consortium|Davies|United Kingdom
>Spike|hCoV-19/Denmark/DCGC-11419/2020|2020-11-02|EPI_ISL_670908|Original|hCoV-19^^Hovedstaden|Human|Department of Virus and Microbiological Special Diagnostics|Albertsen Lab|Michaelsen|Denmark
>Spike|hCoV-19/Japan/PG-7649/2020|2020-08-00|EPI_ISL_686751|Original|hCoV-19^^Japan|Human|Chiba Prefectural Institute of Public Health|Pathogen Genomics Center|Sekizuka|Japan
>Spike|hCoV-19/England/NOTT-112978/2020|2020-04-21|EPI_ISL_493568|Original|hCoV-19^^England|Human|Lincolnshire Hospitals and DeepSeq Nottingham|COVID-19 Genomics UK (COG-UK) Consortium|Loose|United Kingdom
>Spike|hCoV-19/Portugal/PT1797/2020|2020-11-08|EPI_ISL_732024|Original|hCoV-19^^Portugal|Human|Instituto Nacional de Saude (INSA)|Instituto Nacional de Saude (INSA)|Borges|Portugal
>Spike|hCoV-19/USA/TX-HMH-4324/2020|2020-06-20|EPI_ISL_543533|Original|hCoV-19^^Texas|Human|Houston Methodist Hospital|Houston Methodist Hospital|Long|USA

>Spike|hCoV-19/USA/FL-AB4M/2020|2020-06-23|EPI_ISL_513267|Original|hCoV-19^^Florida|Human|University of Miami Immunology and Histocompatibility Laboratory|University of Miami Immunology and Histocompatibility Laboratory|Margolles-Clark|USA
>Spike|hCoV-19/England/SHEF-CCE33/2020|2020-10-14|EPI_ISL_613388|Original|hCoV-19^^England|Human|Virology Department|COVID-19 Genomics UK (COG-UK) Consortium|de Silva|United Kingdom
>Spike|hCoV-19/USA/TX-HMH-MCoV-19217/2020|2020-11-24|EPI_ISL_783696|Original|hCoV-19^^Texas|Human|Houston Methodist Hospital|Houston Methodist Hospital|Long|USA
>Spike|hCoV-19/Singapore/353/2020|2020-05-14|EPI_ISL_469108|Original|hCoV-19^^Singapore|Human|National Public Health Laboratory|National Public Health Laboratory|Mak|Singapore
>Spike|hCoV-19/Scotland/EDB5825/2020|2020-04-27|EPI_ISL_487768|Original|hCoV-19^^Scotland|Human|Virology Department|Wellcome Sanger Institute for the COVID-19 Genomics UK (COG-UK) consortium|Davies|United Kingdom
>Spike|hCoV-19/USA/WA-UW-32819/2020|2020-10-16|EPI_ISL_737089|Original|hCoV-19^^Washington|Human|UW Virology Lab|UW Virology Lab|Roychoudhury|USA
>Spike|hCoV-19/Russia/KDA-RII-MH3351S/2020|2020-10-07|EPI_ISL_733098|Original|hCoV-19^^Krasnodar Krai|Human|HELIX LLC|WHO National Influenza Centre Russian Federation|Komissarov|Russia
>Spike|hCoV-19/Scotland/QEUH-9FA914/2020|2020-09-29|EPI_ISL_600571|Original|hCoV-19^^Scotland|Human|Lighthouse Lab in Glasgow|Wellcome Sanger Institute for the COVID-19 Genomics UK (COG-UK) consortium|Davies|United Kingdom
>Spike|hCoV-19/USA/MN-MDH-1614/2020|2020-04-25|EPI_ISL_530210|Original|hCoV-19^^Minnesota|Human|Minnesota Department of Health|Minnesota Department of Health|Garfin|USA
>Spike|hCoV-19/England/NORW-21F594/2020|2020-12-08|EPI_ISL_725029|Original|hCoV-19^^England|Human|Quadram Institute Bioscience|COVID-19 Genomics UK (COG-UK) Consortium|Alikhan|United Kingdom
>Spike|hCoV-19/England/20169006104/2020|2020-04-17|EPI_ISL_465186|Original|hCoV-19^^England|Human|Respiratory Virus Unit|Respiratory Virus Unit|Platt|United Kingdom
>Spike|hCoV-19/Northern Ireland/NIRE-FB976/2020|2020-03-31|EPI_ISL_585256|Original|hCoV-19^^Northern Ireland|Human|Regional Virus Laboratory|COVID-19 Genomics UK (COG-UK) Consortium|Fairley|United Kingdom
>Spike|hCoV-19/England/C200032709/2020|2020-10-15|EPI_ISL_636707|Original|hCoV-19^^England|Human|Respiratory Virus Unit|Respiratory Virus Unit|Platt|United Kingdom
>Spike|hCoV-19/England/QEUH-A63380/2020|2020-10-12|EPI_ISL_596117|Original|hCoV-19^^England|Human|Quadram Institute Bioscience|COVID-19 Genomics UK (COG-UK) Consortium|Alikhan|United Kingdom
>Spike|hCoV-19/England/IC19/2020|2020-03-17|EPI_ISL_475572|Caco-2 P2|hCoV-19^^England|Human|Imperial College London|Imperial College London|Zhou|United Kingdom
>Spike|hCoV-19/England/ALDP-B5B3B7/2020|2020-11-08|EPI_ISL_655288|Original|hCoV-19^^England|Human|Lighthouse Lab in Alderley Park|Wellcome Sanger Institute for the COVID-19 Genomics UK (COG-UK) Consortium|Davies|United Kingdom
>Spike|hCoV-19/Australia/TAS31/2020|2020-04-10|EPI_ISL_522708|Original|hCoV-19^^Tasmania|Human|Royal Hobart Hospital Microbiology Department|MDU-PHL|Schultz|Australia
>Spike|hCoV-19/Senegal/68488/2020|2020-06-17|EPI_ISL_482877|Original|hCoV-19^^Dakar|Human|Institut Pasteur Dakar|Institut Pasteur de Dakar|Dia|Senegal
>Spike|hCoV-19/England/QEUH-5DABDC/2020|2020-06-17|EPI_ISL_533340|Original|hCoV-19^^England|Human|Lighthouse Lab in Glasgow|Wellcome Sanger Institute for the COVID-19 Genomics UK (COG-UK) consortium|Davies|United Kingdom
>Spike|hCoV-19/Australia/NT47/2020|2020-00-00|EPI_ISL_480694|Original|hCoV-19^^Northern Territory|Human|Royal Darwin Hospital Pathology|MDU-PHL|Schultz|Australia
>Spike|hCoV-19/England/MILK-BB149E/2020|2020-11-18|EPI_ISL_720084|Original|hCoV-19^^England|Human|Lighthouse Lab in Milton Keynes|Wellcome Sanger Institute for the COVID-19 Genomics UK (COG-UK) Consortium|Davies|United Kingdom

>Spike|hCoV-19/Denmark/DCGC-1951/2020|2020-07-27|EPI_ISL_618247|Original|hCoV-19^^Sjaelland|Human|Department of Virus and Microbiological Special Diagnostics|Albertsen lab|Michaelsen|Denmark
>Spike|hCoV-19/USA/TX-DSHS-1184/2020|2020-06-16|EPI_ISL_631646|Original|hCoV-19^^Texas|Human|Texas Department of State Health Services|Texas Department of State Health Services|Rahman|USA
>Spike|hCoV-19/England/QEUH-B31A58/2020|2020-11-04|EPI_ISL_646229|Original|hCoV-19^^England|Human|Lighthouse Lab in Glasgow|Wellcome Sanger Institute for the COVID-19 Genomics UK (COG-UK) Consortium|Davies|United Kingdom
>Spike|hCoV-19/England/NORW-F06D4/2020|2020-10-14|EPI_ISL_595188|Original|hCoV-19^^England|Human|Quadram Institute Bioscience|COVID-19 Genomics UK (COG-UK) Consortium|Alikhan|United Kingdom
>Spike|hCoV-19/USA/WA-S1826/2020|2020-06-02|EPI_ISL_496252|Original|hCoV-19^^Washington|Human|Washington State Department of Health|Seattle Flu Study|Lee|USA
>Spike|hCoV-19/Wales/PHWC-48CA64/2020|2020-11-02|EPI_ISL_666499|Original|hCoV-19^^Wales|Human|Wales Specialist Virology Centre Sequencing lab|COVID-19 Genomics UK (COG-UK) Consortium|Moore|United Kingdom
>Spike|hCoV-19/Switzerland/BS-ETHZ-360010/2020|2020-11-12|EPI_ISL_693870|Original|hCoV-19^^Basel-Stadt|Human|Viollier AG|Department of Biosystems Science and Engineering|Nadeau|Switzerland
>Spike|hCoV-19/USA/WA-UW-21732/2020|2020-08-05|EPI_ISL_570995|Original|hCoV-19^^Washington|Human|UW Virology Lab|UW Virology Lab|Roychoudhury|USA
>Spike|hCoV-19/England/CAMC-B345AF/2020|2020-11-05|EPI_ISL_659591|Original|hCoV-19^^England|Human|Lighthouse Lab in Cambridge|Wellcome Sanger Institute for the COVID-19 Genomics UK (COG-UK) Consortium|Davies|United Kingdom
>Spike|hCoV-19/England/MILK-A23A2D/2020|2020-10-03|EPI_ISL_637371|Original|hCoV-19^^England|Human|Centre for Enzyme Innovation|COVID-19 Genomics UK (COG-UK) Consortium|Robson|United Kingdom
>Spike|hCoV-19/Luxembourg/LNS7986348/2020|2020-11-26|EPI_ISL_744665|Original|hCoV-19^^Luxembourg|Human|Laboratoire national de sant\u00e9|Laboratoire national de sant\u00e9|Wienecke-Baldacchino|Luxembourg
>Spike|hCoV-19/England/ALDP-BC86BC/2020|2020-11-23|EPI_ISL_721217|Original|hCoV-19^^England|Human|Lighthouse Lab in Alderley Park|Wellcome Sanger Institute for the COVID-19 Genomics UK (COG-UK) Consortium|Davies|United Kingdom
>Spike|hCoV-19/Denmark/DCGC-5060/2020|2020-09-21|EPI_ISL_621598|Original|hCoV-19^^Nordjylland|Human|Department of Virus and Microbiological Special Diagnostics|Albertsen lab|Michaelsen|Denmark
>Spike|hCoV-19/England/MILK-8327EA/2020|2020-07-17|EPI_ISL_557344|Original|hCoV-19^^England|Human|Lighthouse Lab in Milton Keynes|Wellcome Sanger Institute for the COVID-19 Genomics UK (COG-UK) consortium|Davies|United Kingdom
>Spike|hCoV-19/England/CAMC-A5844F/2020|2020-10-07|EPI_ISL_596932|Original|hCoV-19^^England|Human|Lighthouse Lab in Cambridge|Wellcome Sanger Institute for the COVID-19 Genomics UK (COG-UK) consortium|Davies|United Kingdom
>Spike|hCoV-19/France/PAC-IHU-0257/2020|2020-03-30|EPI_ISL_569415|Original|hCoV-19^^Provence-Alpes-C\u00f4te-d\u2019Azur|Human|MEPHI|MEPHI|Levasseur|France
>Spike|hCoV-19/England/20136088702/2020|2020-03-25|EPI_ISL_420641|Original|hCoV-19^^England|Human|Respiratory Virus Unit|Respiratory Virus Unit|Platt|United Kingdom
>Spike|hCoV-19/USA/CA-ALSR-2437/2020|2020-07-02|EPI_ISL_635478|Original|hCoV-19^^California|Human|San Diego County Public Health Laboratory|Andersen lab at Scripps Research|Gangavarapu|USA
>Spike|hCoV-19/Sweden/20-22133/2020|2020-04-02|EPI_ISL_660381|Original|hCoV-19^^Orebro|Human|Orebro klinisk mikrobiologi|The Public Health Agency of Sweden|Svartstr\u00f6m|Sweden
>Spike|hCoV-19/Australia/NSW1038/2020|2020-06-28|EPI_ISL_593690|Original|hCoV-19^^New South Wales|Human|South Eastern Area Laboratory Services (SEALS)|NSW Health Pathology - Institute of Clinical Pathology and Medical Research; Westmead Hospital; University of Sydney|Gall|Australia
>Spike|hCoV-19/Brazil/CE-L17-CD362/2020|2020-03-31|EPI_ISL_476308|Original|hCoV-19^^Cear\u00e1|Human|DB Diagn\u00f3sticos do Brasil|Instituto de Medicina Tropical da Univesidade de S\u00e3o Paulo|Faria|Brazil

>Spike|hCoV-19/Scotland/QEUH-964B4C/2020|2020-08-13|EPI_ISL_531083|Original|hCoV-19^^Scotland|Human|NHSGGC West of Scotland Specialist Virology Centre / MRC-University of Glasgow Centre for Virus Research|Wellcome Sanger Institute for the COVID-19 Genomics UK (COG-UK) consortium|Davies|United Kingdom
>Spike|hCoV-19/Denmark/DCGC-15210/2020|2020-11-23|EPI_ISL_713695|Original|hCoV-19^^Hovedstaden|Human|Department of Virus and Microbiological Special Diagnostics|Albertsen Lab|Michaelsen|Denmark
>Spike|hCoV-19/Australia/VIC3983/2020|2020-07-15|EPI_ISL_563989|Original|hCoV-19^^Victoria|Human|Microbiological Diagnostic Unit - Public Health Laboratory (MDU-PHL)|MDU-PHL|Schultz|Australia
>Spike|hCoV-19/England/20134060904/2020|2020-03-21|EPI_ISL_423149|Original|hCoV-19^^England|Human|Respiratory Virus Unit|Respiratory Virus Unit|Platt|United Kingdom
>Spike|hCoV-19/USA/UT-08519/2020|2020-05-17|EPI_ISL_524282|Original|hCoV-19^^Utah|Human|Utah Public Health Laboratory|Utah Public Health Laboratory|Young|USA
>Spike|hCoV-19/Wales/PHWC-48360F/2020|2020-10-22|EPI_ISL_627837|Original|hCoV-19^^Wales|Human|Wales Specialist Virology Centre Sequencing lab|COVID-19 Genomics UK (COG-UK) Consortium|Moore|United Kingdom
>Spike|hCoV-19/England/MILK-467434/2020|2020-05-20|EPI_ISL_559723|Original|hCoV-19^^England|Human|Lighthouse Lab in Milton Keynes|Wellcome Sanger Institute for the COVID-19 Genomics UK (COG-UK) consortium|Davies|United Kingdom
>Spike|hCoV-19/Denmark/DCGC-6375/2020|2020-09-21|EPI_ISL_622290|Original|hCoV-19^^Hovedstaden|Human|Department of Virus and Microbiological Special Diagnostics|Albertsen lab|Michaelsen|Denmark
>Spike|hCoV-19/England/MILK-BB1865/2020|2020-11-18|EPI_ISL_720216|Original|hCoV-19^^England|Human|Lighthouse Lab in Milton Keynes|Wellcome Sanger Institute for the COVID-19 Genomics UK (COG-UK) Consortium|Davies|United Kingdom
>Spike|hCoV-19/USA/OR-OHSU-0534/2020|2020-06-15|EPI_ISL_525834|Original|hCoV-19^^Oregon|Human|OHSU Lab Services Molecular Microbiology Lab|Ginkgo Bioworks Clinical Laboratory|O'Roak|USA
>Spike|hCoV-19/Australia/WA412/2020|2020-05-27|EPI_ISL_672624|Original|hCoV-19^^Western Australia|Human|PathWest Laboratory Medicine WA|PathWest Laboratory Medicine WA Microbial Surveillance Unit|Sikazwe|Australia
>Spike|hCoV-19/Spain/AR-ISCIII-201850/2020|2020-03-09|EPI_ISL_539519|Original|hCoV-19^^Aragon|Human|Hospital Universitario Miguel Servet|Instituto de Salud Carlos III|Caballero|Spain
>Spike|hCoV-19/Netherlands/NA_175/2020|2020-03-22|EPI_ISL_422710|Original|hCoV-19^^Netherlands|Human|Dutch COVID-19 response team|Erasmus Medical Center|Oude Munnink|Netherlands
>Spike|hCoV-19/South Africa/KRISP-K004621/2020|2020-11-03|EPI_ISL_660639|Original|hCoV-19^^KwaZulu-Natal|Human|NHLS-IALCH|KRISP|de Oliveira|South Africa
>Spike|hCoV-19/England/MILK-9A846F/2020|2020-09-02|EPI_ISL_550158|Original|hCoV-19^^England|Human|Lighthouse Lab in Milton Keynes|Wellcome Sanger Institute for the COVID-19 Genomics UK (COG-UK) consortium|Davies|United Kingdom
>Spike|hCoV-19/Netherlands/UT-RIVM-20192/2020|2020-11-30|EPI_ISL_723346|Orignal|hCoV-19^^Utrecht|Human|Dutch COVID-19 response team|National Institute for Public Health and the Environment (RIVM)|Meijer|Netherlands
>Spike|hCoV-19/Denmark/DCGC-8943/2020|2020-10-26|EPI_ISL_626161|Original|hCoV-19^^Syddanmark|Human|Department of Virus and Microbiological Special Diagnostics|Albertsen lab|Michaelsen|Denmark
>Spike|hCoV-19/England/ALDP-AA0C22/2020|2020-10-21|EPI_ISL_624967|Original|hCoV-19^^England|Human|Lighthouse Lab in Alderley Park|Wellcome Sanger Institute for the COVID-19 Genomics UK (COG-UK) consortium|Davies|United Kingdom
>Spike|hCoV-19/USA/VA-DCLS-0033/2020|2020-03-19|EPI_ISL_420019|Original|hCoV-19^^Virginia|Human|Virginia DCLS|Virginia DCLS|St. Jacques|USA
>Spike|hCoV-19/Northern Ireland/NIRE-2331F0/2020|2020-09-23|EPI_ISL_680266|Original|hCoV-19^^Northern Ireland|Human|Regional Virus Laboratory|COVID-19 Genomics UK (COG-UK) Consortium|Fairley|United Kingdom

>Spike|hCoV-19/Latvia/089/2020|2020-07-09|EPI_ISL_512314|Original|hCoV-19^^Latvia|Human|E. Gulbja Laboratorija|Latvian Biomedical Research and Study Centre|Zrelovs|Latvia
>Spike|hCoV-19/USA/OR-OHSU-0235/2020|2020-04-04|EPI_ISL_509077|Original|hCoV-19^^Oregon|Human|OHSU Lab Services Molecular Microbiology Lab|Oregon SARS-CoV-2 Genome Sequencing Center|O'Roak|USA
>Spike|hCoV-19/England/MILK-AC9151/2020|2020-10-22|EPI_ISL_709918|Original|hCoV-19^^England|Human|Lighthouse Lab in Milton Keynes|Wellcome Sanger Institute for the COVID-19 Genomics UK (COG-UK) Consortium|Davies|United Kingdom
>Spike|hCoV-19/England/MILK-BAA1EC/2020|2020-11-18|EPI_ISL_681389|Original|hCoV-19^^England|Human|Lighthouse Lab in Milton Keynes|Wellcome Sanger Institute for the COVID-19 Genomics UK (COG-UK) Consortium|Davies|United Kingdom
>Spike|hCoV-19/Singapore/128/2020|2020-03-21|EPI_ISL_443220|Original|hCoV-19^^Singapore|Human|National Public Health Laboratory|National Public Health Laboratory|Mak|Singapore
>Spike|hCoV-19/USA/UT-UPHL-2009643/2020|2020-07-16|EPI_ISL_605318|Original|hCoV-19^^Utah|Human|Utah Public Health Laboratory|Utah Public Health Laboratory|Young|USA
>Spike|hCoV-19/England/20168035104/2020|2020-04-16|EPI_ISL_466612|Original|hCoV-19^^England|Human|Respiratory Virus Unit|Respiratory Virus Unit|Platt|United Kingdom
>Spike|hCoV-19/England/QEUH-9F7E54/2020|2020-09-27|EPI_ISL_589244|Original|hCoV-19^^England|Human|Lighthouse Lab in Glasgow|Wellcome Sanger Institute for the COVID-19 Genomics UK (COG-UK) consortium|Davies|United Kingdom
>Spike|hCoV-19/USA/CO-CSU-D2373-2/2020|2020-04-22|EPI_ISL_527464|Original|hCoV-19^^Colorado|Human|Colorado State University - Ebel Lab|Colorado State University - Ebel Lab|Cline|USA
>Spike|hCoV-19/England/QEUH-A370F7/2020|2020-10-05|EPI_ISL_706928|Original|hCoV-19^^England|Human|Oxford Viromics|COVID-19 Genomics UK (COG-UK) Consortium|Golubchik|United Kingdom
>Spike|hCoV-19/Japan/PG-8175/2020|2020-07-00|EPI_ISL_687274|Original|hCoV-19^^Japan|Human|Pathogen Genomics Center|Pathogen Genomics Center|Sekizuka|Japan
>Spike|hCoV-19/England/LOND-DA667/2020|2020-11-10|EPI_ISL_724651|Original|hCoV-19^^England|Human|University College London|COVID-19 Genomics UK (COG-UK) Consortium|Roy|United Kingdom
>Spike|hCoV-19/England/NORT-2838DE/2020|2020-04-01|EPI_ISL_488577|Original|hCoV-19^^England|Human|NU-OMICS DNA Sequencing research facility|Wellcome Sanger Institute for the COVID-19 Genomics UK (COG-UK) consortium|Davies|United Kingdom
>Spike|hCoV-19/Netherlands/Utrecht_1/2020|2020-03-03|EPI_ISL_414435|Original|hCoV-19^^Utrecht|Human|Dutch COVID-19 response team|Erasmus Medical Center|Oude Munnink|Netherlands
>Spike|hCoV-19/Scotland/EDB10520/2020|2020-11-30|EPI_ISL_705777|Original|hCoV-19^^Scotland|Human|Virology Department|COVID-19 Genomics UK (COG-UK) Consortium|McHugh|United Kingdom
>Spike|hCoV-19/England/MILK-994080/2020|2020-08-25|EPI_ISL_551652|Original|hCoV-19^^England|Human|Lighthouse Lab in Milton Keynes|Wellcome Sanger Institute for the COVID-19 Genomics UK (COG-UK) consortium|Davies|United Kingdom
>Spike|hCoV-19/USA/NY-NYCPHL-001466/2020|2020-11-27|EPI_ISL_683760|Original|hCoV-19^^New York|Human|DOHMH Morrisania|New York City Public Health Laboratory|Wang|USA
>Spike|hCoV-19/Denmark/DCGC-17509/2020|2020-12-14|EPI_ISL_751032|Original|hCoV-19^^Denmark|Human|Department of Virus and Microbiological Special Diagnostics|Albertsen Lab|Michaelsen|Denmark
>Spike|hCoV-19/USA/UT-UPHL-2012107439/2020|2020-06-18|EPI_ISL_746362|Original|hCoV-19^^Utah|Human|Utah Public Health Laboratory|Utah Public Health Laboratory|Gallagher|USA
>Spike|hCoV-19/Denmark/DCGC-14807/2020|2020-11-23|EPI_ISL_711998|Original|hCoV-19^^Hovedstaden|Human|Department of Virus and Microbiological Special Diagnostics|Albertsen Lab|Michaelsen|Denmark
>Spike|hCoV-19/USA/TX-DSHS-1423/2020|2020-06-22|EPI_ISL_671627|Original|hCoV-19^^Texas|Human|Texas Department of State Health Services|Texas Department of State Health Services|Rahman|USA

>Spike|hCoV-19/USA/MA-MGH-01290/2020|2020-05-05|EPI_ISL_791918|Original|hCoV-19^^Massachusetts|Human|Massachusetts General Hospital|Infectious Disease Program|Park|USA
>Spike|hCoV-19/USA/MN-MDH-2029/2020|2020-11-03|EPI_ISL_654710|Original|hCoV-19^^Minnesota|Human|Minnesota Department of Health|Minnesota Department of Health|Lorentz|USA
>Spike|hCoV-19/Japan/PG-8392/2020|2020-07-00|EPI_ISL_687491|Original|hCoV-19^^Japan|Human|Pathogen Genomics Center|Pathogen Genomics Center|Sekizuka|Japan
>Spike|hCoV-19/England/CAMC-C44C88/2020|2020-12-10|EPI_ISL_718609|Original|hCoV-19^^England|Human|Lighthouse Lab in Cambridge|Wellcome Sanger Institute for the COVID-19 Genomics UK (COG-UK) Consortium|Davies|United Kingdom
>Spike|hCoV-19/USA/TX-HMH-MCoV-15733/2020|2020-07-09|EPI_ISL_785417|Original|hCoV-19^^Texas|Human|Houston Methodist Hospital|Houston Methodist Hospital|Long|USA
>Spike|hCoV-19/England/LEED-2AA367/2020|2020-03-29|EPI_ISL_538819|Original|hCoV-19^^England|Human|Leeds Teaching Hospitals NHS Trust and Public Health England|Wellcome Sanger Institute for the COVID-19 Genomics UK (COG-UK) consortium|Davies|United Kingdom
>Spike|hCoV-19/Greece/NIC_4/2020|2020-04-09|EPI_ISL_476851|Original|hCoV-19^^Thessaloniki|Human|National Influenza Centre for Northern Greece|National Influenza Centre for Northern Greece|Christoforidi|Greece
>Spike|hCoV-19/England/QEUH-9F57B5/2020|2020-09-25|EPI_ISL_588740|Original|hCoV-19^^England|Human|Lighthouse Lab in Glasgow|Wellcome Sanger Institute for the COVID-19 Genomics UK (COG-UK) consortium|Davies|United Kingdom
>Spike|hCoV-19/India/GJ-GBRC354b/2020|2020-06-25|EPI_ISL_514602|Original|hCoV-19^^Gujarat|Human|B.J. Medical College and Civil hospital|Gujarat Biotechnology Research Centre|Joshi|India
>Spike|hCoV-19/Italy/LAZ-INMI-25/2020|2020-07-07|EPI_ISL_590693|Original|hCoV-19^^Lazio|Human|INMI Lazzaro Spallanzani IRCCS|INMI Lazzaro Spallanzani IRCCS|Gruber|Italy
>Spike|hCoV-19/USA/CA-QDX-2039/2020|2020-03-18|EPI_ISL_604319|Original|hCoV-19^^California|Human|Quest Diagnostics|Quest Diagnostics|Kagan|USA
>Spike|hCoV-19/Wales/QEUH-B0DE86/2020|2020-11-02|EPI_ISL_647803|Original|hCoV-19^^Wales|Human|Lighthouse Lab in Glasgow|Wellcome Sanger Institute for the COVID-19 Genomics UK (COG-UK) Consortium|Davies|United Kingdom
>Spike|hCoV-19/Luxembourg/LNS5454756/2020|2020-03-20|EPI_ISL_744233|Original|hCoV-19^^Luxembourg|Human|Laboratoire national de sant\u00e9|Laboratoire national de sant\u00e9|Wienecke-Baldacchino|Luxembourg
>Spike|hCoV-19/Japan/PG-3001/2020|2020-04-00|EPI_ISL_691125|Original|hCoV-19^^Japan|Human|Pathogen Genomics Center|Pathogen Genomics Center|Sekizuka|Japan
>Spike|hCoV-19/Luxembourg/LNS5340325/2020|2020-12-28|EPI_ISL_771115|Original|hCoV-19^^Luxembourg|Human|Laboratoire national de sant\u00e9|Laboratoire national de sant\u00e9|Wienecke-Baldacchino|Luxembourg
>Spike|hCoV-19/USA/TX-HMH-5006/2020|2020-06-18|EPI_ISL_544453|Original|hCoV-19^^Texas|Human|Houston Methodist Hospital|Houston Methodist Hospital|Long|USA
>Spike|hCoV-19/Italy/CAM-IZSM-112/2020|2020-09-12|EPI_ISL_572320|Original|hCoV-19^^Campania|Human|IZSM|IZSM|Viscardi|Italy
>Spike|hCoV-19/Singapore/380/2020|2020-04-05|EPI_ISL_469129|Original|hCoV-19^^Singapore|Human|National Public Health Laboratory|National Public Health Laboratory|Mak|Singapore
>Spike|hCoV-19/Uganda/UG073/2020|2020-08-24|EPI_ISL_737942|Original|hCoV-19^^Uganda|Human|Uganda Central Public Health Lab and Uganda Virus Research Institute|MRC/UVRI & LSHTM Uganda\u00a0Research Unit|Cotten|Uganda
>Spike|hCoV-19/USA/TX-HMH-MCoV-18068/2020|2020-11-13|EPI_ISL_784416|Original|hCoV-19^^Texas|Human|Houston Methodist Hospital|Houston Methodist Hospital|Long|USA
>Spike|hCoV-19/Australia/VIC1615/2020|2020-05-07|EPI_ISL_456524|Original|hCoV-19^^Victoria|Human|Victorian Infectious Diseases Reference Laboratory (VIDRL)|Microbiological Diagnostic Unit Public Health Laboratory and Victorian Infectious Diseases Reference Laboratory|Schultz|Australia
>Spike|hCoV-19/USA/WI-UW-1302/2020|2020-09-01|EPI_ISL_536674|Original|hCoV-19^^Wisconsin|Human|University of Wisconsin-Madison AIDS Vaccine Research

Laboratories|University of Wisconsin-Madison AIDS Vaccine Research Laboratories|Baczenas|USA
>Spike|hCoV-19/United Arab Emirates/L2006030255/2020|2020-06-03|EPI_ISL_520678|Original|hCoV-19^^United Arab Emirates|Human|Mohammed Bin Rashid University of Medicine and Health Sciences|Al Jalila Genomics Center|Ramaswamy|United Arab Emirates
>Spike|hCoV-19/England/ALDP-94DA88/2020|2020-06-17|EPI_ISL_555200|Original|hCoV-19^^England|Human|Lighthouse Lab in Alderley Park|Wellcome Sanger Institute for the COVID-19 Genomics UK (COG-UK) consortium|Davies|United Kingdom
>Spike|hCoV-19/Japan/PG-8233/2020|2020-08-00|EPI_ISL_687332|Original|hCoV-19^^Japan|Human|Pathogen Genomics Center|Pathogen Genomics Center|Sekizuka|Japan
>Spike|hCoV-19/Scotland/QEUH-A21807/2020|2020-10-01|EPI_ISL_598845|Original|hCoV-19^^Scotland|Human|Lighthouse Lab in Glasgow|Wellcome Sanger Institute for the COVID-19 Genomics UK (COG-UK) consortium|Davies|United Kingdom
>Spike|hCoV-19/USA/TX-HMH-MCoV-11777/2020|2020-09-08|EPI_ISL_790108|Original|hCoV-19^^Texas|Human|Houston Methodist Hospital|Houston Methodist Hospital|Long|USA
>Spike|hCoV-19/USA/IL-UW-141/2020|2020-04-07|EPI_ISL_427448|Original|hCoV-19^^Illinois|Human|University of Wisconsin-Madison AIDS Vaccine Research Laboratories|University of Wisconsin-Madison AIDS Vaccine Research Laboratories|Baczenas|USA
>Spike|hCoV-19/Japan/PG-5464/2020|2020-08-00|EPI_ISL_690567|Original|hCoV-19^^Japan|Human|Pathogen Genomics Center|Pathogen Genomics Center|Sekizuka|Japan
>Spike|hCoV-19/England/EXET-13936E/2020|2020-10-26|EPI_ISL_665064|Original|hCoV-19^^England|Human|University of Exeter|COVID-19 Genomics UK (COG-UK) Consortium|Temperton|United Kingdom
>Spike|hCoV-19/Wales/PHWC-49047E/2020|2020-10-30|EPI_ISL_706481|Original|hCoV-19^^Wales|Human|Originating lab|COVID-19 Genomics UK (COG-UK) Consortium|Moore|United Kingdom
>Spike|hCoV-19/Australia/VIC17399/2020|2020-00-00|EPI_ISL_663873|Original|hCoV-19^^Victoria|Human|Microbiological Diagnostic Unit - Public Health Laboratory (MDU-PHL)|MDU-PHL|Schultz|Australia
>Spike|hCoV-19/India/MH-ACTREC-264/2020|2020-07-08|EPI_ISL_699916|Original|hCoV-19^^Maharashtra|Human|Hematopathology Laboratory|Hematopathology Laboratory|Patkar|India
>Spike|hCoV-19/Japan/PG-5279/2020|2020-08-00|EPI_ISL_690382|Original|hCoV-19^^Japan|Human|Pathogen Genomics Center|Pathogen Genomics Center|Sekizuka|Japan
>Spike|hCoV-19/Scotland/CVR2999/2020|2020-04-23|EPI_ISL_461615|Original|hCoV-19^^Scotland|Human|West of Scotland Specialist Virology Centre|COVID-19 Genomics UK (COG-UK) Consortium|Robertson|United Kingdom
>Spike|hCoV-19/Jordan/IR-ALSR-5279/2020|2020-11-03|EPI_ISL_730517|Original|hCoV-19^^Irbid|Human|Biolab Diagnostic Laboratories|Andersen lab at Scripps Research|Gangavarapu|Jordan
>Spike|hCoV-19/Belgium/rega-0317857/2020|2020-03-17|EPI_ISL_734506|Original|hCoV-19^^M\u00e9lin|Human|UZ Leuven|KU Leuven|Maes|Belgium
>Spike|hCoV-19/Argentina/PAIS-A0026/2020|2020-04-18|EPI_ISL_430817|Original|hCoV-19^^Ciudad Autonoma de Buenos Aires|Human|Laboratorio de Virolog\u00eda del Hospital de Ni\u00f1os Dr. Ricardo Gutierrez|\u00c1rea de Secuenciaci\u00f3n del Laboratorio de Virolog\u00eda del Hospital de Ni\u00f1os Dr. Ricardo Gutierrez|Viegas|Argentina
>Spike|hCoV-19/England/CAMC-946199/2020|2020-07-13|EPI_ISL_556655|Original|hCoV-19^^England|Human|Lighthouse Lab in Cambridge|Wellcome Sanger Institute for the COVID-19 Genomics UK (COG-UK) consortium|Davies|United Kingdom
>Spike|hCoV-19/Wales/PHWC-48751D/2020|2020-10-30|EPI_ISL_639206|Original|hCoV-19^^Wales|Human|Wales Specialist Virology Centre Sequencing lab|COVID-19 Genomics UK (COG-UK) Consortium|Moore|United Kingdom
>Spike|hCoV-19/Japan/PG-7534/2020|2020-08-00|EPI_ISL_686636|Original|hCoV-19^^Japan|Human|Pathogen Genomics Center|Pathogen Genomics Center|Sekizuka|Japan
>Spike|hCoV-19/Denmark/DCGC-6274/2020|2020-10-05|EPI_ISL_620034|Original|hCoV-19^^Midtjylland|Human|Department of Virus and Microbiological Special Diagnostics|Albertsen lab|Michaelsen|Denmark

>Spike|hCoV-19/USA/NM-DOH-2020616659/2020|2020-12-12|EPI_ISL_751823|Original|hCoV-19^^New Mexico|Human|New Mexico Department of Health Scientific Laboratory|New Mexico Department of Health Scientific Laboratory|Malone|USA
>Spike|hCoV-19/Japan/PG-2264/2020|2020-04-00|EPI_ISL_685869|Original|hCoV-19^^Japan|Human|Pathogen Genomics Center|Pathogen Genomics Center|Sekizuka|Japan
>Spike|hCoV-19/England/CAMC-B0886F/2020|2020-10-29|EPI_ISL_643954|Original|hCoV-19^^England|Human|Lighthouse Lab in Cambridge|Wellcome Sanger Institute for the COVID-19 Genomics UK (COG-UK) Consortium|Davies|United Kingdom
>Spike|hCoV-19/England/MILK-ABBAEE/2020|2020-10-21|EPI_ISL_631179|Original|hCoV-19^^England|Human|Lighthouse Lab in Milton Keynes|Wellcome Sanger Institute for the COVID-19 Genomics UK (COG-UK) consortium|Davies|United Kingdom
>Spike|hCoV-19/Austria/CeMM0922/2020|2020-03-16|EPI_ISL_583774|Original|hCoV-19^^Austria|Human|Dr. Gernot Walder GmbH|Bergthaler laboratory|Bergthaler|Austria
>Spike|hCoV-19/England/CAMB-7D7CD/2020|2020-04-06|EPI_ISL_441732|Original|hCoV-19^^England|Human|Department of Pathology|Wellcome Sanger Institute for the COVID-19 Genomics UK (COG-UK) consortium|Amato|United Kingdom
>Spike|hCoV-19/England/QEUH-B122FB/2020|2020-11-02|EPI_ISL_642507|Original|hCoV-19^^England|Human|Lighthouse Lab in Glasgow|Wellcome Sanger Institute for the COVID-19 Genomics UK (COG-UK) Consortium|Davies|United Kingdom
>Spike|hCoV-19/England/ALDP-C3BBA5/2020|2020-12-11|EPI_ISL_731013|Original|hCoV-19^^England|Human|Lighthouse Lab in Alderley Park|Wellcome Sanger Institute for the COVID-19 Genomics UK (COG-UK) Consortium|Davies|United Kingdom
>Spike|hCoV-19/Luxembourg/LNS2325120/2020|2020-12-22|EPI_ISL_771053|Original|hCoV-19^^Luxembourg|Human|Laboratoire national de sant\u00e9|Laboratoire national de sant\u00e9|Wienecke-Baldacchino|Luxembourg
>Spike|hCoV-19/England/CAMC-94A326/2020|2020-07-27|EPI_ISL_556508|Original|hCoV-19^^England|Human|Lighthouse Lab in Cambridge|Wellcome Sanger Institute for the COVID-19 Genomics UK (COG-UK) consortium|Davies|United Kingdom
>Spike|hCoV-19/Sweden/20-22297/2020|2020-03-29|EPI_ISL_660394|Original|hCoV-19^^Ostergotland|Human|Klinsisk mikrobiologi Linkoping|The Public Health Agency of Sweden|Svartstr\u00f6m|Sweden
>Spike|hCoV-19/USA/AZ-TG526048/2020|2020-07-20|EPI_ISL_695392|Original|hCoV-19^^Arizona|Human|TGen North|TGen North|French|USA
>Spike|hCoV-19/England/ALDP-B48274/2020|2020-11-07|EPI_ISL_649290|Original|hCoV-19^^England|Human|Lighthouse Lab in Alderley Park|Wellcome Sanger Institute for the COVID-19 Genomics UK (COG-UK) Consortium|Davies|United Kingdom
>Spike|hCoV-19/Zimbabwe/ZW-9521/2020|2020-06-01|EPI_ISL_644815|Original|hCoV-19^^Zimbabwe|Human|National Microbiology Reference Laboratory|Quadram Institute Bioscience|Alikhan|Zimbabwe
>Spike|hCoV-19/Australia/VIC14655/2020|2020-08-13|EPI_ISL_592936|Original|hCoV-19^^Victoria|Human|Microbiological Diagnostic Unit - Public Health Laboratory (MDU-PHL)|MDU-PHL|Schultz|Australia
>Spike|hCoV-19/England/ALDP-BCA0AD/2020|2020-11-23|EPI_ISL_703274|Original|hCoV-19^^England|Human|Lighthouse Lab in Alderley Park|Wellcome Sanger Institute for the COVID-19 Genomics UK (COG-UK) Consortium|Davies|United Kingdom
>Spike|hCoV-19/Japan/PG-7837/2020|2020-08-00|EPI_ISL_686939|Original|hCoV-19^^Japan|Human|Pathogen Genomics Center|Pathogen Genomics Center|Sekizuka|Japan
>Spike|hCoV-19/Wales/PHWC-2E9D4/2020|2020-04-07|EPI_ISL_446096|Original|hCoV-19^^Wales|Human|Wales Specialist Virology Centre|Public Health Wales Microbiology Cardiff|Moore|United Kingdom
>Spike|hCoV-19/USA/WA-S1888/2020|2020-05-28|EPI_ISL_496314|Original|hCoV-19^^Washington|Human|Washington State Department of Health|Seattle Flu Study|Lee|USA
>Spike|hCoV-19/England/MILK-A6BDB7/2020|2020-10-09|EPI_ISL_609063|Original|hCoV-19^^England|Human|Lighthouse Lab in Milton Keynes|Wellcome Sanger Institute for the COVID-19 Genomics UK (COG-UK) consortium|Davies|United Kingdom
>Spike|hCoV-19/Australia/VIC12230/2020|2020-07-21|EPI_ISL_591935|Original|hCoV-19^^Victoria|Human|Microbiological Diagnostic Unit - Public Health Laboratory (MDU-PHL)|MDU-PHL|Schultz|Australia
>Spike|hCoV-19/USA/UT-UPHL-2009381/2020|2020-05-15|EPI_ISL_524973|Original|hCoV-19^^Utah|Human|Utah Public Health Laboratory|Utah Public Health Laboratory|Young|USA

>Spike|hCoV-19/Wales/PHWC-16A328/2020|2020-09-06|EPI_ISL_535234|Original|hCoV-19^^Wales|Human|Wales Specialist Virology Centre Sequencing lab|COVID-19 Genomics UK (COG-UK) Consortium|Moore|United Kingdom
>Spike|hCoV-19/Australia/VIC4250/2020|2020-07-13|EPI_ISL_519578|Original|hCoV-19^^Victoria|Human|Microbiological Diagnostic Unit - Public Health Laboratory (MDU-PHL)|MDU-PHL|Schultz|Australia
>Spike|hCoV-19/Australia/VIC5325/2020|2020-00-00|EPI_ISL_520088|Original|hCoV-19^^Victoria|Human|Microbiological Diagnostic Unit - Public Health Laboratory (MDU-PHL)|MDU-PHL|Schultz|Australia
>Spike|hCoV-19/England/CAMC-A57756/2020|2020-10-07|EPI_ISL_597427|Original|hCoV-19^^England|Human|Lighthouse Lab in Cambridge|Wellcome Sanger Institute for the COVID-19 Genomics UK (COG-UK) consortium|Davies|United Kingdom
>Spike|hCoV-19/Denmark/ALAB-HH-148/2020|2020-03-12|EPI_ISL_452025|Original|hCoV-19^^Denmark|Human|Department of Clinical Microbiology|Albertsen lab|Kirkegaard|Denmark
>Spike|hCoV-19/Scotland/EDB2885/2020|2020-04-01|EPI_ISL_488930|Original|hCoV-19^^Scotland|Human|Virology Department|Wellcome Sanger Institute for the COVID-19 Genomics UK (COG-UK) consortium|Davies|United Kingdom
>Spike|hCoV-19/Denmark/DCGC-5310/2020|2020-09-14|EPI_ISL_615241|Original|hCoV-19^^Nordjylland|Human|Department of Virus and Microbiological Special Diagnostics|Albertsen lab|Michaelsen|Denmark
>Spike|hCoV-19/England/CAMC-B9A52F/2020|2020-11-16|EPI_ISL_673483|Original|hCoV-19^^England|Human|Lighthouse Lab in Cambridge|Wellcome Sanger Institute for the COVID-19 Genomics UK (COG-UK) Consortium|Davies|United Kingdom
>Spike|hCoV-19/England/ALDP-C39BC5/2020|2020-12-09|EPI_ISL_730719|Original|hCoV-19^^England|Human|Lighthouse Lab in Alderley Park|Wellcome Sanger Institute for the COVID-19 Genomics UK (COG-UK) Consortium|Davies|United Kingdom
>Spike|hCoV-19/France/HDF-5666/2020|2020-04-03|EPI_ISL_443288|Original|hCoV-19^^Hauts-de-France|Human|Laboratoire de Microbiologie - B\u00e2t A - CH Ren\u00e9 Dubois|National Reference Center for Viruses of Respiratory Infections|Enouf|France
>Spike|hCoV-19/Australia/VIC11013/2020|2020-09-08|EPI_ISL_563198|Original|hCoV-19^^Victoria|Human|Microbiological Diagnostic Unit - Public Health Laboratory (MDU-PHL)|MDU-PHL|Schultz|Australia
>Spike|hCoV-19/South Africa/N00133/2020|2020-07-06|EPI_ISL_623037|Original|hCoV-19^^Mpumalanga|Human|National Health Laboratory Service|National Institute for Communicable Diseases of the National Health Laboratory Service|Allam|South Africa
>Spike|hCoV-19/England/QEUH-A204E6/2020|2020-10-01|EPI_ISL_587636|Original|hCoV-19^^England|Human|Lighthouse Lab in Glasgow|Wellcome Sanger Institute for the COVID-19 Genomics UK (COG-UK) consortium|Davies|United Kingdom
>Spike|hCoV-19/Norway/5057/2020|2020-10-29|EPI_ISL_635086|Original|hCoV-19^^Oslo|Human|Furst Medical Laboratory|Norwegian Institute of Public Health|Vollan|Norway
>Spike|hCoV-19/England/QEUH-B7F61E/2020|2020-11-14|EPI_ISL_668258|Original|hCoV-19^^England|Human|Lighthouse Lab in Glasgow|Wellcome Sanger Institute for the COVID-19 Genomics UK (COG-UK) Consortium|Davies|United Kingdom
>Spike|hCoV-19/England/201380054/2020|2020-03-25|EPI_ISL_421809|Original|hCoV-19^^England|Human|Respiratory Virus Unit|Respiratory Virus Unit|Platt|United Kingdom
>Spike|hCoV-19/Chile/MA-CADIUMAG-34/2020|2020-08-12|EPI_ISL_681699|Original|hCoV-19^^Magallanes|Human|Molecular Medicine Laboratory|Centro Asistencial Docente y de Investigacion|Navarrete|Chile
>Spike|hCoV-19/Czech Republic/NRL-10715/2020|2020-11-03|EPI_ISL_693684|Original|hCoV-19^^Prague|Human|The National Institute of Public Health|State Veterinary Institute Prague|Nagy|Czech Republic
>Spike|hCoV-19/USA/WA-S2683/2020|2020-06-21|EPI_ISL_497315|Original|hCoV-19^^Washington|Human|Washington State Department of Health|Seattle Flu Study|Lee|USA
>Spike|hCoV-19/England/ALDP-A2FB00/2020|2020-10-05|EPI_ISL_586938|Original|hCoV-19^^England|Human|Lighthouse Lab in Alderley Park|Wellcome Sanger Institute for the COVID-19 Genomics UK (COG-UK) consortium|Davies|United Kingdom

>Spike|hCoV-19/England/MILK-B026A7/2020|2020-10-24|EPI_ISL_642806|Original|hCoV-19^^England|Human|Lighthouse Lab in Milton Keynes|Wellcome Sanger Institute for the COVID-19 Genomics UK (COG-UK) Consortium|Davies|United Kingdom
>Spike|hCoV-19/England/ALDP-A2D847/2020|2020-10-06|EPI_ISL_586637|Original|hCoV-19^^England|Human|Lighthouse Lab in Alderley Park|Wellcome Sanger Institute for the COVID-19 Genomics UK (COG-UK) consortium|Davies|United Kingdom
>Spike|hCoV-19/England/LIVE-A0318/2020|2020-05-23|EPI_ISL_472102|Original|hCoV-19^^England|Human|Liverpool Clinical Laboratories|COVID-19 Genomics UK (COG-UK) Consortium|Haldenby|United Kingdom
>Spike|hCoV-19/Denmark/DCGC-5505/2020|2020-09-21|EPI_ISL_620830|Original|hCoV-19^^Hovedstaden|Human|Department of Virus and Microbiological Special Diagnostics|Albertsen lab|Michaelsen|Denmark
>Spike|hCoV-19/USA/CA-CZB-3671/2020|2020-08-11|EPI_ISL_516746|Original|hCoV-19^^California|Human|UCSF Clinical Microbiology Laboratory|Chan-Zuckerberg Biohub|Kamm|USA
>Spike|hCoV-19/USA/TX-HMH-MCoV-10349/2020|2020-07-08|EPI_ISL_789431|Original|hCoV-19^^Texas|Human|Houston Methodist Hospital|Houston Methodist Hospital|Long|USA
>Spike|hCoV-19/England/QEUH-A8C4D9/2020|2020-10-18|EPI_ISL_643150|Original|hCoV-19^^England|Human|Lighthouse Lab in Glasgow|Wellcome Sanger Institute for the COVID-19 Genomics UK (COG-UK) Consortium|Davies|United Kingdom
>Spike|hCoV-19/Canada/MB-NML-827/2020|2020-07-13|EPI_ISL_582323|Original|hCoV-19^^Manitoba|Human|Cadham Provincial Laboratory|National Microbiology Laboratory (NML)|Majer|Canada
>Spike|hCoV-19/England/NOTT-111DD7/2020|2020-05-03|EPI_ISL_472407|Original|hCoV-19^^England|Human|Queens Medical Centre|COVID-19 Genomics UK (COG-UK) Consortium|Loose|United Kingdom
>Spike|hCoV-19/Wales/PHWC-47EACF/2020|2020-10-08|EPI_ISL_595549|Original|hCoV-19^^Wales|Human|Originating lab|COVID-19 Genomics UK (COG-UK) Consortium|Moore|United Kingdom
>Spike|hCoV-19/England/BRIS-130CF3/2020|2020-03-24|EPI_ISL_492682|Original|hCoV-19^^England|Human|PHE South West Regional Laboratory|Wellcome Sanger Institute for the COVID-19 Genomics UK (COG-UK) consortium|Davies|United Kingdom
>Spike|hCoV-19/England/QEUH-C92D5B/2020|2020-12-17|EPI_ISL_762123|Original|hCoV-19^^England|Human|Lighthouse Lab in Glasgow|Wellcome Sanger Institute for the COVID-19 Genomics UK (COG-UK) Consortium|Davies|United Kingdom
>Spike|hCoV-19/England/QEUH-B9F4F0/2020|2020-11-18|EPI_ISL_720965|Original|hCoV-19^^England|Human|Lighthouse Lab in Glasgow|Wellcome Sanger Institute for the COVID-19 Genomics UK (COG-UK) Consortium|Davies|United Kingdom
>Spike|hCoV-19/USA/CA-CZB-10957/2020|2020-09-16|EPI_ISL_583040|Original|hCoV-19^^California|Human|Orange County Public Health Lab|Chan-Zuckerberg Biohub|Bhatt|USA
>Spike|hCoV-19/USA/CA-CZB-2013/2020|2020-05-01|EPI_ISL_486267|Original|hCoV-19^^California|Human|Orange County Public Health Laboratory|Chan-Zuckerberg Biohub|McGeever|USA
>Spike|hCoV-19/England/CAMC-B33054/2020|2020-11-05|EPI_ISL_659150|Original|hCoV-19^^England|Human|Lighthouse Lab in Cambridge|Wellcome Sanger Institute for the COVID-19 Genomics UK (COG-UK) Consortium|Davies|United Kingdom
>Spike|hCoV-19/Japan/PG-6744/2020|2020-08-00|EPI_ISL_687854|Original|hCoV-19^^Japan|Human|Pathogen Genomics Center|Pathogen Genomics Center|Sekizuka|Japan
>Spike|hCoV-19/Wales/PHWC-495D50/2020|2020-11-26|EPI_ISL_726477|Original|hCoV-19^^Wales|Human|Originating lab|COVID-19 Genomics UK (COG-UK) Consortium|Moore|United Kingdom
>Spike|hCoV-19/England/MILK-992DE5/2020|2020-08-26|EPI_ISL_551977|Original|hCoV-19^^England|Human|Lighthouse Lab in Milton Keynes|Wellcome Sanger Institute for the COVID-19 Genomics UK (COG-UK) consortium|Davies|United Kingdom
>Spike|hCoV-19/Singapore/465/2020|2020-03-05|EPI_ISL_475996|Original|hCoV-19^^Singapore|Human|National Public Health Laboratory|National Public Health Laboratory|Mak|Singapore
>Spike|hCoV-19/England/CAMC-9EBBD4/2020|2020-09-23|EPI_ISL_584404|Original|hCoV-19^^England|Human|Queens Medical Centre|COVID-19 Genomics UK (COG-UK) Consortium|Loose|United Kingdom

>Spike|hCoV-19/England/20138082704/2020|2020-03-26|EPI_ISL_421836|Original|hCoV-
19^^England|Human|Respiratory Virus Unit|Respiratory Virus Unit|Platt|United Kingdom
>Spike|hCoV-19/Scotland/QEUH-9E432E/2020|2020-09-23|EPI_ISL_600995|Original|hCoV-
19^^Scotland|Human|Lighthouse Lab in Glasgow|Wellcome Sanger Institute for the COVID-
19 Genomics UK (COG-UK) consortium|Davies|United Kingdom
>Spike|hCoV-19/Scotland/QEUH-BA371E/2020|2020-11-18|EPI_ISL_757205|Original|hCoV-
19^^Scotland|Human|Lighthouse Lab in Glasgow|Wellcome Sanger Institute for the COVID-
19 Genomics UK (COG-UK) Consortium|Davies|United Kingdom
>Spike|hCoV-19/Australia/VIC9532/2020|2020-08-21|EPI_ISL_562743|Original|hCoV-
19^^Victoria|Human|Microbiological Diagnostic Unit - Public Health Laboratory (MDU-
PHL)|MDU-PHL|Schultz|Australia
>Spike|hCoV-19/Japan/PG-6270/2020|2020-08-00|EPI_ISL_689381|Original|hCoV-
19^^Japan|Human|Pathogen Genomics Center|Pathogen Genomics Center|Sekizuka|Japan
>Spike|hCoV-19/England/QEUH-A8B7FF/2020|2020-10-18|EPI_ISL_721536|Original|hCoV-
19^^England|Human|Lighthouse Lab in Glasgow|Wellcome Sanger Institute for the COVID-
19 Genomics UK (COG-UK) Consortium|Davies|United Kingdom
>Spike|hCoV-19/Denmark/DCGC-7874/2020|2020-10-19|EPI_ISL_618846|Original|hCoV-
19^^Midtjylland|Human|Department of Virus and Microbiological Special
Diagnostics|Albertsen lab|Michaelsen|Denmark
>Spike|hCoV-19/Wales/PHWC-1662E3/2020|2020-06-13|EPI_ISL_490861|Original|hCoV-
19^^Wales|Human|Wales Specialist Virology Centre Sequencing lab|COVID-19 Genomics UK
(COG-UK) Consortium|Moore|United Kingdom
>Spike|hCoV-19/England/MILK-9A854E/2020|2020-09-02|EPI_ISL_550021|Original|hCoV-
19^^England|Human|Lighthouse Lab in Milton Keynes|Wellcome Sanger Institute for the
COVID-19 Genomics UK (COG-UK) consortium|Davies|United Kingdom
>Spike|hCoV-19/Sweden/20-07042/2020|2020-04-04|EPI_ISL_434665|Original|hCoV-
19^^Uppsala|Human|Uppsala Narakut Aleris|The Public Health Agency of
Sweden|Svartstr\u00f6m|Sweden
>Spike|hCoV-19/England/CAMC-B372B4/2020|2020-11-05|EPI_ISL_645372|Original|hCoV-
19^^England|Human|Lighthouse Lab in Cambridge|Wellcome Sanger Institute for the
COVID-19 Genomics UK (COG-UK) Consortium|Davies|United Kingdom
>Spike|hCoV-19/England/QEUH-B6AA83/2020|2020-11-11|EPI_ISL_662611|Original|hCoV-
19^^England|Human|Lighthouse Lab in Glasgow|Wellcome Sanger Institute for the COVID-
19 Genomics UK (COG-UK) Consortium|Davies|United Kingdom
>Spike|hCoV-19/England/CAMC-B21B94/2020|2020-11-01|EPI_ISL_647223|Original|hCoV-
19^^England|Human|Lighthouse Lab in Cambridge|Wellcome Sanger Institute for the
COVID-19 Genomics UK (COG-UK) Consortium|Davies|United Kingdom
>Spike|hCoV-19/England/SHEF-10C903D/2020|2020-12-19|EPI_ISL_763427|Original|hCoV-
19^^England|Human|Virology Department|COVID-19 Genomics UK (COG-UK) Consortium|de
Silva|United Kingdom
>Spike|hCoV-19/Italy/VEN-UniVR-27/2020|2020-08-28|EPI_ISL_751345|Original|hCoV-
19^^Veneto|Human|IRCCS Sacro Cuore Don Calabria Hospital|University of
Verona|Mori|Italy
>Spike|hCoV-19/England/ALDP-CBDC6C/2020|2020-12-19|EPI_ISL_760392|Original|hCoV-
19^^England|Human|Lighthouse Lab in Alderley Park|Wellcome Sanger Institute for the
COVID-19 Genomics UK (COG-UK) Consortium|Davies|United Kingdom
>Spike|hCoV-19/Singapore/348/2020|2020-03-31|EPI_ISL_469103|Original|hCoV-
19^^Singapore|Human|National Public Health Laboratory|National Public Health
Laboratory|Mak|Singapore
>Spike|hCoV-19/England/ALDP-A86536/2020|2020-10-17|EPI_ISL_605985|Original|hCoV-
19^^England|Human|Lighthouse Lab in Alderley Park|Wellcome Sanger Institute for the
COVID-19 Genomics UK (COG-UK) consortium|Davies|United Kingdom
>Spike|hCoV-19/Spain/MD-IBV-99008702/2020|2020-09-08|EPI_ISL_654282|Original|hCoV-
19^^Madrid|Human|Hospital General Universitario Gregorio Mara\u00f1\u00f3n|SeqCOVID-
SPAIN consortium/IBV(CSIC)|Comas|Spain
>Spike|hCoV-19/Wales/PHWC-2CA6A/2020|2020-04-01|EPI_ISL_445940|Original|hCoV-
19^^Wales|Human|Wales Specialist Virology Centre|Public Health Wales Microbiology
Cardiff|Moore|United Kingdom

>Spike|hCoV-19/Wales/PHWC-16483E/2020|2020-04-21|EPI_ISL_479312|Original|hCoV-19^^Wales|Human|Wales Specialist Virology Centre Sequencing lab|COVID-19 Genomics UK (COG-UK) Consortium|Moore|United Kingdom
>Spike|hCoV-19/Spain/VC-IBV-98000423/2020|2020-03-26|EPI_ISL_539238|Original|hCoV-19^^Comunitat Valenciana|Human|Servicio de Microbiologia. Hospital Clinico Universitario de Valencia|SeqCOVID-SPAIN consortium/IBV(CSIC)|Comas|Spain
>Spike|hCoV-19/Gibraltar/204421173/2020|2020-10-27|EPI_ISL_613566|Original|hCoV-19^^Gibraltar|Human|Microbiology|Respiratory Virus Unit|Platt|Gibraltar
>Spike|hCoV-19/USA/ME-HETL-J0078/2020|2020-03-19|EPI_ISL_513489|Original|hCoV-19^^Maine|Human|Maine HETL|Tewhey Lab|Tewhey|USA
>Spike|hCoV-19/England/MILK-C51954/2020|2020-12-11|EPI_ISL_769855|Original|hCoV-19^^England|Human|Lighthouse Lab in Milton Keynes|Wellcome Sanger Institute for the COVID-19 Genomics UK (COG-UK) Consortium|Davies|United Kingdom
>Spike|hCoV-19/USA/MA-QDX-69/2020|2020-03-15|EPI_ISL_467865|Original|hCoV-19^^Massachusetts|Human|Quest Diagnostics|Quest Diagnostics|Kagan|USA
>Spike|hCoV-19/England/QEUH-99041F/2020|2020-08-26|EPI_ISL_539961|Original|hCoV-19^^England|Human|Lighthouse Lab in Glasgow|Wellcome Sanger Institute for the COVID-19 Genomics UK (COG-UK) consortium|Davies|United Kingdom
>Spike|hCoV-19/USA/WI-WSLH-200017/2020|2020-04-06|EPI_ISL_471183|Original|hCoV-19^^Wisconsin|Human|Wisconsin State Laboratory of Hygiene Communicable Disease Division|Wisconsin State Laboratory of Hygiene Communicable Disease Division|Florek|USA
>Spike|hCoV-19/USA/TX-QDX-2111/2020|2020-03-14|EPI_ISL_604485|Original|hCoV-19^^Texas|Human|Quest Diagnostics|Quest Diagnostics|Kagan|USA
>Spike|hCoV-19/England/LOND-D468B/2020|2020-04-08|EPI_ISL_444145|Original|hCoV-19^^England|Human|University College London|COVID-19 Genomics UK (COG-UK) Consortium|Roy|United Kingdom
>Spike|hCoV-19/USA/MI-UM-10036630690/2020|2020-10-26|EPI_ISL_650020|Original|hCoV-19^^Michigan|Human|University of Michigan Clinical Microbiology Laboratory|Lauring Lab|Valesano|USA
>Spike|hCoV-19/USA/TX-HMH-7387/2020|2020-06-29|EPI_ISL_546726|Original|hCoV-19^^Texas|Human|Houston Methodist Hospital|Houston Methodist Hospital|Long|USA
>Spike|hCoV-19/Netherlands/NoordBrabant_94/2020|2020-03-17|EPI_ISL_422888|Original|hCoV-19^^Noord Brabant|Human|Dutch COVID-19 response team|Erasmus Medical Center|Oude Munnink|Netherlands
>Spike|hCoV-19/England/CAMC-B790CB/2020|2020-11-12|EPI_ISL_668069|Original|hCoV-19^^England|Human|Lighthouse Lab in Cambridge|Wellcome Sanger Institute for the COVID-19 Genomics UK (COG-UK) Consortium|Davies|United Kingdom
>Spike|hCoV-19/Australia/VIC6728/2020|2020-07-25|EPI_ISL_562048|Original|hCoV-19^^Victoria|Human|Victorian Infectious Diseases Reference Laboratory (VIDRL)|VIDRL and MDU-PHL|Schultz|Australia
>Spike|hCoV-19/Scotland/CVR2289/2020|2020-04-11|EPI_ISL_490680|Original|hCoV-19^^Scotland|Human|West of Scotland Specialist Virology Centre|COVID-19 Genomics UK (COG-UK) Consortium|Robertson|United Kingdom
>Spike|hCoV-19/Spain/VC-IBV-98003101/2020|2020-03-15|EPI_ISL_538037|Original|hCoV-19^^Comunitat Valenciana|Human|Hospital Universitari i Polit\u00e8cnic La Fe de Val\u00e8ncia|SeqCOVID-SPAIN consortium/IBV(CSIC)|Comas|Spain
>Spike|hCoV-19/England/ALDP-59DF8E/2020|2020-06-14|EPI_ISL_558708|Original|hCoV-19^^England|Human|Lighthouse Lab in Alderley Park|Wellcome Sanger Institute for the COVID-19 Genomics UK (COG-UK) consortium|Davies|United Kingdom
>Spike|hCoV-19/England/MILK-B95C6B/2020|2020-11-14|EPI_ISL_674841|Original|hCoV-19^^England|Human|Lighthouse Lab in Milton Keynes|Wellcome Sanger Institute for the COVID-19 Genomics UK (COG-UK) Consortium|Davies|United Kingdom
>Spike|hCoV-19/USA/WI-UW-1455/2020|2020-09-10|EPI_ISL_541587|Original|hCoV-19^^Wisconsin|Human|University of Wisconsin-Madison AIDS Vaccine Research Laboratories|University of Wisconsin-Madison AIDS Vaccine Research Laboratories|Baczenas|USA
>Spike|hCoV-19/Portugal/PT0874/2020|2020-03-27|EPI_ISL_511133|Original|hCoV-19^^Portugal|Human|Instituto Nacional de Saude (INSA)|Instituto Nacional de Saude (INSA)|Borges|Portugal

>Spike|hCoV-19/Wuhan/HB-WHCM2-274/2020|2020-02-23|EPI_ISL_455008|Original|hCoV-19^^Hubei|Human|Wuhan Chain Medical Labs (CMLabs)|State Key Laboratory of Biotherapy of Sichuan University|Tang|China
>Spike|hCoV-19/USA/MI-MDHHS-SC21665/2020|2020-07-14|EPI_ISL_516392|Original|hCoV-19^^Michigan|Human|Michigan Department of Health and Human Services|Michigan Department of Health and Human Services|Blankenship|USA
>Spike|hCoV-19/Denmark/DCGC-18447/2020|2020-12-07|EPI_ISL_749972|Original|hCoV-19^^Syddanmark|Human|Department of Virus and Microbiological Special Diagnostics|Albertsen Lab|Michaelsen|Denmark
>Spike|hCoV-19/Wales/PHWC-49F002/2020|2020-12-10|EPI_ISL_743156|Original|hCoV-19^^Wales|Human|Wales Specialist Virology Centre Sequencing lab|COVID-19 Genomics UK (COG-UK) Consortium|Moore|United Kingdom
>Spike|hCoV-19/Australia/NSW192/2020|2020-03-25|EPI_ISL_427797|Original|hCoV-19^^New South Wales|Human|Centre for Infectious Diseases and Microbiology Public Health|NSW Health Pathology - Institute of Clinical Pathology and Medical Research; Westmead Hospital; University of Sydney|Gall|Australia
>Spike|hCoV-19/Japan/PG-1494/2020|2020-04-00|EPI_ISL_685101|Original|hCoV-19^^Japan|Human|Saitama Prefectural Institute of Public Health|Pathogen Genomics Center|Sekizuka|Japan
>Spike|hCoV-19/England/ALDP-6819BB/2020|2020-07-03|EPI_ISL_551824|Original|hCoV-19^^England|Human|Lighthouse Lab in Alderley Park|Wellcome Sanger Institute for the COVID-19 Genomics UK (COG-UK) consortium|Davies|United Kingdom
>Spike|hCoV-19/USA/MI-MDHHS-SC21092/2020|2020-03-26|EPI_ISL_507868|Original|hCoV-19^^Michigan|Human|Michigan Department of Health and Human Services|Michigan Department of Health and Human Services|Blankenship|USA
>Spike|hCoV-19/USA/NY-Wadsworth-79903-01/2020|2020-05-13|EPI_ISL_676910|Original|hCoV-19^^New York|Human|Wadsworth Center|Wadsworth Center|Lasek-Nesselquist|USA
>Spike|hCoV-19/England/MILK-9C489E/2020|2020-09-07|EPI_ISL_573258|Original|hCoV-19^^England|Human|Centre for Enzyme Innovation|COVID-19 Genomics UK (COG-UK) Consortium|Robson|United Kingdom
>Spike|hCoV-19/England/CAMC-B534DA/2020|2020-11-08|EPI_ISL_656785|Original|hCoV-19^^England|Human|Lighthouse Lab in Cambridge|Wellcome Sanger Institute for the COVID-19 Genomics UK (COG-UK) Consortium|Davies|United Kingdom
>Spike|hCoV-19/Netherlands/Limburg_11/2020|2020-05-08|EPI_ISL_460999|Original|hCoV-19^^Limburg|Human|Dutch COVID-19 response team|Erasmus Medical Center|Oude Munnink|Netherlands
>Spike|hCoV-19/England/MILK-9A5541/2020|2020-09-03|EPI_ISL_550172|Original|hCoV-19^^England|Human|Lighthouse Lab in Milton Keynes|Wellcome Sanger Institute for the COVID-19 Genomics UK (COG-UK) consortium|Davies|United Kingdom
>Spike|hCoV-19/Wales/PHWC-1650BD/2020|2020-06-12|EPI_ISL_479419|Original|hCoV-19^^Wales|Human|Wales Specialist Virology Centre Sequencing lab|COVID-19 Genomics UK (COG-UK) Consortium|Moore|United Kingdom
>Spike|hCoV-19/Wales/PHWC-4A4AEE/2020|2020-12-14|EPI_ISL_764768|Original|hCoV-19^^Wales|Human|Wales Specialist Virology Centre Sequencing lab|COVID-19 Genomics UK (COG-UK) Consortium|Moore|United Kingdom
>Spike|hCoV-19/England/MILK-9AA3CA/2020|2020-09-01|EPI_ISL_550898|Original|hCoV-19^^England|Human|Lighthouse Lab in Milton Keynes|Wellcome Sanger Institute for the COVID-19 Genomics UK (COG-UK) consortium|Davies|United Kingdom
>Spike|hCoV-19/England/ALDP-9E9ECE/2020|2020-09-24|EPI_ISL_580043|Original|hCoV-19^^England|Human|Lighthouse Lab in Alderley Park|Wellcome Sanger Institute for the COVID-19 Genomics UK (COG-UK) consortium|Davies|United Kingdom
>Spike|hCoV-19/England/MILK-978969/2020|2020-08-01|EPI_ISL_550477|Original|hCoV-19^^England|Human|Lighthouse Lab in Milton Keynes|Wellcome Sanger Institute for the COVID-19 Genomics UK (COG-UK) consortium|Davies|United Kingdom
>Spike|hCoV-19/Gibraltar/205121262/2020|2020-00-00|EPI_ISL_766381|Original|hCoV-19^^Gibraltar|Human|Microbiology|Respiratory Virus Unit|Platt|Gibraltar
>Spike|hCoV-19/England/CAMC-BC05A8/2020|2020-11-23|EPI_ISL_704846|Original|hCoV-19^^England|Human|Lighthouse Lab in Cambridge|Wellcome Sanger Institute for the COVID-19 Genomics UK (COG-UK) Consortium|Davies|United Kingdom

>Spike|hCoV-19/Germany/NW-HHU-298/2020|2020-10-02|EPI_ISL_770773|Original|hCoV-
19^^North Rhine-Westphalia|Human|ZOTZ KLIMAS MVZ D\u00fcsseldorf-Centrum GbR
\u00dcBAG f\u00fcr Labormedizin|Center of Medical Microbiology|Wienemann|Germany
>Spike|hCoV-19/USA/ME-HETL-J0155/2020|2020-09-11|EPI_ISL_755341|Original|hCoV-
19^^Maine|Human|Maine Health and Environmental Testing Laboratory|Tewhey
Lab|Tewhey|USA
>Spike|hCoV-19/England/LIVE-DB3B86/2020|2020-10-21|EPI_ISL_650166|Original|hCoV-
19^^England|Human|Liverpool Clinical Laboratories|COVID-19 Genomics UK (COG-UK)
Consortium|Haldenby|United Kingdom
>Spike|hCoV-19/England/QEUH-AD4C16/2020|2020-10-26|EPI_ISL_634648|Original|hCoV-
19^^England|Human|Lighthouse Lab in Glasgow|Wellcome Sanger Institute for the COVID-
19 Genomics UK (COG-UK) consortium|Davies|United Kingdom
>Spike|hCoV-19/United Arab Emirates/0603/2020|2020-05-
15|EPI_ISL_698761|Original|hCoV-19^^Abu Dhabi|Human|Group 42 (G42) Healthcare|G42
Healthcare|Ogrodzki|United Arab Emirates
>Spike|hCoV-19/England/20099000304/2020|2020-02-28|EPI_ISL_464199|Original|hCoV-
19^^England|Human|Respiratory Virus Unit|Respiratory Virus Unit|Platt|United Kingdom
>Spike|hCoV-19/Hungary/US-32570w/2020|2020-09-02|EPI_ISL_677758|Original|hCoV-
19^^Csongr\u00e1d county|Human|University of Szeged|National Laboratory of
Virology|Kemenesi|Hungary
>Spike|hCoV-19/Japan/PG-4798/2020|2020-08-00|EPI_ISL_689923|Original|hCoV-
19^^Japan|Human|Pathogen Genomics Center|Pathogen Genomics Center|Sekizuka|Japan
>Spike|hCoV-19/England/MILK-5147B4/2020|2020-06-11|EPI_ISL_559017|Original|hCoV-
19^^England|Human|Lighthouse Lab in Milton Keynes|Wellcome Sanger Institute for the
COVID-19 Genomics UK (COG-UK) Consortium|Davies|United Kingdom
>Spike|hCoV-19/USA/TX-HMH-5817/2020|2020-06-28|EPI_ISL_547039|Original|hCoV-
19^^Texas|Human|Houston Methodist Hospital|Houston Methodist Hospital|Long|USA
>Spike|hCoV-19/England/NORW-E73BB/2020|2020-04-20|EPI_ISL_449031|Original|hCoV-
19^^England|Human|Quadram Institute Bioscience|COVID-19 Genomics UK (COG-UK)
Consortium|Alikhan|United Kingdom
>Spike|hCoV-19/England/MILK-83247A/2020|2020-07-16|EPI_ISL_557175|Original|hCoV-
19^^England|Human|Lighthouse Lab in Milton Keynes|Wellcome Sanger Institute for the
COVID-19 Genomics UK (COG-UK) consortium|Davies|United Kingdom
>Spike|hCoV-19/Japan/PG-4076/2020|2020-07-00|EPI_ISL_692317|Original|hCoV-
19^^Japan|Human|Gunma Prefectural Institute of Public Health and Environmental
Sciences|Pathogen Genomics Center|Sekizuka|Japan
>Spike|hCoV-19/England/MILK-B54C2C/2020|2020-11-05|EPI_ISL_657970|Original|hCoV-
19^^England|Human|Lighthouse Lab in Milton Keynes|Wellcome Sanger Institute for the
COVID-19 Genomics UK (COG-UK) Consortium|Davies|United Kingdom
>Spike|hCoV-19/Japan/IC-0185/2020|2020-10-00|EPI_ISL_667614|Original|hCoV-
19^^Japan|Human|Pathogen Genomics Center|Pathogen Genomics Center|Sekizuka|Japan
>Spike|hCoV-19/Canada/ON-S1265/2020|2020-05-05|EPI_ISL_586318|Original|hCoV-
19^^Ontario|Human|Toronto Invasive Bacterial Diseases Network|McMaster
University|McArthur|Canada
>Spike|hCoV-19/Japan/PG-1936/2020|2020-04-00|EPI_ISL_685542|Original|hCoV-
19^^Japan|Human|Pathogen Genomics Center|Pathogen Genomics Center|Sekizuka|Japan
>Spike|hCoV-19/USA/TX-DSHS-1879/2020|2020-07-08|EPI_ISL_751508|Original|hCoV-
19^^Texas|Human|Texas Department of State Health Services|Texas Department of State
Health Services|Rahman|USA
>Spike|hCoV-19/Denmark/DCGC-16149/2020|2020-11-16|EPI_ISL_714605|Original|hCoV-
19^^Midtjylland|Human|Department of Virus and Microbiological Special
Diagnostics|Albertsen Lab|Michaelsen|Denmark
>Spike|hCoV-19/England/LIVE-9C3A6/2020|2020-03-31|EPI_ISL_449534|Original|hCoV-
19^^England|Human|Liverpool Clinical Laboratories|COVID-19 Genomics UK (COG-UK)
Consortium|Haldenby|United Kingdom
>Spike|hCoV-19/England/SHEF-C07F8/2020|2020-03-21|EPI_ISL_420175|Original|hCoV-
19^^England|Human|Virology Department|Department of Infection|de Silva|United Kingdom
>Spike|hCoV-19/England/MILK-AB76B1/2020|2020-10-19|EPI_ISL_623340|Original|hCoV-
19^^England|Human|Lighthouse Lab in Milton Keynes|Wellcome Sanger Institute for the
COVID-19 Genomics UK (COG-UK) consortium|Davies|United Kingdom

>Spike|hCoV-19/South Africa/R12578-20/2020|2020-06-16|EPI_ISL_504194|Original|hCoV-19^^North West|Human|National Institute for Communicable Diseases of the National Health Laboratory Service|National Institute for Communicable Diseases of the National Health Laboratory Service|Allam|South Africa
>Spike|hCoV-19/Denmark/ALAB-SSI-883/2020|2020-03-31|EPI_ISL_444954|Original|hCoV-19^^Denmark|Human|Department of Virus and Microbiological Special Diagnostics|Albertsen lab|Kirkegaard|Denmark
>Spike|hCoV-19/Australia/TAS39/2020|2020-04-12|EPI_ISL_522716|Original|hCoV-19^^Tasmania|Human|Royal Hobart Hospital Microbiology Department|MDU-PHL|Schultz|Australia
>Spike|hCoV-19/Singapore/221/2020|2020-03-23|EPI_ISL_462339|Original|hCoV-19^^Singapore|Human|National Public Health Laboratory|National Public Health Laboratory|Mak|Singapore
>Spike|hCoV-19/Wales/PHWC-48A1BC/2020|2020-11-03|EPI_ISL_652532|Original|hCoV-19^^Wales|Human|Wales Specialist Virology Centre Sequencing lab|COVID-19 Genomics UK (COG-UK) Consortium|Moore|United Kingdom
>Spike|hCoV-19/Wales/PHWC-1644EF/2020|2020-04-29|EPI_ISL_474186|Original|hCoV-19^^Wales|Human|Originating lab|COVID-19 Genomics UK (COG-UK) Consortium|Moore|United Kingdom
>Spike|hCoV-19/England/CAMC-B77A74/2020|2020-11-12|EPI_ISL_783486|Original|hCoV-19^^England|Human|Lighthouse Lab in Cambridge|Wellcome Sanger Institute for the COVID-19 Genomics UK (COG-UK) Consortium|Davies|United Kingdom
>Spike|hCoV-19/Denmark/DCGC-16421/2020|2020-11-16|EPI_ISL_714756|Original|hCoV-19^^Nordjylland|Human|Department of Virus and Microbiological Special Diagnostics|Albertsen Lab|Michaelsen|Denmark
>Spike|hCoV-19/Australia/VIC571/2020|2020-03-25|EPI_ISL_426831|Original|hCoV-19^^Victoria|Human|Victorian Infectious Diseases Reference Laboratory (VIDRL)|Microbiological Diagnostic Unit Public Health Laboratory and Victorian Infectious Diseases Reference Laboratory|Schultz|Australia
>Spike|hCoV-19/Australia/NSW124/2020|2020-03-26|EPI_ISL_427782|Original|hCoV-19^^New South Wales|Human|Centre for Infectious Diseases and Microbiology Public Health|NSW Health Pathology - Institute of Clinical Pathology and Medical Research; Westmead Hospital; University of Sydney|Gall|Australia
>Spike|hCoV-19/Denmark/DCGC-21541/2020|2020-12-21|EPI_ISL_758220|Original|hCoV-19^^Syddanmark|Human|Department of Virus and Microbiological Special Diagnostics|Albertsen Lab|Michaelsen|Denmark
>Spike|hCoV-19/England/ALDP-950ED1/2020|2020-06-14|EPI_ISL_554296|Original|hCoV-19^^England|Human|Lighthouse Lab in Alderley Park|Wellcome Sanger Institute for the COVID-19 Genomics UK (COG-UK) consortium|Davies|United Kingdom
>Spike|hCoV-19/England/CAMB-79433/2020|2020-04-03|EPI_ISL_441140|Original|hCoV-19^^England|Human|Department of Pathology|Wellcome Sanger Institute for the COVID-19 Genomics UK (COG-UK) consortium|Amato|United Kingdom
>Spike|hCoV-19/USA/VA-DCLS-0417/2020|2020-04-28|EPI_ISL_477633|Original|hCoV-19^^Virginia|Human|Virginia DCLS|Virginia DCLS|St. Jacques|USA
>Spike|hCoV-19/USA/MI-MDHHS-SC22606/2020|2020-11-19|EPI_ISL_737179|Original|hCoV-19^^Michigan|Human|Michigan Department of Health and Human Services|Michigan Department of Health and Human Services|Blankenship|USA
>Spike|hCoV-19/England/ALDP-9EA62F/2020|2020-09-24|EPI_ISL_580056|Original|hCoV-19^^England|Human|Lighthouse Lab in Alderley Park|Wellcome Sanger Institute for the COVID-19 Genomics UK (COG-UK) consortium|Davies|United Kingdom
>Spike|hCoV-19/England/MILK-C6398C/2020|2020-12-13|EPI_ISL_735590|Original|hCoV-19^^England|Human|Lighthouse Lab in Milton Keynes|Wellcome Sanger Institute for the COVID-19 Genomics UK (COG-UK) Consortium|Davies|United Kingdom
>Spike|hCoV-19/USA/MI-UM-10036561998/2020|2020-10-21|EPI_ISL_640179|Original|hCoV-19^^Michigan|Human|University of Michigan Clinical Microbiology Laboratory|Lauring Lab|Valesano|USA
>Spike|hCoV-19/USA/TX-HMH-MCoV-9180/2020|2020-07-02|EPI_ISL_780984|Original|hCoV-19^^Texas|Human|Houston Methodist Hospital|Houston Methodist Hospital|Long|USA

>Spike|hCoV-19/Australia/VIC12659/2020|2020-07-31|EPI_ISL_641298|Original|hCoV-
19^^Victoria|Human|Microbiological Diagnostic Unit - Public Health Laboratory (MDU-
PHL)|MDU-PHL|Schultz|Australia
>Spike|hCoV-19/Australia/VIC7923/2020|2020-08-06|EPI_ISL_564745|Original|hCoV-
19^^Victoria|Human|Microbiological Diagnostic Unit - Public Health Laboratory (MDU-
PHL)|MDU-PHL|Schultz|Australia
>Spike|hCoV-19/South Africa/KRISP-0369/2020|2020-06-25|EPI_ISL_487320|Original|hCoV-
19^^KwaZulu-Natal|Human|NHLS-IALCH|KRISP|de Oliveira|South Africa
>Spike|hCoV-19/England/QEUH-89830C/2020|2020-07-24|EPI_ISL_532709|Original|hCoV-
19^^England|Human|Lighthouse Lab in Glasgow|Wellcome Sanger Institute for the COVID-
19 Genomics UK (COG-UK) consortium|Davies|United Kingdom
>Spike|hCoV-19/England/20108004803/2020|2020-03-03|EPI_ISL_417251|Original|hCoV-
19^^England|Human|Respiratory Virus Unit|Respiratory Virus Unit|Platt|United Kingdom
>Spike|hCoV-19/Gibraltar/205121199/2020|2020-00-00|EPI_ISL_766329|Original|hCoV-
19^^Gibraltar|Human|Microbiology|Respiratory Virus Unit|Platt|Gibraltar
>Spike|hCoV-19/USA/TX-HMH-7845/2020|2020-06-30|EPI_ISL_545663|Original|hCoV-
19^^Texas|Human|Houston Methodist Hospital|Houston Methodist Hospital|Long|USA
>Spike|hCoV-19/USA/WI-WSLH-200155/2020|2020-03-30|EPI_ISL_578382|Original|hCoV-
19^^Wisconsin|Human|Wisconsin State Laboratory of Hygiene Communicable Disease
Division|Wisconsin State Laboratory of Hygiene Communicable Disease
Division|Florek|USA
>Spike|hCoV-19/USA/UT-QDX-2090/2020|2020-03-13|EPI_ISL_604490|Original|hCoV-
19^^Utah|Human|Quest Diagnostics|Quest Diagnostics|Kagan|USA
>Spike|hCoV-19/England/MILK-BDC970/2020|2020-11-27|EPI_ISL_709695|Original|hCoV-
19^^England|Human|Lighthouse Lab in Milton Keynes|Wellcome Sanger Institute for the
COVID-19 Genomics UK (COG-UK) Consortium|Davies|United Kingdom
>Spike|hCoV-19/USA/CA-QDX-03/2020|2020-03-13|EPI_ISL_444455|Original|hCoV-
19^^California|Human|Molecular Infectious Disease|Molecular Infectious
Disease|Anderson|USA
>Spike|hCoV-19/England/QEUH-BA0580/2020|2020-11-18|EPI_ISL_719764|Original|hCoV-
19^^England|Human|Lighthouse Lab in Glasgow|Wellcome Sanger Institute for the COVID-
19 Genomics UK (COG-UK) Consortium|Davies|United Kingdom
>Spike|hCoV-19/Shanghai/SH0127/2020|2020-02-12|EPI_ISL_416408|Original|hCoV-
19^^Shanghai|Human|Shanghai Public Health Clinical Center|National Research Center
for Translational Medicine (Shanghai)|Zhang|China
>Spike|hCoV-19/England/QEUH-B80C18/2020|2020-11-14|EPI_ISL_661908|Original|hCoV-
19^^England|Human|Lighthouse Lab in Glasgow|Wellcome Sanger Institute for the COVID-
19 Genomics UK (COG-UK) Consortium|Davies|United Kingdom
>Spike|hCoV-19/France/NAQ-SC203/2020|2020-03-05|EPI_ISL_645169|Original|hCoV-
19^^Nouvelle-Aquitaine|Human|CHU Bordeaux|CNR Virus des Infections Respiratoires -
France SUD|Bal|France
>Spike|hCoV-19/USA/LA-EVTL1144/2020|2020-12-09|EPI_ISL_768413|Original|hCoV-
19^^Louisiana|Human|LSUHS Emerging Viral Threat Laboratory|Microbial Genome
Sequencing Center|Kamil|USA
>Spike|hCoV-19/Australia/VIC16121/2020|2020-08-09|EPI_ISL_641178|Original|hCoV-
19^^Victoria|Human|Microbiological Diagnostic Unit - Public Health Laboratory (MDU-
PHL)|MDU-PHL|Schultz|Australia
>Spike|hCoV-19/England/BRIS-12E9C4/2020|2020-04-23|EPI_ISL_488243|Original|hCoV-
19^^England|Human|PHE South West Regional Laboratory|Wellcome Sanger Institute for
the COVID-19 Genomics UK (COG-UK) consortium|Davies|United Kingdom
>Spike|hCoV-19/Scotland/CVR3109/2020|2020-04-25|EPI_ISL_461682|Original|hCoV-
19^^Scotland|Human|West of Scotland Specialist Virology Centre|COVID-19 Genomics UK
(COG-UK) Consortium|Robertson|United Kingdom
>Spike|hCoV-19/England/ALDP-9BDB17/2020|2020-09-09|EPI_ISL_568193|Original|hCoV-
19^^England|Human|Lighthouse Lab in Alderley Park|Wellcome Sanger Institute for the
COVID-19 Genomics UK (COG-UK) consortium|Davies|United Kingdom
>Spike|hCoV-19/England/204761666/2020|2020-11-18|EPI_ISL_683551|Original|hCoV-
19^^England|Human|Respiratory Virus Unit|COVID-19 Genomics UK (COG-UK)
Consortium|Platt|United Kingdom

>Spike|hCoV-19/Australia/VIC12885/2020|2020-07-29|EPI_ISL_640776|Original|hCoV-19^^Victoria|Human|Microbiological Diagnostic Unit - Public Health Laboratory (MDU-PHL)|MDU-PHL|Schultz|Australia
>Spike|hCoV-19/Russia/Vector_77628/2020|2020-03-22|EPI_ISL_428904|Vero|hCoV-19^^Moscow|Human|State Research Center of Virology and Biotechnology VECTOR|State Research Center of Virology and Biotechnology VECTOR|Shvalov|Russia
>Spike|hCoV-19/USA/TX-HMH-MCoV-15907/2020|2020-07-10|EPI_ISL_785591|Original|hCoV-19^^Texas|Human|Houston Methodist Hospital|Houston Methodist Hospital|Long|USA
>Spike|hCoV-19/USA/WA-UW-12359/2020|2020-06-27|EPI_ISL_570512|Original|hCoV-19^^Washington|Human|UW Virology Lab|UW Virology Lab|Roychoudhury|USA
>Spike|hCoV-19/Oman/205035516/2020|2020-05-13|EPI_ISL_491971|Original|hCoV-19^^Muscat|Human|Oman-NIC|Department of\u00a0Microbiology\u00a0and Immunology-SQUH|AL Zadjali|Oman
>Spike|hCoV-19/Australia/VIC13286/2020|2020-07-28|EPI_ISL_563653|Original|hCoV-19^^Victoria|Human|Microbiological Diagnostic Unit - Public Health Laboratory (MDU-PHL)|MDU-PHL|Schultz|Australia
>Spike|hCoV-19/England/NOTT-110EA8/2020|2020-04-29|EPI_ISL_448431|Original|hCoV-19^^England|Human|Queens Medical Centre|COVID-19 Genomics UK (COG-UK) Consortium|Loose|United Kingdom
>Spike|hCoV-19/Wales/PHWC-49B9A1/2020|2020-12-06|EPI_ISL_742410|Original|hCoV-19^^Wales|Human|Wales Specialist Virology Centre Sequencing lab|COVID-19 Genomics UK (COG-UK) Consortium|Moore|United Kingdom
>Spike|hCoV-19/England/CAMC-B1FF24/2020|2020-11-01|EPI_ISL_647637|Original|hCoV-19^^England|Human|Lighthouse Lab in Cambridge|Wellcome Sanger Institute for the COVID-19 Genomics UK (COG-UK) Consortium|Davies|United Kingdom
>Spike|hCoV-19/Scotland/CVR2366/2020|2020-04-13|EPI_ISL_493792|Original|hCoV-19^^Scotland|Human|West of Scotland Specialist Virology Centre|COVID-19 Genomics UK (COG-UK) Consortium|Robertson|United Kingdom
>Spike|hCoV-19/England/MILK-AC77F7/2020|2020-10-21|EPI_ISL_630258|Original|hCoV-19^^England|Human|Lighthouse Lab in Milton Keynes|Wellcome Sanger Institute for the COVID-19 Genomics UK (COG-UK) consortium|Davies|United Kingdom
>Spike|hCoV-19/Jordan/SEARCH-5457/2020|2020-09-07|EPI_ISL_755245|Original|hCoV-19^^Amman|Human|Biolab Diagnostic Laboratories|Andersen lab at Scripps Research|Gangavarapu|Jordan
>Spike|hCoV-19/Scotland/EDB3976/2020|2020-05-03|EPI_ISL_449268|Original|hCoV-19^^Scotland|Human|Virology Department|COVID-19 Genomics UK (COG-UK) Consortium|Williams|United Kingdom
>Spike|hCoV-19/England/LOND-126368C/2020|2020-11-02|EPI_ISL_679463|Original|hCoV-19^^England|Human|University College London|COVID-19 Genomics UK (COG-UK) Consortium|Roy|United Kingdom
>Spike|hCoV-19/Australia/VIC16474/2020|2020-08-06|EPI_ISL_640879|Original|hCoV-19^^Victoria|Human|Microbiological Diagnostic Unit - Public Health Laboratory (MDU-PHL)|MDU-PHL|Schultz|Australia
>Spike|hCoV-19/USA/WI-UW-331/2020|2020-04-26|EPI_ISL_436625|Original|hCoV-19^^Wisconsin|Human|University of Wisconsin-Madison AIDS Vaccine Research Laboratories|University of Wisconsin-Madison AIDS Vaccine Research Laboratories|Baczenas|USA
>Spike|hCoV-19/England/SHEF-CE88E/2020|2020-05-11|EPI_ISL_475468|Original|hCoV-19^^England|Human|Virology Department|COVID-19 Genomics UK (COG-UK) Consortium|de Silva|United Kingdom
>Spike|hCoV-19/England/MILK-BAC1AE/2020|2020-11-18|EPI_ISL_783234|Original|hCoV-19^^England|Human|Lighthouse Lab in Milton Keynes|Wellcome Sanger Institute for the COVID-19 Genomics UK (COG-UK) Consortium|Davies|United Kingdom
>Spike|hCoV-19/Wales/PHWC-2E0C0/2020|2020-04-10|EPI_ISL_446056|Original|hCoV-19^^Wales|Human|Wales Specialist Virology Centre|Public Health Wales Microbiology Cardiff|Moore|United Kingdom
>Spike|hCoV-19/USA/WA-UW-8941/2020|2020-05-12|EPI_ISL_776737|Original|hCoV-19^^Washington|Human|UW Virology Lab|UW Virology Lab|Roychoudhury|USA
>Spike|hCoV-19/Northern Ireland/NIRE-FACBA/2020|2020-04-15|EPI_ISL_459324|Original|hCoV-19^^Northern Ireland|Human|Regional Virus

Laboratory|Wellcome Sanger Institute for the COVID-19 Genomics UK (COG-UK) consortium|Davies|United Kingdom
>Spike|hCoV-19/England/ALDP-95309C/2020|2020-06-11|EPI_ISL_553685|Original|hCoV-19^^England|Human|Lighthouse Lab in Alderley Park|Wellcome Sanger Institute for the COVID-19 Genomics UK (COG-UK) consortium|Davies|United Kingdom
>Spike|hCoV-19/England/LIVE-DBD472/2020|2020-10-27|EPI_ISL_664345|Original|hCoV-19^^England|Human|Liverpool Clinical Laboratories|COVID-19 Genomics UK (COG-UK) Consortium|Haldenby|United Kingdom
>Spike|hCoV-19/Austria/CeMM0008/2020|2020-03-10|EPI_ISL_419661|Original|hCoV-19^^Austria|Human|Center for Virology|Bergthaler laboratory|Bergthaler|Austria
>Spike|hCoV-19/Switzerland/AG-ETHZ-420679/2020|2020-12-23|EPI_ISL_767975|Original|hCoV-19^^Aargau|Human|Viollier AG|Department of Biosystems Science and Engineering|Nadeau|Switzerland
>Spike|hCoV-19/Scotland/GCVR-171634/2020|2020-03-30|EPI_ISL_469945|Original|hCoV-19^^Scotland|Human|NHSGGC West of Scotland Specialist Virology Centre / MRC-University of Glasgow Centre for Virus Research|Wellcome Sanger Institute for the COVID-19 Genomics UK (COG-UK) consortium|Davies|United Kingdom
>Spike|hCoV-19/Japan/PG-3696/2020|2020-04-00|EPI_ISL_691949|Original|hCoV-19^^Japan|Human|Pathogen Genomics Center|Pathogen Genomics Center|Sekizuka|Japan
>Spike|hCoV-19/USA/NY-NYCPHL-001274/2020|2020-11-09|EPI_ISL_683954|Original|hCoV-19^^New York|Human|DOHMH PHL|New York City Public Health Laboratory|Wang|USA
>Spike|hCoV-19/Hong Kong/HKU-200723-064/2020|2020-03-13|EPI_ISL_497831|Original|hCoV-19^^Hong Kong|Human|Department of Microbiology|Department of Microbiology|Chan|China
>Spike|hCoV-19/Australia/VIC5000/2020|2020-07-18|EPI_ISL_519804|Original|hCoV-19^^Victoria|Human|Microbiological Diagnostic Unit - Public Health Laboratory (MDU-PHL)|MDU-PHL|Schultz|Australia
>Spike|hCoV-19/France/BRE-IPP9980/2020|2020-10-08|EPI_ISL_718237|Original|hCoV-19^^Bretagne|Human|Hospital|National Reference Center for Viruses of Respiratory Infections|Enouf|France
>Spike|hCoV-19/England/QEUH-B10E08/2020|2020-11-02|EPI_ISL_642322|Original|hCoV-19^^England|Human|Lighthouse Lab in Glasgow|Wellcome Sanger Institute for the COVID-19 Genomics UK (COG-UK) Consortium|Davies|United Kingdom
>Spike|hCoV-19/Brazil/RJ-INCA-C194/2020|2020-04-30|EPI_ISL_513525|Original|hCoV-19^^Rio de Janeiro|Human|Programa de Oncovirologia|Programa de Oncovirologia|Ramos Goes|Brazil
>Spike|hCoV-19/USA/TX-HMH-5263/2020|2020-06-22|EPI_ISL_544635|Original|hCoV-19^^Texas|Human|Houston Methodist Hospital|Houston Methodist Hospital|Long|USA
>Spike|hCoV-19/England/QEUH-A1E90A/2020|2020-10-01|EPI_ISL_587445|Original|hCoV-19^^England|Human|Lighthouse Lab in Glasgow|Wellcome Sanger Institute for the COVID-19 Genomics UK (COG-UK) consortium|Davies|United Kingdom
>Spike|hCoV-19/Wales/PHWC-48AD12/2020|2020-11-01|EPI_ISL_666149|Original|hCoV-19^^Wales|Human|Wales Specialist Virology Centre Sequencing lab|COVID-19 Genomics UK (COG-UK) Consortium|Moore|United Kingdom
>Spike|hCoV-19/Denmark/DCGC-2900/2020|2020-08-17|EPI_ISL_616534|Original|hCoV-19^^Nordjylland|Human|Department of Virus and Microbiological Special Diagnostics|Albertsen lab|Michaelsen|Denmark
>Spike|hCoV-19/England/ALDP-AA4B9A/2020|2020-10-21|EPI_ISL_624256|Original|hCoV-19^^England|Human|Lighthouse Lab in Alderley Park|Wellcome Sanger Institute for the COVID-19 Genomics UK (COG-UK) consortium|Davies|United Kingdom
>Spike|hCoV-19/England/QEUH-B12DDC/2020|2020-11-02|EPI_ISL_642494|Original|hCoV-19^^England|Human|Lighthouse Lab in Glasgow|Wellcome Sanger Institute for the COVID-19 Genomics UK (COG-UK) Consortium|Davies|United Kingdom
>Spike|hCoV-19/Indonesia/JK-EIJK-04/2020|2020-04-01|EPI_ISL_437192|Original|hCoV-19^^Jakarta|Human|Mitra Keluarga Kelapa Gading Hospital|Eijkman Institute for Molecular Biology|Soebandrio|Indonesia
>Spike|hCoV-19/England/ALDP-9CDC20/2020|2020-09-16|EPI_ISL_566215|Original|hCoV-19^^England|Human|Lighthouse Lab in Alderley Park|Wellcome Sanger Institute for the COVID-19 Genomics UK (COG-UK) consortium|Davies|United Kingdom

>Spike|hCoV-19/England/CAMB-1AA84B/2020|2020-04-17|EPI_ISL_470093|Original|hCoV-19^^England|Human|Department of Pathology|Wellcome Sanger Institute for the COVID-19 Genomics UK (COG-UK) consortium|Davies|United Kingdom
>Spike|hCoV-19/USA/ME-HETL-J0065/2020|2020-03-19|EPI_ISL_513476|Original|hCoV-19^^Maine|Human|Maine HETL|Tewhey Lab|Tewhey|USA
>Spike|hCoV-19/Wales/PHWC-4A0AE2/2020|2020-12-10|EPI_ISL_743542|Original|hCoV-19^^Wales|Human|Wales Specialist Virology Centre Sequencing lab|COVID-19 Genomics UK (COG-UK) Consortium|Moore|United Kingdom
>Spike|hCoV-19/USA/CO-CDPHE-2009170365/2020|2020-09-16|EPI_ISL_710346|Original|hCoV-19^^Colorado|Human|Colorado Department of Public Health and Environment|Colorado Department of Puplic Health and Environment|Bankers|USA
>Spike|hCoV-19/USA/TX-HMH-MCoV-9712/2020|2020-07-07|EPI_ISL_780237|Original|hCoV-19^^Texas|Human|Houston Methodist Hospital|Houston Methodist Hospital|Long|USA
>Spike|hCoV-19/England/CAMB-1B1243/2020|2020-05-07|EPI_ISL_459197|Original|hCoV-19^^England|Human|Department of Pathology|Wellcome Sanger Institute for the COVID-19 Genomics UK (COG-UK) consortium|Davies|United Kingdom
>Spike|hCoV-19/England/CAMB-7872C/2020|2020-00-00|EPI_ISL_433766|Original|hCoV-19^^England|Human|Department of Pathology|COVID-19 Genomics UK (COG-UK) Consortium|Meredith|United Kingdom
>Spike|hCoV-19/Netherlands/OV-RIVM-21010/2020|2020-12-21|EPI_ISL_791027|Original|hCoV-19^^Overijssel|Human|Dutch COVID-19 response team|National Institute for Public Health and the Environment (RIVM)|Meijer|Netherlands
>Spike|hCoV-19/Denmark/DCGC-11476/2020|2020-11-02|EPI_ISL_670941|Original|hCoV-19^^Hovedstaden|Human|Department of Virus and Microbiological Special Diagnostics|Albertsen Lab|Michaelsen|Denmark
>Spike|hCoV-19/Mexico/BCN-ALSR-3624/2020|2020-06-12|EPI_ISL_636116|Original|hCoV-19^^Sonora|Human|Sharp HealthCare Laboratory|Andersen lab at Scripps Research|Gangavarapu|Mexico
>Spike|hCoV-19/Wales/PHWC-16A425/2020|2020-09-07|EPI_ISL_535249|Original|hCoV-19^^Wales|Human|Wales Specialist Virology Centre Sequencing lab|COVID-19 Genomics UK (COG-UK) Consortium|Moore|United Kingdom
>Spike|hCoV-19/England/ALDP-C39DCF/2020|2020-12-09|EPI_ISL_730764|Original|hCoV-19^^England|Human|Lighthouse Lab in Alderley Park|Wellcome Sanger Institute for the COVID-19 Genomics UK (COG-UK) Consortium|Davies|United Kingdom
>Spike|hCoV-19/Portugal/PT0715/2020|2020-03-24|EPI_ISL_510974|Original|hCoV-19^^Portugal|Human|Instituto Nacional de Saude (INSA)|Instituto Nacional de Saude (INSA)|Borges|Portugal
>Spike|hCoV-19/Norway/4327/2020|2020-10-05|EPI_ISL_775469|Original|hCoV-19^^Vestfold og Telemark|Human|Unilabs Laboratory Medicine|Norwegian Institute of Public Health|Vollan|Norway
>Spike|hCoV-19/USA/TX-HMH-MCoV-18930/2020|2020-11-25|EPI_ISL_785227|Original|hCoV-19^^Texas|Human|Houston Methodist Hospital|Houston Methodist Hospital|Long|USA
>Spike|hCoV-19/Germany/NW-HHU-341/2020|2020-10-13|EPI_ISL_718039|Orginal|hCoV-19^^North Rhine-Westphalia|Human|ZOTZ KLIMAS MVZ D\u00fcsseldorf-Centrum GbR \u00dcBAG f\u00fcr Labormedizin|Center of Medical Microbiology|Wienemann|Germany
>Spike|hCoV-19/Israel/CVL-n17916/2020|2020-06-02|EPI_ISL_516889|Original|hCoV-19^^Tel Aviv|Human|Israel Central Virology laboratory|Israel Central Virology laboratory|Zuckerman|Israel
>Spike|hCoV-19/USA/FL-BPHL-1469/2020|2020-05-09|EPI_ISL_549251|Original|hCoV-19^^Florida|Human|Florida Bureau of Public Health Laboratories|Florida Bureau of Public Health Laboratories|Blanton|USA
>Spike|hCoV-19/Singapore/392/2020|2020-04-17|EPI_ISL_469141|Original|hCoV-19^^Singapore|Human|National Public Health Laboratory|National Public Health Laboratory|Mak|Singapore
>Spike|hCoV-19/Japan/PG-2043/2020|2020-03-00|EPI_ISL_685648|Original|hCoV-19^^Japan|Human|Pathogen Genomics Center|Pathogen Genomics Center|Sekizuka|Japan
>Spike|hCoV-19/England/NORT-29EA9E/2020|2020-05-12|EPI_ISL_490471|Original|hCoV-19^^England|Human|Northumbria University / South Tees Hospitals NHS Foundation Trust / North Cumbria Integrated Care NHS Foundation Trust / North Tees and Hartlepool NHS

Foundation Trust / Newcastle Hospitals NHS Foundation Trust|COVID-19 Genomics UK (COG-UK) Consortium|Bashton|United Kingdom
>Spike|hCoV-19/Scotland/EDB5543/2020|2020-04-01|EPI_ISL_487721|Original|hCoV-19^^Scotland|Human|Virology Department|Wellcome Sanger Institute for the COVID-19 Genomics UK (COG-UK) consortium|Davies|United Kingdom
>Spike|hCoV-19/Iceland/321/2020|2020-03-18|EPI_ISL_417650|Original|hCoV-19^^Reykjavik|Human|The National University Hospital of Iceland|deCODE genetics|J\u00f3nsson|Iceland
>Spike|hCoV-19/Spain/VC-FISABIO-178/2020|2020-03-21|EPI_ISL_436273|Original|hCoV-19^^Comunitat Valenciana|Human|Servicio de Microbiolog\u00eda. Consorcio Hospital General Universitario de Valencia|Sequencing and Bioinformatics Service and Molecular Epidemiology Research Group. FISABIO-Public Health|Gonzalez-Candelas|Spain
>Spike|hCoV-19/USA/SC-DHEC-0203/2020|2020-05-18|EPI_ISL_529190|Original|hCoV-19^^South Carolina|Human|South Carolina Department of Health and Environmental Control|South Carolina Department of Health and Environmental Control|Flores|USA
>Spike|hCoV-19/England/ALDP-B5C79C/2020|2020-11-08|EPI_ISL_655682|Original|hCoV-19^^England|Human|Lighthouse Lab in Alderley Park|Wellcome Sanger Institute for the COVID-19 Genomics UK (COG-UK) Consortium|Davies|United Kingdom
>Spike|hCoV-19/England/CAMB-1B8F63/2020|2020-11-02|EPI_ISL_651693|Original|hCoV-19^^England|Human|Department of Pathology|COVID-19 Genomics UK (COG-UK) Consortium|Goodfellow|United Kingdom
>Spike|hCoV-19/Wales/PHWC-162539/2020|2020-05-19|EPI_ISL_473085|Original|hCoV-19^^Wales|Human|Wales Specialist Virology Centre Sequencing lab|COVID-19 Genomics UK (COG-UK) Consortium|Moore|United Kingdom
>Spike|hCoV-19/Wales/PHWC-4957BC/2020|2020-11-23|EPI_ISL_726396|Original|hCoV-19^^Wales|Human|Originating lab|COVID-19 Genomics UK (COG-UK) Consortium|Moore|United Kingdom
>Spike|hCoV-19/Wales/PHWC-31C20/2020|2020-04-13|EPI_ISL_446546|Original|hCoV-19^^Wales|Human|Wales Specialist Virology Centre|Public Health Wales Microbiology Cardiff|Moore|United Kingdom
>Spike|hCoV-19/USA/LA-GBCL-26629240/2020|2020-04-21|EPI_ISL_485233|Original|hCoV-19^^Louisiana|Human|River Road Testing Lab|Ginkgo Bioworks Clinical Laboratory|Smith|USA
>Spike|hCoV-19/England/MILK-A79E95/2020|2020-10-15|EPI_ISL_606663|Original|hCoV-19^^England|Human|Lighthouse Lab in Milton Keynes|Wellcome Sanger Institute for the COVID-19 Genomics UK (COG-UK) consortium|Davies|United Kingdom
>Spike|hCoV-19/Panama/334898/2020|2020-03-31|EPI_ISL_496797|Original|hCoV-19^^Panama Center|Human|Gorgas Memorial Laboratory of Health Studies|Gorgas Memorial Laboratory of Health Studies|Martinez|Panama
>Spike|hCoV-19/Brazil/RJ-INCA-C22/2020|2020-04-16|EPI_ISL_513530|Original|hCoV-19^^Rio de Janeiro|Human|Programa de Oncovirologia|Programa de Oncovirologia|Ramos Goes|Brazil
>Spike|hCoV-19/Australia/NSW2220/2020|2020-03-20|EPI_ISL_500671|Original|hCoV-19^^New South Wales|Human|Area of Virology|Area of Virology|Bull|Australia
>Spike|hCoV-19/Denmark/DCGC-16341/2020|2020-11-16|EPI_ISL_714714|Original|hCoV-19^^Midtjylland|Human|Department of Virus and Microbiological Special Diagnostics|Albertsen Lab|Michaelsen|Denmark
>Spike|hCoV-19/USA/WI-UW-1152/2020|2020-08-13|EPI_ISL_522908|Original|hCoV-19^^Wisconsin|Human|University of Wisconsin-Madison AIDS Vaccine Research Laboratories|University of Wisconsin-Madison AIDS Vaccine Research Laboratories|Baczenas|USA
>Spike|hCoV-19/England/MILK-A7D5D8/2020|2020-10-12|EPI_ISL_606366|Original|hCoV-19^^England|Human|Lighthouse Lab in Milton Keynes|Wellcome Sanger Institute for the COVID-19 Genomics UK (COG-UK) consortium|Davies|United Kingdom
>Spike|hCoV-19/Hong Kong/HKU-200723-057/2020|2020-03-24|EPI_ISL_497824|Original|hCoV-19^^Hong Kong|Human|Department of Microbiology|Department of Microbiology|Chan|China
>Spike|hCoV-19/England/MILK-B5A3F4/2020|2020-11-06|EPI_ISL_658308|Original|hCoV-19^^England|Human|Lighthouse Lab in Milton Keynes|Wellcome Sanger Institute for the COVID-19 Genomics UK (COG-UK) Consortium|Davies|United Kingdom

>Spike|hCoV-19/Australia/VIC13245/2020|2020-07-25|EPI_ISL_563646|Original|hCoV-
19^^Victoria|Human|Microbiological Diagnostic Unit - Public Health Laboratory (MDU-
PHL)|MDU-PHL|Schultz|Australia
>Spike|hCoV-19/Japan/PG-0591/2020|2020-04-04|EPI_ISL_480223|Original|hCoV-
19^^Kanto|Human|Koshigaya City Public Health Center|Pathogen Genomics
Center|Sekizuka|Japan
>Spike|hCoV-19/England/CAMC-CB79E3/2020|2020-12-19|EPI_ISL_777723|Original|hCoV-
19^^England|Human|Lighthouse Lab in Cambridge|Wellcome Sanger Institute for the
COVID-19 Genomics UK (COG-UK) Consortium|Davies|United Kingdom
>Spike|hCoV-19/USA/TX-HMH-5964/2020|2020-06-26|EPI_ISL_547128|Original|hCoV-
19^^Texas|Human|Houston Methodist Hospital|Houston Methodist Hospital|Long|USA
>Spike|hCoV-19/Japan/PG-4695/2020|2020-08-00|EPI_ISL_689832|Original|hCoV-
19^^Japan|Human|Pathogen Genomics Center|Pathogen Genomics Center|Sekizuka|Japan
>Spike|hCoV-19/USA/MI-UM-10036813449/2020|2020-11-06|EPI_ISL_728411|Original|hCoV-
19^^Michigan|Human|University of Michigan Clinical Microbiology Laboratory|Lauring
Lab|Valesano|USA
>Spike|hCoV-19/England/QEUH-B7ECB3/2020|2020-11-14|EPI_ISL_661645|Original|hCoV-
19^^England|Human|Lighthouse Lab in Glasgow|Wellcome Sanger Institute for the COVID-
19 Genomics UK (COG-UK) Consortium|Davies|United Kingdom
>Spike|hCoV-19/USA/MI-MDHHS-SC21106/2020|2020-03-24|EPI_ISL_507882|Original|hCoV-
19^^Michigan|Human|Michigan Department of Health and Human Services|Michigan
Department of Health and Human Services|Blankenship|USA
>Spike|hCoV-19/Russia/Buryatia-88903/2020|2020-04-01|EPI_ISL_428911|Vero|hCoV-
19^^Buryatia|Human|State Research Center of Virology and Biotechnology VECTOR|State
Research Center of Virology and Biotechnology VECTOR|Shvalov|Russia
>Spike|hCoV-19/Netherlands/ZH-EMC-592/2020|2020-09-18|EPI_ISL_578020|Original|hCoV-
19^^Zuid Holland|Human|Dutch COVID-19 response team|Erasmus Medical Center|Oude
Munnink|Netherlands
>Spike|hCoV-19/USA/MN-MDH-138/2020|2020-03-22|EPI_ISL_427278|Original|hCoV-
19^^Minnesota|Human|Minnesota Department of Health|Minnesota Department of
Health|Wang|USA
>Spike|hCoV-19/USA/WI-UW-2348/2020|2020-11-13|EPI_ISL_728114|Original|hCoV-
19^^Wisconsin|Human|University of Wisconsin-Madison AIDS Vaccine Research
Laboratories|University of Wisconsin-Madison AIDS Vaccine Research
Laboratories|Baczenas|USA
>Spike|hCoV-19/USA/NE-11518/2020|2020-05-05|EPI_ISL_475225|Original|hCoV-
19^^Nebraska|Human|Nebraska Public Health Laboratory|UNMC COVID-19 Response
Team|Pratt|USA
>Spike|hCoV-19/USA/WA-S379/2020|2020-03-27|EPI_ISL_434139|Original|hCoV-
19^^Washington|Human|Washington State Department of Health|Seattle Flu
Study|Bedford|USA
>Spike|hCoV-19/Portugal/PT1325/2020|2020-03-18|EPI_ISL_511284|Original|hCoV-
19^^Portugal|Human|Instituto Nacional de Saude (INSA) and Instituto Gulbenkian de
Ciencia (IGC)|Instituto Nacional de Saude (INSA) and Instituto Gulbenkian de Ciencia
(IGC)|Borges|Portugal
>Spike|hCoV-19/Japan/TK-YE63751d/2020|2020-03-00|EPI_ISL_582145|Original|hCoV-
19^^Tokyo|Human|Tokyo Metropolitan Institute of Public Health|Tokyo Metropolitan
Institute of Public Health|Nagashima|Japan
>Spike|hCoV-19/England/MILK-B56BB4/2020|2020-11-04|EPI_ISL_656860|Original|hCoV-
19^^England|Human|Lighthouse Lab in Milton Keynes|Wellcome Sanger Institute for the
COVID-19 Genomics UK (COG-UK) Consortium|Davies|United Kingdom
>Spike|hCoV-19/Australia/VIC7856/2020|2020-07-29|EPI_ISL_564707|Original|hCoV-
19^^Victoria|Human|Microbiological Diagnostic Unit - Public Health Laboratory (MDU-
PHL)|MDU-PHL|Schultz|Australia
>Spike|hCoV-19/USA/TX-HMH-MCoV-10174/2020|2020-07-06|EPI_ISL_789352|Original|hCoV-
19^^Texas|Human|Houston Methodist Hospital|Houston Methodist Hospital|Long|USA
>Spike|hCoV-19/Netherlands/Limburg_37/2020|2020-04-23|EPI_ISL_461025|Original|hCoV-
19^^Limburg|Human|Dutch COVID-19 response team|Erasmus Medical Center|Oude
Munnink|Netherlands

>Spike|hCoV-19/USA/TX-HMH-MCoV-18190/2020|2020-11-11|EPI_ISL_784538|Original|hCoV-19^^Texas|Human|Houston Methodist Hospital|Houston Methodist Hospital|Long|USA
>Spike|hCoV-19/USA/CA-CZB-14285/2020|2020-08-27|EPI_ISL_739059|Original|hCoV-19^^California|Human|Alameda County Public Health Lab|Chan-Zuckerberg Biohub|Kamm|USA
>Spike|hCoV-19/France/PAC-MEPHI-2086/2020|2020-08-30|EPI_ISL_644492|Original|hCoV-19^^Provence-Alpes-Cote-d\u2019Azur|Human|MEPHI|MEPHI|Levasseur|France
>Spike|hCoV-19/USA/TX-HMH-7759/2020|2020-06-30|EPI_ISL_545621|Original|hCoV-19^^Texas|Human|Houston Methodist Hospital|Houston Methodist Hospital|Long|USA
>Spike|hCoV-19/Scotland/EDB6038/2020|2020-05-07|EPI_ISL_487667|Original|hCoV-19^^Scotland|Human|Virology Department|Wellcome Sanger Institute for the COVID-19 Genomics UK (COG-UK) consortium|Davies|United Kingdom
>Spike|hCoV-19/Northern Ireland/QEUH-963FD8/2020|2020-08-12|EPI_ISL_536906|Original|hCoV-19^^Northern Ireland|Human|Lighthouse Lab in Glasgow|Wellcome Sanger Institute for the COVID-19 Genomics UK (COG-UK) consortium|Davies|United Kingdom
>Spike|hCoV-19/Spain/VC-FISABIO-171/2020|2020-03-20|EPI_ISL_436266|Original|hCoV-19^^Comunitat Valenciana|Human|Servicio de Microbiologia. Hospital Universitario Doctor Peset|Sequencing and Bioinformatics Service and Molecular Epidemiology Research Group. FISABIO-Public Health|Gonzalez-Candelas|Spain
>Spike|hCoV-19/Peru/AMZ-UPCH-0265/2020|2020-09-21|EPI_ISL_729911|Original|hCoV-19^^Amazonas|Human|Instituto de Medicina Tropical|Laboratorio de Gen\u00f3mica Microbiana|Tsukayama|Peru
>Spike|hCoV-19/Wales/PHWC-490757/2020|2020-11-13|EPI_ISL_706507|Original|hCoV-19^^Wales|Human|Originating lab|COVID-19 Genomics UK (COG-UK) Consortium|Moore|United Kingdom
>Spike|hCoV-19/England/NORT-294B4A/2020|2020-03-28|EPI_ISL_490464|Original|hCoV-19^^England|Human|Northumbria University / South Tees Hospitals NHS Foundation Trust / North Cumbria Integrated Care NHS Foundation Trust / North Tees and Hartlepool NHS Foundation Trust / Newcastle Hospitals NHS Foundation Trust|COVID-19 Genomics UK (COG-UK) Consortium|Bashton|United Kingdom
>Spike|hCoV-19/Spain/AR-IBV-98010683/2020|2020-10-02|EPI_ISL_722269|Original|hCoV-19^^Aragon|Human|Servicio de Microbiolog\u00eda|SeqCOVID-SPAIN consortium/IBV(CSIC)|Comas|Spain
>Spike|hCoV-19/England/MILK-9919C4/2020|2020-08-26|EPI_ISL_577010|Original|hCoV-19^^England|Human|Northumbria University / South Tees Hospitals NHS Foundation Trust / North Cumbria Integrated Care NHS Foundation Trust / North Tees and Hartlepool NHS Foundation Trust / Newcastle Hospitals NHS Foundation Trust|COVID-19 Genomics UK (COG-UK) Consortium|Bashton|United Kingdom
>Spike|hCoV-19/England/QEUH-A8C688/2020|2020-10-18|EPI_ISL_643265|Original|hCoV-19^^England|Human|Lighthouse Lab in Glasgow|Wellcome Sanger Institute for the COVID-19 Genomics UK (COG-UK) Consortium|Davies|United Kingdom
>Spike|hCoV-19/England/CAMC-C43F71/2020|2020-12-10|EPI_ISL_719370|Original|hCoV-19^^England|Human|Lighthouse Lab in Cambridge|Wellcome Sanger Institute for the COVID-19 Genomics UK (COG-UK) Consortium|Davies|United Kingdom
>Spike|hCoV-19/Portugal/PT0702/2020|2020-03-17|EPI_ISL_510961|Original|hCoV-19^^Portugal|Human|Instituto Nacional de Saude (INSA)|Instituto Nacional de Saude (INSA)|Borges|Portugal
>Spike|hCoV-19/Finland/8CB1/2020|2020-12-04|EPI_ISL_732620|Original|hCoV-19^^Uusimaa|Human|Department of Virology and Immunology|Department of Virology|Smura|Finland
>Spike|hCoV-19/Denmark/ALAB-SSI-805/2020|2020-03-28|EPI_ISL_444916|Original|hCoV-19^^Denmark|Human|Department of Virus and Microbiological Special Diagnostics|Albertsen lab|Kirkegaard|Denmark
>Spike|hCoV-19/Wales/PHWC-2EAB3/2020|2020-04-07|EPI_ISL_446110|Original|hCoV-19^^Wales|Human|Wales Specialist Virology Centre|Public Health Wales Microbiology Cardiff|Moore|United Kingdom
>Spike|hCoV-19/USA/UT-UPHL-2009691/2020|2020-07-25|EPI_ISL_605365|Original|hCoV-19^^Utah|Human|Utah Public Health Laboratory|Utah Public Health Laboratory|Young|USA

>Spike|hCoV-19/England/QEUH-B1161E/2020|2020-11-02|EPI_ISL_642481|Original|hCoV-19^^England|Human|Lighthouse Lab in Glasgow|Wellcome Sanger Institute for the COVID-19 Genomics UK (COG-UK) Consortium|Davies|United Kingdom
>Spike|hCoV-19/England/ALDP-B1B553/2020|2020-11-01|EPI_ISL_645620|Original|hCoV-19^^England|Human|Lighthouse Lab in Alderley Park|Wellcome Sanger Institute for the COVID-19 Genomics UK (COG-UK) Consortium|Davies|United Kingdom
>Spike|hCoV-19/England/CAMB-80448/2020|2020-04-09|EPI_ISL_470366|Original|hCoV-19^^England|Human|Department of Pathology|Wellcome Sanger Institute for the COVID-19 Genomics UK (COG-UK) consortium|Davies|United Kingdom
>Spike|hCoV-19/England/MILK-A45A8F/2020|2020-10-06|EPI_ISL_598591|Original|hCoV-19^^England|Human|Lighthouse Lab in Milton Keynes|Wellcome Sanger Institute for the COVID-19 Genomics UK (COG-UK) consortium|Davies|United Kingdom
>Spike|hCoV-19/England/CWAR-24FD94D/2020|2020-04-23|EPI_ISL_584453|Original|hCoV-19^^England|Human|UHCW / University of Warwick|COVID-19 Genomics UK (COG-UK) Consortium|Stark|United Kingdom
>Spike|hCoV-19/Switzerland/BS-42210970/2020|2020-03-28|EPI_ISL_581811|Original|hCoV-19^^Basel-Stadt|Human|University Hospital Basel|University Hospital Basel|Roloff Handschin|Switzerland
>Spike|hCoV-19/Switzerland/VD-ETHZ-280168/2020|2020-09-08|EPI_ISL_560518|Original|hCoV-19^^Vaud|Human|Viollier AG|Department of Biosystems Science and Engineering|Nadeau|Switzerland
>Spike|hCoV-19/Switzerland/BE-UNIBE-178-2905/2020|2020-03-20|EPI_ISL_574817|Original|hCoV-19^^Bern|Human|Institute for Infectious Diseases|Institute for Infectious Diseases|Koch|Switzerland
>Spike|hCoV-19/Japan/PG-4426/2020|2020-07-00|EPI_ISL_692650|Original|hCoV-19^^Japan|Human|Pathogen Genomics Center|Pathogen Genomics Center|Sekizuka|Japan
>Spike|hCoV-19/Wales/PHWC-4A5C50/2020|2020-12-13|EPI_ISL_764963|Original|hCoV-19^^Wales|Human|Wales Specialist Virology Centre Sequencing lab|COVID-19 Genomics UK (COG-UK) Consortium|Moore|United Kingdom
>Spike|hCoV-19/USA/CA-ALSR-2691/2020|2020-07-20|EPI_ISL_635634|Original|hCoV-19^^California|Human|San Diego County Public Health Laboratory|Andersen lab at Scripps Research|Gangavarapu|USA
>Spike|hCoV-19/USA/MI-MDHHS-SC22166/2020|2020-10-18|EPI_ISL_614217|Original|hCoV-19^^Michigan|Human|Michigan Department of Health and Human Services|Michigan Department of Health and Human Services|Jacob|USA
>Spike|hCoV-19/England/MILK-A1834E/2020|2020-10-01|EPI_ISL_595024|Original|hCoV-19^^England|Human|Northumbria University / South Tees Hospitals NHS Foundation Trust / North Cumbria Integrated Care NHS Foundation Trust / North Tees and Hartlepool NHS Foundation Trust / Newcastle Hospitals NHS Foundation Trust|COVID-19 Genomics UK (COG-UK) Consortium|Bashton|United Kingdom
>Spike|hCoV-19/Japan/PG-7252/2020|2020-08-00|EPI_ISL_688355|Original|hCoV-19^^Japan|Human|Saitama Prefectural Institute of Public Health|Pathogen Genomics Center|Sekizuka|Japan
>Spike|hCoV-19/Denmark/DCGC-20123/2020|2020-12-07|EPI_ISL_747996|Original|hCoV-19^^Hovedstaden|Human|Department of Virus and Microbiological Special Diagnostics|Albertsen Lab|Michaelsen|Denmark
>Spike|hCoV-19/Denmark/DCGC-2014/2020|2020-07-20|EPI_ISL_618224|Original|hCoV-19^^Hovedstaden|Human|Department of Virus and Microbiological Special Diagnostics|Albertsen lab|Michaelsen|Denmark
>Spike|hCoV-19/USA/IL-QDX-259/2020|2020-04-29|EPI_ISL_498724|Original|hCoV-19^^Illinois|Human|Quest Diagnostics|Quest Diagnostics|Kagan|USA
>Spike|hCoV-19/Scotland/EDB807/2020|2020-04-03|EPI_ISL_433249|Original|hCoV-19^^Scotland|Human|Virology Department|COVID-19 Genomics UK (COG-UK) Consortium|Williams|United Kingdom
>Spike|hCoV-19/Spain/PV-IBV-2111/2020|2020-04-11|EPI_ISL_468880|Original|hCoV-19^^Basque Country|Human|Servicio de Microbiolog\u00eda. Hospital Universitario Donostia. OSI Donostialdea. \u00c1rea de Enfermedades Infecciosas|SeqCOVID-SPAIN consortium/IBV(CSIC)|Comas|Spain

>Spike|hCoV-19/Germany/BY-MVP-0059/2020|2020-03-20|EPI_ISL_437258|Original|hCoV-
19^^Bavaria|Human|Max von Pettenkofer Institute|Laboratory for Functional Genome
Analysis|Graf|Germany
>Spike|hCoV-19/England/MILK-B38945/2020|2020-11-01|EPI_ISL_658761|Original|hCoV-
19^^England|Human|Lighthouse Lab in Milton Keynes|Wellcome Sanger Institute for the
COVID-19 Genomics UK (COG-UK) Consortium|Davies|United Kingdom
>Spike|hCoV-19/England/OXON-B0E7E/2020|2020-04-06|EPI_ISL_478971|Original|hCoV-
19^^England|Human|Oxford Viromics|COVID-19 Genomics UK (COG-UK)
Consortium|Golubchik|United Kingdom
>Spike|hCoV-19/USA/ME-HETL-J0473/2020|2020-12-11|EPI_ISL_755564|Original|hCoV-
19^^Maine|Human|Maine Health and Environmental Testing Laboratory|Tewhey
Lab|Tewhey|USA
>Spike|hCoV-19/Japan/PG-0339/2020|2020-03-29|EPI_ISL_480012|Original|hCoV-
19^^Kanto|Human|Chiba Prefectural Institute of Public Health|Pathogen Genomics
Center|Sekizuka|Japan
>Spike|hCoV-19/USA/MN-MDH-2093/2020|2020-10-23|EPI_ISL_683722|Original|hCoV-
19^^Minnesota|Human|Mayo Clinic & Mayo Clinic Laboratories|Minnesota Department of
Health|Garfin|USA
>Spike|hCoV-19/England/MILK-994105/2020|2020-08-25|EPI_ISL_551610|Original|hCoV-
19^^England|Human|Lighthouse Lab in Milton Keynes|Wellcome Sanger Institute for the
COVID-19 Genomics UK (COG-UK) consortium|Davies|United Kingdom
>Spike|hCoV-19/Denmark/DCGC-6022/2020|2020-09-21|EPI_ISL_621131|Original|hCoV-
19^^Midtjylland|Human|Department of Virus and Microbiological Special
Diagnostics|Albertsen lab|Michaelsen|Denmark
>Spike|hCoV-19/USA/NY-Wadsworth-22874-01/2020|2020-03-
19|EPI_ISL_426315|Original|hCoV-19^^New York|Human|Wadsworth Center|Wadsworth
Center|Lasek-Nesselquist|USA
>Spike|hCoV-19/England/ALDP-9BCF58/2020|2020-09-10|EPI_ISL_573083|Original|hCoV-
19^^England|Human|Oxford Viromics|COVID-19 Genomics UK (COG-UK)
Consortium|Golubchik|United Kingdom
>Spike|hCoV-19/Wales/PHWC-490EF8/2020|2020-11-19|EPI_ISL_706585|Original|hCoV-
19^^Wales|Human|Originating lab|COVID-19 Genomics UK (COG-UK) Consortium|Moore|United
Kingdom
>Spike|hCoV-19/USA/WA-UW-1770/2020|2020-03-22|EPI_ISL_424244|Original|hCoV-
19^^Washington|Human|UW Virology Lab|UW Virology Lab|Roychoudhury|USA
>Spike|hCoV-19/England/OXON-ACF54/2020|2020-00-00|EPI_ISL_448548|Original|hCoV-
19^^England|Human|Oxford Viromics|COVID-19 Genomics UK (COG-UK)
Consortium|Golubchik|United Kingdom
>Spike|hCoV-19/USA/NY-Wadsworth-286247-01/2020|2020-12-
10|EPI_ISL_754565|Original|hCoV-19^^New York|Human|Wadsworth Center|Wadsworth
Center|Lasek-Nesselquist|USA
>Spike|hCoV-19/England/QEUH-96006F/2020|2020-08-05|EPI_ISL_531878|Original|hCoV-
19^^England|Human|Lighthouse Lab in Glasgow|Wellcome Sanger Institute for the COVID-
19 Genomics UK (COG-UK) consortium|Davies|United Kingdom
>Spike|hCoV-19/Canada/ON-PHL-20-02410/2020|2020-09-00|EPI_ISL_671538|Original|hCoV-
19^^Ontario|Human|Public Health Ontario Laboratory|Public Health Ontario
Laboratory|Guthrie|Canada
>Spike|hCoV-19/Wales/PHWC-15E284/2020|2020-04-06|EPI_ISL_472802|Original|hCoV-
19^^Wales|Human|Wales Specialist Virology Centre Sequencing lab|COVID-19 Genomics UK
(COG-UK) Consortium|Moore|United Kingdom
>Spike|hCoV-19/Spain/VC-IBV-98003434/2020|2020-04-02|EPI_ISL_539250|Original|hCoV-
19^^Comunitat Valenciana|Human|Hospital Universitario de La Ribera (Alzira|SeqCOVID-
SPAIN consortium/IBV(CSIC)|Comas|Spain
>Spike|hCoV-19/Denmark/DCGC-14132/2020|2020-11-30|EPI_ISL_711588|Original|hCoV-
19^^Hovedstaden|Human|Department of Virus and Microbiological Special
Diagnostics|Albertsen Lab|Michaelsen|Denmark
>Spike|hCoV-19/USA/WI-UW-2312/2020|2020-11-10|EPI_ISL_728078|Original|hCoV-
19^^Wisconsin|Human|University of Wisconsin-Madison AIDS Vaccine Research
Laboratories|University of Wisconsin-Madison AIDS Vaccine Research
Laboratories|Baczenas|USA

>Spike|hCoV-19/England/LIVE-DB994A/2020|2020-11-09|EPI_ISL_704231|Original|hCoV-19^^England|Human|Liverpool Clinical Laboratories|COVID-19 Genomics UK (COG-UK) Consortium|Haldenby|United Kingdom
>Spike|hCoV-19/Wales/PHWC-16EEE7/2020|2020-10-04|EPI_ISL_585882|Original|hCoV-19^^Wales|Human|Wales Specialist Virology Centre Sequencing lab|COVID-19 Genomics UK (COG-UK) Consortium|Moore|United Kingdom
>Spike|hCoV-19/Netherlands/NA_328/2020|2020-04-03|EPI_ISL_455133|Original|hCoV-19^^Netherlands|Human|Dutch COVID-19 response team|Erasmus Medical Center|Oude Munnink|Netherlands
>Spike|hCoV-19/Scotland/QEUH-9E5299/2020|2020-09-23|EPI_ISL_601016|Original|hCoV-19^^Scotland|Human|Lighthouse Lab in Glasgow|Wellcome Sanger Institute for the COVID-19 Genomics UK (COG-UK) consortium|Davies|United Kingdom
>Spike|hCoV-19/Denmark/DCGC-2058/2020|2020-07-20|EPI_ISL_618299|Original|hCoV-19^^Sjaelland|Human|Department of Virus and Microbiological Special Diagnostics|Albertsen lab|Michaelsen|Denmark
>Spike|hCoV-19/England/NORT-281384/2020|2020-03-13|EPI_ISL_488834|Original|hCoV-19^^England|Human|NU-OMICS DNA Sequencing research facility|Wellcome Sanger Institute for the COVID-19 Genomics UK (COG-UK) consortium|Davies|United Kingdom
>Spike|hCoV-19/England/QEUH-AA0369/2020|2020-10-20|EPI_ISL_611354|Original|hCoV-19^^England|Human|Lighthouse Lab in Glasgow|Wellcome Sanger Institute for the COVID-19 Genomics UK (COG-UK) consortium|Davies|United Kingdom
>Spike|hCoV-19/England/CAMB-1B4BFA/2020|2020-06-10|EPI_ISL_473502|Original|hCoV-19^^England|Human|Department of Pathology|COVID-19 Genomics UK (COG-UK) Consortium|Meredith|United Kingdom
>Spike|hCoV-19/USA/AK-PHL015/2020|2020-03-16|EPI_ISL_420306|Original|hCoV-19^^Alaska|Human|Alaska State Virology Laboratory|Alaska State Virology Laboratory|Chen|USA
>Spike|hCoV-19/Wales/PHWC-495224/2020|2020-11-21|EPI_ISL_726314|Original|hCoV-19^^Wales|Human|Originating lab|COVID-19 Genomics UK (COG-UK) Consortium|Moore|United Kingdom
>Spike|hCoV-19/USA/NY-Wadsworth-233280-01/2020|2020-10-01|EPI_ISL_734309|Original|hCoV-19^^New York|Human|Wadsworth Center|Wadsworth Center|Lasek-Nesselquist|USA
>Spike|hCoV-19/Switzerland/ZH-ETHZ-150001plus/2020|2020-06-12|EPI_ISL_483649|Original|hCoV-19^^Zurich|Human|Viollier AG|Department of Biosystems Science and Engineering|Nadeau|Switzerland
>Spike|hCoV-19/India/MH-ACTREC-579/2020|2020-09-02|EPI_ISL_723059|Original|hCoV-19^^Maharashtra|Human|Hematopathology Laboratory|Hematopathology Laboratory|Patkar|India
>Spike|hCoV-19/Portugal/PT0161/2020|2020-03-28|EPI_ISL_453877|Original|hCoV-19^^Portugal|Human|unknown|Instituto Nacional de Saude (INSA)|Borges|Portugal
>Spike|hCoV-19/Germany/BE-ChVir-W1708-6787/2020|2020-08-05|EPI_ISL_753998|Original|hCoV-19^^Berlin|Human|Charit\u00e9 Universit\u00e4tsmedizin Berlin|Charit\u00e9 Universit\u00e4tsmedizin Berlin|Muehlemann|Germany
>Spike|hCoV-19/USA/AZ-TG547398/2020|2020-07-07|EPI_ISL_695520|Original|hCoV-19^^Arizona|Human|AZ SPHL|TGen North|French|USA
>Spike|hCoV-19/Denmark/DCGC-6160/2020|2020-10-05|EPI_ISL_620063|Original|hCoV-19^^Sjaelland|Human|Department of Virus and Microbiological Special Diagnostics|Albertsen lab|Michaelsen|Denmark
>Spike|hCoV-19/England/CAMB-84523/2020|2020-04-17|EPI_ISL_443569|Original|hCoV-19^^England|Human|Department of Pathology|Wellcome Sanger Institute for the COVID-19 Genomics UK (COG-UK) consortium|Amato|United Kingdom
>Spike|hCoV-19/England/C200044548/2020|2020-11-06|EPI_ISL_664032|Original|hCoV-19^^England|Human|Respiratory Virus Unit|COVID-19 Genomics UK (COG-UK) Consortium|Platt|United Kingdom
>Spike|hCoV-19/USA/TX-HMH-MCoV-15822/2020|2020-07-09|EPI_ISL_785506|Original|hCoV-19^^Texas|Human|Houston Methodist Hospital|Houston Methodist Hospital|Long|USA

>Spike|hCoV-19/Netherlands/NH-RIVM-20133/2020|2020-11-15|EPI_ISL_723304|Orignal|hCoV-
19^^Noord-Holland|Human|Dutch COVID-19 response team|National Institute for Public
Health and the Environment (RIVM)|Meijer|Netherlands
>Spike|hCoV-19/England/NORT-281454/2020|2020-03-15|EPI_ISL_488786|Original|hCoV-
19^^England|Human|NU-OMICS DNA Sequencing research facility|Wellcome Sanger Institute
for the COVID-19 Genomics UK (COG-UK) consortium|Davies|United Kingdom
>Spike|hCoV-19/England/QEUH-B41A55/2020|2020-11-08|EPI_ISL_658632|Original|hCoV-
19^^England|Human|Lighthouse Lab in Glasgow|Wellcome Sanger Institute for the COVID-
19 Genomics UK (COG-UK) Consortium|Davies|United Kingdom
>Spike|hCoV-19/Belgium/ULG-10854/2020|2020-11-10|EPI_ISL_666889|Original|hCoV-
19^^Li\u00e8ge|Human|Department of Clinical Microbiology|GIGA Medical
Genomics|Durkin|Belgium
>Spike|hCoV-19/Spain/MD-IBV-99008736/2020|2020-10-16|EPI_ISL_654304|Original|hCoV-
19^^Madrid|Human|Hospital General Universitario Gregorio Mara\u00f1\u00f3n|SeqCOVID-
SPAIN consortium/IBV(CSIC)|Comas|Spain
>Spike|hCoV-19/India/WB-IK41/2020|2020-05-04|EPI_ISL_508369|Original|hCoV-19^^West
Bengal|Human|Institute of Post Graduate Medical Education & Research|National
Institute of Biomedical Genomics|Maitra|India
>Spike|hCoV-19/Denmark/DCGC-14761/2020|2020-11-23|EPI_ISL_711163|Original|hCoV-
19^^Hovedstaden|Human|Department of Virus and Microbiological Special
Diagnostics|Albertsen Lab|Michaelsen|Denmark
>Spike|hCoV-19/Portugal/PT2033/2020|2020-11-09|EPI_ISL_732249|Original|hCoV-
19^^Portugal|Human|Instituto Nacional de Saude (INSA) and Instituto Gulbenkian de
Ciencia (IGC)|Instituto Nacional de Saude (INSA) and Instituto Gulbenkian de Ciencia
(IGC)|Borges|Portugal
>Spike|hCoV-19/England/ALDP-BB54B8/2020|2020-11-21|EPI_ISL_701677|Original|hCoV-
19^^England|Human|Lighthouse Lab in Alderley Park|Wellcome Sanger Institute for the
COVID-19 Genomics UK (COG-UK) Consortium|Davies|United Kingdom
>Spike|hCoV-19/England/QEUH-9FA1FE/2020|2020-09-27|EPI_ISL_595792|Original|hCoV-
19^^England|Human|Oxford Viromics|COVID-19 Genomics UK (COG-UK)
Consortium|Golubchik|United Kingdom
>Spike|hCoV-19/Singapore/331/2020|2020-04-17|EPI_ISL_469085|Original|hCoV-
19^^Singapore|Human|National Public Health Laboratory|National Public Health
Laboratory|Mak|Singapore
>Spike|hCoV-19/Portugal/PT0027/2020|2020-03-06|EPI_ISL_418012|Original|hCoV-
19^^Portugal|Human|CHULC - H Curry Cabral|Instituto Nacional de Saude
(INSA)|Borges|Portugal
>Spike|hCoV-19/USA/WA-UW-22343/2020|2020-08-05|EPI_ISL_570665|Original|hCoV-
19^^Washington|Human|UW Virology Lab|UW Virology Lab|Roychoudhury|USA
>Spike|hCoV-19/England/LOND-DB183/2020|2020-11-02|EPI_ISL_724740|Original|hCoV-
19^^England|Human|University College London|COVID-19 Genomics UK (COG-UK)
Consortium|Roy|United Kingdom
>Spike|hCoV-19/England/QEUH-A0065F/2020|2020-09-29|EPI_ISL_600144|Original|hCoV-
19^^England|Human|Lighthouse Lab in Glasgow|Wellcome Sanger Institute for the COVID-
19 Genomics UK (COG-UK) consortium|Davies|United Kingdom
>Spike|hCoV-19/England/CAMC-C4705C/2020|2020-12-10|EPI_ISL_718817|Original|hCoV-
19^^England|Human|Lighthouse Lab in Cambridge|Wellcome Sanger Institute for the
COVID-19 Genomics UK (COG-UK) Consortium|Davies|United Kingdom
>Spike|hCoV-19/Canada/BC_01210684/2020|2020-04-00|EPI_ISL_466703|Original|hCoV-
19^^British Columbia|Human|BCCDC Public Health Laboratory|BCCDC Public Health
Laboratory|Harrigan|Canada
>Spike|hCoV-19/England/CAMB-1BACA6/2020|2020-11-12|EPI_ISL_664791|Original|hCoV-
19^^England|Human|Department of Pathology|COVID-19 Genomics UK (COG-UK)
Consortium|Goodfellow|United Kingdom
>Spike|hCoV-19/Japan/PG-3769/2020|2020-04-00|EPI_ISL_692022|Original|hCoV-
19^^Japan|Human|Pathogen Genomics Center|Pathogen Genomics Center|Sekizuka|Japan
>Spike|hCoV-19/Australia/VIC10067/2020|2020-08-21|EPI_ISL_562783|Original|hCoV-
19^^Victoria|Human|Microbiological Diagnostic Unit - Public Health Laboratory (MDU-
PHL)|MDU-PHL|Schultz|Australia

>Spike|hCoV-19/USA/FL-BPHL-0127/2020|2020-04-09|EPI_ISL_450861|Original|hCoV-19^^Florida|Human|Florida Bureau of Public Health Laboratories|Florida Bureau of Public Health Laboratories|Blanton|USA
>Spike|hCoV-19/England/CAMC-B2210A/2020|2020-11-02|EPI_ISL_646102|Original|hCoV-19^^England|Human|Lighthouse Lab in Cambridge|Wellcome Sanger Institute for the COVID-19 Genomics UK (COG-UK) Consortium|Davies|United Kingdom
>Spike|hCoV-19/England/CAMB-83E0B/2020|2020-04-25|EPI_ISL_438696|Original|hCoV-19^^England|Human|Department of Pathology|COVID-19 Genomics UK (COG-UK) Consortium|Meredith|United Kingdom
>Spike|hCoV-19/USA/TX-HMH-7977/2020|2020-06-30|EPI_ISL_545758|Original|hCoV-19^^Texas|Human|Houston Methodist Hospital|Houston Methodist Hospital|Long|USA
>Spike|hCoV-19/Australia/VIC8457/2020|2020-07-27|EPI_ISL_565027|Original|hCoV-19^^Victoria|Human|Microbiological Diagnostic Unit - Public Health Laboratory (MDU-PHL)|MDU-PHL|Schultz|Australia
>Spike|hCoV-19/USA/TX-HMH-MCoV-13324/2020|2020-08-25|EPI_ISL_788472|Original|hCoV-19^^Texas|Human|Houston Methodist Hospital|Houston Methodist Hospital|Long|USA
>Spike|hCoV-19/USA/WI-UW-1033/2020|2020-07-30|EPI_ISL_516459|Original|hCoV-19^^Wisconsin|Human|University of Wisconsin-Madison AIDS Vaccine Research Laboratories|University of Wisconsin-Madison AIDS Vaccine Research Laboratories|Baczenas|USA
>Spike|hCoV-19/Denmark/DCGC-22085/2020|2020-12-14|EPI_ISL_758543|Original|hCoV-19^^Hovedstaden|Human|Department of Virus and Microbiological Special Diagnostics|Albertsen Lab|Michaelsen|Denmark
>Spike|hCoV-19/England/ALDP-B5C4FF/2020|2020-11-10|EPI_ISL_655746|Original|hCoV-19^^England|Human|Lighthouse Lab in Alderley Park|Wellcome Sanger Institute for the COVID-19 Genomics UK (COG-UK) Consortium|Davies|United Kingdom
>Spike|hCoV-19/USA/TX-HMH-MCoV-14864/2020|2020-10-13|EPI_ISL_786348|Original|hCoV-19^^Texas|Human|Houston Methodist Hospital|Houston Methodist Hospital|Long|USA
>Spike|hCoV-19/England/QEUH-B9EB1A/2020|2020-11-18|EPI_ISL_719950|Original|hCoV-19^^England|Human|Lighthouse Lab in Glasgow|Wellcome Sanger Institute for the COVID-19 Genomics UK (COG-UK) Consortium|Davies|United Kingdom
>Spike|hCoV-19/Wales/PHWC-3599E/2020|2020-04-24|EPI_ISL_474505|Original|hCoV-19^^Wales|Human|Originating lab|COVID-19 Genomics UK (COG-UK) Consortium|Moore|United Kingdom
>Spike|hCoV-19/England/NOTT-112F94/2020|2020-08-11|EPI_ISL_526432|Original|hCoV-19^^England|Human|Lincolnshire Hospitals and DeepSeq Nottingham|COVID-19 Genomics UK (COG-UK) Consortium|Loose|United Kingdom
>Spike|hCoV-19/Australia/WA176/2020|2020-03-28|EPI_ISL_596739|Original|hCoV-19^^Western Australia|Human|PathWest Laboratory Medicine WA|PathWest Laboratory Medicine WA Microbial Surveillance Unit|Sikazwe|Australia
>Spike|hCoV-19/Canada/BC_13610520/2020|2020-04-00|EPI_ISL_467341|Original|hCoV-19^^British Columbia|Human|BCCDC Public Health Laboratory|BCCDC Public Health Laboratory|Harrigan|Canada
>Spike|hCoV-19/Scotland/GCVR-1724FD/2020|2020-03-30|EPI_ISL_470011|Original|hCoV-19^^Scotland|Human|NHSGGC West of Scotland Specialist Virology Centre / MRC-University of Glasgow Centre for Virus Research|Wellcome Sanger Institute for the COVID-19 Genomics UK (COG-UK) consortium|Davies|United Kingdom
>Spike|hCoV-19/Wales/PHWC-484D72/2020|2020-10-23|EPI_ISL_628107|Original|hCoV-19^^Wales|Human|Wales Specialist Virology Centre Sequencing lab|COVID-19 Genomics UK (COG-UK) Consortium|Moore|United Kingdom
>Spike|hCoV-19/England/CAMB-1AE55F/2020|2020-04-28|EPI_ISL_459410|Original|hCoV-19^^England|Human|Department of Pathology|Wellcome Sanger Institute for the COVID-19 Genomics UK (COG-UK) consortium|Davies|United Kingdom
>Spike|hCoV-19/Japan/PG-3362/2020|2020-04-00|EPI_ISL_691477|Original|hCoV-19^^Japan|Human|Pathogen Genomics Center|Pathogen Genomics Center|Sekizuka|Japan
>Spike|hCoV-19/USA/CA-CHLA-PLM82118164/2020|2020-11-30|EPI_ISL_753431|Original|hCoV-19^^California|Human|Clinical virology Laboratory|Center for Personalized Medicine|Dien Bard|USA

>Spike|hCoV-19/USA/CA-ALSR-0998-IPL/2020|2020-04-23|EPI_ISL_483452|Original|hCoV-19^^California|Human|UC San Diego Center for Advanced Laboratory Medicine|Andersen lab at Scripps Research|Gangavarapu|USA
>Spike|hCoV-19/England/SHEF-CA372/2020|2020-09-08|EPI_ISL_540748|Original|hCoV-19^^England|Human|Virology Department|COVID-19 Genomics UK (COG-UK) Consortium|de Silva|United Kingdom
>Spike|hCoV-19/Netherlands/NH-RIVM-20058/2020|2020-11-28|EPI_ISL_723242|Orignal|hCoV-19^^Noord-Holland|Human|Dutch COVID-19 response team|National Institute for Public Health and the Environment (RIVM)|Meijer|Netherlands
>Spike|hCoV-19/Australia/VIC6747/2020|2020-07-25|EPI_ISL_564026|Original|hCoV-19^^Victoria|Human|Victorian Infectious Diseases Reference Laboratory (VIDRL)|VIDRL and MDU-PHL|Schultz|Australia
>Spike|hCoV-19/England/CAMC-B99739/2020|2020-11-15|EPI_ISL_673299|Original|hCoV-19^^England|Human|Lighthouse Lab in Cambridge|Wellcome Sanger Institute for the COVID-19 Genomics UK (COG-UK) Consortium|Davies|United Kingdom
>Spike|hCoV-19/England/MILK-AC7618/2020|2020-10-20|EPI_ISL_630069|Original|hCoV-19^^England|Human|Lighthouse Lab in Milton Keynes|Wellcome Sanger Institute for the COVID-19 Genomics UK (COG-UK) consortium|Davies|United Kingdom
>Spike|hCoV-19/Denmark/DCGC-15429/2020|2020-11-23|EPI_ISL_713823|Original|hCoV-19^^Midtjylland|Human|Department of Virus and Microbiological Special Diagnostics|Albertsen Lab|Michaelsen|Denmark
>Spike|hCoV-19/USA/VA-DCLS-1686/2020|2020-09-08|EPI_ISL_609976|Original|hCoV-19^^Virginia|Human|Virginia DCLS|Virginia DCLS|St. Jacques|USA
>Spike|hCoV-19/India/MH-NIV-216641/2020|2020-09-18|EPI_ISL_577662|Original|hCoV-19^^Maharashtra|Human|NIV Influenza|NIV Influenza|Potdar|India
>Spike|hCoV-19/England/BRIS-126FD9/2020|2020-04-22|EPI_ISL_469726|Original|hCoV-19^^England|Human|PHE South West Regional Laboratory|Wellcome Sanger Institute for the COVID-19 Genomics UK (COG-UK) consortium|Davies|United Kingdom
>Spike|hCoV-19/England/BIRM-67BE8/2020|2020-11-04|EPI_ISL_650253|Original|hCoV-19^^England|Human|University of Birmingham|COVID-19 Genomics UK (COG-UK) Consortium|Loman|United Kingdom
>Spike|hCoV-19/Luxembourg/LNS0459171/2020|2020-10-20|EPI_ISL_739778|Original|hCoV-19^^Luxembourg|Human|Laboratoire national de sant\u00e9|Laboratoire national de sant\u00e9|Wienecke-Baldacchino|Luxembourg
>Spike|hCoV-19/England/MILK-B54C77/2020|2020-11-05|EPI_ISL_657918|Original|hCoV-19^^England|Human|Lighthouse Lab in Milton Keynes|Wellcome Sanger Institute for the COVID-19 Genomics UK (COG-UK) Consortium|Davies|United Kingdom
>Spike|hCoV-19/USA/VA-DCLS-2019/2020|2020-10-14|EPI_ISL_677168|Original|hCoV-19^^Virginia|Human|Virginia Division of Consolidated Laboratory Services|Virginia Division of Consolidated Laboratory Services|St. Jacques|USA
>Spike|hCoV-19/England/CAMC-A3F903/2020|2020-10-07|EPI_ISL_609679|Original|hCoV-19^^England|Human|Lighthouse Lab in Cambridge|Wellcome Sanger Institute for the COVID-19 Genomics UK (COG-UK) consortium|Davies|United Kingdom
>Spike|hCoV-19/USA/NY-Wadsworth-013081-01/2020|2020-03-11|EPI_ISL_676731|Original|hCoV-19^^New York|Human|Wadsworth Center|Wadsworth Center|Lasek-Nesselquist|USA
>Spike|hCoV-19/Scotland/MILK-A6C0CC/2020|2020-10-09|EPI_ISL_625430|Original|hCoV-19^^Scotland|Human|Lighthouse Lab in Milton Keynes|Wellcome Sanger Institute for the COVID-19 Genomics UK (COG-UK) consortium|Davies|United Kingdom
>Spike|hCoV-19/Denmark/DCGC-13449/2020|2020-11-30|EPI_ISL_712175|Original|hCoV-19^^Sjaelland|Human|Department of Virus and Microbiological Special Diagnostics|Albertsen Lab|Michaelsen|Denmark
>Spike|hCoV-19/USA/MI-MDHHS-SC21625/2020|2020-03-29|EPI_ISL_516352|Original|hCoV-19^^Michigan|Human|Michigan Department of Health and Human Services|Michigan Department of Health and Human Services|Blankenship|USA
>Spike|hCoV-19/USA/CA-CZB-2119/2020|2020-05-13|EPI_ISL_486349|Original|hCoV-19^^California|Human|UCSF Clinical Microbiology Laboratory|Chan-Zuckerberg Biohub|McGeever|USA

>Spike|hCoV-19/England/ALDP-A85018/2020|2020-10-16|EPI_ISL_610340|Original|hCoV-19^^England|Human|Lighthouse Lab in Alderley Park|Wellcome Sanger Institute for the COVID-19 Genomics UK (COG-UK) consortium|Davies|United Kingdom
>Spike|hCoV-19/Chile/RM-204488/2020|2020-10-12|EPI_ISL_746763|Original|hCoV-19^^Region Metropolitana de Santiago|Human|Genetica Molecular and Subdepartamento de Virologia ISP Chile|Instituto de Salud Publica de Chile|Tognarelli|Chile
>Spike|hCoV-19/Scotland/CVR2153/2020|2020-04-11|EPI_ISL_438877|Original|hCoV-19^^Scotland|Human|West of Scotland Specialist Virology Centre|COVID-19 Genomics UK (COG-UK) Consortium|Robertson|United Kingdom
>Spike|hCoV-19/Australia/VIC6013/2020|2020-07-10|EPI_ISL_518788|Original|hCoV-19^^Victoria|Human|Microbiological Diagnostic Unit - Public Health Laboratory (MDU-PHL)|MDU-PHL|Schultz|Australia
>Spike|hCoV-19/England/ALDP-8E6EE0/2020|2020-06-25|EPI_ISL_556617|Original|hCoV-19^^England|Human|Lighthouse Lab in Alderley Park|Wellcome Sanger Institute for the COVID-19 Genomics UK (COG-UK) consortium|Davies|United Kingdom
>Spike|hCoV-19/Australia/VIC5361/2020|2020-07-18|EPI_ISL_520124|Original|hCoV-19^^Victoria|Human|Microbiological Diagnostic Unit - Public Health Laboratory (MDU-PHL)|MDU-PHL|Schultz|Australia
>Spike|hCoV-19/USA/CA-CZB-11585/2020|2020-07-24|EPI_ISL_625563|Original|hCoV-19^^California|Human|NaN|Chan-Zuckerberg Biohub|Bhatt|USA
>Spike|hCoV-19/England/QEUH-9FFA94/2020|2020-09-30|EPI_ISL_600151|Original|hCoV-19^^England|Human|Lighthouse Lab in Glasgow|Wellcome Sanger Institute for the COVID-19 Genomics UK (COG-UK) consortium|Davies|United Kingdom
>Spike|hCoV-19/Japan/PG-0495/2020|2020-04-06|EPI_ISL_480154|Original|hCoV-19^^HokurikuShinsyu|Human|Fukui Prefectural Institute of Public Health and Environmental Science|Pathogen Genomics Center|Sekizuka|Japan
>Spike|hCoV-19/USA/WA-S2819/2020|2020-09-07|EPI_ISL_574661|Original|hCoV-19^^Washington|Human|Seattle Flu Study|Seattle Flu Study|Lee|USA
>Spike|hCoV-19/Denmark/DCGC-10645/2020|2020-11-02|EPI_ISL_669510|Original|hCoV-19^^Hovedstaden|Human|Department of Virus and Microbiological Special Diagnostics|Albertsen Lab|Michaelsen|Denmark
>Spike|hCoV-19/USA/WI-GMF-30476/2020|2020-10-31|EPI_ISL_661037|Original|hCoV-19^^Wisconsin|Human|Gundersen Molecular Diagnostics Laboratory|Kabara Cancer Research Institute|Kenny|USA
>Spike|hCoV-19/India/BR-NCDC-02251/2020|2020-03-28|EPI_ISL_435112|Original|hCoV-19^^Bihar|Human|National Centre for Disease control (NCDC)|NCDC/CSIR-IGIB|Faruq|India
>Spike|hCoV-19/Sweden/20-50659/2020|2020-04-05|EPI_ISL_475152|Original|hCoV-19^^Stockholm|Human|Folkhalsomyndigheten|The Public Health Agency of Sweden|Svartstr\u00f6m|Sweden
>Spike|hCoV-19/England/CAMB-84918/2020|2020-03-06|EPI_ISL_440491|Original|hCoV-19^^England|Human|Department of Pathology|Wellcome Sanger Institute for the COVID-19 Genomics UK (COG-UK) consortium|Amato|United Kingdom
>Spike|hCoV-19/USA/OR-OHSU-0638/2020|2020-06-13|EPI_ISL_525863|Original|hCoV-19^^Oregon|Human|OHSU Lab Services Molecular Microbiology Lab|Ginkgo Bioworks Clinical Laboratory|O'Roak|USA
>Spike|hCoV-19/France/PAC-IHU-1382/2020|2020-08-08|EPI_ISL_569147|Original|hCoV-19^^Provence-Alpes-C\u00f4te-d\u2019Azur|Human|MEPHI|MEPHI|Levasseur|France
>Spike|hCoV-19/Iceland/587/2020|2020-03-28|EPI_ISL_424607|Original|hCoV-19^^Reykjavik|Human|The National University Hospital of Iceland|deCODE genetics|J\u00f3nsson|Iceland
>Spike|hCoV-19/England/LIVE-9E0E9/2020|2020-04-03|EPI_ISL_453268|Original|hCoV-19^^England|Human|Liverpool Clinical Laboratories|COVID-19 Genomics UK (COG-UK) Consortium|Haldenby|United Kingdom
>Spike|hCoV-19/Denmark/DCGC-22479/2020|2020-12-14|EPI_ISL_757396|Original|hCoV-19^^Sjaelland|Human|Department of Virus and Microbiological Special Diagnostics|Albertsen Lab|Michaelsen|Denmark
>Spike|hCoV-19/Italy/VEN-IZSVe-20RS2160-28/2020|2020-11-26|EPI_ISL_747482|Original|hCoV-19^^Veneto|Human|Ospedale San Bonifacio|Istituto Zooprofilattico Sperimentale delle Venezie|Quaranta|Italy

>Spike|hCoV-19/Japan/PG-1188/2020|2020-03-00|EPI_ISL_684798|Original|hCoV-
19^^Japan|Human|Yamagata Prefectural Institute of Public Health|Pathogen Genomics
Center|Sekizuka|Japan
>Spike|hCoV-19/Wales/PHWC-16429A/2020|2020-05-07|EPI_ISL_474153|Original|hCoV-
19^^Wales|Human|Originating lab|COVID-19 Genomics UK (COG-UK) Consortium|Moore|United
Kingdom
>Spike|hCoV-19/Denmark/DCGC-18859/2020|2020-12-07|EPI_ISL_748601|Original|hCoV-
19^^Midtjylland|Human|Department of Virus and Microbiological Special
Diagnostics|Albertsen Lab|Michaelsen|Denmark
>Spike|hCoV-19/England/QEUH-9D152E/2020|2020-09-14|EPI_ISL_577289|Original|hCoV-
19^^England|Human|University of Exeter|COVID-19 Genomics UK (COG-UK)
Consortium|Temperton|United Kingdom
>Spike|hCoV-19/USA/MI-MDHHS-SC21973/2020|2020-03-15|EPI_ISL_566024|Original|hCoV-
19^^Michigan|Human|Michigan Department of Health and Human Services|Michigan
Department of Health and Human Services|Blankenship|USA
>Spike|hCoV-19/Japan/PG-8429/2020|2020-09-00|EPI_ISL_687528|Original|hCoV-
19^^Japan|Human|Pathogen Genomics Center|Pathogen Genomics Center|Sekizuka|Japan
>Spike|hCoV-19/England/MILK-ACCD78/2020|2020-10-21|EPI_ISL_630187|Original|hCoV-
19^^England|Human|Lighthouse Lab in Milton Keynes|Wellcome Sanger Institute for the
COVID-19 Genomics UK (COG-UK) consortium|Davies|United Kingdom
>Spike|hCoV-19/USA/UT-UPHL-2012416147/2020|2020-08-08|EPI_ISL_745981|Original|hCoV-
19^^Utah|Human|Ginkgo Bioworks Clinical Laboratory|Utah Public Health
Laboratory|Young|USA
>Spike|hCoV-19/Northern Ireland/QEUH-95E84E/2020|2020-08-
09|EPI_ISL_532481|Original|hCoV-19^^Northern Ireland|Human|Lighthouse Lab in
Glasgow|Wellcome Sanger Institute for the COVID-19 Genomics UK (COG-UK)
consortium|Davies|United Kingdom
>Spike|hCoV-19/Japan/PG-4050/2020|2020-07-00|EPI_ISL_692291|Original|hCoV-
19^^Japan|Human|Sakai City Institute of Public Health|Pathogen Genomics
Center|Sekizuka|Japan
>Spike|hCoV-19/Japan/PG-0559/2020|2020-04-07|EPI_ISL_480209|Original|hCoV-
19^^Kansai|Human|Department of Infectious Diseases|Pathogen Genomics
Center|Sekizuka|Japan
>Spike|hCoV-19/England/BRIS-126346/2020|2020-04-10|EPI_ISL_459046|Original|hCoV-
19^^England|Human|PHE South West Regional Laboratory|Wellcome Sanger Institute for
the COVID-19 Genomics UK (COG-UK) consortium|Davies|United Kingdom
>Spike|hCoV-19/England/ALDP-BB5DAE/2020|2020-11-20|EPI_ISL_703019|Original|hCoV-
19^^England|Human|Lighthouse Lab in Alderley Park|Wellcome Sanger Institute for the
COVID-19 Genomics UK (COG-UK) Consortium|Davies|United Kingdom
>Spike|hCoV-19/Wales/PHWC-49B19A/2020|2020-10-27|EPI_ISL_742305|Original|hCoV-
19^^Wales|Human|Wales Specialist Virology Centre Sequencing lab|COVID-19 Genomics UK
(COG-UK) Consortium|Moore|United Kingdom
>Spike|hCoV-19/Denmark/DCGC-17527/2020|2020-12-14|EPI_ISL_751044|Original|hCoV-
19^^Nordjylland|Human|Department of Virus and Microbiological Special
Diagnostics|Albertsen Lab|Michaelsen|Denmark
>Spike|hCoV-19/Scotland/GCVR-172FA1/2020|2020-04-06|EPI_ISL_524542|Original|hCoV-
19^^Scotland|Human|NHSGGC West of Scotland Specialist Virology Centre / MRC-
University of Glasgow Centre for Virus Research|Wellcome Sanger Institute for the
COVID-19 Genomics UK (COG-UK) consortium|Davies|United Kingdom
>Spike|hCoV-19/India/DL-NCDC-3394/2020|2020-04-09|EPI_ISL_436435|Original|hCoV-
19^^Delhi|Human|National Centre for Disease control (NCDC)|NCDC/CSIR-IGIB|Singh|India
>Spike|hCoV-19/Wales/PHWC-48CA82/2020|2020-11-08|EPI_ISL_666501|Original|hCoV-
19^^Wales|Human|Wales Specialist Virology Centre Sequencing lab|COVID-19 Genomics UK
(COG-UK) Consortium|Moore|United Kingdom
>Spike|hCoV-19/USA/TX-HMH-6964/2020|2020-06-28|EPI_ISL_546391|Original|hCoV-
19^^Texas|Human|Houston Methodist Hospital|Houston Methodist Hospital|Long|USA
>Spike|hCoV-19/England/SHEF-C5F73/2020|2020-09-15|EPI_ISL_559909|Original|hCoV-
19^^England|Human|Virology Department|COVID-19 Genomics UK (COG-UK) Consortium|de
Silva|United Kingdom

>Spike|hCoV-19/Russia/SPE-RII-24810V/2020|2020-06-16|EPI_ISL_524047|Vero P1|hCoV-19^^Saint Petersburg|Human|WHO National Influenza Centre Russian Federation|WHO National Influenza Centre Russian Federation|Komissarov|Russia
>Spike|hCoV-19/England/SHEF-D1B61/2020|2020-09-09|EPI_ISL_540769|Original|hCoV-19^^England|Human|Virology Department|COVID-19 Genomics UK (COG-UK) Consortium|de Silva|United Kingdom
>Spike|hCoV-19/England/20169034004/2020|2020-04-18|EPI_ISL_465231|Original|hCoV-19^^England|Human|Respiratory Virus Unit|Respiratory Virus Unit|Platt|United Kingdom
>Spike|hCoV-19/Switzerland/BS-UHB-42190622/2020|2020-03-14|EPI_ISL_528074|Original|hCoV-19^^Basel-Stadt|Human|University Hospital Basel|University Hospital Basel|Roloff Handschin|Switzerland
>Spike|hCoV-19/England/LOND-12F094F/2020|2020-11-22|EPI_ISL_679593|Original|hCoV-19^^England|Human|University College London|COVID-19 Genomics UK (COG-UK) Consortium|Roy|United Kingdom
>Spike|hCoV-19/South Africa/KRISP-K003501/2020|2020-09-16|EPI_ISL_602825|Original|hCoV-19^^KwaZulu-Natal|Human|NHLS-IALCH|KRISP|de Oliveira|South Africa
>Spike|hCoV-19/Wales/PHWC-498C50/2020|2020-11-25|EPI_ISL_727192|Original|hCoV-19^^Wales|Human|Originating lab|COVID-19 Genomics UK (COG-UK) Consortium|Moore|United Kingdom
>Spike|hCoV-19/USA/WA-S2245/2020|2020-06-09|EPI_ISL_497673|Original|hCoV-19^^Washington|Human|Washington State Department of Health|Seattle Flu Study|Lee|USA
>Spike|hCoV-19/Denmark/DCGC-9775/2020|2020-11-09|EPI_ISL_669167|Original|hCoV-19^^Midtjylland|Human|Department of Virus and Microbiological Special Diagnostics|Albertsen Lab|Michaelsen|Denmark
>Spike|hCoV-19/England/OXON-B1411/2020|2020-00-00|EPI_ISL_479013|Original|hCoV-19^^England|Human|Oxford Viromics|COVID-19 Genomics UK (COG-UK) Consortium|Golubchik|United Kingdom
>Spike|hCoV-19/England/201400652/2020|2020-03-30|EPI_ISL_465886|Original|hCoV-19^^England|Human|Respiratory Virus Unit|Respiratory Virus Unit|Platt|United Kingdom
>Spike|hCoV-19/Scotland/CVR3922/2020|2020-06-02|EPI_ISL_512469|Original|hCoV-19^^Scotland|Human|West of Scotland Specialist Virology Centre|COVID-19 Genomics UK (COG-UK) Consortium|Robertson|United Kingdom
>Spike|hCoV-19/Australia/NSW1319/2020|2020-12-20|EPI_ISL_767885|Original|hCoV-19^^New South Wales|Human|South Eastern Area Laboratory Services (SEALS)|NSW Health Pathology - Institute of Clinical Pathology and Medical Research; Westmead Hospital; University of Sydney|Gall|Australia
>Spike|hCoV-19/India/TG-CCMB-M70/2020|2020-06-05|EPI_ISL_495227|Original|hCoV-19^^Telangana|Human|CSIR-Centre for Cellular and Molecular Biology|CSIR-Centre for Cellular and Molecular Biology|Banu|India
>Spike|hCoV-19/Germany/ST-ChVir-W1702-6718/2020|2020-08-09|EPI_ISL_754054|Original|hCoV-19^^Saxony-Anhalt|Human|Charit\u00e9 Universit\u00e4tsmedizin Berlin|Charit\u00e9 Universit\u00e4tsmedizin Berlin|Muehlemann|Germany
>Spike|hCoV-19/Denmark/DCGC-15778/2020|2020-11-23|EPI_ISL_714014|Original|hCoV-19^^Nordjylland|Human|Department of Virus and Microbiological Special Diagnostics|Albertsen Lab|Michaelsen|Denmark
>Spike|hCoV-19/United Arab Emirates/0674/2020|2020-05-15|EPI_ISL_698827|Original|hCoV-19^^Abu Dhabi|Human|Group 42 (G42) Healthcare|G42 Healthcare|Ogrodzki|United Arab Emirates
>Spike|hCoV-19/Belgium/Rega-0329218/2020|2020-03-29|EPI_ISL_458188|Original|hCoV-19^^Mepel|Human|KU Leuven|KU Leuven|Maes|Belgium
>Spike|hCoV-19/India/GJ-GBRC151a/2020|2020-05-02|EPI_ISL_461478|Original|hCoV-19^^Gujarat|Human|Government Medical College|Gujarat Biotechnology Research Centre|Joshi|India
>Spike|hCoV-19/USA/TX-HMH-MCoV-18432/2020|2020-11-19|EPI_ISL_784780|Original|hCoV-19^^Texas|Human|Houston Methodist Hospital|Houston Methodist Hospital|Long|USA
>Spike|hCoV-19/Portugal/PT1854/2020|2020-11-12|EPI_ISL_732073|Original|hCoV-19^^Portugal|Human|Instituto Nacional de Saude (INSA)|Instituto Nacional de Saude (INSA)|Borges|Portugal

>Spike|hCoV-19/Hong Kong/HKU-902b/2020|2020-01-24|EPI_ISL_434564|unknown|hCoV-19^^Hong Kong|Human|unknown|Microbiology|Submitter|China
>Spike|hCoV-19/USA/CA-CSMC64/2020|2020-03-28|EPI_ISL_475678|Original|hCoV-19^^California|Human|Cedars-Sinai Medical Center|Cedars-Sinai Medical Center|Zhang|USA
>Spike|hCoV-19/USA/TX-HMH-MCoV-12495/2020|2020-09-30|EPI_ISL_790365|Original|hCoV-19^^Texas|Human|Houston Methodist Hospital|Houston Methodist Hospital|Long|USA
>Spike|hCoV-19/England/MILK-B54ECC/2020|2020-11-06|EPI_ISL_657984|Original|hCoV-19^^England|Human|Lighthouse Lab in Milton Keynes|Wellcome Sanger Institute for the COVID-19 Genomics UK (COG-UK) Consortium|Davies|United Kingdom
>Spike|hCoV-19/England/CAMB-1B3F95/2020|2020-05-31|EPI_ISL_477794|Original|hCoV-19^^England|Human|Department of Pathology|COVID-19 Genomics UK (COG-UK) Consortium|Meredith|United Kingdom
>Spike|hCoV-19/USA/AZ-TG408211/2020|2020-06-17|EPI_ISL_694947|Original|hCoV-19^^Arizona|Human|AZ SPHL|TGen North|French|USA
>Spike|hCoV-19/Ireland/OY-NVRL-72IRL00307/2020|2020-08-05|EPI_ISL_525410|Original|hCoV-19^^Offaly|Human|National Virus Reference Laboratory|National Virus Reference Laboratory|Gonzalez|Ireland
>Spike|hCoV-19/USA/TX-HMH-MCoV-19679/2020|2020-11-25|EPI_ISL_784036|Original|hCoV-19^^Texas|Human|Houston Methodist Hospital|Houston Methodist Hospital|Long|USA
>Spike|hCoV-19/USA/WA-S1221/2020|2020-05-08|EPI_ISL_495671|Original|hCoV-19^^Washington|Human|Washington State Department of Health|Seattle Flu Study|Lee|USA
>Spike|hCoV-19/Australia/VIC3435/2020|2020-07-05|EPI_ISL_521104|Original|hCoV-19^^Victoria|Human|Victorian Infectious Diseases Reference Laboratory (VIDRL)|VIDRL and MDU-PHL|Schultz|Australia
>Spike|hCoV-19/Wales/PHWC-160C63/2020|2020-05-23|EPI_ISL_473033|Original|hCoV-19^^Wales|Human|Wales Specialist Virology Centre Sequencing lab|COVID-19 Genomics UK (COG-UK) Consortium|Moore|United Kingdom
>Spike|hCoV-19/England/MILK-ACD393/2020|2020-10-21|EPI_ISL_634257|Original|hCoV-19^^England|Human|Lighthouse Lab in Milton Keynes|Wellcome Sanger Institute for the COVID-19 Genomics UK (COG-UK) consortium|Davies|United Kingdom
>Spike|hCoV-19/England/ALDP-A7047E/2020|2020-10-15|EPI_ISL_607966|Original|hCoV-19^^England|Human|Lighthouse Lab in Alderley Park|Wellcome Sanger Institute for the COVID-19 Genomics UK (COG-UK) consortium|Davies|United Kingdom
>Spike|hCoV-19/Switzerland/ZH-UZH-IMV139/2020|2020-12-29|EPI_ISL_766585|Original|hCoV-19^^Zurich|Human|Triemli Hospital|Institute of Medical Virology|Schmutz|Switzerland
>Spike|hCoV-19/United Arab Emirates/0884/2020|2020-05-19|EPI_ISL_699022|Original|hCoV-19^^Abu Dhabi|Human|Group 42 (G42) Healthcare|G42 Healthcare|Ogrodzki|United Arab Emirates
>Spike|hCoV-19/Kenya/C1191/2020|2020-04-19|EPI_ISL_457852|Original|hCoV-19^^Kenya|Human|KEMRI-CGMR-C|KEMRI-Wellcome Trust Research Programme/KEMRI-CGMR-C Kilifi|Githinji|Kenya
>Spike|hCoV-19/Burkina Faso/2163/2020|2020-09-02|EPI_ISL_660512|Original|hCoV-19^^Bobo Dioulasso|Human|Laboratoire de Microbiologie CHU Souro Sanou|Centre Muraz|Ouedraogo|Burkina Faso
>Spike|hCoV-19/Germany/HH-hpi-p2401/2020|2020-04-22|EPI_ISL_776474|Original|hCoV-19^^Hamburg|Human|University Medical Center Hamburg Eppendorf|Heinrich Pette Institute|Robitaille|Germany
>Spike|hCoV-19/USA/FL-BPHL-0876/2020|2020-07-09|EPI_ISL_514222|Original|hCoV-19^^Florida|Human|Florida Bureau of Public Health Laboratories|Florida Bureau of Public Health Laboratories|Blanton|USA
>Spike|hCoV-19/Switzerland/GR-ETHZ-250078/2020|2020-08-18|EPI_ISL_535601|Original|hCoV-19^^Graub\u00fcnden|Human|Viollier AG|Department of Biosystems Science and Engineering|Nadeau|Switzerland
>Spike|hCoV-19/England/MILK-B55757/2020|2020-11-06|EPI_ISL_657438|Original|hCoV-19^^England|Human|Lighthouse Lab in Milton Keynes|Wellcome Sanger Institute for the COVID-19 Genomics UK (COG-UK) Consortium|Davies|United Kingdom

>Spike|hCoV-19/Australia/VIC11378/2020|2020-08-23|EPI_ISL_563348|Original|hCoV-19^^Victoria|Human|Microbiological Diagnostic Unit - Public Health Laboratory (MDU-PHL)|MDU-PHL|Schultz|Australia
>Spike|hCoV-19/England/MILK-99DD62/2020|2020-08-31|EPI_ISL_550776|Original|hCoV-19^^England|Human|Lighthouse Lab in Milton Keynes|Wellcome Sanger Institute for the COVID-19 Genomics UK (COG-UK) consortium|Davies|United Kingdom
>Spike|hCoV-19/Scotland/CVR1926/2020|2020-04-07|EPI_ISL_438856|Original|hCoV-19^^Scotland|Human|West of Scotland Specialist Virology Centre|COVID-19 Genomics UK (COG-UK) Consortium|Robertson|United Kingdom
>Spike|hCoV-19/England/QEUH-B14411/2020|2020-11-03|EPI_ISL_642639|Original|hCoV-19^^England|Human|Lighthouse Lab in Glasgow|Wellcome Sanger Institute for the COVID-19 Genomics UK (COG-UK) Consortium|Davies|United Kingdom
>Spike|hCoV-19/England/QEUH-997BB9/2020|2020-08-28|EPI_ISL_549544|Original|hCoV-19^^England|Human|Oxford Viromics|COVID-19 Genomics UK (COG-UK) Consortium|Golubchik|United Kingdom
>Spike|hCoV-19/England/MILK-9950BC/2020|2020-08-24|EPI_ISL_552322|Original|hCoV-19^^England|Human|Lighthouse Lab in Milton Keynes|Wellcome Sanger Institute for the COVID-19 Genomics UK (COG-UK) consortium|Davies|United Kingdom
>Spike|hCoV-19/USA/UT-QDX-1880/2020|2020-03-13|EPI_ISL_604281|Original|hCoV-19^^Utah|Human|Quest Diagnostics|Quest Diagnostics|Kagan|USA
>Spike|hCoV-19/Scotland/QEUH-B7DF51/2020|2020-11-13|EPI_ISL_742256|Original|hCoV-19^^Scotland|Human|Oxford Viromics|COVID-19 Genomics UK (COG-UK) Consortium|Golubchik|United Kingdom
>Spike|hCoV-19/England/BRIS-129C93/2020|2020-04-15|EPI_ISL_458386|Original|hCoV-19^^England|Human|PHE South West Regional Laboratory|Wellcome Sanger Institute for the COVID-19 Genomics UK (COG-UK) consortium|Davies|United Kingdom
>Spike|hCoV-19/Wales/PHWC-48C660/2020|2020-11-03|EPI_ISL_666452|Original|hCoV-19^^Wales|Human|Wales Specialist Virology Centre Sequencing lab|COVID-19 Genomics UK (COG-UK) Consortium|Moore|United Kingdom
>Spike|hCoV-19/England/ALDP-B1C4DD/2020|2020-11-01|EPI_ISL_645778|Original|hCoV-19^^England|Human|Lighthouse Lab in Alderley Park|Wellcome Sanger Institute for the COVID-19 Genomics UK (COG-UK) Consortium|Davies|United Kingdom
>Spike|hCoV-19/Wales/PHWC-163583/2020|2020-04-25|EPI_ISL_473964|Original|hCoV-19^^Wales|Human|Originating lab|COVID-19 Genomics UK (COG-UK) Consortium|Moore|United Kingdom
>Spike|hCoV-19/USA/CA-CZB-5762/2020|2020-07-12|EPI_ISL_672357|Original|hCoV-19^^California|Human|County of San Luis Obispo Public Health Laboratory|Chan-Zuckerberg Biohub|Bhatt|USA
>Spike|hCoV-19/Scotland/QEUH-98FA99/2020|2020-08-27|EPI_ISL_539980|Original|hCoV-19^^Scotland|Human|Lighthouse Lab in Glasgow|Wellcome Sanger Institute for the COVID-19 Genomics UK (COG-UK) consortium|Davies|United Kingdom
>Spike|hCoV-19/England/CAMB-1B1559/2020|2020-05-07|EPI_ISL_458563|Original|hCoV-19^^England|Human|Department of Pathology|Wellcome Sanger Institute for the COVID-19 Genomics UK (COG-UK) consortium|Davies|United Kingdom
>Spike|hCoV-19/England/CAMC-B073F6/2020|2020-10-30|EPI_ISL_643685|Original|hCoV-19^^England|Human|Lighthouse Lab in Cambridge|Wellcome Sanger Institute for the COVID-19 Genomics UK (COG-UK) Consortium|Davies|United Kingdom
>Spike|hCoV-19/USA/TX-HMH-MCoV-9148/2020|2020-07-02|EPI_ISL_780970|Original|hCoV-19^^Texas|Human|Houston Methodist Hospital|Houston Methodist Hospital|Long|USA
>Spike|hCoV-19/USA/TX-HMH-MCoV-18613/2020|2020-11-20|EPI_ISL_784961|Original|hCoV-19^^Texas|Human|Houston Methodist Hospital|Houston Methodist Hospital|Long|USA
>Spike|hCoV-19/Portugal/PT1271/2020|2020-03-20|EPI_ISL_511232|Original|hCoV-19^^Portugal|Human|Instituto Nacional de Saude (INSA) and Instituto Gulbenkian de Ciencia (IGC)|Instituto Nacional de Saude (INSA) and Instituto Gulbenkian de Ciencia (IGC)|Borges|Portugal
>Spike|hCoV-19/England/GSTT-265E1AA/2020|2020-03-31|EPI_ISL_484164|Original|hCoV-19^^England|Human|Centre for Clinical Infection and Diagnostics Research and Genomics Innovation Unit|COVID-19 Genomics UK (COG-UK) Consortium|Awan|United Kingdom

>Spike|hCoV-19/England/LOND-125F823/2020|2020-04-06|EPI_ISL_516882|Original|hCoV-19^^England|Human|North West London Pathology|Wellcome Sanger Institute for the COVID-19 Genomics UK (COG-UK) consortium|Davies|United Kingdom
>Spike|hCoV-19/Wales/PHWC-490809/2020|2020-11-13|EPI_ISL_703351|Original|hCoV-19^^Wales|Human|Originating lab|COVID-19 Genomics UK (COG-UK) Consortium|Moore|United Kingdom
>Spike|hCoV-19/USA/TX-HMH-1961/2020|2020-05-17|EPI_ISL_542922|Original|hCoV-19^^Texas|Human|Houston Methodist Hospital|Houston Methodist Hospital|Long|USA
>Spike|hCoV-19/Japan/PG-1740/2020|2020-05-00|EPI_ISL_685346|Original|hCoV-19^^Japan|Human|Pathogen Genomics Center|Pathogen Genomics Center|Sekizuka|Japan
>Spike|hCoV-19/Scotland/EDB1811/2020|2020-04-16|EPI_ISL_433135|Original|hCoV-19^^Scotland|Human|Virology Department|COVID-19 Genomics UK (COG-UK) Consortium|Williams|United Kingdom
>Spike|hCoV-19/England/BRIS-12A148/2020|2020-04-16|EPI_ISL_458498|Original|hCoV-19^^England|Human|PHE South West Regional Laboratory|Wellcome Sanger Institute for the COVID-19 Genomics UK (COG-UK) consortium|Davies|United Kingdom
>Spike|hCoV-19/USA/CA-CZB-14818/2020|2020-11-18|EPI_ISL_739114|Original|hCoV-19^^California|Human|Humboldt County Public Health Laboratory|Chan-Zuckerberg Biohub|Kamm|USA
>Spike|hCoV-19/Japan/PG-2038/2020|2020-05-00|EPI_ISL_685643|Original|hCoV-19^^Japan|Human|Pathogen Genomics Center|Pathogen Genomics Center|Sekizuka|Japan
>Spike|hCoV-19/Netherlands/UT-EMC-204/2020|2020-11-16|EPI_ISL_722277|Original|hCoV-19^^Utrecht|Human|Dutch COVID-19 response team|Erasmus Medical Center|Oude Munnink|Netherlands
>Spike|hCoV-19/Spain/AR-IBV-005094/2020|2020-04-23|EPI_ISL_510375|Original|hCoV-19^^Aragon|Human|Servicio de Microbiolog\u00eda|SeqCOVID-SPAIN consortium/IBV(CSIC)|Comas|Spain
>Spike|hCoV-19/Germany/BY-IMB11609/2020|2020-10-08|EPI_ISL_732563|Original|hCoV-19^^Bavaria|Human|Bundeswehr Institute of Microbiology|Bundeswehr Institute of Microbiology|Antwerpen|Germany
>Spike|hCoV-19/Australia/VIC12274/2020|2020-08-07|EPI_ISL_591943|Original|hCoV-19^^Victoria|Human|Victorian Infectious Diseases Reference Laboratory (VIDRL)|VIDRL and MDU-PHL|Schultz|Australia
>Spike|hCoV-19/USA/MI-MDHHS-SC22520/2020|2020-11-17|EPI_ISL_708223|Original|hCoV-19^^Michigan|Human|Michigan Department of Health and Human Services|Michigan Department of Health and Human Services|Jacob|USA
>Spike|hCoV-19/Australia/VIC6828/2020|2020-07-24|EPI_ISL_562152|Original|hCoV-19^^Victoria|Human|Victorian Infectious Diseases Reference Laboratory (VIDRL)|VIDRL and MDU-PHL|Schultz|Australia
>Spike|hCoV-19/Netherlands/Zeeland_6/2020|2020-04-02|EPI_ISL_461288|Original|hCoV-19^^Zeeland|Human|Dutch COVID-19 response team|Erasmus Medical Center|Oude Munnink|Netherlands
>Spike|hCoV-19/Austria/CeMM0210/2020|2020-03-16|EPI_ISL_438047|Original|hCoV-19^^Vienna|Human|Center for Virology|Bergthaler laboratory|Bergthaler|Austria
>Spike|hCoV-19/USA/NM-DOH-2020631352/2020|2020-12-19|EPI_ISL_766891|Original|hCoV-19^^New Mexico|Human|New Mexico Department of Health Scientific Laboratory|New Mexico Department of Health Scientific Laboratory|Malone|USA
>Spike|hCoV-19/England/NOTT-112BEB/2020|2020-04-29|EPI_ISL_493597|Original|hCoV-19^^England|Human|Lincolnshire Hospitals and DeepSeq Nottingham|COVID-19 Genomics UK (COG-UK) Consortium|Loose|United Kingdom
>Spike|hCoV-19/England/NORW-E8D8F/2020|2020-04-26|EPI_ISL_453557|Original|hCoV-19^^England|Human|Quadram Institute Bioscience|COVID-19 Genomics UK (COG-UK) Consortium|Alikhan|United Kingdom
>Spike|hCoV-19/Scotland/QEUH-A793D2/2020|2020-10-14|EPI_ISL_638995|Original|hCoV-19^^Scotland|Human|Oxford Viromics|COVID-19 Genomics UK (COG-UK) Consortium|Golubchik|United Kingdom
>Spike|hCoV-19/England/LIVE-9C6AD/2020|2020-04-01|EPI_ISL_449568|Original|hCoV-19^^England|Human|Liverpool Clinical Laboratories|COVID-19 Genomics UK (COG-UK) Consortium|Haldenby|United Kingdom

>Spike|hCoV-19/USA/TX-HMH-2618/2020|2020-06-05|EPI_ISL_545431|Original|hCoV-19^^Texas|Human|Houston Methodist Hospital|Houston Methodist Hospital|Long|USA
>Spike|hCoV-19/England/ALDP-A8726B/2020|2020-10-17|EPI_ISL_605971|Original|hCoV-19^^England|Human|Lighthouse Lab in Alderley Park|Wellcome Sanger Institute for the COVID-19 Genomics UK (COG-UK) consortium|Davies|United Kingdom
>Spike|hCoV-19/England/QEUH-AD5A75/2020|2020-10-26|EPI_ISL_634549|Original|hCoV-19^^England|Human|Lighthouse Lab in Glasgow|Wellcome Sanger Institute for the COVID-19 Genomics UK (COG-UK) consortium|Davies|United Kingdom
>Spike|hCoV-19/USA/OR-OHSU-1133/2020|2020-07-10|EPI_ISL_509147|Original|hCoV-19^^Oregon|Human|OHSU Lab Services Molecular Microbiology Lab|Oregon SARS-CoV-2 Genome Sequencing Center|O'Roak|USA
>Spike|hCoV-19/Germany/BB-ChVir-W1442-8234/2020|2020-06-19|EPI_ISL_753857|Original|hCoV-19^^Brandenburg|Human|Charit\u00e9 Universit\u00e4tsmedizin Berlin|Charit\u00e9 Universit\u00e4tsmedizin Berlin|Muehlemann|Germany
>Spike|hCoV-19/England/QEUH-BCBB6F/2020|2020-11-27|EPI_ISL_704448|Original|hCoV-19^^England|Human|Lighthouse Lab in Glasgow|Wellcome Sanger Institute for the COVID-19 Genomics UK (COG-UK) Consortium|Davies|United Kingdom
>Spike|hCoV-19/Denmark/DCGC-21558/2020|2020-12-21|EPI_ISL_757749|Original|hCoV-19^^Hovedstaden|Human|Department of Virus and Microbiological Special Diagnostics|Albertsen Lab|Michaelsen|Denmark
>Spike|hCoV-19/USA/CA-CZB-10096/2020|2020-06-21|EPI_ISL_672211|Original|hCoV-19^^California|Human|The Ashley Laboratory|Chan-Zuckerberg Biohub|Bhatt|USA
>Spike|hCoV-19/Bahrain/340860476/2020|2020-10-14|EPI_ISL_632251|Original|hCoV-19^^Bahrain|Human|Communicable Disease Laboratory|Communicable Disease Laboratory|Altaif|Bahrain
>Spike|hCoV-19/Colombia/TOL-GUV-92052/2020|2020-03-31|EPI_ISL_447757|Original|hCoV-19^^Tolima|Human|Instituto Nacional de Salud|Grupo de Investigaciones Microbiol\u00f3gicas-UR (GIMUR)|Ram\u00edrez|Colombia
>Spike|hCoV-19/England/QEUH-9F6453/2020|2020-09-25|EPI_ISL_588891|Original|hCoV-19^^England|Human|Lighthouse Lab in Glasgow|Wellcome Sanger Institute for the COVID-19 Genomics UK (COG-UK) consortium|Davies|United Kingdom
>Spike|hCoV-19/India/DL-56417-CSIR-IGIB/2020|2020-08-01|EPI_ISL_636745|Original|hCoV-19^^Delhi|Human|National Centre for Disease control (NCDC)|NCDC/CSIR-IGIB|Faruq|India
>Spike|hCoV-19/Australia/VIC15249/2020|2020-09-27|EPI_ISL_593218|Original|hCoV-19^^Victoria|Human|Microbiological Diagnostic Unit - Public Health Laboratory (MDU-PHL)|MDU-PHL|Schultz|Australia
>Spike|hCoV-19/England/ALDP-94D903/2020|2020-06-20|EPI_ISL_555196|Original|hCoV-19^^England|Human|Lighthouse Lab in Alderley Park|Wellcome Sanger Institute for the COVID-19 Genomics UK (COG-UK) consortium|Davies|United Kingdom
>Spike|hCoV-19/England/ALDP-A6FB22/2020|2020-10-15|EPI_ISL_607024|Original|hCoV-19^^England|Human|Lighthouse Lab in Alderley Park|Wellcome Sanger Institute for the COVID-19 Genomics UK (COG-UK) consortium|Davies|United Kingdom
>Spike|hCoV-19/USA/TX-HMH-2928/2020|2020-06-09|EPI_ISL_543870|Original|hCoV-19^^Texas|Human|Houston Methodist Hospital|Houston Methodist Hospital|Long|USA
>Spike|hCoV-19/England/MILK-A7B534/2020|2020-10-15|EPI_ISL_607521|Original|hCoV-19^^England|Human|Lighthouse Lab in Milton Keynes|Wellcome Sanger Institute for the COVID-19 Genomics UK (COG-UK) consortium|Davies|United Kingdom
>Spike|hCoV-19/USA/NY-SURV0874/2020|2020-03-09|EPI_ISL_436076|Original|hCoV-19^^New York|Human|NYC Department of Health and Mental Hygiene|Pathogen Discovery|Queen|USA
>Spike|hCoV-19/England/NORW-EB2AB/2020|2020-05-03|EPI_ISL_457415|Original|hCoV-19^^England|Human|Quadram Institute Bioscience|COVID-19 Genomics UK (COG-UK) Consortium|Alikhan|United Kingdom
>Spike|hCoV-19/Costa Rica/INC-0179/2020|2020-11-16|EPI_ISL_770024|Original|hCoV-19^^Alajuela|Human|Hle - Asociacion Hogar De Ancianos Santiago Crespo Calvo|Inciensa|Soto-Garita|Costa Rica
>Spike|hCoV-19/Chile/RM-203885/2020|2020-10-10|EPI_ISL_746757|Original|hCoV-19^^Region Metropolitana de Santiago|Human|Genetica Molecular and Subdepartamento de Virologia ISP Chile|Instituto de Salud Publica de Chile|Tognarelli|Chile

>Spike|hCoV-19/USA/UT-UPHL-201109633/2020|2020-10-05|EPI_ISL_628937|Original|hCoV-
19^^Utah|Human|Utah Public Health Laboratory|Utah Public Health Laboratory|Young|USA
>Spike|hCoV-19/Kenya/C6379/2020|2020-05-15|EPI_ISL_568797|Original|hCoV-
19^^Mombasa|Human|KEMRI-Wellcome Trust Research Programme/KEMRI-CGMR-C Kilifi|KEMRI-
Wellcome Trust Research Programme/KEMRI-CGMR-C Kilifi|Githinji|Kenya
>Spike|hCoV-19/Scotland/QEUH-9B73F7/2020|2020-09-09|EPI_ISL_602089|Original|hCoV-
19^^Scotland|Human|Lighthouse Lab in Glasgow|Wellcome Sanger Institute for the COVID-
19 Genomics UK (COG-UK) consortium|Davies|United Kingdom
>Spike|hCoV-19/Portugal/PT0630/2020|2020-03-24|EPI_ISL_510891|Original|hCoV-
19^^Portugal|Human|Instituto Nacional de Saude (INSA)|Instituto Nacional de Saude
(INSA)|Borges|Portugal
>Spike|hCoV-19/Australia/WA435/2020|2020-09-25|EPI_ISL_672647|Original|hCoV-
19^^Western Australia|Human|PathWest Laboratory Medicine WA|PathWest Laboratory
Medicine WA Microbial Surveillance Unit|Sikazwe|Australia
>Spike|hCoV-19/England/ALDP-B46E81/2020|2020-11-07|EPI_ISL_656129|Original|hCoV-
19^^England|Human|Lighthouse Lab in Alderley Park|Wellcome Sanger Institute for the
COVID-19 Genomics UK (COG-UK) Consortium|Davies|United Kingdom
>Spike|hCoV-19/Panama/338832/2020|2020-04-09|EPI_ISL_496886|Original|hCoV-19^^Panama
Center|Human|Gorgas Memorial Laboratory of Health Studies|Gorgas Memorial Laboratory
of Health Studies|Martinez|Panama
>Spike|hCoV-19/USA/TX-HMH-7013/2020|2020-06-28|EPI_ISL_546432|Original|hCoV-
19^^Texas|Human|Houston Methodist Hospital|Houston Methodist Hospital|Long|USA
>Spike|hCoV-19/Denmark/DCGC-5856/2020|2020-09-28|EPI_ISL_621043|Original|hCoV-
19^^Midtjylland|Human|Department of Virus and Microbiological Special
Diagnostics|Albertsen lab|Michaelsen|Denmark
>Spike|hCoV-19/Scotland/CVR2517/2020|2020-04-15|EPI_ISL_477960|Original|hCoV-
19^^Scotland|Human|West of Scotland Specialist Virology Centre|COVID-19 Genomics UK
(COG-UK) Consortium|Robertson|United Kingdom
>Spike|hCoV-19/England/ALDP-AA219A/2020|2020-10-22|EPI_ISL_625052|Original|hCoV-
19^^England|Human|Lighthouse Lab in Alderley Park|Wellcome Sanger Institute for the
COVID-19 Genomics UK (COG-UK) consortium|Davies|United Kingdom
>Spike|hCoV-19/DRC/3504/2020|2020-04-23|EPI_ISL_447604|Original|hCoV-
19^^Kinshasa|Human|Viral Respiratory Lab|Pathogen Sequencing Lab|Lusamaki|Democratic
Republic of the Congo
>Spike|hCoV-19/USA/MI-MDHHS-SC22647/2020|2020-11-30|EPI_ISL_737198|Original|hCoV-
19^^Michigan|Human|Michigan Department of Health and Human Services|Michigan
Department of Health and Human Services|Blankenship|USA
>Spike|hCoV-19/Belgium/ULG-10405/2020|2020-08-20|EPI_ISL_540572|Original|hCoV-
19^^Li\u00e8ge|Human|Department of Clinical Microbiology|GIGA Medical
Genomics|Durkin|Belgium
>Spike|hCoV-19/Switzerland/ZH-ETHZ-362/2020|2020-04-07|EPI_ISL_483668|Original|hCoV-
19^^Zurich|Human|University Hospital Zurich|Department of Biosystems Science and
Engineering|Nadeau|Switzerland
>Spike|hCoV-19/England/MILK-A46B6D/2020|2020-10-06|EPI_ISL_598461|Original|hCoV-
19^^England|Human|Lighthouse Lab in Milton Keynes|Wellcome Sanger Institute for the
COVID-19 Genomics UK (COG-UK) consortium|Davies|United Kingdom
>Spike|hCoV-19/Scotland/EDB1272/2020|2020-04-09|EPI_ISL_491675|Original|hCoV-
19^^Scotland|Human|Virology Department|Wellcome Sanger Institute for the COVID-19
Genomics UK (COG-UK) consortium|Davies|United Kingdom
>Spike|hCoV-19/England/NORT-2819DD/2020|2020-03-21|EPI_ISL_488815|Original|hCoV-
19^^England|Human|NU-OMICS DNA Sequencing research facility|Wellcome Sanger Institute
for the COVID-19 Genomics UK (COG-UK) consortium|Davies|United Kingdom
>Spike|hCoV-19/South Africa/KRISP-K001100/2020|2020-07-
10|EPI_ISL_509349|Original|hCoV-19^^KwaZulu-Natal|Human|NHLS-IALCH|KRISP|de
Oliveira|South Africa
>Spike|hCoV-19/Denmark/DCGC-16447/2020|2020-11-16|EPI_ISL_714152|Original|hCoV-
19^^Hovedstaden|Human|Department of Virus and Microbiological Special
Diagnostics|Albertsen Lab|Michaelsen|Denmark
>Spike|hCoV-19/England/GSTT-5745/2020|2020-03-23|EPI_ISL_560708|Original|hCoV-
19^^England|Human|Centre for Clinical Infection and Diagnostics Research and Genomics

Innovation Unit|Centre for Clinical Infection and Diagnostics Research and Genomics Innovation Unit|Awan|United Kingdom
>Spike|hCoV-19/USA/WI-UW-1593/2020|2020-09-18|EPI_ISL_605470|Original|hCoV-19^^Wisconsin|Human|University of Wisconsin-Madison AIDS Vaccine Research Laboratories|University of Wisconsin-Madison AIDS Vaccine Research Laboratories|Baczenas|USA
>Spike|hCoV-19/England/MILK-9EE852/2020|2020-09-23|EPI_ISL_580652|Original|hCoV-19^^England|Human|Lighthouse Lab in Milton Keynes|Wellcome Sanger Institute for the COVID-19 Genomics UK (COG-UK) consortium|Davies|United Kingdom
>Spike|hCoV-19/England/GSTT-265D4C0/2020|2020-03-19|EPI_ISL_483967|Original|hCoV-19^^England|Human|Centre for Clinical Infection and Diagnostics Research and Genomics Innovation Unit|COVID-19 Genomics UK (COG-UK) Consortium|Awan|United Kingdom
>Spike|hCoV-19/Israel/CVL-n4115/2020|2020-03-11|EPI_ISL_649106|Original|hCoV-19^^Israel|Human|Israel Central Virology laboratory|Israel Central Virology laboratory|Zuckerman|Israel
>Spike|hCoV-19/USA/FL-BPHL-1069/2020|2020-04-22|EPI_ISL_526555|Original|hCoV-19^^Florida|Human|Florida Bureau of Public Health Laboratories|Florida Bureau of Public Health Laboratories|Blanton|USA
>Spike|hCoV-19/Scotland/CVR3040/2020|2020-04-23|EPI_ISL_449183|Original|hCoV-19^^Scotland|Human|West of Scotland Specialist Virology Centre|COVID-19 Genomics UK (COG-UK) Consortium|Robertson|United Kingdom
>Spike|hCoV-19/USA/MI-QDX-1635/2020|2020-03-18|EPI_ISL_603962|Original|hCoV-19^^Michigan|Human|Quest Diagnostics|Quest Diagnostics|Kagan|USA
>Spike|hCoV-19/Romania/ROSV_8990/2020|2020-05-18|EPI_ISL_491090|Original|hCoV-19^^Suceava|Human|Suceava County Emergency Hospital|\Stefan cel Mare\ University Metagenomics Lab|Lobiuc|Romania
>Spike|hCoV-19/Japan/PG-2538/2020|2020-05-00|EPI_ISL_690673|Original|hCoV-19^^Japan|Human|Saitama Prefectural Institute of Public Health|Pathogen Genomics Center|Sekizuka|Japan
>Spike|hCoV-19/USA/TX-HMH-MCoV-15231/2020|2020-10-20|EPI_ISL_786715|Original|hCoV-19^^Texas|Human|Houston Methodist Hospital|Houston Methodist Hospital|Long|USA
>Spike|hCoV-19/USA/UT-08404/2020|2020-05-13|EPI_ISL_524183|Original|hCoV-19^^Utah|Human|Utah Public Health Laboratory|Utah Public Health Laboratory|Young|USA
>Spike|hCoV-19/England/ALDP-6BC3C8/2020|2020-07-02|EPI_ISL_557912|Original|hCoV-19^^England|Human|Lighthouse Lab in Alderley Park|Wellcome Sanger Institute for the COVID-19 Genomics UK (COG-UK) consortium|Davies|United Kingdom
>Spike|hCoV-19/England/QEUH-CC779B/2020|2020-12-21|EPI_ISL_760452|Original|hCoV-19^^England|Human|Lighthouse Lab in Glasgow|Wellcome Sanger Institute for the COVID-19 Genomics UK (COG-UK) Consortium|Davies|United Kingdom
>Spike|hCoV-19/Japan/PG-5491/2020|2020-08-00|EPI_ISL_690593|Original|hCoV-19^^Japan|Human|Pathogen Genomics Center|Pathogen Genomics Center|Sekizuka|Japan
>Spike|hCoV-19/USA/UT-08496/2020|2020-05-18|EPI_ISL_524263|Original|hCoV-19^^Utah|Human|Utah Public Health Laboratory|Utah Public Health Laboratory|Young|USA
>Spike|hCoV-19/England/QEUH-B4193A/2020|2020-11-08|EPI_ISL_658571|Original|hCoV-19^^England|Human|Lighthouse Lab in Glasgow|Wellcome Sanger Institute for the COVID-19 Genomics UK (COG-UK) Consortium|Davies|United Kingdom
>Spike|hCoV-19/India/GJ-GBRC166a/2020|2020-05-27|EPI_ISL_461501|Original|hCoV-19^^Gujarat|Human|B.J. Medical College and Civil hospital|Gujarat Biotechnology Research Centre|Joshi|India
>Spike|hCoV-19/USA/TX-DSHS-0880/2020|2020-06-16|EPI_ISL_610016|Original|hCoV-19^^Texas|Human|Texas Department of State Health Services|Texas Department of State Health Services|Rahman|USA
>Spike|hCoV-19/Wales/PHWC-34513/2020|2020-04-16|EPI_ISL_446935|Original|hCoV-19^^Wales|Human|Wales Specialist Virology Centre|Public Health Wales Microbiology Cardiff|Moore|United Kingdom
>Spike|hCoV-19/Denmark/DCGC-12780/2020|2020-11-09|EPI_ISL_682961|Original|hCoV-19^^Nordjylland|Human|Department of Virus and Microbiological Special Diagnostics|Albertsen Lab|Michaelsen|Denmark

>Spike|hCoV-19/USA/NY-Wadsworth-289876-01/2020|2020-12-
22|EPI_ISL_767513|Original|hCoV-19^^New York|Human|Wadsworth Center|Wadsworth
Center|Lasek-Nesselquist|USA
>Spike|hCoV-19/England/ALDP-BC86CB/2020|2020-11-23|EPI_ISL_721413|Original|hCoV-
19^^England|Human|Lighthouse Lab in Alderley Park|Wellcome Sanger Institute for the
COVID-19 Genomics UK (COG-UK) Consortium|Davies|United Kingdom
>Spike|hCoV-19/Scotland/EDB2915/2020|2020-04-01|EPI_ISL_488897|Original|hCoV-
19^^Scotland|Human|Virology Department|Wellcome Sanger Institute for the COVID-19
Genomics UK (COG-UK) consortium|Davies|United Kingdom
>Spike|hCoV-19/India/OR-ILSCV35614/2020|2020-06-12|EPI_ISL_481203|Original|hCoV-
19^^Odisha|Human|Immunogenomics lab|Immunogenomics lab|Raghav|India
>Spike|hCoV-19/Australia/VIC8361/2020|2020-08-13|EPI_ISL_562666|Original|hCoV-
19^^Victoria|Human|Microbiological Diagnostic Unit - Public Health Laboratory (MDU-
PHL)|MDU-PHL|Schultz|Australia
>Spike|hCoV-19/Japan/PG-3262/2020|2020-06-00|EPI_ISL_691377|Original|hCoV-
19^^Japan|Human|Saitama Prefectural Institute of Public Health|Pathogen Genomics
Center|Sekizuka|Japan
>Spike|hCoV-19/England/LIVE-9B77E/2020|2020-04-23|EPI_ISL_440875|Original|hCoV-
19^^England|Human|Liverpool Clinical Laboratories|COVID-19 Genomics UK (COG-UK)
Consortium|Haldenby|United Kingdom
>Spike|hCoV-19/England/ALDP-A961F3/2020|2020-10-19|EPI_ISL_624634|Original|hCoV-
19^^England|Human|Lighthouse Lab in Alderley Park|Wellcome Sanger Institute for the
COVID-19 Genomics UK (COG-UK) consortium|Davies|United Kingdom
>Spike|hCoV-19/Ireland/WH-NVRL-78IRL74241/2020|2020-11-
23|EPI_ISL_752568|Original|hCoV-19^^Westmeath|Human|National Virus Reference
Laboratory|National Virus Reference Laboratory|Gonzalez|Ireland
>Spike|hCoV-19/Denmark/DCGC-18789/2020|2020-12-07|EPI_ISL_748557|Original|hCoV-
19^^Syddanmark|Human|Department of Virus and Microbiological Special
Diagnostics|Albertsen Lab|Michaelsen|Denmark
>Spike|hCoV-19/England/ALDP-A50526/2020|2020-10-07|EPI_ISL_597660|Original|hCoV-
19^^England|Human|Lighthouse Lab in Alderley Park|Wellcome Sanger Institute for the
COVID-19 Genomics UK (COG-UK) consortium|Davies|United Kingdom
>Spike|hCoV-19/England/ALDP-BCA526/2020|2020-11-23|EPI_ISL_703785|Original|hCoV-
19^^England|Human|Lighthouse Lab in Alderley Park|Wellcome Sanger Institute for the
COVID-19 Genomics UK (COG-UK) Consortium|Davies|United Kingdom
>Spike|hCoV-19/USA/MA-MGH-00906/2020|2020-04-17|EPI_ISL_791817|Original|hCoV-
19^^Massachusetts|Human|Massachusetts General Hospital|Infectious Disease
Program|Park|USA
>Spike|hCoV-19/England/ALDP-A6D0F8/2020|2020-10-14|EPI_ISL_608298|Original|hCoV-
19^^England|Human|Lighthouse Lab in Alderley Park|Wellcome Sanger Institute for the
COVID-19 Genomics UK (COG-UK) consortium|Davies|United Kingdom
>Spike|hCoV-19/England/QEUH-B6A670/2020|2020-11-11|EPI_ISL_662469|Original|hCoV-
19^^England|Human|Lighthouse Lab in Glasgow|Wellcome Sanger Institute for the COVID-
19 Genomics UK (COG-UK) Consortium|Davies|United Kingdom
>Spike|hCoV-19/England/205260306/2020|2020-12-07|EPI_ISL_766512|Original|hCoV-
19^^England|Human|Respiratory Virus Unit|COVID-19 Genomics UK (COG-UK)
Consortium|Platt|United Kingdom
>Spike|hCoV-19/Spain/AR-IBV-005098/2020|2020-04-25|EPI_ISL_500355|Original|hCoV-
19^^Aragon|Human|Servicio de Microbiolog\u00eda|SeqCOVID-SPAIN
consortium/IBV(CSIC)|Comas|Spain
>Spike|hCoV-19/USA/TX-HMH-MCoV-8617/2020|2020-07-01|EPI_ISL_780655|Original|hCoV-
19^^Texas|Human|Houston Methodist Hospital|Houston Methodist Hospital|Long|USA
>Spike|hCoV-19/England/ALDP-B49282/2020|2020-11-07|EPI_ISL_649481|Original|hCoV-
19^^England|Human|Lighthouse Lab in Alderley Park|Wellcome Sanger Institute for the
COVID-19 Genomics UK (COG-UK) Consortium|Davies|United Kingdom
>Spike|hCoV-19/Scotland/CVR491/2020|2020-03-23|EPI_ISL_433304|Original|hCoV-
19^^Scotland|Human|West of Scotland Specialist Virology Centre|COVID-19 Genomics UK
(COG-UK) Consortium|Robertson|United Kingdom
>Spike|hCoV-19/England/20172045804/2020|2020-04-09|EPI_ISL_465348|Original|hCoV-
19^^England|Human|Respiratory Virus Unit|Respiratory Virus Unit|Platt|United Kingdom

>Spike|hCoV-19/England/LIVE-AACC8/2020|2020-08-03|EPI_ISL_540610|Original|hCoV-19^^England|Human|Liverpool Clinical Laboratories|COVID-19 Genomics UK (COG-UK) Consortium|Haldenby|United Kingdom
>Spike|hCoV-19/Turkey/IST-GLAB-250/2020|2020-05-19|EPI_ISL_735316|Original|hCoV-19^^Istanbul|Human|Genomic Laboratory (GLAB) (Conjoint lab of Health Directorate of Istanbul and Istanbul Technical University)|Genomic Laboratory (GLAB)|Karacan|Turkey
>Spike|hCoV-19/Singapore/1452/2020|2020-12-02|EPI_ISL_728188|Original|hCoV-19^^Singapore|Human|National Public Health Laboratory|National Public Health Laboratory|Mak|Singapore
>Spike|hCoV-19/Switzerland/TI-ETHZ-100337/2020|2020-03-18|EPI_ISL_574965|Original|hCoV-19^^Ticino|Human|Viollier AG|Department of Biosystems Science and Engineering|Nadeau|Switzerland
>Spike|hCoV-19/Luxembourg/LNS4139779/2020|2020-11-18|EPI_ISL_740426|Original|hCoV-19^^Luxembourg|Human|Laboratoire national de sant\u00e9|Laboratoire national de sant\u00e9|Wienecke-Baldacchino|Luxembourg
>Spike|hCoV-19/Denmark/DCGC-12279/2020|2020-11-16|EPI_ISL_682460|Original|hCoV-19^^Midtjylland|Human|Department of Virus and Microbiological Special Diagnostics|Albertsen Lab|Michaelsen|Denmark
>Spike|hCoV-19/South Africa/NHLS-UCT-GS-2214/2020|2020-08-03|EPI_ISL_700466|Original|hCoV-19^^Western Cape|Human|Ladismith (Nissenville) Clinic wc LAF|NHLS/UCT|Iranzadeh|South Africa
>Spike|hCoV-19/Sweden/20-09859/2020|2020-11-04|EPI_ISL_710597|Original|hCoV-19^^Skane|Human|Klinisk mikrobiologi|The Public Health Agency of Sweden|Svartstr\u00f6m|Sweden
>Spike|hCoV-19/USA/UT-UPHL-201013892/2020|2020-04-05|EPI_ISL_594086|Original|hCoV-19^^Utah|Human|Utah Public Health Laboratory|Utah Public Health Laboratory|Young|USA
>Spike|hCoV-19/England/ALDP-9562DF/2020|2020-06-08|EPI_ISL_554046|Original|hCoV-19^^England|Human|Lighthouse Lab in Alderley Park|Wellcome Sanger Institute for the COVID-19 Genomics UK (COG-UK) consortium|Davies|United Kingdom
>Spike|hCoV-19/Denmark/DCGC-20298/2020|2020-11-23|EPI_ISL_748101|Original|hCoV-19^^Sjaelland|Human|Department of Virus and Microbiological Special Diagnostics|Albertsen Lab|Michaelsen|Denmark
>Spike|hCoV-19/South Africa/NHLS-UCT-GS-1990/2020|2020-08-25|EPI_ISL_640049|Original|hCoV-19^^Western Cape|Human|George Road Sat Clinic wc GWM|NHLS/UCT|Iranzadeh|South Africa
>Spike|hCoV-19/England/CAMC-C440AA/2020|2020-12-10|EPI_ISL_719509|Original|hCoV-19^^England|Human|Lighthouse Lab in Cambridge|Wellcome Sanger Institute for the COVID-19 Genomics UK (COG-UK) Consortium|Davies|United Kingdom
>Spike|hCoV-19/USA/UT-UPHL-2008872/2020|2020-05-26|EPI_ISL_524987|Original|hCoV-19^^Utah|Human|Utah Public Health Laboratory|Utah Public Health Laboratory|Young|USA
>Spike|hCoV-19/USA/MA-MGH-00502/2020|2020-03-27|EPI_ISL_460156|Original|hCoV-19^^Massachusetts|Human|Massachusetts General Hospital|Infectious Disease Program|Lemieux|USA
>Spike|hCoV-19/Iceland/11/2020|2020-03-16|EPI_ISL_417539|Original|hCoV-19^^Reykjavik|Human|deCODE genetics|deCODE genetics|J\u00f3nsson|Iceland
>Spike|hCoV-19/England/NOTT-111557/2020|2020-05-05|EPI_ISL_457579|Original|hCoV-19^^England|Human|Queens Medical Centre|COVID-19 Genomics UK (COG-UK) Consortium|Loose|United Kingdom
>Spike|hCoV-19/Netherlands/UT-RIVM-20066/2020|2020-11-24|EPI_ISL_723249|Orignal|hCoV-19^^Utrecht|Human|Dutch COVID-19 response team|National Institute for Public Health and the Environment (RIVM)|Meijer|Netherlands
>Spike|hCoV-19/England/BIRM-61D51/2020|2020-03-25|EPI_ISL_529607|Original|hCoV-19^^England|Human|University of Birmingham|COVID-19 Genomics UK (COG-UK) Consortium|Loman|United Kingdom
>Spike|hCoV-19/Iceland/378/2020|2020-03-19|EPI_ISL_424402|Original|hCoV-19^^Reykjavik|Human|The National University Hospital of Iceland|deCODE genetics|J\u00f3nsson|Iceland
>Spike|hCoV-19/England/CAMC-9EC54E/2020|2020-09-24|EPI_ISL_580286|Original|hCoV-19^^England|Human|Lighthouse Lab in Cambridge|Wellcome Sanger Institute for the COVID-19 Genomics UK (COG-UK) consortium|Davies|United Kingdom

>Spike|hCoV-19/England/CAMC-B337B9/2020|2020-11-05|EPI_ISL_659126|Original|hCoV-19^^England|Human|Lighthouse Lab in Cambridge|Wellcome Sanger Institute for the COVID-19 Genomics UK (COG-UK) Consortium|Davies|United Kingdom
>Spike|hCoV-19/England/MILK-A46A42/2020|2020-10-06|EPI_ISL_598355|Original|hCoV-19^^England|Human|Lighthouse Lab in Milton Keynes|Wellcome Sanger Institute for the COVID-19 Genomics UK (COG-UK) consortium|Davies|United Kingdom
>Spike|hCoV-19/England/QEUH-A00024/2020|2020-10-02|EPI_ISL_600354|Original|hCoV-19^^England|Human|Lighthouse Lab in Glasgow|Wellcome Sanger Institute for the COVID-19 Genomics UK (COG-UK) consortium|Davies|United Kingdom
>Spike|hCoV-19/Australia/VIC5537/2020|2020-07-20|EPI_ISL_518368|Original|hCoV-19^^Victoria|Human|Microbiological Diagnostic Unit - Public Health Laboratory (MDU-PHL)|MDU-PHL|Schultz|Australia
>Spike|hCoV-19/England/ALDP-B748F5/2020|2020-11-12|EPI_ISL_678957|Original|hCoV-19^^England|Human|Queens Medical Centre|COVID-19 Genomics UK (COG-UK) Consortium|Loose|United Kingdom
>Spike|hCoV-19/Germany/HH-hpi-p1502/2020|2020-03-05|EPI_ISL_776008|Original|hCoV-19^^Hamburg|Human|University Medical Center Hamburg Eppendorf|Heinrich Pette Institute|Robitaille|Germany
>Spike|hCoV-19/Denmark/DCGC-19107/2020|2020-12-07|EPI_ISL_748759|Original|hCoV-19^^Hovedstaden|Human|Department of Virus and Microbiological Special Diagnostics|Albertsen Lab|Michaelsen|Denmark
>Spike|hCoV-19/USA/TX-HMH-MCoV-18908/2020|2020-11-25|EPI_ISL_785213|Original|hCoV-19^^Texas|Human|Houston Methodist Hospital|Houston Methodist Hospital|Long|USA
>Spike|hCoV-19/Singapore/1116ilmn/2020|2020-10-26|EPI_ISL_626636|Original|hCoV-19^^Singapore|Human|National Public Health Laboratory|National Public Health Laboratory|Mak|Singapore
>Spike|hCoV-19/USA/TX-HMH-MCoV-13803/2020|2020-08-18|EPI_ISL_788896|Original|hCoV-19^^Texas|Human|Houston Methodist Hospital|Houston Methodist Hospital|Long|USA
>Spike|hCoV-19/Australia/QLDID928/2020|2020-03-19|EPI_ISL_418808|Original|hCoV-19^^Queensland|Human|Pathology Queensland|Public Health Virology Laboratory|Warrilow|Australia
>Spike|hCoV-19/Wales/PHWC-499ADD/2020|2020-12-05|EPI_ISL_727400|Original|hCoV-19^^Wales|Human|Originating lab|COVID-19 Genomics UK (COG-UK) Consortium|Moore|United Kingdom
>Spike|hCoV-19/Belgium/ULG-10577/2020|2020-10-23|EPI_ISL_626333|Original|hCoV-19^^Li\u00e8ge|Human|Department of Clinical Microbiology|GIGA Medical Genomics|Durkin|Belgium
>Spike|hCoV-19/India/MH-NIV-77268/2020|2020-06-23|EPI_ISL_541742|Original|hCoV-19^^Maharashtra|Human|National Institute of Virology|National Institute of Virology|Potdar|India
>Spike|hCoV-19/USA/TX-HMH-MCoV-11179/2020|2020-09-01|EPI_ISL_789725|Original|hCoV-19^^Texas|Human|Houston Methodist Hospital|Houston Methodist Hospital|Long|USA
>Spike|hCoV-19/USA/CT-Yale-065/2020|2020-04-02|EPI_ISL_428392|Original|hCoV-19^^Connecticut|Human|Yale COVID-19 Biorepository|Grubaugh Lab - Yale School of Public Health|Grubaugh|USA
>Spike|hCoV-19/England/CAMB-1AB25B/2020|2020-04-20|EPI_ISL_470201|Original|hCoV-19^^England|Human|Department of Pathology|Wellcome Sanger Institute for the COVID-19 Genomics UK (COG-UK) consortium|Davies|United Kingdom
>Spike|hCoV-19/England/MILK-A8EAC6/2020|2020-10-18|EPI_ISL_652112|Original|hCoV-19^^England|Human|Northumbria University / South Tees Hospitals NHS Foundation Trust / North Cumbria Integrated Care NHS Foundation Trust / North Tees and Hartlepool NHS Foundation Trust / Newcastle Hospitals NHS Foundation Trust|COVID-19 Genomics UK (COG-UK) Consortium|Bashton|United Kingdom
>Spike|hCoV-19/Wales/PHWC-16CFC8/2020|2020-09-18|EPI_ISL_572782|Original|hCoV-19^^Wales|Human|Wales Specialist Virology Centre Sequencing lab|COVID-19 Genomics UK (COG-UK) Consortium|Moore|United Kingdom
>Spike|hCoV-19/Denmark/DCGC-11231/2020|2020-11-02|EPI_ISL_670781|Original|hCoV-19^^Sjaelland|Human|Department of Virus and Microbiological Special Diagnostics|Albertsen Lab|Michaelsen|Denmark

>Spike|hCoV-19/USA/MI-UM-10035772246/2020|2020-09-02|EPI_ISL_583290|Original|hCoV-19^^Michigan|Human|University of Michigan Clinical Microbiology Laboratory|Lauring Lab|Valesano|USA
>Spike|hCoV-19/England/ALDP-9550F4/2020|2020-06-12|EPI_ISL_553544|Original|hCoV-19^^England|Human|Lighthouse Lab in Alderley Park|Wellcome Sanger Institute for the COVID-19 Genomics UK (COG-UK) consortium|Davies|United Kingdom
>Spike|hCoV-19/England/ALDP-9828F6/2020|2020-08-10|EPI_ISL_552618|Original|hCoV-19^^England|Human|Lighthouse Lab in Alderley Park|Wellcome Sanger Institute for the COVID-19 Genomics UK (COG-UK) consortium|Davies|United Kingdom
>Spike|hCoV-19/Japan/PG-3477/2020|2020-07-00|EPI_ISL_691587|Original|hCoV-19^^Japan|Human|Pathogen Genomics Center|Pathogen Genomics Center|Sekizuka|Japan
>Spike|hCoV-19/Denmark/DCGC-15018/2020|2020-11-23|EPI_ISL_713581|Original|hCoV-19^^Syddanmark|Human|Department of Virus and Microbiological Special Diagnostics|Albertsen Lab|Michaelsen|Denmark
>Spike|hCoV-19/England/QEUH-96BC42/2020|2020-08-13|EPI_ISL_531397|Original|hCoV-19^^England|Human|Lighthouse Lab in Glasgow|Wellcome Sanger Institute for the COVID-19 Genomics UK (COG-UK) consortium|Davies|United Kingdom
>Spike|hCoV-19/Slovenia/V0-1288/2020|2020-03-29|EPI_ISL_635269|Original|hCoV-19^^Slovenia|Human|Institute of Microbiology and Immunology|Institute of Microbiology and Immunology|Zakotnik|Slovenia
>Spike|hCoV-19/USA/UT-UPHL-2012388417/2020|2020-08-15|EPI_ISL_746278|Original|hCoV-19^^Utah|Human|Ginkgo Bioworks Clinical Laboratory|Utah Public Health Laboratory|Young|USA
>Spike|hCoV-19/Northern Ireland/QEUH-95FA29/2020|2020-08-06|EPI_ISL_532422|Original|hCoV-19^^Northern Ireland|Human|Lighthouse Lab in Glasgow|Wellcome Sanger Institute for the COVID-19 Genomics UK (COG-UK) consortium|Davies|United Kingdom
>Spike|hCoV-19/Luxembourg/LNS3865386/2020|2020-11-25|EPI_ISL_740385|Original|hCoV-19^^Luxembourg|Human|Laboratoire national de sant\u00e9|Laboratoire national de sant\u00e9|Wienecke-Baldacchino|Luxembourg
>Spike|hCoV-19/England/MILK-A7CADA/2020|2020-10-15|EPI_ISL_606352|Original|hCoV-19^^England|Human|Lighthouse Lab in Milton Keynes|Wellcome Sanger Institute for the COVID-19 Genomics UK (COG-UK) consortium|Davies|United Kingdom
>Spike|hCoV-19/USA/MI-MDHHS-SC20403/2020|2020-04-30|EPI_ISL_452277|Original|hCoV-19^^Michigan|Human|Michigan Department of Health and Human Services|Michigan Department of Health and Human Services|Blankenship|USA
>Spike|hCoV-19/England/QEUH-B2F7FC/2020|2020-11-04|EPI_ISL_646312|Original|hCoV-19^^England|Human|Lighthouse Lab in Glasgow|Wellcome Sanger Institute for the COVID-19 Genomics UK (COG-UK) Consortium|Davies|United Kingdom
>Spike|hCoV-19/USA/NY-NYUMC392/2020|2020-04-13|EPI_ISL_430419|Original|hCoV-19^^New York|Human|NYU Langone Health|Departments of Pathology and Medicine|Maurano|USA
>Spike|hCoV-19/Germany/BY-MVP-V2011675/2020|2020-03-16|EPI_ISL_420907|Original|hCoV-19^^Bavaria|Human|Max von Pettenkofer Institute|Laboratory for Functional Genome Analysis|Graf|Germany
>Spike|hCoV-19/Denmark/DCGC-14048/2020|2020-11-30|EPI_ISL_711235|Original|hCoV-19^^Midtjylland|Human|Department of Virus and Microbiological Special Diagnostics|Albertsen Lab|Michaelsen|Denmark
>Spike|hCoV-19/England/QEUH-9F821B/2020|2020-09-27|EPI_ISL_588955|Original|hCoV-19^^England|Human|Lighthouse Lab in Glasgow|Wellcome Sanger Institute for the COVID-19 Genomics UK (COG-UK) consortium|Davies|United Kingdom
>Spike|hCoV-19/USA/FL-BPHL-1364/2020|2020-07-17|EPI_ISL_541301|Original|hCoV-19^^Florida|Human|Florida Bureau of Public Health Laboratories|Florida Bureau of Public Health Laboratories|Blanton|USA
>Spike|hCoV-19/England/NOTT-10E432/2020|2020-03-22|EPI_ISL_425545|Original|hCoV-19^^England|Human|Queens Medical Centre|COVID-19 Genomics UK (COG-UK) Consortium|Loose|United Kingdom
>Spike|hCoV-19/Netherlands/ZH-RIVM-20737/2020|2020-12-23|EPI_ISL_790803|Original|hCoV-19^^Zuid-holland|Human|Dutch COVID-19 response team|National Institute for Public Health and the Environment (RIVM)|Meijer|Netherlands

>Spike|hCoV-19/USA/CA-CZB-14643/2020|2020-11-16|EPI_ISL_739568|Original|hCoV-19^^California|Human|Orange County Public Health Lab|Chan-Zuckerberg Biohub|Kamm|USA
>Spike|hCoV-19/England/CAMC-B5259C/2020|2020-11-08|EPI_ISL_656854|Original|hCoV-19^^England|Human|Lighthouse Lab in Cambridge|Wellcome Sanger Institute for the COVID-19 Genomics UK (COG-UK) Consortium|Davies|United Kingdom
>Spike|hCoV-19/Denmark/DCGC-6488/2020|2020-10-05|EPI_ISL_620388|Original|hCoV-19^^Midtjylland|Human|Department of Virus and Microbiological Special Diagnostics|Albertsen lab|Michaelsen|Denmark
>Spike|hCoV-19/Japan/PG-0074/2020|2020-02-27|EPI_ISL_479828|Original|hCoV-19^^Hokkaido|Human|Sapporo City Institute of Public Health|Pathogen Genomics Center|Sekizuka|Japan
>Spike|hCoV-19/England/QEUH-AD13D8/2020|2020-10-27|EPI_ISL_633923|Original|hCoV-19^^England|Human|Lighthouse Lab in Glasgow|Wellcome Sanger Institute for the COVID-19 Genomics UK (COG-UK) consortium|Davies|United Kingdom
>Spike|hCoV-19/Denmark/DCGC-4598/2020|2020-09-21|EPI_ISL_621267|Original|hCoV-19^^Nordjylland|Human|Department of Virus and Microbiological Special Diagnostics|Albertsen lab|Michaelsen|Denmark
>Spike|hCoV-19/England/ALDP-AA09C1/2020|2020-10-22|EPI_ISL_630920|Original|hCoV-19^^England|Human|Lighthouse Lab in Alderley Park|Wellcome Sanger Institute for the COVID-19 Genomics UK (COG-UK) consortium|Davies|United Kingdom
>Spike|hCoV-19/USA/WI-GMF-24108/2020|2020-10-05|EPI_ISL_660891|Original|hCoV-19^^Wisconsin|Human|Gundersen Molecular Diagnostics Laboratory|Kabara Cancer Research Institute|Kenny|USA
>Spike|hCoV-19/England/ALDP-9EDFC7/2020|2020-05-06|EPI_ISL_589455|Original|hCoV-19^^England|Human|Lighthouse Lab in Alderley Park|Wellcome Sanger Institute for the COVID-19 Genomics UK (COG-UK) consortium|Davies|United Kingdom
>Spike|hCoV-19/England/CAMC-A64ED9/2020|2020-10-11|EPI_ISL_608666|Original|hCoV-19^^England|Human|Lighthouse Lab in Cambridge|Wellcome Sanger Institute for the COVID-19 Genomics UK (COG-UK) consortium|Davies|United Kingdom
>Spike|hCoV-19/Denmark/DCGC-2057/2020|2020-07-13|EPI_ISL_618292|Original|hCoV-19^^Hovedstaden|Human|Department of Virus and Microbiological Special Diagnostics|Albertsen lab|Michaelsen|Denmark
>Spike|hCoV-19/Australia/VIC12368/2020|2020-07-28|EPI_ISL_591969|Original|hCoV-19^^Victoria|Human|Microbiological Diagnostic Unit - Public Health Laboratory (MDU-PHL)|MDU-PHL|Schultz|Australia
>Spike|hCoV-19/Spain/CL-IBV-97006322/2020|2020-05-08|EPI_ISL_537681|Original|hCoV-19^^Castilla y Leon|Human|Universidad de Le\u00f3n|SeqCOVID-SPAIN consortium/IBV(CSIC)|Comas|Spain
>Spike|hCoV-19/England/SHEF-C291E/2020|2020-04-08|EPI_ISL_441932|Original|hCoV-19^^England|Human|Virology Department|COVID-19 Genomics UK (COG-UK) Consortium|de Silva|United Kingdom
>Spike|hCoV-19/USA/AZ-TG648701/2020|2020-04-13|EPI_ISL_695897|Original|hCoV-19^^Arizona|Human|Sonora Quest Laboratories|TGen North|French|USA
>Spike|hCoV-19/Senegal/139/2020|2020-03-13|EPI_ISL_418217|Original|hCoV-19^^Thies|Human|Institut Pasteur Dakar|Institut Pasteur de Dakar|Dia|Senegal
>Spike|hCoV-19/DRC/16133/2020|2020-06-03|EPI_ISL_591083|Original|hCoV-19^^Kinshasa|Human|Viral Respiratory Lab|Pathogen Sequencing Lab|Lokilo|Democratic Republic of the Congo
>Spike|hCoV-19/England/MILK-ACF267/2020|2020-10-22|EPI_ISL_629740|Original|hCoV-19^^England|Human|Lighthouse Lab in Milton Keynes|Wellcome Sanger Institute for the COVID-19 Genomics UK (COG-UK) consortium|Davies|United Kingdom
>Spike|hCoV-19/Scotland/EDB3373/2020|2020-04-08|EPI_ISL_487700|Original|hCoV-19^^Scotland|Human|Virology Department|Wellcome Sanger Institute for the COVID-19 Genomics UK (COG-UK) consortium|Davies|United Kingdom
>Spike|hCoV-19/Japan/PG-2064/2020|2020-04-00|EPI_ISL_685669|Original|hCoV-19^^Japan|Human|Tokyo Metropolitan Institute of Public Health|Pathogen Genomics Center|Sekizuka|Japan
>Spike|hCoV-19/England/CAMC-B9998E/2020|2020-11-15|EPI_ISL_673830|Original|hCoV-19^^England|Human|Lighthouse Lab in Cambridge|Wellcome Sanger Institute for the COVID-19 Genomics UK (COG-UK) Consortium|Davies|United Kingdom

>Spike|hCoV-19/USA/AZ-TG640858/2020|2020-04-27|EPI_ISL_695798|Original|hCoV-
19^^Arizona|Human|Sonora Quest Laboratories|TGen North|French|USA
>Spike|hCoV-19/England/MILK-A7DCC4/2020|2020-10-13|EPI_ISL_637861|Original|hCoV-
19^^England|Human|Oxford Viromics|COVID-19 Genomics UK (COG-UK)
Consortium|Golubchik|United Kingdom
>Spike|hCoV-19/USA/WA-S2310/2020|2020-06-11|EPI_ISL_496942|Original|hCoV-
19^^Washington|Human|Washington State Department of Health|Seattle Flu Study|Lee|USA
>Spike|hCoV-19/South Korea/KCDC2541/2020|2020-06-21|EPI_ISL_514944|Original|hCoV-
19^^South Korea|Human|Division of Viral Diseases|Division of Viral Diseases|Kim|South
Korea
>Spike|hCoV-19/Denmark/ALAB-SSI263/2020|2020-03-10|EPI_ISL_429425|Original|hCoV-
19^^Denmark|Human|Department of Virus and Microbiological Special
Diagnostics|Albertsen lab|Kirkegaard|Denmark
>Spike|hCoV-19/England/MILK-9AACCF/2020|2020-09-01|EPI_ISL_550915|Original|hCoV-
19^^England|Human|Lighthouse Lab in Milton Keynes|Wellcome Sanger Institute for the
COVID-19 Genomics UK (COG-UK) consortium|Davies|United Kingdom
>Spike|hCoV-19/England/CAMB-1B7AAF/2020|2020-10-20|EPI_ISL_637433|Original|hCoV-
19^^England|Human|Department of Pathology|COVID-19 Genomics UK (COG-UK)
Consortium|Goodfellow|United Kingdom
>Spike|hCoV-19/USA/TX-DSHS-1655/2020|2020-06-24|EPI_ISL_710087|Original|hCoV-
19^^Texas|Human|Texas Department of State Health Services|Texas Department of State
Health Services|Rahman|USA
>Spike|hCoV-19/England/QEUH-9DC68A/2020|2020-09-21|EPI_ISL_580899|Original|hCoV-
19^^England|Human|Lighthouse Lab in Glasgow|Wellcome Sanger Institute for the COVID-
19 Genomics UK (COG-UK) consortium|Davies|United Kingdom
>Spike|hCoV-19/England/MILK-9A5699/2020|2020-09-03|EPI_ISL_550042|Original|hCoV-
19^^England|Human|Lighthouse Lab in Milton Keynes|Wellcome Sanger Institute for the
COVID-19 Genomics UK (COG-UK) consortium|Davies|United Kingdom
>Spike|hCoV-19/Wales/PHWC-165DC5/2020|2020-05-25|EPI_ISL_490802|Original|hCoV-
19^^Wales|Human|Wales Specialist Virology Centre Sequencing lab|COVID-19 Genomics UK
(COG-UK) Consortium|Moore|United Kingdom
>Spike|hCoV-19/Germany/HH-hpi-p2051/2020|2020-03-21|EPI_ISL_776340|Original|hCoV-
19^^Hamburg|Human|University Medical Center Hamburg Eppendorf|Heinrich Pette
Institute|Robitaille|Germany
>Spike|hCoV-19/USA/TX-HMH-4669/2020|2020-06-22|EPI_ISL_544242|Original|hCoV-
19^^Texas|Human|Houston Methodist Hospital|Houston Methodist Hospital|Long|USA
>Spike|hCoV-19/Japan/PG-6726/2020|2020-08-00|EPI_ISL_687837|Original|hCoV-
19^^Japan|Human|Pathogen Genomics Center|Pathogen Genomics Center|Sekizuka|Japan
>Spike|hCoV-19/Denmark/DCGC-16610/2020|2020-11-16|EPI_ISL_714376|Original|hCoV-
19^^Hovedstaden|Human|Department of Virus and Microbiological Special
Diagnostics|Albertsen Lab|Michaelsen|Denmark
>Spike|hCoV-19/England/ALDP-94F50D/2020|2020-06-18|EPI_ISL_555905|Original|hCoV-
19^^England|Human|Lighthouse Lab in Alderley Park|Wellcome Sanger Institute for the
COVID-19 Genomics UK (COG-UK) consortium|Davies|United Kingdom
>Spike|hCoV-19/USA/MA-MGH-00054/2020|2020-03-25|EPI_ISL_460143|Original|hCoV-
19^^Massachusetts|Human|Massachusetts General Hospital|Infectious Disease
Program|Lemieux|USA
>Spike|hCoV-19/USA/LA-EVTL1021/2020|2020-08-25|EPI_ISL_653712|Original|hCoV-
19^^Louisiana|Human|LSUHS Emerging Viral Threat Laboratory|Microbial Genome
Sequencing Center|Kamil|USA
>Spike|hCoV-19/Russia/OMS-ORINFI-4895S/2020|2020-05-03|EPI_ISL_569857|Original|hCoV-
19^^Omsk|Human|Omsk Research Institute of Natural Focal Infections|WHO National
Influenza Centre Russian Federation|Komissarov|Russia
>Spike|hCoV-19/England/QEUH-A4CFB6/2020|2020-10-05|EPI_ISL_596075|Original|hCoV-
19^^England|Human|Quadram Institute Bioscience|COVID-19 Genomics UK (COG-UK)
Consortium|Alikhan|United Kingdom
>Spike|hCoV-19/Canada/ON-S1834/2020|2020-11-24|EPI_ISL_729298|Original|hCoV-
19^^Ontario|Human|Toronto Invasive Bacterial Diseases Network|McMaster
University|McArthur|Canada

>Spike|hCoV-19/Latvia/223/2020|2020-09-14|EPI_ISL_770057|Original|hCoV-19^^Latvia|Human|E. Gulbja Laboratorija|Latvian Biomedical Research and Study Centre|Zrelovs|Latvia
>Spike|hCoV-19/USA/TX-HMH-2732/2020|2020-06-05|EPI_ISL_545511|Original|hCoV-19^^Texas|Human|Houston Methodist Hospital|Houston Methodist Hospital|Long|USA
>Spike|hCoV-19/England/ALDP-AA2604/2020|2020-10-22|EPI_ISL_630897|Original|hCoV-19^^England|Human|Lighthouse Lab in Alderley Park|Wellcome Sanger Institute for the COVID-19 Genomics UK (COG-UK) consortium|Davies|United Kingdom
>Spike|hCoV-19/USA/TX-HMH-MCoV-8543/2020|2020-07-01|EPI_ISL_780619|Original|hCoV-19^^Texas|Human|Houston Methodist Hospital|Houston Methodist Hospital|Long|USA
>Spike|hCoV-19/Switzerland/GE-ETHZ-310472/2020|2020-10-07|EPI_ISL_603543|Original|hCoV-19^^Geneva|Human|Viollier AG|Department of Biosystems Science and Engineering|Nadeau|Switzerland
>Spike|hCoV-19/USA/WI-UW-2497/2020|2020-12-30|EPI_ISL_779329|Original|hCoV-19^^Wisconsin|Human|University of Wisconsin-Madison AIDS Vaccine Research Laboratories|University of Wisconsin-Madison AIDS Vaccine Research Laboratories|Moreno|USA
>Spike|hCoV-19/England/MILK-B94947/2020|2020-11-14|EPI_ISL_673688|Original|hCoV-19^^England|Human|Lighthouse Lab in Milton Keynes|Wellcome Sanger Institute for the COVID-19 Genomics UK (COG-UK) Consortium|Davies|United Kingdom
>Spike|hCoV-19/Australia/NSW942/2020|2020-08-26|EPI_ISL_545014|Original|hCoV-19^^New South Wales|Human|South Eastern Area Laboratory Services (SEALS)|NSW Health Pathology - Institute of Clinical Pathology and Medical Research; Westmead Hospital; University of Sydney|Gall|Australia
>Spike|hCoV-19/USA/CA-SEARCH-5343/2020|2020-12-09|EPI_ISL_755160|Original|hCoV-19^^California|Human|UCSD EXCITE lab|Andersen lab at Scripps Research|Gangavarapu|USA
>Spike|hCoV-19/England/LOND-D5453/2020|2020-04-15|EPI_ISL_444222|Original|hCoV-19^^England|Human|University College London|COVID-19 Genomics UK (COG-UK) Consortium|Roy|United Kingdom
>Spike|hCoV-19/Wales/PHWC-48C4C0/2020|2020-11-08|EPI_ISL_666434|Original|hCoV-19^^Wales|Human|Wales Specialist Virology Centre Sequencing lab|COVID-19 Genomics UK (COG-UK) Consortium|Moore|United Kingdom
>Spike|hCoV-19/Denmark/DCGC-1445/2020|2020-04-27|EPI_ISL_622465|Original|hCoV-19^^Hovedstaden|Human|Department of Virus and Microbiological Special Diagnostics|Albertsen lab|Michaelsen|Denmark
>Spike|hCoV-19/England/SHEF-CE6A2/2020|2020-10-02|EPI_ISL_585069|Original|hCoV-19^^England|Human|Virology Department|COVID-19 Genomics UK (COG-UK) Consortium|de Silva|United Kingdom
>Spike|hCoV-19/England/NORW-EF984/2020|2020-09-22|EPI_ISL_572486|Original|hCoV-19^^England|Human|Quadram Institute Bioscience|COVID-19 Genomics UK (COG-UK) Consortium|Alikhan|United Kingdom
>Spike|hCoV-19/England/CAMC-9D6EE2/2020|2020-09-20|EPI_ISL_584335|Original|hCoV-19^^England|Human|Virology Department|COVID-19 Genomics UK (COG-UK) Consortium|de Silva|United Kingdom
>Spike|hCoV-19/England/QEUH-A31242/2020|2020-10-05|EPI_ISL_595872|Original|hCoV-19^^England|Human|Quadram Institute Bioscience|COVID-19 Genomics UK (COG-UK) Consortium|Alikhan|United Kingdom
>Spike|hCoV-19/Denmark/DCGC-3604/2020|2020-08-31|EPI_ISL_616247|Original|hCoV-19^^Syddanmark|Human|Department of Virus and Microbiological Special Diagnostics|Albertsen lab|Michaelsen|Denmark
>Spike|hCoV-19/USA/MI-QDX-932/2020|2020-03-22|EPI_ISL_571636|Original|hCoV-19^^Michigan|Human|Quest Diagnostics|Quest Diagnostics|Kagan|USA
>Spike|hCoV-19/Scotland/QEUH-A4BBFF/2020|2020-10-08|EPI_ISL_628516|Original|hCoV-19^^Scotland|Human|Oxford Viromics|COVID-19 Genomics UK (COG-UK) Consortium|Golubchik|United Kingdom
>Spike|hCoV-19/Kenya/C8288/2020|2020-05-19|EPI_ISL_568856|Original|hCoV-19^^Mombasa|Human|KEMRI-Wellcome Trust Research Programme/KEMRI-CGMR-C Kilifi|KEMRI-Wellcome Trust Research Programme/KEMRI-CGMR-C Kilifi|Githinji|Kenya

>Spike|hCoV-19/Wales/PHWC-266D5/2020|2020-03-27|EPI_ISL_422158|Original|hCoV-19^^Wales|Human|Wales Specialist Virology Centre|Public Health Wales Microbiology Cardiff|Moore|United Kingdom
>Spike|hCoV-19/England/CAMB-7CB3B/2020|2020-04-06|EPI_ISL_442082|Original|hCoV-19^^England|Human|Department of Pathology|Wellcome Sanger Institute for the COVID-19 Genomics UK (COG-UK) consortium|Amato|United Kingdom
>Spike|hCoV-19/England/PORT-2D3CC4/2020|2020-04-21|EPI_ISL_499321|Original|hCoV-19^^England|Human|Centre for Enzyme Innovation|COVID-19 Genomics UK (COG-UK) Consortium|Robson|United Kingdom
>Spike|hCoV-19/England/ALDP-A870E9/2020|2020-10-17|EPI_ISL_606056|Original|hCoV-19^^England|Human|Lighthouse Lab in Alderley Park|Wellcome Sanger Institute for the COVID-19 Genomics UK (COG-UK) consortium|Davies|United Kingdom
>Spike|hCoV-19/USA/NY-NYCPHL-000412/2020|2020-03-27|EPI_ISL_631896|Original|hCoV-19^^New York|Human|Jamaica Hospital Medical Center|New York City Public Health Laboratory|Wang|USA
>Spike|hCoV-19/Luxembourg/LNS7593998/2020|2020-10-19|EPI_ISL_744595|Original|hCoV-19^^Luxembourg|Human|Laboratoire national de sant\u00e9|Laboratoire national de sant\u00e9|Wienecke-Baldacchino|Luxembourg
>Spike|hCoV-19/England/ALDP-B25064/2020|2020-11-04|EPI_ISL_646513|Original|hCoV-19^^England|Human|Lighthouse Lab in Alderley Park|Wellcome Sanger Institute for the COVID-19 Genomics UK (COG-UK) Consortium|Davies|United Kingdom
>Spike|hCoV-19/England/QEUH-AF4946/2020|2020-10-30|EPI_ISL_663121|Original|hCoV-19^^England|Human|Lighthouse Lab in Glasgow|Wellcome Sanger Institute for the COVID-19 Genomics UK (COG-UK) Consortium|Davies|United Kingdom
>Spike|hCoV-19/England/CAMC-AEB1A4/2020|2020-10-26|EPI_ISL_633223|Original|hCoV-19^^England|Human|Lighthouse Lab in Cambridge|Wellcome Sanger Institute for the COVID-19 Genomics UK (COG-UK) consortium|Davies|United Kingdom
>Spike|hCoV-19/England/20139068504/2020|2020-03-29|EPI_ISL_423357|Original|hCoV-19^^England|Human|Respiratory Virus Unit|Respiratory Virus Unit|Platt|United Kingdom
>Spike|hCoV-19/Australia/VIC3288/2020|2020-07-08|EPI_ISL_520967|Original|hCoV-19^^Victoria|Human|Microbiological Diagnostic Unit - Public Health Laboratory (MDU-PHL)|MDU-PHL|Schultz|Australia
>Spike|hCoV-19/India/MH-NIV-229715/2020|2020-09-29|EPI_ISL_577698|Original|hCoV-19^^Maharashtra|Human|NIV Influenza|NIV Influenza|Potdar|India
>Spike|hCoV-19/England/ALDP-9E9936/2020|2020-09-24|EPI_ISL_584266|Original|hCoV-19^^England|Human|Virology Department|COVID-19 Genomics UK (COG-UK) Consortium|de Silva|United Kingdom
>Spike|hCoV-19/Spain/EX-IBV-004991/2020|2020-05-08|EPI_ISL_510306|Original|hCoV-19^^Extremadura|Human|Hospital San Pedro de Alc\u00e1ntara (C\u00e1ceres)|SeqCOVID-SPAIN consortium/IBV(CSIC)|Comas|Spain
>Spike|hCoV-19/Singapore/390/2020|2020-06-06|EPI_ISL_469139|Original|hCoV-19^^Singapore|Human|National Public Health Laboratory|National Public Health Laboratory|Mak|Singapore
>Spike|hCoV-19/Belgium/ULG-10285/2020|2020-07-23|EPI_ISL_540452|Original|hCoV-19^^Li\u00e8ge|Human|University of Li\u00e8ge COVID-19 testing center|GIGA Medical Genomics|Durkin|Belgium
>Spike|hCoV-19/Ecuador/UEES-7352/2020|2020-00-00|EPI_ISL_671985|Original|hCoV-19^^Ecuador|Human|UEES BioLab|Omics Sciences Laboratory|Fernandez Cadena|Ecuador
>Spike|hCoV-19/England/MILK-97114A/2020|2020-08-17|EPI_ISL_553216|Original|hCoV-19^^England|Human|Lighthouse Lab in Milton Keynes|Wellcome Sanger Institute for the COVID-19 Genomics UK (COG-UK) consortium|Davies|United Kingdom
>Spike|hCoV-19/England/CAMC-B325B0/2020|2020-11-05|EPI_ISL_659237|Original|hCoV-19^^England|Human|Lighthouse Lab in Cambridge|Wellcome Sanger Institute for the COVID-19 Genomics UK (COG-UK) Consortium|Davies|United Kingdom
>Spike|hCoV-19/England/OXON-B2A4B/2020|2020-04-06|EPI_ISL_534788|Original|hCoV-19^^England|Human|Oxford Viromics|COVID-19 Genomics UK (COG-UK) Consortium|Golubchik|United Kingdom
>Spike|hCoV-19/Australia/VIC6038/2020|2020-07-24|EPI_ISL_518079|Original|hCoV-19^^Victoria|Human|Microbiological Diagnostic Unit - Public Health Laboratory (MDU-PHL)|MDU-PHL|Schultz|Australia

>Spike|hCoV-19/USA/FL-BPHL-2191/2020|2020-07-21|EPI_ISL_653344|Original|hCoV-19^^Florida|Human|Florida Bureau of Public Health Laboratories|Florida Bureau of Public Health Laboratories|Blanton|USA
>Spike|hCoV-19/England/LOND-DA588/2020|2020-11-16|EPI_ISL_724641|Original|hCoV-19^^England|Human|University College London|COVID-19 Genomics UK (COG-UK) Consortium|Roy|United Kingdom
>Spike|hCoV-19/England/QEUH-96DCE6/2020|2020-08-20|EPI_ISL_530724|Original|hCoV-19^^England|Human|Lighthouse Lab in Glasgow|Wellcome Sanger Institute for the COVID-19 Genomics UK (COG-UK) consortium|Davies|United Kingdom
>Spike|hCoV-19/Wales/PHWC-342B2/2020|2020-04-20|EPI_ISL_446897|Original|hCoV-19^^Wales|Human|Wales Specialist Virology Centre|Public Health Wales Microbiology Cardiff|Moore|United Kingdom
>Spike|hCoV-19/USA/CA-ALSR-3337/2020|2020-09-17|EPI_ISL_635969|Original|hCoV-19^^California|Human|San Diego County Public Health Laboratory|Andersen lab at Scripps Research|Gangavarapu|USA
>Spike|hCoV-19/USA/CA-IGI-0235/2020|2020-07-02|EPI_ISL_569904|Original|hCoV-19^^California|Human|Innovative Genomics Institute|Innovative Genomics Institute|Wyman|USA
>Spike|hCoV-19/Denmark/DCGC-18032/2020|2020-12-14|EPI_ISL_749714|Original|hCoV-19^^Syddanmark|Human|Department of Virus and Microbiological Special Diagnostics|Albertsen Lab|Michaelsen|Denmark
>Spike|hCoV-19/Wales/PHWC-16E737/2020|2020-10-01|EPI_ISL_585762|Original|hCoV-19^^Wales|Human|Wales Specialist Virology Centre Sequencing lab|COVID-19 Genomics UK (COG-UK) Consortium|Moore|United Kingdom
>Spike|hCoV-19/Chile/RM-127204/2020|2020-07-17|EPI_ISL_746597|Original|hCoV-19^^Region Metropolitana de Santiago|Human|Genetica Molecular and Subdepartamento de Virologia ISP Chile|Instituto de Salud Publica de Chile|Tognarelli|Chile
>Spike|hCoV-19/Wales/PHWC-494656/2020|2020-11-20|EPI_ISL_726148|Original|hCoV-19^^Wales|Human|Originating lab|COVID-19 Genomics UK (COG-UK) Consortium|Moore|United Kingdom
>Spike|hCoV-19/Luxembourg/LNS7948422/2020|2020-10-19|EPI_ISL_744660|Original|hCoV-19^^Luxembourg|Human|Laboratoire national de sant\u00e9|Laboratoire national de sant\u00e9|Wienecke-Baldacchino|Luxembourg
>Spike|hCoV-19/USA/WA-S619/2020|2020-04-26|EPI_ISL_438172|Original|hCoV-19^^Washington|Human|Seattle Flu Study|Seattle Flu Study|Bedford|USA
>Spike|hCoV-19/England/QEUH-B6801A/2020|2020-11-11|EPI_ISL_662188|Original|hCoV-19^^England|Human|Lighthouse Lab in Glasgow|Wellcome Sanger Institute for the COVID-19 Genomics UK (COG-UK) Consortium|Davies|United Kingdom
>Spike|hCoV-19/USA/TX-HMH-4214/2020|2020-06-19|EPI_ISL_543444|Original|hCoV-19^^Texas|Human|Houston Methodist Hospital|Houston Methodist Hospital|Long|USA
>Spike|hCoV-19/Denmark/DCGC-17138/2020|2020-12-14|EPI_ISL_750780|Original|hCoV-19^^Midtjylland|Human|Department of Virus and Microbiological Special Diagnostics|Albertsen Lab|Michaelsen|Denmark
>Spike|hCoV-19/Israel/CVL-45199/2020|2020-12-03|EPI_ISL_745039|Original|hCoV-19^^Tel Aviv|Human|Israel Central Virology laboratory|Israel Central Virology laboratory|Zuckerman|Israel
>Spike|hCoV-19/England/CAMC-A58E50/2020|2020-10-09|EPI_ISL_597061|Original|hCoV-19^^England|Human|Lighthouse Lab in Cambridge|Wellcome Sanger Institute for the COVID-19 Genomics UK (COG-UK) consortium|Davies|United Kingdom
>Spike|hCoV-19/Scotland/EDB8483/2020|2020-10-18|EPI_ISL_651959|Original|hCoV-19^^Scotland|Human|Virology Department|COVID-19 Genomics UK (COG-UK) Consortium|McHugh|United Kingdom
>Spike|hCoV-19/Luxembourg/LNS7510950/2020|2020-11-20|EPI_ISL_744580|Original|hCoV-19^^Luxembourg|Human|Laboratoire national de sant\u00e9|Laboratoire national de sant\u00e9|Wienecke-Baldacchino|Luxembourg
>Spike|hCoV-19/Denmark/DCGC-21900/2020|2020-12-21|EPI_ISL_758442|Original|hCoV-19^^Hovedstaden|Human|Department of Virus and Microbiological Special Diagnostics|Albertsen Lab|Michaelsen|Denmark

>Spike|hCoV-19/Denmark/DCGC-11775/2020|2020-10-26|EPI_ISL_671129|Original|hCoV-19^^Sjaelland|Human|Department of Virus and Microbiological Special Diagnostics|Albertsen Lab|Michaelsen|Denmark
>Spike|hCoV-19/Wales/PHWC-4A1771/2020|2020-12-10|EPI_ISL_743721|Original|hCoV-19^^Wales|Human|Wales Specialist Virology Centre Sequencing lab|COVID-19 Genomics UK (COG-UK) Consortium|Moore|United Kingdom
>Spike|hCoV-19/Australia/VIC6385/2020|2020-07-24|EPI_ISL_521177|Original|hCoV-19^^Victoria|Human|Microbiological Diagnostic Unit - Public Health Laboratory (MDU-PHL)|MDU-PHL|Schultz|Australia
>Spike|hCoV-19/England/LIVE-99EB7/2020|2020-04-14|EPI_ISL_439824|Original|hCoV-19^^England|Human|Liverpool Clinical Laboratories|COVID-19 Genomics UK (COG-UK) Consortium|Haldenby|United Kingdom
>Spike|hCoV-19/Scotland/EDB2354/2020|2020-04-22|EPI_ISL_491613|Original|hCoV-19^^Scotland|Human|Virology Department|Wellcome Sanger Institute for the COVID-19 Genomics UK (COG-UK) consortium|Davies|United Kingdom
>Spike|hCoV-19/Australia/VIC8642/2020|2020-08-18|EPI_ISL_565126|Original|hCoV-19^^Victoria|Human|Microbiological Diagnostic Unit - Public Health Laboratory (MDU-PHL)|MDU-PHL|Schultz|Australia
>Spike|hCoV-19/USA/UT-01979/2020|2020-04-16|EPI_ISL_452166|Original|hCoV-19^^Utah|Human|Utah Public Health Laboratory|Utah Public Health Laboratory|Young|USA
>Spike|hCoV-19/Denmark/DCGC-7447/2020|2020-10-12|EPI_ISL_619331|Original|hCoV-19^^Hovedstaden|Human|Department of Virus and Microbiological Special Diagnostics|Albertsen lab|Michaelsen|Denmark
>Spike|hCoV-19/Denmark/DCGC-16140/2020|2020-11-16|EPI_ISL_714134|Original|hCoV-19^^Sjaelland|Human|Department of Virus and Microbiological Special Diagnostics|Albertsen Lab|Michaelsen|Denmark
>Spike|hCoV-19/England/QEUH-9F6435/2020|2020-09-25|EPI_ISL_588919|Original|hCoV-19^^England|Human|Lighthouse Lab in Glasgow|Wellcome Sanger Institute for the COVID-19 Genomics UK (COG-UK) consortium|Davies|United Kingdom
>Spike|hCoV-19/Scotland/QEUH-A1EA61/2020|2020-10-01|EPI_ISL_598855|Original|hCoV-19^^Scotland|Human|Lighthouse Lab in Glasgow|Wellcome Sanger Institute for the COVID-19 Genomics UK (COG-UK) consortium|Davies|United Kingdom
>Spike|hCoV-19/England/LOND-DAD62/2020|2020-11-08|EPI_ISL_724707|Original|hCoV-19^^England|Human|University College London|COVID-19 Genomics UK (COG-UK) Consortium|Roy|United Kingdom
>Spike|hCoV-19/England/LIVE-9E30E/2020|2020-04-03|EPI_ISL_453287|Original|hCoV-19^^England|Human|Liverpool Clinical Laboratories|COVID-19 Genomics UK (COG-UK) Consortium|Haldenby|United Kingdom
>Spike|hCoV-19/Sweden/20-53269/2020|2020-10-26|EPI_ISL_648161|Original|hCoV-19^^Sweden|Human|Sundsvall|The Public Health Agency of Sweden|Svartstr\u00f6m|Sweden
>Spike|hCoV-19/Australia/VIC1345/2020|2020-04-14|EPI_ISL_430708|Original|hCoV-19^^Victoria|Human|Victorian Infectious Diseases Reference Laboratory (VIDRL)|Microbiological Diagnostic Unit Public Health Laboratory and Victorian Infectious Diseases Reference Laboratory|Schultz|Australia
>Spike|hCoV-19/England/20142023804/2020|2020-03-29|EPI_ISL_423449|Original|hCoV-19^^England|Human|Respiratory Virus Unit|Respiratory Virus Unit|Platt|United Kingdom
>Spike|hCoV-19/England/ALDP-95166F/2020|2020-06-10|EPI_ISL_554449|Original|hCoV-19^^England|Human|Lighthouse Lab in Alderley Park|Wellcome Sanger Institute for the COVID-19 Genomics UK (COG-UK) consortium|Davies|United Kingdom
>Spike|hCoV-19/Denmark/DCGC-13082/2020|2020-11-30|EPI_ISL_712571|Original|hCoV-19^^Hovedstaden|Human|Department of Virus and Microbiological Special Diagnostics|Albertsen Lab|Michaelsen|Denmark
>Spike|hCoV-19/Japan/PG-6513/2020|2020-07-00|EPI_ISL_687627|Original|hCoV-19^^Japan|Human|Pathogen Genomics Center|Pathogen Genomics Center|Sekizuka|Japan
>Spike|hCoV-19/Austria/CeMM0078/2020|2020-03-24|EPI_ISL_437946|Original|hCoV-19^^Innsbruck|Human|Universitaetsklinik f\u00fcr Innere Medizin II Innsbruck|Bergthaler laboratory|Bergthaler|Austria
>Spike|hCoV-19/Russia/OMS-ORINFI-1639S/2020|2020-06-17|EPI_ISL_569842|Original|hCoV-19^^Omsk|Human|Omsk Research Institute of Natural Focal Infections|WHO National Influenza Centre Russian Federation|Komissarov|Russia

>Spike|hCoV-19/Belgium/ULG-10464/2020|2020-08-31|EPI_ISL_581635|Original|hCoV-19^^Li\u00e8ge|Human|Department of Clinical Microbiology|GIGA Medical Genomics|Durkin|Belgium
>Spike|hCoV-19/USA/UT-08524/2020|2020-05-18|EPI_ISL_524287|Original|hCoV-19^^Utah|Human|Utah Public Health Laboratory|Utah Public Health Laboratory|Young|USA
>Spike|hCoV-19/England/ALDP-B1A712/2020|2020-11-01|EPI_ISL_645804|Original|hCoV-19^^England|Human|Lighthouse Lab in Alderley Park|Wellcome Sanger Institute for the COVID-19 Genomics UK (COG-UK) Consortium|Davies|United Kingdom
>Spike|hCoV-19/England/ALDP-B5E28A/2020|2020-11-08|EPI_ISL_723646|Original|hCoV-19^^England|Human|Oxford Viromics|COVID-19 Genomics UK (COG-UK) Consortium|Golubchik|United Kingdom
>Spike|hCoV-19/England/ALDP-B753B7/2020|2020-11-12|EPI_ISL_664922|Original|hCoV-19^^England|Human|Quadram Institute Bioscience|COVID-19 Genomics UK (COG-UK) Consortium|Alikhan|United Kingdom
>Spike|hCoV-19/England/ALDP-B75144/2020|2020-11-12|EPI_ISL_662135|Original|hCoV-19^^England|Human|Lighthouse Lab in Alderley Park|Wellcome Sanger Institute for the COVID-19 Genomics UK (COG-UK) Consortium|Davies|United Kingdom
>Spike|hCoV-19/England/ALDP-AA5021/2020|2020-10-21|EPI_ISL_630882|Original|hCoV-19^^England|Human|Lighthouse Lab in Alderley Park|Wellcome Sanger Institute for the COVID-19 Genomics UK (COG-UK) consortium|Davies|United Kingdom
>Spike|hCoV-19/USA/TX-DSHS-0144/2020|2020-05-28|EPI_ISL_522970|Original|hCoV-19^^Texas|Human|Texas Department of State Health Services|Texas Department of State Health Services|Rahman|USA
>Spike|hCoV-19/England/QEUH-9B8EE6/2020|2020-09-07|EPI_ISL_549784|Original|hCoV-19^^England|Human|Lighthouse Lab in Glasgow|Wellcome Sanger Institute for the COVID-19 Genomics UK (COG-UK) Consortium|Davies|United Kingdom
>Spike|hCoV-19/England/QEUH-95DD04/2020|2020-08-07|EPI_ISL_537095|Original|hCoV-19^^England|Human|Lighthouse Lab in Glasgow|Wellcome Sanger Institute for the COVID-19 Genomics UK (COG-UK) consortium|Davies|United Kingdom
>Spike|hCoV-19/Denmark/DCGC-6142/2020|2020-09-21|EPI_ISL_621176|Original|hCoV-19^^Midtjylland|Human|Department of Virus and Microbiological Special Diagnostics|Albertsen lab|Michaelsen|Denmark
>Spike|hCoV-19/Japan/PG-0386/2020|2020-03-04|EPI_ISL_480055|Original|hCoV-19^^Chubu|Human|Nagoya City Public Health Research Institute|Pathogen Genomics Center|Sekizuka|Japan
>Spike|hCoV-19/England/QEUH-CC336E/2020|2020-12-21|EPI_ISL_760605|Original|hCoV-19^^England|Human|Lighthouse Lab in Glasgow|Wellcome Sanger Institute for the COVID-19 Genomics UK (COG-UK) Consortium|Davies|United Kingdom
>Spike|hCoV-19/Australia/VIC4585/2020|2020-07-12|EPI_ISL_518986|Original|hCoV-19^^Victoria|Human|Victorian Infectious Diseases Reference Laboratory (VIDRL)|VIDRL and MDU-PHL|Schultz|Australia
>Spike|hCoV-19/Vietnam/HCMC-35010/2020|2020-03-18|EPI_ISL_498186|unknown|hCoV-19^^Ho Chi Minh City|Human|OUCRU|OUCRU|Nguyen|Vietnam
>Spike|hCoV-19/Japan/PG-8172/2020|2020-07-00|EPI_ISL_687271|Original|hCoV-19^^Japan|Human|Pathogen Genomics Center|Pathogen Genomics Center|Sekizuka|Japan
>Spike|hCoV-19/India/MH-NIV-65813/2020|2020-06-16|EPI_ISL_479565|Original|hCoV-19^^Maharashtra|Human|NIV Influenza|NIV Influenza|Potdar|India
>Spike|hCoV-19/England/ALDP-A695FD/2020|2020-10-12|EPI_ISL_608998|Original|hCoV-19^^England|Human|Lighthouse Lab in Alderley Park|Wellcome Sanger Institute for the COVID-19 Genomics UK (COG-UK) consortium|Davies|United Kingdom
>Spike|hCoV-19/Canada/ON-UHTC_0381/2020|2020-11-24|EPI_ISL_698103|Original|hCoV-19^^Ontario|Human|Unity Health Toronto|Ontario Institute for Cancer Research|de Borja|Canada
>Spike|hCoV-19/Scotland/QEUH-96114D/2020|2020-08-05|EPI_ISL_532038|Original|hCoV-19^^Scotland|Human|NHSGGC West of Scotland Specialist Virology Centre / MRC-University of Glasgow Centre for Virus Research|Wellcome Sanger Institute for the COVID-19 Genomics UK (COG-UK) consortium|Davies|United Kingdom
>Spike|hCoV-19/England/ALDP-B1F56E/2020|2020-11-01|EPI_ISL_645957|Original|hCoV-19^^England|Human|Lighthouse Lab in Alderley Park|Wellcome Sanger Institute for the COVID-19 Genomics UK (COG-UK) Consortium|Davies|United Kingdom

>Spike|hCoV-19/England/ALDP-A5074E/2020|2020-10-07|EPI_ISL_597865|Original|hCoV-19^^England|Human|Lighthouse Lab in Alderley Park|Wellcome Sanger Institute for the COVID-19 Genomics UK (COG-UK) consortium|Davies|United Kingdom
>Spike|hCoV-19/USA/WA-S12/2020|2020-03-03|EPI_ISL_417065|Original|hCoV-19^^Washington|Human|Washington State Department of Health|Seattle Flu Study|Bedford|USA
>Spike|hCoV-19/Denmark/DCGC-8629/2020|2020-10-26|EPI_ISL_625782|Original|hCoV-19^^Nordjylland|Human|Department of Virus and Microbiological Special Diagnostics|Albertsen lab|Michaelsen|Denmark
>Spike|hCoV-19/India/UT-AR52/2020|2020-05-20|EPI_ISL_511918|Original|hCoV-19^^Uttarakhand|Human|All india institute of Medical Sciences Rishikesh|National Institute of Biomedical Genomics -\u00a0DBT's PAN-INDIA 1000 SARS--CoV-2 RNA Genome Sequencing Consortium|Maitra|India
>Spike|hCoV-19/England/NORW-E9C54/2020|2020-04-30|EPI_ISL_448357|Original|hCoV-19^^England|Human|Quadram Institute Bioscience|COVID-19 Genomics UK (COG-UK) Consortium|Alikhan|United Kingdom
>Spike|hCoV-19/Brazil/RJ-00465/2020|2020-07-06|EPI_ISL_717860|Original|hCoV-19^^Rio de Janeiro|Human|Laboratorio de Virologia Molecular / UFRJ|Bioinformatics Laboratory / LNCC|Almeida|Brazil
>Spike|hCoV-19/England/ALDP-B75EE3/2020|2020-11-12|EPI_ISL_723715|Original|hCoV-19^^England|Human|Oxford Viromics|COVID-19 Genomics UK (COG-UK) Consortium|Golubchik|United Kingdom
>Spike|hCoV-19/Ireland/LH-NVRL-20ILR11478/2020|2020-11-17|EPI_ISL_732505|Original|hCoV-19^^Louth|Human|National Virus Reference Laboratory|National Virus Reference Laboratory|Gonzalez|Ireland
>Spike|hCoV-19/USA/FL-BPHL-1693/2020|2020-08-17|EPI_ISL_594382|Original|hCoV-19^^Florida|Human|Florida Bureau of Public Health Laboratories|Florida Bureau of Public Health Laboratories|Blanton|USA
>Spike|hCoV-19/England/EXET-13A154/2020|2020-10-24|EPI_ISL_664423|Original|hCoV-19^^England|Human|University of Exeter|COVID-19 Genomics UK (COG-UK) Consortium|Temperton|United Kingdom
>Spike|hCoV-19/Australia/VIC15049/2020|2020-07-29|EPI_ISL_593123|Original|hCoV-19^^Victoria|Human|Microbiological Diagnostic Unit - Public Health Laboratory (MDU-PHL)|MDU-PHL|Schultz|Australia
>Spike|hCoV-19/Denmark/DCGC-9879/2020|2020-11-09|EPI_ISL_669249|Original|hCoV-19^^Sjaelland|Human|Department of Virus and Microbiological Special Diagnostics|Albertsen Lab|Michaelsen|Denmark
>Spike|hCoV-19/Australia/NSW823/2020|2020-08-07|EPI_ISL_526165|Original|hCoV-19^^New South Wales|Human|Pathology West - NSW Health Pathology|NSW Health Pathology - Institute of Clinical Pathology and Medical Research; Westmead Hospital; University of Sydney|Gall|Australia
>Spike|hCoV-19/Denmark/DCGC-2378/2020|2020-08-10|EPI_ISL_617558|Original|hCoV-19^^Hovedstaden|Human|Department of Virus and Microbiological Special Diagnostics|Albertsen lab|Michaelsen|Denmark
>Spike|hCoV-19/Japan/PG-4472/2020|2020-07-00|EPI_ISL_692695|Original|hCoV-19^^Japan|Human|Pathogen Genomics Center|Pathogen Genomics Center|Sekizuka|Japan
>Spike|hCoV-19/England/CAMC-AEA6A6/2020|2020-10-26|EPI_ISL_633236|Original|hCoV-19^^England|Human|Lighthouse Lab in Cambridge|Wellcome Sanger Institute for the COVID-19 Genomics UK (COG-UK) consortium|Davies|United Kingdom
>Spike|hCoV-19/England/NORT-2A4B92/2020|2020-09-17|EPI_ISL_573399|Original|hCoV-19^^England|Human|Northumbria University / South Tees Hospitals NHS Foundation Trust / North Cumbria Integrated Care NHS Foundation Trust / North Tees and Hartlepool NHS Foundation Trust / Newcastle Hospitals NHS Foundation Trust|COVID-19 Genomics UK (COG-UK) Consortium|Bashton|United Kingdom
>Spike|hCoV-19/Netherlands/NA_594/2020|2020-04-14|EPI_ISL_460757|Original|hCoV-19^^Netherlands|Human|Dutch COVID-19 response team|Erasmus Medical Center|Oude Munnink|Netherlands
>Spike|hCoV-19/Australia/VIC16742/2020|2020-08-08|EPI_ISL_663562|Original|hCoV-19^^Victoria|Human|Microbiological Diagnostic Unit - Public Health Laboratory (MDU-PHL)|MDU-PHL|Schultz|Australia

>Spike|hCoV-19/USA/CA-QDX-796/2020|2020-03-23|EPI_ISL_571490|Original|hCoV-19^^California|Human|Quest Diagnostics|Quest Diagnostics|Kagan|USA
>Spike|hCoV-19/USA/TX-HMH-MCoV-12973/2020|2020-08-28|EPI_ISL_788125|Original|hCoV-19^^Texas|Human|Houston Methodist Hospital|Houston Methodist Hospital|Long|USA
>Spike|hCoV-19/India/TG-CCMB-M841/2020|2020-06-12|EPI_ISL_495254|Original|hCoV-19^^Telangana|Human|CSIR-Centre for Cellular and Molecular Biology|CSIR-Centre for Cellular and Molecular Biology|Banu|India
>Spike|hCoV-19/USA/UT-QDX-49/2020|2020-03-13|EPI_ISL_467919|Original|hCoV-19^^Utah|Human|Quest Diagnostics|Quest Diagnostics|Kagan|USA
>Spike|hCoV-19/England/QEUH-9B8F5C/2020|2020-09-09|EPI_ISL_549739|Original|hCoV-19^^England|Human|Lighthouse Lab in Glasgow|Wellcome Sanger Institute for the COVID-19 Genomics UK (COG-UK) consortium|Davies|United Kingdom
>Spike|hCoV-19/USA/WI-GMF-00018/2020|2020-03-18|EPI_ISL_418184|Original|hCoV-19^^Wisconsin|Human|Gundersen Molecular Diagnostics Laboratory|Kabara Cancer Research Institute|Kenny|USA
>Spike|hCoV-19/England/LIVE-9EA09/2020|2020-04-11|EPI_ISL_453356|Original|hCoV-19^^England|Human|Liverpool Clinical Laboratories|COVID-19 Genomics UK (COG-UK) Consortium|Haldenby|United Kingdom
>Spike|hCoV-19/England/20110023706/2020|2020-03-08|EPI_ISL_417307|Original|hCoV-19^^England|Human|Respiratory Virus Unit|Respiratory Virus Unit|Platt|United Kingdom
>Spike|hCoV-19/England/QEUH-AF58B1/2020|2020-10-30|EPI_ISL_663058|Original|hCoV-19^^England|Human|Lighthouse Lab in Glasgow|Wellcome Sanger Institute for the COVID-19 Genomics UK (COG-UK) Consortium|Davies|United Kingdom
>Spike|hCoV-19/USA/CA-ALSR-4636/2020|2020-10-22|EPI_ISL_667049|Original|hCoV-19^^California|Human|San Diego County Public Health Laboratory|Andersen lab at Scripps Research|Gangavarapu|USA
>Spike|hCoV-19/Jordan/AM-ALSR-4960/2020|2020-10-03|EPI_ISL_730233|Original|hCoV-19^^Amman|Human|Biolab Diagnostic Laboratories|Andersen lab at Scripps Research|Gangavarapu|Jordan
>Spike|hCoV-19/England/QEUH-9621F1/2020|2020-08-10|EPI_ISL_531800|Original|hCoV-19^^England|Human|Lighthouse Lab in Glasgow|Wellcome Sanger Institute for the COVID-19 Genomics UK (COG-UK) consortium|Davies|United Kingdom
>Spike|hCoV-19/USA/WA-S2435/2020|2020-06-15|EPI_ISL_497067|Original|hCoV-19^^Washington|Human|Washington State Department of Health|Seattle Flu Study|Lee|USA
>Spike|hCoV-19/Scotland/CVR56/2020|2020-03-12|EPI_ISL_425785|Original|hCoV-19^^Scotland|Human|West of Scotland Specialist Virology Centre|COVID-19 Genomics UK (COG-UK) Consortium|Robertson|United Kingdom
>Spike|hCoV-19/USA/CA-CZB-2472/2020|2020-03-30|EPI_ISL_513876|Original|hCoV-19^^California|Human|San Francisco Public Health Laboratory|Chan-Zuckerberg Biohub|Kamm|USA
>Spike|hCoV-19/England/QEUH-BCE258/2020|2020-11-26|EPI_ISL_704249|Original|hCoV-19^^England|Human|Lighthouse Lab in Glasgow|Wellcome Sanger Institute for the COVID-19 Genomics UK (COG-UK) Consortium|Davies|United Kingdom
>Spike|hCoV-19/India/TG-CCMB-L1721/2020|2020-05-30|EPI_ISL_471625|Original|hCoV-19^^Telangana|Human|CSIR-Centre for Cellular and Molecular Biology|CSIR-Centre for Cellular and Molecular Biology|Banu|India
>Spike|hCoV-19/USA/CA-QDX-2068/2020|2020-03-17|EPI_ISL_604323|Original|hCoV-19^^California|Human|Quest Diagnostics|Quest Diagnostics|Kagan|USA
>Spike|hCoV-19/Brazil/MG-0212/2020|2020-03-14|EPI_ISL_470579|Original|hCoV-19^^Minas Gerais|Human|Hermes Pardini|Bioinformatics Laboratory / LNCC|Almeida|Brazil
>Spike|hCoV-19/USA/MN-MDH-1419/2020|2020-03-18|EPI_ISL_514638|Original|hCoV-19^^Minnesota|Human|Mayo Clinic & Mayo Clinic Laboratories|Minnesota Department of Health|Garfin|USA
>Spike|hCoV-19/USA/IL-UW-349/2020|2020-04-15|EPI_ISL_450703|Original|hCoV-19^^Illinois|Human|University of Wisconsin-Madison AIDS Vaccine Research Laboratories|University of Wisconsin-Madison AIDS Vaccine Research Laboratories|Baczenas|USA
>Spike|hCoV-19/USA/CA-ALSR-1049-SAN/2020|2020-06-21|EPI_ISL_483482|Original|hCoV-19^^California|Human|UC San Diego Center for Advanced Laboratory Medicine|Andersen lab at Scripps Research|Gangavarapu|USA

>Spike|hCoV-19/England/QEUH-A5E986/2020|2020-10-12|EPI_ISL_597408|Original|hCoV-19^^England|Human|Lighthouse Lab in Glasgow|Wellcome Sanger Institute for the COVID-19 Genomics UK (COG-UK) consortium|Davies|United Kingdom
>Spike|hCoV-19/Wales/PHWC-24ACC/2020|2020-03-20|EPI_ISL_419480|Original|hCoV-19^^Wales|Human|Wales Specialist Virology Centre|Public Health Wales Microbiology Cardiff|Moore|United Kingdom
>Spike|hCoV-19/Denmark/DCGC-10547/2020|2020-11-02|EPI_ISL_670044|Original|hCoV-19^^Syddanmark|Human|Department of Virus and Microbiological Special Diagnostics|Albertsen Lab|Michaelsen|Denmark
>Spike|hCoV-19/Wales/PHWC-47EB62/2020|2020-09-26|EPI_ISL_595559|Original|hCoV-19^^Wales|Human|Originating lab|COVID-19 Genomics UK (COG-UK) Consortium|Moore|United Kingdom
>Spike|hCoV-19/England/CAMC-AAFC41/2020|2020-10-22|EPI_ISL_623345|Original|hCoV-19^^England|Human|Lighthouse Lab in Cambridge|Wellcome Sanger Institute for the COVID-19 Genomics UK (COG-UK) consortium|Davies|United Kingdom
>Spike|hCoV-19/Japan/Donner4/2020|2020-04-06|EPI_ISL_438949|Original|hCoV-19^^Kanto|Human|Keio University School of Medicine|Keio University School of Medicine|Kosaki|Japan
>Spike|hCoV-19/England/MILK-975E9A/2020|2020-08-20|EPI_ISL_552738|Original|hCoV-19^^England|Human|Lighthouse Lab in Milton Keynes|Wellcome Sanger Institute for the COVID-19 Genomics UK (COG-UK) consortium|Davies|United Kingdom
>Spike|hCoV-19/USA/WA-UW-12796/2020|2020-06-29|EPI_ISL_570841|Original|hCoV-19^^Washington|Human|UW Virology Lab|UW Virology Lab|Roychoudhury|USA
>Spike|hCoV-19/England/NORT-1B8716A/2020|2020-10-27|EPI_ISL_724964|Original|hCoV-19^^England|Human|Northumbria University / South Tees Hospitals NHS Foundation Trust / North Cumbria Integrated Care NHS Foundation Trust / North Tees and Hartlepool NHS Foundation Trust / Newcastle Hospitals NHS Foundation Trust|COVID-19 Genomics UK (COG-UK) Consortium|Bashton|United Kingdom
>Spike|hCoV-19/England/QEUH-B3F4D4/2020|2020-11-08|EPI_ISL_658719|Original|hCoV-19^^England|Human|Lighthouse Lab in Glasgow|Wellcome Sanger Institute for the COVID-19 Genomics UK (COG-UK) Consortium|Davies|United Kingdom
>Spike|hCoV-19/Morocco/FMP-61/2020|2020-12-21|EPI_ISL_775263|Original|hCoV-19^^Rabat|Human|Laboratoire Biolife|Laboratoire de Biotechnologie|Ouadghiri|Morocco
>Spike|hCoV-19/Brazil/SP-L7-CD72/2020|2020-03-24|EPI_ISL_476439|Original|hCoV-19^^S\u00e3o Paulo|Human|Hospital da Cl\u00ednicas da Faculdade de Medicina da Universidade de S\u00e3o Paulo|Instituto de Medicina Tropical da Univesidade de S\u00e3o Paulo|Sabino|Brazil
>Spike|hCoV-19/Denmark/DCGC-9915/2020|2020-11-09|EPI_ISL_668492|Original|hCoV-19^^Hovedstaden|Human|Department of Virus and Microbiological Special Diagnostics|Albertsen Lab|Michaelsen|Denmark
>Spike|hCoV-19/USA/TX-HMH-5878/2020|2020-06-30|EPI_ISL_547081|Original|hCoV-19^^Texas|Human|Houston Methodist Hospital|Houston Methodist Hospital|Long|USA
>Spike|hCoV-19/USA/TX-HMH-870/2020|2020-04-08|EPI_ISL_545887|Original|hCoV-19^^Texas|Human|Houston Methodist Hospital|Houston Methodist Hospital|Long|USA
>Spike|hCoV-19/USA/VA-DCLS-0101/2020|2020-04-10|EPI_ISL_434595|Original|hCoV-19^^Virginia|Human|Virginia DCLS|Virginia DCLS|St. Jacques|USA
>Spike|hCoV-19/USA/NY-Wadsworth-37640-01/2020|2020-04-03|EPI_ISL_525652|Original|hCoV-19^^New York|Human|Wadsworth Center|Wadsworth Center|Plitnick|USA
>Spike|hCoV-19/Northern Ireland/QEUH-95EC06/2020|2020-08-08|EPI_ISL_532502|Original|hCoV-19^^Northern Ireland|Human|Lighthouse Lab in Glasgow|Wellcome Sanger Institute for the COVID-19 Genomics UK (COG-UK) consortium|Davies|United Kingdom
>Spike|hCoV-19/USA/UT-UPHL-2012942531/2020|2020-08-09|EPI_ISL_745802|Original|hCoV-19^^Utah|Human|Ginkgo Bioworks Clinical Laboratory|Utah Public Health Laboratory|Young|USA
>Spike|hCoV-19/Scotland/QEUH-AE2E79/2020|2020-10-27|EPI_ISL_649654|Original|hCoV-19^^Scotland|Human|Lighthouse Lab in Glasgow|Wellcome Sanger Institute for the COVID-19 Genomics UK (COG-UK) Consortium|Davies|United Kingdom

>Spike|hCoV-19/Singapore/455/2020|2020-03-19|EPI_ISL_475986|Original|hCoV-19^^Singapore|Human|National Public Health Laboratory|National Public Health Laboratory|Mak|Singapore
>Spike|hCoV-19/Wales/MILK-9D3FA6/2020|2020-09-18|EPI_ISL_601636|Original|hCoV-19^^Wales|Human|Lighthouse Lab in Milton Keynes|Wellcome Sanger Institute for the COVID-19 Genomics UK (COG-UK) consortium|Davies|United Kingdom
>Spike|hCoV-19/Denmark/DCGC-21417/2020|2020-12-21|EPI_ISL_758096|Original|hCoV-19^^Syddanmark|Human|Department of Virus and Microbiological Special Diagnostics|Albertsen Lab|Michaelsen|Denmark
>Spike|hCoV-19/England/CAMB-1AFCE0/2020|2020-05-04|EPI_ISL_459225|Original|hCoV-19^^England|Human|Department of Pathology|Wellcome Sanger Institute for the COVID-19 Genomics UK (COG-UK) consortium|Davies|United Kingdom
>Spike|hCoV-19/Wales/PHWC-2EDF6/2020|2020-04-07|EPI_ISL_446157|Original|hCoV-19^^Wales|Human|Wales Specialist Virology Centre|Public Health Wales Microbiology Cardiff|Moore|United Kingdom
>Spike|hCoV-19/Switzerland/SO-ETHZ-410345/2020|2020-12-17|EPI_ISL_737799|Original|hCoV-19^^Solothurn|Human|Viollier AG|Department of Biosystems Science and Engineering|Nadeau|Switzerland
>Spike|hCoV-19/USA/NY-Wadsworth-292006-01/2020|2020-00-00|EPI_ISL_767628|Original|hCoV-19^^New York|Human|WHITE PLAINS HOSPITAL CENTER LABORATORY|Wadsworth Center|Lasek-Nesselquist|USA
>Spike|hCoV-19/England/CAMB-1B5476/2020|2020-07-01|EPI_ISL_490328|Original|hCoV-19^^England|Human|Department of Pathology|COVID-19 Genomics UK (COG-UK) Consortium|Meredith|United Kingdom
>Spike|hCoV-19/USA/WI-GMF-19709/2020|2020-09-11|EPI_ISL_547838|Original|hCoV-19^^Wisconsin|Human|Gundersen Molecular Diagnostics Laboratory|Kabara Cancer Research Institute|Kenny|USA
>Spike|hCoV-19/England/204262879/2020|2020-10-15|EPI_ISL_649808|Original|hCoV-19^^England|Human|Respiratory Virus Unit|COVID-19 Genomics UK (COG-UK) Consortium|Platt|United Kingdom
>Spike|hCoV-19/Wales/PHWC-48F4BE/2020|2020-11-10|EPI_ISL_681100|Original|hCoV-19^^Wales|Human|Wales Specialist Virology Centre Sequencing lab|COVID-19 Genomics UK (COG-UK) Consortium|Moore|United Kingdom
>Spike|hCoV-19/England/20132068204/2020|2020-03-23|EPI_ISL_423796|Original|hCoV-19^^England|Human|Respiratory Virus Unit|Respiratory Virus Unit|Platt|United Kingdom
>Spike|hCoV-19/England/ALDP-A85801/2020|2020-10-17|EPI_ISL_610628|Original|hCoV-19^^England|Human|Lighthouse Lab in Alderley Park|Wellcome Sanger Institute for the COVID-19 Genomics UK (COG-UK) consortium|Davies|United Kingdom
>Spike|hCoV-19/Australia/NSW2158/2020|2020-03-12|EPI_ISL_500636|Original|hCoV-19^^New South Wales|Human|Area of Virology|Area of Virology|Bull|Australia
>Spike|hCoV-19/England/20118177604/2020|2020-03-13|EPI_ISL_464998|Original|hCoV-19^^England|Human|Respiratory Virus Unit|Respiratory Virus Unit|Platt|United Kingdom
>Spike|hCoV-19/Peru/LIM-UPCH-0101/2020|2020-06-17|EPI_ISL_540979|Original|hCoV-19^^Lima|Human|Laboratorio de Referencia Nacional de Virus Respiratorios|Laboratorio de Gen\u00f3mica Microbiana|Tsukayama|Peru
>Spike|hCoV-19/England/QEUH-B0DDA7/2020|2020-11-02|EPI_ISL_642090|Original|hCoV-19^^England|Human|Lighthouse Lab in Glasgow|Wellcome Sanger Institute for the COVID-19 Genomics UK (COG-UK) Consortium|Davies|United Kingdom
>Spike|hCoV-19/Wales/PHWC-4A2CF9/2020|2020-12-14|EPI_ISL_744007|Original|hCoV-19^^Wales|Human|Wales Specialist Virology Centre Sequencing lab|COVID-19 Genomics UK (COG-UK) Consortium|Moore|United Kingdom
>Spike|hCoV-19/SaudiArabia/KAUST-JEDDAH469/2020|2020-08-04|EPI_ISL_512924|Original|hCoV-19^^Jeddah|Human|Pathogen Genomics Lab King Abdullah University of Science and Technology(KAUST)|Pathogen Genomics Lab King Abdullah University of Science and Technology(KAUST)|Mohamed Ghazzali|Saudi Arabia
>Spike|hCoV-19/England/BRIS-1853E45/2020|2020-04-27|EPI_ISL_492421|Original|hCoV-19^^England|Human|PHE South West Regional Laboratory|Wellcome Sanger Institute for the COVID-19 Genomics UK (COG-UK) consortium|Davies|United Kingdom
>Spike|hCoV-19/England/LEED-2A8985/2020|2020-04-05|EPI_ISL_539026|Original|hCoV-19^^England|Human|Leeds Teaching Hospitals NHS Trust and Public Health

England|Wellcome Sanger Institute for the COVID-19 Genomics UK (COG-UK) consortium|Davies|United Kingdom
>Spike|hCoV-19/England/CAMC-B09FB4/2020|2020-10-30|EPI_ISL_673047|Original|hCoV-19^^England|Human|Lighthouse Lab in Cambridge|Wellcome Sanger Institute for the COVID-19 Genomics UK (COG-UK) Consortium|Davies|United Kingdom
>Spike|hCoV-19/Australia/VIC15957/2020|2020-07-31|EPI_ISL_640479|Original|hCoV-19^^Victoria|Human|Microbiological Diagnostic Unit - Public Health Laboratory (MDU-PHL)|MDU-PHL|Schultz|Australia
>Spike|hCoV-19/England/MILK-B54B98/2020|2020-11-05|EPI_ISL_657729|Original|hCoV-19^^England|Human|Lighthouse Lab in Milton Keynes|Wellcome Sanger Institute for the COVID-19 Genomics UK (COG-UK) Consortium|Davies|United Kingdom
>Spike|hCoV-19/England/MILK-BB0D1C/2020|2020-11-18|EPI_ISL_720291|Original|hCoV-19^^England|Human|Lighthouse Lab in Milton Keynes|Wellcome Sanger Institute for the COVID-19 Genomics UK (COG-UK) Consortium|Davies|United Kingdom
>Spike|hCoV-19/USA/WI-QDX-1077/2020|2020-03-18|EPI_ISL_571913|Original|hCoV-19^^Wisconsin|Human|Quest Diagnostics|Quest Diagnostics|Kagan|USA
>Spike|hCoV-19/England/MILK-A192D7/2020|2020-09-30|EPI_ISL_612499|Original|hCoV-19^^England|Human|Wales Specialist Virology Centre Sequencing lab|COVID-19 Genomics UK (COG-UK) Consortium|Moore|United Kingdom
>Spike|hCoV-19/Bahrain/341051813_S12_L001/2020|2020-11-22|EPI_ISL_678268|Original|hCoV-19^^Bahrain|Human|Communicable Disease Laboratory|Communicable Disease Laboratory|Altaif|Bahrain
>Spike|hCoV-19/USA/WI-WSLH-200028/2020|2020-04-18|EPI_ISL_471193|Original|hCoV-19^^Wisconsin|Human|Wisconsin State Laboratory of Hygiene Communicable Disease Division|Wisconsin State Laboratory of Hygiene Communicable Disease Division|Florek|USA
>Spike|hCoV-19/Wales/PHWC-49BBC9/2020|2020-12-06|EPI_ISL_742442|Original|hCoV-19^^Wales|Human|Wales Specialist Virology Centre Sequencing lab|COVID-19 Genomics UK (COG-UK) Consortium|Moore|United Kingdom
>Spike|hCoV-19/Denmark/DCGC-20278/2020|2020-11-30|EPI_ISL_748089|Original|hCoV-19^^Syddanmark|Human|Department of Virus and Microbiological Special Diagnostics|Albertsen Lab|Michaelsen|Denmark
>Spike|hCoV-19/Brazil/SP-698/2020|2020-06-30|EPI_ISL_693240|Original|hCoV-19^^S\u00e3o Paulo|Human|Centro de Vigil\u00e2ncia a Saude de Diadema|Instituto Adolfo Lutz|Sacchi|Brazil
>Spike|hCoV-19/Sweden/20-52078/2020|2020-06-15|EPI_ISL_510875|Original|hCoV-19^^Ostergotland|Human|Klinsisk mikrobiologi Linkoping|The Public Health Agency of Sweden|Svartstr\u00f6m|Sweden
>Spike|hCoV-19/England/CAMC-B0897B/2020|2020-10-29|EPI_ISL_643594|Original|hCoV-19^^England|Human|Lighthouse Lab in Cambridge|Wellcome Sanger Institute for the COVID-19 Genomics UK (COG-UK) Consortium|Davies|United Kingdom
>Spike|hCoV-19/Singapore/660/2020|2020-04-21|EPI_ISL_493420|Original|hCoV-19^^Singapore|Human|National Public Health Laboratory|National Public Health Laboratory|Mak|Singapore
>Spike|hCoV-19/England/QEUH-8930A0/2020|2020-07-27|EPI_ISL_532803|Original|hCoV-19^^England|Human|Lighthouse Lab in Glasgow|Wellcome Sanger Institute for the COVID-19 Genomics UK (COG-UK) consortium|Davies|United Kingdom
>Spike|hCoV-19/England/NORT-296C45/2020|2020-04-28|EPI_ISL_484275|Original|hCoV-19^^England|Human|Northumbria University / South Tees Hospitals NHS Foundation Trust / North Cumbria Integrated Care NHS Foundation Trust / North Tees and Hartlepool NHS Foundation Trust / Newcastle Hospitals NHS Foundation Trust|COVID-19 Genomics UK (COG-UK) Consortium|Bashton|United Kingdom
>Spike|hCoV-19/Australia/VIC4713/2020|2020-07-14|EPI_ISL_519101|Original|hCoV-19^^Victoria|Human|Microbiological Diagnostic Unit - Public Health Laboratory (MDU-PHL)|MDU-PHL|Schultz|Australia
>Spike|hCoV-19/Russia/SVE-RII-MH1632S/2020|2020-09-08|EPI_ISL_596302|Original|hCoV-19^^Sverdlovsk|Human|HELIX LCC|WHO National Influenza Centre Russian Federation|Komissarov|Russia

>Spike|hCoV-19/USA/MI-MDHHS-SC21655/2020|2020-07-06|EPI_ISL_516382|Original|hCoV-
19^^Michigan|Human|Michigan Department of Health and Human Services|Michigan
Department of Health and Human Services|Blankenship|USA
>Spike|hCoV-19/Denmark/DCGC-3120/2020|2020-08-31|EPI_ISL_615937|Original|hCoV-
19^^Hovedstaden|Human|Department of Virus and Microbiological Special
Diagnostics|Albertsen lab|Michaelsen|Denmark
>Spike|hCoV-19/Denmark/DCGC-17231/2020|2020-12-14|EPI_ISL_750341|Original|hCoV-
19^^Midtjylland|Human|Department of Virus and Microbiological Special
Diagnostics|Albertsen Lab|Michaelsen|Denmark
>Spike|hCoV-19/USA/FL-BPHL-1262/2020|2020-06-04|EPI_ISL_541169|Original|hCoV-
19^^Florida|Human|Florida Bureau of Public Health Laboratories|Florida Bureau of
Public Health Laboratories|Blanton|USA
>Spike|hCoV-19/England/QEUH-9D0D09/2020|2020-09-13|EPI_ISL_589861|Original|hCoV-
19^^England|Human|Lighthouse Lab in Glasgow|Wellcome Sanger Institute for the COVID-
19 Genomics UK (COG-UK) consortium|Davies|United Kingdom
>Spike|hCoV-19/Scotland/CVR3698/2020|2020-05-25|EPI_ISL_473761|Original|hCoV-
19^^Scotland|Human|West of Scotland Specialist Virology Centre|COVID-19 Genomics UK
(COG-UK) Consortium|Robertson|United Kingdom
>Spike|hCoV-19/Canada/ON-S1237/2020|2020-04-16|EPI_ISL_586308|Original|hCoV-
19^^Ontario|Human|Toronto Invasive Bacterial Diseases Network|McMaster
University|McArthur|Canada
>Spike|hCoV-19/Wales/PHWC-16ECFC/2020|2020-10-03|EPI_ISL_585852|Original|hCoV-
19^^Wales|Human|Wales Specialist Virology Centre Sequencing lab|COVID-19 Genomics UK
(COG-UK) Consortium|Moore|United Kingdom
>Spike|hCoV-19/Denmark/DCGC-11579/2020|2020-11-02|EPI_ISL_670554|Original|hCoV-
19^^Syddanmark|Human|Department of Virus and Microbiological Special
Diagnostics|Albertsen Lab|Michaelsen|Denmark
>Spike|hCoV-19/Gambia/NPHL-3206/2020|2020-00-00|EPI_ISL_561313|Original|hCoV-
19^^Kombo|Human|MRCG at LSHTM Genomics lab|MRCG at LSHTM Genomics lab|Sesay|Gambia
>Spike|hCoV-19/England/QEUH-9B7791/2020|2020-09-08|EPI_ISL_590601|Original|hCoV-
19^^England|Human|Lighthouse Lab in Glasgow|Wellcome Sanger Institute for the COVID-
19 Genomics UK (COG-UK) consortium|Davies|United Kingdom
>Spike|hCoV-19/England/MILK-A79624/2020|2020-10-13|EPI_ISL_606762|Original|hCoV-
19^^England|Human|Lighthouse Lab in Milton Keynes|Wellcome Sanger Institute for the
COVID-19 Genomics UK (COG-UK) consortium|Davies|United Kingdom
>Spike|hCoV-19/England/ALDP-B5BC34/2020|2020-11-09|EPI_ISL_655286|Original|hCoV-
19^^England|Human|Lighthouse Lab in Alderley Park|Wellcome Sanger Institute for the
COVID-19 Genomics UK (COG-UK) Consortium|Davies|United Kingdom
>Spike|hCoV-19/Thailand/Bangkok-CONI-0212/2020|2020-03-
18|EPI_ISL_512862|Original|hCoV-19^^Bangkok|Human|Ramathibodi Hospital|COVID-19
Network Investigations (CONI) Alliance|Batty|Thailand
>Spike|hCoV-19/India/MH-ACTREC-114/2020|2020-05-25|EPI_ISL_699770|Original|hCoV-
19^^Maharashtra|Human|Hematopathology Laboratory|Hematopathology
Laboratory|Patkar|India
>Spike|hCoV-19/England/204420070/2020|2020-10-26|EPI_ISL_637150|Original|hCoV-
19^^England|Human|Respiratory Virus Unit|COVID-19 Genomics UK (COG-UK)
Consortium|Platt|United Kingdom
>Spike|hCoV-19/Singapore/211/2020|2020-03-02|EPI_ISL_462329|Original|hCoV-
19^^Singapore|Human|National Public Health Laboratory|National Public Health
Laboratory|Mak|Singapore
>Spike|hCoV-19/Germany/ST-MD012/2020|2020-04-16|EPI_ISL_728348|Original|hCoV-
19^^Saxony-Anhalt|Human|Institute of medical Microbiology and hospital
Hygiene|Institute of medical Microbiology and hospital Hygiene|Tersteegen|Germany
>Spike|hCoV-19/Wales/PHWC-16B7EF/2020|2020-09-16|EPI_ISL_573904|Original|hCoV-
19^^Wales|Human|Wales Specialist Virology Centre Sequencing lab|COVID-19 Genomics UK
(COG-UK) Consortium|Moore|United Kingdom
>Spike|hCoV-19/Russia/SVE-RII-MH198S/2020|2020-07-17|EPI_ISL_560312|Original|hCoV-
19^^Sverdlovsk|Human|UMMC-Health|WHO National Influenza Centre Russian
Federation|Komissarov|Russia

>Spike|hCoV-19/USA/CA-ALSR-1191/2020|2020-04-02|EPI_ISL_494427|Original|hCoV-19^^California|Human|San Diego County Public Health Laboratory|Andersen lab at Scripps Research|Gangavarapu|USA
>Spike|hCoV-19/Spain/AR-IBV-98008134/2020|2020-10-13|EPI_ISL_654462|Original|hCoV-19^^Aragon|Human|Servicio de Microbiolog\u00eda|SeqCOVID-SPAIN consortium/IBV(CSIC)|Comas|Spain
>Spike|hCoV-19/Wales/PHWC-166E67/2020|2020-06-23|EPI_ISL_494122|Original|hCoV-19^^Wales|Human|Originating lab|COVID-19 Genomics UK (COG-UK) Consortium|Moore|United Kingdom
>Spike|hCoV-19/USA/UT-UPHL-201006281/2020|2020-07-06|EPI_ISL_594048|Original|hCoV-19^^Utah|Human|Utah Public Health Laboratory|Utah Public Health Laboratory|Young|USA
>Spike|hCoV-19/England/20180025604/2020|2020-04-23|EPI_ISL_465430|Original|hCoV-19^^England|Human|Respiratory Virus Unit|Respiratory Virus Unit|Platt|United Kingdom
>Spike|hCoV-19/USA/UT-01677/2020|2020-04-13|EPI_ISL_454707|Original|hCoV-19^^Utah|Human|Utah Public Health Laboratory|Utah Public Health Laboratory|Young|USA
>Spike|hCoV-19/Denmark/DCGC-22581/2020|2020-12-14|EPI_ISL_757458|Original|hCoV-19^^Syddanmark|Human|Department of Virus and Microbiological Special Diagnostics|Albertsen Lab|Michaelsen|Denmark
>Spike|hCoV-19/South Africa/KRISP-K002165/2020|2020-07-22|EPI_ISL_515571|Original|hCoV-19^^KwaZulu-Natal|Human|NHLS-IALCH|KRISP|de Oliveira|South Africa
>Spike|hCoV-19/Wales/PHWC-2CAB5/2020|2020-04-01|EPI_ISL_445945|Original|hCoV-19^^Wales|Human|Wales Specialist Virology Centre|Public Health Wales Microbiology Cardiff|Moore|United Kingdom
>Spike|hCoV-19/England/CAMC-B9A57A/2020|2020-11-15|EPI_ISL_673759|Original|hCoV-19^^England|Human|Lighthouse Lab in Cambridge|Wellcome Sanger Institute for the COVID-19 Genomics UK (COG-UK) Consortium|Davies|United Kingdom
>Spike|hCoV-19/England/QEUH-BF0B52/2020|2020-12-05|EPI_ISL_709133|Original|hCoV-19^^England|Human|Lighthouse Lab in Glasgow|Wellcome Sanger Institute for the COVID-19 Genomics UK (COG-UK) Consortium|Davies|United Kingdom
>Spike|hCoV-19/USA/CA-ALSR-0513-SAN/2020|2020-04-02|EPI_ISL_483192|Original|hCoV-19^^California|Human|UC San Diego Center for Advanced Laboratory Medicine|Andersen lab at Scripps Research|Gangavarapu|USA
>Spike|hCoV-19/England/CAMC-A5903B/2020|2020-10-09|EPI_ISL_597118|Original|hCoV-19^^England|Human|Lighthouse Lab in Cambridge|Wellcome Sanger Institute for the COVID-19 Genomics UK (COG-UK) consortium|Davies|United Kingdom
>Spike|hCoV-19/England/QEUH-AA04FD/2020|2020-10-20|EPI_ISL_650296|Original|hCoV-19^^England|Human|University of Exeter|COVID-19 Genomics UK (COG-UK) Consortium|Temperton|United Kingdom
>Spike|hCoV-19/Scotland/EDB2714/2020|2020-03-27|EPI_ISL_487869|Original|hCoV-19^^Scotland|Human|Virology Department|Wellcome Sanger Institute for the COVID-19 Genomics UK (COG-UK) consortium|Davies|United Kingdom
>Spike|hCoV-19/England/QEUH-A0C037/2020|2020-10-01|EPI_ISL_599274|Original|hCoV-19^^England|Human|Lighthouse Lab in Glasgow|Wellcome Sanger Institute for the COVID-19 Genomics UK (COG-UK) consortium|Davies|United Kingdom
>Spike|hCoV-19/Scotland/QEUH-9B4F23/2020|2020-09-07|EPI_ISL_602078|Original|hCoV-19^^Scotland|Human|Lighthouse Lab in Glasgow|Wellcome Sanger Institute for the COVID-19 Genomics UK (COG-UK) consortium|Davies|United Kingdom
>Spike|hCoV-19/England/OXON-AC6D7/2020|2020-04-05|EPI_ISL_448476|Original|hCoV-19^^England|Human|Oxford Viromics|COVID-19 Genomics UK (COG-UK) Consortium|Golubchik|United Kingdom
>Spike|hCoV-19/USA/MN-QDX-2123/2020|2020-03-16|EPI_ISL_604480|Original|hCoV-19^^Minnesota|Human|Quest Diagnostics|Quest Diagnostics|Kagan|USA
>Spike|hCoV-19/Spain/VC-FISABIO-272/2020|2020-03-25|EPI_ISL_436367|Original|hCoV-19^^Comunitat Valenciana|Human|Servicio de Microbiologia. Hospital Clinico Universitario de Valencia|Sequencing and Bioinformatics Service and Molecular Epidemiology Research Group. FISABIO-Public Health|Gonzalez-Candelas|Spain
>Spike|hCoV-19/England/SHEF-10D3276/2020|2020-12-19|EPI_ISL_765189|Original|hCoV-19^^England|Human|Virology Department|COVID-19 Genomics UK (COG-UK) Consortium|de Silva|United Kingdom

>Spike|hCoV-19/Australia/ACT0089/2020|2020-07-03|EPI_ISL_498547|Original|hCoV-19^^Australian Capital Territory|Human|ACT Pathology|Schwessinger Lab|Hall|Australia
>Spike|hCoV-19/Russia/KL-RII-MH1686S/2020|2020-09-03|EPI_ISL_596317|Original|hCoV-19^^Kalmykia|Human|HELIX LCC|WHO National Influenza Centre Russian Federation|Komissarov|Russia
>Spike|hCoV-19/USA/WI-UW-2616/2021|2021-01-01|EPI_ISL_791133|Original|hCoV-19^^Wisconsin|Human|University of Wisconsin-Madison AIDS Vaccine Research Laboratories|University of Wisconsin-Madison AIDS Vaccine Research Laboratories|Moreno|USA
>Spike|hCoV-19/United Arab Emirates/0140/2020|2020-05-18|EPI_ISL_698327|Original|hCoV-19^^Abu Dhabi|Human|Group 42 (G42) Healthcare|G42 Healthcare|Ogrodzki|United Arab Emirates
>Spike|hCoV-19/England/ALDP-9CAD6C/2020|2020-09-16|EPI_ISL_575618|Original|hCoV-19^^England|Human|Lighthouse Lab in Alderley Park|Wellcome Sanger Institute for the COVID-19 Genomics UK (COG-UK) consortium|Davies|United Kingdom
>Spike|hCoV-19/USA/WA-S2489/2020|2020-06-16|EPI_ISL_497121|Original|hCoV-19^^Washington|Human|Washington State Department of Health|Seattle Flu Study|Lee|USA
>Spike|hCoV-19/USA/CA-ALSR-3396/2020|2020-08-10|EPI_ISL_636002|Original|hCoV-19^^California|Human|San Diego County Public Health Laboratory|Andersen lab at Scripps Research|Gangavarapu|USA
>Spike|hCoV-19/Japan/PG-8451/2020|2020-09-00|EPI_ISL_687550|Original|hCoV-19^^Japan|Human|Fukuoka Institute of Health and Environmental Sciences|Pathogen Genomics Center|Sekizuka|Japan
>Spike|hCoV-19/England/MILK-A69E3E/2020|2020-10-09|EPI_ISL_608753|Original|hCoV-19^^England|Human|Lighthouse Lab in Milton Keynes|Wellcome Sanger Institute for the COVID-19 Genomics UK (COG-UK) consortium|Davies|United Kingdom
>Spike|hCoV-19/England/CAMB-1B2901/2020|2020-05-18|EPI_ISL_456705|Original|hCoV-19^^England|Human|Department of Pathology|COVID-19 Genomics UK (COG-UK) Consortium|Meredith|United Kingdom
>Spike|hCoV-19/England/ALDP-B463DD/2020|2020-11-07|EPI_ISL_656285|Original|hCoV-19^^England|Human|Lighthouse Lab in Alderley Park|Wellcome Sanger Institute for the COVID-19 Genomics UK (COG-UK) Consortium|Davies|United Kingdom
>Spike|hCoV-19/England/LIVE-DC46DC/2020|2020-11-10|EPI_ISL_705904|Original|hCoV-19^^England|Human|Liverpool Clinical Laboratories|COVID-19 Genomics UK (COG-UK) Consortium|Haldenby|United Kingdom
>Spike|hCoV-19/Japan/PG-1497/2020|2020-04-00|EPI_ISL_685104|Original|hCoV-19^^Japan|Human|Saitama Prefectural Institute of Public Health|Pathogen Genomics Center|Sekizuka|Japan
>Spike|hCoV-19/England/CAMB-78CC3/2020|2020-04-03|EPI_ISL_441150|Original|hCoV-19^^England|Human|Department of Pathology|Wellcome Sanger Institute for the COVID-19 Genomics UK (COG-UK) consortium|Amato|United Kingdom
>Spike|hCoV-19/England/ALDP-950BDA/2020|2020-06-14|EPI_ISL_554463|Original|hCoV-19^^England|Human|Lighthouse Lab in Alderley Park|Wellcome Sanger Institute for the COVID-19 Genomics UK (COG-UK) consortium|Davies|United Kingdom
>Spike|hCoV-19/Hong Kong/HKU-200723-012/2020|2020-07-09|EPI_ISL_497779|Original|hCoV-19^^Hong Kong|Human|Department of Microbiology|Department of Microbiology|Chan|China
>Spike|hCoV-19/England/ALDP-A519B0/2020|2020-10-07|EPI_ISL_598195|Original|hCoV-19^^England|Human|Lighthouse Lab in Alderley Park|Wellcome Sanger Institute for the COVID-19 Genomics UK (COG-UK) consortium|Davies|United Kingdom
>Spike|hCoV-19/Beijing/BJ287/2020|2020-01-29|EPI_ISL_430722|Original|hCoV-19^^Beijing|Human|Chinese PLA Institute for Disease Control and Prevention|Chinese PLA Institute for Disease Control and Prevention|Li|China
>Spike|hCoV-19/England/ALDP-4FA375/2020|2020-06-24|EPI_ISL_559209|Original|hCoV-19^^England|Human|Lighthouse Lab in Alderley Park|Wellcome Sanger Institute for the COVID-19 Genomics UK (COG-UK) consortium|Davies|United Kingdom
>Spike|hCoV-19/England/20142082704/2020|2020-03-30|EPI_ISL_423463|Original|hCoV-19^^England|Human|Respiratory Virus Unit|Respiratory Virus Unit|Platt|United Kingdom
>Spike|hCoV-19/England/LIVE-AA080/2020|2020-03-23|EPI_ISL_517396|Original|hCoV-19^^England|Human|Liverpool Clinical Laboratories|COVID-19 Genomics UK (COG-UK) Consortium|Haldenby|United Kingdom

>Spike|hCoV-19/USA/LA-GBCL-26684432/2020|2020-04-14|EPI_ISL_485261|Original|hCoV-19^^Louisiana|Human|River Road Testing Lab|Ginkgo Bioworks Clinical Laboratory|Smith|USA
>Spike|hCoV-19/Russia/StPetersburg-RII15518S/2020|2020-05-15|EPI_ISL_507267|Original|hCoV-19^^Saint Petersburg|Human|WHO National Influenza Centre Russian Federation|WHO National Influenza Centre Russian Federation|Komissarov|Russia
>Spike|hCoV-19/Netherlands/NA_520/2020|2020-03-27|EPI_ISL_460693|Original|hCoV-19^^Netherlands|Human|Dutch COVID-19 response team|Erasmus Medical Center|Oude Munnink|Netherlands
>Spike|hCoV-19/England/QEUH-B2C1A6/2020|2020-11-04|EPI_ISL_680066|Original|hCoV-19^^England|Human|Department of Pathology|COVID-19 Genomics UK (COG-UK) Consortium|Goodfellow|United Kingdom
>Spike|hCoV-19/Belgium/ULG-10224/2020|2020-05-15|EPI_ISL_498130|Original|hCoV-19^^Li\u00e8ge|Human|Department of Clinical Microbiology|GIGA Medical Genomics|Durkin|Belgium
>Spike|hCoV-19/France/ARA-06042/2020|2020-03-04|EPI_ISL_417333|Original|hCoV-19^^Auvergne-Rh\u00f4ne-Alpes|Human|Institut des Agents Infectieux (IAI)|CNR Virus des Infections Respiratoires - France SUD|Josset|France
>Spike|hCoV-19/Denmark/DCGC-19192/2020|2020-12-07|EPI_ISL_748804|Original|hCoV-19^^Midtjylland|Human|Department of Virus and Microbiological Special Diagnostics|Albertsen Lab|Michaelsen|Denmark
>Spike|hCoV-19/USA/VA-DCLS-1235/2020|2020-08-05|EPI_ISL_528654|Original|hCoV-19^^Virginia|Human|Virginia DCLS|Virginia DCLS|St. Jacques|USA
>Spike|hCoV-19/England/MILK-B8F8F7/2020|2020-11-13|EPI_ISL_674639|Original|hCoV-19^^England|Human|Lighthouse Lab in Milton Keynes|Wellcome Sanger Institute for the COVID-19 Genomics UK (COG-UK) Consortium|Davies|United Kingdom
>Spike|hCoV-19/USA/TX-DSHS-0271/2020|2020-06-13|EPI_ISL_527838|Original|hCoV-19^^Texas|Human|Texas Department of State Health Services|Texas Department of State Health Services|Rahman|USA
>Spike|hCoV-19/Wales/PHWC-16BFBA/2020|2020-09-15|EPI_ISL_573998|Original|hCoV-19^^Wales|Human|Wales Specialist Virology Centre Sequencing lab|COVID-19 Genomics UK (COG-UK) Consortium|Moore|United Kingdom
>Spike|hCoV-19/Russia/NVS-RII-MH1097V/2020|2020-07-29|EPI_ISL_596265|V1|hCoV-19^^Novosibirsk|Human|WHO National Influenza Centre Russian Federation|WHO National Influenza Centre Russian Federation|Komissarov|Russia
>Spike|hCoV-19/Scotland/QEUH-B2B6C6/2020|2020-11-03|EPI_ISL_676435|Original|hCoV-19^^Scotland|Human|Lighthouse Lab in Glasgow|Wellcome Sanger Institute for the COVID-19 Genomics UK (COG-UK) Consortium|Davies|United Kingdom
>Spike|hCoV-19/England/ALDP-B5C732/2020|2020-11-08|EPI_ISL_655810|Original|hCoV-19^^England|Human|Lighthouse Lab in Alderley Park|Wellcome Sanger Institute for the COVID-19 Genomics UK (COG-UK) Consortium|Davies|United Kingdom
>Spike|hCoV-19/Denmark/DCGC-13395/2020|2020-11-30|EPI_ISL_712271|Original|hCoV-19^^Sjaelland|Human|Department of Virus and Microbiological Special Diagnostics|Albertsen Lab|Michaelsen|Denmark
>Spike|hCoV-19/England/NORT-29DC4E/2020|2020-05-07|EPI_ISL_484312|Original|hCoV-19^^England|Human|Northumbria University / South Tees Hospitals NHS Foundation Trust / North Cumbria Integrated Care NHS Foundation Trust / North Tees and Hartlepool NHS Foundation Trust / Newcastle Hospitals NHS Foundation Trust|COVID-19 Genomics UK (COG-UK) Consortium|Bashton|United Kingdom
>Spike|hCoV-19/USA/CA-CZB-9529/2020|2020-04-08|EPI_ISL_672121|Original|hCoV-19^^California|Human|The Ashley Laboratory|Chan-Zuckerberg Biohub|Bhatt|USA
>Spike|hCoV-19/Northern Ireland/NIRE-FCC21/2020|2020-04-22|EPI_ISL_470048|Original|hCoV-19^^Northern Ireland|Human|Regional Virus Laboratory|Wellcome Sanger Institute for the COVID-19 Genomics UK (COG-UK) consortium|Davies|United Kingdom
>Spike|hCoV-19/USA/VA-DCLS-0768/2020|2020-06-09|EPI_ISL_526884|Original|hCoV-19^^Virginia|Human|Virginia DCLS|Virginia DCLS|St. Jacques|USA

>Spike|hCoV-19/Denmark/DCGC-13399/2020|2020-11-30|EPI_ISL_712774|Original|hCoV-19^^Hovedstaden|Human|Department of Virus and Microbiological Special Diagnostics|Albertsen Lab|Michaelsen|Denmark
>Spike|hCoV-19/Denmark/DCGC-8852/2020|2020-10-26|EPI_ISL_626081|Original|hCoV-19^^Midtjylland|Human|Department of Virus and Microbiological Special Diagnostics|Albertsen lab|Michaelsen|Denmark
>Spike|hCoV-19/England/ALDP-A67BFD/2020|2020-10-10|EPI_ISL_608621|Original|hCoV-19^^England|Human|Lighthouse Lab in Alderley Park|Wellcome Sanger Institute for the COVID-19 Genomics UK (COG-UK) consortium|Davies|United Kingdom
>Spike|hCoV-19/England/QEUH-960C7A/2020|2020-08-04|EPI_ISL_532033|Original|hCoV-19^^England|Human|Lighthouse Lab in Glasgow|Wellcome Sanger Institute for the COVID-19 Genomics UK (COG-UK) consortium|Davies|United Kingdom
>Spike|hCoV-19/USA/WI-UW-2414/2020|2020-12-07|EPI_ISL_754674|Original|hCoV-19^^Wisconsin|Human|University of Wisconsin-Madison AIDS Vaccine Research Laboratories|University of Wisconsin-Madison AIDS Vaccine Research Laboratories|Moreno|USA
>Spike|hCoV-19/USA/TX-DSHS-1808/2020|2020-06-15|EPI_ISL_738178|Original|hCoV-19^^Texas|Human|Texas Department of State Health Services|Texas Department of State Health Services|Rahman|USA
>Spike|hCoV-19/Wales/QEUH-96BE2E/2020|2020-08-14|EPI_ISL_531283|Original|hCoV-19^^Wales|Human|Lighthouse Lab in Glasgow|Wellcome Sanger Institute for the COVID-19 Genomics UK (COG-UK) consortium|Davies|United Kingdom
>Spike|hCoV-19/England/QEUH-AD487C/2020|2020-10-26|EPI_ISL_634021|Original|hCoV-19^^England|Human|Lighthouse Lab in Glasgow|Wellcome Sanger Institute for the COVID-19 Genomics UK (COG-UK) consortium|Davies|United Kingdom
>Spike|hCoV-19/Australia/VIC16906/2020|2020-00-00|EPI_ISL_663634|Original|hCoV-19^^Victoria|Human|Microbiological Diagnostic Unit - Public Health Laboratory (MDU-PHL)|MDU-PHL|Schultz|Australia
>Spike|hCoV-19/USA/WA-S584/2020|2020-03-24|EPI_ISL_434344|Original|hCoV-19^^Washington|Human|Washington State Department of Health|Seattle Flu Study|Bedford|USA
>Spike|hCoV-19/Wales/PHWC-489EC5/2020|2020-10-27|EPI_ISL_652503|Original|hCoV-19^^Wales|Human|Wales Specialist Virology Centre Sequencing lab|COVID-19 Genomics UK (COG-UK) Consortium|Moore|United Kingdom
>Spike|hCoV-19/USA/CT-Yale-426/2020|2020-04-27|EPI_ISL_738298|Original|hCoV-19^^Connecticut|Human|Connecticut Veterans' Affairs Hospital|Grubaugh Lab - Yale School of Public Health|Grubaugh|USA
>Spike|hCoV-19/England/CAMB-76A34/2020|2020-03-30|EPI_ISL_438377|Original|hCoV-19^^England|Human|Department of Pathology|Wellcome Sanger Institute for the COVID-19 Genomics UK (COG-UK) consortium|Amato|United Kingdom
>Spike|hCoV-19/England/CAMC-B3718A/2020|2020-11-05|EPI_ISL_645356|Original|hCoV-19^^England|Human|Lighthouse Lab in Cambridge|Wellcome Sanger Institute for the COVID-19 Genomics UK (COG-UK) Consortium|Davies|United Kingdom
>Spike|hCoV-19/Germany/ChVir3098/2020|2020-04-09|EPI_ISL_729530|Original|hCoV-19^^Germany|Human|Charit\u00e9 Universit\u00e4tsmedizin Berlin|Charit\u00e9 Universit\u00e4tsmedizin Berlin|Muehlemann|Germany
>Spike|hCoV-19/Wales/PHWC-16619B/2020|2020-06-19|EPI_ISL_490845|Original|hCoV-19^^Wales|Human|Wales Specialist Virology Centre Sequencing lab|COVID-19 Genomics UK (COG-UK) Consortium|Moore|United Kingdom
>Spike|hCoV-19/USA/MI-UM-10036710490/2020|2020-10-30|EPI_ISL_682137|Original|hCoV-19^^Michigan|Human|University of Michigan Clinical Microbiology Laboratory|Lauring Lab|Valesano|USA
>Spike|hCoV-19/USA/MA-MASPHL-00423/2020|2020-04-27|EPI_ISL_693129|Original|hCoV-19^^Massachusetts|Human|Massachusetts State Public Health Laboratory|Massachusetts State Public Health Laboratory|Lang|USA
>Spike|hCoV-19/USA/WA-UW-23371/2020|2020-08-10|EPI_ISL_576434|Original|hCoV-19^^Washington|Human|UW Virology Lab|UW Virology Lab|Roychoudhury|USA
>Spike|hCoV-19/Hong Kong/HKU-200723-090/2020|2020-07-11|EPI_ISL_497857|Original|hCoV-19^^Hong Kong|Human|Department of Microbiology|Department of Microbiology|Chan|China

>Spike|hCoV-19/Japan/PG-6003/2020|2020-07-00|EPI_ISL_689116|Original|hCoV-19^^Japan|Human|Pathogen Genomics Center|Pathogen Genomics Center|Sekizuka|Japan
>Spike|hCoV-19/England/20132087602/2020|2020-03-23|EPI_ISL_420512|Original|hCoV-19^^England|Human|Respiratory Virus Unit|Respiratory Virus Unit|Platt|United Kingdom
>Spike|hCoV-19/Denmark/DCGC-16438/2020|2020-11-16|EPI_ISL_714767|Original|hCoV-19^^Hovedstaden|Human|Department of Virus and Microbiological Special Diagnostics|Albertsen Lab|Michaelsen|Denmark
>Spike|hCoV-19/Wales/ALDP-BC8898/2020|2020-11-22|EPI_ISL_767319|Original|hCoV-19^^Wales|Human|Lighthouse Lab in Alderley Park|Wellcome Sanger Institute for the COVID-19 Genomics UK (COG-UK) Consortium|Davies|United Kingdom
>Spike|hCoV-19/Australia/VIC8897/2020|2020-07-14|EPI_ISL_565270|Original|hCoV-19^^Victoria|Human|Microbiological Diagnostic Unit - Public Health Laboratory (MDU-PHL)|MDU-PHL|Schultz|Australia
>Spike|hCoV-19/Denmark/DCGC-17059/2020|2020-12-14|EPI_ISL_750736|Original|hCoV-19^^Hovedstaden|Human|Department of Virus and Microbiological Special Diagnostics|Albertsen Lab|Michaelsen|Denmark
>Spike|hCoV-19/Denmark/DCGC-12500/2020|2020-11-16|EPI_ISL_682681|Original|hCoV-19^^Sjaelland|Human|Department of Virus and Microbiological Special Diagnostics|Albertsen Lab|Michaelsen|Denmark
>Spike|hCoV-19/England/CAMC-BC1C39/2020|2020-11-22|EPI_ISL_704696|Original|hCoV-19^^England|Human|Lighthouse Lab in Cambridge|Wellcome Sanger Institute for the COVID-19 Genomics UK (COG-UK) Consortium|Davies|United Kingdom
>Spike|hCoV-19/England/ALDP-AA37A6/2020|2020-10-22|EPI_ISL_624969|Original|hCoV-19^^England|Human|Lighthouse Lab in Alderley Park|Wellcome Sanger Institute for the COVID-19 Genomics UK (COG-UK) consortium|Davies|United Kingdom
>Spike|hCoV-19/Australia/VIC16302/2020|2020-10-13|EPI_ISL_641214|Original|hCoV-19^^Victoria|Human|Microbiological Diagnostic Unit - Public Health Laboratory (MDU-PHL)|MDU-PHL|Schultz|Australia
>Spike|hCoV-19/Denmark/DCGC-9332/2020|2020-11-16|EPI_ISL_668615|Original|hCoV-19^^Nordjylland|Human|Department of Virus and Microbiological Special Diagnostics|Albertsen Lab|Michaelsen|Denmark
>Spike|hCoV-19/England/ALDP-9C5842/2020|2020-09-11|EPI_ISL_567579|Original|hCoV-19^^England|Human|Lighthouse Lab in Alderley Park|Wellcome Sanger Institute for the COVID-19 Genomics UK (COG-UK) consortium|Davies|United Kingdom
>Spike|hCoV-19/England/CAMB-8381C/2020|2020-04-15|EPI_ISL_443678|Original|hCoV-19^^England|Human|Department of Pathology|Wellcome Sanger Institute for the COVID-19 Genomics UK (COG-UK) consortium|Amato|United Kingdom
>Spike|hCoV-19/England/PORT-2D17C5/2020|2020-04-12|EPI_ISL_484419|Original|hCoV-19^^England|Human|Centre for Enzyme Innovation|COVID-19 Genomics UK (COG-UK) Consortium|Robson|United Kingdom
>Spike|hCoV-19/England/NORW-EB7BB/2020|2020-05-05|EPI_ISL_472317|Original|hCoV-19^^England|Human|Quadram Institute Bioscience|COVID-19 Genomics UK (COG-UK) Consortium|Alikhan|United Kingdom
>Spike|hCoV-19/USA/TX-HMH-MCoV-15933/2020|2020-07-10|EPI_ISL_785617|Original|hCoV-19^^Texas|Human|Houston Methodist Hospital|Houston Methodist Hospital|Long|USA
>Spike|hCoV-19/Sweden/20-51740/2020|2020-05-25|EPI_ISL_510841|Original|hCoV-19^^Vastra Gotaland|Human|Klinisk mikrobiologi NAL Trollhattan|The Public Health Agency of Sweden|Svartstr\u00f6m|Sweden
>Spike|hCoV-19/Australia/QLD1272/2020|2020-03-20|EPI_ISL_693274|Original|hCoV-19^^Australia|Human|unknown|Public Health Virology Laboratory|Nguyen|Australia
>Spike|hCoV-19/England/NOTT-1157E4/2020|2020-10-14|EPI_ISL_595266|Original|hCoV-19^^England|Human|Queens Medical Centre|COVID-19 Genomics UK (COG-UK) Consortium|Loose|United Kingdom
>Spike|hCoV-19/env/Qingdao/IVDC-08-10/2020|2020-09-24|EPI_ISL_591277|Original|hCoV-19^^Shandong|Environment|National Institute for Viral Disease Control and Prevention|National Institute for Viral Disease Control and Prevention|Chen|China
>Spike|hCoV-19/England/LIVE-9D76D/2020|2020-04-15|EPI_ISL_453231|Original|hCoV-19^^England|Human|Liverpool Clinical Laboratories|COVID-19 Genomics UK (COG-UK) Consortium|Haldenby|United Kingdom

>Spike|hCoV-19/England/MILK-B942F1/2020|2020-11-13|EPI_ISL_676063|Original|hCoV-19^^England|Human|Lighthouse Lab in Milton Keynes|Wellcome Sanger Institute for the COVID-19 Genomics UK (COG-UK) Consortium|Davies|United Kingdom
>Spike|hCoV-19/USA/TX-HMH-1421/2020|2020-04-21|EPI_ISL_542713|Original|hCoV-19^^Texas|Human|Houston Methodist Hospital|Houston Methodist Hospital|Long|USA
>Spike|hCoV-19/USA/WI-GMF-27681/2020|2020-10-25|EPI_ISL_660985|Original|hCoV-19^^Wisconsin|Human|Gundersen Molecular Diagnostics Laboratory|Kabara Cancer Research Institute|Kenny|USA
>Spike|hCoV-19/Spain/PV-IBV-2099/2020|2020-04-11|EPI_ISL_468922|Original|hCoV-19^^Basque Country|Human|Servicio de Microbiolog\u00eda. Hospital Universitario Donostia. OSI Donostialdea. \u00c1rea de Enfermedades Infecciosas|SeqCOVID-SPAIN consortium/IBV(CSIC)|Comas|Spain
>Spike|hCoV-19/South Korea/KDCA0319/2020|2020-10-16|EPI_ISL_760105|Original|hCoV-19^^South Korea|Human|Division of Emerging Infectious Diseases|Division of Emerging Infectious Diseases|Park|South Korea
>Spike|hCoV-19/Wales/PHWC-16C4C9/2020|2020-05-30|EPI_ISL_574060|Original|hCoV-19^^Wales|Human|Wales Specialist Virology Centre Sequencing lab|COVID-19 Genomics UK (COG-UK) Consortium|Moore|United Kingdom
>Spike|hCoV-19/USA/TX-HMH-MCoV-14757/2020|2020-10-12|EPI_ISL_786242|Original|hCoV-19^^Texas|Human|Houston Methodist Hospital|Houston Methodist Hospital|Long|USA
>Spike|hCoV-19/USA/MI-UM-10036599140/2020|2020-10-23|EPI_ISL_640215|Original|hCoV-19^^Michigan|Human|University of Michigan Clinical Microbiology Laboratory|Lauring Lab|Valesano|USA
>Spike|hCoV-19/Denmark/DCGC-18198/2020|2020-12-14|EPI_ISL_749810|Original|hCoV-19^^Nordjylland|Human|Department of Virus and Microbiological Special Diagnostics|Albertsen Lab|Michaelsen|Denmark
>Spike|hCoV-19/USA/MA-MGH-00031/2020|2020-03-25|EPI_ISL_460111|Original|hCoV-19^^Massachusetts|Human|Massachusetts General Hospital|Infectious Disease Program|Lemieux|USA
>Spike|hCoV-19/USA/CA-ALSR-2167/2020|2020-06-01|EPI_ISL_635368|Original|hCoV-19^^California|Human|San Diego County Public Health Laboratory|Andersen lab at Scripps Research|Gangavarapu|USA
>Spike|hCoV-19/England/ALDP-BCA605/2020|2020-11-23|EPI_ISL_703571|Original|hCoV-19^^England|Human|Lighthouse Lab in Alderley Park|Wellcome Sanger Institute for the COVID-19 Genomics UK (COG-UK) Consortium|Davies|United Kingdom
>Spike|hCoV-19/England/QEUH-B9E1CD/2020|2020-11-16|EPI_ISL_720954|Original|hCoV-19^^England|Human|Lighthouse Lab in Glasgow|Wellcome Sanger Institute for the COVID-19 Genomics UK (COG-UK) Consortium|Davies|United Kingdom
>Spike|hCoV-19/Sweden/20-50107/2020|2020-03-03|EPI_ISL_454881|Original|hCoV-19^^Stockholm|Human|Karolinska Universitetslaboratoriet|The Public Health Agency of Sweden|Svartstr\u00f6m|Sweden
>Spike|hCoV-19/Denmark/DCGC-11554/2020|2020-11-02|EPI_ISL_670680|Original|hCoV-19^^Hovedstaden|Human|Department of Virus and Microbiological Special Diagnostics|Albertsen Lab|Michaelsen|Denmark
>Spike|hCoV-19/USA/TX-HMH-625/2020|2020-04-11|EPI_ISL_547340|Original|hCoV-19^^Texas|Human|Houston Methodist Hospital|Houston Methodist Hospital|Long|USA
>Spike|hCoV-19/Netherlands/NA_435/2020|2020-04-07|EPI_ISL_455237|Original|hCoV-19^^Netherlands|Human|Dutch COVID-19 response team|Erasmus Medical Center|Oude Munnink|Netherlands
>Spike|hCoV-19/Wales/PHWC-48C0AE/2020|2020-11-07|EPI_ISL_666389|Original|hCoV-19^^Wales|Human|Wales Specialist Virology Centre Sequencing lab|COVID-19 Genomics UK (COG-UK) Consortium|Moore|United Kingdom
>Spike|hCoV-19/England/LCST-24D6A47/2020|2020-06-04|EPI_ISL_478322|Original|hCoV-19^^England|Human|University Hospitals Of Leicester NHS Trust and DeepSeq Nottingham|COVID-19 Genomics UK (COG-UK) Consortium|Loose|United Kingdom
>Spike|hCoV-19/Sweden/20-22153/2020|2020-06-02|EPI_ISL_623084|Original|hCoV-19^^Skane|Human|Klinisk mikrobiologi|The Public Health Agency of Sweden|Svartstr\u00f6m|Sweden

>Spike|hCoV-19/Netherlands/ZH-EMC-492/2020|2020-00-00|EPI_ISL_577921|Original|hCoV-19^^Zuid Holland|Human|Dutch COVID-19 response team|Erasmus Medical Center|Oude Munnink|Netherlands
>Spike|hCoV-19/Australia/VIC5779/2020|2020-07-20|EPI_ISL_518577|Original|hCoV-19^^Victoria|Human|Microbiological Diagnostic Unit - Public Health Laboratory (MDU-PHL)|MDU-PHL|Schultz|Australia
>Spike|hCoV-19/England/ALDP-A4FA3A/2020|2020-10-07|EPI_ISL_597774|Original|hCoV-19^^England|Human|Lighthouse Lab in Alderley Park|Wellcome Sanger Institute for the COVID-19 Genomics UK (COG-UK) consortium|Davies|United Kingdom
>Spike|hCoV-19/Spain/CT-IBV-99009877/2020|2020-08-01|EPI_ISL_660274|Original|hCoV-19^^Catalunya|Human|Servicio de Microbiolog\u00eda|SeqCOVID-SPAIN consortium/IBV(CSIC)|Comas|Spain
>Spike|hCoV-19/England/ALDP-A66B94/2020|2020-10-12|EPI_ISL_630378|Original|hCoV-19^^England|Human|Lighthouse Lab in Alderley Park|Wellcome Sanger Institute for the COVID-19 Genomics UK (COG-UK) consortium|Davies|United Kingdom
>Spike|hCoV-19/Denmark/DCGC-11003/2020|2020-11-02|EPI_ISL_670338|Original|hCoV-19^^Hovedstaden|Human|Department of Virus and Microbiological Special Diagnostics|Albertsen Lab|Michaelsen|Denmark
>Spike|hCoV-19/USA/RI-CDC-2-3714588/2020|2020-12-03|EPI_ISL_751671|Original|hCoV-19^^Rhode Island|Human|RI State Health Laboratories|Genomics and Discovery|Queen|USA
>Spike|hCoV-19/England/ALDP-BBABFA/2020|2020-11-21|EPI_ISL_702570|Original|hCoV-19^^England|Human|Lighthouse Lab in Alderley Park|Wellcome Sanger Institute for the COVID-19 Genomics UK (COG-UK) Consortium|Davies|United Kingdom
>Spike|hCoV-19/England/QEUH-B6A1F7/2020|2020-11-11|EPI_ISL_662620|Original|hCoV-19^^England|Human|Lighthouse Lab in Glasgow|Wellcome Sanger Institute for the COVID-19 Genomics UK (COG-UK) Consortium|Davies|United Kingdom
>Spike|hCoV-19/England/QEUH-A8B9CC/2020|2020-10-16|EPI_ISL_643203|Original|hCoV-19^^England|Human|Lighthouse Lab in Glasgow|Wellcome Sanger Institute for the COVID-19 Genomics UK (COG-UK) Consortium|Davies|United Kingdom
>Spike|hCoV-19/Italy/TUS-UOS-21/2020|2020-04-27|EPI_ISL_591331|Original|hCoV-19^^Tuscany|Human|Dipartimento di Biotecnologie Mediche|Dipartimento di Biotecnologie Mediche|Cusi|Italy
>Spike|hCoV-19/England/LIVE-9A593/2020|2020-03-25|EPI_ISL_449348|Original|hCoV-19^^England|Human|Liverpool Clinical Laboratories|COVID-19 Genomics UK (COG-UK) Consortium|Haldenby|United Kingdom
>Spike|hCoV-19/Denmark/DCGC-15875/2020|2020-11-23|EPI_ISL_714079|Original|hCoV-19^^Nordjylland|Human|Department of Virus and Microbiological Special Diagnostics|Albertsen Lab|Michaelsen|Denmark
>Spike|hCoV-19/England/LOND-126018E/2020|2020-04-07|EPI_ISL_524672|Original|hCoV-19^^England|Human|North West London Pathology|Wellcome Sanger Institute for the COVID-19 Genomics UK (COG-UK) consortium|Davies|United Kingdom
>Spike|hCoV-19/England/ALDP-954329/2020|2020-06-13|EPI_ISL_553817|Original|hCoV-19^^England|Human|Lighthouse Lab in Alderley Park|Wellcome Sanger Institute for the COVID-19 Genomics UK (COG-UK) consortium|Davies|United Kingdom
>Spike|hCoV-19/USA/WA-UW-11533/2020|2020-06-21|EPI_ISL_570432|Original|hCoV-19^^Washington|Human|UW Virology Lab|UW Virology Lab|Roychoudhury|USA
>Spike|hCoV-19/Netherlands/ZH-EMC-393/2020|2020-07-22|EPI_ISL_523733|Original|hCoV-19^^Zuid Holland|Human|Dutch COVID-19 response team|Erasmus Medical Center|Oude Munnink|Netherlands
>Spike|hCoV-19/Wales/PHWC-4812D0/2020|2020-10-16|EPI_ISL_613052|Original|hCoV-19^^Wales|Human|Wales Specialist Virology Centre Sequencing lab|COVID-19 Genomics UK (COG-UK) Consortium|Moore|United Kingdom
>Spike|hCoV-19/Wales/MILK-9E2065/2020|2020-09-20|EPI_ISL_581120|Original|hCoV-19^^Wales|Human|Lighthouse Lab in Milton Keynes|Wellcome Sanger Institute for the COVID-19 Genomics UK (COG-UK) consortium|Davies|United Kingdom
>Spike|hCoV-19/USA/WI-WSLH-200716/2020|2020-06-28|EPI_ISL_631472|Original|hCoV-19^^Wisconsin|Human|Wisconsin State Laboratory of Hygiene Communicable Disease Division|Wisconsin State Laboratory of Hygiene Communicable Disease Division|Florek|USA

>Spike|hCoV-19/England/CAMB-7EB75/2020|2020-04-07|EPI_ISL_442963|Original|hCoV-19^^England|Human|Department of Pathology|Wellcome Sanger Institute for the COVID-19 Genomics UK (COG-UK) consortium|Amato|United Kingdom
>Spike|hCoV-19/England/ALDP-94F4A6/2020|2020-06-19|EPI_ISL_556003|Original|hCoV-19^^England|Human|Lighthouse Lab in Alderley Park|Wellcome Sanger Institute for the COVID-19 Genomics UK (COG-UK) consortium|Davies|United Kingdom
>Spike|hCoV-19/France/ARA-16665/2020|2020-03-30|EPI_ISL_508987|Original|hCoV-19^^Auvergne-Rh\u00f4ne-Alpes|Human|CNR Virus des Infections Respiratoires - France SUD|CNR Virus des Infections Respiratoires - France SUD|Josset|France
>Spike|hCoV-19/England/ALDP-B75584/2020|2020-11-12|EPI_ISL_662276|Original|hCoV-19^^England|Human|Lighthouse Lab in Alderley Park|Wellcome Sanger Institute for the COVID-19 Genomics UK (COG-UK) Consortium|Davies|United Kingdom
>Spike|hCoV-19/Switzerland/BS-UHB-42197732/2020|2020-03-18|EPI_ISL_528217|Original|hCoV-19^^Basel-Stadt|Human|University Hospital Basel|University Hospital Basel|Roloff Handschin|Switzerland
>Spike|hCoV-19/England/BIRM-6AECE/2020|2020-11-13|EPI_ISL_679115|Original|hCoV-19^^England|Human|University of Birmingham|COVID-19 Genomics UK (COG-UK) Consortium|Loman|United Kingdom
>Spike|hCoV-19/Japan/PG-4200/2020|2020-07-00|EPI_ISL_692438|Original|hCoV-19^^Japan|Human|Pathogen Genomics Center|Pathogen Genomics Center|Sekizuka|Japan
>Spike|hCoV-19/Spain/MD-IBV-99012346/2020|2020-11-20|EPI_ISL_779995|Original|hCoV-19^^Madrid|Human|Hospital General Universitario Gregorio Mara\u00f1\u00f3n|SeqCOVID-SPAIN consortium/IBV(CSIC)|Comas|Spain
>Spike|hCoV-19/Wales/PHWC-4909AC/2020|2020-11-16|EPI_ISL_703870|Original|hCoV-19^^Wales|Human|Originating lab|COVID-19 Genomics UK (COG-UK) Consortium|Moore|United Kingdom
>Spike|hCoV-19/England/LIVE-DCB9AF/2020|2020-11-25|EPI_ISL_724444|Original|hCoV-19^^England|Human|Liverpool Clinical Laboratories|COVID-19 Genomics UK (COG-UK) Consortium|Haldenby|United Kingdom
>Spike|hCoV-19/Denmark/DCGC-7989/2020|2020-10-26|EPI_ISL_617110|Original|hCoV-19^^Syddanmark|Human|Department of Virus and Microbiological Special Diagnostics|Albertsen lab|Michaelsen|Denmark
>Spike|hCoV-19/Egypt/MASRI-3/2020|2020-05-00|EPI_ISL_475746|Original|hCoV-19^^Egypt|Human|Medical Ain Shams Research Institute (MASRI)|Medical Ain Shams Research Institute (MASRI)|Moustafa|Egypt
>Spike|hCoV-19/USA/WI-UW-2589/2021|2021-01-03|EPI_ISL_791107|Original|hCoV-19^^Wisconsin|Human|University of Wisconsin-Madison AIDS Vaccine Research Laboratories|University of Wisconsin-Madison AIDS Vaccine Research Laboratories|Moreno|USA
>Spike|hCoV-19/USA/UT-UPHL-2012190312/2020|2020-08-15|EPI_ISL_746297|Original|hCoV-19^^Utah|Human|Ginkgo Bioworks Clinical Laboratory|Utah Public Health Laboratory|Young|USA
>Spike|hCoV-19/Singapore/865/2020|2020-08-20|EPI_ISL_527374|Original|hCoV-19^^Singapore|Human|National Public Health Laboratory|National Public Health Laboratory|Mak|Singapore
>Spike|hCoV-19/England/203480317/2020|2020-08-19|EPI_ISL_566068|Original|hCoV-19^^England|Human|Respiratory Virus Unit|Respiratory Virus Unit|Platt|United Kingdom
>Spike|hCoV-19/England/QEUH-88DA9C/2020|2020-07-27|EPI_ISL_532822|Original|hCoV-19^^England|Human|Lighthouse Lab in Glasgow|Wellcome Sanger Institute for the COVID-19 Genomics UK (COG-UK) consortium|Davies|United Kingdom
>Spike|hCoV-19/Scotland/QEUH-A9EE4F/2020|2020-10-20|EPI_ISL_625377|Original|hCoV-19^^Scotland|Human|Lighthouse Lab in Glasgow|Wellcome Sanger Institute for the COVID-19 Genomics UK (COG-UK) consortium|Davies|United Kingdom
>Spike|hCoV-19/England/QEUH-9963C1/2020|2020-08-27|EPI_ISL_549529|Original|hCoV-19^^England|Human|Oxford Viromics|COVID-19 Genomics UK (COG-UK) Consortium|Golubchik|United Kingdom
>Spike|hCoV-19/Denmark/DCGC-4342/2020|2020-09-07|EPI_ISL_615544|Original|hCoV-19^^Hovedstaden|Human|Department of Virus and Microbiological Special Diagnostics|Albertsen lab|Michaelsen|Denmark

>Spike|hCoV-19/Saudi Arabia/KAUST-MAKKAH1562/2020|2020-04-14|EPI_ISL_678114|Original|hCoV-19^^Makkah|Human|Pathogen Genomics Lab King Abdullah University of Science and Technology(KAUST)|Pathogen Genomics Lab King Abdullah University of Science and Technology(KAUST)|Mohamed Ghazzali|Saudi Arabia
>Spike|hCoV-19/USA/DE-DHSS-F918046/2020|2020-03-27|EPI_ISL_593939|Original|hCoV-19^^Delaware|Human|Delaware Public Health Lab|Delaware Public Health Lab|Bajwa|USA
>Spike|hCoV-19/Australia/VIC2900/2020|2020-07-08|EPI_ISL_521731|Original|hCoV-19^^Victoria|Human|Microbiological Diagnostic Unit - Public Health Laboratory (MDU-PHL)|MDU-PHL|Schultz|Australia
>Spike|hCoV-19/USA/MI-MDHHS-SC20302/2020|2020-03-25|EPI_ISL_447099|Original|hCoV-19^^Michigan|Human|Michigan Department of Health and Human Services|Michigan Department of Health and Human Services|Blankenship|USA
>Spike|hCoV-19/South Africa/LR00149/2020|2020-07-21|EPI_ISL_514417|Original|hCoV-19^^Free State|Human|National Institute for Communicable Diseases of the National Health Laboratory Service|National Institute for Communicable Diseases of the National Health Laboratory Service|Allam|South Africa
>Spike|hCoV-19/USA/VA-DCLS-0077/2020|2020-04-04|EPI_ISL_429976|Original|hCoV-19^^Virginia|Human|Virginia DCLS|Virginia DCLS|St. Jacques|USA
>Spike|hCoV-19/England/QEUH-9904B5/2020|2020-08-29|EPI_ISL_530379|Original|hCoV-19^^England|Human|Lighthouse Lab in Glasgow|Wellcome Sanger Institute for the COVID-19 Genomics UK (COG-UK) consortium|Davies|United Kingdom
>Spike|hCoV-19/Luxembourg/LNS7147879/2020|2020-11-22|EPI_ISL_744523|Original|hCoV-19^^Luxembourg|Human|Laboratoire national de sant\u00e9|Laboratoire national de sant\u00e9|Wienecke-Baldacchino|Luxembourg
>Spike|hCoV-19/Spain/CT-IBV-97009563/2020|2020-07-30|EPI_ISL_671860|Original|hCoV-19^^Catalunya|Human|Servicio de Microbiolog\u00eda|SeqCOVID-SPAIN consortium/IBV(CSIC)|Comas|Spain
>Spike|hCoV-19/Australia/QLD1121/2020|2020-09-23|EPI_ISL_593636|Original|hCoV-19^^Australia|Human|unknown|Public Health Virology Laboratory|Nguyen|Australia
>Spike|hCoV-19/England/ALDP-B1CD78/2020|2020-11-01|EPI_ISL_645715|Original|hCoV-19^^England|Human|Lighthouse Lab in Alderley Park|Wellcome Sanger Institute for the COVID-19 Genomics UK (COG-UK) Consortium|Davies|United Kingdom
>Spike|hCoV-19/USA/CA-CHLA-PLM95751496/2020|2020-11-18|EPI_ISL_753376|Original|hCoV-19^^California|Human|Clinical virology Laboratory|Center for Personalized Medicine|Dien Bard|USA
>Spike|hCoV-19/England/GSTT-265DDF2/2020|2020-03-28|EPI_ISL_484109|Original|hCoV-19^^England|Human|Centre for Clinical Infection and Diagnostics Research and Genomics Innovation Unit|COVID-19 Genomics UK (COG-UK) Consortium|Awan|United Kingdom
>Spike|hCoV-19/SaudiArabia/KAUST-JEDDAH1055/2020|2020-06-17|EPI_ISL_512905|Original|hCoV-19^^Jeddah|Human|Pathogen Genomics Lab King Abdullah University of Science and Technology(KAUST)|Pathogen Genomics Lab King Abdullah University of Science and Technology(KAUST)|Mohamed Ghazzali|Saudi Arabia
>Spike|hCoV-19/England/ALDP-C48C1B/2020|2020-12-12|EPI_ISL_736704|Original|hCoV-19^^England|Human|Lighthouse Lab in Alderley Park|Wellcome Sanger Institute for the COVID-19 Genomics UK (COG-UK) Consortium|Davies|United Kingdom
>Spike|hCoV-19/Northern Ireland/NIRE-10336D/2020|2020-04-17|EPI_ISL_489266|Original|hCoV-19^^Northern Ireland|Human|Regional Virus Laboratory|Wellcome Sanger Institute for the COVID-19 Genomics UK (COG-UK) consortium|Davies|United Kingdom
>Spike|hCoV-19/USA/CA-ALSR-4221/2020|2020-08-18|EPI_ISL_648895|Original|hCoV-19^^California|Human|San Diego County Public Health Laboratory|Andersen lab at Scripps Research|Gangavarapu|USA
>Spike|hCoV-19/Switzerland/BS-UHB-42201473/2020|2020-03-21|EPI_ISL_528295|Original|hCoV-19^^Basel-Stadt|Human|University Hospital Basel|University Hospital Basel|Roloff Handschin|Switzerland
>Spike|hCoV-19/England/MILK-9F18A7/2020|2020-09-25|EPI_ISL_589636|Original|hCoV-19^^England|Human|Lighthouse Lab in Milton Keynes|Wellcome Sanger Institute for the COVID-19 Genomics UK (COG-UK) consortium|Davies|United Kingdom
>Spike|hCoV-19/USA/NY-NYCPHL-000994/2020|2020-10-01|EPI_ISL_633019|Original|hCoV-19^^New York|Human|DOHMH Morrisania|New York City Public Health Laboratory|Wang|USA

>Spike|hCoV-19/USA/FL-BPHL-1761/2020|2020-10-02|EPI_ISL_613766|Original|hCoV-19^^Florida|Human|Florida Bureau of Public Health Laboratories|Florida Bureau of Public Health Laboratories|Blanton|USA
>Spike|hCoV-19/England/CAMB-7BFD6/2020|2020-04-13|EPI_ISL_433976|Original|hCoV-19^^England|Human|Department of Pathology|COVID-19 Genomics UK (COG-UK) Consortium|Meredith|United Kingdom
>Spike|hCoV-19/England/BHRT-24B3925/2020|2020-05-21|EPI_ISL_528790|Original|hCoV-19^^England|Human|Microbiology Department|Wellcome Sanger Institute for the COVID-19 Genomics UK (COG-UK) consortium|Davies|United Kingdom
>Spike|hCoV-19/DRC/11376/2020|2020-05-25|EPI_ISL_527513|Original|hCoV-19^^Kinshasa|Human|Viral Respiratory Lab|Pathogen Sequencing Lab|Lokilo|Democratic Republic of the Congo
>Spike|hCoV-19/Australia/NSW895/2020|2020-08-26|EPI_ISL_544976|Original|hCoV-19^^New South Wales|Human|Pathology North - Royal North Shore Hospital - NSW Health Pathology|NSW Health Pathology - Institute of Clinical Pathology and Medical Research; Westmead Hospital; University of Sydney|Gall|Australia
>Spike|hCoV-19/South Korea/KDCA0408/2020|2020-11-10|EPI_ISL_760192|Original|hCoV-19^^South Korea|Human|Division of Emerging Infectious Diseases|Division of Emerging Infectious Diseases|Park|South Korea
>Spike|hCoV-19/England/BRIS-122D79/2020|2020-04-02|EPI_ISL_440702|Original|hCoV-19^^England|Human|PHE South West Regional Laboratory|Wellcome Sanger Institute for the COVID-19 Genomics UK (COG-UK) consortium|Amato|United Kingdom
>Spike|hCoV-19/Hungary/UD-6858/2020|2020-04-08|EPI_ISL_476071|Original|hCoV-19^^Debrecen|Human|University of Debrecen|National Laboratory of Virology|Kemenesi|Hungary
>Spike|hCoV-19/England/EXET-13EB25/2020|2020-12-14|EPI_ISL_764183|Original|hCoV-19^^England|Human|University of Exeter|COVID-19 Genomics UK (COG-UK) Consortium|Temperton|United Kingdom
>Spike|hCoV-19/England/ALDP-BB357B/2020|2020-11-19|EPI_ISL_702741|Original|hCoV-19^^England|Human|Lighthouse Lab in Alderley Park|Wellcome Sanger Institute for the COVID-19 Genomics UK (COG-UK) Consortium|Davies|United Kingdom
>Spike|hCoV-19/Mexico/TAM-InDRE_38/2020|2020-06-19|EPI_ISL_516624|Original|hCoV-19^^Tamaulipas|Human|Instituto de Diagnostico y Referencia Epidemiologicos (INDRE)|Instituto de Diagnostico y Referencia Epidemiologicos (INDRE)|Rodriguez|Mexico
>Spike|hCoV-19/England/20149027404/2020|2020-04-03|EPI_ISL_466180|Original|hCoV-19^^England|Human|Respiratory Virus Unit|Respiratory Virus Unit|Platt|United Kingdom
>Spike|hCoV-19/Ukraine/203100356/2020|2020-05-28|EPI_ISL_512635|Original|hCoV-19^^Ukraine|Human|National Laboratory for Influenza/Virology reference laboratory|Respiratory Virus Unit|Platt|Ukraine
>Spike|hCoV-19/USA/WY-WPHL-20149634/2020|2020-11-24|EPI_ISL_770063|Original|hCoV-19^^Wyoming|Human|Wyoming Public Health Laboratory|Wyoming Public Health Laboratory|Hull|USA
>Spike|hCoV-19/Spain/MD-IBV-99009620/2020|2020-08-06|EPI_ISL_654362|Original|hCoV-19^^Madrid|Human|Hospital General Universitario Gregorio Mara\u00f1\u00f3n|SeqCOVID-SPAIN consortium/IBV(CSIC)|Comas|Spain
>Spike|hCoV-19/Wales/PHWC-49298C/2020|2020-11-20|EPI_ISL_725727|Original|hCoV-19^^Wales|Human|Originating lab|COVID-19 Genomics UK (COG-UK) Consortium|Moore|United Kingdom
>Spike|hCoV-19/Japan/PG-2253/2020|2020-04-00|EPI_ISL_685858|Original|hCoV-19^^Japan|Human|Pathogen Genomics Center|Pathogen Genomics Center|Sekizuka|Japan
>Spike|hCoV-19/England/CAMC-B510AB/2020|2020-11-08|EPI_ISL_657885|Original|hCoV-19^^England|Human|Lighthouse Lab in Cambridge|Wellcome Sanger Institute for the COVID-19 Genomics UK (COG-UK) Consortium|Davies|United Kingdom
>Spike|hCoV-19/England/ALDP-AA1A18/2020|2020-10-22|EPI_ISL_624511|Original|hCoV-19^^England|Human|Lighthouse Lab in Alderley Park|Wellcome Sanger Institute for the COVID-19 Genomics UK (COG-UK) consortium|Davies|United Kingdom
>Spike|hCoV-19/Greece/44/2020|2020-03-23|EPI_ISL_434465|Original|hCoV-19^^Athens|Human|Laboratory of Microbiology|Laboratory of Biology|Karakasiliotis|Greece

>Spike|hCoV-19/Wuhan/HB-WHCM2-271/2020|2020-02-17|EPI_ISL_455006|Original|hCoV-19^^Hubei|Human|Wuhan Chain Medical Labs (CMLabs)|State Key Laboratory of Biotherapy of Sichuan University|Tang|China
>Spike|hCoV-19/Denmark/DCGC-18970/2020|2020-12-07|EPI_ISL_748672|Original|hCoV-19^^Hovedstaden|Human|Department of Virus and Microbiological Special Diagnostics|Albertsen Lab|Michaelsen|Denmark
>Spike|hCoV-19/USA/MI-UM-10036361683/2020|2020-10-09|EPI_ISL_596619|Original|hCoV-19^^Michigan|Human|University of Michigan Clinical Microbiology Laboratory|Lauring Lab|Valesano|USA
>Spike|hCoV-19/Australia/VIC2578/2020|2020-07-03|EPI_ISL_521453|Original|hCoV-19^^Victoria|Human|Microbiological Diagnostic Unit - Public Health Laboratory (MDU-PHL)|MDU-PHL|Schultz|Australia
>Spike|hCoV-19/Japan/PG-7806/2020|2020-07-00|EPI_ISL_686908|Original|hCoV-19^^Japan|Human|Pathogen Genomics Center|Pathogen Genomics Center|Sekizuka|Japan
>Spike|hCoV-19/England/QEUH-5317C0/2020|2020-05-18|EPI_ISL_532217|Original|hCoV-19^^England|Human|Lighthouse Lab in Glasgow|Wellcome Sanger Institute for the COVID-19 Genomics UK (COG-UK) consortium|Davies|United Kingdom
>Spike|hCoV-19/USA/VA-DCLS-1317/2020|2020-07-09|EPI_ISL_572257|Original|hCoV-19^^Virginia|Human|Virginia DCLS|Virginia DCLS|St. Jacques|USA
>Spike|hCoV-19/USA/UT-UPHL-01963/2020|2020-04-15|EPI_ISL_575245|Original|hCoV-19^^Utah|Human|Utah Public Health Laboratory|Utah Public Health Laboratory|Young|USA
>Spike|hCoV-19/France/PAC-IHU-0224/2020|2020-03-29|EPI_ISL_569387|Original|hCoV-19^^Provence-Alpes-C\u00f4te-d\u2019Azur|Human|MEPHI|MEPHI|Levasseur|France
>Spike|hCoV-19/New Zealand/20VR2531/2020|2020-00-00|EPI_ISL_579276|Original|hCoV-19^^Auckland|Human|LabPLUS|Institute of Environmental Science and Research (ESR)|Wang|New Zealand
>Spike|hCoV-19/Netherlands/Gelderland_118/2020|2020-05-22|EPI_ISL_460879|Original|hCoV-19^^Gelderland|Human|Dutch COVID-19 response team|Erasmus Medical Center|Oude Munnink|Netherlands
>Spike|hCoV-19/England/LIVE-AA75E/2020|2020-03-24|EPI_ISL_517496|Original|hCoV-19^^England|Human|Liverpool Clinical Laboratories|COVID-19 Genomics UK (COG-UK) Consortium|Haldenby|United Kingdom
>Spike|hCoV-19/Norway/4917/2020|2020-10-25|EPI_ISL_668431|Original|hCoV-19^^Nordland|Human|Nordland Hospital - Bodo|Norwegian Institute of Public Health|Kopperud Riis|Norway
>Spike|hCoV-19/Netherlands/NA_350/2020|2020-04-06|EPI_ISL_455155|Original|hCoV-19^^Netherlands|Human|Dutch COVID-19 response team|Erasmus Medical Center|Oude Munnink|Netherlands
>Spike|hCoV-19/England/20130072404/2020|2020-03-23|EPI_ISL_423666|Original|hCoV-19^^England|Human|Respiratory Virus Unit|Respiratory Virus Unit|Platt|United Kingdom
>Spike|hCoV-19/USA/WY-WPHL-20172574/2020|2020-12-31|EPI_ISL_770488|Original|hCoV-19^^Wyoming|Human|Wyoming Public Health Laboratory|Wyoming Public Health Laboratory|Hull|USA
>Spike|hCoV-19/Austria/CeMM0617/2020|2020-04-22|EPI_ISL_583616|Original|hCoV-19^^Ischgl|Human|Institut f\u00fcr Virologie am Department f\u00fcr Hygiene|Bergthaler laboratory|Bergthaler|Austria
>Spike|hCoV-19/England/205160919/2020|2020-12-11|EPI_ISL_766261|Original|hCoV-19^^England|Human|Respiratory Virus Unit|COVID-19 Genomics UK (COG-UK) Consortium|Platt|United Kingdom
>Spike|hCoV-19/USA/CA-CZB-15238/2020|2020-11-24|EPI_ISL_739456|Original|hCoV-19^^California|Human|Santa Clara County Public Health Laboratory|Chan-Zuckerberg Biohub|Kamm|USA
>Spike|hCoV-19/South Africa/KRISP-K005332/2020|2020-11-14|EPI_ISL_678620|Original|hCoV-19^^KwaZulu-Natal|Human|NHLS-IALCH|KRISP|de Oliveira|South Africa
>Spike|hCoV-19/USA/CO-CSU-D1490-2/2020|2020-04-15|EPI_ISL_527440|Original|hCoV-19^^Colorado|Human|Colorado State University - Ebel Lab|Colorado State University - Ebel Lab|Cline|USA

>Spike|hCoV-19/India/GJ-GBRC163a/2020|2020-05-27|EPI_ISL_461497|Original|hCoV-
19^^Gujarat|Human|B.J. Medical College and Civil hospital|Gujarat Biotechnology
Research Centre|Joshi|India
>Spike|hCoV-19/England/CAMB-1AFFF6/2020|2020-05-07|EPI_ISL_448096|Original|hCoV-
19^^England|Human|Department of Pathology|COVID-19 Genomics UK (COG-UK)
Consortium|Meredith|United Kingdom
>Spike|hCoV-19/USA/TX-HMH-MCoV-19546/2020|2020-11-17|EPI_ISL_783921|Original|hCoV-
19^^Texas|Human|Houston Methodist Hospital|Houston Methodist Hospital|Long|USA
>Spike|hCoV-19/Scotland/EDB5348/2020|2020-05-23|EPI_ISL_457004|Original|hCoV-
19^^Scotland|Human|Virology Department|COVID-19 Genomics UK (COG-UK)
Consortium|Williams|United Kingdom
>Spike|hCoV-19/England/CAMC-B22E7C/2020|2020-11-02|EPI_ISL_647170|Original|hCoV-
19^^England|Human|Lighthouse Lab in Cambridge|Wellcome Sanger Institute for the
COVID-19 Genomics UK (COG-UK) Consortium|Davies|United Kingdom
>Spike|hCoV-19/England/BRIS-126F15/2020|2020-04-23|EPI_ISL_469755|Original|hCoV-
19^^England|Human|PHE South West Regional Laboratory|Wellcome Sanger Institute for
the COVID-19 Genomics UK (COG-UK) consortium|Davies|United Kingdom
>Spike|hCoV-19/USA/TX-HMH-MCoV-15749/2020|2020-07-09|EPI_ISL_785433|Original|hCoV-
19^^Texas|Human|Houston Methodist Hospital|Houston Methodist Hospital|Long|USA
>Spike|hCoV-19/Australia/VIC684/2020|2020-03-29|EPI_ISL_426979|Original|hCoV-
19^^Victoria|Human|Victorian Infectious Diseases Reference Laboratory
(VIDRL)|Microbiological Diagnostic Unit Public Health Laboratory and Victorian
Infectious Diseases Reference Laboratory|Schultz|Australia
>Spike|hCoV-19/England/ALDP-B73FD3/2020|2020-11-12|EPI_ISL_662047|Original|hCoV-
19^^England|Human|Lighthouse Lab in Alderley Park|Wellcome Sanger Institute for the
COVID-19 Genomics UK (COG-UK) Consortium|Davies|United Kingdom
>Spike|hCoV-19/England/MILK-ACE480/2020|2020-10-21|EPI_ISL_629295|Original|hCoV-
19^^England|Human|Lighthouse Lab in Milton Keynes|Wellcome Sanger Institute for the
COVID-19 Genomics UK (COG-UK) consortium|Davies|United Kingdom
>Spike|hCoV-19/Hangzhou/HZCDC0048L/2020|2020-01-21|EPI_ISL_421232|Original|hCoV-
19^^Hangzhou|Human|Hangzhou Center for Diseases Control and Prevention|Hangzhou
Center for Diseases Control and Prevention|Li|China
>Spike|hCoV-19/USA/MD-MDH-0526/2020|2020-12-01|EPI_ISL_778903|Original|hCoV-
19^^Maryland|Human|Maryland Public Health Laboratory|Maryland Public Health
Laboratory|Keller|USA
>Spike|hCoV-19/England/MILK-99D1EE/2020|2020-08-30|EPI_ISL_550731|Original|hCoV-
19^^England|Human|Lighthouse Lab in Milton Keynes|Wellcome Sanger Institute for the
COVID-19 Genomics UK (COG-UK) consortium|Davies|United Kingdom
>Spike|hCoV-19/England/CAMB-1BA101/2020|2020-11-06|EPI_ISL_651582|Original|hCoV-
19^^England|Human|Department of Pathology|COVID-19 Genomics UK (COG-UK)
Consortium|Goodfellow|United Kingdom
>Spike|hCoV-19/England/QEUH-9F3ACD/2020|2020-09-24|EPI_ISL_588622|Original|hCoV-
19^^England|Human|Lighthouse Lab in Glasgow|Wellcome Sanger Institute for the COVID-
19 Genomics UK (COG-UK) consortium|Davies|United Kingdom
>Spike|hCoV-19/Australia/VIC3108/2020|2020-07-06|EPI_ISL_520815|Original|hCoV-
19^^Victoria|Human|Victorian Infectious Diseases Reference Laboratory (VIDRL)|VIDRL
and MDU-PHL|Schultz|Australia
>Spike|hCoV-19/England/NORW-222D2D/2020|2020-12-19|EPI_ISL_741519|Original|hCoV-
19^^England|Human|Quadram Institute Bioscience|COVID-19 Genomics UK (COG-UK)
Consortium|Alikhan|United Kingdom
>Spike|hCoV-19/USA/CA-CZB-11820/2020|2020-10-02|EPI_ISL_625658|Original|hCoV-
19^^California|Human|Orange County Public Health Lab|Chan-Zuckerberg Biohub|Bhatt|USA
>Spike|hCoV-19/Russia/SVE-RII-MH2038S/2020|2020-09-21|EPI_ISL_602386|Original|hCoV-
19^^Sverdlovsk|Human|HELIX LLC|WHO National Influenza Centre Russian
Federation|Komissarov|Russia
>Spike|hCoV-19/USA/ID-UW-2047/2020|2020-03-22|EPI_ISL_477680|Original|hCoV-
19^^Idaho|Human|UW Virology Lab|UW Virology Lab|Roychoudhury|USA
>Spike|hCoV-19/England/MILK-A05766/2020|2020-09-30|EPI_ISL_599860|Original|hCoV-
19^^England|Human|Lighthouse Lab in Milton Keynes|Wellcome Sanger Institute for the
COVID-19 Genomics UK (COG-UK) consortium|Davies|United Kingdom

>Spike|hCoV-19/USA/LA-GBCL-26544541/2020|2020-04-18|EPI_ISL_485122|Original|hCoV-19^^Louisiana|Human|River Road Testing Lab|Ginkgo Bioworks Clinical Laboratory|Smith|USA
>Spike|hCoV-19/Denmark/DCGC-18836/2020|2020-12-07|EPI_ISL_748587|Original|hCoV-19^^Midtjylland|Human|Department of Virus and Microbiological Special Diagnostics|Albertsen Lab|Michaelsen|Denmark
>Spike|hCoV-19/England/QEUH-B14068/2020|2020-11-03|EPI_ISL_641793|Original|hCoV-19^^England|Human|Lighthouse Lab in Glasgow|Wellcome Sanger Institute for the COVID-19 Genomics UK (COG-UK) Consortium|Davies|United Kingdom
>Spike|hCoV-19/USA/TX-HMH-MCoV-12736/2020|2020-08-31|EPI_ISL_790494|Original|hCoV-19^^Texas|Human|Houston Methodist Hospital|Houston Methodist Hospital|Long|USA
>Spike|hCoV-19/USA/OR-OHSU-2081/2020|2020-08-04|EPI_ISL_667111|Original|hCoV-19^^Oregon|Human|OHSU Lab Services Molecular Microbiology Lab|Oregon SARS-CoV-2 Genome Sequencing Center|O'Roak|USA
>Spike|hCoV-19/USA/WI-GMF-18735/2020|2020-09-04|EPI_ISL_547781|Original|hCoV-19^^Wisconsin|Human|Gundersen Molecular Diagnostics Laboratory|Kabara Cancer Research Institute|Kenny|USA
>Spike|hCoV-19/USA/FL-BPHL-0427/2020|2020-03-21|EPI_ISL_495333|Original|hCoV-19^^Florida|Human|Florida Bureau of Public Health Laboratories|Florida Bureau of Public Health Laboratories|Blanton|USA
>Spike|hCoV-19/USA/AZ-TG408144/2020|2020-06-12|EPI_ISL_694900|Original|hCoV-19^^Arizona|Human|AZ SPHL|TGen North|French|USA
>Spike|hCoV-19/Australia/VIC17907/2020|2020-08-21|EPI_ISL_779550|Original|hCoV-19^^Victoria|Human|Microbiological Diagnostic Unit - Public Health Laboratory (MDU-PHL)|MDU-PHL|Beck|Australia
>Spike|hCoV-19/England/CAMB-80BBC/2020|2020-04-11|EPI_ISL_442949|Original|hCoV-19^^England|Human|Department of Pathology|Wellcome Sanger Institute for the COVID-19 Genomics UK (COG-UK) consortium|Amato|United Kingdom
>Spike|hCoV-19/Australia/VIC12651/2020|2020-07-26|EPI_ISL_592045|Original|hCoV-19^^Victoria|Human|Microbiological Diagnostic Unit - Public Health Laboratory (MDU-PHL)|MDU-PHL|Schultz|Australia
>Spike|hCoV-19/USA/WI-UW-2154/2020|2020-10-15|EPI_ISL_677558|Original|hCoV-19^^Wisconsin|Human|University of Wisconsin-Madison AIDS Vaccine Research Laboratories|University of Wisconsin-Madison AIDS Vaccine Research Laboratories|Baczenas|USA
>Spike|hCoV-19/England/GSTT-265DEFF/2020|2020-03-28|EPI_ISL_484123|Original|hCoV-19^^England|Human|Centre for Clinical Infection and Diagnostics Research and Genomics Innovation Unit|COVID-19 Genomics UK (COG-UK) Consortium|Awan|United Kingdom
>Spike|hCoV-19/England/QEUH-96C86B/2020|2020-08-21|EPI_ISL_530656|Original|hCoV-19^^England|Human|Lighthouse Lab in Glasgow|Wellcome Sanger Institute for the COVID-19 Genomics UK (COG-UK) consortium|Davies|United Kingdom
>Spike|hCoV-19/England/BRIS-12EB73/2020|2020-04-24|EPI_ISL_488415|Original|hCoV-19^^England|Human|PHE South West Regional Laboratory|Wellcome Sanger Institute for the COVID-19 Genomics UK (COG-UK) consortium|Davies|United Kingdom
>Spike|hCoV-19/England/20134079504/2020|2020-03-24|EPI_ISL_423178|Original|hCoV-19^^England|Human|Respiratory Virus Unit|Respiratory Virus Unit|Platt|United Kingdom
>Spike|hCoV-19/England/CAMB-8373D/2020|2020-04-15|EPI_ISL_443647|Original|hCoV-19^^England|Human|Department of Pathology|Wellcome Sanger Institute for the COVID-19 Genomics UK (COG-UK) consortium|Amato|United Kingdom
>Spike|hCoV-19/Russia/CRIE4980432/2020|2020-07-16|EPI_ISL_534342|Original|hCoV-19^^Moscow|Human|Molecular diagnostic laboratory of Federal Budget Institution of Science \Central Research Institute of Epidemiology\ of The Federal Service on Customers' Rights Protection and Human Well-being Surveillance|Group of Genomics and Postgenomic Technologies of Central Research Institute of Epidemilology|Sper
>anskaya|Russia
>Spike|hCoV-19/USA/WA-UW-5166/2020|2020-04-02|EPI_ISL_429653|Original|hCoV-19^^Washington|Human|UW Virology Lab|UW Virology Lab|Roychoudhury|USA
>Spike|hCoV-19/Ireland/D-NVRL-AIIDV0081v2/2020|2020-04-15|EPI_ISL_644286|Original|hCoV-19^^Dublin|Human|CEPHR / Vincent's Hospital|Irish

Coronavirus Sequencing Consortium - National Virus Reference Laboratory|Gonzalez|Ireland
>Spike|hCoV-19/Australia/VIC7442/2020|2020-07-23|EPI_ISL_564416|Original|hCoV-19^^Victoria|Human|Microbiological Diagnostic Unit - Public Health Laboratory (MDU-PHL)|MDU-PHL|Schultz|Australia
>Spike|hCoV-19/SaudiArabia/KAUST-MADINAH1048/2020|2020-06-13|EPI_ISL_513079|Original|hCoV-19^^Madinah|Human|Pathogen Genomics Lab King Abdullah University of Science and Technology(KAUST)|Pathogen Genomics Lab King Abdullah University of Science and Technology(KAUST)|Mohamed Ghazzali|Saudi Arabia
>Spike|hCoV-19/USA/IL-NM-0552/2020|2020-04-30|EPI_ISL_626455|Original|hCoV-19^^Illinois|Human|Northwestern Memorial Hospital|Ozer Lab|Ozer|USA
>Spike|hCoV-19/USA/AK-PHL602/2020|2020-06-24|EPI_ISL_586245|Original|hCoV-19^^Alaska|Human|Alaska State Virology Laboratory|Alaska State Virology Laboratory|Chen|USA
>Spike|hCoV-19/England/QEUH-B3F744/2020|2020-11-08|EPI_ISL_658641|Original|hCoV-19^^England|Human|Lighthouse Lab in Glasgow|Wellcome Sanger Institute for the COVID-19 Genomics UK (COG-UK) Consortium|Davies|United Kingdom
>Spike|hCoV-19/England/CAMB-1AA9A2/2020|2020-04-18|EPI_ISL_470199|Original|hCoV-19^^England|Human|Department of Pathology|Wellcome Sanger Institute for the COVID-19 Genomics UK (COG-UK) consortium|Davies|United Kingdom
>Spike|hCoV-19/England/NORT-298751/2020|2020-05-18|EPI_ISL_469316|Original|hCoV-19^^England|Human|NU-OMICS DNA Sequencing research facility|Wellcome Sanger Institute for the COVID-19 Genomics UK (COG-UK) consortium|Davies|United Kingdom
>Spike|hCoV-19/Denmark/DCGC-1138/2020|2020-03-23|EPI_ISL_614632|Original|hCoV-19^^Nordjylland|Human|Department of Virus and Microbiological Special Diagnostics|Albertsen lab|Michaelsen|Denmark
>Spike|hCoV-19/USA/DE-DHSS-F1010662/2020|2020-10-01|EPI_ISL_708429|Original|hCoV-19^^Delaware|Human|Delaware Public Health Lab|Delaware Public Health Lab|Bajwa|USA
>Spike|hCoV-19/Vietnam/VNHN_4958/2020|2020-04-10|EPI_ISL_455718|Original|hCoV-19^^Hanoi|Human|National Hospital of Tropical Diseases|Oxford University Clinical Research Unit|Nguyen|Vietnam
>Spike|hCoV-19/India/MH-NCCS-NR2099/2020|2020-05-21|EPI_ISL_496568|Original|hCoV-19^^Maharashtra|Human|National Centre For Cell Science|National Centre For Cell Science|Paul|India
>Spike|hCoV-19/South Africa/KRISP-K002241/2020|2020-07-21|EPI_ISL_515632|Original|hCoV-19^^KwaZulu-Natal|Human|NHLS-IALCH|KRISP|de Oliveira|South Africa
>Spike|hCoV-19/Denmark/DCGC-18390/2020|2020-12-07|EPI_ISL_749230|Original|hCoV-19^^Hovedstaden|Human|Department of Virus and Microbiological Special Diagnostics|Albertsen Lab|Michaelsen|Denmark
>Spike|hCoV-19/Norway/3272/2020|2020-08-24|EPI_ISL_590910|original|hCoV-19^^Oslo|Human|Oslo University Hospital|Norwegian Institute of Public Health|Vollan|Norway
>Spike|hCoV-19/USA/CA-CDPH168/2020|2020-07-19|EPI_ISL_754944|Original|hCoV-19^^California|Human|California Department of Public Health|California Department of Public Health|Bell|USA
>Spike|hCoV-19/Belgium/Rega-0328202/2020|2020-03-28|EPI_ISL_458172|Original|hCoV-19^^Colfontaine|Human|KU Leuven|KU Leuven|Maes|Belgium
>Spike|hCoV-19/England/MILK-B390F4/2020|2020-11-02|EPI_ISL_651289|Original|hCoV-19^^England|Human|University of Exeter|COVID-19 Genomics UK (COG-UK) Consortium|Temperton|United Kingdom
>Spike|hCoV-19/Japan/PG-8146/2020|2020-08-00|EPI_ISL_687245|Original|hCoV-19^^Japan|Human|Pathogen Genomics Center|Pathogen Genomics Center|Sekizuka|Japan
>Spike|hCoV-19/Sweden/20-51681/2020|2020-05-25|EPI_ISL_476137|Original|hCoV-19^^Vastra Gotaland|Human|Wasterlakarna|The Public Health Agency of Sweden|Svartstr\u00f6m|Sweden
>Spike|hCoV-19/Germany/NW-HHU-265/2020|2020-09-22|EPI_ISL_631353|Orginal|hCoV-19^^North Rhine-Westphalia|Human|ZOTZ KLIMAS MVZ D\u00fcsseldorf-Centrum GbR \u00dcBAG f\u00fcr Labormedizin|Center of Medical Microbiology|Wienemann|Germany

>Spike|hCoV-19/France/ARA-SC316/2020|2020-04-18|EPI_ISL_649950|Original|hCoV-19^^Auvergne-Rh\u00f4ne-Alpes|Human|CHU de Saint-\u00c9tienne H\u00f4pital Nord|CNR Virus des Infections Respiratoires - France SUD|Bal|France
>Spike|hCoV-19/Australia/VIC3797/2020|2020-07-12|EPI_ISL_520514|Original|hCoV-19^^Victoria|Human|Microbiological Diagnostic Unit - Public Health Laboratory (MDU-PHL)|MDU-PHL|Schultz|Australia
>Spike|hCoV-19/England/ALDP-A92AED/2020|2020-10-19|EPI_ISL_611129|Original|hCoV-19^^England|Human|Lighthouse Lab in Alderley Park|Wellcome Sanger Institute for the COVID-19 Genomics UK (COG-UK) consortium|Davies|United Kingdom
>Spike|hCoV-19/England/CAMB-1B1B48/2020|2020-05-13|EPI_ISL_453000|Original|hCoV-19^^England|Human|Department of Pathology|COVID-19 Genomics UK (COG-UK) Consortium|Meredith|United Kingdom
>Spike|hCoV-19/England/EXET-13AFE1/2020|2020-11-10|EPI_ISL_664417|Original|hCoV-19^^England|Human|University of Exeter|COVID-19 Genomics UK (COG-UK) Consortium|Temperton|United Kingdom
>Spike|hCoV-19/India/TG-CCMB-L1999/2020|2020-06-04|EPI_ISL_495180|Original|hCoV-19^^Telangana|Human|CSIR-Centre for Cellular and Molecular Biology|CSIR-Centre for Cellular and Molecular Biology|Banu|India
>Spike|hCoV-19/England/LIVE-A85CF/2020|2020-03-26|EPI_ISL_517186|Original|hCoV-19^^England|Human|Liverpool Clinical Laboratories|COVID-19 Genomics UK (COG-UK) Consortium|Haldenby|United Kingdom
>Spike|hCoV-19/Japan/PG-2990/2020|2020-04-00|EPI_ISL_691114|Original|hCoV-19^^Japan|Human|Pathogen Genomics Center|Pathogen Genomics Center|Sekizuka|Japan
>Spike|hCoV-19/England/CAMB-78874/2020|2020-04-06|EPI_ISL_433782|Original|hCoV-19^^England|Human|Department of Pathology|COVID-19 Genomics UK (COG-UK) Consortium|Meredith|United Kingdom
>Spike|hCoV-19/England/ALDP-9C601C/2020|2020-09-10|EPI_ISL_567748|Original|hCoV-19^^England|Human|Lighthouse Lab in Alderley Park|Wellcome Sanger Institute for the COVID-19 Genomics UK (COG-UK) consortium|Davies|United Kingdom
>Spike|hCoV-19/India/GJ-GBRC269/2020|2020-06-13|EPI_ISL_483866|Original|hCoV-19^^Gujarat|Human|Department of Microbiology|Gujarat Biotechnology Research Centre|Joshi|India
>Spike|hCoV-19/England/QEUH-AE4F74/2020|2020-10-27|EPI_ISL_633202|Original|hCoV-19^^England|Human|Lighthouse Lab in Glasgow|Wellcome Sanger Institute for the COVID-19 Genomics UK (COG-UK) consortium|Davies|United Kingdom
>Spike|hCoV-19/Wales/PHWC-15FF04/2020|2020-04-23|EPI_ISL_472871|Original|hCoV-19^^Wales|Human|Wales Specialist Virology Centre Sequencing lab|COVID-19 Genomics UK (COG-UK) Consortium|Moore|United Kingdom
>Spike|hCoV-19/Canada/BC-BCCDC-7553/2020|2020-11-00|EPI_ISL_740716|Original|hCoV-19^^British Columbia|Human|BCCDC Public Health Laboratory|BCCDC Public Health Laboratory|Prystajecky|Canada
>Spike|hCoV-19/England/ALDP-9CDC99/2020|2020-09-16|EPI_ISL_566111|Original|hCoV-19^^England|Human|Lighthouse Lab in Alderley Park|Wellcome Sanger Institute for the COVID-19 Genomics UK (COG-UK) consortium|Davies|United Kingdom
>Spike|hCoV-19/England/LEED-2AA1D6/2020|2020-04-04|EPI_ISL_538895|Original|hCoV-19^^England|Human|Leeds Teaching Hospitals NHS Trust and Public Health England|Wellcome Sanger Institute for the COVID-19 Genomics UK (COG-UK) consortium|Davies|United Kingdom
>Spike|hCoV-19/England/BRIS-1297CF/2020|2020-03-31|EPI_ISL_458825|Original|hCoV-19^^England|Human|PHE South West Regional Laboratory|Wellcome Sanger Institute for the COVID-19 Genomics UK (COG-UK) consortium|Davies|United Kingdom
>Spike|hCoV-19/Scotland/EDB3123/2020|2020-04-04|EPI_ISL_488914|Original|hCoV-19^^Scotland|Human|Virology Department|Wellcome Sanger Institute for the COVID-19 Genomics UK (COG-UK) consortium|Davies|United Kingdom
>Spike|hCoV-19/Netherlands/ZH-RIVM-20890/2020|2020-10-02|EPI_ISL_790928|Original|hCoV-19^^Zuid-holland|Human|Dutch COVID-19 response team|National Institute for Public Health and the Environment (RIVM)|Meijer|Netherlands

>Spike|hCoV-19/England/204761672/2020|2020-11-19|EPI_ISL_766274|Original|hCoV-19^^England|Human|Respiratory Virus Unit|COVID-19 Genomics UK (COG-UK) Consortium|Platt|United Kingdom
>Spike|hCoV-19/Luxembourg/LNS9024343/2020|2020-12-28|EPI_ISL_771124|Original|hCoV-19^^Luxembourg|Human|Laboratoire national de sant\u00e9|Laboratoire national de sant\u00e9|Wienecke-Baldacchino|Luxembourg
>Spike|hCoV-19/USA/WI-UW-1197/2020|2020-07-29|EPI_ISL_527807|Original|hCoV-19^^Wisconsin|Human|University of Wisconsin-Madison AIDS Vaccine Research Laboratories|University of Wisconsin-Madison AIDS Vaccine Research Laboratories|Baczenas|USA
>Spike|hCoV-19/Canada/ON-PHL-20-03678/2020|2020-11-00|EPI_ISL_775162|Original|hCoV-19^^Ontario|Human|Public Health Ontario Laboratory|Public Health Ontario Laboratory|Guthrie|Canada
>Spike|hCoV-19/USA/TX-DSHS-0714/2020|2020-09-09|EPI_ISL_576357|Original|hCoV-19^^Texas|Human|Texas Department of State Health Services|Texas Department of State Health Services|Rahman|USA
>Spike|hCoV-19/England/BIRM-646BD/2020|2020-09-23|EPI_ISL_594504|Original|hCoV-19^^England|Human|University of Birmingham|COVID-19 Genomics UK (COG-UK) Consortium|Loman|United Kingdom
>Spike|hCoV-19/England/SHEF-C0075/2020|2020-03-23|EPI_ISL_420271|Original|hCoV-19^^England|Human|Virology Department|Department of Infection|de Silva|United Kingdom
>Spike|hCoV-19/USA/TX-HMH-1970/2020|2020-05-15|EPI_ISL_542927|Original|hCoV-19^^Texas|Human|Houston Methodist Hospital|Houston Methodist Hospital|Long|USA
>Spike|hCoV-19/USA/UT-UPHL-2012303126/2020|2020-08-17|EPI_ISL_746059|Original|hCoV-19^^Utah|Human|Ginkgo Bioworks Clinical Laboratory|Utah Public Health Laboratory|Young|USA
>Spike|hCoV-19/England/QEUH-B6C483/2020|2020-11-11|EPI_ISL_655272|Original|hCoV-19^^England|Human|Lighthouse Lab in Glasgow|Wellcome Sanger Institute for the COVID-19 Genomics UK (COG-UK) Consortium|Davies|United Kingdom
>Spike|hCoV-19/USA/TX-HMH-4125/2020|2020-06-18|EPI_ISL_543391|Original|hCoV-19^^Texas|Human|Houston Methodist Hospital|Houston Methodist Hospital|Long|USA
>Spike|hCoV-19/USA/OR-OHSU-3702/2020|2020-10-19|EPI_ISL_667510|Original|hCoV-19^^Oregon|Human|OHSU Lab Services Molecular Microbiology Lab|Oregon SARS-CoV-2 Genome Sequencing Center|O'Roak|USA
>Spike|hCoV-19/England/ALDP-9E9392/2020|2020-09-24|EPI_ISL_584210|Original|hCoV-19^^England|Human|Virology Department|COVID-19 Genomics UK (COG-UK) Consortium|de Silva|United Kingdom
>Spike|hCoV-19/England/ALDP-B5BCCB/2020|2020-11-09|EPI_ISL_655878|Original|hCoV-19^^England|Human|Lighthouse Lab in Alderley Park|Wellcome Sanger Institute for the COVID-19 Genomics UK (COG-UK) Consortium|Davies|United Kingdom
>Spike|hCoV-19/USA/TX-HMH-MCoV-8256/2020|2020-07-07|EPI_ISL_784295|Original|hCoV-19^^Texas|Human|Houston Methodist Hospital|Houston Methodist Hospital|Long|USA
>Spike|hCoV-19/England/QEUH-B0DE1D/2020|2020-11-02|EPI_ISL_641998|Original|hCoV-19^^England|Human|Lighthouse Lab in Glasgow|Wellcome Sanger Institute for the COVID-19 Genomics UK (COG-UK) Consortium|Davies|United Kingdom
>Spike|hCoV-19/England/205260463/2020|2020-12-20|EPI_ISL_754319|Original|hCoV-19^^England|Human|Respiratory Virus Unit|COVID-19 Genomics UK (COG-UK) Consortium|Platt|United Kingdom
>Spike|hCoV-19/England/CAMB-7874A/2020|2020-04-06|EPI_ISL_433768|Original|hCoV-19^^England|Human|Department of Pathology|COVID-19 Genomics UK (COG-UK) Consortium|Meredith|United Kingdom
>Spike|hCoV-19/South Korea/KUMC01/2020|2020-02-06|EPI_ISL_413017|Original|hCoV-19^^South Korea|Human|Department of Microbiology|Department of Microbiology|Park|South Korea
>Spike|hCoV-19/Denmark/DCGC-1485/2020|2020-06-01|EPI_ISL_617969|Original|hCoV-19^^Hovedstaden|Human|Department of Virus and Microbiological Special Diagnostics|Albertsen lab|Michaelsen|Denmark
>Spike|hCoV-19/Netherlands/Gelderland_125/2020|2020-05-22|EPI_ISL_460886|Original|hCoV-19^^Gelderland|Human|Dutch COVID-19 response team|Erasmus Medical Center|Oude Munnink|Netherlands

>Spike|hCoV-19/England/SHEF-D1D6B/2020|2020-04-18|EPI_ISL_462080|Original|hCoV-19^^England|Human|Virology Department|COVID-19 Genomics UK (COG-UK) Consortium|de Silva|United Kingdom
>Spike|hCoV-19/England/CAMC-B38031/2020|2020-11-05|EPI_ISL_658745|Original|hCoV-19^^England|Human|Lighthouse Lab in Cambridge|Wellcome Sanger Institute for the COVID-19 Genomics UK (COG-UK) Consortium|Davies|United Kingdom
>Spike|hCoV-19/England/CAMB-79EBD/2020|2020-04-01|EPI_ISL_440613|Original|hCoV-19^^England|Human|Department of Pathology|Wellcome Sanger Institute for the COVID-19 Genomics UK (COG-UK) consortium|Amato|United Kingdom
>Spike|hCoV-19/USA/IL-NM0119/2020|2020-03-21|EPI_ISL_444602|Original|hCoV-19^^Illinois|Human|Northwestern Memorial Hospital|Ozer Lab|Ozer|USA
>Spike|hCoV-19/Denmark/DCGC-8784/2020|2020-10-26|EPI_ISL_626054|Original|hCoV-19^^Midtjylland|Human|Department of Virus and Microbiological Special Diagnostics|Albertsen lab|Michaelsen|Denmark
>Spike|hCoV-19/England/MILK-A7BA35/2020|2020-10-15|EPI_ISL_607477|Original|hCoV-19^^England|Human|Lighthouse Lab in Milton Keynes|Wellcome Sanger Institute for the COVID-19 Genomics UK (COG-UK) consortium|Davies|United Kingdom
>Spike|hCoV-19/Russia/NGR-RII-MH6330S/2020|2020-11-12|EPI_ISL_733382|Original|hCoV-19^^Novgorod|Human|HELIX LLC|WHO National Influenza Centre Russian Federation|Komissarov|Russia
>Spike|hCoV-19/England/ALDP-B24706/2020|2020-11-04|EPI_ISL_646864|Original|hCoV-19^^England|Human|Lighthouse Lab in Alderley Park|Wellcome Sanger Institute for the COVID-19 Genomics UK (COG-UK) Consortium|Davies|United Kingdom
>Spike|hCoV-19/Netherlands/ZH-EMC-867/2020|2020-10-16|EPI_ISL_632751|Original|hCoV-19^^Zuid Holland|Human|Dutch COVID-19 response team|Erasmus Medical Center|Oude Munnink|Netherlands
>Spike|hCoV-19/England/ALDP-952CB7/2020|2020-06-13|EPI_ISL_555320|Original|hCoV-19^^England|Human|Lighthouse Lab in Alderley Park|Wellcome Sanger Institute for the COVID-19 Genomics UK (COG-UK) consortium|Davies|United Kingdom
>Spike|hCoV-19/Wales/PHWC-242E2/2020|2020-03-16|EPI_ISL_418165|Original|hCoV-19^^Wales|Human|Wales Specialist Virology Centre|Public Health Wales Microbiology Cardiff|Moore|United Kingdom
>Spike|hCoV-19/USA/CA-CZB-2350/2020|2020-05-13|EPI_ISL_513798|Original|hCoV-19^^California|Human|Orange County Public Health Laboratory|Chan-Zuckerberg Biohub|Kamm|USA
>Spike|hCoV-19/England/ALDP-AA21E5/2020|2020-10-22|EPI_ISL_625104|Original|hCoV-19^^England|Human|Lighthouse Lab in Alderley Park|Wellcome Sanger Institute for the COVID-19 Genomics UK (COG-UK) consortium|Davies|United Kingdom
>Spike|hCoV-19/England/MILK-9942D5/2020|2020-08-26|EPI_ISL_551634|Original|hCoV-19^^England|Human|Lighthouse Lab in Milton Keynes|Wellcome Sanger Institute for the COVID-19 Genomics UK (COG-UK) consortium|Davies|United Kingdom
>Spike|hCoV-19/England/ALDP-BB54D6/2020|2020-11-21|EPI_ISL_701700|Original|hCoV-19^^England|Human|Lighthouse Lab in Alderley Park|Wellcome Sanger Institute for the COVID-19 Genomics UK (COG-UK) Consortium|Davies|United Kingdom
>Spike|hCoV-19/Scotland/EDB7121/2020|2020-08-05|EPI_ISL_514539|Original|hCoV-19^^Scotland|Human|Virology Department|COVID-19 Genomics UK (COG-UK) Consortium|Williams|United Kingdom
>Spike|hCoV-19/Wales/PHWC-497E79/2020|2020-12-01|EPI_ISL_726987|Original|hCoV-19^^Wales|Human|Originating lab|COVID-19 Genomics UK (COG-UK) Consortium|Moore|United Kingdom
>Spike|hCoV-19/Denmark/DCGC-5585/2020|2020-09-28|EPI_ISL_620910|Original|hCoV-19^^Sjaelland|Human|Department of Virus and Microbiological Special Diagnostics|Albertsen lab|Michaelsen|Denmark
>Spike|hCoV-19/Canada/BC_23052516/2020|2020-03-00|EPI_ISL_462839|Original|hCoV-19^^British Columbia|Human|BCCDC Public Health Laboratory|BCCDC Public Health Laboratory|Harrigan|Canada
>Spike|hCoV-19/USA/NY-PV09121/2020|2020-03-19|EPI_ISL_422496|Original|hCoV-19^^New York|Human|MSHS Clinical Microbiology Laboratories|MSHS Pathogen Surveillance Program|van Bakel|USA

>Spike|hCoV-19/India/GJ-GBRC297b/2020|2020-06-15|EPI_ISL_495039|Original|hCoV-
19^^Gujarat|Human|GMERS Medical College & Hospital|Gujarat Biotechnology Research
Centre|Joshi|India
>Spike|hCoV-19/England/MILK-B3D096/2020|2020-11-01|EPI_ISL_706217|Original|hCoV-
19^^England|Human|Oxford Viromics|COVID-19 Genomics UK (COG-UK)
Consortium|Golubchik|United Kingdom
>Spike|hCoV-19/USA/VA-DCLS-0511/2020|2020-05-29|EPI_ISL_485822|Original|hCoV-
19^^Virginia|Human|Virginia DCLS|Virginia DCLS|St. Jacques|USA
>Spike|hCoV-19/Australia/WA350/2020|2020-04-06|EPI_ISL_605827|Original|hCoV-
19^^Western Australia|Human|PathWest Laboratory Medicine WA|PathWest Laboratory
Medicine WA Microbial Surveillance Unit|Sikazwe|Australia
>Spike|hCoV-19/Scotland/CVR787/2020|2020-03-27|EPI_ISL_439110|Original|hCoV-
19^^Scotland|Human|West of Scotland Specialist Virology Centre|COVID-19 Genomics UK
(COG-UK) Consortium|Robertson|United Kingdom
>Spike|hCoV-19/Denmark/DCGC-8163/2020|2020-10-26|EPI_ISL_617230|Original|hCoV-
19^^Hovedstaden|Human|Department of Virus and Microbiological Special
Diagnostics|Albertsen lab|Michaelsen|Denmark
>Spike|hCoV-19/England/MILK-9A9774/2020|2020-09-01|EPI_ISL_551262|Original|hCoV-
19^^England|Human|Lighthouse Lab in Milton Keynes|Wellcome Sanger Institute for the
COVID-19 Genomics UK (COG-UK) consortium|Davies|United Kingdom
>Spike|hCoV-19/Portugal/PT1261/2020|2020-03-19|EPI_ISL_511222|Original|hCoV-
19^^Portugal|Human|Instituto Nacional de Saude (INSA) and Instituto Gulbenkian de
Ciencia (IGC)|Instituto Nacional de Saude (INSA) and Instituto Gulbenkian de Ciencia
(IGC)|Borges|Portugal
>Spike|hCoV-19/England/ALDP-95028A/2020|2020-06-17|EPI_ISL_555273|Original|hCoV-
19^^England|Human|Lighthouse Lab in Alderley Park|Wellcome Sanger Institute for the
COVID-19 Genomics UK (COG-UK) consortium|Davies|United Kingdom
>Spike|hCoV-19/Finland/5LS16H9/2020|2020-10-05|EPI_ISL_756046|Original|hCoV-
19^^Uusimaa|Human|Department of Virology and Immunology|Department of
Virology|Smura|Finland
>Spike|hCoV-19/USA/WA-UW-2743/2020|2020-04-15|EPI_ISL_486025|Original|hCoV-
19^^Washington|Human|UW Virology Lab|UW Virology Lab|Roychoudhury|USA
>Spike|hCoV-19/England/MILK-9AA1B1/2020|2020-09-01|EPI_ISL_550869|Original|hCoV-
19^^England|Human|Lighthouse Lab in Milton Keynes|Wellcome Sanger Institute for the
COVID-19 Genomics UK (COG-UK) consortium|Davies|United Kingdom
>Spike|hCoV-19/USA/VA-DCLS-1776/2020|2020-09-16|EPI_ISL_644866|Original|hCoV-
19^^Virginia|Human|Virginia DCLS|Virginia DCLS|St. Jacques|USA
>Spike|hCoV-19/England/QEUH-A9F118/2020|2020-10-20|EPI_ISL_611370|Original|hCoV-
19^^England|Human|Lighthouse Lab in Glasgow|Wellcome Sanger Institute for the COVID-
19 Genomics UK (COG-UK) consortium|Davies|United Kingdom
>Spike|hCoV-19/United Arab Emirates/0825/2020|2020-05-
19|EPI_ISL_698964|Original|hCoV-19^^Abu Dhabi|Human|Group 42 (G42) Healthcare|G42
Healthcare|Ogrodzki|United Arab Emirates
>Spike|hCoV-19/Switzerland/VD-ETHZ-240074/2020|2020-08-
10|EPI_ISL_523912|Original|hCoV-19^^Vaud|Human|Viollier AG|Department of Biosystems
Science and Engineering|Nadeau|Switzerland
>Spike|hCoV-19/England/ALDP-9521A9/2020|2020-06-09|EPI_ISL_553636|Original|hCoV-
19^^England|Human|Lighthouse Lab in Alderley Park|Wellcome Sanger Institute for the
COVID-19 Genomics UK (COG-UK) consortium|Davies|United Kingdom
>Spike|hCoV-19/USA/UT-08491/2020|2020-05-21|EPI_ISL_524259|Original|hCoV-
19^^Utah|Human|Utah Public Health Laboratory|Utah Public Health Laboratory|Young|USA
>Spike|hCoV-19/Switzerland/BE-ETHZ-321444/2020|2020-10-
14|EPI_ISL_603668|Original|hCoV-19^^Bern|Human|Viollier AG|Department of Biosystems
Science and Engineering|Nadeau|Switzerland
>Spike|hCoV-19/USA/TX-HMH-MCoV-18175/2020|2020-11-14|EPI_ISL_784523|Original|hCoV-
19^^Texas|Human|Houston Methodist Hospital|Houston Methodist Hospital|Long|USA
>Spike|hCoV-19/England/CAMC-BFB498/2020|2020-12-04|EPI_ISL_709029|Original|hCoV-
19^^England|Human|Lighthouse Lab in Cambridge|Wellcome Sanger Institute for the
COVID-19 Genomics UK (COG-UK) Consortium|Davies|United Kingdom

>Spike|hCoV-19/Malaysia/IMR-WC124489/2020|2020-07-25|EPI_ISL_718284|Original|hCoV-19^^Malaysia|Human|Institute for Medical Research|Institute for Medical Research|Suppiah|Malaysia
>Spike|hCoV-19/England/MILK-99A80A/2020|2020-08-29|EPI_ISL_550263|Original|hCoV-19^^England|Human|Lighthouse Lab in Milton Keynes|Wellcome Sanger Institute for the COVID-19 Genomics UK (COG-UK) consortium|Davies|United Kingdom
>Spike|hCoV-19/England/LOND-124DCE0/2020|2020-00-00|EPI_ISL_584646|Original|hCoV-19^^England|Human|University College London Hospital|COVID-19 Genomics UK (COG-UK) Consortium|Byott|United Kingdom
>Spike|hCoV-19/Scotland/EDB1481/2020|2020-04-12|EPI_ISL_491590|Original|hCoV-19^^Scotland|Human|Virology Department|Wellcome Sanger Institute for the COVID-19 Genomics UK (COG-UK) consortium|Davies|United Kingdom
>Spike|hCoV-19/Australia/VIC15918/2020|2020-07-31|EPI_ISL_640778|Original|hCoV-19^^Victoria|Human|Microbiological Diagnostic Unit - Public Health Laboratory (MDU-PHL)|MDU-PHL|Schultz|Australia
>Spike|hCoV-19/England/MILK-99C5C5/2020|2020-08-30|EPI_ISL_550766|Original|hCoV-19^^England|Human|Lighthouse Lab in Milton Keynes|Wellcome Sanger Institute for the COVID-19 Genomics UK (COG-UK) consortium|Davies|United Kingdom
>Spike|hCoV-19/England/ALDP-B48010/2020|2020-11-07|EPI_ISL_649406|Original|hCoV-19^^England|Human|Lighthouse Lab in Alderley Park|Wellcome Sanger Institute for the COVID-19 Genomics UK (COG-UK) Consortium|Davies|United Kingdom
>Spike|hCoV-19/Australia/VIC2323/2020|2020-06-26|EPI_ISL_522044|Original|hCoV-19^^Victoria|Human|Microbiological Diagnostic Unit - Public Health Laboratory (MDU-PHL)|MDU-PHL|Schultz|Australia
>Spike|hCoV-19/England/QEUH-CC2280/2020|2020-12-21|EPI_ISL_778145|Original|hCoV-19^^England|Human|Lighthouse Lab in Glasgow|Wellcome Sanger Institute for the COVID-19 Genomics UK (COG-UK) Consortium|Davies|United Kingdom
>Spike|hCoV-19/Wales/PHWC-1658C4/2020|2020-05-11|EPI_ISL_490737|Original|hCoV-19^^Wales|Human|Wales Specialist Virology Centre Sequencing lab|COVID-19 Genomics UK (COG-UK) Consortium|Moore|United Kingdom
>Spike|hCoV-19/Chile/RM-128080/2020|2020-07-18|EPI_ISL_746614|Original|hCoV-19^^Region Metropolitana de Santiago|Human|Genetica Molecular and Subdepartamento de Virologia ISP Chile|Instituto de Salud Publica de Chile|Tognarelli|Chile
>Spike|hCoV-19/France/OCC-SC493/2020|2020-03-26|EPI_ISL_644692|Original|hCoV-19^^Occitanie|Human|CHU Montpellier|CNR Virus des Infections Respiratoires - France SUD|Bal|France
>Spike|hCoV-19/Wales/PHWC-4A0C0A/2020|2020-12-11|EPI_ISL_743559|Original|hCoV-19^^Wales|Human|Wales Specialist Virology Centre Sequencing lab|COVID-19 Genomics UK (COG-UK) Consortium|Moore|United Kingdom
>Spike|hCoV-19/Denmark/DCGC-18362/2020|2020-12-07|EPI_ISL_749497|Original|hCoV-19^^Syddanmark|Human|Department of Virus and Microbiological Special Diagnostics|Albertsen Lab|Michaelsen|Denmark
>Spike|hCoV-19/Japan/PG-7261/2020|2020-08-00|EPI_ISL_688364|Original|hCoV-19^^Japan|Human|Saitama Prefectural Institute of Public Health|Pathogen Genomics Center|Sekizuka|Japan
>Spike|hCoV-19/USA/OR-OHSU-1181/2020|2020-07-11|EPI_ISL_509157|Original|hCoV-19^^Oregon|Human|OHSU Lab Services Molecular Microbiology Lab|Oregon SARS-CoV-2 Genome Sequencing Center|O'Roak|USA
>Spike|hCoV-19/USA/MO-WUSTL063/2020|2020-06-19|EPI_ISL_493082|Original|hCoV-19^^Missouri|Human|Washington University in St. Louis|Washington University in St. Louis|Tahan|USA
>Spike|hCoV-19/England/ALDP-63CBF5/2020|2020-07-04|EPI_ISL_556265|Original|hCoV-19^^England|Human|Lighthouse Lab in Alderley Park|Wellcome Sanger Institute for the COVID-19 Genomics UK (COG-UK) consortium|Davies|United Kingdom
>Spike|hCoV-19/Wales/PHWC-16D219/2020|2020-09-26|EPI_ISL_574213|Original|hCoV-19^^Wales|Human|Wales Specialist Virology Centre Sequencing lab|COVID-19 Genomics UK (COG-UK) Consortium|Moore|United Kingdom
>Spike|hCoV-19/USA/AZ-TG359176/2020|2020-06-08|EPI_ISL_694443|Original|hCoV-19^^Arizona|Human|TGen North|TGen North|French|USA

>Spike|hCoV-19/USA/UT-UPHL-2012874225/2020|2020-08-03|EPI_ISL_745744|Original|hCoV-19^^Utah|Human|Ginkgo Bioworks Clinical Laboratory|Utah Public Health Laboratory|Young|USA
>Spike|hCoV-19/USA/WI-GMF-32385/2020|2020-11-09|EPI_ISL_661140|Original|hCoV-19^^Wisconsin|Human|Gundersen Molecular Diagnostics Laboratory|Kabara Cancer Research Institute|Kenny|USA
>Spike|hCoV-19/England/MILK-9F2958/2020|2020-09-22|EPI_ISL_589237|Original|hCoV-19^^England|Human|Lighthouse Lab in Milton Keynes|Wellcome Sanger Institute for the COVID-19 Genomics UK (COG-UK) consortium|Davies|United Kingdom
>Spike|hCoV-19/Portugal/PT0077/2020|2020-03-21|EPI_ISL_421480|Original|hCoV-19^^Portugal|Human|CH Barreiro Montijo|Instituto Nacional de Saude (INSA)|Borges|Portugal
>Spike|hCoV-19/Spain/MD-IBV-99008607/2020|2020-06-17|EPI_ISL_654186|Original|hCoV-19^^Madrid|Human|Hospital General Universitario Gregorio Mara\u00f1\u00f3n|SeqCOVID-SPAIN consortium/IBV(CSIC)|Comas|Spain
>Spike|hCoV-19/USA/NY-PV09435/2020|2020-03-13|EPI_ISL_450112|Original|hCoV-19^^New York|Human|MSHS Clinical Microbiology Laboratories|MSHS Pathogen Surveillance Program|van Bakel|USA
>Spike|hCoV-19/USA/NE-11323/2020|2020-05-04|EPI_ISL_466661|Original|hCoV-19^^Nebraska|Human|Nebraska Public Health Laboratory|UNMC COVID-19 Response Team|Pratt|USA
>Spike|hCoV-19/Wales/PHWC-49A0CB/2020|2020-11-30|EPI_ISL_727492|Original|hCoV-19^^Wales|Human|Originating lab|COVID-19 Genomics UK (COG-UK) Consortium|Moore|United Kingdom
>Spike|hCoV-19/USA/CA-CZB-15442/2020|2020-12-03|EPI_ISL_739401|Original|hCoV-19^^California|Human|Orange County Public Health Lab|Chan-Zuckerberg Biohub|Kamm|USA
>Spike|hCoV-19/USA/MA-MGH-02662/2020|2020-09-23|EPI_ISL_765583|Original|hCoV-19^^Massachusetts|Human|Brigham and Womens Hospital|Infectious Disease Program|Park|USA
>Spike|hCoV-19/England/201140388/2020|2020-03-10|EPI_ISL_464730|Original|hCoV-19^^England|Human|Respiratory Virus Unit|Respiratory Virus Unit|Platt|United Kingdom
>Spike|hCoV-19/England/ALDP-A86E2C/2020|2020-10-16|EPI_ISL_606166|Original|hCoV-19^^England|Human|Lighthouse Lab in Alderley Park|Wellcome Sanger Institute for the COVID-19 Genomics UK (COG-UK) consortium|Davies|United Kingdom
>Spike|hCoV-19/USA/TX-HMH-MCoV-15482/2020|2020-10-25|EPI_ISL_786966|Original|hCoV-19^^Texas|Human|Houston Methodist Hospital|Houston Methodist Hospital|Long|USA
>Spike|hCoV-19/France/PAC-IHU-0471/2020|2020-04-05|EPI_ISL_569535|Original|hCoV-19^^Provence-Alpes-C\u00f4te-d\u2019Azur|Human|MEPHI|MEPHI|Levasseur|France
>Spike|hCoV-19/Denmark/DCGC-19115/2020|2020-12-07|EPI_ISL_748193|Original|hCoV-19^^Hovedstaden|Human|Department of Virus and Microbiological Special Diagnostics|Albertsen Lab|Michaelsen|Denmark
>Spike|hCoV-19/England/CAMC-B77A0B/2020|2020-11-11|EPI_ISL_661817|Original|hCoV-19^^England|Human|Lighthouse Lab in Cambridge|Wellcome Sanger Institute for the COVID-19 Genomics UK (COG-UK) Consortium|Davies|United Kingdom
>Spike|hCoV-19/Denmark/DCGC-4285/2020|2020-09-07|EPI_ISL_615722|Original|hCoV-19^^Hovedstaden|Human|Department of Virus and Microbiological Special Diagnostics|Albertsen lab|Michaelsen|Denmark
>Spike|hCoV-19/England/NORT-283355/2020|2020-00-00|EPI_ISL_472239|Original|hCoV-19^^England|Human|Northumbria University / South Tees Hospitals NHS Foundation Trust / North Cumbria Integrated Care NHS Foundation Trust / North Tees and Hartlepool NHS Foundation Trust / Newcastle Hospitals NHS Foundation Trust|COVID-19 Genomics UK (COG-UK) Consortium|Bashton|United Kingdom
>Spike|hCoV-19/England/QEUH-C321D9/2020|2020-12-11|EPI_ISL_782598|Original|hCoV-19^^England|Human|Lighthouse Lab in Glasgow|Wellcome Sanger Institute for the COVID-19 Genomics UK (COG-UK) Consortium|Davies|United Kingdom
>Spike|hCoV-19/Canada/ON-PHL-20-03694/2020|2020-11-00|EPI_ISL_775177|Original|hCoV-19^^Ontario|Human|Public Health Ontario Laboratory|Public Health Ontario Laboratory|Guthrie|Canada

>Spike|hCoV-19/England/ALDP-9C5BD0/2020|2020-09-10|EPI_ISL_567805|Original|hCoV-19^^England|Human|Lighthouse Lab in Alderley Park|Wellcome Sanger Institute for the COVID-19 Genomics UK (COG-UK) consortium|Davies|United Kingdom
>Spike|hCoV-19/Sweden/20-07896/2020|2020-04-27|EPI_ISL_455102|Original|hCoV-19^^Vastmanland|Human|Kungsors VC|The Public Health Agency of Sweden|Svartstr\u00f6m|Sweden
>Spike|hCoV-19/Denmark/DCGC-22337/2020|2020-12-14|EPI_ISL_758687|Original|hCoV-19^^Sjaelland|Human|Department of Virus and Microbiological Special Diagnostics|Albertsen Lab|Michaelsen|Denmark
>Spike|hCoV-19/England/ALDP-B1D050/2020|2020-11-01|EPI_ISL_645573|Original|hCoV-19^^England|Human|Lighthouse Lab in Alderley Park|Wellcome Sanger Institute for the COVID-19 Genomics UK (COG-UK) Consortium|Davies|United Kingdom
>Spike|hCoV-19/South Africa/R13564-20/2020|2020-07-13|EPI_ISL_622907|Original|hCoV-19^^North West|Human|National Institute for Communicable Diseases of the National Health Laboratory Service|National Institute for Communicable Diseases of the National Health Laboratory Service|Allam|South Africa
>Spike|hCoV-19/England/LIVE-DB2CB1/2020|2020-10-27|EPI_ISL_637908|Original|hCoV-19^^England|Human|Liverpool Clinical Laboratories|COVID-19 Genomics UK (COG-UK) Consortium|Haldenby|United Kingdom
>Spike|hCoV-19/USA/MD-HP00093/2020|2020-03-31|EPI_ISL_457758|Original|hCoV-19^^Maryland|Human|Johns Hopkins Hospital Department of Pathology|Johns Hopkins Hospital Department of Pathology|Ramakrishnan|USA
>Spike|hCoV-19/Gibraltar/205121243/2020|2020-00-00|EPI_ISL_766365|Original|hCoV-19^^Gibraltar|Human|Microbiology|Respiratory Virus Unit|Platt|Gibraltar
>Spike|hCoV-19/England/MILK-A7BEFD/2020|2020-10-15|EPI_ISL_607369|Original|hCoV-19^^England|Human|Lighthouse Lab in Milton Keynes|Wellcome Sanger Institute for the COVID-19 Genomics UK (COG-UK) consortium|Davies|United Kingdom
>Spike|hCoV-19/England/ALDP-A8667F/2020|2020-10-16|EPI_ISL_606035|Original|hCoV-19^^England|Human|Lighthouse Lab in Alderley Park|Wellcome Sanger Institute for the COVID-19 Genomics UK (COG-UK) consortium|Davies|United Kingdom
>Spike|hCoV-19/England/NORW-FA2C7/2020|2020-12-08|EPI_ISL_725490|Original|hCoV-19^^England|Human|Quadram Institute Bioscience|COVID-19 Genomics UK (COG-UK) Consortium|Alikhan|United Kingdom
>Spike|hCoV-19/USA/FL-BPHL-0176/2020|2020-04-03|EPI_ISL_480827|Original|hCoV-19^^Florida|Human|Florida Bureau of Public Health Laboratories|Florida Bureau of Public Health Laboratories|Blanton|USA
>Spike|hCoV-19/England/ALDP-C3AA5E/2020|2020-12-09|EPI_ISL_731570|Original|hCoV-19^^England|Human|Lighthouse Lab in Alderley Park|Wellcome Sanger Institute for the COVID-19 Genomics UK (COG-UK) Consortium|Davies|United Kingdom
>Spike|hCoV-19/USA/WA-UW-2767/2020|2020-03-27|EPI_ISL_486030|Original|hCoV-19^^Washington|Human|UW Virology Lab|UW Virology Lab|Roychoudhury|USA
>Spike|hCoV-19/Canada/BC-BCCDC-6460/2020|2020-10-00|EPI_ISL_740607|Original|hCoV-19^^British Columbia|Human|BCCDC Public Health Laboratory|BCCDC Public Health Laboratory|Prystajecky|Canada
>Spike|hCoV-19/England/ALDP-B48F9B/2020|2020-11-07|EPI_ISL_649413|Original|hCoV-19^^England|Human|Lighthouse Lab in Alderley Park|Wellcome Sanger Institute for the COVID-19 Genomics UK (COG-UK) Consortium|Davies|United Kingdom
>Spike|hCoV-19/England/204590546/2020|2020-11-02|EPI_ISL_700647|Original|hCoV-19^^England|Human|Respiratory Virus Unit|COVID-19 Genomics UK (COG-UK) Consortium|Platt|United Kingdom
>Spike|hCoV-19/USA/MI-MDHHS-SC22654/2020|2020-11-16|EPI_ISL_737268|Original|hCoV-19^^Michigan|Human|Michigan Department of Health and Human Services|Michigan Department of Health and Human Services|Blankenship|USA
>Spike|hCoV-19/Luxembourg/LNS7693582/2020|2020-10-30|EPI_ISL_744616|Original|hCoV-19^^Luxembourg|Human|Laboratoire national de sant\u00e9|Laboratoire national de sant\u00e9|Wienecke-Baldacchino|Luxembourg
>Spike|hCoV-19/Japan/PG-3385/2020|2020-05-00|EPI_ISL_691500|Original|hCoV-19^^Japan|Human|Pathogen Genomics Center|Pathogen Genomics Center|Sekizuka|Japan

>Spike|hCoV-19/Wales/PHWC-16443A/2020|2020-05-10|EPI_ISL_474177|Original|hCoV-
19^^Wales|Human|Originating lab|COVID-19 Genomics UK (COG-UK) Consortium|Moore|United
Kingdom
>Spike|hCoV-19/USA/MN-MDH-120/2020|2020-03-20|EPI_ISL_427276|Original|hCoV-
19^^Minnesota|Human|Minnesota Department of Health|Minnesota Department of
Health|Wang|USA
>Spike|hCoV-19/Spain/MD-IBV-003720/2020|2020-04-03|EPI_ISL_467236|Original|hCoV-
19^^Madrid|Human|Hospital General Universitario Gregorio Mara\u00f1\u00f3n|SeqCOVID-
SPAIN consortium/IBV(CSIC)|Comas|Spain
>Spike|hCoV-19/Wales/PHWC-499ABF/2020|2020-12-05|EPI_ISL_727399|Original|hCoV-
19^^Wales|Human|Originating lab|COVID-19 Genomics UK (COG-UK) Consortium|Moore|United
Kingdom
>Spike|hCoV-19/USA/WA-UW-12352/2020|2020-06-27|EPI_ISL_570509|Original|hCoV-
19^^Washington|Human|UW Virology Lab|UW Virology Lab|Roychoudhury|USA
>Spike|hCoV-19/England/QEUH-A4C8E8/2020|2020-10-05|EPI_ISL_628575|Original|hCoV-
19^^England|Human|Oxford Viromics|COVID-19 Genomics UK (COG-UK)
Consortium|Golubchik|United Kingdom
>Spike|hCoV-19/England/CAMC-A6461D/2020|2020-10-11|EPI_ISL_609156|Original|hCoV-
19^^England|Human|Lighthouse Lab in Cambridge|Wellcome Sanger Institute for the
COVID-19 Genomics UK (COG-UK) consortium|Davies|United Kingdom
>Spike|hCoV-19/England/MILK-ACF12E/2020|2020-10-22|EPI_ISL_629629|Original|hCoV-
19^^England|Human|Lighthouse Lab in Milton Keynes|Wellcome Sanger Institute for the
COVID-19 Genomics UK (COG-UK) consortium|Davies|United Kingdom
>Spike|hCoV-19/England/ALDP-A6F17B/2020|2020-10-15|EPI_ISL_607963|Original|hCoV-
19^^England|Human|Lighthouse Lab in Alderley Park|Wellcome Sanger Institute for the
COVID-19 Genomics UK (COG-UK) consortium|Davies|United Kingdom
>Spike|hCoV-19/USA/TX-HMH-MCoV-12172/2020|2020-09-21|EPI_ISL_790233|Original|hCoV-
19^^Texas|Human|Houston Methodist Hospital|Houston Methodist Hospital|Long|USA
>Spike|hCoV-19/USA/FL-BPHL-1976/2020|2020-04-16|EPI_ISL_653129|Original|hCoV-
19^^Florida|Human|Florida Bureau of Public Health Laboratories|Florida Bureau of
Public Health Laboratories|Blanton|USA
>Spike|hCoV-19/SaudiArabia/KAUST-MADINAH1061/2020|2020-06-
13|EPI_ISL_513086|Original|hCoV-19^^Madinah|Human|Pathogen Genomics Lab King Abdullah
University of Science and Technology(KAUST)|Pathogen Genomics Lab King Abdullah
University of Science and Technology(KAUST)|Mohamed Ghazzali|Saudi Arabia
>Spike|hCoV-19/Scotland/QEUH-9DC4DB/2020|2020-09-22|EPI_ISL_601174|Original|hCoV-
19^^Scotland|Human|Lighthouse Lab in Glasgow|Wellcome Sanger Institute for the COVID-
19 Genomics UK (COG-UK) consortium|Davies|United Kingdom
>Spike|hCoV-19/Germany/BW-MVZ-LS-51/2020|2020-04-15|EPI_ISL_640268|Original|hCoV-
19^^Baden-W\u00fcrttemberg|Human|MVZ Labor\u00e4rzte Singen|MVZ Labor\u00e4rzte
Singen|Schmidt|Germany
>Spike|hCoV-19/South Africa/KRISP-K001408/2020|2020-07-
18|EPI_ISL_515891|Original|hCoV-19^^KwaZulu-Natal|Human|Medical Disagnostics Services
(MDS)|KRISP|de Oliveira|South Africa
>Spike|hCoV-19/France/ARA-9476/2020|2020-03-16|EPI_ISL_509004|Original|hCoV-
19^^Auvergne-Rh\u00f4ne-Alpes|Human|GH Les Portes du Sud|CNR Virus des Infections
Respiratoires - France SUD|Josset|France
>Spike|hCoV-19/USA/TX-HMH-MCoV-19413/2020|2020-11-30|EPI_ISL_783825|Original|hCoV-
19^^Texas|Human|Houston Methodist Hospital|Houston Methodist Hospital|Long|USA
>Spike|hCoV-19/USA/MI-MDHHS-SC20761/2020|2020-04-22|EPI_ISL_471782|Original|hCoV-
19^^Michigan|Human|Michigan Department of Health and Human Services|Michigan
Department of Health and Human Services|Blankenship|USA
>Spike|hCoV-19/England/MILK-C908B6/2020|2020-12-09|EPI_ISL_777295|Original|hCoV-
19^^England|Human|Lighthouse Lab in Milton Keynes|Wellcome Sanger Institute for the
COVID-19 Genomics UK (COG-UK) Consortium|Davies|United Kingdom
>Spike|hCoV-19/Wales/PHWC-497EC4/2020|2020-11-30|EPI_ISL_726992|Original|hCoV-
19^^Wales|Human|Originating lab|COVID-19 Genomics UK (COG-UK) Consortium|Moore|United
Kingdom

>Spike|hCoV-19/England/ALDP-B5B8B8/2020|2020-11-08|EPI_ISL_655389|Original|hCoV-19^^England|Human|Lighthouse Lab in Alderley Park|Wellcome Sanger Institute for the COVID-19 Genomics UK (COG-UK) Consortium|Davies|United Kingdom
>Spike|hCoV-19/France/BRE-IPP11309/2020|2020-12-16|EPI_ISL_754855|Original|hCoV-19^^Bretagne|Human|CHU - H\u00f4pital Cavale Blanche - Labo. de Virologie|National Reference Center for Viruses of Respiratory Infections|Enouf|France
>Spike|hCoV-19/England/LIVE-DC9BD8/2020|2020-12-06|EPI_ISL_705352|Original|hCoV-19^^England|Human|Liverpool Clinical Laboratories|COVID-19 Genomics UK (COG-UK) Consortium|Haldenby|United Kingdom
>Spike|hCoV-19/Denmark/DCGC-14814/2020|2020-11-23|EPI_ISL_712002|Original|hCoV-19^^Syddanmark|Human|Department of Virus and Microbiological Special Diagnostics|Albertsen Lab|Michaelsen|Denmark
>Spike|hCoV-19/Wales/PHWC-4A4F1C/2020|2020-12-09|EPI_ISL_763590|Original|hCoV-19^^Wales|Human|Wales Specialist Virology Centre Sequencing lab|COVID-19 Genomics UK (COG-UK) Consortium|Moore|United Kingdom
>Spike|hCoV-19/England/QEUH-9DE59A/2020|2020-09-21|EPI_ISL_581346|Original|hCoV-19^^England|Human|Lighthouse Lab in Glasgow|Wellcome Sanger Institute for the COVID-19 Genomics UK (COG-UK) consortium|Davies|United Kingdom
>Spike|hCoV-19/Denmark/DCGC-4870/2020|2020-09-21|EPI_ISL_621556|Original|hCoV-19^^Hovedstaden|Human|Department of Virus and Microbiological Special Diagnostics|Albertsen lab|Michaelsen|Denmark
>Spike|hCoV-19/England/LOND-124C434/2020|2020-04-10|EPI_ISL_492887|Original|hCoV-19^^England|Human|Royal Free Hospital / Health Services Laboratories|Wellcome Sanger Institute for the COVID-19 Genomics UK (COG-UK) consortium|Davies|United Kingdom
>Spike|hCoV-19/Denmark/DCGC-12983/2020|2020-12-07|EPI_ISL_712507|Original|hCoV-19^^Hovedstaden|Human|Department of Virus and Microbiological Special Diagnostics|Albertsen Lab|Michaelsen|Denmark
>Spike|hCoV-19/Scotland/CVR763/2020|2020-03-26|EPI_ISL_439105|Original|hCoV-19^^Scotland|Human|West of Scotland Specialist Virology Centre|COVID-19 Genomics UK (COG-UK) Consortium|Robertson|United Kingdom
>Spike|hCoV-19/England/QEUH-C53FBA/2020|2020-12-12|EPI_ISL_736269|Original|hCoV-19^^England|Human|Lighthouse Lab in Glasgow|Wellcome Sanger Institute for the COVID-19 Genomics UK (COG-UK) Consortium|Davies|United Kingdom
>Spike|hCoV-19/England/MILK-46667B/2020|2020-05-27|EPI_ISL_559750|Original|hCoV-19^^England|Human|Lighthouse Lab in Milton Keynes|Wellcome Sanger Institute for the COVID-19 Genomics UK (COG-UK) consortium|Davies|United Kingdom
>Spike|hCoV-19/United Arab Emirates/0348/2020|2020-05-15|EPI_ISL_698523|Original|hCoV-19^^Abu Dhabi|Human|Group 42 (G42) Healthcare|G42 Healthcare|Ogrodzki|United Arab Emirates
>Spike|hCoV-19/Russia/OMS-ORINFI-3961S/2020|2020-07-08|EPI_ISL_569834|Original|hCoV-19^^Omsk|Human|Omsk Research Institute of Natural Focal Infections|WHO National Influenza Centre Russian Federation|Komissarov|Russia
>Spike|hCoV-19/Thailand/Bangkok-0056/2020|2020-03-28|EPI_ISL_446998|Original|hCoV-19^^Bangkok|Human|Ramathibodi Hospital|COVID-19 Network Investigations (CONI) Alliance|Batty|Thailand
>Spike|hCoV-19/Australia/SAP387/2020|2020-04-01|EPI_ISL_492152|Original|hCoV-19^^South Australia|Human|SA Pathology|SA Pathology|Leong|Australia
>Spike|hCoV-19/USA/FL-BPHL-0799/2020|2020-07-01|EPI_ISL_514145|Original|hCoV-19^^Florida|Human|Florida Bureau of Public Health Laboratories|Florida Bureau of Public Health Laboratories|Blanton|USA
>Spike|hCoV-19/England/ALDP-B47D38/2020|2020-11-07|EPI_ISL_649274|Original|hCoV-19^^England|Human|Lighthouse Lab in Alderley Park|Wellcome Sanger Institute for the COVID-19 Genomics UK (COG-UK) Consortium|Davies|United Kingdom
>Spike|hCoV-19/England/ALDP-9D9B7F/2020|2020-09-18|EPI_ISL_576796|Original|hCoV-19^^England|Human|Oxford Viromics|COVID-19 Genomics UK (COG-UK) Consortium|Golubchik|United Kingdom
>Spike|hCoV-19/France/OCC-SC473/2020|2020-03-16|EPI_ISL_660675|Original|hCoV-19^^Occitanie|Human|CHU Montpellier|CNR Virus des Infections Respiratoires - France SUD|Bal|France

>Spike|hCoV-19/England/CAMB-76278/2020|2020-03-29|EPI_ISL_438266|Original|hCoV-19^^England|Human|Department of Pathology|Wellcome Sanger Institute for the COVID-19 Genomics UK (COG-UK) consortium|Amato|United Kingdom
>Spike|hCoV-19/USA/CA-LACPHL-AD00010/2020|2020-10-02|EPI_ISL_708507|Original|hCoV-19^^California|Human|Los Angeles County Public Health Laboratory|Los Angeles County Public Health Laboratory|Hemarajata|USA
>Spike|hCoV-19/England/QEUH-CDF8BB/2020|2020-12-26|EPI_ISL_782449|Original|hCoV-19^^England|Human|Lighthouse Lab in Glasgow|Wellcome Sanger Institute for the COVID-19 Genomics UK (COG-UK) Consortium|Davies|United Kingdom
>Spike|hCoV-19/England/20134019502/2020|2020-03-23|EPI_ISL_423105|Original|hCoV-19^^England|Human|Respiratory Virus Unit|Respiratory Virus Unit|Platt|United Kingdom
>Spike|hCoV-19/England/ALDP-9E6CA9/2020|2020-09-24|EPI_ISL_580520|Original|hCoV-19^^England|Human|Lighthouse Lab in Alderley Park|Wellcome Sanger Institute for the COVID-19 Genomics UK (COG-UK) consortium|Davies|United Kingdom
>Spike|hCoV-19/Gibraltar/204462846/2020|2020-10-29|EPI_ISL_615016|Original|hCoV-19^^Gibraltar|Human|Microbiology|Respiratory Virus Unit|Platt|Gibraltar
>Spike|hCoV-19/Australia/VIC5016/2020|2020-07-19|EPI_ISL_519820|Original|hCoV-19^^Victoria|Human|Microbiological Diagnostic Unit - Public Health Laboratory (MDU-PHL)|MDU-PHL|Schultz|Australia
>Spike|hCoV-19/England/QEUH-A31497/2020|2020-10-05|EPI_ISL_595893|Original|hCoV-19^^England|Human|Quadram Institute Bioscience|COVID-19 Genomics UK (COG-UK) Consortium|Alikhan|United Kingdom
>Spike|hCoV-19/England/QEUH-AD1BC1/2020|2020-10-23|EPI_ISL_633694|Original|hCoV-19^^England|Human|Lighthouse Lab in Glasgow|Wellcome Sanger Institute for the COVID-19 Genomics UK (COG-UK) consortium|Davies|United Kingdom
>Spike|hCoV-19/USA/NY-Wadsworth-142783-01/2020|2020-04-10|EPI_ISL_676976|Original|hCoV-19^^New York|Human|Wadsworth Center|Wadsworth Center|Lasek-Nesselquist|USA
>Spike|hCoV-19/England/CAMB-1B633C/2020|2020-09-21|EPI_ISL_576899|Original|hCoV-19^^England|Human|Department of Pathology|COVID-19 Genomics UK (COG-UK) Consortium|Goodfellow|United Kingdom
>Spike|hCoV-19/USA/TX-HMH-5232/2020|2020-06-19|EPI_ISL_544611|Original|hCoV-19^^Texas|Human|Houston Methodist Hospital|Houston Methodist Hospital|Long|USA
>Spike|hCoV-19/USA/WA-UW-3600/2020|2020-03-25|EPI_ISL_570033|Original|hCoV-19^^Washington|Human|UW Virology Lab|UW Virology Lab|Roychoudhury|USA
>Spike|hCoV-19/England/MILK-A6B2B8/2020|2020-10-09|EPI_ISL_608730|Original|hCoV-19^^England|Human|Lighthouse Lab in Milton Keynes|Wellcome Sanger Institute for the COVID-19 Genomics UK (COG-UK) consortium|Davies|United Kingdom
>Spike|hCoV-19/Japan/PG-1173/2020|2020-04-00|EPI_ISL_684783|Original|hCoV-19^^Japan|Human|Pathogen Genomics Center|Pathogen Genomics Center|Sekizuka|Japan
>Spike|hCoV-19/Netherlands/NH-RIVM-20633/2020|2020-12-14|EPI_ISL_790709|Original|hCoV-19^^Noord-Holland|Human|Dutch COVID-19 response team|National Institute for Public Health and the Environment (RIVM)|Meijer|Netherlands
>Spike|hCoV-19/USA/NJ-QDX-2400/2020|2020-03-16|EPI_ISL_604703|Original|hCoV-19^^New Jersey|Human|Quest Diagnostics|Quest Diagnostics|Kagan|USA
>Spike|hCoV-19/Denmark/DCGC-4535/2020|2020-09-21|EPI_ISL_621331|Original|hCoV-19^^Hovedstaden|Human|Department of Virus and Microbiological Special Diagnostics|Albertsen lab|Michaelsen|Denmark
>Spike|hCoV-19/USA/OR-OHSU-0267/2020|2020-04-11|EPI_ISL_509086|Original|hCoV-19^^Oregon|Human|OHSU Lab Services Molecular Microbiology Lab|Oregon SARS-CoV-2 Genome Sequencing Center|O'Roak|USA
>Spike|hCoV-19/Australia/NSW1188/2020|2020-10-28|EPI_ISL_667789|Original|hCoV-19^^New South Wales|Human|South Eastern Area Laboratory Services (SEALS)|NSW Health Pathology - Institute of Clinical Pathology and Medical Research; Westmead Hospital; University of Sydney|Gall|Australia
>Spike|hCoV-19/England/SHEF-C9197/2020|2020-09-17|EPI_ISL_585035|Original|hCoV-19^^England|Human|Virology Department|COVID-19 Genomics UK (COG-UK) Consortium|de Silva|United Kingdom

>Spike|hCoV-19/England/201640232/2020|2020-04-14|EPI_ISL_466450|Original|hCoV-
19^^England|Human|Respiratory Virus Unit|Respiratory Virus Unit|Platt|United Kingdom
>Spike|hCoV-19/England/CAMC-B0B18F/2020|2020-10-29|EPI_ISL_643602|Original|hCoV-
19^^England|Human|Lighthouse Lab in Cambridge|Wellcome Sanger Institute for the
[truncated: 490,035 more chars]
